# Global fertility in 204 countries and territories, 1950–2021, with forecasts to 2100: a comprehensive demographic analysis for the Global Burden of Disease Study 2021

**DOI:** 10.1016/S0140-6736(24)00550-6

**Published:** 2024-05-18

**Authors:** GBD 2021 Fertility and Forecasting Collaborators, Natalia V Bhattacharjee, Austin E Schumacher, Amirali Aali, Yohannes Habtegiorgis Abate, Rouzbeh Abbasgholizadeh, Mohammadreza Abbasian, Mohsen Abbasi-Kangevari, Hedayat Abbastabar, Samar Abd ElHafeez, Sherief Abd-Elsalam, Mohammad Abdollahi, Mohammad-Amin Abdollahifar, Meriem Abdoun, Auwal Abdullahi, Mesfin Abebe, Samrawit Shawel Abebe, Olumide Abiodun, Hassan Abolhassani, Meysam Abolmaali, Mohamed Abouzid, Girma Beressa Aboye, Lucas Guimarães Abreu, Woldu Aberhe Abrha, Michael R M Abrigo, Dariush Abtahi, Hasan Abualruz, Bilyaminu Abubakar, Eman Abu-Gharbieh, Niveen ME Abu-Rmeileh, Tadele Girum Girum Adal, Mesafint Molla Adane, Oluwafemi Atanda Adeagbo Adeagbo, Rufus Adesoji Adedoyin, Victor Adekanmbi, Bashir Aden, Abiola Victor Adepoju, Olatunji O Adetokunboh, Juliana Bunmi Adetunji, Daniel Adedayo Adeyinka, Olorunsola Israel Adeyomoye, Qorinah Estiningtyas Sakilah Adnani, Saryia Adra, Rotimi Felix Afolabi, Shadi Afyouni, Muhammad Sohail Afzal, Saira Afzal, Shahin Aghamiri, Antonella Agodi, Williams Agyemang-Duah, Bright Opoku Ahinkorah, Austin J Ahlstrom, Aqeel Ahmad, Danish Ahmad, Firdos Ahmad, Muayyad M Ahmad, Sajjad Ahmad, Tauseef Ahmad, Ali Ahmed, Ayman Ahmed, Haroon Ahmed, Luai A Ahmed, Meqdad Saleh Ahmed, Syed Anees Ahmed, Marjan Ajami, Budi Aji, Gizachew Taddesse Akalu, Hossein Akbarialiabad, Rufus Olusola Akinyemi, Mohammed Ahmed Akkaif, Sreelatha Akkala, Hanadi Al Hamad, Syed Mahfuz Al Hasan, Mohammad Al Qadire, Tareq Mohammed Ali AL-Ahdal, Samer O Alalalmeh, Tariq A Alalwan, Ziyad Al-Aly, Khurshid Alam, Rasmieh Mustafa Al-amer, Fahad Mashhour Alanezi, Turki M Alanzi, Almaza Albakri, Mohammed Albashtawy, Mohammad T AlBataineh, Hediyeh Alemi, Sharifullah Alemi, Yihun Mulugeta Alemu, Ayman Al-Eyadhy, Adel Ali Saeed Al-Gheethi, Khalid F Alhabib, Noora Alhajri, Fadwa Alhalaiqa Naji Alhalaiqa, Robert Kaba Alhassan, Abid Ali, Beriwan Abdulqadir Ali, Liaqat Ali, Mohammed Usman Ali, Rafat Ali, Syed Shujait Shujait Ali, Sheikh Mohammad Alif, Mohammad Aligol, Mehran Alijanzadeh, Mohammad A M Aljasir, Syed Mohamed Aljunid, Sabah Al-Marwani, Joseph Uy Almazan, Hesham M Al-Mekhlafi, Omar Almidani, Mahmoud A Alomari, Basem Al-Omari, Jaber S Alqahtani, Ahmed Yaseen Alqutaibi, Rajaa M Al-Raddadi, Salman Khalifah Al-Sabah, Awais Altaf, Jaffar A Al-Tawfiq, Khalid A Altirkawi, Deborah Oyine Aluh, Farrukh Jawad Alvi, Nelson Alvis-Guzman, Hassan Alwafi, Yaser Mohammed Al-Worafi, Hany Aly, Safwat Aly, Karem H Alzoubi, Edward Kwabena Ameyaw, Tarek Tawfik Amin, Alireza Amindarolzarbi, Mostafa Amini-Rarani, Sohrab Amiri, Irene Gyamfuah Ampomah, Dickson A Amugsi, Ganiyu Adeniyi Amusa, Robert Ancuceanu, Deanna Anderlini, Pedro Prata Andrade, Catalina Liliana Andrei, Tudorel Andrei, Abhishek Anil, Sneha Anil, Adnan Ansar, Alireza Ansari-Moghaddam, Catherine M Antony, Ernoiz Antriyandarti, Saeid Anvari, SALEHA ANWAR, Razique Anwer, Anayochukwu Edward Anyasodor, Jalal Arabloo, Razman Arabzadeh Bahri, Elshaimaa A Arafa, Mosab Arafat, Ana Margarida Araújo, Aleksandr Y Aravkin, Abdulfatai Aremu, Timur Aripov, Mesay Arkew, Benedetta Armocida, Johan Ärnlöv, Mahwish Arooj, Anton A Artamonov, Judie Arulappan, Raphael Taiwo Aruleba, Ashokan Arumugam, Mohsen Asadi-Lari, Zatollah Asemi, Saeed Asgary, Mona Asghariahmadabad, Mohammad Asghari-Jafarabadi, Mubarek Yesse Ashemo, Muhammad Ashraf, Tahira Ashraf, Marvellous O Asika, Seyyed Shamsadin Athari, Maha Moh'd Wahbi Atout, Alok Atreya, Avinash Aujayeb, Marcel Ausloos, Abolfazl Avan, Amlaku Mulat Aweke, Getnet Melaku Ayele, Seyed Mohammad Ayyoubzadeh, Sina Azadnajafabad, Rui M S Azevedo, Ahmed Y Azzam, Muhammad Badar, Ashish D Badiye, Soroush Baghdadi, Nasser Bagheri, Sara Bagherieh, Najmeh Bahmanziari, Ruhai Bai, Atif Amin Baig, Jennifer L Baker, Abdulaziz T Bako, Ravleen Kaur Bakshi, Madhan Balasubramanian, Ovidiu Constantin Baltatu, Kiran Bam, Maciej Banach, Soham Bandyopadhyay, Biswajit Banik, Palash Chandra Banik, Hansi Bansal, Mehmet Firat Baran, Martina Barchitta, Mainak Bardhan, Erfan Bardideh, Suzanne Lyn Barker-Collo, Till Winfried Bärnighausen, Francesco Barone-Adesi, Hiba Jawdat Barqawi, Amadou Barrow, Sandra Barteit, Zarrin Basharat, Asma'u I J Bashir, Hameed Akande Bashiru, Afisu Basiru, João Diogo Basso, Sanjay Basu, Abdul-Monim Mohammad Batiha, Kavita Batra, Bernhard T Baune, Mohsen Bayati, Tahmina Begum, Emad Behboudi, Amir Hossein Behnoush, Maryam Beiranvand, Diana Fernanda Bejarano Ramirez, Alehegn Bekele, Sefealem Assefa Belay, Uzma Iqbal Belgaumi, Michelle L Bell, Olorunjuwon Omolaja Bello, Apostolos Beloukas, Isabela M Bensenor, Zombor Berezvai, Alemshet Yirga Berhie, Amiel Nazer C Bermudez, Paulo J G Bettencourt, Akshaya Srikanth Bhagavathula, Nikha Bhardwaj, Pankaj Bhardwaj, Prarthna V Bhardwaj, Sonu Bhaskar, Vivek Bhat, Gurjit Kaur Bhatti, Jasvinder Singh Bhatti, Manpreet S Bhatti, Rajbir Bhatti, Antonio Biondi, Catherine Bisignano, Atanu Biswas, Raaj Kishore Biswas, Veera R Bitra, Tone Bjørge, Elye Bliss, Micheal Kofi Boachie, Anca Vasilica Bobirca, Virginia Bodolica, Aadam Olalekan Bodunrin, Eyob Ketema Bogale, Kassawmar Angaw Bogale, Milad Bonakdar Hashemi, Berrak Bora Basara, Souad Bouaoud, Dejana Braithwaite, Michael Brauer, Nicholas J K Breitborde, Dana Bryazka, Norma B Bulamu, Danilo Buonsenso, Katrin Burkart, Richard A Burns, Yasser Bustanji, Nadeem Shafique Butt, Zahid A Butt, Florentino Luciano Caetano dos Santos, Daniela Calina, Ismael R Campos-Nonato, Fan Cao, Shujin Cao, Angelo Capodici, Giulia Carreras, Andrea Carugno, Carlos A Castañeda-Orjuela, Giulio Castelpietra, Maria Sofia Cattaruzza, Arthur Caye, Luca Cegolon, Francieli Cembranel, Ester Cerin, Joshua Chadwick, Yaacoub Chahine, Chiranjib Chakraborty, Julian Chalek, Jeffrey Shi Kai Chan, Periklis Charalampous, Vijay Kumar Chattu, Sarika Chaturvedi, Malizgani Paul Chavula, An-Tian Chen, Haowei Chen, Simiao Chen, Gerald Chi, Fatemeh Chichagi, Ju-Huei Chien, Patrick R Ching, William C S Cho, Sungchul Choi, Bryan Chong, Hitesh Chopra, Sonali Gajanan Choudhari, Devasahayam J Christopher, Dinh-Toi Chu, Isaac Sunday Chukwu, Eric Chung, Sheng-Chia Chung, Zinhle Cindi, Iolanda Cioffi, Raffaela Ciuffreda, Rafael M Claro, Kaleb Coberly, Alyssa Columbus, Haley Comfort, Joao Conde, Michael H Criqui, Natália Cruz-Martins, Silvia Magali Cuadra-Hernández, Sriharsha Dadana, Omid Dadras, Tukur Dahiru, Zhaoli Dai, Bronte Dalton, Giovanni Damiani, Aso Mohammad Darwesh, Jai K Das, Saswati Das, Mohsen Dashti, Anna Dastiridou, Claudio Alberto Dávila-Cervantes, Kairat Davletov, Aklilu Tamire Debele, Shayom Debopadhaya, Somayeh Delavari, Ivan Delgado-Enciso, Dessalegn Demeke, Berecha Hundessa Demessa, Xinlei Deng, Edgar Denova-Gutiérrez, Kebede Deribe, Nikolaos Dervenis, Hardik Dineshbhai Desai, Rupak Desai, Vinoth Gnana Chellaiyan Devanbu, Arkadeep Dhali, Kuldeep Dhama, Meghnath Dhimal, Vishal R Dhulipala, Diana Dias da Silva, Daniel Diaz, Michael J Diaz, Adriana Dima, Delaney D Ding, M Ashworth Dirac, Thanh Chi Do, Thao Huynh Phuong Do, Camila Bruneli do Prado, Sushil Dohare, Wanyue Dong, Mario D'Oria, Wendel Mombaque dos Santos, Leila Doshmangir, Robert Kokou Dowou, Ashel Chelsea Dsouza, Haneil Larson Dsouza, Viola Dsouza, John Dube, Joe Duprey, Andre Rodrigues Duraes, Senbagam Duraisamy, Oyewole Christopher Durojaiye, Sulagna Dutta, Laura Dwyer-Lindgren, Paulina Agnieszka Dzianach, Arkadiusz Marian Dziedzic, Alireza Ebrahimi, Hisham Atan Edinur, Kristina Edvardsson, Ferry Efendi, Terje Andreas Eikemo, Michael Ekholuenetale, Maha El Tantawi, Noha Mousaad Elemam, Ghada Metwally Tawfik ElGohary, Muhammed Elhadi, Legesse Tesfaye Elilo, Omar Abdelsadek Abdou Elmeligy, Mohamed A Elmonem, Mohammed Elshaer, Ibrahim Elsohaby, Amir Emami Zeydi, Luchuo Engelbert Bain, Sharareh Eskandarieh, Francesco Esposito, Kara Estep, Farshid Etaee, Natalia Fabin, Adeniyi Francis Fagbamigbe, Saman Fahimi, Aliasghar Fakhri-Demeshghieh, Luca Falzone, Ali Faramarzi, MoezAlIslam Ezzat Mahmoud Faris, Sam Farmer, Andre Faro, Abidemi Omolara Fasanmi, Ali Fatehizadeh, Nelsensius Klau Fauk, Pooria Fazeli, Valery L Feigin, Seyed-Mohammad Fereshtehnejad, Abdullah Hamid Feroze, Pietro Ferrara, Nuno Ferreira, Getahun Fetensa, Irina Filip, Florian Fischer, Joanne Flavel, Nataliya A Foigt, Morenike Oluwatoyin Folayan, Artem Alekseevich Fomenkov, Behzad Foroutan, Matteo Foschi, Kayode Raphael Fowobaje, Kate Louise Francis, Alberto Freitas, Takeshi Fukumoto, John E Fuller, Blima Fux, Peter Andras Gaal, Muktar A Gadanya, Abhay Motiramji Gaidhane, Yaseen Galali, Silvano Gallus, Aravind P Gandhi, Balasankar Ganesan, Mohammad Arfat Ganiyani, M.A. Garcia-Gordillo, Naval Garg, Rupesh K Gautam, Federica Gazzelloni, Semiu Olatunde Gbadamosi, Miglas W Gebregergis, Mesfin Gebrehiwot, Tesfay Brhane Gebremariam, Tesfay B B Gebremariam, Teferi Gebru Gebremeskel, Yohannes Fikadu Geda, Simona Roxana Georgescu, Urge Gerema, Habtamu Geremew, Motuma Erena Getachew, Peter W Gething, MohammadReza Ghasemi, Ghazal Ghasempour Dabaghi, Afsaneh Ghasemzadeh, Fariba Ghassemi, Ramy Mohamed Ghazy, Sailaja Ghimire, Asadollah Gholamian, Ali Gholamrezanezhad, Mahsa Ghorbani, Aloke Gopal Ghoshal, Arun Digambarrao Ghuge, Artyom Urievich Gil, Tiffany K Gill, Matteo Giorgi, Alem Girmay, James C Glasbey, Laszlo Göbölös, Amit Goel, Ali Golchin, Mahaveer Golechha, Pouya Goleij, Sameer Vali Gopalani, Houman Goudarzi, Alessandra C Goulart, Anmol Goyal, Simon Matthew Graham, Michal Grivna, Shi-Yang Guan, Giovanni Guarducci, Mohammed Ibrahim Mohialdeen Gubari, Mesay Dechasa Gudeta, Stefano Guicciardi, Snigdha Gulati, David Gulisashvili, Damitha Asanga Gunawardane, Cui Guo, Anish Kumar Gupta, Bhawna Gupta, Manoj Kumar Gupta, Mohak Gupta, Sapna Gupta, Veer Bala Gupta, Vijai Kumar Gupta, Vivek Kumar Gupta, Annie Haakenstad, Farrokh Habibzadeh, Najah R Hadi, Nils Haep, Ramtin Hajibeygi, Sebastian Haller, Rabih Halwani, Randah R Hamadeh, Nadia M Hamdy, Sajid Hameed, Samer Hamidi, Qiuxia Han, Alexis J Handal, Graeme J Hankey, Md Nuruzzaman Haque, Josep Maria Haro, Ahmed I Hasaballah, Ikramul Hasan, Mohammad Jahid Hasan, S.M. Mahmudul Hasan, Hamidreza Hasani, Md Saquib Hasnain, Amr Hassan, Ikrama Hassan, Soheil Hassanipour, Hadi Hassankhani, Simon I Hay, Jeffrey J Hebert, Omar E Hegazi, Mohammad Heidari, Bartosz Helfer, Mehdi Hemmati, Brenda Yuliana Herrera-Serna, Claudiu Herteliu, Kamran Hessami, Kamal Hezam, Yuta Hiraike, Nguyen Quoc Hoan, Ramesh Holla, Nobuyuki Horita, Md Mahbub Hossain, Mohammad Bellal Hossain Hossain, Hassan Hosseinzadeh, Mehdi Hosseinzadeh, Mihaela Hostiuc, Sorin Hostiuc, Mohamed Hsairi, Vivian Chia-rong Hsieh, Chengxi Hu, Junjie Huang, M Mamun Huda, Ayesha Humayun, Javid Hussain, Nawfal R Hussein, Hong-Han Huynh, Bing-Fang Hwang, Segun Emmanuel Ibitoye, Pulwasha Maria Iftikhar, Olayinka Stephen Ilesanmi, Irena M Ilic, Milena D Ilic, Mustapha Immurana, Leeberk Raja Inbaraj, Afrin Iqbal, Md. Rabiul Islam, Nahlah Elkudssiah Ismail, Hiroyasu Iso, Gaetano Isola, Masao Iwagami, Mahalaxmi Iyer, Linda Merin J, Jalil Jaafari, Louis Jacob, Farhad Jadidi-Niaragh, Khushleen Jaggi, Kasra Jahankhani, Nader Jahanmehr, Haitham Jahrami, Akhil Jain, Nityanand Jain, Ammar Abdulrahman Jairoun, Mihajlo Jakovljevic, Elham Jamshidi, Sabzali Javadov, Tahereh Javaheri, Sathish Kumar Jayapal, Shubha Jayaram, Sun Ha Jee, Jayakumar Jeganathan, Anil K Jha, Ravi Prakash Jha, Heng Jiang, Mohammad Jokar, Jost B Jonas, Tamas Joo, Nitin Joseph, Charity Ehimwenma Joshua, Farahnaz Joukar, Jacek Jerzy Jozwiak, Mikk Jürisson, Vaishali K, Billingsley Kaambwa, Abdulkareem Kabir, Ali Kabir, Hannaneh Kabir, Zubair Kabir, Rizwan Kalani, Leila R Kalankesh, Feroze Kaliyadan, Sanjay Kalra, Rajesh Kamath, Sagarika Kamath, Tanuj Kanchan, Edmund Wedam Kanmiki, Kehinde Kazeem Kanmodi, Suthanthira Kannan S, Sushil Kumar Kansal, Rami S Kantar, Neeti Kapoor, Mehrdad Karajizadeh, Manoochehr Karami, Ibraheem M Karaye, Faizan Zaffar Kashoo, Hengameh Kasraei, Nicholas J Kassebaum, Molly B Kassel, Joonas H Kauppila, Foad Kazemi, sara Kazeminia, John H Kempen, Evie Shoshannah Kendal, Kamyab Keshtkar, Mohammad Keykhaei, Himanshu Khajuria, Amirmohammad Khalaji, Nauman Khalid, Anees Ahmed Khalil, Alireza Khalilian, Faham Khamesipour, Ajmal Khan, Asaduzzaman Khan, Ikramullah Khan, M Nuruzzaman Khan, Maseer Khan, Mohammad Jobair Khan, Moien AB Khan, Young-Ho Khang, Shaghayegh Khanmohammadi, Khaled Khatab, Armin Khavandegar, Hamid Reza Khayat Kashani, Feriha Fatima Khidri, Moein Khormali, Mohammad Ali Khosravi, Mahmood Khosrowjerdi, Wondwosen Teklesilasie Kidane, Zemene Demelash Kifle, Julie Sojin Kim, Min Seo Kim, Ruth W Kimokoti, Kasey E Kinzel, Girmay Tsegay Kiross, Adnan Kisa, Sezer Kisa, Ali-Asghar Kolahi, Farzad Kompani, Gerbrand Koren, Oleksii Korzh, Soewarta Kosen, Sindhura Lakshmi Koulmane Laxminarayana, Kewal Krishan, Varun Krishna, Vijay Krishnamoorthy, Barthelemy Kuate Defo, Connor M Kubeisy, Burcu Kucuk Bicer, Md Abdul Kuddus, Mohammed Kuddus, Ilari Kuitunen, Mukhtar Kulimbet, Harish Kumar, Satyajit Kundu, Kunle Rotimi Kunle, Om P Kurmi, Asep Kusnali, Dian Kusuma, Evans F Kyei, Ilias Kyriopoulos, Carlo La Vecchia, Ben Lacey, Muhammad Awwal Ladan, Lucie Laflamme, Chandrakant Lahariya, Daphne Teck Ching Lai, Dharmesh Kumar Lal, Ratilal Lalloo, Judit Lám, Demetris Lamnisos, Iván Landires, Francesco Lanfranchi, Berthold Langguth, Ariane Laplante-Lévesque, Heidi Jane Larson, Anders O Larsson, Savita Lasrado, Kamaluddin Latief, Kaveh Latifinaibin, Long Khanh Dao Le, Nhi Huu Hanh Le, Trang Diep Thanh Le, Caterina Ledda, Munjae Lee, Paul H Lee, Seung Won Lee, Yo Han Lee, Gebretsadik Kiros Lema, Elvynna Leong, Temesgen L Lerango, An Li, Ming-Chieh Li, Shanshan Li, Wei Li, Xiaopan Li, Virendra S Ligade, Stephen S Lim, Ro-Ting Lin, Paulina A Lindstedt, Stefan Listl, Gang Liu, Jue Liu, Xiaofeng Liu, Xuefeng Liu, Yuewei Liu, Erand Llanaj, Rubén López-Bueno, Platon D Lopukhov, László Lorenzovici, Paulo A Lotufo, Jailos Lubinda, Giancarlo Lucchetti, Alessandra Lugo, Raimundas Lunevicius, Hengliang Lv, Zheng Feei Ma, Kelsey Lynn Maass, Monika Machoy, Áurea M Madureira-Carvalho, Mohammed Magdy Abd El Razek, Azzam A Maghazachi, Soleiman Mahjoub, Mansour Adam Mahmoud, Azeem Majeed, Jeadran N Malagón-Rojas, Elaheh Malakan Rad, Kashish Malhotra, Ahmad Azam Malik, Iram Malik, Deborah Carvalho Malta, Abdullah A Mamun, Yosef Manla, Yasaman Mansoori, Ali Mansour, Borhan Mansouri, Zeinab Mansouri, Mohammad Ali Mansournia, Joemer C Maravilla, Mirko Marino, Abdoljalal Marjani, Gabriel Martinez, Ramon Martinez-Piedra, Francisco Rogerlândio Martins-Melo, Miquel Martorell, Sharmeen Maryam, Roy Rillera Marzo, Alireza Masoudi, Jishanth Mattumpuram, Richard James Maude, Andrea Maugeri, Erin A May, Mahsa Mayeli, Maryam Mazaheri, John J McGrath, Martin McKee, Anna Laura Wensel McKowen, Susan A McLaughlin, Steven M McPhail, Rahul Mehra, Kamran Mehrabani-Zeinabad, Entezar Mehrabi Nasab, Tesfahun Mekene Meto, Max Alberto Mendez Mendez-Lopez, Walter Mendoza, Ritesh G Menezes, George A Mensah, Alexios-Fotios A Mentis, Sultan Ayoub Meo, Mohsen Merati, Atte Meretoja, Tuomo J Meretoja, Abera M Mersha, Tomislav Mestrovic, Pouya Metanat, Kukulege Chamila Dinushi Mettananda, Sachith Mettananda, Adquate Mhlanga, Laurette Mhlanga, Tianyue Mi, Tomasz Miazgowski, Georgia Micha, Irmina Maria Michalek, Ted R Miller, Le Huu Nhat Minh, Mojgan Mirghafourvand, Erkin M Mirrakhimov, Mizan Kiros Mirutse, Moonis Mirza, Roya Mirzaei, Ashim Mishra, Sanjeev Misra, Philip B Mitchell, Chaitanya Mittal, Babak Moazen, Abdalla Z Mohamed, Ahmed Ismail Mohamed, Jama Mohamed, Mouhand F H Mohamed, Nouh Saad Mohamed, Sakineh Mohammad-Alizadeh-Charandabi, Soheil Mohammadi, Abdollah Mohammadian-Hafshejani, Mustapha Mohammed, Salahuddin Mohammed, Shafiu Mohammed, Ali H Mokdad, Peyman Mokhtarzadehazar, Hossein Molavi Vardanjani, Sabrina Molinaro, Lorenzo Monasta, Mohammad Ali Moni, Maryam Moradi, Yousef Moradi, Paula Moraga, Rafael Silveira Moreira, Negar Morovatdar, Shane Douglas Morrison, Jakub Morze, Abbas Mosapour, Elias Mossialos, Rohith Motappa, Parsa Mousavi, Amin Mousavi Khaneghah, Christine Mpundu-Kaambwa, Sumaira Mubarik, Lorenzo Muccioli, Francesk Mulita, Kavita Munjal, Efrén Murillo-Zamora, Jonah Musa, Fungai Musaigwa, Ana-Maria Musina, Sathish Muthu, Saravanan Muthupandian, Muhammad Muzaffar, Woojae Myung, Ahamarshan Jayaraman Nagarajan, Gabriele Nagel, Pirouz Naghavi, Ganesh R Naik, Gurudatta Naik, Mukhammad David Naimzada, Firzan Nainu, Vinay Nangia, Sreenivas Narasimha Swamy, Bruno Ramos Nascimento, Gustavo G Nascimento, Abdallah Y Naser, Mohammad Javad Nasiri, Zuhair S Natto, Javaid Nauman, Muhammad Naveed, Biswa Prakash Nayak, Vinod C Nayak, Rawlance Ndejjo, Sabina Onyinye Nduaguba, Hadush Negash, Chernet Tafere Negesse, Ionut Negoi, Ruxandra Irina Negoi, Seyed Aria Nejadghaderi, Chakib Nejjari, Samata Nepal, Henok Biresaw Netsere, Georges Nguefack-Tsague, Josephine W. Ngunjiri, Dang H Nguyen, Hau Thi Hien Nguyen, Phuong The Nguyen, QuynhAnh P Nguyen, Van Thanh Nguyen, Robina Khan Niazi, Yeshambel T Nigatu, Taxiarchis Konstantinos Nikolouzakis, Ali Nikoobar, Amin Reza Nikpoor, Chukwudi A Nnaji, Lawrence Achilles Nnyanzi, Efaq Ali Noman, Shuhei Nomura, Mamoona Noreen, Nafise Noroozi, Chisom Adaobi Nri-Ezedi, Mengistu H Nunemo, Virginia Nuñez-Samudio, Dieta Nurrika, Jerry John Nutor, Bogdan Oancea, Kehinde O Obamiro, Ismail A Odetokun, Nkechi Martina Odogwu, Martin James O'Donnell, Oluwakemi Ololade Odukoya, Ayodipupo Sikiru Oguntade, James Odhiambo Oguta, In-Hwan Oh, Sylvester Reuben Okeke, Akinkunmi Paul Okekunle, Osaretin Christabel Okonji, Patrick Godwin Okwute, Andrew T Olagunju, Omotola O Olasupo, Matthew Idowu Olatubi, Gláucia Maria Moraes Oliveira, Bolajoko Olubukunola Olusanya, Jacob Olusegun Olusanya, Gideon Olamilekan Oluwatunase, Hany A Omar, Goran Latif Omer, Obinna E Onwujekwe, Michal Ordak, Orish Ebere Orisakwe, Verner N Orish, Doris V Ortega-Altamirano, Alberto Ortiz, Esteban Ortiz-Prado, Wael M S Osman, Uchechukwu Levi Osuagwu, Olayinka Osuolale, Adrian Otoiu, Stanislav S Otstavnov, Amel Ouyahia, Guoqing Ouyang, Mayowa O Owolabi, Yaz Ozten, Mahesh Padukudru P A, Mohammad Taha Pahlevan Fallahy, Feng Pan, Hai-Feng Pan, Adrian Pana, Paramjot Panda, Songhomitra Panda-Jonas, Helena Ullyartha Pangaribuan, Georgios D Panos, Leonidas D Panos, Ioannis Pantazopoulos, Anca Mihaela Pantea Stoian, Romil R Parikh, Seoyeon Park, Ashwaghosha Parthasarathi, Ava Pashaei, Roberto Passera, Hemal M Patel, Jay Patel, Shankargouda Patil, Dimitrios Patoulias, Venkata Suresh Patthipati, Uttam Paudel, Mihaela Paun, Hamidreza Pazoki Toroudi, Spencer A Pease, Amy E Peden, Paolo Pedersini, Minjin Peng, Umberto Pensato, Veincent Christian Filipino Pepito, Prince Peprah, Gavin Pereira, Mario F P Peres, Arokiasamy Perianayagam, Norberto Perico, Simone Perna, Richard G Pestell, Fanny Emily Petermann-Rocha, Hoang Tran Pham, Anil K Philip, Daniela Pierannunzio, Manon Pigeolet, David M Pigott, Evgenii Plotnikov, Dimitri Poddighe, Peter Pollner, Ramesh Poluru, Maarten J Postma, Ghazaleh Pourali, Akram Pourshams, Naeimeh Pourtaheri, Disha Prabhu, Sergio I Prada, Pranil Man Singh Pradhan, Manya Prasad, Akila Prashant, Bharathi M Purohit, Jagadeesh Puvvula, Nameer Hashim Qasim, Ibrahim Qattea, Deepthi R, Mehrdad Rabiee Rad, Amir Radfar, Venkatraman Radhakrishnan, Pourya Raee, Hadi Raeisi Shahraki, Alireza Rafiei, Seyedeh Niloufar Rafiei Alavi, Cat Raggi, Pankaja Raghav Raghav, Fakher Rahim, Md Jillur Rahim, Md. Mosfequr Rahman, Mohammad Hifz Ur Rahman, Mosiur Rahman, Muhammad Aziz Rahman, Vahid Rahmanian, Masoud Rahmati, Niloufar Rahnavard, Pramila Rai, Diego Raimondo, Ali Rajabpour-Sanati, Prashant Rajput, Prasanna Ram, Shakthi Kumaran Ramasamy, Juwel Rana, Kritika Rana, Shailendra Singh Rana, Chhabi Lal Ranabhat, Nemanja Rancic, Amey Rane, Shubham Ranjan, Chythra R Rao, Indu Ramachandra Rao, Deepthi Rapaka, Davide Rasella, Sina Rashedi, Vahid Rashedi, Mohammad-Mahdi Rashidi, Azad Rasul, Zubair Ahmed Ratan, Giridhara Rathnaiah Babu, Santosh Kumar Rauniyar, Nakul Ravikumar, David Laith Rawaf, Salman Rawaf, Reza Rawassizadeh, Bharat Rawlley, Murali Mohan Rama Krishna Reddy, Elrashdy Moustafa Mohamed Redwan, Giuseppe Remuzzi, Bhageerathy Reshmi, Nazila Rezaei, Aida Rezaei Nejad, Mohsen Rezaeian, Abanoub Riad, Mavra A Riaz, Jennifer Rickard, Reza Rikhtegar, Hannah Elizabeth Robinson-Oden, Célia Fortuna Rodrigues, Jefferson Antonio Buendia Rodriguez, Ravi Rohilla, Debby Syahru Romadlon, Luca Ronfani, Himanshu Sekhar Rout, Bedanta Roy, Nitai Roy, Priyanka Roy, Enrico Rubagotti, Guilherme de Andrade Ruela, Susan Fred Rumisha, Tilleye Runghien, Manjula S, Chandan S N, Aly M A Saad, Zahra Saadatian, Maha Mohamed Saber-Ayad, Morteza SaberiKamarposhti, Siamak Sabour, Fatos Sada, Basema Saddik, Bashdar Abuzed Sadee, Ehsan Sadeghi, Erfan Sadeghi, Mohammad Reza Saeb, Umar Saeed, Sher Zaman Safi, Dominic Sagoe, Manika Saha, Amirhossein Sahebkar, Soumya Swaroop Sahoo, Monalisha Sahu, Zahra Saif, Joseph W Sakshaug, Payman Salamati, Afeez Abolarinwa Salami, Mohamed A Saleh, Marwa Rashad Salem, Mohammed Z Y Salem, Sohrab Salimi, Sara Samadzadeh, Yoseph Leonardo Samodra, Vijaya Paul Samuel, Abdallah M Samy, Juan Sanabria, Nima Sanadgol, Francesca Sanna, Milena M Santric-Milicevic, Haaris Saqib, Sivan Yegnanarayana Iyer Saraswathy, Aswini Saravanan, Babak Saravi, Yaser Sarikhani, Tanmay Sarkar, Rodrigo Sarmiento-Suárez, Gargi Sachin Sarode, Sachin C Sarode, Arash Sarveazad, Brijesh Sathian, Thirunavukkarasu Sathish, Anudeep Sathyanarayan, Abu Sayeed, Md Abu Sayeed, Nikolaos Scarmeas, Winfried Schlee, Art Schuermans, David C Schwebel, Falk Schwendicke, Siddharthan Selvaraj, Pallav Sengupta, Subramanian Senthilkumaran, Sadaf G Sepanlou, Dragos Serban, Edson Serván-Mori, Yashendra Sethi, SeyedAhmad SeyedAlinaghi, Seyed Arsalan Seyedi, Allen Seylani, Mahan Shafie, Jaffer Shah, Pritik A Shah, Ataollah Shahbandi, Samiah Shahid, Moyad Jamal Shahwan, Ahmed Shaikh, Masood Ali Shaikh, Muhammad Aaqib Shamim, Mehran Shams-Beyranvand, Mohammad Anas Shamsi, Mohd Shanawaz, Abhishek Shankar, Mohammed Shannawaz, Medha Sharath, Sadaf Sharfaei, Amin Sharifan, Javad Sharifi-Rad, Manoj Sharma, Rajesh Sharma, Ujjawal Sharma, Vishal Sharma, Rajesh P Shastry, Amin Shavandi, David H Shaw, Amir Mehdi Shayan, Maryam Shayan, Amr Mohamed Elsayed Shehabeldine, Aziz Sheikh, Rahim Ali Sheikhi, Manjunath Mala Shenoy, Pavanchand H Shetty, Peilin Shi, Desalegn Shiferaw, Mika Shigematsu, Rahman Shiri, Reza Shirkoohi, Aminu Shittu, Velizar Shivarov, Farhad Shokraneh, Sina Shool, Seyed Afshin Shorofi, Kanwar Hamza Shuja, Kerem Shuval, Emmanuel Edwar Siddig, João Pedro Silva, Luís Manuel Lopes Rodrigues Silva, Soraia Silva, Biagio Simonetti, Anjali Singal, Abhinav Singh, Balbir Bagicha Singh, Jasvinder A Singh, Md Shahjahan Siraj, Georgia Smith, Bogdan Socea, Anton Sokhan, Ranjan Solanki, Shipra Solanki, Hamidreza Soleimani, Sameh S M Soliman, Yonatan Solomon, Yimeng Song, Reed J D Sorensen, Michael Spartalis, Chandrashekhar T Sreeramareddy, Vijay Kumar Srivastava, Muhammad Haroon Stanikzai, Vladimir I Starodubov, Antonina V Starodubova, Simona Cătălina Stefan, Paschalis Steiropoulos, Mark A Stokes, Vetriselvan Subramaniyan, Muhammad Suleman, Rizwan Suliankatchi Abdulkader, Abida Sultana, Jing Sun, Chandan Kumar Swain, Bryan L Sykes, Lukasz Szarpak, Mindy D Szeto, Miklós Szócska, Payam Tabaee Damavandi, Rafael Tabarés-Seisdedos, Ozra Tabatabaei Malazy, Seyed-Amir Tabatabaeizadeh, Shima Tabatabai, Karen M Tabb, Mohammad Tabish, Moslem Taheri Soodejani, Jabeen Taiba, Ardeshir Tajbakhsh, Iman M Talaat, Ashis Talukder, Mircea Tampa, Jacques Lukenze Tamuzi, Ker-Kan Tan, Haosu Tang, Derbie Alemu DA Tareke, Mengistie Kassahun Tariku, Vivian Y Tat, Seyed Mohammad Tavangar, Mojtaba Teimoori, Mohamad-Hani Temsah, Reem Mohamad Hani Temsah, Masayuki Teramoto, Dufera Rikitu Terefa, Riki Tesler, Enoch Teye-Kwadjo, Ramna Thakur, Pugazhenthan Thangaraju, Kavumpurathu Raman Thankappan, Rekha Thapar, Samar Tharwat, Rasiah Thayakaran, Nihal Thomas, Ales Tichopad, Jansje Henny Vera Ticoalu, Tenaw Yimer Tiruye, Mariya Vladimirovna Titova, Marcello Tonelli, Marcos Roberto Tovani-Palone, Eugenio Traini, Jasmine T Tran, Nghia Minh Tran, Indang Trihandini, Samuel Joseph Tromans, Thien Tan Tri Tai Truyen, Aristidis Tsatsakis, Evangelia Eirini Tsermpini, Munkhtuya Tumurkhuu, Stefanos Tyrovolas, Sayed Mohammad Nazim Uddin, Aniefiok John Udoakang, Arit Udoh, Atta Ullah, Saeed Ullah, Sana Ullah, Srikanth Umakanthan, Chukwuma David Umeokonkwo, Brigid Unim, Bhaskaran Unnikrishnan, Era Upadhyay, Jibrin Sammani Usman, Marco Vacante, Seyed Mohammad Vahabi, Asokan Govindaraj Vaithinathan, Rohollah Valizadeh, Jef Van den Eynde, Elena Varavikova, Orsolya Varga, Priya Vart, Shoban Babu Varthya, Tommi Juhani Vasankari, Balachandar Vellingiri, Deneshkumar Venugopal, Nicholas Alexander Verghese, Madhur Verma, Massimiliano Veroux, Georgios-Ioannis Verras, Dominique Vervoort, Jorge Hugo Villafañe, Manish Vinayak, Francesco S Violante, Mukesh Vishwakarma, Sergey Konstantinovitch Vladimirov, Vasily Vlassov, Bay Vo, Simona Ruxandra Volovat, Theo Vos, Isidora S Vujcic, Hatem A Wafa, Yasir Waheed, Elias Bekele Wakwoya, Cong Wang, Denny Wang, Fang Wang, Shu Wang, Yanzhong Wang, Yuan-Pang Wang, Paul Ward, Emebet Gashaw Wassie, Stefanie Watson, Marcia R Weaver, Kosala Gayan Weerakoon, Daniel J Weiss, Katherine M Wells, Yi Feng Wen, Ronny Westerman, Taweewat Wiangkham, Dakshitha Praneeth Wickramasinghe, Nuwan Darshana Wickramasinghe, Peter Willeit, Yohannes Addisu Wondimagegene, Felicia Wu, Juan Xia, Hong Xiao, Gelin Xu, Suowen Xu, Xiaoyue Xu, Ali Yadollahpour, Shirin Yaghoobpoor, Tina Yaghoobpour, Sajad Yaghoubi, Zwanden Sule Yahaya, Danting Yang, Lin Yang, Yuichiro Yano, Habib Yaribeygi, Pengpeng Ye, Renjulal Yesodharan, Subah Abderehim Yesuf, Saber Yezli, Amanuel Yigezu, Paul Yip, Dong Keon Yon, Naohiro Yonemoto, Yuyi You, Mustafa Z Younis, Zabihollah Yousefi, Chuanhua Yu, Yong Yu, Chun-Wei Yuan, Nima Zafari, Fathiah Zakham, Nazar Zaki, Giulia Zamagni, Milad Zandi, Ghazal G Z Zandieh, Moein Zangiabadian, Mikhail Sergeevich Zastrozhin, Haijun Zhang, Meixin Zhang, Yunquan Zhang, Chenwen Zhong, Juexiao Zhou, Bin Zhu, Lei Zhu, Magdalena Zielińska, Zhiyong Zou, Samer H Zyoud, Christopher J L Murray, Amanda E Smith, Stein Emil Vollset

## Abstract

**Background:**

Accurate assessments of current and future fertility—including overall trends and changing population age structures across countries and regions—are essential to help plan for the profound social, economic, environmental, and geopolitical challenges that these changes will bring. Estimates and projections of fertility are necessary to inform policies involving resource and health-care needs, labour supply, education, gender equality, and family planning and support. The Global Burden of Diseases, Injuries, and Risk Factors Study (GBD) 2021 produced up-to-date and comprehensive demographic assessments of key fertility indicators at global, regional, and national levels from 1950 to 2021 and forecast fertility metrics to 2100 based on a reference scenario and key policy-dependent alternative scenarios.

**Methods:**

To estimate fertility indicators from 1950 to 2021, mixed-effects regression models and spatiotemporal Gaussian process regression were used to synthesise data from 8709 country-years of vital and sample registrations, 1455 surveys and censuses, and 150 other sources, and to generate age-specific fertility rates (ASFRs) for 5-year age groups from age 10 years to 54 years. ASFRs were summed across age groups to produce estimates of total fertility rate (TFR). Livebirths were calculated by multiplying ASFR and age-specific female population, then summing across ages 10–54 years. To forecast future fertility up to 2100, our Institute for Health Metrics and Evaluation (IHME) forecasting model was based on projections of completed cohort fertility at age 50 years (CCF50; the average number of children born over time to females from a specified birth cohort), which yields more stable and accurate measures of fertility than directly modelling TFR. CCF50 was modelled using an ensemble approach in which three sub-models (with two, three, and four covariates variously consisting of female educational attainment, contraceptive met need, population density in habitable areas, and under-5 mortality) were given equal weights, and analyses were conducted utilising the MR-BRT (meta-regression—Bayesian, regularised, trimmed) tool. To capture time-series trends in CCF50 not explained by these covariates, we used a first-order autoregressive model on the residual term. CCF50 as a proportion of each 5-year ASFR was predicted using a linear mixed-effects model with fixed-effects covariates (female educational attainment and contraceptive met need) and random intercepts for geographical regions. Projected TFRs were then computed for each calendar year as the sum of single-year ASFRs across age groups. The reference forecast is our estimate of the most likely fertility future given the model, past fertility, forecasts of covariates, and historical relationships between covariates and fertility. We additionally produced forecasts for multiple alternative scenarios in each location: the UN Sustainable Development Goal (SDG) for education is achieved by 2030; the contraceptive met need SDG is achieved by 2030; pro-natal policies are enacted to create supportive environments for those who give birth; and the previous three scenarios combined. Uncertainty from past data inputs and model estimation was propagated throughout analyses by taking 1000 draws for past and present fertility estimates and 500 draws for future forecasts from the estimated distribution for each metric, with 95% uncertainty intervals (UIs) given as the 2·5 and 97·5 percentiles of the draws. To evaluate the forecasting performance of our model and others, we computed skill values—a metric assessing gain in forecasting accuracy—by comparing predicted versus observed ASFRs from the past 15 years (2007–21). A positive skill metric indicates that the model being evaluated performs better than the baseline model (here, a simplified model holding 2007 values constant in the future), and a negative metric indicates that the evaluated model performs worse than baseline.

**Findings:**

During the period from 1950 to 2021, global TFR more than halved, from 4·84 (95% UI 4·63–5·06) to 2·23 (2·09–2·38). Global annual livebirths peaked in 2016 at 142 million (95% UI 137–147), declining to 129 million (121–138) in 2021. Fertility rates declined in all countries and territories since 1950, with TFR remaining above 2·1—canonically considered replacement-level fertility—in 94 (46·1%) countries and territories in 2021. This included 44 of 46 countries in sub-Saharan Africa, which was the super-region with the largest share of livebirths in 2021 (29·2% [28·7–29·6]). 47 countries and territories in which lowest estimated fertility between 1950 and 2021 was below replacement experienced one or more subsequent years with higher fertility; only three of these locations rebounded above replacement levels. Future fertility rates were projected to continue to decline worldwide, reaching a global TFR of 1·83 (1·59–2·08) in 2050 and 1·59 (1·25–1·96) in 2100 under the reference scenario. The number of countries and territories with fertility rates remaining above replacement was forecast to be 49 (24·0%) in 2050 and only six (2·9%) in 2100, with three of these six countries included in the 2021 World Bank-defined low-income group, all located in the GBD super-region of sub-Saharan Africa. The proportion of livebirths occurring in sub-Saharan Africa was forecast to increase to more than half of the world's livebirths in 2100, to 41·3% (39·6–43·1) in 2050 and 54·3% (47·1–59·5) in 2100. The share of livebirths was projected to decline between 2021 and 2100 in most of the six other super-regions—decreasing, for example, in south Asia from 24·8% (23·7–25·8) in 2021 to 16·7% (14·3–19·1) in 2050 and 7·1% (4·4–10·1) in 2100—but was forecast to increase modestly in the north Africa and Middle East and high-income super-regions. Forecast estimates for the alternative combined scenario suggest that meeting SDG targets for education and contraceptive met need, as well as implementing pro-natal policies, would result in global TFRs of 1·65 (1·40–1·92) in 2050 and 1·62 (1·35–1·95) in 2100. The forecasting skill metric values for the IHME model were positive across all age groups, indicating that the model is better than the constant prediction.

**Interpretation:**

Fertility is declining globally, with rates in more than half of all countries and territories in 2021 below replacement level. Trends since 2000 show considerable heterogeneity in the steepness of declines, and only a small number of countries experienced even a slight fertility rebound after their lowest observed rate, with none reaching replacement level. Additionally, the distribution of livebirths across the globe is shifting, with a greater proportion occurring in the lowest-income countries. Future fertility rates will continue to decline worldwide and will remain low even under successful implementation of pro-natal policies. These changes will have far-reaching economic and societal consequences due to ageing populations and declining workforces in higher-income countries, combined with an increasing share of livebirths among the already poorest regions of the world.

**Funding:**

Bill & Melinda Gates Foundation.

## Introduction

Characterising trends in key demographic indicators of fertility and projecting estimates into the future are essential to understand the impact of changing birth rates on social, economic, and geopolitical systems, both now and in the coming century. Dynamics in fertility patterns are central to the well established concept of the demographic transition,^1^, ^2^ which classically holds that societies will passage from a condition of high fertility and high mortality with more young than old people to a state of low fertility and low mortality with an increasingly older population. Some theorists have proposed the concept of a demographic dividend, whereby declining fertility rates lead temporarily to higher proportions of working adults available to generate resources and capital, potentially stimulating economic growth and eventual rebounds in fertility rates.^3^ Demographic data in the 5 years preceding 2021 demonstrate that the total fertility rate (TFR) in some countries has fallen below replacement levels—the minimum rate necessary for generational replacement of the population assuming no migration—with no evidence of this predicted rebound.^4^, ^5^, ^6^, ^7^ The replacement level is generally accepted to be a TFR of at least 2·1, although the true replacement level depends on the specific mortality rate and sex ratio at birth in a population.^8^ Low levels of fertility have the potential over time to result in inverted population pyramids with growing numbers of older people and declining working-age populations. These changes are likely to place increasing burdens on health care and social systems, transform labour and consumer markets, and alter patterns of resource use. Accurate estimates and future forecasts of fertility rates and their impact on population age structures are therefore essential to anticipate potential economic and geopolitical consequences and to inform the development of effective health, environmental, and economic policies.

At present, an important source of fertility estimates and future forecasts for countries and areas throughout the world has been the Population Division of the UN Department of Economic and Social Affairs, which most recently produced the 2022 Revision of World Population Prospects (WPP 2022).^5^ The UN Population Division estimates of past fertility are not compliant with the Guidelines on Accurate and Transparent Health Estimates Reporting (GATHER) statement in important respects; notably, they do not provide all code for statistical models or explicit details on criteria for exclusion or adjustment of primary data sources. Furthermore, the validity of UN Population Division projections has been questioned due to the assumptions applied in countries experiencing low post-transition fertility dropping below replacement level.^9^, ^10^ Previous UN Population Division forecasts have assumed that, in such circumstances, fertility rates will increase towards replacement levels,^11^, ^12^, ^13^ and WPP 2022 assumes convergence to a rate that is a combination of country-specific historical rates and the mean rate in low-fertility countries that have experienced fertility increases.^14^ The WPP 2022 projects gradual increases in TFR even in countries that have shown no evidence of fertility rate increases, such as South Korea and Thailand.^6^, ^14^, ^15^, ^16^, ^17^ Additionally, UN Population Division models are based on TFR, which is a period measure and therefore does not account for change over time in fertility behaviours. For example, in settings where fertility rates in older women increase due to choices to delay births, TFR would underestimate fertility forecasts. Reliance on TFR can also lead to short-term fluctuations in estimated fertility forecasts that are especially impactful in countries with low fertility rates.^18^ Moreover, their projections forecast TFR solely as a function of time and do not include other covariates to inform the models, which disregards potentially explanatory data and precludes investigating the effects of alternative policy-related scenarios or other drivers of fertility. The US Census Bureau International Database has also provided worldwide fertility estimates and projections, currently in 227 countries, since the 1960s, but country-specific updates are not performed on a regular basis.^19^ Since the 1990s, global and regional fertility forecasts have also been generated by the World Population Program of the International Institute for Applied Systems Analysis,^20^ with country-level projections more recently produced by an affiliated group, the Wittgenstein Centre for Demography and Global Human Capital.^21^, ^22^ These forecasts rely on assumptions informed by expert opinions from demographic scientists to predict future fertility rates.^21^, ^22^, ^23^

**Research in context**

**Evidence before this study**

Since the 1950s, global and national estimates and projections of key fertility indicators have been produced and regularly updated by the Population Division of the UN Department of Economic and Social Affairs, with the most recent iteration being the 2022 Revision of World Population Prospects. Assessments of fertility at national and subnational levels worldwide have also been conducted by the US Census Bureau since the 1960s, with estimates reported in the Bureau's International Database. More recently, fertility estimates and projections have been generated by the Wittgenstein Centre for Demography and Global Human Capital and by the Global Burden of Diseases, Injuries, and Risk Factors Study (GBD), an ongoing, large-scale research enterprise that systematically analyses worldwide data to assess global health trends. Past estimates of fertility have been produced as part of GBD since 2017, and future forecasts based on GBD findings were first published in 2020.

**Added value of this study**

Of the existing large-scale efforts to estimate worldwide trends in fertility, only GBD analyses are compliant with the Guidelines on Accurate and Transparent Health Estimates Reporting (GATHER) statement. This study also incorporates several important innovations introduced by the GBD population forecasting study by Vollset and colleagues in 2020 that support forecasting accuracy assessment and provide a framework by which to explore the impact of various policy scenarios on fertility patterns. These methods include: basing the GBD forecasting model on a measure of cohort fertility (completed cohort fertility at age 50 years, CCF50) that reflects the number of children born over time to females from a specific cohort, which better captures long-term choices people make about childbearing—such as delaying having children—than does the classic period-based measure of total fertility rate; and incorporating measures of female education and met need for modern contraceptives as covariates, which improves accuracy and allows for modelling alternative scenarios by changing levels of these indicators. In contrast to other models that assume rates in countries currently experiencing low fertility will tend to increase over time, or those that base their projections on expert judgements, GBD fertility forecasting methods are grounded in existing, real-world evidence about fertility patterns in long-term cohorts of females and in data on related evidence-based covariates such as education and contraception. GBD 2021 has further improved the estimation of past, current, and future fertility in four important ways. First, an additional 147 surveys, 21 censuses, and 634 country-years of vital and sample registration data were added for estimation of past fertility trends. Second, smoothing parameters for estimating past fertility trends were updated to better fit available data. Third, to further improve specificity and accuracy of future fertility projections, two additional covariates were included that account for urbanicity (defined here as population density in habitable areas) and under-5 mortality in the CCF50 model. Fourth, we added a pro-natal alternative scenario to help policy makers plan interventions in countries with fertility rates below replacement level. Based on a skill metric designed to evaluate forecasting accuracy, the model presented here performed better across all age groups compared with a constant prediction.

**Implications of all the available evidence**

Our past estimates and future forecasts indicate that fertility rates are declining everywhere and are projected to continue to decrease over the coming century. By 2100, we estimate that fertility rates will be below replacement level in more than 95% of the world's countries and territories but that marked disparities in rates will remain. Our forecasts also suggest that, by 2100, the largest concentrations of livebirths will shift to low-income settings, particularly a subset of countries and territories in sub-Saharan Africa, which are among the most vulnerable to economic and environmental challenges. Extreme shifts in the global distribution of livebirths can be partially ameliorated by improved female education and met need for modern contraception. Outside of this subset of low-income areas, most of the world's countries will experience the repercussions of low fertility, with ageing populations, declining workforces, and inverted population pyramids, which are likely to lead to profound fiscal, economic, and social consequences. National policy makers and the global health community must plan to address these divided sets of demographic challenges emerging worldwide.

The Global Burden of Diseases, Injuries, and Risk Factors Study (GBD) is an ongoing, large-scale research enterprise that characterises the state of global health by estimating key health metrics at global, regional, and national levels.^24^ Beginning with the 2017 GBD cycle, past and current fertility estimates generated as part of the GBD analytic framework were published;^25^ before that, estimates from the UN Population Division were used as inputs to GBD analytic processes.^26^, ^27^ For GBD 2019, past and current fertility estimates were reported jointly with mortality, life expectancy, and population measures in a publication focused on overall demographic estimates,^28^ and GBD-based forecasts of population and fertility up to 2100 were reported separately by Vollset and colleagues in 2020.^5^ GBD fertility estimates are based on clear data and methods applying a standardised approach, providing publicly available code. Vollset and colleagues addressed some of the existing issues regarding the use of TFR in the modelling process by developing an Institute for Health Metrics and Evaluation (IHME) forecasting model based instead on completed cohort fertility at age 50 years (CCF50: the average number of children born over time to females from a specified birth cohort)^29^ to capture change over time in fertility behaviours, which yields more stable and accurate fertility estimates. Vollset and colleagues^4^ also included covariates representing female educational attainment and contraceptive met need (a measure of the proportion of females of reproductive age whose need for contraception has been met with modern contraceptive methods) to better inform fertility estimates and facilitate exploration of alternative future scenarios associated with achievement of UN Sustainable Development Goals (SDGs) related to education and contraception.

The present GBD 2021 study focuses on fertility metrics, presenting past estimates (1950–2021) along with forecasts up to 2100. Results were improved since GBD 2019 and the 2020 study by Vollset and colleagues by incorporating newly available demographic data and through key methodological advances. This paper provides a high-level overview of our findings. We anticipate that the results will provide insights for policy makers and will be used as a tool to help plan and shape future policies to better prepare for profound changes in global fertility.

This paper was produced as part of the GBD Collaborator Network and in accordance with the GBD Protocol.^30^

## Methods

### Overview

For each subsequent GBD round, newly available data and updated methods are used to update the full time series of estimates from 1950 up to the latest year of analysis. As a consequence, GBD 2021 estimates supersede all previous estimates. GBD 2021 estimated key fertility metrics in females between ages 10 years and 54 years in 204 countries and territories grouped into 21 regions and seven super-regions. GBD regions are made up of countries and territories that are geographically close and epidemiologically similar.^31^ These regions are then grouped into super-regions based on cause of death patterns. The full GBD location hierarchy is shown in appendix 1 (table S1). GBD 2021 drew on the expertise of more than 11 000 collaborators across more than 160 countries and territories. The GBD 2021 fertility analysis framework produced estimates for every year from 1950 to 2021 and forecasts up to 2100.

The methods used to produce fertility estimates from 1950 to 2021 closely followed those of GBD 2019.^28^ Methods used to generate fertility forecasts to 2100 were based on a modified and revised version of the modelling approach used in the 2020 study by Vollset and colleagues.^4^ These methods have been peer-reviewed over previous GBD rounds and as part of the peer-review process for GBD 2021. Here we provide an overview of the methods with an emphasis on the main changes since GBD 2019 and the 2020 study by Vollset and colleagues;^4^, ^28^ a more comprehensive description of the analytical methods for GBD 2021 is provided in appendix 1. Additional details on specific data inputs are accessible through the GBD Sources Tool.

### Data sources and processing

We systematically searched for accurate and complete data on livebirths reported according to the age of mothers. In many high-income countries and territories, these data were available from high-quality vital registration systems, but in many lower-income countries, birth registries were incomplete, interrupted, or delayed; in these instances, we instead relied on complete and summary birth histories in censuses and household surveys. Fertility rates from vital registration data were calculated as observed births divided by population estimates. Complete and summary birth history data were collapsed from the available microdata and sample weights applied to calculated age-specific fertility rates (ASFRs) and number of children ever born, respectively. A full description of data seeking and synthesis is provided in appendix 1 (section 2.1). In total, we compiled 58 072 unique location-source-years of data for females aged 10–54 years for the period between 1950 and 2021 (number of sources by location and by year can be found in appendix 1 tables S3 and S4). At the national level, we obtained 8680 unique country-source-years of vital registration data, with an additional 29 country-source-years of data from sample registration systems. We additionally extracted data on period ASFR, or average number of children ever born from surveys and censuses that yielded 735 complete birth histories, 879 summary birth histories, and 28 unclassified forms of birth histories (details are in appendix 1 tables S3 and S4).

Throughout the forecasting modelling processes, we used female education, under-5 mortality, met need for contraceptive use, and population density in habitable areas as covariates. Details of these covariates are available in appendix 1 (section 3.1).

### Fertility from 1950 to 2021

GBD 2021 estimates of fertility metrics between 1950 and 2021 were based on a systematic synthesis of all available data for all GBD locations. The fertility estimation process was closely connected to parallel, concordant modelling of population and mortality, with population estimates used iteratively to generate inputs to fertility estimation models and vice versa.^28^ GBD methods are designed to account for the diversity of data available and the different biases inherent in various data sources, with customised data processing and data synthesis steps implemented to account for known biases, missing data, and heterogeneous measurement metrics used across data sources. Estimation of fertility rates between 1950 and 2021 for females ages 10–54 years largely followed the methods used in GBD 2019.^28^ First, ASFRs were estimated for 5-year age groups between 15 years and 49 years only using age-specific vital registration and complete birth history data. These results were used to split all-age data from vital registration, summary birth history, and other sources into ASFRs, and then another model was fit to estimate ASFRs using the original age-specific ASFR data from vital registration and complete birth history along with these age-split data. Next, we extended these estimates to the age groups of 10–14 years and 50–54 years using data from these ages and adjacent age groups. Finally, ASFR estimates were used to calculate TFR. A summary of these methods follows, with a comprehensive description provided in appendix 1 (section 2).

To estimate ASFRs by 5-year age groups for females aged 15–49 years, we implemented mixed-effects regression models using bounded logit(ASFR) as the outcome. The 20–24-years age group was estimated first, and these estimates were used to model the remaining age groups. Both sets of models were fit separately for the high-income, sub-Saharan Africa, and central Europe, eastern Europe, and central Asia super-regions to account for differences in the relationships between the ASFR of the 20–24-years age group and that of other age groups. ASFRs in the 20–24-years age group were modelled with female educational attainment as a covariate and random intercepts for each location source. Then, we separately modelled ASFRs in the remaining age groups between 15 years and 49 years using a linear spline on the logit(ASFR) in the 20–24-years age group. The selection of spline knots varied by super-region and age group. These models also included female educational attainment as a covariate, except in the high-income super-region, and random intercepts for each location source. After running these mixed-effects models, we corrected for systematic differences across data sources by selecting a reference source for each location and adjusting other sources based on their discrepancy from the reference source. Last, a spatiotemporal Gaussian process regression (ST-GPR) was used to smooth ASFRs across location and time, producing final point estimates and uncertainty intervals (UIs).

First-round ASFR estimates were generated from this modelling approach using age-specific vital registration and complete birth history data. To split total birth data from vital registration data, summary birth histories, and other sources into ASFRs, we calculated the ratio of the parity implied by each total birth data source to the parity estimated in this first-round ASFR model. This ratio was then multiplied by the estimated ASFRs from the first-round model. These age-split data were incorporated into a second round of estimation for each location using the same modelling approach described earlier. To generate estimates for ages 10–14 years and 50–54 years, we estimated the ratio of ASFR to the adjacent age group using all available data, then applied these ratios to the second-round ASFR estimates. We used a mixed-effects regression model to estimate location-specific ratios for ages 10–14 years, whereas we calculated the average ratio across all locations for ages 50–54 years. Finally, TFR was calculated by multiplying the ASFRs from each 5-year age group by five and summing.

### Fertility forecasting

We produced forecasts of fertility using an updated modelling framework (appendix 1 section 3) that improved on the methods in the 2020 study by Vollset and colleagues.^4^ In our updated methods, we used not only estimates of female educational attainment and contraceptive met need as covariates, but also estimates of under-5 mortality and population density in habitable areas to account for a larger variation in CCF50 across all countries in the sub-models (appendix 1 section 3.1, appendix 2 figure S2). Similar to Vollset and colleagues, we continued to forecast fertility with CCF50 rather than TFR, because modelling in cohort space is more stable than in period space. For this analysis, we used past CCF50 estimates for birth cohorts from 1945 to 1972 to forecast CCF50 up to the 2085 birth cohort of females, followed by predicting ASFR for each 5-year age interval as a proportion of CCF50. CCF50 was defined as the average number of children born to an individual female from an observed birth cohort (indexed by year of birth) if she lived to the end of her reproductive lifespan (ages 15–49 years). CCF50 was forecast using an ensemble modelling approach with three equally weighted sub-models (with two, three, and four covariates) in which each sub-model utilised the MR-BRT (meta-regression—Bayesian, regularised, trimmed) tool.^32^ For example, the four-covariate sub-model was represented by the following equation:

$$\begin{array}{c} \log it_{\left(0.7,10\right)}(CCF50_{lc})=\beta_{0}+spline(education_{lc})\times \beta_{1}\\ +met\;need_{lc}\times \beta_{2}\\ +under-5\;mortality_{lc}\times \beta_{3}\\ +population\;density\;per\;habitable\;area_{lc}\times \beta_{4}\\ +{ɛ}_{lc} \end{array}$$

where CCF50 is scaled from more than 0·7 to less than 10 and modelled in logit space for location (*l*) and cohort (*c*), β_0_ is an intercept, **β**_1_ is a vector of the spline coefficients of female educational attainment covariate, β_2_ is a slope on proportion of met need for contraception, β_3_ is a slope on under-5 mortality, β_4_ is a slope on population density in habitable areas, and ε is a residual term. Further details are provided in appendix 1 (section 3.2, figure S1).

From forecast CCF50, we then derived ASFR forecasts for the years 2022 to 2100 using a combination of a linear mixed-effects model, spline interpolation, and an autoregressive integrated moving average (ARIMA) model (1,0,0) on residuals to estimate the age pattern of fertility for each cohort. Once ASFR values for ages 15–49 years were obtained, we inferred the ASFR values for the 10–14-years and 50–54-years age groups based on their ratios to the rest of the age pattern during the last observed year (2021). Single-year age interval ASFRs were summed over all ages to yield the TFR for each calendar year (appendix 1 section 3.3).

We also produced fertility forecasts for four alternative scenarios applied to all 204 countries and territories. These scenarios explore shifting forecast values of two known drivers of fertility (education and met need for contraceptives) as well as a proxy pro-natal policy. More specifically, the scenarios included were: the UN SDG target 4.1 for education is achieved by 2030; the contraceptive met need SDG target 3.7 is achieved by 2030; pro-natal policies are enacted that create supportive environments for those who give birth; and the previous three scenarios combined (more details are provided in appendix 1 section 3.4). For the education SDG scenario, the forecasts assume that by 2030, all people will have 12 years or more of education by the age of 25 years and then maintains the same rate of change as the reference scenario up to 2100. For the contraceptive met need scenario, to reflect the SDG scenario of universal access, the forecasts assumed a linear increase in contraceptive coverage to reach 100% by 2030 and then stay constant up to 2100.

In the pro-natal scenario, we assumed a country will introduce pro-natal policies, such as childcare subsidies, extended parental leave, insurance coverage expansion for infertility treatment,^33^ and other forms of support for parents to afford high-quality child-care services, once TFR decreases to less than 1·75. We then made three assumptions on the effects of such policies. First, we assumed the full effect of pro-natal policies will be to increase TFR by 0·2. Second, it will take 5 years after the policy is introduced for the full increase in TFR to occur, and TFR will rise linearly over that time span. Last, we assumed that both the policies and the increase in TFR by 0·2 will endure for the remainder of the century. For each pro-natal year, the TFR increase was distributed proportionally among the single-year ages according to their reference forecast ASFR values. The pro-natal scenario parameters were drawn from previously observed increases in TFR that coincided with pro-natal policies and broader empirical evidence regarding effects of pro-natal policies in low-fertility contexts. Further details on the pro-natal scenario can be found in appendix 1 (section 3.4.3).

In the combined scenario, we applied the aforementioned changes to the covariate forecasts simultaneously without assigning any weights because these covariates were already embedded in our model and the coefficients for each covariate were calculated based on the observed data.

### GBD 2021 updates

To estimate ASFRs from 1950 to 2021, GBD 2021 added 147 surveys, 21 censuses, and 634 country-years of vital and sample registration data compared with GBD 2019, for a total of 1455 surveys and censuses, 8709 country-years of vital and sample registration data, and 150 other sources. Methods were updated for GBD 2021 by changing the time weight in ST-GPR to use a beta density function, in which hyperparameters were assigned based on quality of available data sources and the number of available datapoints. This better accounted for increased data availability, which improved precision and produced more plausible time trends compared with GBD 2019.

Updates to the fertility forecasting methods first introduced in the 2020 study by Vollset and colleagues^4^ included the incorporation of two new covariates in the CCF50 model—namely, under-5 mortality and population density in habitable areas—in addition to those previously used (ie, female educational attainment and contraceptive met need). Furthermore, the current iteration of the IHME model employed a linear fixed-effect model to forecast 5-year ASFRs, which were interpolated to 1-year estimates using an ARIMA model on the residuals to quantify variation not explained by the covariates.

### Comparison with other models

We evaluated the IHME fertility forecasting model performance based on out-of-sample predictions during the validation period 2007–21. We used the following skill metric^34^ for model evaluation and comparison (see appendix 1 section 3.6 for more details):

$$skill=1-\frac{RMSE(Model)}{RMSE(Baseline\;Model),}$$

where Model is our IHME model, and Baseline Model is a simplistic model in which ASFR of the most recent past year is held constant in the future.^34^ Out-of-sample predicted values for our forecasts were based on the GBD fertility model fit using a dataset in which data sources from 2007 to 2021 were excluded, and these were compared to our final GBD 2021 estimates to compute root mean square error (RMSE) values. This skill metric was calculated across locations and reported for each 5-year age group. A positive skill metric indicates that a model being evaluated performs better than the baseline model, whereas a negative skill metric suggests the opposite.

### GBD research and reporting practices

Point estimates were computed using the mean across 1000 draws from the estimated distribution of the given metric for past and present fertility estimates and 500 draws for future forecasts (see appendix 1 section 2 and 3.5, respectively, for details), and 95% UIs were obtained by taking the 2·5 and 97·5 percentile values from the draws. UIs were computed for forecast alternative scenarios but are only reported in the text and tables. For readability, figures only include UIs for the past and for the future reference scenario. GBD 2021 complies with the GATHER statement (appendix 1 table S2).^35^

Analyses were completed with Python version 3.10.12, Stata 15, and R version 3.5.1. Statistical code used for GBD estimation is publicly available online.

### Role of the funding source

The funder of this study had no role in study design, data collection, data analysis, data interpretation, or writing of the report.

## Results

Additional results and forecasts from the analysis are presented in appendix 2 and are also available in downloadable form through the GBD Results tool.

### Fertility estimates 1950–2021

**Figure 1:**
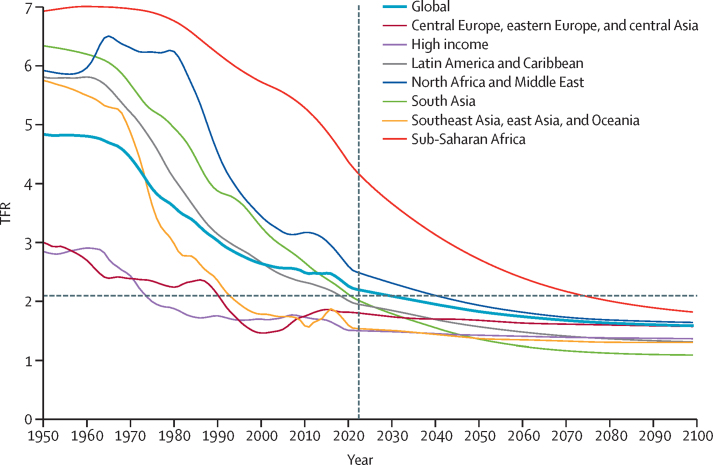
TFR, globally and by GBD super-region, 1950–2100 The dashed horizontal line indicates replacement TFR (2·1), and the dashed vertical line indicates the year 2022 (the first forecast year). GBD=Global Burden of Diseases, Injuries, and Risk Factors Study. TFR=total fertility rate.

There were 129 million (95% UI 121–138) livebirths globally in 2021 (table 1). This is an increase from 92·7 million (88·7–96·6) livebirths in 1950, but a decline from the peak of 142 million (137–147) in 2016 (appendix 2 table S1). The global TFR was 2·23 (95% UI 2·09–2·38) in 2021, a decrease from 4·84 (4·63–5·06) in 1950 and 3·61 (3·53–3·69) in 1980 (table 1, figure 1). This approximate halving constitutes an annualised rate of decline in TFR of 1·1% (1·0–1·2). Across GBD super-regions, the distribution of livebirths changed substantially over the previous seven decades, as did relative levels of TFR. More than one-third of global livebirths in 1950 occurred in southeast Asia, east Asia, and Oceania, which was the largest proportion across super-regions (for livebirth counts, see table 1), corresponding to a TFR of 5·76 (5·44–6·09). This proportion decreased to less than 20% of global livebirths in 2021, with a TFR of 1·55 (1·44–1·66). By contrast, livebirths in south Asia increased from approximately 20% to 25% of global livebirths between 1950 and 2021, and contributed the largest proportion from 1981 to 2011. TFR in this super-region decreased from 6·35 (5·95–6·75) in 1950 to 2·07 (1·89–2·28) in 2021. After 2011, sub-Saharan Africa contributed the largest share of livebirths, up to approximately 30% by 2021 (up from 8% in 1950). Large numbers of livebirths in sub-Saharan Africa in 2021 resulted from a much less steep decrease in TFR over the study period compared with other super-regions, falling from 6·94 (6·62–7·25) in 1950 to 4·29 (4·03–4·58) in 2021. Livebirths and TFRs over time for all locations are presented in table 1.

**Table 1:** Total fertility rate and number of livebirths (thousands) by location in 1950, 1980, and 2021, and for the reference scenario in 2050 and 2100; and net reproductive rate in 2021

			**Total fertility rate**					**Livebirths (thousands)**					**Net reproductive rate, 2021**
			1950	1980	2021	2050	2100	1950	1980	2021	2050	2100	
**Global**			**4·84 (4·63–5·06)**	**3·61 (3·53–3·69)**	**2·23 (2·09–2·38)**	**1·83 (1·59–2·08)**	**1·59 (1·25–1·96)**	**92 675·8 (88 663·5–96 630·8)**	**122 023·7 (119 441·0–124 623·8)**	**129 383·6 (121 382·9–138 206·0)**	**112 073·6 (93 698·4–133 329·8)**	**723 86·8 (40 812·5–118 843·5)**	**1·0 (1·0–1·1)**
**Central Europe, eastern Europe, and central Asia**			**3·01 (2·91–3·11)**	**2·24 (2·21–2·28)**	**1·81 (1·72–1·92)**	**1·68 (1·56–1·81)**	**1·57 (1·42–1·73)**	**7452·5 (7227·2–7699·6)**	**7113·3 (7020·5–7211·4)**	**4906·1 (4635·7–5195·7)**	**3874·8 (3409·2–4396·4)**	**2344·9 (1739·5–3067·1)**	**0·9 (0·8–0·9)**
	Central Asia		4·45 (4·32–4·59)	3·73 (3·65–3·80)	2·79 (2·68–2·91)	2·31 (2·16–2·47)	1·95 (1·76–2·13)	1016·0 (986·0–1046·1)	1676·9 (1642·3–1709·2)	2073·1 (1990·1–2158·2)	1913·2 (1629·7–2201·3)	1418·0 (1007·6–1927·4)	1·3 (1·3–1·4)
		Armenia	4·14 (3·95–4·33)	2·49 (2·39–2·61)	1·68 (1·53–1·84)	1·45 (1·27–1·65)	1·24 (1·01–1·49)	49·0 (46·8–51·1)	76·5 (73·2–79·9)	35·0 (31·6–38·5)	16·9 (11·9–22·2)	6·7 (3·5–11·3)	0·8 (0·7–0·9)
		Azerbaijan	4·38 (4·09–4·69)	3·33 (3·18–3·48)	1·75 (1·55–1·95)	1·51 (1·27–1·76)	1·29 (1·01–1·59)	107·5 (100·2–114·9)	162·2 (155·0–169·6)	138·7 (123·5–154·9)	93·5 (69·0–122·7)	38·3 (17·9–69·8)	0·8 (0·7–0·9)
		Georgia	2·60 (2·42–2·79)	2·21 (2·07–2·33)	2·05 (1·92–2·18)	1·80 (1·65–1·96)	1·52 (1·34–1·71)	84·2 (78·4–90·3)	91·8 (86·3–96·9)	45·2 (42·4–48·1)	36·1 (28·7–43·7)	21·3 (14·2–30·4)	1·0 (0·9–1·0)
		Kazakhstan	3·94 (3·79–4·11)	3·02 (2·93–3·11)	3·02 (2·85–3·20)	2·43 (2·21–2·65)	1·94 (1·69–2·19)	253·8 (244·5–264·1)	365·1 (353·6–376·9)	424·9 (400·9–448·7)	392·9 (325·0–461·4)	261·4 (147·7–409·6)	1·4 (1·4–1·5)
		Kyrgyzstan	4·19 (4·03–4·35)	4·12 (3·98–4·27)	2·92 (2·66–3·21)	2·35 (2·05–2·70)	1·95 (1·63–2·33)	57·9 (55·6–60·2)	111·9 (107·3–116·3)	159·2 (145·4–175·1)	139·1 (104·2–181·6)	72·0 (21·5–145·6)	1·4 (1·3–1·5)
		Mongolia	5·09 (4·78–5·41)	5·76 (5·55–5·97)	3·16 (2·86–3·49)	2·46 (2·02–2·88)	1·87 (1·35–2·35)	30·2 (28·4–32·1)	61·9 (59·7–64·1)	80·0 (72·4–88·0)	100·9 (76·4–124·5)	104·2 (46·7–179·1)	1·5 (1·4–1·6)
		Tajikistan	6·65 (6·33–6·96)	5·65 (5·47–5·84)	3·40 (3·17–3·64)	2·66 (2·33–2·97)	2·13 (1·75–2·49)	86·2 (82·6–89·9)	159·7 (154·8–164·5)	286·4 (268·0–306·6)	301·0 (229·2–381·8)	243·2 (101·7–421·6)	1·6 (1·5–1·7)
		Turkmenistan	4·82 (4·63–5·01)	4·75 (4·55–4·94)	2·83 (2·54–3·15)	2·25 (1·87–2·66)	1·81 (1·38–2·28)	48·7 (46·7–50·5)	96·4 (92·9–99·8)	110·5 (99·4–122·7)	105·2 (80·2–139·0)	75·9 (33·1–146·2)	1·3 (1·2–1·5)
		Uzbekistan	5·68 (5·32–6·06)	4·58 (4·47–4·69)	2·87 (2·66–3·10)	2·34 (2·08–2·62)	1·97 (1·69–2·27)	298·6 (280·9–316·8)	551·5 (538·2–564·1)	793·1 (733·9–854·3)	727·6 (491·2–993·0)	595·0 (307·1–992·9)	1·3 (1·2–1·4)
	Central Europe		3·22 (3·13–3·30)	2·21 (2·18–2·24)	1·48 (1·36–1·61)	1·34 (1·19–1·50)	1·21 (1·03–1·41)	2336·9 (2276·3–2399·1)	2085·5 (2056·7–2113·8)	1038·3 (954·3–1129·1)	668·1 (567·7–786·3)	283·6 (185·8–412·4)	0·7 (0·7–0·8)
		Albania	5·88 (5·63–6·13)	3·44 (3·31–3·58)	1·50 (1·33–1·69)	1·34 (1·10–1·61)	1·17 (0·86–1·50)	48·0 (45·9–50·0)	71·4 (68·8–74·3)	27·9 (24·7–31·4)	16·4 (11·6–22·4)	6·3 (2·9–12·3)	0·7 (0·6–0·8)
		Bosnia and Herzegovina	3·68 (3·31–4·08)	2·21 (1·97–2·46)	1·33 (1·20–1·46)	1·16 (0·99–1·35)	0·95 (0·71–1·19)	92·6 (83·8–102·1)	79·0 (70·7–87·8)	26·7 (24·2–29·4)	12·4 (8·8–16·8)	3·5 (1·7–6·2)	0·6 (0·6–0·7)
		Bulgaria	2·77 (2·65–2·91)	2·07 (2·02–2·12)	1·58 (1·47–1·70)	1·43 (1·29–1·59)	1·26 (1·08–1·45)	167·6 (160·5–175·7)	127·8 (124·7–130·8)	58·1 (53·7–62·4)	36·6 (29·6–44·0)	13·9 (8·5–21·6)	0·8 (0·7–0·8)
		Croatia	2·89 (2·78–3·00)	1·83 (1·78–1·87)	1·37 (1·22–1·53)	1·27 (1·08–1·46)	1·14 (0·92–1·38)	90·2 (87·0–93·5)	67·4 (65·9–69·2)	34·8 (31·0–38·9)	16·8 (12·3–22·0)	3·2 (0·3–7·2)	0·7 (0·6–0·7)
		Czechia	2·81 (2·69–2·94)	2·08 (2·03–2·13)	1·74 (1·57–1·93)	1·54 (1·34–1·76)	1·36 (1·13–1·60)	186·6 (178·6–194·9)	152·3 (148·6–155·8)	105·9 (95·4–117·6)	82·1 (67·2–98·5)	44·9 (27·3–68·3)	0·8 (0·8–0·9)
		Hungary	2·58 (2·43–2·72)	1·89 (1·84–1·94)	1·56 (1·40–1·75)	1·42 (1·22–1·65)	1·29 (1·06–1·55)	193·1 (182·7–203·9)	147·0 (143·1–151·2)	87·9 (78·5–98·4)	77·0 (61·9–94·7)	51·6 (32·1–78·9)	0·8 (0·7–0·8)
		Montenegro	4·12 (3·89–4·37)	2·22 (2·13–2·30)	1·72 (1·61–1·83)	1·56 (1·43–1·70)	1·40 (1·23–1·58)	12·5 (11·8–13·2)	10·5 (10·1–10·9)	7·0 (6·5–7·4)	4·5 (3·7–5·4)	1·6 (0·9–2·5)	0·8 (0·8–0·9)
		North Macedonia	3·62 (3·26–4·02)	2·45 (2·21–2·71)	1·23 (1·16–1·30)	1·10 (1·01–1·20)	0·97 (0·84–1·09)	37·6 (34·0–41·5)	39·9 (36·0–44·0)	18·7 (17·7–19·8)	9·4 (7·3–11·9)	1·8 (0·9–3·0)	0·6 (0·6–0·6)
		Poland	3·63 (3·53–3·72)	2·28 (2·24–2·32)	1·37 (1·22–1·53)	1·21 (1·04–1·40)	1·07 (0·87–1·29)	757·4 (737·3–776·6)	693·3 (680·1–706·4)	342·0 (304·9–381·1)	206·2 (163·3–254·7)	74·4 (43·4–116·7)	0·7 (0·6–0·7)
		Romania	3·02 (2·84–3·22)	2·32 (2·25–2·39)	1·70 (1·57–1·84)	1·48 (1·32–1·66)	1·26 (1·06–1·48)	415·0 (390·9–442·4)	387·3 (375·7–399·2)	177·6 (163·6–192·6)	114·9 (89·2–143·0)	37·7 (18·8–62·7)	0·8 (0·8–0·9)
		Serbia	3·31 (3·22–3·40)	2·22 (2·17–2·28)	1·08 (0·99–1·16)	1·01 (0·90–1·11)	0·96 (0·82–1·09)	183·9 (179·1–188·1)	157·5 (153·3–161·7)	61·6 (56·8–66·7)	34·0 (27·8–40·6)	10·5 (7·1–14·6)	0·5 (0·5–0·6)
		Slovakia	3·65 (3·56–3·75)	2·32 (2·27–2·36)	1·63 (1·53–1·73)	1·46 (1·34–1·59)	1·31 (1·16–1·46)	99·6 (97·2–102·2)	94·6 (92·9–96·3)	56·3 (52·9–59·8)	40·0 (34·2–45·7)	20·9 (15·1–27·4)	0·8 (0·7–0·8)
		Slovenia	2·86 (2·54–3·20)	2·01 (1·97–2·06)	1·63 (1·53–1·74)	1·51 (1·39–1·64)	1·38 (1·24–1·54)	32·3 (28·8–36·2)	29·5 (28·8–30·1)	18·8 (17·6–20·0)	17·9 (15·7–20·5)	13·2 (10·3–16·9)	0·8 (0·7–0·8)
	Eastern Europe		2·70 (2·59–2·82)	1·91 (1·88–1·95)	1·38 (1·27–1·49)	1·28 (1·15–1·42)	1·19 (1·05–1·35)	4099·6 (3935·2–4285·2)	3350·8 (3289·2–3418·5)	1794·7 (1651·3–1949·2)	1293·5 (1082·3–1534·3)	643·2 (456·2–881·7)	0·7 (0·6–0·7)
		Belarus	3·00 (2·83–3·18)	2·01 (1·94–2·09)	1·42 (1·23–1·64)	1·29 (1·06–1·55)	1·19 (0·95–1·47)	192·3 (181·8–203·2)	156·4 (150·9–162·4)	82·5 (70·6–95·6)	59·8 (43·0–80·3)	30·5 (16·3–53·1)	0·7 (0·6–0·8)
		Estonia	2·30 (2·18–2·43)	2·06 (2·01–2·11)	1·60 (1·49–1·71)	1·37 (1·24–1·50)	1·21 (1·06–1·36)	20·1 (19·1–21·2)	22·5 (21·9–23·1)	13·1 (12·2–14·0)	9·4 (8·0–11·2)	4·2 (2·8–5·9)	0·8 (0·7–0·8)
		Latvia	1·98 (1·84–2·14)	1·90 (1·86–1·94)	1·52 (1·35–1·71)	1·35 (1·16–1·56)	1·22 (1·01–1·49)	32·9 (30·7–35·5)	35·6 (34·9–36·4)	16·8 (14·9–18·9)	9·7 (7·5–12·4)	3·9 (2·2–6·6)	0·7 (0·6–0·8)
		Lithuania	2·92 (2·78–3·10)	2·00 (1·95–2·05)	1·40 (1·30–1·51)	1·23 (1·11–1·35)	1·09 (0·96–1·25)	58·6 (55·7–62·0)	51·3 (49·9–52·6)	23·6 (21·9–25·4)	12·4 (10·0–15·4)	4·2 (2·7–6·1)	0·7 (0·6–0·7)
		Moldova	3·77 (3·61–3·92)	2·46 (2·38–2·54)	1·18 (1·06–1·33)	1·09 (0·94–1·25)	1·03 (0·87–1·24)	84·7 (81·4–88·0)	86·1 (83·4–88·9)	28·4 (25·4–32·0)	9·5 (5·6–13·4)	2·7 (1·3–4·7)	0·6 (0·5–0·6)
		Russia	2·77 (2·62–2·95)	1·87 (1·83–1·93)	1·48 (1·37–1·60)	1·33 (1·20–1·47)	1·21 (1·06–1·37)	2819·9 (2671·0–2995·4)	2237·6 (2184·0–2299·0)	1352·4 (1251·9–1464·1)	1053·8 (871·5–1239·4)	562·8 (408·0–758·1)	0·7 (0·7–0·8)
		Ukraine	2·44 (2·33–2·55)	1·95 (1·90–2·01)	1·05 (0·94–1·18)	1·01 (0·88–1·16)	0·98 (0·83–1·16)	891·1 (853·1–930·3)	761·3 (739·2–783·2)	277·9 (246·7–311·8)	138·8 (104·8–185·4)	34·9 (19·2–60·5)	0·5 (0·4–0·6)
**High income**			**2·85 (2·78–2·92)**	**1·88 (1·86–1·90)**	**1·51 (1·41–1·61)**	**1·43 (1·30–1·56)**	**1·37 (1·22–1·53)**	**13 626·1 (13 275·0–13 959·1)**	**12 483·6 (12 339·3–12 633·7)**	**10 399·8 (9728·0–11 116·3)**	**9387·4 (8381·2–10 552·0)**	**6961·9 (5348·6–8941·5)**	**0·7 (0·7–0·8)**
	Australasia		3·13 (3·06–3·21)	1·93 (1·90–1·97)	1·64 (1·48–1·80)	1·45 (1·25–1·68)	1·33 (1·08–1·59)	251·9 (246·1–258·0)	278·2 (273·0–283·7)	357·9 (324·6–393·9)	404·9 (338·5–481·9)	363·9 (250·1–516·0)	0·8 (0·7–0·9)
		Australia	3·06 (2·98–3·14)	1·92 (1·88–1·97)	1·64 (1·47–1·82)	1·45 (1·23–1·70)	1·32 (1·06–1·61)	202·2 (196·9–207·7)	227·7 (222·7–232·9)	299·3 (268·5–332·1)	339·1 (278·3–411·8)	307·8 (204·0–447·8)	0·8 (0·7–0·9)
		New Zealand	3·49 (3·41–3·57)	1·96 (1·92–2·00)	1·62 (1·53–1·72)	1·45 (1·33–1·58)	1·35 (1·20–1·51)	49·7 (48·7–50·8)	50·5 (49·4–51·6)	58·6 (55·2–62·0)	65·7 (57·4–74·2)	56·1 (42·5–72·1)	0·8 (0·7–0·8)
	High-income Asia Pacific		3·72 (3·59–3·86)	1·94 (1·89–2·00)	1·12 (1·03–1·22)	1·14 (1·00–1·30)	1·14 (0·96–1·35)	3059·8 (2947·5–3174·6)	2467·7 (2400·6–2541·4)	1169·5 (1075·7–1275·1)	908·3 (784·1–1047·7)	499·8 (348·5–707·9)	0·5 (0·5–0·6)
		Brunei	6·41 (6·24–6·57)	3·87 (3·65–4·10)	1·65 (1·43–1·88)	1·40 (1·08–1·78)	1·25 (0·87–1·70)	2·7 (2·6–2·7)	5·8 (5·4–6·1)	6·4 (5·6–7·3)	3·4 (2·4–4·7)	1·0 (0·2–2·5)	0·8 (0·7–0·9)
		Japan	3·27 (3·12–3·42)	1·69 (1·64–1·76)	1·26 (1·14–1·41)	1·26 (1·09–1·45)	1·21 (1·00–1·43)	2188·1 (2087·3–2289·4)	1573·3 (1518·2–1636·1)	838·0 (754·0–933·3)	667·4 (555·1–790·8)	387·8 (259·2–572·2)	0·6 (0·5–0·7)
		Singapore	6·03 (5·75–6·31)	1·77 (1·66–1·88)	1·20 (1·05–1·39)	1·15 (0·93–1·41)	1·12 (0·88–1·41)	48·0 (45·7–50·3)	42·0 (39·2–44·8)	55·5 (48·5–64·0)	56·0 (44·0–70·8)	45·3 (28·5–69·5)	0·6 (0·5–0·7)
		South Korea	5·72 (5·37–6·08)	2·56 (2·48–2·64)	0·82 (0·75–0·89)	0·82 (0·73–0·92)	0·82 (0·71–0·95)	821·1 (774·1–869·0)	846·6 (822·3–873·2)	269·6 (246·4–294·2)	181·5 (155·9–209·6)	65·6 (45·4–90·3)	0·4 (0·4–0·4)
	High-income North America		3·10 (3·03–3·18)	1·78 (1·75–1·81)	1·63 (1·53–1·73)	1·51 (1·38–1·64)	1·43 (1·27–1·60)	4023·2 (3927·9–4124·2)	3948·6 (3866·5–4026·4)	4014·6 (3772·1–4278·3)	3732·8 (3300·8–4245·6)	2967·3 (2256·1–3805·1)	0·8 (0·7–0·8)
		Canada	3·31 (3·23–3·40)	1·65 (1·62–1·69)	1·46 (1·31–1·62)	1·39 (1·21–1·58)	1·32 (1·12–1·54)	361·9 (353·0–370·7)	357·7 (350·4–365·1)	361·6 (324·1–401·3)	442·0 (374·4–521·9)	415·5 (299·8–570·6)	0·7 (0·6–0·8)
		Greenland	5·62 (5·37–5·87)	2·32 (2·21–2·43)	1·94 (1·78–2·13)	1·84 (1·60–2·10)	1·67 (1·36–2·00)	1·0 (0·9–1·0)	1·0 (0·9–1·0)	0·8 (0·7–0·9)	0·7 (0·5–0·8)	0·5 (0·3–0·7)	0·9 (0·8–1·0)
		USA	3·08 (3·01–3·17)	1·79 (1·76–1·83)	1·64 (1·55–1·75)	1·52 (1·40–1·65)	1·45 (1·30–1·60)	3660·2 (3569·5–3758·9)	3589·8 (3510·8–3663·5)	3652·2 (3445·3–3878·2)	3290·0 (2928·9–3719·1)	2551·2 (1954·8–3256·2)	0·8 (0·7–0·8)
	Southern Latin America		3·20 (3·11–3·30)	2·97 (2·93–3·01)	1·49 (1·32–1·67)	1·32 (1·10–1·57)	1·23 (0·97–1·53)	673·6 (653·6–693·9)	987·3 (972·7–1001·9)	769·3 (683·8–862·6)	584·3 (452·6–745·4)	293·8 (159·6–482·9)	0·7 (0·6–0·8)
		Argentina	3·03 (2·92–3·15)	3·17 (3·11–3·23)	1·52 (1·34–1·72)	1·33 (1·09–1·60)	1·22 (0·91–1·56)	440·7 (424·2–457·7)	683·1 (669·7–696·0)	536·7 (473·3–606·6)	389·6 (284·0–511·3)	173·1 (80·2–310·7)	0·7 (0·6–0·8)
		Chile	4·05 (3·94–4·16)	2·59 (2·53–2·65)	1·39 (1·23–1·54)	1·29 (1·09–1·51)	1·24 (0·99–1·51)	188·2 (183·3–193·1)	250·2 (244·2–256·4)	197·2 (175·7–219·6)	169·6 (136·6–210·7)	109·6 (69·1–169·7)	0·7 (0·6–0·7)
		Uruguay	2·44 (2·31–2·57)	2·53 (2·47–2·60)	1·47 (1·30–1·64)	1·36 (1·14–1·60)	1·25 (0·97–1·56)	44·8 (42·3–47·1)	53·9 (52·5–55·4)	35·4 (31·5–39·6)	25·1 (19·6–32·2)	11·1 (5·8–19·3)	0·7 (0·6–0·8)
	Western Europe		2·41 (2·34–2·47)	1·79 (1·77–1·81)	1·53 (1·44–1·63)	1·44 (1·32–1·57)	1·37 (1·23–1·52)	5617·5 (5459·1–5774·9)	4801·7 (4750·3–4856·9)	4088·4 (3844·3–4353·4)	3757·3 (3360·8–4160·6)	2837·2 (2247·8–3546·1)	0·7 (0·7–0·8)
		Andorra	2·79 (2·44–3·18)	1·59 (1·51–1·66)	0·98 (0·91–1·05)	1·02 (0·92–1·11)	1·01 (0·89–1·13)	0·1 (0·1–0·1)	0·5 (0·5–0·5)	0·5 (0·5–0·6)	0·3 (0·3–0·3)	0·1 (0·1–0·1)	0·5 (0·4–0·5)
		Austria	2·08 (2·01–2·15)	1·67 (1·63–1·70)	1·46 (1·37–1·55)	1·42 (1·29–1·55)	1·34 (1·18–1·51)	105·8 (102·3–109·7)	91·3 (89·3–93·3)	85·7 (80·5–91·1)	81·0 (71·2–91·5)	63·4 (48·1–80·5)	0·7 (0·7–0·7)
		Belgium	2·30 (2·22–2·38)	1·70 (1·66–1·73)	1·56 (1·41–1·72)	1·43 (1·24–1·63)	1·34 (1·13–1·57)	142·4 (137·8–147·2)	123·3 (120·9–125·7)	113·3 (102·2–124·9)	114·6 (95·9–137·7)	99·9 (69·0–140·3)	0·8 (0·7–0·8)
		Cyprus	3·96 (3·80–4·11)	2·42 (2·35–2·49)	1·33 (1·15–1·53)	1·18 (0·97–1·43)	1·13 (0·89–1·40)	13·9 (13·4–14·5)	13·4 (13·0–13·8)	15·1 (13·1–17·4)	11·0 (8·5–13·8)	7·6 (4·7–11·6)	0·6 (0·6–0·7)
		Denmark	2·54 (2·46–2·63)	1·49 (1·45–1·52)	1·73 (1·63–1·83)	1·57 (1·46–1·69)	1·47 (1·34–1·60)	78·2 (75·8–81·1)	55·3 (53·9–56·7)	63·2 (59·7–66·7)	62·4 (55·2–70·2)	58·0 (46·1–70·9)	0·8 (0·8–0·9)
		Finland	3·08 (2·99–3·19)	1·65 (1·60–1·70)	1·44 (1·35–1·53)	1·36 (1·24–1·49)	1·32 (1·18–1·48)	95·6 (92·8–98·9)	63·9 (62·1–65·7)	48·5 (45·3–51·7)	41·9 (36·2–48·5)	30·1 (23·3–38·7)	0·7 (0·7–0·7)
		France	2·80 (2·72–2·87)	1·90 (1·85–1·95)	1·75 (1·57–1·93)	1·56 (1·35–1·79)	1·43 (1·19–1·69)	840·4 (817·3–862·6)	795·3 (774·5–816·9)	693·1 (623·0–766·7)	561·9 (448·3–683·9)	348·5 (214·5–542·2)	0·8 (0·8–0·9)
		Germany	2·09 (1·94–2·24)	1·52 (1·48–1·55)	1·53 (1·44–1·62)	1·47 (1·35–1·58)	1·40 (1·27–1·53)	1105·0 (1023·7–1188·6)	852·3 (833·8–873·0)	790·3 (742·4–837·6)	742·6 (647·9–832·5)	609·5 (489·2–740·4)	0·7 (0·7–0·8)
		Greece	2·50 (2·41–2·60)	2·07 (2·03–2·12)	1·40 (1·25–1·56)	1·36 (1·17–1·57)	1·28 (1·06–1·54)	154·7 (148·6–160·8)	143·4 (140·4–146·8)	82·3 (73·4–92·1)	52·3 (39·8–66·9)	26·1 (15·2–42·2)	0·7 (0·6–0·8)
		Iceland	3·81 (3·57–4·08)	2·40 (2·32–2·49)	1·97 (1·81–2·13)	1·73 (1·54–1·93)	1·58 (1·36–1·82)	4·0 (3·8–4·3)	4·3 (4·2–4·5)	4·7 (4·3–5·1)	5·4 (4·5–6·4)	5·4 (3·8–7·5)	0·9 (0·9–1·0)
		Ireland	3·18 (3·09–3·28)	3·14 (3·08–3·20)	1·76 (1·65–1·88)	1·54 (1·40–1·70)	1·40 (1·22–1·58)	63·9 (62·1–66·0)	74·1 (72·8–75·4)	57·7 (54·0–61·4)	61·5 (52·5–70·8)	46·8 (34·4–61·9)	0·9 (0·8–0·9)
		Israel	3·79 (3·68–3·90)	3·14 (3·08–3·20)	2·90 (2·76–3·05)	2·38 (2·20–2·59)	2·09 (1·86–2·34)	47·0 (45·6–48·4)	92·9 (91·1–94·7)	183·2 (174·1–192·7)	208·6 (174·7–248·4)	231·4 (167·3–315·1)	1·4 (1·3–1·5)
		Italy	2·45 (2·37–2·53)	1·63 (1·60–1·66)	1·21 (1·08–1·36)	1·18 (1·00–1·37)	1·09 (0·88–1·32)	883·2 (855·0–912·8)	640·5 (628·7–652·9)	398·2 (354·1–445·2)	285·5 (236·9–343·9)	136·4 (84·0–209·0)	0·6 (0·5–0·7)
		Luxembourg	2·00 (1·87–2·15)	1·51 (1·46–1·55)	1·38 (1·28–1·48)	1·30 (1·17–1·44)	1·24 (1·09–1·40)	4·5 (4·2–4·9)	4·2 (4·0–4·3)	6·6 (6·1–7·1)	8·8 (7·7–10·1)	8·8 (6·8–11·1)	0·7 (0·6–0·7)
		Malta	4·04 (3·88–4·22)	1·98 (1·91–2·05)	1·53 (1·37–1·71)	1·39 (1·18–1·64)	1·26 (1·01–1·55)	9·8 (9·4–10·2)	5·7 (5·5–6·0)	4·3 (3·9–4·8)	4·8 (3·9–5·9)	4·0 (2·6–6·0)	0·7 (0·7–0·8)
		Monaco	2·21 (1·91–2·55)	1·64 (1·43–1·88)	1·52 (1·29–1·80)	1·44 (1·16–1·76)	1·37 (1·06–1·73)	0·3 (0·3–0·4)	0·3 (0·3–0·3)	0·3 (0·2–0·3)	0·2 (0·2–0·3)	0·1 (0·1–0·2)	0·7 (0·6–0·8)
		Netherlands	3·12 (3·04–3·22)	1·60 (1·56–1·63)	1·68 (1·58–1·78)	1·54 (1·41–1·67)	1·42 (1·27–1·57)	229·6 (223·4–236·8)	178·6 (174·7–182·6)	177·7 (167·2–188·3)	165·6 (146·4–186·7)	142·0 (112·1–177·0)	0·8 (0·8–0·9)
		Norway	2·52 (2·44–2·60)	1·61 (1·57–1·65)	1·55 (1·46–1·64)	1·43 (1·32–1·54)	1·36 (1·24–1·49)	61·9 (60·1–63·9)	47·8 (46·8–48·9)	55·9 (52·8–59·1)	54·8 (48·5–61·5)	46·2 (37·2–57·5)	0·7 (0·7–0·8)
		Portugal	3·04 (2·94–3·16)	2·13 (2·09–2·18)	1·30 (1·22–1·39)	1·27 (1·13–1·42)	1·17 (1·00–1·37)	206·3 (199·2–214·4)	154·5 (151·0–158·2)	80·6 (75·2–86·2)	70·9 (59·7–83·9)	46·0 (32·1–63·2)	0·6 (0·6–0·7)
		San Marino	2·47 (2·14–2·84)	1·58 (1·48–1·69)	1·30 (1·15–1·48)	1·27 (1·09–1·49)	1·20 (0·99–1·46)	0·3 (0·2–0·3)	0·2 (0·2–0·3)	0·2 (0·2–0·3)	0·2 (0·1–0·2)	0·1 (0·0–0·1)	0·6 (0·5–0·7)
		Spain	2·47 (2·38–2·55)	2·13 (2·09–2·17)	1·26 (1·17–1·35)	1·23 (1·10–1·38)	1·11 (0·93–1·30)	560·3 (542·4–579·3)	549·2 (538·5–559·9)	336·7 (312·8–362·0)	377·6 (319·2–439·7)	248·3 (169·5–331·8)	0·6 (0·6–0·7)
		Sweden	2·27 (2·20–2·36)	1·65 (1·62–1·69)	1·71 (1·61–1·81)	1·51 (1·39–1·64)	1·38 (1·24–1·53)	113·9 (110·3–118·3)	95·5 (93·4–97·5)	113·9 (107·4–120·7)	135·0 (119·4–151·5)	136·8 (108·5–166·8)	0·8 (0·8–0·9)
		Switzerland	2·35 (2·28–2·43)	1·58 (1·54–1·61)	1·48 (1·39–1·59)	1·40 (1·28–1·52)	1·33 (1·20–1·47)	83·2 (80·7–85·8)	75·8 (74·2–77·4)	89·1 (83·5–95·3)	83·8 (74·0–94·7)	68·5 (54·8–84·4)	0·7 (0·7–0·8)
		UK	2·19 (2·13–2·25)	1·85 (1·80–1·90)	1·49 (1·33–1·67)	1·38 (1·18–1·58)	1·30 (1·08–1·53)	809·2 (786·1–832·5)	735·5 (715·4–755·5)	683·8 (608·2–762·0)	623·3 (504·8–743·2)	470·8 (303·6–664·1)	0·7 (0·6–0·8)
**Latin America and Caribbean**			**5·82 (5·58–6·06)**	**4·09 (4·01–4·18)**	**1·98 (1·83–2·13)**	**1·57 (1·38–1·79)**	**1·31 (1·08–1·57)**	**6278·2 (6037·0–6525·3)**	**10 310·9 (10 111·1–10 514·4)**	**9377·7 (8692·9–10 090·5)**	**6763·5 (5627·9–8076·1)**	**3002·6 (1769·5–4786·3)**	**0·9 (0·9–1·0)**
	Andean Latin America		6·72 (6·47–6·95)	4·97 (4·86–5·08)	2·32 (2·14–2·51)	1·80 (1·58–2·05)	1·45 (1·19–1·73)	679·8 (655·9–702·4)	1115·9 (1089·4–1141·9)	1242·6 (1146·2–1345·6)	961·5 (782·6–1184·9)	457·7 (242·3–768·2)	1·1 (1·0–1·2)
		Bolivia	6·84 (6·49–7·19)	5·65 (5·45–5·85)	2·53 (2·31–2·77)	1·84 (1·55–2·17)	1·40 (1·07–1·77)	150·5 (143·0–157·8)	216·0 (208·8–223·4)	245·0 (223·7–270·0)	208·5 (162·6–277·0)	113·0 (53·3–210·1)	1·2 (1·1–1·3)
		Ecuador	6·09 (5·78–6·44)	4·24 (4·10–4·40)	2·20 (1·95–2·50)	1·74 (1·42–2·10)	1·45 (1·10–1·86)	149·7 (142·2–157·9)	249·6 (241·3–259·0)	321·5 (285·0–363·6)	269·3 (194·5–367·0)	152·8 (66·7–298·3)	1·1 (0·9–1·2)
		Peru	6·95 (6·68–7·22)	5·10 (4·96–5·24)	2·30 (2·08–2·55)	1·83 (1·56–2·13)	1·44 (1·14–1·80)	379·7 (366·2–392·8)	650·3 (630·8–669·5)	676·2 (611·2–748·3)	483·7 (374·1–630·9)	192·0 (79·6–384·3)	1·1 (1·0–1·2)
	Caribbean		5·02 (4·90–5·14)	3·39 (3·31–3·47)	2·19 (2·02–2·39)	1·77 (1·50–2·08)	1·43 (1·11–1·88)	699·5 (682·6–715·4)	837·0 (818·1–856·6)	797·4 (734·1–868·9)	570·0 (432·5–738·2)	241·7 (98·1–477·6)	1·0 (0·9–1·1)
		Antigua and Barbuda	4·63 (4·38–4·88)	2·33 (2·24–2·42)	1·49 (1·33–1·68)	1·30 (1·11–1·52)	1·15 (0·93–1·41)	1·7 (1·6–1·7)	1·2 (1·2–1·3)	1·0 (0·9–1·2)	0·7 (0·5–0·9)	0·3 (0·1–0·5)	0·7 (0·6–0·8)
		The Bahamas	3·97 (3·77–4·17)	2·65 (2·56–2·76)	1·23 (1·05–1·45)	1·24 (1·02–1·49)	1·24 (0·99–1·52)	2·6 (2·5–2·7)	5·0 (4·8–5·2)	3·9 (3·3–4·5)	3·1 (2·3–4·2)	1·9 (1·0–3·2)	0·6 (0·5–0·7)
		Barbados	3·54 (3·36–3·73)	1·94 (1·86–2·04)	1·30 (1·09–1·56)	1·18 (0·95–1·47)	1·10 (0·85–1·42)	6·7 (6·4–7·1)	4·3 (4·1–4·5)	2·6 (2·2–3·1)	1·7 (1·1–2·3)	0·7 (0·3–1·3)	0·6 (0·5–0·7)
		Belize	5·47 (5·21–5·76)	5·42 (5·27–5·57)	1·96 (1·74–2·20)	1·58 (1·28–1·90)	1·28 (0·93–1·67)	2·8 (2·7–3·0)	5·7 (5·5–5·8)	7·6 (6·7–8·5)	7·5 (5·5–9·8)	5·2 (2·4–9·0)	0·9 (0·8–1·1)
		Bermuda	3·58 (3·38–3·81)	1·62 (1·55–1·70)	1·28 (1·15–1·43)	1·19 (1·04–1·36)	1·07 (0·88–1·28)	1·1 (1·1–1·2)	0·8 (0·8–0·9)	0·5 (0·4–0·5)	0·3 (0·2–0·4)	0·1 (0·1–0·2)	0·6 (0·6–0·7)
		Cuba	3·29 (3·13–3·45)	1·65 (1·59–1·71)	1·44 (1·34–1·55)	1·31 (1·18–1·44)	1·23 (1·07–1·39)	151·3 (144·0–158·5)	141·1 (136·2–146·2)	99·6 (92·7–107·0)	58·5 (46·8–71·0)	19·4 (11·9–29·3)	0·7 (0·6–0·7)
		Dominica	5·12 (4·88–5·37)	3·50 (3·32–3·69)	1·29 (1·09–1·52)	1·18 (0·96–1·45)	1·13 (0·89–1·42)	1·9 (1·8–2·0)	1·9 (1·8–2·0)	0·6 (0·5–0·7)	0·4 (0·3–0·6)	0·2 (0·1–0·3)	0·6 (0·5–0·7)
		Dominican Republic	7·83 (7·60–8·04)	4·72 (4·50–4·94)	2·32 (2·10–2·56)	1·84 (1·55–2·15)	1·51 (1·19–1·86)	132·5 (129·1–135·6)	210·1 (199·5–220·2)	213·5 (192·9–236·2)	159·1 (118·5–211·1)	70·4 (27·8–142·4)	1·1 (1·0–1·2)
		Grenada	5·27 (5·05–5·48)	3·57 (3·39–3·75)	1·74 (1·49–2·05)	1·41 (1·09–1·79)	1·19 (0·81–1·62)	3·5 (3·3–3·6)	2·7 (2·5–2·8)	1·4 (1·2–1·6)	0·8 (0·5–1·1)	0·1 (0·0–0·4)	0·8 (0·7–1·0)
		Guyana	6·17 (5·96–6·41)	3·91 (3·73–4·09)	2·35 (2·06–2·67)	1·91 (1·54–2·32)	1·58 (1·15–2·04)	19·4 (18·7–20·2)	26·1 (24·8–27·3)	15·3 (13·5–17·4)	8·6 (5·7–12·8)	5·5 (2·0–11·1)	1·1 (1·0–1·2)
		Haiti	6·67 (6·36–6·96)	5·98 (5·76–6·18)	3·16 (2·82–3·55)	2·10 (1·68–2·61)	1·44 (0·92–2·05)	175·3 (167·4–182·9)	228·7 (221·2–235·7)	344·4 (308·5–386·7)	271·1 (179·5–381·4)	120·8 (30·5–290·6)	1·4 (1·2–1·5)
		Jamaica	4·06 (3·78–4·34)	3·28 (3·18–3·39)	1·37 (1·18–1·56)	1·16 (0·93–1·39)	1·04 (0·79–1·31)	50·1 (46·9–53·6)	58·2 (56·3–60·2)	32·8 (28·4–37·5)	15·8 (11·3–21·6)	2·9 (0·7–6·8)	0·7 (0·6–0·7)
		Puerto Rico	5·20 (5·10–5·31)	2·60 (2·53–2·66)	0·90 (0·84–0·97)	0·84 (0·76–0·92)	0·81 (0·72–0·93)	84·9 (83·2–86·8)	71·7 (69·8–73·7)	19·0 (17·7–20·5)	6·7 (5·0–8·5)	1·4 (0·8–2·0)	0·4 (0·4–0·5)
		Saint Kitts and Nevis	3·90 (3·73–4·06)	3·32 (3·14–3·52)	1·27 (1·13–1·42)	1·08 (0·92–1·27)	1·00 (0·81–1·20)	1·9 (1·8–1·9)	1·2 (1·1–1·3)	0·6 (0·5–0·6)	0·3 (0·2–0·4)	0·1 (0·0–0·1)	0·6 (0·5–0·7)
		Saint Lucia	5·03 (4·80–5·26)	4·24 (4·10–4·38)	1·28 (1·08–1·51)	1·04 (0·79–1·32)	0·87 (0·58–1·19)	2·9 (2·8–3·1)	3·9 (3·8–4·0)	1·7 (1·4–2·0)	1·0 (0·7–1·4)	0·3 (0·1–0·7)	0·6 (0·5–0·7)
		Saint Vincent and the Grenadines	4·83 (4·66–4·99)	3·89 (3·76–4·05)	1·60 (1·41–1·82)	1·35 (1·10–1·64)	1·16 (0·87–1·51)	2·8 (2·7–2·9)	3·2 (3·1–3·3)	1·3 (1·1–1·5)	0·8 (0·6–1·1)	0·3 (0·1–0·6)	0·8 (0·7–0·9)
		Suriname	5·56 (5·33–5·78)	3·76 (3·65–3·88)	2·09 (1·87–2·33)	1·73 (1·41–2·05)	1·39 (1·02–1·78)	7·8 (7·5–8·1)	10·3 (10·0–10·7)	9·0 (8·1–10·0)	7·4 (5·4–9·8)	3·3 (1·1–6·7)	1·0 (0·9–1·1)
		Trinidad and Tobago	4·60 (4·44–4·78)	3·29 (3·18–3·40)	1·52 (1·34–1·72)	1·35 (1·13–1·60)	1·19 (0·94–1·49)	23·7 (23·0–24·6)	31·0 (29·9–32·1)	14·7 (13·0–16·7)	6·6 (4·5–8·9)	0·5 (0·0–2·0)	0·7 (0·6–0·8)
		Virgin Islands	4·85 (4·61–5·09)	2·91 (2·73–3·08)	1·68 (1·46–1·94)	1·49 (1·28–1·77)	1·37 (1·13–1·69)	0·9 (0·8–0·9)	2·5 (2·3–2·6)	0·8 (0·7–0·9)	0·5 (0·3–0·6)	0·2 (0·1–0·4)	0·8 (0·7–0·9)
	Central Latin America		5·73 (5·49–5·99)	4·32 (4·22–4·42)	1·87 (1·68–2·08)	1·47 (1·23–1·74)	1·21 (0·93–1·53)	2327·7 (2229·7–2431·0)	4477·0 (4375·7–4578·5)	3877·3 (3483·8–4308·1)	2704·0 (2091·0–3465·5)	1064·5 (501·2–1993·8)	0·9 (0·8–1·0)
		Colombia	5·67 (5·27–6·12)	3·62 (3·41–3·84)	1·67 (1·43–1·96)	1·35 (1·02–1·71)	1·14 (0·77–1·54)	499·9 (466·3–537·1)	823·8 (774·8–875·5)	675·0 (576·7–788·6)	444·7 (296·9–633·1)	176·8 (59·7–379·0)	0·8 (0·7–0·9)
		Costa Rica	6·04 (5·87–6·22)	3·60 (3·52–3·70)	1·38 (1·26–1·51)	1·18 (1·02–1·36)	1·03 (0·84–1·26)	38·2 (37·1–39·4)	69·7 (68·0–71·6)	54·6 (49·8–59·7)	33·8 (26·6–42·3)	11·3 (6·2–18·4)	0·7 (0·6–0·7)
		El Salvador	6·47 (6·31–6·64)	5·14 (5·02–5·26)	2·05 (1·80–2·32)	1·58 (1·23–1·93)	1·28 (0·86–1·72)	97·8 (95·4–100·5)	181·2 (177·2–185·3)	115·9 (101·7–130·8)	50·9 (30·9–74·0)	0·2 (0·0–1·7)	1·0 (0·9–1·1)
		Guatemala	6·47 (6·38–6·56)	6·66 (6·56–6·77)	2·41 (2·16–2·68)	1·62 (1·26–1·98)	1·16 (0·73–1·61)	146·1 (144·2–148·2)	303·8 (298·8–308·6)	344·7 (309·1–383·9)	228·2 (158·2–314·1)	66·4 (12·7–166·4)	1·1 (1·0–1·3)
		Honduras	6·84 (6·49–7·18)	6·38 (6·23–6·52)	2·40 (2·11–2·75)	1·71 (1·35–2·11)	1·27 (0·82–1·76)	73·7 (70·0–77·2)	154·7 (151·1–158·0)	220·5 (195·1–252·5)	175·5 (119·4–238·7)	76·9 (23·0–174·0)	1·1 (1·0–1·3)
		Mexico	5·66 (5·39–5·95)	4·29 (4·18–4·40)	1·77 (1·61–1·94)	1·39 (1·19–1·62)	1·15 (0·91–1·41)	1172·2 (1115·3–1232·6)	2275·4 (2222·6–2330·8)	1857·4 (1689·0–2038·3)	1343·9 (1065·2–1696·2)	564·3 (296·0–956·2)	0·9 (0·8–0·9)
		Nicaragua	6·12 (5·82–6·43)	6·14 (6·00–6·28)	2·20 (1·93–2·49)	1·65 (1·30–2·01)	1·29 (0·86–1·73)	54·6 (52·0–57·5)	127·7 (125·0–130·3)	126·9 (111·4–143·2)	84·4 (56·0–123·5)	22·2 (1·3–66·1)	1·0 (0·9–1·2)
		Panama	4·04 (3·90–4·20)	3·54 (3·43–3·65)	2·13 (1·91–2·37)	1·76 (1·49–2·05)	1·49 (1·20–1·81)	26·2 (25·3–27·2)	53·9 (52·2–55·5)	69·8 (62·7–77·3)	70·4 (53·4–93·0)	55·5 (31·8–93·2)	1·0 (0·9–1·1)
		Venezuela	5·38 (5·22–5·54)	4·09 (3·99–4·20)	2·13 (1·84–2·43)	1·79 (1·43–2·19)	1·51 (1·10–1·97)	219·1 (212·3–225·6)	486·7 (472·9–501·5)	412·7 (355·6–471·4)	272·2 (186·9–399·4)	90·9 (4·0–255·5)	1·0 (0·9–1·1)
	Tropical Latin America		5·95 (5·54–6·36)	3·83 (3·72–3·94)	1·94 (1·79–2·12)	1·57 (1·36–1·81)	1·32 (1·08–1·59)	2571·2 (2402·6–2735·9)	3881·0 (3774·3–3985·9)	3460·4 (3188·0–3775·6)	2528·0 (1966·2–3181·6)	1238·7 (691·6–2043·1)	0·9 (0·9–1·0)
		Brazil	5·93 (5·51–6·36)	3·81 (3·70–3·92)	1·93 (1·78–2·12)	1·57 (1·35–1·81)	1·31 (1·06–1·59)	2504·7 (2335·7–2669·2)	3773·8 (3670·2–3875·7)	3332·2 (3059·7–3648·5)	2440·4 (1881·9–3096·7)	1207·6 (654·5–2015·8)	0·9 (0·8–1·0)
		Paraguay	6·62 (6·35–6·90)	4·90 (4·50–5·29)	2·15 (1·83–2·50)	1·66 (1·25–2·11)	1·39 (0·93–1·89)	66·5 (63·7–69·6)	107·1 (98·7–115·7)	128·2 (109·5–148·5)	87·5 (53·2–131·0)	31·1 (3·6–88·8)	1·0 (0·9–1·2)
**North Africa and Middle East**			**5·93 (5·56–6·31)**	**6·25 (6·14–6·37)**	**2·53 (2·33–2·76)**	**1·94 (1·62–2·28)**	**1·64 (1·28–2·06)**	**4777·2 (4479·4–5080·5)**	**10 964·6 (10 754·6–11 168·9)**	**12 137·4 (11 185·8–13 232·1)**	**11 415·2 (8900·7–14 116·1)**	**8157·0 (4021·1–14 491·3)**	**1·2 (1·1–1·3)**
		Afghanistan	6·94 (6·60–7·27)	7·25 (7·04–7·46)	5·39 (5·10–5·72)	3·34 (2·78–3·89)	1·61 (0·90–2·32)	353·5 (336·6–370·1)	616·1 (597·7–632·9)	1211·5 (1147·5–1283·0)	1785·5 (1104·3–2491·9)	1349·7 (348·0–2742·3)	2·3 (2·2–2·4)
		Algeria	6·55 (6·18–6·91)	6·82 (6·72–6·91)	2·64 (2·38–2·91)	1·79 (1·37–2·18)	1·48 (1·04–1·89)	415·5 (392·0–438·9)	793·9 (781·7–807·2)	907·7 (818·7–1000·0)	704·6 (502·0–930·0)	342·7 (128·4–657·3)	1·2 (1·1–1·4)
		Bahrain	6·15 (5·75–6·56)	4·41 (4·31–4·52)	1·71 (1·52–1·91)	1·39 (1·10–1·68)	1·26 (0·92–1·57)	4·0 (3·7–4·3)	10·0 (9·8–10·3)	16·9 (15·0–18·8)	17·6 (13·1–22·1)	13·3 (6·9–21·5)	0·8 (0·7–0·9)
		Egypt	3·54 (3·12–4·02)	5·91 (5·73–6·10)	3·16 (2·82–3·54)	2·38 (1·97–2·82)	2·05 (1·62–2·50)	557·4 (491·3–629·7)	1850·4 (1789·0–1913·1)	2611·0 (2335·5–2921·4)	2968·5 (2235·8–3819·0)	3117·2 (1456·3–5603·2)	1·5 (1·3–1·7)
		Iran	6·21 (5·79–6·62)	7·41 (7·32–7·51)	1·52 (1·33–1·73)	1·31 (1·03–1·58)	1·28 (0·97–1·58)	772·2 (721·6–822·6)	1948·0 (1921·2–1973·2)	1027·8 (897·0–1168·0)	776·4 (560·5–1032·3)	456·1 (215·5–786·6)	0·7 (0·6–0·8)
		Iraq	6·37 (5·98–6·76)	7·06 (6·83–7·26)	2·87 (2·63–3·16)	1·95 (1·63–2·35)	1·59 (1·25–2·01)	223·0 (209·4–236·5)	606·4 (587·4–624·5)	932·7 (853·6–1028·2)	873·0 (596·0–1223·7)	626·4 (284·1–1129·5)	1·3 (1·2–1·5)
		Jordan	8·53 (8·41–8·64)	7·05 (6·96–7·14)	2·33 (2·06–2·62)	1·78 (1·44–2·10)	1·57 (1·20–1·93)	23·4 (23·0–23·7)	91·6 (90·1–93·1)	211·7 (187·4–238·2)	289·6 (219·8–367·5)	362·8 (205·1–553·3)	1·1 (1·0–1·2)
		Kuwait	5·21 (4·77–5·67)	5·24 (5·11–5·37)	1·13 (0·98–1·30)	1·07 (0·89–1·30)	1·14 (0·93–1·39)	2·7 (2·5–2·9)	50·2 (48·9–51·6)	50·9 (44·1–59·0)	47·2 (37·1–60·8)	40·3 (26·2–60·8)	0·5 (0·5–0·6)
		Lebanon	6·90 (6·55–7·24)	4·25 (3·95–4·55)	1·76 (1·49–2·09)	1·44 (1·12–1·80)	1·33 (0·98–1·73)	59·0 (56·0–61·9)	79·4 (74·3–84·5)	81·3 (69·4–96·1)	49·3 (34·2–68·0)	24·2 (8·6–49·8)	0·8 (0·7–1·0)
		Libya	7·60 (7·39–7·81)	7·10 (6·92–7·25)	1·37 (1·14–1·63)	1·13 (0·87–1·43)	1·03 (0·75–1·37)	52·0 (50·6–53·3)	121·6 (118·8–124·4)	79·1 (66·1–94·5)	45·8 (31·7–66·0)	14·9 (4·8–34·7)	0·6 (0·5–0·8)
		Morocco	7·18 (6·86–7·49)	5·71 (5·45–5·97)	2·26 (2·05–2·49)	1·36 (1·05–1·70)	1·02 (0·67–1·40)	463·2 (443·0–482·8)	806·7 (771·6–842·5)	646·6 (586·7–714·3)	373·2 (262·4–500·7)	90·9 (20·3–206·9)	1·1 (1·0–1·2)
		Oman	7·48 (7·25–7·71)	7·57 (7·39–7·76)	2·48 (2·24–2·73)	1·64 (1·27–2·02)	1·29 (0·88–1·72)	23·9 (23·1–24·7)	59·1 (57·5–60·7)	81·7 (74·1–89·8)	76·9 (56·8–99·4)	53·4 (23·6–95·4)	1·2 (1·1–1·3)
		Palestine	7·61 (7·37–7·84)	6·75 (6·50–6·98)	2·88 (2·64–3·16)	2·08 (1·79–2·44)	1·77 (1·43–2·15)	36·8 (35·7–38·0)	62·8 (60·4–65·1)	119·8 (109·5–132·2)	108·4 (82·3–139·7)	66·4 (26·4–129·3)	1·4 (1·3–1·5)
		Qatar	7·04 (6·75–7·33)	5·35 (5·17–5·54)	1·95 (1·75–2·16)	1·43 (1·17–1·69)	1·29 (1·01–1·57)	1·2 (1·2–1·3)	8·2 (8·0–8·5)	38·0 (34·4–42·0)	59·8 (47·0–72·7)	58·2 (35·0–85·0)	0·9 (0·9–1·0)
		Saudi Arabia	6·84 (6·51–7·15)	6·79 (6·48–7·09)	1·44 (1·24–1·68)	1·09 (0·80–1·39)	0·97 (0·65–1·31)	131·3 (124·7–137·8)	396·7 (377·1–416·4)	461·9 (396·5–535·4)	291·3 (195·0–395·8)	128·2 (48·7–242·9)	0·7 (0·6–0·8)
		Sudan	6·63 (6·35–6·90)	6·72 (6·51–6·92)	3·38 (3·08–3·72)	1·93 (1·48–2·44)	1·40 (0·93–1·95)	238·8 (229·1–248·2)	702·4 (679·3–723·8)	1168·4 (1067·7–1281·7)	1011·1 (689·4–1360·9)	504·2 (148·5–1154·4)	1·5 (1·4–1·7)
		Syria	7·68 (7·47–7·88)	6·79 (6·60–6·98)	2·06 (1·76–2·42)	1·57 (1·21–1·98)	1·39 (1·01–1·84)	165·5 (160·5–170·3)	373·0 (360·4–385·6)	196·6 (165·8–234·5)	167·9 (110·2–241·9)	100·5 (38·4–217·1)	1·0 (0·8–1·1)
		Tunisia	6·48 (6·15–6·82)	5·07 (4·90–5·23)	1·82 (1·59–2·09)	1·36 (1·02–1·73)	1·19 (0·82–1·59)	166·5 (157·7–175·1)	222·0 (214·0–230·2)	166·5 (145·6–191·5)	99·5 (68·0–136·0)	28·7 (6·9–67·4)	0·9 (0·7–1·0)
		Türkiye	5·73 (5·31–6·16)	4·81 (4·61–5·01)	1·67 (1·52–1·85)	1·32 (1·13–1·56)	1·17 (0·95–1·42)	859·4 (797·8–921·8)	1611·4 (1541·0–1680·6)	1052·9 (955·7–1164·2)	634·5 (497·4–793·2)	218·5 (114·1–382·4)	0·8 (0·7–0·9)
		United Arab Emirates	7·14 (6·83–7·46)	5·88 (5·69–6·07)	1·90 (1·68–2·14)	1·53 (1·26–1·81)	1·31 (1·00–1·64)	3·6 (3·4–3·7)	35·5 (34·6–36·5)	74·0 (64·1–84·7)	160·2 (122·7–198·1)	155·8 (88·6–234·7)	0·9 (0·8–1·0)
		Yemen	7·36 (7·07–7·64)	7·91 (7·77–8·04)	3·87 (3·47–4·31)	1·91 (1·32–2·54)	1·22 (0·54–1·95)	224·0 (214·9–232·8)	513·9 (504·7–522·7)	989·2 (886·4–1105·7)	863·3 (536·7–1288·7)	396·3 (56·2–1154·2)	1·8 (1·6–1·9)
**South Asia**			**6·35 (5·95–6·75)**	**4·96 (4·74–5·16)**	**2·07 (1·89–2·28)**	**1·36 (1·09–1·64)**	**1·10 (0·80–1·43)**	**20 472·6 (19 194·5–21 717·6)**	**31 555·9 (30 245·2–32 782·2)**	**32 043·4 (29 175·4–35 206·1)**	**18 743·1 (13 775·0–24 181·6)**	**5272·8 (1922·0–10 462·8)**	**0·9 (0·9–1·0)**
		Bangladesh	7·30 (6·99–7·59)	6·03 (5·87–6·18)	1·90 (1·68–2·14)	1·20 (0·84–1·54)	0·97 (0·57–1·37)	2067·5 (1988·9–2140·7)	3641·1 (3563·4–3718·0)	2806·8 (2477·5–3152·8)	1370·6 (828·0–1977·2)	224·3 (6·5–644·2)	0·9 (0·8–1·0)
		Bhutan	6·70 (6·35–7·04)	5·89 (5·59–6·18)	1·92 (1·74–2·09)	1·07 (0·73–1·34)	0·69 (0·33–1·00)	8·5 (8·1–8·9)	19·4 (18·5–20·2)	12·6 (11·5–13·8)	6·1 (3·9–8·2)	1·1 (0·2–2·3)	0·9 (0·8–1·0)
		India	6·18 (5·75–6·59)	4·60 (4·35–4·83)	1·91 (1·69–2·13)	1·29 (0·97–1·62)	1·04 (0·67–1·42)	16 366·5 (15 255·4–17 463·8)	23 512·7 (22 306·6–24 656·5)	22 393·2 (19 926·1–25 068·0)	13 026·2 (8946·1–17 555·0)	3792·9 (1086·0–7903·8)	0·9 (0·8–1·0)
		Nepal	6·33 (6·03–6·60)	6·17 (5·93–6·41)	2·14 (1·92–2·38)	1·18 (0·80–1·53)	0·82 (0·40–1·22)	416·7 (397·7–436·2)	727·6 (702·1–752·3)	642·2 (576·4–711·9)	273·0 (160·3–402·5)	14·6 (0·0–69·7)	1·0 (0·9–1·1)
		Pakistan	7·26 (6·95–7·56)	6·75 (6·54–6·96)	3·22 (2·87–3·62)	1·76 (1·25–2·28)	1·16 (0·59–1·77)	1613·4 (1542·5–1682·1)	3655·1 (3533·0–3773·2)	6188·5 (5530·6–6957·2)	4067·1 (2636·1–5810·4)	1239·9 (85·1–3558·0)	1·4 (1·3–1·6)
**Southeast Asia, east Asia, and Oceania**			**5·76 (5·44–6·09)**	**2·99 (2·89–3·08)**	**1·55 (1·44–1·66)**	**1·37 (1·22–1·54)**	**1·30 (1·11–1·53)**	**31 218·7 (29 613·6–32 875·9)**	**31 743·5 (30 751·9–32 760·5)**	**22 805·8 (21 221·6–24 442·9)**	**15 544·8 (13 337·3–18 252·3)**	**6819·6 (4409·9–10 247·2)**	**0·7 (0·7–0·8)**
	East Asia		5·57 (5·25–5·90)	2·46 (2·35–2·56)	1·23 (1·12–1·34)	1·14 (0·99–1·30)	1·16 (0·99–1·34)	22 400·1 (21 213·3–23 644·8)	18 856·4 (18 088·5–19 630·1)	11 202·9 (10 243·8–12 246·4)	6621·8 (5397·9–8088·3)	2201·4 (1341·4–3394·0)	0·6 (0·5–0·6)
		China	5·55 (5·24–5·89)	2·44 (2·33–2·55)	1·23 (1·12–1·34)	1·14 (0·99–1·31)	1·16 (0·99–1·35)	21 609·2 (20 451·1–22 827·1)	18 000·3 (17 240·4–18 768·2)	10 747·2 (9807·6–11 774·1)	6360·0 (5139·5–7778·2)	2105·2 (1273·0–3268·7)	0·6 (0·5–0·6)
		North Korea	5·41 (5·06–5·77)	3·25 (2·96–3·59)	1·51 (1·32–1·71)	1·24 (1·00–1·48)	1·16 (0·90–1·42)	424·7 (399·0–451·2)	450·7 (408·9–500·1)	299·0 (263·4–339·7)	172·2 (119·0–227·4)	63·4 (27·8–111·8)	0·7 (0·6–0·8)
		Taiwan (province of China)	6·82 (6·66–6·98)	2·42 (2·34–2·51)	0·98 (0·87–1·09)	0·90 (0·78–1·04)	0·90 (0·77–1·05)	366·2 (357·7–375·6)	405·5 (391·4–420·2)	156·8 (139·6–175·5)	89·6 (73·8–107·7)	32·8 (22·0–46·8)	0·5 (0·4–0·5)
	Oceania		6·63 (6·37–6·90)	5·36 (5·18–5·53)	4·02 (3·68–4·41)	2·93 (2·45–3·46)	1·67 (1·01–2·35)	121·4 (116·7–126·1)	193·5 (186·9–199·5)	430·7 (393·9–472·2)	590·1 (457·1–736·3)	566·7 (224·7–1096·8)	1·8 (1·6–1·9)
		American Samoa	5·97 (5·71–6·25)	4·24 (4·08–4·40)	2·42 (2·14–2·71)	1·98 (1·67–2·31)	1·68 (1·34–2·05)	0·8 (0·8–0·8)	1·1 (1·1–1·1)	0·8 (0·7–0·9)	0·8 (0·6–1·0)	0·6 (0·3–1·0)	1·1 (1·0–1·3)
		Cook Islands	5·79 (5·49–6·08)	3·66 (3·48–3·84)	1·74 (1·52–2·00)	1·40 (1·14–1·72)	1·25 (0·96–1·58)	0·6 (0·6–0·6)	0·4 (0·4–0·5)	0·2 (0·2–0·3)	0·2 (0·1–0·2)	0·1 (0·0–0·1)	0·8 (0·7–0·9)
		Federated States of Micronesia	7·69 (7·48–7·88)	5·87 (5·58–6·14)	2·37 (2·04–2·78)	1·82 (1·39–2·33)	1·46 (0·96–2·05)	2·0 (2·0–2·1)	3·2 (3·1–3·4)	1·9 (1·6–2·2)	1·4 (0·8–2·0)	0·8 (0·2–1·9)	1·1 (1·0–1·3)
		Fiji	5·64 (5·38–5·90)	3·34 (3·21–3·46)	2·42 (2·15–2·73)	1·95 (1·61–2·31)	1·64 (1·28–2·04)	11·5 (11·0–12·0)	18·8 (18·0–19·6)	16·7 (14·8–18·8)	12·6 (9·3–17·0)	8·7 (4·2–15·8)	1·1 (1·0–1·3)
		Guam	5·38 (5·12–5·68)	3·06 (2·94–3·17)	2·59 (2·30–2·86)	2·07 (1·76–2·38)	1·76 (1·42–2·09)	1·8 (1·7–1·8)	2·9 (2·8–3·1)	2·7 (2·4–3·0)	1·9 (1·4–2·4)	1·4 (0·7–2·4)	1·2 (1·1–1·3)
		Kiribati	6·40 (6·25–6·54)	4·81 (4·50–5·12)	2·95 (2·63–3·32)	2·13 (1·70–2·62)	1·67 (1·19–2·21)	1·3 (1·3–1·3)	2·3 (2·1–2·4)	2·9 (2·6–3·3)	2·3 (1·7–3·1)	1·0 (0·2–2·3)	1·3 (1·2–1·5)
		Marshall Islands	7·08 (6·77–7·38)	5·25 (5·02–5·50)	2·56 (2·31–2·84)	1·98 (1·67–2·32)	1·65 (1·31–2·02)	0·6 (0·6–0·6)	1·3 (1·3–1·4)	1·2 (1·0–1·3)	1·1 (0·8–1·4)	0·7 (0·4–1·3)	1·2 (1·1–1·3)
		Nauru	6·62 (6·45–6·80)	4·95 (4·54–5·35)	3·24 (2·86–3·67)	2·40 (1·93–2·95)	1·91 (1·39–2·51)	0·1 (0·1–0·1)	0·3 (0·3–0·4)	0·3 (0·3–0·3)	0·3 (0·2–0·4)	0·3 (0·1–0·6)	1·5 (1·3–1·6)
		Niue	6·36 (6·07–6·69)	4·10 (3·87–4·32)	2·09 (1·84–2·38)	1·71 (1·37–2·08)	1·50 (1·13–1·90)	0·2 (0·2–0·2)	0·1 (0·1–0·1)	0·0 (0·0–0·0)	0·0 (0·0–0·0)	0·0 (0·0–0·0)	0·9 (0·8–1·0)
		Northern Mariana Islands	6·06 (5·66–6·46)	2·90 (2·58–3·28)	1·93 (1·68–2·21)	1·67 (1·36–2·01)	1·50 (1·17–1·88)	0·2 (0·2–0·2)	0·4 (0·3–0·4)	0·6 (0·5–0·7)	0·5 (0·4–0·7)	0·3 (0·2–0·6)	0·9 (0·8–1·0)
		Palau	5·86 (5·44–6·27)	2·75 (2·44–3·09)	1·93 (1·72–2·15)	1·65 (1·37–1·92)	1·44 (1·14–1·74)	0·3 (0·3–0·3)	0·2 (0·2–0·3)	0·2 (0·2–0·2)	0·1 (0·1–0·2)	0·1 (0·0–0·1)	0·9 (0·8–1·0)
		Papua New Guinea	6·73 (6·39–7·06)	5·81 (5·59–6·02)	4·26 (3·86–4·71)	3·03 (2·50–3·58)	1·64 (0·94–2·35)	81·5 (77·4–85·4)	125·1 (120·4–129·4)	345·1 (312·9–381·6)	500·2 (374·6–636·3)	491·1 (180·0–958·2)	1·9 (1·7–2·0)
		Samoa	7·45 (7·24–7·64)	5·99 (5·70–6·26)	4·25 (3·91–4·61)	3·18 (2·69–3·68)	2·57 (1·98–3·14)	4·5 (4·4–4·6)	5·8 (5·5–6·0)	6·2 (5·7–6·8)	7·8 (6·1–9·6)	11·5 (5·6–19·0)	2·0 (1·8–2·1)
		Solomon Islands	7·10 (6·89–7·30)	6·51 (6·25–6·76)	3·90 (3·61–4·20)	2·51 (2·10–2·90)	1·70 (1·21–2·21)	4·8 (4·7–4·9)	9·9 (9·5–10·2)	20·5 (19·0–22·1)	19·7 (14·8–25·6)	10·5 (3·6–21·0)	1·8 (1·7–1·9)
		Tokelau	6·71 (6·46–6·97)	4·18 (3·87–4·49)	1·89 (1·61–2·20)	1·54 (1·17–1·94)	1·34 (0·94–1·78)	0·1 (0·1–0·1)	0·0 (0·0–0·0)	0·0 (0·0–0·0)	0·0 (0·0–0·0)	0·0 (0·0–0·0)	0·8 (0·7–0·9)
		Tonga	6·66 (6·47–6·86)	5·88 (5·73–6·03)	4·08 (3·75–4·42)	3·04 (2·58–3·50)	2·45 (1·93–2·98)	2·2 (2·1–2·3)	3·6 (3·5–3·7)	3·0 (2·7–3·2)	3·2 (2·5–4·1)	4·3 (2·3–7·1)	1·9 (1·7–2·0)
		Tuvalu	6·69 (6·37–7·01)	5·98 (5·67–6·28)	3·08 (2·74–3·45)	2·20 (1·71–2·70)	1·71 (1·17–2·26)	0·2 (0·2–0·2)	0·4 (0·4–0·4)	0·3 (0·2–0·3)	0·3 (0·2–0·4)	0·2 (0·1–0·4)	1·4 (1·3–1·6)
		Vanuatu	7·08 (6·77–7·38)	6·10 (5·88–6·31)	3·52 (3·23–3·84)	2·44 (2·06–2·87)	1·79 (1·33–2·31)	2·1 (2·0–2·2)	5·1 (4·9–5·3)	8·7 (8·0–9·5)	9·8 (7·7–12·5)	8·2 (3·8–15·6)	1·6 (1·5–1·8)
	Southeast Asia		6·40 (6·08–6·72)	4·31 (4·20–4·43)	2·05 (1·89–2·23)	1·60 (1·40–1·83)	1·35 (1·14–1·60)	8697·2 (8276·7–9096·0)	12 693·6 (12 352·2–13 033·5)	11 172·2 (10 297·5–12 144·2)	8332·9 (7018·9–10 074·5)	4051·6 (2523·7–6469·1)	1·0 (0·9–1·0)
		Cambodia	6·60 (6·26–6·93)	5·89 (5·64–6·13)	2·61 (2·39–2·82)	1·65 (1·32–1·93)	1·10 (0·71–1·45)	211·5 (200·4–222·5)	351·8 (337·4–366·1)	372·7 (341·8–403·6)	267·3 (193·9–342·9)	102·9 (35·5–192·8)	1·2 (1·1–1·3)
		Indonesia	6·07 (5·71–6·42)	4·28 (4·15–4·41)	1·97 (1·77–2·22)	1·53 (1·25–1·84)	1·29 (0·99–1·63)	3626·7 (3445·3–3802·8)	5175·0 (5017·4–5342·6)	4393·8 (3937·5–4935·6)	3147·3 (2402·1–4049·5)	1453·3 (684·5–2718·1)	0·9 (0·8–1·0)
		Laos	6·65 (6·27–7·03)	6·22 (5·88–6·56)	2·76 (2·54–2·98)	1·61 (1·29–1·88)	1·09 (0·73–1·40)	84·4 (79·7–89·0)	147·1 (139·3–154·9)	177·4 (162·5–192·1)	116·7 (88·2–149·0)	32·4 (10·7–63·5)	1·3 (1·2–1·4)
		Malaysia	6·89 (6·56–7·22)	3·98 (3·88–4·08)	1·81 (1·62–2·05)	1·39 (1·11–1·70)	1·17 (0·86–1·52)	303·5 (289·8–317·0)	421·1 (409·4–432·6)	474·2 (424·4–534·7)	364·6 (271·6–471·1)	203·4 (106·1–366·8)	0·9 (0·8–1·0)
		Maldives	4·97 (4·69–5·25)	6·46 (6·35–6·57)	1·64 (1·47–1·84)	1·07 (0·79–1·34)	0·77 (0·42–1·11)	2·7 (2·5–2·9)	6·8 (6·6–6·9)	6·0 (5·4–6·7)	5·4 (3·9–7·0)	2·6 (1·1–4·8)	0·8 (0·7–0·9)
		Mauritius	6·31 (6·17–6·45)	2·61 (2·52–2·71)	1·39 (1·23–1·57)	1·17 (0·94–1·42)	1·03 (0·77–1·32)	23·3 (22·7–23·8)	23·4 (22·6–24·2)	12·7 (11·2–14·3)	6·7 (5·0–9·1)	1·8 (0·6–3·8)	0·7 (0·6–0·8)
		Myanmar	6·45 (6·08–6·82)	5·44 (5·18–5·67)	2·40 (2·20–2·62)	1·69 (1·42–1·97)	1·22 (0·89–1·57)	869·3 (819·0–919·4)	1328·9 (1263·1–1389·9)	1073·6 (983·0–1169·8)	754·4 (596·8–943·5)	248·2 (96·8–474·8)	1·1 (1·0–1·2)
		Philippines	6·75 (6·43–7·05)	4·77 (4·57–4·98)	2·40 (2·21–2·60)	1·84 (1·61–2·11)	1·50 (1·23–1·79)	937·1 (891·5–982·6)	1767·1 (1681·8–1852·7)	2185·7 (2000·9–2368·9)	1967·3 (1575·7–2425·6)	1254·1 (747·7–2010·5)	1·1 (1·0–1·2)
		Seychelles	4·00 (3·75–4·27)	3·55 (3·44–3·68)	2·31 (2·10–2·53)	1·86 (1·60–2·14)	1·60 (1·31–1·91)	1·0 (1·0–1·1)	1·7 (1·6–1·8)	1·6 (1·4–1·7)	1·7 (1·4–2·2)	1·6 (1·0–2·4)	1·1 (1·0–1·2)
		Sri Lanka	5·19 (4·94–5·46)	3·39 (3·24–3·53)	1·85 (1·64–2·08)	1·50 (1·24–1·81)	1·30 (0·99–1·66)	306·2 (290·5–323·7)	421·2 (402·2–441·1)	298·6 (265·2–335·8)	179·5 (124·6–245·5)	45·9 (5·8–121·4)	0·9 (0·8–1·0)
		Thailand	6·89 (6·58–7·19)	3·15 (3·02–3·27)	1·32 (1·20–1·46)	1·13 (0·96–1·31)	1·04 (0·86–1·24)	1003·1 (956·7–1048·8)	1181·9 (1131·0–1233·1)	573·1 (520·0–635·5)	300·0 (225·5–379·9)	87·3 (44·3–144·5)	0·6 (0·6–0·7)
		Timor-Leste	7·00 (6·76–7·26)	6·72 (6·54–6·90)	3·85 (3·43–4·29)	2·27 (1·62–2·90)	1·58 (0·85–2·30)	22·5 (21·6–23·4)	28·8 (27·9–29·6)	41·0 (36·7–45·6)	35·7 (22·7–51·3)	17·5 (1·7–48·6)	1·8 (1·6–1·9)
		Viet Nam	6·72 (6·40–7·04)	4·40 (4·25–4·55)	2·06 (1·88–2·28)	1·63 (1·38–1·93)	1·38 (1·10–1·70)	1293·3 (1232·9–1353·8)	1819·8 (1761·6–1875·2)	1546·1 (1416·2–1708·8)	1175·5 (928·2–1496·6)	595·3 (314·7–1084·8)	1·0 (0·9–1·1)
**Sub-Saharan Africa**			**6·94 (6·62–7·25)**	**6·78 (6·60–6·94)**	**4·29 (4·03–4·58)**	**2·72 (2·32–3·15)**	**1·82 (1·35–2·32)**	**8850·5 (8455·5–9233·0)**	**17 852·0 (17 412·0–18 264·8)**	**37 713·3 (35 513·3–40 151·9)**	**46 344·7 (37 509·8–55 394·7)**	**39 828·0 (19 422·1–69 747·3)**	**1·9 (1·8–2·0)**
	Central sub-Saharan Africa		7·19 (6·93–7·42)	7·07 (6·84–7·28)	4·44 (4·15–4·72)	2·52 (2·05–2·94)	1·86 (1·37–2·31)	1010·4 (975·5–1042·7)	2065·0 (1997·0–2125·8)	4459·3 (4183·3–4730·0)	5233·7 (3753·5–6919·5)	4873·6 (2153·5–8575·8)	2·0 (1·9–2·1)
		Angola	6·94 (6·61–7·26)	7·29 (7·06–7·52)	5·02 (4·66–5·38)	2·76 (2·21–3·35)	1·97 (1·37–2·62)	241·6 (230·2–252·6)	374·6 (361·9–386·9)	1202·7 (1115·3–1294·1)	1594·1 (1152·6–2079·7)	1735·5 (690·4–3385·4)	2·2 (2·1–2·4)
		Central African Republic	5·79 (5·46–6·12)	6·47 (6·18–6·74)	4·36 (4·00–4·74)	2·36 (1·86–2·88)	1·35 (0·77–2·03)	59·7 (56·4–62·7)	110·6 (105·9–115·3)	191·1 (176·5–205·8)	142·5 (93·6–203·7)	29·6 (0·0–98·2)	1·8 (1·7–1·9)
		Congo (Brazzaville)	6·65 (6·25–7·03)	6·16 (5·88–6·42)	2·95 (2·69–3·23)	1·90 (1·54–2·32)	1·49 (1·11–1·93)	40·8 (38·4–43·1)	76·4 (73·1–79·5)	128·6 (117·3–141·1)	118·9 (86·6–159·7)	78·2 (34·3–146·1)	1·3 (1·2–1·5)
		Democratic Republic of the Congo	7·56 (7·27–7·83)	7·16 (6·90–7·40)	4·40 (4·02–4·77)	2·46 (1·82–3·03)	1·76 (1·09–2·38)	639·8 (615·5–662·2)	1459·4 (1404·9–1507·8)	2856·0 (2620·9–3089·5)	3277·7 (1995·4–4613·9)	2910·6 (858·8–5975·8)	2·0 (1·8–2·1)
		Equatorial Guinea	7·18 (6·84–7·50)	6·83 (6·50–7·15)	3·09 (2·69–3·57)	2·19 (1·70–2·75)	1·83 (1·29–2·43)	9·6 (9·1–10·0)	14·4 (13·7–15·0)	37·4 (32·6–42·9)	55·9 (39·6–75·8)	82·7 (37·9–154·9)	1·4 (1·2–1·5)
		Gabon	6·51 (6·10–6·91)	5·74 (5·37–6·10)	2·84 (2·46–3·30)	1·93 (1·41–2·52)	1·56 (1·02–2·19)	18·9 (17·7–20·0)	29·6 (27·7–31·4)	43·5 (37·7–50·3)	44·7 (29·8–63·8)	36·9 (12·5–80·6)	1·3 (1·1–1·5)
	Eastern sub-Saharan Africa		7·15 (6·86–7·45)	7·02 (6·84–7·17)	4·09 (3·80–4·39)	2·50 (2·04–2·96)	1·68 (1·17–2·22)	3378·3 (3239·4–3512·6)	7091·2 (6932·8–7242·3)	13 778·4 (12 785·2–14 858·1)	15 968·4 (12 317·1–19 784·1)	12 206·9 (4940·5–23 355·8)	1·8 (1·7–1·9)
		Burundi	7·14 (6·84–7·43)	6·86 (6·72–6·99)	4·93 (4·55–5·35)	2·74 (2·16–3·31)	1·55 (0·83–2·25)	124·8 (119·4–130·0)	213·9 (209·1–218·4)	468·8 (431·5–510·0)	552·4 (389·3–723·9)	381·8 (97·3–874·8)	2·2 (2·1–2·3)
		Comoros	5·53 (5·12–5·90)	7·20 (6·95–7·44)	2·93 (2·55–3·35)	1·73 (1·17–2·33)	1·23 (0·60–1·92)	6·5 (6·0–6·9)	17·2 (16·6–17·7)	17·3 (15·1–19·8)	11·5 (6·5–17·6)	3·5 (0·0–11·1)	1·3 (1·2–1·5)
		Djibouti	5·75 (5·35–6·19)	5·18 (4·92–5·44)	2·52 (2·22–2·85)	1·41 (0·92–1·88)	0·95 (0·38–1·51)	2·4 (2·3–2·6)	9·5 (9·0–10·0)	25·2 (22·2–28·4)	22·5 (13·9–31·8)	12·8 (2·9–28·1)	1·1 (1·0–1·3)
		Eritrea	6·88 (6·52–7·22)	6·64 (6·35–6·90)	3·85 (3·41–4·34)	2·20 (1·52–2·86)	1·28 (0·49–2·09)	54·3 (51·4–57·2)	118·9 (113·7–123·7)	195·7 (173·8–220·4)	163·7 (88·6–262·8)	66·9 (2·5–221·8)	1·7 (1·5–1·9)
		Ethiopia	6·73 (6·35–7·08)	6·93 (6·67–7·17)	4·10 (3·79–4·43)	2·40 (1·86–2·89)	1·29 (0·64–1·87)	868·1 (819·0–914·9)	1786·6 (1715·1–1853·9)	3498·1 (3239·1–3788·0)	3957·1 (2829·4–5150·9)	2375·2 (637·3–4829·0)	1·8 (1·7–2·0)
		Kenya	7·64 (7·41–7·86)	7·03 (6·84–7·21)	2·75 (2·43–3·13)	1·84 (1·39–2·35)	1·45 (0·96–2·01)	310·2 (302·7–317·4)	811·8 (792·2–830·5)	1186·7 (1053·2–1340·0)	1050·1 (715·7–1486·9)	551·7 (134·8–1299·0)	1·3 (1·1–1·4)
		Madagascar	7·44 (7·12–7·73)	6·72 (6·54–6·89)	3·77 (3·48–4·07)	2·33 (1·91–2·78)	1·70 (1·23–2·22)	230·8 (221·6–239·4)	427·6 (416·9–438·2)	877·1 (815·6–945·5)	958·9 (730·1–1221·2)	735·6 (307·9–1382·5)	1·7 (1·6–1·8)
		Malawi	6·17 (5·76–6·57)	7·62 (7·42–7·80)	3·46 (3·07–3·87)	2·03 (1·46–2·57)	1·55 (0·94–2·14)	130·2 (121·2–138·9)	348·4 (339·0–356·9)	574·2 (511·5–639·8)	570·3 (363·3–776·9)	417·2 (95·9–891·8)	1·5 (1·4–1·7)
		Mozambique	6·95 (6·69–7·19)	6·67 (6·49–6·85)	4·50 (4·15–4·88)	2·44 (1·91–2·93)	1·55 (0·95–2·14)	337·7 (324·7–348·9)	596·5 (581·4–612·7)	1104·9 (1015·0–1202·2)	1221·8 (875·5–1614·2)	790·1 (242·4–1762·7)	1·9 (1·8–2·1)
		Rwanda	7·39 (7·15–7·61)	7·45 (7·35–7·55)	3·55 (3·28–3·84)	1·97 (1·54–2·42)	1·24 (0·76–1·77)	137·3 (132·6–141·8)	267·5 (263·5–271·5)	373·6 (345·4–404·5)	405·7 (303·0–518·7)	298·1 (121·9–552·8)	1·6 (1·5–1·7)
		Somalia	7·77 (7·51–8·01)	7·68 (7·50–7·84)	6·54 (6·27–6·81)	4·30 (3·92–4·68)	2·45 (1·92–3·00)	109·7 (105·8–113·3)	334·0 (326·2–341·2)	960·1 (919·1–1004·4)	1464·1 (1008·1–2005·2)	1425·7 (492·7–2833·8)	2·7 (2·6–2·8)
		South Sudan	6·13 (5·69–6·56)	6·12 (5·79–6·45)	5·45 (5·04–5·87)	4·09 (3·59–4·64)	1·98 (1·22–2·75)	111·3 (103·6–118·7)	208·7 (196·9–219·7)	384·1 (353·8–414·9)	690·1 (519·9–872·8)	791·2 (304·1–1536·2)	2·2 (2·1–2·3)
		Tanzania	7·60 (7·30–7·86)	6·94 (6·71–7·14)	4·04 (3·74–4·37)	2·42 (2·02–2·86)	1·70 (1·23–2·20)	481·7 (463·9–498·1)	943·5 (913·1–972·0)	1902·1 (1762·2–2057·9)	2091·0 (1582·8–2666·8)	1577·3 (661·3–2941·2)	1·8 (1·7–1·9)
		Uganda	7·86 (7·60–8·09)	7·53 (7·36–7·69)	4·76 (4·47–5·07)	2·72 (2·26–3·19)	1·98 (1·48–2·50)	339·2 (329·0–348·4)	703·0 (688·9–716·8)	1591·6 (1490·7–1704·3)	2049·6 (1533·6–2610·7)	2014·5 (858·0–3620·6)	2·1 (2·0–2·2)
		Zambia	7·58 (7·29–7·86)	7·22 (7·01–7·41)	3·84 (3·52–4·21)	2·39 (1·88–2·91)	1·83 (1·28–2·40)	132·3 (127·4–136·7)	300·2 (292·5–307·8)	606·9 (552·7–668·9)	747·0 (534·7–1007·7)	755·7 (306·5–1475·5)	1·7 (1·6–1·9)
	Southern sub- Saharan Africa		6·20 (5·77–6·63)	4·86 (4·69–5·05)	2·42 (2·25–2·61)	1·94 (1·67–2·21)	1·63 (1·29–1·99)	792·2 (739·1–844·6)	1517·8 (1465·6–1572·3)	1636·8 (1518·7–1764·9)	1416·1 (1138·4–1739·9)	876·3 (453·7–1528·0)	1·1 (1·0–1·2)
		Botswana	7·01 (6·65–7·35)	6·06 (5·78–6·33)	2·31 (2·05–2·61)	1·70 (1·31–2·12)	1·38 (0·94–1·84)	19·4 (18·4–20·4)	40·8 (38·8–42·7)	48·7 (43·1–55·2)	37·7 (26·2–50·8)	21·1 (7·3–43·8)	1·0 (0·9–1·2)
		Eswatini	7·19 (6·84–7·52)	6·17 (5·96–6·38)	2·89 (2·65–3·15)	1·98 (1·62–2·36)	1·53 (1·13–1·95)	12·5 (12·0–13·1)	27·7 (26·8–28·5)	29·4 (27·0–32·1)	21·3 (15·1–28·1)	6·7 (0·7–16·1)	1·3 (1·2–1·3)
		Lesotho	6·54 (6·14–6·93)	5·66 (5·46–5·84)	2·61 (2·31–2·93)	1·88 (1·50–2·34)	1·47 (1·03–1·98)	29·9 (28·1–31·7)	55·8 (54·2–57·4)	42·5 (37·8–48·1)	34·6 (23·5–48·4)	16·1 (4·1–39·0)	1·1 (1·0–1·2)
		Namibia	7·16 (6·81–7·49)	5·63 (5·42–5·84)	2·80 (2·58–3·08)	2·03 (1·70–2·40)	1·62 (1·24–2·03)	22·1 (21·0–23·1)	40·6 (39·2–41·9)	58·0 (53·3–63·7)	54·7 (40·5–71·4)	39·4 (18·8–69·9)	1·3 (1·2–1·4)
		South Africa	5·91 (5·46–6·37)	4·35 (4·16–4·56)	2·07 (1·92–2·23)	1·69 (1·46–1·89)	1·45 (1·20–1·67)	572·2 (529·6–614·5)	1007·6 (963·8–1054·9)	988·1 (913·4–1065·5)	785·7 (619·2–968·1)	470·7 (286·1–706·9)	0·9 (0·9–1·0)
		Zimbabwe	7·24 (6·90–7·56)	6·63 (6·50–6·75)	3·60 (3·34–3·89)	2·56 (2·12–3·01)	2·01 (1·52–2·51)	136·0 (129·9–141·8)	345·3 (339·1–351·1)	470·0 (437·0–506·3)	482·1 (360·8–626·6)	322·3 (97·3–699·7)	1·6 (1·5–1·7)
	Western sub-Saharan Africa		6·87 (6·54–7·17)	7·03 (6·83–7·22)	4·79 (4·51–5·09)	3·03 (2·60–3·48)	1·89 (1·39–2·44)	3669·7 (3499·6–3827·7)	7177·9 (6992·5–7355·4)	17 838·8 (16 834·6–18 900·8)	23 726·4 (19 422·7–28 301·3)	21 871·2 (10 771·0–37 177·5)	2·1 (2·0–2·2)
		Benin	6·52 (6·12–6·92)	7·00 (6·79–7·19)	5·17 (4·86–5·50)	3·12 (2·65–3·60)	1·58 (0·95–2·18)	92·8 (87·0–98·3)	171·2 (166·5–175·6)	522·3 (488·8–559·3)	724·6 (552·9–910·7)	568·0 (197·5–1084·8)	2·3 (2·2–2·4)
		Burkina Faso	6·30 (5·93–6·65)	7·32 (7·17–7·46)	5·52 (5·18–5·86)	3·76 (3·23–4·28)	1·62 (0·89–2·26)	187·8 (178·1–197·5)	379·6 (371·6–386·8)	950·8 (893·8–1010·0)	1519·4 (1216·6–1833·9)	1193·7 (384·0–2296·1)	2·4 (2·3–2·5)
		Cabo Verde	5·08 (4·78–5·37)	5·14 (4·99–5·28)	1·78 (1·53–2·04)	1·09 (0·73–1·48)	0·91 (0·51–1·34)	7·0 (6·5–7·4)	10·9 (10·5–11·3)	8·5 (7·4–9·7)	4·3 (2·5–6·6)	1·0 (0·1–2·7)	0·8 (0·7–1·0)
		Cameroon	6·40 (5·98–6·80)	6·73 (6·56–6·89)	4·13 (3·76–4·54)	2·44 (1·92–3·03)	1·71 (1·13–2·36)	216·3 (202·6–229·5)	394·4 (385·4–402·9)	1032·5 (940·4–1137·6)	1138·8 (768·0–1579·8)	811·5 (241·3–1828·0)	1·8 (1·7–2·0)
		Chad	7·36 (7·03–7·66)	7·60 (7·39–7·80)	6·99 (6·75–7·24)	4·81 (4·45–5·18)	2·15 (1·65–2·71)	127·4 (121·9–132·5)	242·8 (236·2–248·8)	860·2 (829·3–892·5)	1841·1 (1531·6–2177·8)	2491·7 (1487·4–3800·2)	2·9 (2·8–3·0)
		Côte d'Ivoire	6·89 (6·52–7·25)	6·95 (6·77–7·11)	4·54 (4·23–4·90)	2·57 (2·11–3·04)	1·44 (0·87–1·99)	127·5 (120·7–133·9)	405·3 (395·4–414·8)	950·1 (879·5–1027·5)	1000·2 (744·1–1262·9)	521·1 (150·7–1089·8)	2·0 (1·9–2·1)
		The Gambia	5·90 (5·45–6·35)	6·57 (6·39–6·76)	4·12 (3·89–4·38)	2·21 (1·81–2·61)	1·37 (0·92–1·87)	10·7 (10·0–11·4)	34·0 (33·2–34·9)	77·9 (73·1–83·1)	67·4 (50·8–86·7)	29·8 (9·4–59·0)	1·9 (1·8–1·9)
		Ghana	5·37 (4·92–5·84)	6·71 (6·49–6·90)	3·40 (3·01–3·81)	2·12 (1·57–2·71)	1·57 (0·97–2·20)	204·3 (187·1–223·2)	551·2 (532·4–567·7)	966·8 (855·7–1082·9)	922·4 (602·0–1318·0)	636·0 (168·0–1417·0)	1·5 (1·4–1·7)
		Guinea	7·05 (6·76–7·34)	6·81 (6·59–7·01)	4·67 (4·33–5·00)	3·02 (2·58–3·43)	1·42 (0·81–2·00)	127·4 (122·3–132·2)	245·1 (238·0–251·7)	495·4 (462·6–528·8)	633·0 (506·6–787·0)	366·2 (114·2–728·3)	2·0 (1·9–2·1)
		Guinea-Bissau	7·11 (6·82–7·38)	6·13 (5·86–6·41)	4·42 (4·08–4·78)	2·41 (1·88–2·91)	1·26 (0·63–1·86)	31·2 (29·9–32·4)	37·4 (35·6–39·0)	72·0 (66·1–78·3)	75·6 (52·9–99·7)	35·5 (8·0–81·5)	1·9 (1·8–2·1)
		Liberia	6·78 (6·40–7·15)	6·93 (6·74–7·12)	3·81 (3·39–4·26)	2·10 (1·52–2·71)	1·47 (0·85–2·14)	44·2 (41·8–46·6)	100·9 (98·2–103·2)	163·5 (145·6–183·0)	157·0 (100·9–229·0)	101·5 (22·7–247·4)	1·7 (1·5–1·8)
		Mali	7·33 (7·00–7·64)	7·51 (7·29–7·71)	6·15 (5·85–6·46)	4·21 (3·83–4·63)	1·85 (1·30–2·42)	182·8 (174·7–190·4)	389·4 (379·0–398·7)	1064·4 (1007·3–1123·0)	1863·5 (1522·4–2237·3)	1675·3 (779·9–2834·3)	2·6 (2·5–2·7)
		Mauritania	6·68 (6·29–7·06)	6·70 (6·53–6·88)	4·22 (3·86–4·64)	2·50 (1·98–3·08)	1·66 (1·04–2·34)	33·5 (31·5–35·4)	71·1 (69·1–72·9)	135·3 (122·6–149·5)	153·2 (109·3–206·1)	113·3 (32·7–258·4)	1·9 (1·8–2·1)
		Niger	7·64 (7·36–7·89)	7·98 (7·82–8·12)	6·97 (6·71–7·24)	5·15 (4·68–5·64)	2·24 (1·48–2·92)	144·4 (139·4–149·1)	349·4 (342·8–355·8)	1174·8 (1126·7–1224·8)	2766·0 (2211·9–3290·7)	3891·3 (1737·1–6484·1)	3·0 (2·9–3·1)
		Nigeria	7·08 (6·72–7·42)	6·99 (6·71–7·24)	4·75 (4·35–5·14)	2·69 (2·06–3·31)	1·87 (1·19–2·54)	1826·3 (1735·3–1911·0)	3200·3 (3085·6–3310·7)	8333·3 (7671·1–8973·3)	9845·1 (6861·8–13037·8)	8949·4 (2670·4–18113·0)	2·0 (1·9–2·2)
		São Tomé and Príncipe	6·22 (6·01–6·42)	6·24 (6·03–6·44)	2·84 (2·51–3·19)	1·77 (1·29–2·28)	1·37 (0·83–1·94)	2·4 (2·3–2·5)	3·7 (3·6–3·9)	4·9 (4·3–5·6)	3·1 (1·9–4·6)	0·3 (0·0–1·9)	1·3 (1·2–1·5)
		Senegal	7·17 (6·82–7·49)	7·39 (7·27–7·51)	4·02 (3·72–4·34)	2·32 (1·79–2·79)	1·25 (0·60–1·83)	135·9 (129·3–142·0)	303·8 (298·8–308·7)	479·3 (441·8–520·9)	489·6 (354·1–621·1)	233·5 (50·6–499·9)	1·8 (1·7–2·0)
		Sierra Leone	6·60 (6·19–7·00)	6·55 (6·27–6·81)	4·20 (3·86–4·56)	2·43 (1·99–2·85)	1·31 (0·78–1·82)	91·7 (86·2–97·0)	159·2 (152·9–164·8)	302·8 (278·5–327·5)	303·3 (228·2–373·7)	144·7 (42·3–292·0)	1·8 (1·7–1·9)
		Togo	7·46 (7·16–7·74)	6·93 (6·76–7·08)	3·72 (3·44–4·02)	2·01 (1·61–2·42)	1·24 (0·79–1·72)	75·8 (72·8–78·7)	127·9 (124·9–130·7)	243·6 (225·8–263·1)	218·3 (157·3–281·1)	107·1 (35·6–216·8)	1·7 (1·6–1·8)

Numbers in parentheses are 95% uncertainty intervals. Super-regions, regions, and countries are listed in alphabetical order.

**Figure 2:**
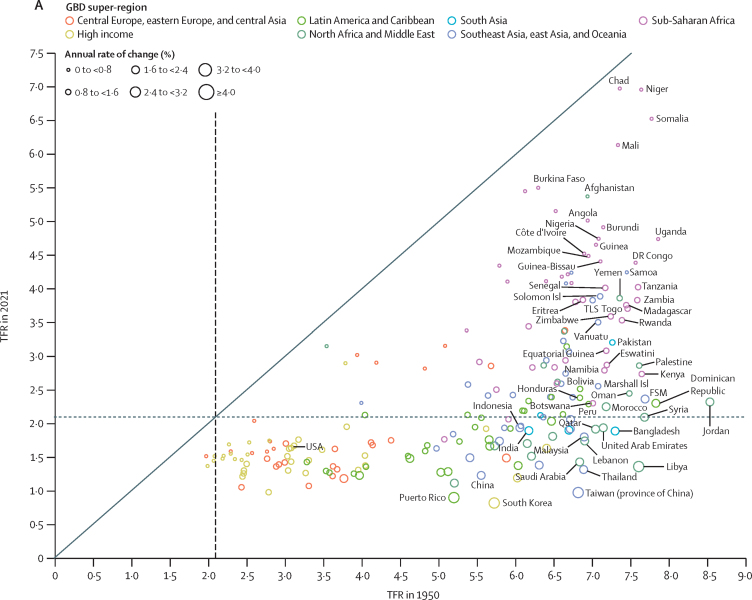

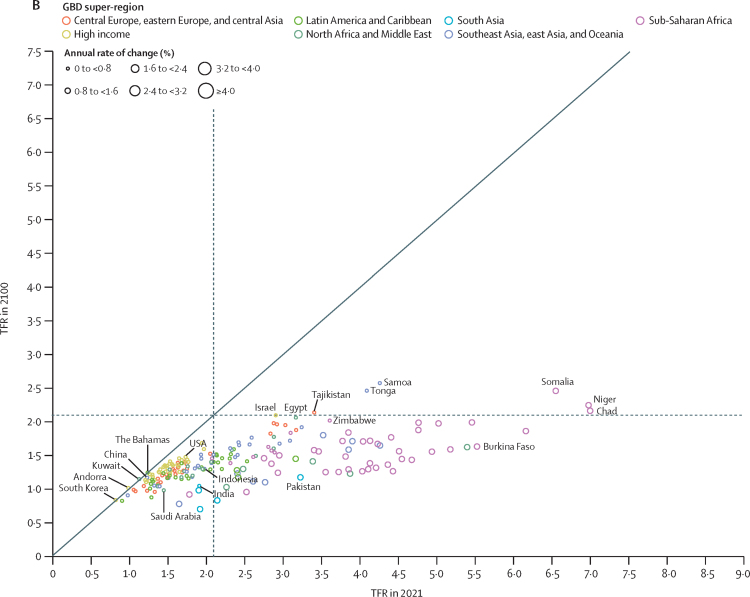
TFR by country or territory, 1950 *vs* 2021 (A) and 2021 *vs* 2100 (B) Each circle represents the TFR for a country or territory in 1950 and 2021 (A) or in 2021 and 2100 (B). The size of the circle indicates the absolute annual rate of change in TFR between the two years. Circles above the diagonal line show countries or territories that have seen an increase in TFR over the study period, whereas those below the diagonal had a decline in TFR over the study period. The horizontal and vertical dashed lines indicate replacement TFR (2·1). Country name labels are provided for locations that have the largest TFR values, those with large TFR values and annualised rates of change, and the five most populous countries with a low TFR value. FSM= Federated States of Micronesia. GBD=Global Burden of Diseases, Injuries, and Risk Factors Study. Isl=Islands. TLS=Timor-Leste. TFR=total fertility rate.

At the national level, estimates of TFR in 2021 ranged from 0·82 (95% UI 0·75–0·89) in South Korea to 6·99 (6·75–7·24) in Chad, with below-replacement levels of fertility (TFR <2·1) in 110 of 204 countries and territories (table 1, figures 2A, 3). This was an increase from 82 countries and territories below replacement level in 2000. Since 2000, the steepest declines in TFR were seen in São Tomé and Príncipe, Puerto Rico, and Kuwait, all of which had declines of more than 36%. Overall, fertility has declined steadily at the global level and across nearly all countries and territories since 1950, with rebounds in low fertility levels (ie, below-replacement fertility in the year with lowest TFR followed by higher fertility in at least one subsequent year) observed in 47 countries and territories (appendix 2 figure S1). The magnitude of these rebounds was small, with an average increase of 0·20 from lowest estimated TFR to the TFR in 2021, and only three countries rebounded above replacement levels: Georgia, Kazakhstan, and Seychelles. Cohort-completed fertility is a better indicator of fertility trends due to controlling for general shifts in the timing of childbearing. Since the 1940 birth cohort, CCF50 declined from peak levels in all countries and territories (the 1971 birth cohort is the last one for which we have complete age-specific fertility estimates up to age 50 years). Temporal rebounds from below-replacement CCF50 are less common compared with TFR, with only 15 countries or territories having a cohort that dropped below replacement fertility followed by a subsequent cohort with higher CCF50. These rebounds were also of smaller magnitude, with an average increase of 0·02 from lowest estimated CCF50 to the CCF50 in the 1971 birth cohort, and no rebounds exceeded replacement levels.

### Reference scenario fertility forecasts

Our forecasting estimates suggest that fertility rates will continue to decline worldwide from a global TFR of 2·21 (95% UI 2·06–2·36) in 2022 to 1·83 (1·59–2·08) in 2050 and 1·59 (1·25–1·96) in 2100 (table 1, figure 1). Except for four locations (South Korea, Andorra, The Bahamas, and Kuwait), we project that every country and territory will experience a decrease in TFR between 2021 and 2100 (figure 2B). Across GBD super-regions, forecasts of TFR in 2050 range from 1·36 (1·09–1·64) in south Asia to 2·72 (2·32–3·15) in sub-Saharan Africa; in 2100, TFRs in these two super-regions are forecast to be 1·10 (0·80–1·43) and 1·82 (1·35–2·32), respectively (table 1, figure 1). Countries projected to have the highest fertility rates in 2050 are Chad (4·81 [4·45–5·18]) and Niger (5·15 [4·68–5·64]); in 2100, the highest rates are forecast in Tonga (2·45 [1·93–2·98]) and Samoa (2·57 [1·98–3·14]; table 1). Countries projected to have the lowest fertility rates in 2050 are South Korea (0·82 [0·73–0·92]) and Puerto Rico (0·84 [0·76–0·92]), with the lowest rates in 2100 forecast in Bhutan (0·69 [0·33–1·00]) and the Maldives (0·77 [0·42–1·11]; table 1). TFRs are projected to decline between 2021 and 2100 at rates of more than 1% per year in 45 (22·1%) of 204 countries and territories. Burkina Faso and Guinea-Bissau are forecast to have the fastest annualised declines at rates of around 1·6% per year (figure 2B).

**Figure 4:**
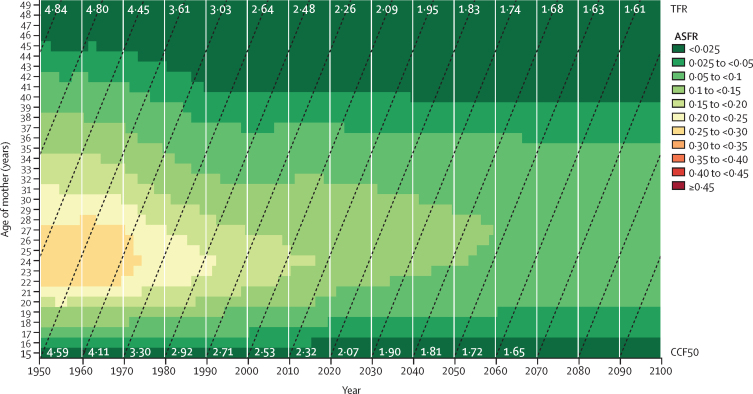
Lexis diagram of CCF50, single-year age interval ASFR, and TFR by age of mother, globally, 1950–2100 These Lexis diagram heatmaps simultaneously display single-year ASFR (colour fill), TFR (numbers at the top), and CCF50 estimates (white numbers at the bottom). The horizontal axis of the Lexis diagrams extends from 1950 (the beginning year of data availability) to 2100 (the final year of our forecasts), and the vertical axis indicates age of mother. CCF50 estimates are shown for each 10-year birth cohort. CCF50 is the sum of ASFR cells on the diagonal (ie, representing birth cohort), whereas TFR is the sum of ASFR cells vertically (ie, ASFR values from the same calendar year by age of mother). CCF50 is a cohort measure and years labelled on the x-axis are in period space. CCF50 values correspond to those entering their reproductive age (15–49 years) at that year (in other words, the birth cohort 15 years earlier). For example, the CCF50 value shown in 1950 represents CCF50 for those born in 1935. The vertical white lines indicate each decade, and the diagonal black dashed lines assist with visualising cohort space. ASFR=age-specific fertility rate. CCF50=completed cohort fertility at age 50 years. TFR=total fertility rate.

Global CCF50 is projected to decrease from 3·59 (95% UI 3·52–3·66) for females who were born in 1950 to 1·64 (1·33–1·97) for females born in 2050 who will have transitioned out of their reproductive years by 2100 (see figure 4, a Lexis diagram heatmap that simultaneously displays single-year ASFR, TFR, and CCF50 estimates for comparison). Globally, ASFRs are forecast to decline from 2022 to 2100, especially between ages 22 years and 32 years (as reflected in the change in pixel colours from lighter green to darker green moving rightward across the horizontal axis). This reflects that in some areas—particularly countries in the high-income GBD super-region and some in the central Europe, eastern Europe, and central Asia super-region—maternal age structure has shifted and will continue to shift dramatically towards females being older when they have children. Country-specific heatmaps that provide insight into different fertility patterns for a range of countries are shown in appendix 2 (figure S3).

**Figure 3:**
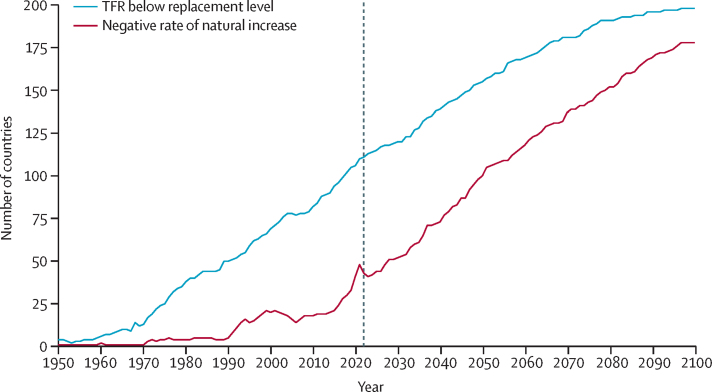
Number of countries and territories with TFR below replacement level (2·1) and with a negative rate of natural increase, 1950–2100 The number of countries and territories is out of a total of 204. The rate of natural increase is calculated as the birth rate minus the death rate; a negative rate indicates more deaths than births in a particular year and location. The vertical dashed line is at year 2022 (the first forecast year). TFR=total fertility rate.

103 (50·5%) of the 204 countries and territories included in the study had already reached TFRs below replacement level in 2018, and we project that 100 (49·0%) countries will have negative rates of natural increase (ie, the number of deaths will exceed the number of livebirths) by 2050 (figure 3). The concept of population momentum is illustrated in figure 3, which is the tendency of a population to continue to grow beyond the time it falls below replacement-level fertility. Population growth is heavily influenced by the age structure of the population; in locations with relatively large cohorts of young people (particularly younger than 15 years), the population will continue to grow well beyond the first year of below-replacement fertility as these young cohorts move through their reproductive years.^36^, ^37^ Our estimates indicate that there is approximately a 30-year gap between the time when TFR falls below 2·1 and when the natural rate of population increase turns negative. We forecast that 155 (76·0%) countries and territories will have fertility rates below replacement level in 2050; by 2100, we project this number will increase to 198 (97·1%), with 178 (87·3%) having a negative natural rate of increase (figure 3).

**Figure 5:**
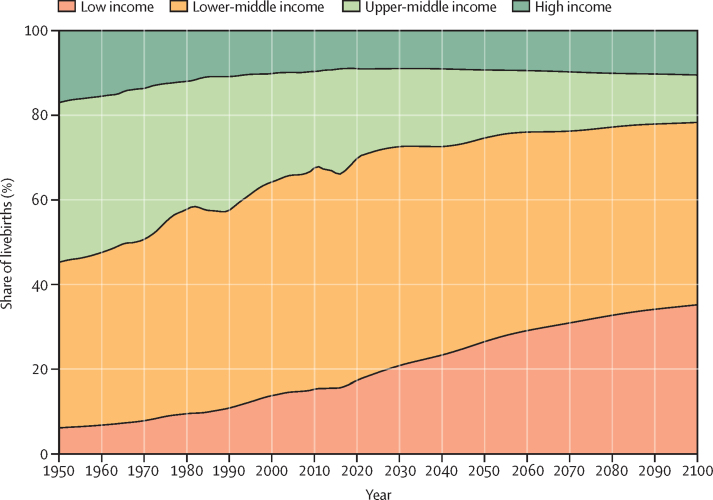
Share of livebirths by 2021 World Bank income group The World Bank income group is explicitly chosen to highlight the share of births according to resources per person. Resources are defined as gross national income (gross domestic product plus net income) per person.

We project that the 2021 World Bank low-income and lower-middle-income groups^38^ combined will contribute the majority of the global share of livebirths in 2100, at 77·4% (95% UI 69·1–83·6; figure 5, appendix 1 table S6, figure S2). Our results indicate that the proportion of global livebirths in the World Bank low-income group will increase from 17·8% (17·3–18·2) in 2021 to 26·5% (24·4–28·5) in 2050 and 34·6% (26·4–40·5) in 2100. In 1992, the number of births in the low-income group (14·9 million [14·6–15·3]) surpassed the number of births in the high-income group (14·0 million [13·8–14·2]; appendix 2 figure S4). We forecast that the number of births in the low-income group will exceed the number in the upper-middle-income group in 2026. In 1972, the number of births in the lower-middle-income group exceeded the number of births in the upper-middle-income group. We project that the lower-middle-income group will have the highest number of births among the four income groups during the 2022–2100 period even though it will decrease from 52·7% (51·7–53·7) of births in 2021 to 48·1% (45·2–50·5) in 2050 and 42·7% (37·5–48·7) in 2100 (figure 5). We forecast that the number of births in the lower-middle-income group will reach 53·9 million (43·3–67·1) by 2050 and 31·2 million (16·2–53·8) by 2100 (appendix 2 figure S4). By contrast, the share of livebirths contributed by the upper-middle-income group will decrease from 20·4% (19·8–21·2) in 2021 to 16·1% (14·7–17·8) in 2050 and 11·6% (8·9–15·3) in 2100 (figure 5). The proportion of the world's livebirths in the high-income group will remain relatively stable at 9·3% (8·3–10·6) in 2050 and 11·1% (7·2–16·2) in 2100 (figure 5). We also computed the global proportion of livebirths projected by GBD super-region (appendix 1 table S5), showing that the share of livebirths in sub-Saharan Africa is forecast to increase from 29·2% (28·7–29·6) in 2021 to 41·3% (39·6–43·1) in 2050 and 54·3% (47·1–59·5) in 2100. The two super-regions of south Asia and southeast Asia, east Asia, and Oceania—which were the primary sources of livebirths in the 1950s and 1980s—are projected to contribute only 7·1% (4·4–10·1) and 9·6% (7·9–12·0) of livebirths, respectively, in 2100. Considerable heterogeneity in livebirth counts exists at the regional and country level within each super-region. For example, the increase in livebirth counts between 2021 and 2100 is most notable in western and eastern sub-Saharan Africa, especially countries in the Sahel along with Somalia and South Sudan (table 1).

### Alternative scenario fertility forecasts

We developed four alternative scenarios based on independent drivers that are included in our model: educational attainment, contraceptive met need, implementation of pro-natal policies, and a combination of these three drivers (appendix 1 section 3.4). The first scenario, which assumes meeting the SDG education target by 2030, is estimated to result in global TFRs of 1·65 (95% UI 1·40–1·92) in 2050 and 1·56 (1·26–1·92) in 2100 (table 2). The second scenario, which assumes meeting the SDG contraceptive met need target by 2030, will produce global TFRs of 1·64 (1·39–1·89) in 2050 and 1·52 (1·21–1·87) in 2100. The third scenario, which incorporates pro-natal policy implementation, is forecast to yield global TFRs of 1·93 (1·69–2·19) in 2050 and 1·68 (1·36–2·04) in 2100. The combined scenario, in which all three other alternative scenarios are applied, is projected to result in a global TFR of 1·65 (1·40–1·92) in 2050 and 1·62 (1·35–1·95) in 2100. We also projected country-level future fertility rates based on the reference (presented here) and four alternative (presented in appendix 1 section 3.8) scenarios for the world's ten most populous countries in 2021. Among these countries, only Nigeria and Pakistan will remain above 1·75 (our threshold for the pro-natal scenario implementation) well into the future in our reference scenario (beyond 2100 for Nigeria and 2050 in Pakistan). TFRs in both Brazil and Indonesia are forecast to decrease below 1·75 in 2036. Reference TFR values in Bangladesh and India are projected to decrease below 1·75 by 2026 and 2027, respectively. Mexico, Russia, the USA, and China already experienced TFR values below 1·75 in or before 2021 and are forecast to maintain flat future trends. Detailed results on the alternative scenarios in the world's ten most populous countries are shown in appendix 1 (section 3.8). Projections in all 204 countries and territories are shown in appendix 2 (figure S5) and table 2.

**Table 2:** Total fertility rate for the reference scenario and four alternative scenarios by location, 2050 and 2100

			**Reference scenario**		**Education SDG achieved**		**Contraceptive met need SDG achieved**		**Pro-natal policies enacted**		**Combined scenario**	
			2050	2100	2050	2100	2050	2100	2050	2100	2050	2100
**Global**			**1·83 (1·59–2·08)**	**1·59 (1·25–1·96)**	**1·65 (1·40–1·92)**	**1·56 (1·26–1·92)**	**1·64 (1·39–1·89)**	**1·52 (1·21–1·87)**	**1·93 (1·69–2·19)**	**1·68 (1·36–2·04)**	**1·65 (1·40–1·92)**	**1·62 (1·35–1·95)**
**Central Europe, eastern Europe, and central Asia**			**1·68 (1·56–1·81)**	**1·57 (1·42–1·73)**	**1·65 (1·52–1·78)**	**1·56 (1·41–1·72)**	**1·52 (1·39–1·66)**	**1·51 (1·36–1·65)**	**1·80 (1·68–1·93)**	**1·64 (1·49–1·79)**	**1·62 (1·49–1·75)**	**1·57 (1·42–1·71)**
	Central Asia		2·31 (2·16–2·47)	1·95 (1·76–2·13)	2·27 (2·11–2·43)	1·93 (1·75–2·11)	2·11 (1·95–2·26)	1·88 (1·70–2·07)	2·33 (2·18–2·48)	1·95 (1·77–2·14)	2·09 (1·94–2·25)	1·89 (1·70–2·08)
		Armenia	1·45 (1·27–1·65)	1·24 (1·01–1·49)	1·41 (1·23–1·62)	1·21 (0·98–1·46)	1·18 (0·94–1·42)	1·11 (0·87–1·37)	1·65 (1·47–1·85)	1·44 (1·21–1·69)	1·35 (1·12–1·60)	1·29 (1·05–1·55)
		Azerbaijan	1·51 (1·27–1·76)	1·29 (1·01–1·59)	1·48 (1·24–1·74)	1·28 (1·00–1·57)	1·15 (0·84–1·44)	1·04 (0·73–1·35)	1·71 (1·47–1·96)	1·49 (1·21–1·79)	1·34 (1·03–1·64)	1·23 (0·92–1·54)
		Georgia	1·80 (1·65–1·96)	1·52 (1·34–1·71)	1·71 (1·57–1·88)	1·49 (1·31–1·68)	1·53 (1·34–1·72)	1·39 (1·20–1·60)	1·80 (1·65–1·96)	1·72 (1·54–1·91)	1·67 (1·49–1·87)	1·57 (1·38–1·78)
		Kazakhstan	2·43 (2·21–2·65)	1·94 (1·69–2·19)	2·38 (2·16–2·60)	1·92 (1·68–2·16)	2·27 (2·05–2·49)	1·89 (1·64–2·13)	2·43 (2·21–2·65)	1·94 (1·69–2·19)	2·23 (2·01–2·46)	1·87 (1·62–2·11)
		Kyrgyzstan	2·35 (2·05–2·70)	1·95 (1·63–2·33)	2·32 (2·01–2·67)	1·94 (1·62–2·31)	2·01 (1·68–2·38)	1·73 (1·40–2·10)	2·35 (2·05–2·70)	1·95 (1·63–2·33)	1·99 (1·65–2·37)	1·92 (1·59–2·29)
		Mongolia	2·46 (2·02–2·88)	1·87 (1·35–2·35)	2·36 (1·90–2·80)	1·83 (1·32–2·32)	2·22 (1·79–2·64)	1·77 (1·28–2·24)	2·46 (2·02–2·88)	1·87 (1·35–2·35)	2·15 (1·70–2·58)	1·79 (1·29–2·26)
		Tajikistan	2·66 (2·33–2·97)	2·13 (1·75–2·49)	2·59 (2·25–2·91)	2·10 (1·73–2·45)	2·18 (1·83–2·50)	1·84 (1·48–2·18)	2·66 (2·33–2·97)	2·13 (1·75–2·49)	2·13 (1·79–2·46)	1·81 (1·46–2·15)
		Turkmenistan	2·25 (1·87–2·66)	1·81 (1·38–2·28)	2·10 (1·68–2·54)	1·74 (1·29–2·22)	2·02 (1·65–2·44)	1·72 (1·30–2·18)	2·25 (1·87–2·66)	1·81 (1·38–2·28)	1·91 (1·51–2·34)	1·86 (1·43–2·32)
		Uzbekistan	2·34 (2·08–2·62)	1·97 (1·69–2·27)	2·32 (2·06–2·60)	1·97 (1·69–2·26)	2·27 (2·02–2·54)	1·97 (1·69–2·27)	2·34 (2·08–2·62)	1·97 (1·69–2·27)	2·26 (2·00–2·53)	1·97 (1·70–2·26)
	Central Europe		1·34 (1·19–1·50)	1·21 (1·03–1·41)	1·33 (1·17–1·49)	1·21 (1·03–1·41)	1·18 (1·01–1·35)	1·15 (0·97–1·35)	1·54 (1·39–1·70)	1·41 (1·23–1·61)	1·37 (1·20–1·55)	1·34 (1·16–1·55)
		Albania	1·34 (1·10–1·61)	1·17 (0·86–1·50)	1·29 (1·04–1·57)	1·15 (0·83–1·48)	0·86 (0·52–1·20)	0·80 (0·44–1·15)	1·54 (1·30–1·81)	1·37 (1·06–1·70)	1·04 (0·69–1·38)	0·99 (0·63–1·35)
		Bosnia and Herzegovina	1·16 (0·99–1·35)	0·95 (0·71–1·19)	1·15 (0·98–1·35)	0·96 (0·73–1·20)	0·85 (0·61–1·08)	0·80 (0·55–1·05)	1·36 (1·19–1·55)	1·15 (0·91–1·39)	1·05 (0·80–1·28)	1·01 (0·75–1·25)
		Bulgaria	1·43 (1·29–1·59)	1·26 (1·08–1·45)	1·40 (1·25–1·56)	1·26 (1·08–1·45)	1·25 (1·08–1·42)	1·18 (0·99–1·37)	1·63 (1·49–1·79)	1·46 (1·28–1·65)	1·43 (1·26–1·61)	1·38 (1·20–1·57)
		Croatia	1·27 (1·08–1·46)	1·14 (0·92–1·38)	1·26 (1·08–1·46)	1·14 (0·92–1·38)	1·05 (0·83–1·26)	1·01 (0·78–1·25)	1·47 (1·28–1·66)	1·34 (1·12–1·58)	1·25 (1·03–1·46)	1·21 (0·98–1·45)
		Czechia	1·54 (1·34–1·76)	1·36 (1·13–1·60)	1·53 (1·33–1·75)	1·35 (1·13–1·60)	1·44 (1·23–1·66)	1·31 (1·09–1·55)	1·74 (1·54–1·96)	1·56 (1·33–1·80)	1·62 (1·41–1·84)	1·50 (1·28–1·74)
		Hungary	1·42 (1·22–1·65)	1·29 (1·06–1·55)	1·42 (1·22–1·65)	1·29 (1·06–1·55)	1·34 (1·13–1·57)	1·25 (1·02–1·51)	1·62 (1·42–1·85)	1·49 (1·26–1·75)	1·53 (1·33–1·77)	1·44 (1·22–1·71)
		Montenegro	1·56 (1·43–1·70)	1·40 (1·23–1·58)	1·55 (1·42–1·69)	1·40 (1·23–1·58)	1·18 (0·95–1·39)	1·12 (0·89–1·34)	1·76 (1·63–1·90)	1·60 (1·43–1·78)	1·38 (1·15–1·58)	1·32 (1·09–1·53)
		North Macedonia	1·10 (1·01–1·20)	0·97 (0·84–1·09)	1·08 (0·98–1·18)	0·97 (0·84–1·09)	0·80 (0·66–0·93)	0·76 (0·62–0·90)	1·30 (1·21–1·40)	1·17 (1·04–1·29)	0·99 (0·85–1·11)	0·96 (0·82–1·10)
		Poland	1·21 (1·04–1·40)	1·07 (0·87–1·29)	1·21 (1·04–1·39)	1·07 (0·87–1·29)	1·06 (0·86–1·27)	1·00 (0·79–1·22)	1·41 (1·24–1·60)	1·27 (1·07–1·49)	1·26 (1·06–1·47)	1·20 (0·99–1·42)
		Romania	1·48 (1·32–1·66)	1·26 (1·06–1·48)	1·45 (1·28–1·63)	1·26 (1·06–1·47)	1·34 (1·15–1·52)	1·23 (1·03–1·44)	1·68 (1·52–1·86)	1·46 (1·26–1·68)	1·51 (1·32–1·70)	1·43 (1·23–1·64)
		Serbia	1·01 (0·90–1·11)	0·96 (0·82–1·09)	1·00 (0·90–1·11)	0·96 (0·82–1·09)	0·81 (0·66–0·94)	0·82 (0·66–0·96)	1·21 (1·10–1·31)	1·16 (1·02–1·29)	1·01 (0·85–1·13)	1·02 (0·86–1·16)
		Slovakia	1·46 (1·34–1·59)	1·31 (1·16–1·46)	1·45 (1·32–1·58)	1·30 (1·15–1·46)	1·30 (1·15–1·44)	1·22 (1·06–1·38)	1·66 (1·54–1·79)	1·51 (1·36–1·66)	1·49 (1·34–1·63)	1·41 (1·26–1·57)
		Slovenia	1·51 (1·39–1·64)	1·38 (1·24–1·54)	1·50 (1·38–1·64)	1·38 (1·24–1·54)	1·32 (1·16–1·47)	1·25 (1·08–1·42)	1·71 (1·59–1·84)	1·58 (1·44–1·74)	1·51 (1·35–1·67)	1·45 (1·28–1·62)
	Eastern Europe		1·28 (1·15–1·42)	1·19 (1·05–1·35)	1·24 (1·12–1·39)	1·18 (1·04–1·34)	1·16 (1·02–1·32)	1·13 (0·99–1·30)	1·48 (1·35–1·62)	1·39 (1·24–1·55)	1·34 (1·20–1·49)	1·32 (1·18–1·49)
		Belarus	1·29 (1·06–1·55)	1·19 (0·95–1·47)	1·28 (1·05–1·54)	1·19 (0·94–1·47)	1·14 (0·89–1·41)	1·09 (0·83–1·36)	1·49 (1·26–1·75)	1·39 (1·15–1·67)	1·33 (1·09–1·60)	1·28 (1·03–1·56)
		Estonia	1·37 (1·24–1·50)	1·21 (1·06–1·36)	1·30 (1·16–1·44)	1·19 (1·04–1·35)	1·30 (1·17–1·44)	1·18 (1·04–1·33)	1·57 (1·44–1·70)	1·41 (1·26–1·56)	1·44 (1·31–1·58)	1·37 (1·22–1·52)
		Latvia	1·35 (1·16–1·56)	1·22 (1·01–1·49)	1·29 (1·10–1·52)	1·22 (1·00–1·48)	1·25 (1·04–1·47)	1·18 (0·96–1·44)	1·55 (1·36–1·76)	1·42 (1·21–1·69)	1·41 (1·20–1·63)	1·37 (1·15–1·64)
		Lithuania	1·23 (1·11–1·35)	1·09 (0·96–1·25)	1·21 (1·09–1·33)	1·08 (0·95–1·24)	1·12 (0·99–1·26)	1·05 (0·91–1·20)	1·43 (1·31–1·55)	1·29 (1·16–1·45)	1·30 (1·18–1·44)	1·24 (1·10–1·40)
		Moldova	1·09 (0·94–1·25)	1·03 (0·87–1·24)	1·07 (0·92–1·23)	1·03 (0·87–1·23)	0·95 (0·78–1·13)	0·95 (0·77–1·15)	1·29 (1·14–1·45)	1·23 (1·07–1·44)	1·13 (0·96–1·31)	1·15 (0·97–1·35)
		Russia	1·33 (1·20–1·47)	1·21 (1·06–1·37)	1·29 (1·16–1·44)	1·20 (1·06–1·36)	1·21 (1·07–1·37)	1·15 (1·01–1·31)	1·53 (1·40–1·67)	1·41 (1·26–1·57)	1·38 (1·24–1·54)	1·34 (1·20–1·50)
		Ukraine	1·01 (0·88–1·16)	0·98 (0·83–1·16)	0·99 (0·85–1·14)	0·97 (0·82–1·15)	0·92 (0·77–1·07)	0·93 (0·77–1·11)	1·21 (1·08–1·36)	1·18 (1·03–1·36)	1·10 (0·95–1·25)	1·12 (0·97–1·30)
**High income**			**1·43 (1·30–1·56)**	**1·37 (1·22–1·53)**	**1·42 (1·29–1·55)**	**1·36 (1·22–1·52)**	**1·38 (1·24–1·52)**	**1·34 (1·19–1·50)**	**1·62 (1·50–1·76)**	**1·56 (1·41–1·72)**	**1·57 (1·43–1·71)**	**1·52 (1·38–1·68)**
	Australasia		1·45 (1·25–1·68)	1·33 (1·08–1·59)	1·46 (1·26–1·67)	1·33 (1·11–1·57)	1·40 (1·18–1·64)	1·31 (1·06–1·58)	1·65 (1·45–1·88)	1·53 (1·28–1·79)	1·60 (1·39–1·84)	1·51 (1·28–1·76)
		Australia	1·45 (1·23–1·70)	1·32 (1·06–1·61)	1·46 (1·24–1·69)	1·32 (1·09–1·58)	1·41 (1·16–1·67)	1·31 (1·03–1·59)	1·65 (1·43–1·90)	1·52 (1·26–1·81)	1·61 (1·38–1·86)	1·51 (1·25–1·78)
		New Zealand	1·45 (1·33–1·58)	1·35 (1·20–1·51)	1·45 (1·34–1·57)	1·35 (1·22–1·49)	1·39 (1·25–1·54)	1·31 (1·15–1·48)	1·65 (1·53–1·78)	1·55 (1·40–1·71)	1·59 (1·45–1·73)	1·51 (1·37–1·67)
	High-income Asia Pacific		1·14 (1·00–1·30)	1·14 (0·96–1·35)	1·12 (0·99–1·28)	1·13 (0·97–1·33)	1·07 (0·91–1·24)	1·08 (0·89–1·30)	1·34 (1·20–1·49)	1·33 (1·15–1·54)	1·25 (1·10–1·42)	1·27 (1·10–1·48)
		Brunei	1·40 (1·08–1·78)	1·25 (0·87–1·70)	1·38 (1·08–1·72)	1·26 (0·91–1·65)	1·28 (0·94–1·68)	1·19 (0·81–1·64)	1·60 (1·28–1·98)	1·45 (1·07–1·90)	1·45 (1·13–1·82)	1·39 (1·04–1·80)
		Japan	1·26 (1·09–1·45)	1·21 (1·00–1·43)	1·24 (1·07–1·43)	1·20 (1·02–1·41)	1·16 (0·96–1·38)	1·14 (0·92–1·38)	1·46 (1·29–1·65)	1·41 (1·20–1·63)	1·35 (1·15–1·56)	1·33 (1·13–1·56)
		Singapore	1·15 (0·93–1·41)	1·12 (0·88–1·41)	1·14 (0·94–1·39)	1·12 (0·90–1·38)	1·14 (0·92–1·39)	1·12 (0·89–1·40)	1·35 (1·13–1·61)	1·32 (1·08–1·61)	1·33 (1·13–1·57)	1·32 (1·11–1·58)
		South Korea	0·82 (0·73–0·92)	0·82 (0·71–0·95)	0·82 (0·73–0·91)	0·82 (0·71–0·94)	0·79 (0·67–0·92)	0·82 (0·68–0·95)	1·02 (0·93–1·12)	1·02 (0·91–1·15)	0·99 (0·87–1·11)	1·01 (0·88–1·15)
	High-income North America		1·51 (1·38–1·64)	1·43 (1·27–1·60)	1·50 (1·38–1·63)	1·43 (1·28–1·58)	1·44 (1·31–1·58)	1·38 (1·22–1·54)	1·71 (1·58–1·84)	1·63 (1·47–1·80)	1·64 (1·50–1·77)	1·57 (1·42–1·73)
		Canada	1·39 (1·21–1·58)	1·32 (1·12–1·54)	1·39 (1·22–1·58)	1·32 (1·13–1·53)	1·36 (1·17–1·56)	1·30 (1·10–1·53)	1·59 (1·41–1·78)	1·52 (1·32–1·74)	1·56 (1·37–1·76)	1·50 (1·30–1·72)
		Greenland	1·84 (1·60–2·10)	1·67 (1·36–2·00)	1·74 (1·46–2·02)	1·62 (1·28–1·94)	1·77 (1·52–2·03)	1·62 (1·30–1·94)	1·84 (1·60–2·10)	1·87 (1·56–2·20)	1·88 (1·60–2·16)	1·76 (1·44–2·09)
		USA	1·52 (1·40–1·65)	1·45 (1·30–1·60)	1·51 (1·40–1·64)	1·44 (1·30–1·59)	1·46 (1·33–1·59)	1·39 (1·24–1·54)	1·72 (1·60–1·85)	1·65 (1·50–1·80)	1·65 (1·52–1·77)	1·58 (1·44–1·73)
	Southern Latin America		1·32 (1·10–1·57)	1·23 (0·97–1·53)	1·30 (1·09–1·54)	1·22 (0·99–1·50)	1·27 (1·04–1·53)	1·22 (0·96–1·52)	1·52 (1·30–1·77)	1·43 (1·17–1·73)	1·45 (1·23–1·71)	1·41 (1·16–1·70)
		Argentina	1·33 (1·09–1·60)	1·22 (0·91–1·56)	1·31 (1·07–1·57)	1·21 (0·93–1·51)	1·28 (1·01–1·55)	1·20 (0·89–1·54)	1·53 (1·29–1·80)	1·42 (1·11–1·76)	1·45 (1·20–1·73)	1·39 (1·10–1·71)
		Chile	1·29 (1·09–1·51)	1·24 (0·99–1·51)	1·29 (1·10–1·50)	1·24 (1·01–1·50)	1·26 (1·05–1·50)	1·23 (0·98–1·51)	1·49 (1·29–1·71)	1·44 (1·19–1·71)	1·46 (1·25–1·70)	1·43 (1·19–1·70)
		Uruguay	1·36 (1·14–1·60)	1·25 (0·97–1·56)	1·27 (1·05–1·52)	1·20 (0·95–1·50)	1·29 (1·05–1·56)	1·22 (0·93–1·55)	1·56 (1·34–1·80)	1·45 (1·17–1·76)	1·42 (1·16–1·69)	1·38 (1·10–1·70)
	Western Europe		1·44 (1·32–1·57)	1·37 (1·23–1·52)	1·43 (1·32–1·56)	1·37 (1·24–1·51)	1·42 (1·30–1·55)	1·36 (1·23–1·51)	1·63 (1·52–1·76)	1·55 (1·42–1·70)	1·60 (1·49–1·73)	1·54 (1·42–1·68)
		Andorra	1·02 (0·92–1·11)	1·01 (0·89–1·13)	1·01 (0·92–1·11)	1·01 (0·90–1·12)	1·00 (0·91–1·10)	1·00 (0·89–1·12)	1·22 (1·12–1·31)	1·21 (1·09–1·33)	1·20 (1·11–1·30)	1·20 (1·10–1·32)
		Austria	1·42 (1·29–1·55)	1·34 (1·18–1·51)	1·41 (1·29–1·55)	1·34 (1·18–1·50)	1·40 (1·27–1·54)	1·34 (1·18–1·51)	1·62 (1·49–1·75)	1·54 (1·38–1·71)	1·60 (1·48–1·73)	1·53 (1·39–1·69)
		Belgium	1·43 (1·24–1·63)	1·34 (1·13–1·57)	1·43 (1·25–1·63)	1·34 (1·15–1·56)	1·41 (1·23–1·61)	1·34 (1·13–1·57)	1·63 (1·44–1·83)	1·54 (1·33–1·77)	1·61 (1·43–1·81)	1·54 (1·35–1·76)
		Cyprus	1·18 (0·97–1·43)	1·13 (0·89–1·40)	1·18 (0·97–1·41)	1·12 (0·90–1·38)	1·17 (0·96–1·41)	1·12 (0·89–1·38)	1·38 (1·17–1·63)	1·33 (1·09–1·60)	1·36 (1·16–1·60)	1·31 (1·09–1·57)
		Denmark	1·57 (1·46–1·69)	1·47 (1·34–1·60)	1·56 (1·45–1·68)	1·47 (1·34–1·59)	1·55 (1·44–1·67)	1·46 (1·33–1·59)	1·77 (1·66–1·89)	1·67 (1·54–1·80)	1·75 (1·63–1·86)	1·66 (1·54–1·79)
		Finland	1·36 (1·24–1·49)	1·32 (1·18–1·48)	1·36 (1·25–1·49)	1·32 (1·19–1·47)	1·35 (1·23–1·48)	1·31 (1·18–1·46)	1·56 (1·44–1·69)	1·52 (1·38–1·68)	1·55 (1·43–1·67)	1·51 (1·38–1·66)
		France	1·56 (1·35–1·79)	1·43 (1·19–1·69)	1·56 (1·36–1·78)	1·42 (1·20–1·67)	1·54 (1·33–1·77)	1·43 (1·19–1·69)	1·76 (1·55–1·99)	1·63 (1·39–1·89)	1·74 (1·53–1·97)	1·63 (1·40–1·87)
		Germany	1·47 (1·35–1·58)	1·40 (1·27–1·53)	1·46 (1·35–1·58)	1·41 (1·28–1·53)	1·45 (1·33–1·57)	1·39 (1·26–1·52)	1·67 (1·55–1·78)	1·60 (1·47–1·73)	1·64 (1·52–1·77)	1·59 (1·47–1·72)
		Greece	1·36 (1·17–1·57)	1·28 (1·06–1·54)	1·35 (1·17–1·56)	1·28 (1·07–1·52)	1·34 (1·15–1·55)	1·28 (1·06–1·52)	1·56 (1·37–1·77)	1·48 (1·26–1·74)	1·53 (1·35–1·74)	1·48 (1·27–1·71)
		Iceland	1·73 (1·54–1·93)	1·58 (1·36–1·82)	1·73 (1·54–1·92)	1·58 (1·38–1·80)	1·71 (1·52–1·92)	1·59 (1·37–1·82)	1·89 (1·70–2·09)	1·78 (1·56–2·02)	1·91 (1·72–2·11)	1·78 (1·58–2·00)
		Ireland	1·54 (1·40–1·70)	1·40 (1·22–1·58)	1·54 (1·40–1·69)	1·40 (1·24–1·57)	1·53 (1·38–1·68)	1·40 (1·23–1·57)	1·74 (1·60–1·90)	1·60 (1·42–1·78)	1·72 (1·58–1·87)	1·59 (1·44–1·76)
		Israel	2·38 (2·20–2·59)	2·09 (1·86–2·34)	2·33 (2·15–2·54)	2·08 (1·87–2·31)	2·35 (2·16–2·56)	2·09 (1·87–2·34)	2·38 (2·20–2·59)	2·09 (1·86–2·34)	2·31 (2·12–2·52)	2·08 (1·88–2·31)
		Italy	1·18 (1·00–1·37)	1·09 (0·88–1·32)	1·18 (1·00–1·37)	1·09 (0·90–1·32)	1·16 (0·99–1·35)	1·09 (0·89–1·32)	1·38 (1·20–1·57)	1·29 (1·08–1·52)	1·36 (1·19–1·55)	1·29 (1·10–1·51)
		Luxembourg	1·30 (1·17–1·44)	1·24 (1·09–1·40)	1·30 (1·17–1·43)	1·24 (1·10–1·39)	1·28 (1·16–1·42)	1·24 (1·09–1·39)	1·50 (1·37–1·64)	1·44 (1·29–1·60)	1·48 (1·36–1·61)	1·44 (1·30–1·59)
		Malta	1·39 (1·18–1·64)	1·26 (1·01–1·55)	1·39 (1·19–1·64)	1·26 (1·03–1·54)	1·38 (1·16–1·62)	1·26 (1·02–1·55)	1·59 (1·38–1·84)	1·46 (1·21–1·75)	1·58 (1·37–1·82)	1·46 (1·23–1·74)
		Monaco	1·44 (1·16–1·76)	1·37 (1·06–1·73)	1·42 (1·14–1·75)	1·36 (1·06–1·72)	1·43 (1·15–1·75)	1·37 (1·06–1·72)	1·64 (1·36–1·96)	1·57 (1·26–1·93)	1·61 (1·33–1·94)	1·56 (1·27–1·92)
		Netherlands	1·54 (1·41–1·67)	1·42 (1·27–1·57)	1·54 (1·41–1·67)	1·42 (1·28–1·57)	1·50 (1·37–1·64)	1·40 (1·25–1·56)	1·74 (1·61–1·87)	1·62 (1·47–1·77)	1·70 (1·57–1·83)	1·60 (1·46–1·75)
		Norway	1·43 (1·32–1·54)	1·36 (1·24–1·49)	1·43 (1·33–1·54)	1·36 (1·25–1·48)	1·42 (1·31–1·53)	1·36 (1·24–1·48)	1·63 (1·52–1·74)	1·56 (1·44–1·69)	1·62 (1·52–1·73)	1·56 (1·44–1·68)
		Portugal	1·27 (1·13–1·42)	1·17 (1·00–1·37)	1·27 (1·14–1·42)	1·17 (1·00–1·36)	1·23 (1·08–1·39)	1·17 (0·99–1·36)	1·47 (1·33–1·62)	1·37 (1·20–1·57)	1·43 (1·28–1·59)	1·37 (1·19–1·56)
		San Marino	1·27 (1·09–1·49)	1·20 (0·99–1·46)	1·26 (1·08–1·48)	1·20 (1·00–1·44)	1·25 (1·07–1·47)	1·20 (0·99–1·45)	1·47 (1·29–1·69)	1·40 (1·19–1·66)	1·44 (1·26–1·66)	1·40 (1·20–1·64)
		Spain	1·23 (1·10–1·38)	1·11 (0·93–1·30)	1·21 (1·07–1·35)	1·10 (0·93–1·29)	1·21 (1·07–1·35)	1·11 (0·93–1·30)	1·43 (1·30–1·58)	1·31 (1·13–1·50)	1·38 (1·24–1·53)	1·30 (1·13–1·49)
		Sweden	1·51 (1·39–1·64)	1·38 (1·24–1·53)	1·48 (1·35–1·61)	1·37 (1·23–1·51)	1·47 (1·34–1·61)	1·36 (1·22–1·51)	1·71 (1·59–1·84)	1·58 (1·44–1·73)	1·64 (1·51–1·78)	1·54 (1·40–1·69)
		Switzerland	1·40 (1·28–1·52)	1·33 (1·20–1·47)	1·39 (1·28–1·51)	1·33 (1·21–1·47)	1·38 (1·27–1·50)	1·33 (1·20–1·46)	1·60 (1·48–1·72)	1·53 (1·40–1·67)	1·58 (1·46–1·70)	1·53 (1·41–1·66)
		UK	1·38 (1·18–1·58)	1·30 (1·08–1·53)	1·37 (1·18–1·56)	1·30 (1·09–1·52)	1·36 (1·17–1·56)	1·29 (1·07–1·51)	1·58 (1·38–1·78)	1·50 (1·28–1·73)	1·55 (1·36–1·75)	1·49 (1·29–1·70)
**Latin America and Caribbean**			**1·57 (1·38–1·79)**	**1·31 (1·08–1·57)**	**1·50 (1·30–1·73)**	**1·31 (1·09–1·56)**	**1·49 (1·30–1·71)**	**1·31 (1·08–1·55)**	**1·72 (1·54–1·95)**	**1·50 (1·28–1·76)**	**1·64 (1·45–1·86)**	**1·50 (1·29–1·74)**
	Andean Latin America		1·80 (1·58–2·05)	1·45 (1·19–1·73)	1·75 (1·52–2·00)	1·46 (1·20–1·73)	1·66 (1·44–1·91)	1·44 (1·18–1·71)	1·82 (1·59–2·06)	1·65 (1·39–1·93)	1·82 (1·60–2·08)	1·64 (1·39–1·91)
		Bolivia	1·84 (1·55–2·17)	1·40 (1·07–1·77)	1·80 (1·50–2·12)	1·44 (1·12–1·80)	1·67 (1·38–1·99)	1·40 (1·08–1·76)	1·84 (1·55–2·17)	1·60 (1·27–1·97)	1·84 (1·55–2·16)	1·63 (1·32–1·98)
		Ecuador	1·74 (1·42–2·10)	1·45 (1·10–1·86)	1·73 (1·41–2·10)	1·45 (1·10–1·86)	1·65 (1·34–2·01)	1·44 (1·10–1·83)	1·78 (1·46–2·14)	1·65 (1·30–2·06)	1·85 (1·53–2·21)	1·64 (1·30–2·03)
		Peru	1·83 (1·56–2·13)	1·44 (1·14–1·80)	1·74 (1·47–2·06)	1·43 (1·13–1·79)	1·67 (1·41–1·97)	1·42 (1·13–1·77)	1·83 (1·56–2·13)	1·64 (1·34–2·00)	1·80 (1·54–2·12)	1·62 (1·33–1·96)
	Caribbean		1·77 (1·50–2·08)	1·43 (1·11–1·88)	1·67 (1·39–2·00)	1·45 (1·15–1·88)	1·57 (1·29–1·89)	1·37 (1·09–1·79)	1·82 (1·56–2·12)	1·61 (1·31–2·05)	1·71 (1·43–2·02)	1·58 (1·31–1·98)
		Antigua and Barbuda	1·30 (1·11–1·52)	1·15 (0·93–1·41)	1·26 (1·06–1·48)	1·15 (0·93–1·41)	1·25 (1·06–1·46)	1·15 (0·93–1·40)	1·50 (1·31–1·72)	1·35 (1·13–1·61)	1·41 (1·22–1·64)	1·35 (1·13–1·60)
		The Bahamas	1·24 (1·02–1·49)	1·24 (0·99–1·52)	1·21 (0·99–1·47)	1·24 (0·99–1·52)	1·19 (0·98–1·44)	1·21 (0·97–1·50)	1·44 (1·22–1·69)	1·44 (1·19–1·72)	1·37 (1·16–1·62)	1·41 (1·17–1·69)
		Barbados	1·18 (0·95–1·47)	1·10 (0·85–1·42)	1·17 (0·94–1·46)	1·10 (0·85–1·42)	1·10 (0·85–1·39)	1·06 (0·81–1·38)	1·38 (1·15–1·67)	1·30 (1·05–1·62)	1·29 (1·04–1·58)	1·26 (1·01–1·58)
		Belize	1·58 (1·28–1·90)	1·28 (0·93–1·67)	1·46 (1·14–1·81)	1·25 (0·90–1·64)	1·38 (1·08–1·71)	1·18 (0·85–1·56)	1·78 (1·48–2·10)	1·48 (1·13–1·87)	1·49 (1·18–1·83)	1·36 (1·02–1·73)
		Bermuda	1·19 (1·04–1·36)	1·07 (0·88–1·28)	1·13 (0·97–1·32)	1·06 (0·88–1·28)	1·15 (0·99–1·31)	1·05 (0·87–1·26)	1·39 (1·24–1·56)	1·27 (1·08–1·48)	1·29 (1·13–1·47)	1·25 (1·07–1·46)
		Cuba	1·31 (1·18–1·44)	1·23 (1·07–1·39)	1·29 (1·16–1·43)	1·23 (1·07–1·38)	1·27 (1·15–1·40)	1·22 (1·06–1·37)	1·51 (1·38–1·64)	1·43 (1·27–1·59)	1·46 (1·33–1·59)	1·42 (1·27–1·57)
		Dominica	1·18 (0·96–1·45)	1·13 (0·89–1·42)	1·15 (0·93–1·42)	1·13 (0·89–1·42)	1·13 (0·91–1·39)	1·11 (0·87–1·39)	1·38 (1·16–1·65)	1·33 (1·09–1·62)	1·30 (1·09–1·57)	1·31 (1·08–1·59)
		Dominican Republic	1·84 (1·55–2·15)	1·51 (1·19–1·86)	1·80 (1·52–2·11)	1·53 (1·22–1·87)	1·72 (1·45–2·03)	1·47 (1·16–1·81)	1·84 (1·55–2·15)	1·71 (1·39–2·06)	1·90 (1·63–2·20)	1·69 (1·39–2·02)
		Grenada	1·41 (1·09–1·79)	1·19 (0·81–1·62)	1·37 (1·05–1·76)	1·19 (0·82–1·63)	1·32 (0·99–1·70)	1·17 (0·81–1·60)	1·61 (1·29–1·99)	1·39 (1·01–1·82)	1·49 (1·16–1·87)	1·38 (1·02–1·81)
		Guyana	1·91 (1·54–2·32)	1·58 (1·15–2·04)	1·87 (1·48–2·28)	1·58 (1·16–2·04)	1·63 (1·25–2·05)	1·43 (1·02–1·88)	1·91 (1·54–2·32)	1·78 (1·35–2·24)	1·81 (1·42–2·23)	1·63 (1·22–2·07)
		Haiti	2·10 (1·68–2·61)	1·44 (0·92–2·05)	1·89 (1·43–2·44)	1·46 (0·95–2·06)	1·73 (1·27–2·28)	1·35 (0·84–1·94)	2·10 (1·68–2·61)	1·64 (1·12–2·25)	1·82 (1·34–2·37)	1·58 (1·07–2·17)
		Jamaica	1·16 (0·93–1·39)	1·04 (0·79–1·31)	1·15 (0·93–1·39)	1·05 (0·80–1·32)	1·12 (0·90–1·35)	1·05 (0·80–1·31)	1·36 (1·13–1·59)	1·24 (0·99–1·51)	1·32 (1·10–1·55)	1·25 (1·00–1·51)
		Puerto Rico	0·84 (0·76–0·92)	0·81 (0·72–0·93)	0·83 (0·75–0·92)	0·81 (0·72–0·93)	0·81 (0·73–0·89)	0·81 (0·71–0·93)	1·04 (0·96–1·12)	1·01 (0·92–1·13)	1·00 (0·92–1·09)	1·01 (0·91–1·13)
		Saint Kitts and Nevis	1·08 (0·92–1·27)	1·00 (0·81–1·20)	1·08 (0·91–1·26)	1·01 (0·83–1·21)	1·05 (0·89–1·23)	1·00 (0·82–1·19)	1·28 (1·12–1·47)	1·20 (1·01–1·40)	1·24 (1·08–1·42)	1·20 (1·03–1·40)
		Saint Lucia	1·04 (0·79–1·32)	0·87 (0·58–1·19)	1·03 (0·78–1·31)	0·87 (0·59–1·19)	0·96 (0·70–1·24)	0·86 (0·57–1·17)	1·24 (0·99–1·52)	1·07 (0·78–1·39)	1·15 (0·90–1·43)	1·06 (0·79–1·38)
		Saint Vincent and the Grenadines	1·35 (1·10–1·64)	1·16 (0·87–1·51)	1·30 (1·04–1·60)	1·16 (0·88–1·50)	1·30 (1·06–1·59)	1·17 (0·88–1·51)	1·55 (1·30–1·84)	1·36 (1·07–1·71)	1·46 (1·21–1·76)	1·37 (1·09–1·70)
		Suriname	1·73 (1·41–2·05)	1·39 (1·02–1·78)	1·64 (1·31–1·98)	1·39 (1·02–1·77)	1·48 (1·18–1·81)	1·26 (0·90–1·63)	1·77 (1·45–2·09)	1·59 (1·22–1·98)	1·62 (1·31–1·97)	1·46 (1·11–1·83)
		Trinidad and Tobago	1·35 (1·13–1·60)	1·19 (0·94–1·49)	1·33 (1·10–1·58)	1·19 (0·94–1·49)	1·16 (0·92–1·43)	1·10 (0·84–1·39)	1·55 (1·33–1·80)	1·39 (1·14–1·69)	1·35 (1·11–1·61)	1·30 (1·04–1·59)
		Virgin Islands	1·49 (1·28–1·77)	1·37 (1·13–1·69)	1·44 (1·21–1·72)	1·36 (1·12–1·67)	1·44 (1·22–1·72)	1·35 (1·10–1·66)	1·69 (1·48–1·97)	1·57 (1·33–1·89)	1·59 (1·36–1·88)	1·54 (1·29–1·85)
	Central Latin America		1·47 (1·23–1·74)	1·21 (0·93–1·53)	1·38 (1·13–1·66)	1·20 (0·93–1·52)	1·40 (1·16–1·67)	1·21 (0·94–1·52)	1·64 (1·40–1·91)	1·40 (1·13–1·72)	1·53 (1·28–1·81)	1·40 (1·14–1·71)
		Colombia	1·35 (1·02–1·71)	1·14 (0·77–1·54)	1·32 (0·98–1·68)	1·14 (0·78–1·54)	1·32 (1·00–1·67)	1·16 (0·80–1·56)	1·55 (1·22–1·91)	1·34 (0·97–1·74)	1·49 (1·17–1·85)	1·37 (1·01–1·76)
		Costa Rica	1·18 (1·02–1·36)	1·03 (0·84–1·26)	1·15 (0·98–1·34)	1·02 (0·83–1·25)	1·11 (0·96–1·29)	1·01 (0·83–1·23)	1·38 (1·22–1·56)	1·23 (1·04–1·46)	1·29 (1·13–1·47)	1·20 (1·02–1·42)
		El Salvador	1·58 (1·23–1·93)	1·28 (0·86–1·72)	1·47 (1·10–1·85)	1·29 (0·88–1·72)	1·52 (1·18–1·88)	1·29 (0·89–1·72)	1·78 (1·43–2·13)	1·48 (1·06–1·92)	1·63 (1·28–2·01)	1·50 (1·10–1·92)
		Guatemala	1·62 (1·26–1·98)	1·16 (0·73–1·61)	1·40 (1·02–1·80)	1·15 (0·74–1·58)	1·49 (1·13–1·87)	1·17 (0·76–1·59)	1·82 (1·46–2·18)	1·36 (0·93–1·81)	1·53 (1·16–1·93)	1·37 (0·98–1·79)
		Honduras	1·71 (1·35–2·11)	1·27 (0·82–1·76)	1·48 (1·07–1·94)	1·25 (0·80–1·73)	1·63 (1·27–2·04)	1·29 (0·87–1·76)	1·79 (1·43–2·19)	1·47 (1·02–1·96)	1·65 (1·25–2·09)	1·48 (1·06–1·95)
		Mexico	1·39 (1·19–1·62)	1·15 (0·91–1·41)	1·31 (1·09–1·54)	1·14 (0·90–1·39)	1·33 (1·13–1·55)	1·15 (0·92–1·41)	1·59 (1·39–1·82)	1·35 (1·11–1·61)	1·46 (1·25–1·69)	1·35 (1·12–1·59)
		Nicaragua	1·65 (1·30–2·01)	1·29 (0·86–1·73)	1·51 (1·13–1·91)	1·29 (0·87–1·73)	1·64 (1·29–1·99)	1·33 (0·92–1·76)	1·85 (1·50–2·21)	1·49 (1·06–1·93)	1·72 (1·35–2·11)	1·54 (1·13–1·96)
		Panama	1·76 (1·49–2·05)	1·49 (1·20–1·81)	1·72 (1·45–2·02)	1·49 (1·21–1·81)	1·64 (1·38–1·93)	1·45 (1·17–1·76)	1·76 (1·49–2·05)	1·69 (1·40–2·01)	1·82 (1·55–2·11)	1·65 (1·37–1·97)
		Venezuela	1·79 (1·43–2·19)	1·51 (1·10–1·97)	1·72 (1·35–2·14)	1·52 (1·12–1·97)	1·65 (1·29–2·05)	1·45 (1·04–1·89)	1·79 (1·43–2·19)	1·71 (1·30–2·17)	1·80 (1·44–2·21)	1·66 (1·27–2·10)
	Tropical Latin America		1·57 (1·36–1·81)	1·32 (1·08–1·59)	1·52 (1·30–1·76)	1·32 (1·08–1·58)	1·52 (1·31–1·76)	1·32 (1·08–1·59)	1·77 (1·56–2·01)	1·52 (1·28–1·79)	1·68 (1·47–1·92)	1·52 (1·29–1·79)
		Brazil	1·57 (1·35–1·81)	1·31 (1·06–1·59)	1·52 (1·29–1·77)	1·31 (1·06–1·59)	1·52 (1·31–1·76)	1·32 (1·07–1·60)	1·77 (1·55–2·01)	1·51 (1·26–1·79)	1·68 (1·46–1·93)	1·52 (1·27–1·79)
		Paraguay	1·66 (1·25–2·11)	1·39 (0·93–1·89)	1·61 (1·19–2·06)	1·39 (0·94–1·88)	1·62 (1·21–2·06)	1·41 (0·96–1·90)	1·86 (1·45–2·31)	1·59 (1·13–2·09)	1·77 (1·36–2·22)	1·61 (1·17–2·09)
**North Africa and Middle East**			**1·94 (1·62–2·28)**	**1·64 (1·28–2·06)**	**1·80 (1·48–2·14)**	**1·67 (1·35–2·06)**	**1·72 (1·40–2·06)**	**1·63 (1·30–2·04)**	**2·00 (1·68–2·34)**	**1·75 (1·40–2·15)**	**1·76 (1·44–2·10)**	**1·73 (1·42–2·12)**
		Afghanistan	3·34 (2·78–3·89)	1·61 (0·90–2·32)	2·30 (1·67–2·92)	1·62 (0·98–2·28)	2·63 (2·00–3·24)	1·52 (0·87–2·18)	3·34 (2·78–3·89)	1·81 (1·10–2·52)	1·95 (1·31–2·57)	1·75 (1·16–2·37)
		Algeria	1·79 (1·37–2·18)	1·48 (1·04–1·89)	1·74 (1·32–2·13)	1·49 (1·06–1·89)	1·59 (1·19–1·96)	1·37 (0·96–1·77)	1·79 (1·37–2·18)	1·68 (1·24–2·09)	1·75 (1·36–2·12)	1·58 (1·17–1·97)
		Bahrain	1·39 (1·10–1·68)	1·26 (0·92–1·57)	1·37 (1·07–1·65)	1·27 (0·94–1·57)	1·22 (0·91–1·51)	1·17 (0·84–1·49)	1·59 (1·30–1·88)	1·46 (1·12–1·77)	1·41 (1·10–1·69)	1·38 (1·06–1·69)
		Egypt	2·38 (1·97–2·82)	2·05 (1·62–2·50)	2·38 (1·97–2·80)	2·06 (1·65–2·50)	2·35 (1·94–2·78)	2·07 (1·64–2·51)	2·38 (1·97–2·82)	2·05 (1·62–2·50)	2·34 (1·95–2·77)	2·07 (1·66–2·51)
		Iran	1·31 (1·03–1·58)	1·28 (0·97–1·58)	1·30 (1·02–1·58)	1·28 (0·98–1·58)	1·18 (0·91–1·44)	1·20 (0·90–1·49)	1·51 (1·23–1·78)	1·48 (1·17–1·78)	1·38 (1·11–1·63)	1·40 (1·11–1·68)
		Iraq	1·95 (1·63–2·35)	1·59 (1·25–2·01)	1·92 (1·61–2·31)	1·60 (1·28–2·01)	1·69 (1·36–2·09)	1·50 (1·15–1·90)	1·95 (1·63–2·35)	1·79 (1·45–2·21)	1·88 (1·56–2·26)	1·71 (1·38–2·10)
		Jordan	1·78 (1·44–2·10)	1·57 (1·20–1·93)	1·75 (1·41–2·08)	1·57 (1·21–1·93)	1·50 (1·09–1·88)	1·40 (0·99–1·80)	1·78 (1·44–2·10)	1·77 (1·40–2·13)	1·68 (1·27–2·06)	1·60 (1·20–1·99)
		Kuwait	1·07 (0·89–1·30)	1·14 (0·93–1·39)	1·05 (0·87–1·28)	1·14 (0·93–1·39)	1·04 (0·86–1·26)	1·13 (0·92–1·37)	1·27 (1·09–1·50)	1·34 (1·13–1·59)	1·22 (1·04–1·44)	1·33 (1·13–1·57)
		Lebanon	1·44 (1·12–1·80)	1·33 (0·98–1·73)	1·40 (1·09–1·77)	1·33 (0·99–1·73)	1·28 (0·93–1·66)	1·24 (0·88–1·65)	1·64 (1·32–2·00)	1·53 (1·18–1·93)	1·45 (1·11–1·84)	1·45 (1·09–1·85)
		Libya	1·13 (0·87–1·43)	1·03 (0·75–1·37)	1·09 (0·84–1·40)	1·03 (0·75–1·36)	0·91 (0·62–1·23)	0·90 (0·60–1·24)	1·33 (1·07–1·63)	1·23 (0·95–1·57)	1·09 (0·80–1·41)	1·10 (0·80–1·44)
		Morocco	1·36 (1·05–1·70)	1·02 (0·67–1·40)	1·22 (0·88–1·57)	1·04 (0·69–1·42)	1·28 (0·97–1·61)	1·03 (0·69–1·39)	1·56 (1·25–1·90)	1·22 (0·87–1·60)	1·37 (1·04–1·72)	1·26 (0·92–1·62)
		Oman	1·64 (1·27–2·02)	1·29 (0·88–1·72)	1·59 (1·23–1·97)	1·31 (0·92–1·72)	1·30 (0·87–1·73)	1·18 (0·76–1·62)	1·84 (1·47–2·22)	1·49 (1·08–1·92)	1·47 (1·05–1·90)	1·40 (0·98–1·83)
		Palestine	2·08 (1·79–2·44)	1·77 (1·43–2·15)	2·05 (1·75–2·40)	1·77 (1·45–2·15)	1·82 (1·47–2·22)	1·65 (1·30–2·05)	2·08 (1·79–2·44)	1·77 (1·43–2·15)	1·80 (1·46–2·19)	1·85 (1·51–2·25)
		Qatar	1·43 (1·17–1·69)	1·29 (1·01–1·57)	1·40 (1·15–1·66)	1·28 (1·01–1·57)	1·25 (0·97–1·52)	1·20 (0·91–1·49)	1·63 (1·37–1·89)	1·49 (1·21–1·77)	1·43 (1·15–1·70)	1·40 (1·11–1·68)
		Saudi Arabia	1·09 (0·80–1·39)	0·97 (0·65–1·31)	1·06 (0·77–1·37)	0·98 (0·66–1·31)	0·92 (0·62–1·23)	0·89 (0·57–1·22)	1·29 (1·00–1·59)	1·17 (0·85–1·51)	1·11 (0·80–1·41)	1·10 (0·78–1·42)
		Sudan	1·93 (1·48–2·44)	1·40 (0·93–1·95)	1·83 (1·37–2·34)	1·42 (0·96–1·96)	1·54 (1·06–2·07)	1·30 (0·83–1·85)	1·93 (1·48–2·44)	1·60 (1·13–2·15)	1·69 (1·22–2·22)	1·53 (1·07–2·07)
		Syria	1·57 (1·21–1·98)	1·39 (1·01–1·84)	1·55 (1·20–1·96)	1·40 (1·03–1·84)	1·40 (1·03–1·83)	1·33 (0·95–1·77)	1·77 (1·41–2·18)	1·59 (1·21–2·04)	1·59 (1·22–2·01)	1·53 (1·16–1·97)
		Tunisia	1·36 (1·02–1·73)	1·19 (0·82–1·59)	1·33 (0·99–1·71)	1·19 (0·83–1·59)	1·22 (0·88–1·59)	1·14 (0·78–1·53)	1·56 (1·22–1·93)	1·39 (1·02–1·79)	1·40 (1·06–1·76)	1·34 (0·98–1·73)
		Türkiye	1·32 (1·13–1·56)	1·17 (0·95–1·42)	1·29 (1·09–1·52)	1·17 (0·96–1·41)	1·15 (0·95–1·39)	1·10 (0·88–1·34)	1·52 (1·33–1·76)	1·37 (1·15–1·62)	1·33 (1·12–1·57)	1·30 (1·09–1·54)
		United Arab Emirates	1·53 (1·26–1·81)	1·31 (1·00–1·64)	1·52 (1·26–1·79)	1·31 (1·00–1·63)	1·44 (1·13–1·73)	1·30 (0·97–1·62)	1·73 (1·46–2·01)	1·51 (1·20–1·84)	1·63 (1·33–1·92)	1·50 (1·18–1·82)
		Yemen	1·91 (1·32–2·54)	1·22 (0·54–1·95)	1·66 (1·04–2·32)	1·29 (0·64–2·00)	1·63 (1·03–2·26)	1·23 (0·59–1·93)	1·91 (1·32–2·54)	1·42 (0·74–2·15)	1·67 (1·07–2·33)	1·49 (0·88–2·18)
**South Asia**			**1·36 (1·09–1·64)**	**1·10 (0·80–1·43)**	**1·27 (1·00–1·56)**	**1·10 (0·80–1·43)**	**1·24 (0·97–1·53)**	**1·09 (0·78–1·41)**	**1·53 (1·26–1·81)**	**1·28 (0·97–1·61)**	**1·38 (1·10–1·67)**	**1·29 (0·98–1·61)**
		Bangladesh	1·20 (0·84–1·54)	0·97 (0·57–1·37)	1·17 (0·80–1·52)	1·03 (0·64–1·41)	1·18 (0·83–1·52)	1·02 (0·63–1·41)	1·40 (1·04–1·74)	1·17 (0·77–1·57)	1·36 (1·01–1·71)	1·26 (0·88–1·64)
		Bhutan	1·07 (0·73–1·34)	0·69 (0·33–1·00)	0·93 (0·62–1·20)	0·80 (0·48–1·09)	1·05 (0·76–1·30)	0·75 (0·44–1·03)	1·27 (0·93–1·54)	0·89 (0·53–1·20)	1·15 (0·86–1·40)	1·06 (0·76–1·33)
		India	1·29 (0·97–1·62)	1·04 (0·67–1·42)	1·22 (0·89–1·57)	1·04 (0·67–1·41)	1·20 (0·88–1·53)	1·04 (0·67–1·41)	1·49 (1·17–1·82)	1·24 (0·87–1·62)	1·35 (1·01–1·69)	1·24 (0·87–1·62)
		Nepal	1·18 (0·80–1·53)	0·82 (0·40–1·22)	1·13 (0·77–1·49)	0·94 (0·55–1·32)	1·04 (0·66–1·39)	0·82 (0·43–1·20)	1·38 (1·00–1·73)	1·02 (0·60–1·42)	1·22 (0·86–1·57)	1·11 (0·75–1·48)
		Pakistan	1·76 (1·25–2·28)	1·16 (0·59–1·77)	1·56 (1·04–2·11)	1·18 (0·64–1·77)	1·47 (0·93–2·04)	1·12 (0·58–1·72)	1·76 (1·25–2·28)	1·36 (0·79–1·97)	1·54 (1·01–2·11)	1·34 (0·81–1·92)
**Southeast Asia, east Asia, and Oceania**			**1·37 (1·22–1·54)**	**1·30 (1·11–1·53)**	**1·32 (1·16–1·49)**	**1·29 (1·11–1·51)**	**1·31 (1·15–1·47)**	**1·27 (1·09–1·50)**	**1·53 (1·38–1·70)**	**1·49 (1·30–1·71)**	**1·45 (1·30–1·62)**	**1·46 (1·28–1·66)**
	East Asia		1·14 (0·99–1·30)	1·16 (0·99–1·34)	1·11 (0·96–1·27)	1·16 (0·99–1·34)	1·13 (0·99–1·29)	1·16 (1·00–1·34)	1·34 (1·19–1·50)	1·36 (1·19–1·54)	1·31 (1·16–1·47)	1·36 (1·20–1·54)
		China	1·14 (0·99–1·31)	1·16 (0·99–1·35)	1·12 (0·97–1·28)	1·16 (1·00–1·35)	1·14 (0·99–1·30)	1·16 (1·00–1·35)	1·34 (1·19–1·51)	1·36 (1·19–1·55)	1·31 (1·17–1·48)	1·37 (1·20–1·55)
		North Korea	1·24 (1·00–1·48)	1·16 (0·90–1·42)	1·19 (0·95–1·43)	1·15 (0·90–1·41)	1·18 (0·94–1·41)	1·13 (0·88–1·39)	1·44 (1·20–1·68)	1·36 (1·10–1·62)	1·34 (1·10–1·58)	1·33 (1·08–1·59)
		Taiwan (province of China)	0·90 (0·78–1·04)	0·90 (0·77–1·05)	0·89 (0·77–1·03)	0·90 (0·77–1·05)	0·89 (0·77–1·03)	0·89 (0·76–1·05)	1·10 (0·98–1·24)	1·10 (0·97–1·25)	1·08 (0·96–1·22)	1·10 (0·97–1·25)
	Oceania		2·93 (2·45–3·46)	1·67 (1·01–2·35)	2·09 (1·51–2·70)	1·58 (0·98–2·22)	2·38 (1·83–2·96)	1·55 (0·93–2·20)	2·93 (2·45–3·46)	1·86 (1·20–2·54)	1·81 (1·22–2·43)	1·68 (1·10–2·32)
		American Samoa	1·98 (1·67–2·31)	1·68 (1·34–2·05)	1·84 (1·50–2·19)	1·68 (1·34–2·04)	1·84 (1·51–2·18)	1·61 (1·27–1·97)	1·98 (1·67–2·31)	1·88 (1·54–2·25)	1·89 (1·56–2·24)	1·81 (1·48–2·17)
		Cook Islands	1·40 (1·14–1·72)	1·25 (0·96–1·58)	1·36 (1·10–1·68)	1·24 (0·96–1·57)	1·37 (1·11–1·67)	1·25 (0·97–1·58)	1·60 (1·34–1·92)	1·45 (1·16–1·78)	1·53 (1·27–1·84)	1·45 (1·17–1·77)
		Federated States of Micronesia	1·82 (1·39–2·33)	1·46 (0·96–2·05)	1·74 (1·30–2·27)	1·46 (0·97–2·04)	1·56 (1·11–2·09)	1·35 (0·87–1·93)	1·82 (1·39–2·33)	1·66 (1·16–2·25)	1·71 (1·26–2·24)	1·56 (1·07–2·13)
		Fiji	1·95 (1·61–2·31)	1·64 (1·28–2·04)	1·92 (1·58–2·30)	1·65 (1·30–2·04)	1·84 (1·52–2·20)	1·62 (1·27–2·01)	1·95 (1·61–2·31)	1·84 (1·48–2·24)	1·82 (1·50–2·19)	1·83 (1·48–2·21)
		Guam	2·07 (1·76–2·38)	1·76 (1·42–2·09)	2·01 (1·70–2·33)	1·73 (1·40–2·07)	1·98 (1·66–2·30)	1·71 (1·38–2·05)	2·07 (1·76–2·38)	1·76 (1·42–2·09)	1·93 (1·62–2·25)	1·89 (1·56–2·22)
		Kiribati	2·13 (1·70–2·62)	1·67 (1·19–2·21)	2·12 (1·69–2·60)	1·68 (1·22–2·21)	1·78 (1·34–2·27)	1·52 (1·06–2·05)	2·13 (1·70–2·62)	1·87 (1·39–2·41)	1·78 (1·34–2·26)	1·74 (1·28–2·26)
		Marshall Islands	1·98 (1·67–2·32)	1·65 (1·31–2·02)	1·86 (1·53–2·20)	1·64 (1·31–2·00)	1·94 (1·64–2·27)	1·66 (1·33–2·03)	1·98 (1·67–2·32)	1·85 (1·51–2·22)	1·84 (1·52–2·18)	1·86 (1·53–2·22)
		Nauru	2·40 (1·93–2·95)	1·91 (1·39–2·51)	2·27 (1·79–2·84)	1·90 (1·40–2·49)	2·04 (1·53–2·62)	1·76 (1·25–2·36)	2·40 (1·93–2·95)	1·91 (1·39–2·51)	1·95 (1·45–2·54)	1·76 (1·26–2·35)
		Niue	1·71 (1·37–2·08)	1·50 (1·13–1·90)	1·66 (1·32–2·04)	1·50 (1·13–1·90)	1·61 (1·28–1·97)	1·47 (1·10–1·86)	1·87 (1·53–2·24)	1·70 (1·33–2·10)	1·78 (1·44–2·14)	1·67 (1·31–2·06)
		Northern Mariana Islands	1·67 (1·36–2·01)	1·50 (1·17–1·88)	1·58 (1·27–1·94)	1·49 (1·17–1·86)	1·57 (1·26–1·91)	1·44 (1·11–1·81)	1·87 (1·56–2·21)	1·70 (1·37–2·08)	1·70 (1·38–2·06)	1·64 (1·31–2·00)
		Palau	1·65 (1·37–1·92)	1·44 (1·14–1·74)	1·58 (1·29–1·85)	1·43 (1·14–1·72)	1·51 (1·24–1·76)	1·38 (1·09–1·66)	1·85 (1·57–2·12)	1·64 (1·34–1·94)	1·66 (1·38–1·92)	1·57 (1·29–1·85)
		Papua New Guinea	3·03 (2·50–3·58)	1·64 (0·94–2·35)	2·08 (1·45–2·72)	1·52 (0·87–2·22)	2·45 (1·84–3·06)	1·53 (0·88–2·22)	3·03 (2·50–3·58)	1·84 (1·14–2·55)	1·79 (1·15–2·46)	1·65 (1·02–2·33)
		Samoa	3·18 (2·69–3·68)	2·57 (1·98–3·14)	3·07 (2·56–3·59)	2·54 (1·95–3·12)	2·51 (1·90–3·07)	2·17 (1·55–2·75)	3·18 (2·69–3·68)	2·57 (1·98–3·14)	2·45 (1·83–3·03)	2·16 (1·54–2·75)
		Solomon Islands	2·51 (2·10–2·90)	1·70 (1·21–2·21)	2·19 (1·73–2·67)	1·74 (1·25–2·26)	2·01 (1·53–2·47)	1·49 (1·00–1·99)	2·51 (2·10–2·90)	1·90 (1·41–2·41)	1·82 (1·34–2·31)	1·73 (1·26–2·24)
		Tokelau	1·54 (1·17–1·94)	1·34 (0·94–1·78)	1·46 (1·09–1·88)	1·33 (0·94–1·76)	1·42 (1·07–1·82)	1·30 (0·92–1·73)	1·74 (1·37–2·14)	1·54 (1·14–1·98)	1·56 (1·21–1·97)	1·49 (1·12–1·92)
		Tonga	3·04 (2·58–3·50)	2·45 (1·93–2·98)	2·90 (2·43–3·39)	2·43 (1·92–2·95)	2·47 (1·91–3·00)	2·14 (1·58–2·68)	3·04 (2·58–3·50)	2·45 (1·93–2·98)	2·38 (1·83–2·91)	2·13 (1·59–2·65)
		Tuvalu	2·20 (1·71–2·70)	1·71 (1·17–2·26)	2·15 (1·65–2·65)	1·72 (1·19–2·25)	1·84 (1·34–2·35)	1·58 (1·06–2·11)	2·20 (1·71–2·70)	1·91 (1·37–2·46)	1·81 (1·31–2·31)	1·79 (1·28–2·31)
		Vanuatu	2·44 (2·06–2·87)	1·79 (1·33–2·31)	2·21 (1·79–2·69)	1·79 (1·34–2·30)	2·04 (1·63–2·50)	1·62 (1·18–2·13)	2·44 (2·06–2·87)	1·79 (1·33–2·31)	1·90 (1·48–2·38)	1·84 (1·40–2·33)
	Southeast Asia		1·60 (1·40–1·83)	1·35 (1·14–1·60)	1·54 (1·34–1·78)	1·35 (1·13–1·60)	1·48 (1·28–1·71)	1·31 (1·10–1·57)	1·74 (1·54–1·98)	1·54 (1·32–1·80)	1·63 (1·43–1·87)	1·51 (1·29–1·76)
		Cambodia	1·65 (1·32–1·93)	1·10 (0·71–1·45)	1·36 (1·00–1·68)	1·08 (0·70–1·43)	1·49 (1·14–1·78)	1·12 (0·75–1·44)	1·77 (1·44–2·05)	1·30 (0·91–1·65)	1·47 (1·11–1·78)	1·30 (0·93–1·62)
		Indonesia	1·53 (1·25–1·84)	1·29 (0·99–1·63)	1·51 (1·23–1·82)	1·30 (1·00–1·63)	1·44 (1·17–1·74)	1·26 (0·97–1·60)	1·73 (1·45–2·04)	1·49 (1·19–1·83)	1·62 (1·35–1·92)	1·47 (1·18–1·80)
		Laos	1·61 (1·29–1·88)	1·09 (0·73–1·40)	1·43 (1·08–1·72)	1·14 (0·78–1·44)	1·53 (1·22–1·79)	1·14 (0·79–1·44)	1·81 (1·49–2·08)	1·29 (0·93–1·60)	1·60 (1·25–1·89)	1·39 (1·02–1·70)
		Malaysia	1·39 (1·11–1·70)	1·17 (0·86–1·52)	1·35 (1·07–1·67)	1·19 (0·88–1·53)	1·32 (1·06–1·62)	1·17 (0·87–1·50)	1·59 (1·31–1·90)	1·37 (1·06–1·72)	1·49 (1·23–1·80)	1·38 (1·09–1·71)
		Maldives	1·07 (0·79–1·34)	0·77 (0·42–1·11)	0·97 (0·67–1·27)	0·79 (0·45–1·13)	0·84 (0·52–1·15)	0·71 (0·37–1·05)	1·27 (0·99–1·54)	0·97 (0·62–1·31)	0·98 (0·67–1·30)	0·93 (0·60–1·27)
		Mauritius	1·17 (0·94–1·42)	1·03 (0·77–1·32)	1·16 (0·93–1·41)	1·04 (0·78–1·33)	0·97 (0·72–1·24)	0·92 (0·66–1·21)	1·37 (1·14–1·62)	1·23 (0·97–1·52)	1·16 (0·92–1·43)	1·13 (0·87–1·42)
		Myanmar	1·69 (1·42–1·97)	1·22 (0·89–1·57)	1·48 (1·17–1·81)	1·22 (0·88–1·56)	1·63 (1·37–1·91)	1·27 (0·95–1·61)	1·77 (1·50–2·05)	1·42 (1·09–1·77)	1·66 (1·37–1·98)	1·47 (1·14–1·81)
		Philippines	1·84 (1·61–2·11)	1·50 (1·23–1·79)	1·78 (1·54–2·05)	1·48 (1·21–1·77)	1·62 (1·36–1·90)	1·43 (1·16–1·72)	1·84 (1·61–2·11)	1·70 (1·43–1·99)	1·78 (1·52–2·06)	1·62 (1·35–1·91)
		Seychelles	1·86 (1·60–2·14)	1·60 (1·31–1·91)	1·85 (1·58–2·13)	1·61 (1·33–1·91)	1·83 (1·57–2·10)	1·61 (1·33–1·91)	1·86 (1·60–2·14)	1·80 (1·51–2·11)	1·81 (1·55–2·09)	1·82 (1·54–2·12)
		Sri Lanka	1·50 (1·24–1·81)	1·30 (0·99–1·66)	1·47 (1·20–1·79)	1·29 (0·99–1·66)	1·40 (1·14–1·72)	1·28 (0·99–1·63)	1·70 (1·44–2·01)	1·50 (1·19–1·86)	1·58 (1·31–1·89)	1·48 (1·19–1·83)
		Thailand	1·13 (0·96–1·31)	1·04 (0·86–1·24)	1·13 (0·97–1·31)	1·04 (0·86–1·24)	1·08 (0·92–1·25)	1·02 (0·83–1·21)	1·33 (1·16–1·51)	1·24 (1·06–1·44)	1·28 (1·12–1·45)	1·22 (1·04–1·41)
		Timor-Leste	2·27 (1·62–2·90)	1·58 (0·85–2·30)	2·18 (1·54–2·83)	1·61 (0·92–2·32)	1·86 (1·18–2·53)	1·45 (0·75–2·15)	2·27 (1·62–2·90)	1·78 (1·05–2·50)	1·83 (1·14–2·50)	1·70 (1·01–2·39)
		Viet Nam	1·63 (1·38–1·93)	1·38 (1·10–1·70)	1·59 (1·34–1·88)	1·36 (1·09–1·69)	1·53 (1·29–1·82)	1·35 (1·07–1·67)	1·83 (1·58–2·13)	1·58 (1·30–1·90)	1·70 (1·44–1·99)	1·53 (1·26–1·85)
**Sub-Saharan Africa**			**2·72 (2·32–3·15)**	**1·82 (1·35–2·32)**	**2·27 (1·83–2·74)**	**1·80 (1·37–2·28)**	**2·29 (1·88–2·73)**	**1·73 (1·29–2·22)**	**2·73 (2·33–3·16)**	**1·89 (1·42–2·40)**	**2·03 (1·60–2·49)**	**1·82 (1·41–2·31)**
	Central sub-Saharan Africa		2·52 (2·05–2·94)	1·86 (1·37–2·31)	2·38 (1·91–2·82)	1·89 (1·42–2·34)	2·06 (1·61–2·48)	1·77 (1·31–2·21)	2·52 (2·05–2·94)	1·86 (1·38–2·31)	2·01 (1·55–2·43)	1·93 (1·48–2·36)
		Angola	2·76 (2·21–3·35)	1·97 (1·37–2·62)	2·61 (2·06–3·22)	2·03 (1·46–2·67)	2·30 (1·74–2·91)	1·87 (1·30–2·51)	2·76 (2·21–3·35)	1·97 (1·37–2·62)	2·23 (1·69–2·83)	1·93 (1·39–2·55)
		Central African Republic	2·36 (1·86–2·88)	1·35 (0·77–2·03)	1·94 (1·38–2·54)	1·41 (0·81–2·10)	1·74 (1·22–2·29)	1·17 (0·59–1·83)	2·36 (1·86–2·88)	1·55 (0·97–2·23)	1·73 (1·17–2·35)	1·45 (0·85–2·13)
		Congo (Brazzaville)	1·90 (1·54–2·32)	1·49 (1·11–1·93)	1·86 (1·51–2·27)	1·49 (1·13–1·92)	1·64 (1·29–2·05)	1·45 (1·08–1·88)	1·90 (1·54–2·32)	1·69 (1·31–2·13)	1·81 (1·46–2·22)	1·65 (1·29–2·07)
		Democratic Republic of the Congo	2·46 (1·82–3·03)	1·76 (1·09–2·38)	2·34 (1·69–2·93)	1·78 (1·13–2·40)	2·00 (1·37–2·56)	1·67 (1·02–2·27)	2·46 (1·82–3·03)	1·76 (1·09–2·38)	1·93 (1·31–2·49)	1·89 (1·26–2·49)
		Equatorial Guinea	2·19 (1·70–2·75)	1·83 (1·29–2·43)	2·19 (1·71–2·74)	1·83 (1·32–2·42)	1·90 (1·35–2·49)	1·76 (1·23–2·37)	2·19 (1·70–2·75)	1·83 (1·29–2·43)	1·90 (1·37–2·49)	1·77 (1·25–2·36)
		Gabon	1·93 (1·41–2·52)	1·56 (1·02–2·19)	1·88 (1·37–2·47)	1·56 (1·03–2·17)	1·68 (1·17–2·27)	1·51 (0·97–2·12)	1·93 (1·41–2·52)	1·76 (1·22–2·39)	1·85 (1·34–2·44)	1·71 (1·18–2·31)
	Eastern sub-Saharan Africa		2·50 (2·04–2·96)	1·68 (1·17–2·22)	2·11 (1·64–2·61)	1·70 (1·22–2·21)	2·17 (1·72–2·65)	1·61 (1·12–2·13)	2·50 (2·04–2·96)	1·80 (1·29–2·34)	1·98 (1·51–2·48)	1·77 (1·30–2·27)
		Burundi	2·74 (2·16–3·31)	1·55 (0·83–2·25)	2·11 (1·45–2·77)	1·55 (0·86–2·24)	2·20 (1·53–2·84)	1·48 (0·80–2·18)	2·74 (2·16–3·31)	1·75 (1·03–2·45)	1·80 (1·11–2·48)	1·69 (1·03–2·39)
		Comoros	1·73 (1·17–2·33)	1·23 (0·60–1·92)	1·66 (1·10–2·26)	1·26 (0·66–1·92)	1·42 (0·82–2·05)	1·17 (0·56–1·84)	1·73 (1·17–2·33)	1·43 (0·80–2·12)	1·58 (1·00–2·21)	1·40 (0·81–2·05)
		Djibouti	1·41 (0·92–1·88)	0·95 (0·38–1·51)	1·26 (0·78–1·76)	1·00 (0·47–1·54)	1·27 (0·79–1·75)	0·96 (0·43–1·50)	1·61 (1·12–2·08)	1·15 (0·58–1·71)	1·36 (0·89–1·86)	1·20 (0·71–1·73)
		Eritrea	2·20 (1·52–2·86)	1·28 (0·49–2·09)	1·84 (1·15–2·55)	1·32 (0·59–2·09)	1·62 (0·87–2·35)	1·15 (0·41–1·92)	2·20 (1·52–2·86)	1·48 (0·69–2·29)	1·63 (0·91–2·37)	1·39 (0·69–2·13)
		Ethiopia	2·40 (1·86–2·89)	1·29 (0·64–1·87)	1·73 (1·15–2·27)	1·30 (0·72–1·86)	2·14 (1·60–2·63)	1·32 (0·75–1·86)	2·40 (1·86–2·89)	1·49 (0·84–2·07)	1·83 (1·28–2·35)	1·53 (1·00–2·06)
		Kenya	1·84 (1·39–2·35)	1·45 (0·96–2·01)	1·80 (1·34–2·31)	1·48 (1·00–2·03)	1·77 (1·33–2·26)	1·46 (0·98–2·01)	1·84 (1·39–2·35)	1·65 (1·16–2·21)	1·77 (1·34–2·28)	1·68 (1·22–2·23)
		Madagascar	2·33 (1·91–2·78)	1·70 (1·23–2·22)	2·14 (1·69–2·61)	1·71 (1·24–2·21)	2·19 (1·77–2·62)	1·73 (1·25–2·25)	2·33 (1·91–2·78)	1·90 (1·43–2·42)	2·04 (1·60–2·50)	1·93 (1·46–2·45)
		Malawi	2·03 (1·46–2·57)	1·55 (0·94–2·14)	1·94 (1·36–2·48)	1·59 (1·01–2·16)	2·00 (1·42–2·53)	1·62 (1·02–2·22)	2·03 (1·46–2·57)	1·75 (1·14–2·34)	1·92 (1·35–2·47)	1·86 (1·27–2·45)
		Mozambique	2·44 (1·91–2·93)	1·55 (0·95–2·14)	2·09 (1·52–2·65)	1·64 (1·07–2·21)	2·16 (1·62–2·67)	1·56 (0·99–2·13)	2·44 (1·91–2·93)	1·75 (1·15–2·34)	1·93 (1·37–2·49)	1·85 (1·29–2·42)
		Rwanda	1·97 (1·54–2·42)	1·24 (0·76–1·77)	1·60 (1·15–2·08)	1·22 (0·76–1·73)	1·76 (1·35–2·20)	1·21 (0·77–1·70)	1·97 (1·54–2·42)	1·44 (0·96–1·97)	1·67 (1·25–2·14)	1·40 (0·97–1·89)
		Somalia	4·30 (3·92–4·68)	2·45 (1·92–3·00)	3·15 (2·68–3·62)	2·37 (1·87–2·90)	2·73 (2·15–3·28)	1·69 (1·14–2·24)	4·30 (3·92–4·68)	2·45 (1·92–3·00)	2·15 (1·59–2·69)	1·91 (1·39–2·44)
		South Sudan	4·09 (3·59–4·64)	1·98 (1·22–2·75)	2·67 (1·98–3·34)	1·91 (1·18–2·65)	2·54 (1·77–3·26)	1·35 (0·58–2·11)	4·09 (3·59–4·64)	1·98 (1·22–2·75)	1·77 (1·00–2·53)	1·56 (0·82–2·32)
		Tanzania	2·42 (2·02–2·86)	1·70 (1·23–2·20)	2·25 (1·81–2·72)	1·74 (1·28–2·24)	2·20 (1·79–2·64)	1·70 (1·24–2·20)	2·42 (2·02–2·86)	1·90 (1·43–2·40)	2·08 (1·66–2·54)	1·75 (1·30–2·25)
		Uganda	2·72 (2·26–3·19)	1·98 (1·48–2·50)	2·59 (2·13–3·07)	2·01 (1·54–2·51)	2·49 (2·05–2·96)	2·00 (1·51–2·51)	2·72 (2·26–3·19)	1·98 (1·48–2·50)	2·40 (1·95–2·88)	2·03 (1·56–2·53)
		Zambia	2·39 (1·88–2·91)	1·83 (1·28–2·40)	2·31 (1·80–2·83)	1·83 (1·31–2·39)	2·25 (1·77–2·77)	1·85 (1·32–2·43)	2·39 (1·88–2·91)	1·83 (1·28–2·40)	2·19 (1·70–2·72)	1·85 (1·33–2·43)
	Southern sub-Saharan Africa		1·94 (1·67–2·21)	1·63 (1·29–1·99)	1·92 (1·66–2·20)	1·63 (1·30–2·00)	1·86 (1·61–2·13)	1·62 (1·29–2·00)	2·07 (1·80–2·34)	1·76 (1·44–2·09)	1·99 (1·73–2·25)	1·75 (1·44–2·10)
		Botswana	1·70 (1·31–2·12)	1·38 (0·94–1·84)	1·70 (1·32–2·12)	1·39 (0·96–1·85)	1·68 (1·30–2·10)	1·41 (0·98–1·88)	1·82 (1·43–2·24)	1·58 (1·14–2·04)	1·88 (1·51–2·30)	1·62 (1·20–2·08)
		Eswatini	1·98 (1·62–2·36)	1·53 (1·13–1·95)	1·93 (1·56–2·32)	1·55 (1·16–1·97)	1·96 (1·60–2·34)	1·59 (1·20–2·03)	1·98 (1·62–2·36)	1·73 (1·33–2·15)	1·92 (1·55–2·30)	1·81 (1·42–2·24)
		Lesotho	1·88 (1·50–2·34)	1·47 (1·03–1·98)	1·78 (1·38–2·25)	1·45 (1·01–1·95)	1·82 (1·45–2·27)	1·49 (1·06–2·01)	1·88 (1·50–2·34)	1·67 (1·23–2·18)	1·78 (1·39–2·24)	1·67 (1·25–2·18)
		Namibia	2·03 (1·70–2·40)	1·62 (1·24–2·03)	2·00 (1·67–2·37)	1·63 (1·26–2·04)	1·96 (1·63–2·32)	1·63 (1·26–2·04)	2·03 (1·70–2·40)	1·82 (1·44–2·23)	1·93 (1·60–2·29)	1·84 (1·47–2·25)
		South Africa	1·69 (1·46–1·89)	1·45 (1·20–1·67)	1·67 (1·45–1·88)	1·44 (1·19–1·66)	1·60 (1·39–1·79)	1·41 (1·17–1·62)	1·89 (1·66–2·09)	1·65 (1·40–1·87)	1·79 (1·58–1·98)	1·60 (1·37–1·82)
		Zimbabwe	2·56 (2·12–3·01)	2·01 (1·52–2·51)	2·55 (2·11–3·00)	2·04 (1·55–2·53)	2·51 (2·09–2·95)	2·05 (1·57–2·56)	2·56 (2·12–3·01)	2·01 (1·52–2·51)	2·50 (2·08–2·94)	2·07 (1·59–2·58)
	Western sub-Saharan Africa		3·03 (2·60–3·48)	1·89 (1·39–2·44)	2·40 (1·93–2·91)	1·85 (1·38–2·39)	2·49 (2·05–2·95)	1·80 (1·32–2·33)	3·04 (2·60–3·48)	1·94 (1·44–2·49)	2·08 (1·61–2·57)	1·83 (1·38–2·36)
		Benin	3·12 (2·65–3·60)	1·58 (0·95–2·18)	2·26 (1·70–2·80)	1·55 (0·96–2·14)	2·33 (1·75–2·87)	1·46 (0·88–2·03)	3·12 (2·65–3·60)	1·78 (1·15–2·38)	1·81 (1·21–2·36)	1·66 (1·08–2·22)
		Burkina Faso	3·76 (3·23–4·28)	1·62 (0·89–2·26)	2·12 (1·42–2·73)	1·53 (0·84–2·15)	3·20 (2·61–3·75)	1·62 (0·93–2·24)	3·76 (3·23–4·28)	1·82 (1·09–2·46)	1·95 (1·27–2·56)	1·75 (1·08–2·38)
		Cabo Verde	1·09 (0·73–1·48)	0·91 (0·51–1·34)	1·07 (0·71–1·46)	0·94 (0·55–1·35)	1·08 (0·73–1·46)	0·95 (0·57–1·37)	1·29 (0·93–1·68)	1·11 (0·71–1·54)	1·26 (0·92–1·65)	1·17 (0·80–1·58)
		Cameroon	2·44 (1·92–3·03)	1·71 (1·13–2·36)	2·35 (1·82–2·94)	1·74 (1·19–2·36)	2·12 (1·62–2·69)	1·69 (1·13–2·31)	2·44 (1·92–3·03)	1·91 (1·33–2·56)	2·06 (1·55–2·64)	1·92 (1·38–2·53)
		Chad	4·81 (4·45–5·18)	2·15 (1·65–2·71)	3·04 (2·57–3·57)	2·10 (1·63–2·65)	3·28 (2·77–3·82)	1·73 (1·26–2·28)	4·81 (4·45–5·18)	2·15 (1·65–2·71)	2·26 (1·73–2·83)	1·77 (1·30–2·33)
		Côte d'Ivoire	2·57 (2·11–3·04)	1·44 (0·87–1·99)	2·03 (1·53–2·53)	1·45 (0·93–1·98)	2·13 (1·65–2·63)	1·42 (0·91–1·94)	2·57 (2·11–3·04)	1·64 (1·07–2·19)	1·78 (1·28–2·29)	1·65 (1·15–2·18)
		The Gambia	2·21 (1·81–2·61)	1·37 (0·92–1·87)	1·88 (1·49–2·33)	1·41 (0·98–1·88)	1·73 (1·31–2·18)	1·25 (0·81–1·73)	2·21 (1·81–2·61)	1·57 (1·12–2·07)	1·75 (1·33–2·21)	1·50 (1·08–1·97)
		Ghana	2·12 (1·57–2·71)	1·57 (0·97–2·20)	2·04 (1·48–2·63)	1·57 (0·98–2·19)	1·81 (1·26–2·39)	1·51 (0·92–2·13)	2·12 (1·57–2·71)	1·77 (1·17–2·40)	1·76 (1·20–2·34)	1·71 (1·14–2·32)
		Guinea	3·02 (2·58–3·43)	1·42 (0·81–2·00)	2·02 (1·44–2·54)	1·40 (0·81–1·95)	2·20 (1·62–2·70)	1·28 (0·70–1·80)	3·02 (2·58–3·43)	1·62 (1·01–2·20)	1·81 (1·23–2·33)	1·49 (0·95–2·01)
		Guinea-Bissau	2·41 (1·88–2·91)	1·26 (0·63–1·86)	1·80 (1·23–2·35)	1·30 (0·72–1·87)	2·01 (1·47–2·54)	1·25 (0·68–1·82)	2·41 (1·88–2·91)	1·46 (0·83–2·06)	1·81 (1·26–2·37)	1·51 (0·97–2·07)
		Liberia	2·10 (1·52–2·71)	1·47 (0·85–2·14)	1·94 (1·34–2·58)	1·52 (0·91–2·18)	1·83 (1·25–2·45)	1·45 (0·85–2·11)	2·10 (1·52–2·71)	1·67 (1·05–2·34)	1·77 (1·18–2·41)	1·70 (1·11–2·36)
		Mali	4·21 (3·83–4·63)	1·85 (1·30–2·42)	2·42 (1·86–3·01)	1·70 (1·15–2·28)	3·37 (2·87–3·88)	1·79 (1·26–2·33)	4·21 (3·83–4·63)	1·85 (1·30–2·42)	2·12 (1·56–2·70)	1·89 (1·35–2·49)
		Mauritania	2·50 (1·98–3·08)	1·66 (1·04–2·34)	2·24 (1·68–2·85)	1·66 (1·07–2·32)	1·97 (1·40–2·57)	1·54 (0·95–2·19)	2·50 (1·98–3·08)	1·86 (1·24–2·54)	1·81 (1·23–2·43)	1·75 (1·17–2·39)
		Niger	5·15 (4·68–5·64)	2·24 (1·48–2·92)	2·74 (2·00–3·40)	1·99 (1·28–2·64)	4·34 (3·75–4·89)	2·20 (1·50–2·85)	5·15 (4·68–5·64)	2·24 (1·48–2·92)	2·52 (1·83–3·17)	2·02 (1·36–2·67)
		Nigeria	2·69 (2·06–3·31)	1·87 (1·19–2·54)	2·54 (1·88–3·17)	1·90 (1·22–2·56)	2·25 (1·63–2·82)	1·78 (1·12–2·43)	2·69 (2·06–3·31)	1·87 (1·19–2·54)	2·16 (1·53–2·76)	1·81 (1·15–2·46)
		São Tomé and Príncipe	1·77 (1·29–2·28)	1·37 (0·83–1·94)	1·67 (1·18–2·19)	1·38 (0·87–1·93)	1·61 (1·12–2·12)	1·35 (0·83–1·90)	1·77 (1·29–2·28)	1·57 (1·03–2·14)	1·74 (1·25–2·26)	1·57 (1·06–2·11)
		Senegal	2·32 (1·79–2·79)	1·25 (0·60–1·83)	1·66 (1·08–2·18)	1·24 (0·62–1·80)	1·98 (1·44–2·48)	1·26 (0·67–1·79)	2·32 (1·79–2·79)	1·45 (0·80–2·03)	1·70 (1·14–2·22)	1·45 (0·88–1·97)
		Sierra Leone	2·43 (1·99–2·85)	1·31 (0·78–1·82)	1·78 (1·27–2·28)	1·28 (0·76–1·80)	2·04 (1·59–2·47)	1·29 (0·77–1·80)	2·43 (1·99–2·85)	1·51 (0·98–2·02)	1·79 (1·29–2·29)	1·49 (0·97–2·02)
		Togo	2·01 (1·61–2·42)	1·24 (0·79–1·72)	1·75 (1·32–2·18)	1·28 (0·84–1·74)	1·65 (1·24–2·08)	1·21 (0·78–1·67)	2·01 (1·61–2·42)	1·44 (0·99–1·92)	1·69 (1·26–2·14)	1·45 (1·03–1·90)

Numbers in parentheses are 95% uncertainty intervals. Super-regions, regions, and countries are listed in alphabetical order. SDG=Sustainable Development Goal.

### Comparison with other models

The WPP 2022 revision projected a global TFR of 2·15 in 2050 and 1·84 in 2100 compared with the global TFRs forecast by our model of 1·83 (95% UI 1·59–2·08) and 1·59 (1·25–1·96) in the same years (appendix 2 figure S6). In 2100, WPP's TFR forecasts converge to a narrower range of 1·38–2·22 than do our model's reference scenario TFR values, which range from 0·69 to 2·57. Additionally, for countries with low fertility levels, WPP predicts that fertility rates will rebound to a much larger extent than in our projections, which suggest they will remain low or decline (appendix 2 figure S6). We forecast that global TFR will fall below the replacement level of 2·1 after 2030, in contrast to WPP's forecast that global TFR will fall below replacement level in 2056.

Time-series country-level comparisons between ASFR and TFR predictions from our model and WPP 2022 are presented in appendix 2 (figures S5, S6). For example, our model predicts that reference TFR in South Korea will be low throughout the forecast period (0·82 [95% UI 0·73–0·92] in 2050 and 0·82 [0·71–0·95] in 2100), but WPP 2022 forecasts an increase in TFR in South Korea (1·17 in 2050 and 1·43 in 2100; appendix 2 figure S5). These forecasting differences are apparent in other countries and territories, including in Taiwan (province of China), where we forecast almost no change in TFR from 2050 to 2100 (0·90 [0·78–1·04] and 0·90 [0·77–1·05], respectively), compared with the WPP projection of a rebound in TFR to 1·41 in 2050 and 1·53 in 2100. We forecast that TFR will be lower in 200 of 204 countries and territories in 2100, including those that already have very low fertility rates, and projected a much steeper decline in TFR in many African countries than did WPP. For example, our model projected that TFR in Sudan will fall below replacement in approximately 2045, whereas the WPP model forecast that this will happen after 2095. Overall, the WPP model predicted that 142 (69·6%) of 204 countries and territories will have fertility rates below replacement level in 2050, whereas our model forecast that the number will be 155 (76·0%). By 2100, the two models reach similar conclusions globally, with 197 countries and territories (96·6%) projected by WPP to reach TFR below replacement level and 198 (97·1%) predicted by our model.

We validated the IHME model over the period 2007–21 using a forecasting skill metric based on RMSE values. The predicted values for our ASFR forecasts were compared with our final GBD 2021 estimates to compute RMSE values across locations for each 5-year age group. Our model had a positive skill value across all age groups, indicating that it is better than the baseline model (here, simply holding 2007 ASFR values constant over the period 2007–21). The lowest skill value was 0·15 (age 30–34 years), and the highest skill value was 0·46 (age 45–49 years; appendix 1 figure S3).

## Discussion

### Main findings

This study presents comprehensive estimates of past and future trends in fertility in 204 countries and territories from 1950 to 2100. We broadly found that human civilisation is rapidly converging on a sustained low-fertility reality, although comparatively high fertility rates in low-income regions, particularly in a subset of countries and territories in western and eastern sub-Saharan Africa, will result in a demographically divided world. As much of the planet contends with challenges related to low fertility, many low-income countries will still be facing issues associated with high fertility during the 21st century. Overall, fertility has declined steadily at the global level and across almost all countries and territories since 1950 and is likely to continue to do so until 2100, from a global TFR of more than 4·8 births per female in 1950 to approximately 2·2 in 2021, with TFRs of approximately 1·8 and 1·6 projected in our reference scenario in 2050 and 2100, respectively. Only six of 204 countries and territories (Samoa, Somalia, Tonga, Niger, Chad, and Tajikistan) are projected to have above-replacement levels of fertility by 2100, and only 26 will still have a positive rate of natural increase (ie, the number of births will exceed the number of deaths).

Historically, fertility rates have varied dramatically between GBD super-regions, with the highest rates in sub-Saharan Africa and the lowest in the high-income super-region (eg, TFR of approximately 4·3 *vs* 1·5 in 2021), driven by many factors, such as wealth, education, and sociocultural behaviours and practices.^39^, ^40^ By 2100, TFRs will continue to differ, but to a smaller extent, from just over 1·8 in sub-Saharan Africa to approximately 1·1 in south Asia, all converging well below replacement levels. Patterns in livebirths will shift dramatically over the coming century, with the proportion of livebirths occurring in sub-Saharan Africa increasing from less than 30% in 2021 to almost 55% in 2100. Likewise, we forecast that the proportion of livebirths occurring in the World Bank low-income group will increase from just under 18% in 2021 to 35% in 2100. The proportion of global livebirths in the low-income and lower-middle-income groups combined will surpass 77% by 2100.

### Implications of sustained low fertility

The aforementioned changes in fertility over the coming century will have profound effects on populations, economies, geopolitics, food security, health, and the environment, with a clear demographic divide between the impacts on many middle-to-high-income locations versus many low-income locations. For nearly all countries and territories outside of sub-Saharan Africa, sustained low fertility will produce a contracting population with fewer young people relative to older people before the end of the 21st century. These changes in age structure are likely to present considerable economic challenges caused by a growing dependency ratio of older to working-age population and a shrinking labour force.^41^, ^42^ Unless governments identify unforeseen innovations or funding sources that address the challenges of population ageing, this demographic shift will put increasing pressure on national health insurance, social security programmes, and health-care infrastructure. These same programmes will receive less funding as working-age, tax-paying populations decline, further exacerbating the problem.^43^, ^44^

Sustained low fertility rates might likewise lead to labour shortages in some sectors, potentially hindering economic growth. If productivity per working-aged adult does not increase in accordance with declines in the working-age population, growth in gross domestic product will slow.^4^ Reliance on immigrants will become increasingly necessary to sustain economic growth in low-fertility countries.^45^ The shifting global distribution of livebirths, with a higher proportion occurring in current lower-income countries, could make immigration a viable way to address these issues. However, this approach will only work if there is a shift in current public and political attitudes towards immigration in many lower-fertility countries and if there are sufficient incentives in place for people to migrate from higher-fertility countries. Continued skilled worker migration to high-income, low-fertility economies—a concept referred to as brain drain—can also have devastating effects on the economies these workers leave behind.^46^, ^47^ This underscores the importance of developing ethical and effective immigration policies with global cooperation. Aside from immigration, innovations to the labour force, such as advancements in artificial intelligence and robotics, could reduce the economic effects of changes in age structure, but the potential landscape is difficult to predict and would undoubtedly vary between nations.^48^ Furthermore, shifts in productivity in older ages, years of education required to participate fully in certain sectors of the workforce, the proportion of people who could give birth in the workforce, the ability to fulfil fertility intentions in older ages, and other factors could also affect the impact of ageing on economic growth, but these are likewise complex, and the impacts are largely unknown and beyond the scope of this study to consider.

To date, one strategy to reverse declining fertility in low-fertility settings has been to implement pro-natal policies, such as child-related cash transfers and tax incentives, childcare subsidies, extended parental leave, re-employment rights, and other forms of support for parents to care and pay for their children.^49^, ^50^ Yet there are few data to show that such policies have led to strong, sustained rebounds in fertility, with empirical evidence suggesting an effect size of no more than 0·2 additional livebirths per female.^51^, ^52^ The pro-natal alternative scenario we present here thus assumes that pro-natal policies will be implemented once the TFR of a country or territory falls below 1·75 and that the effect will be to increase TFR by 0·2 births per female 5 years later. Under this scenario, we project a global TFR of 1·68 in 2100 compared with 1·59 in the reference scenario. This modest increase suggests that even under optimistic assumptions on the impact of pro-natal policies based on current data, global TFR will remain low—and well below replacement level—up to 2100. Nevertheless, our pro-natal scenario forecasts also suggest that pro-natal policies might prevent some countries from dropping below very-low (<1·6 TFR) or the lowest-low (<1·3 TFR) fertility in the future. We projected that 64 fewer countries and territories would fall below lowest-low fertility levels in 2100 in the pro-natal scenario compared with the reference scenario (30 *vs* 94). Moreover, although pro-natal policies primarily aim to increase births, they also offer additional benefits to society, including better quality of life, greater household gender equality (ie, more equal division of household labour),^53^ higher rates of female labour force participation,^54^ lower child-care costs,^55^ and better maternal health outcomes,^56^ depending on policy design and contextual factors. In the future, it will be beneficial to perform an in-depth analysis on varying impacts of pro-natal policies in selected countries that have a meaningful impact on population.

Importantly, low fertility rates and the modest effects that pro-natal policies might have on them should not be used to justify more draconian measures that limit reproductive rights, such as restricting access to modern contraceptives or abortions. For example, in Romania during and in the aftermath of severely restricting abortions and the sale of contraceptives in the 1960s–80s, coercive policies led to dramatic increases in maternal mortality rates from illegal abortions; large numbers of children placed in orphanages; harmful, long-lasting effects on the labour market and educational outcomes for the population born under the restrictions; and other negative impacts, including long-term trauma to women and children.^6^, ^57^, ^58^ Access to modern contraceptives is not only fundamental to the principles of basic human rights and reproductive justice, but also has demonstrated positive effects on the economy; contraceptive access and use is positively associated with formal labour force participation and higher incomes.^59^, ^60^, ^61^, ^62^, ^63^

Although sustained below-replacement fertility will pose serious potential challenges for much of the world over the course of the century, it also presents opportunities for environmental progress. Alongside strong pro-environmental regulations, a smaller global population in the future could alleviate some strain on global food systems, fragile environments, and other finite resources, and also reduce carbon emissions.^64^, ^65^, ^66^, ^67^, ^68^, ^69^ A 2012 study suggests that if global population were to follow a low-growth rather than a medium-growth path, worldwide carbon emissions would be 15% lower by 2050 and 40% lower by 2100.^70^ The 2023 Intergovernmental Panel on Climate Change (IPCC) report likewise suggests that low population growth (a result of low fertility) is an important factor in limiting global warming.^71^ However, increasing consumption per capita due to economic development could offset the benefits of smaller populations.^72^, ^73^

### Implications of the changing global distribution of livebirths

While the world faces the challenges that arise from sustained low fertility in most locations, it will simultaneously be confronted with challenges that arise from the concentration of the world's livebirths shifting from middle-to-high-income towards low-income countries and territories. In the coming decades, the majority of children will be born in some of the poorest regions of the world, with the proportion of global livebirths almost doubling in low-income countries and territories (as defined by the World Bank) between 2021 and 2100, from 18% to 35%. Sub-Saharan Africa is projected to contribute over half of the world's livebirths—more than 54%—by 2100, up from approximately 29% in 2021. Countries in eastern and western sub-Saharan Africa, many in the Sahel, are projected to be primary drivers of livebirths by 2100, but considerable heterogeneity exists across countries within these regions. Child mortality rates are disproportionately high in low-income settings, with the highest rates in western sub-Saharan Africa (at more than 85 deaths per 1000 among children younger than 5 years in 2021 compared with approximately 35 per 1000 at the global level).^74^ Thus, this shift in fertility and livebirths from higher-income to lower-income settings will make the challenge of continued progress on improving health outcomes—particularly child mortality—even more difficult.

Many higher-fertility, low-income countries will also face increasingly frequent droughts, flooding, and extreme heat as climate change worsens.^71^ All of these aspects of climate change threaten food, water, and other resource security and dramatically increase the risk of heat-related illness and death.^71^, ^75^, ^76^, ^77^, ^78^ For example, the IPCC projects substantial declines in crop yields across many low-income settings due to climate change, including a 20–40% decrease in millet yield in the Sahel region in response to a potential 30-year mean increase of 2–3°C in maximum temperature.^79^, ^80^ Population growth will only worsen the growing strain on food supplies in this region in the future.^81^ Food and resource scarcity, along with several other issues including the long legacy of colonialism, contribute to political instability and security issues in some vulnerable areas. Between January, 2020, and July, 2023, there were coups in six Sahel nations^82^ and, in 2022, 43% of all global terrorism deaths occurred in this region.^83^ Broadly, over the coming decades, the majority of livebirths will become concentrated in the areas of the world that are most vulnerable to climate change, resource insecurity, political instability, poverty, and child mortality. High numbers of births in these regions will further strain all areas of vulnerability.

Our projections suggest that improving access to modern contraceptives and female education—the two primary drivers of fertility^4^, ^84^, ^85^, ^86^—would reduce fertility rates in higher-fertility countries and territories, limiting the increasing concentration of livebirths in these areas. We project that in sub-Saharan Africa, achieving universal female education or universal contraceptive met need by 2030 would each result in TFRs of approximately 2·3 in 2050, compared with approximately 2·7 in the reference scenario. By combining universal access to both drivers of fertility, plus an increase in TFR of 0·2 in locations with a TFR less than 1·75 from the pro-natal scenario (which will not apply to most locations in this super-region until almost 2100, if at all), our combined scenario highlights opportunities for even larger declines in fertility to 2·03 in sub-Saharan Africa in 2050. Although we project that global TFRs will eventually converge to 1·52–1·68 in 2100 across the reference and three alternative scenarios, the considerably steeper fertility declines in the next several decades achieved through the rapid scale-up of education and contraceptive access would reduce the number of livebirths in sub-Saharan Africa in 2100. For the highest-fertility countries, the opportunities are even greater; in Niger, for example, our reference scenario forecasts a TFR of 5·15 (95% UI 4·68–5·64) in 2050 versus 2·74 (2·00–3·40) in the education SDG target scenario, 4·34 (3·75–4·89) in the contraceptive SDG target scenario, and 2·52 (1·83–3·17) in the combined scenario. Although achieving both SDG targets in all locations by 2030 is likely to be unattainable, our SDG-related scenarios demonstrate that increasing levels of access to female education and contraceptives in higher-fertility countries will result in fewer individuals in the future being born into severely heat-stressed, politically fragile, economically weak environments. Policy makers can and should use these projections to inform priorities.

In addition to its direct impact on fertility rates, expanding female access to education and contraceptives has important societal benefits. First, access to education contributes to women's empowerment: the process through which women gain the freedom to make their own choices and the opportunity to participate fully in society.^87^ Quality education increases the knowledge, skills, and self-confidence needed to challenge traditional gender roles, and equips women to make more informed decisions about their health, careers, and lives as a whole.^88^, ^89^, ^90^ It also improves women's decision-making power in the household and lowers their risk of exposure to abuse in the home.^87^, ^90^, ^91^, ^92^ Furthermore, higher female educational attainment is associated with higher paid labour-force participation and higher wages. In fact, the financial returns on female education exceed those of males (which is not to say that earnings are higher for females; they remain lower for females at the same level of education), as do the returns in low-income settings compared with high-income settings, making female education a valuable personal and societal investment.^93^ Finally, universal access to modern contraceptives and education are fundamental human rights that the world should be working towards for all populations regardless of their outcomes on fertility, society, and the economy.

### Comparisons with estimates from WPP 2022

TFR estimates from 1950 to 2021 generated by UN Population Division WPP 2022 generally align with the estimates produced by our model in countries with high-quality vital registration data (appendix 2 figure S5). However, estimates differ in some locations with less reliable data sources, particularly in the sub-Saharan Africa and north Africa and the Middle East super-regions. This is mostly due to differences in data sources used and data processing steps. For example, in locations without vital registration data, our methods use complete birth histories as a reference source and correspondingly adjust the estimates of sources such as summary birth histories that do not give information on fertility by mother's age. By contrast, estimates from WPP 2022 more closely follow estimates from these less reliable sources. This process affects estimates in countries for which the most recent data come from summary birth histories, leading to differences in recent time trends that can heavily influence forecasts. For example, in South Sudan, we estimated a decline in TFR from 5·98 (95% UI 5·61–6·33) in 2000 to 5·45 (5·04–5·87) in 2021, compared with the WPP estimates of a decline from 7·51 (6·99–8·09) in 2000 to 4·47 (3·42–5·76) in 2021. Other countries in which there are large discrepancies in 2021 estimates from the two models include the Democratic Republic of the Congo and Central African Republic.

In a comparison between our IHME model forecasts and those of the WPP 2022 revision, we found that the WPP global TFR forecasts were higher throughout the 2022–2100 period and the country-level TFR forecasts converged to a much narrower range in 2100 than the projections from our model (appendix 2 figure S6). Broadly, for countries with low fertility levels, WPP predicts that fertility rates will rebound, whereas our projections suggest they will remain low or decline. The higher TFRs predicted by WPP are primarily a reflection of differences in how post-transition countries were modelled. The WPP forecasting methodology is based in demographic transition theory and assumes that all countries follow the historically observed three-phase pattern of fertility.^14^ However, there is some evidence that current higher-fertility countries—especially those in sub-Saharan Africa—have experienced fertility patterns that do not perfectly reflect these phases, such as periods with stalling declines.^94^, ^95^ Our method does not make this structural assumption. Furthermore, WPP denotes a country moving from phase II (fertility decline) to phase III (low-fertility post-transition) when it experiences two successive periods of TFR increase after falling below a TFR of 2·0. This threshold of 2·0 might not hold in the future for all countries. In fact, our estimates in Seychelles show a transition into phase III after a TFR of 2·04 (95% UI 1·95–2·12). Due to these assumptions, WPP reference forecasts in all locations might rely too heavily on the fertility patterns of a subset of low-fertility countries that have experienced increases in TFR.

### Limitations

This study has a number of limitations, many of which are related to data availability and quality. First, sparsity of recent census data and lags between censuses affected the availability of birth history data for certain locations. This absence of data means that past fertility estimates in some locations—particularly countries in sub-Saharan Africa as well as others such as Afghanistan, Haiti, Syria, and Yemen—were based on modelled projections. Similarly, the absence of high-quality vital registration systems affected the precision of fertility estimates in many locations, resulting in large UIs. This effect was especially apparent during the 2020–21 COVID-19 pandemic period, during which reporting from vital registration systems was particularly limited and delayed. Furthermore, our forecasts rely on past time-series data not only for fertility, but also the drivers of fertility, such as met need for contraceptives. These data were not always available for each location, and even when they were, they were only available as far back as 1970.

Second, we made several simplifying assumptions in the modelling of past fertility. Due to sparse data, we estimated fertility in the age groups of 10–14 years and 50–54 years solely based on neighbouring age groups using data from locations with complete vital registration data. Other factors could be driving fertility rates in these age groups that are not captured in the available data, but the effect of our models for these age groups on TFR is minimal. We compared the TFR in 2021 calculated with and without these age groups across GBD regions and found that the maximum difference was 0·03 (95% UI 0·02–0·04; appendix 2 table S3). Additionally, estimates of uncertainty were simplified due to computational resource constraints. Uncertainty for some covariates was not propagated through our analytical process, including female educational attainment and lag-distributed income in our first-stage regression model. Furthermore, we do not account for correlations between locations when producing geographical aggregate values, which might underestimate uncertainty because estimates in nearby locations are likely to have positive correlation. We could improve accuracy of UIs in the future by accounting for correlations when aggregating estimates.

Third, our forecasting analysis modelled four covariates as potential drivers of fertility: female educational attainment, contraceptive met need, population density in habitable areas, and under-5 mortality. This method contrasts with non-causal time-series models by UN Population Division, in which time is the only driver of fertility and no covariates are used. Our inclusion of covariates can be considered both a strength and a limitation.^13^, ^96^, ^97^ Explicitly modelling associations between drivers and outcomes requires us to separately forecast future trends for each driver, which has advantages in that we capture potentially important effects and can vary covariate levels to model fertility outcomes of policy choices related, for example, to education or contraception. However, modelling such associations also presents a challenge in that accurate fertility forecasts rely heavily on accurately forecasting each of these independent drivers into the future. Although we have not studied mechanisms by which our covariates impact fertility, living in urban areas may give better access to education, family planning services, and employment opportunities for women, all of which are associated with lower fertility.^98^

Fourth, more research is needed on determinants of fertility in low-fertility locations; most of the difference between our forecasts and those produced by the UN Population Division WPP 2022 are due to the projected level of fertility to which locations ultimately converge after dropping below replacement levels. Our use of CCF50 captures the effects of age-related declines in each cohort of females.

Fifth, we encountered a range of additional limitations specific to our forecasting models. All long-range forecasting models, regardless of the modelling strategy, face the challenge that the past is not always predictive of the future—ie, there will be potential changes in the future that cannot be predicted. Also, we applied the pro-natal scenario to all locations even if the location has already implemented pro-natal policies, such as Australia, Japan, South Korea, and countries in Scandinavia. We further describe this in appendix 1 (section 3.2–3.3). Additionally, we did not incorporate into any scenarios the possibility that certain locations could exceed their capacity to feed their people. In most locations, any insufficiencies in domestic food production could be solved by importing food. But for several countries with growing food production concerns^71^, ^99^ that are forecast to have large population growth and remain low income—including Niger, Chad, and South Sudan—it is possible that our forecast levels of fertility will be unsustainable due to food insecurity. Finally, due to paucity of data, we did not define the pro-natal scenario based on a specific policy or policies that have a known impact on fertility rates. Rather, we considered policies such as paid parental leave, the right to return to work, and subsidised or universal childcare as pro-natal—in other words, policies that have been enacted in countries such as Australia, Sweden, Denmark, Norway, Finland, and elsewhere that are thought of as making it more financially feasible to have children.

### Conclusions

Fertility rates have declined dramatically around the world since 1950 and will continue to decline in almost all countries and territories up to 2100. While human civilisation is converging on a sustained low-fertility reality, comparatively high fertility rates in some low-income countries and territories will result in a clear demographic divide between a subset of low-income countries and the rest of the world. On one side, sustained low fertility rates—and a resulting contraction and ageing of the population—will lead to serious economic challenges and increasing pressure on health systems, social security programmes, and the labour force. On the other hand, a dramatic shift in the concentration of livebirths from middle-income and high-income settings to low-income settings will lead to serious challenges related to sustaining and supporting a growing young population in some of the most heat-stressed, politically unstable, economically vulnerable, health system-strained locations. In low-fertility settings, implementing pro-natal policies that support parents and children might provide a small boost to fertility rates, whereas in higher-fertility settings, rapidly expanding access to female education and contraceptives will accelerate declines in fertility and lessen the concentration of livebirths in these locations. Future trends in fertility rates and livebirths will propagate shifts in global population dynamics, driving changes to international relations and a geopolitical environment, and highlighting new challenges in migration and global aid networks. All of these issues will necessitate focused and collaborative work to address.

For the **GBD Sources Tool** see https://ghdx.healthdata.org/gbd-2021/sources

For the **GBD 2021 statistical code** see http://ghdx.healthdata.org/gbd-2021/code

## Data sharing

To download the data used in these analyses, please visit the Global Health Data Exchange GBD 2021 website (http://ghdx.healthdata.org/gbd-2021/sources).

## Declaration of interests

S Afzal reports payment or honoraria for lectures, presentations, speakers bureaus, manuscript writing, or educational events and webinars with King Edward Medical University and collaborative partners including University of Johns Hopkins, University of California, and University of Massachusetts; participation on a Data Safety Monitoring Board or Advisory Board with the National Bioethics Committee Pakistan, King Edward Medical University Institutional Ethical Review Board, and the Ethical Review Board Fatima Jinnah Medical University and Sir Ganga Ram Hospital; leadership or fiduciary roles in board, society, committee, or advocacy groups, paid or unpaid, with Pakistan Association of Medical Editors, Fellow of Faculty of Public Health Royal Colleges UK (FFPH), the Society of Prevention, Advocacy and Research of King Edward Medical University (SPARK), and as a Member of the Pakistan Society of Infectious Diseases; and other financial support as Dean of Public Health and Preventive Medicine, King Edward Medical University, as the Chief Editor of *Annals of King Edward Medical University* since 2014, as the Director of Quality Enhancement Cell, King Edward Medical University, and with the Member Research and Publications Higher Education Commission Pakistan; all outside the submitted work. R Ancuceanu reports consulting fees from AbbVie, Sandoz, B Braun, and Laropharm, all outside the submitted work. J Ärnlöv reports payment or honoraria for lectures, presentations, speakers bureaus, manuscript writing, or educational events from AstraZeneca and Novartis; participation on a Data Safety Monitoring Board or Advisory Board with AstraZeneca, Astella, and Boehringer Ingelheim; all outside the submitted work. M Ausloos reports grants or contracts from the Romanian National Authority for Scientific Research and Innovation, CNDS-UEFISCDI (project number PN-III-P4-ID-PCCF-2016-0084), outside the submitted work. R Bai reports support for the present manuscript from the Social Science Fund of Jiangsu Province (grant number 21GLD008). O C Baltatu reports support for the present manuscript from the National Council for Scientific and Technological Development (CNPq, 304224/2022-7) and from Anima Institute (AI research professor fellowship); leadership or fiduciary roles in board, society, committee, or advocacy groups, paid or unpaid, as a Biotech Board Member at São José dos Campos Technology Park, outside the submitted work. T Bärnighausen reports grants or contracts from the National Institutes of Health (NIH), Alexander von Humboldt Foundation, German National Research Foundation (DFG), EU, German Ministry of Education and Research, German Ministry of the Environment, Wellcome Trust, and KfW; payment or honoraria for lectures, presentations, speakers bureaus, manuscript writing, or educational events from *PLOS Medicine* as the Editor-in-Chief; participation on a Data Safety Monitoring Board or Advisory Board for NIH-funded research projects in Africa on climate change and health; and stock ownership in CHEERS; outside the submitted work. S Barteit reports support for attending meetings or travel from the Wellcome Trust, and stocks or stock options with Climate Change and Health Evaluation and Response System; all outside the submitted work. M L Bell reports grants or contracts from US EPA, NIH, High Tide Foundation, Health Effects Institute, Yale Women Faculty Forum, the Environmental Defense Fund, Wellcome Trust Foundation, Yale Climate Change and Health Center, Robert Wood Johnson Foundation, and the Hutchinson Postdoctoral Fellowship; consulting fees from Clinique; honoraria for lectures, presentations, or speakers bureaus from Colorado School of Public Health, Duke University, University of Texas, Data4Justice, Korea University, Organization of Teratology Information Specialists, UPenn, and Boston University; honorarium for editorial duties from IOP Publishing; honorarium for grant review from NIH, Health Canada, PAC-10, UK Research and Innovation, and AXA Research Fund Fellowship; honorarium for external advisory committee from Harvard University and the University of Montana; support for attending meetings or travel from Colorado School of Public Health, University of Texas, Duke University, Boston University, UPenn, Harvard University, and the *American Journal of Public Health*; a leadership or fiduciary role in a board, society, committee, or advocacy group, unpaid, with Fifth National Climate Assessment, the *Lancet* Countdown, Johns Hopkins EHE Advisory Board, a Harvard external advisory committee for training grants, WHO Global Air Pollution and Health Technical Advisory group, and the National Academies Panels and Committees; leadership or fiduciary role in a board, society, committee, or advocacy group, paid, from the US EPA Clean Air Scientific Advisory Committee (CASAC); all outside the submitted work. P J G Bettencourt reports other financial or non-financial support as a Project Reviewer at Botnar Foundation; outside the submitted work. P V Bhardwaj reports stock or stock options with Doximity, outside the submitted work. S Bhaskar reports grants or contracts from Japan Society for the Promotion of Science (JSPS), Japanese Ministry of Education, Culture, Sports, Science and Technology (MEXT), Grant-in-Aid for Scientific Research (KAKENHI), and JSPS and the Australian Academy of Science (JSPS International Fellowship); leadership or fiduciary roles in board, society, committee, or advocacy groups, paid or unpaid, with Rotary District 9675 as the District Chair, Diversity, Equity, and Inclusion, the Global Health and Migration Hub Community, Global Health Hub Germany (Berlin, Germany) as the Chair and Manager, *PLOS One, BMC Neurology, Frontiers in Neurology, Frontiers in Stroke, Frontiers in Public Health*, and *BMC Medical Research Methodology* as an Editorial Board Member; and the College of Reviewers, Canadian Institutes of Health Research (CIHR), Government of Canada as a Member; outside the submitted work. A Biswas reports consulting fees from Lupin Pharmaceuticals (India), Alkem Laboratories (India), and Intas Pharmaceuticals (India); payment or honoraria for lectures, presentations, speakers bureaus, manuscript writing, or educational events from Roche Diagnostics (India); outside the submitted work. J Conde reports grants or contracts from the European Research Council Starting Grant (ERC-StG-2019-848325), outside the submitted work. S Das reports a leadership or fiduciary role in a board, society, committee, or advocacy group, unpaid, as an executive voluntary member and leadership position in the Association for Diagnostic and Laboratory Medicine and Women in Global Health India Chapter; outside the submitted work. A Dastiridou reports payment or honoraria for lectures, presentations, speakers bureaus, manuscript writing, or educational events from AbbVie and Thea; outside the submitted work. A Faro reports support for the present manuscript from Coordination of Superior Level Staff Improvement (CNPq, Brazil) Productivity in Research Scholarship (PQ Scholarship). A A Fomenkov reports support for the present manuscript from the Ministry of Science and Higher Education of the Russian Federation (theme number 121050500047-5). M Foschi reports consulting fees from Roche, Biogen, Merck, Sanofi, and Novartis; support for attending meetings or travel from Roche, Novartis, Biogen, Sanofi, and Merck; leadership or fiduciary role in a board, society, committee, or advocacy group, paid or unpaid, as a current member of the MSBase collaboration scientific leadership group; outside the submitted work. I Filip reports support for the present manuscript from Avicenna Medical and Clinical Research Institute. I Ilic and M Ilic report support for the present manuscript from the Ministry of Science, Technological Development and Innovation of the Republic of Serbia (grants 451-03-47/2023-01/200111 and 175042, 2011-2023). N E Ismail reports a leadership or fiduciary role in a board, society, committee, or advocacy group, unpaid, with The Bursar as a Council Member of the Malaysian Academy of Pharmacy; outside the submitted work. A Hassan reports consulting fees from Novartis, Sanofi Genzyme, Biologix, Merck, Hikma Pharma, Janssen, Inspire Pharma, Future Pharma, and Elixir Pharma; payment or honoraria for lectures, presentations, speakers bureaus, manuscript writing, or educational events from Novartis, Allergan, Merck, Biologix, Janssen, Roche, Sanofi Genzyme, Bayer, Hikma Pharma, Al Andalus, Chemipharm, Lundbeck, Inspire Pharma, Future Pharma and Habib Scientific Office, and Everpharma; support for attending meetings or travel from Novartis, Allergan, Merck, Biologix, Roche, Sanofi Genzyme, Bayer, Hikma Pharma, Chemipharm, Al Andalus Pharmaceuticals, and Clavita Pharmaceuticals; a leadership or fiduciary role in a board, society, committee, or advocacy group, paid or unpaid, as a member of educational, membership, and regional committees of international headache societies; outside the submitted work. C Herteliu reports grants or contracts from the Romanian Ministry of Research Innovation and Digitalization (MCID, project number ID-585-CTR-42-PFE-2021), a grant of the European Commission Horizon 4P-CAN Personalised Cancer Primary Prevention Research through Citizen Participation and Digitally Enabled Social Innovation Project “Societal and Economic Resilience within multi-hazards environment in Romania” funded by EU—NextgenerationEU and Romanian Government, under National Recovery and Resilience Plan for Romania (contract number 760050/23.05.2023, cod PNRR-C9-I8-CF 267/29.11.2022), through the Romanian Ministry of Research, Innovation and Digitalization, within Component 9, Investment I8, and Project “A better understanding of socio-economic systems using quantitative methods from Physics” funded by EU—NextgenerationEU and Romanian Government, under National Recovery and Resilience Plan for Romania (contract number 760034/23.05.2023, cod PNRR-C9-I8-CF 255/29.11.2022), through the Romanian Ministry of Research, Innovation and Digitalization, within Component 9, Investment I8; outside the submitted work. T Joo reports support for the present manuscript from the National Research, Development and Innovation Office in Hungary (RRF-2.3.1-21-2022-00006), Data-Driven Health Division of National Laboratory for Health Security. J Jozwiak reports payment or honoraria for lectures, presentations, speakers bureaus, manuscript writing, or educational events from Novartis, ADAMed, and Amgen, outside the submitted work. J H Kempen reports support for the present manuscript from Sight for Souls and the Mass Eye and Ear Global Surgery Program; participation on a Data Safety Monitoring Board or Advisory Board with Gilead; leadership or fiduciary role in a board, society, committee, or advocacy group, unpaid, on the board of Sight for Souls; stock or stock options with Betaliq and Tarsier; outside the submitted work. K Krishan reports other non-financial support from the UGC Centre of Advanced Study, CAS II, awarded to the Department of Anthropology, Panjab University (Chandigarh, India); outside the submitted work. B Lacey reports support for the present manuscript from UK Biobank, funded largely by the UK Medical Research Council and Wellcome Trust. J Lam reports support for the present manuscript from the National Research, Development and Innovation Fund (project number TKP2021-NVA-11). H J Larson reports grants or contracts from GSK and Moderna; consulting fees from the Gates Medical Research Institute and Apiject; payment or honoraria for lectures, presentations, speakers bureaus, manuscript writing, or educational events as a 2022 Merrimon Lecturer (UNC); outside the submitted work. M Lee reports support for the present manuscript from the Ministry of Education of the Republic of Korea and the National Research Foundation of Korea (NRF-2021R1I1A4A01057428) and Bio-convergence Technology Education Program through the Korea Institute for Advancement Technology (KIAT) funded by the Ministry of Trade, Industry and Energy (P0017805). M-C Li reports grant support from the National Science and Technology Council in Taiwan (NSTC 112-2410-H-003-031); leadership or fiduciary role in a board, society, committee or advocacy group, paid or unpaid, as the Technical Editor of the *Journal of the American Heart Association*. M A Mahmoud reports grants or contracts from Deputyship for Research & Innovation, Ministry of Education in Saudi Arabia for funding this research work through the project number 445-5-762; outside the submitted work. R J Maude reports support for the present manuscript from Wellcome Trust (grant number 220211) because it provides core funding for Mahidol Oxford Tropical Medicine Research. A-F A Mentis reports grants or contracts from MilkSafe: a novel pipeline to enrich formula milk using omics technologies, a research co-financed by the European Regional Development Fund of the EU and Greek national funds through the Operational Program Competitiveness, Entrepreneurship and Innovation, under the call RESEARCH–CREATE–INNOVATE (project code T2EDK-02222), as well as from ELIDEK (Hellenic Foundation for Research and Innovation, MIMS-860), both outside of the present manuscript; payment or expert testimony as a peer reviewer for Fondazione Cariplo, Italy; participation on a Data Safety Monitoring Board or Advisory Board as Editorial Board Member for *Systematic Reviews* and *Annals of Epidemiology*, and as Associate Editor for *Translational Psychiatry*; stock or stock options on a family winery; other financial interests as a scientific officer as part of the BGI Group; outside the submitted work. P B Mentis reports payment or honoraria for lectures, presentations, speakers bureaus, manuscript writing, or educational events from Janssen (Australia) and Sanofi (Hangzhou); participation on a Data Safety Monitoring Board or Advisory Board from Janssen (Australia); outside the submitted work. L Monasta and L Ronfani report support for the present manuscript from the Italian Ministry of Health (Ricerca Corrente 34/2017) via payments made to the Institute for Maternal and Child Health Istituto di Ricovero e Cura a Carattere Scientifico (IRCCS) Burlo Garofolo. R S Moreira reports grants or contracts from the National Council for Scientific and Technological Development CNPq Research Productivity Scholarship (scholarship registration number 316607/2021-5). J Morze reports grants or contracts from SciLifeLab and Wallenberg Data Driven Life Science Program (KAW 2020.0239); consulting fees from ALAB Laboratoria; outside the submitted work. S Muthu reports leadership or fiduciary role in other board, society, committee, or advocacy group, paid or unpaid, on the NEXTGen Committee (ICRS), Grants Committee (SICOT), and with the Knowledge Forum Degenerative Associate as a member (AO Spine); outside the submitted work. S Nomura reports support for the present manuscript from the Ministry of Education, Culture, Sports, Science and Technology of Japan (21H03203) and Precursory Research for Embryonic Science and Technology from the Japan Science and Technology Agency (JPMJPR22R8). A P Okekunle reports support for the present manuscript from the National Research Foundation of Korea funded by the Ministry of Science and ICT (2020H1D3A1A04081265); support for attending meetings or travel from the National Research Foundation of Korea funded by the Ministry of Science and ICT (2020H1D3A1A04081265), outside the submitted work. A Pantea Stoian reports payment or honoraria for lectures, presentations, speakers bureaus, manuscript writing, or educational events from AstraZeneca, Eli Lilly, Merck, Medtronic, Novo Nordisk, Servier, and Sanofi; support for attending meetings or travel from Sanofi, Novo Nordisk, and Eli Lilly; participation on a Data Safety Monitoring Board or Advisory Board with Eli Lilly, Novo Nordisk, and Sanofi; leadership or fiduciary role in other board, society, committee or advocacy group, paid or unpaid, as the Vice-president of the Central European Diabetes Association and as the President of the Romanian National Diabetes Committee; outside the submitted work. R Passera reports participation on a Data Safety Monitoring Board or Advisory Board as the expert biostatistician member for the clinical trial Consolidation with ADCT-402 (loncastuximab tesirine) after immunochemotherapy: a phase II study in BTKi-treated/ineligible Relapse/Refractory Mantle Cell Lymphoma (MCL) patients, sponsor FIL, Fondazione Italiana Linfomi, Alessandria-I; outside the submitted work. M Pigeolet reports grants or contracts from the Belgian Kids' Fund for Pediatric Research; outside the submitted work. A E Peden reports support for the present manuscript from the Australian National Health and Medical Research Council (grant number APP2009306). V C F Pepito reports grants or contracts from Sanofi Consumer Healthcare and International Initiative for Impact Evaluation; outside the submitted work. A Radfar reports support for the present manuscript from Avicenna Medical and Clinical Research Institute. A Rane reports support for the present manuscript from The Bill & Melinda Gates Foundation; stock or stock options as a full-time employee of Agios Pharmaceuticals; outside the submitted work. J Sanabria reports a pending grant award from the NIH, National Cancer Institute (NCI), and DoD; support for attending meetings or travel from Marshall University Medical School; three patents pending; participation on a Data Safety Monitoring Board or Advisory Board as the Chair of quality assessment and assurance for the Marshall University Department of Surgery; leadership or fiduciary role in other board, society, committee, or advocacy group, paid or unpaid with the American Society of Transplant Surgeons, the American Association for the Study of Liver Diseases, International Hepato-Pancreato Biliary Association, the Americas Hepato-Pancreato-Biliary Association, the Society for Surgery of the Alimentary Tract, and the Society of Surgical Oncology; outside the submitted work. N Scarmeas reports grants or contracts from Novo Nordisk; participation on a Data Safety Monitoring Board or Advisory Board with the NIH; outside the submitted work. A Sharifan reports leadership or fiduciary role in other board, society, committee, or advocacy group, unpaid, with Cochrane as a steering member of the Cochrane Early Career Professionals Network; receipt of equipment, materials, drugs, medical writing, gifts, or other services from Elsevier; outside the submitted work. V Sharma acknowledges support from DFSS (MHA)'s research project (DFSS28[1]2019/EMR/6) at Institute of Forensic Science and Criminology, Panjab University (Chandigarh, India); outside the submitted work. V Shivarov reports one patent and one utility model with the Bulgarian Patent Office; stock or stock options from ICONplc (RSUs); and other financial interests from an ICONplc salary; outside the submitted work. J P Silva reports support for the present manuscript from the Portuguese Foundation for Science and Technology. J A Singh reports consulting fees from AstraZeneca, Crealta/Horizon, Medisys, Fidia, PK Med, Two Labs, Adept Field Solutions, Clinical Care Options, Clearview Healthcare Partners, Putnam Associates, Focus Forward, Navigant Consulting, Spherix, MedIQ, Jupiter Life Science, UBM, Trio Health, Medscape, WebMD, and Practice Point Communications; and the NIH and the American College of Rheumatology; payment for lectures, presentations, speakers bureaus, manuscript writing, or educational events as a member of the speakers bureau Simply Speaking; support for attending meetings or travel as a past steering committee member of OMERACT; participation on a Data Safety Monitoring Board or Advisory Board for the FDA Arthritis Advisory Committee; leadership or fiduciary role in a board, society, committee, or advocacy group, paid or unpaid, as a past steering committee member of the OMERACT, an international organisation that develops measures for clinical trials and receives arm's length funding from 12 pharmaceutical companies, as Co-Chair of the Veterans Affairs Rheumatology Field Advisory Committee, and as Editor and Director of the UAB Cochrane Musculoskeletal Group Satellite Center on Network Meta-analysis; stock or stock options in Atai Life Sciences, Kintara Therapeutics, Intelligent Biosolutions, Acumen Pharmaceutical, TPT Global Tech, Vaxart Pharmaceuticals, Atyu Biopharma, Adaptimmune Therapeutics, GeoVax Labs, Pieris Pharmaceuticals, Enzolytics, Seres Therapeutics, Tonix Pharmaceuticals Holding, and Charlotte's Web Holdings, and previously owned stock options in Amarin, Viking, and Moderna Pharmaceuticals; outside the submitted work. M V Titova reports support for the present manuscript from the state assignment of the Ministry of Science and Higher Education of the Russian Federation (theme number 121050500047-5). H Zhang reports grants or contracts from World Health Organization (WHO) funding; outside the submitted work. M Zielinska reports other financial support as an AstraZeneca employee; outside the submitted work. All other authors declare no competing interests.
