# Supplementary material for: Global fertility in 204 countries and territories, 1950–2021, with forecasts to 2100: a comprehensive demographic analysis for the Global Burden of Disease Study 2021
Source: Lancet. 2024 May 18;403(10440):2057–99. doi: 10.1016/S0140-6736(24)00550-6 (PMC11122687; doi:10.1016/S0140-6736(24)00550-6)
Supplement: Supplementary appendix 1 [file mmc1.pdf]

THE LANCET

Supplementary appendix 1

This appendix formed part of the original submission and has been peer reviewed. We post it as supplied by the authors.

Supplement to: GBD 2021 Fertility and Forecasting Collaborators. Global fertility in 204 countries and territories, 1950–2021, with forecasts to 2100: a comprehensive demographic analysis for the Global Burden of Disease Study 2021. *Lancet* 2024; **403:** 2057–99.

Appendix 1: methods appendix to "Global fertility in 204 countries and territories, 1950–2021 with forecasts to 2100: a comprehensive demographic analysis for the Global Burden of Disease Study 2021"

This appendix provides further methodological detail for "Global fertility in 204 countries and territories, 1950–2021 with forecasts to 2100: a comprehensive demographic analysis for the Global Burden of Disease Study 2021."

Preamble

This appendix provides further methodological detail for "Global fertility in 204 countries and territories, 1950–2021 with forecasts to 2100: a comprehensive demographic analysis for the Global Burden of Disease Study 2021." This study complies with the Guidelines for Accurate and Transparent Health Estimates Reporting (GATHER) recommendations.[1] It includes detailed tables and information on data in an effort to maximise transparency in our estimation processes and provide a comprehensive description of analytical steps. We intend this appendix to be a living document, to be updated with each iteration of the Global Burden of Disease Study.

Portions of this appendix have been reproduced or adapted from appendices for Wang et al.[2], Foreman et al.[3], and Vollset et al.[4] References are provided for reproduced or adapted sections.

Table of contents

List of appendix figures and tables 5
Appendix figures 5
Appendix tables 5
Section 1: GBD overview 6
Section 1.1: Study 2021 6
Section 1.2: Geographical locations of the analysis 6
Section 1.3: Time period of the analysis 6
Section 1.4: Statement of GATHER compliance 6
Section 1.5: List of abbreviations 6
Section 2: Past and current fertility estimates overview 7
Section 2.1: Data sources 7
Section 2.1.1: Fertility data source types 7
Section 2.1.2: Fertility data identification and synthesis 8
Section 2.2: Modelling strategy 8
Section 2.2.1: Age-specific fertility rate estimation for 15 to 49 years 8
Section 2.2.2: Data source adjustment 10
Section 2.2.3: Hyperparameter selection 10
Section 2.2.4: SBH methods 12
Section 2.2.5: Age-specific fertility rate estimation for 10- to 14-year-olds and 50- to 54-year-olds 12
Section 2.2.6: Fertility metrics 13
Section 2.3: Sex ratio at birth 13
Section 2.3.1: Overview 13
Section 2.3.2: Modelling approach 13
Section 3: Forecasting fertility methods 13
Section 3.1: Covariate forecasts 14
Section 3.1.1: Contraceptive met need 14
Section 3.1.2: Educational attainment 14
Section 3.1.3: Under-5 mortality 15
Section 3.1.4: Population in habitable areas 15
Section 3.2: CCF50 forecasts 15
Section 3.3: Forecasting age patterns of fertility 16
Section 3.4: Alternative scenarios based on met need for contraception, educational attainment, and pro-natal policies 17
Section 3.4.1: Met need sustainable development goal scenario 17
Section 3.4.2: Educational attainment sustainable development goal scenario 17
Section 3.4.3: Pro-natal policy scenario 18
Section 3.4.4: Combined scenario 19

Section 3.5: Uncertainty interval evaluation 19
Section 3.6: Comparing our forecasts to other models 19
Section 3.7: A tutorial to Lexis Diagram heatmaps – country results annotated 20
Section 3.8: Illustrating fertility alternative scenarios with examples of their country specific impacts 20
Section 4: References 22
Section 5: Tables and figures 24
Section 6: Author contributions 6

List of appendix figures and tables

See section 5 for all appendix figures and tables listed here.

Appendix figures

Appendix Figure S1: MR-BRT model fit for the two-covariate CCF50 model with spline on education covariate with respect to quantiles (0.1, 0.25, 0.5, 0.75, 0.99) of the proportion of met need for contraception covariate

Appendix Figure S2: Map of 2021 World Bank income groups

Appendix Figure S3: Age-specific fertility rate skill metric comparison for IHME and WPP models

Appendix tables

Appendix Table S1. GBD location hierarchy with levels

Appendix Table S2. GATHER checklist

Appendix Table S3: Number of sources used for the analysis of age-specific fertility (VR, CBH-survey, SBH-survey, SBH-census) for each location

Appendix Table S4: Number of sources used for the analysis of age-specific fertility by year (VR, CBH-survey, SBH-survey, SBH-census)

Appendix Table S5: Livebirths share by GBD super region, 1950, 1980, 2021, 2050, 2100

Appendix Table S6: Livebirths share by World Back income group, 1950, 1980, 2021, 2050, 2100

Section 1: GBD overview

Section 1.1: Study 2021

The Global Burden of Diseases, Injuries, and Risk Factors Study (GBD) is a collaborative research effort aimed at estimating worldwide population, fertility, morbidity, and mortality. The GBD collaborator network draws on the expertise of over 10 000 contributors from around the world. For this paper, we estimated fertility by age and location from 1950 to 2100.

Section 1.2: Geographical locations of the analysis

We produced estimates for 204 countries and territories that were grouped into 21 regions and seven super-regions. A list of locations can be found in appendix 1 table S1 (section 5).

Section 1.3: Time period of the analysis

We estimated age-specific fertility rates (ASFRs), total fertility rates (TFRs), and live births for the years 1950-2100. Additionally, we estimated cumulative cohort fertility by age 50 (CCF50), defined as the average number of children born to an individual female from an observed birth cohort (indexed by year of birth) if she lived to the end of her modelled reproductive lifespan (from age 15 to 49) out to the 2085 birth cohort. We forecast CCF50 to the 2085 birth cohort to be able to report ASFR, TFR and live births for the year 2100.

Section 1.4: Statement of GATHER compliance

This study complies with the Guidelines for Accurate and Transparent Health Estimates Reporting (GATHER)[1] recommendations. We have documented the steps involved in our analytical procedures and detailed the data sources used. See appendix 1 table S2 (section 5) for the GATHER checklist. The GATHER recommendations can be found here: http://gather-statement.org/

Section 1.5: List of abbreviations

Abbreviation	Full phrase
ASFR	Age-specific fertility rate
ARIMA	Autoregressive integrated moving average
AROC	Annualised rates of change
CBH	Complete birth histories
CCF50	Cumulative cohort fertility by age 50
CEB	Children ever born
DYB	UN Demographic Yearbook
GBD	Global Burden of Disease Study
GHDx	Global Health Data Exchange
HFC	Human Fertility Collection
HFD	Human Fertility Database
MPIDR	Max Planck Institute for Demographic Research

MR-BRT	Meta-regression—Bayesian, regularised, trimmed
RMSE	Root mean-squared error
SBH	Summary birth histories
SRB	Sex ratio at birth
TFR	Total fertility rate
TFO30	Total fertility over age 30
TFU25	Total fertility under-age 25
UNPD	Population Division of the Department of Economic and Social Affairs of the United Nations Secretariat
UNSD	UN Statistical Division
VR	Vital registration
WPP	United Nations World Population Prospects

Section 2: Past and current fertility estimates overview

Prior to GBD 2016, we used United Nations World Population Prospects (WPP) fertility estimates for all countries and territories.[5] Starting in GBD 2016, we estimated TFRs on based on a systematic synthesis of available data for all GBD 2016 locations and then used the age-specific fertility pattern from WPP.[6] By GBD 2017, we were estimating age-specific fertility rates for ages 10–54 years based on a systematic synthesis of all available data for all GBD locations and calculating TFR as a function of the age-specific fertility rates.[7]

Section 2.1: Data sources

Fertility estimates are based on three main types of data sources: (1) the number of livebirths reported in vital registration (VR) systems; (2) complete birth histories (CBH); and (3) summary birth histories (SBH). We compiled a total of 37,286 unique location-source-years of data for women aged 10 to 54 for the period between 1950 and 2021. Appendix 1 tables S3 and S4 provide the number of data sources by location and year.

Section 2.1.1: Fertility data source types

We sought to use accurate and complete accounts of livebirths reported by age of mother. Complete livebirth registration reports are designed to account for all births in a single country, territory, or subnational location in a single year, which makes them the gold standard for fertility data. Most high-income countries and territories have high-quality VR systems with information on dates and locations for all births as well as demographic characteristics of each mother. Many lower-income countries and territories, however, had birth registries with incomplete data coverage or interrupted and/or delayed reporting. In these locations, we utilised birth history information from household surveys conducted among women aged 15 to 49 at the time of the survey but had to use birth registries for females aged 10 to 14 and 50 to 54 since most household surveys do not collect birth histories from those particular age groups.

For triangulating the level and age-pattern of fertility, we had to rely on other types of data sources, primarily household surveys and censuses, where birth registration data quality and completeness were low. Household

surveys and censuses had two primary types of fertility information: CBHs and SBHs. See section 2.2.4 for more information about CBHs and SBHs.

Section 2.1.2: Fertility data identification and synthesis

We used VR data from the UN Demographic Yearbook (DYB) published by the UN Statistical Division (UNSD),[8] the Human Fertility Database (HFD) and Human Fertility Collection (HFC) from the Max Planck Institute for Demographic Research (MPIDR)[9] the WHO mortality database[10], official publications, online data portals of national statistical offices, and international collaborators. The HFD/HFC and DYB are both compilations of registry-based fertility data from national statistical offices and research institutes. We obtained DYB data on live births by age of mother for every year available from 1950 to 2021. We obtained the complete set of age-specific HFD and HFC empirical data up to 2021 but excluded country-year-ages already accounted for by the DYB and prioritised HFD over HFC. In addition, we obtained data from SRSs where available, primarily in South Asian countries such as India, Pakistan, and Bangladesh. In total, we had 8046 unique country-source-years of VR data, with 2577 from before 1970 and 1864 from after 2000. We also had 32 country-source-years of data from SRS.

We identified fertility data from censuses and household surveys using the Global Health Data Exchange (GHDx) by searching for "complete birth history," "summary birth history," "fertility," and "live births" keywords among records categorised as "survey" and "census." Research team members reviewed these data to verify whether they included the necessary information for GBD analysis. We then conducted additional research to identify and fill gaps in data, primarily through country statistical office websites and surveys such as DHS, MICS, WFS, and RHS. In-country collaborators also assisted in acquiring data that were not publicly available. For low-income locations (especially in sub-Saharan Africa), we sought out colonial censuses from the 1950s and 1960s with SBH data. For survey sources that included microdata, we computed period ASFR every three years over a 15-year recall using CBH data and calculated the average number of children ever born (CEB) for each year of mother's age, which would later be split by cohort age patterns from the first modelling stage (see section 2.2.4), using SBH data. For sources that did not contain microdata, we extracted period ASFR or average CEB by mother's age from reports or other publications. In total, we used 439 CBH and 628 SBH sources from surveys and 349 censuses. We were occasionally unable to identify whether a survey that had tabulated period ASFRs was a CBH or SBH survey, but these data only accounted for 156 country-source-years from 45 sources. We have provided details on the nature and quantity of identified sources in appendix 1 tables S3 and S4. We then estimated fertility rates for the 10–14-year age group as a function of estimated fertility in the 15–19-year age group and for the 50–54-year age group as a function of the estimated fertility in the 45–49-year group. We provide more information about the age-specific fertility estimation process below. After estimating ASFRs, we computed summary measures of fertility including TFR, total fertility under age 25 (TFU25), and total fertility over age 30 (TFO30).

Section 2.2: Modelling strategy

Section 2.2.1: Age-specific fertility rate estimation for 15 to 49 years

As stated above, we used ST-GPR to estimate ASFR for age groups 15–19, 20–24, 25–29, 30–34, 35–39, 40–44, and 45–49. The methods for this process have been described in full elsewhere,[2,7] but in short, we did the following:

(1) estimated ASFR for the 20–24 age group using age-specific data from CBH and VR sources, using mean years of education in the corresponding age group as a covariate; (2) estimated ASFR for the other age groups using age-specific data from CBH and VR sources as well as age-specific mean years of education and the 20–24 age group ASFR as covariates; (3) split SBH and other total births data by age and period using estimated location, time, and ASFR for each age group; (4) re-estimated ASFR for the 20–24 age group using CBH, VR, and period-age-split SBH data; and (5) re-estimated ASFR for the other age groups using CBH, VR, and the period-age-split SBH data.

We implemented the ST-GPR models for ASFR as explained below. The first stage of our mixed effect regression was fit in bounded logit space:

$$Logit\left(\frac{ASFR\ data - lower\ bound_{age}}{upper\ bound_{age} - lower\ bound_{age}}\right)$$

We set the lower bound as the minimum fertility by age across time and location and the upper bound, after dropping implausibly high ASFRs over 0.5, as the 99.3 percentile of fertility by age across time and location. The upper bound set an implied maximum TFR of 9.35.

We used the following formula for our mixed effects regression:

$$logit_{bound}(ASFR_{20-24})_{c,t,s,i} = \beta_0 + \beta_1 * female\ education_{c,t} + \gamma_{cs} + \varepsilon_{c,t,s,i}$$

$$logit_{bound}(ASFR_{n-n+4})_{c,t,s,i} = \beta_0 + \beta_1 * fem\ edu_{c,t} + spline(ASFR_{20-24,c,t}) + \gamma_{cs} + \varepsilon_{c,t,s,i}$$

$$\gamma_{cs} \sim N(0, \sigma^2_{\gamma_{cs}})$$

$$\varepsilon_{c,t,s,i} \sim N(0, \sigma^2_{\varepsilon})$$

Where

c is location, t is time, s is source of datapoint i

n is between 15 and 45

β_0 is the intercept

β_1 is the coefficient on female education

γ_{cs} is a location-source random intercept

ε is the residual

Female education and the 20-24 age group ASFR estimates were specific to each country or territory and year.

We only used female education as a covariate in high-income locations for the 20-24 age group, not for the other age groups. We fit separate models for high-income, sub-Saharan Africa, central Europe, eastern Europe, and central Asia to factor in the differences in the relationships between 20-24 age group ASFR and the ASFR of other age groups. We selected the knots in the linear spline (in logit space) by super-region and age group, as outlined in Table A below.

Table A. Knots on ASFR 20–24

Region	Age	Knot
Central Europe, eastern Europe, and central Asia	15	NA
Central Europe, eastern Europe, and central Asia	25	-1.5
Central Europe, eastern Europe, and central Asia	30	-2
Central Europe, eastern Europe, and central Asia	35	-1.75
Central Europe, eastern Europe, and central Asia	40	-1.75
Central Europe, eastern Europe, and central Asia	45	-2
High-income	15	NA
High-income	25	NA
High-income	30	-2.25
High-income	35	-2
High-income	40	-2.25
High-income	45	-2.25
Others	15	NA
Others	25	-1.5
Others	30	-1.3
Others	35	-1.3
Others	40	-2
Others	45	-2.5
Sub-Saharan Africa	15	NA
Sub-Saharan Africa	25	-1.75

Sub-Saharan Africa	30	-1.25
Sub-Saharan Africa	35	-1.3
Sub-Saharan Africa	40	-1.5
Sub-Saharan Africa	45	-1.75

We outliered data that reported improbably high ASFR (ie, ASFR over 0.5); had 0 values as a result of sampling error, particularly in the 45-to-49-year age group; reflected an undercounting of births, when we could not adjust the data using other sources; or reported implausibly high fertility levels or trends compared to complete VR data or other more reliable sources.

Section 2.2.2: Data source adjustment

After running the mixed effects model, we adjusted data to a reference source using the random intercept on the concatenation of location and source. To get the adjustment factor, we did the following using the equation below: (1) calculated the difference between the fixed and random effects of the reference source, (2) calculated the difference between the fixed and random effects of the datapoint for the specific source, and (3) added the two differences together. We then added the adjustment factor to the data to get an adjusted value.

$$Adjustment\ Factor = (Location\ Source\ RE_{ref} - Location\ Source\ RE_{datapoint})$$

where RE represents a random intercept of either a reference source or a datapoint-specific location-source.

When we had more than one reference source for a single location, we averaged the values of the location source random effects from all the reference sources and used that for the $Location\ Source\ RE_{ref}$ part of the equation.

We primarily chose reference sources which met one of the following criteria: (1) complete VR for locations with complete VR, (2) an average of complete birth history sources for locations with one or more complete birth histories, and (3) an average of all the sources for each location for locations without complete VR or complete birth histories. We considered VR to be complete if the median completeness of child death registration in the location over all available years was over 95% according to the previous round of GBD.[2] We also chose reference sources for some locations using expert judgement. For example, the 1950s and 1960s censuses in sub-Saharan Africa are widely viewed as an accurate reflection of depressed fertility in the region at that time, so the censuses were used as reference sources.

Section 2.2.3: Hyperparameter selection

We used the outputs of the previous processes to implement residual smoothing and GPR. We chose hyperparameters for these steps based on a location- and age-specific data density score. We calculated data density scores based on the years for which VR sources were available plus the number of unique CBH and SBH sources available for the given location, using the following computational methods for each type of data source:

1. **Complete VR sources:** calculated as the number of years for which VR data were available. If the number of births in the age group was below 100, this part of the score was down-weighted by the difference between the number of births and 100.
2. **Incomplete VR sources:** calculated the same way as with complete VR data, but down-weighted by 0.5.
3. **Total CBH sources:** the number of unique complete birth histories for a single location.
4. **Total SBH sources:** the number of unique summary birth histories for a single location.

We calculated the data density score using the following equation:

$$DD\ Score_{loc,age} = Complete\ VR\ years_{loc,age} + (2 * Number\ CBH\ Sources_{loc,age}) + (0.25 * Number\ SBH\ sources_{loc,age}) + (0.5 * Incomplete\ VR\ years_{loc,age}) + Number\ Other\ Sources_{loc,age}$$

Where

DD is the data density

CBH is complete birth history

SBH is summary birth history

In this round of GBD, we updated our time weights to use a beta density function. We assigned hyperparameters α and β for the beta density function, generally based on final data densities, as shown in Table B. However, there were exceptions where we manually assigned a different set of hyperparameters.

Table B. Hyperparameter values by data density

Data density	Alpha	Beta	Zeta	Scale
Over 50	500	500	0·99	5
Between 30 and 50	100	100	0·9	10
Between 20 and 29	20	20	0·8	15
Between 10 and 19	15	15	0·7	15
Under 10	10	10	0·6	15

Where the time weights were calculated as:

$$w_t = \frac{\frac{\Gamma(\alpha+\beta)}{\Gamma(\alpha)\Gamma(\beta)} x^{\alpha-1}(1-x)^{\beta-1}}{\frac{\Gamma(\alpha+\beta)}{\Gamma(\alpha)\Gamma(\beta)} 0.5^{\alpha-1}(1-0.5)^{\beta-1}} = \frac{x^{\alpha-1}(1-x)^{\beta-1}}{0.5^{\alpha+\beta-2}}$$

And:

$$x = \frac{(t+72)}{144}$$

In cases of incomplete VR sources, we defined data variance as the difference between the spatiotemporal prediction and the unadjusted data. Some location-ages had very little data, resulting in implausible variance. As such, for location-ages with fewer than five datapoints, we used the maximum data variance in the location's GBD region.

For complete VR sources, we assumed that non-sampling variance was 0. We calculated sampling variance for these sources using the following binomial equation:

$$Sampling\ Variance = \frac{ASFR * (1-ASFR)}{Births}$$

We then calculated amplitude and applied it to all locations other than high-income VR-only national locations with 40+ years of VR data. For these locations, we took the mean of the location-specific variance of the difference between the data and the spatiotemporal smoothing. However, we only included national locations from 1990 to 2021.

The adjusted data, sampling variance, amplitude and smoothed estimates based on spatiotemporal smoothing are input to GPR to generate 1000 draws of each location-time-year-age group. GPR is our implementation of the PyMC python package. The smoothed estimates are used as the mean function, and we use a Matern covariance function made up of three parameters; the amplitude (described above and calculated based on the available data), the scale (values displayed in Table B and also determined by the availability of data) and the degree of

differentiability (which is currently set to 1). A posterior is calculated based on the mean and variance of the observations and the prior and draws are taken from this posterior to get our final estimates with uncertainty.[2]

Section 2.2.4: SBH methods

For the earlier years of the study period, CEB data were more readily available from SBHs than from CBHs. While there are numerous methods for calculating period- and age-specific fertility from SBH information, the Brass Parity/Fertility ratio method is used most often. However, this method assumes a constant ASFR over time. We wanted a dynamic measure of cohort age patterns over time, so we instead used ASFR estimates from CBH and VR data from the first run-through of ST-GPR described above to split SBH into period ASFR. We used these estimates to compute implied annualised fertility for all the five-year birth cohorts represented in a given SBH from age 10 through whichever came sooner: age 54 or the year of the survey. To account for births that occurred in years when part of the initial age group had moved into the next age group, we calculated the weighted average of estimated ASFRs in the age groups on either side of the selected age group and took that average as the fertility experienced by that hypothetical cohort in that year, assuming a uniform age distribution within the age group. For example, for a cohort of women aged 20 to 24 in 1984 with ASFR F, we would compute the ASFR experienced by this cohort as women aged 21 to 25 in 1985 as:

$$.8 *_5 F_{20}^{1984} + .2 *_5 F_{25}^{1989}$$

since 20% of the cohort had aged into the 25 to 29 age group by the following year.

We then used the implied annualised cohort ASFR to calculate cumulative cohort fertility up to the age of each cohort at the time of the survey. We compared implied cumulative fertility to observed cumulative fertility (average CEB from SBHs) by each cohort to get a scaling factor. We then applied this scaling factor to the original implied cohort age pattern, which distributed CEB back across time and age. This method only covered birth cohorts between 1940 (who began to experience ASFR 10-14 in 1950, the first year of our study period) and 2009 (who began to experience ASFR 10-14 in 2019, at the end of our study period).

Splitting of total birth and historic location aggregate data

A large portion of the data could only be obtained already aggregated by age and/or location (eg, total live births instead of births by mother's age; former USSR prior to its dissolution). For these data, we split just the CBH and VR data using the age and location proportions designated in the first ST-GPR run-through. After splitting the data, we reran the estimation process describe above using all CBH, VR, and period and age-split data as well as location and age-split miscellany. This improved the availability of past data and gave us more information about aggregate levels of fertility over time.

Section 2.2.5: Age-specific fertility rate estimation for 10- to 14-year-olds and 50- to 54-year-olds

We estimated ASFR for the 10 to 14 and 50 to 54 year age groups separately because data for these age groups in locations without VR systems were scarce. We used the relationship between ASFR in sequential age groups to estimate both age groups. For the 10 to 14 age group, we ran a mixed effects regression on the log of the ratio of ASFR 10-14 over ASFR 15-19, and used ASFR 15-19 and nested random intercepts by super-region, region, and location as predictors, as defined by the equation below:

$$\log\left(\frac{ASFR\ 10-14}{ASFR\ 15-19}\right) = \beta_0 + \beta_1 \log(\text{ASFR } 15-19) + \gamma_{k[j]} + \gamma_{jk[i]} + \gamma_{ijk}$$

$$\gamma_{k[j]} \sim N\left(0, \sigma^2_{\gamma_{k[j]}}\right)$$

$$\gamma_{jk[i]} \sim N\left(0, \sigma^2_{\gamma_{jk[i]}}\right)$$

$$\gamma_{ijk} \sim N(0, \sigma^2_{\gamma_{ijk}})$$

Where

i is location, j is region, k is super-region

β_i is a fixed covariate coefficient

$\gamma_{k[j]}$, $\gamma_{jk[i]}$, and γ_{ijk} are nested super-region, region, and location random intercepts

We then calculate draws based on uncertainty in the random effects and variance-covariance matrix of the fixed effects. 1000 draws of the fixed covariate are taken from the mean estimate of the coefficient and the variance-covariance matrix assuming a normal distribution. Similarly, we assume the random effects follow a normal distribution and are all independent and identically distributed. 1000 draws for the random effects are taken from a normal distribution of the mean random effect and the standard error. These draws are combined to make final predictions at the draw level and summarised.

For ASFR in the 50 to 54 age group, we instead estimated a regression on the log ratio of ASFR 50–54 over ASFR 45–49 with a constant. We did this because there was not a clear relationship between this ratio and ASFR 45–49. We produced 1000 draws from the variance-covariance matrix to generate uncertainty,

Section 2.2.6: Fertility metrics

We calculated TFR as the time-weighted sum of the ASFRs from each age group. To do this, we added ASFR for each five-year age group and multiplied that by the five years spent in each age group. We calculated TFU25 and TFO30 in the same way, but with only the relevant age groups included. We defined livebirths as the sum of ASFR from all age groups multiplied by the 10- to 54-year-old female population from the population model described previously.[2]

Section 2.3: Sex ratio at birth

Section 2.3.1: Overview

Another component of population structures and reproductive capacity is the sex ratio at birth (SRB). For the GBD analysis, we defined SRB as the ratio of total male to total female livebirths in each location in a given calendar year. The naturally occurring SRB generally hovers around 1.05 males per female, with some location-specific variation.[11] Since the introduction of ultrasound technologies and the ability to conduct sex-selective abortions, previously stable SRBs have shifted in some locations as a result of systematic sex preferences for children.[11] SRBs in recent years are particularly skewed in the Caucasus, south Asia, and east Asia. To reflect historic equilibria and recent shifts in SRB, we developed a model to estimate SRB in all GBD countries and territories, Hong Kong, and Macau from 1950 to 2021.

Section 2.3.2: Modelling approach

For GBD 2021, we updated our sex ratio at birth estimates. We implemented ST-GPR to estimate a complete time series of the sex ratio at birth for all GBD locations and years. The main differences from previous GBD rounds are additional VR and CBH data, updated outliering decisions following thorough model review, and the use of higher values of the beta hyperparameter for locations with more data. The beta hyperparameter change lowers the smoothness of the model fit, allowing the model to follow the data more closely. See the fertility section for more details on the methodology.

Section 3: Forecasting fertility methods

We produced forecasts of fertility using an updated modelling framework which modified our previously published methods in Vollset et al.[4] We continued to forecast CCF50 rather than directly modelling TFR. However, we did make a few changes to our modelling framework including no longer completing incomplete birth cohorts but using complete observed past cohort data only, adding two new covariates (under-5 mortality and population in habitable areas), as well as an additional alternative scenario regarding pro-natal policies.

Briefly, we forecast CCF50 out to the 2085 cohort, followed by unpacking it into ASFRs. CCF50 was defined as the average number of children born to an individual female from an observed birth cohort (indexed by year of birth) if she lived to the end of her modelled reproductive lifespan (from age 15 to 49). The observed past CCF50 estimates for birth cohorts from 1945 to 1972 were used to forecast CCF50 for birth cohorts from 1973 to 2085. We only forecast CCF50 to 2085 (not 2100) because it is a cohort not period measure. In other words, to report ASFR and TFR (period measures) at 2100 we need to account for all women who will have entered their reproductive years by then (2100-15 = 2085). We utilised not only estimates of female education and proportion of met need for

contraception as covariates in the CCF50 sub-models, but also estimates of under-5 mortality and population in habitable areas. We forecast CCF50 using three sub-models (with 2, 3 and 4 covariates) to generate an ensemble model forecast where all three sub-models were equally weighted (appendix 1 section 3.2).From forecasted CCF50, we then derived ASFR forecasts from the year 2022 to 2100 using a combination of a linear mixed effects model, spline interpolation, and Autoregressive integrated moving average (ARIMA) (1,0,0) on residuals to estimate the age pattern of fertility for each cohort (appendix 1 section 3.3). Once the 15-49 ASFR values were obtained, we inferred the 10-14 and 50-54 ASFR values based on their ratios to the rest of the age pattern during the last observed year (2021).

Section 3.1: Covariate forecasts

We used four covariates in our forecasting model including, contraceptive met need, educational attainment, under-5 mortality, and population in habitable areas. As we needed to complete the reproductive years of the youngest birth cohort by 2100 (those born in 2085) to report ASFR and TFR at 2100, all covariates needed to be forecast through 2134 (when those born in 2085 turn 49 years old). We present visuals of each covariate through 2135 in appendix 2 figure S2.

Section 3.1.1: Contraceptive met need

We forecasted contraceptive met need by age and locations using an ensemble model. Met need was defined as the proportion of women—from among those aged 25–29 who are fertile and sexually active, who report not wanting children or more children or wanting to delay having a child—who are using or whose sexual partner is using a method of modern contraception. The ensemble model was comprised of six annualised rate of change (ARC) sub-models with varying recency-weighting parameters (the higher the weight, the more weight given to recent years).

We calculated the age-standardised and location-specific annual change of the logit-transformed met need values. To account for the effect of noisy data, we replaced annual changes outside the 2.5th and 97.5th percentiles with those corresponding percentile-values. The weight of each sub-model was determined by running out-of-sample predictive validity, training each sub-model on data from 1990-2009 and validated based on 2010-2019 GBD estimates. We measured each child model's performance using root mean-squared error (RMSE) based on which we determined sampling weights of each child model. Then we produced the sub-model forecasts based on the 1990-2019 training dataset with 500 draws for each sub-model and sampled the draws according to the RMSE in the training dataset to obtain the final ensemble forecasts.

Section 3.1.2: Educational attainment

Educational attainment was forecasted using the methodology described in Foreman et al, 2018[3] with an added assumption that educational attainment (up to a maximum of 18 years of education) does not change after age 25 as in Vollset et al, 2020.[4] After age 25, we held forecasted education constant within each location- and sex-specific birth cohort (all individuals born in a certain year). This prevented implausible within-cohort changes in education during older age and was more congruent with our cohort-specific modelling approach for fertility forecasting (section 5), for which education was a key input.

Briefly, for age groups with a starting interval of 25 years or below, we computed age-, sex-, and location-specific annualised rates of change (AROCs) by a recency-weighted average of annual differences in logit space after scaling mean years of education (based on GBD 2019 estimates[2]) by 18 years. The recency-weighting parameters were chosen using cross-validation, where to reduce the potential for overfitting, we selected the parameter producing the smallest root-mean square error at least 5% greater than the minimum. These AROCs were applied to GBD 2019 draws to produce forecast draws, denoted $\widehat{EDU}_{lastd}$, of mean years of education for location l, age $a \leq 25$, sex s, future years $t = 2020, \ldots, 2100$, and draw d. For age groups with interval starts $a > 25$, the forecasted value was set to the previous value on the cohort trajectory, which is lagged in time by the age-group interval (5 years) due to the relationship

$$cohort\ birth = time - age.$$

Specifically, for age groups indexed by the interval start $a = 30, 35, \ldots, 95$ this is given by

$$\widehat{EDU}_{lastd} = \begin{cases} EDU_{l(a-5)s(t-5)d}, & t \leq 2019 + 5 \\ \widehat{EDU}_{l(a-5)s(t-5)d}, & t > 2019 + 5 \end{cases}$$

where $EDU_{l(a-5)s(t-5)d}$ and $\widehat{EDU}_{l(a-5)s(t-5)d}$ denote draws of past GBD and future forecasts, respectively.

Section 3.1.3: Under-5 mortality

Forecasts for 2017 - 2100 under-5 mortality rates were derived from mortality forecasts published in Vollset et al, 2020.[4] These values were only available for 195 of the 204 locations modelled, so the remaining 9 were filled in with the under-5 mortality rates of their corresponding regions. Additionally, subnational values were available until 2017 but not for any future years. We calculated the sex specific, subnational to national ratios in 2017 and applied these ratios to future national values to get the subnational estimates, assuming that the ratio remains constant after 2017.

Values for 2100 – 2125 were forecasted on a sex and location specific level using AROCs in logit space. These AROCs were weighted using different recency parameters and selected using an out-of-sample RMSE. The selected annual rates of change were applied to each year after 2100, then converted back to normal space to get the values for the final 25 years.

Section 3.1.4: Population in habitable areas

We defined this covariate as population divided by habitable area,

$$\log_{10} \frac{population}{habitable\ area}$$

We used an updated population reference forecast based on GBD 2019 past estimates. Details on our population forecasting methods can be found in Vollset et al.[4] In brief, we forecast population to 2100 for all 204 locations, but due to time horizon constraints on inputs to our population forecasting model we could not produce results further into the future. Due to this constraint, we held population in each location constant from 2101-2035. Habitable area was defined as a country's area in km^2 that excluded areas with fewer than ten people per 1 × 1 km and classified as barren or sparse[12]. The same habitable area value was held constant for each location from 1970-2135. Of note, our uninhabitable area did not include desert or high elevation locations.

Section 3.2: CCF50 forecasts

For modelling the single-age group CCF50, we used a combination of four covariates: female education (25-29 years), proportion of met need for contraception (25-29 years), population per habitable area) and under-5 mortality (further described in appendix 1 section 3.1). The relationship between CCF50 and the covariates was modelled with the Meta-Regression Bayesian, Regularized, Trimmed tool (MR-BRT)[13], after transformation to scaled logit space.

The scaled logit space is defined by the logit transformation of CCF50 scaled from (0.7, 10) to (0, 1):

$$logit_{(0.7,10)}(CCF50) = \ln\left(\frac{CCF50 - 0.7}{10 - CCF50}\right)$$

Appendix 1 figure S1 shows an example plot visualising the relationship between CCF50 (after the inverse scaled logit transformation) and two covariates at a time, ie, education and proportion of met need for contraception covariates, in this case.

We used equally weighted three sub-model forecasts for each location and birth cohort to generate the ensemble CCF50 forecasts. The three sub-models are represented by the following equations:

(1) Two covariates sub-model:

$$logit_{(0.7,10)}(CCF50_{lc}) = \beta_0 + spline(education_{lc}) * \boldsymbol{\beta}_1 + met\ need_{lc} * \beta_2 + \varepsilon_{lc}$$

(2) Three covariates sub-model:

$$logit_{(0.7,10)}(CCF50_{lc}) = \beta_0 + spline(education_{lc}) * \boldsymbol{\beta}_1 + met\ need_{lc} * \beta_2 + under{-}5\ mortality_{lc} * \beta_3 + \varepsilon_{lc}$$

(3) Four covariates sub-model:

$$logit_{(0.7,10)}(CCF50_{lc}) = \beta_0 + spline(education_{lc}) * \boldsymbol{\beta}_1 + met\ need_{lc} * \beta_2 + under - 5\ mortality_{lc} * \beta_3 + urbanicity_{lc} * \beta_4 + \varepsilon_{lc}$$

where CCF50 is modelled in scaled logit space for location (l) and cohort (c) , β_0 is an intercept, $\boldsymbol{\beta}_1$ is a vector of the spline coefficients of female education (25-29 years) covariate, β_2 is a slope on proportion of met need for contraception (25-29 years), β_3 is a slope on under-5 mortality, β_4 is a slope on population per habitable area and ε is a residual term.

To capture time-series trends in CCF50 not explained by these covariates, we used a first-order autoregressive model $ARIMA_{(1,0,0)}$ on the scaled logit residual term to produce forecasts on residuals for each sub-model:

$$logit_{(0.7,10)}(\widehat{\varepsilon_{lc}}) = \varphi * logit(\widehat{\varepsilon_{l(c-1)}}) + z_{lc}$$

where $\widehat{\varepsilon_{lc}}$ is the estimated residuals for location (l) and cohort (c), φ is an autoregressive model constant, $\widehat{\varepsilon_{l(c-1)}}$ is the estimated residuals for location (l) and cohort $(c-1)$, z_{xlc} is a white noise term.

Afterwards, we added these forecasted residuals to forecasted draws of CCF50. These draws were based on covariates mean values from a multivariate normal distribution with mean and estimated variance-covariance matrix. To run each of the sub-models we used the following configurations of MR-BRT models:

- Cubic spline with left and right linear tails
- Knots placement chosen based on quantiles: 10th, 36.5^{th}, and 75th of education covariate
- No random effects by location

We equally weighted all three sub-models to generate the ensemble model forecasts of CCF50 for a given location (l) and cohort (c).

Section 3.3: Forecasting age patterns of fertility

The resulting CCF50 forecasts from the previous stage were then "unpacked" to produce forecasts of ASFR (period space). To do so, we forecasted cohort age-specific fertility patterns using a linear mixed effects regression in five-year age intervals with female education (25-29 years) and proportion of met need for contraception (25-29 years) as fixed effects covariates, while accounting for geographical regions as random intercepts. This gave us cohort-ASFR with 5-year age intervals. We then interpolated the five-year age interval cohort-ASFR to a single-year age cohort-ASFR, which was then converted to period space, followed by $ARIMA_{(1,0,0)}$ model on the logit residuals to capture ASFR time-trends not explained by the covariates, similar to the prior stage described in Section 3.2.

In the linear mixed effect regression model, we first stratified our data based on GBD's super-regions as "high-income" and "not high-income". For each of the two super-regions, we modelled $\left(\frac{ASFR_{xlc}}{CCF50_{lc}}\right)$ proportion in logit space:

$$logit\left(\frac{ASFR_{xlc}}{CCF50_{lc}}\right) = \beta_1 * education_{xlc} + \beta_2 * \ met\ need_{xlc} + \tau * region + \varepsilon_{xlc}$$

where ASFR is age-specific fertility rate for age-interval (x), location (l) and cohort (c), CCF50 is complete cohort fertility for location (l) and cohort (c), region is an indicator variable for a region name, and ε_{xlc} is a residual term. Within either super-region, the above equation is solved for every 5-year age group within CCF50.

We then used the estimated proportion $logit\left(\frac{\widehat{ASFR}_{xlc}}{CCF50_{lc}}\right)$ to calculate the values of $\widehat{ASFR}_{xlc}$ in five-year age intervals (after taking the inverse logit (*expit*) transformation of the proportion and multiplying by $\widehat{CCF50}_{lc}$ produced in the previous stage (Section 3.2)):

$$\widehat{ASFR}_{xlc} = expit\left[logit\left(\frac{\widehat{ASFR}_{xlc}}{CCF50_{lc}}\right)\right] * \widehat{CCF50}_{lc}$$

Afterwards, we used the interpolation method "*slinear*" in ***interp1d*** from python's SciPy package "interpolate" to estimate a single-year age group ASFR from a five-year age group ASFR.

We then utilised $ARIMA_{(1,0,0)}$ on logit residual term to capture single-year ASFR trends unexplained by education and contraceptive met need:

$$logit(\widehat{\varepsilon_{xlc}}) = \varphi * logit(\widehat{\varepsilon_{xl(c-1)}}) + z_{xlc}$$

where $\widehat{\varepsilon_{xlc}}$ is the estimated residuals for age (x), location (l) and cohort (c), φ is an autoregressive model constant, $\widehat{\varepsilon_{xl(c-1)}}$ is the estimated residuals for age (x), location (l) and cohort $(c-1)$, z_{xlc} is a white noise term. We then added these forecasted residuals to the future draws of single-year age group ASFR. Afterwards, we inferred the 10-14 and 50-54 single year ASFR values based on their ratios to the rest of the age pattern during the last observed year (2021).

At the end, we aggregated our single-year ASFR to five-year ASFR for each calendar year (t) by taking the mean over the five single-years within each age group. Sum of the single-year ASFR over all ages gave us the TFR for each calendar year. A new cumulative cohort fertility forecast is calculated, this time summing over ages 15-49 for each birth cohort.

Section 3.4: Alternative scenarios based on met need for contraception, educational attainment, and pro-natal policies

In addition to a reference scenario, we produced four alternative scenarios based on hypothetical rates of change to met need of contraception, educational attainment, and pro-natal policies. These alternative scenarios illustrate the potential impacts of shifts in these drivers on fertility rates throughout this century. Changes in contraceptive met need and educational attainment impact our fertility forecasts through their direct effects as covariates in the model, while our pro-natal scenario was applied in within or forecasting framework (more details can be found in appendix 1 sections 3.4.1 – 3.4.3. These scenarios were applied to all locations. In instances where the reference forecast for a given location was more optimistic than the defined alternative scenario, the reference forecast was used.

Section 3.4.1: Met need sustainable development goal scenario

This scenario assumes all locations reach SDG target 3.7.1[14], that by 2030, the proportion of women of reproduction age (15-49 years) have their need for family planning met with modern methods. To implement this scenario, we altered our contraceptive met need covariate, described above in appendix 1 section 3.1.1, according to the methods described in Vollset et al.[4] using 2021 as the last past year. Briefly, for each location modelled, contraceptive met need was taken from the last measured value in 2021 and linearly increased to 100% need met by 2030 and then held constant at 100% through 2135 (appendix 2 figure S2).

Section 3.4.2: Educational attainment sustainable development goal scenario

We based the SDG pace scenario for education on Target 4.1[15], which specifies "by 2030, ensure that all girls and boys complete free, equitable and quality primary and secondary education leading to relevant and effective learning outcomes." Similar to Vollset et al.[4], we defined this scenario as 100% of the population completing at least 12 years of education by age group 20-24. We utilised forecasts of single-year education distributions in 2030 for each sex and location for age group 20-24 years to imply the smallest mean years of education required to meet the SDG Target 4.1 without changing the trajectory of those that would already attain 12 or more years absent this scenario. We let $\hat{p}_{ls}(y)$ denote the forecasted proportion of individuals in country or territory l of sex s that have y years of education in 2030 at age 20-24 years. The most conservative adjustment to this distribution to meet SDG Target 4.1

sets all individuals with <12 years of education to instead have 12 years, accomplished by the following single-year education distribution adjustment:

$$\hat{p}_{ls}^{[SDG]}(y) = \begin{cases} 0, & y = 0, 1, \dots, 11 \\ \sum_{i=0}^{12} \hat{p}_{ls}(i)\ , & y = 12 \\ \hat{p}_{ls}(y)\ , & y = 13, 14, \dots, 18 \end{cases}$$

The mean years of education of this adjusted distribution, $EDU_{ls}^{[SDG]}$, represents the minimum mean required to meet SDG 4.1 in 2030 given the forecasted distribution, and is computed by

$$EDU_{ls}^{[SDG]} = \sum_{i=12}^{18} i \times \hat{p}_{ls}^{[SDG]}(i)$$

For each location and sex, we set the SDG pace by deriving the AROC required to reach $EDU_{ls}^{[SDG]}$ in 2030 for 20–24-year-olds starting from the corresponding value in 2021. We assumed a constant rate of change over the 9-year timespan in logit space (after scaling by 18 years to represent proportion out of maximum educational attainment) and computed the SDG AROC by

$$\delta_{ls}^{[SDG-EDU]} = \frac{\text{logit}\left(EDU_{ls}^{[SDG]}/18\right) - \text{logit}\left(EDU_{l(20-24)s(2021)}/18\right)}{2030 - 2021}$$

where $EDU_{l(20-24)s(2021)}$ is the mean years of education for 20–24-year-olds of sex s at location l in 2021.

Some locations may have high AROCs under the reference scenario for a particular sex and age group. To avoid having the SDG pace scenario slow down progress relative to the reference scenario, the location-, age-, and sex-specific AROCs for the SDG pace were taken to be

$$\Delta_{las}^{[SDG-EDU]} = \max\left(\delta_{ls}^{[SDG-EDU]}, \Delta_{las}^{[REF-EDU]}\right)$$

where $\Delta_{las}^{[REF-EDU]}$ is the education AROC under the reference scenario for location l, age a, and sex s (obtained using methods in Foreman et al, 2018).[3] Additionally, the AROC used to compute the scenario was set to the reference scenario AROC after the year 2030. Estimates are shown in appendix 2 figure S2.

Section 3.4.3: Pro-natal policy scenario

Due to lack of data, we did not define this scenario based on a specific policy or policies that have a known impact on fertility rates. Rather, we considered policies such as paid parental leave, the right to return to work, and subsidised or universal childcare as pro-natal. In other words, policies that have been enacted in countries such as Australia, Sweden, Denmark, Norway, and Finland that are thought of as making it more financially feasible to have children.

The pro-natal scenario parameters were drawn from previously observed increases in TFR that coincided with pro-natal policies and broader empirical evidence regarding effects of pro-natal policies in low-fertility contexts. Australia implemented Maternity Payment (also known as Baby Bonus) coupled with public childcare expansion in July 2004. Since this implementation, TFR increased from 1.78 to 1.97 from 2004 to 2009.[16] Although how much of this increase can be explained with the policy change remains unclear,[17–19] additional empirical evidence suggests that the effect size of family policies on fertility was 0.2 or less child per female.[20] Furthermore, it is worth noting that while the effects of cash incentives for more children often diminish over time, other dimensions of pro-natal policies, such as public childcare and parental leave, tend to have a more sustaining influence.[21] Therefore, in our analysis, we assumed that the impact of the pro-natal package would not diminish over time, aligning with the evidence that certain aspects of such policies can sustain a long-lasting increase in fertility. We note that these pro-

natal policy impact estimates were taken at face value and have not been controlled for possible confounders such as immigration.

In our implementation of the pro-natal scenario, we assumed that once a country's TFR dropped to 1.75 there would be a pro-natal policy in place to encourage fertility (ie, increase TFR). The effect was applied in period space and assumed to require a 5-year linear ramp-up to reach a final TFR increment of 0.2 in the fifth year. This final effect persisted for the remainder of the forecast. For each pro-natal year, the TFR increase was distributed proportionally amongst the single year ages according to their reference forecast ASFR values.

Section 3.4.4: Combined scenario

This scenario combines all scenarios explained in sections 3.4.1-3.4.3. In the combined scenario, we applied the above changes to the covariate forecasts at the same time without assigning any weights since these covariates are already embedded in our model and the coefficients for each covariate are calculated based on the observed data. Appendix 2 figure S4 visualises TFR estimates for all scenarios for each GBD super region, region and country/territory from 1950-2100. The combined scenario looks different across locations. Pakistan is one example where the combined scenario accelerates the decrease in TFR compared to other scenarios, bringing the TFR value to our 1.75 pro-natal scenario threshold by 2035, about 20 years prior to the pro-natal scenario alone. In appendix 2 figure S4 you can see the yellow line, indicating the combined scenario, getting the prenatal TFR bump earlier than the green line, indicating the pro-natal scenario alone. In contrast, Niger's TFR never drops below our 1.75 TFR threshold, so none of the scenario lines in appendix 2 figure S4 have the pro-natal scenario bump in TFR.

Section 3.5: Uncertainty interval evaluation

Uncertainty intervals (UIs) were estimated using the 0.025 and 0.095 quantiles of the 500-draw distribution for each measure of interest. When TFR, ASFR, CCF50 and live birth forecasts were aggregated over geographical locations (eg, global TFR), we performed an ad-hoc adjustment to account for unmodelled spatial correlation. More detailed methods of this ad-hoc adjustment can be found in Vollset et al.[4] UIs were only computed for the reference scenario in the future and were not computed for forecasted alternative scenarios as they are target and policy scenarios rather than probabilistic forecasts.

Section 3.6: Comparing our forecasts to other models

We evaluated our model performance based on out-of-sample predictions and compared against the Population Division of the Department of Economic and Social Affairs of the United Nations Secretariat (UNPD) WPP 2006[22] predictions. Specifically, we used the following skill metric[23] for model evaluation and comparison for validation period 2007–2021:

$$skill = 1 - \frac{RMSE(Model)}{RMSE(Baseline\ Model)}$$

where $Model$ is the ASFR model from IHME and $Baseline\ Model$ is a simplistic model where ASFR of 2007 year was held constant over 2007–2021. For each model, we calculated squared errors between observed and predicted mean values for each age/location/year and winsorized the errors at the 95% level to remove outliers. To calculate RMSE values, we took a square root of an average of the winsorized squared errors across location/year. This skill metric was reported for each five-year age group (appendix 1 figure S3). A positive skill metric indicates that a model being evaluated performs better than the baseline model whereas a negative skill suggests the opposite.

We then repeated the same calculation for UNPD WPP 2006. We had to use WPP 2006 for this purpose since we wanted a long validation period for this analysis. Since we are unable to the WPP 2022[24] model ourselves on their past data, we had to use WPP 2006 in order to meet our validation period requirements of 2007–2021. For a fair comparison, out-of-sample predicted values for our forecasts were based on the GBD fertility model fit using a dataset where data sources from 2007–2021 were excluded, and these were compared to our final GBD 2021 estimates to compute RMSE values. When evaluating skill for WPP 2006 forecasts, we used WPP 2022 estimates as true values to calculate RMSE values.

Our model had a positive skill value across all age groups, while WPP 2006 skill values were negative for age groups 15–19, 35–39, and 40–44. For example, IHME model skill was 0.19 and WPP 2006 skill was -0.11 for the

age group 35–39 (appendix 1 figure S3). The lowest skill values were 0.15 (30–34 age group) for the IHME model and -0.17 (40-44 age group) for the WPP 2006 model. The highest skill values were 0.46 (45–49 age group) and 0.17 (both 25–29 and 45–49 age groups) for the IHME and WPP 2006 models, respectively.

A similar analysis was performed on WPP 2012 forecasts[25], which use probabilistic projection methods[26], yielded similar results for validation period 2011–2021. The skill metric for WPP 2012 was in the range of -0.13–0.08 with negative skill for age groups 15–19, 35–39 and 40–44.

Section 3.7: A tutorial to Lexis Diagram heatmaps – country results annotated

To complement the presentation of TFR values, we created Lexis diagram heatmaps that simultaneously display single-year ASFR, TFR, and CCF50 estimates (appendix 2 figure S3). The horizontal axis of the Lexis diagrams extends from 1950 (the beginning year of data availability) to 2100 (the final year of our forecasts), and the vertical axis indicates mother's age. The colour value in each cell represents ASFR estimated for a given calendar year, ranging from purple and red for higher ASFRs to yellow and green for lower ASFRs. The numbers in black at the bottom of the figure present estimates of CCF50 for each ten-year birth cohort. CCF50 is the sum of ASFR cells on the diagonal (ie, representing birth cohort), whereas TFR is the sum of ASFR cells vertically (ie, ASFR values from the same calendar year by age of mother).

Country specific heatmaps bring insights into each country's fertility patterns. Here, we provide a tutorial to Lexis Diagram heatmaps by presenting fertility estimates in four countries: South Korea, Tajikistan, Australia, and Bangladesh. Broadly, the diagrams show that South Korea and Tajikistan had high fertility rates (TFR >5.5) in the 1950s, but after that period their trajectories diverged with respect to both fertility rates and age patterns reflecting female age when giving birth. The heatmaps for Australia and Bangladesh show that both countries currently have low fertility rates (TFR <2.1), but the former has experienced females delaying births to older ages, while the latter has not.

More specifically, ASFR was high (0.30 to 0.40) in South Korea for females aged 22 to 28 years in 1950 and declined over time to a range of 0.005 to 0.035 for the same ages in 2021 (appendix 2 figure S3). The CCF50 value indicates that females born in 1950 had, on average, 2·64 (95% UI 2·57–2·72) children before they reached the age of 50. Over the period covered by our analysis, CCF50 rates in South Korea were highest in 1950 and are declining over time; we project that females in South Korea born in 2050 will have 0·82 (0·71–0·93) children before they turn 50. The South Korea Lexis diagram also reveals a shift in mothers' age of childbearing from 1990 to 2020; during this period, females in South Korea started to delay childbirth until they were older than females who gave birth from 1950 to 1980. In contrast, the Lexis diagram for Tajikistan (appendix 2 figure S3) shows little change over time in mothers' age at childbearing: most females in Tajikistan continued and will continue to give birth at ages 20–29. ASFR was high (0.30 to 0.40) for females in Tajikistan aged 20–29 years in 1950 and declined over time to a range of 0.17 to 0.28 in 2021. The Lexis diagram shows that in Tajikistan, females born in 1950 had 5·43 (5·30–5·58) children before they reached age 50, and we project that those born in 2050 will have 2·32 (1·96–2·67) children before they turn 50 (appendix 2 figure S3).

In Australia, the Lexis heatmap shows that the highest ASFRs over the study period were observed for females ages 21–28 at 0.20 to 0.26 in 1960, and in 2021, the highest ASFRs are observed for ages 29–34, ranging from 0.10 to 0.12 (appendix 2 figure S3). This demonstrates a shift in Australian female age at giving birth from 21–28 years during the period between 1950 and 2000 to 29–34 years between 2000 and 2021. Females born in Australia in 1950 had 2·35 children (95% UI 2·32–2·37) children before they turned 50, and we project that females born in 2050 will have 1·36 (1·11–1·64) children before age 50. Conversely, the heatmap for Bangladesh does not show a similar shift over time in female age at childbirth; from 1950 to 2021, it was primarily females aged 20–29 who continued to have the highest ASFRs in Bangladesh (appendix 2 figure S3). These age groups had ASFRs of 0.30 to 0.35 in 1950, with rates declining to 0.09 to 0.12 in 2021. Females in Bangladesh born in 1950 had 6·13 (6·03–6·24) children before they reached age 50, and we project that females born in 2050 will have 1·01 (0·62–1·39) children before they turn 50.

Section 3.8: Illustrating fertility alternative scenarios with examples of their country specific impacts

We projected country level future fertility rates based on the reference and four alternative scenarios and present them here for world's ten most populous countries. Based on our forecasts, the reference scenario TFR in Nigeria stays above 1·75 (our threshold for pro-natal scenario implementation) throughout our forecast and will be 2·69 (95% UI 2·06–3·31) and 1·87 (1·19–2·54) in 2050 and 2100, respectively. Therefore, we do not anticipate

differences between reference and pro-natal policy scenario forecasts in Nigeria. However, we project faster declines of TFR in the education, met need, and combined alternative scenarios; for example, by 2050, TFR in Nigeria is forecasted to be 2·54 (1·88–3·17) for the education scenario, 2·25 (1·63–2·82) for the met need scenario, and 2·16 (1·53–2·76) for the combined scenario. TFR mean values across these scenarios are projected to converge by 2100 in the range of 1·78 to 1·90 (appendix 2 figure S5, table 2).

The reference scenario TFR in Pakistan is forecasted to be 1·76 (95% UI 1·25–2·28) in 2050 and 1·16 (0·59–1·77) in 2100 (appendix 2 figure S5). If pro-natal policies are implemented in 2051, we project (assuming a 0·2 bump) the final number to be 1·36 (0·79–1·97) instead. On the other hand, the TFR in Pakistan is projected to exponentially decline to 1·56 (1·04–2·11) and 1·47 (0·93–2·04) by 2050 for the education and met need scenarios, respectively, and then to roughly converge to the projected reference scenario TFR by 2100 (1·18 [0·64–1·77] and 1·12 [0·58–1·72]). The combined alternative scenario TFR in Pakistan will reach a threshold of 1·75 (1·22–2·28) by 2035, with this downward trajectory slowing by 2040 and remaining at 1·54 (1·01–2·11) in 2050 and 1·34 (0·81–1·92) in 2100.

Reference scenario TFR values in Brazil are forecasted to decline below 1·75 in 2036. In the years following, TFR will reach 1·57 (95% UI 1·35–1·81) by 2050 and 1·31 (1·06–1·59) by 2100 (appendix 2 figure S5, table 2). The effect of policies in the pro-natal scenario is projected to yield TFR values of 1·77 (1·55–2·01) by 2050 and 1·51 (1·26–1·79) in Brazil by 2100. Under both education and met need scenarios, TFRs will remain within the range of reference scenario TFR values in 2050 and 2100 (1·52 [1·29–1·77] to 1·31 [1·06–1·59] and 1·52 [1·31–1·76] to 1·32 [1·07–1·60], respectively). The combined scenario in Brazil is projected to yield TFRs below a 1·75 threshold by 2030, with rates dropping to 1·68 (1·46–1·93) and 1·52 (1·27–1·79) by 2050 and 2100, respectively; as such, this scenario will lead to declines in fertility later than in the reference scenario.

As in Brazil, reference scenario TFR values in Indonesia are also forecasted to decline below 1·75 in 2036. Reference TFR values are projected to be 1·53 (95% UI 1·25–1·84) and 1·29 (0·99–1·63) by 2050 and 2100, respectively (appendix 2 figure S5, table 2). Similarly, TFR values under the pro-natal scenario in Indonesia are forecasted to reach 1·73 (1·45–2·04) by 2050 and 1·49 (1·19–1·83) by 2100. TFRs under both the education and met need scenarios will remain within the range of reference scenario TFRs in 2050 and 2100 (1·51 [1·23–1·82] to 1·30 [1·00–1·63] and 1·44 [1·17–1·74] to 1·26 [0·97–1·60], respectively). TFR in the combined scenario in Indonesia is projected to drop below a 1·75 threshold by 2030, and this scenario will have an earlier effect on fertility trajectory than the reference scenario, with combined-scenario TFR values reaching 1·62 (1·35–1·92) in 2050 and 1·47 (1·18–1·80) in 2100.

Reference TFR values in Bangladesh and India are projected to decline below the 1·75 threshold by 2026 and 2027, respectively. TFR in Bangladesh in the reference scenario is projected to be 1·20 (95% UI 0·84–1·54) by 2050 and 0·97 (0·57–1·37) by 2100 (appendix 2 figure S5, table 2). Similarly, reference scenario TFR in India will reach 1·29 (0·97–1·62) and 1·04 (0·67–1·42) by 2050 and 2100, respectively (appendix 2 figure S5, table 2). The pro-natal scenario TFR is projected to slow the fertility decline starting in 2027 in Bangladesh and 2028 in India. For example, TFR in Bangladesh under the pro-natal scenario will reach 1·40 (1·04–1·74) in 2050 and 1·17 (0·77–1·57) in 2100. The education and met need scenarios in Bangladesh ultimately converge to the reference scenario TFR both in 2050 and 2100. The combined scenario in Bangladesh will slow down the declines in TFR value (which stays above the reference scenario after 2027) earlier than will the combined scenario in India (which will stay above the reference scenario after 2042).

Mexico, Russia, the USA, and China already experienced TFR values below 1·75 in 2021; thus, we assume that the full effect of the pro-natal scenario will occur in 2026 in these countries. For instance, under the pro-natal scenario, we project TFR values in Mexico to be 1·59 (95% UI 1·39–1·82) in 2050 and 1·35 (1·11–1·61) in 2100. Across all four countries, mean TFR values in the reference scenario values range from 1·14 to 1·52 in 2050 and 1·15 to 1·45 in 2100; mean TFR values in the education scenario range from 1·12 to 1·51 in 2050 and 1·14 to 1·44 in 2100; mean TFR values in the met need scenario range from 1·14 to 1·46 in 2050 and 1·15 to 1·39 in 2100; and mean TFR values in the combined scenario also range from 1·31 to 1·65 in 2050 and 1·34 to 1·58 in 2100 (appendix 2 figure S5, table 2).

Section 4: References

1 Stevens GA, Alkema L, Black RE, *et al.* Guidelines for accurate and transparent health estimates reporting: the GATHER statement. *PLoS Med* 2016; **13**: e1002056.

2 Wang H, Abbas KM, Abbasifard M, *et al.* Global age-sex-specific fertility, mortality, healthy life expectancy (HALE), and population estimates in 204 countries and territories, 1950–2019: a comprehensive demographic analysis for the Global Burden of Disease Study 2019. *The Lancet* 2020; **396**: 1160–203.

3 Foreman KJ, Marquez N, Dolgert A, *et al.* Forecasting life expectancy, years of life lost, and all-cause and cause-specific mortality for 250 causes of death: reference and alternative scenarios for 2016–40 for 195 countries and territories. *Lancet* 2018; **392**: 2052–90.

4 Vollset SE, Goren E, Yuan C-W, *et al.* Fertility, mortality, migration, and population scenarios for 195 countries and territories from 2017 to 2100: a forecasting analysis for the Global Burden of Disease Study. *Lancet* 2020; **396**: 1285–306.

5 Wang H, Naghavi M, Allen C, *et al.* Global, regional, and national life expectancy, all-cause mortality, and cause-specific mortality for 249 causes of death, 1980–2015: a systematic analysis for the Global Burden of Disease Study 2015. *The Lancet* 2016; **388**: 1459–544.

6 Wang H, Abajobir AA, Abate KH, *et al.* Global, regional, and national under-5 mortality, adult mortality, age-specific mortality, and life expectancy, 1970–2016: a systematic analysis for the Global Burden of Disease Study 2016. *The Lancet* 2017; **390**: 1084–150.

7 GBD 2017 Population and Fertility Collaborators. Population and fertility by age and sex for 195 countries and territories, 1950–2017: a systematic analysis for the Global Burden of Disease Study 2017. *The Lancet* 2018; **392**: 1995–2051.

8 United Nations Department of Economic and Social Affairs. United Nations Demographic Yearbook. New York: United Nations, 2020 https://unstats.un.org/unsd/demographic-social/products/dyb/dyb_2020/.

9 Max Planck Institute for Demographic Research (Germany) and Vienna Institute of Demography (Austria). Human Fertility Collection. www.fertilitydata.org.

10 World Health Organization. WHO Mortality Database. https://www.who.int/data/data-collection-tools/who-mortality-database (accessed Sept 1, 2023).

11 Preston S, Heuveline P, Guillot M. Demography: measuring and modeling population processes, 1 edition. Wiley-Blackwell, 2000.

12 Copernicus Climate Change Service Global Land Cover Maps, Version 2.1.1 | GHDx. https://internal-ghdx.healthdata.org/record/copernicus-climate-change-service-global-land-cover-maps-version-211 (accessed Oct 27, 2023).

13 Zheng P, Barber R, Sorensen RJ, Murray CJ, Aravkin AY. Trimmed constrained mixed effects models: formulations and algorithms. *J Comput Graph Stat* 2021; **30**: 544–56.

14 United Nations. Good health and well-being. https://www.un.org/sustainabledevelopment/health/ (accessed June 12, 2019).

15 United Nations. Quality education. https://www.un.org/sustainabledevelopment/education/ (accessed June 12, 2019).

16 Australian Bureau of Statistics. Births, Australia, 2021. 2022; published online Oct 25. https://www.abs.gov.au/statistics/people/population/births-australia/2021 (accessed Oct 24, 2023).

17 Drago R, Sawyer K, Shreffler KM, Warren D, Wooden M. Did australia's baby bonus increase fertility intentions and births? *Popul Res Policy Rev* 2011; **30**: 381–97.

18 Einarsdóttir K, Langridge A, Hammond G, Gunnell AS, Haggar FA, Stanley FJ. The Australian baby bonus maternity payment and birth characteristics in western Australia. *PLOS ONE* 2012; **7**: e48885.

19 Parr N, Guest R. The contribution of increases in family benefits to Australia's early 21st-century fertility increase: An empirical analysis. *Demogr Res* 2011; **25**: 215–44.

20 Gauthier AH. The impact of family policies on fertility in industrialized countries: a review of the literature. *Popul Res Policy Rev* 2007; **26**: 323–46.

21 Bergsvik J, Fauske A, Hart RK. Can policies stall the fertility fall? A systematic review of the (quasi-) experimental literature. *Popul Dev Rev* 2021; **47**: 913–64.

22 Division UP. World population prospects :: the 2006 revision.: Vol. 3,: Analytical report. UN, 2010 https://digitallibrary.un.org/record/3924313 (accessed Oct 22, 2023).

23 Murphy AH. Skill Scores Based on the Mean Square Error and Their Relationships to the Correlation Coefficient. *Mon Weather Rev* 1988; **116**: 2417–24.

24 United Nations Department of Economic and Social Affairs, Population Division. World Population Prospects 2022: summary of results. 2022.

25 United Nations, Department of Economic and Social Affairs, Population Division. World Population Prospects: The 2012 revision, methodology of the United Nations population estimates and projections. New York: United Nations, 2014.

26 Ševčíková H, Alkema L, Raftery A. bayesTFR: An R package for probabilistic projections of the total fertility rate. *J Stat Softw* 2011; **43**: 1–29.

Section 5: Tables and figures

Appendix figure S1:
MR-BRT model fit for the two-covariate CCF50 model with spline on education covariate with respect to quantiles (0.1, 0.25, 0.5, 0.75, 0.99) of the proportion of met need for contraception covariate

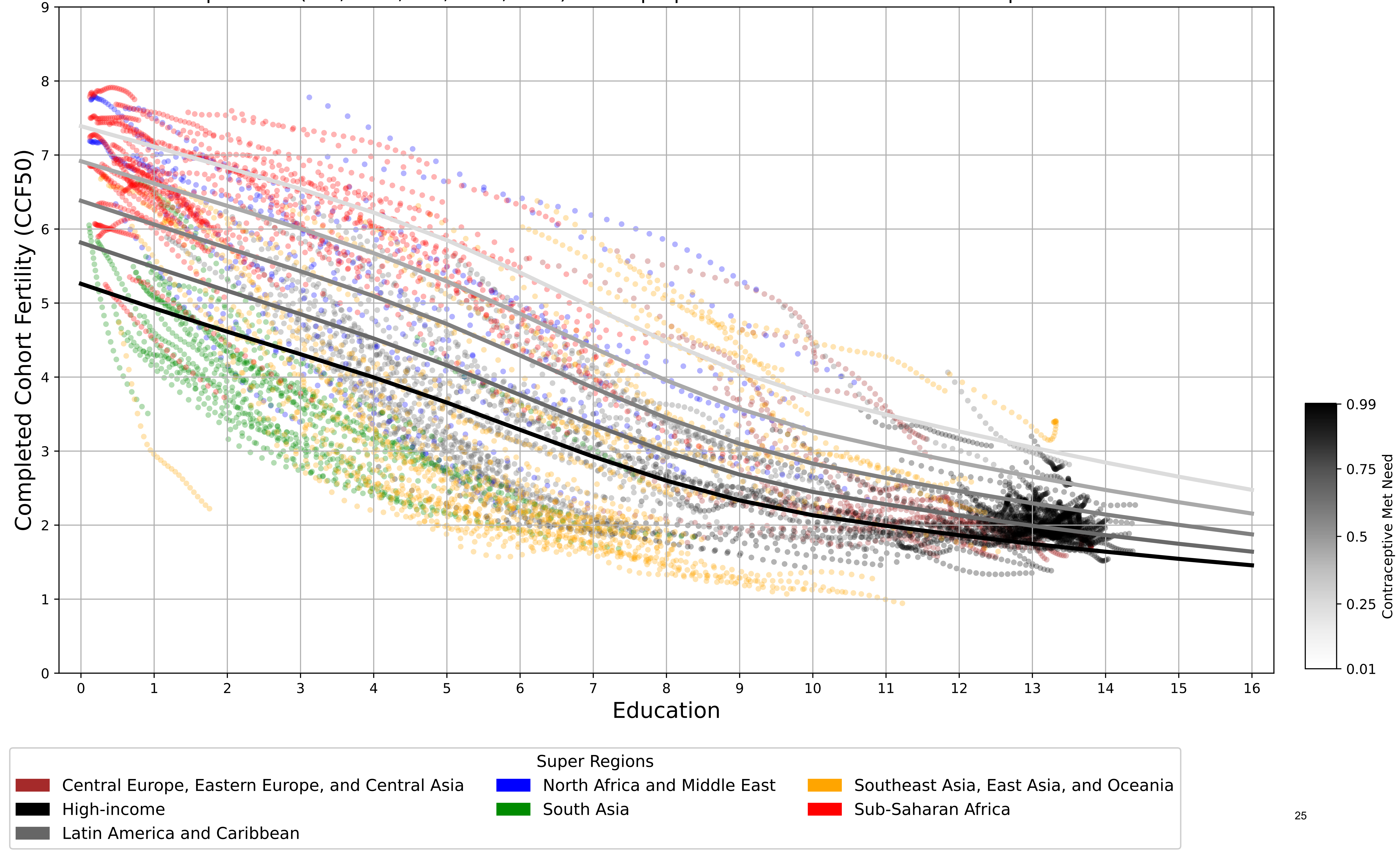

Appendix figure S2: Map of 2021 World Bank income groups

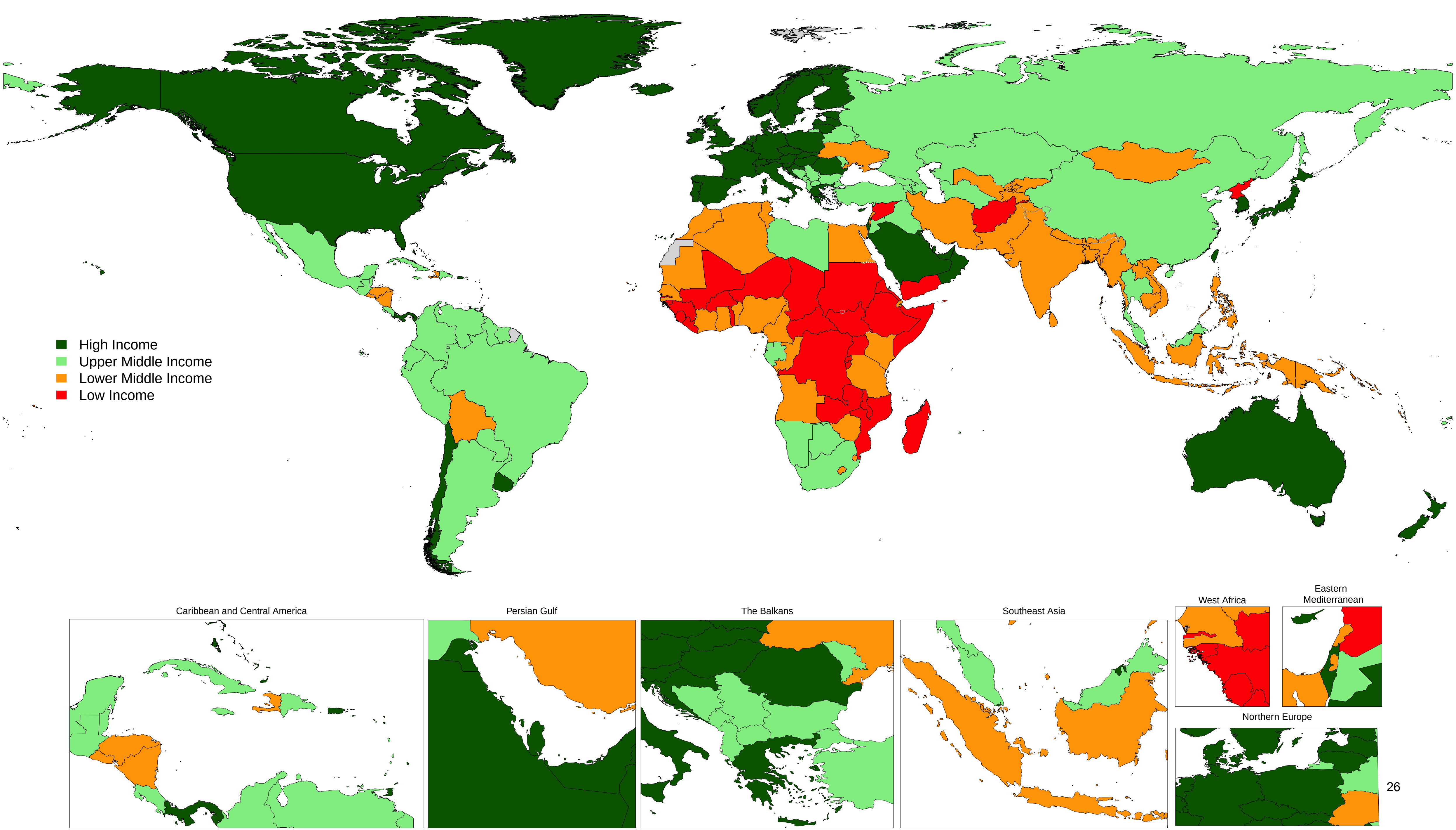

Appendix figure S3: Age-specfic fertility rate skill metric comparison for IHME and WPP models

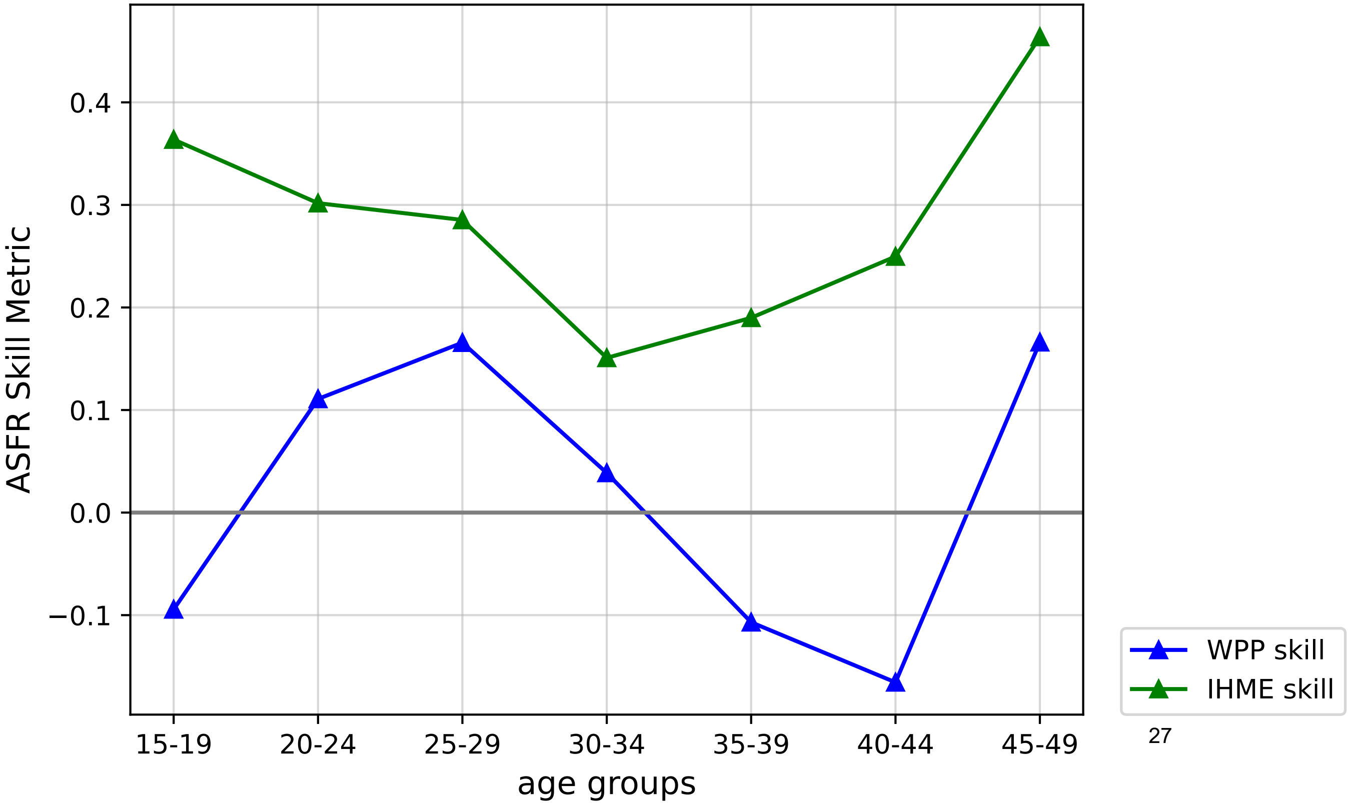

Appendix Table S1: GBD location hierarchy with levels

Location	Level
Global	0
Central Europe, eastern Europe, and central Asia	1
Central Asia	2
Armenia	3
Azerbaijan	3
Georgia	3
Kazakhstan	3
Kyrgyzstan	3
Mongolia	3
Tajikistan	3
Turkmenistan	3
Uzbekistan	3
Central Europe	2
Albania	3
Bosnia and Herzegovina	3
Bulgaria	3
Croatia	3
Czechia	3
Hungary	3
Montenegro	3
North Macedonia	3
Poland	3
Romania	3
Serbia	3
Slovakia	3
Slovenia	3
Eastern Europe	2
Belarus	3
Estonia	3
Latvia	3
Lithuania	3
Moldova	3
Russia	3
Ukraine	3
High income	1
Australasia	2
Australia	3
New Zealand	3
High-income Asia Pacific	2
Brunei	3
Japan	3
Aichi	4
Akita	4
Aomori	4
Chiba	4

Ehime	4
Fukui	4
Fukuoka	4
Fukushima	4
Gifu	4
Gunma	4
Hiroshima	4
Hokkaidō	4
Hyōgo	4
Ibaraki	4
Ishikawa	4
Iwate	4
Kagawa	4
Kagoshima	4
Kanagawa	4
Kōchi	4
Kumamoto	4
Kyōto	4
Mie	4
Miyagi	4
Miyazaki	4
Nagano	4
Nagasaki	4
Nara	4
Niigata	4
Ōita	4
Okayama	4
Okinawa	4
Ōsaka	4
Saga	4
Saitama	4
Shiga	4
Shimane	4
Shizuoka	4
Tochigi	4
Tokushima	4
Tōkyō	4
Tottori	4
Toyama	4
Wakayama	4
Yamagata	4
Yamaguchi	4
Yamanashi	4
South Korea	3
Singapore	3
High-income North America	2
Canada	3

Greenland	3
USA	3
Alabama	4
Alaska	4
Arizona	4
Arkansas	4
California	4
Colorado	4
Connecticut	4
Delaware	4
Washington, DC	4
Florida	4
Georgia	4
Hawaii	4
Idaho	4
Illinois	4
Indiana	4
Iowa	4
Kansas	4
Kentucky	4
Louisiana	4
Maine	4
Maryland	4
Massachusetts	4
Michigan	4
Minnesota	4
Mississippi	4
Missouri	4
Montana	4
Nebraska	4
Nevada	4
New Hampshire	4
New Jersey	4
New Mexico	4
New York	4
North Carolina	4
North Dakota	4
Ohio	4
Oklahoma	4
Oregon	4
Pennsylvania	4
Rhode Island	4
South Carolina	4
South Dakota	4
Tennessee	4
Texas	4
Utah	4

Vermont	4
Virginia	4
Washington	4
West Virginia	4
Wisconsin	4
Wyoming	4
Southern Latin America	2
Argentina	3
Chile	3
Uruguay	3
Western Europe	2
Andorra	3
Austria	3
Belgium	3
Cyprus	3
Denmark	3
Finland	3
France	3
Germany	3
Greece	3
Iceland	3
Ireland	3
Israel	3
Italy	3
Abruzzo	4
Basilicata	4
Calabria	4
Campania	4
Emilia-Romagna	4
Friuli-Venezia Giulia	4
Lazio	4
Liguria	4
Lombardia	4
Marche	4
Molise	4
Piemonte	4
Provincia autonoma di Bolzano	4
Provincia autonoma di Trento	4
Puglia	4
Sardegna	4
Sicilia	4
Toscana	4
Umbria	4
Valle d'Aosta	4
Veneto	4
Luxembourg	3
Malta	3

Monaco	3
Netherlands	3
Norway	3
Agder	4
Innlandet	4
Møre og Romsdal	4
Nordland	4
Oslo	4
Rogaland	4
Troms og Finnmark	4
Trøndelag	4
Vestfold og Telemark	4
Vestland	4
Viken	4
Portugal	3
San Marino	3
Spain	3
Sweden	3
Stockholm	4
Sweden except Stockholm	4
Switzerland	3
UK	3
England	4
East Midlands	5
Derby	6
Derbyshire	6
Leicester	6
Leicestershire	6
Lincolnshire	6
Northamptonshire	6
Nottingham	6
Nottinghamshire	6
Rutland	6
East of England	5
Bedford	6
Cambridgeshire	6
Central Bedfordshire	6
Essex	6
Hertfordshire	6
Luton	6
Norfolk	6
Peterborough	6
Southend-on-Sea	6
Suffolk	6
Thurrock	6
Greater London	5
Barking and Dagenham	6

Barnet	6
Bexley	6
Brent	6
Bromley	6
Camden	6
Croydon	6
Ealing	6
Enfield	6
Greenwich	6
Hackney	6
Hammersmith and Fulham	6
Haringey	6
Harrow	6
Havering	6
Hillingdon	6
Hounslow	6
Islington	6
Kensington and Chelsea	6
Kingston upon Thames	6
Lambeth	6
Lewisham	6
Merton	6
Newham	6
Redbridge	6
Richmond upon Thames	6
Southwark	6
Sutton	6
Tower Hamlets	6
Waltham Forest	6
Wandsworth	6
Westminster	6
North East England	5
County Durham	6
Darlington	6
Gateshead	6
Hartlepool	6
Middlesbrough	6
Newcastle upon Tyne	6
North Tyneside	6
Northumberland	6
Redcar and Cleveland	6
South Tyneside	6
Stockton-on-Tees	6
Sunderland	6
North West England	5
Blackburn with Darwen	6
Blackpool	6

Bolton	6
Bury	6
Cheshire East	6
Cheshire West and Chester	6
Cumbria	6
Halton	6
Knowsley	6
Lancashire	6
Liverpool	6
Manchester	6
Oldham	6
Rochdale	6
Salford	6
Sefton	6
St Helens	6
Stockport	6
Tameside	6
Trafford	6
Warrington	6
Wigan	6
Wirral	6
South East England	5
Bracknell Forest	6
Brighton and Hove	6
Buckinghamshire	6
East Sussex	6
Hampshire	6
Isle of Wight	6
Kent	6
Medway	6
Milton Keynes	6
Oxfordshire	6
Portsmouth	6
Reading	6
Slough	6
Southampton	6
Surrey	6
West Berkshire	6
West Sussex	6
Windsor and Maidenhead	6
Wokingham	6
South West England	5
Bath and North East Somerset	6
Bournemouth	6
Bristol, City of	6
Cornwall	6
Devon	6

Dorset	6
Gloucestershire	6
North Somerset	6
Plymouth	6
Poole	6
Somerset	6
South Gloucestershire	6
Swindon	6
Torbay	6
Wiltshire	6
West Midlands	5
Birmingham	6
Coventry	6
Dudley	6
Herefordshire, County of	6
Sandwell	6
Shropshire	6
Solihull	6
Staffordshire	6
Stoke-on-Trent	6
Telford and Wrekin	6
Walsall	6
Warwickshire	6
Wolverhampton	6
Worcestershire	6
Yorkshire and the Humber	5
Barnsley	6
Bradford	6
Calderdale	6
Doncaster	6
East Riding of Yorkshire	6
Kingston upon Hull, City of	6
Kirklees	6
Leeds	6
North East Lincolnshire	6
North Lincolnshire	6
North Yorkshire	6
Rotherham	6
Sheffield	6
Wakefield	6
York	6
Northern Ireland	4
Scotland	4
Wales	4
Latin America and Caribbean	1
Andean Latin America	2
Bolivia	3

Ecuador	3
Peru	3
Caribbean	2
Antigua and Barbuda	3
The Bahamas	3
Barbados	3
Belize	3
Bermuda	3
Cuba	3
Dominica	3
Dominican Republic	3
Grenada	3
Guyana	3
Haiti	3
Jamaica	3
Puerto Rico	3
Saint Kitts and Nevis	3
Saint Lucia	3
Saint Vincent and the Grenadines	3
Suriname	3
Trinidad and Tobago	3
Virgin Islands	3
Central Latin America	2
Colombia	3
Costa Rica	3
El Salvador	3
Guatemala	3
Honduras	3
Mexico	3
Aguascalientes	4
Baja California	4
Baja California Sur	4
Campeche	4
Chiapas	4
Chihuahua	4
Coahuila	4
Colima	4
Durango	4
Guanajuato	4
Guerrero	4
Hidalgo	4
Jalisco	4
México	4
Mexico City	4
Michoacán de Ocampo	4
Morelos	4
Nayarit	4

Nuevo León	4
Oaxaca	4
Puebla	4
Querétaro	4
Quintana Roo	4
San Luis Potosí	4
Sinaloa	4
Sonora	4
Tabasco	4
Tamaulipas	4
Tlaxcala	4
Veracruz de Ignacio de la Llave	4
Yucatán	4
Zacatecas	4
Nicaragua	3
Panama	3
Venezuela	3
Tropical Latin America	2
Brazil	3
Acre	4
Alagoas	4
Amapá	4
Amazonas	4
Bahia	4
Ceará	4
Distrito Federal	4
Espírito Santo	4
Goiás	4
Maranhão	4
Mato Grosso	4
Mato Grosso do Sul	4
Minas Gerais	4
Pará	4
Paraíba	4
Paraná	4
Pernambuco	4
Piauí	4
Rio de Janeiro	4
Rio Grande do Norte	4
Rio Grande do Sul	4
Rondônia	4
Roraima	4
Santa Catarina	4
São Paulo	4
Sergipe	4
Tocantins	4
Paraguay	3

North Africa and Middle East	1
North Africa and Middle East	2
Afghanistan	3
Algeria	3
Bahrain	3
Egypt	3
Iran	3
Alborz	4
Ardebil	4
Bushehr	4
Chahar Mahaal and Bakhtiari	4
East Azarbayejan	4
Fars	4
Gilan	4
Golestan	4
Hamadan	4
Hormozgan	4
Ilam	4
Isfahan	4
Kerman	4
Kermanshah	4
Khorasan-e-Razavi	4
Khuzestan	4
Kohgiluyeh and Boyer-Ahmad	4
Kurdistan	4
Lorestan	4
Markazi	4
Mazandaran	4
North Khorasan	4
Qazvin	4
Qom	4
Semnan	4
Sistan and Baluchistan	4
South Khorasan	4
Tehran	4
West Azarbayejan	4
Yazd	4
Zanjan	4
Iraq	3
Jordan	3
Kuwait	3
Lebanon	3
Libya	3
Morocco	3
Oman	3
Palestine	3
Qatar	3

Saudi Arabia	3
Sudan	3
Syria	3
Tunisia	3
Türkiye	3
United Arab Emirates	3
Yemen	3
South Asia	1
South Asia	2
Bangladesh	3
Bhutan	3
India	3
Nepal	3
Pakistan	3
Azad Jammu & Kashmir	4
Balochistan	4
Gilgit-Baltistan	4
Islamabad Capital Territory	4
Khyber Pakhtunkhwa	4
Punjab	4
Sindh	4
Southeast Asia, east Asia, and Oceania	1
East Asia	2
China	3
North Korea	3
Taiwan (province of China)	3
Oceania	2
American Samoa	3
Cook Islands	3
Fiji	3
Guam	3
Kiribati	3
Marshall Islands	3
Federated States of Micronesia	3
Nauru	3
Niue	3
Northern Mariana Islands	3
Palau	3
Papua New Guinea	3
Samoa	3
Solomon Islands	3
Tokelau	3
Tonga	3
Tuvalu	3
Vanuatu	3
Southeast Asia	2
Cambodia	3

Indonesia	3
Aceh	4
Bali	4
Bangka-Belitung Islands	4
Banten	4
Bengkulu	4
Gorontalo	4
Jakarta	4
Jambi	4
West Java	4
Central Java	4
East Java	4
West Kalimantan	4
South Kalimantan	4
Central Kalimantan	4
East Kalimantan	4
North Kalimantan	4
Riau Islands	4
Lampung	4
Maluku	4
North Maluku	4
West Nusa Tenggara	4
East Nusa Tenggara	4
Papua	4
West Papua	4
Riau	4
West Sulawesi	4
South Sulawesi	4
Central Sulawesi	4
Southeast Sulawesi	4
North Sulawesi	4
West Sumatra	4
South Sumatra	4
North Sumatra	4
Yogyakarta	4
Laos	3
Malaysia	3
Maldives	3
Mauritius	3
Myanmar	3
Philippines	3
Abra	4
Agusan Del Norte	4
Agusan Del Sur	4
Aklan	4
Albay	4
Antique	4

Apayao	4
Aurora	4
Basilan	4
Bataan	4
Batanes	4
Batangas	4
Benguet	4
Biliran	4
Bohol	4
Bukidnon	4
Bulacan	4
Cagayan	4
Camarines Norte	4
Camarines Sur	4
Camiguin	4
Capiz	4
Catanduanes	4
Cavite	4
Cebu	4
Cotabato (North Cotabato)	4
Davao de Oro	4
Davao Del Norte	4
Davao Del Sur	4
Davao Occidental	4
Davao Oriental	4
Dinagat Islands	4
Eastern Samar	4
Guimaras	4
Ifugao	4
Ilocos Norte	4
Ilocos Sur	4
Iloilo	4
Isabela	4
Kalinga	4
La Union	4
Laguna	4
Lanao Del Norte	4
Lanao Del Sur	4
Leyte	4
Maguindanao	4
Marinduque	4
Masbate	4
Misamis Occidental	4
Misamis Oriental	4
Mountain Province	4
National Capital Region	4
Negros Occidental	4

Negros Oriental	4
Northern Samar	4
Nueva Ecija	4
Nueva Vizcaya	4
Occidental Mindoro	4
Oriental Mindoro	4
Palawan	4
Pampanga	4
Pangasinan	4
Quezon	4
Quirino	4
Rizal	4
Romblon	4
Samar (Western Samar)	4
Sarangani	4
Siquijor	4
Sorsogon	4
South Cotabato	4
Southern Leyte	4
Sultan Kudarat	4
Sulu	4
Surigao Del Norte	4
Surigao Del Sur	4
Tarlac	4
Tawi-Tawi	4
Zambales	4
Zamboanga Del Norte	4
Zamboanga Del Sur	4
Zamboanga Sibugay	4
Seychelles	3
Sri Lanka	3
Thailand	3
Timor-Leste	3
Viet Nam	3
Sub-Saharan Africa	1
Central sub-Saharan Africa	2
Angola	3
Central African Republic	3
Congo (Brazzaville)	3
DR Congo	3
Equatorial Guinea	3
Gabon	3
Eastern sub-Saharan Africa	2
Burundi	3
Comoros	3
Djibouti	3
Eritrea	3

Ethiopia	3
Addis Ababa	4
Afar	4
Amhara	4
Benishangul-Gumuz	4
Dire Dawa	4
Gambella	4
Harari	4
Oromia	4
Somali	4
Southern Nations, Nationalities, and Peoples	4
Tigray	4
Kenya	3
Baringo	4
Bomet	4
Bungoma	4
Busia	4
Elgeyo Marakwet	4
Embu	4
Garissa	4
Homa Bay	4
Isiolo	4
Kajiado	4
Kakamega	4
Kericho	4
Kiambu	4
Kilifi	4
Kirinyaga	4
Kisii	4
Kisumu	4
Kitui	4
Kwale	4
Laikipia	4
Lamu	4
Machakos	4
Makueni	4
Mandera	4
Marsabit	4
Meru	4
Migori	4
Mombasa	4
Murang'a	4
Nairobi	4
Nakuru	4
Nandi	4
Narok	4
Nyamira	4

Nyandarua	4
Nyeri	4
Samburu	4
Siaya	4
Taita Taveta	4
Tana River	4
Tharaka Nithi	4
Trans Nzoia	4
Turkana	4
Uasin Gishu	4
Vihiga	4
Wajir	4
West Pokot	4
Madagascar	3
Malawi	3
Mozambique	3
Rwanda	3
Somalia	3
South Sudan	3
Uganda	3
Tanzania	3
Zambia	3
Southern sub-Saharan Africa	2
Botswana	3
Eswatini	3
Lesotho	3
Namibia	3
South Africa	3
Eastern Cape	4
Free State	4
Gauteng	4
KwaZulu-Natal	4
Limpopo	4
Mpumalanga	4
North West	4
Northern Cape	4
Western Cape	4
Zimbabwe	3
Western sub-Saharan Africa	2
Benin	3
Burkina Faso	3
Cabo Verde	3
Cameroon	3
Chad	3
Côte d'Ivoire	3
The Gambia	3
Ghana	3

Guinea	3
Guinea-Bissau	3
Liberia	3
Mali	3
Mauritania	3
Niger	3
Nigeria	3
São Tomé and Príncipe	3
Senegal	3
Sierra Leone	3
Togo	3

Gather Compliance

This study complies with the Guidelines for Accurate and Transparent Health Estimates Reporting (GATHER) recommendations. We have documented the steps involved in our analytical procedures and detailed the data sources in the GATHER checklist below. The GATHER recommendations can be found here: http://gather-statement.org/

Appendix Table S2. GATHER compliance

#	GATHER checklist item	Description of compliance	Reference
	Objectives and funding		
**1**	Define the indicator(s), populations (including age, sex, and geographic entities), and time period(s) for which estimates were made.	Narrative provided in the paper and appendix describing indicators, definitions, and populations	Main text (Methods - Overview) and appendix
**2**	List the funding sources for the work.	Funding sources listed in paper	Main text (Summary – Funding)
	Data Inputs		
	For all data inputs from multiple sources that are synthesized as part of the study:		
**3**	Describe how the data were identified and how the data were accessed.	Narrative provided in paper and appendix describing data seeking methods	Main text (Methods) and appendix
**4**	Specify the inclusion and exclusion criteria. Identify all ad-hoc exclusions.	Narrative provided in paper and appendix describing inclusion and exclusion criteria by data type	Main text (Methods) and appendix
**5**	Provide information on all included data sources and their main characteristics. For each data source used, report reference information or contact name/institution, population represented, data collection method, year(s) of data collection, sex and age range, diagnostic criteria or measurement method, and sample size, as relevant.	Narrative for data sources is provided in paper and appendix. Metadata for sources by geography are available through an online data source tool; information on metadata for UNPD data available in the appendix	Main text (Methods), appendix, and through the online data citation tool: https://ghdx.healthdata.org/gbd-2021/sources; UNPD data from: https://population.un.org/wpp/Download/Standard/Fertility/
**6**	Identify and describe any categories of input data that have potentially important biases (e.g., based on characteristics listed in item 5).	Limitations of and biases in data included in paper	Main text (Discussion – Limitations)
	For data inputs that contribute to the analysis but were not synthesized as part of the study:		
**7**	Describe and give sources for any other data inputs.	Included in online data source tools	UNPD data from: https://population.un.org/wpp/Download/Standard/Fertility/; online data citation tool: https://ghdx.healthdata.org/gbd-2021/sources
	For all data inputs:		
**8**	Provide all data inputs in a file format from which data can be efficiently extracted (e.g., a spreadsheet rather than a PDF), including all relevant meta-data listed in item 5. For any data inputs that cannot be shared because of ethical or legal reasons, such as third-party ownership, provide a contact name or the name of the	Downloads of input data are available through online data query tools	Global Health Data Exchange: https://ghdx.healthdata.org/gbd-2021/sources

	institution that retains the right to the data.		
	Data analysis		
**9**	Provide a conceptual overview of the data analysis method. A diagram may be helpful.	A brief overview of the overall methodological processes have been provided	Main text (Methods) and appendix
**10**	Provide a detailed description of all steps of the analysis, including mathematical formulae. This description should cover, as relevant, data cleaning, data pre-processing, data adjustments and weighting of data sources, and mathematical or statistical model(s).	Detailed descriptions of all steps of the analysis, as well as relevant mathematical formulae, have been provided	Main text (Methods) and appendix
**11**	Describe how candidate models were evaluated and how the final model(s) were selected.	Details on model evaluation and finalisation have been provided	Main text (Comparison with other models) and appendix
**12**	Provide the results of an evaluation of model performance, if done, as well as the results of any relevant sensitivity analysis.	Details on evaluation of model performance have been provided	Main text (Comparison with other models) and appendix
**13**	Describe methods for calculating uncertainty of the estimates. State which sources of uncertainty were, and were not, accounted for in the uncertainty analysis.	Details on uncertainty calculations have been provided	Main text (Methods) and appendix
**14**	State how analytic or statistical source code used to generate estimates can be accessed.	Access statement provided; online repository provides code access	Code is provided in an online repository: https://ghdx.healthdata.org/gbd-2021/code
	Results and Discussion		
**15**	Provide published estimates in a file format from which data can be efficiently extracted.	Results are available through online tools	Datasets of results are available for download on the GHDx: https://ghdx.healthdata.org/gbd-2021
**16**	Report a quantitative measure of the uncertainty of the estimates (e.g. uncertainty intervals).	Uncertainty intervals are provided with results	Main text (Results and Discussion), appendix 2, and online results: https://ghdx.healthdata.org/gbd-2021
**17**	Interpret results in light of existing evidence. If updating a previous set of estimates, describe the reasons for changes in estimates.	Discussion of methodological differences between this and existing evidence (by IHME, UNPD, and Wittgenstein Centre)	Main text (Research in Context, Introduction, Methods, Discussion) and appendix
**18**	Discuss limitations of the estimates. Include a discussion of any modelling assumptions or data limitations that affect interpretation of the estimates.	Discussion of limitations was provided	Main text (Discussion – Limitations)

Appendix Table S3. Number of sources used for the analysis of age-specific fertility by location

Location	Vital Registrations	Complete Birth Histories	Summary Birth Histories	Censuses	Other	Sample Registrations
Afghanistan	0	3	5	0	0	0
Albania	64	2	5	1	0	0
Algeria	61	5	0	0	0	0
American Samoa	69	0	0	2	0	0
Andorra	50	0	0	0	0	0
Angola	23	5	4	1	0	0
Antigua and Barbuda	62	0	0	1	0	0
Argentina	66	0	0	4	0	0
Armenia	61	4	4	2	0	0
Australia	71	0	0	1	0	0
Austria	71	0	0	0	25	0
Azerbaijan	66	3	2	1	0	0
Bahrain	52	1	0	2	0	0
Bangladesh	41	15	16	2	0	0
Barbados	64	0	0	3	0	0
Belarus	70	0	2	2	0	0
Belgium	69	0	0	0	0	0
Belize	69	2	5	4	0	0
Benin	2	6	7	2	0	0
Bermuda	67	0	0	2	0	0
Bhutan	5	1	3	2	0	0
Bolivia	41	7	22	4	0	0
Bosnia and Herzegovina	32	1	1	0	1	0
Botswana	20	5	6	5	0	0
Brazil	57	4	25	7	0	0
Brunei	61	0	0	0	0	0
Bulgaria	70	0	2	0	0	0
Burkina Faso	0	6	9	4	0	0
Burundi	9	6	7	1	0	0
Cabo Verde	47	1	2	1	0	0
Cambodia	1	6	8	2	0	0
Cameroon	0	8	8	1	0	0
Canada	69	0	0	1	0	0
Central African Republic	0	2	4	2	0	0
Chad	0	5	5	0	0	0
Chile	69	0	16	4	3	0
Colombia	65	9	9	4	0	0
Comoros	9	2	3	3	0	0
Congo (Brazzaville)	1	3	4	1	0	0
Cook Islands	46	0	0	3	0	0
Costa Rica	72	1	3	3	0	0
Croatia	71	0	0	2	1	0
Cuba	69	1	3	1	2	0
Cyprus	69	0	0	2	0	0
Czechia	71	0	0	2	0	0
Côte d'Ivoire	5	6	8	2	0	0
DR Congo	0	2	12	0	0	0
Denmark	72	0	0	0	0	0
Djibouti	28	3	3	0	0	0
Dominica	68	0	0	2	0	0
Dominican Republic	55	13	10	4	0	0
Ecuador	70	5	5	5	0	0

Egypt	67	13	14	1	2	0
El Salvador	60	5	6	2	0	0
Equatorial Guinea	17	1	1	0	0	0
Eritrea	0	3	2	0	0	0
Estonia	72	0	0	2	0	0
Eswatini	6	3	3	4	0	0
Ethiopia	0	10	6	2	0	0
Federated States of Micronesia	8	0	0	4	0	0
Fiji	58	2	1	5	0	0
Finland	72	0	0	2	0	0
France	71	0	0	0	0	0
Gabon	0	1	2	1	0	0
Georgia	58	1	5	0	0	0
Germany	70	0	0	0	2	0
Ghana	28	14	22	3	0	0
Greece	71	0	0	1	4	0
Greenland	72	0	0	0	0	0
Grenada	43	0	0	2	0	0
Guam	70	0	0	0	0	0
Guatemala	50	6	8	0	3	0
Guinea	2	7	5	2	0	0
Guinea-Bissau	21	2	5	0	0	0
Guyana	39	6	7	3	0	0
Haiti	0	8	6	2	0	0
Honduras	40	8	6	3	0	0
Hungary	68	0	0	2	13	0
Iceland	72	0	0	1	0	0
India	67	31	14	4	2	28
Indonesia	14	14	42	5	1	0
Iran	70	0	2	4	0	0
Iraq	43	6	5	2	0	0
Ireland	72	0	0	1	0	0
Israel	71	0	0	0	0	0
Italy	71	0	0	0	0	0
Jamaica	61	3	6	3	2	0
Japan	68	0	0	0	0	0
Jordan	52	20	7	0	0	0
Kazakhstan	58	2	6	2	0	0
Kenya	52	12	16	6	1	0
Kiribati	18	2	1	5	0	0
Kuwait	62	2	0	1	0	0
Kyrgyzstan	58	4	6	2	0	0
Laos	1	4	3	1	0	0
Latvia	71	0	0	2	0	0
Lebanon	30	2	4	0	0	0
Lesotho	8	6	6	4	0	0
Liberia	4	8	6	2	0	0
Libya	48	1	2	0	0	0
Lithuania	72	0	0	1	0	0
Luxembourg	72	0	0	1	0	0
Madagascar	24	7	9	0	0	0
Malawi	6	13	12	4	0	0
Malaysia	63	1	1	2	4	0
Maldives	46	2	2	4	0	0
Mali	2	9	8	3	0	0

Malta	71	0	0	0	0	0
Marshall Islands	26	1	0	2	0	0
Mauritania	1	7	6	1	0	0
Mauritius	71	0	0	2	0	0
Mexico	69	4	22	7	0	0
Moldova	57	2	3	1	0	0
Monaco	55	0	0	0	0	0
Mongolia	49	6	13	0	0	0
Montenegro	31	1	2	0	1	0
Morocco	27	10	7	3	0	0
Mozambique	29	6	7	5	0	0
Myanmar	10	6	2	1	0	0
Namibia	4	4	4	3	0	0
Nauru	31	1	0	2	0	0
Nepal	0	11	9	4	0	0
Netherlands	72	0	0	0	0	0
New Zealand	72	0	0	1	0	0
Nicaragua	56	5	7	3	0	0
Niger	2	5	7	1	0	0
Nigeria	2	14	22	2	0	0
Niue	68	0	0	4	0	0
North Korea	2	2	0	1	0	0
North Macedonia	36	0	2	2	1	0
Northern Mariana Islands	14	0	0	0	0	0
Norway	71	0	0	0	0	0
Oman	18	2	0	0	0	0
Pakistan	18	36	12	1	1	1
Palau	11	0	0	3	0	0
Palestine	24	4	6	1	0	0
Panama	70	1	4	5	3	0
Papua New Guinea	4	4	2	3	0	0
Paraguay	43	6	8	4	6	0
Peru	65	15	21	1	2	0
Philippines	70	18	7	3	3	0
Poland	71	0	0	0	6	0
Portugal	68	1	0	1	2	0
Puerto Rico	60	0	0	1	0	0
Qatar	51	2	0	1	0	0
Romania	70	0	0	3	17	0
Russia	72	0	0	1	0	0
Rwanda	18	11	10	3	0	0
Saint Kitts and Nevis	62	1	0	2	0	0
Saint Lucia	55	0	0	2	0	0
Saint Vincent and the Grenadines	66	0	0	2	0	0
Samoa	38	5	0	1	0	0
San Marino	57	0	0	0	0	0
Saudi Arabia	11	1	1	0	0	0
Senegal	3	16	14	3	0	0
Serbia	64	0	7	0	1	0
Seychelles	69	0	0	1	0	0
Sierra Leone	13	6	6	1	0	0
Singapore	72	0	0	1	0	0
Slovakia	72	0	1	1	0	0
Slovenia	69	0	0	2	1	0
Solomon Islands	1	2	0	4	0	0

Somalia	0	2	3	0	0	0
South Africa	30	3	13	2	0	0
South Korea	52	1	1	8	22	0
South Sudan	0	1	1	1	0	0
Spain	71	0	0	0	0	0
Sri Lanka	70	11	3	2	12	0
Sudan	4	6	5	3	0	0
Suriname	57	0	2	1	0	0
Sweden	72	0	0	0	0	0
Switzerland	71	0	0	0	0	0
Syria	56	3	6	1	0	0
São Tomé and Príncipe	44	2	4	2	0	0
Taiwan (province of China)	72	0	0	0	0	0
Tajikistan	56	3	5	1	0	0
Tanzania	1	10	12	1	1	0
Thailand	68	9	9	4	1	0
The Bahamas	68	0	0	3	0	0
The Gambia	0	4	5	3	0	0
Timor-Leste	19	5	16	0	0	0
Togo	1	4	7	1	0	0
Tokelau	18	0	0	1	0	0
Tonga	52	1	1	3	0	0
Trinidad and Tobago	67	2	5	2	2	0
Tunisia	70	6	7	0	0	0
Turkmenistan	35	3	3	0	0	0
Tuvalu	9	2	0	1	0	0
Türkiye	31	10	8	6	0	0
UK	71	0	0	0	0	0
USA	70	0	0	1	0	0
Uganda	1	8	17	2	1	0
Ukraine	71	2	4	2	0	0
United Arab Emirates	44	2	0	1	0	0
Uruguay	60	0	5	5	0	0
Uzbekistan	54	3	4	0	0	0
Vanuatu	3	1	1	4	0	0
Venezuela	65	2	0	2	0	0
Viet Nam	5	11	10	3	0	0
Virgin Islands	49	0	0	0	0	0
Yemen	9	6	6	1	0	0
Zambia	3	6	10	5	1	0
Zimbabwe	1	10	9	3	0	0

Appendix Table S4. Number of sources used for the analysis of age-specific fertility by year

Year	Vital Registrations	Complete Birth Histories	Summary Birth Histories	Censuses	Other	Sample Registrations
1950	116	0	0	4	4	0
1951	123	0	0	0	2	0
1952	122	0	0	0	1	0
1953	122	0	0	1	3	0
1954	121	0	0	1	1	0
1955	125	0	0	0	2	0
1956	120	0	0	0	2	0
1957	125	0	0	1	3	0
1958	126	0	0	0	3	0
1959	128	0	0	0	3	0
1960	113	0	0	7	11	0
1961	120	0	0	2	5	0
1962	126	0	0	1	5	0
1963	128	1	0	1	5	0
1964	130	1	0	1	7	0
1965	123	1	0	0	8	0
1966	117	1	0	4	5	0
1967	120	0	0	1	5	0
1968	119	1	0	1	6	0
1969	117	0	0	2	10	0
1970	112	2	0	10	16	0
1971	121	3	0	6	4	1
1972	118	0	0	1	10	0
1973	114	1	0	7	2	0
1974	118	5	3	6	1	0
1975	116	9	7	5	3	0
1976	115	9	5	5	1	1
1977	114	7	4	1	1	0
1978	118	10	5	1	1	0
1979	128	6	2	1	1	0
1980	120	5	3	18	2	0
1981	123	4	5	7	1	1
1982	122	2	0	6	1	0
1983	125	4	3	3	1	0
1984	124	8	1	2	1	0
1985	129	5	3	6	2	0
1986	129	8	7	9	1	1
1987	128	20	13	4	1	0
1988	125	10	14	6	1	0
1989	123	5	4	11	1	0
1990	128	10	7	28	1	0
1991	127	19	12	23	1	1
1992	127	13	17	8	1	0
1993	122	12	16	5	1	0

1994	118	11	16	5	1	0
1995	113	13	17	4	1	0
1996	112	24	19	9	0	1
1997	104	19	21	4	0	0
1998	101	18	28	4	0	0
1999	102	17	22	9	1	1
2000	109	20	63	21	0	1
2001	108	13	20	20	0	1
2002	116	12	16	13	0	1
2003	119	17	22	2	0	1
2004	114	15	21	2	0	1
2005	120	22	37	6	0	2
2006	115	33	55	6	0	1
2007	119	25	30	6	0	1
2008	121	21	27	7	0	1
2009	119	18	22	7	0	1
2010	121	22	38	11	0	1
2011	122	22	36	14	0	1
2012	144	28	39	5	0	1
2013	140	21	30	2	0	1
2014	140	35	44	1	0	1
2015	140	22	32	2	0	1
2016	145	23	37	1	0	1
2017	141	25	31	2	0	1
2018	134	19	16	1	0	1
2019	123	22	9	0	0	1
2020	107	7	0	0	0	1
2021	46	9	0	0	0	0
2022	0	2	0	0	0	0

Appendix Table S5. Share of livebirths by GBD super region, 1950, 1980, 2021, 2050, and 2100 for the reference scenario.

	Share of livebirths (%)				
	1950	1980	2021	2050	2100
Central Europe, Eastern Europe, and	8·0 (7·7 - 8·4)	5·8 (5·7 - 5·9)	3·8 (3·6 - 4·0)	3·5 (3·0 - 4·1)	3·4 (2·2 - 5·2)
High-income	14·7 (14·2 - 15·2)	10·2 (10·0 - 10·4)	8·0 (7·6 - 8·5)	8·4 (7·4 - 9·6)	10·2 (6·5 - 15·1)
Latin America and Caribbean	6·8 (6·7 - 6·9)	8·5 (8·3 - 8·6)	7·2 (7·0 - 7·6)	6·0 (5·4 - 6·8)	4·2 (3·3 - 5·7)
North Africa and Middle East	5·2 (5·0 - 5·3)	9·0 (8·9 - 9·1)	9·4 (9·1 - 9·7)	10·2 (9·2 - 11·2)	11·2 (8·7 - 13·8)
South Asia	22·1 (21·6 - 22·5)	25·9 (25·2 - 26·5)	24·8 (23·7 - 25·8)	16·7 (14·3 - 19·1)	7·1 (4·4 - 10·1)
Southeast Asia, East Asia, and Oceania	33·7 (33·3 - 34·0)	26·0 (25·5 - 26·6)	17·6 (17·1 - 18·2)	13·9 (12·8 - 15·1)	9·6 (7·9 - 12·0)
Sub-Saharan Africa	9·6 (9·5 - 9·6)	14·6 (14·4 - 14·8)	29·2 (28·7 - 29·6)	41·3 (39·6 - 43·1)	54·3 (47·1 - 59·5)

Appendix Table S6. Share of livebirths by World Bank income region, 1950, 1980, 2021, 2050, and 2100 for the reference scenario.

	Share of livebirths (%)				
	1950	1980	2021	2050	2100
High Income	17·0 (16·4 - 17·6)	12·0 (11·8 - 12·2)	9·1 (8·6 - 9·5)	9·3 (8·3 - 10·6)	11·1 (7·2 - 16·2)
Upper Middle Income	37·7 (37·6 - 37·9)	30·2 (29·7 - 30·8)	20·4 (19·8 - 21·2)	16·1 (14·7 - 17·8)	11·6 (8·9 - 15·3)
Lower Middle Income	39·1 (38·6 - 39·6)	48·3 (47·6 - 48·9)	52·7 (51·7 - 53·7)	48·1 (45·2 - 50·5)	42·7 (37·5 - 48·7)
Low Income	6·2 (6·1 - 6·2)	9·5 (9·4 - 9·6)	17·8 (17·3 - 18·2)	26·5 (24·4 - 28·5)	34·6 (26·4 - 40·5)

Section 6: Author contributions

Managing the overall research enterprise

Stein Emil Vollset, Natalia V Bhattacharjee, Austin E Schumacher, Amanda E Smith, Paulina A Lindstedt, Kasey E Kinzel, Simon I Hay, Christopher J L Murray

Writing the first draft of the manuscript

Natalia V Bhattacharjee, Austin E Schumacher, Stein Emil Vollset, Catherine Bisignano, Susan A McLaughlin, Amanda E Smith, Paulina A Lindstedt, Simon I Hay, Christopher J L Murray

Primary responsibility for applying analytical methods to produce estimates

Natalia V Bhattacharjee, Chun-Wei Yuan, Cat Raggi, Julie Sojin Kim, Shujin Cao, Julian Chalek, Erin A May

Primary responsibility for seeking, cataloguing, extracting, or cleaning data; designing or coding figures and tables

Haley Comfort, John E Fuller, Denny Wang, Stefanie Watson, Nicholas Alexander Verghese, Julie Sojin Kim, Chun-Wei Yuan, Cat Raggi, QuynhAnh P Nguyen, Austin J Ahlstrom, Sam Farmer, Julian Chalek

Providing data or critical feedback on data sources

Yohannes Habtegiorgis Abate, Hedayat Abbastabar, Sherief Abd-Elsalam, Meriem Abdoun, Auwal Abdullahi, Mesfin Abebe, Samrawit Shawel Abebe, Hassan Abolhassani, Meysam Abolmaali, Lucas Guimarães Abreu, Michael R M Abrigo, Niveen ME Abu-Rmeileh, Tadele Girum Adal, Mesafint Molla Adane, Oluwafemi Atanda Adeagbo Adeagbo, Victor Adekanmbi, Olatunji O Adetokunboh, Qorinah Estiningtyas Sakilah Adnani, Muhammad Sohail Afzal, Saira Afzal, Antonella Agodi, Bright Opoku Ahinkorah, Sajjad Ahmad, Muayyad M Ahmad, Tauseef Ahmad, Ali Ahmed, Ayman Ahmed, Haroon Ahmed, Meqdad Saleh Ahmed, Budi Aji, Sreelatha Akkala, Khurshid Alam, Fahad Mashhour Alanezi, Turki M Alanzi, Almaza Albakri, Mohammed Albashtawy, Mohammad T. AlBataineh, Yihun Mulugeta Alemu, Khalid F Alhabib, Hanadi Al Hamad, Abid Ali, Rafat Ali, Liaqat Ali, Beriwan Abdulqadir Ali, Syed Shujait Shujait Ali, Sheikh Mohammad Alif, Syed Mohamed Aljunid, Joseph Uy Almazan, Omar Almidani, Salman Khalifah Al-Sabah, Awais Altaf, Farrukh Jawad Alvi, Nelson Alvis-Guzman, Hassan Alwafi, Hany Aly, Edward Kwabena Ameyaw, Dickson A Amugsi, Ganiyu Adeniyi Amusa, Deanna Anderlini, Pedro Prata Andrade, Tudorel Andrei, Ernoiz Antriyandarti, Saeid Anvari, SALEHA ANWAR, Razique Anwer, Jalal Arabloo, Timur Aripov, Benedetta Armocida, Johan Ärnlöv, Anton A Artamonov, Judie Arulappan, Zatollah Asemi, Tahira Ashraf, Seyyed Shamsadin Athari, Alok Atreya, Getnet Melaku Ayele, Ahmed Y Azzam, Soroush Baghdadi, Sara Bagherieh, Atif Amin Baig, Abdulaziz T. Bako, Ovidiu Constantin Baltatu, Maciej Banach, Palash Chandra Banik, Mehmet Firat Baran, Martina Barchitta, Mainak Bardhan, Till Winfried Bärnighausen, Amadou Barrow, Zarrin Basharat, Sanjay Basu, Abdul-Monim Mohammad Batiha, Emad Behboudi, Diana Fernanda Bejarano Ramirez, Alehegn Bekele, Sefealem Assefa Belay, Uzma Iqbal Belgaumi, Michelle L Bell, Olorunjuwon Omolaja Bello, Akshaya Srikanth Bhagavathula, Vivek Bhat, Jasvinder Singh Bhatti, Gurjit Kaur Bhatti, Virginia Bodolica, Aadam Olalekan Bodunrin, Milad Bonakdar Hashemi, Berrak Bora Basara, Souad Bouaoud, Dejana Braithwaite, Danilo Buonsenso, Florentino Luciano Caetano dos Santos, Carlos A Castañeda-Orjuela, Francieli Cembranel, Chiranjib Chakraborty, Periklis Charalampous, Vijay Kumar Chattu, Ju-Huei Chien, William C S Cho, Sungchul Choi, Bryan Chong, Hitesh Chopra, Dinh-Toi Chu, Eric Chung, Zinhle Cindi, Rafael M Claro, Alyssa Columbus, Haley Comfort, Joao Conde, Michael H Criqui, Natália Cruz-Martins, Zhaoli Dai, Giovanni Damiani, Aso Mohammad Darwesh, Saswati Das, Claudio Alberto Dávila-Cervantes, Aklilu Tamire Debele, Shayom Debopadhaya, Ivan Delgado-Enciso, Berecha Hundessa Demessa, Kebede Deribe, Nikolaos Dervenis, Hardik Dineshbhai Desai, Rupak Desai, Vinoth Gnana Chellaiyan Devanbu, Vishal R Dhulipala, Diana Dias da Silva, Daniel Diaz, Michael J Diaz, M Ashworth Dirac, Thao Huynh Phuong Do, Thanh Chi Do, Leila Doshmangir, Wendel Mombaque dos Santos, Robert Kokou Dowou, Haneil Larson Dsouza, John Dube, Joe Duprey, Andre Rodrigues Duraes, Senbagam Duraisamy, Paulina Agnieszka Dzianach, Michael Ekholuenetale, Legesse Tesfaye Elilo, Mohammed Elshaer, Amir Emami Zeydi, Sharareh Eskandarieh, Adeniyi Francis Fagbamigbe, Andre Faro, Abidemi Omolara Fasanmi, Ali Fatehizadeh, Seyed-Mohammad Fereshtehnejad, Artem Alekseevich Fomenkov, Kayode Raphael Fowobaje, Takeshi Fukumoto, John E Fuller, Peter Andras Gaal, Muktar A. Gadanya, Yaseen Galali, Mohammad Arfat Ganiyani, Tesfay B B Gebremariam, Simona Roxana Georgescu, Peter W Gething, Arun Digambarrao Ghuge, Alem Girmay, Laszlo Göbölös, Ali Golchin, Mahaveer Golechha, Pouya Goleij, Sameer Vali Gopalani, Houman Goudarzi, Shi-Yang Guan, Mesay Dechasa Gudeta, Vijai Kumar Gupta, Manoj Kumar Gupta, Sapna Gupta, Bhawna Gupta, Rabih Halwani, Nadia M Hamdy, Josep Maria Haro, Mohammad Jahid Hasan, Soheil Hassanipour, Hadi Hassankhani, Bartosz Helfer, Claudiu Herteliu, Kamran Hessami, Nguyen Quoc Hoan, Nobuyuki

Horita, Md Mahbub Hossain, Mehdi Hosseinzadeh, Chengxi Hu, Javid Hussain, Nawfal R Hussein, Hong-Han Huynh, Segun Emmanuel Ibitoye, Olayinka Stephen Ilesanmi, Nahlah Elkudssiah Ismail, Gaetano Isola, Mahalaxmi Iyer, Linda Merin J, Khushleen Jaggi, Nader Jahanmehr, Haitham Jahrami, Nityanand Jain, Mihajlo Jakovljevic, Tahereh Javaheri, Sathish Kumar Jayapal, Shubha Jayaram, Heng Jiang, Jost B. Jonas, Tamas Joo, Charity Ehimwenma Joshua, Farahnaz Joukar, Jacek Jerzy Jozwiak, Mikk Jürisson, Billingsley Kaambwa, Zubair Kabir, Leila R Kalankesh, Tanuj Kanchan, Rami S. Kantar, Mehrdad Karajizadeh, Manoochehr Karami, Faizan Zaffar Kashoo, Nicholas J Kassebaum, Evie Shoshannah Kendal, Himanshu Khajuria, Nauman Khalid, Faham Khamesipour, Maseer Khan, M Nuruzzaman Khan, Ikramullah Khan, Moien AB Khan, Khaled Khatab, Feriha Fatima Khidri, Min Seo Kim, Adnan Kisa, Sezer Kisa, Gerbrand Koren, Oleksii Korzh, Soewarta Kosen, Sindhura Lakshmi Koulmane Laxminarayana, Kewal Krishan, Varun Krishna, Vijay Krishnamoorthy, Barthelemy Kuate Defo, Burcu Kucuk Bicer, Ilari Kuitunen, Harish Kumar, Kunle Rotimi Kunle, Asep Kusnali, Dian Kusuma, Muhammad Awwal Ladan, Chandrakant Lahariya, Daphne Teck Ching Lai, Dharmesh Kumar Lal, Judit Lám, Iván Landires, Savita Lasrado, Kamaluddin Latief, Kaveh Latifinaibin, Trang Diep Thanh Le, Nhi Huu Hanh Le, Caterina Ledda, Seung Won Lee, Munjae Lee, An Li, Stephen S Lim, Stefan Listl, Jue Liu, Gang Liu, Xuefeng Liu, Erand Llanaj, Rubén López-Bueno, László Lorenzovici, Paulo A. Lotufo, Zheng Feei Ma, Mohammed Magdy Abd El Razek, Azzam A Maghazachi, Jeadran N. Malagón-Rojas, Elaheh Malakan Rad, Kashish Malhotra, Deborah Carvalho Malta, Abdullah A Mamun, Yasaman Mansoori, Borhan Mansouri, Mohammad Ali Mansournia, Joemer C. Maravilla, Abdoljalal Marjani, Francisco Rogerlândio Martins-Melo, Sharmeen Maryam, Roy Rillera Marzo, Alireza Masoudi, Jishanth Mattumpuram, Andrea Maugeri, Erin A May, Maryam Mazaheri, Anna Laura Wensel McKowen, Entezar Mehrabi Nasab, Tesfahun Mekene Meto, Walter Mendoza, Ritesh G Menezes, Sultan Ayoub Meo, Atte Meretoja, Sachith Mettananda, Kukulege Chamila Dinushi Mettananda, Irmina Maria Michalek, Ted R Miller, Le Huu Nhat Minh, Erkin M Mirrakhimov, Chaitanya Mittal, Babak Moazen, Soheil Mohammadi, Abdollah Mohammadian-Hafshejani, Mustapha Mohammed, Shafiu Mohammed, Salahuddin Mohammed, Ali H Mokdad, Peyman Mokhtarzadehazar, Lorenzo Monasta, Mohammad Ali Moni, Maryam Moradi, Yousef Moradi, Shane Douglas Morrison, Jakub Morze, Sumaira Mubarik, Francesk Mulita, Christopher J L Murray, Fungai Musaigwa, Ana-Maria Musina, Saravanan Muthupandian, Ahamarshan Jayaraman Nagarajan, Gabriele Nagel, Ganesh R Naik, Mukhammad David Naimzada, Sreenivas Narasimha Swamy, Bruno Ramos Nascimento, Abdallah Y Naser, Mohammad Javad Nasiri, Zuhair S Natto, Biswa Prakash Nayak, Vinod C Nayak, Ionut Negoi, Ruxandra Irina Negoi, Henok Biresaw Netsere, Josephine W. Ngunjiri, Van Thanh Nguyen, Dang H Nguyen, QuynhAnh P Nguyen, Robina Khan Niazi, Taxiarchis Konstantinos Nikolouzakis, Amin Reza Nikpoor, Chukwudi A Nnaji, Lawrence Achilles Nnyanzi, Shuhei Nomura, Mamoona Noreen, Dieta Nurrika, Bogdan Oancea, Kehinde O Obamiro, Ismail A. Odetokun, Nkechi Martina Odogwu, Ayodipupo Sikiru Oguntade, Osaretin Christabel Okonji, Andrew T Olagunju, Bolajoko Olubukunola Olusanya, Jacob Olusegun Olusanya, Hany A Omar, Obinna E Onwujekwe, Uchechukwu Levi Osuagwu, Adrian Otoiu, Stanislav S Otstavnov, Amel Ouyahia, Mayowa O Owolabi, Mahesh Padukudru P A, Mohammad Taha Pahlevan Fallahy, Adrian Pana, Paramjot Panda, Songhomitra Panda-Jonas, Anca Mihaela Pantea Stoian, Romil R Parikh, Seoyeon Park, Ashwaghosha Parthasarathi, Hemal M Patel, Jay Patel, Shankargouda Patil, Uttam Paudel, Mihaela Paun, Veincent Christian Filipino Pepito, Gavin Pereira, Arokiasamy Perianayagam, Simone Perna, Hoang Tran Pham, Anil K. Philip, Daniela Pierannunzio, David M Pigott, Peter Pollner, Ramesh Poluru, Maarten J Postma, Naeimeh Pourtaheri, Sergio I Prada, Jagadeesh Puvvula, Fakher Rahim, Masoud Rahmati, Prasanna Ram, Shakthi Kumaran Ramasamy, Juwel Rana, Chhabi Lal Ranabhat, Nemanja Rancic, Amey Rane, Chythra R Rao, Vahid Rashedi, Sina Rashedi, Zubair Ahmed Ratan, Giridhara Rathnaiah Babu, Santosh Kumar Rauniyar, Nakul Ravikumar, Salman Rawaf, Reza Rawassizadeh, Bhageerathy Reshmi, Aida Rezaei nejad, Hannah Elizabeth Robinson-Oden, Jefferson Antonio Buendia Rodriguez, Luca Ronfani, Himanshu Sekhar Rout, Priyanka Roy, Enrico Rubagotti, Tilleye Runghien, Aly M A Saad, Zahra Saadatian, Siamak Sabour, Basema Saddik, Bashdar Abuzed Sadee, Ehsan Sadeghi, Umar Saeed, Sher Zaman Safi, Manika Saha, Zahra Saif, Joseph W Sakshaug, Afeez Abolarinwa Salami, Marwa Rashad Salem, Sara Samadzadeh, Vijaya Paul Samuel, Abdallah M Samy, Nima Sanadgol, Francesca Sanna, Milena M. Santric-Milicevic, Sivan Yegnanarayana Iyer Saraswathy, Babak Saravi, Brijesh Sathian, Anudeep Sathyanarayan, Md Abu Sayeed, David C Schwebel, Subramanian Senthilkumaran, Edson Serván-Mori, Yashendra Sethi, Jaffer Shah, Ahmed Shaikh, Masood Ali Shaikh, Mehran Shams-Beyranvand, Mohammad Anas Shamsi, Abhishek Shankar, Mohammed Shannawaz, Medha Sharath, Javad Sharifi-Rad, Vishal Sharma, Rajesh Sharma, Rajesh P. Shastry, David H Shaw, Maryam Shayan, Pavanchand H Shetty, Peilin Shi, Aminu Shittu, Farhad Shokraneh, Sina Shool, Kanwar Hamza Shuja, Jasvinder A. Singh, Abhinav Singh, Sameh S M Soliman, Yonatan Solomon, Michael Spartalis, Chandrashekhar T Sreeramareddy, Muhammad Haroon Stanikzai, Muhammad Suleman, Rizwan Suliankatchi Abdulkader, Abida Sultana, Chandan Kumar Swain, Lukasz Szarpak, Mindy D Szeto, Miklós Szócska, Payam Tabaee Damavandi, Rafael Tabarés-Seisdedos, Shima Tabatabai, Karen M Tabb, Mohammad Tabish, Ardeshir

Tajbakhsh, Mircea Tampa, Ker-Kan Tan, Mengistie Kassahun Tariku, Mohamad-Hani Temsah, Pugazhenthan Thangaraju, Nihal Thomas, Marcos Roberto Tovani-Palone, Jasmine T Tran, Indang Trihandini, Samuel Joseph Tromans, Aristidis Tsatsakis, Sana Ullah, Srikanth Umakanthan, Bhaskaran Unnikrishnan, Era Upadhyay, Jibrin Sammani Usman, Jef Van den Eynde, Shoban Babu Varthya, Tommi Juhani Vasankari, Balachandar Vellingiri, Massimiliano Veroux, Georgios-Ioannis Verras, Sergey Konstantinovitch Vladimirov, Vasily Vlassov, Bay Vo, Simona Ruxandra Volovat, Theo Vos, Yasir Waheed, Elias Bekele Wakwoya, Shu Wang, Denny Wang, Stefanie Watson, Kosala Gayan Weerakoon, Ronny Westerman, Peter Willeit, Felicia Wu, Juan Xia, Hong Xiao, Suowen Xu, Gelin Xu, Ali Yadollahpour, Shirin Yaghoobpoor, Tina Yaghoobpour, Sajad Yaghoubi, Pengpeng Ye, Renjulal Yesodharan, Paul Yip, Dong Keon Yon, Naohiro Yonemoto, Mustafa Z Younis, Chuanhua Yu, Yong Yu, Mikhail Sergeevich Zastrozhin, Magdalena Zielińska, Zhiyong Zou.

Developing methods or computational machinery

Samrawit Shawel Abebe, Rufus Adesoji Adedoyin, Olatunji O Adetokunboh, Qorinah Estiningtyas Sakilah Adnani, Saira Afzal, Austin J Ahlstrom, Mohammed Albashtawy, Liaqat Ali, Jalal Arabloo, Aleksandr Y Aravkin, Judie Arulappan, Ahmed Y Azzam, Akshaya Srikanth Bhagavathula, Natalia V Bhattacharjee, Aadam Olalekan Bodunrin, Souad Bouaoud, Fan Cao, Shujin Cao, Julian Chalek, Hitesh Chopra, Kaleb Coberly, Haley Comfort, Aso Mohammad Darwesh, Hardik Dineshbhai Desai, Thanh Chi Do, Joe Duprey, Adeniyi Francis Fagbamigbe, Samuel Farmer, Ali Fatehizadeh, Peter W Gething, Alem Girmay, Manoj Kumar Gupta, Simon I Hay, Mohammad Heidari, Mehdi Hosseinzadeh, Hong-Han Huynh, Gaetano Isola, Linda Merin J, Tahereh Javaheri, Sathish Kumar Jayapal, Faizan Zaffar Kashoo, Nicholas J Kassebaum, Julie Sojin Kim, Adnan Kisa, Chandrakant Lahariya, Nhi Huu Hanh Le, An Li, Mohammed Magdy Abd El Razek, Yasaman Mansoori, Borhan Mansouri, Kelsey Lynn Maass, Alireza Masoudi, Erin A May, Le Huu Nhat Minh, Abdollah Mohammadian-Hafshejani, Ali H Mokdad, Mohammad Ali Moni, Yousef Moradi, Rohith Motappa, Francesk Mulita, Christopher J L Murray, Henok Biresaw Netsere, Josephine W. Ngunjiri, Van Thanh Nguyen, Dang H Nguyen, Andrew T Olagunju, Michal Ordak, Yaz Ozten, Adrian Pana, Spencer A Pease, Hoang Tran Pham, Hadi Raeisi Shahraki, Cat Raggi, Chhabi Lal Ranabhat, Santosh Kumar Rauniyar, Reza Rawassizadeh, Himanshu Sekhar Rout, Enrico Rubagotti, Susan Fred Rumisha, Tilleye Runghien, Umar Saeed, Zahra Saif, Abdallah M Samy, Austin E Schumacher, Yashendra Sethi, Mohammad Anas Shamsi, Javad Sharifi-Rad, Vishal Sharma, David H Shaw, Amanda E Smith, Georgia Smith, Reed J D Sorensen, Michael Spartalis, Muhammad Haroon Stanikzai, Muhammad Suleman, Chandan Kumar Swain, Nicholas Alexander Verghese, Bay Vo, Stein Emil Vollset, Ronny Westerman, Ali Yadollahpour, Chun-Wei Yuan, Milad Zandi, Ghazal G Z Zandieh.

Providing critical feedback on methods or results

Amirali Aali, Yohannes Habtegiorgis Abate, Rouzbeh Abbasgholizadeh, Hedayat Abbastabar, Samar Abd ElHafeez, Sherief Abd-Elsalam, Mohammad Abdollahi, Meriem Abdoun, Auwal Abdullahi, Mesfin Abebe, Samrawit Shawel Abebe, Hassan Abolhassani, Meysam Abolmaali, Mohamed Abouzid, Girma Beressa Aboye, Lucas Guimarães Abreu, Woldu Aberhe Abrha, Michael R M Abrigo, Dariush Abtahi, Hasan Abualruz, Bilyaminu Abubakar, Eman Abu-Gharbieh, Tadele Girum Adal, Mesafint Molla Adane, Oluwafemi Atanda Adeagbo Adeagbo, Rufus Adesoji Adedoyin, Victor Adekanmbi, Bashir Aden, Abiola Victor Adepoju, Olatunji O Adetokunboh, Juliana Bunmi Adetunji, Daniel Adedayo Adeyinka, Olorunsola Israel Adeyomoye, Qorinah Estiningtyas Sakilah Adnani, Saryia Adra, Rotimi Felix Afolabi, Shadi Afyouni, Muhammad Sohail Afzal, Saira Afzal, Shahin Aghamiri, Antonella Agodi, Williams Agyemang-Duah, Bright Opoku Ahinkorah, Sajjad Ahmad, Muayyad M Ahmad, Tauseef Ahmad, Danish Ahmad, Firdos Ahmad, Aqeel Ahmad, Ayman Ahmed, Haroon Ahmed, Syed Anees Ahmed, Luai A. Ahmed, Budi Aji, Gizachew Taddesse Akalu, Hossein Akbarialiabad, Rufus Olusola Akinyemi, Sreelatha Akkala, Tareq Mohammed Ali AL-Ahdal, Samer O. Alalalmeh, Ziyad Al-Aly, Khurshid Alam, Rasmieh Mustafa Al-amer, Fahad Mashhour Alanezi, Turki M Alanzi, Almaza Albakri, Mohammed Albashtawy, Mohammad T. AlBataineh, Sharifullah Alemi, Hediyeh Alemi, Yihun Mulugeta Alemu, Adel Ali Saeed Al-Gheethi, Khalid F Alhabib, Hanadi Al Hamad, Syed Mahfuz Al Hasan, Robert Kaba Alhassan, Mohammed Usman Ali, Abid Ali, Rafat Ali, Liaqat Ali, Syed Shujait Shujait Ali, Sheikh Mohammad Alif, Mohammad Aligol, Mehran Alijanzadeh, Mohammad A M Aljasir, Syed Mohamed Aljunid, Sabah Al-Marwani, Joseph Uy Almazan, Hesham M Al-Mekhlafi, Omar Almidani, Mahmoud A. Alomari, Basem Al-Omari, Mohammad Al Qadire, Jaber S Alqahtani, Ahmed Yaseen Alqutaibi, Rajaa M Al-Raddadi, Salman Khalifah Al-Sabah, Awais Altaf, Jaffar A. Al-Tawfiq, Khalid A Altirkawi, Nelson Alvis-Guzman, Hassan Alwafi, Yaser Mohammed Al-Worafi, Hany Aly, Safwat Aly, Karem H Alzoubi, Edward Kwabena Ameyaw, Tarek Tawfik Amin, Alireza Amindarolzarbi, Mostafa Amini-Rarani, Sohrab Amiri, Dickson A

Amugsi, Ganiyu Adeniyi Amusa, Robert Ancuceanu, Deanna Anderlini, Pedro Prata Andrade, Tudorel Andrei, Catalina Liliana Andrei, Alireza Ansari-Moghaddam, Ernoiz Antriyandarti, SALEHA ANWAR, Razique Anwer, Anayochukwu Edward Anyasodor, Jalal Arabloo, Elshaimaa A Arafa, Mosab Arafat, Timur Aripov, Mesay Arkew, Benedetta Armocida, Mahwish Arooj, Anton A Artamonov, Judie Arulappan, Raphael Taiwo Aruleba, Ashokan Arumugam, Mohsen Asadi-Lari, Saeed Asgary, Mohammad Asghari-Jafarabadi, Mubarek Yesse Ashemo, Tahira Ashraf, Muhammad Ashraf, Marvellous O Asika, Seyyed Shamsadin Athari, Maha Moh'd Wahbi Atout, Alok Atreya, Avinash Aujayeb, Abolfazl Avan, Amlaku Mulat Aweke, Getnet Melaku Ayele, Sina Azadnajafabad, Rui M S Azevedo, Ahmed Y Azzam, Muhammad Badar, Ashish D Badiye, Soroush Baghdadi, Nasser Bagheri, Sara Bagherieh, Najmeh Bahmanziari, Ruhai Bai, Atif Amin Baig, Jennifer L Baker, Abdulaziz T. Bako, Ravleen Kaur Bakshi, Madhan Balasubramanian, Ovidiu Constantin Baltatu, Maciej Banach, Soham Bandyopadhyay, Palash Chandra Banik, Biswajit Banik, Hansi Bansal, Mehmet Firat Baran, Martina Barchitta, Mainak Bardhan, Till Winfried Bärnighausen, Hiba Jawdat Barqawi, Amadou Barrow, Sandra Barteit, Zarrin Basharat, Asma'u I J Bashir, Hameed Akande Bashiru, Afisu Basiru, João Diogo Basso, Sanjay Basu, Abdul-Monim Mohammad Batiha, Kavita Batra, Bernhard T Baune, Mohsen Bayati, Tahmina Begum, Emad Behboudi, Amir Hossein Behnoush, Diana Fernanda Bejarano Ramirez, Alehegn Bekele, Sefealem Assefa Belay, Uzma Iqbal Belgaumi, Michelle L Bell, Olorunjuwon Omolaja Bello, Apostolos Beloukas, Isabela M Bensenor, Zombor Berezvai, Alemshet Yirga Berhie, Amiel Nazer C Bermudez, Paulo J G Bettencourt, Akshaya Srikanth Bhagavathula, Nikha Bhardwaj, Pankaj Bhardwaj, Vivek Bhat, Natalia V Bhattacharjee, Jasvinder Singh Bhatti, Gurjit Kaur Bhatti, Manpreet S Bhatti, Rajbir Bhatti, Atanu Biswas, Raaj Kishore Biswas, Veera R Bitra, Micheal Kofi Boachie, Virginia Bodolica, Aadam Olalekan Bodunrin, Eyob Ketema Bogale, Milad Bonakdar Hashemi, Souad Bouaoud, Dejana Braithwaite, Nicholas J K Breitborde, Dana Bryazka, Norma B Bulamu, Danilo Buonsenso, Katrin Burkart, Richard A Burns, Yasser Bustanji, Zahid A Butt, Nadeem Shafique Butt, Florentino Luciano Caetano dos Santos, Ismael R Campos-Nonato, Fan Cao, Angelo Capodici, Carlos A Castañeda-Orjuela, Giulio Castelpietra, Luca Cegolon, Francieli Cembranel, Ester Cerin, Yaacoub Chahine, Chiranjib Chakraborty, Periklis Charalampous, Vijay Kumar Chattu, Malizgani Paul Chavula, An-Tian Chen, Haowei Chen, William C S Cho, Sungchul Choi, Bryan Chong, Hitesh Chopra, Sonali Gajanan Choudhari, Devasahayam J Christopher, Dinh-Toi Chu, Isaac Sunday Chukwu, Eric Chung, Sheng-Chia Chung, Zinhle Cindi, Iolanda Cioffi, Raffaela Ciuffreda, Alyssa Columbus, Haley Comfort, Joao Conde, Michael H Criqui, Natália Cruz-Martins, Silvia Magali Cuadra-Hernández, Mario D'Oria, Omid Dadras, Zhaoli Dai, Giovanni Damiani, Aso Mohammad Darwesh, Jai K Das, Saswati Das, Mohsen Dashti, Anna Dastiridou, Claudio Alberto Dávila-Cervantes, Kairat Davletov, Aklilu Tamire Debele, Shayom Debopadhaya, Ivan Delgado-Enciso, Dessalegn Demeke, Berecha Hundessa Demessa, Xinlei Deng, Kebede Deribe, Nikolaos Dervenis, Hardik Dineshbhai Desai, Rupak Desai, Vinoth Gnana Chellaiyan Devanbu, Arkadeep Dhali, Kuldeep Dhama, Meghnath Dhimal, Vishal R Dhulipala, Diana Dias da Silva, Daniel Diaz, Michael J Diaz, Adriana Dima, Delaney D Ding, M Ashworth Dirac, Thao Huynh Phuong Do, Thanh Chi Do, Leila Doshmangir, Wendel Mombaque dos Santos, Robert Kokou Dowou, Viola Dsouza, Haneil Larson Dsouza, John Dube, Senbagam Duraisamy, Oyewole Christopher Durojaiye, Sulagna Dutta, Arkadiusz Marian Dziedzic, Alireza Ebrahimi, Hisham Atan Edinur, Kristina Edvardsson, Ferry Efendi, Terje Andreas Eikemo, Michael Ekholuenetale, Noha Mousaad Elemam, Ghada Metwally Tawfik ElGohary, Muhammed Elhadi, Legesse Tesfaye Elilo, Omar Abdelsadek Abdou Elmeligy, Mohamed A Elmonem, Mohammed Elshaer, Ibrahim Elsohaby, Amir Emami Zeydi, Luchuo Engelbert Bain, Sharareh Eskandarieh, Farshid Etaee, Natalia Fabin, Adeniyi Francis Fagbamigbe, Saman Fahimi, Aliasghar Fakhri-Demeshghieh, Luca Falzone, Ali Faramarzi, MoezAlIslam Ezzat Mahmoud Faris, Samuel Farmer, Andre Faro, Abidemi Omolara Fasanmi, Ali Fatehizadeh, Pooria Fazeli, Valery L. Feigin, Seyed-Mohammad Fereshtehnejad, Abdullah Hamid Feroze, Pietro Ferrara, Getahun Fetensa, Irina Filip, Florian Fischer, Joanne Flavel, Nataliya A. Foigt, Morenike Oluwatoyin Folayan, Artem Alekseevich Fomenkov, Matteo Foschi, Kayode Raphael Fowobaje, Kate Louise Francis, Alberto Freitas, Takeshi Fukumoto, Peter Andras Gaal, Muktar A. Gadanya, Abhay Motiramji Gaidhane, Yaseen Galali, Aravind P Gandhi, Balasankar Ganesan, Mohammad Arfat Ganiyani, M.A. Garcia-Gordillo, Naval Garg, Rupesh K. Gautam, Federica Gazzelloni, Semiu Olatunde Gbadamosi, Miglas W Gebregergis, Mesfin Gebrehiwot, Tesfay Brhane Gebremariam, Tesfay B B Gebremariam, Teferi Gebru Gebremeskel, Yohannes Fikadu Geda, Simona Roxana Georgescu, Urge Gerema, Habtamu Geremew, Motuma Erena Getachew, MohammadReza Ghasemi, Afsaneh Ghasemzadeh, Fariba Ghassemi, Ramy Mohamed Ghazy, Sailaja Ghimire, Asadollah Gholamian, Ali Gholamrezanezhad, Arun Digambarrao Ghuge, Tiffany K Gill, Matteo Giorgi, Alem Girmay, James C Glasbey, Laszlo Göbölös, Amit Goel, Ali Golchin, Mahaveer Golechha, Alessandra C Goulart, Anmol Goyal, Simon

Matthew Graham, Michal Grivna, Shi-Yang Guan, Mohammed Ibrahim Mohialdeen Gubari, Mesay Dechasa Gudeta, Stefano Guicciardi, David Gulisashvili, Damitha Asanga Gunawardane, Cui Guo, Vijai Kumar Gupta, Manoj Kumar Gupta, Sapna Gupta, Anish Kumar Gupta, Bhawna Gupta, Veer Bala Gupta, Vivek Kumar Gupta, Annie Haakenstad, Farrokh Habibzadeh, Najah R Hadi, Nils Haep, Ramtin Hajibeygi, Sebastian Haller, Rabih Halwani, Randah R Hamadeh, Nadia M Hamdy, Samer Hamidi, Qiuxia Han, Md Nuruzzaman Haque, Josep Maria Haro, Mohammad Jahid Hasan, Ikramul Hasan, Hamidreza Hasani, Md Saquib Hasnain, Ikrama Hassan, Soheil Hassanipour, Hadi Hassankhani, Simon I Hay, Jeffrey J. Hebert, Omar E. Hegazi, Mohammad Heidari, Bartosz Helfer, Mehdi Hemmati, Brenda Yuliana Herrera-Serna, Claudiu Herteliu, Kamran Hessami, Kamal Hezam, Yuta Hiraike, Nguyen Quoc Hoan, Ramesh Holla, Nobuyuki Horita, Md Mahbub Hossain, Mohammad Bellal Hossain Hossain, Hassan Hosseinzadeh, Mehdi Hosseinzadeh, Mihaela Hostiuc, Mohamed Hsairi, Chengxi Hu, M Mamun Huda, Ayesha Humayun, Nawfal R Hussein, Hong-Han Huynh, Bing-Fang Hwang, Segun Emmanuel Ibitoye, Pulwasha Maria Iftikhar, Olayinka Stephen Ilesanmi, Milena D. Ilic, Irena M. Ilic, Mustapha Immurana, Leeberk Raja Inbaraj, Afrin Iqbal, Md. Rabiul Islam, Nahlah Elkudssiah Ismail, Hiroyasu Iso, Gaetano Isola, Masao Iwagami, Mahalaxmi Iyer, Linda Merin J, Jalil Jaafari, Louis Jacob, Farhad Jadidi-Niaragh, Khushleen Jaggi, Nader Jahanmehr, Haitham Jahrami, Nityanand Jain, Akhil Jain, Ammar Abdulrahman Jairoun, Mihajlo Jakovljevic, Elham Jamshidi, Sabzali Javadov, Tahereh Javaheri, Sathish Kumar Jayapal, Shubha Jayaram, Sun Ha Jee, Jayakumar Jeganathan, Anil K Jha, Ravi Prakash Jha, Heng Jiang, Mohammad Jokar, Jost B. Jonas, Tamas Joo, Nitin Joseph, Charity Ehimwenma Joshua, Farahnaz Joukar, Jacek Jerzy Jozwiak, Mikk Jürisson, Vaishali K, Billingsley Kaambwa, Abdulkareem Kabir, Ali Kabir, Zubair Kabir, Hannaneh Kabir, Rizwan Kalani, Leila R Kalankesh, Feroze Kaliyadan, Sanjay Kalra, Rajesh Kamath, Sagarika Kamath, Tanuj Kanchan, Kehinde Kazeem Kanmodi, Sushil Kumar Kansal, Rami S. Kantar, Neeti Kapoor, Mehrdad Karajizadeh, Manoochehr Karami, Ibraheem M Karaye, Faizan Zaffar Kashoo, Nicholas J Kassebaum, Joonas H Kauppila, Foad Kazemi, sara Kazeminia, John H Kempen, Evie Shoshannah Kendal, Kamyab Keshtkar, Mohammad Keykhaei, Himanshu Khajuria, Amirmohammad Khalaji, Nauman Khalid, Anees Ahmed Khalil, Alireza Khalilian, Faham Khamesipour, Ajmal Khan, Mohammad Jobair Khan, Asaduzzaman Khan, Maseer Khan, M Nuruzzaman Khan, Ikramullah Khan, Moien AB Khan, Young-Ho Khang, Shaghayegh Khanmohammadi, Khaled Khatab, Feriha Fatima Khidri, Moein Khormali, Wondwosen Teklesilasie Kidane, Zemene Demelash Kifle, Julie Sojin Kim, Ruth W Kimokoti, Girmay Tsegay Kiross, Adnan Kisa, Sezer Kisa, Ali-Asghar Kolahi, Farzad Kompani, Gerbrand Koren, Oleksii Korzh, Sindhura Lakshmi Koulmane Laxminarayana, Kewal Krishan, Varun Krishna, Vijay Krishnamoorthy, Barthelemy Kuate Defo, Burcu Kucuk Bicer, Mohammed Kuddus, Md Abdul Kuddus, Ilari Kuitunen, Satyajit Kundu, Kunle Rotimi Kunle, Om P Kurmi, Asep Kusnali, Dian Kusuma, Evans F Kyei, Ilias Kyriopoulos, Ben Lacey, Muhammad Awwal Ladan, Chandrakant Lahariya, Daphne Teck Ching Lai, Dharmesh Kumar Lal, Judit Lám, Demetris Lamnisos, Iván Landires, Francesco Lanfranchi, Berthold Langguth, Savita Lasrado, Kamaluddin Latief, Kaveh Latifinaibin, Carlo La Vecchia, Long Khanh Dao Le, Trang Diep Thanh Le, Nhi Huu Hanh Le, Caterina Ledda, Seung Won Lee, Munjae Lee, Yo Han Lee, Gebretsadik Kiros Lema, Elvynna Leong, Temesgen L. Lerango, Xiaopan Li, Ming-Chieh Li, Wei Li, Shanshan Li, Virendra S Ligade, Stephen S Lim, Ro-Ting Lin, Stefan Listl, Xiaofeng Liu, Yuewei Liu, Jue Liu, Gang Liu, Xuefeng Liu, Erand Llanaj, Rubén López-Bueno, László Lorenzovici, Paulo A. Lotufo, Jailos Lubinda, Giancarlo Lucchetti, Zheng Feei Ma, Monika Machoy, Áurea M. Madureira-Carvalho, Mohammed Magdy Abd El Razek, Azzam A Maghazachi, Soleiman Mahjoub, Mansour Adam Mahmoud, Azeem Majeed, Elaheh Malakan Rad, Kashish Malhotra, Iram Malik, Ahmad Azam Malik, Deborah Carvalho Malta, Abdullah A Mamun, Yosef Manla, Yasaman Mansoori, Ali Mansour, Borhan Mansouri, Zeinab Mansouri, Mohammad Ali Mansournia, Joemer C. Maravilla, Mirko Marino, Abdoljalal Marjani, Ramon Martinez-Piedra, Francisco Rogerlândio Martins-Melo, Miquel Martorell, Sharmeen Maryam, Roy Rillera Marzo, Kelsey Lynn Maass, Alireza Masoudi, Jishanth Mattumpuram, Richard James Maude, Andrea Maugeri, Erin A May, Mahsa Mayeli, John J. McGrath, Martin McKee, Anna Laura Wensel McKowen, Steven M McPhail, Rahul Mehra, Kamran Mehrabani-Zeinabad, Entezar Mehrabi Nasab, Tesfahun Mekene Meto, Max Alberto Mendez Mendez-Lopez, Walter Mendoza, Ritesh G Menezes, Alexios-Fotios A. Mentis, Sultan Ayoub Meo, Mohsen Merati, Tuomo J Meretoja, Atte Meretoja, Abera M Mersha, Tomislav Mestrovic, Pouya Metanat, Sachith Mettananda, Kukulege Chamila Dinushi Mettananda, Laurette Mhlanga, Adquate Mhlanga, Tianyue Mi, Tomasz Miazgowski, Georgia Micha, Irmina Maria Michalek, Ted R Miller, Le Huu Nhat Minh, Erkin M Mirrakhimov, Mizan Kiros Mirutse, Moonis Mirza, Sanjeev Misra, Chaitanya Mittal, Babak Moazen, Nouh Saad Mohamed, Ahmed Ismail Mohamed, Jama Mohamed, Mouhand F H Mohamed, Abdalla Z Mohamed, Sakineh Mohammad-Alizadeh-Charandabi, Soheil

Mohammadi, Abdollah Mohammadian-Hafshejani, Mustapha Mohammed, Shafiu Mohammed, Salahuddin Mohammed, Ali H Mokdad, Peyman Mokhtarzadehazar, Hossein Molavi Vardanjani, Sabrina Molinaro, Mohammad Ali Moni, Maryam Moradi, Yousef Moradi, Paula Moraga, Rafael Silveira Moreira, Negar Morovatdar, Elias Mossialos, Rohith Motappa, Amin Mousavi Khaneghah, Christine Mpundu-Kaambwa, Sumaira Mubarik, Lorenzo Muccioli, Francesk Mulita, Kavita Munjal, Efrén Murillo-Zamora, Christopher J L Murray, Fungai Musaigwa, Ana-Maria Musina, Sathish Muthu, Saravanan Muthupandian, Muhammad Muzaffar, Woojae Myung, Ahamarshan Jayaraman Nagarajan, Gabriele Nagel, Pirouz Naghavi, Ganesh R Naik, Gurudatta Naik, Firzan Nainu, Vinay Nangia, Sreenivas Narasimha Swamy, Bruno Ramos Nascimento, Gustavo G Nascimento, Abdallah Y Naser, Mohammad Javad Nasiri, Zuhair S Natto, Javaid Nauman, Muhammad Naveed, Biswa Prakash Nayak, Rawlance Ndejjo, Sabina Onyinye Nduaguba, Hadush Negash, Chernet Tafere Negesse, Ionut Negoi, Ruxandra Irina Negoi, Seyed Aria Nejadghaderi, Chakib Nejjari, Henok Biresaw Netsere, Georges Nguefack-Tsague, Josephine W. Ngunjiri, Van Thanh Nguyen, Dang H Nguyen, Phuong The Nguyen, Robina Khan Niazi, Yeshambel T Nigatu, Taxiarchis Konstantinos Nikolouzakis, Ali Nikoobar, Amin Reza Nikpoor, Chukwudi A Nnaji, Lawrence Achilles Nnyanzi, Efaq Ali Noman, Shuhei Nomura, Mamoona Noreen, Nafise Noroozi, Chisom Adaobi Nri-Ezedi, Mengistu H Nunemo, Dieta Nurrika, Jerry John Nutor, Martin James O'Donnell, Bogdan Oancea, Kehinde O Obamiro, Ismail A. Odetokun, Nkechi Martina Odogwu, Oluwakemi Ololade Odukoya, Ayodipupo Sikiru Oguntade, James Odhiambo Oguta, Akinkunmi Paul Okekunle, Osaretin Christabel Okonji, Andrew T Olagunju, Omotola O. Olasupo, Matthew Idowu Olatubi, Gláucia Maria Moraes Oliveira, Bolajoko Olubukunola Olusanya, Jacob Olusegun Olusanya, Gideon Olamilekan Oluwatunase, Hany A Omar, Goran Latif Omer, Obinna E Onwujekwe, Michal Ordak, Orish Ebere Orisakwe, Verner N. Orish, Doris V. Ortega-Altamirano, Alberto Ortiz, Wael M S Osman, Uchechukwu Levi Osuagwu, Olayinka Osuolale, Adrian Otoiu, Stanislav S Otstavnov, Amel Ouyahia, Mayowa O Owolabi, Mahesh Padukudru P A, Mohammad Taha Pahlevan Fallahy, Feng Pan, Hai-Feng Pan, Adrian Pana, Paramjot Panda, Songhomitra Panda-Jonas, Helena Ullyartha Pangaribuan, Georgios D Panos, Leonidas D Panos, Ioannis Pantazopoulos, Anca Mihaela Pantea Stoian, Romil R Parikh, Seoyeon Park, Ashwaghosha Parthasarathi, Ava Pashaei, Roberto Passera, Jay Patel, Shankargouda Patil, Dimitrios Patoulias, Uttam Paudel, Mihaela Paun, Spencer A Pease, Amy E Peden, Paolo Pedersini, Minjin Peng, Veincent Christian Filipino Pepito, Prince Peprah, Gavin Pereira, Arokiasamy Perianayagam, Richard G. Pestell, Fanny Emily Petermann-Rocha, Hoang Tran Pham, Anil K. Philip, Manon Pigeolet, David M Pigott, Evgenii Plotnikov, Peter Pollner, Ramesh Poluru, Maarten J Postma, Ghazaleh Pourali, Akram Pourshams, Disha Prabhu, Sergio I Prada, Pranil Man Singh Pradhan, Manya Prasad, Akila Prashant, Bharathi M Purohit, Jagadeesh Puvvula, Nameer Hashim Qasim, Ibrahim Qattea, Deepthi R, Mehrdad Rabiee Rad, Amir Radfar, Venkatraman Radhakrishnan, Pourya Raee, Hadi Raeisi Shahraki, Alireza Rafiei, Seyedeh Niloufar Rafiei Alavi, Pankaja Raghav Raghav, Fakher Rahim, Md Jillur Rahim, Muhammad Aziz Rahman, Md. Mosfequr Rahman, Mosiur Rahman, Vahid Rahmanian, Masoud Rahmati, Niloufar Rahnavard, Pramila Rai, Diego Raimondo, Ali Rajabpour-Sanati, Prashant Rajput, Prasanna Ram, Shakthi Kumaran Ramasamy, Kritika Rana, Juwel Rana, Chhabi Lal Ranabhat, Nemanja Rancic, Amey Rane, Shubham Ranjan, Chythra R Rao, Indu Ramachandra Rao, Vahid Rashedi, Sina Rashedi, Mohammad-Mahdi Rashidi, Azad Rasul, Zubair Ahmed Ratan, Giridhara Rathnaiah Babu, Santosh Kumar Rauniyar, Nakul Ravikumar, Salman Rawaf, David Laith Rawaf, Reza Rawassizadeh, Bharat Rawlley, Murali Mohan Rama Krishna Reddy, Elrashdy Moustafa Mohamed Redwan, Bhageerathy Reshmi, Nazila Rezaei, Mohsen Rezaeian, Aida Rezaei nejad, Abanoub Riad, Mavra A Riaz, Jennifer Rickard, Reza Rikhtegar, Célia Fortuna Rodrigues, Jefferson Antonio Buendia Rodriguez, Debby Syahru Romadlon, Himanshu Sekhar Rout, Nitai Roy, Priyanka Roy, Enrico Rubagotti, Tilleye Runghien, Aly M A Saad, Zahra Saadatian, Maha Mohamed Saber-Ayad, Morteza SaberiKamarposhti, Siamak Sabour, Fatos Sada, Basema Saddik, Bashdar Abuzed Sadee, Ehsan Sadeghi, Erfan Sadeghi, Mohammad Reza Saeb, Umar Saeed, Sher Zaman Safi, Manika Saha, Soumya Swaroop Sahoo, Monalisha Sahu, Zahra Saif, Joseph W Sakshaug, Payman Salamati, Afeez Abolarinwa Salami, Mohamed A. Saleh, Marwa Rashad Salem, Sara Samadzadeh, Yoseph Leonardo Samodra, Vijaya Paul Samuel, Abdallah M Samy, Juan Sanabria, Nima Sanadgol, Milena M. Santric-Milicevic, Sivan Yegnanarayana Iyer Saraswathy, Babak Saravi, Yaser Sarikhani, Tanmay Sarkar, Rodrigo Sarmiento-Suárez, Sachin C Sarode, Gargi Sachin Sarode, Arash Sarveazad, Brijesh Sathian, Thirunavukkarasu Sathish, Anudeep Sathyanarayan, Md Abu Sayeed, Abu Sayeed, Nikolaos Scarmeas, Winfried Schlee, Art Schuermans, David C Schwebel, Falk Schwendicke, Pallav Sengupta, Subramanian Senthilkumaran, Sadaf G Sepanlou, Dragos Serban, Edson Serván-Mori, SeyedAhmad SeyedAlinaghi, Seyed Arsalan Seyedi, Mahan Shafie, Jaffer Shah, Pritik A Shah, Ataollah Shahbandi, Samiah Shahid, Moyad Jamal Shahwan, Ahmed Shaikh,

Masood Ali Shaikh, Muhammad Aaqib Shamim, Mehran Shams-Beyranvand, Mohammad Anas Shamsi, Mohd Shanawaz, Abhishek Shankar, Mohammed Shannawaz, Medha Sharath, Sadaf Sharfaei, Amin Sharifan, Javad Sharifi-Rad, Vishal Sharma, Ujjawal Sharma, Rajesh Sharma, Rajesh P. Shastry, Amin Shavandi, Maryam Shayan, Amir Mehdi Shayan, Amr Mohamed Elsayed Shehabeldine, Aziz Sheikh, Rahim Ali Sheikhi, Pavanchand H Shetty, Desalegn Shiferaw, Mika Shigematsu, Rahman Shiri, Aminu Shittu, Velizar Shivarov, Farhad Shokraneh, Sina Shool, Seyed Afshin Shorofi, Kanwar Hamza Shuja, Kerem Shuval, Emmanuel Edwar Siddig, Luís Manuel Lopes Rodrigues Silva, Soraia Silva, João Pedro Silva, Biagio Simonetti, Jasvinder A. Singh, Abhinav Singh, Md Shahjahan Siraj, Amanda E Smith, Chandan S N, Bogdan Socea, Hamidreza Soleimani, Sameh S M Soliman, Yonatan Solomon, Yimeng Song, Reed J D Sorensen, Michael Spartalis, Chandrashekhar T Sreeramareddy, Vijay Kumar Srivastava, Muhammad Haroon Stanikzai, Antonina V Starodubova, Simona Cătălina Stefan, Mark A Stokes, Vetriselvan Subramaniyan, Muhammad Suleman, Rizwan Suliankatchi Abdulkader, Abida Sultana, Jing Sun, Chandan Kumar Swain, Bryan L. Sykes, Lukasz Szarpak, Mindy D Szeto, Miklós Szócska, Payam Tabaee Damavandi, Rafael Tabarés-Seisdedos, Ozra Tabatabaei Malazy, Seyed-Amir Tabatabaeizadeh, Shima Tabatabai, Karen M Tabb, Mohammad Tabish, Moslem Taheri Soodejani, Jabeen Taiba, Iman M. Talaat, Mircea Tampa, Jacques Lukenze Tamuzi, Ker-Kan Tan, Haosu Tang, Derbie Alemu DA Tareke, Mengistie Kassahun Tariku, Seyed Mohammad Tavangar, Mohamad-Hani Temsah, Reem Mohamad Hani Temsah, Masayuki Teramoto, Dufera Rikitu Terefa, Enoch Teye-Kwadjo, Ramna Thakur, Pugazhenthan Thangaraju, Rekha Thapar, Samar Tharwat, Rasiah Thayakaran, Nihal Thomas, Jansje Henny Ver Ticoalu, Tenaw Yimer Tiruye, Mariya Vladimirovna Titova, Marcello Tonelli, Marcos Roberto Tovani-Palone, Eugenio Traini, Nghia Minh Tran, Jasmine T Tran, Indang Trihandini, Samuel Joseph Tromans, Thien Tan Tri Tai Truyen, Evangelia Eirini Tsermpini, Munkhtuya Tumurkhuu, Stefanos Tyrovolas, Sayed Mohammad Nazim Uddin, Arit Udoh, Saeed Ullah, Sana Ullah, Srikanth Umakanthan, Chukwuma David Umeokonkwo, Bhaskaran Unnikrishnan, Era Upadhyay, Jibrin Sammani Usman, Seyed Mohammad Vahabi, Rohollah Valizadeh, Jef Van den Eynde, Orsolya Varga, Priya Vart, Shoban Babu Varthya, Balachandar Vellingiri, Deneshkumar Venugopal, Madhur Verma, Massimiliano Veroux, Georgios-Ioannis Verras, Dominique Vervoort, Jorge Hugo Villafañe, Manish Vinayak, Francesco S Violante, Mukesh Vishwakarma, Bay Vo, Stein Emil Vollset, Simona Ruxandra Volovat, Theo Vos, Isidora S Vujcic, Hatem A Wafa, Yasir Waheed, Elias Bekele Wakwoya, Yuan-Pang Wang, Yanzhong Wang, Cong Wang, Shu Wang, Fang Wang, Paul Ward, Emebet Gashaw Wassie, Kosala Gayan Weerakoon, Yi Feng Wen, Ronny Westerman, Nuwan Darshana Wickramasinghe, Dakshitha Praneeth Wickramasinghe, Peter Willeit, Yohannes Addisu Wondimagegene, Felicia Wu, Juan Xia, Hong Xiao, Suowen Xu, Xiaoyue Xu, Gelin Xu, Ali Yadollahpour, Shirin Yaghoobpoor, Tina Yaghoobpour, Zwanden Sule Yahaya, Danting Yang, Yuichiro Yano, Habib Yaribeygi, Pengpeng Ye, Renjulal Yesodharan, Subah Abderehim Yesuf, Saber Yezli, Amanuel Yigezu, Dong Keon Yon, Naohiro Yonemoto, Yuyi You, Mustafa Z Younis, Zabihollah Yousefi, Chuanhua Yu, Yong Yu, Chun-Wei Yuan, Nima Zafari, Fathiah Zakham, Nazar Zaki, Giulia Zamagni, Ghazal G Z Zandieh, Moein Zangiabadian, Mikhail Sergeevich Zastrozhin, Haijun Zhang, Yunquan Zhang, Meixin Zhang, Chenwen Zhong, Juexiao Zhou, Bin Zhu, Lei Zhu, Magdalena Zielińska, Samer H. Zyoud.

Drafting the work or revising it critically for important intellectual content

Amirali Aali, Yohannes Habtegiorgis Abate, Mohammadreza Abbasian, Mohsen Abbasi-Kangevari, Hedayat Abbastabar, Samar Abd ElHafeez, Sherief Abd-Elsalam, Mohammad Abdollahi, Mohammad-Amin Abdollahifar, Auwal Abdullahi, Mesfin Abebe, Samrawit Shawel Abebe, Olumide Abiodun, Hassan Abolhassani, Mohamed Abouzid, Girma Beressa Aboye, Lucas Guimarães Abreu, Hasan Abualruz, Eman Abu-Gharbieh, Niveen ME Abu-Rmeileh, Tadele Girum Adal, Mesafint Molla Adane, Oluwafemi Atanda Adeagbo Adeagbo, Rufus Adesoji Adedoyin, Victor Adekanmbi, Abiola Victor Adepoju, Olatunji O Adetokunboh, Juliana Bunmi Adetunji, Daniel Adedayo Adeyinka, Olorunsola Israel Adeyomoye, Qorinah Estiningtyas Sakilah Adnani, Saryia Adra, Rotimi Felix Afolabi, Saira Afzal, Antonella Agodi, Bright Opoku Ahinkorah, Muayyad M Ahmad, Danish Ahmad, Firdos Ahmad, Ali Ahmed, Ayman Ahmed, Syed Anees Ahmed, Luai A. Ahmed, Marjan Ajami, Hossein Akbarialiabad, Rufus Olusola Akinyemi, Mohammed Ahmed Akkaif, Samer O. Alalalmeh, Tariq A. Alalwan, Khurshid Alam, Rasmieh Mustafa Al-amer, Almaza Albakri, Mohammed Albashtawy, Mohammad T. AlBataineh, Hediyeh Alemi, Yihun Mulugeta Alemu, Ayman Al-Eyadhy, Khalid F Alhabib, Noora Alhajri, Fadwa Alhalaiqa Naji Alhalaiqa, Robert Kaba Alhassan, Mohammed Usman Ali, Abid Ali, Syed Shujait Shujait Ali, Sheikh Mohammad Alif, Mohammad Aligol, Omar Almidani, Mahmoud A. Alomari, Mohammad Al Qadire, Jaber S Alqahtani, Ahmed Yaseen Alqutaibi, Jaffar A. Al-Tawfiq, Deborah Oyine Aluh, Nelson Alvis-Guzman, Hassan Alwafi, Yaser

Mohammed Al-Worafi, Hany Aly, Safwat Aly, Karem H Alzoubi, Tarek Tawfik Amin, Alireza Amindarolzarbi, Mostafa Amini-Rarani, Sohrab Amiri, Irene Gyamfuah Ampomah, Dickson A Amugsi, Ganiyu Adeniyi Amusa, Robert Ancuceanu, Deanna Anderlini, Pedro Prata Andrade, Tudorel Andrei, Catalina Liliana Andrei, Abhishek Anil, Sneha Anil, Adnan Ansar, Ernoiz Antriyandarti, SALEHA ANWAR, Anayochukwu Edward Anyasodor, Jalal Arabloo, Razman Arabzadeh Bahri, Elshaimaa A Arafa, Mosab Arafat, Ana Margarida Araújo, Abdulfatai Aremu, Timur Aripov, Mesay Arkew, Benedetta Armocida, Johan Ärnlöv, Mahwish Arooj, Judie Arulappan, Raphael Taiwo Aruleba, Ashokan Arumugam, Mohsen Asadi-Lari, Saeed Asgary, Mona Asghariahmadabad, Muhammad Ashraf, Marvellous O Asika, Seyyed Shamsadin Athari, Maha Moh'd Wahbi Atout, Alok Atreya, Avinash Aujayeb, Abolfazl Avan, Amlaku Mulat Aweke, Getnet Melaku Ayele, Seyed Mohammad Ayyoubzadeh, Sina Azadnajafabad, Rui M S Azevedo, Ahmed Y Azzam, Muhammad Badar, Ashish D Badiye, Soroush Baghdadi, Sara Bagherieh, Atif Amin Baig, Jennifer L Baker, Abdulaziz T. Bako, Madhan Balasubramanian, Ovidiu Constantin Baltatu, Kiran Bam, Maciej Banach, Soham Bandyopadhyay, Hansi Bansal, Mehmet Firat Baran, Martina Barchitta, Mainak Bardhan, Erfan Bardideh, Suzanne Lyn Barker-Collo, Till Winfried Bärnighausen, Francesco Barone-Adesi, Hiba Jawdat Barqawi, Amadou Barrow, Sandra Barteit, Asma'u I J Bashir, Hameed Akande Bashiru, Afisu Basiru, João Diogo Basso, Sanjay Basu, Bernhard T Baune, Tahmina Begum, Amir Hossein Behnoush, Maryam Beiranvand, Alehegn Bekele, Sefealem Assefa Belay, Uzma Iqbal Belgaumi, Michelle L Bell, Olorunjuwon Omolaja Bello, Apostolos Beloukas, Isabela M Bensenor, Zombor Berezvai, Paulo J G Bettencourt, Akshaya Srikanth Bhagavathula, Prarthna V Bhardwaj, Sonu Bhaskar, Vivek Bhat, Natalia V Bhattacharjee, Jasvinder Singh Bhatti, Gurjit Kaur Bhatti, Manpreet S Bhatti, Rajbir Bhatti, Antonio Biondi, Catherine Bisignano, Atanu Biswas, Raaj Kishore Biswas, Veera R Bitra, Tone Bjørge, Anca Vasilica Bobirca, Virginia Bodolica, Aadam Olalekan Bodunrin, Kassawmar Angaw Bogale, Milad Bonakdar Hashemi, Souad Bouaoud, Dejana Braithwaite, Michael Brauer, Nicholas J K Breitborde, Norma B Bulamu, Danilo Buonsenso, Richard A Burns, Yasser Bustanji, Florentino Luciano Caetano dos Santos, Daniela Calina, Ismael R Campos-Nonato, Giulia Carreras, Andrea Carugno, Carlos A Castañeda-Orjuela, Giulio Castelpietra, Maria Sofia Cattaruzza, Arthur Caye, Luca Cegolon, Francieli Cembranel, Ester Cerin, Joshua Chadwick, Yaacoub Chahine, Chiranjib Chakraborty, Jeffrey Shi Kai Chan, Vijay Kumar Chattu, Malizgani Paul Chavula, Simiao Chen, An-Tian Chen, Haowei Chen, Gerald Chi, Fatemeh Chichagi, Ju-Huei Chien, Patrick R Ching, William C S Cho, Sungchul Choi, Bryan Chong, Devasahayam J Christopher, Dinh-Toi Chu, Eric Chung, Iolanda Cioffi, Raffaela Ciuffreda, Rafael M Claro, Alyssa Columbus, Haley Comfort, Joao Conde, Michael H Criqui, Natália Cruz-Martins, Silvia Magali Cuadra-Hernández, Mario D'Oria, Sriharsha Dadana, Tukur Dahiru, Zhaoli Dai, Giovanni Damiani, Claudio Alberto Dávila-Cervantes, Aklilu Tamire Debele, Shayom Debopadhaya, Somayeh Delavari, Ivan Delgado-Enciso, Berecha Hundessa Demessa, Edgar Denova-Gutiérrez, Kebede Deribe, Nikolaos Dervenis, Hardik Dineshbhai Desai, Rupak Desai, Arkadeep Dhali, Meghnath Dhimal, Vishal R Dhulipala, Diana Dias da Silva, Daniel Diaz, Michael J Diaz, Adriana Dima, Delaney D Ding, M Ashworth Dirac, Thanh Chi Do, Sushil Dohare, Camila Bruneli do Prado, Leila Doshmangir, Wendel Mombaque dos Santos, Robert Kokou Dowou, Viola Dsouza, Haneil Larson Dsouza, Ashel Chelsea Dsouza, John Dube, Senbagam Duraisamy, Oyewole Christopher Durojaiye, Sulagna Dutta, Laura Dwyer-Lindgren, Arkadiusz Marian Dziedzic, Michael Ekholuenetale, Noha Mousaad Elemam, Ghada Metwally Tawfik ElGohary, Muhammed Elhadi, Omar Abdelsadek Abdou Elmeligy, Mohamed A Elmonem, Ibrahim Elsohaby, Maha El Tantawi, Amir Emami Zeydi, Sharareh Eskandarieh, Francesco Esposito, Farshid Etaee, Natalia Fabin, Adeniyi Francis Fagbamigbe, Saman Fahimi, Aliasghar Fakhri-Demeshghieh, Luca Falzone, Ali Faramarzi, MoezAlIslam Ezzat Mahmoud Faris, Samuel Farmer, Andre Faro, Ali Fatehizadeh, Nelsensius Klau Fauk, Pooria Fazeli, Valery L. Feigin, Seyed-Mohammad Fereshtehnejad, Abdullah Hamid Feroze, Pietro Ferrara, Nuno Ferreira, Getahun Fetensa, Irina Filip, Florian Fischer, Joanne Flavel, Morenike Oluwatoyin Folayan, Behzad Foroutan, Matteo Foschi, Kate Louise Francis, Alberto Freitas, Takeshi Fukumoto, Blima Fux, Peter Andras Gaal, Muktar A. Gadanya, Yaseen Galali, Silvano Gallus, Aravind P Gandhi, Balasankar Ganesan, Mohammad Arfat Ganiyani, M.A. Garcia-Gordillo, Rupesh K. Gautam, Federica Gazzelloni, Semiu Olatunde Gbadamosi, Miglas W Gebregergis, Mesfin Gebrehiwot, Teferi Gebru Gebremeskel, Yohannes Fikadu Geda, Simona Roxana Georgescu, Urge Gerema, Habtamu Geremew, Motuma Erena Getachew, MohammadReza Ghasemi, Ghazal Ghasempour Dabaghi, Fariba Ghassemi, Ramy Mohamed Ghazy, Sailaja Ghimire, Mahsa Ghorbani, Aloke Gopal Ghoshal, Artyom Urievich Gil, Tiffany K Gill, Matteo Giorgi, Alem Girmay, James C Glasbey, Laszlo Göbölös, Ali Golchin, Sameer Vali Gopalani, Alessandra C Goulart, Anmol Goyal, Michal Grivna, Shi-Yang Guan, Giovanni Guarducci, Mesay Dechasa Gudeta, Stefano Guicciardi, Snigdha Gulati, David Gulisashvili, Damitha Asanga Gunawardane, Cui Guo, Mohak Gupta, Manoj Kumar Gupta, Sapna Gupta, Bhawna Gupta, Veer Bala Gupta, Vivek Kumar Gupta, Annie Haakenstad, Farrokh Habibzadeh, Najah R Hadi, Nils Haep, Ramtin Hajibeygi, Rabih Halwani, Randah R Hamadeh, Nadia M Hamdy, Sajid Hameed, Alexis J Handal, Graeme J Hankey, Josep Maria Haro, Ahmed I. Hasaballah, Mohammad Jahid Hasan, S. M. Mahmudul Hasan, Hamidreza Hasani, Md Saquib Hasnain, Amr

Hassan, Simon I Hay, Jeffrey J. Hebert, Omar E. Hegazi, Bartosz Helfer, Mehdi Hemmati, Claudiu Herteliu, Kamran Hessami, Kamal Hezam, Yuta Hiraike, Nguyen Quoc Hoan, Ramesh Holla, Nobuyuki Horita, Md Mahbub Hossain, Mohammad Bellal Hossain Hossain, Sorin Hostiuc, Vivian Chia-rong Hsieh, Junjie Huang, M Mamun Huda, Ayesha Humayun, Javid Hussain, Hong-Han Huynh, Segun Emmanuel Ibitoye, Pulwasha Maria Iftikhar, Olayinka Stephen Ilesanmi, Milena D. Ilic, Irena M. Ilic, Mustapha Immurana, Md. Rabiul Islam, Nahlah Elkudssiah Ismail, Gaetano Isola, Mahalaxmi Iyer, Linda Merin J, Louis Jacob, Khushleen Jaggi, Kasra Jahankhani, Nader Jahanmehr, Haitham Jahrami, Akhil Jain, Mihajlo Jakovljevic, Sabzali Javadov, Sathish Kumar Jayapal, Shubha Jayaram, Ravi Prakash Jha, Jost B. Jonas, Tamas Joo, Nitin Joseph, Charity Ehimwenma Joshua, Jacek Jerzy Jozwiak, Mikk Jürisson, Abdulkareem Kabir, Ali Kabir, Hannaneh Kabir, Rizwan Kalani, Feroze Kaliyadan, Sanjay Kalra, Rajesh Kamath, Sagarika Kamath, Edmund Wedam Kanmiki, Kehinde Kazeem Kanmodi, Suthanthira Kannan S, Sushil Kumar Kansal, Rami S. Kantar, Neeti Kapoor, Mehrdad Karajizadeh, Faizan Zaffar Kashoo, Hengameh Kasraei, Joonas H Kauppila, Foad Kazemi, sara Kazeminia, John H Kempen, Himanshu Khajuria, Amirmohammad Khalaji, Nauman Khalid, Anees Ahmed Khalil, Alireza Khalilian, Ajmal Khan, Mohammad Jobair Khan, Maseer Khan, M Nuruzzaman Khan, Ikramullah Khan, Moien AB Khan, Shaghayegh Khanmohammadi, Khaled Khatab, Armin Khavandegar, Hamid Reza Khayat Kashani, Feriha Fatima Khidri, Mohammad Ali Khosravi, Mahmood Khosrowjerdi, Wondwosen Teklesilasie Kidane, Min Seo Kim, Julie Sojin Kim, Adnan Kisa, Sezer Kisa, Ali-Asghar Kolahi, Farzad Kompani, Oleksii Korzh, Sindhura Lakshmi Koulmane Laxminarayana, Kewal Krishan, Varun Krishna, Barthelemy Kuate Defo, Connor M Kubeisy, Mohammed Kuddus, Md Abdul Kuddus, Ilari Kuitunen, Mukhtar Kulimbet, Satyajit Kundu, Kunle Rotimi Kunle, Om P Kurmi, Asep Kusnali, Dian Kusuma, Ben Lacey, Muhammad Awwal Ladan, Lucie Laflamme, Chandrakant Lahariya, Ratilal Lalloo, Judit Lám, Iván Landires, Berthold Langguth, Ariane Laplante-Lévesque, Heidi Jane Larson, Anders O Larsson, Savita Lasrado, Kamaluddin Latief, Kaveh Latifinaibin, Carlo La Vecchia, Nhi Huu Hanh Le, Caterina Ledda, Paul H Lee, Gebretsadik Kiros Lema, Elvynna Leong, Wei Li, Shanshan Li, An Li, Paulina A Lindstedt, Stefan Listl, Xuefeng Liu, Erand Llanaj, Rubén López-Bueno, Platon D Lopukhov, László Lorenzovici, Paulo A. Lotufo, Jailos Lubinda, Giancarlo Lucchetti, Alessandra Lugo, Raimundas Lunevicius, hengliang lv, Zheng Feei Ma, Monika Machoy, Áurea M. Madureira-Carvalho, Mohammed Magdy Abd El Razek, Soleiman Mahjoub, Mansour Adam Mahmoud, Jeadran N. Malagón-Rojas, Elaheh Malakan Rad, Kashish Malhotra, Ahmad Azam Malik, Deborah Carvalho Malta, Abdullah A Mamun, Yasaman Mansoori, Borhan Mansouri, Gabriel Martinez, Ramon Martinez-Piedra, Francisco Rogerlândio Martins-Melo, Miquel Martorell, Sharmeen Maryam, Roy Rillera Marzo, Alireza Masoudi, Jishanth Mattumpuram, Andrea Maugeri, Erin A May, Mahsa Mayeli, John J. McGrath, Anna Laura Wensel McKowen, Susan A McLaughlin, Steven M McPhail, Kamran Mehrabani-Zeinabad, Entezar Mehrabi Nasab, Max Alberto Mendez Mendez-Lopez, Walter Mendoza, Ritesh G Menezes, George A Mensah, Alexios-Fotios A. Mentis, Sultan Ayoub Meo, Mohsen Merati, Tuomo J Meretoja, Atte Meretoja, Tomislav Mestrovic, Sachith Mettananda, Kukulege Chamila Dinushi Mettananda, Tomasz Miazgowski, Georgia Micha, Irmina Maria Michalek, Ted R Miller, Le Huu Nhat Minh, Mojgan Mirghafourvand, Moonis Mirza, Roya Mirzaei, Ashim Mishra, Philip B Mitchell, Chaitanya Mittal, Babak Moazen, Nouh Saad Mohamed, Mouhand F H Mohamed, Abdalla Z Mohamed, Sakineh Mohammad-Alizadeh-Charandabi, Soheil Mohammadi, Abdollah Mohammadian-Hafshejani, Mustapha Mohammed, Shafiu Mohammed, Salahuddin Mohammed, Ali H Mokdad, Hossein Molavi Vardanjani, Sabrina Molinaro, Lorenzo Monasta, Mohammad Ali Moni, Maryam Moradi, Yousef Moradi, Paula Moraga, Rafael Silveira Moreira, Shane Douglas Morrison, Jakub Morze, Abbas Mosapour, Rohith Motappa, Parsa Mousavi, Amin Mousavi Khaneghah, Efrén Murillo-Zamora, Christopher J L Murray, Jonah Musa, Fungai Musaigwa, Sathish Muthu, Saravanan Muthupandian, Muhammad Muzaffar, Woojae Myung, Ahamarshan Jayaraman Nagarajan, Mukhammad David Naimzada, Firzan Nainu, Sreenivas Narasimha Swamy, Bruno Ramos Nascimento, Gustavo G Nascimento, Abdallah Y Naser, Zuhair S Natto, Javaid Nauman, Biswa Prakash Nayak, Vinod C Nayak, Sabina Onyinye Nduaguba, Hadush Negash, Chernet Tafere Negesse, Ionut Negoi, Ruxandra Irina Negoi, Seyed Aria Nejadghaderi, Samata Nepal, Henok Biresaw Netsere, Georges Nguefack-Tsague, Josephine W. Ngunjiri, Van Thanh Nguyen, Hau Thi Hien Nguyen, Dang H Nguyen, Phuong The Nguyen, Robina Khan Niazi, Taxiarchis Konstantinos Nikolouzakis, Amin Reza Nikpoor, Lawrence Achilles Nnyanzi, Mamoona Noreen, Chisom Adaobi Nri-Ezedi, Mengistu H Nunemo, Virginia Nuñez-Samudio, Jerry John Nutor, Martin James O'Donnell, Bogdan Oancea, Kehinde O Obamiro, Ismail A. Odetokun, Nkechi Martina Odogwu, Oluwakemi Ololade Odukoya, Ayodipupo Sikiru Oguntade, In-Hwan Oh, Sylvester Reuben Okeke, Akinkunmi Paul Okekunle, Osaretin Christabel Okonji, Patrick Godwin Okwute, Andrew T Olagunju, Matthew Idowu Olatubi, Gláucia Maria Moraes Oliveira, Bolajoko Olubukunola Olusanya, Jacob Olusegun Olusanya, Gideon Olamilekan Oluwatunase, Hany A Omar, Obinna E Onwujekwe, Michal Ordak, Verner N. Orish, Doris V. Ortega-Altamirano, Alberto Ortiz, Esteban Ortiz-Prado, Wael M S Osman, Uchechukwu Levi Osuagwu, Olayinka Osuolale, Adrian Otoiu, Stanislav S Otstavnov, Guoqing Ouyang, Mayowa O Owolabi, Mahesh Padukudru P A, Mohammad Taha Pahlevan Fallahy, Feng Pan, Paramjot

Panda, Songhomitra Panda-Jonas, Ioannis Pantazopoulos, Romil R Parikh, Ashwaghosha Parthasarathi, Ava Pashaei, Roberto Passera, Hemal M Patel, Jay Patel, Shankargouda Patil, Dimitrios Patoulias, Venkata Suresh Patthipati, Uttam Paudel, Hamidreza Pazoki Toroudi, Amy E Peden, Paolo Pedersini, Umberto Pensato, Veincent Christian Filipino Pepito, Gavin Pereira, Mario F P Peres, Arokiasamy Perianayagam, Norberto Perico, Richard G. Pestell, Fanny Emily Petermann-Rocha, Hoang Tran Pham, Daniela Pierannunzio, Dimitri Poddighe, Peter Pollner, Maarten J Postma, Ghazaleh Pourali, Naeimeh Pourtaheri, Disha Prabhu, Sergio I Prada, Pranil Man Singh Pradhan, Manya Prasad, Akila Prashant, Nameer Hashim Qasim, Ibrahim Qattea, Deepthi R, Mehrdad Rabiee Rad, Amir Radfar, Venkatraman Radhakrishnan, Hadi Raeisi Shahraki, Seyedeh Niloufar Rafiei Alavi, Cat Raggi, Pankaja Raghav Raghav, Fakher Rahim, Mohammad Hifz Ur Rahman, Masoud Rahmati, Niloufar Rahnavard, Diego Raimondo, Ali Rajabpour-Sanati, Prashant Rajput, Shakthi Kumaran Ramasamy, Kritika Rana, Shailendra Singh Rana, Chhabi Lal Ranabhat, Nemanja Rancic, Shubham Ranjan, Chythra R Rao, Indu Ramachandra Rao, Deepthi Rapaka, Davide Rasella, Vahid Rashedi, Giridhara Rathnaiah Babu, Nakul Ravikumar, Salman Rawaf, David Laith Rawaf, Bharat Rawlley, Elrashdy Moustafa Mohamed Redwan, Giuseppe Remuzzi, Bhageerathy Reshmi, Nazila Rezaei, Aida Rezaei nejad, Abanoub Riad, Mavra A Riaz, Jennifer Rickard, Reza Rikhtegar, Célia Fortuna Rodrigues, Jefferson Antonio Buendia Rodriguez, Ravi Rohilla, Debby Syahru Romadlon, Luca Ronfani, Himanshu Sekhar Rout, Nitai Roy, Bedanta Roy, Enrico Rubagotti, Guilherme de Andrade Ruela, Susan Fred Rumisha, Manjula S, Aly M A Saad, Zahra Saadatian, Maha Mohamed Saber-Ayad, Siamak Sabour, Fatos Sada, Basema Saddik, Bashdar Abuzed Sadee, Ehsan Sadeghi, Umar Saeed, Dominic Sagoe, Manika Saha, Amirhossein Sahebkar, Soumya Swaroop Sahoo, Monalisha Sahu, Zahra Saif, Joseph W Sakshaug, Afeez Abolarinwa Salami, Marwa Rashad Salem, Mohammed Z Y Salem, Sohrab Salimi, Sara Samadzadeh, Vijaya Paul Samuel, Abdallah M Samy, Juan Sanabria, Milena M. Santric-Milicevic, Haaris Saqib, Aswini Saravanan, Babak Saravi, Yaser Sarikhani, Tanmay Sarkar, Rodrigo Sarmiento-Suárez, Sachin C Sarode, Gargi Sachin Sarode, Arash Sarveazad, Thirunavukkarasu Sathish, Anudeep Sathyanarayan, Md Abu Sayeed, Abu Sayeed, Nikolaos Scarmeas, Winfried Schlee, Art Schuermans, Austin E Schumacher, David C Schwebel, Falk Schwendicke, Siddharthan Selvaraj, Pallav Sengupta, Sadaf G Sepanlou, Dragos Serban, Yashendra Sethi, Allen Seylani, Mahan Shafie, Jaffer Shah, Pritik A Shah, Samiah Shahid, Moyad Jamal Shahwan, Ahmed Shaikh, Muhammad Aaqib Shamim, Mehran Shams-Beyranvand, Mohd Shanawaz, Abhishek Shankar, Mohammed Shannawaz, Medha Sharath, Amin Sharifan, Javad Sharifi-Rad, Manoj Sharma, Ujjawal Sharma, Rajesh P. Shastry, Amr Mohamed Elsayed Shehabeldine, Manjunath Mala Shenoy, Pavanchand H Shetty, Mika Shigematsu, Reza Shirkoohi, Aminu Shittu, Farhad Shokraneh, Seyed Afshin Shorofi, Kerem Shuval, Emmanuel Edwar Siddig, Luís Manuel Lopes Rodrigues Silva, Soraia Silva, João Pedro Silva, Biagio Simonetti, Anjali Singal, Jasvinder A. Singh, Abhinav Singh, Balbir Bagicha Singh, Amanda E Smith, Chandan S N, Bogdan Socea, Anton Sokhan, Ranjan Solanki, Shipra Solanki, Hamidreza Soleimani, Yonatan Solomon, Michael Spartalis, Chandrashekhar T Sreeramareddy, Muhammad Haroon Stanikzai, Vladimir I Starodubov, Antonina V Starodubova, Simona Cătălina Stefan, Paschalis Steiropoulos, Vetriselvan Subramaniyan, Muhammad Suleman, Abida Sultana, Chandan Kumar Swain, Bryan L. Sykes, Lukasz Szarpak, Mindy D Szeto, Miklós Szócska, Payam Tabaee Damavandi, Rafael Tabarés-Seisdedos, Seyed-Amir Tabatabaeizadeh, Shima Tabatabai, Karen M Tabb, Mohammad Tabish, Ardeshir Tajbakhsh, Iman M. Talaat, Ashis Talukder, Mircea Tampa, Jacques Lukenze Tamuzi, Ker-Kan Tan, Derbie Alemu DA Tareke, Vivian Y Tat, Seyed Mohammad Tavangar, Mojtaba Teimoori, Mohamad-Hani Temsah, Reem Mohamad Hani Temsah, Masayuki Teramoto, Dufera Rikitu Terefa, Riki Tesler, Ramna Thakur, Pugazhenthan Thangaraju, Kavumpurathu Raman Thankappan, Samar Tharwat, Nihal Thomas, Ales Tichopad, Tenaw Yimer Tiruye, Marcello Tonelli, Marcos Roberto Tovani-Palone, Nghia Minh Tran, Jasmine T Tran, Samuel Joseph Tromans, Thien Tan Tri Tai Truyen, Evangelia Eirini Tsermpini, Stefanos Tyrovolas, Aniefiok John Udoakang, Arit Udoh, Atta Ullah, Srikanth Umakanthan, Chukwuma David Umeokonkwo, Brigid Unim, Bhaskaran Unnikrishnan, Era Upadhyay, Jibrin Sammani Usman, Marco Vacante, Asokan Govindaraj Vaithinathan, Jef Van den Eynde, Elena Varavikova, Orsolya Varga, Priya Vart, Shoban Babu Varthya, Tommi Juhani Vasankari, Balachandar Vellingiri, Madhur Verma, Massimiliano Veroux, Georgios-Ioannis Verras, Dominique Vervoort, Jorge Hugo Villafañe, Vasily Vlassov, Stein Emil Vollset, Simona Ruxandra Volovat, Theo Vos, Yuan-Pang Wang, Yanzhong Wang, Cong Wang, Shu Wang, Paul Ward, Emebet Gashaw Wassie, Marcia R Weaver, Ronny Westerman, Taweewat Wiangkham, Nuwan Darshana Wickramasinghe, Dakshitha Praneeth Wickramasinghe, Peter Willeit, Yohannes Addisu Wondimagegene, Juan Xia, Shirin Yaghoobpoor, Sajad Yaghoubi, Zwanden Sule Yahaya, Lin Yang, Habib Yaribeygi, Saber Yezli, Dong Keon Yon, Naohiro Yonemoto, Chun-Wei Yuan, Nima Zafari, Mikhail Sergeevich Zastrozhin, Haijun Zhang, Chenwen Zhong, Magdalena Zielińska, Samer H. Zyoud.

Managing the estimation or publications process

Saira Afzal, Mohammed Albashtawy, Mohammed Usman Ali, Catherine M Antony, Ahmed Y Azzam, Natalia V Bhattacharjee, Catherine Bisignano, Milad Bonakdar Hashemi, Thanh Chi Do, Kara Estep, Ali Fatehizadeh, Alem Girmay, Manoj Kumar Gupta, Simon I Hay, Hong-Han Huynh, Faizan Zaffar Kashoo, Nicholas J Kassebaum, Molly B Kassel, M Nuruzzaman Khan, Kasey E Kinzel, Chandrakant Lahariya, Nhi Huu Hanh Le, Paulina A Lindstedt, Mohammed Magdy Abd El Razek, Borhan Mansouri, Alireza Masoudi, Anna Laura Wensel McKowen, Le Huu Nhat Minh, Salahuddin Mohammed, Ali H Mokdad, Christopher J L Murray, Van Thanh Nguyen, Mahesh Padukudru P A, Paramjot Panda, Hoang Tran Pham, David M Pigott, Nemanja Rancic, Aly M A Saad, Abdallah M Samy, Md Abu Sayeed, Mohammad Anas Shamsi, Vishal Sharma, Amanda E Smith, Michael Spartalis, Derbie Alemu DA Tareke, Nicholas Alexander Verghese, Stein Emil Vollset, Katherine M Wells, Yohannes Addisu Wondimagegene, Mikhail Sergeevich Zastrozhin.
