# Supplementary material for: Global fertility in 204 countries and territories, 1950–2021, with forecasts to 2100: a comprehensive demographic analysis for the Global Burden of Disease Study 2021
Source: Lancet. 2024 May 18;403(10440):2057–99. doi: 10.1016/S0140-6736(24)00550-6 (PMC11122687; doi:10.1016/S0140-6736(24)00550-6)
Supplement: Supplementary appendix 3 [file mmc3.pdf]

THE LANCET

Supplementary appendix 3

This appendix formed part of the original submission and has been peer reviewed. We post it as supplied by the authors.

Supplement to: GBD 2021 Fertility and Forecasting Collaborators. Global fertility in 204 countries and territories, 1950–2021, with forecasts to 2100: a comprehensive demographic analysis for the Global Burden of Disease Study 2021. *Lancet* 2024; **403:** 2057–99.

Appendix 3: "Global fertility in 204 countries and territories, 1950–2021 with forecasts to 2100: a comprehensive demographic analysis for the Global Burden of Disease Study 2021"

This appendix provides further authorship detail for "Global fertility in 204 countries and territories, 1950–2021 with forecasts to 2100: a comprehensive demographic analysis for the Global Burden of Disease Study 2021."

Table of Contents

Author Names 2

Author Affiliations 9

Author Contributions 43

GBD 2021 Fertility Capstone Collaborators

Natalia V Bhattacharjee*, Austin E Schumacher*, Amirali Aali, Yohannes Habtegiorgis Abate, Rouzbeh Abbasgholizadeh, Mohammadreza Abbasian, Mohsen Abbasi-Kangevari, Hedayat Abbastabar, Samar Abd ElHafeez, Sherief Abd-Elsalam, Mohammad Abdollahi, Mohammad-Amin Abdollahifar, Meriem Abdoun, Auwal Abdullahi, Mesfin Abebe, Samrawit Shawel Abebe, Olumide Abiodun, Hassan Abolhassani, Meysam Abolmaali, Mohamed Abouzid, Girma Beressa Aboye, Lucas Guimarães Abreu, Woldu Aberhe Abrha, Michael R M Abrigo, Dariush Abtahi, Hasan Abualruz, Bilyaminu Abubakar, Eman Abu-Gharbieh, Niveen ME Abu-Rmeileh, Tadele Girum Girum Adal, Mesafint Molla Adane, Oluwafemi Atanda Adeagbo Adeagbo, Rufus Adesoji Adedoyin, Victor Adekanmbi, Bashir Aden, Abiola Victor Adepoju, Olatunji O Adetokunboh, Juliana Bunmi Adetunji, Daniel Adedayo Adeyinka, Olorunsola Israel Adeyomoye, Qorinah Estiningtyas Sakilah Adnani, Saryia Adra, Rotimi Felix Afolabi, Shadi Afyouni, Muhammad Sohail Afzal, Saira Afzal, Shahin Aghamiri, Antonella Agodi, Williams Agyemang-Duah, Bright Opoku Ahinkorah, Austin J Ahlstrom, Aqeel Ahmad, Danish Ahmad, Firdos Ahmad, Muayyad M Ahmad, Sajjad Ahmad, Tauseef Ahmad, Ali Ahmed, Ayman Ahmed, Haroon Ahmed, Luai A Ahmed, Meqdad Saleh Ahmed, Syed Anees Ahmed, Marjan Ajami, Budi Aji, Gizachew Taddesse Akalu, Hossein Akbarialiabad, Rufus Olusola Akinyemi, Mohammed Ahmed Akkaif, Sreelatha Akkala, Hanadi Al Hamad, Syed Mahfuz Al Hasan, Mohammad Al Qadire, Tareq Mohammed Ali AL-Ahdal, Samer O Alalalmeh, Tariq A Alalwan, Ziyad Al-Aly, Khurshid Alam, Rasmieh Mustafa Al-amer, Fahad Mashhour Alanezi, Turki M Alanzi, Almaza Albakri, Mohammed Albashtawy, Mohammad T AlBataineh, Hediyeh Alemi, Sharifullah Alemi, Yihun Mulugeta Alemu, Ayman Al-Eyadhy, Adel Ali Saeed Al-Gheethi, Khalid F Alhabib, Noora Alhajri, Fadwa Alhalaiqa Naji Alhalaiqa, Robert Kaba Alhassan, Abid Ali, Beriwan Abdulqadir Ali, Liaqat Ali, Mohammed Usman Ali, Rafat Ali, Syed Shujait Shujait Ali, Sheikh Mohammad Alif, Mohammad Aligol, Mehran Alijanzadeh, Mohammad A M Aljasir, Syed Mohamed Aljunid, Sabah Al-Marwani, Joseph Uy Almazan, Hesham M Al-Mekhlafi, Omar Almidani, Mahmoud A Alomari, Basem Al-Omari, Jaber S Alqahtani, Ahmed Yaseen Alqutaibi, Rajaa M Al-Raddadi, Salman Khalifah Al-Sabah, Awais Altaf, Jaffar A Al-Tawfiq, Khalid A Altirkawi, Deborah Oyine Aluh, Farrukh Jawad Alvi, Nelson Alvis-Guzman, Hassan Alwafi, Yaser Mohammed Al-Worafi, Hany Aly, Safwat Aly, Karem H Alzoubi, Edward Kwabena Ameyaw, Tarek Tawfik Amin, Alireza Amindarolzarbi, Mostafa Amini-Rarani, Sohrab Amiri, Irene Gyamfuah Ampomah, Dickson A Amugsi, Ganiyu Adeniyi Amusa, Robert Ancuceanu, Deanna Anderlini, Pedro Prata Andrade, Catalina Liliana Andrei, Tudorel Andrei, Abhishek Anil, Sneha Anil, Adnan Ansar, Alireza Ansari-Moghaddam, Catherine M Antony, Ernoiz Antriyandarti, Saeid Anvari, SALEHA ANWAR, Razique Anwer, Anayochukwu Edward Anyasodor, Jalal Arabloo, Razman Arabzadeh Bahri, Elshaimaa A Arafa, Mosab Arafat, Ana Margarida Araújo, Aleksandr Y Aravkin, Abdulfatai Aremu, Timur Aripov, Mesay Arkew, Benedetta Armocida, Johan Ärnlöv, Mahwish Arooj, Anton A Artamonov, Judie Arulappan, Raphael Taiwo Aruleba, Ashokan Arumugam, Mohsen Asadi-Lari, Zatollah Asemi, Saeed Asgary, Mona Asghariahmadabad, Mohammad Asghari-Jafarabadi, Mubarek Yesse Ashemo, Muhammad Ashraf, Tahira Ashraf, Marvellous O Asika, Seyyed Shamsadin Athari, Maha Moh'd Wahbi Atout, Alok Atreya, Avinash Aujayeb, Marcel Ausloos, Abolfazl Avan, Amlaku Mulat Aweke, Getnet Melaku Ayele, Seyed Mohammad Ayyoubzadeh, Sina Azadnajafabad, Rui M S Azevedo, Ahmed Y Azzam, Muhammad Badar, Ashish D Badiye, Soroush Baghdadi, Nasser Bagheri, Sara Bagherieh, Najmeh Bahmanziari, Ruhai Bai, Atif Amin Baig, Jennifer L Baker, Abdulaziz T Bako, Ravleen Kaur Bakshi, Madhan Balasubramanian, Ovidiu Constantin Baltatu, Kiran Bam, Maciej Banach, Soham Bandyopadhyay, Biswajit Banik, Palash Chandra Banik, Hansi Bansal, Mehmet Firat Baran, Martina Barchitta, Mainak Bardhan, Erfan Bardideh, Suzanne Lyn Barker-Collo, Till Winfried Bärnighausen, Francesco Barone-Adesi, Hiba Jawdat Barqawi, Amadou Barrow, Sandra Barteit, Zarrin Basharat, Asma'u I J Bashir, Hameed Akande Bashiru, Afisu Basiru, João Diogo Basso, Sanjay Basu,

Abdul-Monim Mohammad Batiha, Kavita Batra, Bernhard T Baune, Mohsen Bayati, Tahmina Begum, Emad Behboudi, Amir Hossein Behnoush, Maryam Beiranvand, Diana Fernanda Bejarano Ramirez, Alehegn Bekele, Sefealem Assefa Belay, Uzma Iqbal Belgaumi, Michelle L Bell, Olorunjuwon Omolaja Bello, Apostolos Beloukas, Isabela M Bensenor, Zombor Berezvai, Alemshet Yirga Berhie, Amiel Nazer C Bermudez, Paulo J G Bettencourt, Akshaya Srikanth Bhagavathula, Nikha Bhardwaj, Pankaj Bhardwaj, Prarthna V Bhardwaj, Sonu Bhaskar, Vivek Bhat, Gurjit Kaur Bhatti, Jasvinder Singh Bhatti, Manpreet S Bhatti, Rajbir Bhatti, Antonio Biondi, Catherine Bisignano, Atanu Biswas, Raaj Kishore Biswas, Veera R Bitra, Tone Bjørge, Elye Bliss, Micheal Kofi Boachie, Anca Vasilica Bobirca, Virginia Bodolica, Aadam Olalekan Bodunrin, Eyob Ketema Bogale, Kassawmar Angaw Bogale, Milad Bonakdar Hashemi, Berrak Bora Basara, Souad Bouaoud, Dejana Braithwaite, Michael Brauer, Nicholas J K Breitborde, Dana Bryazka, Norma B Bulamu, Danilo Buonsenso, Katrin Burkart, Richard A Burns, Yasser Bustanji, Nadeem Shafique Butt, Zahid A Butt, Florentino Luciano Caetano dos Santos, Daniela Calina, Ismael R Campos-Nonato, Fan Cao, Shujin Cao, Angelo Capodici, Giulia Carreras, Andrea Carugno, Carlos A Castañeda-Orjuela, Giulio Castelpietra, Maria Sofia Cattaruzza, Arthur Caye, Luca Cegolon, Francieli Cembranel, Ester Cerin, Joshua Chadwick, Yaacoub Chahine, Chiranjib Chakraborty, Julian Chalek, Jeffrey Shi Kai Chan, Periklis Charalampous, Vijay Kumar Chattu, Sarika Chaturvedi, Malizgani Paul Chavula, An-Tian Chen, Haowei Chen, Simiao Chen, Gerald Chi, Fatemeh Chichagi, Ju-Huei Chien, Patrick R Ching, William C S Cho, Sungchul Choi, Bryan Chong, Hitesh Chopra, Sonali Gajanan Choudhari, Devasahayam J Christopher, Dinh-Toi Chu, Isaac Sunday Chukwu, Eric Chung, Sheng-Chia Chung, Zinhle Cindi, Iolanda Cioffi, Raffaela Ciuffreda, Rafael M Claro, Kaleb Coberly, Alyssa Columbus, Haley Comfort, Joao Conde, Michael H Criqui, Natália Cruz-Martins, Silvia Magali Cuadra-Hernández, Sriharsha Dadana, Omid Dadras, Tukur Dahiru, Zhaoli Dai, Bronte Dalton, Giovanni Damiani, Aso Mohammad Darwesh, Jai K Das, Saswati Das, Mohsen Dashti, Anna Dastiridou, Claudio Alberto Dávila-Cervantes, Kairat Davletov, Aklilu Tamire Debele, Shayom Debopadhaya, Somayeh Delavari, Ivan Delgado-Enciso, Dessalegn Demeke, Berecha Hundessa Demessa, Xinlei Deng, Edgar Denova-Gutiérrez, Kebede Deribe, Nikolaos Dervenis, Hardik Dineshbhai Desai, Rupak Desai, Vinoth Gnana Chellaiyan Devanbu, Arkadeep Dhali, Kuldeep Dhama, Meghnath Dhimal, Vishal R Dhulipala, Diana Dias da Silva, Daniel Diaz, Michael J Diaz, Adriana Dima, Delaney D Ding, M Ashworth Dirac, Thanh Chi Do, Thao Huynh Phuong Do, Camila Bruneli do Prado, Sushil Dohare, Wanyue Dong, Mario D'Oria, Wendel Mombaque dos Santos, Leila Doshmangir, Robert Kokou Dowou, Ashel Chelsea Dsouza, Haneil Larson Dsouza, Viola Dsouza, John Dube, Joe Duprey, Andre Rodrigues Duraes, Senbagam Duraisamy, Oyewole Christopher Durojaiye, Sulagna Dutta, Laura Dwyer-Lindgren, Paulina Agnieszka Dzianach, Arkadiusz Marian Dziedzic, Alireza Ebrahimi, Hisham Atan Edinur, Kristina Edvardsson, Ferry Efendi, Terje Andreas Eikemo, Michael Ekholuenetale, Maha El Tantawi, Noha Mousaad Elemam, Ghada Metwally Tawfik ElGohary, Muhammed Elhadi, Legesse Tesfaye Elilo, Omar Abdelsadek Abdou Elmeligy, Mohamed A Elmonem, Mohammed Elshaer, Ibrahim Elsohaby, Amir Emami Zeydi, Luchuo Engelbert Bain, Sharareh Eskandarieh, Francesco Esposito, Kara Estep, Farshid Etaee, Natalia Fabin, Adeniyi Francis Fagbamigbe, Saman Fahimi, Aliasghar Fakhri-Demeshghieh, Luca Falzone, Ali Faramarzi, MoezAlIslam Ezzat Mahmoud Faris, Sam Farmer, Andre Faro, Abidemi Omolara Fasanmi, Ali Fatehizadeh, Nelsensius Klau Fauk, Pooria Fazeli, Valery L Feigin, Seyed-Mohammad Fereshtehnejad, Abdullah Hamid Feroze, Pietro Ferrara, Nuno Ferreira, Getahun Fetensa, Irina Filip, Florian Fischer, Joanne Flavel, Nataliya A Foigt, Morenike Oluwatoyin Folayan, Artem Alekseevich Fomenkov, Behzad Foroutan, Matteo Foschi, Kayode Raphael Fowobaje, Kate Louise Francis, Alberto Freitas, Takeshi Fukumoto, John E Fuller, Blima Fux, Peter Andras Gaal, Muktar A Gadanya, Abhay Motiramji Gaidhane, Yaseen Galali, Silvano Gallus, Aravind P Gandhi, Balasankar Ganesan, Mohammad Arfat Ganiyani, M.A. Garcia-Gordillo, Naval Garg, Rupesh K Gautam, Federica Gazzelloni, Semiu Olatunde Gbadamosi, Miglas

W Gebregergis, Mesfin Gebrehiwot, Tesfay Brhane Gebremariam, Tesfay B B Gebremariam, Teferi Gebru Gebremeskel, Yohannes Fikadu Geda, Simona Roxana Georgescu, Urge Gerema, Habtamu Geremew, Motuma Erena Getachew, Peter W Gething, MohammadReza Ghasemi, Ghazal Ghasempour Dabaghi, Afsaneh Ghasemzadeh, Fariba Ghassemi, Ramy Mohamed Ghazy, Sailaja Ghimire, Asadollah Gholamian, Ali Gholamrezanezhad, Mahsa Ghorbani, Aloke Gopal Ghoshal, Arun Digambarrao Ghuge, Artyom Urievich Gil, Tiffany K Gill, Matteo Giorgi, Alem Girmay, James C Glasbey, Laszlo Göbölös, Amit Goel, Ali Golchin, Mahaveer Golechha, Pouya Goleij, Sameer Vali Gopalani, Houman Goudarzi, Alessandra C Goulart, Anmol Goyal, Simon Matthew Graham, Michal Grivna, Shi-Yang Guan, Giovanni Guarducci, Mohammed Ibrahim Mohialdeen Gubari, Mesay Dechasa Gudeta, Stefano Guicciardi, Snigdha Gulati, David Gulisashvili, Damitha Asanga Gunawardane, Cui Guo, Anish Kumar Gupta, Bhawna Gupta, Manoj Kumar Gupta, Mohak Gupta, Sapna Gupta, Veer Bala Gupta, Vijai Kumar Gupta, Vivek Kumar Gupta, Annie Haakenstad, Farrokh Habibzadeh, Najah R Hadi, Nils Haep, Ramtin Hajibeygi, Sebastian Haller, Rabih Halwani, Randah R Hamadeh, Nadia M Hamdy, Sajid Hameed, Samer Hamidi, Qiuxia Han, Alexis J Handal, Graeme J Hankey, Md Nuruzzaman Haque, Josep Maria Haro, Ahmed I Hasaballah, Ikramul Hasan, Mohammad Jahid Hasan, S. M. Mahmudul Hasan, Hamidreza Hasani, Md Saquib Hasnain, Amr Hassan, Ikrama Hassan, Soheil Hassanipour, Hadi Hassankhani, Simon I Hay, Jeffrey J Hebert, Omar E Hegazi, Mohammad Heidari, Bartosz Helfer, Mehdi Hemmati, Brenda Yuliana Herrera-Serna, Claudiu Herteliu, Kamran Hessami, Kamal Hezam, Yuta Hiraike, Nguyen Quoc Hoan, Ramesh Holla, Nobuyuki Horita, Md Mahbub Hossain, Mohammad Bellal Hossain Hossain, Hassan Hosseinzadeh, Mehdi Hosseinzadeh, Mihaela Hostiuc, Sorin Hostiuc, Mohamed Hsairi, Vivian Chia-rong Hsieh, Chengxi Hu, Junjie Huang, M Mamun Huda, Ayesha Humayun, Javid Hussain, Nawfal R Hussein, Hong-Han Huynh, Bing-Fang Hwang, Segun Emmanuel Ibitoye, Pulwasha Maria Iftikhar, Olayinka Stephen Ilesanmi, Irena M Ilic, Milena D Ilic, Mustapha Immurana, Leeberk Raja Inbaraj, Afrin Iqbal, Md. Rabiul Islam, Nahlah Elkudssiah Ismail, Hiroyasu Iso, Gaetano Isola, Masao Iwagami, Mahalaxmi Iyer, Linda Merin J, Jalil Jaafari, Louis Jacob, Farhad Jadidi-Niaragh, Khushleen Jaggi, Kasra Jahankhani, Nader Jahanmehr, Haitham Jahrami, Akhil Jain, Nityanand Jain, Ammar Abdulrahman Jairoun, Mihajlo Jakovljevic, Elham Jamshidi, Sabzali Javadov, Tahereh Javaheri, Sathish Kumar Jayapal, Shubha Jayaram, Sun Ha Jee, Jayakumar Jeganathan, Anil K Jha, Ravi Prakash Jha, Heng Jiang, Mohammad Jokar, Jost B Jonas, Tamas Joo, Nitin Joseph, Charity Ehimwenma Joshua, Farahnaz Joukar, Jacek Jerzy Jozwiak, Mikk Jürisson, Vaishali K, Billingsley Kaambwa, Abdulkareem Kabir, Ali Kabir, Hannaneh Kabir, Zubair Kabir, Rizwan Kalani, Leila R Kalankesh, Feroze Kaliyadan, Sanjay Kalra, Rajesh Kamath, Sagarika Kamath, Tanuj Kanchan, Edmund Wedam Kanmiki, Kehinde Kazeem Kanmodi, Suthanthira Kannan S, Sushil Kumar Kansal, Rami S Kantar, Neeti Kapoor, Mehrdad Karajizadeh, Manoochehr Karami, Ibraheem M Karaye, Faizan Zaffar Kashoo, Hengameh Kasraei, Nicholas J Kassebaum, Molly B Kassel, Joonas H Kauppila, Foad Kazemi, sara Kazeminia, John H Kempen, Evie Shoshannah Kendal, Kamyab Keshtkar, Mohammad Keykhaei, Himanshu Khajuria, Amirmohammad Khalaji, Nauman Khalid, Anees Ahmed Khalil, Alireza Khalilian, Faham Khamesipour, Ajmal Khan, Asaduzzaman Khan, Ikramullah Khan, M Nuruzzaman Khan, Maseer Khan, Mohammad Jobair Khan, Moien AB Khan, Young-Ho Khang, Shaghayegh Khanmohammadi, Khaled Khatab, Armin Khavandegar, Hamid Reza Khayat Kashani, Feriha Fatima Khidri, Moein Khormali, Mohammad Ali Khosravi, Mahmood Khosrowjerdi, Wondwosen Teklesilasie Kidane, Zemene Demelash Kifle, Julie Sojin Kim, Min Seo Kim, Ruth W Kimokoti, Kasey E Kinzel, Girmay Tsegay Kiross, Adnan Kisa, Sezer Kisa, Ali-Asghar Kolahi, Farzad Kompani, Gerbrand Koren, Oleksii Korzh, Soewarta Kosen, Sindhura Lakshmi Koulmane Laxminarayana, Kewal Krishan, Varun Krishna, Vijay Krishnamoorthy, Barthelemy Kuate Defo, Connor M Kubeisy, Burcu Kucuk Bicer, Md Abdul Kuddus, Mohammed Kuddus, Ilari Kuitunen, Mukhtar Kulimbet, Harish Kumar, Satyajit Kundu, Kunle Rotimi

Kunle, Om P Kurmi, Asep Kusnali, Dian Kusuma, Evans F Kyei, Ilias Kyriopoulos, Carlo La Vecchia, Ben Lacey, Muhammad Awwal Ladan, Lucie Laflamme, Chandrakant Lahariya, Daphne Teck Ching Lai, Dharmesh Kumar Lal, Ratilal Lalloo, Judit Lám, Demetris Lamnisos, Iván Landires, Francesco Lanfranchi, Berthold Langguth, Ariane Laplante-Lévesque, Heidi Jane Larson, Anders O Larsson, Savita Lasrado, Kamaluddin Latief, Kaveh Latifinaibin, Long Khanh Dao Le, Nhi Huu Hanh Le, Trang Diep Thanh Le, Caterina Ledda, Munjae Lee, Paul H Lee, Seung Won Lee, Yo Han Lee, Gebretsadik Kiros Lema, Elvynna Leong, Temesgen L Lerango, An Li, Ming-Chieh Li, Shanshan Li, Wei Li, Xiaopan Li, Virendra S Ligade, Stephen S Lim, Ro-Ting Lin, Paulina A Lindstedt, Stefan Listl, Gang Liu, Jue Liu, Xiaofeng Liu, Xuefeng Liu, Yuewei Liu, Erand Llanaj, Rubén López-Bueno, Platon D Lopukhov, László Lorenzovici, Paulo A Lotufo, Jailos Lubinda, Giancarlo Lucchetti, Alessandra Lugo, Raimundas Lunevicius, Hengliang Lv, Zheng Feei Ma, Kelsey Lynn Maass, Monika Machoy, Áurea M Madureira-Carvalho, Mohammed Magdy Abd El Razek, Azzam A Maghazachi, Soleiman Mahjoub, Mansour Adam Mahmoud, Azeem Majeed, Jeadran N Malagón-Rojas, Elaheh Malakan Rad, Kashish Malhotra, Ahmad Azam Malik, Iram Malik, Deborah Carvalho Malta, Abdullah A Mamun, Yosef Manla, Yasaman Mansoori, Ali Mansour, Borhan Mansouri, Zeinab Mansouri, Mohammad Ali Mansournia, Joemer C Maravilla, Mirko Marino, Abdoljalal Marjani, Gabriel Martinez, Ramon Martinez-Piedra, Francisco Rogerlândio Martins-Melo, Miquel Martorell, Sharmeen Maryam, Roy Rillera Marzo, Alireza Masoudi, Jishanth Mattumpuram, Richard James Maude, Andrea Maugeri, Erin A May, Mahsa Mayeli, Maryam Mazaheri, John J McGrath, Martin McKee, Anna Laura Wensel McKowen, Susan A McLaughlin, Steven M McPhail, Rahul Mehra, Kamran Mehrabani-Zeinabad, Entezar Mehrabi Nasab, Tesfahun Mekene Meto, Max Alberto Mendez Mendez-Lopez, Walter Mendoza, Ritesh G Menezes, George A Mensah, Alexios-Fotios A Mentis, Sultan Ayoub Meo, Mohsen Merati, Atte Meretoja, Tuomo J Meretoja, Abera M Mersha, Tomislav Mestrovic, Pouya Metanat, Kukulege Chamila Dinushi Mettananda, Sachith Mettananda, Adquate Mhlanga, Laurette Mhlanga, Tianyue Mi, Tomasz Miazgowski, Georgia Micha, Irmina Maria Michalek, Ted R Miller, Le Huu Nhat Minh, Mojgan Mirghafourvand, Erkin M Mirrakhimov, Mizan Kiros Mirutse, Moonis Mirza, Roya Mirzaei, Ashim Mishra, Sanjeev Misra, Philip B Mitchell, Chaitanya Mittal, Babak Moazen, Abdalla Z Mohamed, Ahmed Ismail Mohamed, Jama Mohamed, Mouhand F H Mohamed, Nouh Saad Mohamed, Sakineh Mohammad-Alizadeh-Charandabi, Soheil Mohammadi, Abdollah Mohammadian-Hafshejani, Mustapha Mohammed, Salahuddin Mohammed, Shafiu Mohammed, Ali H Mokdad, Peyman Mokhtarzadehazar, Hossein Molavi Vardanjani, Sabrina Molinaro, Lorenzo Monasta, Mohammad Ali Moni, Maryam Moradi, Yousef Moradi, Paula Moraga, Rafael Silveira Moreira, Negar Morovatdar, Shane Douglas Morrison, Jakub Morze, Abbas Mosapour, Elias Mossialos, Rohith Motappa, Parsa Mousavi, Amin Mousavi Khaneghah, Christine Mpundu-Kaambwa, Sumaira Mubarik, Lorenzo Muccioli, Francesk Mulita, Kavita Munjal, Efrén Murillo-Zamora, Jonah Musa, Fungai Musaigwa, Ana-Maria Musina, Sathish Muthu, Saravanan Muthupandian, Muhammad Muzaffar, Woojae Myung, Ahamarshan Jayaraman Nagarajan, Gabriele Nagel, Pirouz Naghavi, Ganesh R Naik, Gurudatta Naik, Mukhammad David Naimzada, Firzan Nainu, Vinay Nangia, Sreenivas Narasimha Swamy, Bruno Ramos Nascimento, Gustavo G Nascimento, Abdallah Y Naser, Mohammad Javad Nasiri, Zuhair S Natto, Javaid Nauman, Muhammad Naveed, Biswa Prakash Nayak, Vinod C Nayak, Rawlance Ndejjo, Sabina Onyinye Nduaguba, Hadush Negash, Chernet Tafere Negesse, Ionut Negoi, Ruxandra Irina Negoi, Seyed Aria Nejadghaderi, Chakib Nejjari, Samata Nepal, Henok Biresaw Netsere, Georges Nguefack-Tsague, Josephine W. Ngunjiri, Dang H Nguyen, Hau Thi Hien Nguyen, Phuong The Nguyen, QuynhAnh P Nguyen, Van Thanh Nguyen, Robina Khan Niazi, Yeshambel T Nigatu, Taxiarchis Konstantinos Nikolouzakis, Ali Nikoobar, Amin Reza Nikpoor, Chukwudi A Nnaji, Lawrence Achilles Nnyanzi, Efaq Ali Noman, Shuhei Nomura, Mamoona Noreen, Nafise Noroozi, Chisom Adaobi Nri-Ezedi, Mengistu H Nunemo, Virginia Nuñez-Samudio, Dieta Nurrika, Jerry John Nutor,

Bogdan Oancea, Kehinde O Obamiro, Ismail A Odetokun, Nkechi Martina Odogwu, Martin James O'Donnell, Oluwakemi Ololade Odukoya, Ayodipupo Sikiru Oguntade, James Odhiambo Oguta, In-Hwan Oh, Sylvester Reuben Okeke, Akinkunmi Paul Okekunle, Osaretin Christabel Okonji, Patrick Godwin Okwute, Andrew T Olagunju, Omotola O Olasupo, Matthew Idowu Olatubi, Gláucia Maria Moraes Oliveira, Bolajoko Olubukunola Olusanya, Jacob Olusegun Olusanya, Gideon Olamilekan Oluwatunase, Hany A Omar, Goran Latif Omer, Obinna E Onwujekwe, Michal Ordak, Orish Ebere Orisakwe, Verner N Orish, Doris V Ortega-Altamirano, Alberto Ortiz, Esteban Ortiz-Prado, Wael M S Osman, Uchechukwu Levi Osuagwu, Olayinka Osuolale, Adrian Otoiu, Stanislav S Otstavnov, Amel Ouyahia, Guoqing Ouyang, Mayowa O Owolabi, Yaz Ozten, Mahesh Padukudru P A, Mohammad Taha Pahlevan Fallahy, Feng Pan, Hai-Feng Pan, Adrian Pana, Paramjot Panda, Songhomitra Panda-Jonas, Helena Ullyartha Pangaribuan, Georgios D Panos, Leonidas D Panos, Ioannis Pantazopoulos, Anca Mihaela Pantea Stoian, Romil R Parikh, Seoyeon Park, Ashwaghosha Parthasarathi, Ava Pashaei, Roberto Passera, Hemal M Patel, Jay Patel, Shankargouda Patil, Dimitrios Patoulias, Venkata Suresh Patthipati, Uttam Paudel, Mihaela Paun, Hamidreza Pazoki Toroudi, Spencer A Pease, Amy E Peden, Paolo Pedersini, Minjin Peng, Umberto Pensato, Veincent Christian Filipino Pepito, Prince Peprah, Gavin Pereira, Mario F P Peres, Arokiasamy Perianayagam, Norberto Perico, Simone Perna, Richard G Pestell, Fanny Emily Petermann-Rocha, Hoang Tran Pham, Anil K Philip, Daniela Pierannunzio, Manon Pigeolet, David M Pigott, Evgenii Plotnikov, Dimitri Poddighe, Peter Pollner, Ramesh Poluru, Maarten J Postma, Ghazaleh Pourali, Akram Pourshams, Naeimeh Pourtaheri, Disha Prabhu, Sergio I Prada, Pranil Man Singh Pradhan, Manya Prasad, Akila Prashant, Bharathi M Purohit, Jagadeesh Puvvula, Nameer Hashim Qasim, Ibrahim Qattea, Deepthi R, Mehrdad Rabiee Rad, Amir Radfar, Venkatraman Radhakrishnan, Pourya Raee, Hadi Raeisi Shahraki, Alireza Rafiei, Seyedeh Niloufar Rafiei Alavi, Cat Raggi, Pankaja Raghav Raghav, Fakher Rahim, Md Jillur Rahim, Md. Mosfequr Rahman, Mohammad Hifz Ur Rahman, Mosiur Rahman, Muhammad Aziz Rahman, Vahid Rahmanian, Masoud Rahmati, Niloufar Rahnavard, Pramila Rai, Diego Raimondo, Ali Rajabpour-Sanati, Prashant Rajput, Prasanna Ram, Shakthi Kumaran Ramasamy, Juwel Rana, Kritika Rana, Shailendra Singh Rana, Chhabi Lal Ranabhat, Nemanja Rancic, Amey Rane, Shubham Ranjan, Chythra R Rao, Indu Ramachandra Rao, Deepthi Rapaka, Davide Rasella, Sina Rashedi, Vahid Rashedi, Mohammad-Mahdi Rashidi, Azad Rasul, Zubair Ahmed Ratan, Giridhara Rathnaiah Babu, Santosh Kumar Rauniyar, Nakul Ravikumar, David Laith Rawaf, Salman Rawaf, Reza Rawassizadeh, Bharat Rawlley, Murali Mohan Rama Krishna Reddy, Elrashdy Moustafa Mohamed Redwan, Giuseppe Remuzzi, Bhageerathy Reshmi, Nazila Rezaei, Aida Rezaei Nejad, Mohsen Rezaeian, Abanoub Riad, Mavra A Riaz, Jennifer Rickard, Reza Rikhtegar, Hannah Elizabeth Robinson-Oden, Célia Fortuna Rodrigues, Jefferson Antonio Buendia Rodriguez, Ravi Rohilla, Debby Syahru Romadlon, Luca Ronfani, Himanshu Sekhar Rout, Bedanta Roy, Nitai Roy, Priyanka Roy, Enrico Rubagotti, Guilherme de Andrade Ruela, Susan Fred Rumisha, Tilleye Runghien, Manjula S, Chandan S N, Aly M A Saad, Zahra Saadatian, Maha Mohamed Saber-Ayad, Morteza SaberiKamarposhti, Siamak Sabour, Fatos Sada, Basema Saddik, Bashdar Abuzed Sadee, Ehsan Sadeghi, Erfan Sadeghi, Mohammad Reza Saeb, Umar Saeed, Sher Zaman Safi, Dominic Sagoe, Manika Saha, Amirhossein Sahebkar, Soumya Swaroop Sahoo, Monalisha Sahu, Zahra Saif, Joseph W Sakshaug, Payman Salamati, Afeez Abolarinwa Salami, Mohamed A Saleh, Marwa Rashad Salem, Mohammed Z Y Salem, Sohrab Salimi, Sara Samadzadeh, Yoseph Leonardo Samodra, Vijaya Paul Samuel, Abdallah M Samy, Juan Sanabria, Nima Sanadgol, Francesca Sanna, Milena M Santric-Milicevic, Haaris Saqib, Sivan Yegnanarayana Iyer Saraswathy, Aswini Saravanan, Babak Saravi, Yaser Sarikhani, Tanmay Sarkar, Rodrigo Sarmiento-Suárez, Gargi Sachin Sarode, Sachin C Sarode, Arash Sarveazad, Brijesh Sathian, Thirunavukkarasu Sathish, Anudeep Sathyanarayan, Abu Sayeed, Md Abu Sayeed, Nikolaos Scarmeas, Winfried Schlee, Art Schuermans, David C Schwebel, Falk Schwendicke, Siddharthan Selvaraj, Pallav

Sengupta, Subramanian Senthilkumaran, Sadaf G Sepanlou, Dragos Serban, Edson Serván-Mori, Yashendra Sethi, SeyedAhmad SeyedAlinaghi, Seyed Arsalan Seyedi, Allen Seylani, Mahan Shafie, Jaffer Shah, Pritik A Shah, Ataollah Shahbandi, Samiah Shahid, Moyad Jamal Shahwan, Ahmed Shaikh, Masood Ali Shaikh, Muhammad Aaqib Shamim, Mehran Shams-Beyranvand, Mohammad Anas Shamsi, Mohd Shanawaz, Abhishek Shankar, Mohammed Shannawaz, Medha Sharath, Sadaf Sharfaei, Amin Sharifan, Javad Sharifi-Rad, Manoj Sharma, Rajesh Sharma, Ujjawal Sharma, Vishal Sharma, Rajesh P Shastry, Amin Shavandi, David H Shaw, Amir Mehdi Shayan, Maryam Shayan, Amr Mohamed Elsayed Shehabeldine, Aziz Sheikh, Rahim Ali Sheikhi, Manjunath Mala Shenoy, Pavanchand H Shetty, Peilin Shi, Desalegn Shiferaw, Mika Shigematsu, Rahman Shiri, Reza Shirkoohi, Aminu Shittu, Velizar Shivarov, Farhad Shokraneh, Sina Shool, Seyed Afshin Shorofi, Kanwar Hamza Shuja, Kerem Shuval, Emmanuel Edwar Siddig, João Pedro Silva, Luís Manuel Lopes Rodrigues Silva, Soraia Silva, Biagio Simonetti, Anjali Singal, Abhinav Singh, Balbir Bagicha Singh, Jasvinder A Singh, Md Shahjahan Siraj, Georgia Smith, Bogdan Socea, Anton Sokhan, Ranjan Solanki, Shipra Solanki, Hamidreza Soleimani, Sameh S M Soliman, Yonatan Solomon, Yimeng Song, Reed J D Sorensen, Michael Spartalis, Chandrashekhar T Sreeramareddy, Vijay Kumar Srivastava, Muhammad Haroon Stanikzai, Vladimir I Starodubov, Antonina V Starodubova, Simona Cătălina Stefan, Paschalis Steiropoulos, Mark A Stokes, Vetriselvan Subramaniyan, Muhammad Suleman, Rizwan Suliankatchi Abdulkader, Abida Sultana, Jing Sun, Chandan Kumar Swain, Bryan L Sykes, Lukasz Szarpak, Mindy D Szeto, Miklós Szócska, Payam Tabaee Damavandi, Rafael Tabarés-Seisdedos, Ozra Tabatabaei Malazy, Seyed-Amir Tabatabaeizadeh, Shima Tabatabai, Karen M Tabb, Mohammad Tabish, Moslem Taheri Soodejani, Jabeen Taiba, Ardeshir Tajbakhsh, Iman M Talaat, Ashis Talukder, Mircea Tampa, Jacques Lukenze Tamuzi, Ker-Kan Tan, Haosu Tang, Derbie Alemu DA Tareke, Mengistie Kassahun Tariku, Vivian Y Tat, Seyed Mohammad Tavangar, Mojtaba Teimoori, Mohamad-Hani Temsah, Reem Mohamad Hani Temsah, Masayuki Teramoto, Dufera Rikitu Terefa, Riki Tesler, Enoch Teye-Kwadjo, Ramna Thakur, Pugazhenthan Thangaraju, Kavumpurathu Raman Thankappan, Rekha Thapar, Samar Tharwat, Rasiah Thayakaran, Nihal Thomas, Ales Tichopad, Jansje Henny Vera Ticoalu, Tenaw Yimer Tiruye, Mariya Vladimirovna Titova, Marcello Tonelli, Marcos Roberto Tovani-Palone, Eugenio Traini, Jasmine T Tran, Nghia Minh Tran, Indang Trihandini, Samuel Joseph Tromans, Thien Tan Tri Tai Truyen, Aristidis Tsatsakis, Evangelia Eirini Tsermpini, Munkhtuya Tumurkhuu, Stefanos Tyrovolas, Sayed Mohammad Nazim Uddin, Aniefiok John Udoakang, Arit Udoh, Atta Ullah, Saeed Ullah, Sana Ullah, Srikanth Umakanthan, Chukwuma David Umeokonkwo, Brigid Unim, Bhaskaran Unnikrishnan, Era Upadhyay, Jibrin Sammani Usman, Marco Vacante, Seyed Mohammad Vahabi, Asokan Govindaraj Vaithinathan, Rohollah Valizadeh, Jef Van den Eynde, Elena Varavikova, Orsolya Varga, Priya Vart, Shoban Babu Varthya, Tommi Juhani Vasankari, Balachandar Vellingiri, Deneshkumar Venugopal, Nicholas Alexander Verghese, Madhur Verma, Massimiliano Veroux, Georgios-Ioannis Verras, Dominique Vervoort, Jorge Hugo Villafañe, Manish Vinayak, Francesco S Violante, Mukesh Vishwakarma, Sergey Konstantinovitch Vladimirov, Vasily Vlassov, Bay Vo, Simona Ruxandra Volovat, Theo Vos, Isidora S Vujcic, Hatem A Wafa, Yasir Waheed, Elias Bekele Wakwoya, Cong Wang, Denny Wang, Fang Wang, Shu Wang, Yanzhong Wang, Yuan-Pang Wang, Paul Ward, Emebet Gashaw Wassie, Stefanie Watson, Marcia R Weaver, Kosala Gayan Weerakoon, Daniel J Weiss, Katherine M Wells, Yi Feng Wen, Ronny Westerman, Taweewat Wiangkham, Dakshitha Praneeth Wickramasinghe, Nuwan Darshana Wickramasinghe, Peter Willeit, Yohannes Addisu Wondimagegene, Felicia Wu, Juan Xia, Hong Xiao, Gelin Xu, Suowen Xu, Xiaoyue Xu, Ali Yadollahpour, Shirin Yaghoobpoor, Tina Yaghoobpour, Sajad Yaghoubi, Zwanden Sule Yahaya, Danting Yang, Lin Yang, Yuichiro Yano, Habib Yaribeygi, Pengpeng Ye, Renjulal Yesodharan, Subah Abderehim Yesuf, Saber Yezli, Amanuel Yigezu, Paul Yip, Dong Keon Yon, Naohiro Yonemoto, Yuyi You, Mustafa Z Younis, Zabihollah Yousefi, Chuanhua Yu, Yong Yu, Chun-Wei Yuan, Nima

Zafari, Fathiah Zakham, Nazar Zaki, Giulia Zamagni, Milad Zandi, Ghazal G Z Zandieh, Moein Zangiabadian, Mikhail Sergeevich Zastrozhin, Haijun Zhang, Meixin Zhang, Yunquan Zhang, Chenwen Zhong, Juexiao Zhou, Bin Zhu, Lei Zhu, Magdalena Zielińska, Zhiyong Zou, Samer H Zyoud, Christopher J L Murray#, Amanda E Smith#, Stein Emil Vollset#.

* Joint first authors
# Joint senior authors

Affiliations

Institute for Health Metrics and Evaluation (N V Bhattacharjee PhD, A E Schumacher PhD, Prof A J Ahlstrom MSc, C M Antony MA, A Y Aravkin PhD, C Bisignano MPH, E Bliss MSc, Prof M Brauer DSc, D Bryazka BA, K Burkart PhD, S Cao MS, J Chalek BS, K Coberly BS, H Comfort MPH, B Dalton BA, M A Dirac MD, J Duprey MS, L Dwyer-Lindgren PhD, K Estep MPA, S Farmer BA, Prof V L Feigin PhD, J E Fuller MLIS, A Haakenstad ScD, Prof S I Hay FMedSci, N J Kassebaum MD, M B Kassel BA, J S Kim MS, K E Kinzel MSPH, Prof H J Larson PhD, Prof S S Lim PhD, P A Lindstedt MPH, K L Maass PhD, E A May, A W McKowen, S A McLaughlin PhD, T Mestrovic PhD, A H Mokdad PhD, Q P Nguyen BS, Y Ozten MS, S A Pease BS, D M Pigott PhD, C Raggi MS, H E Robinson-Oden MLIS, H Saqib MA, D H Shaw BA, G Smith MS, R J D Sorensen PhD, N A Verghese BA, Prof T Vos PhD, D Wang BA, S Watson MS, Prof M R Weaver PhD, K M Wells BA, C Yuan PhD, M Zhang MS, Prof C J L Murray DPhil, A E Smith MPA, Prof S Vollset DrPH), Department of Applied Mathematics (Prof A J Ahlstrom MSc, A Y Aravkin PhD), Department of Health Metrics Sciences, School of Medicine (A Y Aravkin PhD, K Burkart PhD, M A Dirac MD, L Dwyer-Lindgren PhD, Prof S I Hay FMedSci, N J Kassebaum MD, J S Kim MS, Prof S S Lim PhD, A H Mokdad PhD, D M Pigott PhD, Prof T Vos PhD, Prof M R Weaver PhD, Prof C J L Murray DPhil, Prof S Vollset DrPH), Department of Internal Medicine (Y Chahine MD), Department of Cardiology (Y Chahine MD), Department of Family Medicine (M A Dirac MD), Department of Neurology (R Kalani MD), Department of Anesthesiology & Pain Medicine (N J Kassebaum MD, V Krishnamoorthy MD), Division of Plastic and Reconstructive Surgery (S D Morrison MD), Department of Global Health (R J D Sorensen PhD), University of Washington, Seattle, WA, USA; Faculty of Medicine (A Aali MD, N Rahnavard MD), Dental Research Center (E Bardideh DDS), Orthodontics Department (M Ghorbani DDS), Clinical Research Development Unit (N Morovatdar MD), Metabolic Syndrome Research Center (G Pourali MD), International UNESCO Center for Health-related Basic Sciences and Human Nutrition (G Pourali MD), Applied Biomedical Research Center (A Sahebkar PhD), Biotechnology Research Center (A Sahebkar PhD), Department of Medical Genetics (N Zafari MD), Mashhad University of Medical Sciences, Mashhad, Iran; Department of Clinical Governance and Quality Improvement (Y H Abate MSc), Aleta Wondo Hospital, Aleta Wondo, Ethiopia; Doheny Eye Institute (R Abbasgholizadeh MD), University of California Los Angeles, Pasadena, CA, USA; Department of Orthopedic Surgery (M Abbasian MD), Department of Pediatrics (S Aly MD), T.H. Chan School of Public Health (Prof T W Bärnighausen MD), Center for Primary Care (S Basu PhD), Harvard Business School (F Caetano dos Santos PhD), Division of Cardiovascular Medicine (G Chi MD), Department of Neurological Surgery at Brigham and Women's Hospital (A H Feroze MD), Maternal Fetal Care Center (K Hessami MD), Department of Ophthalmology (Prof J H Kempen MD), Department of Health Policy and Management (C M Kubeisy BA), Radiology and Data Science Department (X Liu PhD), Department of Health Policy and Oral Epidemiology (Z S Natto DrPH), Department of Global Health and Social Medicine (M Pigeolet MD), Harvard T.H. Chan School of Public Health (P M S Pradhan MD), Beth Israel Deaconess Medical Center (S Sharfaei MD), Division of General Internal Medicine (Prof A Sheikh MD), Harvard University, Boston, MA, USA; Department of Orthopaedic Surgery (M Abbasian MD), Department of Anesthesiology (D Abtahi MD, S Salimi MD, A Tajbakhsh MD, A Tajbakhsh MD), Department of Biotechnology (S Aghamiri PhD), National Nutrition and Food Technology Research Institute (M Ajami PhD), Research Institute of Dental Sciences (Prof S Asgary MSc), Urology Department (M Bonakdar Hashemi MD), Department of Medical Genetics (M Ghasemi PhD), Center for Comprehensive Genetic Services (M Ghasemi PhD), Department of Immunology (K Jahankhani MSc), Department of Health Policy and Management (N Jahanmehr PhD), Safety Promotion and Injury Prevention Research Center (N Jahanmehr PhD), Department of Epidemiology (M Karami PhD, S Sabour PhD), Department of Neurosurgery (H Khayat Kashani MD), Social Determinants of Health Research Center (A Kolahi MD, A Nikoobar Dipl Experimental Sciences, M

Rashidi MD), Department of Microbiology and Infectious Diseases (M Nasiri PhD), School of Medicine (S Nejadghaderi MD, M Zangiabadian MD), Department of Biology and Anatomical Sciences (P Raee PhD), Ophthalmic Research Center (ORC) (M Shayan MD), Emergency Department (S Shool MD), Department of Medical Education (S Tabatabai PhD), Shahid Beheshti University of Medical Sciences, Tehran, Iran (S Yaghoobpoor MD); Non-communicable Diseases Research Center (M Abbasi-Kangevari MD, S Azadnajafabad MD, M Keykhaei MD, P Mousavi MD, M Rashidi MD, N Rezaei MD), Advanced Diagnostic and Interventional Radiology Research Center (H Abbastabar PhD), The Institute of Pharmaceutical Sciences (TIPS) (Prof M Abdollahi PhD), School of Pharmacy (Prof M Abdollahi PhD), Research Center for Immunodeficiencies (H Abolhassani PhD), Hematology, Oncology and Stem Cell Transplantation Research Center (H Alemi MD), Urology Research Center (R Arabzadeh Bahri MD), Department of Health Information Management (S Ayyoubzadeh PhD), Translational Ophthalmology Research Center (N Bahmanziari PhD), School of Medicine (A Behnoush BS, A Khalaji BS, S Khanmohammadi MD, M Mayeli MD, M Merati MD, S Mohammadi MD), Department of Scientific Research (F Chichagi MD), Multiple Sclerosis Research Center (S Eskandarieh PhD), Digestive Diseases Research Institute (S Fahimi MD, Prof A Pourshams MD, S G Sepanlou MD), Ophthalmology Department (Prof F Ghassemi MD), Department of Radiology (R Hajibeygi MD), Students’ Scientific Research Center (SSRC) (M Keykhaei MD), Center for Research and Training in Skin Diseases and Leprosy (F Khamesipour PhD), Sina Trauma and Surgery Research Center (A Khavandegar MD, M Khormali MD, Prof P Salamati MD, S Shool MD), Children's Medical Center (F Kompani MD), Department of Pediatric Cardiology (Prof E Malakan Rad MD), Department of Obstetrics and Gynecology (Z Mansouri MD), Department of Epidemiology and Biostatistics (M Mansournia PhD), Tehran Heart Center (E Mehrabi Nasab MD), Water Quality Research Center (R Mirzaei PhD), Department of Pharmacology (N Noroozi DVM), Department of Medicine (M Pahlevan Fallahy MD, A Shahbandi MD), Department of Cardiology (S Rashedi MD), Stem Cell and Center of Regenerative Medicine (A Rezaei Nejad MD), Iranian Research Center for HIV/AIDS (S SeyedAlinaghi PhD), Endocrinology and Metabolism Research Center (EMRC) (S Seyedi MD, O Tabatabaei Malazy PhD), Department of Neurology (M Shafie MD), Department of Pharmaceutical Care (A Sharifan PharmD), Research Center for Rational Use of Drugs (A Sharifan PharmD), Cancer Research Center (R Shirkoohi PhD), Cancer Biology Research Center (R Shirkoohi PhD), Department of Pathology (Prof S Tavangar MD), Faculty of Medicine (S Vahabi MD), Tehran University of Medical Sciences, Tehran, Iran; Epidemiology Department (S Abd ElHafeez DrPH), Pediatric Dentistry and Dental Public Health Department (Prof M El Tantawi PhD, Prof O A A Elmeligy PhD), Tropical Health Department (R M Ghazy PhD), Pathology Department (Prof I M Talaat PhD), Alexandria University, Alexandria, Egypt; Tropical Medicine Department (S Abd-Elsalam PhD), Tanta University, Tanta, Egypt; Department of Small Animal Clinical Sciences (M Abdollahifar PhD), Department of Community Health and Epidemiology (D A Adeyinka PhD), University of Saskatchewan, Saskatoon, SK, Canada; Department of Medicine (Prof M Abdoun BMedSc), University of Setif Algeria, Sétif, Algeria; Department of Physiotherapy (A Abdullahi PhD, J S Usman PhD), Community Medicine Department (Prof M A Gadanya FMCPH), Department of Nursing Science (M Ladan PhD), Bayero University Kano, Kano, Nigeria; Department of Rehabilitation Sciences (A Abdullahi PhD, M U Ali MSc, M Khan MPH, J S Usman PhD), School of Nursing (S Tyrovolas PhD), Hong Kong Polytechnic University, Hong Kong, China; Department of Midwifery (M Abebe MSc, G M Ayele MSc), Department of Public Health (T L Lerango MPH, Y A Wondimagegene PhD), Dilla University, Dilla, Ethiopia; Department of Public Health (S S Abebe MPH), Department of Medical Laboratory Sciences (M Arkew MSc), Department of Health Policy and Management (A T Debele MSc), Department of Clinical Pharmacy (M D Gudeta MSc), Haramaya University, Harar, Ethiopia; Department of Community Medicine (O Abiodun MPH), Department of Medical Physiology (P G Okwute MSc),

Babcock University, Ilishan-Remo, Nigeria; Department of Medical Biochemistry and Biophysics (H Abolhassani PhD), Department of Neurobiology, Care Sciences, and Society (Prof J Ärnlöv PhD, S Fereshtehnejad PhD), Department of Molecular Medicine and Surgery (Prof J H Kauppila MD), Department of Global Public Health (Prof L Laflamme PhD), Karolinska Institute, Stockholm, Sweden; Department of Neurosurgery (M Abolmaali MD), Health Management and Economics Research Center (J Arabloo PhD), Department of Epidemiology (M Asadi-Lari PhD), Center for Educational Research in Medical Educaion (CERMS) (S Delavari PhD), Minimally Invasive Surgery Research Center (A Kabir MD), Eye Research Center (H Kasraei MD), Department of Anesthesiology (K Latifinaibin MD), Comprehensive Research Laboratory (R Mirzaei PhD), Department of Physiology (H Pazoki Toroudi PhD), Physiology Research Center (H Pazoki Toroudi PhD), Colorectal Research Center (A Sarveazad PhD), Iran University of Medical Sciences, Tehran, Iran (M Moradi MD); Khatam Al-anbia Hospital (M Abolmaali MD), Shefa Neuroscience Research Center, Tehran, Iran; Department of Physical Pharmacy and Pharmacokinetics (M Abouzid PharmD), Poznan University of Medical Sciences, Poznan, Poland; Department of Public Health (G B Aboye MSc), Madda Walabu University, Addis Ababa, Ethiopia; Nutrition and Dietetics Department (G B Aboye MSc), USAID-JSI (B H Demessa MPH), Jimma University, Addis Ababa, Ethiopia; Department of Pediatric Dentistry (Prof L G Abreu PhD), Department of Nutrition (Prof R M Claro PhD), Department of Maternal and Child Nursing and Public Health (Prof D C Malta PhD), Department of Clinical Medicine (Prof B R Nascimento PhD), Clinical Hospital (Prof B R Nascimento PhD), Federal University of Minas Gerais, Belo Horizonte, Brazil; Department of Adult Health Nursing (W A Abrha MSc), Department of Nursing (A Girmay MSc), Aksum University, Aksum, Ethiopia; Department of Research (M R M Abrigo PhD), Philippine Institute for Development Studies, Quezon City, Philippines; Department of Nursing (H Abualruz PhD), Al Zaytoonah University of Jordan, Amman, Jordan; Department of Pharmacology and Toxicology (B Abubakar PhD), Department of Veterinary Public Health and Preventive Medicine (A Shittu MSc), Usmanu Danfodiyo University, Sokoto, Sokoto, Nigeria; Nigerian Institute of Medical Research (B Abubakar PhD), Nigerian Institute of Medical Research, Lagos, Nigeria; Clinical Sciences Department (E Abu-Gharbieh PhD, S Adra MD, H J Barqawi MPhil, Prof R Halwani PhD, Prof A A Maghazachi PhD, M M Saber-Ayad MD, Prof I M Talaat PhD), College of Medicine (F Ahmad PhD, Prof R Halwani PhD, Prof B Saddik PhD, M A Saleh PhD), Department of Pharmacy Practice and Pharmacotherapeutics (Prof K H Alzoubi PhD, Prof H A Omar PhD), Department of Physiotherapy (A Arumugam PhD), Department of Basic Biomedical Sciences (Y Bustanji PhD), Sharjah Institute for Medical Research (N M Elemam PhD), Department of Clinical Nutrition and Dietetics (M E M Faris PhD), Department of Medicinal Chemistry (S S M Soliman PhD), University of Sharjah, Sharjah, United Arab Emirates (K A Altirkawi MD); Institute of Community and Public Health (Prof N M Abu-Rmeileh PhD), Birzeit University, Ramallah, Palestine; Department of Public Health (T G G Adal MPH), Midwifery Department (Y F Geda MSc), Wolkite University, Wolkite, Ethiopia; College of Medicine and Health Sciences (M M Adane PhD, S A Belay MSc), Department of Epidemiology and Biostatistics (Y Alemu MPH, K A Bogale MPH), Department of Midwifery (A M Aweke MSc), School of Health Science (A Y Berhie MSc), Health Promotion and Behavioural Science Department (E K Bogale MPH), Department of Physiology (D Demeke MSc), Department of Pharmacy (C T Negesse MSc), Bahir Dar University, Bahir Dar, Ethiopia; Department of Health Promotion, Education, and Behavior (O A A Adeagbo PhD, T Mi PhD), University of South Carolina, Columbia, SC, USA; Public Health Department (O A A Adeagbo PhD), University of KwaZulu-Natal, Durban, South Africa; Department of Medical Rehabilitation (Prof R A Adedoyin PhD), Department of Animal Sciences (H A Bashiru MSc), Department of Child Dental Health (Prof M O Folayan FWACS), Obafemi Awolowo University, Ile-Ife, Nigeria; Department of Obstetrics and Gynecology (V Adekanmbi PhD), University of Texas Medical Branch, Galveston, TX, USA; Department of

Molecular Biology and Genetics (Prof M T AlBataineh PhD), Department of Biology (W M S Osman PhD), Khalifa University, Abu Dhabi, United Arab Emirates (B Aden PhD); Institute of Public Health (B Aden PhD), Walden University, Al Ain, United Arab Emirates; HIV and Infectious Diseases Department (A V Adepoju MD), Jhpiego, Abuja, Nigeria; Department of Adolescent Research and Care (A V Adepoju MD), Adolescent Friendly Research Initiative and Care, Ado Ekiti, Nigeria; DSI-NRF Centre of Excellence for Epidemiological Modelling and Analysis (SACEMA) (O O Adetokunboh PhD), Stellenbosch University, Stellenbosch, South Africa; Division of Epidemiology & Biostatistics (O O Adetokunboh PhD), South African Centre for Epidemiological Modelling and Analysis (SACEMA) (L Mhlanga PhD), Department of Epidemiology (J L Tamuzi MSc), Department of Industrial Psychology (E Teye-Kwadjo PhD), Stellenbosch University, Cape Town, South Africa; Department of Biochemistry (J B Adetunji PhD), Osun State University, Osogbo, Nigeria; Department of Public Health (D A Adeyinka PhD), Federal Ministry of Health, Abuja, Nigeria; Department of Physiology (O I Adeyomoye PhD), Department of Microbiology (O O Bello PhD), Department of Anatomy (G O Oluwatunase MSc), Department of Biosciences and Biotechnology (A J Udoakang PhD), University of Medical Sciences, Ondo, Ondo, Nigeria; Faculty of Medicine (Q E S Adnani PhD), Center of Excellence in Higher Education for Pharmaceutical Care Innovation (Prof M J Postma PhD), Universitas Padjadjaran (Padjadjaran University), Bandung, Indonesia; Department of Epidemiology and Medical Statistics (R F Afolabi PhD, M Ekholuenetale MSc, A F Fagbamigbe PhD, K R Fowobaje MSc), Institute for Advanced Medical Research and Training (R O Akinyemi PhD), Faculty of Public Health (M Ekholuenetale MSc), Department of Health Promotion and Education (S E Ibitoye MPH), Department of Community Medicine (O S Ilesanmi PhD), College of Medicine (A P Okekunle PhD), Department of Medicine (Prof M O Owolabi DrM), University of Ibadan, Ibadan, Nigeria; Department of Radiology (S Afyouni PhD, A Amindarolzarbi MD, G G Z Zandieh MD), Department of Epidemiology and Population Health (N Alhajri MD), Department of Biostatistics (A Columbus MS), Department of Neurosurgery (F Kazemi MD), Department of Health Policy and Management (D Vervoort MD), Department of International Health (H Zhang MS), Johns Hopkins University, Baltimore, MD, USA (E Jamshidi PharmD); Department of Life Sciences (M S Afzal PhD), University of Management and Technology, Lahore, Pakistan; Department of Community Medicine (Prof S Afzal PhD), King Edward Memorial Hospital, Lahore, Pakistan; Department of Public Health (Prof S Afzal PhD), Public Health Institute, Lahore, Pakistan; Department of Medical and Surgical Sciences and Advanced Technologies "GF Ingrassia" (Prof A Agodi PhD, M Barchitta PhD, A Maugeri PhD, Prof M Veroux PhD), Department of General Surgery and Medical-Surgical Specialties (Prof A Biondi PhD, Prof G Isola PhD, M Vacante PhD), Department of Biomedical and Biotechnological Sciences (L Falzone PhD), Department of Clinical and Experimental Medicine (C Ledda PhD), University of Catania, Catania, Italy; Department of Geography and Planning (W Agyemang-Duah MSc), Department of Biomedical and Molecular Sciences (A Nikpoor PhD), Queen's University, Kingston, ON, Canada; School of Public Health (B O Ahinkorah MPhil), University of Technology Sydney, Sydney, NSW, Australia; Department of Medical Biochemistry (A Ahmad PhD), Department of Pharmacology (M Tabish MPharm), Shaqra University, Shaqra, Saudi Arabia; School of Medicine and Psychology (D Ahmad PhD), National Centre of Epidemiology and Population Health (Y Alemu MPH), Research School of Population Health (N Bagheri PhD, R A Burns PhD), Australian National University, Canberra, ACT, Australia; Public Health Foundation of India, Gandhinagar, India (D Ahmad PhD); Department of Clinical Nursing (Prof M M Ahmad PhD), University of Jordan, Amman, Jordan; Department of Health and Biological Sciences (S Ahmad PhD), Abasyn University, Peshawar, Pakistan; Department of Natural Sciences (S Ahmad PhD), Labanese American University, Beirut, Lebanon; Department of Epidemiology and Health Statistics (T Ahmad MS), Southeast University, Nanjing, China; Department of Pharmacy Practice (A Ahmed PhD), Riphah Institute

of Pharmaceutical Sciences, Islamabad, Pakistan; Division of Infectious Diseases and Global Public Health (IDGPH) (A Ahmed PhD), University of California, San Diego, CA, USA; Institute of Endemic Diseases (A Ahmed MSc), Unit of Basic Medical Sciences (E E Siddig MD), University of Khartoum, Khartoum, Sudan; Swiss Tropical and Public Health Institute (A Ahmed MSc), University of Basel, Basel, Switzerland; Department of Biosciences (H Ahmed PhD), COMSATS Institute of Information Technology, Islamabad, Pakistan; Institute of Public Health (L A Ahmed PhD), College of Medicine and Health Sciences (Prof M Grivna PhD, J Nauman PhD), Family Medicine Department (M A Khan MSc), Department of Computer Science and Software Engineering (Prof N Zaki PhD), United Arab Emirates University, Al Ain, United Arab Emirates; Department of Pathology and Microbiology (M S Ahmed MSc), University of Duhok, Duhok, Iraq; Brody School of Medicine (S Ahmed PhD), Department of Computer Science (A O Bodunrin MSc), Department of Physiology (M Tumurkhuu PhD), East Carolina University, Greenville, NC, USA (R T Aruleba PhD); Department of Food and Nutrition Policy and Planning Research (M Ajami PhD), National Institute of Nutrition, Tehran, Iran; Faculty of Medicine and Public Health (B Aji DrPH), Jenderal Soedirman University, Purwokerto, Indonesia; Microbiology, Immunology and Parasitology Department (G T Akalu MSc), St. Paul's Hospital Millennium Medical College, Addis Ababa, Ethiopia; Microbial, Cellular and Molecular Biology Department (G T Akalu MSc), School of Public Health (K Deribe PhD), Addis Ababa University, Addis Ababa, Ethiopia; St George and Sutherland Clinical School (H Akbarialiabad MD), School of Population Health (Z Dai PhD, X Xu PhD), School of Psychiatry (Prof P B Mitchell MD), Centre for Social Research in Health (S R Okeke PhD), School of Public Health and Community Medicine (A E Peden PhD), The George Institute for Global Health (P Ye MPH), University of New South Wales, Sydney, NSW, Australia; Institute of Neuroscience (R O Akinyemi PhD), Newcastle University, Newcastle upon Tyne, UK; Department of Cardiology (M A Akkaif PhD), Department of Health Management Center (X Li PhD), Fudan University, Shanghai, China; Department of Management, Policy, and Community Health (S Akkala MPH), University of Texas, Houston, TX, USA; Geriatric and Long Term Care Department (H Al Hamad MD, B Sathian PhD), Rumailah Hospital (H Al Hamad MD), Hamad Medical Corporation, Doha, Qatar; Department of Surgery (S Al Hasan PhD), Washington University School of Medicine, St. Louis, MO, USA; Department of Nursing (Prof M Al Qadire PhD), Community and Mental Health Department (Prof M Albashtawy PhD), Al Al-Bayt University, Mafraq, Jordan; Institute of Global Health (T M A AL-Ahdal MPH), Heidelberg Institute of Global Health (HIGH) (Prof T W Bärnighausen MD, S Chen DSc, B Moazen MSc), Heidelberg University, Heidelberg, Germany; Department of Clinical Sciences (S O Alalalmeh Bpharm, Prof E A Arafa PhD, O E Hegazi BPharm), Center for Medical and Bio-Allied Health Sciences Research (Prof M J Shahwan PhD, M A Shamsi PhD, S H Zyoud PhD), Ajman University, Ajman, United Arab Emirates; Department of Biology (T A Alalwan PhD, Prof S Perna PhD), University of Bahrain, Sakhir, Bahrain; John T. Milliken Department of Internal Medicine (Z Al-Aly MD), Brown School (C Wang MPH), Department of Surgery (C Wang MPH), Washington University in St. Louis, St. Louis, MO, USA; Clinical Epidemiology Center (Z Al-Aly MD), US Department of Veterans Affairs (VA), St. Louis, MO, USA; Murdoch Business School (K Alam PhD), Murdoch University, Perth, WA, Australia; School of Nursing (R M Al-amer PhD), Yarmouk University, Irbid, Jordan; School of Nursing and Midwifery (R M Al-amer PhD), Department of Engineering (G R Naik PhD), Western Sydney University, Sydney, NSW, Australia; Health Information Management and Technology Department (T M Alanzi PhD), Forensic Medicine Division (Prof R G Menezes MD), Imam Abdulrahman Bin Faisal University, Dammam, Saudi Arabia (F M Alanezi PhD); Department of Medicine (A Albakri MD), Royal Jordanian Medical Services, Amman, Jordan; Global Health Entrepreneurship (S Alemi PhD), Tokyo Medical and Dental University, Tokyo, Japan; Pediatric Intensive Care Unit (A Al-Eyadhy MD, M Temsah MD), Department of Cardiac Sciences (Prof K F Alhabib MD), Section of Adult Hematology (Prof G M T ElGohary MD),

Department of Physiology (Prof S A Meo PhD), King Saud University, Riyadh, Saudi Arabia; Global Centre for Environmental Remediation (A A S Al-Gheethi PhD), Department of Women's Health (G T Kiross MPH), University of Newcastle, Newcastle, NSW, Australia; Cooperative Research Centre for Contamination Assessment and Remediation of the Environment, Newcastle, NSW, Australia (A A S Al-Gheethi PhD); College of Nursing (Prof F A N Alhalaiqa PhD), Department of Physical Education (Prof M A Alomari PhD), QU Health (M Mohammed PhD), Department of Population Medicine (Prof G Rathnaiah Babu PhD), Qatar University, Doha, Qatar; Psychological Sciences Association, Amman, Jordan (Prof F A N Alhalaiqa PhD); Institute of Health Research (R K Alhassan PhD, M Immurana PhD), Department of Health Policy Planning and Management (M K Boachie PhD), Department of Epidemiology and Biostatistics (R K Dowou MPhil), Department of Microbiology and Immunology (V N Orish PhD), University of Health and Allied Sciences, Ho, Ghana; Department of Zoology (A Ali PhD), Department of Botany (Prof I Khan PhD), Abdul Wali Khan University Mardan, Mardan, Pakistan; Erbil Technical Health College (B A Ali PhD), Erbil Polytechnic University, Erbil, Iraq; School of Pharmacy (B A Ali PhD), Tishk International University, Erbil, Iraq; Department of Biological Sciences (L Ali PhD), National University of Medical Sciences (NUMS), Rawalpindi, Pakistan; Department of Medical Rehabilitation (Physiotherapy) (M U Ali MSc), University of Maiduguri, Maiduguri, Nigeria; Department of Biosciences (R Ali MPhil), Centre for Interdisciplinary Research In Basic Sciences (CIRBSc) (M A Shamsi PhD), Jamia Millia Islamia, New Delhi, India; Center for Biotechnology and Microbiology (S S Ali PhD), University of Swat, Swat, Pakistan; School of Public Health and Preventive Medicine (S M Alif PhD, S Li PhD, P Rai MPH), School of Public Health and Preventative Medicine (Prof M Asghari-Jafarabadi PhD), Department of Human-Centred Computing, Faculty of Information Technology (M Saha MSc), Monash University, Melbourne, VIC, Australia; Department of Public Health (M Aligol PhD), Qom University of Medical Sciences, Qom, Iran; Social Determinants of Health Research Center (M Alijanzadeh PhD), Qazvin University of Medical Sciences, Qazvin, Iran; Medical Laboratories (M A M Aljasir PhD), Qassim University, Buraydah, Saudi Arabia; Department of Molecular and Clinical Pharmacology (M A M Aljasir PhD), Institute of Infection and Global Health (Prof A Beloukas PhD), Liverpool Orthopaedic and Trauma Service (S M Graham PhD), Department of Surgery (Prof R Lunevicius DSc), University of Liverpool, Liverpool, UK; Department of Health Policy and Management (Prof S M Aljunid PhD), Department of Surgery (S K Al-Sabah MD), Kuwait University, Kuwait, Kuwait; International Centre for Casemix and Clinical Coding (Prof S M Aljunid PhD), National University of Malaysia, Bandar Tun Razak, Malaysia; Department of Dentistry (S Al-Marwani MSc), Independent Consultant, Sana'a, Yemen; Department of Public Health and Community Medicine (S Al-Marwani MSc), Independent Consultant, Irbid, Jordan; Department of Medicine (J U Almazan PhD, Prof D Poddighe PhD), Nazarbayev University, Astana, Kazakhstan; Department of Parasitology (Prof H M Al-Mekhlafi PhD), University of Malaya, Kuala Lumpur, Malaysia; Department of Parasitology (Prof H M Al-Mekhlafi PhD), Sana'a University, Sana'a, Yemen; Department of Urology (O Almidani MSc), Department of Cardiac Surgery (L Göbölös PhD), Cleveland Clinic Abu Dhabi, Abu Dhabi, United Arab Emirates; Nuffield Department of Surgical Sciences (O Almidani MSc, S Bandyopadhyay BA), Nuffield Department of Orthopaedics (S M Graham PhD), Nuffield Department of Population Health (B Lacey PhD), Nuffield Department of Medicine (Prof R J Maude PhD, T Runghien MSc), Health Economics Research Centre (Prof J A B Rodriguez PhD), University of Oxford, Oxford, UK; Department of Rehabilitation Sciences and Physical Therapy (Prof M A Alomari PhD), Department of Clinical Pharmacy (Prof K H Alzoubi PhD), Jordan University of Science and Technology, Irbid, Jordan; Department of Epidemiology and Population Health (B Al-Omari PhD), Khalifa University of Science, Technology & Research, Abu Dhabi, United Arab Emirates; Department of Respiratory Care (J S Alqahtani PhD), Prince Sultan Military College of Health Sciences, Dammam, Saudi Arabia; Department of Prosthodontics and

Implant Dentistry (A Alqutaibi PhD), Taibah University, Medinah, Saudi Arabia; Department of Prosthodontics (A Alqutaibi PhD), Ibb University, Ibb, Yemen; Department of Community Medicine (R M Al-Raddadi PhD), Department of Family and Community Medicine (N S Butt PhD), Pediatric Dentistry Department (Prof O A A Elmeligy PhD), Rabigh Faculty of Medicine (A A Malik PhD), Department of Dental Public Health (Z S Natto DrPH), King Abdulaziz University, Jeddah, Saudi Arabia; Jaber Al Ahmad Al Sabah Hospital (S K Al-Sabah MD), Ministry of Health, Kuwait, Kuwait; Institute of Molecular Biology and Biotechnology (A Altaf PhD, S Shahid PhD), University Institute of Public Health (F J Alvi MPH, S Hameed MPH, A A Malik PhD), University College of Medicine & Dentistry (Prof M Arooj PhD), University Institute of Radiological Sciences and Medical Imaging Technology (T Ashraf MS), University Institute of Diet and Nutritional Sciences (A Khalil PhD), Department of Technology (M Muzaffar MBA), Research Centre for Health Sciences (RCHS) (M Muzaffar MBA, S Shahid PhD), The University of Lahore, Lahore, Pakistan (Prof M Ashraf PhD, M A Riaz Mcom); Department of Specialty Internal Medicine (Prof J A Al-Tawfiq MD), Johns Hopkins Aramco Healthcare, Dhahran, Saudi Arabia; Medicine Department (Prof J A Al-Tawfiq MD), Indiana University School of Medicine, Indianapolis, IN, USA; Lisbon Institute of Global Mental Health (D O Aluh MSc), Nova University of Lisbon, Lisboa, Nigeria; Department of Clinical Pharmacy and Pharmacy Management (D O Aluh MSc), University of Nigeria Nsukka, Nsukka, Nigeria; Research Group in Hospital Management and Health Policies (Prof N Alvis-Guzman PhD), Universidad de la Costa (University of the Coast), Barranquilla, Colombia; Research Group in Health Economics (Prof N Alvis-Guzman PhD), University of Cartagena, Cartagena, Colombia; Department of Clinical Pharmacology and Toxicology (H Alwafi PhD), Umm Al-Qura University, Makkah, Saudi Arabia; Department of Medical Sciences (Prof Y M Al-Worafi PhD), Azal University for Human Development, Sana'a, Yemen; Department of Clinical Sciences (Prof Y M Al-Worafi PhD), University of Science and Technology of Fujairah, Fujairah, United Arab Emirates; Department of Pediatrics (Prof H Aly MD), Department of Internal Medicine (M Gupta MD), Lerner Research Institute (X Liu PhD), Cleveland Clinic, Cleveland, OH, USA; Department of Pediatric Cardiology (S Aly MD), Boston Children's Hospital, Boston, MA, USA (T Aripov PhD); School of Graduate Studies (E K Ameyaw MPhil), Lingnan University, Hong Kong, China; Public Heath Department (Prof T T Amin MD), Department of Neurology (A Hassan MD), Cairo University, Cairo, Egypt; Social Determinants of Health Research Center (M Amini-Rarani PhD), School of Medicine (S Bagherieh BSc, G Ghasempour Dabaghi MD, M Rabiee Rad MD), Department of Environmental Health Engineering (A Fatehizadeh PhD), Cardiac Rehabilitation Research Center (K Mehrabani-Zeinabad PhD), Isfahan University of Medical Sciences, Isfahan, Iran; Medicine, Quran and Hadith Research Center (S Amiri PhD), Baqiyatallah University of Medical Sciences, Tehran, Iran; College of Public Health, Medical and Veterinary Sciences (I G Ampomah MPhil), College of Public Health, Medical, and Veterinary Sciences (A E Peden PhD), James Cook University, Townsville, QLD, Australia (K O Obamiro PhD); Department of Maternal and Child Wellbeing (D A Amugsi PhD), African Population and Health Research Center, Nairobi, Kenya; Department of Medicine (G A Amusa MD), Department of Obstetrics and Gynecology (J Musa MD), University of Jos, Jos, Nigeria; Department of Internal Medicine (G A Amusa MD), Jos University Teaching Hospital, Jos, Nigeria; Faculty of Pharmacy (Prof R Ancuceanu PhD), Department of Cardiology (C Andrei PhD), Department of Internal Medicine and Rheumatology (A V Bobirca PhD), Department of Dermatology and Venereology (Prof S R Georgescu PhD), Internal Medicine Department (M Hostiuc PhD), Department of Legal Medicine and Bioethics (S Hostiuc PhD), Department of General Surgery (I Negoi PhD, D Serban PhD, B Socea PhD), Department of Anatomy and Embryology (R I Negoi PhD), Department of Diabetes, Nutrition and Metabolic Diseases (A Pantea Stoian PhD), Department of Dermatology (M Tampa PhD), Carol Davila University of Medicine and Pharmacy, Bucharest, Romania; Centre for Sensorimotor Performance (D Anderlini MD), Center of Research Excellence in Stillbirth (T

Begum MPH), Department of Urology (Prof E Chung MD), Institute for Social Science Research (M Huda MSc, E Kanmiki MPH, J C Maravilla PhD), School of Health and Rehabilitation Sciences (A Khan PhD, M Moni PhD), School of Dentistry (R Lalloo PhD), Queensland Brain Institute (Prof J J McGrath MD), The University of Queensland, Brisbane, QLD, Australia; Neurology Department (D Anderlini MD), Royal Brisbane and Women's Hospital, Brisbane, QLD, Australia; Department of Health Care Management (P P Andrade MD, S Mohammed PhD), Technical University of Berlin, Berlin, Germany; European University, Lisbon, Portugal (P P Andrade MD); Department of Statistics and Econometrics (Prof T Andrei PhD, Prof C Herteliu PhD, A Otoiu PhD), Department of Statistics and Economics (Prof M Ausloos PhD), Management Department (A Dima PhD, S Stefan PhD), Bucharest University of Economic Studies, Bucharest, Romania; Department of Pharmacology (A Anil MD, M Shamim MBBS, S B Varthya MD), Department of Anatomy (Prof N Bhardwaj MD), Department of Community Medicine and Family Medicine (P Bhardwaj MD, M K Gupta MD, Prof P R Raghav MD), School of Public Health (P Bhardwaj MD), Department of Forensic Medicine and Toxicology (T Kanchan MD), Department of Surgical Oncology (Prof S Misra MCh), Department of Pharmacology and Research (A Saravanan MD), All India Institute of Medical Sciences, Jodhpur, India; Department of Urology (P Ram MS), All India Institute of Medical Sciences, Bhubaneswar, India (A Anil MD); Department of Obstetrics and Gynecology (S Anil MBBS), Ernakulam Medical Centre, Palarivattom, Kochi, India; School of Nursing and Midwifery (A Ansar PhD, F Efendi PhD, M Rahman PhD), Department of Public Health (H Jiang PhD), La Trobe University, Melbourne, VIC, Australia; Special Interest Group International Health (A Ansar PhD), Public Health Association of Australia, Canberra, ACT, Australia; Department of Epidemiology and Biostatistics (Prof A Ansari-Moghaddam PhD), Zahedan University of Medical Sciences, Zahedan, Iran; Agribusiness Study Program (E Antriyandarti DrAgrSc), Sebelas Maret University, Surakarta, Indonesia; Regenerative Medicine, Organ Procurement and Transplantation Multi-diciplinary Center (S Anvari MD), Gastrointestinal and Liver Diseases Research Center (S Hassanipour PhD, F Joukar PhD), Caspian Digestive Disease Research Center (S Hassanipour PhD, F Joukar PhD), Department of Environmental Health Engineering (J Jaafari PhD), Guilan University of Medical Sciences, Rasht, Iran; Centre for Interdisciplinary Research in Basic Sciences (CIRBSc) (S Anwar PhD), Jamia Millia Islamia, new delhi, India; SCLS (S Anwar PhD), Jamia Hamdard, New Delhi, India; Department of Pathology (R Anwer PhD), Imam Mohammad Ibn Saud Islamic University, Riyadh, Saudi Arabia; School of Dentistry and Medical Sciences (A E Anyasodor PhD), Charles Sturt University, Orange, NSW, Australia; Faculty of Pharmacy, Department of Pharmacology and Toxicology (Prof E A Arafa PhD, Prof H A Omar PhD), Beni-Suef University, Beni-Suef, Egypt; College of Pharmacy (M Arafat PhD), Al Ain University, Abu Dhabi, United Arab Emirates; Associated Laboratory for Green Chemistry (LAQV) (A M Araújo PhD), Institute for Research and Innovation in Health (Prof N Cruz-Martins PhD), Research Unit on Applied Molecular Biosciences (UCIBIO) (Prof D Dias da Silva PhD, J P Silva PhD), Department of Community Medicine, Information and Health Decision Sciences (A Freitas PhD), Department of Chemical Engineering (Prof C F Rodrigues PhD), University of Porto, Porto, Portugal; Department of Veterinary Pharmacology and Toxicology (A Aremu PhD), Department of Veterinary Physiology and Biochemistry (A Basiru PhD), Department of Veterinary Public Health and Preventive Medicine (I A Odetokun PhD), University of Ilorin, Ilorin, Nigeria; Public Health and Healthcare Management Department (T Aripov PhD), Tashkent Institute of Postgraduate Medical Education, Tashkent, Uzbekistan; Department of Cardiovascular, Endocrine-metabolic Diseases and Aging (B Armocida MSc, B Unim PhD), National Institute of Health, Rome, Italy; Division of Tropical and Humanitarian Medicine (B Armocida MSc), University of Geneva, Geneva, Switzerland; School of Health and Social Studies (Prof J Ärnlöv PhD), Dalarna University, Falun, Sweden; Department of Biophysics (A A Artamonov PhD), K.A. Timiryazev Institute of Plant Physiology

(M V Titova PhD), Russian Academy of Sciences, Moscow, Russia; Department of Maternal and Child Health (J Arulappan DSc), Sultan Qaboos University, Muscat, Oman; Community Medicine and Rehabilitation - Physiotherapy Section (A Arumugam PhD), Department of Epidemiology and Global Health (M P Chavula MPH), Umeå University, Umea, Sweden; International Relations Department (M Asadi-Lari PhD), National Agency for Strategic Research in Medical Education (NASRME) (Prof S Asgary MSc), Ministry of Health and Medical Education, Tehran, Iran; Research Center for Biochemistry and Nutrition in Metabolic Diseases (Z Asemi PhD), Kashan University of Medical Sciences, Kashan, Iran; Neurological Surgery Department (M Asghariahmadabad MD), School of Nursing, Family Health Care Department (J Nutor PhD), Department of Epidemiology and Biostatistics (M Teramoto MD), Department of Bioengineering and Therapeutic Sciences (Prof M S Zastrozhin PhD), University of California San Francisco, San Francisco, CA, USA; Cabrini Research (Prof M Asghari-Jafarabadi PhD), Cabrini Health, Malvern, VIC, Australia; Department of Public Health (M Y Ashemo PhD, U Gerema MSc, M E Getachew MPH), Institute of Health Science (A I Mohamed MSc), Department of Epidemiology (D Shiferaw MPH), Jimma University, Jimma, Ethiopia; Department of Public Health (M Y Ashemo PhD, L T Elilo MPH), Wachemo University, Hossana, Ethiopia; Department of Medical Laboratory Sciences (M O Asika BMLS), Department of Pharmacology and Therapeutics (Prof O E Onwujekwe PhD), University of Nigeria Nsukka, Enugu, Nigeria; Telemedicine Department (M O Asika BMLS), Society For Disease Prevention, Inc., Hummelstown, PA, USA; Department of Immunology (S Athari PhD), Zanjan University of Medical Sciences, Zanjan, Iran; Faculty of Nursing (M M W Atout PhD, Prof A M Batiha PhD), Philadelphia University, Amman, Jordan; Department of Forensic Medicine (A Atreya MD), Lumbini Medical College, Palpa, Nepal; Northumbria HealthCare NHS Foundation Trust, Newcastle upon Tyne, UK (A Aujayeb MBBS); School of Business (Prof M Ausloos PhD), Department of Health Sciences (P H Lee PhD, S J Tromans PhD), University of Leicester, Leicester, UK; Robarts Research Institute (A Avan MD), The University of Western Ontario, London, ON, Canada; Department of Sciences (Prof R M S Azevedo PhD), Instituto Universitário de Ciências da Saúde (IUCS/CESPU) (University Institute of Health Sciences), Gandra, Portugal; Department of Neurovascular Research (A Y Azzam MBBCh), Nested Knowledge, Inc., Saint Paul, MN, USA; Faculty of Medicine (A Y Azzam MBBCh), October 6 University, 6th of October City, Egypt; Gomal Center of Biochemistry and Biotechnology (M Badar PhD), Gomal University, Dera Ismail Khan, Pakistan; Department of Forensic Science (A D Badiye PhD, H Bansal MSc, N Kapoor PhD), Government Institute of Forensic Science, Nagpur, India; Division of Orthopaedics (S Baghdadi MD), Children's Hospital of Philadelphia, Philadelphia, PA, USA; Health Research Institute (N Bagheri PhD), University of Canberra, Canberra, ACT, Australia; School of Public Affairs (R Bai MD), Nanjing University of Science and Technology, Nanjing, China; International Medical School (A A Baig PhD), Management and Science University, Alam, Malaysia; Center for Clinical Research and Prevention (J L Baker PhD), Bispebjerg University Hospital, Frederiksberg, Denmark; Department of Neurosurgery (A T Bako PhD), Houston Methodist Hospital, Houston, TX, USA; Maternal and Child Health Unit (R K Bakshi MD), Indian Council of Medical Research, New Delhi, India (D K Lal MD); Health Care Management Department (M Balasubramanian PhD), Flinders Health and Medical Research Institute (N B Bulamu PhD), Population Health Department (T G Gebremeskel PhD), Health Economics Unit (B Kaambwa PhD), College of Medicine and Public Health (B Kaambwa PhD, G R Naik PhD), Health and Social Care Economics Group (C Mpundu-Kaambwa PhD), Department of Nursing and Health Sciences (S Shorofi PhD), Flinders University, Adelaide, SA, Australia; Menzies Centre for Health Policy and Economics (M Balasubramanian PhD), Charles Perkins Centre (R Biswas PhD), School of Pharmacy and Charles Perkins Centre (Z Dai PhD), Department of Public Health (M Khan PhD), School of Veterinary Science (B B Singh PhD), Save Sight Institute (Y You PhD), University of Sydney, Sydney, NSW, Australia; Center of Innovation, Technology

and Education (CITE) (Prof O C Baltatu PhD), Anhembi Morumbi University, Sao Jose dos Campos, Brazil; Department of Medicine (K Bam MPH), Monash University, Clayton, VIC, Australia; Department of Hypertension (Prof M Banach PhD), Medical University of Lodz, Lodz, Poland; Polish Mothers' Memorial Hospital Research Institute, Lodz, Poland (Prof M Banach PhD); Department of Neurosurgery (S Bandyopadhyay BA), Faculty of Medicine (R Thayakaran PhD), University of Southampton, Southampton, UK; Institute of Health and Wellbeing (B Banik PhD), Federation University Australia, Melbourne, VIC, Australia; Manna Institute (B Banik PhD), University of New England, Armidale, NSW, Australia; Department of Non-communicable Diseases (P C Banik MPhil), Bangladesh University of Health Sciences, Dhaka, Bangladesh; Vocational School of Technical Sciences (M Baran PhD), Batman University, Batman, Türkiye; Miami Cancer Institute (M Bardhan MD), Baptist Health South Florida, Miami, FL, USA; School of Psychology (Prof S L Barker-Collo PhD), University of Auckland, Auckland, New Zealand; Department of Translational Medicine (F Barone-Adesi PhD), University of Eastern Piedmont, Novara, Italy; Department of Epidemiology (A Barrow MPH, D Braithwaite PhD, D D Ding BS, D Yang MPH), College of Medicine (M J Diaz BS), Department of Computer and Information Science and Engineering (P Naghavi MSc), University of Florida, Gainesville, FL, USA; Department of Public & Environmental Health (A Barrow MPH), University of The Gambia, Brikama, The Gambia; Heidelberg Institute of Global Health (S Barteit PhD), Department of Translational Health Economics (Prof S Listl PhD), Heidelberg University Hospital, Heidelberg, Germany; Alpha Genomics, Islamabad, Pakistan (Z Basharat PhD); Department of Pharmacology and Toxicology (A I J Bashir PhD), Department of Pharmaceutics and Industrial Pharmacy (Z S Yahaya PhD), Kaduna State University, Kaduna, Nigeria; Faculty of Pharmacy (J D Basso PharmD, S Silva MSc), Coimbra Chemistry Centre (J D Basso PharmD), Coimbra Institute for Biomedical Imaging and Translational Research (S Silva MSc), University of Coimbra, Coimbra, Portugal; School of Public Health (S Basu PhD), Department of Primary Care and Public Health (Prof A Majeed MD, Prof S Rawaf MD), Department of Surgery and Cancer (Prof E Mossialos PhD), WHO Collaborating Centre for Public Health Education and Training (D L Rawaf MRCS), Imperial College London, London, UK; Department of Medical Education (K Batra PhD), Department of Social and Behavioral Health (Prof M Sharma PhD), University of Nevada, Las Vegas, Las Vegas, NV, USA; Department of Psychiatry (Prof B T Baune PhD), University of Münster, Münster, Germany; Department of Psychiatry (Prof B T Baune PhD), Melbourne Medical School, Melbourne, VIC, Australia; Health Human Resources Research Center (M Bayati PhD), Student Research Committee (A Faramarzi MD), Department of Otolaryngology (A Faramarzi MD), Trauma Research Center (P Fazeli MSc, M Karajizadeh PhD), Department of Medical Immunology (P Fazeli MSc), Maternal Fetal Medicine Research Center (K Hessami MD), Health Policy Research Center (H Kasraei MD, Y Sarikhani PhD), Department of Biostatistics (H Molavi Vardanjani PhD, E Sadeghi PhD), Non-communicable Disease Research Center (S G Sepanlou MD), Department of Clinical Science (T Yaghoobpour DVM), Shiraz University of Medical Sciences, Shiraz, Iran (Y Mansoori MD); Health System and Population Studies Division (T Begum MPH), Maternal and Child Health Division (A Iqbal MPH, A Sayeed MSc, M Siraj MSc), International Centre for Diarrhoeal Disease Research, Dhaka, Bangladesh; Department of Basic Sciences (E Behboudi PhD), Khoy University of Medical Sciences, Khoy, Iran; Department of Epidemiology (S Khanmohammadi MD, S Nejadghaderi MD, S Rashedi MD, H Soleimani MD), Non-Communicable Diseases Research Center (NCDRC), Tehran, Iran (A Behnoush BS, A Khalaji BS); Division of Pulmonary, Critical Care, and Sleep (M Beiranvand PhD), University of Florida, Jacksonville, FL, USA; Department of Medicine (D F Bejarano Ramirez BN), Faculty of Medicine (J N Malagón-Rojas MSc), El Bosque University, Bogota, Colombia; Transplant Service Department (D F Bejarano Ramirez BN), University Hospital Foundation Santa Fe de Bogotá, Bogota, Colombia; Department of Medical Anatomy (A Bekele MSc), Department of Nursing (A M Mersha MSc), Medical Laboratory Sciences (D A D

Tareke MSc), Arba Minch University, Arba Minch, Ethiopia; Department of Oral Pathology and Microbiology (U I Belgaumi MD), Krishna Vishwa Vidyapeeth Deemed to be University, Karad, India; School of the Environment (Prof M L Bell PhD, Y Song PhD), Department of Internal Medicine (F Etaee MD), Department of Psychiatry (W Li PhD), Yale University, New Haven, CT, USA; Department of Biomedical Sciences (Prof A Beloukas PhD), University of West Attica, Athens, Greece; Department of Internal Medicine (I M Bensenor PhD), Department of Medicine (Prof P A Lotufo DrPH), Department of Psychiatry (Prof M F P Peres MD, Y Wang PhD), University of São Paulo, São Paulo, Brazil; Institute of Marketing (Z Berezvai PhD), Corvinus University of Budapest, Budapest, Hungary; Competition Economics and Market Research Section (Z Berezvai PhD), Hungarian Competition Authority, Budapest, Hungary; Department of Epidemiology and Biostatistics (A C Bermudez MD), University of the Philippines Manila, Manila, Philippines; Department of Epidemiology (A C Bermudez MD), Department of Internal Medicine (M F H Mohamed MSc), Brown University, Providence, RI, USA; Faculty of Medicine (P J G Bettencourt PhD), Catholic University of Portugal, Rio de Mouro, Portugal; Department of Public Health (A S Bhagavathula PhD), North Dakota State University, Fargo, ND, USA; Department of Hematology Oncology (P V Bhardwaj MD), University of Massachusetts Medical School, Springfield, MA, USA; Global Health Neurology Lab (S Bhaskar PhD), NSW Brain Clot Bank, Sydney, NSW, Australia; Department of Neurology and Neurophysiology (S Bhaskar PhD), South West Sydney Local Heath District and Liverpool Hospital, Sydney, NSW, Australia; Department of Internal Medicine (V Bhat MBBS), St. John's National Academy of Health Sciences, Bangalore, India; Medical Lab Technology (G K Bhatti PhD), University Centre for Research and Development (S Kalra DM), Chandigarh University, Mohali, India; Human Genetics and Molecular Medicine Department (Prof J S Bhatti PhD, U Sharma PhD), Department of Zoology (B Vellingiri PhD), Central University of Punjab, Bathinda, India; Department of Botanical and Environmental Sciences (Prof M S Bhatti PhD), Department of Pharmaceutical Sciences (R Bhatti PhD), Guru Nanak Dev University, Amritsar, India; Department of Neurology (Prof A Biswas DM), Department of GI Surgery (A Dhali MBBS), Institute of Post-Graduate Medical Education and Research and Seth Sukhlal Karnani Memorial Hospital, Kolkata, India; Clinical Research Centre (R Biswas PhD), Sydney Local Health District, Sydney, NSW, Australia; Faculty of Health Sciences (V R Bitra PhD), University of Botswana, Gaborone, Botswana; Department of Global Public Health and Primary Care (Prof T Bjørge PhD, O Dadras DrPH), Department of Psychosocial Science (D Sagoe PhD), University of Bergen, Bergen, Norway; Cancer Registry of Norway, Oslo, Norway (Prof T Bjørge PhD); SAMRC Centre for Health Economics and Decision Science (PRICELESS SA) (M K Boachie PhD), University of the Witwatersrand, Johannesburg, South Africa; School of Business Administration (Prof V Bodolica PhD), American University of Sharjah, Sharjah, United Arab Emirates; General Directorate of Health Information Systems (B Bora Basara PhD), Ministry of Health, Ankara, Türkiye; Department of Medicine (Prof S Bouaoud MD), Faculty of Medicine (Prof A Ouyahia PhD), University Ferhat Abbas of Setif, Sétif, Algeria; Department of Epidemiology and Preventive Medicine (Prof S Bouaoud MD), University Hospital Saadna Abdenour, Setif, Algeria; Cancer Population Sciences Program (D Braithwaite PhD), University of Florida Health Cancer Center, Gainesville, FL, USA; School of Population and Public Health (Prof M Brauer DSc), School of Nursing (A Pashaei MSc), University of British Columbia, Vancouver, BC, Canada; Psychiatry and Behavioral Health Department (Prof N J K Breitborde PhD), Department of Psychology (Prof N J K Breitborde PhD), Ohio State University, Columbus, OH, USA; Department of Woman and Child Health and Public Health (D Buonsenso MD), Fondazione Policlinico Universitario A. Gemelli IRCCS (Agostino Gemelli University Polyclinic IRCCS), Roma, Italy; Global Health Research Institute (D Buonsenso MD), Università Cattolica del Sacro Cuore (Catholic University of Sacred Heart), Roma, Italy; Department of Biopharmaceutics and Clinical Pharmacy (Y Bustanji PhD), The University of Jordan, Amman, Jordan;

School of Public Health and Health Systems (Z A Butt PhD), University of Waterloo, Waterloo, ON, Canada; Al Shifa School of Public Health (Z A Butt PhD), Al Shifa Trust Eye Hospital, Rawalpindi, Pakistan; Department of Clinical Pharmacy (Prof D Calina PhD), University of Medicine and Pharmacy of Craiova, Craiova, Romania; Center for Nutrition and Health Research (I R Campos-Nonato PhD, E Denova-Gutiérrez DSc), Center for Health Systems Research (S M Cuadra-Hernández PhD, D V Ortega-Altamirano DrPH, E Serván-Mori PhD), National Institute of Public Health, Cuernavaca, Mexico; Department of Ophthalmology (F Cao MD), Beijing Institute of Ophthalmology, Beijing, China; Department of Biomedical and Neuromotor Sciences (A Capodici MD, S Guicciardi MD, L Muccioli MD), Dipartimento di Scienze Biomediche e Neuromotorie (DIBINEM) (F Esposito MD), Department of Medical and Surgical Sciences (Prof F S Violante MD), University of Bologna, Bologna, Italy; Management and Healthcare (EMbeDS) (A Capodici MD), Sant'Anna School of Advanced Studies, Pisa, Italy; Institute for Cancer Research, Prevention and Clinical Network, Florence, Italy (G Carreras PhD); Dermatology Unit (A Carugno MD), Azienda Socio Sanitaria Territoriale Papa Giovanni XXIII (Territorial Healthcare Company Pope John XXIII), Bergamo, Italy; Colombian National Health Observatory (C A Castañeda-Orjuela MD), Department of Public Health Research (J N Malagón-Rojas MSc), National Institute of Health, Bogota, Colombia; Epidemiology and Public Health Evaluation Group (C A Castañeda-Orjuela MD), National University of Colombia, Bogota, Colombia; Department of Medicine (G Castelpietra PhD), University of Udine, Udine, Italy; Department of Mental Health (G Castelpietra PhD), Healthcare Agency "Friuli Occidentale", Pordenone, Italy; Department of Public Health and Infectious Diseases (M S Cattaruzza PhD), La Sapienza University, Rome, Italy; Department of Psychiatry (A Caye PhD), Federal University of Rio Grande do Sul, Porto Alegre, Brazil; Department of Medical, Surgical, and Health Sciences (L Cegolon PhD, Prof M D'Oria MD), University of Trieste, Trieste, Italy; Public Health Unit (L Cegolon PhD), University Health Agency Giuliano-Isontina (ASUGI), Trieste, Italy; Department of Nutrition (Prof F Cembranel DSc), Federal University of Santa Catarina, Florianópolis, Brazil; Mary MacKillop Institute for Health Research (Prof E Cerin PhD), Australian Catholic University, Melbourne, VIC, Australia; School of Public Health (Prof E Cerin PhD), Department of Urban Planning and Design (C Guo PhD), Centre for Suicide Research and Prevention (Prof P Yip PhD), Department of Social Work and Social Administration (Prof P Yip PhD), University of Hong Kong, Hong Kong, China; ICMR School of Public Health (J Chadwick MD), National Institute of Epidemiology, Chennai, India; Department of Biotechnology (Prof C Chakraborty PhD), Adamas University, Kolkata, India; Department of Skeletal Aging and Orthopedic Surgery (Prof C Chakraborty PhD), Hallym University, Chuncheon, South Korea; Heart Failure and Structural Heart Disease Unit (J Chan MBChB), Cardiovascular Analytics Group, Hong Kong, China; Department of Public Health (P Charalampous PhD), Erasmus University Medical Center, Rotterdam, Netherlands; Temerty Faculty of Medicine (V Chattu MD), University of Toronto, Toronto, ON, Canada; Department of Community Medicine (V Chattu MD), Datta Meghe Institute of Medical Sciences, Sawangi, India; Dr D Y Patil Medical College Hospital and Research Centre (S Chaturvedi PhD), Dr D Y Patil Vidyapeeth, Pune, India; School of Public Health (M P Chavula MPH), University of Zambia, Lusaka, Zambia; Fuwai Hospital (A Chen PhD), Chinese Academy of Medical Sciences & Peking Union Medical College, Beijing, China; Department of Computer Science (A Chen PhD), University of Texas Austin, Austin, TX, USA; Clinical Research Center (H Chen MB), Stomatological Hospital (A Li PhD), Southern Medical University, Guangzhou, China; Department of Laboratory Medicine (J Chien PhD), Taichung Tzu-Chi Hospital Buddhist Tzu-Chi Medical Foundation, Tanzih, Taiwan; Department of Medical Laboratory Science and Biotechnology (J Chien PhD), Central Taiwan University of Science and Technology, Taiwan; Division of Infectious Diseases (P R Ching MD), Virginia Commonwealth University, Richmond, VA, USA; Department of Clinical Oncology (W C S Cho PhD), Queen Elizabeth Hospital, Hong Kong, China; College

of Medicine (S Choi MD Cand), Department of Epidemiology and Health Promotion (Prof S Jee PhD), Yonsei University, Seoul, South Korea; Department of Medicine (B Chong MBBS), Department of Surgery (K Tan PhD), National University of Singapore, Singapore, Singapore; Department of Biosciences (H Chopra PhD), Saveetha Dental College and Hospitals (M R Tovani-Palone PhD), Saveetha Institute of Medical and Technical Sciences, Chennai, India; Department of Community Medicine (Prof S G Choudhari MD, Prof A M Gaidhane MD), Datta Meghe Institute of Medical Sciences, Wardha, India; Department of Pulmonary Medicine (Prof D J Christopher MD), Department of Endocrinology, Diabetes and Metabolism (Prof N Thomas PhD), Christian Medical College and Hospital (CMC), Vellore, India; Center for Biomedicine and Community Health (D Chu PhD), VNU-International School, Hanoi, Viet Nam; Department of Paediatric Surgery (I S Chukwu BMedSc), Federal Medical Centre, Umuahia, Nigeria; Department of AndroUrology (Prof E Chung MD), AndroUrology Centre, Brisbane, QLD, Australia; Department of Health Informatics (S Chung PhD), Institute of Cardiovascular Science (A S Oguntade MSc), University College London, London, UK; Health Data Research UK, London, UK (S Chung PhD); Department of Genetics (Z Cindi PhD), School of Veterinary Medicine (F Musaigwa PhD), Department of Biostatistics Epidemiology and Informatics (J Puvvula PhD), University of Pennsylvania, Philadelphia, PA, USA; Department of Food, Environmental and Nutritional Sciences (I Cioffi PhD), University of Milan, Università degli Studi di Milano, Italy; Department of Law, Economics, Management and Quantitative Methods (R Ciuffreda MSc, Prof B Simonetti PhD), University of Sannio, Benevento, Italy; Nova Medical School (J Conde PhD), Nova University of Lisbon, Lisbon, Portugal; Department of Family Medicine and Public Health (Prof M H Criqui MD), University of California San Diego, La Jolla, CA, USA; Department of Therapeutic and Diagnostic Technologies (Prof N Cruz-Martins PhD), Toxicology Research Unit (TOXRUN) (Prof D Dias da Silva PhD, Á M Madureira-Carvalho PhD), Cooperativa de Ensino Superior Politécnico e Universitário (CESPU) (University Polytechnic Higher Education Cooperative), Gandra, Portugal; Department of Internal Medicine (S Dadana MD), Cheyenne Regional Medical Center, Cheyenne, WY, USA; Department of Addiction Medicine (O Dadras DrPH), Haukland University Hospital, Bergen, Norway; Department of Community Medicine (Prof T Dahiru MA), Department of Surgery (A Kabir MD), Health Systems and Policy Research Unit (S Mohammed PhD), Ahmadu Bello University, Zaria, Nigeria; IRCCS Istituto Ortopedico Galeazzi (G Damiani MD), Galeazzi Orthopedic Institute IRCCS (University of Milan), Milan, Italy; Department of Dermatology (G Damiani MD), Lerner College of Medicine (L Göbölös PhD), Department of Quantitative Health Science (X Liu PhD), Department of Neonatology (I Qattea MD), Department of Nutrition and Preventive Medicine (Prof J Sanabria MD), Case Western Reserve University, Cleveland, OH, USA; Department of Information Technology (A M Darwesh PhD), Department of Computer Science (Prof M Hosseinzadeh PhD), University of Human Development, Sulaymaniyah, Iraq; Division of Women and Child Health (J K Das MD), Aga Khan University, Karachi, Pakistan; Department of Biochemistry (S Das MD), Ministry of Health and Welfare, New Delhi, India; Department of Radiology (M Dashti MD, A Ghasemzadeh MD), Department of Health Policy and Management (L Doshmangir PhD), School of Nursing and Midwifery (H Hassankhani PhD), Department of Immunology (F Jadidi-Niaragh PhD), School of Management and Medical Informatics (L R Kalankesh PhD), Midwifery Department (Prof M Mirghafourvand PhD, Prof S Mohammad-Alizadeh-Charandabi PhD), Social Determinants of Health Research Center (Prof S Mohammad-Alizadeh-Charandabi PhD), Tabriz University of Medical Sciences, Tabriz, Iran; 2nd University Ophthalmology Department (A Dastiridou MD), Department of Ophthalmology (N Dervenis MD), Second Department of Cardiology (D Patoulias PhD), Aristotle University of Thessaloniki, Thessaloniki, Greece; Ophthalmology Department (A Dastiridou MD), University of Thessaly, Greece; Department of Population and Development (C A Dávila-Cervantes PhD), Latin American Faculty of Social Sciences Mexico, Mexico City, Mexico; Health Research

Institute (K Davletov PhD), Asfendiyarov Kazakh National Medical University, Almaty, Kazakhstan; Medical College (S Debopadhaya BS), Albany Medical College, Albany, NY, USA; School of Medicine (I Delgado-Enciso DSc), University of Colima, Colima, Mexico; Department of Research (I Delgado-Enciso DSc), Colima State Health Services, Colima, Mexico; Epidemiology Branch (X Deng PhD), National Institute of Health, Durham, NC, USA; Wellcome Trust Brighton and Sussex Centre for Global Health Research (K Deribe PhD), Brighton and Sussex Medical School, Brighton, UK; St Paul's Eye Unit (N Dervenis MD), Royal Liverpool University Hospital, Liverpool, UK; Graduate Medical Education (H D Desai MD), Gujarat Adani Institute of Medical Sciences, Bhuj, India; Division of Cardiology (R Desai MBBS), Atlanta Veterans Affairs Medical Center, Decatur, GA, USA; Department of Community Medicine (V G C Devanbu MD), Chettinad Academy of Research and Education, Chennai, India; Division of Pathology (K Dhama PhD), ICAR-Indian Veterinary Research Institute, Bareilly, India; Research Department (M Dhimal PhD, S Ghimire MPH, U Paudel PhD), Nepal Health Research Council, Kathmandu, Nepal; The Zena and Michael A. Wiener Cardiovascular Institute (V R Dhulipala MD), Institute of Critical Care Medicine (A Shaikh MD), Department of Cardiology (M Vinayak MD), Icahn School of Medicine at Mount Sinai, New York, NY, USA (A Shaikh MD); Faculty of Science (Prof D Diaz PhD), National Autonomous University of Mexico, Mexico City, Mexico; Department of Medicine (T C Do MD), Medical School (H Pham MD), Pham Ngoc Thach University of Medicine, Ho Chi Minh City, Viet Nam; Department of Medicine (T H Do MD), Can Tho University of Medicine and Pharmacy, Can Tho, Viet Nam; Center for Health Sciences (C B do Prado MSc), Department of Pathology (Prof B Fux PhD), Federal University of Espirito Santo, Vitória, Brazil; Department of Epidemiology (S Dohare MD, M Khan MD), Department of Health Education and Promotion (M Shanawaz MD), Jazan University, Jazan, Saudi Arabia; School of Elderly Care Services and Management (W Dong MD), Nanjing University of Chinese Medicine, Nanjing, China; Cardio-Thoraco-Vascular Department (Prof M D'Oria MD), Azienda Sanitaria Universitaria Giuliano Isontina, Trieste, Italy; Responsabilidade Social (W M dos Santos PhD), Hospital Alemão Oswaldo Cruz (Oswaldo Cruz German Hospital), São Paulo, Brazil; Brazilian Centre for Evidence-based Healthcare (W M dos Santos PhD), Joanna Briggs Institute, São Paulo, Brazil; Department of Medicine (A C Dsouza MBBS, A Sathyanarayan MD, M Sharath MD), Bangalore Medical College and Research Institute, Bangalore, India; Department of Forensic Medicine and Toxicology (H L Dsouza MD, V Krishna MD), Department of General Medicine (J Jeganathan MD), Department of Community Medicine (N Joseph MD, R Motappa MD, R Thapar MD), Department of Internal Medicine (M M R Reddy MD), Department of Forensic Medicine (P H Shetty MD), Kasturba Medical College (Prof B Unnikrishnan MD), Manipal Academy of Higher Education, Mangalore, India; Forensic Medicine and Toxicology Department (H L Dsouza MD), Kasturba Medical College Mangalore, Mangalore, India; Health Policy Department (V Dsouza MSc), Kasturba Medical College, Mangalore (R Holla MD), Department of Physiotherapy (Prof V K PhD), Prasanna School of Public health (R Kamath MHA), Manipal Institute of Management (S Kamath MHA), Department of Pharmacy Management (V S Ligade PhD), Department of Forensic Medicine (A Mishra MD), Department of Forensic Medicine and Toxicology (Prof V C Nayak MD), Manipal TATA Medical College (M Rahman PhD), Department of Community Medicine (C R Rao MD), Department of Nephrology (I Rao DM), Department of Health Information Management (B Reshmi PhD), Manipal Academy of Higher Education, Manipal, India (B Reshmi PhD); Office of Institutional Analysis (J Dube MA), University of Windsor, Windsor, ON, Canada; School of Medicine (Prof A R Duraes PhD), Institute of Collective Health (Prof D Rasella PhD), Federal University of Bahia, Salvador, Brazil; Department of Internal Medicine (Prof A R Duraes PhD), Escola Bahiana de Medicina e Saúde Pública (Bahiana School of Medicine and Public Health), Salvador, Brazil; Department of Biotechnology (S Duraisamy PhD), SRM College of Pharmacy (M R Tovani-Palone PhD), SRM Institute of Science and Technology (SRMIST), Chennai, India; Department of

Infection and Tropical Medicine (O C Durojaiye MPH), School of Health and Related Research (J O Oguta MSc), Psychology Department (A Yadollahpour PhD), University of Sheffield, Sheffield, UK; School of Life Sciences (S Dutta PhD), Manipal Academy of Higher Education, Dubai, United Arab Emirates; Child Health Analytics Research Program (P A Dzianach PhD, Prof P W Gething PhD, F Sanna PhD, D J Weiss PhD), Geospatial Health and Development Team (J Lubinda PhD), The Malaria Atlas Project (S F Rumisha PhD), Telethon Kids Institute, Perth, WA, Australia; Department of Conservative Dentistry with Endodontics (A M Dziedzic DSc), Medical University of Silesia, Katowice, Poland; Department of Orthopaedic Surgery (A Ebrahimi MD), Department of Radiology (X Liu PhD), Division of Cardiology (D H Nguyen BS), Massachusetts General Hospital, Boston, MA, USA; School of Health Sciences (H A Edinur PhD), Universiti Sains Malaysia (University of Science Malaysia), Kubang Kerian, Malaysia; College of Science, Health and Engineering (K Edvardsson PhD), La Trobe University, Bundoora, VIC, Australia; Department of Community Health Nursing (F Efendi PhD), Universitas Airlangga (Airlangga University), Surabaya, Indonesia; Centre for Global Health Inequalities Research (CHAIN) (Prof T Eikemo PhD), Department of Circulation and Medical Imaging (J Nauman PhD), Norwegian University of Science and Technology, Trondheim, Norway; Department of Internal Medicine and Hematology Unit (Prof G M T ElGohary MD), Biochemistry Department (Prof N M Hamdy PhD), Department of Entomology (A M Samy PhD), Medical Ain Shams Research Institute (MASRI) (A M Samy PhD), Ain Shams University, Cairo, Egypt; Faculty of Medicine (M Elhadi MD), University of Tripoli, Tripoli, Libya; Egypt Center for Research and Regenerative Medicine (ECRRM), Cairo, Egypt (M A Elmonem PhD); Clinical Pathology Department (M Elshaer MD), Faculty of Pharmacy (M A Saleh PhD), Rheumatology and Immunology Unit (S Tharwat MD), Mansoura University, Mansoura, Egypt; Department of Infectious Diseases and Public Health (I Elsohaby PhD), City University of Hong Kong, Hong Kong, China; Department of Animal Medicine (I Elsohaby PhD), Cardiovascular Department (Prof A M A Saad MD), Zagazig University, Zagazig, Egypt; Department of Medical-Surgical Nursing (A Emami Zeydi PhD, S Shorofi PhD), Department of Immunology (Prof A Rafiei PhD), Molecular and Cell Biology Research Center (Prof A Rafiei PhD), Department of Environmental Health (Prof Z Yousefi PhD), Mazandaran University of Medical Sciences, Sari, Iran; Lincoln International Institute for Rural Health (L Engelbert Bain PhD), University of Lincoln, Lincoln, UK; Independent Consultant, Bologna, Italy (N Fabin MD); Research Centre for Healthcare and Community (A F Fagbamigbe PhD), Faculty of Health and Life Sciences (O P Kurmi PhD), Coventry University, Coventry, UK; Department of Food Hygiene and Quality Control (A Fakhri-Demeshghieh DVM), University of Tehran, Tehran, Iran; Epidemiology and Biostatistics Unit IRCCS Pascale (L Falzone PhD), IRCCS, Naples, Italy; Department of Psychology (Prof A Faro PhD), Federal University of Sergipe, São Cristóvão, Brazil; Satcher Health Leadership Institute (A O Fasanmi PhD), Morehouse School of Medicine, Atlanta, GA, USA; School of Medicine (A O Fasanmi PhD), Department of Family and Preventive Medicine (T Sathish PhD), Emory University, Atlanta, GA, USA; Centre for Health Policy Research (Prof P Ward PhD), Torrens University Australia, Adelaide, SA, Australia (N K Fauk MSc); Institute of Resource Governance and Social Change, Kupang, Indonesia (N K Fauk MSc); National Institute for Stroke and Applied Neurosciences (Prof V L Feigin PhD), Auckland University of Technology, Auckland, New Zealand; Research Center of Neurology, Moscow, Russia (Prof V L Feigin PhD); Division of Neurology (S Fereshtehnejad PhD), University of Ottawa, Ottawa, ON, Canada; Research Center on Public Health (P Ferrara MD), University of Milan Bicocca, Monza, Italy; Department of Social Sciences (Prof N Ferreira PhD), University of Nicosia, Nicosia, Cyprus; Department of Nursing (G Fetensa MSc), Department of Public Health (M E Getachew MPH, D R Terefa MSc), Wollega University, Nekemte, Ethiopia; Psychiatry Department (I Filip MD), Kaiser Permanente, Fontana, CA, USA; School of Health Sciences (I Filip MD), A.T. Still University, Mesa, AZ, USA; Institute of Public Health (F Fischer PhD),

Departement of Surgery (N Haep MD), Department of Neurology (S Samadzadeh MD), Charité Universitätsmedizin Berlin (Charité Medical University Berlin), Berlin, Germany; School of Social Sciences (J Flavel PhD), Stretton Health Equity, Adelaide, SA, Australia; Institute of Gerontology (N A Foigt PhD), National Academy of Medical Sciences of Ukraine, Kyiv, Ukraine; Clinical Science Department (Prof M O Folayan FWACS), Nigerian Institute of Medical Research, Yaba, Nigeria; Department of Cell Biology and Biotechnology (A A Fomenkov PhD), K.A. Timiryazev Institute of Plant Physiology, Moscow, Russia; Department of Pharmacology (Prof B Foroutan PhD), Iranshahr University of Medical Sciences, Iranshahr, Iran; Department of Biotechnological and Applied Clinical Sciences (DISCAB) (M Foschi MD), University of L'Aquila, L'Aquila, Italy; Department of Neuroscience (M Foschi MD), Hospital Santa Maria delle Croci, Ravenna, Italy; Child Survival Unit (K R Fowobaje MSc), Centre for African Newborn Health and Nutrition, Ibadan, Nigeria; Centre for Adolescent Health (K L Francis MBiostat), Murdoch Childrens Research Institute, Melbourne, VIC, Australia; Center for Health Technology and Services Research (CINTESIS), Porto, Portugal (A Freitas PhD); Department of Dermatology (T Fukumoto PhD), Kobe University, Kobe, Japan; Health Services Management Training Centre (P A Gaal PhD, T Joo PhD, J Lám PhD), Institute of Digital Health Sciences (P Pollner PhD), Faculty of Health and Public Administration (M Szócska PhD), Semmelweis University, Budapest, Hungary; Department of Applied Social Sciences (P A Gaal PhD), Sapientia Hungarian University of Transylvania, Targu Mures, Romania; Department of Community Medicine (Prof M A Gadanya FMCPH), Aminu Kano Teaching Hospital, Kano, Nigeria; Food Technology Department (Y Galali ResM, B A Sadee PhD), Salahaddin University-Erbil, Erbil, Iraq; Department of Nutrition and Dietetics (Y Galali ResM, B A Sadee PhD), Cihan University-Erbil, Erbil, Iraq; Department of Environmental Health Sciences (S Gallus DSc, A Lugo PhD), Mario Negri Institute for Pharmacological Research, Milan, Italy; Department of Community Medicine and Family Medicine (A P Gandhi MD), All India Institute of Medical Sciences, Nagpur, India; Institute of Health and Wellbeing (B Ganesan PhD), Federation University, Churchill, VIC, Australia; Department of General Medicine (M Ganiyani MD), Grant Medical College & Sir J.J. Group of Hospitals, Mumbai, India; Department of Medicine (M Ganiyani MD), Miami Cancer Institute, Miami, FL, USA; Faculty of Business and Management (M Garcia-Gordillo PhD), Universidad Autonóma de Chile (Autonomous University of Chile), Talca, Chile; University School of Management and Entrepreneurship (N Garg PhD, R Sharma PhD), Delhi Technological University, Delhi, India; Department of Pharmacology (Prof R K Gautam PhD), Indore Institute of Pharmacy, Indore, India; Institute and Faculty of Actuaries, London, UK (F Gazzelloni BSc); Department of Epidemiology (S O Gbadamosi MD), Florida International University, Miami, FL, USA; Department of Midwifery (M W Gebregergis MSc), Department of Medical Laboratory Sciences (H Negash MSc), Adigrat University, Adigrat, Ethiopia; Department of Environmental Health (M Gebrehiwot DSc), Wollo University, Dessie, Ethiopia; Department of Public Health (T B Gebremariam MPH), Debre Berhan University, Debre Berhan, Ethiopia; Public Health Department (T B Gebremariam MPH), Independent Consultant, Addis Ababa, Ethiopia (S A Yesuf MSc); Department of Public Health Nutrition (T B B Gebremariam MPH), Aksum University, Mekelle, Ethiopia; Reproductive and Family Health Department (T G Gebremeskel PhD), Axum College of Health Science, Axum, Ethiopia; School of Population Health (Prof P W Gething PhD), School of Public Health (T R Miller PhD), Curtin School of Population Health (D J Weiss PhD), Curtin University, Perth, WA, Australia; Department of Dermatology (Prof S R Georgescu PhD), "Victor Babes" Clinical Hospital of Infectious and Tropical Diseases, Bucharest, Romania; College of Health Science (H Geremew MPH), Oda Bultum University, Chiro, Ethiopia; Young Researchers and Elite Club (A Gholamian MSc), Islamic Azad University, Rasht, Iran; Department of Biology (A Gholamian MSc), Islamic Azad University, Tehran, Iran; Department of Radiology (A Gholamrezanezhad MD), University of Southern California, Los Angeles, CA, USA; Department of

Respiratory Medicine (Prof A G Ghoshal MD), National Allergy Asthma Bronchitis Institute, Kolkata, India; Department of Respiratory Medicine (Prof A G Ghoshal MD), Fortis Hospital, Kolkata, India; Department of Forensic Biology (A D Ghuge MPhil), Government Institute of Forensic Science, Aurangabad, India; Department of Clinical Research 1 (A D Ghuge MPhil), National Institute For Research In Reproductive and Child Health, Mumbai, India; NCD Surveillance Unit (A U Gil PhD), World Health Organization (WHO), Moscow, Russia; Institute for Leadership and Health Management (A U Gil PhD), Moscow Medical Academy, Moscow, Russia; Adelaide Medical School (T K Gill PhD), University of Adelaide, Adelaide, SA, Australia; Department of Molecular and Developmental Medicine (M Giorgi MD), Post Graduate School of Public Health (G Guarducci MD), University of Siena, Siena, Italy; NIHR Global Health Research Unit on Global Surgery (J C Glasbey MSc), Institute of Applied Health Research (R Thayakaran PhD), University of Birmingham, Birmingham, UK; Department of Hepatology (Prof A Goel DM), Sanjay Gandhi Postgraduate Institute of Medical Sciences, Lucknow, India; Department of Applied Cell Sciences (A Golchin PhD), Cellular and Molecular Medicine Institute (A Golchin PhD), School of Medicine (P Mokhtarzadehazar MD), Urmia University of Medical Sciences, Urmia, Iran (R Valizadeh PhD); Health Systems and Policy Research Department (M Golechha PhD), Indian Institute of Public Health, Gandhinagar, India; Department of Genetics (P Goleij MSc), Sana Institute of Higher Education, Sari, Iran; Universal Scientific Education and Research Network (USERN) (P Goleij MSc), Substance Abuse Prevention Research Center (B Mansouri PhD), Research Center for Environmental Determinants of Health (Prof E Sadeghi PhD), Kermanshah University of Medical Sciences, Kermanshah, Iran; Hudson College of Public Health (S V Gopalani MPH), University of Oklahoma Health Sciences Center, Oklahoma City, OK, USA; Department of Health and Social Affairs (S V Gopalani MPH), Government of the Federated States of Micronesia, Palikir, Federated States of Micronesia; Department of Respiratory Medicine (H Goudarzi PhD), Center for Environmental and Health Sciences (H Goudarzi PhD), Hokkaido University, Sapporo, Japan; Department of Epidemiology (Prof A C Goulart PhD), Universidade de São Paulo (University of São Paulo), São Paulo, Brazil; Blood and Marrow Transplantation and Cellular Therapy Program (A Goyal MD), Stanford University, Palo Alto, CA, USA; Department of Public Health and Preventive Medicine (Prof M Grivna PhD), Charles University, Prague, Czech Republic; Department of Epidemiology and Biostatistics (S Guan MD, Prof H Pan PhD), Anhui Medical University, Hefei, China; Department of Family and Community Medicine (M I M Gubari PhD), University Of Sulaimani, Sulaimani, Iraq; Health Directorate (S Guicciardi MD), Local Health Authority of Bologna, Bologna, Italy; Department of General Surgery (S Gulati MD), Dignity Health, Phoenix, AZ, USA; Diagnostic Radiology and Nuclear Medicine (D Gulisashvili MD), University of Maryland, Baltimore, MD, USA; Department of Community Medicine (D A Gunawardane MD), University of Peradeniya, Kandy, Sri Lanka; Department of Internal Medicine (A K Gupta PharmD), Shree Guru Gobind Singh Tricentenary University, Gurugram, India; Non-communicable Division (NCD) (A K Gupta PharmD), Indian Council of Medical Research, Delhi, India; Department of Public Health (B Gupta PhD), Torrens University Australia, Melbourne, VIC, Australia; Toxicology Department (S Gupta MSc), Shriram Institute for Industrial Research, Delhi, India; School of Medicine (V Gupta PhD), Deakin University, Geelong, VIC, Australia; School of Biotechnology (V Gupta PhD), Dublin City University, Glasnevin, Ireland; Faculty of Medicine Health and Human Sciences (Prof V K Gupta PhD), Macquarie Medical School (Y You PhD), Macquarie University, Sydney, NSW, Australia; Department of Global Health and Population (A Haakenstad ScD), T.H. Chan School of Public Health, Boston, MA, USA; Global Virus Network, Middle East Region, Shiraz, Iran (F Habibzadeh MD); Department of Clinical Pharmacology and Medicine (Prof N R Hadi PhD), University of Kufa, Najaf, Iraq; Clinician Scientist Program (N Haep MD), Berlin Institute of Health, Berlin, Germany; Department of Infectious Disease Epidmiology (S Haller MD), Robert Koch Institute, Berlin, Germany; Department of

Public Health (S Haller MD), Charité Insitute of Public Health, Berlin, Germany; Department of Family and Community Medicine (Prof R R Hamadeh PhD), College of Medicine and Medical Sciences (H Jahrami PhD), Arabian Gulf University, Manama, Bahrain; School of Health and Environmental Studies (Prof S Hamidi DrPH), Hamdan Bin Mohammed Smart University, Dubai, United Arab Emirates; Department of Nephrology (Q Han PhD), Beijing Chao-yang Hospital, Beijing, China; Department of Epidemiology (A J Handal PhD), University of Michigan, Ann Arbor, MI, USA; Centre for Neuromuscular and Neurological Disorders (Prof G J Hankey MD), University of Western Australia, Perth, WA, Australia; Perron Institute for Neurological and Translational Science, Perth, WA, Australia (Prof G J Hankey MD); Department of Population Science and Human Resource Development (Prof M Haque PhD, Prof M Rahman PhD, M Rahman DrPH), Department of Mathematics (M Kuddus PhD), University of Rajshahi, Rajshahi, Bangladesh; Research Unit (J M Haro MD), University of Barcelona, Barcelona, Spain; Biomedical Research Networking Center for Mental Health Network (CiberSAM), Barcelona, Spain (J M Haro MD); Department of Zoology and Entomology (A I Hasaballah PhD), Al Azhar University, Cairo, Egypt; Department of Pharmaceutical Technology (I Hasan MPharm), Department of Population Sciences (Prof M B H Hossain PhD), University of Dhaka, Dhaka, Bangladesh; Health Administration, Policy & Leadership Program (M Hasan MPH), Murdoch University, Australia, Perth, Western Australia, Australia; Department of public health (M Hasan MPH), Tropical Disease and Health Research Center, Bangladesh, Dhaka, Bangladesh; Department of Biomedical Engineering and Public Health (S Hasan PhD), World University of Bangladesh, Dhaka, Bangladesh; Department of Ophthalmology (H Hasani MD), Iran University of Medical Sciences, Karaj, Iran; Department of Pharmacy (Prof M S Hasnain PhD), Palamau Institute of Pharmacy, Daltonganj, India; Department of Public Health (I Hassan MPH), Dalhatu Araf Specialist Hospital, Lafia, Nigeria; Public Health Department (I Hassan MPH), Federal University of Lafia, Lafia, Nigeria; Independent Consultant, Tabriz, Iran (H Hassankhani PhD); Faculty of Kinesiology (Prof J J Hebert PhD), University of New Brunswick, Fredericton, NB, Canada; School of Allied Health (Prof J J Hebert PhD), Murdoch University, Murdoch, WA, Australia; Community-Oriented Nursing Midwifery Research Center (M Heidari PhD), Department of Epidemiology and Biostatistics (A Mohammadian-Hafshejani PhD), Department of Health in Disasters and Emergencies (R Sheikhi BHlthSci), Shahrekord University of Medical Sciences, Shahrekord, Iran; Institute of Psychology (B Helfer PhD), University of Wroclaw, Wroclaw, Poland; Meta Research Centre (B Helfer PhD), University of Wrocław, Wroclaw, Poland; Department of Medicine (M Hemmati MD), MedStar Health, Columbia, MD, USA; Department of Medicine (M Hemmati MD), Georgetown University, Washington, DC, USA; Departamento de Salud Oral (Department of Oral Health) (B Y Herrera-Serna PhD), Universidad Autónoma de Manizales (Autonomous University of Manizales), Manizales, Colombia; School of Business (Prof C Herteliu PhD), London South Bank University, London, UK; Department of Microbiology (K Hezam PhD), Department of Applied Microbiology (E A Noman PhD), Taiz University, Taiz, Yemen; School of Medicine (K Hezam PhD), Nankai University, Tianjin, China; Division for Health Service Promotion (Y Hiraike PhD), Department of Global Health Policy (S Nomura PhD, S K Rauniyar PhD), University of Tokyo, Tokyo, Japan; School of Dentistry (N Q Hoan DDS), Hanoi Medical University, Hanoi, Viet Nam; Department of Pulmonology (N Horita PhD), Yokohama City University, Yokohama, Japan; National Human Genome Research Institute (NHGRI) (N Horita PhD), Center for Translation Research and Implementation Science (G A Mensah MD), National Institutes of Health, Bethesda, MD, USA; Social and Environmental Health Research (M Hossain MPH), Nature Study Society of Bangladesh, Khulna, Bangladesh; Department of Health Promotion and Community Health Sciences (M Hossain MPH), Texas A&M University, College Station, TX, USA; School of Health and Society (H Hosseinzadeh PhD, Z Ratan MSc), University of Wollongong, Wollongong, NSW, Australia; Institute of Research and Development

(Prof M Hosseinzadeh PhD), Faculty of Medicine (H T H Nguyen MD), Institue for Research and Training in Medicine, Biology and Pharmacy (H T H Nguyen MD), Duy Tan University, Da Nang, Viet Nam; Clinical Legal Medicine Department (S Hostiuc PhD), National Institute of Legal Medicine Mina Minovici, Bucharest, Romania; Faculty of Medicine of Tunis (Prof M Hsairi MPH), University Tunis El Manar, Tunis, Tunisia; Department of Health Services Administration (V Hsieh PhD), Department of Occupational Safety and Health (Prof B Hwang PhD), College of Public Health (R Lin PhD), China Medical University, Taichung, Taiwan; Department of Psychology (C Hu PhD), Tsinghua University, Beijing, China; Jockey Club School of Public Health and Primary Care (J Huang MD, C Zhong MD), The Chinese University of Hong Kong, Hong Kong, China; Department of Public Health and Community Medicine (Prof A Humayun PhD), Shaikh Khalifa Bin Zayed Al-Nahyan Medical College, Lahore, Pakistan; Department of Biological Sciences and Chemistry (Prof J Hussain PhD), Natural and Medical Sciences Research Center (A Khan PhD, A Ullah MS, S Ullah MSc), School of Pharmacy (A K Philip PhD), University of Nizwa Oman, Nizwa, Oman; Department of Biomolecular Sciences (N R Hussein PhD), University of Zakho, Zakho, Iraq; International Master Program for Translational Science (H Huynh BS), Department of Global Health and Health Security (K Latief MS), International Ph.D. Program in Medicine (L Minh MD), Research Center for Artificial Intelligence in Medicine (L Minh MD), School of Public Health (Y L Samodra MPH, Y L Samodra MPH), Taipei Medical University, Taipei, Taiwan; Department of Occupational Therapy (Prof B Hwang PhD), Asia University, Taiwan, Taichung, Taiwan; Health Policy and Management Department (P M Iftikhar MD), City University of New York, New York, NY, USA; Department of Community Medicine (O S Ilesanmi PhD), Department of Medicine (A S Oguntade MSc, Prof M O Owolabi DrM), Department of Oral and Maxillofacial Surgery (A A Salami BDS), University College Hospital, Ibadan, Ibadan, Nigeria; Faculty of Medicine (I M Ilic PhD, Prof M M Santric-Milicevic PhD), School of Public Health and Health Management (Prof M M Santric-Milicevic PhD), Faculty of Medicine Institute of Epidemiology (I S Vujcic PhD), University of Belgrade, Belgrade, Serbia; Department of Epidemiology (Prof M D Ilic PhD), University of Kragujevac, Kragujevac, Serbia; Department of Health Research (L R Inbaraj MD), ICMR National Institute for Research in Tuberculosis, Chennai, India; Department of Pharmacy (M R Islam PhD), University of Asia Pacific, Dhaka, Bangladesh; Department of Clinical Pharmacy & Pharmacy Practice (Prof N Ismail PhD), Asian Institute of Medicine, Science and Technology, Kedah, Malaysia; Malaysian Academy of Pharmacy, Puchong, Malaysia (Prof N Ismail PhD); Public Health Department of Social Medicine (Prof H Iso MD), Osaka University, Suita, Japan; Department of Health Services Research (M Iwagami PhD), University of Tsukuba, Tsukuba, Japan; Department of Non-Communicable Disease Epidemiology (M Iwagami PhD), Department of Infectious Disease Epidemiology (Prof H J Larson PhD), Department of Health Services Research and Policy (Prof M McKee DSc), London School of Hygiene & Tropical Medicine, London, UK; Department of Biotechnology (M Iyer PhD, S Muthu MS), Karpagam Academy of Higher Education (Deemed to be University), Coimbatore, India; Department of Orthodontics & Dentofacial Orthopedics (L J BDS), Department of Oral Pathology and Microbiology (Prof G S Sarode PhD, Prof S C Sarode PhD), Dr. D. Y. Patil University, Pune, India; Research and Development Unit (L Jacob MD), Biomedical Research Networking Center for Mental Health Network (CiberSAM), Sant Boi de Llobregat, Spain; Faculty of Medicine (L Jacob MD), University of Versailles Saint-Quentin-en-Yvelines, Montigny-le-Bretonneux, France; Department of Nephrology (K Jaggi MD), San Mateo Medical Center, San Mateo, CA, USA; Department of Nephrology, Internal Medicine (K Jaggi MD), Mills Peninsula Medical Center, Burlingame, CA, USA; Ministry of Health (H Jahrami PhD), Department of Psychiatry (Z Saif MBA), Ministry of Health, Manama, Bahrain; Department of Leukemia (A Jain MD), The University of MD Anderson Cancer Center, Houston, TX, USA; Statistics Unit (N Jain MD), Riga Stradins University, Riga, Latvia; Health and Safety Department (A A Jairoun PhD), Dubai Municipality, Dubai, United Arab

Emirates; The World Academy of Sciences UNESCO, Trieste, Italy (Prof M Jakovljevic PhD); Shaanxi University of Technology, Hanzhong, China (Prof M Jakovljevic PhD); Department of Physiology (Prof S Javadov PhD), University of Puerto Rico Medical Sciences Campus, San Juan, Puerto Rico; Health Informatic Lab (T Javaheri PhD), Department of Computer Science (R Rawassizadeh PhD), Boston University, Boston, MA, USA; Centre of Studies and Research (S Jayapal PhD), Ministry of Health, Muscat, Oman; Department of Biochemistry (Prof S Jayaram MD), Government Medical College, Mysuru, India; Department of Cardiovascular Medicine (A K Jha MD), Saint Vincent Hospital, Worcester, MA, USA; Department of Community Medicine (R P Jha MSc), Dr. Baba Saheb Ambedkar Medical College & Hospital, Delhi, India; Department of Community Medicine (R P Jha MSc), Banaras Hindu University, Varanasi, India; Melbourne School of Population and Global Health (H Jiang PhD), School of Health Sciences (A Meretoja MD), University of Melbourne, Melbourne, VIC, Australia; Zoonoses Research Center (M Jokar DVM), Islamic Azad University, Karaj, Iran; Department of Clinical Sciences (M Jokar DVM), Department of Public Health (Y Sarikhani PhD), Jahrom University of Medical Sciences, Jahrom, Iran; Institute of Molecular and Clinical Ophthalmology Basel, Basel, Switzerland (Prof J B Jonas MD); Department of Ophthalmology (Prof J B Jonas MD), Heidelberg University, Mannheim, Germany; Hungarian Health Management Association, Budapest, Hungary (T Joo PhD); Department of Economics (C E Joshua BSc), National Open University, Benin City, Nigeria; Department of Family Medicine and Public Health (J J Jozwiak PhD), University of Opole, Opole, Poland; Institute of Family Medicine and Public Health (M Jürisson PhD), University of Tartu, Tartu, Estonia; Department of Bioengineering (H Kabir MSc), University of California Berkeley, Berkeley, CA, USA; School of Public Health (Z Kabir PhD), University College Cork, Cork, Ireland; Dermatology Department (F Kaliyadan MD), King Faisal University, Hofuf, Saudi Arabia; Department of Endocrinology (S Kalra DM), Bharti Hospital Karnal, Karnal, India; Care and Public Health Research Institute (CAPHRI) (R Kamath MHA), Maastricht University, Maastricht, Netherlands; Regional Institute for Population Studies (E Kanmiki MPH), University of Ghana, Accra, Ghana; Faculty of Dentistry (K K Kanmodi MPH), University of Puthisastra, Phnom Penh, Cambodia; Office of the Executive Director (K K Kanmodi MPH), Campaign for Health and Neck Cancer Education (CHANCE) Programme (A A Salami BDS), Cephas Health Research Initiative Inc, Ibadan, Nigeria; Department of Community Medicine (S Kannan S MD), ESIC Medical College and Hospital Chennai, Chennai, India; Dr. S S Bhatnagar University Institute of Chemical Engg. & Technology (Prof S K Kansal PhD), Department of Anthropology (Prof K Krishan PhD), Department of Community Medicine (R Rohilla MD), Institute of Forensic Science & Criminology (V Sharma PhD), Panjab University, Chandigarh, India; The Hansjörg Wyss Department of Plastic and Reconstructive Surgery (R S Kantar MD), Nab'a Al-Hayat Foundation for Medical Sciences and Health Care, New York, NY, USA; Cleft Lip and Palate Surgery Division (R S Kantar MD), Global Smile Foundation, Norwood, MA, USA; School of Health Professions and Human Services (I M Karaye MD), Hofstra University, Hempstead, NY, USA; Department of Anesthesiology (I M Karaye MD), Montefiore Medical Center, Bronx, NY, USA; Department of Physical Therapy and Health Rehabilitation (F Z Kashoo MPT), Majmaah University, Majmaah, Saudi Arabia; Surgery Research Unit (Prof J H Kauppila MD), University of Oulu, Oulu, Finland; Division of Nephrology and Hypertension (s Kazeminia MD), Radiology Department (P Metanat MD), Division of General Internal Medicine (N M Odogwu PhD), Mayo Clinic, Rochester, MN, USA; Eye Unit (Prof J H Kempen MD), MyungSung Medical College, Addis Ababa, Ethiopia; Department of Health Sciences and Biostatistics (E S Kendal PhD), Swinburne University of Technology, Hawthorn, VIC, Australia; Biomedical Informatics department (K Keshtkar BSc), Arizona State University, Phoenix, AZ, USA; Amity Institute of Forensic Sciences (H Khajuria PhD, B P Nayak PhD), Amity Institute of Pharmacy (K Munjal PhD), Amity Institute of Public Health (M Shannawaz PhD), Amity University, Noida, India; College of Health Sciences (N Khalid

PhD), Abu Dhabi University, Adu Dhabi, United Arab Emirates; Department of Biostatistics (Prof A Khalilian PhD), Mazandaran University of Medical Sciences, Mazandaran, Iran; Research Center for Hydatid Disease (F Khamesipour PhD), Kerman University of Medical Sciences, Kerman, Iran; Population Science Department (M Khan PhD), Jatiya Kabi Kazi Nazrul Islam University, Mymensingh, Bangladesh; Primary Care Department (M A Khan MSc), NHS North West London, London, UK; Department of Health Policy and Management (Prof Y Khang MD), Institute of Health Policy and Management (Prof Y Khang MD), Department of Food and Nutrition (A P Okekunle PhD), Seoul National University, Seoul, South Korea; College of Health, Wellbeing and Life Sciences (Prof K Khatab PhD), Sheffield Hallam University, Sheffield, UK; College of Arts and Sciences (Prof K Khatab PhD), Ohio University, Zanesville, OH, USA; Department of Biochemistry (F Khidri PhD), Liaquat University Of Medical and Health Sciences, Jamshoro, Pakistan; Molecular Medicine Department (M Khosravi PhD), Pasteur Institute of Iran, Tehran, Iran; Research Department (M Khosrowjerdi PhD), Inland Norway University of Applied Sciences, Elverum, Norway; School of Public Health (W T Kidane PhD), Hawassa University, Hawassa, Ethiopia; Department of Pharmacology (Z D Kifle MSc), School of Nursing (H B Netsere MS), University of Gondar, Gondar, Ethiopia; Cardiovascular Disease Initiative (M Kim MD), Broad Institute of MIT and Harvard, Cambridge, MA, USA; Millennium Prevention, Westwood, MA, USA (R W Kimokoti MD); Department of Public Health (G T Kiross MPH), Debre Markos University, East Gojjam, Ethiopia; School of Health Sciences (Prof A Kisa PhD), Kristiania University College, Oslo, Norway; Department of International Health and Sustainable Development (Prof A Kisa PhD), Tulane University, New Orleans, LA, USA; Department of Nursing and Health Promotion (S Kisa PhD), Oslo Metropolitan University, Oslo, Norway; Copernicus Institute of Sustainable Development (G Koren PhD), Institute for Risk Assessment Sciences (IRAS) (E Traini MSc), Utrecht University, Utrecht, Netherlands; Department of General Practice (Prof O Korzh DSc), Department of Infectious Diseases (A Sokhan PhD), Kharkiv National Medical University, Kharkiv, Ukraine; Independent Consultant, Jakarta, Indonesia (S Kosen MD); Kasturba Medical College (S Koulmane Laxminarayana MD), Manipal College of Nursing (R Yesodharan MSc), Manipal Academy of Higher Education, Udupi, India; Department of Anesthesiology (V Krishnamoorthy MD), Duke University, Durham, NC, USA; Department of Demography (Prof B Kuate Defo PhD), Department of Social and Preventive Medicine (Prof B Kuate Defo PhD), University of Montreal, Montreal, QC, Canada; Smart Approaches to Marijuana/Foundation for Drug Policy Solutions, Washington, DC, USA (C M Kubeisy BA); Faculty of Medicine (B Kucuk Bicer PhD), Gazi University, Ankara, Türkiye; Department of Biochemistry (Prof M Kuddus PhD), University of Hail, Hail, Saudi Arabia; Pediatrics Department (I Kuitunen PhD), Kuopio University Hospital, Kuopio, Finland; Institute of Clinical Medicine (I Kuitunen PhD), University of Eastern Finland, Kuopio, Finland; Department of Health Research (M Kulimbet MSc), Atchabarov Scientific Research Institute of Fundamental and Applied Medicine (M Kulimbet MSc), Kazakh National Medical University, Almaty, Kazakhstan; Department of Food Technology (Prof H Kumar PhD), Shri Vishwakarma Skill University, Palwal, India; Department of Biotechnology (Prof H Kumar PhD), Amity University Rajasthan India, Jaipur, India; Global Health Institute (S Kundu MPH), North South University, Dhaka, Bangladesh; Department of Nutrition and Food Science (S Kundu MPH), Department of Biochemistry and Food Analysis (N Roy PhD), Department of Post-Harvest Technology and Marketing (A Sayeed MSc), Patuakhali Science and Technology University, Patuakhali, Bangladesh; Department of Public Health/Malaria (K R Kunle MSc), Malaria Consortium, Abuja, Nigeria; Public Health Pharmacy Department (K R Kunle MSc), West African Postgraduate College of Pharmacists, Lagos, Nigeria; Department of Medicine (O P Kurmi PhD), Department of Psychiatry and Behavioural Neurosciences (A T Olagunju MD), McMaster University, Hamilton, ON, Canada; National Research and Innovation Agency, Jakarta, Indonesia (A Kusnali LLB, H U Pangaribuan MSc); Department

of Health Services Research and Management (D Kusuma DSc), City University of London, London, UK; Faculty of Public Health (D Kusuma DSc, Prof I Trihandini PhD), Centre for Family Welfare (K Latief MS), University of Indonesia, Depok, Indonesia; Department of Nursing (E F Kyei MSc), University of Massachusetts Boston, Boston, MA, USA; Department of Health Policy (I Kyriopoulos PhD, Prof E Mossialos PhD), London School of Economics and Political Science, London, UK; Department of Clinical Sciences and Community Health (Prof C La Vecchia MD), University of Milan, Milan, Italy; National Institute for Health Research (NIHR) Oxford Biomedical Research Centre, Oxford, UK (B Lacey PhD); Institute for Social and Health Sciences (Prof L Laflamme PhD), University of South Africa, Pretoria, South Africa; Department of Health Policy and Strategy (Prof C Lahariya MD), Foundation for People-centric Health Systems, New Delhi, India; SD Gupta School of Public Health (Prof C Lahariya MD), Indian Institute of Health Management Research University, Jaipur, India; School of Digital Science (D T C Lai PhD), Institute of Applied Data Analytics (D T C Lai PhD), Universiti Brunei Darussalam (University of Brunei Darussalam), Gadong, Brunei; NEVES Society for Patient Safety, Budapest, Hungary (J Lám PhD); Department of Health Sciences (D Lamnisos PhD), European University Cyprus, Nicosia, Cyprus; Unit of Genetics and Public Health (Prof I Landires MD), Unit of Microbiology and Public Health (V Nuñez-Samudio PhD), Institute of Medical Sciences, Las Tablas, Panama; Department of Public Health (V Nuñez-Samudio PhD), Ministry of Health, Herrera, Panama (Prof I Landires MD); Department of Health Sciences (DISSAL) (F Lanfranchi MD), University of Genoa, Genoa, Italy; Department of Psychiatry and Psychotherapy (B Langguth PhD, W Schlee PhD), University of Regensburg, Regensburg, Germany; Department of Behavioural Sciences and Learning (A Laplante-Lévesque PhD), Linköping University, Linköping, Sweden; Department of Medical Sciences (Prof A O Larsson PhD), Uppsala University, Uppsala, Sweden; Department of Clinical Chemistry and Pharmacology (Prof A O Larsson PhD), Uppsala University Hospital, Uppsala, Sweden; Department of Otorhinolaryngology (S Lasrado MS), Father Muller Medical College, Mangalore, India; Health Economcs Division (L K D Le PhD), Monash University, Burwood, VIC, Australia; Faculty of Medicine (N Le MD), Department of General Medicine (V T Nguyen MD), University of Medicine and Pharmacy at Ho Chi Minh City, Ho Chi Minh City, Viet Nam (T D T Le MD); Cardiovascular Research Department (N Le MD), Methodist Hospital, Merrillville, IN, USA; Independent Consultant, Ho Chi Minh City, Viet Nam (T D T Le MD); Department of Medical Science (M Lee PhD), Ajou University School of Medicine, Suwon, South Korea; Department of Precision Medicine (Prof S W Lee MD), Sungkyunkwan University, Suwon-si, South Korea; Department of Preventive Medicine (Prof Y Lee PhD), Korea University, Seoul, South Korea; Department of Reproductive Health (G Lema MPH), Department of Medical Microbiology and Immunology (S Muthupandian PhD), Mekelle University, Mekelle, Ethiopia; Faculty of Science (E Leong PhD), Universiti Brunei Darussalam (University of Brunei Darussalam), Bandar Seri Begawan, Brunei; Center for Dentistry and Oral Hygiene (A Li PhD), Unit of Pharmacotherapy, Epidemiology and Economy (S Mubarik MS), University Medical Center Groningen (Prof M J Postma PhD), Department of Internal Medicine (P Vart PhD), University of Groningen, Groningen, Netherlands; Department of Health Promotion and Health Education (M Li PhD), National Taiwan Normal University, Taipei, Taiwan; Asbestos Diseases Research Institute, Concord, NSW, Australia (R Lin PhD); Department of Dentistry- Quality and Safety of Oral Health Care (Prof S Listl PhD), Radboud University, Nijmegen, Netherlands; School of Life Sciences (G Liu PhD), University of Technology Sydney, Ultimo, NSW, Australia; Centre for Inflammation (G Liu PhD), Centenary Institute, Camperdown, NSW, Australia; Department of Epidemiology and Biostatistics (Prof J Liu PhD), School of Public Health (H Zhang MS), Institute of Child and Adolescent Health (Z Zou MD), Peking University, Beijing, China; School of Public Health (Y Liu PhD), Sun Yat-sen University, Guangzhou, China; Department of Molecular Epidemiology (E Llanaj PhD), German Institute of Human Nutrition Potsdam-

Rehbrücke, Potsdam, Germany; German Center for Diabetes Research (DZD), München-Neuherberg, Germany (E Llanaj PhD); Department of Physical Medicine and Nursing (R López-Bueno PhD), University of Zaragoza, Zaragoza, Spain; Department of Musculoskeletal Disorders (R López-Bueno PhD), National Research Centre for the Working Environment, Copenhagen, Denmark; Department of Epidemiology and Evidence-Based Medicine (P D Lopukhov PhD), Department of Information and Internet Technologies (S K Vladimirov PhD), I.M. Sechenov First Moscow State Medical University, Moscow, Russia; Department of Health Economics (L Lorenzovici MSc), Syreon Research Romania, Targu Mures, Romania; Department of Doctoral Studies (L Lorenzovici MSc), George Emil Palade University of Medicine, Pharmacy, Science, and Technology of Targu Mures, Targu Mures, Romania; School of Medicine (Prof G Lucchetti PhD), Federal University of Juiz de Fora, Juiz de Fora, Brazil; Department of General Surgery (Prof R Lunevicius DSc), Liverpool University Hospitals NHS Foundation Trust, Liverpool, UK; Department of Epidemiology (H Lv BA), Chinese Center for Disease Control and Prevention, Shenyang, China; Centre for Public Health and Wellbeing (Z Ma PhD), University of the West of England, Bristol, UK; Periodontal Department, Faculty of Medicin and Dentistry (Prof M Machoy PhD), Department of Propedeutics of Internal Diseases & Arterial Hypertension (Prof T Miazgowski MD), Pomeranian Medical University, Szczecin, Poland; Laboratório de Farmacognosia (LAQV) (Associated Laboratory for Green Chemistry (Á M Madureira-Carvalho PhD), Universidade do Porto (University of Porto), Porto, Portugal; Ophthalmology Department (M Magdy Abd El Razek MSc), Ministry of Health & Population, Aswan, Egypt; Cellular and Molecular Biology Research Center (Prof S Mahjoub PhD), Department of Clinical Biochemistry (Prof S Mahjoub PhD, A Mosapour PhD), Babol University of Medical Sciences, Babol, Iran; Department of Clinical and Hospital Pharmacy (M A Mahmoud PhD), Taibah University, Al-Madinah Al-Munawarrah, Saudi Arabia; Department of Internal Medicine (K Malhotra MBBS), Dayanand Medical College and Hospital, Ludhiana, India; Electrical Engineering Department (I Malik PhD), Prince Sattam bin Abdulaziz University, Al Kharj, Saudi Arabia; Institute for Social Science Research (A A Mamun PhD), The University of Queensland, Indooroopilly, QLD, Australia; Smidt Heart Institute (Y Manla MD), Cedars-Sinai Medical Center, Los Angeles, CA, USA; STIC (Prof A Mansour PhD), ENSTA Bretagne, Brest, France; Security, Intelligence and Integrity of Information Team (SI3) (Prof A Mansour PhD), Laboratoire des Sciences et Techniques de l'Information de la Communication et de la Connaissance (LABSTICC), Brest, France; Department of Food, Environmental and Nutritional Sciences (M Marino PhD), University of Milan, Milano, Italy; Department of Biochemistry (A Marjani PhD), Golestan University of Medical Sciences, Gorgan, Iran; Department of Economics (Prof G Martinez PhD), Autonomous Technology Institute of Mexico, Mexico City, Mexico; Noncommunicable Diseases and Mental Health Department (R Martinez-Piedra BSc), Pan American Health Organization, Washington, DC, USA; Campus Fortaleza (F R Martins-Melo PhD), Federal Institute of Education, Science and Technology of Ceará, Fortaleza, Brazil; Department of Nutrition and Dietetics (M Martorell PhD), Centre for Healthy Living (M Martorell PhD), University of Concepción, Concepción, Chile; Department of Pharmacy (S Maryam PharmD), Bahauddin Zakariya University, Multan, Pakistan; Faculty of Humanities and Health Sciences (Prof R R Marzo MD), Curtin University, Sarawak, Malaysia; Jeffrey Cheah School of Medicine and Health Sciences (Prof R R Marzo MD), Monash University, Subang Jaya, Malaysia; Department of Pharmacology (A Masoudi PhD), Shahroud University of Medical Sciences, Shahroud, Iran; Department of Medicine (J Mattumpuram MD), University of Louisville, Louisville, KY, USA; Epidemiology Department (Prof R J Maude PhD), Mahidol Oxford Tropical Medicine Research Unit, Bangkok, Thailand; Department of Social Medicine and Family (M Mazaheri PhD), Dezful University of Medical Sciences, Dezful, Iran; National Centre for Register-based Research (Prof J J McGrath MD), Aarhus University, Aarhus, Denmark; Australian Centre for Health Services Innovation (Prof S M McPhail

PhD), Queensland University of Technology, Kelvin Grove, QLD, Australia; Digital Health and Informatics Directorate (Prof S M McPhail PhD), Queensland Health, Brisbane, QLD, Australia; Department of Food Science and Technology (R Mehra PhD), Maharishi Markandeshwar (Deemed to be University), Ambala, India; Department of Public Health (T Mekene Meto MPH), Arba Minch University, Arbaminch, Ethiopia; Department of Medical Oncology and Hematology (M A M Mendez-Lopez PhD), Kantonsspital St. Gallen, St. Gallen, Switzerland; Peru Country Office (W Mendoza MD), United Nations Population Fund (UNFPA), Lima, Peru; Department of Medicine (G A Mensah MD), School of Public Health and Family Medicine (C A Nnaji MPH), University of Cape Town, Cape Town, South Africa; International Dx Department (A A Mentis MD), BGI Genomics, Copenhagen, Denmark; Neurology Unit (A Meretoja MD), Breast Surgery Unit (T J Meretoja MD), Helsinki University Hospital, Helsinki, Finland; Department of Virology (F Zakham PhD), University of Helsinki, Helsinki, Finland (T J Meretoja MD); University Centre Varazdin (T Mestrovic PhD), University North, Varazdin, Croatia; Department of Pharmacology (Prof K D Mettananda PhD), Department of Paediatrics (Prof S Mettananda DPhil), University of Kelaniya, Ragama, Sri Lanka; Clinical Medicine Department (Prof K D Mettananda PhD), North Colombo Teadhing Hospital, Ragama, Sri Lanka; University Paediatrics Unit (Prof S Mettananda DPhil), Colombo North Teaching Hospital, Ragama, Sri Lanka; Stritch School of Medicine (A Mhlanga PhD), Loyola University Chicago, Chicago, IL, USA; Department of Preventive Medicine (L Mhlanga PhD), Center for Global Health (J Musa MD), Northwestern University, Chicago, IL, USA; Anaesthesiology Department (G Micha PhD), "Helena Venizelou" General and Maternity Hospital, Athens, Greece; Department of Epidemiology (I Michalek PhD), National Cancer Registry (I Michalek PhD), Maria Sklodowska-Curie National Research Institute of Oncology, Warsaw, Poland; Pacific Institute for Research & Evaluation, Calverton, MD, USA (T R Miller PhD); Internal Medicine Programme (Prof E M Mirrakhimov PhD), Kyrgyz State Medical Academy, Bishkek, Kyrgyzstan; Department of Atherosclerosis and Coronary Heart Disease (Prof E M Mirrakhimov PhD), National Center of Cardiology and Internal Disease, Bishkek, Kyrgyzstan; Office of the Minster (M K Mirutse MPH), Federal Ministry of Health, Addis Ababa, Ethiopia; Department of Hospital Administration (M Mirza MD), Department of Dentistry (S S Rana MDS), Department of Community Medicine and Family Medicine (S S Sahoo MD, M Verma MD), Department of Anatomy (A Singal PhD), All India Institute of Medical Sciences, Bathinda, India; Department of Forensic Medicine and Toxicology (C Mittal MD), Dr. B. C. Roy Multi-Specialty Medical Research Centre, Kharagpur, India; Institute of Addiction Research (ISFF) (B Moazen MSc), Frankfurt University of Applied Sciences, Frankfurt, Germany; Thompson Institute (A Z Mohamed PhD), University of the Sunshine Coast, Birtinya, QLD, Australia; College of Health Science (A I Mohamed MSc), College of Applied and Natural Science (J Mohamed MSc), University of Hargeisa, Hargeisa, Somalia; Molecular Biology Unit (N S Mohamed MSc), Bio-Statistical and Molecular Biology Department (N S Mohamed MSc), Sirius Training and Research Centre, Khartoum, Sudan; Department of Pharmaceutical Sciences (S Mohammed PhD), Notre Dame of Maryland University, Baltimore, MD, USA; Department of Pharmacy (S Mohammed PhD), Mizan-Tepi University, Mizan, Ethiopia; Institute of Clinical Physiology (S Molinaro PhD), National Research Council, Pisa, Italy; Clinical Epidemiology and Public Health Research Unit (L Monasta DSc, L Ronfani PhD, E Traini MSc, G Zamagni MSc), Burlo Garofolo Institute for Maternal and Child Health, Trieste, Italy; Department of Epidemiology and Biostatistics (Y Moradi PhD), Kurdistan University of Medical Sciences, Sanandaj, Iran; Computer, Electrical, and Mathematical Sciences and Engineering Division (P Moraga PhD), King Abdullah University of Science and Technology, Thuwal, Saudi Arabia; Department of Public Health (Prof R S Moreira PhD), Oswaldo Cruz Foundation, Recife, Brazil; Department of Public Health (Prof R S Moreira PhD), Federal University of Pernambuco, Recife, Brazil; Department of Biology and Biological Engineering (J Morze PhD), Chalmers University of Technology, Gothenburg, Sweden; College of Medical

Sciences (J Morze PhD), SGMK Copernicus University, Warsaw, Poland; Department of Clinical Biochemistry (A Mosapour PhD), Tarbiat Modares University, Tehran, Iran; Department of Fruit and Vegetable Product Technology (Prof A Mousavi Khaneghah PhD), Prof. Wacław Dąbrowski Institute of Agricultural and Food Biotechnology State Research Institute, Warsaw, Poland; Department of Surgery (F Mulita PhD, G Verras MD), General University Hospital of Patras, Patras, Greece; Faculty of Medicine (F Mulita PhD), Department of Emergency Medicine (I Pantazopoulos PhD), University of Thessaly, Larissa, Greece; Clinical Epidemiology Research Unit (E Murillo-Zamora PhD), Mexican Institute of Social Security, Villa de Alvarez, Mexico; Postgraduate in Medical Sciences (E Murillo-Zamora PhD), Universidad de Colima, Colima, Mexico; Surgery Department (A Musina MD), Department of Medical Oncology (S R Volovat PhD), University of Medicine and Pharmacy Grigore T. Popa, Iaşi, Romania; Second Surgical Unit (A Musina MD), Department of Medical Oncology (S R Volovat PhD), Regional Institute of Oncology, Iaşi, Romania; Department of Research Methodology (S Muthu MS), Orthopaedic Research Group, Coimbatore, India; Saveetha Dental College (S Muthupandian PhD), Saveetha Institute of Medical and Technical Sciences (SIMATS), Chennai, India; Department of Neuropsychiatry (W Myung PhD), Seoul National University, Seongnam-si, South Korea; Research and Analytics Department (A J Nagarajan MTech), Initiative for Financing Health and Human Development, Chennai, India; Department of Research and Analytics (A J Nagarajan MTech), Bioinsilico Technologies, Chennai, India; Institute of Epidemiology and Medical Biometry (Prof G Nagel PhD), Ulm University, Ulm, Germany; Comprehensive Cancer Center (G Naik MPH), Department of Health Policy & Organization (M Rahim MA), Department of Health Services Administration (M Rahim MA), Department of Psychology (D C Schwebel PhD), School of Medicine (Prof J A Singh MD), University of Alabama at Birmingham, Birmingham, AL, USA; Laboratory of Public Health Indicators Analysis and Health Digitalization (M Naimzada MD, S S Otstavnov PhD), Department of Information Technologies and Management (S K Vladimirov PhD), Moscow Institute of Physics and Technology, Dolgoprudny, Russia; Experimental Surgery and Oncology Laboratory (M Naimzada MD), Kursk State Medical University, Kursk, Russia; Faculty of Pharmacy (F Nainu PhD), Hasanuddin University, Makassar, Indonesia; Suraj Eye Institute, Nagpur, India (V Nangia MD); Mysore Medical College and Research Institute (Prof S Narasimha Swamy MD), Government Medical College, Mysore, India; National Dental Research Institute Singapore (G G Nascimento PhD), Duke-NUS Medical School, Singapore, Singapore; Department of Applied Pharmaceutical Sciences and Clinical Pharmacy (A Y Naser PhD), Isra University, Amman, Jordan; Department of Biotechnology (M Naveed PhD), University of Central Punjab, Lahore, Pakistan; Department of Disease Control and Environmental Health (R Ndejjo MSc), Makerere University, Kampala, Uganda; School of Pharmacy (S O Nduaguba PhD), West Virginia University, Morgantown, WV, USA; Department of General Surgery (I Negoi PhD), Fourth Department of General Surgery (D Serban PhD), Emergency University Hospital Bucharest, Bucharest, Romania; Department of Cardiology (R I Negoi PhD), Cardio-Aid, Bucharest, Romania; Faculty of Medicine (Prof C Nejjari PhD), Euromed University of Fes, Fez, Morocco; Faculty of Medicine (Prof C Nejjari PhD), University Sidi Mohammed Ben Abdellah, Fez, Morocco; Department of Community Medicine (S Nepal MD), Kathmandu University, Palpa, Nepal; College of Medicine and Health Sciences (H B Netsere MS), Bahir Dar University, Gondar, Ethiopia; Department of Public Health (G Nguefack-Tsague PhD), University of Yaoundé I, Yaoundé, Cameroon; Department of Biological Sciences (J W Ngunjiri DrPH), University of Embu, Embu, Kenya; Department of Medical Engineering (D H Nguyen BS), University of South Florida, Tampa, FL, USA; Institute for Cancer Control (P T Nguyen MPH), National Cancer Center, Tokyo, Japan; Graduate School of Public Health (P T Nguyen MPH), St. Luke's International University, Chuo-ku, Japan; International Islamic University Islamabad, Islamabad, Pakistan (R K Niazi PhD); Institute for Mental Health and Policy (Y T Nigatu PhD), Centre for Addiction and Mental Health, Toronto, ON,

Canada; Department of General Surgery (T K Nikolouzakis PhD), University Hospital of Heraklion, Heraklion, Greece; Laboratory of Toxicology (T K Nikolouzakis PhD), Department of Medicine (Prof A Tsatsakis DSc), University of Crete, Heraklion, Greece; South African Medical Research Council, Cape Town, South Africa (C A Nnaji MPH); Center for Public Health (L A Nnyanzi PhD), Teesside University, Middlesbrough, UK; Faculty of Applied Sciences and Technology (E A Noman PhD), Universiti Tun Hussein Onn Malaysia, Johor, Malaysia; Department of Health Policy and Management (S Nomura PhD), Keio University, Tokyo, Japan; Department of Microbiology and Molecular Genetics (M Noreen PhD), The Women University Multan, Multan, Pakistan; Department of Paediatrics (C A Nri-Ezedi MD), Nnamdi Azikiwe University, Awka, Nigeria; Department of Public Health (M H Nunemo MPH), Wachemo University, Addis Ababa, Ethiopia; Public Health Department (D Nurrika PhD), Banten School of Health Science, South Tangerang, Indonesia; Ministry of Research, Technology and Higher Education, Higher Education Service Institutions (LL-DIKTI) Region IV, Bandung, Indonesia (D Nurrika PhD); Department of Applied Economics and Quantitative Analysis (Prof B Oancea PhD), Department of Statistics and Cybernetics (Prof M Paun PhD), University of Bucharest, Bucharest, Romania; School of Public Health (N M Odogwu PhD), Department of Epidemiology and Community Health (R R Parikh MD), Department of Surgery (J Rickard MD), University of Minnesota, Minneapolis, MN, USA; Department of Medicine (M J O'Donnell PhD), National University of Ireland, Galway, Ireland; Department of Community Health and Primary Care (O O Odukoya MSc), University of Lagos, Idi Araba, Nigeria; Department of Family and Preventive Medicine (O O Odukoya MSc), University of Utah, Salt Lake City, UT, USA; Department of Preventive Medicine (I Oh PhD), Department of Pediatrics (Prof D Yon MD), Kyung Hee University, Seoul, South Korea; Independent Consultant, Sydney, NSW, Australia (S R Okeke PhD); School of Pharmacy (O C Okonji MSc), University of the Western Cape, Cape Town, South Africa; Department of Medical Physiology (P G Okwute MSc), Department of Psychiatry (A T Olagunju MD), University of Lagos, Lagos, Nigeria; Helath Information Research Unit (O O Olasupo PhD), McMaster University, Hamilton, Ontario, Canada; Department of Nursing Science (M I Olatubi PhD), Bowen University, Iwo, Nigeria; Cardiology Department (G M M Oliveira PhD), Federal University of Rio de Janeiro, Rio de Janeiro, Brazil; Centre for Healthy Start Initiative, Lagos, Nigeria (B O Olusanya PhD, J O Olusanya MBA); Department of Anatomy (G O Oluwatunase MSc), Olabisi Onabanjo University, Sagamu, Nigeria; Surgery Department (G L Omer MD), Sulaimani University, Sulaimani, Iraq; ENT Department (G L Omer MD), Tor Vergata University of Rome, Rome, Italy; Department of Pharmacotherapy and Pharmaceutical Care (M Ordak PhD), Department of Biochemistry and Pharmacogenomics (M Zielińska MPharm), Medical University of Warsaw, Warsaw, Poland; University of Port Harcourt, Port Harcourt, Nigeria (Prof O E Orisakwe PhD); Sickle Cell Unit (V N Orish PhD), Ho Teaching Hospital, Ho Municipality, Ghana; Department of Medicine (Prof A Ortiz MD), Universidad Autónoma de Madrid (Autonomous University of Madrid), Madrid, Spain; Department of Nephrology and Hypertension (Prof A Ortiz MD), The Institute for Health Research Foundation Jiménez Díaz University Hospital, Madrid, Spain; One Health Global Research Group (Prof E Ortiz-Prado PhD), Universidad de las Americas, Quito, Ecuador; School of Medicine (U L Osuagwu PhD), Translation Health Research Institute (K Rana PhD), Western Sydney University, Campbelltown, NSW, Australia; Department of Optometry and Vision Science (U L Osuagwu PhD), University of KwaZulu-Natal, KwaZulu-Natal, South Africa; Department of Biological Sciences (O Osuolale PhD), Elizade University, Ilara-Mokin, Nigeria; Department of Project Management (S S Otstavnov PhD), Department of Health Care Administration and Economics (Prof V Vlassov MD), National Research University Higher School of Economics, Moscow, Russia; Division of Infectious Diseases (Prof A Ouyahia PhD), University Hospital of Setif, Setif, Algeria; Department of General Surgery (G Ouyang MD), Central South University, ChangSha, China; Department of Respiratory Medicine (Prof M P P A DNB), Department of Oral and

Maxillofacial Surgery (M S MDS, C S N PhD), Jagadguru Sri Shivarathreeswara University, Mysore, India; Menzies Institute for Medical Research (F Pan PhD), University of Tasmania, Hobart, TAS, Australia; Department of Public Health (A Pana PhD), Babes Bolyai University, Cluj Napoca, Romania; Department of Health Metrics (A Pana PhD), Center for Health Outcomes & Evaluation, Bucharest, Romania; School of Public Health (P Panda MPH), Asian Institute of Public Health University, Bhubaneswar, India; Privatpraxis, Heidelberg, Germany (S Panda-Jonas MD); Department of Ophthalmology (G D Panos PhD), Nottingham University Hospitals - QMC Campus, Nottingham, UK; Division of Ophthalmology & Visual Sciences (G D Panos PhD), People in Psychiatry and Applied Psychology (F Shokraneh PhD), University of Nottingham, Nottingham, UK; Department of Neurology (L D Panos MD), Department of Emergency Medicine (I Pantazopoulos PhD), University of Bern, Bern, Switzerland; Yonsei University College of Medicine, Seodaemun-gu, South Korea (S Park MD); Center for Pharmacoepidemiology and Treatment Science (A Parthasarathi MD), Rutgers University, New Brunswick, NJ, USA; Research Center (A Parthasarathi MD), Allergy Asthma and Chest Center, Mysore, India; Department of Medical Sciences (R Passera PhD), University of Torino, Torino, Italy; Department of Imaging (R Passera PhD), AOU Città della Salute e della Scienza di Torino, Torino, Italy; Department of Physiotherapy (H M Patel PhD), Charotar University of Science and Technology, Anand, India; Global Health Governance Programme (J Patel BSc), Centre for Medical Informatics (Prof A Sheikh MD), College of Medicine and Veterinary Medicine (G Verras MD), University of Edinburgh, Edinburgh, UK; School of Dentistry (J Patel BSc), University of Leeds, Leeds, UK; College of Dental Medicine (Prof S Patil PhD), Roseman University of Health Sciences, South Jordan, UT, USA; Centre of Molecular Medicine and Diagnostics (COMManD) (Prof S Patil PhD), Saveetha University, Chennai, India; Second Department of Internal Medicine (D Patoulias PhD), European Interbalkan Medical Center, Thessaloniki, Greece; Department of Internal Medicine (V Patthipati MD), Advent Health, Palm Coast, FL, USA; Department of Hospital Medicine (V Patthipati MD), Sound Physicians, Palm Coast, FL, USA; Faculty of Humanities and Social Sciences (U Paudel PhD), Department of Community Medicine (P M S Pradhan MD), Tribhuvan University, Kathmandu, Nepal; Department of Bioinformatics and Biostatistics (Prof M Paun PhD), National Institute of Research and Development for Biological Sciences, Bucharest, Romania; Clinical Research Department (P Pedersini MSc, J H Villafañe PhD), IRCCS Fondazione Don Carlo Gnocchi, Milan, Italy; Department of Outpatient (M Peng MPH), Taihe Hospital, Shiyan, China; The First Clinical College (M Peng MPH), School of Public Health and Management (Y Yu MS), Hubei University of Medicine, Shiyan, China; Department of Neurology (U Pensato MD), IRCCS Humanitas Research Hospital, Milan, Italy; Center for Research and Innovation (V F Pepito MSc), Ateneo De Manila University, Pasig City, Philippines; Centre for Primary Health Care and Equity (P Peprah MSc), University of New South Wales, Kensington, Australia; Curtin School of Population Health (Prof G Pereira PhD), Curtin University, Bentley, WA, Australia; Centre for Fertility and Health (Prof G Pereira PhD), Norwegian Institute of Public Health, Oslo, Norway; International Institute for Educational Planning (IIEP) (Prof M F P Peres MD), Albert Einstein Hospital, São Paulo, Brazil; Department of Development Studies (Prof A Perianayagam PhD), International Institute for Population Sciences, Mumbai, India; Mario Negri Institute for Pharmacological Research, Bergamo, Italy (N Perico MD, Prof G Remuzzi MD); Pennsylvania Cancer and Regenerative Medicine Center (R G Pestell MD), Baruch S Blumberg Institute, Doylestown, PA, USA; Department of Medicine (R G Pestell MD), Xavier University School of Medicine, Woodbury, NY, USA; Facultad de Medicina (F E Petermann-Rocha PhD), Universidad Diego Portales (Diego Portales University), Santiago, Chile; School of Cardiovascular and Metabolic Health (F E Petermann-Rocha PhD), University of Glasgow, Glasgow, UK; National Centre for Disease Prevention and Health Promotion (D Pierannunzio PhD), National Institute of Health, Roma, Italy; Department of Pediatric Orthopedic Surgery (M Pigeolet MD), Hôpital

Necker - Enfants Malades, Paris, France; Research School of Chemistry and Applied Biomedical Sciences (E Plotnikov PhD), Tomsk Polytechnic University, Tomsk, Russia; Mental Health Research Institute (E Plotnikov PhD), Tomsk National Research Medical Center of the Russian Academy of Sciences, Tomsk, Russia; Clinical Academic Department of Pediatrics (Prof D Poddighe PhD), University Medical Center (UMC), Astana, Kazakhstan; Data Driven Health Division (P Pollner PhD), Hungarian Healthcare Management Association, Budapest, Hungary; Department of Data Management and Analysis (R Poluru PhD), The INCLEN Trust International, New Delhi, India; Non-communicable Diseases Research Center (N Pourtaheri PhD), Bam University of Medical Sciences, Bam, Iran; University of Mississippi, MS, USA (D Prabhu PhD); Centro de Investigaciones Clinicas (Clinical Research Center) (S I Prada PhD), Fundación Valle del Lili (Valle del Lili Foundation), Cali, Colombia; Centro de Estudios en Protección Social y Economía de la Salud (PROESA) (Research Center for Social and Health Economics) (S I Prada PhD), Universidad Icesi (ICESI University), Cali, Colombia; Department of Clinical Research and Epidemiology (M Prasad MD), Institute of Liver and Biliary Sciences, New Delhi, India; Department of Biochemistry (Prof A Prashant PhD), Jagadguru Sri Shivarathreeswara University, Mysuru, India; Centre for Dental Education and Research (B M Purohit MDS), Department of Radiation Oncology (A Shankar MD), All India Institute of Medical Sciences, New Delhi, India; Department of Computer Science (N H Qasim PhD), Department of Health Sciences (Prof F Rahim PhD), Cihan University Sulaimaniya, Sulaymaniyah, Iraq; Department of Community Medicine (D R MD), Government Medical College, Bengaluru, India; Cardiovascular Research Center (M Rabiee Rad MD), Isfahan Cariovascular Research Institute, Isfahan, Iran; College of Medicine (A Radfar MD), University of Central Florida, Orlando, FL, USA; Department of Medical Oncology (Prof V Radhakrishnan MD), Cancer Institute (W.I.A), Chennai, India; Department of Epidemiology and Biostatistics (H Raeisi Shahraki PhD), Shiraz University of Medical Sciences, Shahrekord, Iran; Department of Radiology (S Rafiei Alavi MD), University of California Irvine, Irvine, CA, USA; Cihan University Sulaimaniya Research Center (CUSRC), Sulaymaniyah, Iraq (Prof F Rahim PhD); Institute of Health and Wellbeing (M Rahman PhD), Federation University Australia, Berwick, VIC, Australia; Department of Public Health (V Rahmanian PhD), Torbat Jam Faculty of Medical Sciences, Torbat Jam, Iran; Department of Physical Education and Sport Sciences (Prof M Rahmati PhD), Hepatitis Research Center (M Zandi PhD), Lorestan University of Medical Sciences, Khorramabad, Iran; Department "Hospital of women and children", IRCCS Azienda Ospedaliero (D Raimondo PhD), Occupational Health Unit (Prof F S Violante MD), Sant'Orsola Malpighi Hospital, Bologna, Italy; Faculty of Medicine (A Rajabpour-Sanati MD), Birjand University of Medical Sciences, Birjand, Iran; Centre for Chronic Disease Control, New Delhi, India (P Rajput PhD); Department of Radiology (S Ramasamy MD), Stanford University, Stanford, CA, USA; Department of Epidemiology, Biostatistics and Occupational Health (J Rana MPH), McGill University, Montreal, QC, Canada; Research and Innovation Division (J Rana MPH), South Asian Institute for Social Transformation (SAIST), Dhaka, Bangladesh; Research Department (C L Ranabhat PhD), Policy Research Institute, Kathmandu, Nepal; Health and Public Policy Department (C L Ranabhat PhD), Global Center for Research and Development, Kathmandu, Nepal; Centre for Clinical Pharmacology (N Rancic PhD), University of Defence in Belgrade, Belgrade, Serbia; Centre for Clinical Pharmacology (N Rancic PhD), Medical College of Georgia at Augusta University, Belgrade, Serbia; Department of Health Economics and Outcomes Research (A Rane MS), Agios Pharmaceuticals, Cambridge, MA, USA; Department of Pharmaceutical Economics and Policy (A Rane MS), Massachusetts College of Pharmacy and Health Sciences, Boston, MA, USA; School of Humanities and Social Sciences (S Ranjan MA, R Thakur PhD), Indian Institute of Technology Mandi, Mandi, India; College of Pharmaceutical Sciences (D Rapaka PhD), Andhra University, Visakhapatnam, India; University of Social Welfare and Rehabilitation Sciences, Tehran, Iran (V Rashedi PhD); Department of Geography (A Rasul

PhD), Soran University, Soran, Iraq; Department of Biomedical Engineering (Z Ratan MSc), Khulna University of Engineering and Technology, Khulna, Bangladesh; Section of Pulmonary and Critical Care Medicine (N Ravikumar MD), University of Chicago, Chicago, IL, USA; Inovus Medical, St Helens, UK (D L Rawaf MRCS); Academic Public Health England (Prof S Rawaf MD), Public Health England, London, UK; Department of Internal Medicine (B Rawlley MD), State University of New York, Syracuse, NY, USA; Department Biological Sciences (Prof E M M Redwan PhD), King Abdulaziz University, Jeddah, Egypt; Department of Protein Research (Prof E M M Redwan PhD), Research and Academic Institution, Alexandria, Egypt; Department of Epidemiology and Biostatistics (Prof M Rezaeian PhD), Rafsanjan University of Medical Sciences, Rafsanjan, Iran; Department of Public Health (A Riad DDS), Czech National Centre for Evidence-based Healthcare and Knowledge Translation (A Riad DDS), Masaryk University, Brno, Czech Republic; Department of Surgery (J Rickard MD), University Teaching Hospital of Kigali, Kigali, Rwanda; Institute of Diagnostic and Interventional Radiology and Neuroradiology (R Rikhtegar MD), Essen University Hospital, Essen, Germany; One Health Toxicology Research Unit (1H-TOXRUN) (Prof C F Rodrigues PhD), Instituto Universitário de Ciências da Saúde (CESPU), Porto, Portugal; Department of Pharmacology and Toxicology (Prof J A B Rodriguez PhD), Universidad de Antioquia (University of Antioquia), Medellin, Colombia; Faculty of Nursing (D S Romadlon PhD), Chulalongkorn University, Bangkok, Thailand; Department of Analytical and Applied Economics (Prof H S Rout PhD, C K Swain MPhil), Utkal University, Bhubaneswar, India; Faculty of Medicine (B Roy PhD), Quest International University Perak, Ipoh, Malaysia; Department of Labour (P Roy PhD), Directorate of Factories, Government of West Bengal, Kolkata, India; Centro de Investigación Palmira (Palmira Research Center) (E Rubagotti PhD), Corporación Colombiana de Investigación Agropecuaria AGROSAVIA (Colombian Agricultural Research Corporation), Bogota, Colombia; Advanced Campus Governador Valadares (Prof G d Ruela MSc), Juiz de For a Federal University, Governador Valadares, Brazil; Nursing Department (Prof G d Ruela MSc), Universidade Presidente Antônio Carlos (President Antônio Carlos University), Governador Valadares, Brazil; Department of Health Statistics (S F Rumisha PhD), National Institute for Medical Research, Dar es Salaam, Tanzania; Institute for Health Metrics and Evaluation (T Runghien MSc), University of Washington, Seattle, USA; Faculty of Medicine (Z Saadatian PhD), Infectious Diseases Research Center (Z Saadatian PhD), Gonabad University of Medical Sciences, Gonabad, Iran; Department of Medical Pharmacology (M M Saber-Ayad MD), Public Health and Community Medicine Department (M R Salem MD), Cairo University, Giza, Egypt; Faculty of Computing and Informatics (M SaberiKamarposhti PhD), Multimedia University, Cyberjaya, Malaysia; Department of Anesthesiology and Reanimation (F Sada PhD), University of Pristina, Pristina, Kosovo; Department of Anesthesiology and Intensive Care (F Sada PhD), University Clinical Center of Kosovo, Pristina, Kosovo; Department of Pharmaceutical Chemistry (Prof M R Saeb PhD), Medical University of Gdańsk, Gdańsk, Poland; Multidisciplinary Laboratory Foundation University School of Health Sciences (FUSH) (Prof U Saeed PhD), Foundation University, Islamabad, Pakistan; International Center of Medical Sciences Research (ICMSR), Islamabad, Pakistan (Prof U Saeed PhD); Faculty of Medicine, Bioscience and Nursing (S Z Safi PhD), MAHSA University, Selangor, Malaysia; Interdisciplinary Research Centre in Biomedical Materials (IRCBM) (S Z Safi PhD), COMSATS Institute of Information Technology, Lahore, Pakistan; Department of Humanities and Social Sciences (M Saha MSc), Deakin University, Melbourne, VIC, Australia; Department of Preventive & Social Medicine (M Sahu MD), Independent Consultant, Kolkata, India; Ludwig Maximilian University of Munich, Munich, Germany (J W Sakshaug PhD); Institute for Employment Research, Nuremberg, Germany (J W Sakshaug PhD); Technology Management Department (Prof M Z Y Salem PhD), University College of Applied Sciences, Gaza, Palestine; School of Economics and Management (Prof M Z Y Salem PhD), University of Kassel, Kassel, Germany; Department

of Neurology (S Samadzadeh MD), University of Southern Denmark, Odense, Denmark; Department of Anatomy (Prof V P Samuel PhD), Ras Al Khaimah Medical and Health Sciences University, Ras Al Khaimah, United Arab Emirates; Department of Surgery (Prof J Sanabria MD), Marshall University, Huntington, WV, USA; Institute of Neuroanatomy (N Sanadgol PhD), Uniklinik Rhine-Westphalia Technical University of Aachen, Aachen, Germany; Independent Consultant, Thiruvananthapuram, India (S Y Saraswathy PhD); Indira Gandhi Medical College and Research Institute, Puducherry, India (A Saravanan MD); Department of Orthpaedics and Trauma Surgery (B Saravi PhD), University of Freiburg, Freiburg, Germany; Department of Orthopaedics (B Saravi PhD), Loretto Hospital Freiburg, Freiburg, Germany; Department of Food Processing Technology (T Sarkar PhD), West Bengal State Council of Technical Education, Malda, India; Department of Health and Society (Prof R Sarmiento-Suárez MPH), University of Applied and Environmental Sciences, Bogota, Colombia; National School of Public Health (Prof R Sarmiento-Suárez MPH), Carlos III Health Institute, Madrid, Spain; Faculty of Health & Social Sciences (B Sathian PhD), Bournemouth University, Bournemouth, UK; National Centre for Epidemiology and Population Health (M Sayeed MS, A Talukder MSc), Australian National University, Acton, ACT, Australia; Department of Neurology (Prof N Scarmeas PhD), National and Kapodistrian University of Athens, Athens, Greece; Department of Neurology (Prof N Scarmeas PhD), Columbia University, New York, NY, USA; Cardiovascular Research Center (A Schuermans BSc), Massachusetts General Hospital, Cambridge, MA, USA; Department of Cardiovascular Sciences (A Schuermans BSc, J Van den Eynde BSc), Katholieke Universiteit Leuven, Leuven, Belgium; Clinic for Conservative Dentistry and Periodontology (Prof F Schwendicke PhD), University Hospital of the Ludwig-Maximilians-University Munich, Munich, Germany; Faculty of Dentistry (S Selvaraj PhD), AIMST University, Bedong, Malaysia; Department of Biomedical Sciences (P Sengupta PhD), Gulf Medical University, Ajman, United Arab Emirates; Emergency Department (S Senthilkumaran MD), Manian Medical Centre, Erode, India; Department of Medicine and Surgery (Y Sethi MBBS), Government Doon Medical College, Dehradun, India; National Heart, Lung, and Blood Institute (A Seylani BS), National Institute of Health, Rockville, MD, USA; Medical Research Center (J Shah BS), Kateb University, Kabul, Afghanistan; Department of Infectious Diseases and Microbiology (P A Shah MBBS), Rajiv Gandhi University of Health Sciences, Bangalore, India; HepatoPancreatoBiliary Surgery and Liver Transplant Department (P A Shah MBBS), Healthcare Global Limited Cancer Care Hospital, Bangalore, India; Independent Consultant, Karachi, Pakistan (M A Shaikh MD); School of Medicine (M Shams-Beyranvand MSc), Alborz University of Medical Sciences, Karaj, Iran; Department of Clinical Review and Safety (S Sharfaei MD), Baim Institute for Clinical Research, Boston, MA, USA; Facultad de Medicina (Faculty of Medicine) (J Sharifi-Rad PhD), Universidad del Azuay (University of Azuay), Cuenca, Ecuador; Department of Microbiology (R P Shastry PhD), Yenepoya University, Mangalore, India; Department of Engineering (A Shavandi PhD), Free University of Brussels (ULB), Bruxelles, Belgium; Bioengineering Department (A Shayan BS), Clemson University, Clemson, SC, USA; Department of Ophthalmology (M Shayan MD), Harvard Medical School, Boston, MA, USA; Botany and Microbiology Department (A M E Shehabeldine PhD), Al-Azhar University, Cairo, Egypt; Department of Dermatology, Venereology and Leprosy (Prof M M Shenoy MD), Yenepoya Medical College, Mangalore, India; Friedman School of Nutrition Science and Policy (P Shi PhD), Tufts University, Boston, MA, USA; Department of Public Health (D Shiferaw MPH), Dambi Dollo University, Dembi Dollo, Ethiopia; National Institute of Infectious Diseases, Tokyo, Japan (M Shigematsu PhD); Finnish Institute of Occupational Health, Helsinki, Finland (R Shiri PhD); Department of Clinical Immunology and Hematology (V Shivarov PhD), Sofiamed University Hospital, Sofia, Bulgaria; Department of Genetics (V Shivarov PhD), Sofia University "St. Kliment Ohridiski", Sofia, Bulgaria; Department of Public Health and Primary Care (F Shokraneh PhD, Prof P Willeit PhD), University of Cambridge, Cambridge, UK; National Institute

of Psychology (K Shuja MS), Quaid-i-Azam University, Islamabad, Pakistan; The Cooper Institute, Dallas, TX, USA (K Shuval PhD); Department of Medical Microbiology and Infectious Diseases (E E Siddig MD), Erasmus University, Rotterdam, Netherlands; Center of Potential and Innovation of Natural Resources (Prof L M R Silva PhD), Polytechnic Institute of Guarda, Guarda, Portugal; Health Sciences Research Centre (Prof L M R Silva PhD), University of Beira Interior, Covilhã, Portugal; WSB University in Gdańsk, Gdańsk, Poland (Prof B Simonetti PhD); Department of Dentistry (A Singh MD), All India Institute of Medical Sciences, Bhopal, India; School of Public Health & Zoonoses (B B Singh PhD), Guru Angad Dev Veterinary & Animal Sciences University, Ludhiana, India; Medicine Service Department (Prof J A Singh MD), US Department of Veterans Affairs (VA), Birmingham, AL, USA; Department of Surgery (B Socea PhD), "Sf. Pantelimon" Emergency Clinical Hospital Bucharest, Bucharest, Romania; Department of Systemic Pathology (R Solanki MD), Touro College of Osteopathic Medicine, Middletown, NY, USA; Department of Pathology (R Solanki MD), American University of the Caribbean School of Medicine, Cupecoy, Saint Martin; Department of Biochemistry (S Solanki MD), American University of Integrative Sciences, Barbados, Barbados; Department of Nursing (Y Solomon MSc), Dire Dawa University, Dire Dawa, Ethiopia; 3rd Department of Cardiology (M Spartalis PhD), University of Athens, Athens, Greece; Division of Community Medicine (C T Sreeramareddy MD), International Medical University, Kuala Lumpur, Malaysia; Amity Institute of Biotechnology (V K Srivastava PhD, E Upadhyay PhD), Amity University Rajasthan, Jaipur, India; Department of Biosciences (V K Srivastava PhD), Indian Institute of Science Education and Research, Bhopal, Bhopal, India; Department of Public Health (M Stanikzai MPH), Kandahar University, Kandahar, Afghanistan; Central Research Institute of Cytology and Genetics (E Varavikova PhD), Federal Research Institute for Health Organization and Informatics of the Ministry of Health (FRIHOI), Moscow, Russia (Prof V I Starodubov DSc); Nutrition and Dietetics Department (A V Starodubova DSc), Federal Research Institute of Nutrition, Biotechnology and Food Safety, Moscow, Russia; Department of Internal Disease (A V Starodubova DSc), Pirogov Russian National Research Medical University, Moscow, Russia; Department of Medicine (P Steiropoulos MD), Democritus University of Thrace, Alexandroupolis, Greece; Department of Psychology (M A Stokes PhD), Deakin University, Burwood, VIC, Australia; School of Medicine (V Subramaniyan PhD), Monash University, Sunway, Malaysia; Center for Biotechnology and Microbiology (M Suleman PhD), University of Swat, Mingora, Pakistan; School of Life Sciences (M Suleman PhD), Xiamen University, Xiamen, China; National Institute of Epidemiology (R Suliankatchi Abdulkader MD), Indian Council of Medical Research, Chennai, India; Mental Health Research (A Sultana MD), Independent Consultant, Khulna, Bangladesh; Division of Global Mental Health (A Sultana MD), EviSyn Health, Khulna, Bangladesh; Rural Health Research Institute (Prof J Sun PhD), Charles Sturt University, Bathurst, NSW, Australia; Institute of Integrated Intelligence and Systems (Prof J Sun PhD), Griffith University, QLD, Australia; Jeb E. Brooks School of Public Policy and the Department of Sociology (Prof B L Sykes PhD), Cornell University, Ithaca, NY, USA; Department of Clinical Outcomes (Prof L Szarpak PhD), Maria Sklodowska-Curie Medical Academy, Warsaw, Poland; Department of Clinical Research and Development (Prof L Szarpak PhD), LUXMED Group, Warsaw, Poland; Department of Dermatology (M D Szeto BS), University of Colorado, Aurora, CO, USA; Department of Neurology (P Tabaee Damavandi MD), Neurocenter of Southern Switzerland (NSI), Lugano, Switzerland; Department of Medicine (Prof R Tabarés-Seisdedos PhD), University of Valencia, Valencia, Spain; Carlos III Health Institute (Prof R Tabarés-Seisdedos PhD), Biomedical Research Networking Center for Mental Health Network (CiberSAM), Madrid, Spain; Department of Basic Medical Sciences (S Tabatabaeizadeh PhD), Department of Internal Medicine (S Tabatabaeizadeh PhD), Islamic Azad University, Mashhad, Iran; School of Social Work (Prof K M Tabb PhD), University of Illinois, Urbana, IL, USA; Department of Biostatistics and Epidemiology (M Taheri Soodejani PhD), Shahid

Sadoughi University of Medical Sciences, Yazd, Iran; Department of Environmental, Agricultural and Occupational Health (J Taiba MPH), University of Nebraska Medical Center, Omaha, NE, USA; Statistics Discipline (A Talukder MSc), Khulna University, Khulna, Bangladesh; Department of Dermato-Venereology (M Tampa PhD), Dr. Victor Babes Clinical Hospital of Infectious Diseases and Tropical Diseases, Bucharest, Romania; Department of Medicine (J L Tamuzi MSc), Northlands Medical Group, Omuthiya, Namibia; State key Laboratory of Numerical Modeling for Atmospheric Sciences and Geophysical Fluid Dynamics (LASG) (H Tang PhD), Chinese Academy of Sciences, Beijing, China; Department of Epidemiology and Biostatistics (M K Tariku MPH), Department of Public Health (T Y Tiruye PhD), Department of Human Nutrition and Food Sciences (E G Wassie MSc), Debre Markos University, Debre Markos, Ethiopia; Department of Pathology (V Y Tat BS), University of Texas, Galveston, TX, USA; Department of Urology (M Teimoori MD), Sabzevar University of Medical Sciences, Sabzevar, Iran; College of Pharmacy (R M H Temsah PharmD), Alfaisal University, Riyadh, Saudi Arabia; Outpatient Department (D R Terefa MSc), Wollega University, Bedele town, Ethiopia; Health Management Department (R Tesler PhD), Ariel University, Ariel, Israel; Psychology Department (E Teye-Kwadjo PhD), University of Ghana, Legon, Accra, Ghana; Department of Pharmacology (P Thangaraju MD), All India Institute of Medical Sciences, Raipur, India; Public Health Department (Prof K R Thankappan MD), Amrita Institute of Medical Sciences, Kochi, India; Faculty of Biomedical Engeneering (A Tichopad PhD), Czech Technical University, Prague, Czech Republic; Faculty of Public Health (J H V Ticoalu MPH), Universitas Sam Ratulangi, Manado, Indonesia; Department of Allied Health and Human Performance (T Y Tiruye PhD), University of South Australia, Adelaide, SA, Australia; Laboratory of Public Health Indicators Analysis and Health Digitalization (M V Titova PhD), Moscow Institute of Physics and Technology, Moscow, Russia; Department of Medicine (Prof M Tonelli MD), Department of Oncology (L Yang PhD), University of Calgary, Calgary, AB, Canada; School of Medicine (J T Tran BS), Indiana University, Indianapolis, IN, USA; Department of Health (N M Tran MD), Children's Hospital 1, Ho Chi Minh City, Viet Nam; Adult Learning Disability Service (S J Tromans PhD), Leicestershire Partnership National Health Service Trust, Leicester, UK; School of Medicine (T T Truyen MD), Nam Can Tho University, Can Tho, Viet Nam; Department of Psychiatry (E Tsermpini PhD), Dalhousie University, Halifax, NS, Canada; Department of Nutrition and Food Studies (S Tyrovolas PhD), George Mason University, Fairfax, VA, USA; Environmental Sciences Program (S Uddin PhD), Asian University for Women, Chittagong, Bangladesh; Department of Geography (S Uddin PhD), University of Victoria, Victoria, BC, Canada; Department of Health and Community Sciences (A Udoh PhD), University of Exeter, Exeter, UK; International Center for Chemical and Biological Sciences (S Ullah MSc), University of Karachi, Karachi, Pakistan; Department of Zoology (S Ullah PhD), Division of Science and Technology (S Ullah PhD), University of Education, Lahore, Pakistan; Department of Paraclinical Sciences (S Umakanthan MD), The University of the West Indies, St. Augustine, Trinidad and Tobago; Department of Community Medicine (C D Umeokonkwo MPH), Alex Ekwueme Federal University Teaching Hospital Abakaliki, Abakaliki, Nigeria; College of Health and Sport Sciences (A G Vaithinathan MSc), University of Bahrain, Salmanya, Bahrain; Department. of Public Health and Epidemiology (O Varga PhD), University of Debrecen, Debrecen, Hungary; UKK Institute, Tampere, Finland (Prof T J Vasankari MD); Faculty of Medicine and Health Technology (Prof T J Vasankari MD), Tampere University, Tampere, Finland; Department of Human Genetics & Molecular Biology (B Vellingiri PhD), Bharathiar University, Coimbatore, India; Department of Statistics (D Venugopal PhD), Manonmaniam Sundaranar University, Tirunelveil, India; Department of Mathematics and Statistics (M Vishwakarma MSc), Banasthali Vidyapith, Tonk, India; Department of Community Medicine (M Vishwakarma MSc), Government Medical College, Barmer, India; Faculty of Information Technology (B Vo PhD), HUTECH University, Ho

Chi Minh City, Viet Nam; School of Population Health and Environmental Sciences (H A Wafa MPH, Y Wang PhD), King's College London, London, UK; Office of Research, Innovation, and Commercialization (ORIC) (Prof Y Waheed PhD), Shaheed Zulfiqar Ali Bhutto Medical University (SZABMU), Islamabad, Pakistan; Gilbert and Rose-Marie Chagoury School of Medicine (Prof Y Waheed PhD), Lebanese American University, Byblos, Lebanon; Department of Midwifery (E B Wakwoya MSc), Arsi University, Asella, Ethiopia; School of Public Health (F Wang PhD), Xuzhou Medical University, Xuzhou, China; Department of Neurosurgery (S Wang MD), School of Public Health (J Xia PhD), Capital Medical University, Beijing, China; Department of Parasitology (Prof K G Weerakoon PhD), Department of Community Medicine (N D Wickramasinghe MD), Rajarata University of Sri Lanka, Anuradhapura, Sri Lanka; Key Laboratory of Shaanxi Province for Craniofacial Precision Medicine Research (Y Wen PhD), Stomatological Hospital (College) of Xi'an Jiaotong University, Xi'an, China; Competence Center of Mortality-Follow-Up of the German National Cohort (R Westerman DSc), Federal Institute for Population Research, Wiesbaden, Germany; Department of Physical Therapy (T Wiangkham PhD), Naresuan University, Phitsanulok, Thailand; Department of Surgery (D P Wickramasinghe MD), University of Colombo, Colombo, Sri Lanka; Department of Medical Statistics, Informatics and Health Economics (Prof P Willeit PhD), Medical University Innsbruck, Innsbruck, Austria; Department of Food Science and Human Nutrition (Prof F Wu PhD), Michigan State University, East Lansing, MI, USA; School of Public Health (H Xiao PhD), Zhejiang University, Zhejiang, China; Department of Public Health Science (H Xiao PhD), Fred Hutchinson Cancer Research Center, Seattle, WA, USA; School of Medicine (Prof G Xu MD), Nanjing University, Nanjing, China; Department of Endocrinology (Prof S Xu PhD), University of Science and Technology of China, Hefei, China; School of Medicine (Prof S Xu PhD), University of Rochester, Rochester, NY, USA; Cardiovascular Program (X Xu PhD), The George Institute for Global Health, Sydney, NSW, Australia; Department of Basic Medical Sciences (S Yaghoubi PhD), Neyshabur University of Medical Sciences, Neyshabur, Iran; Department of Cancer Epidemiology and Prevention Research (L Yang PhD), Alberta Health Services, Calgary, AB, Canada; Faculty of Medicine (Y Yano MD), Department of Public Health (N Yonemoto PhD), Juntendo University, Tokyo, Japan; Research Center of Physiology (H Yaribeygi PhD), Semnan University of Medical Sciences, Semnan, Iran; National Center for Chronic and Noncommunicable Disease Control and Prevention (P Ye MPH), Chinese Center for Disease Control and Prevention, Beijing, China; Department of Family Medicine (S A Yesuf MSc), St. Peter's Specialized Hospital, Addis Ababa, Ethiopia; Biostatics, Epidemiology, and Science Computing Department (S Yezli PhD), King Faisal Specialist Hospital & Research Center, Riyadh, Saudi Arabia; Trinity College Institute for Neuroscience (A Yigezu MPH), School of Medicine (A Yigezu MPH), Trinity College Dublin, Dublin, Ireland; Department of Neuropsychopharmacology (N Yonemoto PhD), National Center of Neurology and Psychiatry, Kodaira, Japan; Department of Health Policy and Management (Prof M Z Younis PhD), Jackson State University, Jackson, MS, USA; School of Business & Economics (Prof M Z Younis PhD), Universiti Putra Malaysia (University of Putra Malaysia), Kuala Lumpur, Malaysia; Department of Epidemiology and Biostatistics (Prof C Yu PhD), Wuhan University, Wuhan, China; Faculty of Medicine and Health Sciences (F Zakham PhD), Hodeidah University, Hodeidah, Yemen; Addictology Department (Prof M S Zastrozhin PhD), Russian Medical Academy of Continuous Professional Education, Moscow, Russia; School of Public Health (Y Zhang PhD), Hubei Province Key Laboratory of Occupational Hazard Identification and Control (Y Zhang PhD), Wuhan University of Science and Technology, Wuhan, China; Computational Bioscience Research Center, CEMSE (J Zhou PhD), King Abdullah University of Science and Technology, Jeddah, Saudi Arabia; School of Public Health and Emergency Management (B Zhu PhD), Southern University of Science and Technology, Shenzhen, China; School of Life Sciences (L Zhu

PhD), Yunnan University, Kunming, China; School of Physics (S H Zyoud PhD), Universiti Sains Malaysia, Penang, Malaysia.

Authors' Contributions

Contributions to be included in appendix at the above location:

Managing the overall research enterprise

Stein Emil Vollset, Natalia V Bhattacharjee, Austin E Schumacher, Amanda E Smith, Paulina A Lindstedt, Kasey E Kinzel, Simon I Hay, Christopher J L Murray

Writing the first draft of the manuscript

Natalia V Bhattacharjee, Austin E Schumacher, Stein Emil Vollset, Catherine Bisignano, Susan A McLaughlin, Amanda E Smith, Paulina A Lindstedt, Simon I Hay, Christopher J L Murray

Primary responsibility for applying analytical methods to produce estimates

Natalia V Bhattacharjee, Chun-Wei Yuan, Cat Raggi, Julie Sojin Kim, Shujin Cao, Julian Chalek, Erin A May

Primary responsibility for seeking, cataloguing, extracting, or cleaning data; designing or coding figures and tables

Haley Comfort, John E Fuller, Denny Wang, Stefanie Watson, Nicholas Alexander Verghese, Julie Sojin Kim, Chun-Wei Yuan, Cat Raggi, QuynhAnh P Nguyen, Austin J Ahlstrom, Sam Farmer, Julian Chalek

Providing data or critical feedback on data sources

Yohannes Habtegiorgis Abate, Hedayat Abbastabar, Sherief Abd-Elsalam, Meriem Abdoun, Auwal Abdullahi, Mesfin Abebe, Samrawit Shawel Abebe, Hassan Abolhassani, Meysam Abolmaali, Lucas Guimarães Abreu, Michael R M Abrigo, Niveen ME Abu-Rmeileh, Tadele Girum Adal, Mesafint Molla Adane, Oluwafemi Atanda Adeagbo Adeagbo, Victor Adekanmbi, Olatunji O Adetokunboh, Qorinah Estiningtyas Sakilah Adnani, Muhammad Sohail Afzal, Saira Afzal, Antonella Agodi, Bright Opoku Ahinkorah, Sajjad Ahmad, Muayyad M Ahmad, Tauseef Ahmad, Ali Ahmed, Ayman Ahmed, Haroon Ahmed, Meqdad Saleh Ahmed, Budi Aji, Sreelatha Akkala, Khurshid Alam, Fahad Mashhour Alanezi, Turki M Alanzi, Almaza Albakri, Mohammed Albashtawy, Mohammad T. AlBataineh, Yihun Mulugeta Alemu, Khalid F Alhabib, Hanadi Al Hamad, Abid Ali, Rafat Ali, Liaqat Ali, Beriwan Abdulqadir Ali, Syed Shujait Shujait Ali, Sheikh Mohammad Alif, Syed Mohamed Aljunid, Joseph Uy Almazan, Omar Almidani, Salman Khalifah Al-Sabah, Awais Altaf, Farrukh Jawad Alvi, Nelson Alvis-Guzman, Hassan Alwafi, Hany Aly, Edward Kwabena Ameyaw, Dickson A Amugsi, Ganiyu Adeniyi Amusa, Deanna Anderlini, Pedro Prata Andrade, Tudorel Andrei, Ernoiz Antriyandarti, Saeid Anvari, SALEHA ANWAR, Razique Anwer, Jalal Arabloo, Timur Aripov, Benedetta Armocida, Johan Ärnlöv, Anton A Artamonov, Judie Arulappan, Zatollah Asemi, Tahira Ashraf, Seyyed Shamsadin Athari, Alok Atreya, Getnet Melaku Ayele, Ahmed Y Azzam, Soroush Baghdadi, Sara Bagherieh, Atif Amin Baig, Abdulaziz T. Bako, Ovidiu Constantin Baltatu, Maciej Banach, Palash Chandra Banik, Mehmet Firat Baran, Martina Barchitta, Mainak Bardhan, Till Winfried Bärnighausen, Amadou Barrow, Zarrin Basharat, Sanjay Basu, Abdul-Monim Mohammad Batiha, Emad Behboudi, Diana Fernanda Bejarano Ramirez, Alehegn Bekele, Sefealem Assefa Belay, Uzma Iqbal Belgaumi, Michelle L Bell, Olorunjuwon Omolaja Bello, Akshaya Srikanth Bhagavathula, Vivek Bhat, Jasvinder Singh Bhatti, Gurjit Kaur Bhatti, Virginia Bodolica, Aadam Olalekan Bodunrin, Milad Bonakdar Hashemi, Berrak Bora Basara, Souad Bouaoud, Dejana Braithwaite, Danilo Buonsenso, Florentino Luciano Caetano dos Santos, Carlos A Castañeda-Orjuela, Francieli Cembranel, Chiranjib Chakraborty, Periklis Charalampous, Vijay Kumar Chattu, Ju-Huei Chien, William C S Cho, Sungchul Choi, Bryan Chong, Hitesh Chopra, Dinh-Toi Chu, Eric Chung, Zinhle Cindi, Rafael M Claro, Alyssa Columbus, Haley Comfort, Joao Conde, Michael H Criqui, Natália Cruz-Martins, Zhaoli Dai, Giovanni Damiani, Aso Mohammad Darwesh, Saswati Das, Claudio Alberto Dávila-Cervantes, Aklilu Tamire Debele, Shayom

Debopadhaya, Ivan Delgado-Enciso, Berecha Hundessa Demessa, Kebede Deribe, Nikolaos Dervenis, Hardik Dineshbhai Desai, Rupak Desai, Vinoth Gnana Chellaiyan Devanbu, Vishal R Dhulipala, Diana Dias da Silva, Daniel Diaz, Michael J Diaz, M Ashworth Dirac, Thao Huynh Phuong Do, Thanh Chi Do, Leila Doshmangir, Wendel Mombaque dos Santos, Robert Kokou Dowou, Haneil Larson Dsouza, John Dube, Joe Duprey, Andre Rodrigues Duraes, Senbagam Duraisamy, Paulina Agnieszka Dzianach, Michael Ekholuenetale, Legesse Tesfaye Elilo, Mohammed Elshaer, Amir Emami Zeydi, Sharareh Eskandarieh, Adeniyi Francis Fagbamigbe, Andre Faro, Abidemi Omolara Fasanmi, Ali Fatehizadeh, Seyed-Mohammad Fereshtehnejad, Artem Alekseevich Fomenkov, Kayode Raphael Fowobaje, Takeshi Fukumoto, John E Fuller, Peter Andras Gaal, Muktar A. Gadanya, Yaseen Galali, Mohammad Arfat Ganiyani, Tesfay B B Gebremariam, Simona Roxana Georgescu, Peter W Gething, Arun Digambarrao Ghuge, Alem Girmay, Laszlo Göbölös, Ali Golchin, Mahaveer Golechha, Pouya Goleij, Sameer Vali Gopalani, Houman Goudarzi, Shi-Yang Guan, Mesay Dechasa Gudeta, Vijai Kumar Gupta, Manoj Kumar Gupta, Sapna Gupta, Bhawna Gupta, Rabih Halwani, Nadia M Hamdy, Josep Maria Haro, Mohammad Jahid Hasan, Soheil Hassanipour, Hadi Hassankhani, Bartosz Helfer, Claudiu Herteliu, Kamran Hessami, Nguyen Quoc Hoan, Nobuyuki Horita, Md Mahbub Hossain, Mehdi Hosseinzadeh, Chengxi Hu, Javid Hussain, Nawfal R Hussein, Hong-Han Huynh, Segun Emmanuel Ibitoye, Olayinka Stephen Ilesanmi, Nahlah Elkudssiah Ismail, Gaetano Isola, Mahalaxmi Iyer, Linda Merin J, Khushleen Jaggi, Nader Jahanmehr, Haitham Jahrami, Nityanand Jain, Mihajlo Jakovljevic, Tahereh Javaheri, Sathish Kumar Jayapal, Shubha Jayaram, Heng Jiang, Jost B. Jonas, Tamas Joo, Charity Ehimwenma Joshua, Farahnaz Joukar, Jacek Jerzy Jozwiak, Mikk Jürisson, Billingsley Kaambwa, Zubair Kabir, Leila R Kalankesh, Tanuj Kanchan, Rami S. Kantar, Mehrdad Karajizadeh, Manoochehr Karami, Faizan Zaffar Kashoo, Nicholas J Kassebaum, Evie Shoshannah Kendal, Himanshu Khajuria, Nauman Khalid, Faham Khamesipour, Maseer Khan, M Nuruzzaman Khan, Ikramullah Khan, Moien AB Khan, Khaled Khatab, Feriha Fatima Khidri, Min Seo Kim, Adnan Kisa, Sezer Kisa, Gerbrand Koren, Oleksii Korzh, Soewarta Kosen, Sindhura Lakshmi Koulmane Laxminarayana, Kewal Krishan, Varun Krishna, Vijay Krishnamoorthy, Barthelemy Kuate Defo, Burcu Kucuk Bicer, Ilari Kuitunen, Harish Kumar, Kunle Rotimi Kunle, Asep Kusnali, Dian Kusuma, Muhammad Awwal Ladan, Chandrakant Lahariya, Daphne Teck Ching Lai, Dharmesh Kumar Lal, Judit Lám, Iván Landires, Savita Lasrado, Kamaluddin Latief, Kaveh Latifinaibin, Trang Diep Thanh Le, Nhi Huu Hanh Le, Caterina Ledda, Seung Won Lee, Munjae Lee, An Li, Stephen S Lim, Stefan Listl, Jue Liu, Gang Liu, Xuefeng Liu, Erand Llanaj, Rubén López-Bueno, László Lorenzovici, Paulo A. Lotufo, Zheng Feei Ma, Mohammed Magdy Abd El Razek, Azzam A Maghazachi, Jeadran N. Malagón-Rojas, Elaheh Malakan Rad, Kashish Malhotra, Deborah Carvalho Malta, Abdullah A Mamun, Yasaman Mansoori, Borhan Mansouri, Mohammad Ali Mansournia, Joemer C. Maravilla, Abdoljalal Marjani, Francisco Rogerlândio Martins-Melo, Sharmeen Maryam, Roy Rillera Marzo, Alireza Masoudi, Jishanth Mattumpuram, Andrea Maugeri, Erin A May, Maryam Mazaheri, Anna Laura Wensel McKowen, Entezar Mehrabi Nasab, Tesfahun Mekene Meto, Walter Mendoza, Ritesh G Menezes, Sultan Ayoub Meo, Atte Meretoja, Sachith Mettananda, Kukulege Chamila Dinushi Mettananda, Irmina Maria Michalek, Ted R Miller, Le Huu Nhat Minh, Erkin M Mirrakhimov, Chaitanya Mittal, Babak Moazen, Soheil Mohammadi, Abdollah Mohammadian-Hafshejani, Mustapha Mohammed, Shafiu Mohammed, Salahuddin Mohammed, Ali H Mokdad, Peyman Mokhtarzadehazar, Lorenzo Monasta, Mohammad Ali Moni, Maryam Moradi, Yousef Moradi, Shane Douglas Morrison, Jakub Morze, Sumaira Mubarik, Francesk Mulita, Christopher J L Murray, Fungai Musaigwa, Ana-Maria Musina, Saravanan Muthupandian, Ahamarshan Jayaraman Nagarajan, Gabriele Nagel, Ganesh R Naik, Mukhammad David Naimzada, Sreenivas Narasimha Swamy, Bruno Ramos Nascimento, Abdallah Y Naser, Mohammad Javad Nasiri, Zuhair S Natto, Biswa Prakash Nayak, Vinod C Nayak, Ionut Negoi, Ruxandra Irina Negoi, Henok Biresaw Netsere, Josephine W. Ngunjiri, Van Thanh Nguyen, Dang H Nguyen, QuynhAnh P Nguyen, Robina Khan Niazi, Taxiarchis Konstantinos Nikolouzakis, Amin Reza Nikpoor, Chukwudi A Nnaji, Lawrence Achilles Nnyanzi, Shuhei Nomura, Mamoona Noreen, Dieta Nurrika, Bogdan Oancea, Kehinde O Obamiro, Ismail A. Odetokun, Nkechi Martina Odogwu,

Ayodipupo Sikiru Oguntade, Osaretin Christabel Okonji, Andrew T Olagunju, Bolajoko Olubukunola Olusanya, Jacob Olusegun Olusanya, Hany A Omar, Obinna E Onwujekwe, Uchechukwu Levi Osuagwu, Adrian Otoiu, Stanislav S Otstavnov, Amel Ouyahia, Mayowa O Owolabi, Mahesh Padukudru P A, Mohammad Taha Pahlevan Fallahy, Adrian Pana, Paramjot Panda, Songhomitra Panda-Jonas, Anca Mihaela Pantea Stoian, Romil R Parikh, Seoyeon Park, Ashwaghosha Parthasarathi, Hemal M Patel, Jay Patel, Shankargouda Patil, Uttam Paudel, Mihaela Paun, Veincent Christian Filipino Pepito, Gavin Pereira, Arokiasamy Perianayagam, Simone Perna, Hoang Tran Pham, Anil K. Philip, Daniela Pierannunzio, David M Pigott, Peter Pollner, Ramesh Poluru, Maarten J Postma, Naeimeh Pourtaheri, Sergio I Prada, Jagadeesh Puvvula, Fakher Rahim, Masoud Rahmati, Prasanna Ram, Shakthi Kumaran Ramasamy, Juwel Rana, Chhabi Lal Ranabhat, Nemanja Rancic, Amey Rane, Chythra R Rao, Vahid Rashedi, Sina Rashedi, Zubair Ahmed Ratan, Giridhara Rathnaiah Babu, Santosh Kumar Rauniyar, Nakul Ravikumar, Salman Rawaf, Reza Rawassizadeh, Bhageerathy Reshmi, Aida Rezaei nejad, Hannah Elizabeth Robinson-Oden, Jefferson Antonio Buendia Rodriguez, Luca Ronfani, Himanshu Sekhar Rout, Priyanka Roy, Enrico Rubagotti, Tilleye Runghien, Aly M A Saad, Zahra Saadatian, Siamak Sabour, Basema Saddik, Bashdar Abuzed Sadee, Ehsan Sadeghi, Umar Saeed, Sher Zaman Safi, Manika Saha, Zahra Saif, Joseph W Sakshaug, Afeez Abolarinwa Salami, Marwa Rashad Salem, Sara Samadzadeh, Vijaya Paul Samuel, Abdallah M Samy, Nima Sanadgol, Francesca Sanna, Milena M. Santric-Milicevic, Sivan Yegnanarayana Iyer Saraswathy, Babak Saravi, Brijesh Sathian, Anudeep Sathyanarayan, Md Abu Sayeed, David C Schwebel, Subramanian Senthilkumaran, Edson Serván-Mori, Yashendra Sethi, Jaffer Shah, Ahmed Shaikh, Masood Ali Shaikh, Mehran Shams-Beyranvand, Mohammad Anas Shamsi, Abhishek Shankar, Mohammed Shannawaz, Medha Sharath, Javad Sharifi-Rad, Vishal Sharma, Rajesh Sharma, Rajesh P. Shastry, David H Shaw, Maryam Shayan, Pavanchand H Shetty, Peilin Shi, Aminu Shittu, Farhad Shokraneh, Sina Shool, Kanwar Hamza Shuja, Jasvinder A. Singh, Abhinav Singh, Sameh S M Soliman, Yonatan Solomon, Michael Spartalis, Chandrashekhar T Sreeramareddy, Muhammad Haroon Stanikzai, Muhammad Suleman, Rizwan Suliankatchi Abdulkader, Abida Sultana, Chandan Kumar Swain, Lukasz Szarpak, Mindy D Szeto, Miklós Szócska, Payam Tabaee Damavandi, Rafael Tabarés-Seisdedos, Shima Tabatabai, Karen M Tabb, Mohammad Tabish, Ardeshir Tajbakhsh, Mircea Tampa, Ker-Kan Tan, Mengistie Kassahun Tariku, Mohamad-Hani Temsah, Pugazhenthan Thangaraju, Nihal Thomas, Marcos Roberto Tovani-Palone, Jasmine T Tran, Indang Trihandini, Samuel Joseph Tromans, Aristidis Tsatsakis, Sana Ullah, Srikanth Umakanthan, Bhaskaran Unnikrishnan, Era Upadhyay, Jibrin Sammani Usman, Jef Van den Eynde, Shoban Babu Varthya, Tommi Juhani Vasankari, Balachandar Vellingiri, Massimiliano Veroux, Georgios-Ioannis Verras, Sergey Konstantinovitch Vladimirov, Vasily Vlassov, Bay Vo, Simona Ruxandra Volovat, Theo Vos, Yasir Waheed, Elias Bekele Wakwoya, Shu Wang, Denny Wang, Stefanie Watson, Kosala Gayan Weerakoon, Ronny Westerman, Peter Willeit, Felicia Wu, Juan Xia, Hong Xiao, Suowen Xu, Gelin Xu, Ali Yadollahpour, Shirin Yaghoobpoor, Tina Yaghoobpour, Sajad Yaghoubi, Pengpeng Ye, Renjulal Yesodharan, Paul Yip, Dong Keon Yon, Naohiro Yonemoto, Mustafa Z Younis, Chuanhua Yu, Yong Yu, Mikhail Sergeevich Zastrozhin, Magdalena Zielińska, Zhiyong Zou.

Developing methods or computational machinery

Samrawit Shawel Abebe, Rufus Adesoji Adedoyin, Olatunji O Adetokunboh, Qorinah Estiningtyas Sakilah Adnani, Saira Afzal, Austin J Ahlstrom, Mohammed Albashtawy, Liaqat Ali, Jalal Arabloo, Aleksandr Y Aravkin, Judie Arulappan, Ahmed Y Azzam, Akshaya Srikanth Bhagavathula, Natalia V Bhattacharjee, Aadam Olalekan Bodunrin, Souad Bouaoud, Fan Cao, Shujin Cao, Julian Chalek, Hitesh Chopra, Kaleb Coberly, Haley Comfort, Aso Mohammad Darwesh, Hardik Dineshbhai Desai, Thanh Chi Do, Joe Duprey, Adeniyi Francis Fagbamigbe, Samuel Farmer, Ali Fatehizadeh, Peter W Gething, Alem Girmay, Manoj Kumar Gupta, Simon I Hay, Mohammad Heidari, Mehdi Hosseinzadeh, Hong-Han Huynh, Gaetano Isola, Linda Merin J, Tahereh Javaheri, Sathish Kumar Jayapal, Faizan Zaffar Kashoo, Nicholas J Kassebaum,

Julie Sojin Kim, Adnan Kisa, Chandrakant Lahariya, Nhi Huu Hanh Le, An Li, Mohammed Magdy Abd El Razek, Yasaman Mansoori, Borhan Mansouri, Kelsey Lynn Maass, Alireza Masoudi, Erin A May, Le Huu Nhat Minh, Abdollah Mohammadian-Hafshejani, Ali H Mokdad, Mohammad Ali Moni, Yousef Moradi, Rohith Motappa, Francesk Mulita, Christopher J L Murray, Henok Biresaw Netsere, Josephine W. Ngunjiri, Van Thanh Nguyen, Dang H Nguyen, Andrew T Olagunju, Michal Ordak, Yaz Ozten, Adrian Pana, Spencer A Pease, Hoang Tran Pham, Hadi Raeisi Shahraki, Cat Raggi, Chhabi Lal Ranabhat, Santosh Kumar Rauniyar, Reza Rawassizadeh, Himanshu Sekhar Rout, Enrico Rubagotti, Susan Fred Rumisha, Tilleye Runghien, Umar Saeed, Zahra Saif, Abdallah M Samy, Austin E Schumacher, Yashendra Sethi, Mohammad Anas Shamsi, Javad Sharifi-Rad, Vishal Sharma, David H Shaw, Amanda E Smith, Georgia Smith, Reed J D Sorensen, Michael Spartalis, Muhammad Haroon Stanikzai, Muhammad Suleman, Chandan Kumar Swain, Nicholas Alexander Verghese, Bay Vo, Stein Emil Vollset, Ronny Westerman, Ali Yadollahpour, Chun-Wei Yuan, Milad Zandi, Ghazal G Z Zandieh.

Providing critical feedback on methods or results

Amirali Aali, Yohannes Habtegiorgis Abate, Rouzbeh Abbasgholizadeh, Hedayat Abbastabar, Samar Abd ElHafeez, Sherief Abd-Elsalam, Mohammad Abdollahi, Meriem Abdoun, Auwal Abdullahi, Mesfin Abebe, Samrawit Shawel Abebe, Hassan Abolhassani, Meysam Abolmaali, Mohamed Abouzid, Girma Beressa Aboye, Lucas Guimarães Abreu, Woldu Aberhe Abrha, Michael R M Abrigo, Dariush Abtahi, Hasan Abualruz, Bilyaminu Abubakar, Eman Abu-Gharbieh, Tadele Girum Adal, Mesafint Molla Adane, Oluwafemi Atanda Adeagbo Adeagbo, Rufus Adesoji Adedoyin, Victor Adekanmbi, Bashir Aden, Abiola Victor Adepoju, Olatunji O Adetokunboh, Juliana Bunmi Adetunji, Daniel Adedayo Adeyinka, Olorunsola Israel Adeyomoye, Qorinah Estiningtyas Sakilah Adnani, Saryia Adra, Rotimi Felix Afolabi, Shadi Afyouni, Muhammad Sohail Afzal, Saira Afzal, Shahin Aghamiri, Antonella Agodi, Williams Agyemang-Duah, Bright Opoku Ahinkorah, Sajjad Ahmad, Muayyad M Ahmad, Tauseef Ahmad, Danish Ahmad, Firdos Ahmad, Aqeel Ahmad, Ayman Ahmed, Haroon Ahmed, Syed Anees Ahmed, Luai A. Ahmed, Budi Aji, Gizachew Taddesse Akalu, Hossein Akbarialiabad, Rufus Olusola Akinyemi, Sreelatha Akkala, Tareq Mohammed Ali AL-Ahdal, Samer O. Alalalmeh, Ziyad Al-Aly, Khurshid Alam, Rasmieh Mustafa Al-amer, Fahad Mashhour Alanezi, Turki M Alanzi, Almaza Albakri, Mohammed Albashtawy, Mohammad T. AlBataineh, Sharifullah Alemi, Hediyeh Alemi, Yihun Mulugeta Alemu, Adel Ali Saeed Al-Gheethi, Khalid F Alhabib, Hanadi Al Hamad, Syed Mahfuz Al Hasan, Robert Kaba Alhassan, Mohammed Usman Ali, Abid Ali, Rafat Ali, Liaqat Ali, Syed Shujait Shujait Ali, Sheikh Mohammad Alif, Mohammad Aligol, Mehran Alijanzadeh, Mohammad A M Aljasir, Syed Mohamed Aljunid, Sabah Al-Marwani, Joseph Uy Almazan, Hesham M Al-Mekhlafi, Omar Almidani, Mahmoud A. Alomari, Basem Al-Omari, Mohammad Al Qadire, Jaber S Alqahtani, Ahmed Yaseen Alqutaibi, Rajaa M Al-Raddadi, Salman Khalifah Al-Sabah, Awais Altaf, Jaffar A. Al-Tawfiq, Khalid A Altirkawi, Nelson Alvis-Guzman, Hassan Alwafi, Yaser Mohammed Al-Worafi, Hany Aly, Safwat Aly, Karem H Alzoubi, Edward Kwabena Ameyaw, Tarek Tawfik Amin, Alireza Amindarolzarbi, Mostafa Amini-Rarani, Sohrab Amiri, Dickson A Amugsi, Ganiyu Adeniyi Amusa, Robert Ancuceanu, Deanna Anderlini, Pedro Prata Andrade, Tudorel Andrei, Catalina Liliana Andrei, Alireza Ansari-Moghaddam, Ernoiz Antriyandarti, SALEHA ANWAR, Razique Anwer, Anayochukwu Edward Anyasodor, Jalal Arabloo, Elshaimaa A Arafa, Mosab Arafat, Timur Aripov, Mesay Arkew, Benedetta Armocida, Mahwish Arooj, Anton A Artamonov, Judie Arulappan, Raphael Taiwo Aruleba, Ashokan Arumugam, Mohsen Asadi-Lari, Saeed Asgary, Mohammad Asghari-Jafarabadi, Mubarek Yesse Ashemo, Tahira Ashraf, Muhammad Ashraf, Marvellous O Asika, Seyyed Shamsadin Athari, Maha Moh'd Wahbi Atout, Alok Atreya, Avinash Aujayeb, Abolfazl Avan, Amlaku Mulat Aweke, Getnet Melaku Ayele, Sina Azadnajafabad, Rui M S Azevedo, Ahmed Y Azzam, Muhammad Badar, Ashish D Badiye, Soroush Baghdadi, Nasser Bagheri, Sara Bagherieh, Najmeh Bahmanziari, Ruhai Bai, Atif Amin Baig, Jennifer L Baker, Abdulaziz T. Bako, Ravleen Kaur Bakshi, Madhan Balasubramanian, Ovidiu Constantin Baltatu,

Maciej Banach, Soham Bandyopadhyay, Palash Chandra Banik, Biswajit Banik, Hansi Bansal, Mehmet Firat Baran, Martina Barchitta, Mainak Bardhan, Till Winfried Bärnighausen, Hiba Jawdat Barqawi, Amadou Barrow, Sandra Barteit, Zarrin Basharat, Asma'u I J Bashir, Hameed Akande Bashiru, Afisu Basiru, João Diogo Basso, Sanjay Basu, Abdul-Monim Mohammad Batiha, Kavita Batra, Bernhard T Baune, Mohsen Bayati, Tahmina Begum, Emad Behboudi, Amir Hossein Behnoush, Diana Fernanda Bejarano Ramirez, Alehegn Bekele, Sefealem Assefa Belay, Uzma Iqbal Belgaumi, Michelle L Bell, Olorunjuwon Omolaja Bello, Apostolos Beloukas, Isabela M Bensenor, Zombor Berezvai, Alemshet Yirga Berhie, Amiel Nazer C Bermudez, Paulo J G Bettencourt, Akshaya Srikanth Bhagavathula, Nikha Bhardwaj, Pankaj Bhardwaj, Vivek Bhat, Natalia V Bhattacharjee, Jasvinder Singh Bhatti, Gurjit Kaur Bhatti, Manpreet S Bhatti, Rajbir Bhatti, Atanu Biswas, Raaj Kishore Biswas, Veera R Bitra, Micheal Kofi Boachie, Virginia Bodolica, Aadam Olalekan Bodunrin, Eyob Ketema Bogale, Milad Bonakdar Hashemi, Souad Bouaoud, Dejana Braithwaite, Nicholas J K Breitborde, Dana Bryazka, Norma B Bulamu, Danilo Buonsenso, Katrin Burkart, Richard A Burns, Yasser Bustanji, Zahid A Butt, Nadeem Shafique Butt, Florentino Luciano Caetano dos Santos, Ismael R Campos-Nonato, Fan Cao, Angelo Capodici, Carlos A Castañeda-Orjuela, Giulio Castelpietra, Luca Cegolon, Francieli Cembranel, Ester Cerin, Yaacoub Chahine, Chiranjib Chakraborty, Periklis Charalampous, Vijay Kumar Chattu, Malizgani Paul Chavula, An-Tian Chen, Haowei Chen, William C S Cho, Sungchul Choi, Bryan Chong, Hitesh Chopra, Sonali Gajanan Choudhari, Devasahayam J Christopher, Dinh-Toi Chu, Isaac Sunday Chukwu, Eric Chung, Sheng-Chia Chung, Zinhle Cindi, Iolanda Cioffi, Raffaela Ciuffreda, Alyssa Columbus, Haley Comfort, Joao Conde, Michael H Criqui, Natália Cruz-Martins, Silvia Magali Cuadra-Hernández, Mario D'Oria, Omid Dadras, Zhaoli Dai, Giovanni Damiani, Aso Mohammad Darwesh, Jai K Das, Saswati Das, Mohsen Dashti, Anna Dastiridou, Claudio Alberto Dávila-Cervantes, Kairat Davletov, Aklilu Tamire Debele, Shayom Debopadhaya, Ivan Delgado-Enciso, Dessalegn Demeke, Berecha Hundessa Demessa, Xinlei Deng, Kebede Deribe, Nikolaos Dervenis, Hardik Dineshbhai Desai, Rupak Desai, Vinoth Gnana Chellaiyan Devanbu, Arkadeep Dhali, Kuldeep Dhama, Meghnath Dhimal, Vishal R Dhulipala, Diana Dias da Silva, Daniel Diaz, Michael J Diaz, Adriana Dima, Delaney D Ding, M Ashworth Dirac, Thao Huynh Phuong Do, Thanh Chi Do, Leila Doshmangir, Wendel Mombaque dos Santos, Robert Kokou Dowou, Viola Dsouza, Haneil Larson Dsouza, John Dube, Senbagam Duraisamy, Oyewole Christopher Durojaiye, Sulagna Dutta, Arkadiusz Marian Dziedzic, Alireza Ebrahimi, Hisham Atan Edinur, Kristina Edvardsson, Ferry Efendi, Terje Andreas Eikemo, Michael Ekholuenetale, Noha Mousaad Elemam, Ghada Metwally Tawfik ElGohary, Muhammed Elhadi, Legesse Tesfaye Elilo, Omar Abdelsadek Abdou Elmeligy, Mohamed A Elmonem, Mohammed Elshaer, Ibrahim Elsohaby, Amir Emami Zeydi, Luchuo Engelbert Bain, Sharareh Eskandarieh, Farshid Etaee, Natalia Fabin, Adeniyi Francis Fagbamigbe, Saman Fahimi, Aliasghar Fakhri-Demeshghieh, Luca Falzone, Ali Faramarzi, MoezAlIslam Ezzat Mahmoud Faris, Samuel Farmer, Andre Faro, Abidemi Omolara Fasanmi, Ali Fatehizadeh, Pooria Fazeli, Valery L. Feigin, Seyed-Mohammad Fereshtehnejad, Abdullah Hamid Feroze, Pietro Ferrara, Getahun Fetensa, Irina Filip, Florian Fischer, Joanne Flavel, Nataliya A. Foigt, Morenike Oluwatoyin Folayan, Artem Alekseevich Fomenkov, Matteo Foschi, Kayode Raphael Fowobaje, Kate Louise Francis, Alberto Freitas, Takeshi Fukumoto, Peter Andras Gaal, Muktar A. Gadanya, Abhay Motiramji Gaidhane, Yaseen Galali, Aravind P Gandhi, Balasankar Ganesan, Mohammad Arfat Ganiyani, M.A. Garcia-Gordillo, Naval Garg, Rupesh K. Gautam, Federica Gazzelloni, Semiu Olatunde Gbadamosi, Miglas W Gebregergis, Mesfin Gebrehiwot, Tesfay Brhane Gebremariam, Tesfay B B Gebremariam, Teferi Gebru Gebremeskel, Yohannes Fikadu Geda, Simona Roxana Georgescu, Urge Gerema, Habtamu Geremew, Motuma Erena Getachew, MohammadReza Ghasemi, Afsaneh Ghasemzadeh, Fariba Ghassemi, Ramy Mohamed Ghazy, Sailaja Ghimire, Asadollah Gholamian, Ali Gholamrezanezhad, Arun Digambarrao Ghuge, Tiffany K Gill, Matteo Giorgi, Alem Girmay, James C Glasbey, Laszlo Göbölös, Amit Goel, Ali Golchin, Mahaveer Golechha, Alessandra C Goulart, Anmol Goyal, Simon Matthew Graham, Michal Grivna, Shi-Yang Guan, Mohammed Ibrahim Mohialdeen Gubari, Mesay Dechasa Gudeta, Stefano Guicciardi, David Gulisashvili, Damitha Asanga

Gunawardane, Cui Guo, Vijai Kumar Gupta, Manoj Kumar Gupta, Sapna Gupta, Anish Kumar Gupta, Bhawna Gupta, Veer Bala Gupta, Vivek Kumar Gupta, Annie Haakenstad, Farrokh Habibzadeh, Najah R Hadi, Nils Haep, Ramtin Hajibeygi, Sebastian Haller, Rabih Halwani, Randah R Hamadeh, Nadia M Hamdy, Samer Hamidi, Qiuxia Han, Md Nuruzzaman Haque, Josep Maria Haro, Mohammad Jahid Hasan, Ikramul Hasan, Hamidreza Hasani, Md Saquib Hasnain, Ikrama Hassan, Soheil Hassanipour, Hadi Hassankhani, Simon I Hay, Jeffrey J. Hebert, Omar E. Hegazi, Mohammad Heidari, Bartosz Helfer, Mehdi Hemmati, Brenda Yuliana Herrera-Serna, Claudiu Herteliu, Kamran Hessami, Kamal Hezam, Yuta Hiraike, Nguyen Quoc Hoan, Ramesh Holla, Nobuyuki Horita, Md Mahbub Hossain, Mohammad Bellal Hossain Hossain, Hassan Hosseinzadeh, Mehdi Hosseinzadeh, Mihaela Hostiuc, Mohamed Hsairi, Chengxi Hu, M Mamun Huda, Ayesha Humayun, Nawfal R Hussein, Hong-Han Huynh, Bing-Fang Hwang, Segun Emmanuel Ibitoye, Pulwasha Maria Iftikhar, Olayinka Stephen Ilesanmi, Milena D. Ilic, Irena M. Ilic, Mustapha Immurana, Leeberk Raja Inbaraj, Afrin Iqbal, Md. Rabiul Islam, Nahlah Elkudssiah Ismail, Hiroyasu Iso, Gaetano Isola, Masao Iwagami, Mahalaxmi Iyer, Linda Merin J, Jalil Jaafari, Louis Jacob, Farhad Jadidi-Niaragh, Khushleen Jaggi, Nader Jahanmehr, Haitham Jahrami, Nityanand Jain, Akhil Jain, Ammar Abdulrahman Jairoun, Mihajlo Jakovljevic, Elham Jamshidi, Sabzali Javadov, Tahereh Javaheri, Sathish Kumar Jayapal, Shubha Jayaram, Sun Ha Jee, Jayakumar Jeganathan, Anil K Jha, Ravi Prakash Jha, Heng Jiang, Mohammad Jokar, Jost B. Jonas, Tamas Joo, Nitin Joseph, Charity Ehimwenma Joshua, Farahnaz Joukar, Jacek Jerzy Jozwiak, Mikk Jürisson, Vaishali K, Billingsley Kaambwa, Abdulkareem Kabir, Ali Kabir, Zubair Kabir, Hannaneh Kabir, Rizwan Kalani, Leila R Kalankesh, Feroze Kaliyadan, Sanjay Kalra, Rajesh Kamath, Sagarika Kamath, Tanuj Kanchan, Kehinde Kazeem Kanmodi, Sushil Kumar Kansal, Rami S. Kantar, Neeti Kapoor, Mehrdad Karajizadeh, Manoochehr Karami, Ibraheem M Karaye, Faizan Zaffar Kashoo, Nicholas J Kassebaum, Joonas H Kauppila, Foad Kazemi, sara Kazeminia, John H Kempen, Evie Shoshannah Kendal, Kamyab Keshtkar, Mohammad Keykhaei, Himanshu Khajuria, Amirmohammad Khalaji, Nauman Khalid, Anees Ahmed Khalil, Alireza Khalilian, Faham Khamesipour, Ajmal Khan, Mohammad Jobair Khan, Asaduzzaman Khan, Maseer Khan, M Nuruzzaman Khan, Ikramullah Khan, Moien AB Khan, Young-Ho Khang, Shaghayegh Khanmohammadi, Khaled Khatab, Feriha Fatima Khidri, Moein Khormali, Wondwosen Teklesilasie Kidane, Zemene Demelash Kifle, Julie Sojin Kim, Ruth W Kimokoti, Girmay Tsegay Kiross, Adnan Kisa, Sezer Kisa, Ali-Asghar Kolahi, Farzad Kompani, Gerbrand Koren, Oleksii Korzh, Sindhura Lakshmi Koulmane Laxminarayana, Kewal Krishan, Varun Krishna, Vijay Krishnamoorthy, Barthelemy Kuate Defo, Burcu Kucuk Bicer, Mohammed Kuddus, Md Abdul Kuddus, Ilari Kuitunen, Satyajit Kundu, Kunle Rotimi Kunle, Om P Kurmi, Asep Kusnali, Dian Kusuma, Evans F Kyei, Ilias Kyriopoulos, Ben Lacey, Muhammad Awwal Ladan, Chandrakant Lahariya, Daphne Teck Ching Lai, Dharmesh Kumar Lal, Judit Lám, Demetris Lamnisos, Iván Landires, Francesco Lanfranchi, Berthold Langguth, Savita Lasrado, Kamaluddin Latief, Kaveh Latifinaibin, Carlo La Vecchia, Long Khanh Dao Le, Trang Diep Thanh Le, Nhi Huu Hanh Le, Caterina Ledda, Seung Won Lee, Munjae Lee, Yo Han Lee, Gebretsadik Kiros Lema, Elvynna Leong, Temesgen L. Lerango, Xiaopan Li, Ming-Chieh Li, Wei Li, Shanshan Li, Virendra S Ligade, Stephen S Lim, Ro-Ting Lin, Stefan Listl, Xiaofeng Liu, Yuewei Liu, Jue Liu, Gang Liu, Xuefeng Liu, Erand Llanaj, Rubén López-Bueno, László Lorenzovici, Paulo A. Lotufo, Jailos Lubinda, Giancarlo Lucchetti, Zheng Feei Ma, Monika Machoy, Áurea M. Madureira-Carvalho, Mohammed Magdy Abd El Razek, Azzam A Maghazachi, Soleiman Mahjoub, Mansour Adam Mahmoud, Azeem Majeed, Elaheh Malakan Rad, Kashish Malhotra, Iram Malik, Ahmad Azam Malik, Deborah Carvalho Malta, Abdullah A Mamun, Yosef Manla, Yasaman Mansoori, Ali Mansour, Borhan Mansouri, Zeinab Mansouri, Mohammad Ali Mansournia, Joemer C. Maravilla, Mirko Marino, Abdoljalal Marjani, Ramon Martinez-Piedra, Francisco Rogerlândio Martins-Melo, Miquel Martorell, Sharmeen Maryam, Roy Rillera Marzo, Kelsey Lynn Maass, Alireza Masoudi, Jishanth Mattumpuram, Richard James Maude, Andrea Maugeri, Erin A May, Mahsa Mayeli, John J. McGrath, Martin McKee, Anna Laura Wensel McKowen, Steven M McPhail, Rahul Mehra, Kamran Mehrabani-Zeinabad, Entezar Mehrabi Nasab, Tesfahun Mekene Meto, Max Alberto Mendez Mendez-Lopez, Walter Mendoza, Ritesh G Menezes,

Alexios-Fotios A. Mentis, Sultan Ayoub Meo, Mohsen Merati, Tuomo J Meretoja, Atte Meretoja, Abera M Mersha, Tomislav Mestrovic, Pouya Metanat, Sachith Mettananda, Kukulege Chamila Dinushi Mettananda, Laurette Mhlanga, Adquate Mhlanga, Tianyue Mi, Tomasz Miazgowski, Georgia Micha, Irmina Maria Michalek, Ted R Miller, Le Huu Nhat Minh, Erkin M Mirrakhimov, Mizan Kiros Mirutse, Moonis Mirza, Sanjeev Misra, Chaitanya Mittal, Babak Moazen, Nouh Saad Mohamed, Ahmed Ismail Mohamed, Jama Mohamed, Mouhand F H Mohamed, Abdalla Z Mohamed, Sakineh Mohammad-Alizadeh-Charandabi, Soheil Mohammadi, Abdollah Mohammadian-Hafshejani, Mustapha Mohammed, Shafiu Mohammed, Salahuddin Mohammed, Ali H Mokdad, Peyman Mokhtarzadehazar, Hossein Molavi Vardanjani, Sabrina Molinaro, Mohammad Ali Moni, Maryam Moradi, Yousef Moradi, Paula Moraga, Rafael Silveira Moreira, Negar Morovatdar, Elias Mossialos, Rohith Motappa, Amin Mousavi Khaneghah, Christine Mpundu-Kaambwa, Sumaira Mubarik, Lorenzo Muccioli, Francesk Mulita, Kavita Munjal, Efrén Murillo-Zamora, Christopher J L Murray, Fungai Musaigwa, Ana-Maria Musina, Sathish Muthu, Saravanan Muthupandian, Muhammad Muzaffar, Woojae Myung, Ahamarshan Jayaraman Nagarajan, Gabriele Nagel, Pirouz Naghavi, Ganesh R Naik, Gurudatta Naik, Firzan Nainu, Vinay Nangia, Sreenivas Narasimha Swamy, Bruno Ramos Nascimento, Gustavo G Nascimento, Abdallah Y Naser, Mohammad Javad Nasiri, Zuhair S Natto, Javaid Nauman, Muhammad Naveed, Biswa Prakash Nayak, Rawlance Ndejjo, Sabina Onyinye Nduaguba, Hadush Negash, Chernet Tafere Negesse, Ionut Negoi, Ruxandra Irina Negoi, Seyed Aria Nejadghaderi, Chakib Nejjari, Henok Biresaw Netsere, Georges Nguefack-Tsague, Josephine W. Ngunjiri, Van Thanh Nguyen, Dang H Nguyen, Phuong The Nguyen, Robina Khan Niazi, Yeshambel T Nigatu, Taxiarchis Konstantinos Nikolouzakis, Ali Nikoobar, Amin Reza Nikpoor, Chukwudi A Nnaji, Lawrence Achilles Nnyanzi, Efaq Ali Noman, Shuhei Nomura, Mamoona Noreen, Nafise Noroozi, Chisom Adaobi Nri-Ezedi, Mengistu H Nunemo, Dieta Nurrika, Jerry John Nutor, Martin James O'Donnell, Bogdan Oancea, Kehinde O Obamiro, Ismail A. Odetokun, Nkechi Martina Odogwu, Oluwakemi Ololade Odukoya, Ayodipupo Sikiru Oguntade, James Odhiambo Oguta, Akinkunmi Paul Okekunle, Osaretin Christabel Okonji, Andrew T Olagunju, Omotola O. Olasupo, Matthew Idowu Olatubi, Gláucia Maria Moraes Oliveira, Bolajoko Olubukunola Olusanya, Jacob Olusegun Olusanya, Gideon Olamilekan Oluwatunase, Hany A Omar, Goran Latif Omer, Obinna E Onwujekwe, Michal Ordak, Orish Ebere Orisakwe, Verner N. Orish, Doris V. Ortega-Altamirano, Alberto Ortiz, Wael M S Osman, Uchechukwu Levi Osuagwu, Olayinka Osuolale, Adrian Otoiu, Stanislav S Otstavnov, Amel Ouyahia, Mayowa O Owolabi, Mahesh Padukudru P A, Mohammad Taha Pahlevan Fallahy, Feng Pan, Hai-Feng Pan, Adrian Pana, Paramjot Panda, Songhomitra Panda-Jonas, Helena Ullyartha Pangaribuan, Georgios D Panos, Leonidas D Panos, Ioannis Pantazopoulos, Anca Mihaela Pantea Stoian, Romil R Parikh, Seoyeon Park, Ashwaghosha Parthasarathi, Ava Pashaei, Roberto Passera, Jay Patel, Shankargouda Patil, Dimitrios Patoulias, Uttam Paudel, Mihaela Paun, Spencer A Pease, Amy E Peden, Paolo Pedersini, Minjin Peng, Veincent Christian Filipino Pepito, Prince Peprah, Gavin Pereira, Arokiasamy Perianayagam, Richard G. Pestell, Fanny Emily Petermann-Rocha, Hoang Tran Pham, Anil K. Philip, Manon Pigeolet, David M Pigott, Evgenii Plotnikov, Peter Pollner, Ramesh Poluru, Maarten J Postma, Ghazaleh Pourali, Akram Pourshams, Disha Prabhu, Sergio I Prada, Pranil Man Singh Pradhan, Manya Prasad, Akila Prashant, Bharathi M Purohit, Jagadeesh Puvvula, Nameer Hashim Qasim, Ibrahim Qattea, Deepthi R, Mehrdad Rabiee Rad, Amir Radfar, Venkatraman Radhakrishnan, Pourya Raee, Hadi Raeisi Shahraki, Alireza Rafiei, Seyedeh Niloufar Rafiei Alavi, Pankaja Raghav Raghav, Fakher Rahim, Md Jillur Rahim, Muhammad Aziz Rahman, Md. Mosfequr Rahman, Mosiur Rahman, Vahid Rahmanian, Masoud Rahmati, Niloufar Rahnavard, Pramila Rai, Diego Raimondo, Ali Rajabpour-Sanati, Prashant Rajput, Prasanna Ram, Shakthi Kumaran Ramasamy, Kritika Rana, Juwel Rana, Chhabi Lal Ranabhat, Nemanja Rancic, Amey Rane, Shubham Ranjan, Chythra R Rao, Indu Ramachandra Rao, Vahid Rashedi, Sina Rashedi, Mohammad-Mahdi Rashidi, Azad Rasul, Zubair Ahmed Ratan, Giridhara Rathnaiah Babu, Santosh Kumar Rauniyar, Nakul Ravikumar, Salman Rawaf, David Laith Rawaf, Reza Rawassizadeh, Bharat Rawlley, Murali Mohan Rama Krishna Reddy, Elrashdy Moustafa Mohamed Redwan, Bhageerathy Reshmi, Nazila Rezaei, Mohsen Rezaeian,

Aida Rezaei nejad, Abanoub Riad, Mavra A Riaz, Jennifer Rickard, Reza Rikhtegar, Célia Fortuna Rodrigues, Jefferson Antonio Buendia Rodriguez, Debby Syahru Romadlon, Himanshu Sekhar Rout, Nitai Roy, Priyanka Roy, Enrico Rubagotti, Tilleye Runghien, Aly M A Saad, Zahra Saadatian, Maha Mohamed Saber-Ayad, Morteza SaberiKamarposhti, Siamak Sabour, Fatos Sada, Basema Saddik, Bashdar Abuzed Sadee, Ehsan Sadeghi, Erfan Sadeghi, Mohammad Reza Saeb, Umar Saeed, Sher Zaman Safi, Manika Saha, Soumya Swaroop Sahoo, Monalisha Sahu, Zahra Saif, Joseph W Sakshaug, Payman Salamati, Afeez Abolarinwa Salami, Mohamed A. Saleh, Marwa Rashad Salem, Sara Samadzadeh, Yoseph Leonardo Samodra, Vijaya Paul Samuel, Abdallah M Samy, Juan Sanabria, Nima Sanadgol, Milena M. Santric-Milicevic, Sivan Yegnanarayana Iyer Saraswathy, Babak Saravi, Yaser Sarikhani, Tanmay Sarkar, Rodrigo Sarmiento-Suárez, Sachin C Sarode, Gargi Sachin Sarode, Arash Sarveazad, Brijesh Sathian, Thirunavukkarasu Sathish, Anudeep Sathyanarayan, Md Abu Sayeed, Abu Sayeed, Nikolaos Scarmeas, Winfried Schlee, Art Schuermans, David C Schwebel, Falk Schwendicke, Pallav Sengupta, Subramanian Senthilkumaran, Sadaf G Sepanlou, Dragos Serban, Edson Serván-Mori, SeyedAhmad SeyedAlinaghi, Seyed Arsalan Seyedi, Mahan Shafie, Jaffer Shah, Pritik A Shah, Ataollah Shahbandi, Samiah Shahid, Moyad Jamal Shahwan, Ahmed Shaikh, Masood Ali Shaikh, Muhammad Aaqib Shamim, Mehran Shams-Beyranvand, Mohammad Anas Shamsi, Mohd Shanawaz, Abhishek Shankar, Mohammed Shannawaz, Medha Sharath, Sadaf Sharfaei, Amin Sharifan, Javad Sharifi-Rad, Vishal Sharma, Ujjawal Sharma, Rajesh Sharma, Rajesh P. Shastry, Amin Shavandi, Maryam Shayan, Amir Mehdi Shayan, Amr Mohamed Elsayed Shehabeldine, Aziz Sheikh, Rahim Ali Sheikhi, Pavanchand H Shetty, Desalegn Shiferaw, Mika Shigematsu, Rahman Shiri, Aminu Shittu, Velizar Shivarov, Farhad Shokraneh, Sina Shool, Seyed Afshin Shorofi, Kanwar Hamza Shuja, Kerem Shuval, Emmanuel Edwar Siddig, Luís Manuel Lopes Rodrigues Silva, Soraia Silva, João Pedro Silva, Biagio Simonetti, Jasvinder A. Singh, Abhinav Singh, Md Shahjahan Siraj, Amanda E Smith, Chandan S N, Bogdan Socea, Hamidreza Soleimani, Sameh S M Soliman, Yonatan Solomon, Yimeng Song, Reed J D Sorensen, Michael Spartalis, Chandrashekhar T Sreeramareddy, Vijay Kumar Srivastava, Muhammad Haroon Stanikzai, Antonina V Starodubova, Simona Cătălina Stefan, Mark A Stokes, Vetriselvan Subramaniyan, Muhammad Suleman, Rizwan Suliankatchi Abdulkader, Abida Sultana, Jing Sun, Chandan Kumar Swain, Bryan L. Sykes, Lukasz Szarpak, Mindy D Szeto, Miklós Szócska, Payam Tabaee Damavandi, Rafael Tabarés-Seisdedos, Ozra Tabatabaei Malazy, Seyed-Amir Tabatabaeizadeh, Shima Tabatabai, Karen M Tabb, Mohammad Tabish, Moslem Taheri Soodejani, Jabeen Taiba, Iman M. Talaat, Mircea Tampa, Jacques Lukenze Tamuzi, Ker-Kan Tan, Haosu Tang, Derbie Alemu DA Tareke, Mengistie Kassahun Tariku, Seyed Mohammad Tavangar, Mohamad-Hani Temsah, Reem Mohamad Hani Temsah, Masayuki Teramoto, Dufera Rikitu Terefa, Enoch Teye-Kwadjo, Ramna Thakur, Pugazhenthan Thangaraju, Rekha Thapar, Samar Tharwat, Rasiah Thayakaran, Nihal Thomas, Jansje Henny Ver Ticoalu, Tenaw Yimer Tiruye, Mariya Vladimirovna Titova, Marcello Tonelli, Marcos Roberto Tovani-Palone, Eugenio Traini, Nghia Minh Tran, Jasmine T Tran, Indang Trihandini, Samuel Joseph Tromans, Thien Tan Tri Tai Truyen, Evangelia Eirini Tsermpini, Munkhtuya Tumurkhuu, Stefanos Tyrovolas, Sayed Mohammad Nazim Uddin, Arit Udoh, Saeed Ullah, Sana Ullah, Srikanth Umakanthan, Chukwuma David Umeokonkwo, Bhaskaran Unnikrishnan, Era Upadhyay, Jibrin Sammani Usman, Seyed Mohammad Vahabi, Rohollah Valizadeh, Jef Van den Eynde, Orsolya Varga, Priya Vart, Shoban Babu Varthya, Balachandar Vellingiri, Deneshkumar Venugopal, Madhur Verma, Massimiliano Veroux, Georgios-Ioannis Verras, Dominique Vervoort, Jorge Hugo Villafañe, Manish Vinayak, Francesco S Violante, Mukesh Vishwakarma, Bay Vo, Stein Emil Vollset, Simona Ruxandra Volovat, Theo Vos, Isidora S Vujcic, Hatem A Wafa, Yasir Waheed, Elias Bekele Wakwoya, Yuan-Pang Wang, Yanzhong Wang, Cong Wang, Shu Wang, Fang Wang, Paul Ward, Emebet Gashaw Wassie, Kosala Gayan Weerakoon, Yi Feng Wen, Ronny Westerman, Nuwan Darshana Wickramasinghe, Dakshitha Praneeth Wickramasinghe, Peter Willeit, Yohannes Addisu Wondimagegene, Felicia Wu, Juan Xia, Hong Xiao, Suowen Xu, Xiaoyue Xu, Gelin Xu, Ali Yadollahpour, Shirin Yaghoobpoor, Tina Yaghoobpour, Zwanden Sule Yahaya, Danting Yang, Yuichiro Yano, Habib Yaribeygi, Pengpeng Ye, Renjulal Yesodharan, Subah Abderehim Yesuf, Saber Yezli,

Amanuel Yigezu, Dong Keon Yon, Naohiro Yonemoto, Yuyi You, Mustafa Z Younis, Zabihollah Yousefi, Chuanhua Yu, Yong Yu, Chun-Wei Yuan, Nima Zafari, Fathiah Zakham, Nazar Zaki, Giulia Zamagni, Ghazal G Z Zandieh, Moein Zangiabadian, Mikhail Sergeevich Zastrozhin, Haijun Zhang, Yunquan Zhang, Meixin Zhang, Chenwen Zhong, Juexiao Zhou, Bin Zhu, Lei Zhu, Magdalena Zielińska, Samer H. Zyoud.

Drafting the work or revising it critically for important intellectual content

Amirali Aali, Yohannes Habtegiorgis Abate, Mohammadreza Abbasian, Mohsen Abbasi-Kangevari, Hedayat Abbastabar, Samar Abd ElHafeez, Sherief Abd-Elsalam, Mohammad Abdollahi, Mohammad-Amin Abdollahifar, Auwal Abdullahi, Mesfin Abebe, Samrawit Shawel Abebe, Olumide Abiodun, Hassan Abolhassani, Mohamed Abouzid, Girma Beressa Aboye, Lucas Guimarães Abreu, Hasan Abualruz, Eman Abu-Gharbieh, Niveen ME Abu-Rmeileh, Tadele Girum Adal, Mesafint Molla Adane, Oluwafemi Atanda Adeagbo Adeagbo, Rufus Adesoji Adedoyin, Victor Adekanmbi, Abiola Victor Adepoju, Olatunji O Adetokunboh, Juliana Bunmi Adetunji, Daniel Adedayo Adeyinka, Olorunsola Israel Adeyomoye, Qorinah Estiningtyas Sakilah Adnani, Saryia Adra, Rotimi Felix Afolabi, Saira Afzal, Antonella Agodi, Bright Opoku Ahinkorah, Muayyad M Ahmad, Danish Ahmad, Firdos Ahmad, Ali Ahmed, Ayman Ahmed, Syed Anees Ahmed, Luai A. Ahmed, Marjan Ajami, Hossein Akbarialiabad, Rufus Olusola Akinyemi, Mohammed Ahmed Akkaif, Samer O. Alalalmeh, Tariq A. Alalwan, Khurshid Alam, Rasmieh Mustafa Al-amer, Almaza Albakri, Mohammed Albashtawy, Mohammad T. AlBataineh, Hediyeh Alemi, Yihun Mulugeta Alemu, Ayman Al-Eyadhy, Khalid F Alhabib, Noora Alhajri, Fadwa Alhalaiqa Naji Alhalaiqa, Robert Kaba Alhassan, Mohammed Usman Ali, Abid Ali, Syed Shujait Shujait Ali, Sheikh Mohammad Alif, Mohammad Aligol, Omar Almidani, Mahmoud A. Alomari, Mohammad Al Qadire, Jaber S Alqahtani, Ahmed Yaseen Alqutaibi, Jaffar A. Al-Tawfiq, Deborah Oyine Aluh, Nelson Alvis-Guzman, Hassan Alwafi, Yaser Mohammed Al-Worafi, Hany Aly, Safwat Aly, Karem H Alzoubi, Tarek Tawfik Amin, Alireza Amindarolzarbi, Mostafa Amini-Rarani, Sohrab Amiri, Irene Gyamfuah Ampomah, Dickson A Amugsi, Ganiyu Adeniyi Amusa, Robert Ancuceanu, Deanna Anderlini, Pedro Prata Andrade, Tudorel Andrei, Catalina Liliana Andrei, Abhishek Anil, Sneha Anil, Adnan Ansar, Ernoiz Antriyandarti, SALEHA ANWAR, Anayochukwu Edward Anyasodor, Jalal Arabloo, Razman Arabzadeh Bahri, Elshaimaa A Arafa, Mosab Arafat, Ana Margarida Araújo, Abdulfatai Aremu, Timur Aripov, Mesay Arkew, Benedetta Armocida, Johan Ärnlöv, Mahwish Arooj, Judie Arulappan, Raphael Taiwo Aruleba, Ashokan Arumugam, Mohsen Asadi-Lari, Saeed Asgary, Mona Asghariahmadabad, Muhammad Ashraf, Marvellous O Asika, Seyyed Shamsadin Athari, Maha Moh'd Wahbi Atout, Alok Atreya, Avinash Aujayeb, Abolfazl Avan, Amlaku Mulat Aweke, Getnet Melaku Ayele, Seyed Mohammad Ayyoubzadeh, Sina Azadnajafabad, Rui M S Azevedo, Ahmed Y Azzam, Muhammad Badar, Ashish D Badiye, Soroush Baghdadi, Sara Bagherieh, Atif Amin Baig, Jennifer L Baker, Abdulaziz T. Bako, Madhan Balasubramanian, Ovidiu Constantin Baltatu, Kiran Bam, Maciej Banach, Soham Bandyopadhyay, Hansi Bansal, Mehmet Firat Baran, Martina Barchitta, Mainak Bardhan, Erfan Bardideh, Suzanne Lyn Barker-Collo, Till Winfried Bärnighausen, Francesco Barone-Adesi, Hiba Jawdat Barqawi, Amadou Barrow, Sandra Barteit, Asma'u I J Bashir, Hameed Akande Bashiru, Afisu Basiru, João Diogo Basso, Sanjay Basu, Bernhard T Baune, Tahmina Begum, Amir Hossein Behnoush, Maryam Beiranvand, Alehegn Bekele, Sefealem Assefa Belay, Uzma Iqbal Belgaumi, Michelle L Bell, Olorunjuwon Omolaja Bello, Apostolos Beloukas, Isabela M Bensenor, Zombor Berezvai, Paulo J G Bettencourt, Akshaya Srikanth Bhagavathula, Prarthna V Bhardwaj, Sonu Bhaskar, Vivek Bhat, Natalia V Bhattacharjee, Jasvinder Singh Bhatti, Gurjit Kaur Bhatti, Manpreet S Bhatti, Rajbir Bhatti, Antonio Biondi, Catherine Bisignano, Atanu Biswas, Raaj Kishore Biswas, Veera R Bitra, Tone Bjørge, Anca Vasilica Bobirca, Virginia Bodolica, Aadam Olalekan Bodunrin, Kassawmar Angaw Bogale, Milad Bonakdar Hashemi, Souad Bouaoud, Dejana Braithwaite, Michael Brauer, Nicholas J K Breitborde, Norma B Bulamu, Danilo Buonsenso, Richard A Burns, Yasser Bustanji, Florentino Luciano Caetano dos Santos, Daniela Calina, Ismael R Campos-Nonato, Giulia Carreras, Andrea Carugno, Carlos A Castañeda-Orjuela, Giulio Castelpietra, Maria Sofia Cattaruzza, Arthur Caye, Luca Cegolon, Francieli

Cembranel, Ester Cerin, Joshua Chadwick, Yaacoub Chahine, Chiranjib Chakraborty, Jeffrey Shi Kai Chan, Vijay Kumar Chattu, Malizgani Paul Chavula, Simiao Chen, An-Tian Chen, Haowei Chen, Gerald Chi, Fatemeh Chichagi, Ju-Huei Chien, Patrick R Ching, William C S Cho, Sungchul Choi, Bryan Chong, Devasahayam J Christopher, Dinh-Toi Chu, Eric Chung, Iolanda Cioffi, Raffaela Ciuffreda, Rafael M Claro, Alyssa Columbus, Haley Comfort, Joao Conde, Michael H Criqui, Natália Cruz-Martins, Silvia Magali Cuadra-Hernández, Mario D'Oria, Sriharsha Dadana, Tukur Dahiru, Zhaoli Dai, Giovanni Damiani, Claudio Alberto Dávila-Cervantes, Aklilu Tamire Debele, Shayom Debopadhaya, Somayeh Delavari, Ivan Delgado-Enciso, Berecha Hundessa Demessa, Edgar Denova-Gutiérrez, Kebede Deribe, Nikolaos Dervenis, Hardik Dineshbhai Desai, Rupak Desai, Arkadeep Dhali, Meghnath Dhimal, Vishal R Dhulipala, Diana Dias da Silva, Daniel Diaz, Michael J Diaz, Adriana Dima, Delaney D Ding, M Ashworth Dirac, Thanh Chi Do, Sushil Dohare, Camila Bruneli do Prado, Leila Doshmangir, Wendel Mombaque dos Santos, Robert Kokou Dowou, Viola Dsouza, Haneil Larson Dsouza, Ashel Chelsea Dsouza, John Dube, Senbagam Duraisamy, Oyewole Christopher Durojaiye, Sulagna Dutta, Laura Dwyer-Lindgren, Arkadiusz Marian Dziedzic, Michael Ekholuenetale, Noha Mousaad Elemam, Ghada Metwally Tawfik ElGohary, Muhammed Elhadi, Omar Abdelsadek Abdou Elmeligy, Mohamed A Elmonem, Ibrahim Elsohaby, Maha El Tantawi, Amir Emami Zeydi, Sharareh Eskandarieh, Francesco Esposito, Farshid Etaee, Natalia Fabin, Adeniyi Francis Fagbamigbe, Saman Fahimi, Aliasghar Fakhri-Demeshghieh, Luca Falzone, Ali Faramarzi, MoezAlIslam Ezzat Mahmoud Faris, Samuel Farmer, Andre Faro, Ali Fatehizadeh, Nelsensius Klau Fauk, Pooria Fazeli, Valery L. Feigin, Seyed-Mohammad Fereshtehnejad, Abdullah Hamid Feroze, Pietro Ferrara, Nuno Ferreira, Getahun Fetensa, Irina Filip, Florian Fischer, Joanne Flavel, Morenike Oluwatoyin Folayan, Behzad Foroutan, Matteo Foschi, Kate Louise Francis, Alberto Freitas, Takeshi Fukumoto, Blima Fux, Peter Andras Gaal, Muktar A. Gadanya, Yaseen Galali, Silvano Gallus, Aravind P Gandhi, Balasankar Ganesan, Mohammad Arfat Ganiyani, M.A. Garcia-Gordillo, Rupesh K. Gautam, Federica Gazzelloni, Semiu Olatunde Gbadamosi, Miglas W Gebregergis, Mesfin Gebrehiwot, Teferi Gebru Gebremeskel, Yohannes Fikadu Geda, Simona Roxana Georgescu, Urge Gerema, Habtamu Geremew, Motuma Erena Getachew, MohammadReza Ghasemi, Ghazal Ghasempour Dabaghi, Fariba Ghassemi, Ramy Mohamed Ghazy, Sailaja Ghimire, Mahsa Ghorbani, Aloke Gopal Ghoshal, Artyom Urievich Gil, Tiffany K Gill, Matteo Giorgi, Alem Girmay, James C Glasbey, Laszlo Göbölös, Ali Golchin, Sameer Vali Gopalani, Alessandra C Goulart, Anmol Goyal, Michal Grivna, Shi-Yang Guan, Giovanni Guarducci, Mesay Dechasa Gudeta, Stefano Guicciardi, Snigdha Gulati, David Gulisashvili, Damitha Asanga Gunawardane, Cui Guo, Mohak Gupta, Manoj Kumar Gupta, Sapna Gupta, Bhawna Gupta, Veer Bala Gupta, Vivek Kumar Gupta, Annie Haakenstad, Farrokh Habibzadeh, Najah R Hadi, Nils Haep, Ramtin Hajibeygi, Rabih Halwani, Randah R Hamadeh, Nadia M Hamdy, Sajid Hameed, Alexis J Handal, Graeme J Hankey, Josep Maria Haro, Ahmed I. Hasaballah, Mohammad Jahid Hasan, S. M. Mahmudul Hasan, Hamidreza Hasani, Md Saquib Hasnain, Amr Hassan, Simon I Hay, Jeffrey J. Hebert, Omar E. Hegazi, Bartosz Helfer, Mehdi Hemmati, Claudiu Herteliu, Kamran Hessami, Kamal Hezam, Yuta Hiraike, Nguyen Quoc Hoan, Ramesh Holla, Nobuyuki Horita, Md Mahbub Hossain, Mohammad Bellal Hossain Hossain, Sorin Hostiuc, Vivian Chia-rong Hsieh, Junjie Huang, M Mamun Huda, Ayesha Humayun, Javid Hussain, Hong-Han Huynh, Segun Emmanuel Ibitoye, Pulwasha Maria Iftikhar, Olayinka Stephen Ilesanmi, Milena D. Ilic, Irena M. Ilic, Mustapha Immurana, Md. Rabiul Islam, Nahlah Elkudssiah Ismail, Gaetano Isola, Mahalaxmi Iyer, Linda Merin J, Louis Jacob, Khushleen Jaggi, Kasra Jahankhani, Nader Jahanmehr, Haitham Jahrami, Akhil Jain, Mihajlo Jakovljevic, Sabzali Javadov, Sathish Kumar Jayapal, Shubha Jayaram, Ravi Prakash Jha, Jost B. Jonas, Tamas Joo, Nitin Joseph, Charity Ehimwenma Joshua, Jacek Jerzy Jozwiak, Mikk Jürisson, Abdulkareem Kabir, Ali Kabir, Hannaneh Kabir, Rizwan Kalani, Feroze Kaliyadan, Sanjay Kalra, Rajesh Kamath, Sagarika Kamath, Edmund Wedam Kanmiki, Kehinde Kazeem Kanmodi, Suthanthira Kannan S, Sushil Kumar Kansal, Rami S. Kantar, Neeti Kapoor, Mehrdad Karajizadeh, Faizan Zaffar Kashoo, Hengameh Kasraei, Joonas H Kauppila, Foad Kazemi, sara Kazeminia, John H Kempen, Himanshu Khajuria, Amirmohammad Khalaji, Nauman Khalid, Anees Ahmed Khalil, Alireza Khalilian, Ajmal Khan,

Mohammad Jobair Khan, Maseer Khan, M Nuruzzaman Khan, Ikramullah Khan, Moien AB Khan, Shaghayegh Khanmohammadi, Khaled Khatab, Armin Khavandegar, Hamid Reza Khayat Kashani, Feriha Fatima Khidri, Mohammad Ali Khosravi, Mahmood Khosrowjerdi, Wondwosen Teklesilasie Kidane, Min Seo Kim, Julie Sojin Kim, Adnan Kisa, Sezer Kisa, Ali-Asghar Kolahi, Farzad Kompani, Oleksii Korzh, Sindhura Lakshmi Koulmane Laxminarayana, Kewal Krishan, Varun Krishna, Barthelemy Kuate Defo, Connor M Kubeisy, Mohammed Kuddus, Md Abdul Kuddus, Ilari Kuitunen, Mukhtar Kulimbet, Satyajit Kundu, Kunle Rotimi Kunle, Om P Kurmi, Asep Kusnali, Dian Kusuma, Ben Lacey, Muhammad Awwal Ladan, Lucie Laflamme, Chandrakant Lahariya, Ratilal Lalloo, Judit Lám, Iván Landires, Berthold Langguth, Ariane Laplante-Lévesque, Heidi Jane Larson, Anders O Larsson, Savita Lasrado, Kamaluddin Latief, Kaveh Latifinaibin, Carlo La Vecchia, Nhi Huu Hanh Le, Caterina Ledda, Paul H Lee, Gebretsadik Kiros Lema, Elvynna Leong, Wei Li, Shanshan Li, An Li, Paulina A Lindstedt, Stefan Listl, Xuefeng Liu, Erand Llanaj, Rubén López-Bueno, Platon D Lopukhov, László Lorenzovici, Paulo A. Lotufo, Jailos Lubinda, Giancarlo Lucchetti, Alessandra Lugo, Raimundas Lunevicius, hengliang lv, Zheng Feei Ma, Monika Machoy, Áurea M. Madureira-Carvalho, Mohammed Magdy Abd El Razek, Soleiman Mahjoub, Mansour Adam Mahmoud, Jeadran N. Malagón-Rojas, Elaheh Malakan Rad, Kashish Malhotra, Ahmad Azam Malik, Deborah Carvalho Malta, Abdullah A Mamun, Yasaman Mansoori, Borhan Mansouri, Gabriel Martinez, Ramon Martinez-Piedra, Francisco Rogerlândio Martins-Melo, Miquel Martorell, Sharmeen Maryam, Roy Rillera Marzo, Alireza Masoudi, Jishanth Mattumpuram, Andrea Maugeri, Erin A May, Mahsa Mayeli, John J. McGrath, Anna Laura Wensel McKowen, Susan A McLaughlin, Steven M McPhail, Kamran Mehrabani-Zeinabad, Entezar Mehrabi Nasab, Max Alberto Mendez Mendez-Lopez, Walter Mendoza, Ritesh G Menezes, George A Mensah, Alexios-Fotios A. Mentis, Sultan Ayoub Meo, Mohsen Merati, Tuomo J Meretoja, Atte Meretoja, Tomislav Mestrovic, Sachith Mettananda, Kukulege Chamila Dinushi Mettananda, Tomasz Miazgowski, Georgia Micha, Irmina Maria Michalek, Ted R Miller, Le Huu Nhat Minh, Mojgan Mirghafourvand, Moonis Mirza, Roya Mirzaei, Ashim Mishra, Philip B Mitchell, Chaitanya Mittal, Babak Moazen, Nouh Saad Mohamed, Mouhand F H Mohamed, Abdalla Z Mohamed, Sakineh Mohammad-Alizadeh-Charandabi, Soheil Mohammadi, Abdollah Mohammadian-Hafshejani, Mustapha Mohammed, Shafiu Mohammed, Salahuddin Mohammed, Ali H Mokdad, Hossein Molavi Vardanjani, Sabrina Molinaro, Lorenzo Monasta, Mohammad Ali Moni, Maryam Moradi, Yousef Moradi, Paula Moraga, Rafael Silveira Moreira, Shane Douglas Morrison, Jakub Morze, Abbas Mosapour, Rohith Motappa, Parsa Mousavi, Amin Mousavi Khaneghah, Efrén Murillo-Zamora, Christopher J L Murray, Jonah Musa, Fungai Musaigwa, Sathish Muthu, Saravanan Muthupandian, Muhammad Muzaffar, Woojae Myung, Ahamarshan Jayaraman Nagarajan, Mukhammad David Naimzada, Firzan Nainu, Sreenivas Narasimha Swamy, Bruno Ramos Nascimento, Gustavo G Nascimento, Abdallah Y Naser, Zuhair S Natto, Javaid Nauman, Biswa Prakash Nayak, Vinod C Nayak, Sabina Onyinye Nduaguba, Hadush Negash, Chernet Tafere Negesse, Ionut Negoi, Ruxandra Irina Negoi, Seyed Aria Nejadghaderi, Samata Nepal, Henok Biresaw Netsere, Georges Nguefack-Tsague, Josephine W. Ngunjiri, Van Thanh Nguyen, Hau Thi Hien Nguyen, Dang H Nguyen, Phuong The Nguyen, Robina Khan Niazi, Taxiarchis Konstantinos Nikolouzakis, Amin Reza Nikpoor, Lawrence Achilles Nnyanzi, Mamoona Noreen, Chisom Adaobi Nri-Ezedi, Mengistu H Nunemo, Virginia Nuñez-Samudio, Jerry John Nutor, Martin James O'Donnell, Bogdan Oancea, Kehinde O Obamiro, Ismail A. Odetokun, Nkechi Martina Odogwu, Oluwakemi Ololade Odukoya, Ayodipupo Sikiru Oguntade, In-Hwan Oh, Sylvester Reuben Okeke, Akinkunmi Paul Okekunle, Osaretin Christabel Okonji, Patrick Godwin Okwute, Andrew T Olagunju, Matthew Idowu Olatubi, Gláucia Maria Moraes Oliveira, Bolajoko Olubukunola Olusanya, Jacob Olusegun Olusanya, Gideon Olamilekan Oluwatunase, Hany A Omar, Obinna E Onwujekwe, Michal Ordak, Verner N. Orish, Doris V. Ortega-Altamirano, Alberto Ortiz, Esteban Ortiz-Prado, Wael M S Osman, Uchechukwu Levi Osuagwu, Olayinka Osuolale, Adrian Otoiu, Stanislav S Otstavnov, Guoqing Ouyang, Mayowa O Owolabi, Mahesh Padukudru P A, Mohammad Taha Pahlevan Fallahy, Feng Pan, Paramjot Panda, Songhomitra Panda-Jonas, Ioannis Pantazopoulos, Romil R Parikh, Ashwaghosha Parthasarathi, Ava Pashaei, Roberto

Passera, Hemal M Patel, Jay Patel, Shankargouda Patil, Dimitrios Patoulias, Venkata Suresh Patthipati, Uttam Paudel, Hamidreza Pazoki Toroudi, Amy E Peden, Paolo Pedersini, Umberto Pensato, Veincent Christian Filipino Pepito, Gavin Pereira, Mario F P Peres, Arokiasamy Perianayagam, Norberto Perico, Richard G. Pestell, Fanny Emily Petermann-Rocha, Hoang Tran Pham, Daniela Pierannunzio, Dimitri Poddighe, Peter Pollner, Maarten J Postma, Ghazaleh Pourali, Naeimeh Pourtaheri, Disha Prabhu, Sergio I Prada, Pranil Man Singh Pradhan, Manya Prasad, Akila Prashant, Nameer Hashim Qasim, Ibrahim Qattea, Deepthi R, Mehrdad Rabiee Rad, Amir Radfar, Venkatraman Radhakrishnan, Hadi Raeisi Shahraki, Seyedeh Niloufar Rafiei Alavi, Cat Raggi, Pankaja Raghav Raghav, Fakher Rahim, Mohammad Hifz Ur Rahman, Masoud Rahmati, Niloufar Rahnavard, Diego Raimondo, Ali Rajabpour-Sanati, Prashant Rajput, Shakthi Kumaran Ramasamy, Kritika Rana, Shailendra Singh Rana, Chhabi Lal Ranabhat, Nemanja Rancic, Shubham Ranjan, Chythra R Rao, Indu Ramachandra Rao, Deepthi Rapaka, Davide Rasella, Vahid Rashedi, Giridhara Rathnaiah Babu, Nakul Ravikumar, Salman Rawaf, David Laith Rawaf, Bharat Rawlley, Elrashdy Moustafa Mohamed Redwan, Giuseppe Remuzzi, Bhageerathy Reshmi, Nazila Rezaei, Aida Rezaei nejad, Abanoub Riad, Mavra A Riaz, Jennifer Rickard, Reza Rikhtegar, Célia Fortuna Rodrigues, Jefferson Antonio Buendia Rodriguez, Ravi Rohilla, Debby Syahru Romadlon, Luca Ronfani, Himanshu Sekhar Rout, Nitai Roy, Bedanta Roy, Enrico Rubagotti, Guilherme de Andrade Ruela, Susan Fred Rumisha, Manjula S, Aly M A Saad, Zahra Saadatian, Maha Mohamed Saber-Ayad, Siamak Sabour, Fatos Sada, Basema Saddik, Bashdar Abuzed Sadee, Ehsan Sadeghi, Umar Saeed, Dominic Sagoe, Manika Saha, Amirhossein Sahebkar, Soumya Swaroop Sahoo, Monalisha Sahu, Zahra Saif, Joseph W Sakshaug, Afeez Abolarinwa Salami, Marwa Rashad Salem, Mohammed Z Y Salem, Sohrab Salimi, Sara Samadzadeh, Vijaya Paul Samuel, Abdallah M Samy, Juan Sanabria, Milena M. Santric-Milicevic, Haaris Saqib, Aswini Saravanan, Babak Saravi, Yaser Sarikhani, Tanmay Sarkar, Rodrigo Sarmiento-Suárez, Sachin C Sarode, Gargi Sachin Sarode, Arash Sarveazad, Thirunavukkarasu Sathish, Anudeep Sathyanarayan, Md Abu Sayeed, Abu Sayeed, Nikolaos Scarmeas, Winfried Schlee, Art Schuermans, Austin E Schumacher, David C Schwebel, Falk Schwendicke, Siddharthan Selvaraj, Pallav Sengupta, Sadaf G Sepanlou, Dragos Serban, Yashendra Sethi, Allen Seylani, Mahan Shafie, Jaffer Shah, Pritik A Shah, Samiah Shahid, Moyad Jamal Shahwan, Ahmed Shaikh, Muhammad Aaqib Shamim, Mehran Shams-Beyranvand, Mohd Shanawaz, Abhishek Shankar, Mohammed Shannawaz, Medha Sharath, Amin Sharifan, Javad Sharifi-Rad, Manoj Sharma, Ujjawal Sharma, Rajesh P. Shastry, Amr Mohamed Elsayed Shehabeldine, Manjunath Mala Shenoy, Pavanchand H Shetty, Mika Shigematsu, Reza Shirkoohi, Aminu Shittu, Farhad Shokraneh, Seyed Afshin Shorofi, Kerem Shuval, Emmanuel Edwar Siddig, Luís Manuel Lopes Rodrigues Silva, Soraia Silva, João Pedro Silva, Biagio Simonetti, Anjali Singal, Jasvinder A. Singh, Abhinav Singh, Balbir Bagicha Singh, Amanda E Smith, Chandan S N, Bogdan Socea, Anton Sokhan, Ranjan Solanki, Shipra Solanki, Hamidreza Soleimani, Yonatan Solomon, Michael Spartalis, Chandrashekhar T Sreeramareddy, Muhammad Haroon Stanikzai, Vladimir I Starodubov, Antonina V Starodubova, Simona Cătălina Stefan, Paschalis Steiropoulos, Vetriselvan Subramaniyan, Muhammad Suleman, Abida Sultana, Chandan Kumar Swain, Bryan L. Sykes, Lukasz Szarpak, Mindy D Szeto, Miklós Szócska, Payam Tabaee Damavandi, Rafael Tabarés-Seisdedos, Seyed-Amir Tabatabaeizadeh, Shima Tabatabai, Karen M Tabb, Mohammad Tabish, Ardeshir Tajbakhsh, Iman M. Talaat, Ashis Talukder, Mircea Tampa, Jacques Lukenze Tamuzi, Ker-Kan Tan, Derbie Alemu DA Tareke, Vivian Y Tat, Seyed Mohammad Tavangar, Mojtaba Teimoori, Mohamad-Hani Temsah, Reem Mohamad Hani Temsah, Masayuki Teramoto, Dufera Rikitu Terefa, Riki Tesler, Ramna Thakur, Pugazhenthan Thangaraju, Kavumpurathu Raman Thankappan, Samar Tharwat, Nihal Thomas, Ales Tichopad, Tenaw Yimer Tiruye, Marcello Tonelli, Marcos Roberto Tovani-Palone, Nghia Minh Tran, Jasmine T Tran, Samuel Joseph Tromans, Thien Tan Tri Tai Truyen, Evangelia Eirini Tsermpini, Stefanos Tyrovolas, Aniefiok John Udoakang, Arit Udoh, Atta Ullah, Srikanth Umakanthan, Chukwuma David Umeokonkwo, Brigid Unim, Bhaskaran Unnikrishnan, Era Upadhyay, Jibrin Sammani Usman, Marco Vacante, Asokan Govindaraj Vaithinathan, Jef Van den Eynde, Elena Varavikova, Orsolya Varga, Priya Vart, Shoban Babu Varthya, Tommi Juhani Vasankari, Balachandar Vellingiri, Madhur Verma,

Massimiliano Veroux, Georgios-Ioannis Verras, Dominique Vervoort, Jorge Hugo Villafañe, Vasily Vlassov, Stein Emil Vollset, Simona Ruxandra Volovat, Theo Vos, Yuan-Pang Wang, Yanzhong Wang, Cong Wang, Shu Wang, Paul Ward, Emebet Gashaw Wassie, Marcia R Weaver, Ronny Westerman, Taweewat Wiangkham, Nuwan Darshana Wickramasinghe, Dakshitha Praneeth Wickramasinghe, Peter Willeit, Yohannes Addisu Wondimagegene, Juan Xia, Shirin Yaghoobpoor, Sajad Yaghoubi, Zwanden Sule Yahaya, Lin Yang, Habib Yaribeygi, Saber Yezli, Dong Keon Yon, Naohiro Yonemoto, Chun-Wei Yuan, Nima Zafari, Mikhail Sergeevich Zastrozhin, Haijun Zhang, Chenwen Zhong, Magdalena Zielińska, Samer H. Zyoud.

Managing the estimation or publications process

Saira Afzal, Mohammed Albashtawy, Mohammed Usman Ali, Catherine M Antony, Ahmed Y Azzam, Natalia V Bhattacharjee, Catherine Bisignano, Milad Bonakdar Hashemi, Thanh Chi Do, Kara Estep, Ali Fatehizadeh, Alem Girmay, Manoj Kumar Gupta, Simon I Hay, Hong-Han Huynh, Faizan Zaffar Kashoo, Nicholas J Kassebaum, Molly B Kassel, M Nuruzzaman Khan, Kasey E Kinzel, Chandrakant Lahariya, Nhi Huu Hanh Le, Paulina A Lindstedt, Mohammed Magdy Abd El Razek, Borhan Mansouri, Alireza Masoudi, Anna Laura Wensel McKowen, Le Huu Nhat Minh, Salahuddin Mohammed, Ali H Mokdad, Christopher J L Murray, Van Thanh Nguyen, Mahesh Padukudru P A, Paramjot Panda, Hoang Tran Pham, David M Pigott, Nemanja Rancic, Aly M A Saad, Abdallah M Samy, Md Abu Sayeed, Mohammad Anas Shamsi, Vishal Sharma, Amanda E Smith, Michael Spartalis, Derbie Alemu DA Tareke, Nicholas Alexander Verghese, Stein Emil Vollset, Katherine M Wells, Yohannes Addisu Wondimagegene, Mikhail Sergeevich Zastrozhin.
