# Supplementary material for: Global fertility in 204 countries and territories, 1950–2021, with forecasts to 2100: a comprehensive demographic analysis for the Global Burden of Disease Study 2021
Source: Lancet. 2024 May 18;403(10440):2057–99. doi: 10.1016/S0140-6736(24)00550-6 (PMC11122687; doi:10.1016/S0140-6736(24)00550-6)
Supplement: Supplementary appendix 2 [file mmc2.pdf]

THE LANCET

Supplementary appendix 2

This appendix formed part of the original submission and has been peer reviewed. We post it as supplied by the authors.

Supplement to: GBD 2021 Fertility and Forecasting Collaborators. Global fertility in 204 countries and territories, 1950–2021, with forecasts to 2100: a comprehensive demographic analysis for the Global Burden of Disease Study 2021. *Lancet* 2024; **403:** 2057–99.

Appendix 2: supplementary results appendix to "Global fertility in 204 countries and territories, 1950–2021 with forecasts to 2100: a comprehensive demographic analysis for the Global Burden of Disease Study 2021"

This supplement provides additional figures and tables containing more detailed results for "Global fertility in 204 countries and territories, 1950–2021 with forecasts to 2100: a comprehensive demographic analysis for the Global Burden of Disease Study 2021".

Results are also available for download in the Global Health Data Exchange (GHDx): https://ghdx.healthdata.org/gbd-2021.

Figures

Supplemental Results Figure S1: Age−specific fertility fitted trend lines, 1950−2021……………………………….2

Supplemental Results Figure S2: Covariates used in fertility forecasts by location, 1970-2135………………….7782

Supplemental Results Figure S3: Lexis diagram of CCF50, single-year age interval ASFR, and TFR by mother's age and location, 1950–2100…………………………………………………………………………………...7986

Supplemental Results Figure S4: Number of livebirths (millions) by 2021 World Bank income group…………8219

Supplemental Results Figure S5: TFR for reference and alternative scenario forecasts by location, 1950– 2100..8220

Supplemental Results Figure S6: Forecasts of TFR, globally and by country and territory, for the IHME reference scenario and the UNPD medium variant scenario, 1950–2100……………………………………………….8453

Supplemental Results Figure S7: TFR for IHME reference scenario and the UNPD medium variant scenario by location, 1950– 2100……………………………………………………………………………………….8454

Supplemental Results Figure S8: Five-year interval ASFR from IHME reference scenario and the UNPD medium variant scenario by location, 1950-2100………………………………………………………………...8659

Tables

Supplemental Results Table S1a: Live births (in thousands) by location, both sexes combined, 1950, 1960, 1970, 1980, 1990, 2000, 2010, 2019, 2021)…………………………………………………………………...8863

Supplemental Results Table S1b: Live births (in thousands) by location, females, 1950, 1960, 1970, 1980, 1990, 2000, 2010, 2019, 2021)..……………………………………………………………………………….8875

Supplemental Results Table S1c: Live births (in thousands) by location, males, 1950, 1960, 1970, 1980, 1990, 2000, 2010, 2019, 2021)………………………………………………………………………………….8888

Supplemental Results Table S2: Replacement total fertility rate by location, 2021...……………………...…8901

Supplemental Results Table S3: Total Fertility Rate in 2021 with and without Ages 10-14 and 50-54…………...8909

Supplemental Results Figure S1: Age−specific fertility fitted trend lines, 1950−2021: Armenia, Ages: 10−14 Data points enclosed with a diamond are reference sources

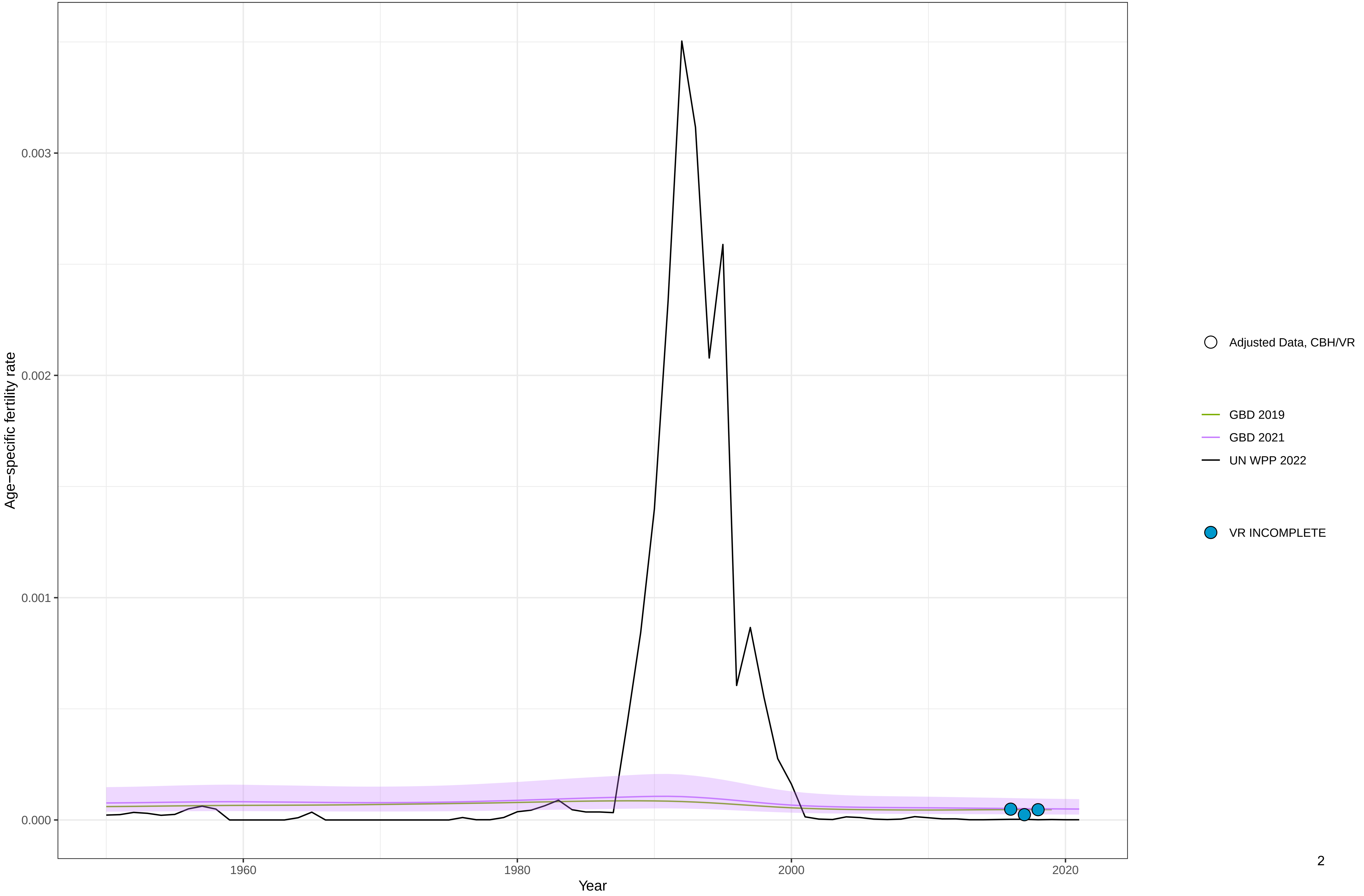

Age−specific fertility fitted trend lines, 1950−2021: Armenia, Ages: 15−19
Data points enclosed with a diamond are reference sources

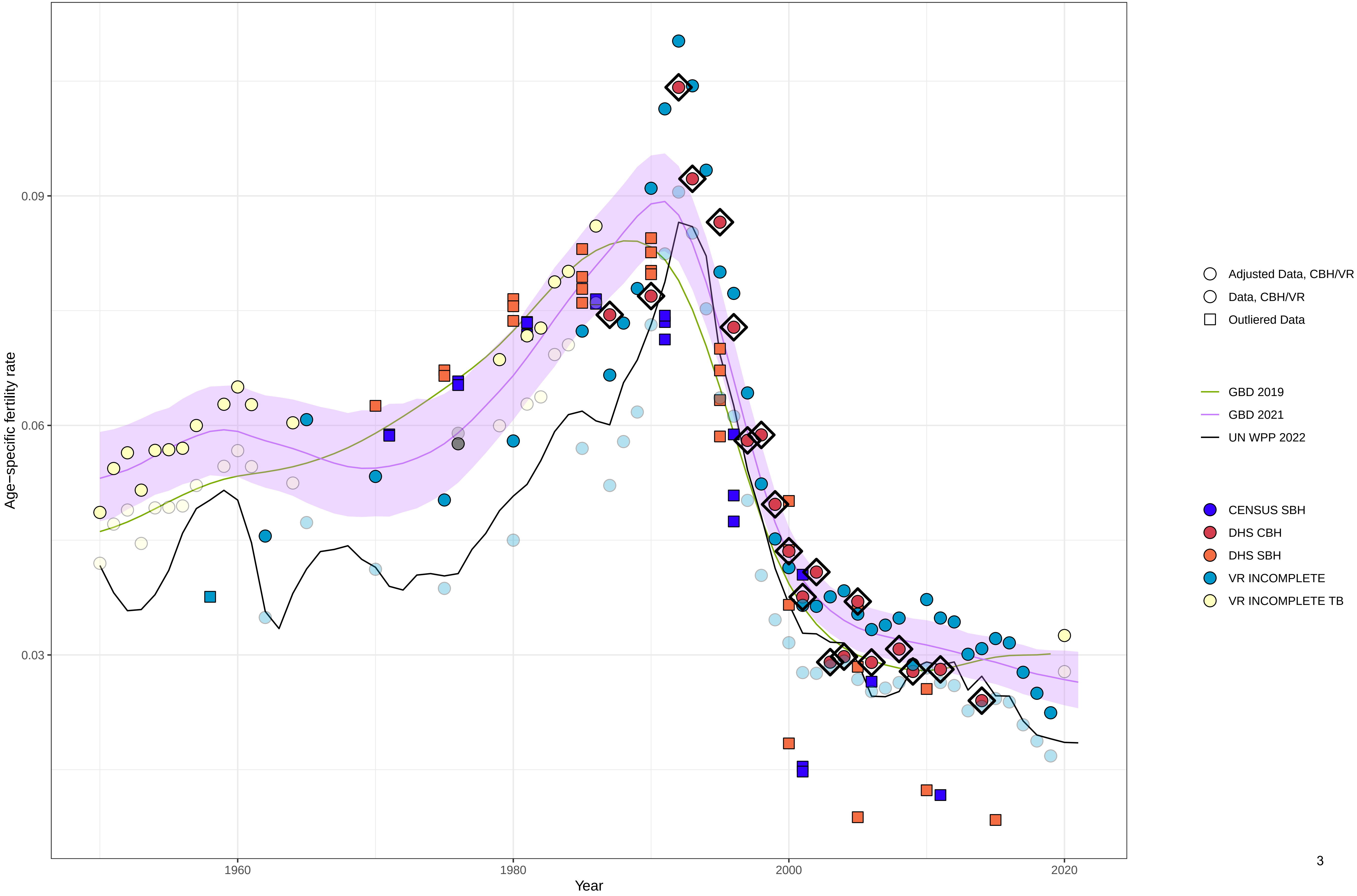

Age−specific fertility fitted trend lines, 1950−2021: Armenia, Ages: 20−24
Data points enclosed with a diamond are reference sources

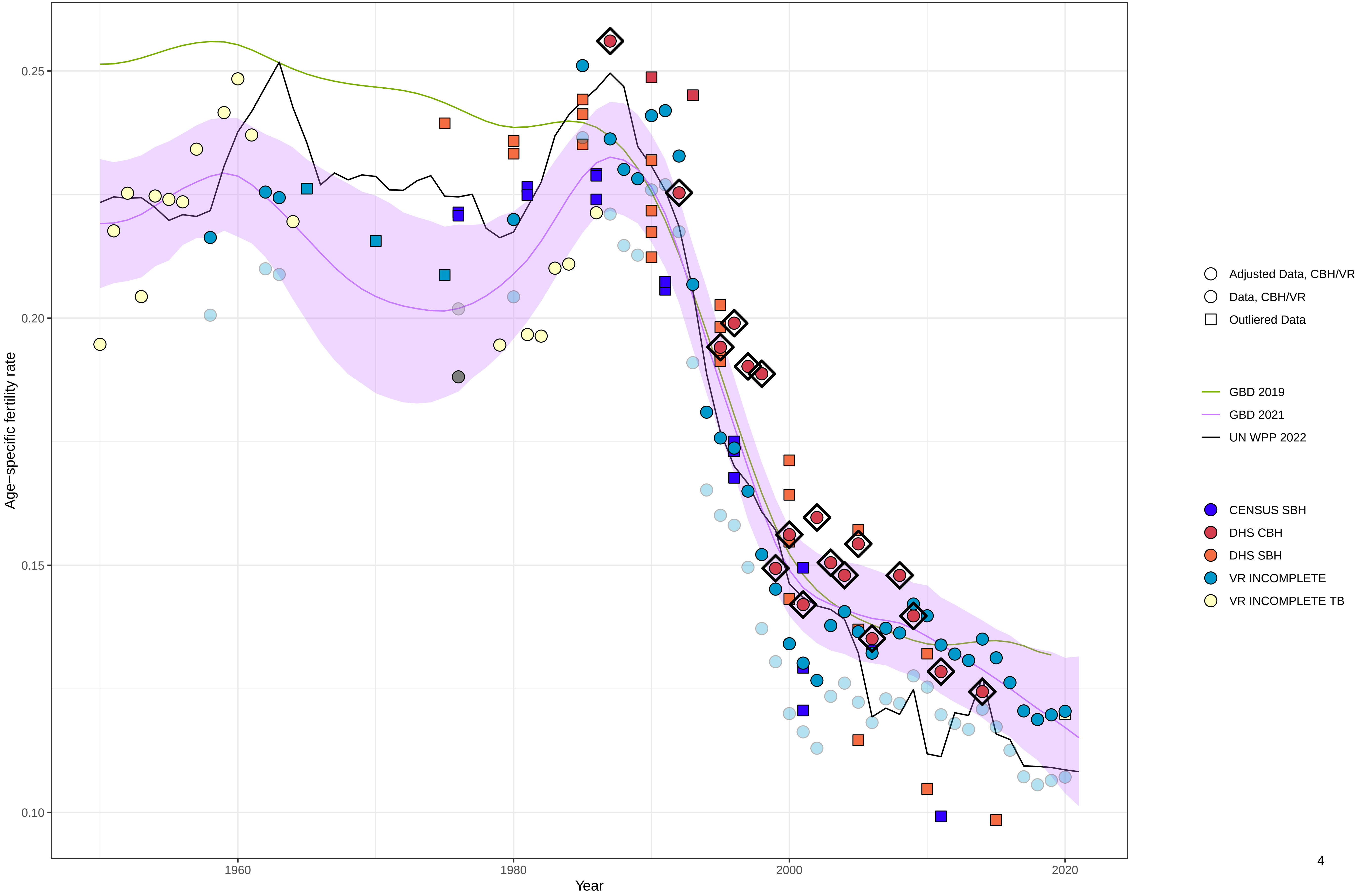

Age−specific fertility fitted trend lines, 1950−2021: Armenia, Ages: 25−29
Data points enclosed with a diamond are reference sources

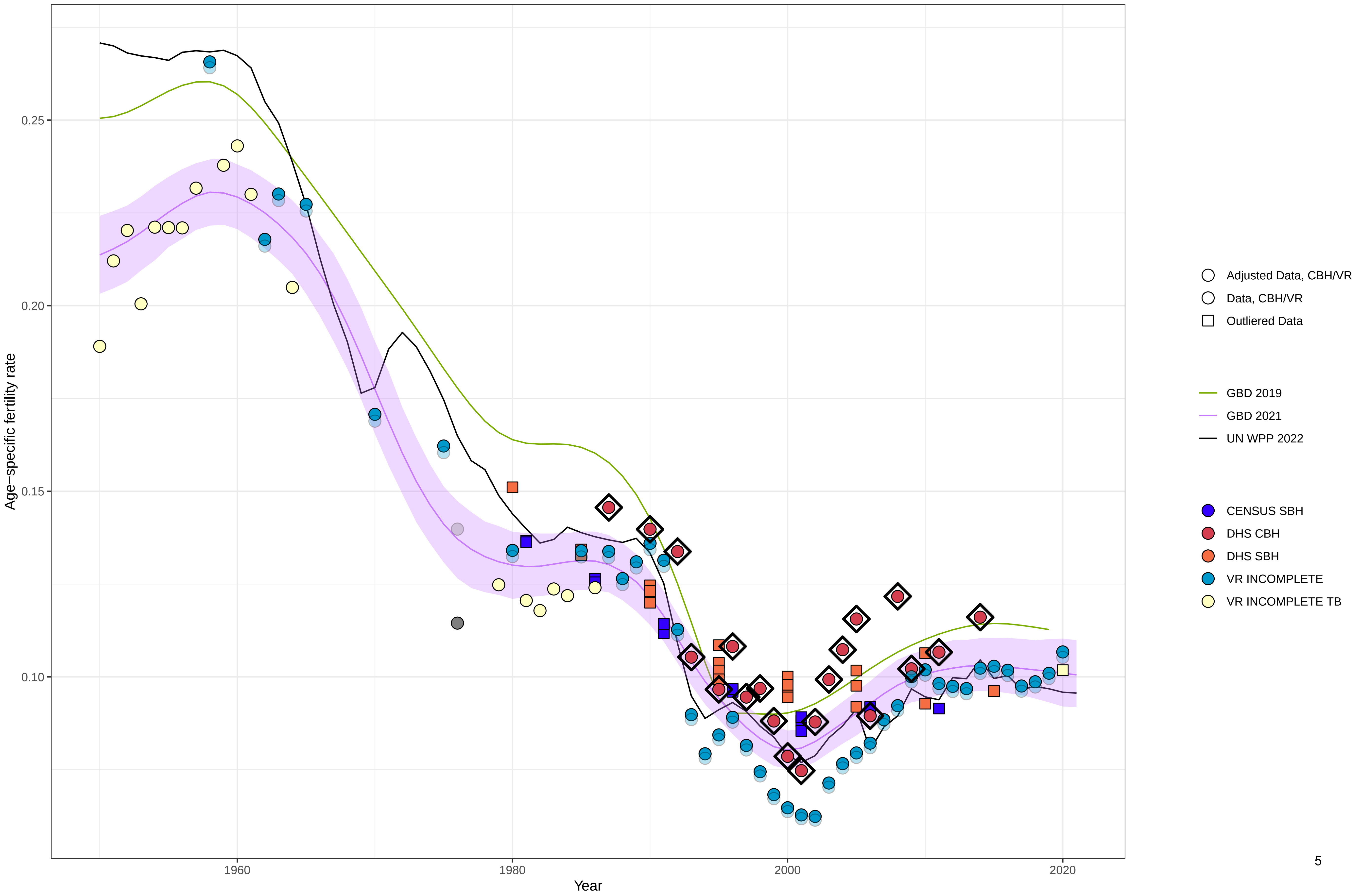

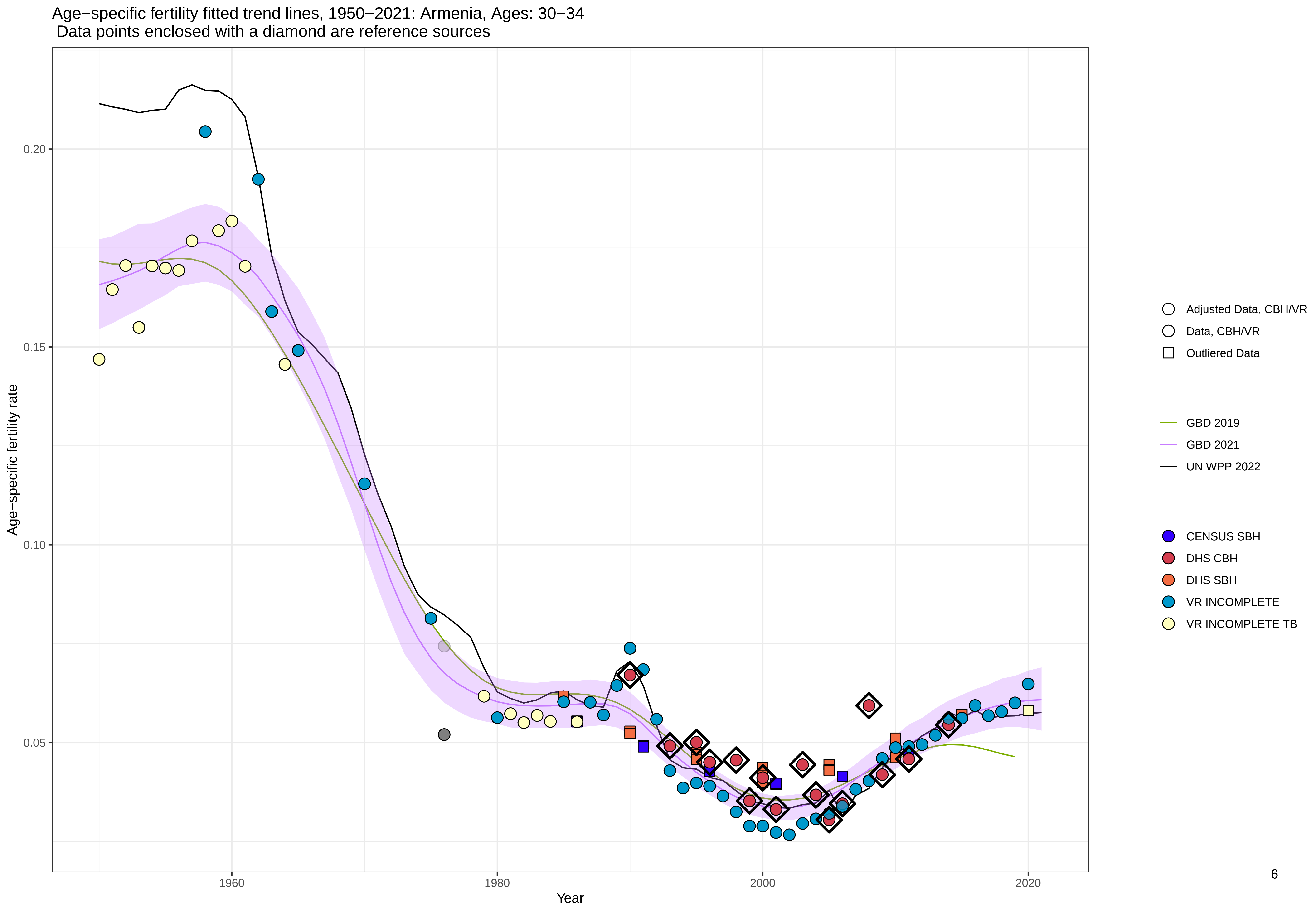
Age−specific fertility fitted trend lines, 1950−2021: Armenia, Ages: 30−34
Data points enclosed with a diamond are reference sources
Age−specific fertility rate
0.20
0.15
0.10
0.05
1960
1980
2000
2020
Year
Adjusted Data, CBH/VR
Data, CBH/VR
Outliered Data
GBD 2019
GBD 2021
UN WPP 2022
CENSUS SBH
DHS CBH
DHS SBH
VR INCOMPLETE
VR INCOMPLETE TB

Age−specific fertility fitted trend lines, 1950−2021: Armenia, Ages: 35−39
Data points enclosed with a diamond are reference sources

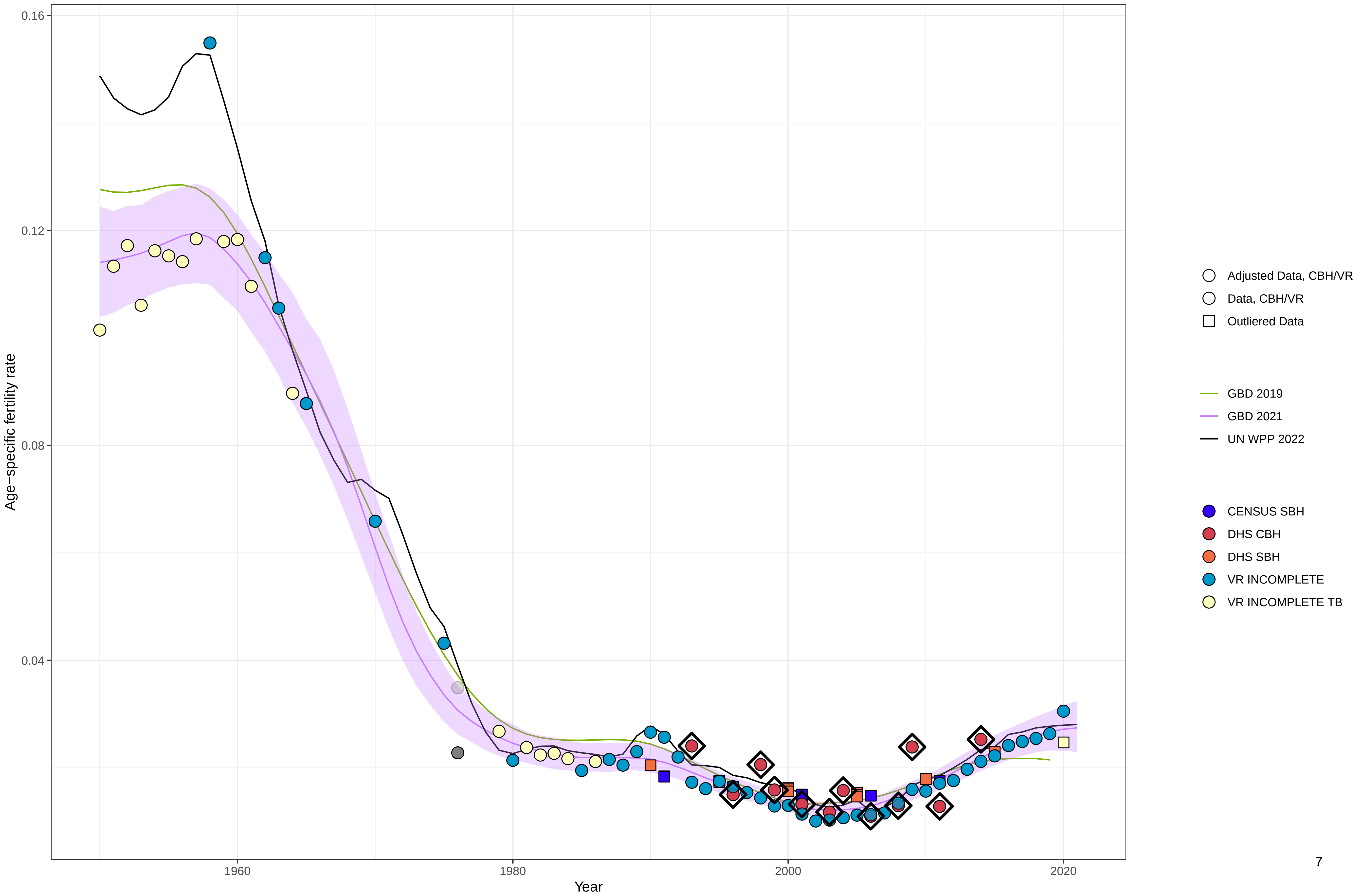

Age−specific fertility fitted trend lines, 1950−2021: Armenia, Ages: 40−44
Data points enclosed with a diamond are reference sources

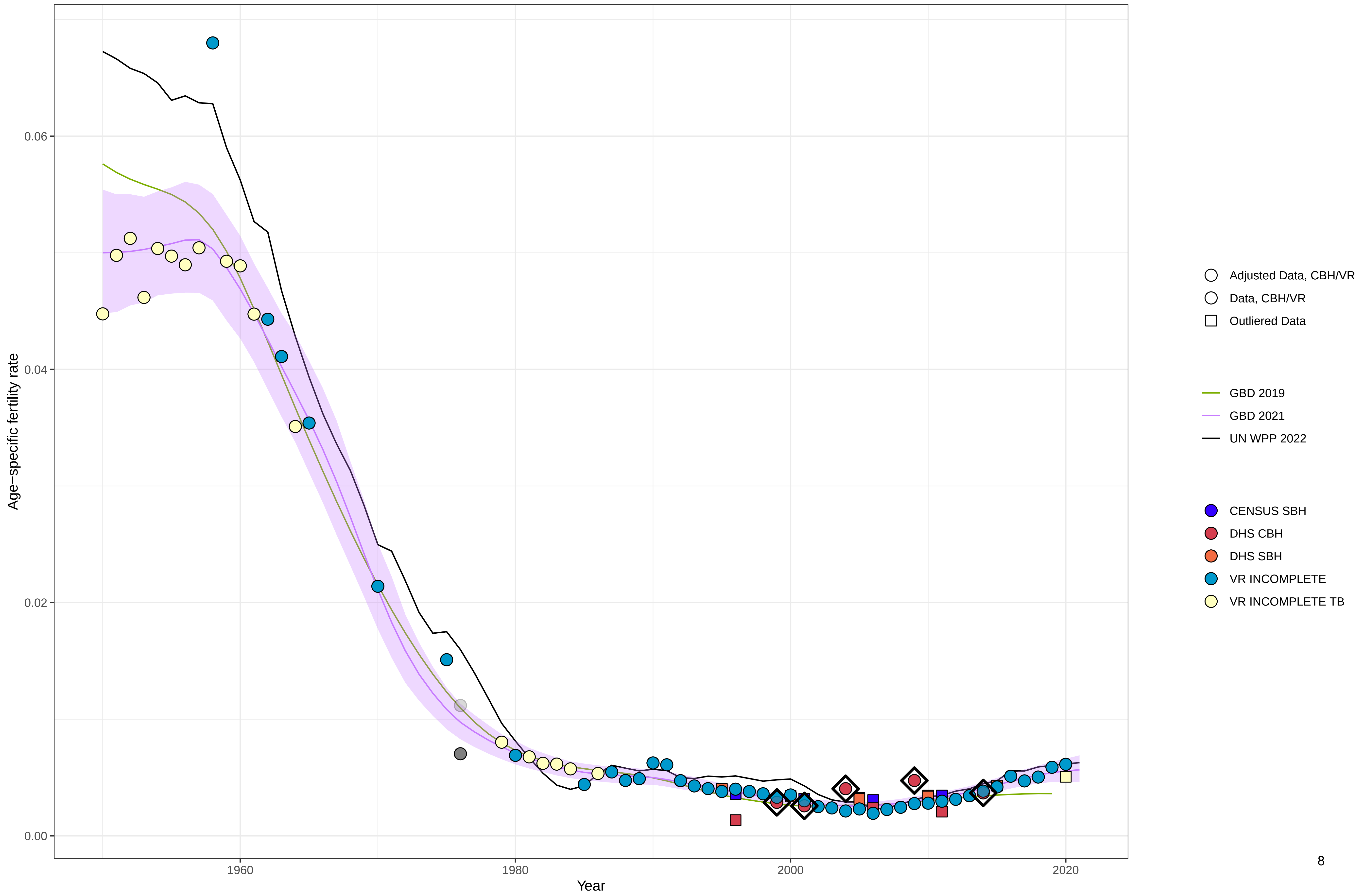

Age−specific fertility fitted trend lines, 1950−2021: Armenia, Ages: 45−49
Data points enclosed with a diamond are reference sources

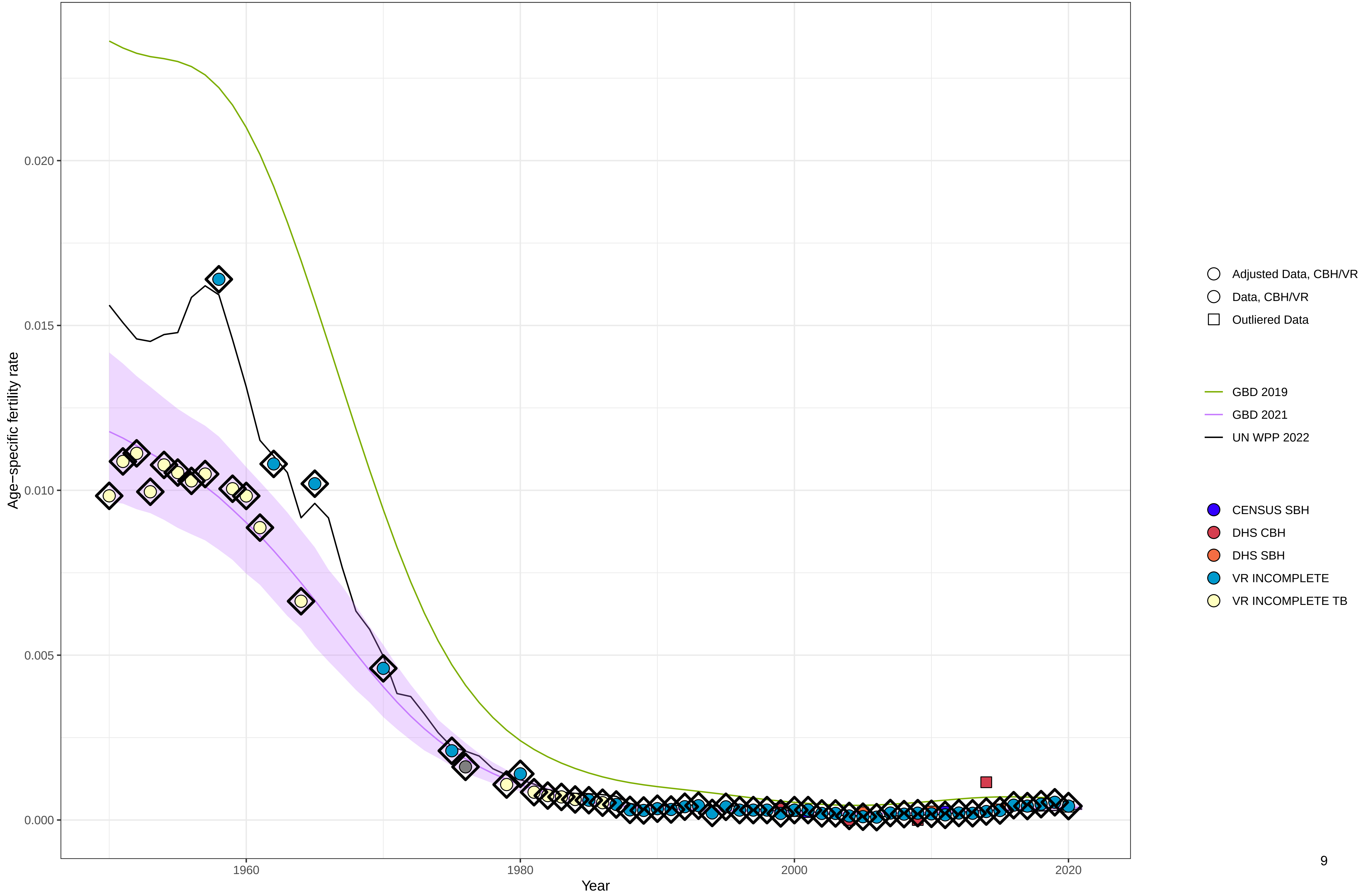

Age−specific fertility fitted trend lines, 1950−2021: Armenia, Ages: 50−54
Data points enclosed with a diamond are reference sources

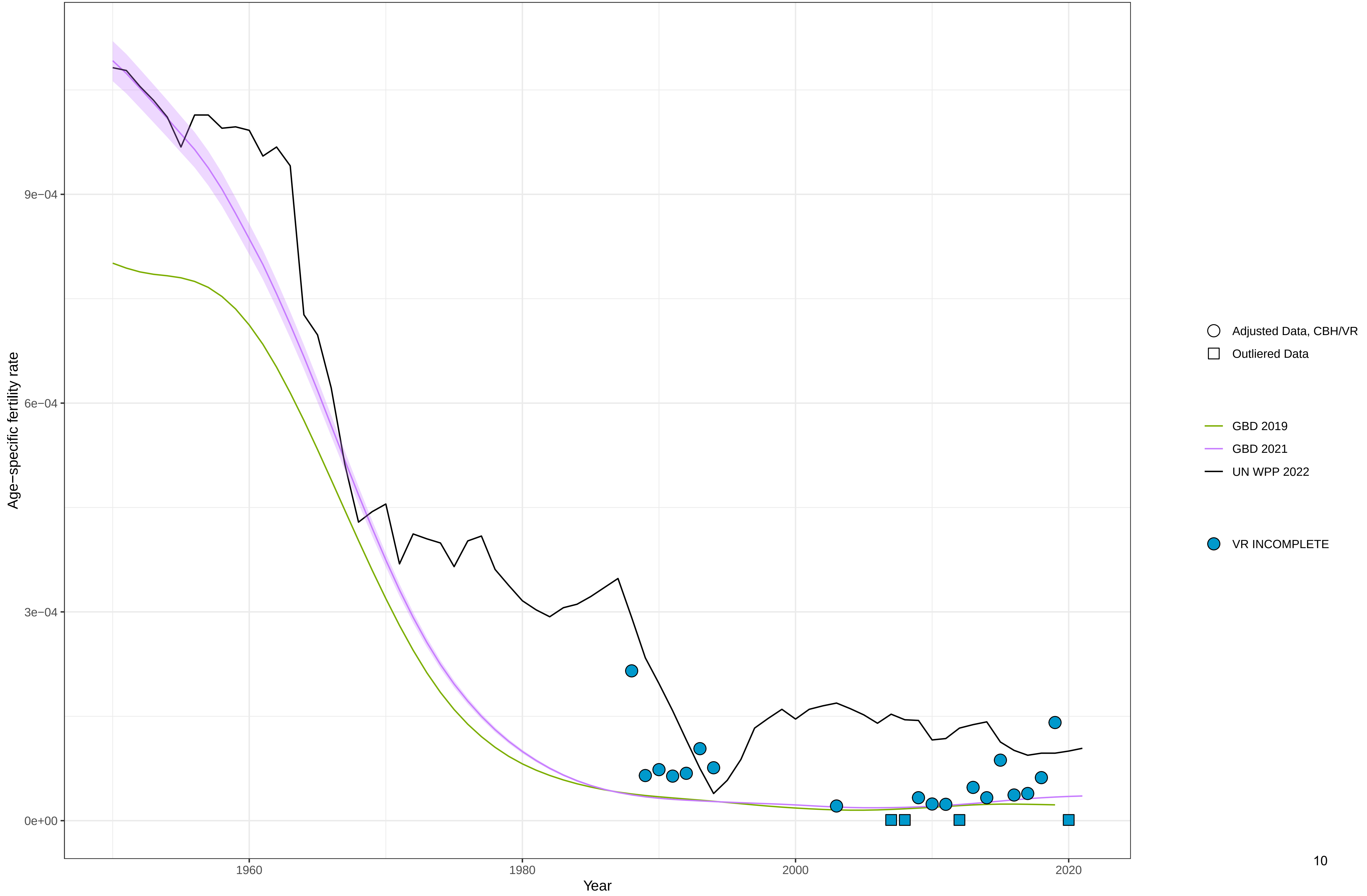

Total fertility rate fitted trend lines, 1950−2021: Armenia TFR
Data points enclosed with a diamond are reference sources

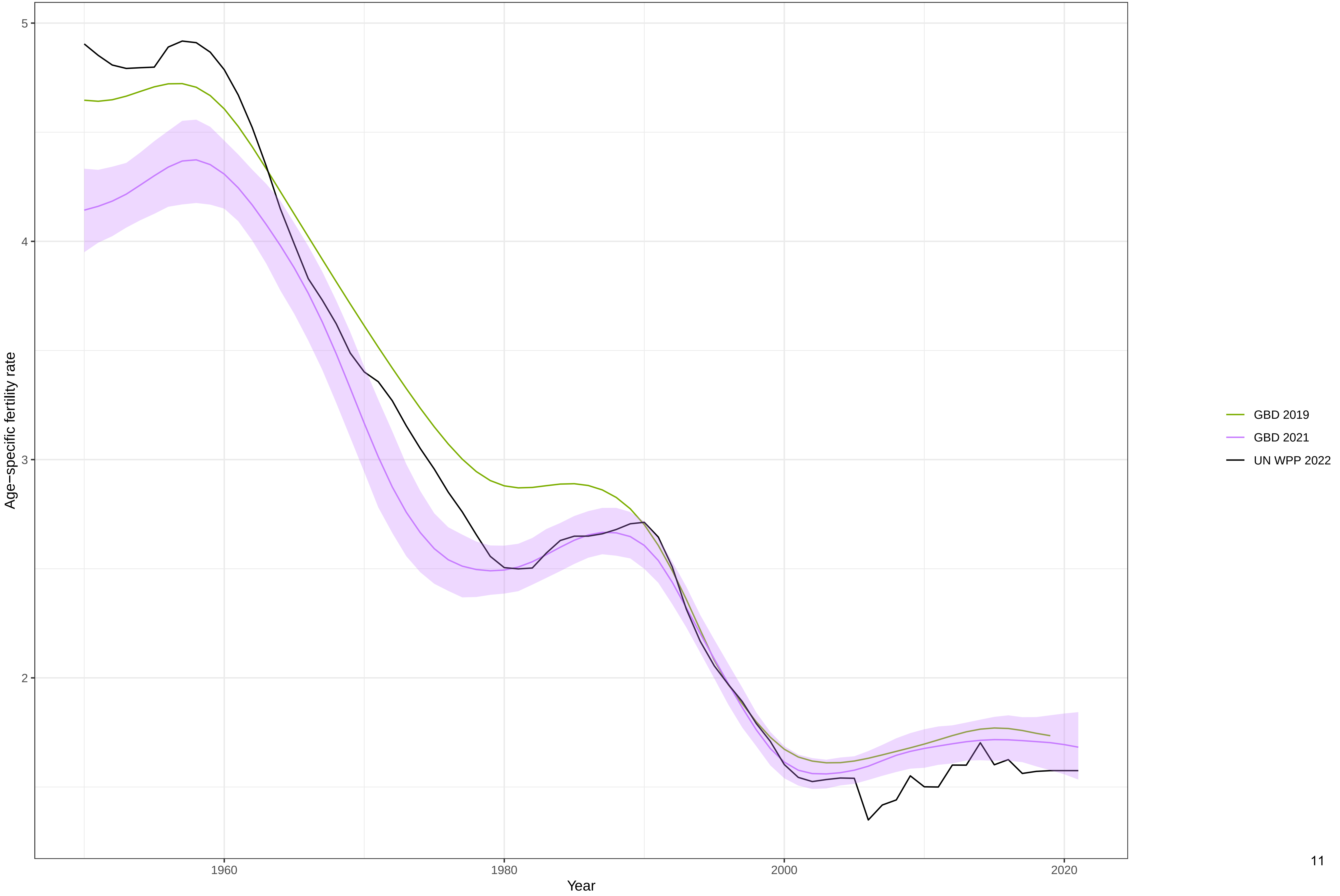

Age−specific fertility fitted trend lines, 1950−2021: Azerbaijan, Ages: 10−14
Data points enclosed with a diamond are reference sources

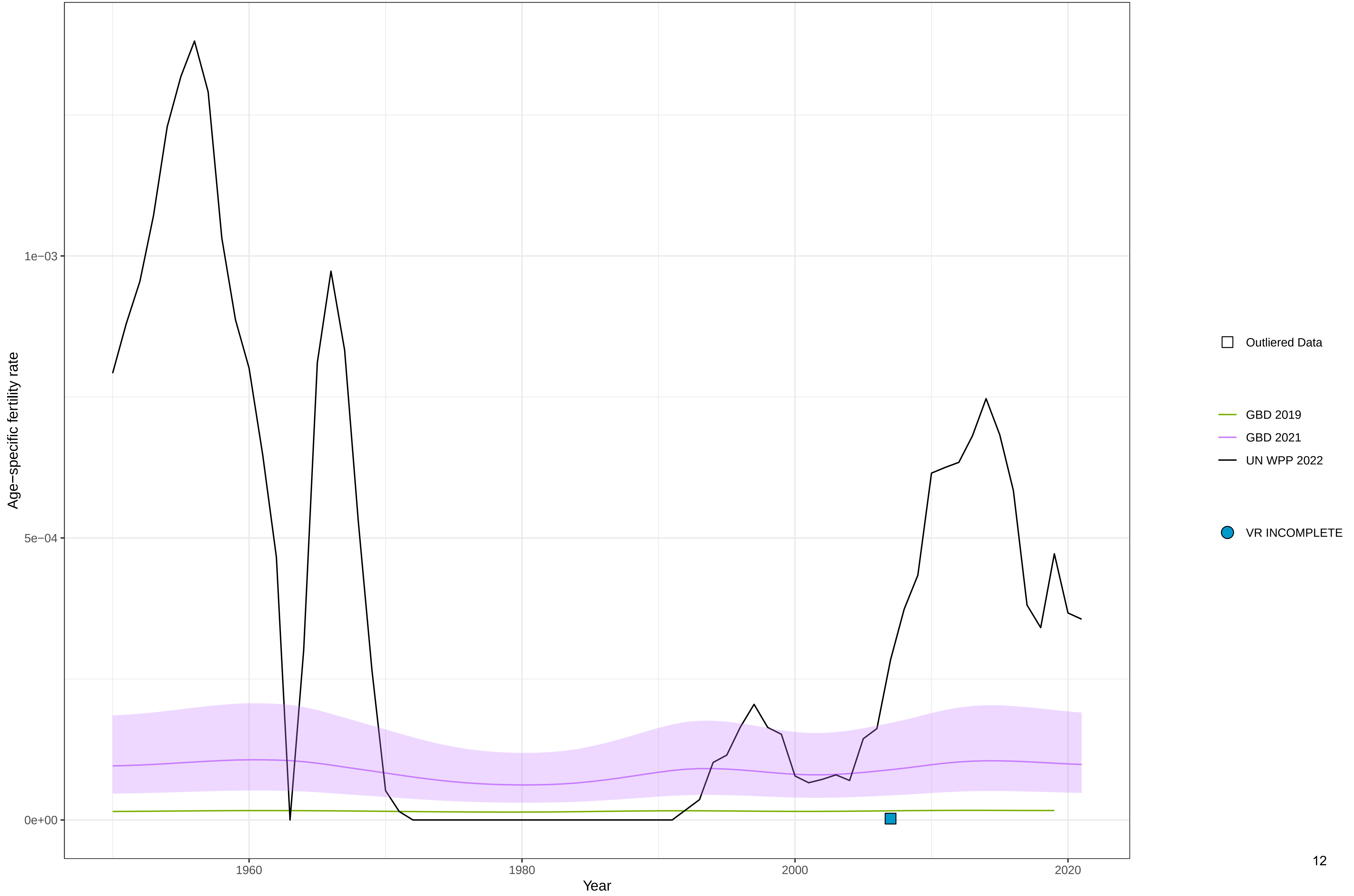

Age−specific fertility fitted trend lines, 1950−2021: Azerbaijan, Ages: 15−19
Data points enclosed with a diamond are reference sources

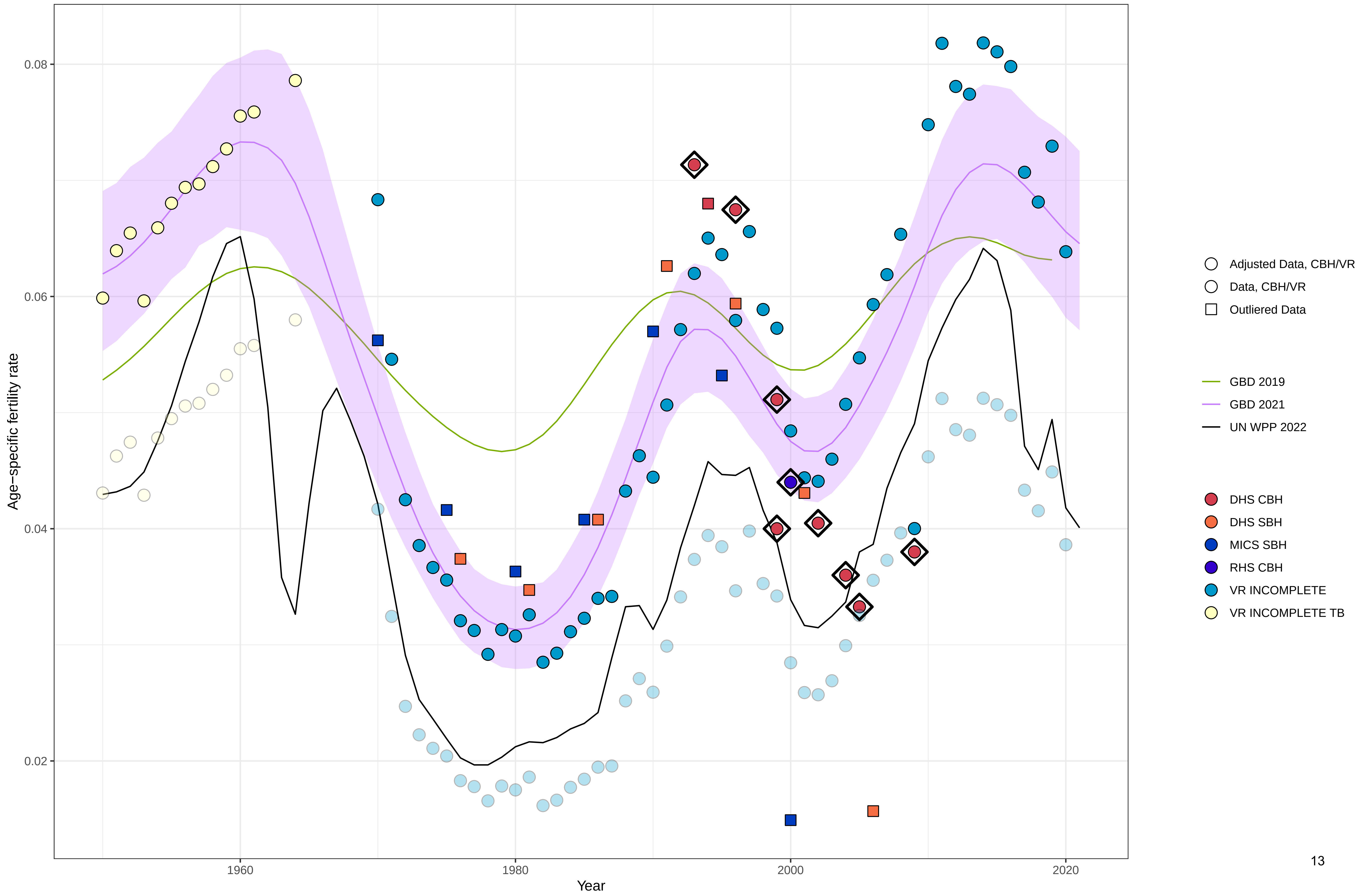

Age−specific fertility fitted trend lines, 1950−2021: Azerbaijan, Ages: 20−24
Data points enclosed with a diamond are reference sources

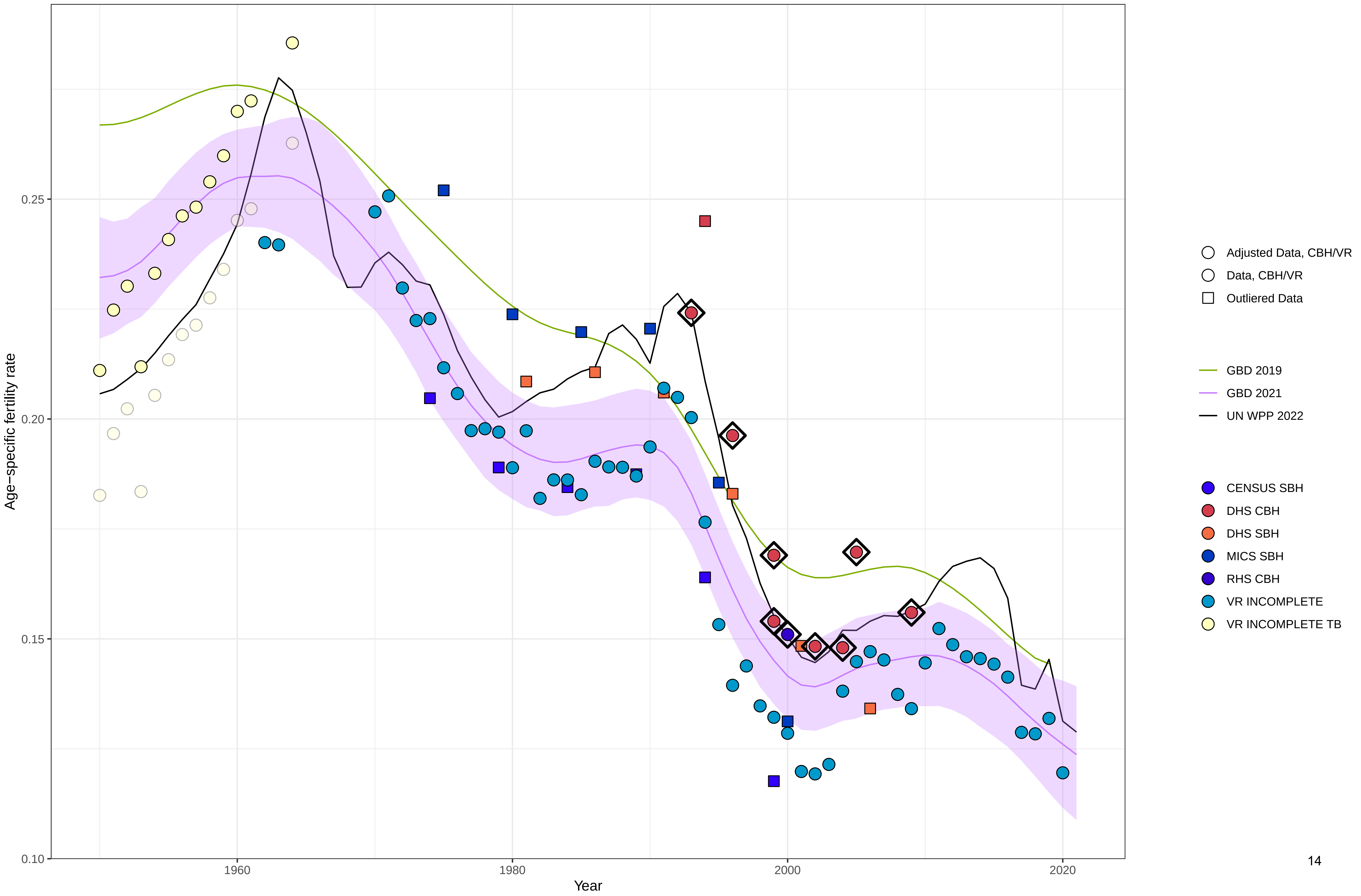

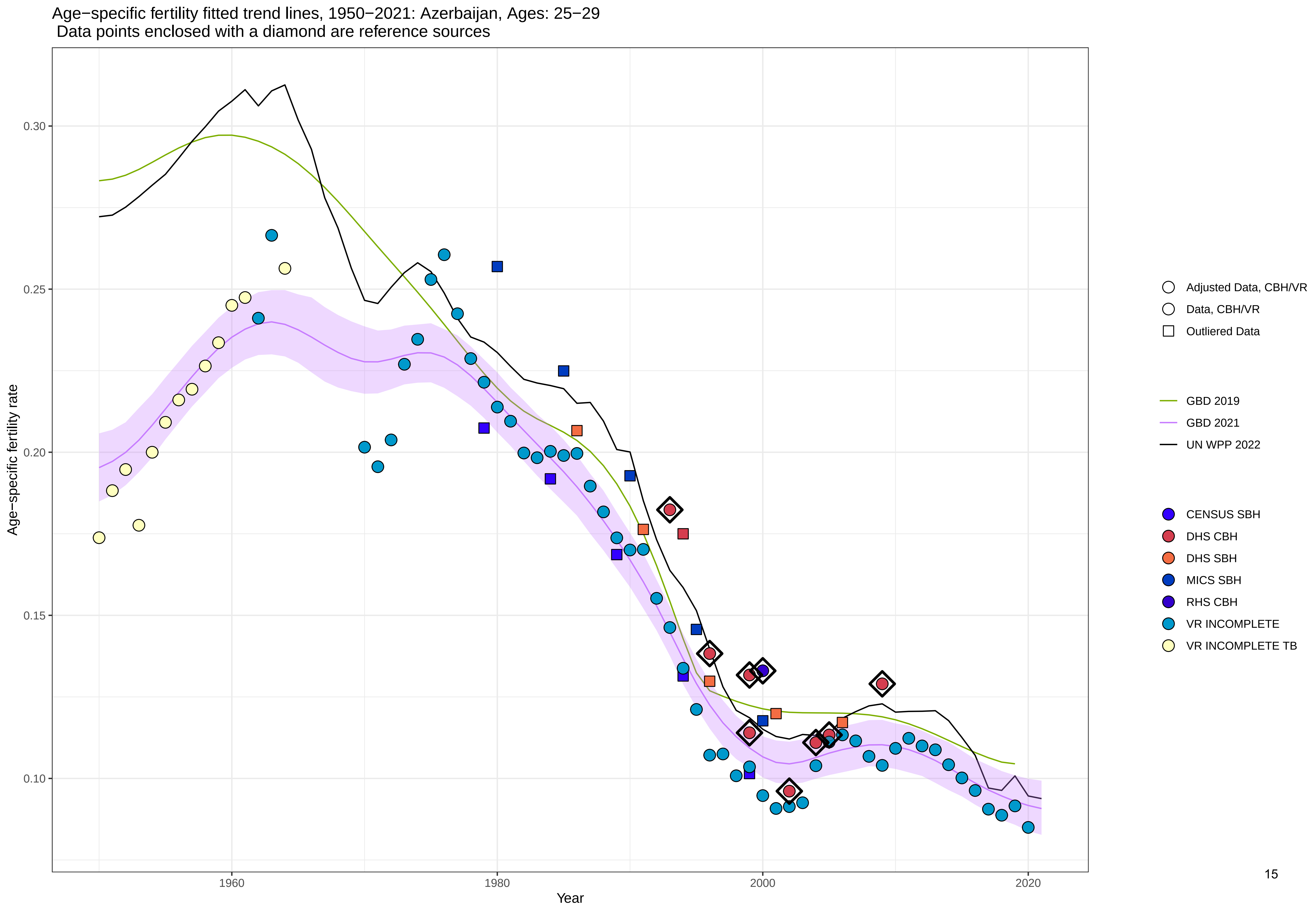
Age−specific fertility fitted trend lines, 1950−2021: Azerbaijan, Ages: 25−29
Data points enclosed with a diamond are reference sources
Age−specific fertility rate
0.30
0.25
0.20
0.15
0.10
1960
1980
2000
2020
Year
Adjusted Data, CBH/VR
Data, CBH/VR
Outliered Data
GBD 2019
GBD 2021
UN WPP 2022
CENSUS SBH
DHS CBH
DHS SBH
MICS SBH
RHS CBH
VR INCOMPLETE
VR INCOMPLETE TB

Age−specific fertility fitted trend lines, 1950−2021: Azerbaijan, Ages: 30−34
Data points enclosed with a diamond are reference sources

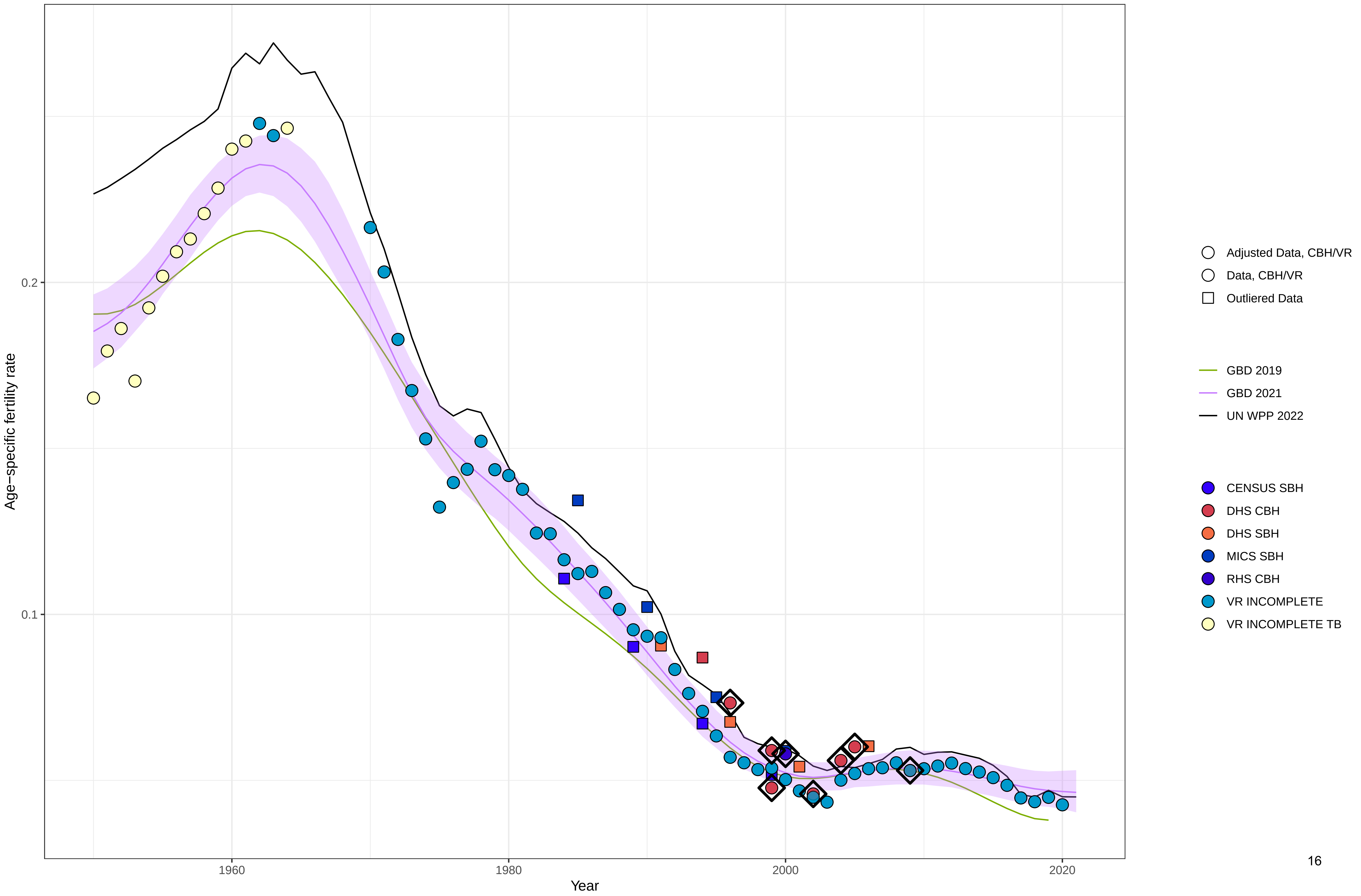

Age−specific fertility fitted trend lines, 1950−2021: Azerbaijan, Ages: 35−39
Data points enclosed with a diamond are reference sources

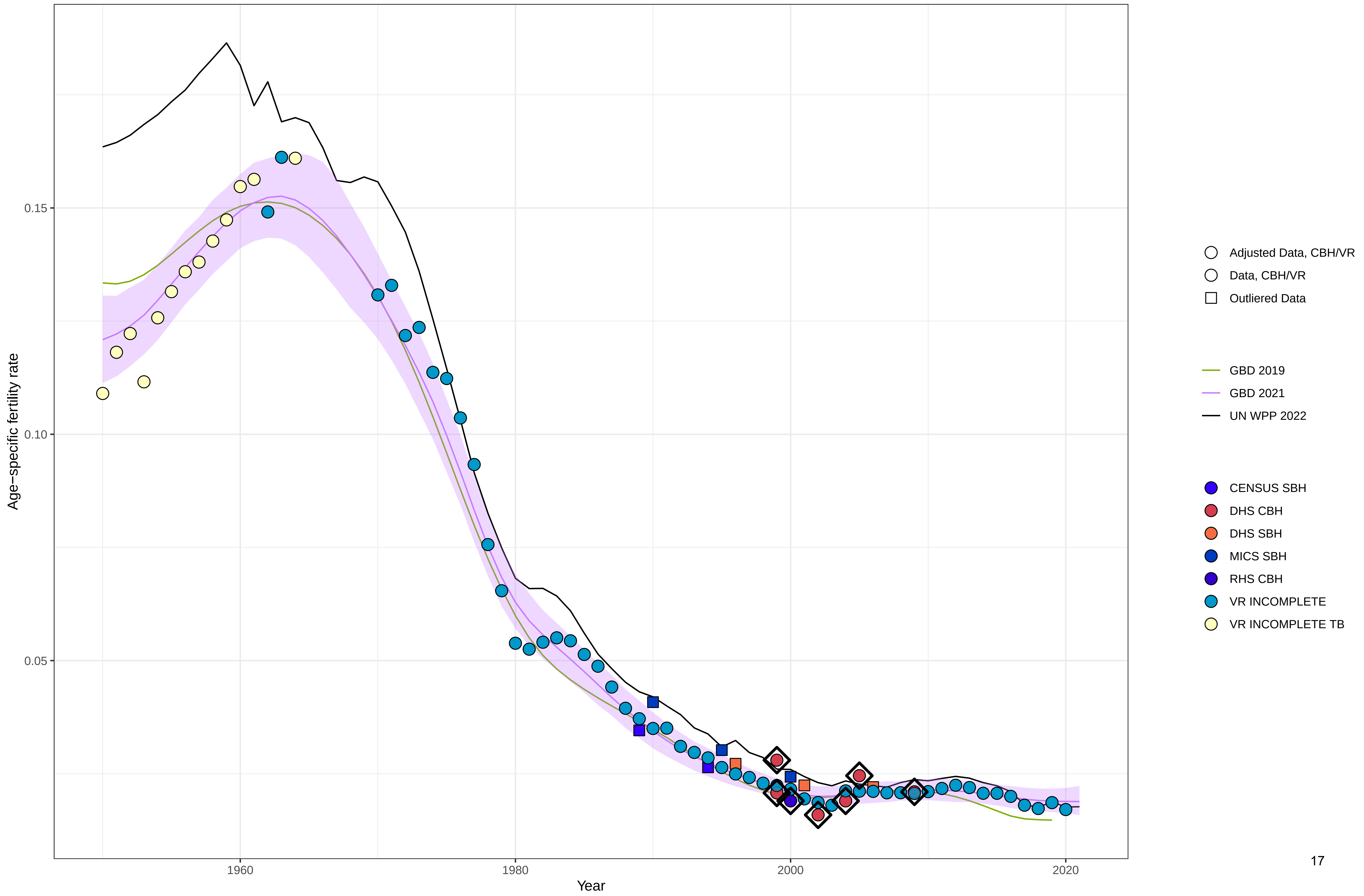

Age−specific fertility fitted trend lines, 1950−2021: Azerbaijan, Ages: 40−44
Data points enclosed with a diamond are reference sources

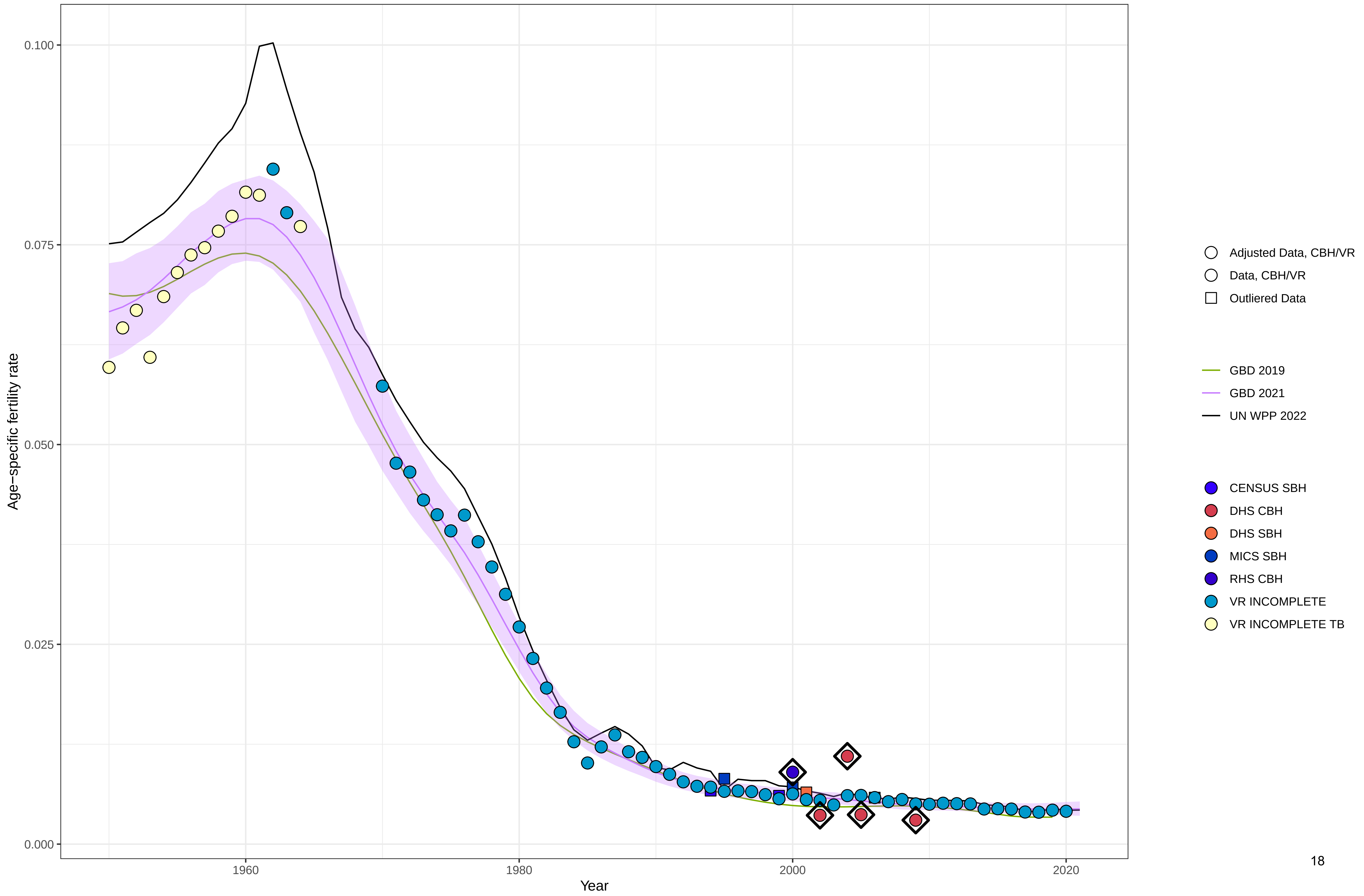

Age−specific fertility fitted trend lines, 1950−2021: Azerbaijan, Ages: 45−49
Data points enclosed with a diamond are reference sources

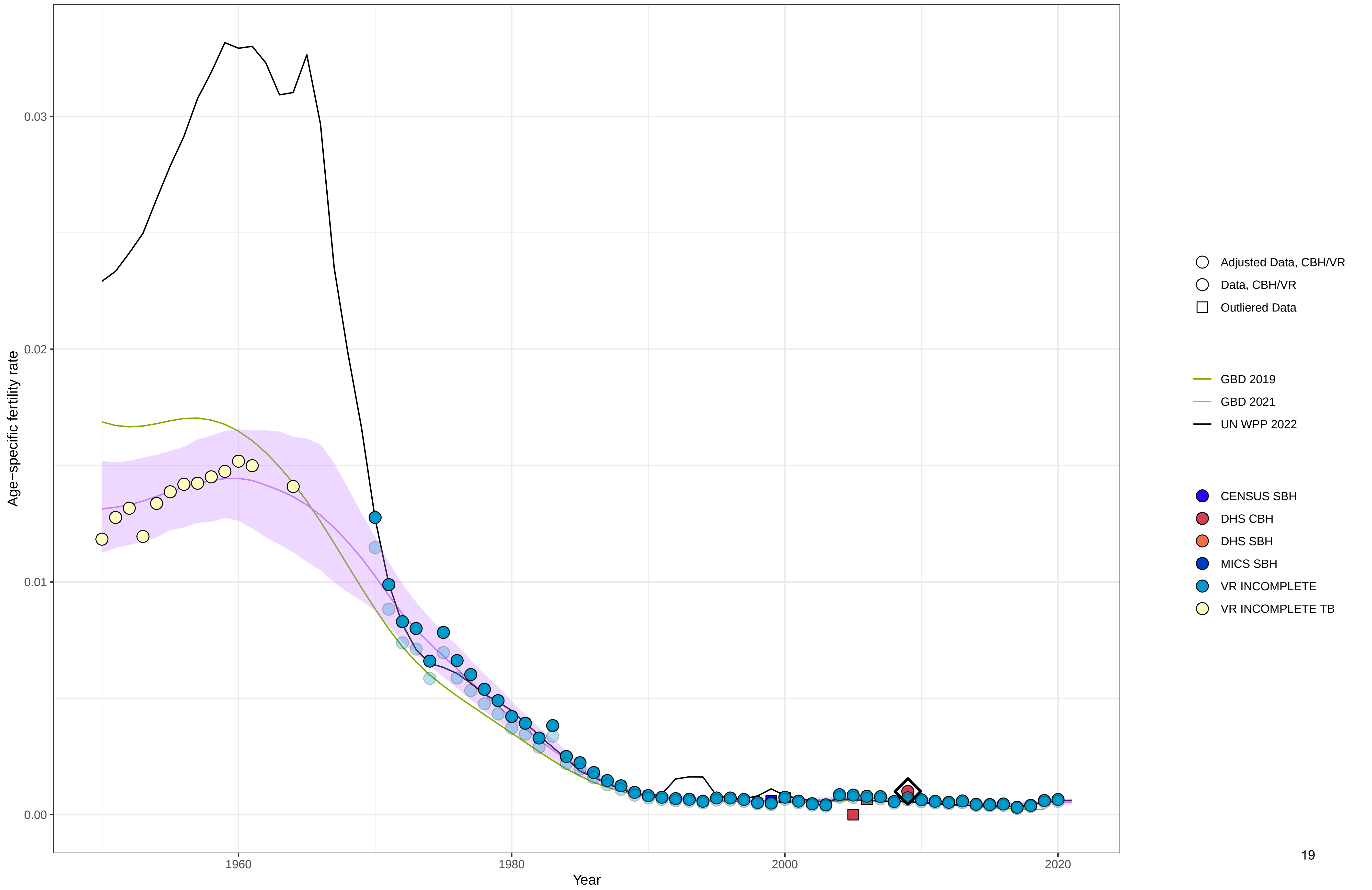

Age−specific fertility fitted trend lines, 1950−2021: Azerbaijan, Ages: 50−54
Data points enclosed with a diamond are reference sources

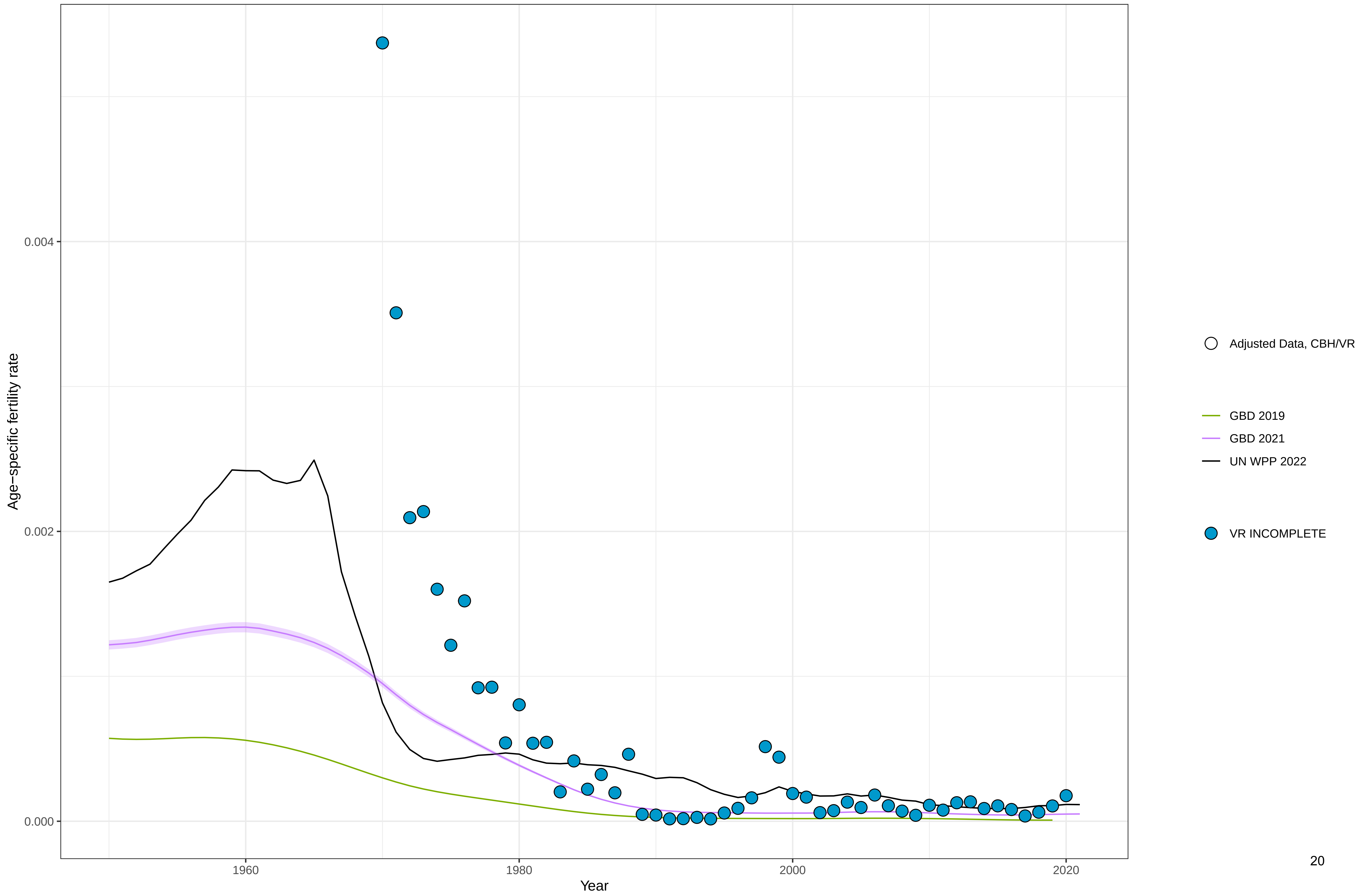

Total fertility rate fitted trend lines, 1950−2021: Azerbaijan TFR
Data points enclosed with a diamond are reference sources

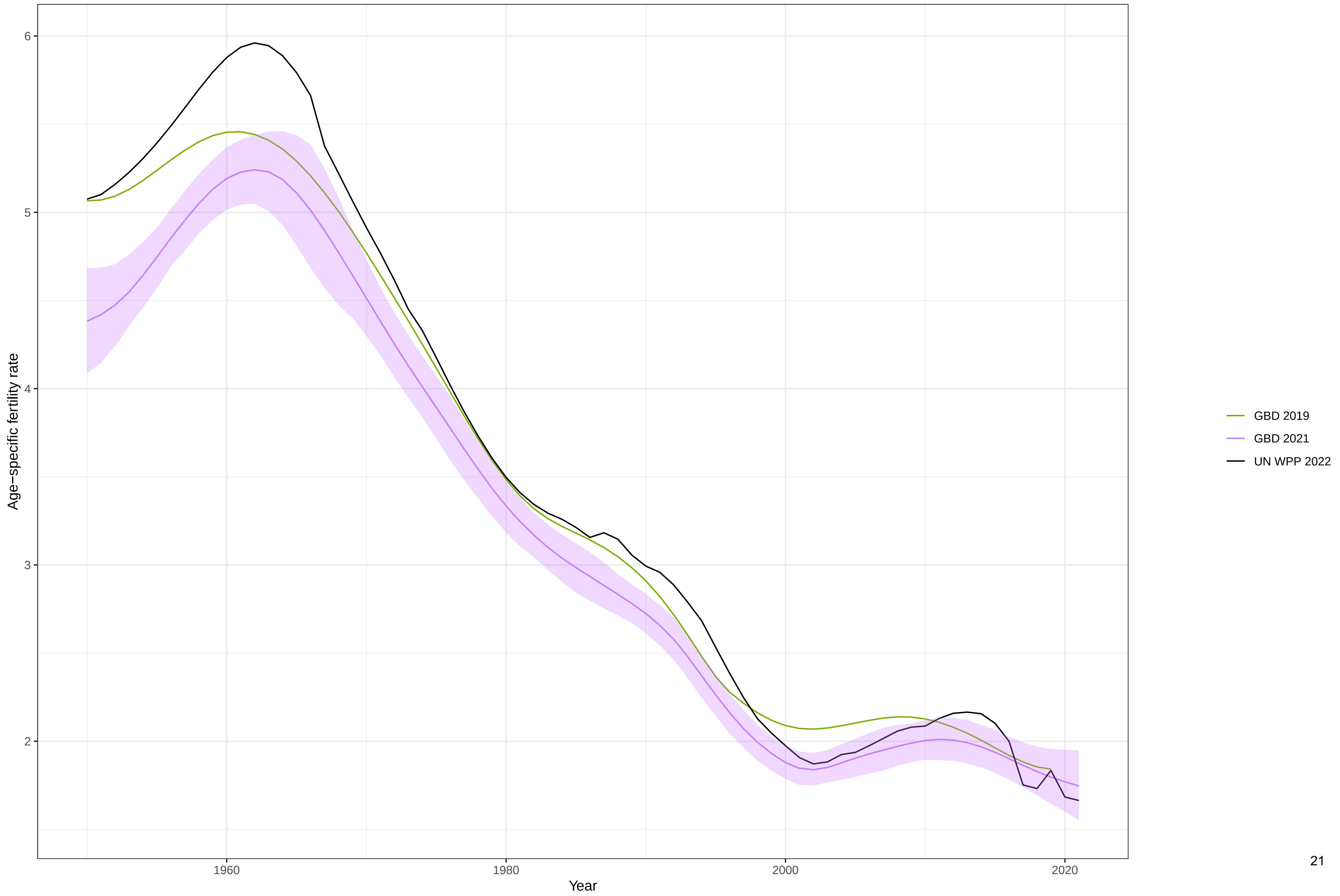

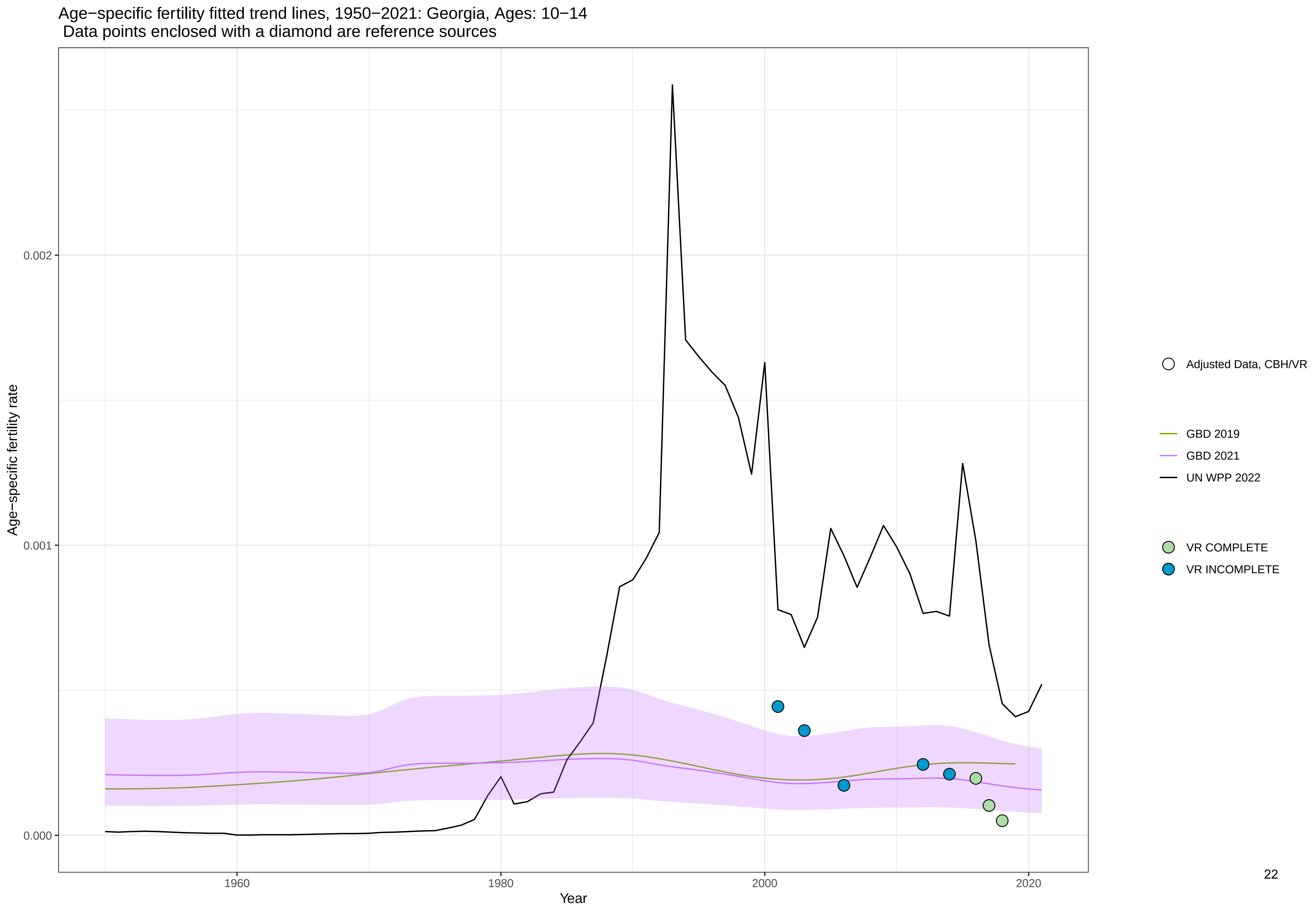
Age−specific fertility fitted trend lines, 1950−2021: Georgia, Ages: 10−14
Data points enclosed with a diamond are reference sources
Age−specific fertility rate
0.000
0.001
0.002
Year
1960
1980
2000
2020
Adjusted Data, CBH/VR
GBD 2019
GBD 2021
UN WPP 2022
VR COMPLETE
VR INCOMPLETE

Age−specific fertility fitted trend lines, 1950−2021: Georgia, Ages: 15−19
Data points enclosed with a diamond are reference sources

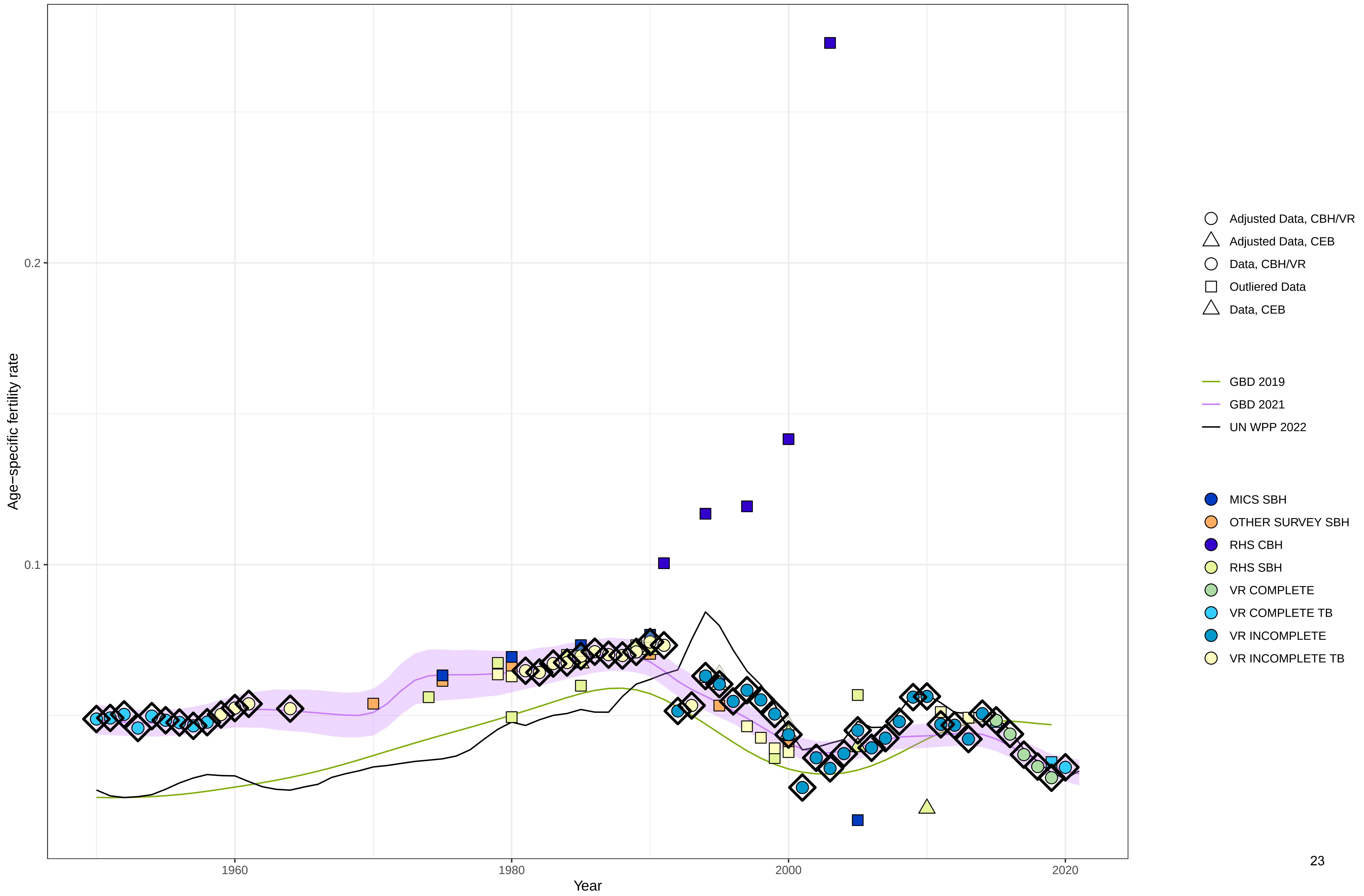

Age−specific fertility fitted trend lines, 1950−2021: Georgia, Ages: 20−24
Data points enclosed with a diamond are reference sources

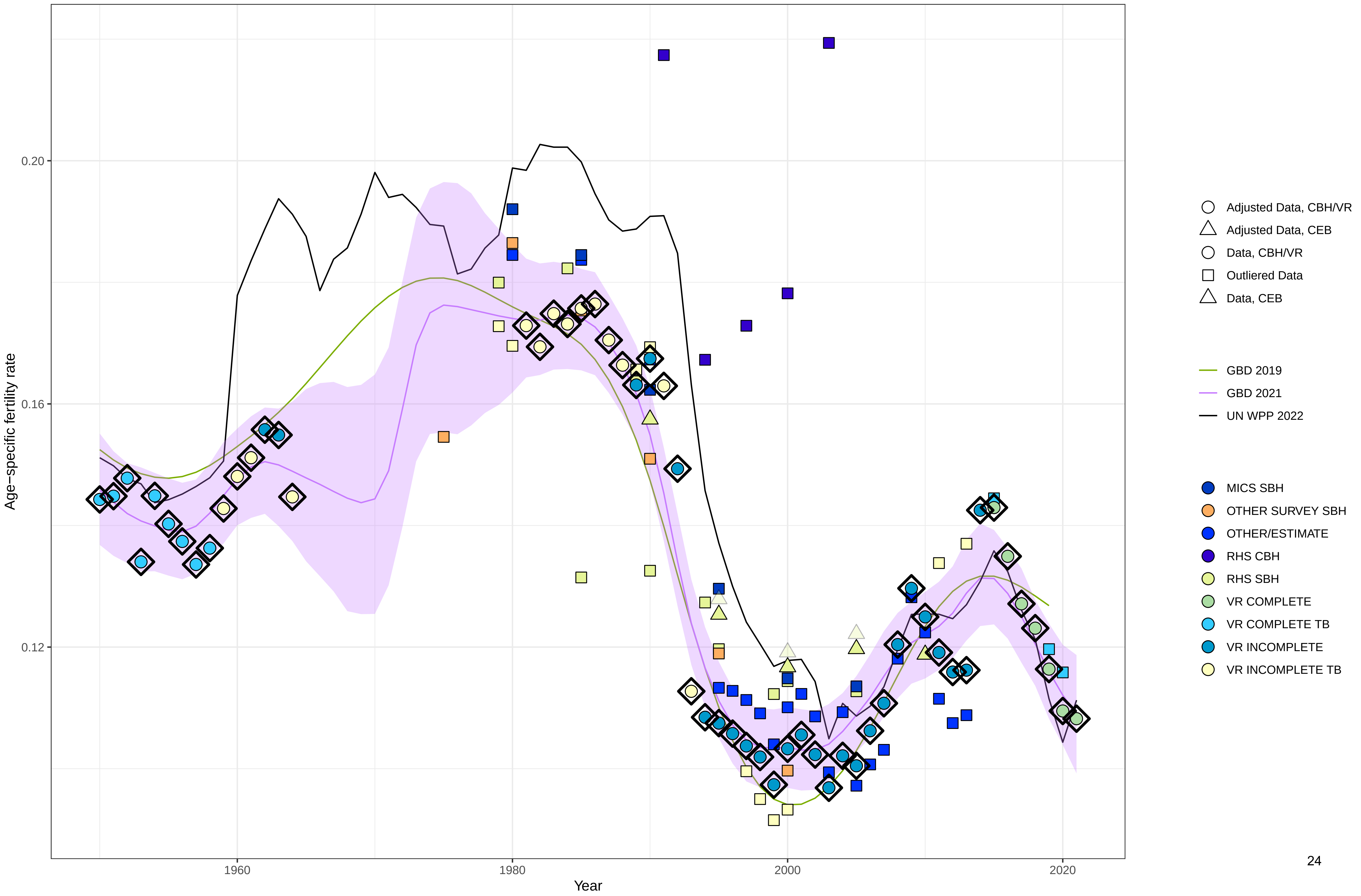

Age−specific fertility fitted trend lines, 1950−2021: Georgia, Ages: 25−29
Data points enclosed with a diamond are reference sources

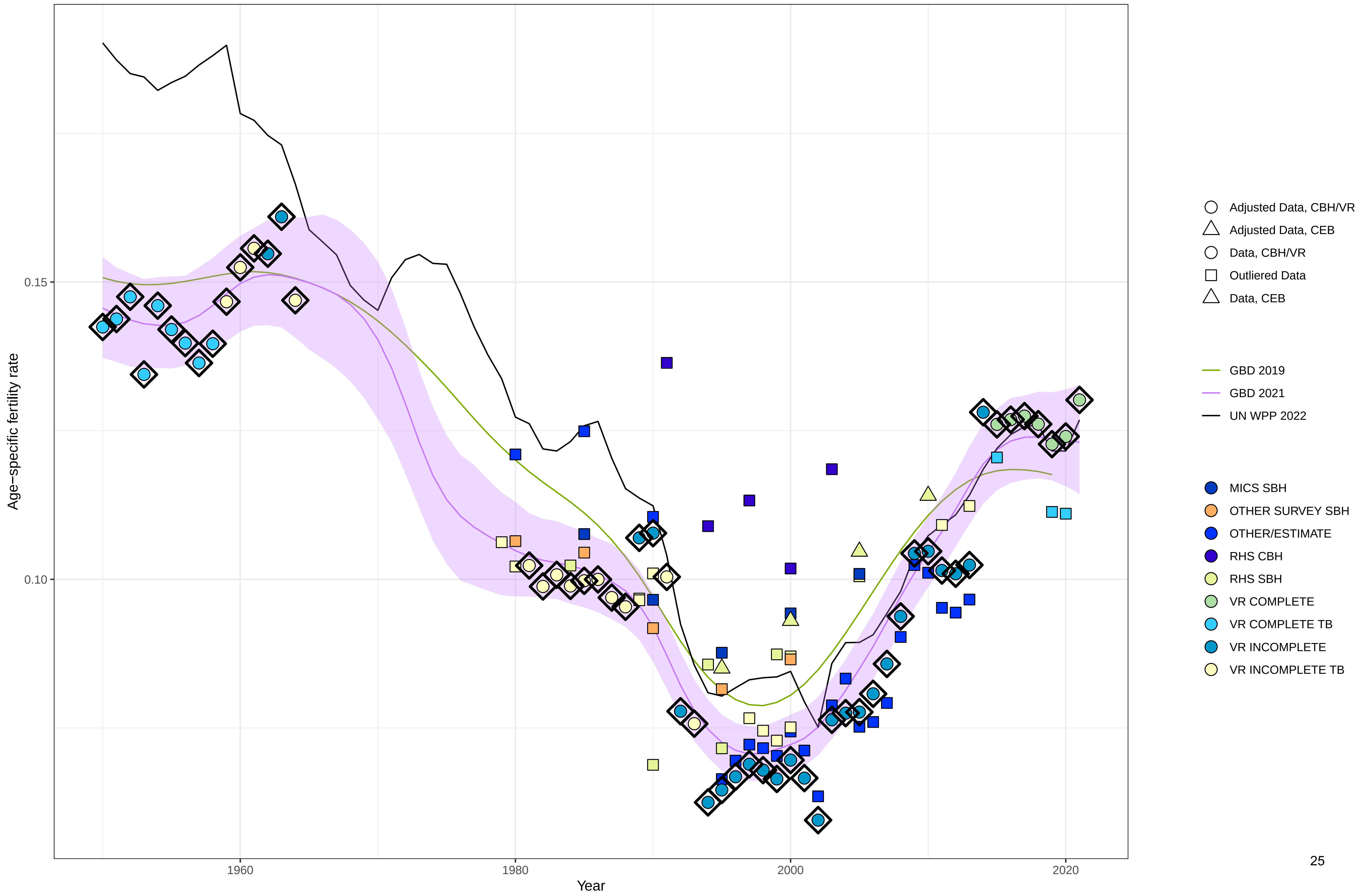

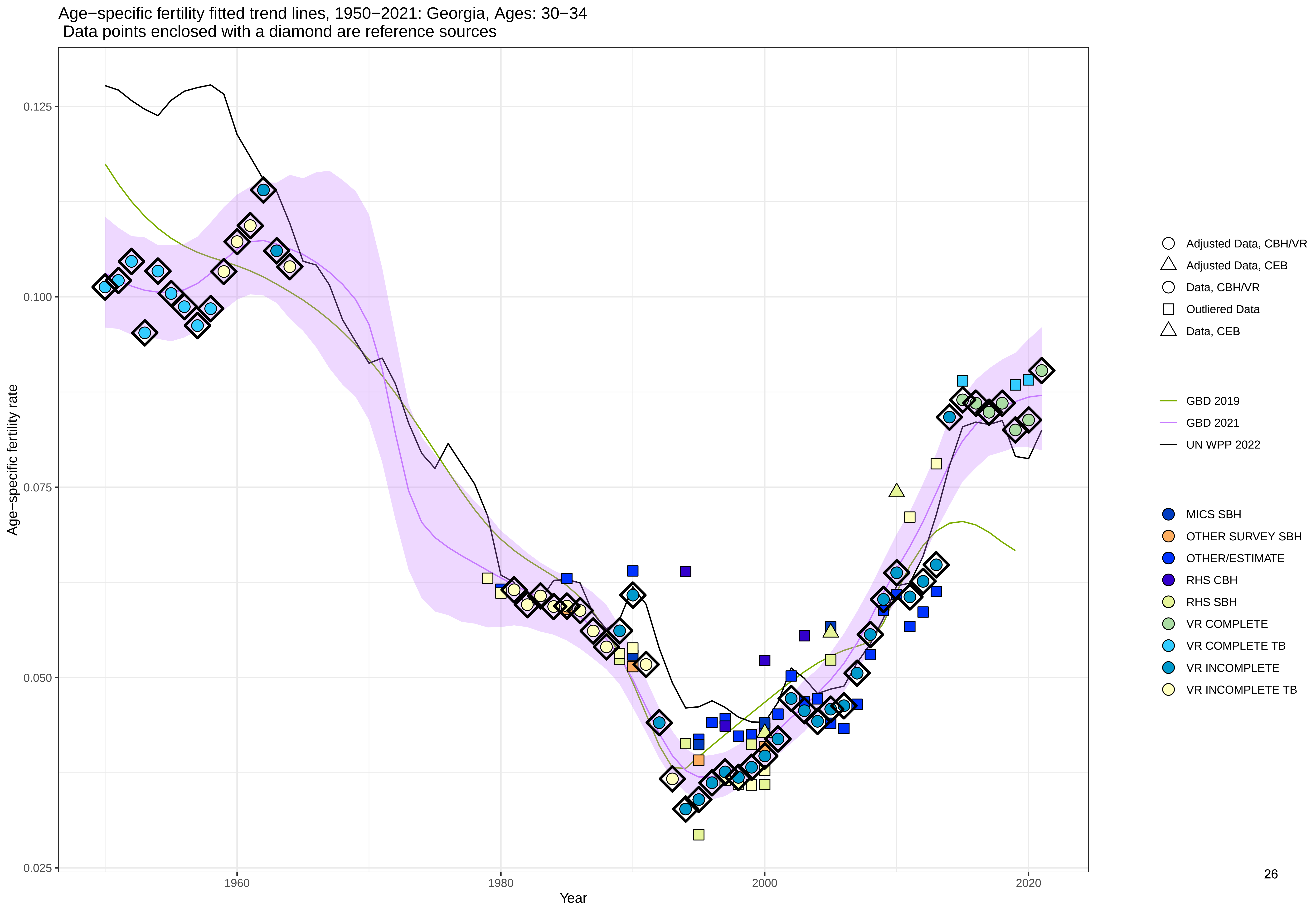

Age−specific fertility fitted trend lines, 1950−2021: Georgia, Ages: 30−34
Data points enclosed with a diamond are reference sources
Age−specific fertility rate
0.125
0.100
0.075
0.050
0.025
1960
1980
2000
2020
Year
Adjusted Data, CBH/VR
Adjusted Data, CEB
Data, CBH/VR
Outliered Data
Data, CEB
GBD 2019
GBD 2021
UN WPP 2022
MICS SBH
OTHER SURVEY SBH
OTHER/ESTIMATE
RHS CBH
RHS SBH
VR COMPLETE
VR COMPLETE TB
VR INCOMPLETE
VR INCOMPLETE TB

Age−specific fertility fitted trend lines, 1950−2021: Georgia, Ages: 35−39
Data points enclosed with a diamond are reference sources

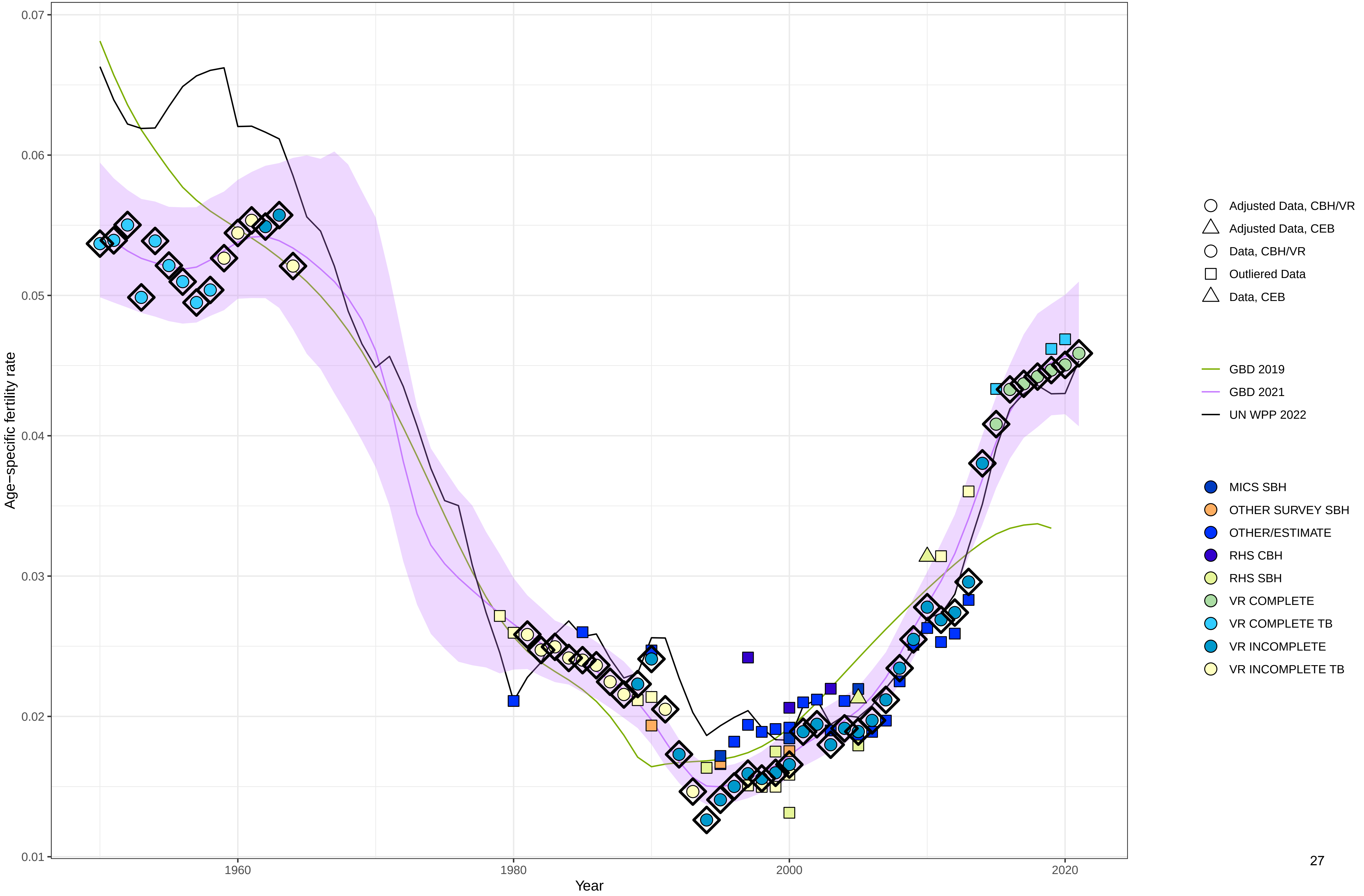

Age−specific fertility fitted trend lines, 1950−2021: Georgia, Ages: 40−44
Data points enclosed with a diamond are reference sources

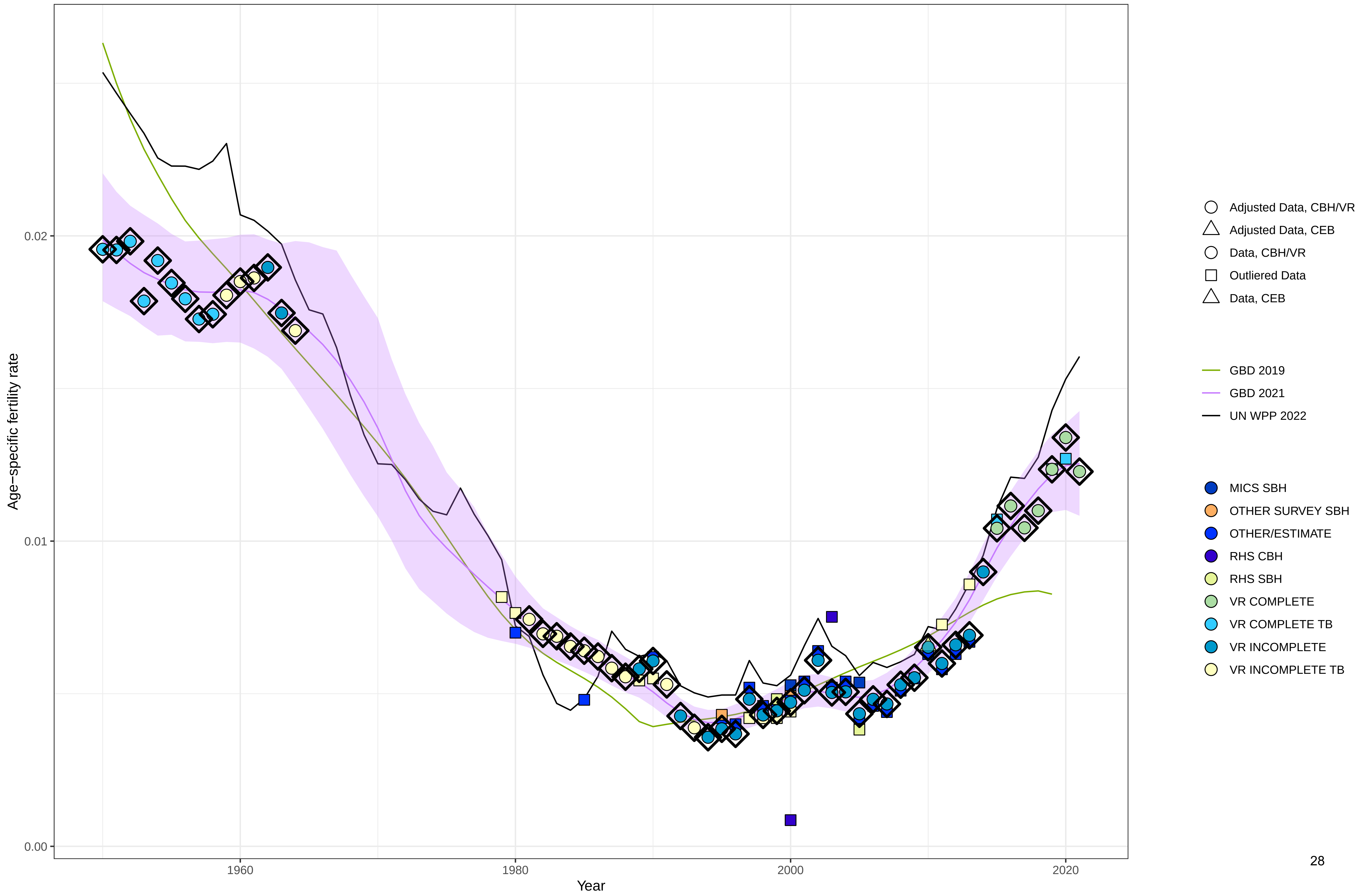

Age−specific fertility fitted trend lines, 1950−2021: Georgia, Ages: 45−49
Data points enclosed with a diamond are reference sources

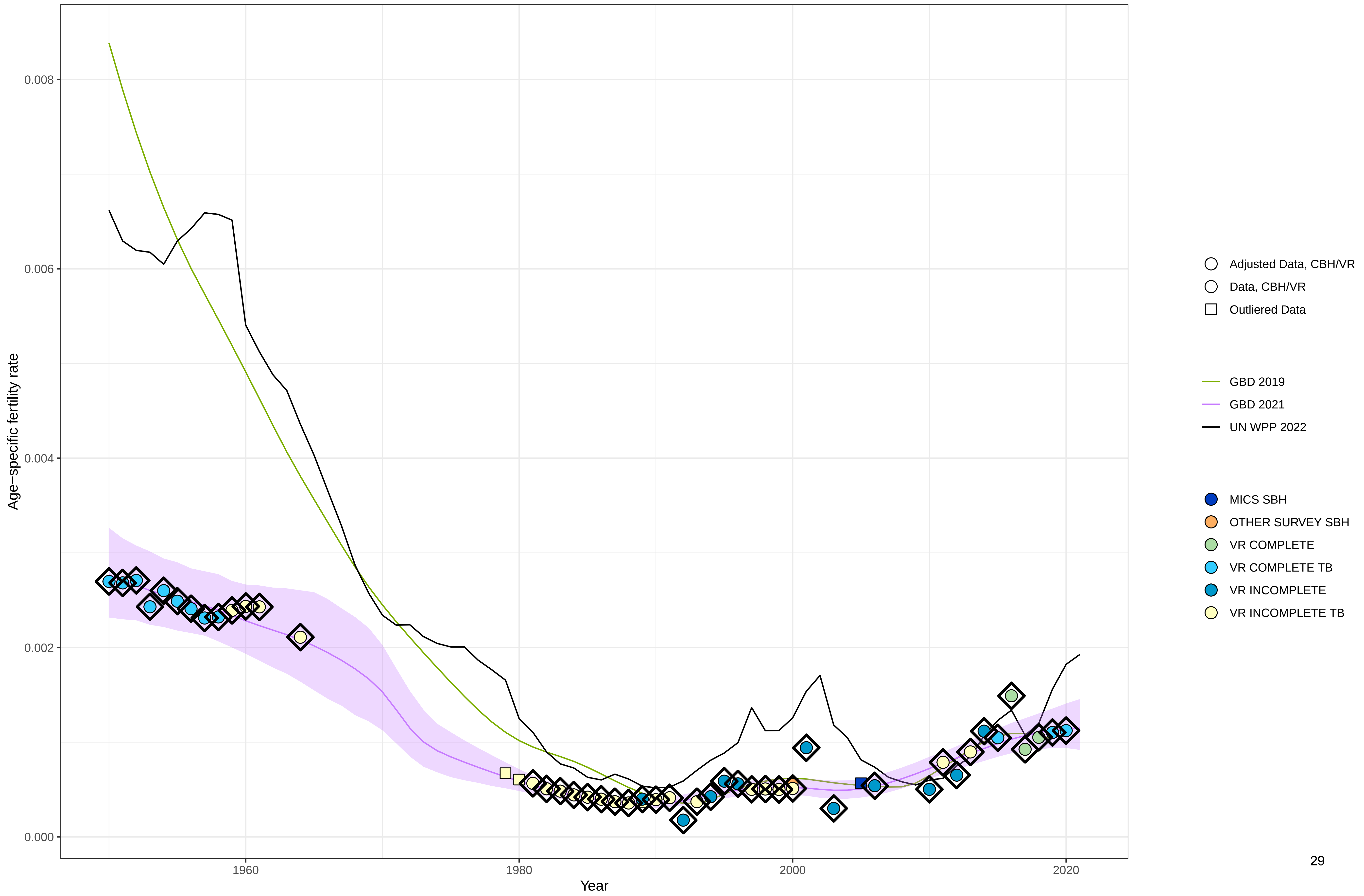

Age−specific fertility fitted trend lines, 1950−2021: Georgia, Ages: 50−54
Data points enclosed with a diamond are reference sources

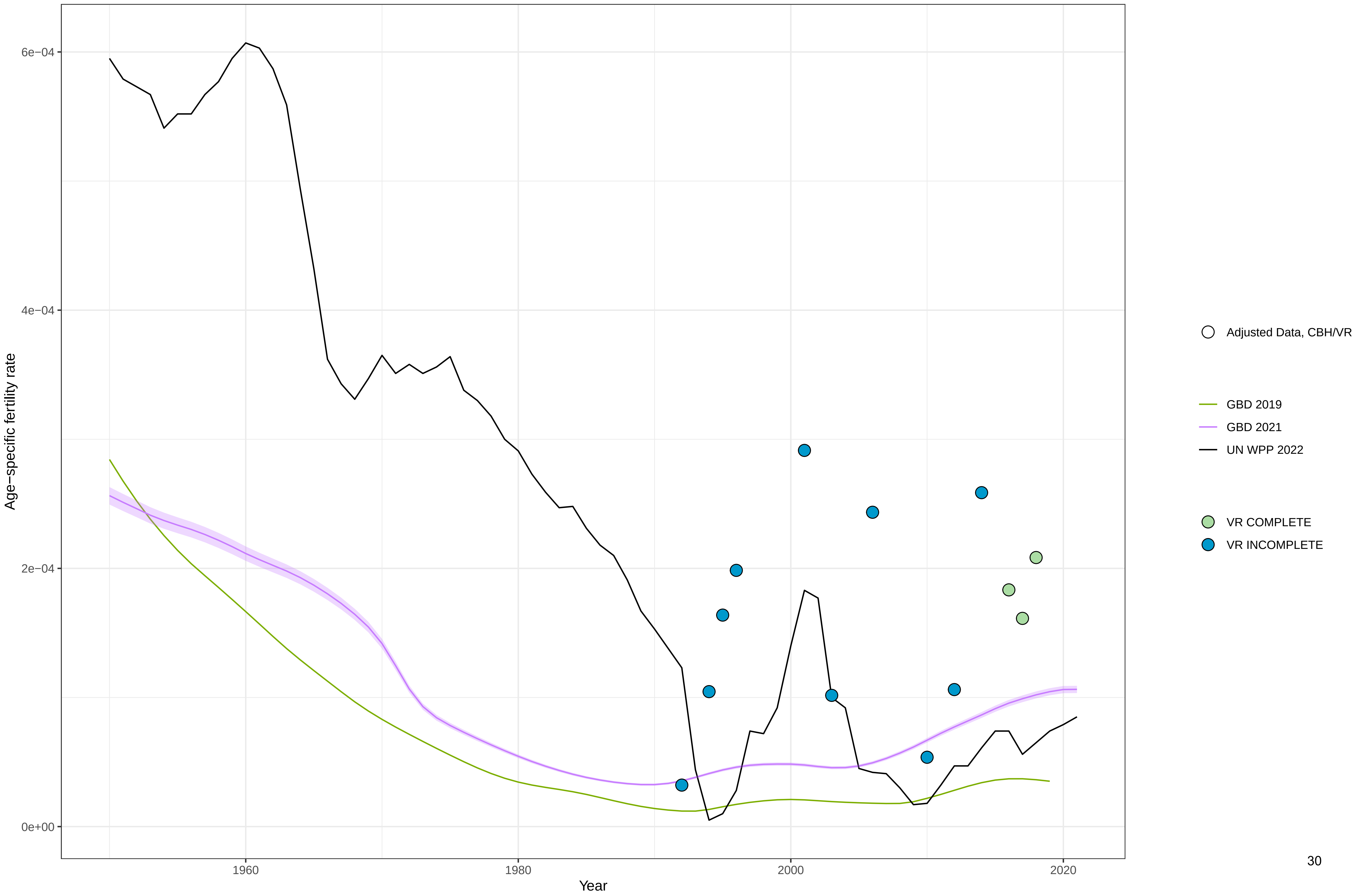

Total fertility rate fitted trend lines, 1950−2021: Georgia TFR
Data points enclosed with a diamond are reference sources

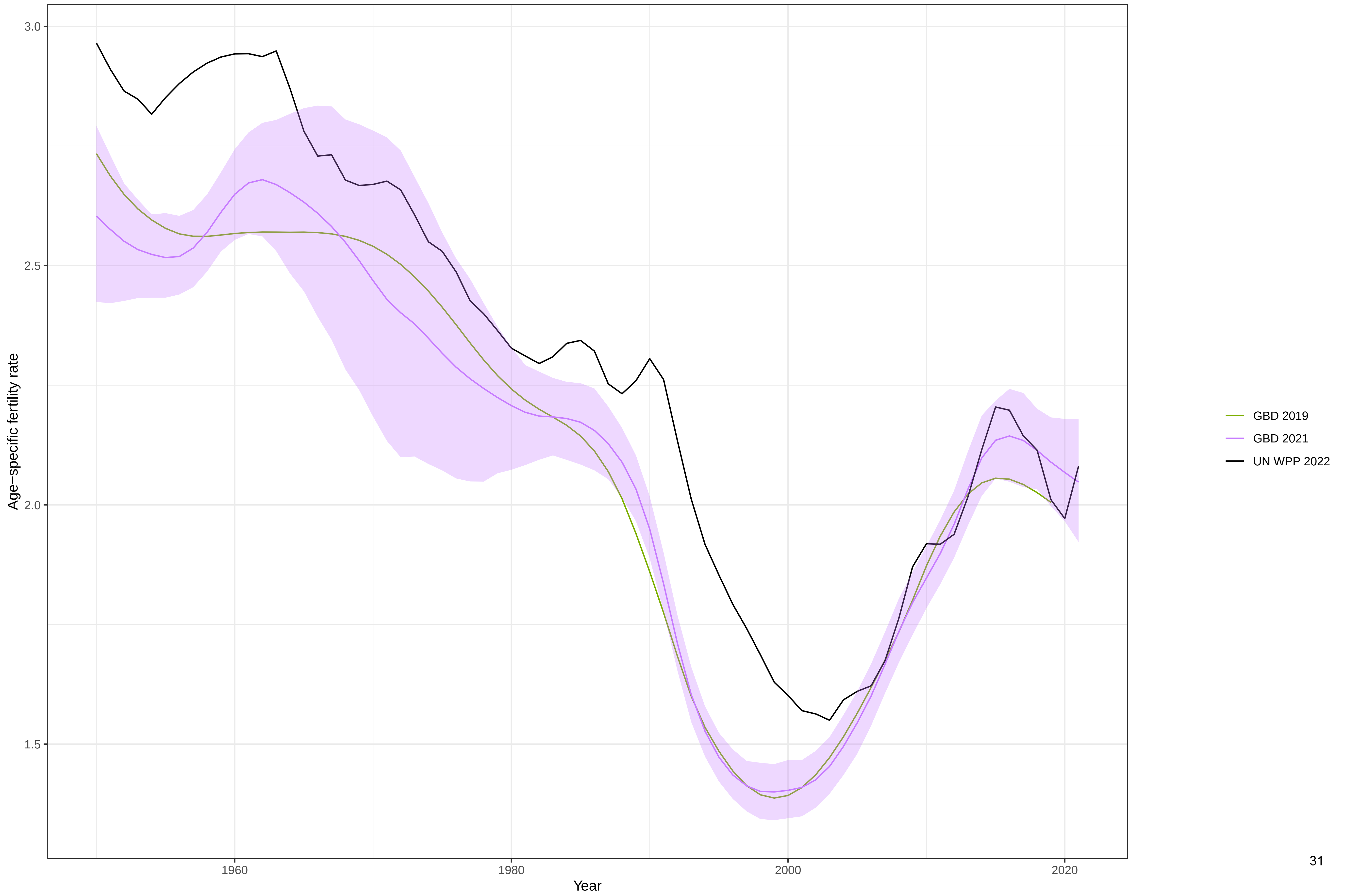

Age−specific fertility fitted trend lines, 1950−2021: Kazakhstan, Ages: 10−14
Data points enclosed with a diamond are reference sources

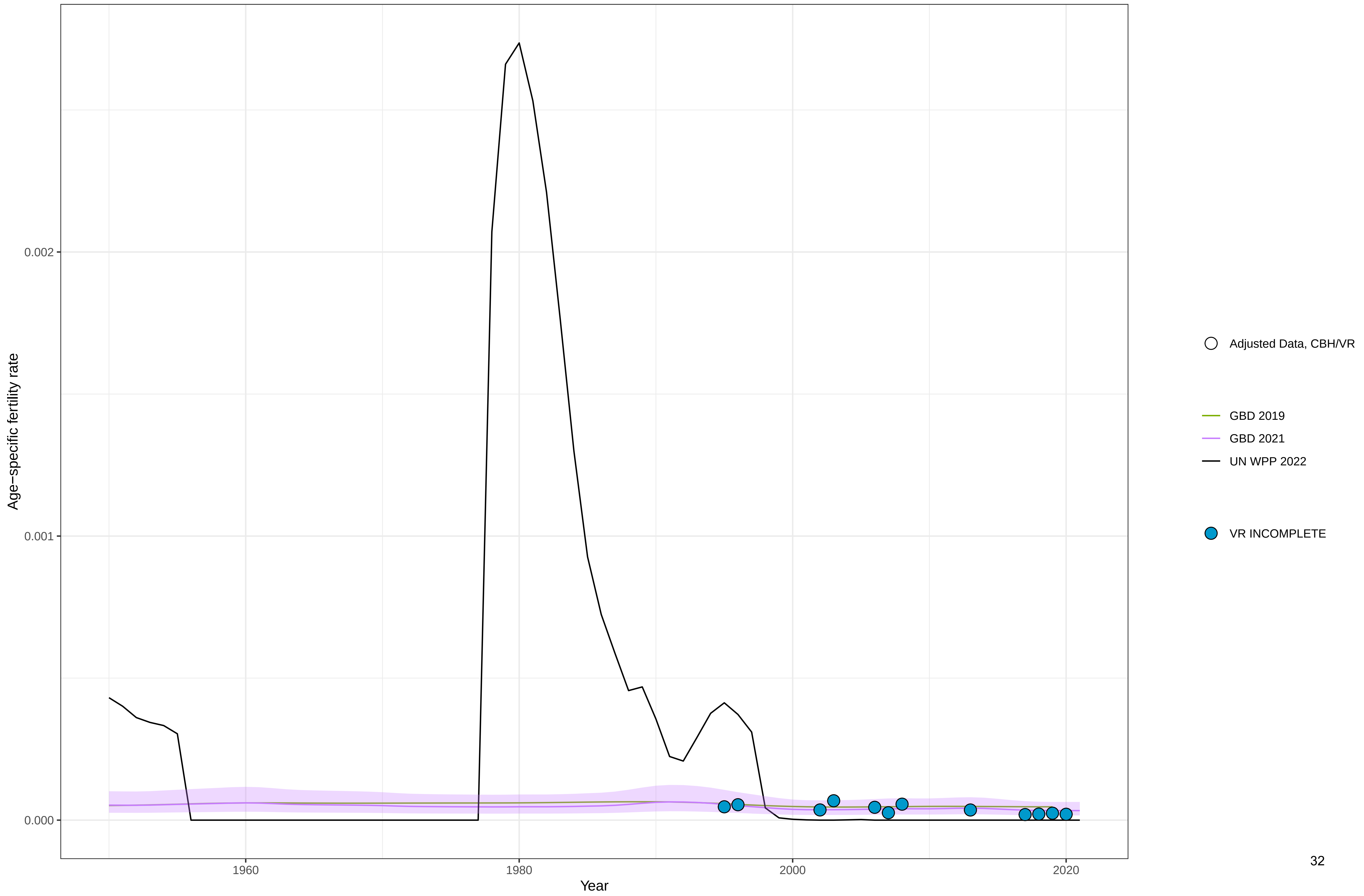

Age−specific fertility fitted trend lines, 1950−2021: Kazakhstan, Ages: 15−19
Data points enclosed with a diamond are reference sources

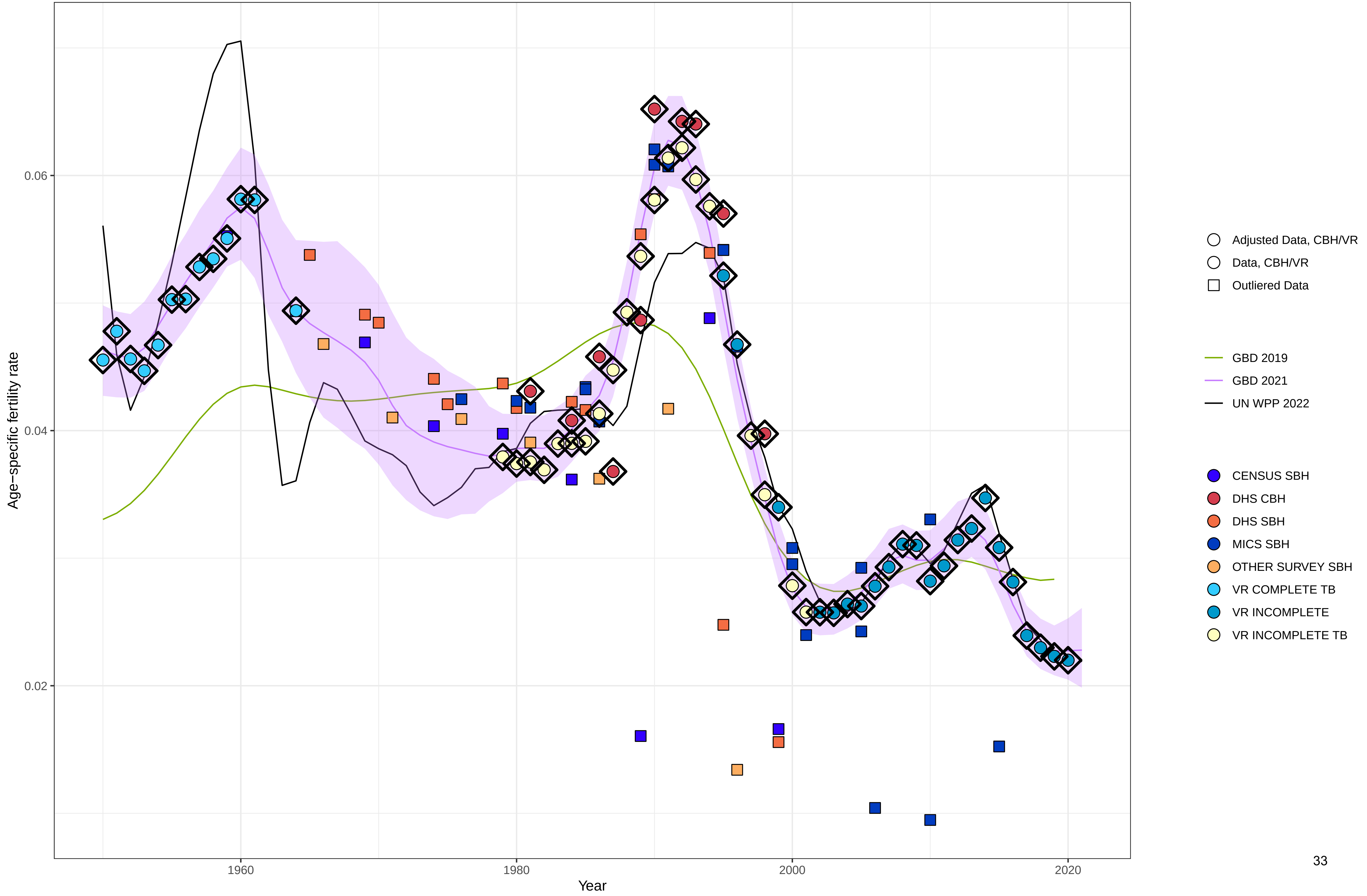

Age−specific fertility fitted trend lines, 1950−2021: Kazakhstan, Ages: 20−24
Data points enclosed with a diamond are reference sources

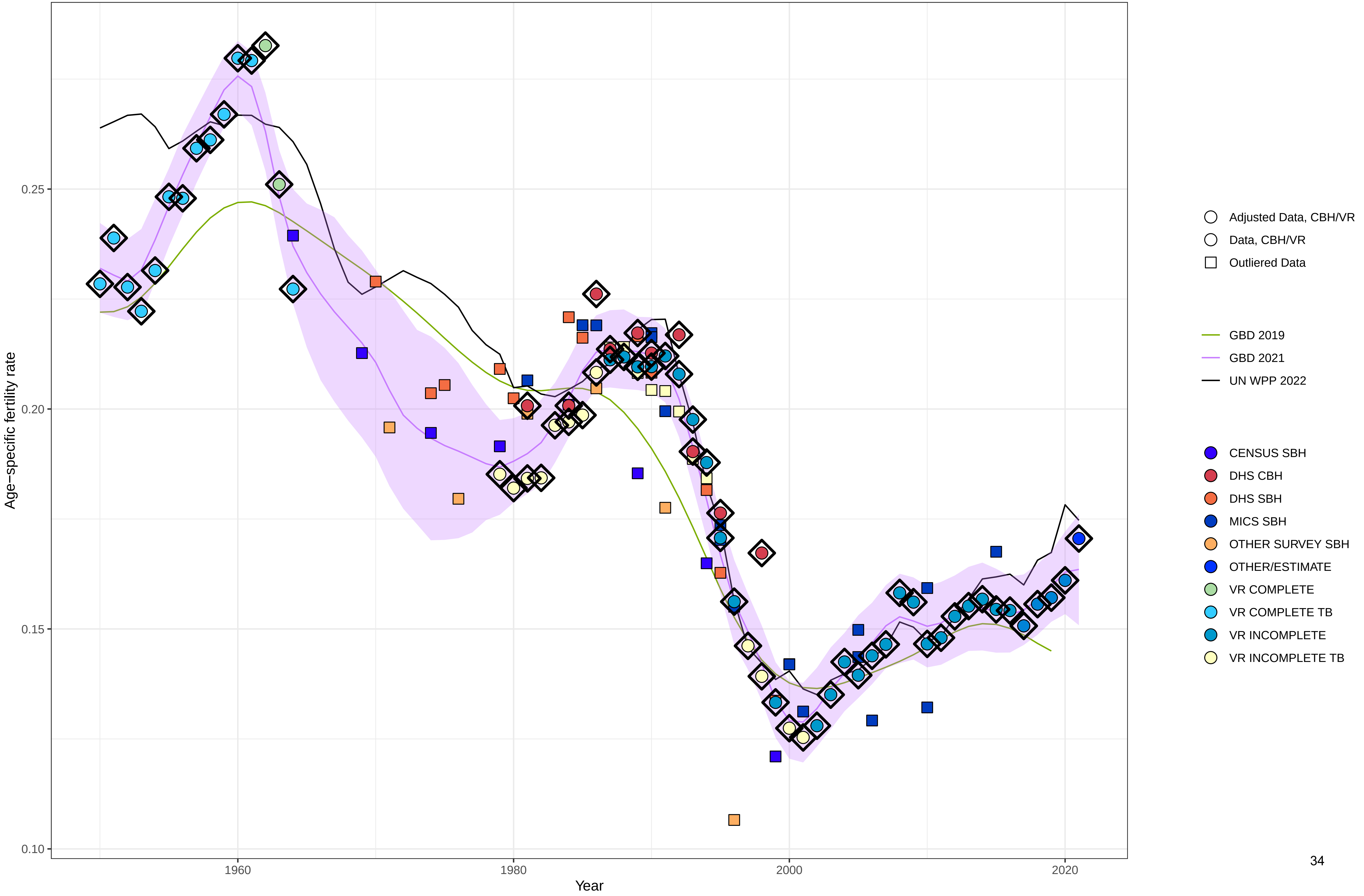

Age−specific fertility fitted trend lines, 1950−2021: Kazakhstan, Ages: 25−29
Data points enclosed with a diamond are reference sources

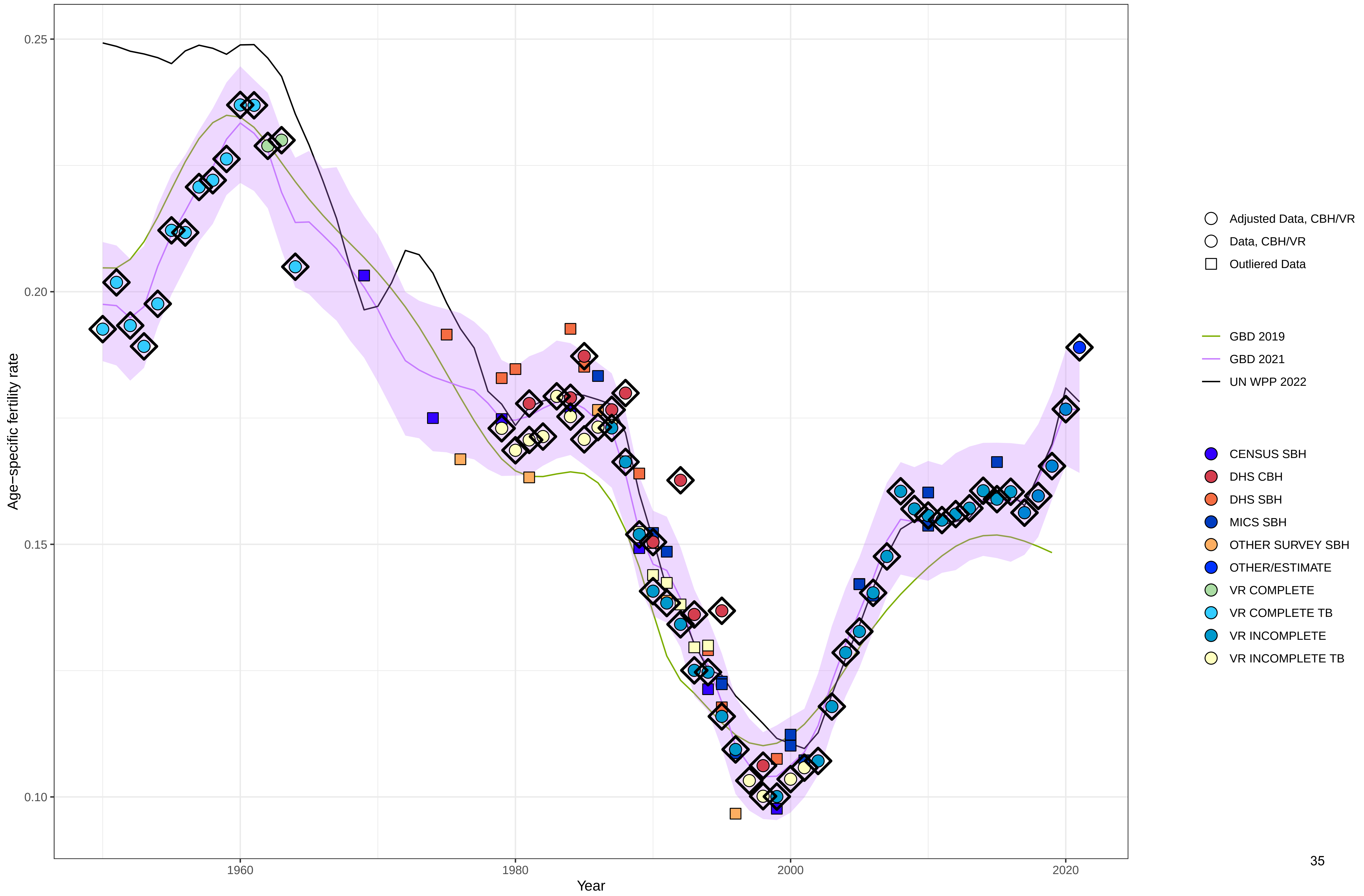

Age−specific fertility fitted trend lines, 1950−2021: Kazakhstan, Ages: 30−34
Data points enclosed with a diamond are reference sources

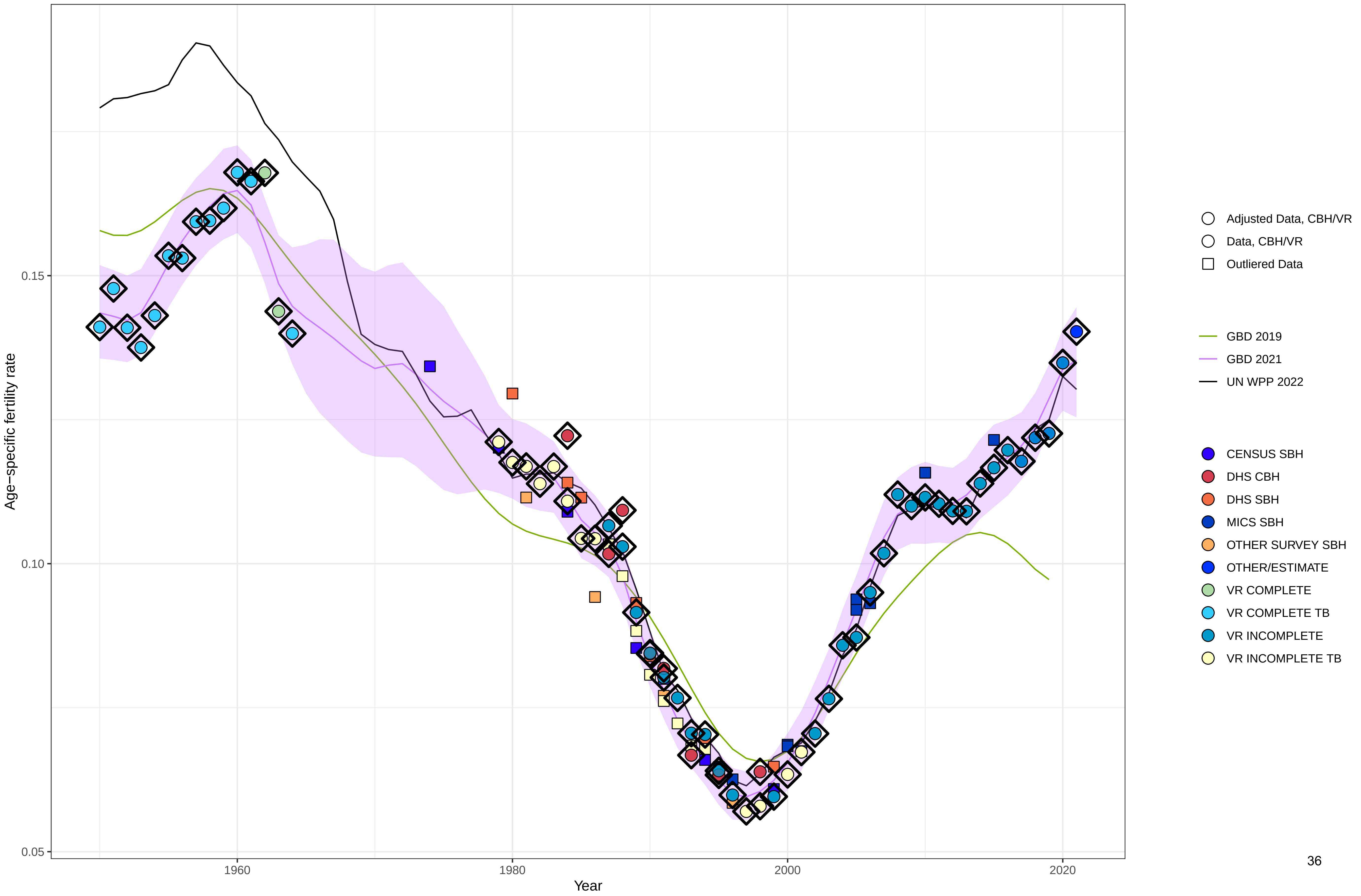

Age−specific fertility fitted trend lines, 1950−2021: Kazakhstan, Ages: 35−39
Data points enclosed with a diamond are reference sources

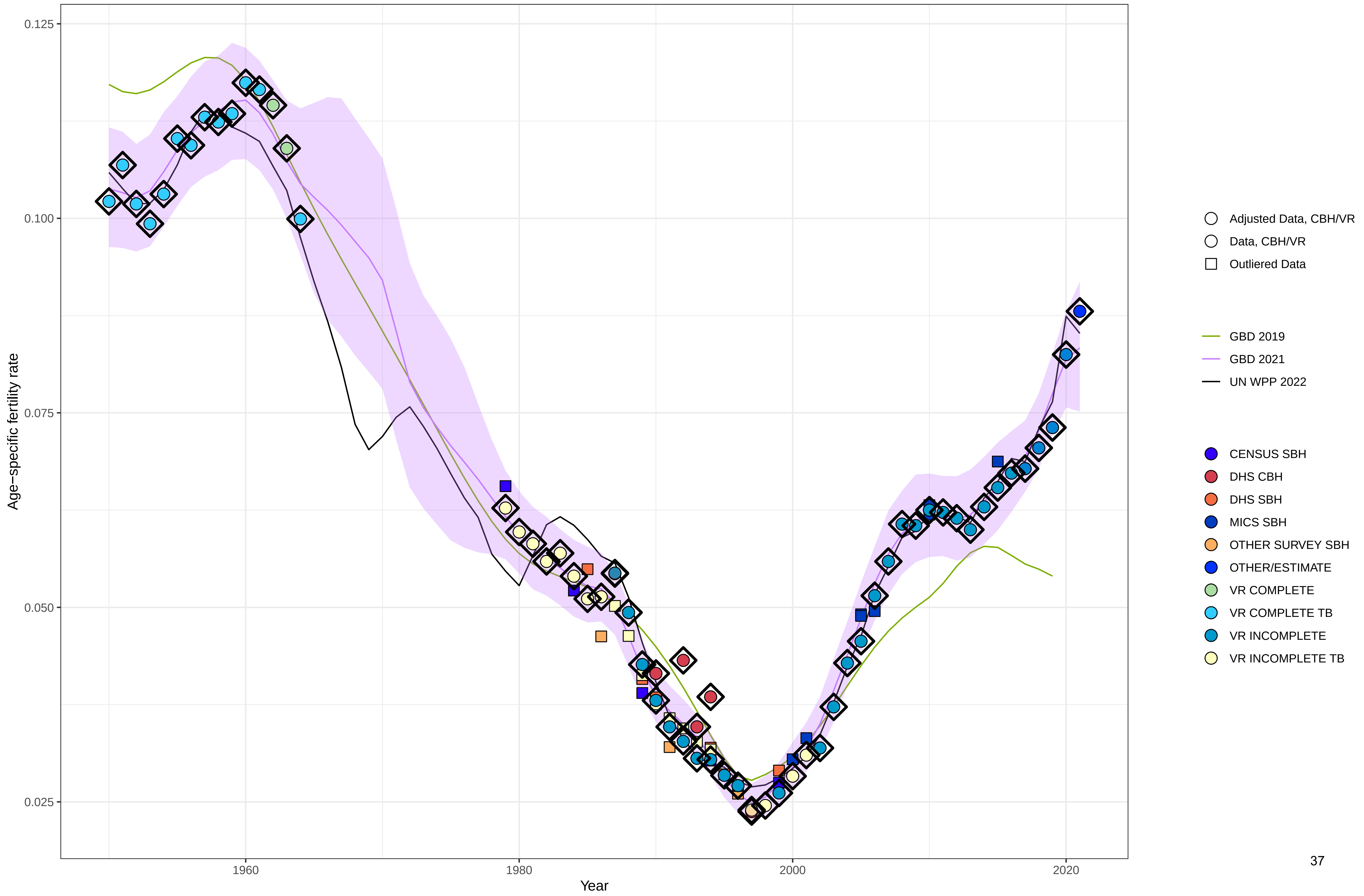

Age−specific fertility fitted trend lines, 1950−2021: Kazakhstan, Ages: 40−44
Data points enclosed with a diamond are reference sources

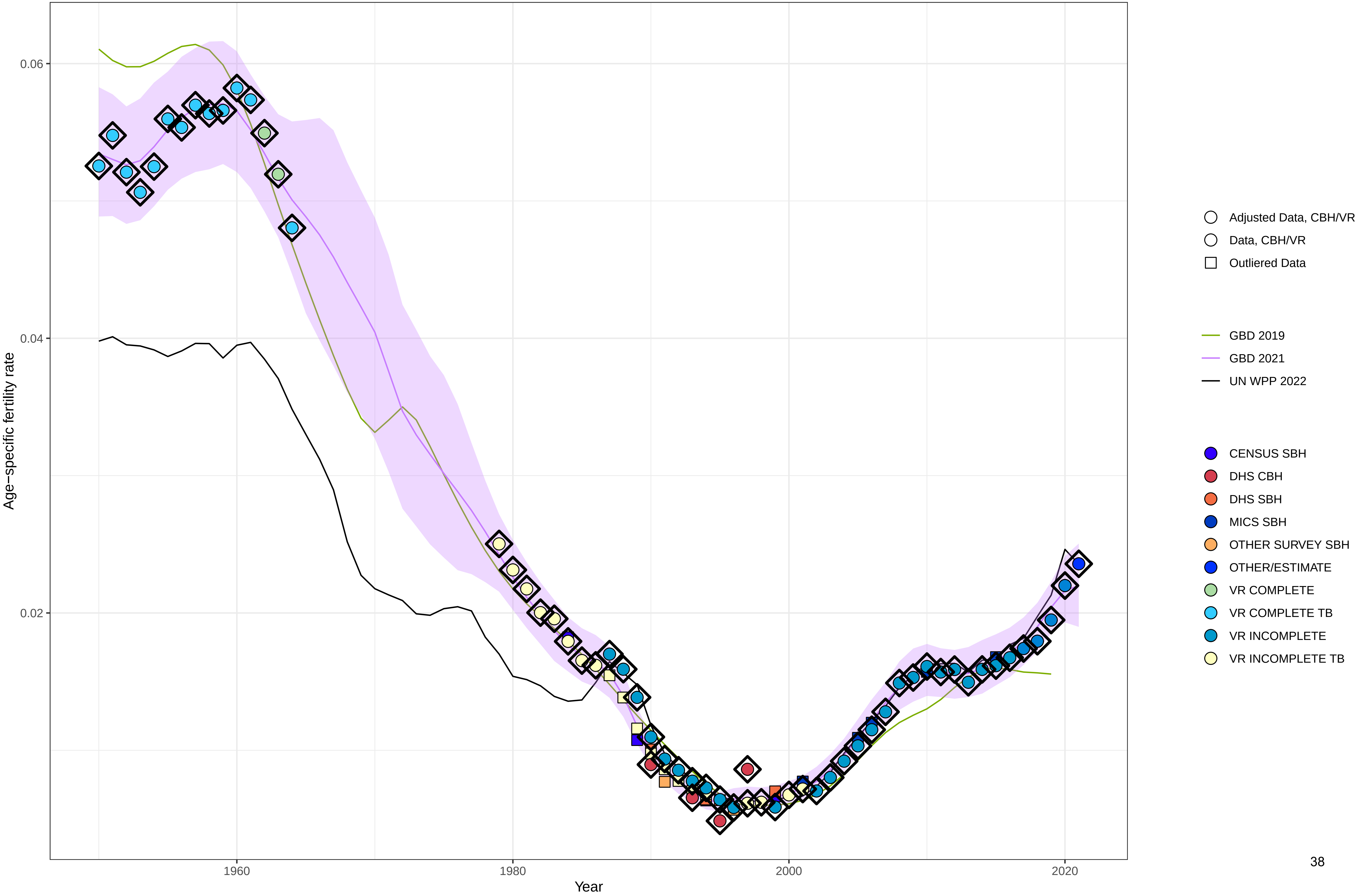

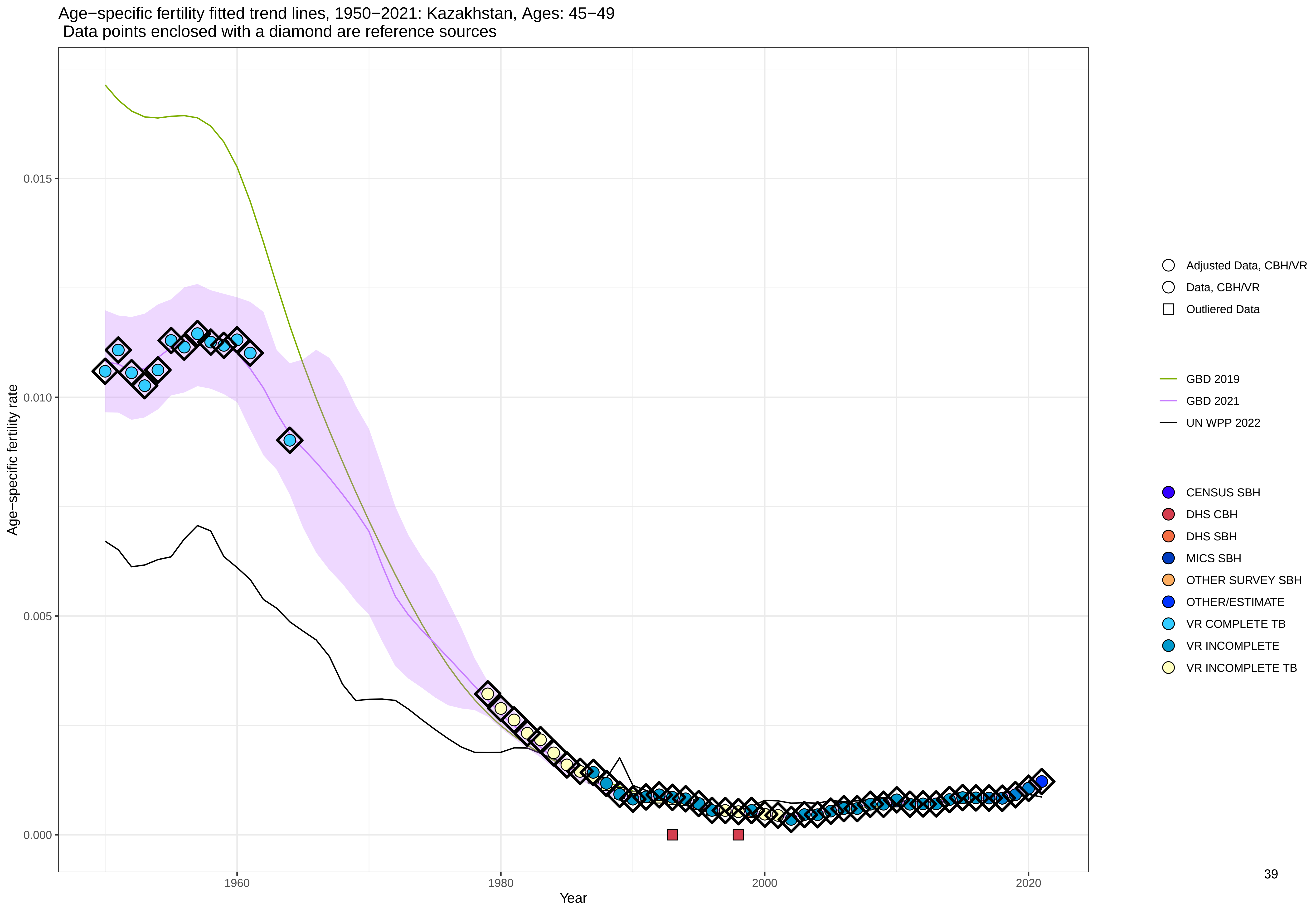
Age−specific fertility fitted trend lines, 1950−2021: Kazakhstan, Ages: 45−49
Data points enclosed with a diamond are reference sources
Age−specific fertility rate
0.015
0.010
0.005
0.000
1960
1980
2000
2020
Year
Adjusted Data, CBH/VR
Data, CBH/VR
Outliered Data
GBD 2019
GBD 2021
UN WPP 2022
CENSUS SBH
DHS CBH
DHS SBH
MICS SBH
OTHER SURVEY SBH
OTHER/ESTIMATE
VR COMPLETE TB
VR INCOMPLETE
VR INCOMPLETE TB

Age−specific fertility fitted trend lines, 1950−2021: Kazakhstan, Ages: 50−54
Data points enclosed with a diamond are reference sources

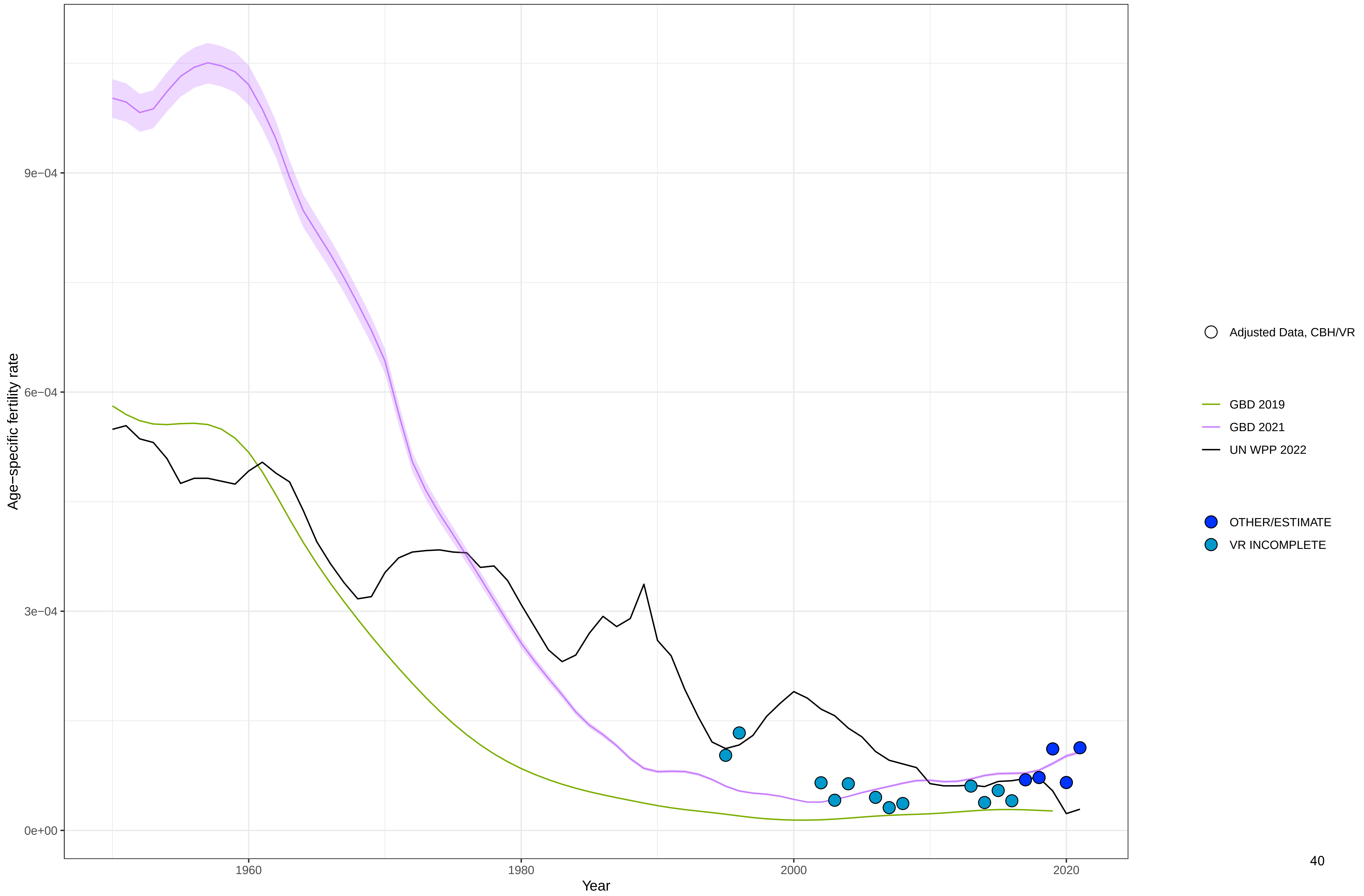

Total fertility rate fitted trend lines, 1950−2021: Kazakhstan TFR
Data points enclosed with a diamond are reference sources

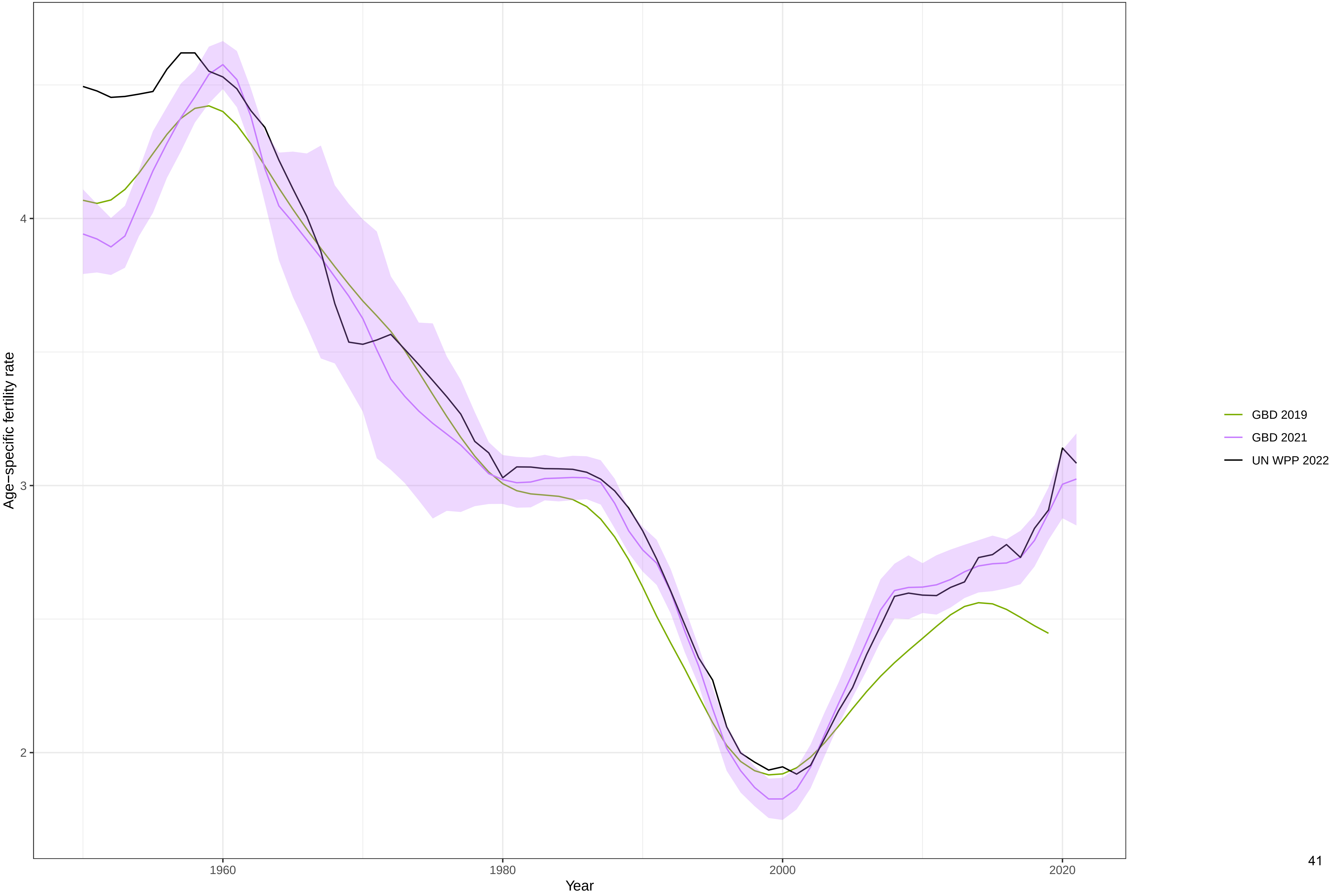

Age−specific fertility fitted trend lines, 1950−2021: Kyrgyzstan, Ages: 10−14
Data points enclosed with a diamond are reference sources

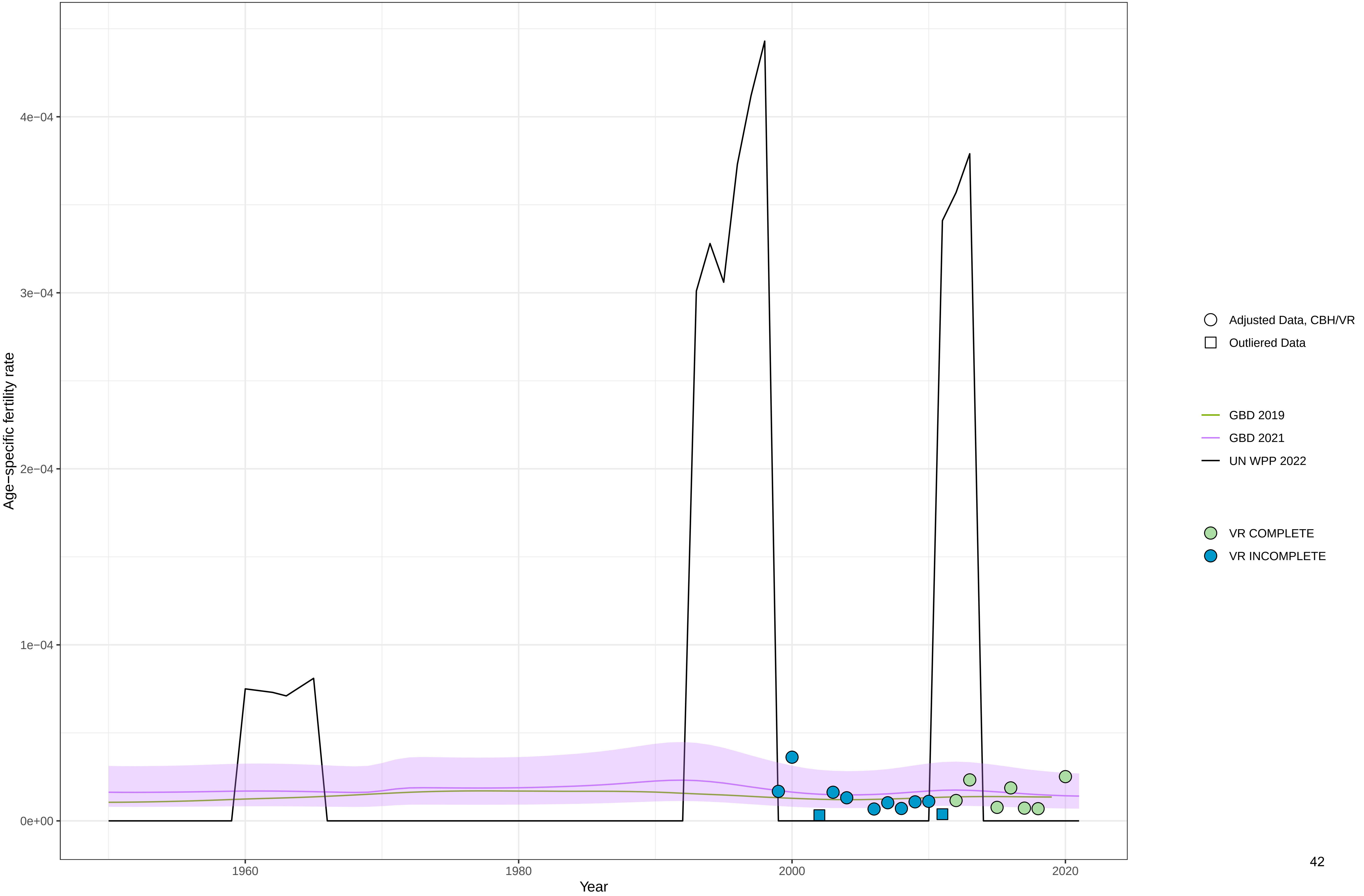

Age−specific fertility fitted trend lines, 1950−2021: Kyrgyzstan, Ages: 15−19
Data points enclosed with a diamond are reference sources

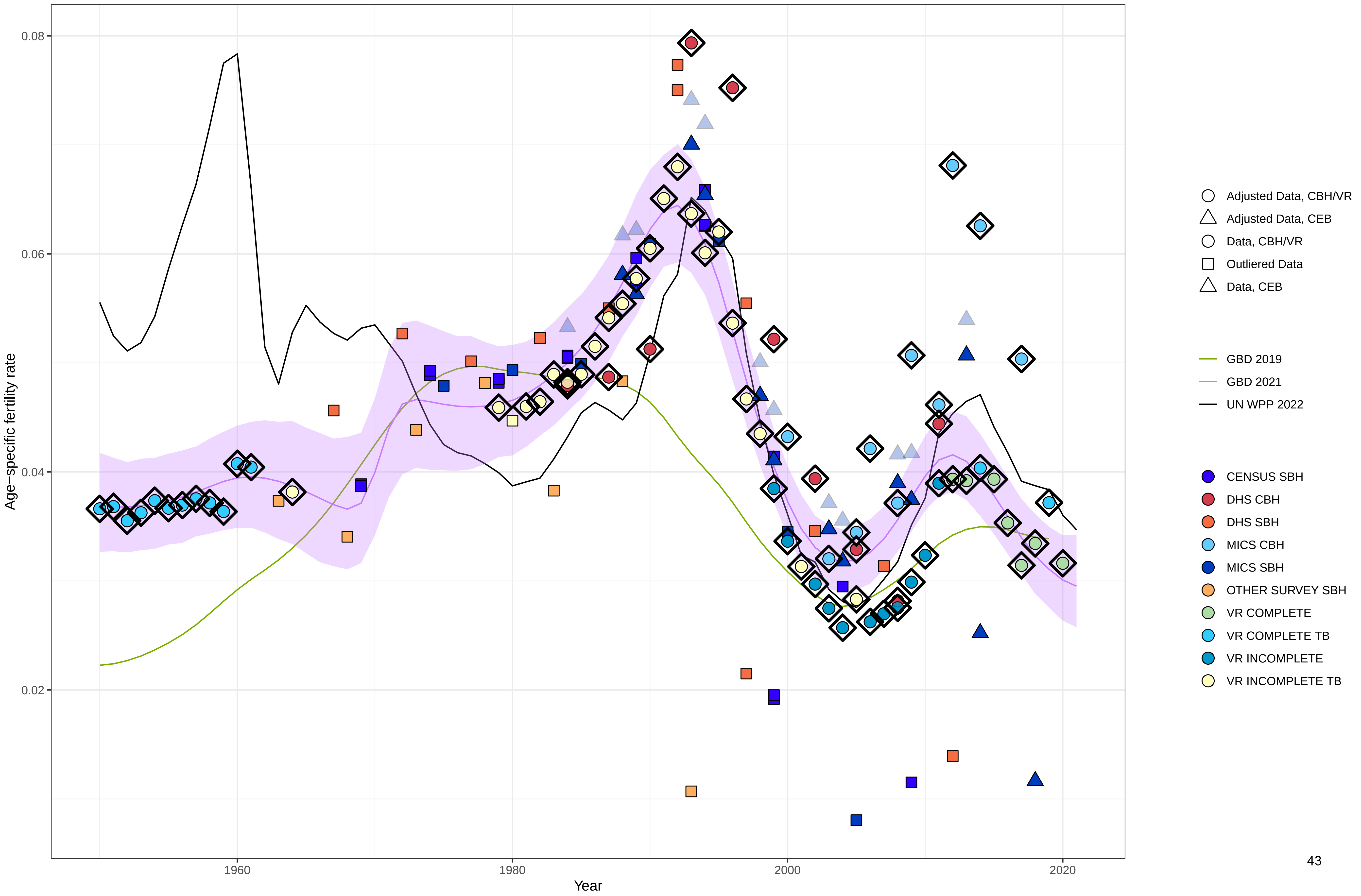

Age−specific fertility fitted trend lines, 1950−2021: Kyrgyzstan, Ages: 20−24
Data points enclosed with a diamond are reference sources

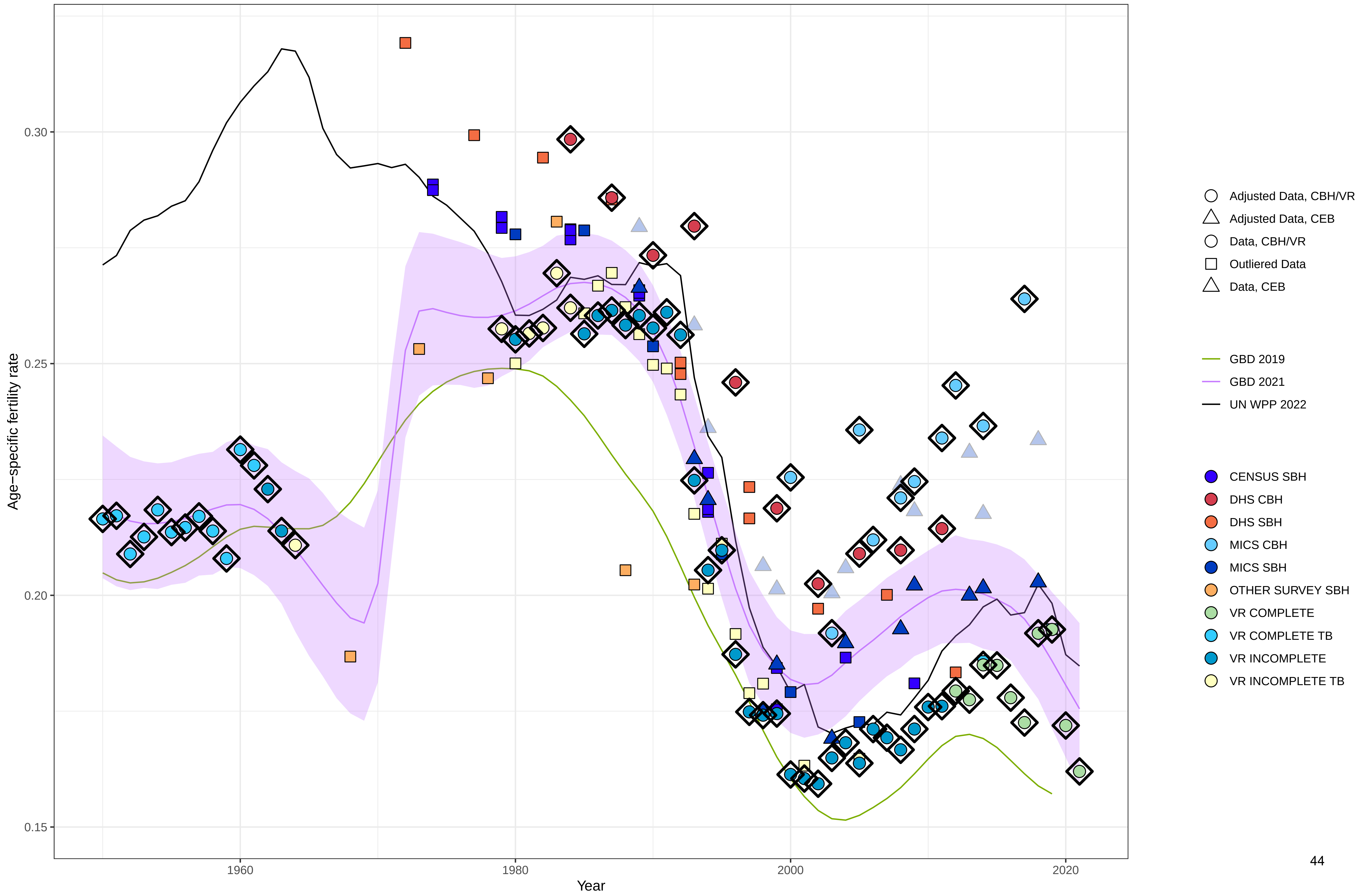

Age−specific fertility fitted trend lines, 1950−2021: Kyrgyzstan, Ages: 25−29
Data points enclosed with a diamond are reference sources

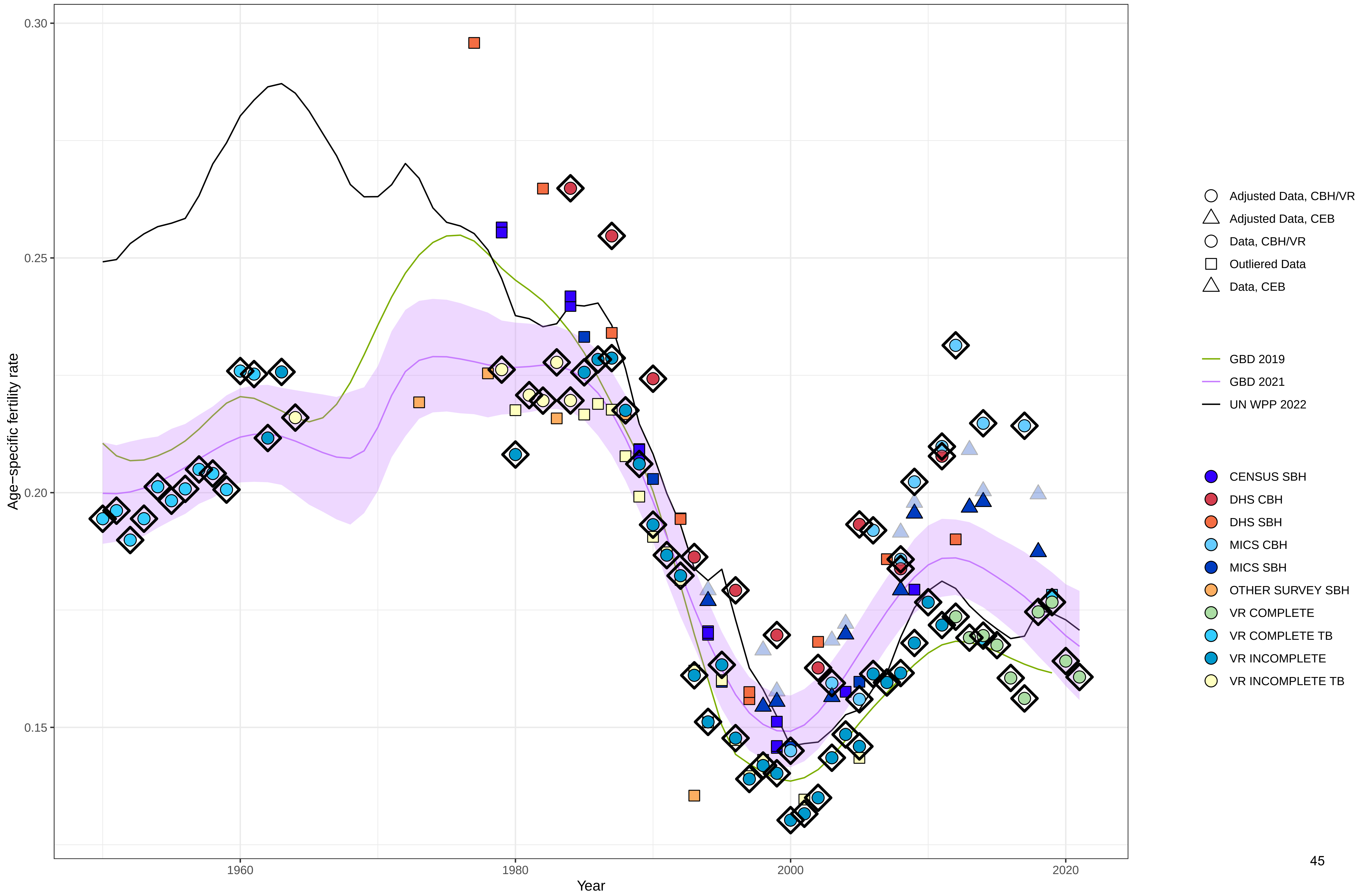

Age−specific fertility fitted trend lines, 1950−2021: Kyrgyzstan, Ages: 30−34
Data points enclosed with a diamond are reference sources

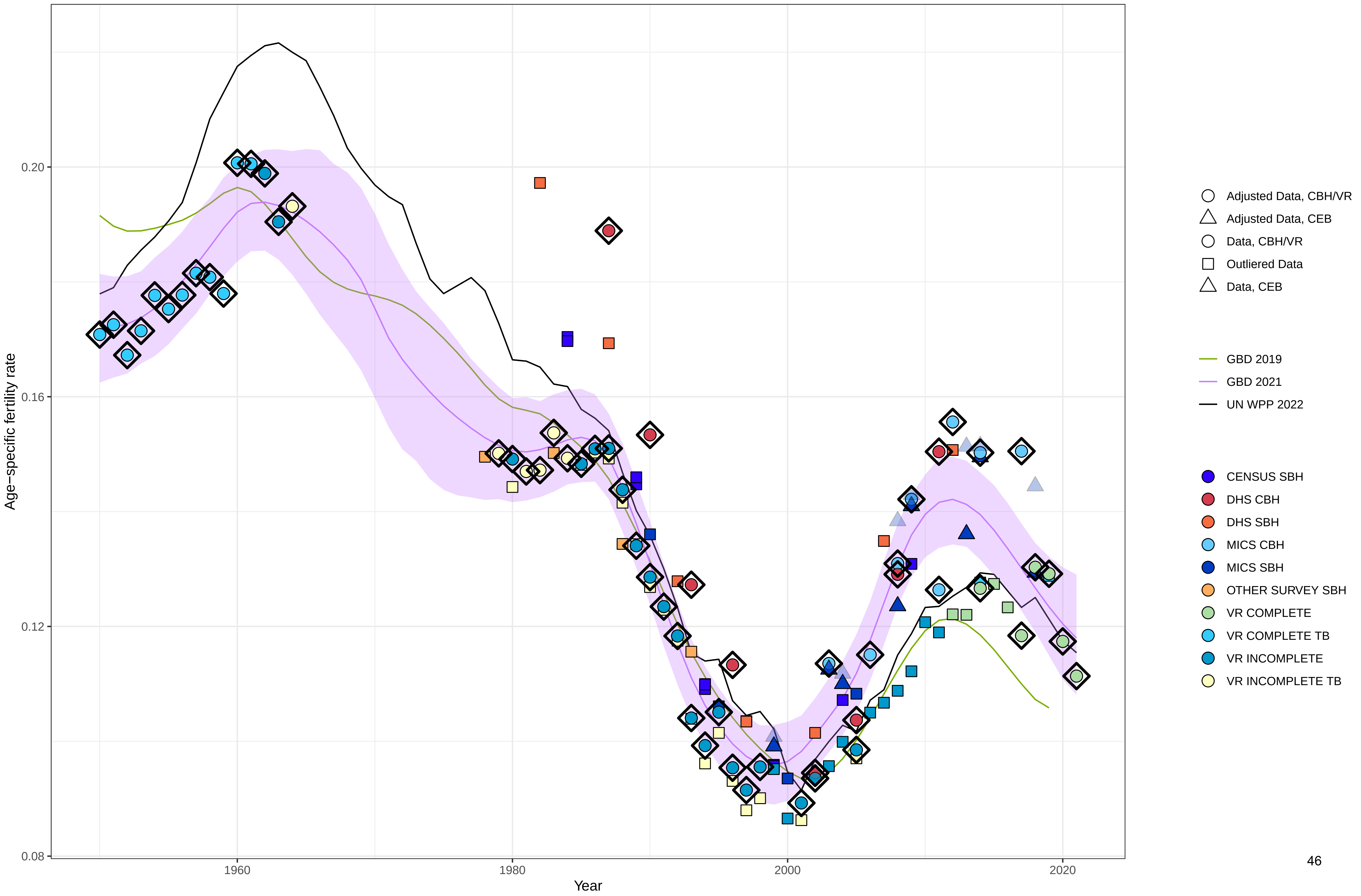

Age−specific fertility fitted trend lines, 1950−2021: Kyrgyzstan, Ages: 35−39
Data points enclosed with a diamond are reference sources

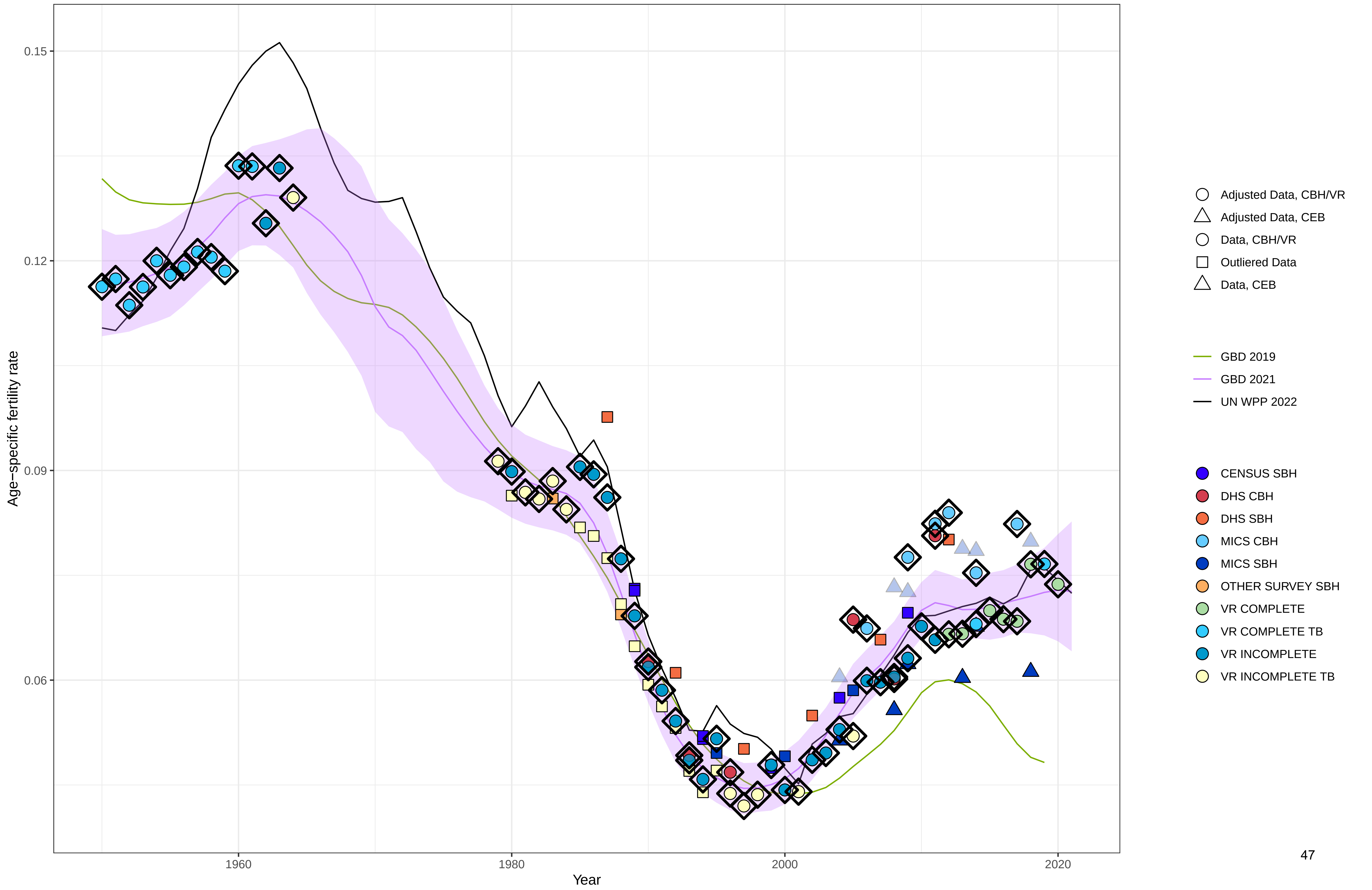

Age−specific fertility fitted trend lines, 1950−2021: Kyrgyzstan, Ages: 40−44
Data points enclosed with a diamond are reference sources

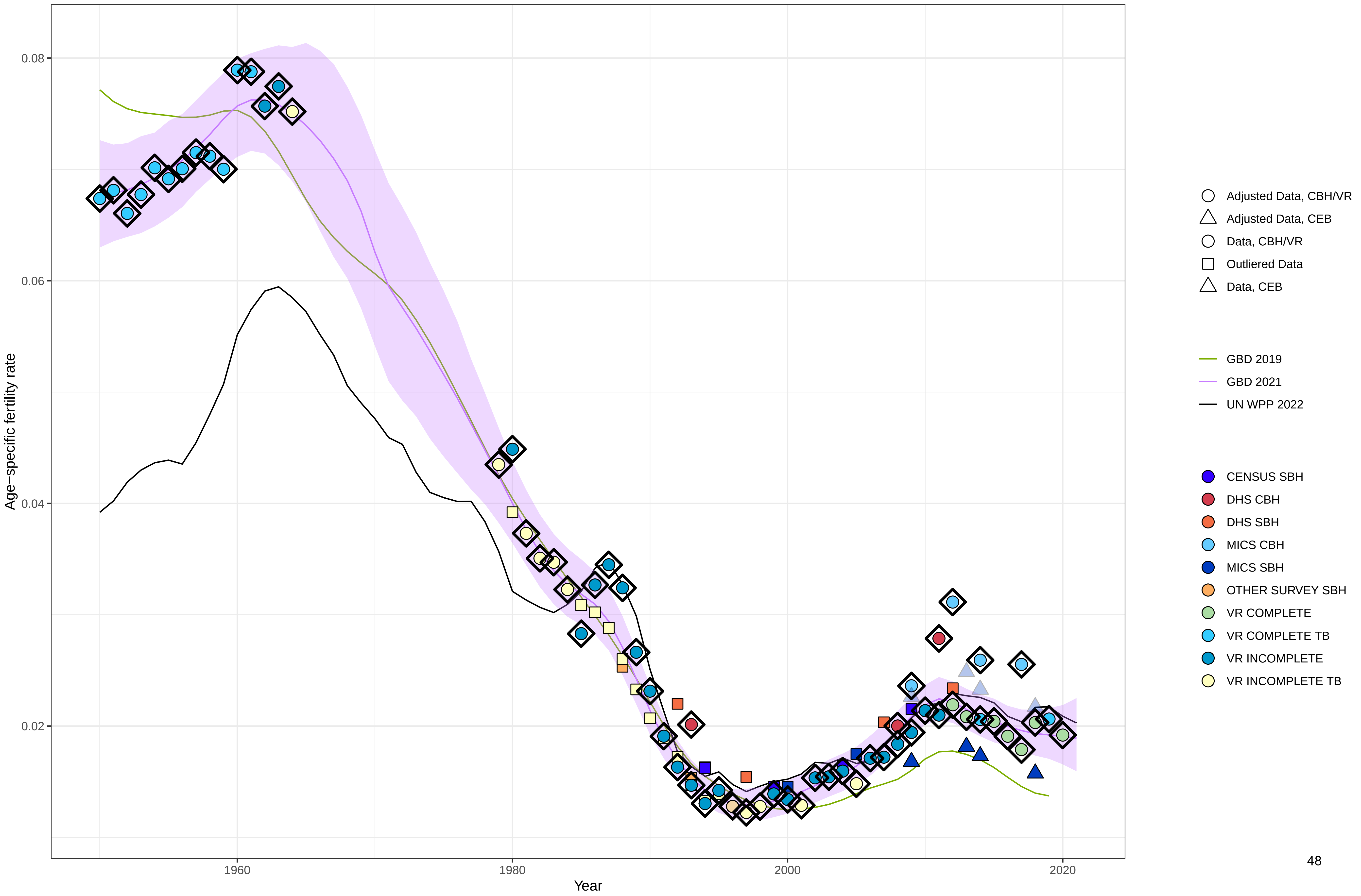

Age−specific fertility fitted trend lines, 1950−2021: Kyrgyzstan, Ages: 45−49
Data points enclosed with a diamond are reference sources

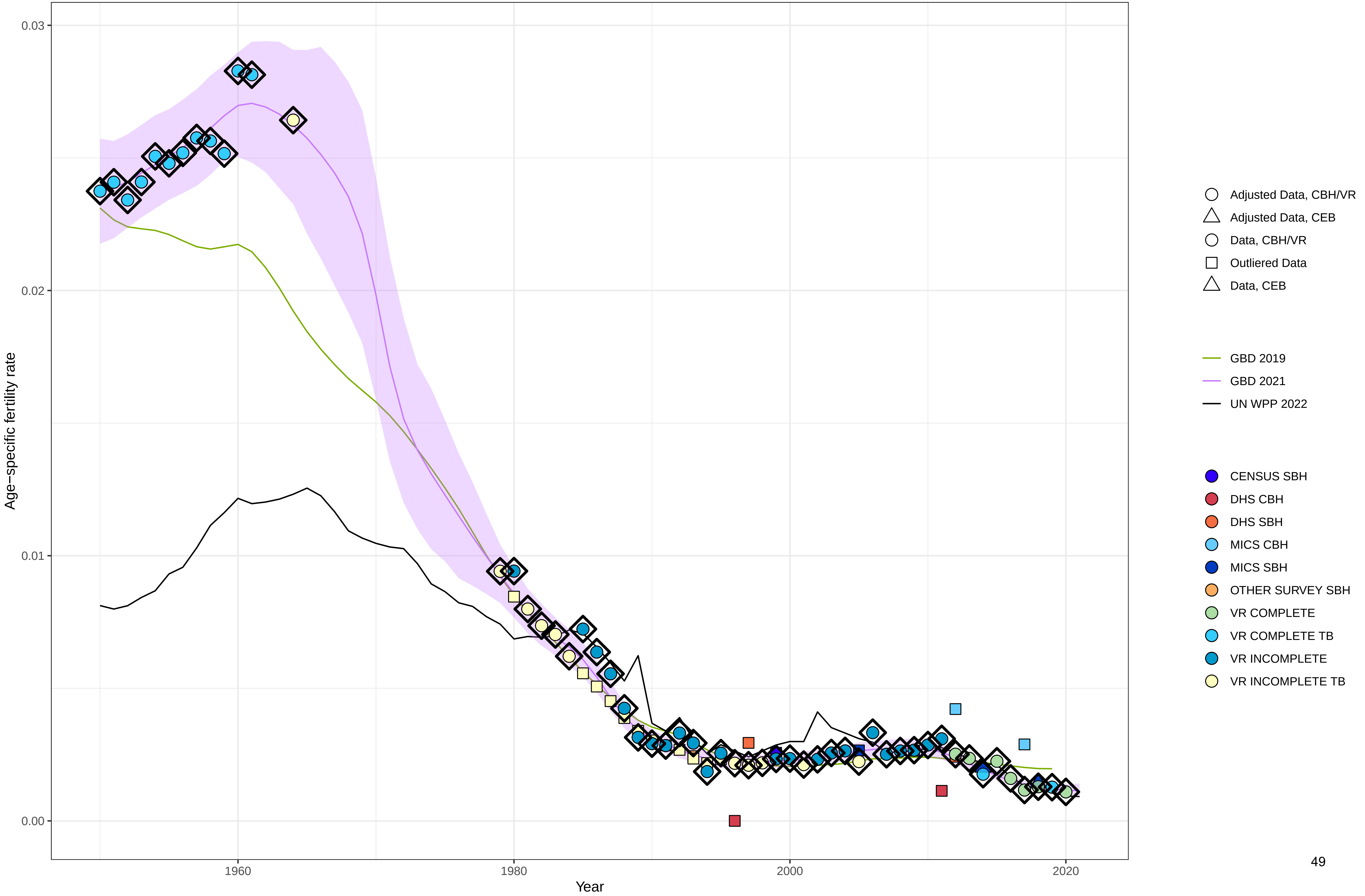

Age−specific fertility fitted trend lines, 1950−2021: Kyrgyzstan, Ages: 50−54
Data points enclosed with a diamond are reference sources

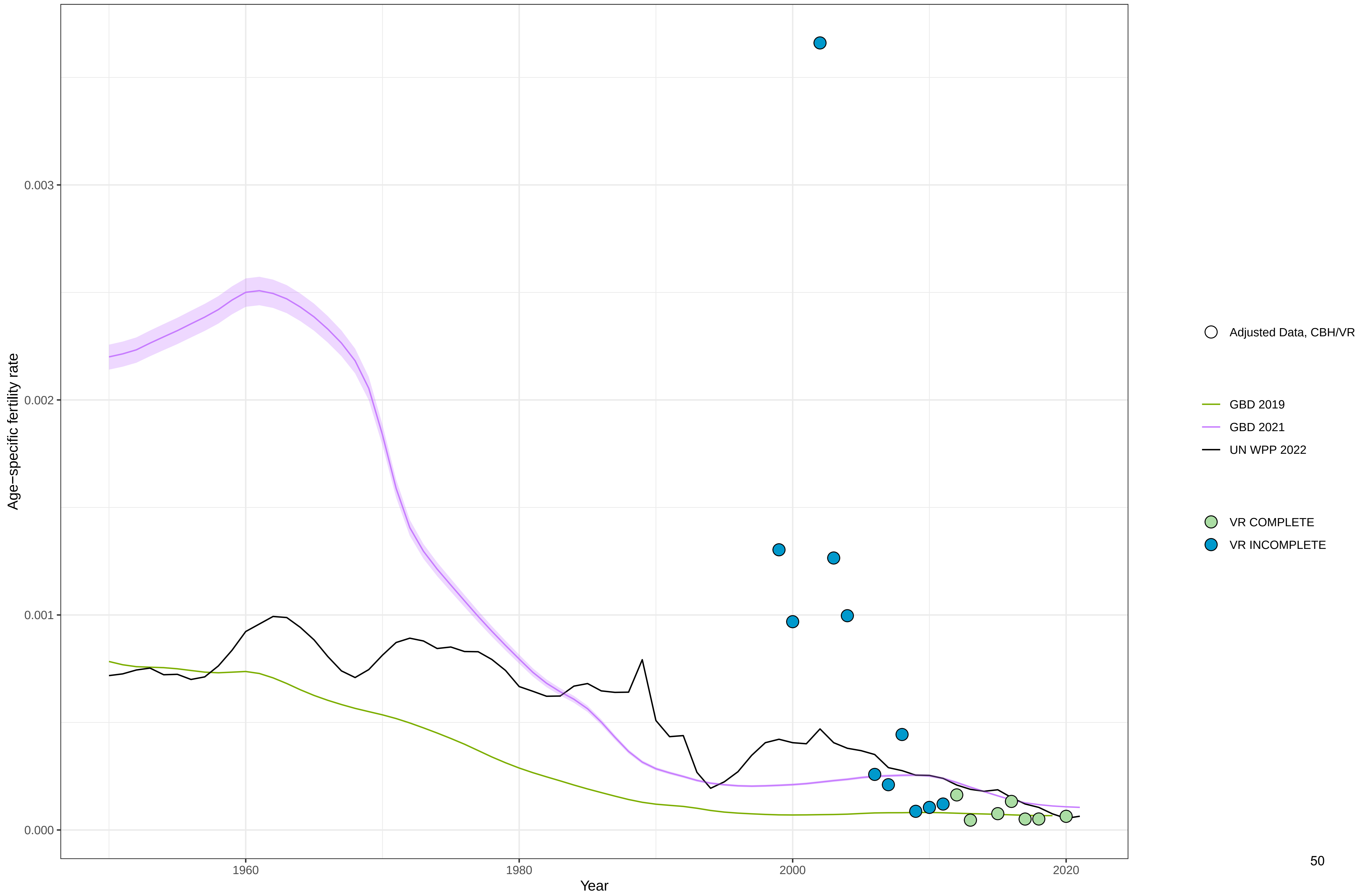

Total fertility rate fitted trend lines, 1950−2021: Kyrgyzstan TFR
Data points enclosed with a diamond are reference sources

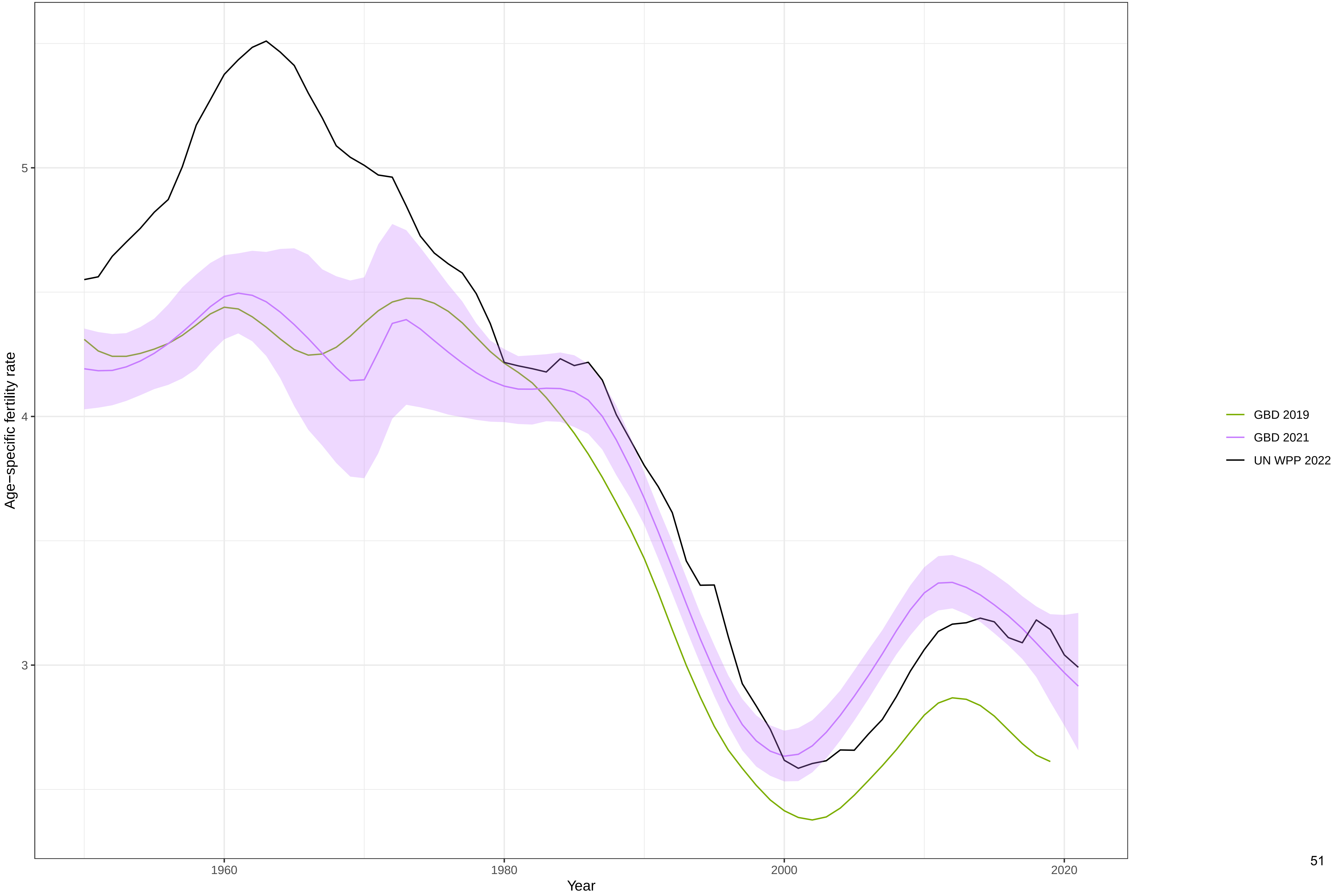

Age−specific fertility fitted trend lines, 1950−2021: Mongolia, Ages: 10−14
Data points enclosed with a diamond are reference sources

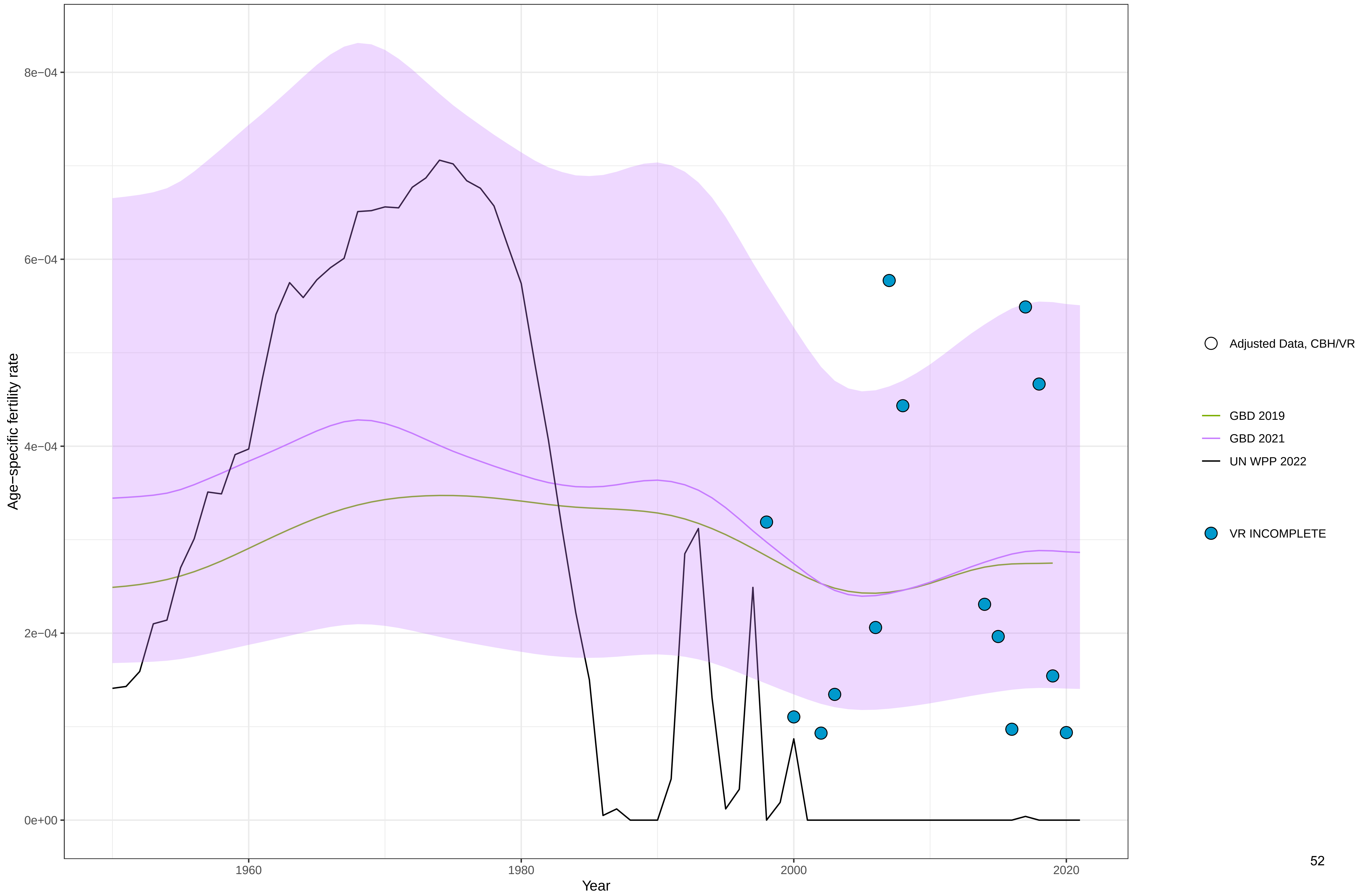

Age−specific fertility fitted trend lines, 1950−2021: Mongolia, Ages: 15−19
Data points enclosed with a diamond are reference sources

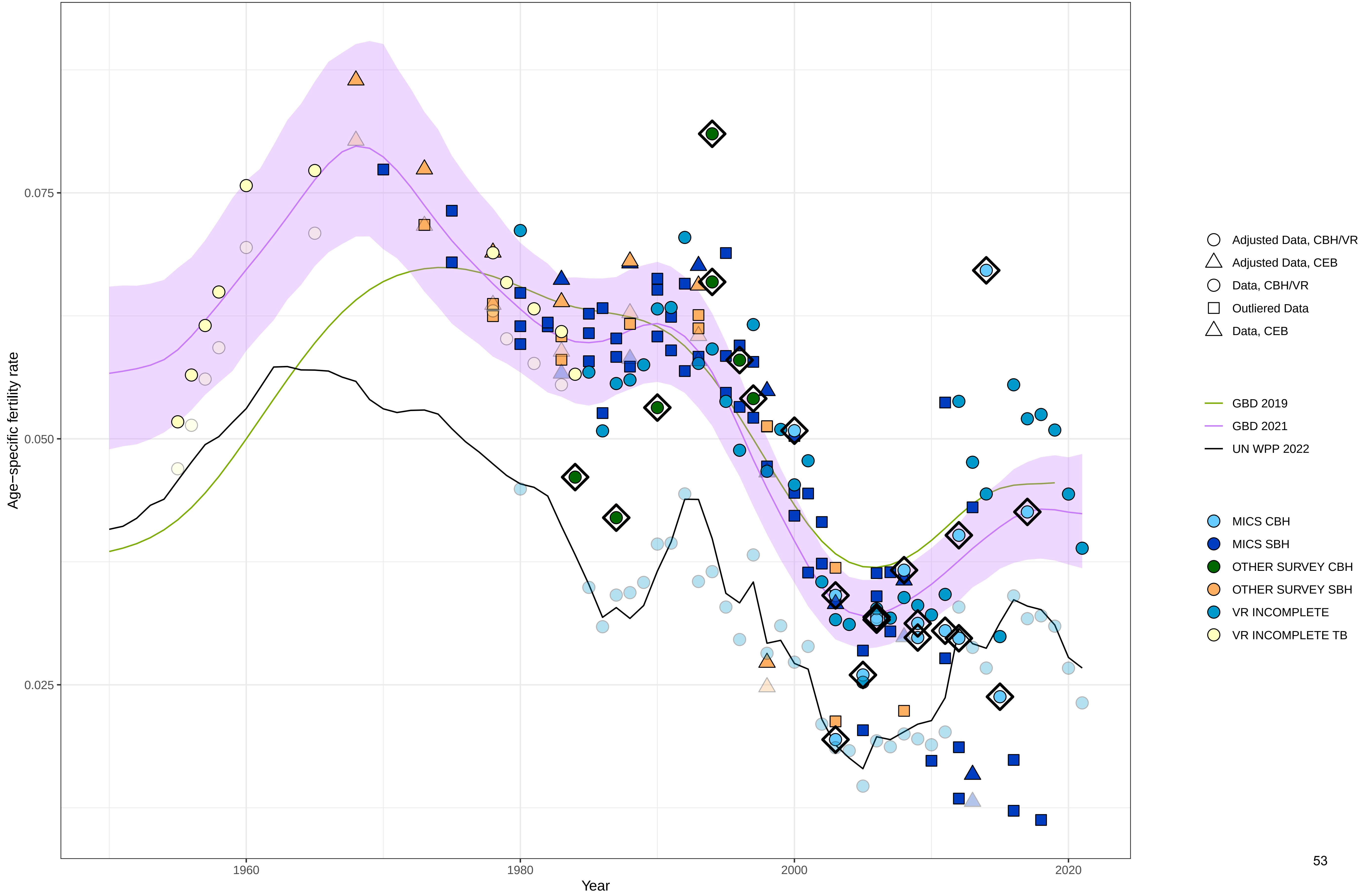

Age−specific fertility fitted trend lines, 1950−2021: Mongolia, Ages: 20−24
Data points enclosed with a diamond are reference sources

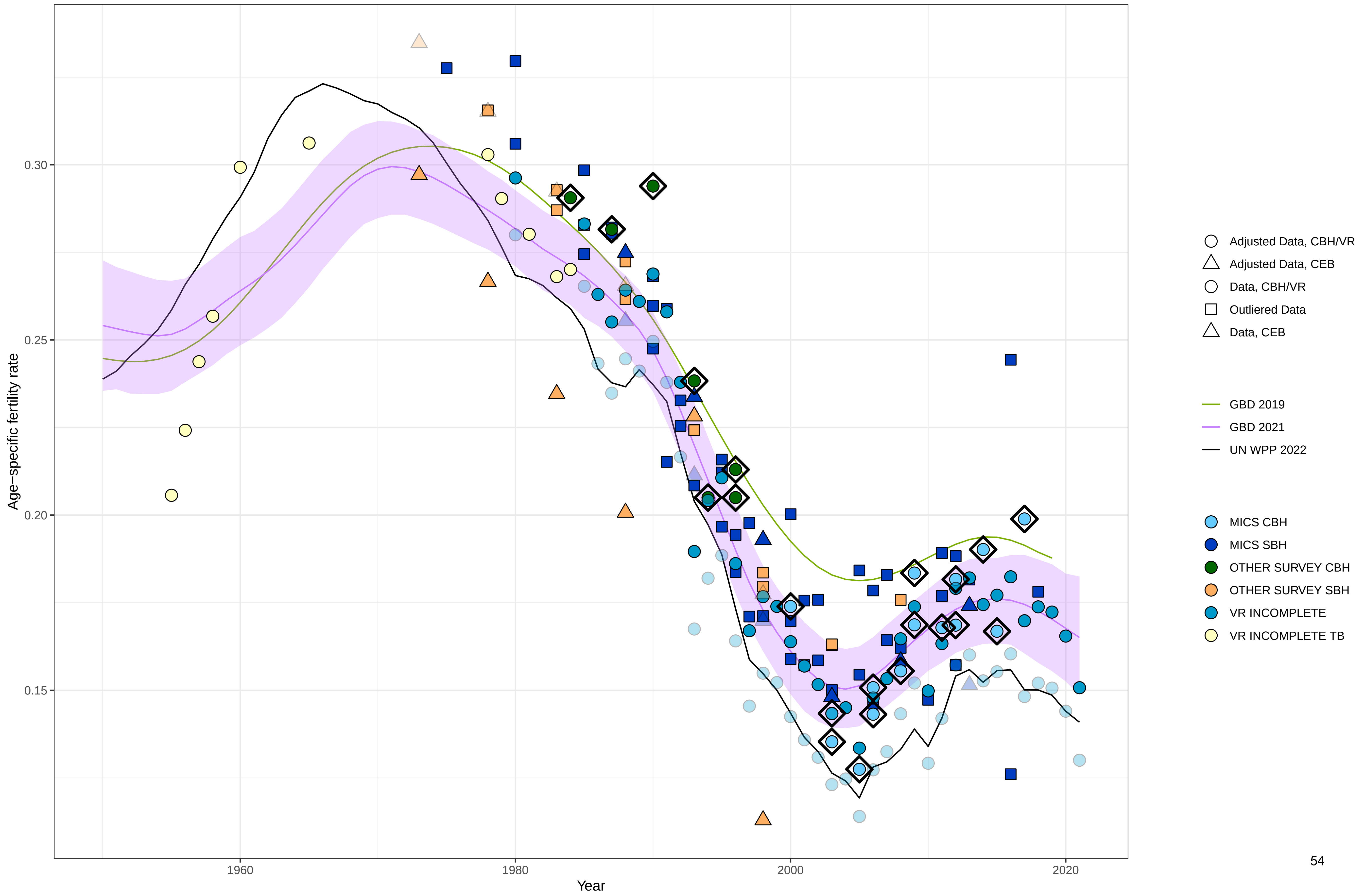

Age−specific fertility fitted trend lines, 1950−2021: Mongolia, Ages: 25−29
Data points enclosed with a diamond are reference sources

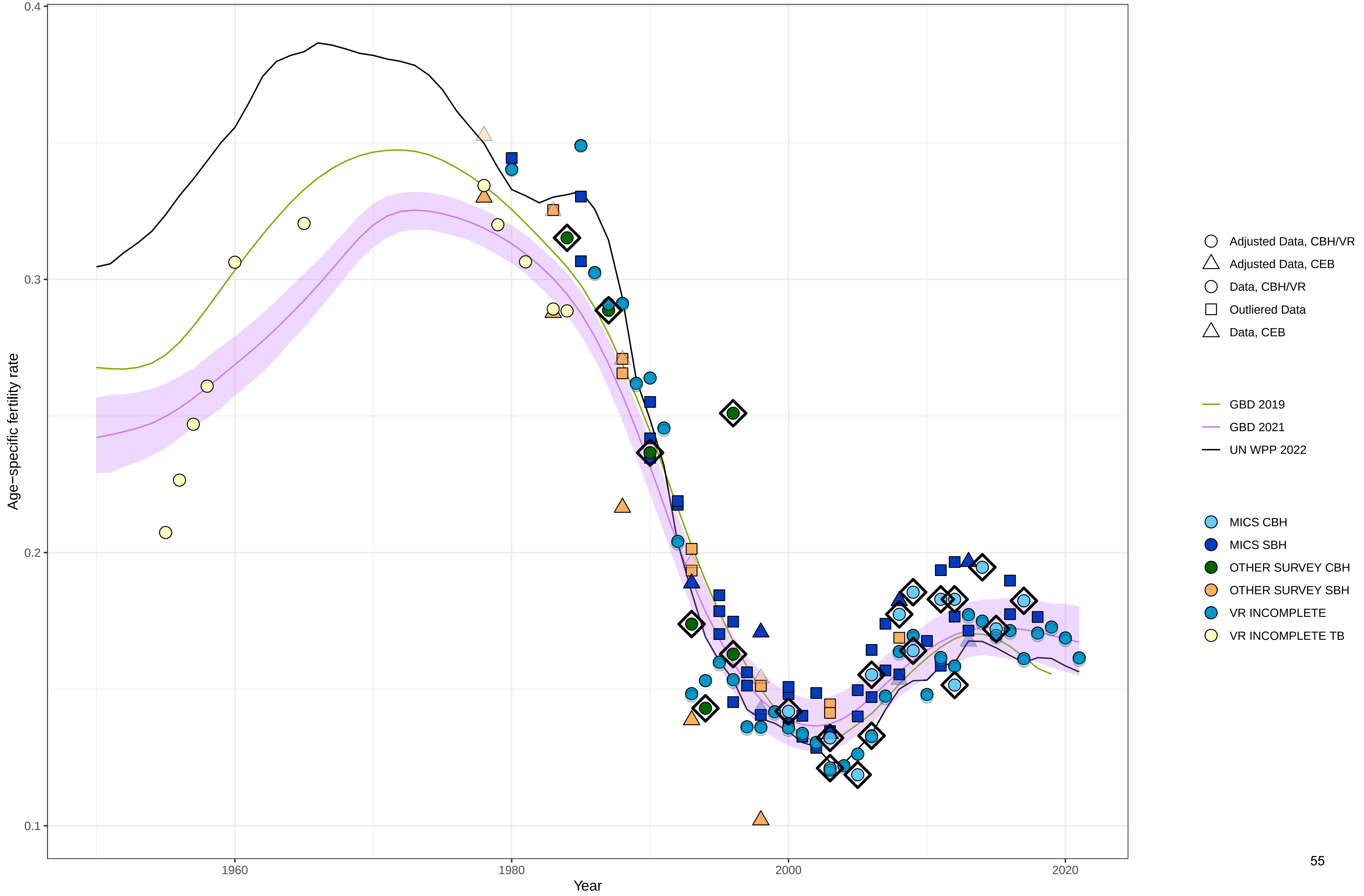

Age−specific fertility fitted trend lines, 1950−2021: Mongolia, Ages: 30−34
Data points enclosed with a diamond are reference sources

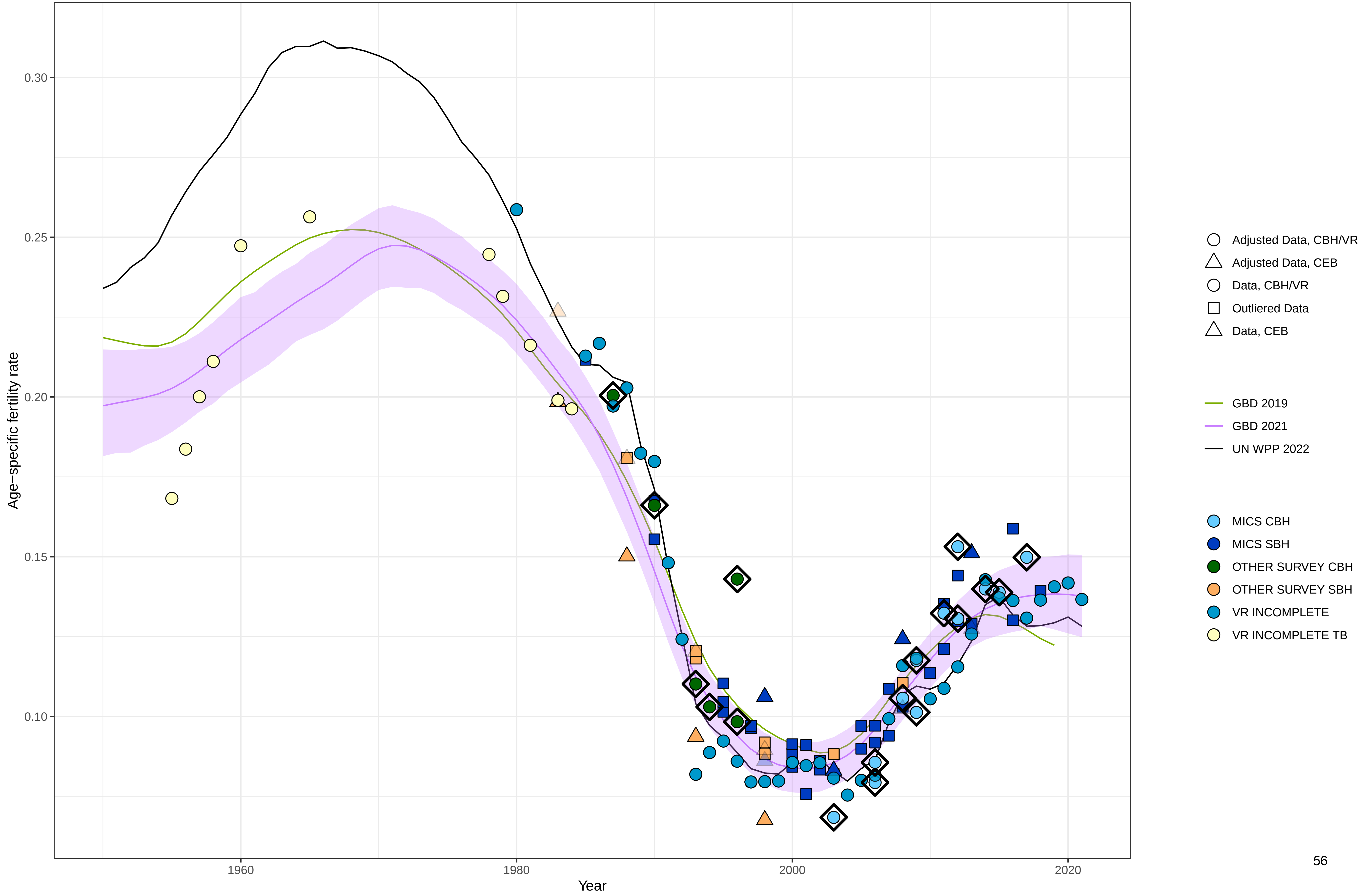

Age−specific fertility fitted trend lines, 1950−2021: Mongolia, Ages: 35−39
Data points enclosed with a diamond are reference sources

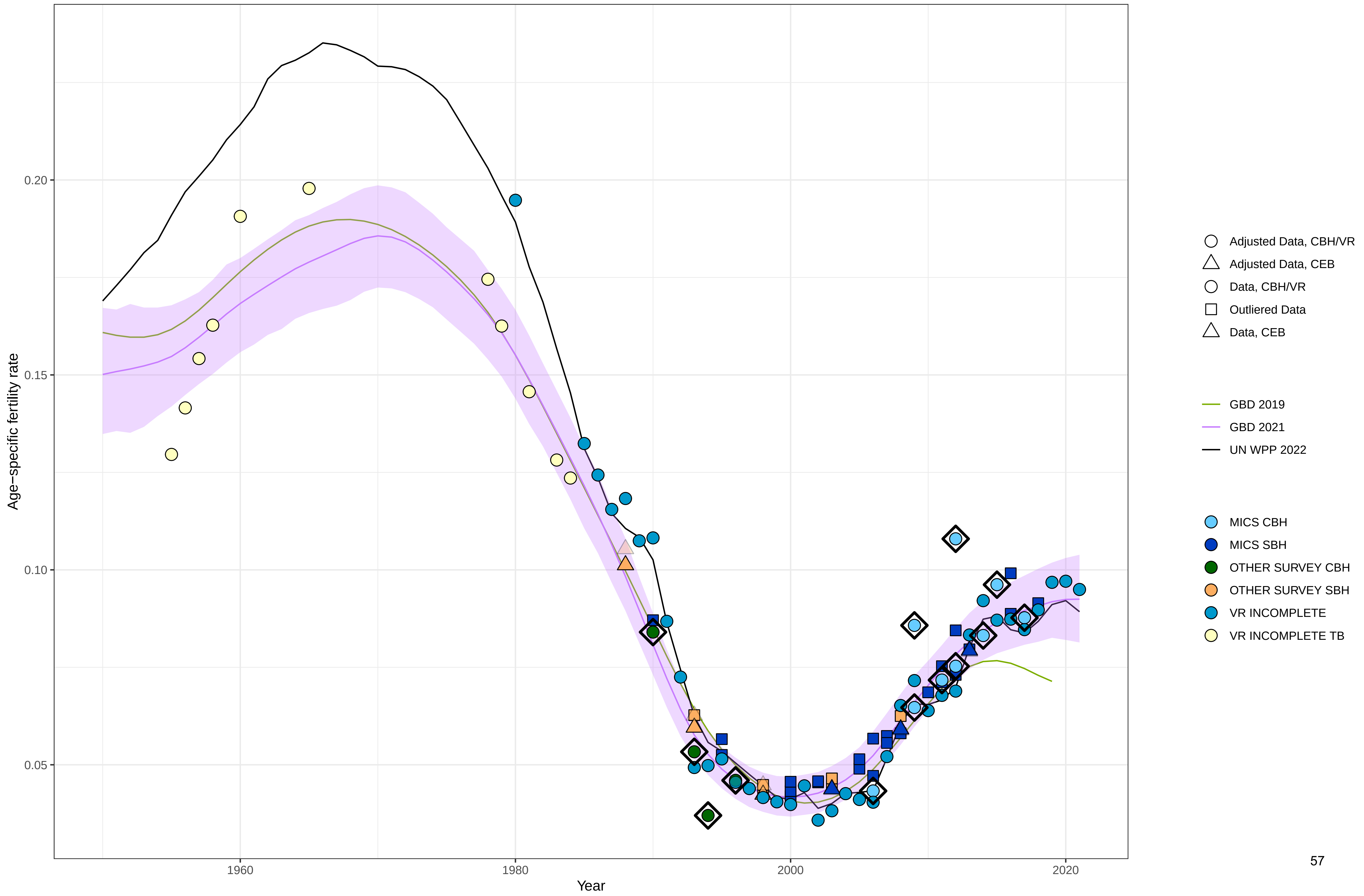

Age−specific fertility fitted trend lines, 1950−2021: Mongolia, Ages: 40−44
Data points enclosed with a diamond are reference sources

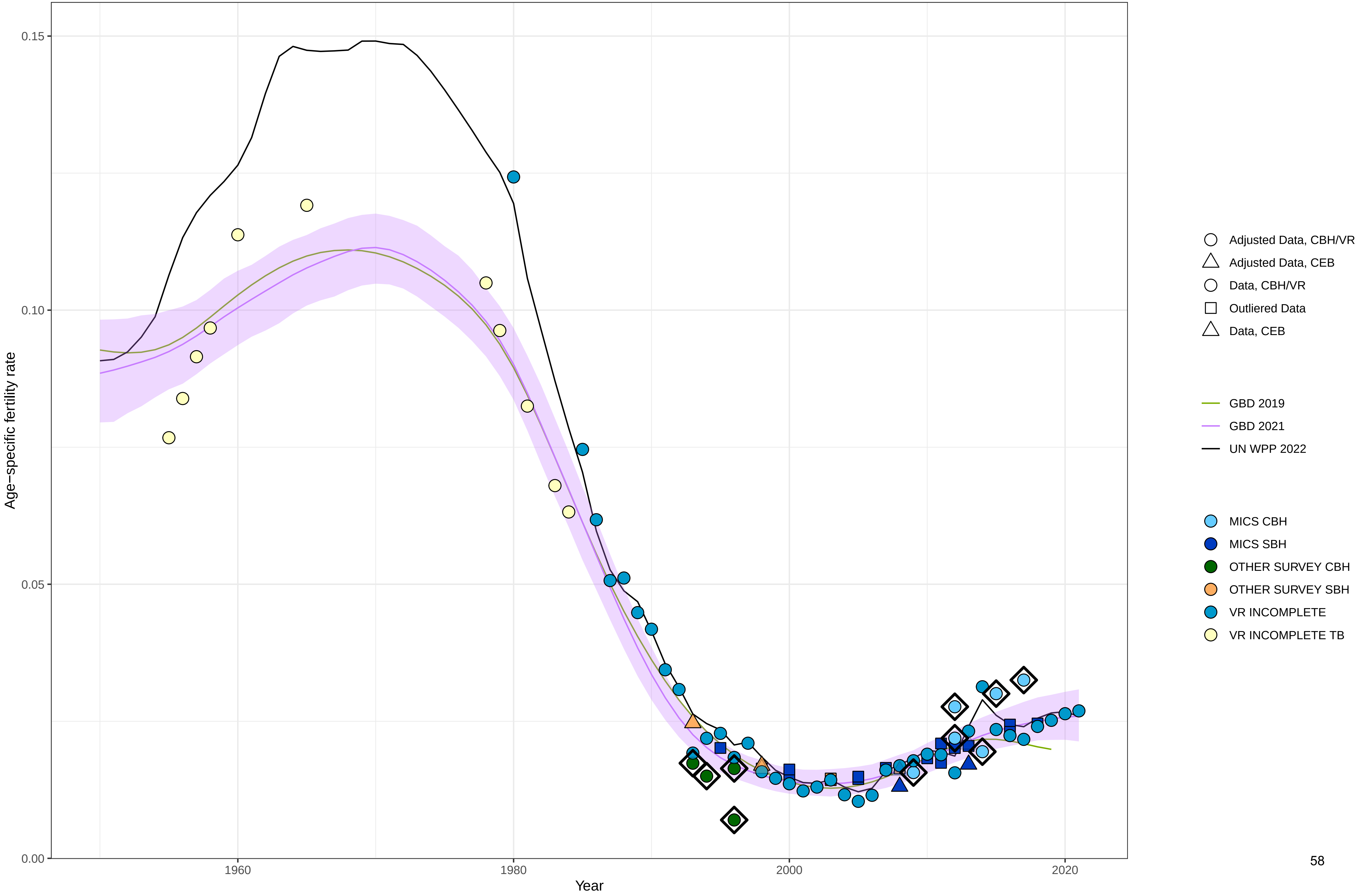

Age−specific fertility fitted trend lines, 1950−2021: Mongolia, Ages: 45−49
Data points enclosed with a diamond are reference sources

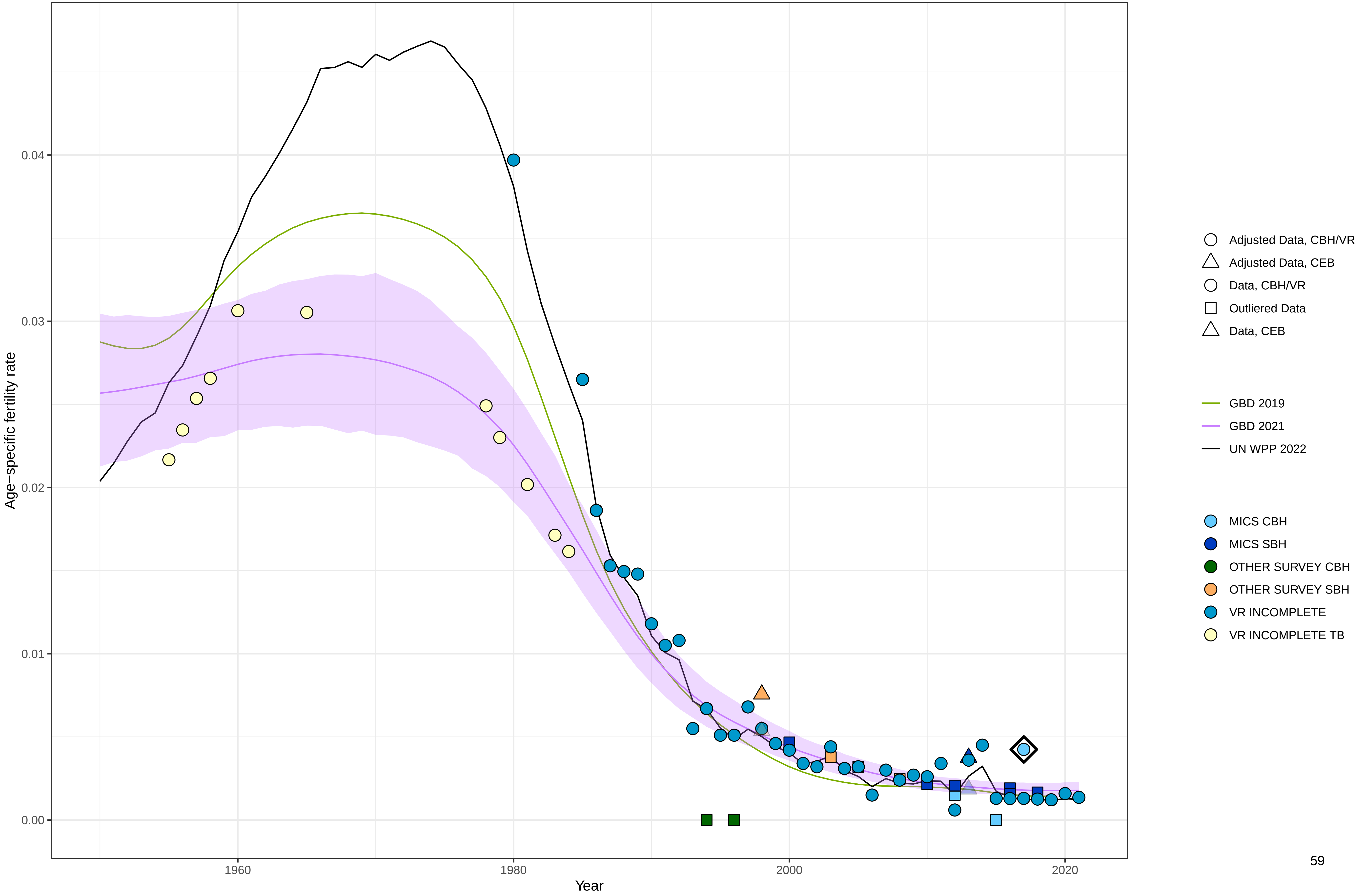

Age−specific fertility fitted trend lines, 1950−2021: Mongolia, Ages: 50−54
Data points enclosed with a diamond are reference sources

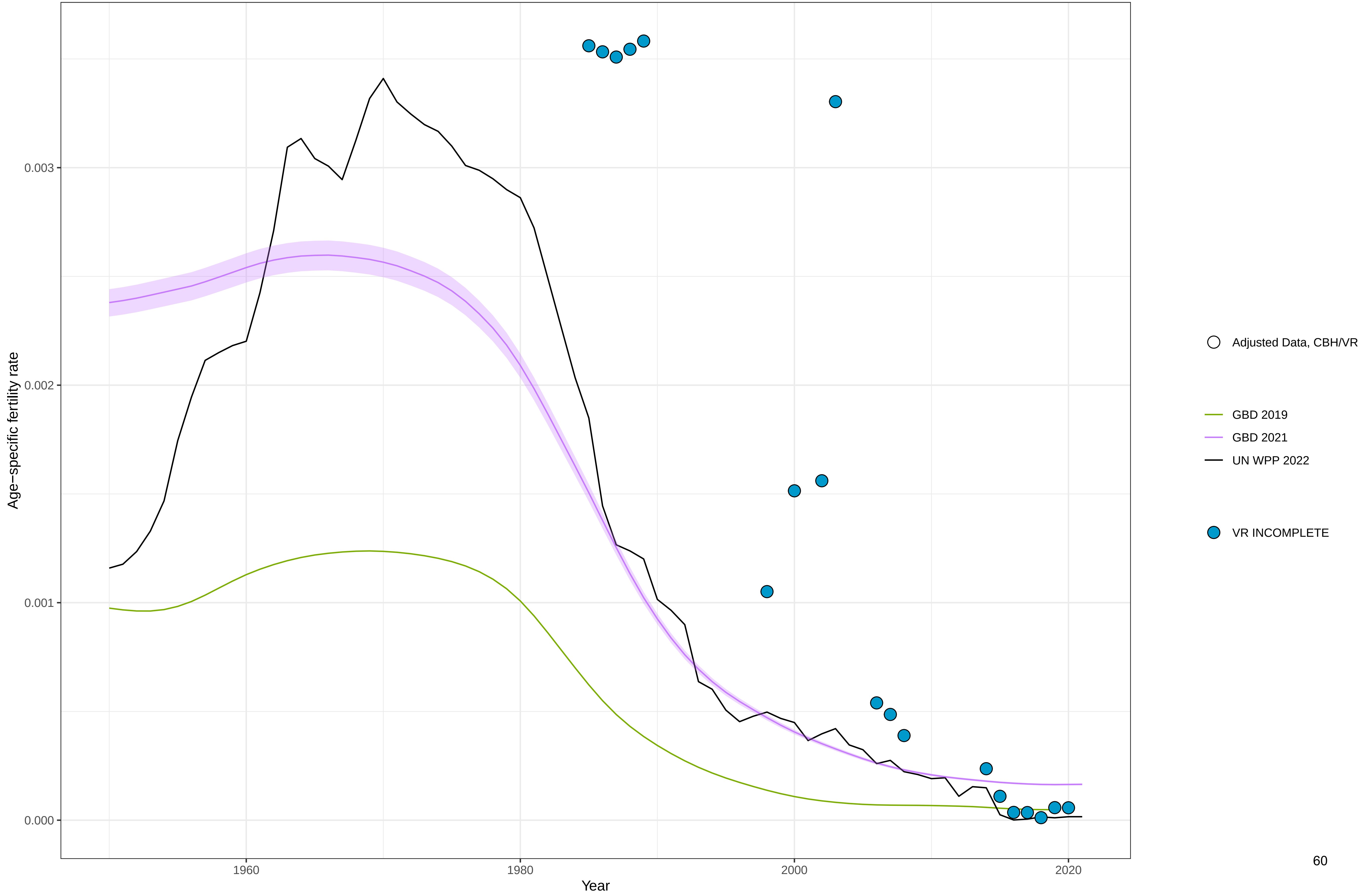

Total fertility rate fitted trend lines, 1950−2021: Mongolia TFR
Data points enclosed with a diamond are reference sources

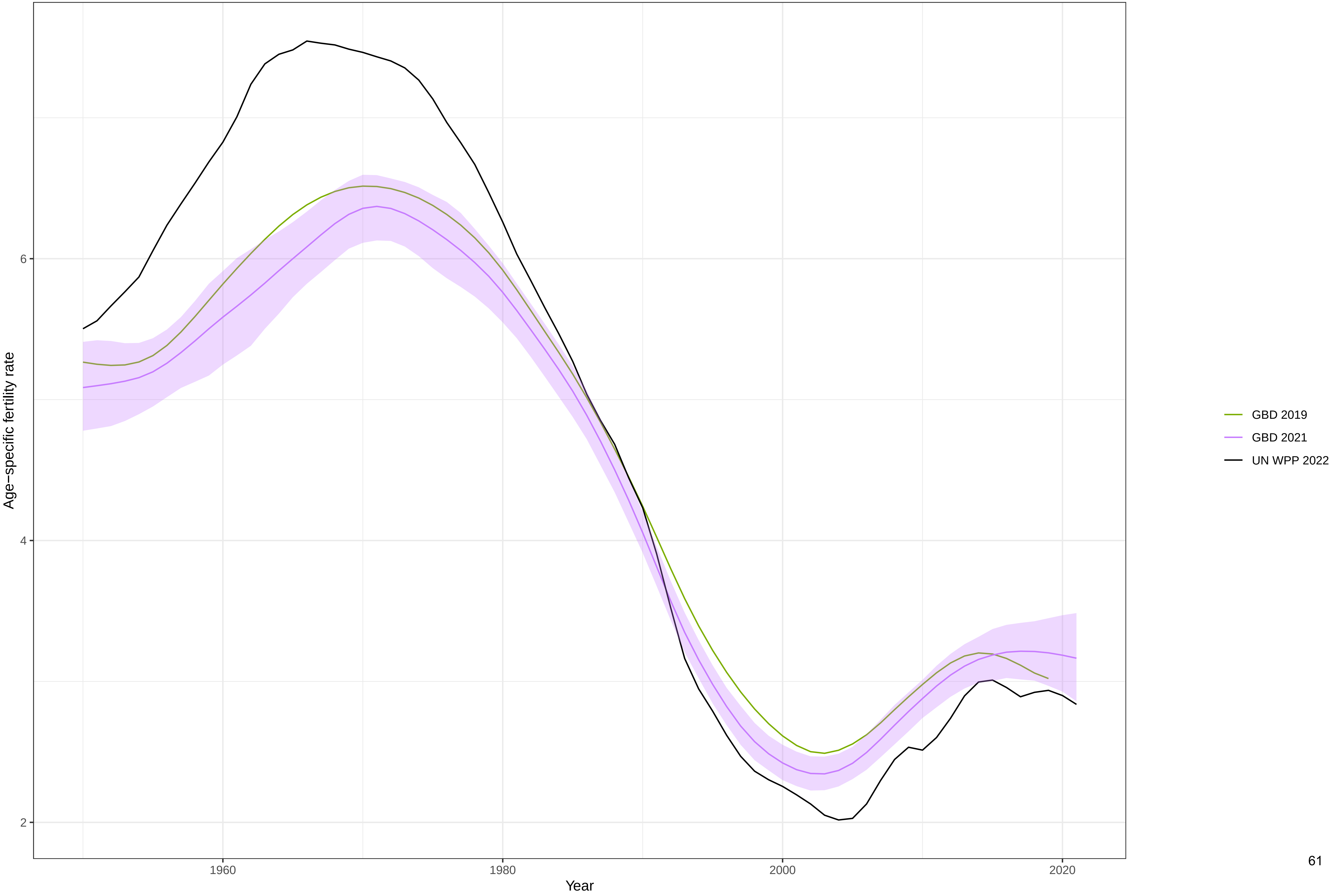

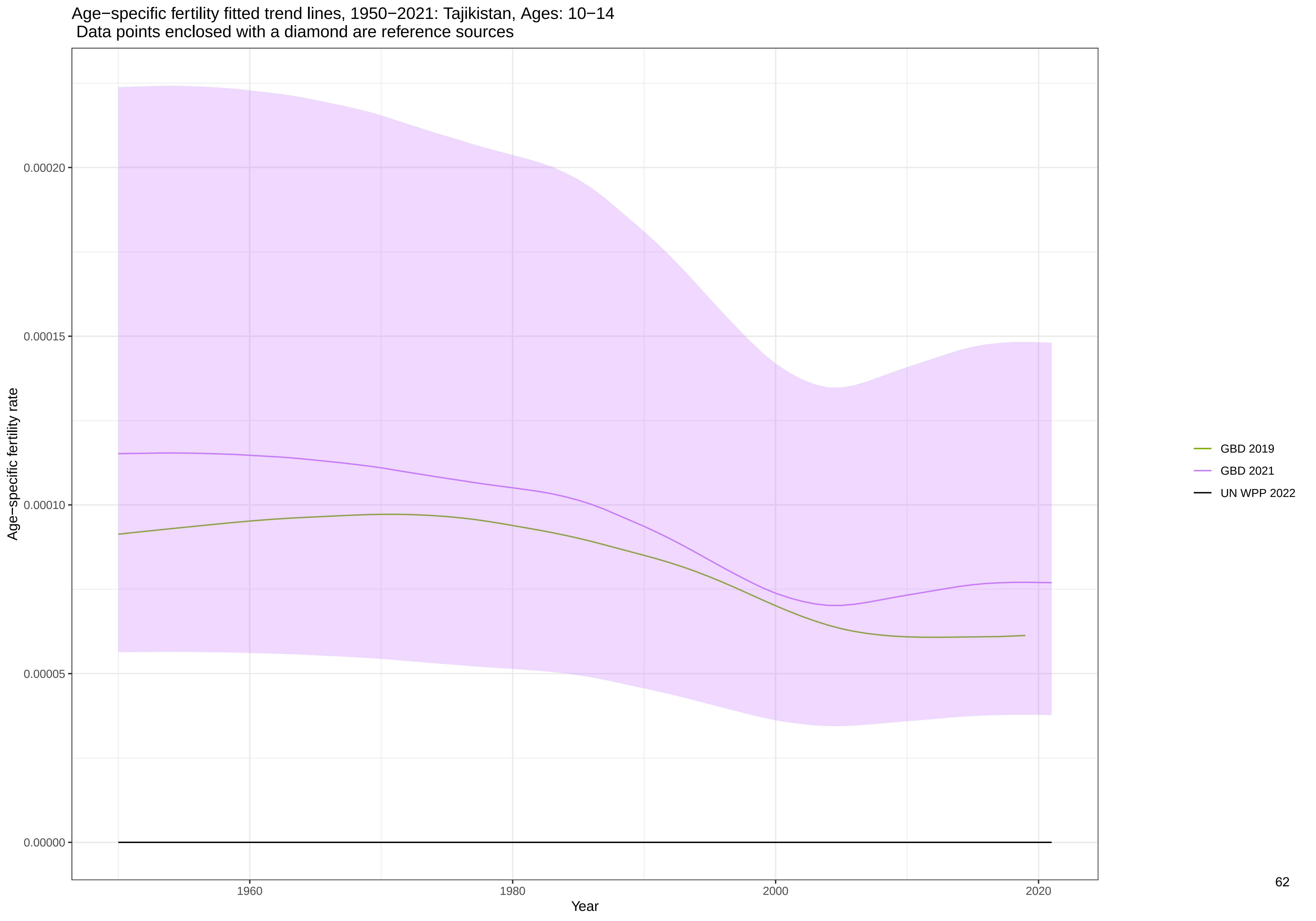
Age−specific fertility fitted trend lines, 1950−2021: Tajikistan, Ages: 10−14
Data points enclosed with a diamond are reference sources
Age−specific fertility rate
0.00020
0.00015
0.00010
0.00005
0.00000
1960
1980
2000
2020
Year
GBD 2019
GBD 2021
UN WPP 2022

Age−specific fertility fitted trend lines, 1950−2021: Tajikistan, Ages: 15−19
Data points enclosed with a diamond are reference sources

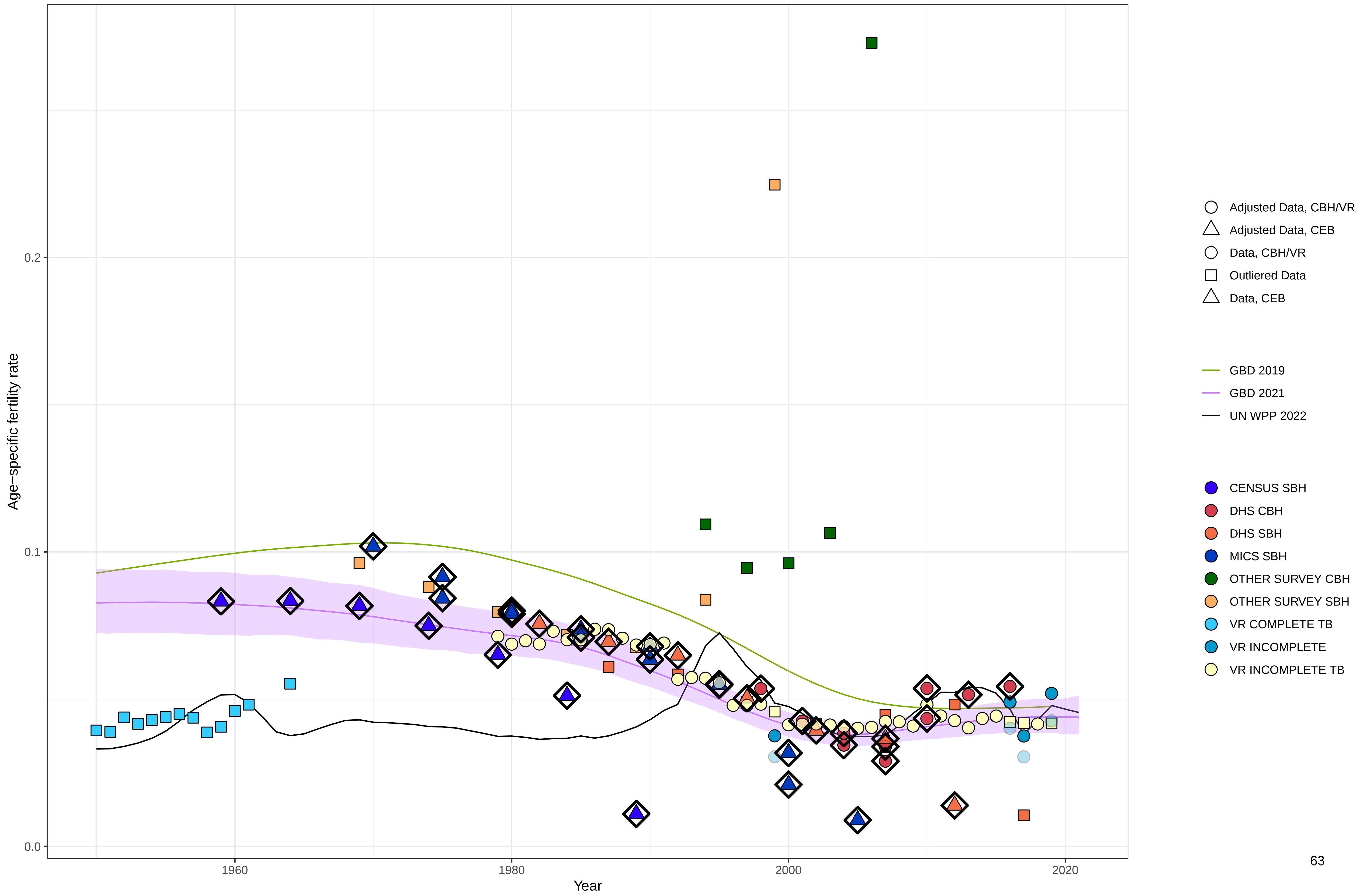

Age−specific fertility fitted trend lines, 1950−2021: Tajikistan, Ages: 20−24
Data points enclosed with a diamond are reference sources

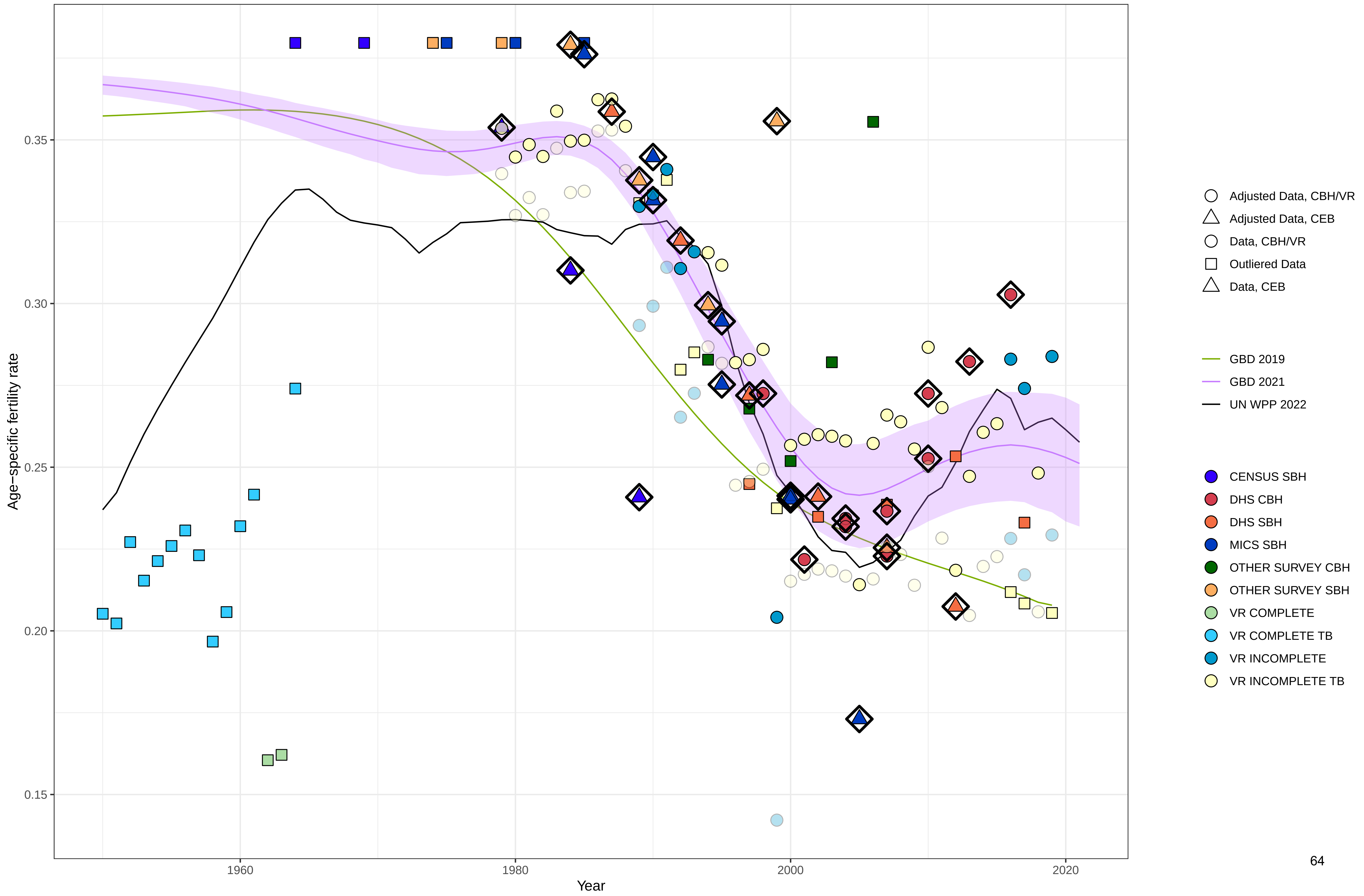

Age−specific fertility fitted trend lines, 1950−2021: Tajikistan, Ages: 25−29
Data points enclosed with a diamond are reference sources

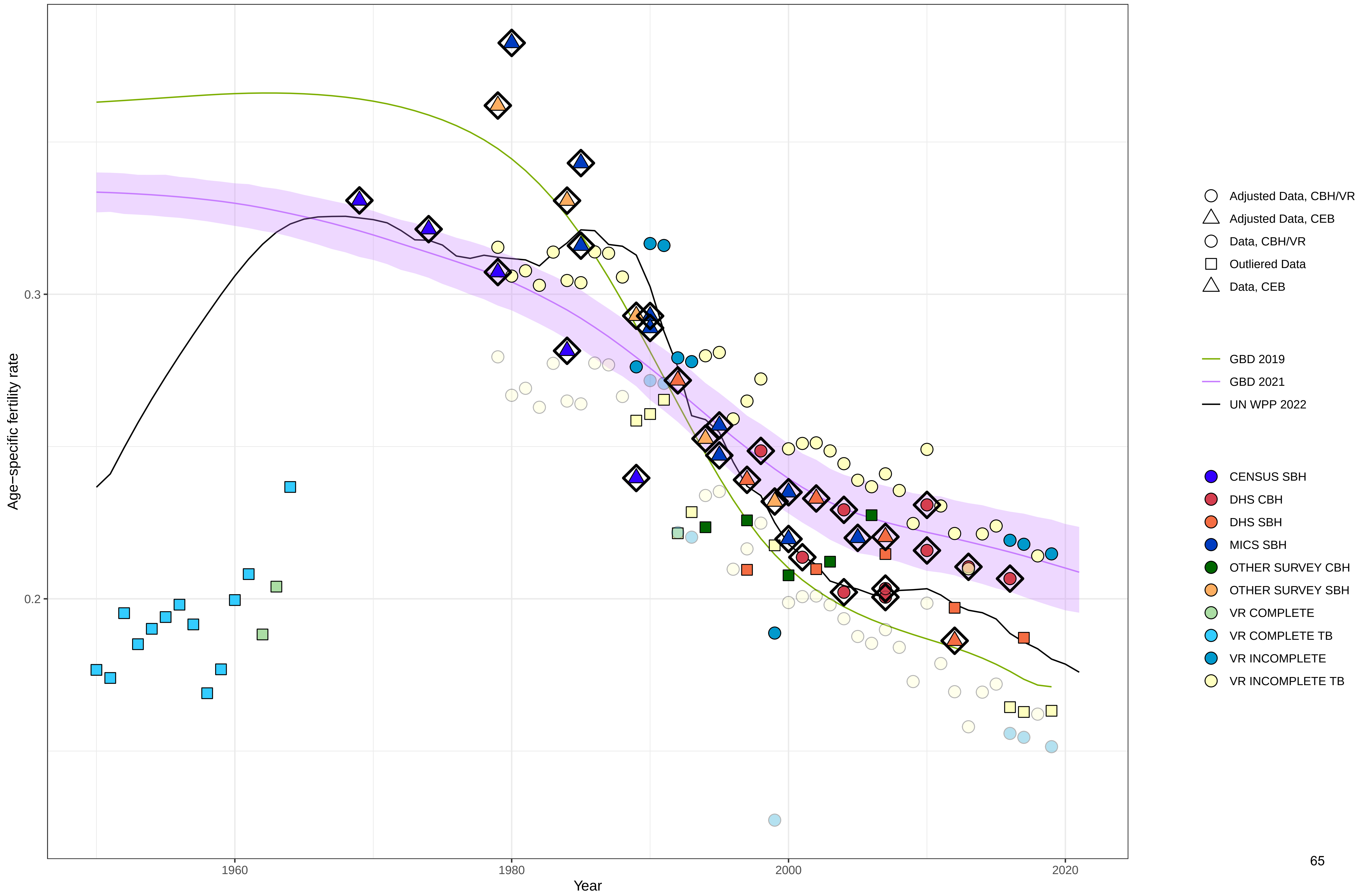

Age−specific fertility fitted trend lines, 1950−2021: Tajikistan, Ages: 30−34
Data points enclosed with a diamond are reference sources

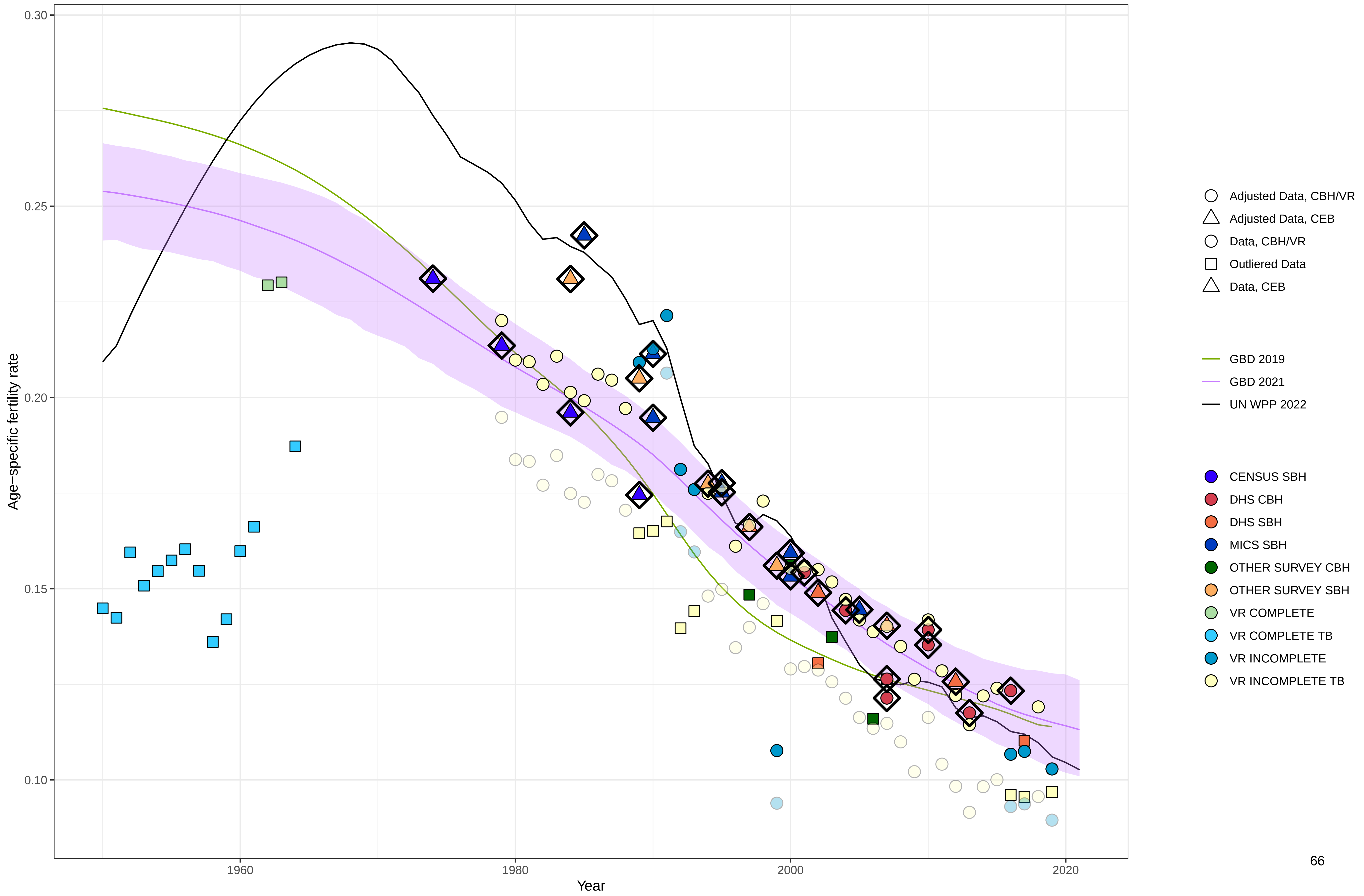

Age−specific fertility fitted trend lines, 1950−2021: Tajikistan, Ages: 35−39
Data points enclosed with a diamond are reference sources

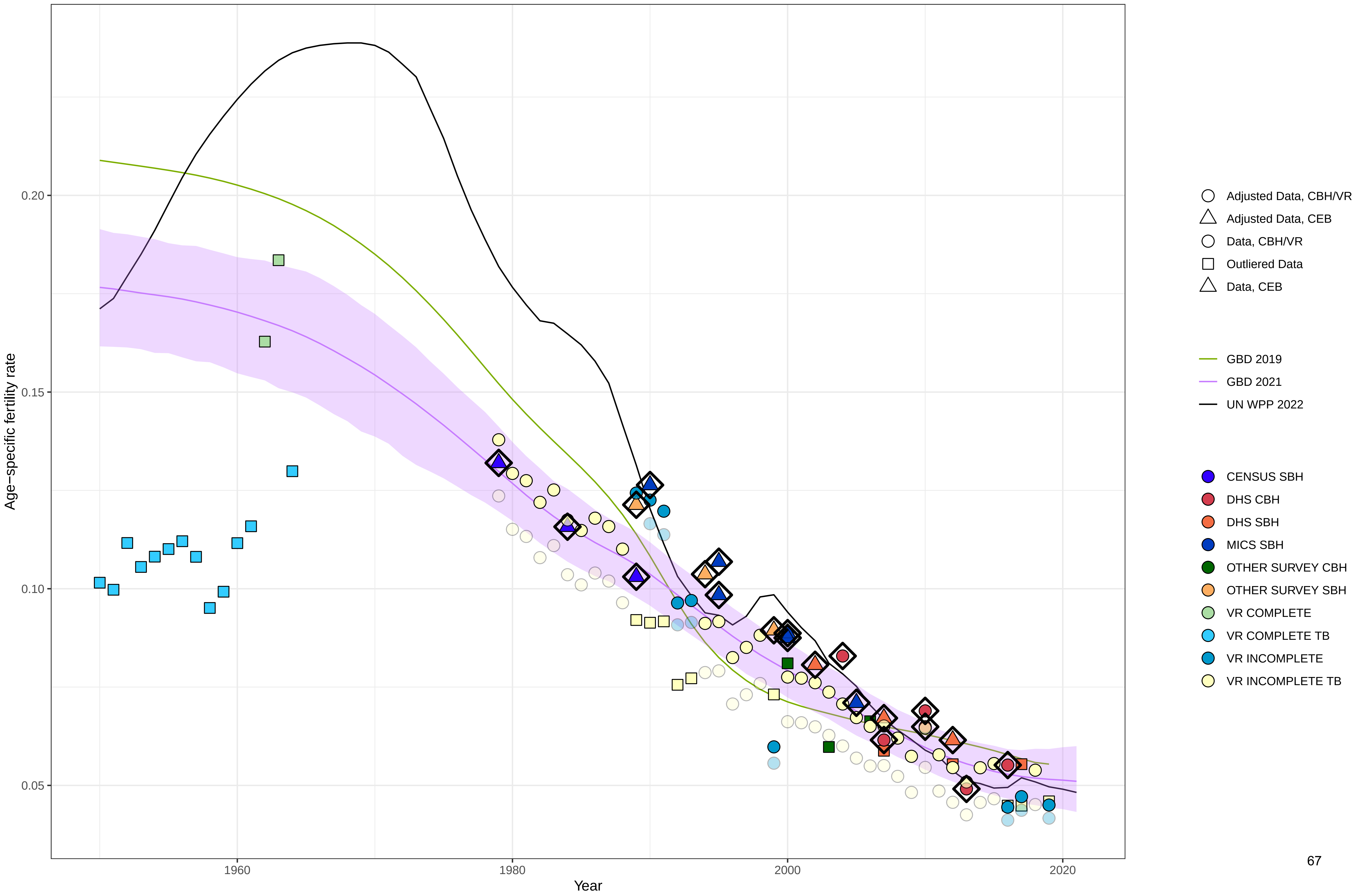

Age−specific fertility fitted trend lines, 1950−2021: Tajikistan, Ages: 40−44
Data points enclosed with a diamond are reference sources

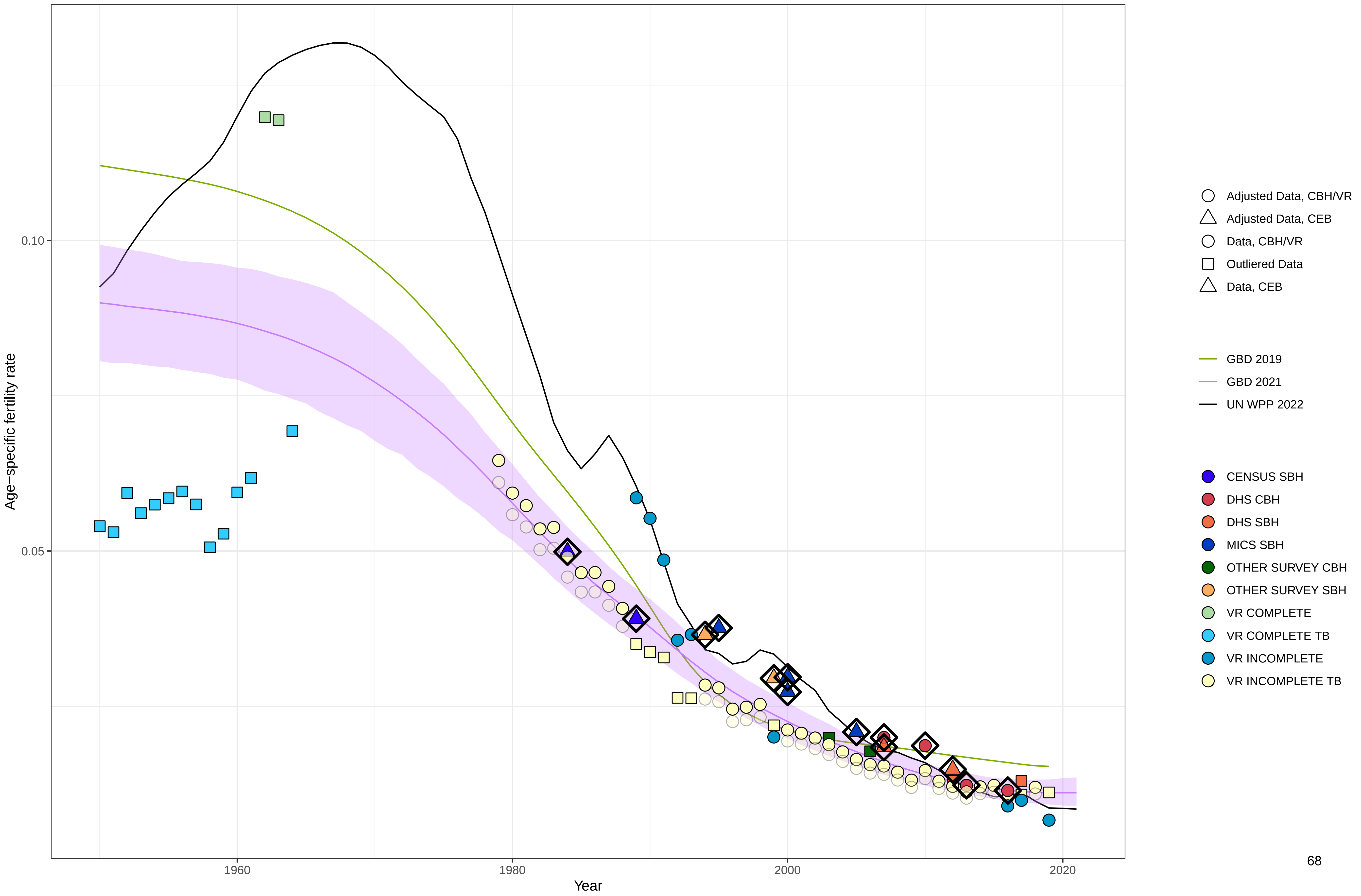

Age−specific fertility fitted trend lines, 1950−2021: Tajikistan, Ages: 45−49
Data points enclosed with a diamond are reference sources

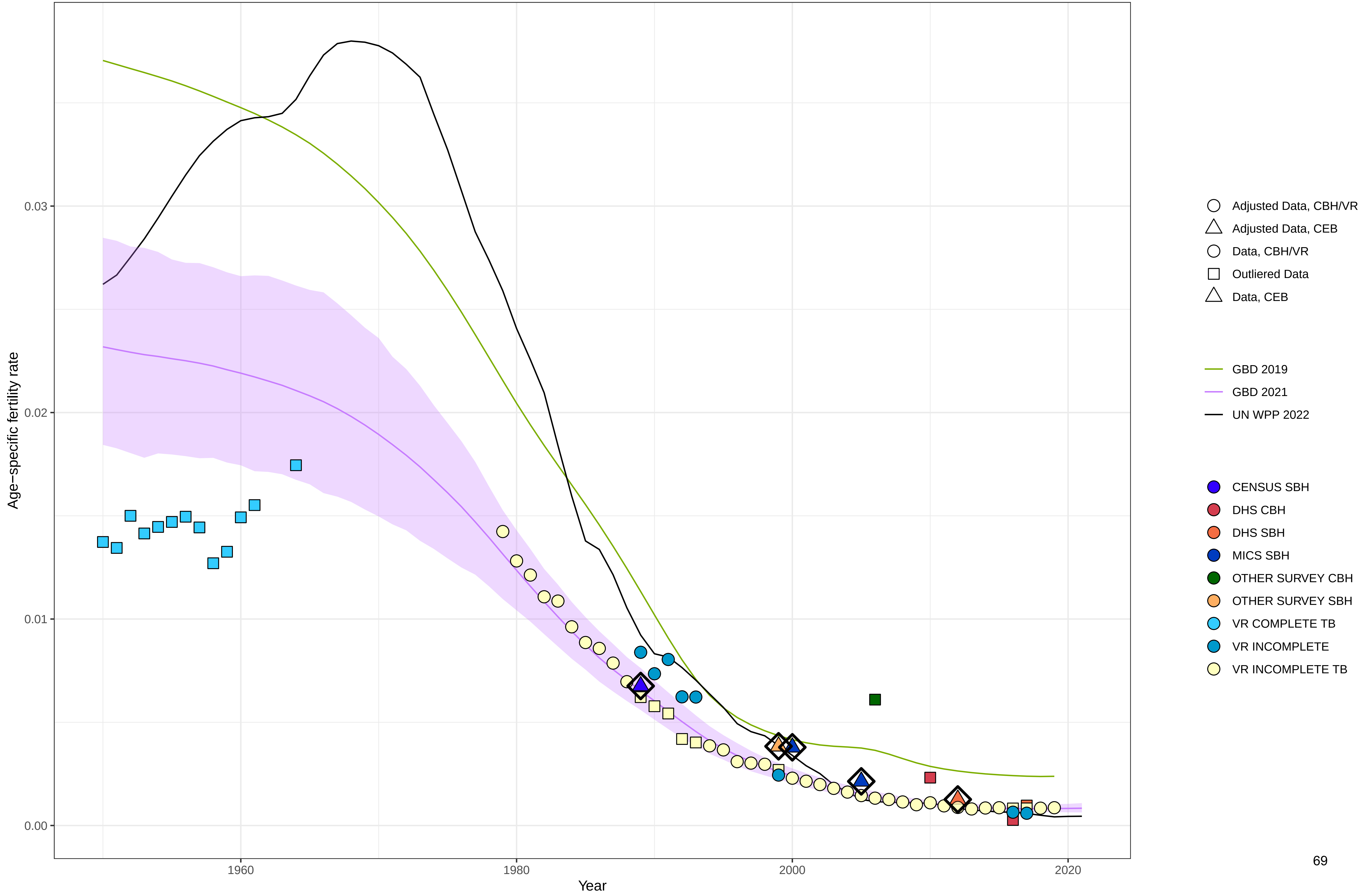

Age−specific fertility fitted trend lines, 1950−2021: Tajikistan, Ages: 50−54
Data points enclosed with a diamond are reference sources

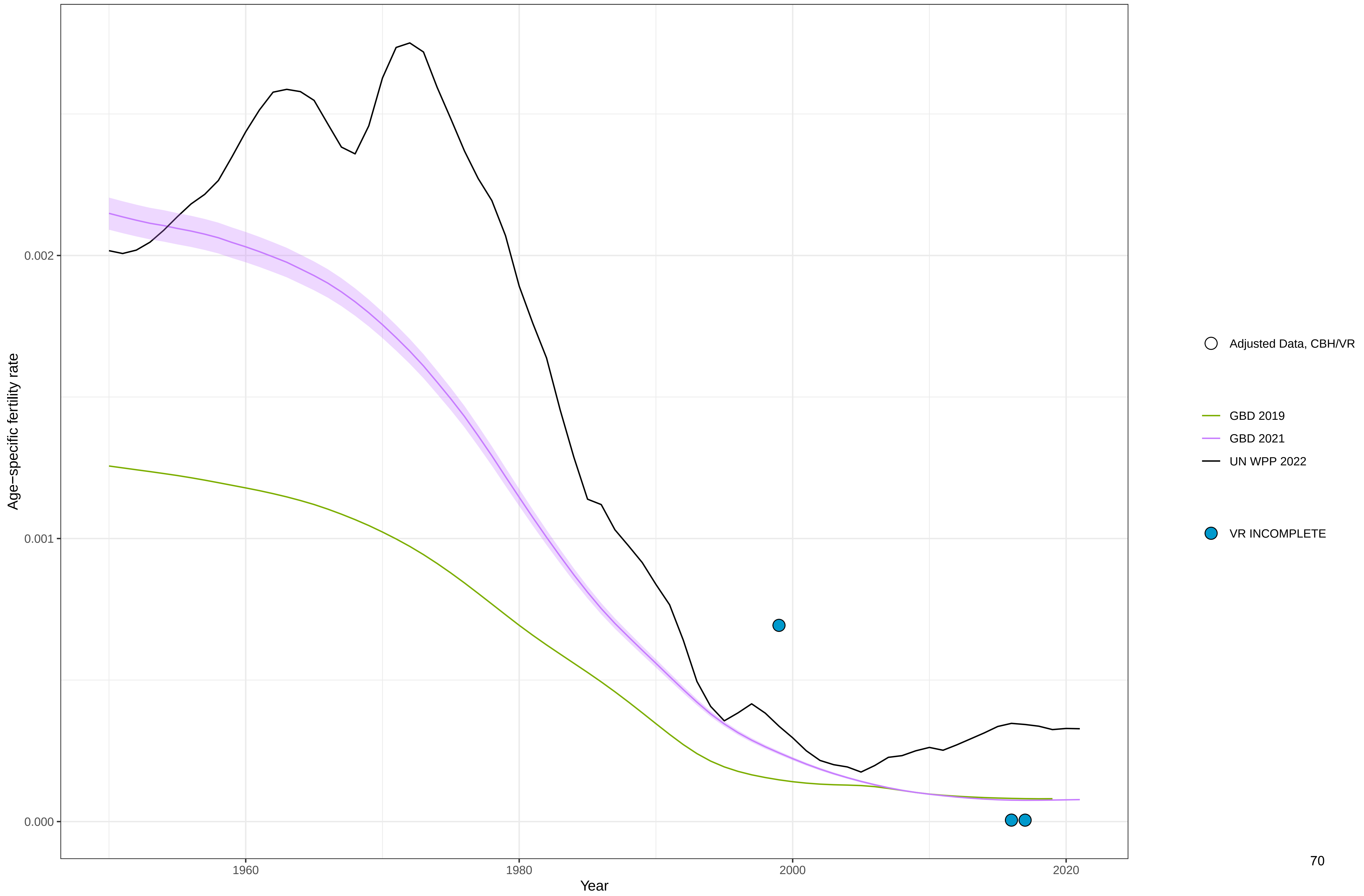

Total fertility rate fitted trend lines, 1950−2021: Tajikistan TFR
Data points enclosed with a diamond are reference sources

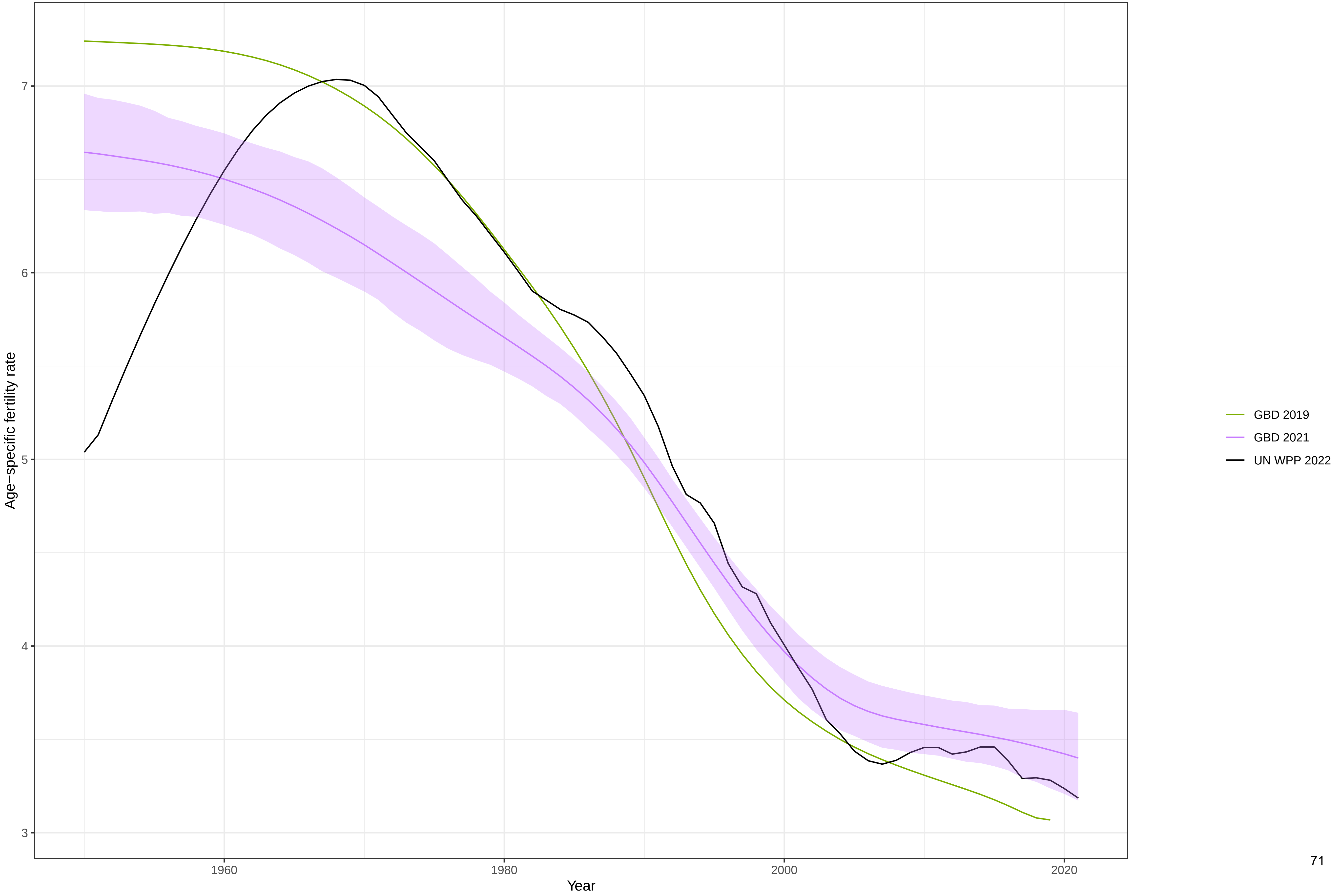

Age−specific fertility fitted trend lines, 1950−2021: Turkmenistan, Ages: 10−14
Data points enclosed with a diamond are reference sources

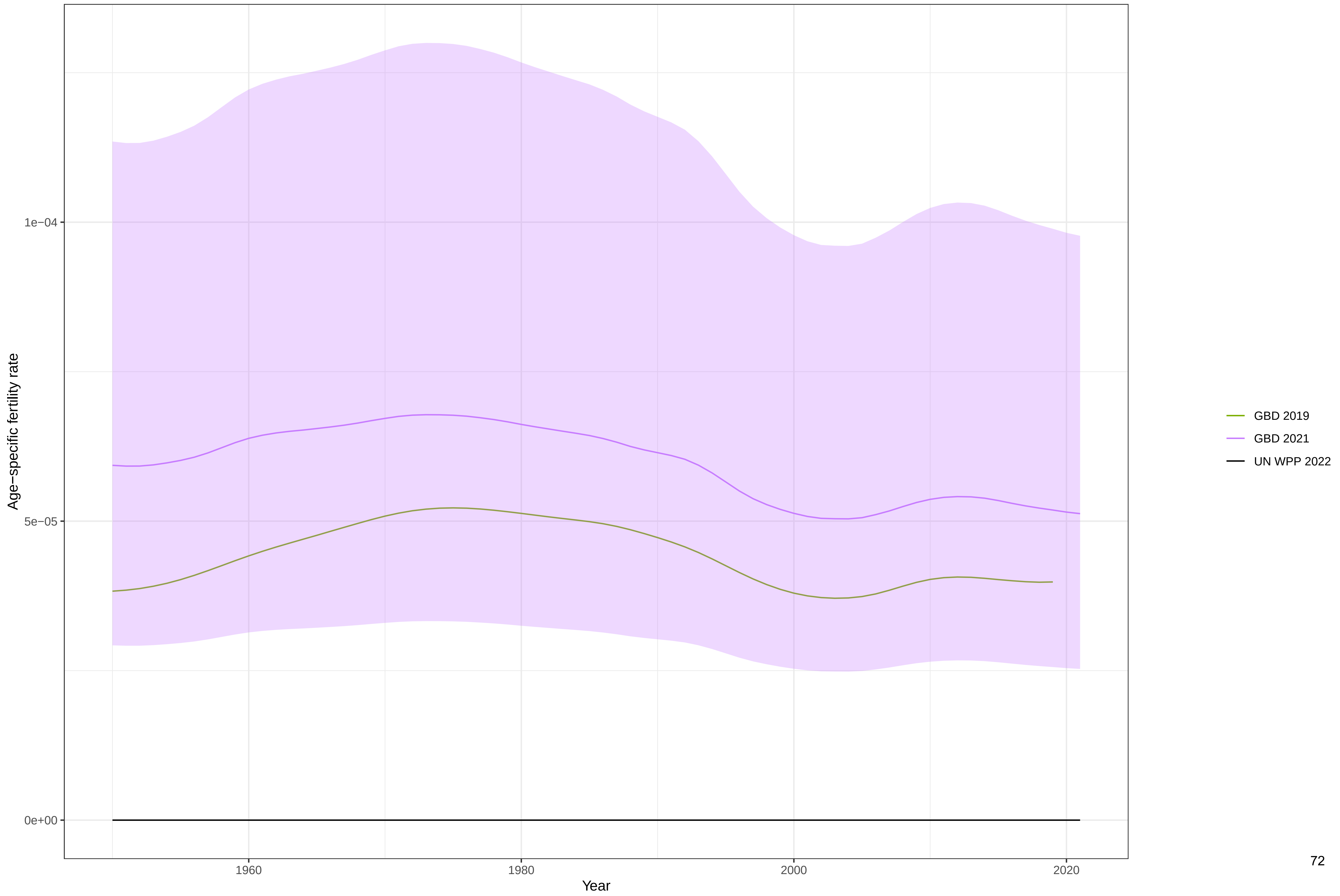

Age−specific fertility fitted trend lines, 1950−2021: Turkmenistan, Ages: 15−19
Data points enclosed with a diamond are reference sources

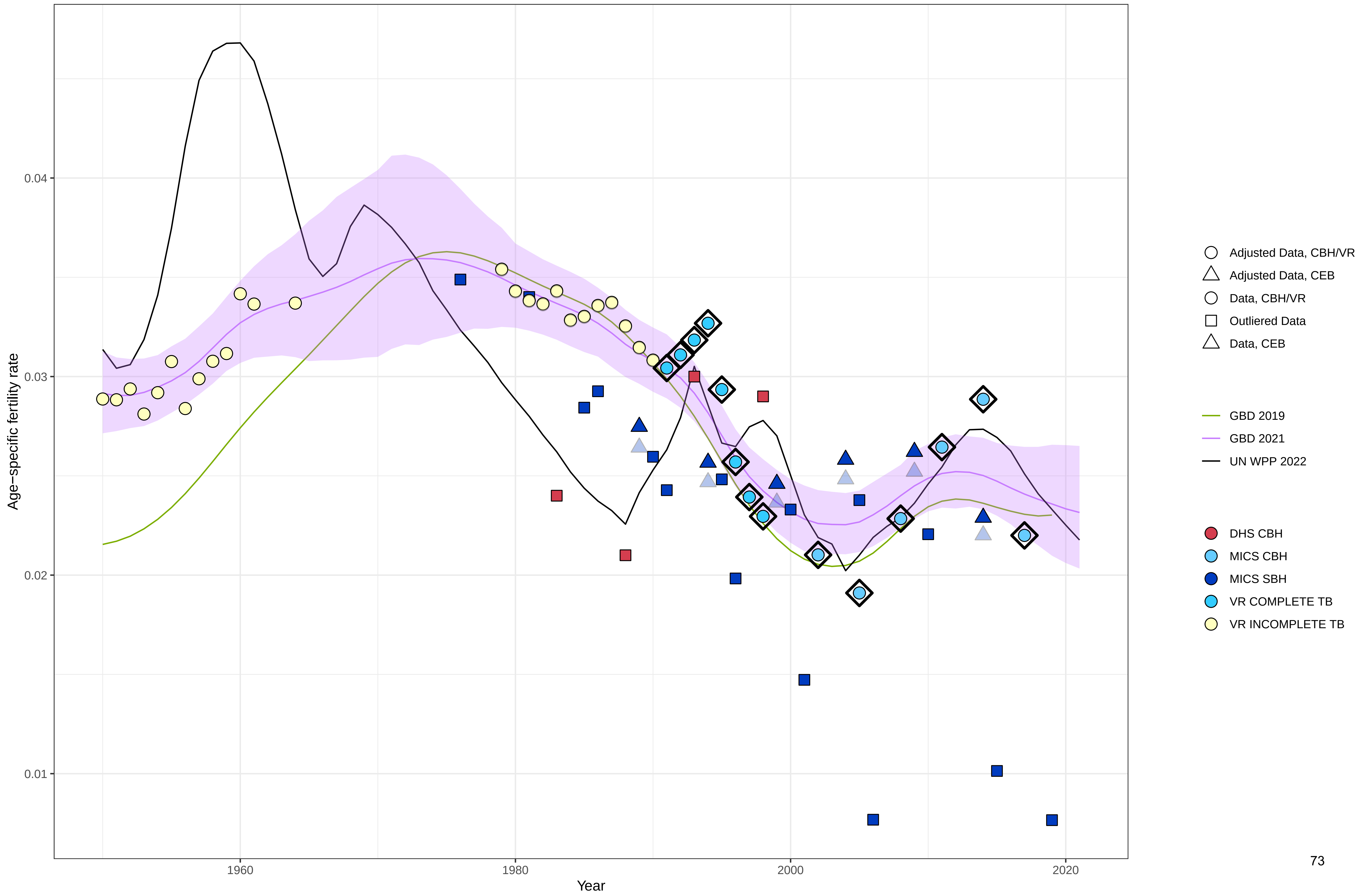

Age−specific fertility fitted trend lines, 1950−2021: Turkmenistan, Ages: 20−24
Data points enclosed with a diamond are reference sources

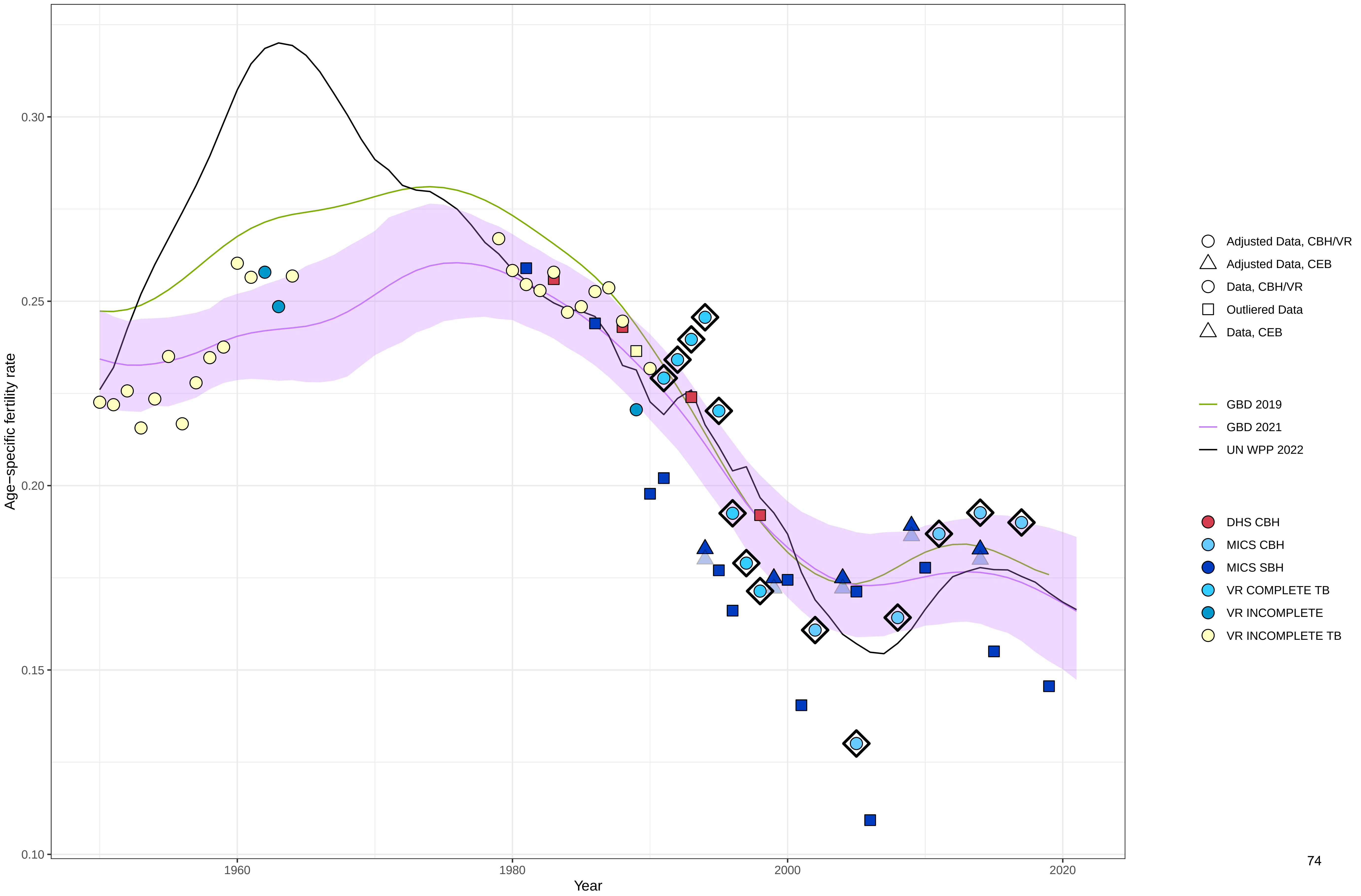

Age−specific fertility fitted trend lines, 1950−2021: Turkmenistan, Ages: 25−29
Data points enclosed with a diamond are reference sources

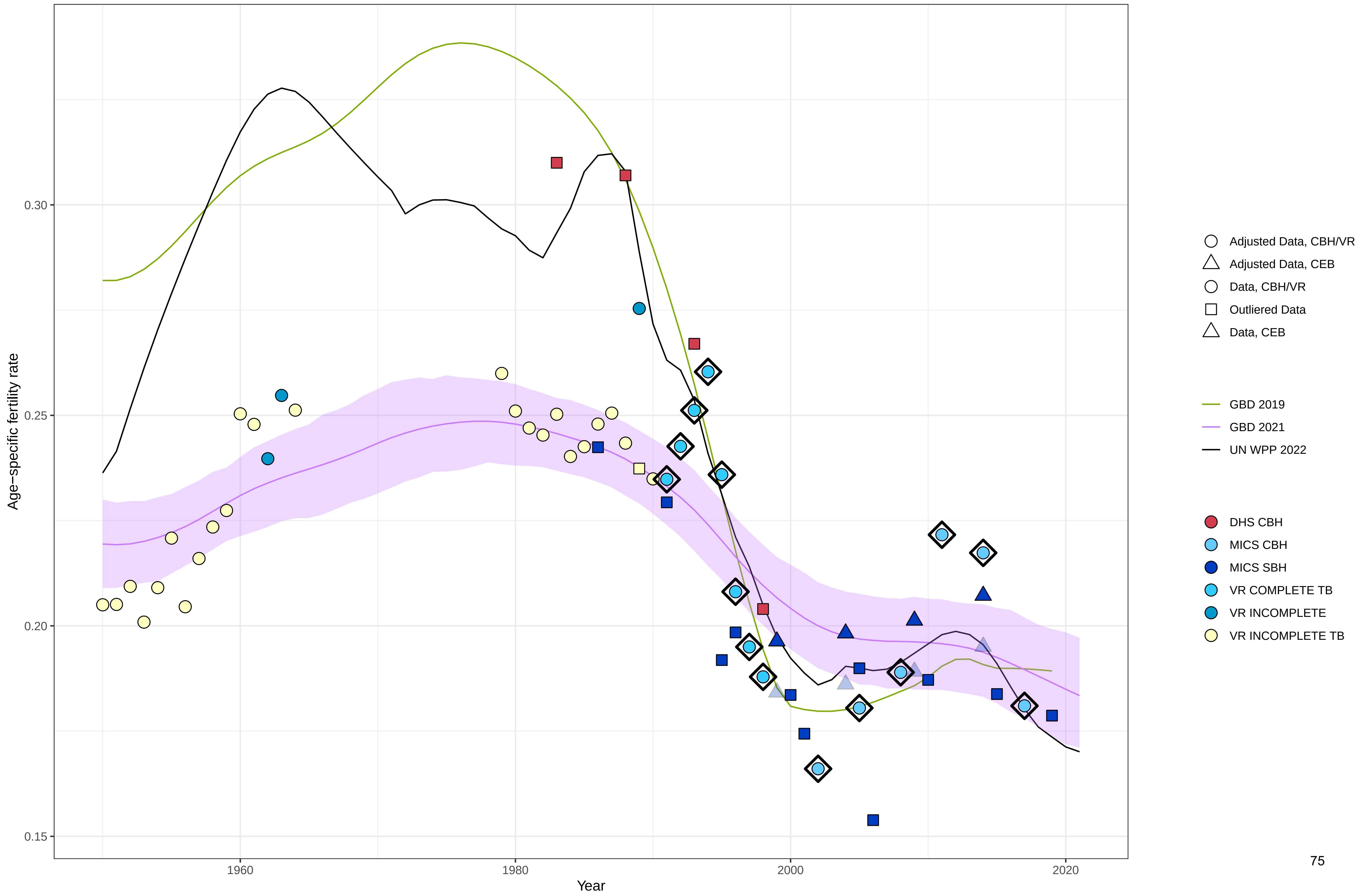

Age−specific fertility fitted trend lines, 1950−2021: Turkmenistan, Ages: 30−34
Data points enclosed with a diamond are reference sources

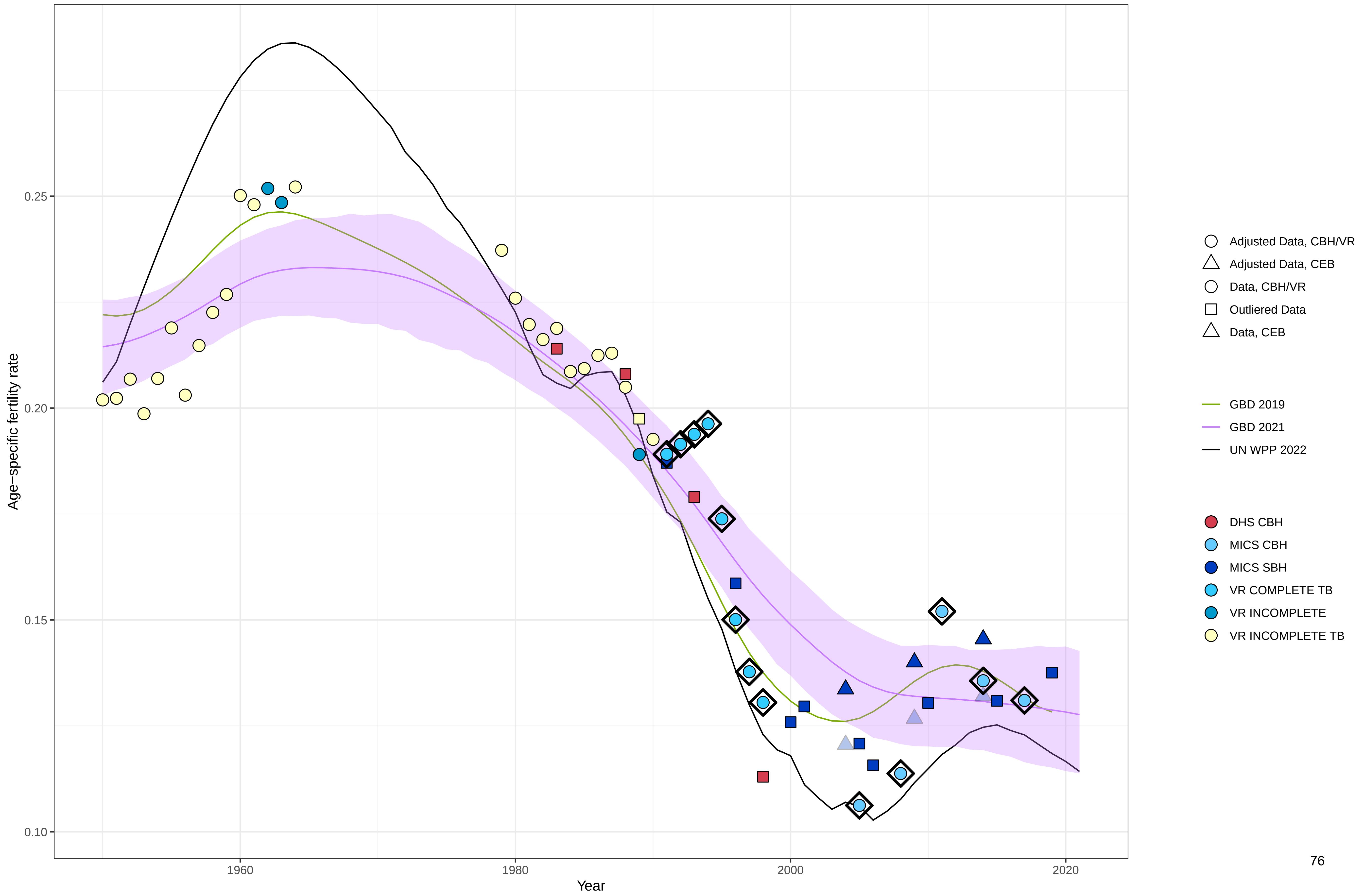

Age−specific fertility fitted trend lines, 1950−2021: Turkmenistan, Ages: 35−39
Data points enclosed with a diamond are reference sources

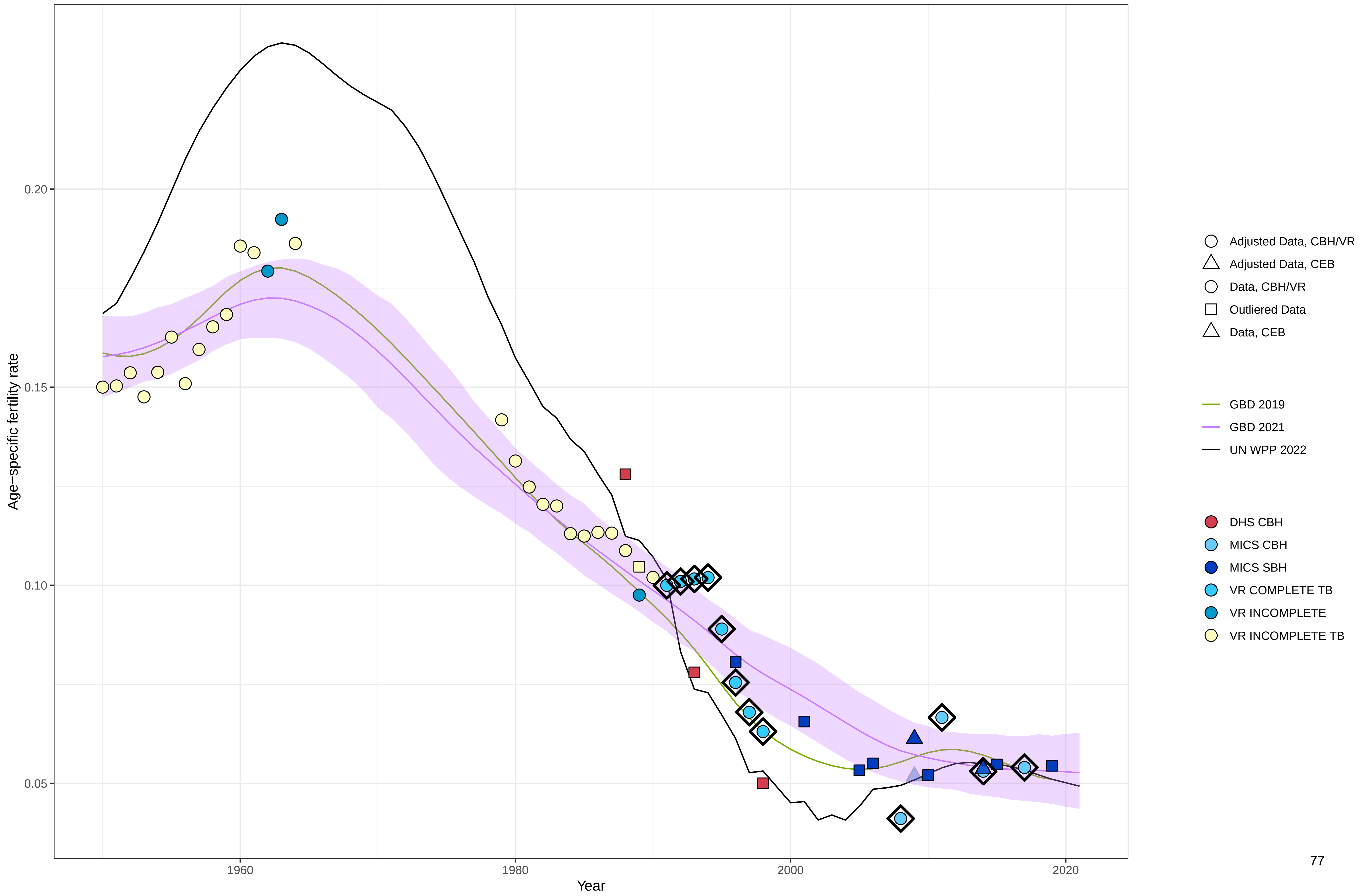

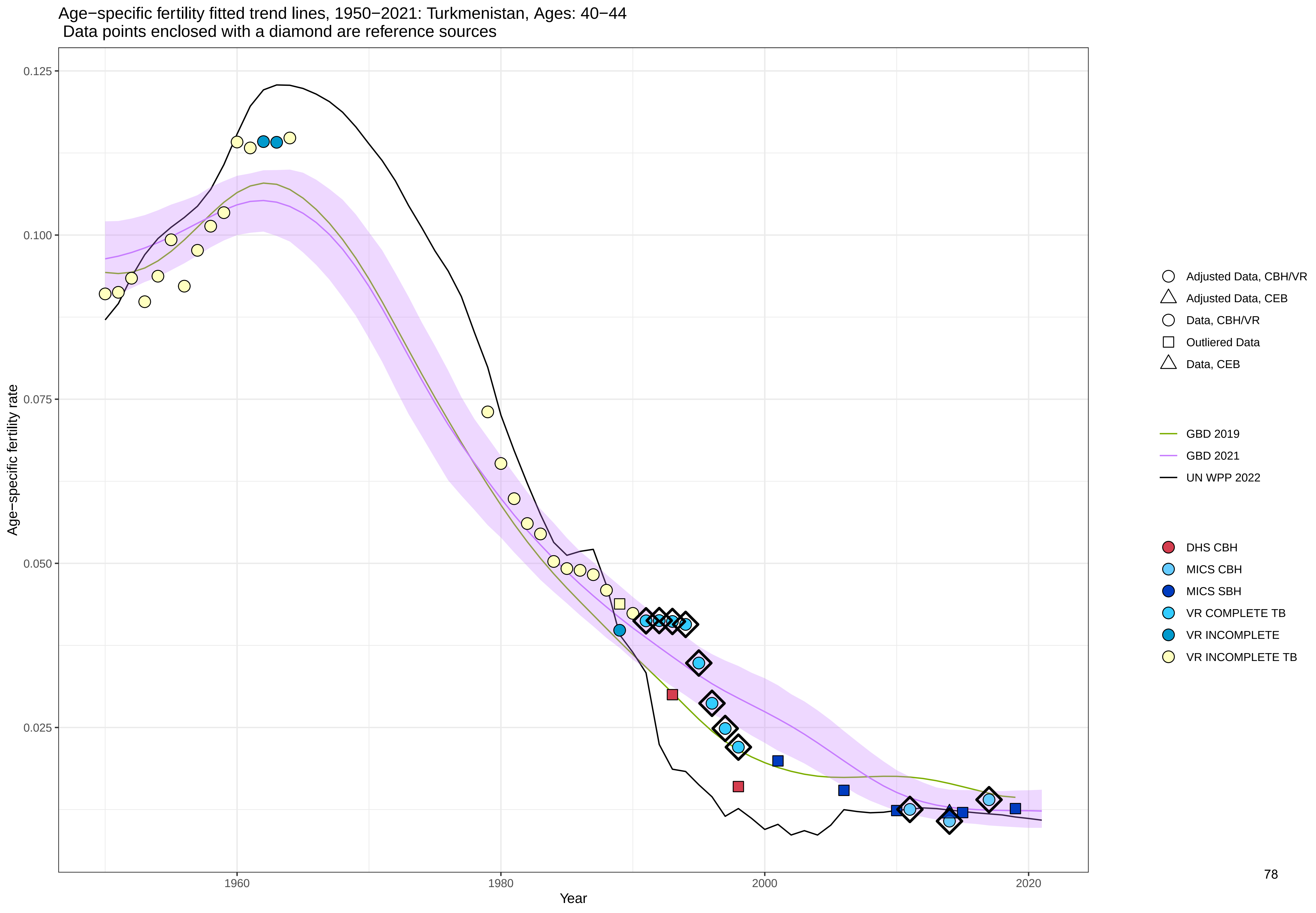

Age−specific fertility fitted trend lines, 1950−2021: Turkmenistan, Ages: 40−44
Data points enclosed with a diamond are reference sources
Age−specific fertility rate
Year
0.125
0.100
0.075
0.050
0.025
1960
1980
2000
2020
Adjusted Data, CBH/VR
Adjusted Data, CEB
Data, CBH/VR
Outliered Data
Data, CEB
GBD 2019
GBD 2021
UN WPP 2022
DHS CBH
MICS CBH
MICS SBH
VR COMPLETE TB
VR INCOMPLETE
VR INCOMPLETE TB

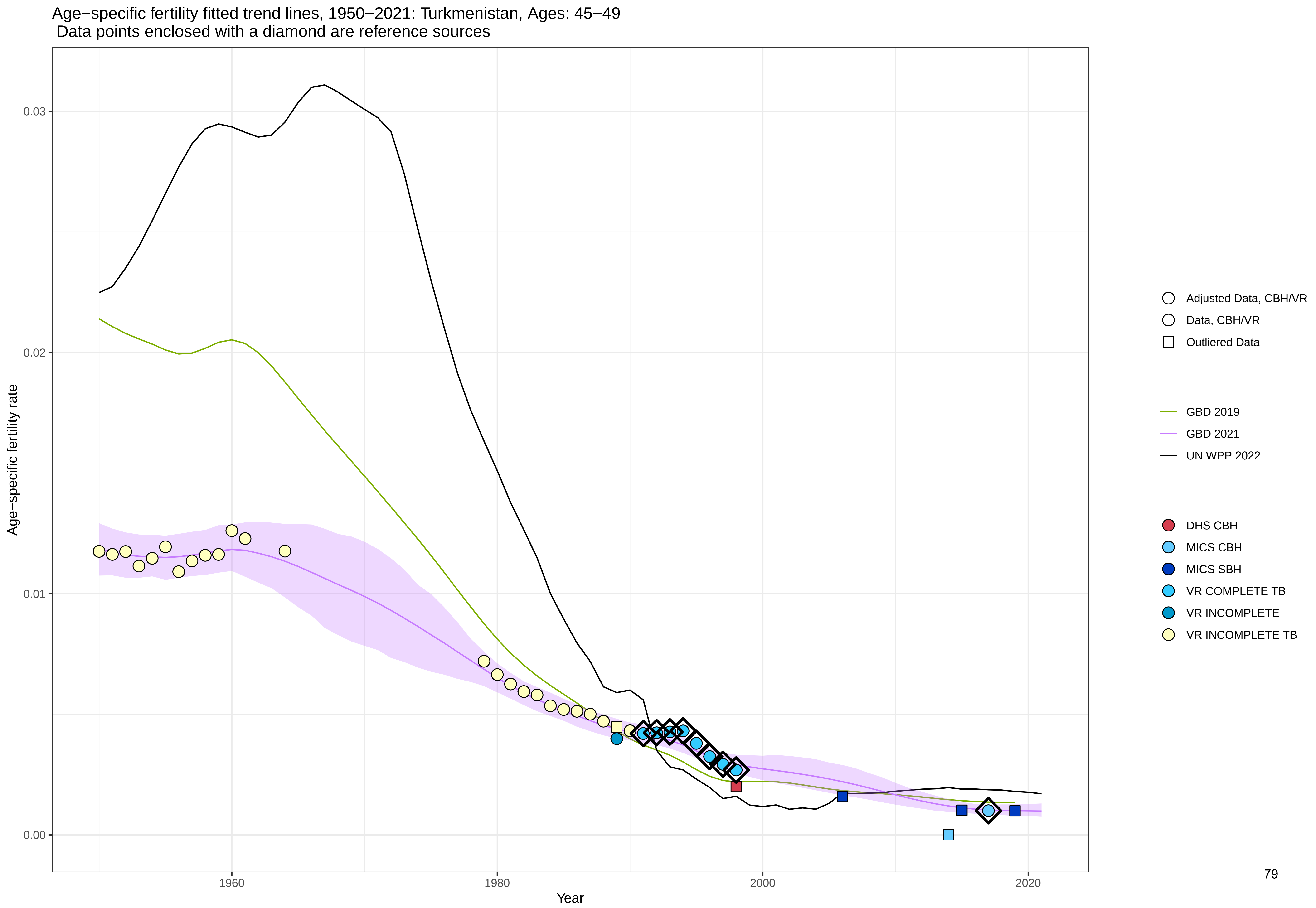

Age−specific fertility fitted trend lines, 1950−2021: Turkmenistan, Ages: 45−49
Data points enclosed with a diamond are reference sources
Age−specific fertility rate
0.00
0.01
0.02
0.03
1960
1980
2000
2020
Year
Adjusted Data, CBH/VR
Data, CBH/VR
Outliered Data
GBD 2019
GBD 2021
UN WPP 2022
DHS CBH
MICS CBH
MICS SBH
VR COMPLETE TB
VR INCOMPLETE
VR INCOMPLETE TB

Age−specific fertility fitted trend lines, 1950−2021: Turkmenistan, Ages: 50−54
Data points enclosed with a diamond are reference sources

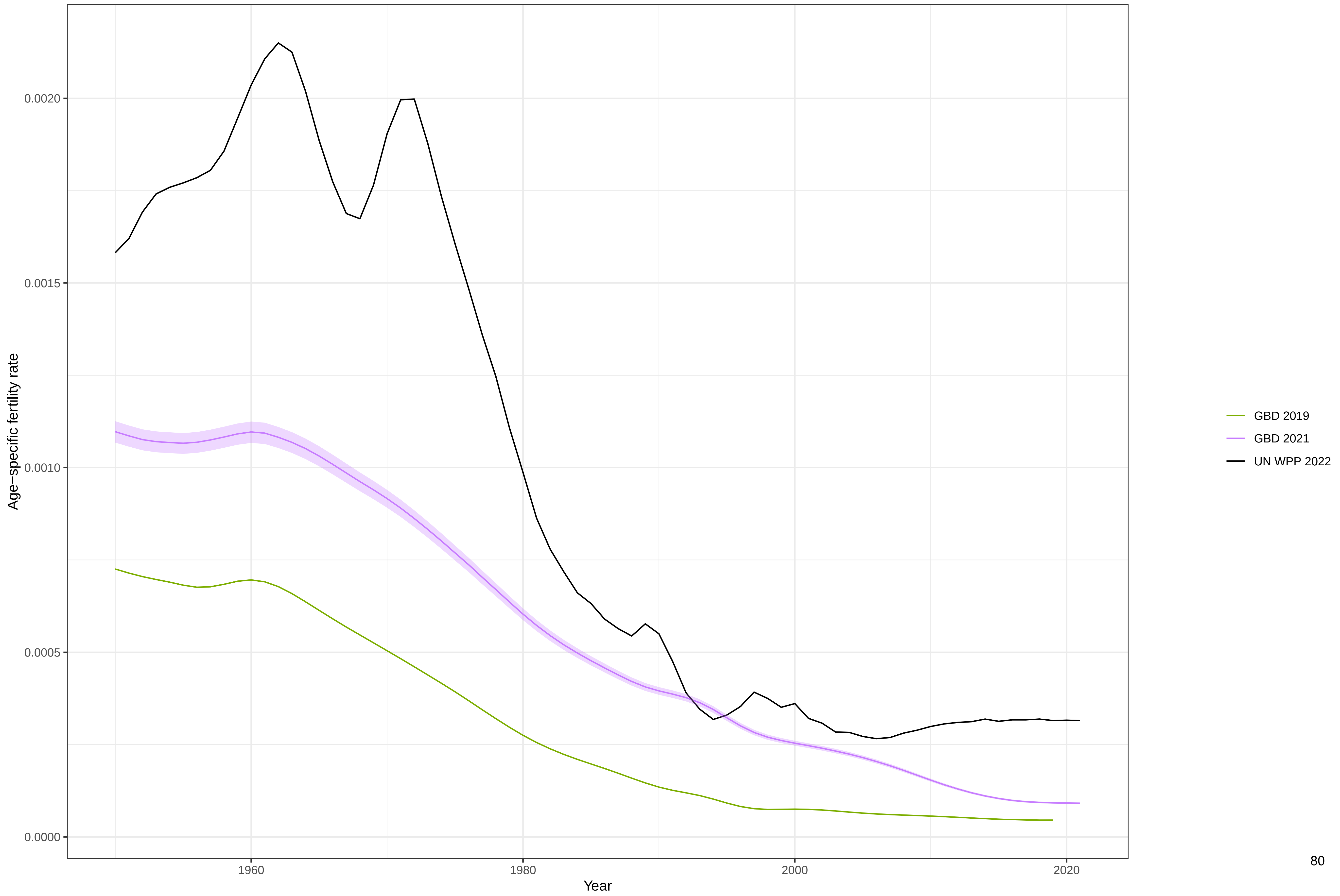

Total fertility rate fitted trend lines, 1950−2021: Turkmenistan TFR
Data points enclosed with a diamond are reference sources

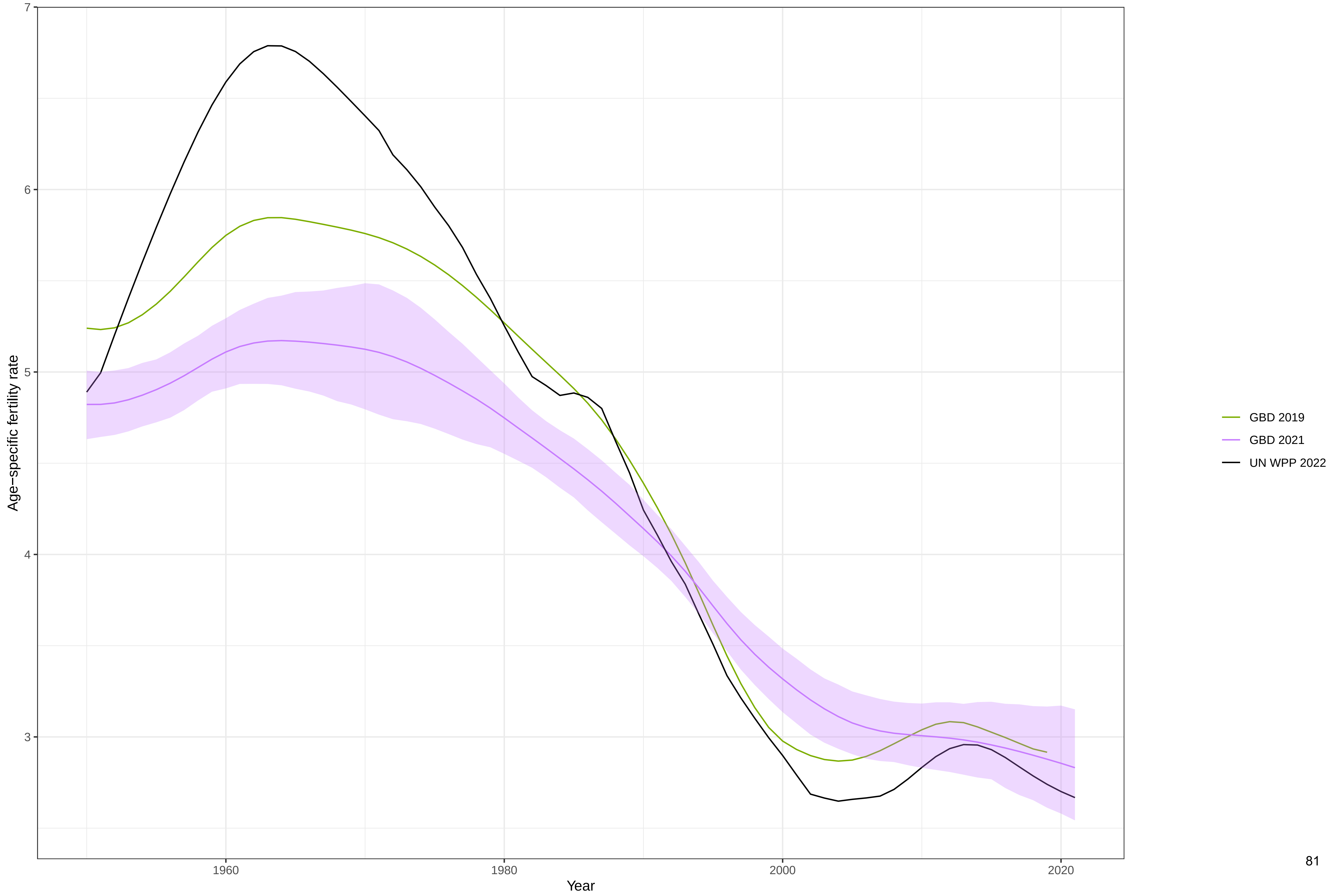

Age−specific fertility fitted trend lines, 1950−2021: Uzbekistan, Ages: 10−14
Data points enclosed with a diamond are reference sources

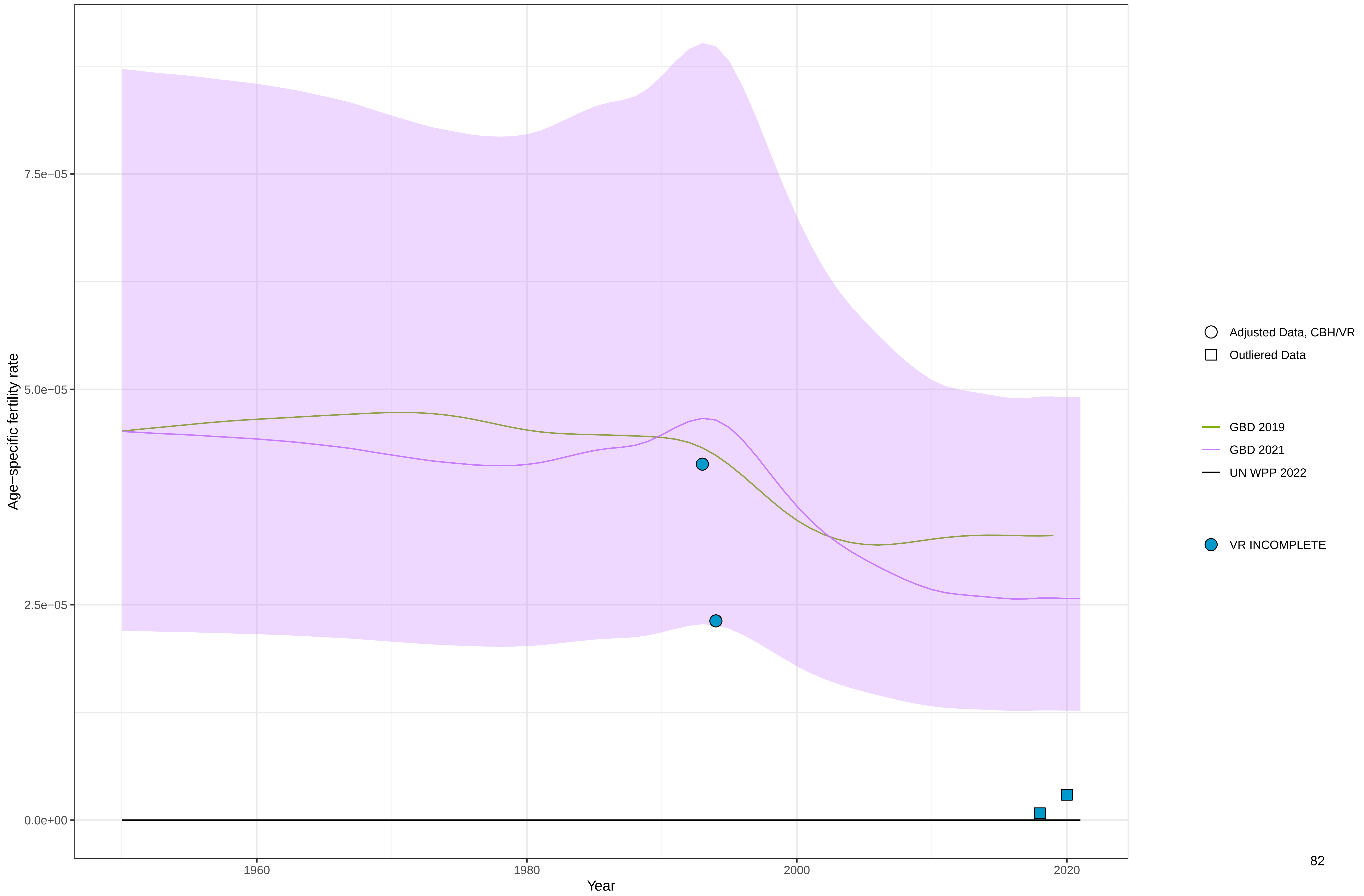

Age−specific fertility fitted trend lines, 1950−2021: Uzbekistan, Ages: 15−19
Data points enclosed with a diamond are reference sources

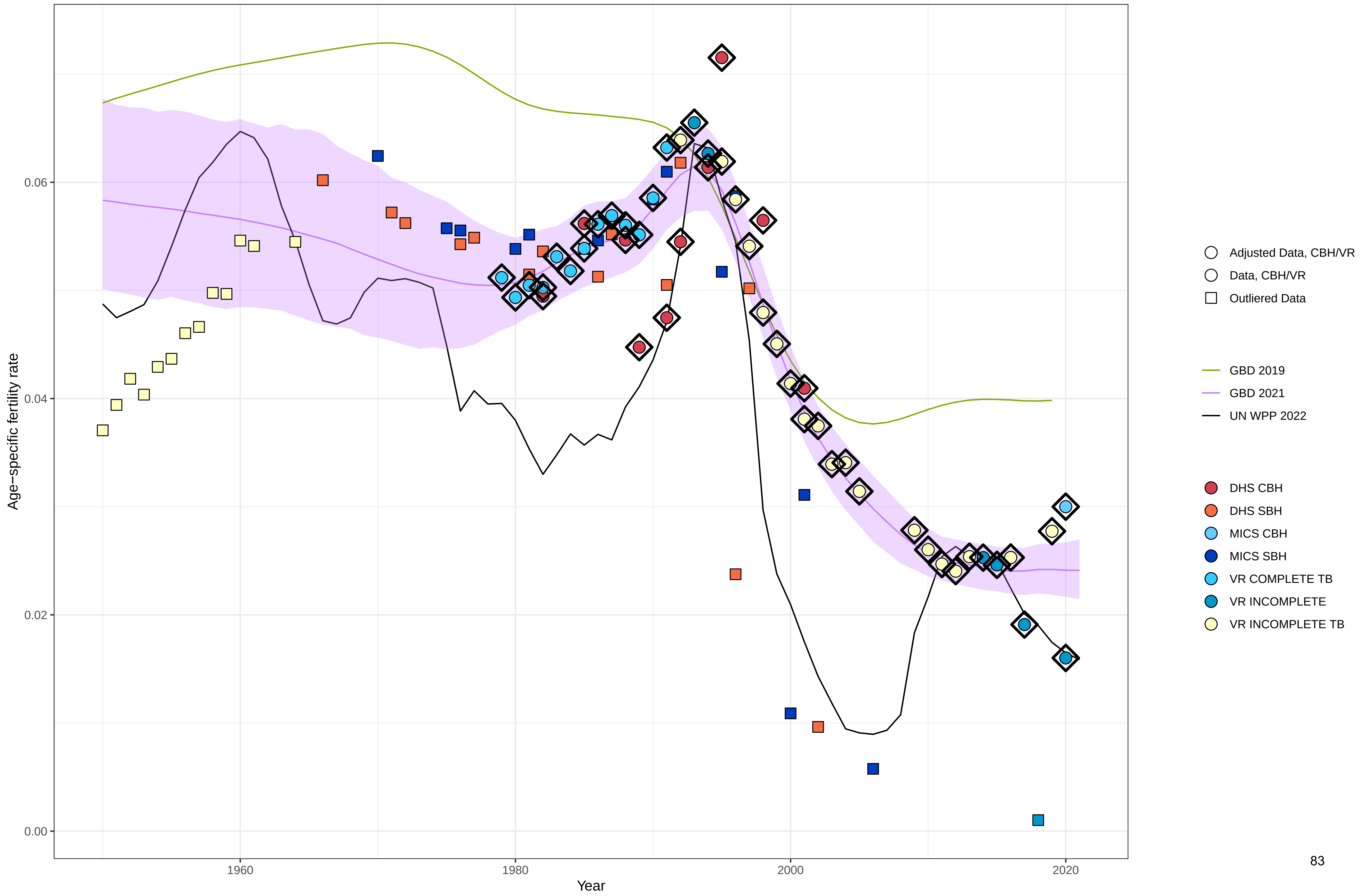

Age−specific fertility fitted trend lines, 1950−2021: Uzbekistan, Ages: 20−24
Data points enclosed with a diamond are reference sources

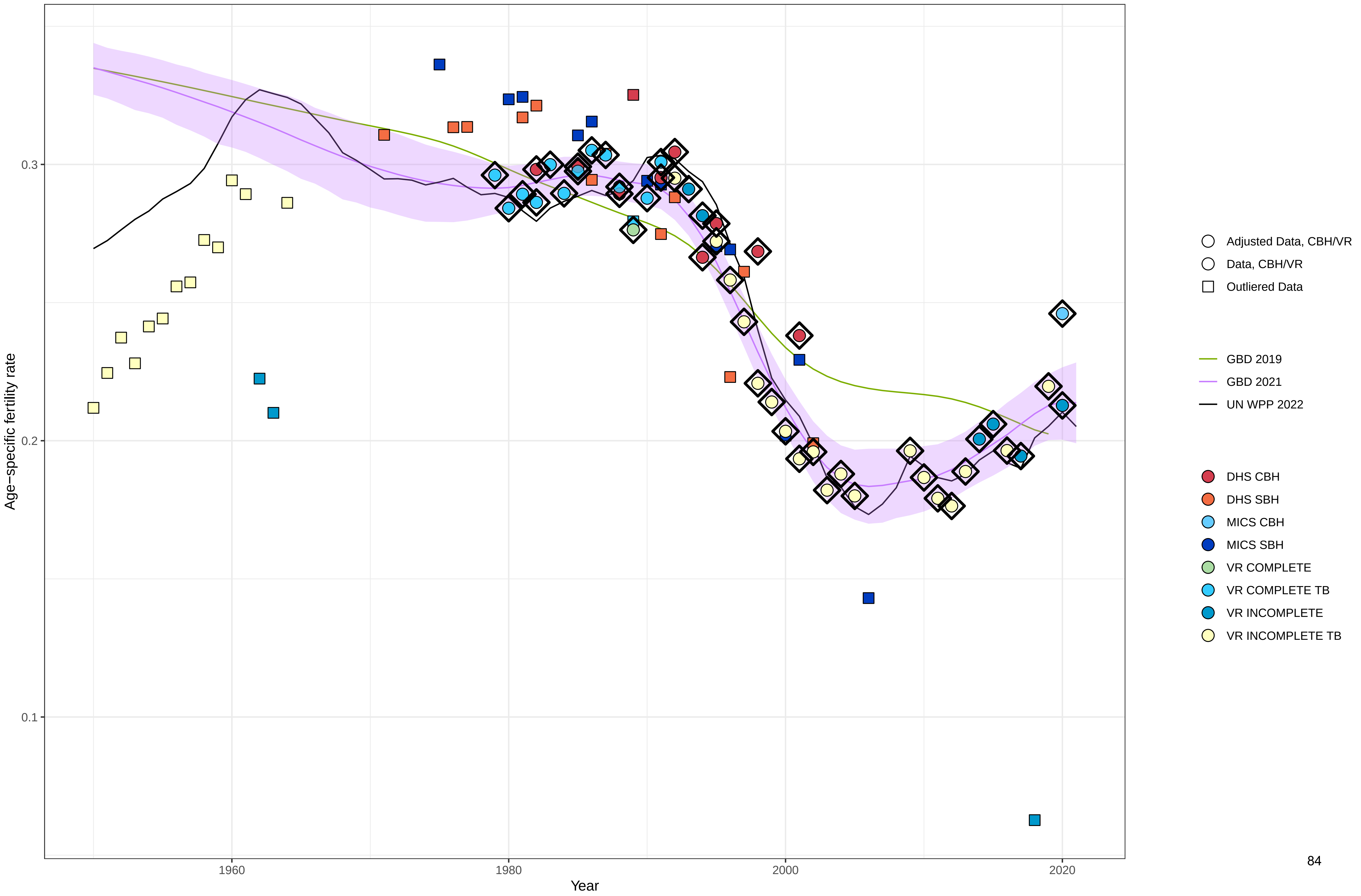

Age−specific fertility fitted trend lines, 1950−2021: Uzbekistan, Ages: 25−29
Data points enclosed with a diamond are reference sources

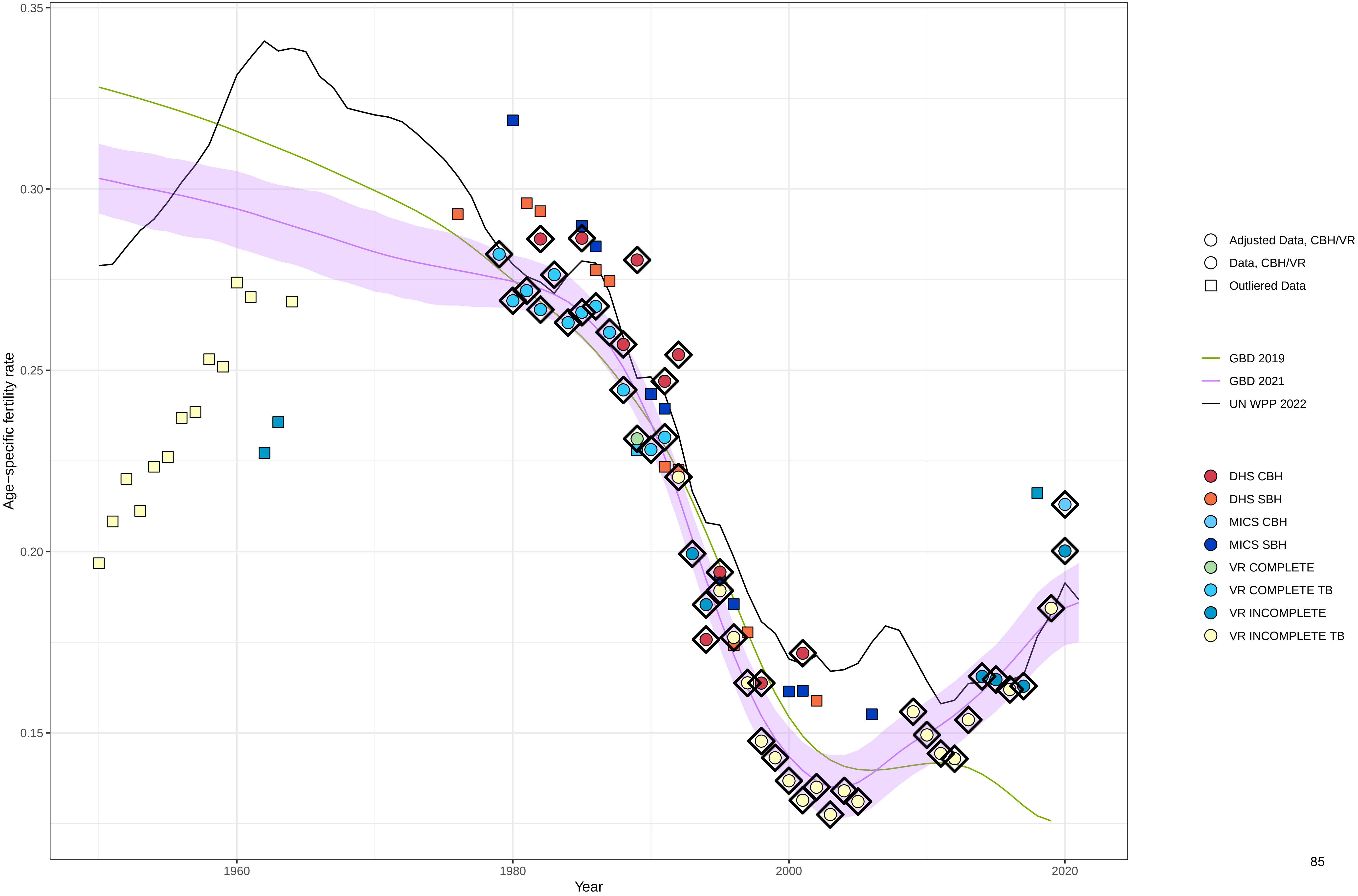

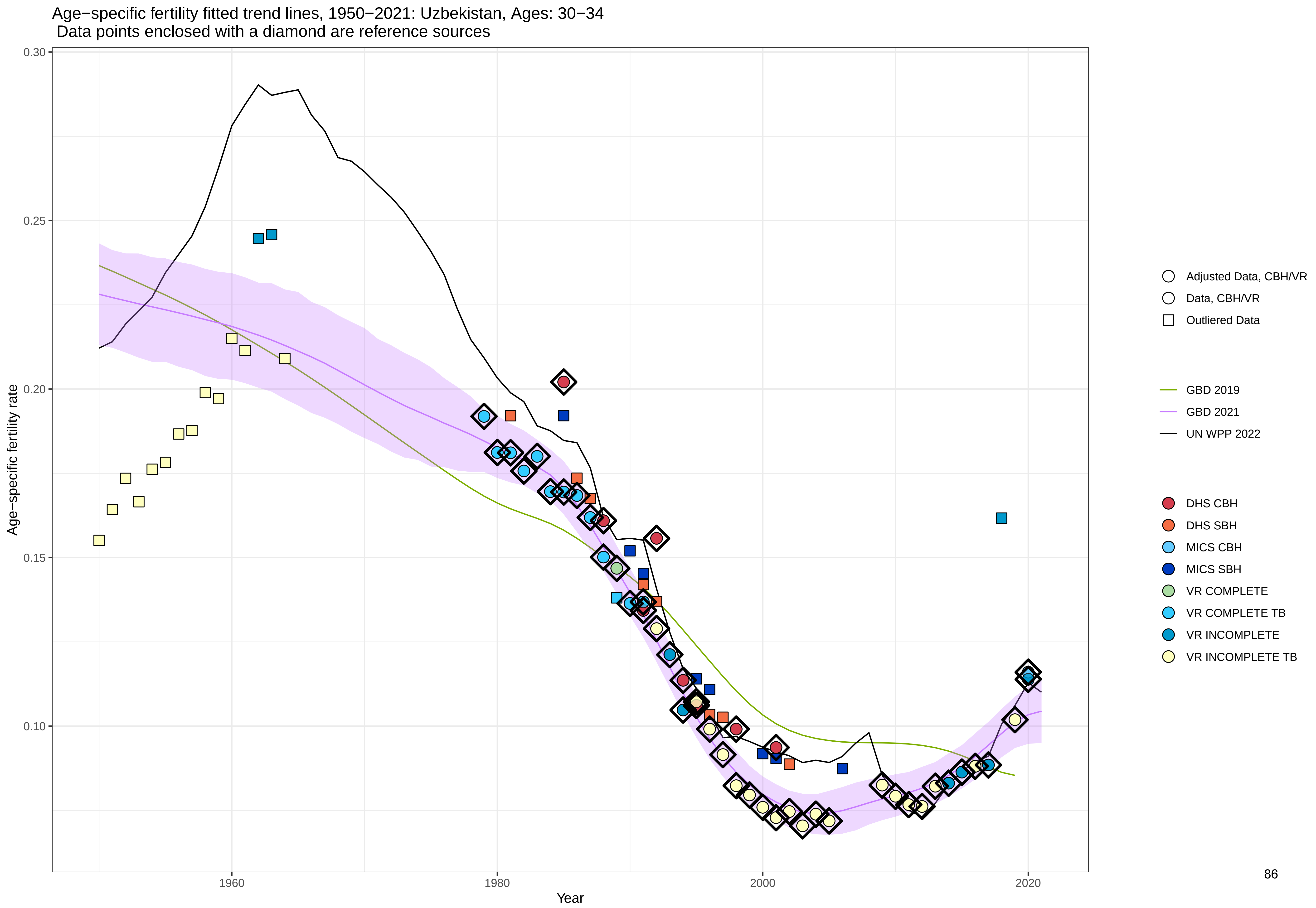

Age−specific fertility fitted trend lines, 1950−2021: Uzbekistan, Ages: 30−34
Data points enclosed with a diamond are reference sources
0.30
0.25
0.20
0.15
0.10
Age−specific fertility rate
1960
1980
2000
2020
Year
Adjusted Data, CBH/VR
Data, CBH/VR
Outliered Data
GBD 2019
GBD 2021
UN WPP 2022
DHS CBH
DHS SBH
MICS CBH
MICS SBH
VR COMPLETE
VR COMPLETE TB
VR INCOMPLETE
VR INCOMPLETE TB

Age−specific fertility fitted trend lines, 1950−2021: Uzbekistan, Ages: 35−39
Data points enclosed with a diamond are reference sources

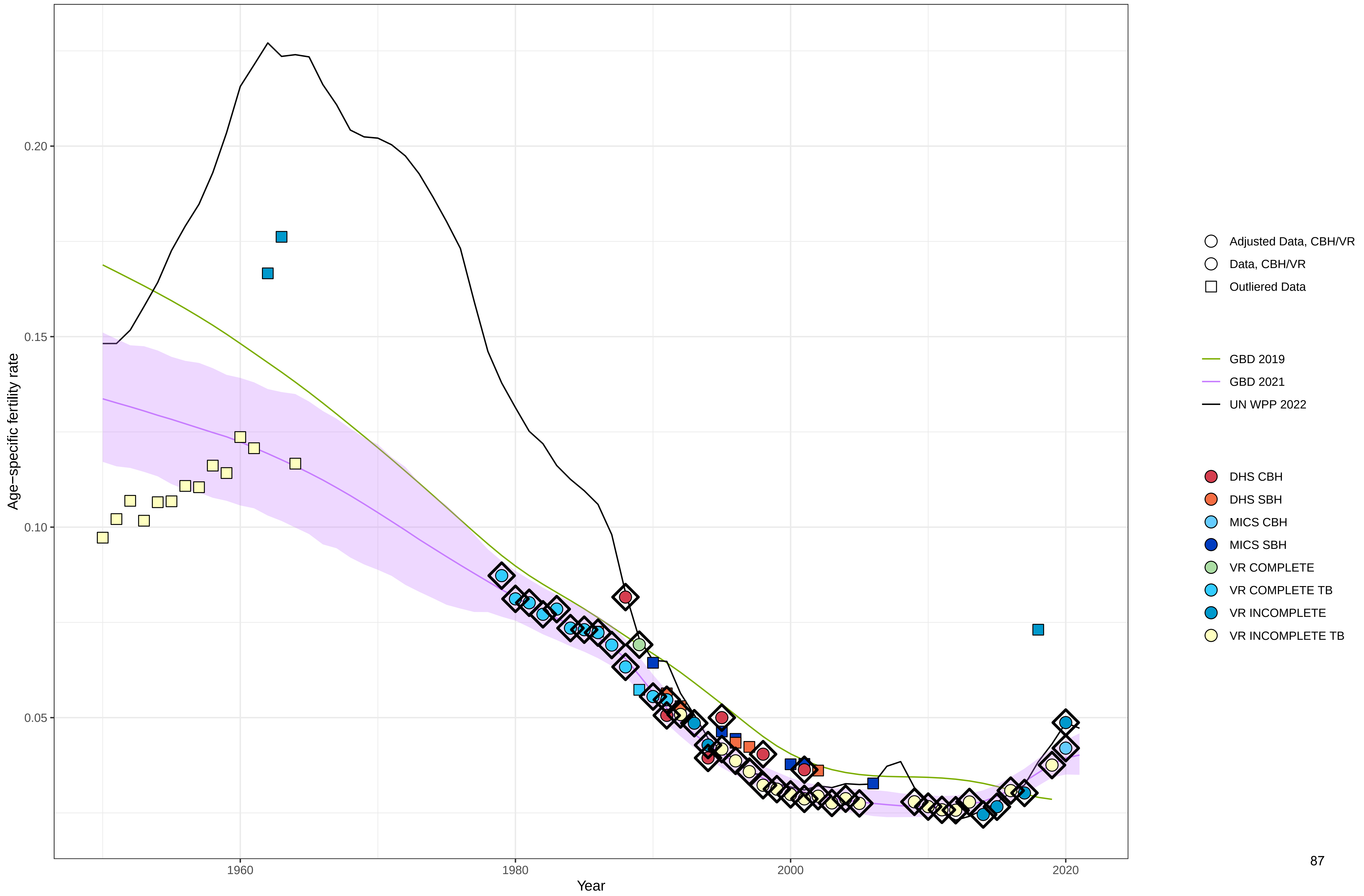

Age−specific fertility fitted trend lines, 1950−2021: Uzbekistan, Ages: 40−44
Data points enclosed with a diamond are reference sources

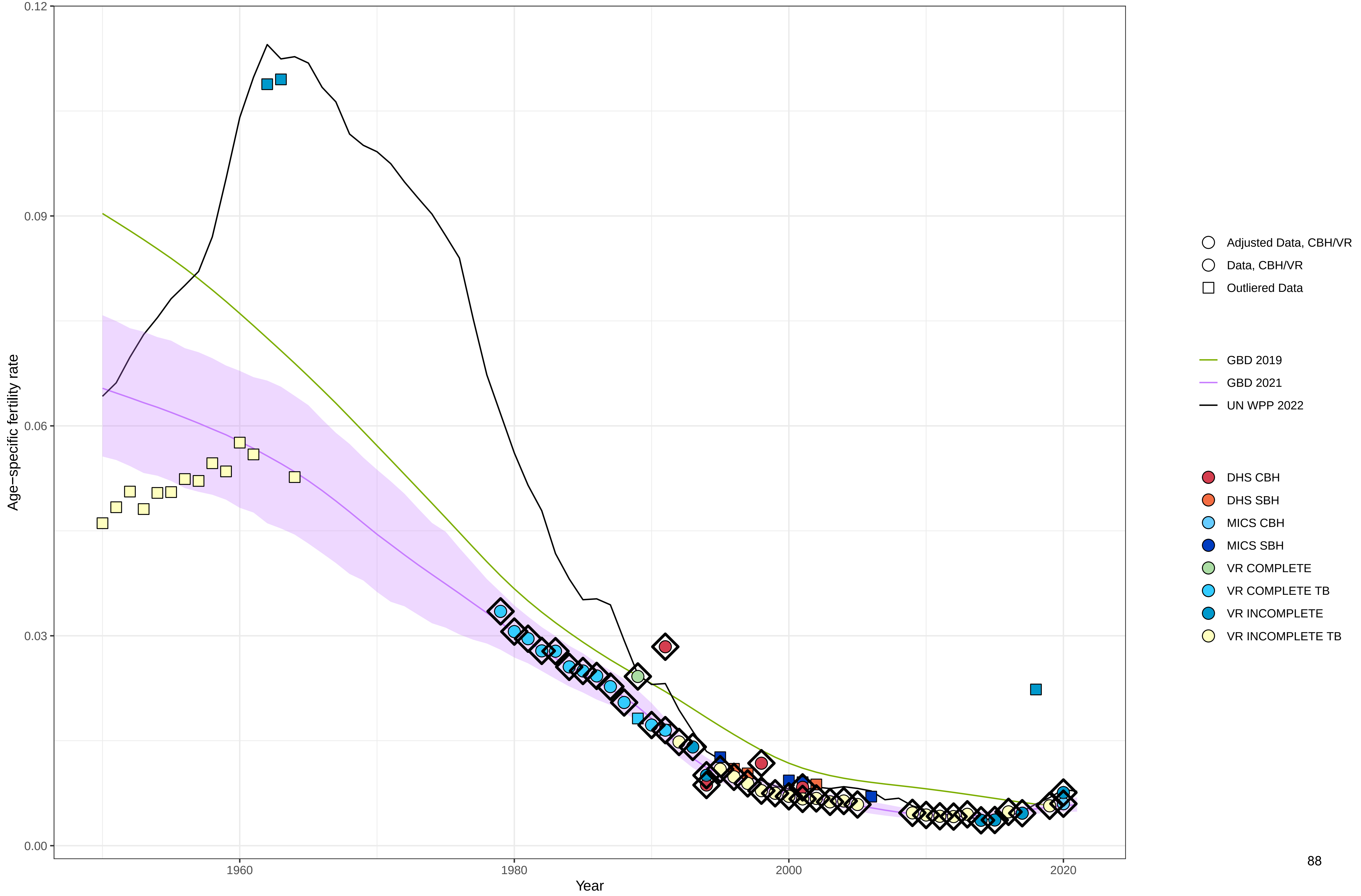

Age−specific fertility fitted trend lines, 1950−2021: Uzbekistan, Ages: 45−49
Data points enclosed with a diamond are reference sources

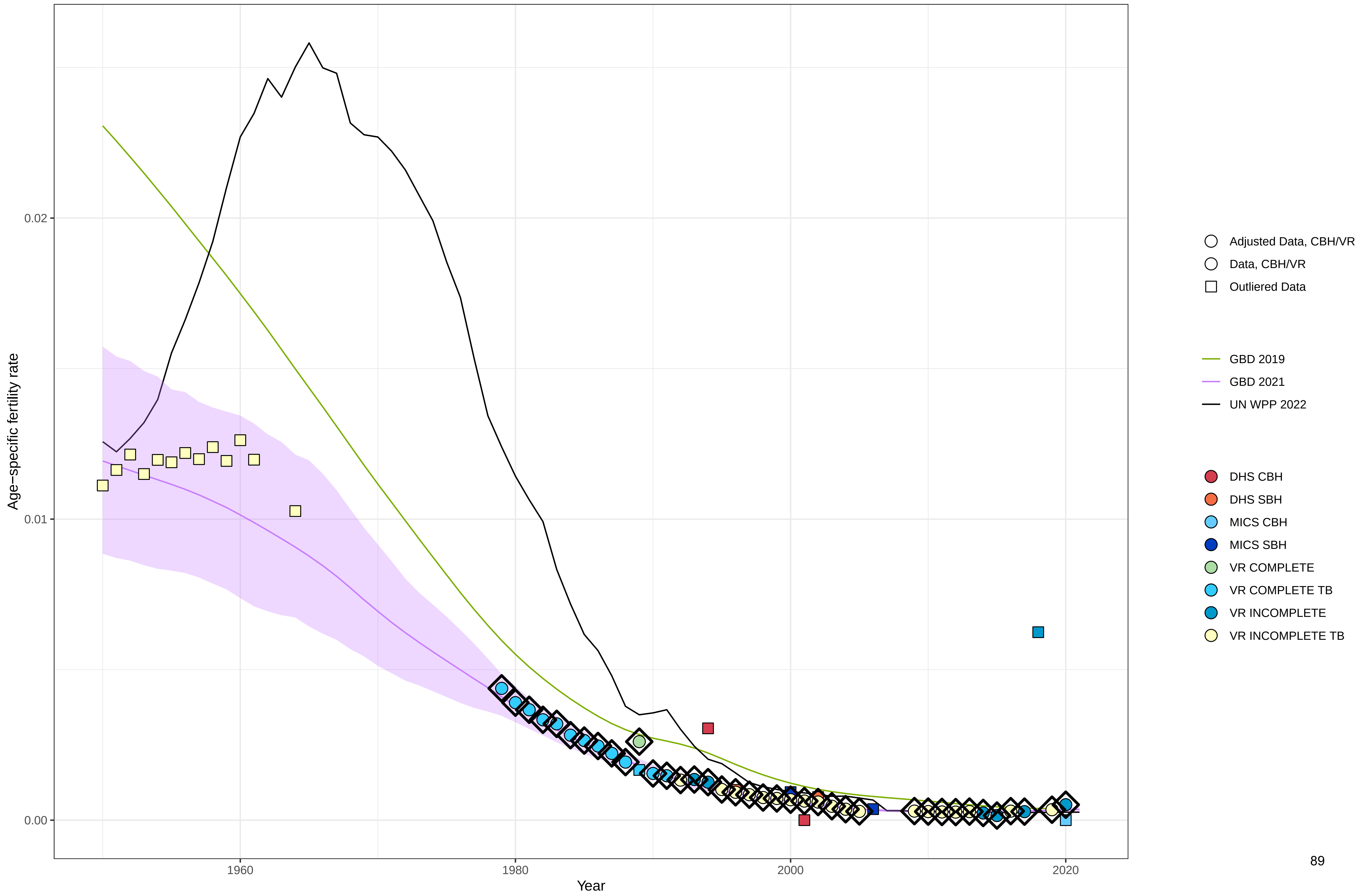

Age−specific fertility fitted trend lines, 1950−2021: Uzbekistan, Ages: 50−54
Data points enclosed with a diamond are reference sources

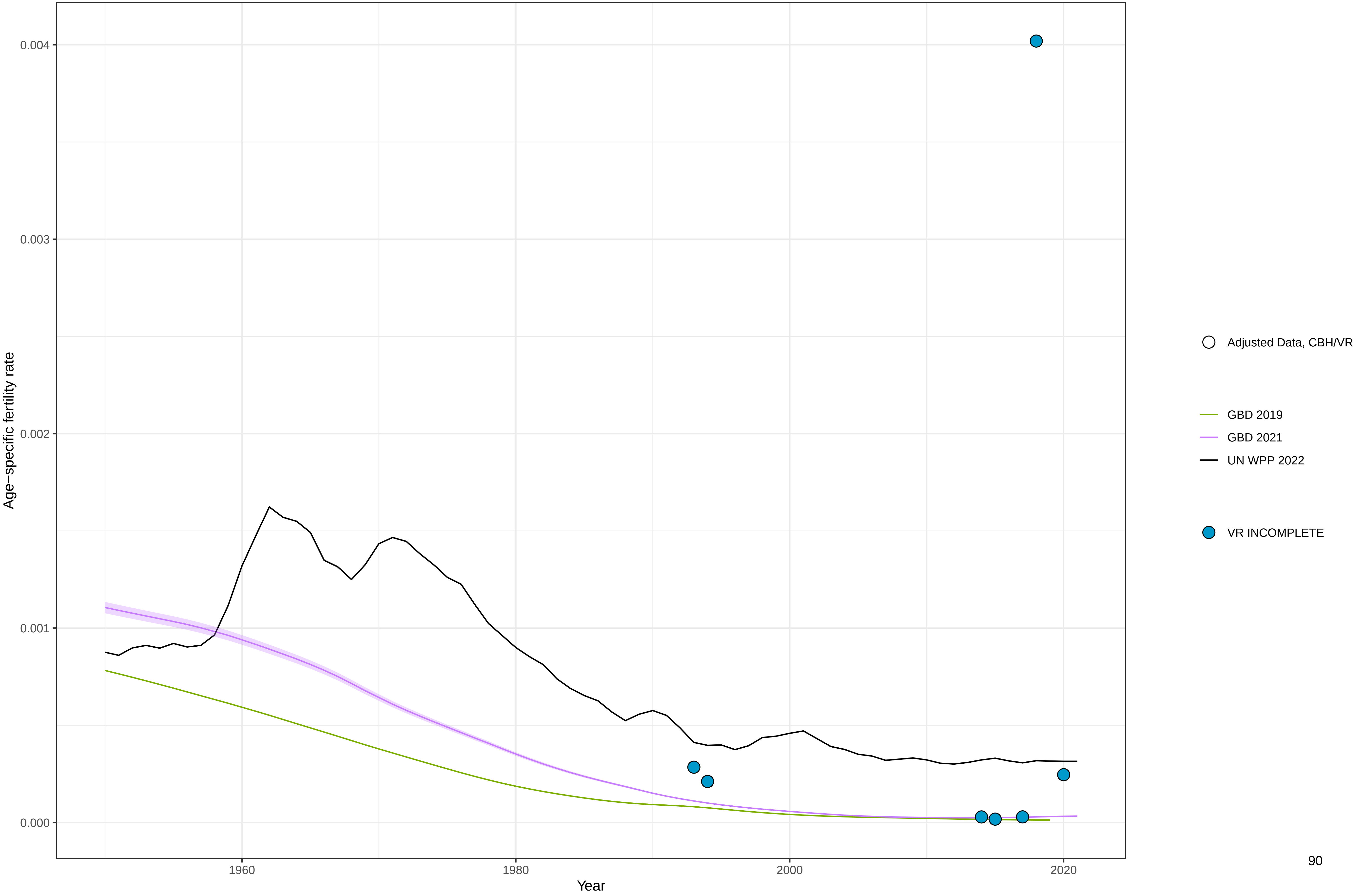

Total fertility rate fitted trend lines, 1950−2021: Uzbekistan TFR
Data points enclosed with a diamond are reference sources

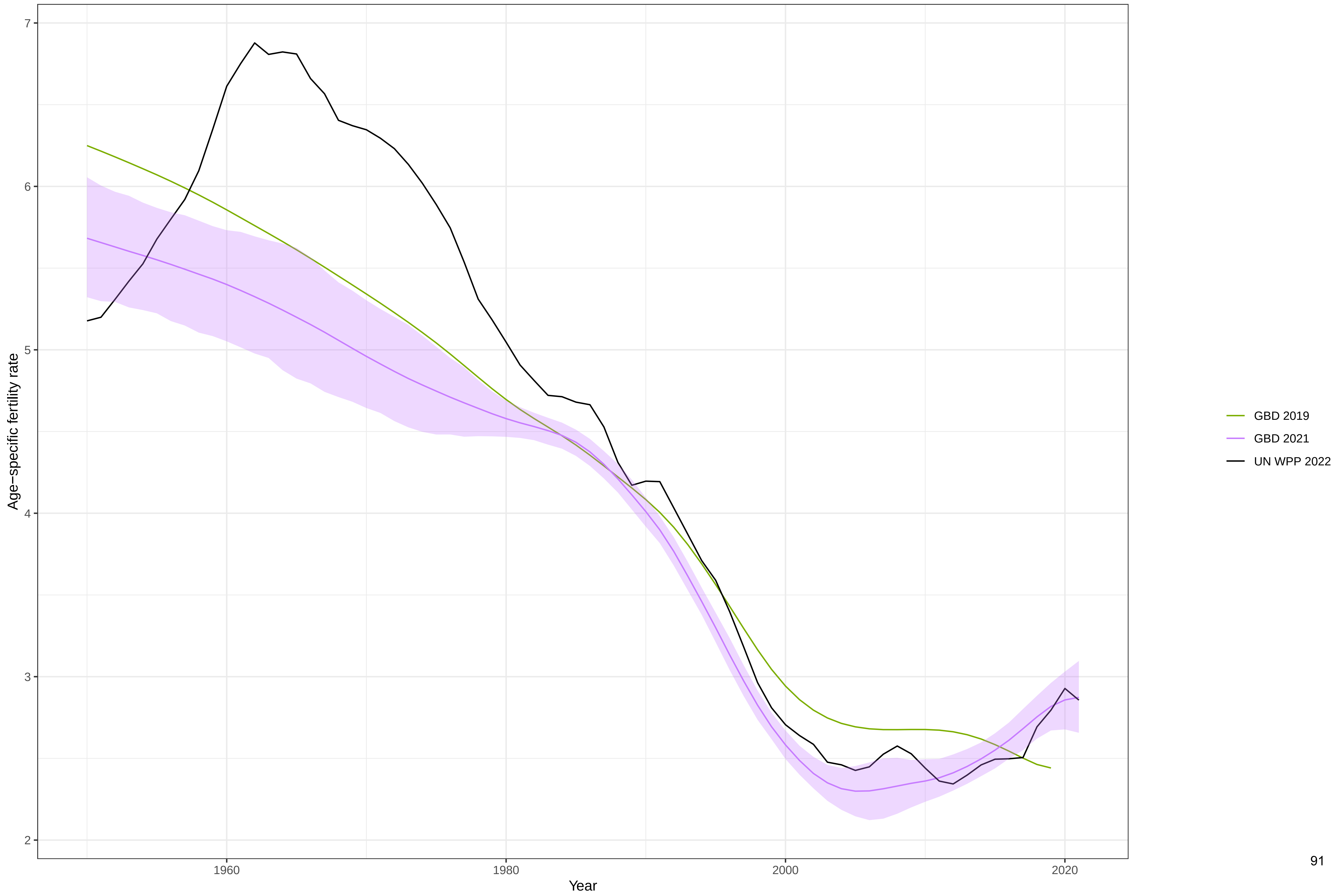

Age−specific fertility fitted trend lines, 1950−2021: Albania, Ages: 10−14
Data points enclosed with a diamond are reference sources

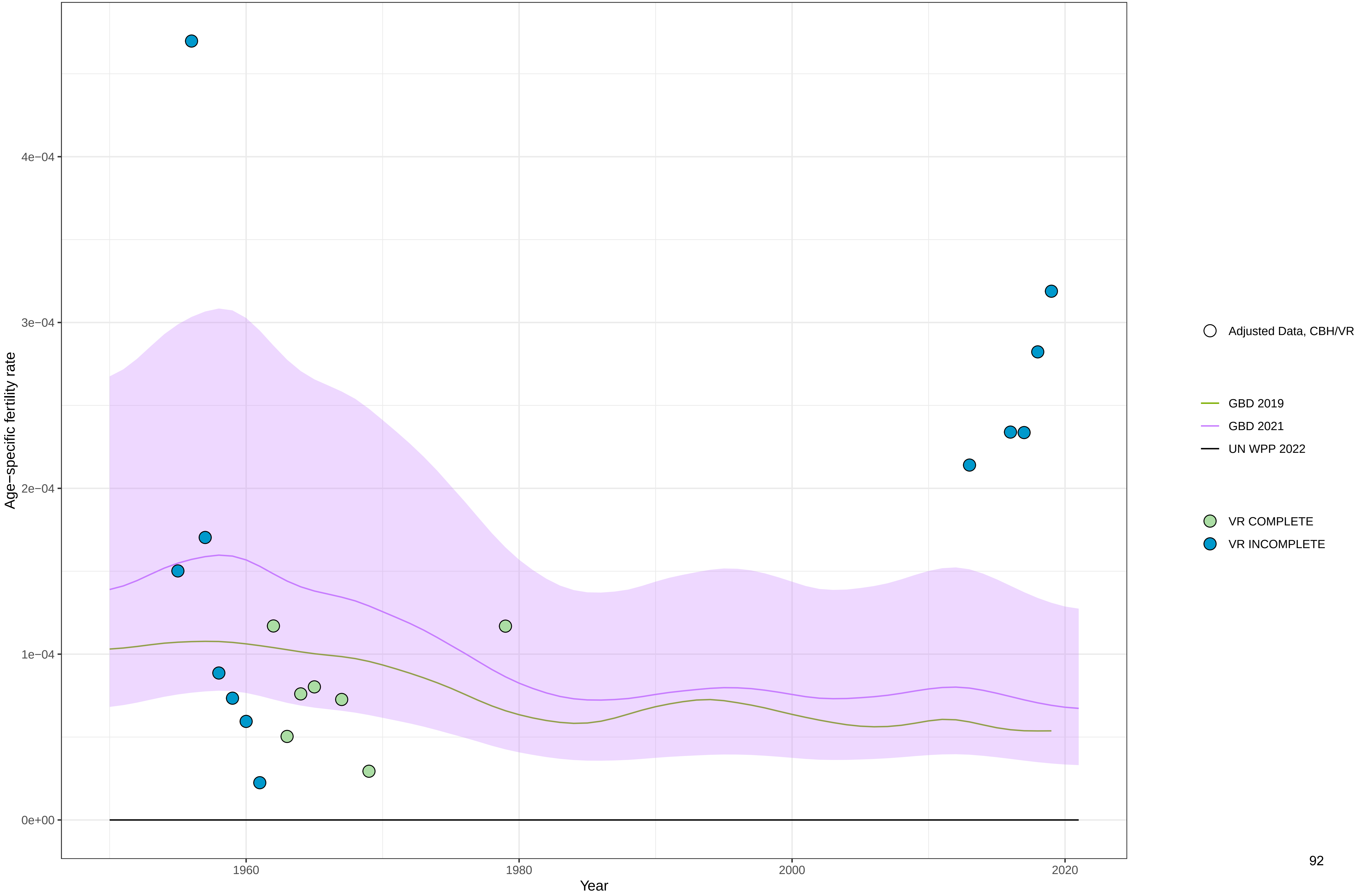

Age−specific fertility fitted trend lines, 1950−2021: Albania, Ages: 15−19
Data points enclosed with a diamond are reference sources

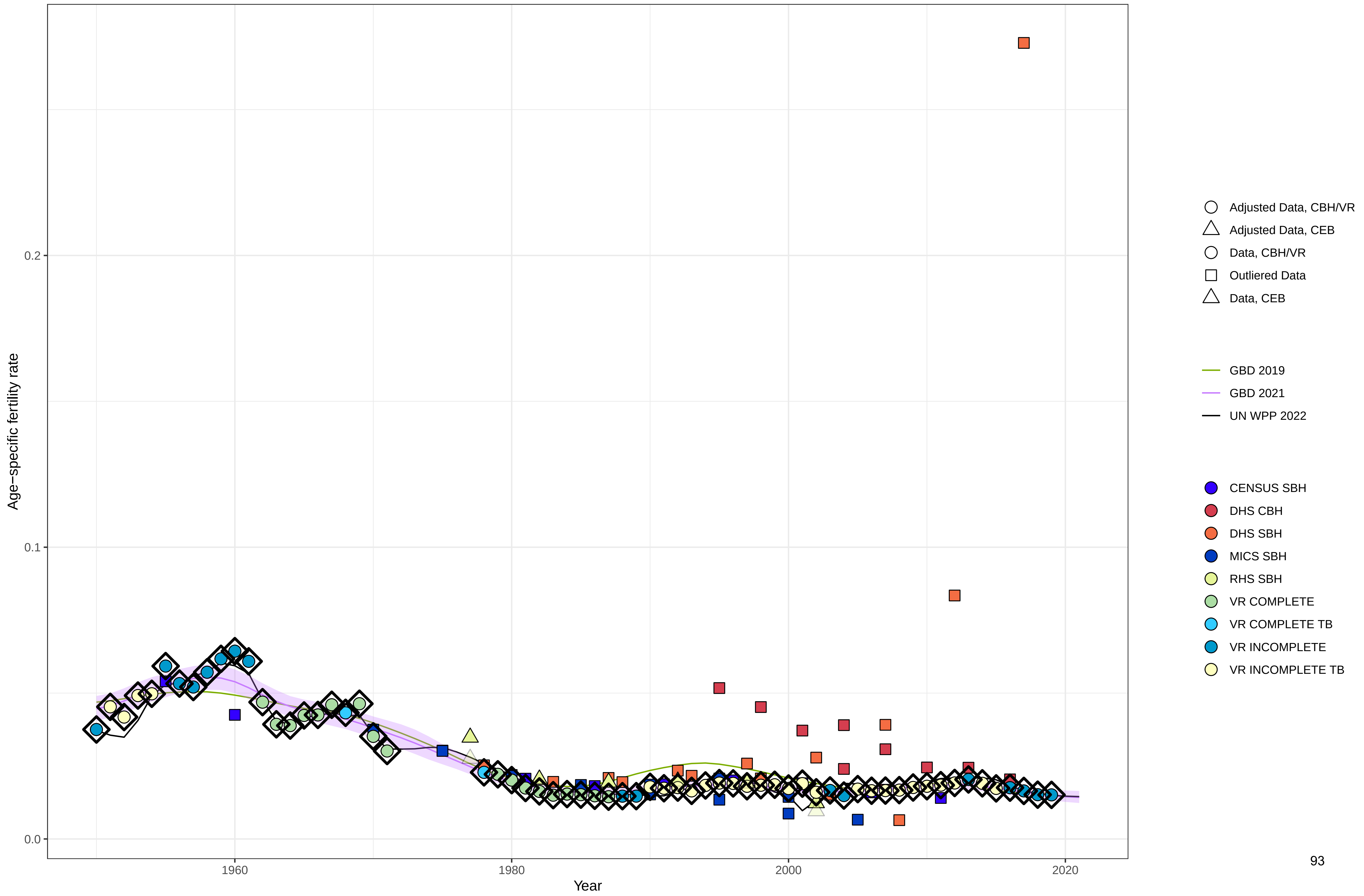

Age−specific fertility fitted trend lines, 1950−2021: Albania, Ages: 20−24
Data points enclosed with a diamond are reference sources

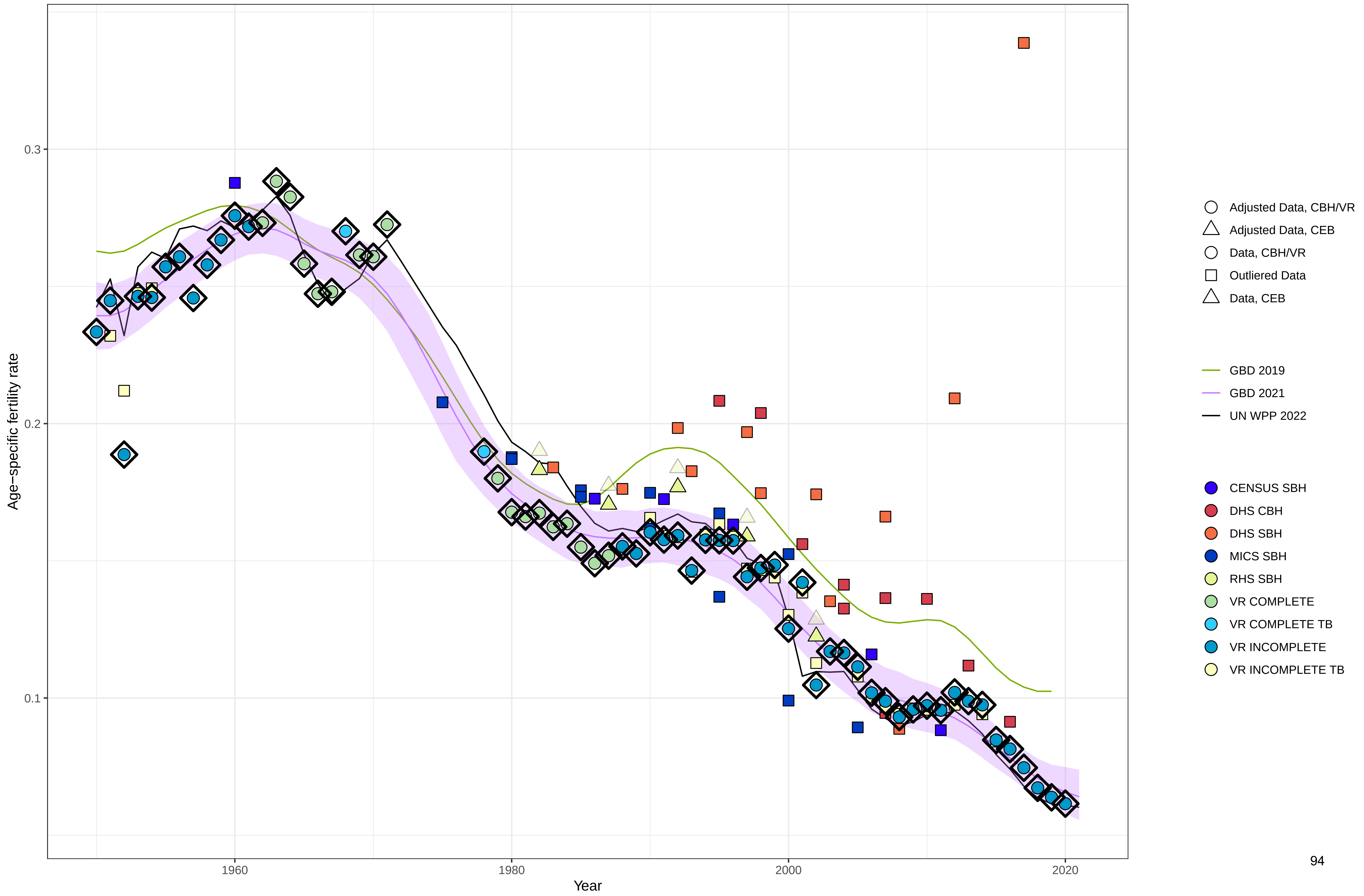

Age−specific fertility fitted trend lines, 1950−2021: Albania, Ages: 25−29
Data points enclosed with a diamond are reference sources

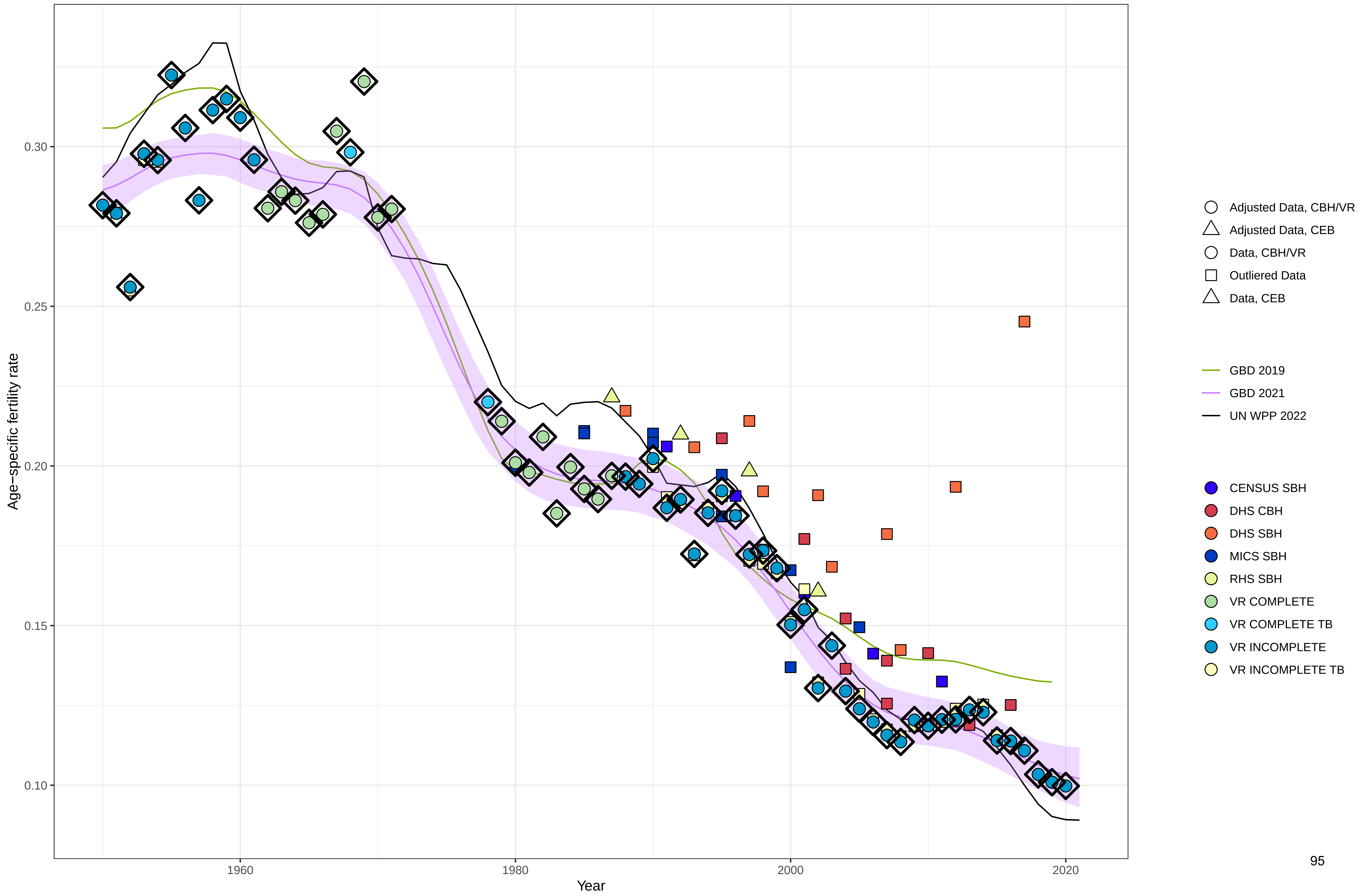

Age−specific fertility fitted trend lines, 1950−2021: Albania, Ages: 30−34
Data points enclosed with a diamond are reference sources

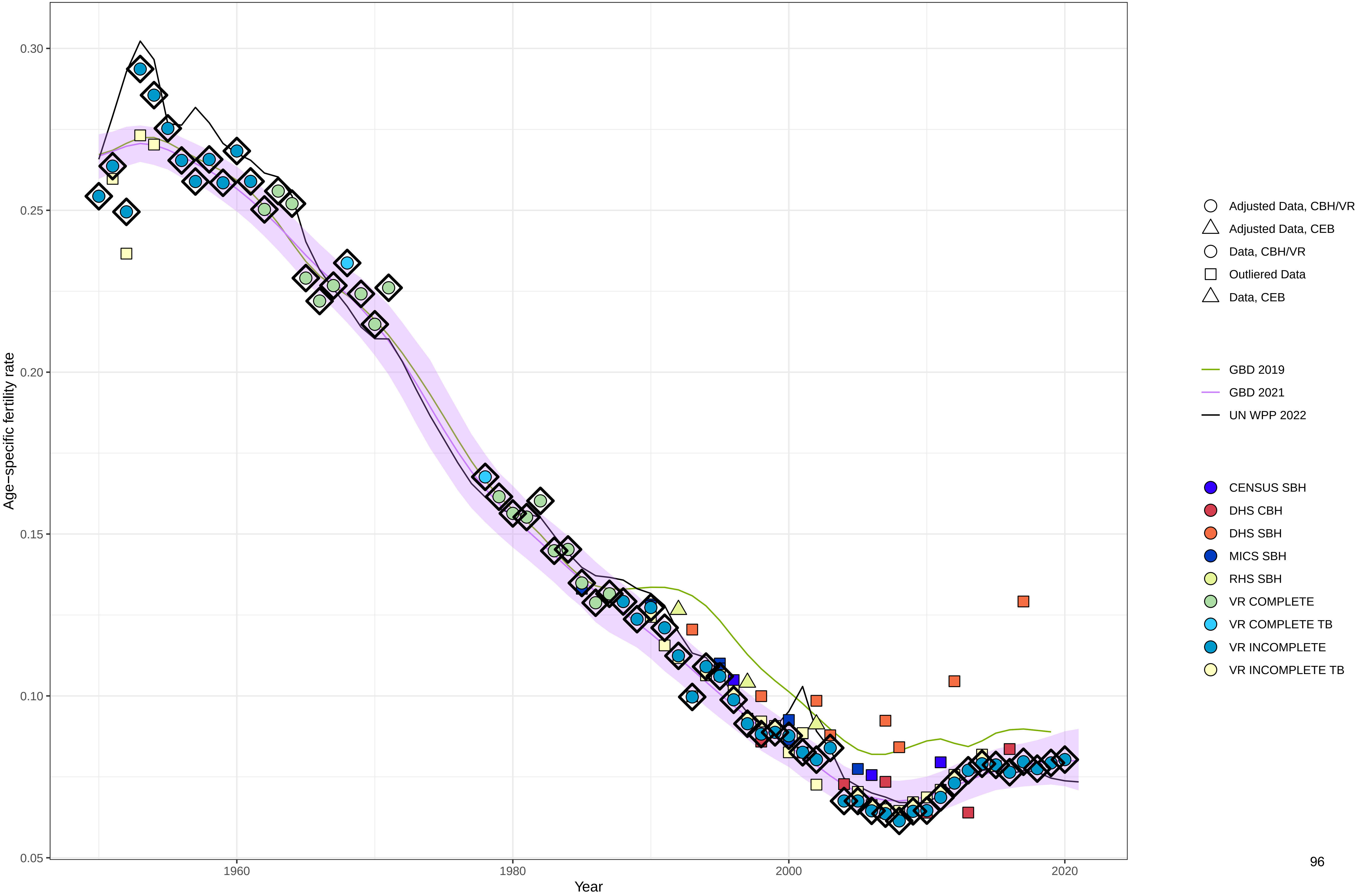

Age−specific fertility fitted trend lines, 1950−2021: Albania, Ages: 35−39
Data points enclosed with a diamond are reference sources

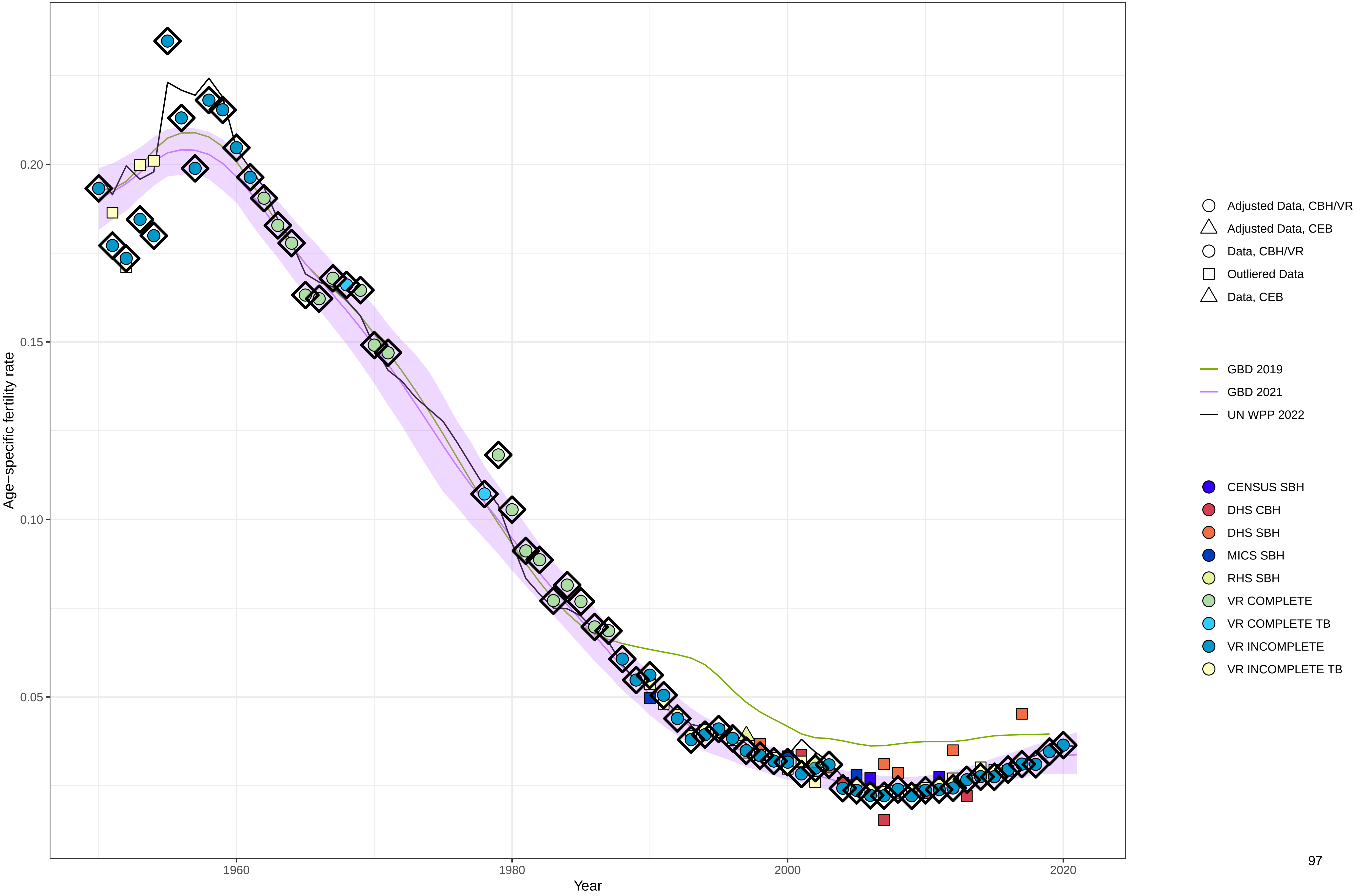

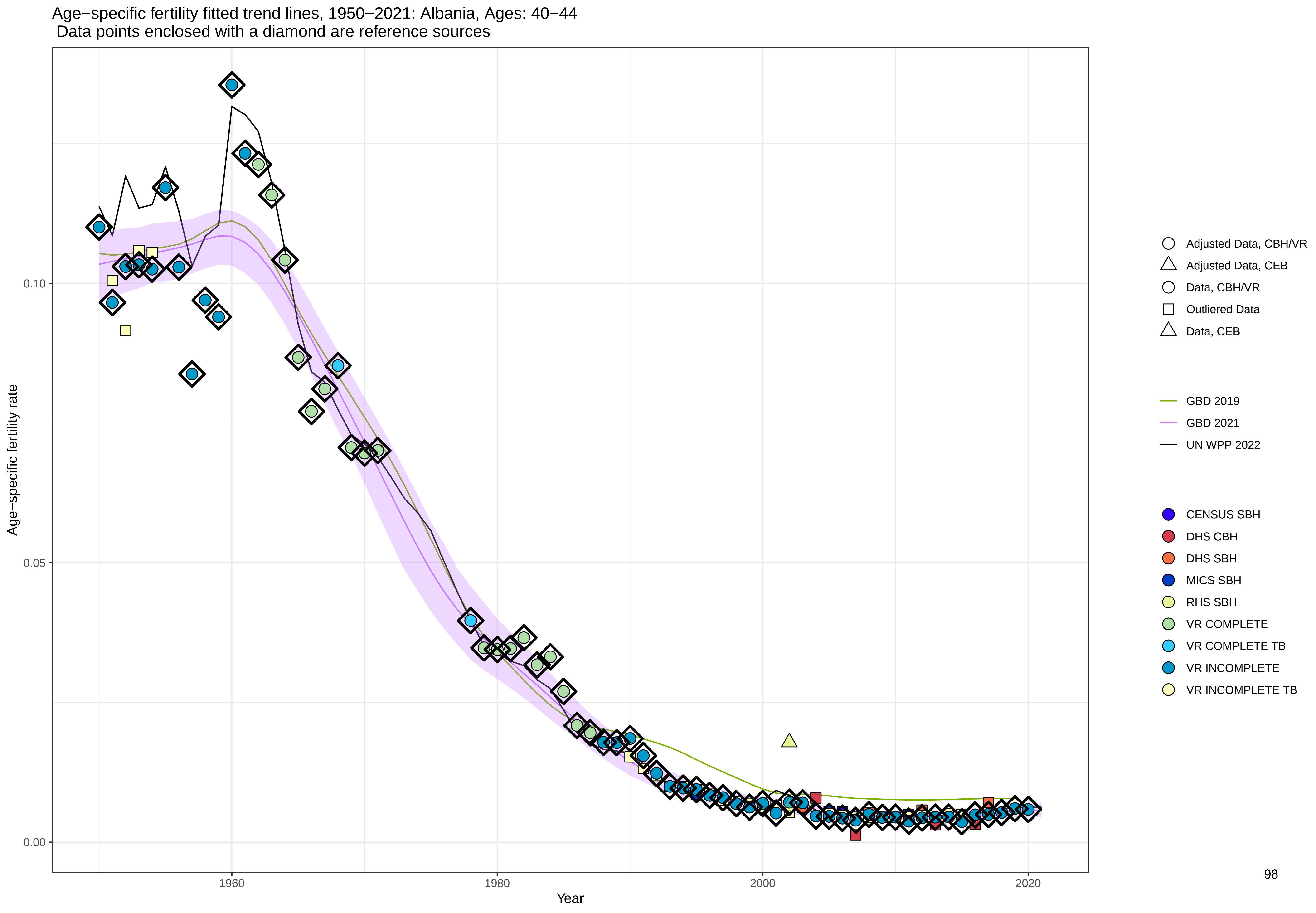

Age−specific fertility fitted trend lines, 1950−2021: Albania, Ages: 40−44
Data points enclosed with a diamond are reference sources
Age−specific fertility rate
0.00
0.05
0.10
1960
1980
2000
2020
Year
Adjusted Data, CBH/VR
Adjusted Data, CEB
Data, CBH/VR
Outliered Data
Data, CEB
GBD 2019
GBD 2021
UN WPP 2022
CENSUS SBH
DHS CBH
DHS SBH
MICS SBH
RHS SBH
VR COMPLETE
VR COMPLETE TB
VR INCOMPLETE
VR INCOMPLETE TB

Age−specific fertility fitted trend lines, 1950−2021: Albania, Ages: 45−49
Data points enclosed with a diamond are reference sources

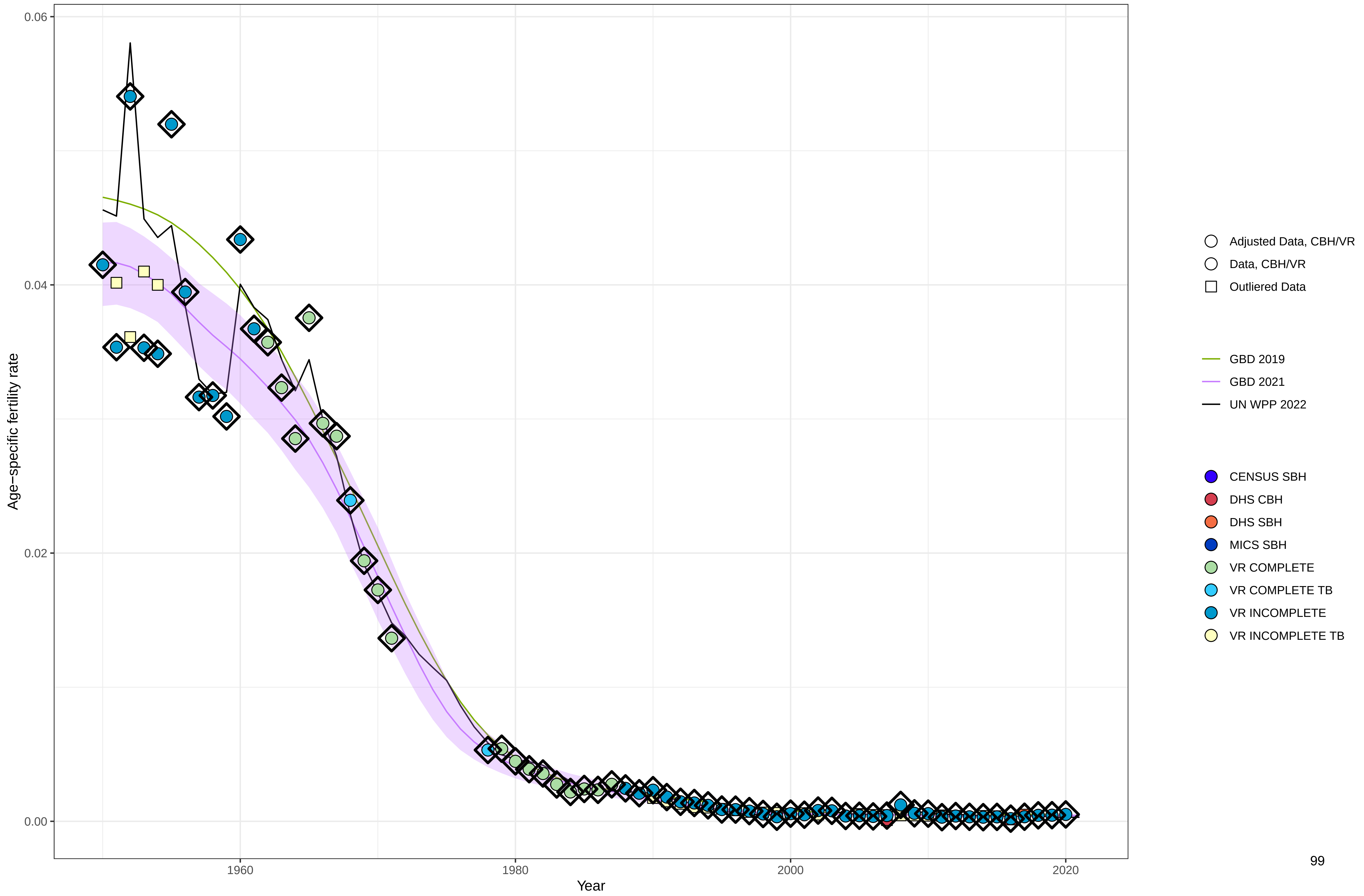

Age−specific fertility fitted trend lines, 1950−2021: Albania, Ages: 50−54
Data points enclosed with a diamond are reference sources

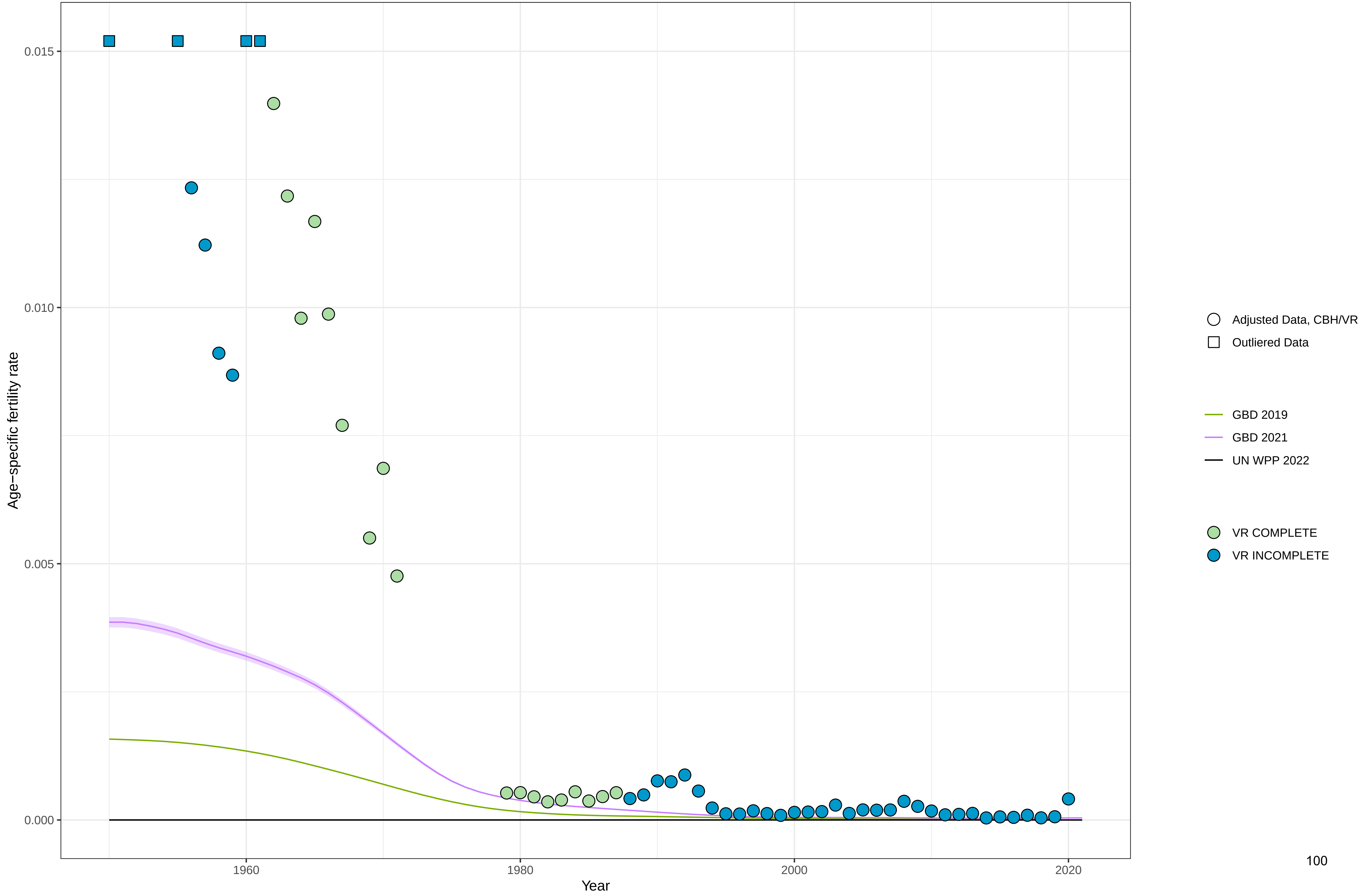

Total fertility rate fitted trend lines, 1950−2021: Albania TFR
Data points enclosed with a diamond are reference sources

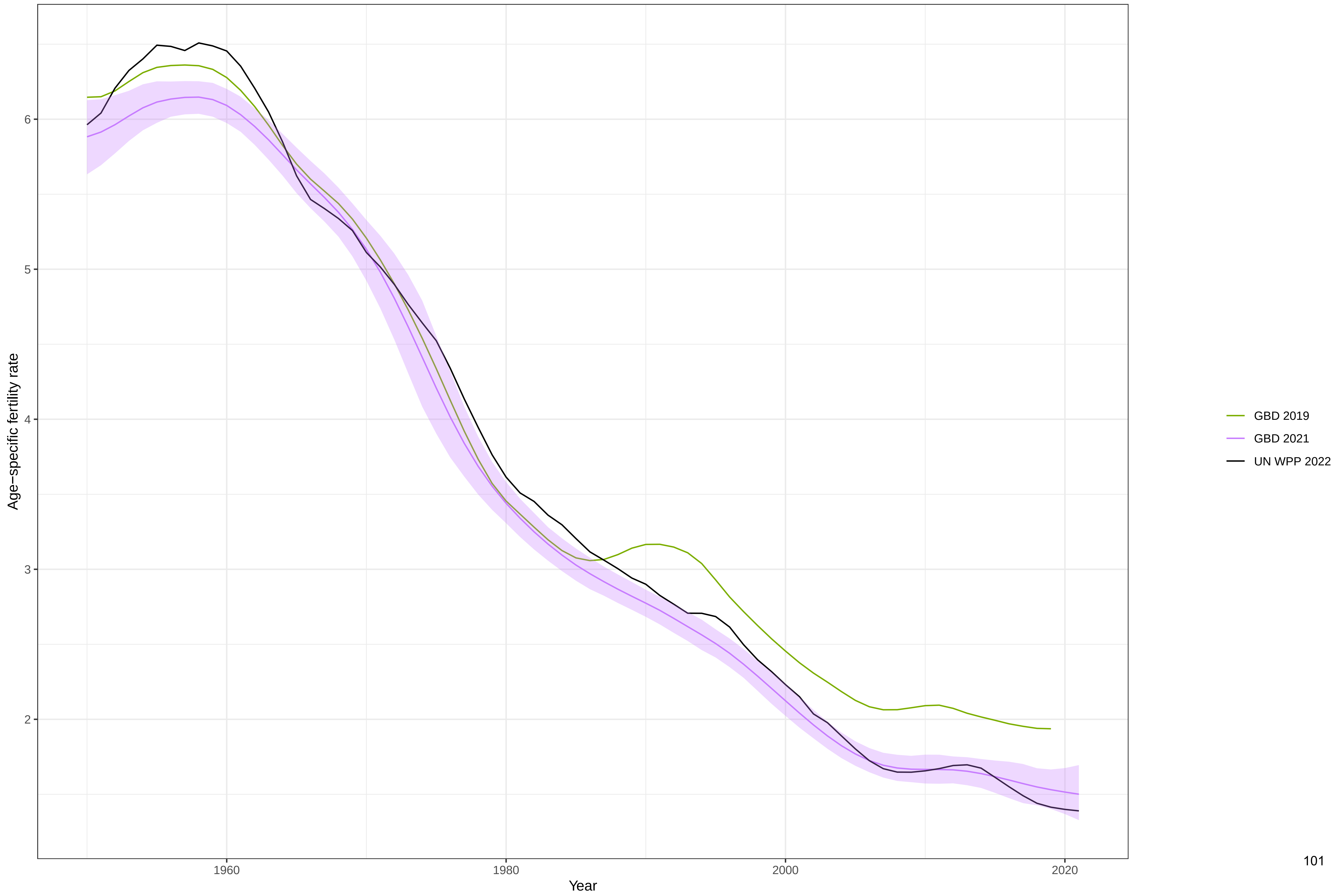

Age−specific fertility fitted trend lines, 1950−2021: Bosnia and Herzegovina, Ages: 10−14
Data points enclosed with a diamond are reference sources

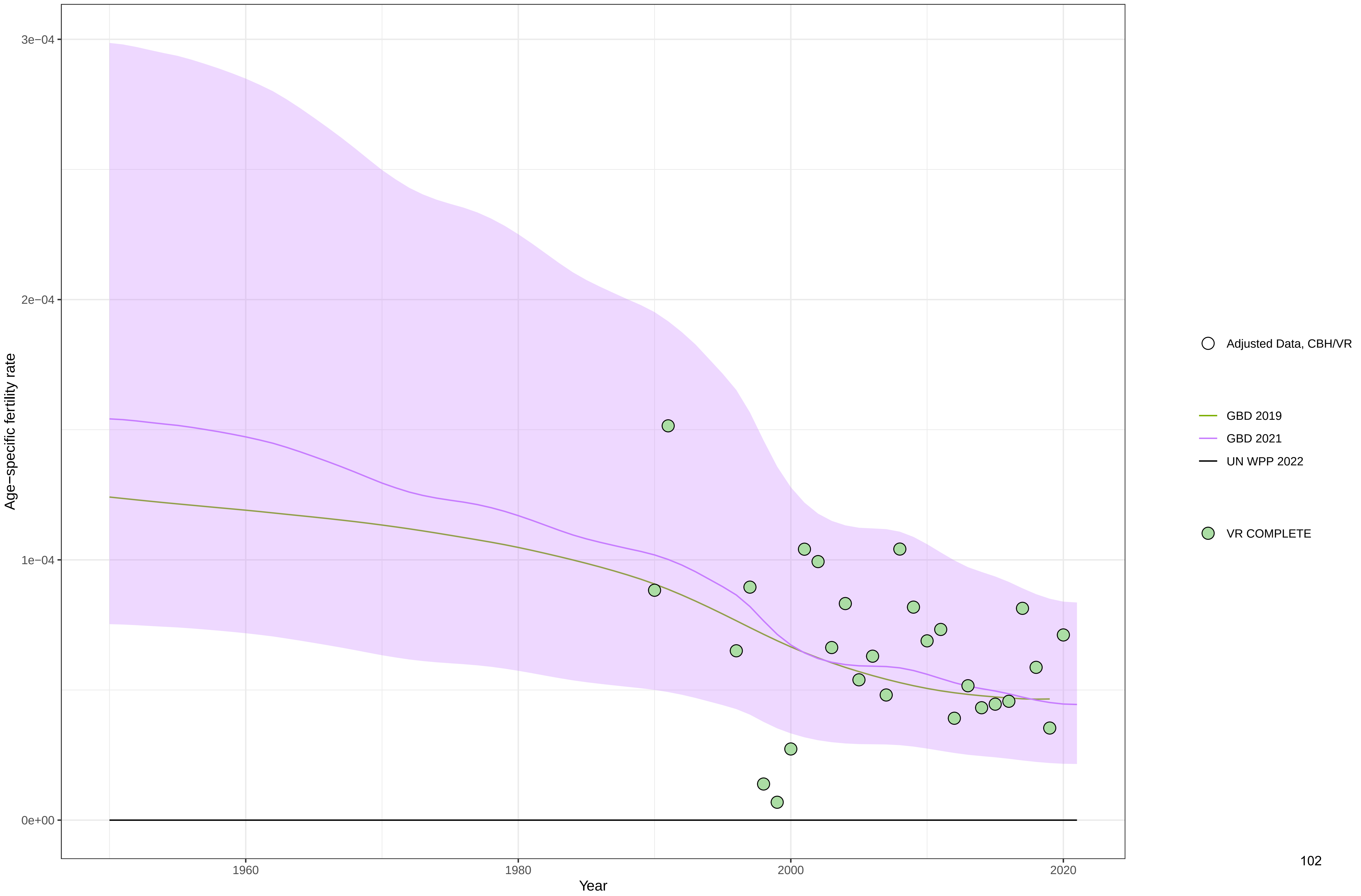

Age−specific fertility fitted trend lines, 1950−2021: Bosnia and Herzegovina, Ages: 15−19
Data points enclosed with a diamond are reference sources

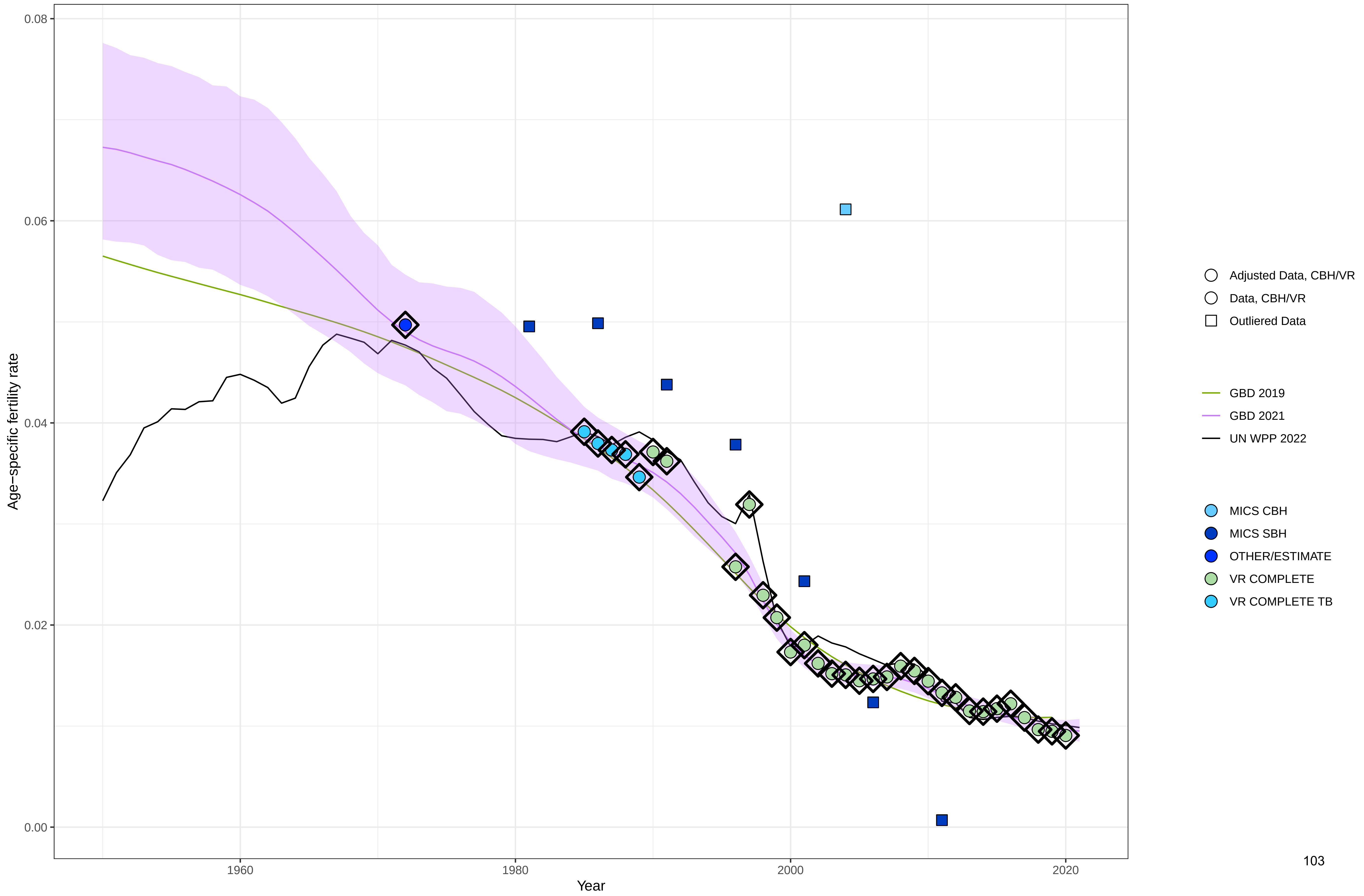

Age−specific fertility fitted trend lines, 1950−2021: Bosnia and Herzegovina, Ages: 20−24
Data points enclosed with a diamond are reference sources

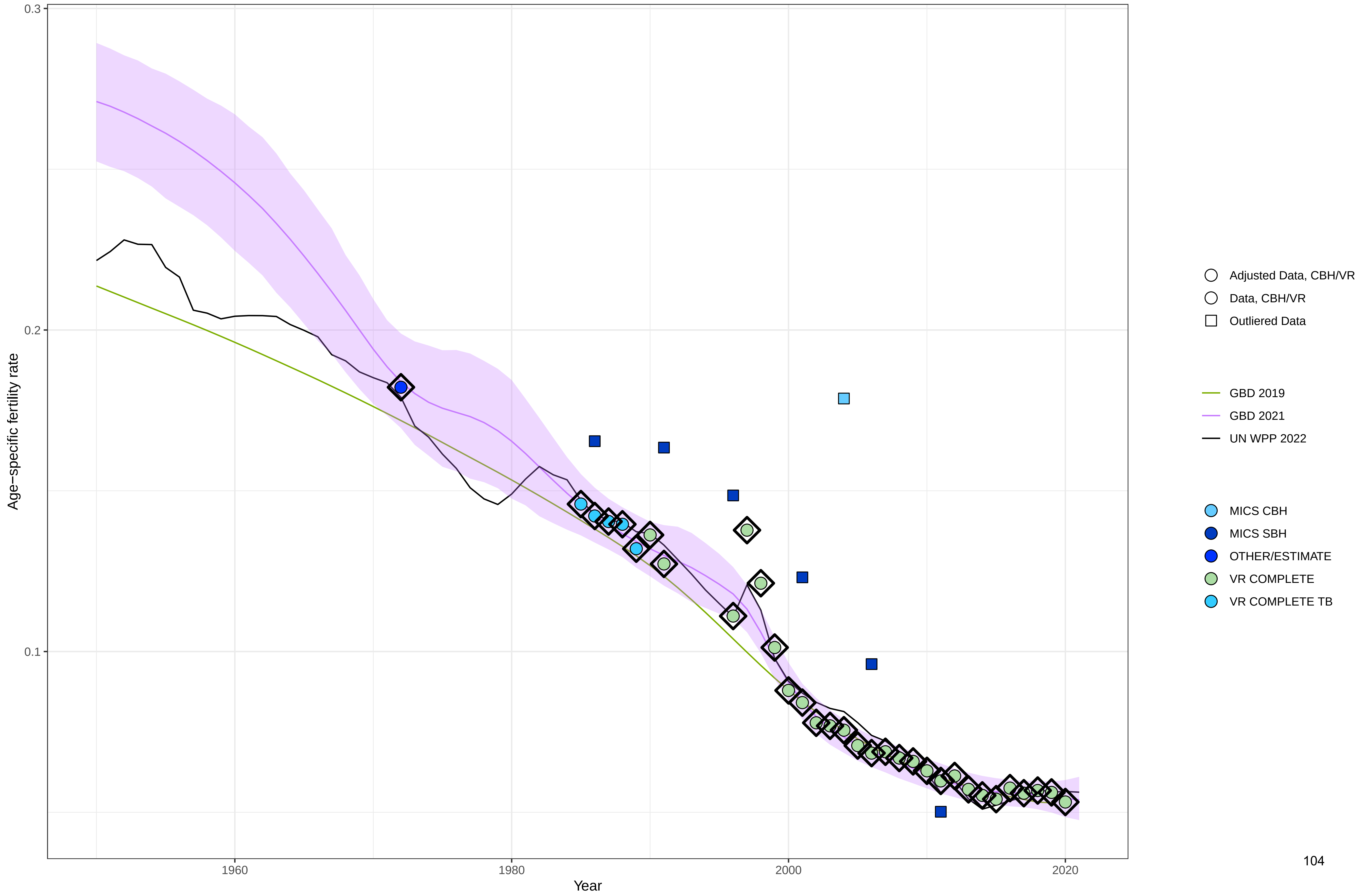

Age−specific fertility fitted trend lines, 1950−2021: Bosnia and Herzegovina, Ages: 25−29
Data points enclosed with a diamond are reference sources

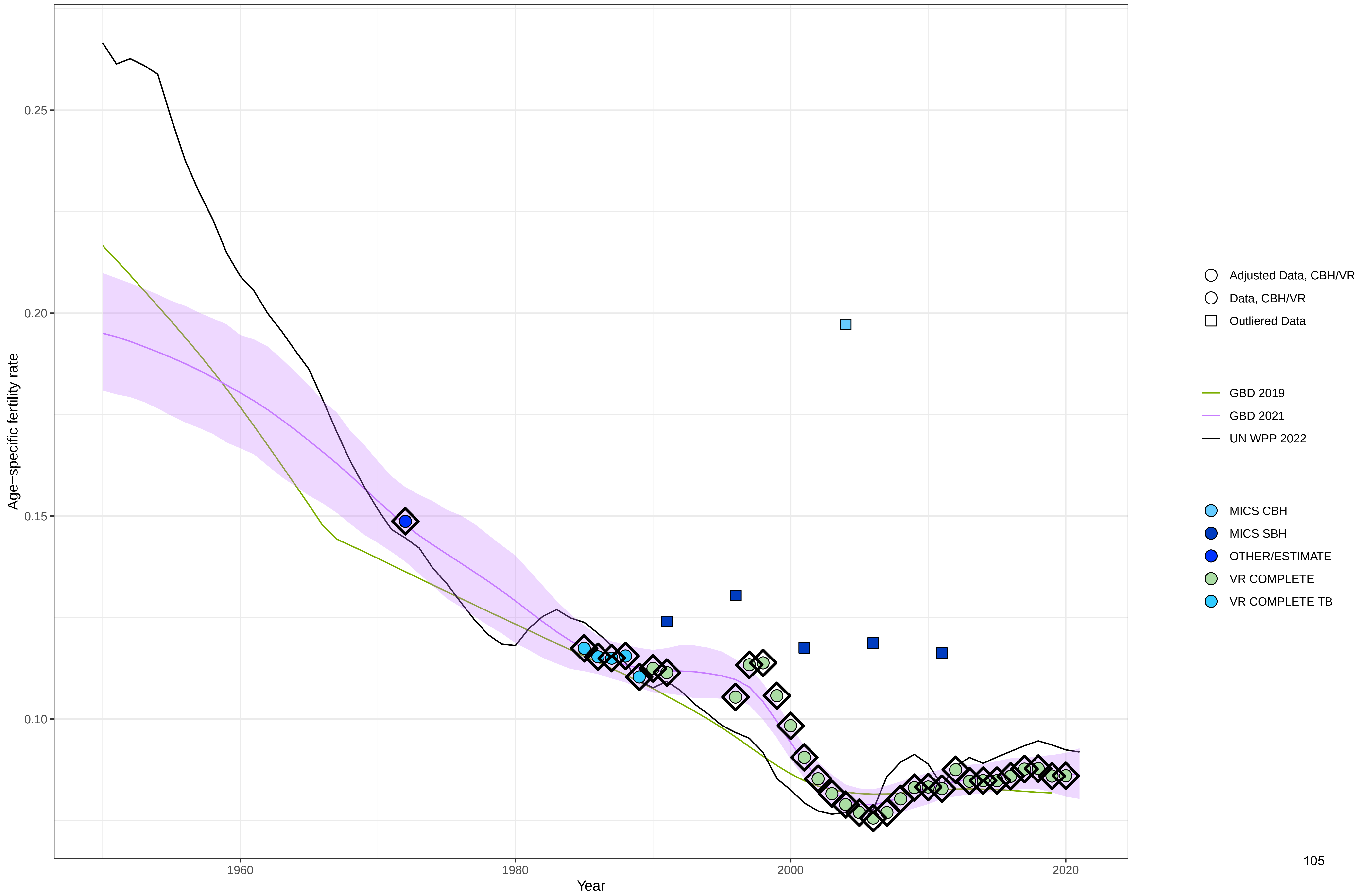

Age−specific fertility fitted trend lines, 1950−2021: Bosnia and Herzegovina, Ages: 30−34
Data points enclosed with a diamond are reference sources

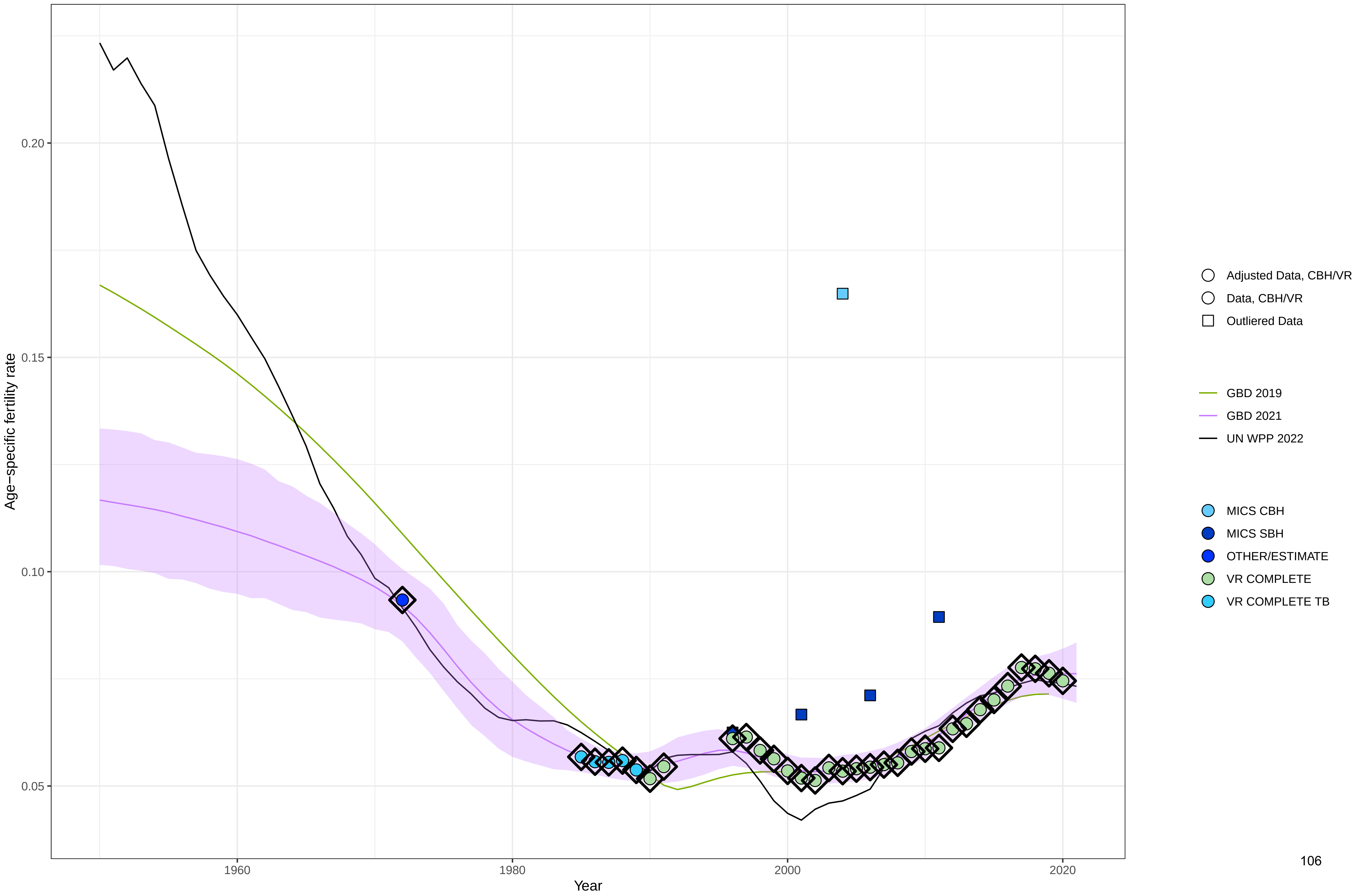

Age−specific fertility fitted trend lines, 1950−2021: Bosnia and Herzegovina, Ages: 35−39
Data points enclosed with a diamond are reference sources

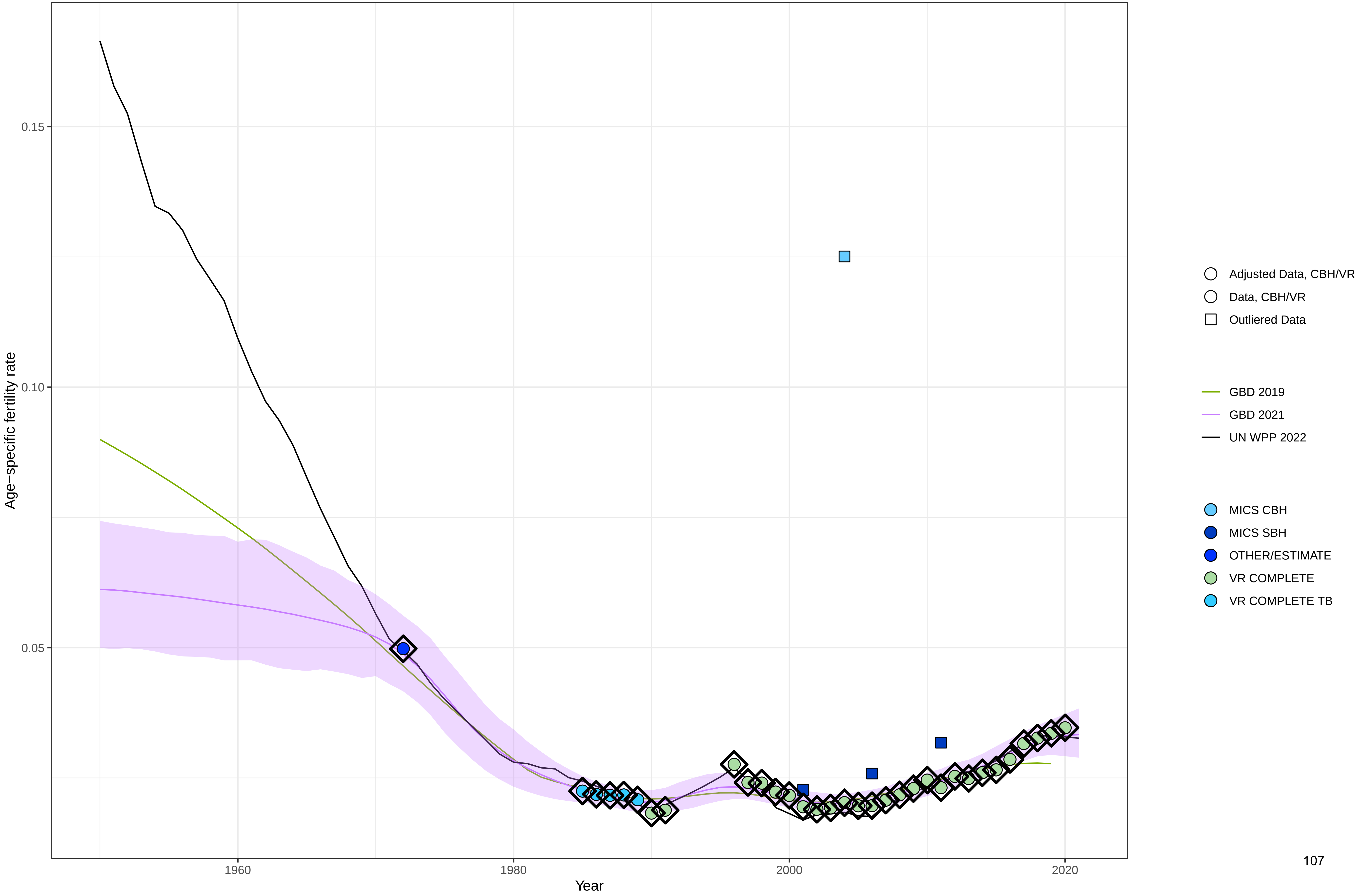

Age−specific fertility fitted trend lines, 1950−2021: Bosnia and Herzegovina, Ages: 40−44
Data points enclosed with a diamond are reference sources

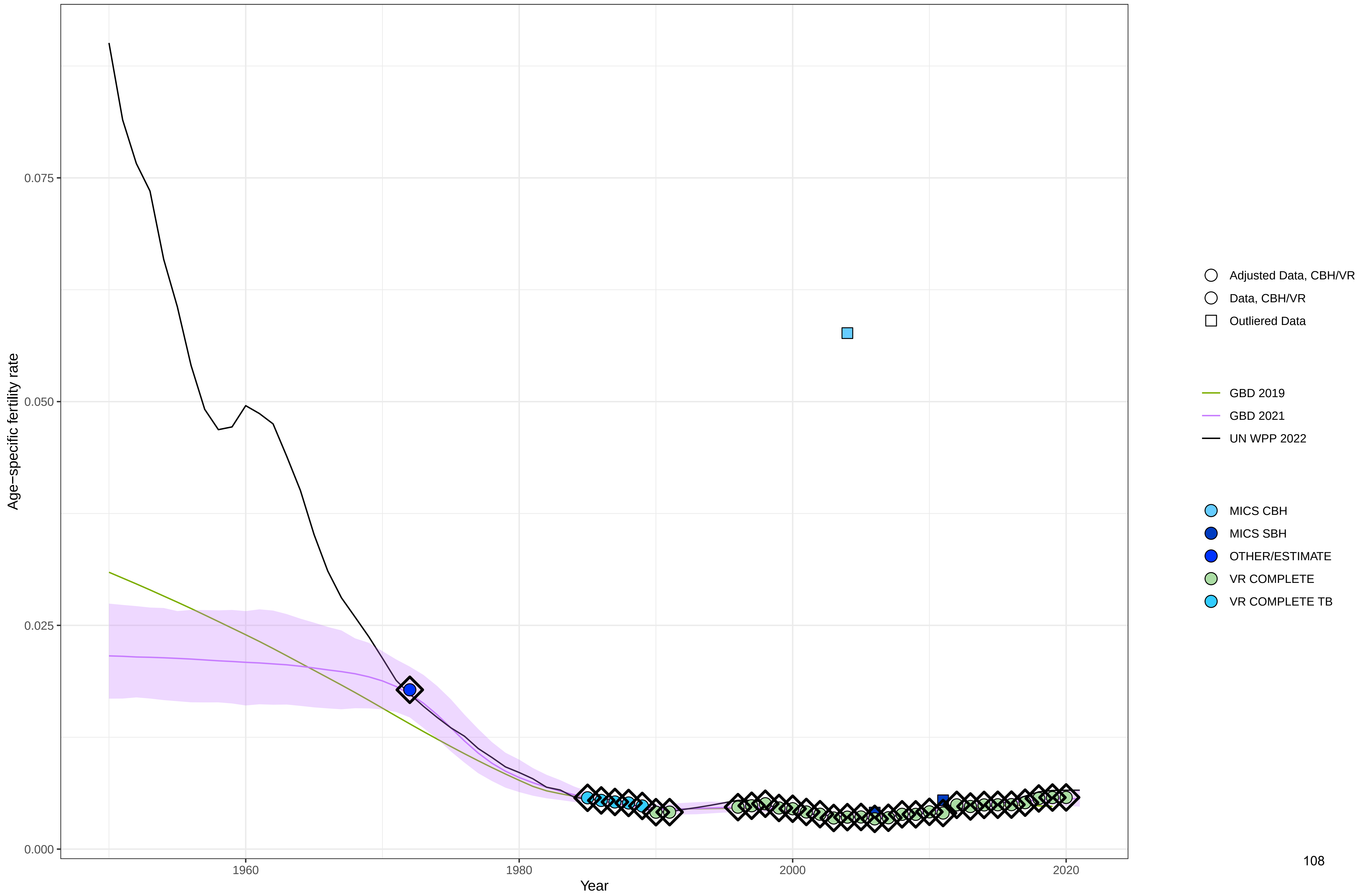

Age−specific fertility fitted trend lines, 1950−2021: Bosnia and Herzegovina, Ages: 45−49
Data points enclosed with a diamond are reference sources

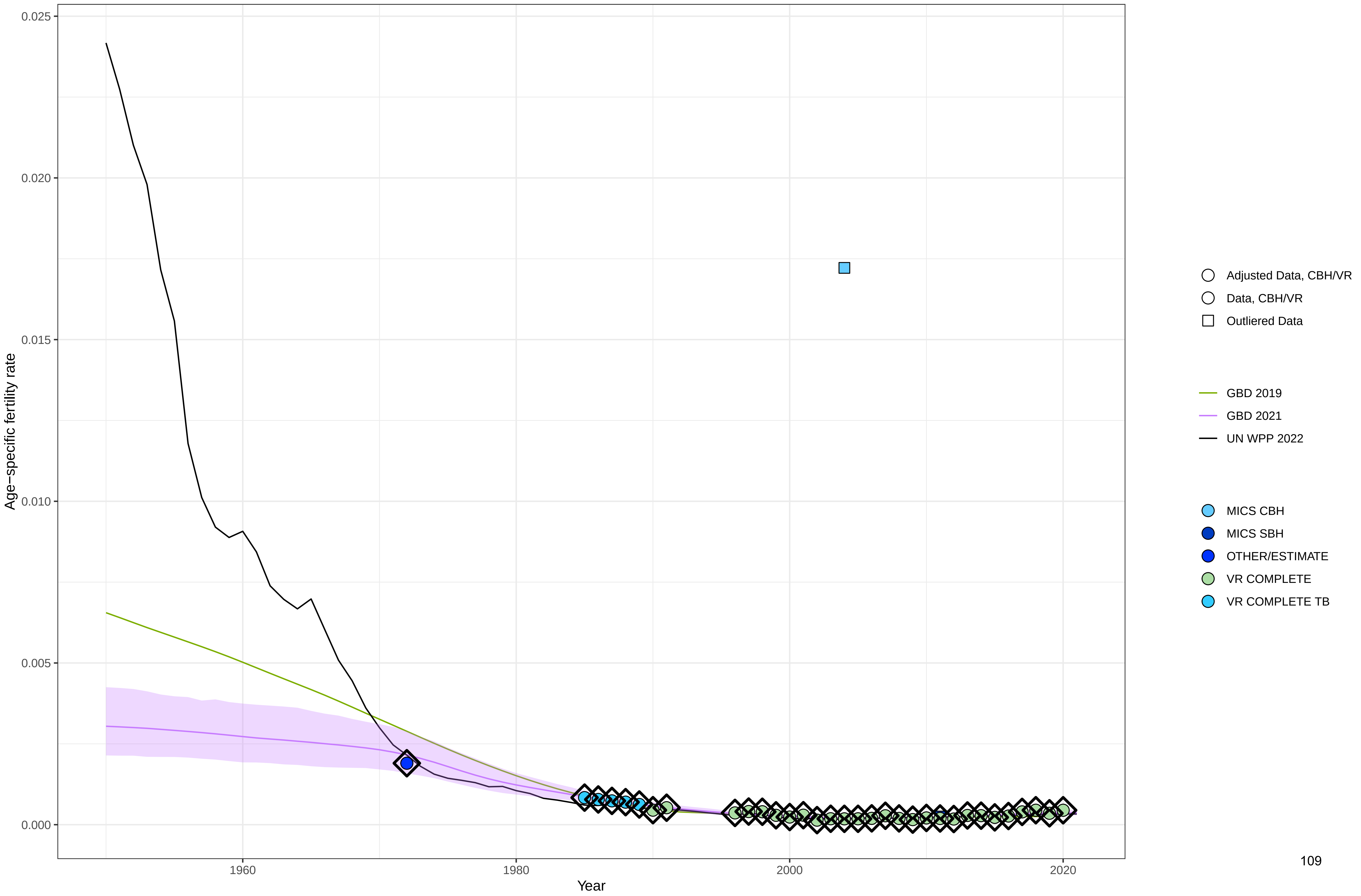

Age−specific fertility fitted trend lines, 1950−2021: Bosnia and Herzegovina, Ages: 50−54
Data points enclosed with a diamond are reference sources

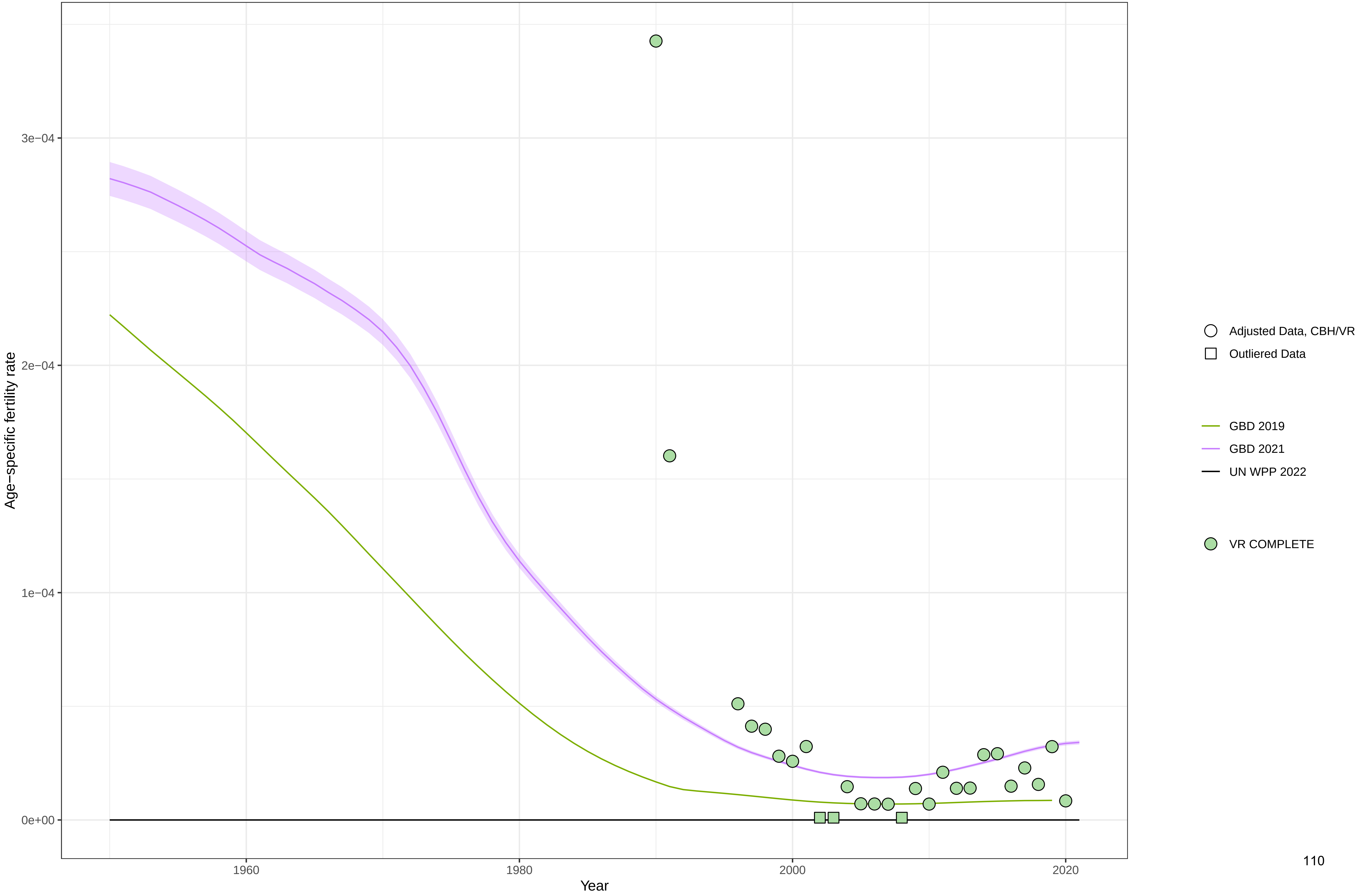

Total fertility rate fitted trend lines, 1950−2021: Bosnia and Herzegovina TFR
Data points enclosed with a diamond are reference sources

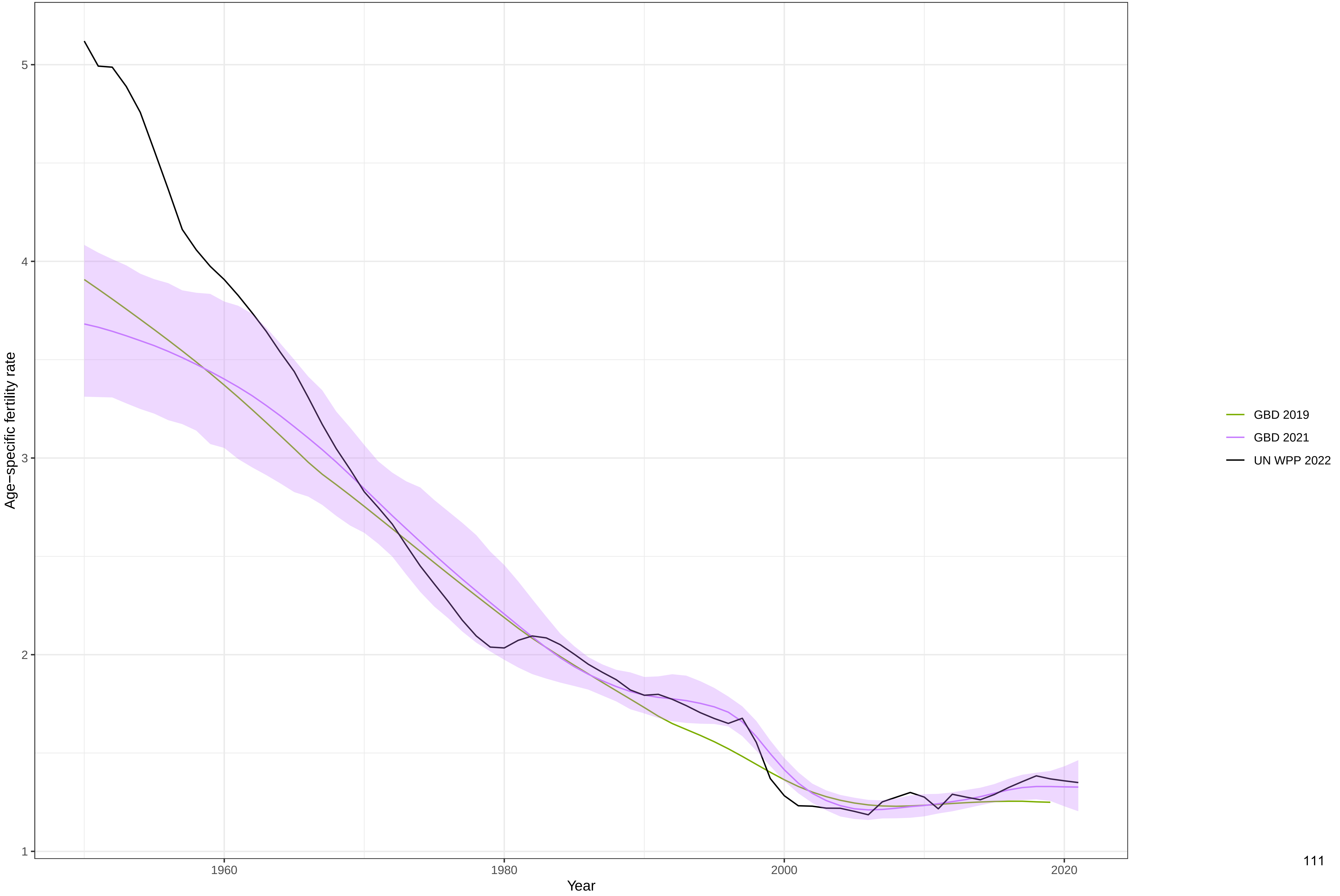

Age−specific fertility fitted trend lines, 1950−2021: Bulgaria, Ages: 10−14
Data points enclosed with a diamond are reference sources

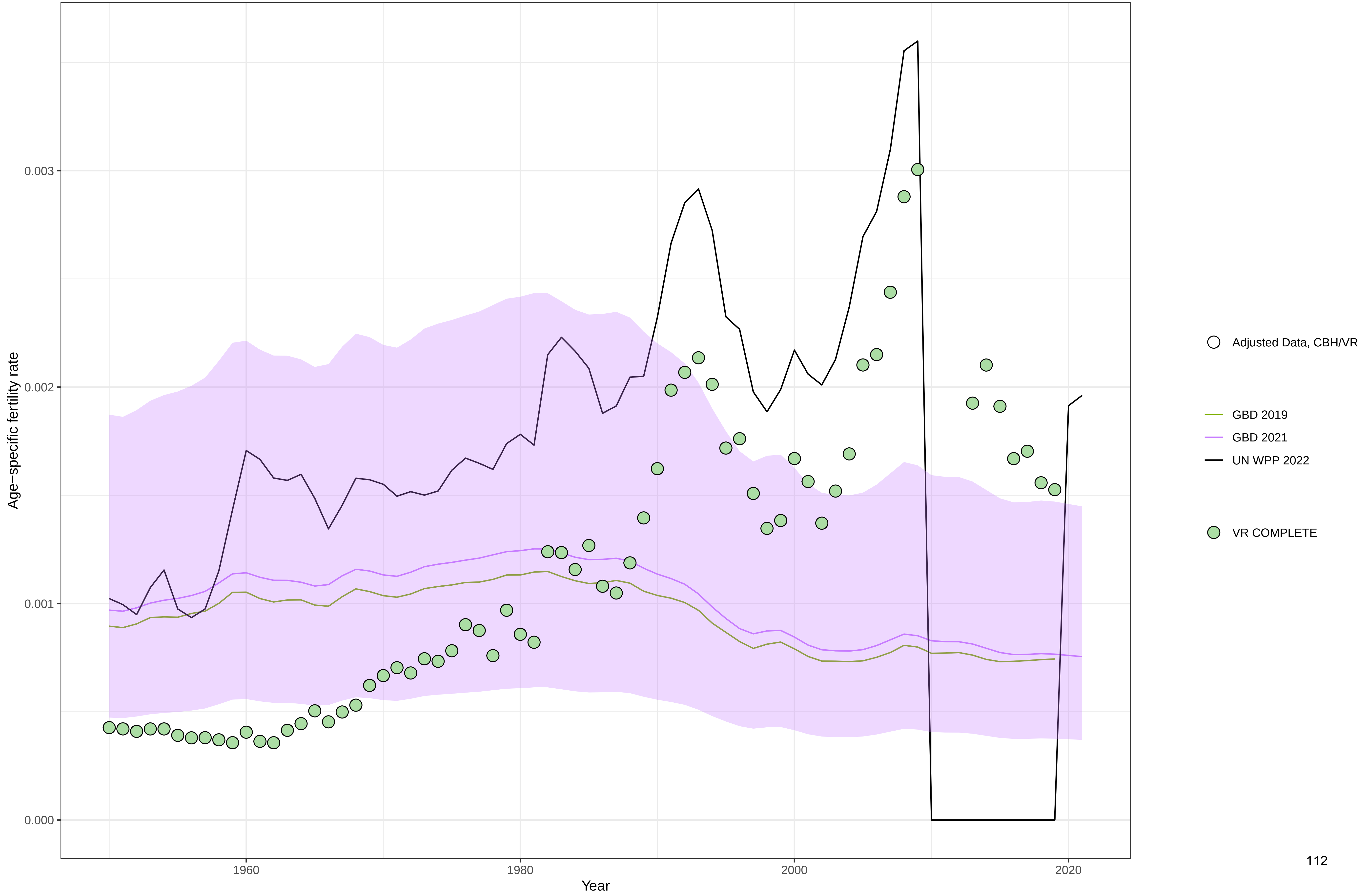

Age−specific fertility fitted trend lines, 1950−2021: Bulgaria, Ages: 15−19
Data points enclosed with a diamond are reference sources

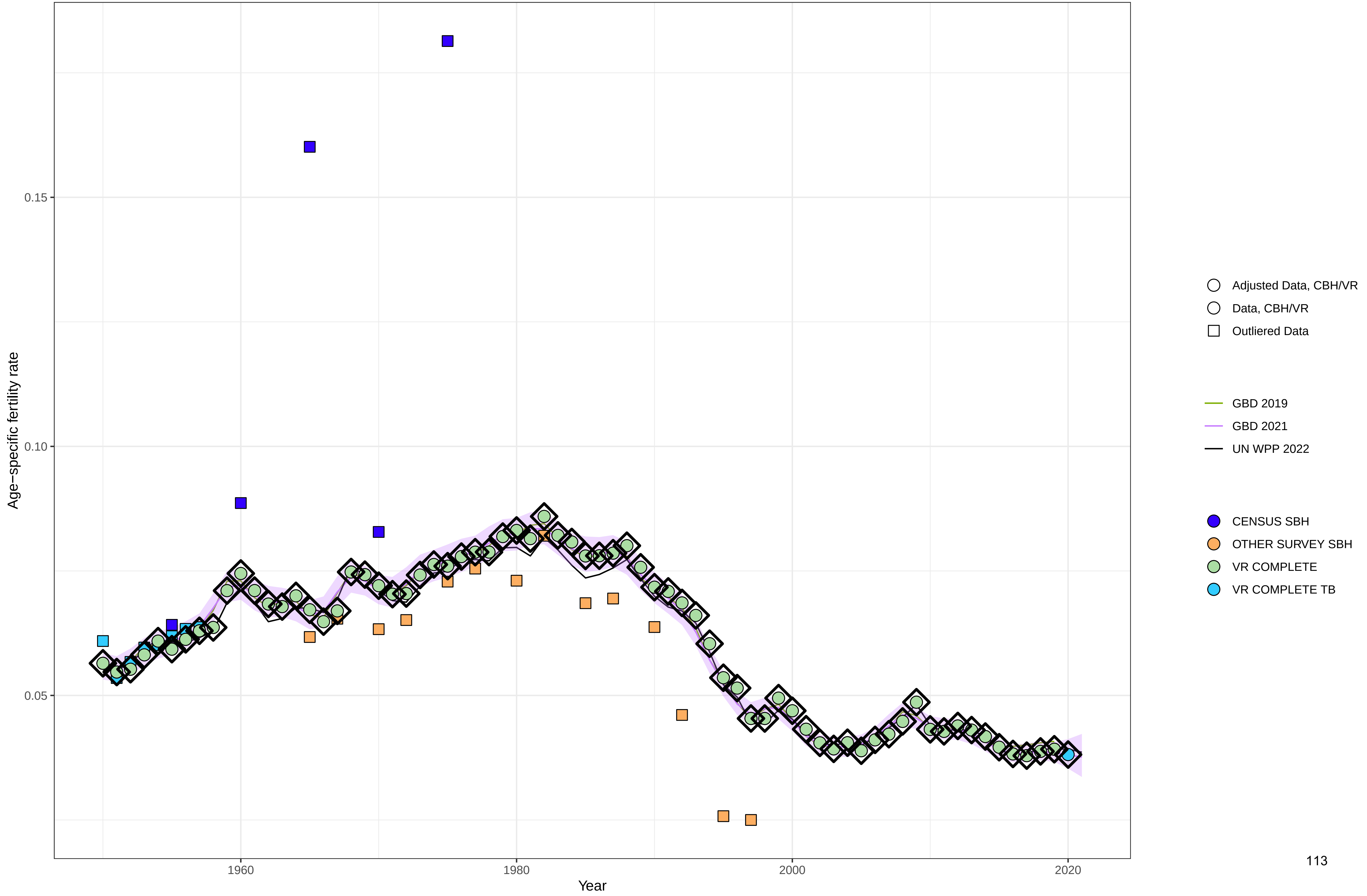

Age−specific fertility fitted trend lines, 1950−2021: Bulgaria, Ages: 20−24
Data points enclosed with a diamond are reference sources

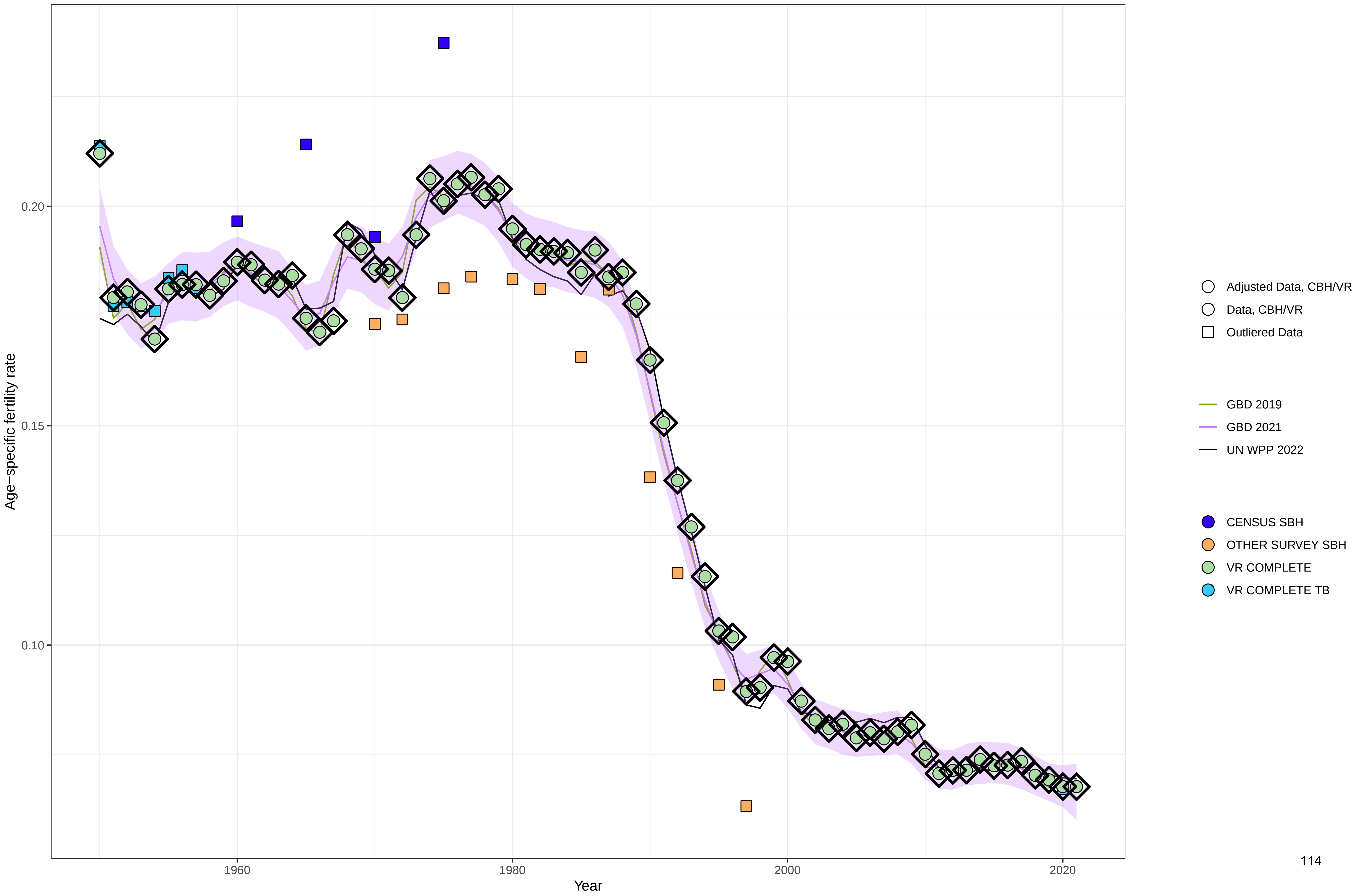

Age−specific fertility fitted trend lines, 1950−2021: Bulgaria, Ages: 25−29
Data points enclosed with a diamond are reference sources

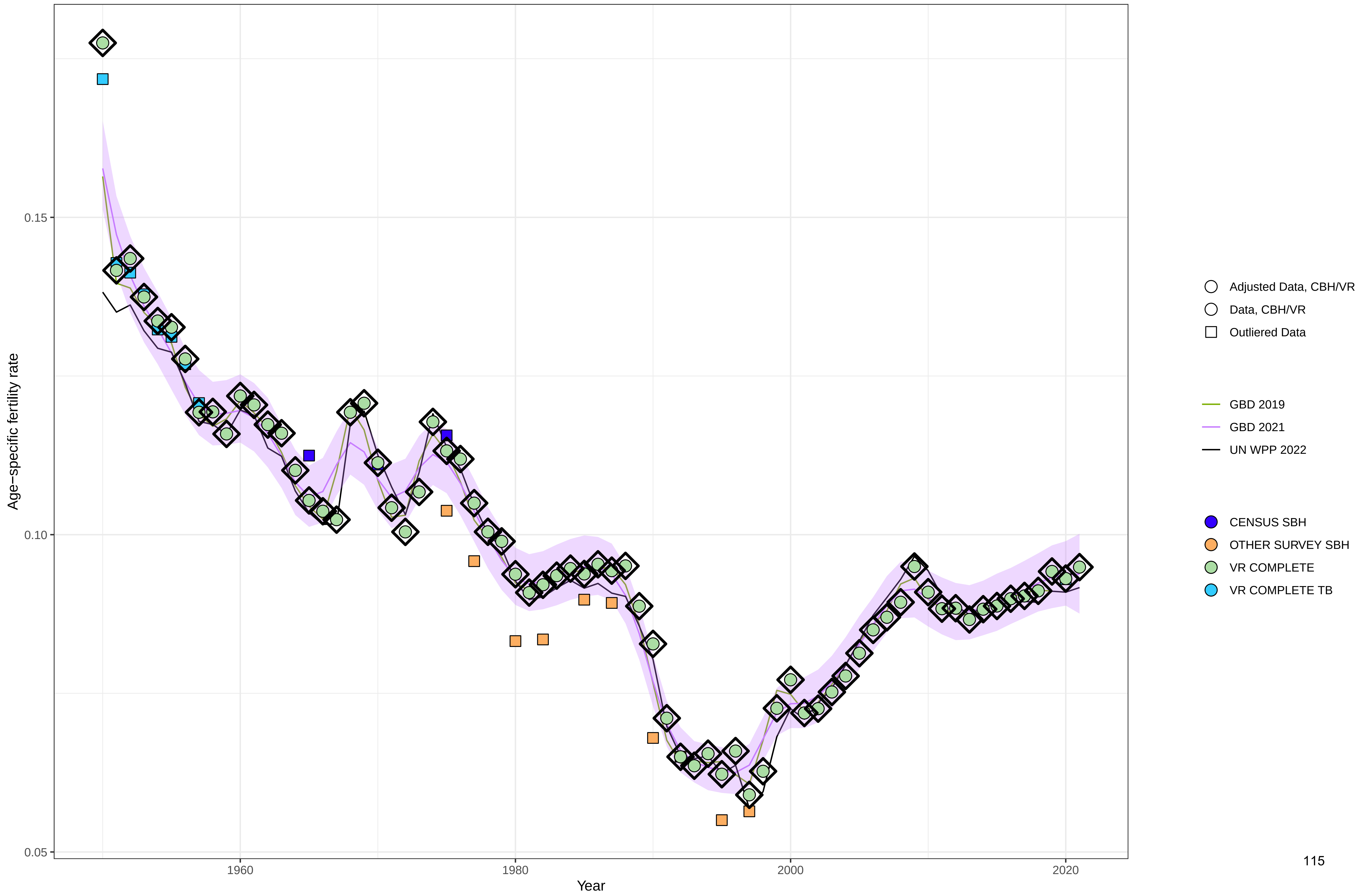

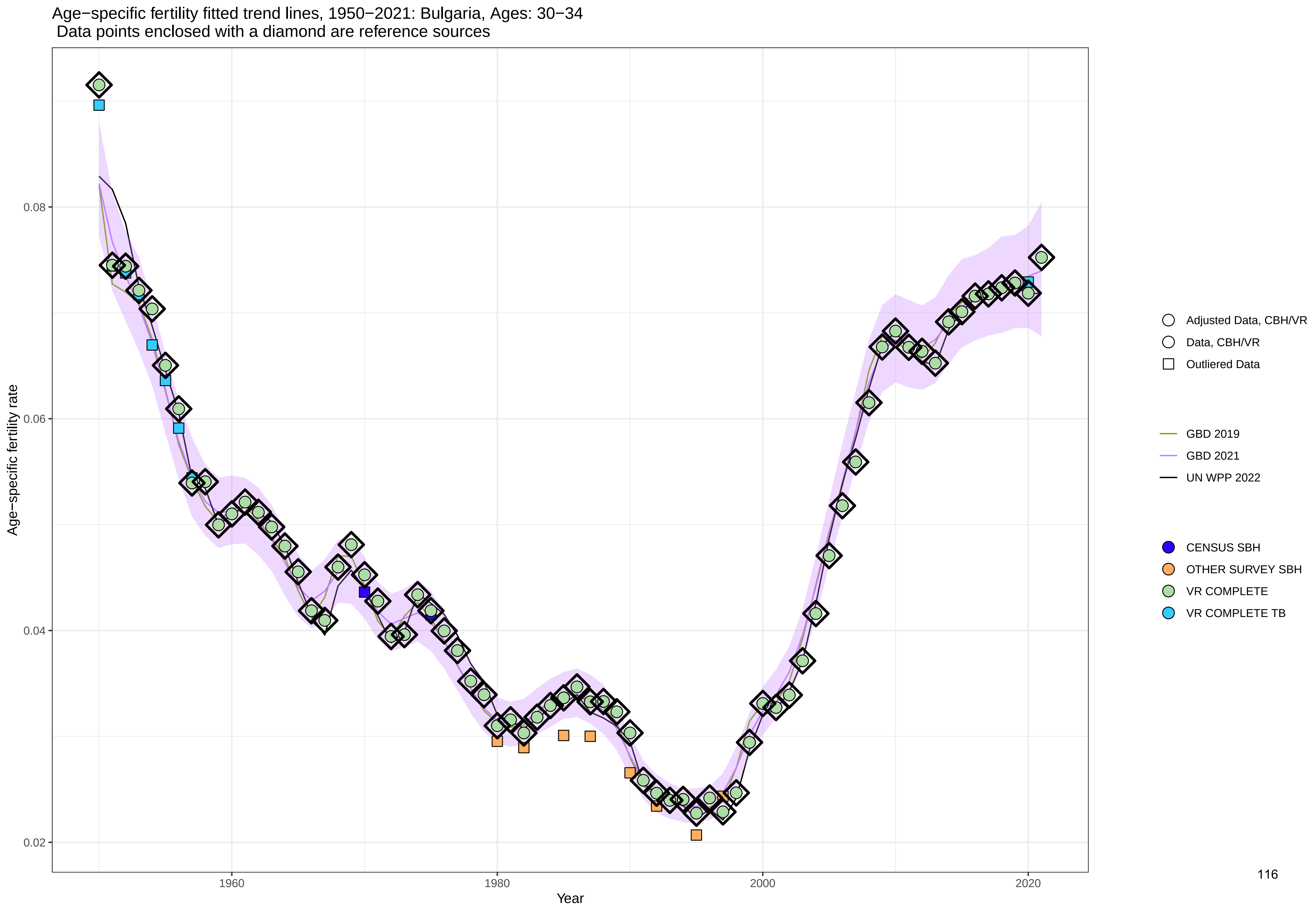

Age−specific fertility fitted trend lines, 1950−2021: Bulgaria, Ages: 30−34
Data points enclosed with a diamond are reference sources
Age−specific fertility rate
0.08
0.06
0.04
0.02
1960
1980
2000
2020
Year
Adjusted Data, CBH/VR
Data, CBH/VR
Outliered Data
GBD 2019
GBD 2021
UN WPP 2022
CENSUS SBH
OTHER SURVEY SBH
VR COMPLETE
VR COMPLETE TB

Age−specific fertility fitted trend lines, 1950−2021: Bulgaria, Ages: 35−39
Data points enclosed with a diamond are reference sources

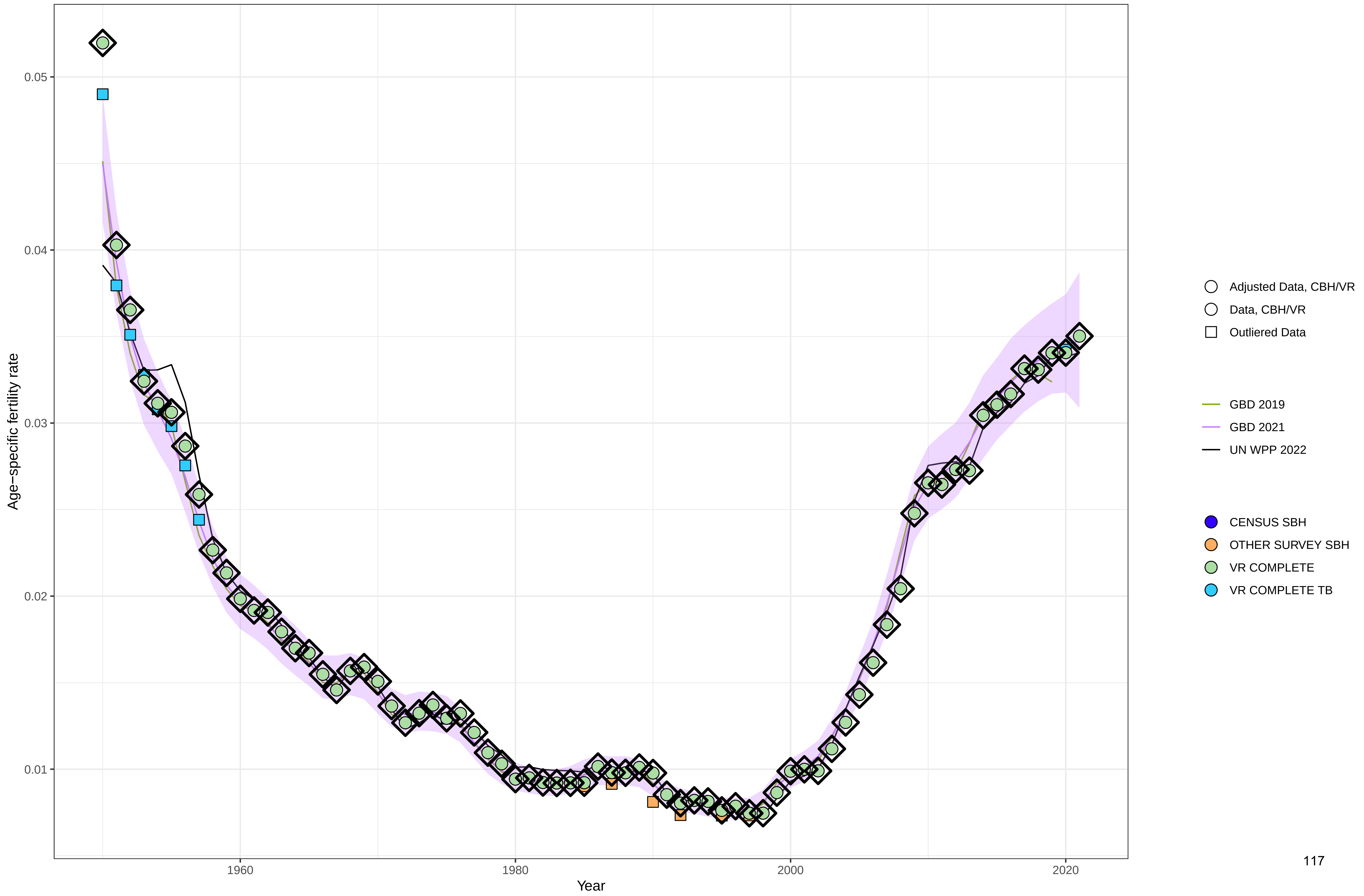

Age−specific fertility fitted trend lines, 1950−2021: Bulgaria, Ages: 40−44
Data points enclosed with a diamond are reference sources

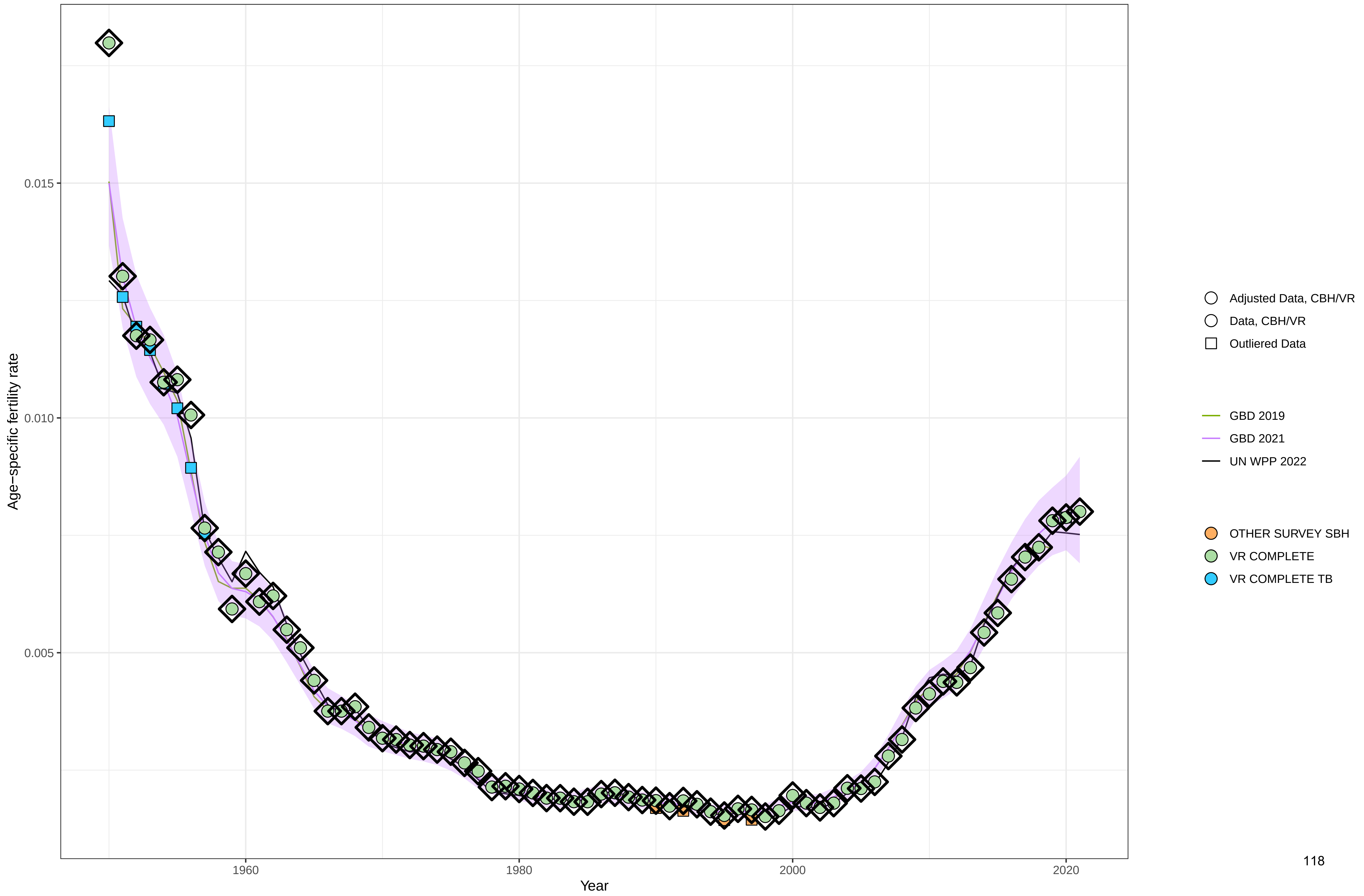

Age−specific fertility fitted trend lines, 1950−2021: Bulgaria, Ages: 45−49
Data points enclosed with a diamond are reference sources

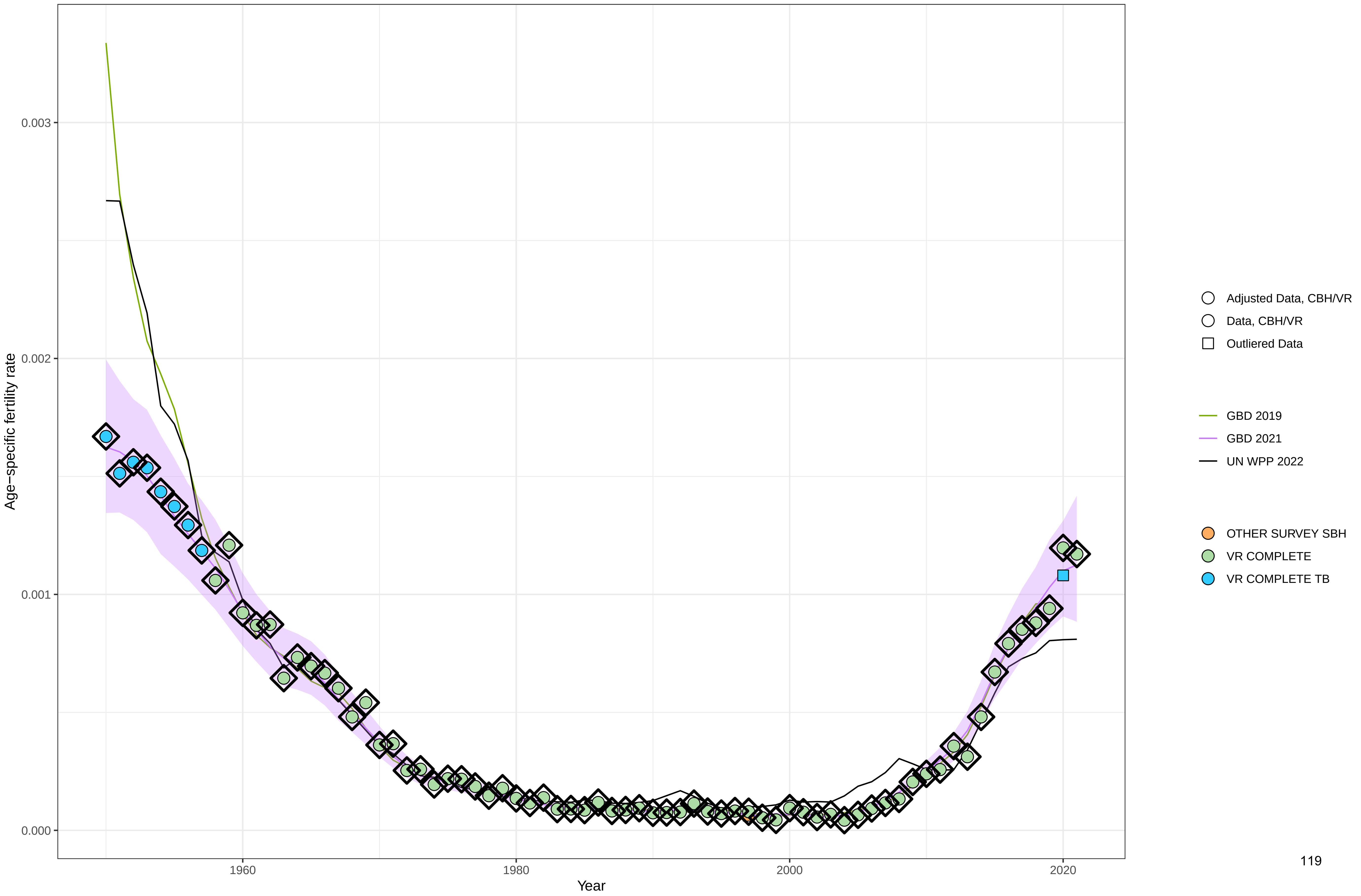

Age−specific fertility fitted trend lines, 1950−2021: Bulgaria, Ages: 50−54
Data points enclosed with a diamond are reference sources

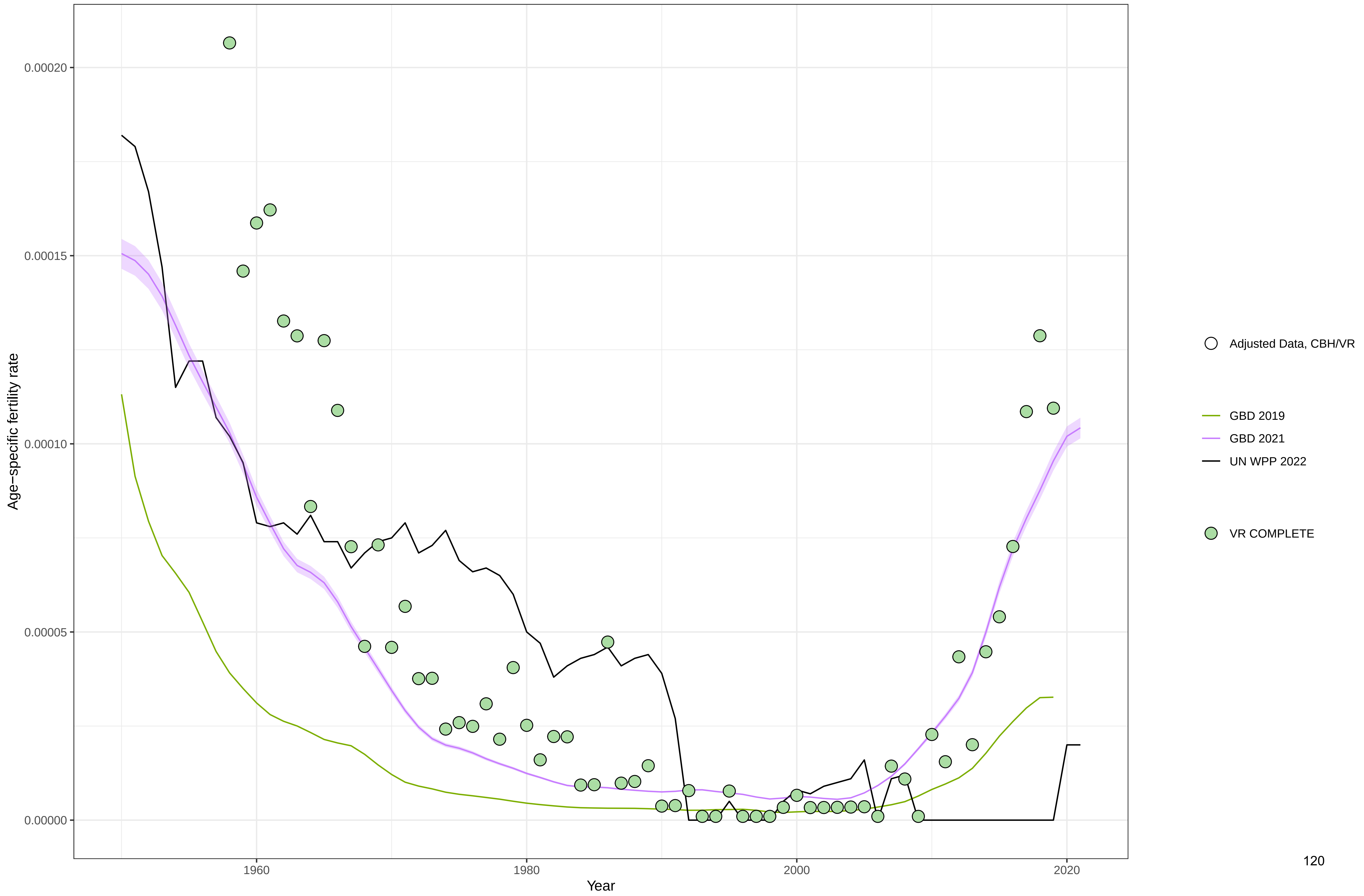

Total fertility rate fitted trend lines, 1950−2021: Bulgaria TFR
Data points enclosed with a diamond are reference sources

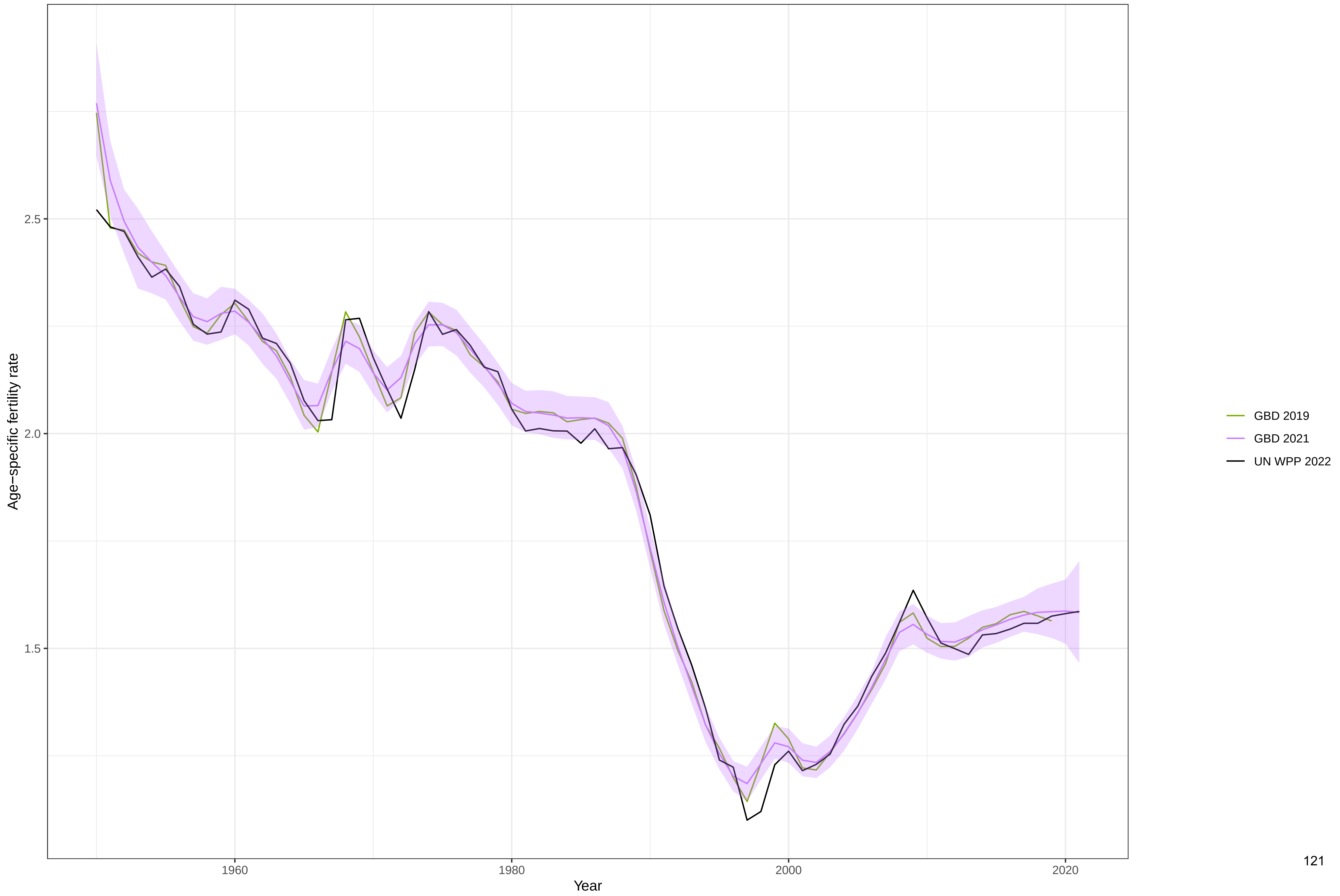

Age−specific fertility fitted trend lines, 1950−2021: Croatia, Ages: 10−14
Data points enclosed with a diamond are reference sources

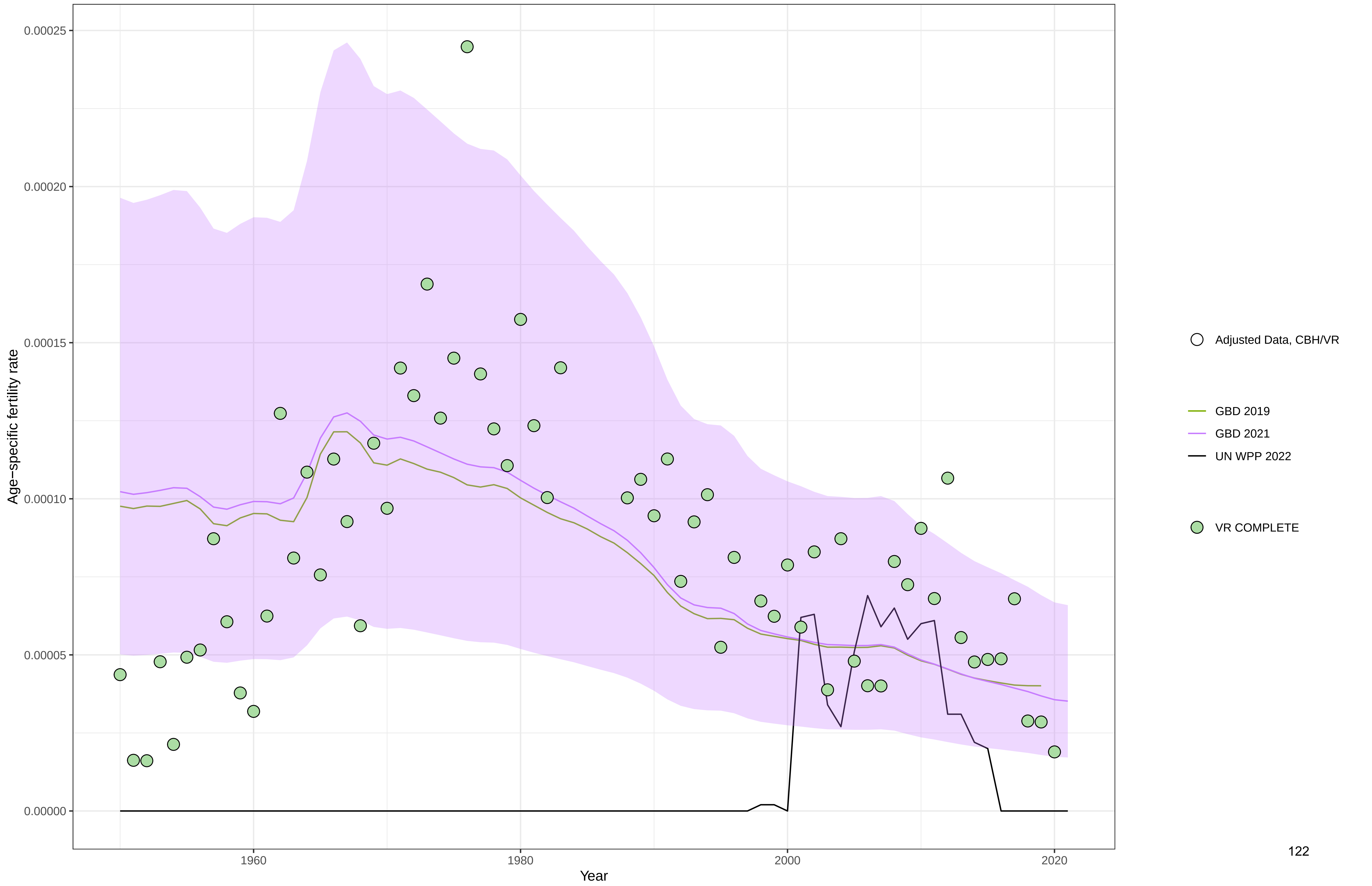

Age−specific fertility fitted trend lines, 1950−2021: Croatia, Ages: 15−19
Data points enclosed with a diamond are reference sources

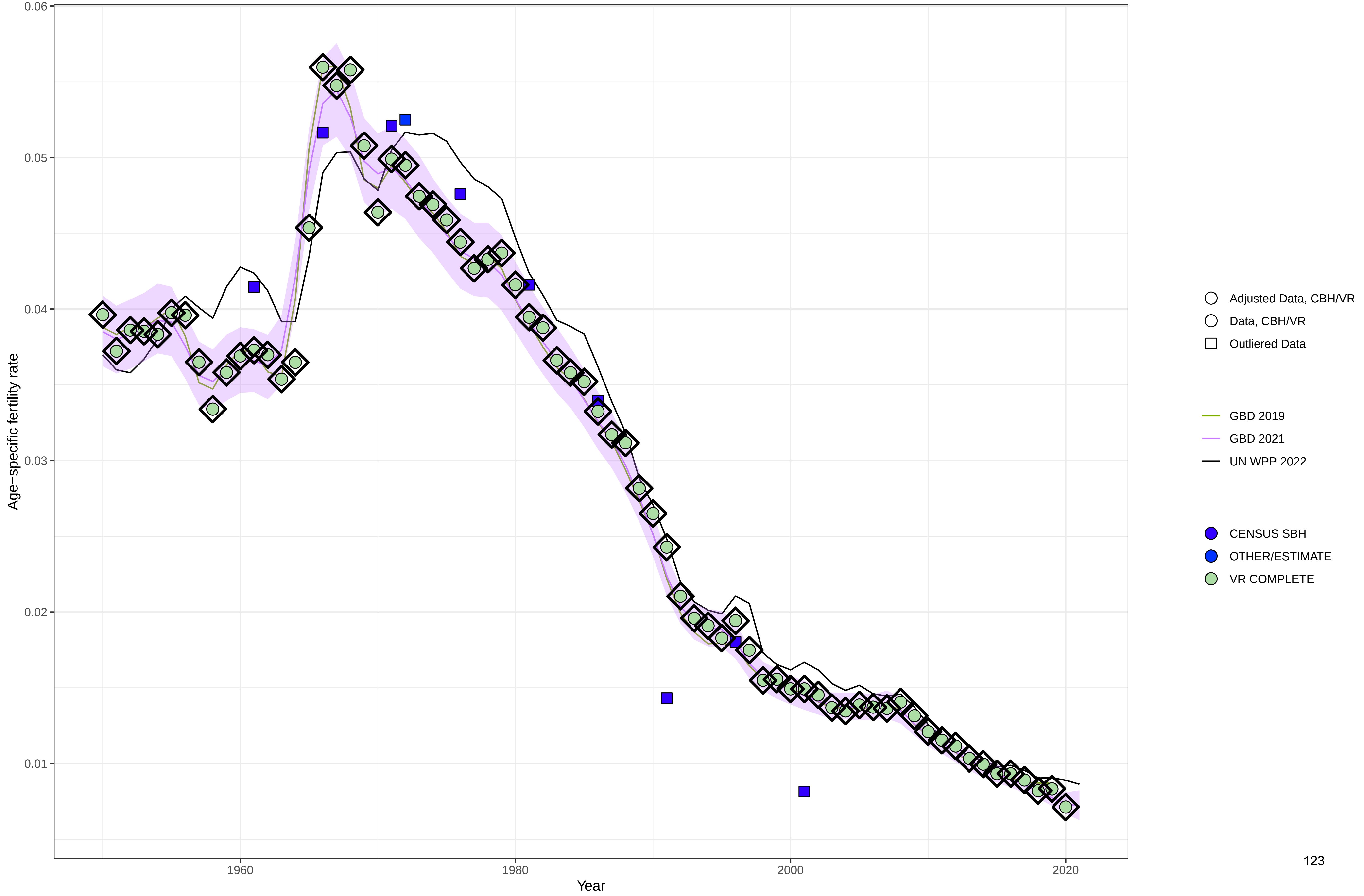

Age−specific fertility fitted trend lines, 1950−2021: Croatia, Ages: 20−24
Data points enclosed with a diamond are reference sources

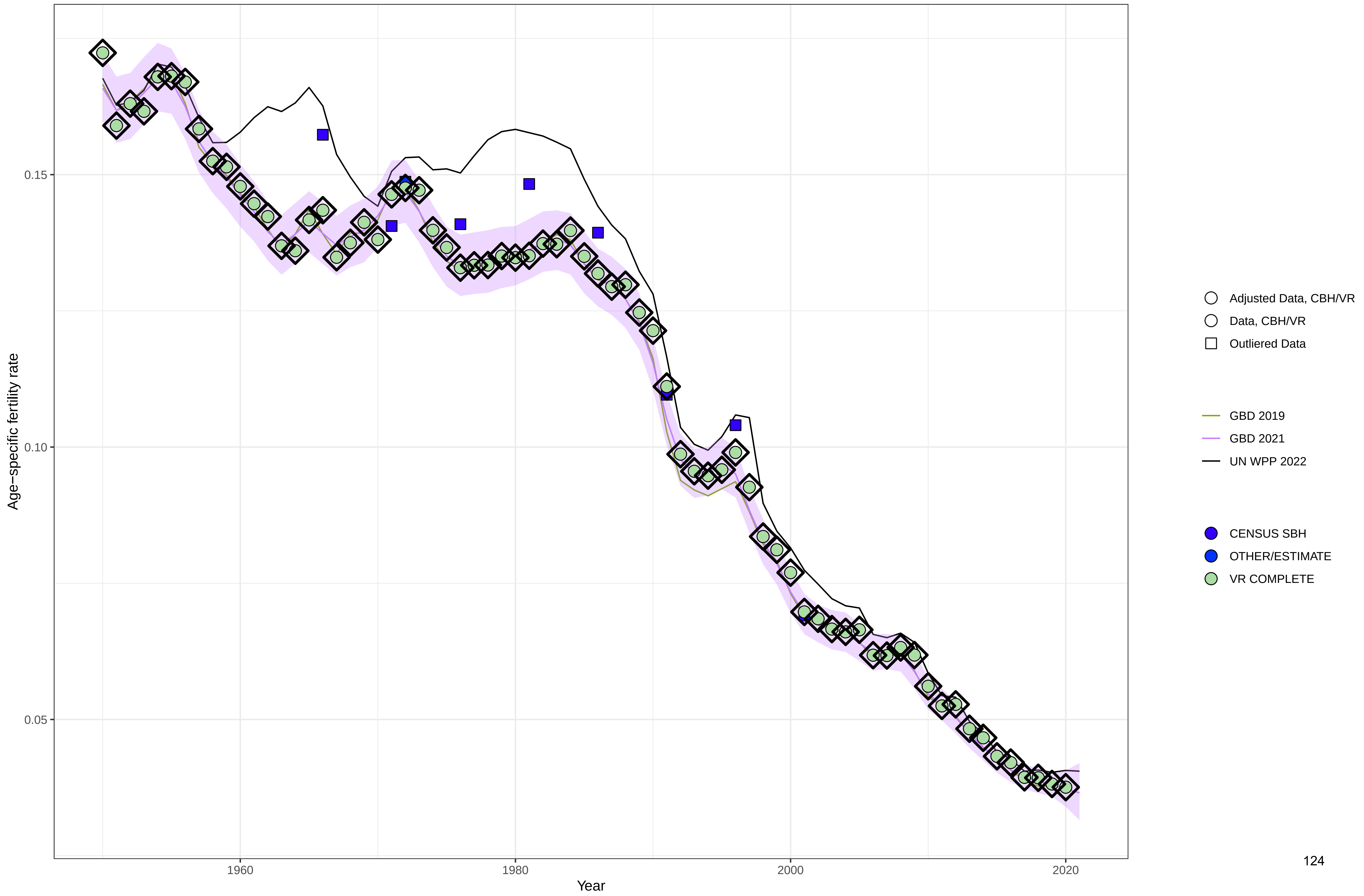

Age−specific fertility fitted trend lines, 1950−2021: Croatia, Ages: 25−29
Data points enclosed with a diamond are reference sources

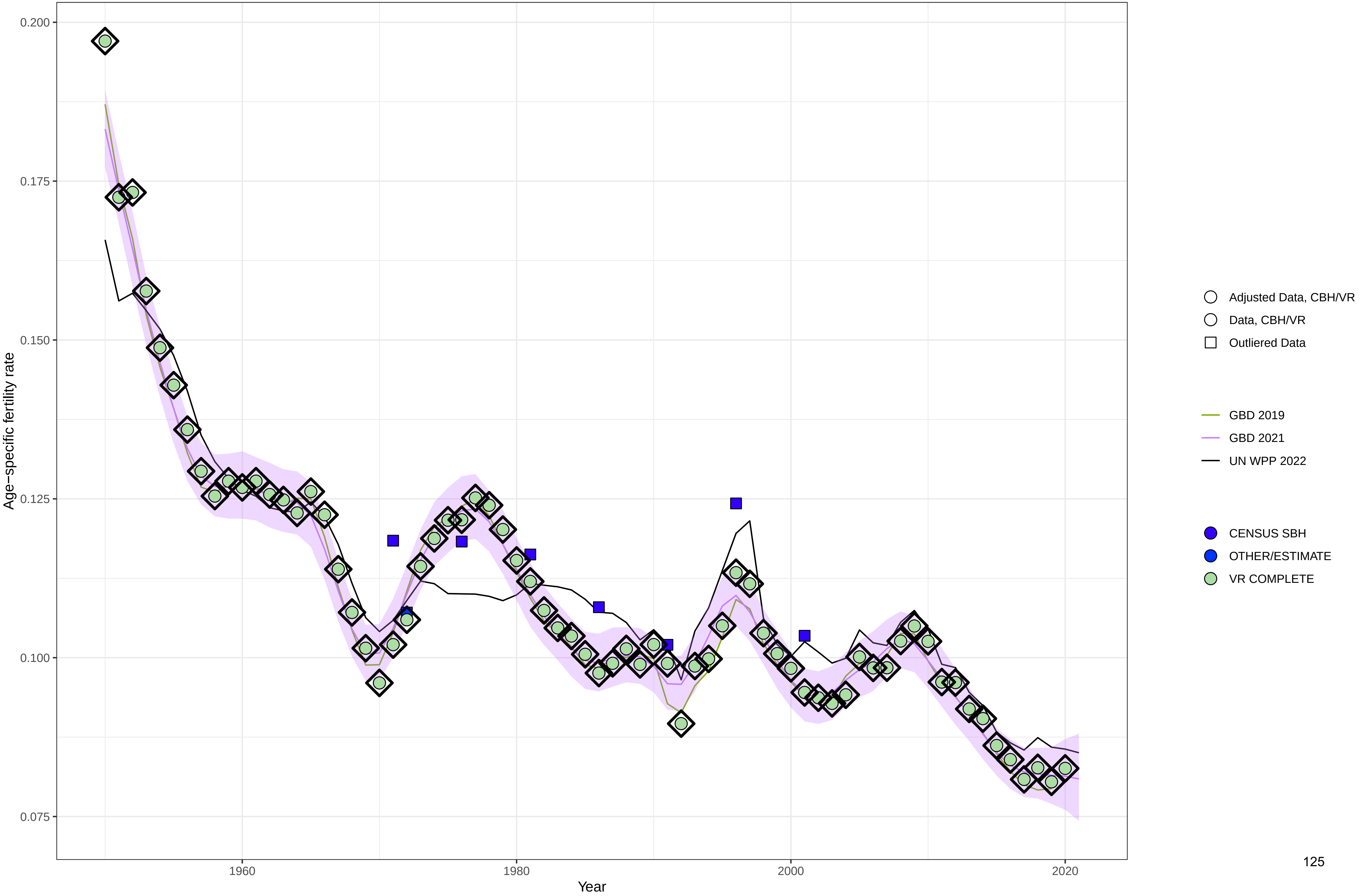

Age−specific fertility fitted trend lines, 1950−2021: Croatia, Ages: 30−34
Data points enclosed with a diamond are reference sources

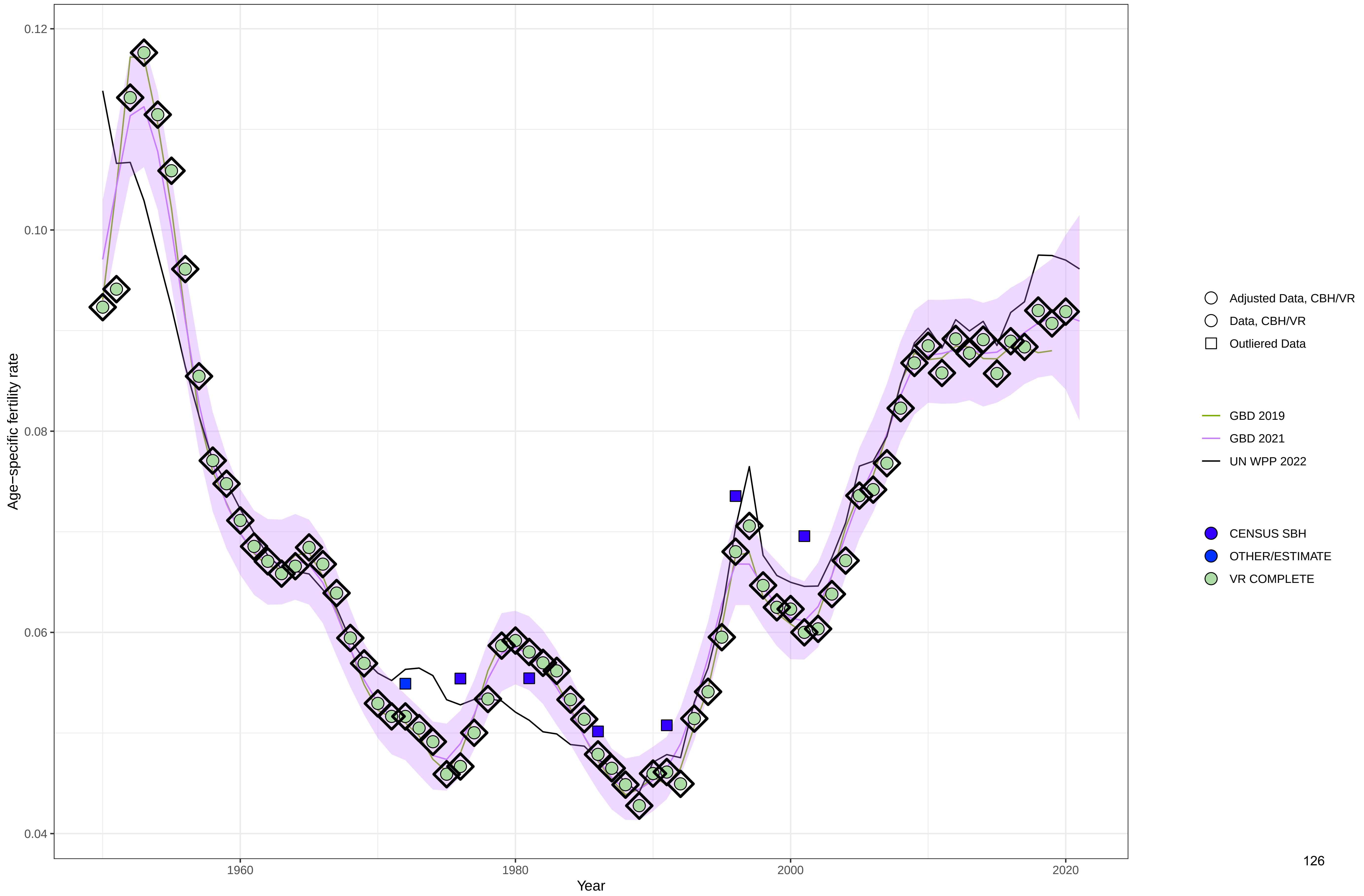

Age−specific fertility fitted trend lines, 1950−2021: Croatia, Ages: 35−39
Data points enclosed with a diamond are reference sources

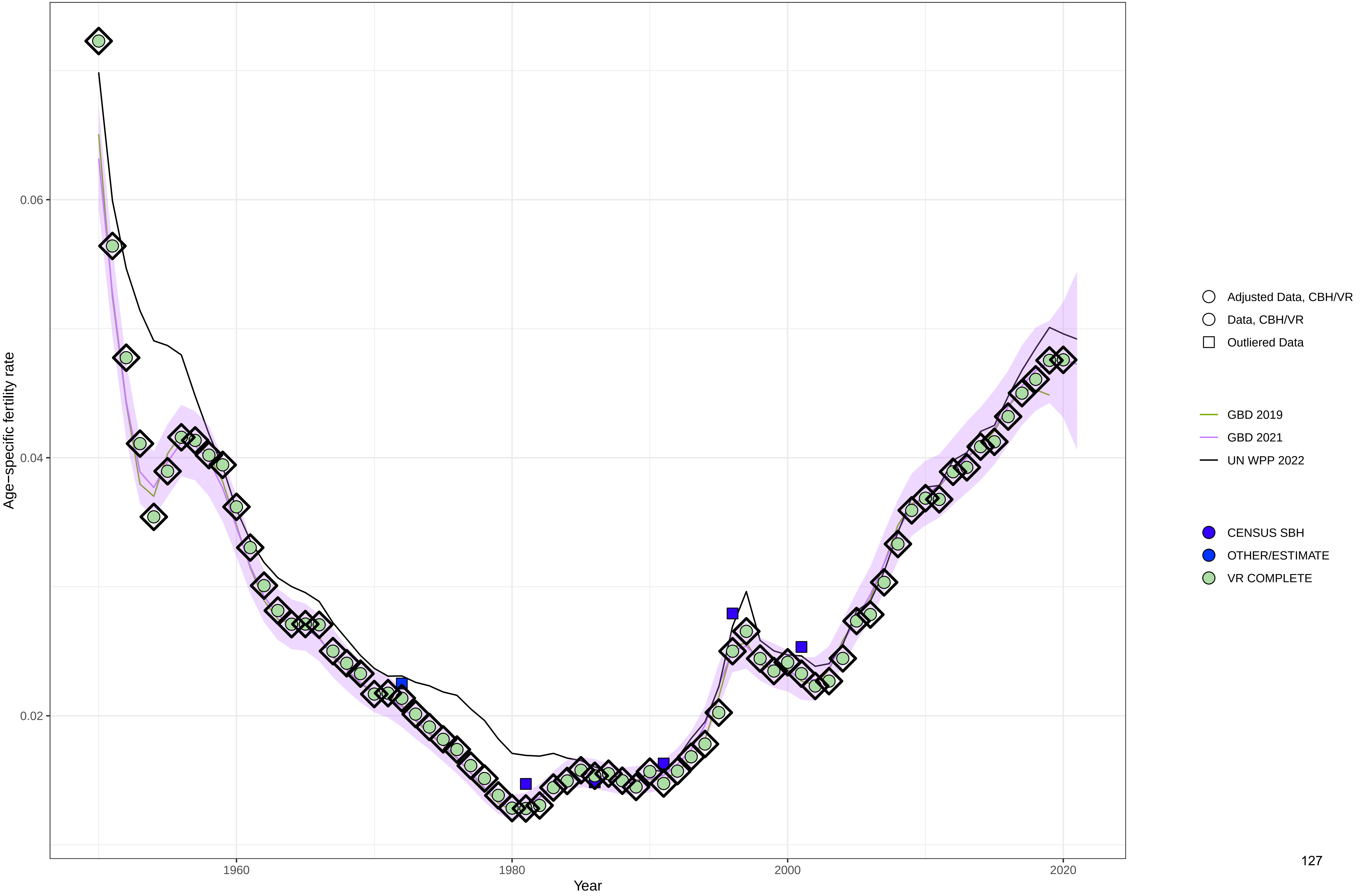

Age−specific fertility fitted trend lines, 1950−2021: Croatia, Ages: 40−44
Data points enclosed with a diamond are reference sources

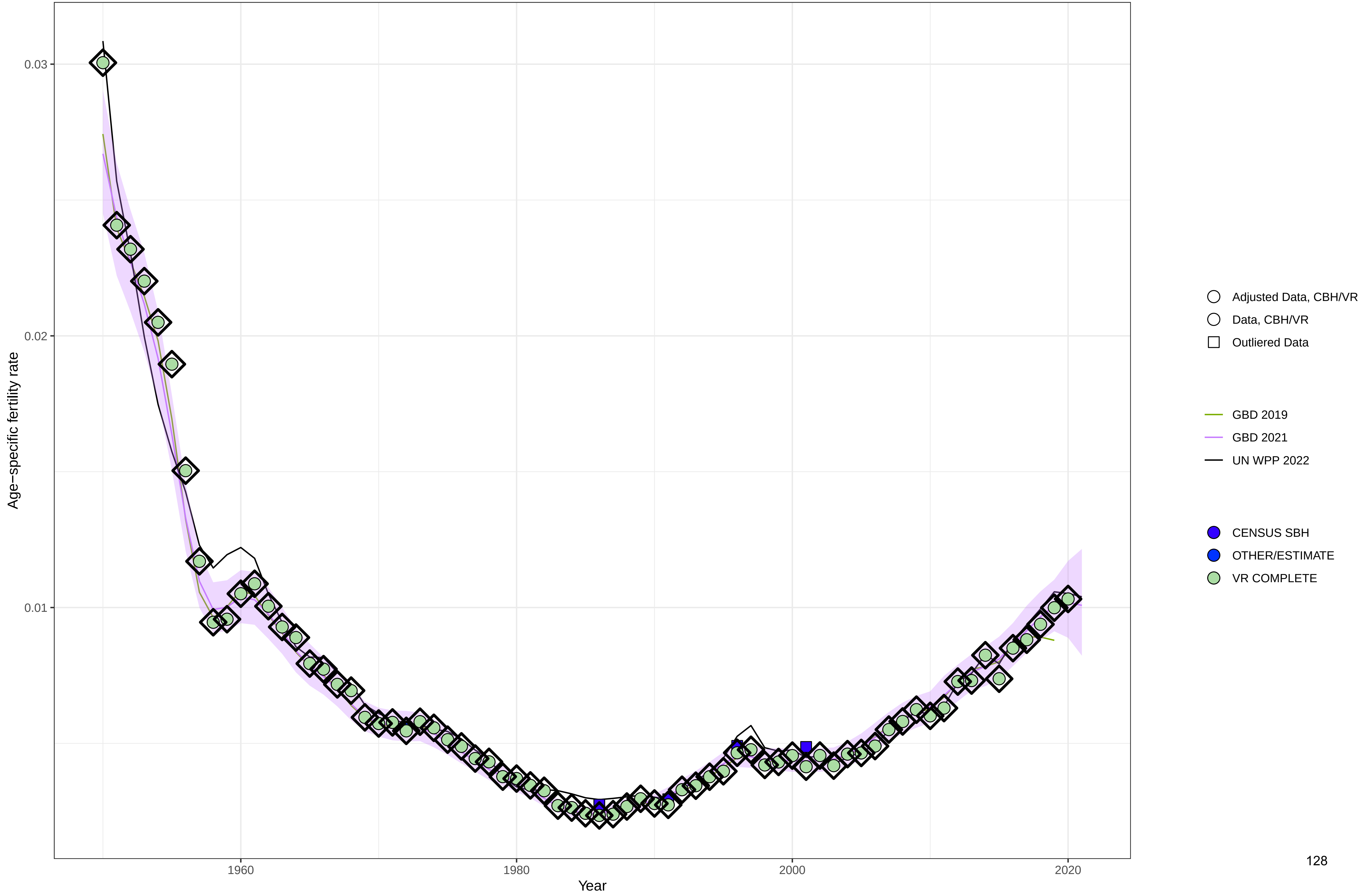

Age−specific fertility fitted trend lines, 1950−2021: Croatia, Ages: 45−49
Data points enclosed with a diamond are reference sources

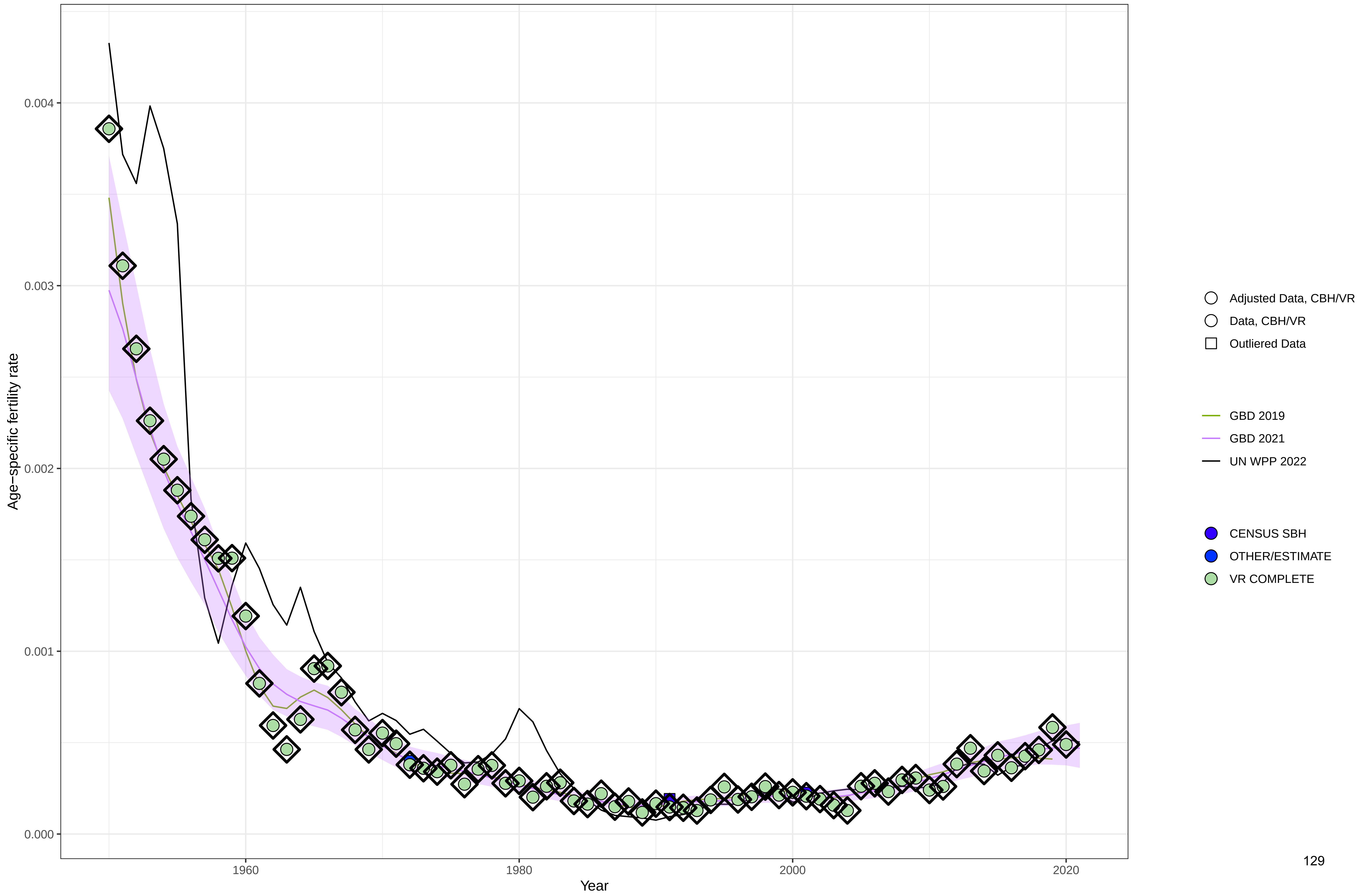

Age−specific fertility fitted trend lines, 1950−2021: Croatia, Ages: 50−54
Data points enclosed with a diamond are reference sources

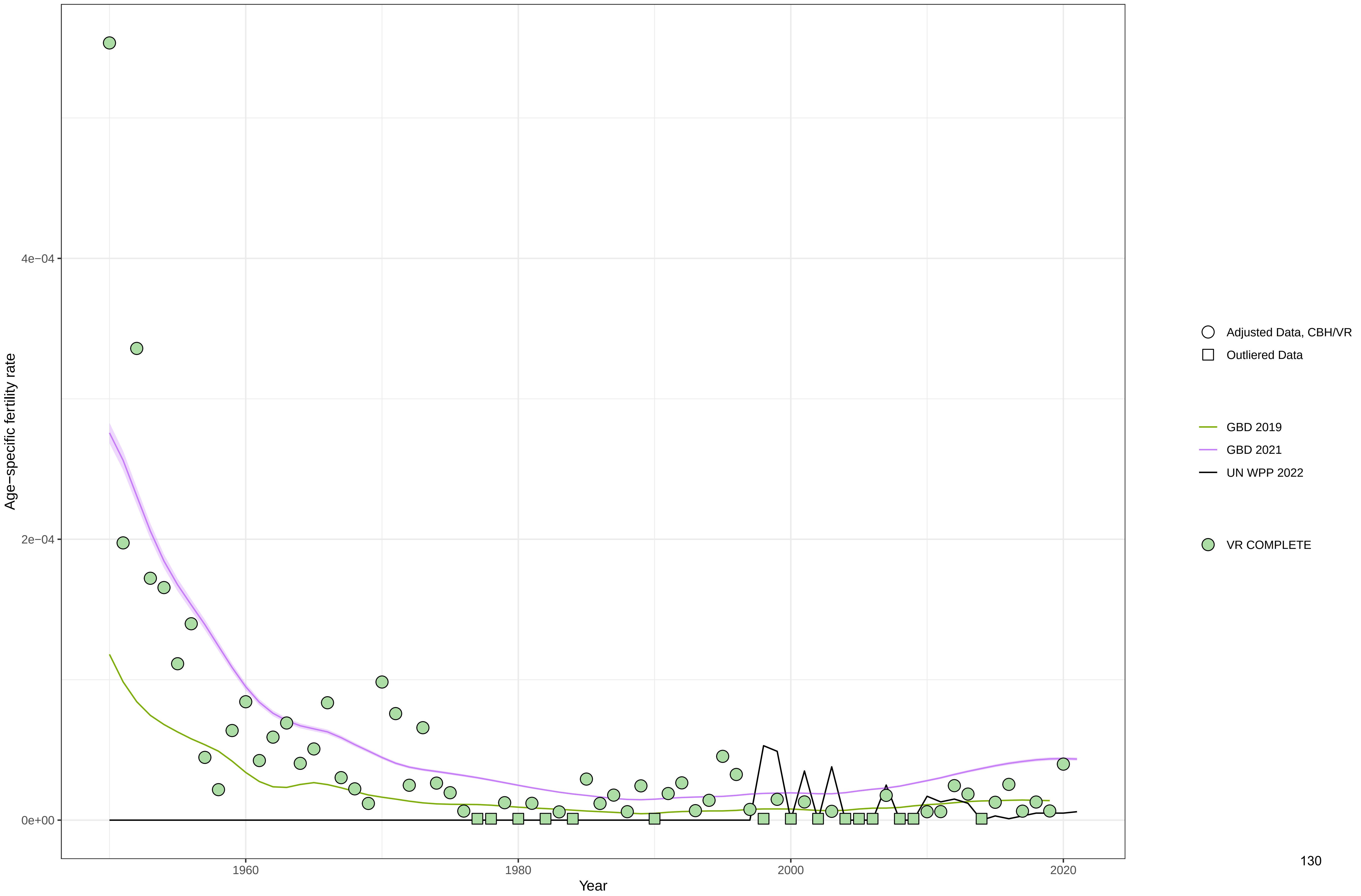

Total fertility rate fitted trend lines, 1950−2021: Croatia TFR
Data points enclosed with a diamond are reference sources

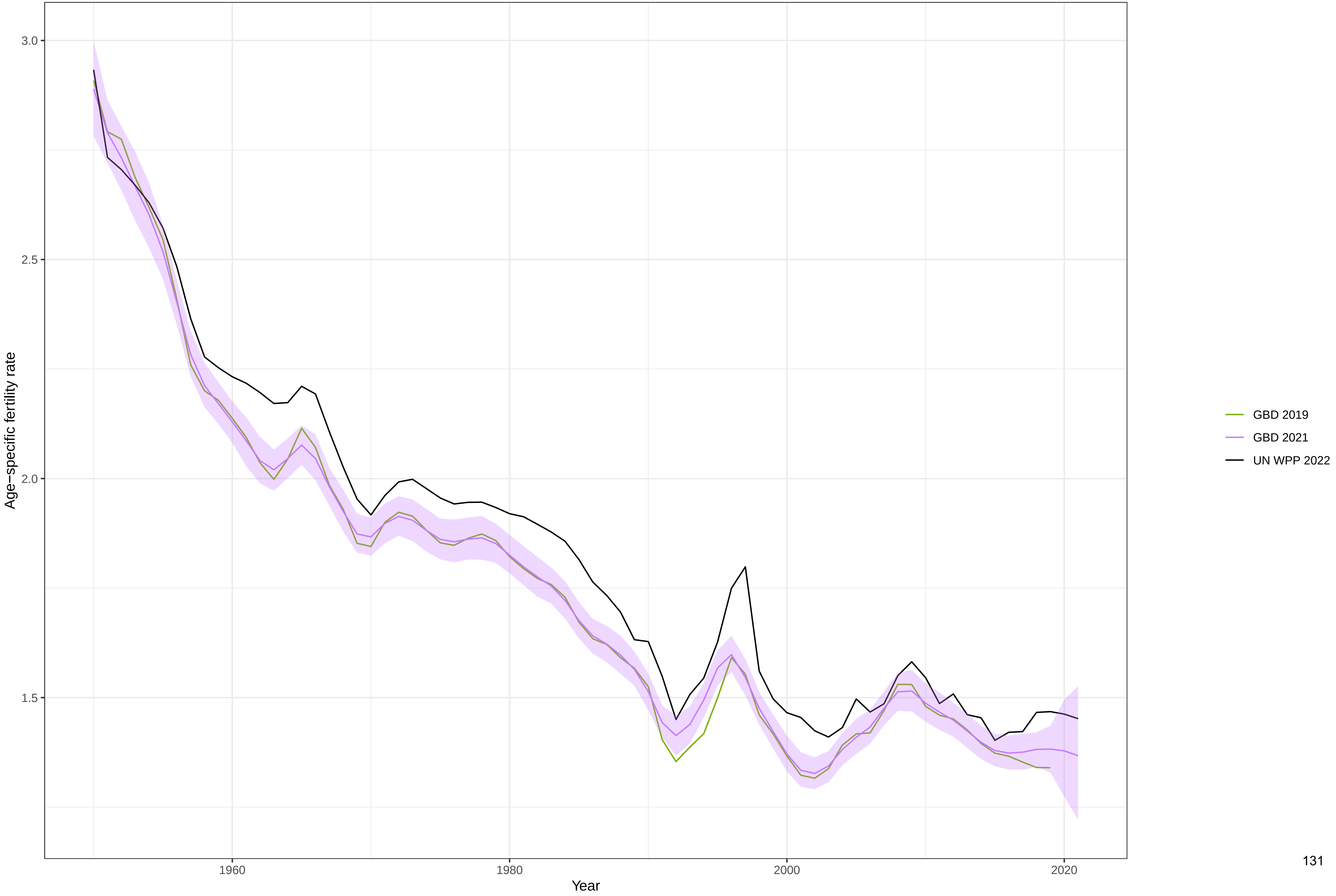

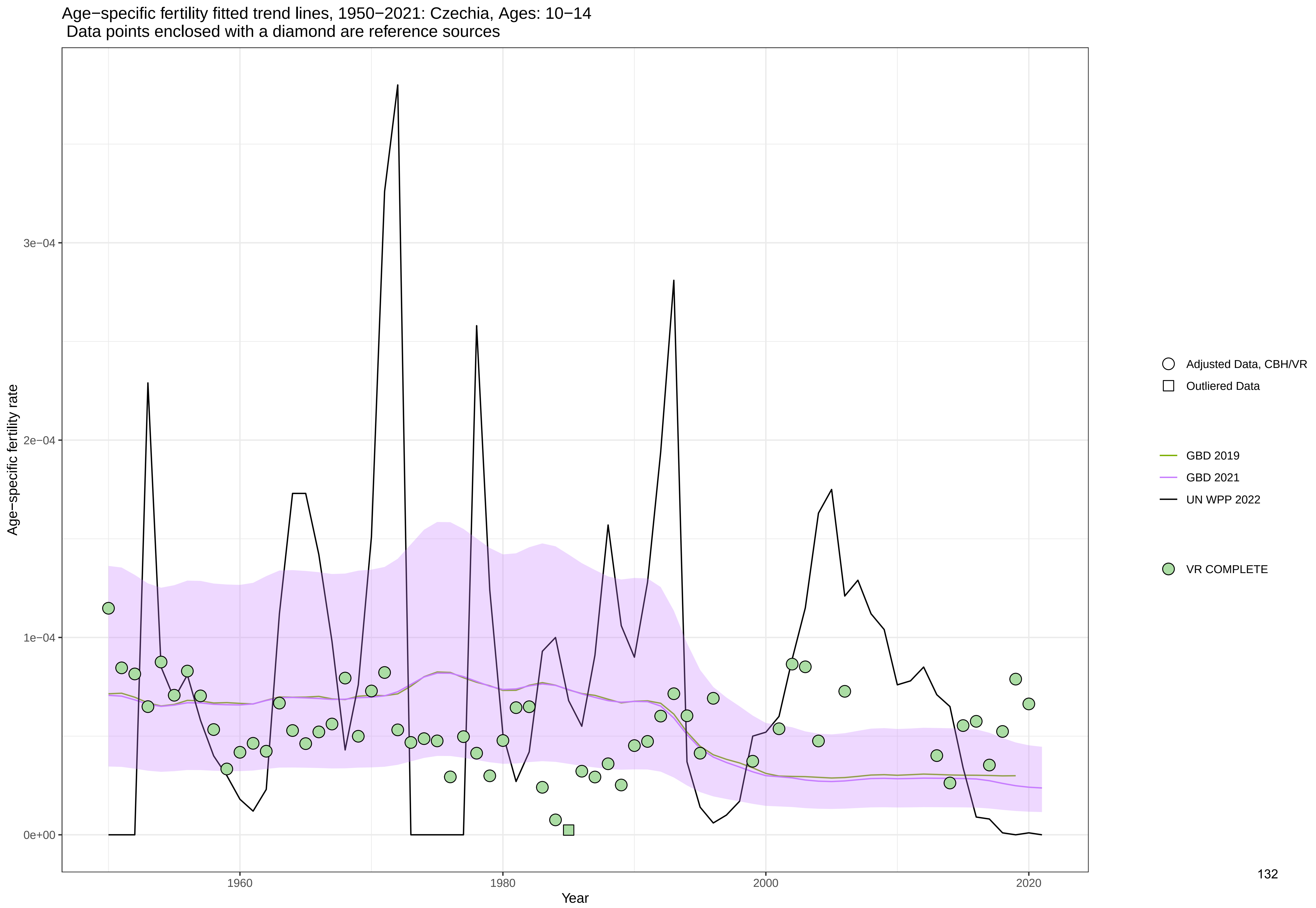

Age−specific fertility fitted trend lines, 1950−2021: Czechia, Ages: 10−14
Data points enclosed with a diamond are reference sources
Age−specific fertility rate
3e−04
2e−04
1e−04
0e+00
1960
1980
2000
2020
Year
Adjusted Data, CBH/VR
Outliered Data
GBD 2019
GBD 2021
UN WPP 2022
VR COMPLETE

Age−specific fertility fitted trend lines, 1950−2021: Czechia, Ages: 15−19
Data points enclosed with a diamond are reference sources

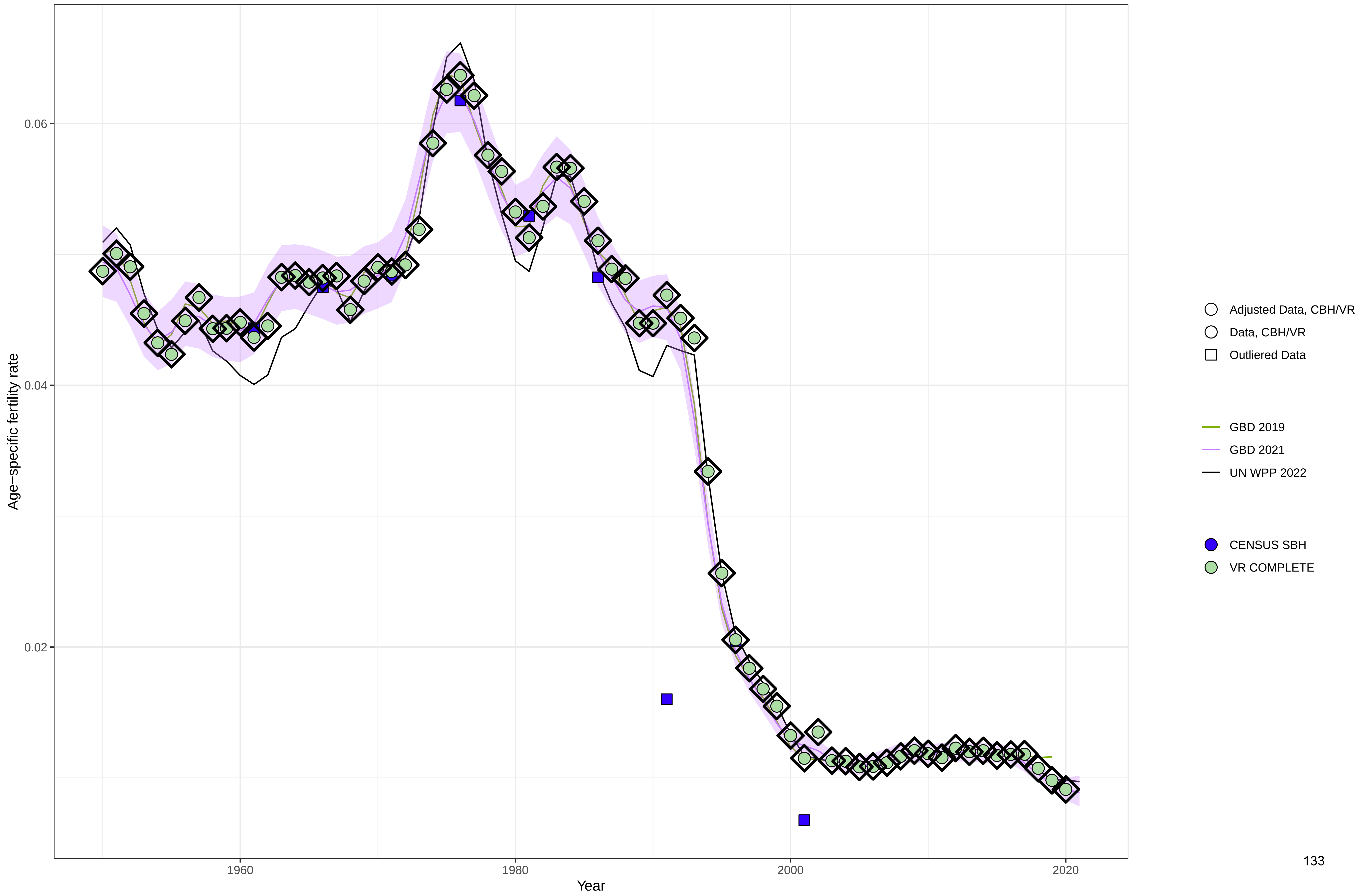

Age−specific fertility fitted trend lines, 1950−2021: Czechia, Ages: 20−24
Data points enclosed with a diamond are reference sources

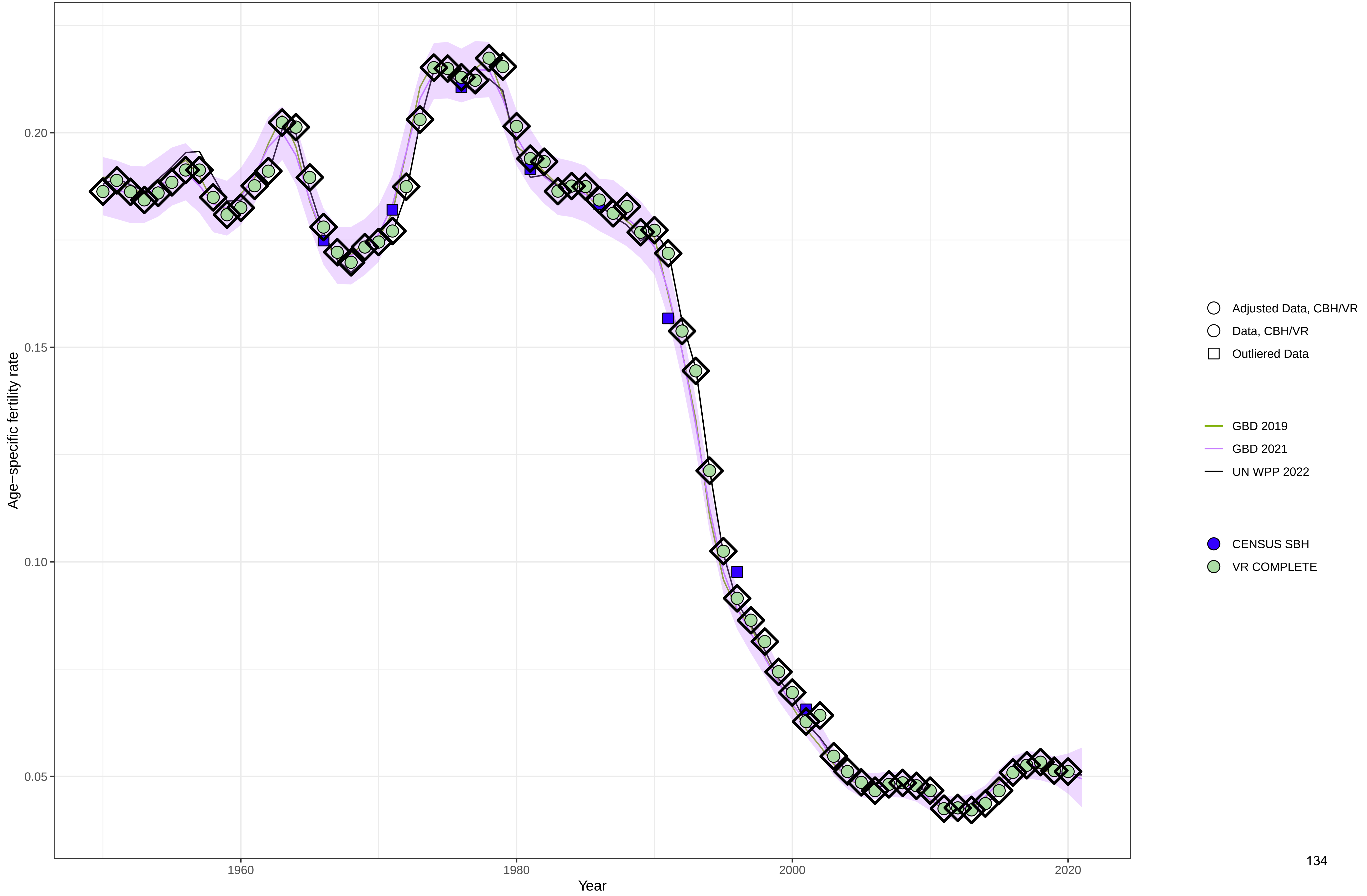

Age−specific fertility fitted trend lines, 1950−2021: Czechia, Ages: 25−29
Data points enclosed with a diamond are reference sources

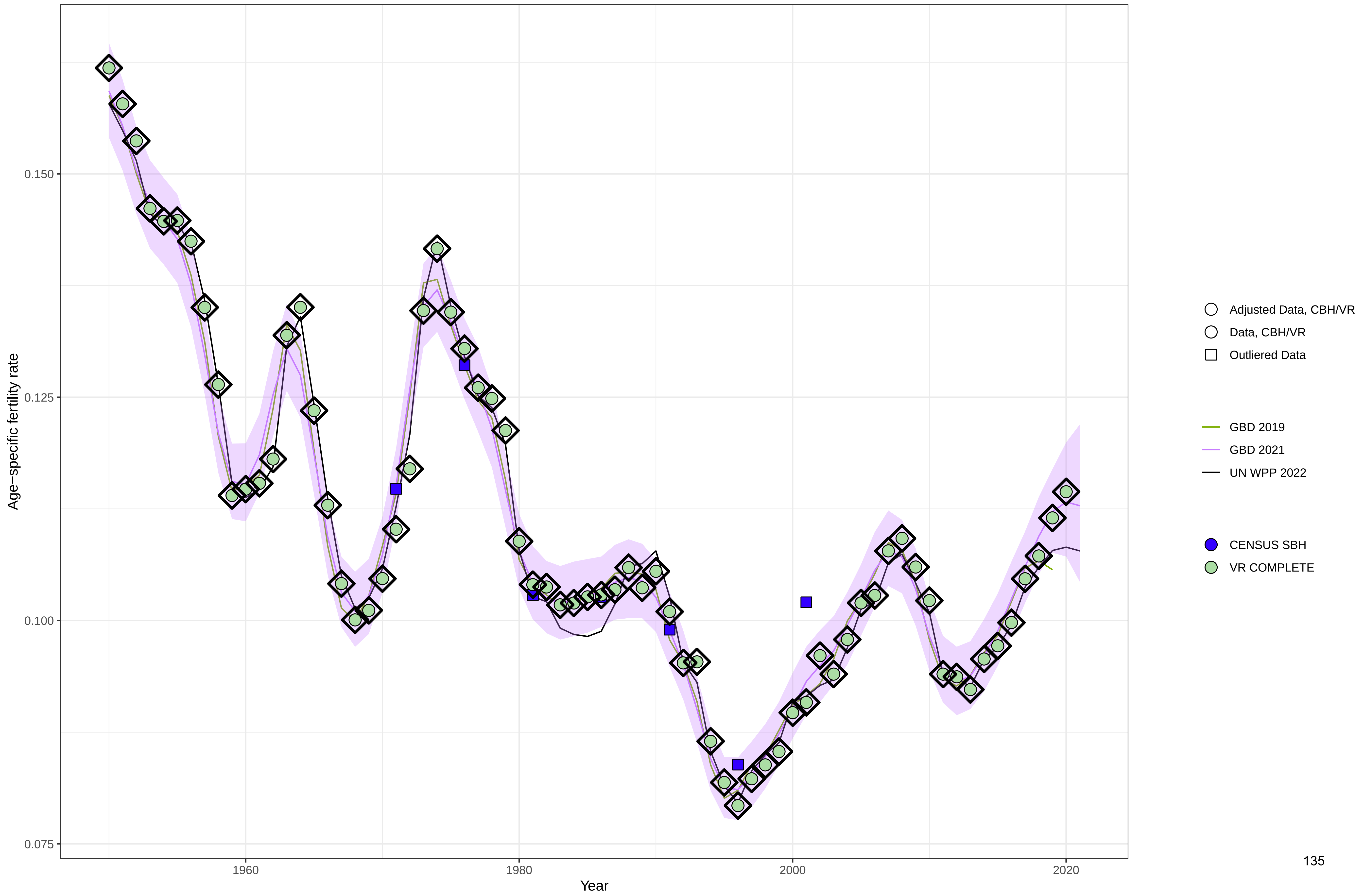

Age−specific fertility fitted trend lines, 1950−2021: Czechia, Ages: 30−34
Data points enclosed with a diamond are reference sources

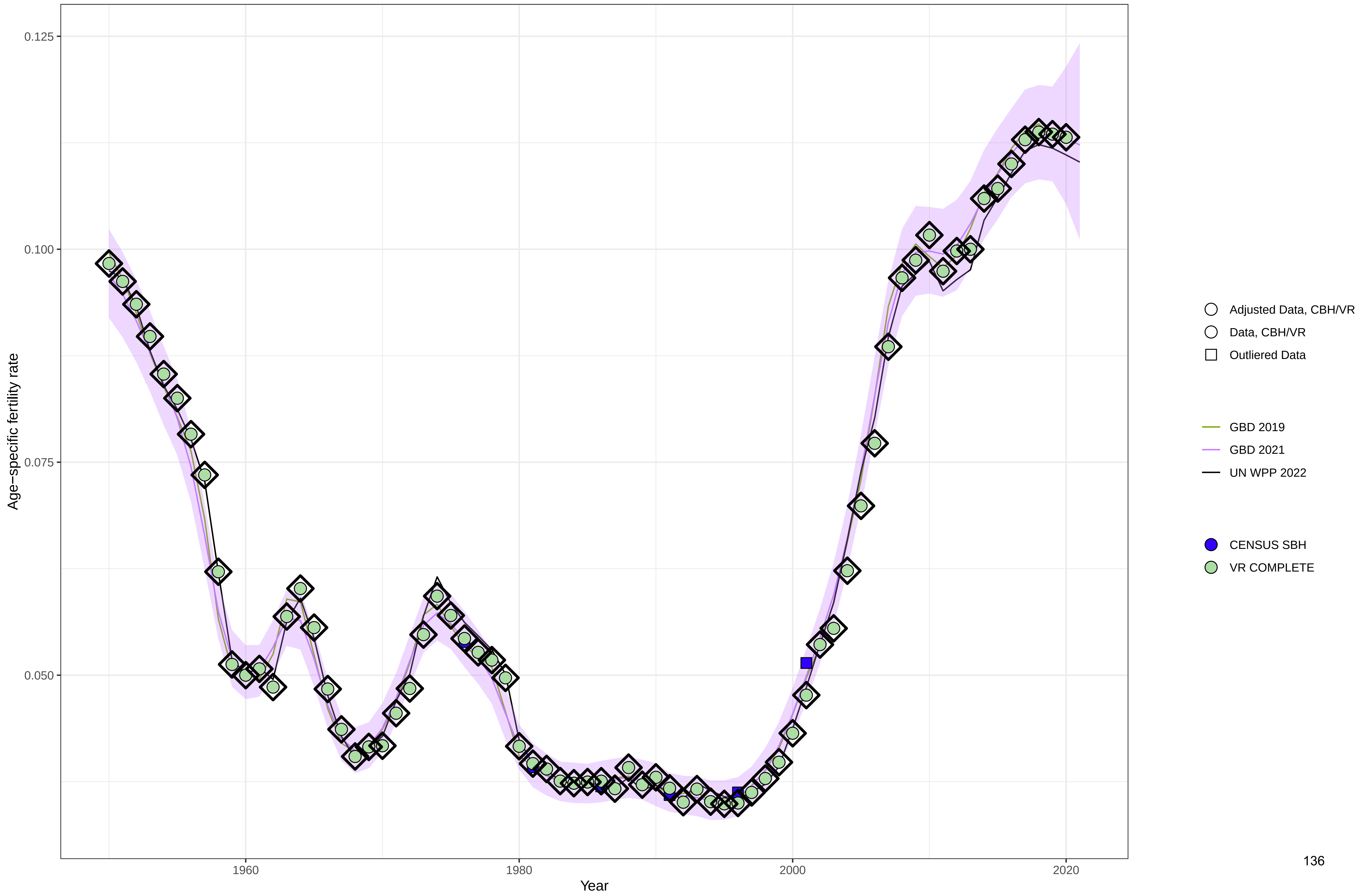

Age−specific fertility fitted trend lines, 1950−2021: Czechia, Ages: 35−39
Data points enclosed with a diamond are reference sources

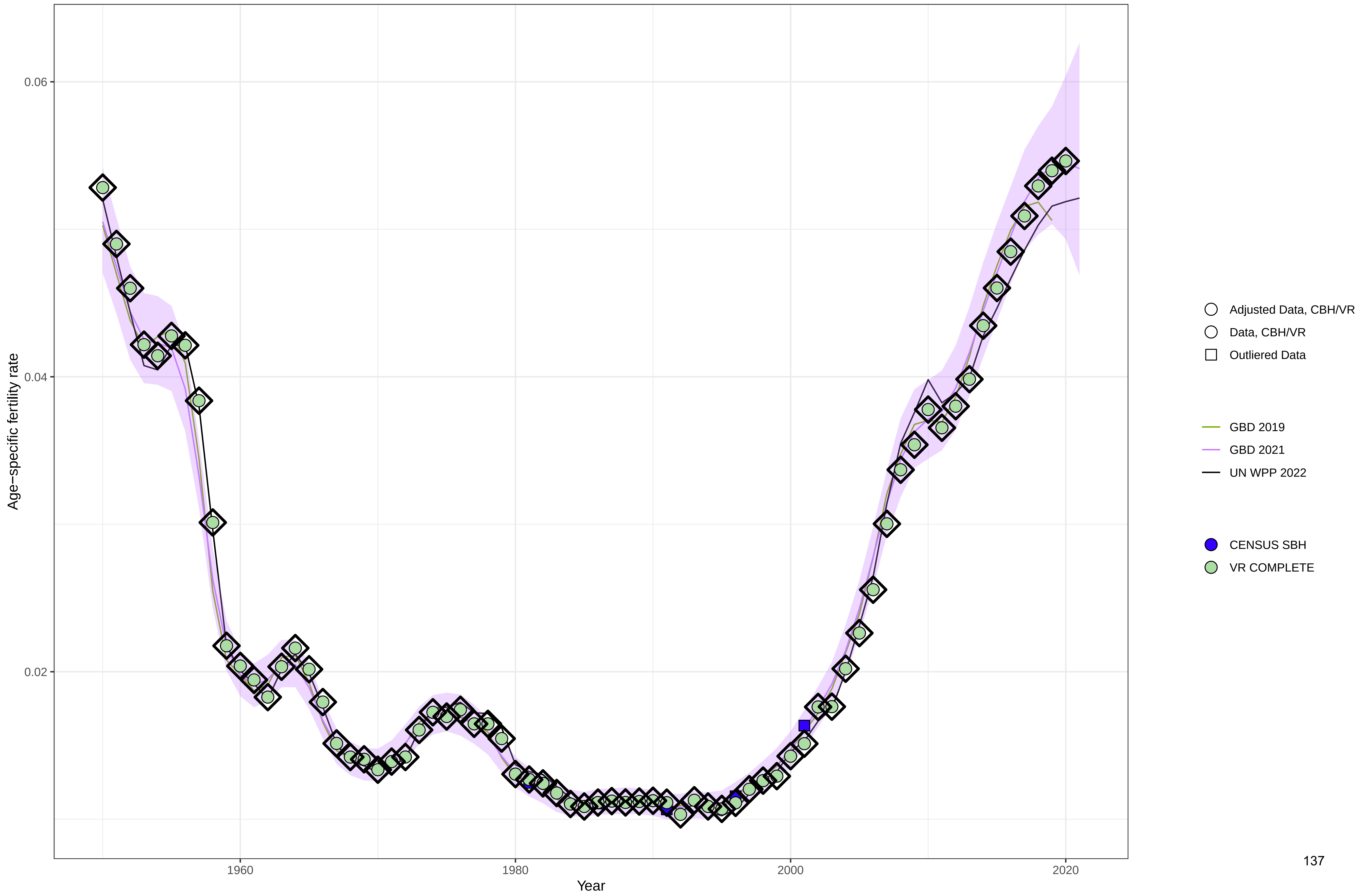

Age−specific fertility fitted trend lines, 1950−2021: Czechia, Ages: 40−44
Data points enclosed with a diamond are reference sources

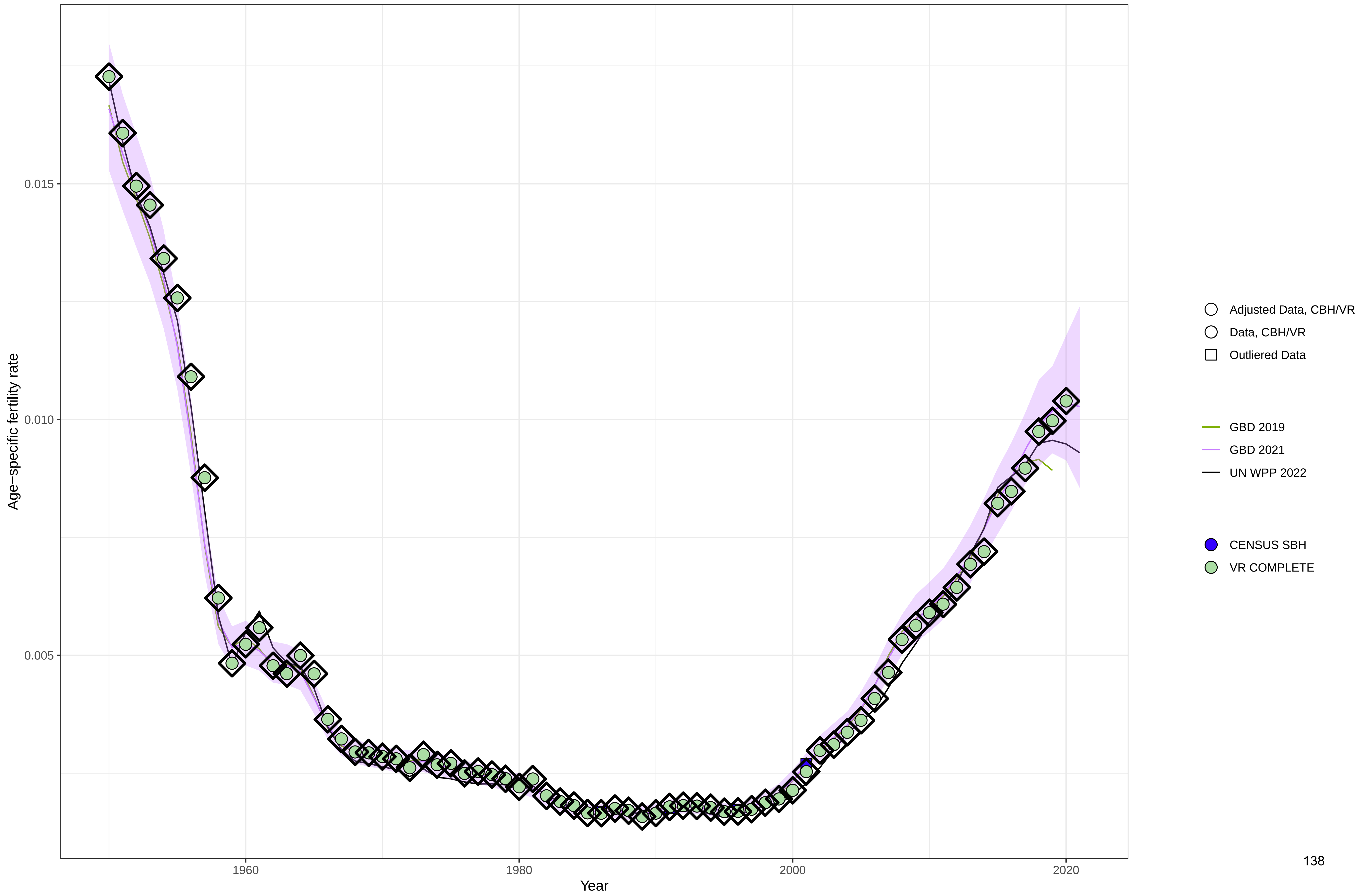

Age−specific fertility fitted trend lines, 1950−2021: Czechia, Ages: 45−49
Data points enclosed with a diamond are reference sources

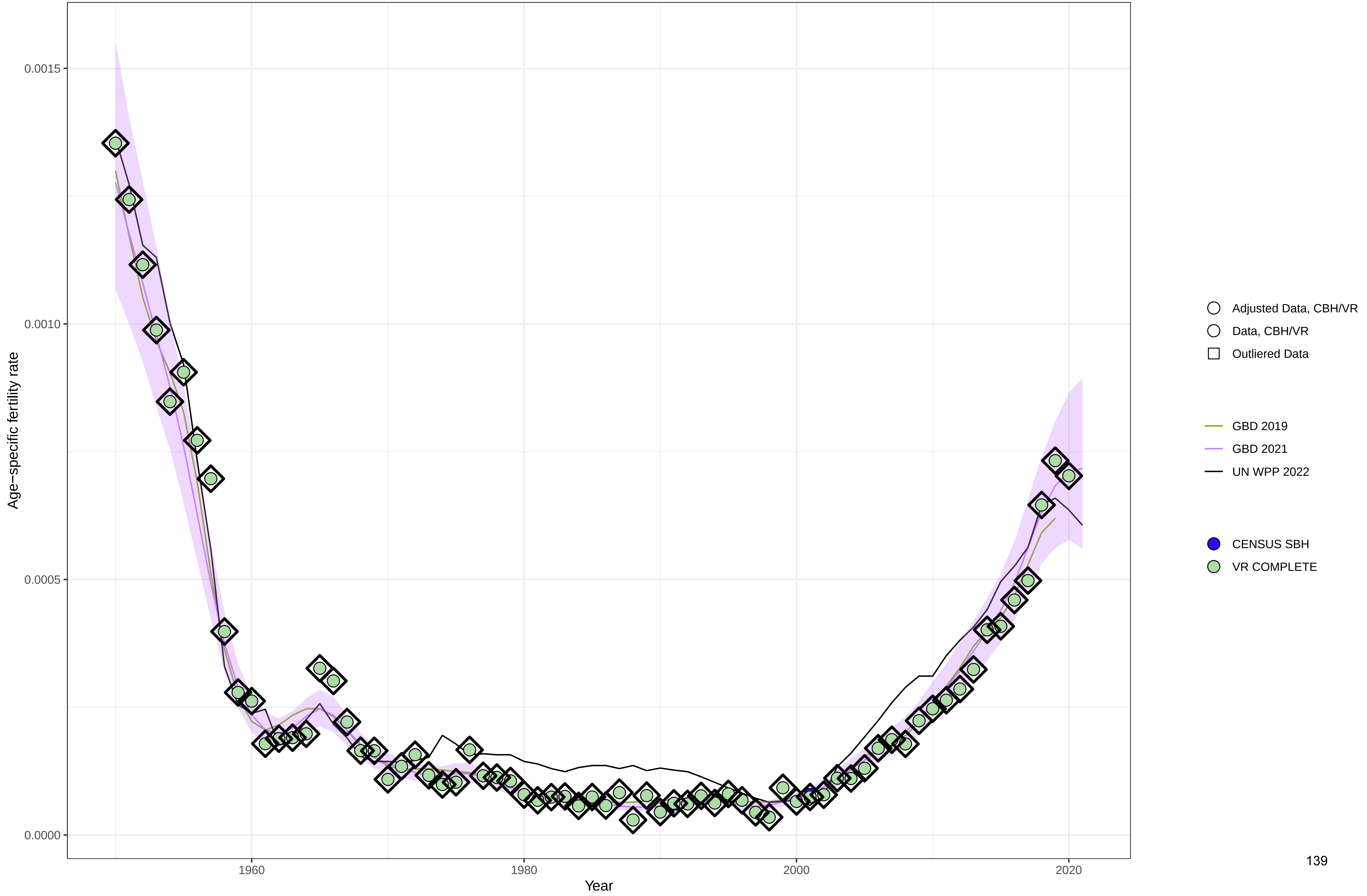

Age−specific fertility fitted trend lines, 1950−2021: Czechia, Ages: 50−54
Data points enclosed with a diamond are reference sources

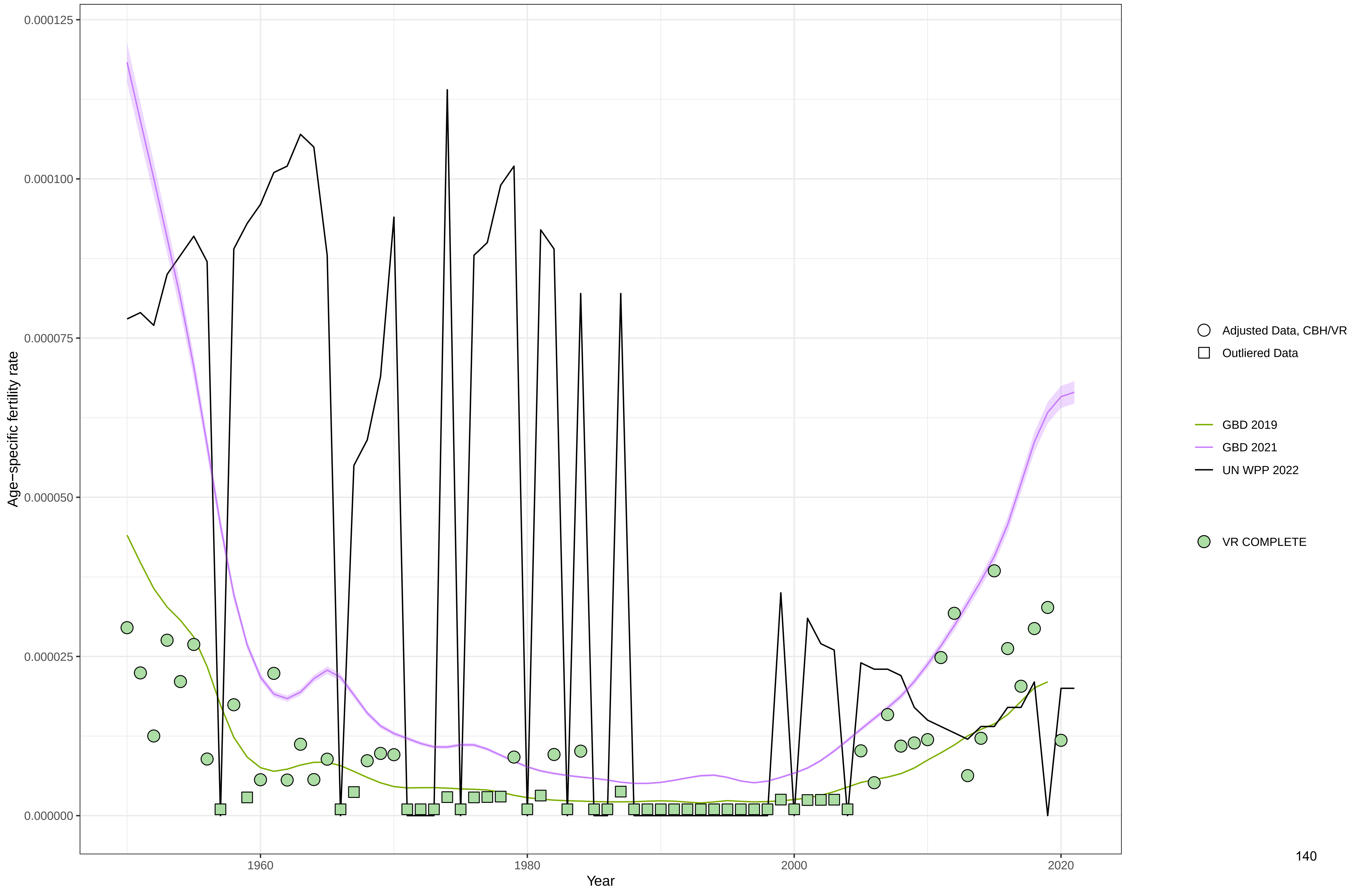

Total fertility rate fitted trend lines, 1950−2021: Czechia TFR
Data points enclosed with a diamond are reference sources

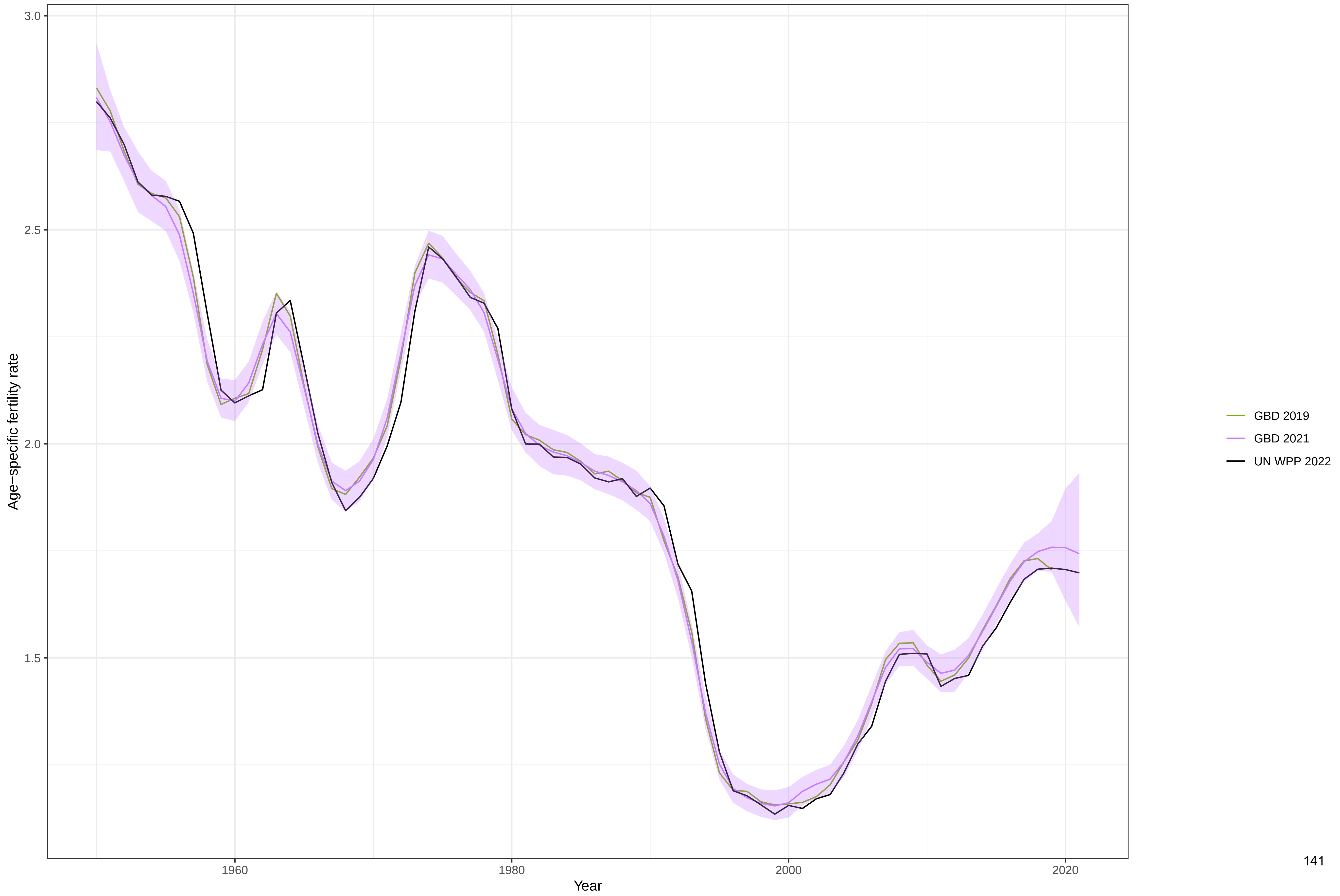

Age−specific fertility fitted trend lines, 1950−2021: Hungary, Ages: 10−14
Data points enclosed with a diamond are reference sources

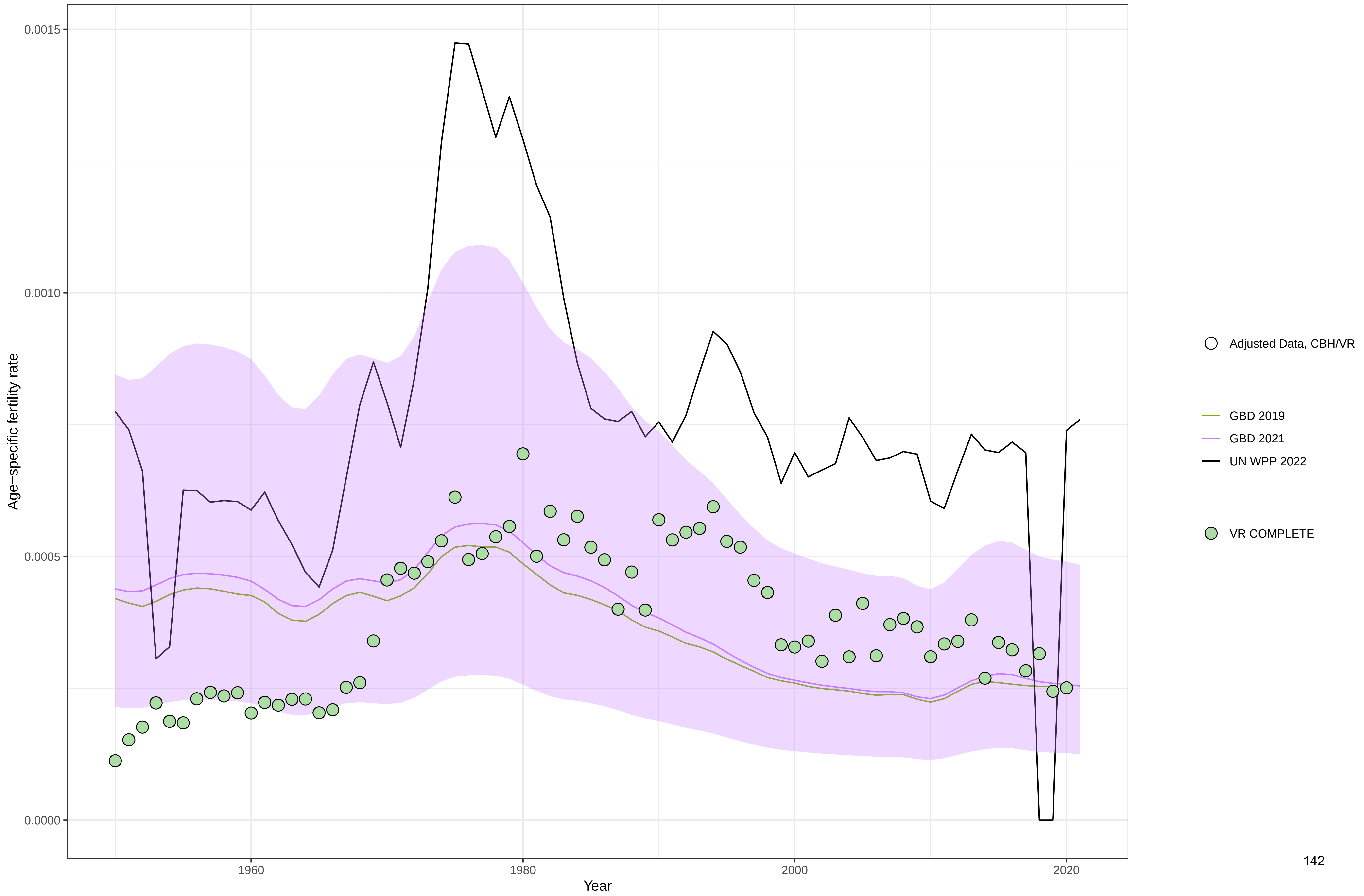

Age−specific fertility fitted trend lines, 1950−2021: Hungary, Ages: 15−19
Data points enclosed with a diamond are reference sources

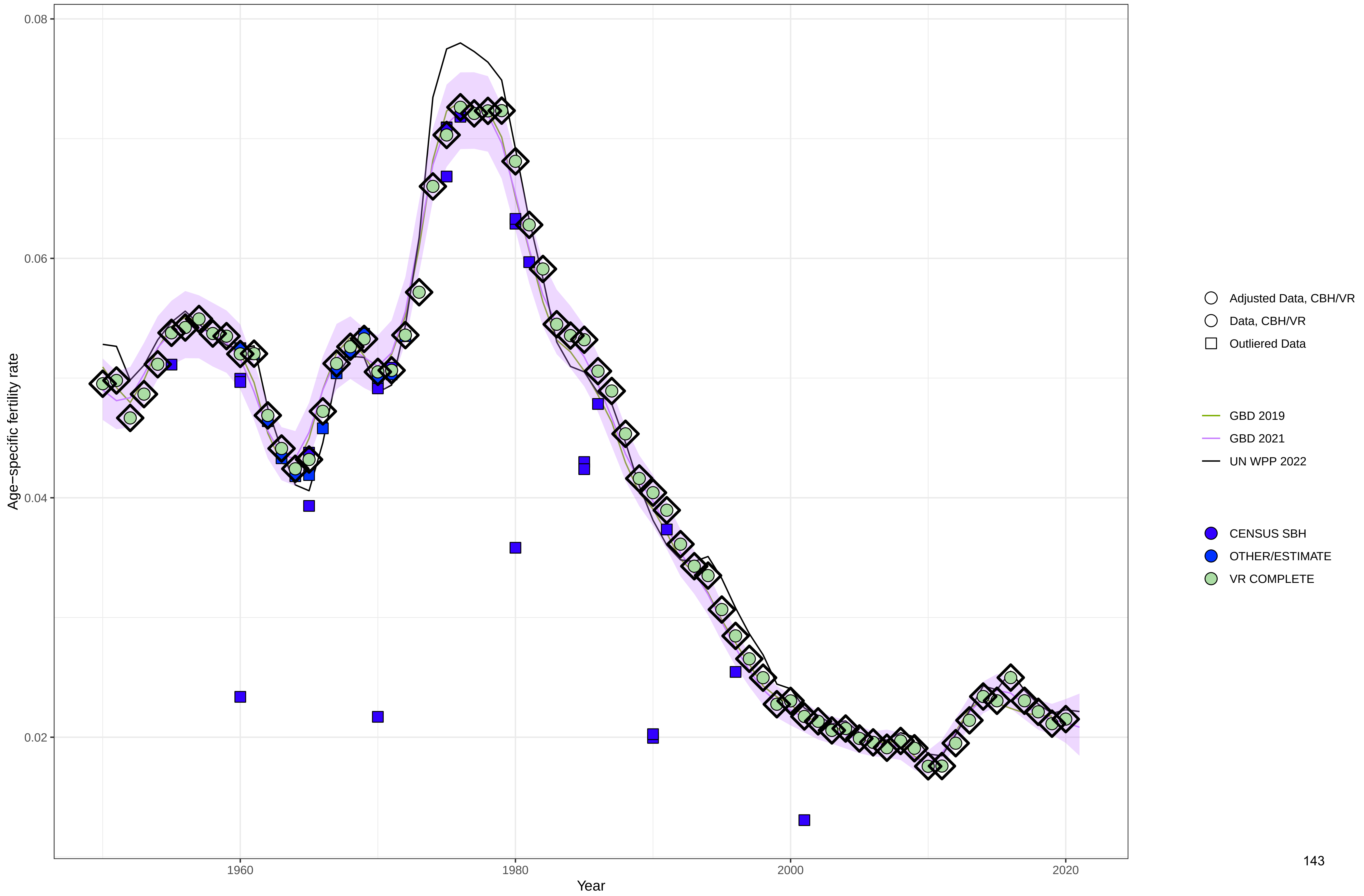

Age−specific fertility fitted trend lines, 1950−2021: Hungary, Ages: 20−24
Data points enclosed with a diamond are reference sources

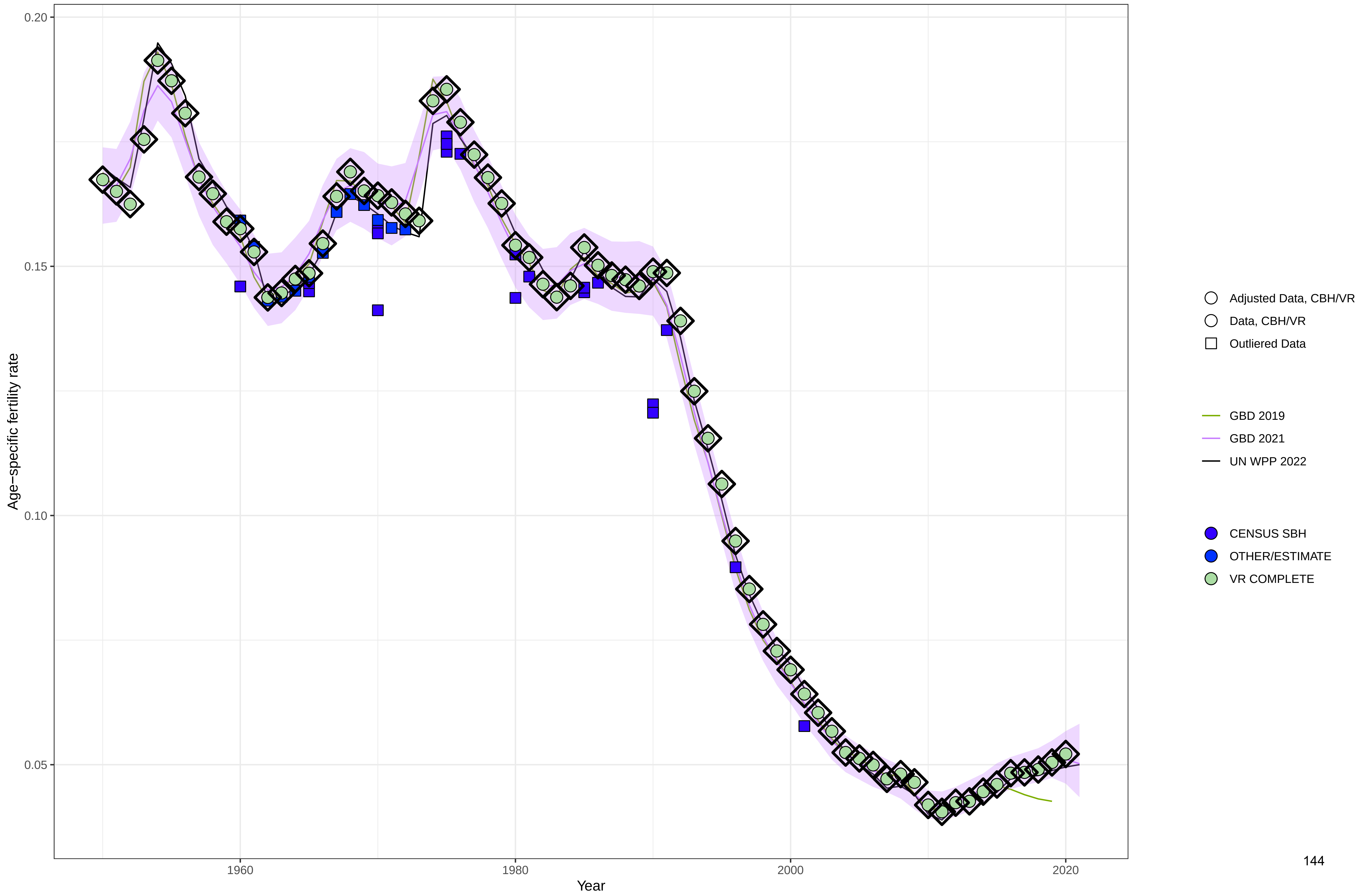

Age−specific fertility fitted trend lines, 1950−2021: Hungary, Ages: 25−29
Data points enclosed with a diamond are reference sources

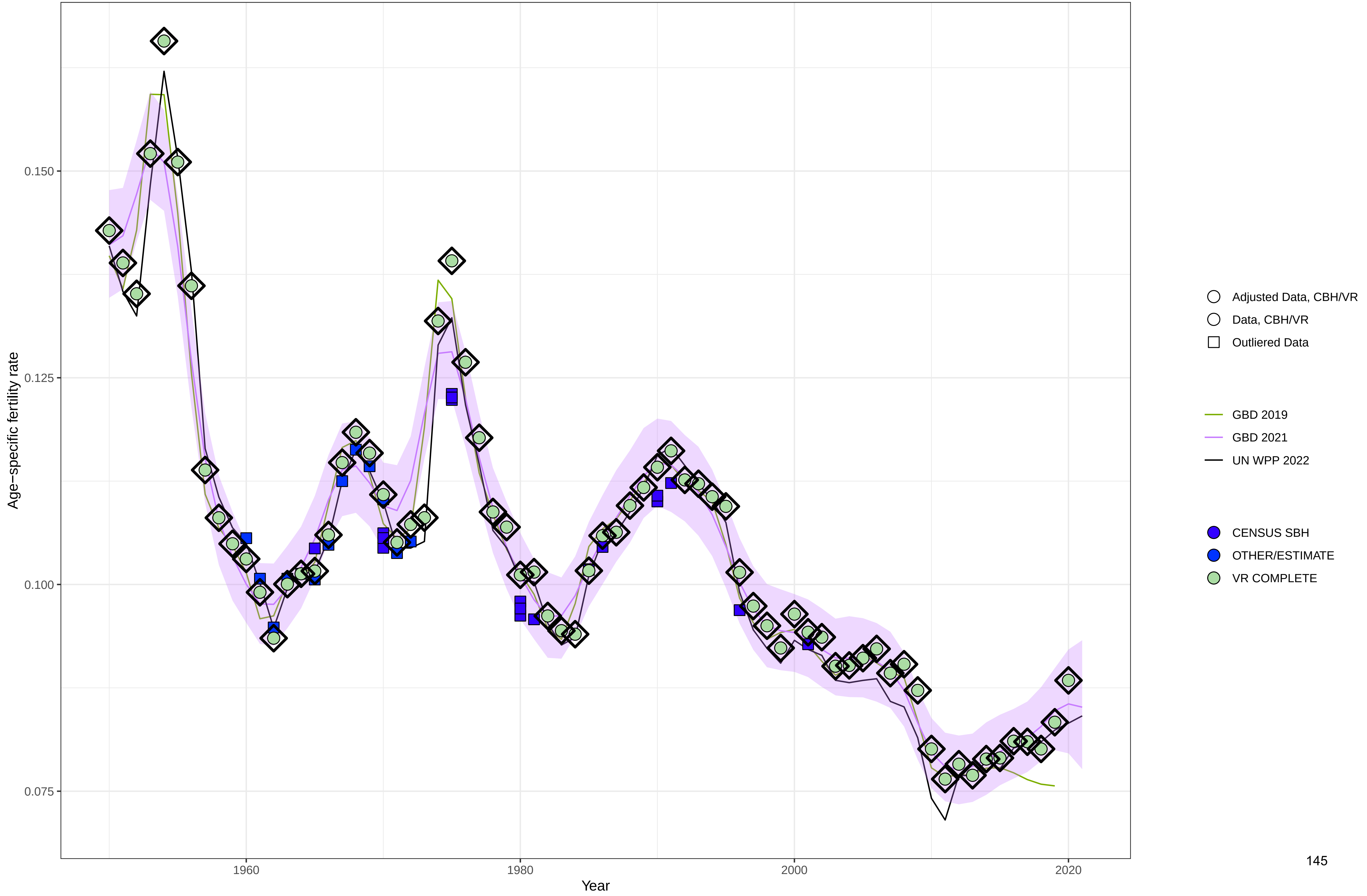

Age−specific fertility fitted trend lines, 1950−2021: Hungary, Ages: 30−34
Data points enclosed with a diamond are reference sources

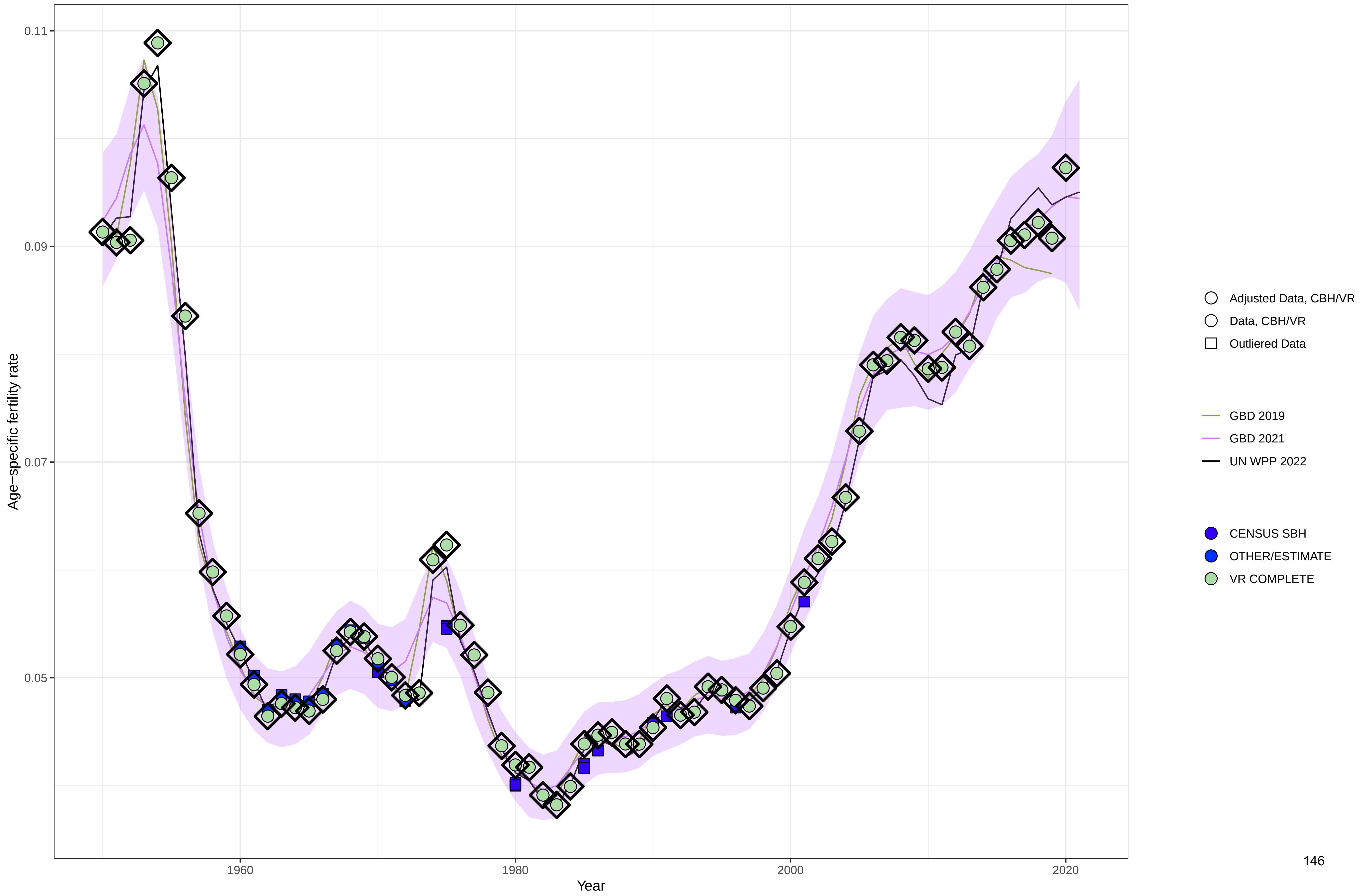

Age−specific fertility fitted trend lines, 1950−2021: Hungary, Ages: 35−39
Data points enclosed with a diamond are reference sources

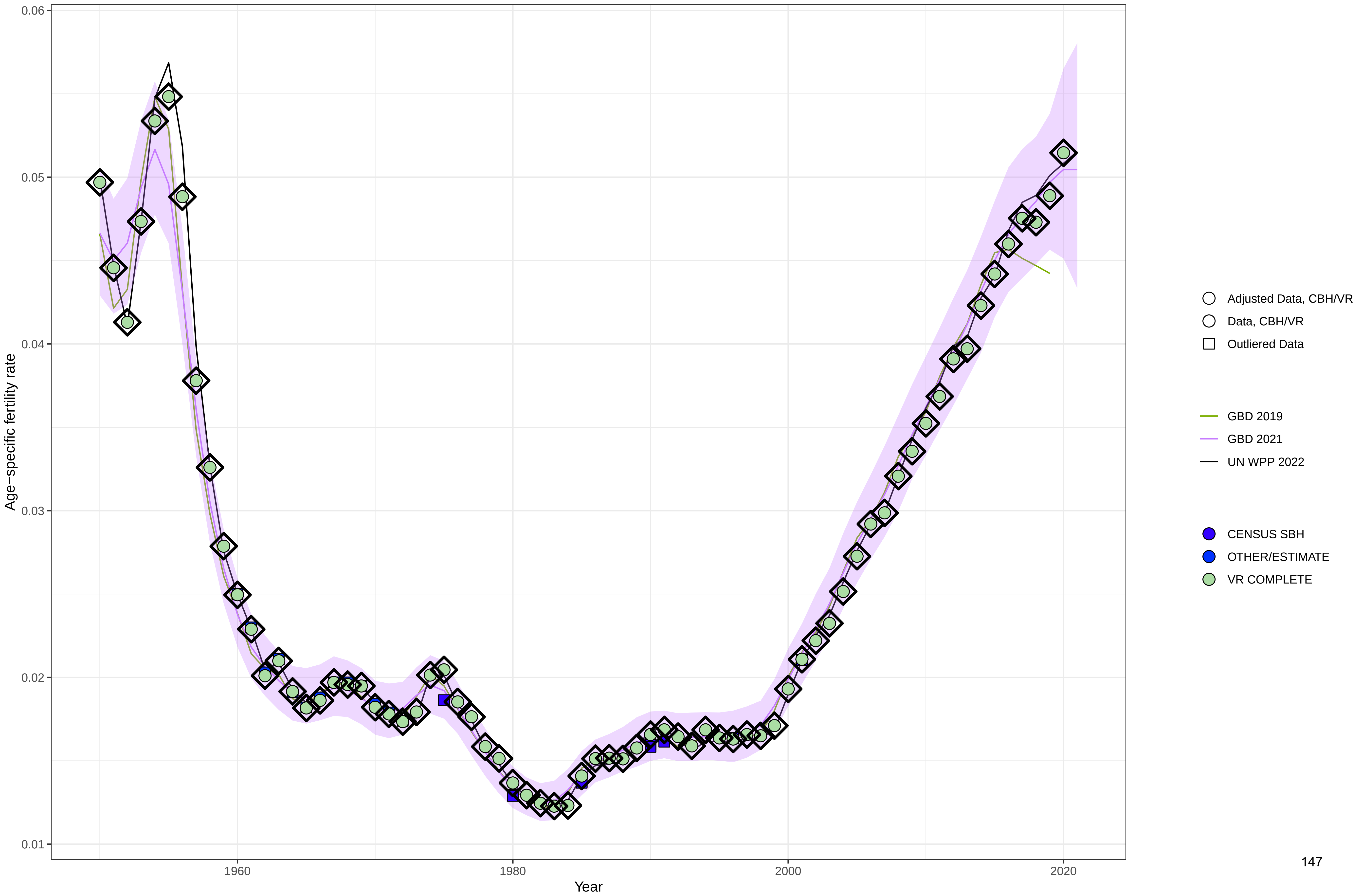

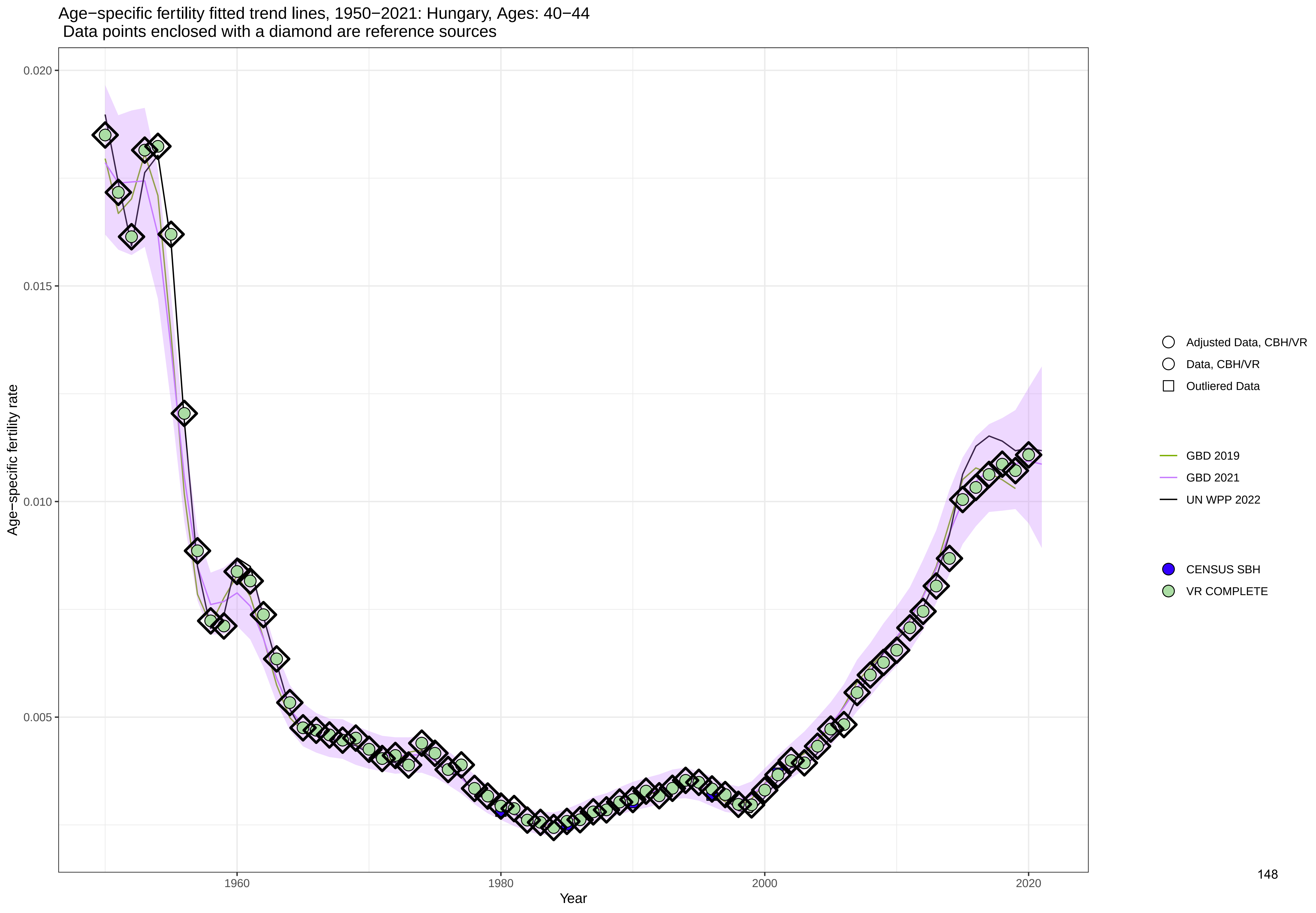

Age−specific fertility fitted trend lines, 1950−2021: Hungary, Ages: 40−44
Data points enclosed with a diamond are reference sources
Age−specific fertility rate
0.020
0.015
0.010
0.005
1960
1980
2000
2020
Year
Adjusted Data, CBH/VR
Data, CBH/VR
Outliered Data
GBD 2019
GBD 2021
UN WPP 2022
CENSUS SBH
VR COMPLETE

Age−specific fertility fitted trend lines, 1950−2021: Hungary, Ages: 45−49
Data points enclosed with a diamond are reference sources

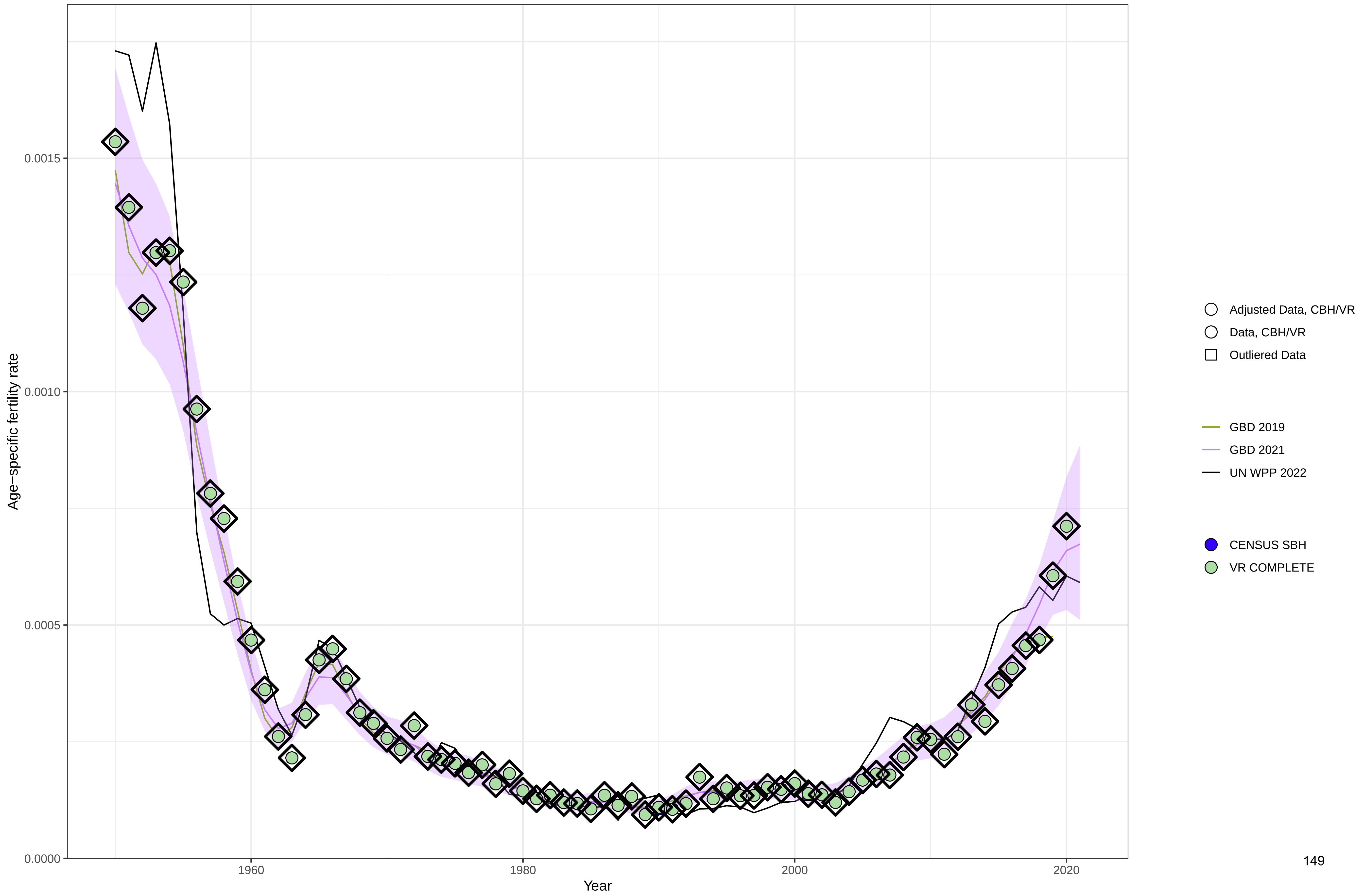

Age−specific fertility fitted trend lines, 1950−2021: Hungary, Ages: 50−54
Data points enclosed with a diamond are reference sources

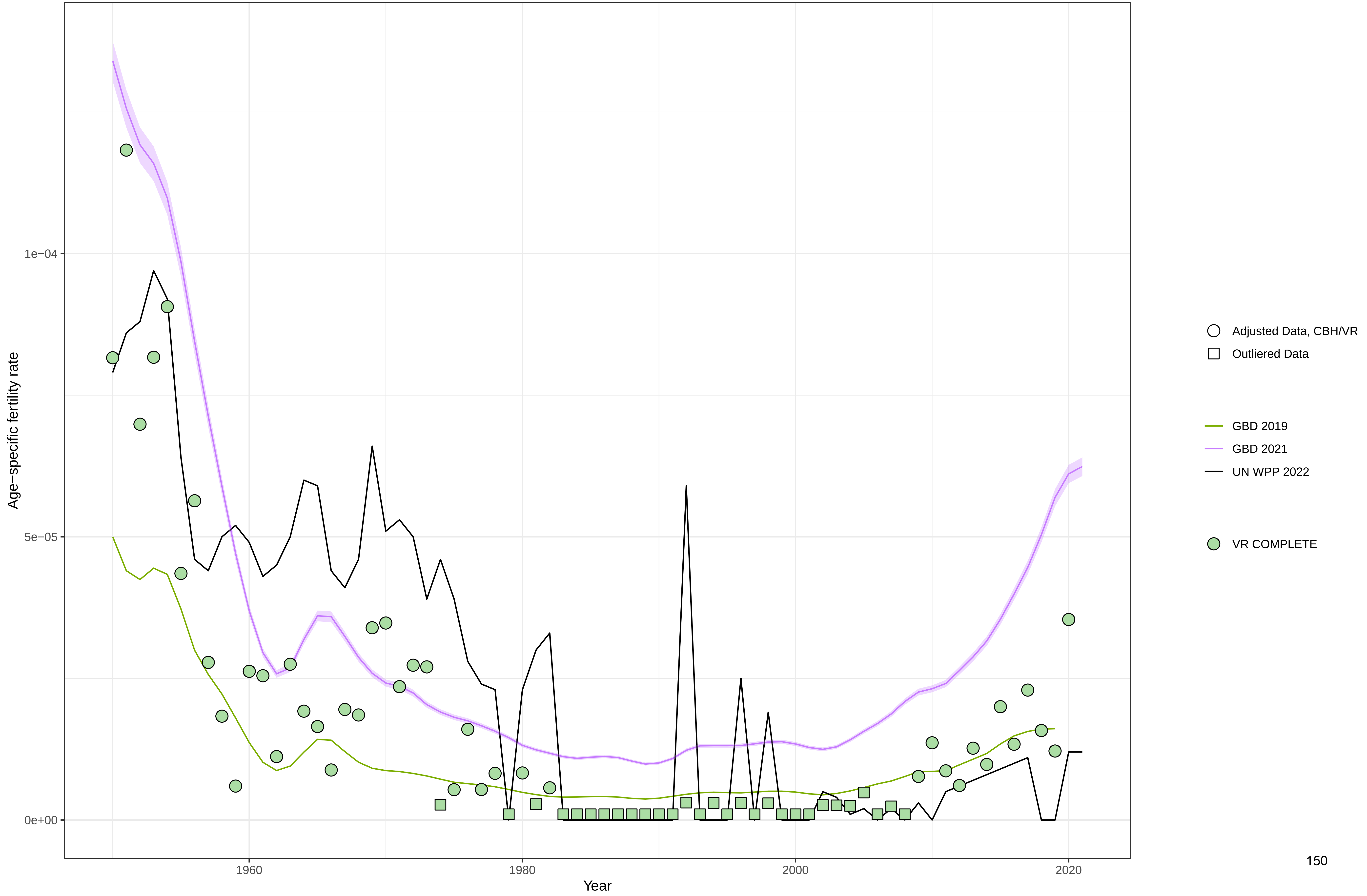

Total fertility rate fitted trend lines, 1950−2021: Hungary TFR
Data points enclosed with a diamond are reference sources

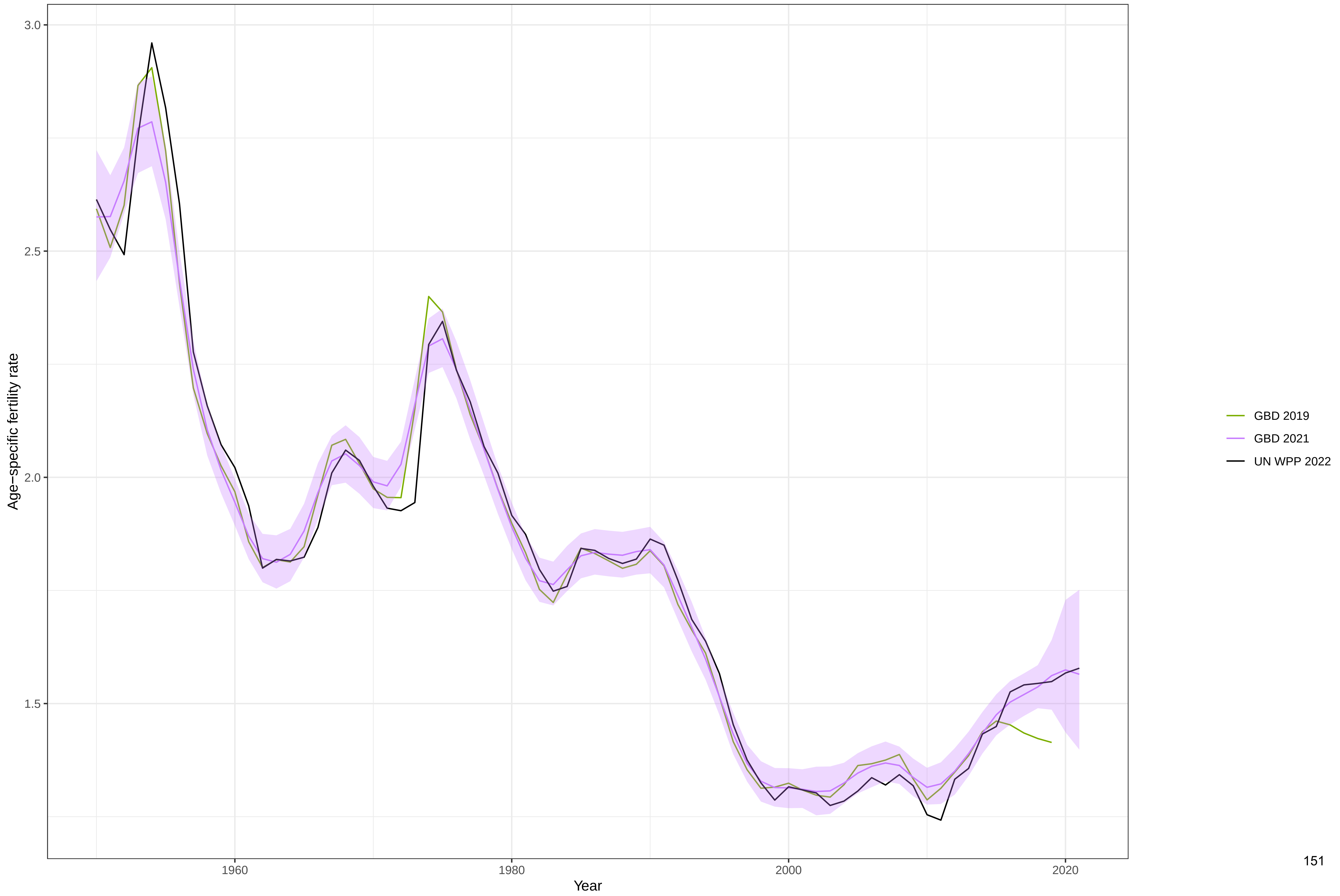

Age−specific fertility fitted trend lines, 1950−2021: Montenegro, Ages: 10−14
Data points enclosed with a diamond are reference sources

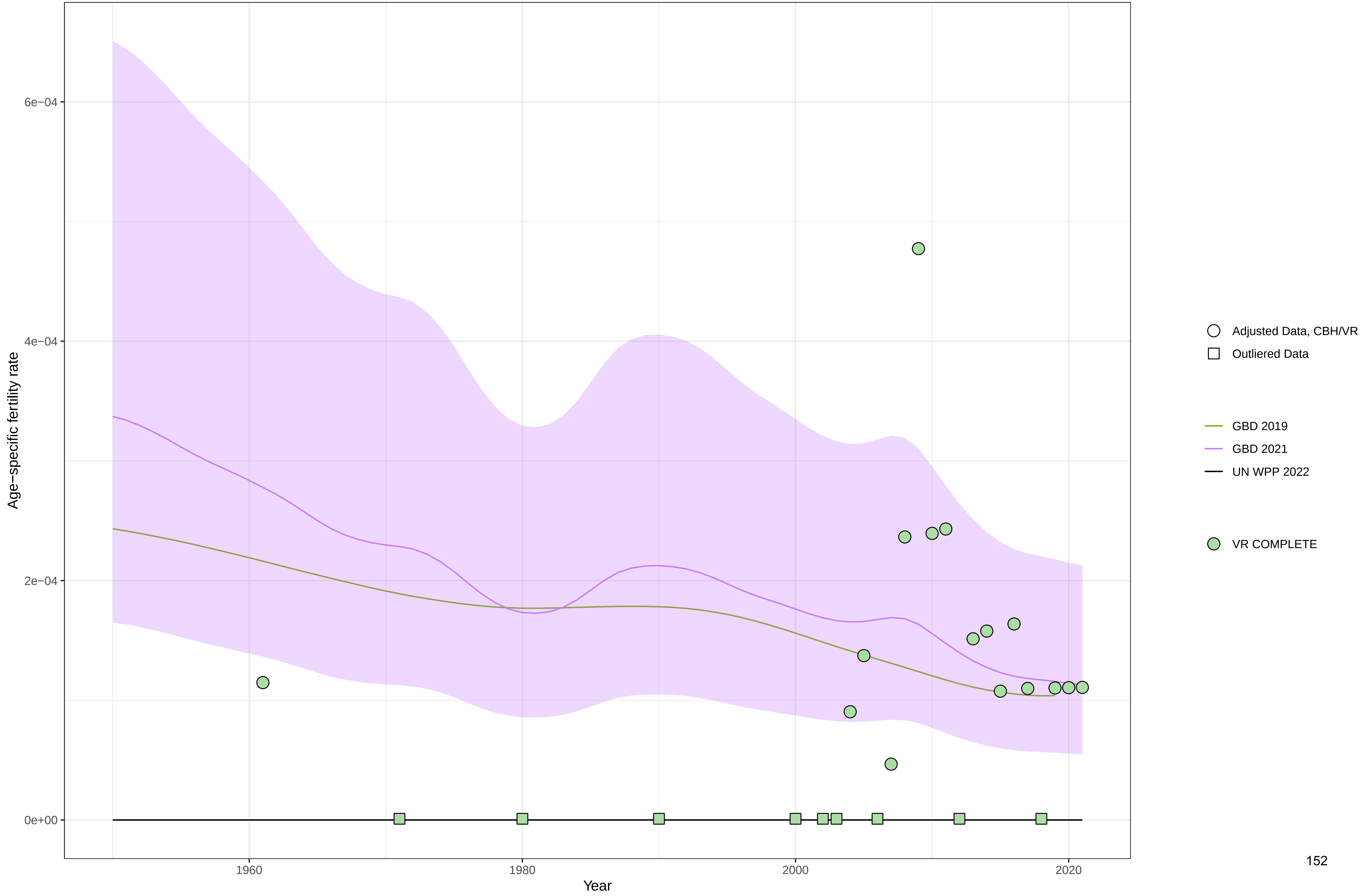

Age−specific fertility fitted trend lines, 1950−2021: Montenegro, Ages: 15−19
Data points enclosed with a diamond are reference sources

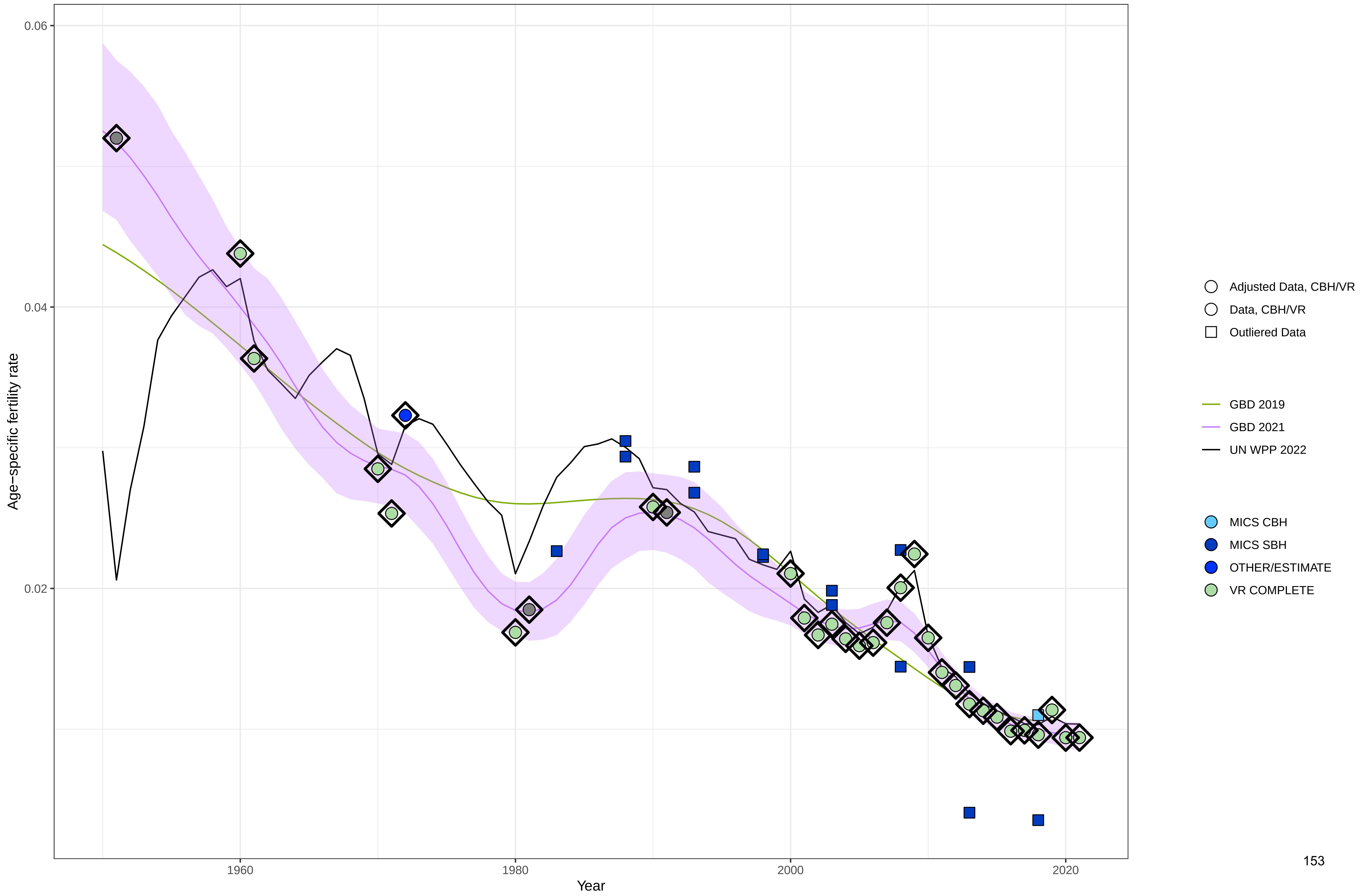

Age−specific fertility fitted trend lines, 1950−2021: Montenegro, Ages: 20−24
Data points enclosed with a diamond are reference sources

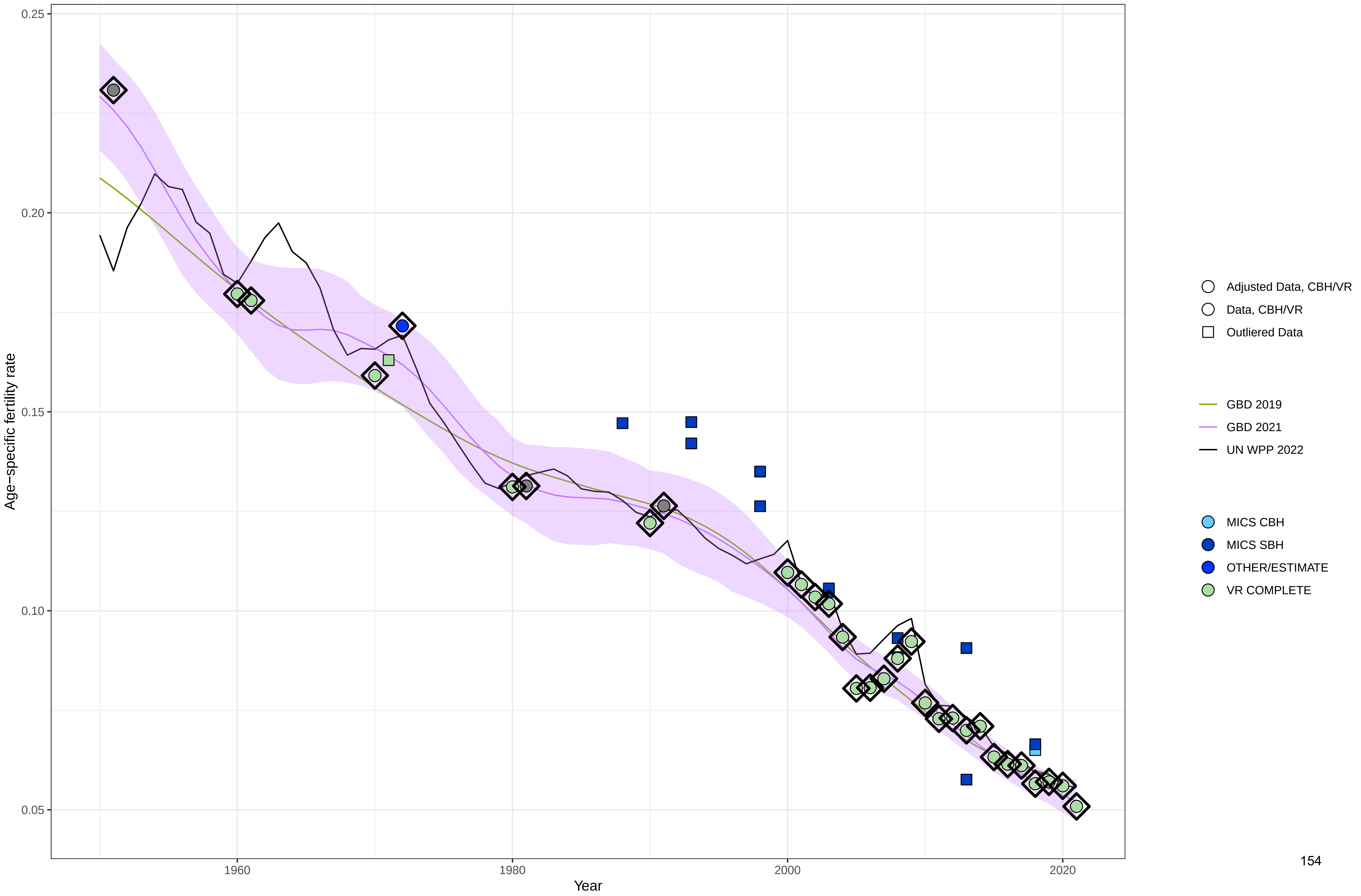

Age−specific fertility fitted trend lines, 1950−2021: Montenegro, Ages: 25−29
Data points enclosed with a diamond are reference sources

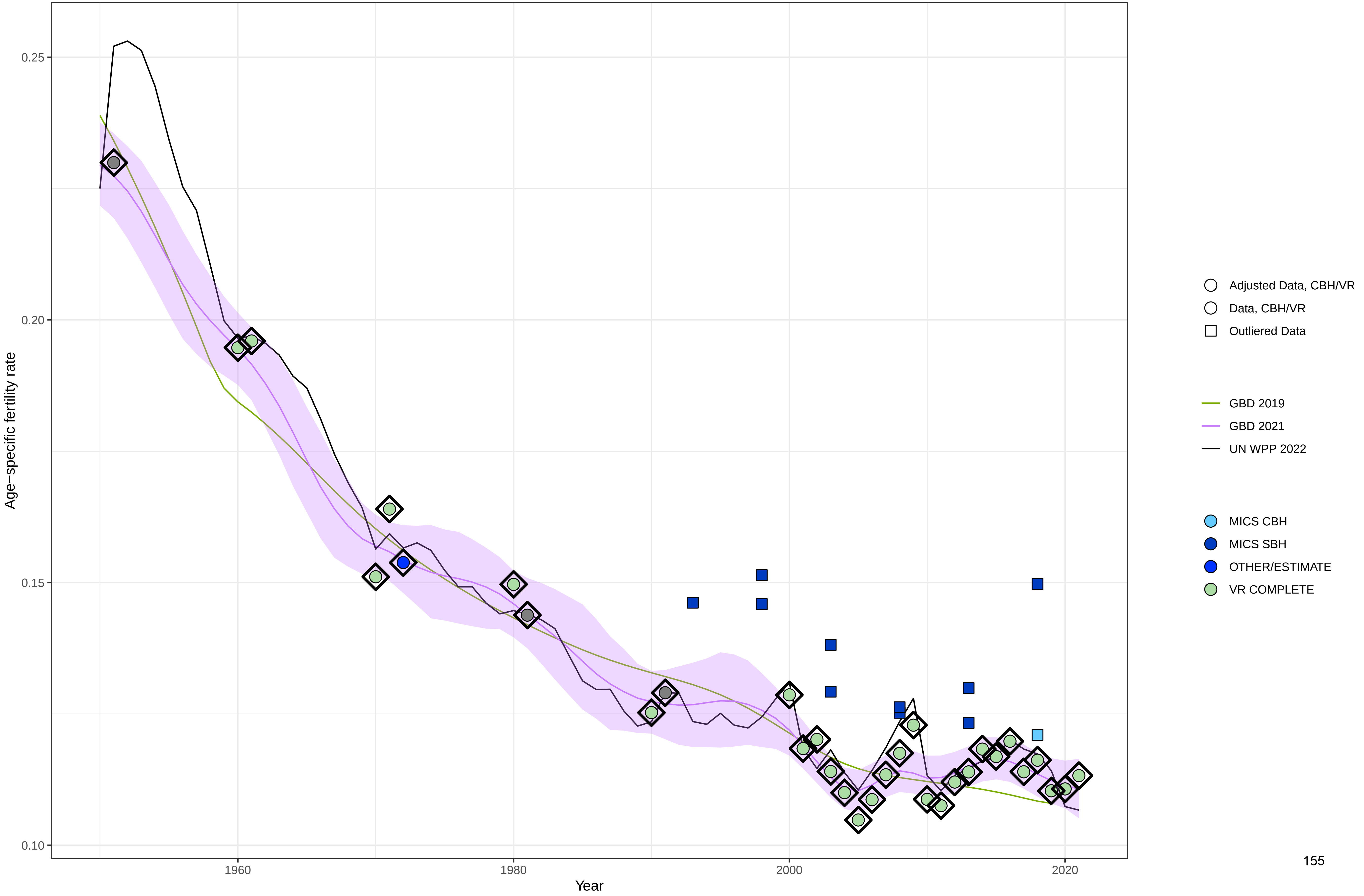

Age−specific fertility fitted trend lines, 1950−2021: Montenegro, Ages: 30−34
Data points enclosed with a diamond are reference sources

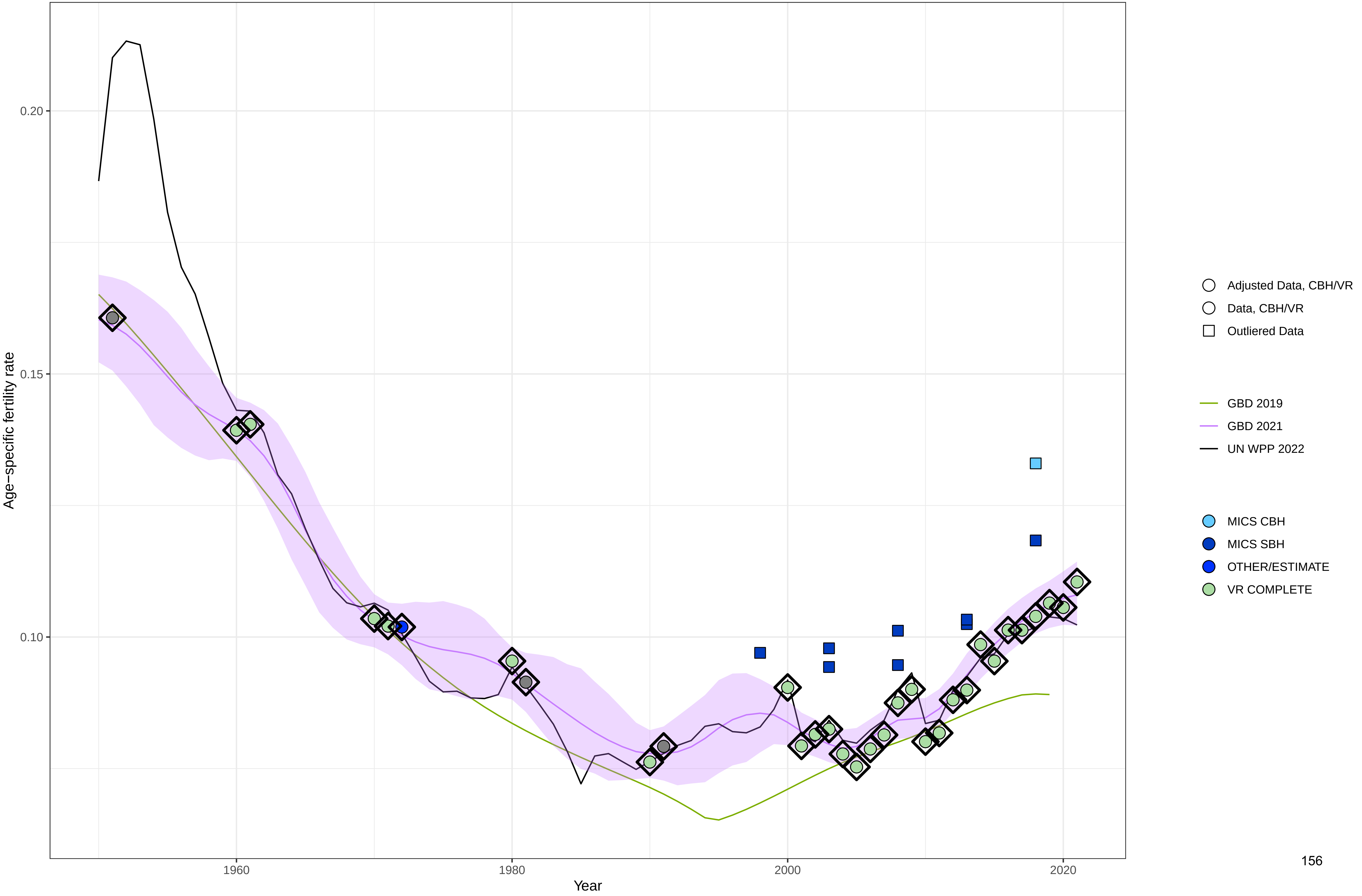

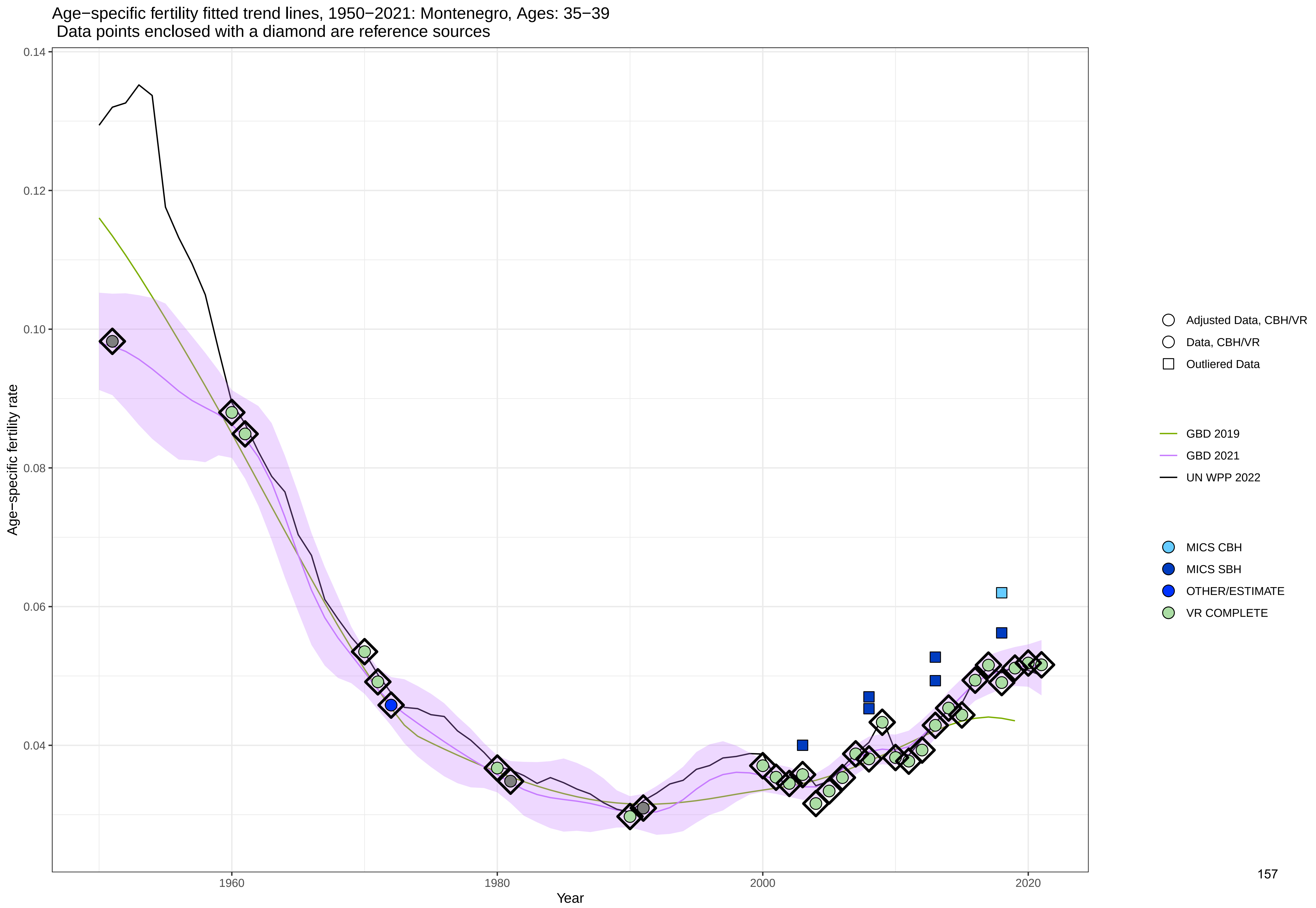
Age−specific fertility fitted trend lines, 1950−2021: Montenegro, Ages: 35−39
Data points enclosed with a diamond are reference sources
Age−specific fertility rate
Year
0.14
0.12
0.10
0.08
0.06
0.04
1960
1980
2000
2020
Adjusted Data, CBH/VR
Data, CBH/VR
Outliered Data
GBD 2019
GBD 2021
UN WPP 2022
MICS CBH
MICS SBH
OTHER/ESTIMATE
VR COMPLETE

Age−specific fertility fitted trend lines, 1950−2021: Montenegro, Ages: 40−44
Data points enclosed with a diamond are reference sources

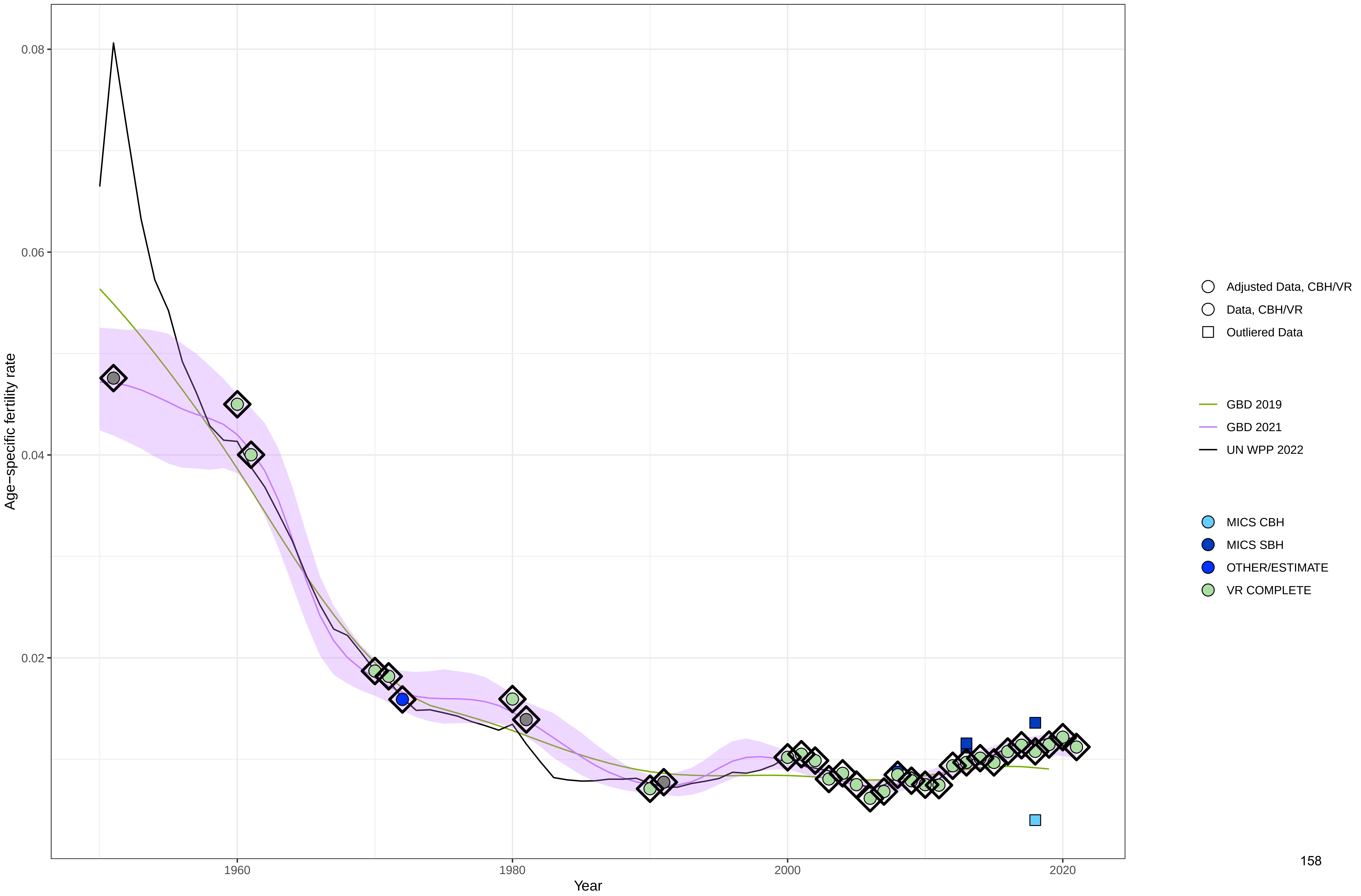

Age−specific fertility fitted trend lines, 1950−2021: Montenegro, Ages: 45−49
Data points enclosed with a diamond are reference sources

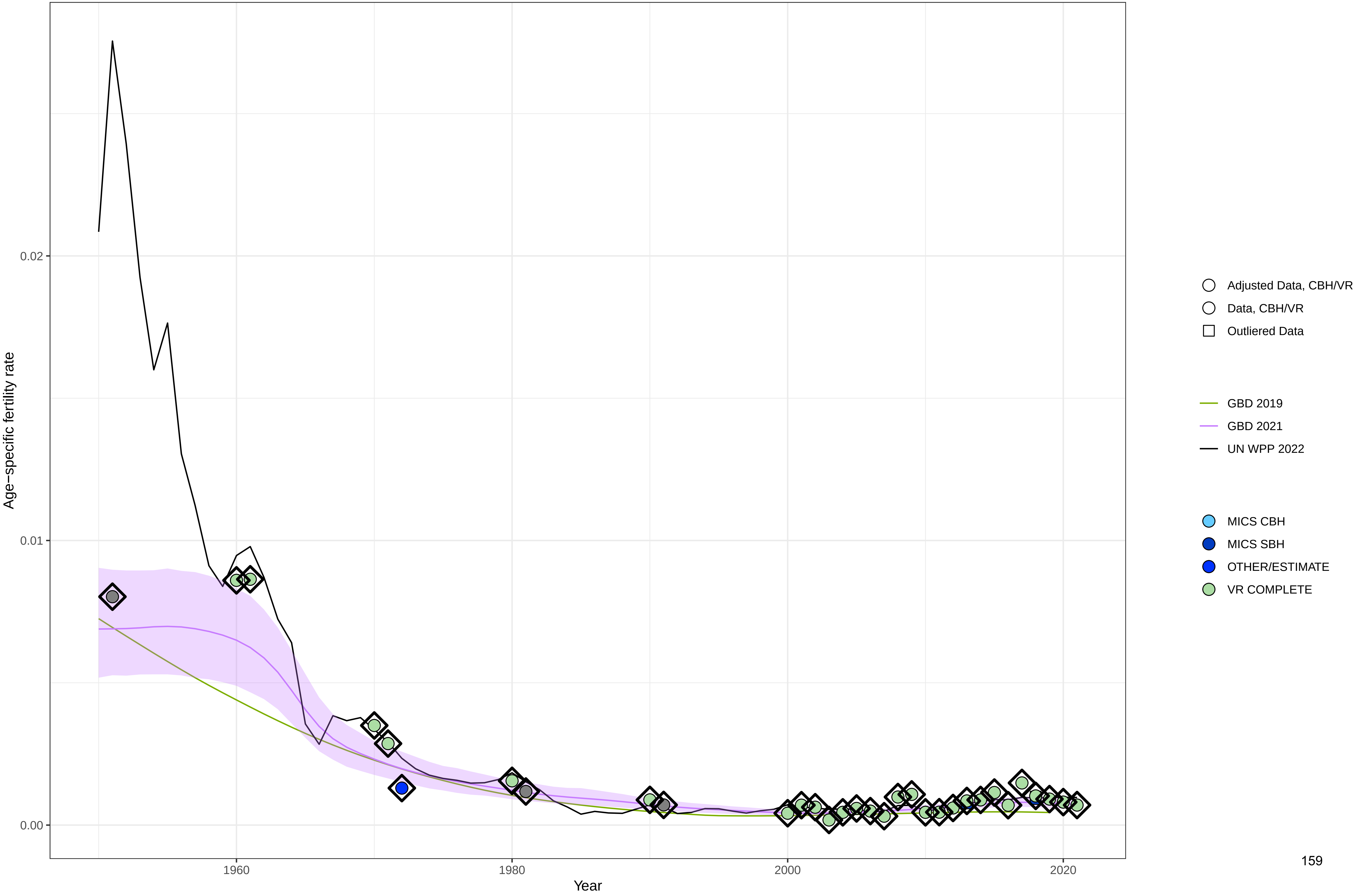

Age−specific fertility fitted trend lines, 1950−2021: Montenegro, Ages: 50−54
Data points enclosed with a diamond are reference sources

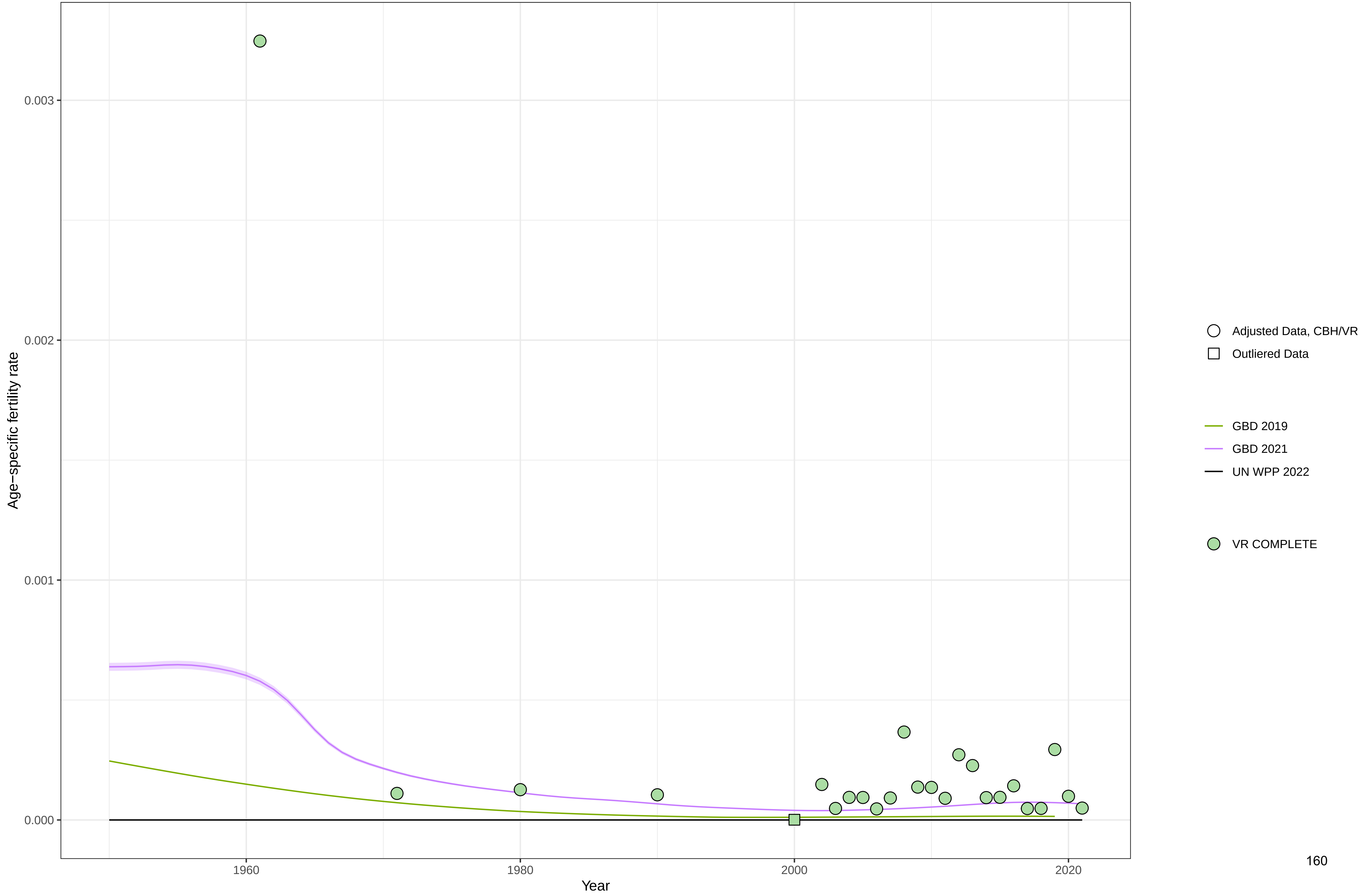

Total fertility rate fitted trend lines, 1950−2021: Montenegro TFR
Data points enclosed with a diamond are reference sources

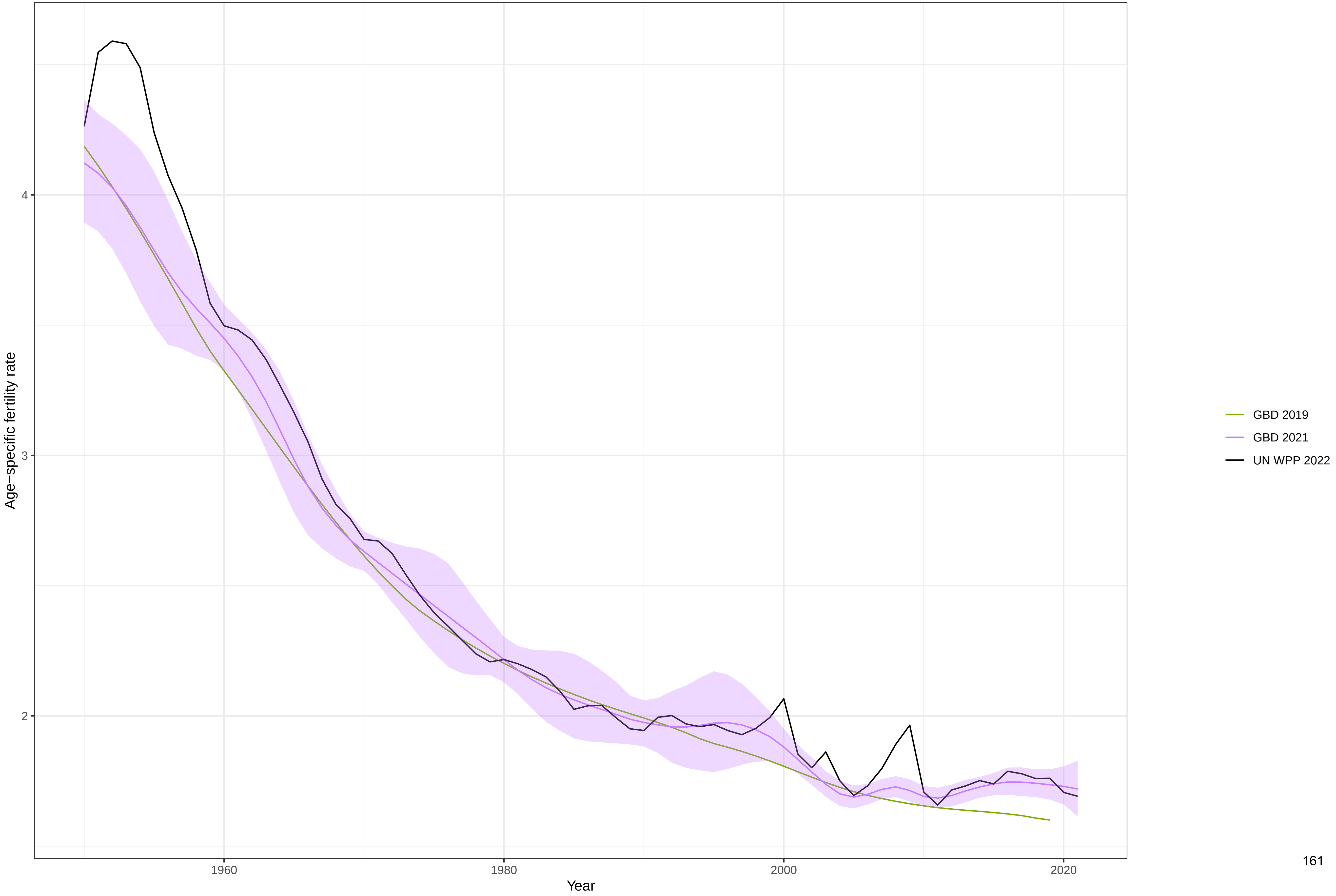

Age−specific fertility fitted trend lines, 1950−2021: North Macedonia, Ages: 10−14
Data points enclosed with a diamond are reference sources

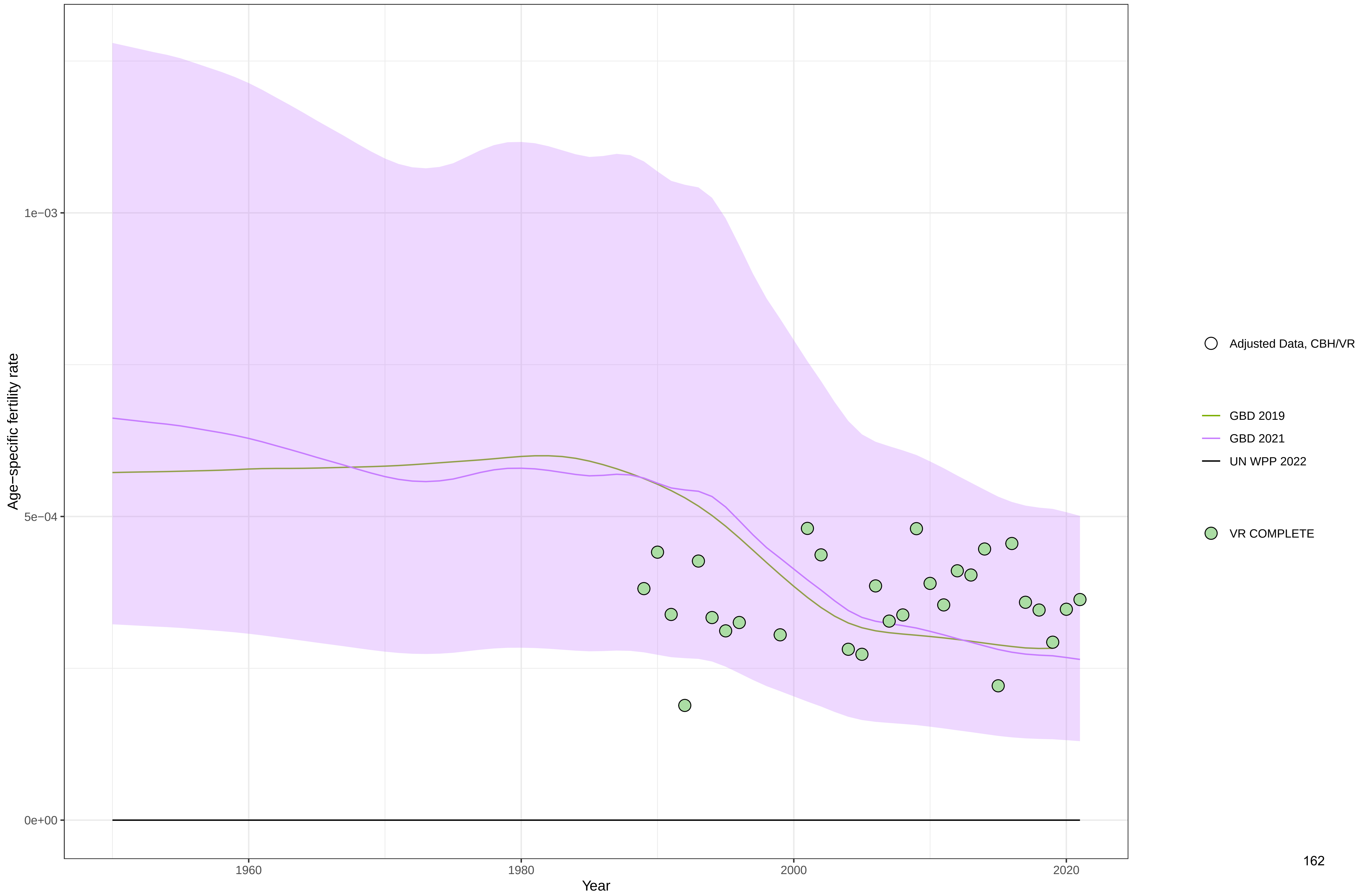

Age−specific fertility fitted trend lines, 1950−2021: North Macedonia, Ages: 15−19
Data points enclosed with a diamond are reference sources

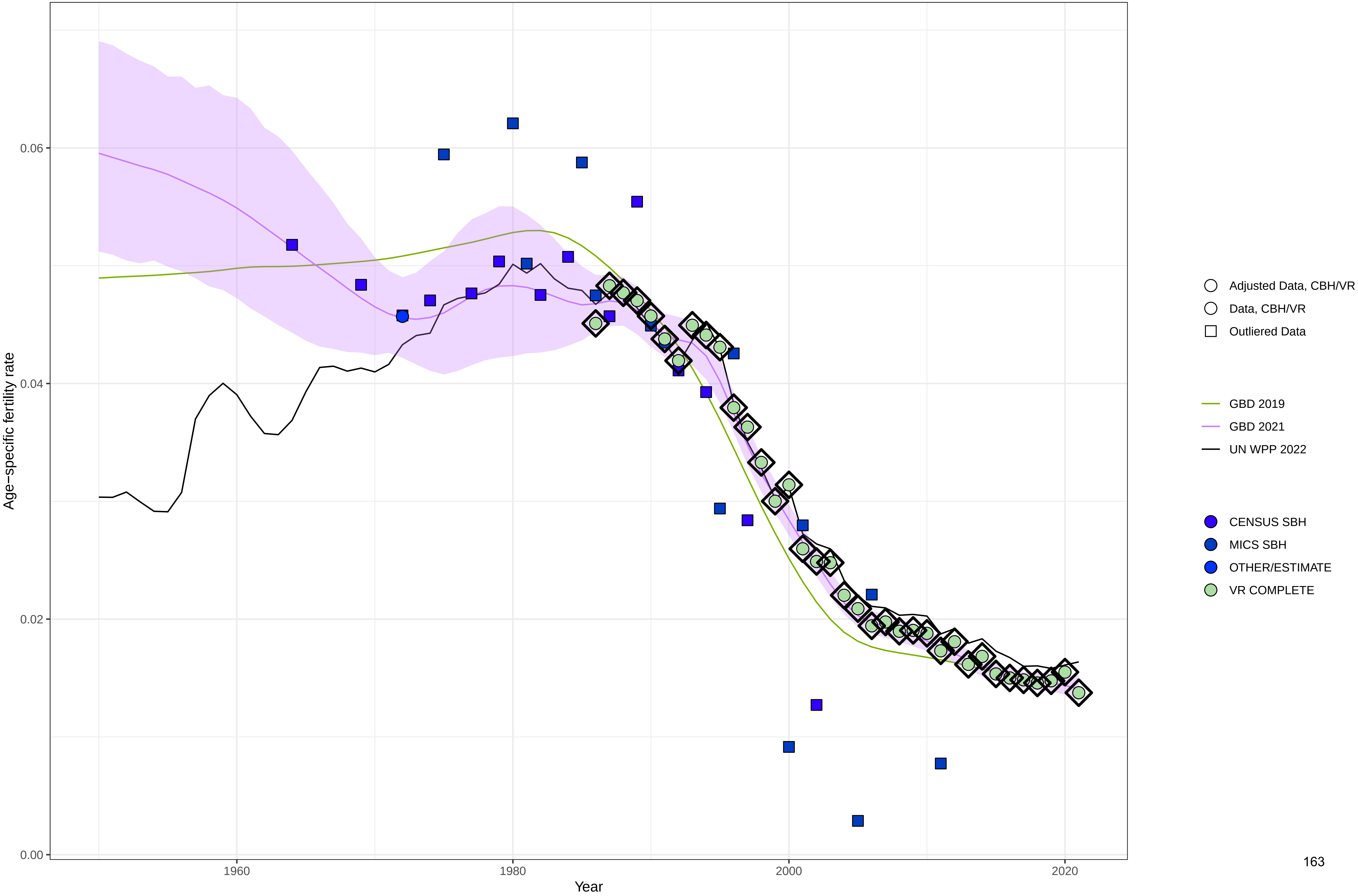

Age−specific fertility fitted trend lines, 1950−2021: North Macedonia, Ages: 20−24
Data points enclosed with a diamond are reference sources

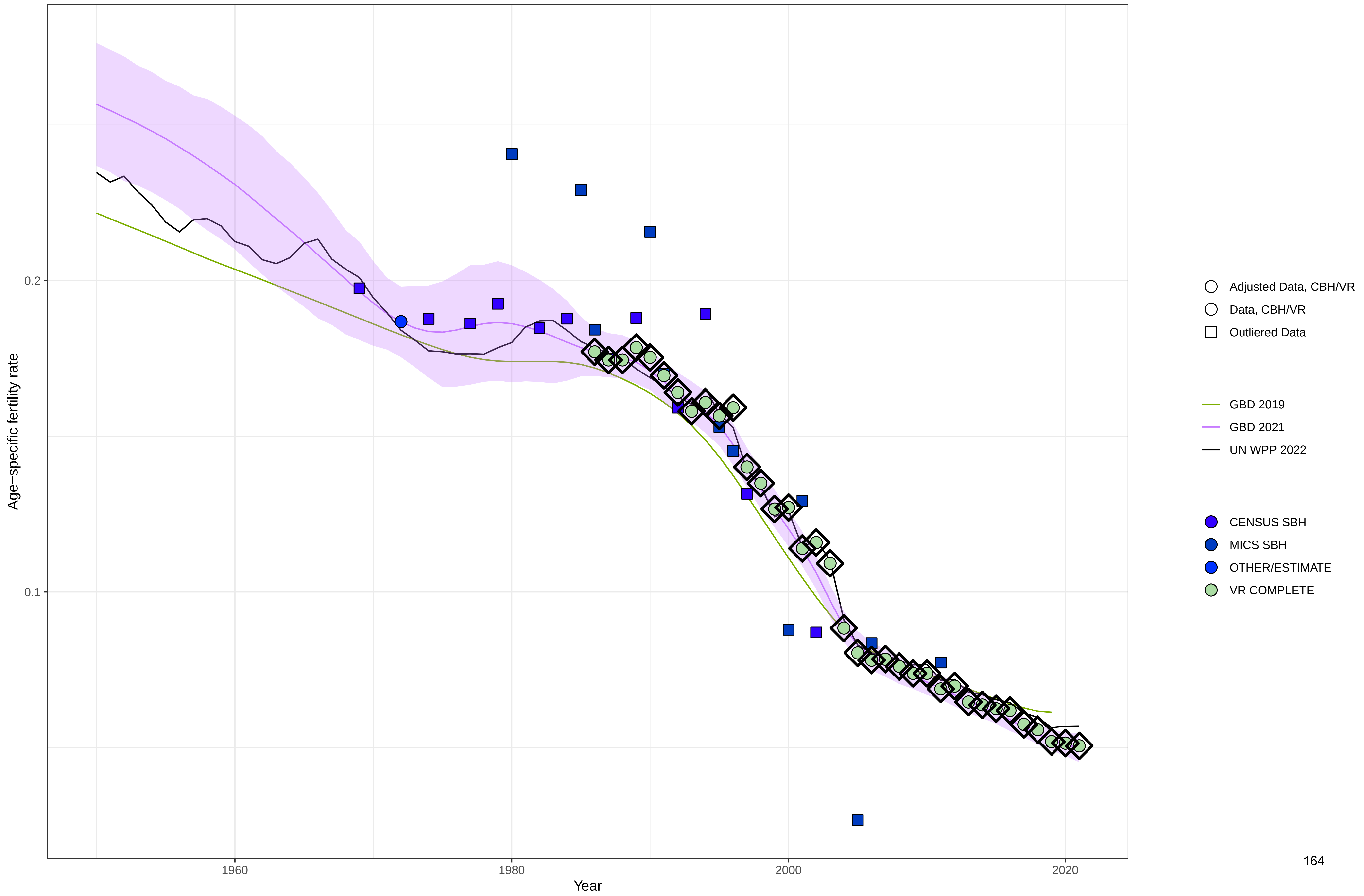

Age−specific fertility fitted trend lines, 1950−2021: North Macedonia, Ages: 25−29
Data points enclosed with a diamond are reference sources

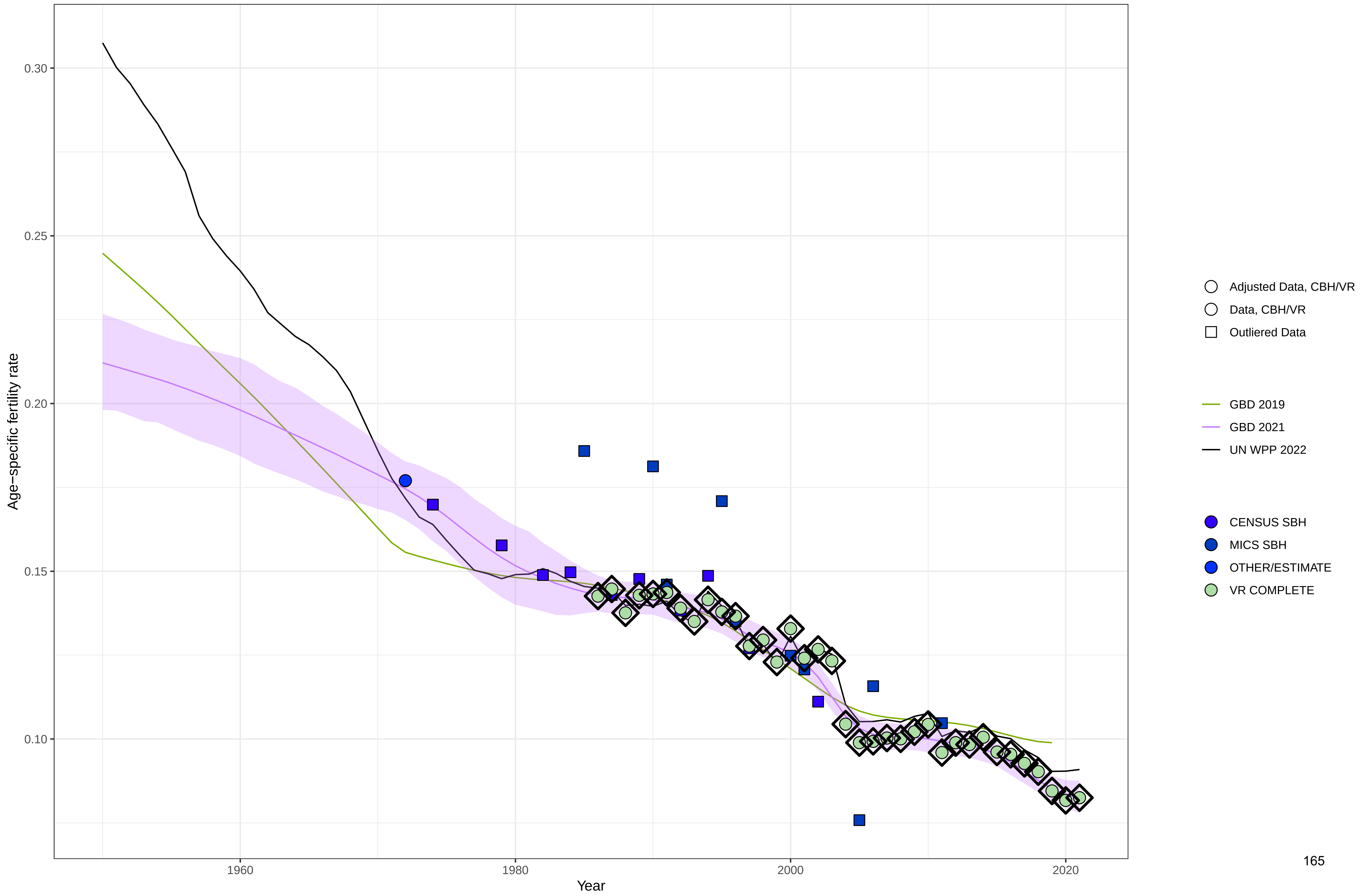

Age−specific fertility fitted trend lines, 1950−2021: North Macedonia, Ages: 30−34
Data points enclosed with a diamond are reference sources

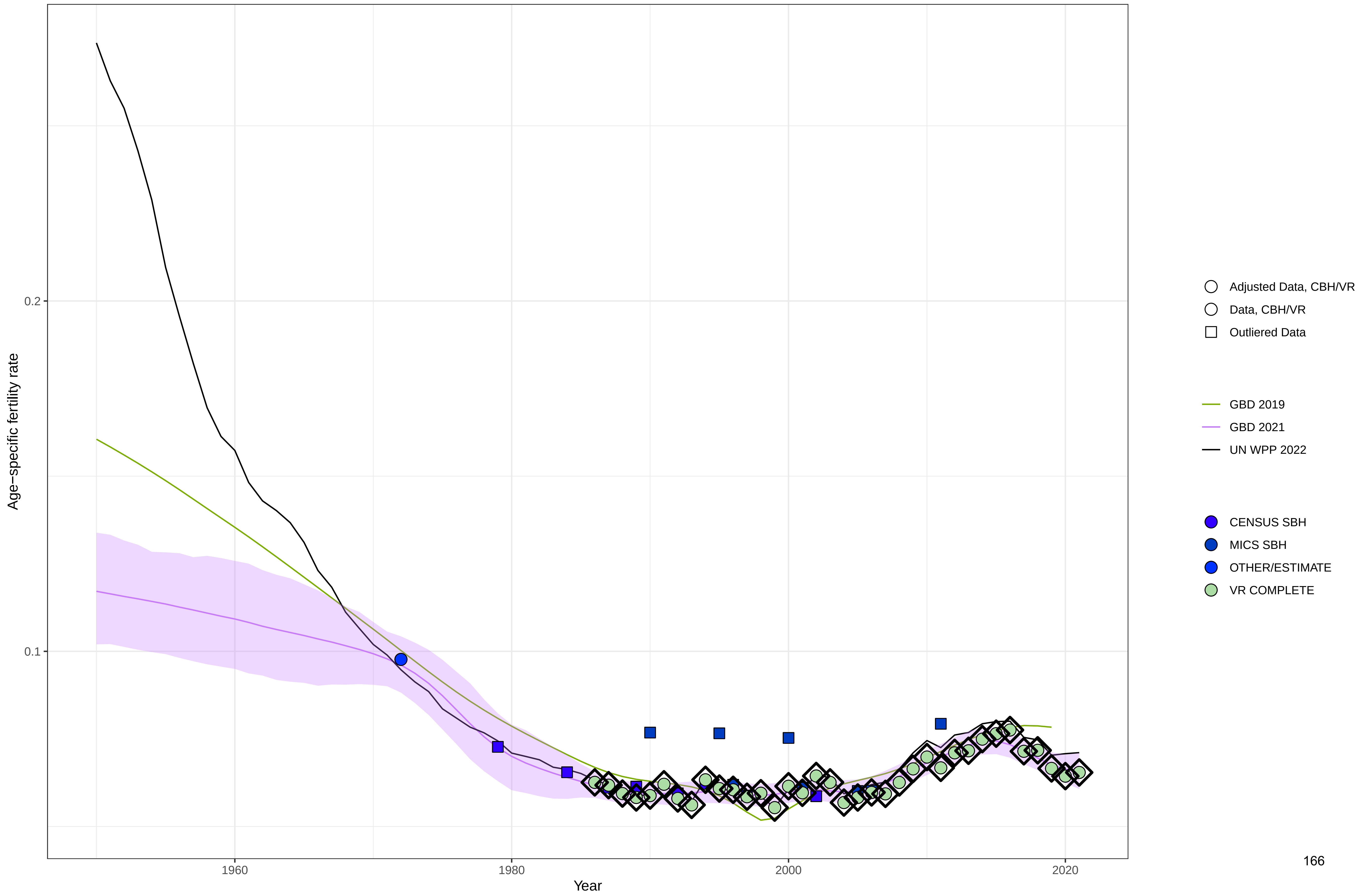

Age−specific fertility fitted trend lines, 1950−2021: North Macedonia, Ages: 35−39
Data points enclosed with a diamond are reference sources

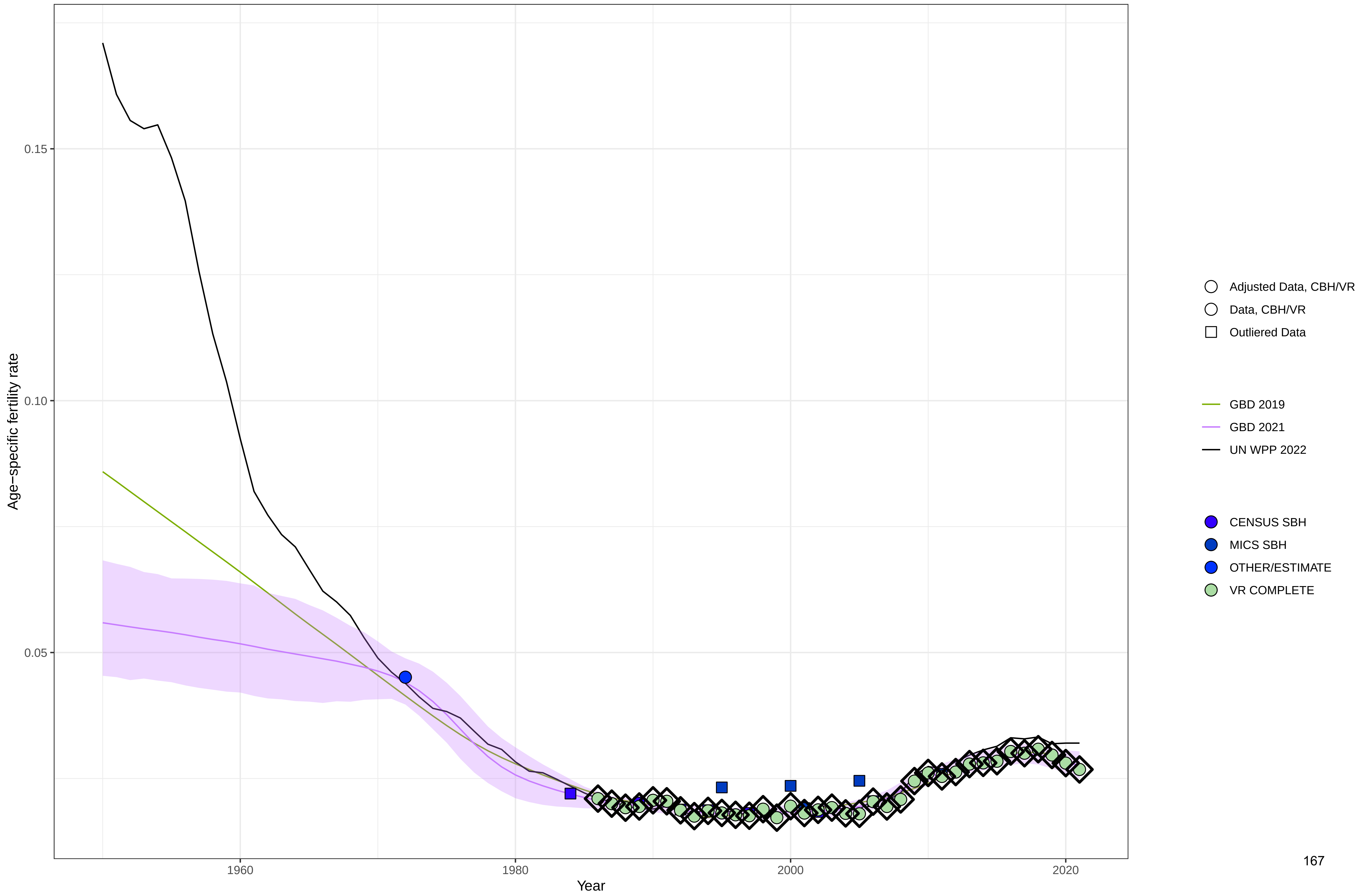

Age−specific fertility fitted trend lines, 1950−2021: North Macedonia, Ages: 40−44
Data points enclosed with a diamond are reference sources

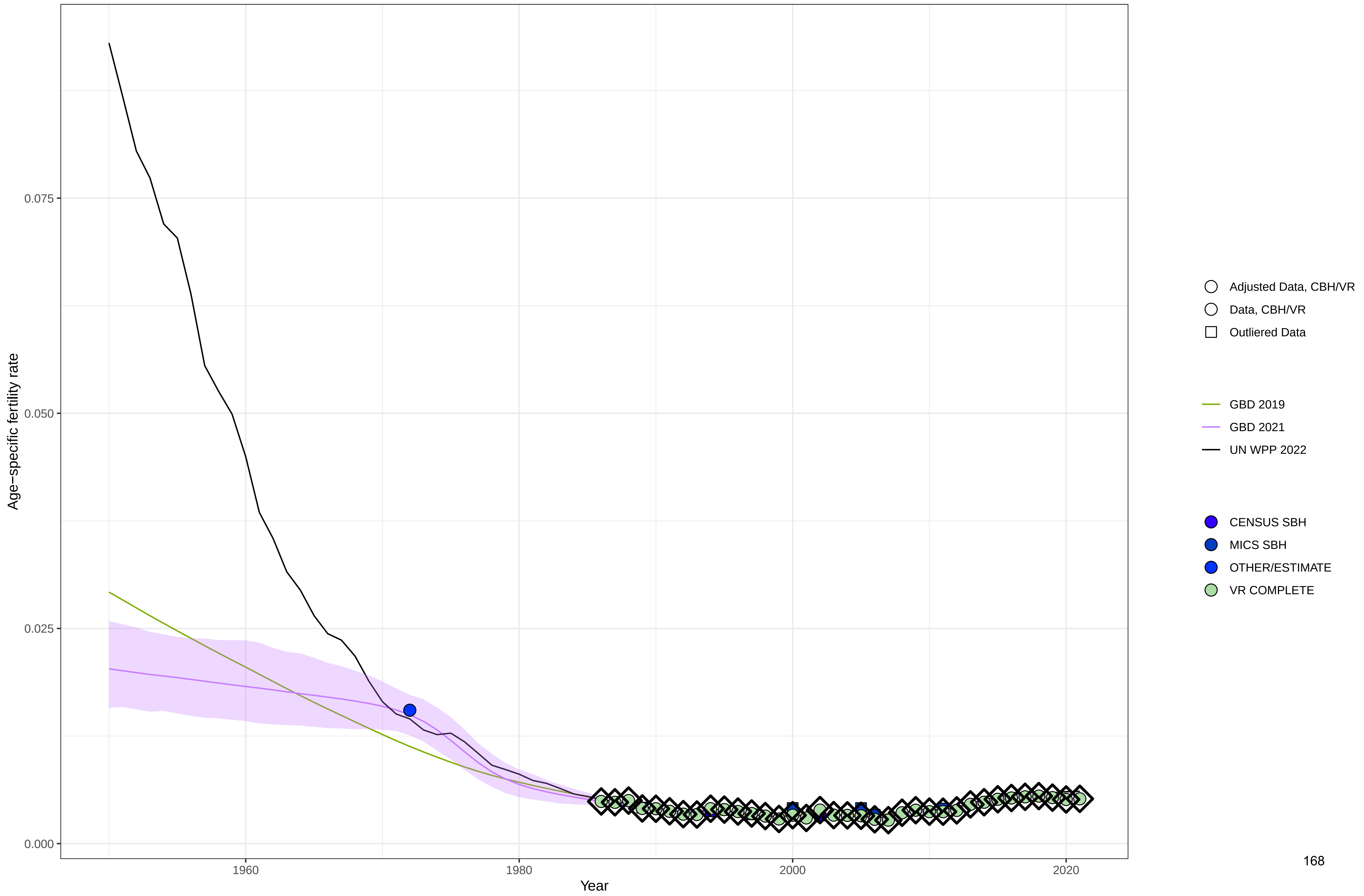

Age−specific fertility fitted trend lines, 1950−2021: North Macedonia, Ages: 45−49
Data points enclosed with a diamond are reference sources

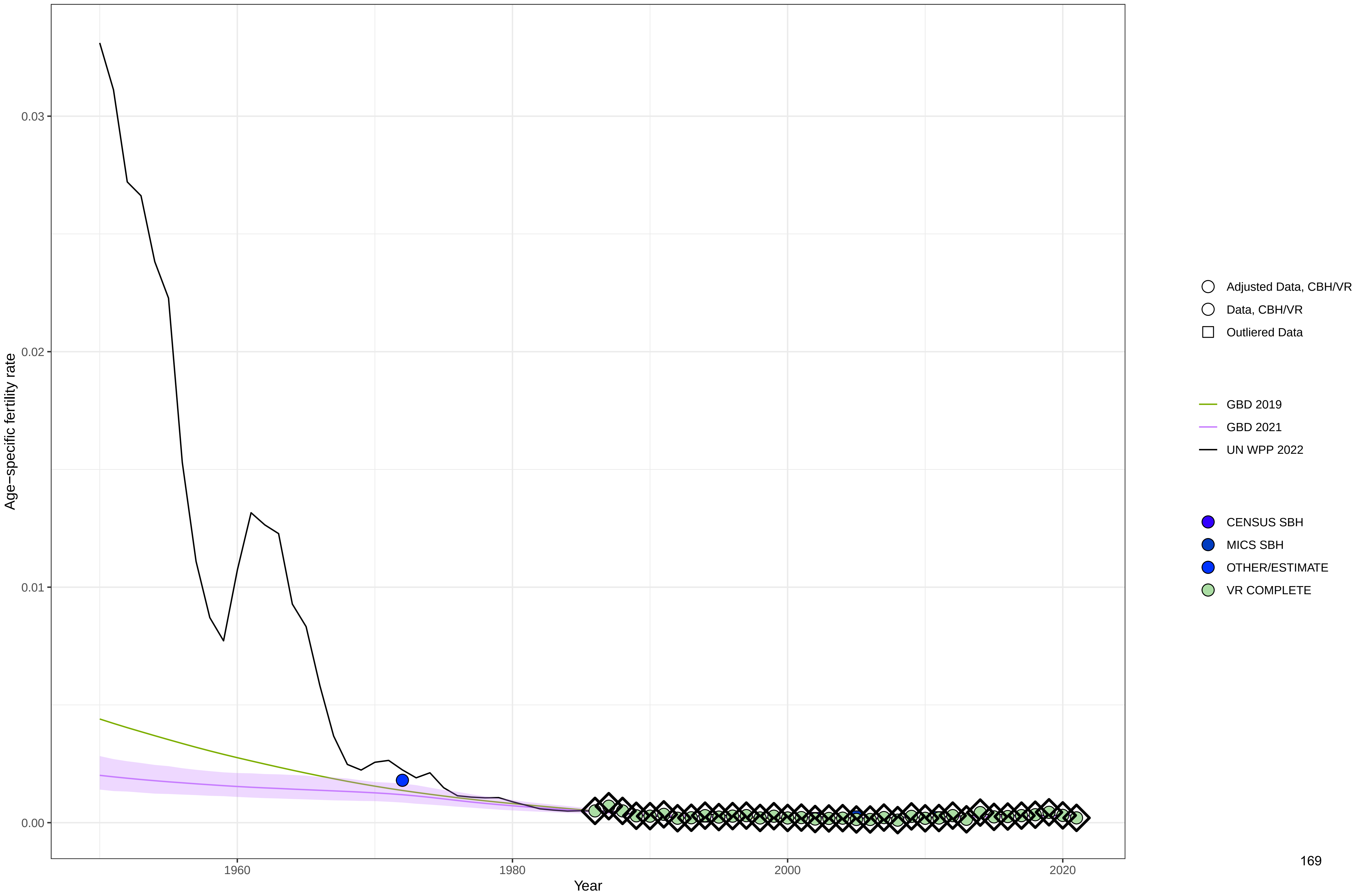

Age−specific fertility fitted trend lines, 1950−2021: North Macedonia, Ages: 50−54
Data points enclosed with a diamond are reference sources

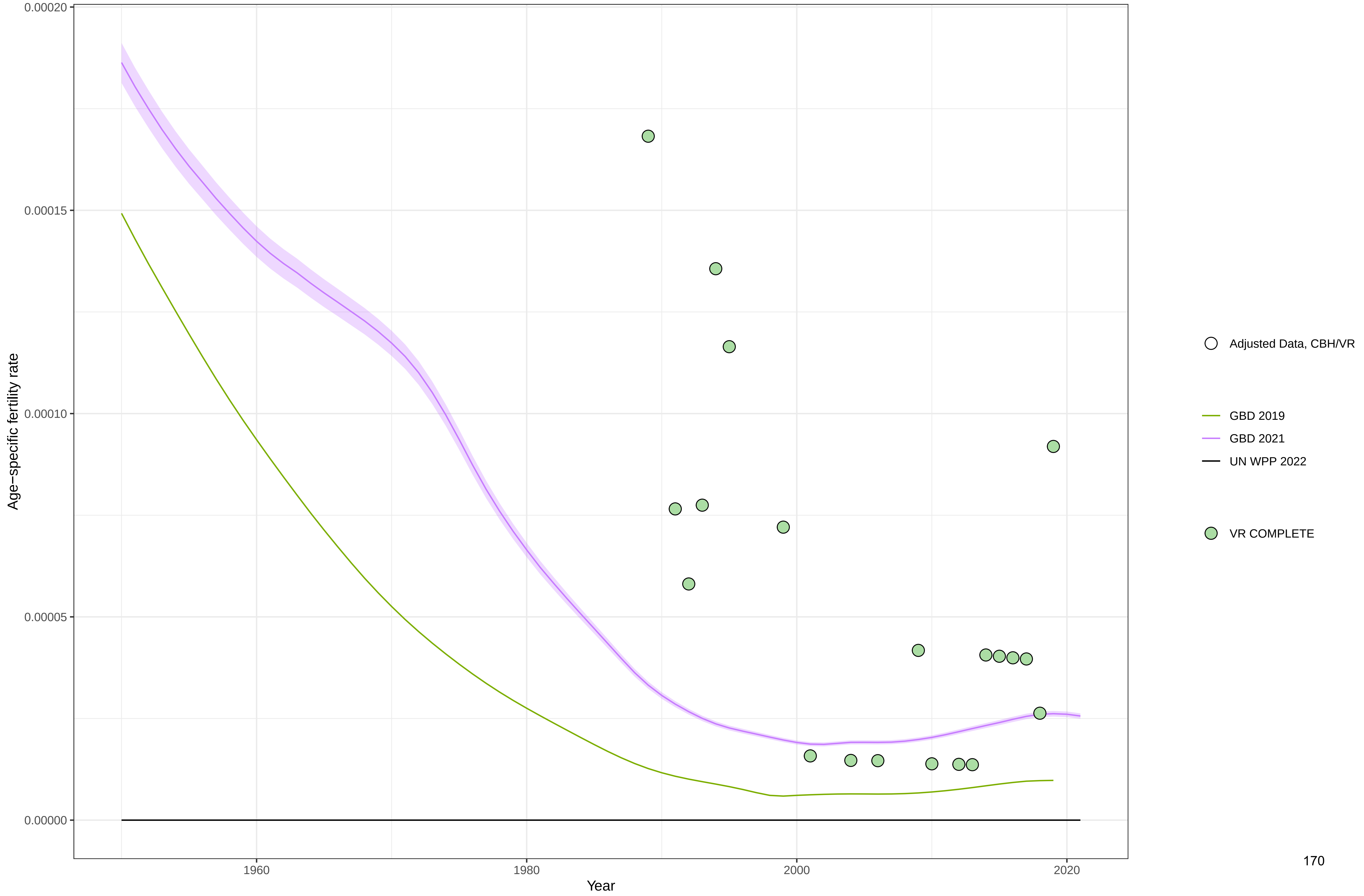

Total fertility rate fitted trend lines, 1950−2021: North Macedonia TFR
Data points enclosed with a diamond are reference sources

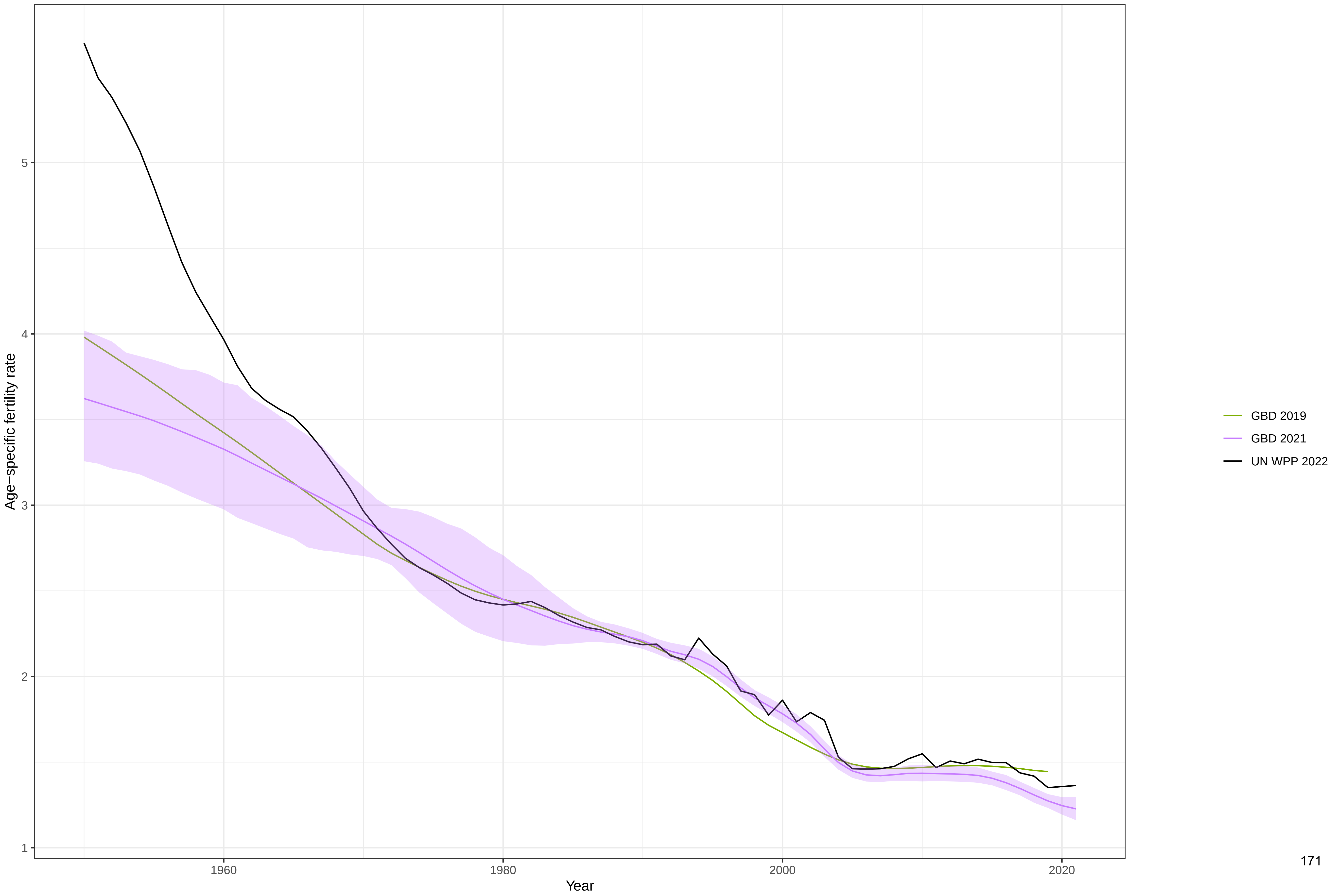

Age−specific fertility fitted trend lines, 1950−2021: Poland, Ages: 10−14
 Data points enclosed with a diamond are reference sources

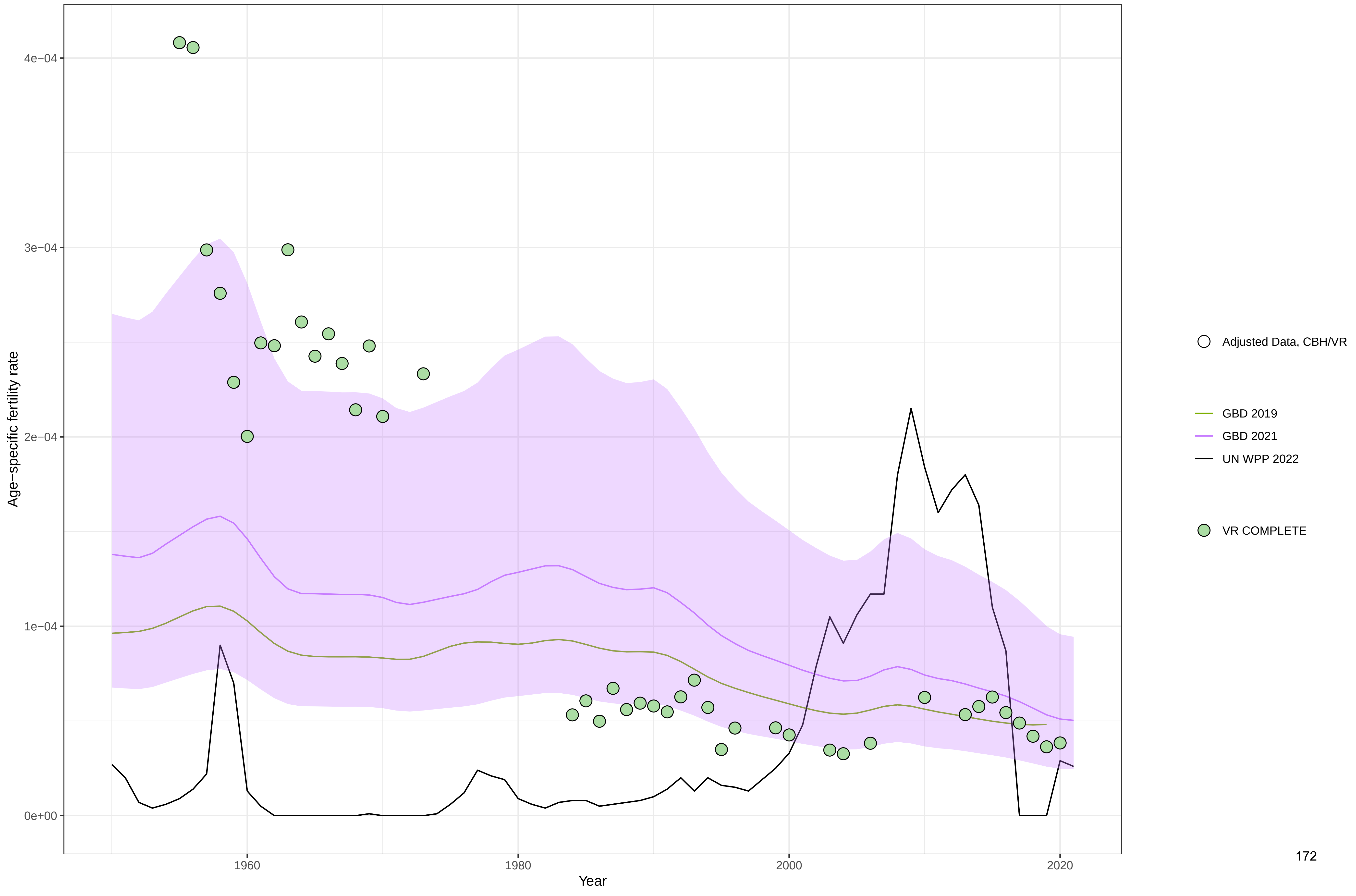

Age−specific fertility fitted trend lines, 1950−2021: Poland, Ages: 15−19
Data points enclosed with a diamond are reference sources

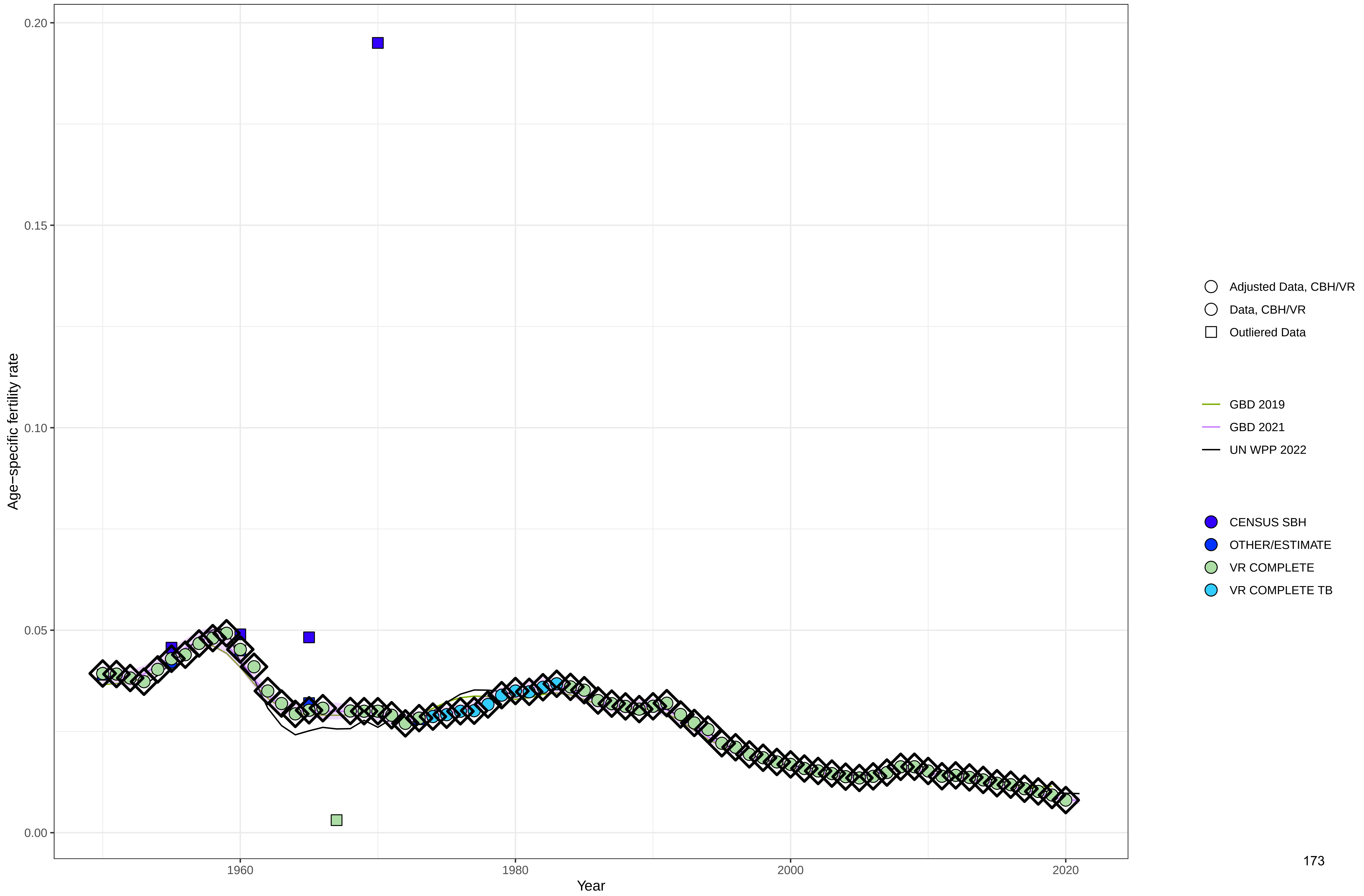

Age−specific fertility fitted trend lines, 1950−2021: Poland, Ages: 20−24
Data points enclosed with a diamond are reference sources

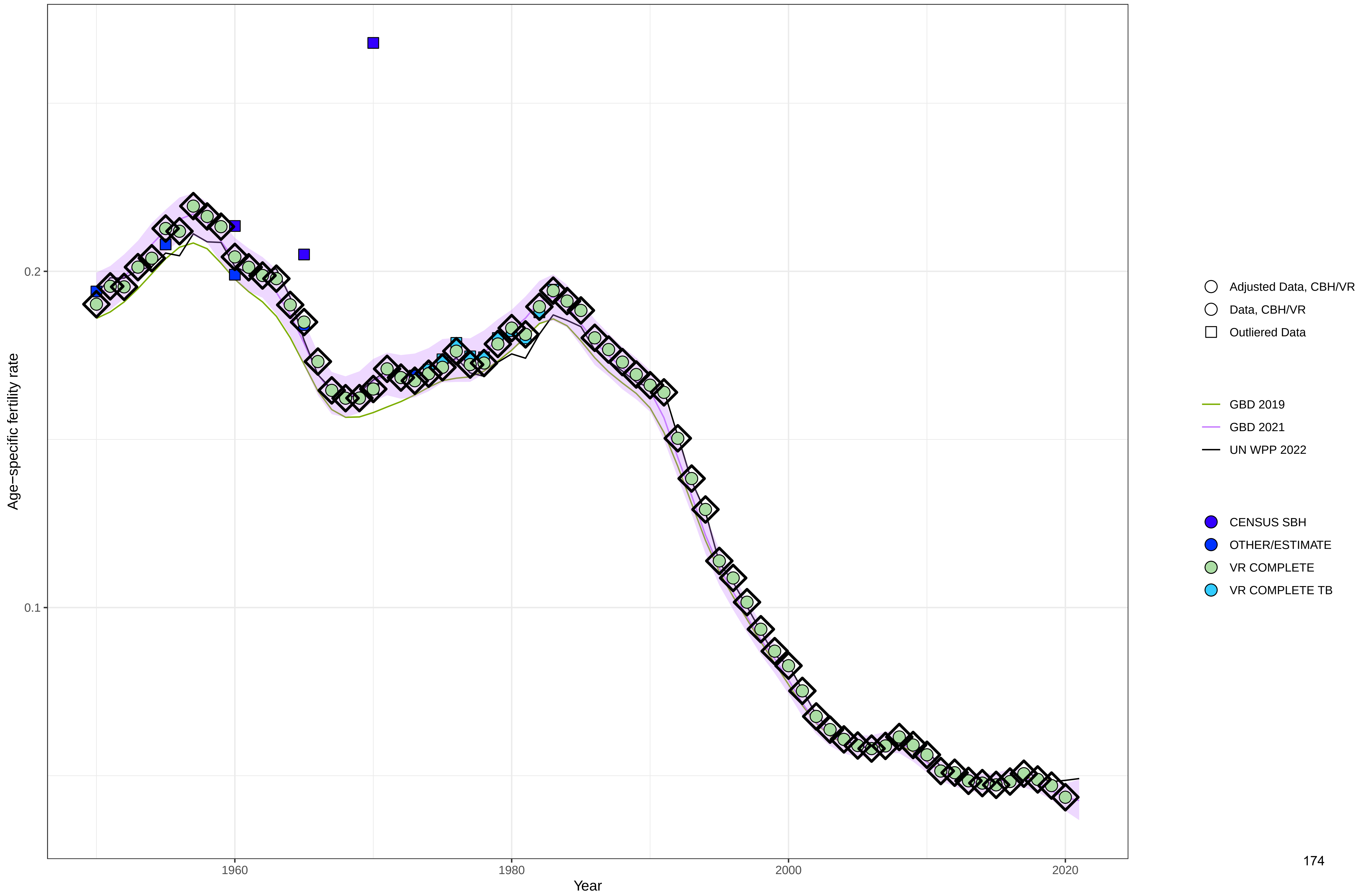

Age−specific fertility fitted trend lines, 1950−2021: Poland, Ages: 25−29
Data points enclosed with a diamond are reference sources

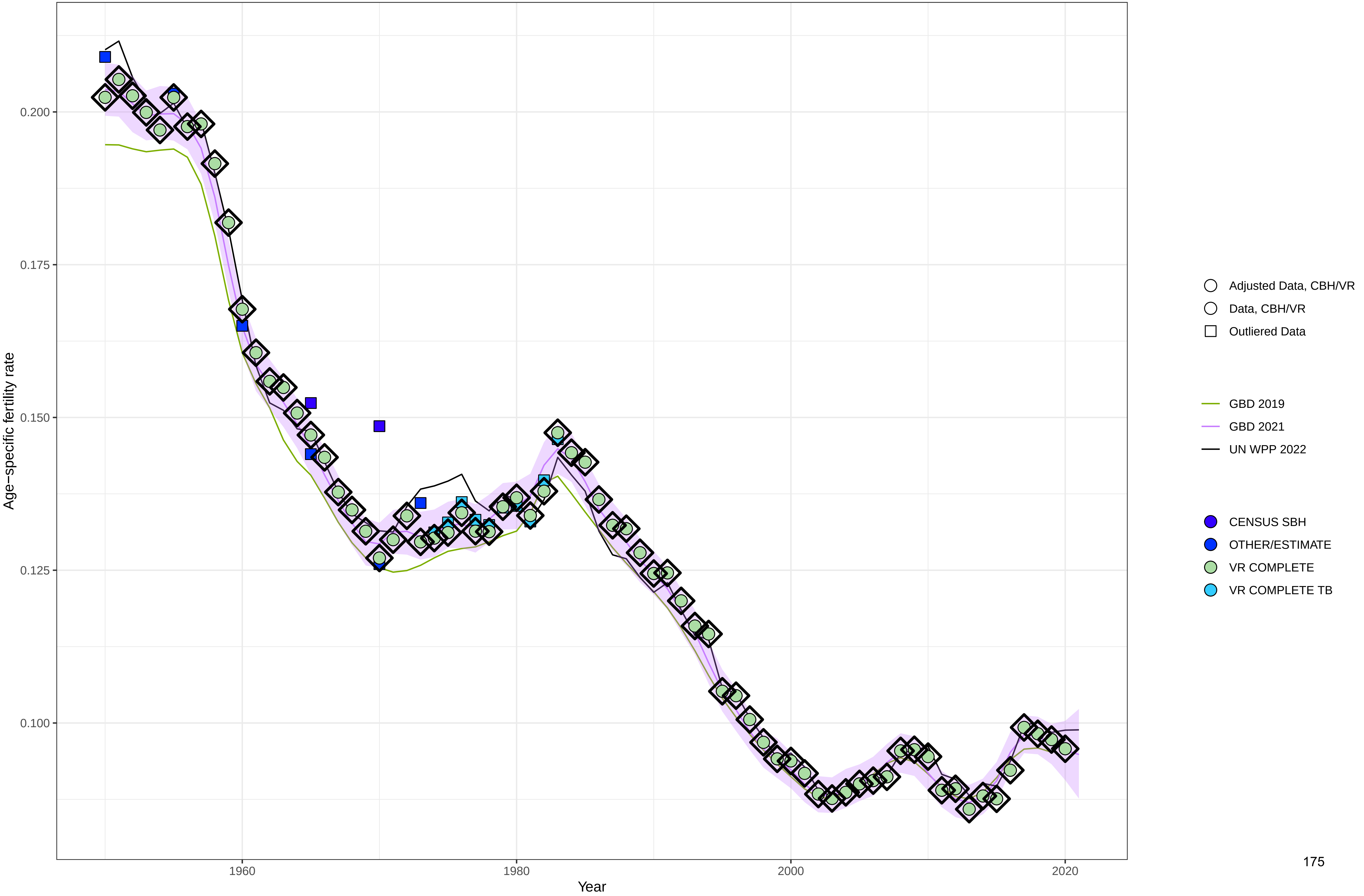

Age−specific fertility fitted trend lines, 1950−2021: Poland, Ages: 30−34
Data points enclosed with a diamond are reference sources

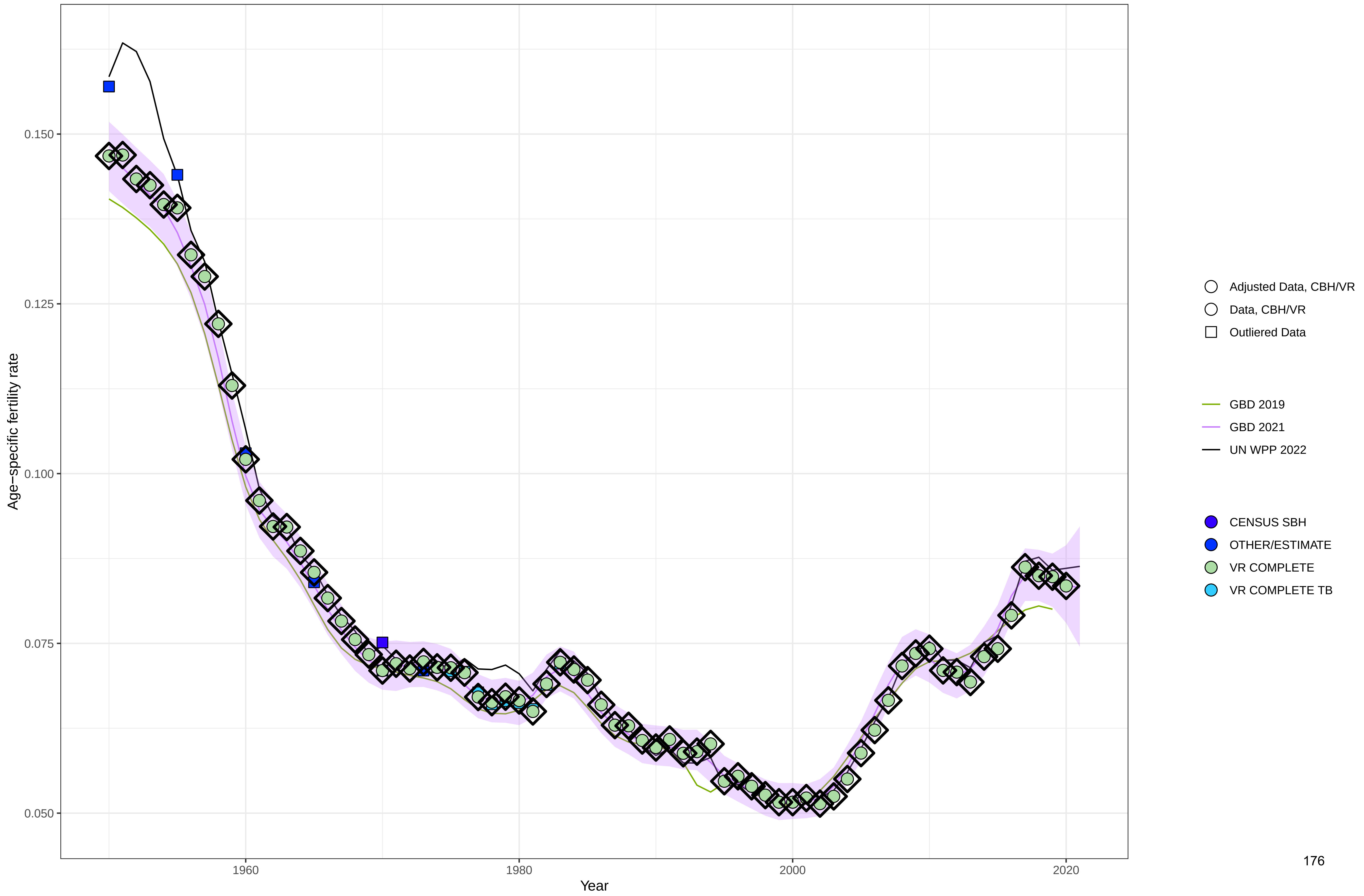

Age−specific fertility fitted trend lines, 1950−2021: Poland, Ages: 35−39
Data points enclosed with a diamond are reference sources

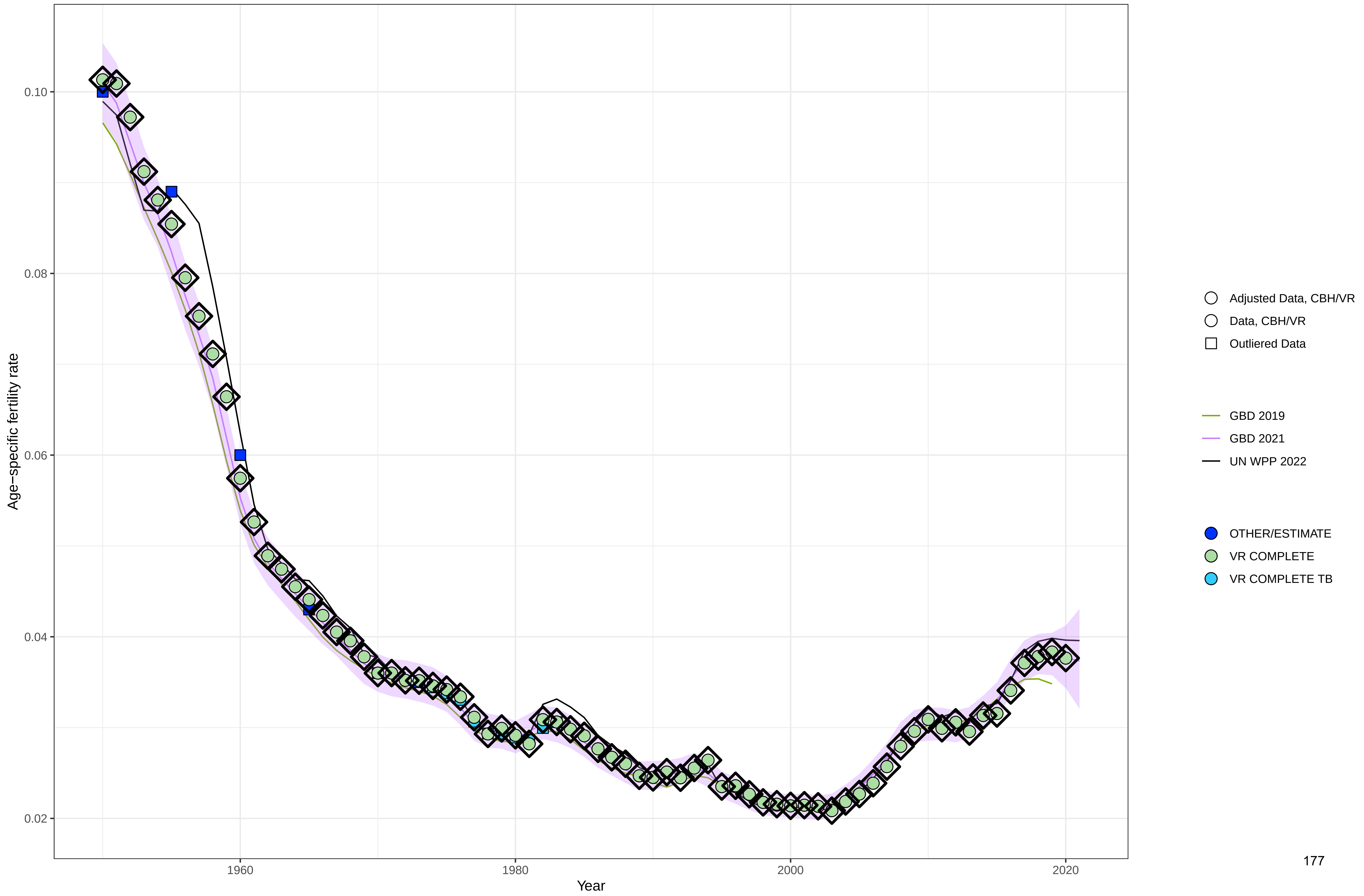

Age−specific fertility fitted trend lines, 1950−2021: Poland, Ages: 40−44
Data points enclosed with a diamond are reference sources

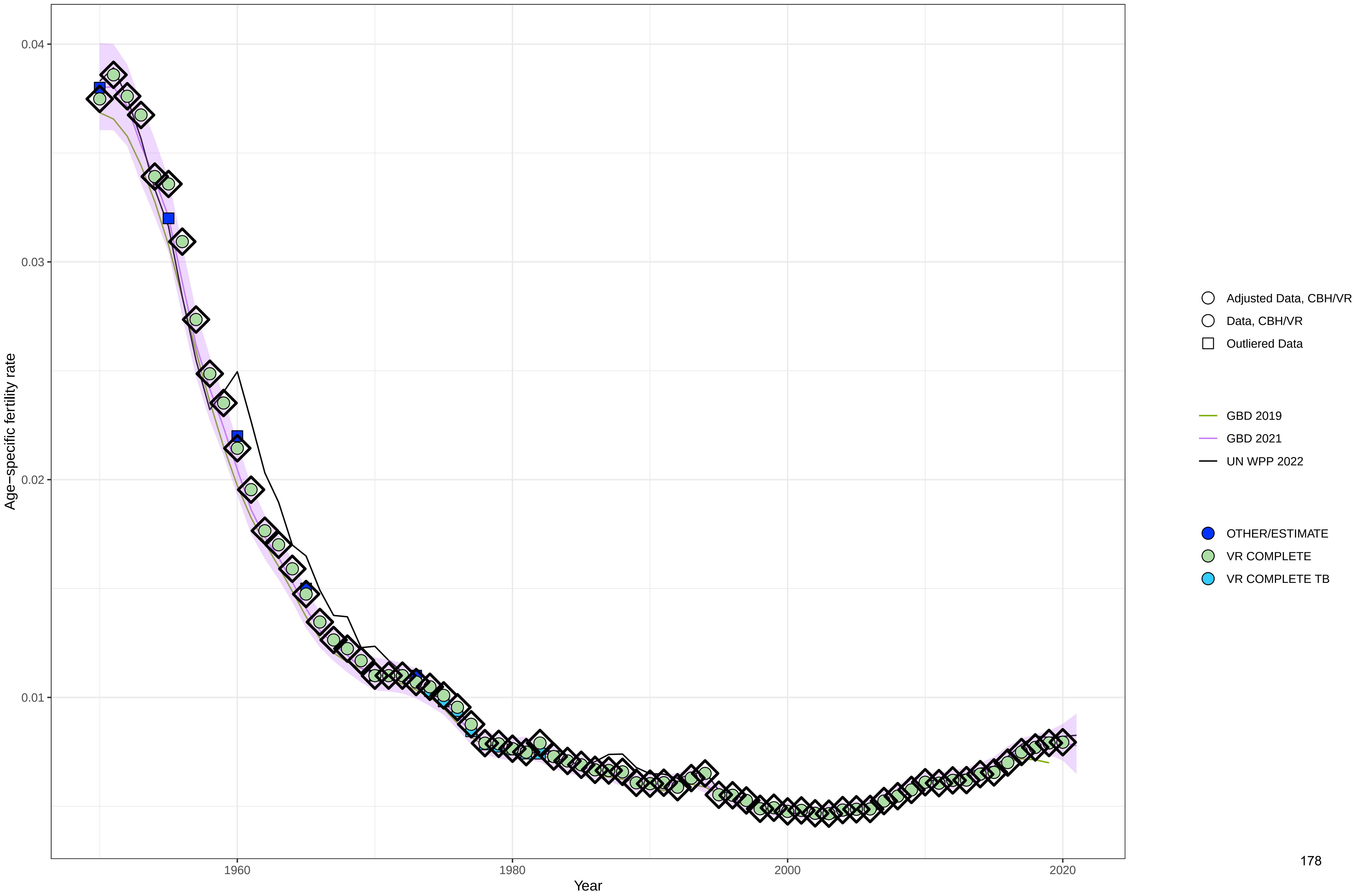

Age−specific fertility fitted trend lines, 1950−2021: Poland, Ages: 45−49
Data points enclosed with a diamond are reference sources

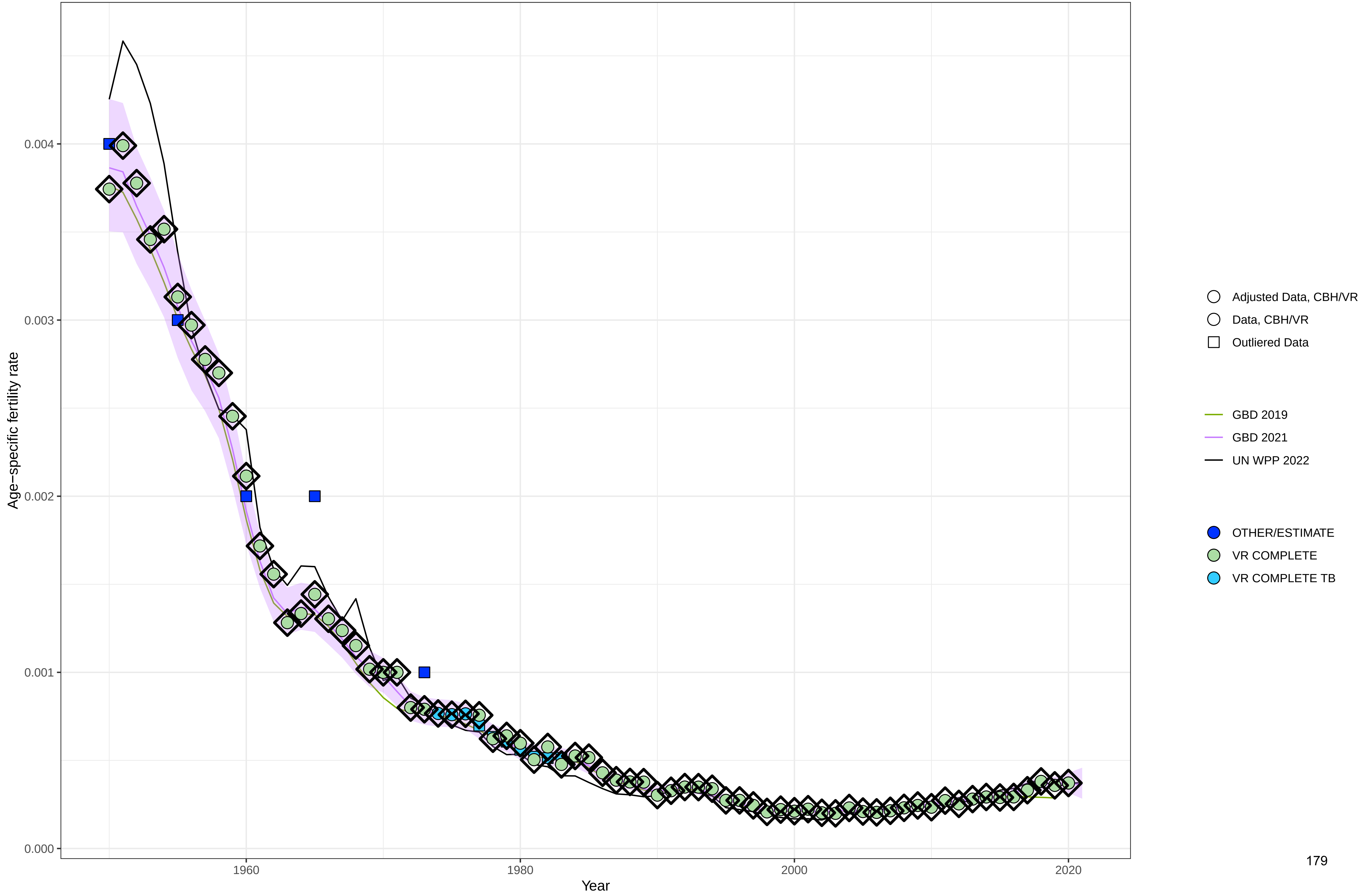

Age−specific fertility fitted trend lines, 1950−2021: Poland, Ages: 50−54
Data points enclosed with a diamond are reference sources

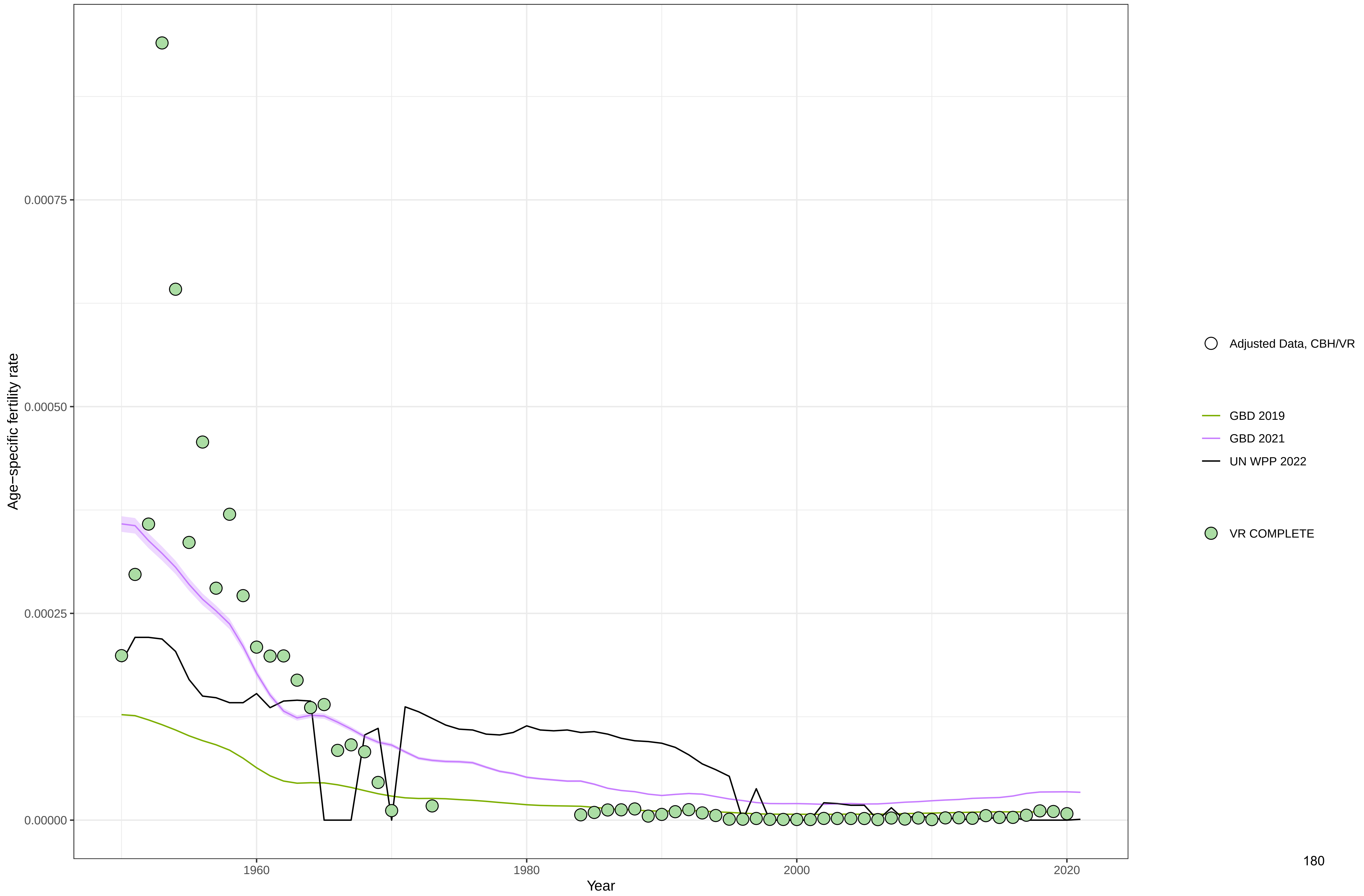

Total fertility rate fitted trend lines, 1950−2021: Poland TFR
Data points enclosed with a diamond are reference sources

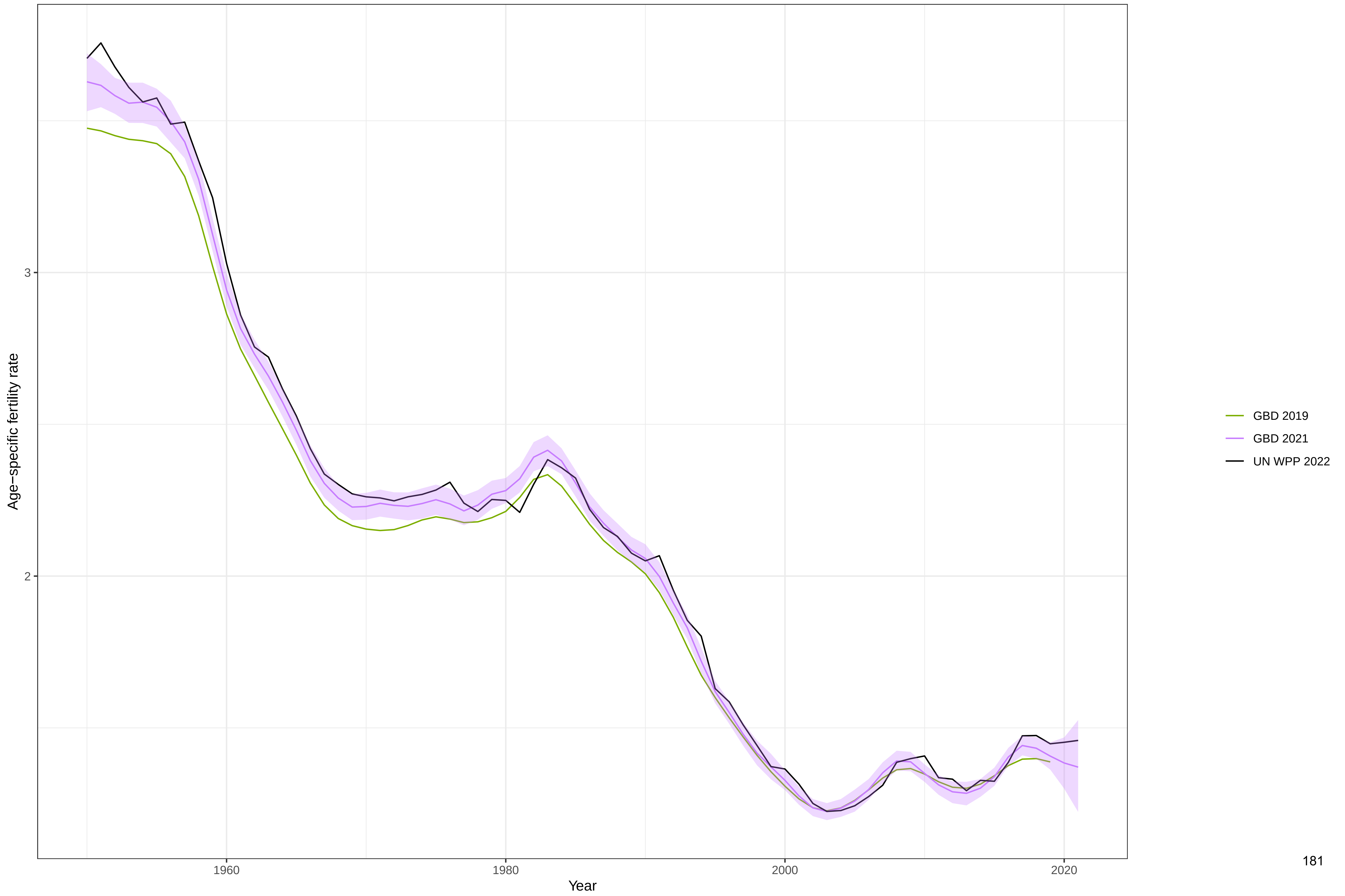

Age−specific fertility fitted trend lines, 1950−2021: Romania, Ages: 10−14
Data points enclosed with a diamond are reference sources

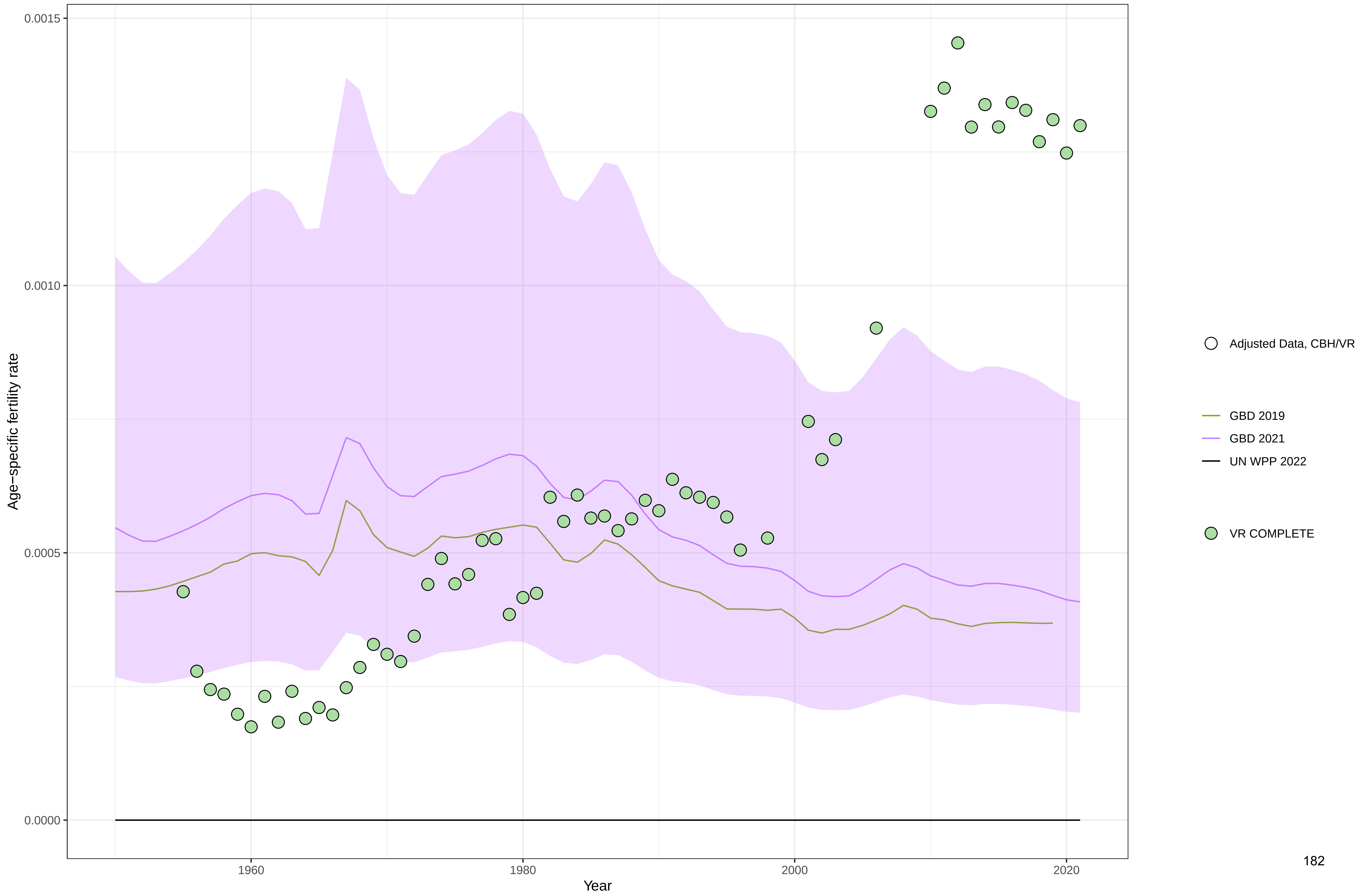

Age−specific fertility fitted trend lines, 1950−2021: Romania, Ages: 15−19
Data points enclosed with a diamond are reference sources

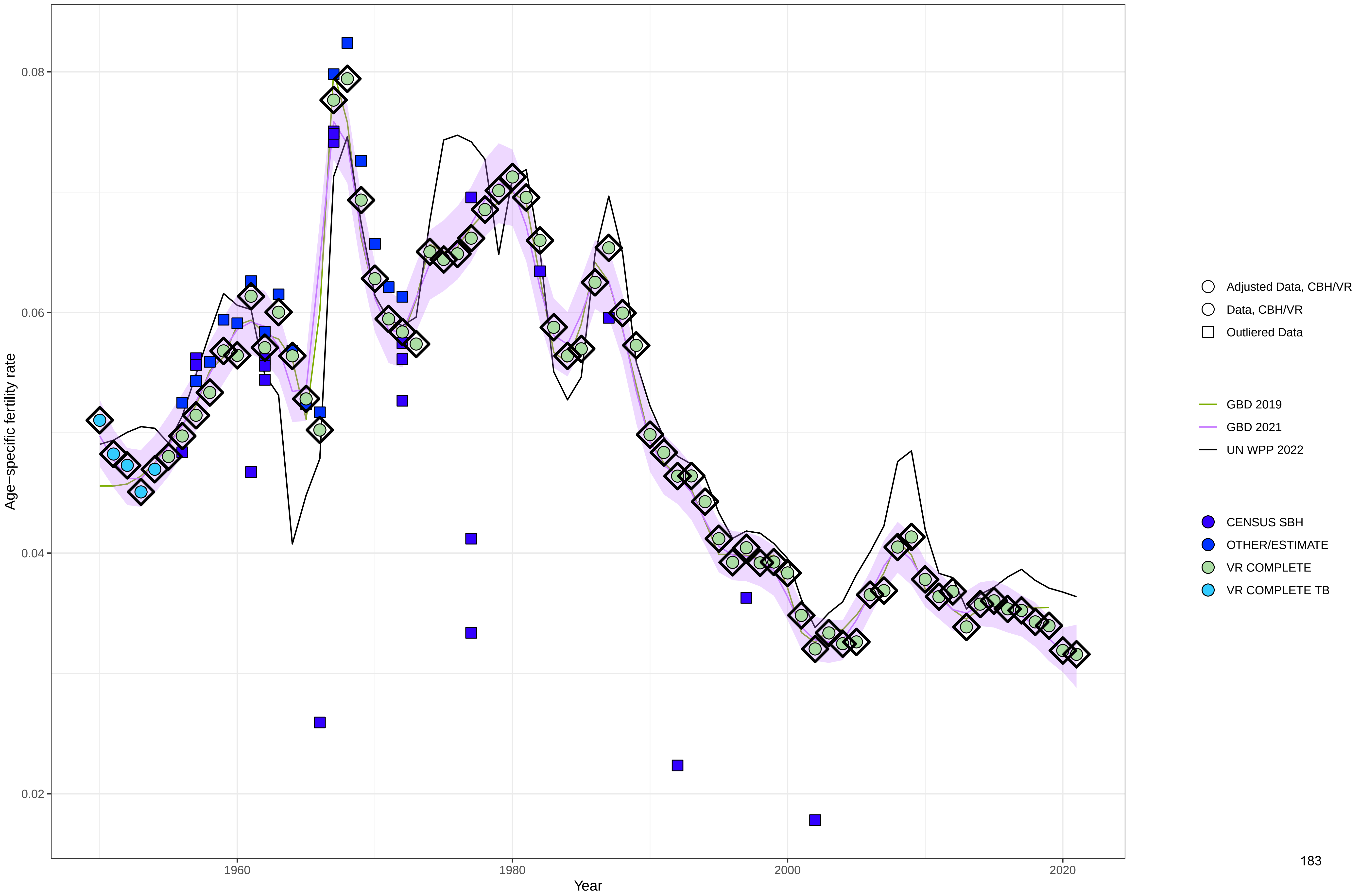

Age−specific fertility fitted trend lines, 1950−2021: Romania, Ages: 20−24
Data points enclosed with a diamond are reference sources

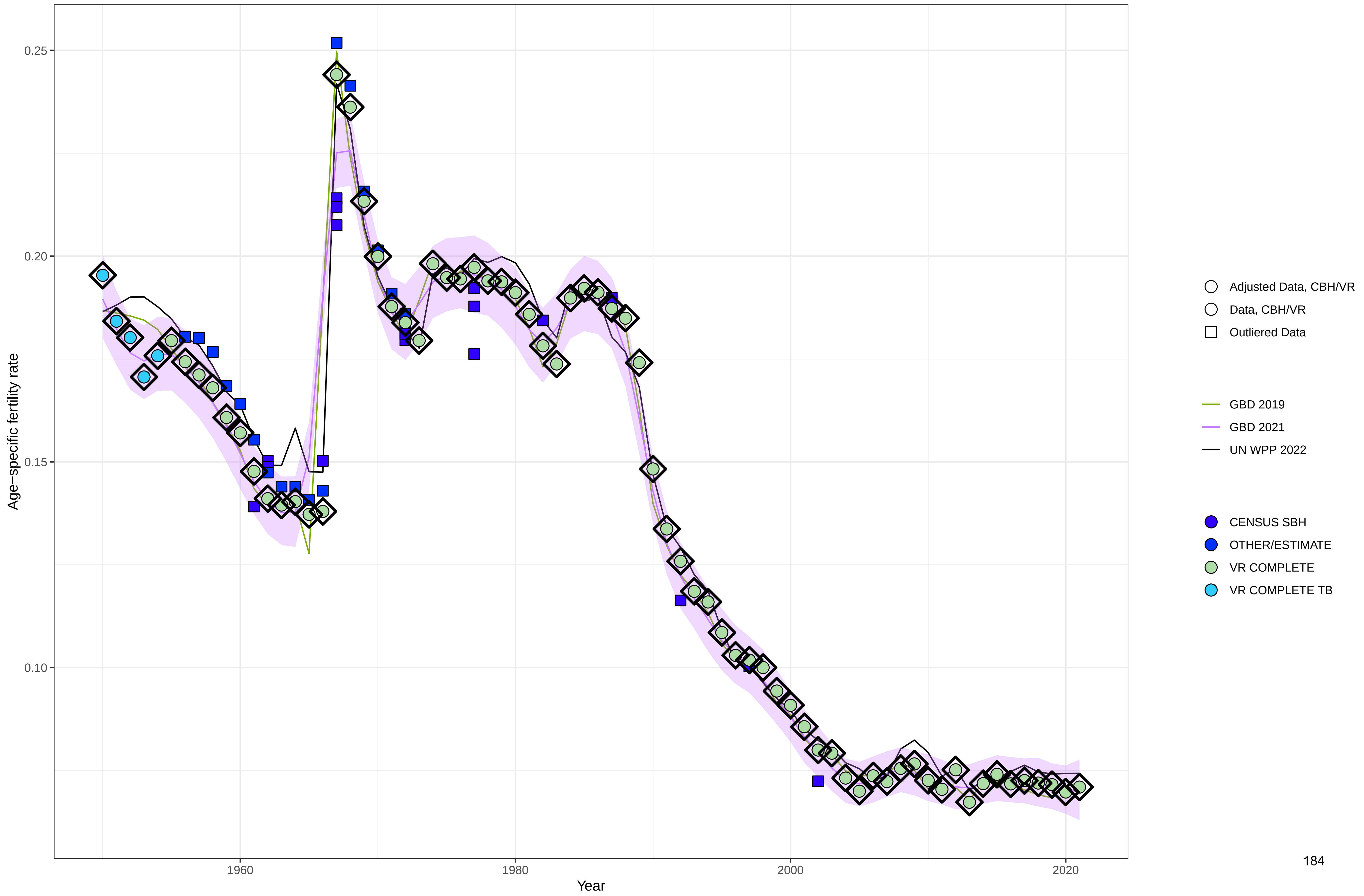

Age−specific fertility fitted trend lines, 1950−2021: Romania, Ages: 25−29
Data points enclosed with a diamond are reference sources

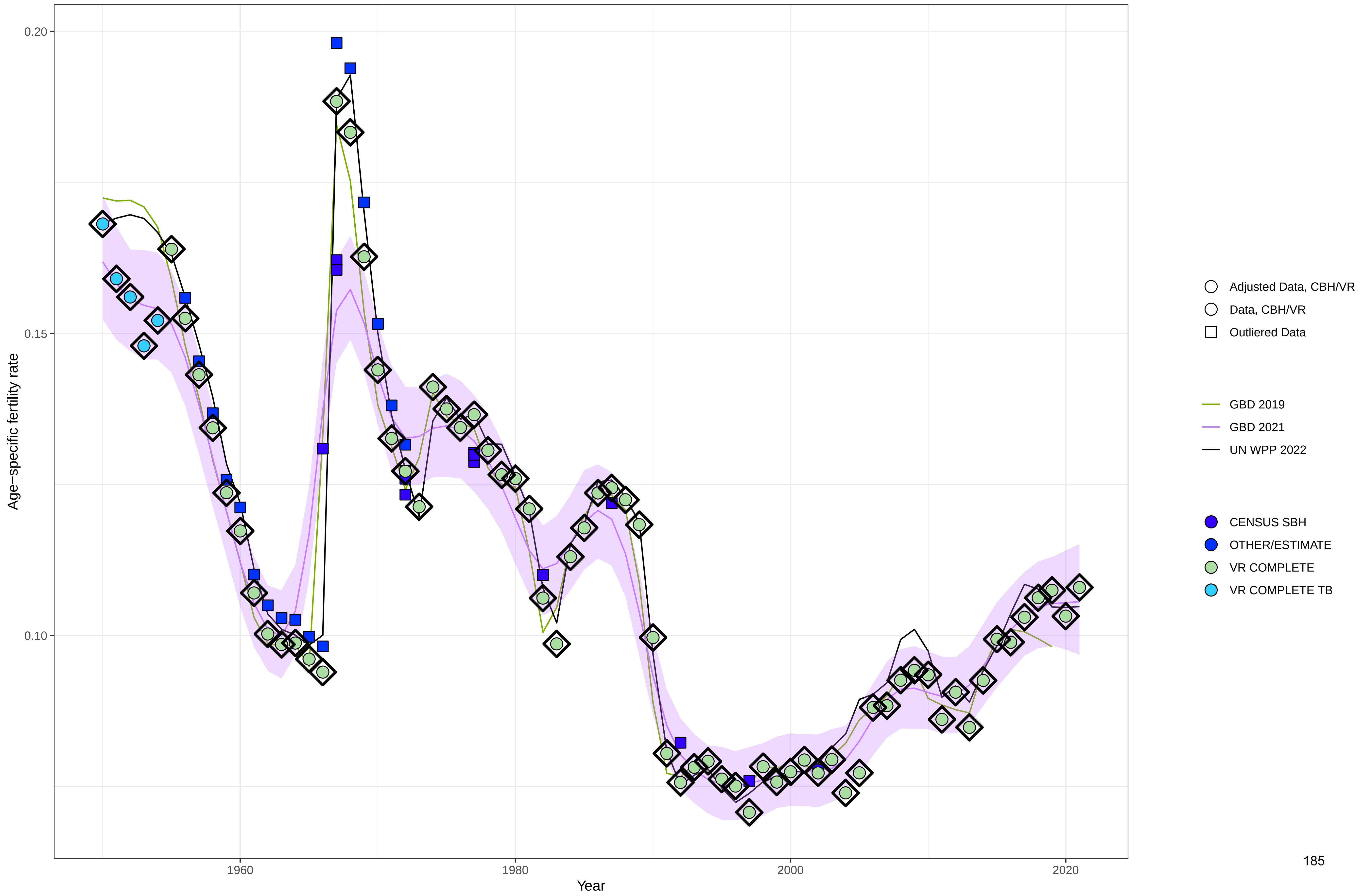

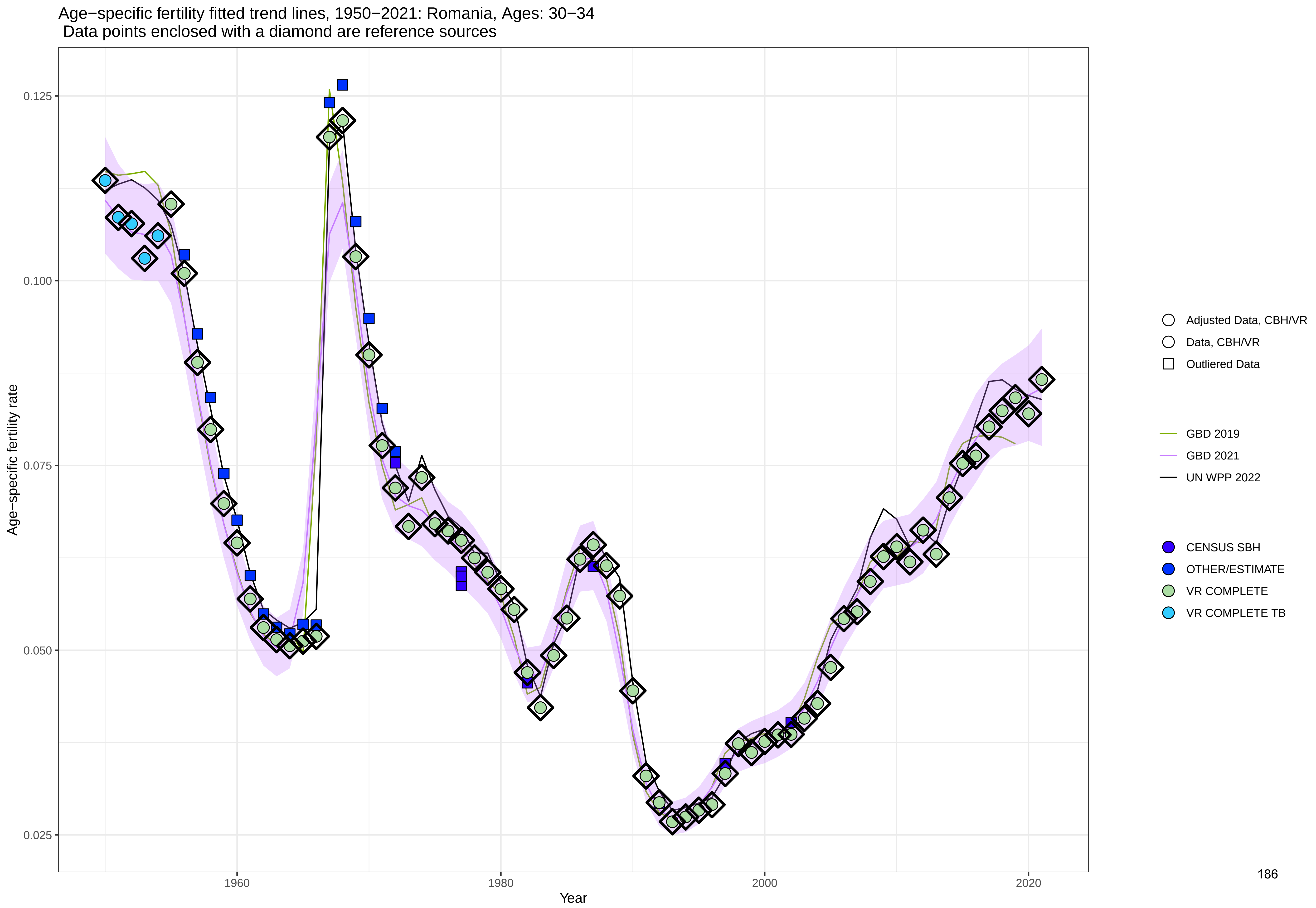
Age−specific fertility fitted trend lines, 1950−2021: Romania, Ages: 30−34
Data points enclosed with a diamond are reference sources
Age−specific fertility rate
0.125
0.100
0.075
0.050
0.025
Year
1960
1980
2000
2020
Adjusted Data, CBH/VR
Data, CBH/VR
Outliered Data
GBD 2019
GBD 2021
UN WPP 2022
CENSUS SBH
OTHER/ESTIMATE
VR COMPLETE
VR COMPLETE TB

Age−specific fertility fitted trend lines, 1950−2021: Romania, Ages: 35−39
Data points enclosed with a diamond are reference sources

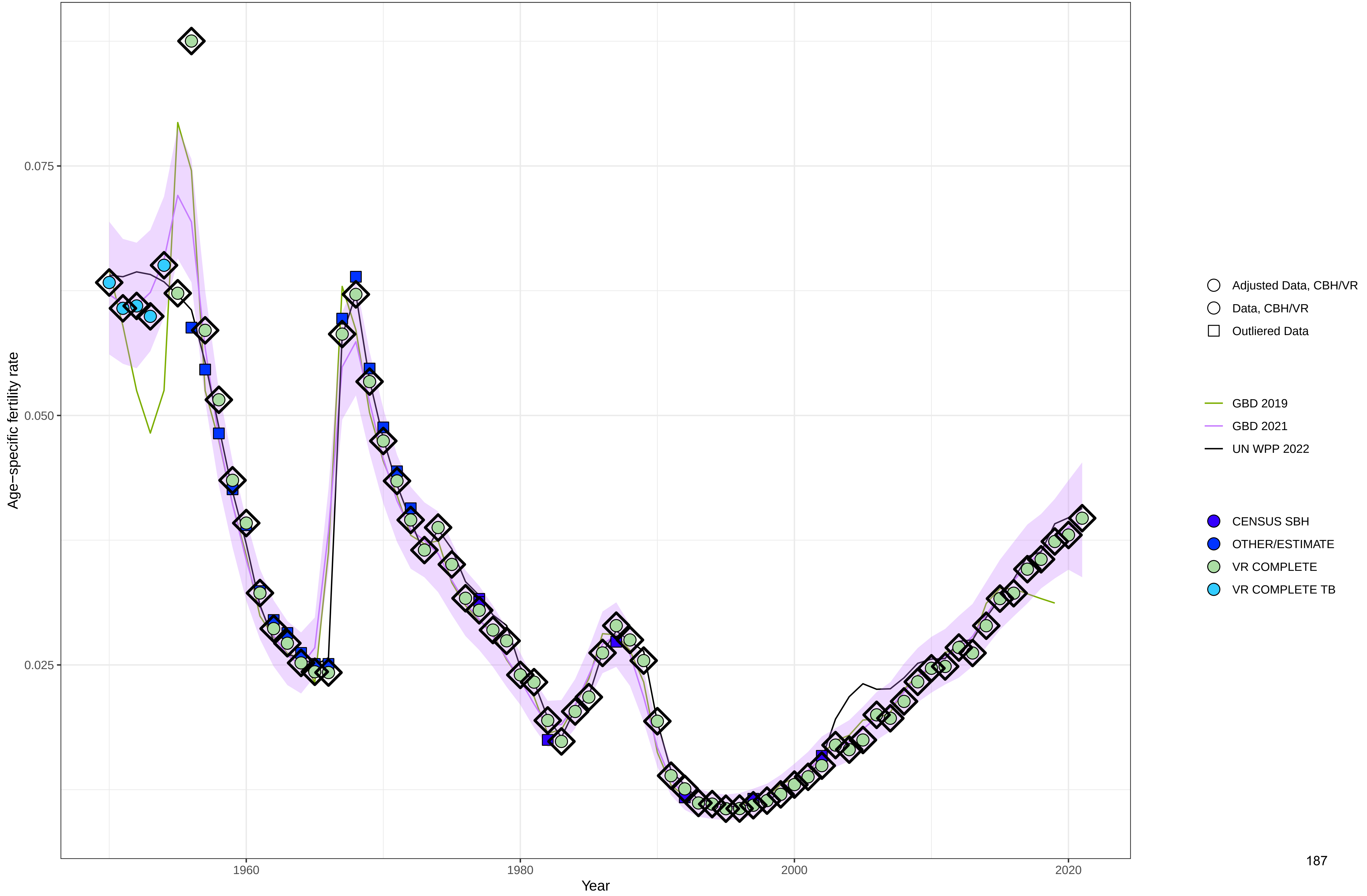

Age−specific fertility fitted trend lines, 1950−2021: Romania, Ages: 40−44
Data points enclosed with a diamond are reference sources

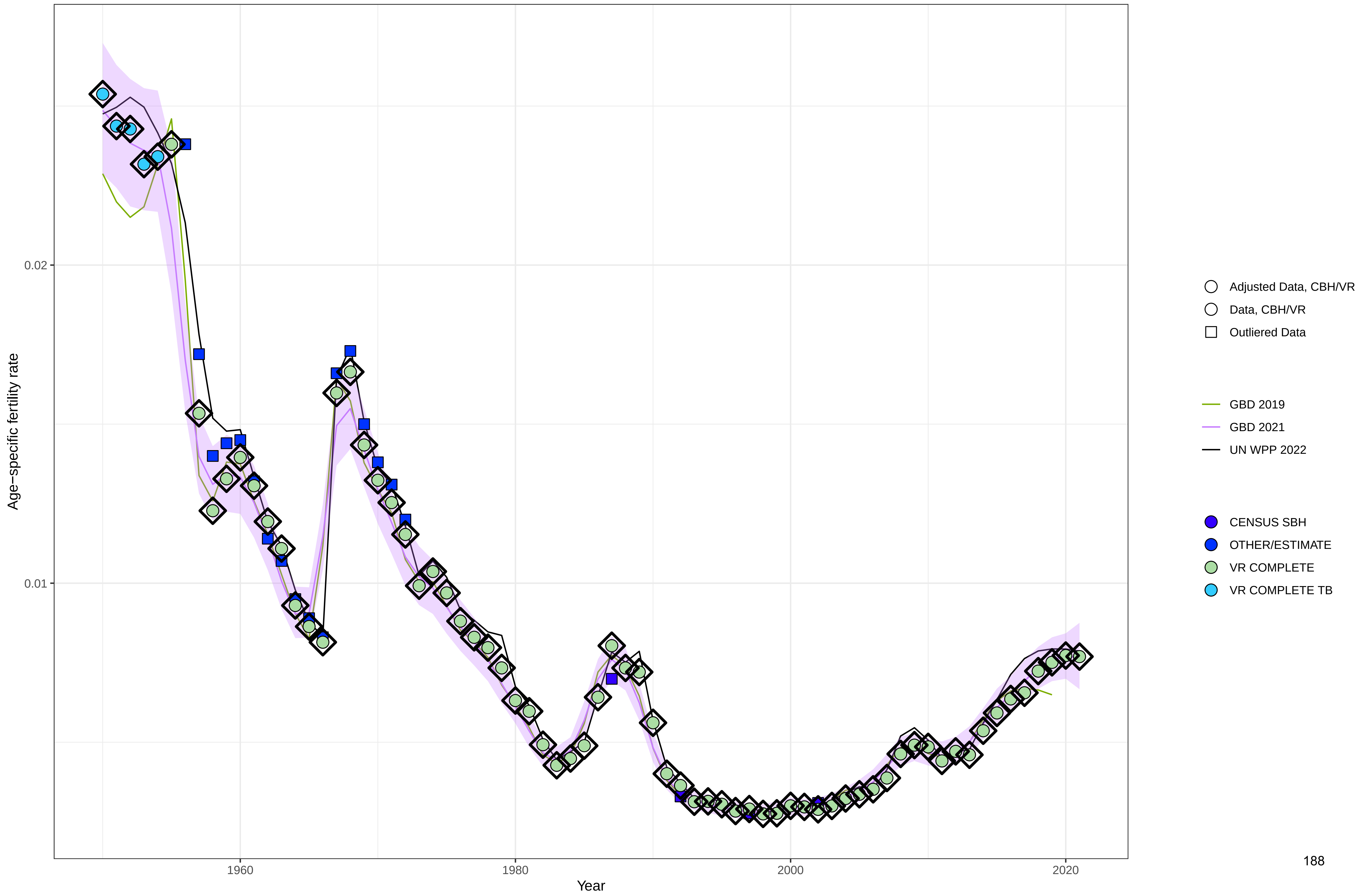

Age−specific fertility fitted trend lines, 1950−2021: Romania, Ages: 45−49
Data points enclosed with a diamond are reference sources

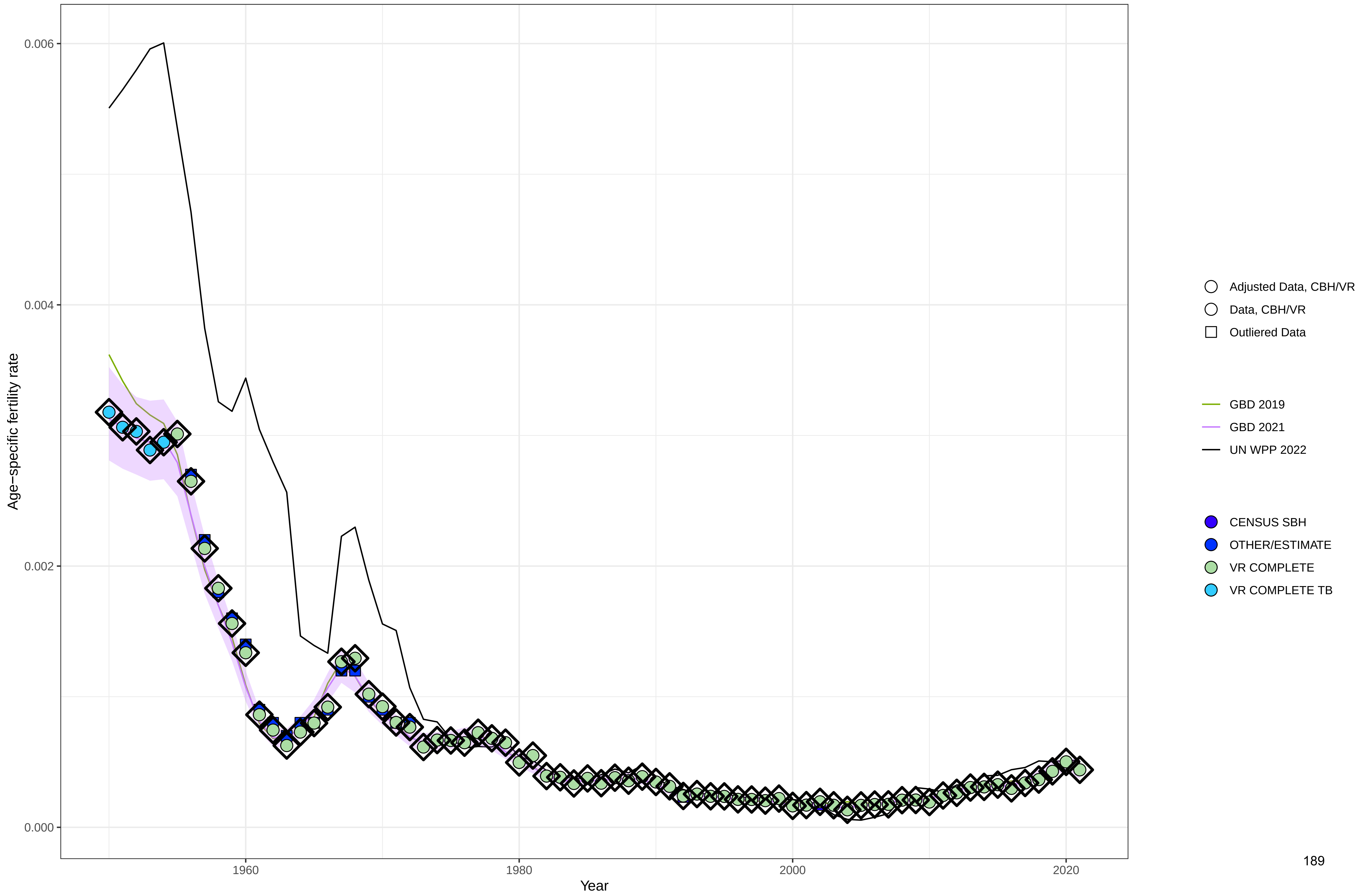

Age−specific fertility fitted trend lines, 1950−2021: Romania, Ages: 50−54
Data points enclosed with a diamond are reference sources

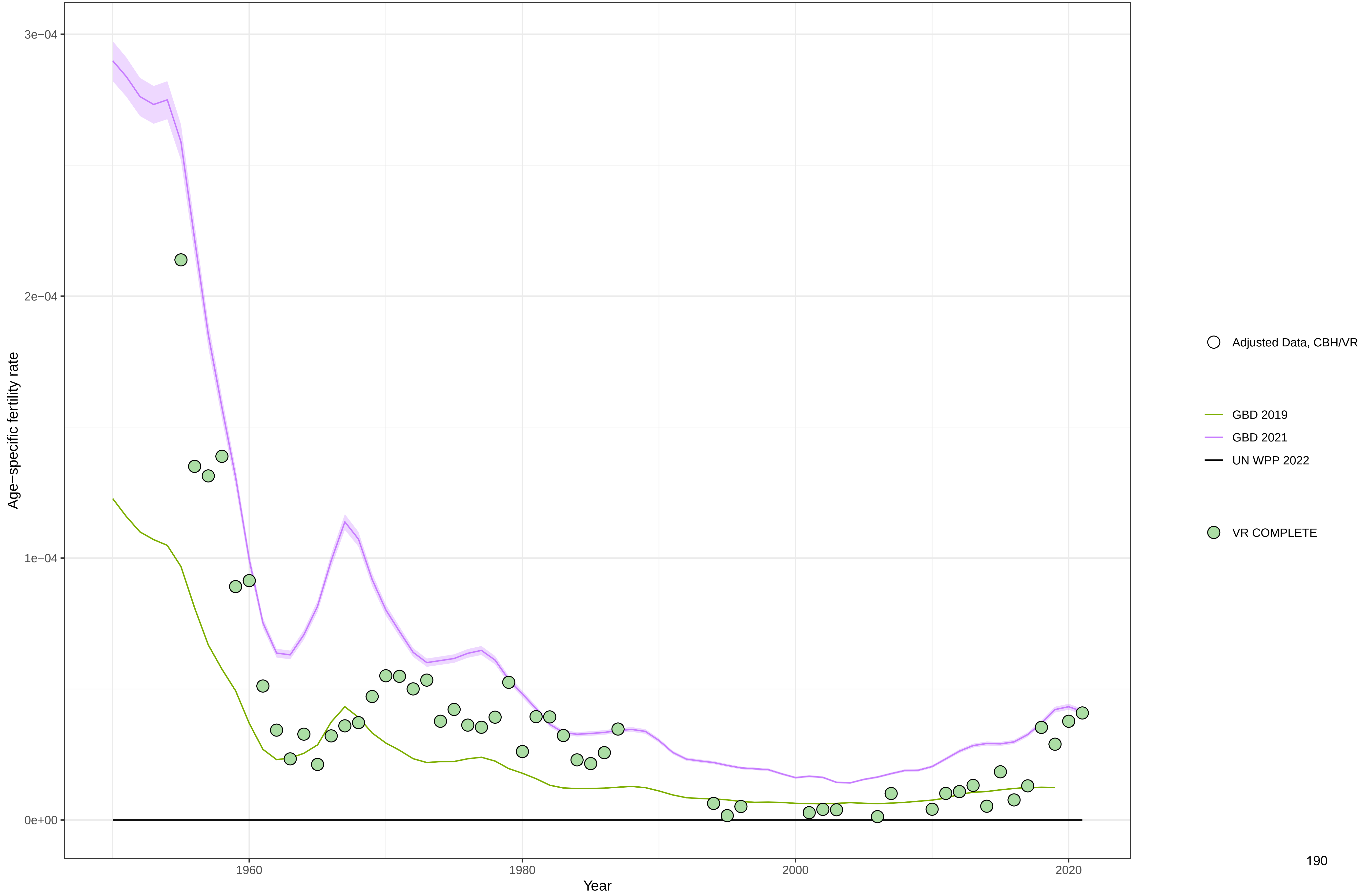

Total fertility rate fitted trend lines, 1950−2021: Romania TFR
Data points enclosed with a diamond are reference sources

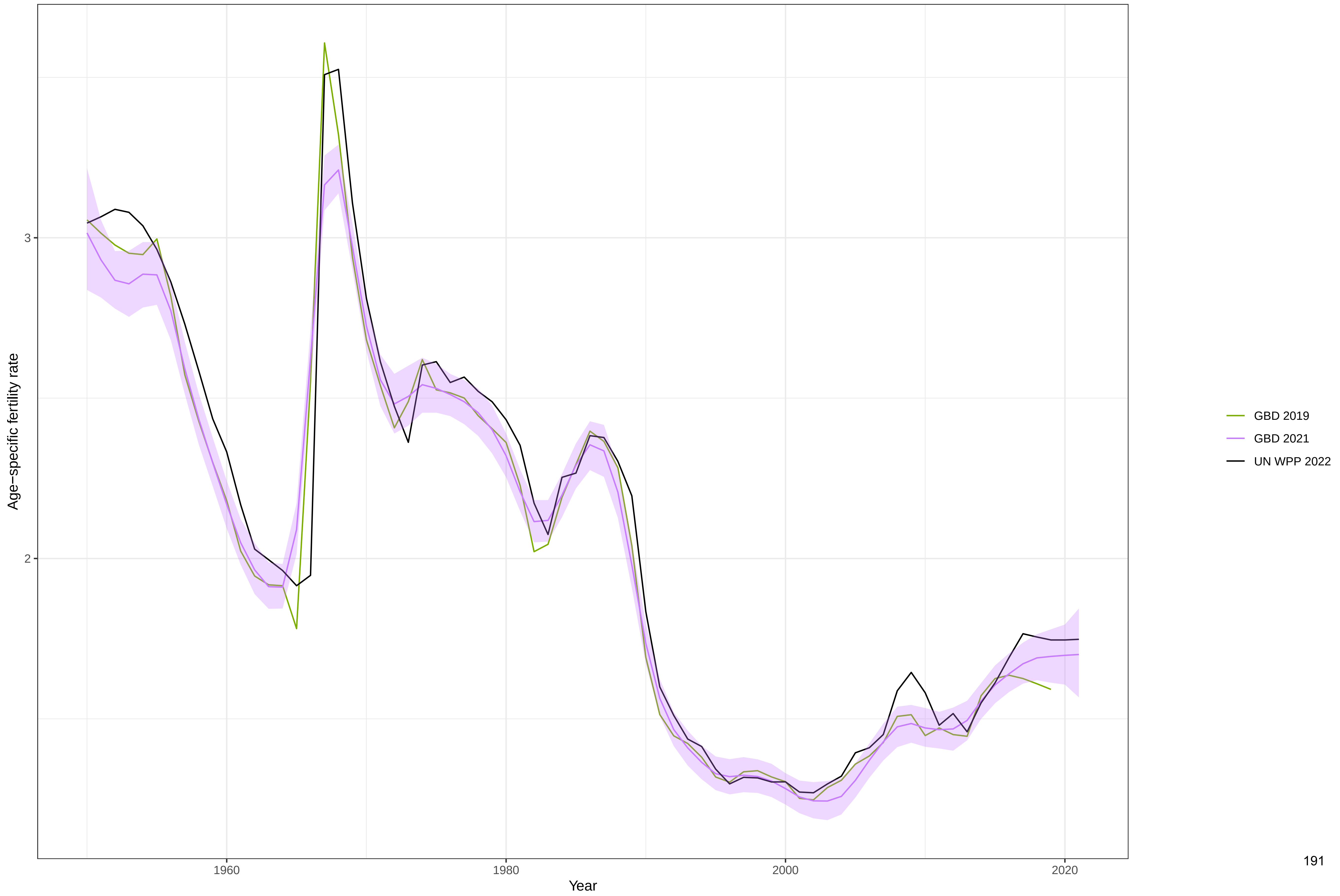

Age−specific fertility fitted trend lines, 1950−2021: Serbia, Ages: 10−14
Data points enclosed with a diamond are reference sources

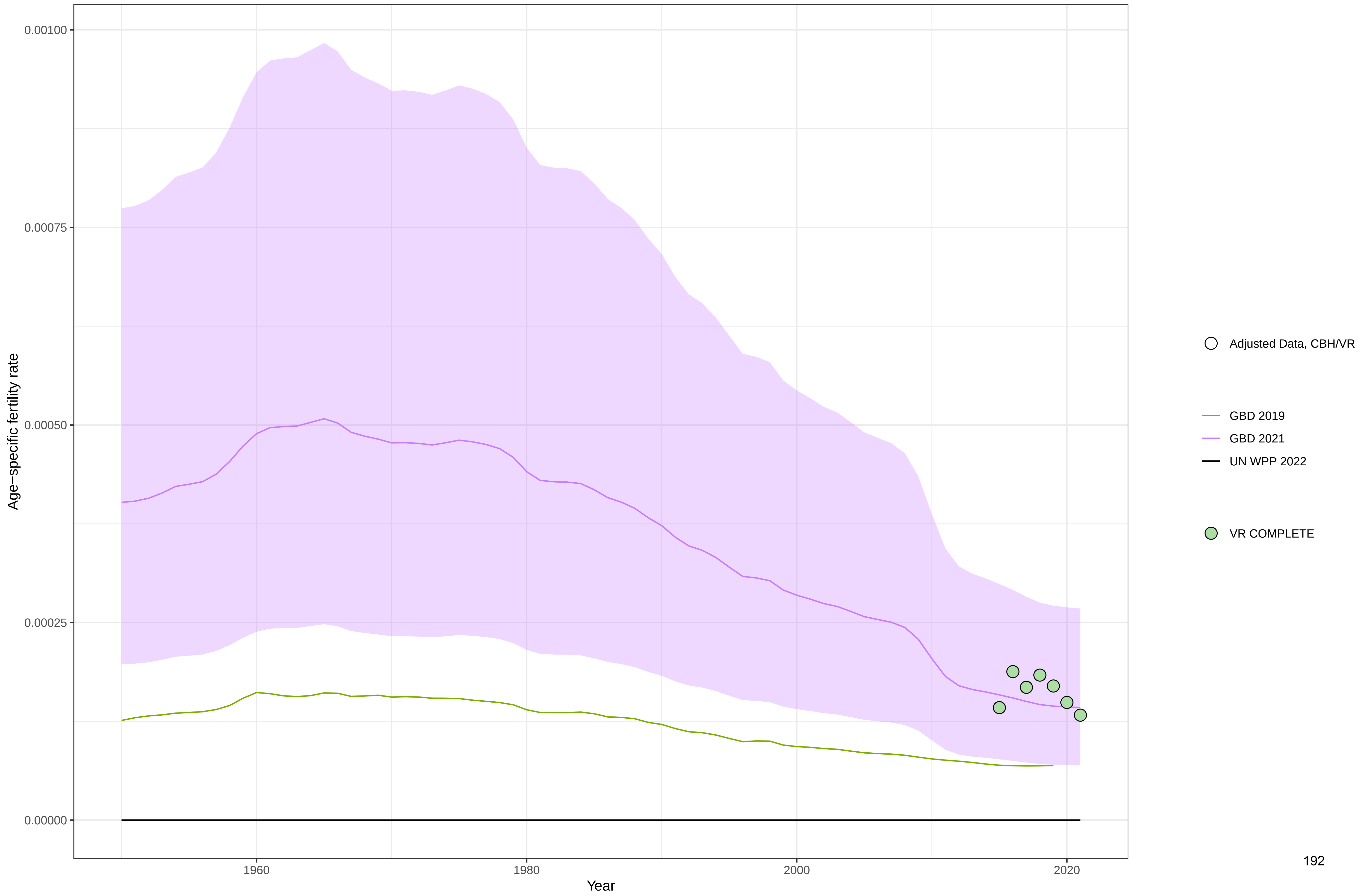

Age−specific fertility fitted trend lines, 1950−2021: Serbia, Ages: 15−19
Data points enclosed with a diamond are reference sources

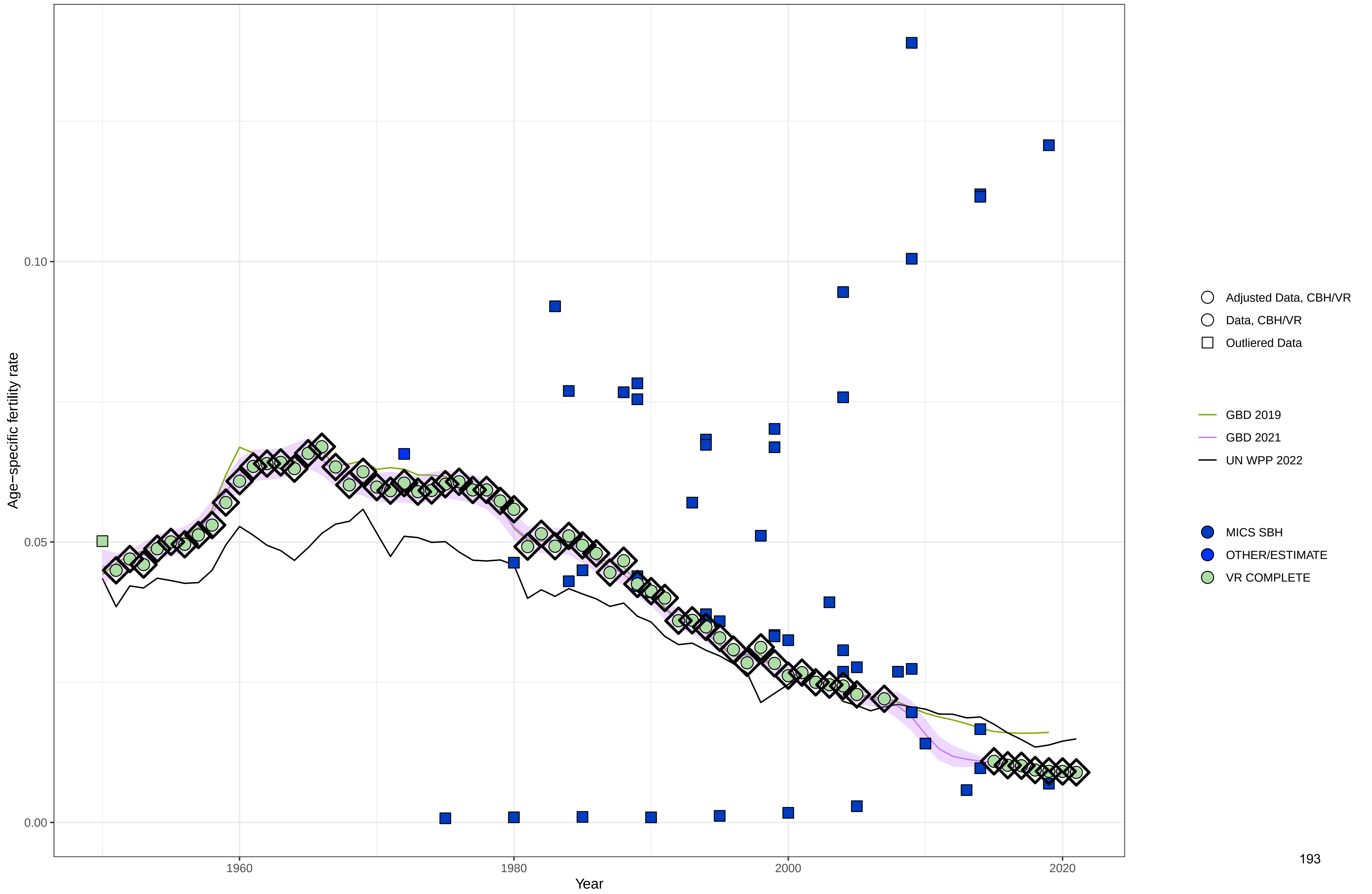

Age−specific fertility fitted trend lines, 1950−2021: Serbia, Ages: 20−24
Data points enclosed with a diamond are reference sources

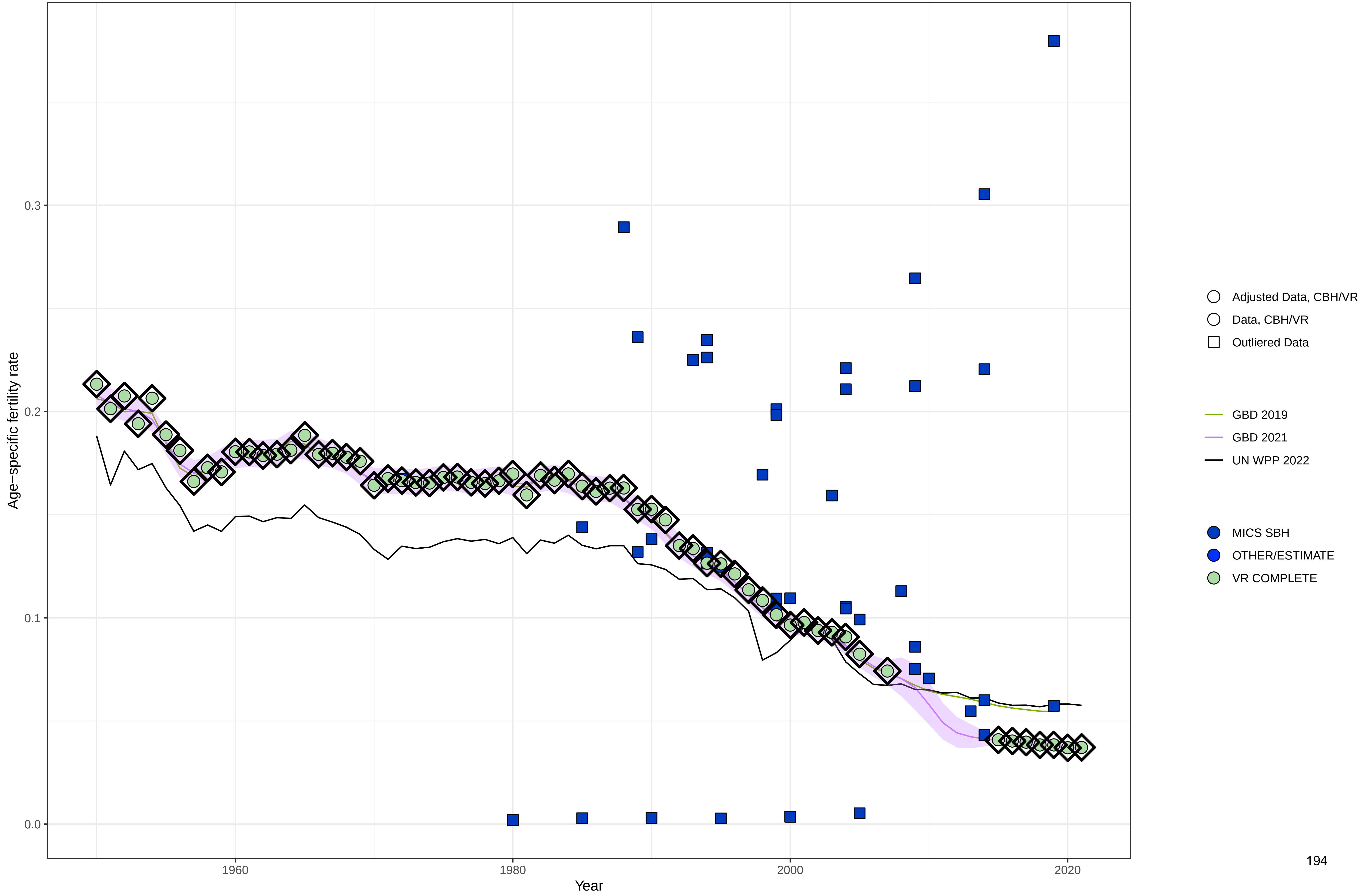

Age−specific fertility fitted trend lines, 1950−2021: Serbia, Ages: 25−29
Data points enclosed with a diamond are reference sources

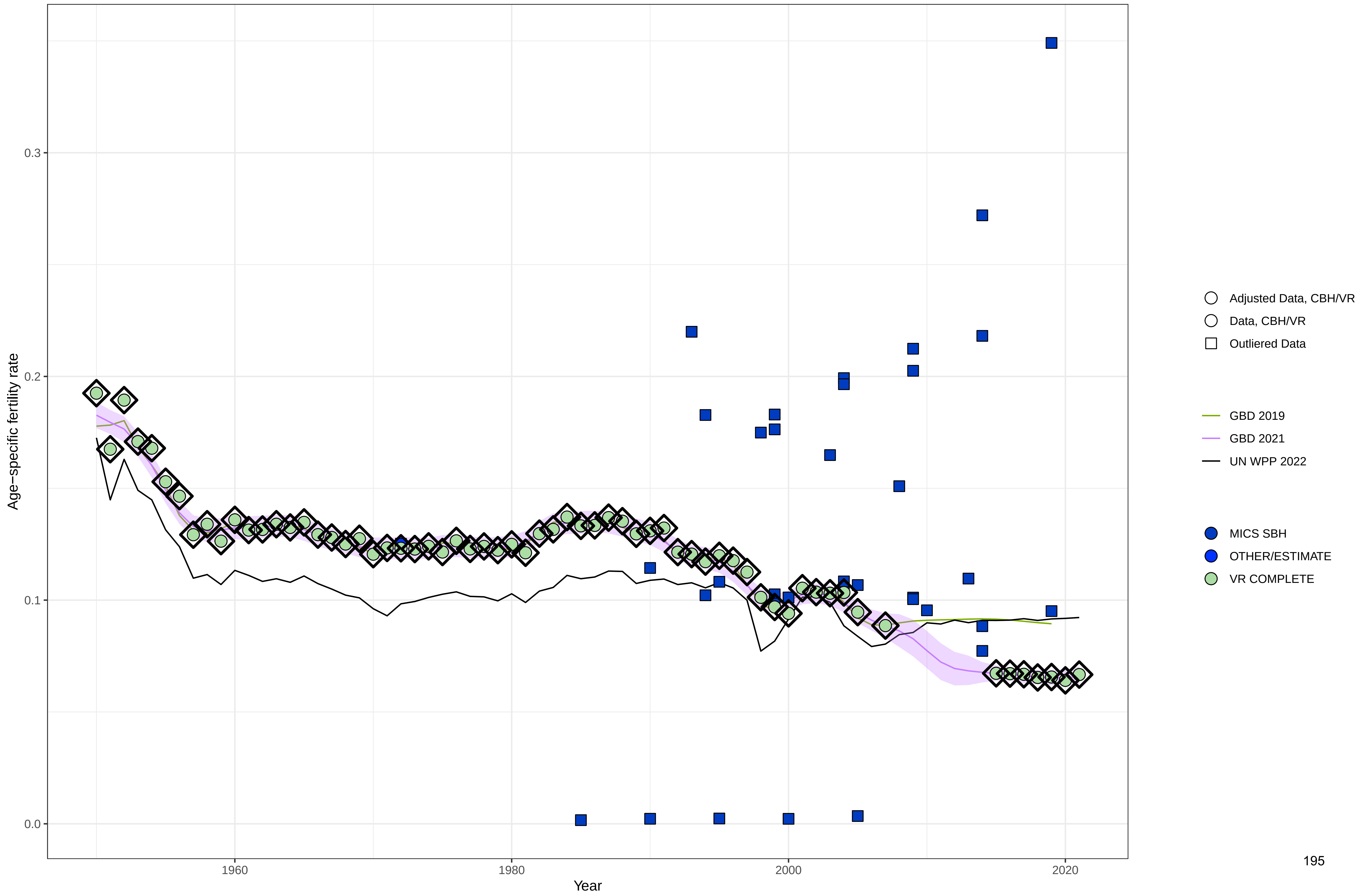

Age−specific fertility fitted trend lines, 1950−2021: Serbia, Ages: 30−34
Data points enclosed with a diamond are reference sources

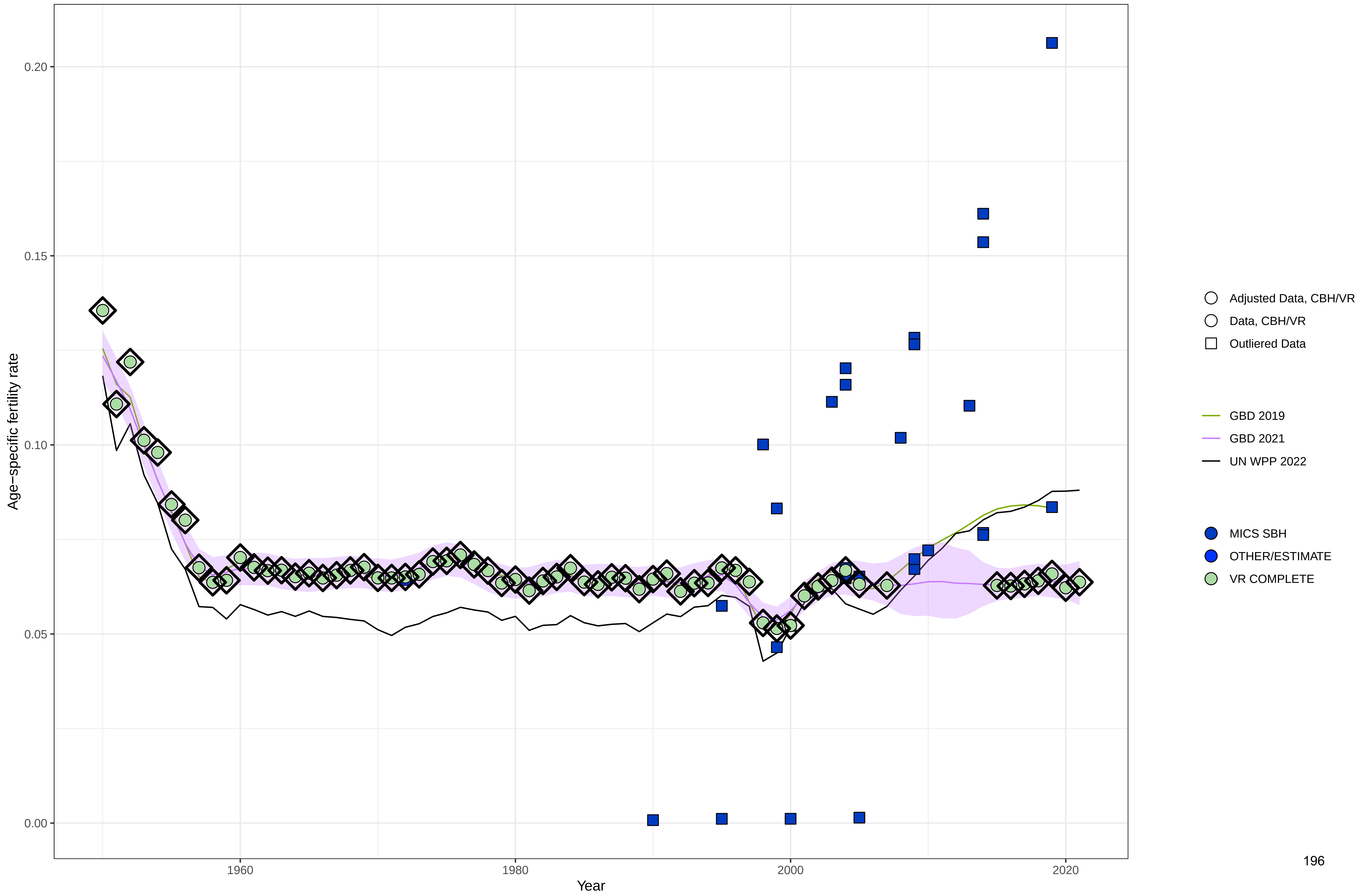

Age−specific fertility fitted trend lines, 1950−2021: Serbia, Ages: 35−39
Data points enclosed with a diamond are reference sources

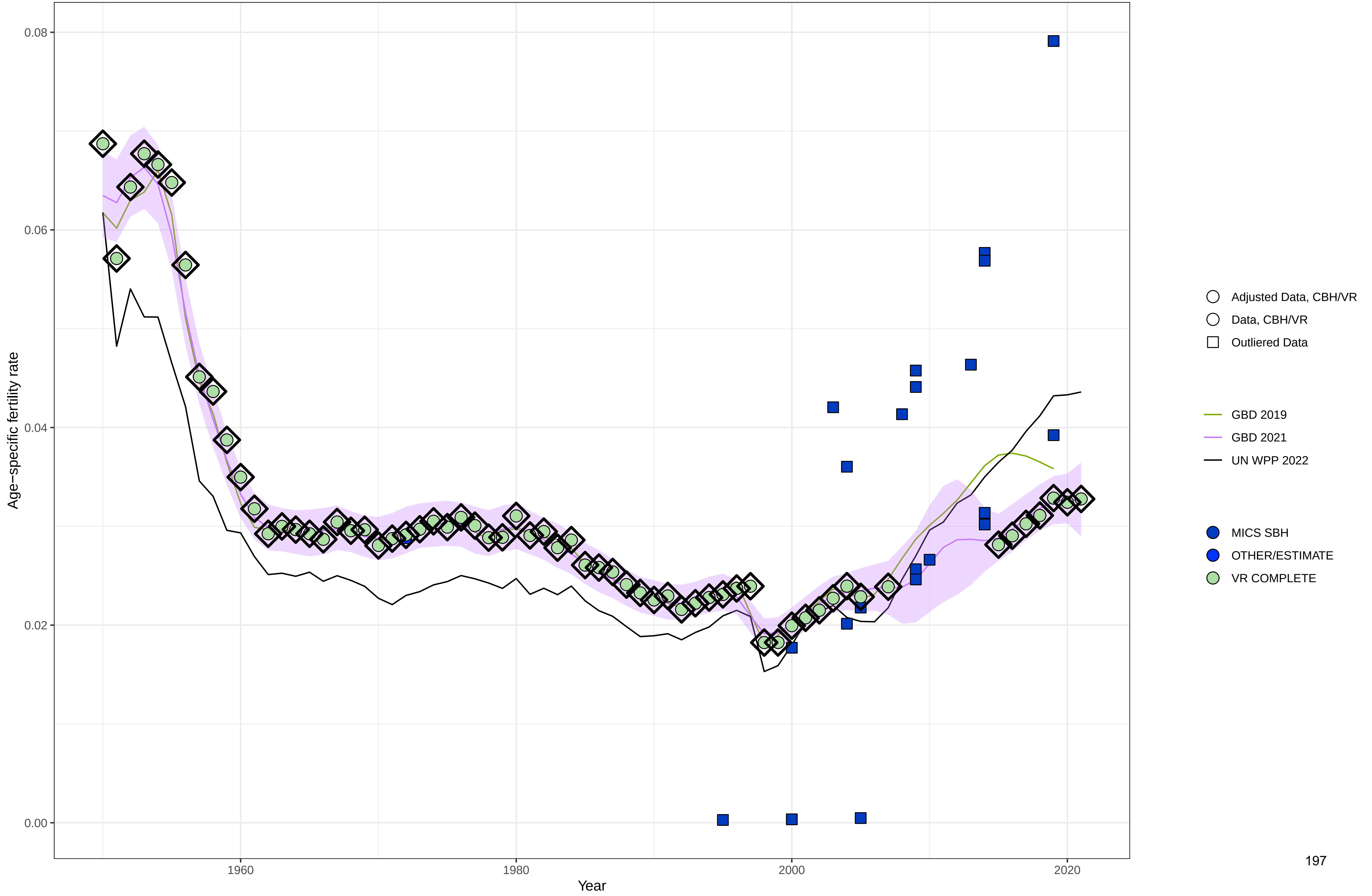

Age−specific fertility fitted trend lines, 1950−2021: Serbia, Ages: 40−44
Data points enclosed with a diamond are reference sources

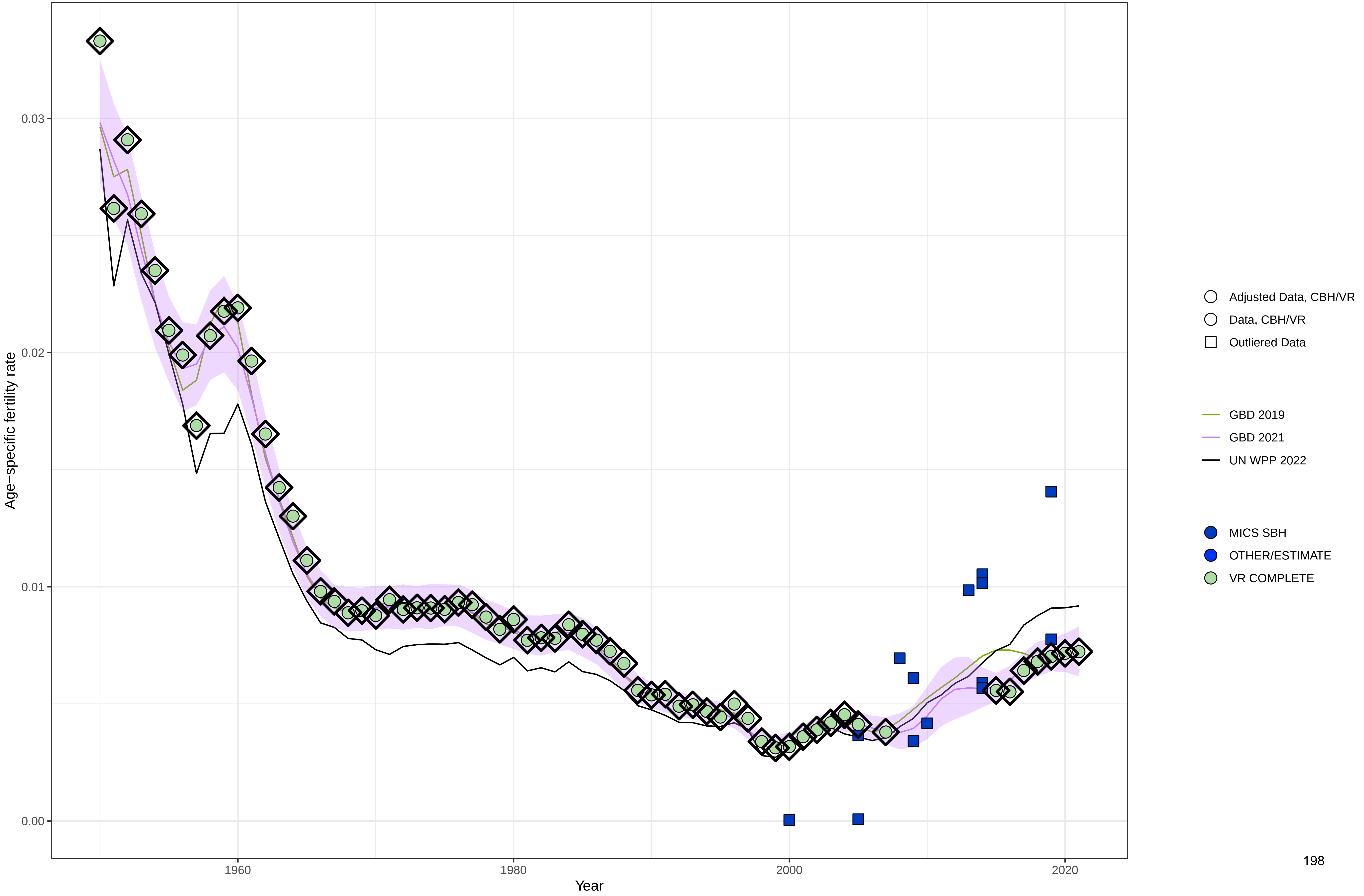

Age−specific fertility fitted trend lines, 1950−2021: Serbia, Ages: 45−49
Data points enclosed with a diamond are reference sources

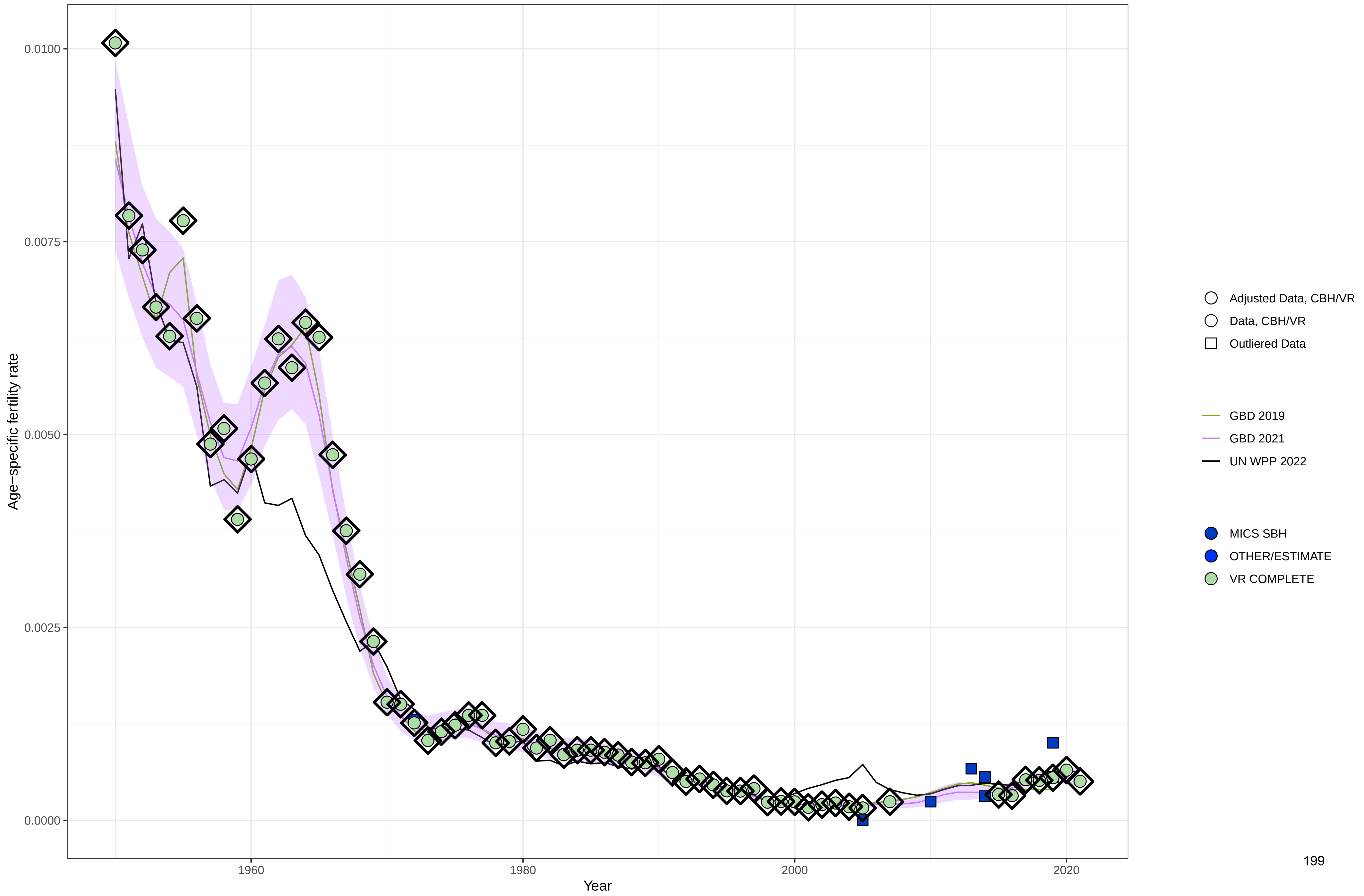

Age−specific fertility fitted trend lines, 1950−2021: Serbia, Ages: 50−54
Data points enclosed with a diamond are reference sources

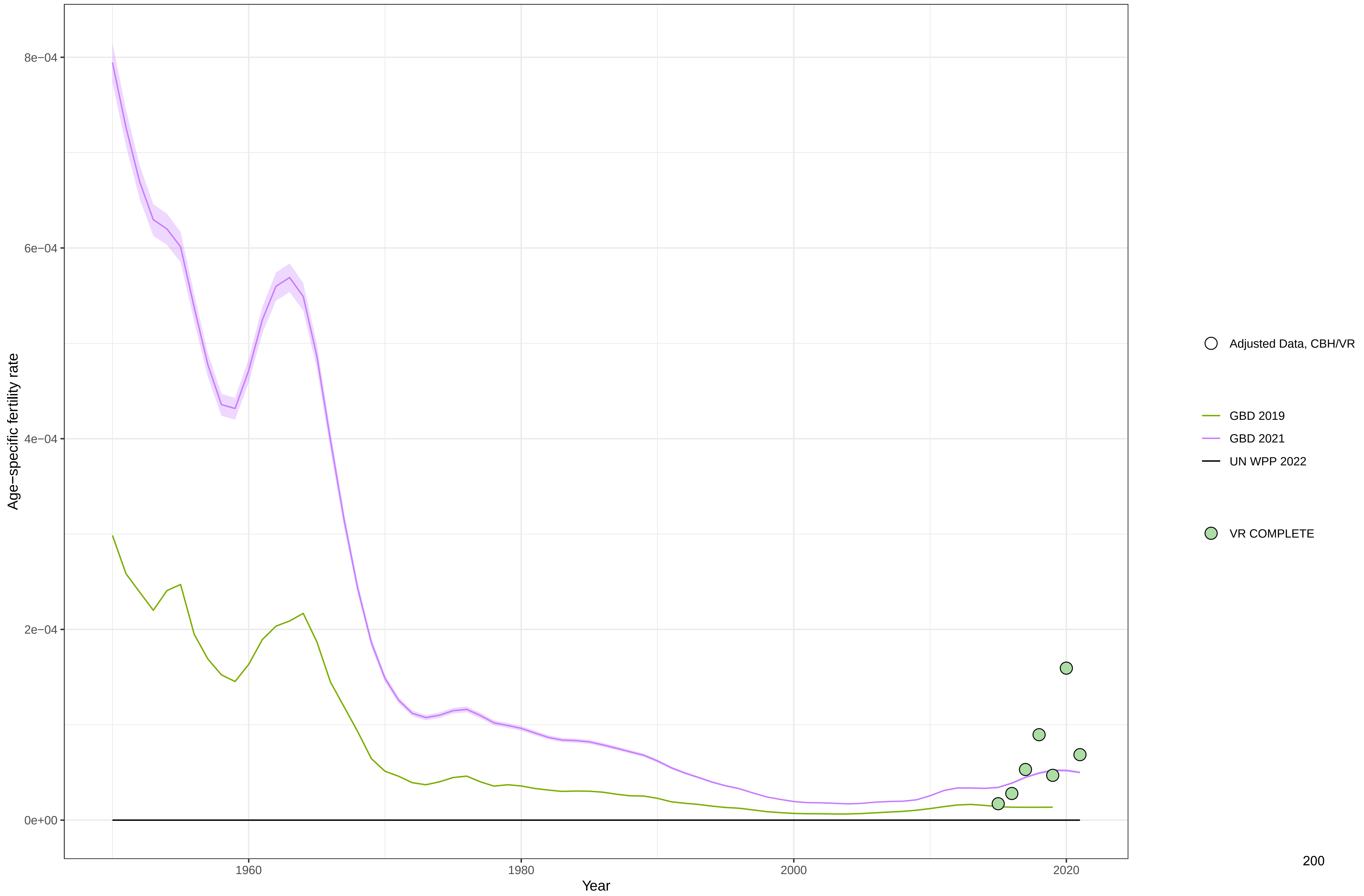

Total fertility rate fitted trend lines, 1950−2021: Serbia TFR
Data points enclosed with a diamond are reference sources

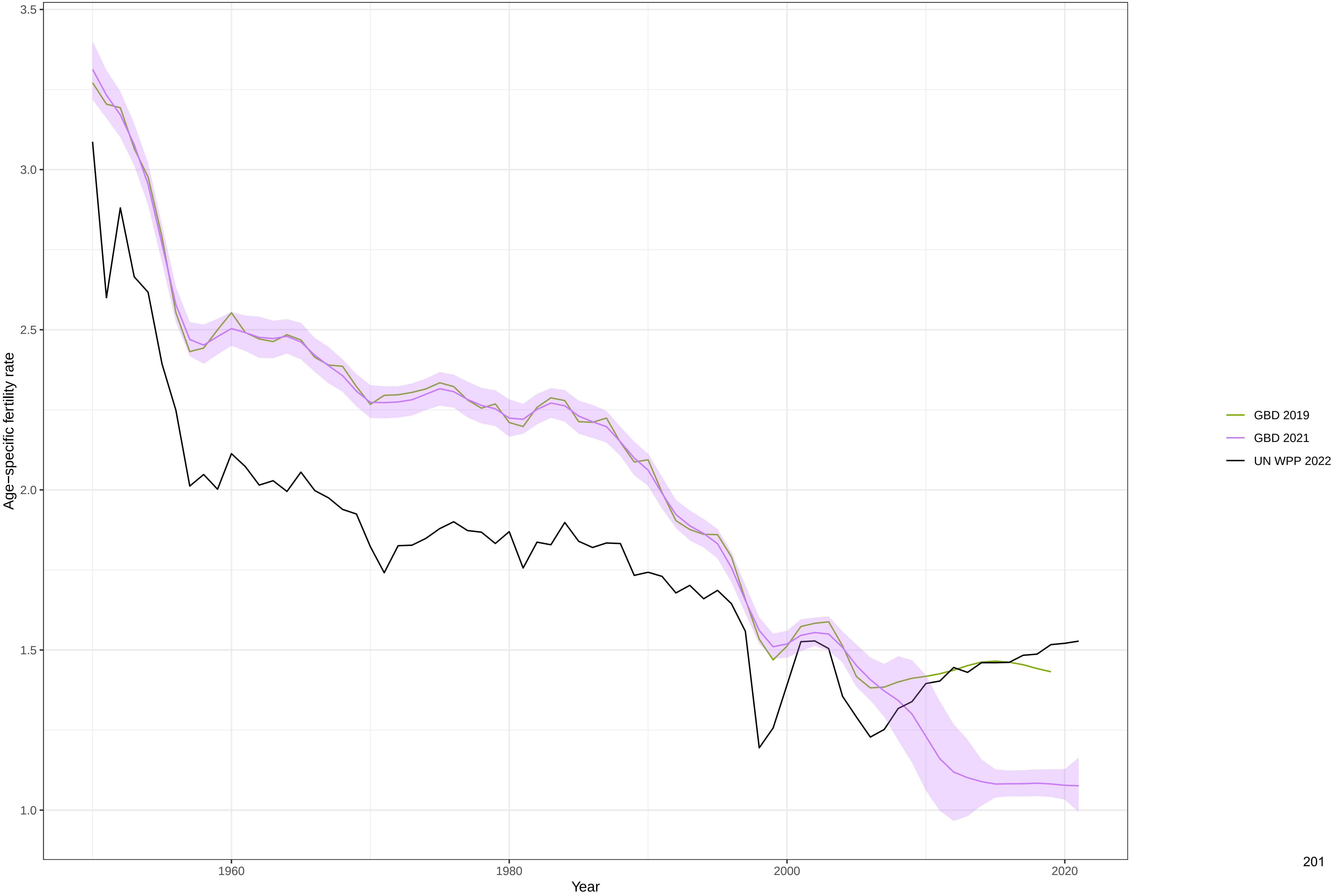

Age−specific fertility fitted trend lines, 1950−2021: Slovakia, Ages: 10−14
Data points enclosed with a diamond are reference sources

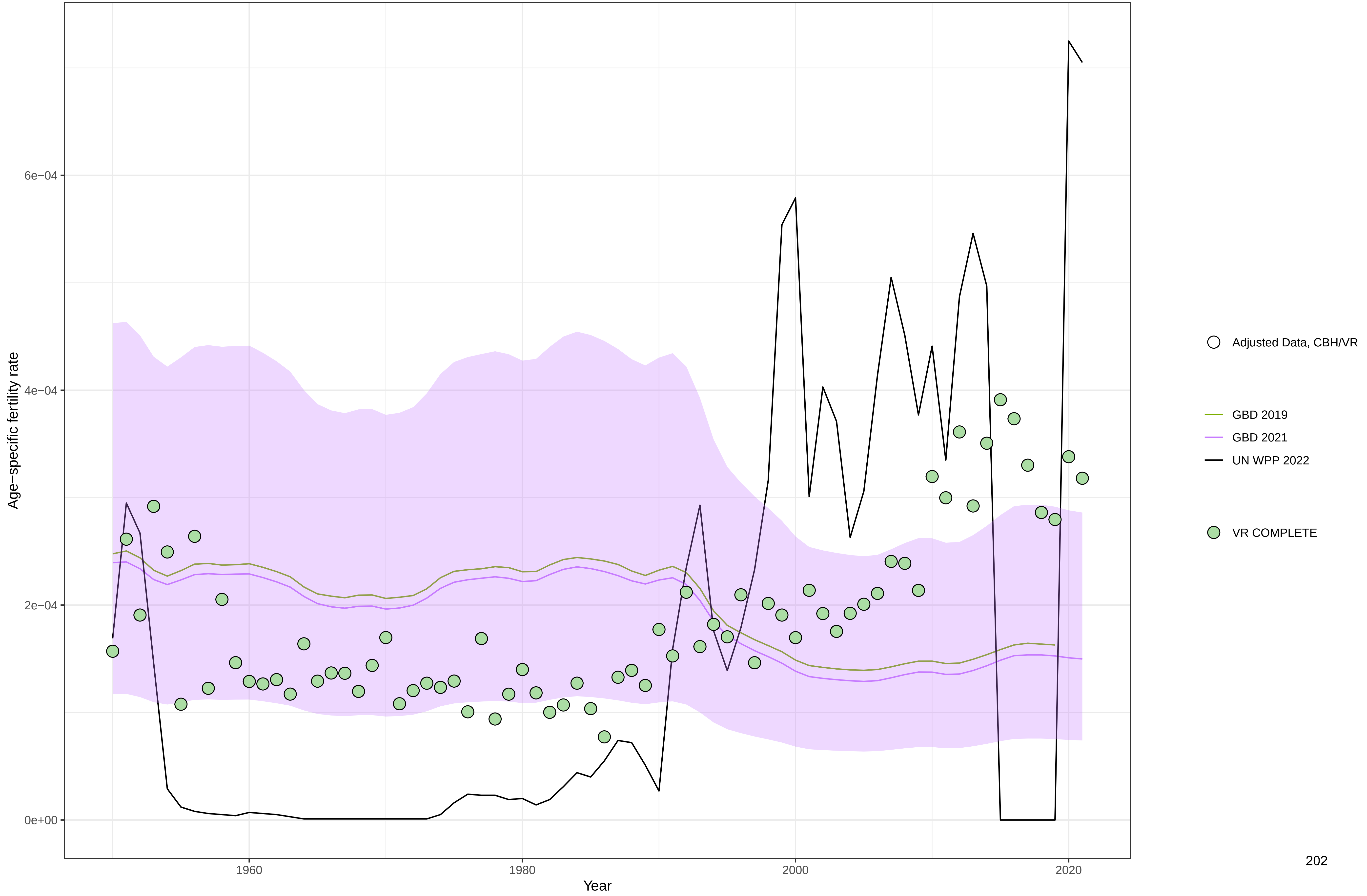

Age−specific fertility fitted trend lines, 1950−2021: Slovakia, Ages: 15−19
Data points enclosed with a diamond are reference sources

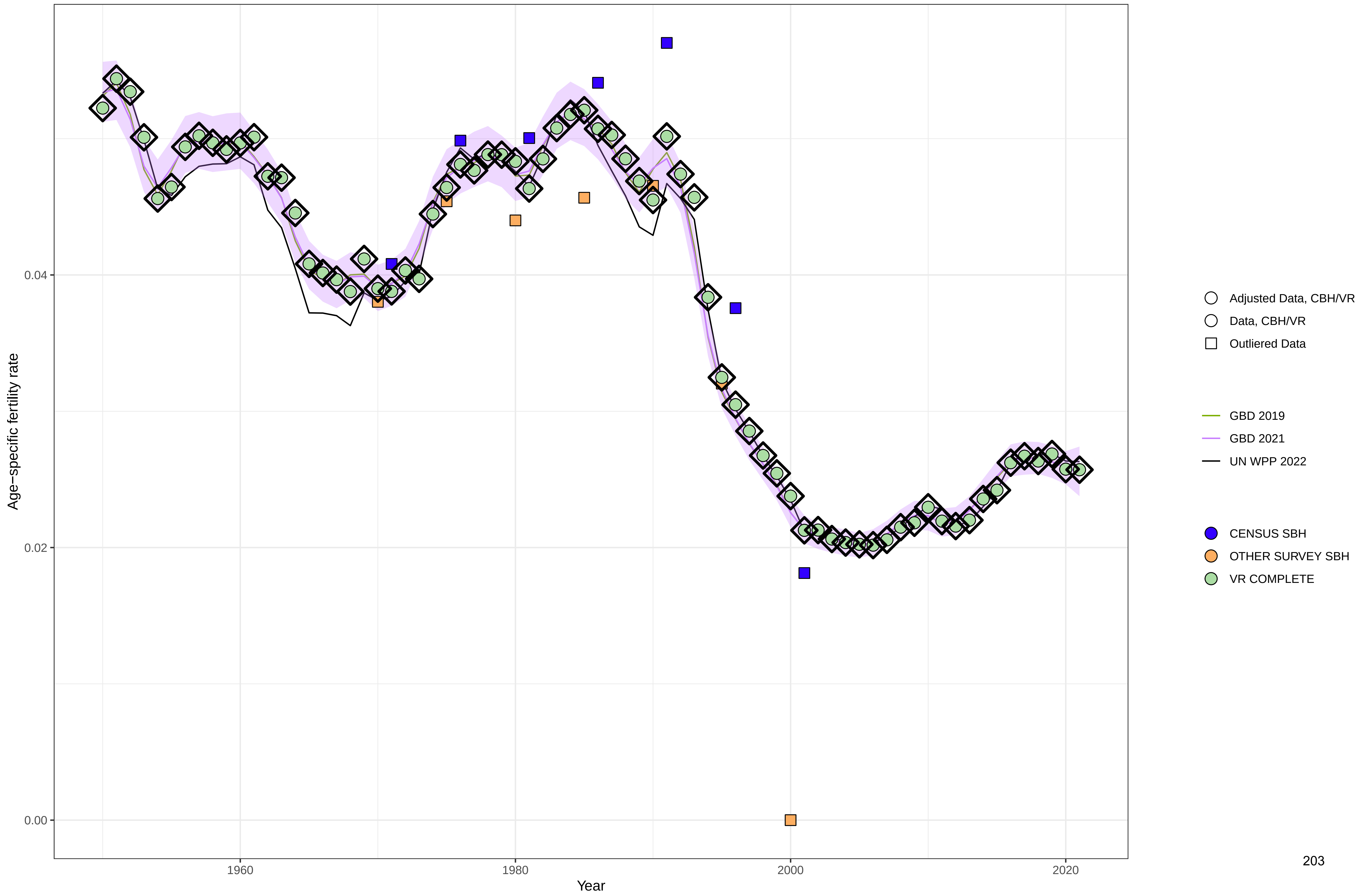

Age−specific fertility fitted trend lines, 1950−2021: Slovakia, Ages: 20−24
Data points enclosed with a diamond are reference sources

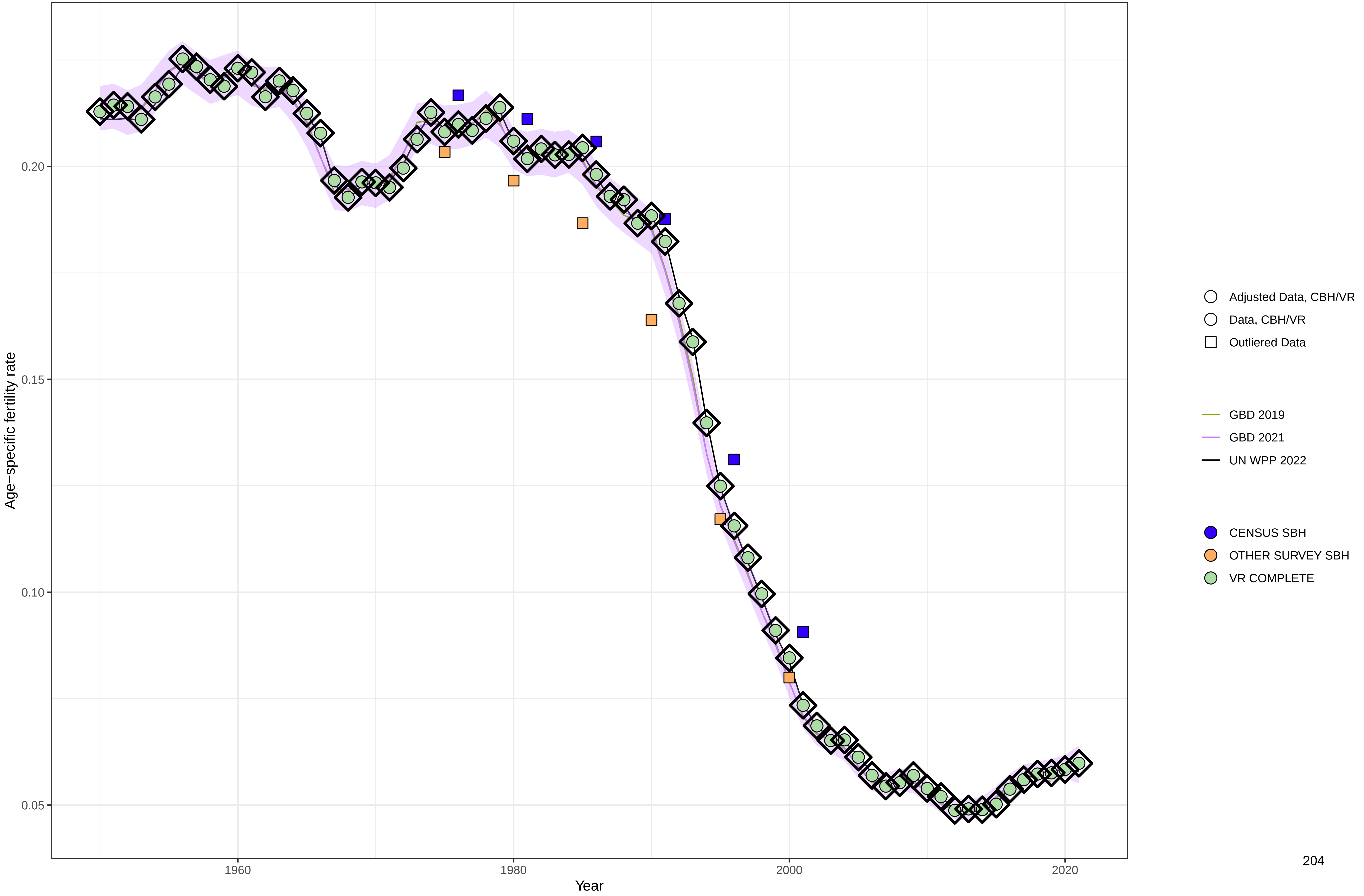

Age−specific fertility fitted trend lines, 1950−2021: Slovakia, Ages: 25−29
Data points enclosed with a diamond are reference sources

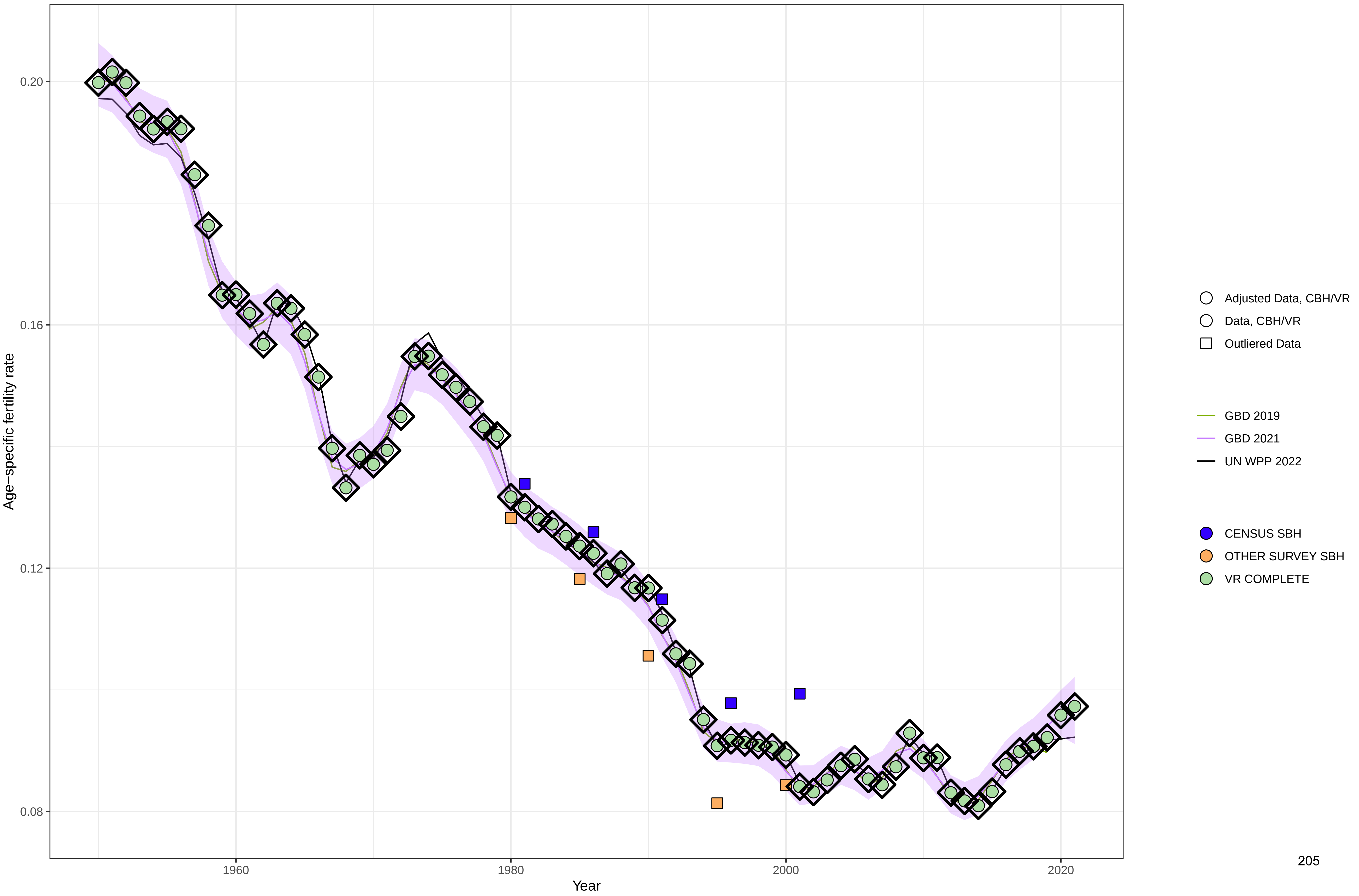

Age−specific fertility fitted trend lines, 1950−2021: Slovakia, Ages: 30−34
Data points enclosed with a diamond are reference sources

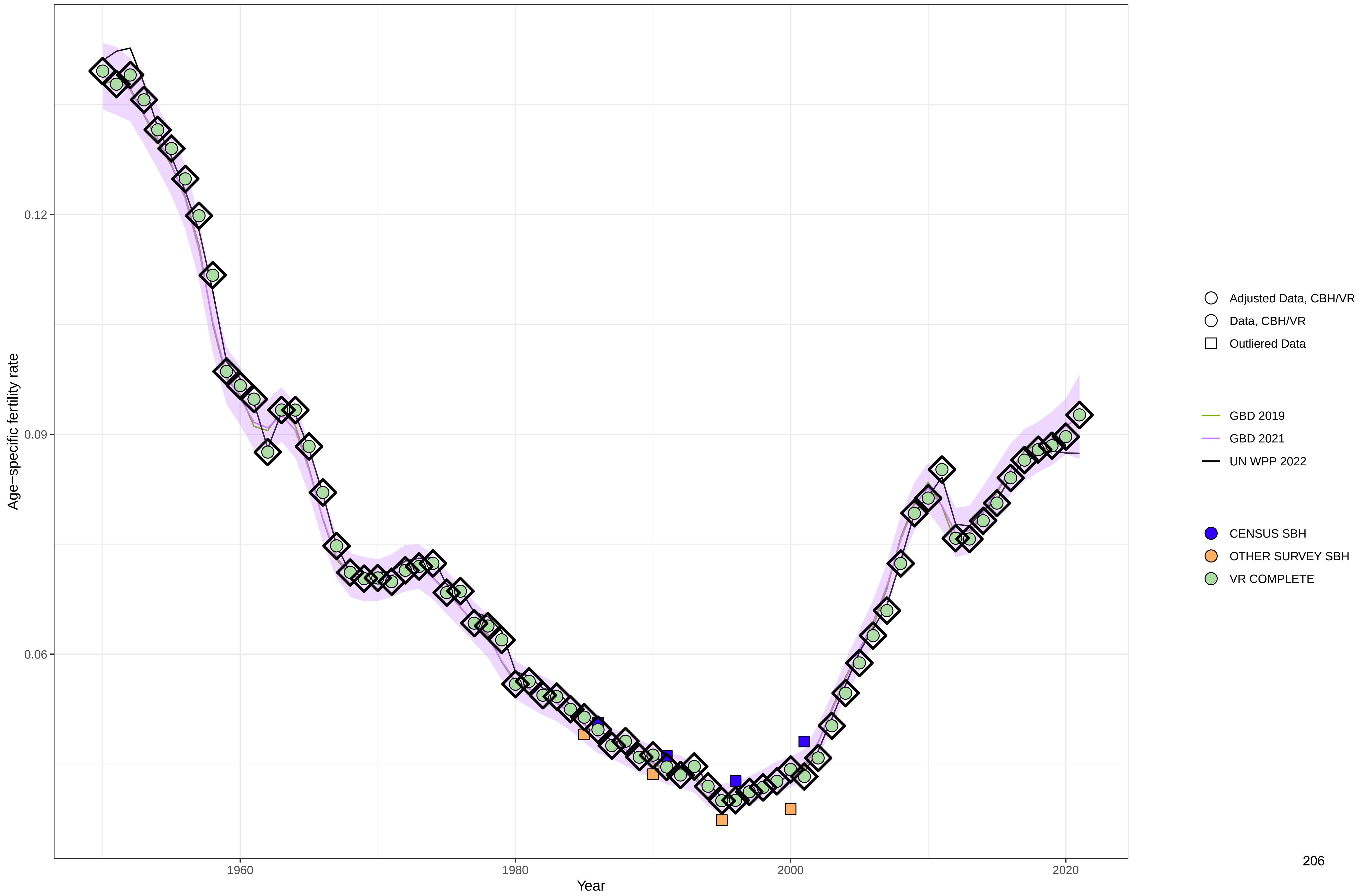

Age−specific fertility fitted trend lines, 1950−2021: Slovakia, Ages: 35−39
Data points enclosed with a diamond are reference sources

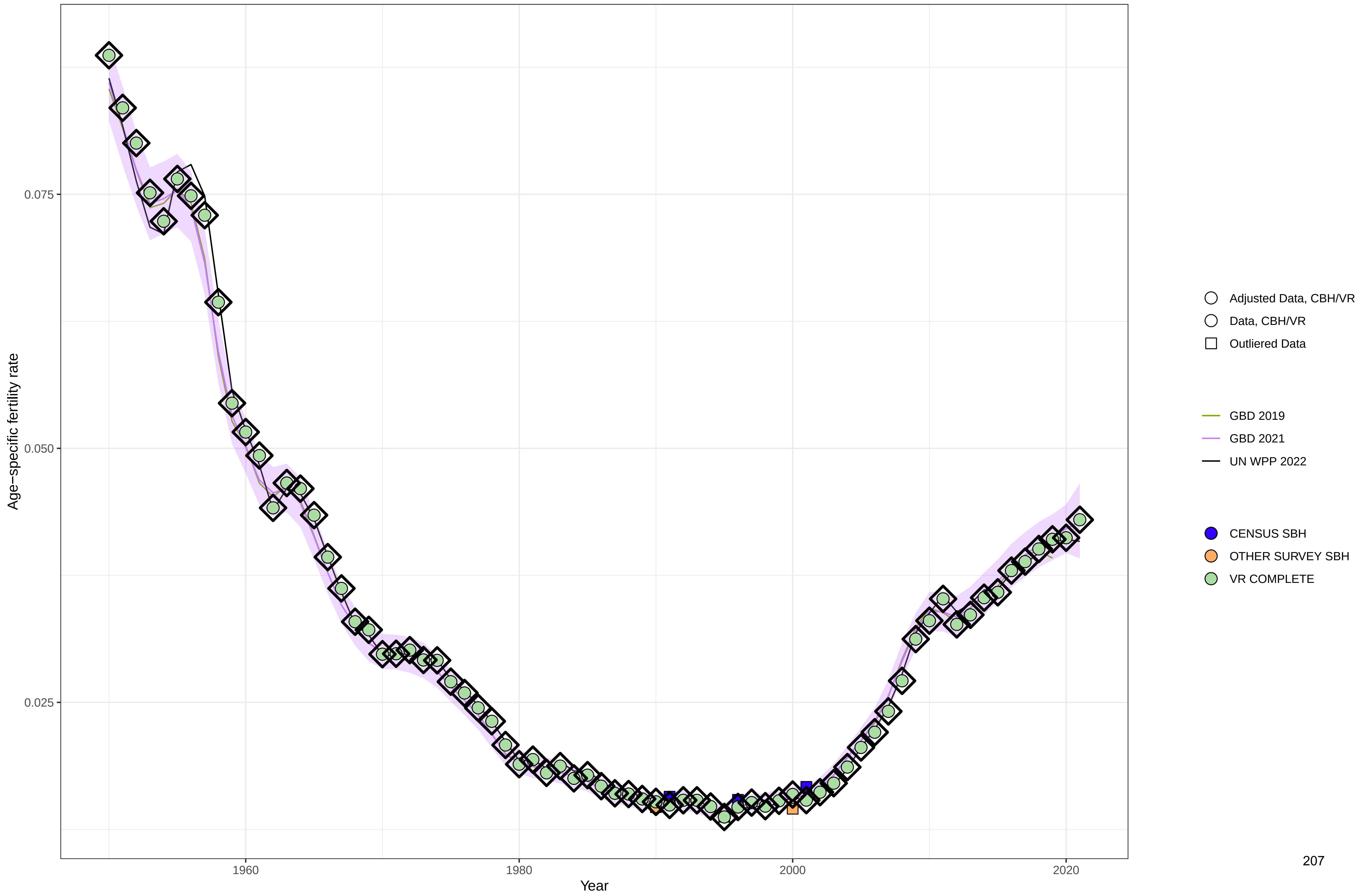

Age−specific fertility fitted trend lines, 1950−2021: Slovakia, Ages: 40−44
Data points enclosed with a diamond are reference sources

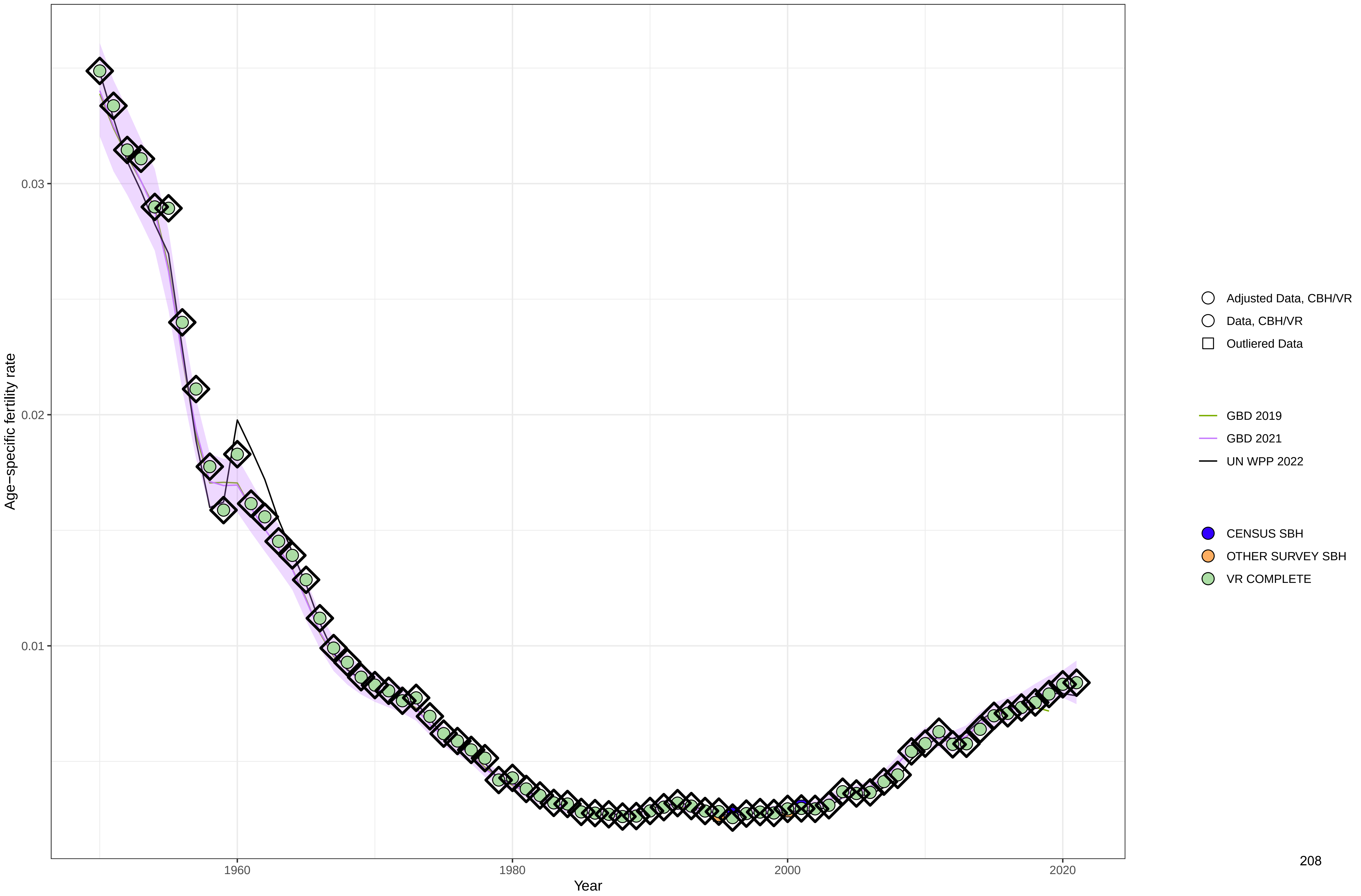

Age−specific fertility fitted trend lines, 1950−2021: Slovakia, Ages: 45−49
Data points enclosed with a diamond are reference sources

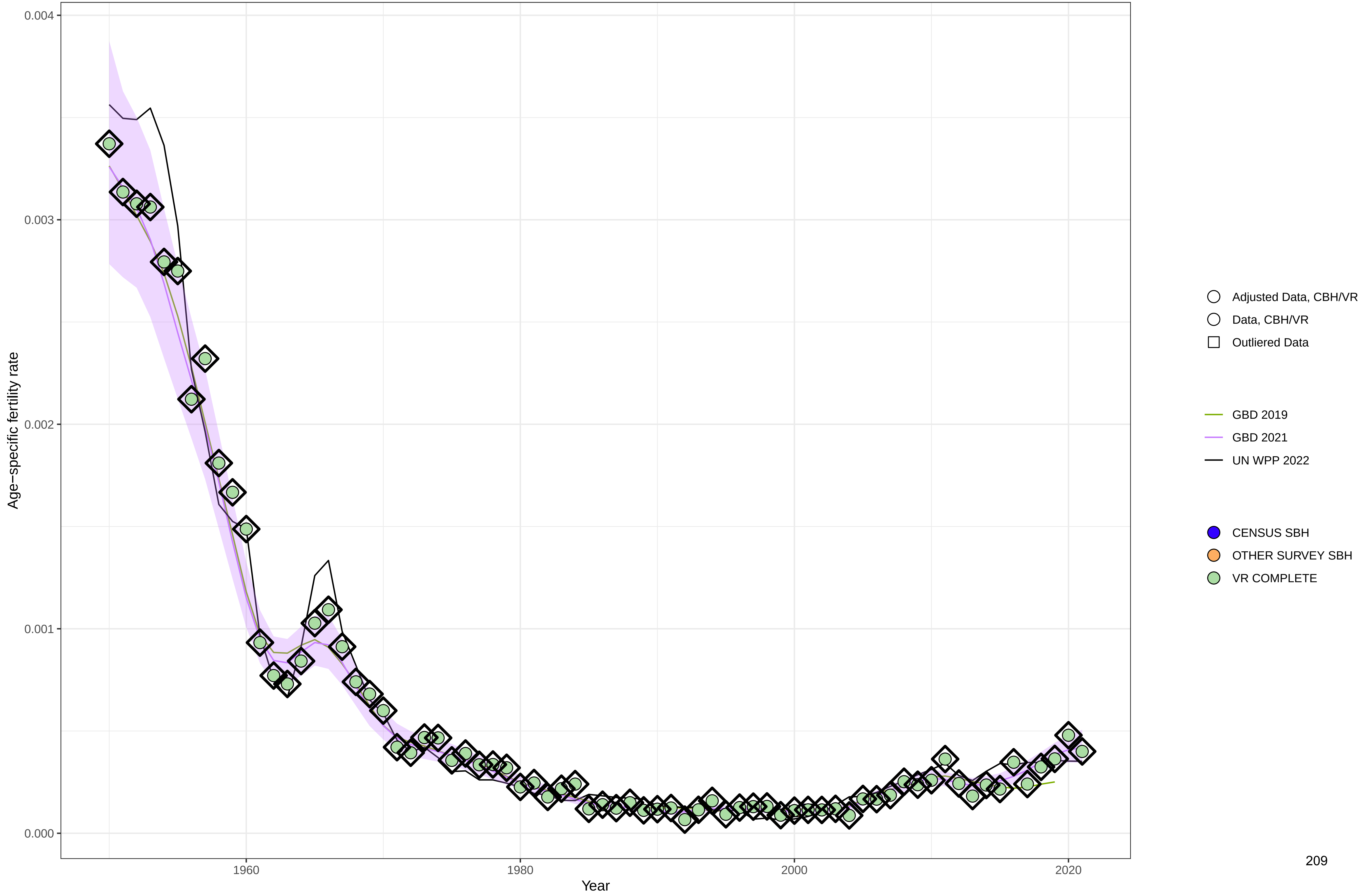

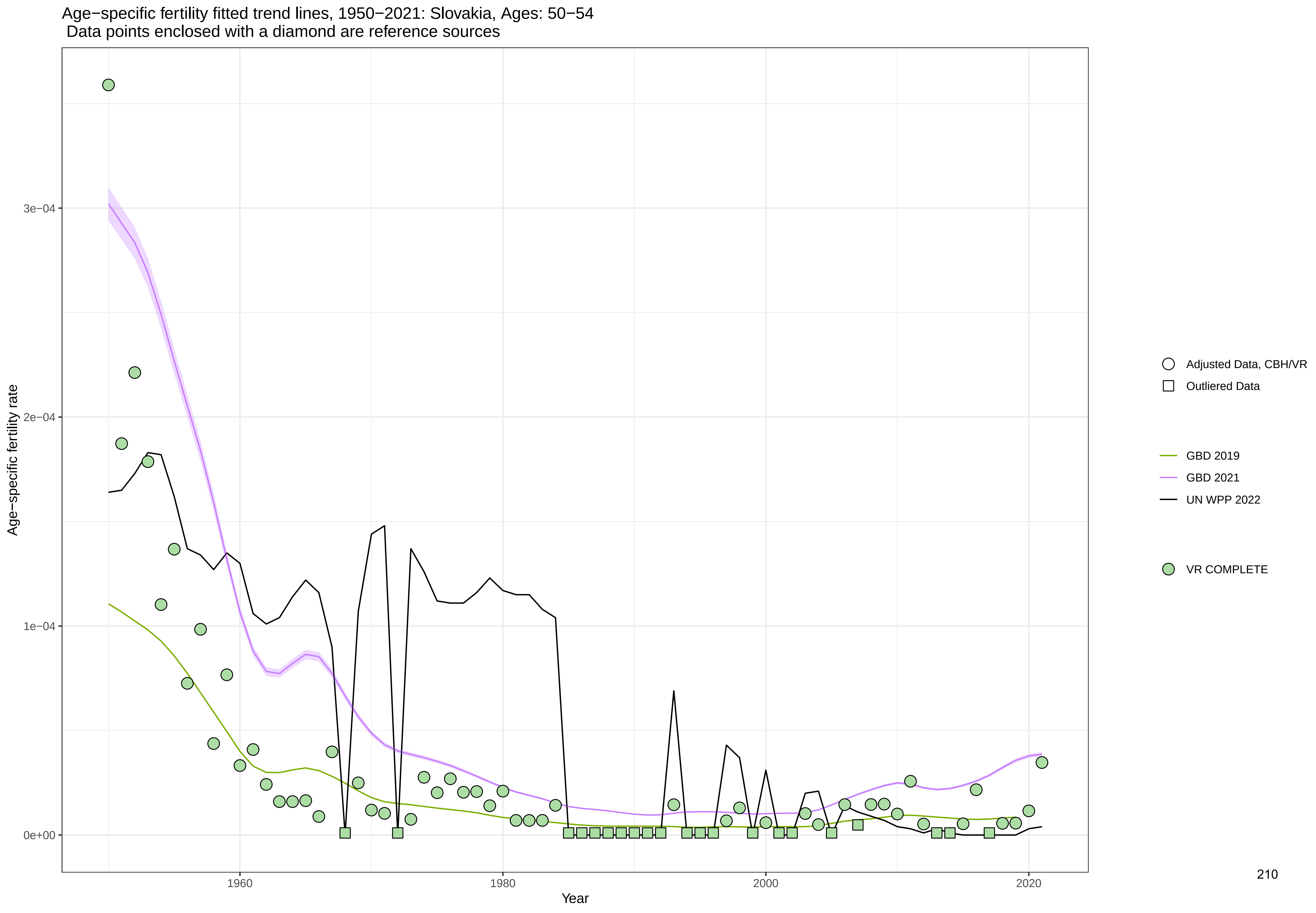
Age−specific fertility fitted trend lines, 1950−2021: Slovakia, Ages: 50−54
Data points enclosed with a diamond are reference sources
Age−specific fertility rate
3e−04
2e−04
1e−04
0e+00
1960
1980
2000
2020
Year
Adjusted Data, CBH/VR
Outliered Data
GBD 2019
GBD 2021
UN WPP 2022
VR COMPLETE

Total fertility rate fitted trend lines, 1950−2021: Slovakia TFR
Data points enclosed with a diamond are reference sources

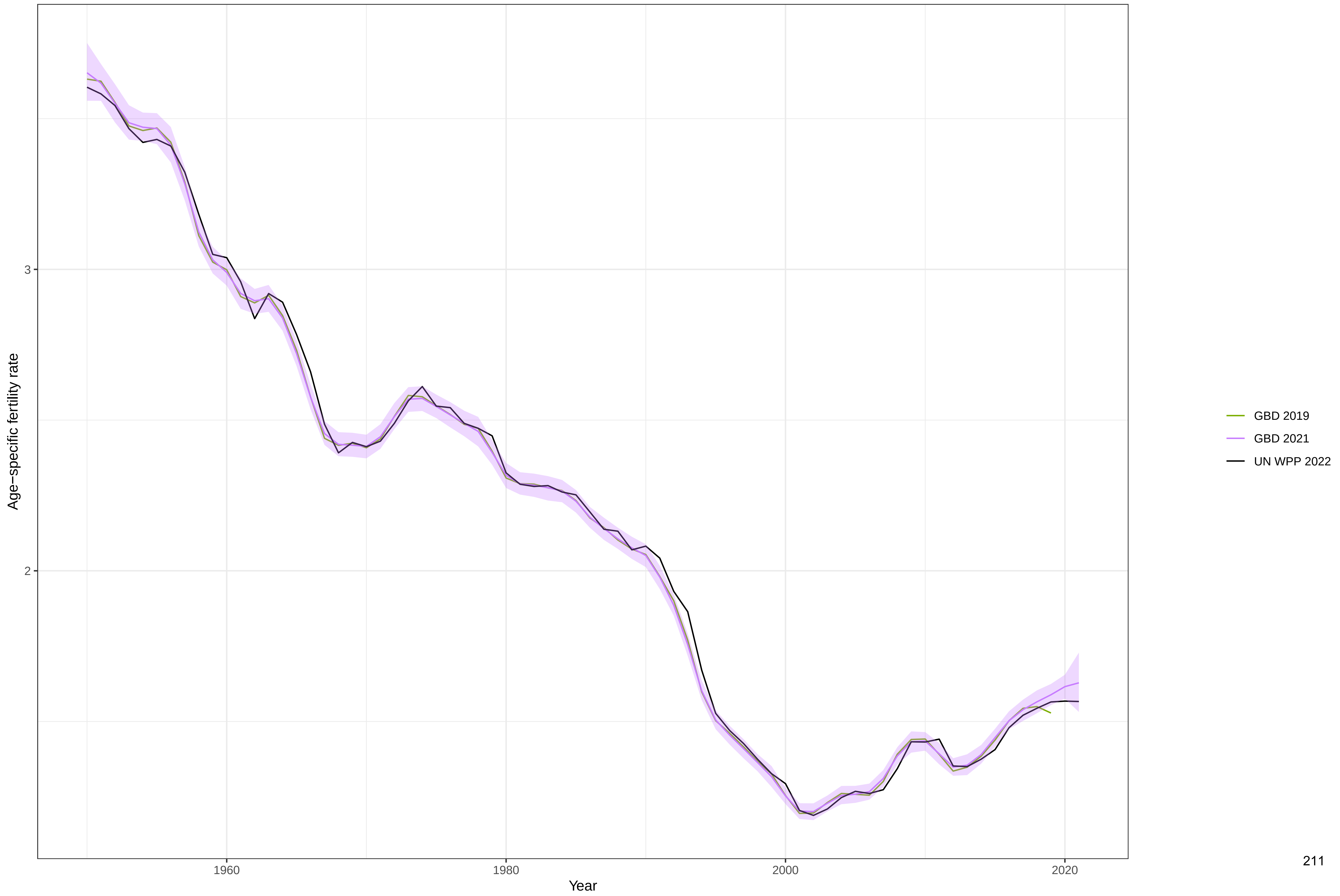

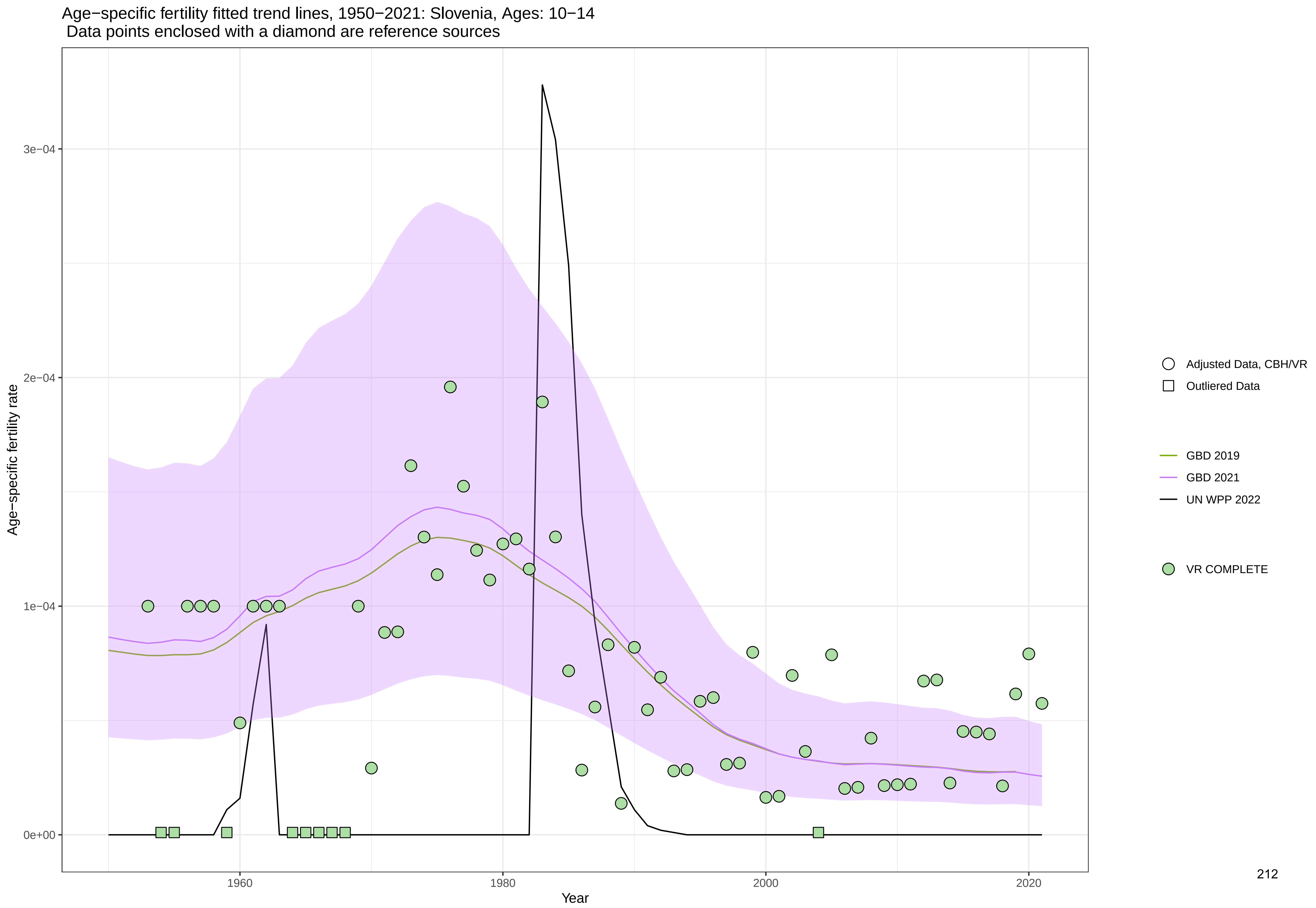
Age−specific fertility fitted trend lines, 1950−2021: Slovenia, Ages: 10−14
Data points enclosed with a diamond are reference sources
Age−specific fertility rate
3e−04
2e−04
1e−04
0e+00
1960
1980
2000
2020
Year
Adjusted Data, CBH/VR
Outliered Data
GBD 2019
GBD 2021
UN WPP 2022
VR COMPLETE

Age−specific fertility fitted trend lines, 1950−2021: Slovenia, Ages: 15−19
Data points enclosed with a diamond are reference sources

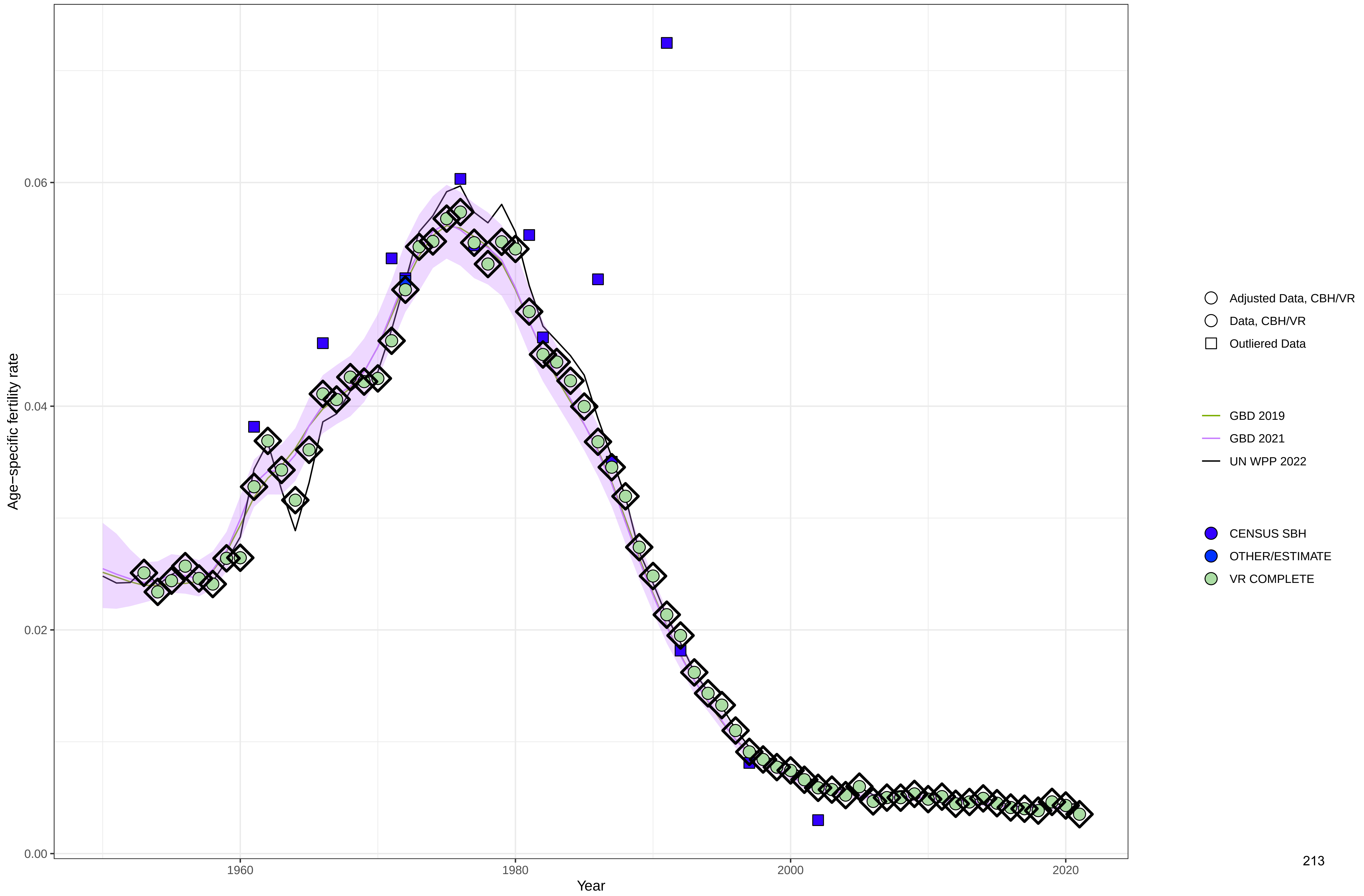

Age−specific fertility fitted trend lines, 1950−2021: Slovenia, Ages: 20−24
Data points enclosed with a diamond are reference sources

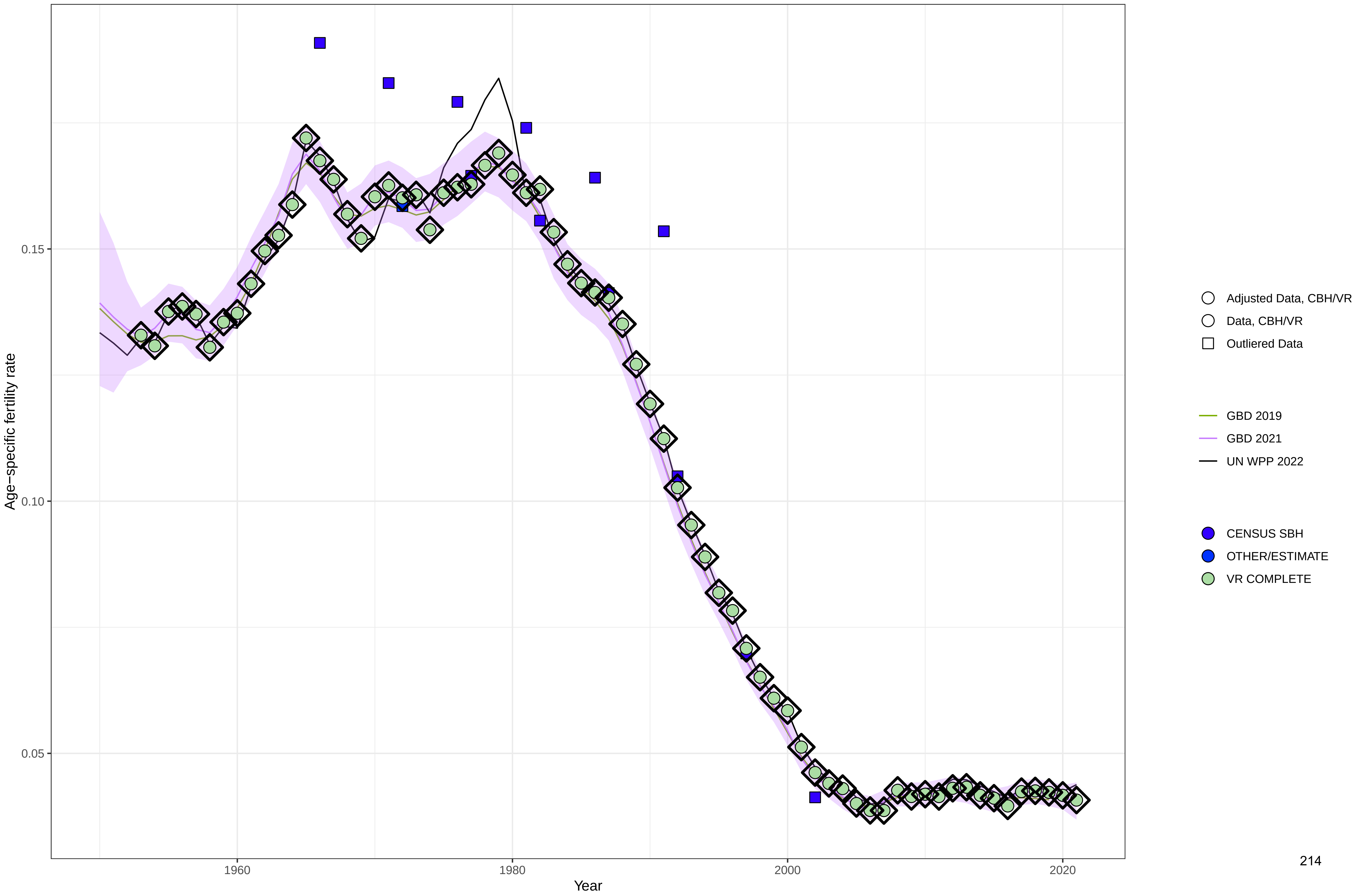

Age−specific fertility fitted trend lines, 1950−2021: Slovenia, Ages: 25−29
Data points enclosed with a diamond are reference sources

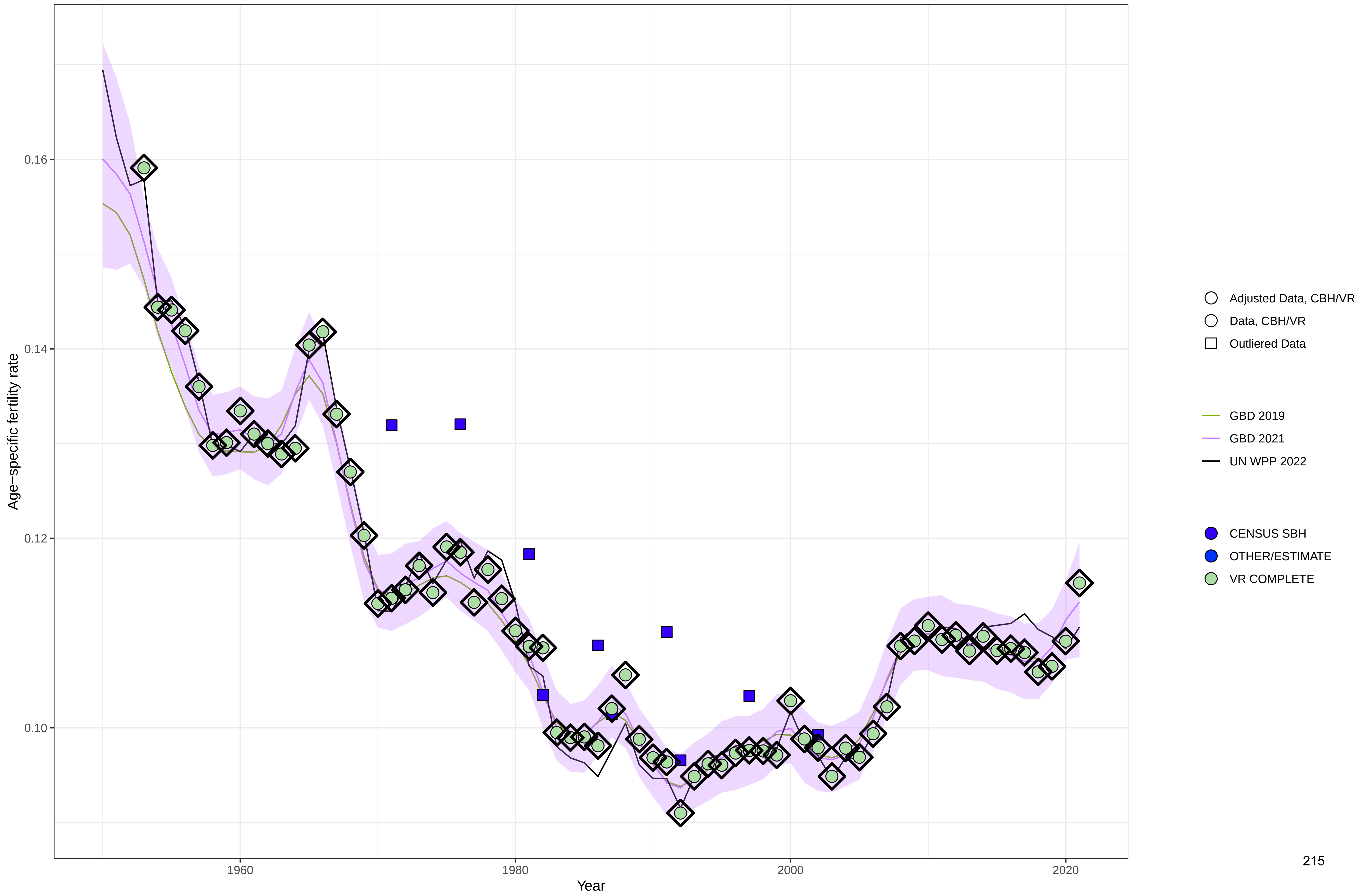

Age−specific fertility fitted trend lines, 1950−2021: Slovenia, Ages: 30−34
Data points enclosed with a diamond are reference sources

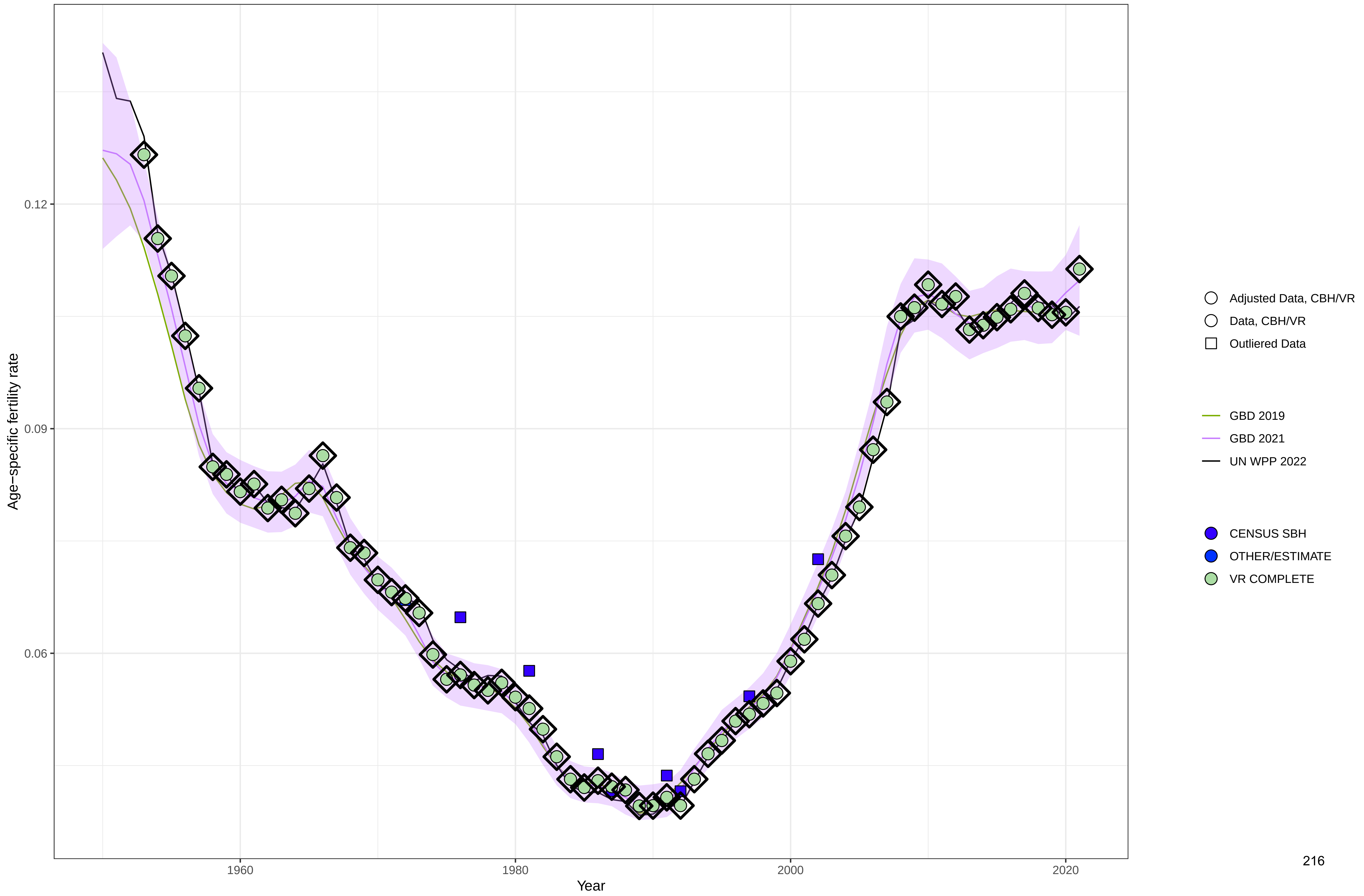

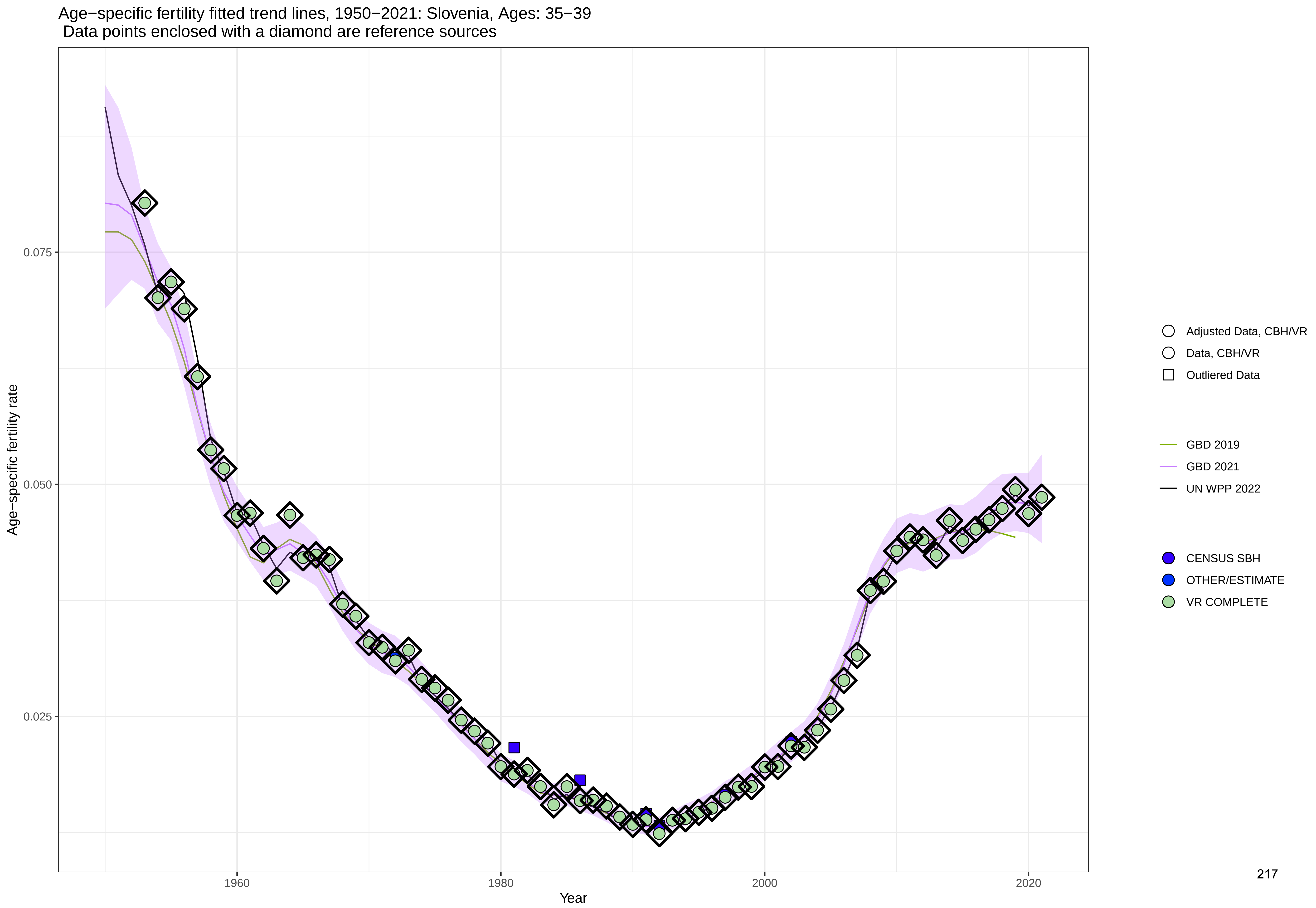
Age−specific fertility fitted trend lines, 1950−2021: Slovenia, Ages: 35−39
Data points enclosed with a diamond are reference sources
Age−specific fertility rate
0.075
0.050
0.025
1960
1980
2000
2020
Year
Adjusted Data, CBH/VR
Data, CBH/VR
Outliered Data
GBD 2019
GBD 2021
UN WPP 2022
CENSUS SBH
OTHER/ESTIMATE
VR COMPLETE

Age−specific fertility fitted trend lines, 1950−2021: Slovenia, Ages: 40−44
Data points enclosed with a diamond are reference sources

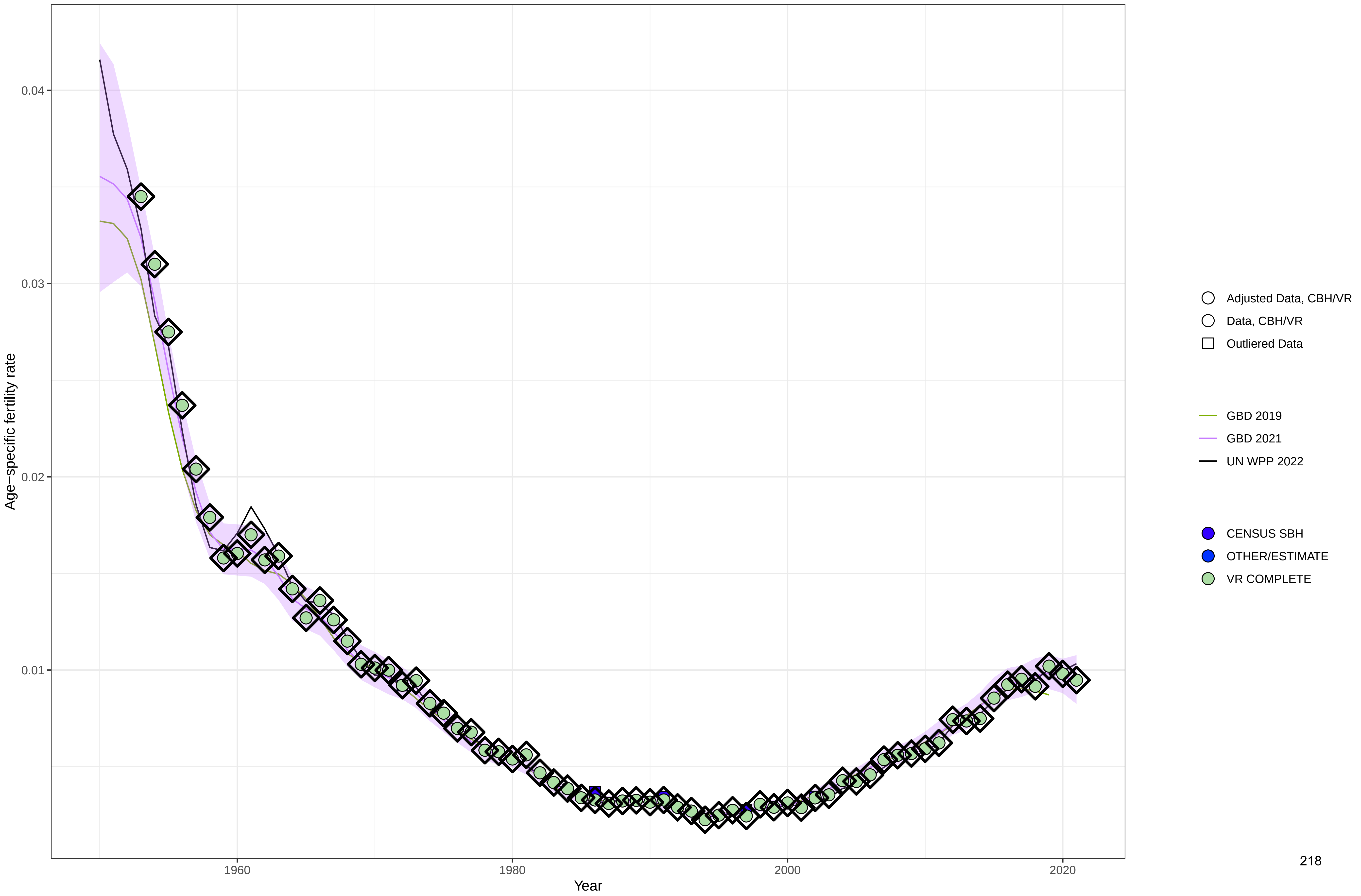

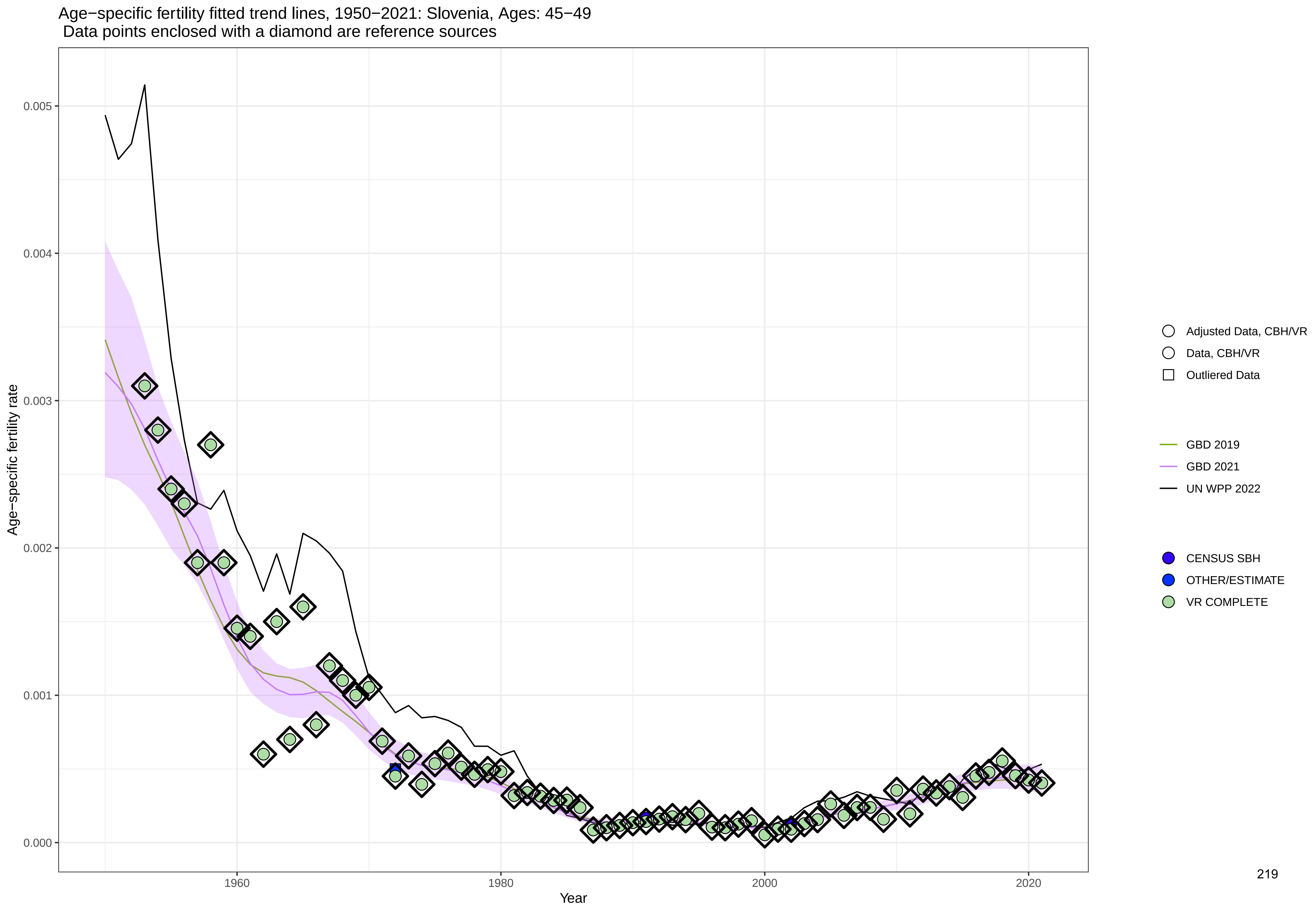

Age−specific fertility fitted trend lines, 1950−2021: Slovenia, Ages: 45−49
Data points enclosed with a diamond are reference sources
Age−specific fertility rate
0.000
0.001
0.002
0.003
0.004
0.005
1960
1980
2000
2020
Year
Adjusted Data, CBH/VR
Data, CBH/VR
Outliered Data
GBD 2019
GBD 2021
UN WPP 2022
CENSUS SBH
OTHER/ESTIMATE
VR COMPLETE

Age−specific fertility fitted trend lines, 1950−2021: Slovenia, Ages: 50−54
Data points enclosed with a diamond are reference sources

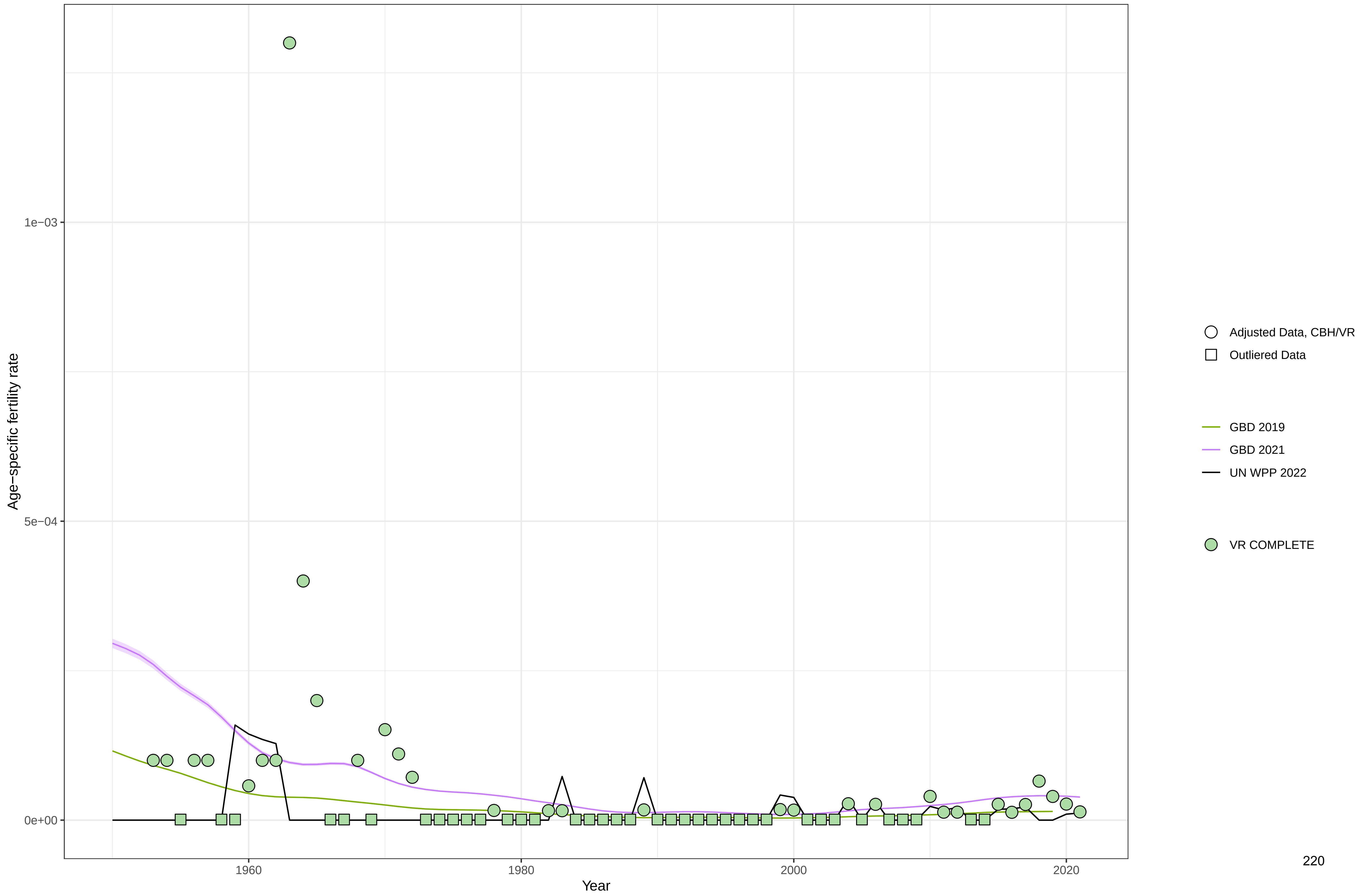

Total fertility rate fitted trend lines, 1950−2021: Slovenia TFR
Data points enclosed with a diamond are reference sources

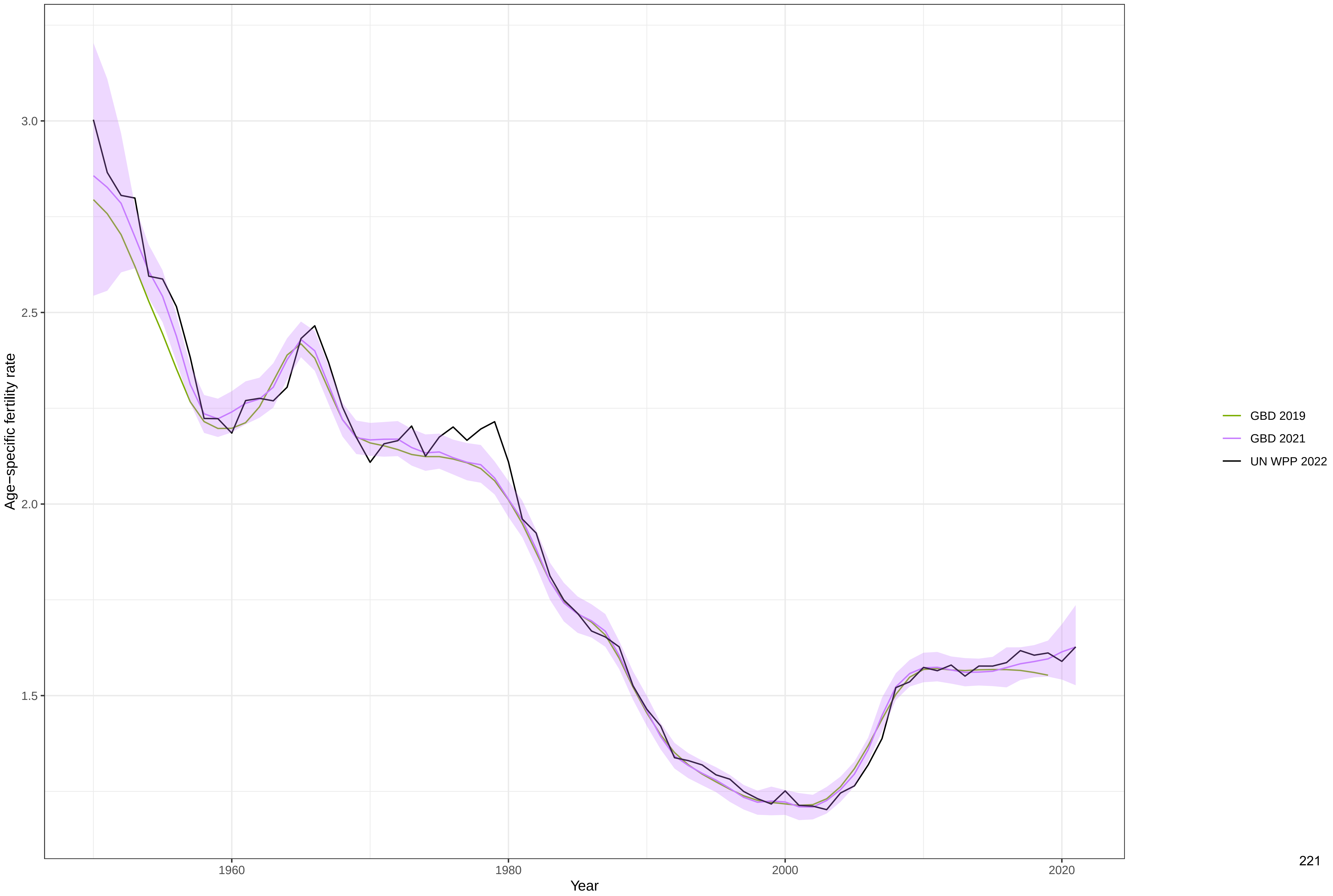

Age−specific fertility fitted trend lines, 1950−2021: Belarus, Ages: 10−14
Data points enclosed with a diamond are reference sources

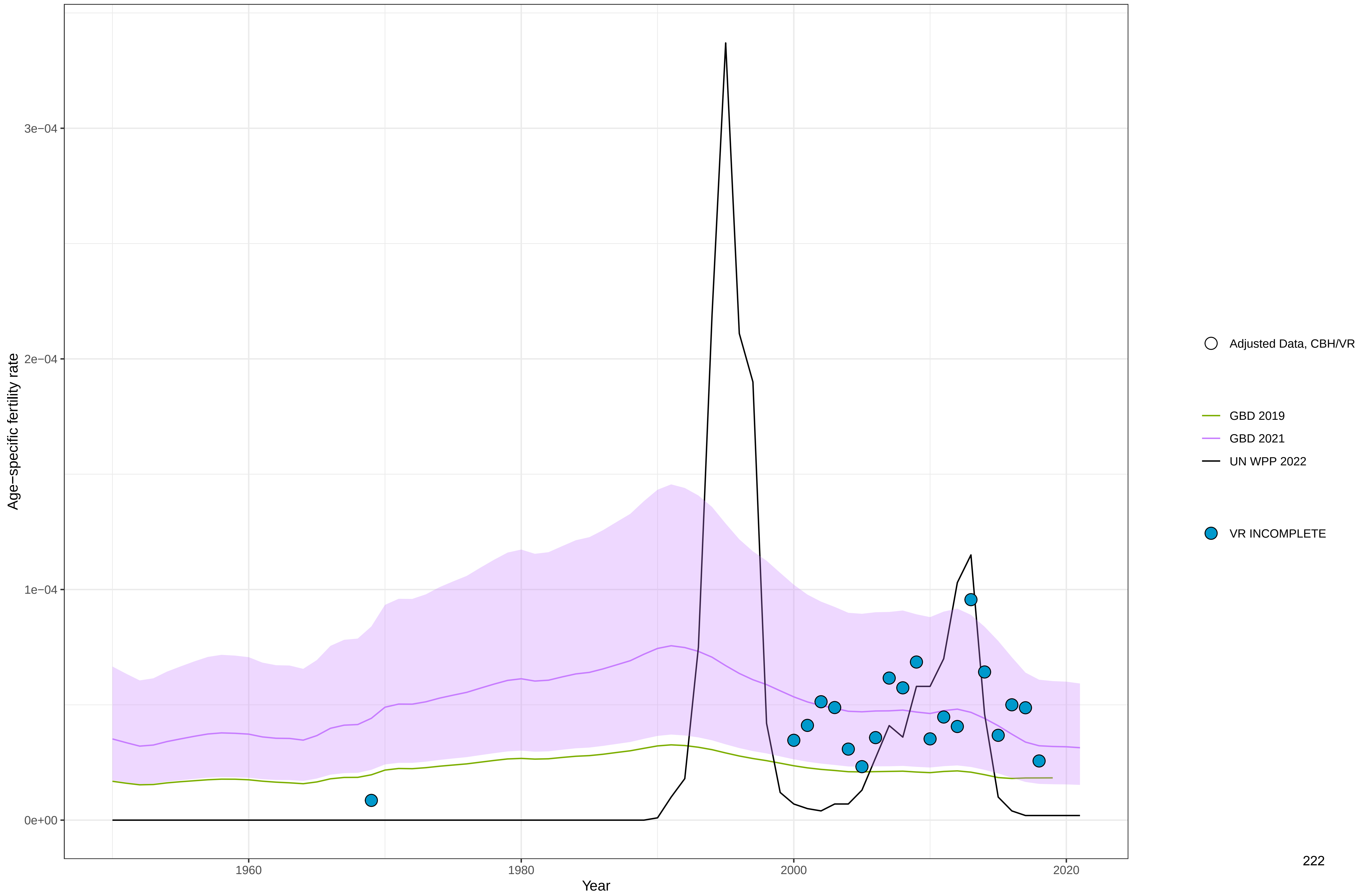

Age−specific fertility fitted trend lines, 1950−2021: Belarus, Ages: 15−19
Data points enclosed with a diamond are reference sources

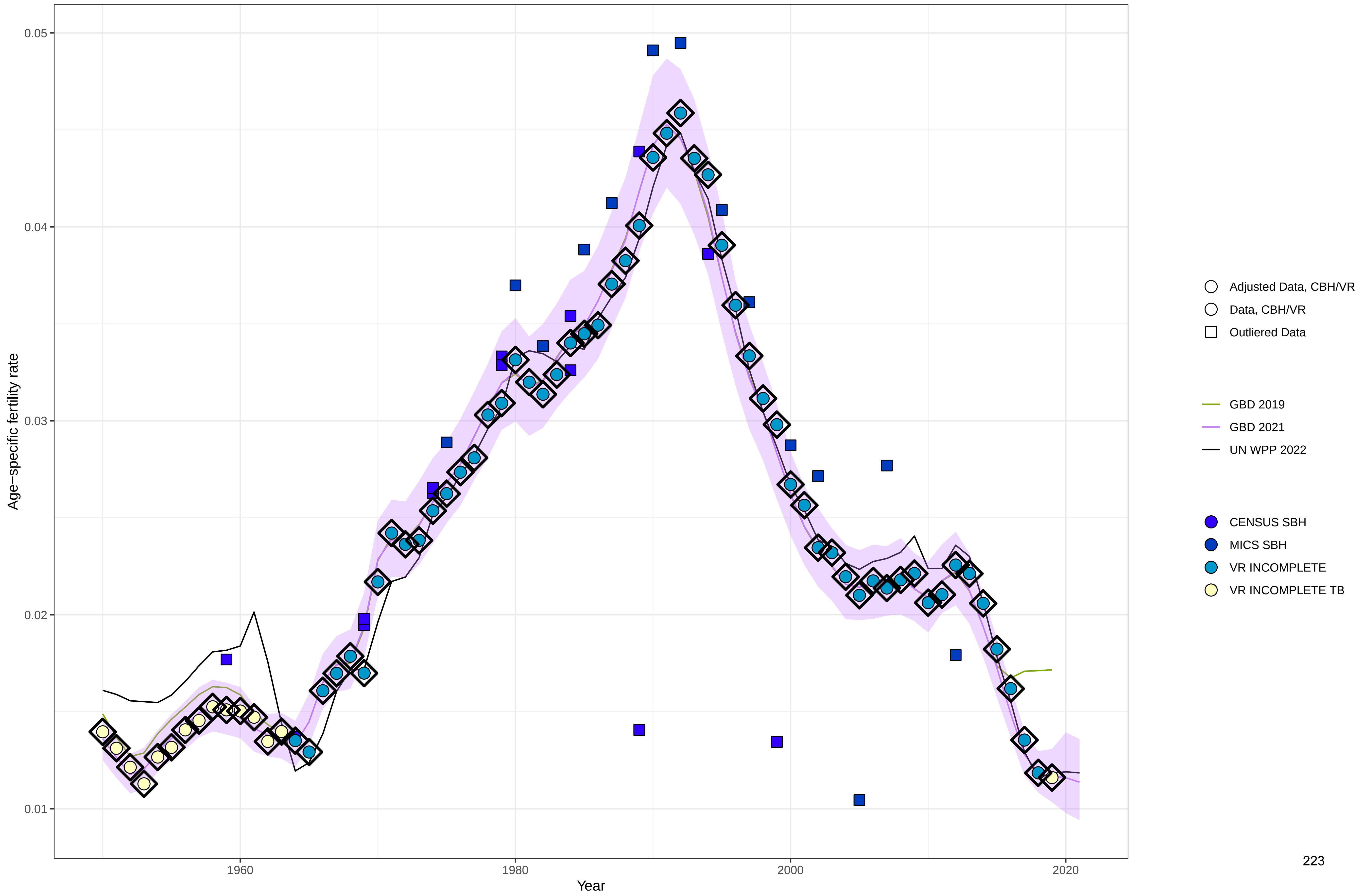

Age−specific fertility fitted trend lines, 1950−2021: Belarus, Ages: 20−24
Data points enclosed with a diamond are reference sources

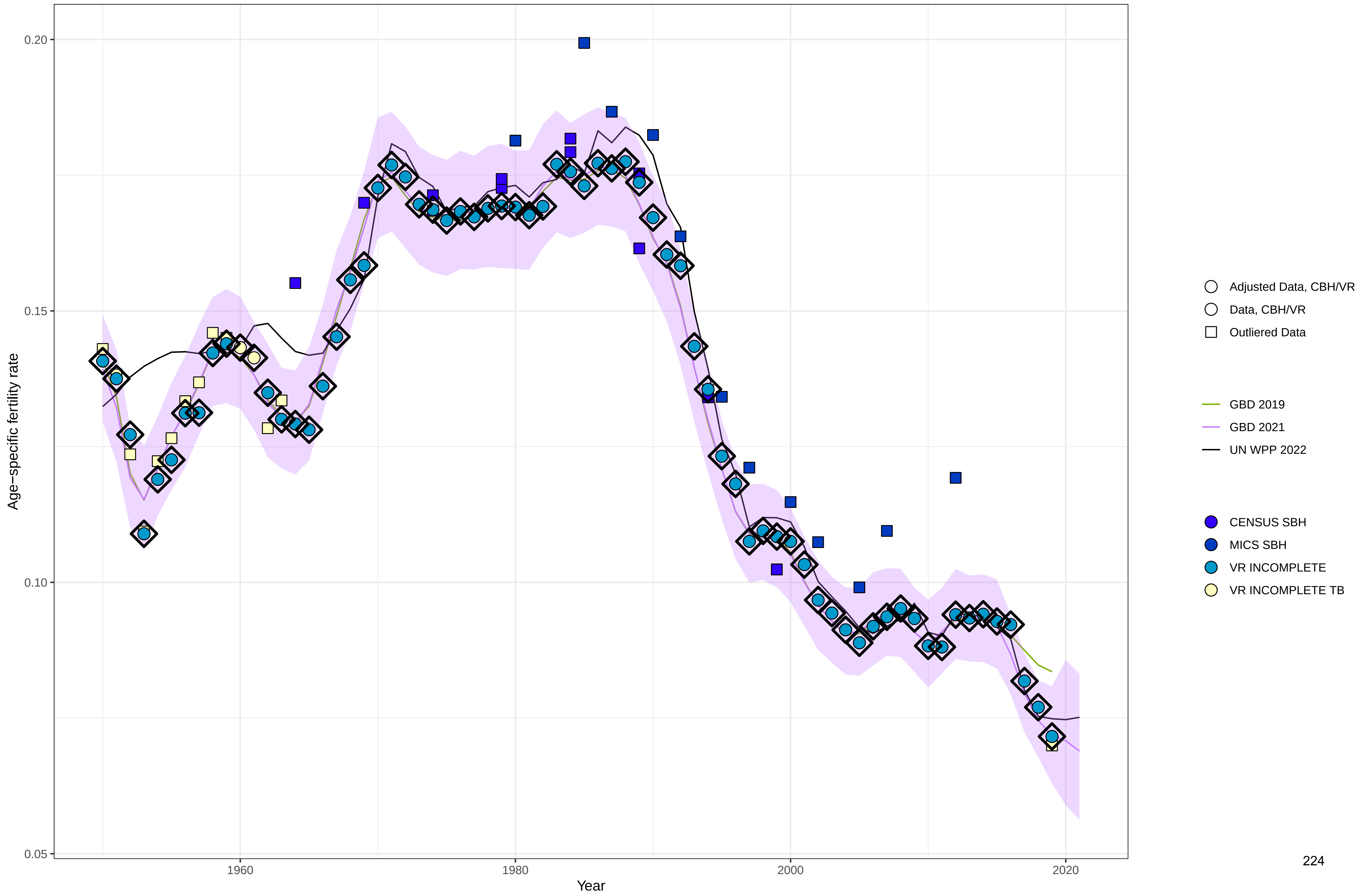

Age−specific fertility fitted trend lines, 1950−2021: Belarus, Ages: 25−29
Data points enclosed with a diamond are reference sources

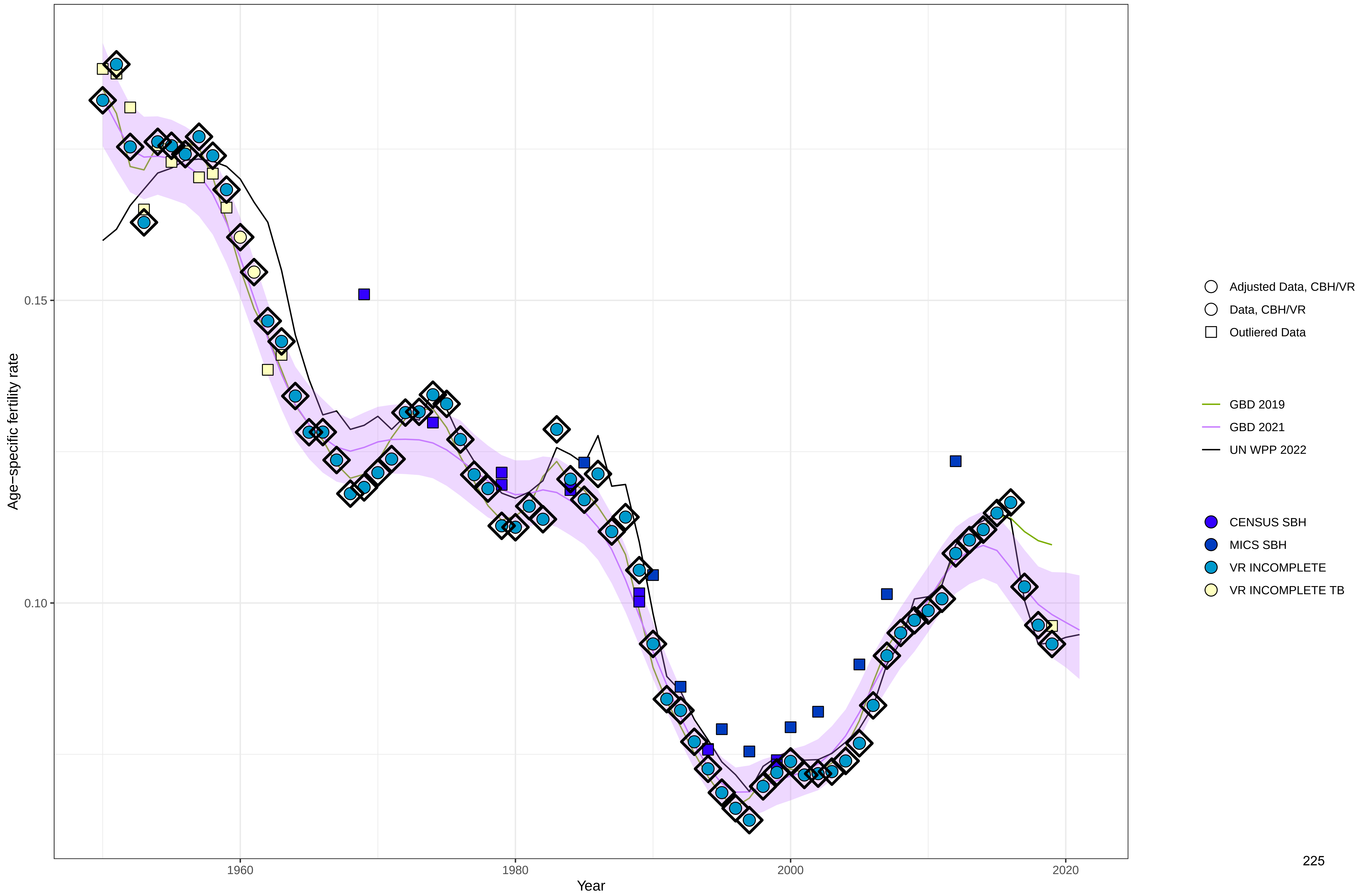

Age−specific fertility fitted trend lines, 1950−2021: Belarus, Ages: 30−34
Data points enclosed with a diamond are reference sources

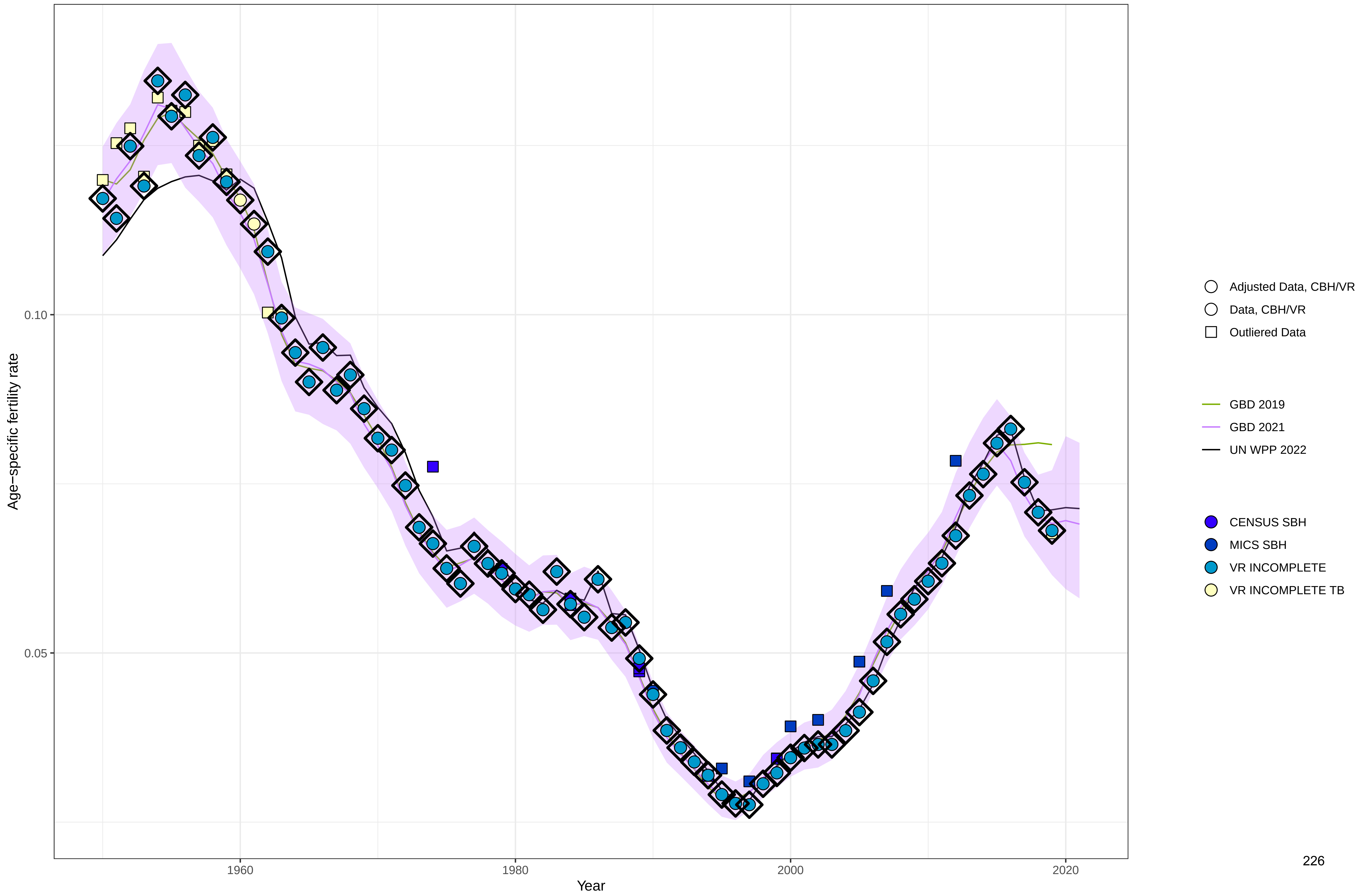

Age−specific fertility fitted trend lines, 1950−2021: Belarus, Ages: 35−39
Data points enclosed with a diamond are reference sources

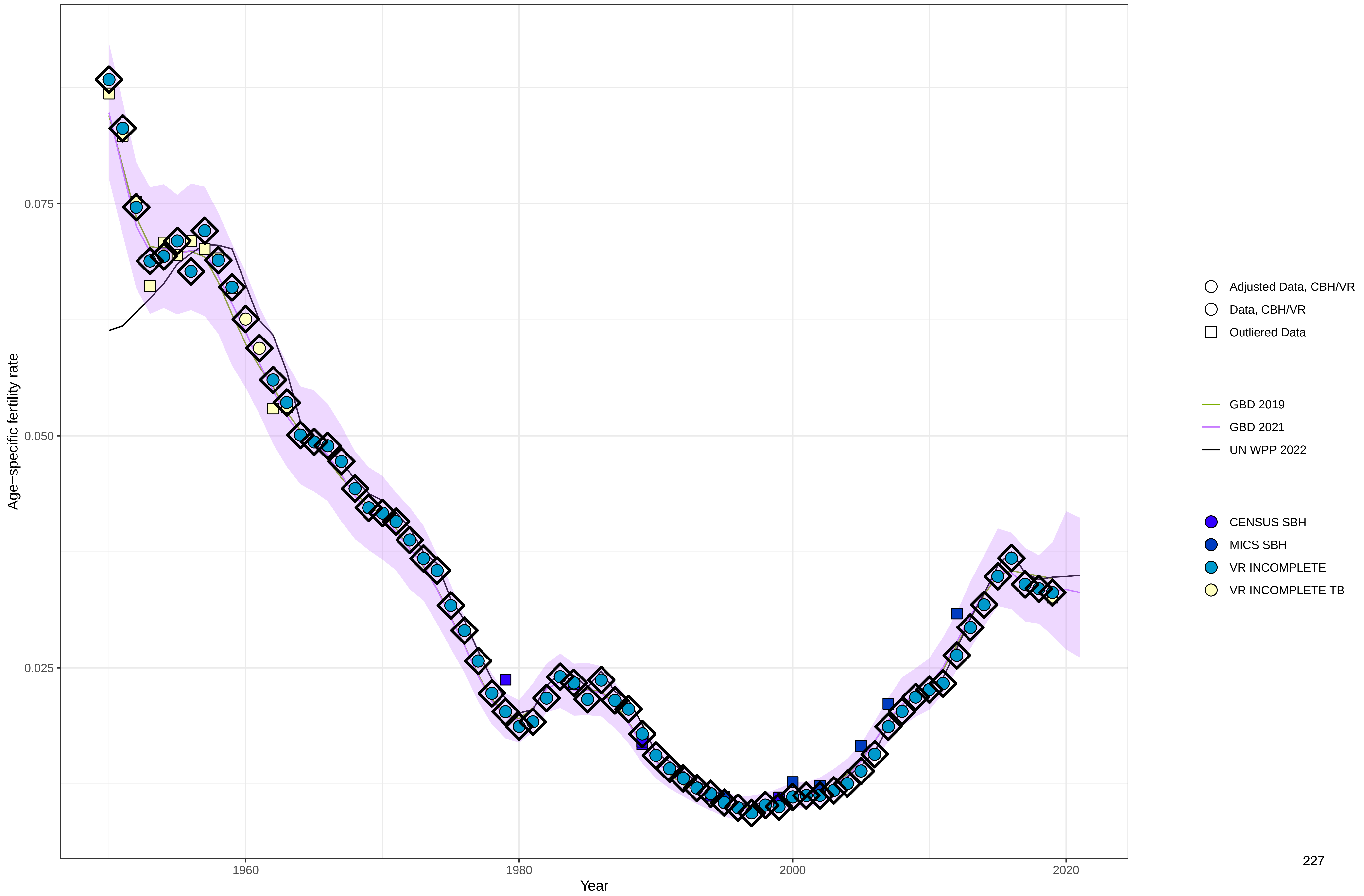

Age−specific fertility fitted trend lines, 1950−2021: Belarus, Ages: 40−44
Data points enclosed with a diamond are reference sources

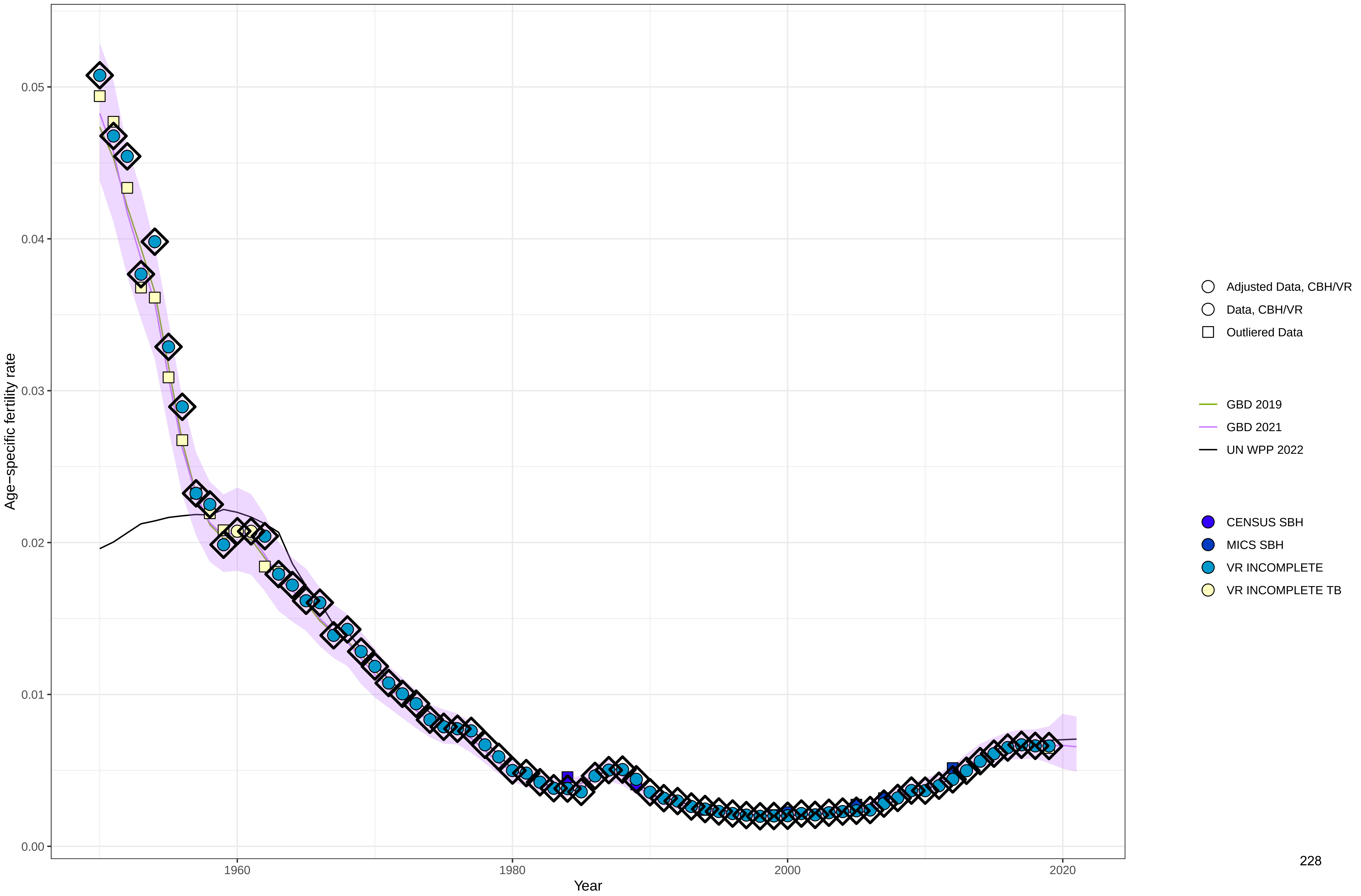

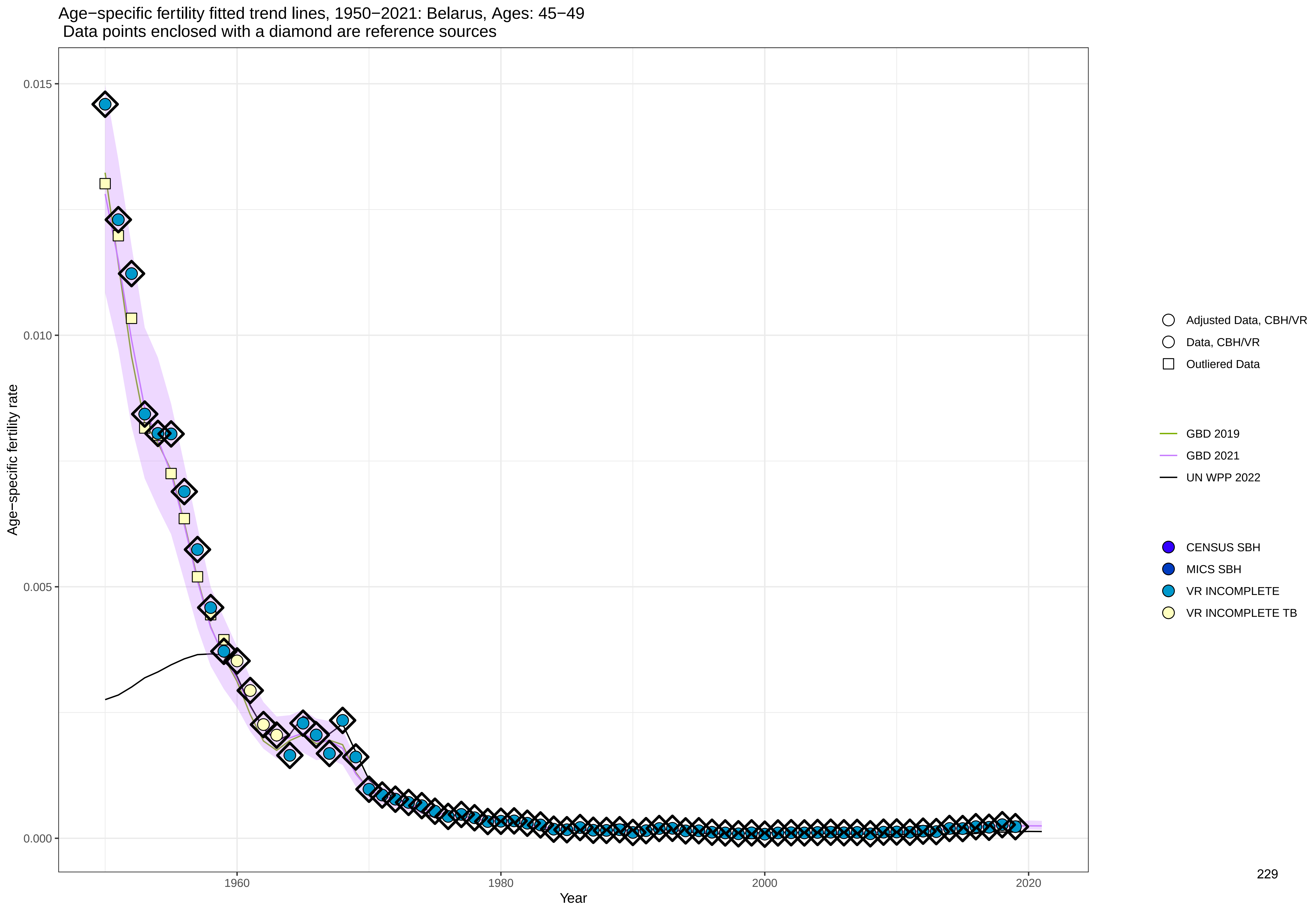
Age−specific fertility fitted trend lines, 1950−2021: Belarus, Ages: 45−49
Data points enclosed with a diamond are reference sources
Age−specific fertility rate
0.015
0.010
0.005
0.000
1960
1980
2000
2020
Year
Adjusted Data, CBH/VR
Data, CBH/VR
Outliered Data
GBD 2019
GBD 2021
UN WPP 2022
CENSUS SBH
MICS SBH
VR INCOMPLETE
VR INCOMPLETE TB

Age−specific fertility fitted trend lines, 1950−2021: Belarus, Ages: 50−54
Data points enclosed with a diamond are reference sources

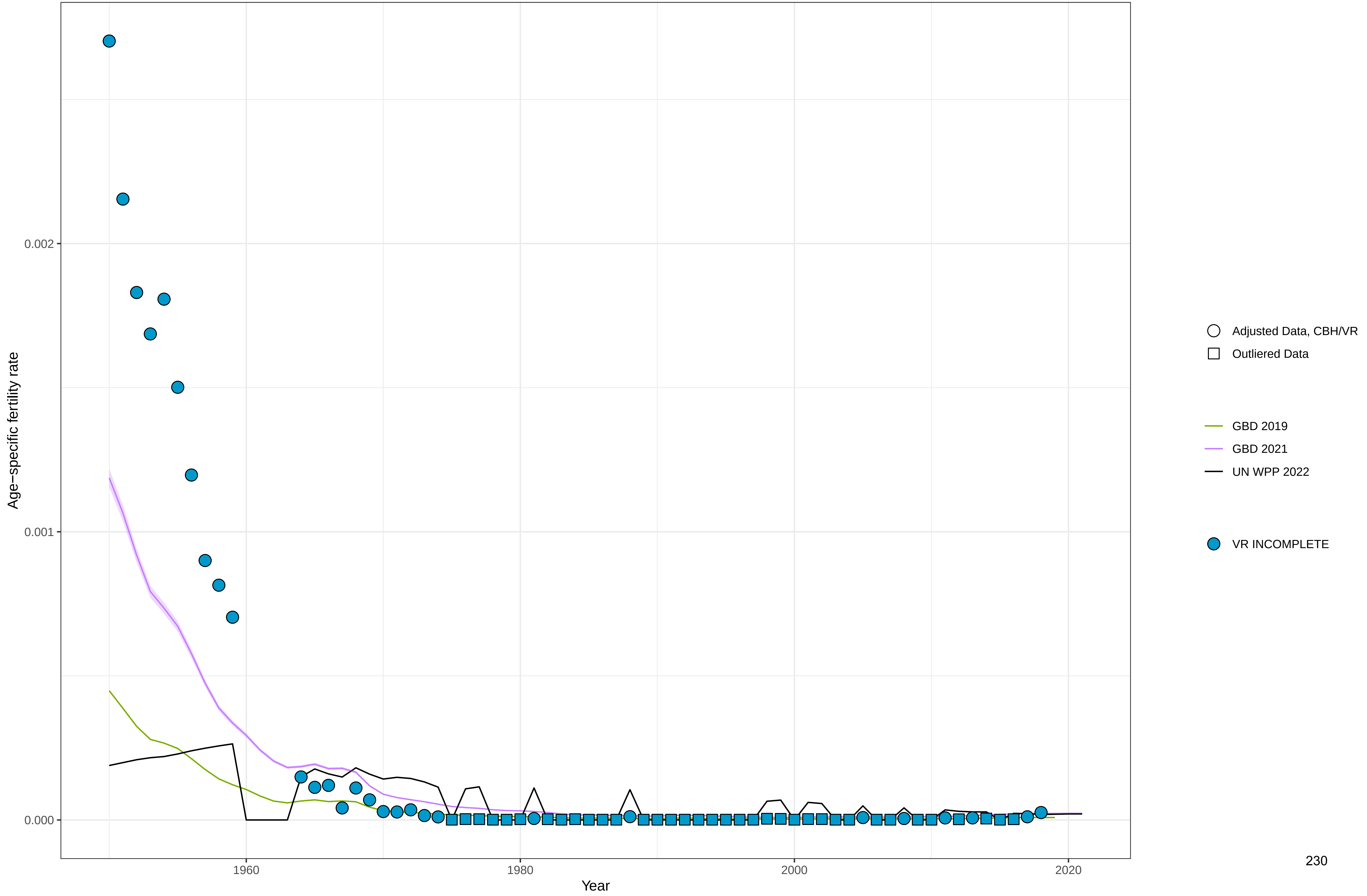

Total fertility rate fitted trend lines, 1950−2021: Belarus TFR
Data points enclosed with a diamond are reference sources

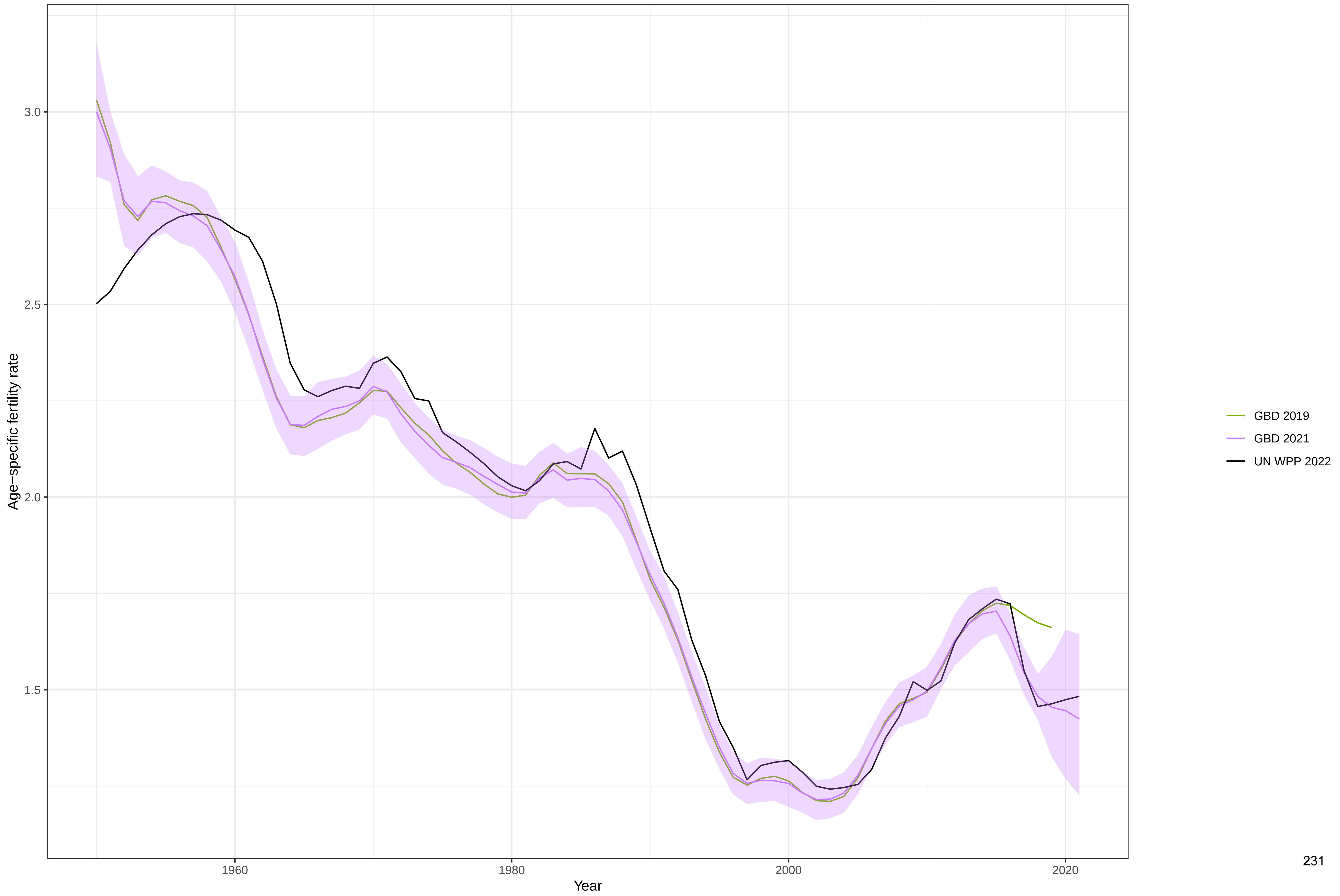

Age−specific fertility fitted trend lines, 1950−2021: Estonia, Ages: 10−14
Data points enclosed with a diamond are reference sources

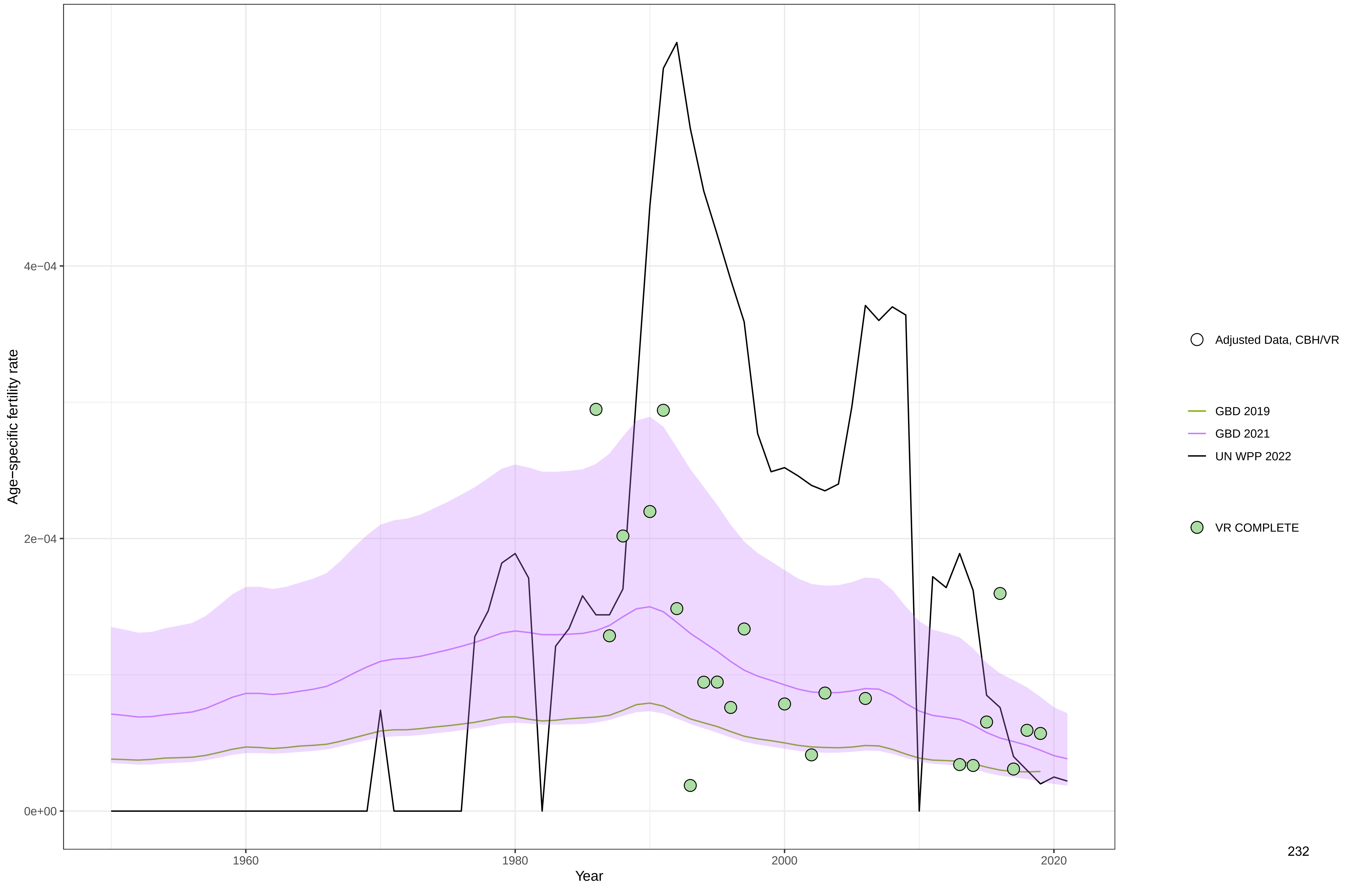

Age−specific fertility fitted trend lines, 1950−2021: Estonia, Ages: 15−19
Data points enclosed with a diamond are reference sources

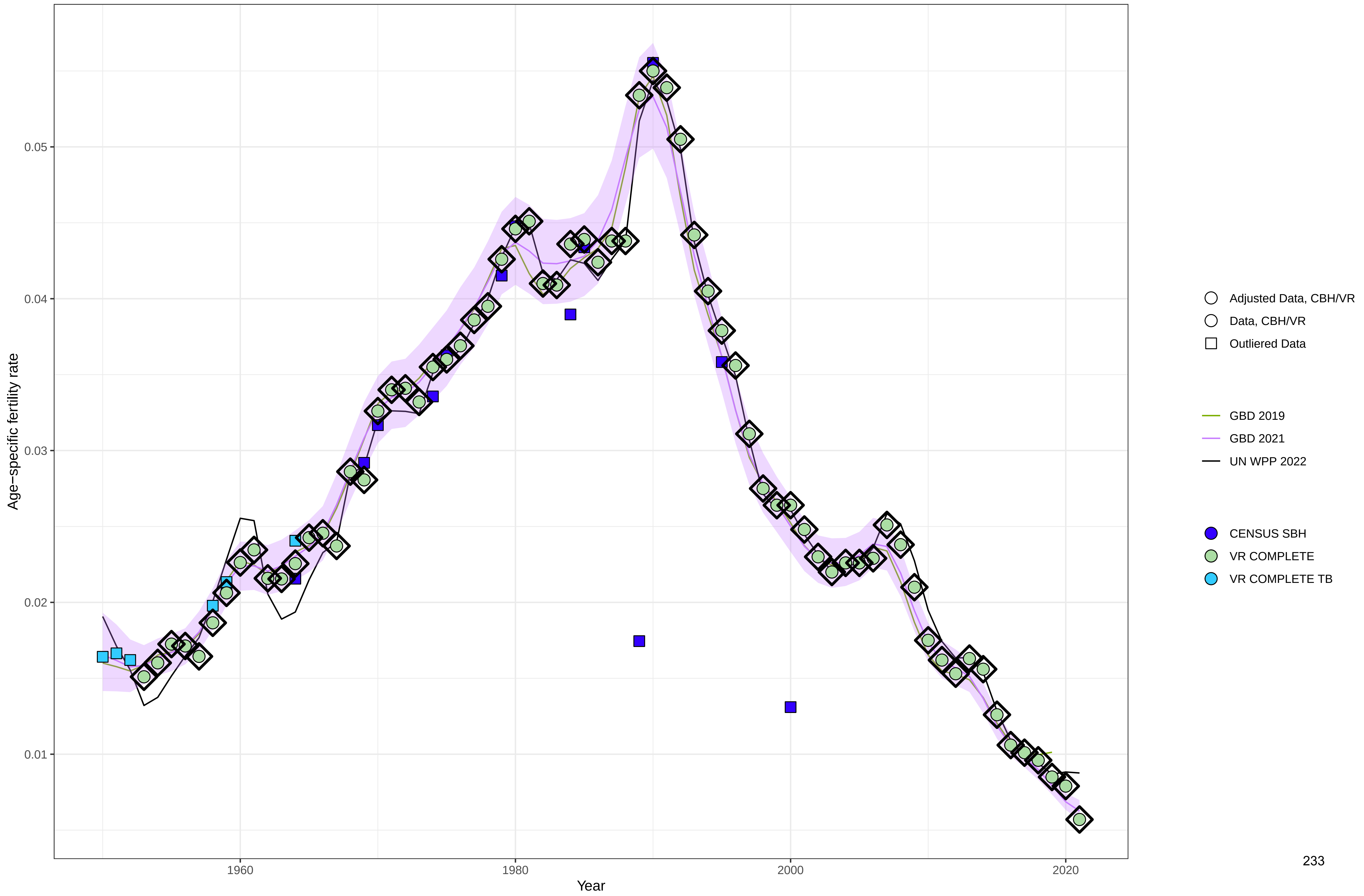

Age−specific fertility fitted trend lines, 1950−2021: Estonia, Ages: 20−24
Data points enclosed with a diamond are reference sources

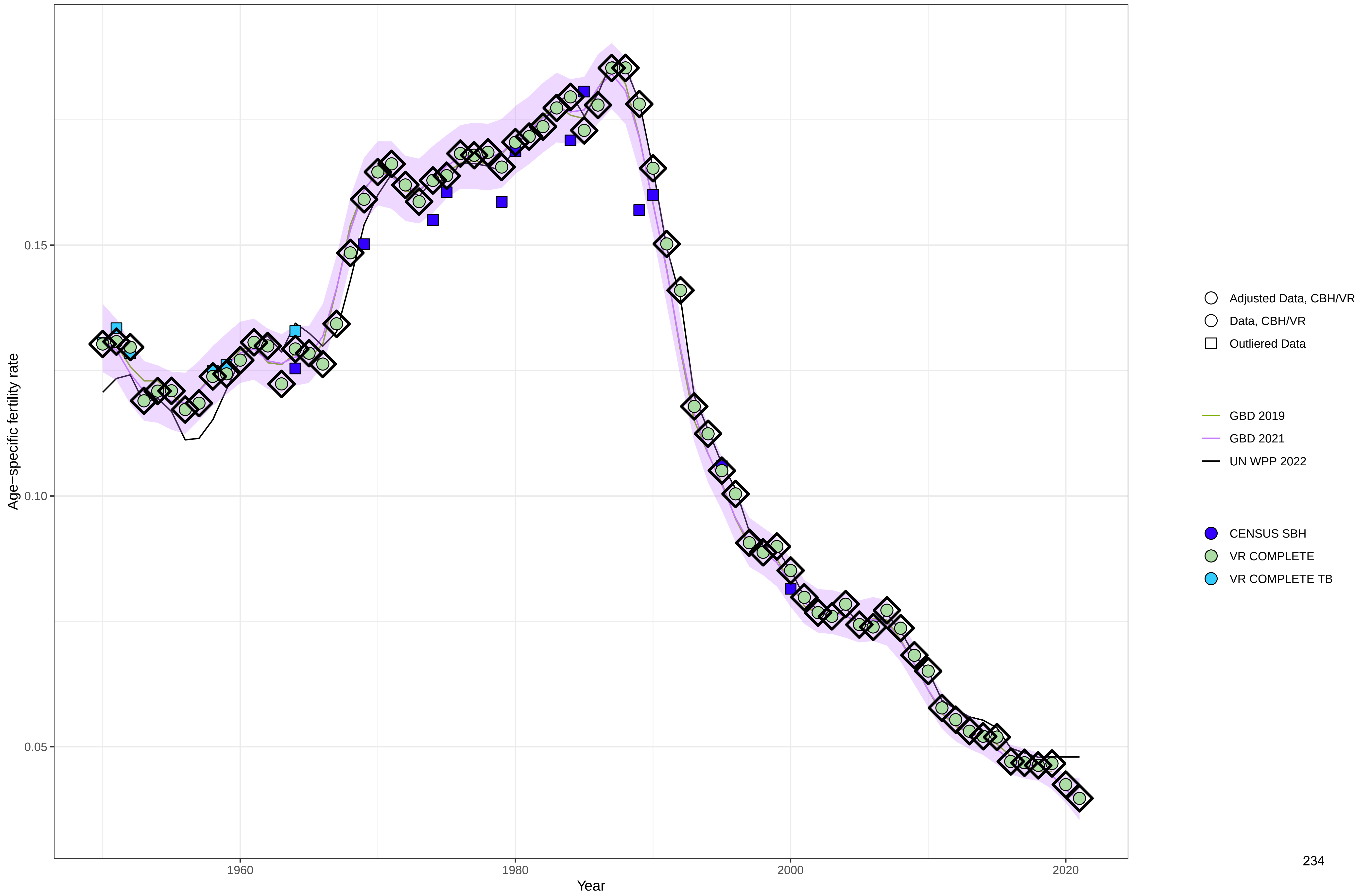

Age−specific fertility fitted trend lines, 1950−2021: Estonia, Ages: 25−29
Data points enclosed with a diamond are reference sources

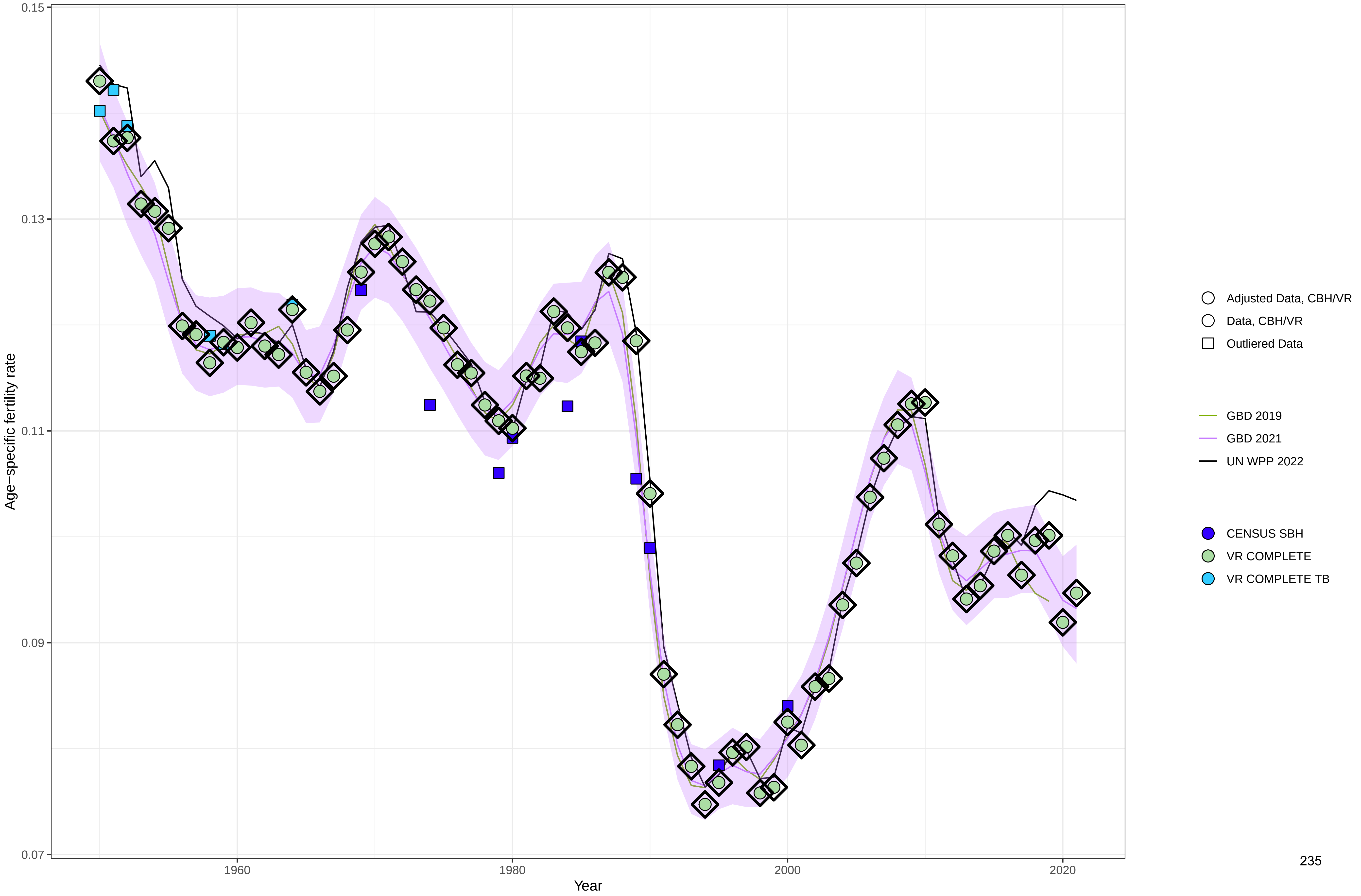

Age−specific fertility fitted trend lines, 1950−2021: Estonia, Ages: 30−34
Data points enclosed with a diamond are reference sources

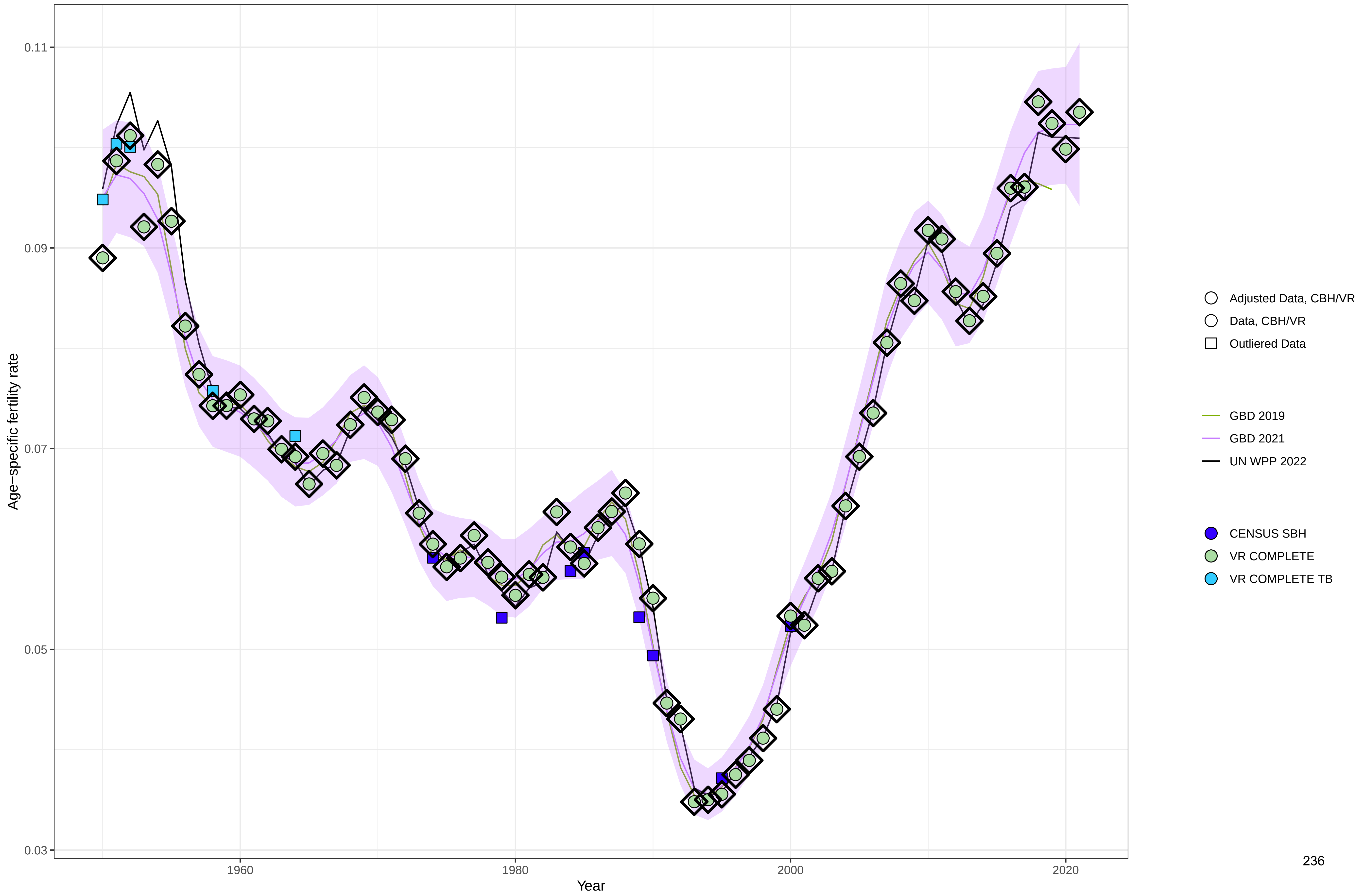

Age−specific fertility fitted trend lines, 1950−2021: Estonia, Ages: 35−39
Data points enclosed with a diamond are reference sources

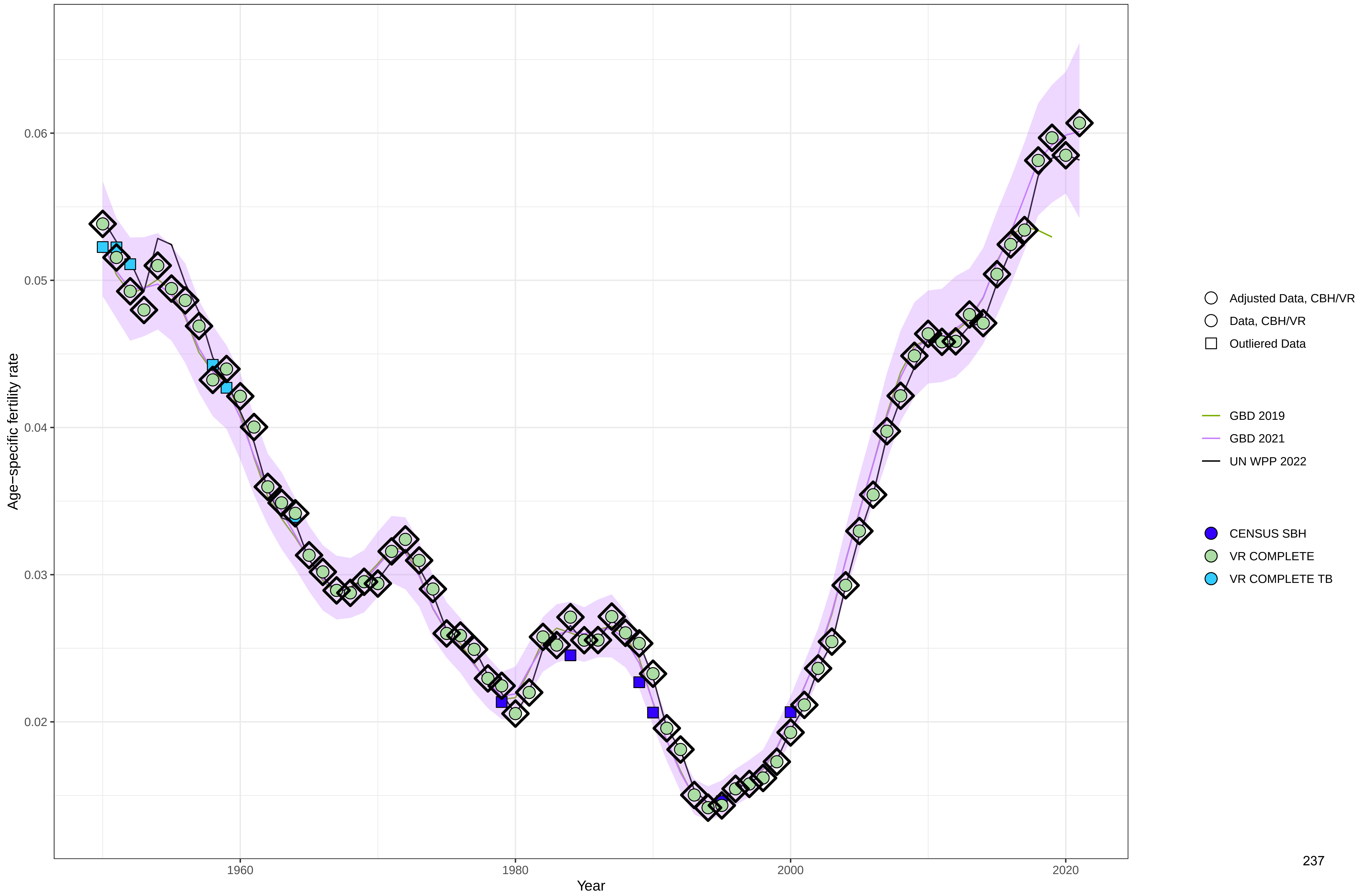

Age−specific fertility fitted trend lines, 1950−2021: Estonia, Ages: 40−44
Data points enclosed with a diamond are reference sources

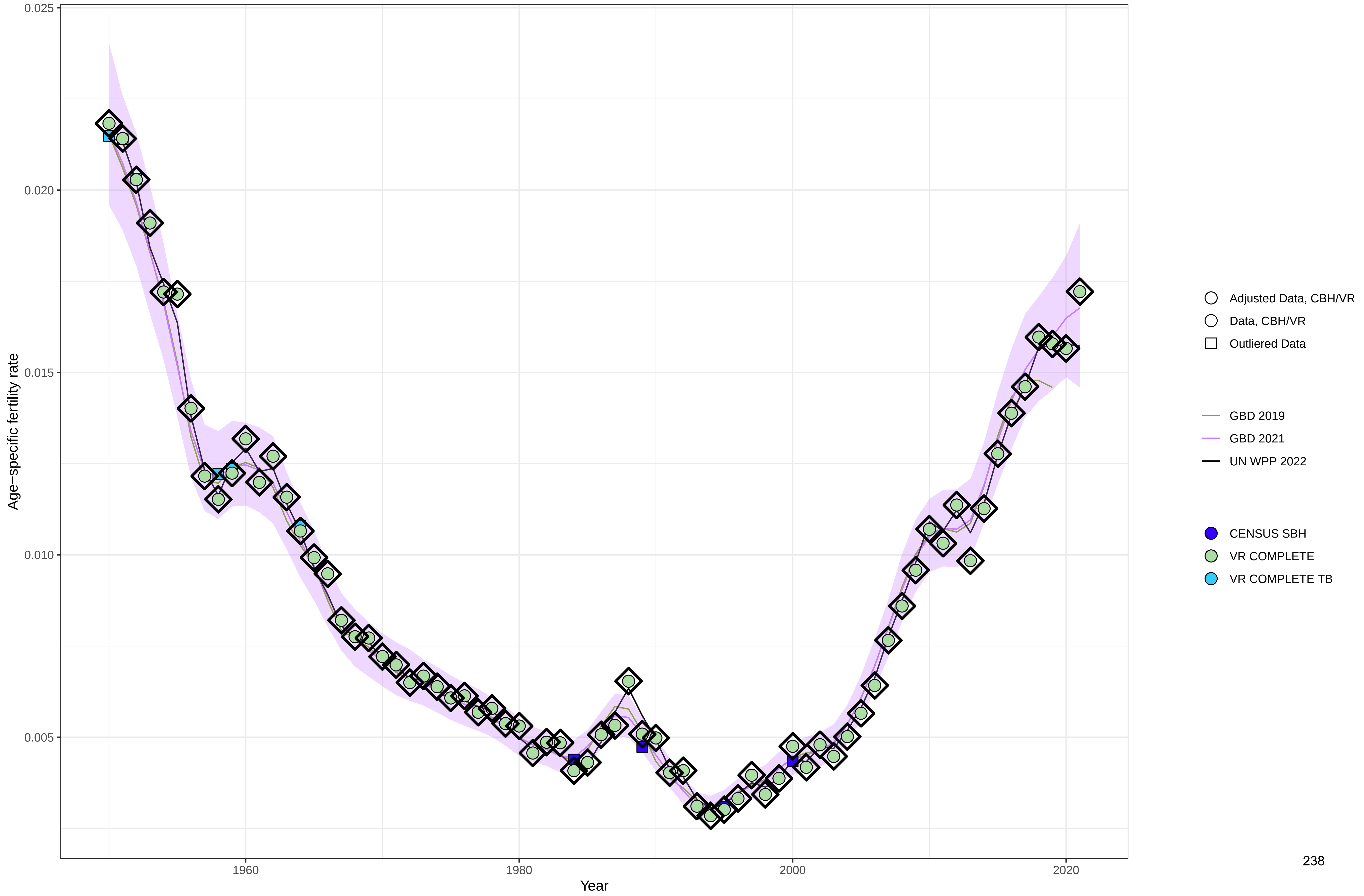

Age−specific fertility fitted trend lines, 1950−2021: Estonia, Ages: 45−49
Data points enclosed with a diamond are reference sources

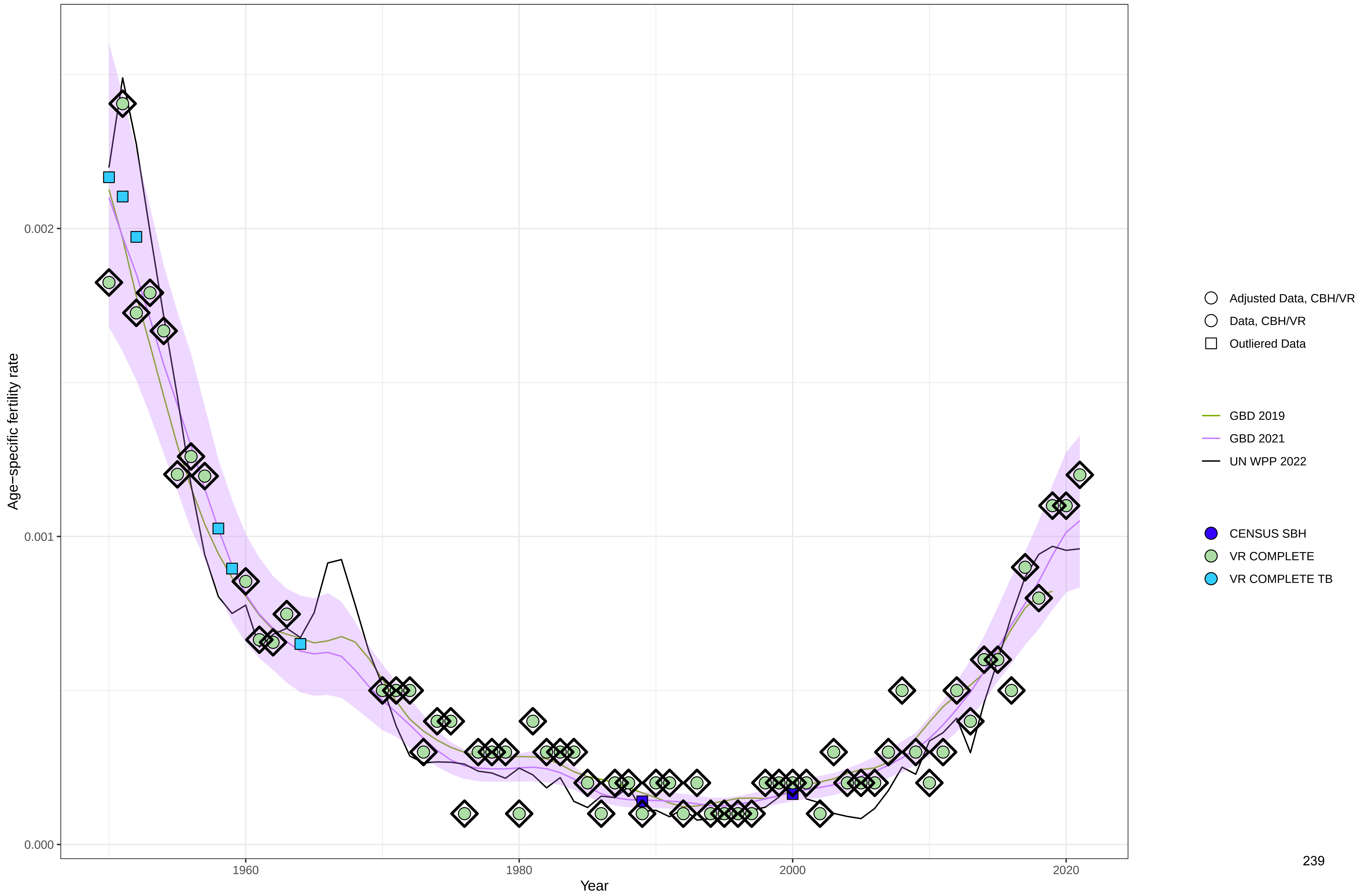

Age−specific fertility fitted trend lines, 1950−2021: Estonia, Ages: 50−54
Data points enclosed with a diamond are reference sources

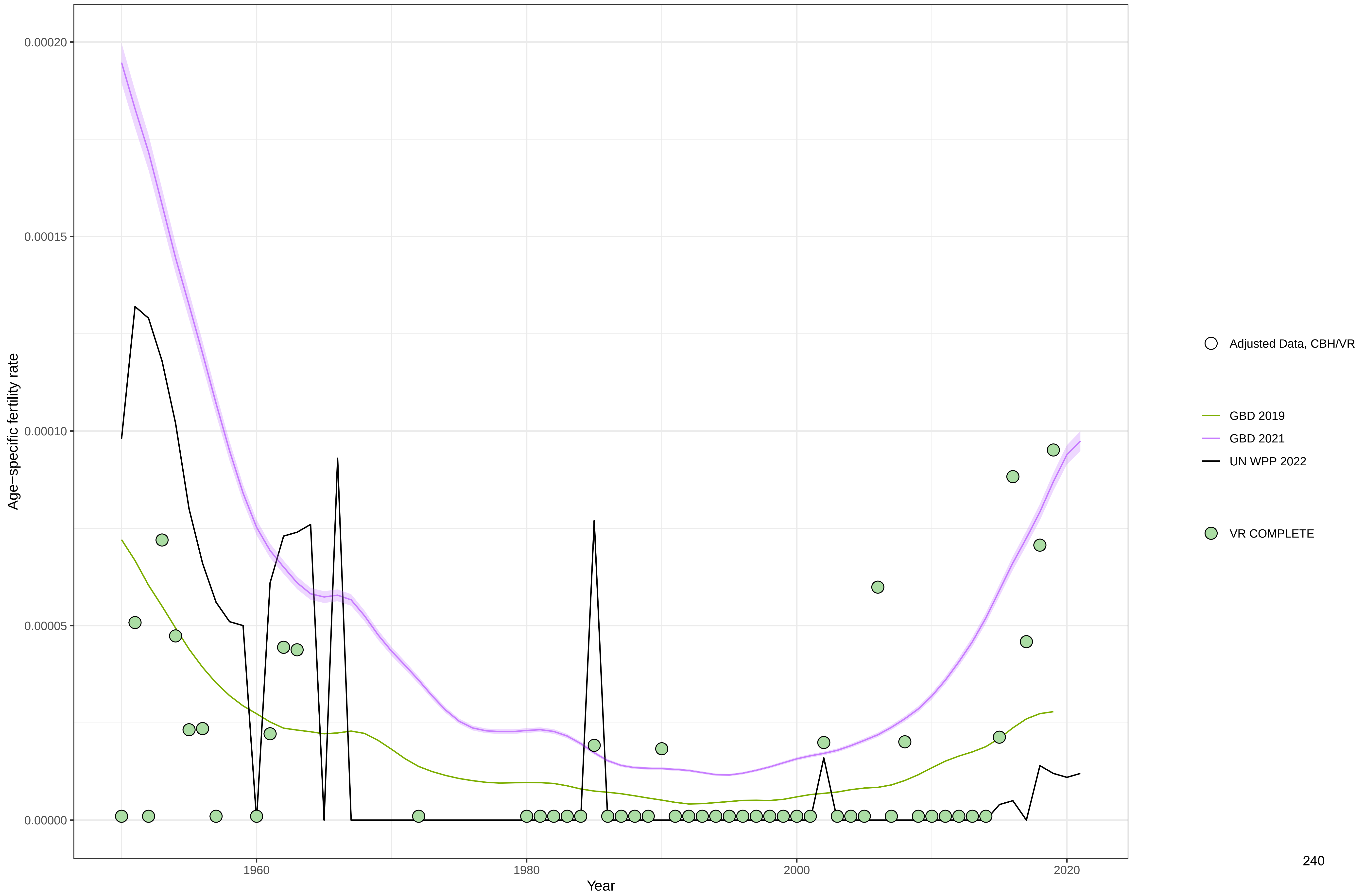

Total fertility rate fitted trend lines, 1950−2021: Estonia TFR
Data points enclosed with a diamond are reference sources

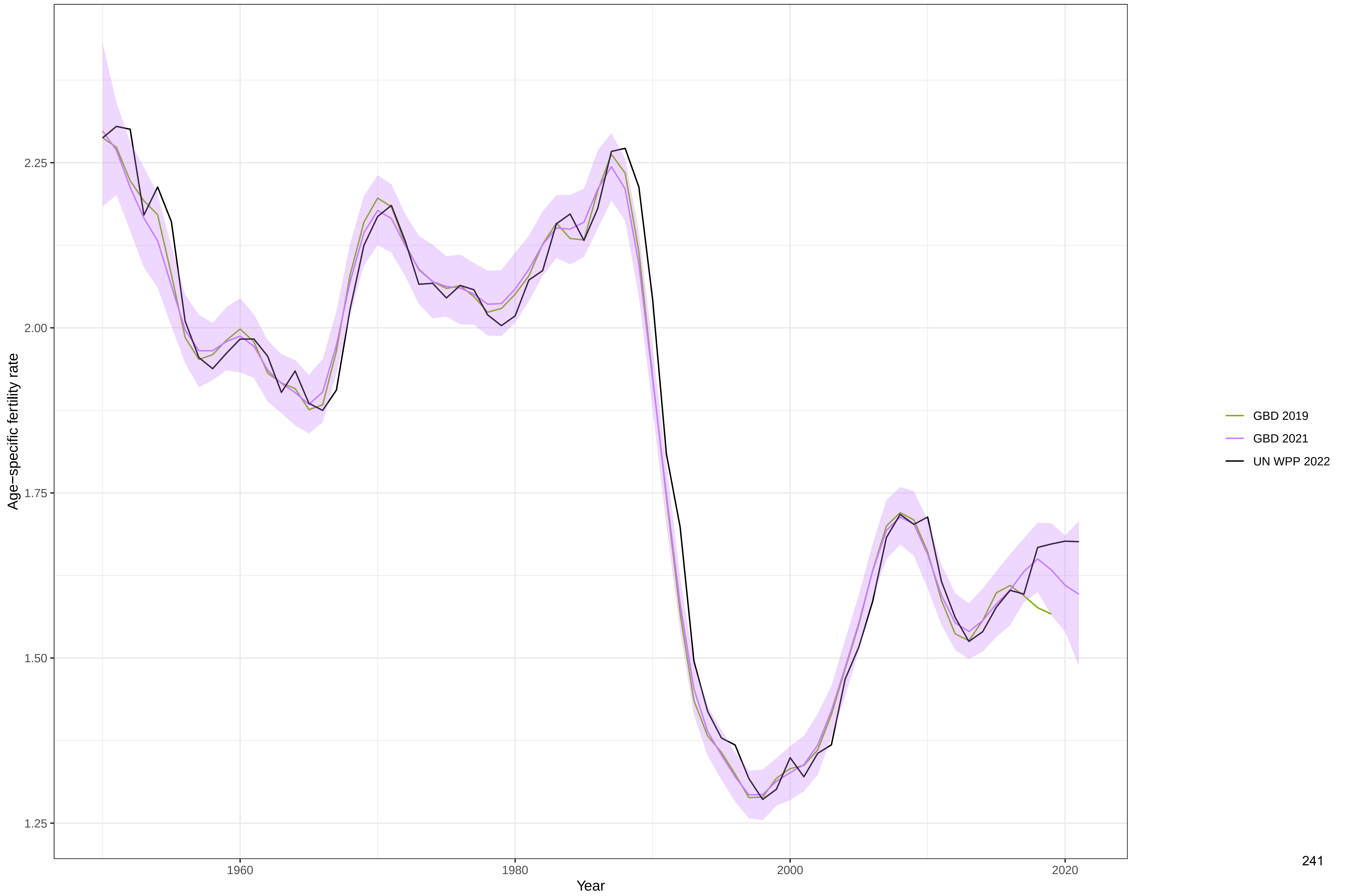

Age−specific fertility fitted trend lines, 1950−2021: Latvia, Ages: 10−14
Data points enclosed with a diamond are reference sources

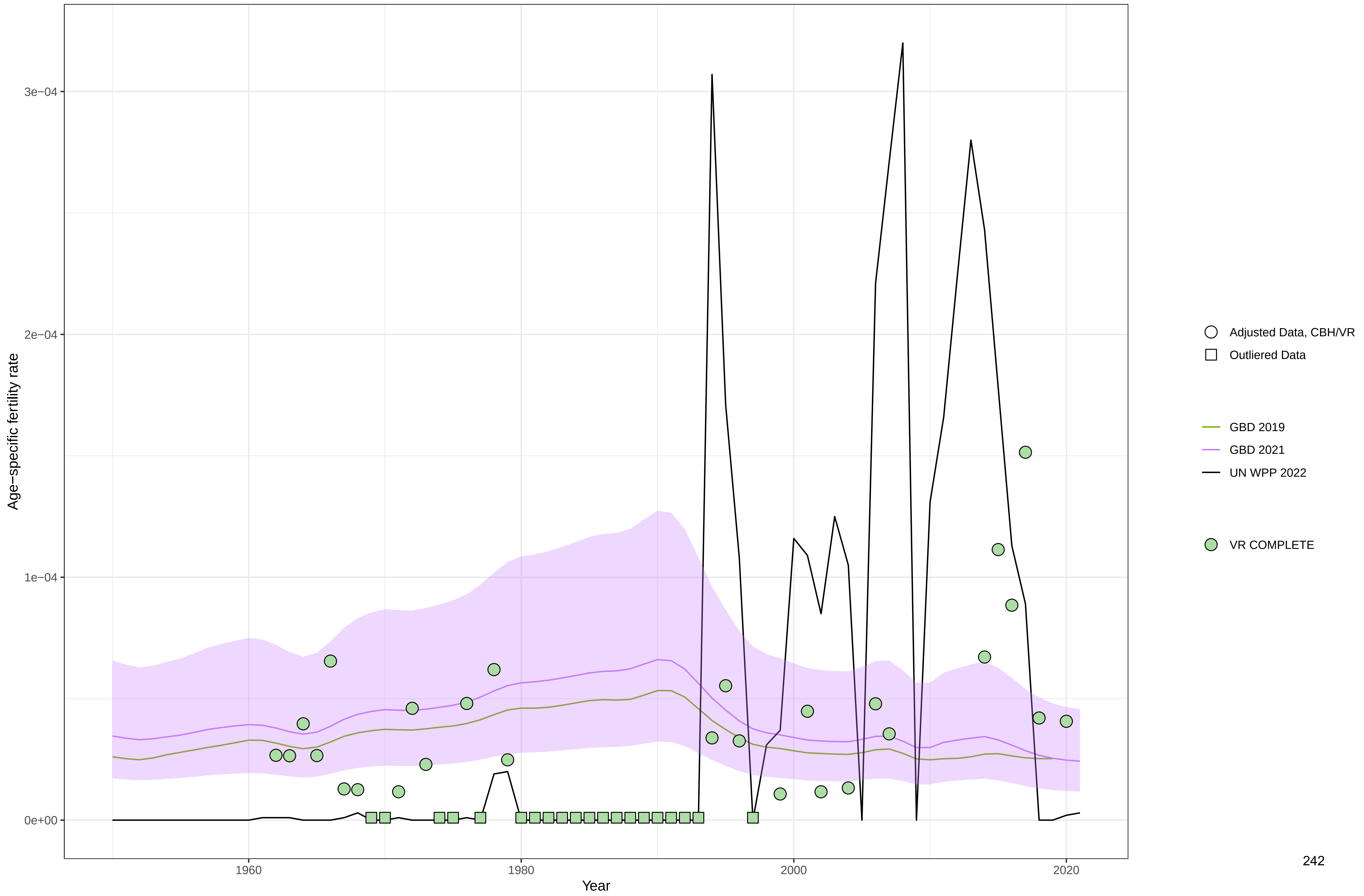

Age−specific fertility fitted trend lines, 1950−2021: Latvia, Ages: 15−19
Data points enclosed with a diamond are reference sources

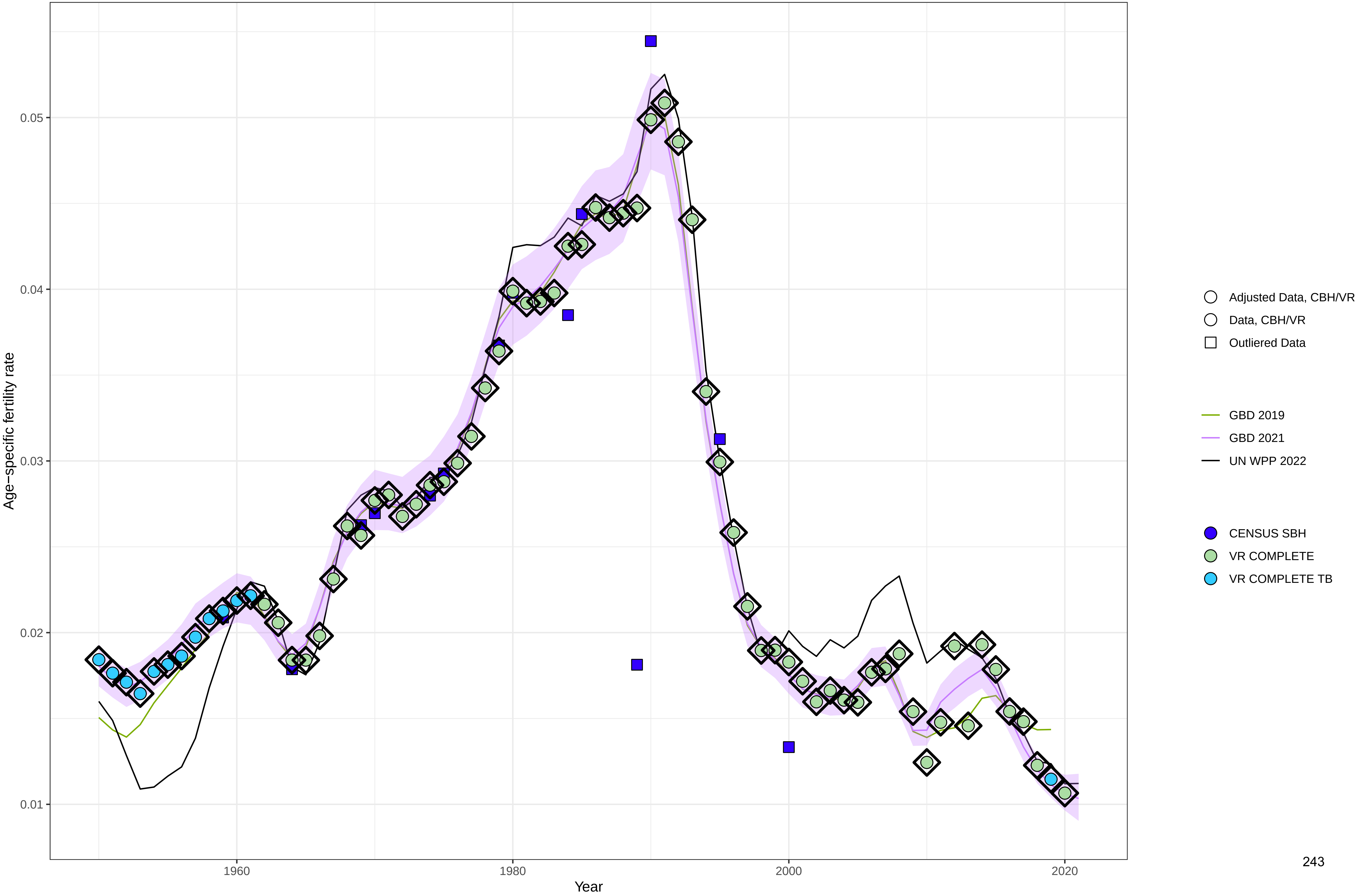

Age−specific fertility fitted trend lines, 1950−2021: Latvia, Ages: 20−24
Data points enclosed with a diamond are reference sources

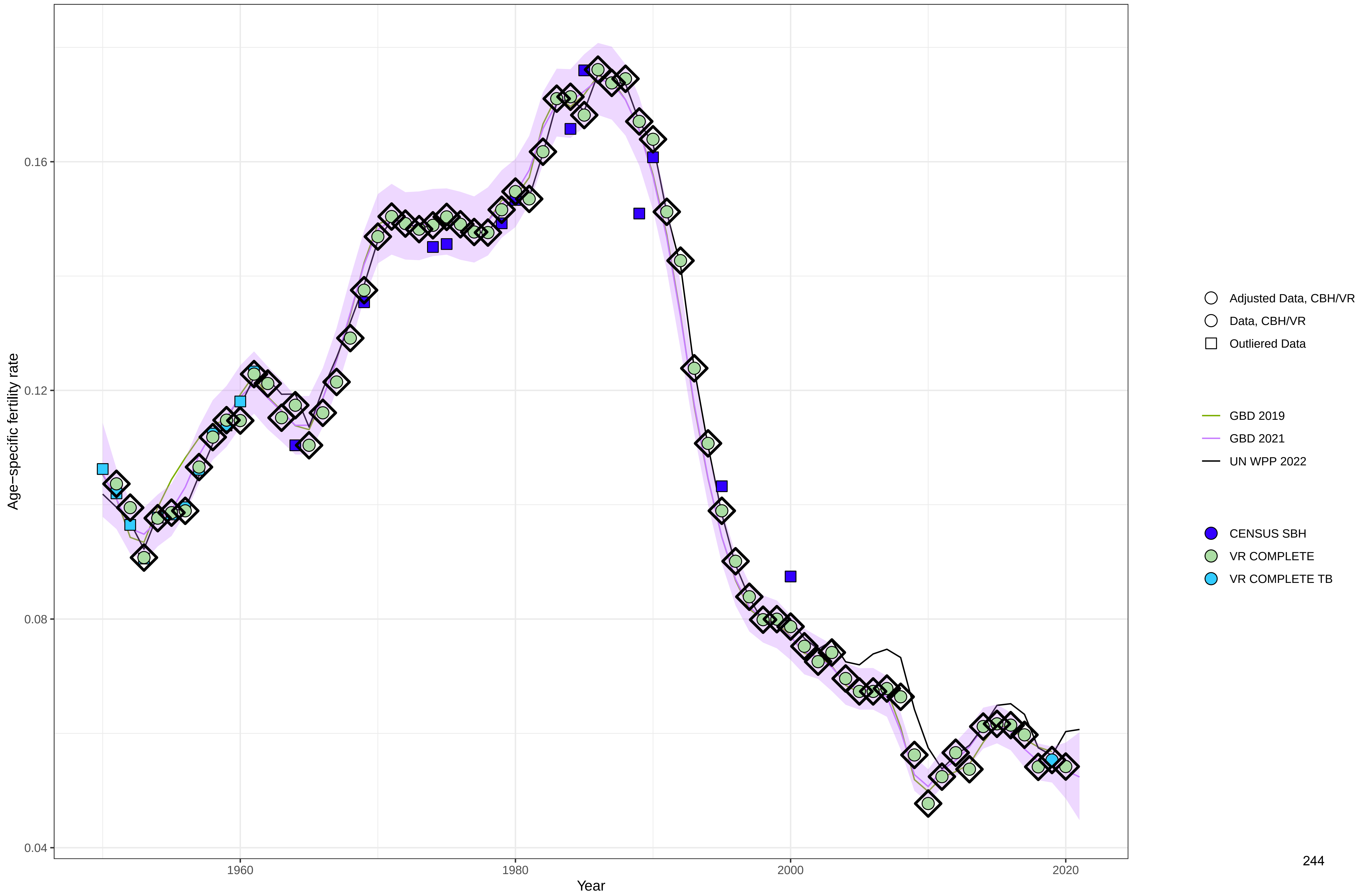

Age−specific fertility fitted trend lines, 1950−2021: Latvia, Ages: 25−29
Data points enclosed with a diamond are reference sources

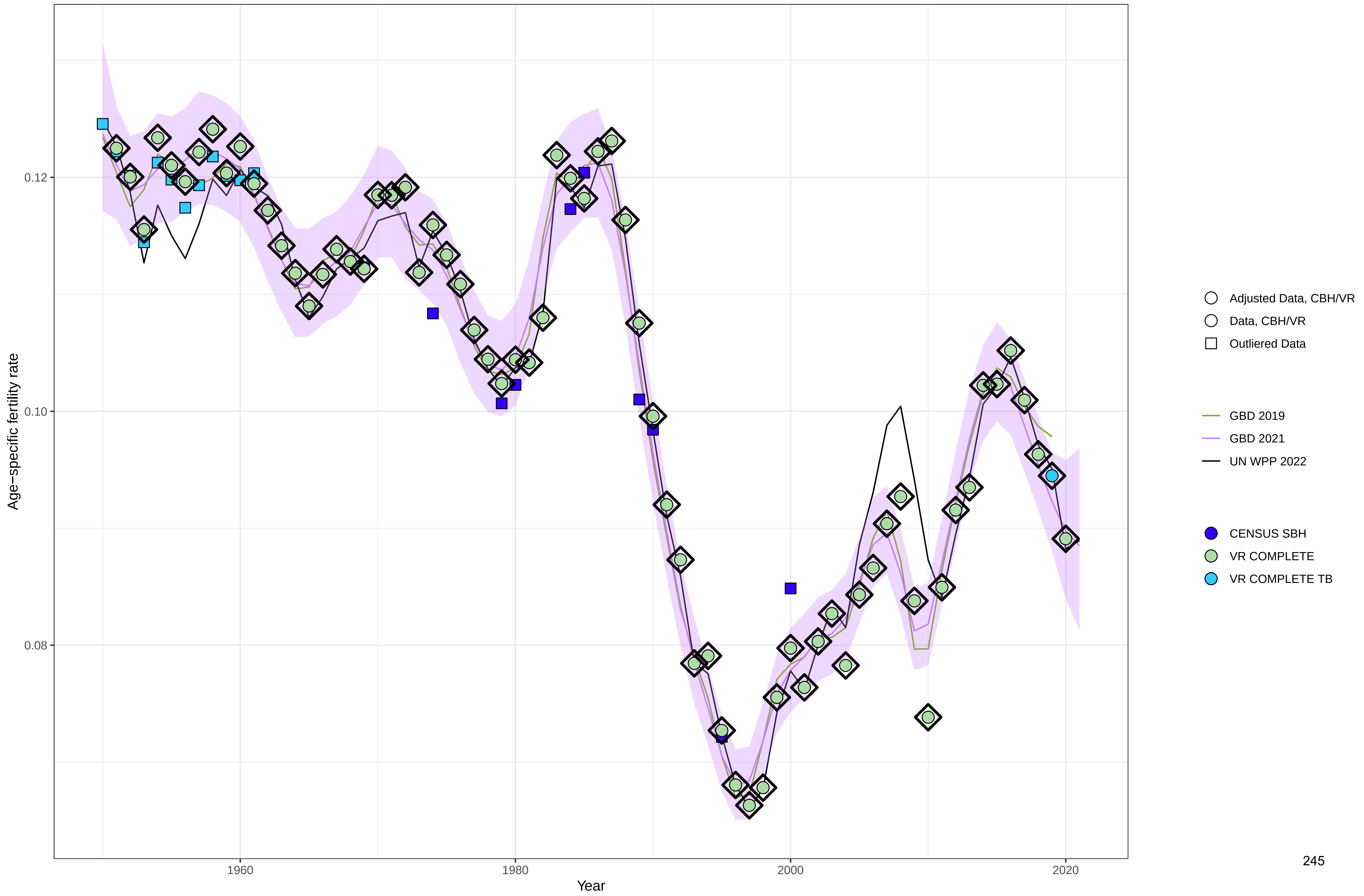

Age−specific fertility fitted trend lines, 1950−2021: Latvia, Ages: 30−34
Data points enclosed with a diamond are reference sources

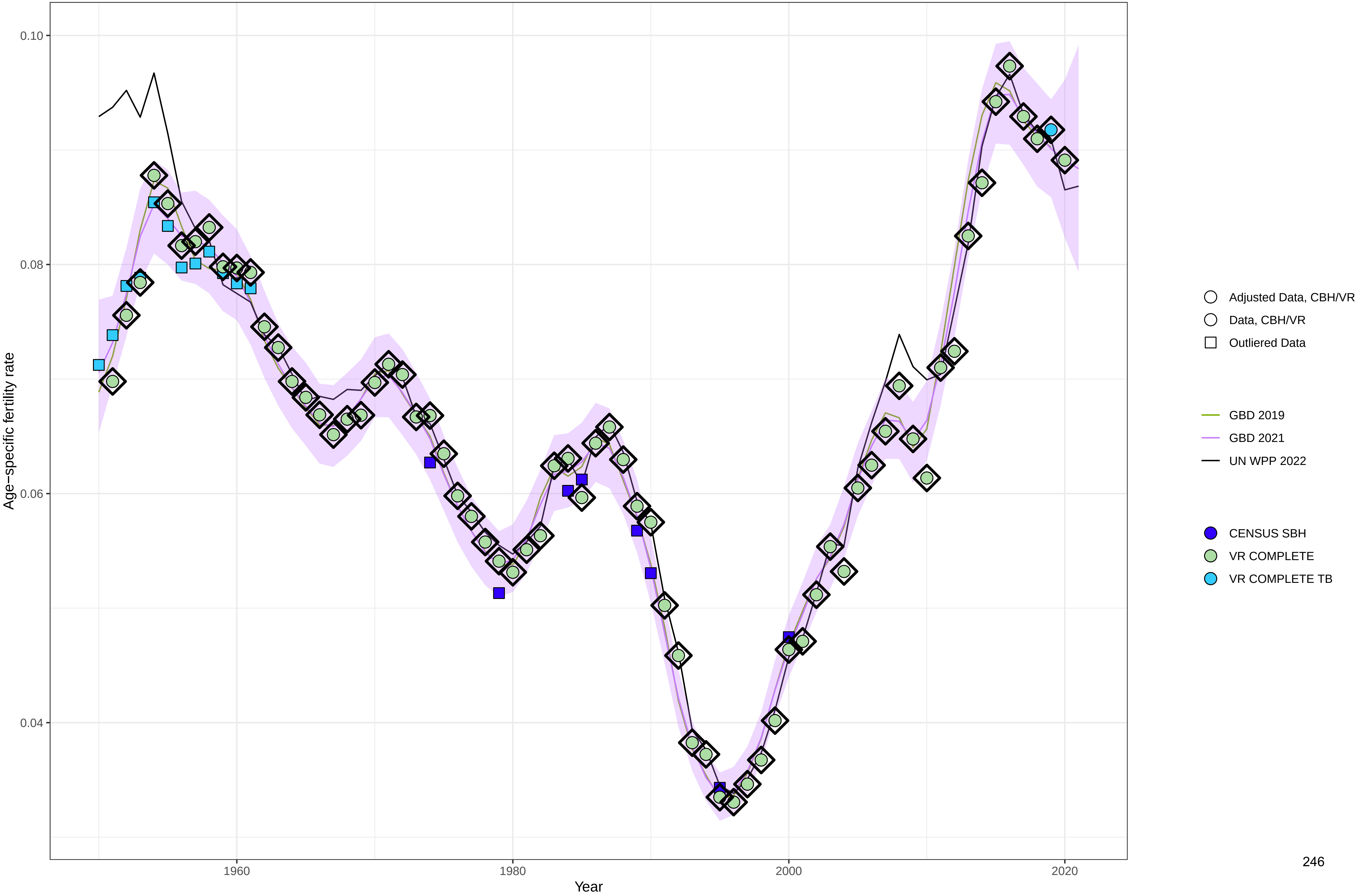

Age−specific fertility fitted trend lines, 1950−2021: Latvia, Ages: 35−39
Data points enclosed with a diamond are reference sources

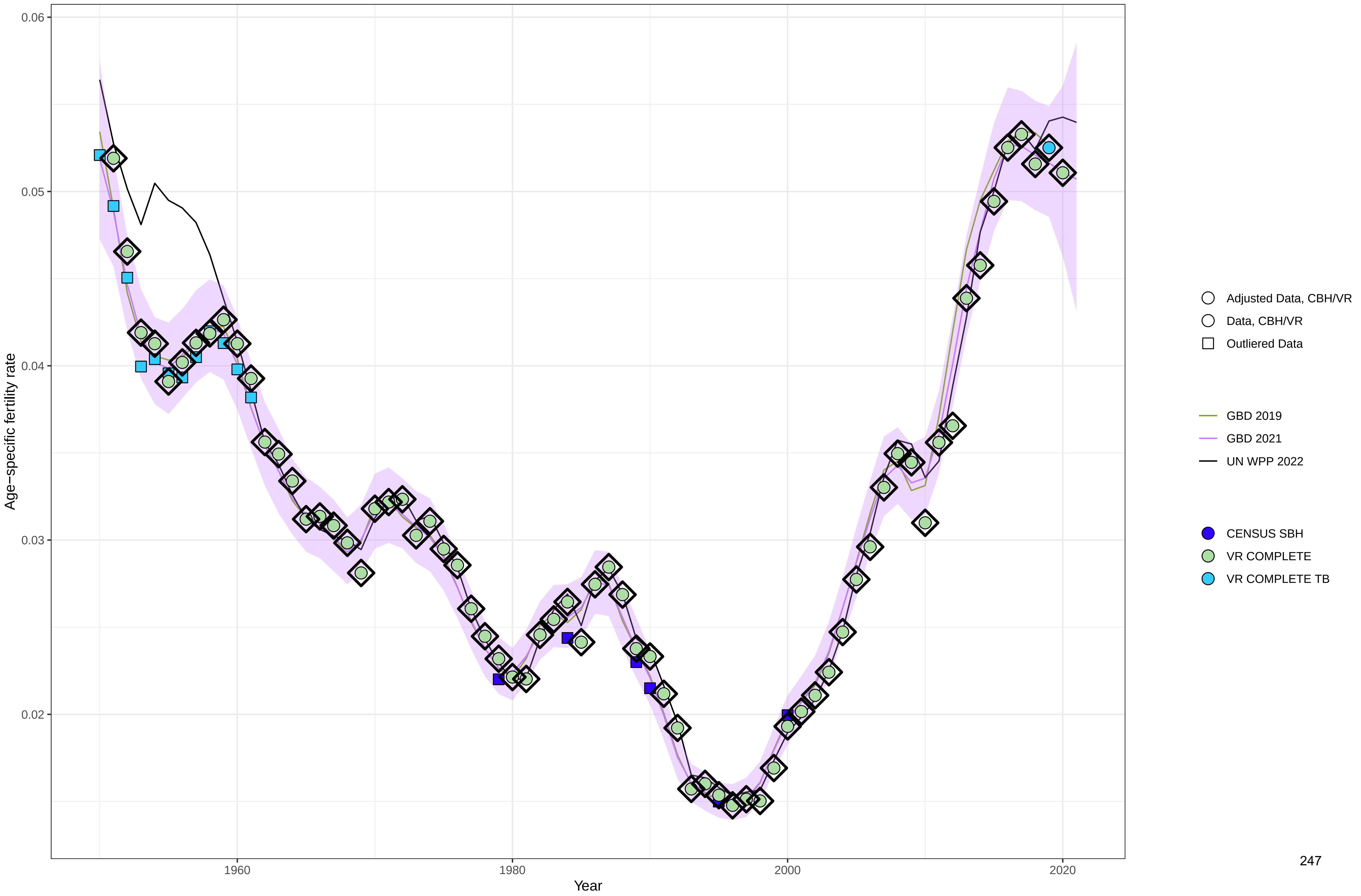

Age−specific fertility fitted trend lines, 1950−2021: Latvia, Ages: 40−44
Data points enclosed with a diamond are reference sources

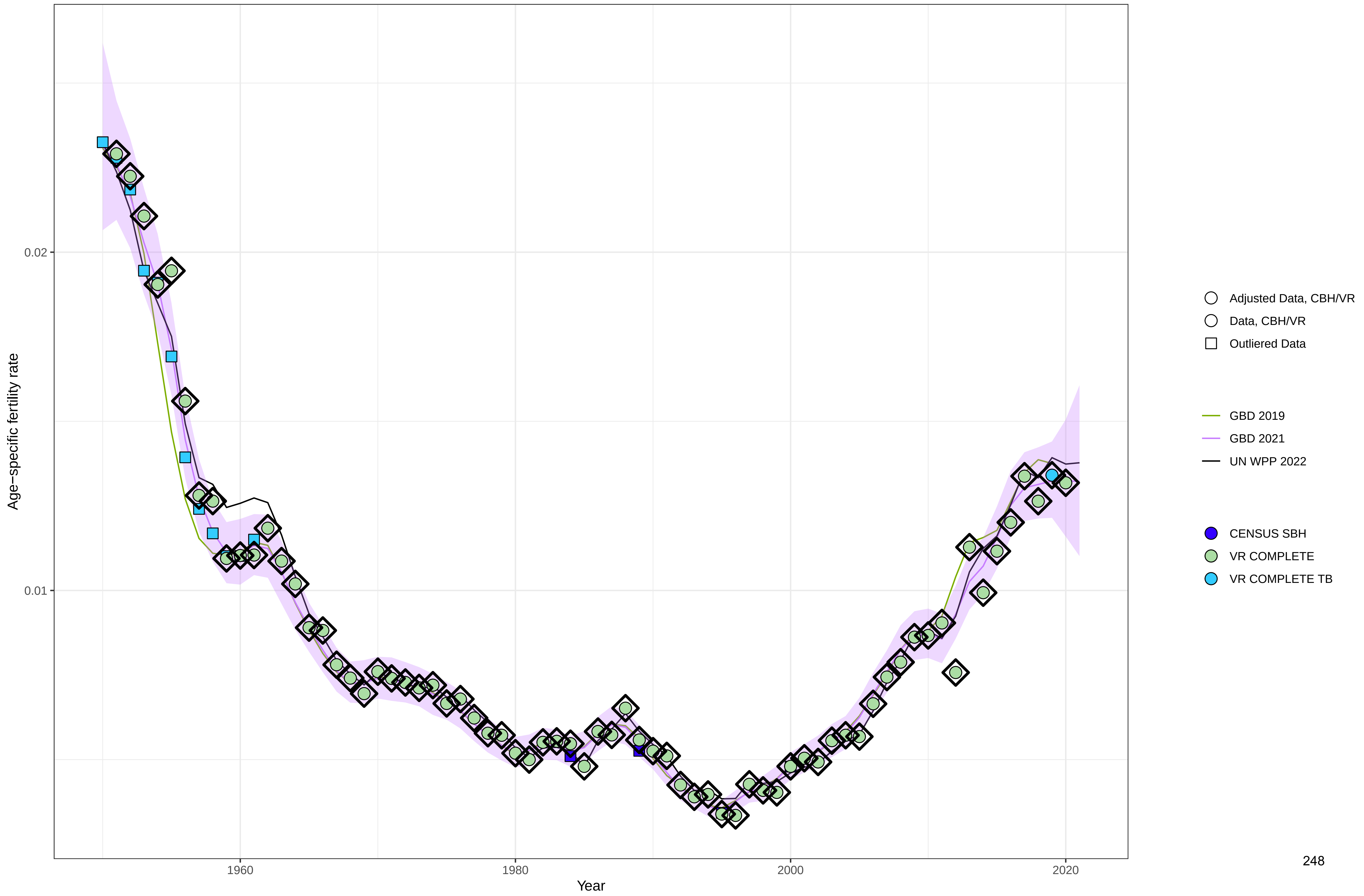

Age−specific fertility fitted trend lines, 1950−2021: Latvia, Ages: 45−49
Data points enclosed with a diamond are reference sources

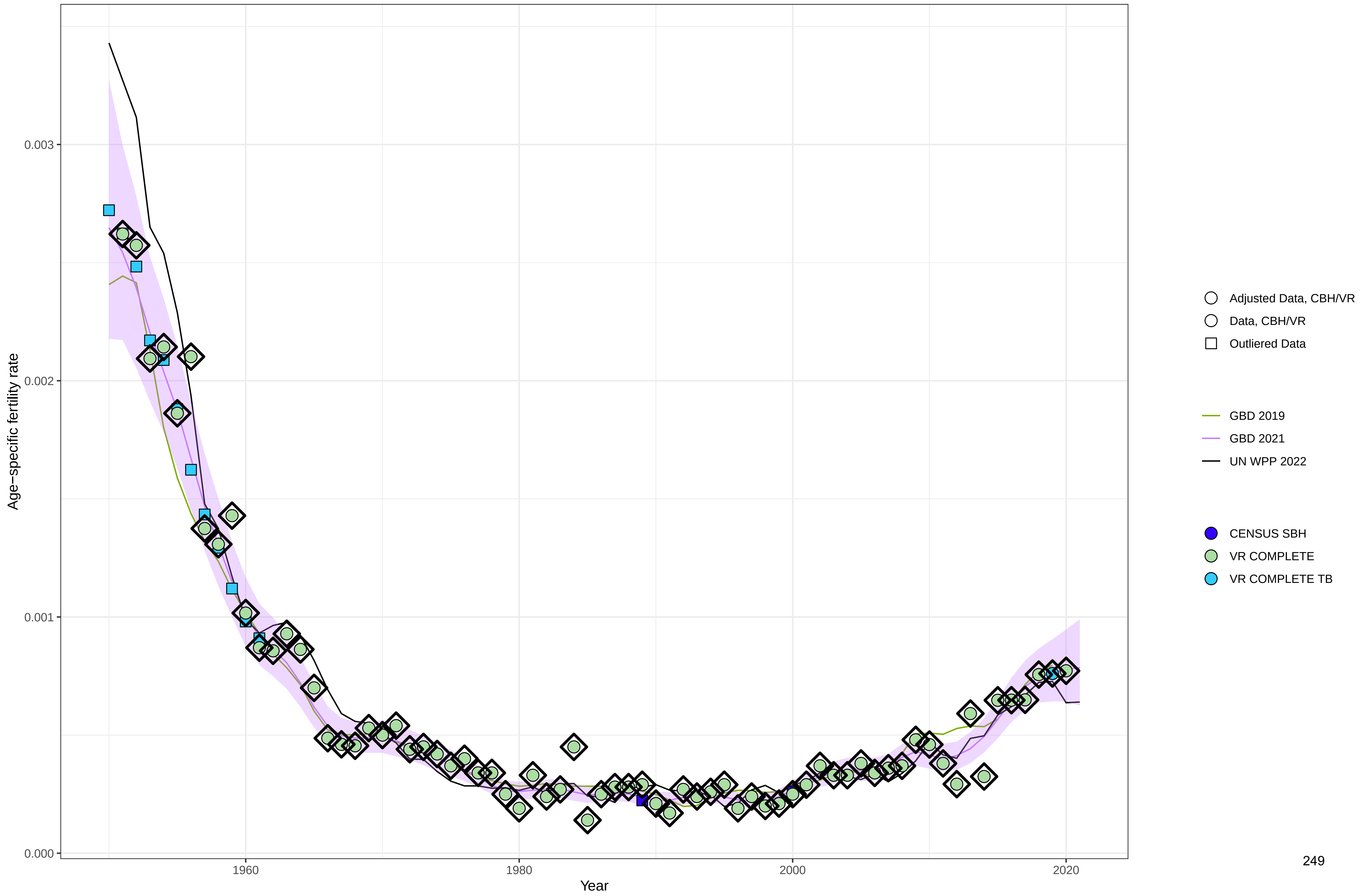

Age−specific fertility fitted trend lines, 1950−2021: Latvia, Ages: 50−54
Data points enclosed with a diamond are reference sources

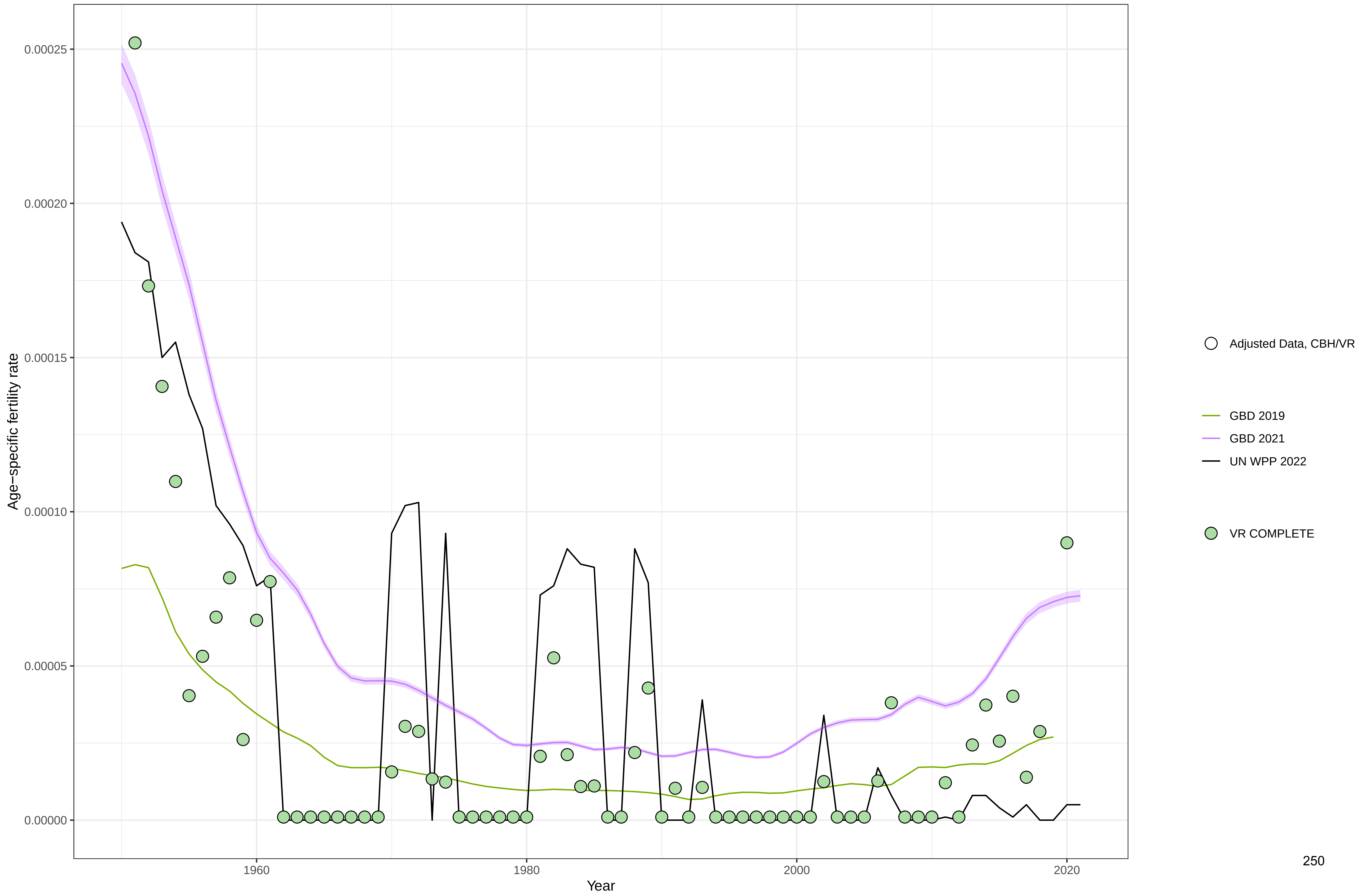

Total fertility rate fitted trend lines, 1950−2021: Latvia TFR
Data points enclosed with a diamond are reference sources

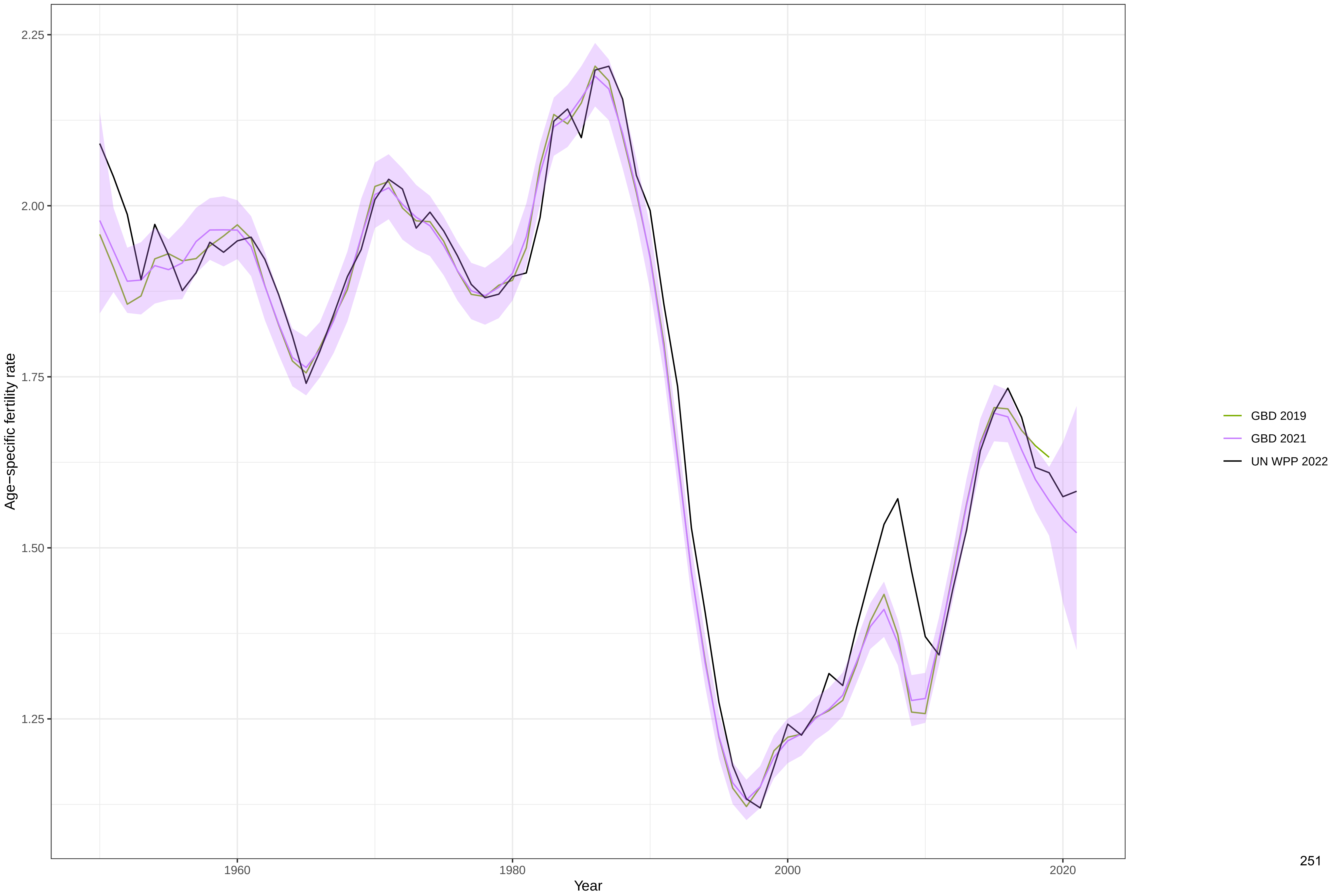

Age−specific fertility fitted trend lines, 1950−2021: Lithuania, Ages: 10−14
Data points enclosed with a diamond are reference sources

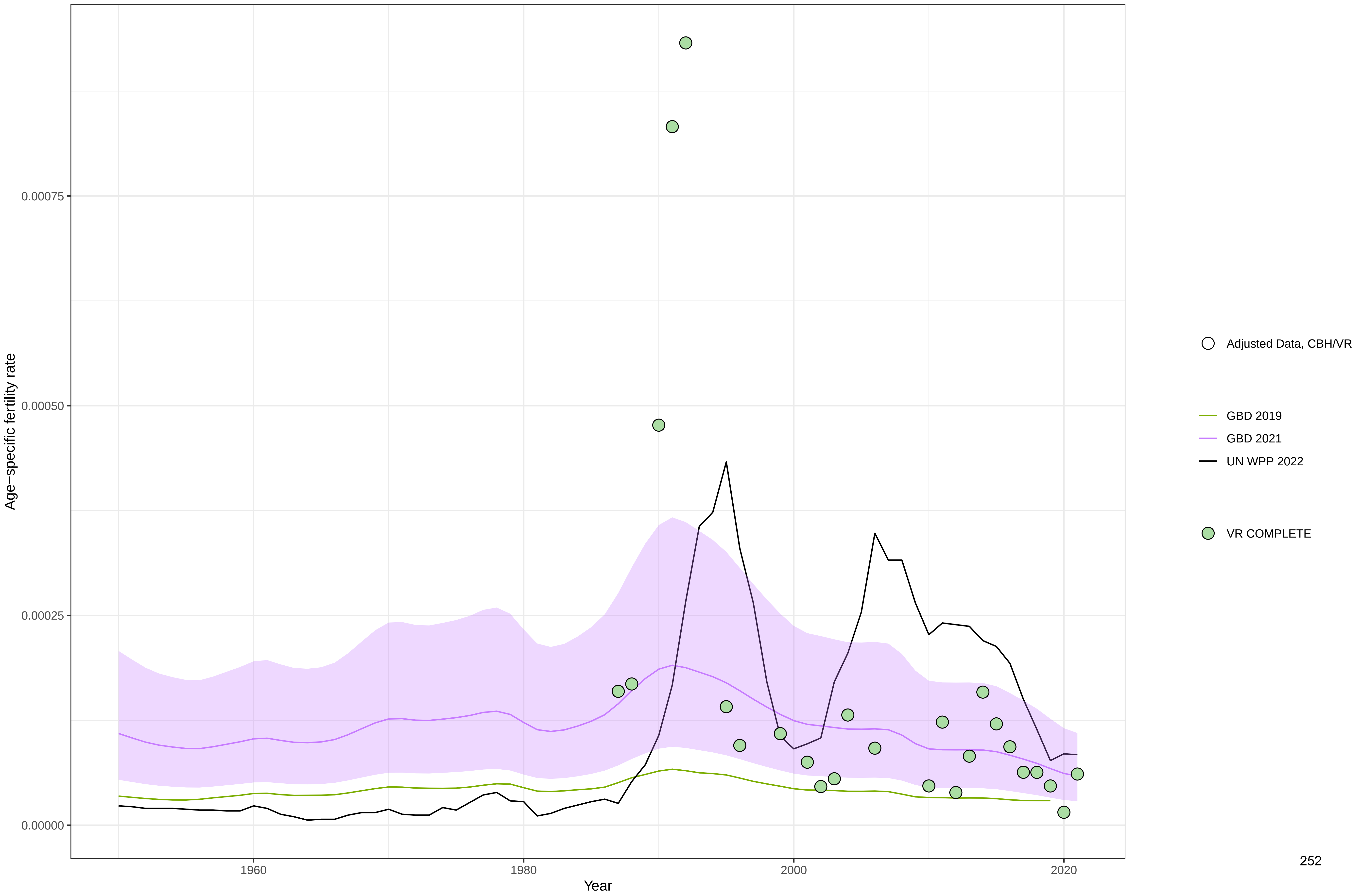

Age−specific fertility fitted trend lines, 1950−2021: Lithuania, Ages: 15−19
Data points enclosed with a diamond are reference sources

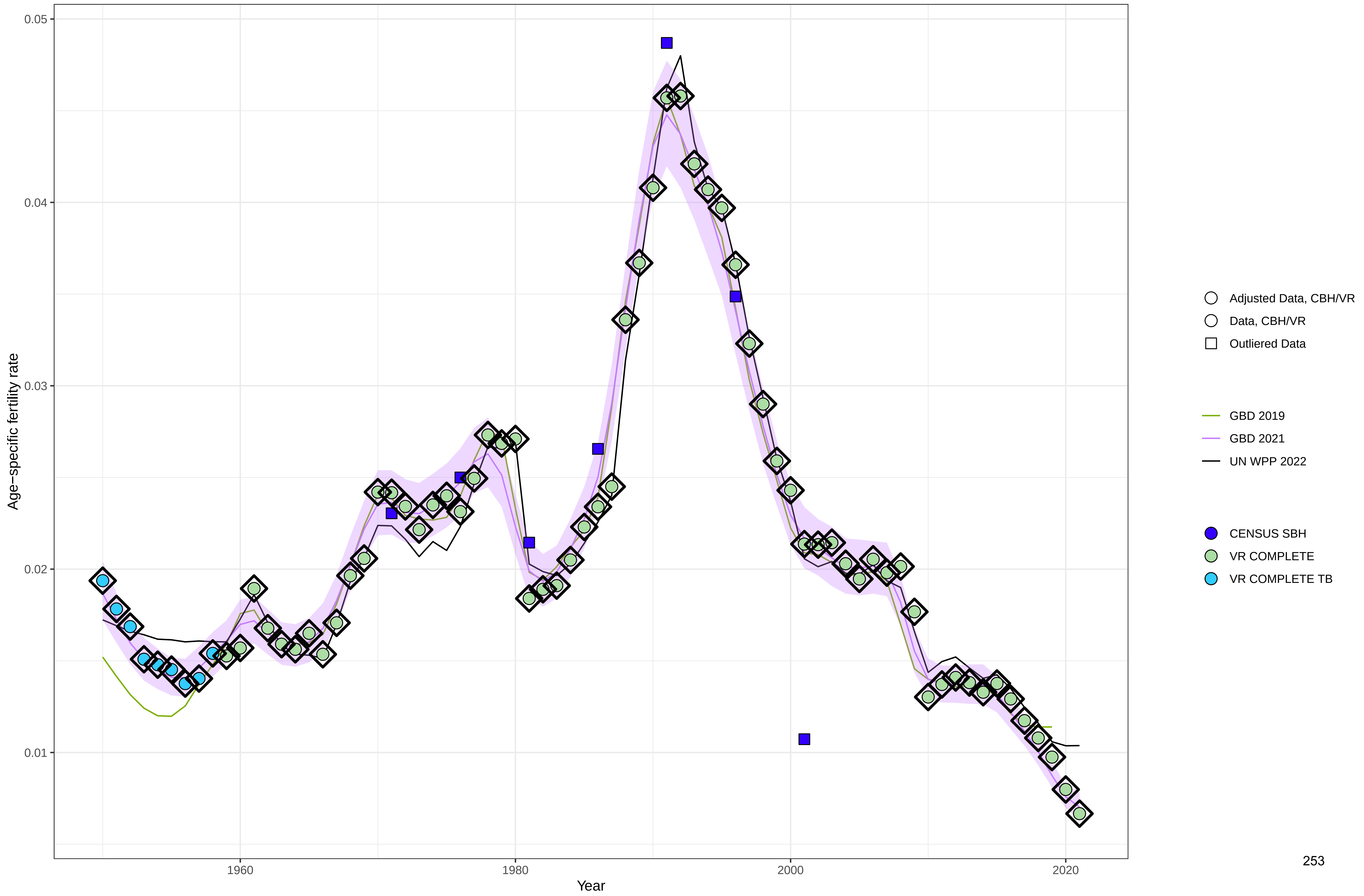

Age−specific fertility fitted trend lines, 1950−2021: Lithuania, Ages: 20−24
Data points enclosed with a diamond are reference sources

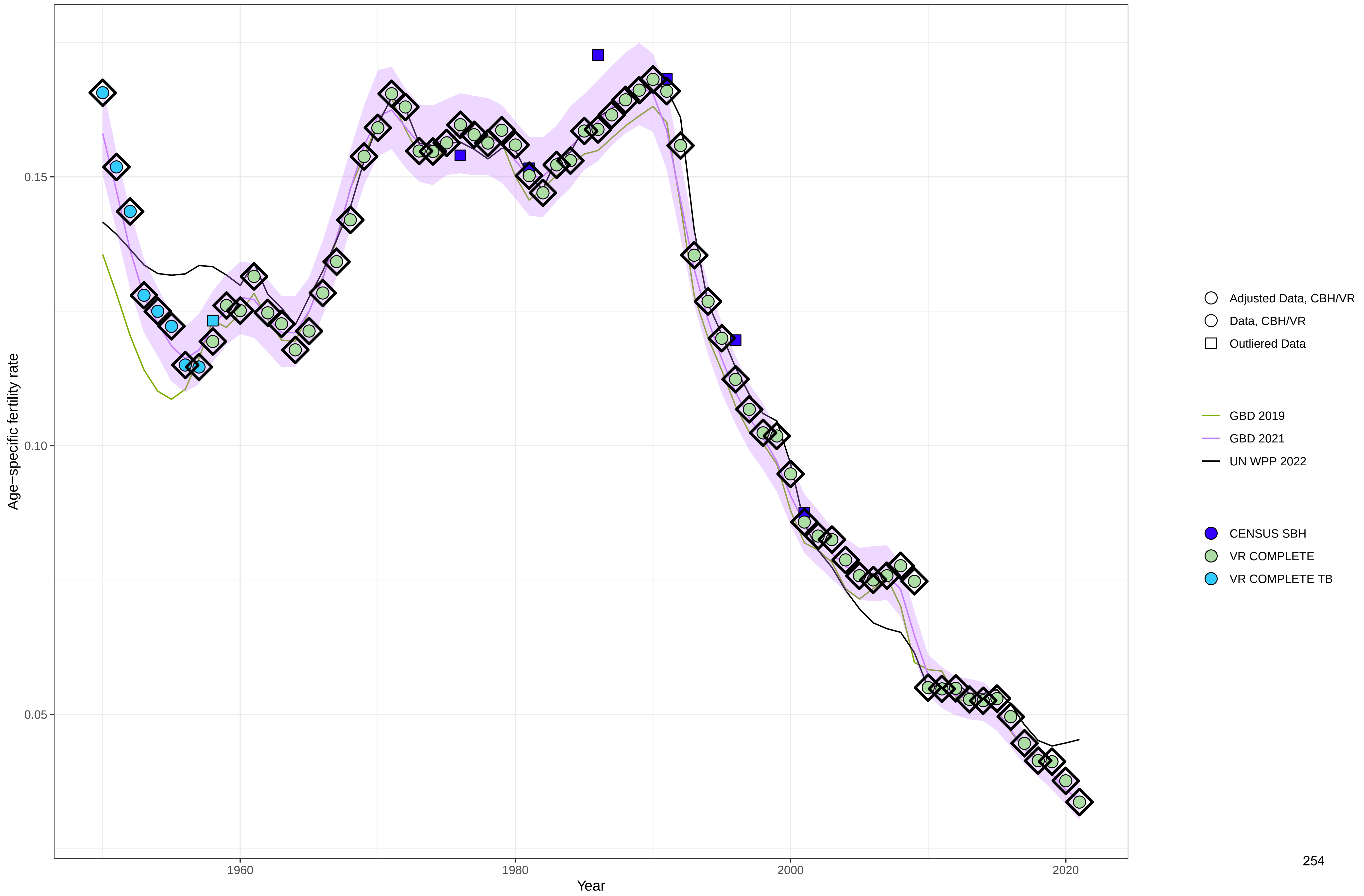

Age−specific fertility fitted trend lines, 1950−2021: Lithuania, Ages: 25−29
Data points enclosed with a diamond are reference sources

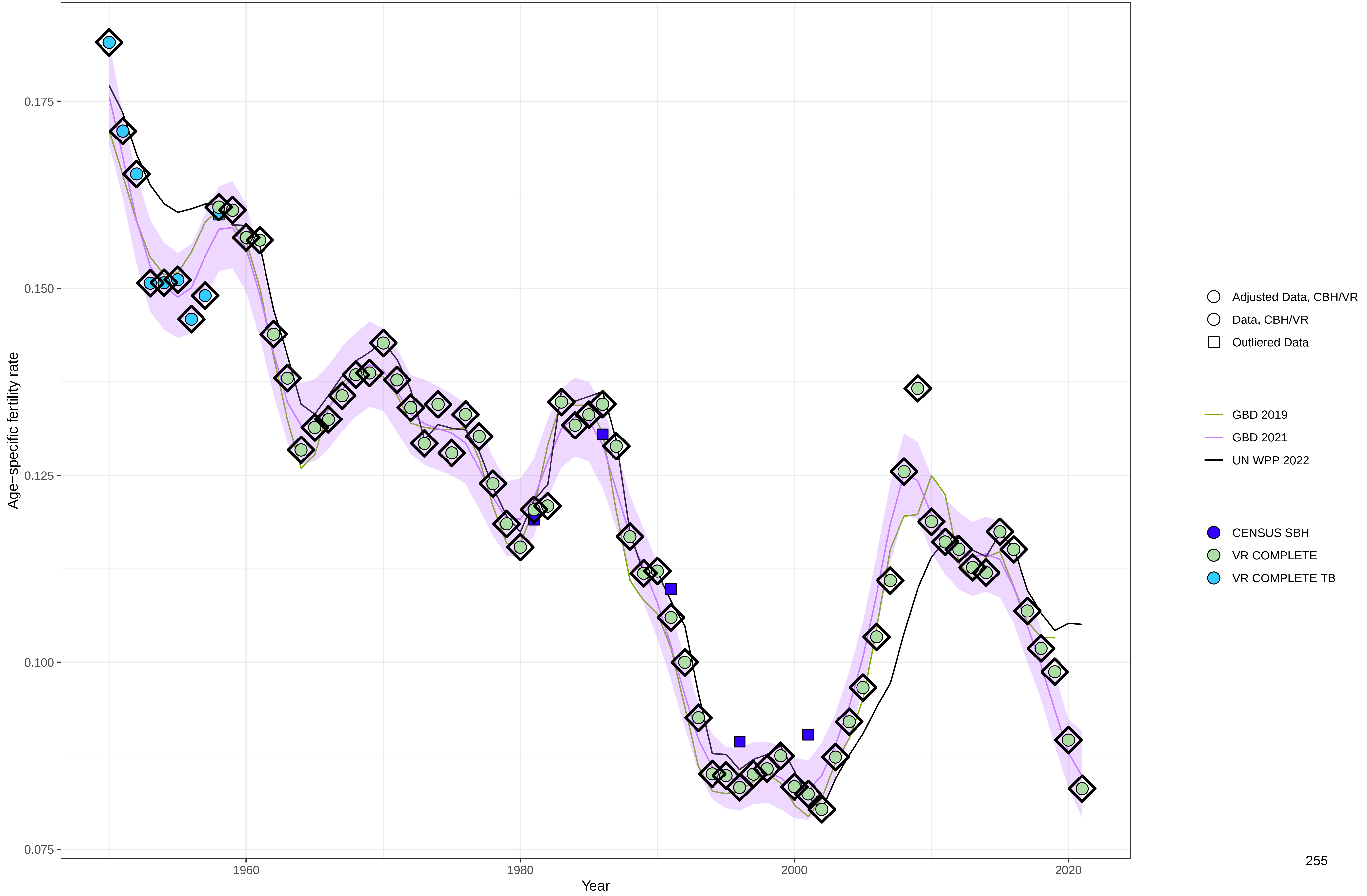

Age−specific fertility fitted trend lines, 1950−2021: Lithuania, Ages: 30−34
Data points enclosed with a diamond are reference sources

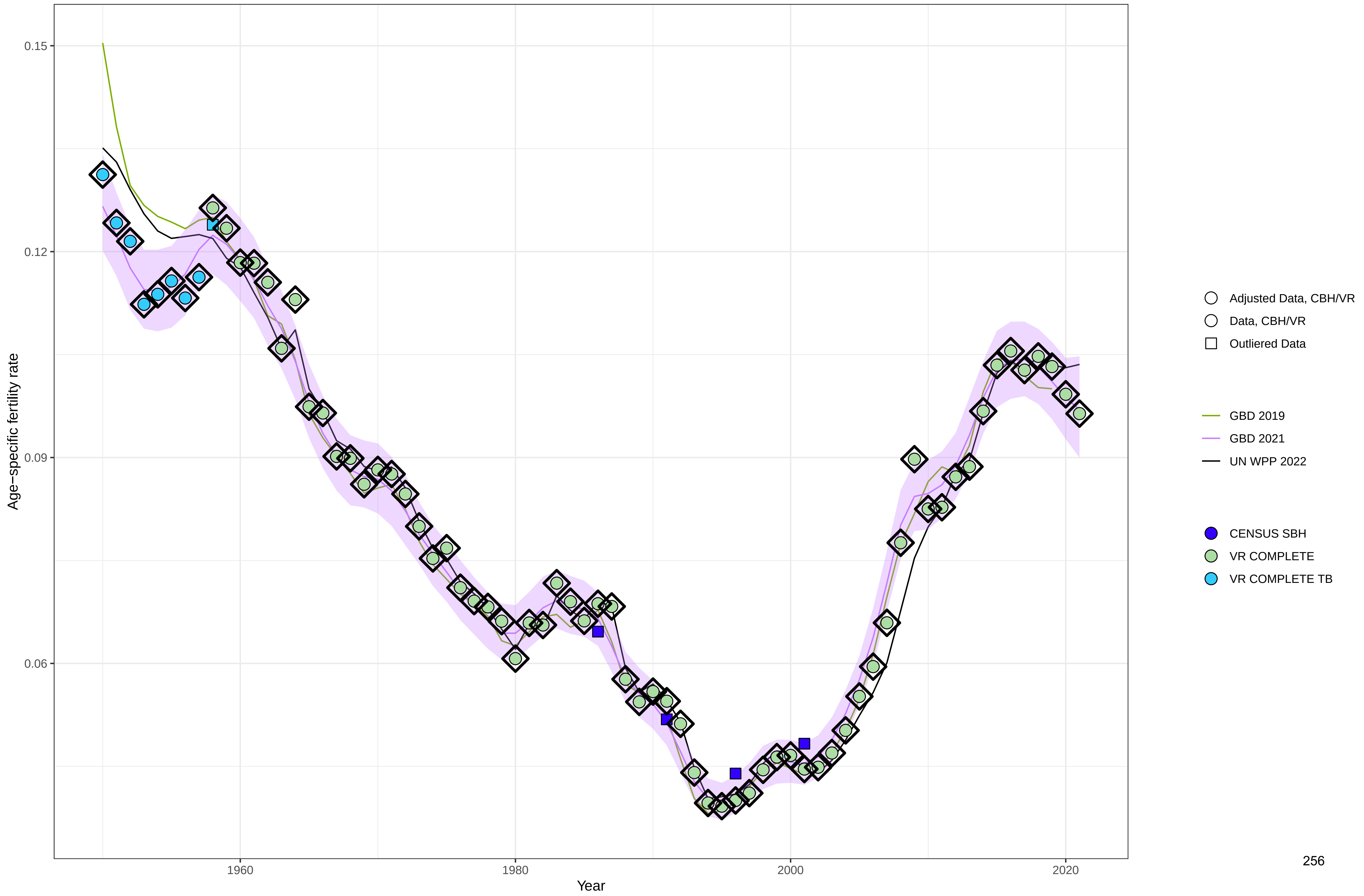

Age−specific fertility fitted trend lines, 1950−2021: Lithuania, Ages: 35−39
Data points enclosed with a diamond are reference sources

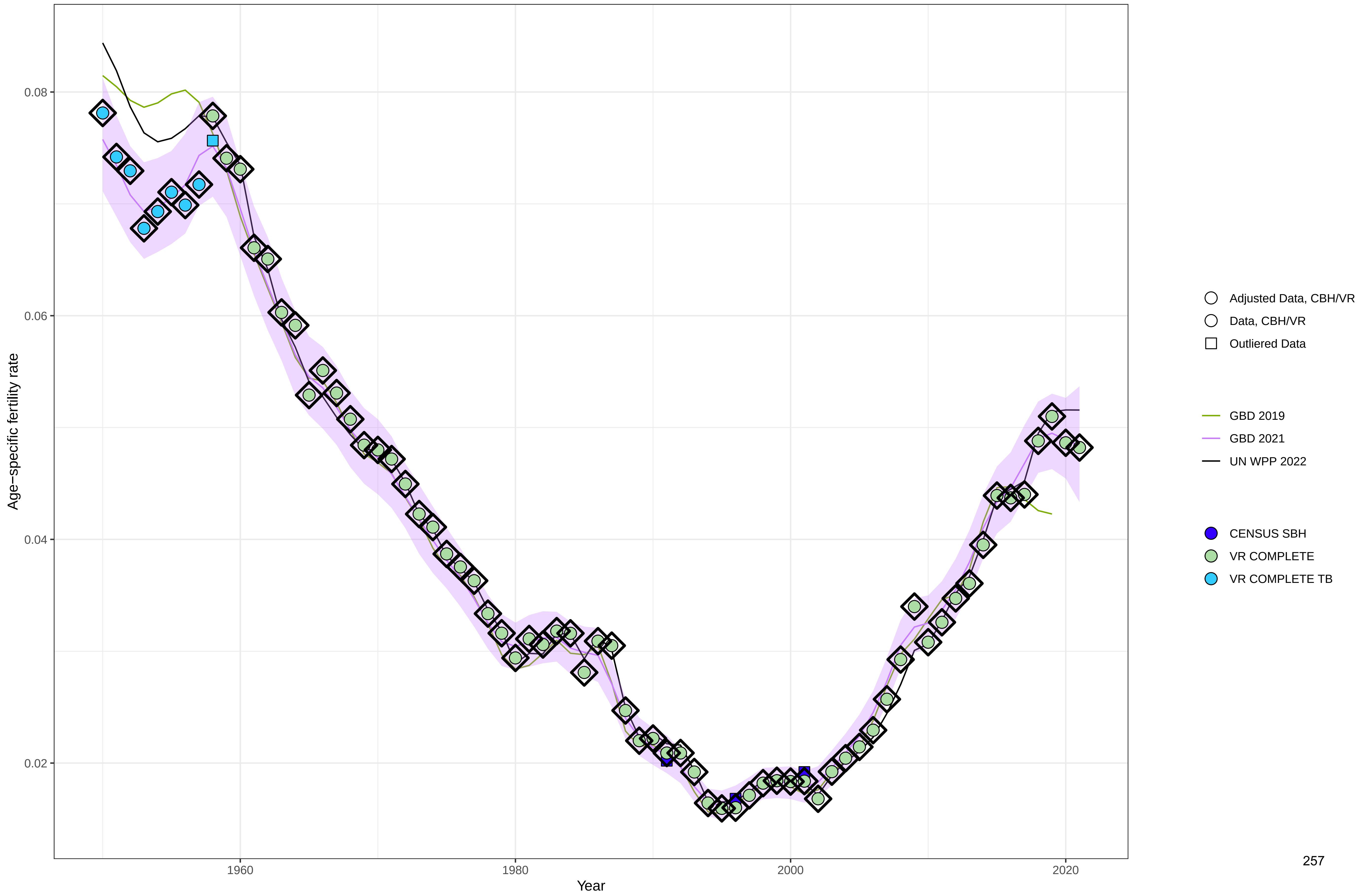

Age−specific fertility fitted trend lines, 1950−2021: Lithuania, Ages: 40−44
Data points enclosed with a diamond are reference sources

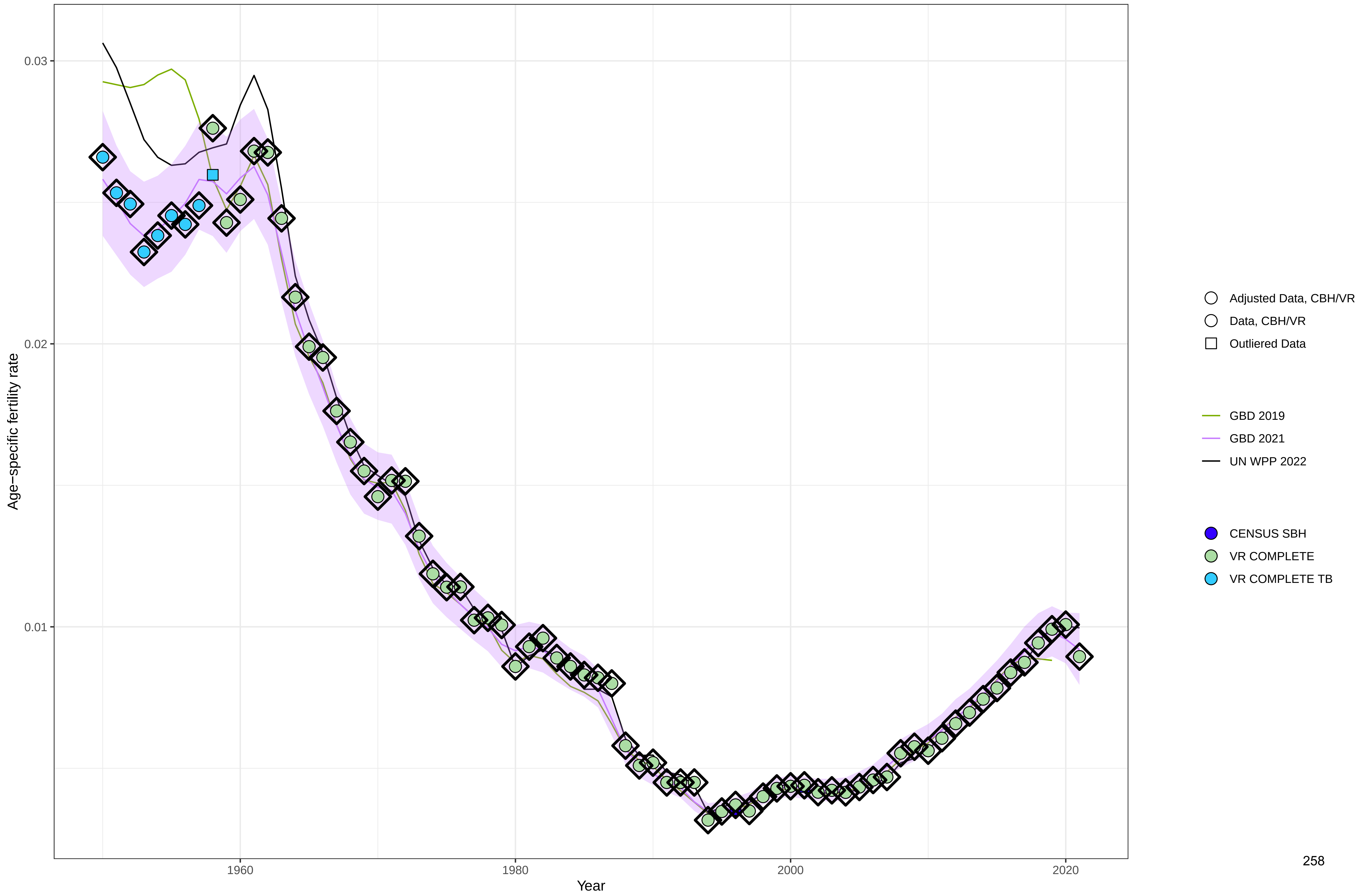

Age−specific fertility fitted trend lines, 1950−2021: Lithuania, Ages: 45−49
Data points enclosed with a diamond are reference sources

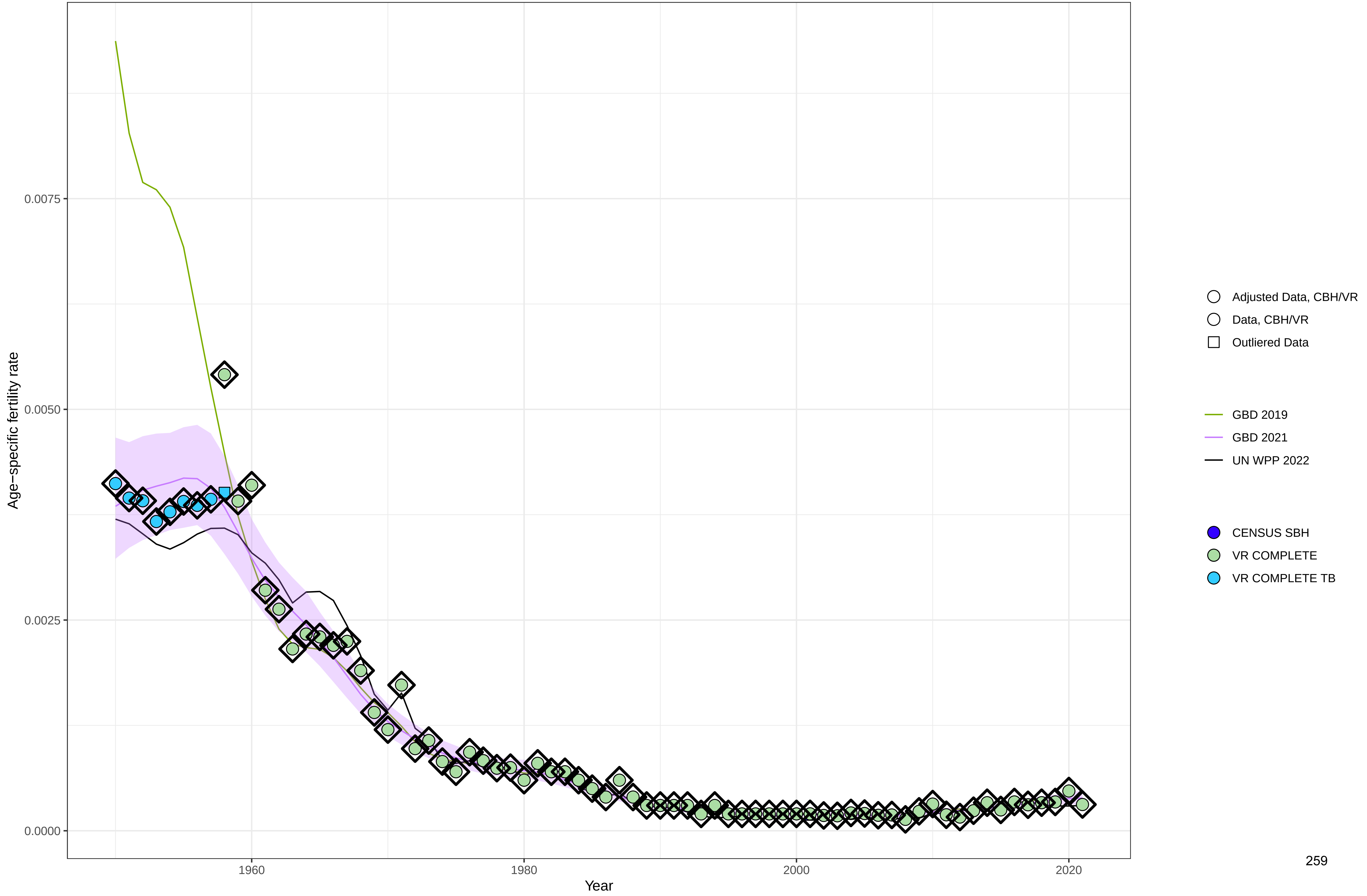

Age−specific fertility fitted trend lines, 1950−2021: Lithuania, Ages: 50−54
Data points enclosed with a diamond are reference sources

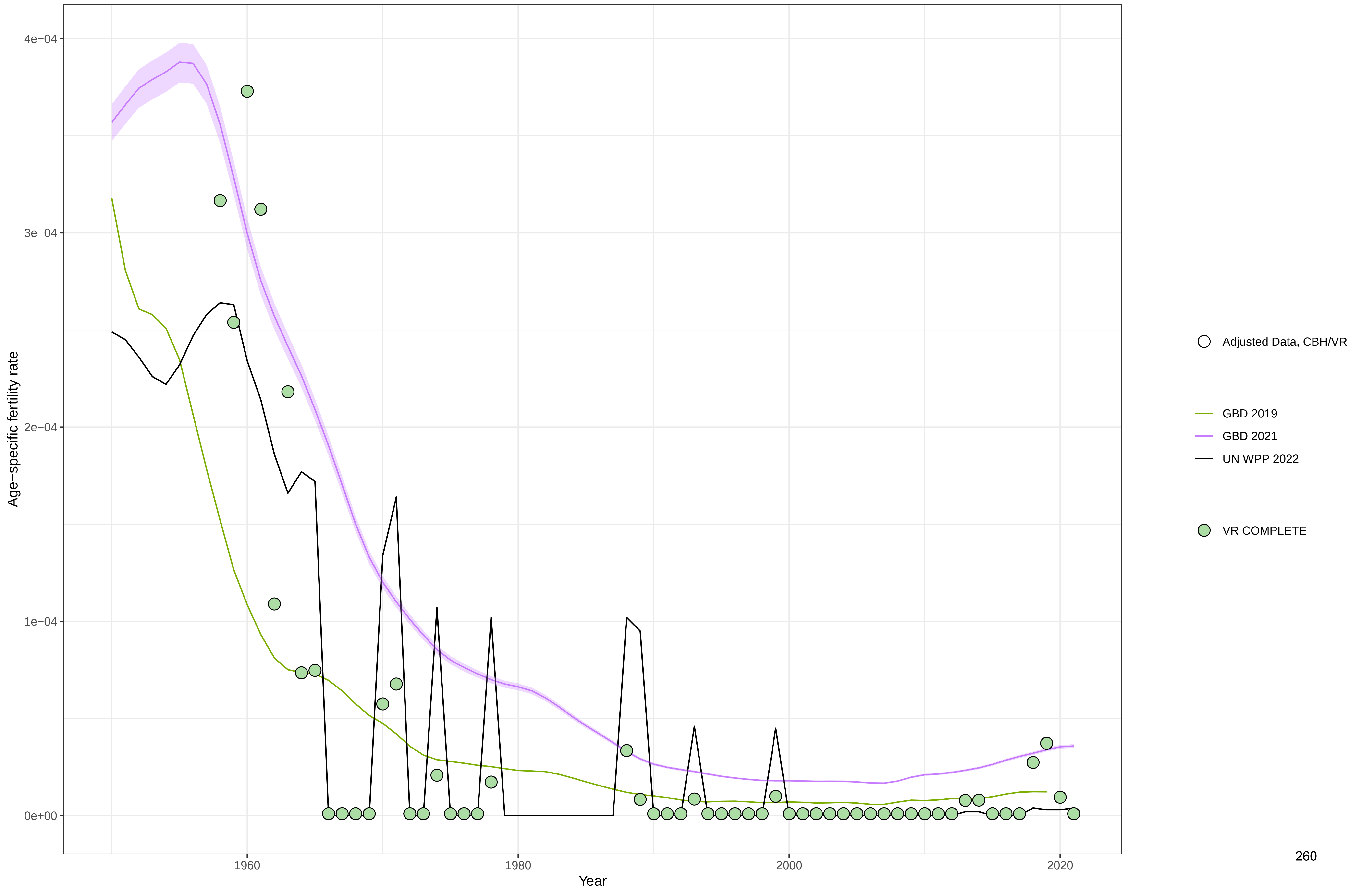

Total fertility rate fitted trend lines, 1950−2021: Lithuania TFR
Data points enclosed with a diamond are reference sources

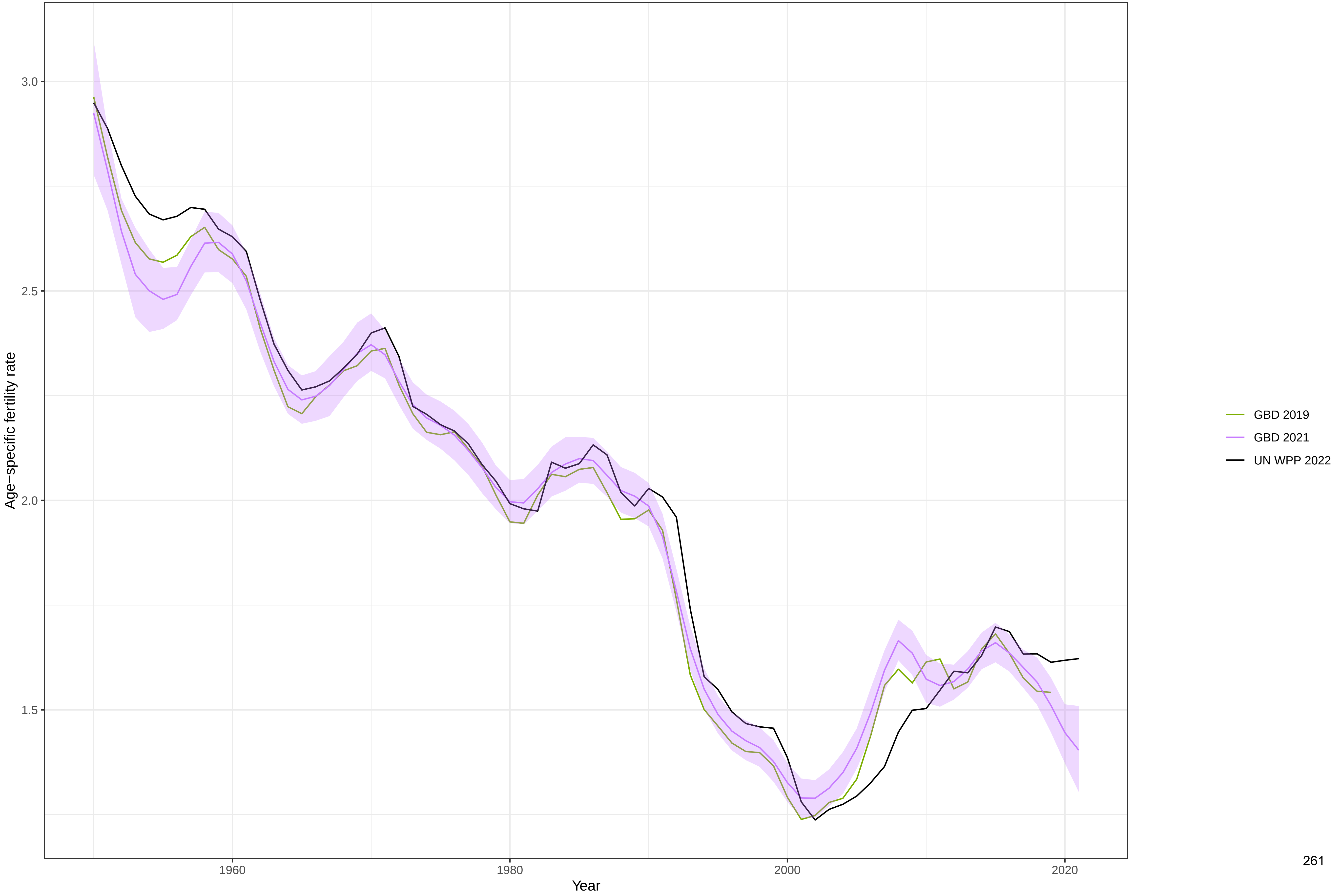

Age−specific fertility fitted trend lines, 1950−2021: Moldova, Ages: 10−14
Data points enclosed with a diamond are reference sources

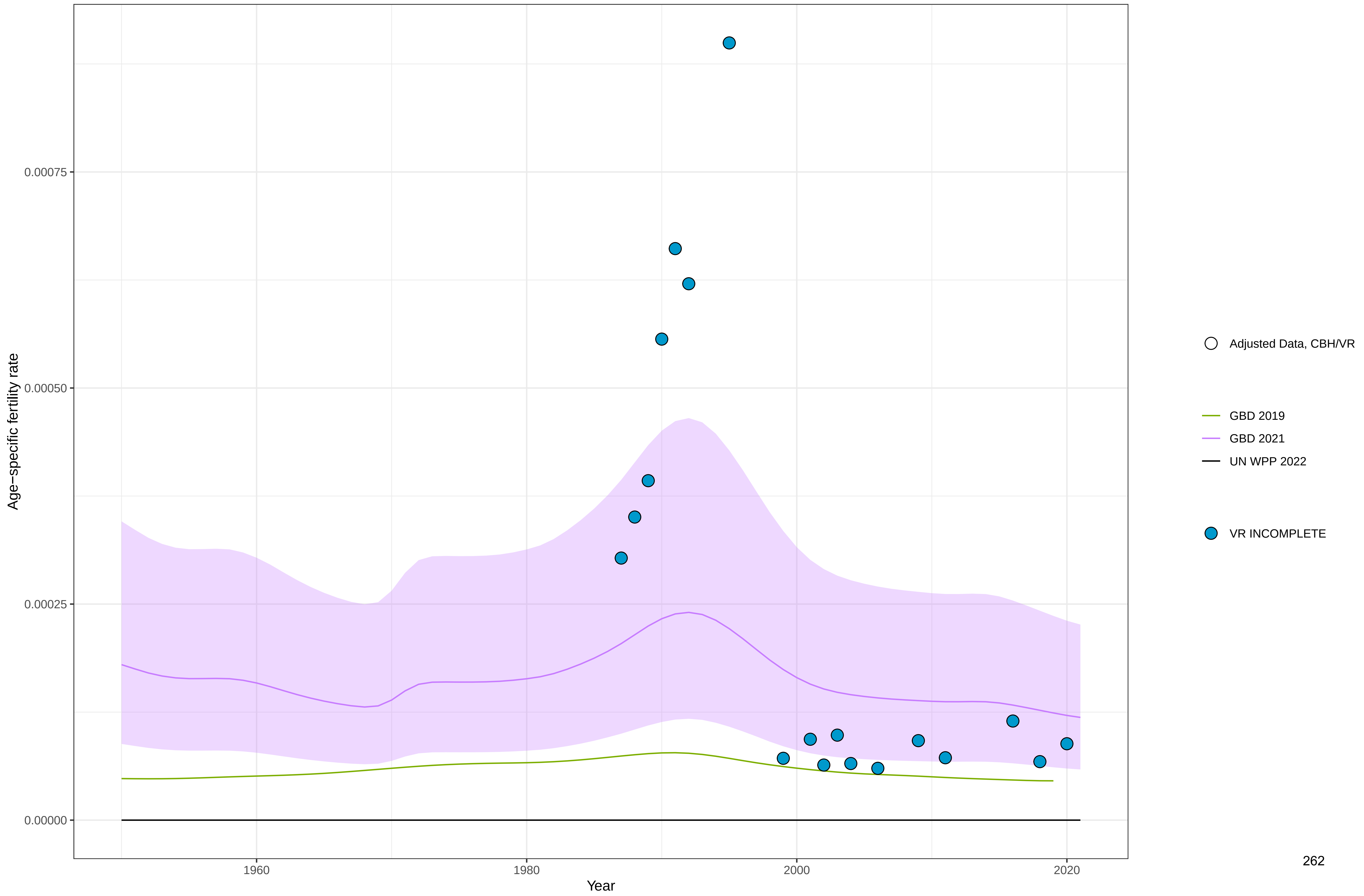

Age−specific fertility fitted trend lines, 1950−2021: Moldova, Ages: 15−19
Data points enclosed with a diamond are reference sources

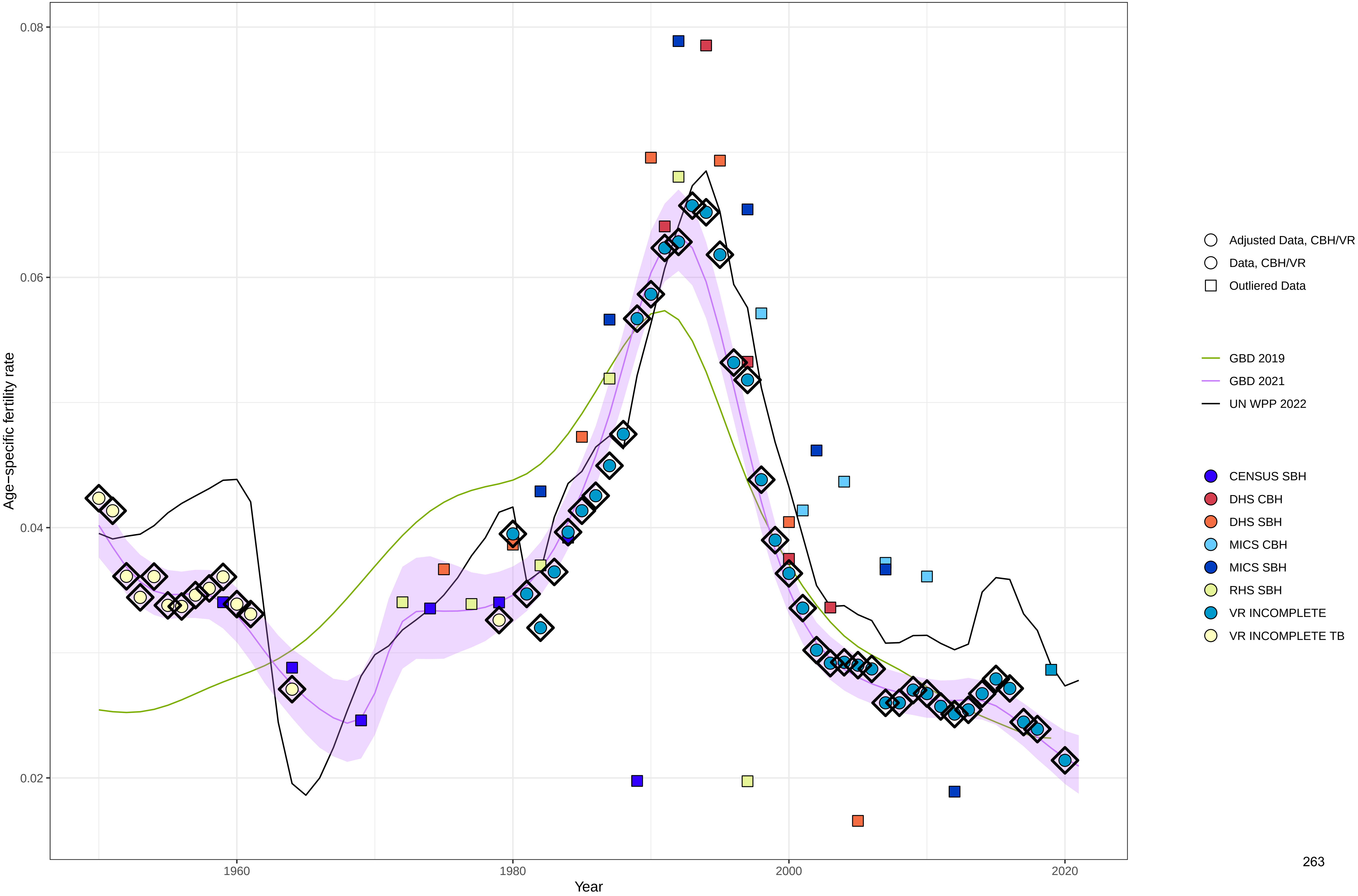

Age−specific fertility fitted trend lines, 1950−2021: Moldova, Ages: 20−24
Data points enclosed with a diamond are reference sources

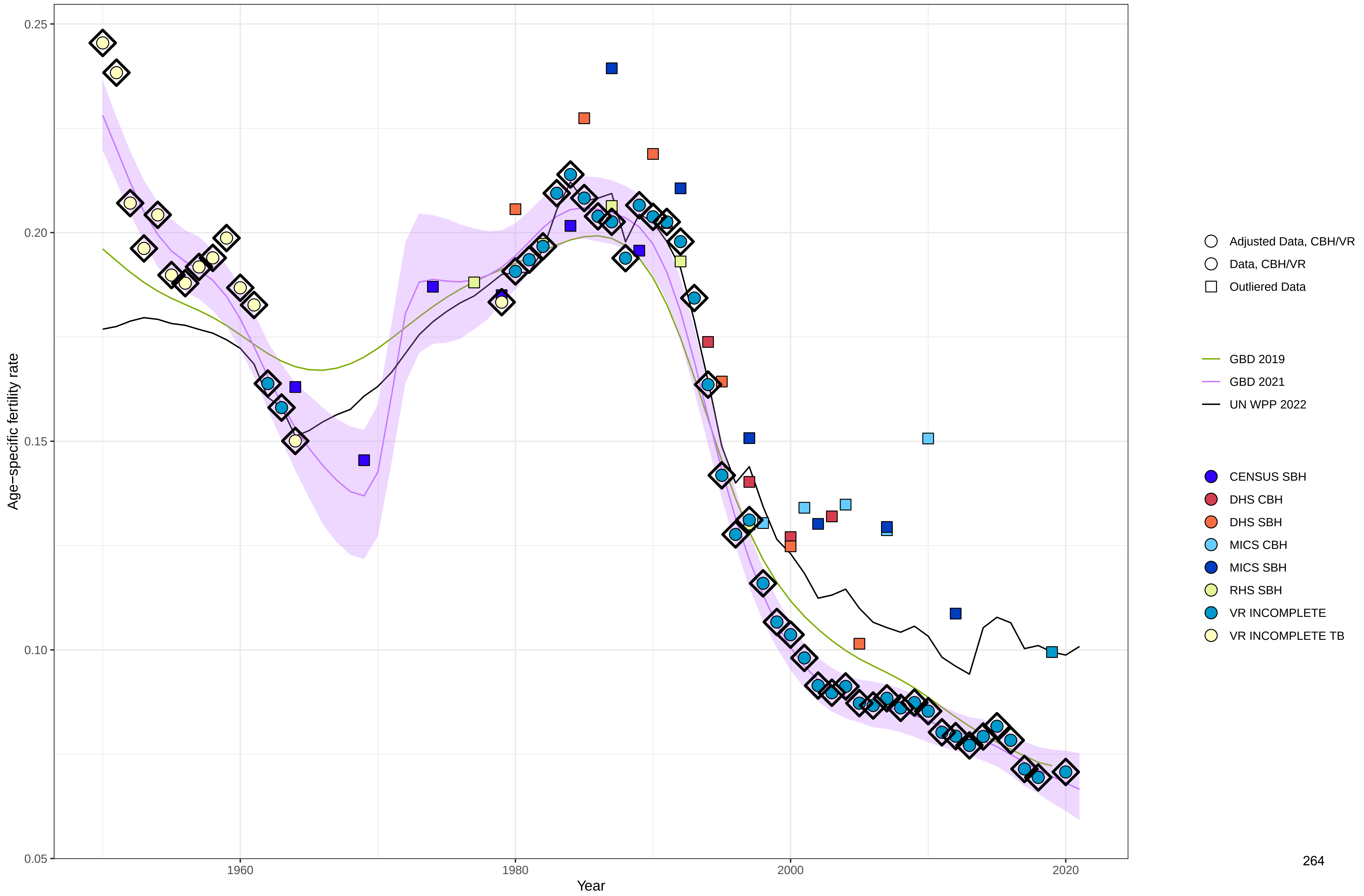

Age−specific fertility fitted trend lines, 1950−2021: Moldova, Ages: 25−29
Data points enclosed with a diamond are reference sources

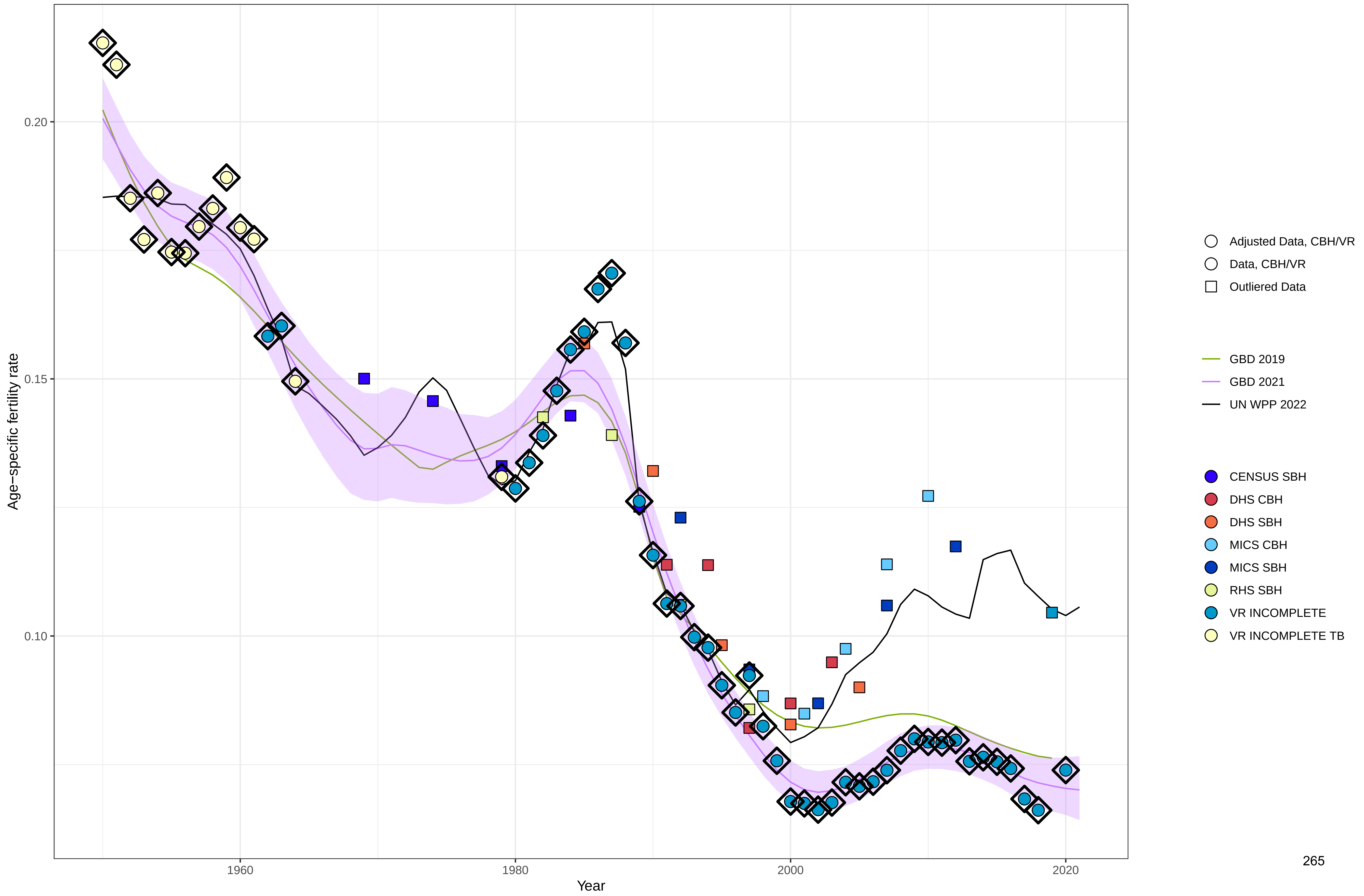

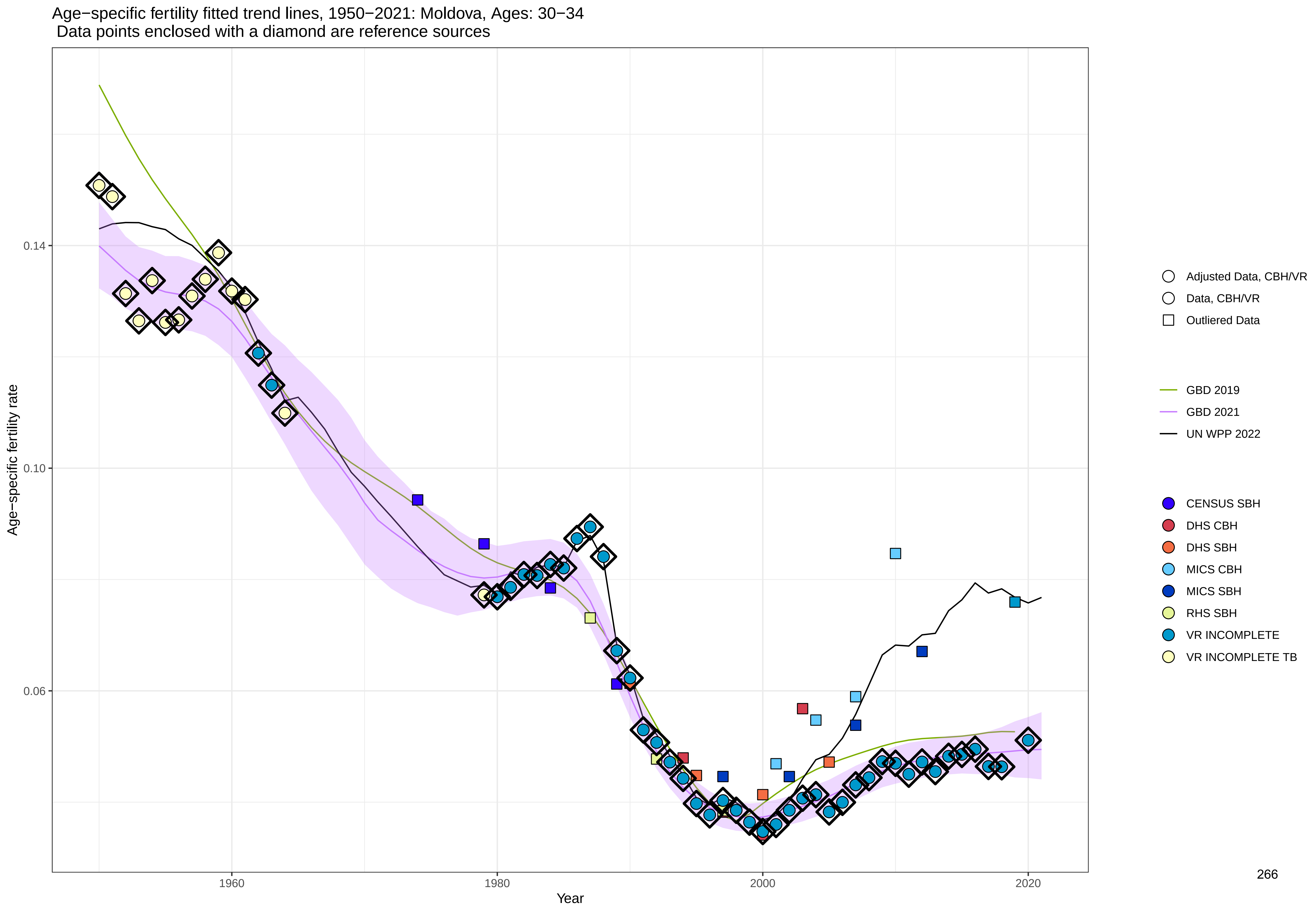

Age−specific fertility fitted trend lines, 1950−2021: Moldova, Ages: 30−34
Data points enclosed with a diamond are reference sources
Age−specific fertility rate
0.14
0.10
0.06
1960
1980
2000
2020
Year
Adjusted Data, CBH/VR
Data, CBH/VR
Outliered Data
GBD 2019
GBD 2021
UN WPP 2022
CENSUS SBH
DHS CBH
DHS SBH
MICS CBH
MICS SBH
RHS SBH
VR INCOMPLETE
VR INCOMPLETE TB

Age−specific fertility fitted trend lines, 1950−2021: Moldova, Ages: 35−39
Data points enclosed with a diamond are reference sources

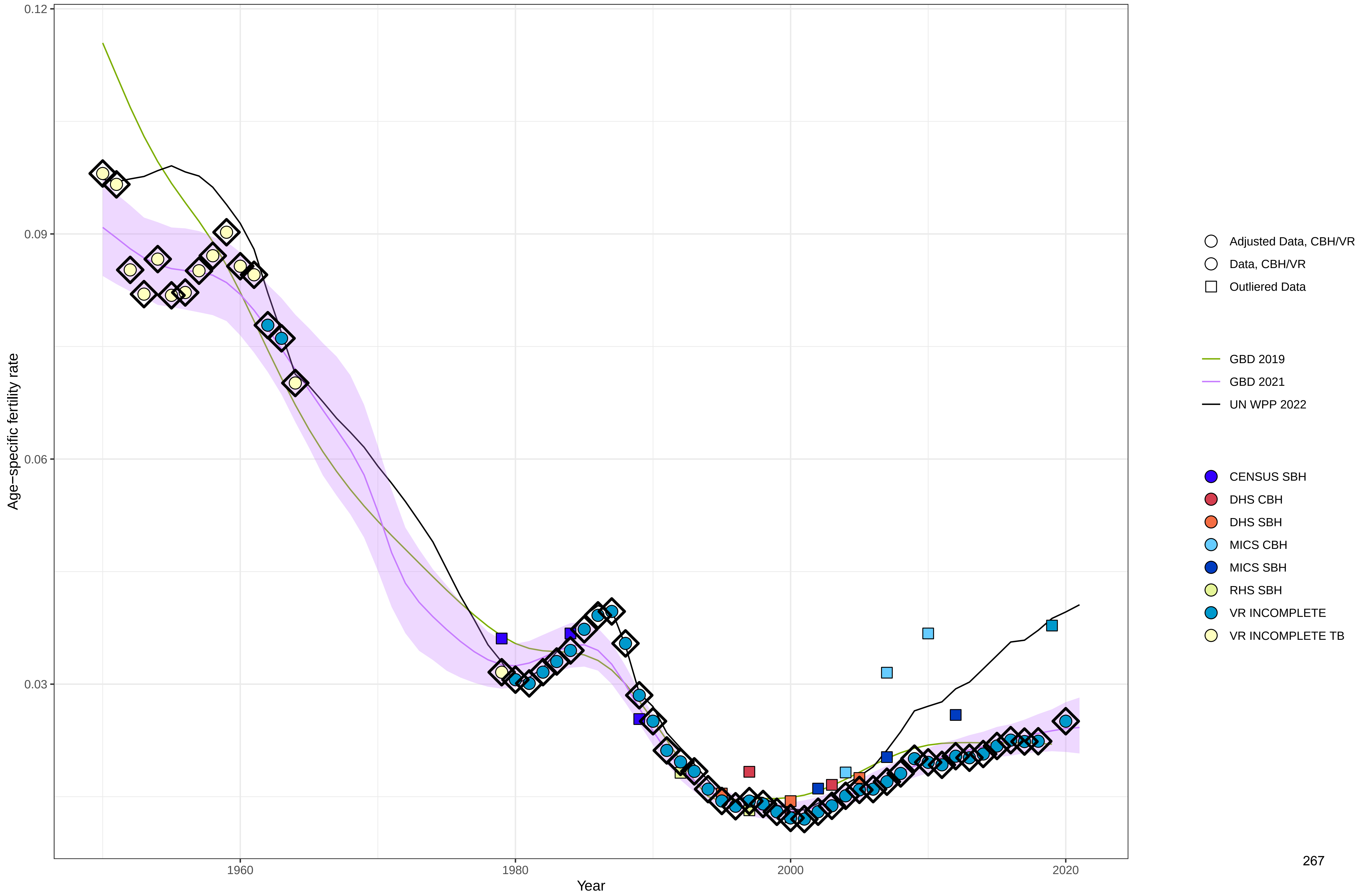

Age−specific fertility fitted trend lines, 1950−2021: Moldova, Ages: 40−44
Data points enclosed with a diamond are reference sources

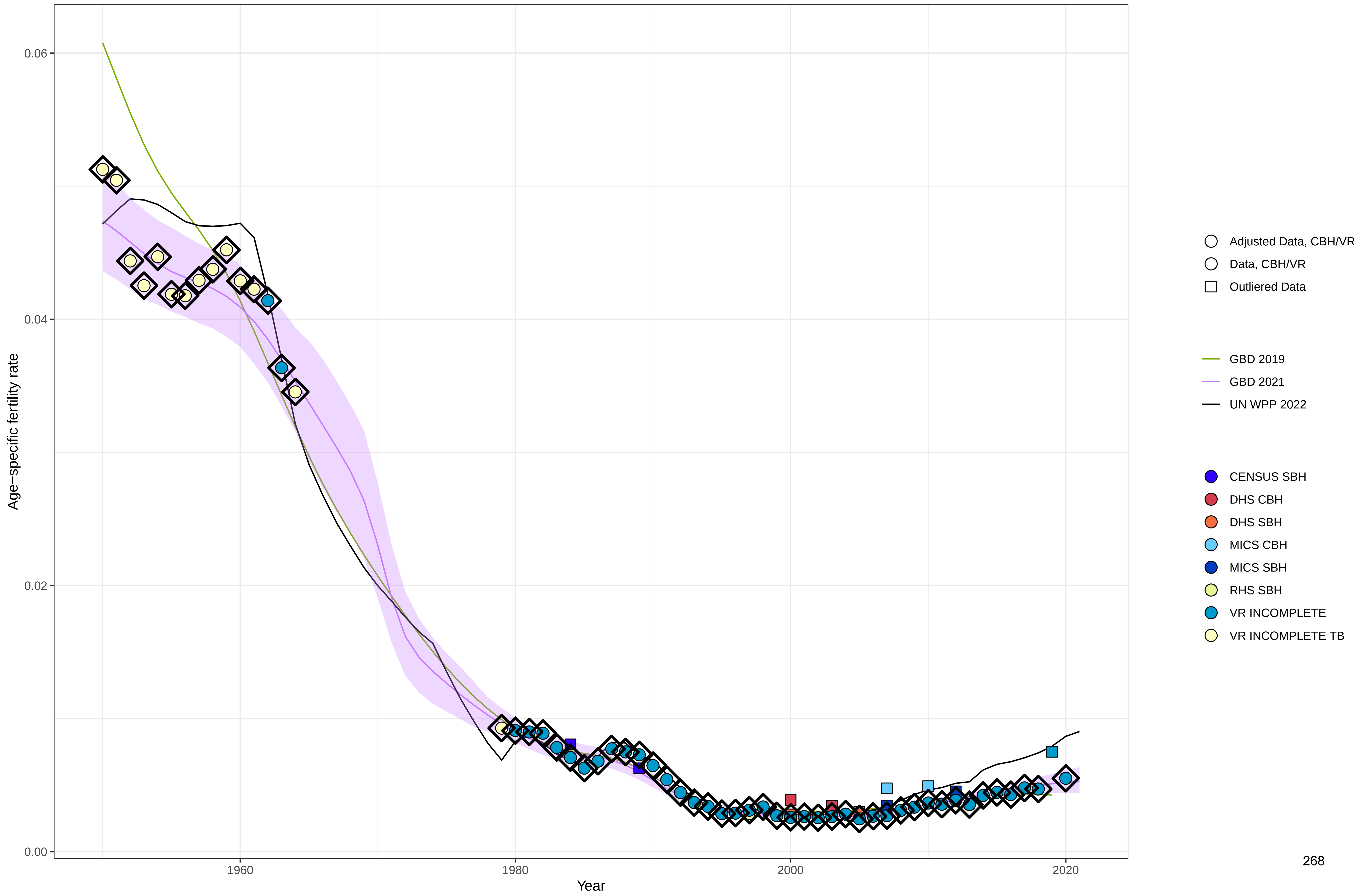

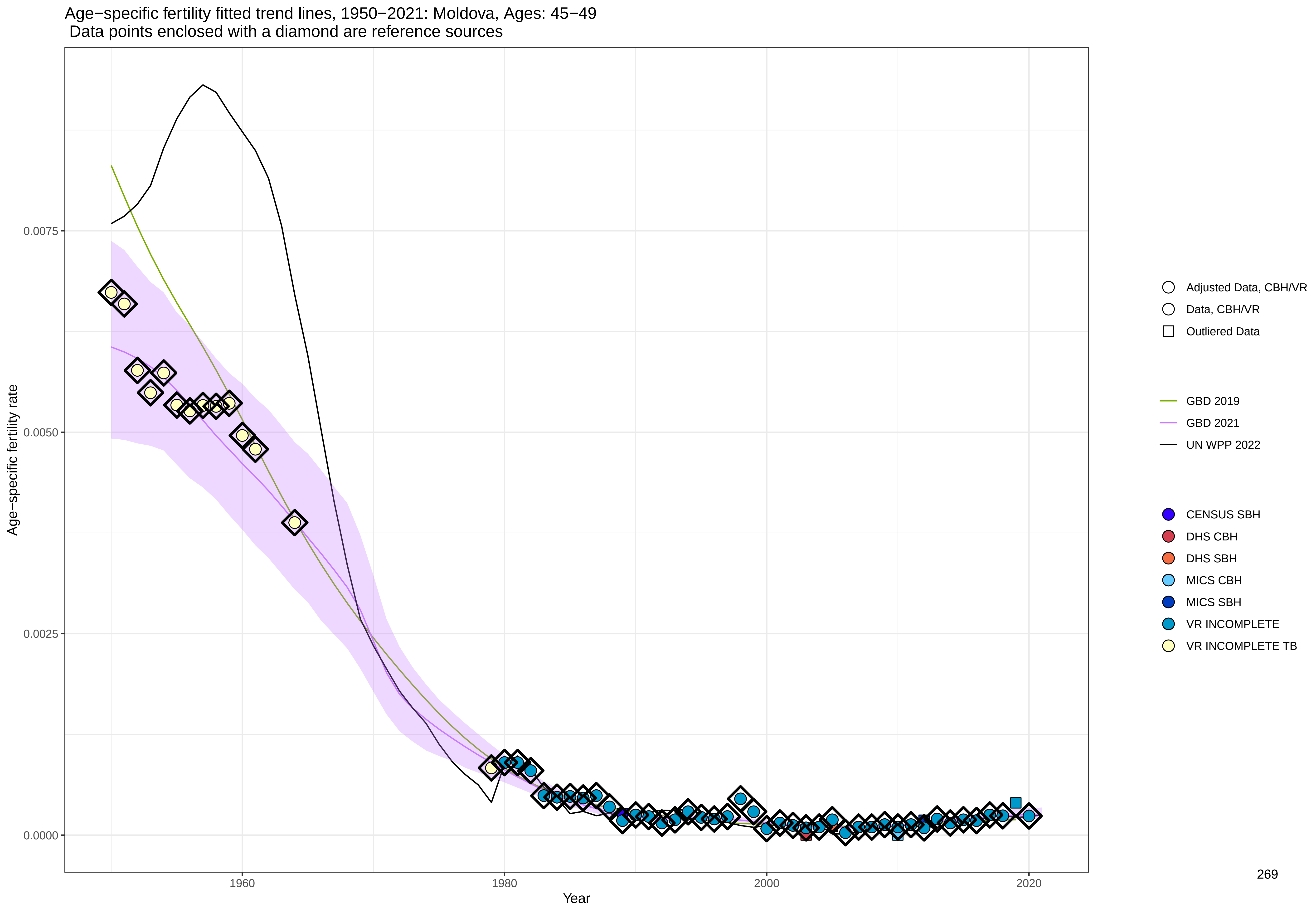

Age−specific fertility fitted trend lines, 1950−2021: Moldova, Ages: 45−49
Data points enclosed with a diamond are reference sources
Age−specific fertility rate
0.0075
0.0050
0.0025
0.0000
1960
1980
2000
2020
Year
Adjusted Data, CBH/VR
Data, CBH/VR
Outliered Data
GBD 2019
GBD 2021
UN WPP 2022
CENSUS SBH
DHS CBH
DHS SBH
MICS CBH
MICS SBH
VR INCOMPLETE
VR INCOMPLETE TB

Age−specific fertility fitted trend lines, 1950−2021: Moldova, Ages: 50−54
Data points enclosed with a diamond are reference sources

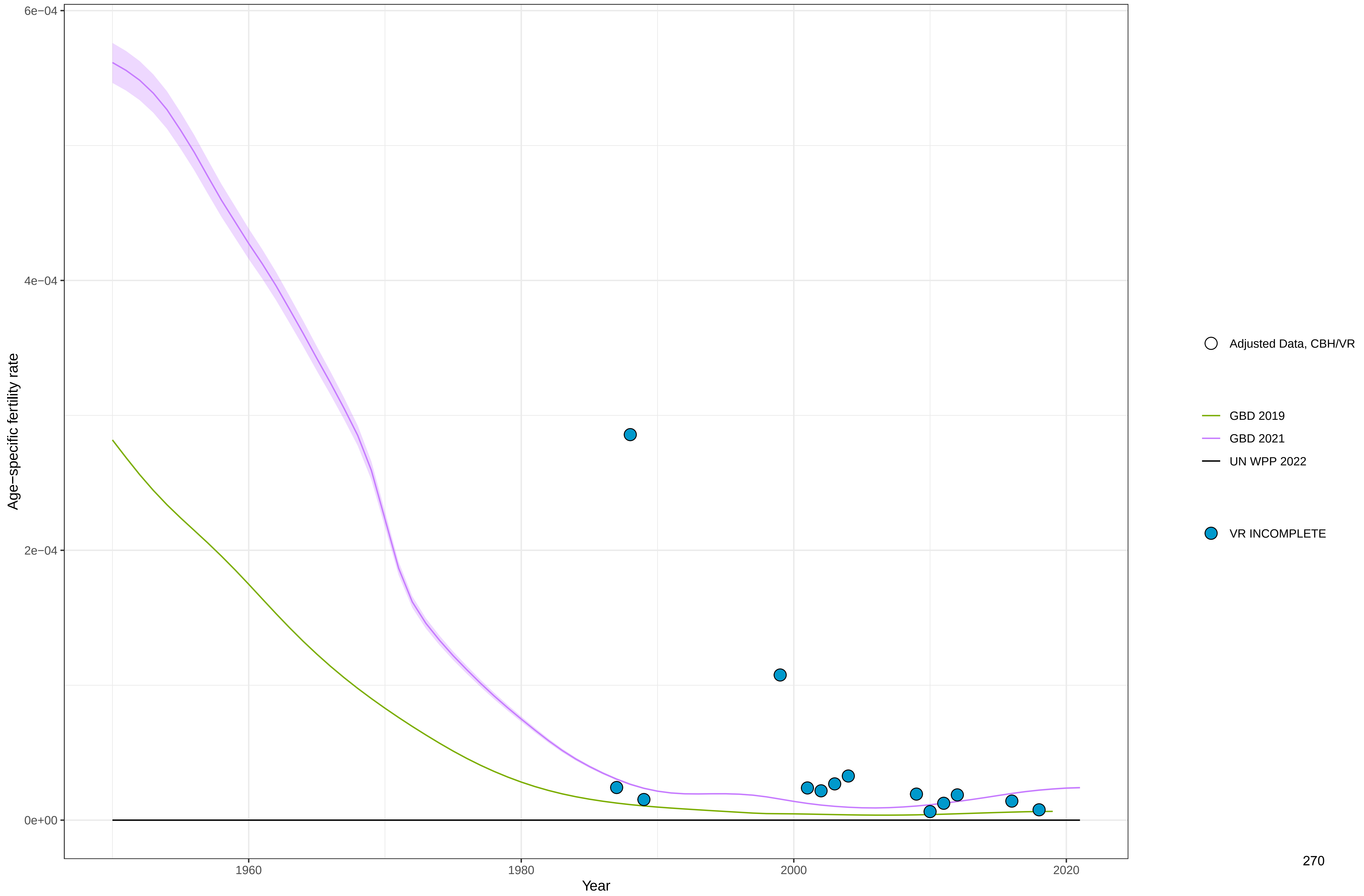

Total fertility rate fitted trend lines, 1950−2021: Moldova TFR
Data points enclosed with a diamond are reference sources

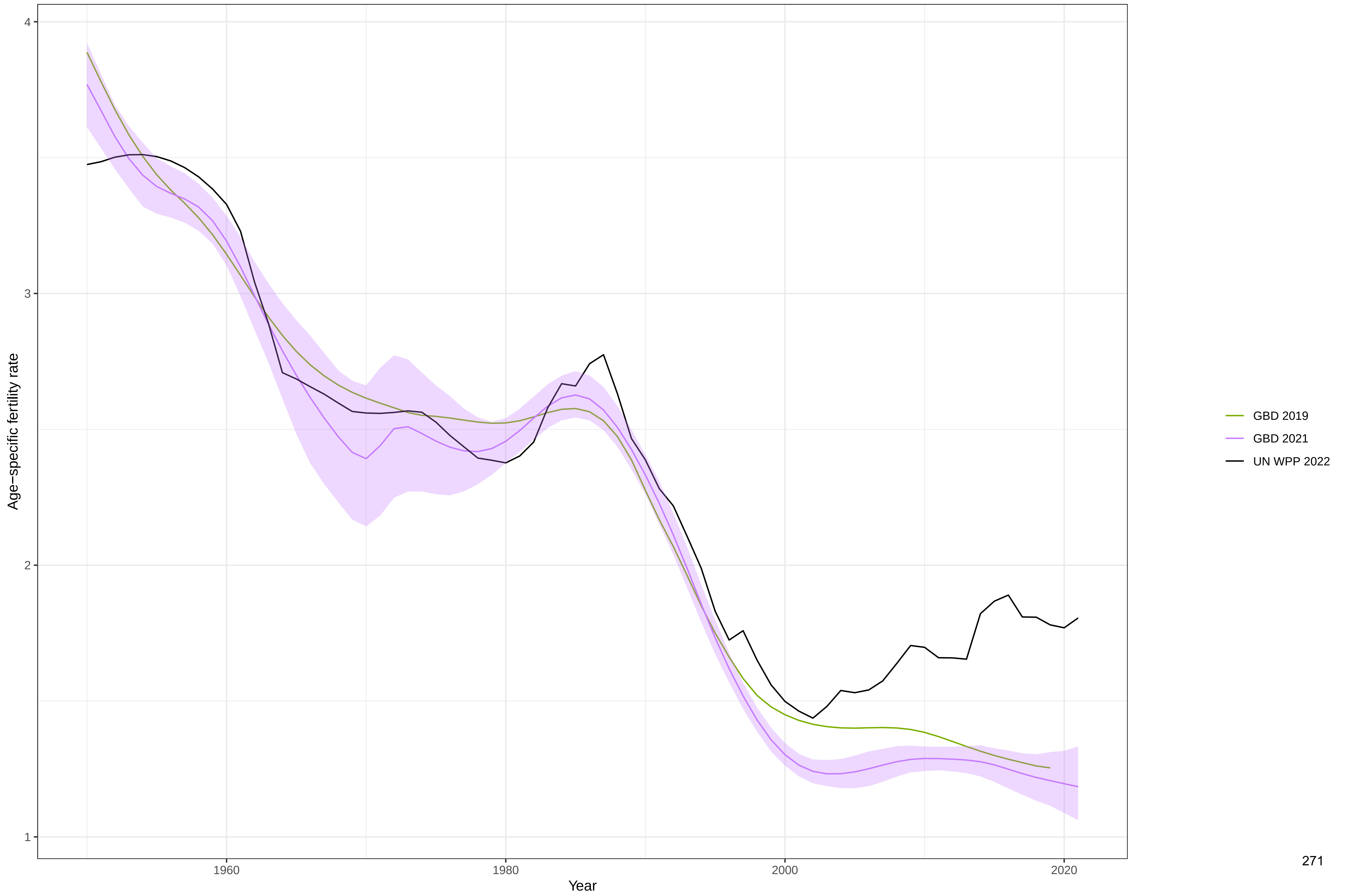

Age−specific fertility fitted trend lines, 1950−2021: Russia, Ages: 10−14
Data points enclosed with a diamond are reference sources

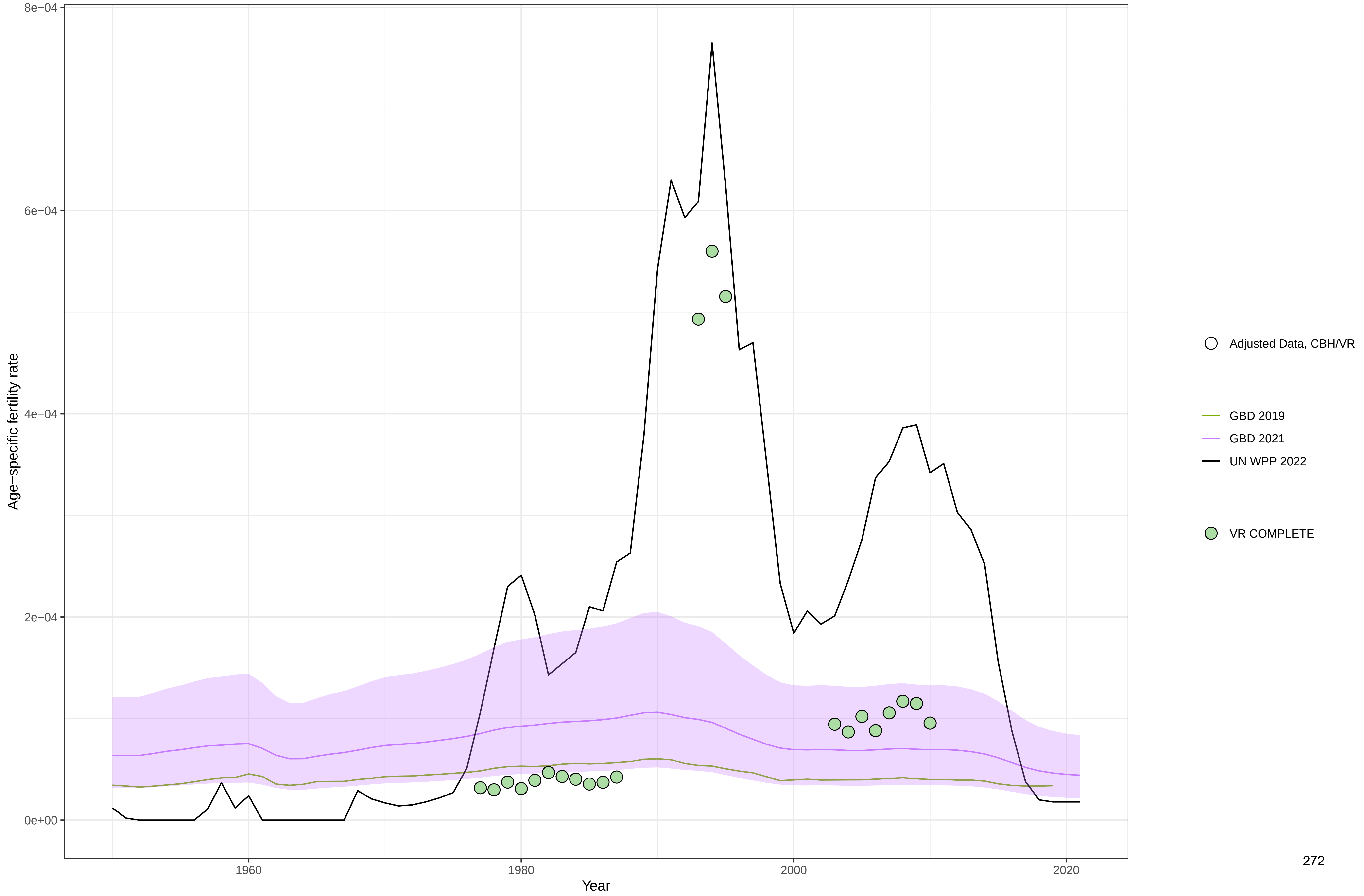

Age−specific fertility fitted trend lines, 1950−2021: Russia, Ages: 15−19
Data points enclosed with a diamond are reference sources

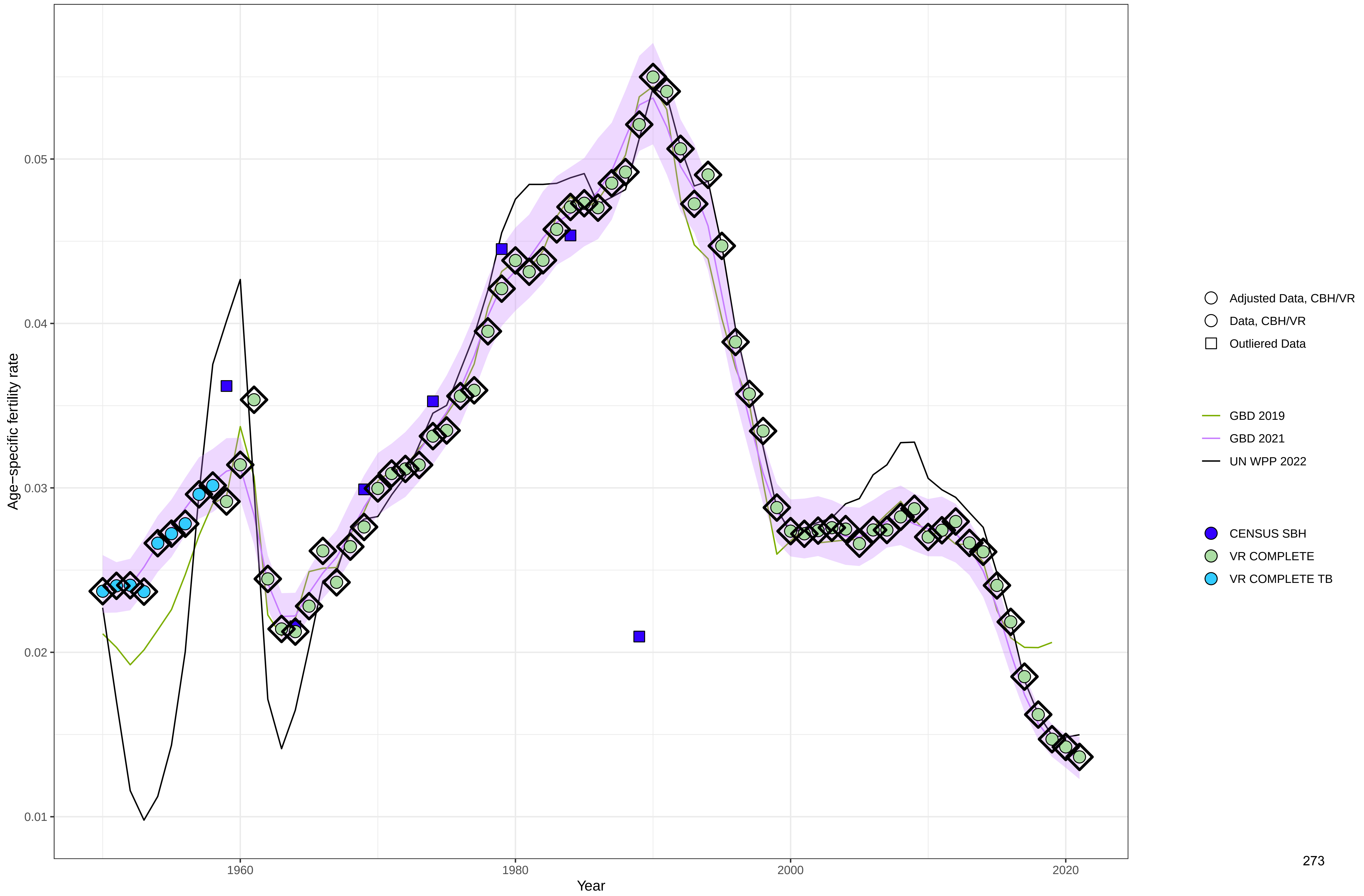

Age−specific fertility fitted trend lines, 1950−2021: Russia, Ages: 20−24
Data points enclosed with a diamond are reference sources

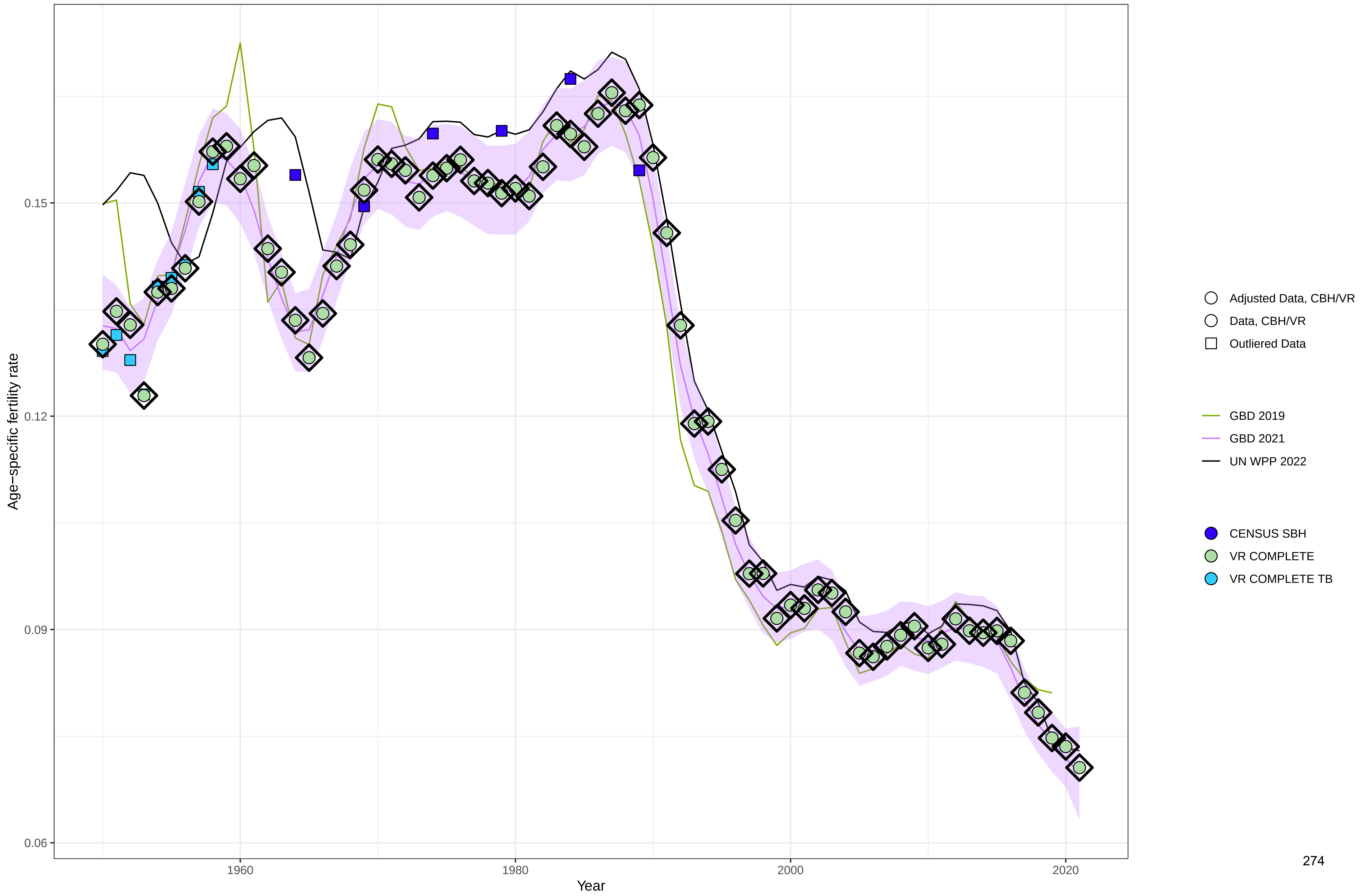

Age−specific fertility fitted trend lines, 1950−2021: Russia, Ages: 25−29
Data points enclosed with a diamond are reference sources

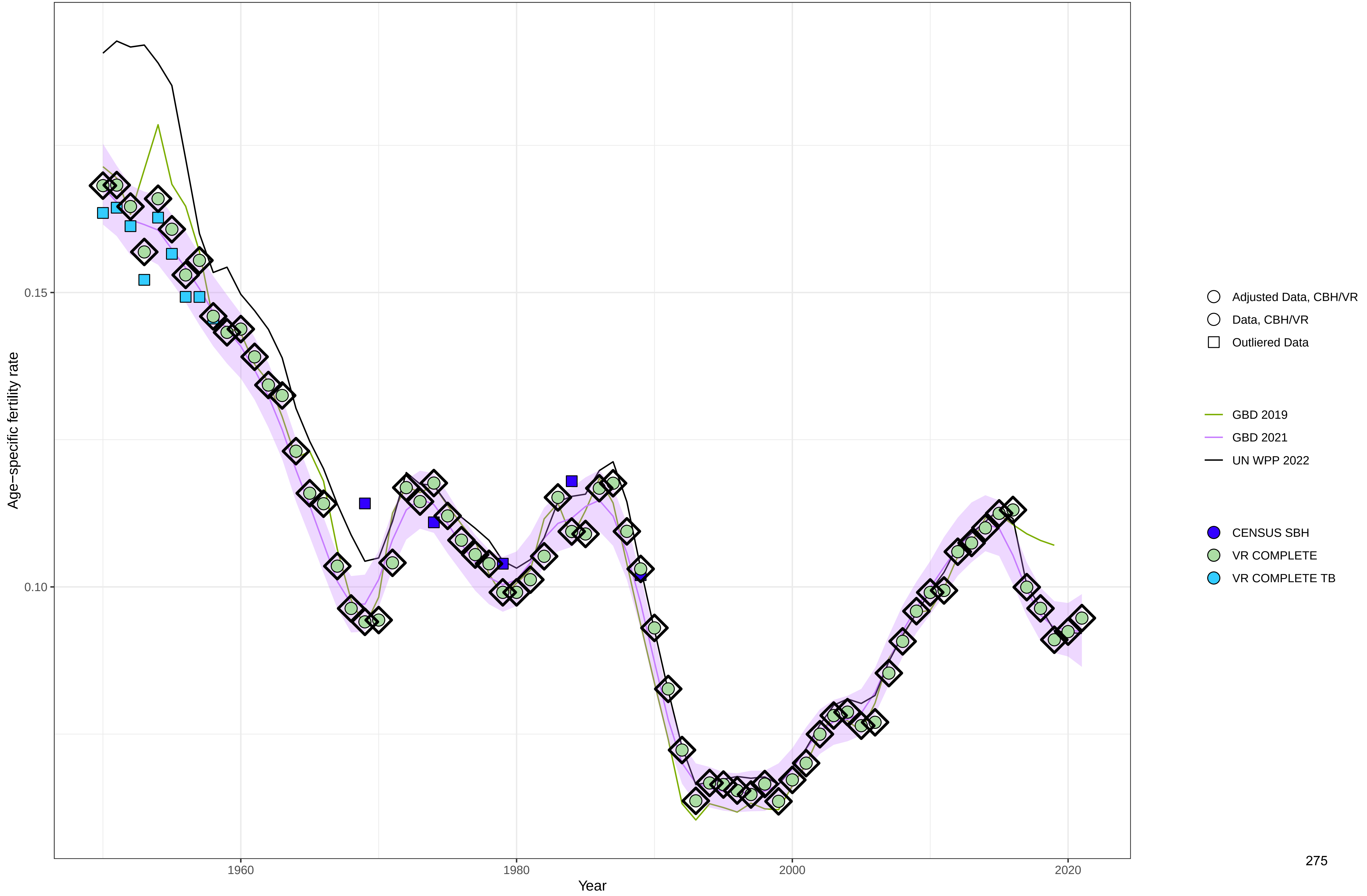

Age−specific fertility fitted trend lines, 1950−2021: Russia, Ages: 30−34
Data points enclosed with a diamond are reference sources

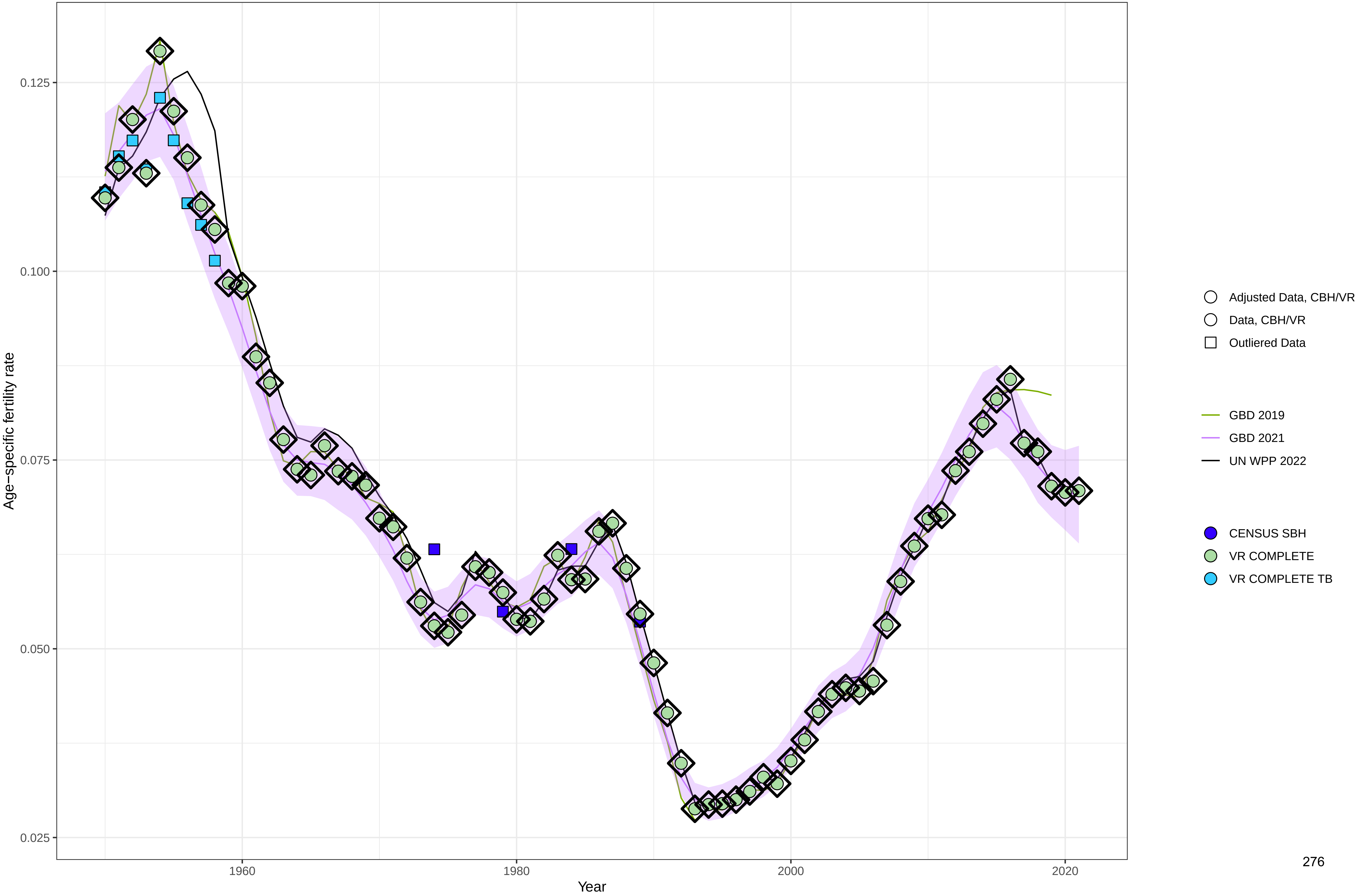

Age−specific fertility fitted trend lines, 1950−2021: Russia, Ages: 35−39
Data points enclosed with a diamond are reference sources

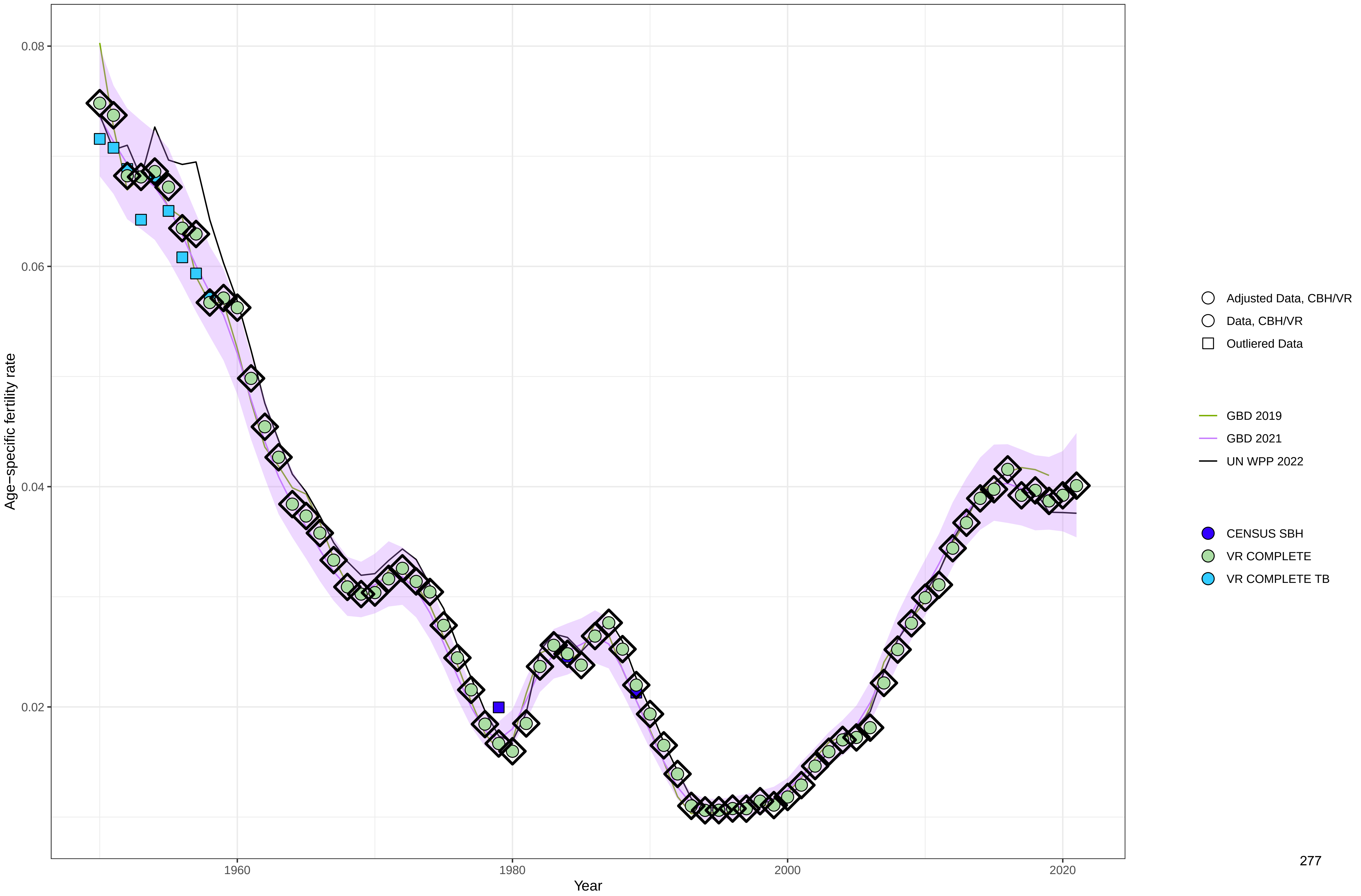

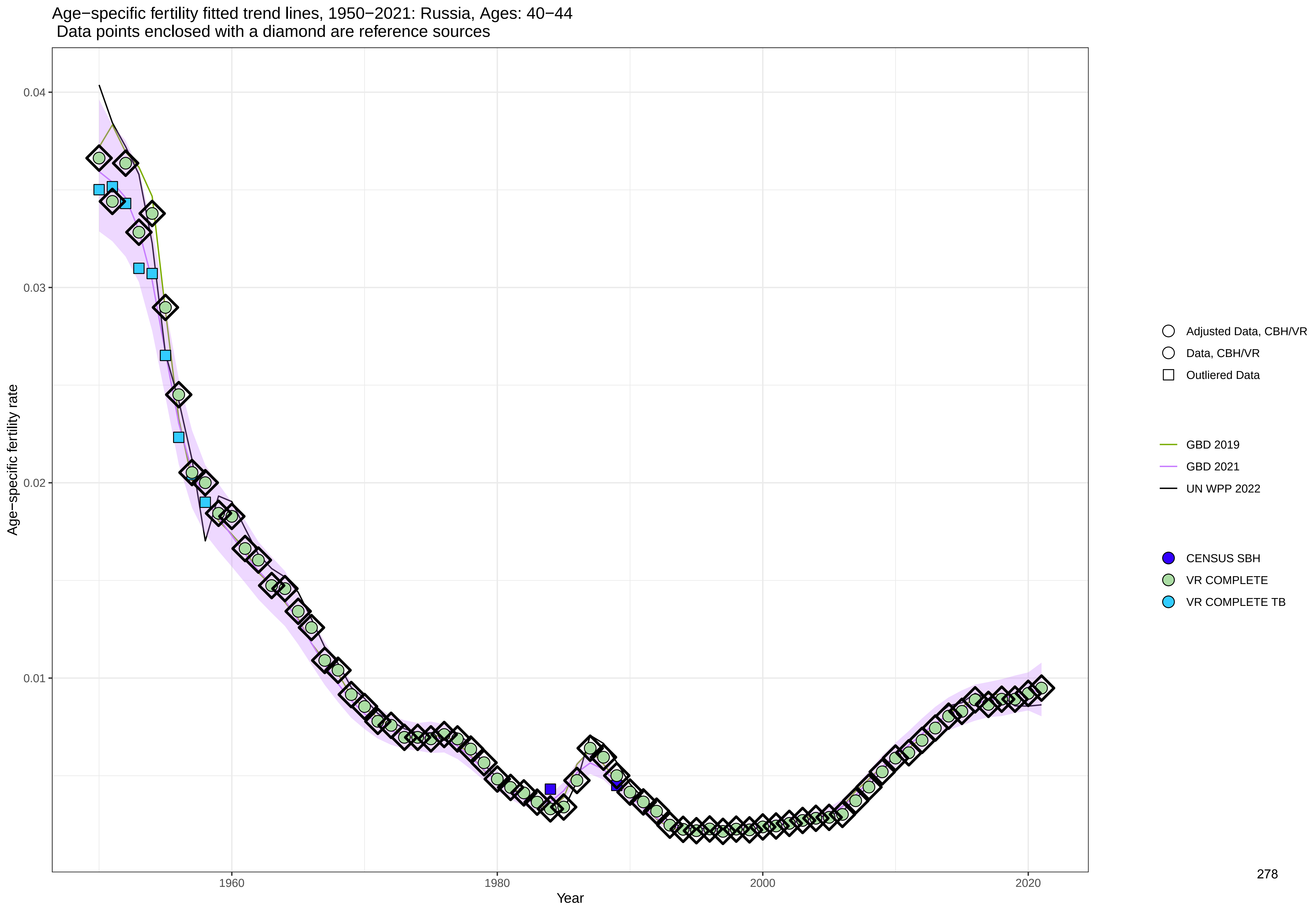
Age−specific fertility fitted trend lines, 1950−2021: Russia, Ages: 40−44
Data points enclosed with a diamond are reference sources
Age−specific fertility rate
0.04
0.03
0.02
0.01
1960
1980
2000
2020
Year
Adjusted Data, CBH/VR
Data, CBH/VR
Outliered Data
GBD 2019
GBD 2021
UN WPP 2022
CENSUS SBH
VR COMPLETE
VR COMPLETE TB

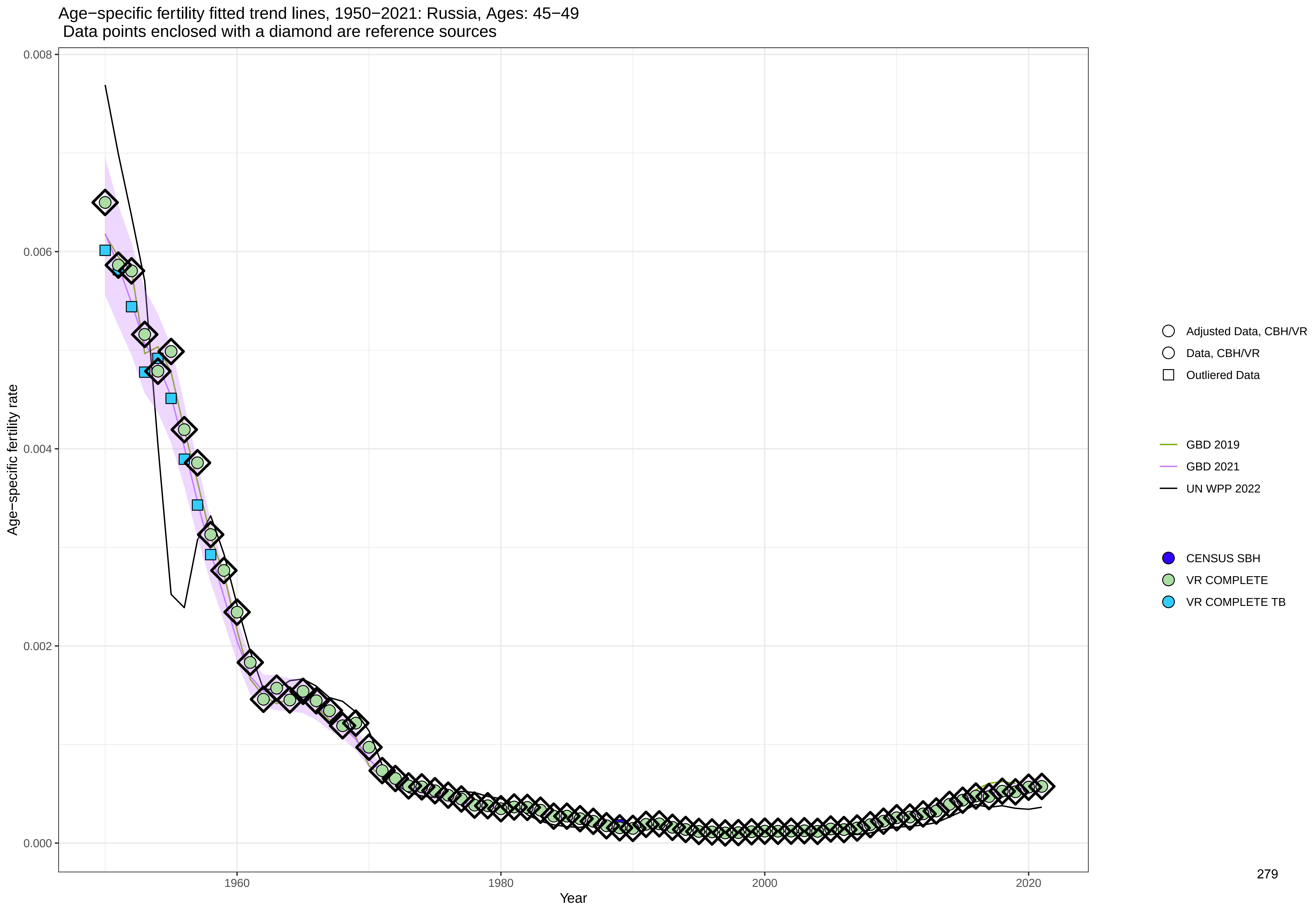

Age−specific fertility fitted trend lines, 1950−2021: Russia, Ages: 45−49
Data points enclosed with a diamond are reference sources
0.008
0.006
0.004
0.002
0.000
Age−specific fertility rate
1960
1980
2000
2020
Year
Adjusted Data, CBH/VR
Data, CBH/VR
Outliered Data
GBD 2019
GBD 2021
UN WPP 2022
CENSUS SBH
VR COMPLETE
VR COMPLETE TB

Age−specific fertility fitted trend lines, 1950−2021: Russia, Ages: 50−54
Data points enclosed with a diamond are reference sources

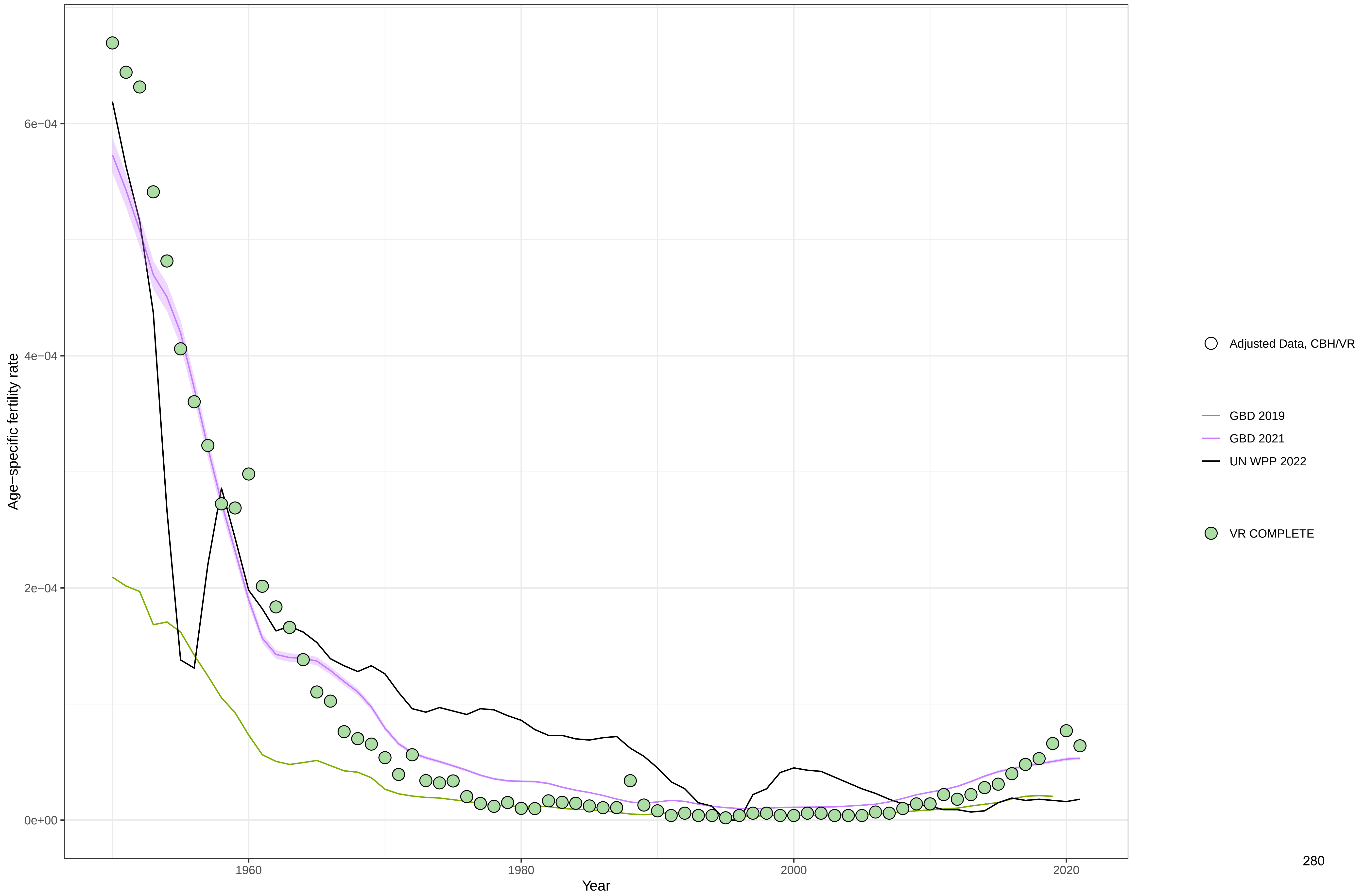

Total fertility rate fitted trend lines, 1950−2021: Russia TFR
Data points enclosed with a diamond are reference sources

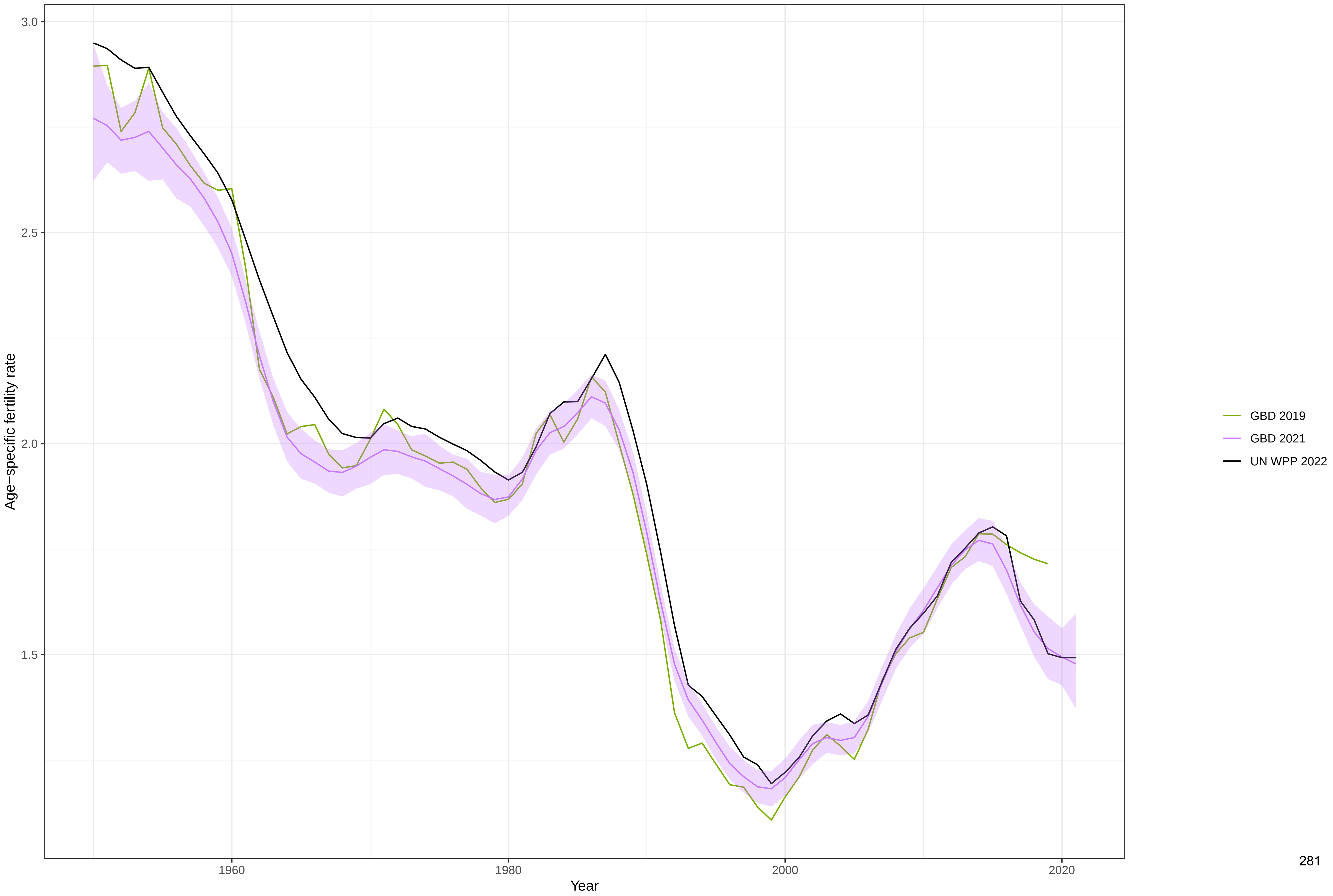

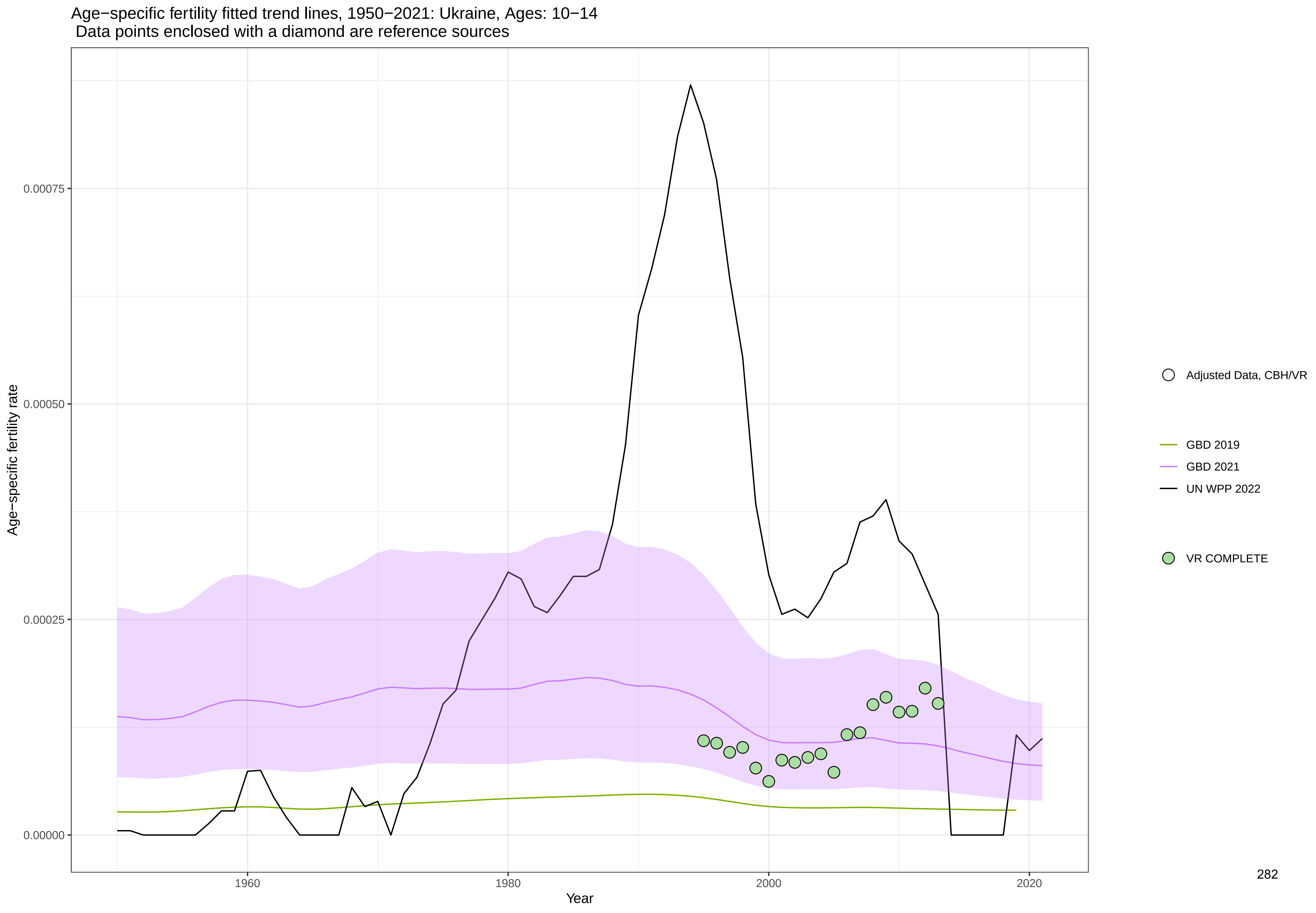
Age−specific fertility fitted trend lines, 1950−2021: Ukraine, Ages: 10−14
Data points enclosed with a diamond are reference sources
Age−specific fertility rate
0.00075
0.00050
0.00025
0.00000
1960
1980
2000
2020
Year
Adjusted Data, CBH/VR
GBD 2019
GBD 2021
UN WPP 2022
VR COMPLETE

Age−specific fertility fitted trend lines, 1950−2021: Ukraine, Ages: 15−19
Data points enclosed with a diamond are reference sources

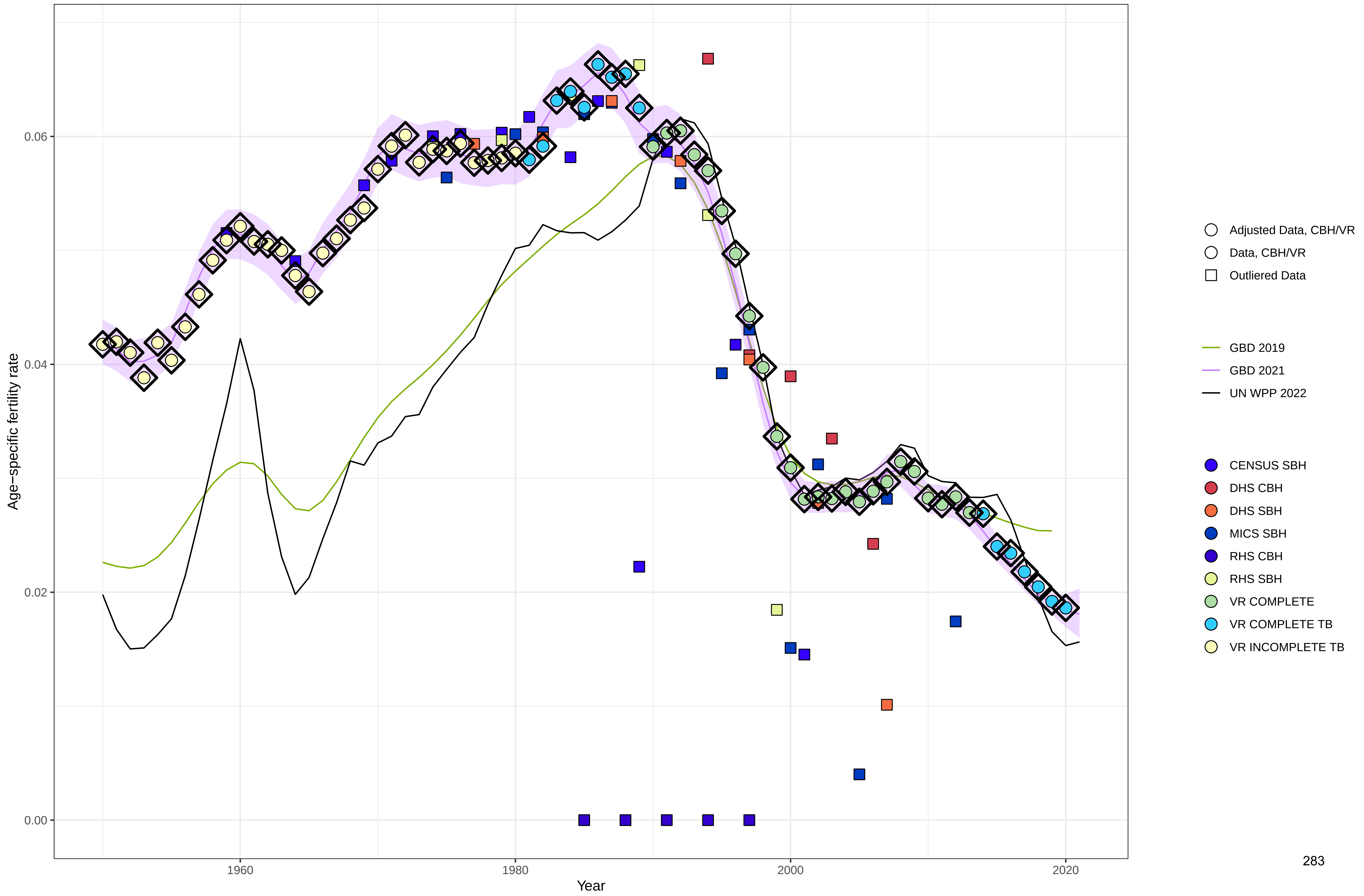

Age−specific fertility fitted trend lines, 1950−2021: Ukraine, Ages: 20−24
Data points enclosed with a diamond are reference sources

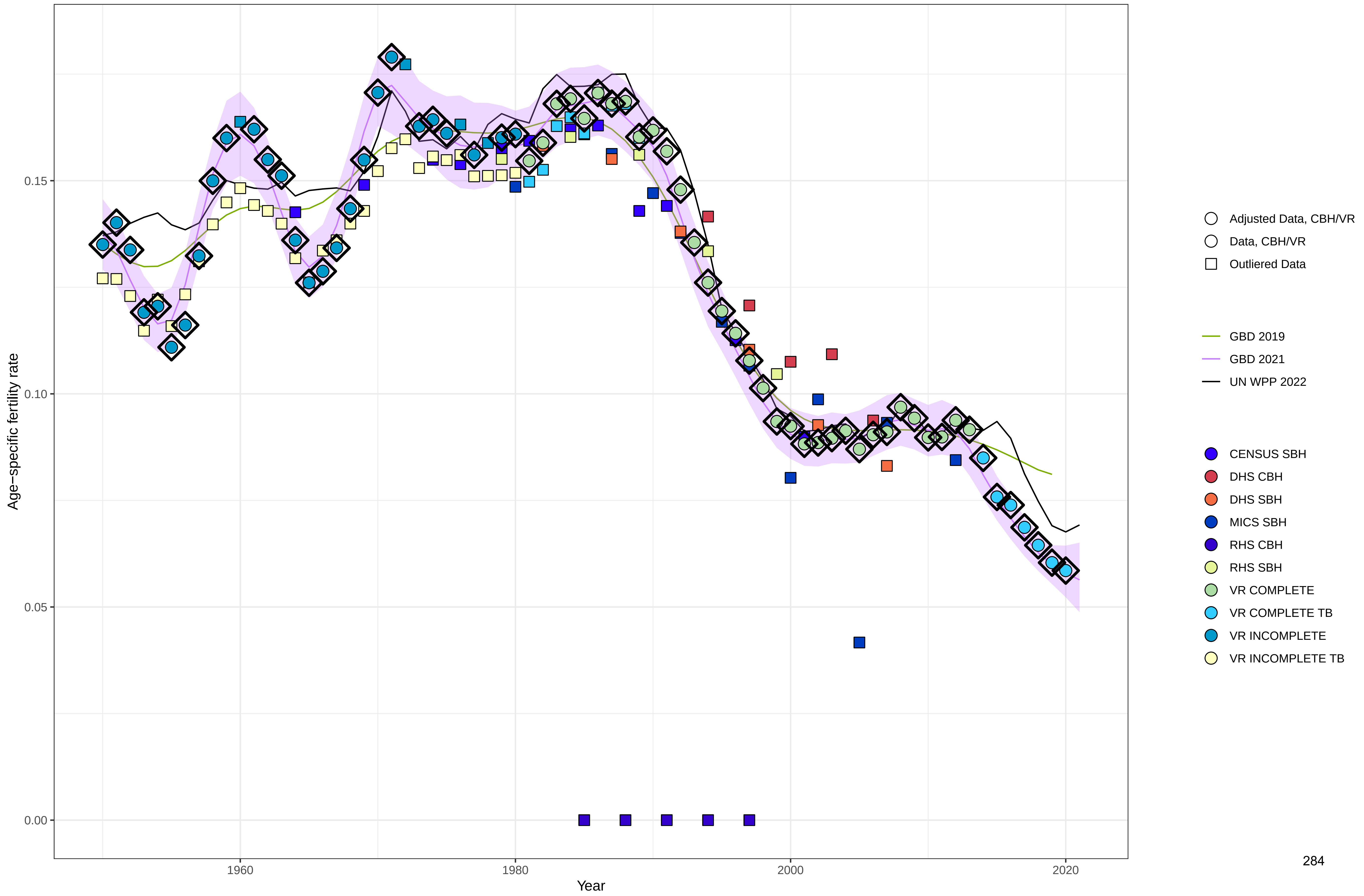

Age−specific fertility fitted trend lines, 1950−2021: Ukraine, Ages: 25−29
Data points enclosed with a diamond are reference sources

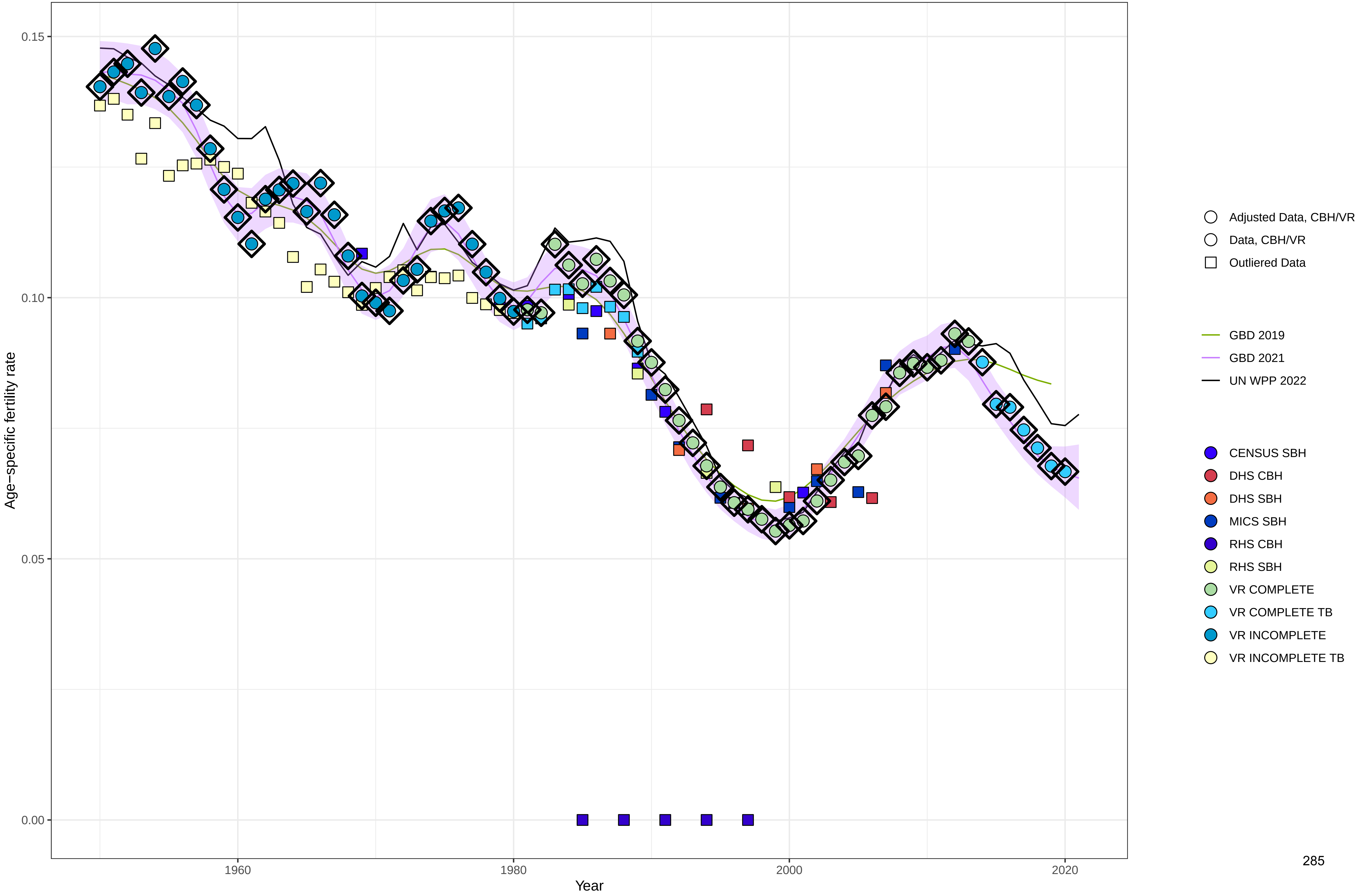

Age−specific fertility fitted trend lines, 1950−2021: Ukraine, Ages: 30−34
Data points enclosed with a diamond are reference sources

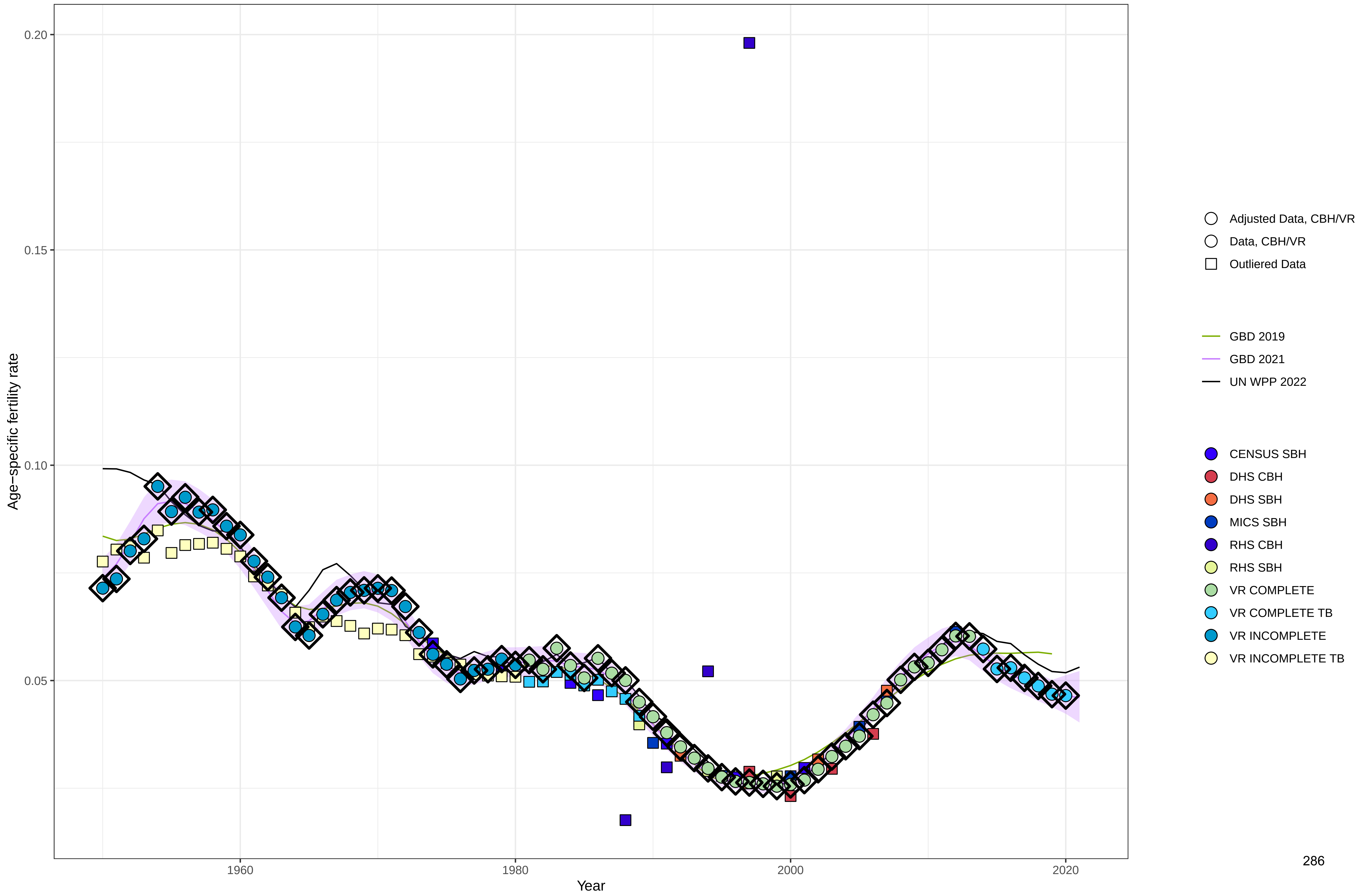

Age−specific fertility fitted trend lines, 1950−2021: Ukraine, Ages: 35−39
Data points enclosed with a diamond are reference sources

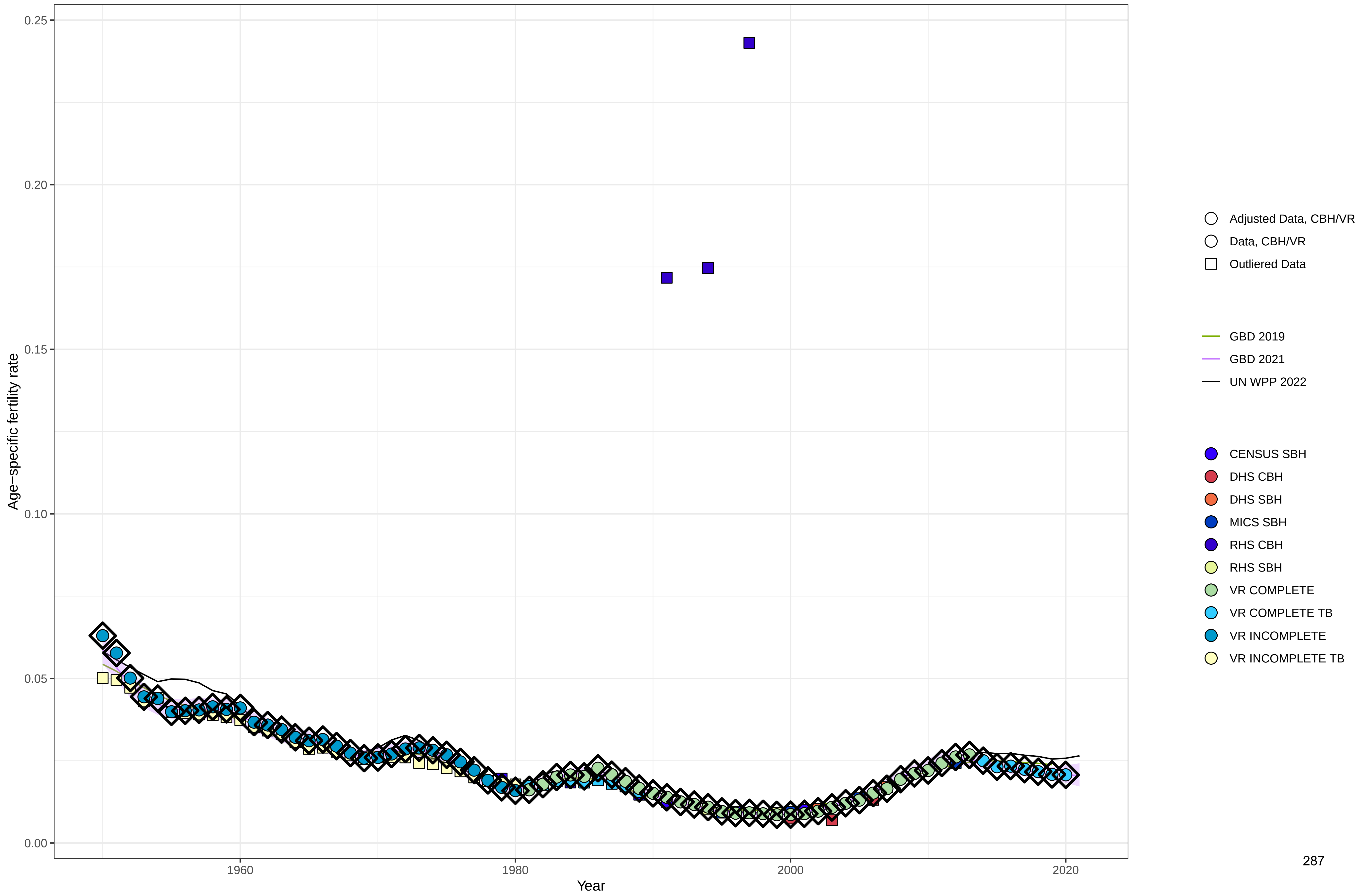

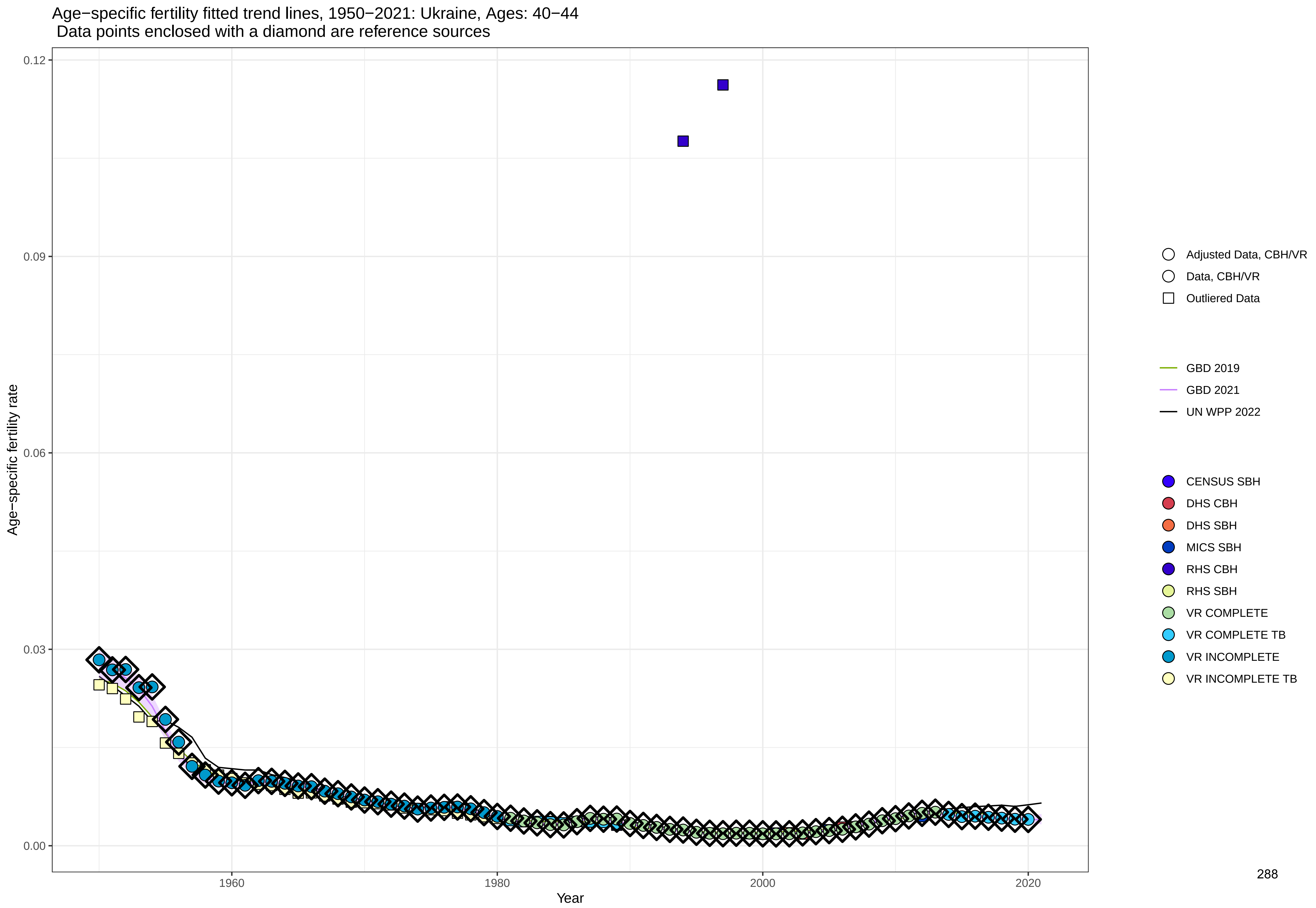

Age−specific fertility fitted trend lines, 1950−2021: Ukraine, Ages: 40−44
Data points enclosed with a diamond are reference sources
Age−specific fertility rate
Year
0.00
0.03
0.06
0.09
0.12
1960
1980
2000
2020
Adjusted Data, CBH/VR
Data, CBH/VR
Outliered Data
GBD 2019
GBD 2021
UN WPP 2022
CENSUS SBH
DHS CBH
DHS SBH
MICS SBH
RHS CBH
RHS SBH
VR COMPLETE
VR COMPLETE TB
VR INCOMPLETE
VR INCOMPLETE TB

Age−specific fertility fitted trend lines, 1950−2021: Ukraine, Ages: 45−49
Data points enclosed with a diamond are reference sources

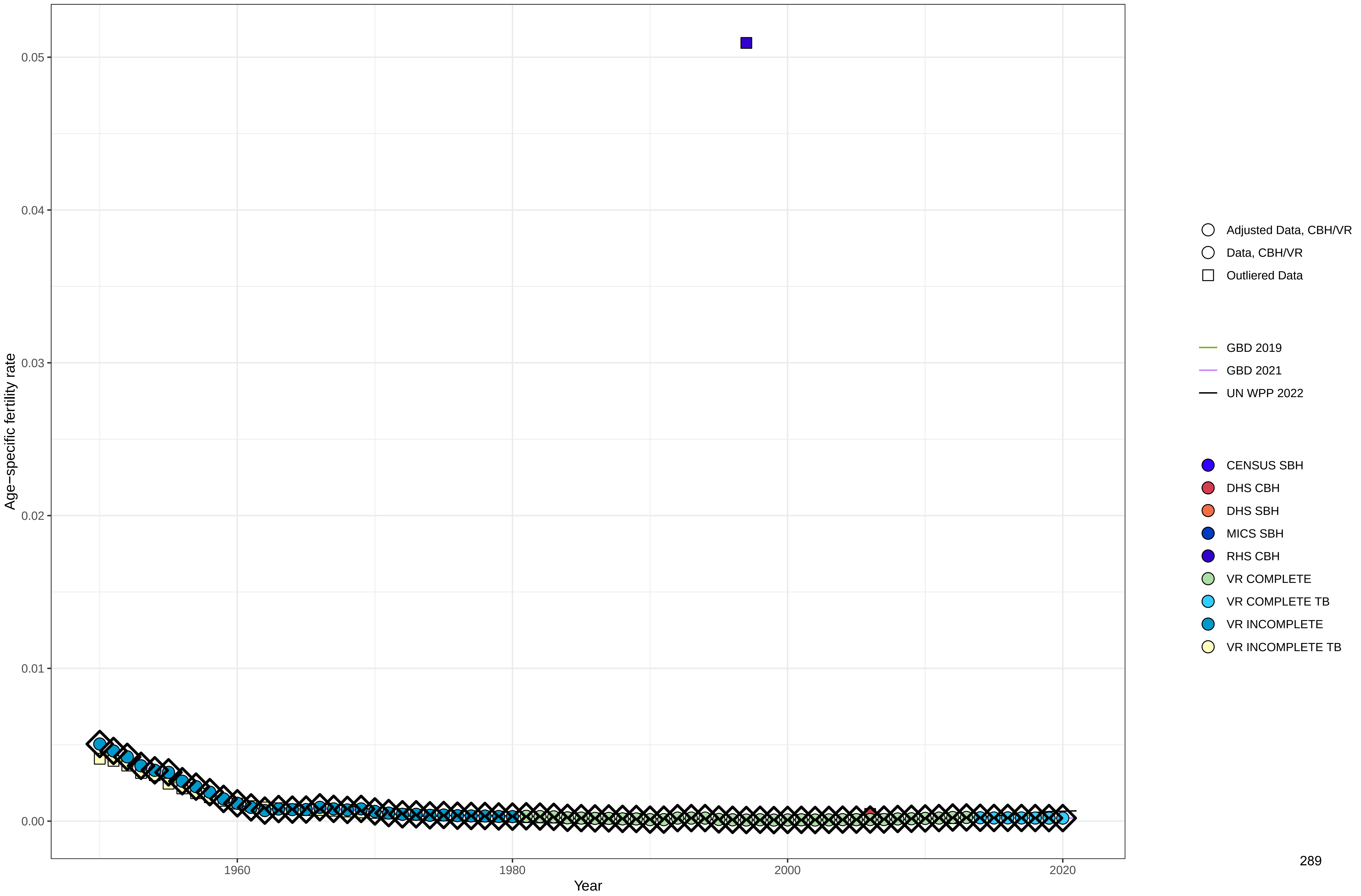

Age−specific fertility fitted trend lines, 1950−2021: Ukraine, Ages: 50−54
Data points enclosed with a diamond are reference sources

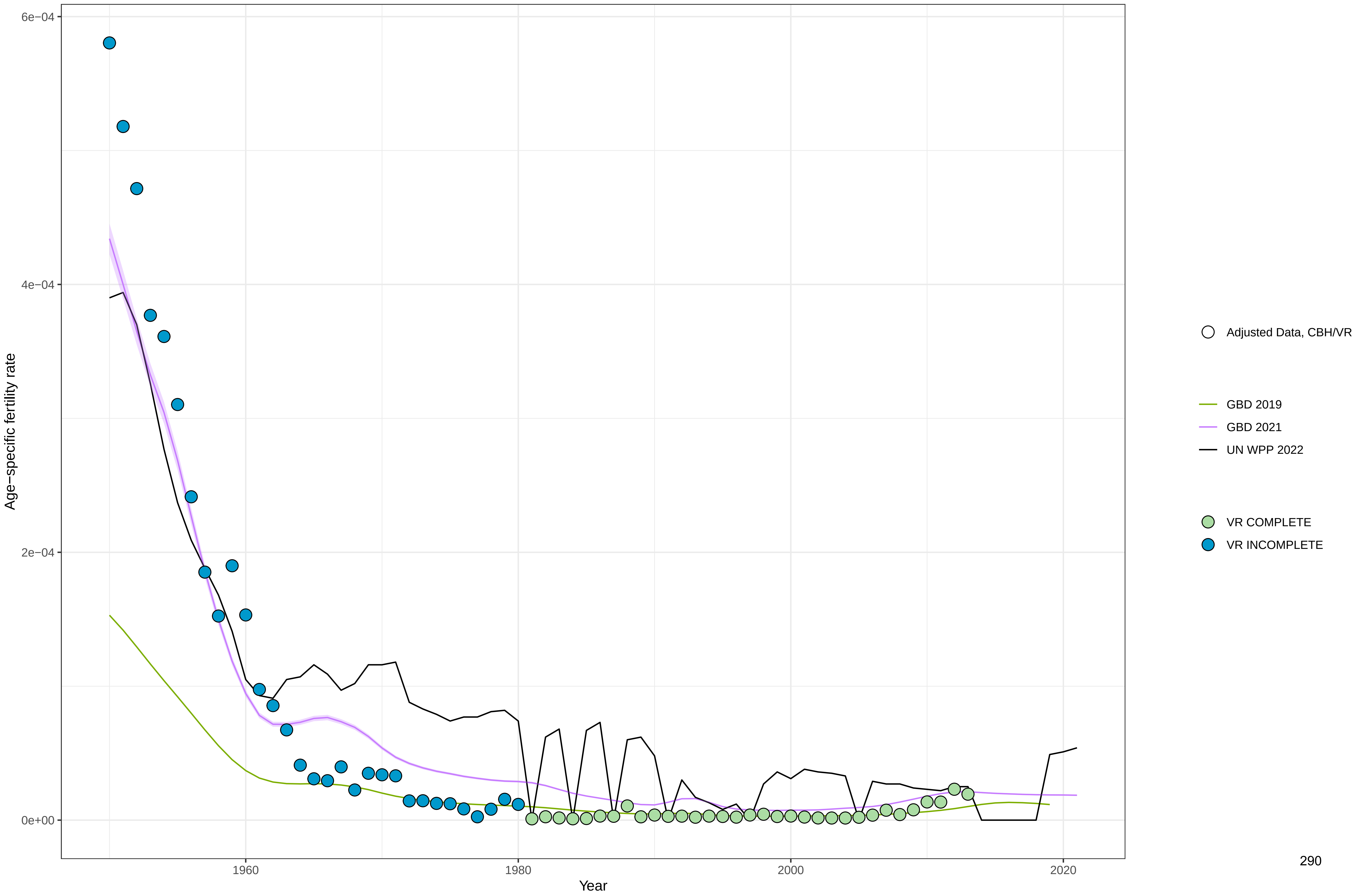

Total fertility rate fitted trend lines, 1950−2021: Ukraine TFR
Data points enclosed with a diamond are reference sources

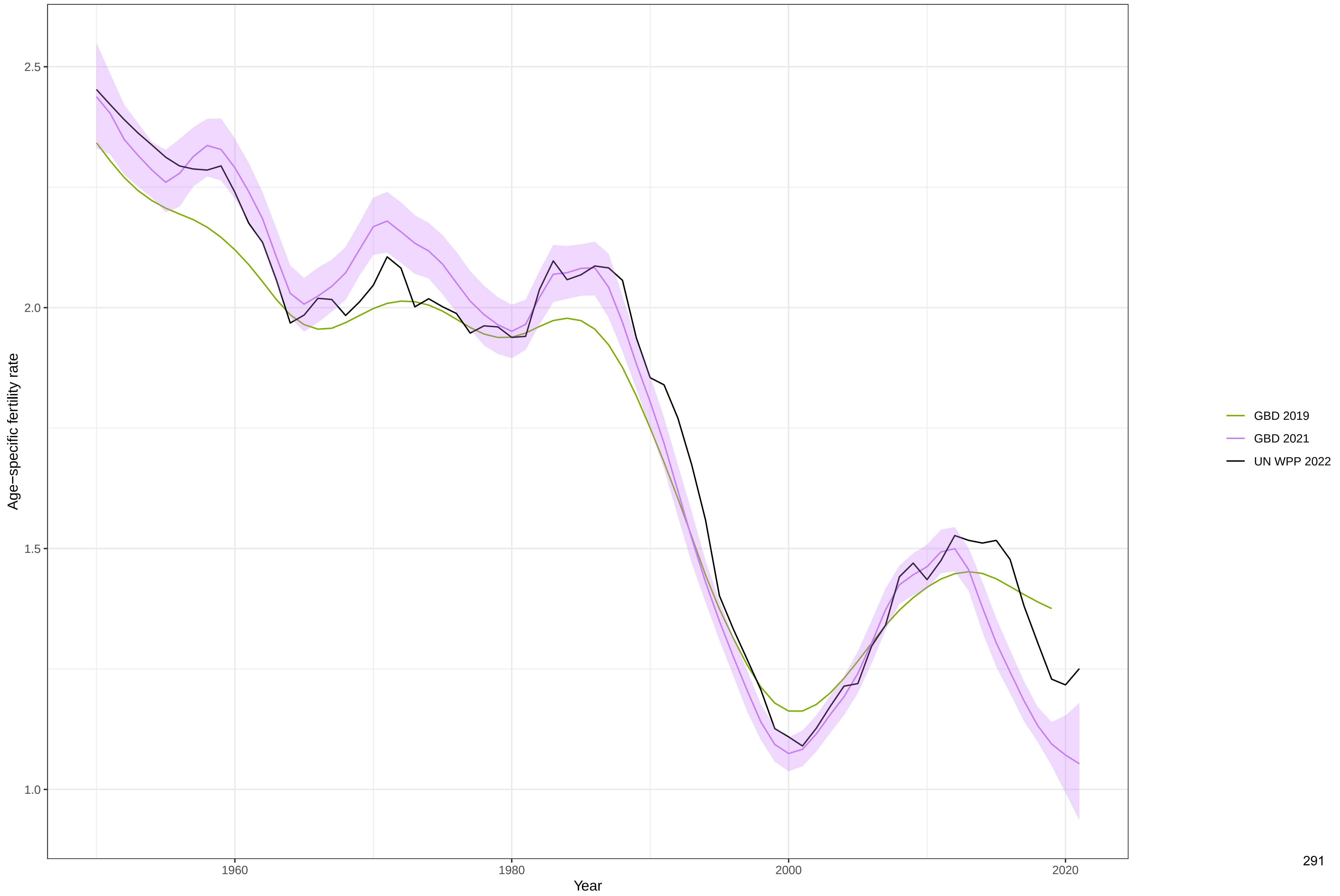

Age−specific fertility fitted trend lines, 1950−2021: Australia, Ages: 10−14
Data points enclosed with a diamond are reference sources

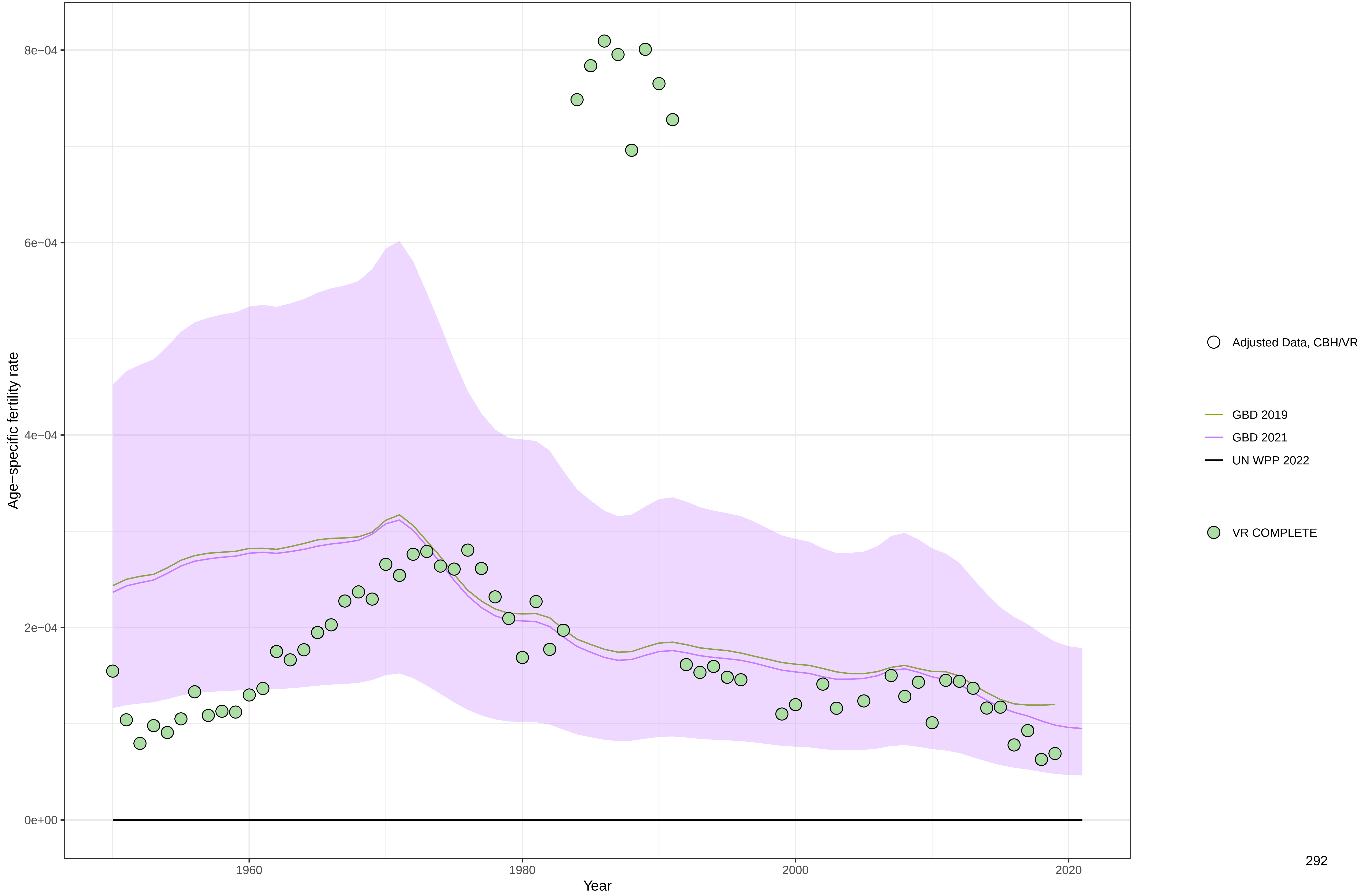

Age−specific fertility fitted trend lines, 1950−2021: Australia, Ages: 15−19
Data points enclosed with a diamond are reference sources

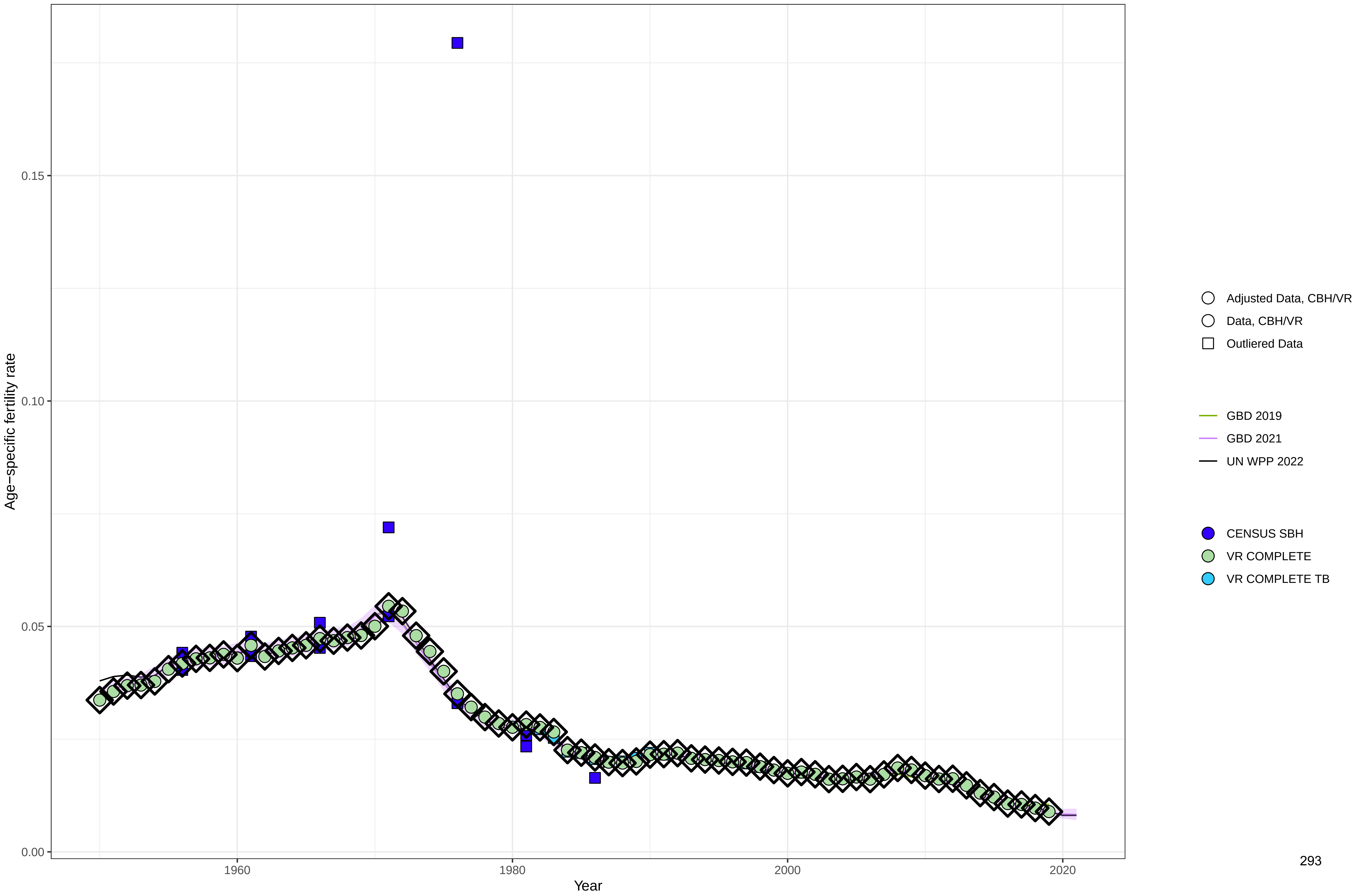

Age−specific fertility fitted trend lines, 1950−2021: Australia, Ages: 20−24
Data points enclosed with a diamond are reference sources

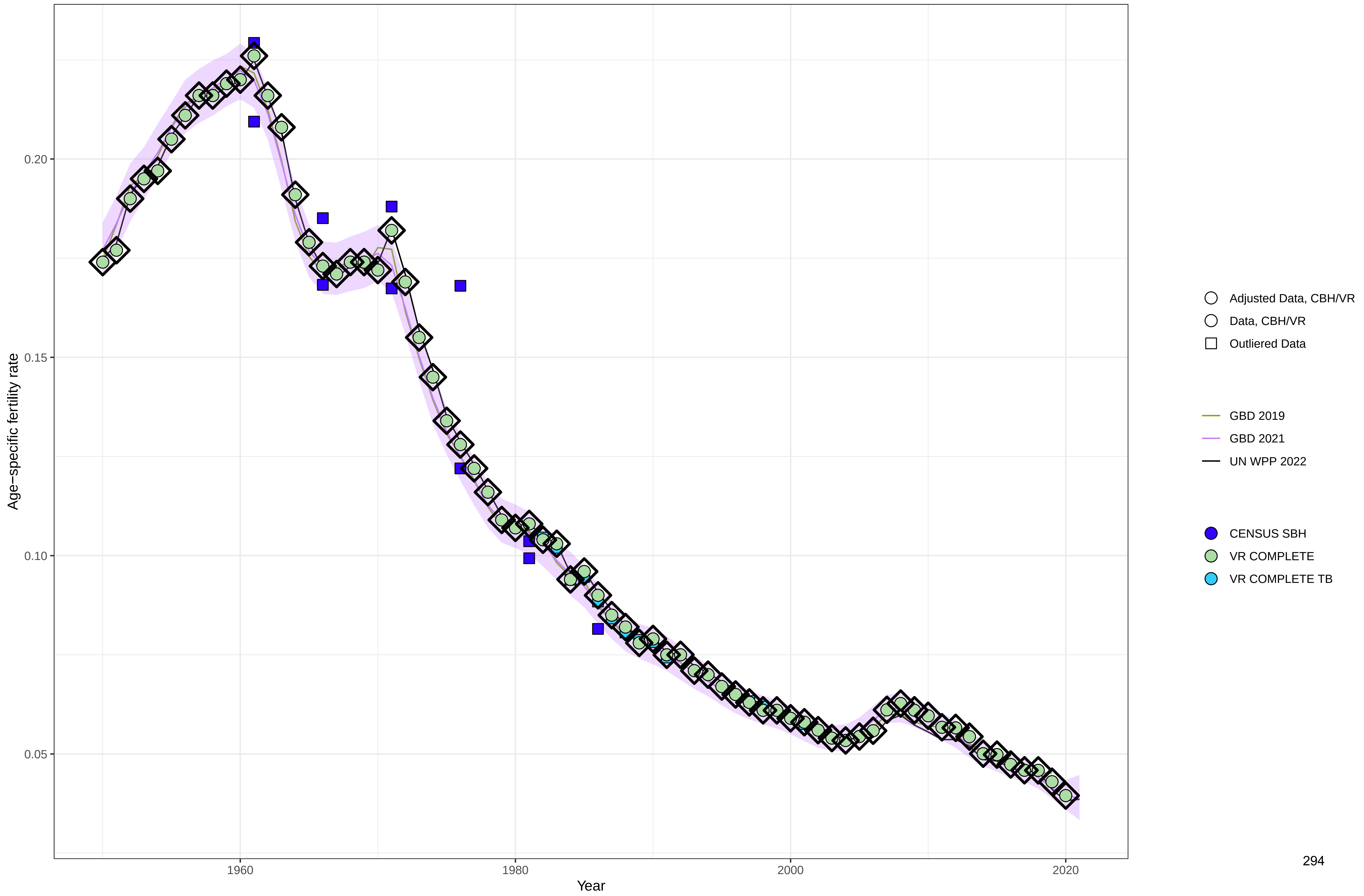

Age−specific fertility fitted trend lines, 1950−2021: Australia, Ages: 25−29
Data points enclosed with a diamond are reference sources

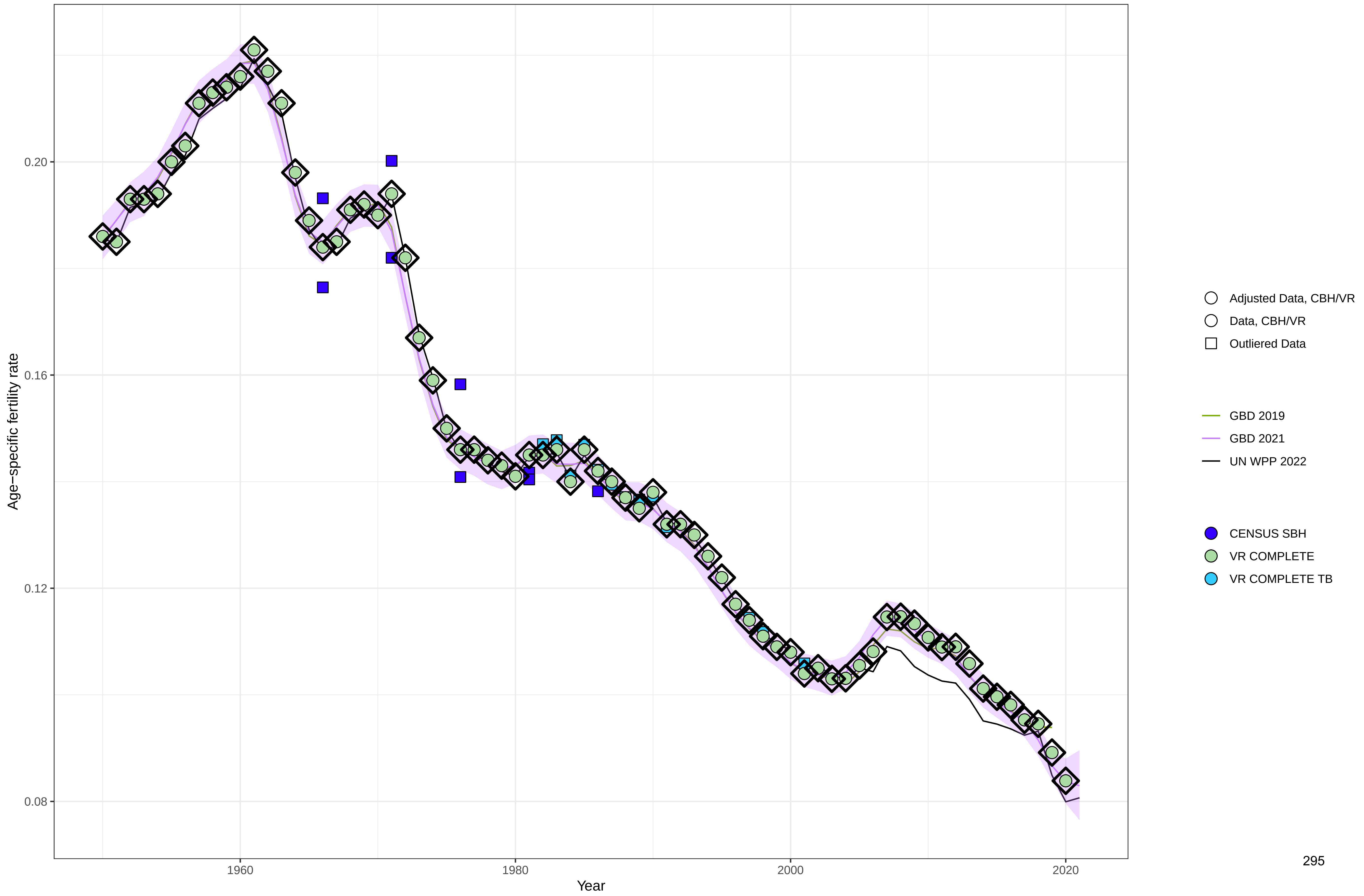

Age−specific fertility fitted trend lines, 1950−2021: Australia, Ages: 30−34
Data points enclosed with a diamond are reference sources

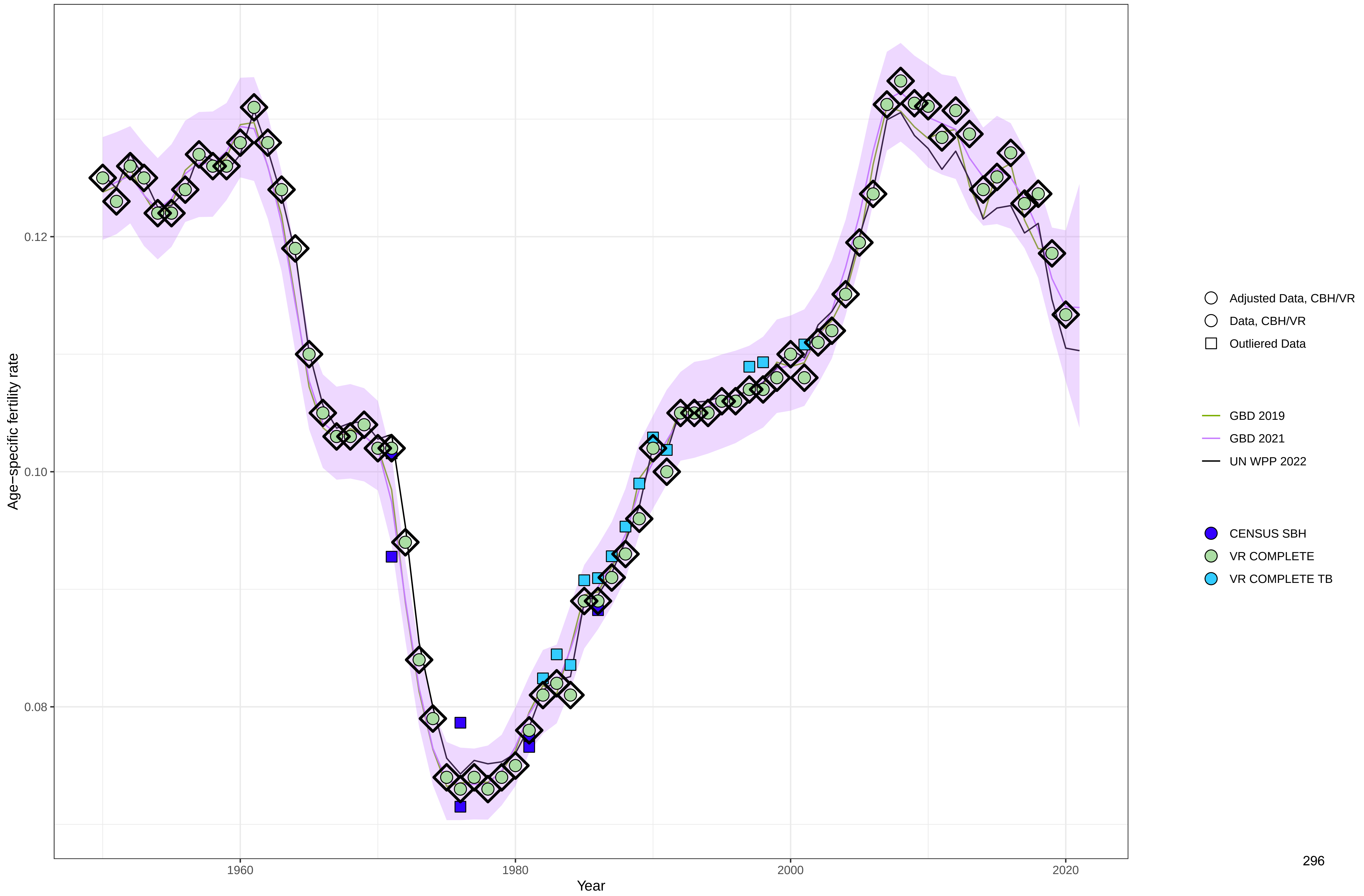

Age−specific fertility fitted trend lines, 1950−2021: Australia, Ages: 35−39
 Data points enclosed with a diamond are reference sources

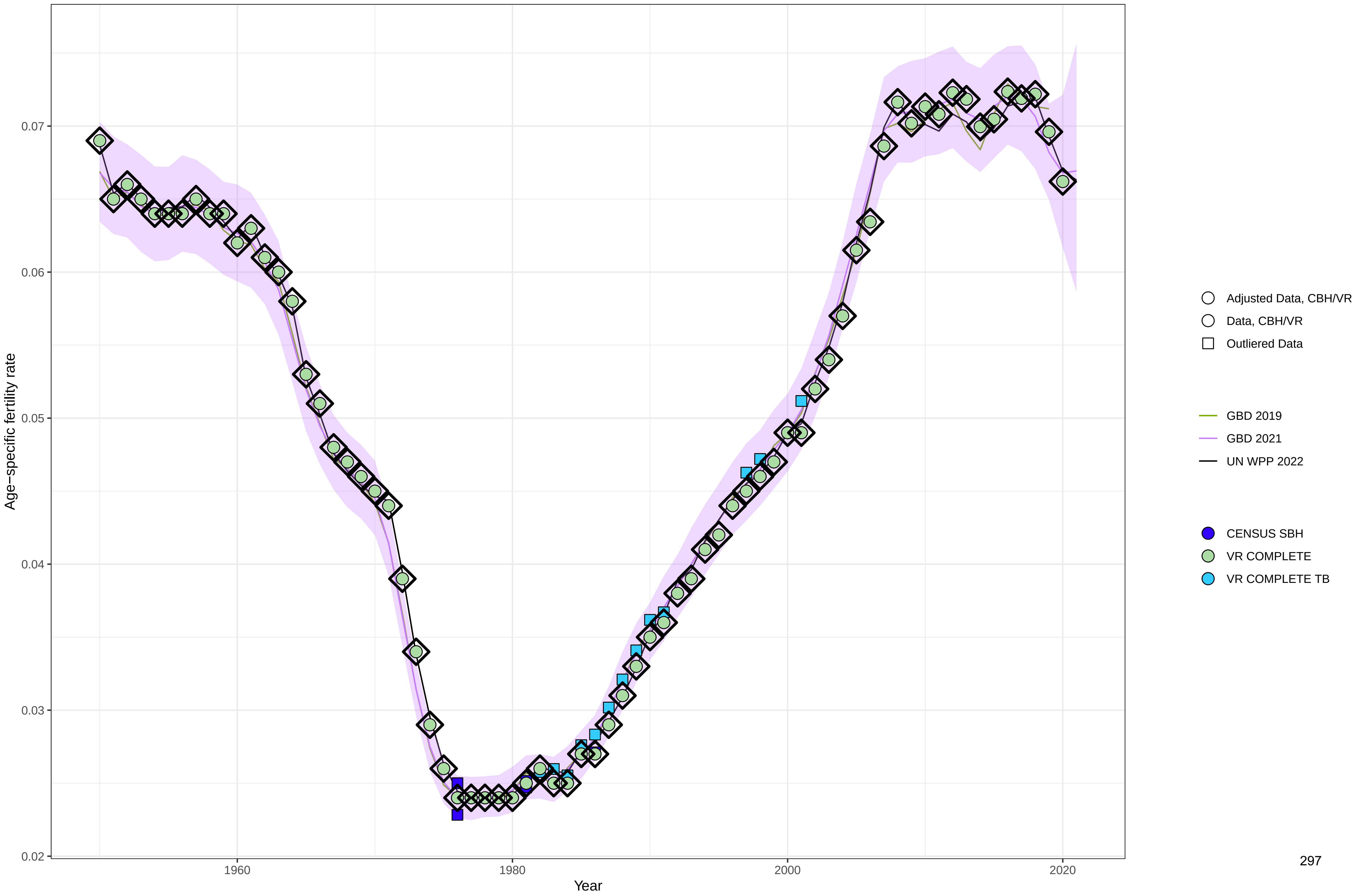

Age−specific fertility fitted trend lines, 1950−2021: Australia, Ages: 40−44
Data points enclosed with a diamond are reference sources

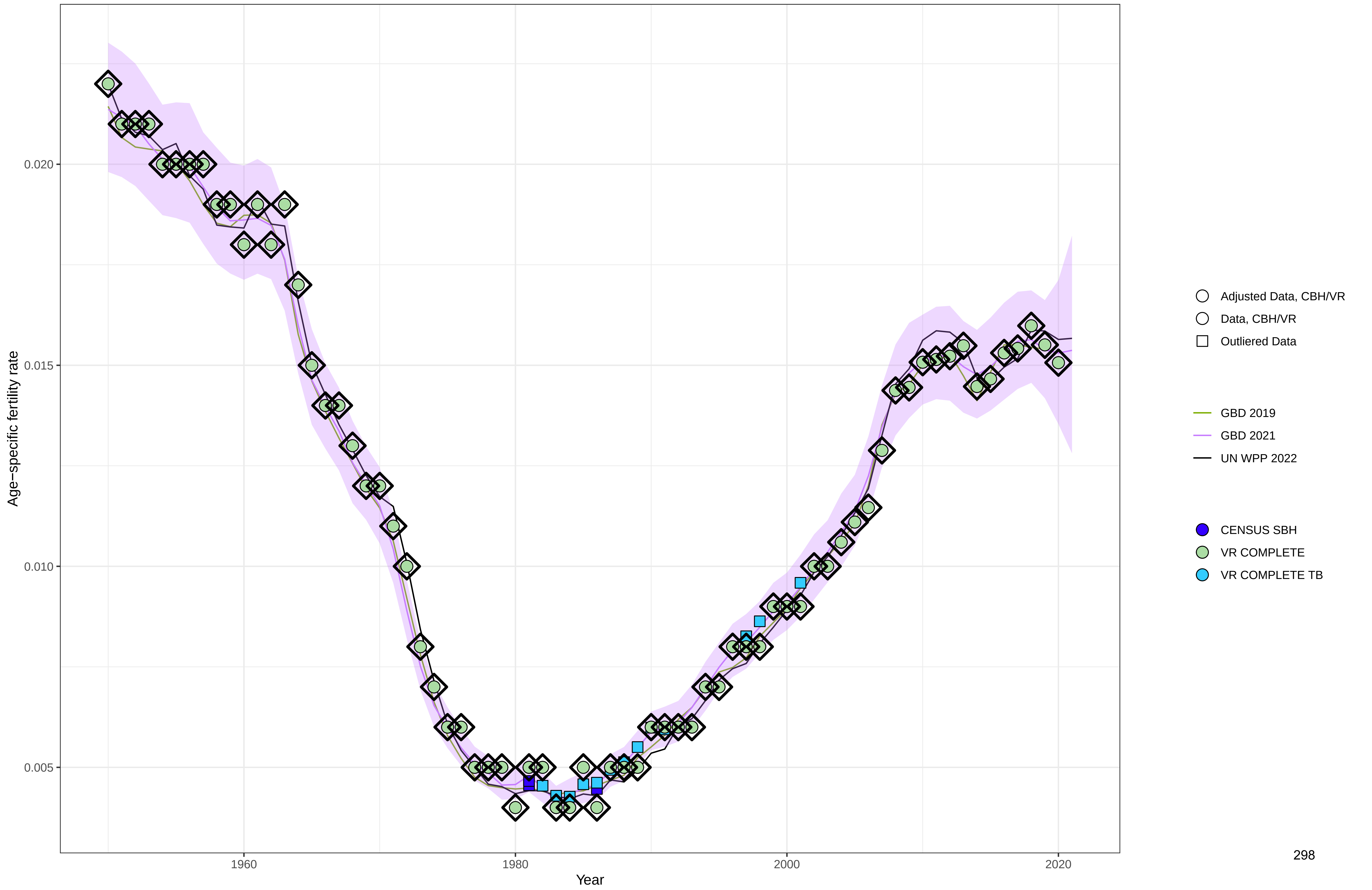

Age−specific fertility fitted trend lines, 1950−2021: Australia, Ages: 45−49
Data points enclosed with a diamond are reference sources

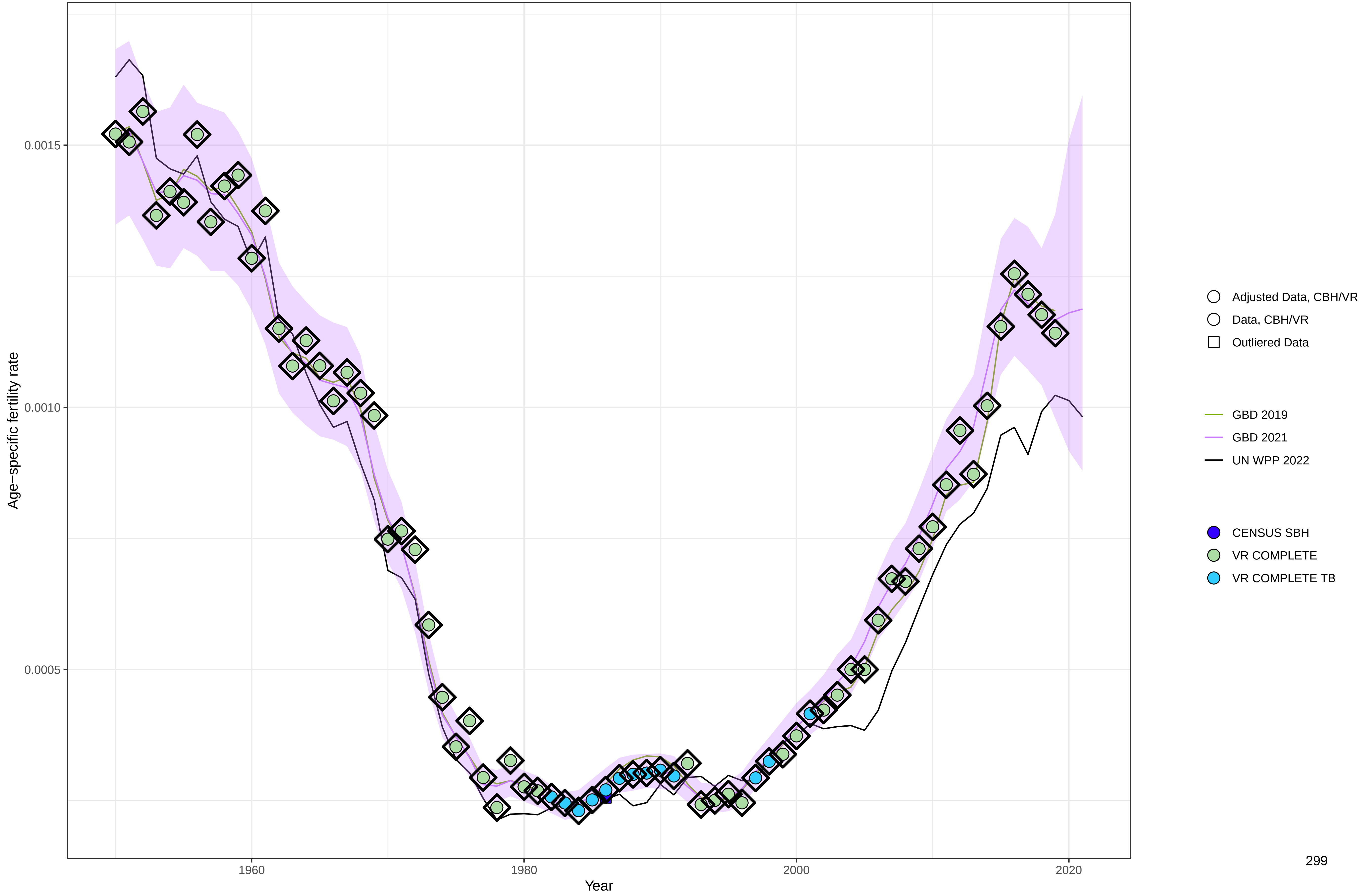

Age−specific fertility fitted trend lines, 1950−2021: Australia, Ages: 50−54
Data points enclosed with a diamond are reference sources

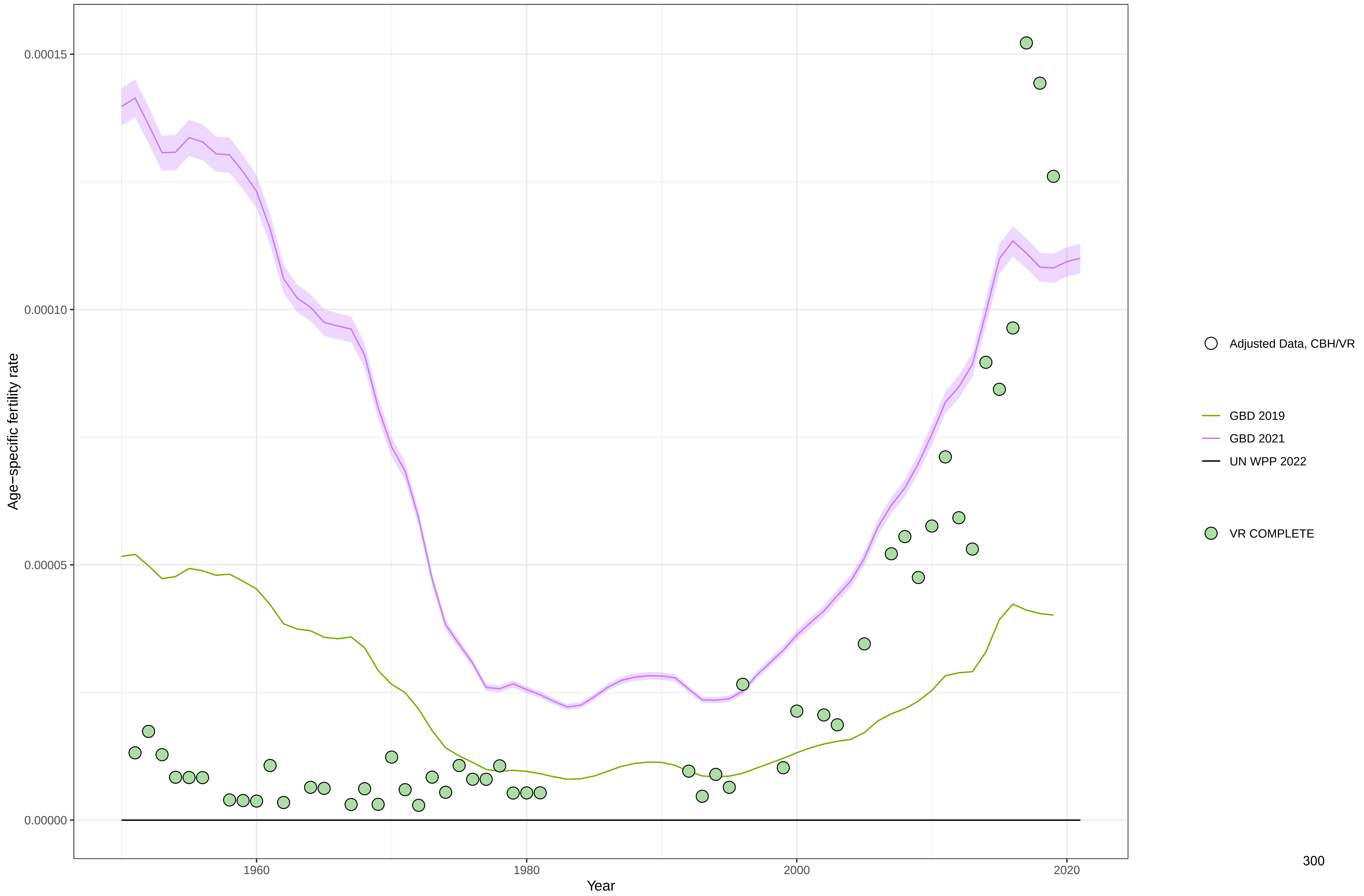

Total fertility rate fitted trend lines, 1950−2021: Australia TFR
Data points enclosed with a diamond are reference sources

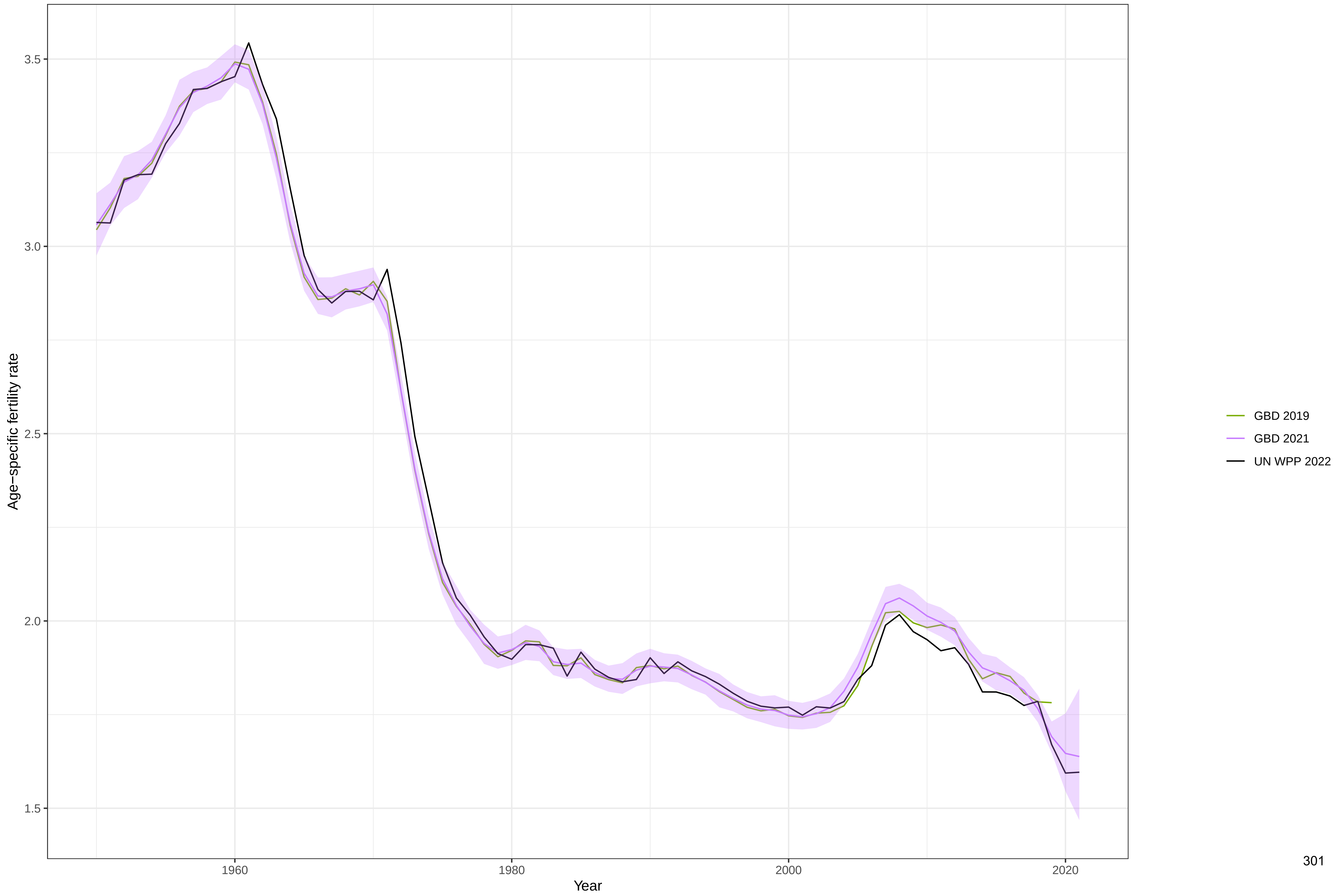

Age−specific fertility fitted trend lines, 1950−2021: New Zealand, Ages: 10−14
Data points enclosed with a diamond are reference sources

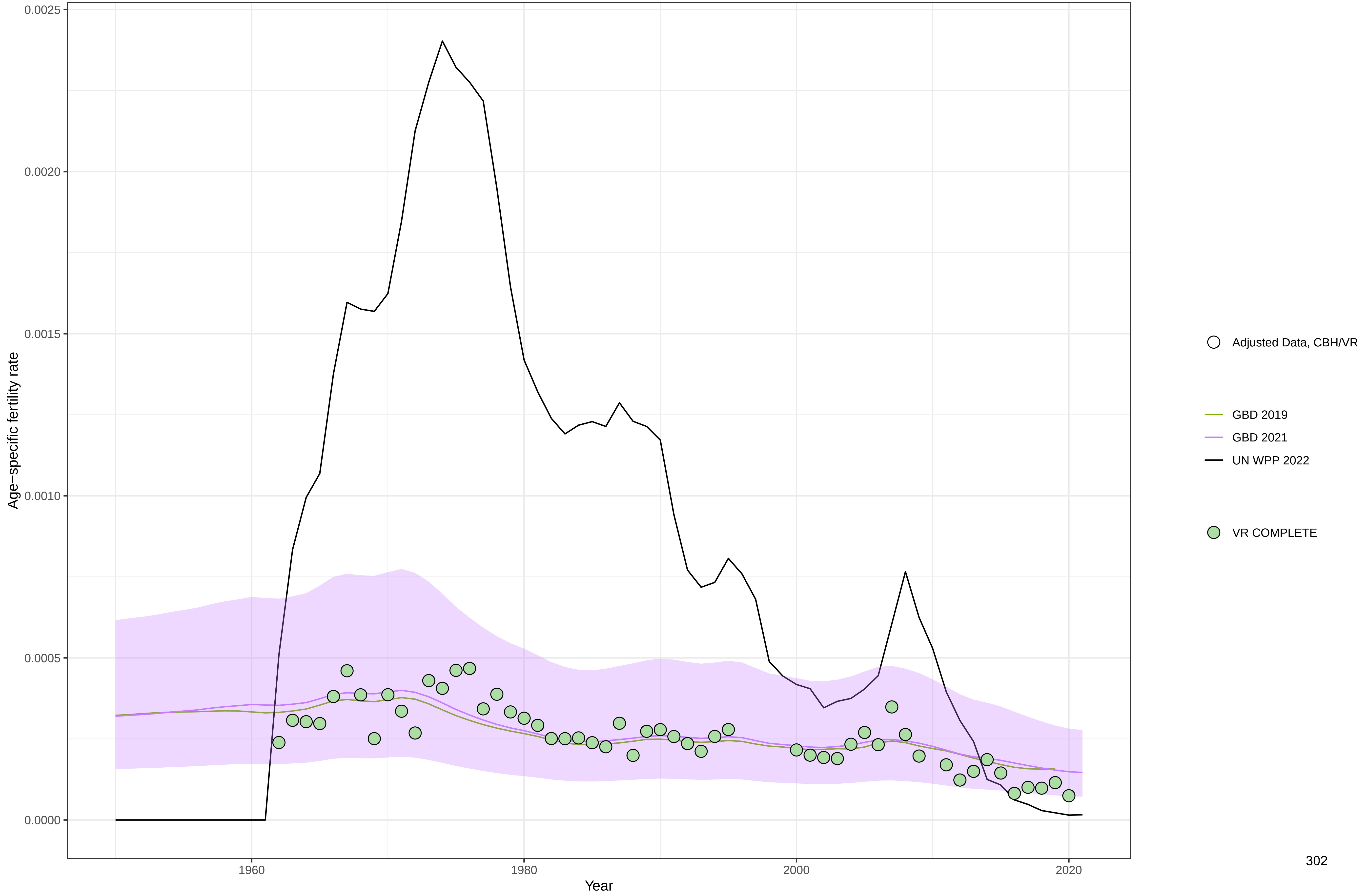

Age−specific fertility fitted trend lines, 1950−2021: New Zealand, Ages: 15−19
Data points enclosed with a diamond are reference sources

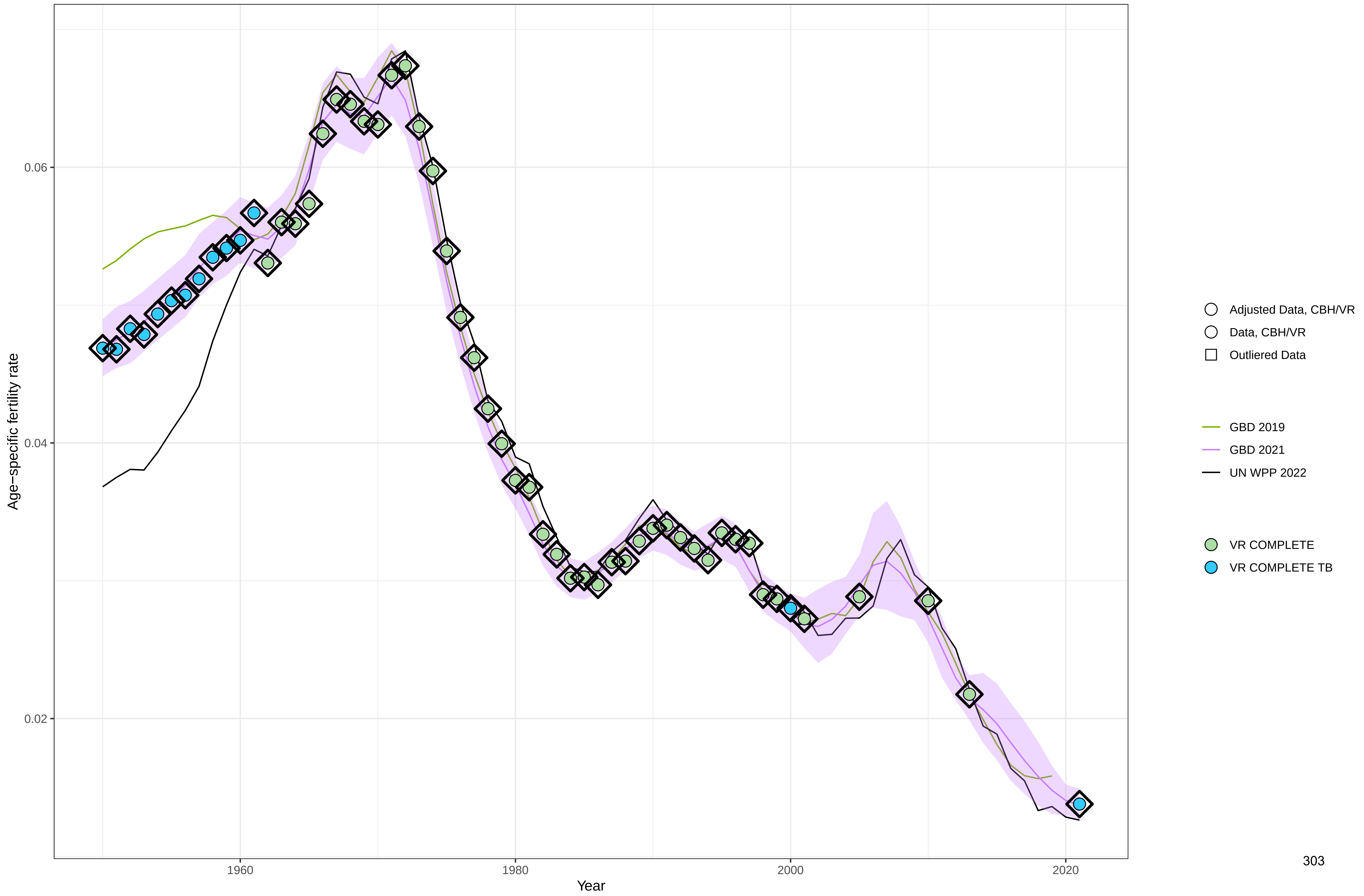

Age−specific fertility fitted trend lines, 1950−2021: New Zealand, Ages: 20−24
Data points enclosed with a diamond are reference sources

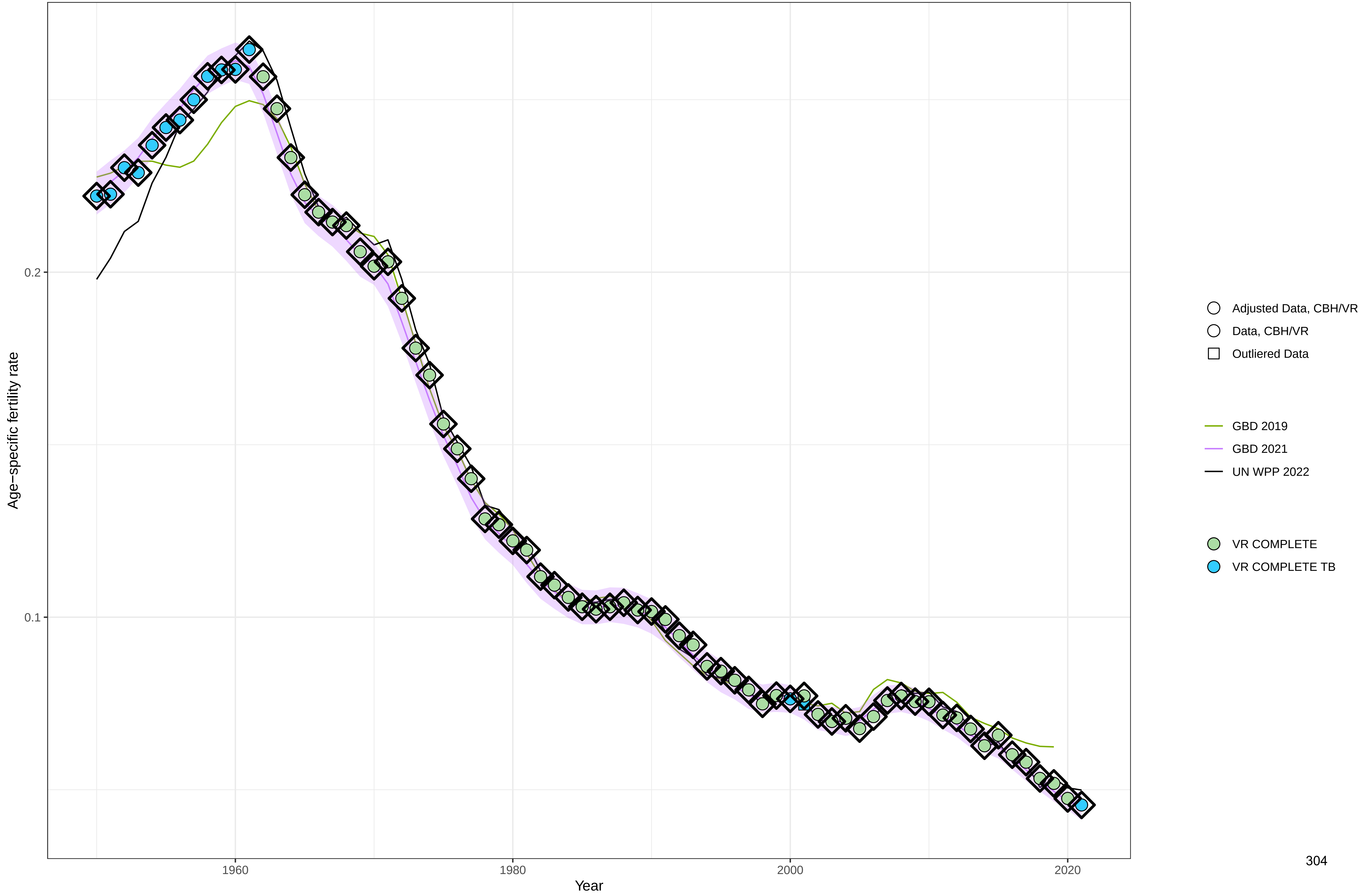

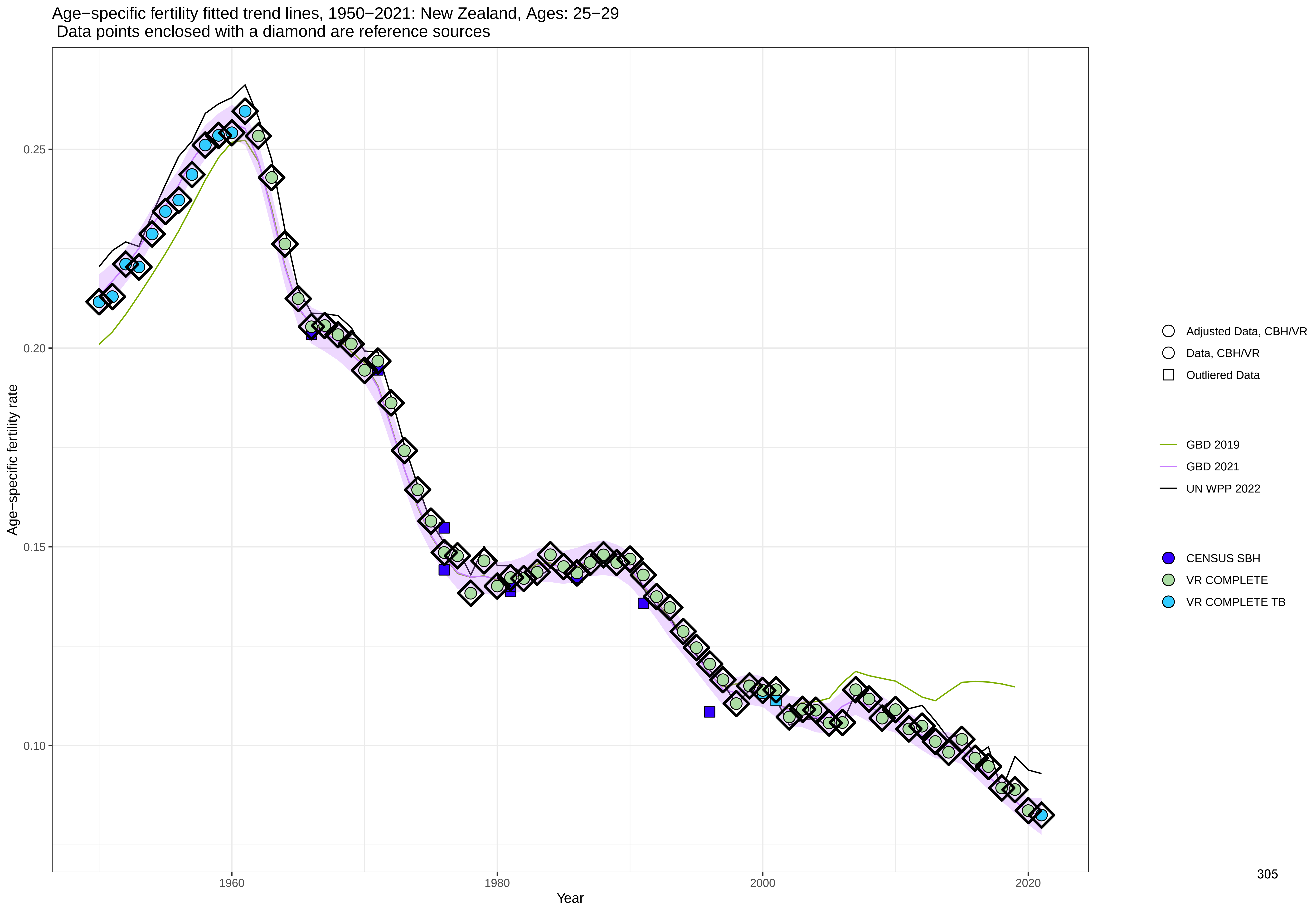
Age−specific fertility fitted trend lines, 1950−2021: New Zealand, Ages: 25−29
Data points enclosed with a diamond are reference sources
Age−specific fertility rate
0.25
0.20
0.15
0.10
1960
1980
2000
2020
Year
Adjusted Data, CBH/VR
Data, CBH/VR
Outliered Data
GBD 2019
GBD 2021
UN WPP 2022
CENSUS SBH
VR COMPLETE
VR COMPLETE TB

Age−specific fertility fitted trend lines, 1950−2021: New Zealand, Ages: 30−34
Data points enclosed with a diamond are reference sources

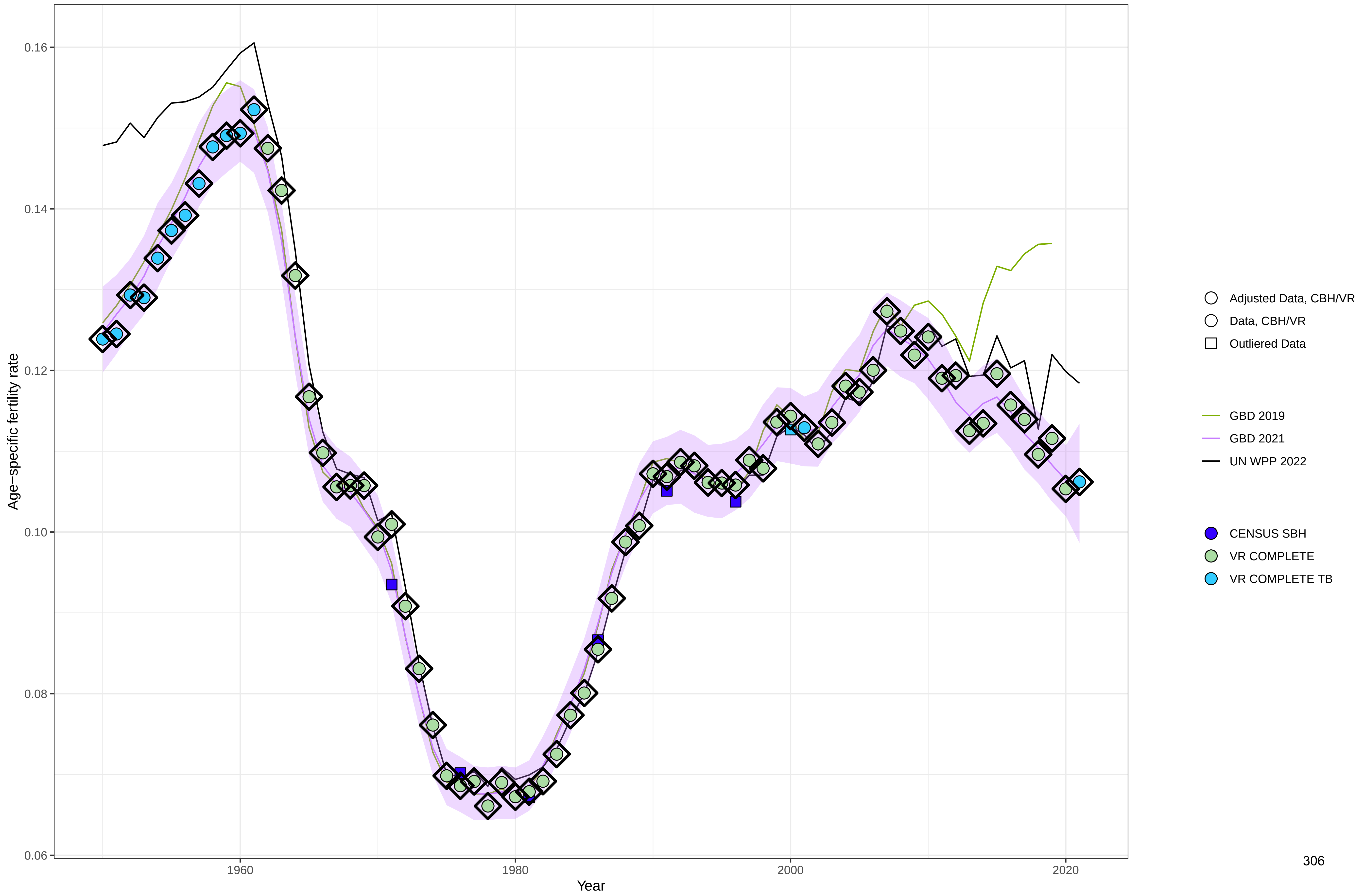

Age−specific fertility fitted trend lines, 1950−2021: New Zealand, Ages: 35−39
Data points enclosed with a diamond are reference sources

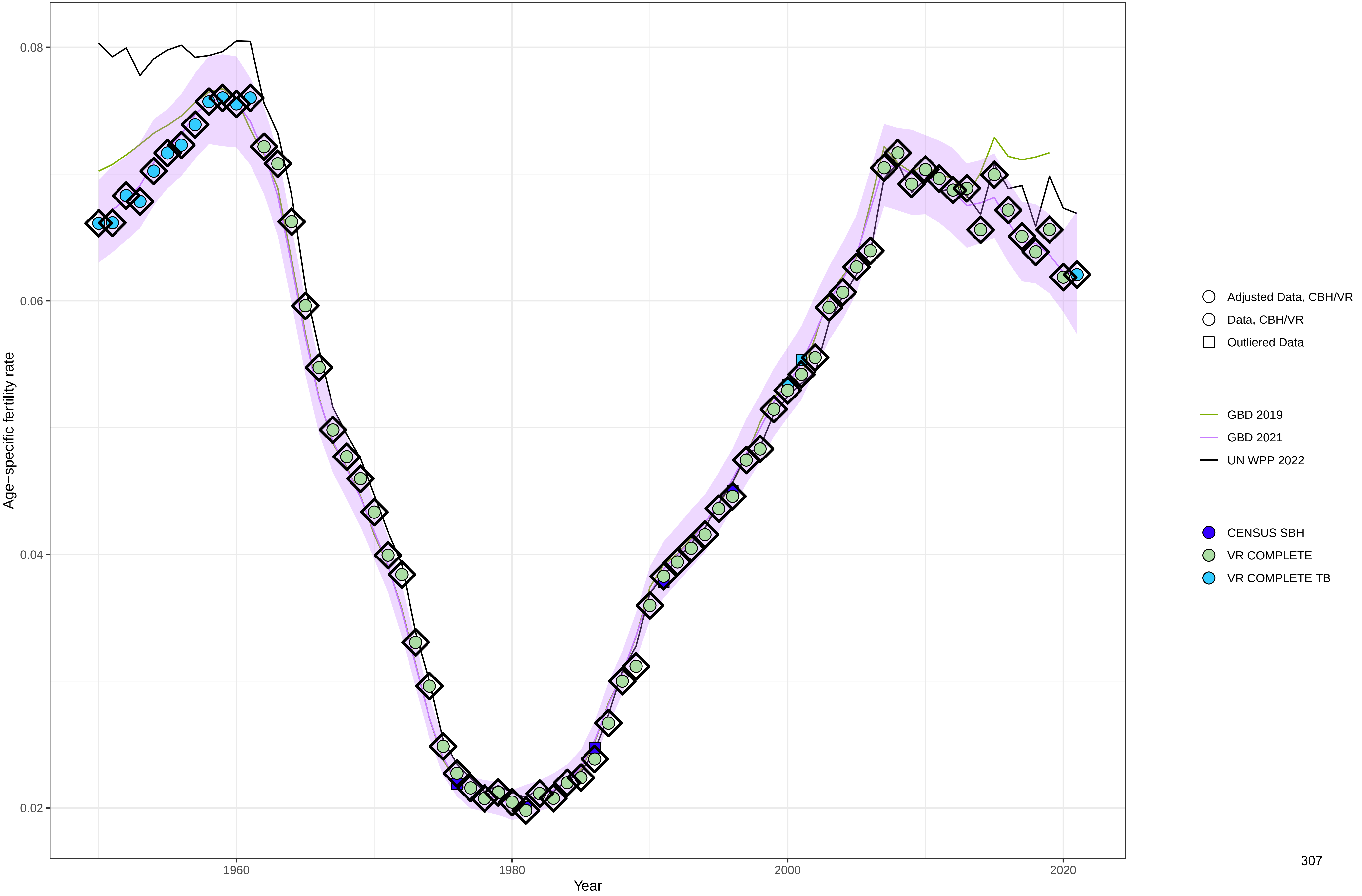

Age−specific fertility fitted trend lines, 1950−2021: New Zealand, Ages: 40−44
Data points enclosed with a diamond are reference sources

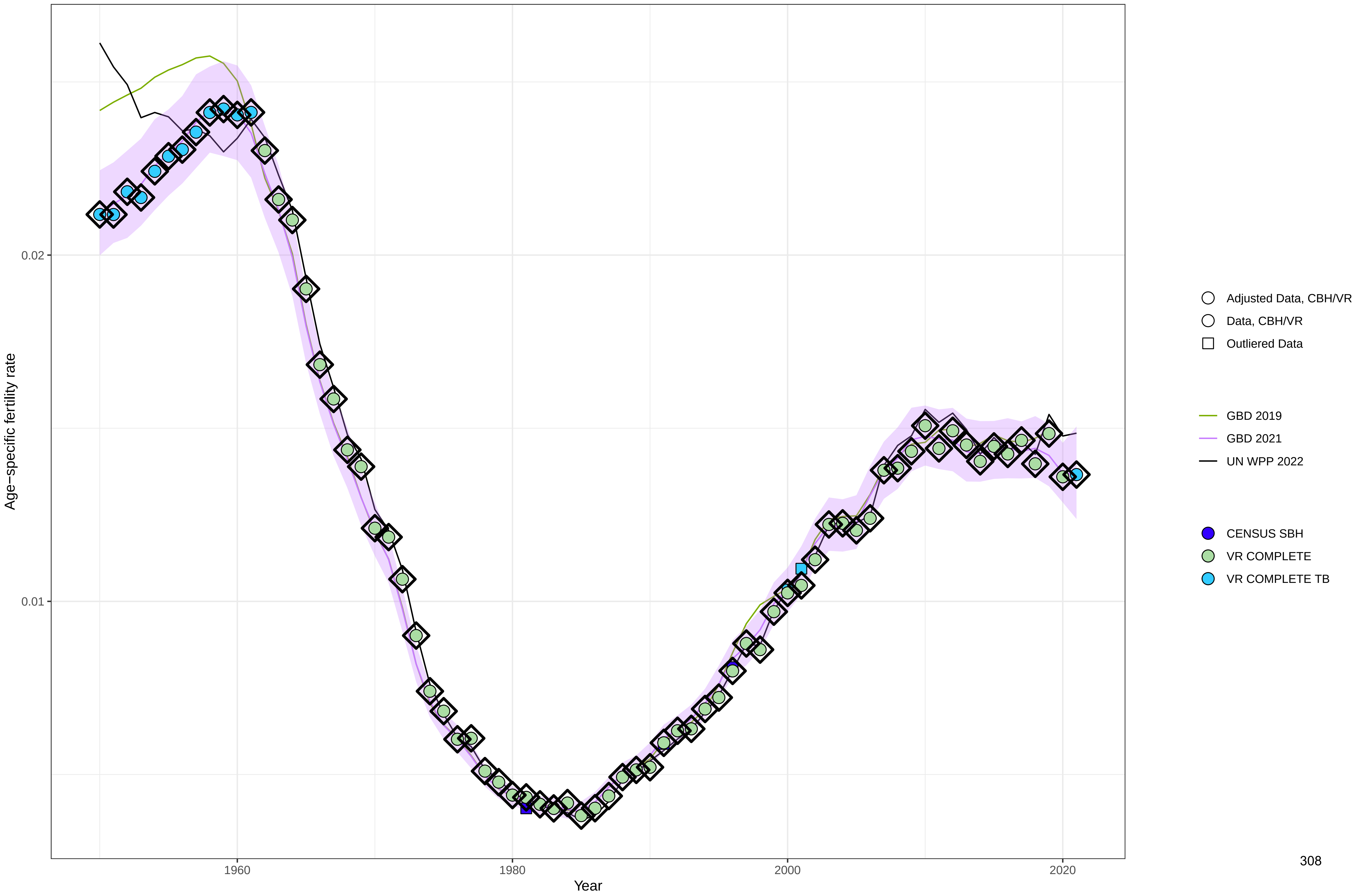

Age−specific fertility fitted trend lines, 1950−2021: New Zealand, Ages: 45−49
Data points enclosed with a diamond are reference sources

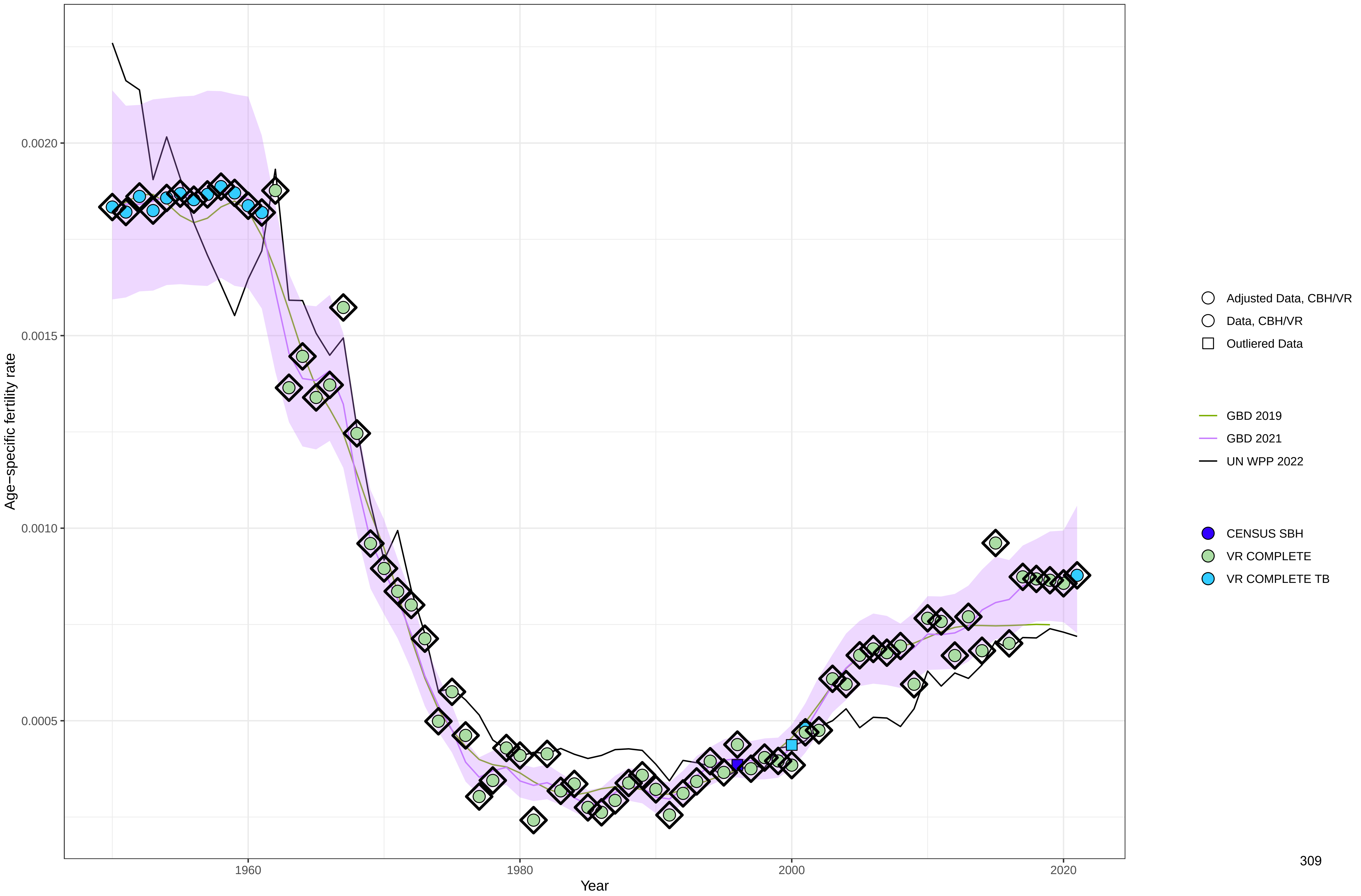

Age−specific fertility fitted trend lines, 1950−2021: New Zealand, Ages: 50−54
Data points enclosed with a diamond are reference sources

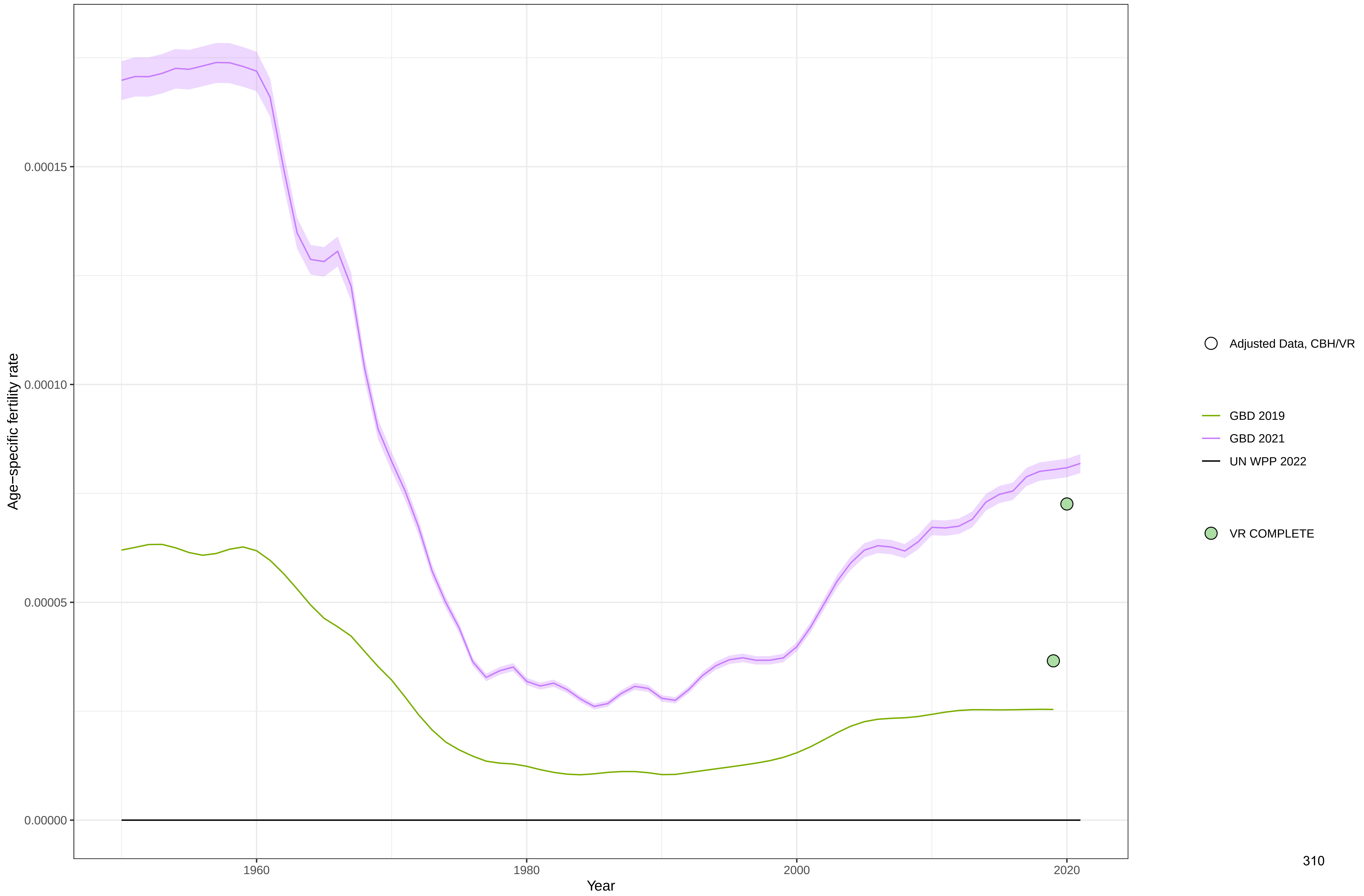

Total fertility rate fitted trend lines, 1950−2021: New Zealand TFR
Data points enclosed with a diamond are reference sources

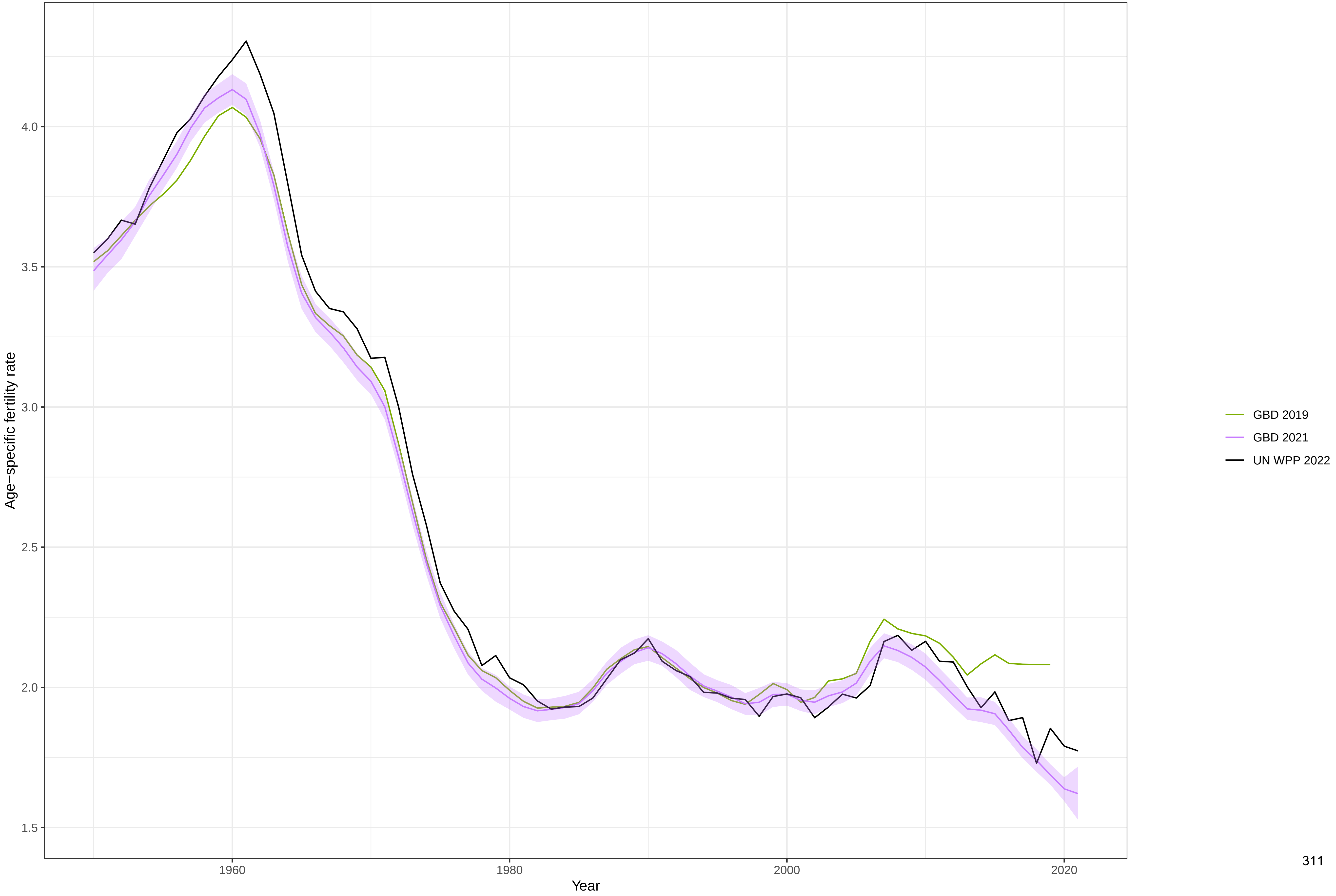

Age−specific fertility fitted trend lines, 1950−2021: Brunei, Ages: 10−14
Data points enclosed with a diamond are reference sources

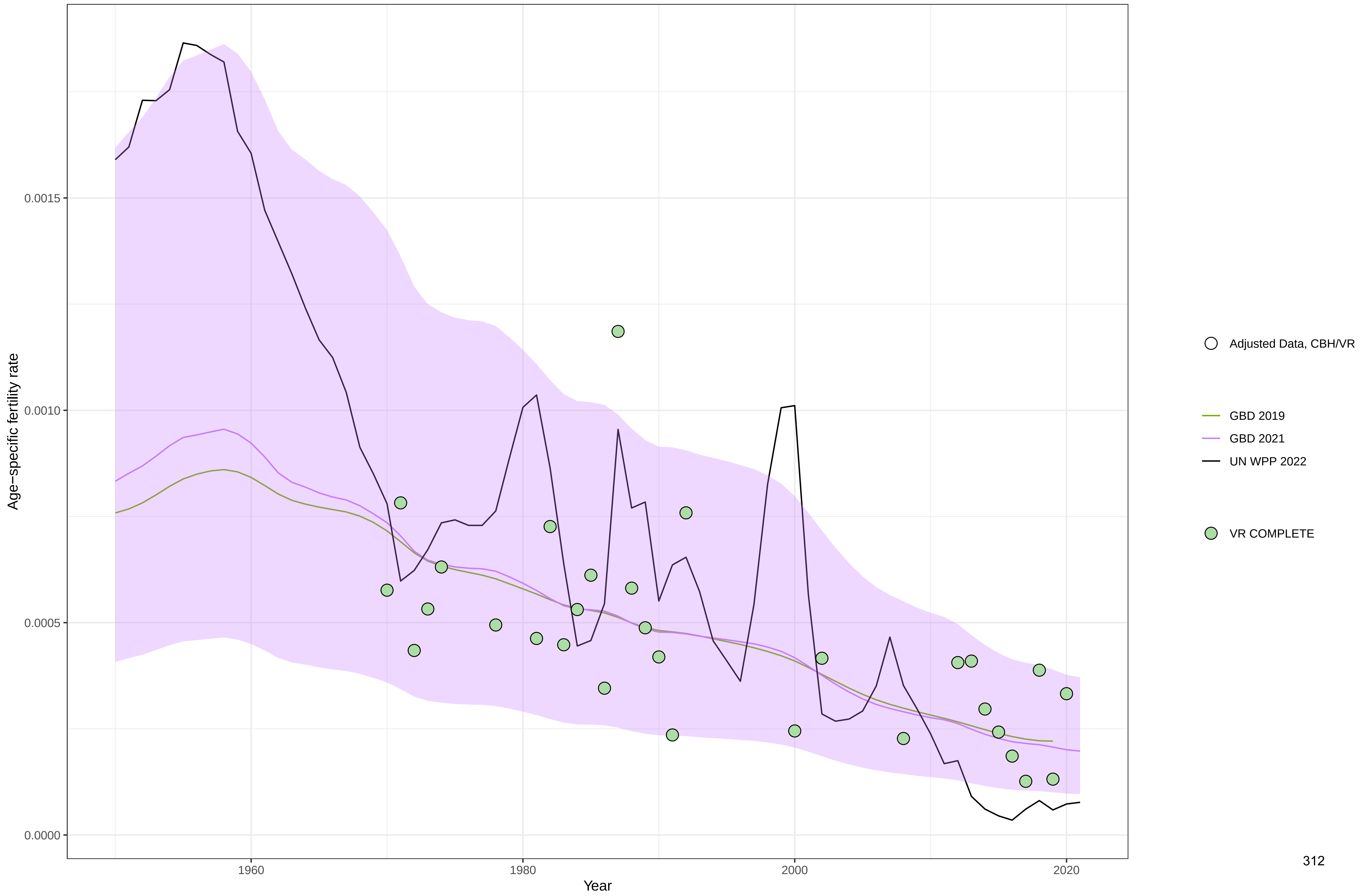

Age−specific fertility fitted trend lines, 1950−2021: Brunei, Ages: 15−19
Data points enclosed with a diamond are reference sources

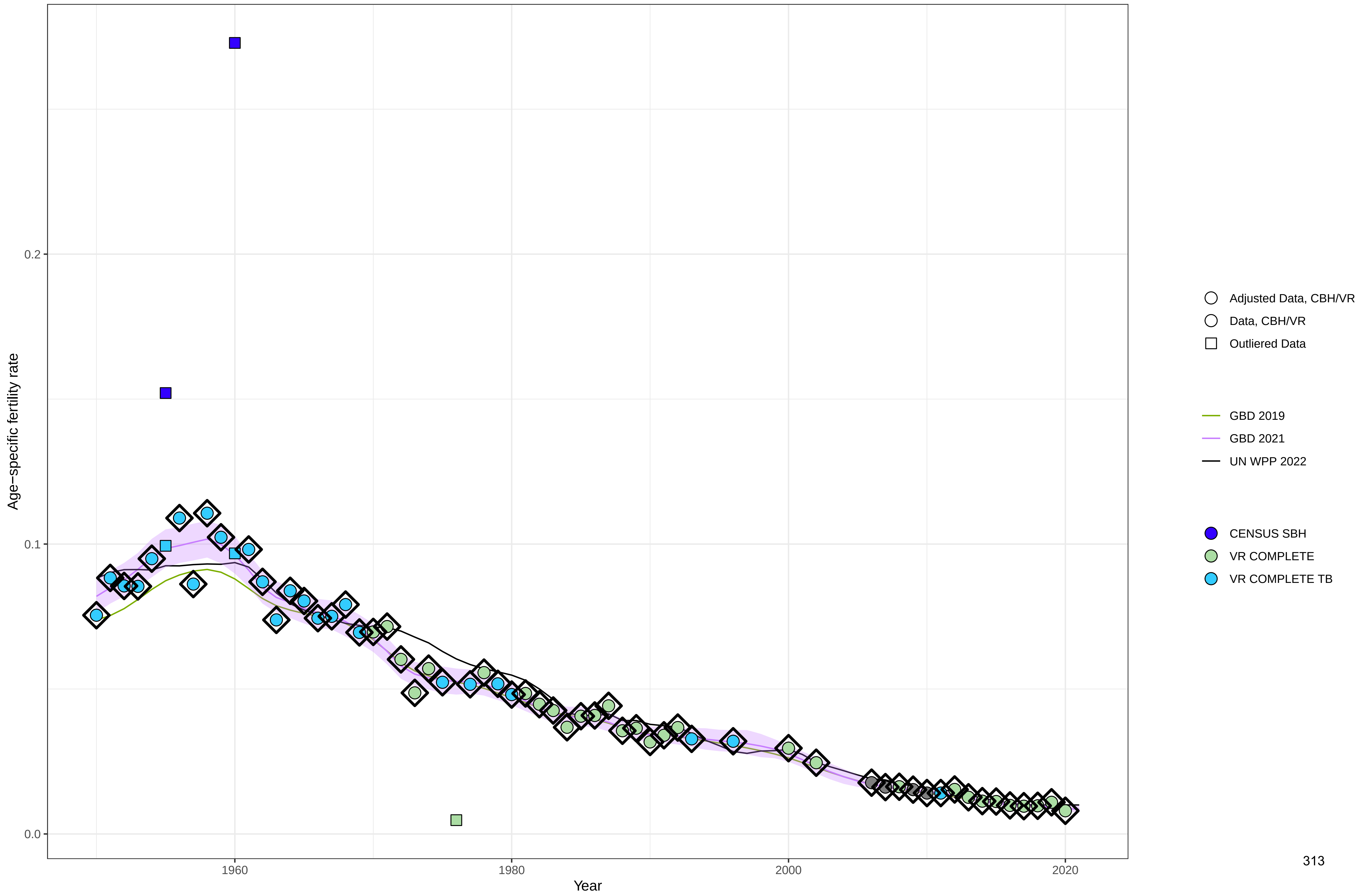

Age−specific fertility fitted trend lines, 1950−2021: Brunei, Ages: 20−24
Data points enclosed with a diamond are reference sources

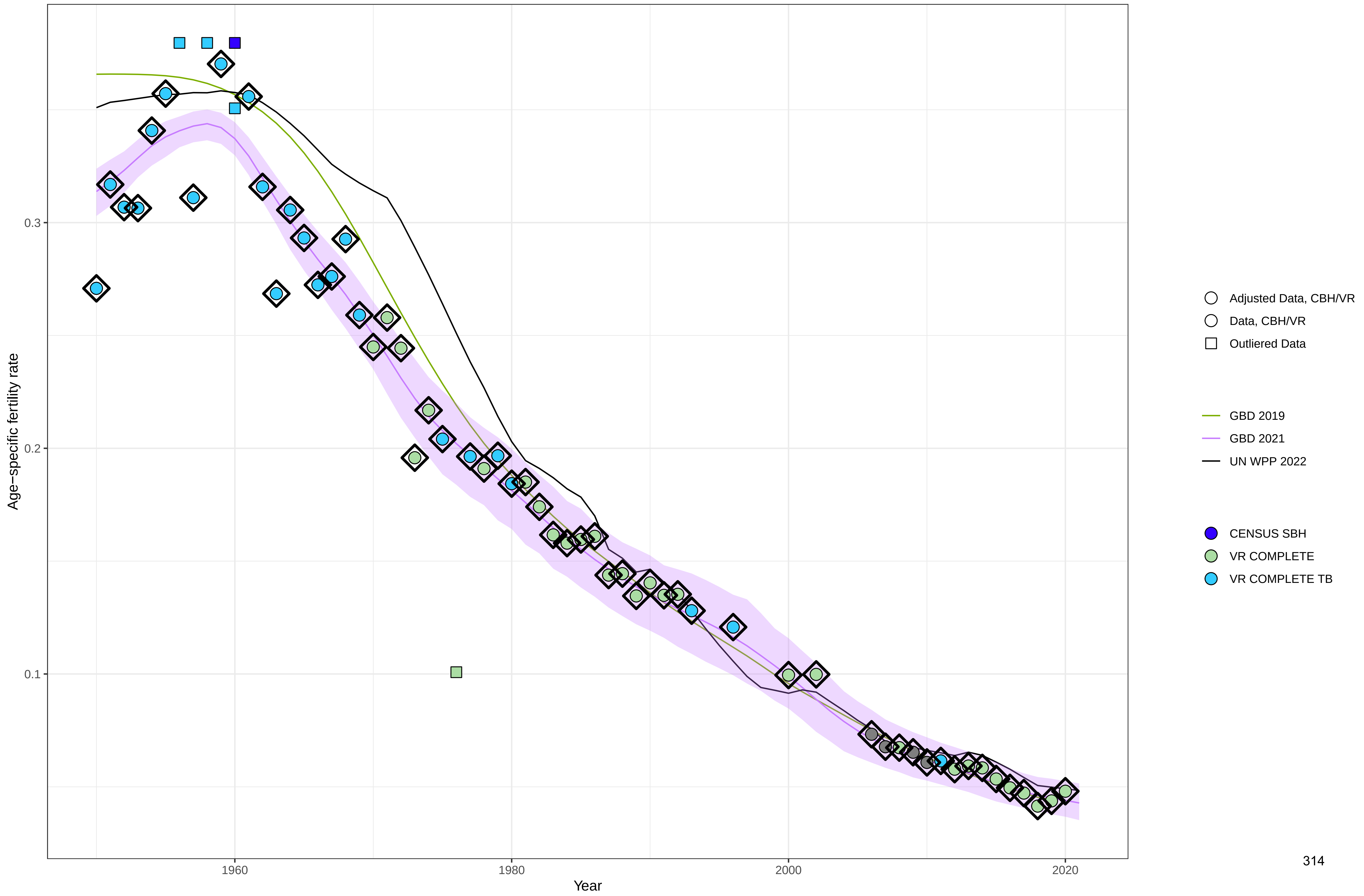

Age−specific fertility fitted trend lines, 1950−2021: Brunei, Ages: 25−29
Data points enclosed with a diamond are reference sources

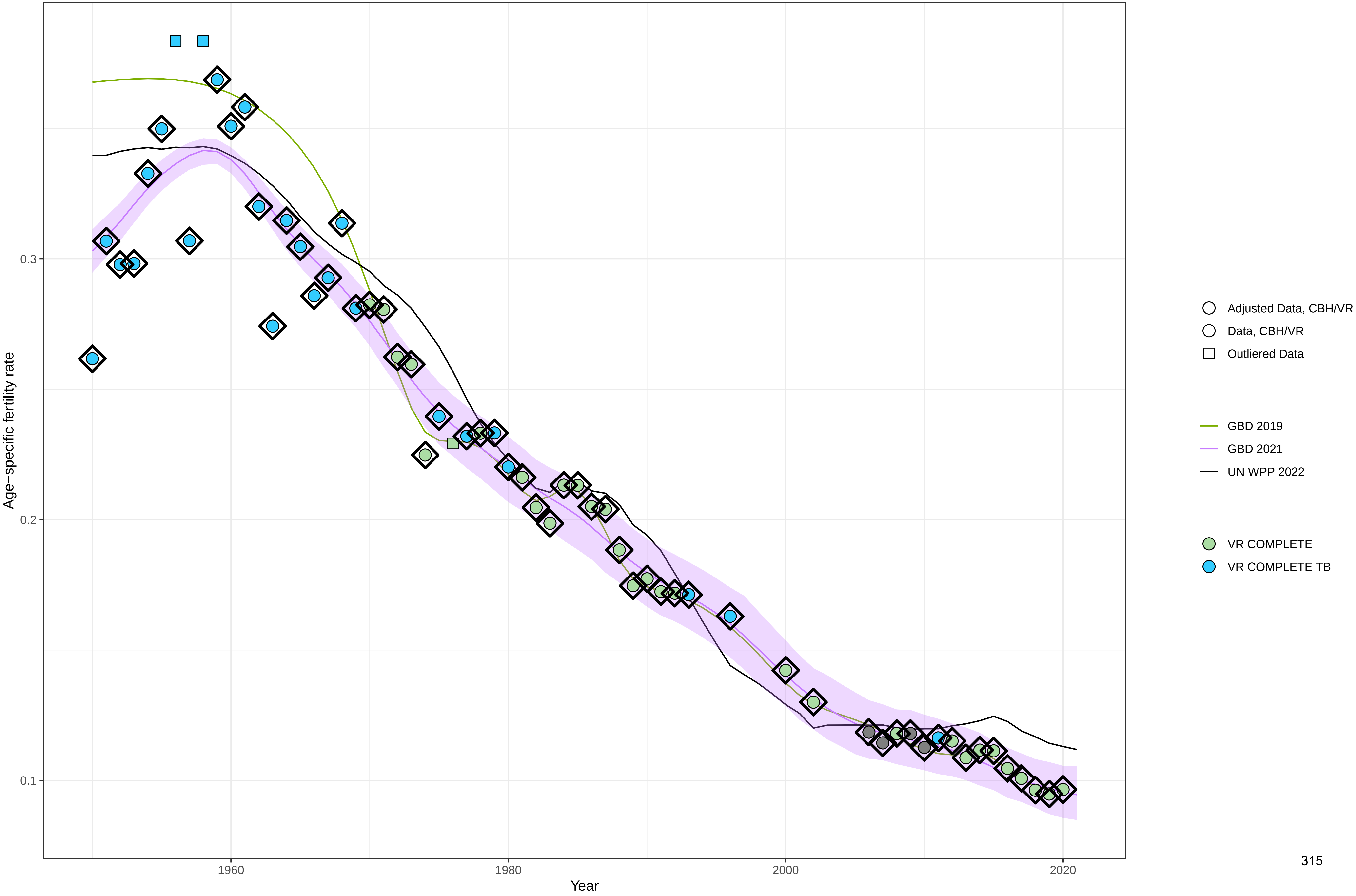

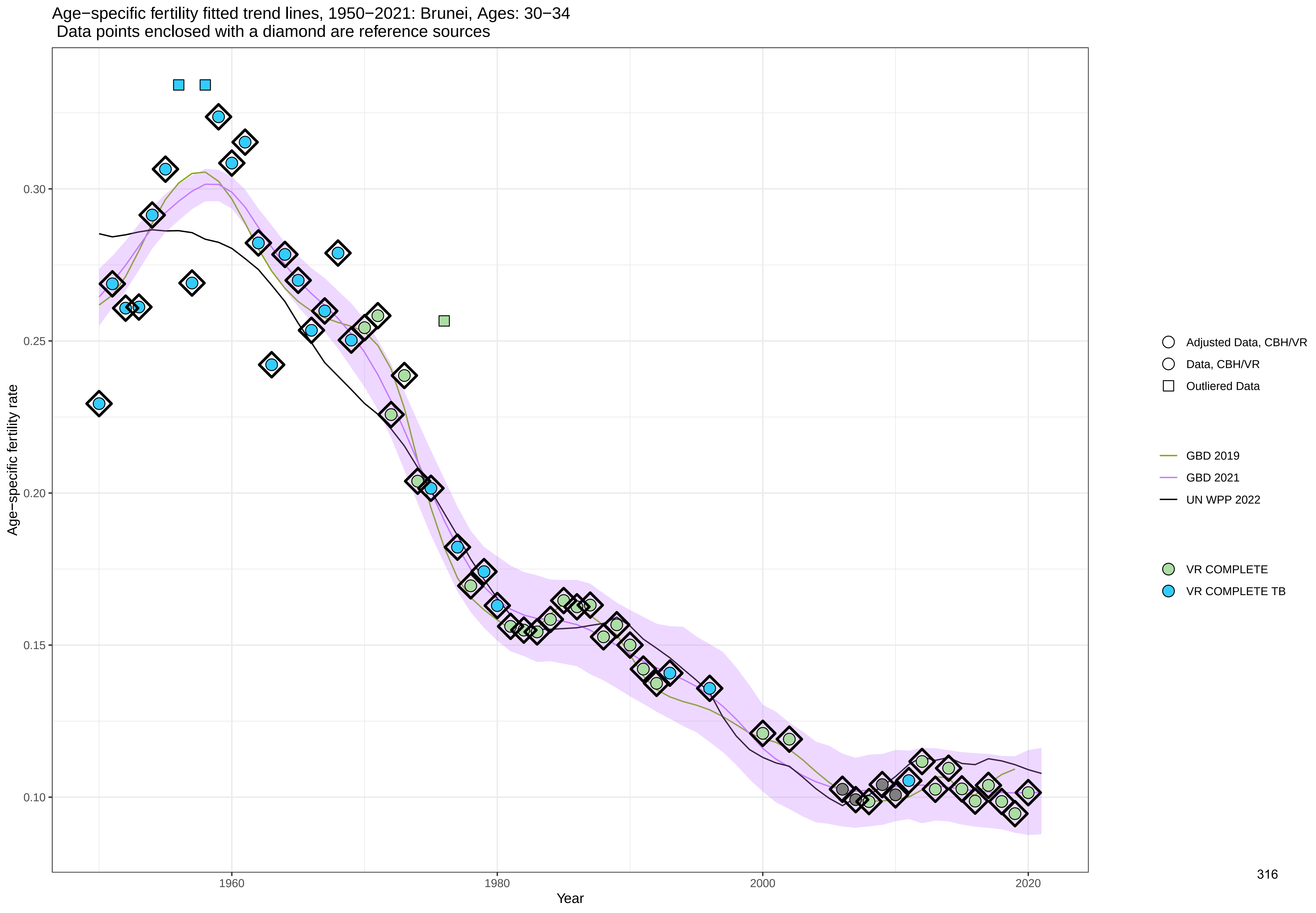
Age−specific fertility fitted trend lines, 1950−2021: Brunei, Ages: 30−34
Data points enclosed with a diamond are reference sources
Age−specific fertility rate
Year
0.30
0.25
0.20
0.15
0.10
1960
1980
2000
2020
Adjusted Data, CBH/VR
Data, CBH/VR
Outliered Data
GBD 2019
GBD 2021
UN WPP 2022
VR COMPLETE
VR COMPLETE TB

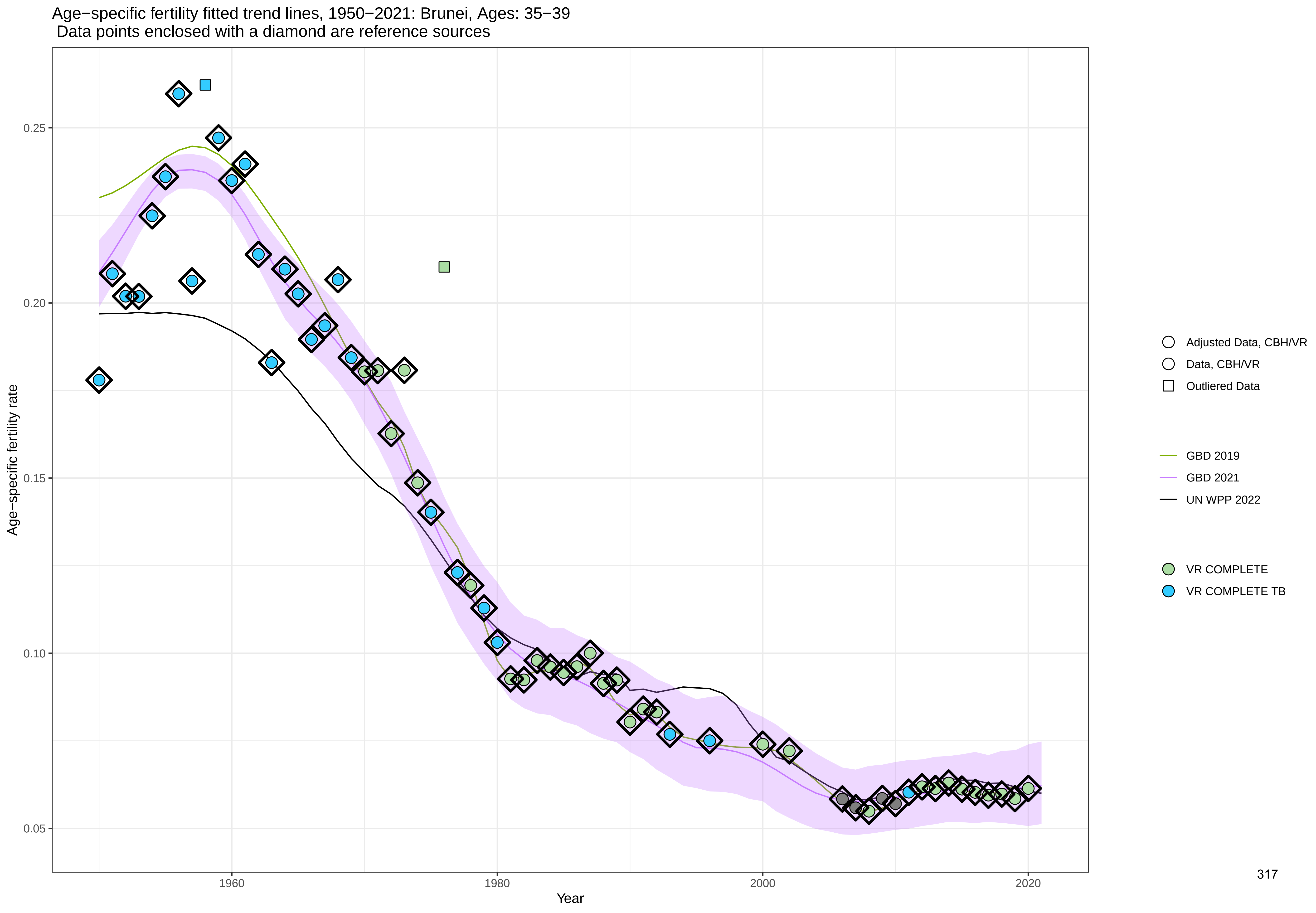

Age−specific fertility fitted trend lines, 1950−2021: Brunei, Ages: 35−39
Data points enclosed with a diamond are reference sources
Age−specific fertility rate
Year
0.25
0.20
0.15
0.10
0.05
1960
1980
2000
2020
Adjusted Data, CBH/VR
Data, CBH/VR
Outliered Data
GBD 2019
GBD 2021
UN WPP 2022
VR COMPLETE
VR COMPLETE TB

Age−specific fertility fitted trend lines, 1950−2021: Brunei, Ages: 40−44
Data points enclosed with a diamond are reference sources

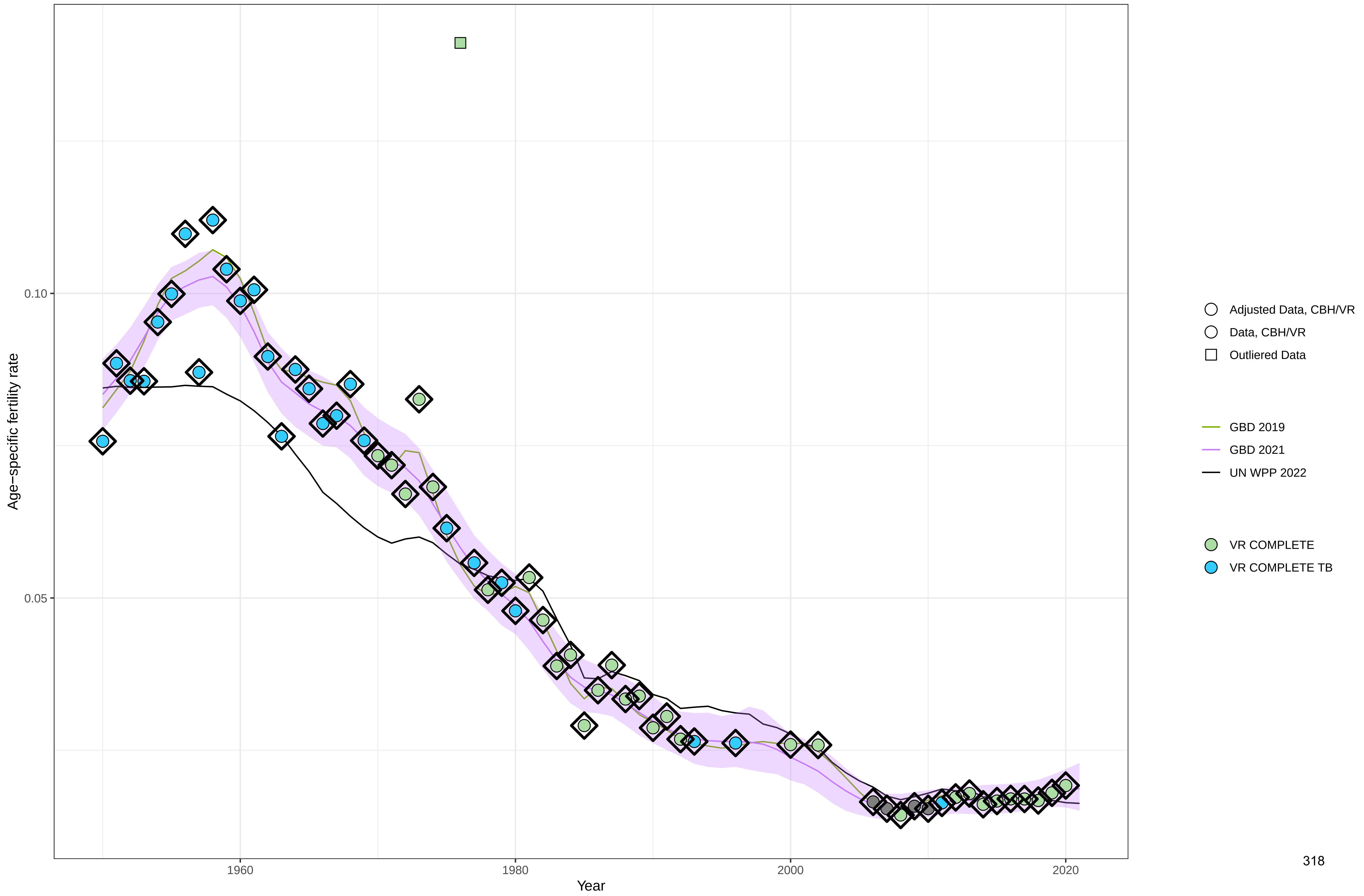

Age−specific fertility fitted trend lines, 1950−2021: Brunei, Ages: 45−49
Data points enclosed with a diamond are reference sources

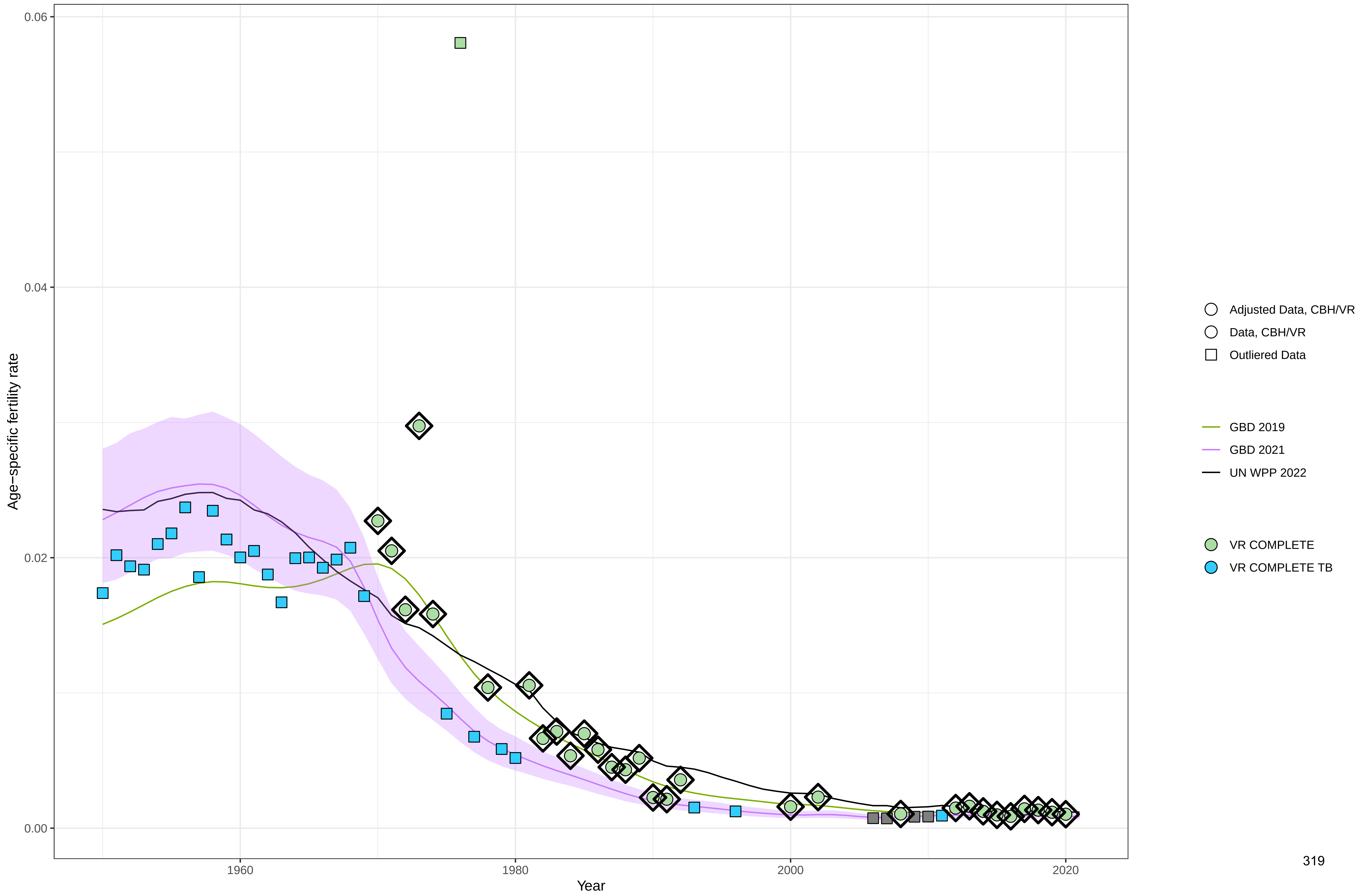

Age−specific fertility fitted trend lines, 1950−2021: Brunei, Ages: 50−54
Data points enclosed with a diamond are reference sources

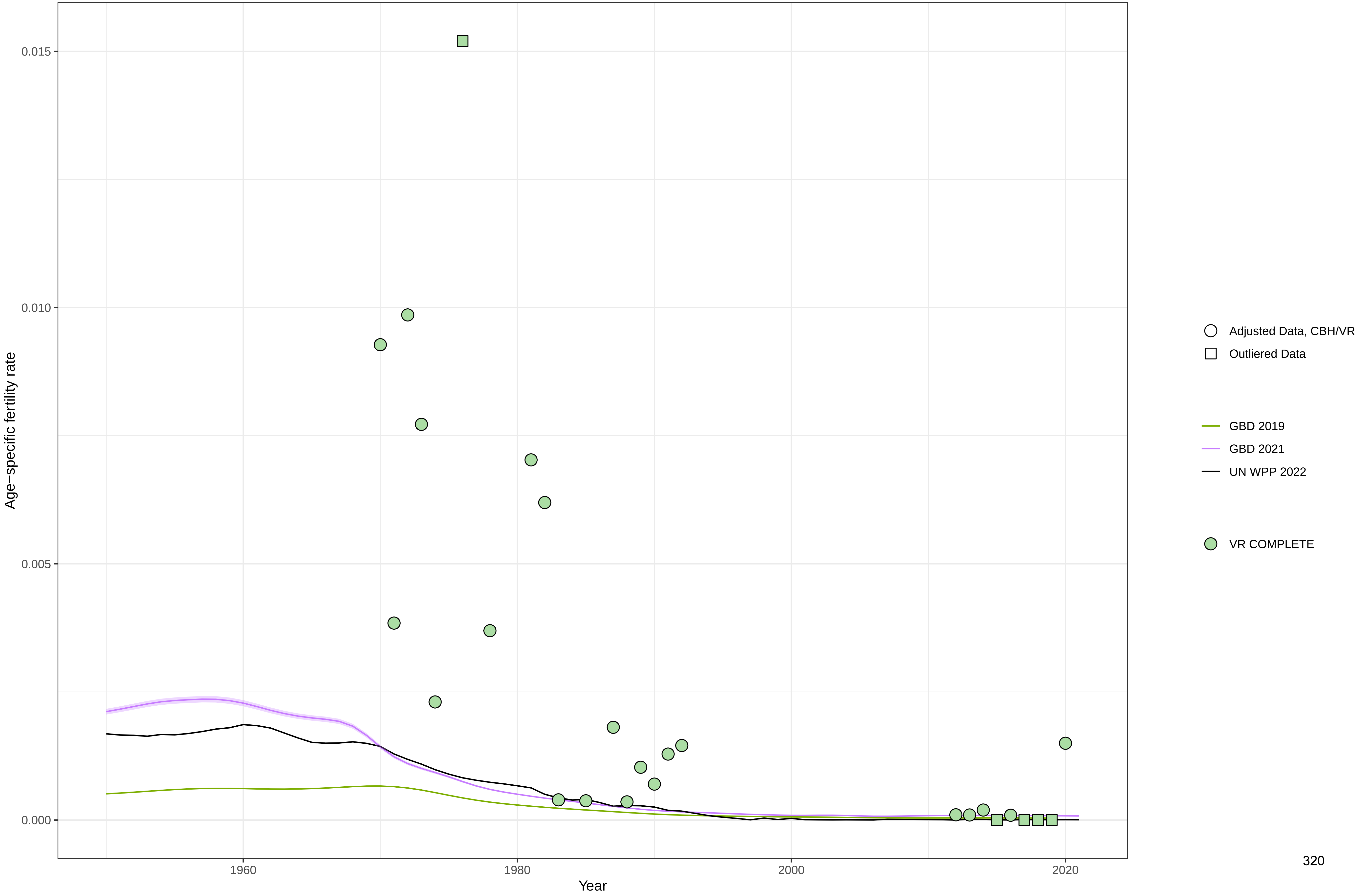

Total fertility rate fitted trend lines, 1950−2021: Brunei TFR
Data points enclosed with a diamond are reference sources

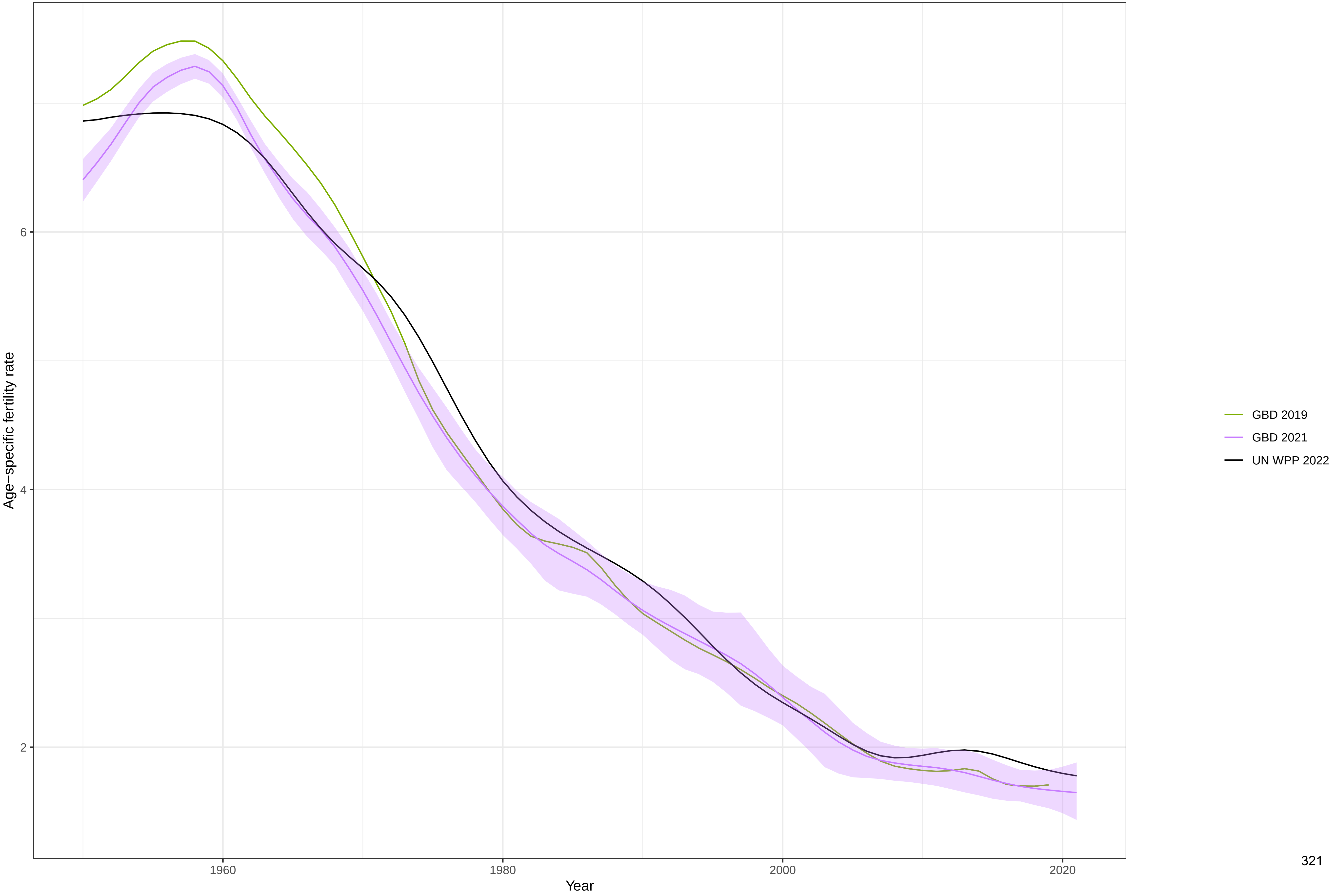

Age−specific fertility fitted trend lines, 1950−2021: Japan, Ages: 10−14
Data points enclosed with a diamond are reference sources

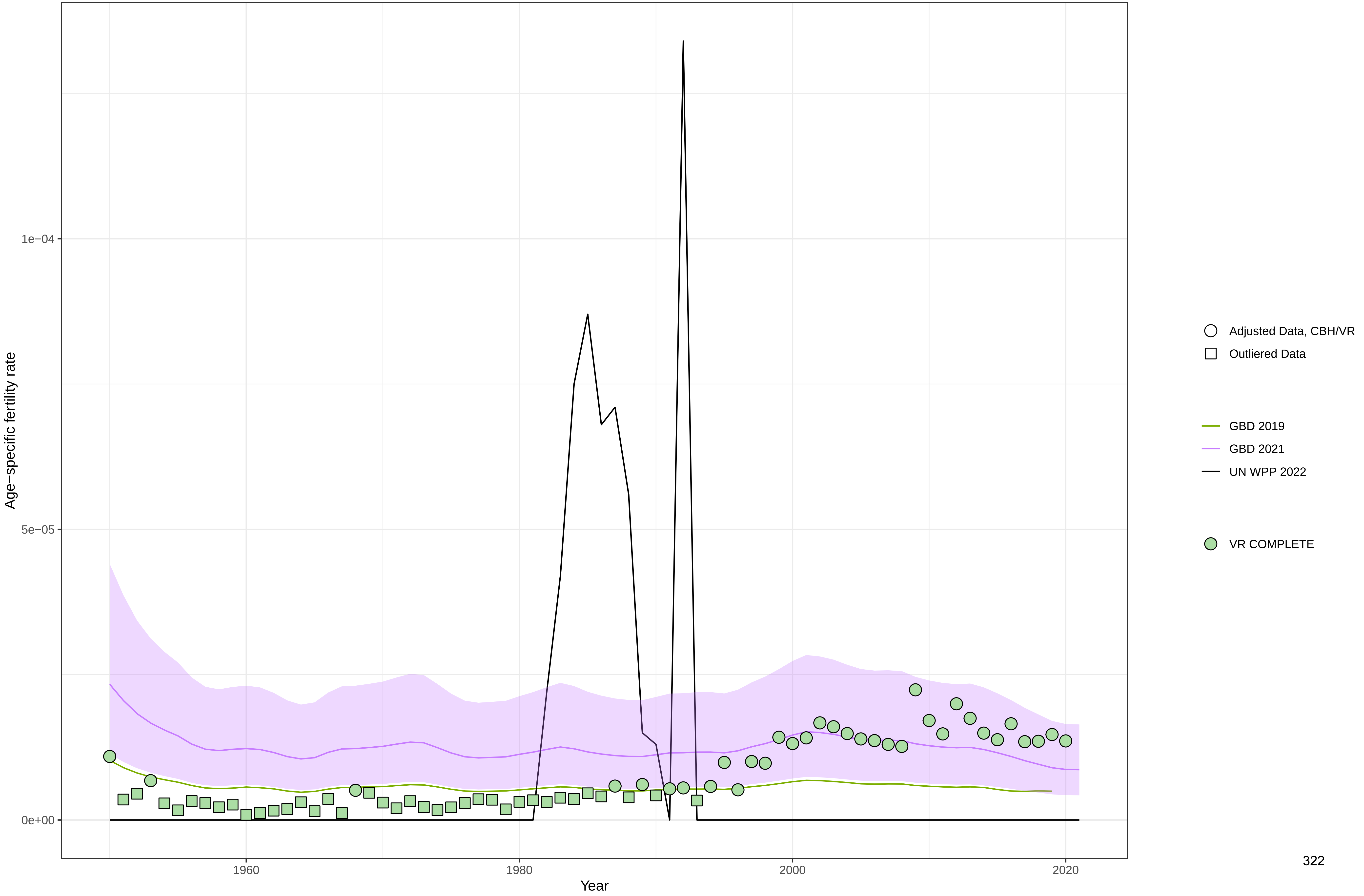

Age−specific fertility fitted trend lines, 1950−2021: Japan, Ages: 15−19
Data points enclosed with a diamond are reference sources

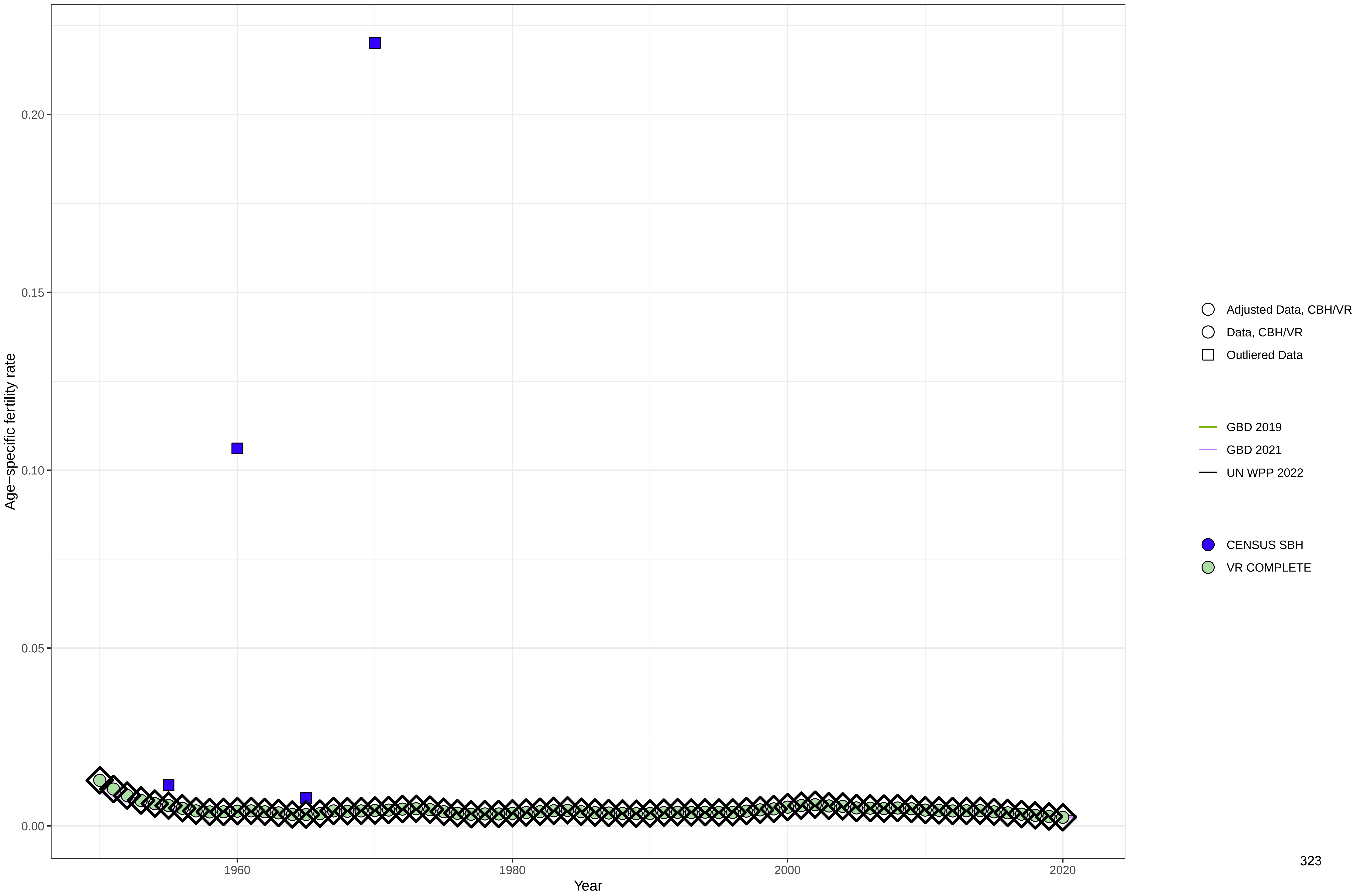

Age−specific fertility fitted trend lines, 1950−2021: Japan, Ages: 20−24
Data points enclosed with a diamond are reference sources

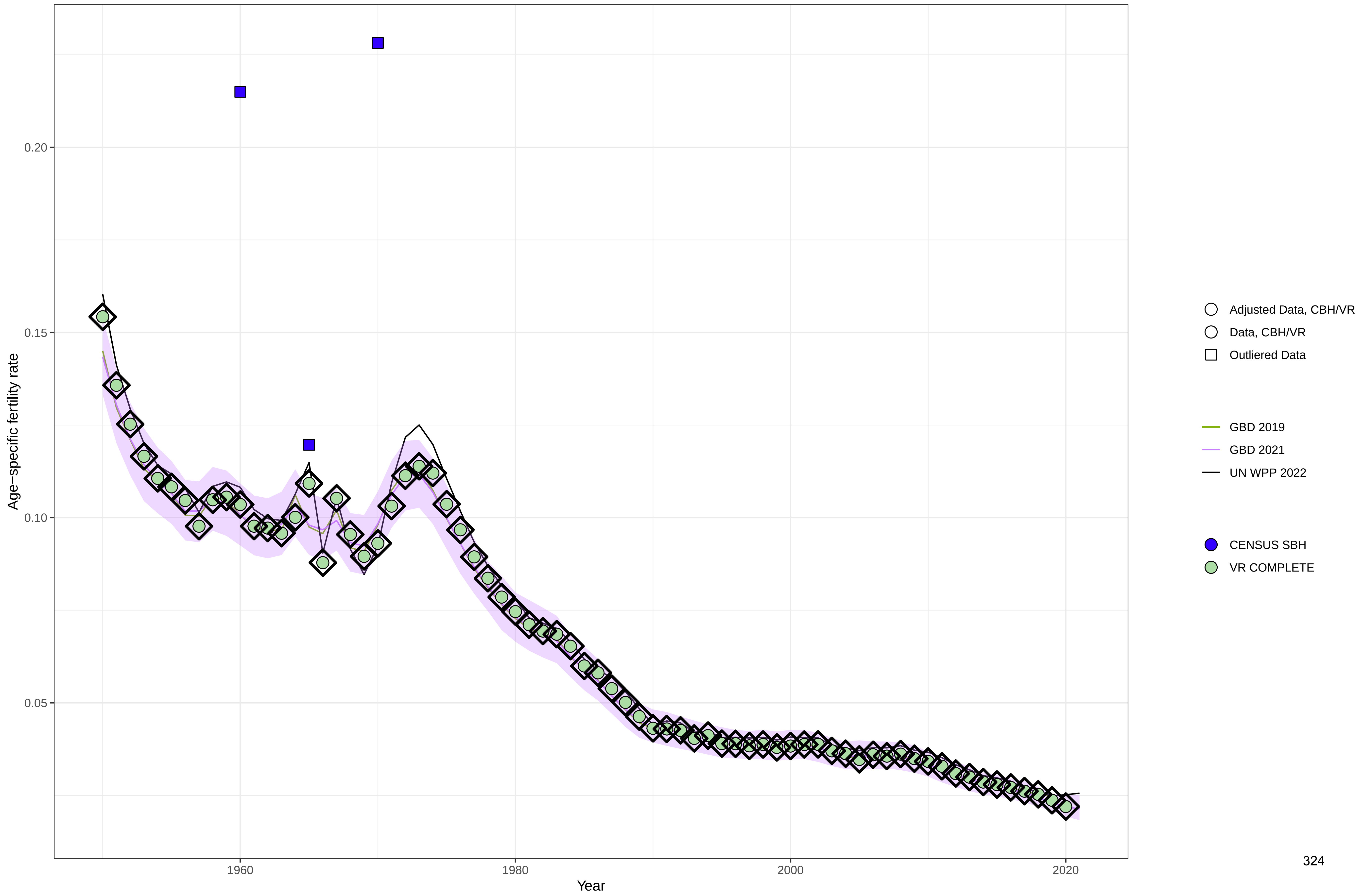

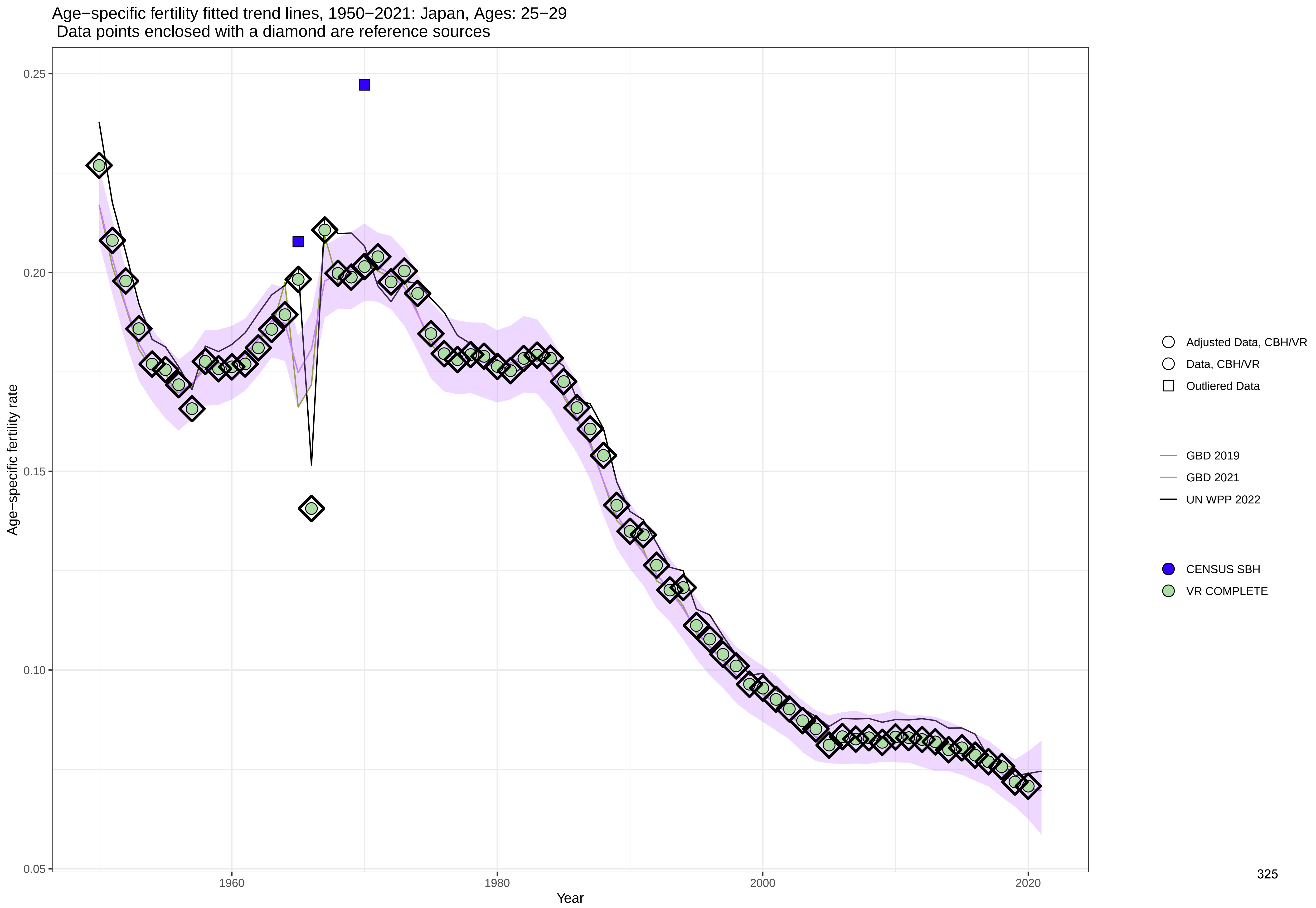

Age−specific fertility fitted trend lines, 1950−2021: Japan, Ages: 25−29
Data points enclosed with a diamond are reference sources
Age−specific fertility rate
Year
0.05
0.10
0.15
0.20
0.25
1960
1980
2000
2020
Adjusted Data, CBH/VR
Data, CBH/VR
Outliered Data
GBD 2019
GBD 2021
UN WPP 2022
CENSUS SBH
VR COMPLETE

Age−specific fertility fitted trend lines, 1950−2021: Japan, Ages: 30−34
Data points enclosed with a diamond are reference sources

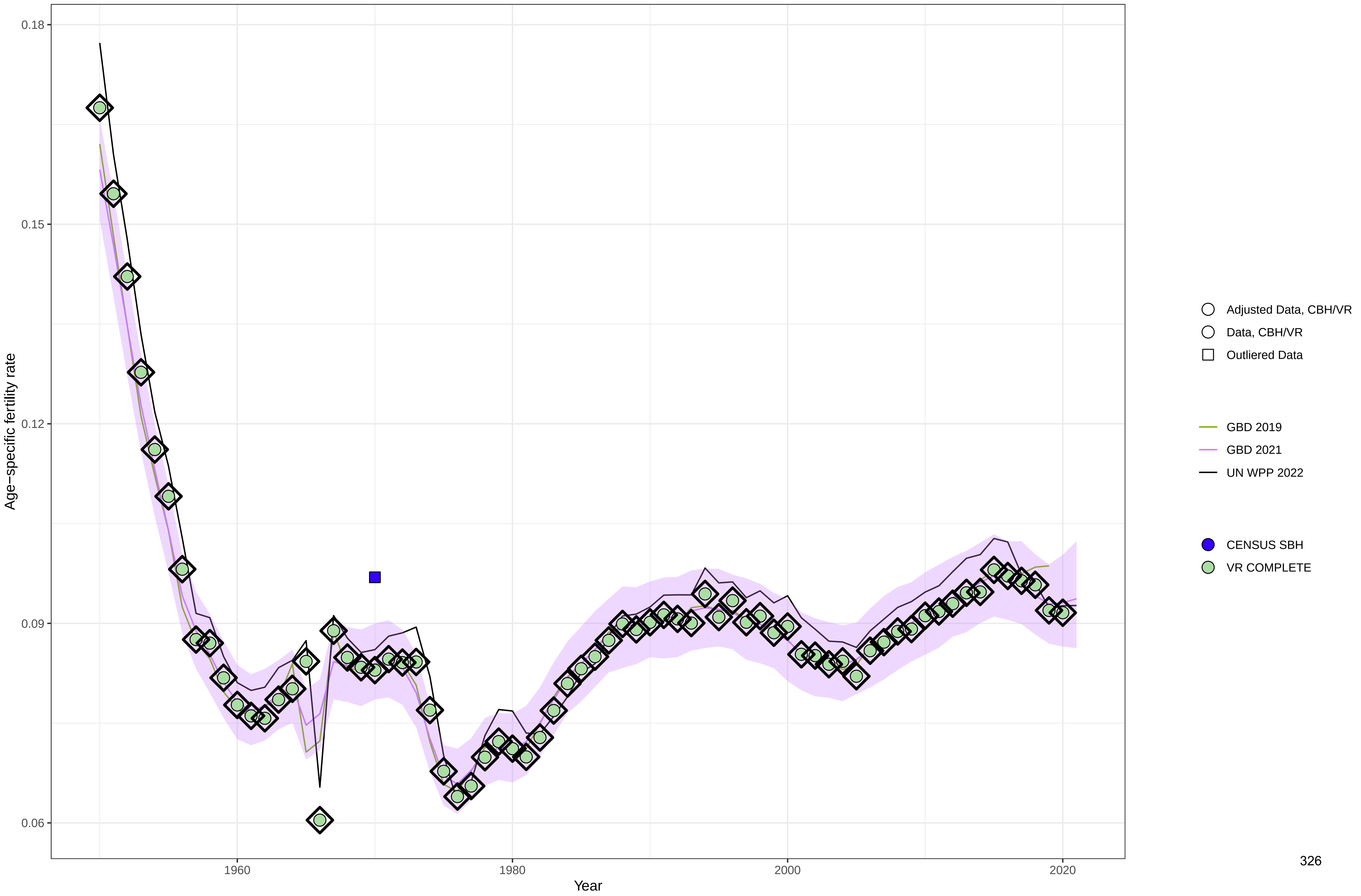

Age−specific fertility fitted trend lines, 1950−2021: Japan, Ages: 35−39
Data points enclosed with a diamond are reference sources

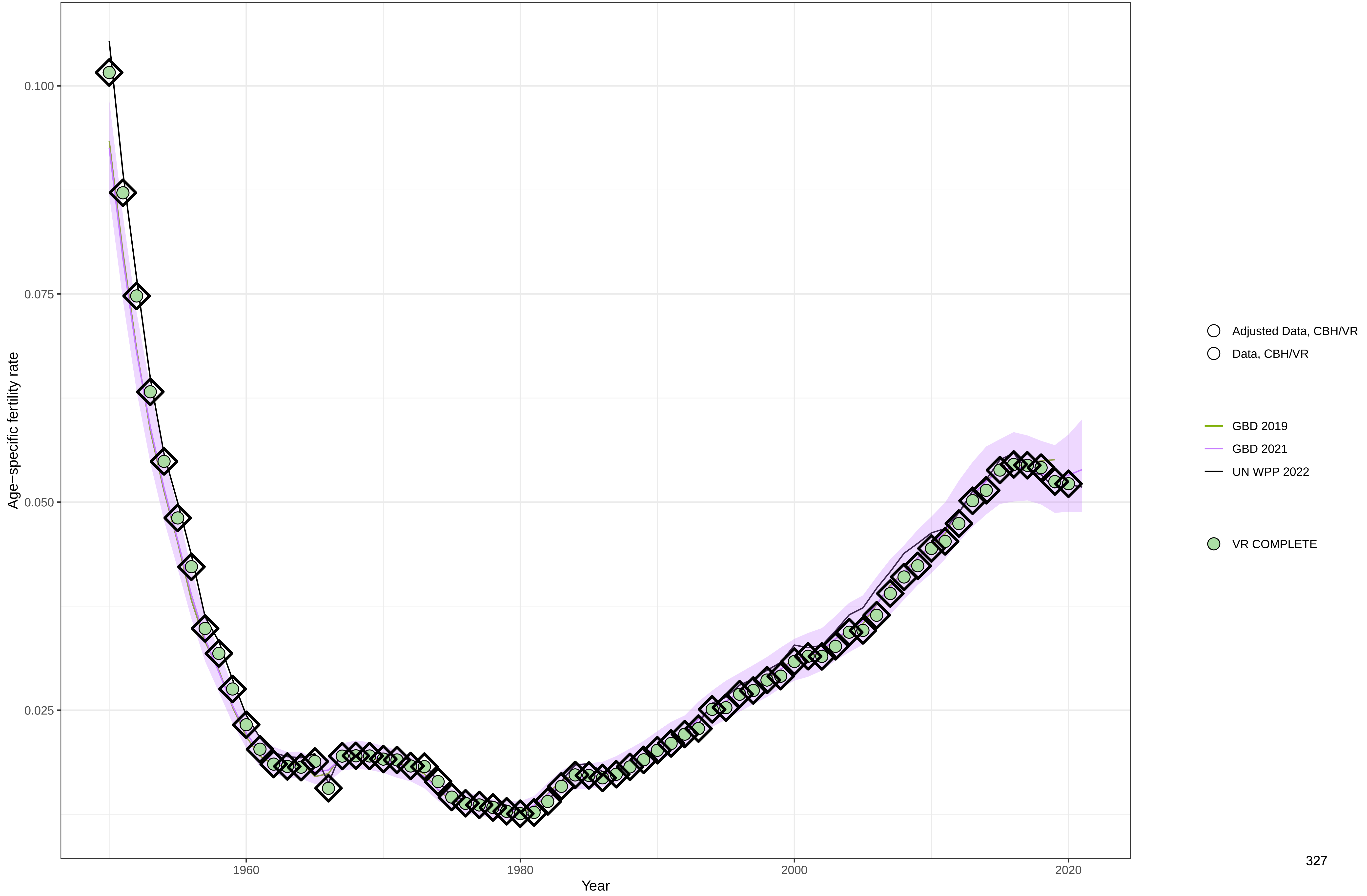

Age−specific fertility fitted trend lines, 1950−2021: Japan, Ages: 40−44
Data points enclosed with a diamond are reference sources

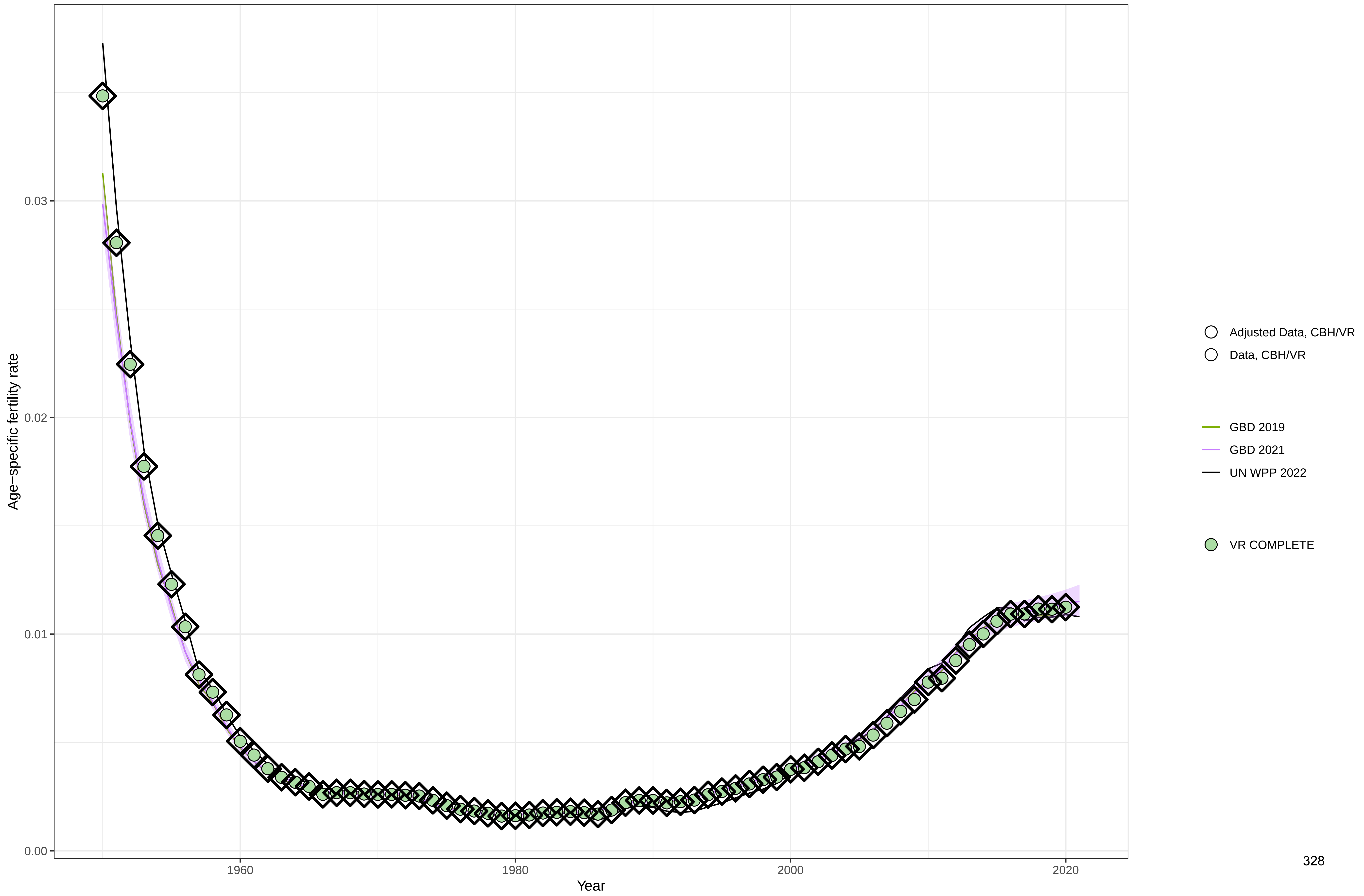

Age−specific fertility fitted trend lines, 1950−2021: Japan, Ages: 45−49
Data points enclosed with a diamond are reference sources

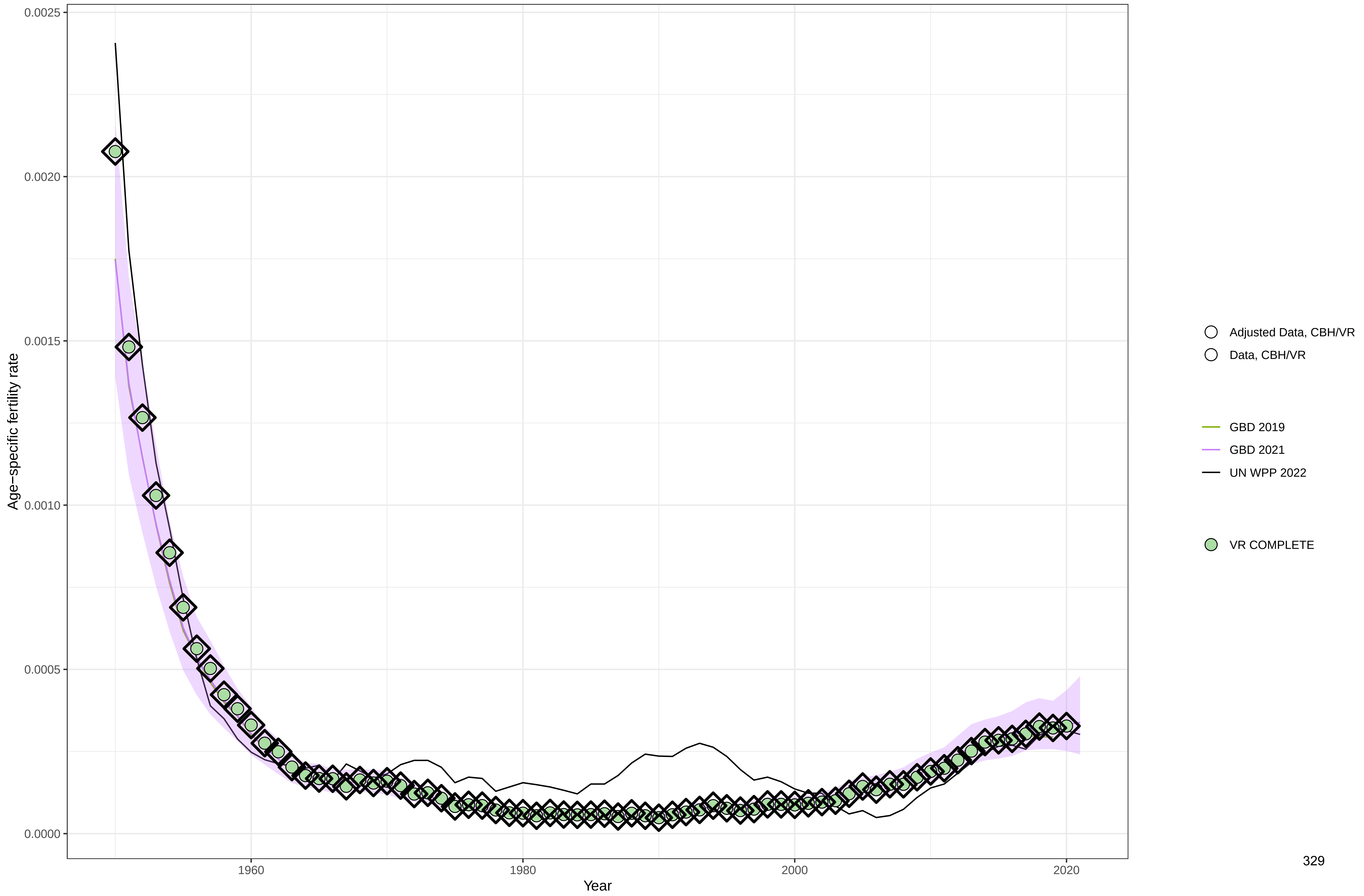

Age−specific fertility fitted trend lines, 1950−2021: Japan, Ages: 50−54
Data points enclosed with a diamond are reference sources

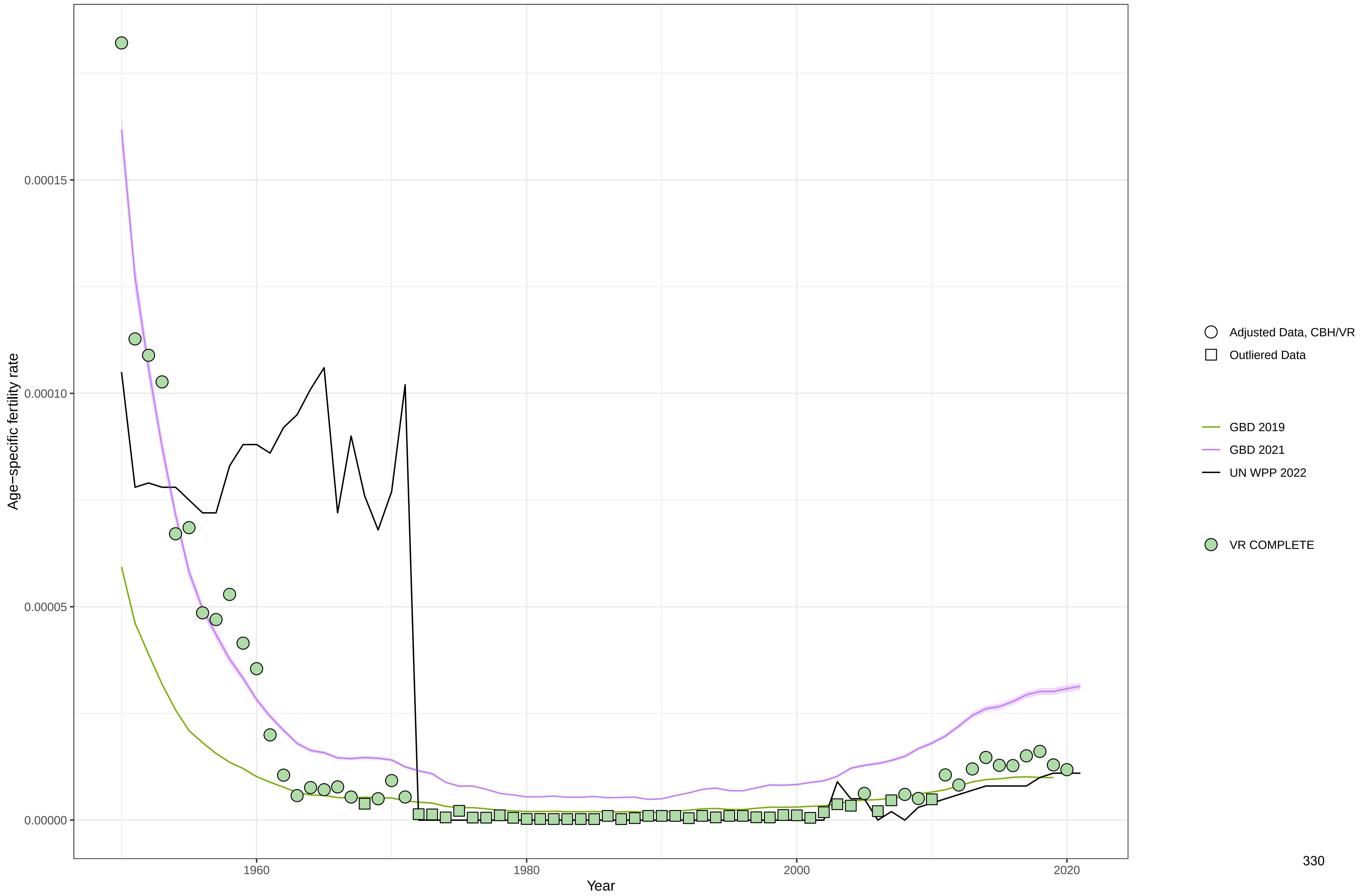

Total fertility rate fitted trend lines, 1950−2021: Japan TFR
Data points enclosed with a diamond are reference sources

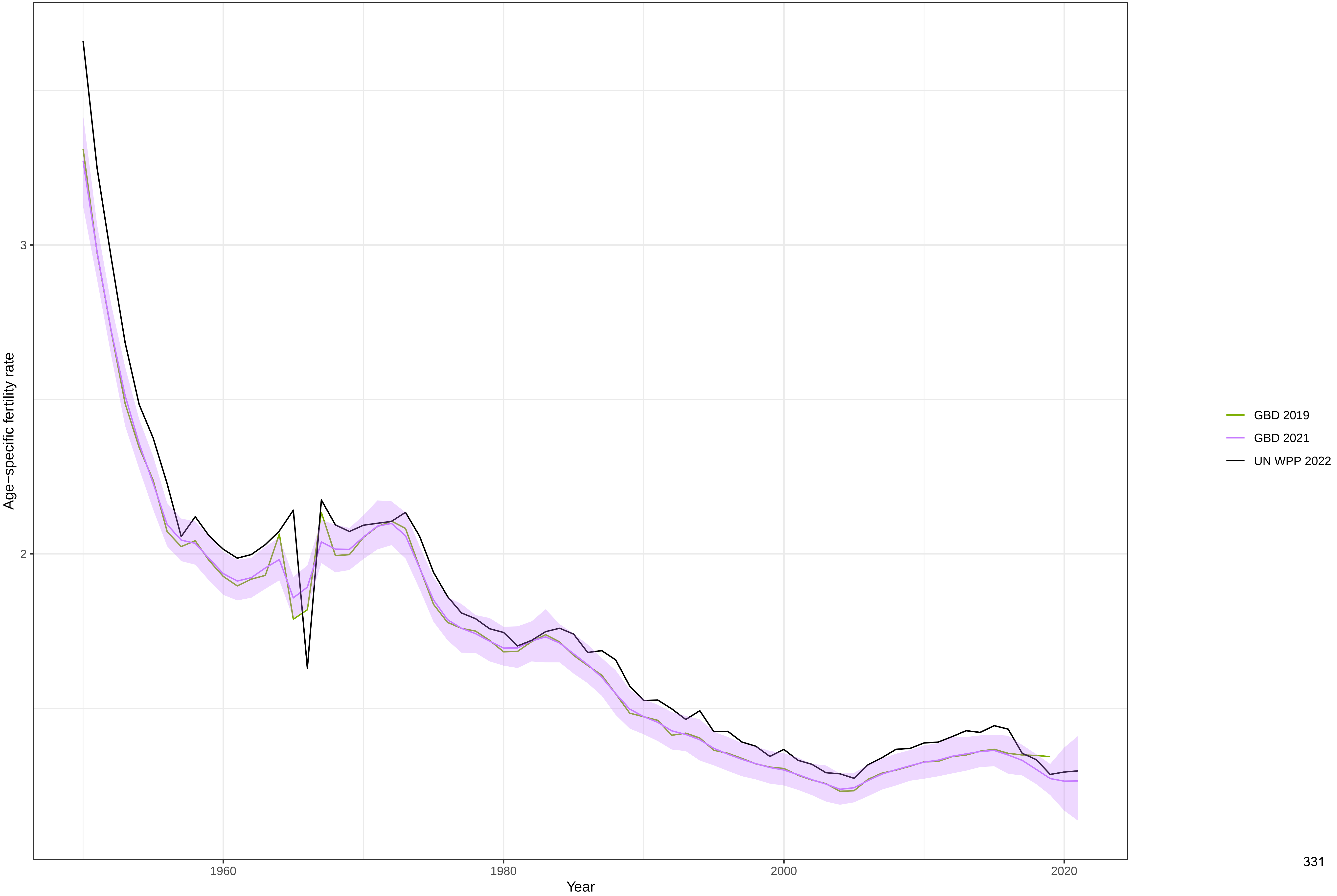

Age−specific fertility fitted trend lines, 1950−2021: Aichi, Ages: 10−14
Data points enclosed with a diamond are reference sources

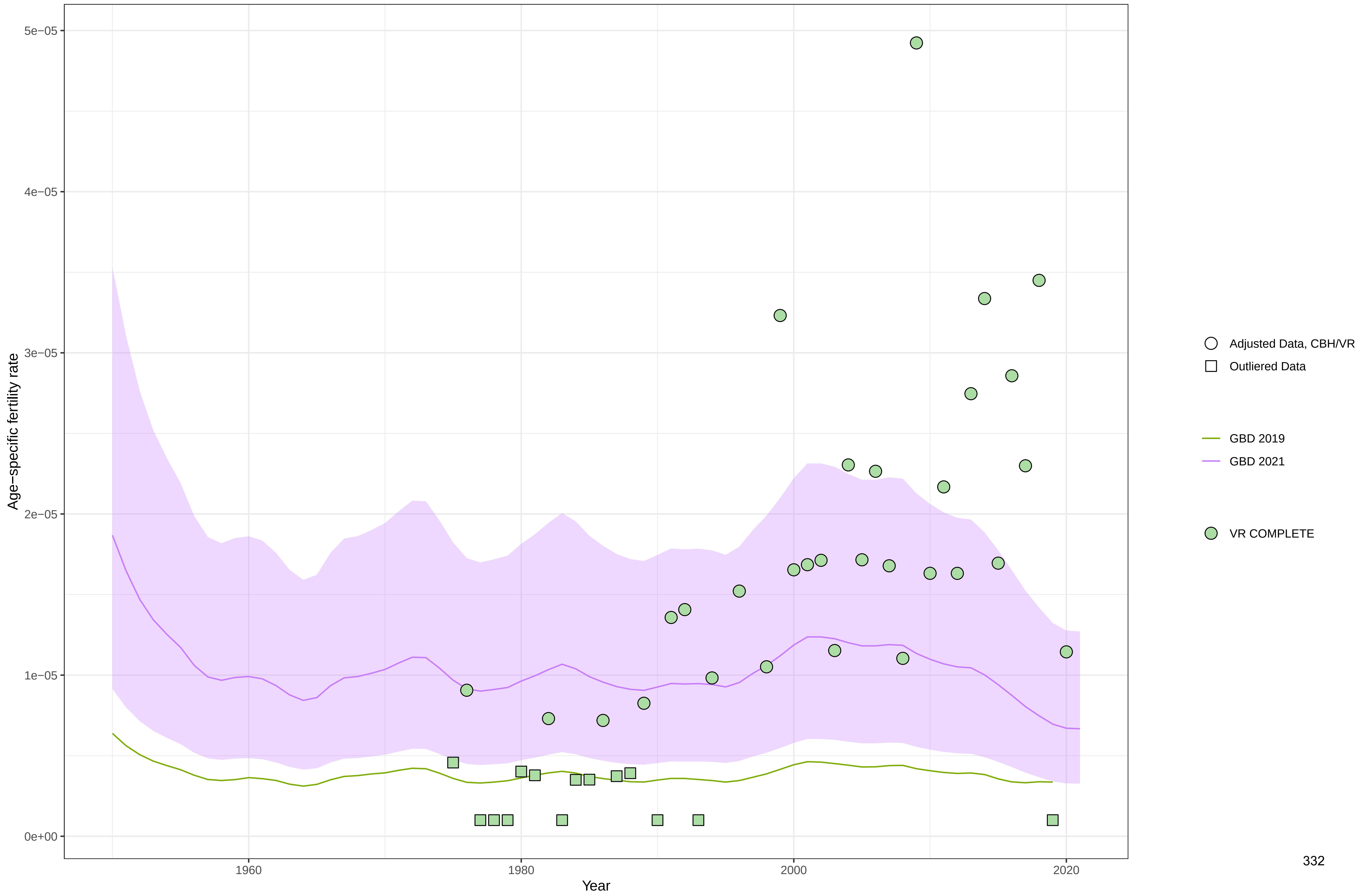

Age−specific fertility fitted trend lines, 1950−2021: Aichi, Ages: 15−19
Data points enclosed with a diamond are reference sources

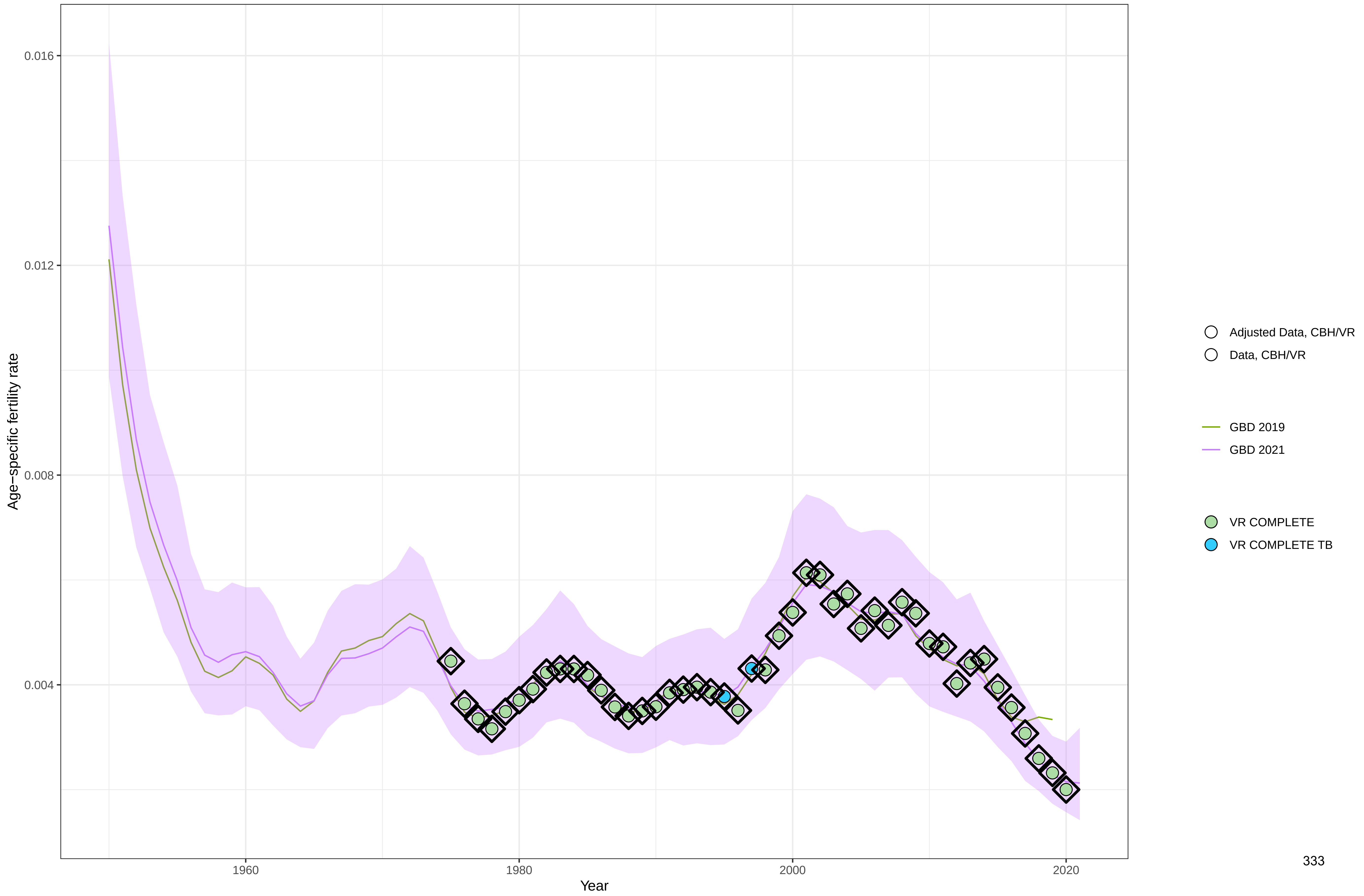

Age−specific fertility fitted trend lines, 1950−2021: Aichi, Ages: 20−24
Data points enclosed with a diamond are reference sources

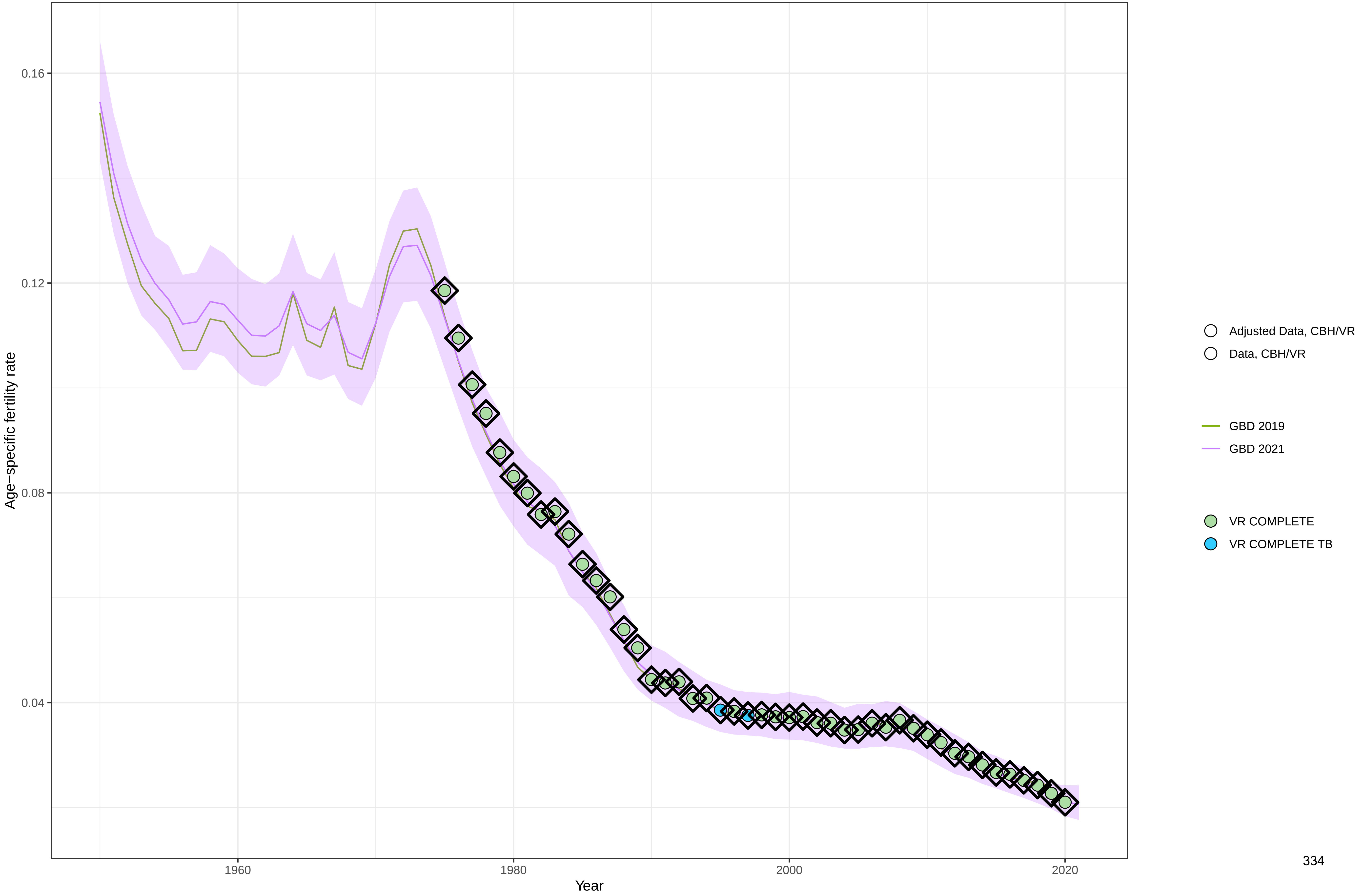

Age−specific fertility fitted trend lines, 1950−2021: Aichi, Ages: 25−29
Data points enclosed with a diamond are reference sources

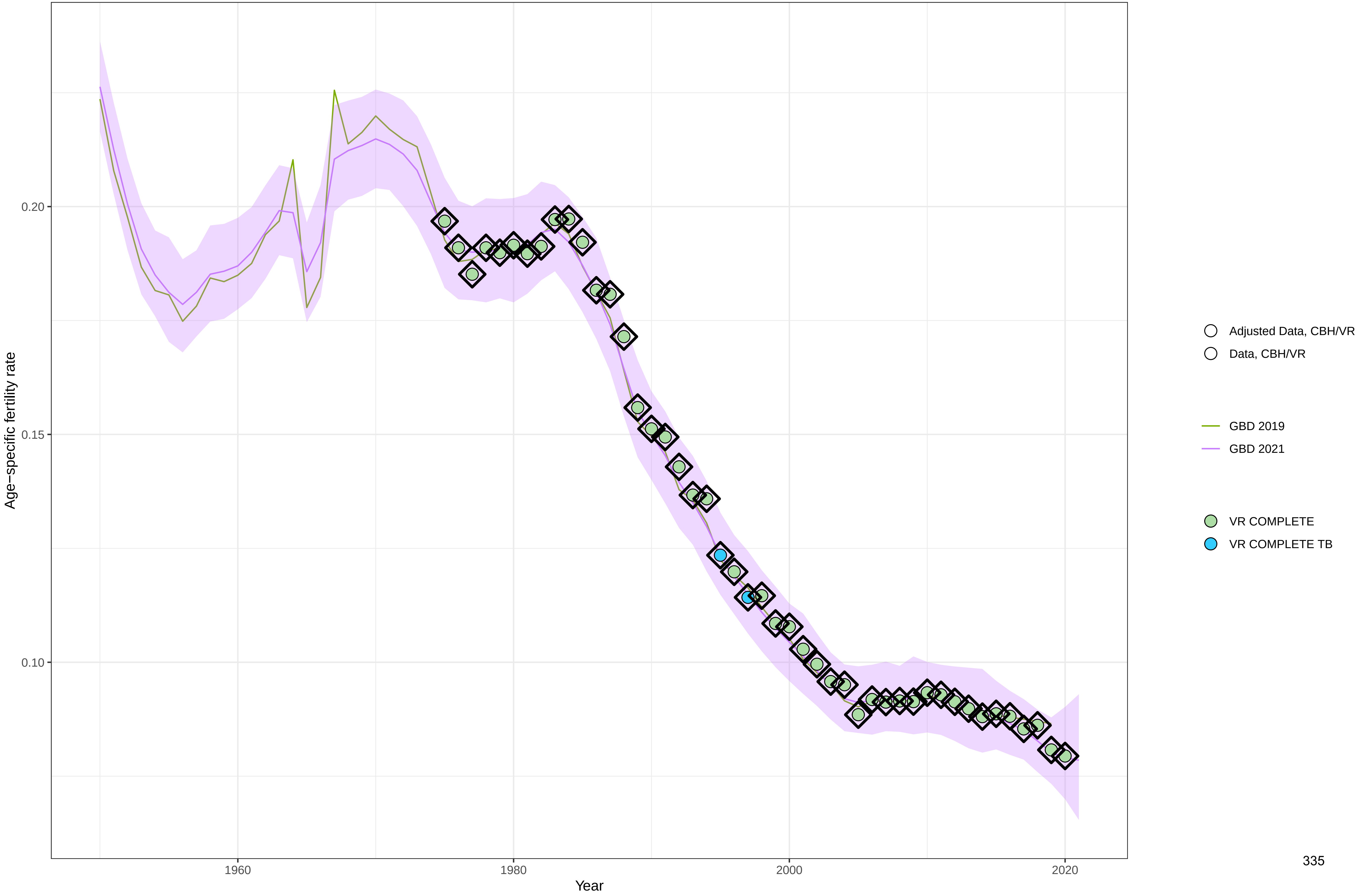

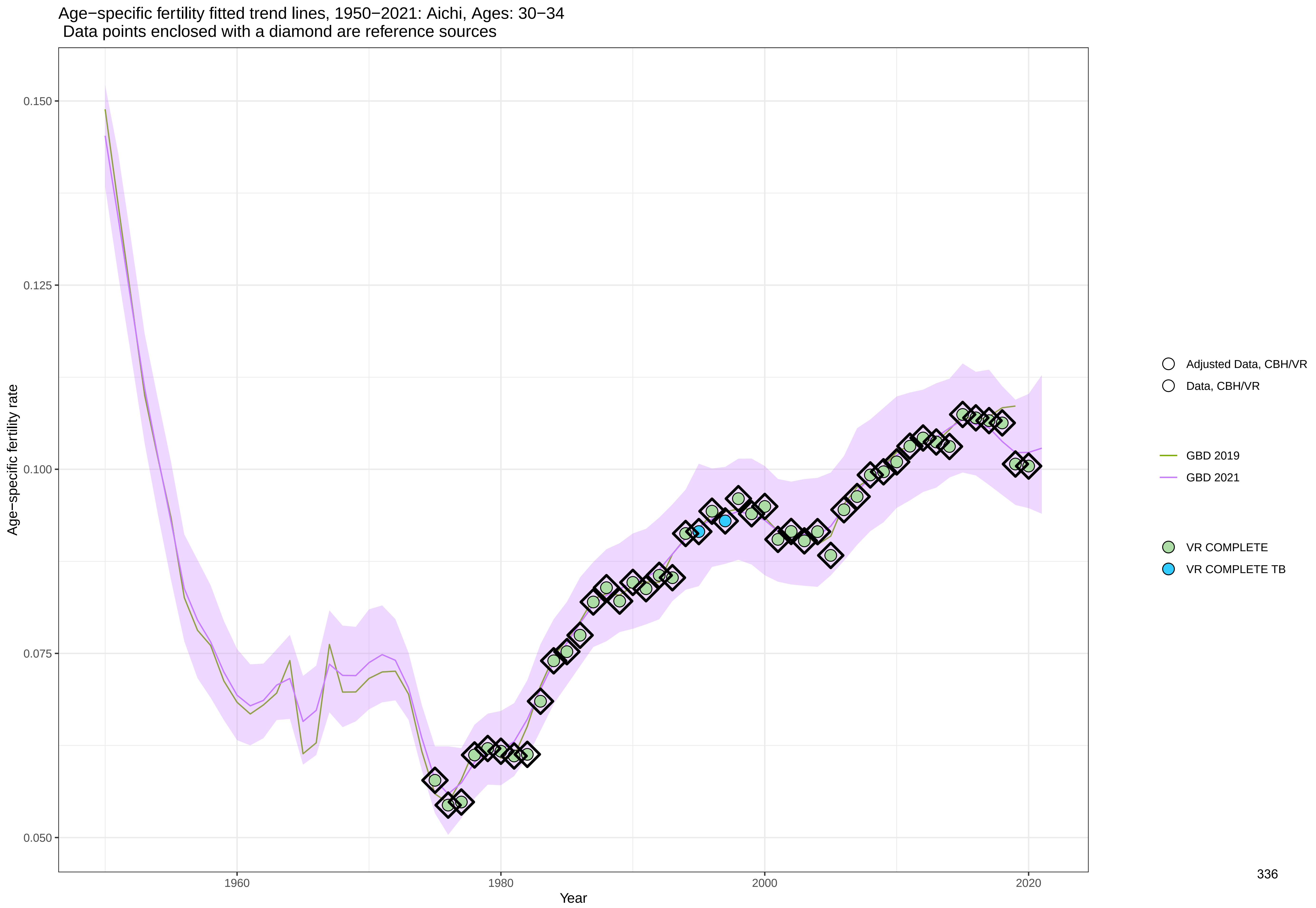

Age−specific fertility fitted trend lines, 1950−2021: Aichi, Ages: 30−34
Data points enclosed with a diamond are reference sources
Age−specific fertility rate
Year
0.050
0.075
0.100
0.125
0.150
1960
1980
2000
2020
Adjusted Data, CBH/VR
Data, CBH/VR
GBD 2019
GBD 2021
VR COMPLETE
VR COMPLETE TB

Age−specific fertility fitted trend lines, 1950−2021: Aichi, Ages: 35−39
Data points enclosed with a diamond are reference sources

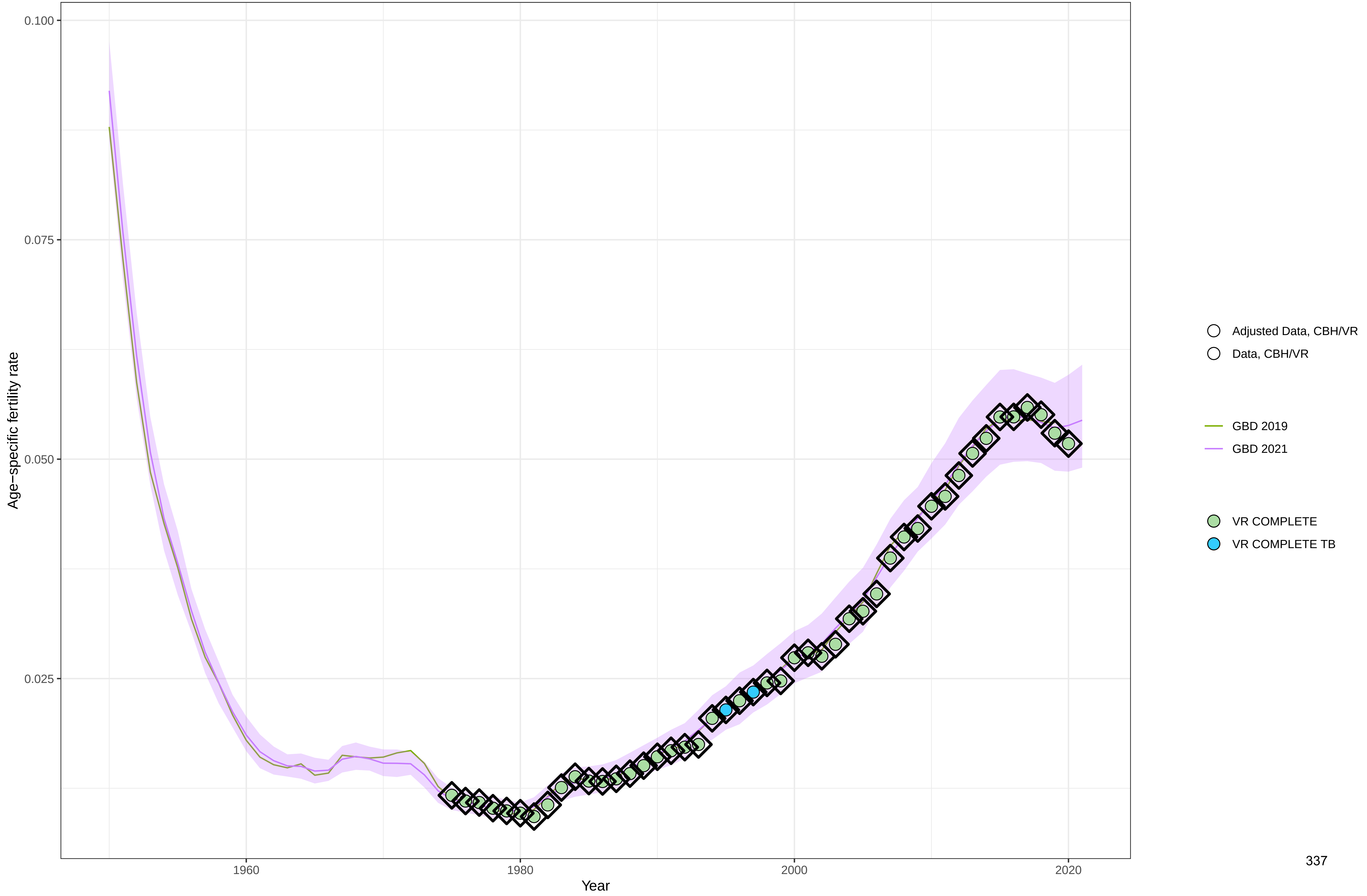

Age−specific fertility fitted trend lines, 1950−2021: Aichi, Ages: 40−44
Data points enclosed with a diamond are reference sources

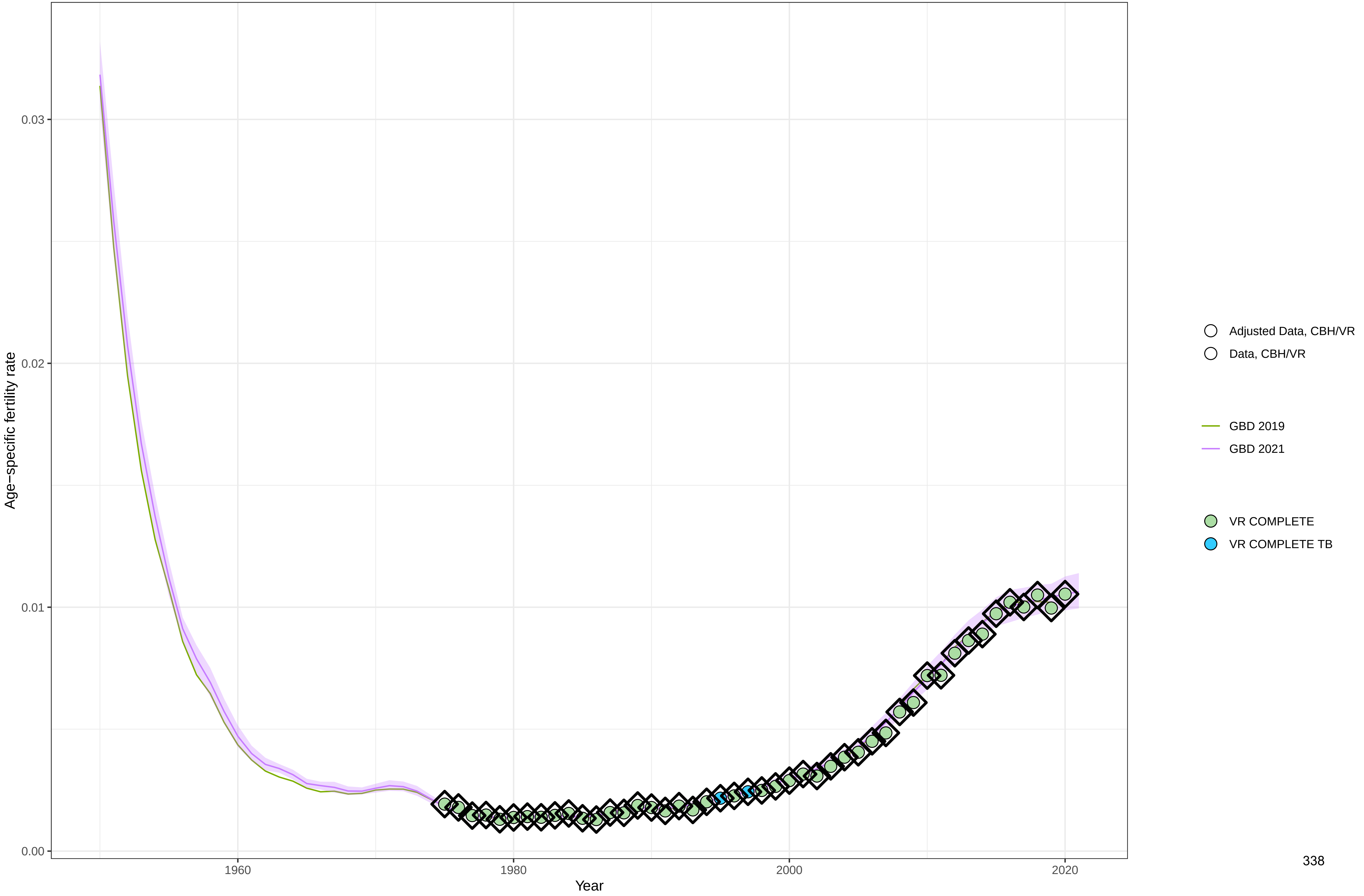

Age−specific fertility fitted trend lines, 1950−2021: Aichi, Ages: 45−49
Data points enclosed with a diamond are reference sources

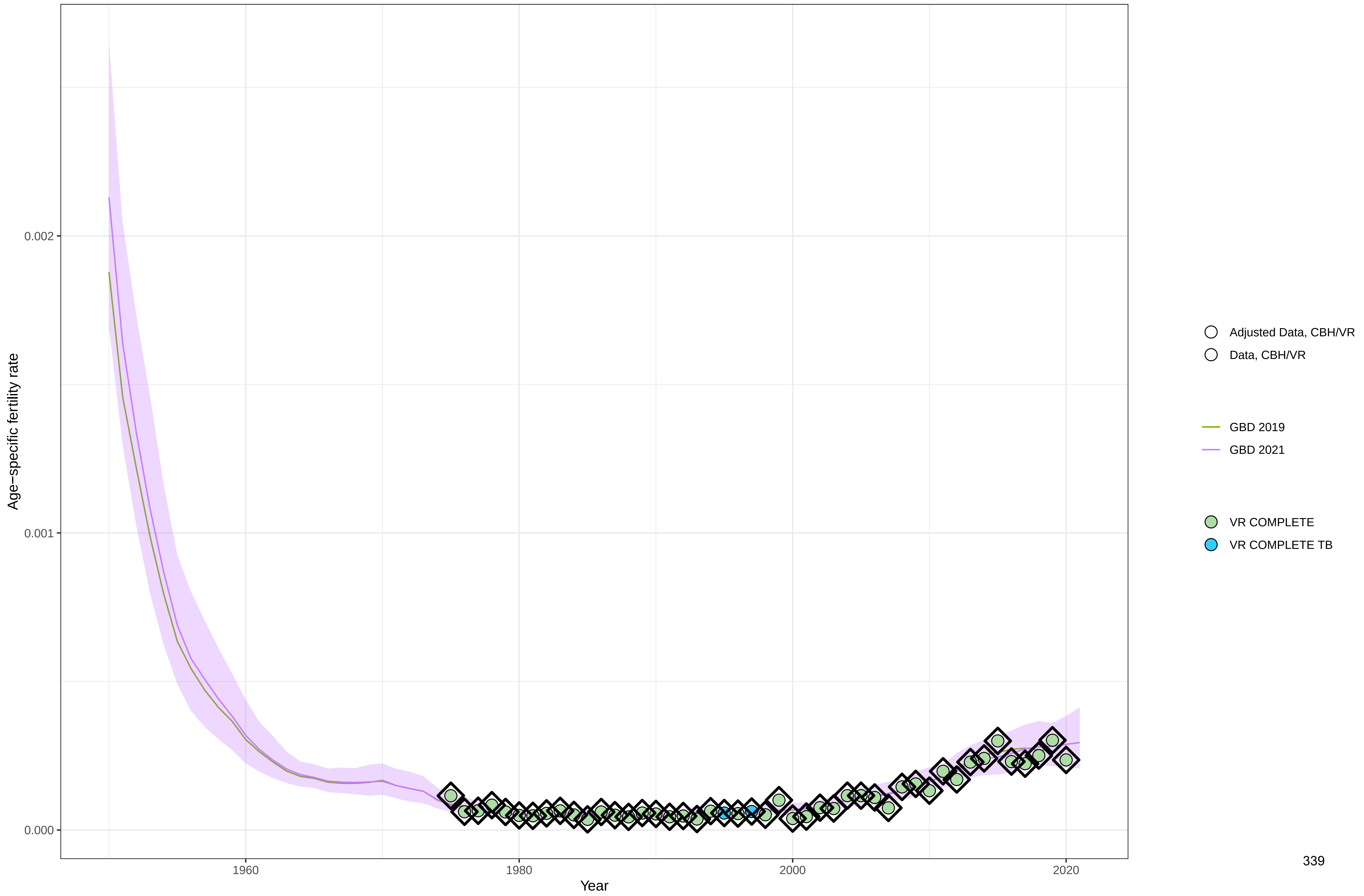

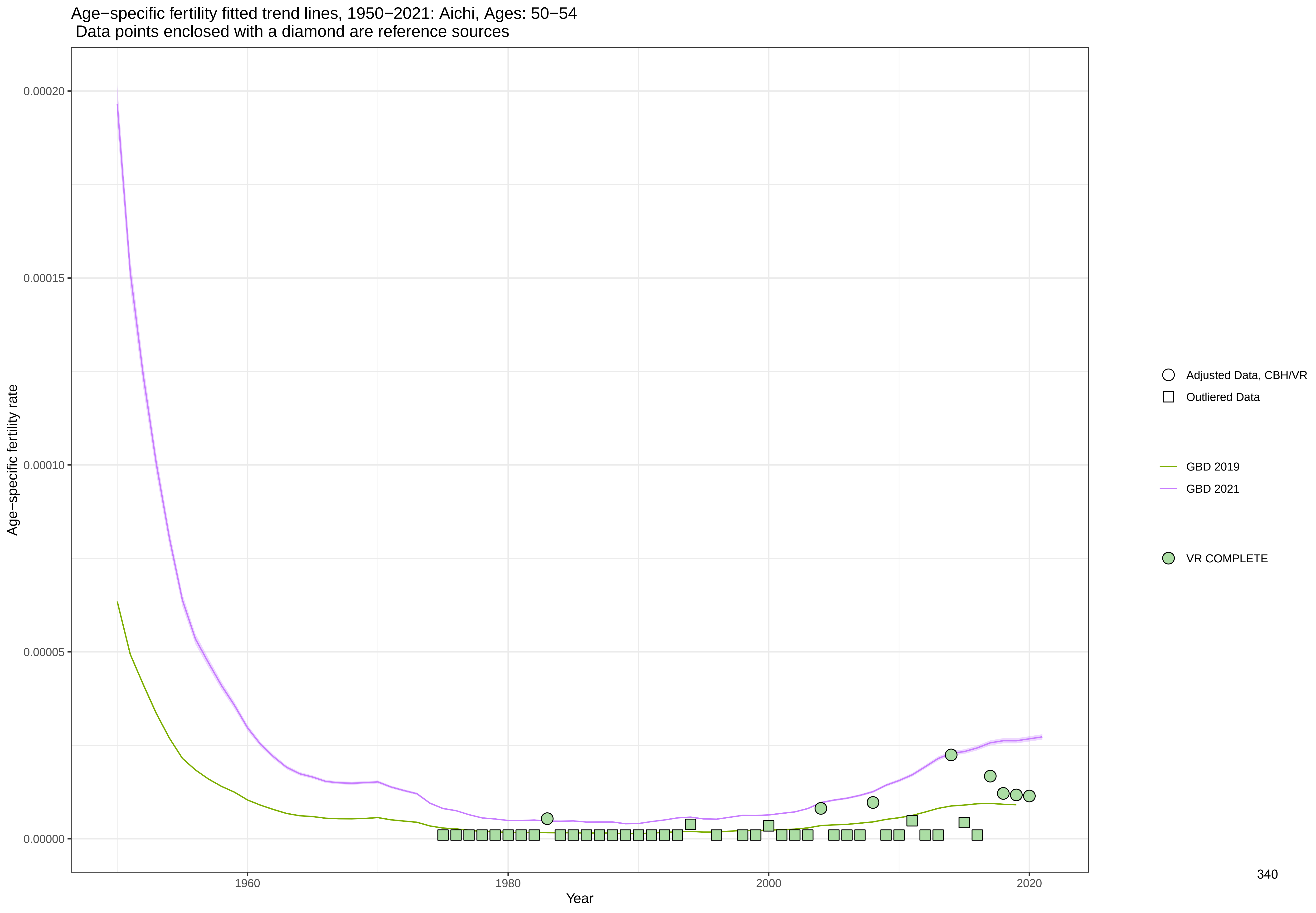
Age−specific fertility fitted trend lines, 1950−2021: Aichi, Ages: 50−54
Data points enclosed with a diamond are reference sources
0.00020
0.00015
0.00010
0.00005
0.00000
Age−specific fertility rate
1960
1980
2000
2020
Year
Adjusted Data, CBH/VR
Outliered Data
GBD 2019
GBD 2021
VR COMPLETE

Total fertility rate fitted trend lines, 1950−2021: Aichi TFR
Data points enclosed with a diamond are reference sources

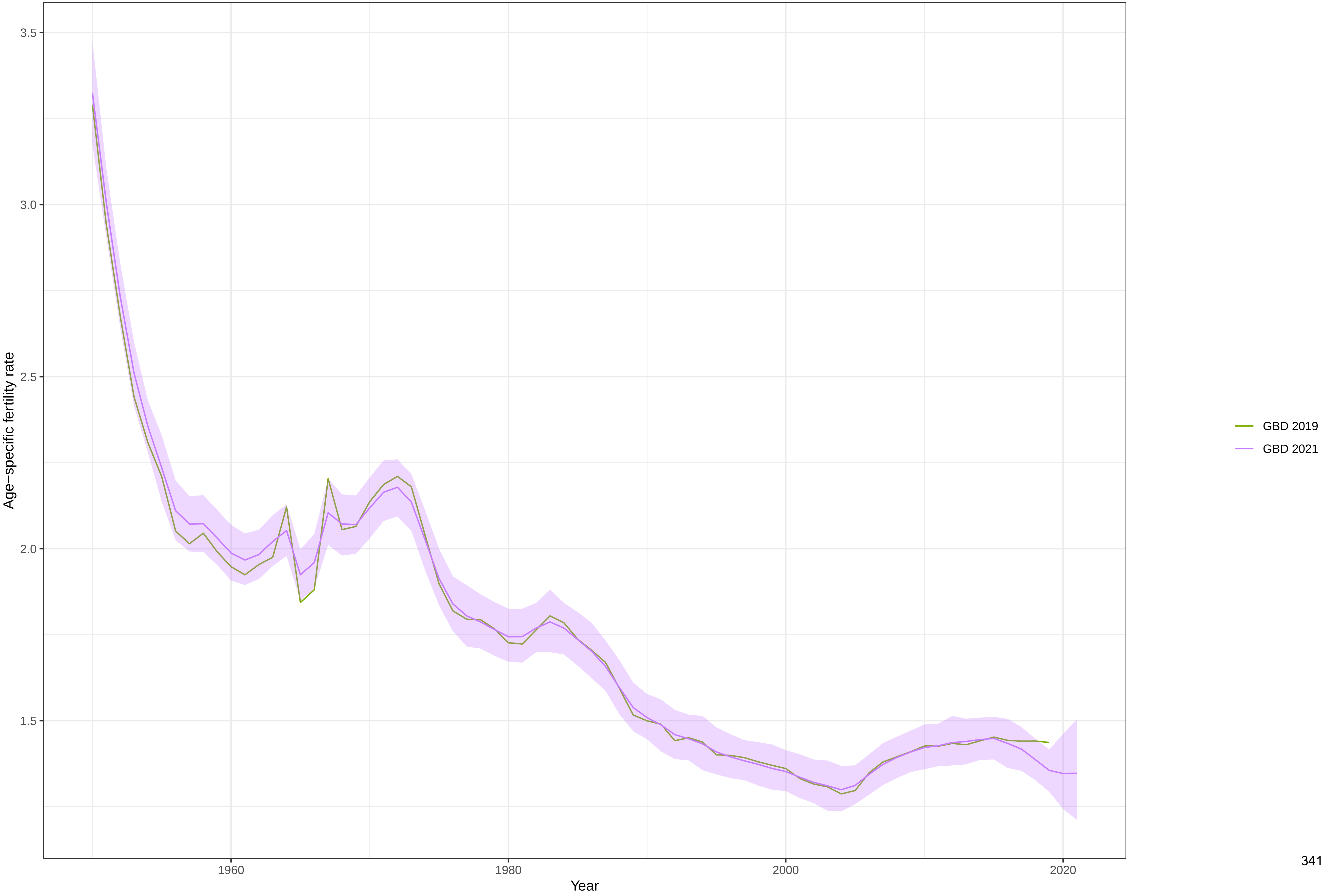

Age−specific fertility fitted trend lines, 1950−2021: Akita, Ages: 10−14
Data points enclosed with a diamond are reference sources

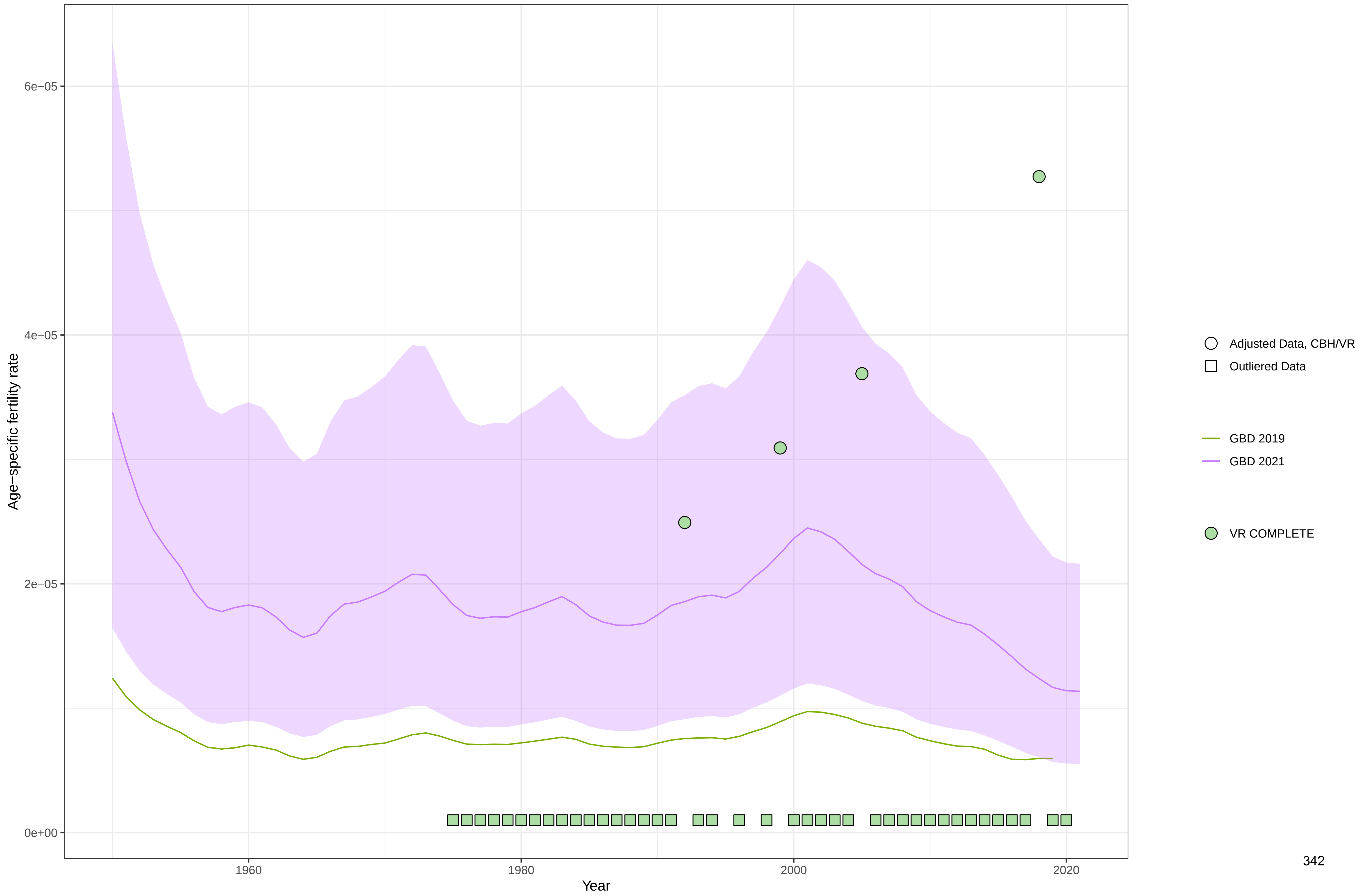

Age−specific fertility fitted trend lines, 1950−2021: Akita, Ages: 15−19
Data points enclosed with a diamond are reference sources

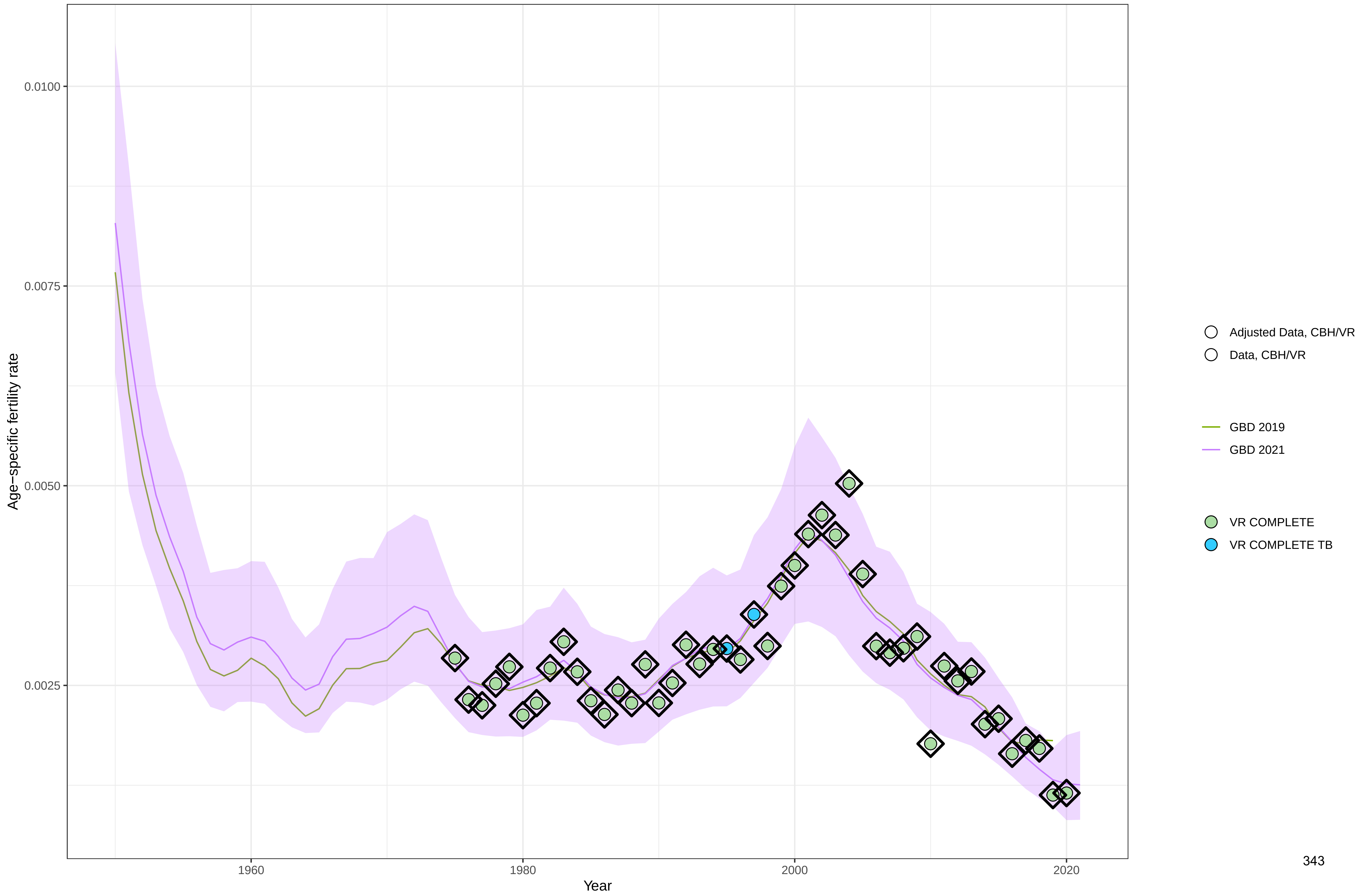

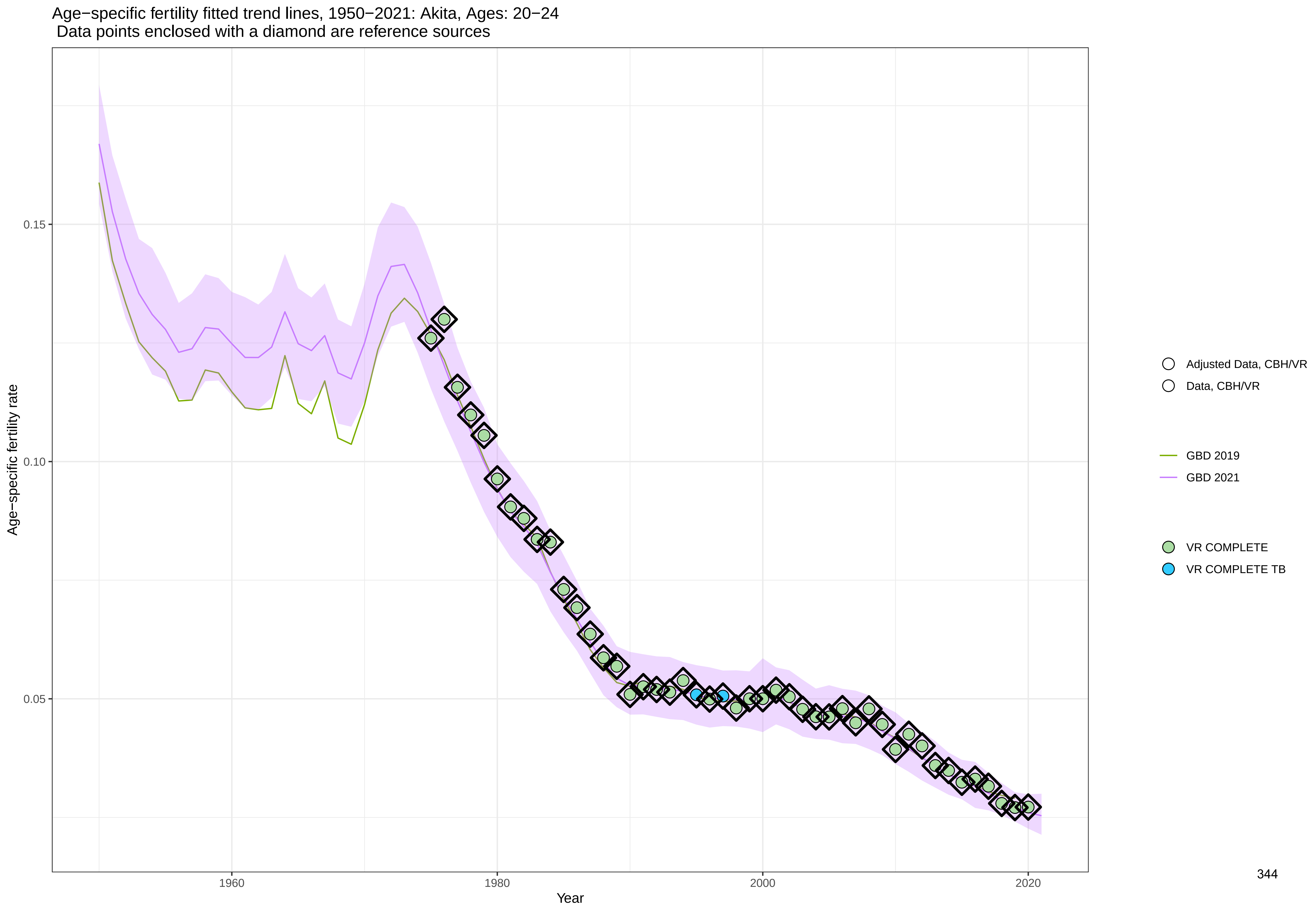

Age−specific fertility fitted trend lines, 1950−2021: Akita, Ages: 20−24
Data points enclosed with a diamond are reference sources
Age−specific fertility rate
0.15
0.10
0.05
1960
1980
2000
2020
Year
Adjusted Data, CBH/VR
Data, CBH/VR
GBD 2019
GBD 2021
VR COMPLETE
VR COMPLETE TB

Age−specific fertility fitted trend lines, 1950−2021: Akita, Ages: 25−29
Data points enclosed with a diamond are reference sources

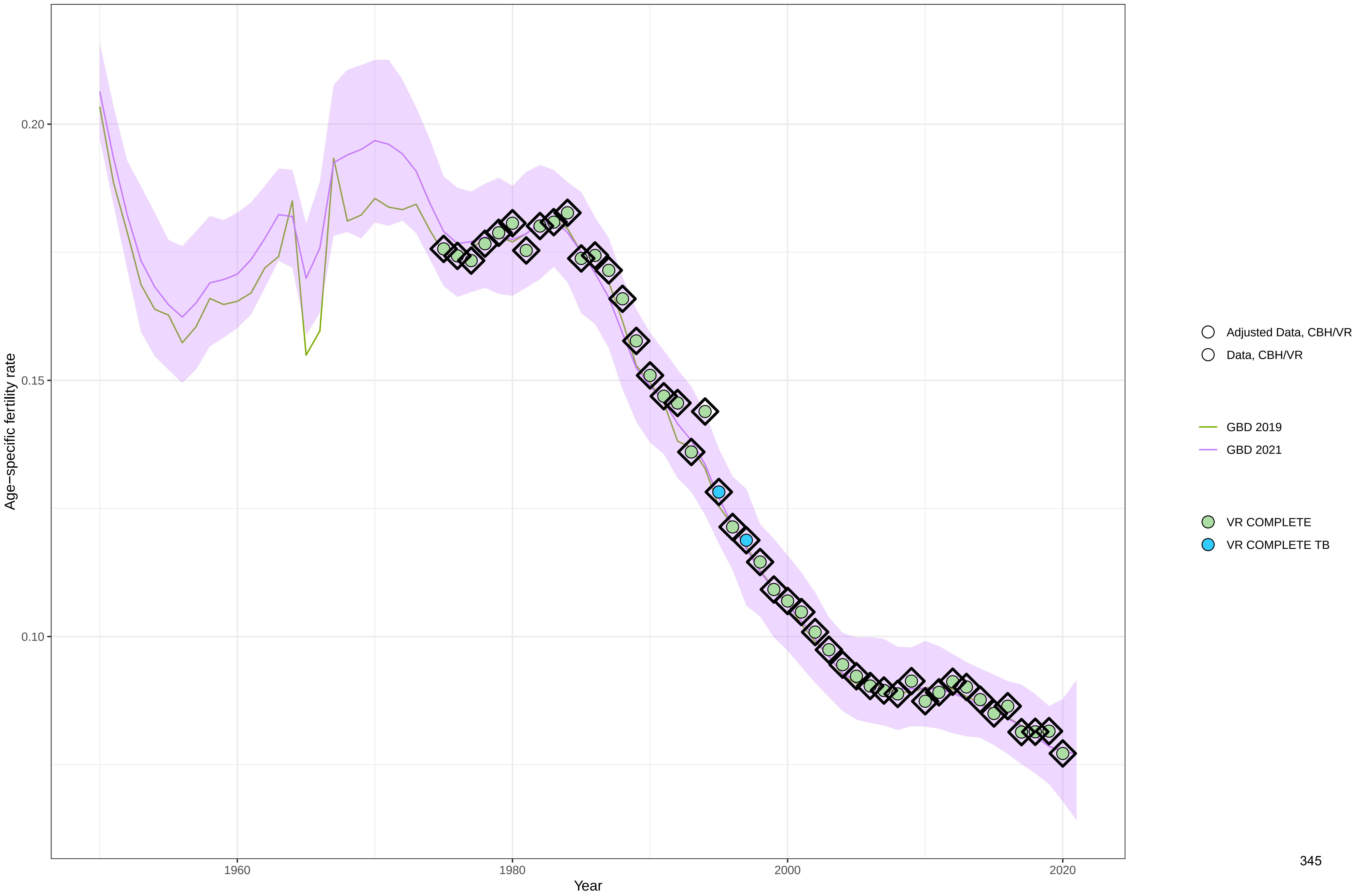

Age−specific fertility fitted trend lines, 1950−2021: Akita, Ages: 30−34
Data points enclosed with a diamond are reference sources

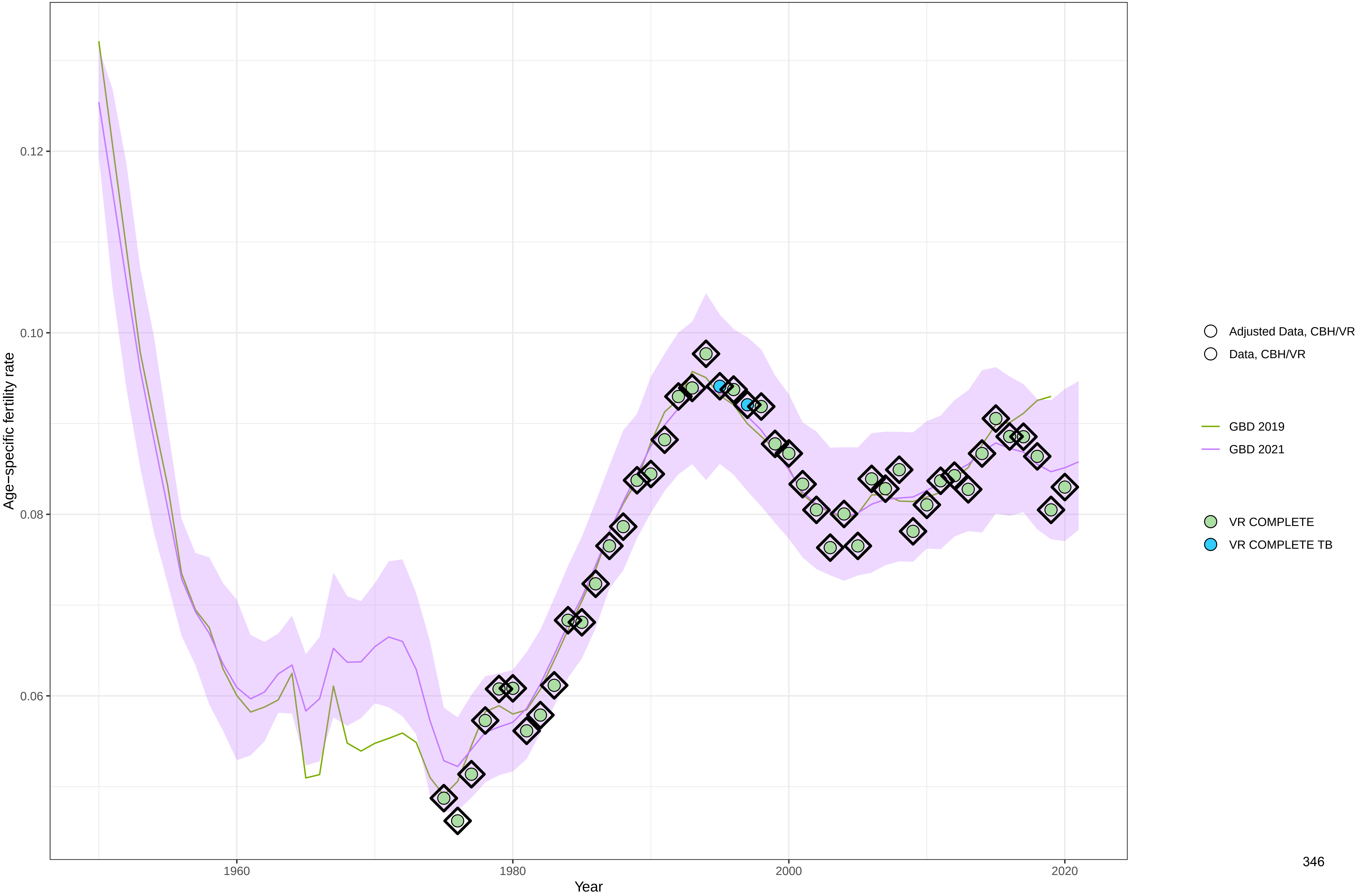

Age−specific fertility fitted trend lines, 1950−2021: Akita, Ages: 35−39
Data points enclosed with a diamond are reference sources

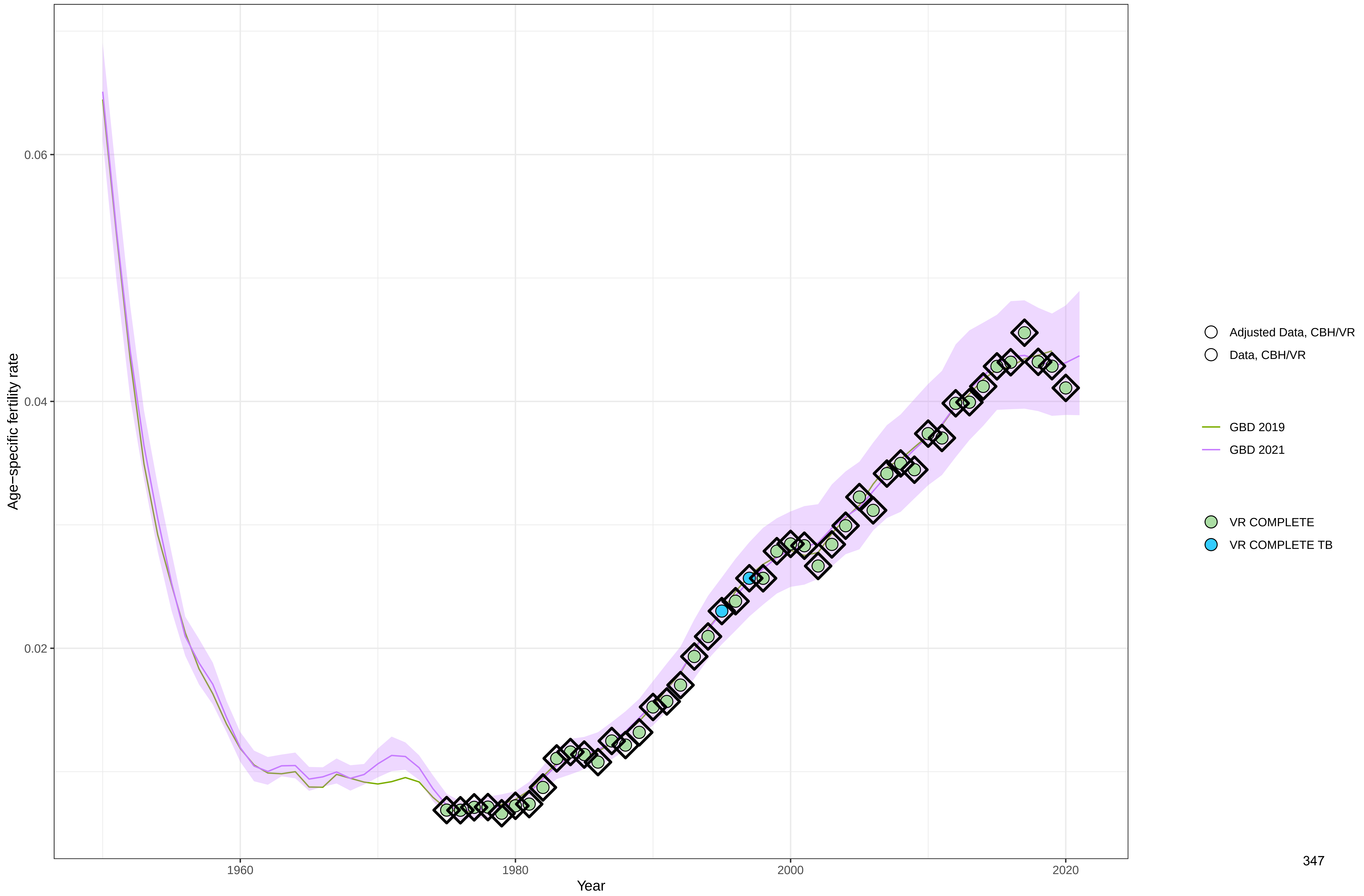

Age−specific fertility fitted trend lines, 1950−2021: Akita, Ages: 40−44
Data points enclosed with a diamond are reference sources

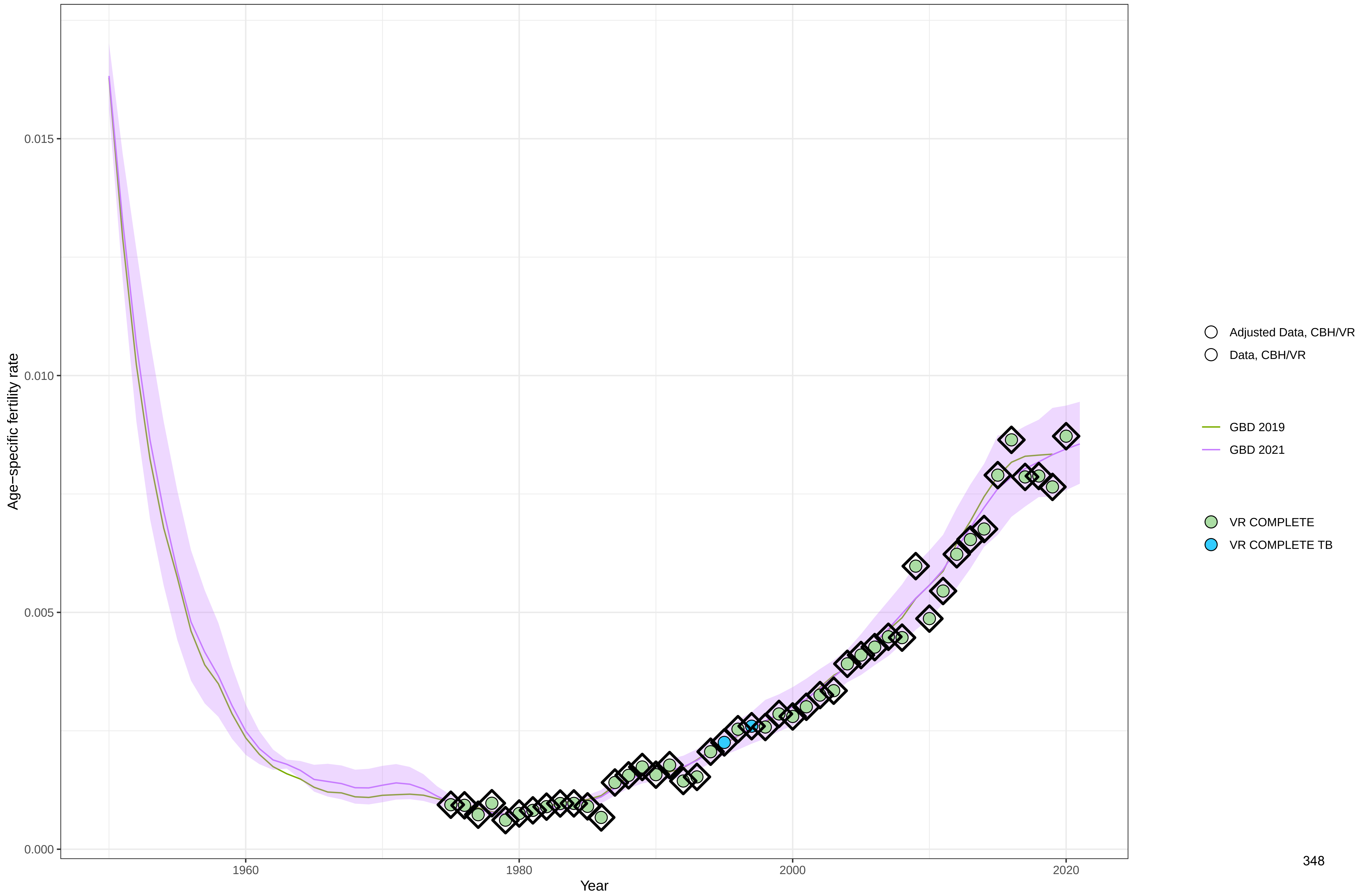

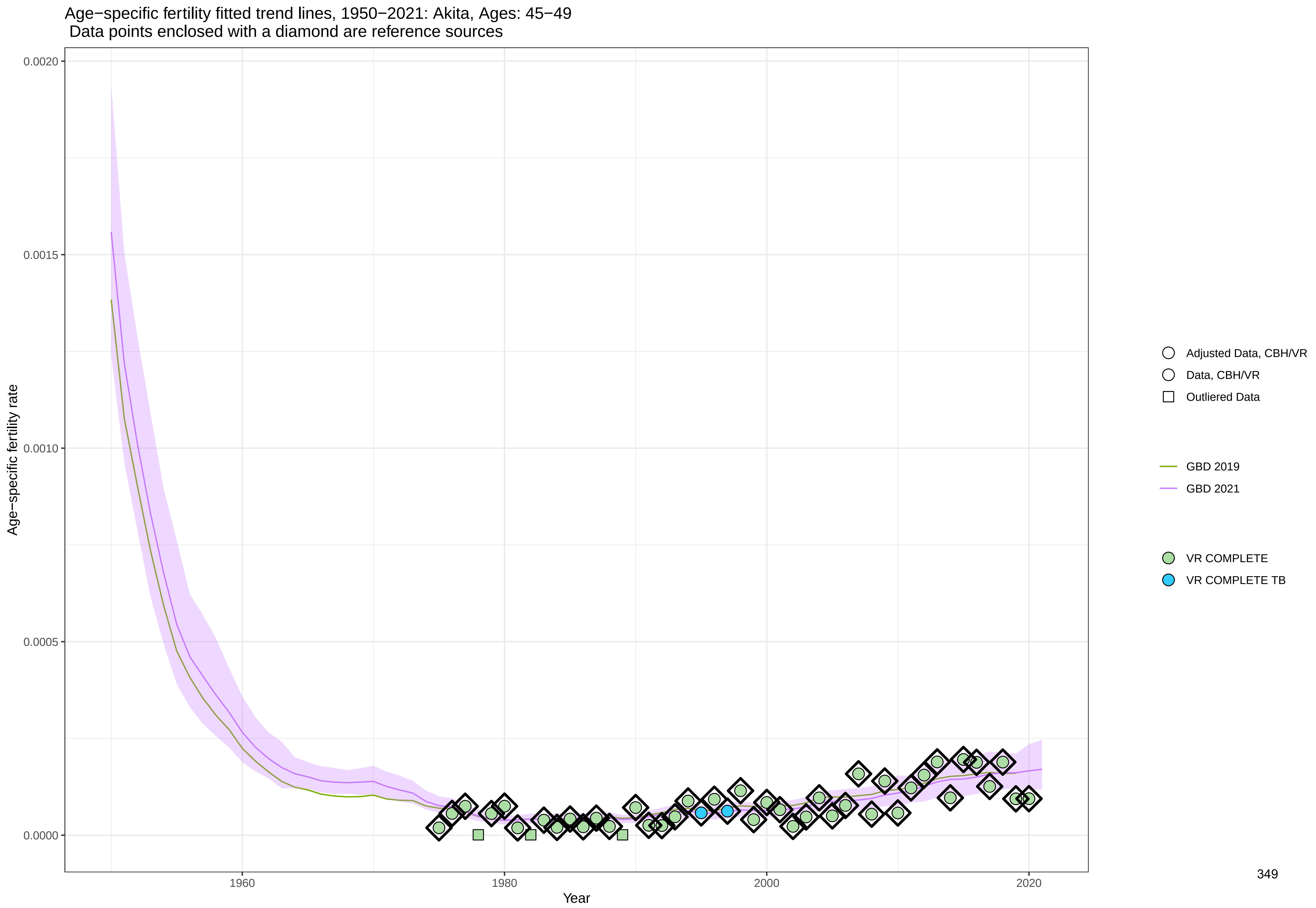

Age−specific fertility fitted trend lines, 1950−2021: Akita, Ages: 45−49
Data points enclosed with a diamond are reference sources
0.0020
0.0015
0.0010
0.0005
0.0000
Age−specific fertility rate
1960
1980
2000
2020
Year
Adjusted Data, CBH/VR
Data, CBH/VR
Outliered Data
GBD 2019
GBD 2021
VR COMPLETE
VR COMPLETE TB

Age−specific fertility fitted trend lines, 1950−2021: Akita, Ages: 50−54
Data points enclosed with a diamond are reference sources

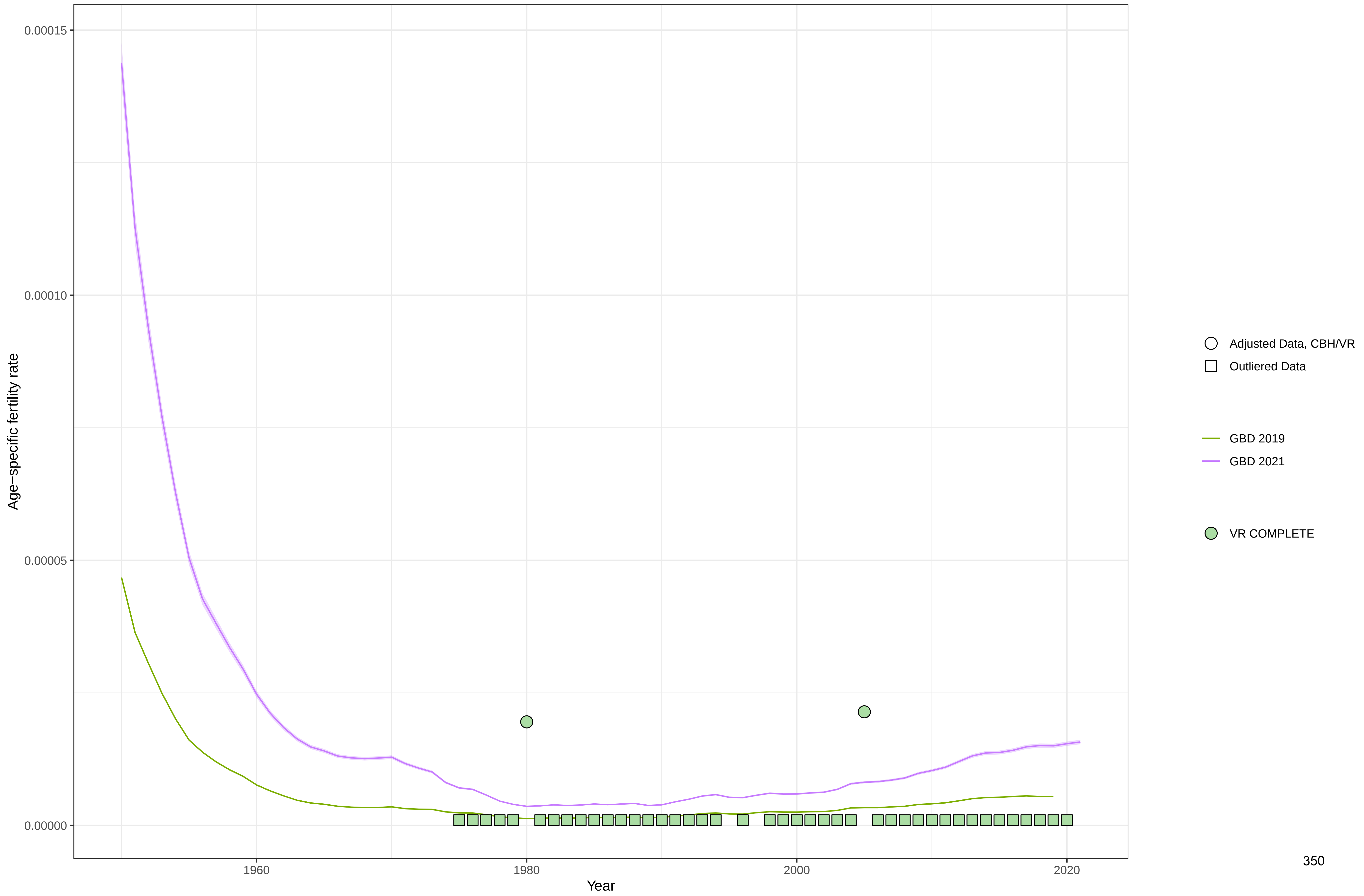

Total fertility rate fitted trend lines, 1950−2021: Akita TFR
Data points enclosed with a diamond are reference sources

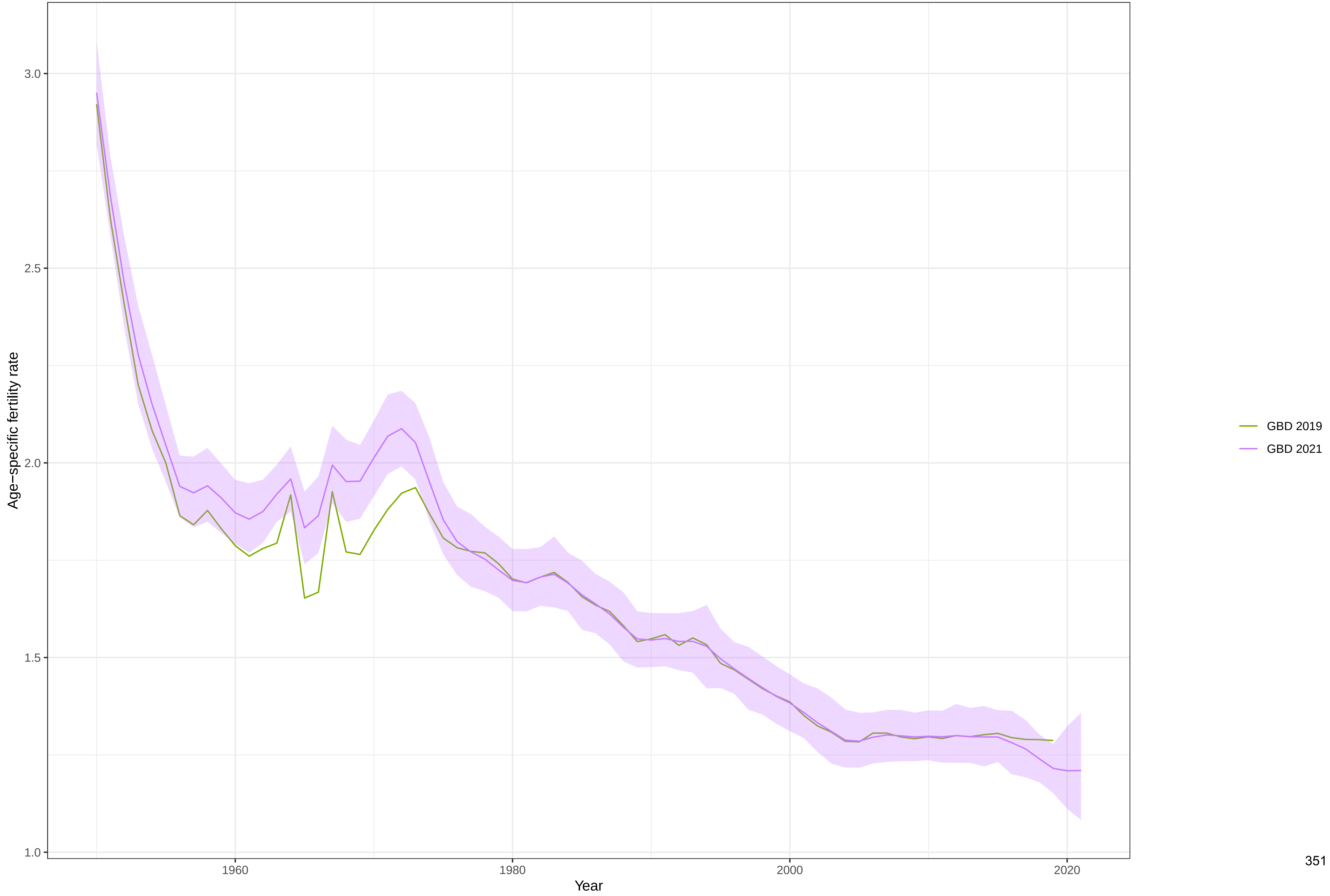

Age−specific fertility fitted trend lines, 1950−2021: Aomori, Ages: 10−14
Data points enclosed with a diamond are reference sources

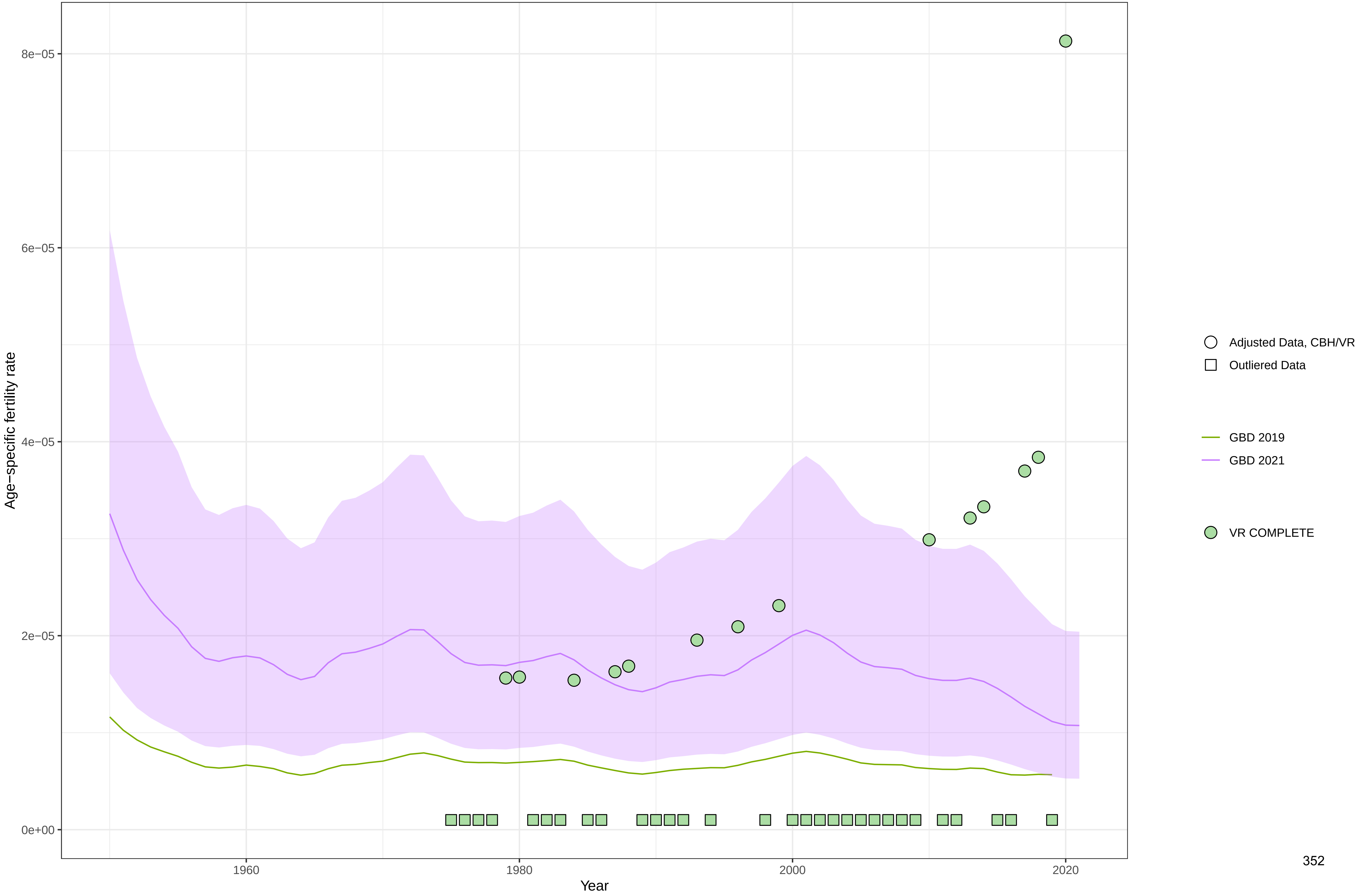

Age−specific fertility fitted trend lines, 1950−2021: Aomori, Ages: 15−19
Data points enclosed with a diamond are reference sources

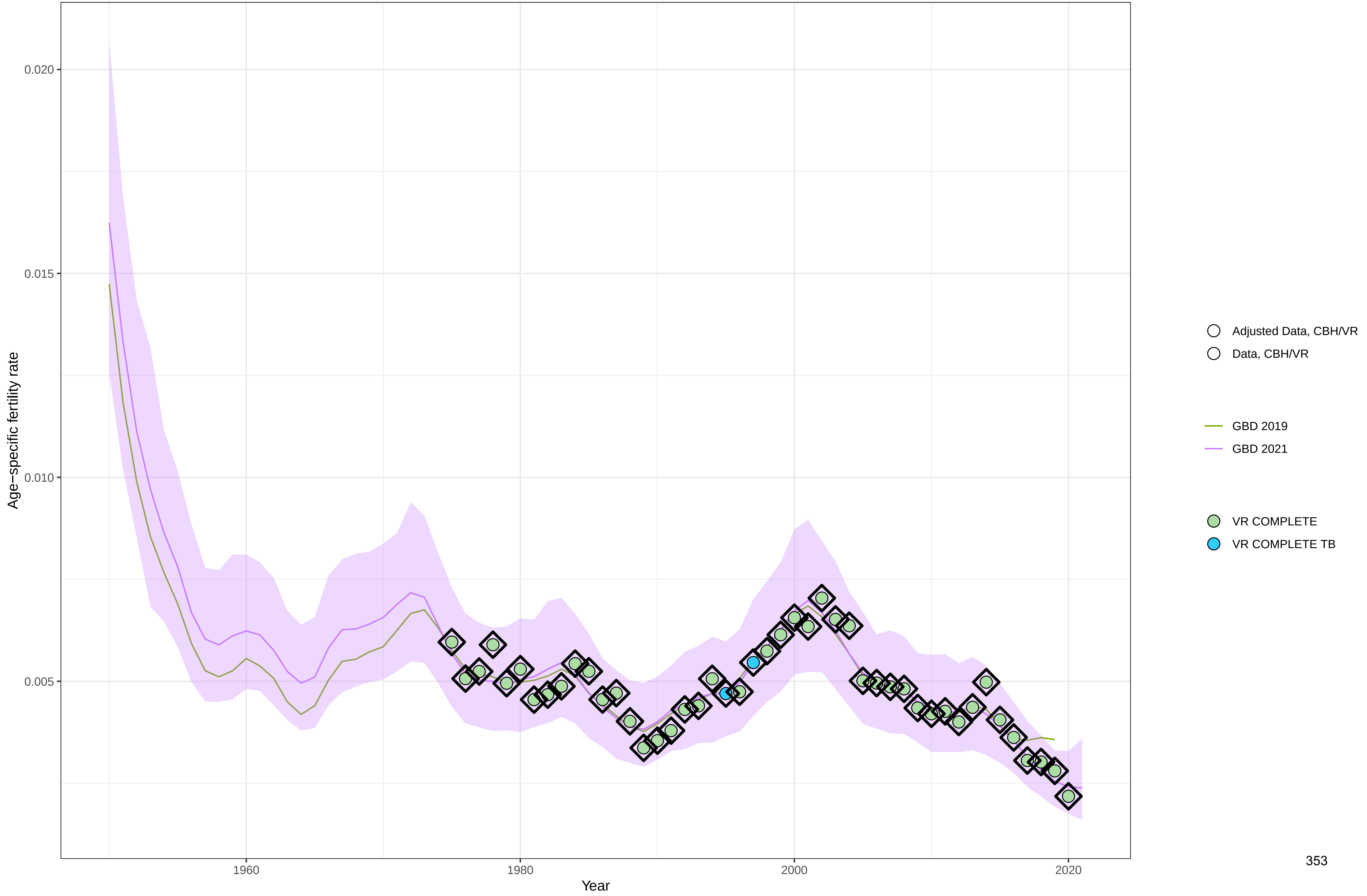

Age−specific fertility fitted trend lines, 1950−2021: Aomori, Ages: 20−24
Data points enclosed with a diamond are reference sources

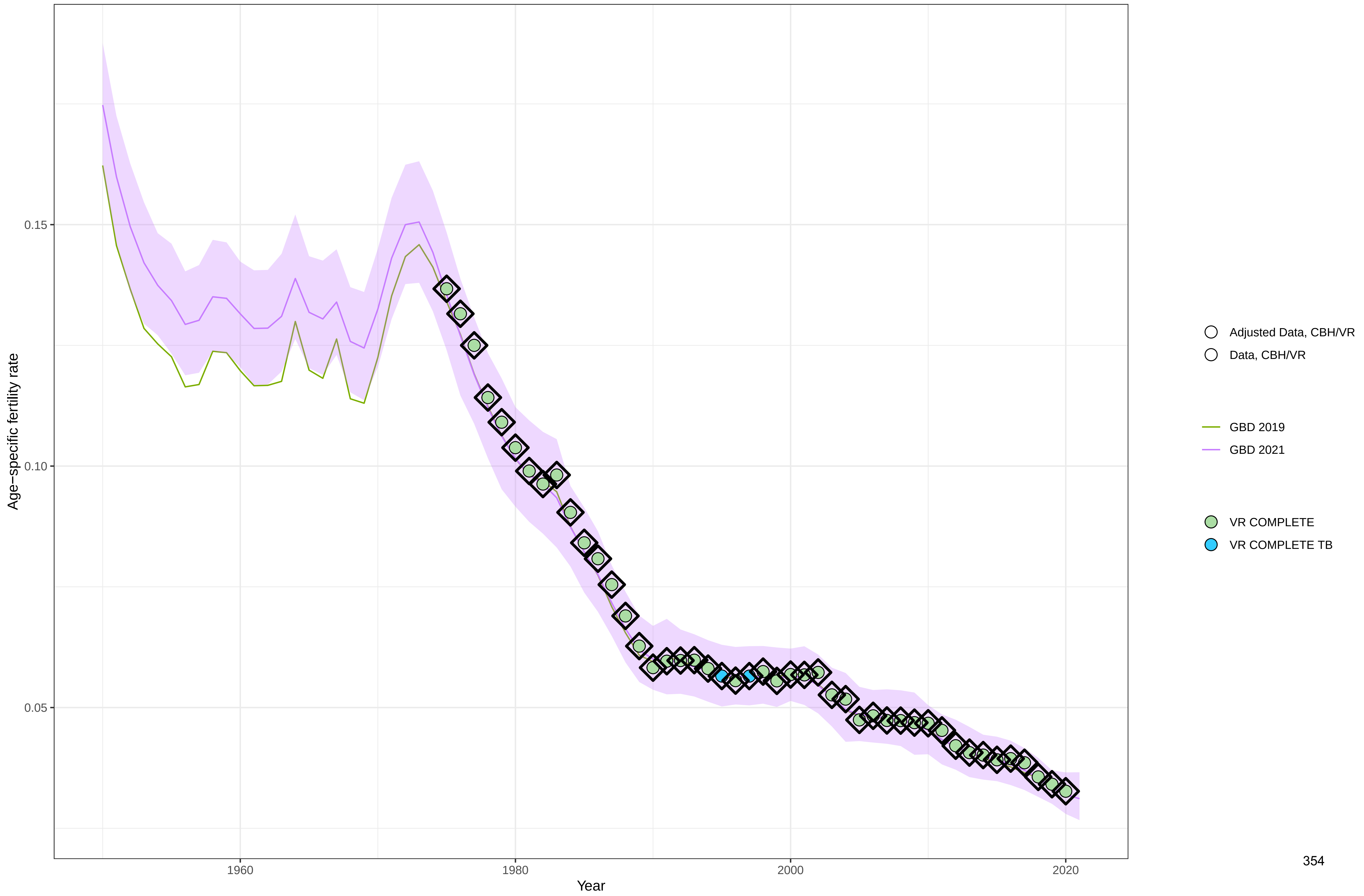

Age−specific fertility fitted trend lines, 1950−2021: Aomori, Ages: 25−29
Data points enclosed with a diamond are reference sources

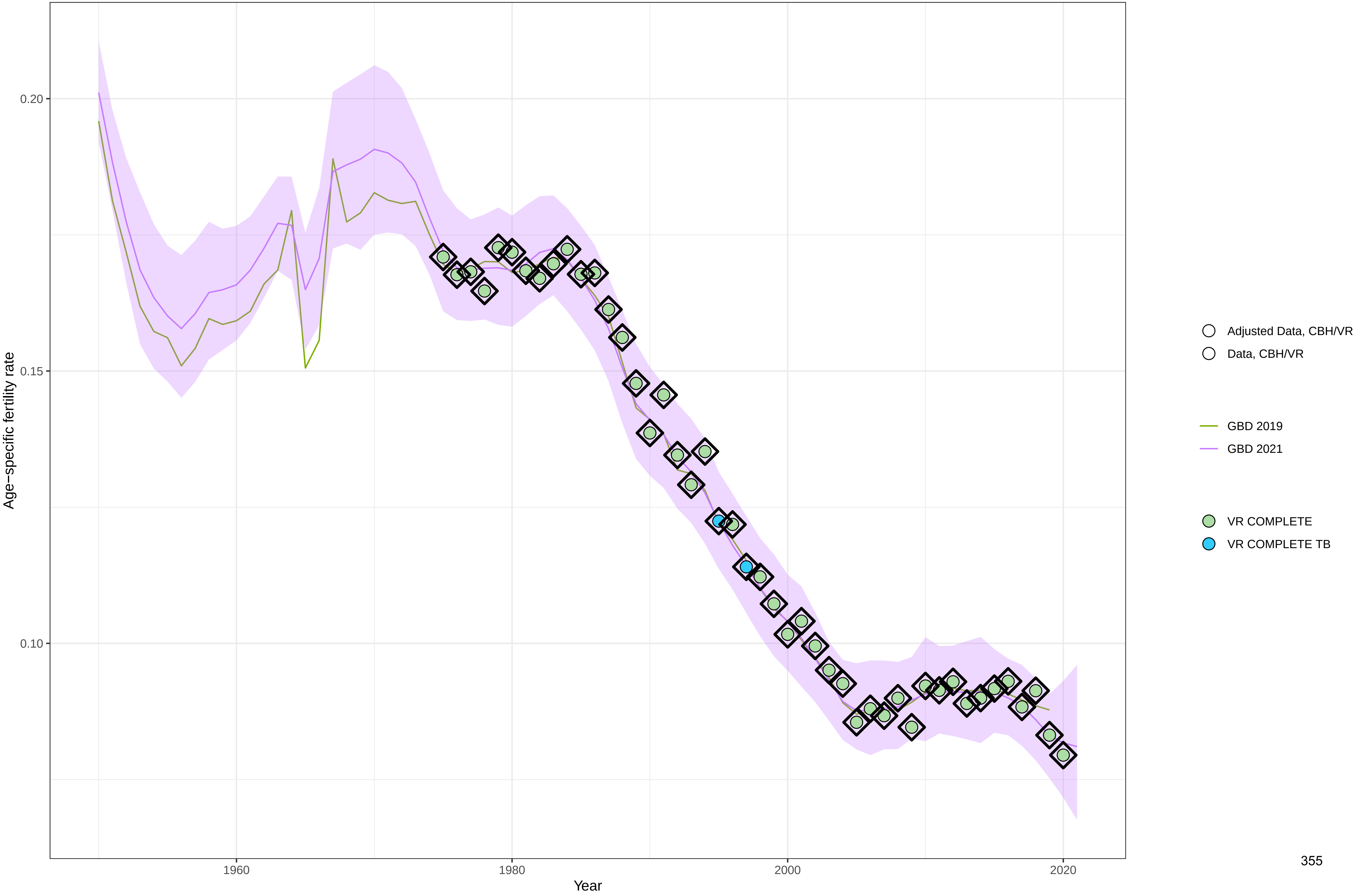

Age−specific fertility fitted trend lines, 1950−2021: Aomori, Ages: 30−34
Data points enclosed with a diamond are reference sources

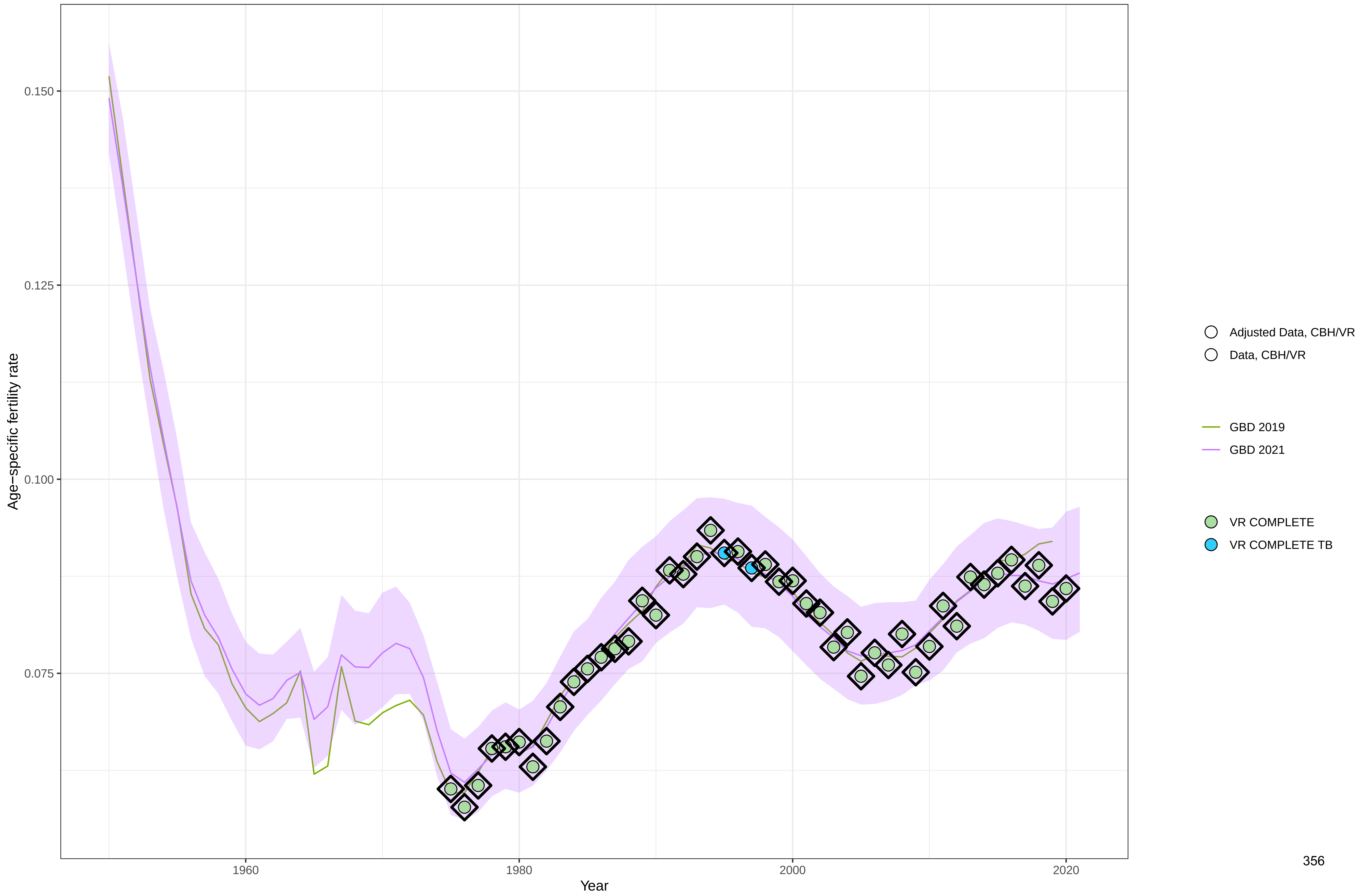

Age−specific fertility fitted trend lines, 1950−2021: Aomori, Ages: 35−39
Data points enclosed with a diamond are reference sources

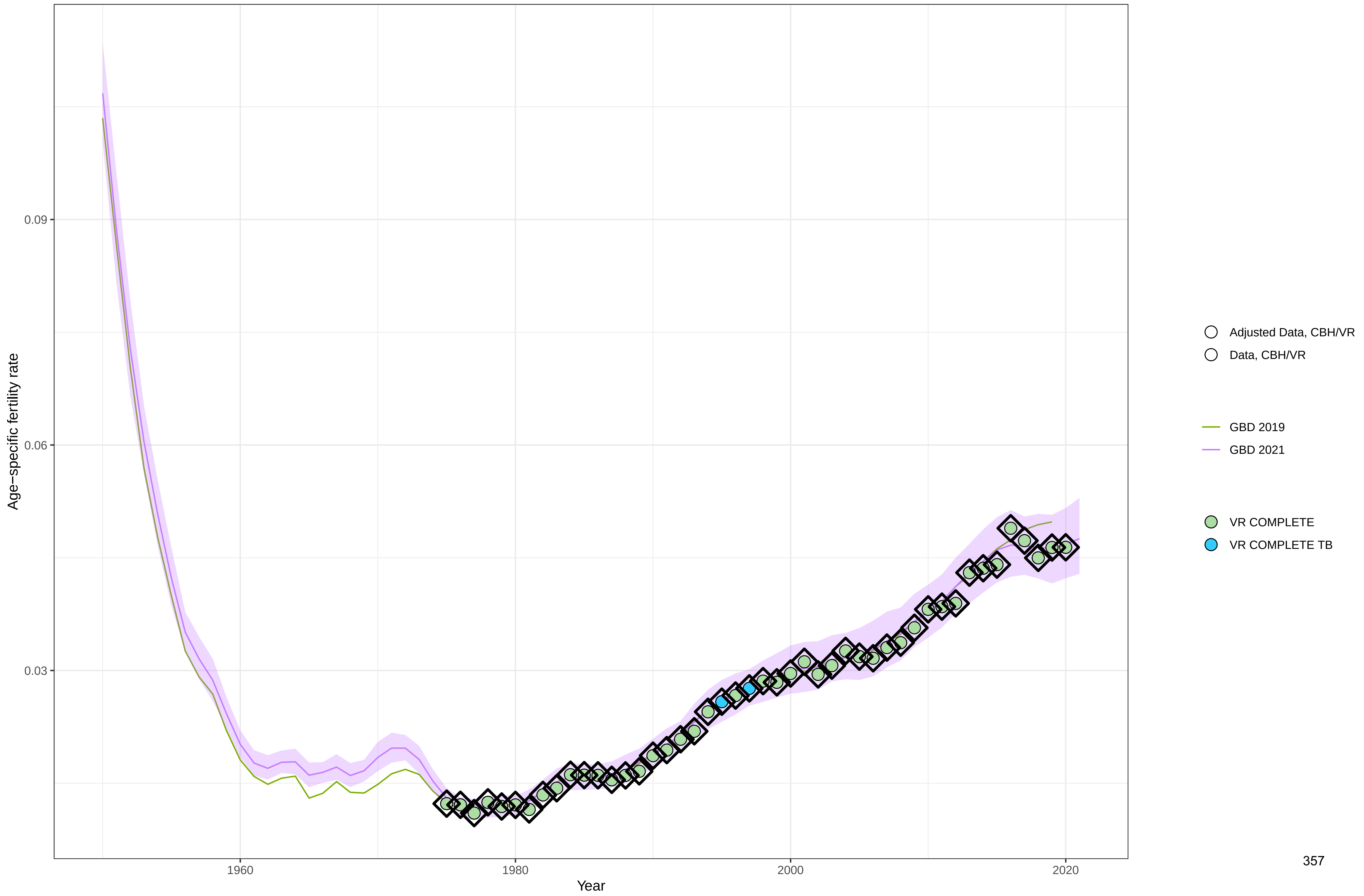

Age−specific fertility fitted trend lines, 1950−2021: Aomori, Ages: 40−44
Data points enclosed with a diamond are reference sources

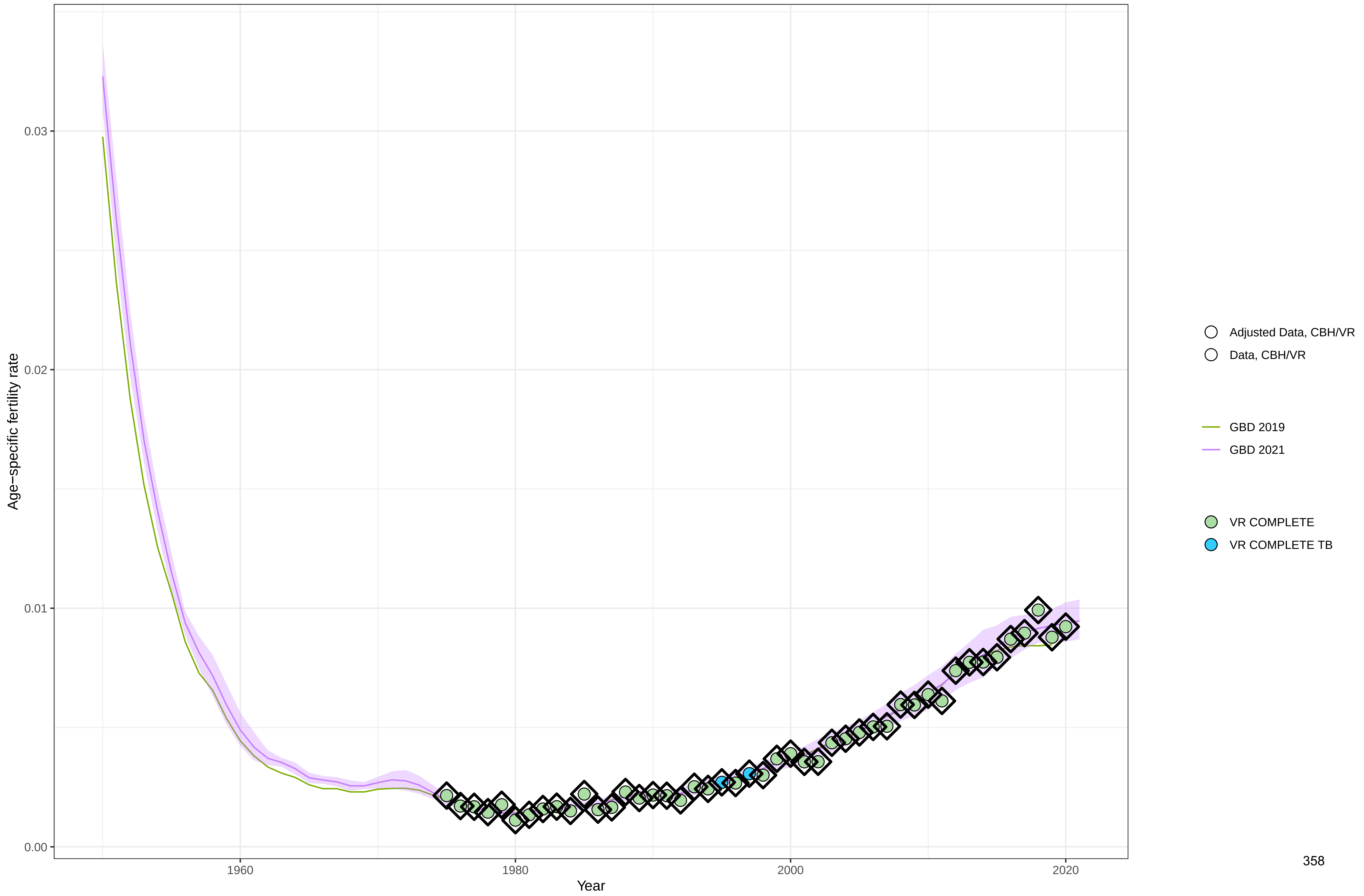

Age−specific fertility fitted trend lines, 1950−2021: Aomori, Ages: 45−49
Data points enclosed with a diamond are reference sources

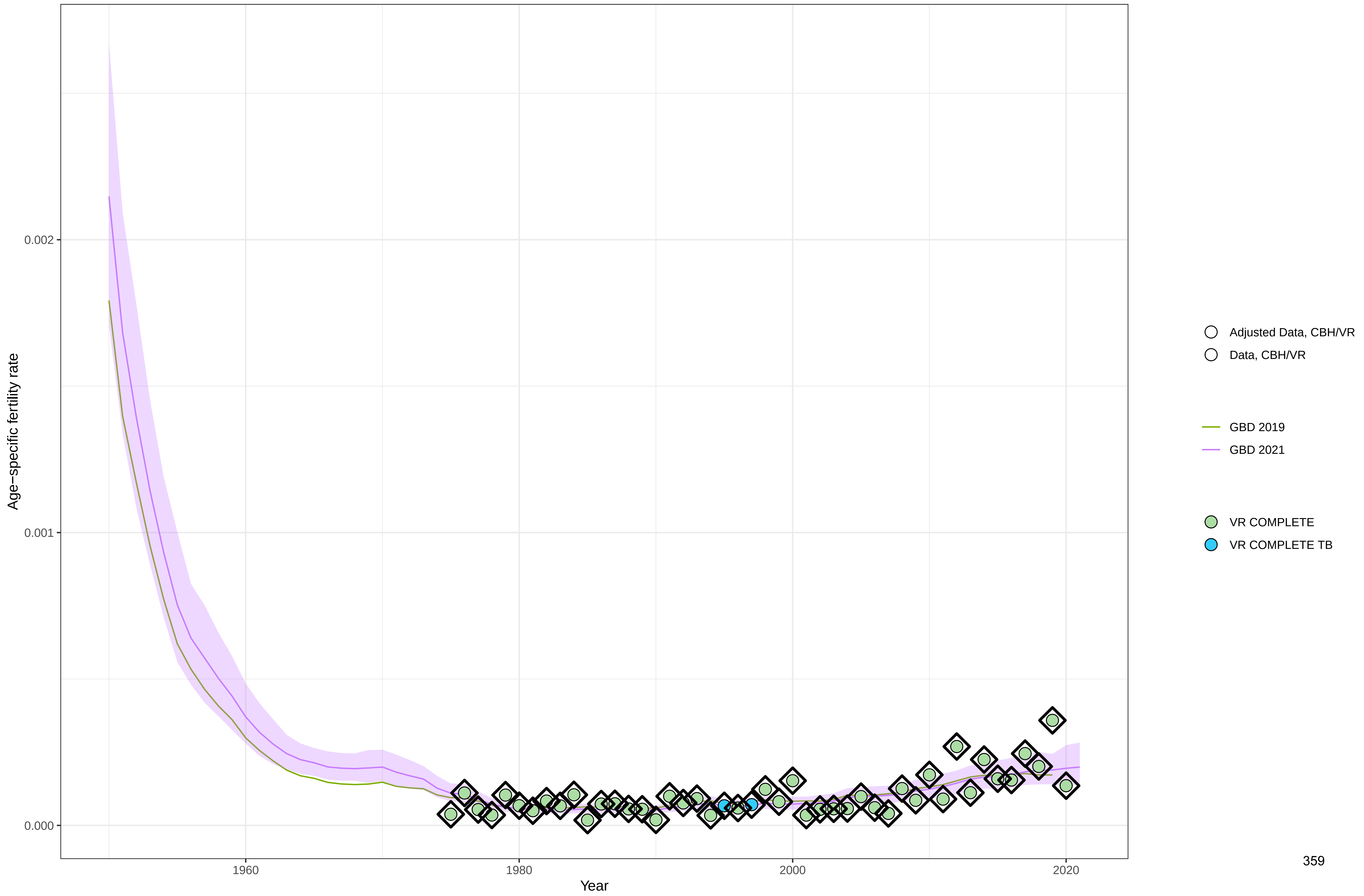

Age−specific fertility fitted trend lines, 1950−2021: Aomori, Ages: 50−54
Data points enclosed with a diamond are reference sources

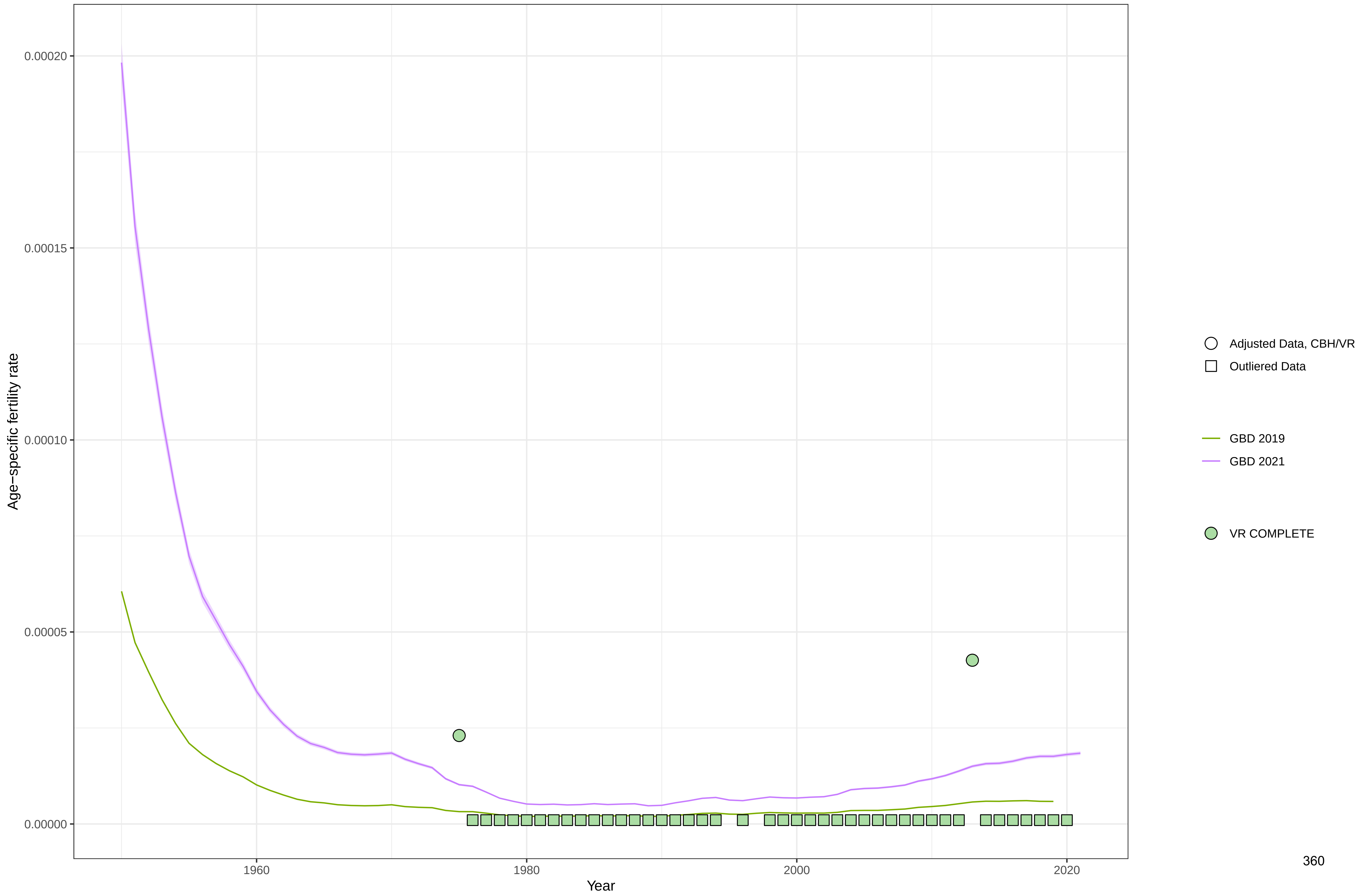

Total fertility rate fitted trend lines, 1950−2021: Aomori TFR
Data points enclosed with a diamond are reference sources

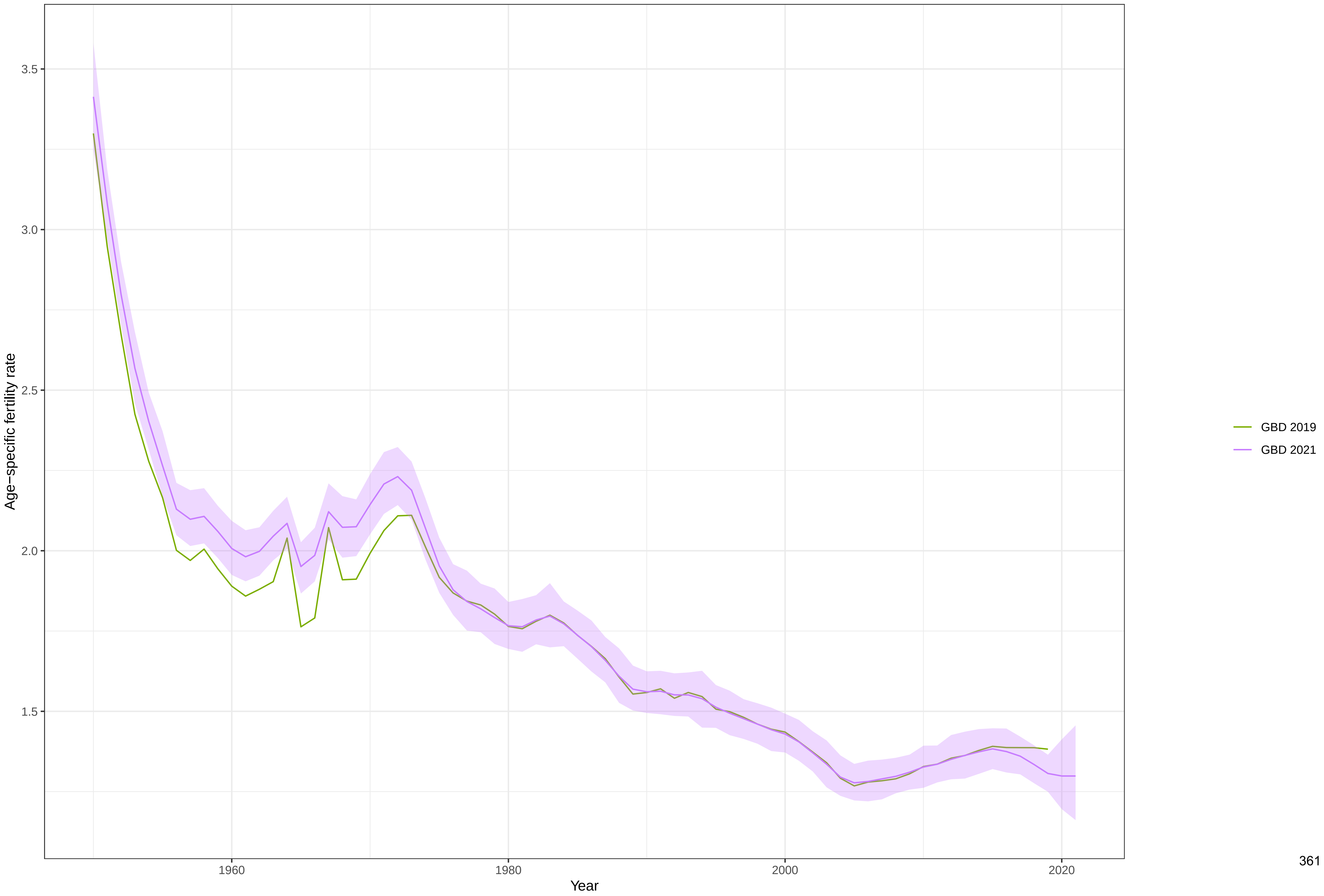

Age−specific fertility fitted trend lines, 1950−2021: Chiba, Ages: 10−14
Data points enclosed with a diamond are reference sources

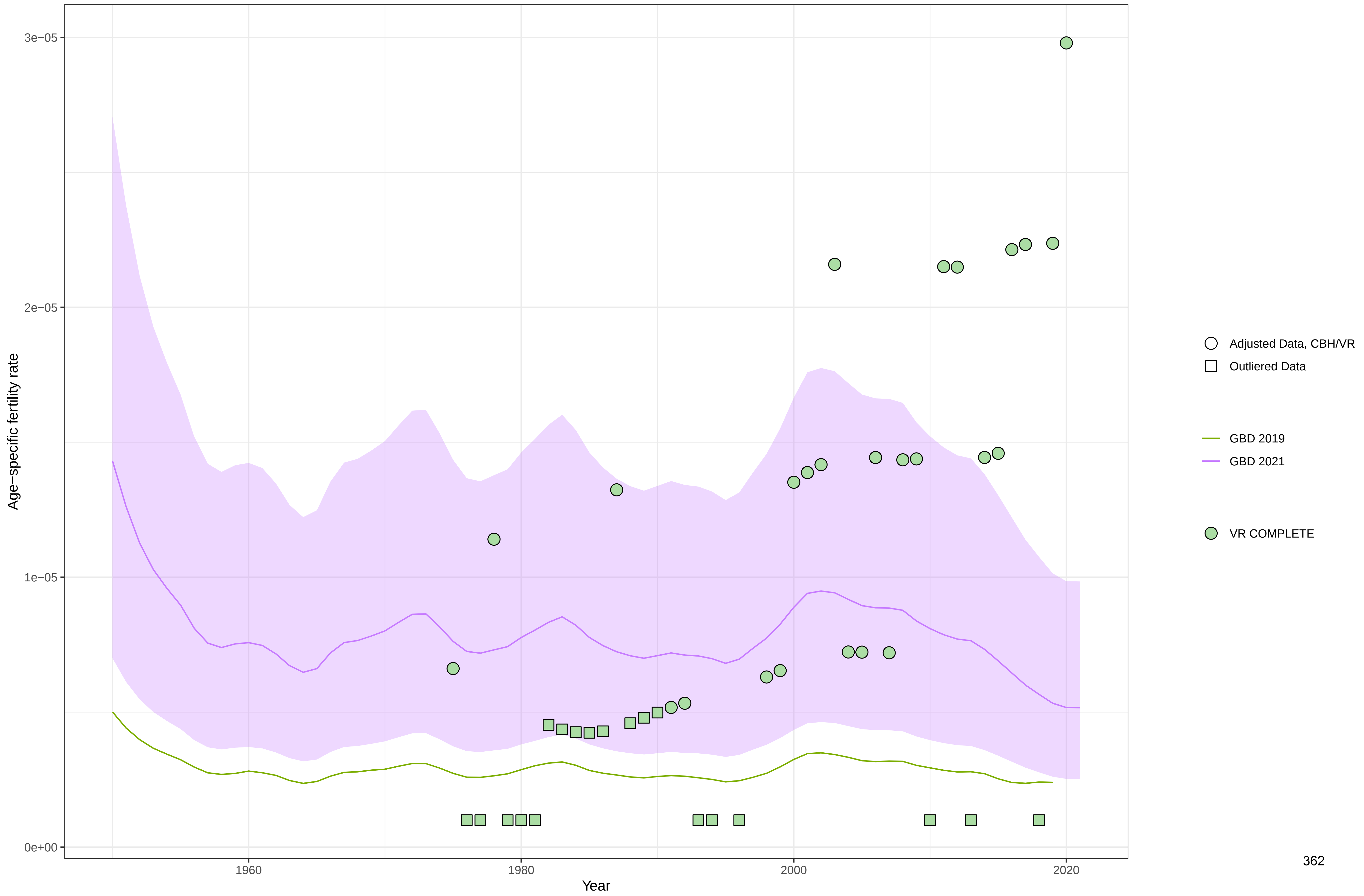

Age−specific fertility fitted trend lines, 1950−2021: Chiba, Ages: 15−19
Data points enclosed with a diamond are reference sources

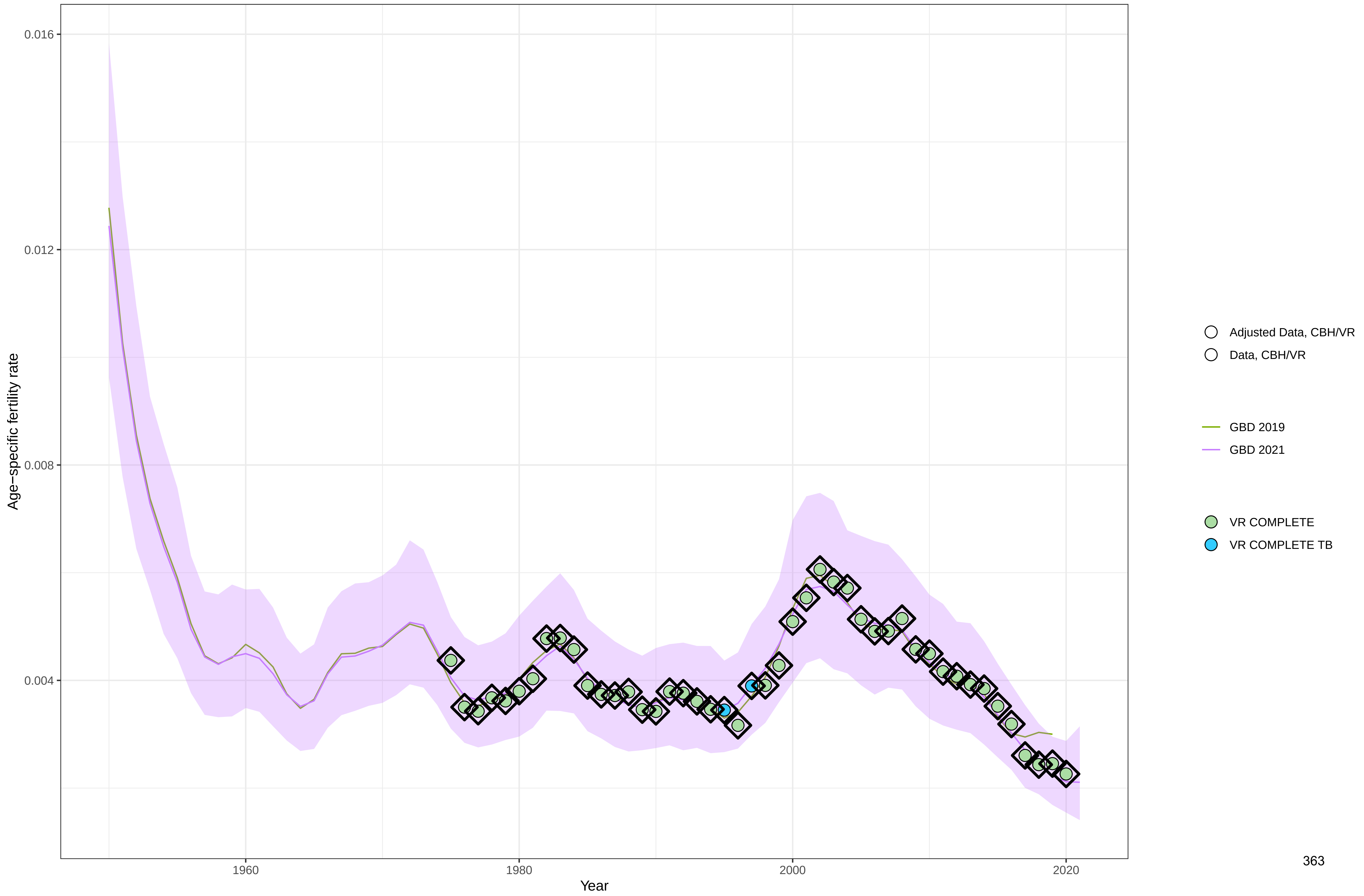

Age−specific fertility fitted trend lines, 1950−2021: Chiba, Ages: 20−24
Data points enclosed with a diamond are reference sources

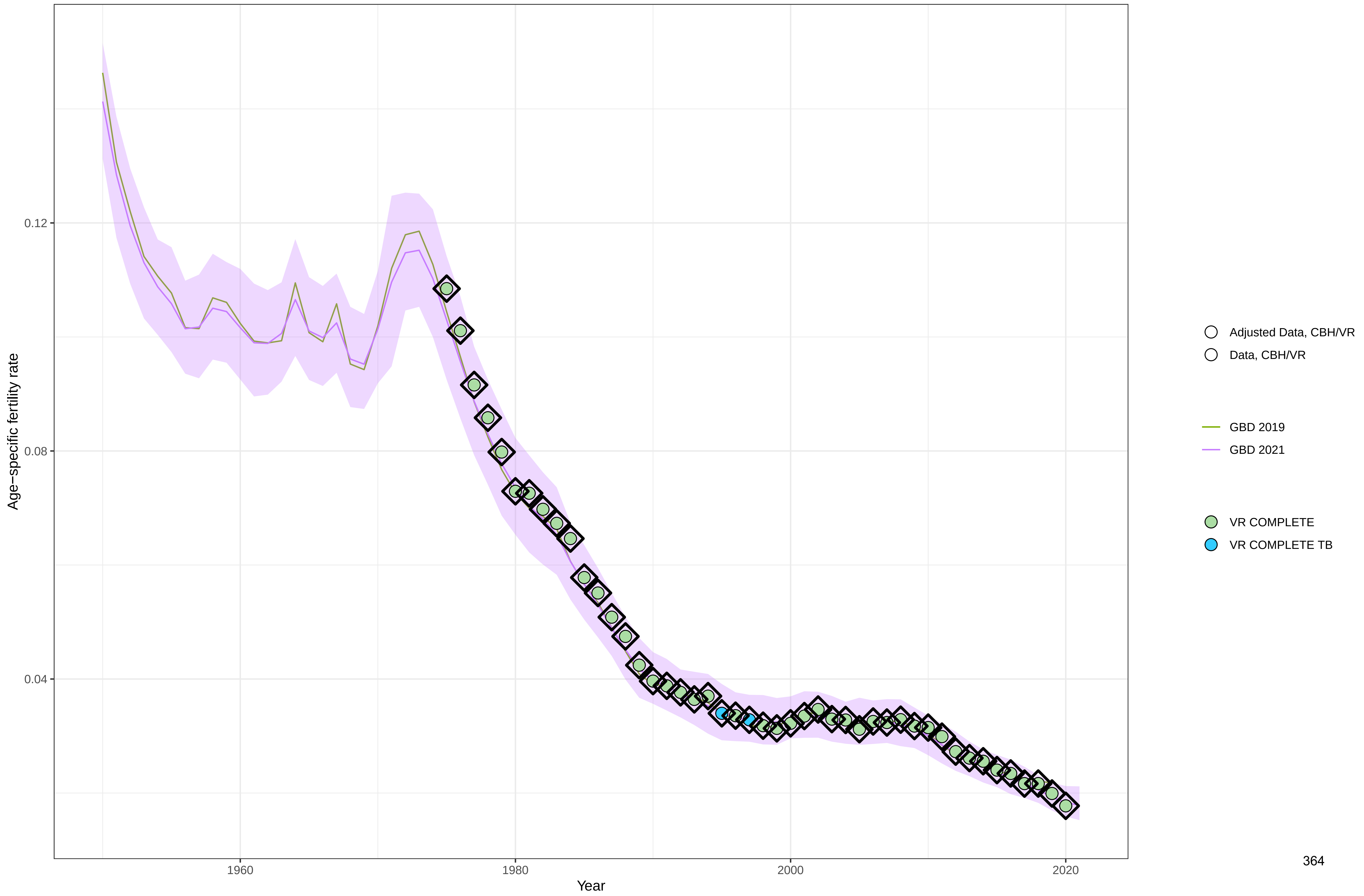

Age−specific fertility fitted trend lines, 1950−2021: Chiba, Ages: 25−29
Data points enclosed with a diamond are reference sources

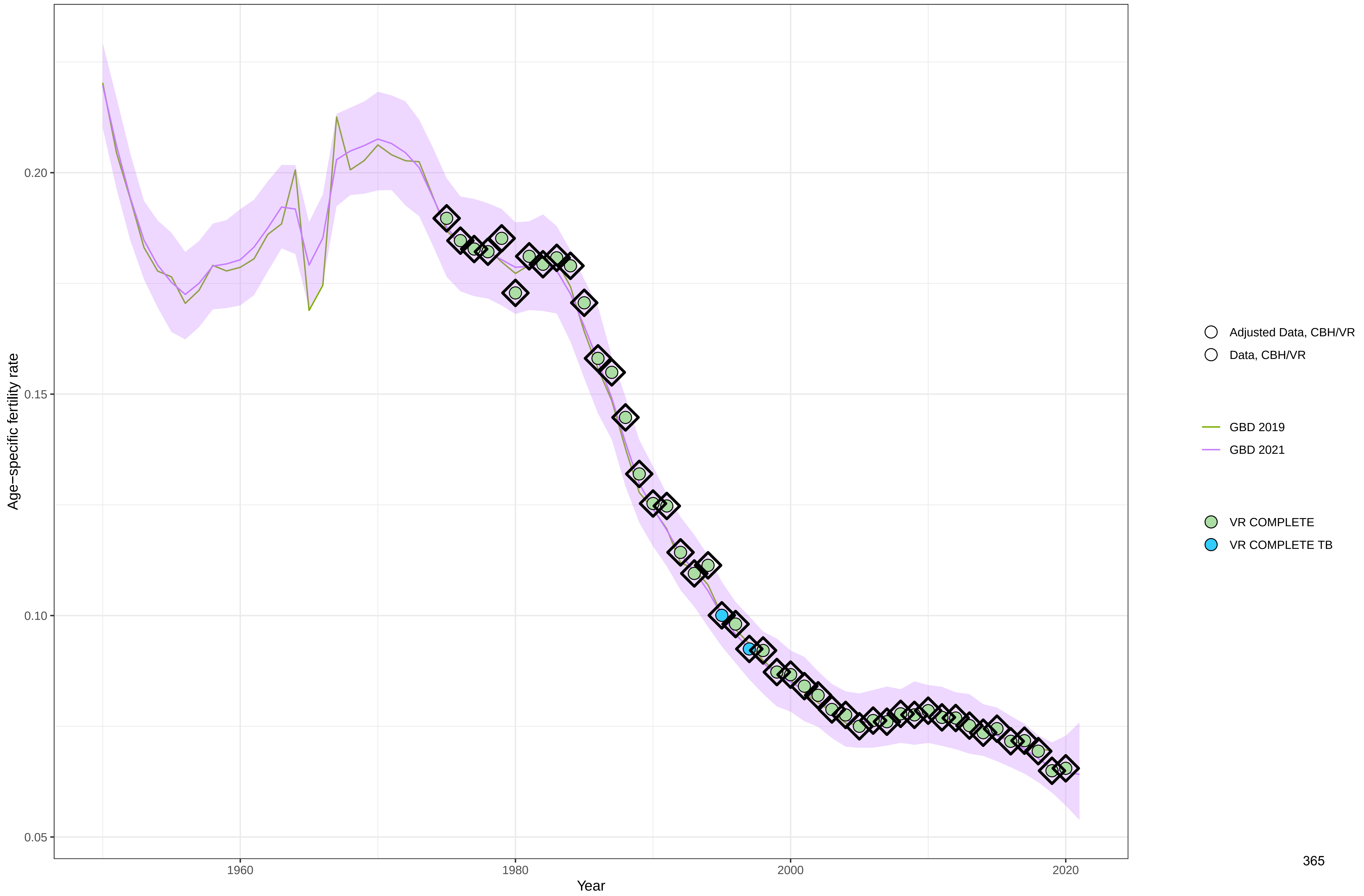

Age−specific fertility fitted trend lines, 1950−2021: Chiba, Ages: 30−34
Data points enclosed with a diamond are reference sources

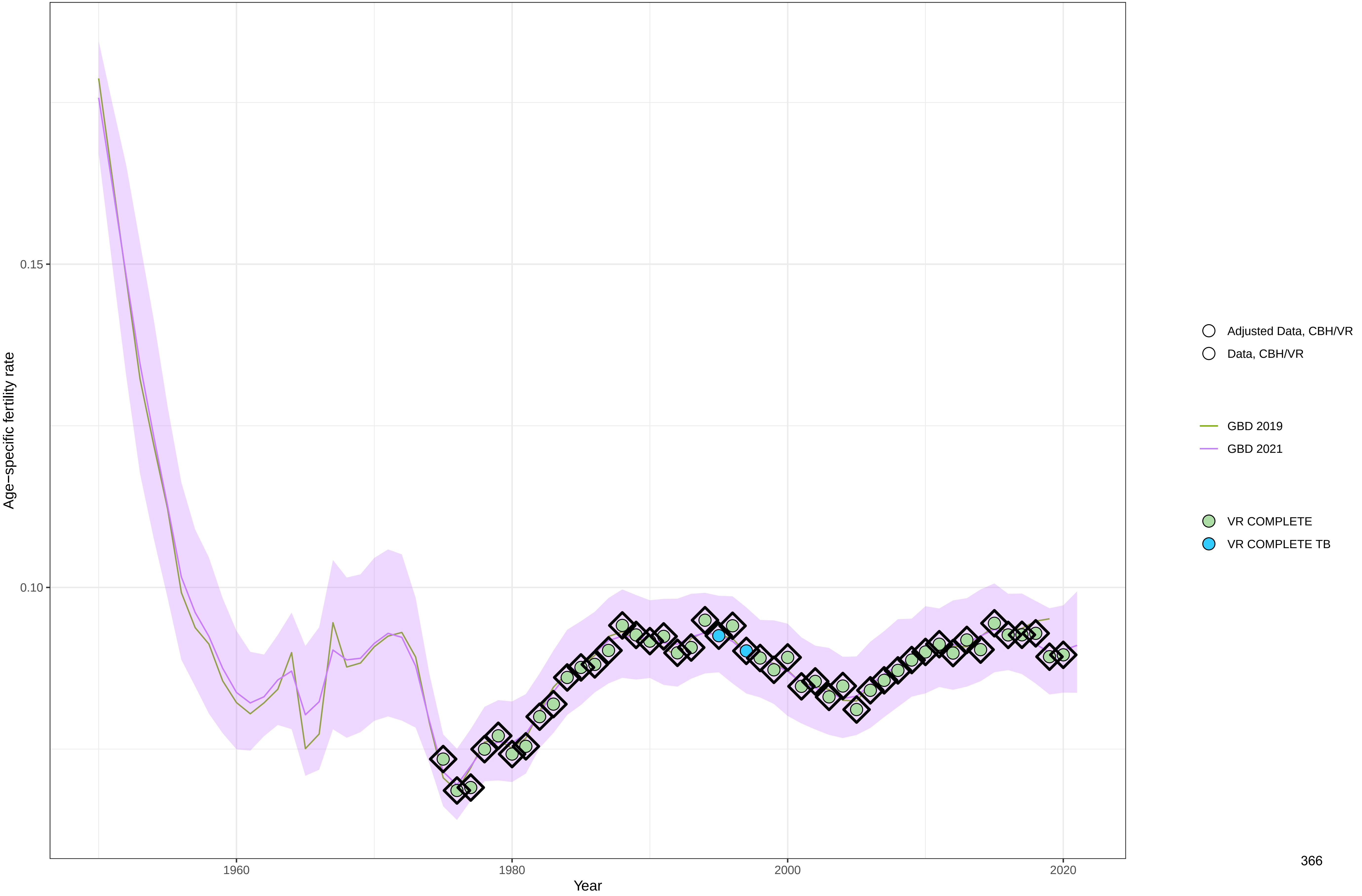

Age−specific fertility fitted trend lines, 1950−2021: Chiba, Ages: 35−39
Data points enclosed with a diamond are reference sources

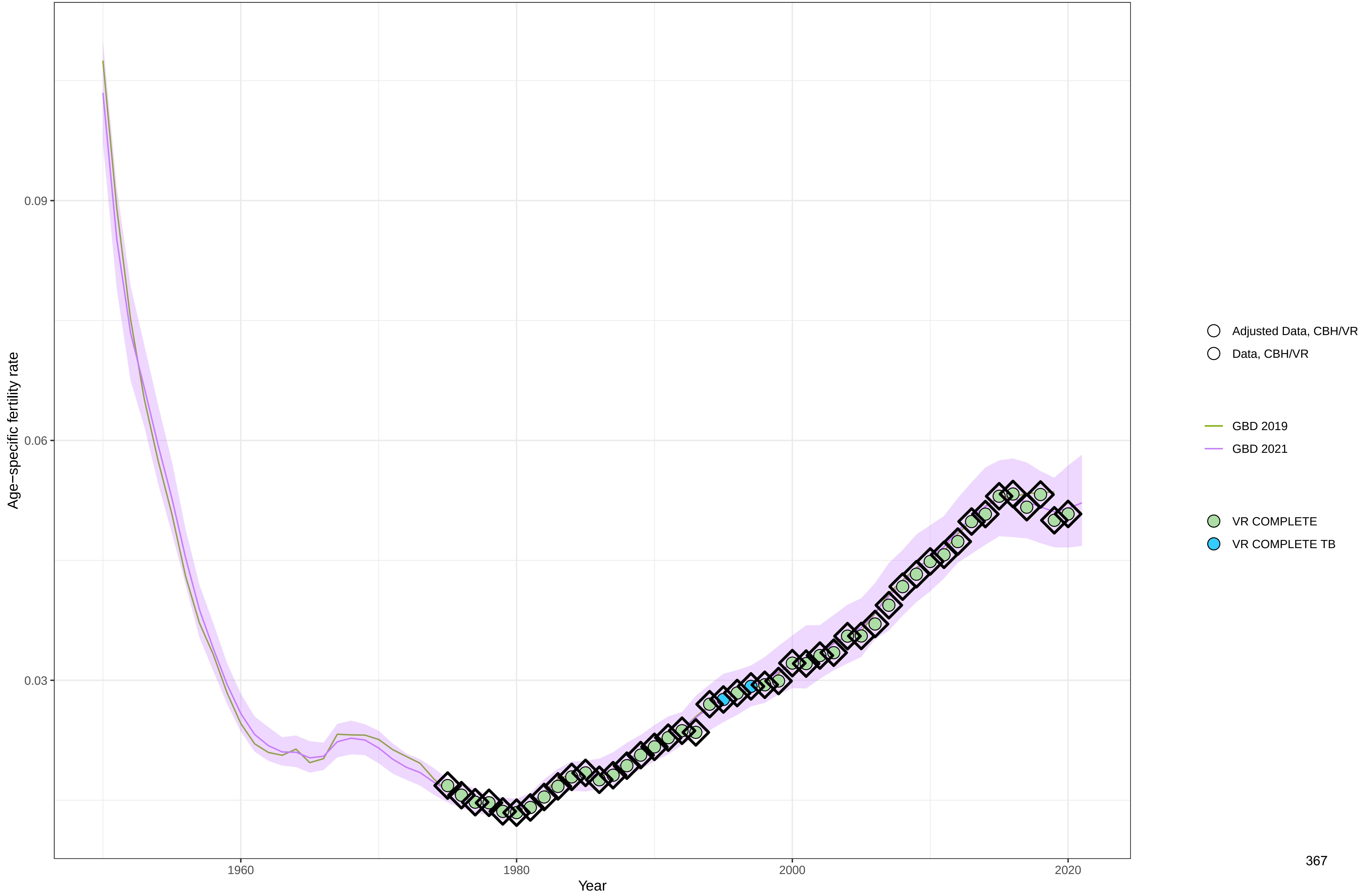

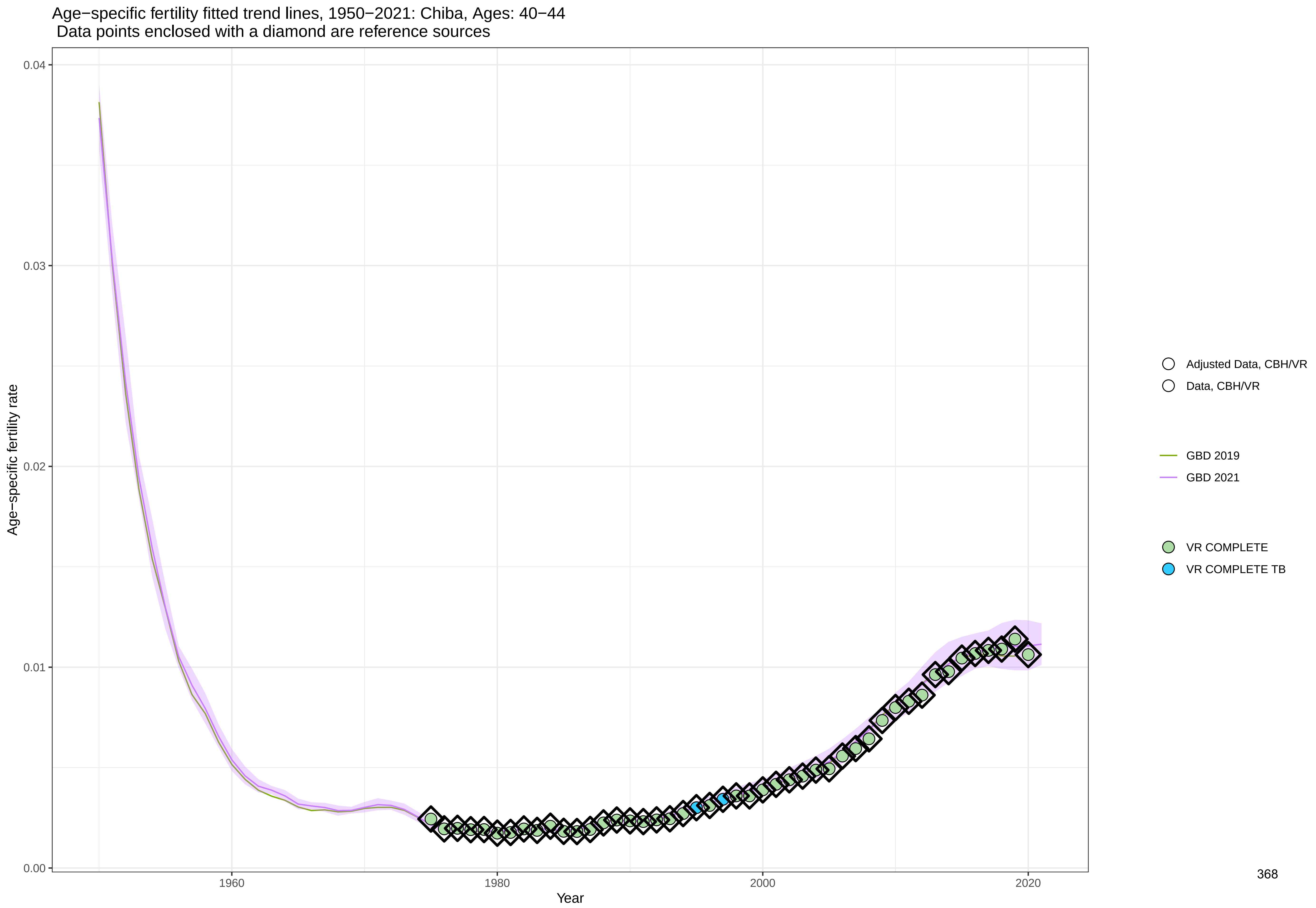

Age−specific fertility fitted trend lines, 1950−2021: Chiba, Ages: 40−44
Data points enclosed with a diamond are reference sources
0.04
0.03
0.02
0.01
0.00
Age−specific fertility rate
1960
1980
2000
2020
Year
Adjusted Data, CBH/VR
Data, CBH/VR
GBD 2019
GBD 2021
VR COMPLETE
VR COMPLETE TB

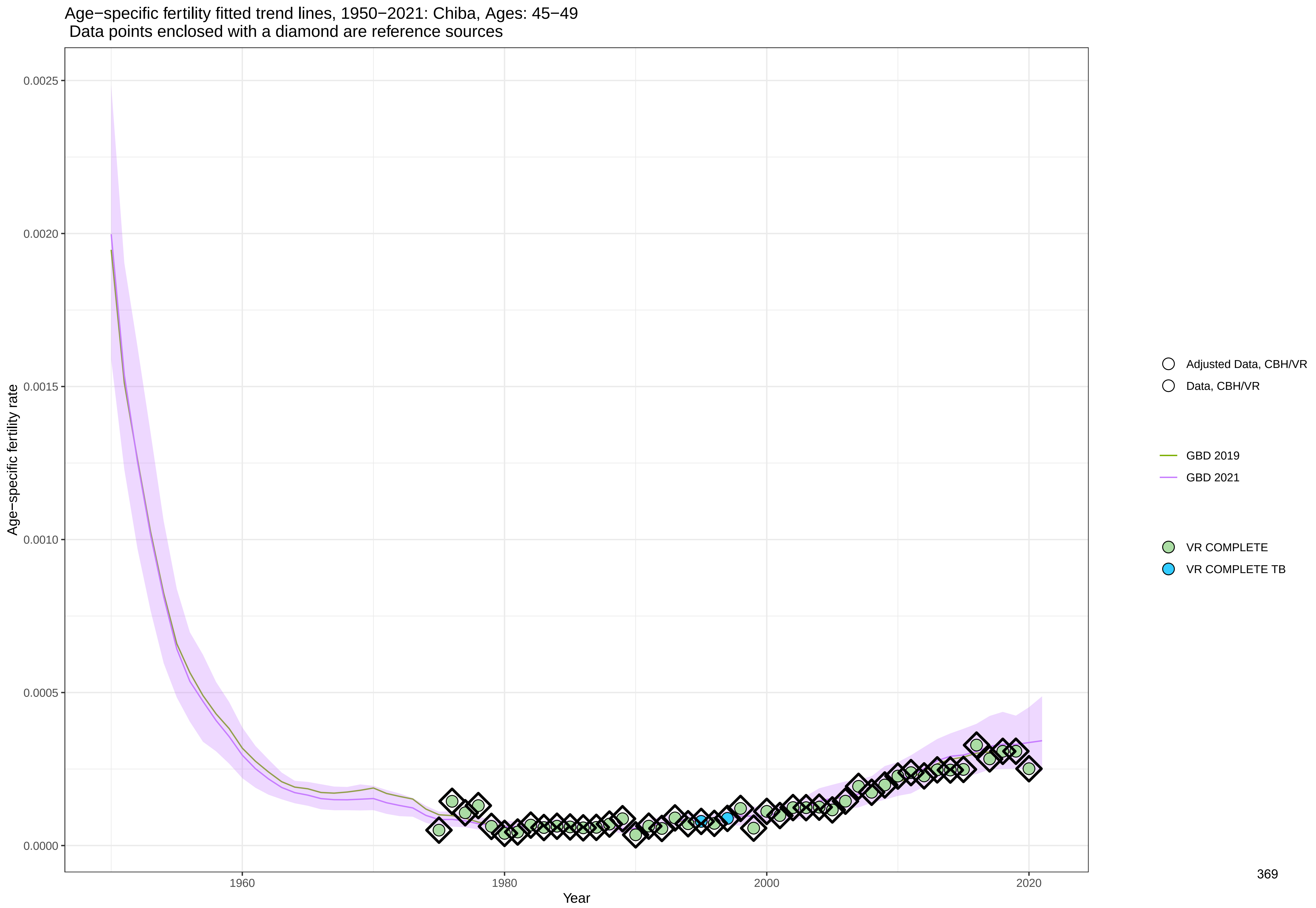

Age−specific fertility fitted trend lines, 1950−2021: Chiba, Ages: 45−49
Data points enclosed with a diamond are reference sources
Age−specific fertility rate
0.0025
0.0020
0.0015
0.0010
0.0005
0.0000
1960
1980
2000
2020
Year
Adjusted Data, CBH/VR
Data, CBH/VR
GBD 2019
GBD 2021
VR COMPLETE
VR COMPLETE TB

Age−specific fertility fitted trend lines, 1950−2021: Chiba, Ages: 50−54
Data points enclosed with a diamond are reference sources

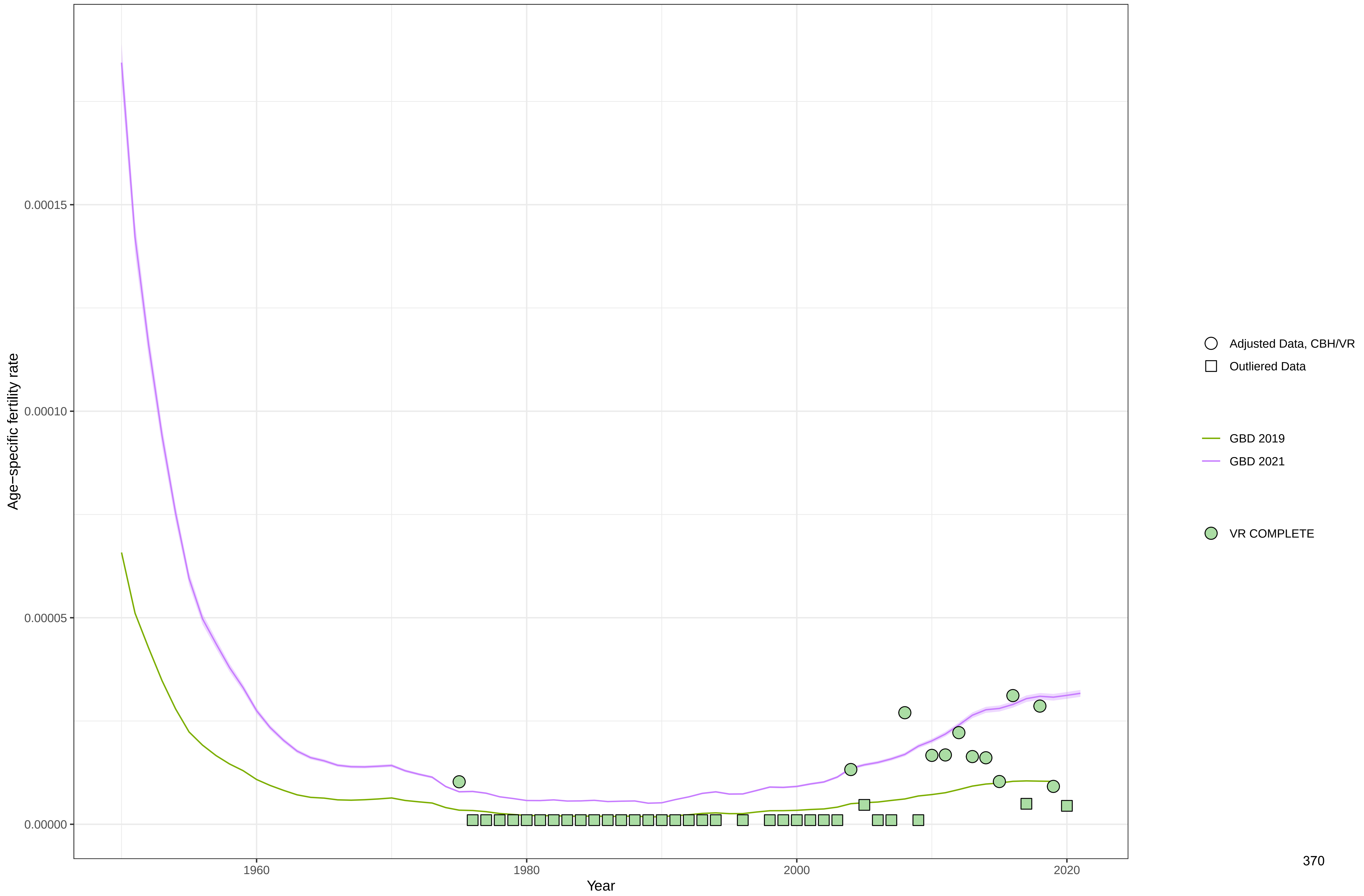

Total fertility rate fitted trend lines, 1950−2021: Chiba TFR
Data points enclosed with a diamond are reference sources

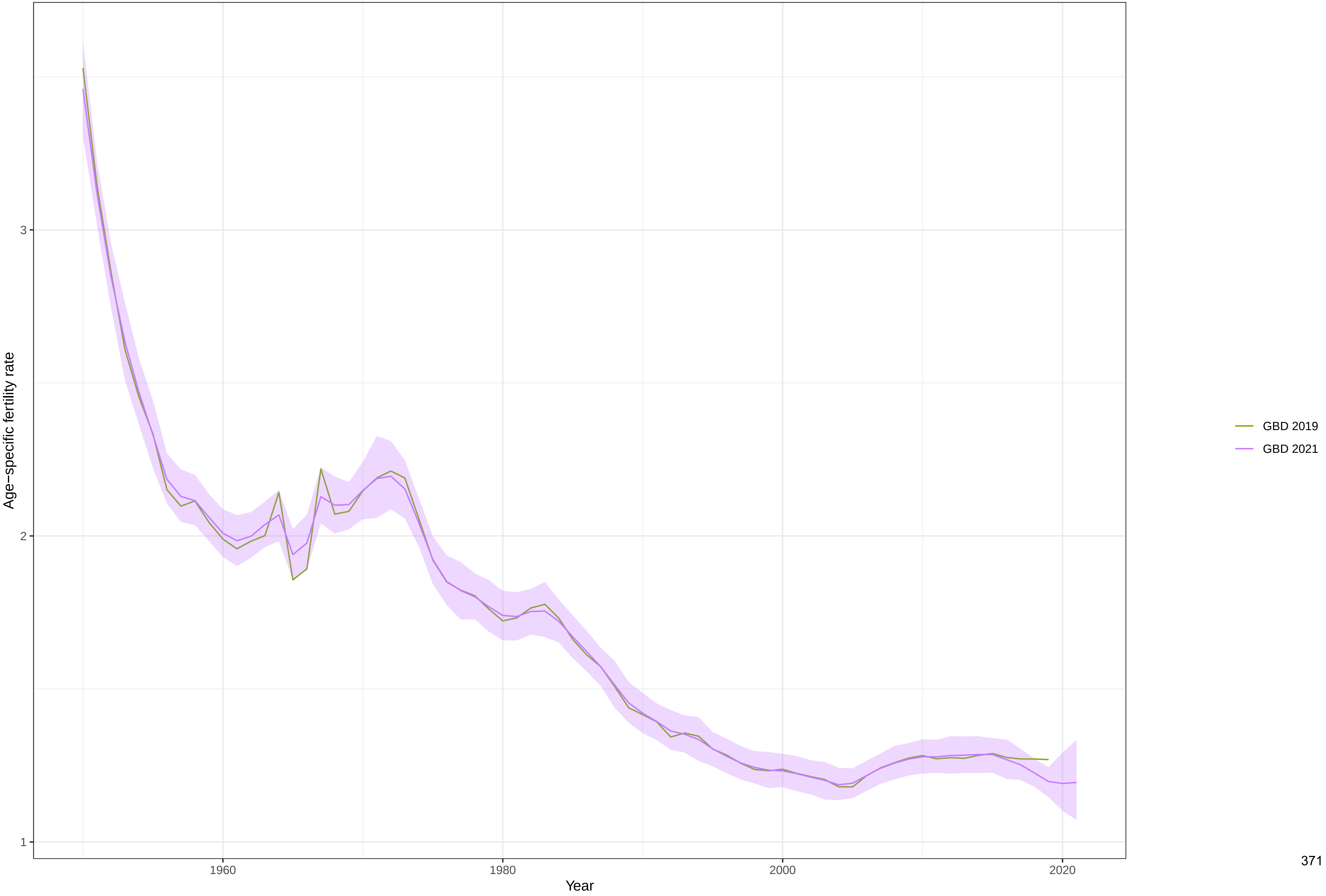

Age−specific fertility fitted trend lines, 1950−2021: Ehime, Ages: 10−14
 Data points enclosed with a diamond are reference sources

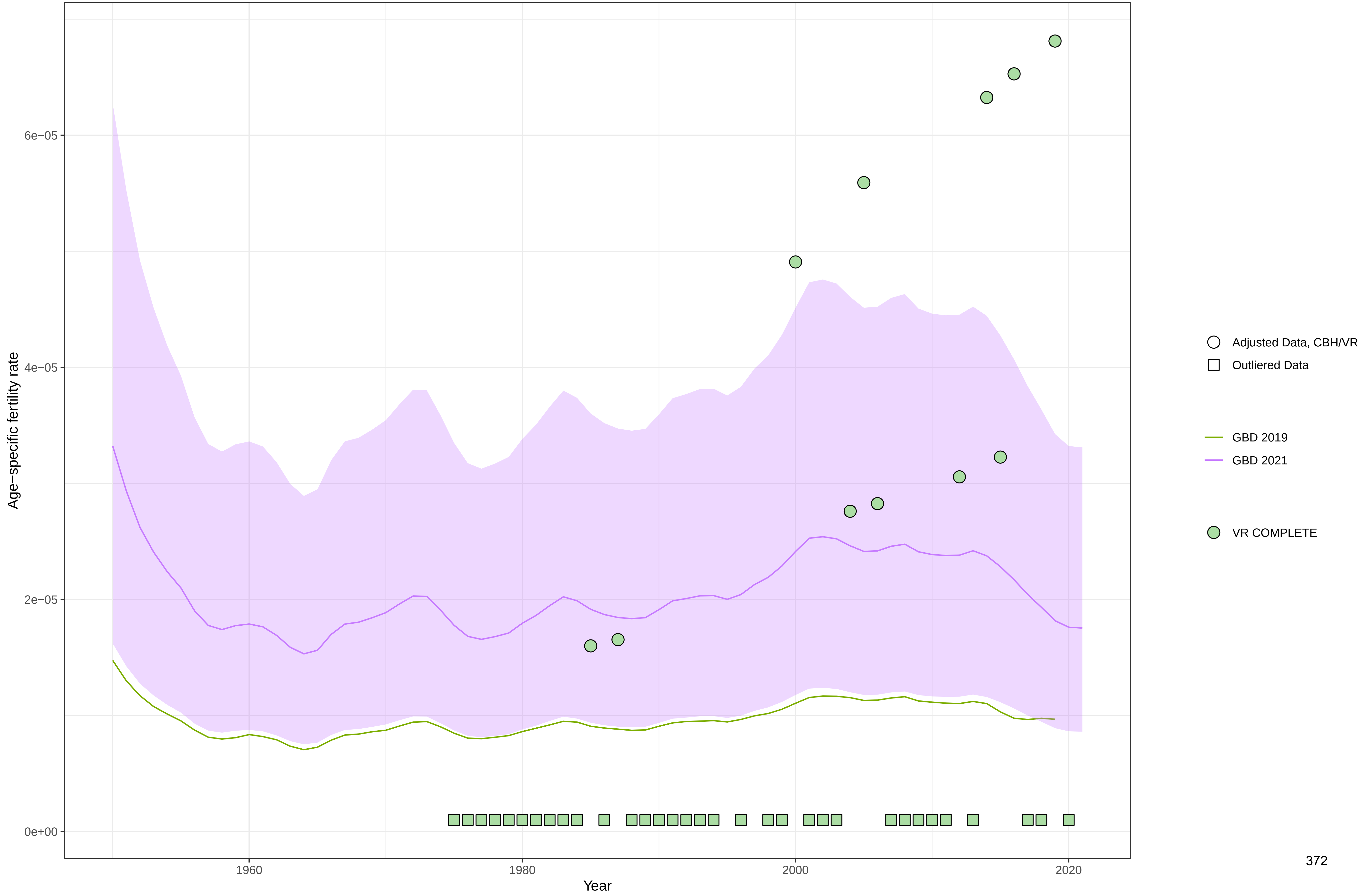

Age−specific fertility fitted trend lines, 1950−2021: Ehime, Ages: 15−19
Data points enclosed with a diamond are reference sources

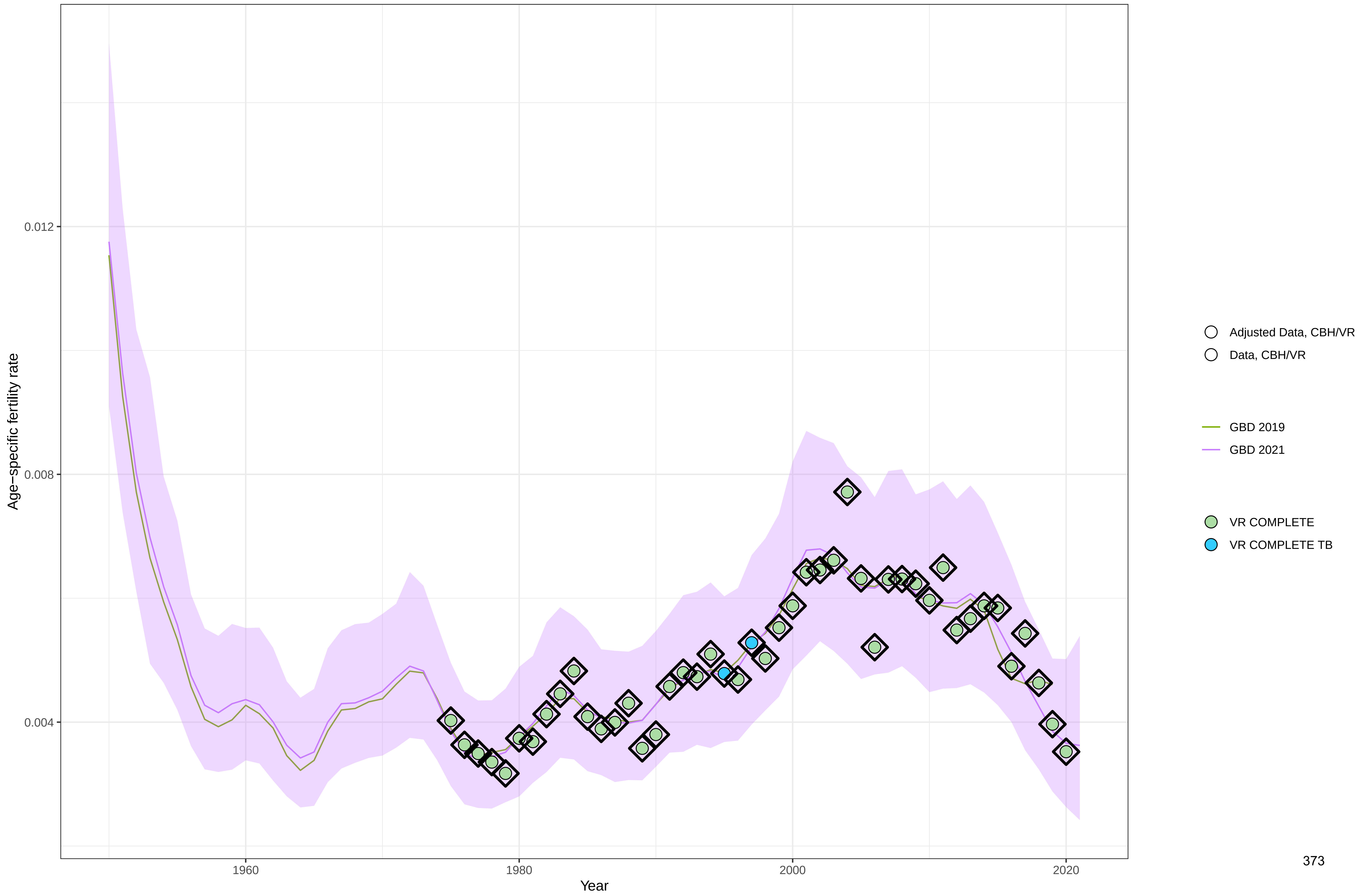

Age−specific fertility fitted trend lines, 1950−2021: Ehime, Ages: 20−24
Data points enclosed with a diamond are reference sources

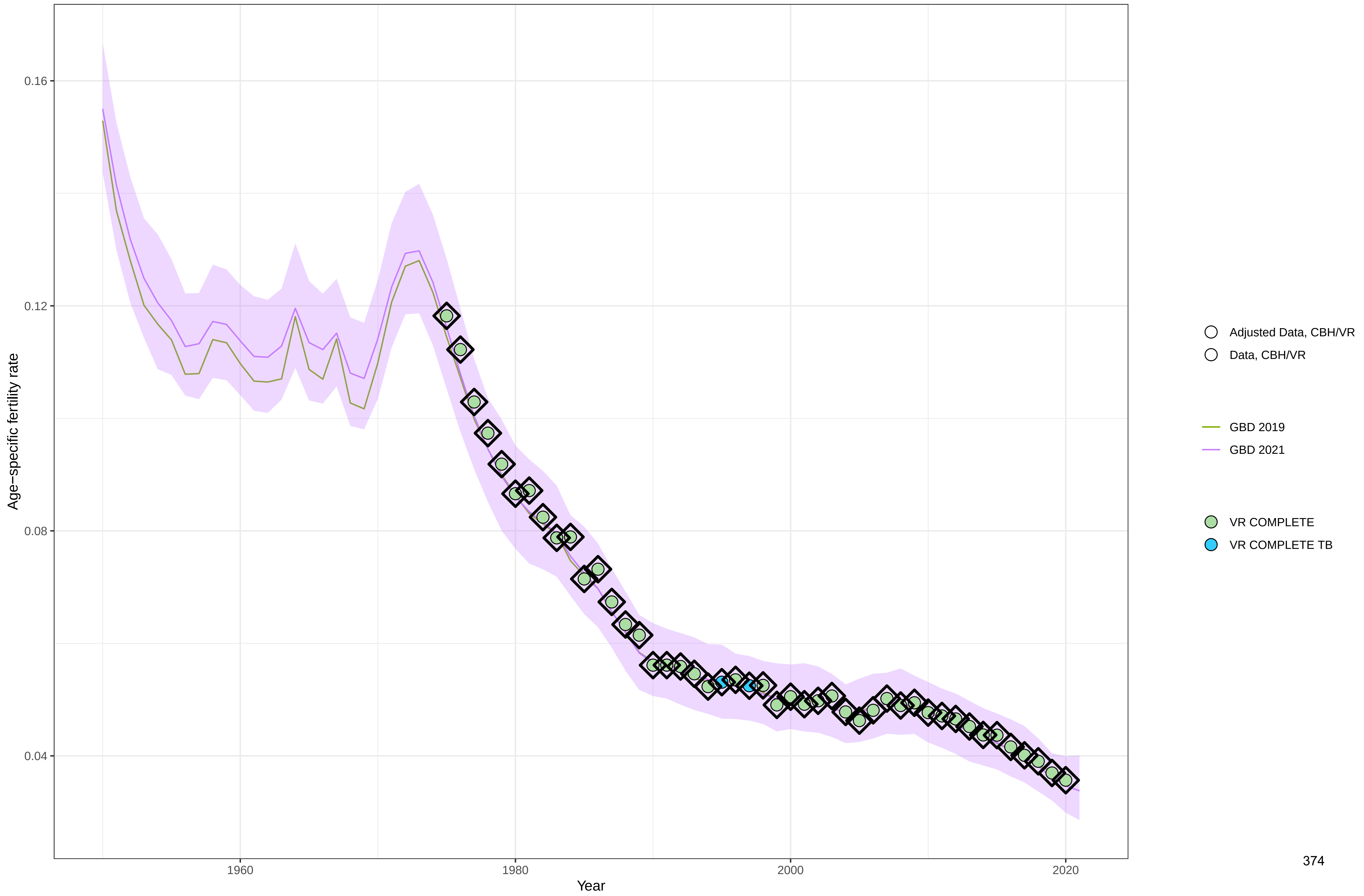

Age−specific fertility fitted trend lines, 1950−2021: Ehime, Ages: 25−29
Data points enclosed with a diamond are reference sources

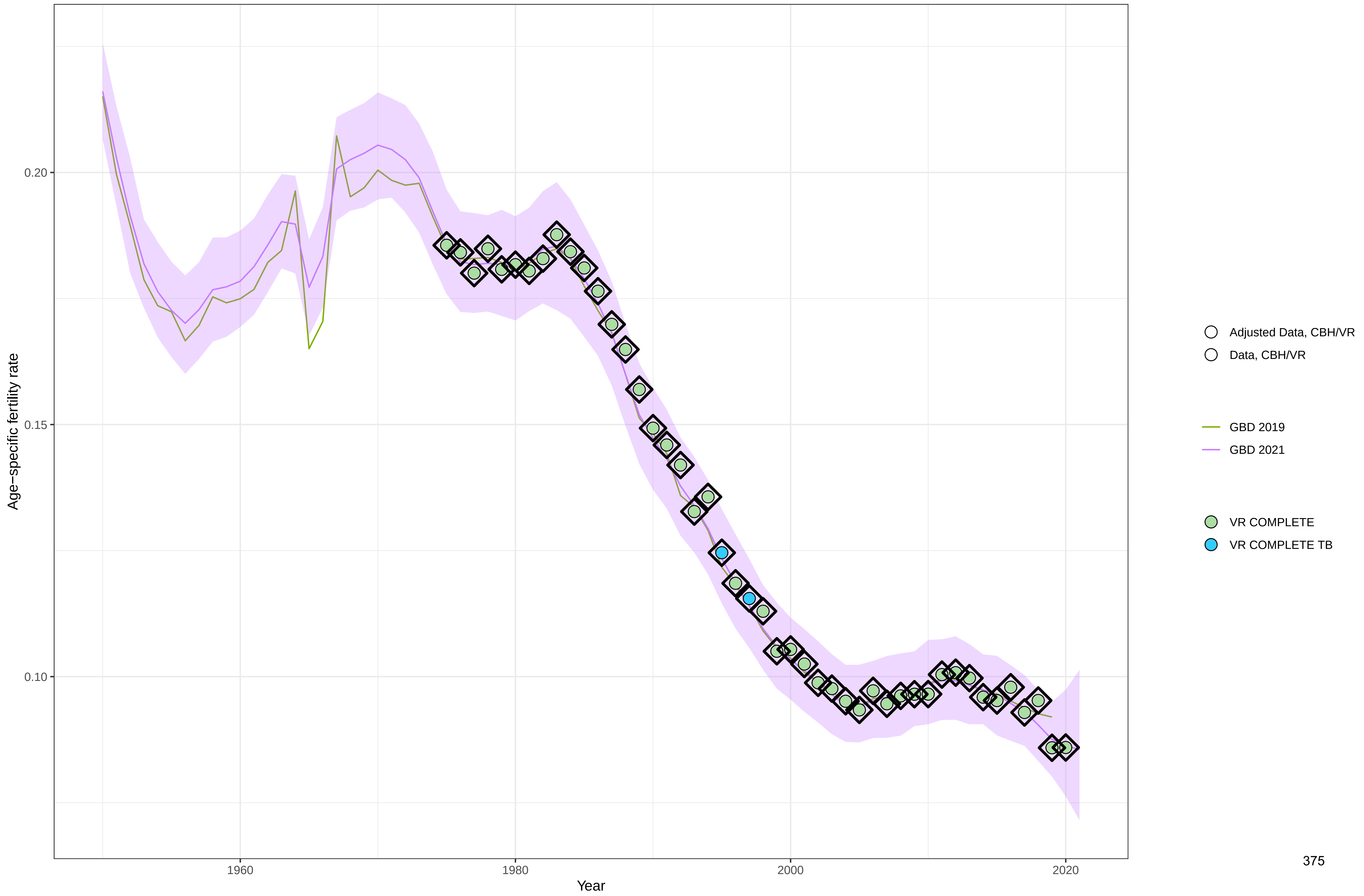

Age−specific fertility fitted trend lines, 1950−2021: Ehime, Ages: 30−34
Data points enclosed with a diamond are reference sources

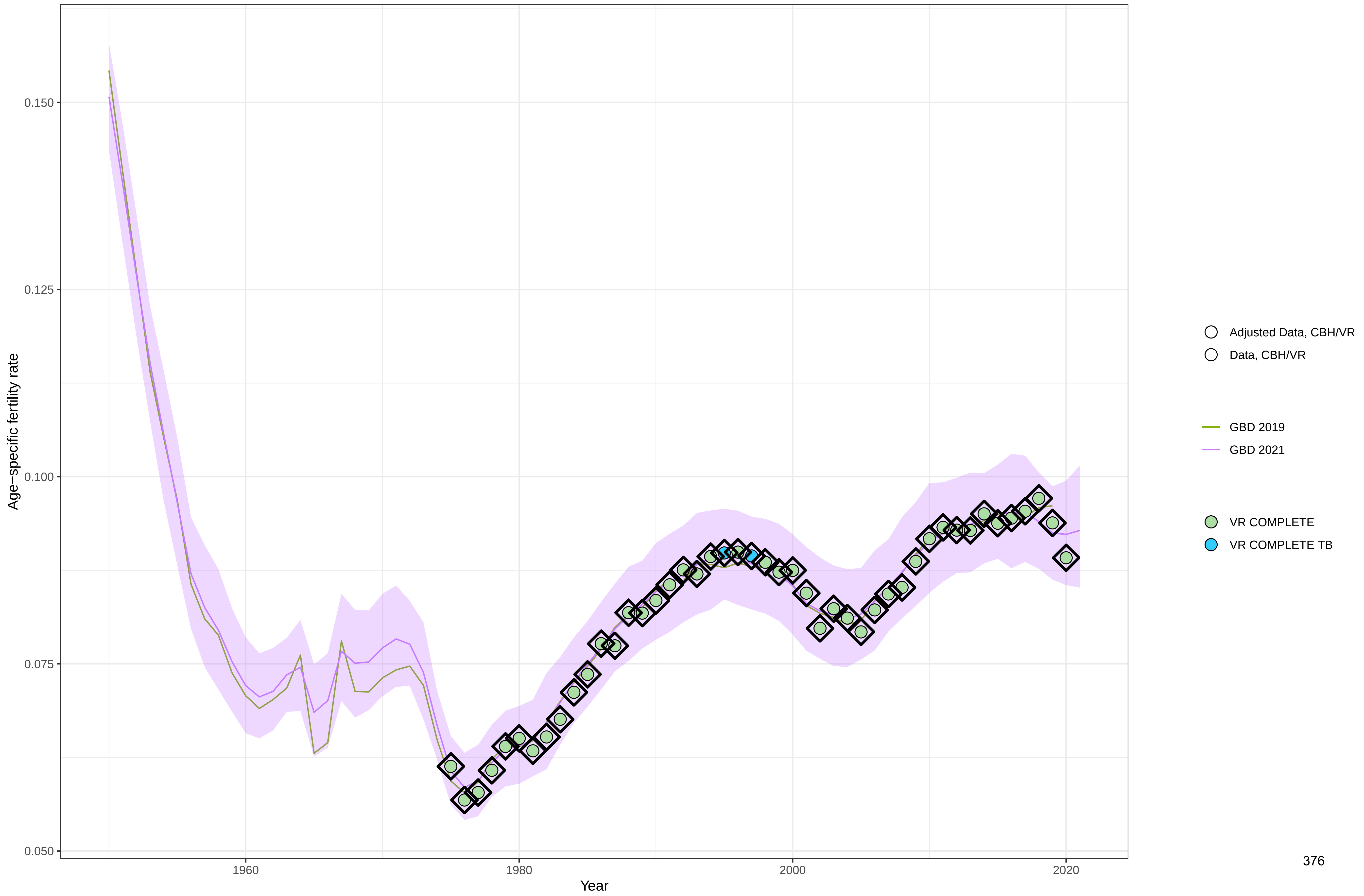

Age−specific fertility fitted trend lines, 1950−2021: Ehime, Ages: 35−39
Data points enclosed with a diamond are reference sources

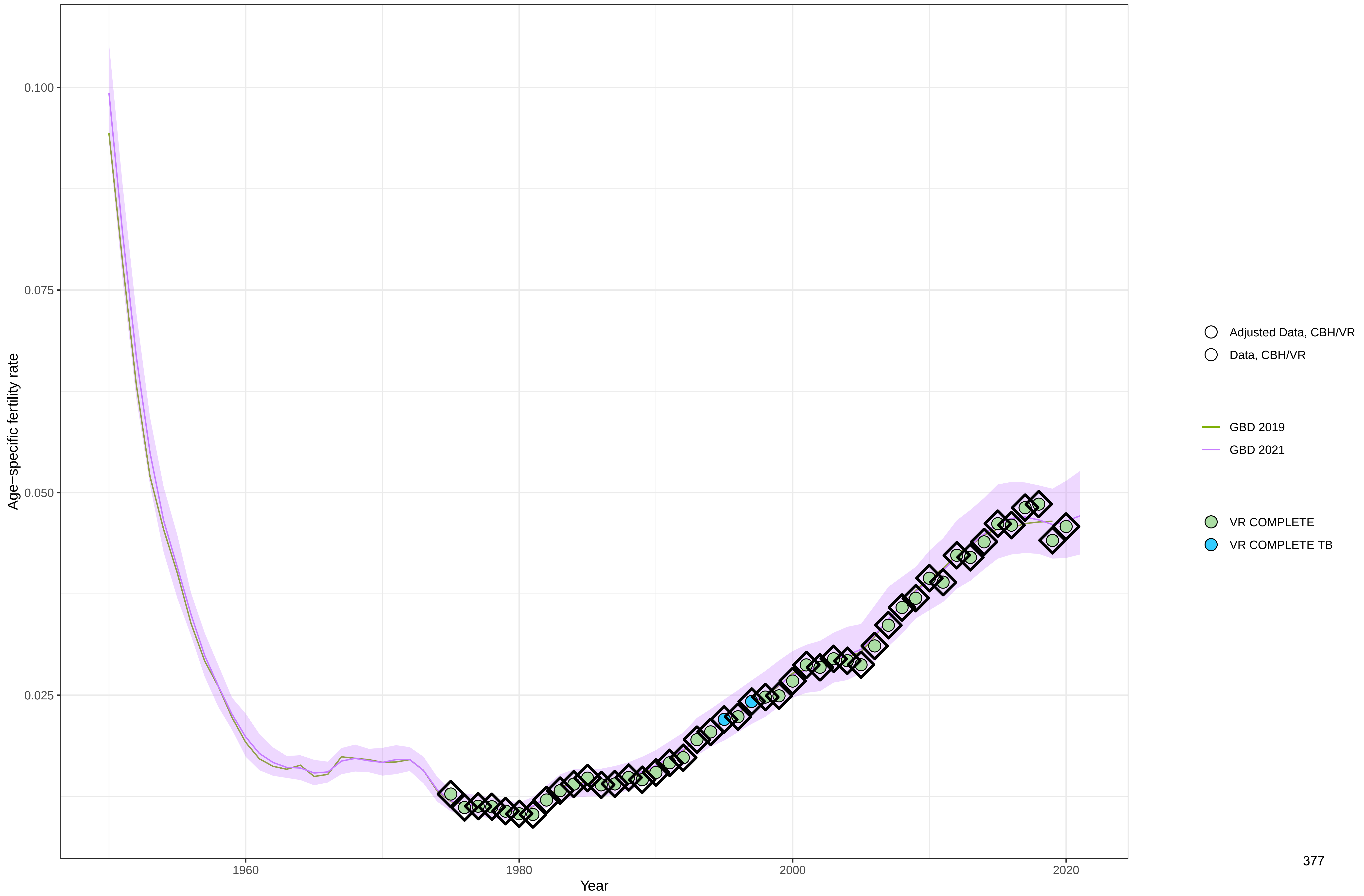

Age−specific fertility fitted trend lines, 1950−2021: Ehime, Ages: 40−44
Data points enclosed with a diamond are reference sources

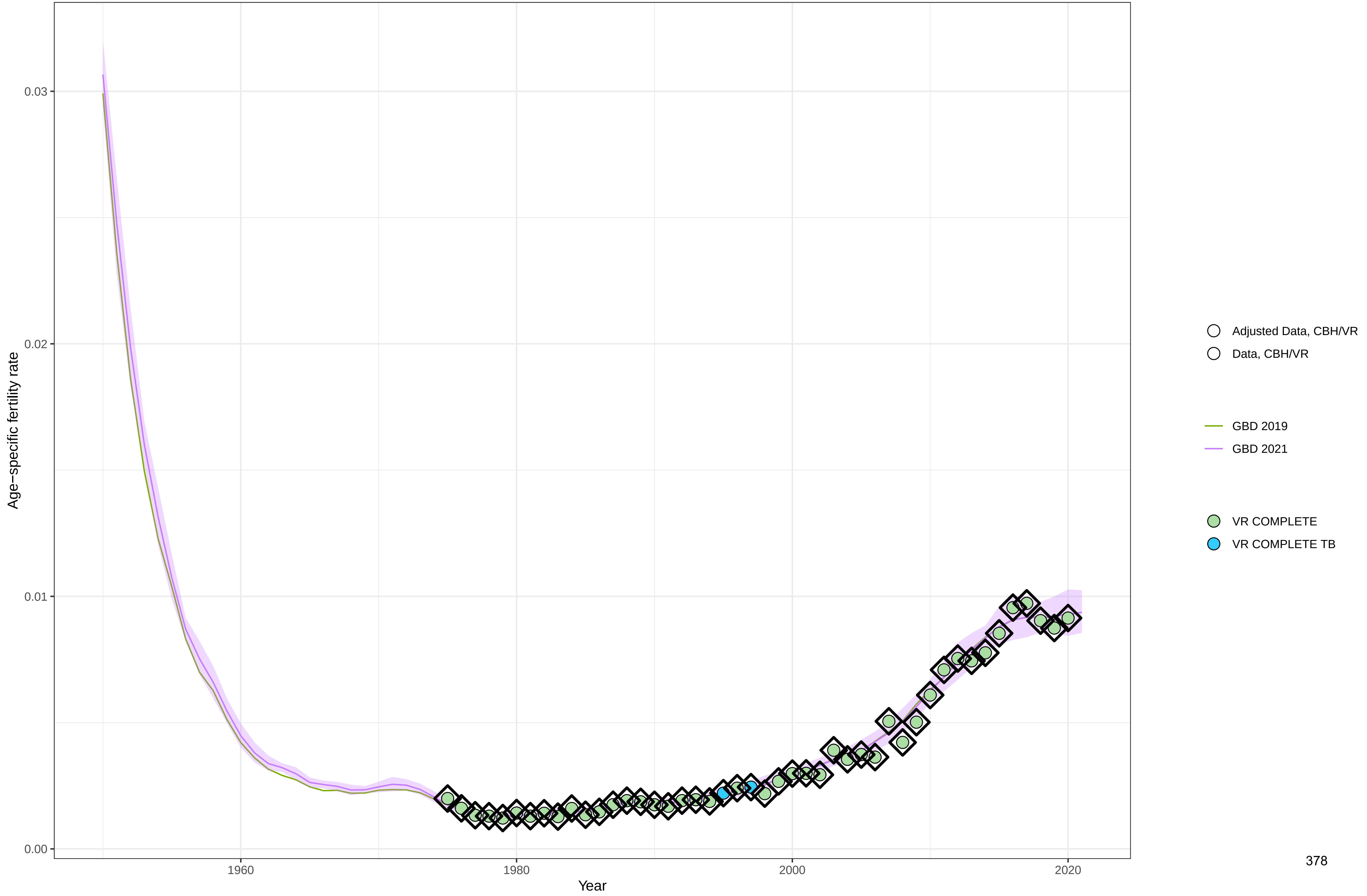

Age−specific fertility fitted trend lines, 1950−2021: Ehime, Ages: 45−49
Data points enclosed with a diamond are reference sources

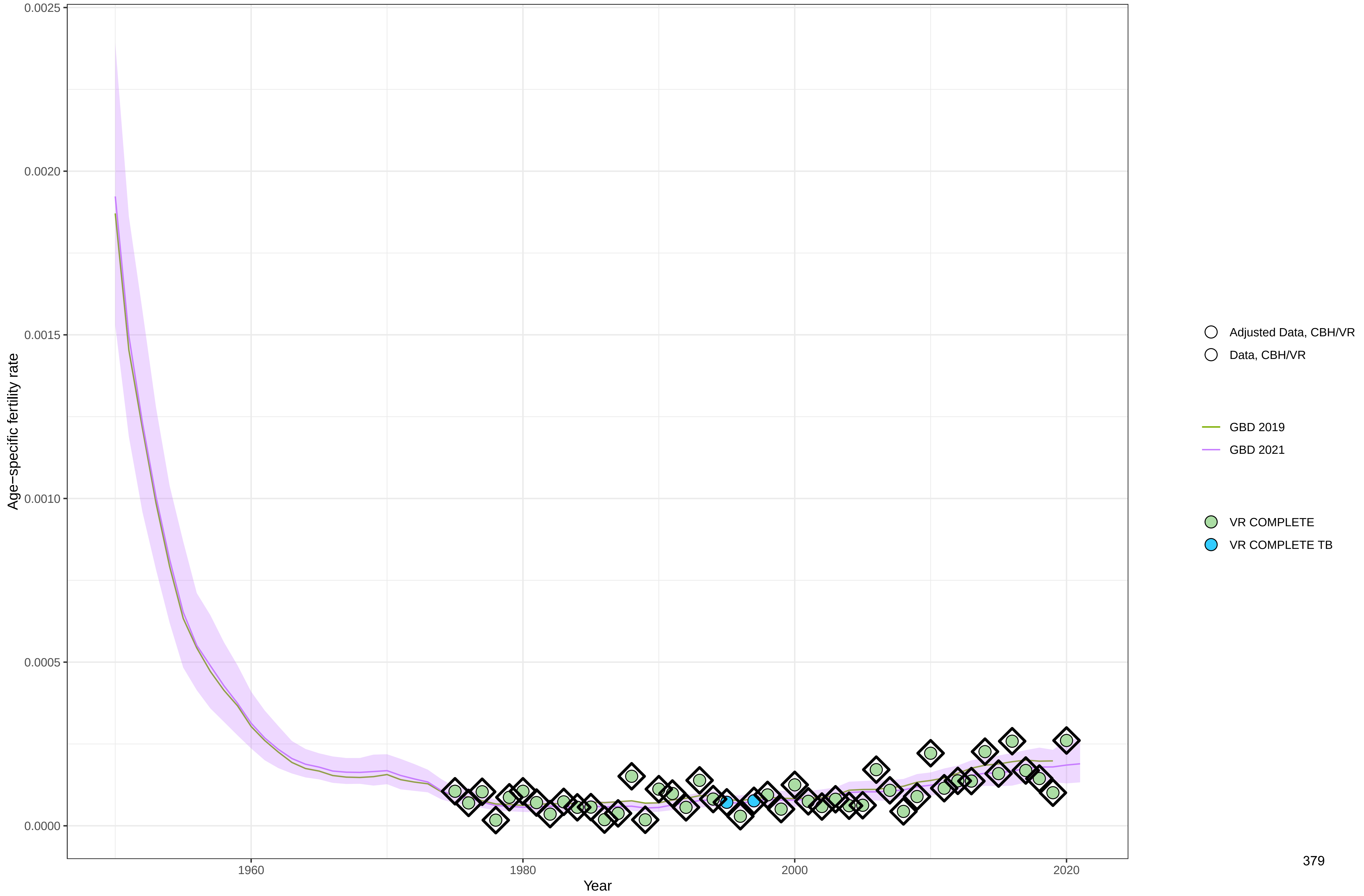

Age−specific fertility fitted trend lines, 1950−2021: Ehime, Ages: 50−54
Data points enclosed with a diamond are reference sources

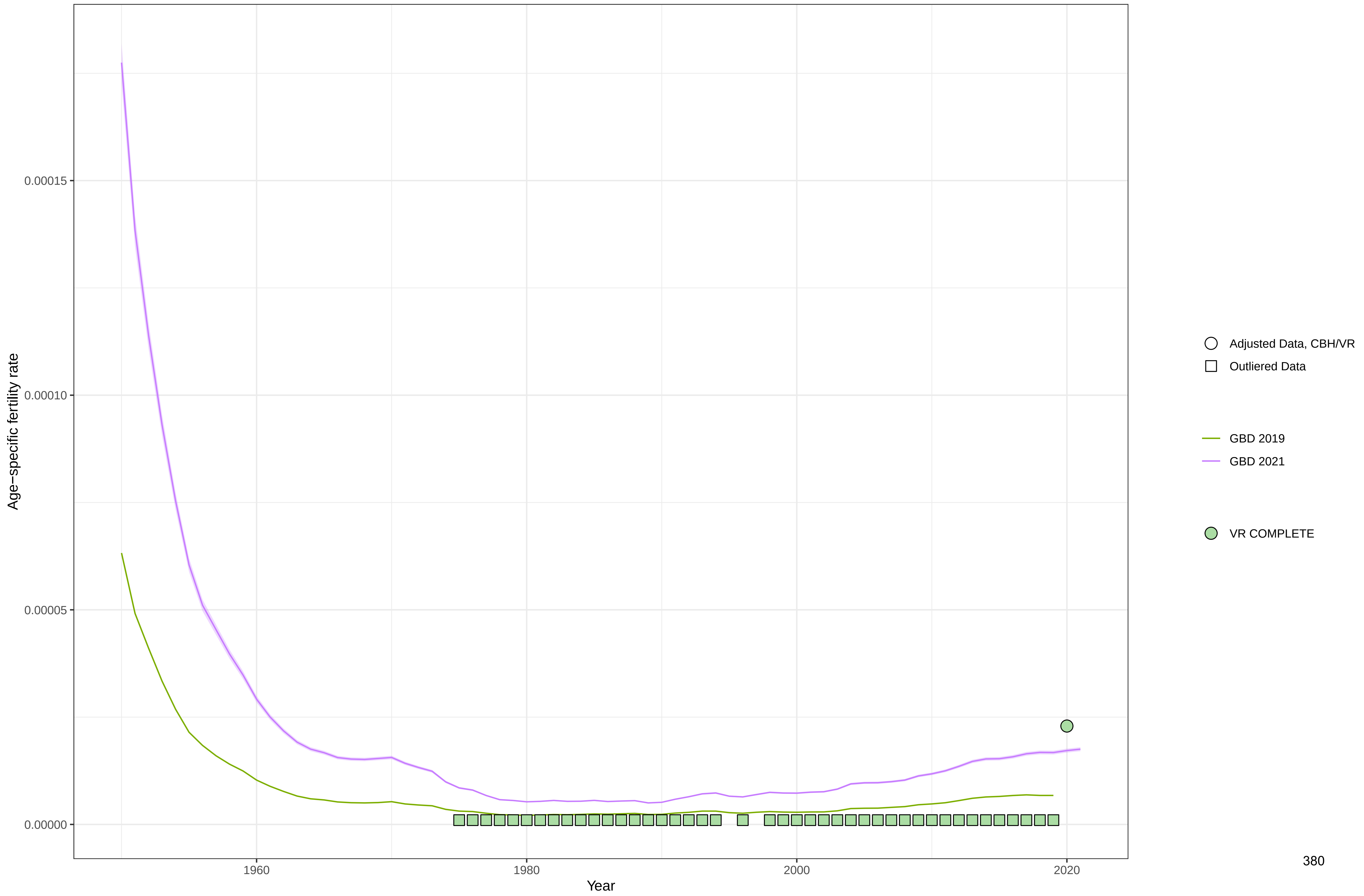

Total fertility rate fitted trend lines, 1950−2021: Ehime TFR
Data points enclosed with a diamond are reference sources

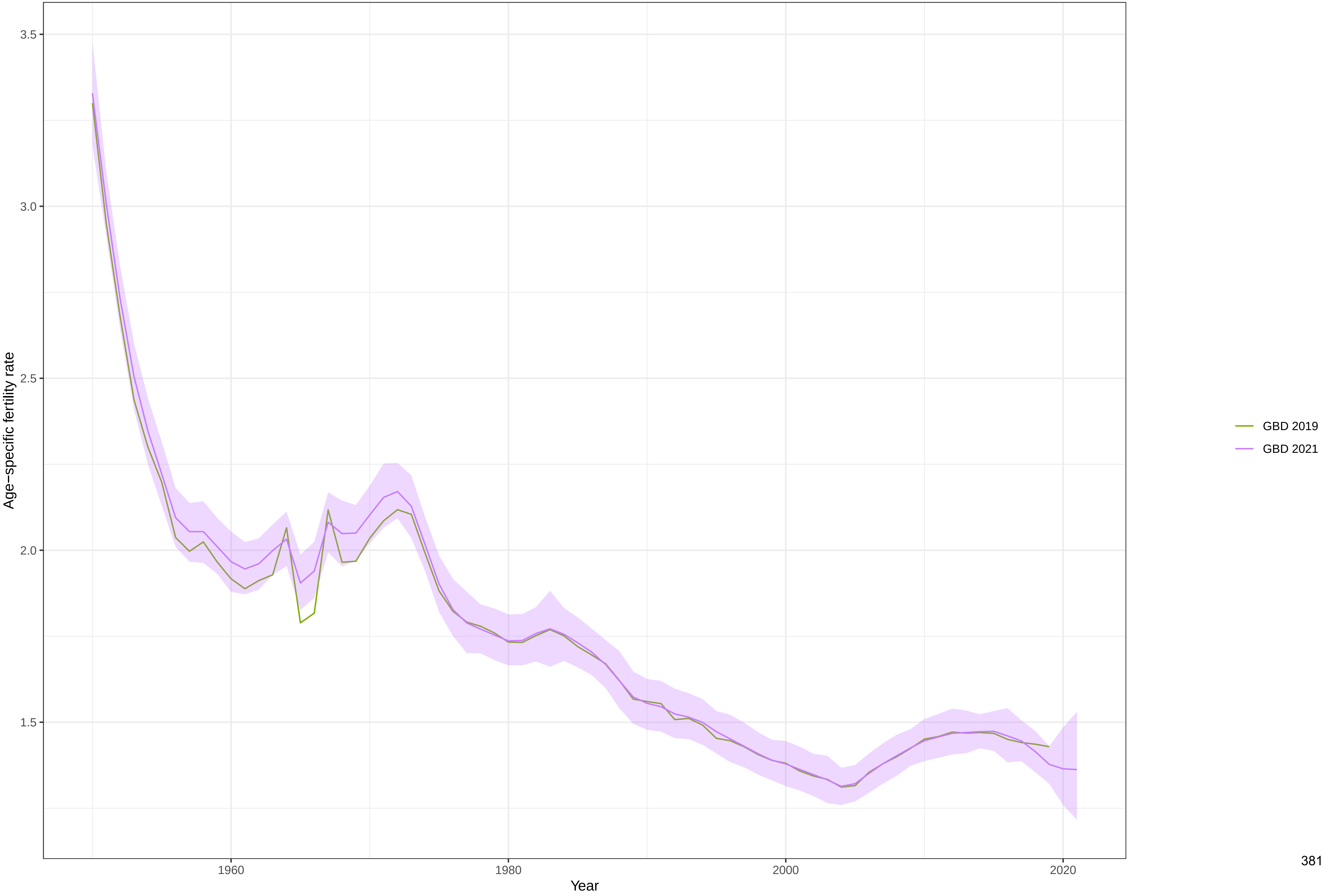

Age−specific fertility fitted trend lines, 1950−2021: Fukui, Ages: 10−14
Data points enclosed with a diamond are reference sources

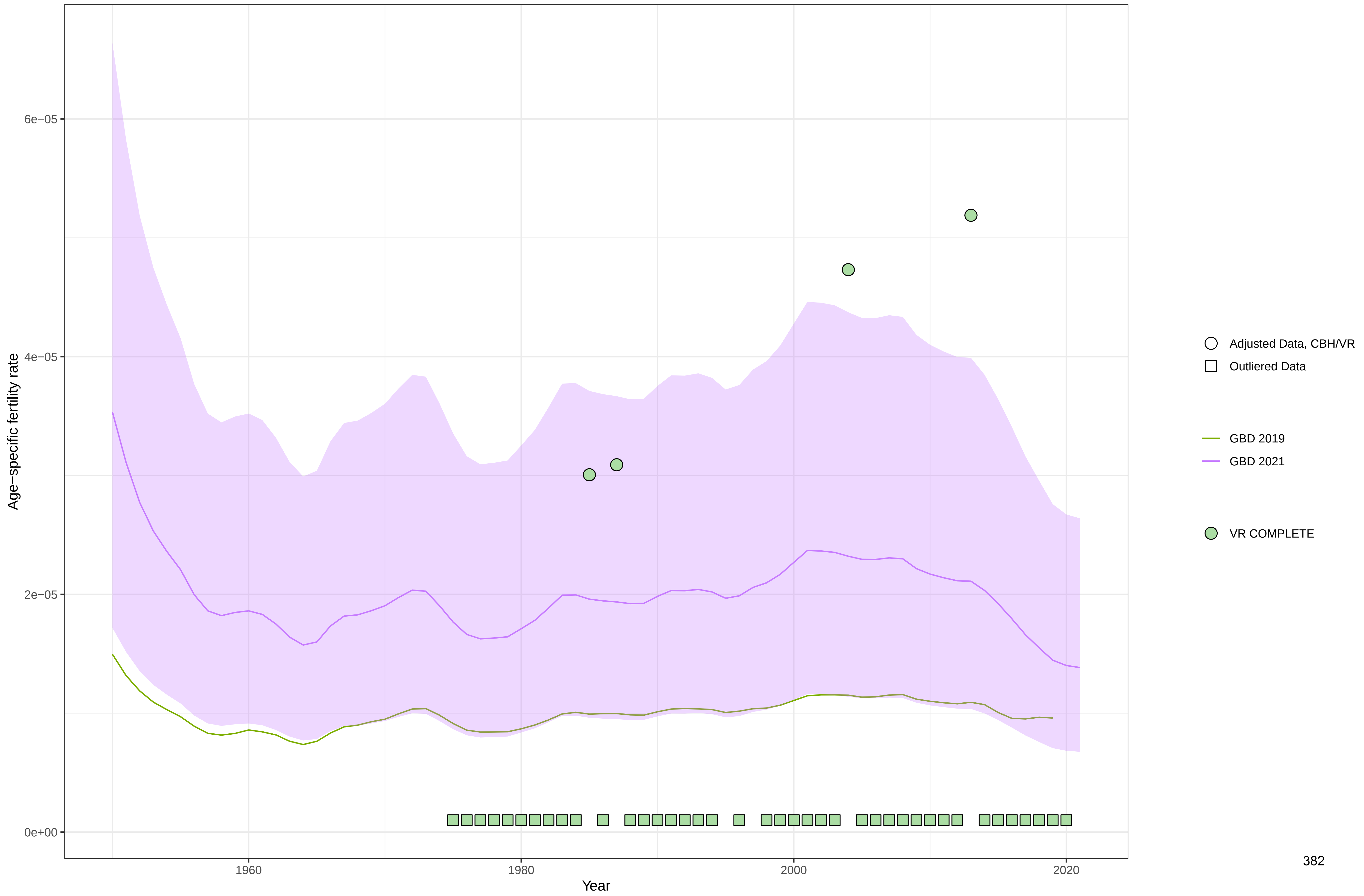

Age−specific fertility fitted trend lines, 1950−2021: Fukui, Ages: 15−19
 Data points enclosed with a diamond are reference sources

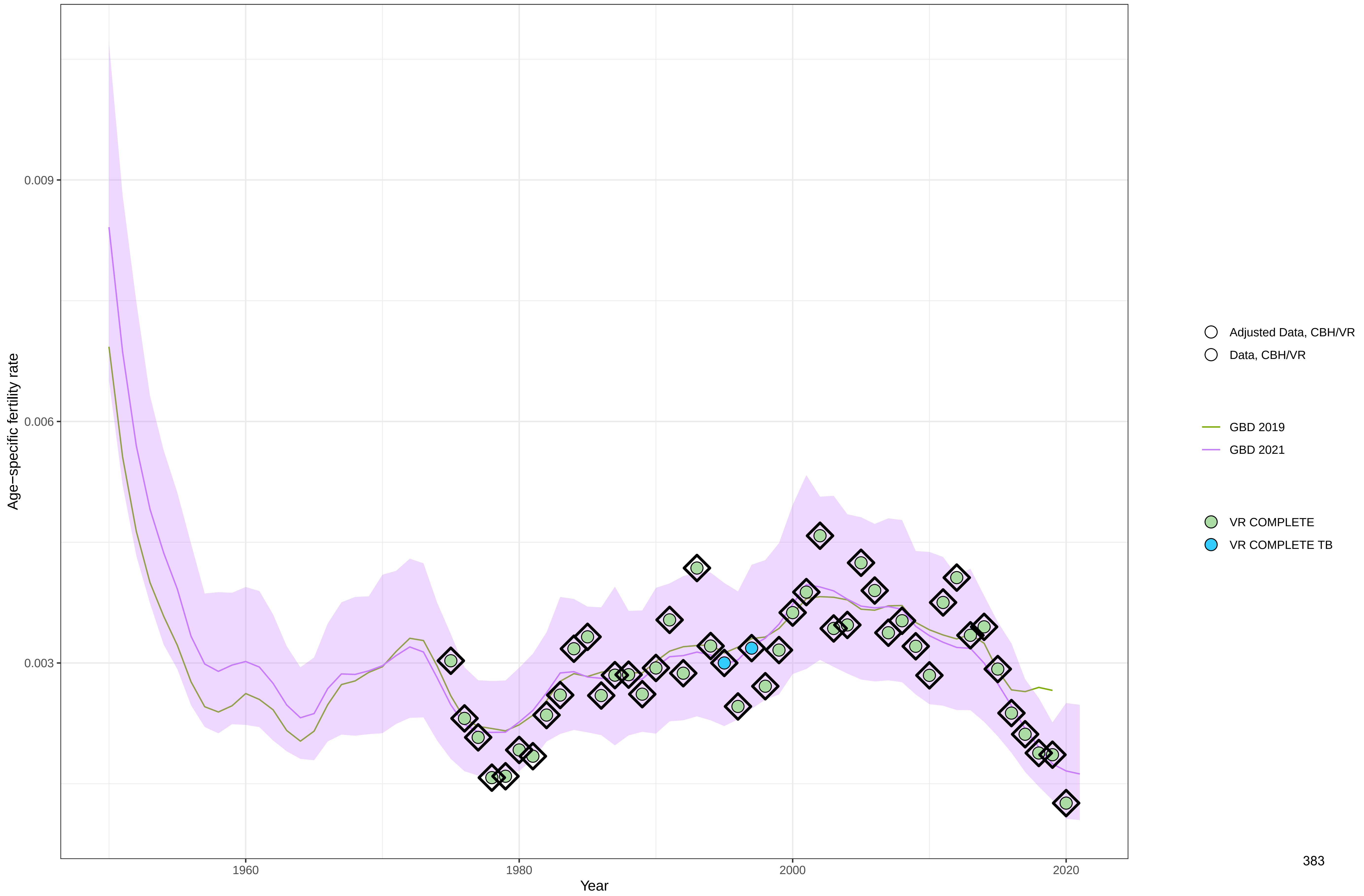

Age−specific fertility fitted trend lines, 1950−2021: Fukui, Ages: 20−24
Data points enclosed with a diamond are reference sources

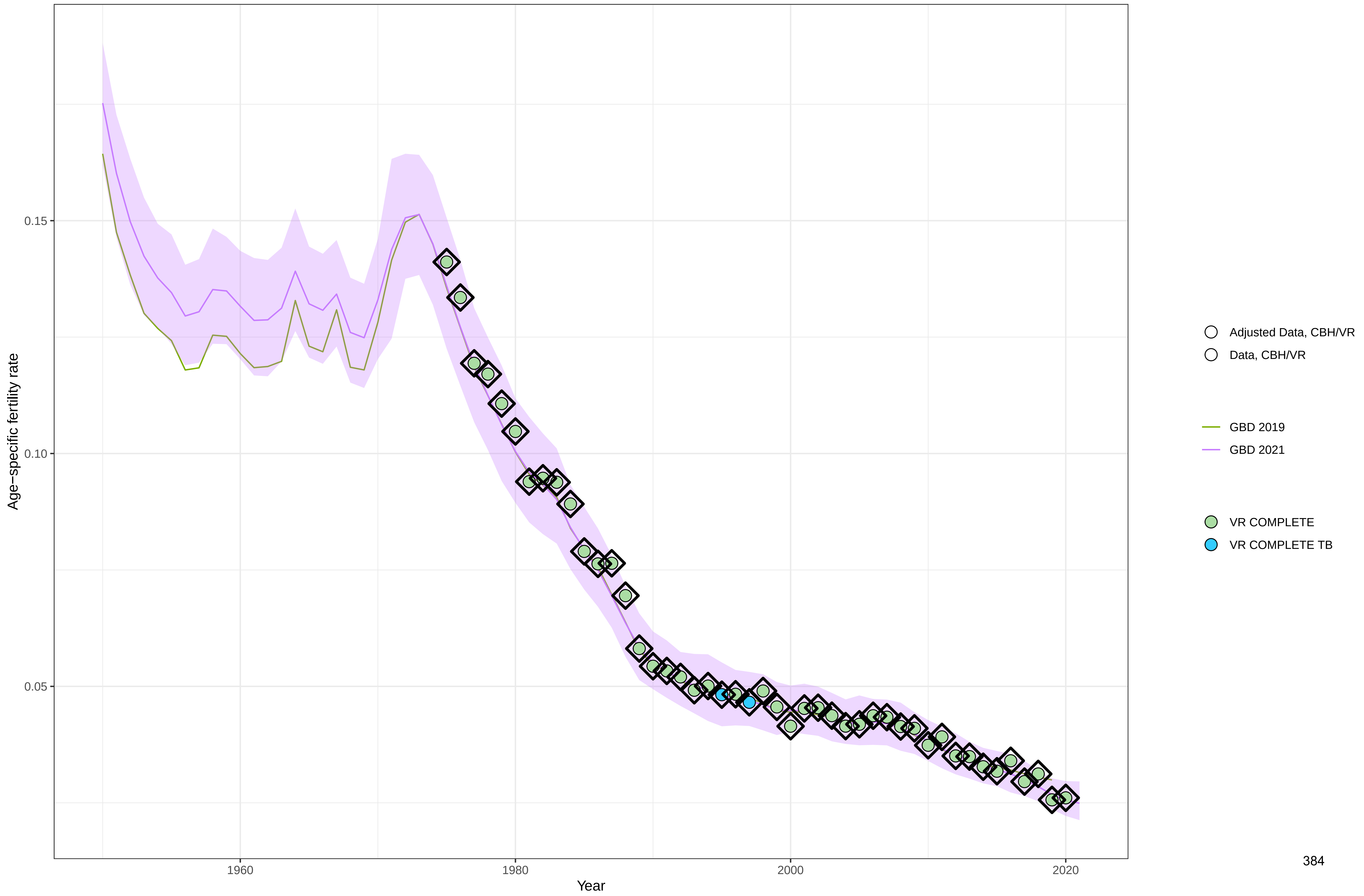

Age−specific fertility fitted trend lines, 1950−2021: Fukui, Ages: 25−29
Data points enclosed with a diamond are reference sources

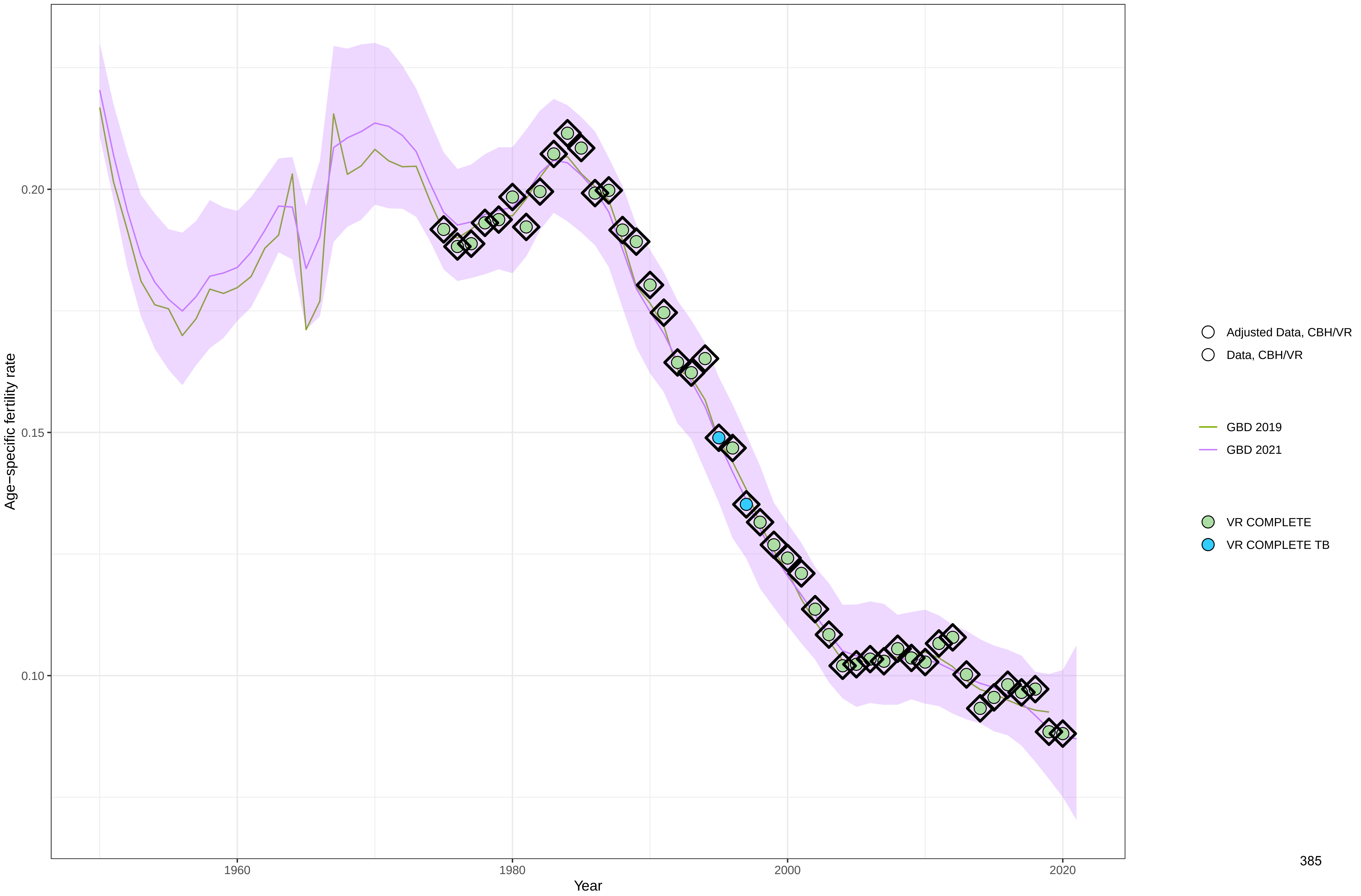

Age−specific fertility fitted trend lines, 1950−2021: Fukui, Ages: 30−34
Data points enclosed with a diamond are reference sources

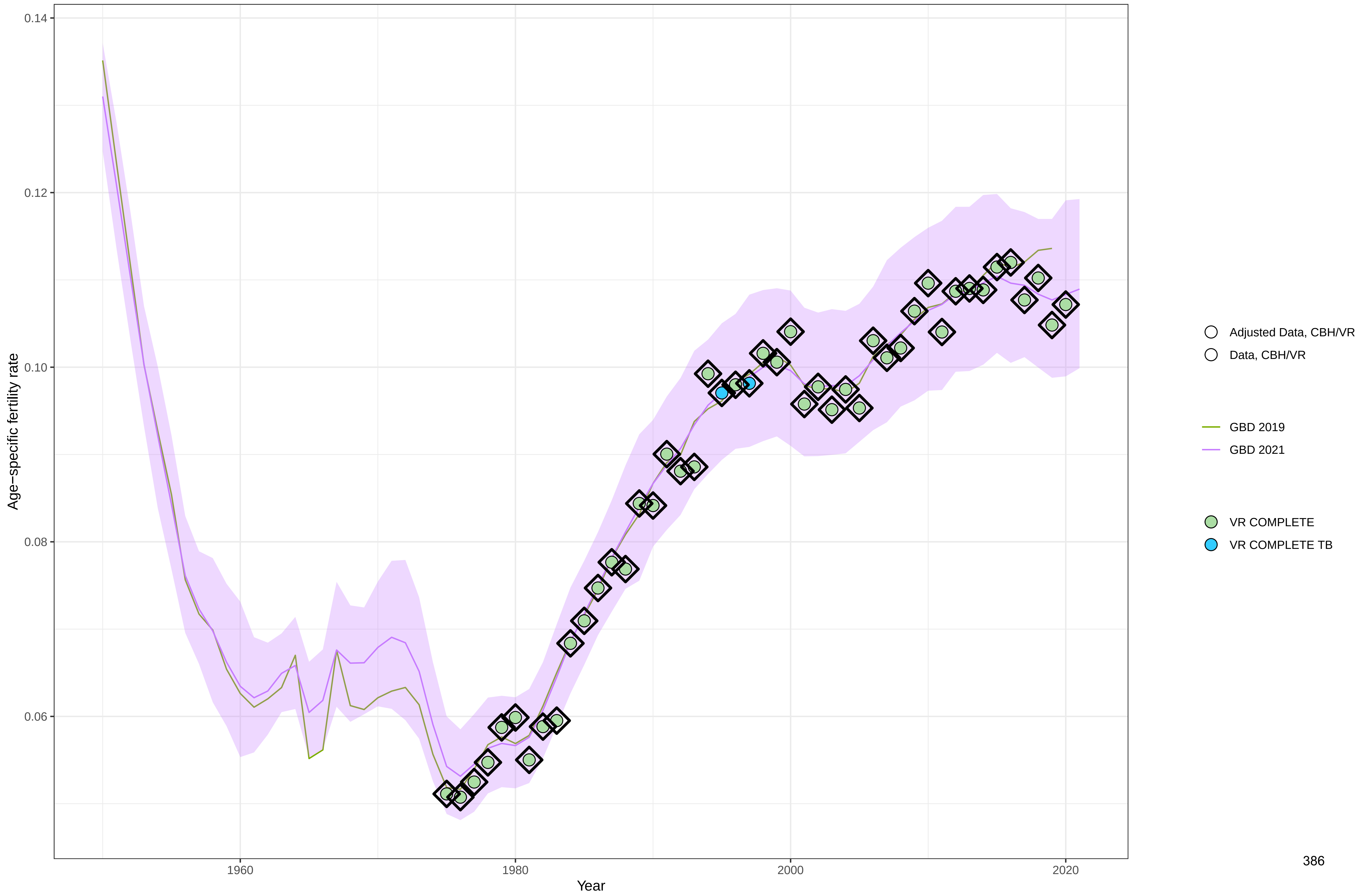

Age−specific fertility fitted trend lines, 1950−2021: Fukui, Ages: 35−39
Data points enclosed with a diamond are reference sources

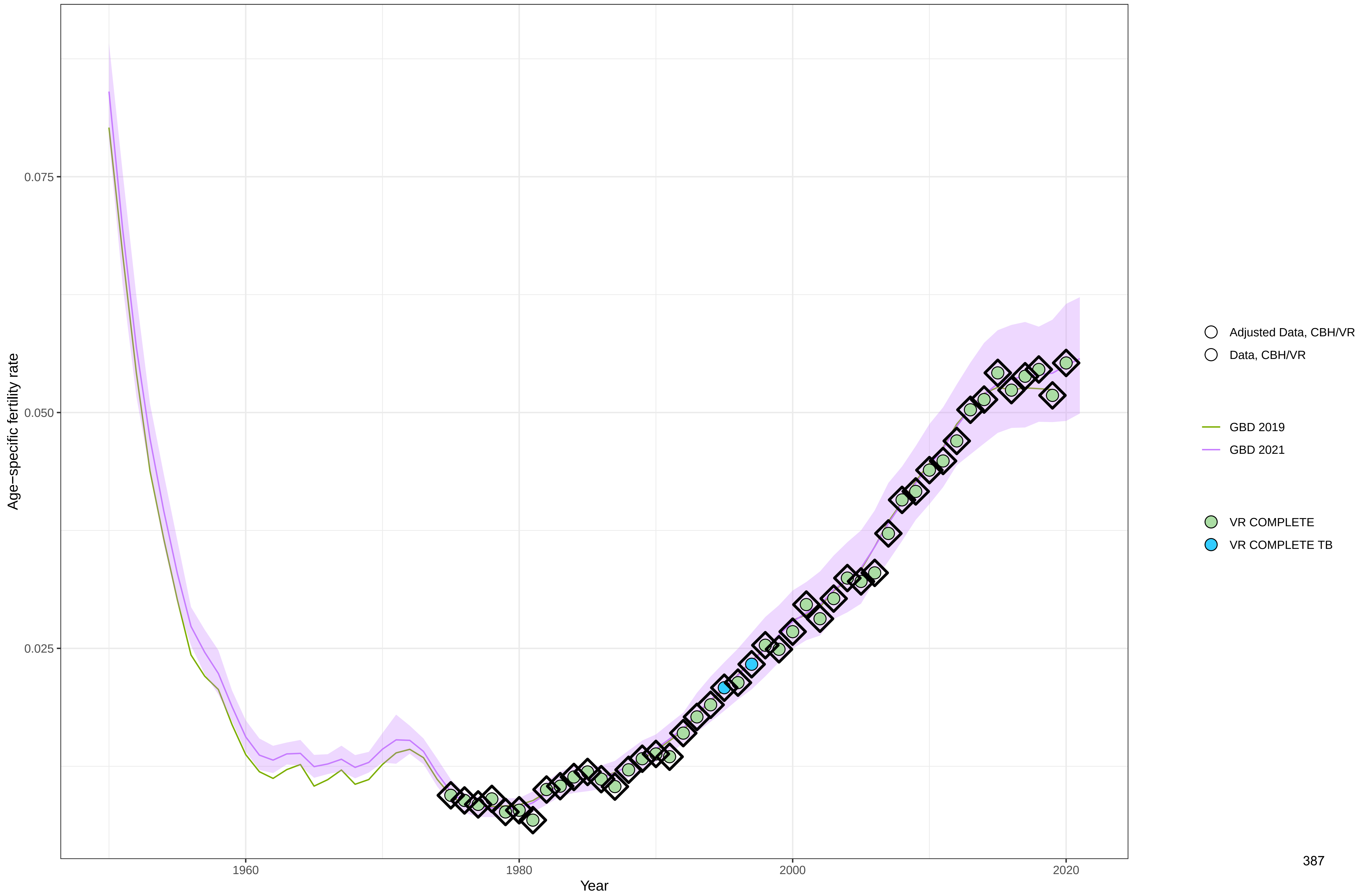

Age−specific fertility fitted trend lines, 1950−2021: Fukui, Ages: 40−44
Data points enclosed with a diamond are reference sources

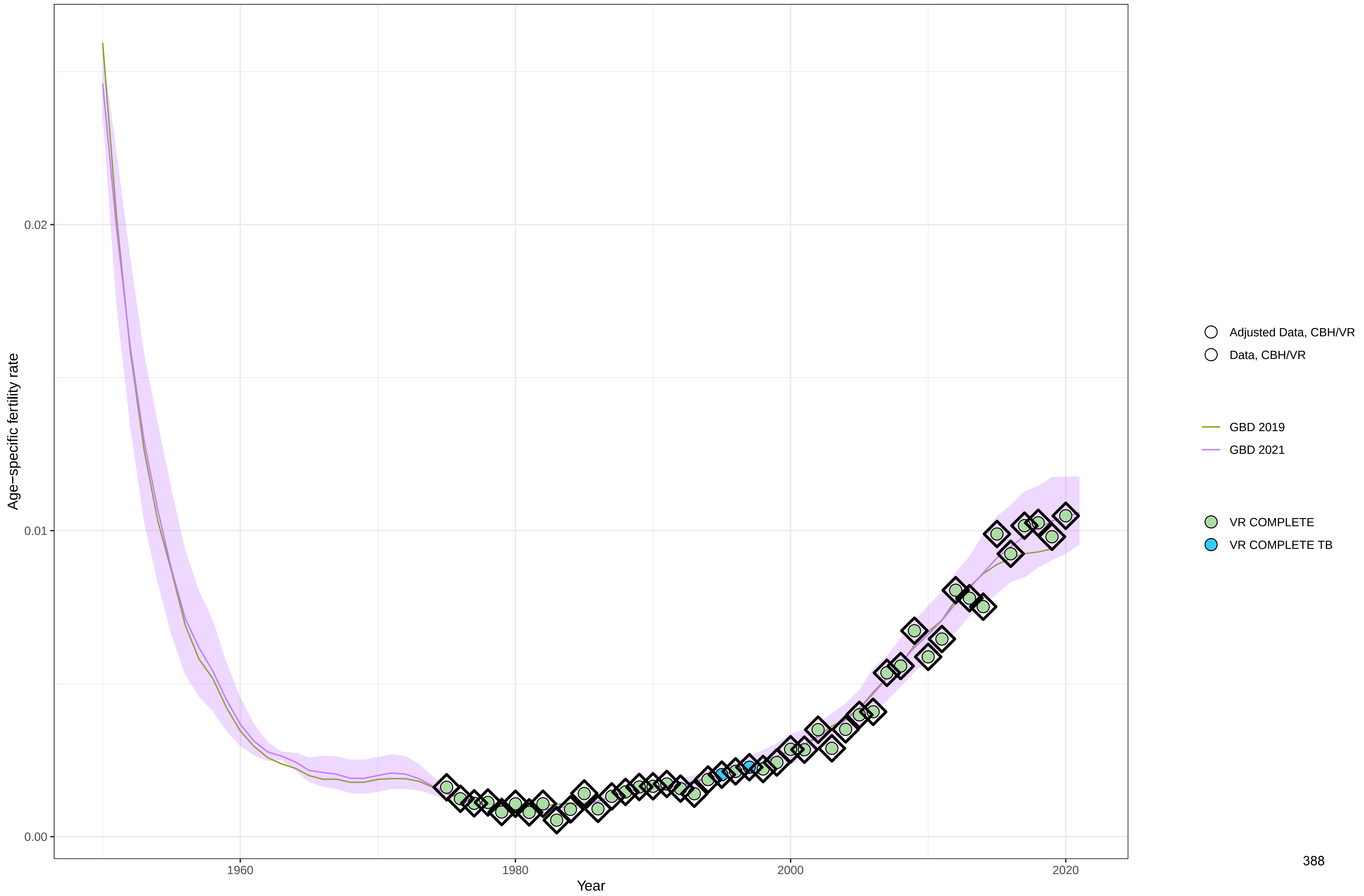

Age−specific fertility fitted trend lines, 1950−2021: Fukui, Ages: 45−49
Data points enclosed with a diamond are reference sources

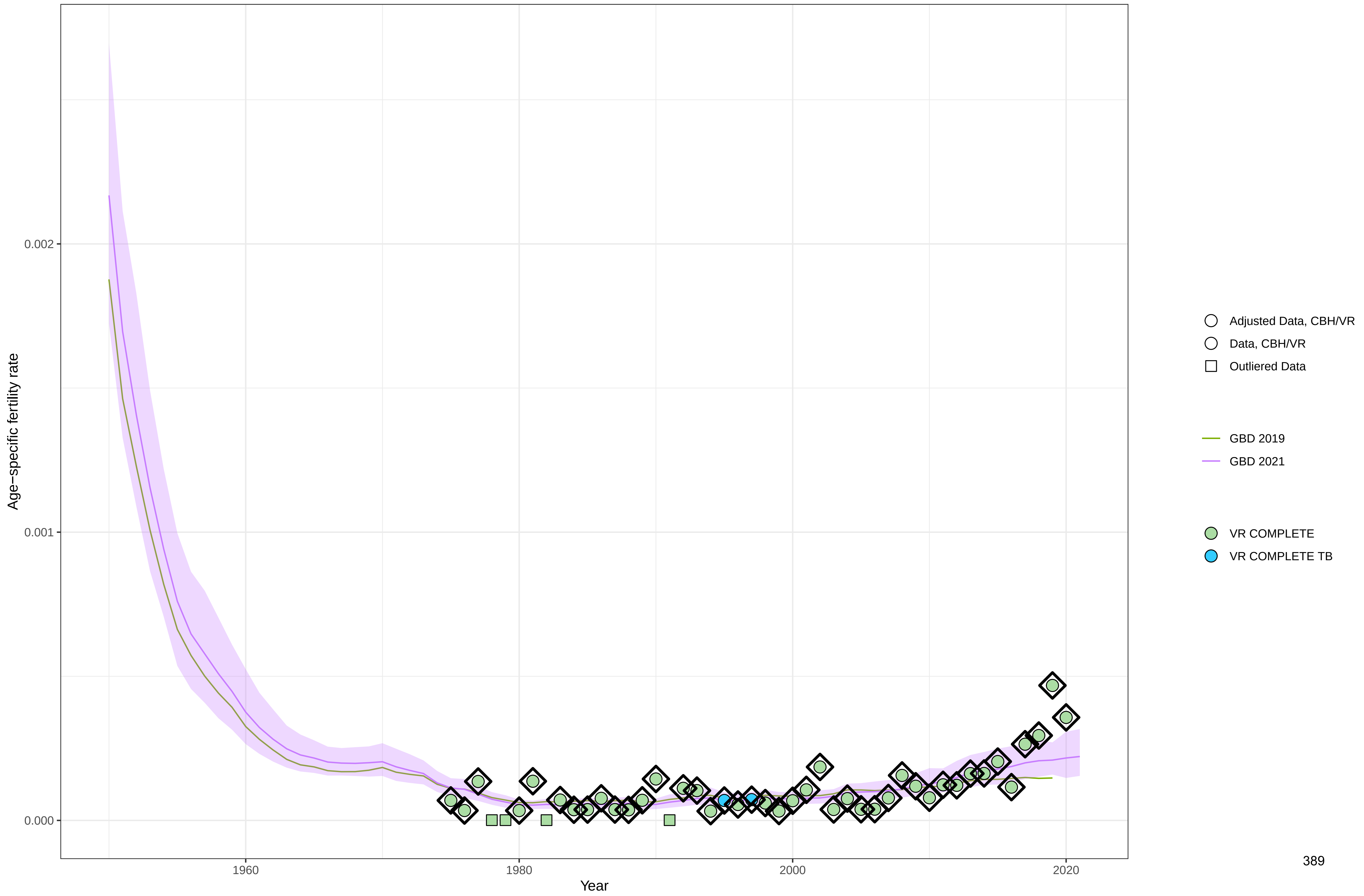

Age−specific fertility fitted trend lines, 1950−2021: Fukui, Ages: 50−54
Data points enclosed with a diamond are reference sources

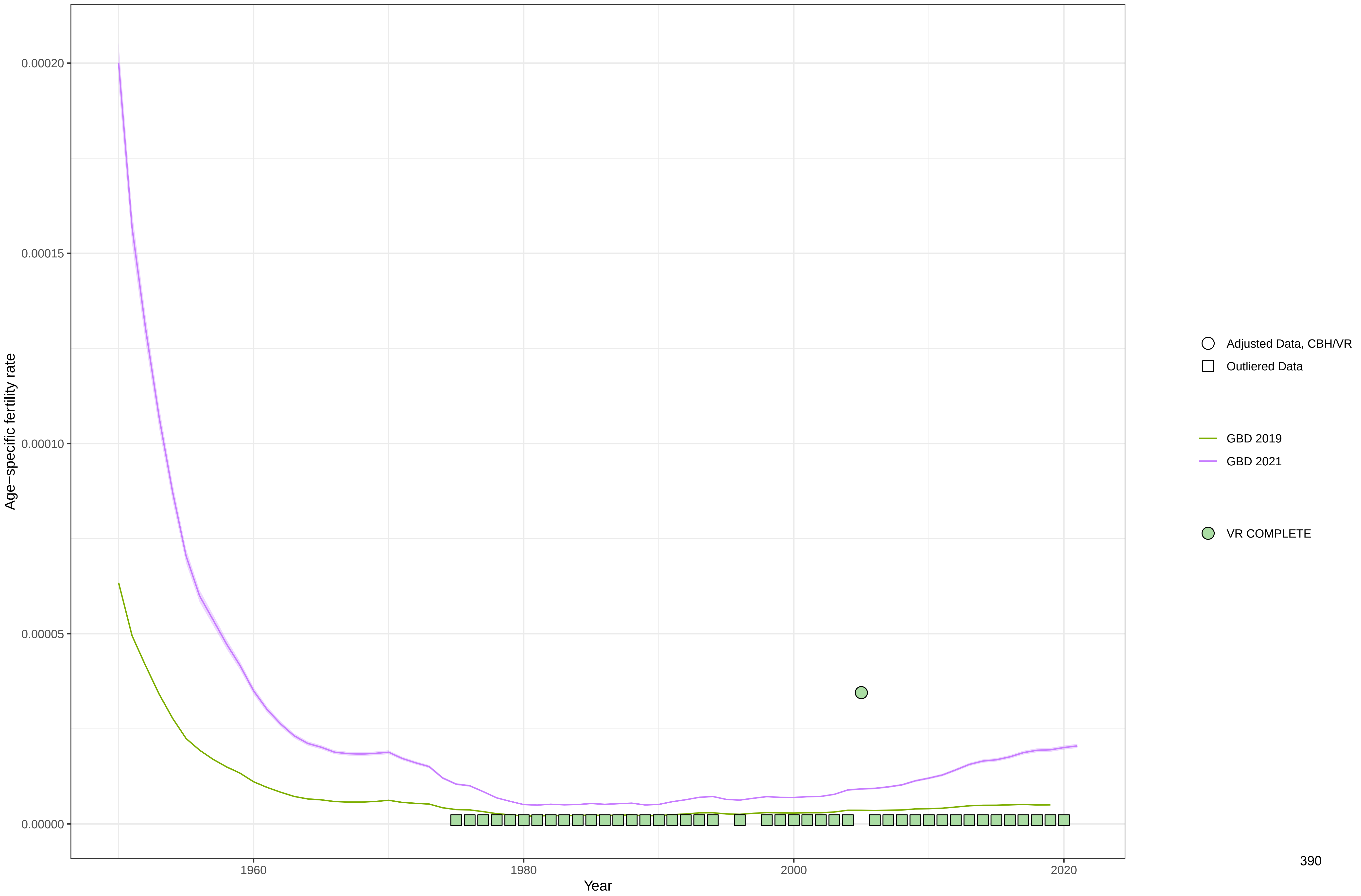

Total fertility rate fitted trend lines, 1950−2021: Fukui TFR
Data points enclosed with a diamond are reference sources

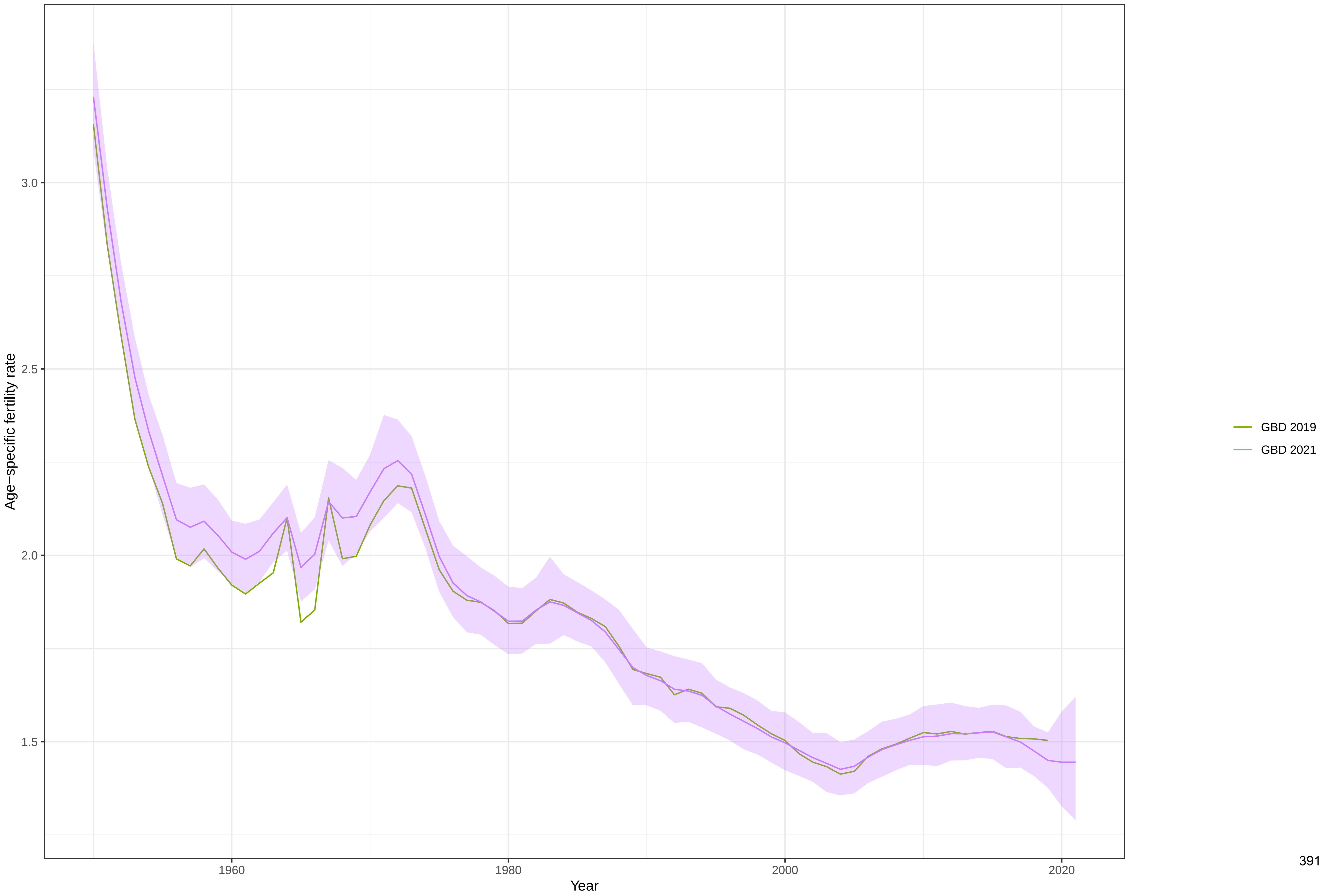

Age−specific fertility fitted trend lines, 1950−2021: Fukuoka, Ages: 10−14
Data points enclosed with a diamond are reference sources

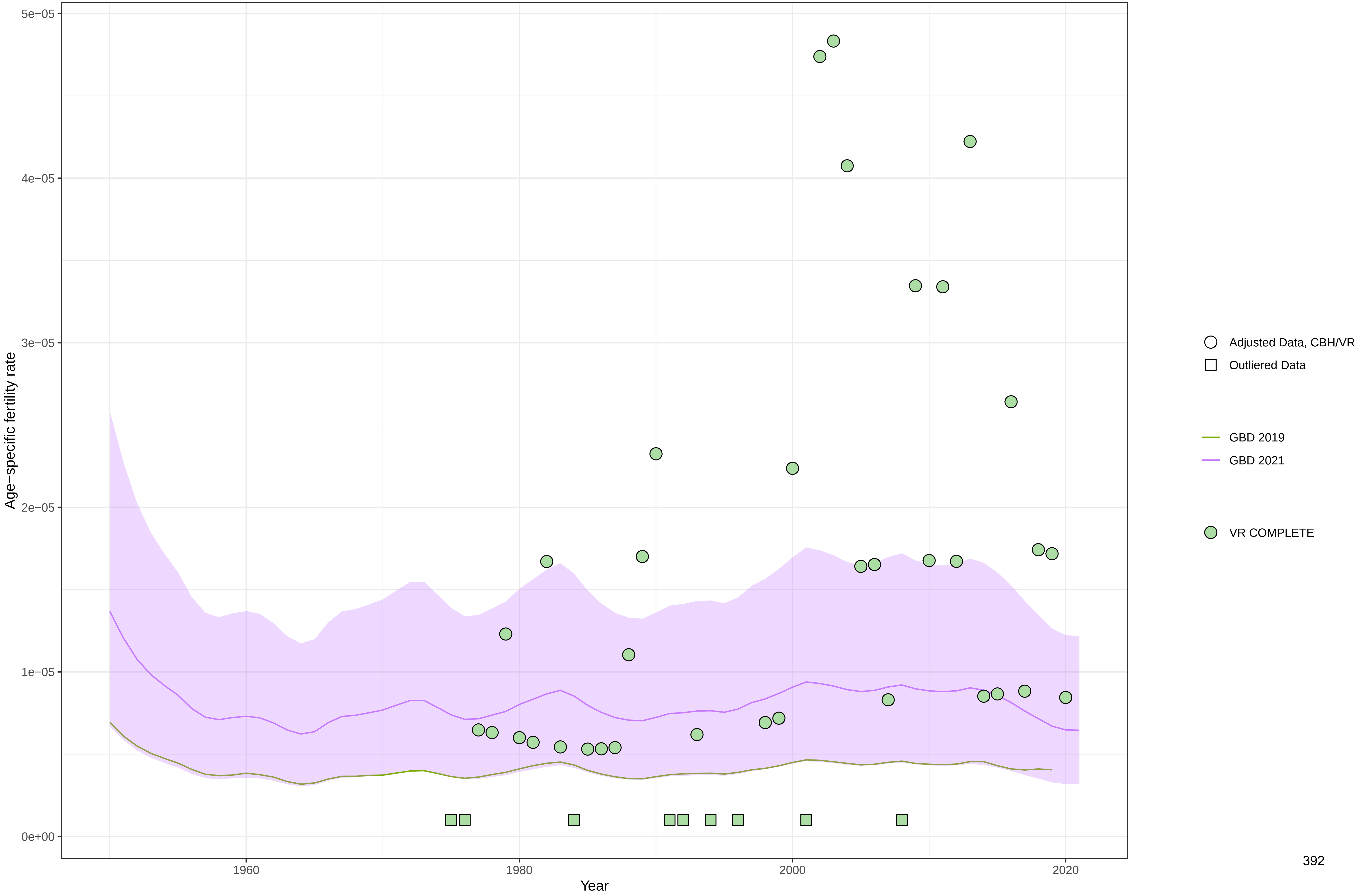

Age−specific fertility fitted trend lines, 1950−2021: Fukuoka, Ages: 15−19
Data points enclosed with a diamond are reference sources

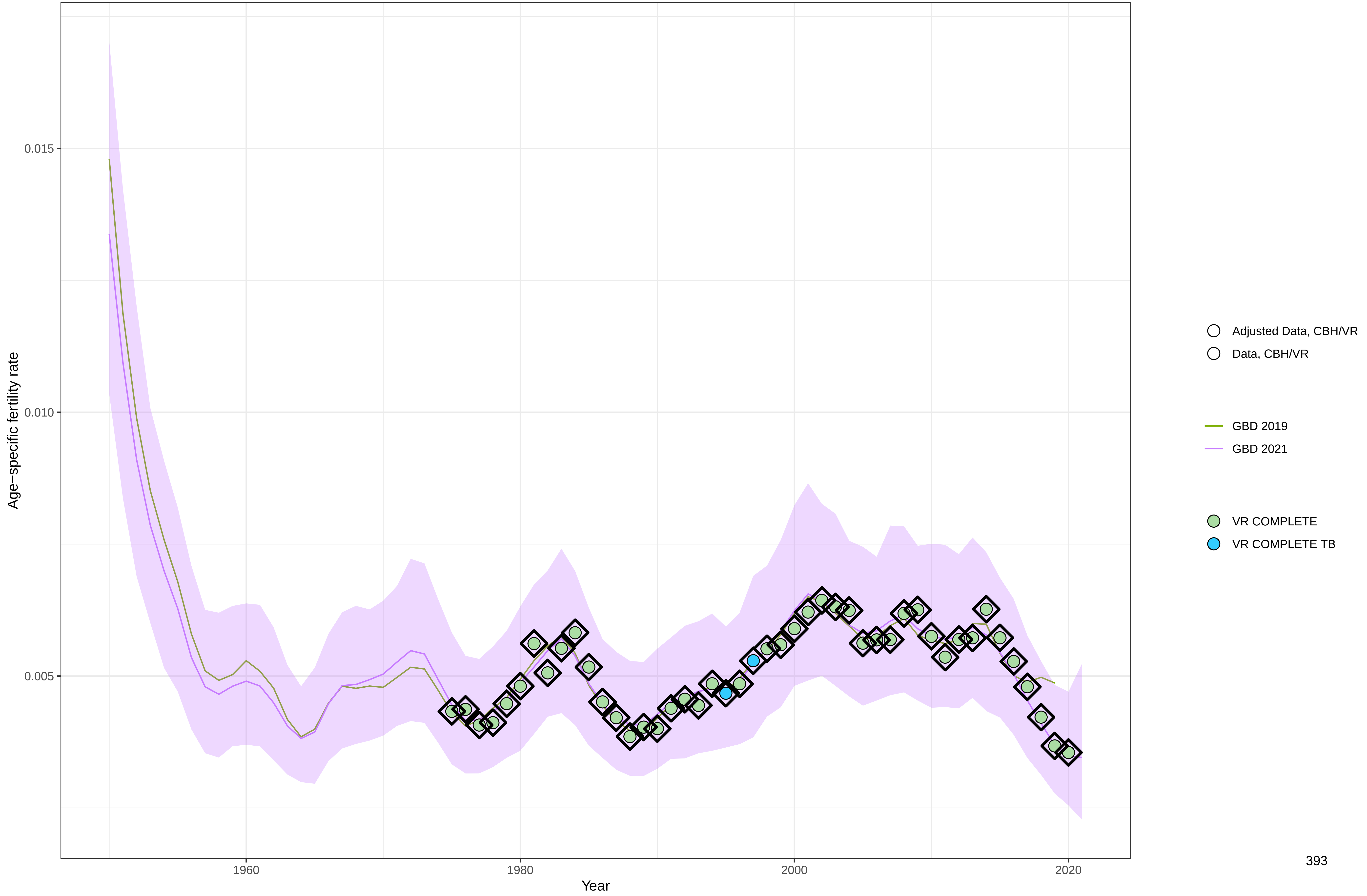

Age−specific fertility fitted trend lines, 1950−2021: Fukuoka, Ages: 20−24
Data points enclosed with a diamond are reference sources

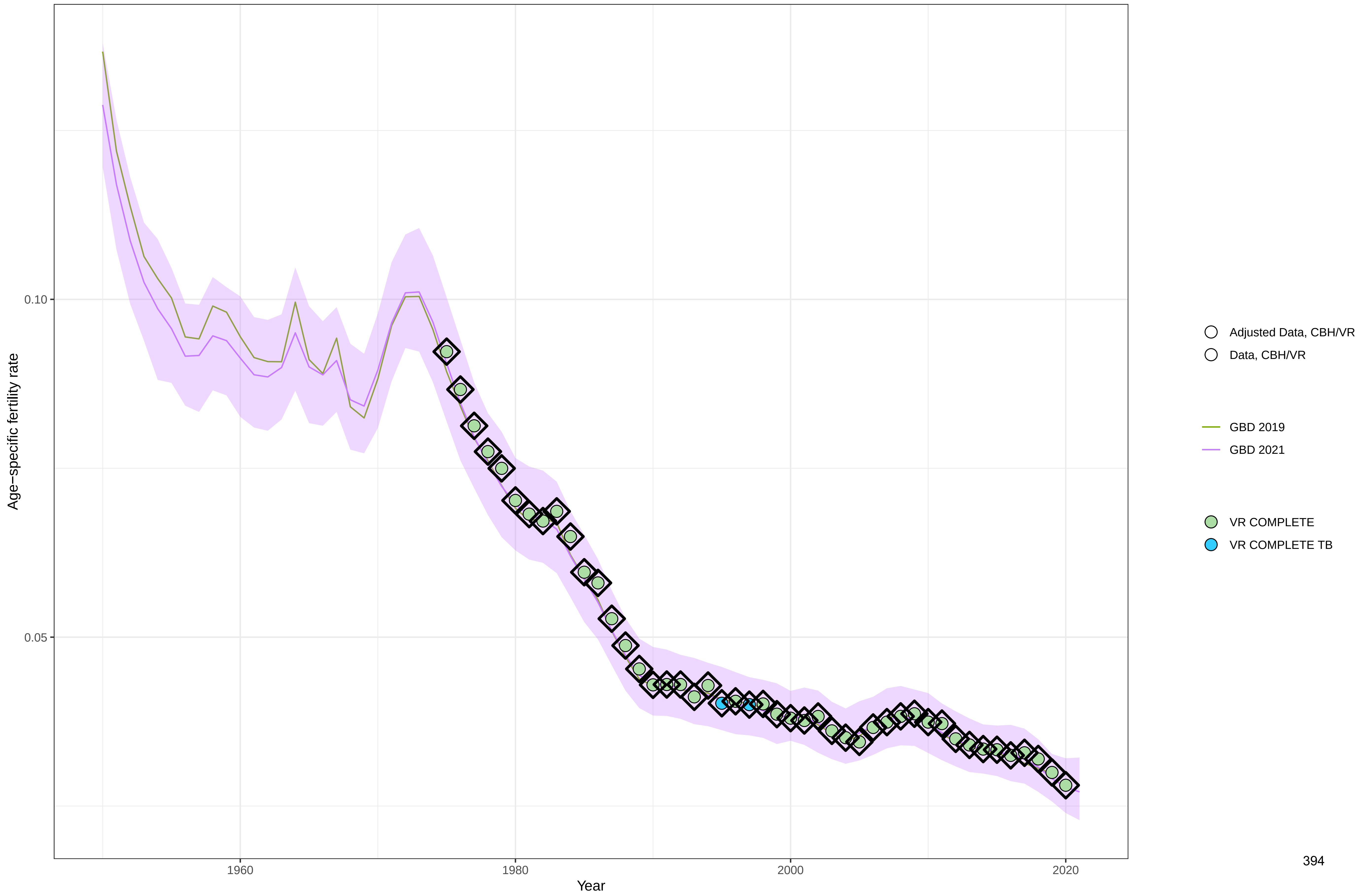

Age−specific fertility fitted trend lines, 1950−2021: Fukuoka, Ages: 25−29
Data points enclosed with a diamond are reference sources

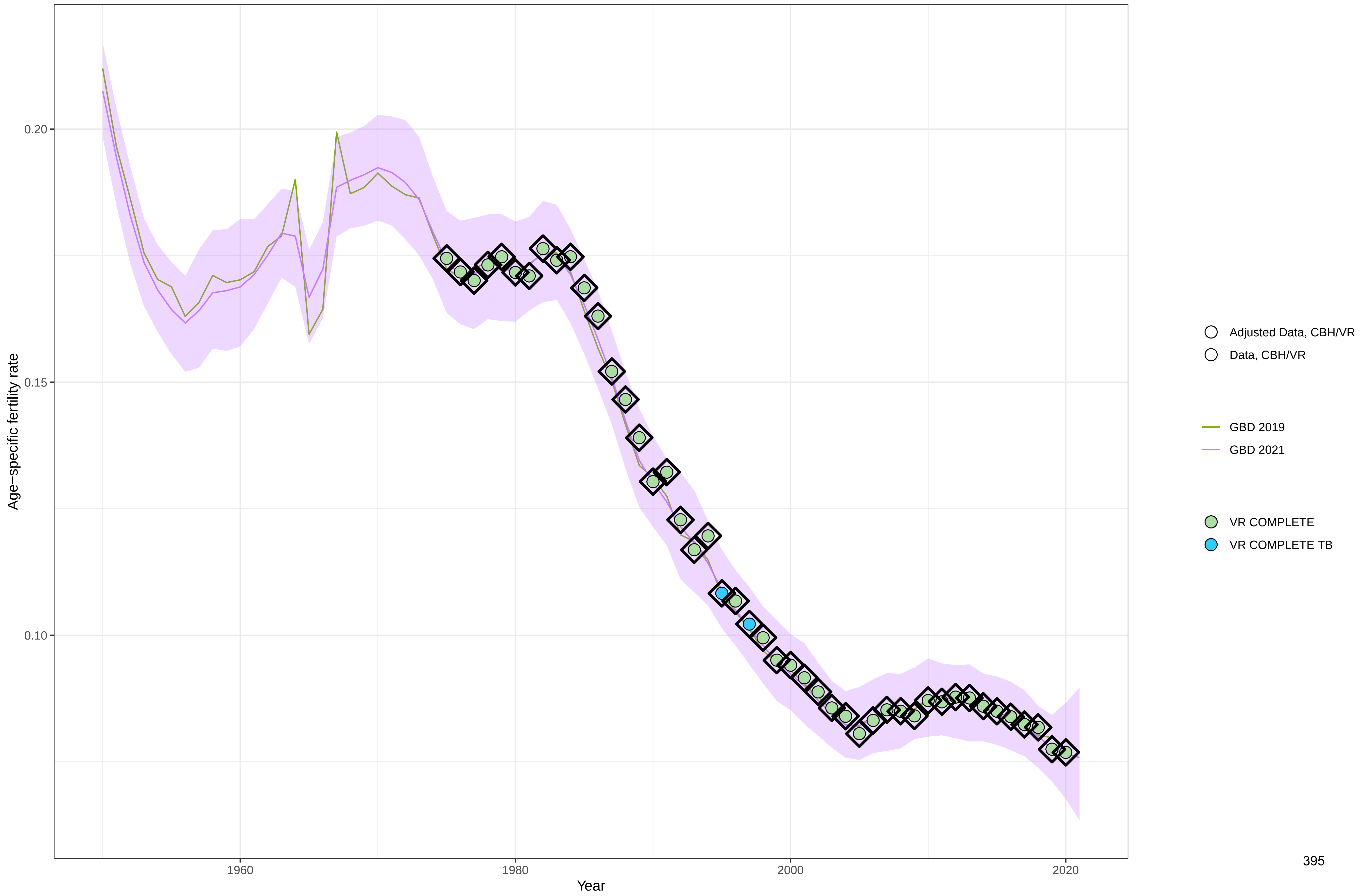

Age−specific fertility fitted trend lines, 1950−2021: Fukuoka, Ages: 30−34
Data points enclosed with a diamond are reference sources

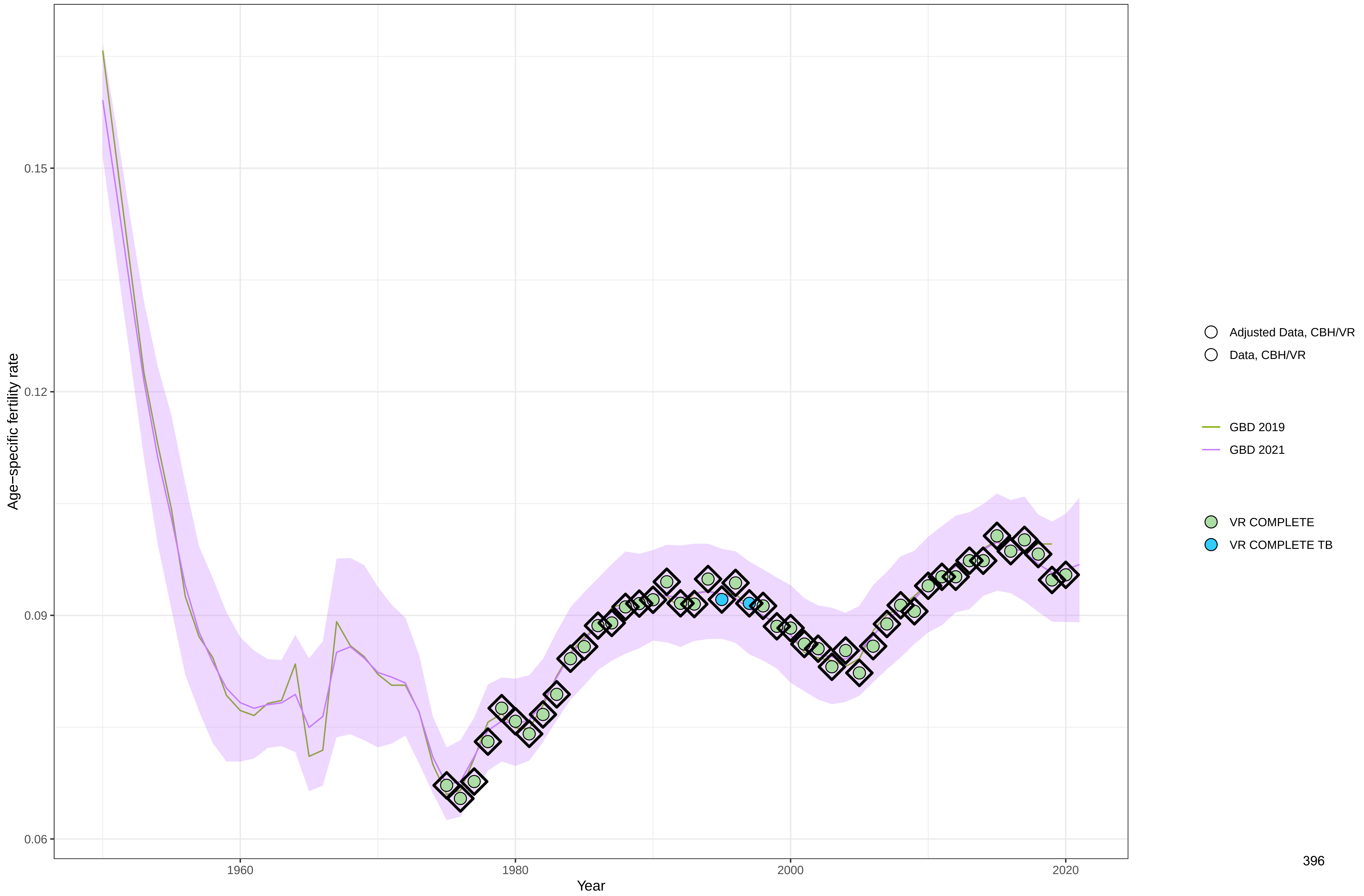

Age−specific fertility fitted trend lines, 1950−2021: Fukuoka, Ages: 35−39
Data points enclosed with a diamond are reference sources

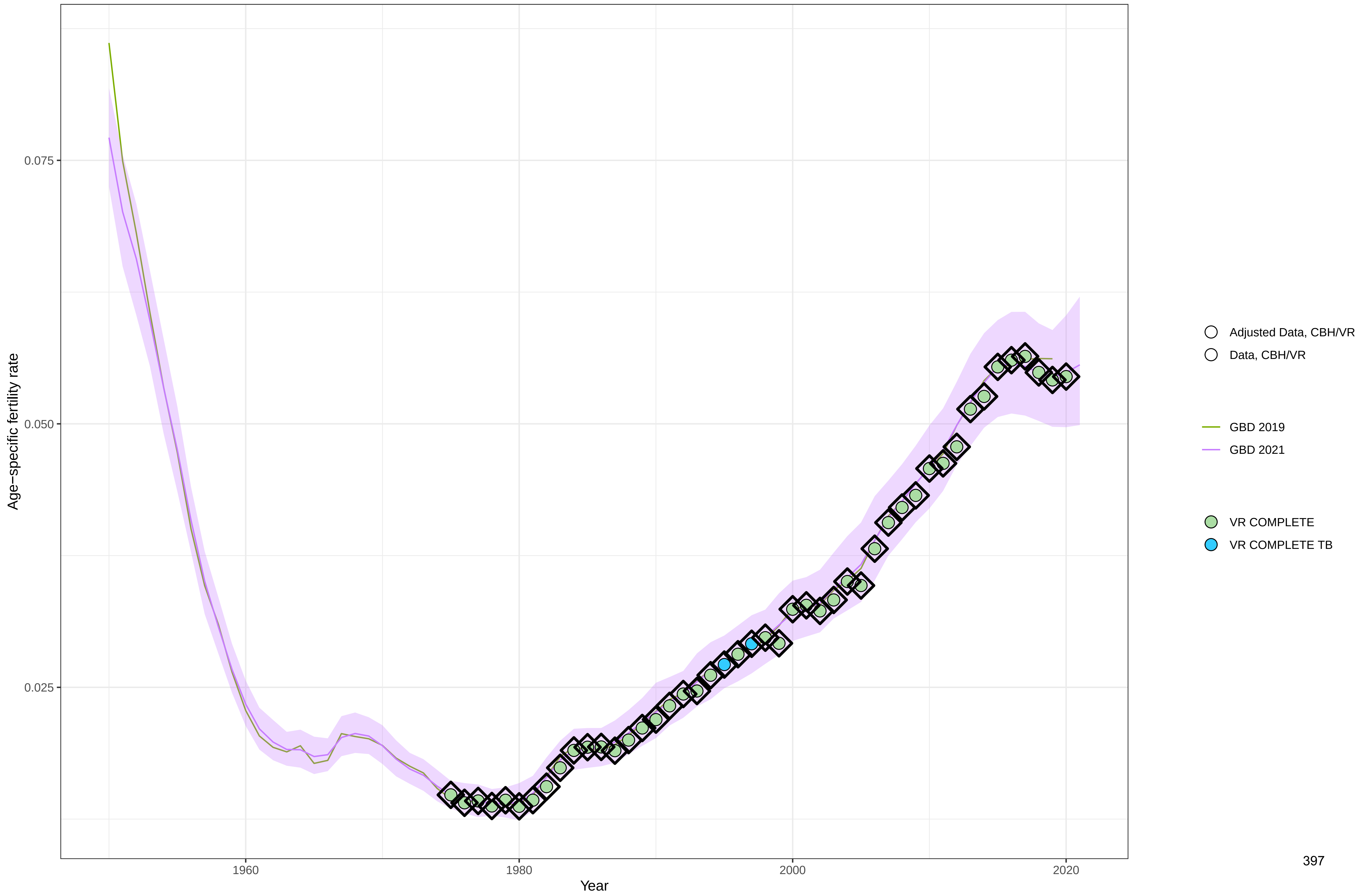

Age−specific fertility fitted trend lines, 1950−2021: Fukuoka, Ages: 40−44
Data points enclosed with a diamond are reference sources

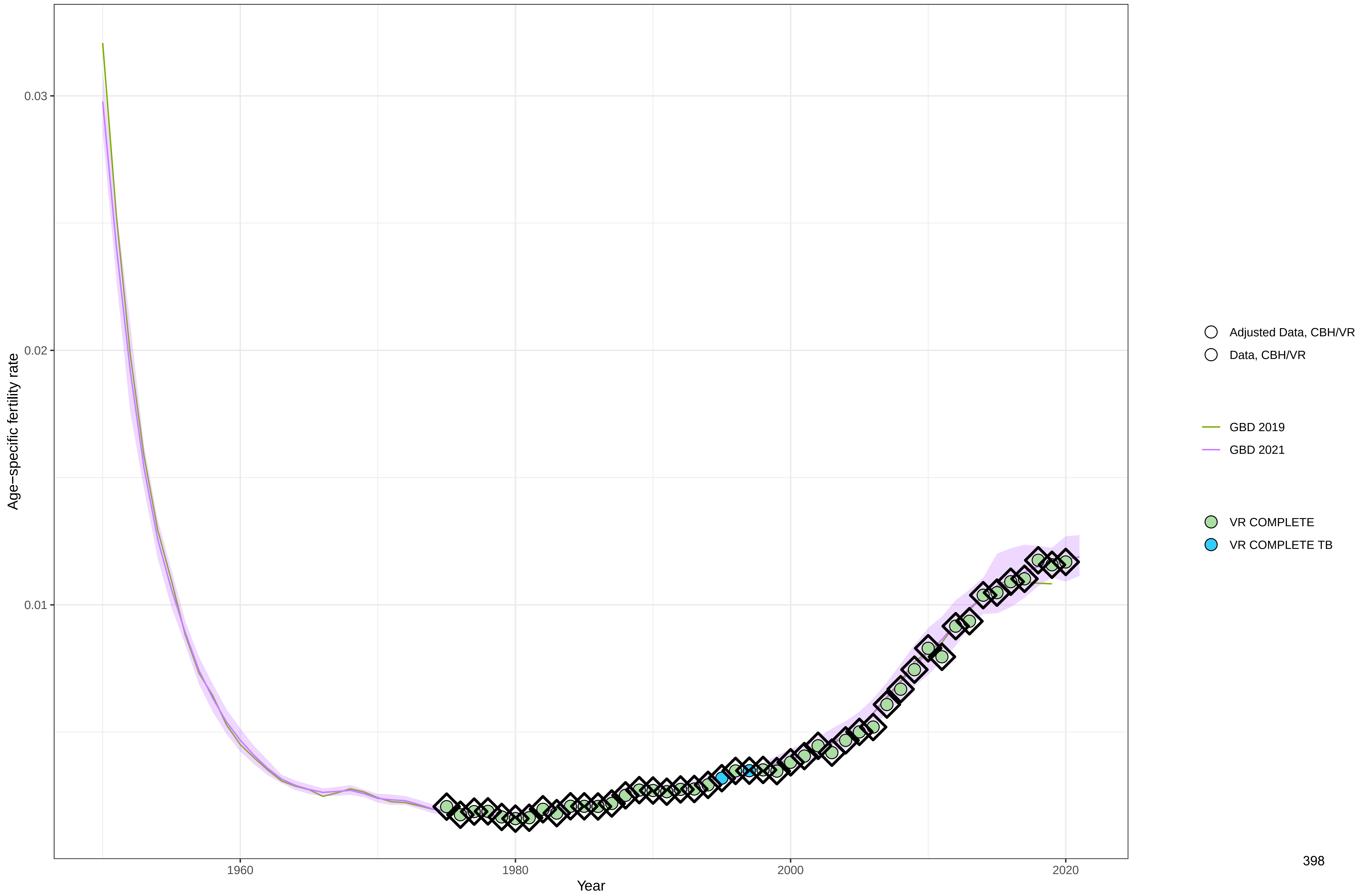

Age−specific fertility fitted trend lines, 1950−2021: Fukuoka, Ages: 45−49
Data points enclosed with a diamond are reference sources

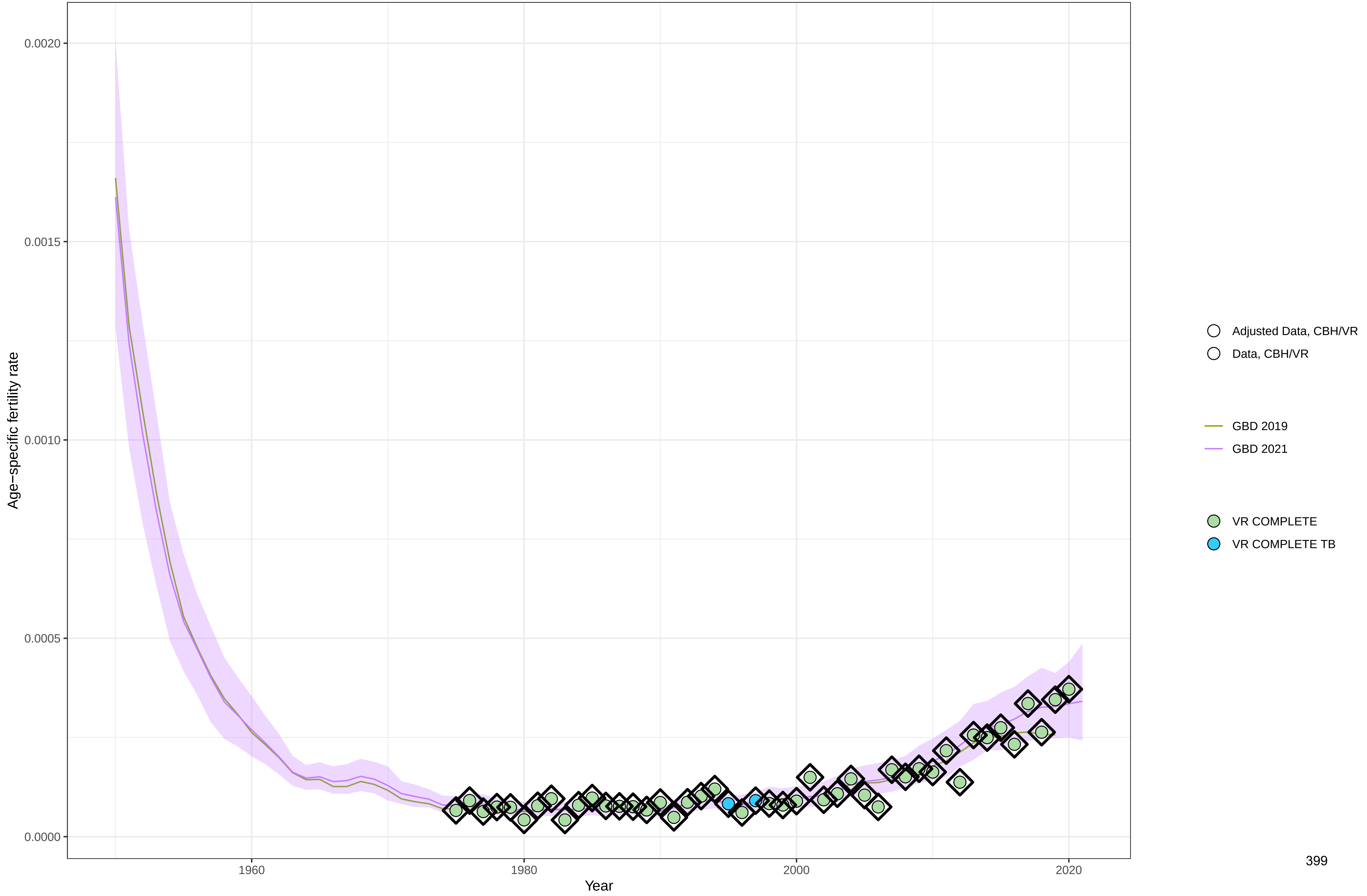

Age−specific fertility fitted trend lines, 1950−2021: Fukuoka, Ages: 50−54
Data points enclosed with a diamond are reference sources

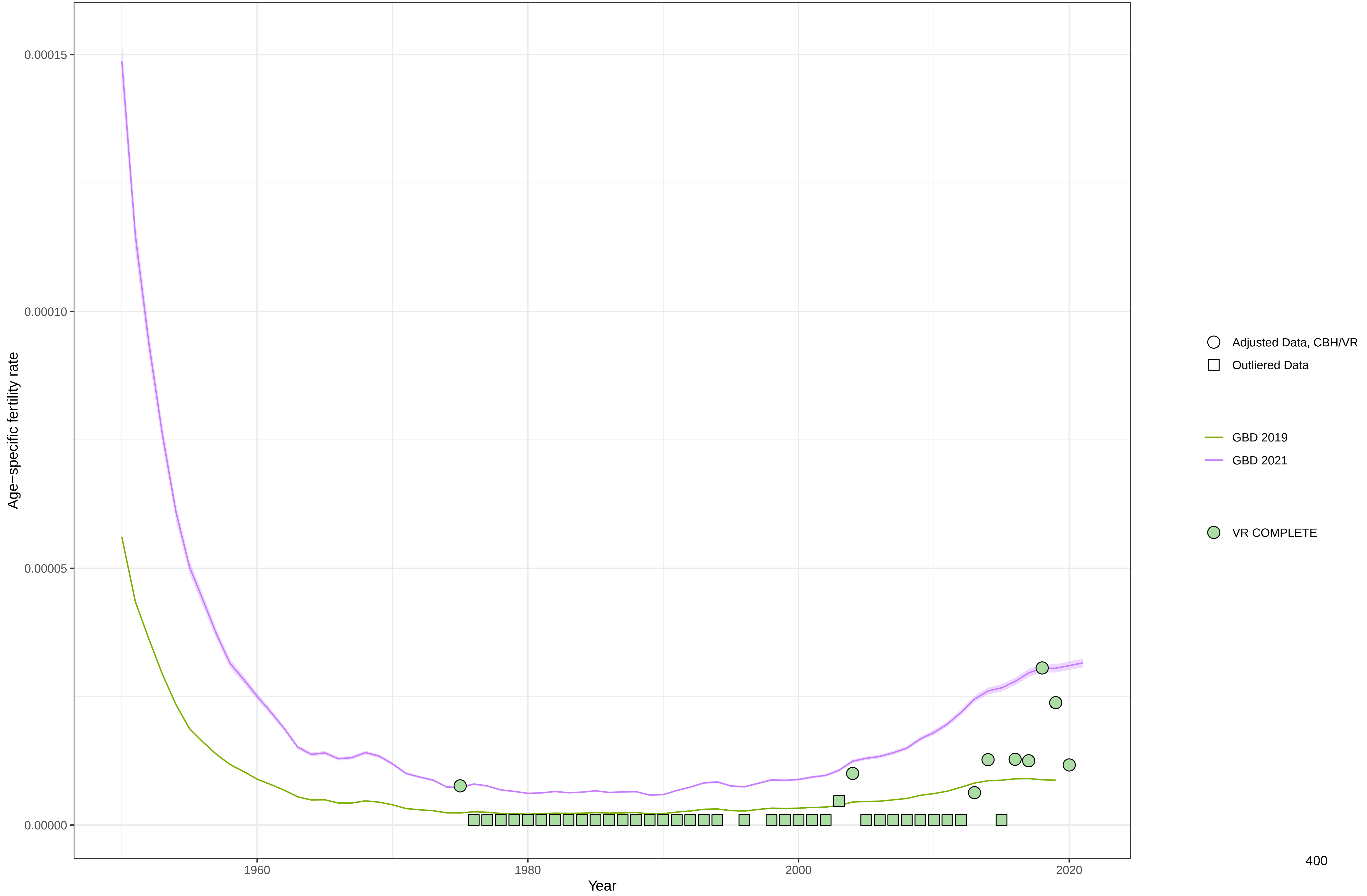

Total fertility rate fitted trend lines, 1950−2021: Fukuoka TFR
Data points enclosed with a diamond are reference sources

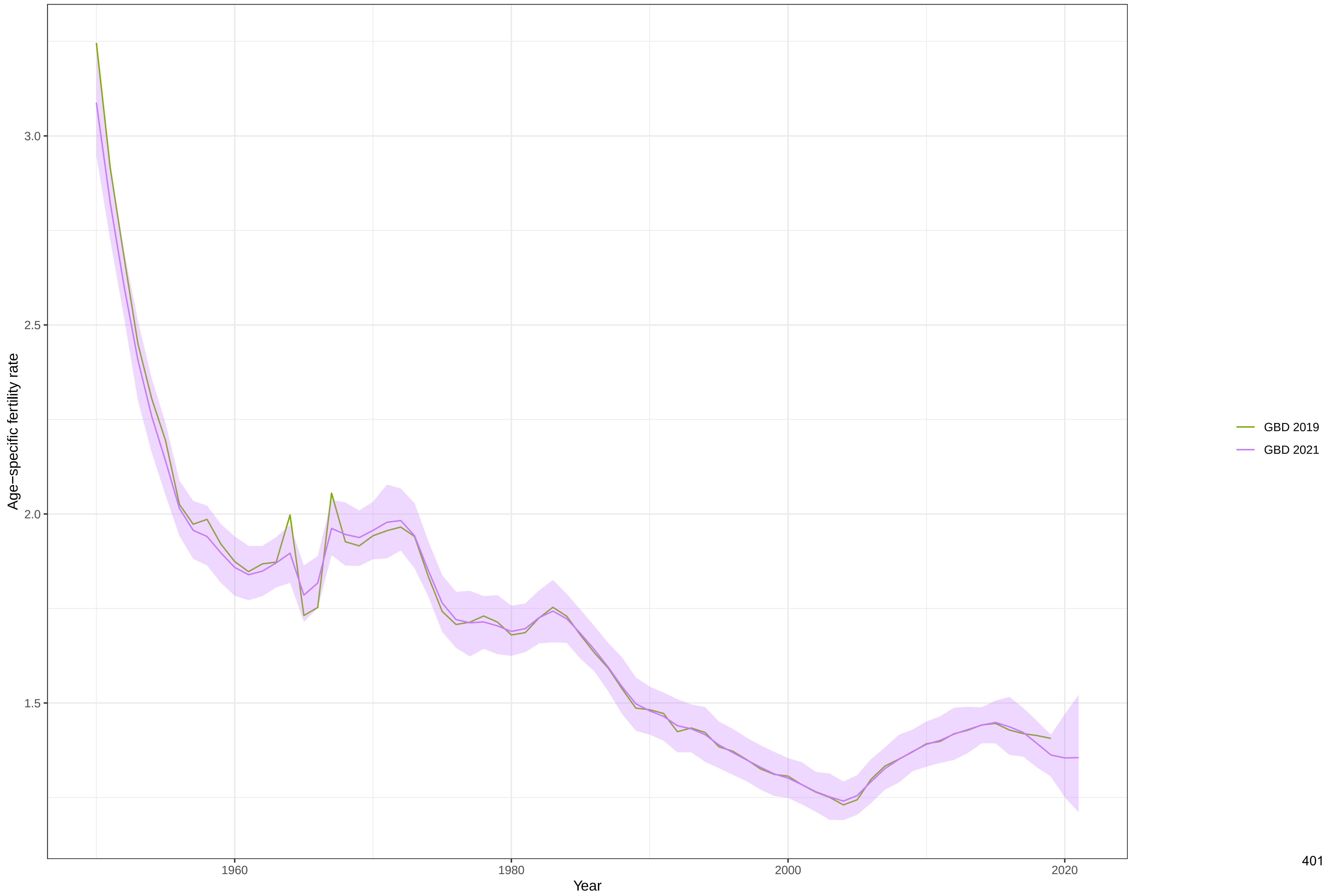

Age−specific fertility fitted trend lines, 1950−2021: Fukushima, Ages: 10−14
Data points enclosed with a diamond are reference sources

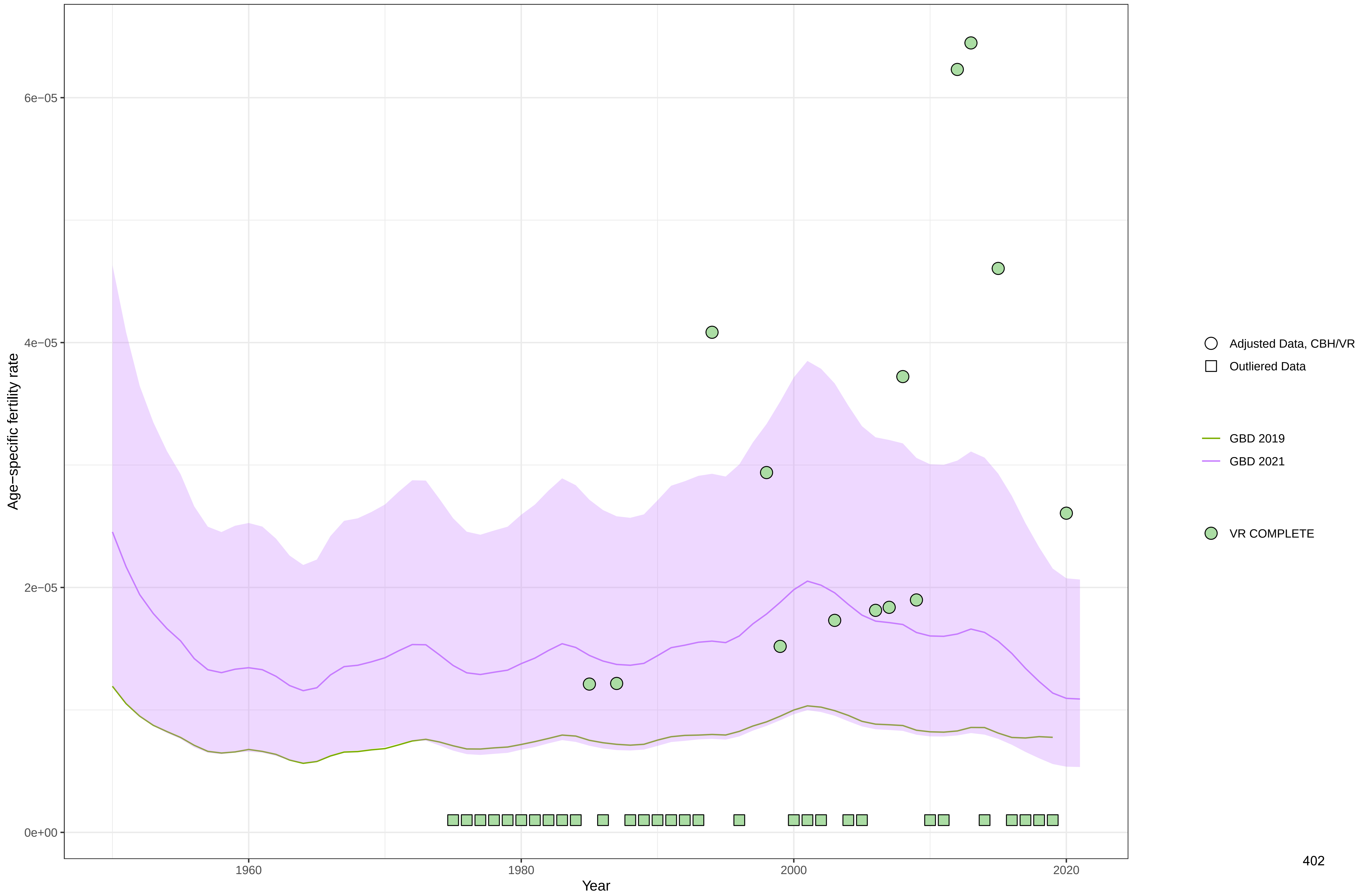

Age−specific fertility fitted trend lines, 1950−2021: Fukushima, Ages: 15−19
Data points enclosed with a diamond are reference sources

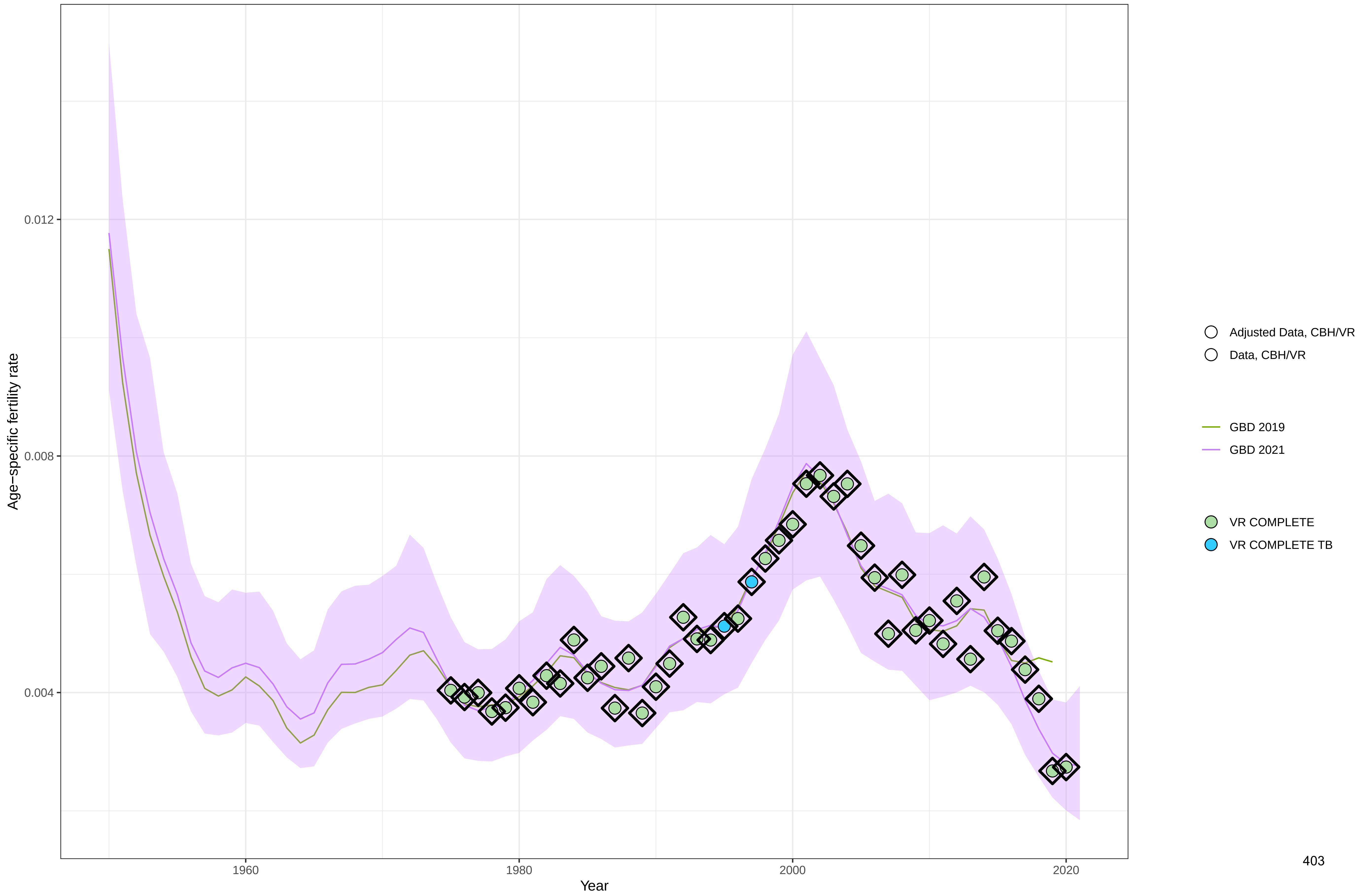

Age−specific fertility fitted trend lines, 1950−2021: Fukushima, Ages: 20−24
Data points enclosed with a diamond are reference sources

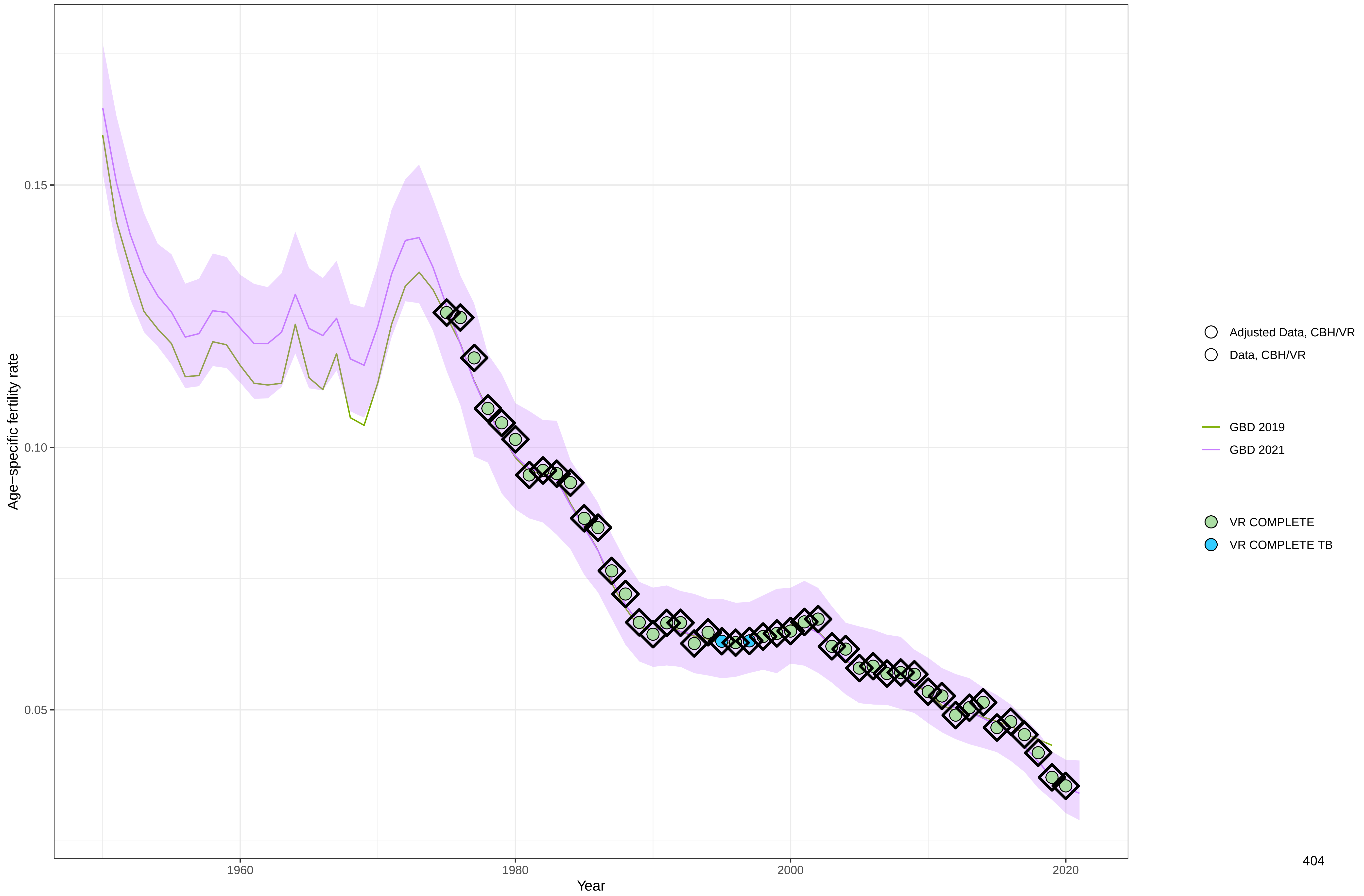

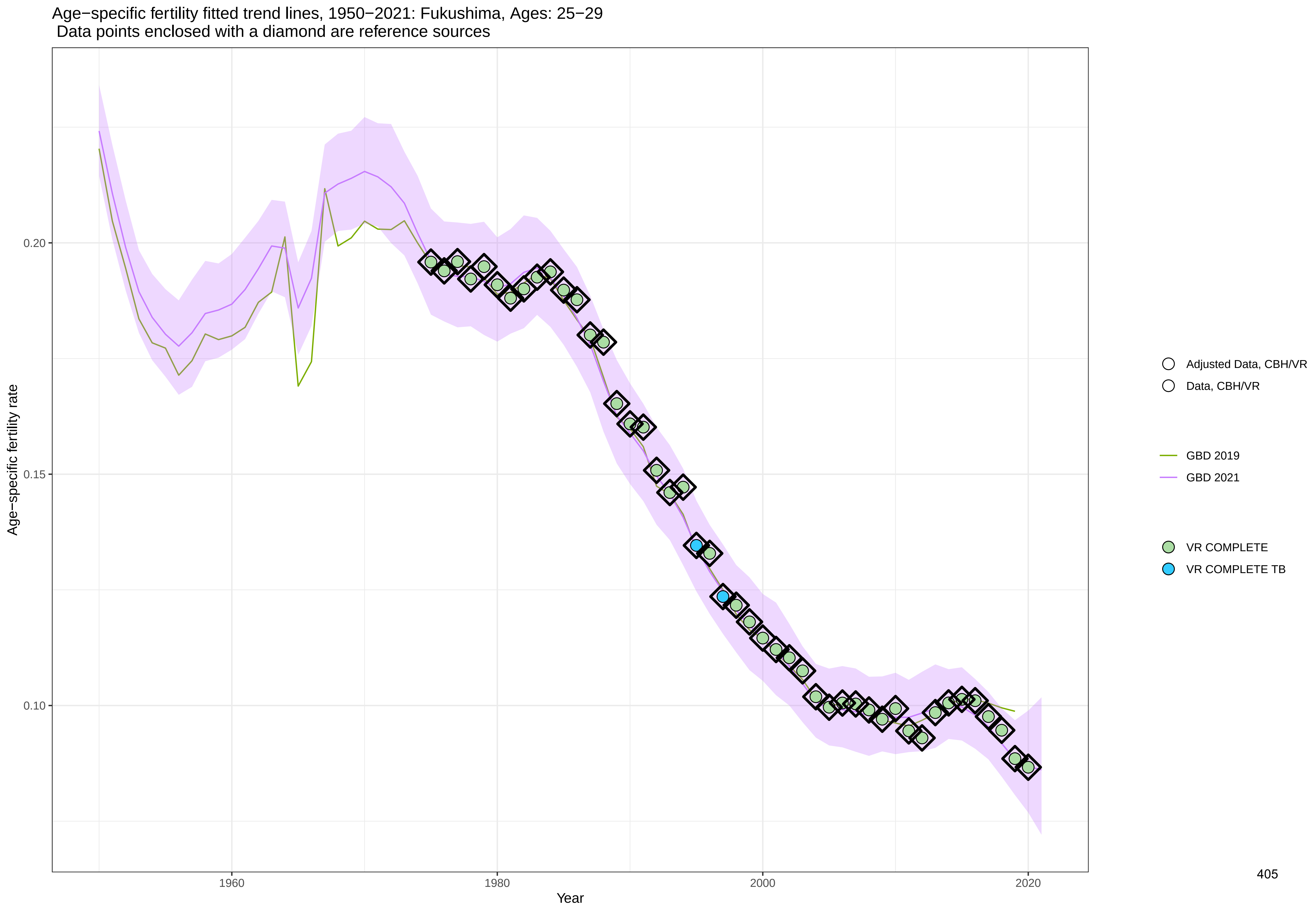
Age−specific fertility fitted trend lines, 1950−2021: Fukushima, Ages: 25−29
Data points enclosed with a diamond are reference sources
Age−specific fertility rate
0.20
0.15
0.10
1960
1980
2000
2020
Year
Adjusted Data, CBH/VR
Data, CBH/VR
GBD 2019
GBD 2021
VR COMPLETE
VR COMPLETE TB

Age−specific fertility fitted trend lines, 1950−2021: Fukushima, Ages: 30−34
Data points enclosed with a diamond are reference sources

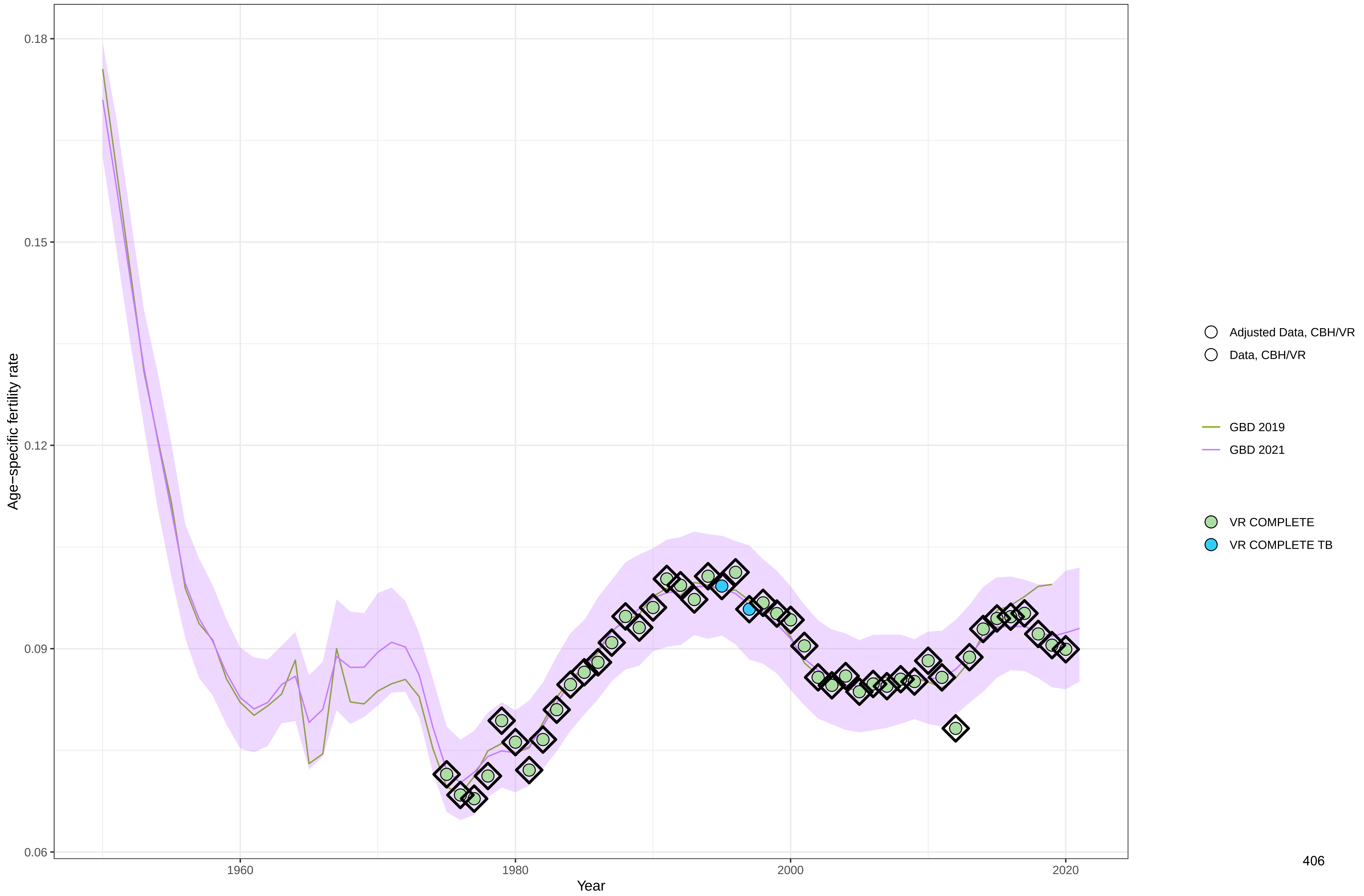

Age−specific fertility fitted trend lines, 1950−2021: Fukushima, Ages: 35−39
Data points enclosed with a diamond are reference sources

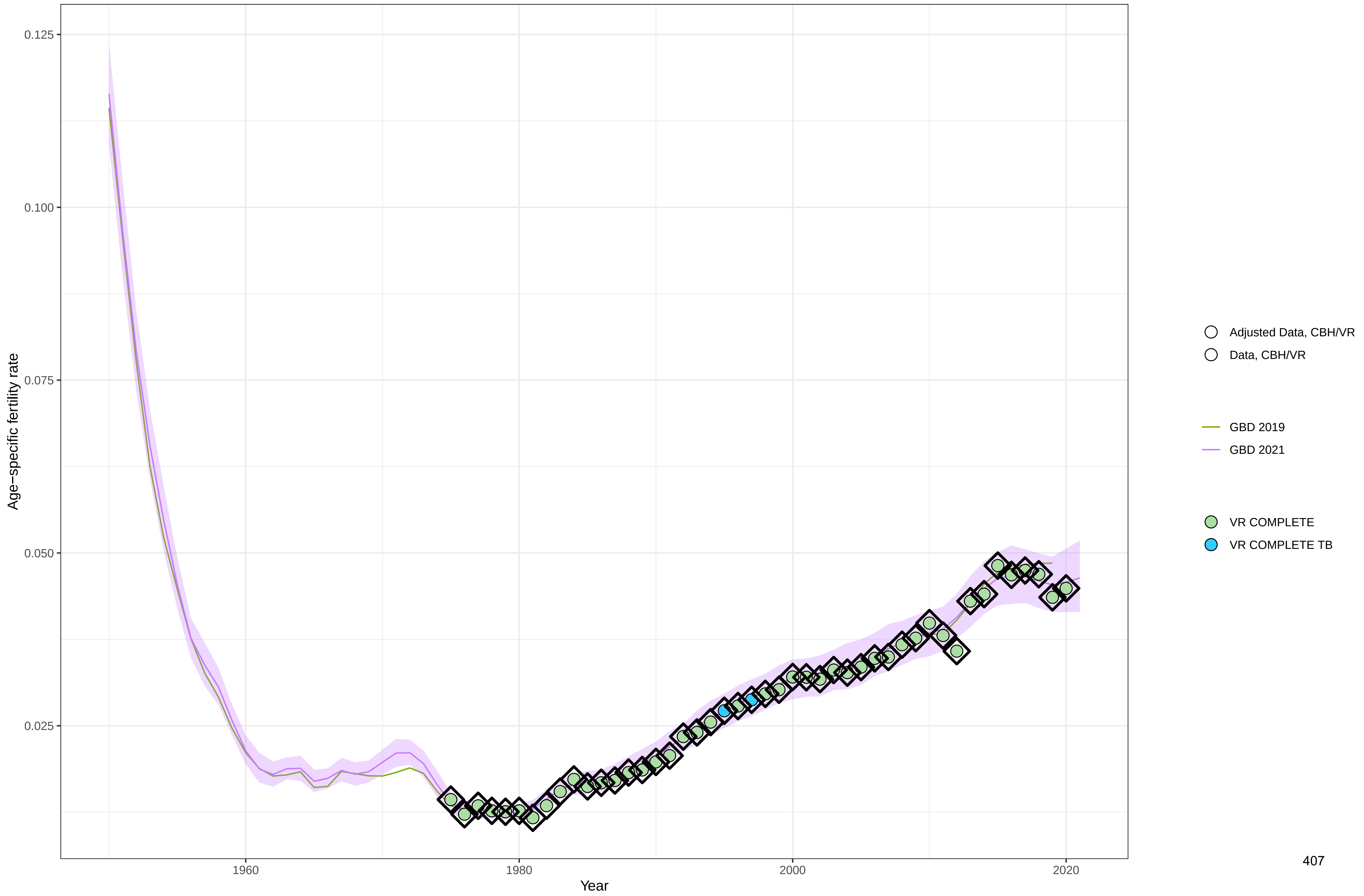

Age−specific fertility fitted trend lines, 1950−2021: Fukushima, Ages: 40−44
Data points enclosed with a diamond are reference sources

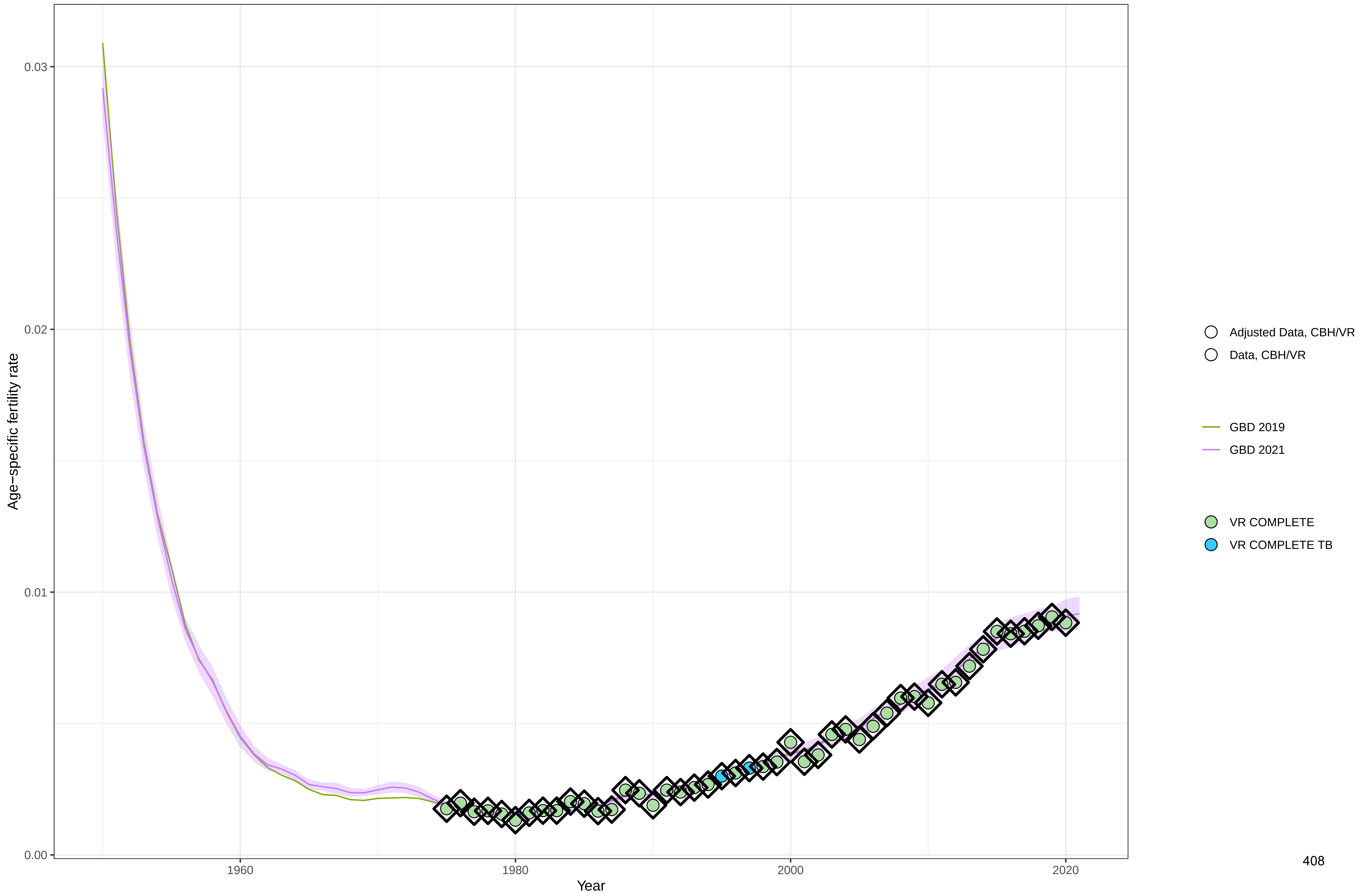

Age−specific fertility fitted trend lines, 1950−2021: Fukushima, Ages: 45−49
Data points enclosed with a diamond are reference sources

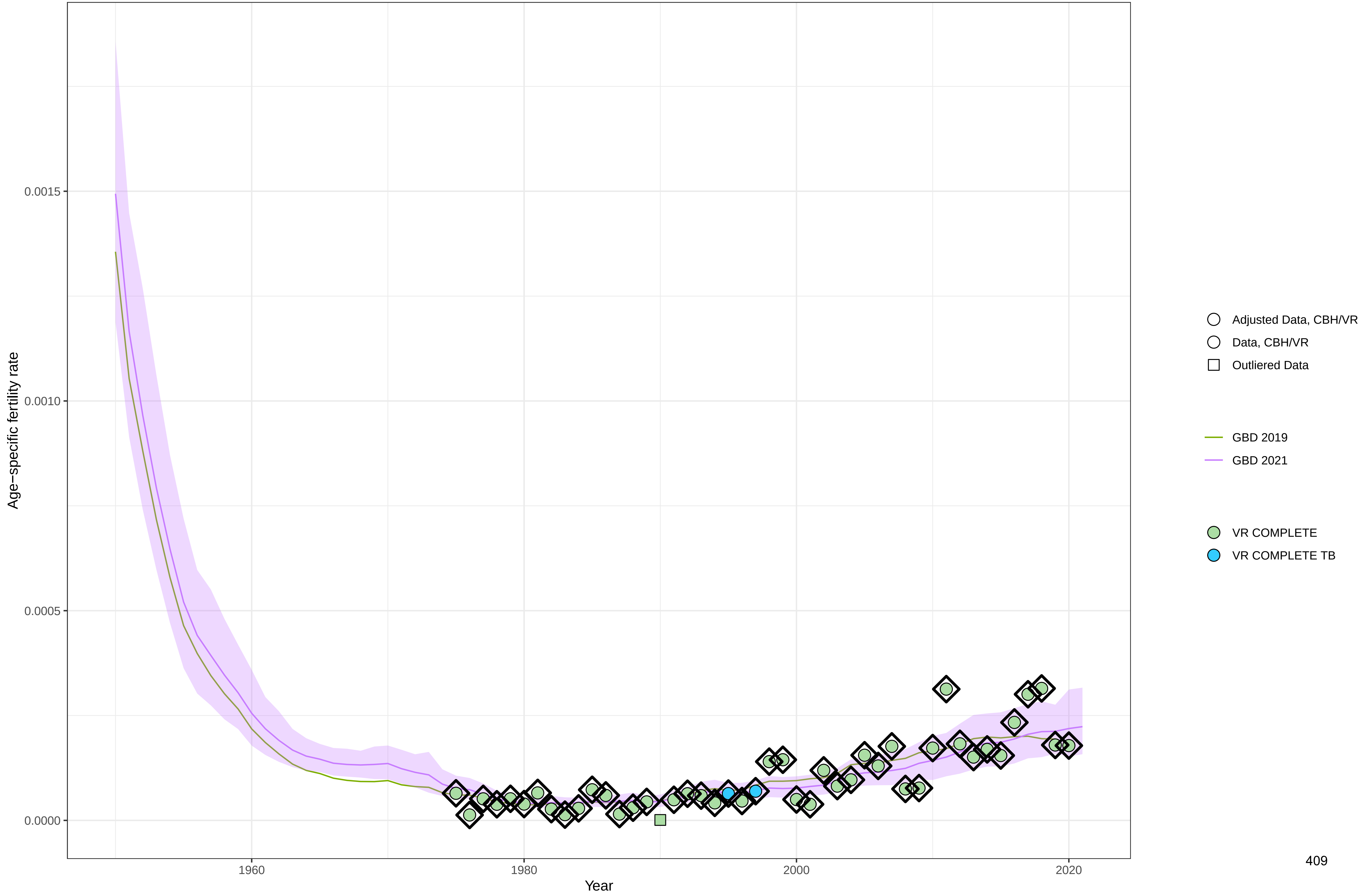

Age−specific fertility fitted trend lines, 1950−2021: Fukushima, Ages: 50−54
Data points enclosed with a diamond are reference sources

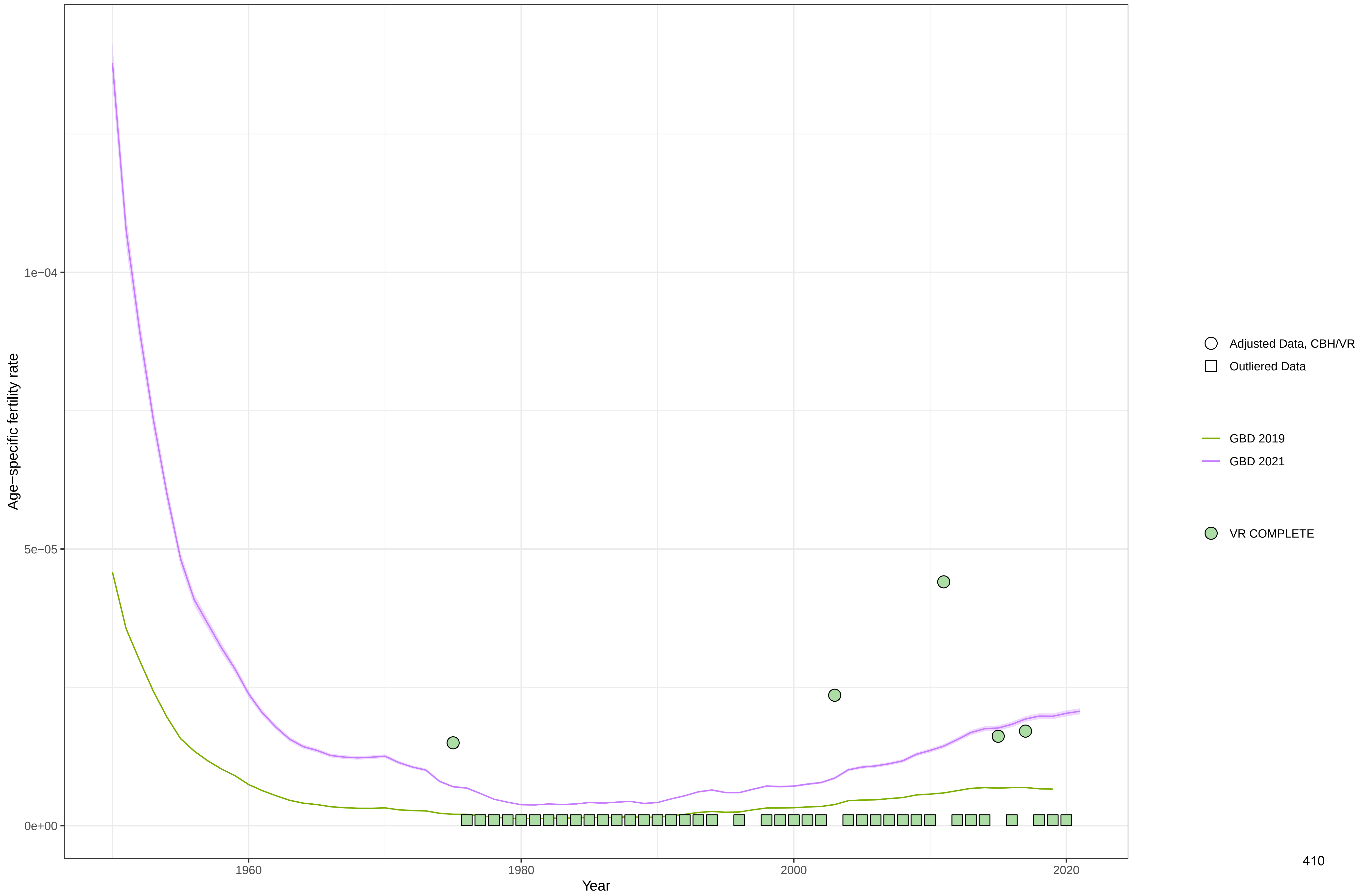

Total fertility rate fitted trend lines, 1950−2021: Fukushima TFR
Data points enclosed with a diamond are reference sources

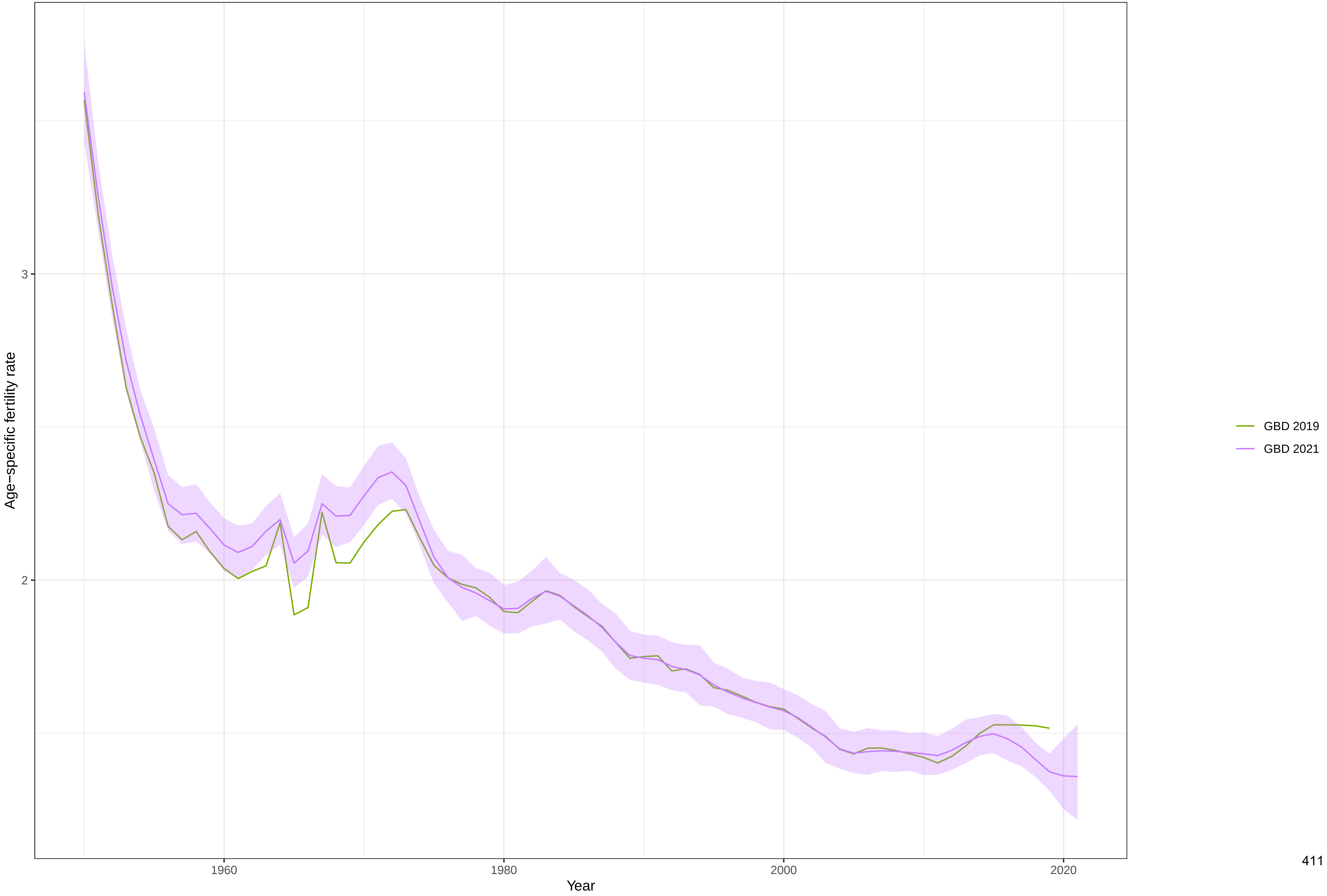

Age−specific fertility fitted trend lines, 1950−2021: Gifu, Ages: 10−14
Data points enclosed with a diamond are reference sources

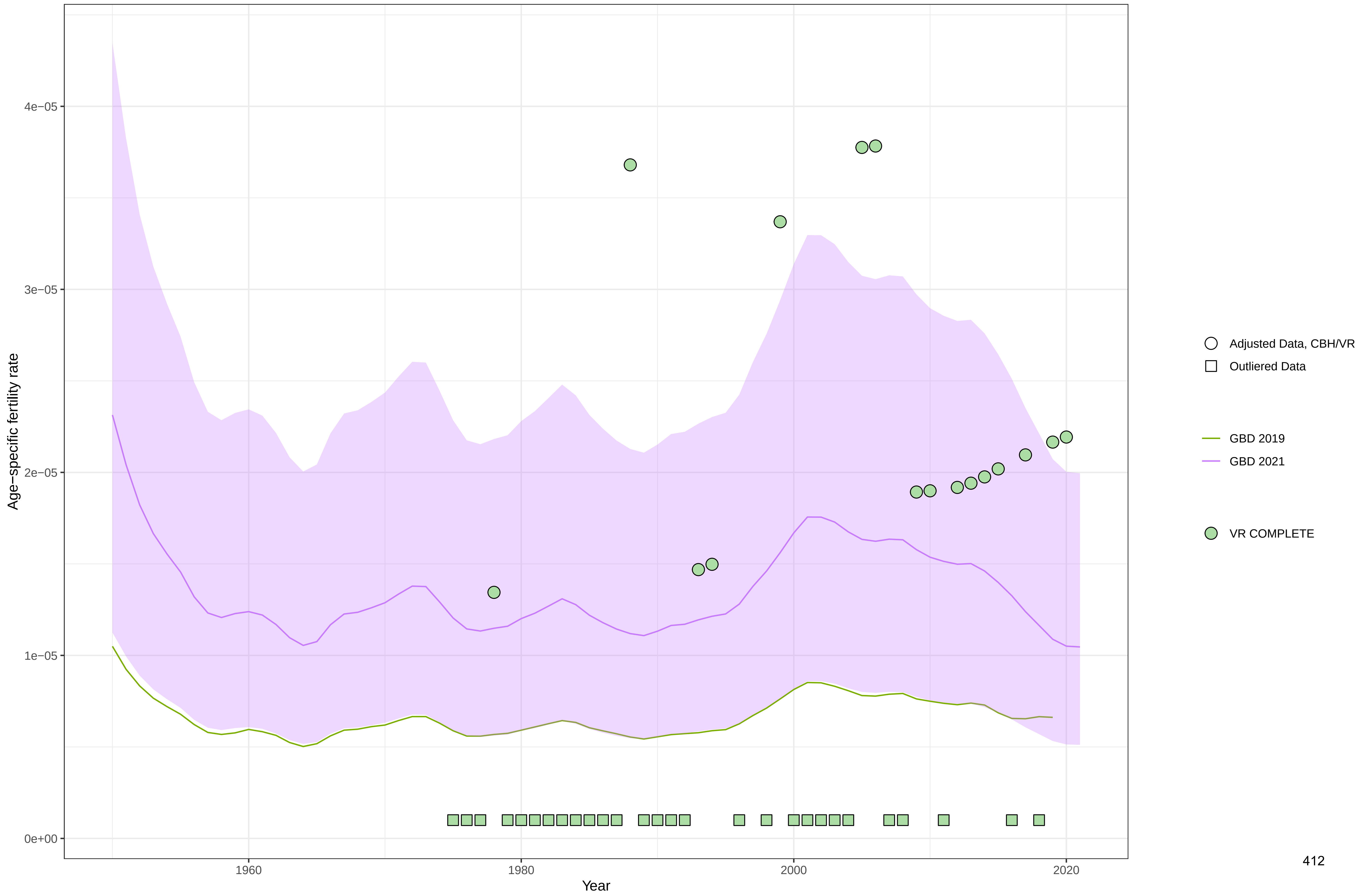

Age−specific fertility fitted trend lines, 1950−2021: Gifu, Ages: 15−19
Data points enclosed with a diamond are reference sources

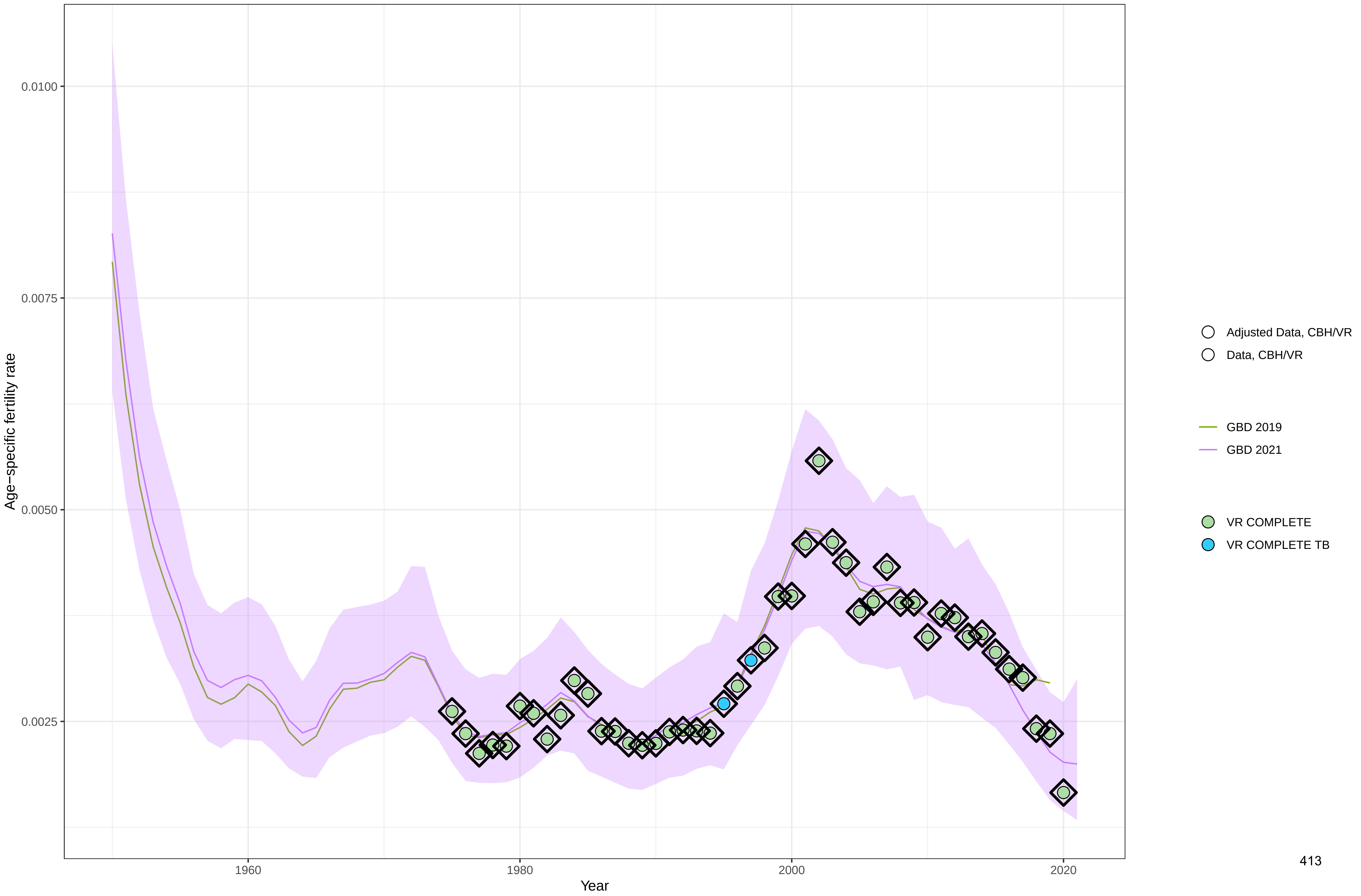

Age−specific fertility fitted trend lines, 1950−2021: Gifu, Ages: 20−24
Data points enclosed with a diamond are reference sources

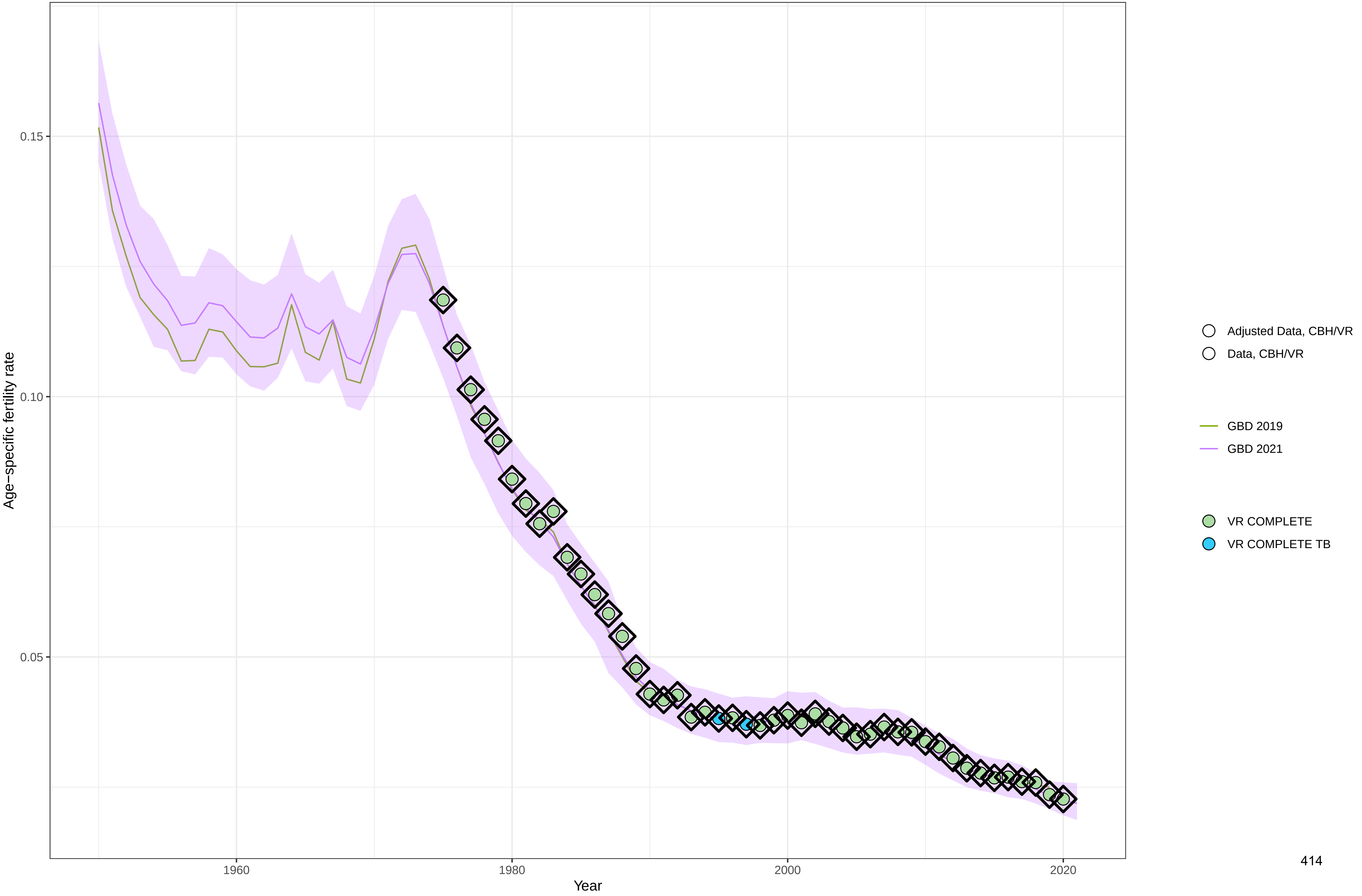

Age−specific fertility fitted trend lines, 1950−2021: Gifu, Ages: 25−29
Data points enclosed with a diamond are reference sources

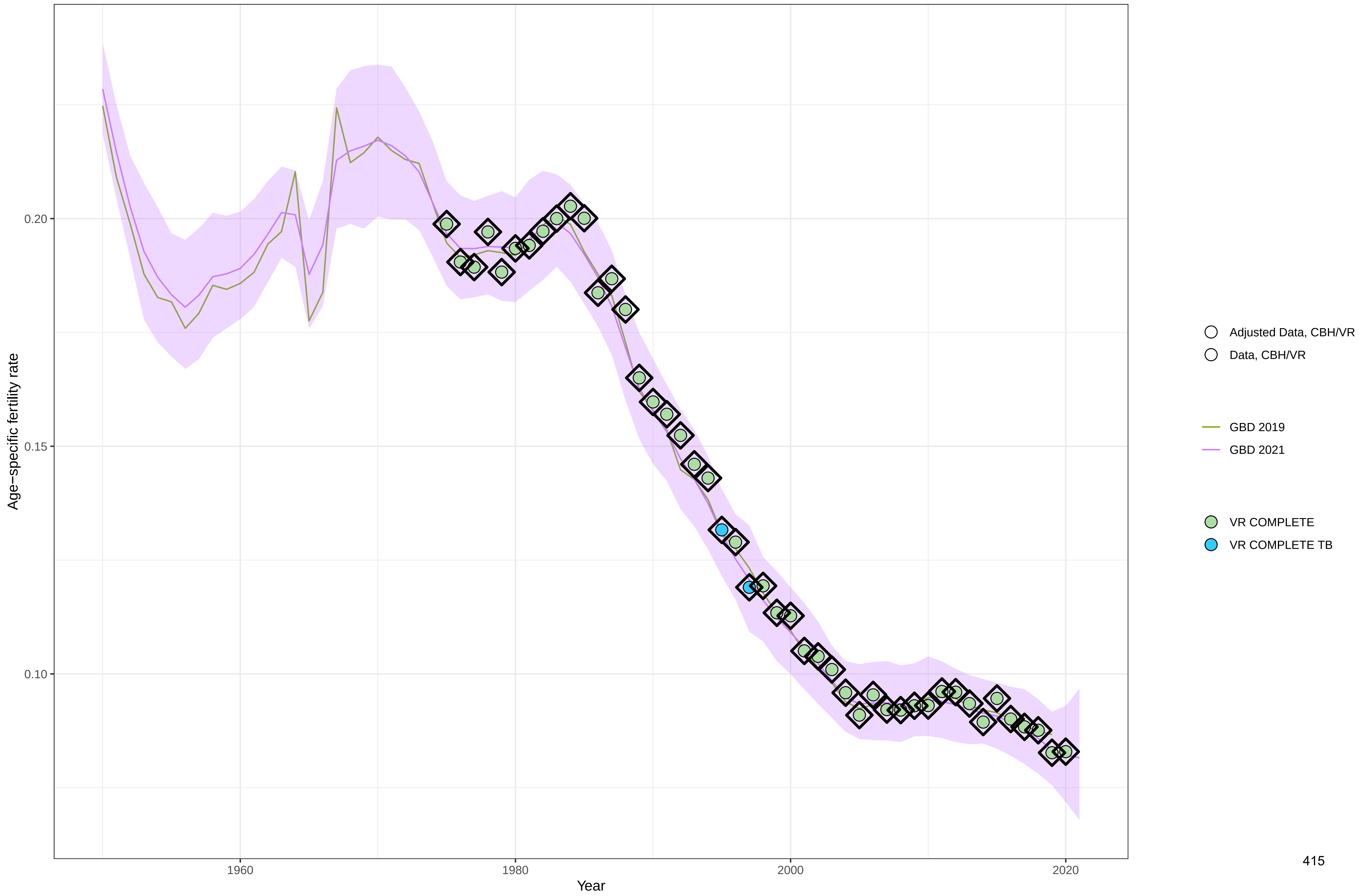

Age−specific fertility fitted trend lines, 1950−2021: Gifu, Ages: 30−34
Data points enclosed with a diamond are reference sources

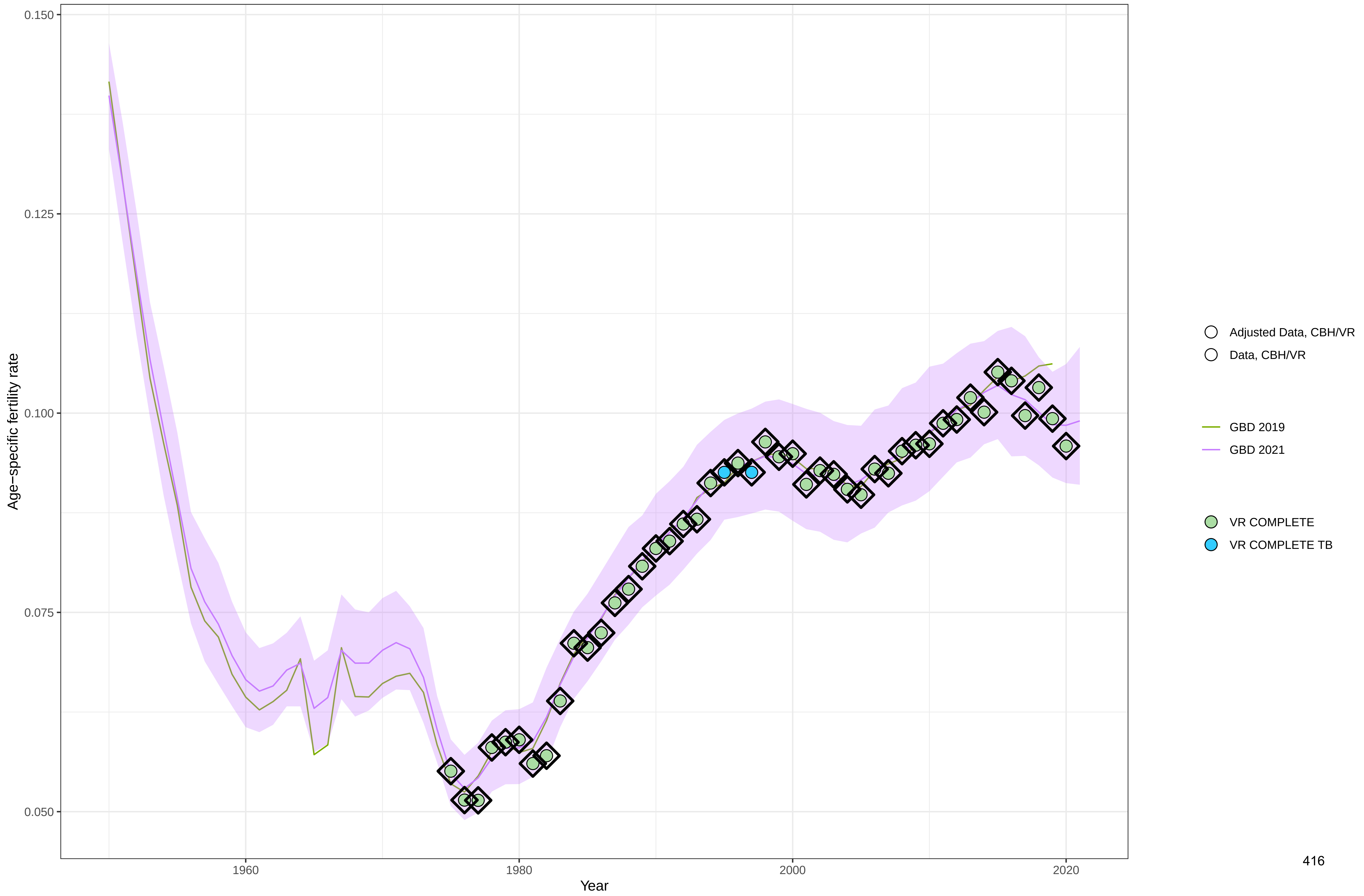

Age−specific fertility fitted trend lines, 1950−2021: Gifu, Ages: 35−39
Data points enclosed with a diamond are reference sources

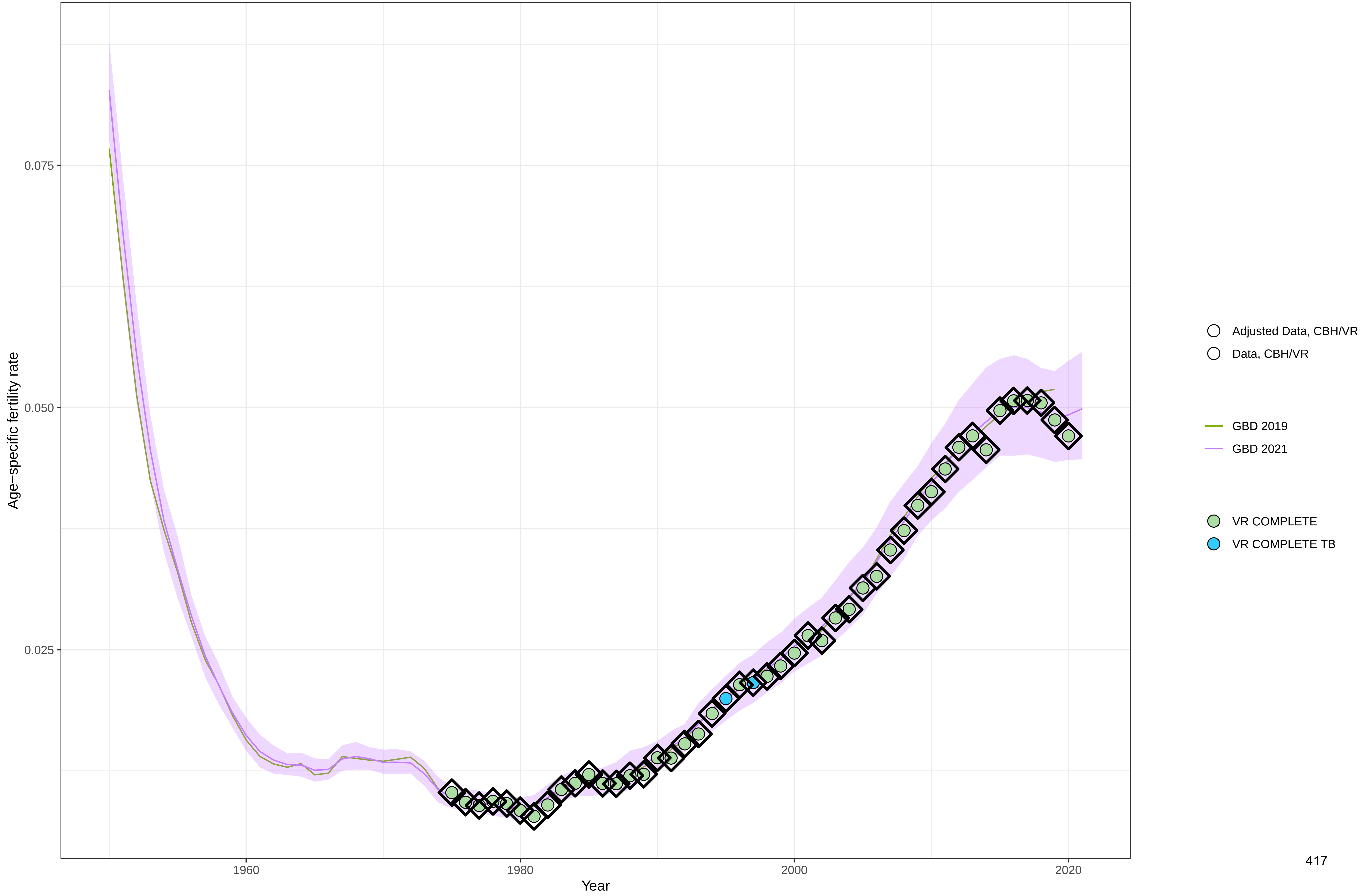

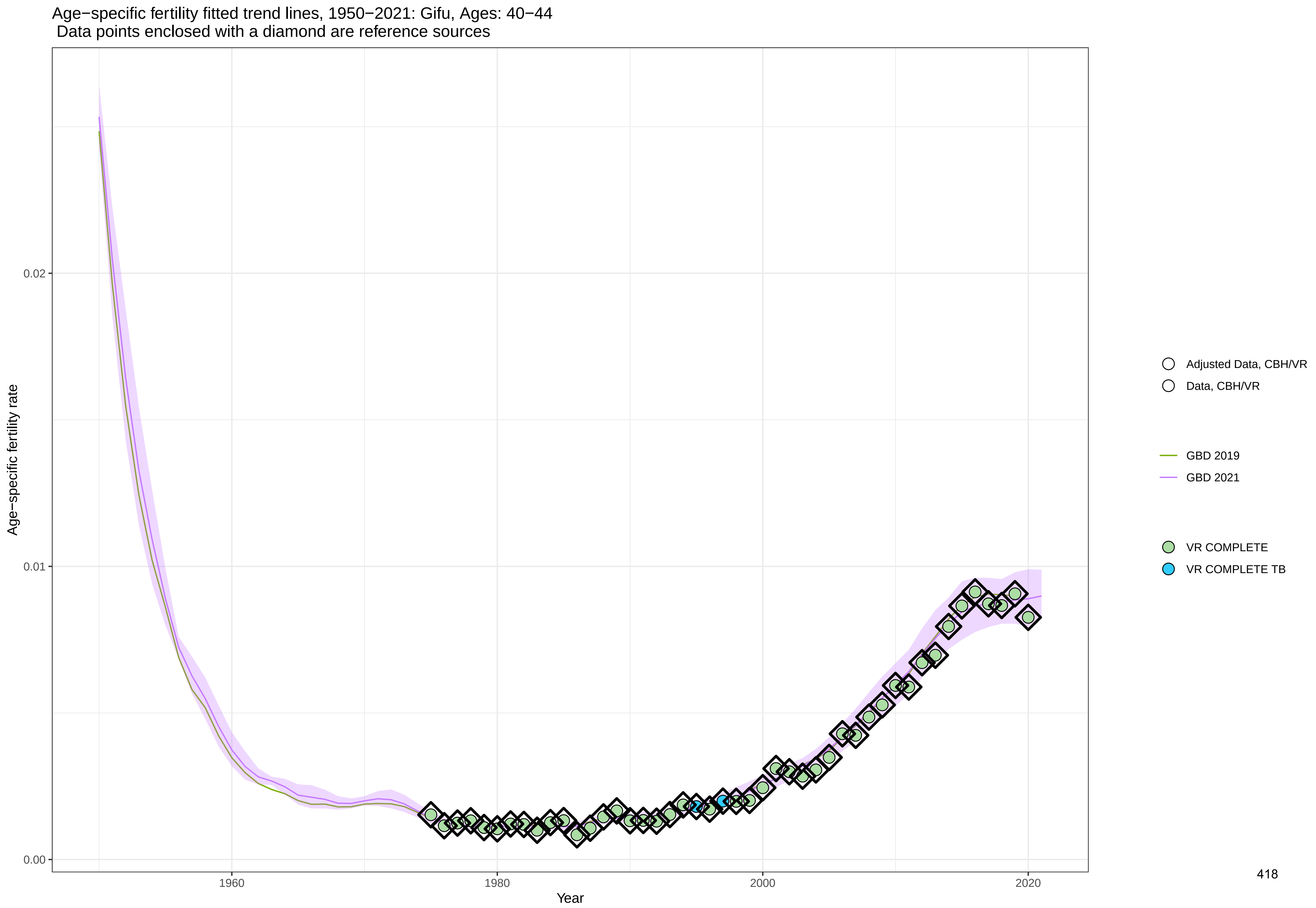

Age−specific fertility fitted trend lines, 1950−2021: Gifu, Ages: 40−44
Data points enclosed with a diamond are reference sources
Age−specific fertility rate
0.00
0.01
0.02
1960
1980
2000
2020
Year
Adjusted Data, CBH/VR
Data, CBH/VR
GBD 2019
GBD 2021
VR COMPLETE
VR COMPLETE TB

Age−specific fertility fitted trend lines, 1950−2021: Gifu, Ages: 45−49
Data points enclosed with a diamond are reference sources

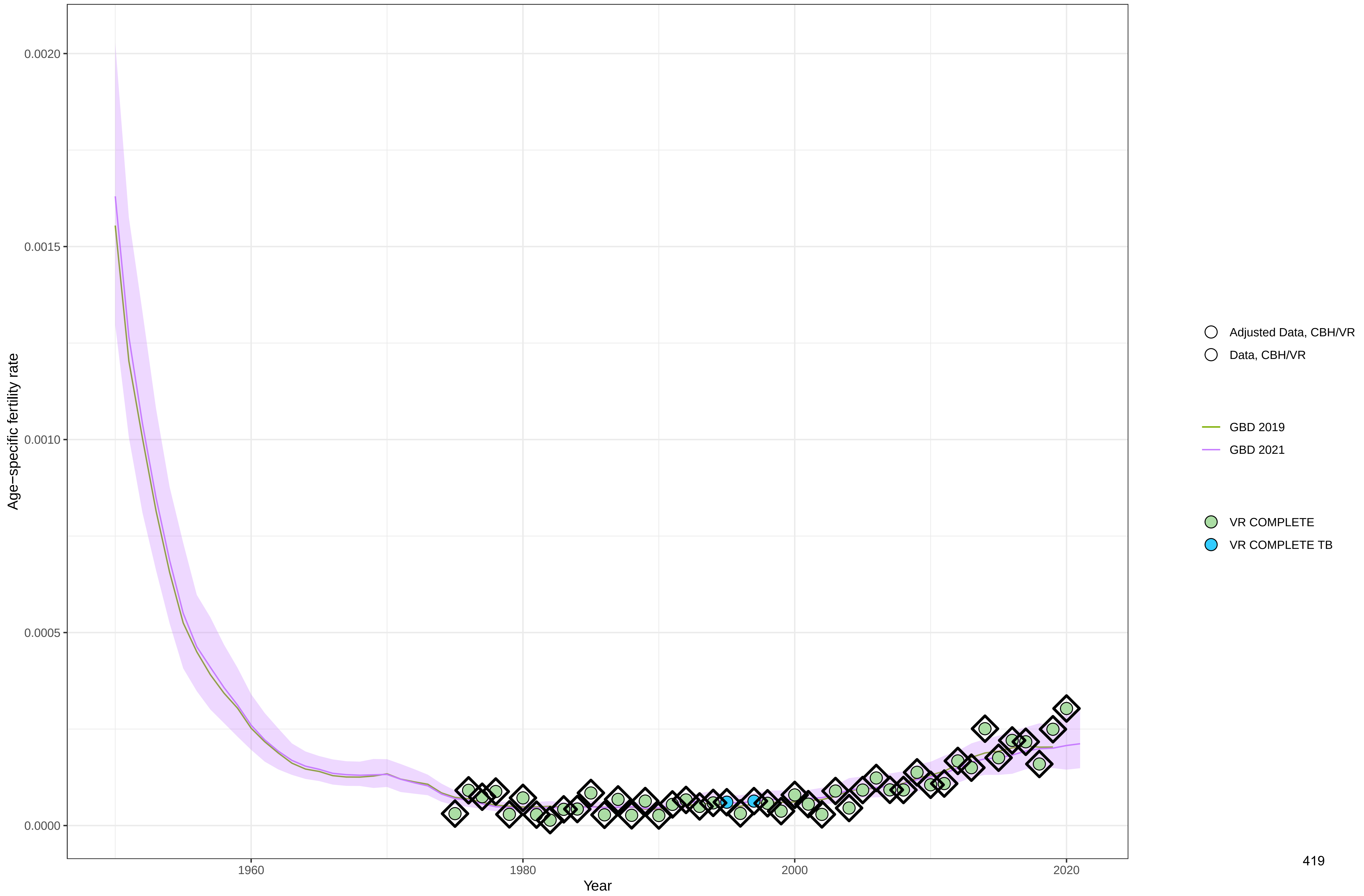

Age−specific fertility fitted trend lines, 1950−2021: Gifu, Ages: 50−54
Data points enclosed with a diamond are reference sources

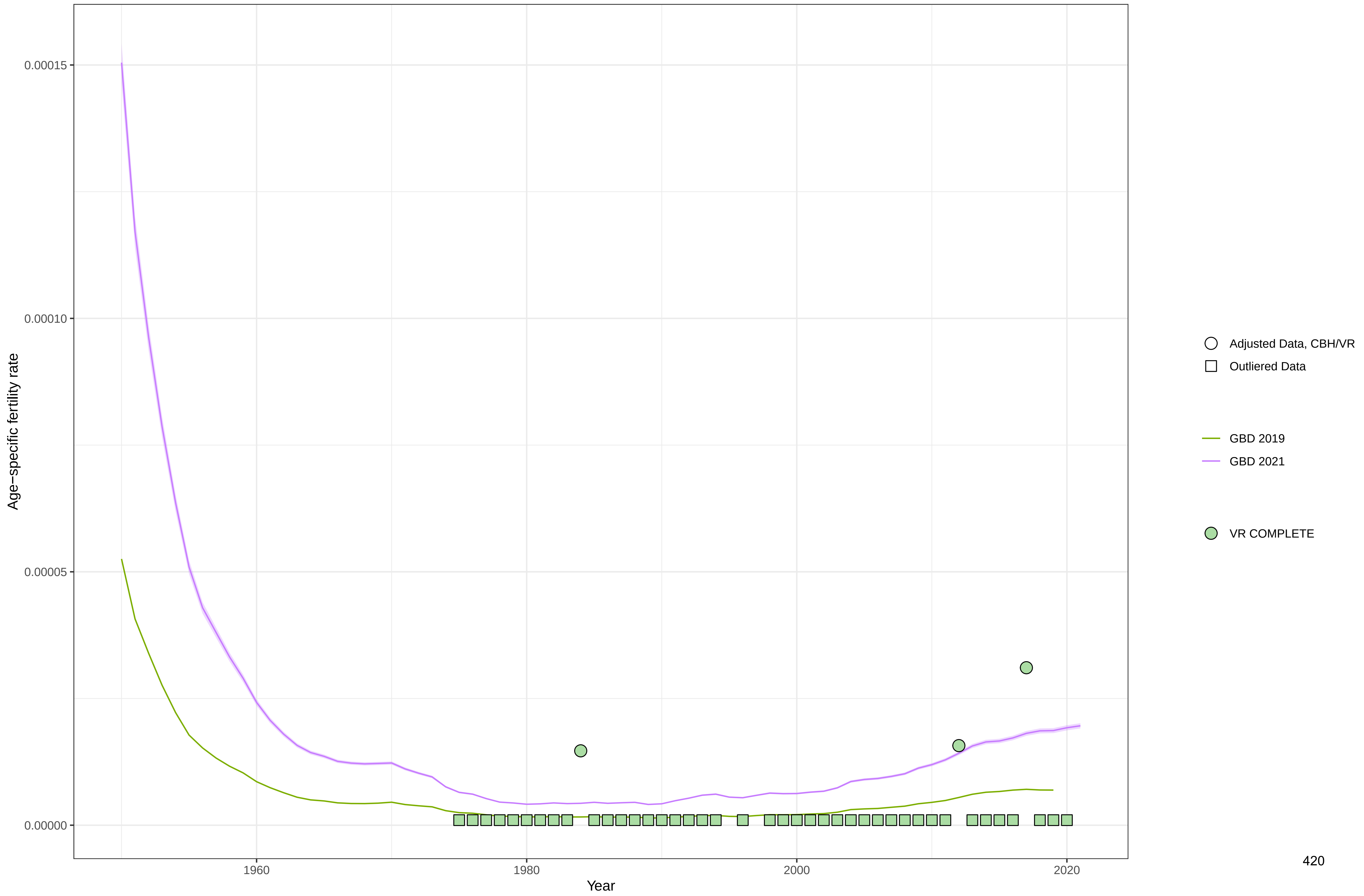

Total fertility rate fitted trend lines, 1950−2021: Gifu TFR
Data points enclosed with a diamond are reference sources

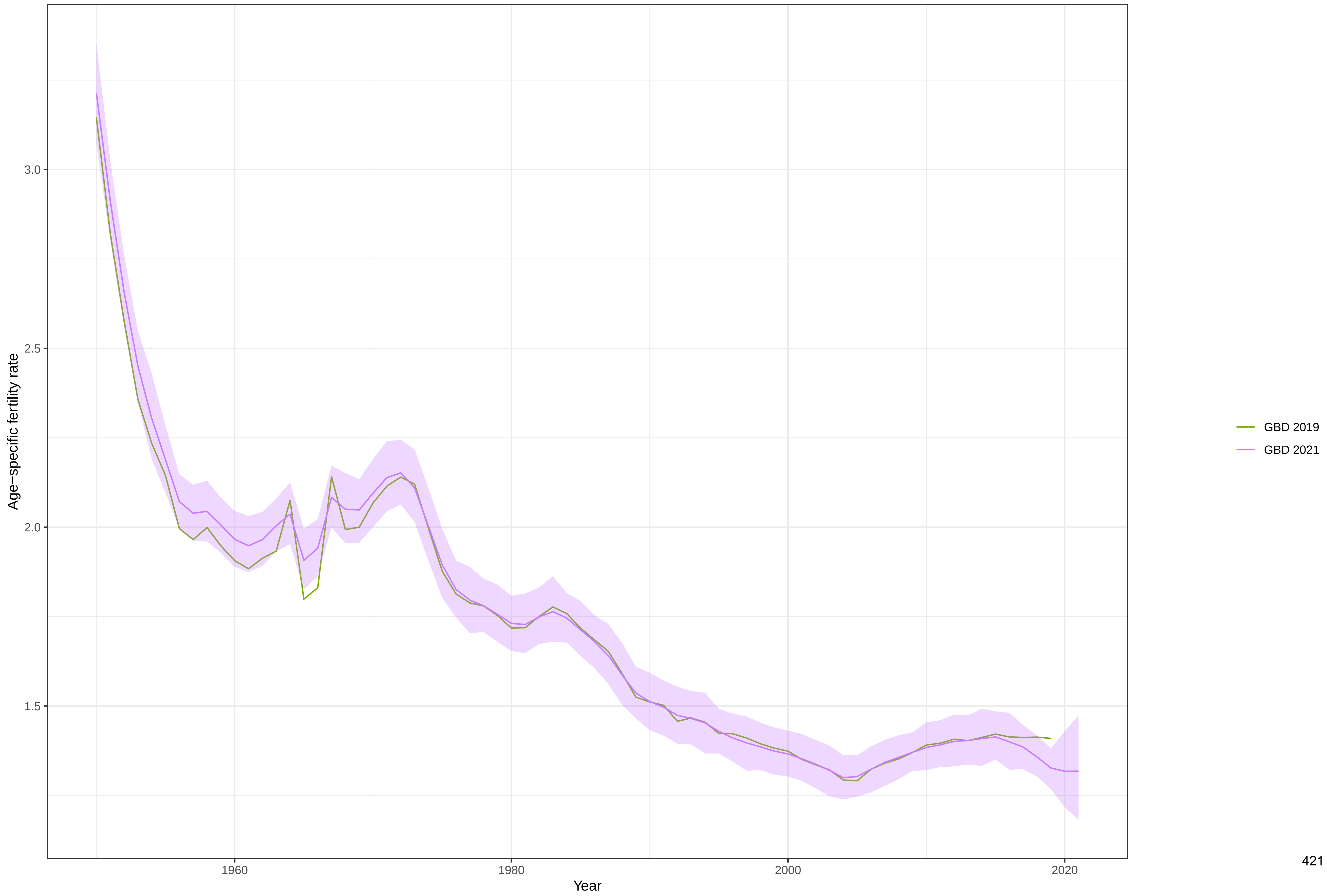

Age−specific fertility fitted trend lines, 1950−2021: Gunma, Ages: 10−14
Data points enclosed with a diamond are reference sources

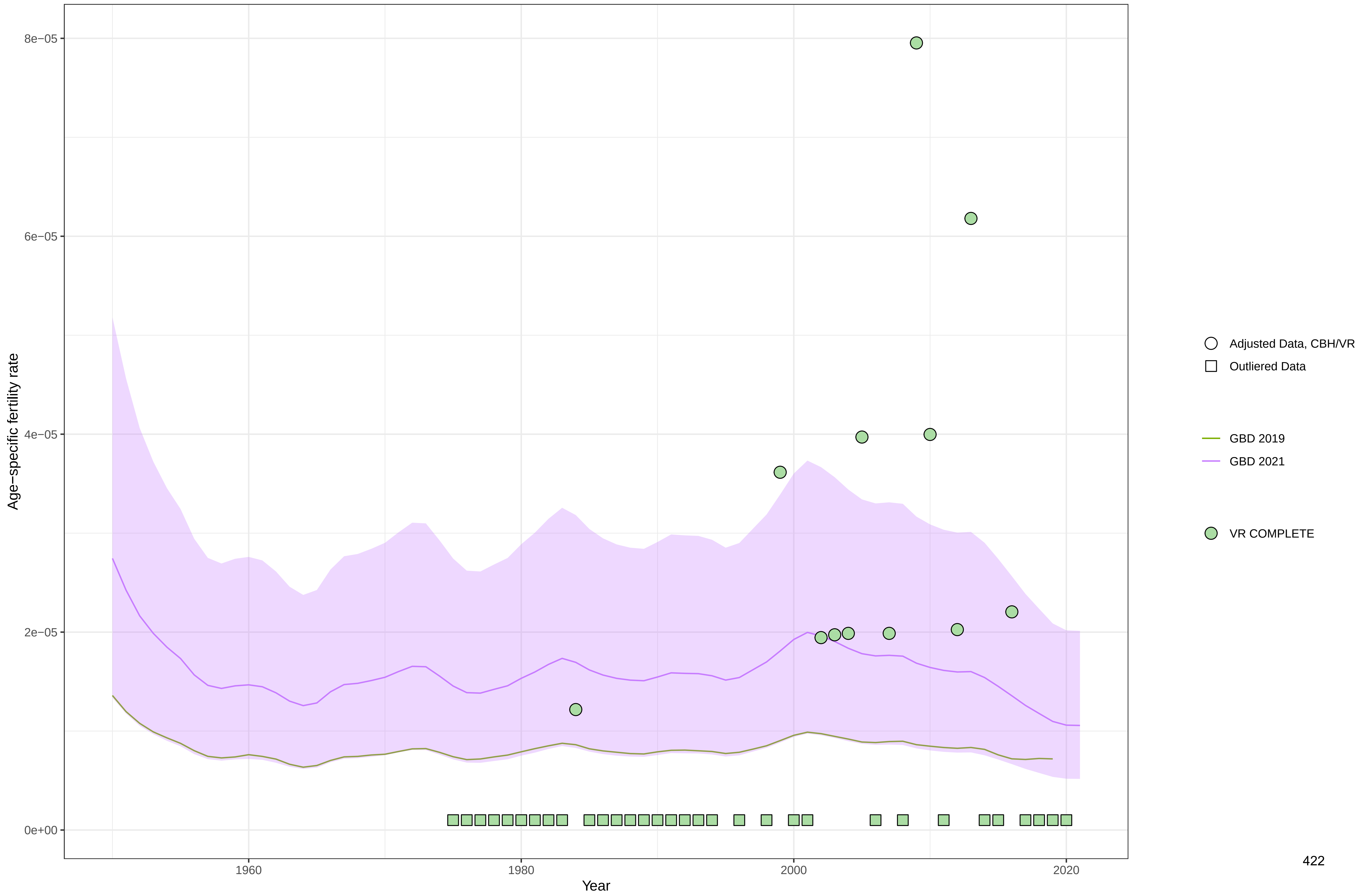

Age−specific fertility fitted trend lines, 1950−2021: Gunma, Ages: 15−19
Data points enclosed with a diamond are reference sources

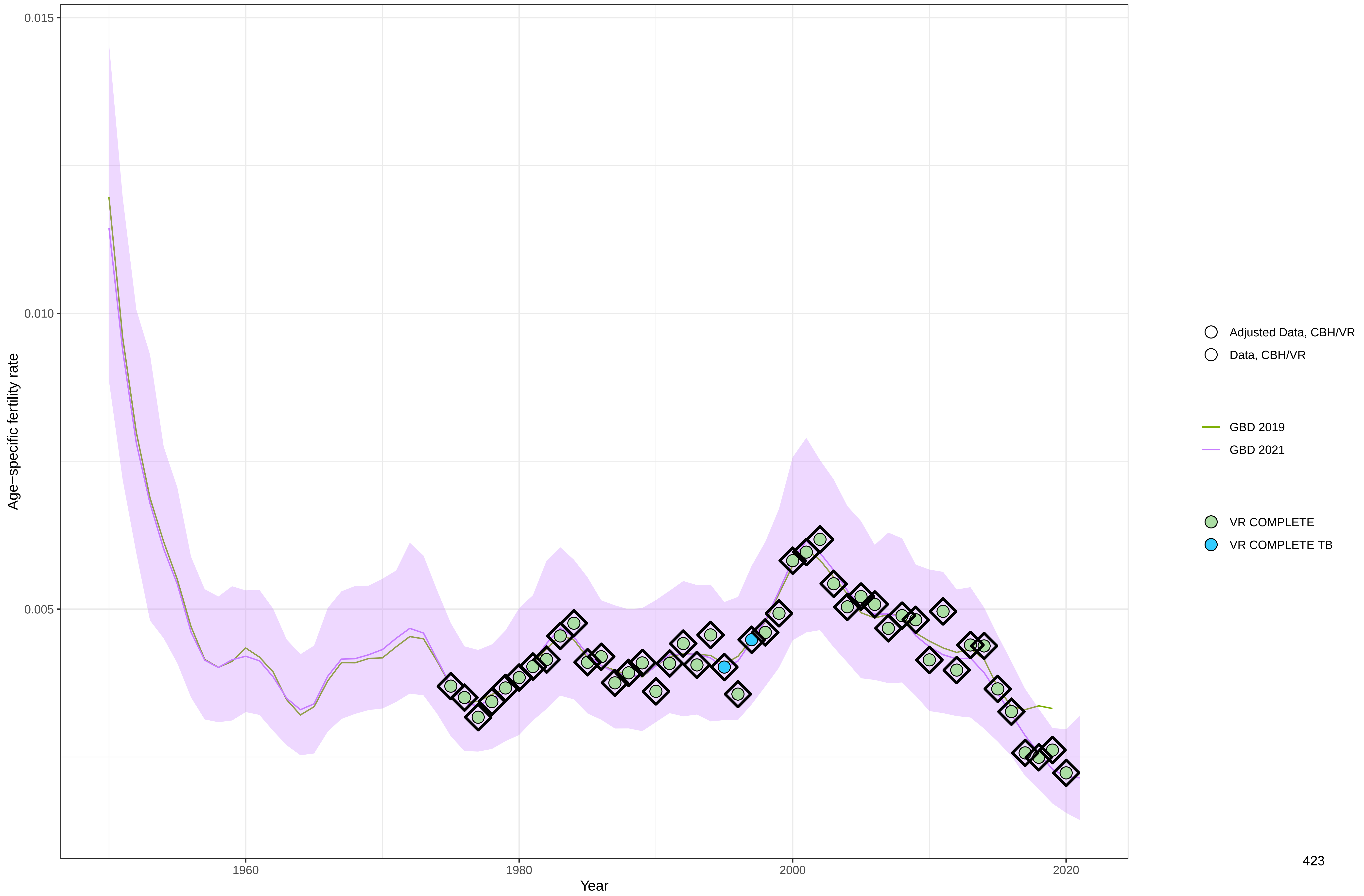

Age−specific fertility fitted trend lines, 1950−2021: Gunma, Ages: 20−24
Data points enclosed with a diamond are reference sources

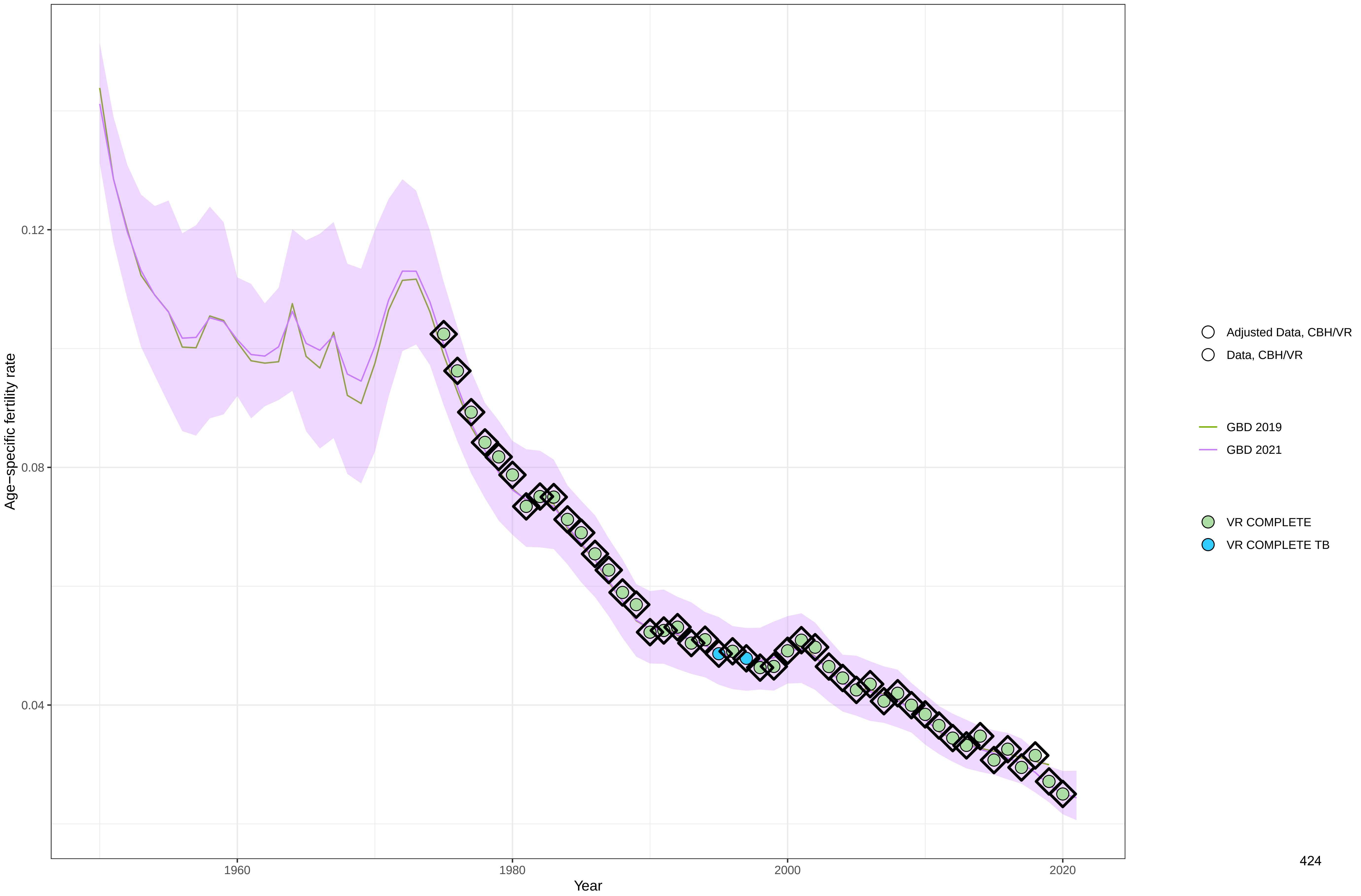

Age−specific fertility fitted trend lines, 1950−2021: Gunma, Ages: 25−29
Data points enclosed with a diamond are reference sources

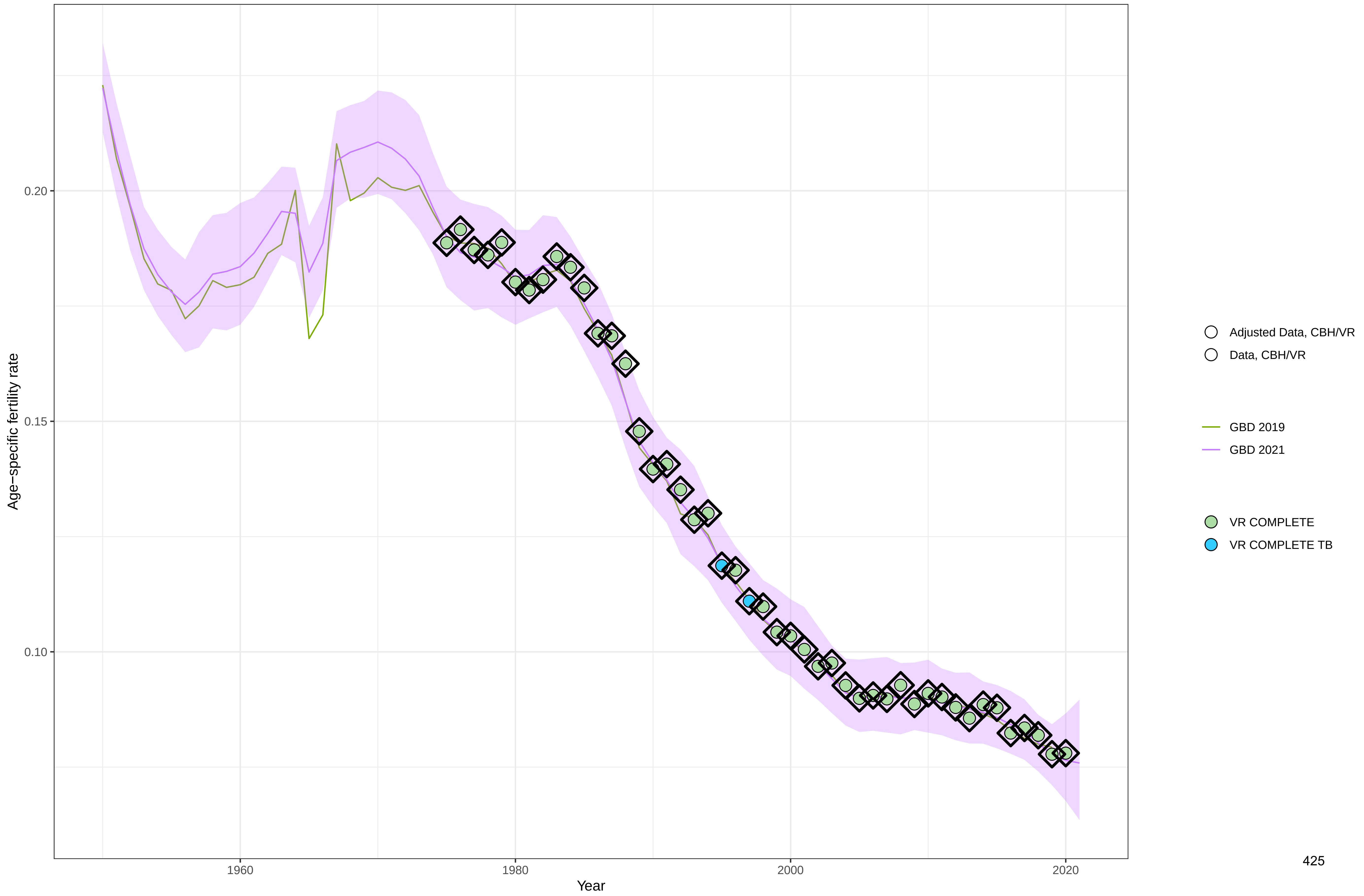

Age−specific fertility fitted trend lines, 1950−2021: Gunma, Ages: 30−34
Data points enclosed with a diamond are reference sources

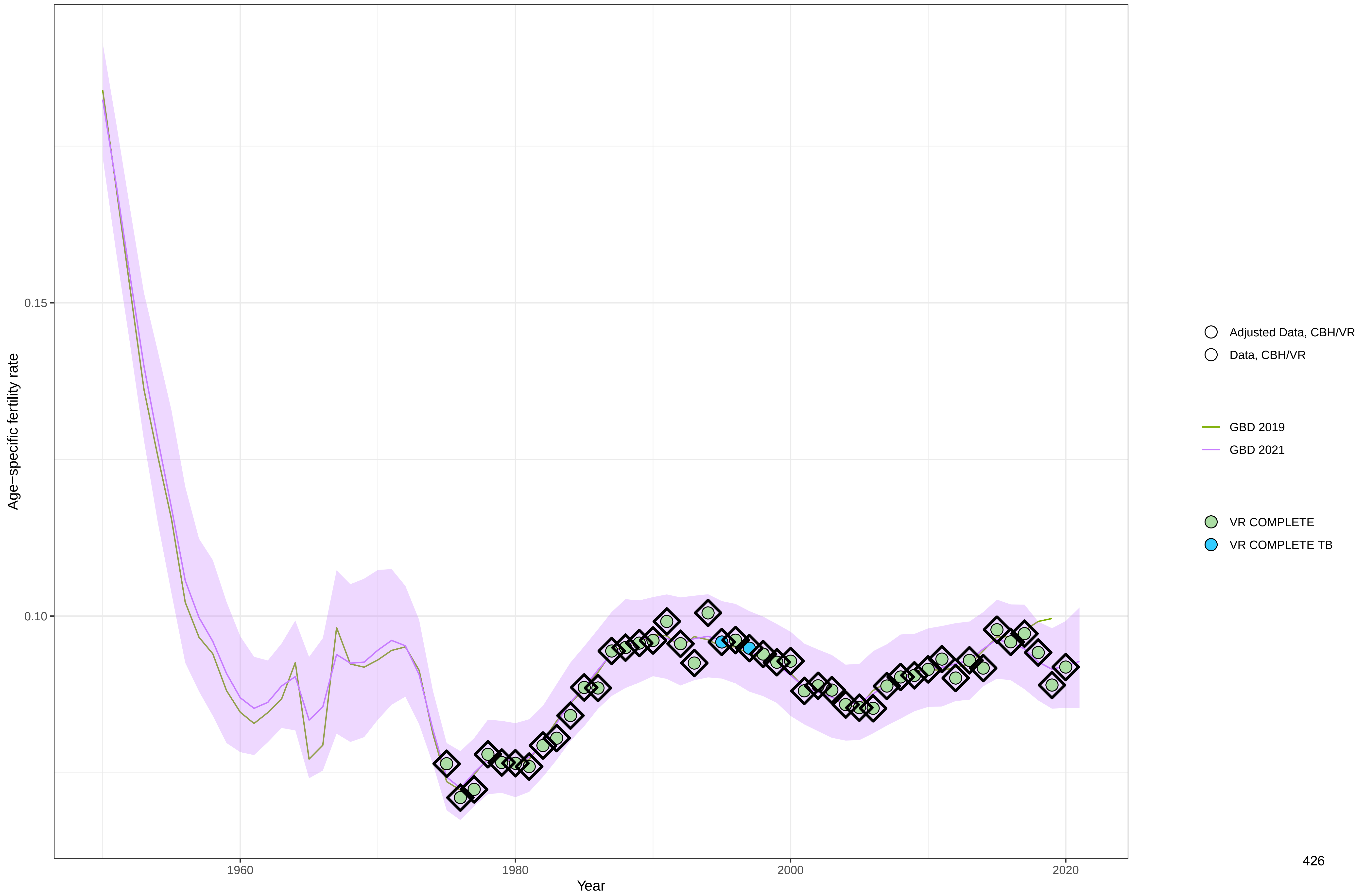

Age−specific fertility fitted trend lines, 1950−2021: Gunma, Ages: 35−39
Data points enclosed with a diamond are reference sources

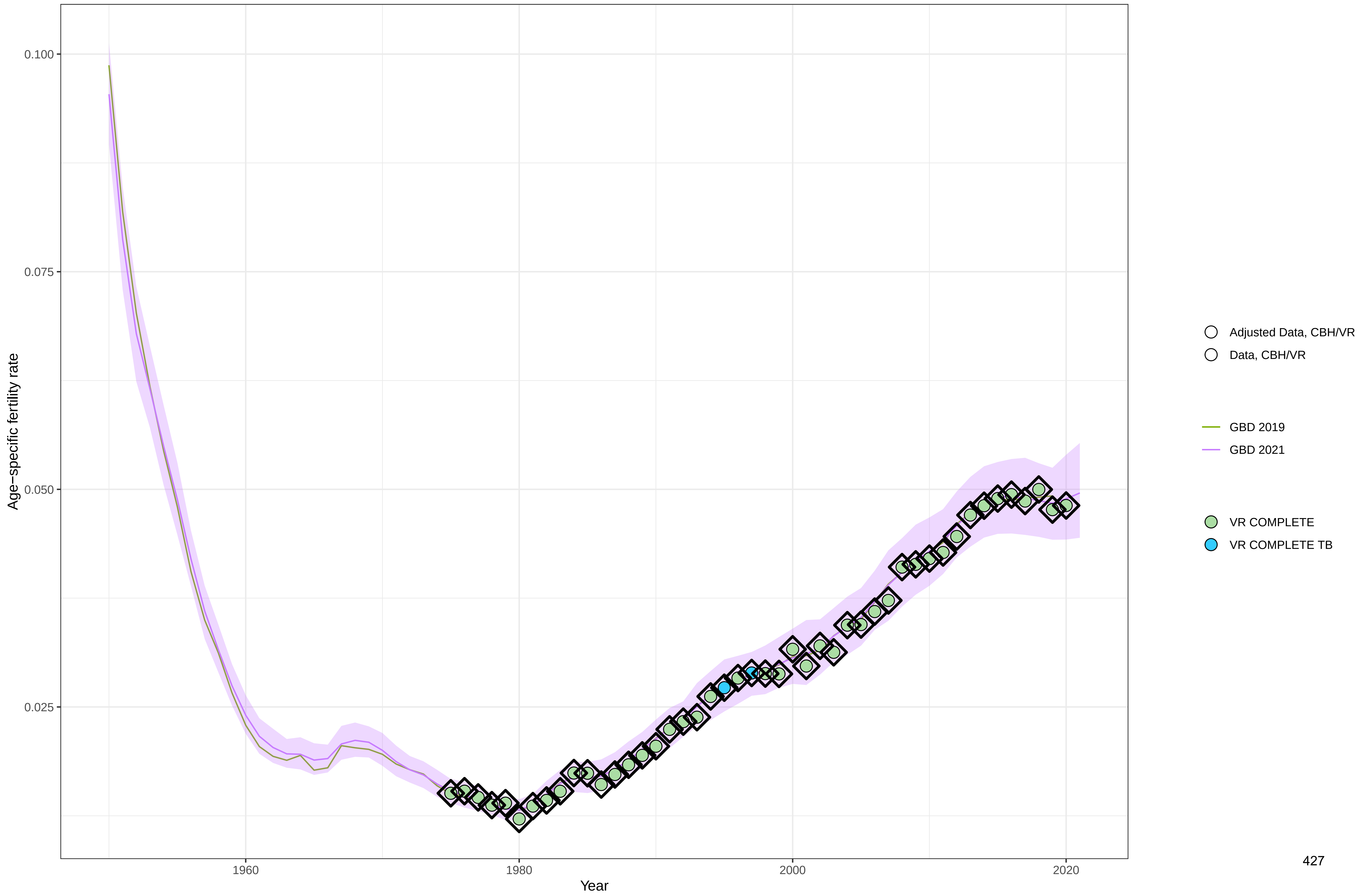

Age−specific fertility fitted trend lines, 1950−2021: Gunma, Ages: 40−44
Data points enclosed with a diamond are reference sources

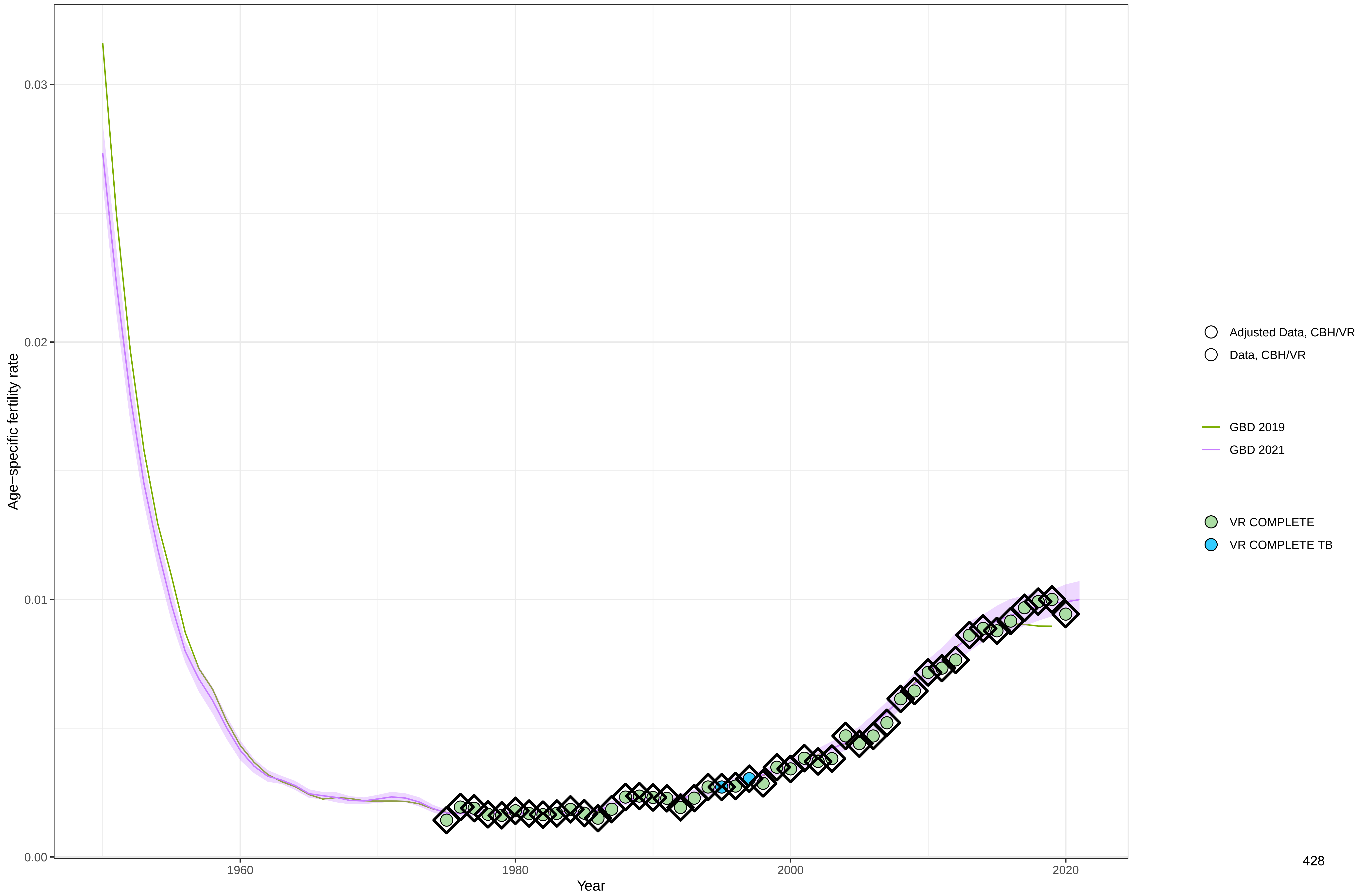

Age−specific fertility fitted trend lines, 1950−2021: Gunma, Ages: 45−49
Data points enclosed with a diamond are reference sources

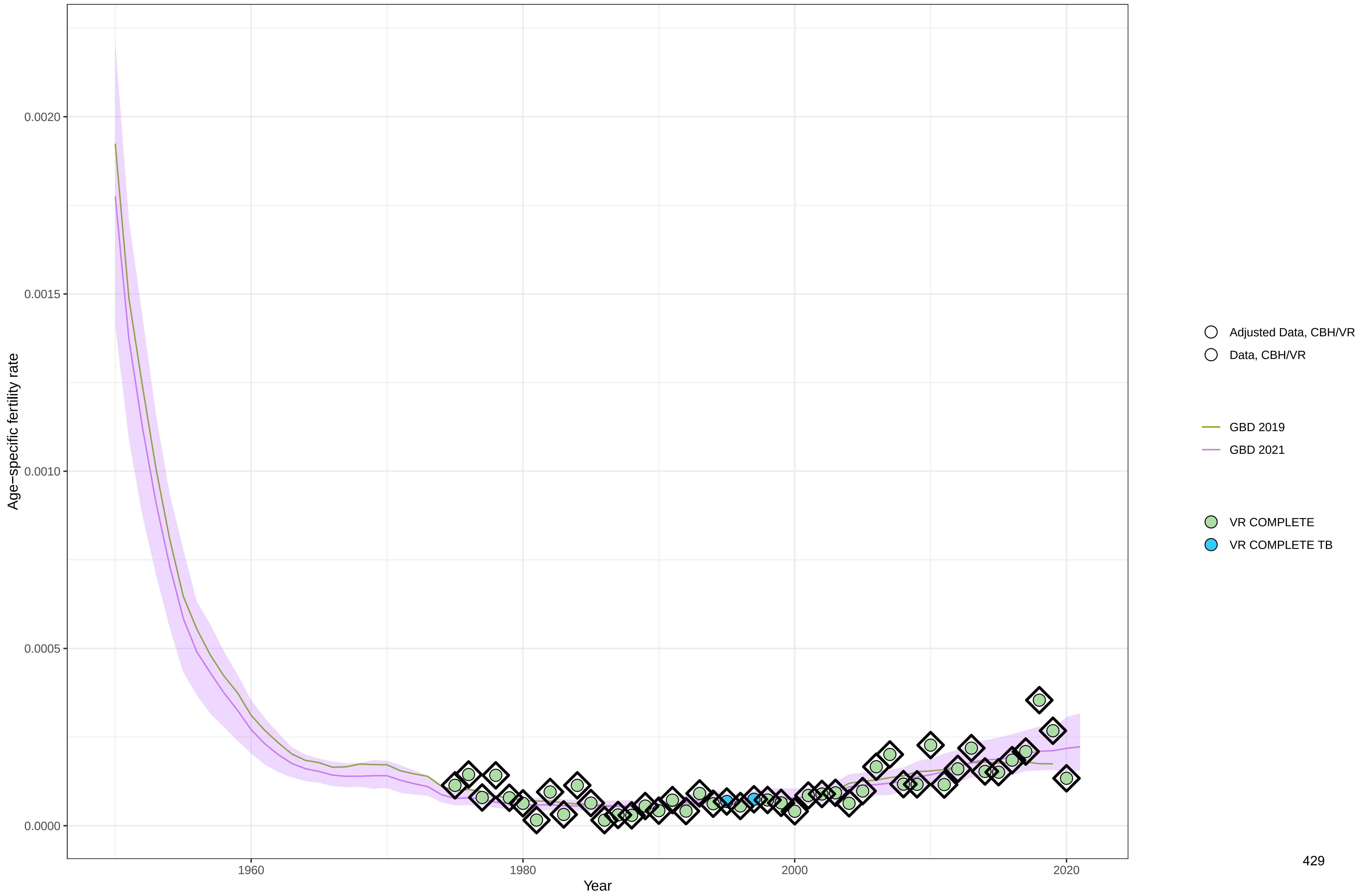

Age−specific fertility fitted trend lines, 1950−2021: Gunma, Ages: 50−54
Data points enclosed with a diamond are reference sources

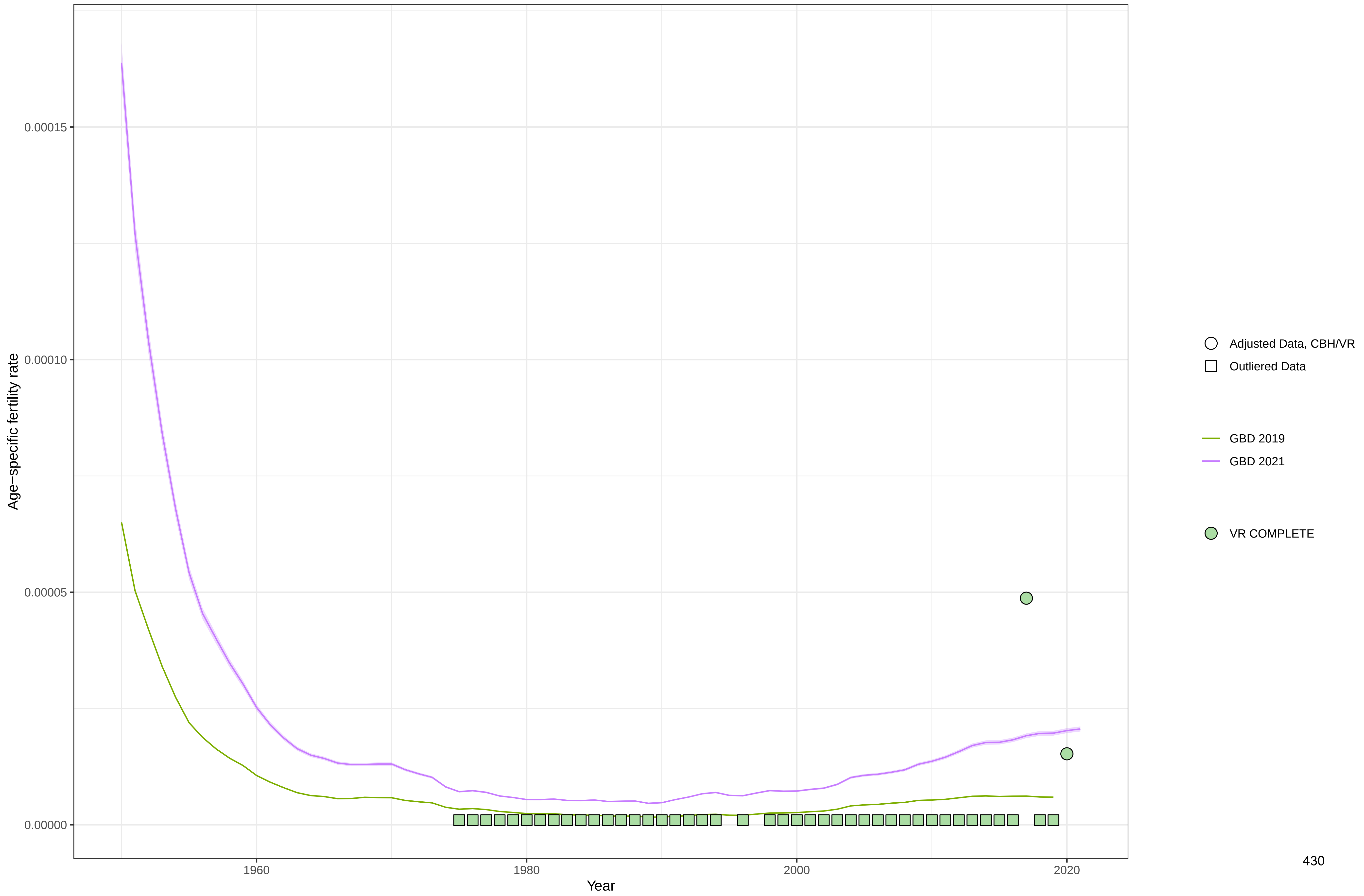

Total fertility rate fitted trend lines, 1950−2021: Gunma TFR
Data points enclosed with a diamond are reference sources

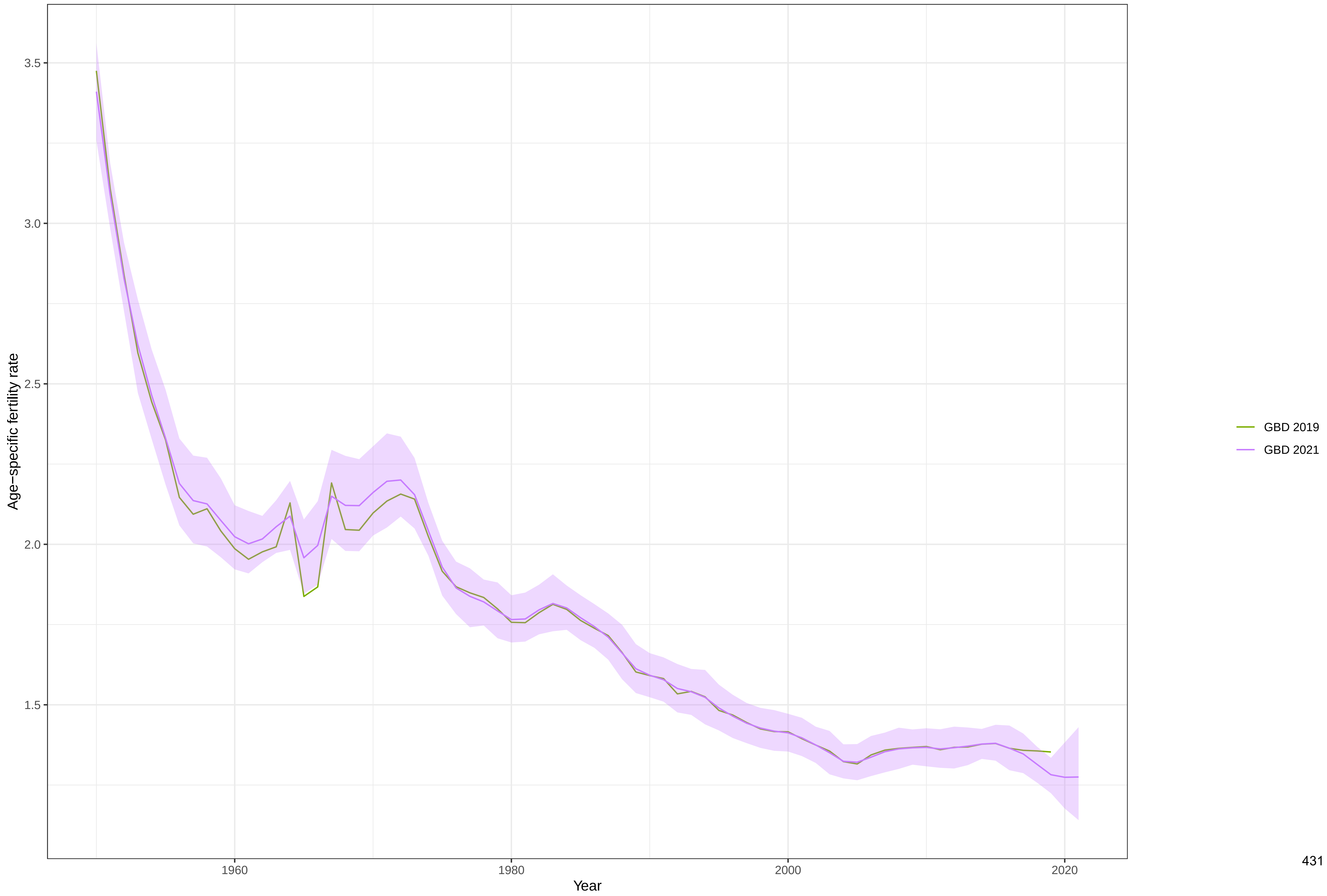

Age−specific fertility fitted trend lines, 1950−2021: Hiroshima, Ages: 10−14
Data points enclosed with a diamond are reference sources

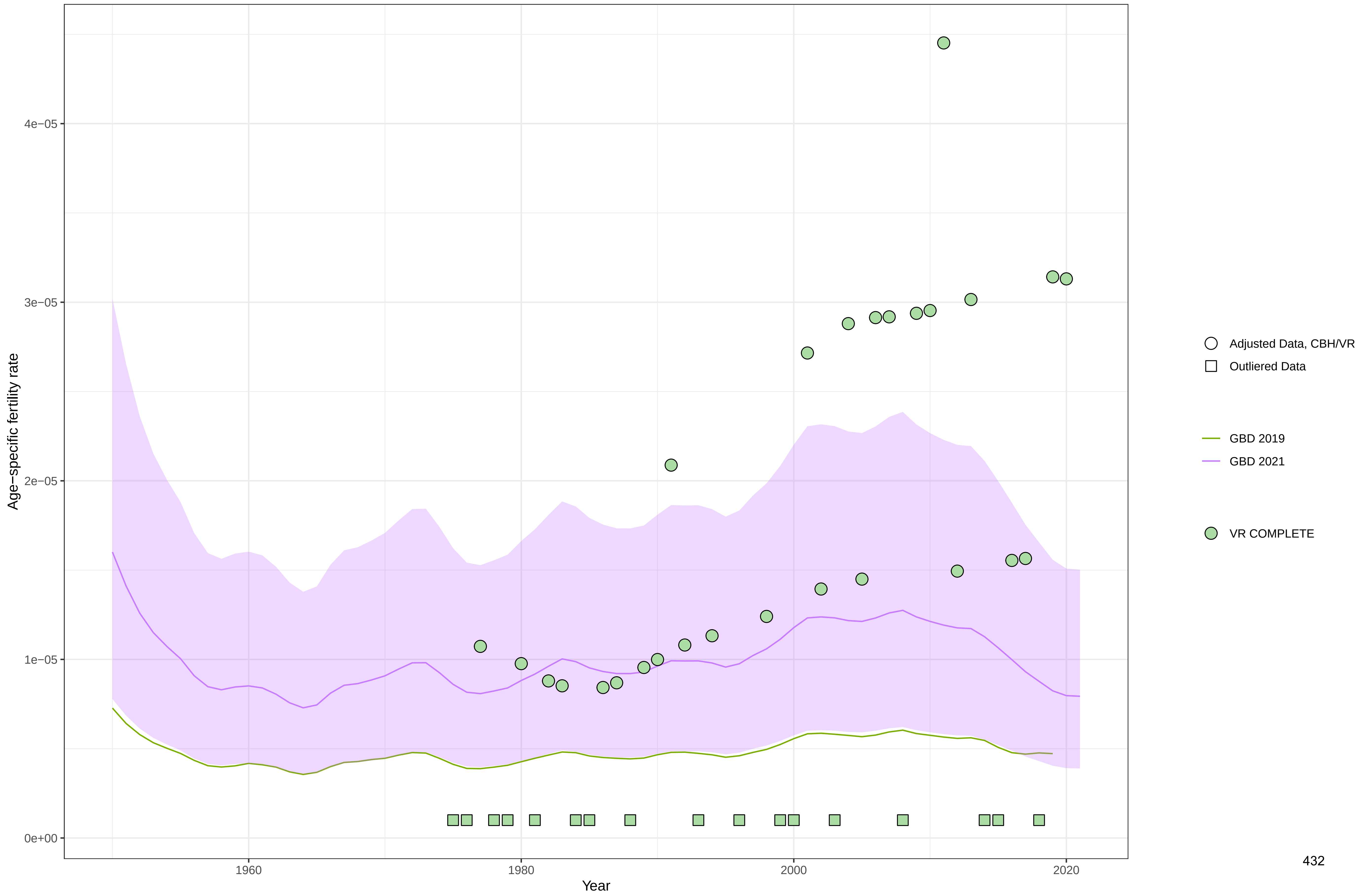

Age−specific fertility fitted trend lines, 1950−2021: Hiroshima, Ages: 15−19
Data points enclosed with a diamond are reference sources

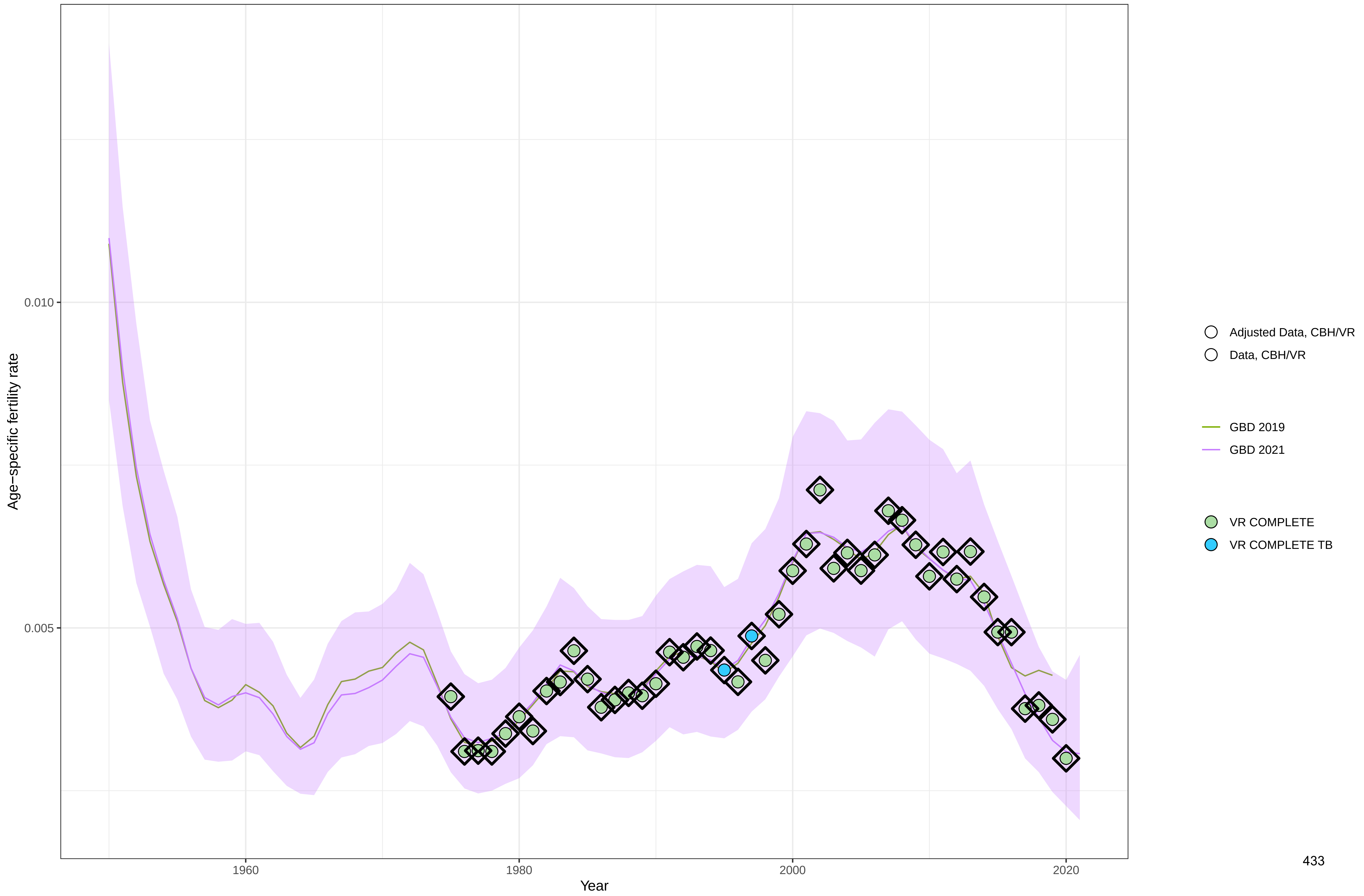

Age−specific fertility fitted trend lines, 1950−2021: Hiroshima, Ages: 20−24
Data points enclosed with a diamond are reference sources

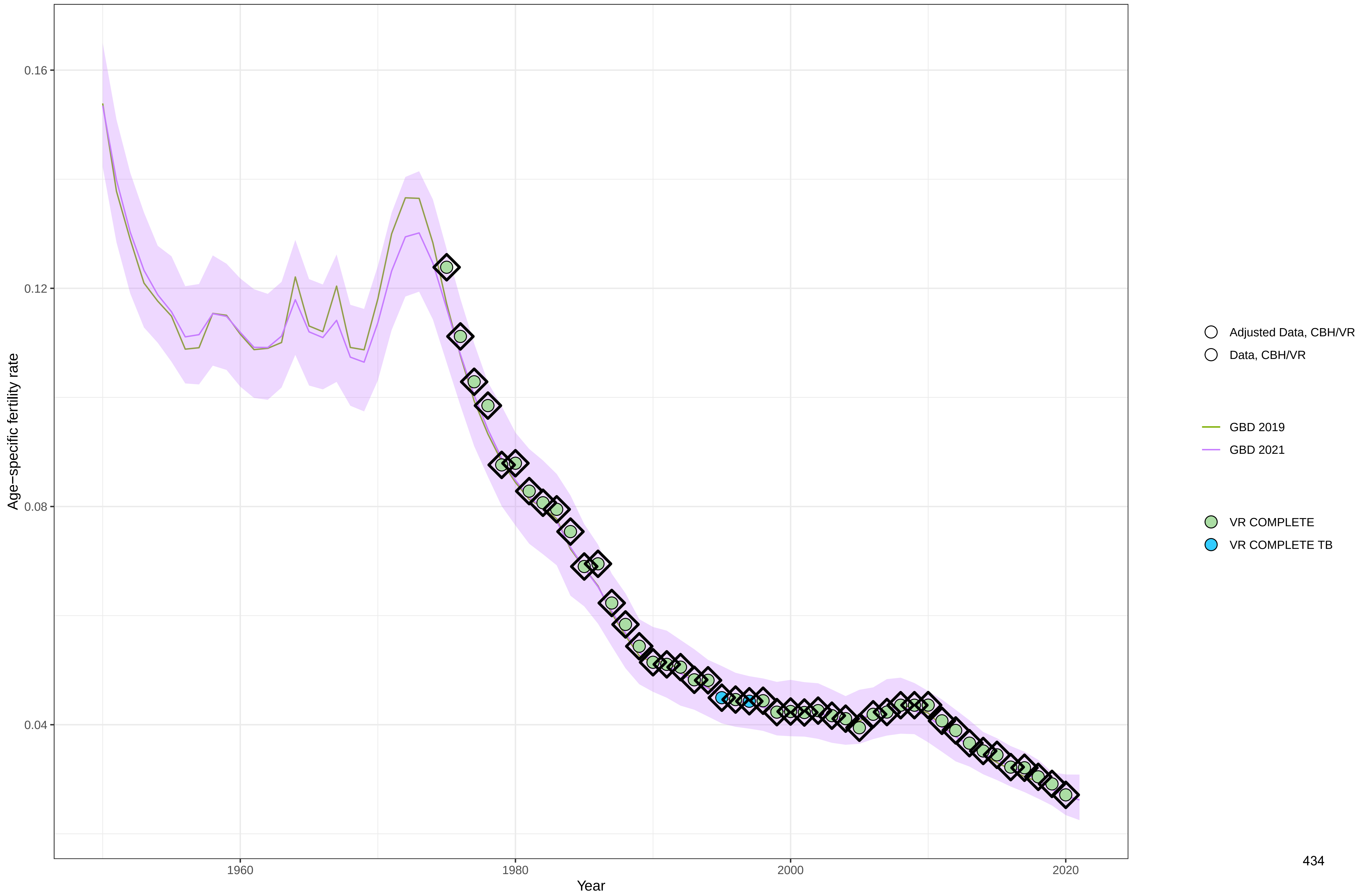

Age−specific fertility fitted trend lines, 1950−2021: Hiroshima, Ages: 25−29
Data points enclosed with a diamond are reference sources

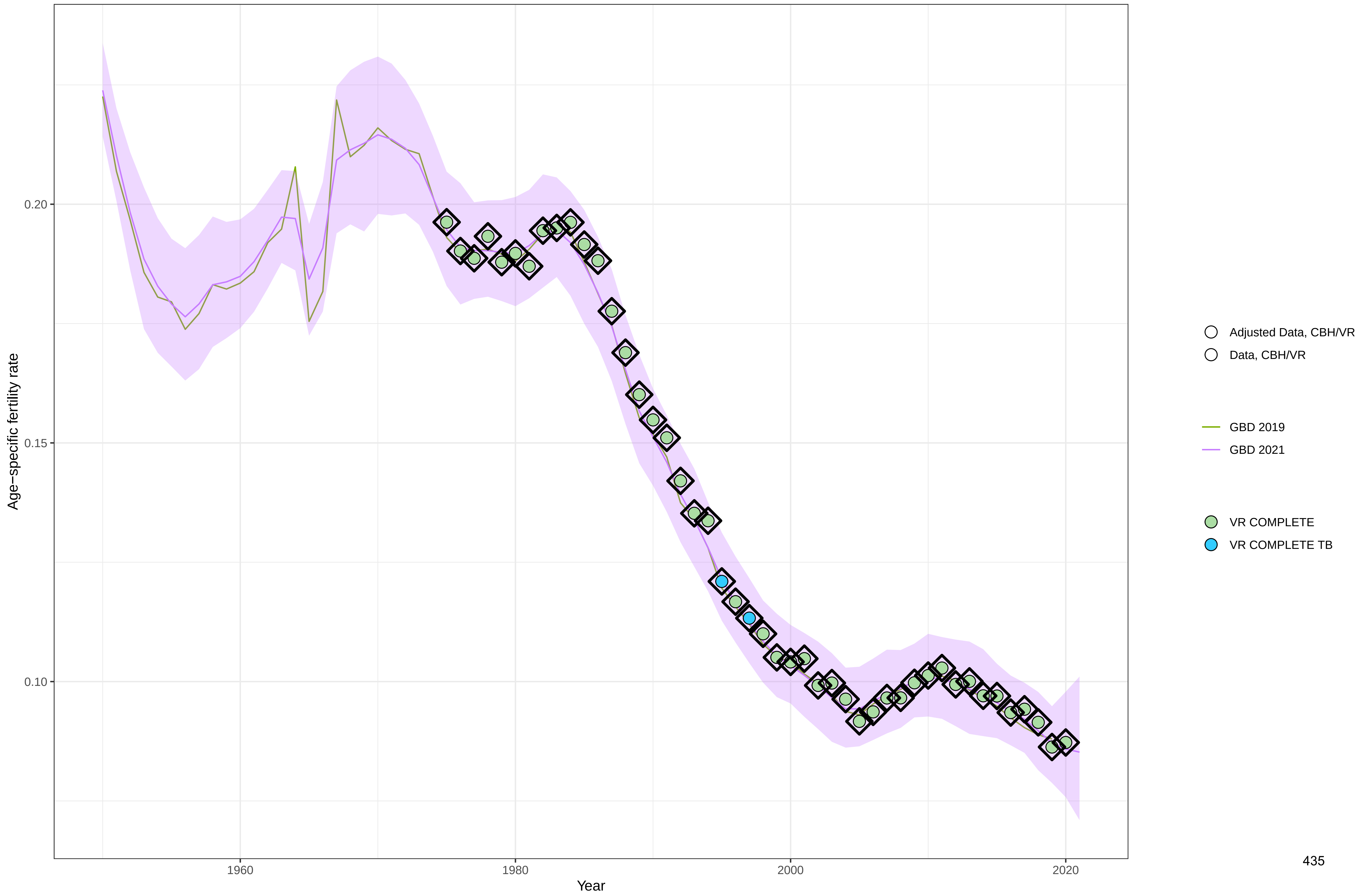

Age−specific fertility fitted trend lines, 1950−2021: Hiroshima, Ages: 30−34
Data points enclosed with a diamond are reference sources

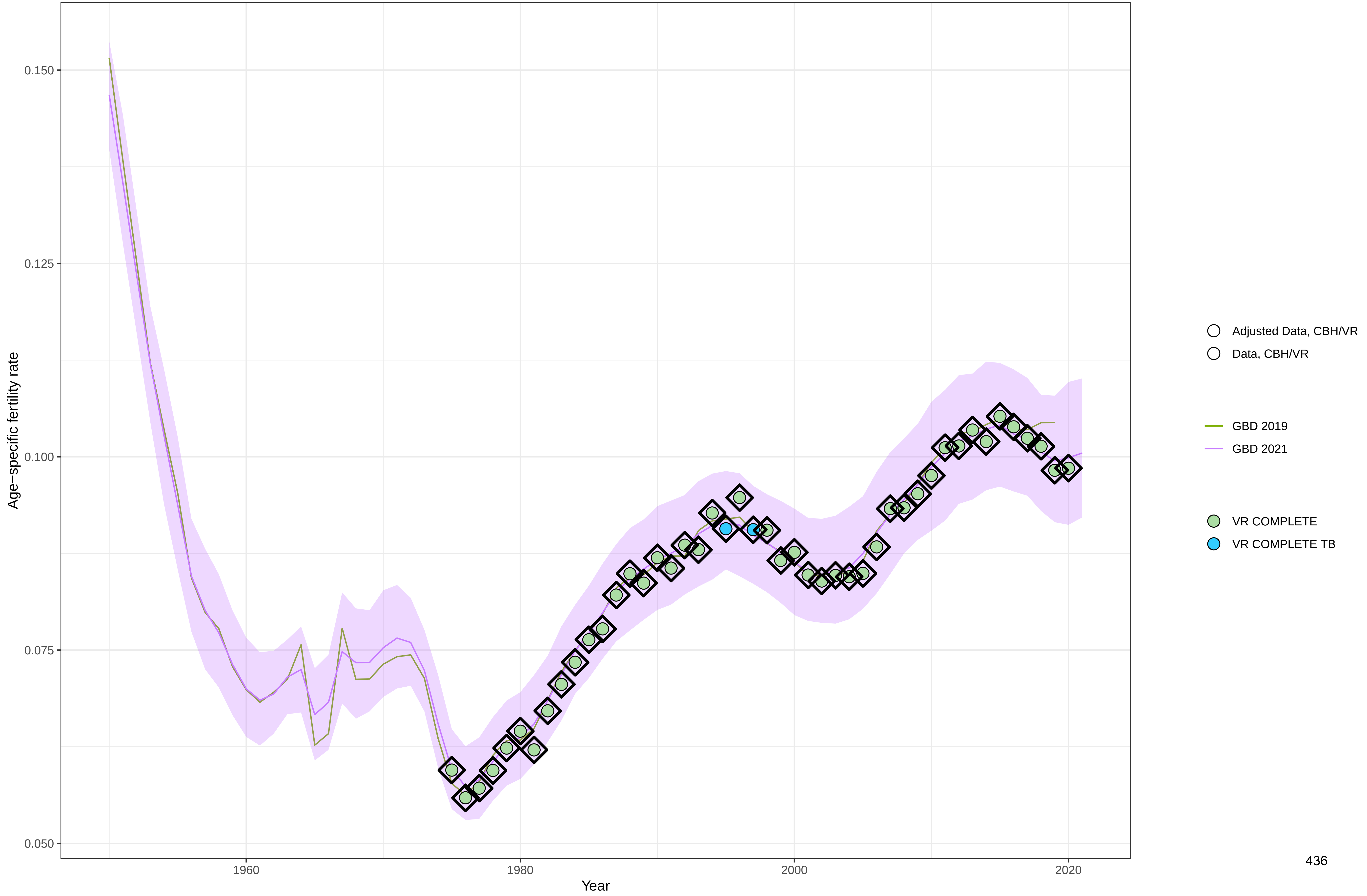

Age−specific fertility fitted trend lines, 1950−2021: Hiroshima, Ages: 35−39
Data points enclosed with a diamond are reference sources

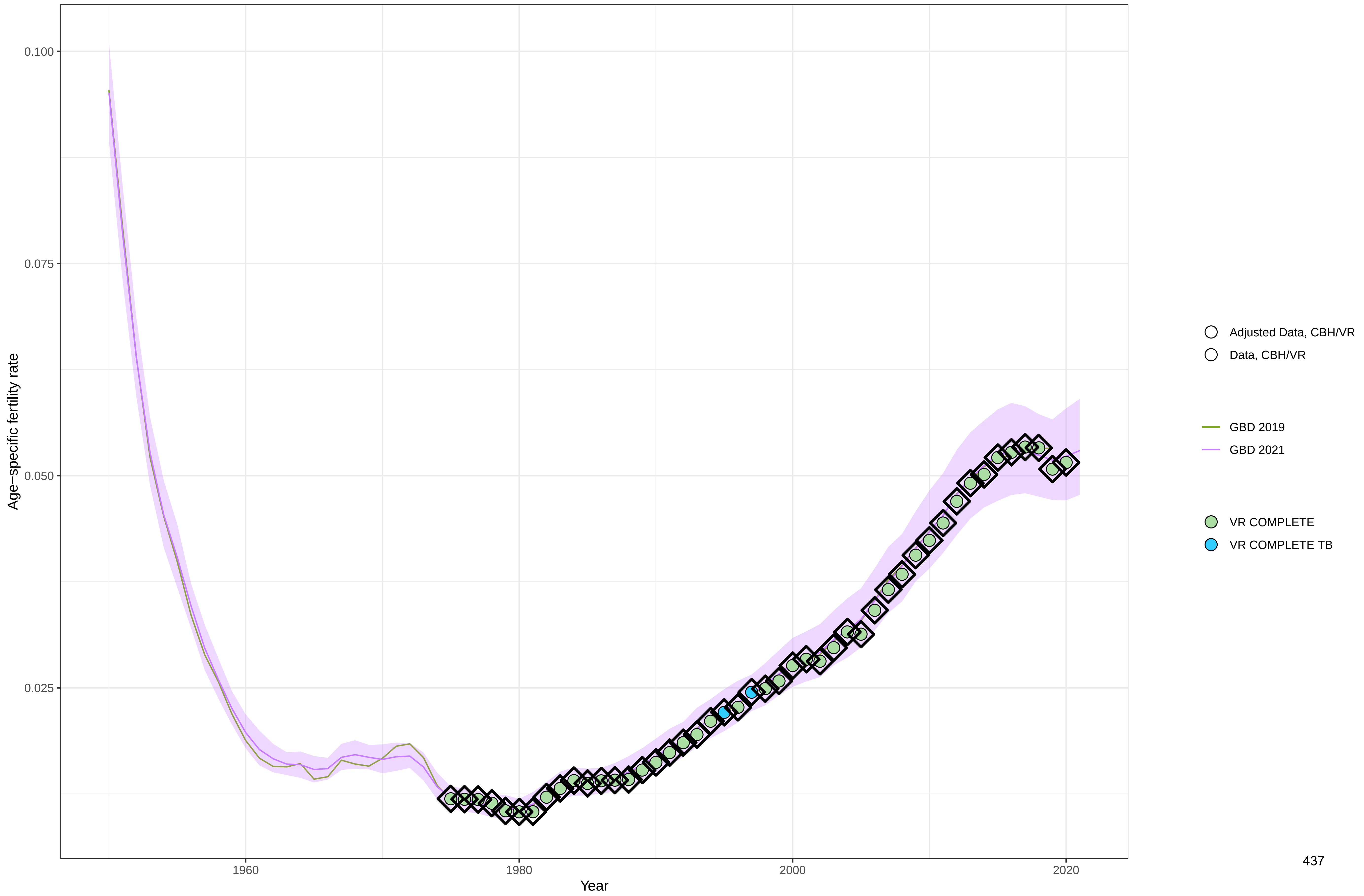

Age−specific fertility fitted trend lines, 1950−2021: Hiroshima, Ages: 40−44
Data points enclosed with a diamond are reference sources

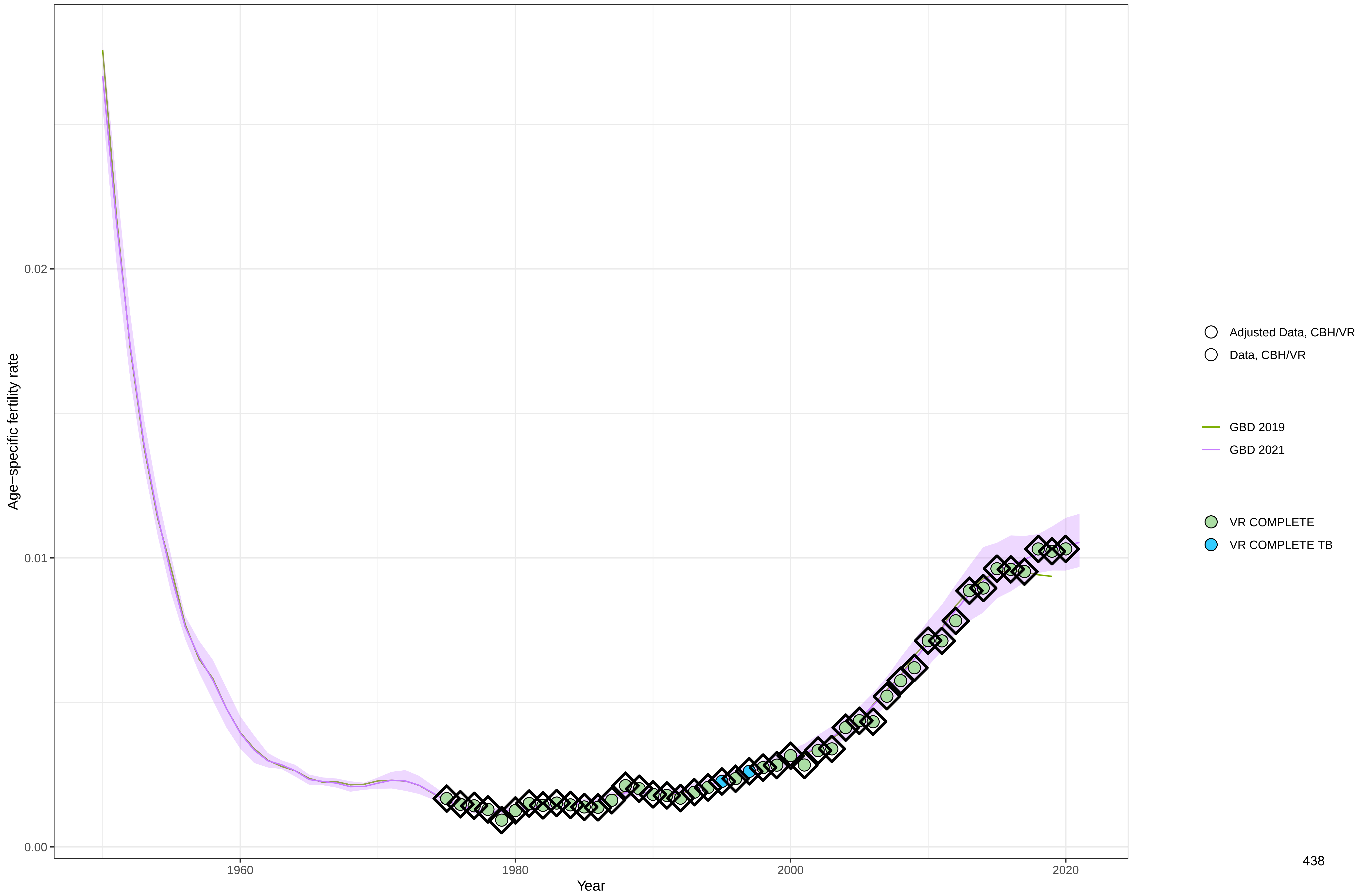

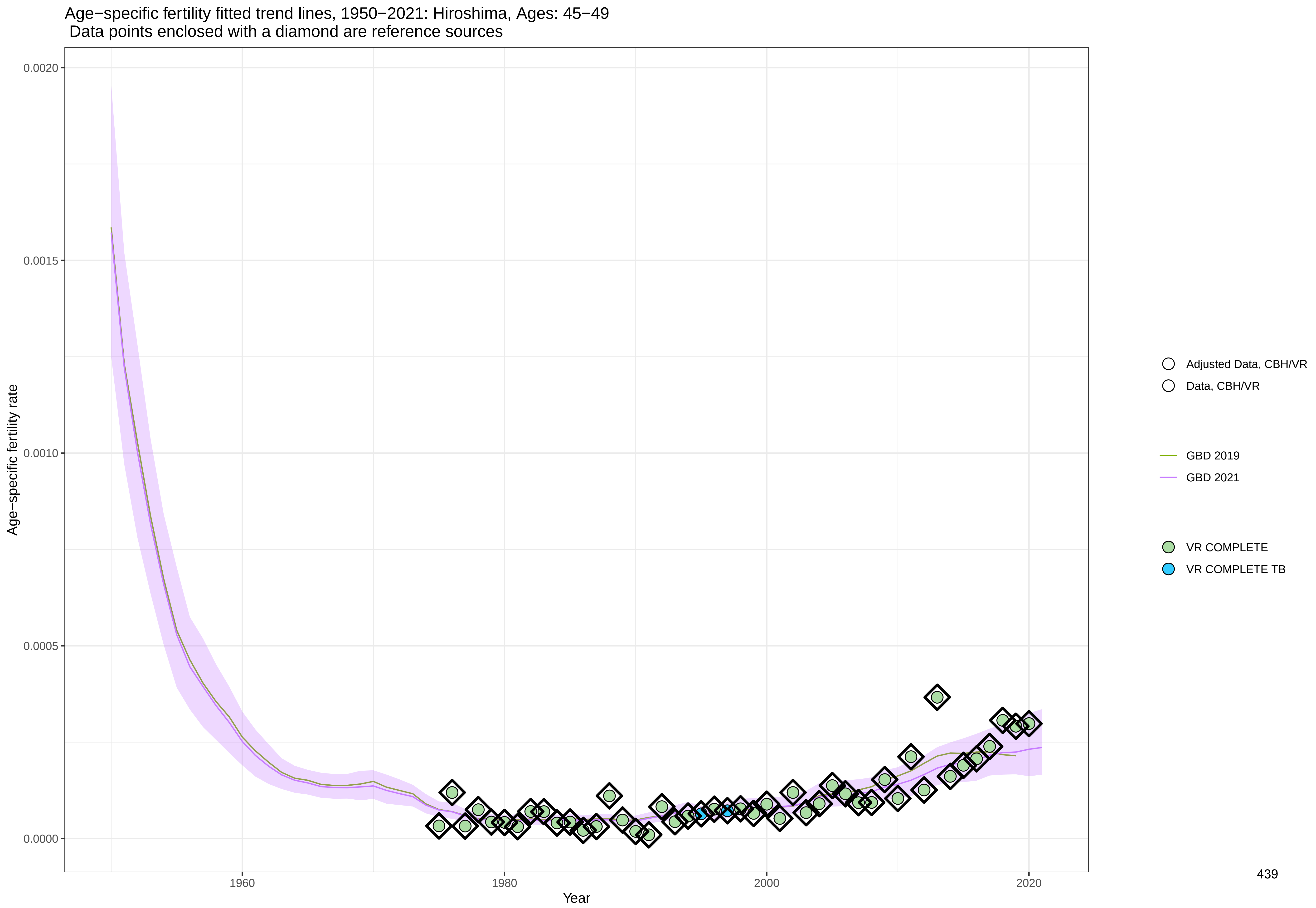
Age−specific fertility fitted trend lines, 1950−2021: Hiroshima, Ages: 45−49
Data points enclosed with a diamond are reference sources
Age−specific fertility rate
0.0020
0.0015
0.0010
0.0005
0.0000
1960
1980
2000
2020
Year
Adjusted Data, CBH/VR
Data, CBH/VR
GBD 2019
GBD 2021
VR COMPLETE
VR COMPLETE TB

Age−specific fertility fitted trend lines, 1950−2021: Hiroshima, Ages: 50−54
Data points enclosed with a diamond are reference sources

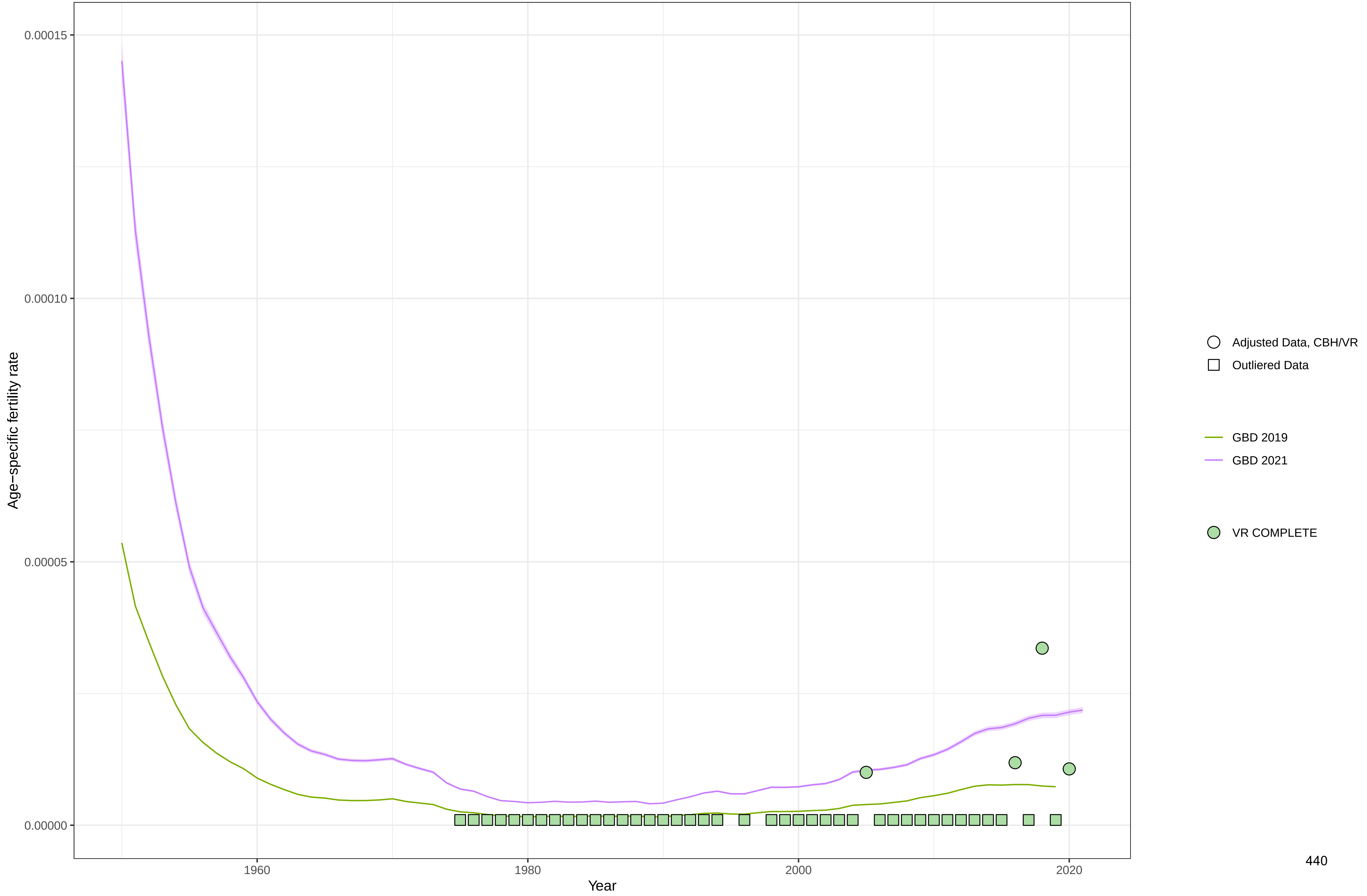

Total fertility rate fitted trend lines, 1950−2021: Hiroshima TFR
Data points enclosed with a diamond are reference sources

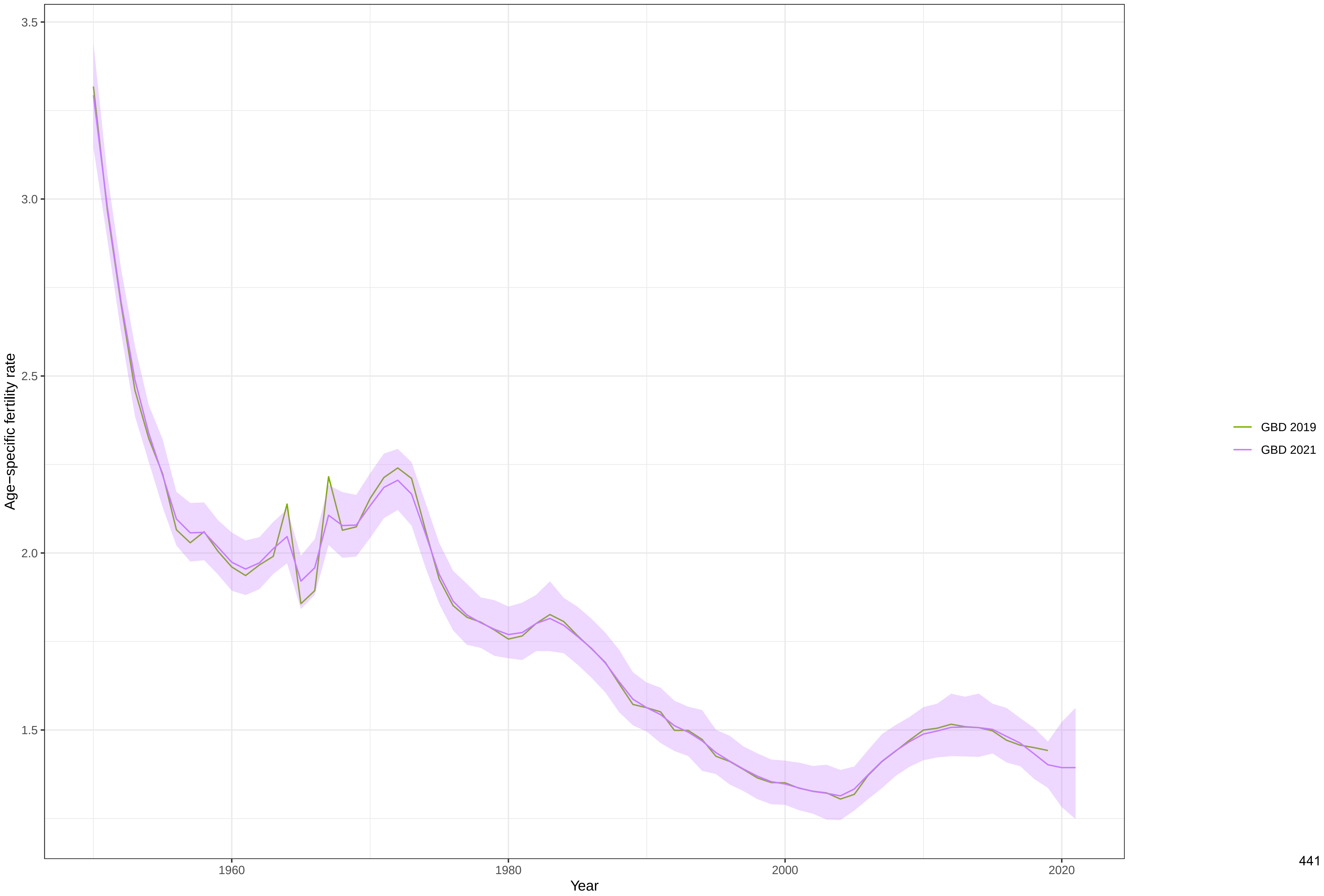

Age−specific fertility fitted trend lines, 1950−2021: Hokkaid.., Ages: 10−14
Data points enclosed with a diamond are reference sources

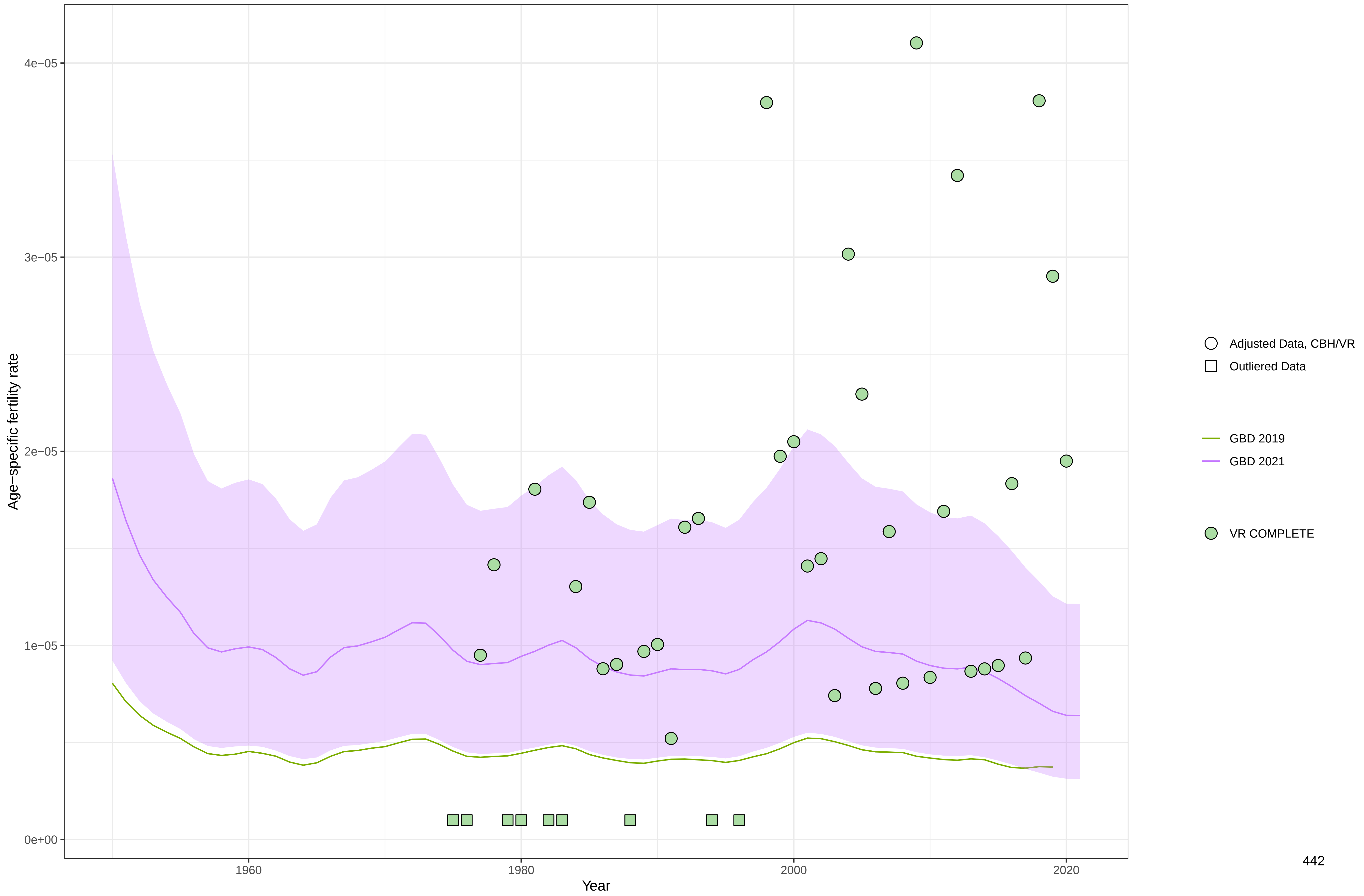

Age−specific fertility fitted trend lines, 1950−2021: Hokkaid.., Ages: 15−19
Data points enclosed with a diamond are reference sources

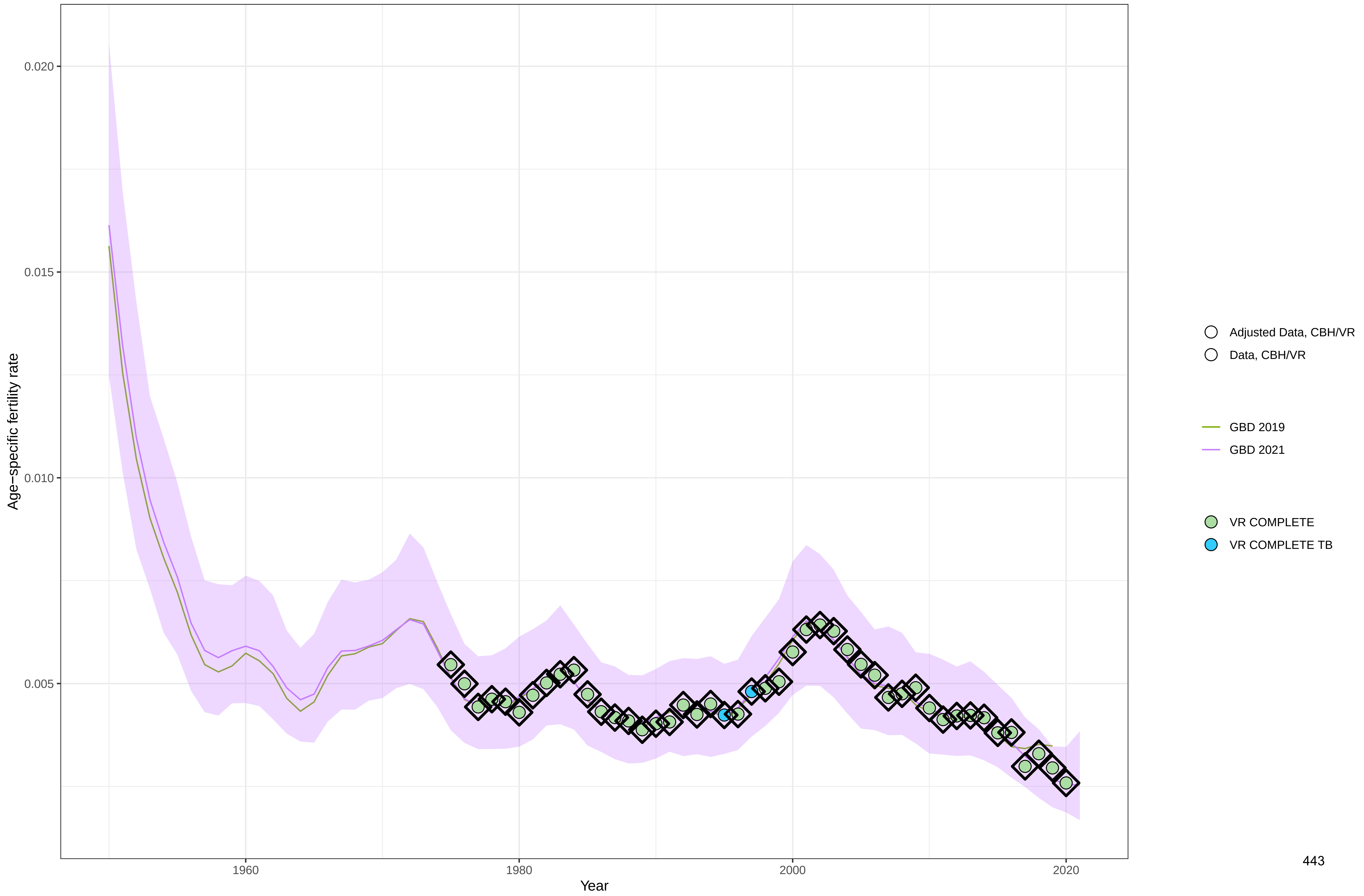

Age−specific fertility fitted trend lines, 1950−2021: Hokkaid.., Ages: 20−24
Data points enclosed with a diamond are reference sources

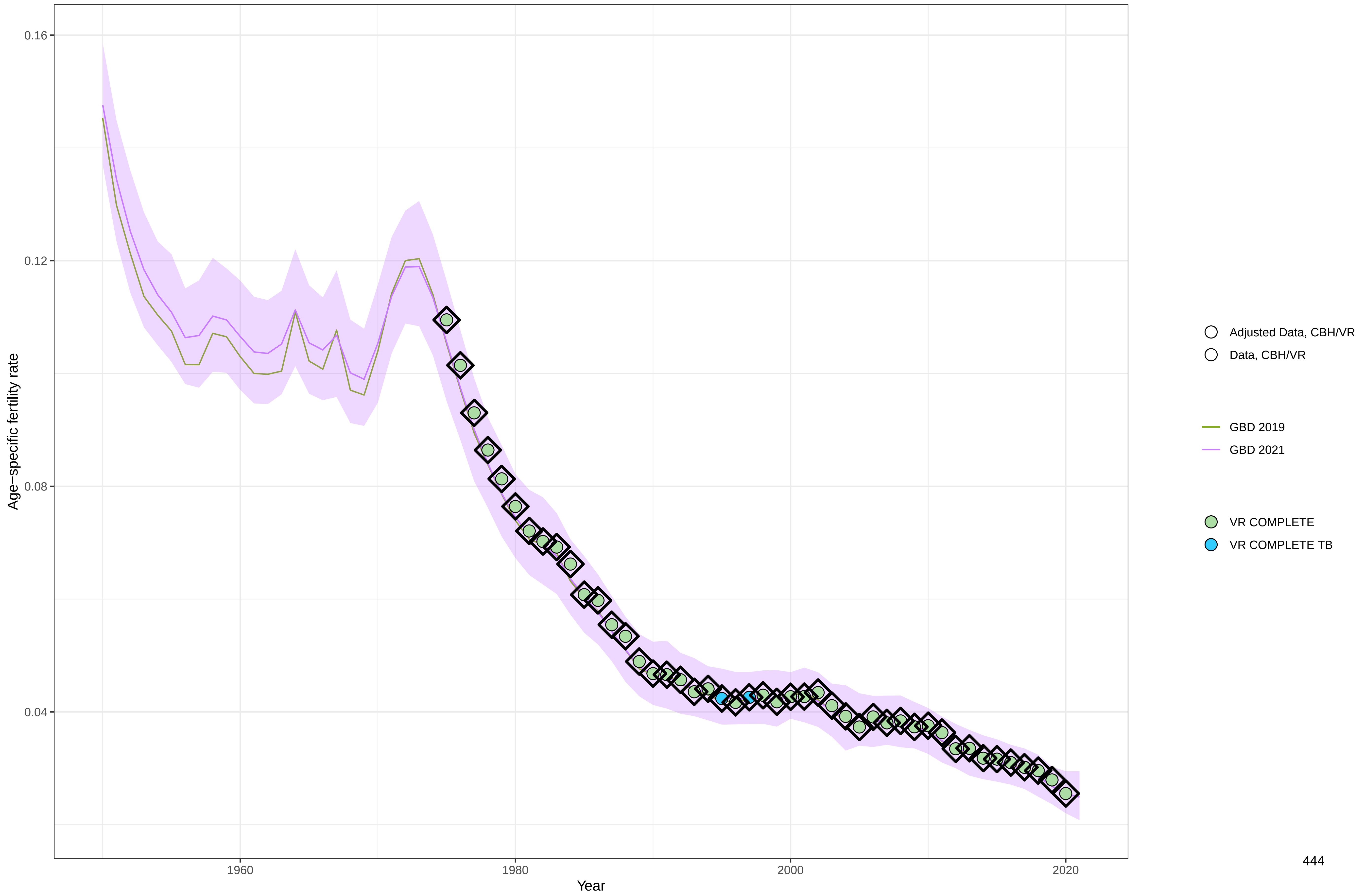

Age−specific fertility fitted trend lines, 1950−2021: Hokkaid.., Ages: 25−29
Data points enclosed with a diamond are reference sources

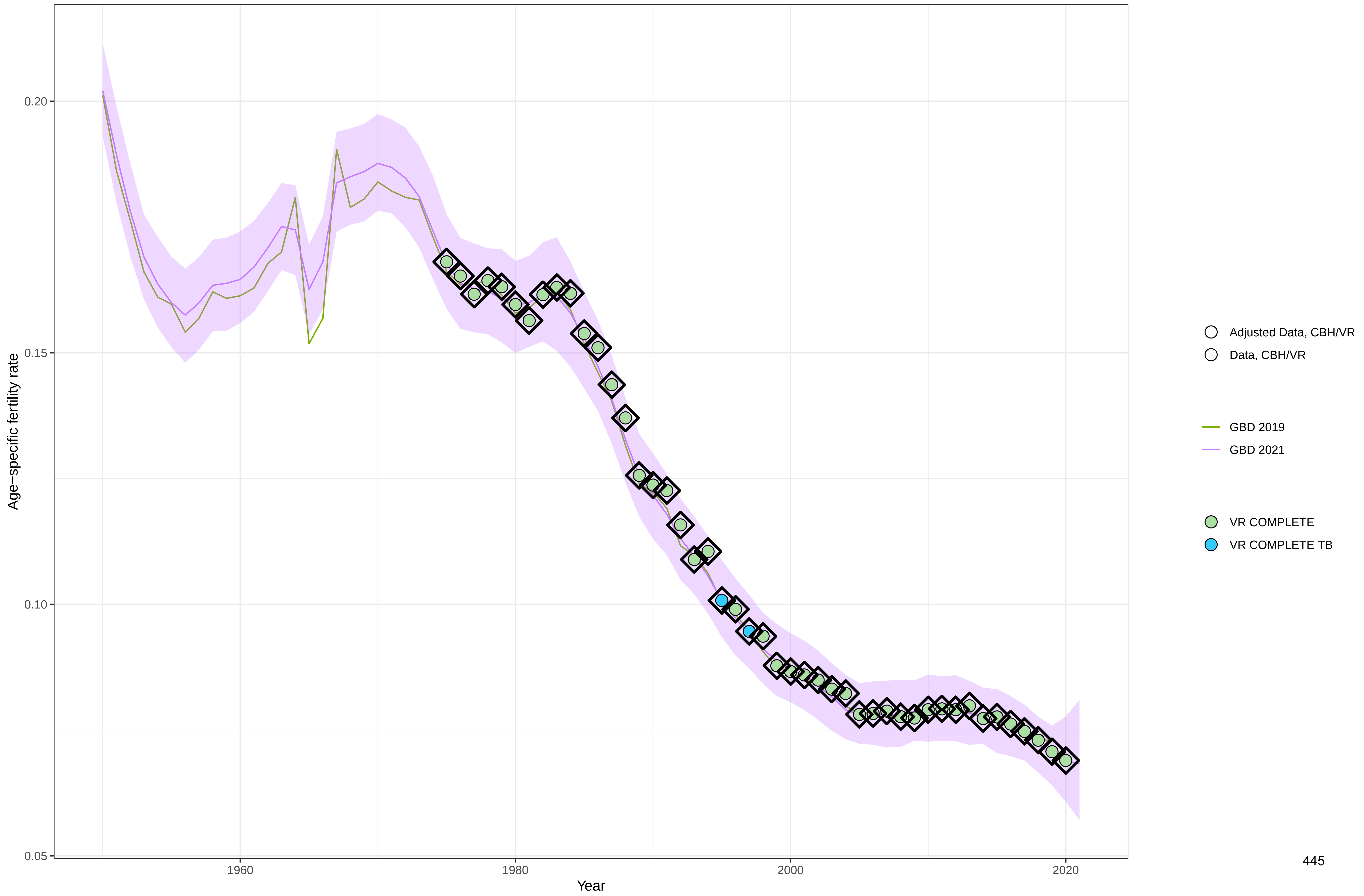

Age−specific fertility fitted trend lines, 1950−2021: Hokkaid.., Ages: 30−34
Data points enclosed with a diamond are reference sources

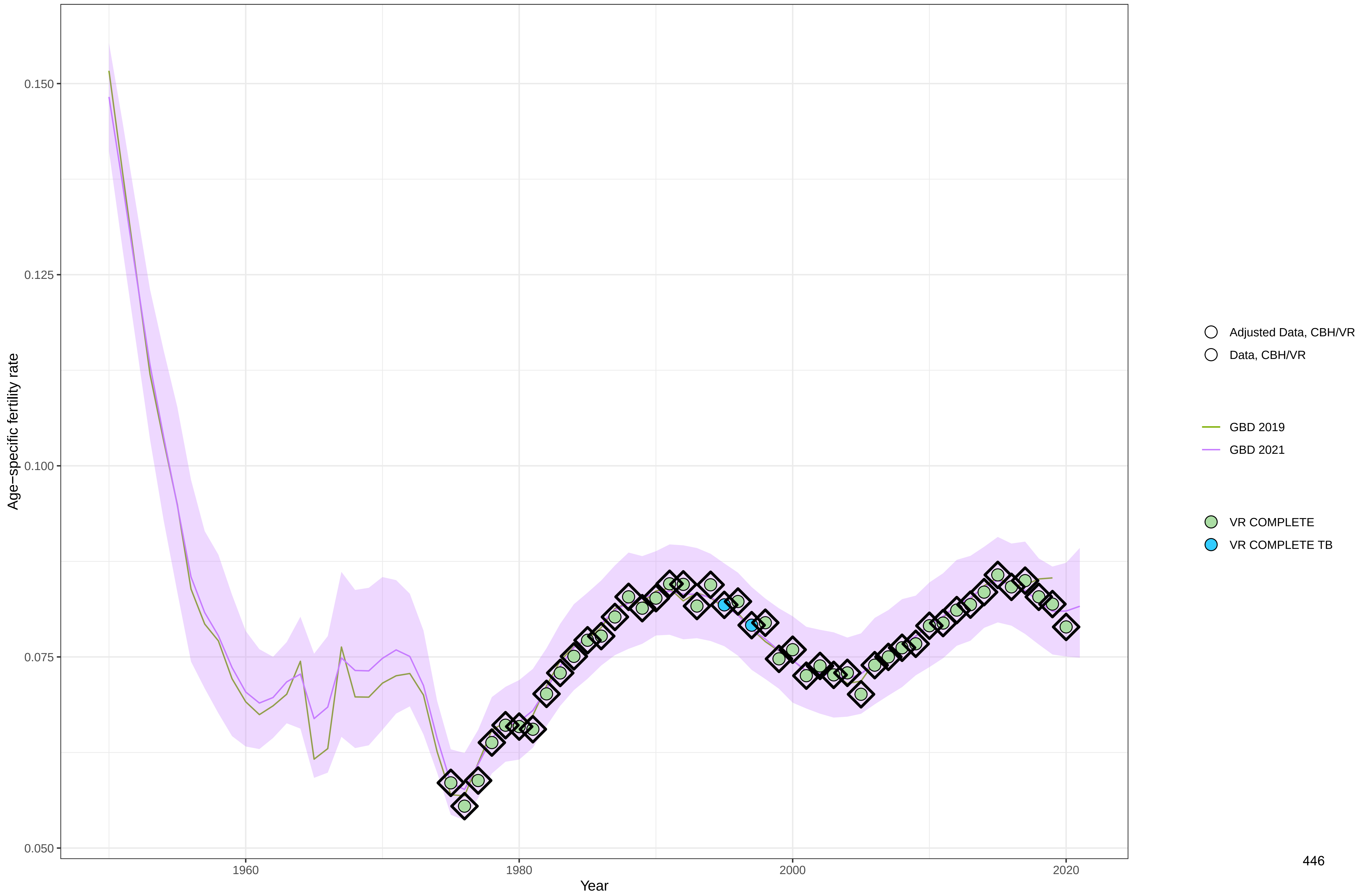

Age−specific fertility fitted trend lines, 1950−2021: Hokkaid.., Ages: 35−39
Data points enclosed with a diamond are reference sources

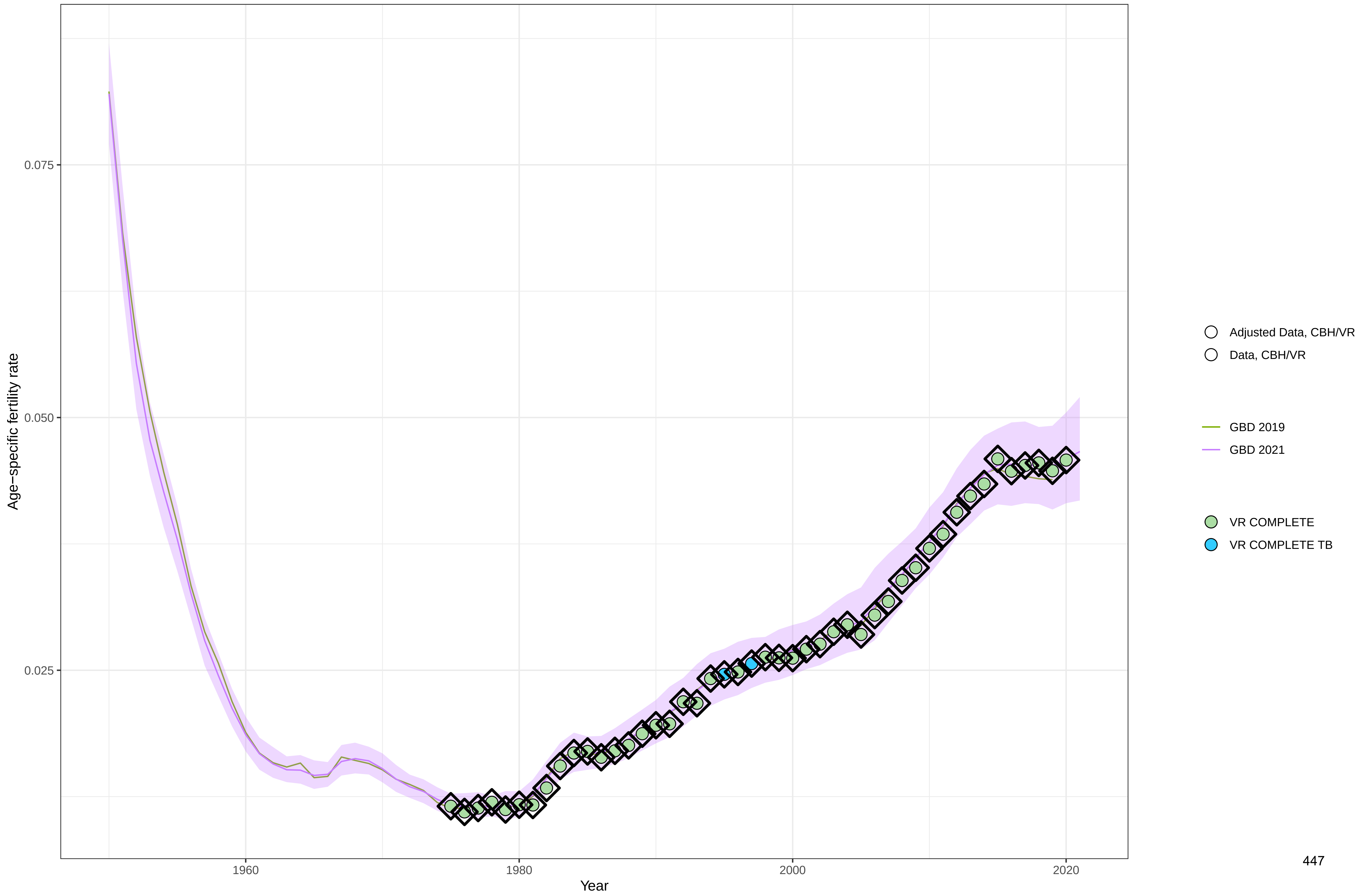

Age−specific fertility fitted trend lines, 1950−2021: Hokkaid.., Ages: 40−44
Data points enclosed with a diamond are reference sources

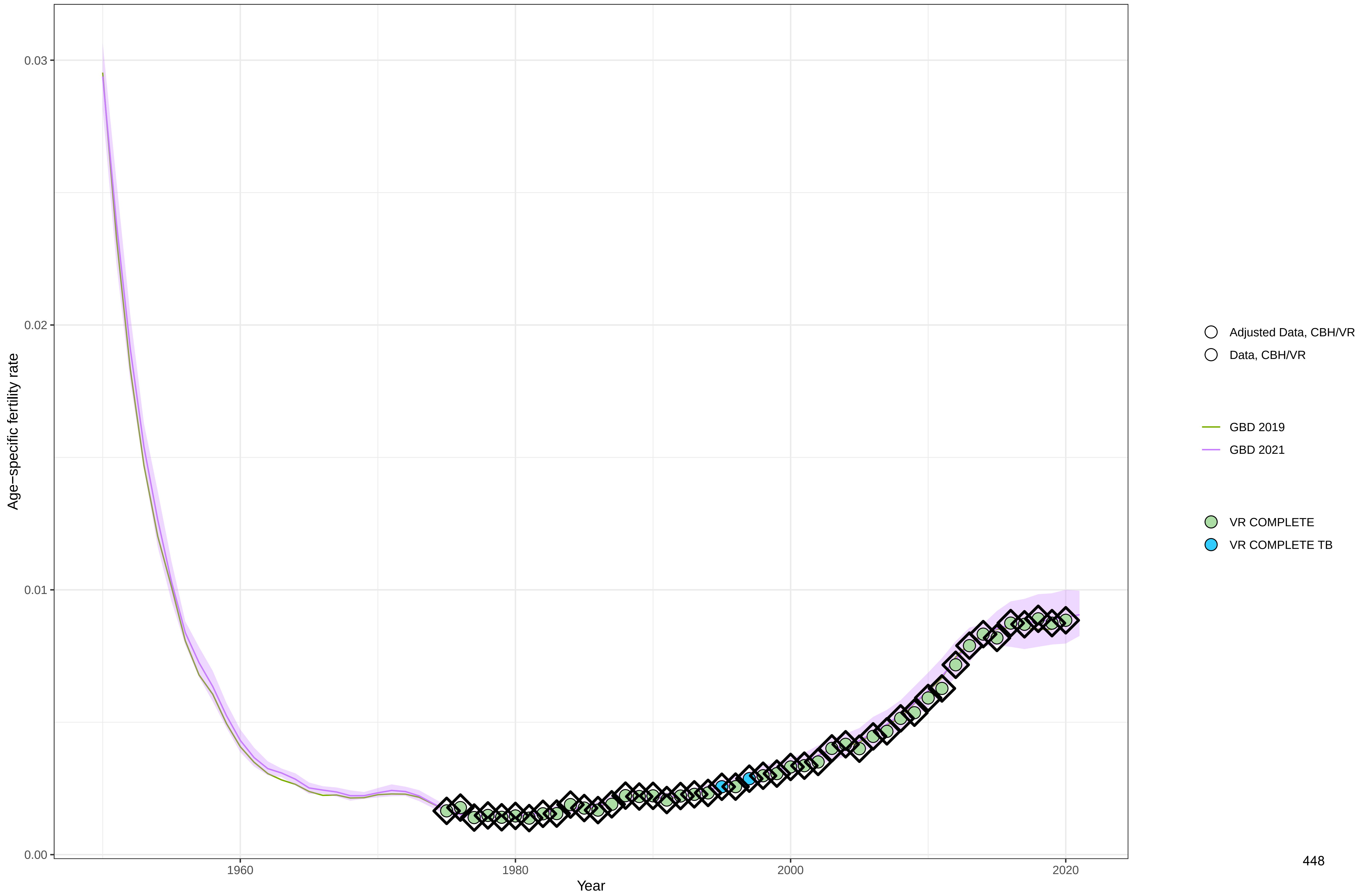

Age−specific fertility fitted trend lines, 1950−2021: Hokkaid.., Ages: 45−49
Data points enclosed with a diamond are reference sources

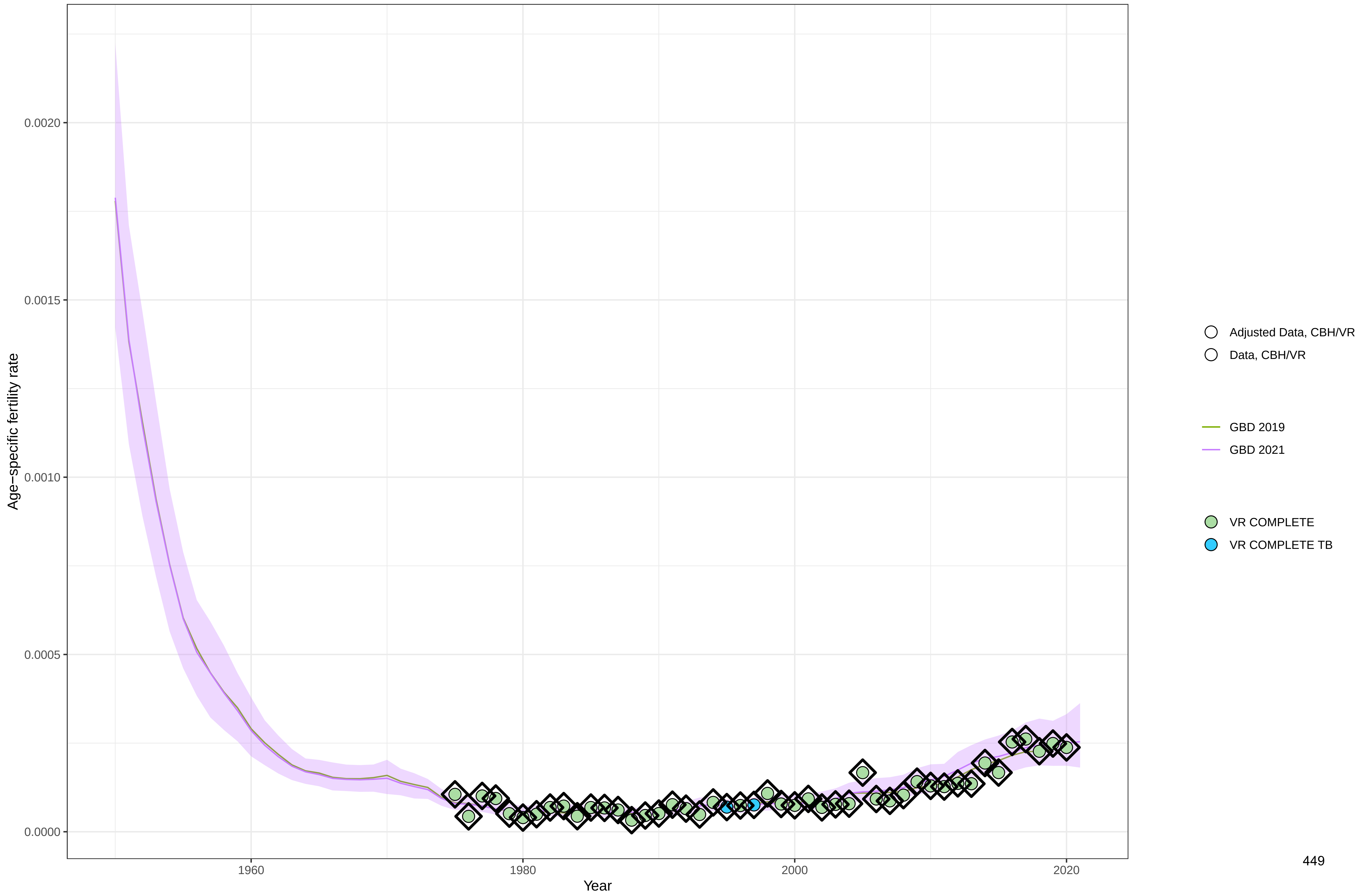

Age−specific fertility fitted trend lines, 1950−2021: Hokkaid.., Ages: 50−54
Data points enclosed with a diamond are reference sources

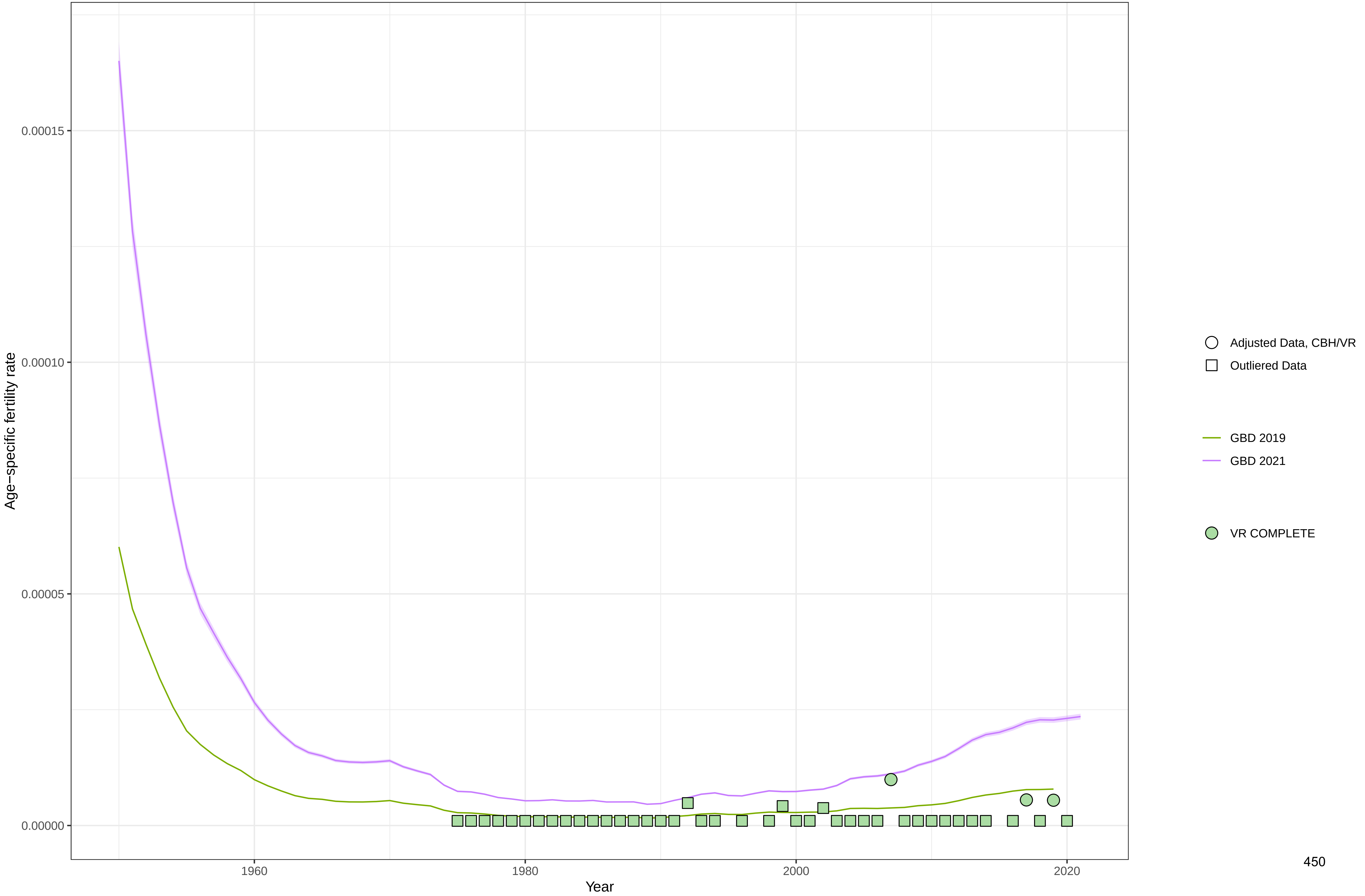

Total fertility rate fitted trend lines, 1950−2021: Hokkaid.. TFR
Data points enclosed with a diamond are reference sources

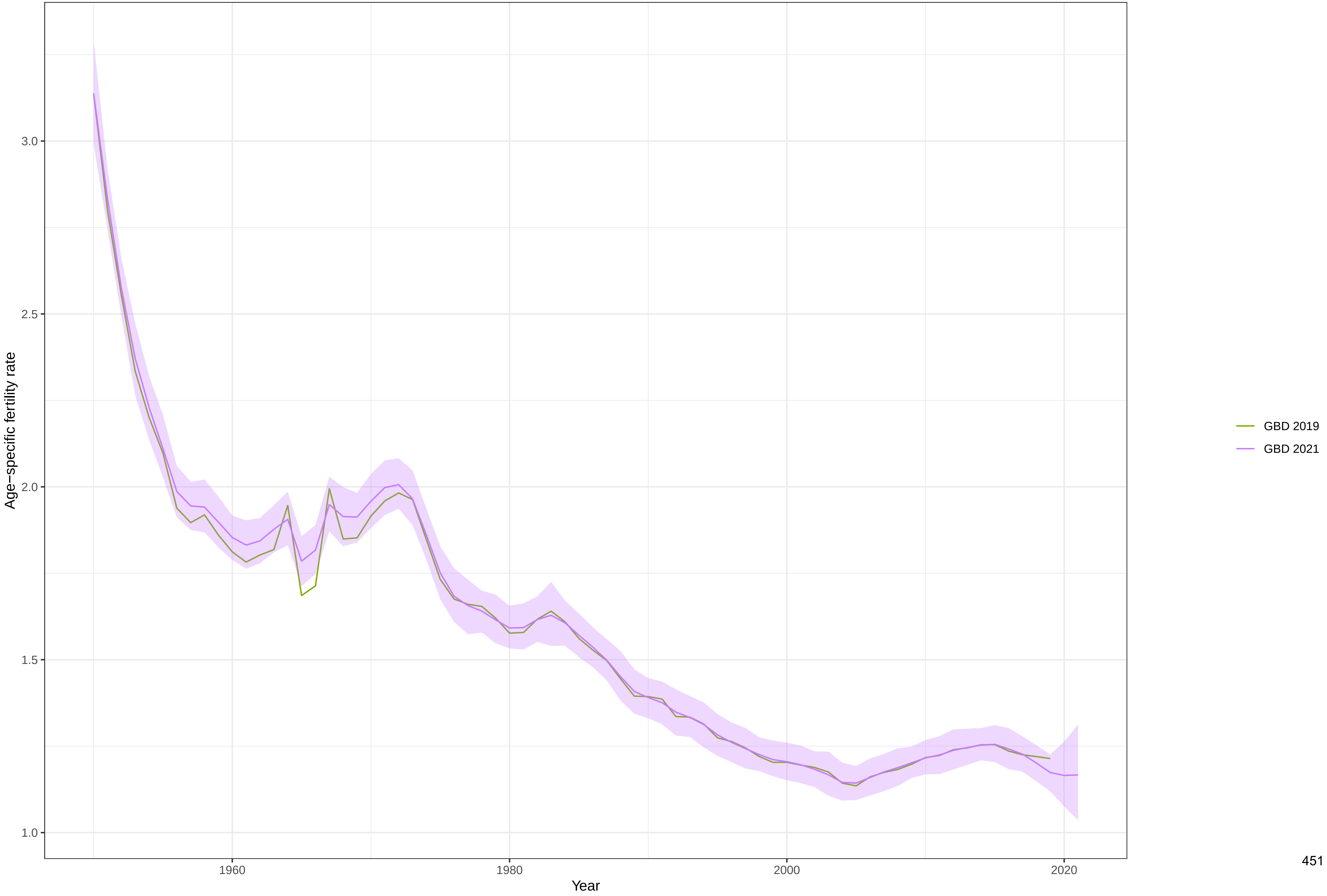

Age−specific fertility fitted trend lines, 1950−2021: Hy..go, Ages: 10−14
Data points enclosed with a diamond are reference sources

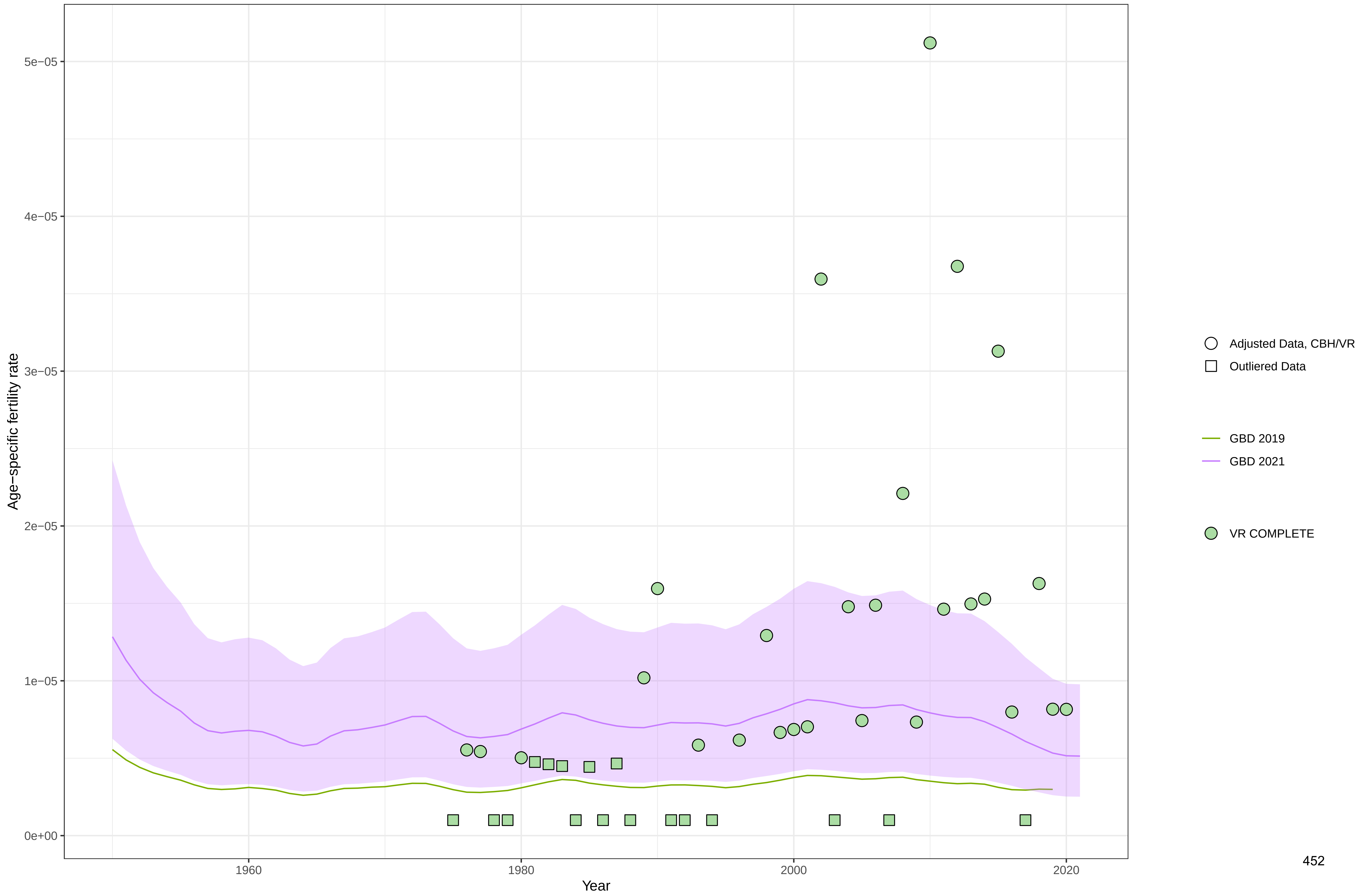

Age−specific fertility fitted trend lines, 1950−2021: Hy..go, Ages: 15−19
Data points enclosed with a diamond are reference sources

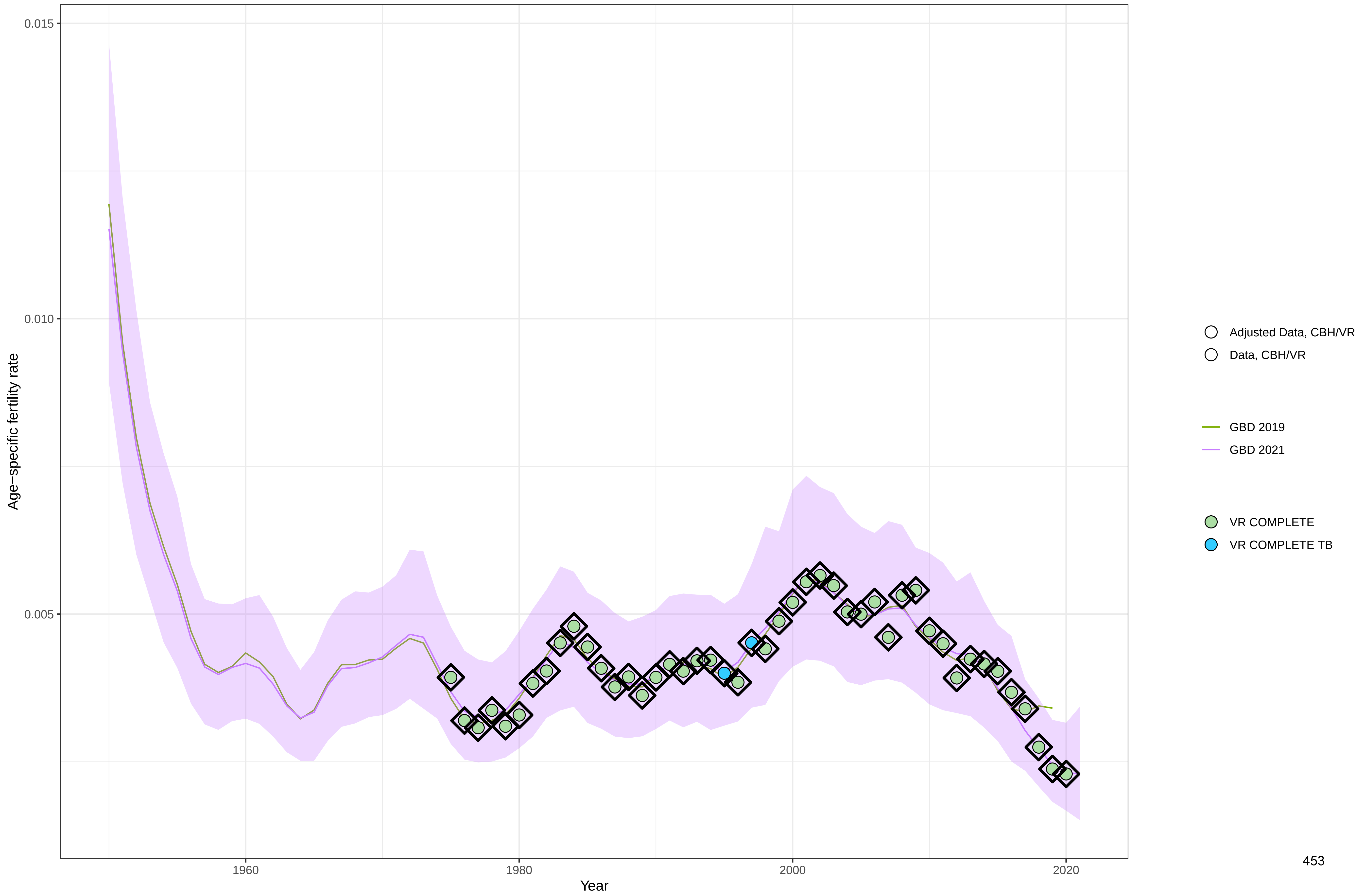

Age−specific fertility fitted trend lines, 1950−2021: Hy..go, Ages: 20−24
Data points enclosed with a diamond are reference sources

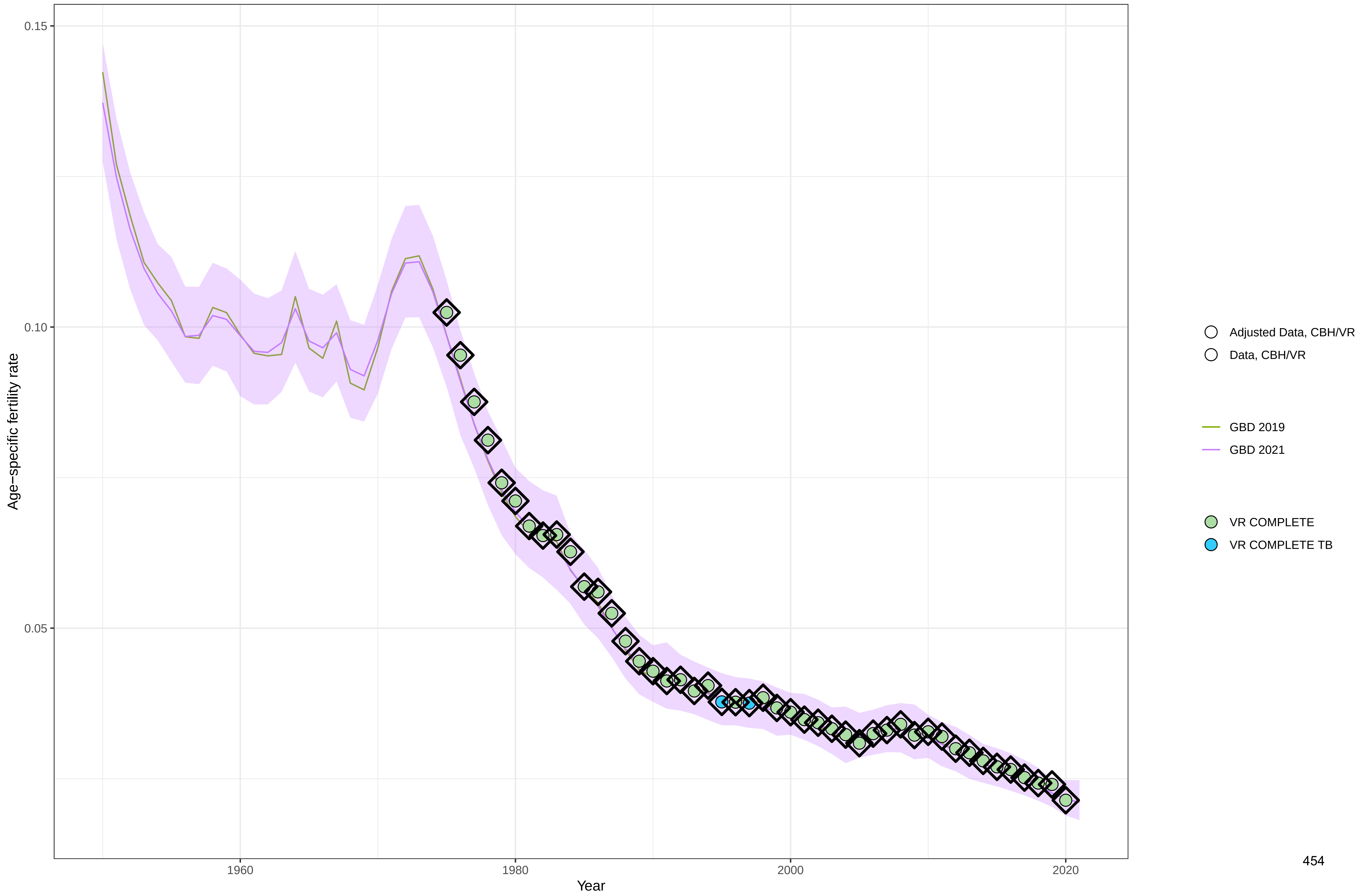

Age−specific fertility fitted trend lines, 1950−2021: Hy..go, Ages: 25−29
Data points enclosed with a diamond are reference sources

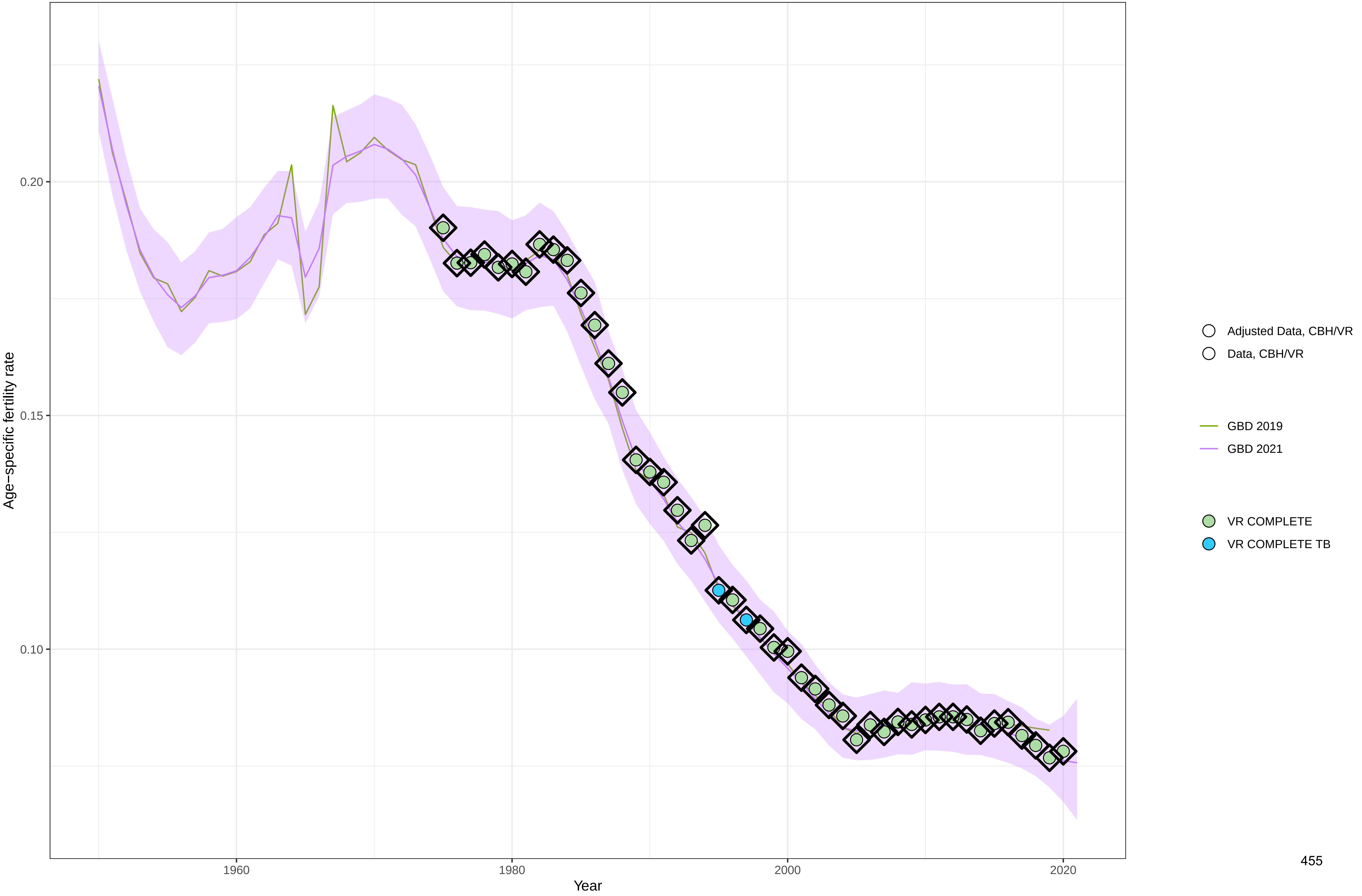

Age−specific fertility fitted trend lines, 1950−2021: Hy..go, Ages: 30−34
Data points enclosed with a diamond are reference sources

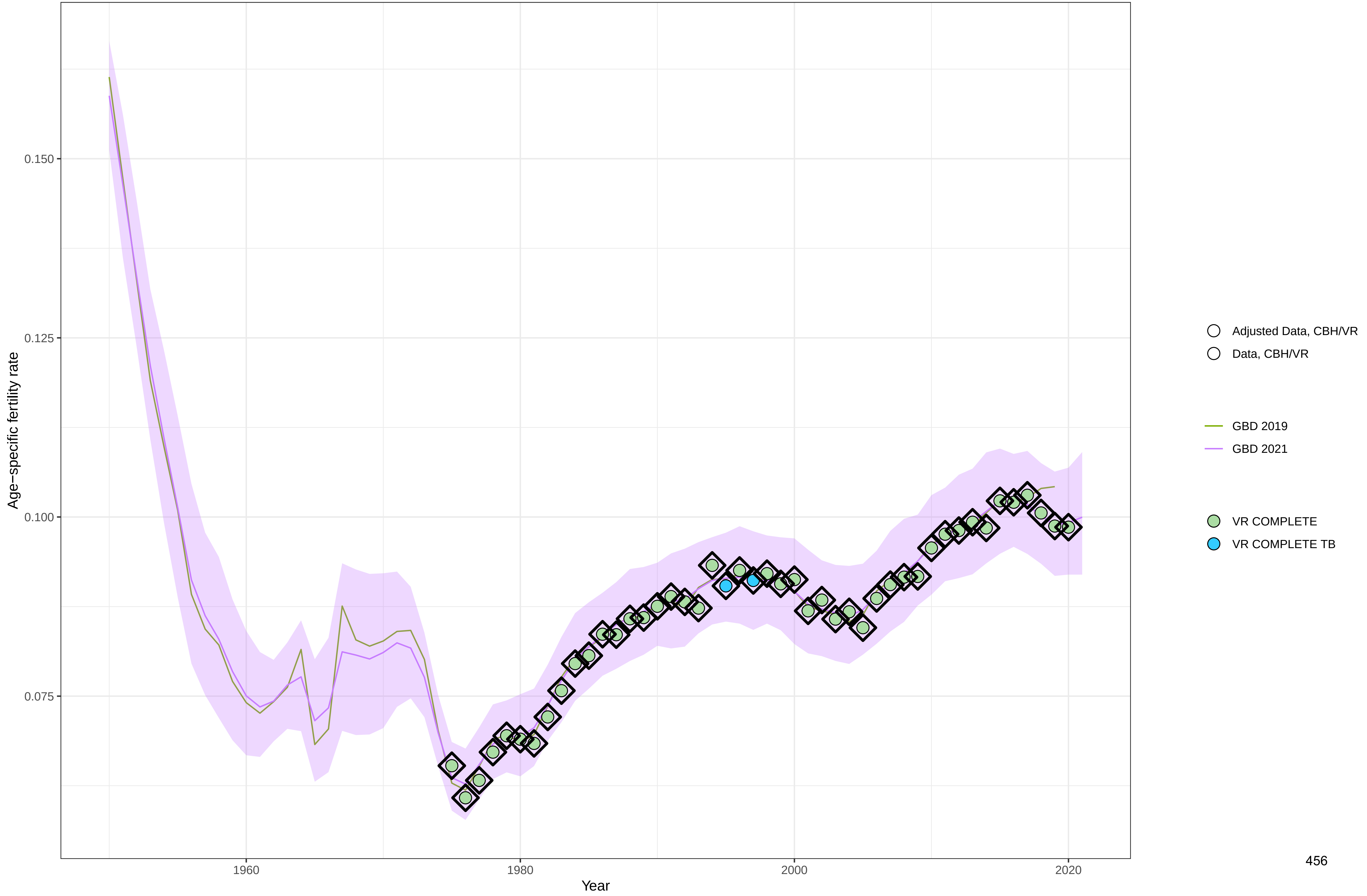

Age−specific fertility fitted trend lines, 1950−2021: Hy..go, Ages: 35−39
Data points enclosed with a diamond are reference sources

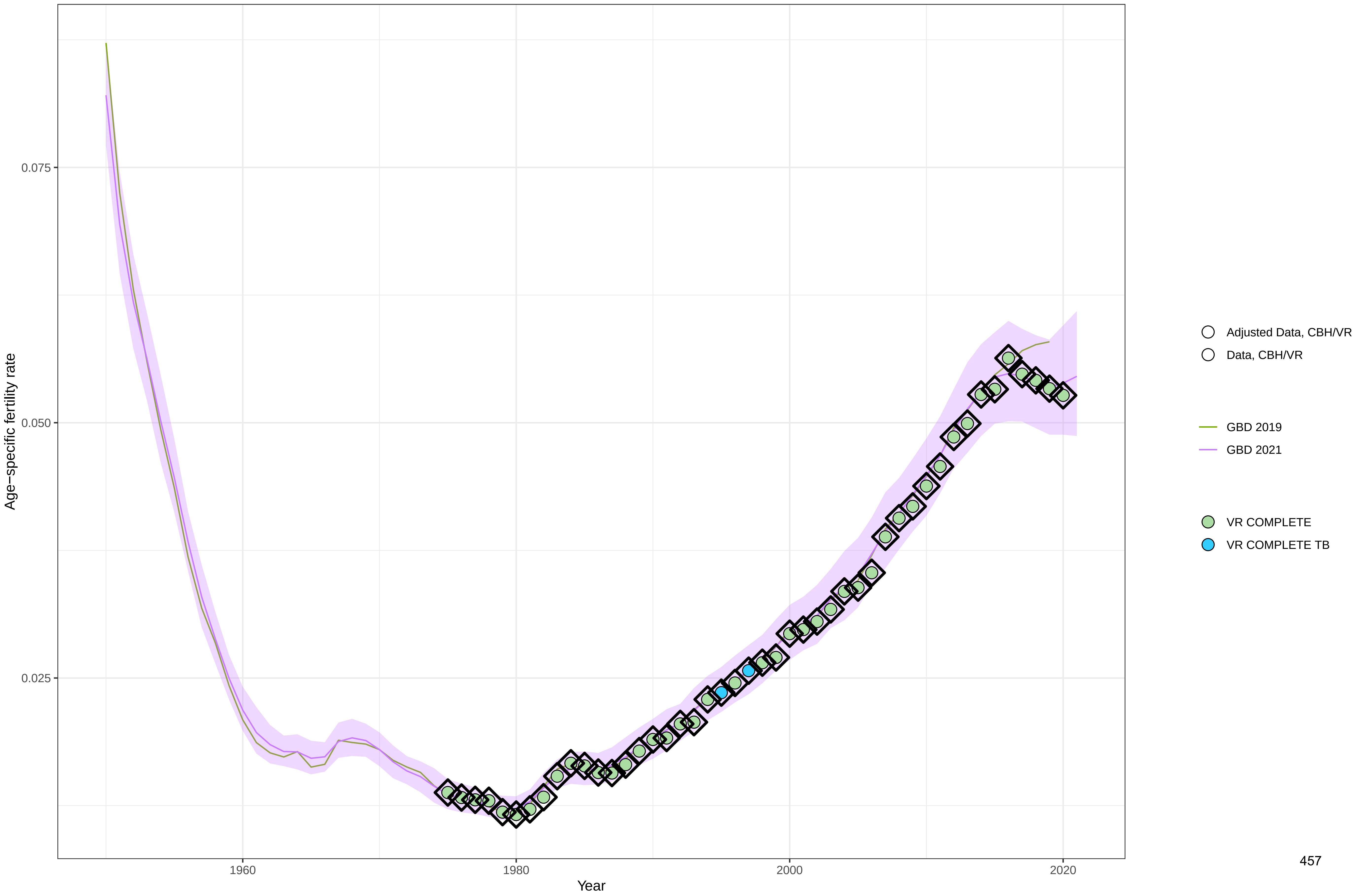

Age−specific fertility fitted trend lines, 1950−2021: Hy..go, Ages: 40−44
Data points enclosed with a diamond are reference sources

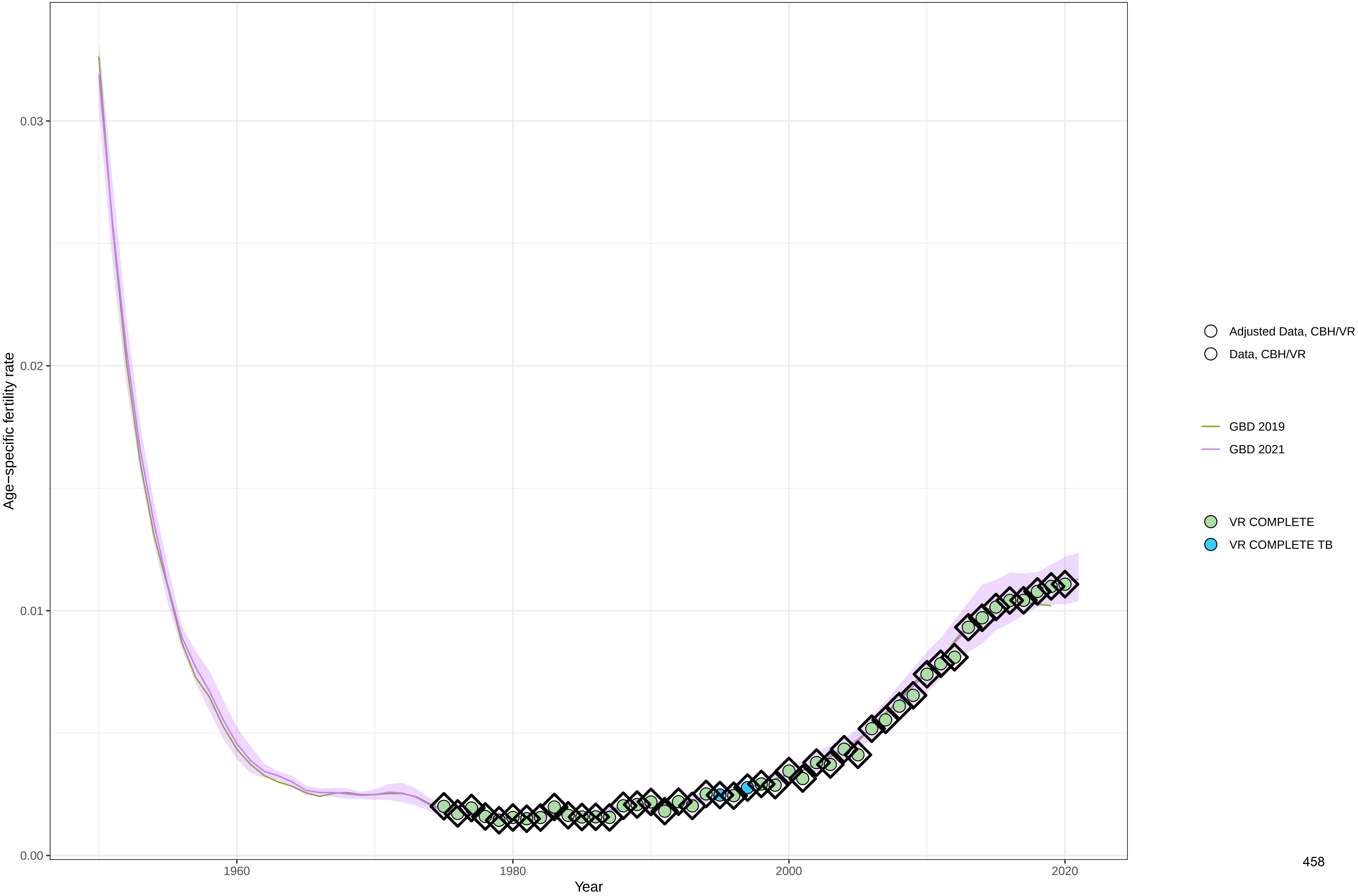

Age−specific fertility fitted trend lines, 1950−2021: Hy..go, Ages: 45−49
Data points enclosed with a diamond are reference sources

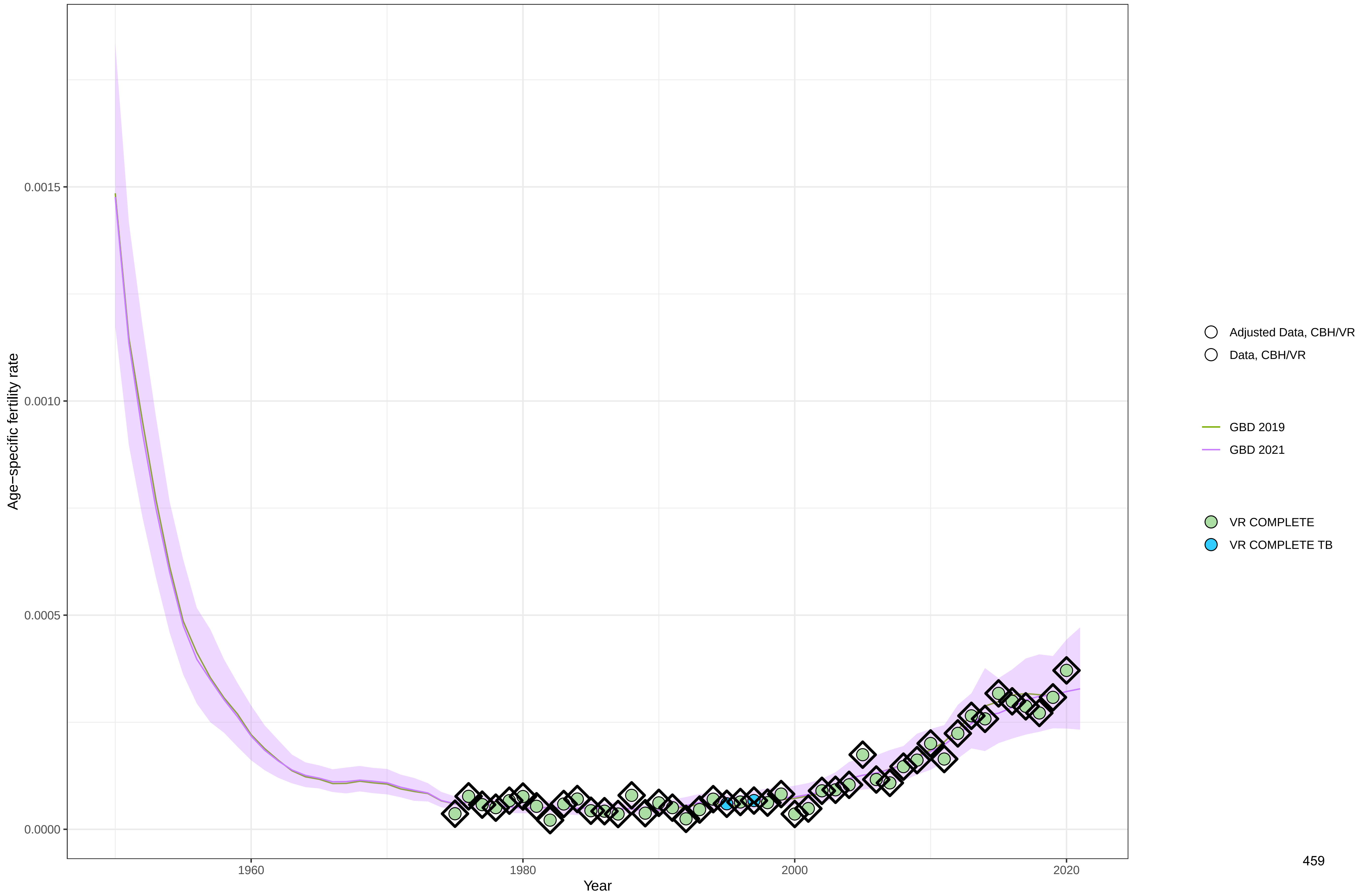

Age−specific fertility fitted trend lines, 1950−2021: Hy..go, Ages: 50−54
Data points enclosed with a diamond are reference sources

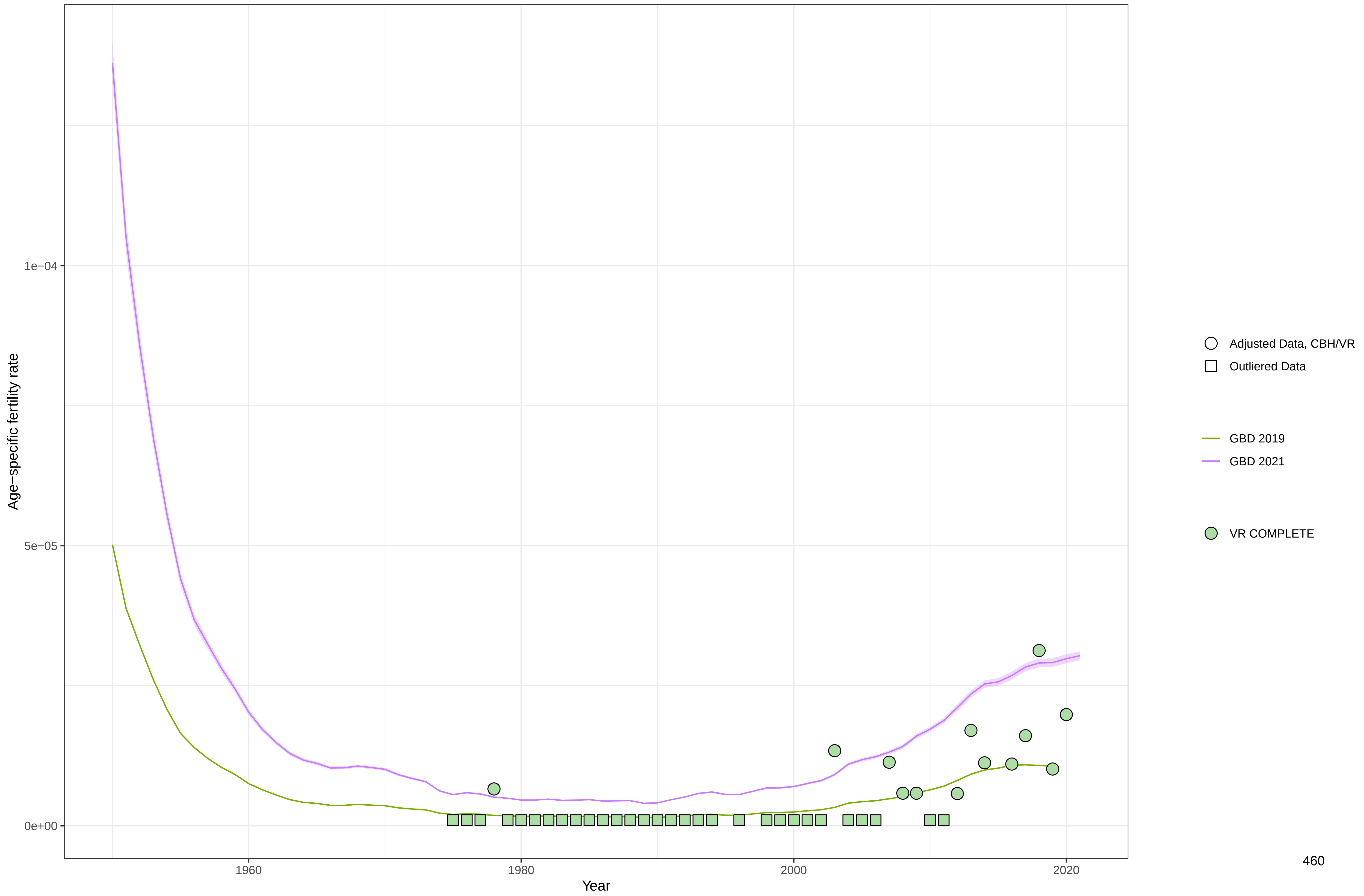

Total fertility rate fitted trend lines, 1950−2021: Hy..go TFR
Data points enclosed with a diamond are reference sources

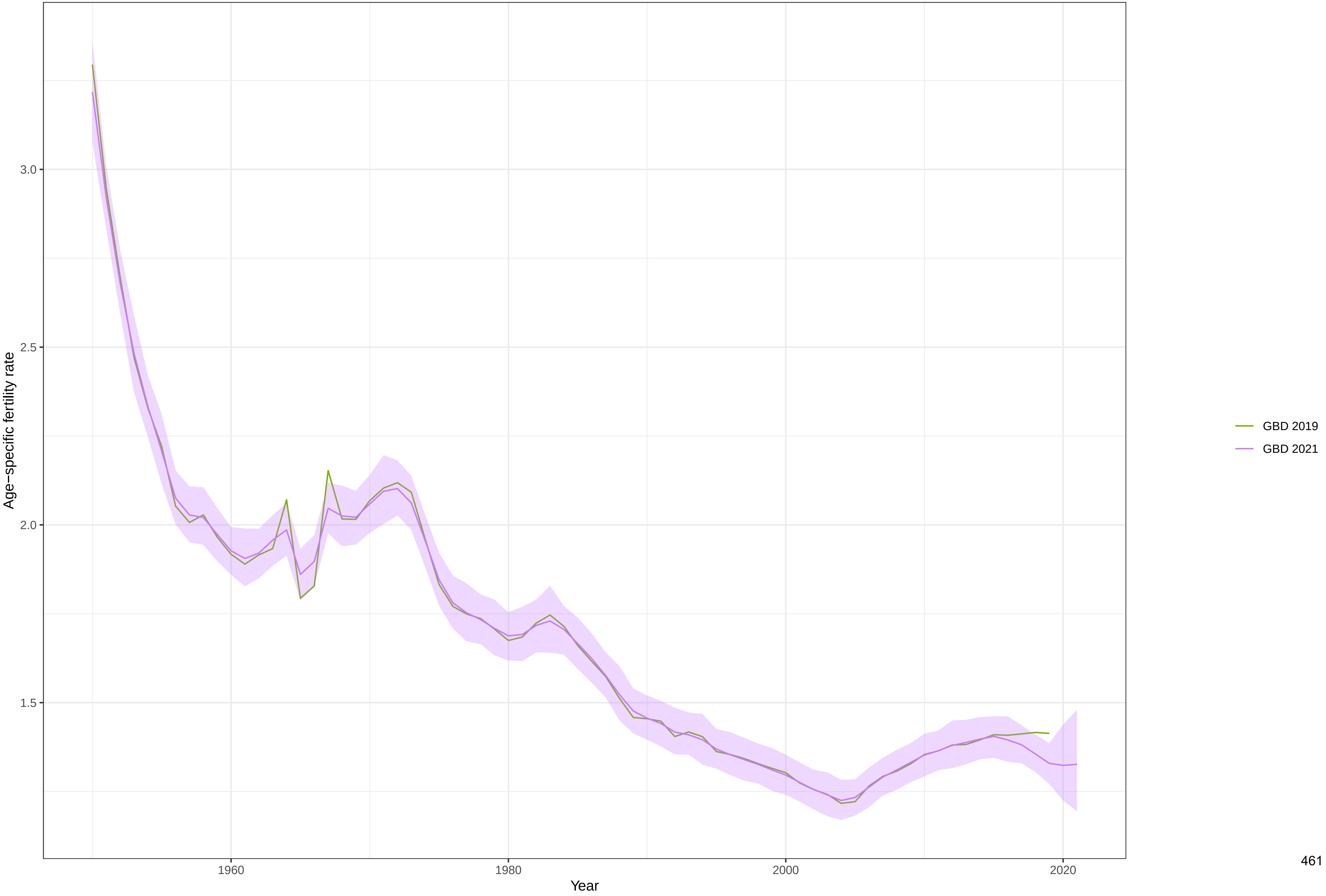

Age−specific fertility fitted trend lines, 1950−2021: Ibaraki, Ages: 10−14
Data points enclosed with a diamond are reference sources

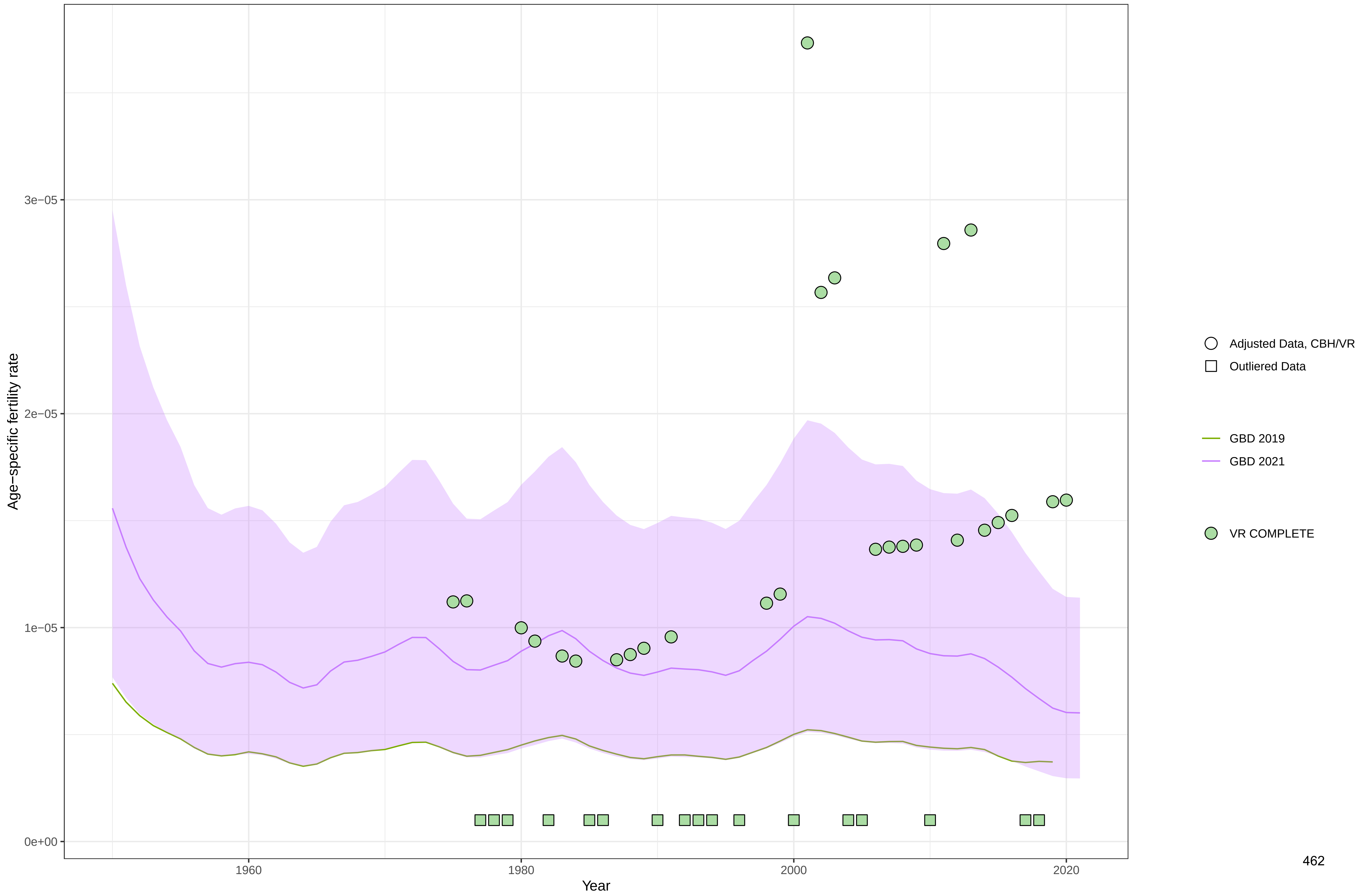

Age−specific fertility fitted trend lines, 1950−2021: Ibaraki, Ages: 15−19
Data points enclosed with a diamond are reference sources

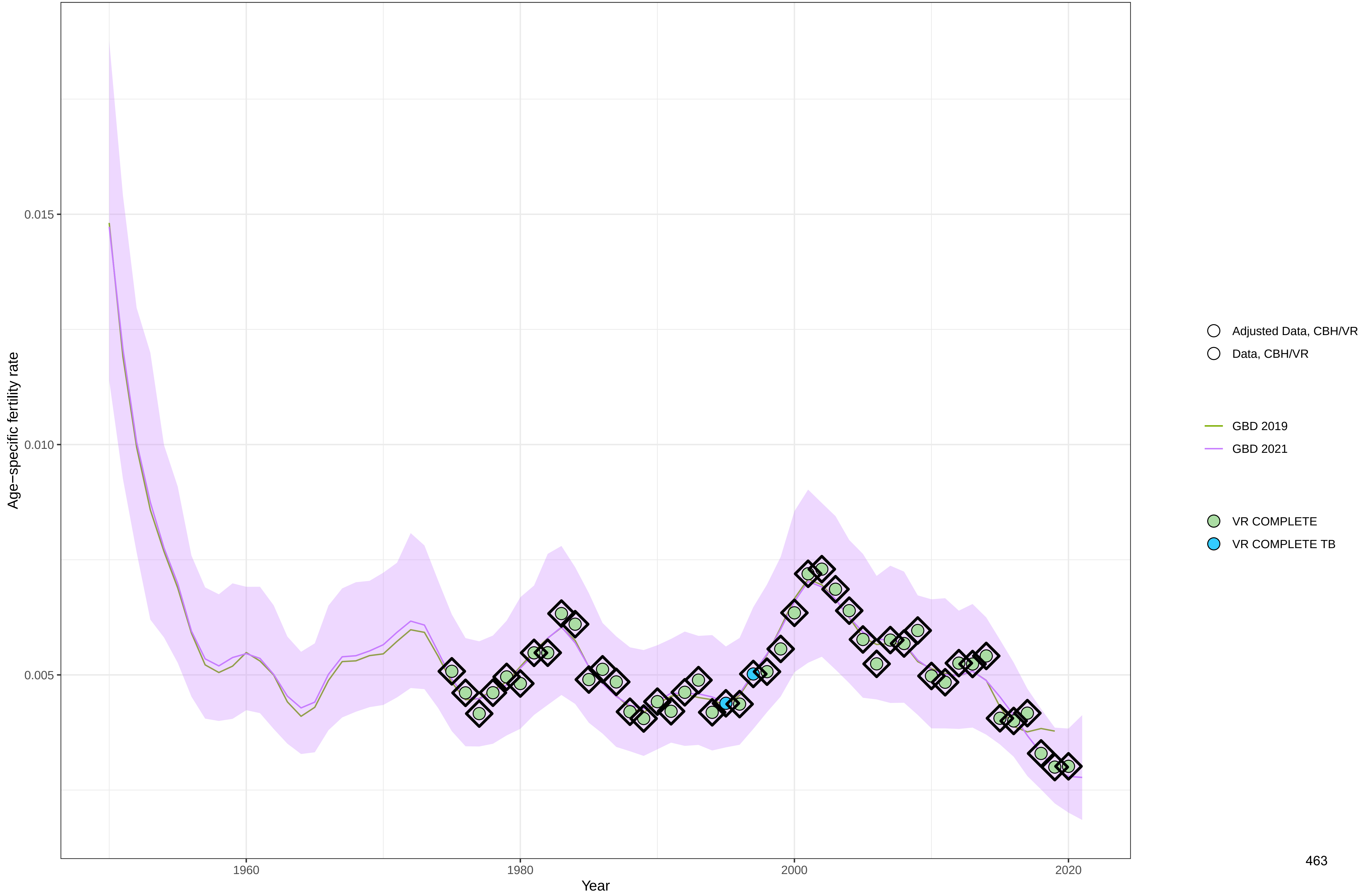

Age−specific fertility fitted trend lines, 1950−2021: Ibaraki, Ages: 20−24
Data points enclosed with a diamond are reference sources

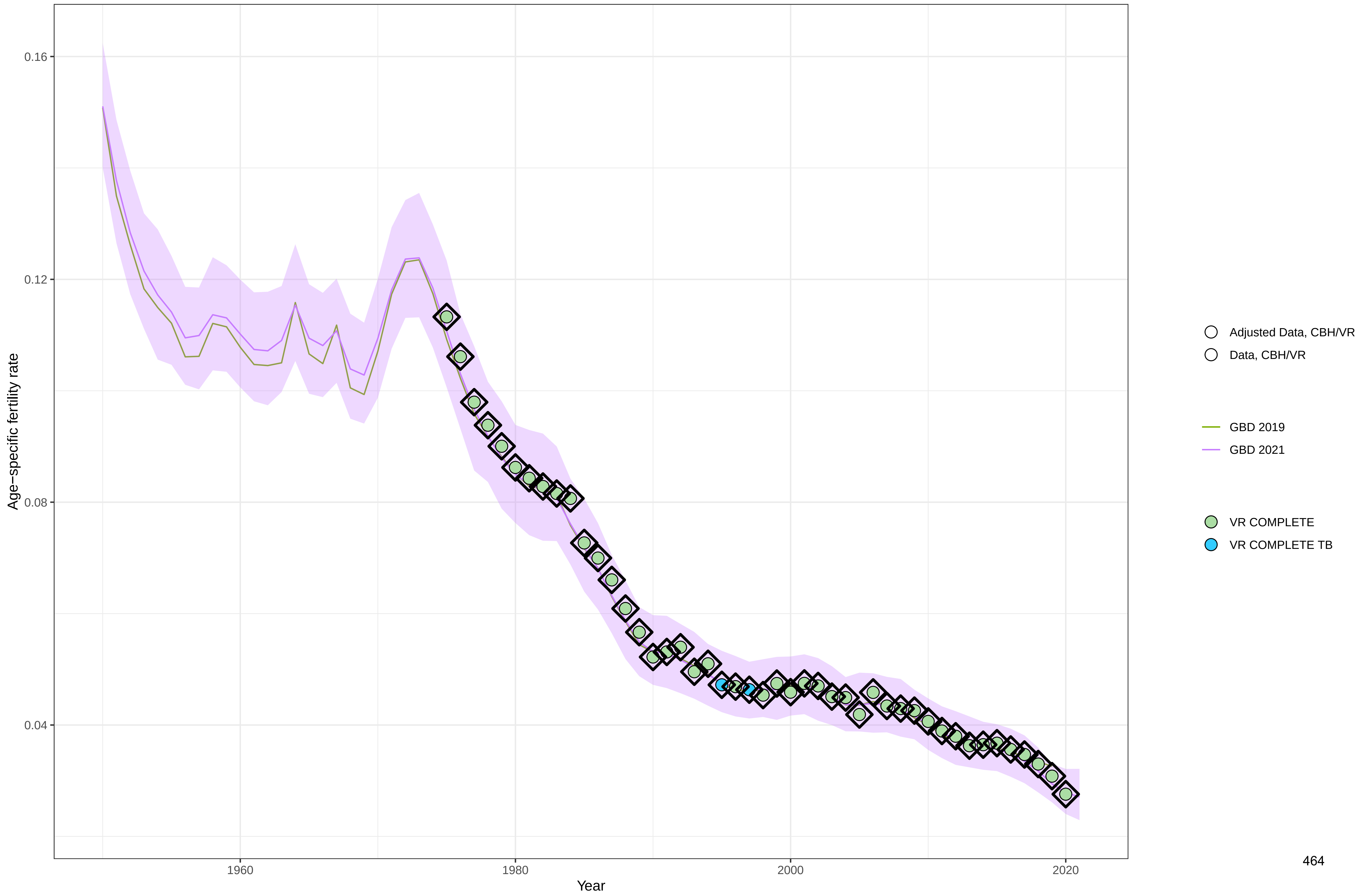

Age−specific fertility fitted trend lines, 1950−2021: Ibaraki, Ages: 25−29
Data points enclosed with a diamond are reference sources

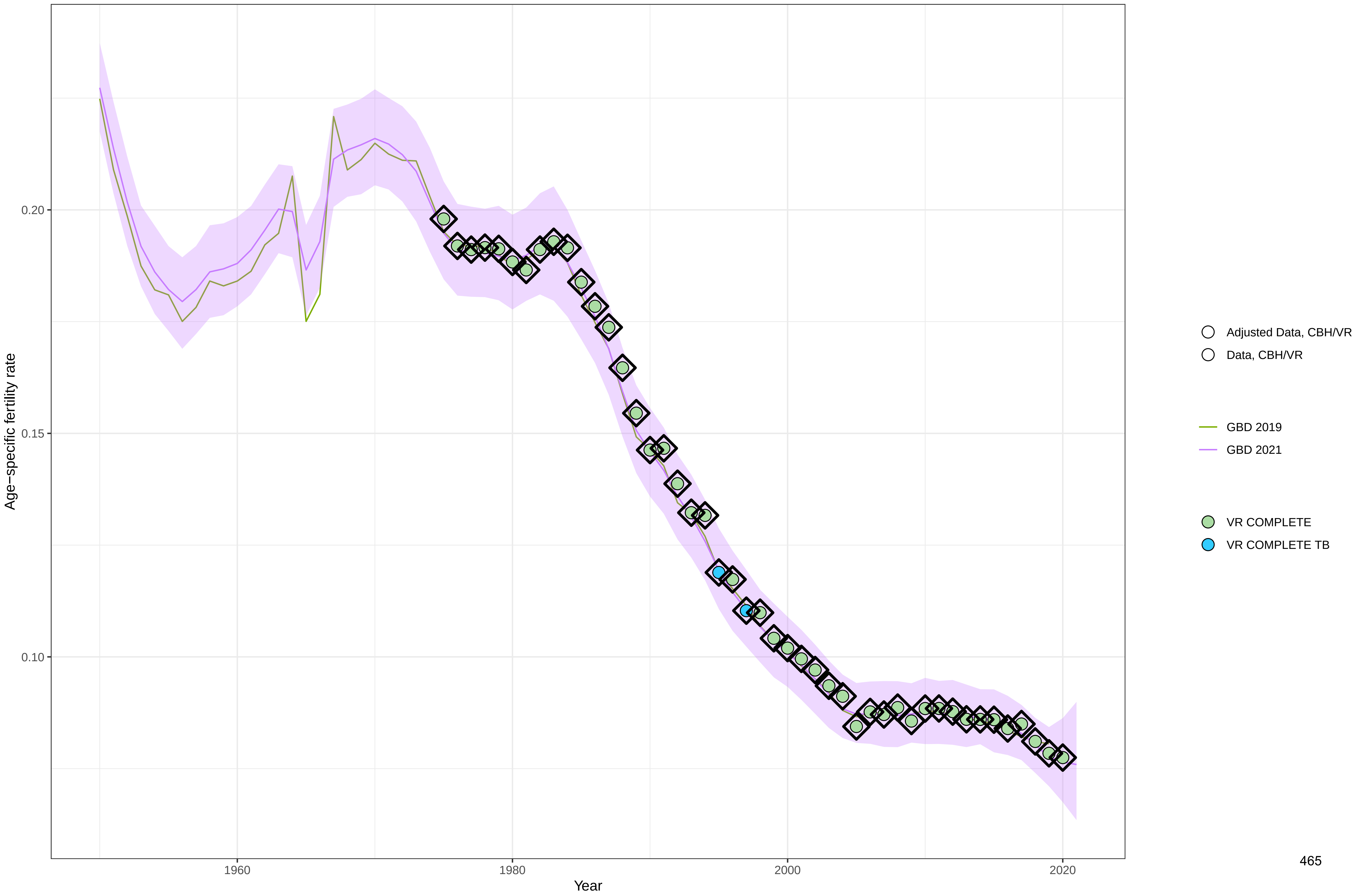

Age−specific fertility fitted trend lines, 1950−2021: Ibaraki, Ages: 30−34
Data points enclosed with a diamond are reference sources

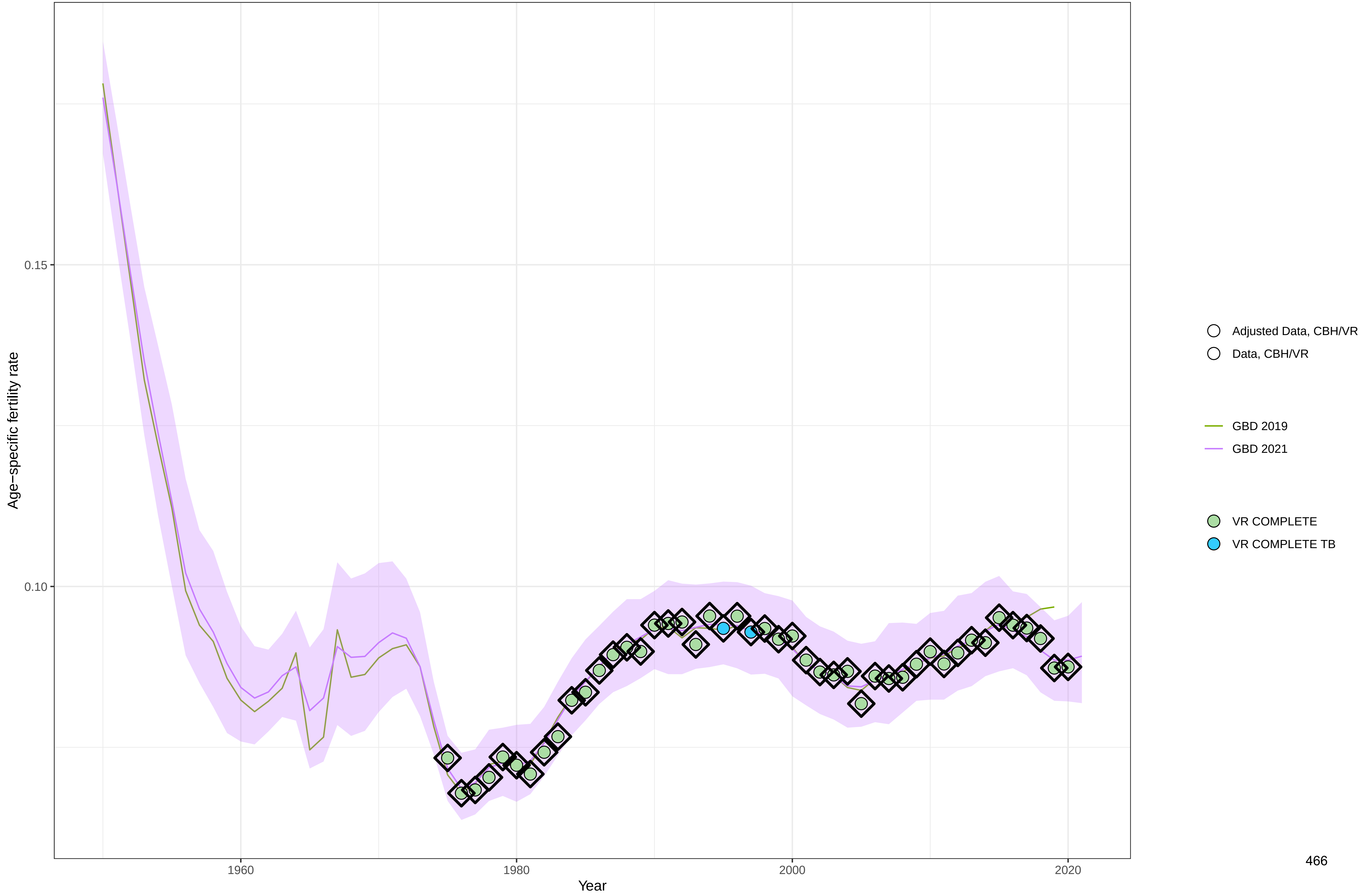

Age−specific fertility fitted trend lines, 1950−2021: Ibaraki, Ages: 35−39
Data points enclosed with a diamond are reference sources

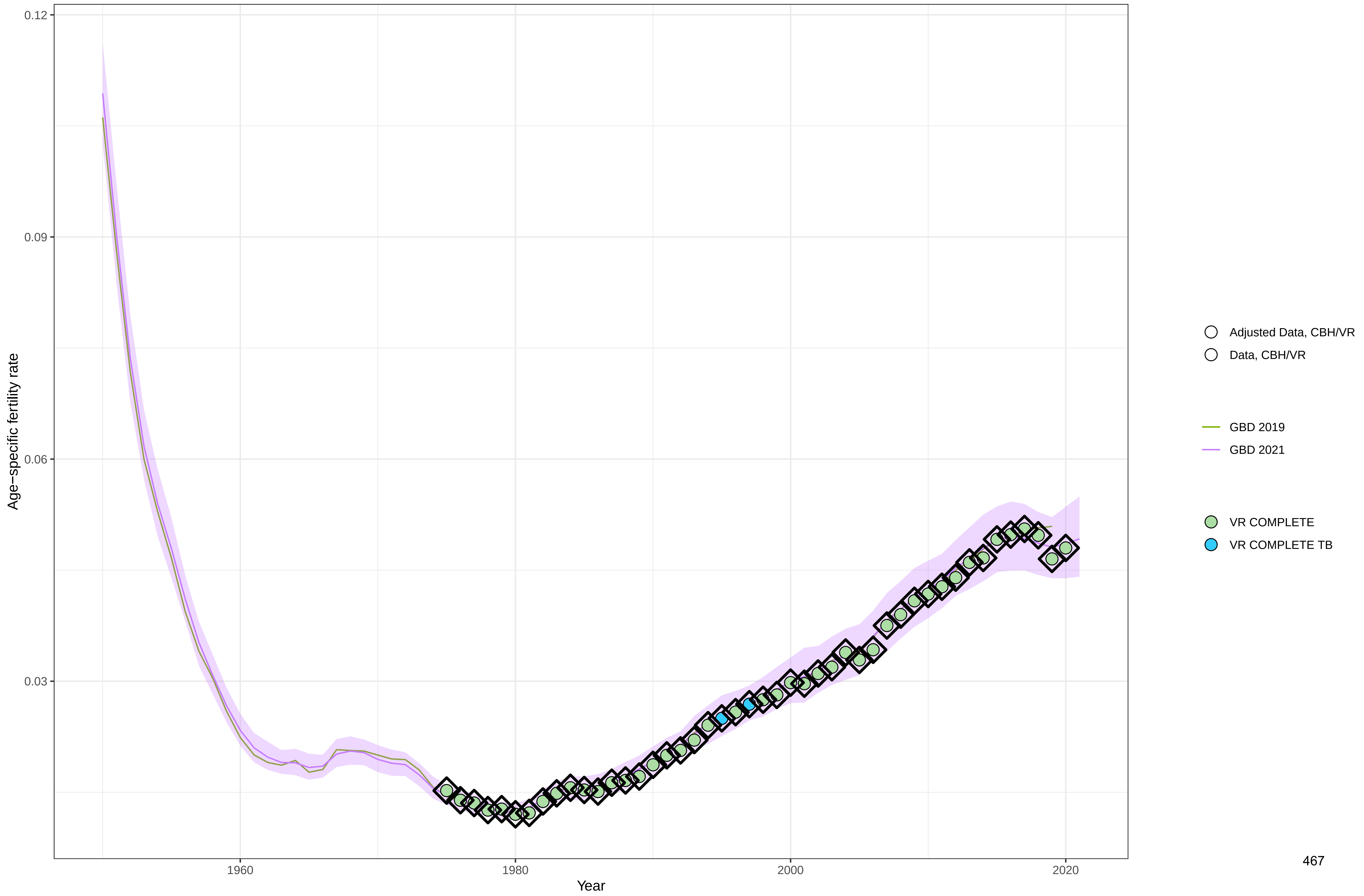

Age−specific fertility fitted trend lines, 1950−2021: Ibaraki, Ages: 40−44
Data points enclosed with a diamond are reference sources

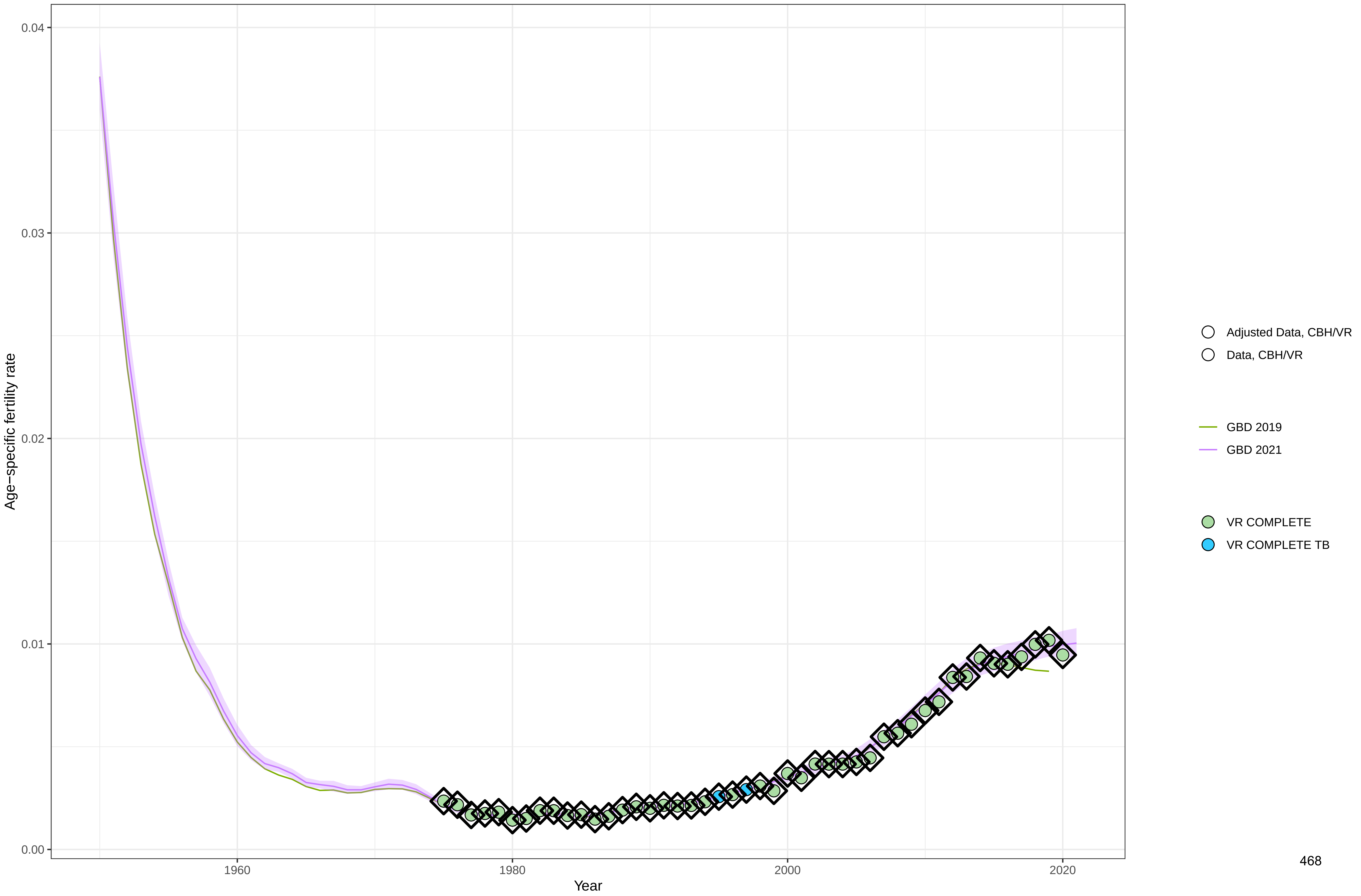

Age−specific fertility fitted trend lines, 1950−2021: Ibaraki, Ages: 45−49
Data points enclosed with a diamond are reference sources

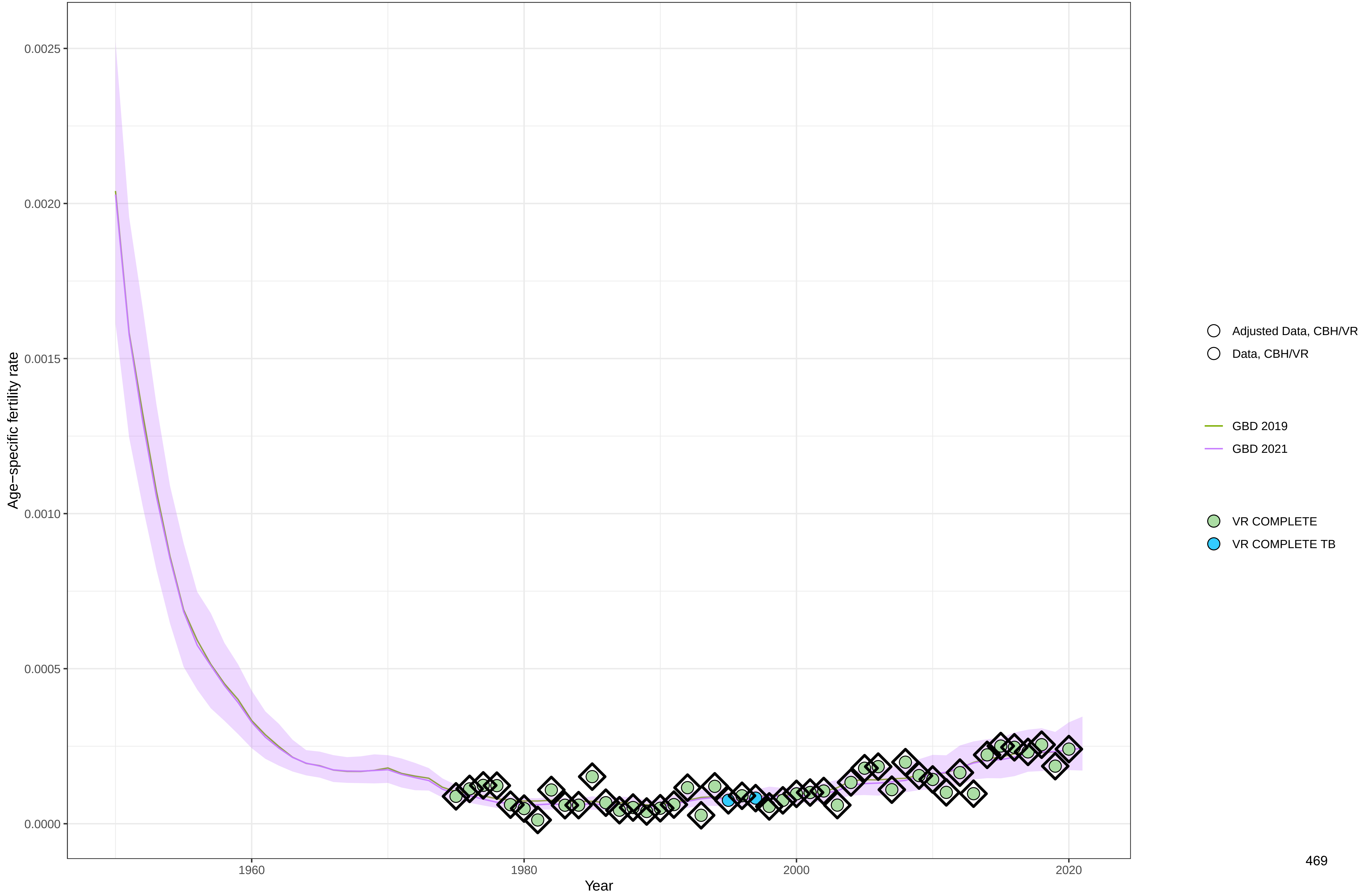

Age−specific fertility fitted trend lines, 1950−2021: Ibaraki, Ages: 50−54
Data points enclosed with a diamond are reference sources

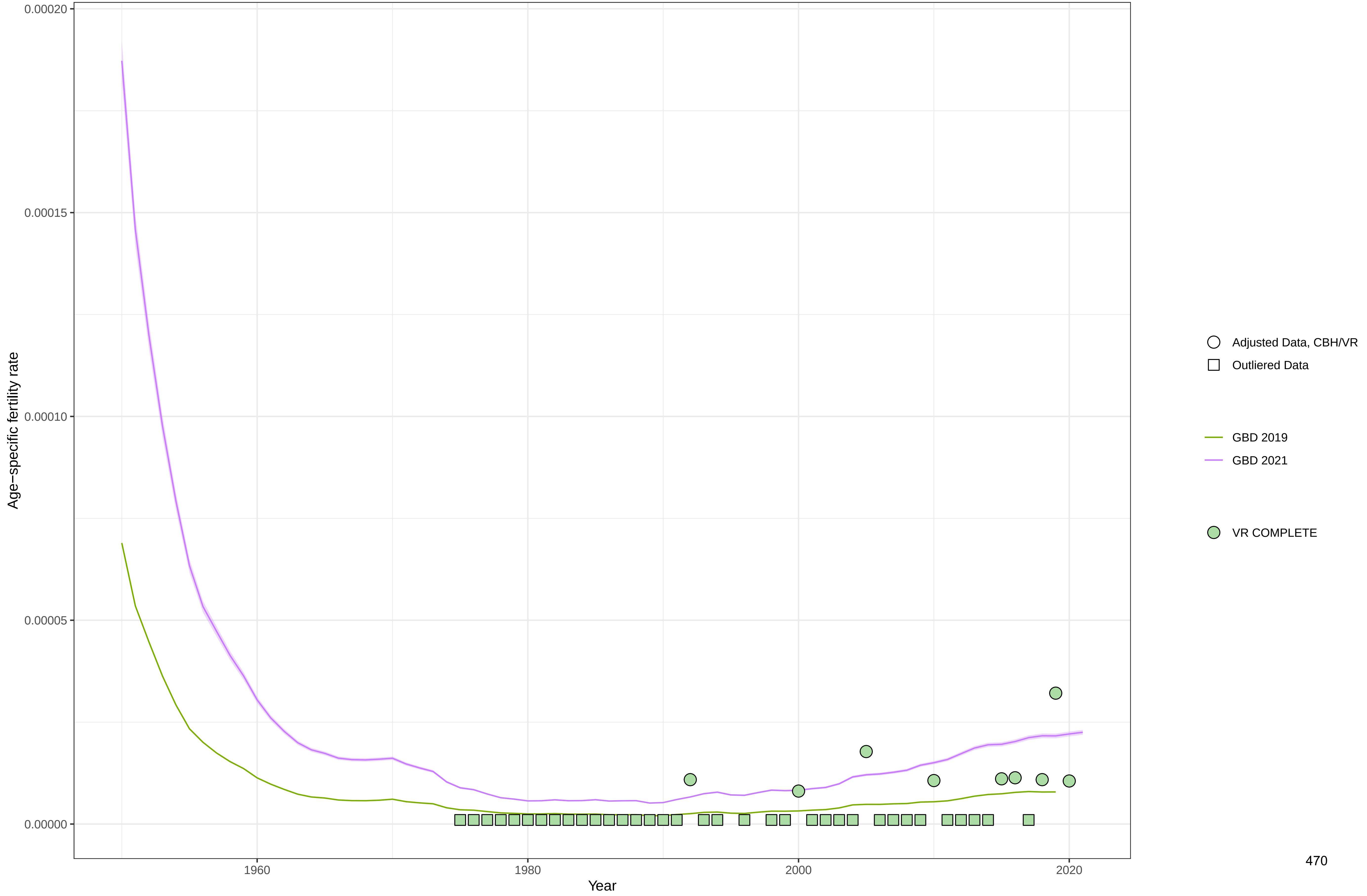

Total fertility rate fitted trend lines, 1950−2021: Ibaraki TFR
Data points enclosed with a diamond are reference sources

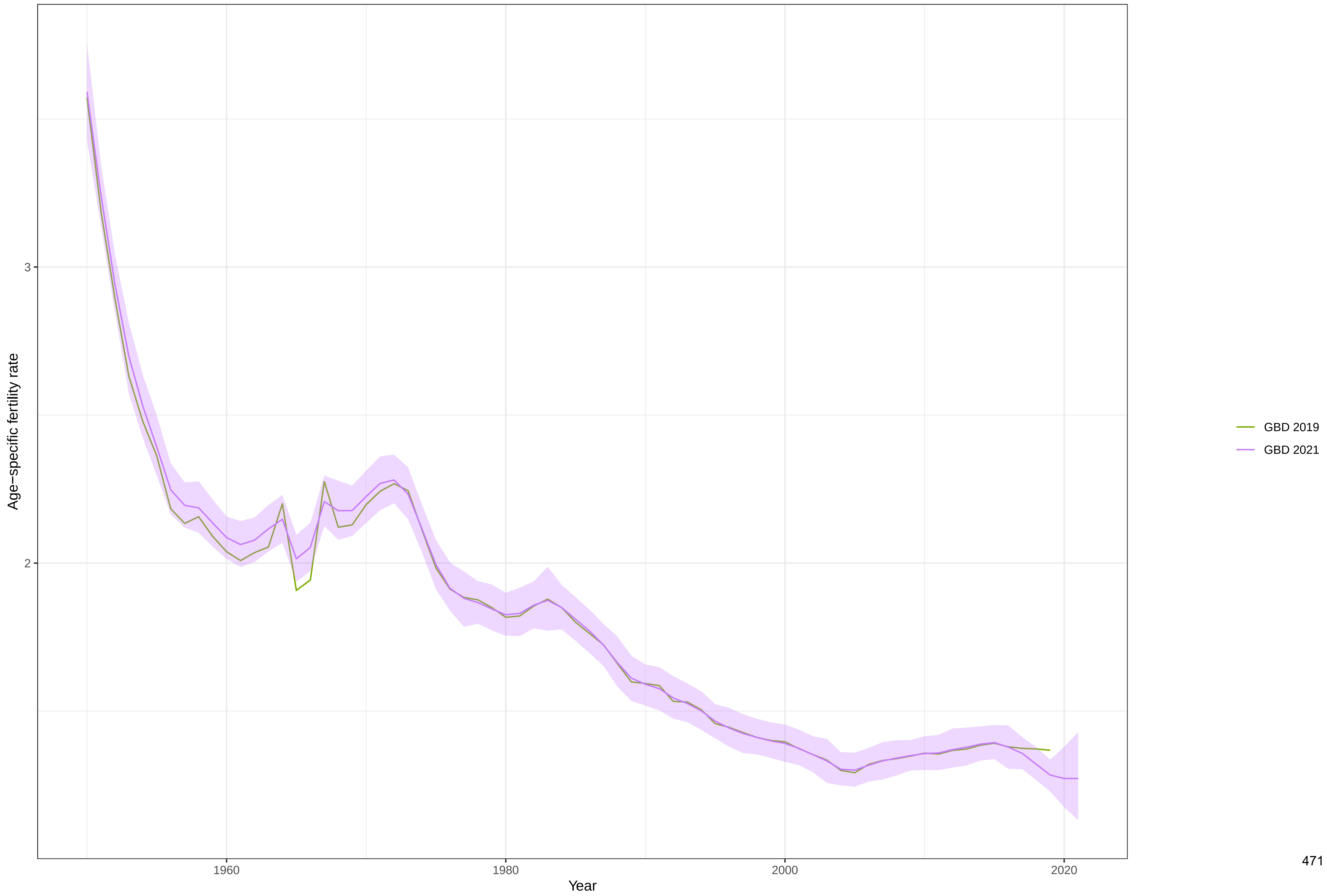

Age−specific fertility fitted trend lines, 1950−2021: Ishikawa, Ages: 10−14
Data points enclosed with a diamond are reference sources

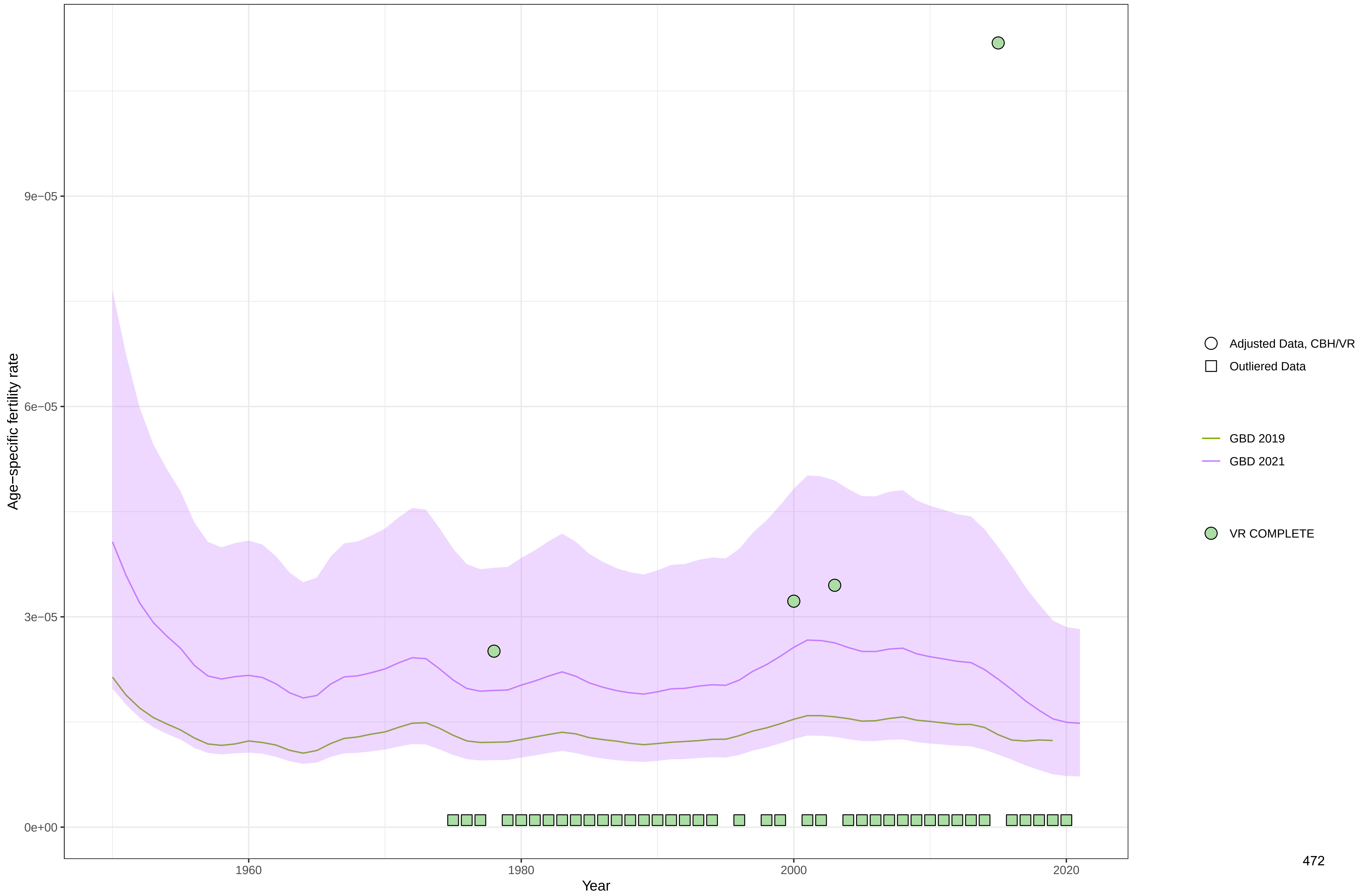

Age−specific fertility fitted trend lines, 1950−2021: Ishikawa, Ages: 15−19
Data points enclosed with a diamond are reference sources

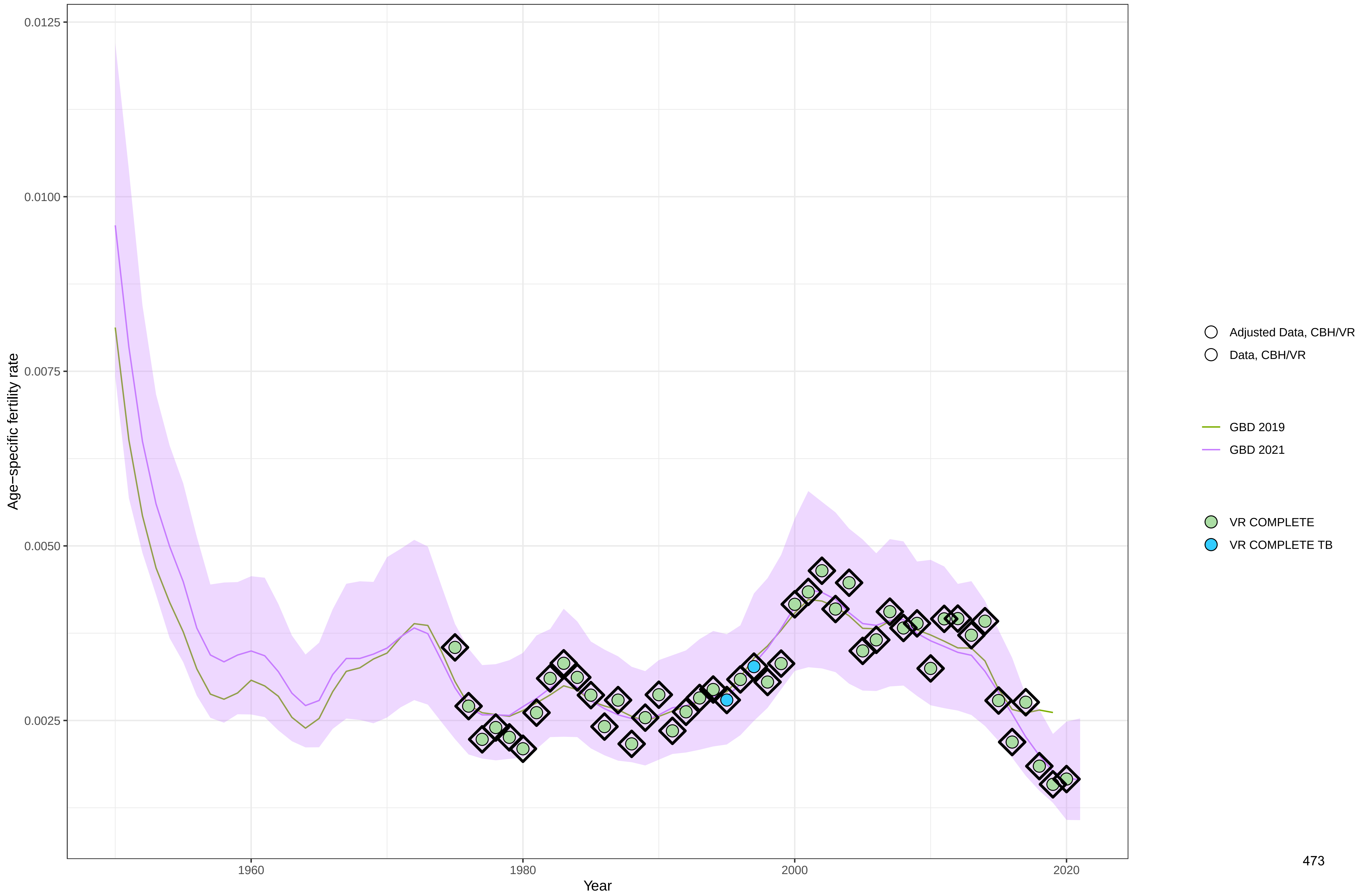

Age−specific fertility fitted trend lines, 1950−2021: Ishikawa, Ages: 20−24
Data points enclosed with a diamond are reference sources

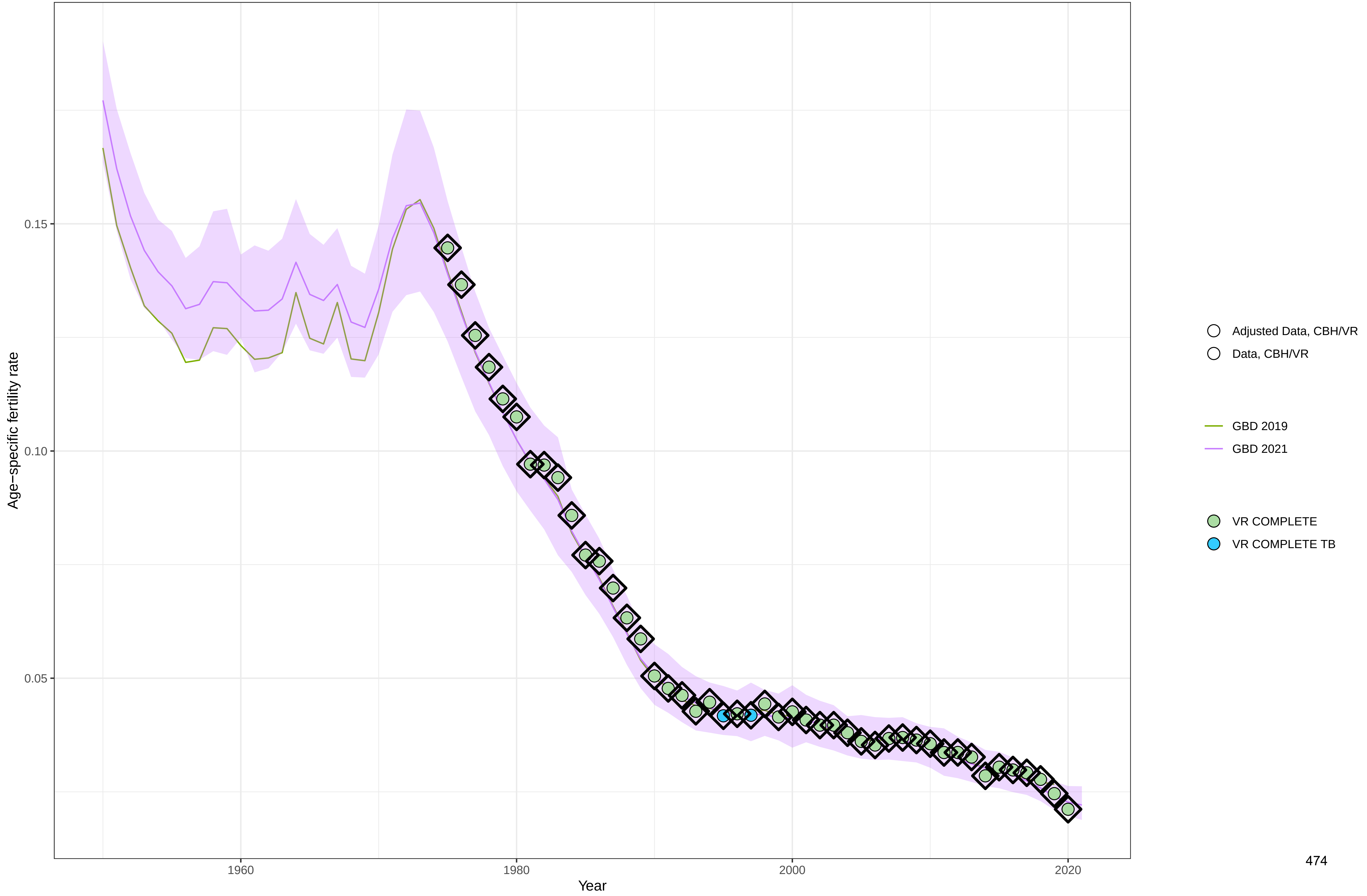

Age−specific fertility fitted trend lines, 1950−2021: Ishikawa, Ages: 25−29
Data points enclosed with a diamond are reference sources

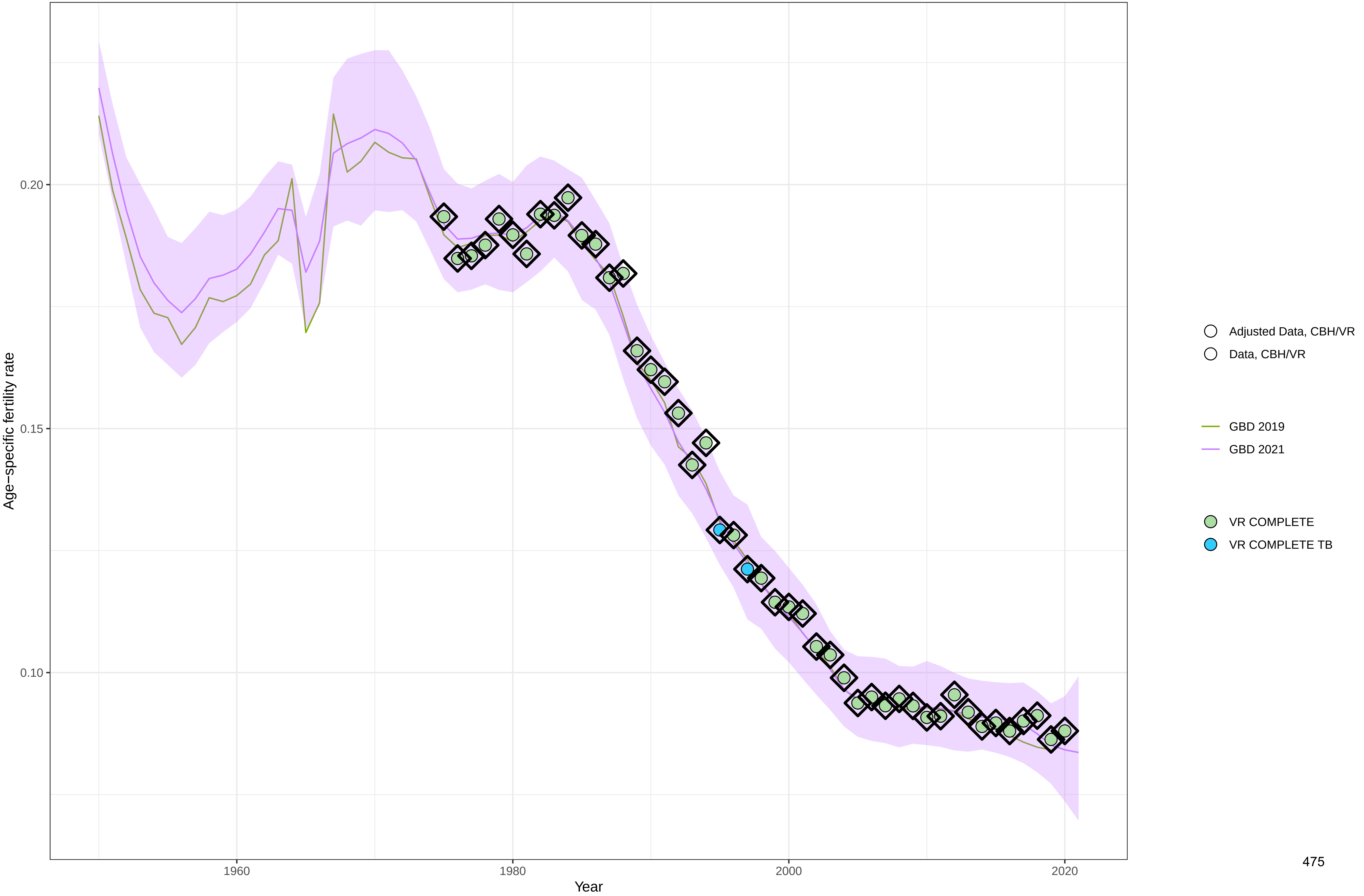

Age−specific fertility fitted trend lines, 1950−2021: Ishikawa, Ages: 30−34
Data points enclosed with a diamond are reference sources

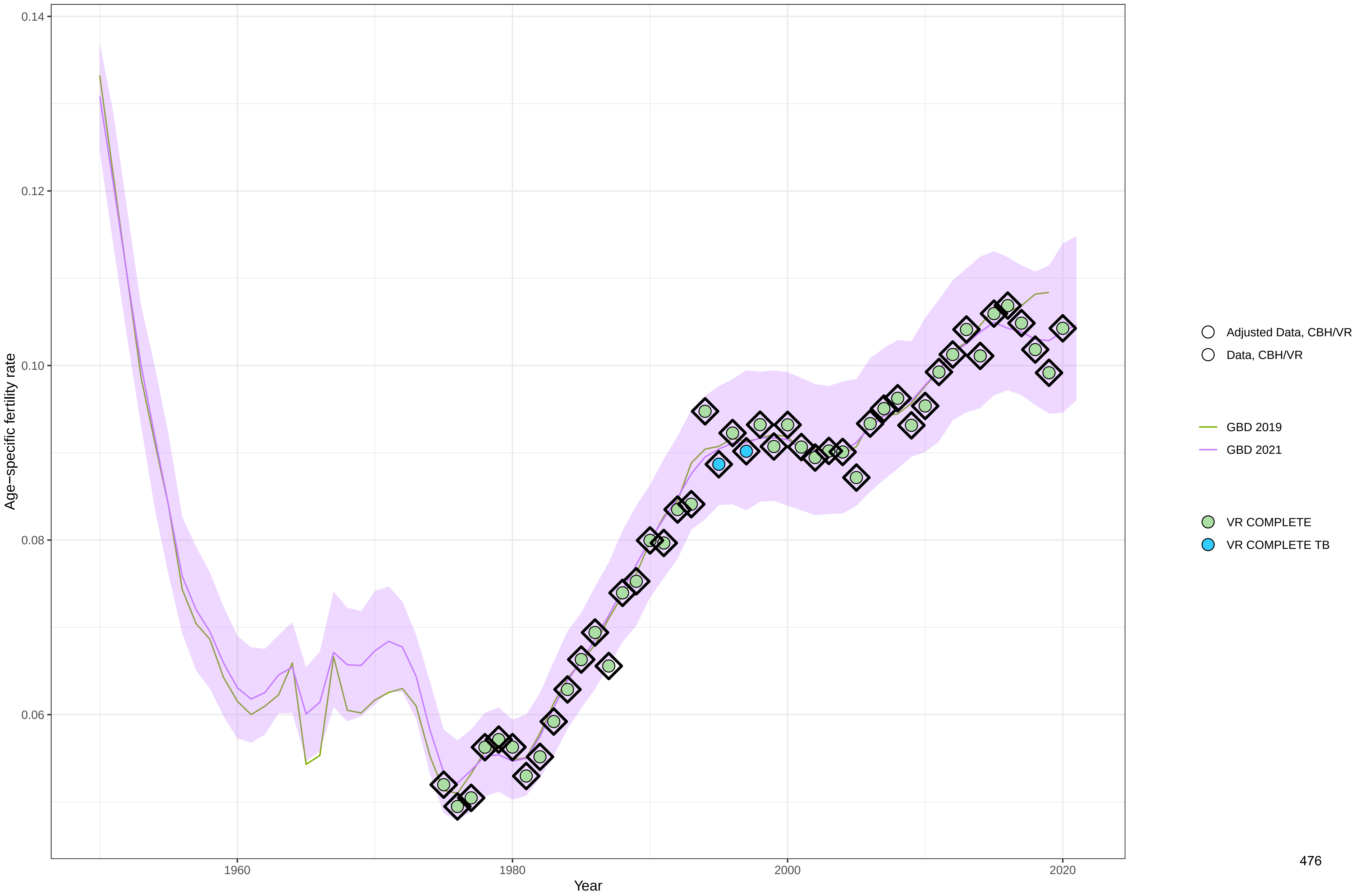

Age−specific fertility fitted trend lines, 1950−2021: Ishikawa, Ages: 35−39
Data points enclosed with a diamond are reference sources

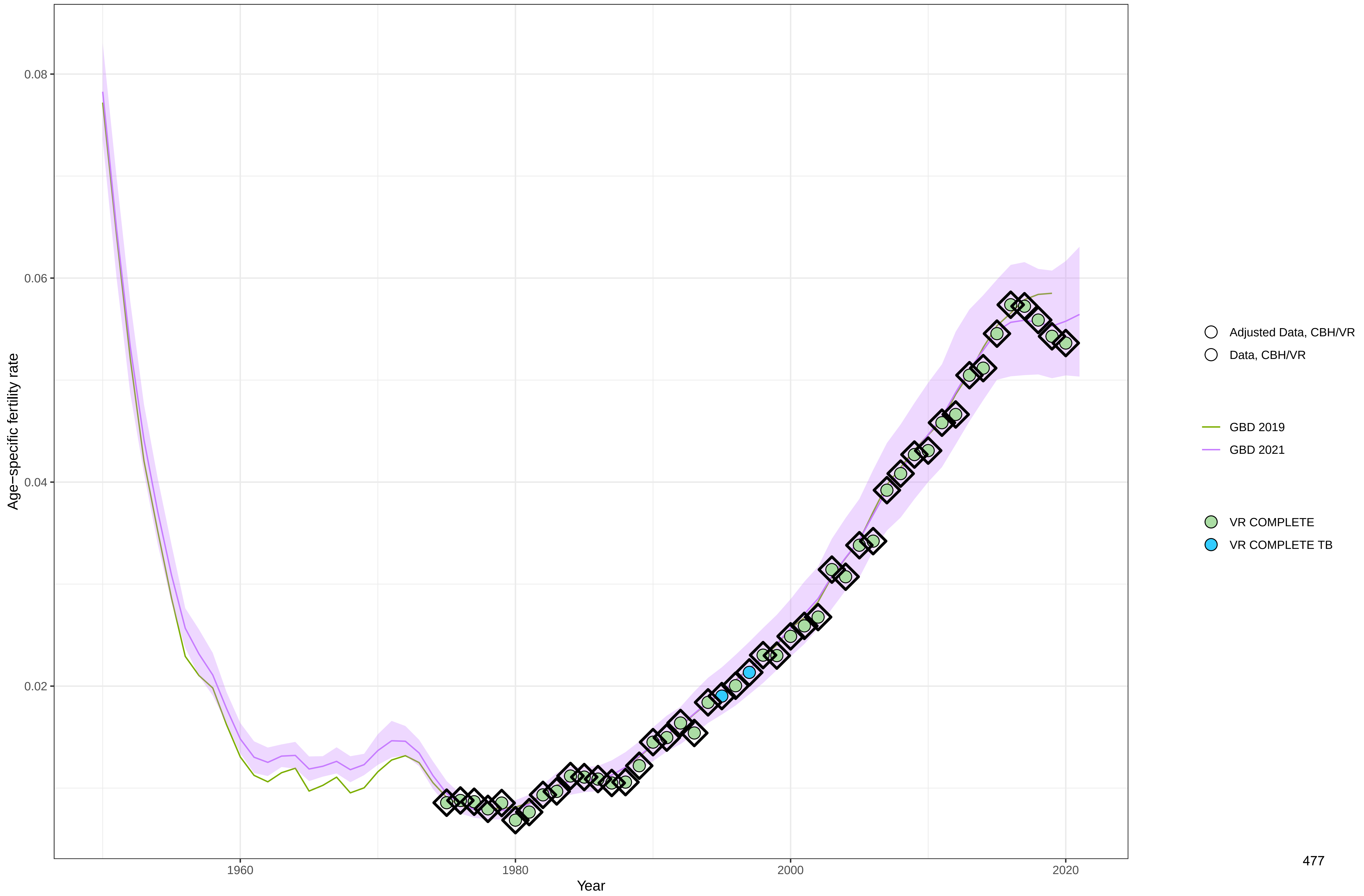

Age−specific fertility fitted trend lines, 1950−2021: Ishikawa, Ages: 40−44
Data points enclosed with a diamond are reference sources

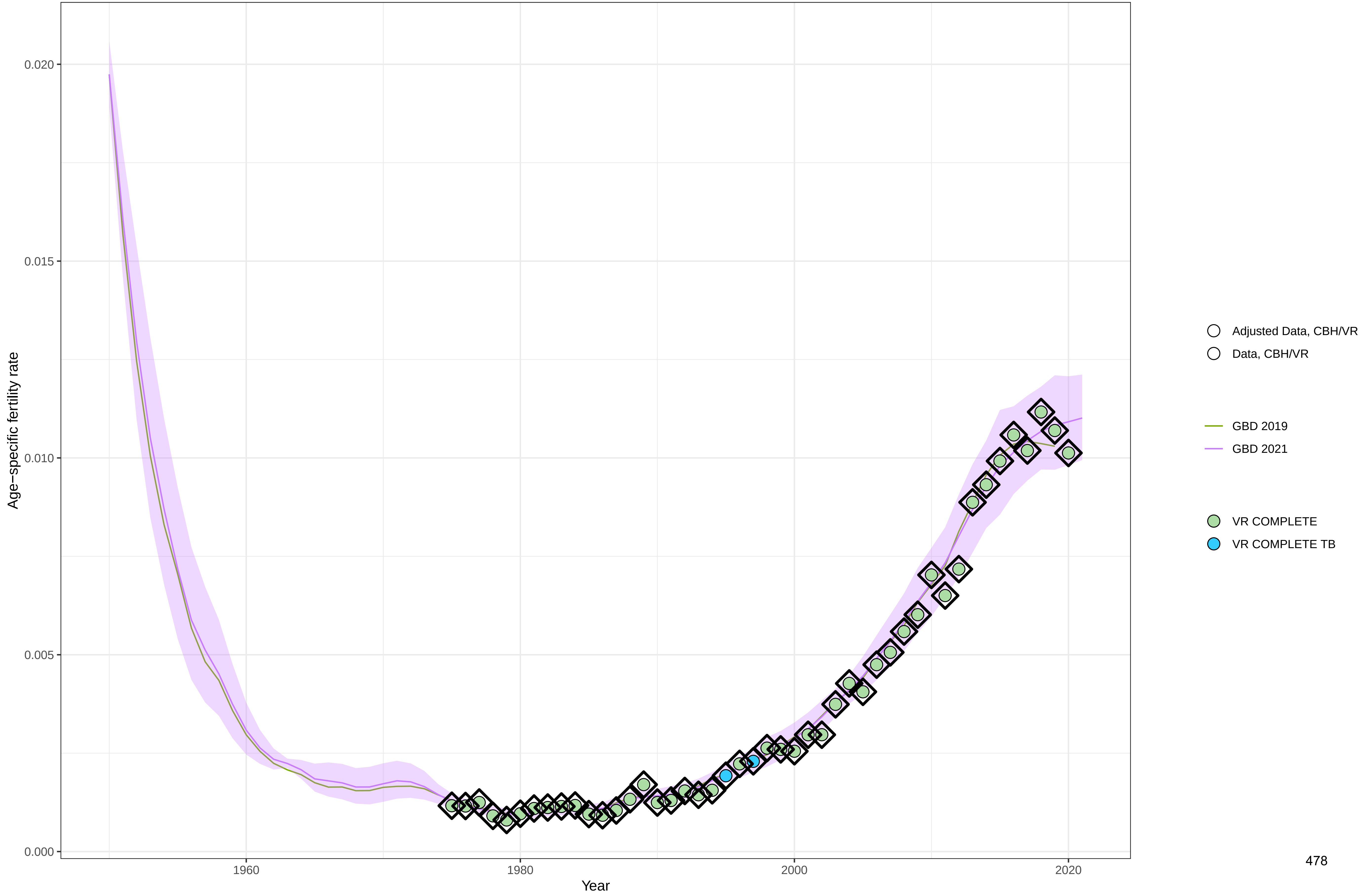

Age−specific fertility fitted trend lines, 1950−2021: Ishikawa, Ages: 45−49
Data points enclosed with a diamond are reference sources

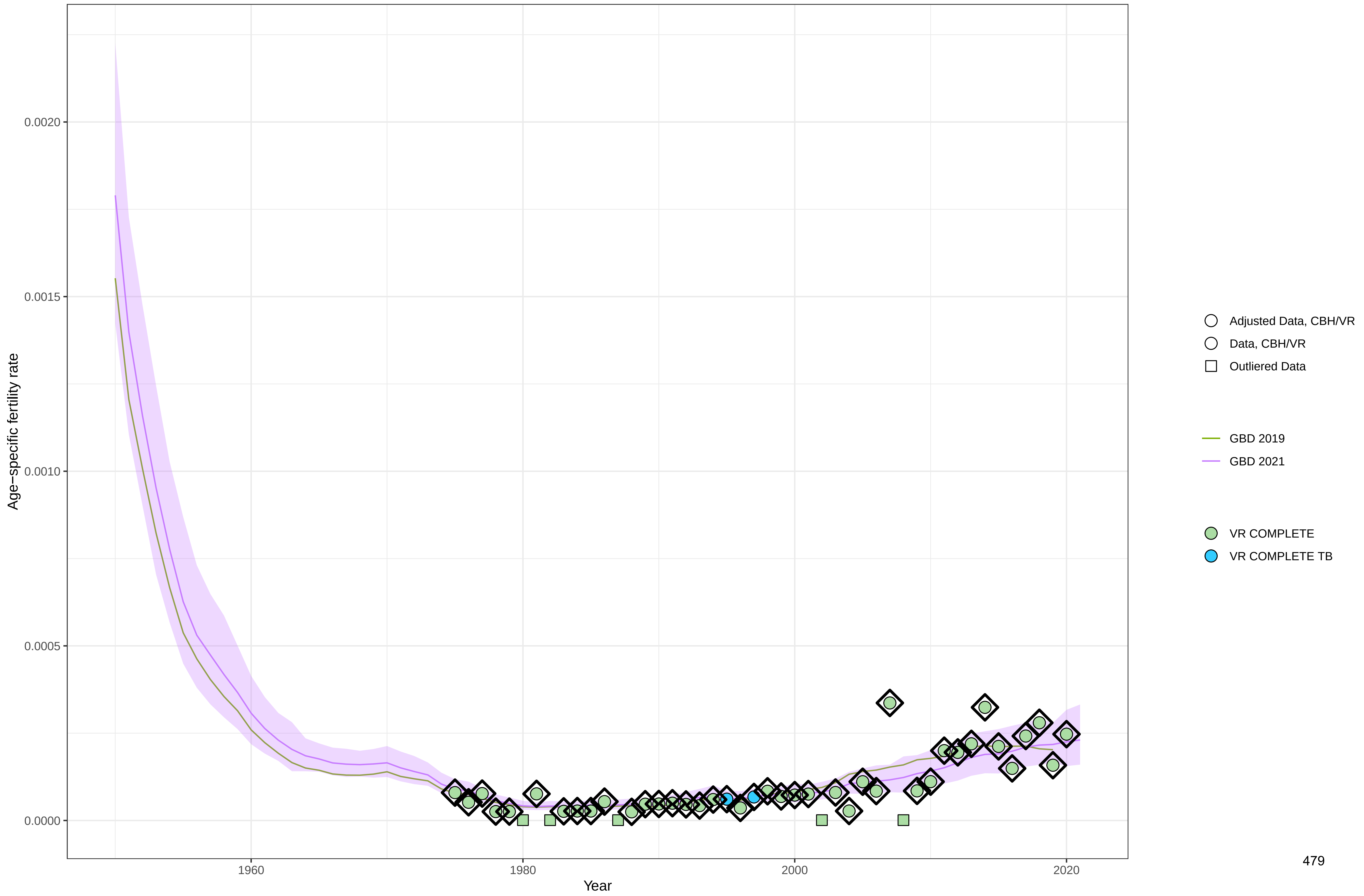

Age−specific fertility fitted trend lines, 1950−2021: Ishikawa, Ages: 50−54
Data points enclosed with a diamond are reference sources

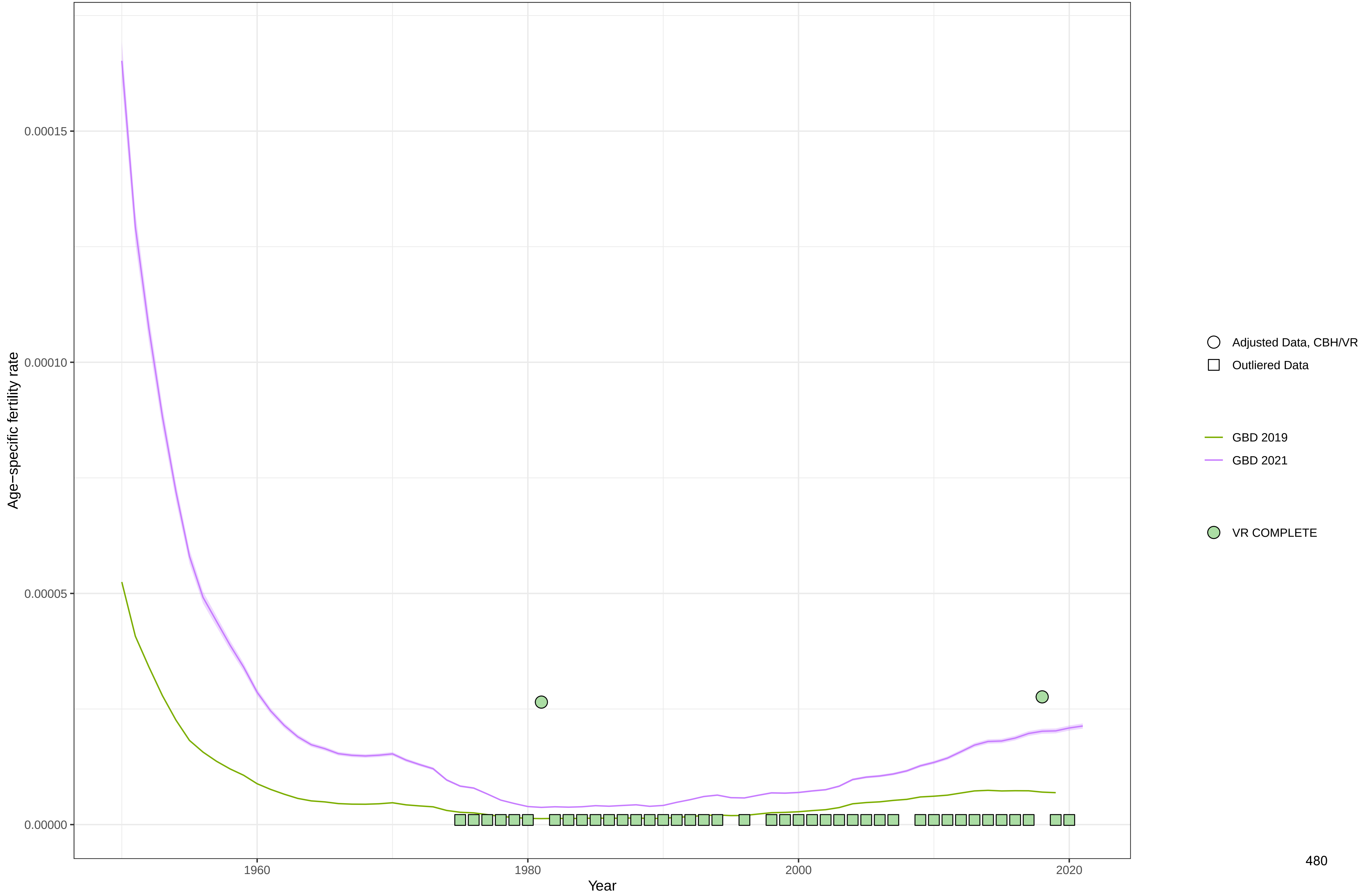

Total fertility rate fitted trend lines, 1950−2021: Ishikawa TFR
Data points enclosed with a diamond are reference sources

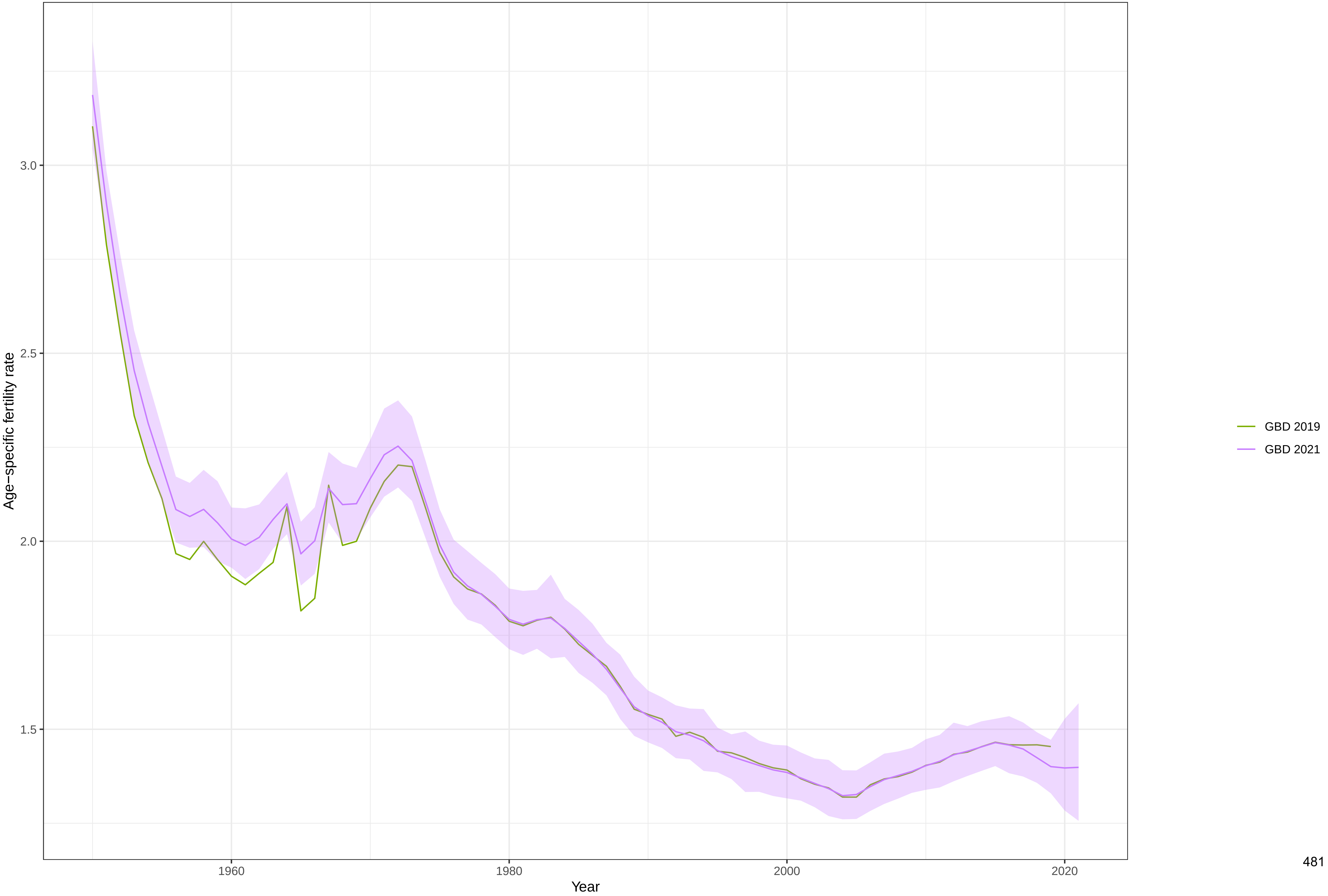

Age−specific fertility fitted trend lines, 1950−2021: Iwate, Ages: 10−14
Data points enclosed with a diamond are reference sources

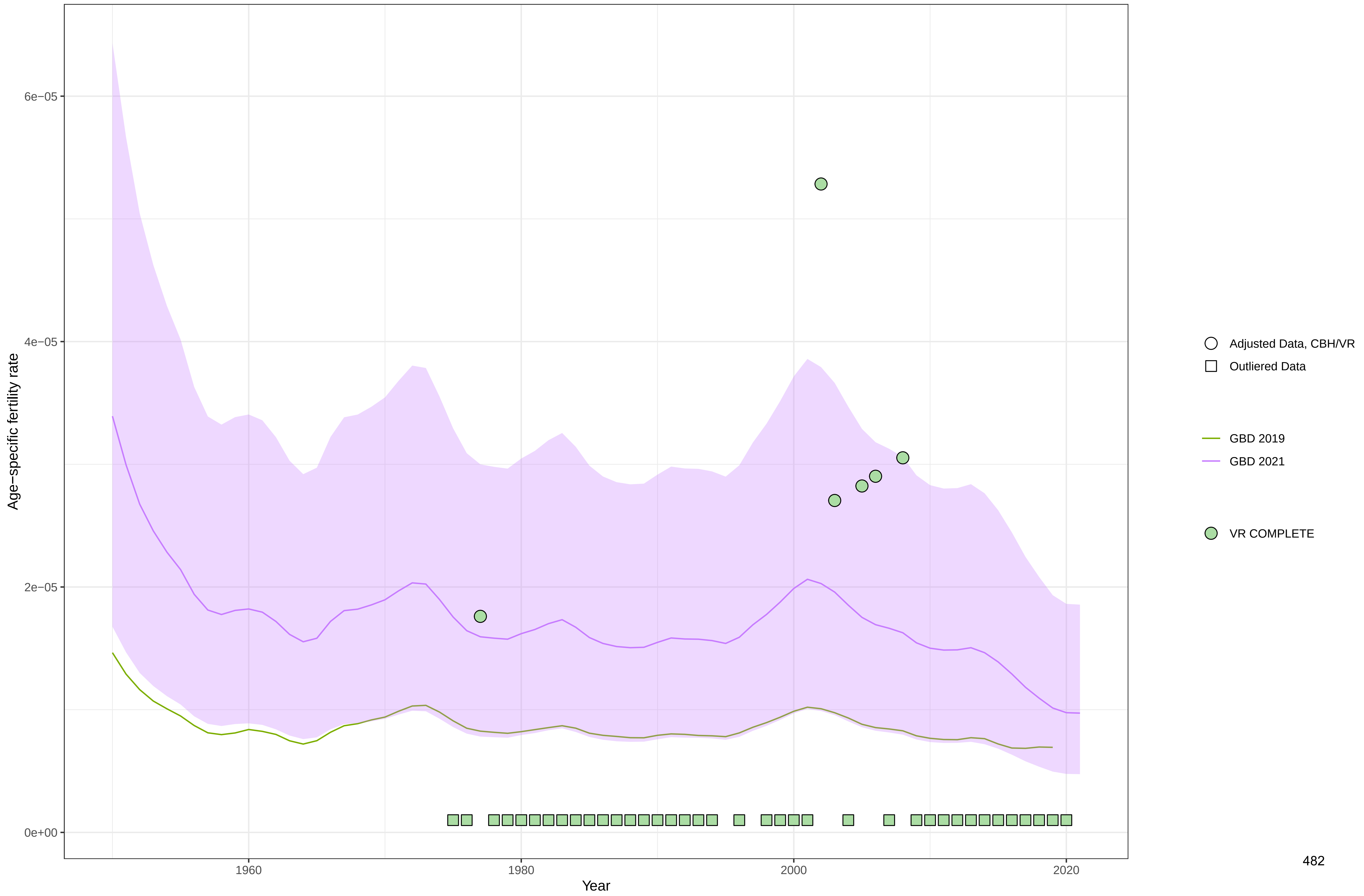

Age−specific fertility fitted trend lines, 1950−2021: Iwate, Ages: 15−19
Data points enclosed with a diamond are reference sources

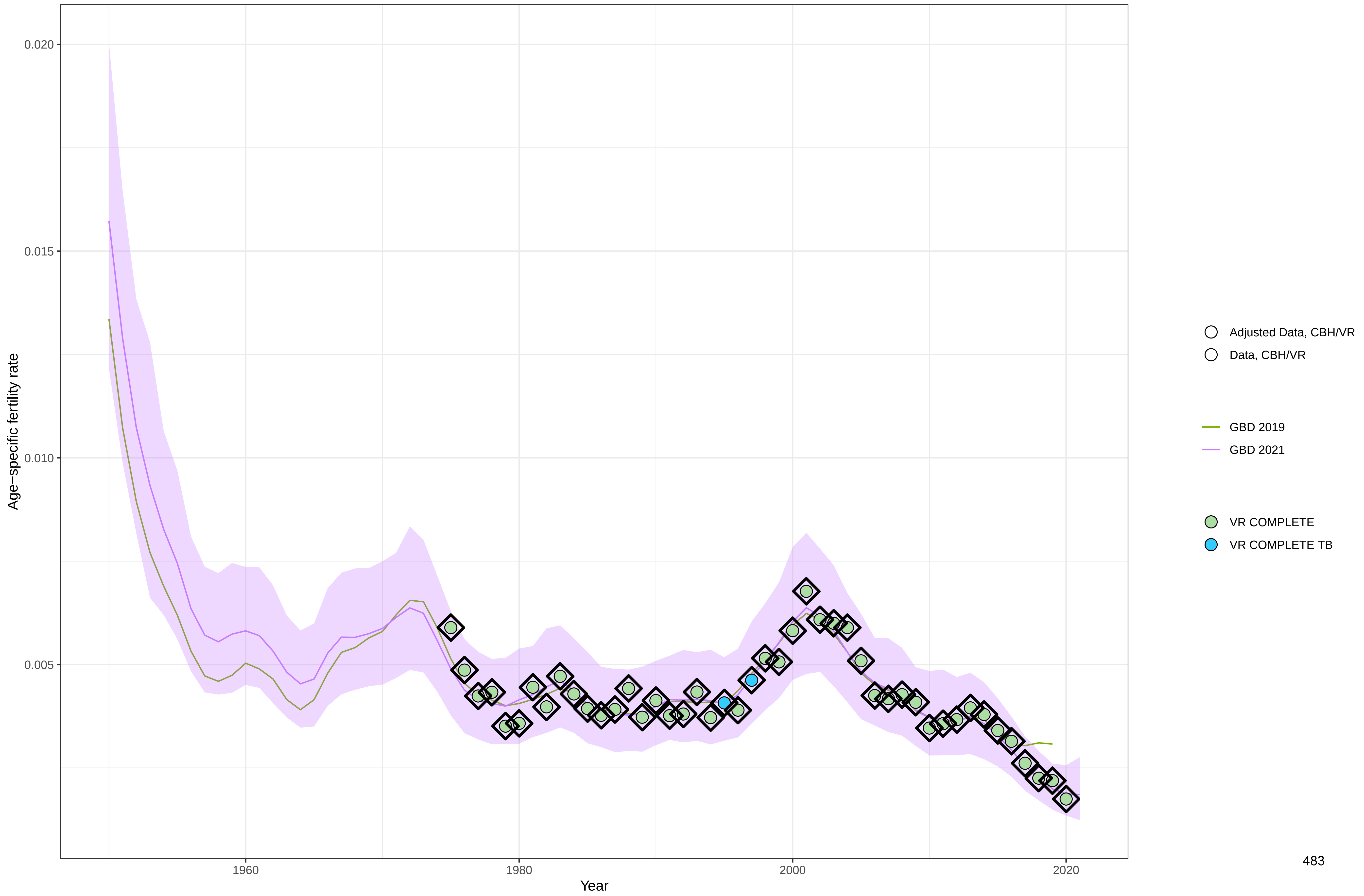

Age−specific fertility fitted trend lines, 1950−2021: Iwate, Ages: 20−24
Data points enclosed with a diamond are reference sources

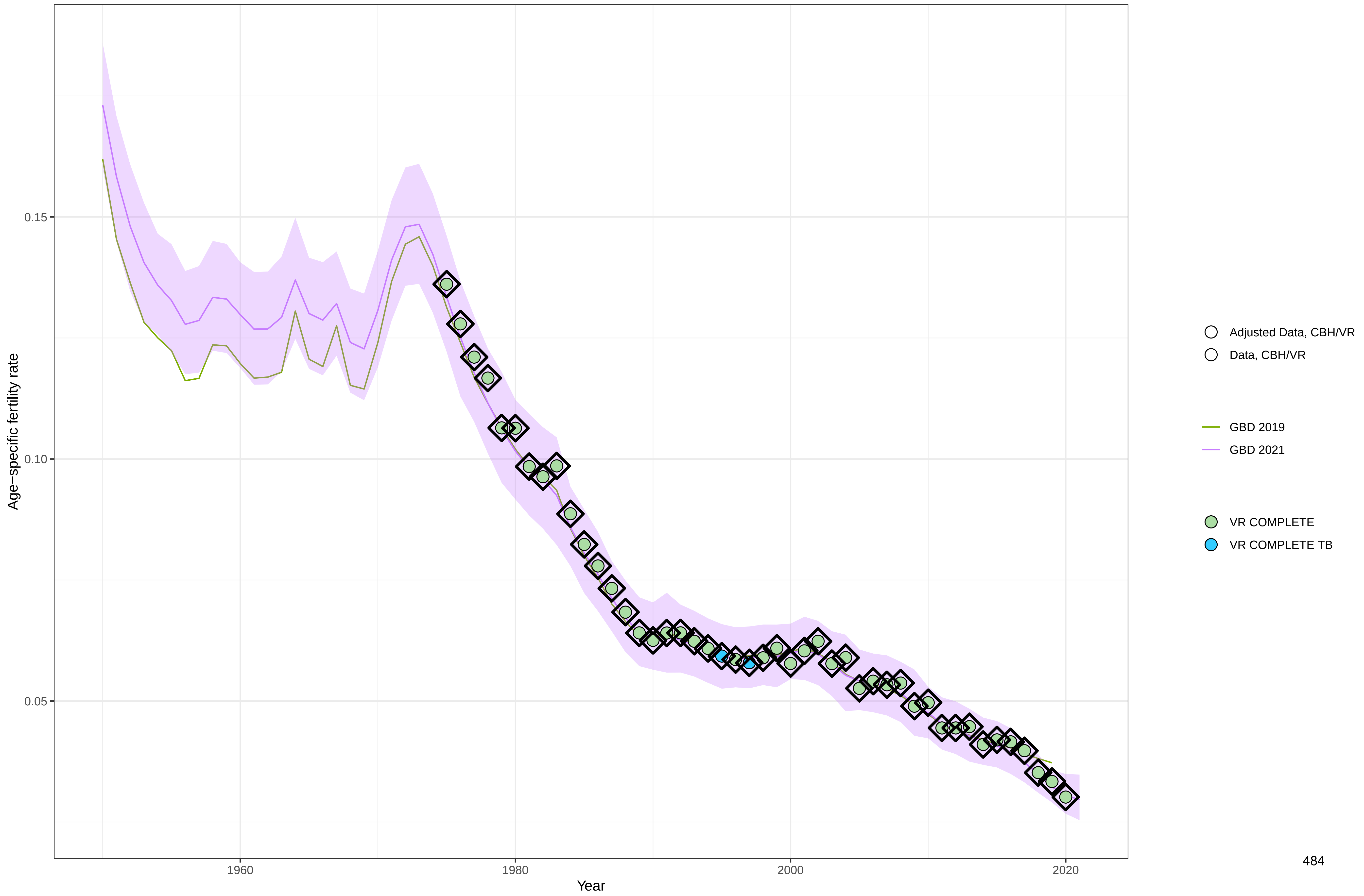

Age−specific fertility fitted trend lines, 1950−2021: Iwate, Ages: 25−29
Data points enclosed with a diamond are reference sources

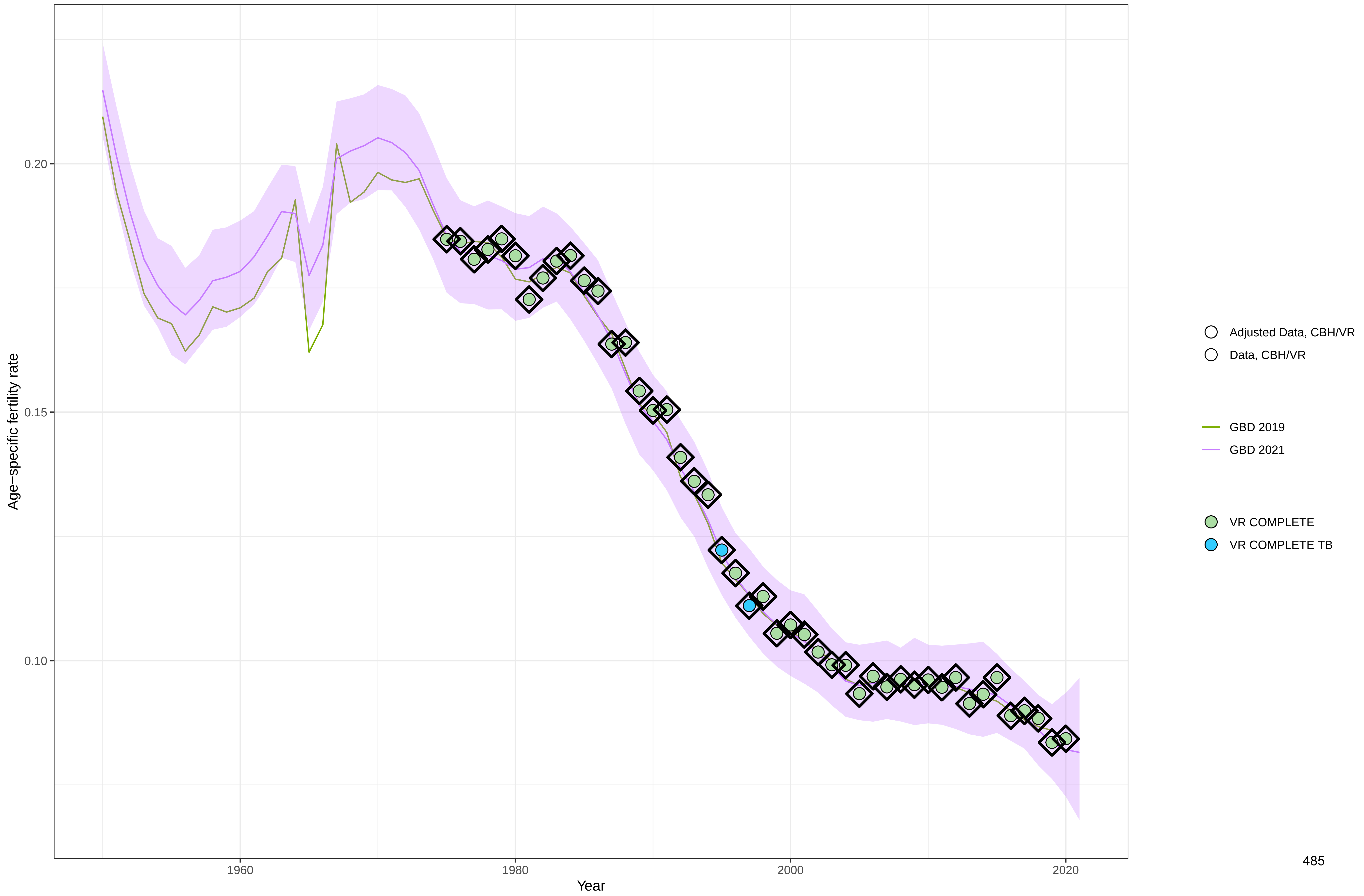

Age−specific fertility fitted trend lines, 1950−2021: Iwate, Ages: 30−34
Data points enclosed with a diamond are reference sources

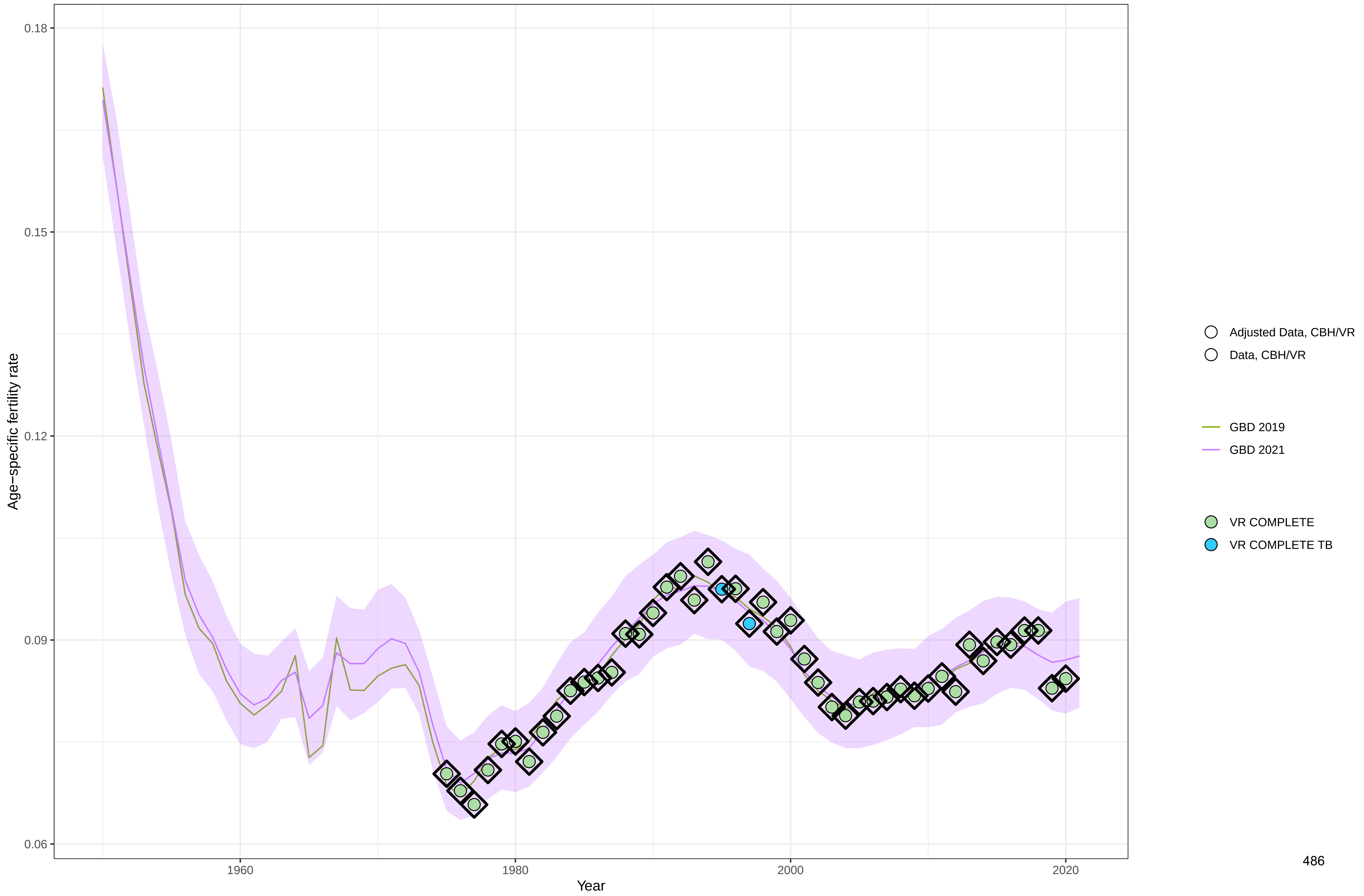

Age−specific fertility fitted trend lines, 1950−2021: Iwate, Ages: 35−39
Data points enclosed with a diamond are reference sources

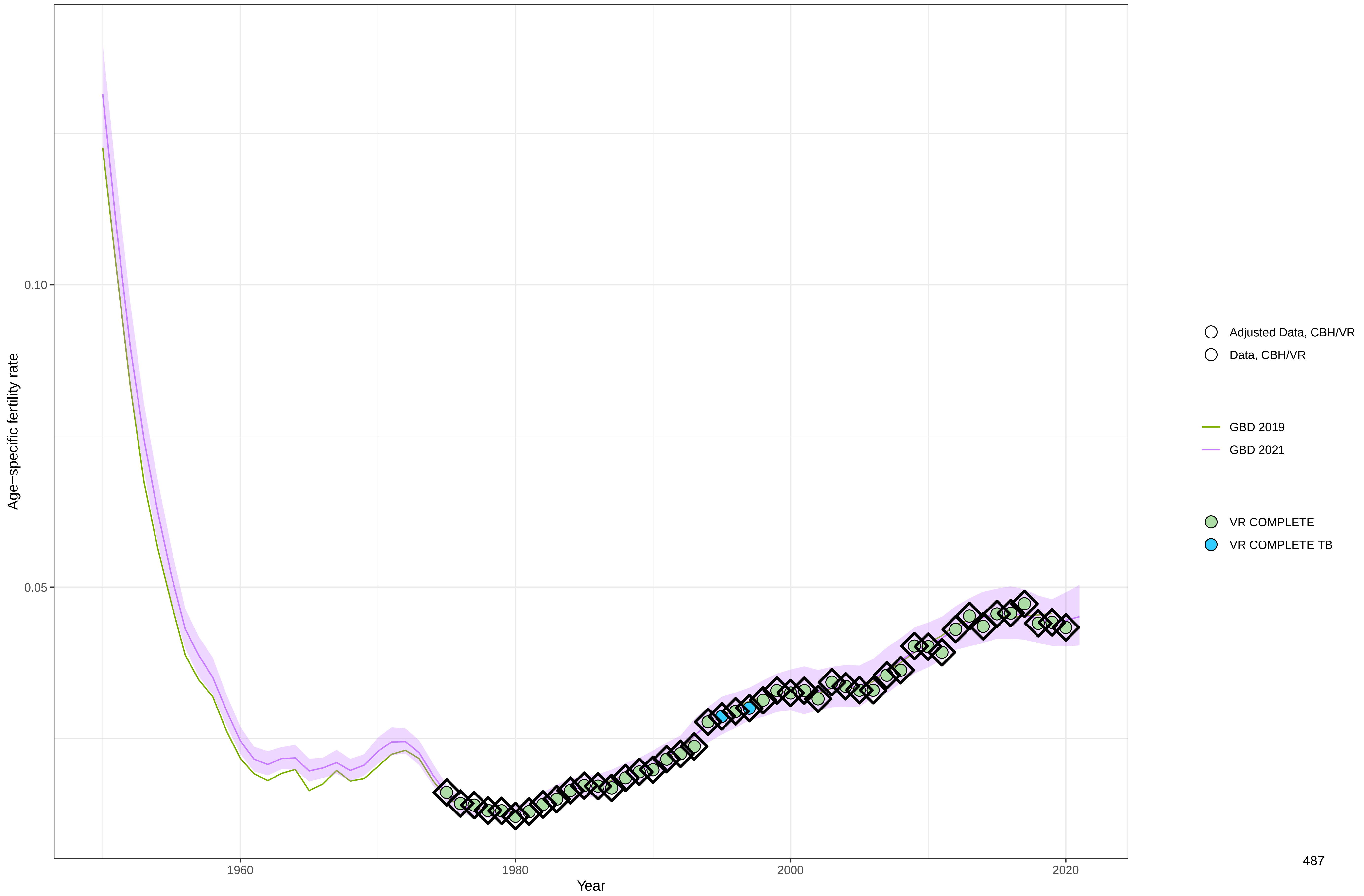

Age−specific fertility fitted trend lines, 1950−2021: Iwate, Ages: 40−44
Data points enclosed with a diamond are reference sources

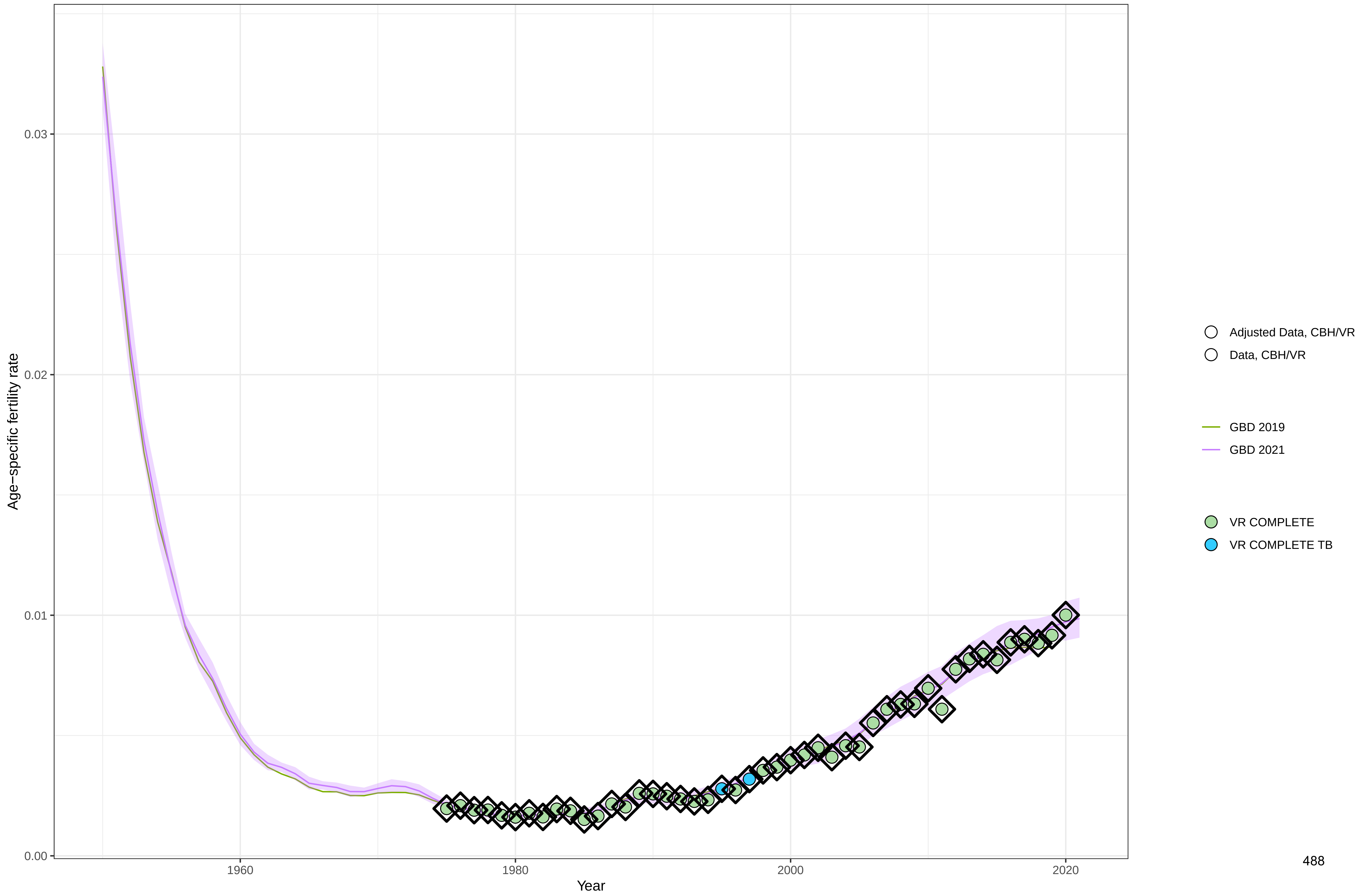

Age−specific fertility fitted trend lines, 1950−2021: Iwate, Ages: 45−49
Data points enclosed with a diamond are reference sources

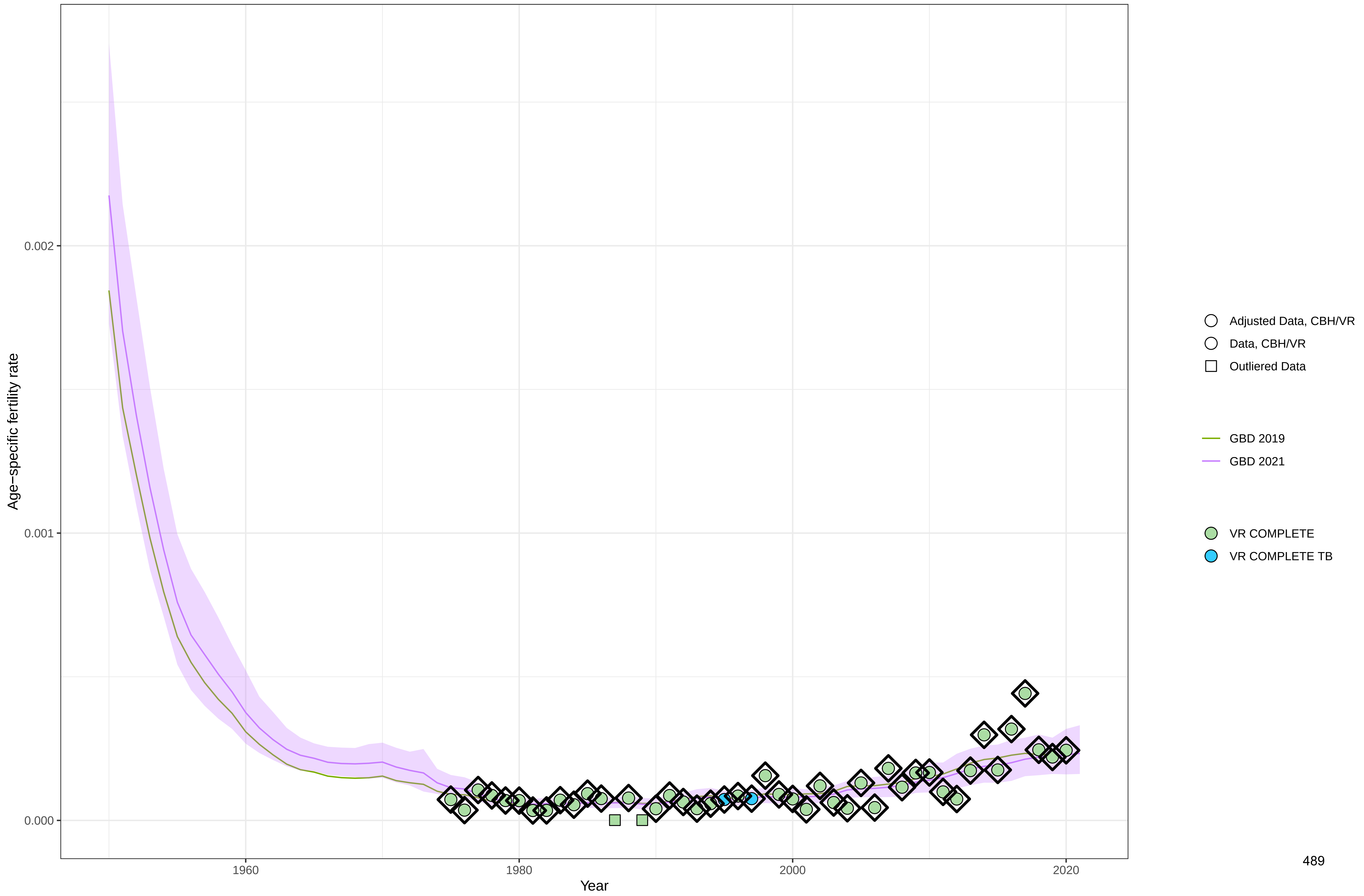

Age−specific fertility fitted trend lines, 1950−2021: Iwate, Ages: 50−54
Data points enclosed with a diamond are reference sources

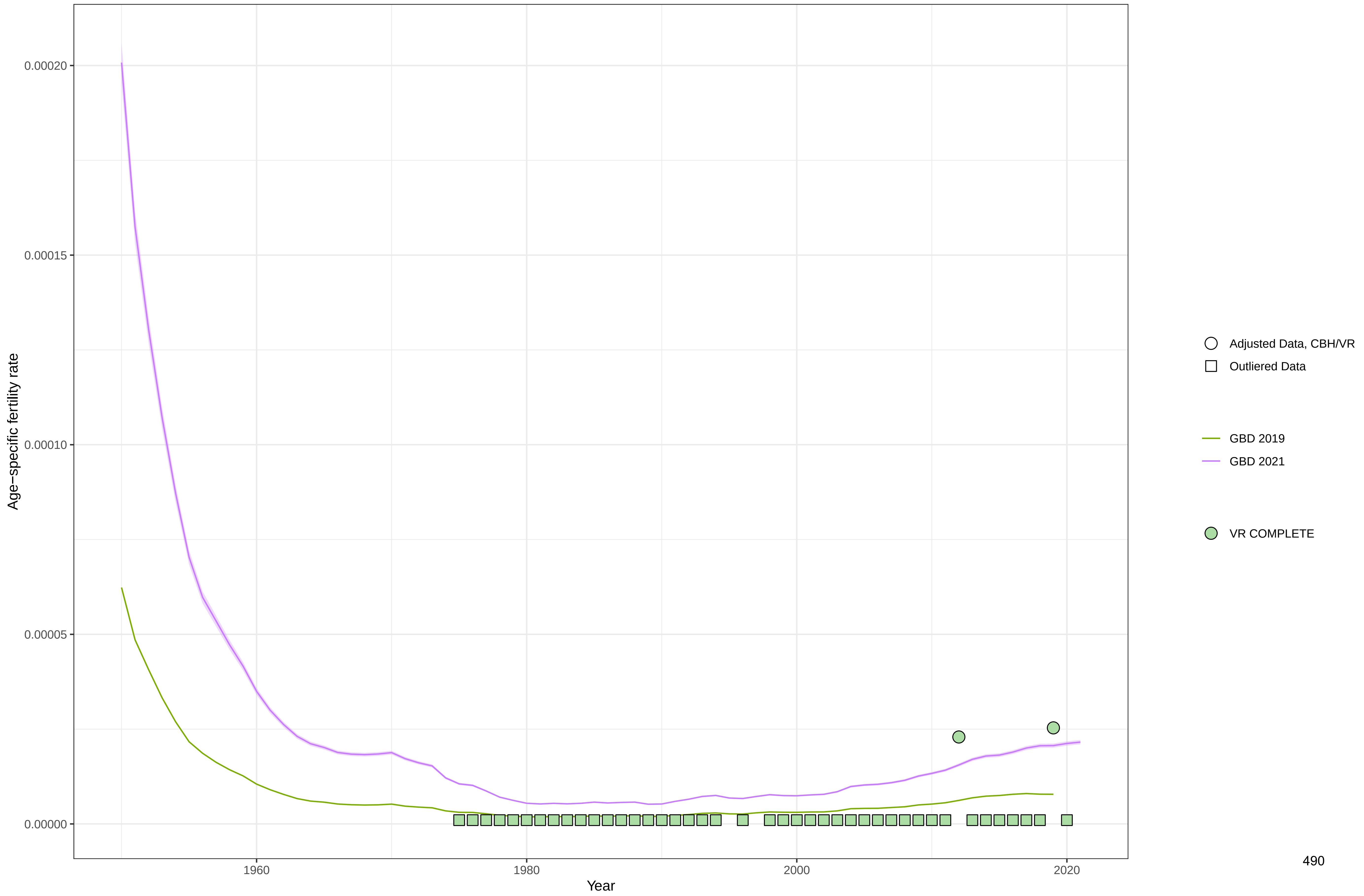

Total fertility rate fitted trend lines, 1950−2021: Iwate TFR
Data points enclosed with a diamond are reference sources

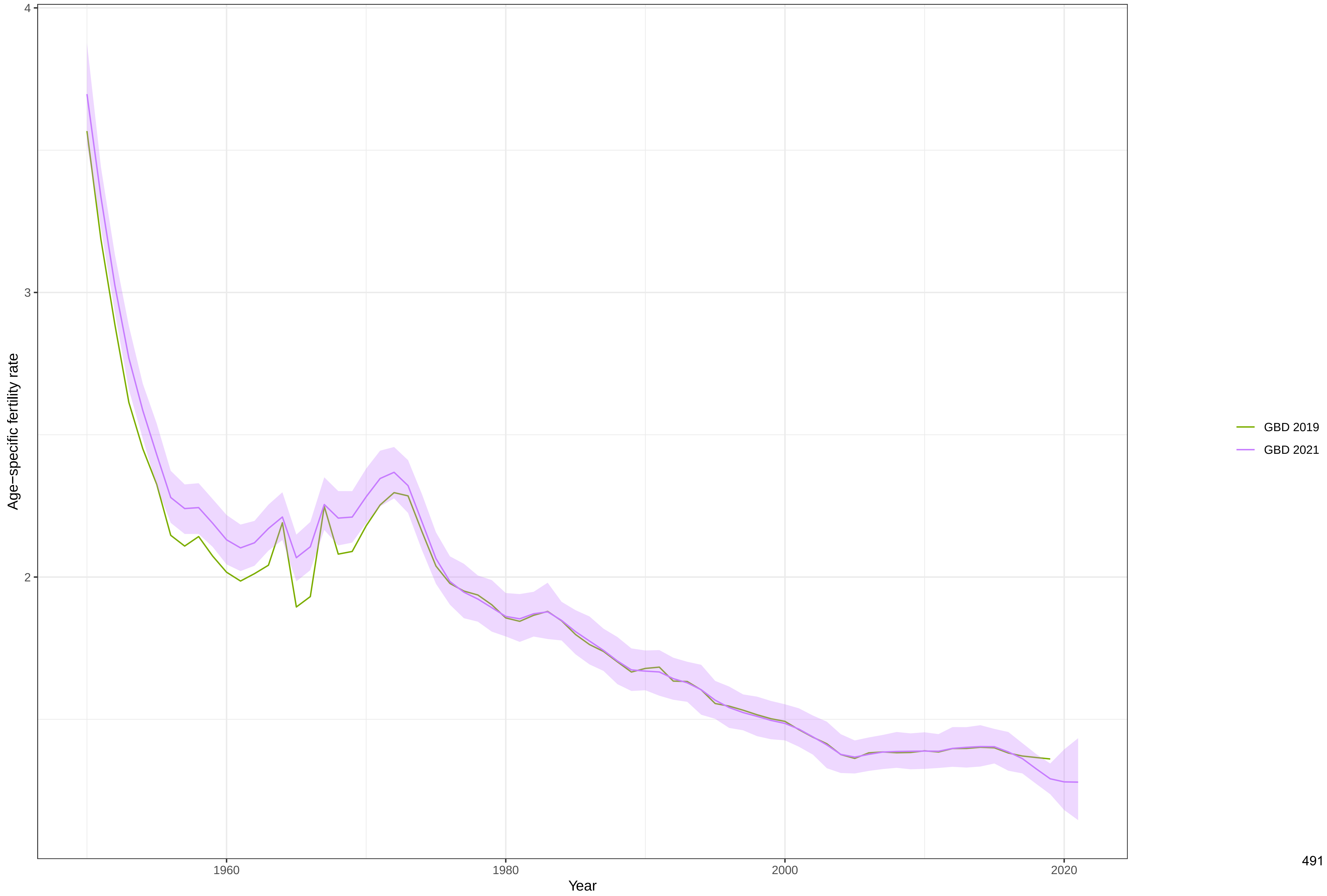

Age−specific fertility fitted trend lines, 1950−2021: Kagawa, Ages: 10−14
Data points enclosed with a diamond are reference sources

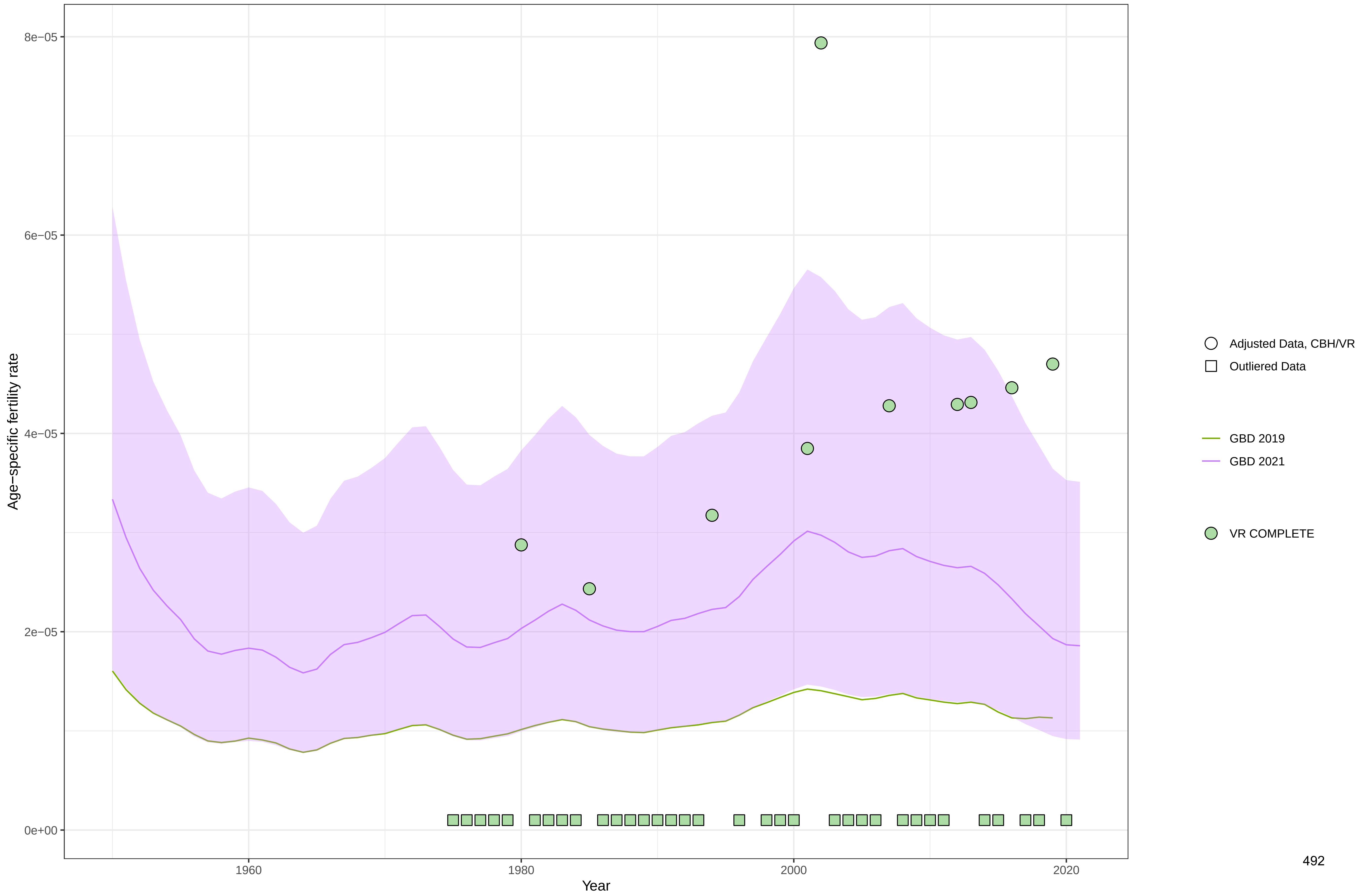

Age−specific fertility fitted trend lines, 1950−2021: Kagawa, Ages: 15−19
Data points enclosed with a diamond are reference sources

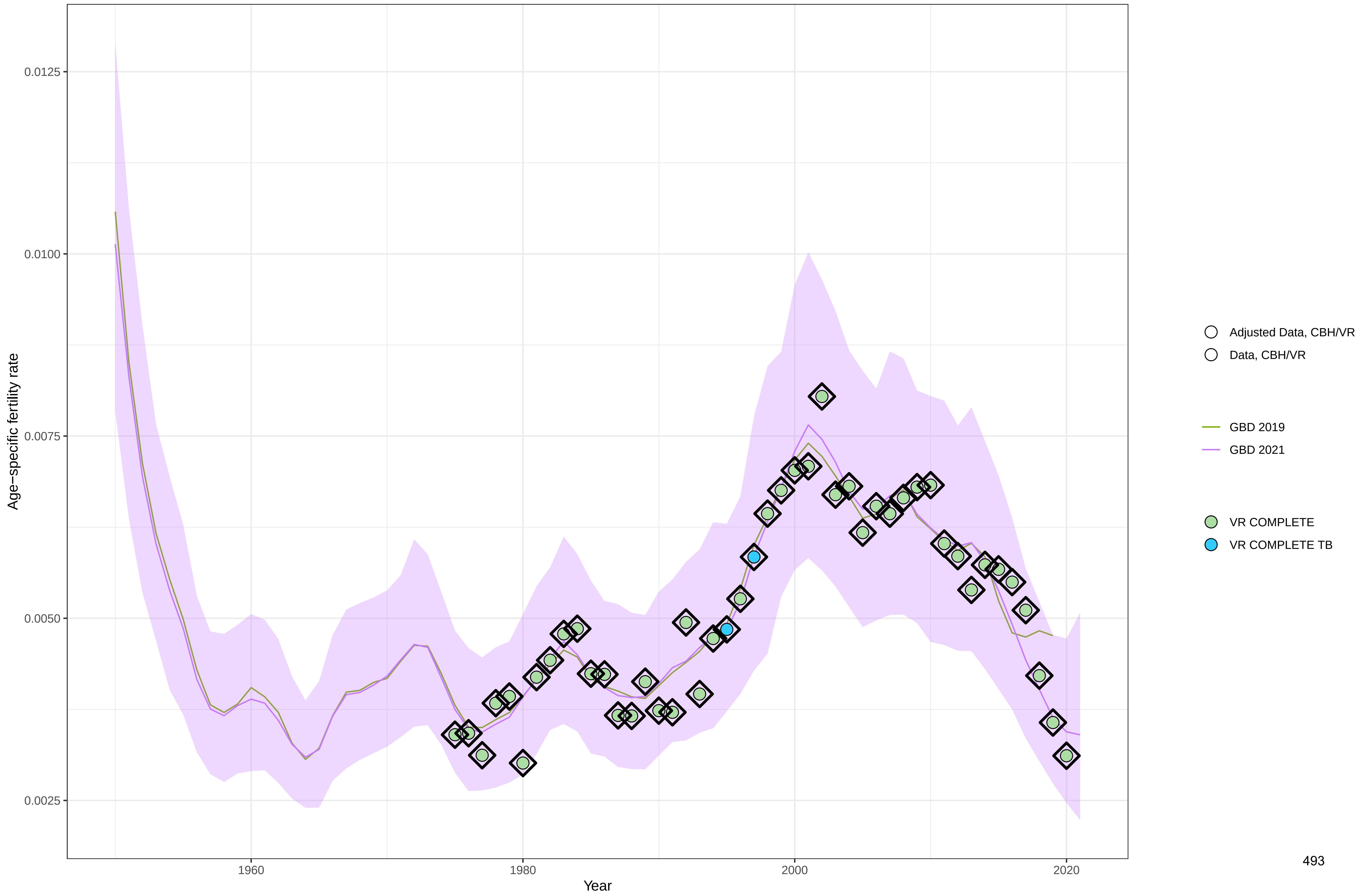

Age−specific fertility fitted trend lines, 1950−2021: Kagawa, Ages: 20−24
Data points enclosed with a diamond are reference sources

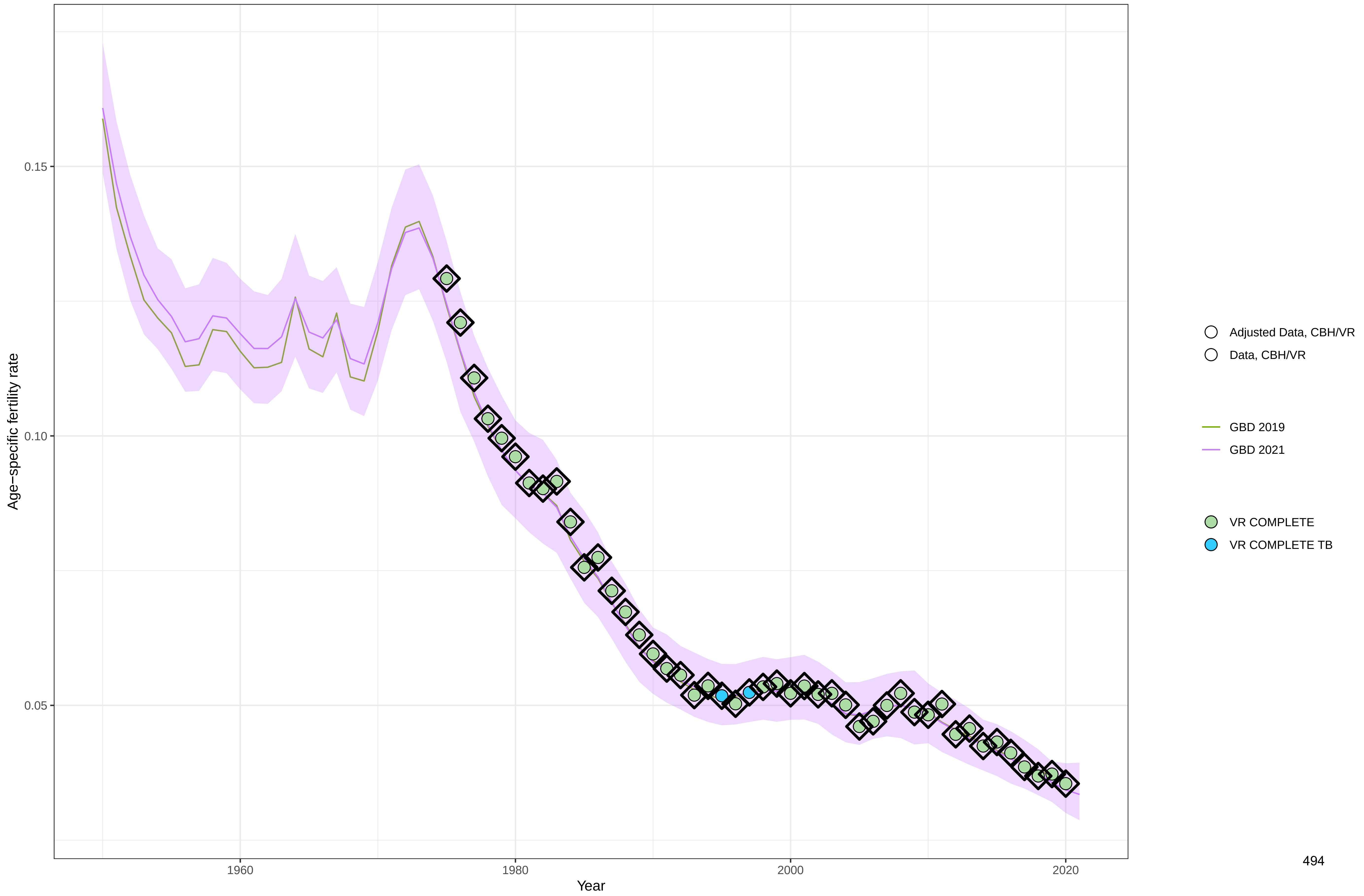

Age−specific fertility fitted trend lines, 1950−2021: Kagawa, Ages: 25−29
Data points enclosed with a diamond are reference sources

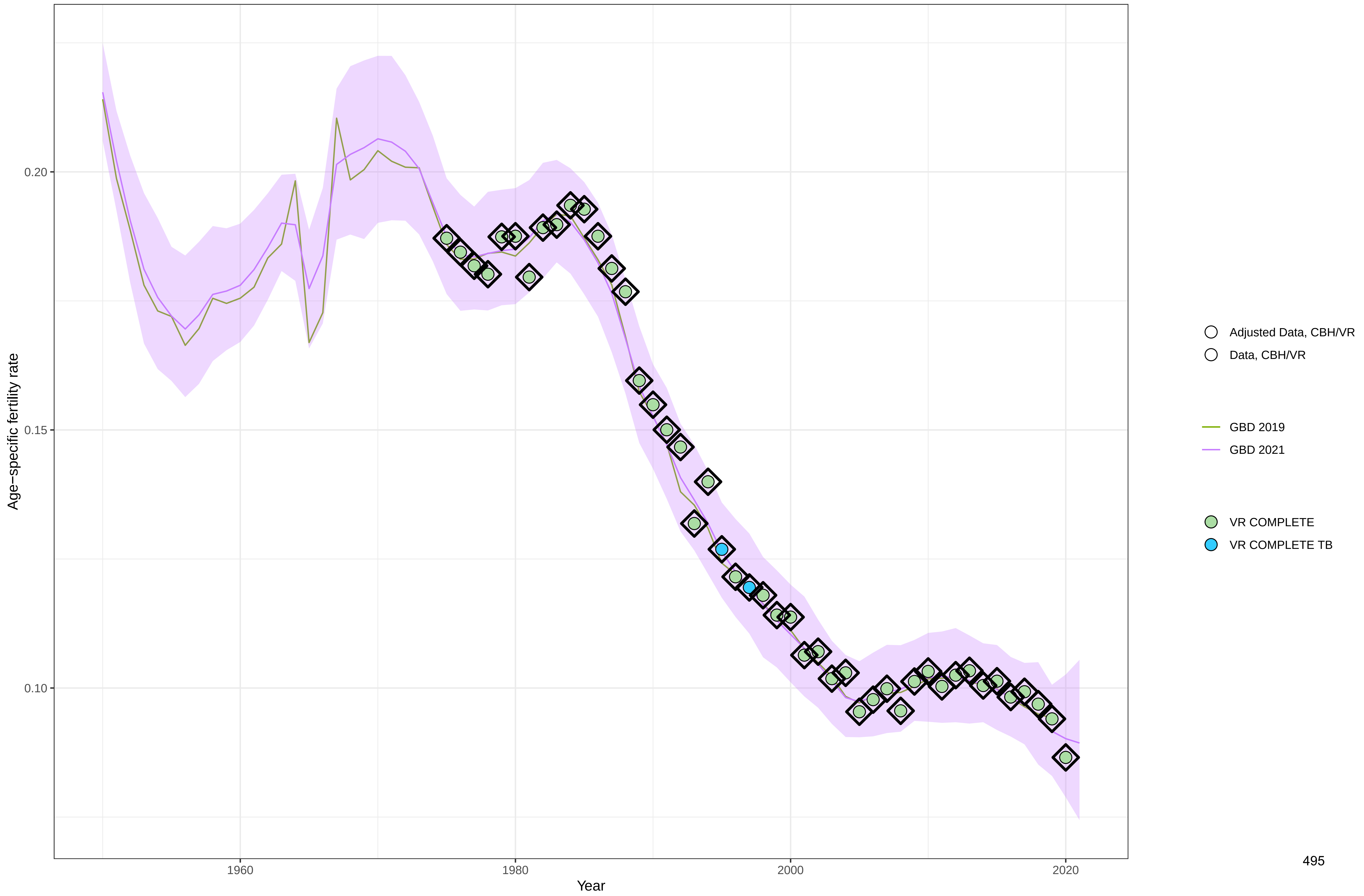

Age−specific fertility fitted trend lines, 1950−2021: Kagawa, Ages: 30−34
Data points enclosed with a diamond are reference sources

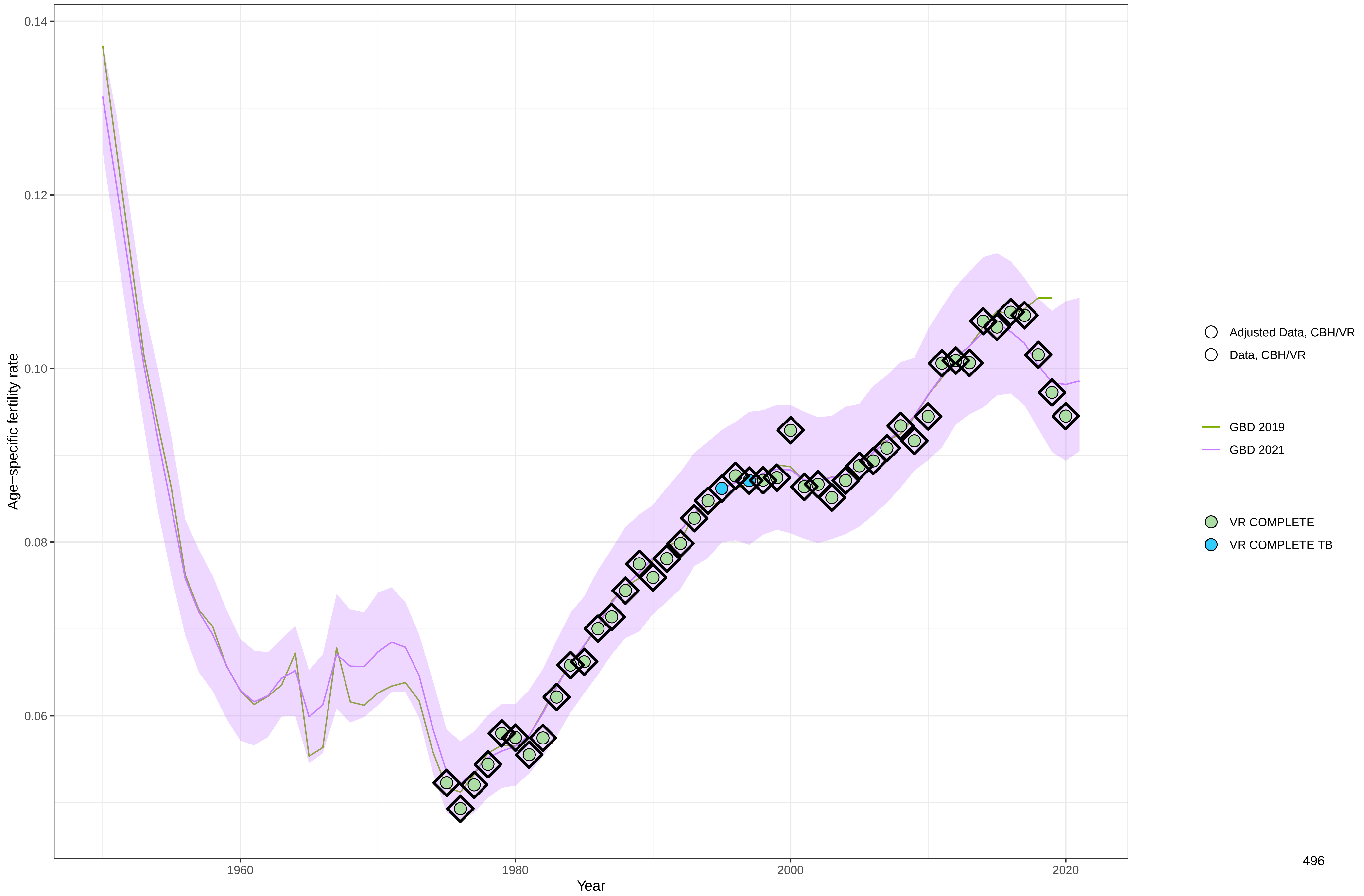

Age−specific fertility fitted trend lines, 1950−2021: Kagawa, Ages: 35−39
Data points enclosed with a diamond are reference sources

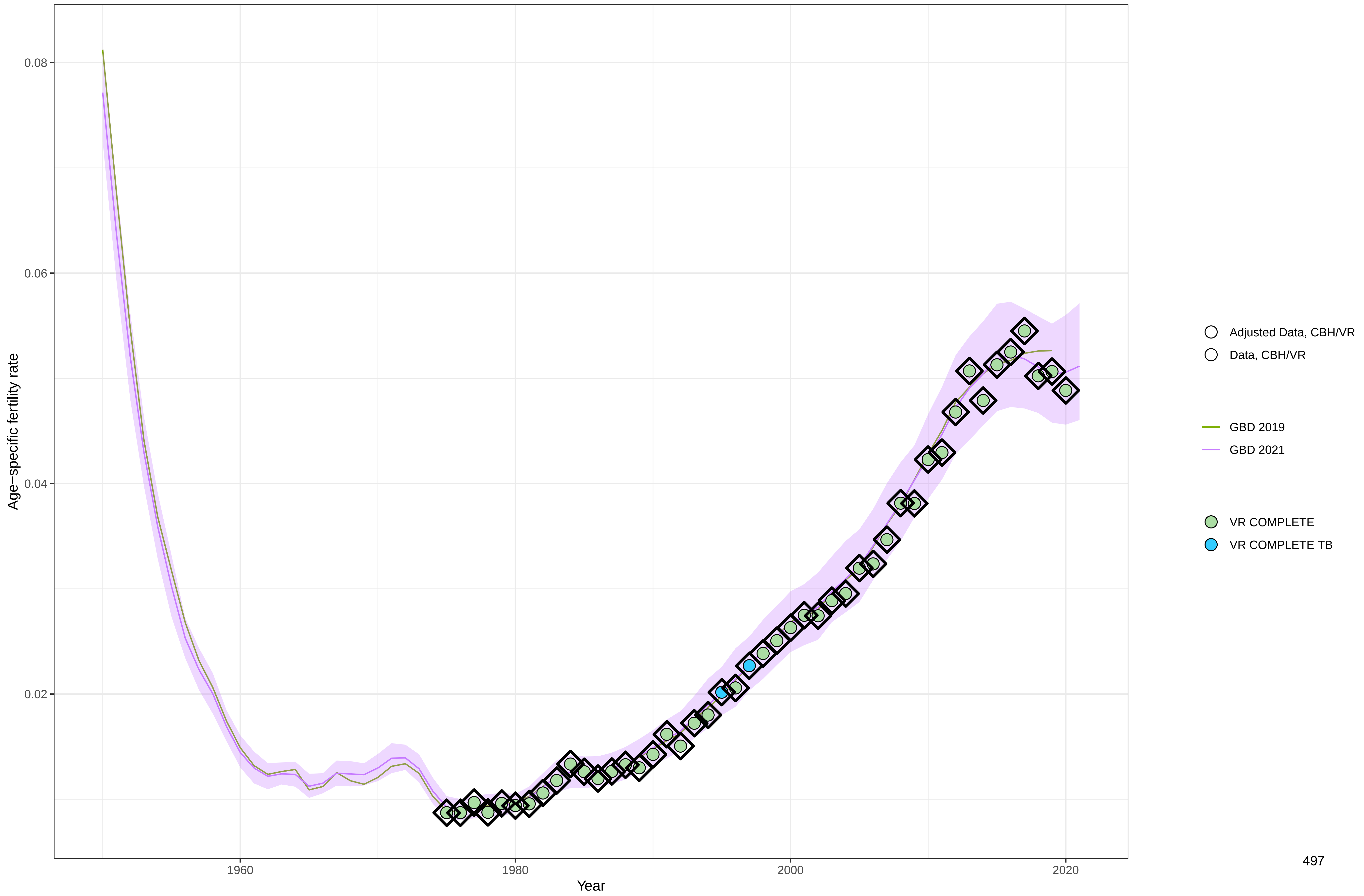

Age−specific fertility fitted trend lines, 1950−2021: Kagawa, Ages: 40−44
Data points enclosed with a diamond are reference sources

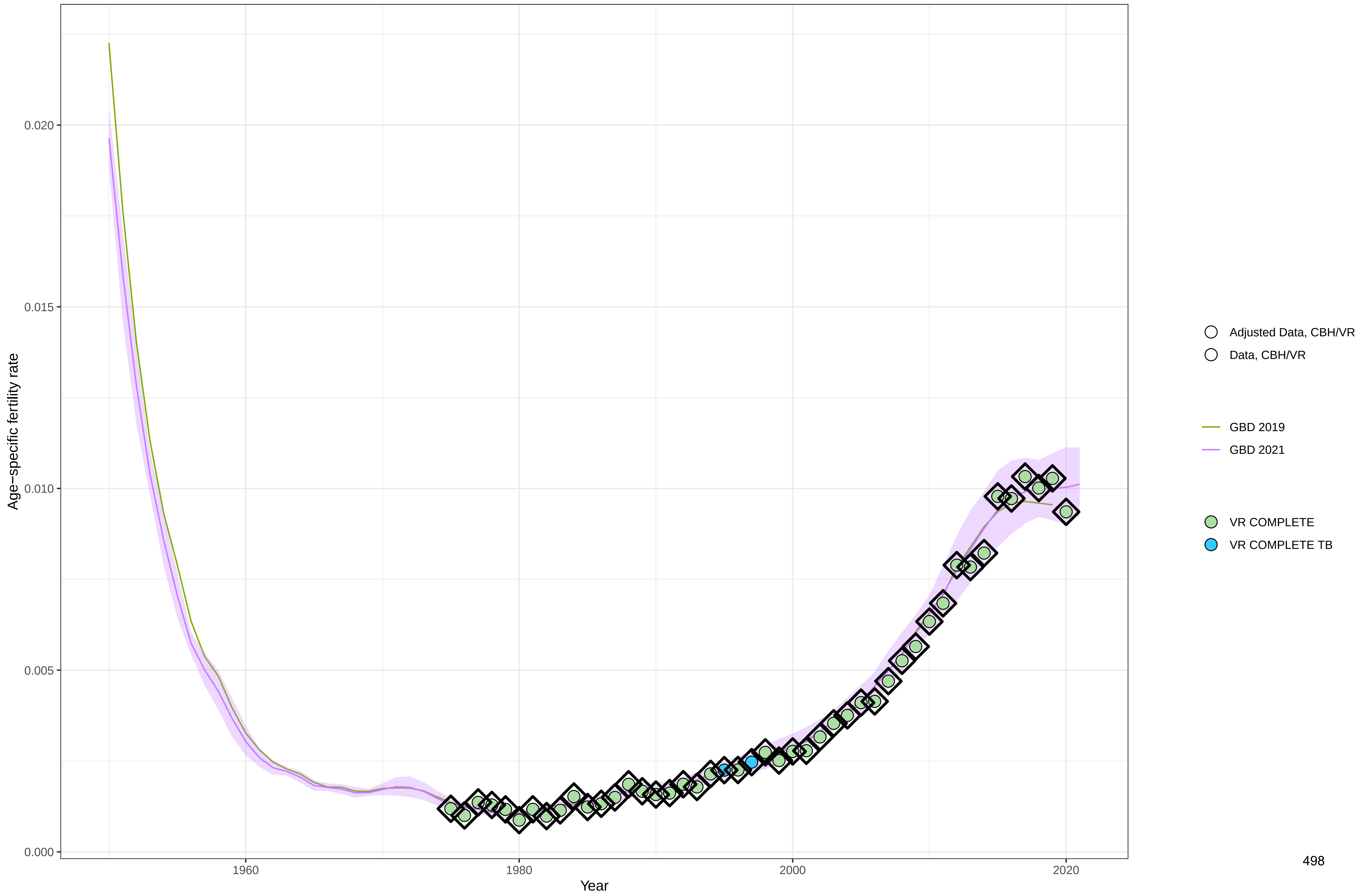

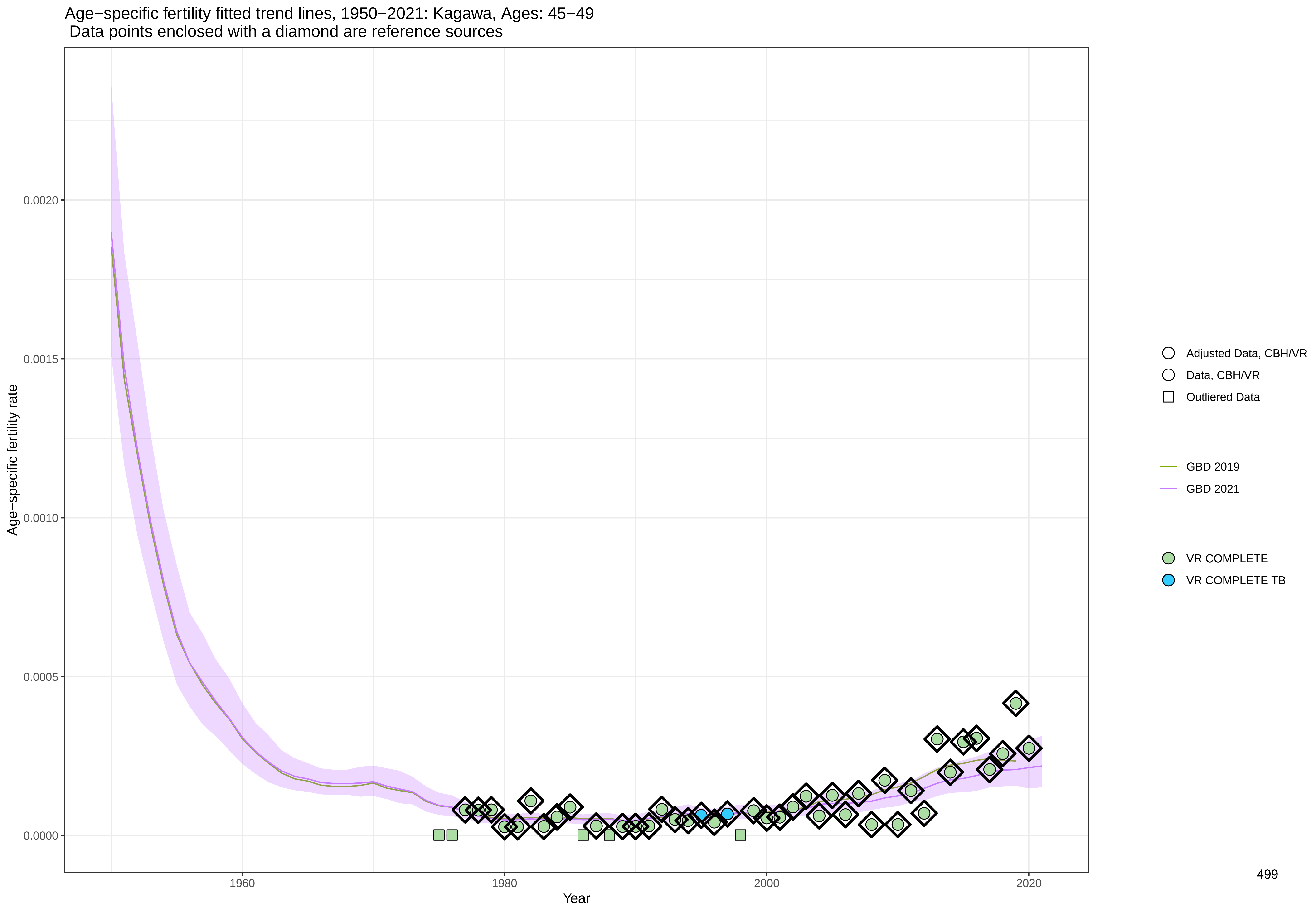

Age−specific fertility fitted trend lines, 1950−2021: Kagawa, Ages: 45−49
Data points enclosed with a diamond are reference sources
Age−specific fertility rate
0.0000
0.0005
0.0010
0.0015
0.0020
1960
1980
2000
2020
Year
Adjusted Data, CBH/VR
Data, CBH/VR
Outliered Data
GBD 2019
GBD 2021
VR COMPLETE
VR COMPLETE TB

Age−specific fertility fitted trend lines, 1950−2021: Kagawa, Ages: 50−54
Data points enclosed with a diamond are reference sources

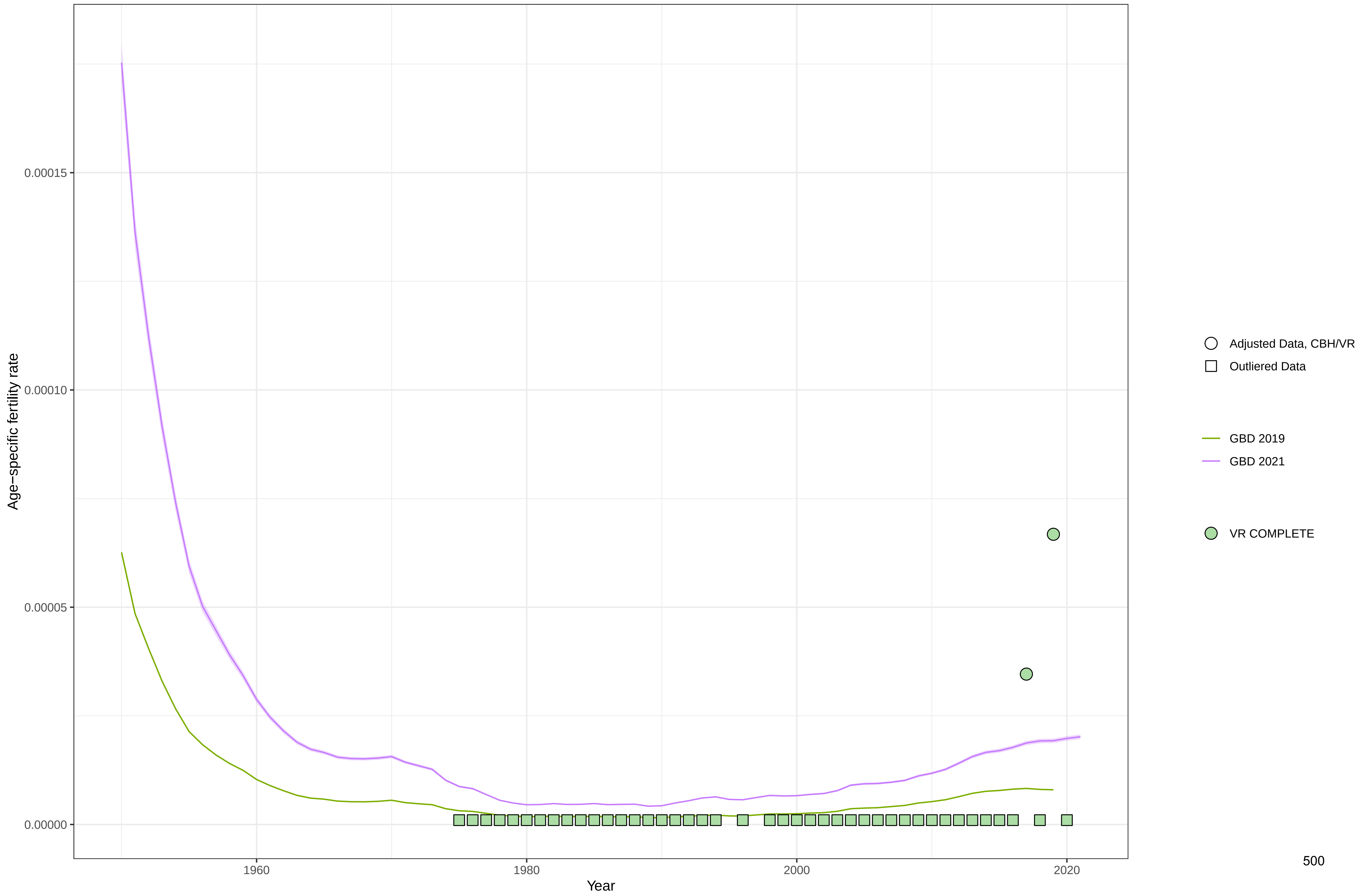

Total fertility rate fitted trend lines, 1950−2021: Kagawa TFR
Data points enclosed with a diamond are reference sources

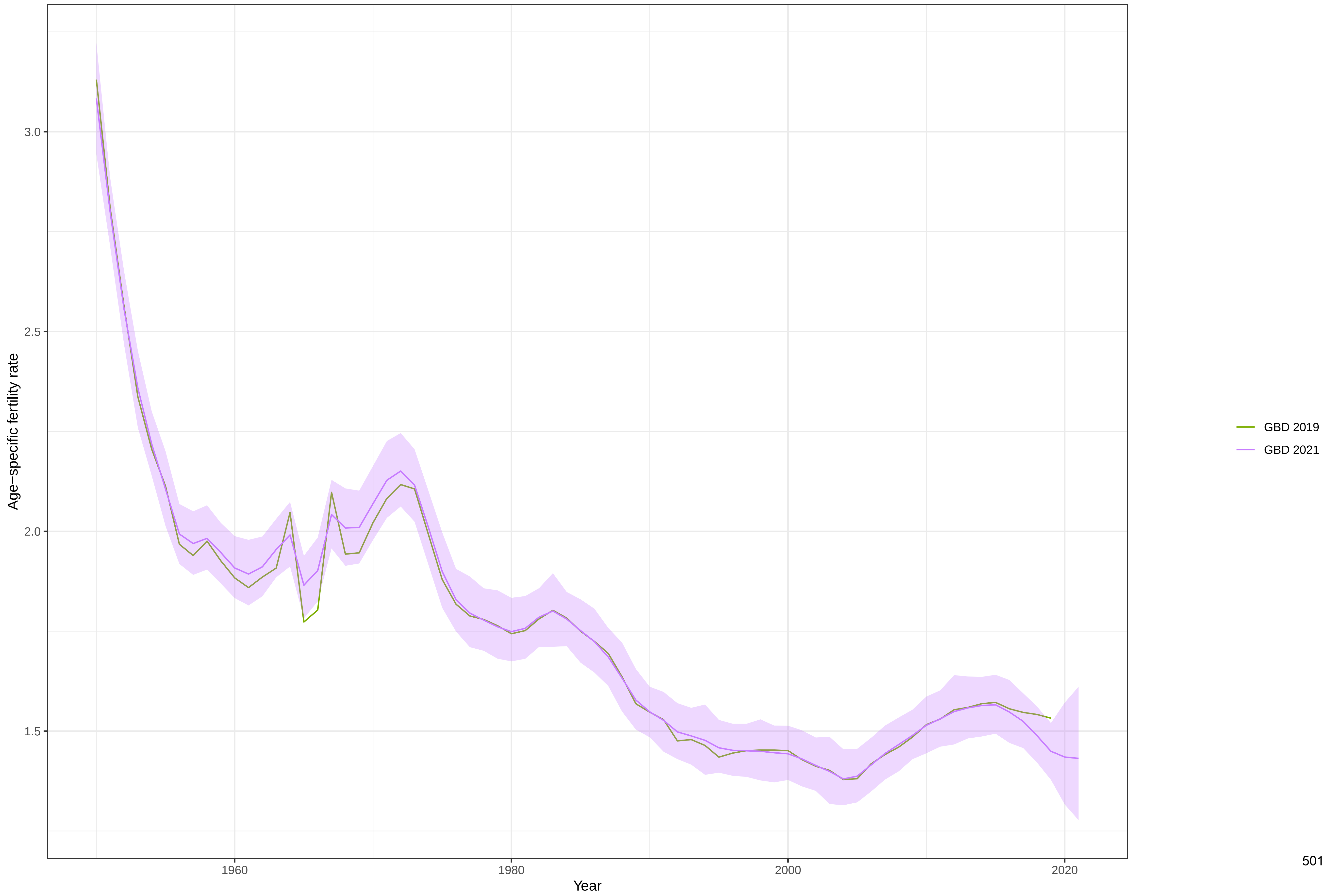

Age−specific fertility fitted trend lines, 1950−2021: Kagoshima, Ages: 10−14
Data points enclosed with a diamond are reference sources

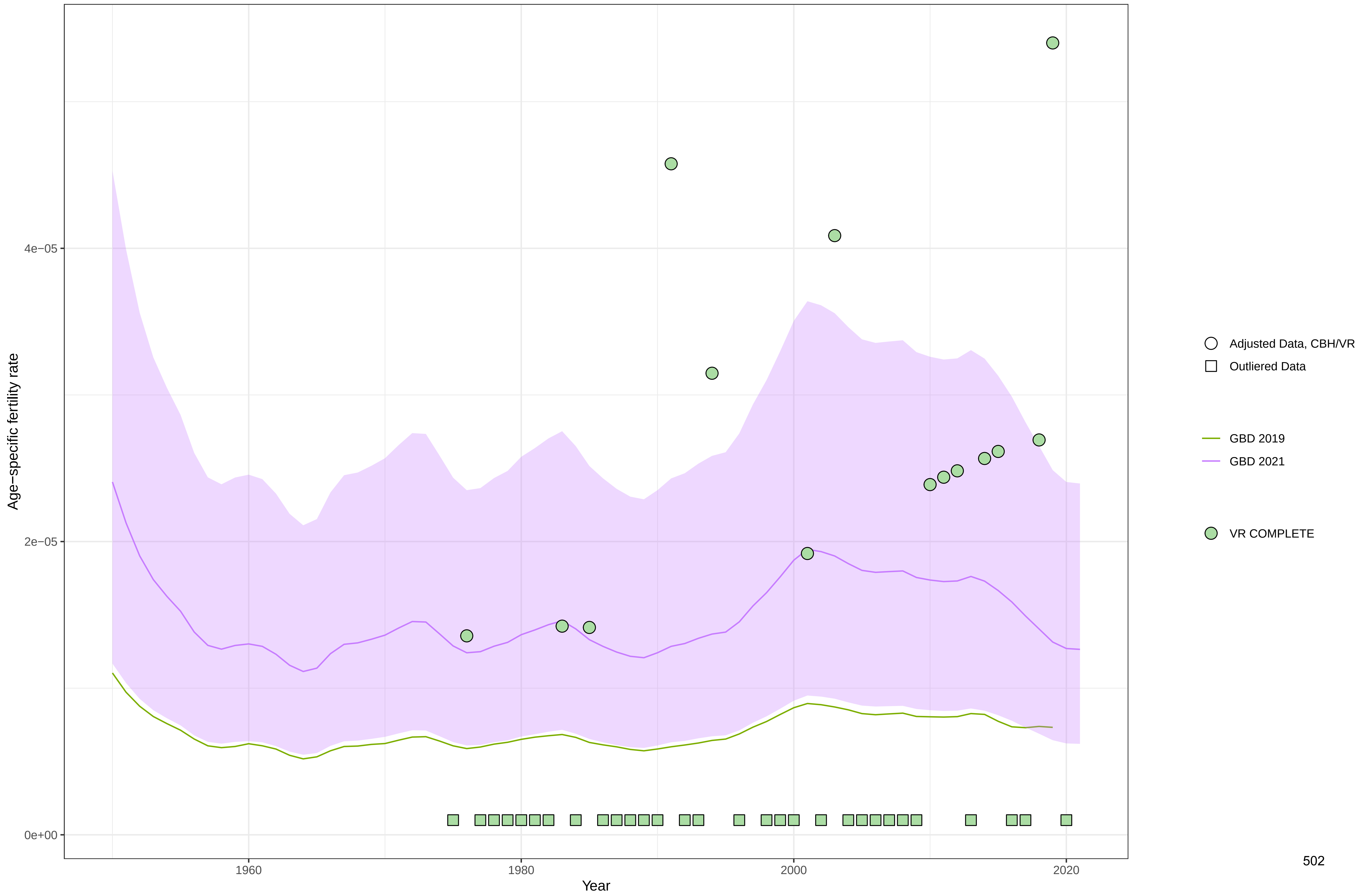

Age−specific fertility fitted trend lines, 1950−2021: Kagoshima, Ages: 15−19
Data points enclosed with a diamond are reference sources

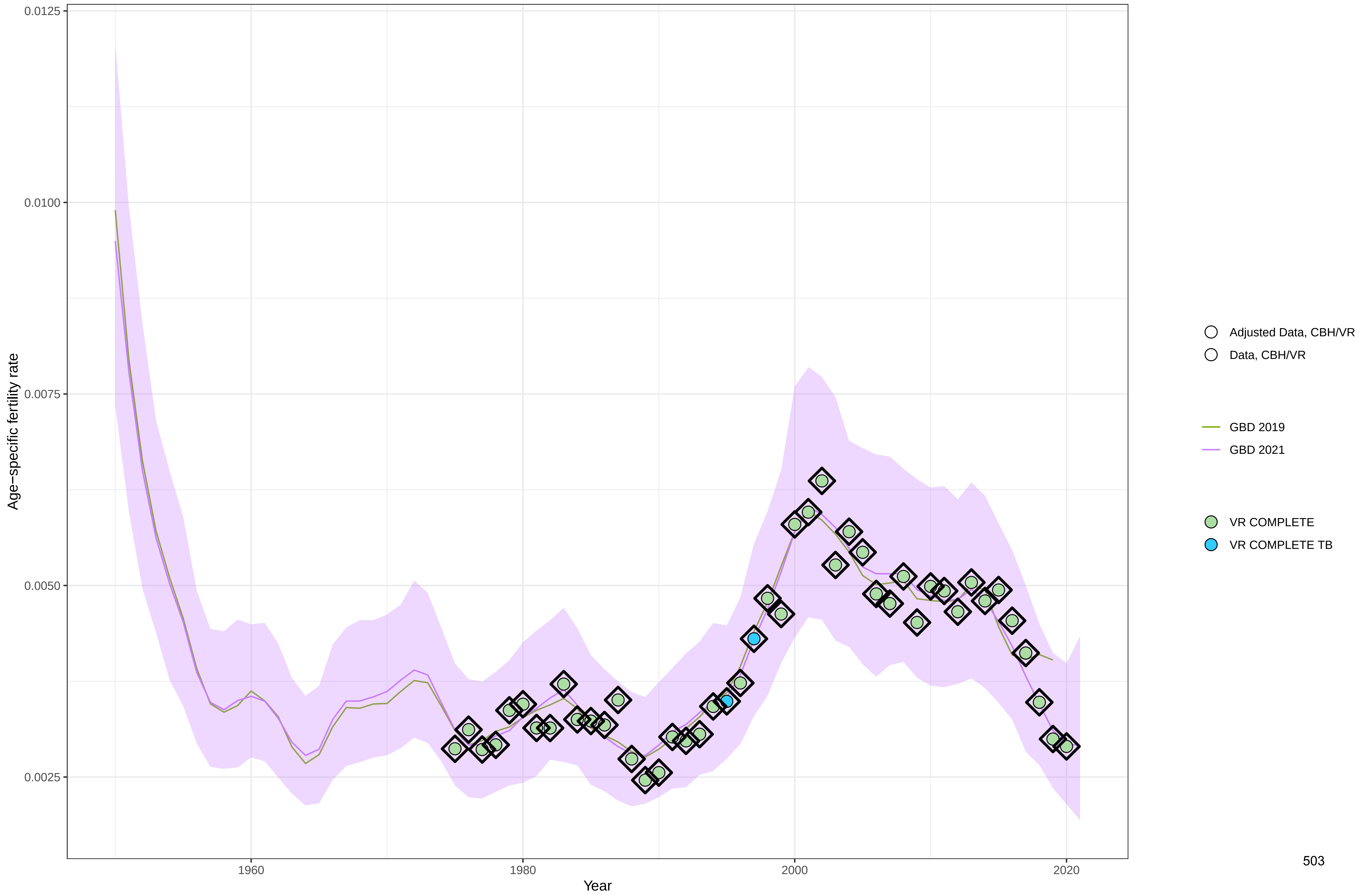

Age−specific fertility fitted trend lines, 1950−2021: Kagoshima, Ages: 20−24
Data points enclosed with a diamond are reference sources

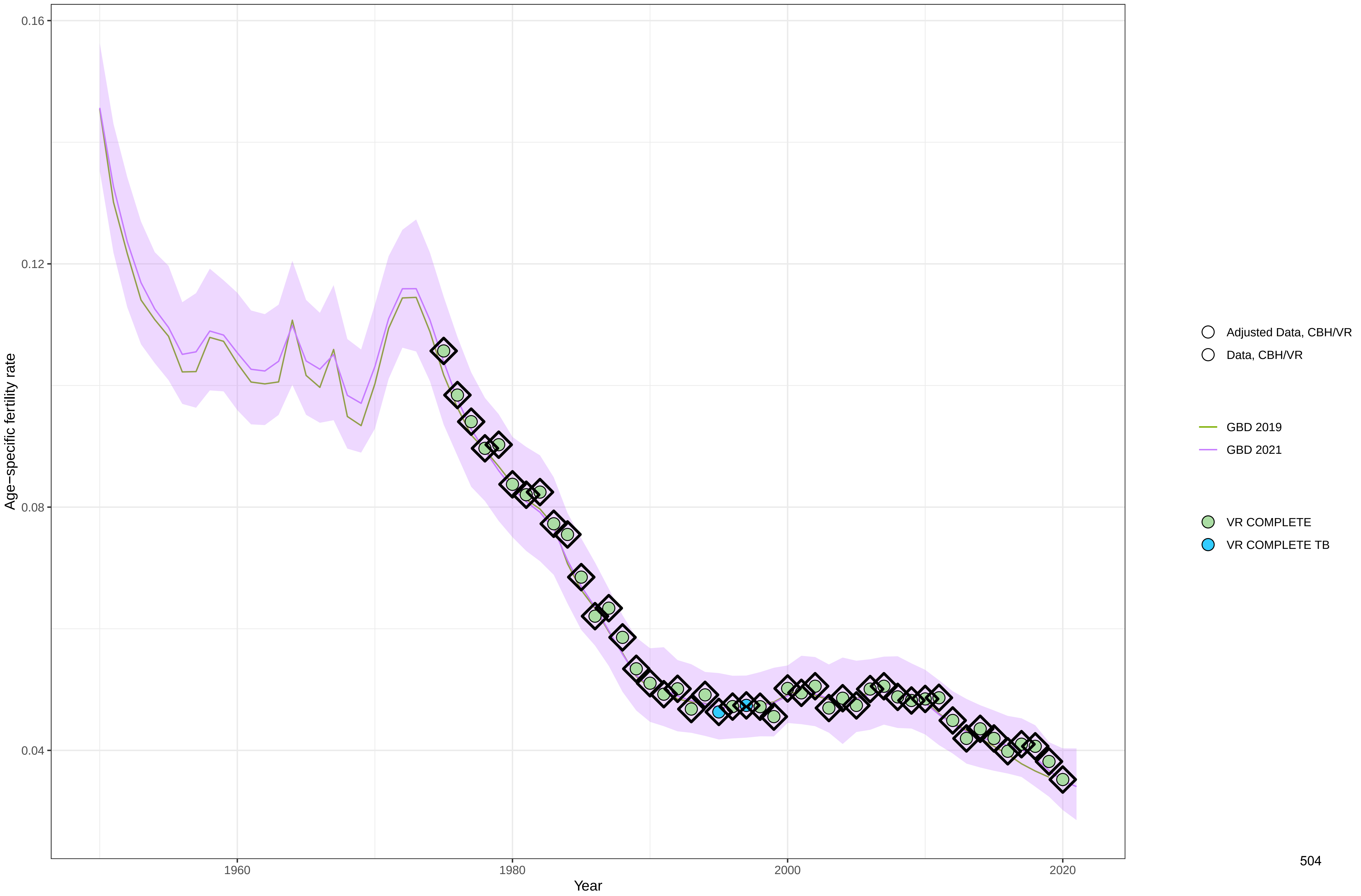

Age−specific fertility fitted trend lines, 1950−2021: Kagoshima, Ages: 25−29
Data points enclosed with a diamond are reference sources

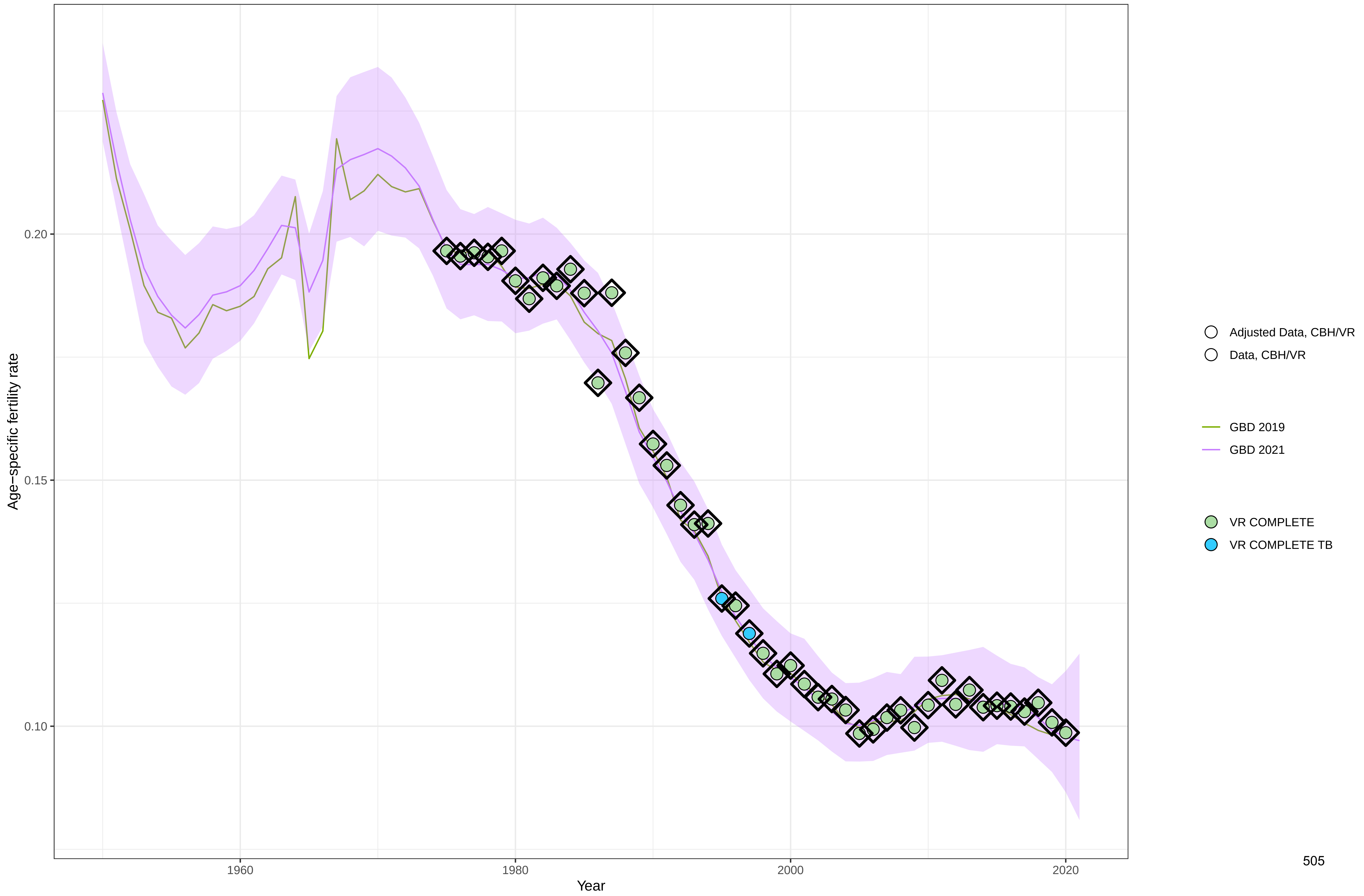

Age−specific fertility fitted trend lines, 1950−2021: Kagoshima, Ages: 30−34
Data points enclosed with a diamond are reference sources

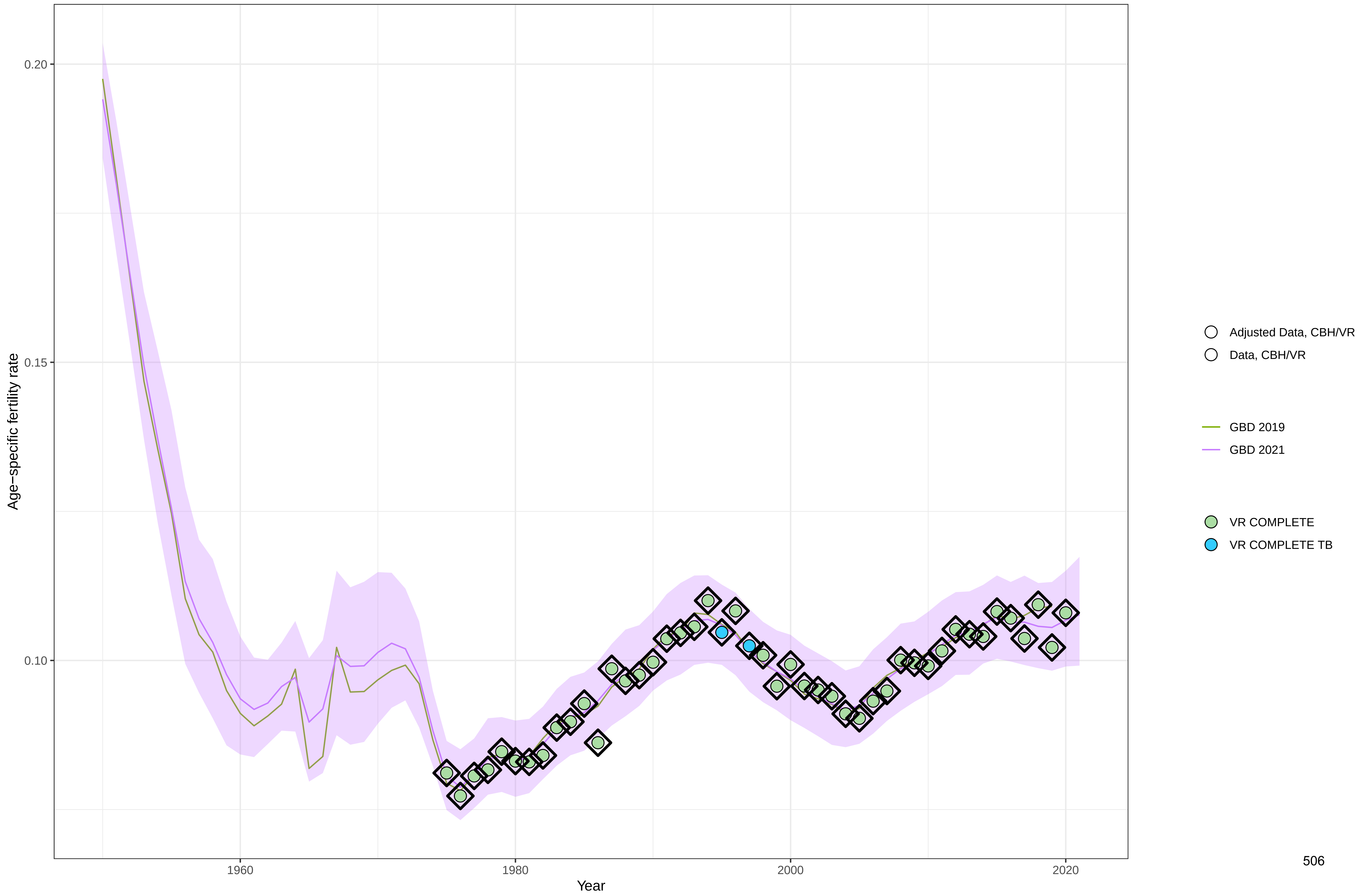

Age−specific fertility fitted trend lines, 1950−2021: Kagoshima, Ages: 35−39
Data points enclosed with a diamond are reference sources

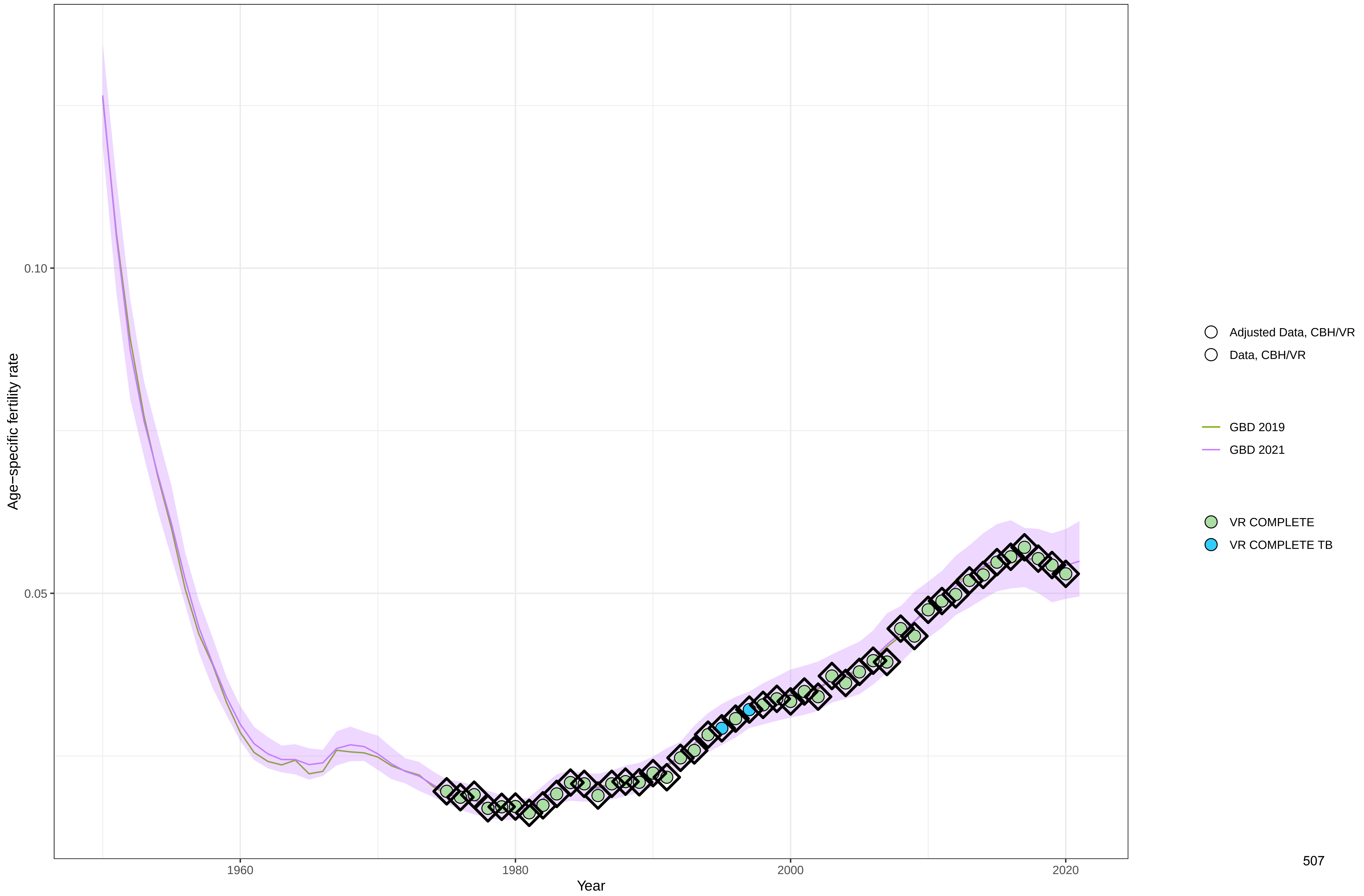

Age−specific fertility fitted trend lines, 1950−2021: Kagoshima, Ages: 40−44
Data points enclosed with a diamond are reference sources

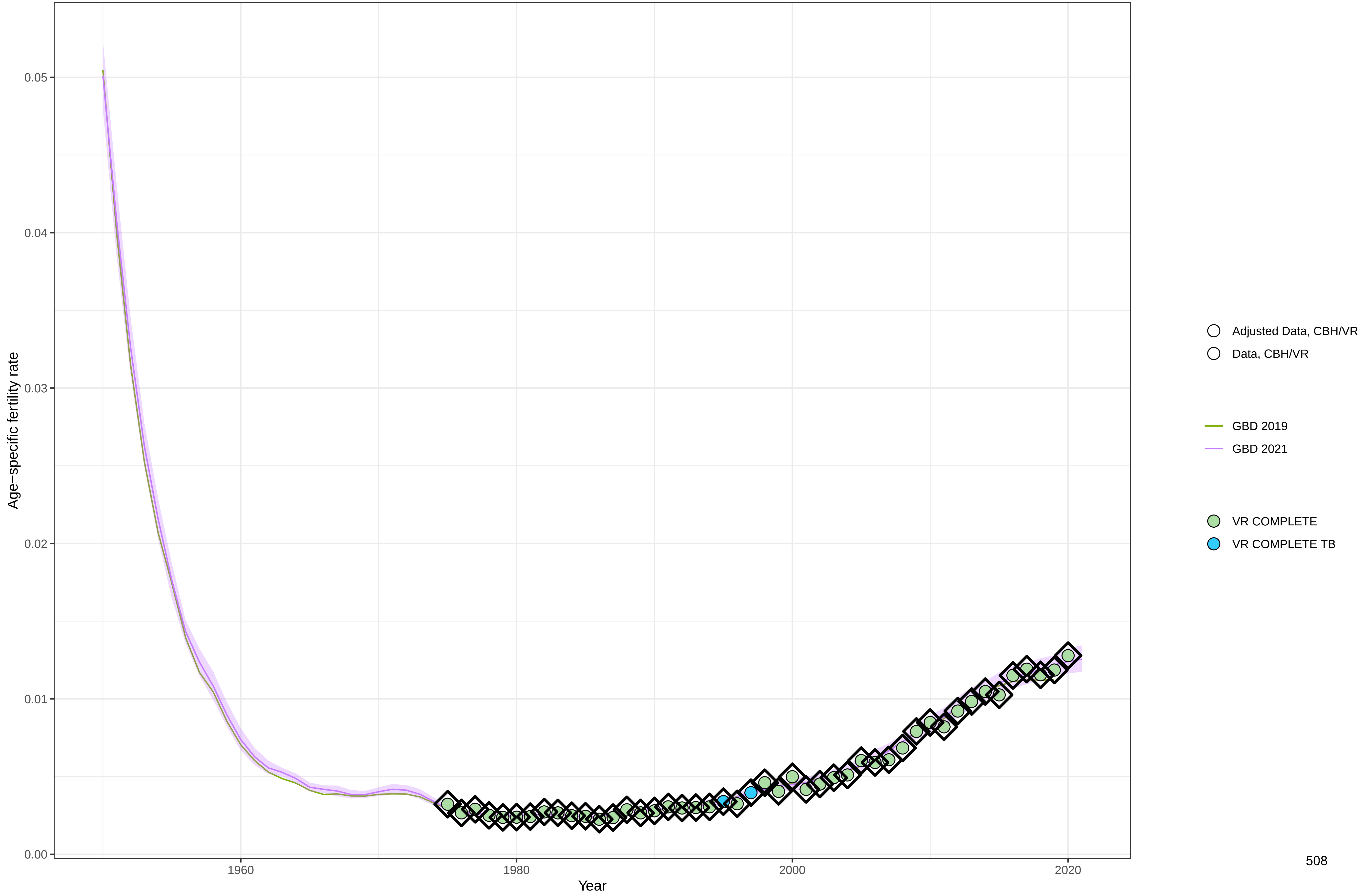

Age−specific fertility fitted trend lines, 1950−2021: Kagoshima, Ages: 45−49
Data points enclosed with a diamond are reference sources

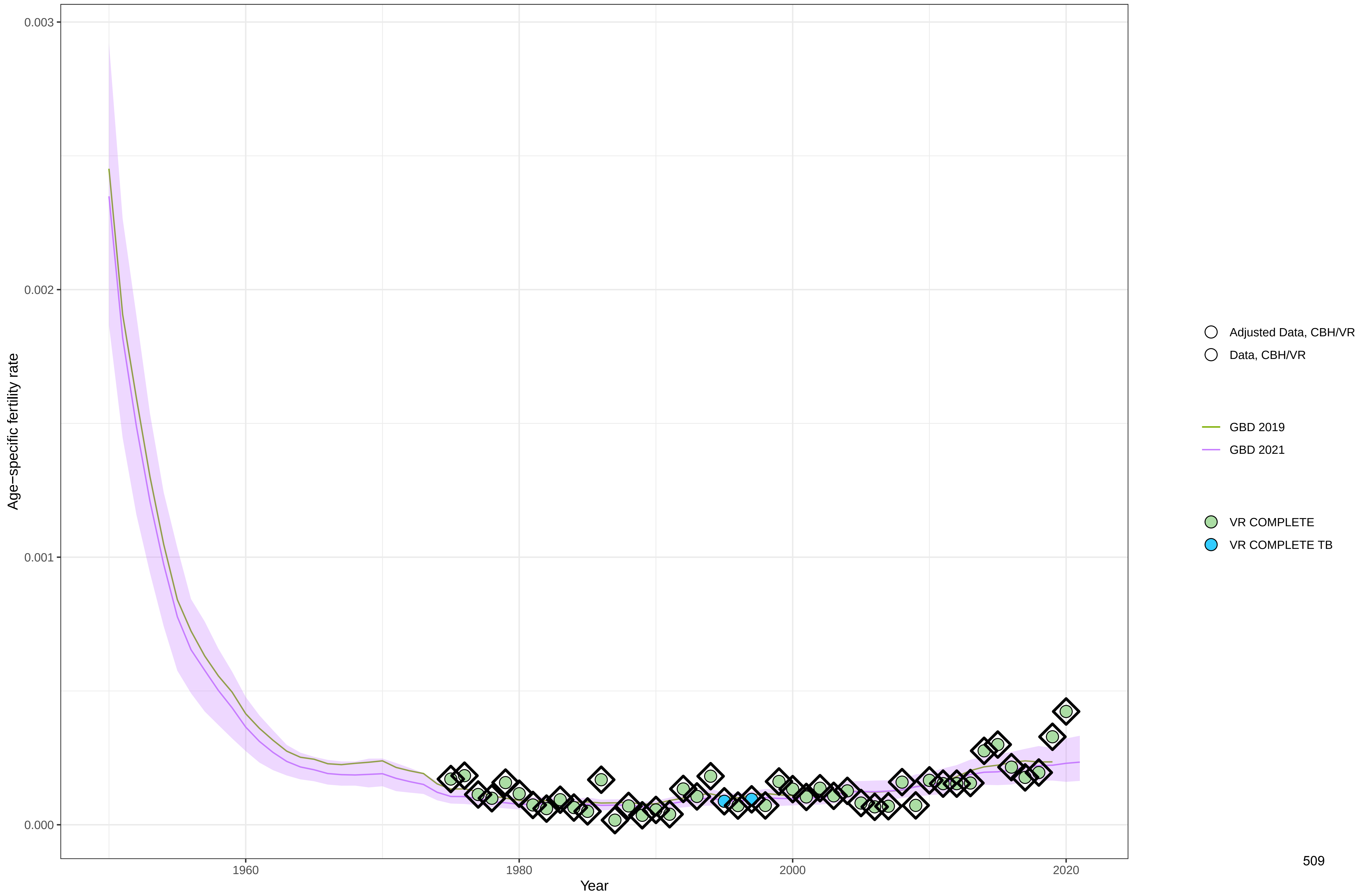

Age−specific fertility fitted trend lines, 1950−2021: Kagoshima, Ages: 50−54
Data points enclosed with a diamond are reference sources

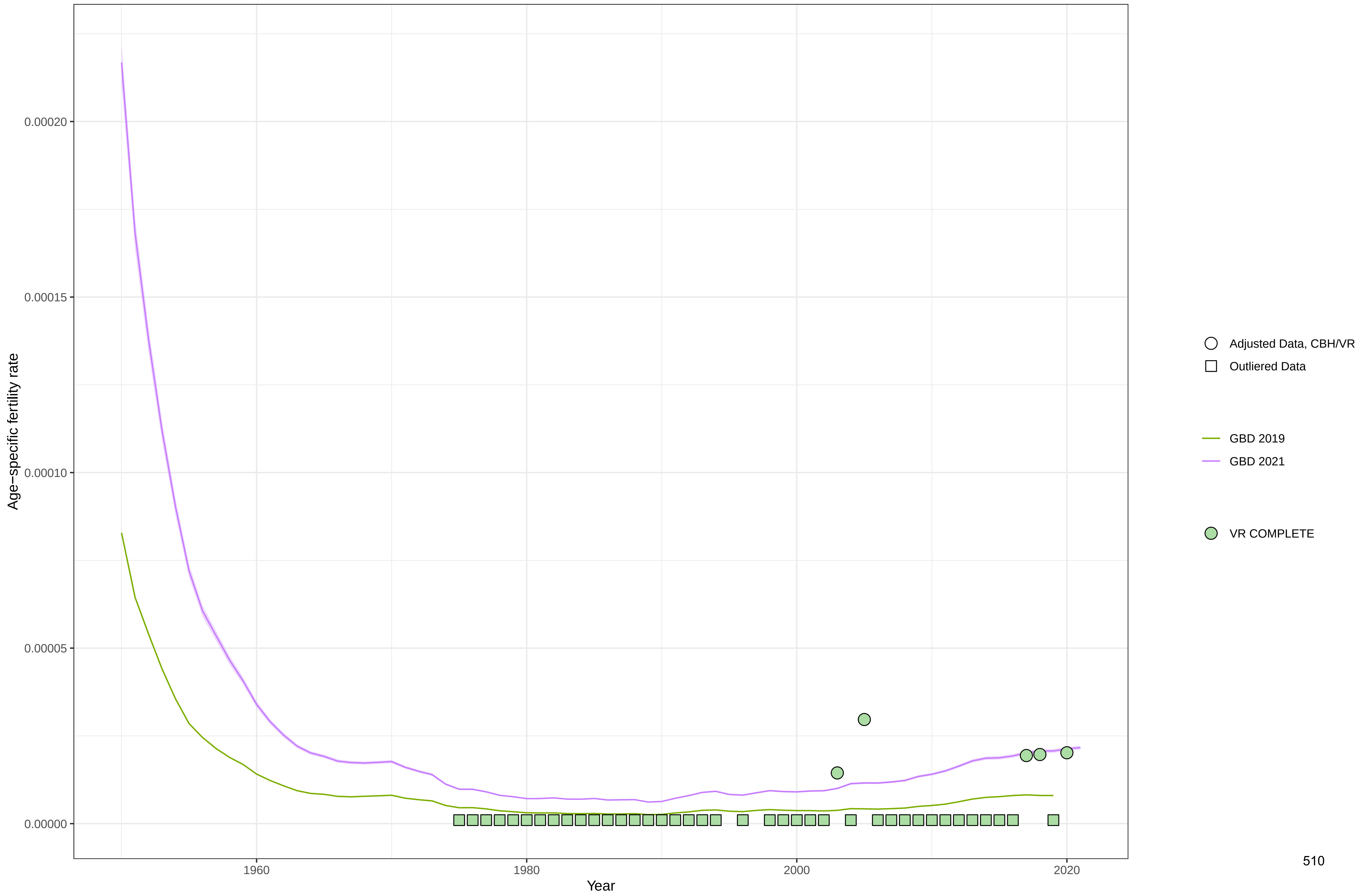

Total fertility rate fitted trend lines, 1950−2021: Kagoshima TFR
Data points enclosed with a diamond are reference sources

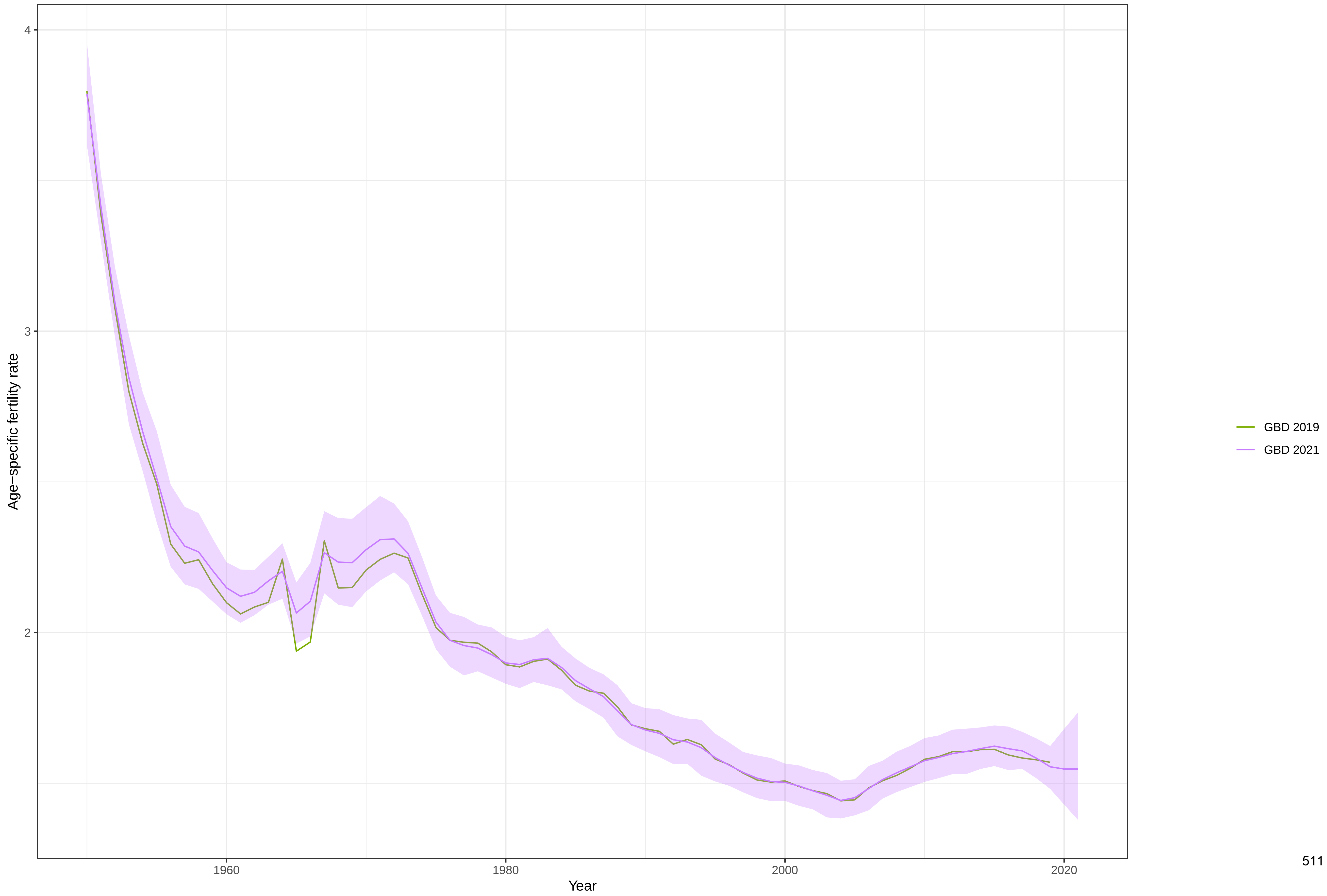

Age−specific fertility fitted trend lines, 1950−2021: Kanagawa, Ages: 10−14
Data points enclosed with a diamond are reference sources

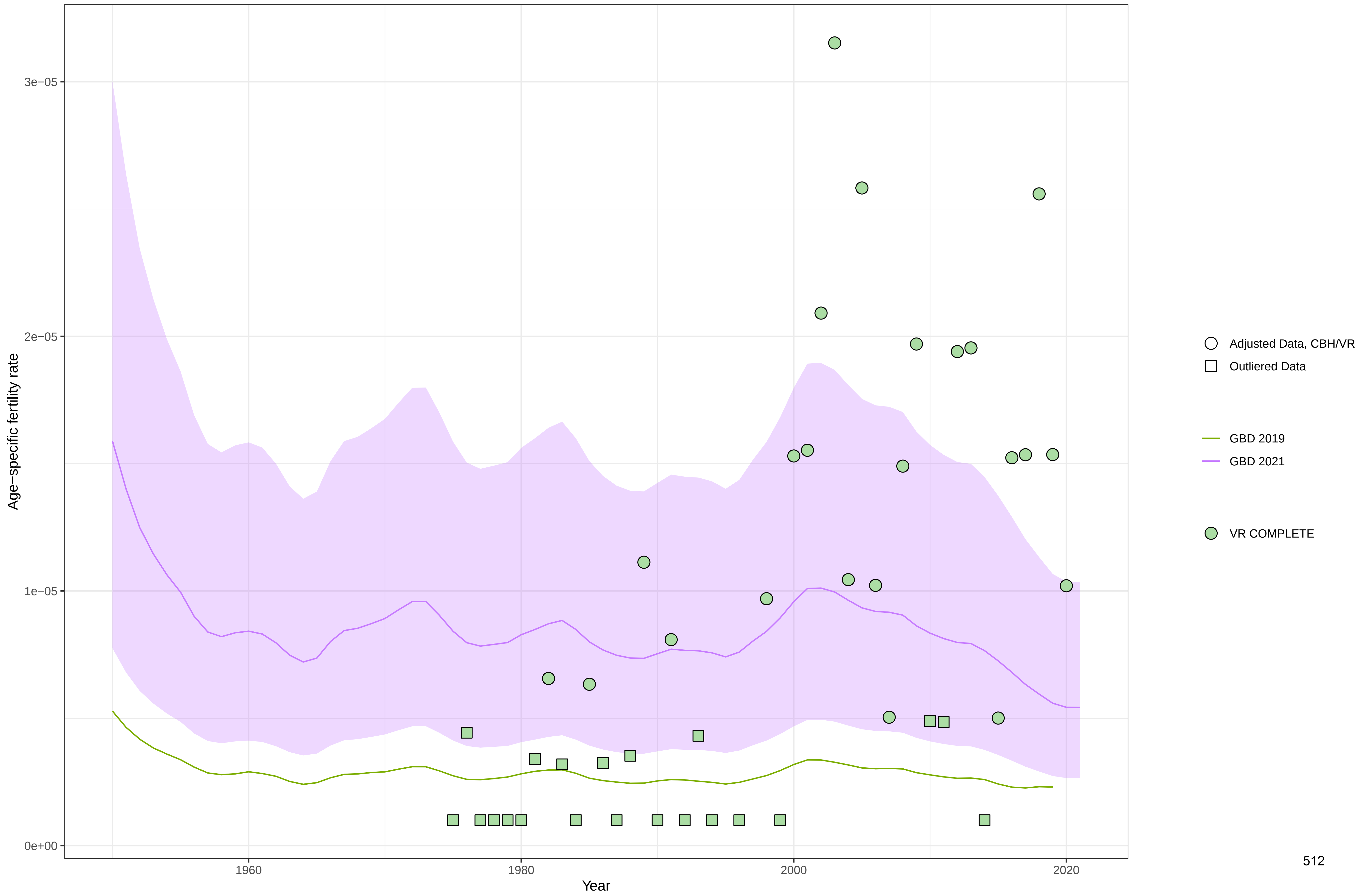

Age−specific fertility fitted trend lines, 1950−2021: Kanagawa, Ages: 15−19
Data points enclosed with a diamond are reference sources

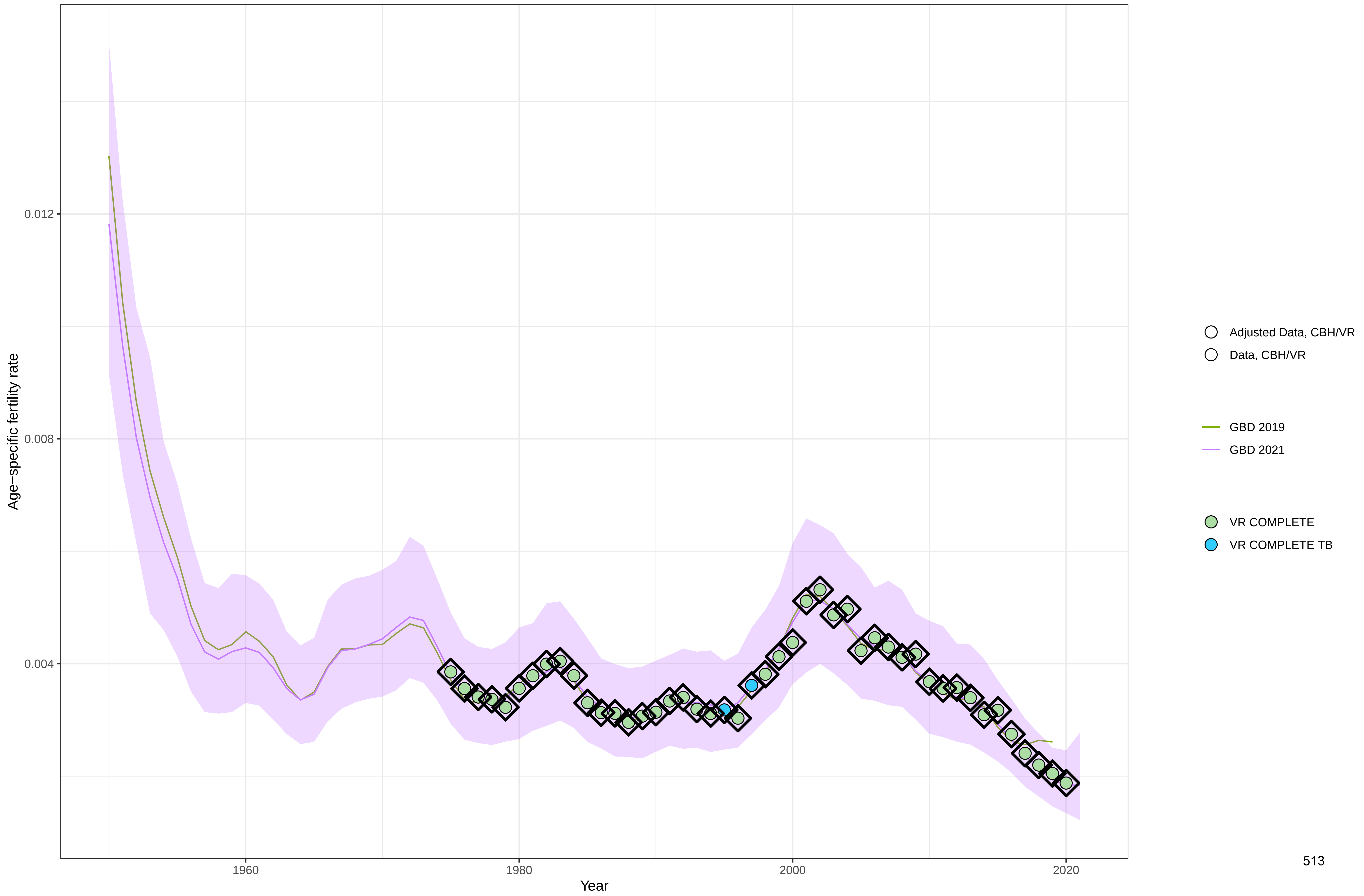

Age−specific fertility fitted trend lines, 1950−2021: Kanagawa, Ages: 20−24
Data points enclosed with a diamond are reference sources

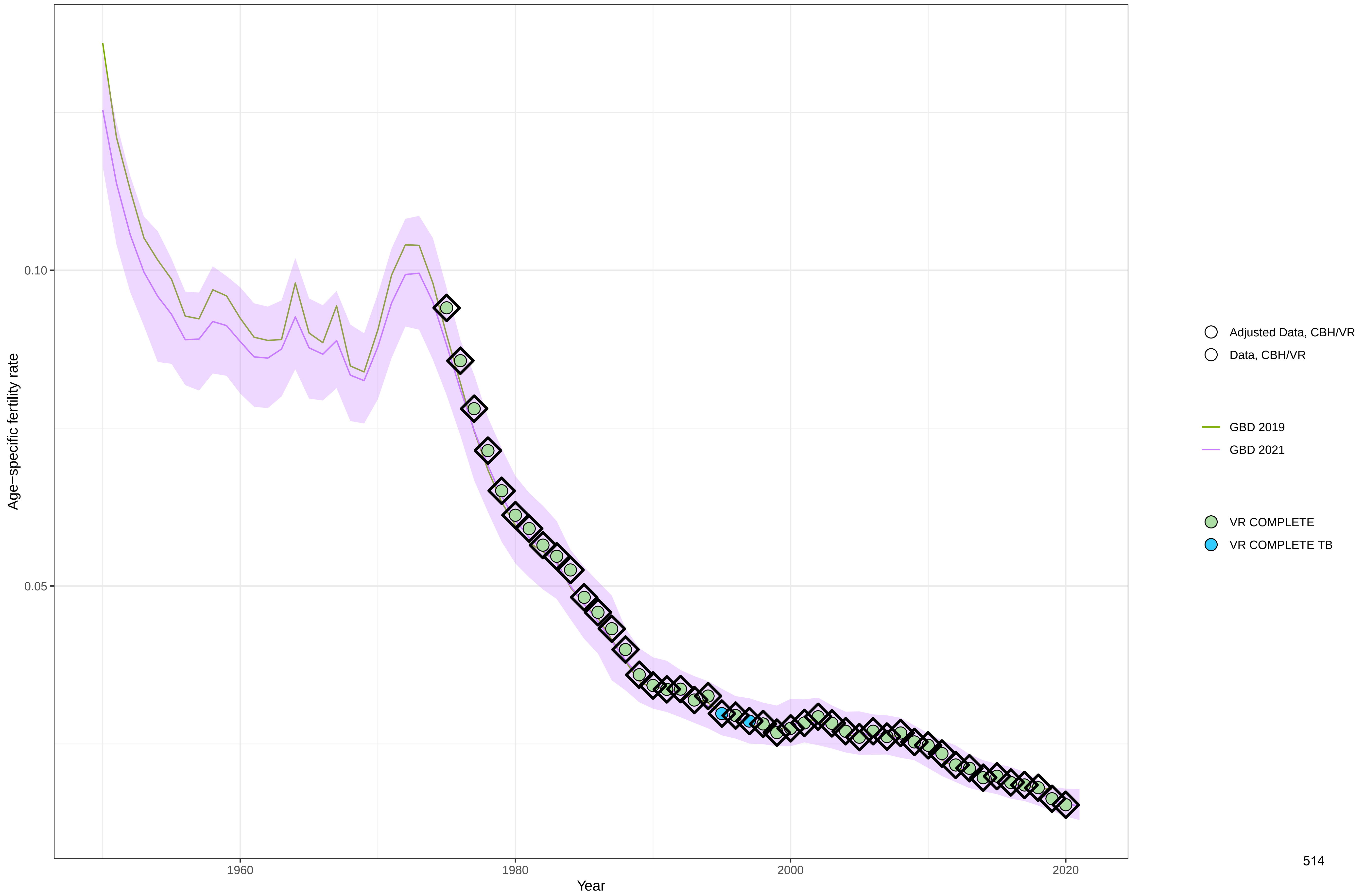

Age−specific fertility fitted trend lines, 1950−2021: Kanagawa, Ages: 25−29
Data points enclosed with a diamond are reference sources

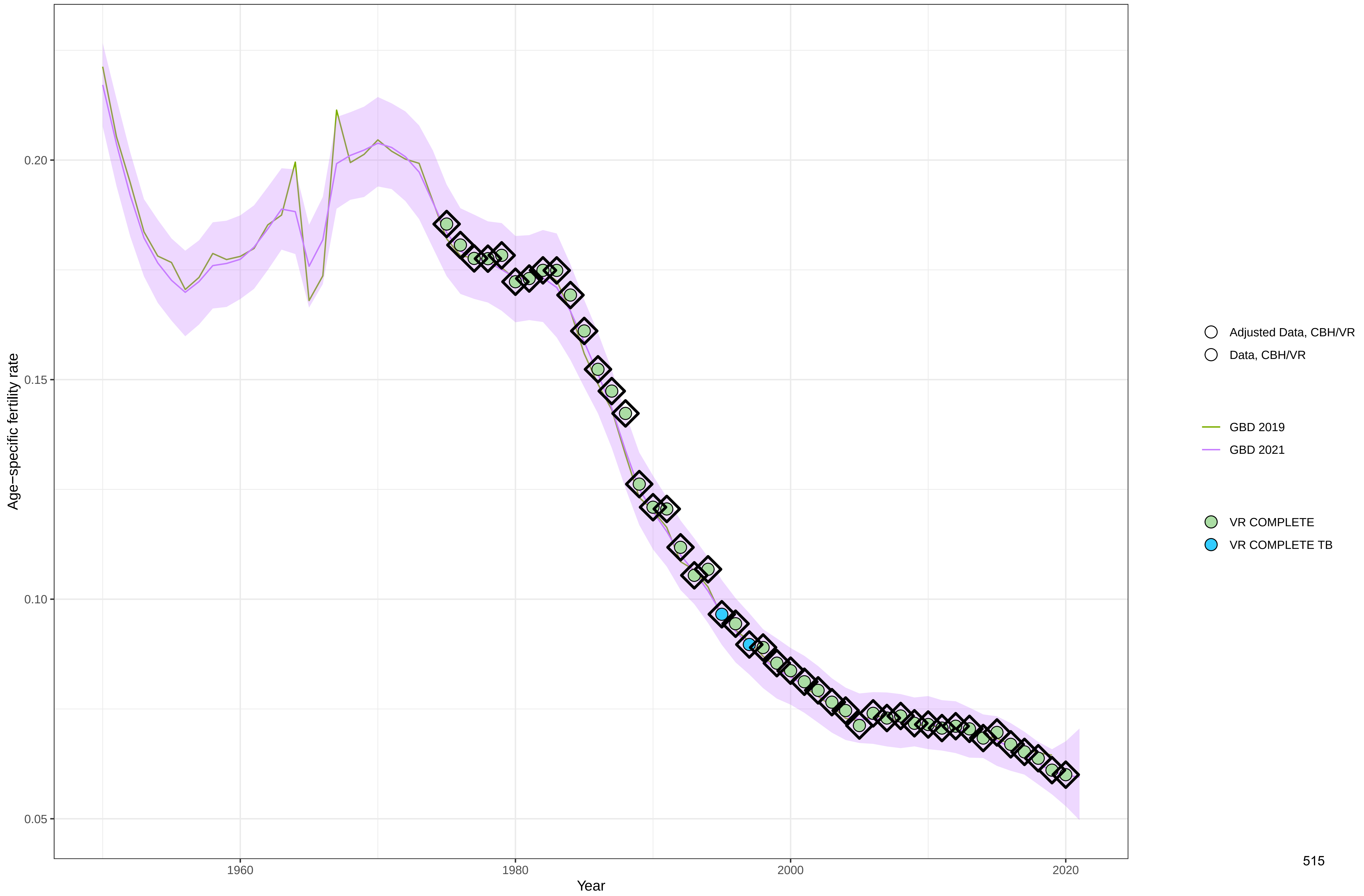

Age−specific fertility fitted trend lines, 1950−2021: Kanagawa, Ages: 30−34
Data points enclosed with a diamond are reference sources

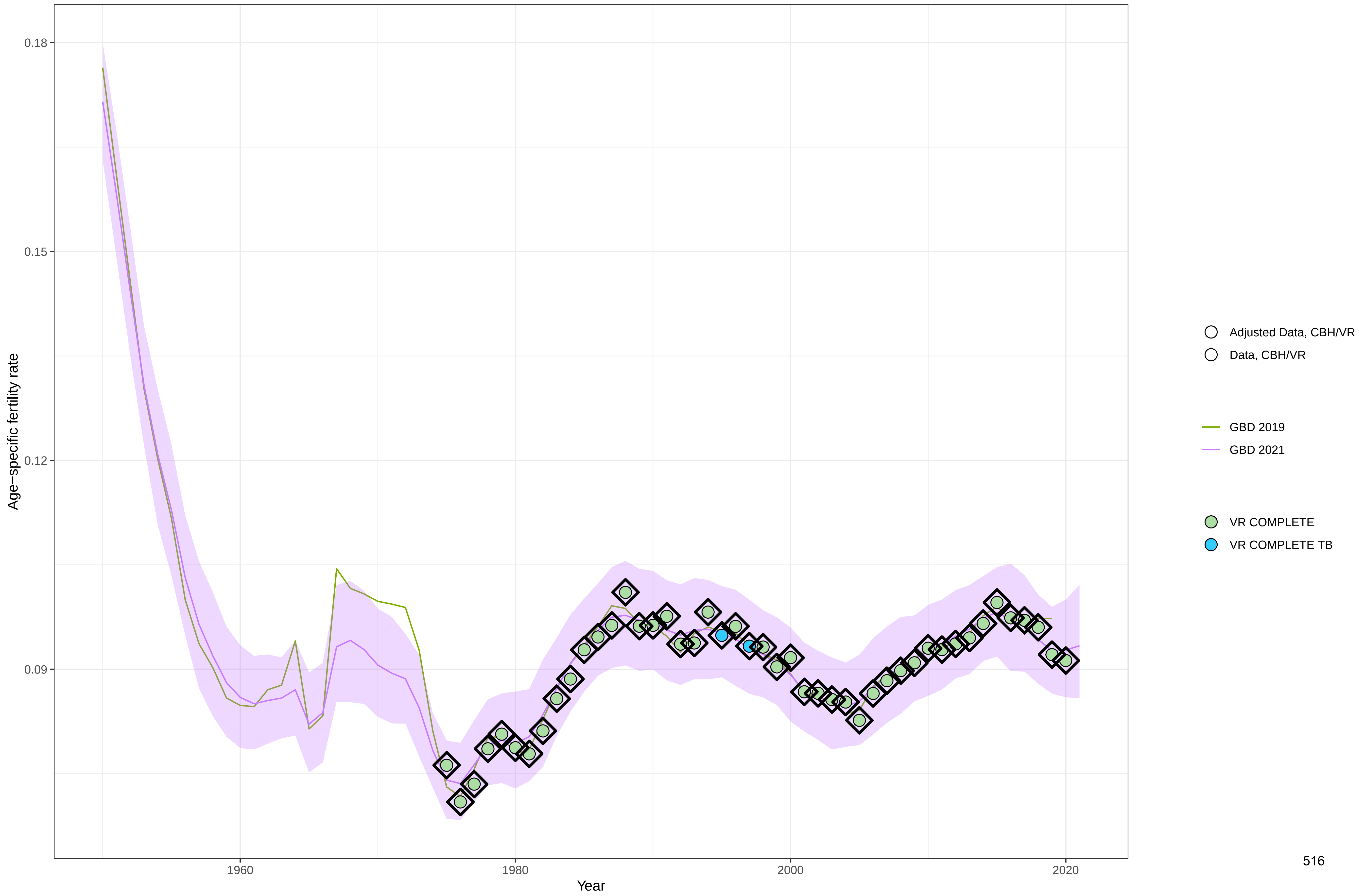

Age−specific fertility fitted trend lines, 1950−2021: Kanagawa, Ages: 35−39
Data points enclosed with a diamond are reference sources

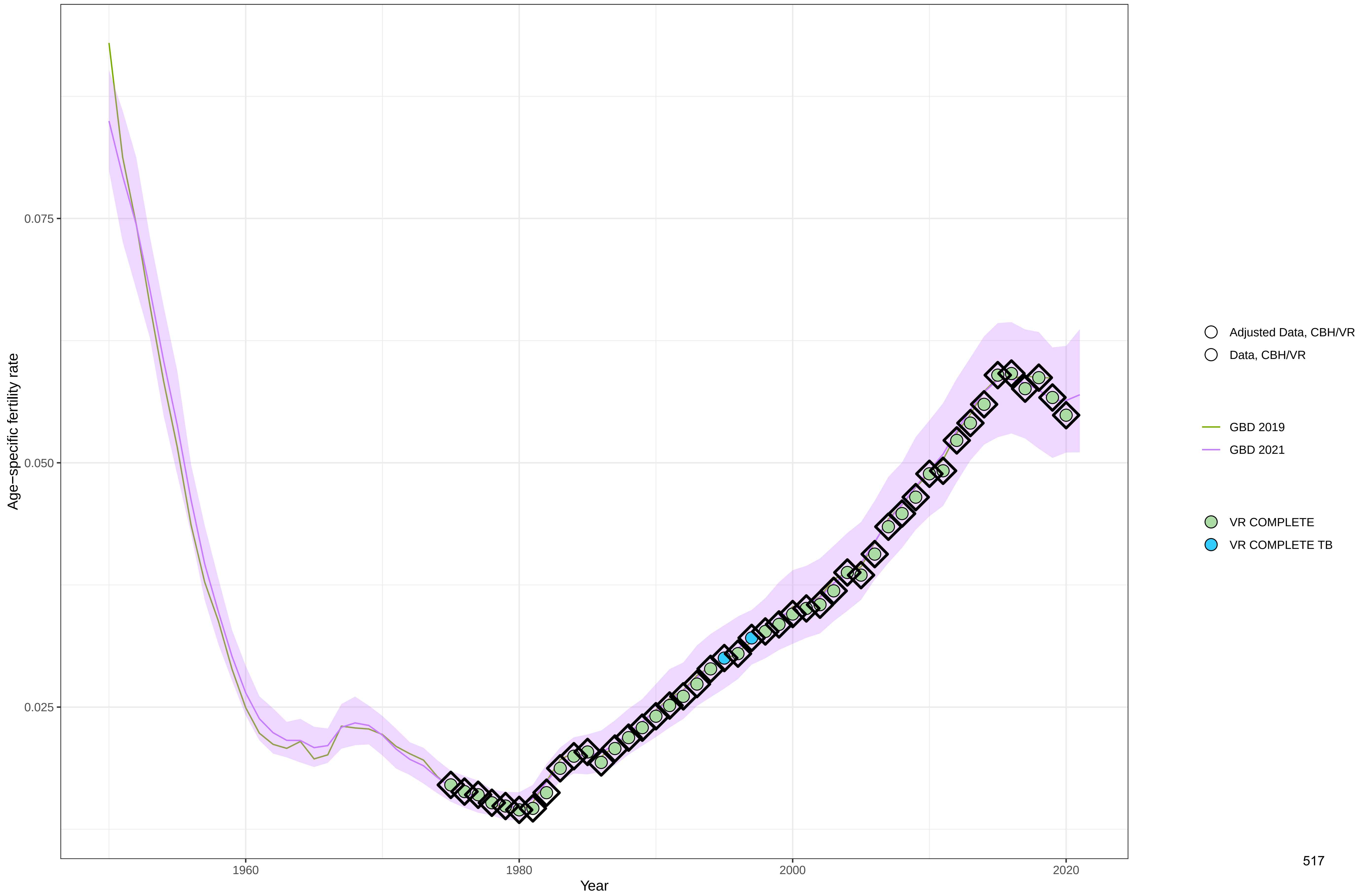

Age−specific fertility fitted trend lines, 1950−2021: Kanagawa, Ages: 40−44
Data points enclosed with a diamond are reference sources

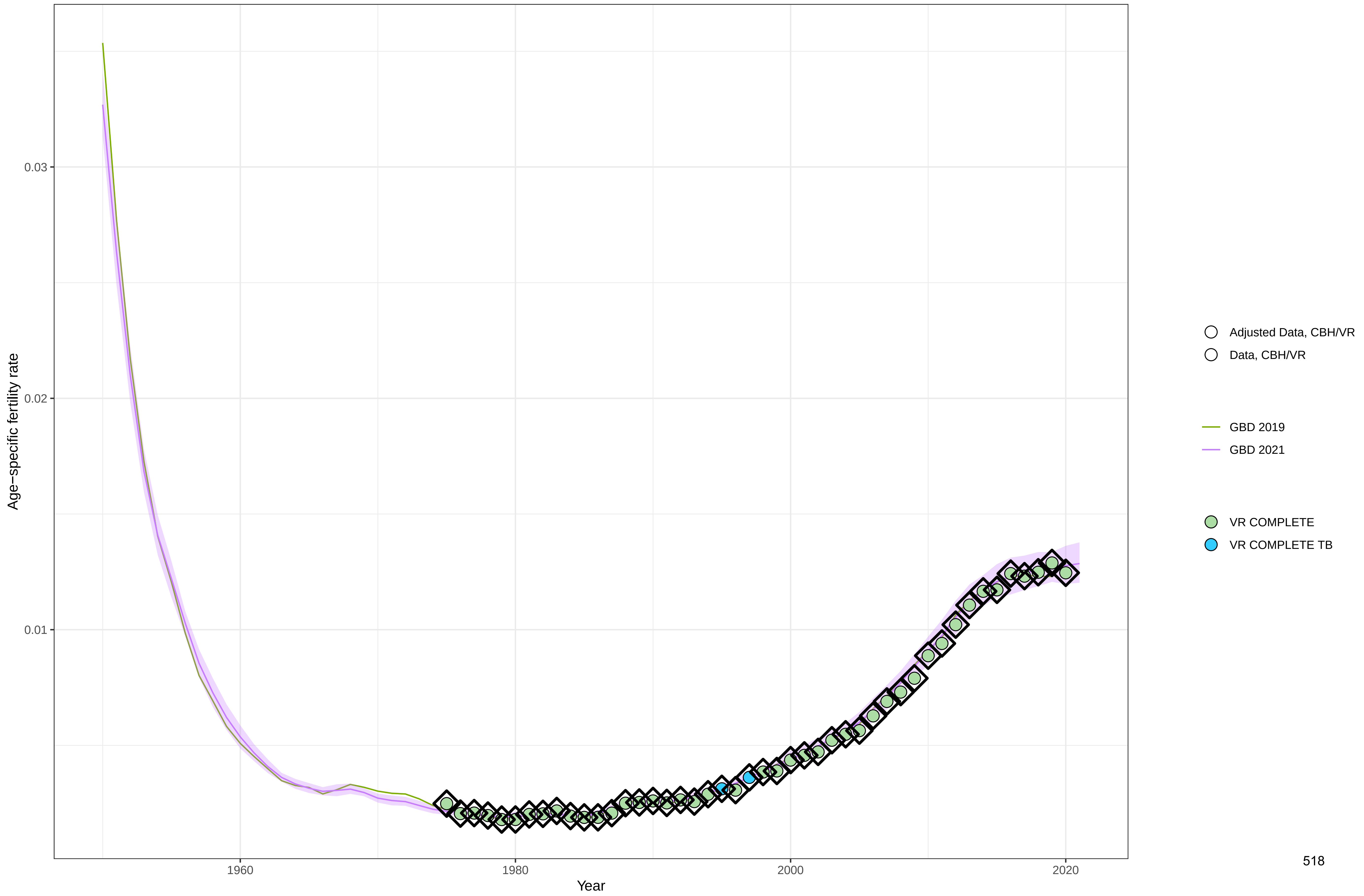

Age−specific fertility fitted trend lines, 1950−2021: Kanagawa, Ages: 45−49
Data points enclosed with a diamond are reference sources

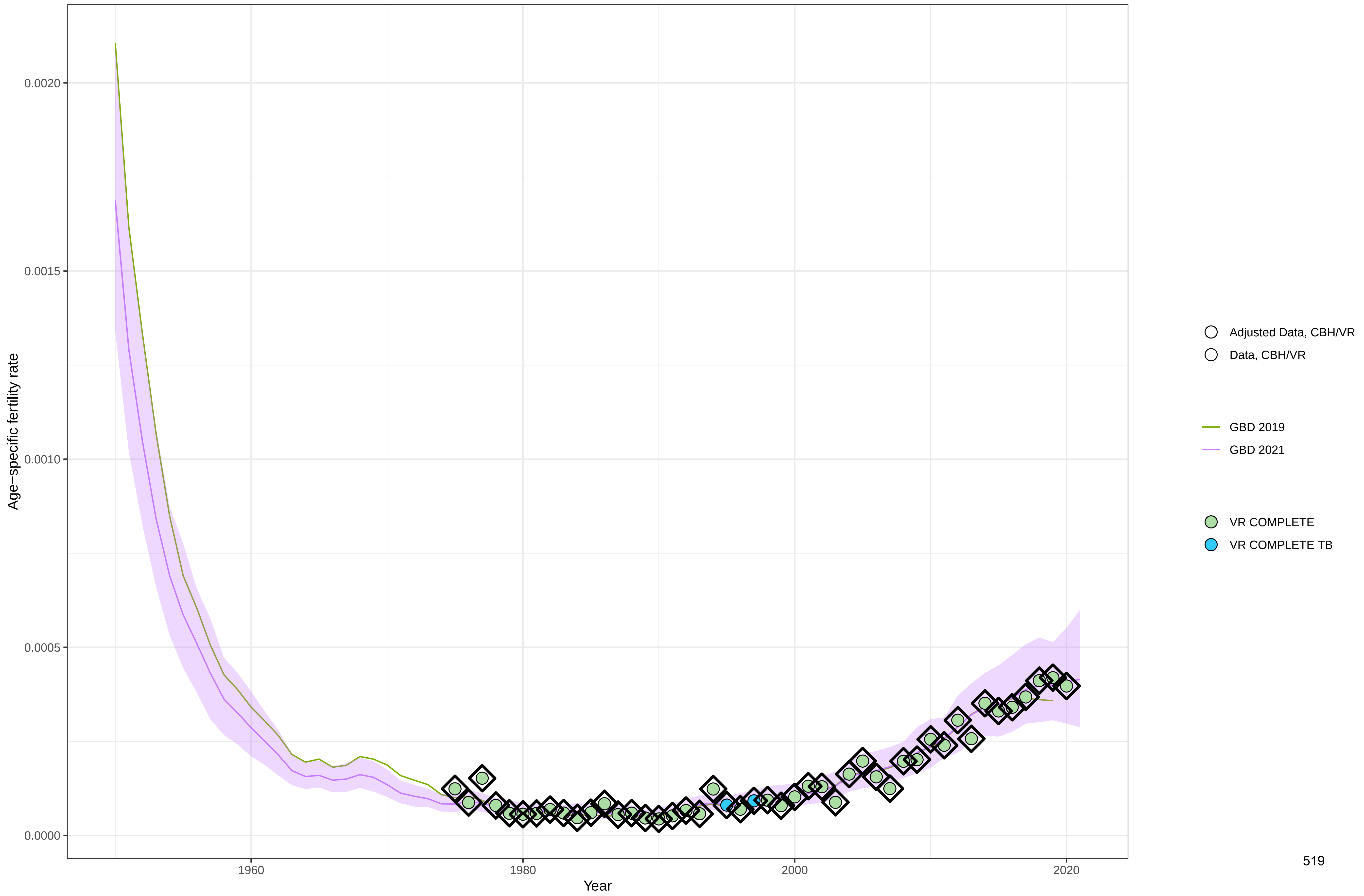

Age−specific fertility fitted trend lines, 1950−2021: Kanagawa, Ages: 50−54
Data points enclosed with a diamond are reference sources

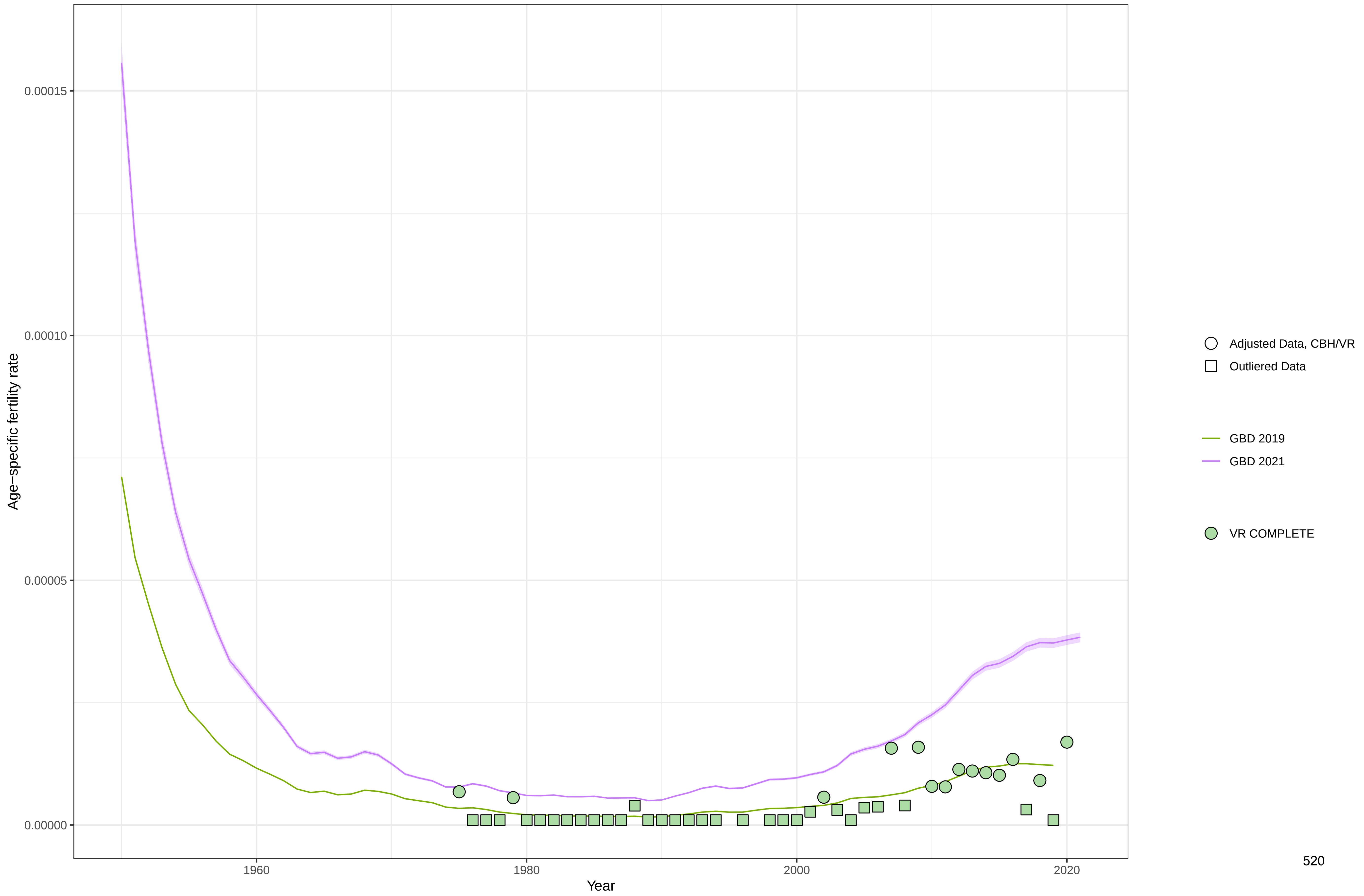

Total fertility rate fitted trend lines, 1950−2021: Kanagawa TFR
Data points enclosed with a diamond are reference sources

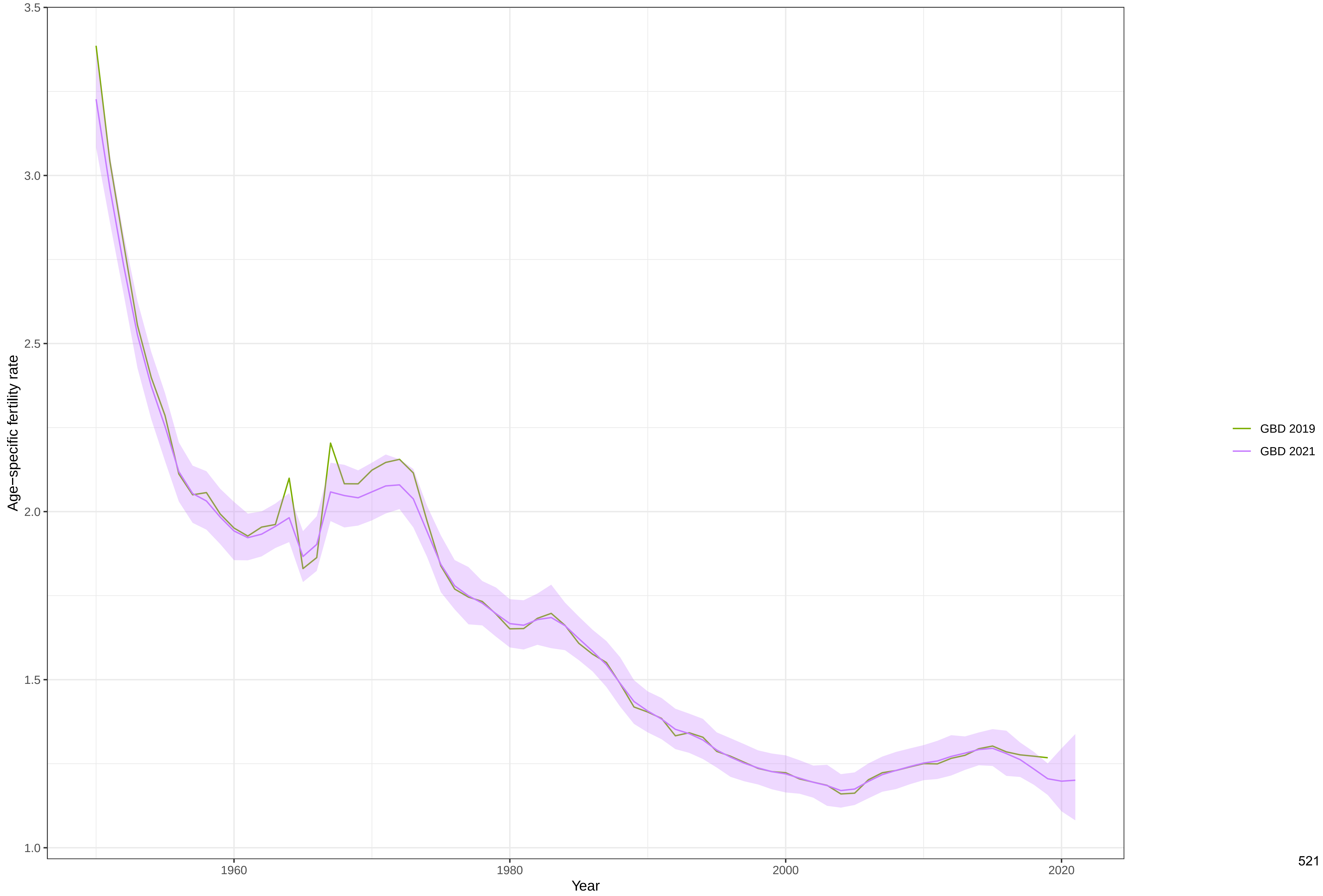

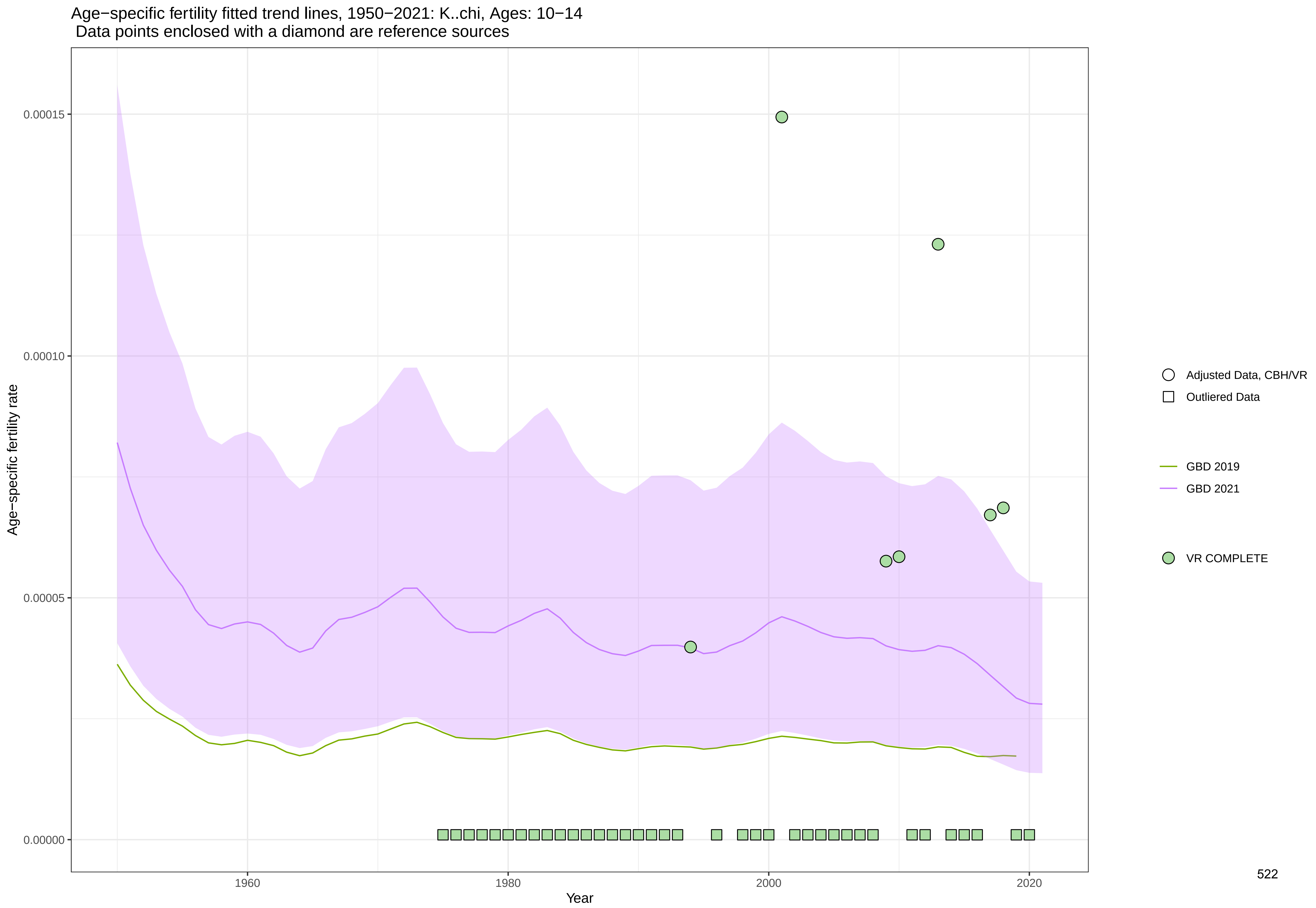

Age−specific fertility fitted trend lines, 1950−2021: K..chi, Ages: 10−14
Data points enclosed with a diamond are reference sources
Age−specific fertility rate
0.00015
0.00010
0.00005
0.00000
1960
1980
2000
2020
Year
Adjusted Data, CBH/VR
Outliered Data
GBD 2019
GBD 2021
VR COMPLETE

Age−specific fertility fitted trend lines, 1950−2021: K..chi, Ages: 15−19
Data points enclosed with a diamond are reference sources

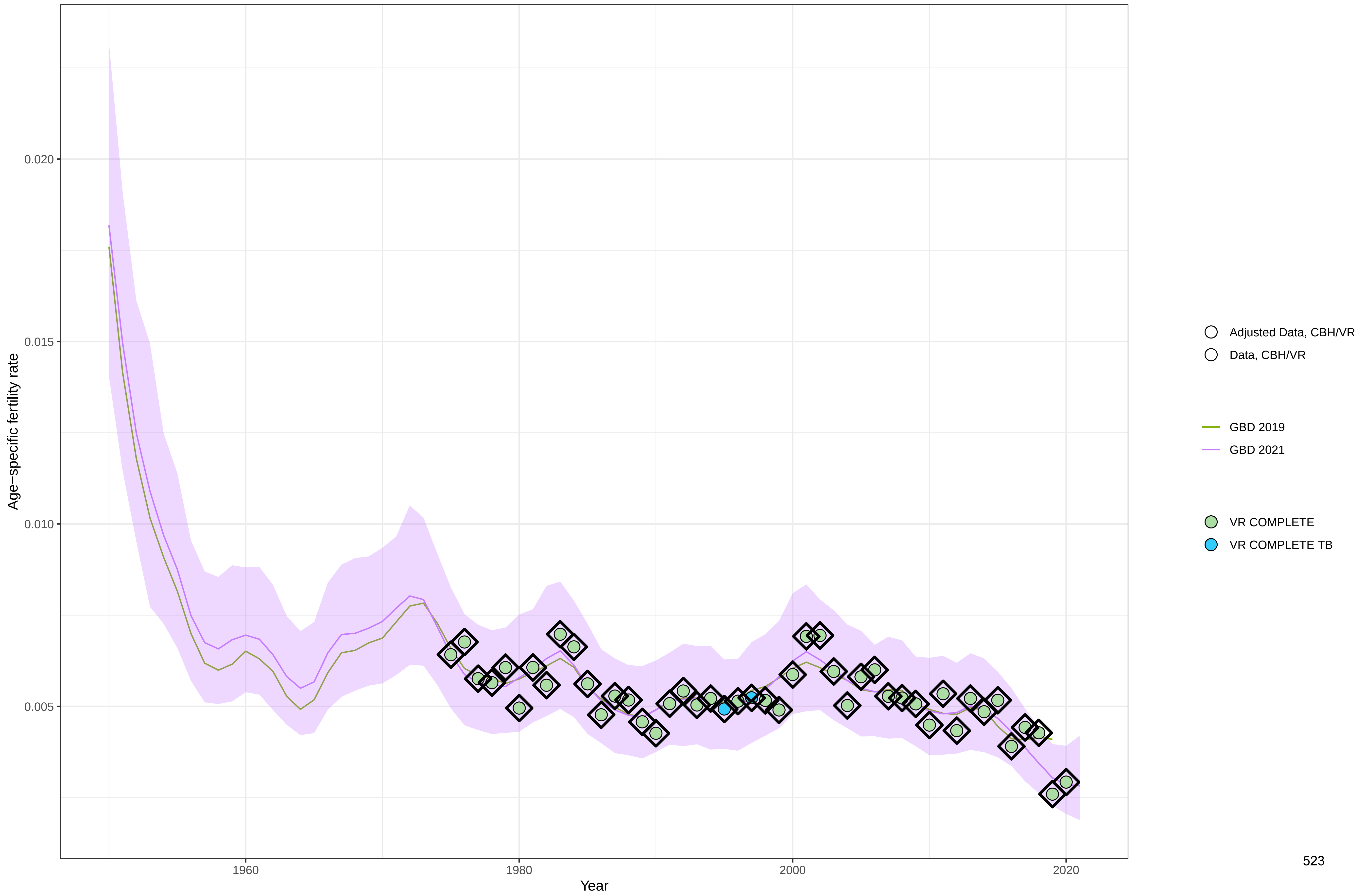

Age−specific fertility fitted trend lines, 1950−2021: K..chi, Ages: 20−24
Data points enclosed with a diamond are reference sources

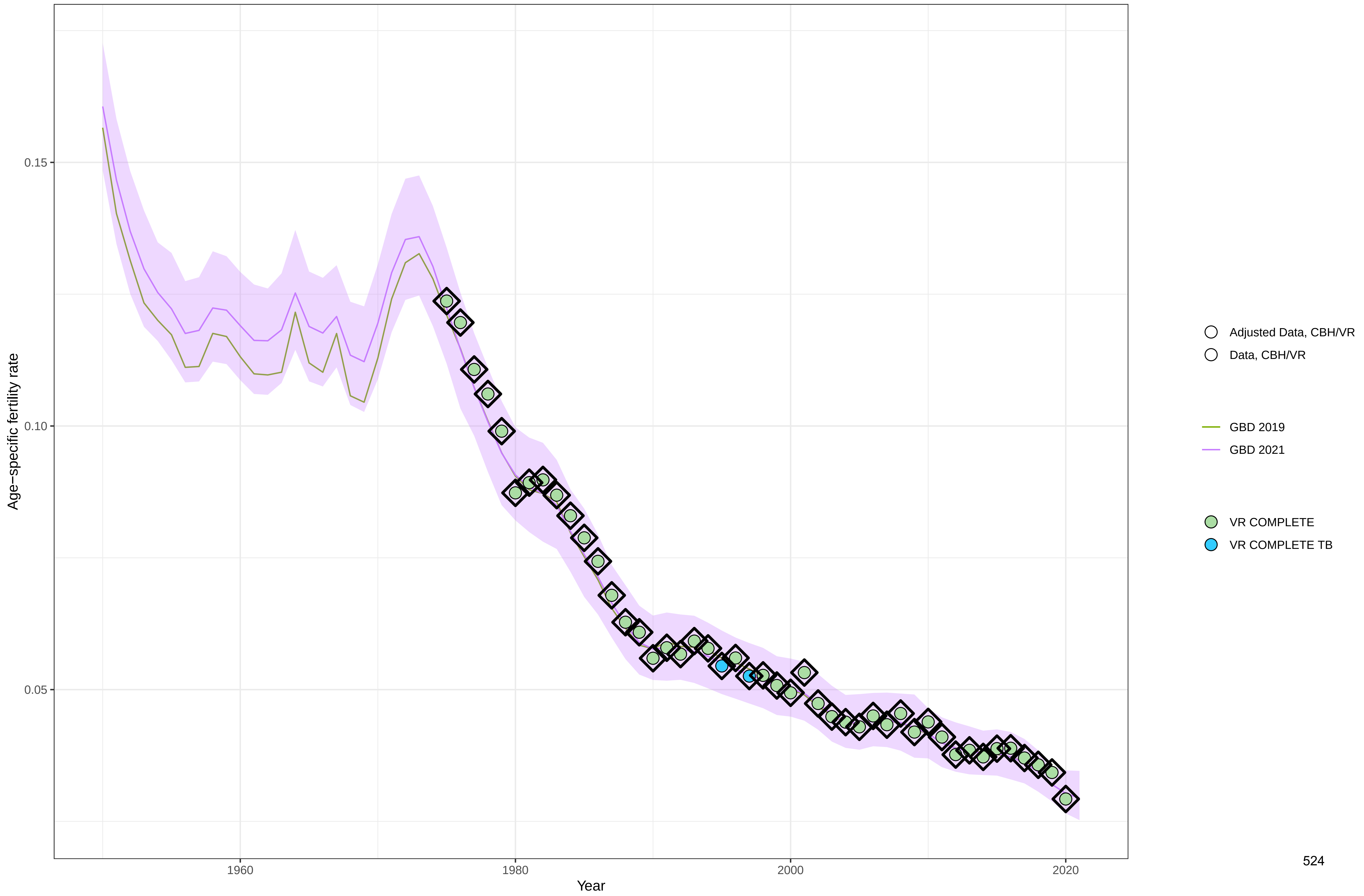

Age−specific fertility fitted trend lines, 1950−2021: K..chi, Ages: 25−29
 Data points enclosed with a diamond are reference sources

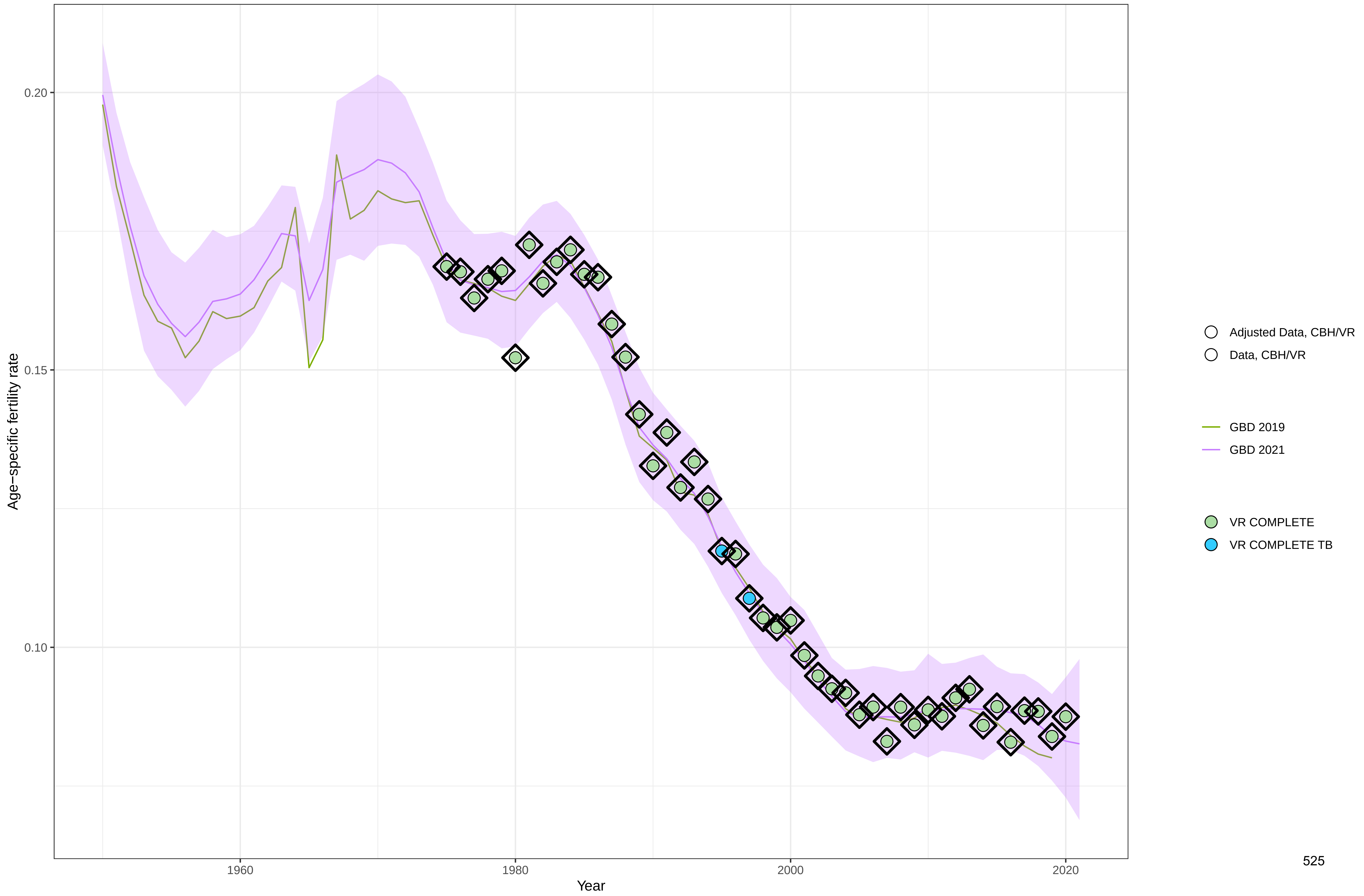

Age−specific fertility fitted trend lines, 1950−2021: K..chi, Ages: 30−34
Data points enclosed with a diamond are reference sources

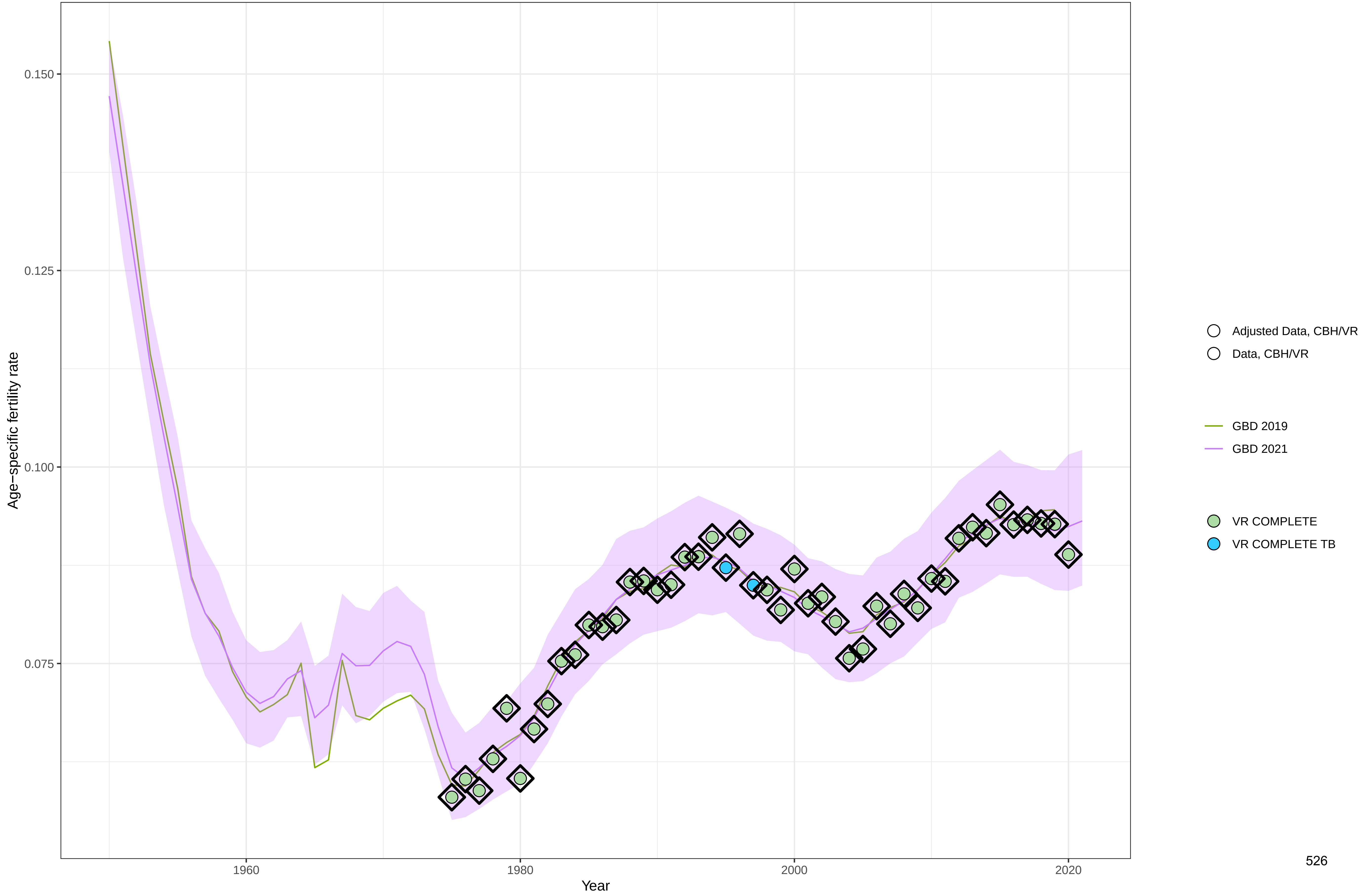

Age−specific fertility fitted trend lines, 1950−2021: K..chi, Ages: 35−39
Data points enclosed with a diamond are reference sources

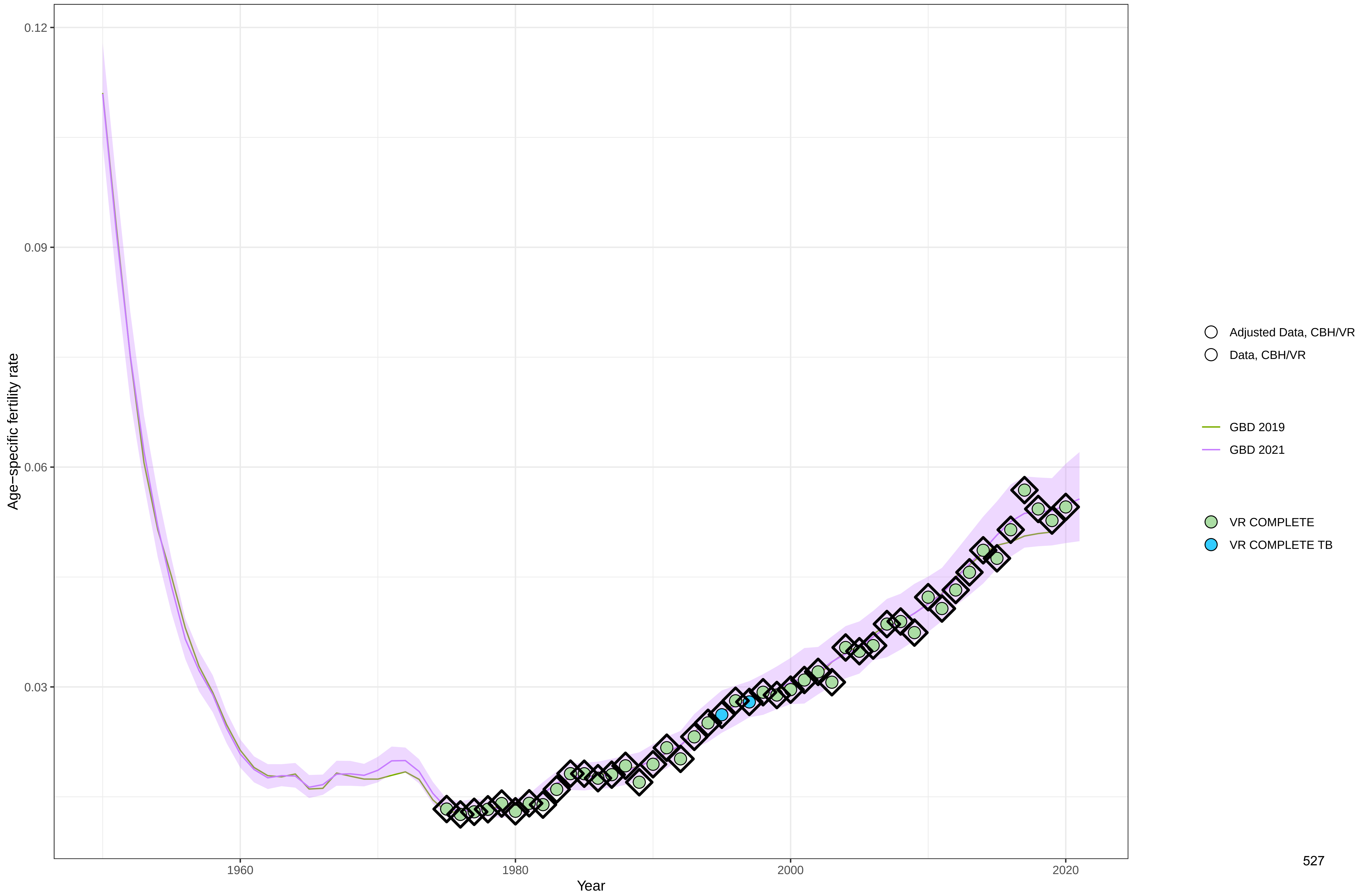

Age−specific fertility fitted trend lines, 1950−2021: K..chi, Ages: 40−44
Data points enclosed with a diamond are reference sources

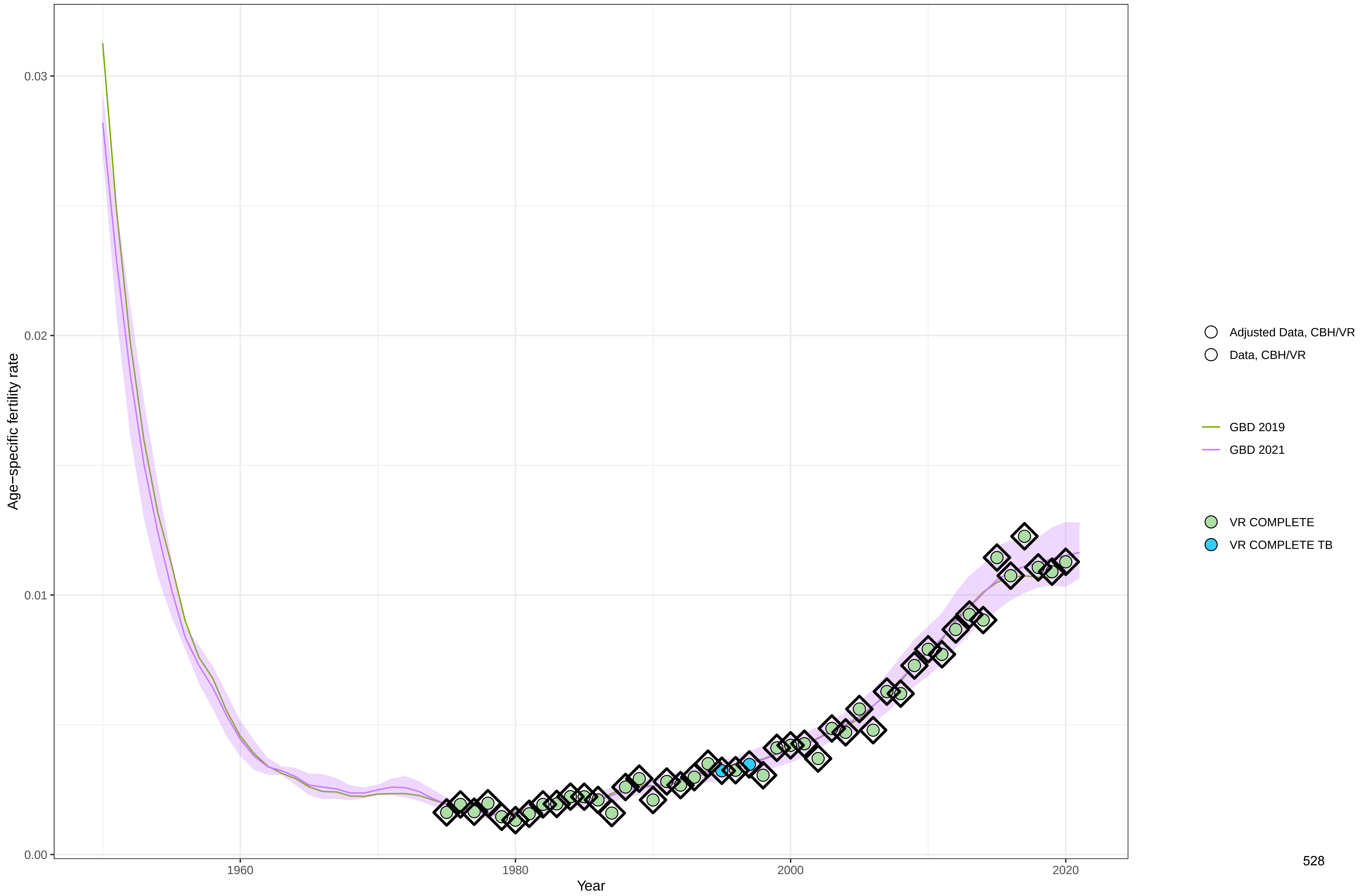

Age−specific fertility fitted trend lines, 1950−2021: K..chi, Ages: 45−49
Data points enclosed with a diamond are reference sources

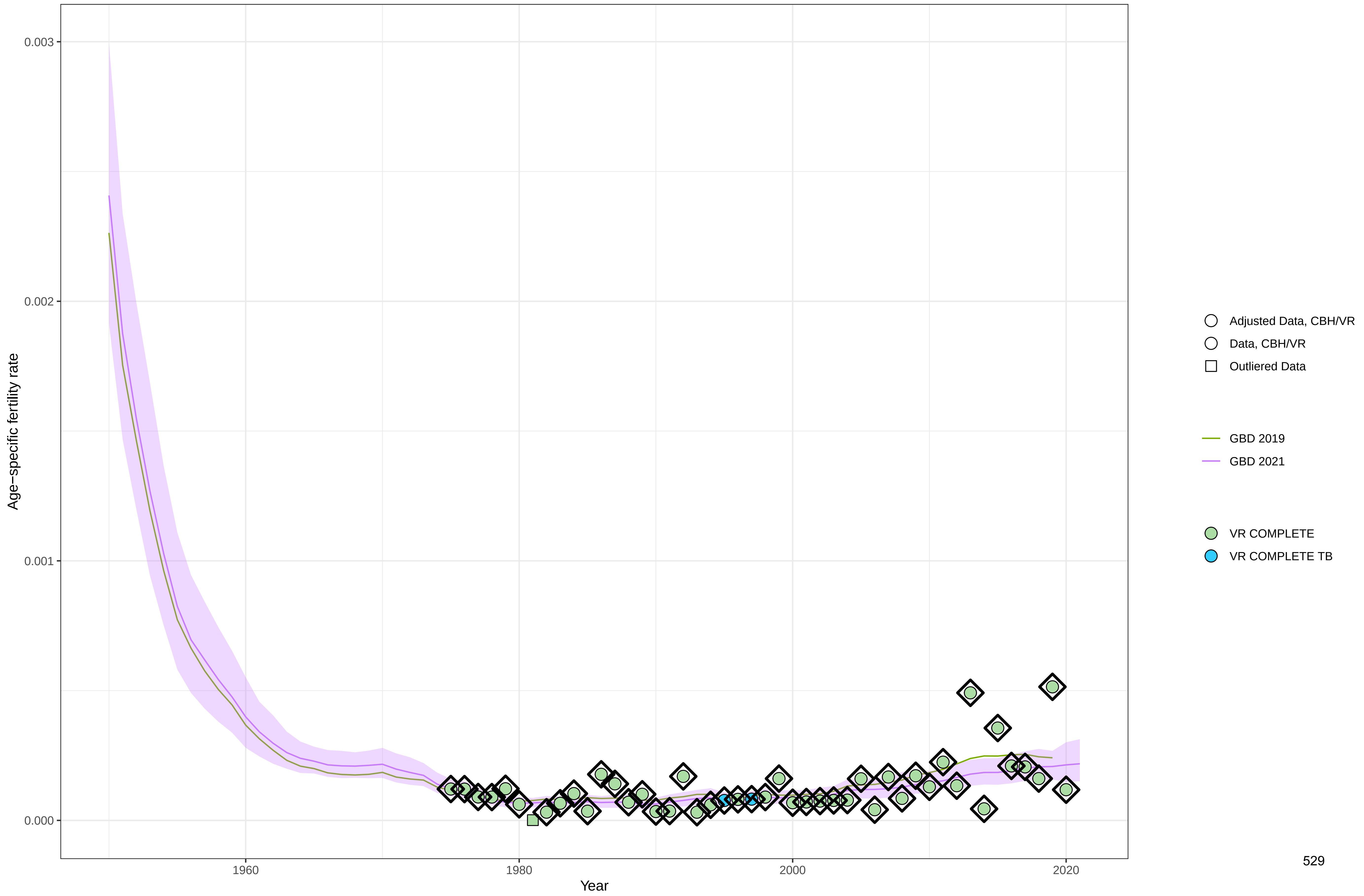

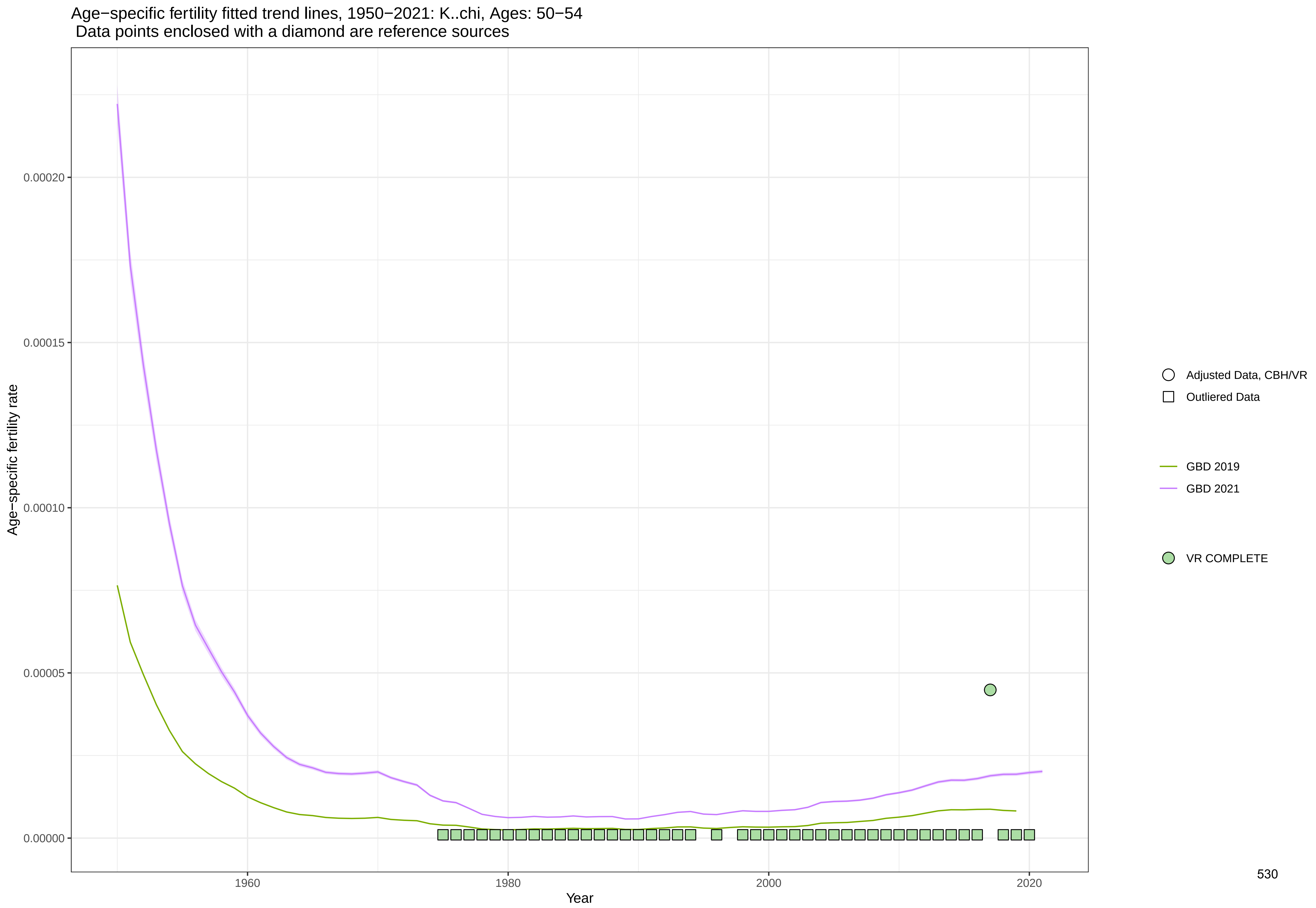
Age−specific fertility fitted trend lines, 1950−2021: K..chi, Ages: 50−54
Data points enclosed with a diamond are reference sources
Age−specific fertility rate
0.00020
0.00015
0.00010
0.00005
0.00000
1960
1980
2000
2020
Year
Adjusted Data, CBH/VR
Outliered Data
GBD 2019
GBD 2021
VR COMPLETE

Total fertility rate fitted trend lines, 1950−2021: K..chi TFR
Data points enclosed with a diamond are reference sources

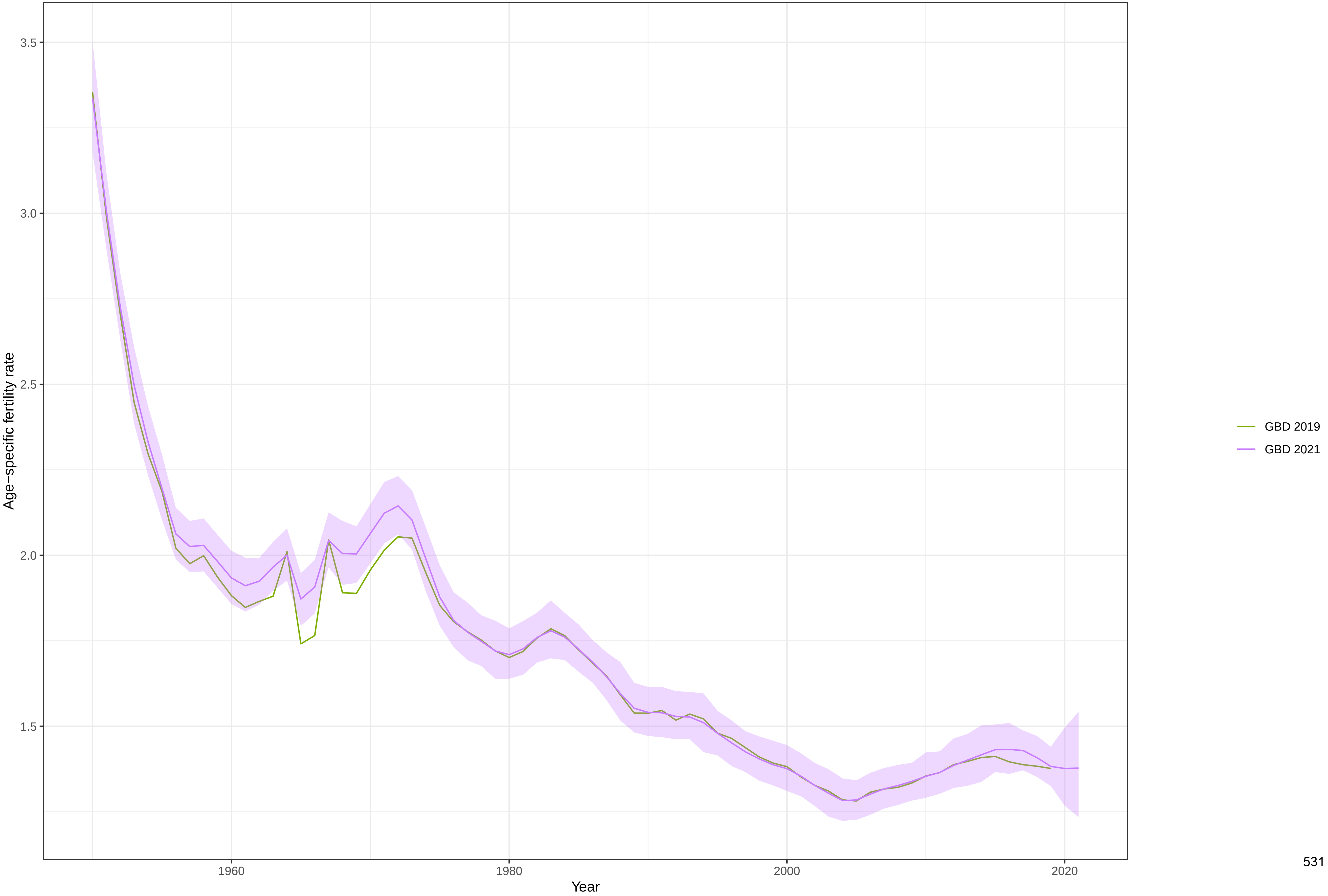

Age−specific fertility fitted trend lines, 1950−2021: Kumamoto, Ages: 10−14
Data points enclosed with a diamond are reference sources

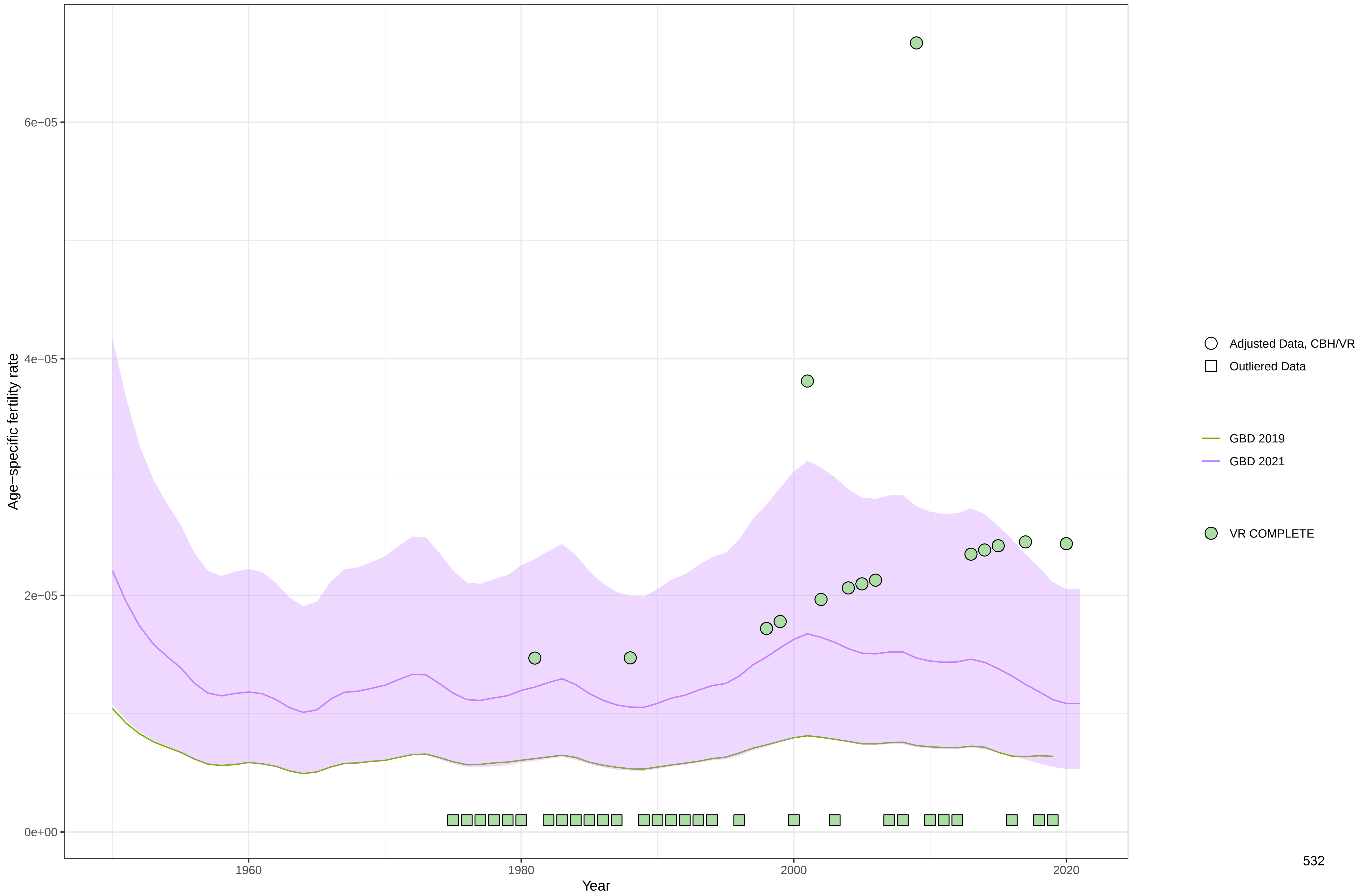

Age−specific fertility fitted trend lines, 1950−2021: Kumamoto, Ages: 15−19
Data points enclosed with a diamond are reference sources

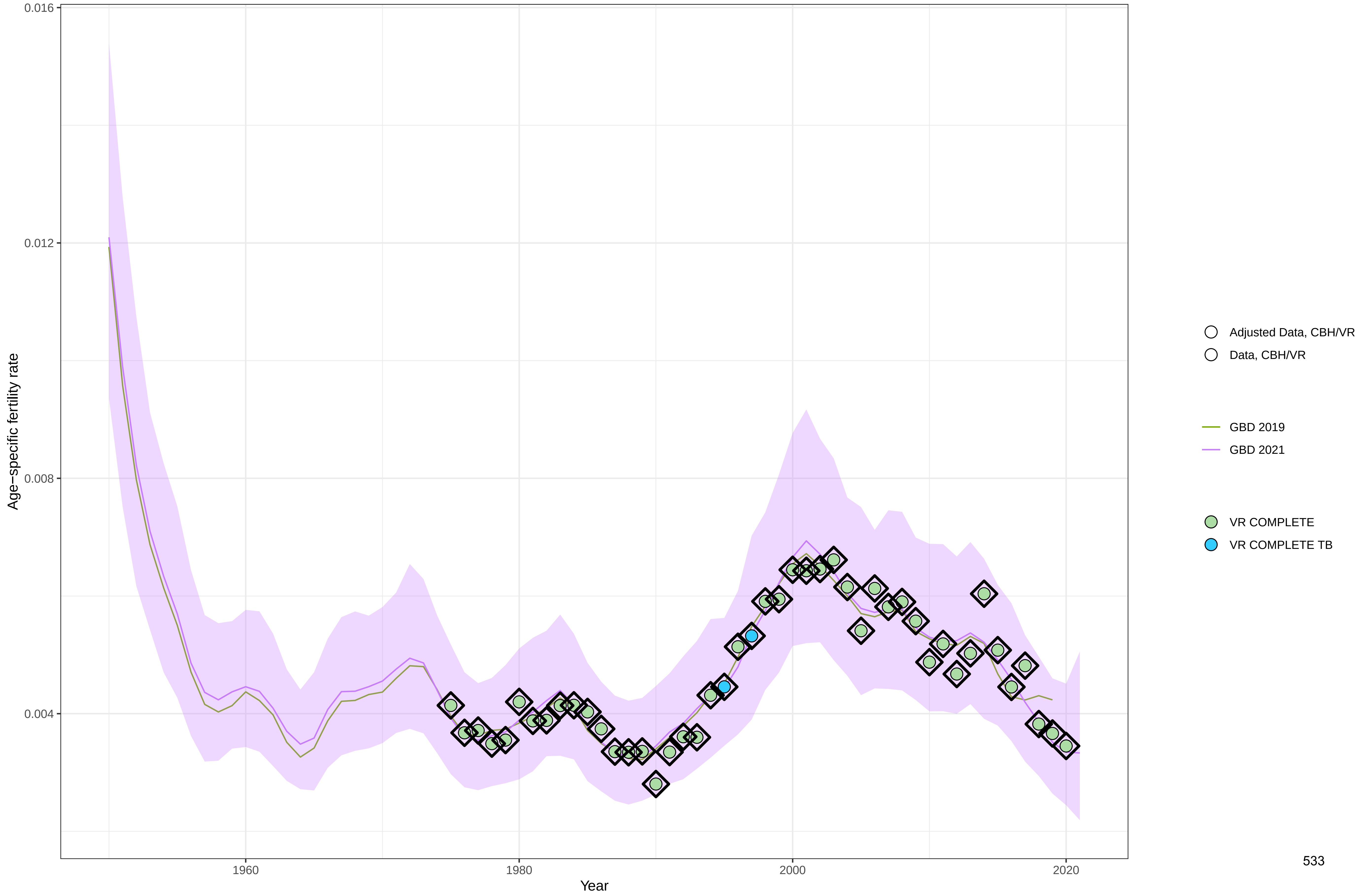

Age−specific fertility fitted trend lines, 1950−2021: Kumamoto, Ages: 20−24
Data points enclosed with a diamond are reference sources

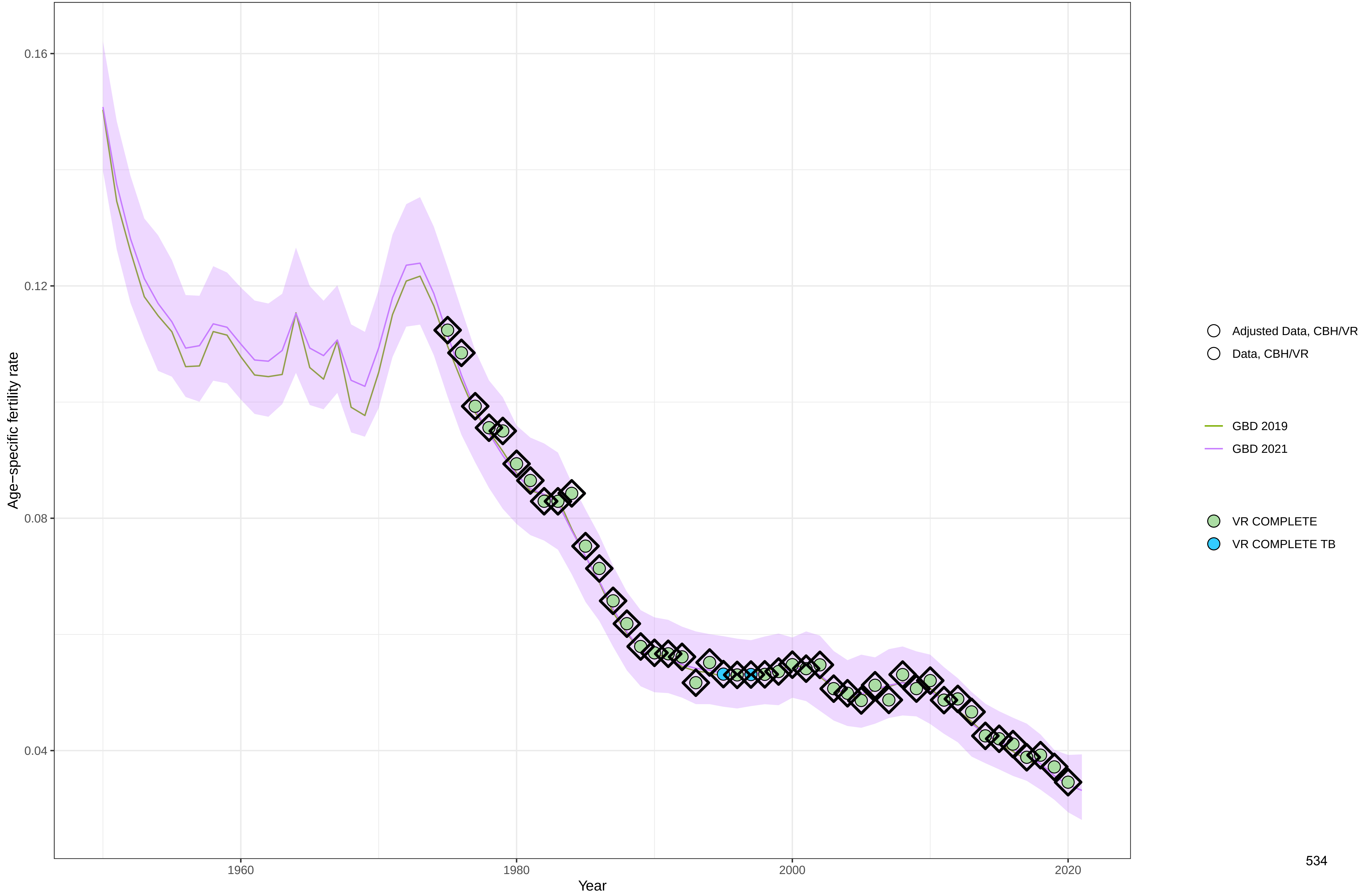

Age−specific fertility fitted trend lines, 1950−2021: Kumamoto, Ages: 25−29
Data points enclosed with a diamond are reference sources

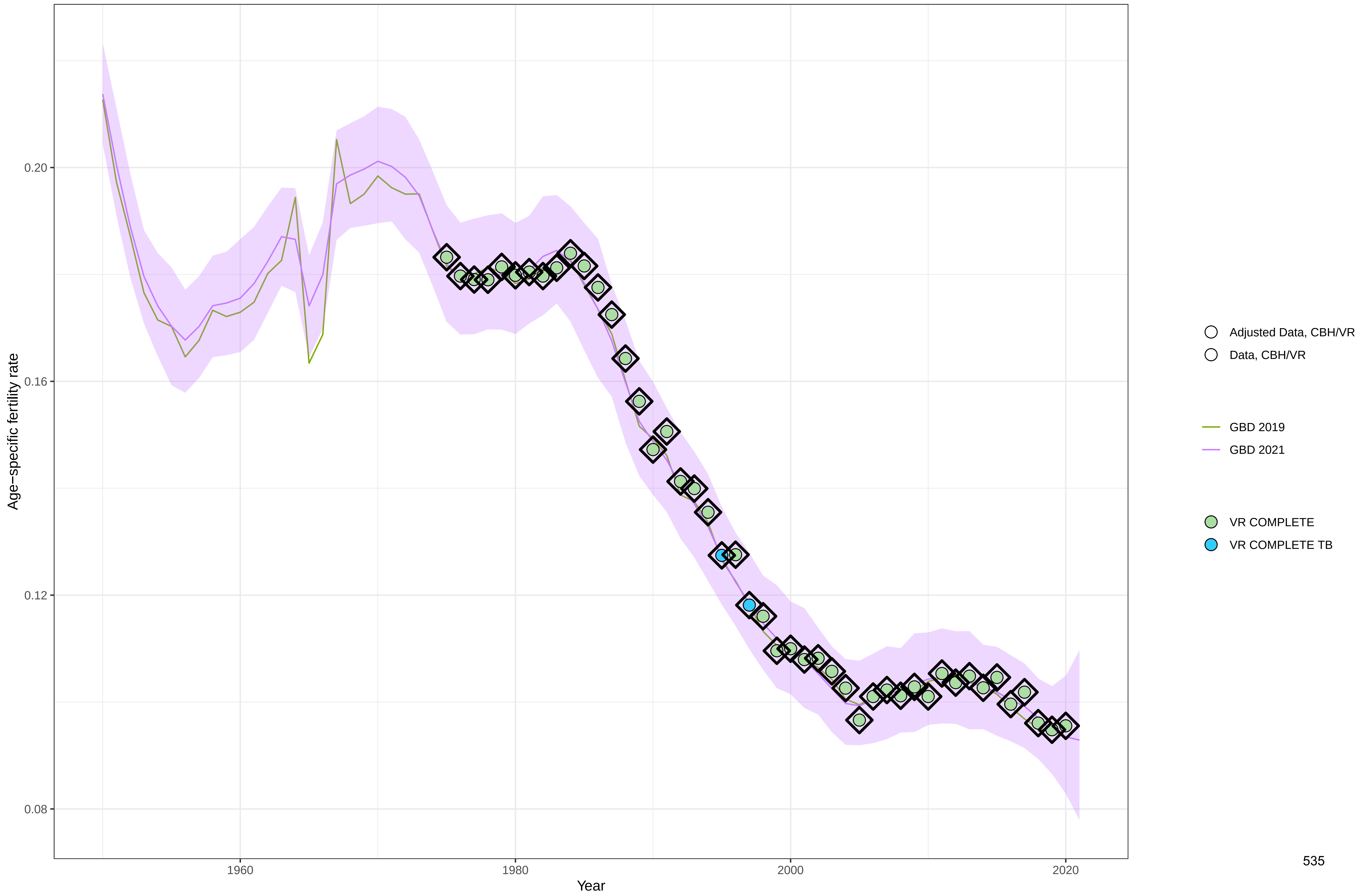

Age−specific fertility fitted trend lines, 1950−2021: Kumamoto, Ages: 30−34
Data points enclosed with a diamond are reference sources

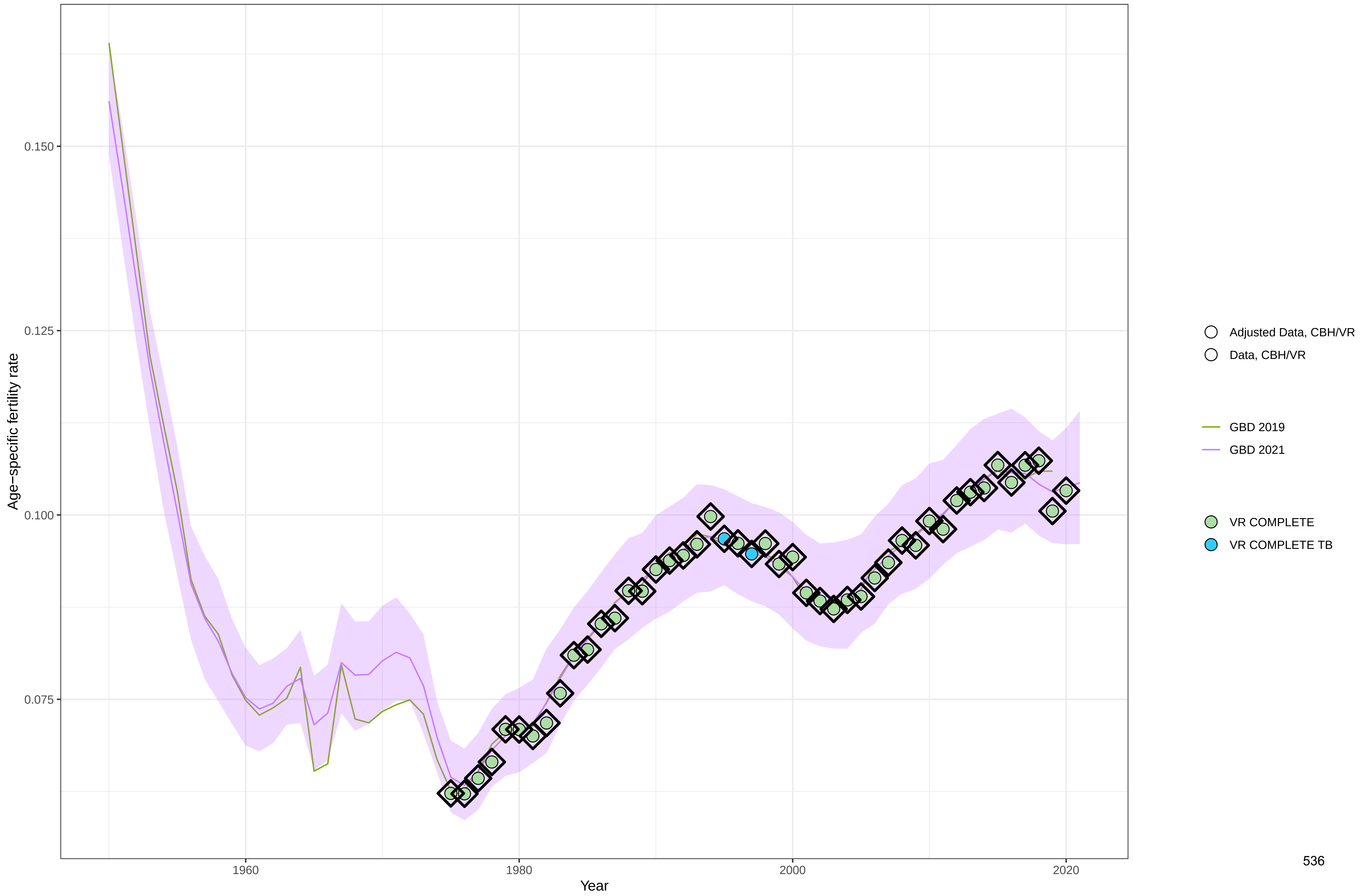

Age−specific fertility fitted trend lines, 1950−2021: Kumamoto, Ages: 35−39
Data points enclosed with a diamond are reference sources

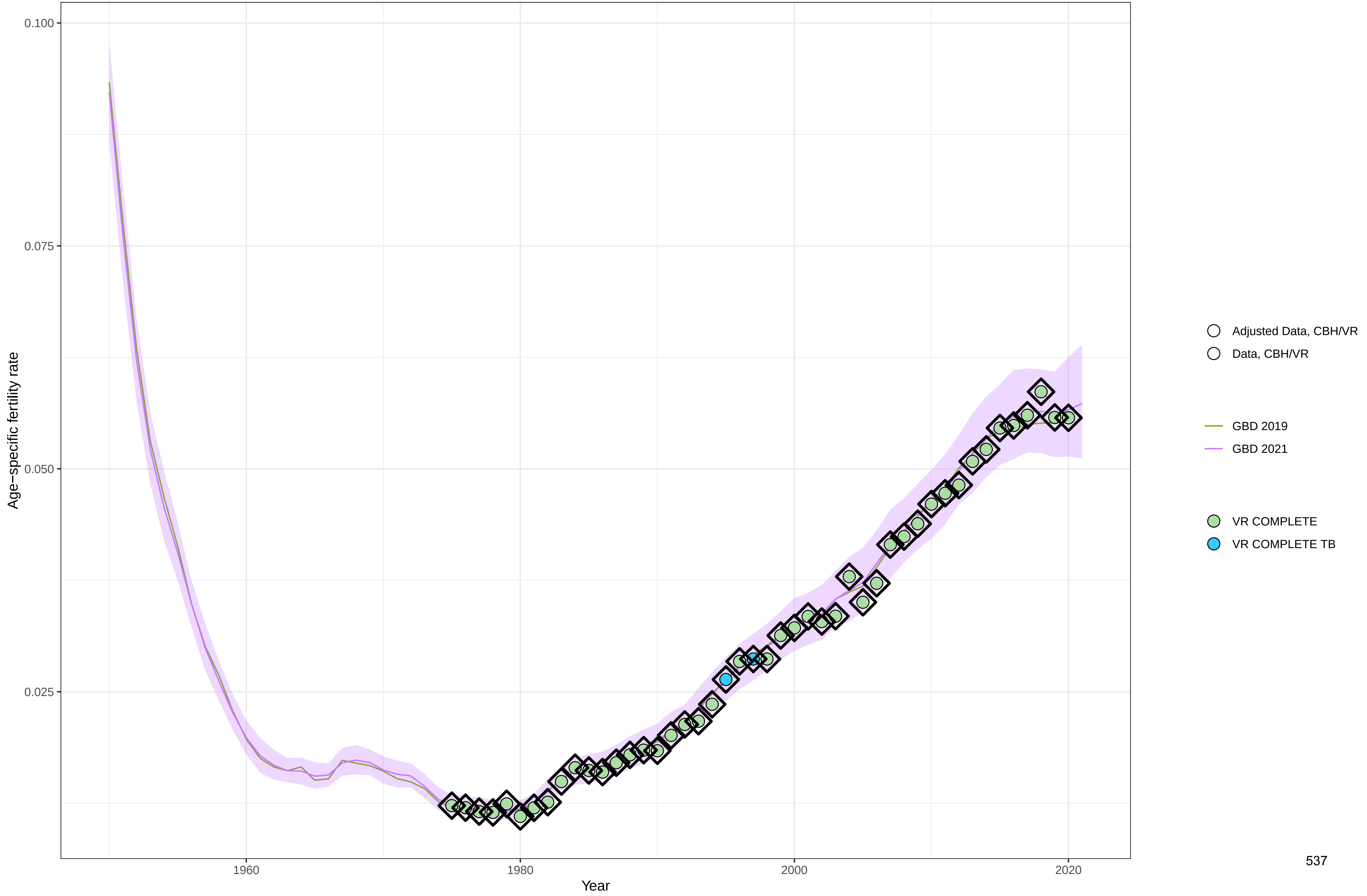

Age−specific fertility fitted trend lines, 1950−2021: Kumamoto, Ages: 40−44
Data points enclosed with a diamond are reference sources

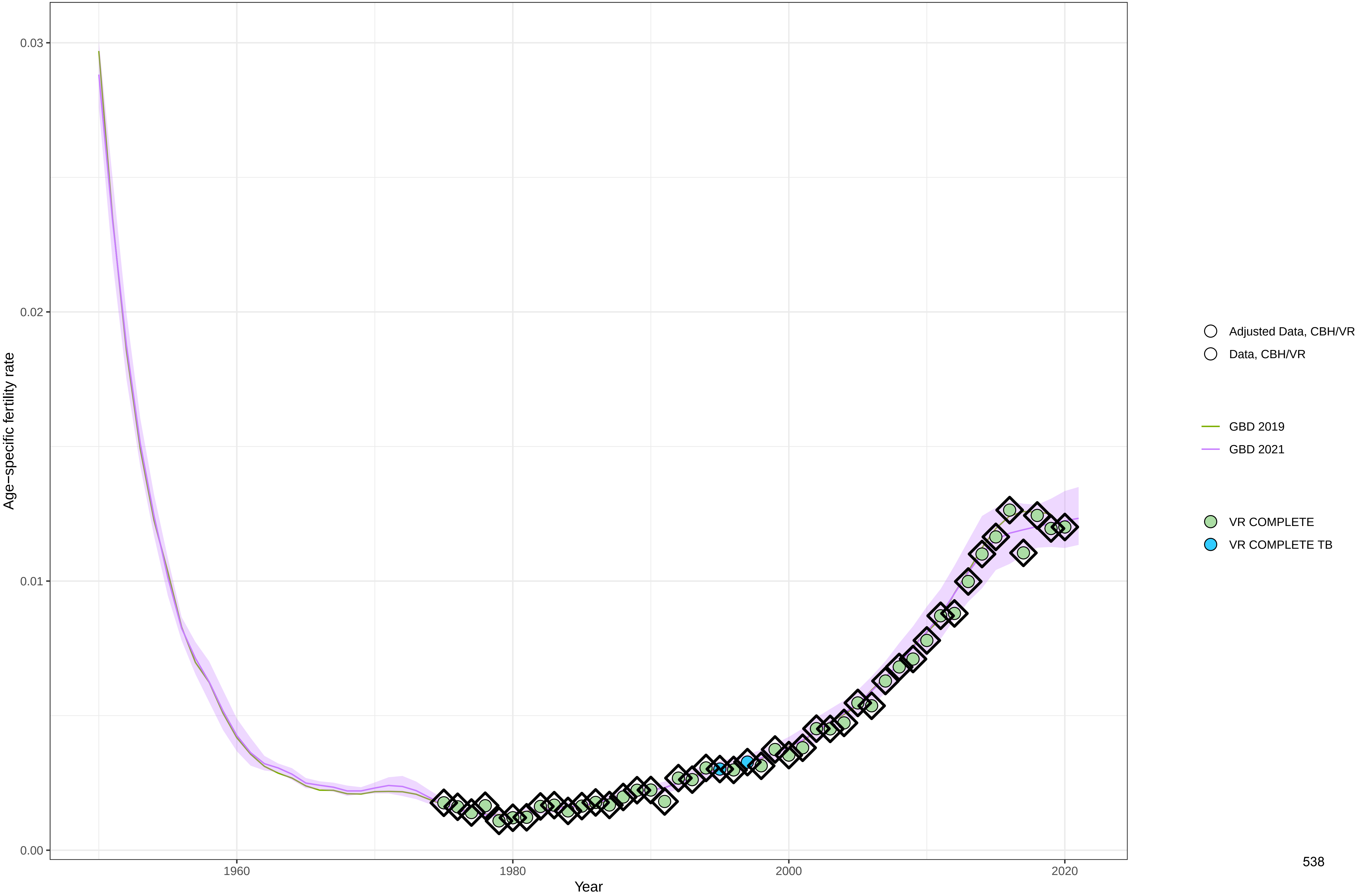

Age−specific fertility fitted trend lines, 1950−2021: Kumamoto, Ages: 45−49
Data points enclosed with a diamond are reference sources

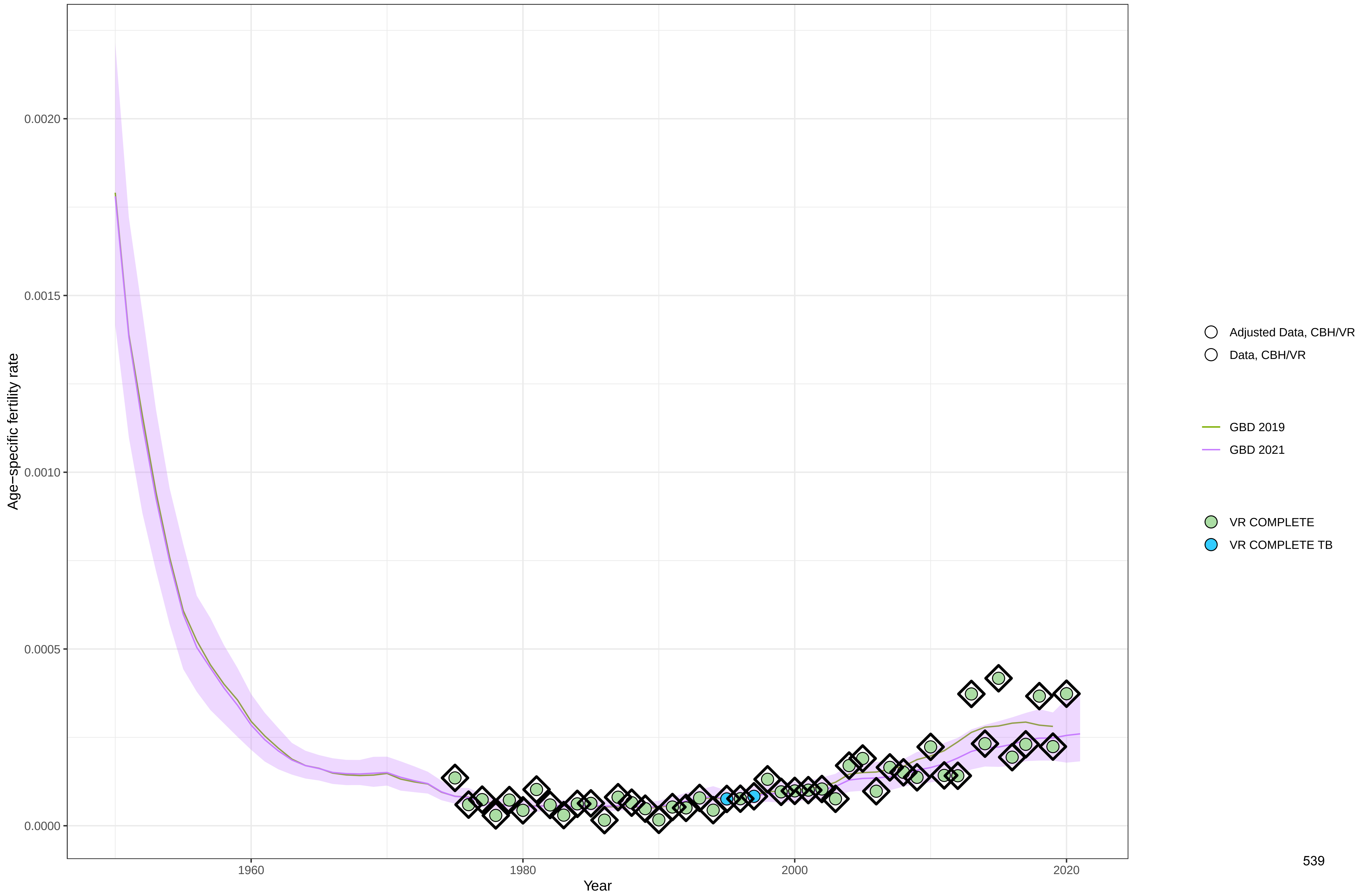

Age−specific fertility fitted trend lines, 1950−2021: Kumamoto, Ages: 50−54
Data points enclosed with a diamond are reference sources

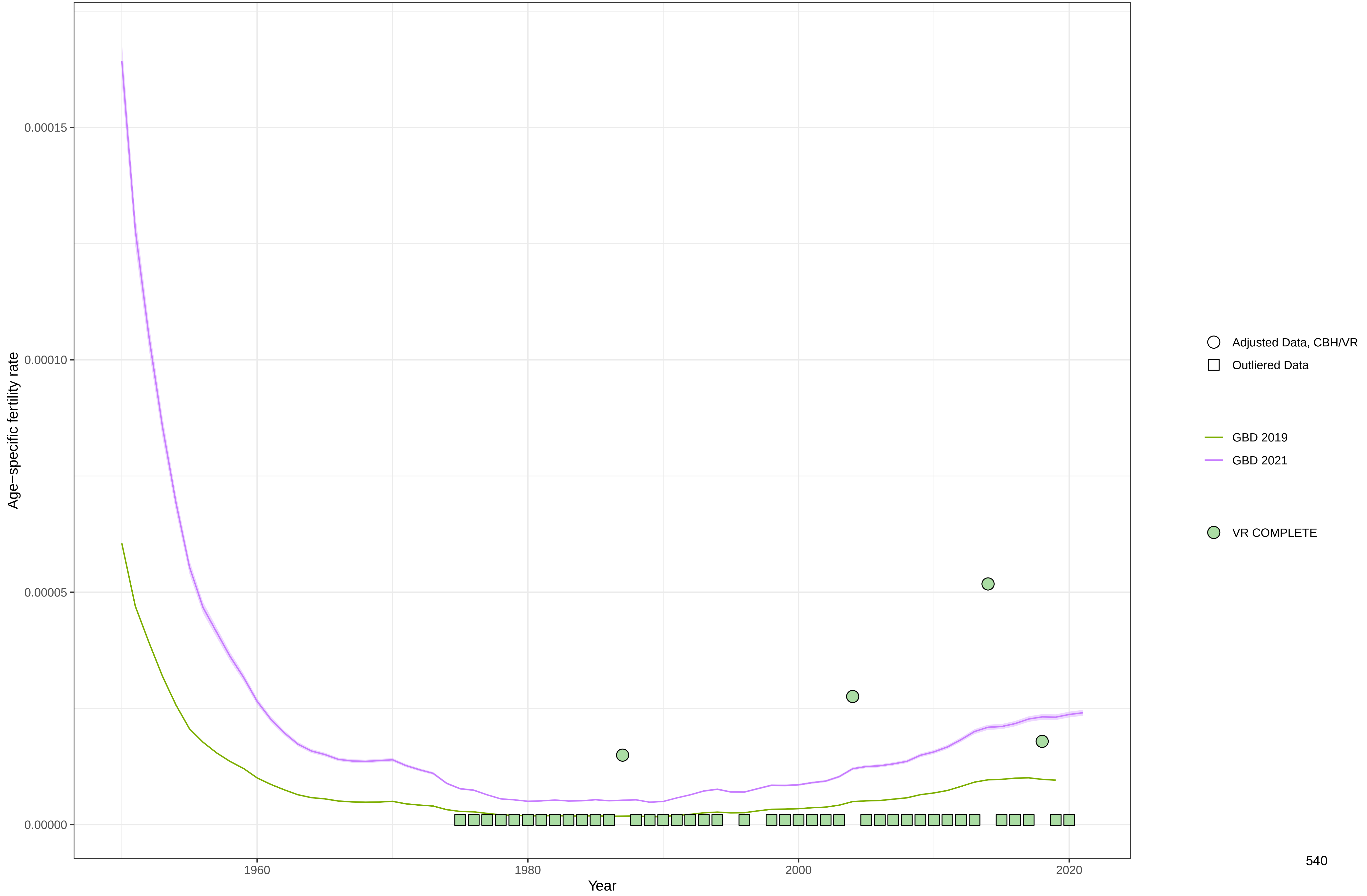

Total fertility rate fitted trend lines, 1950−2021: Kumamoto TFR
Data points enclosed with a diamond are reference sources

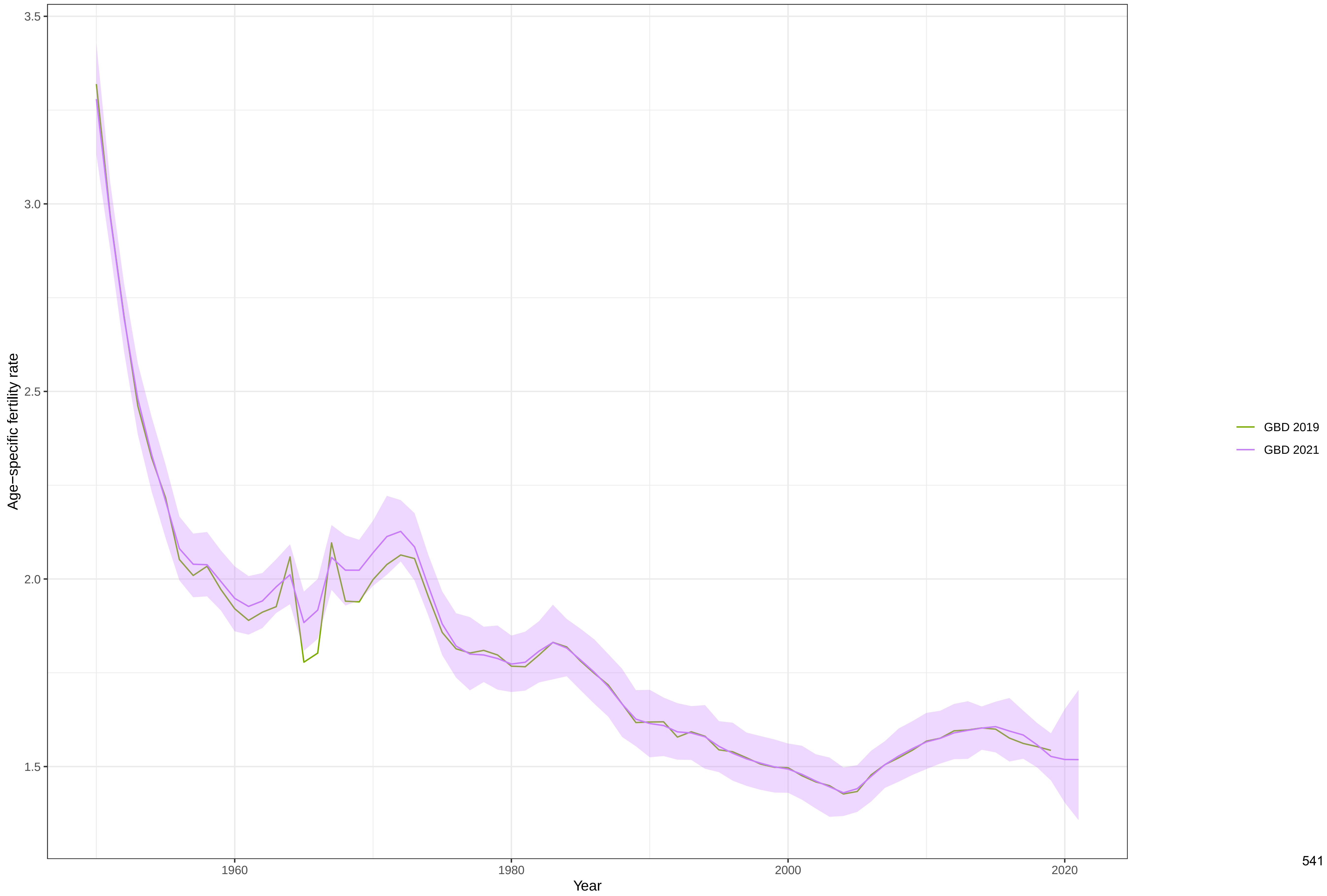

Age−specific fertility fitted trend lines, 1950−2021: Ky..to, Ages: 10−14
Data points enclosed with a diamond are reference sources

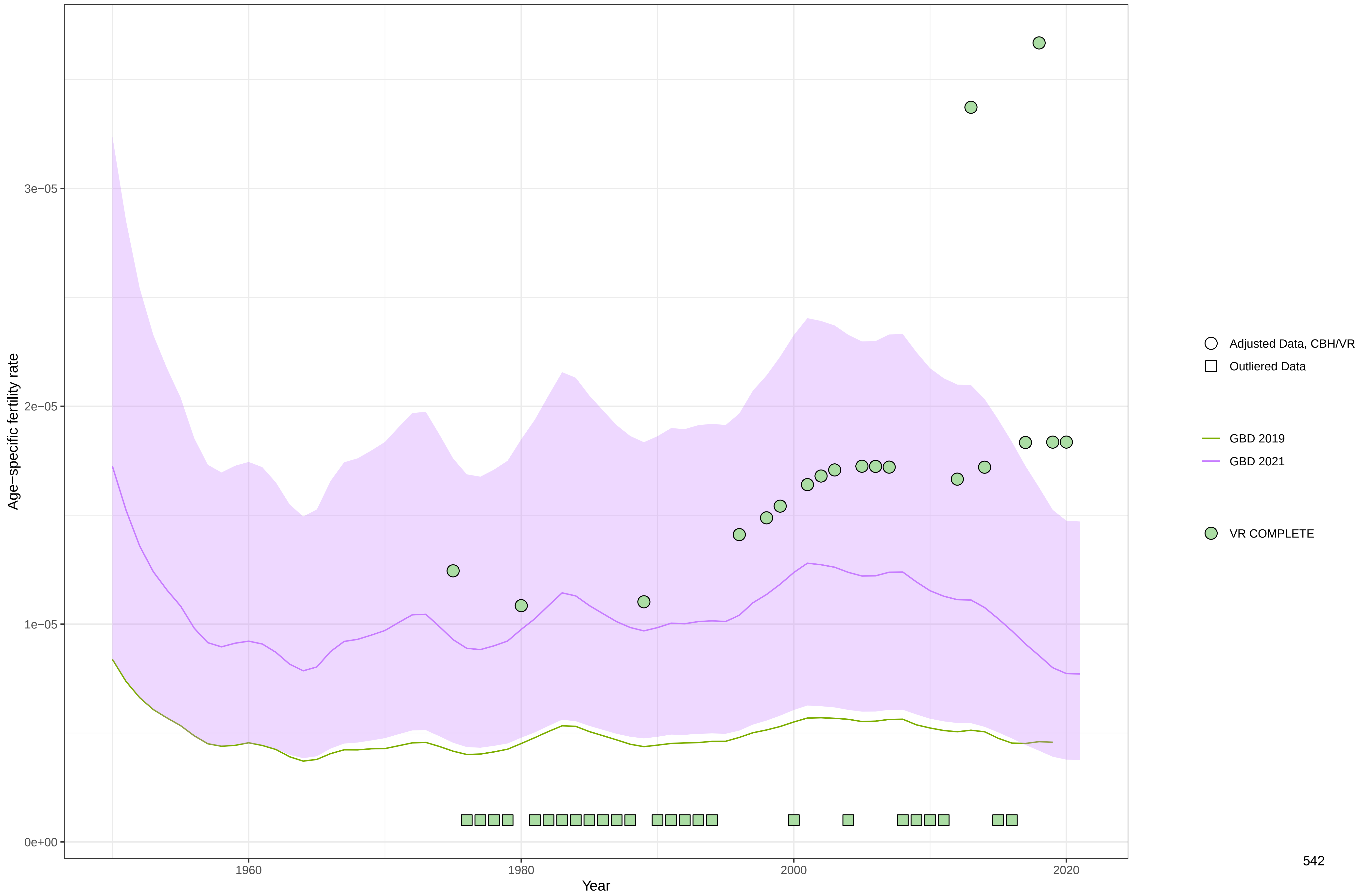

Age−specific fertility fitted trend lines, 1950−2021: Ky..to, Ages: 15−19
Data points enclosed with a diamond are reference sources

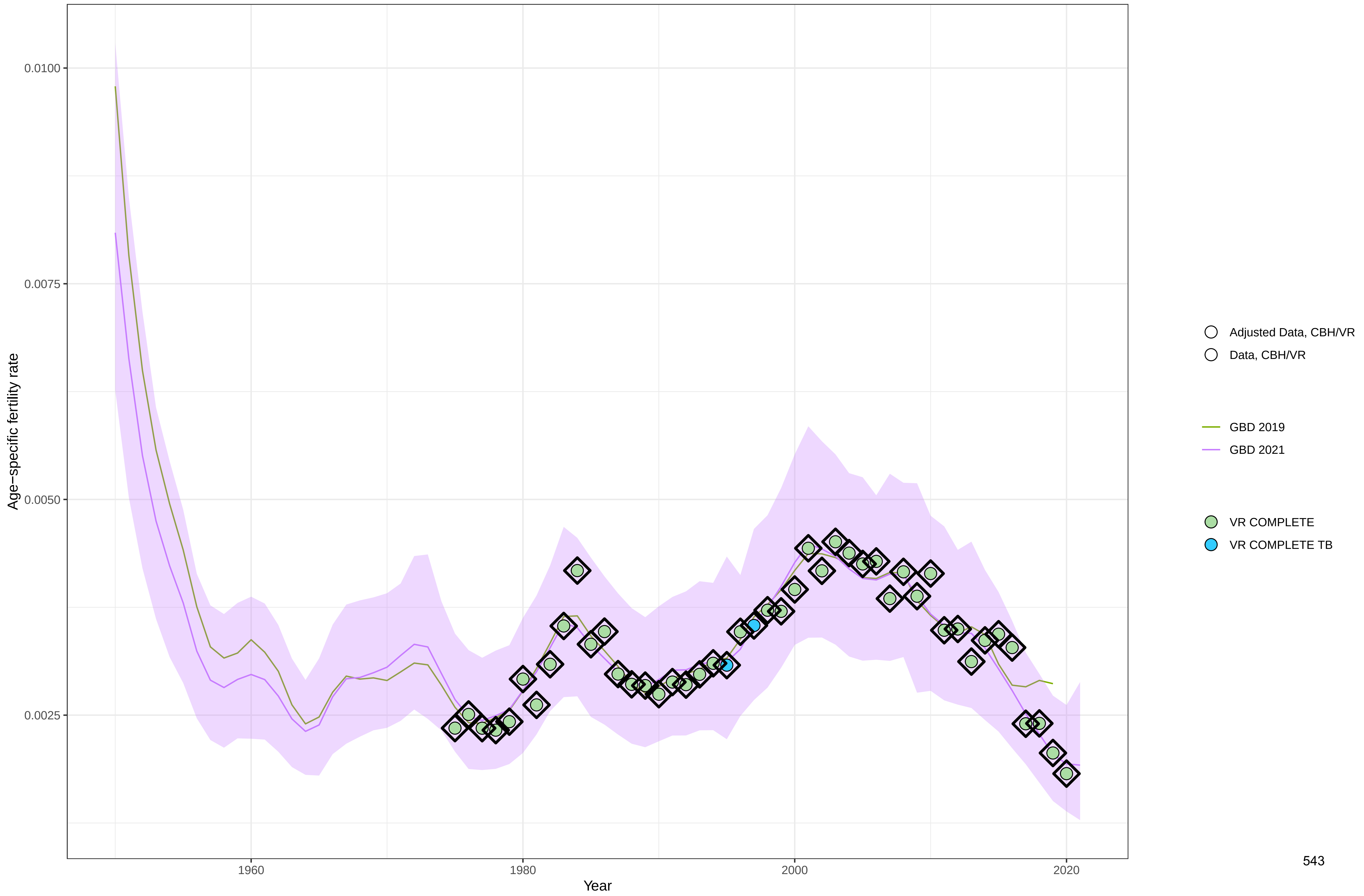

Age−specific fertility fitted trend lines, 1950−2021: Ky..to, Ages: 20−24
Data points enclosed with a diamond are reference sources

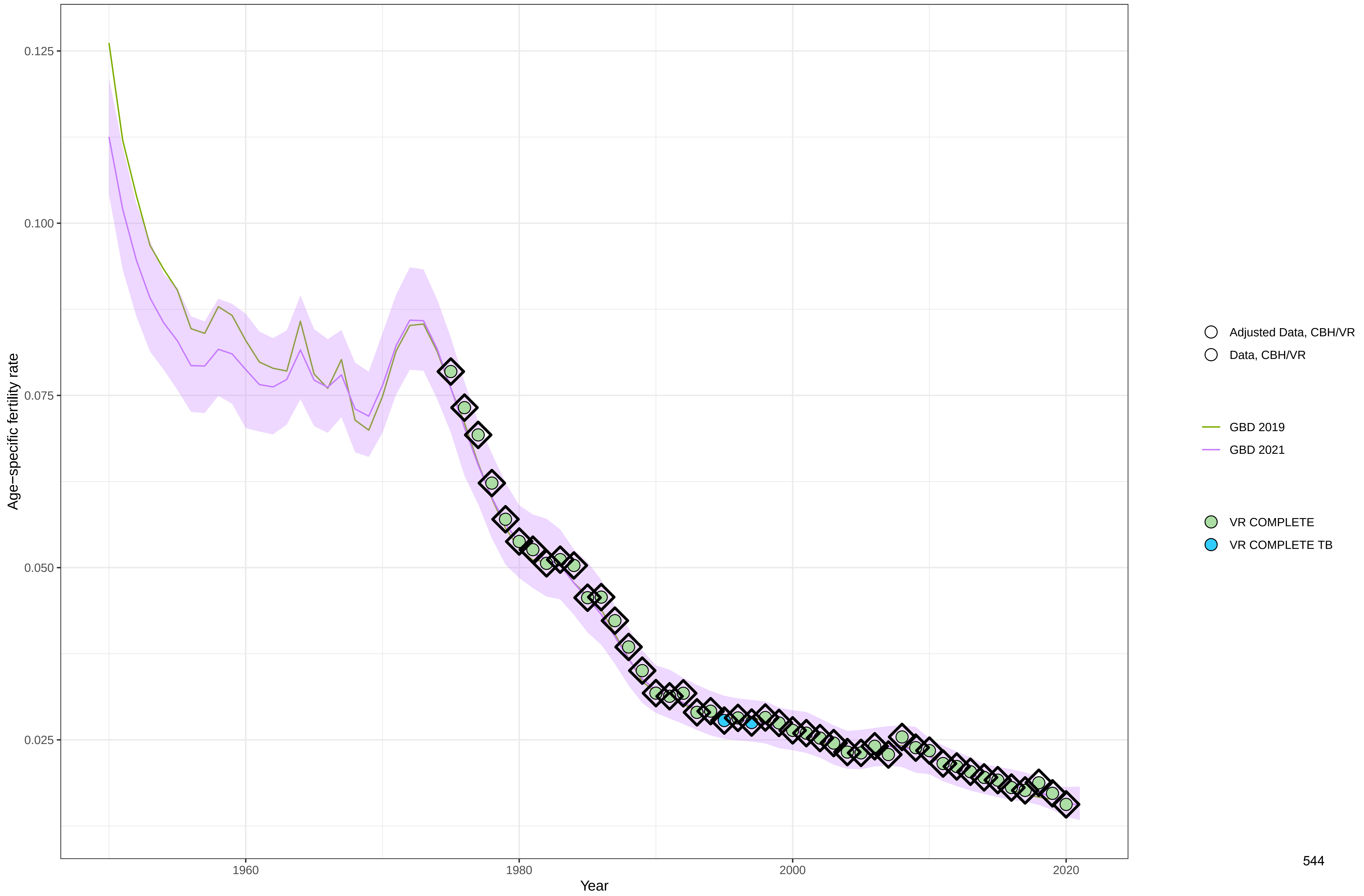

Age−specific fertility fitted trend lines, 1950−2021: Ky..to, Ages: 25−29
Data points enclosed with a diamond are reference sources

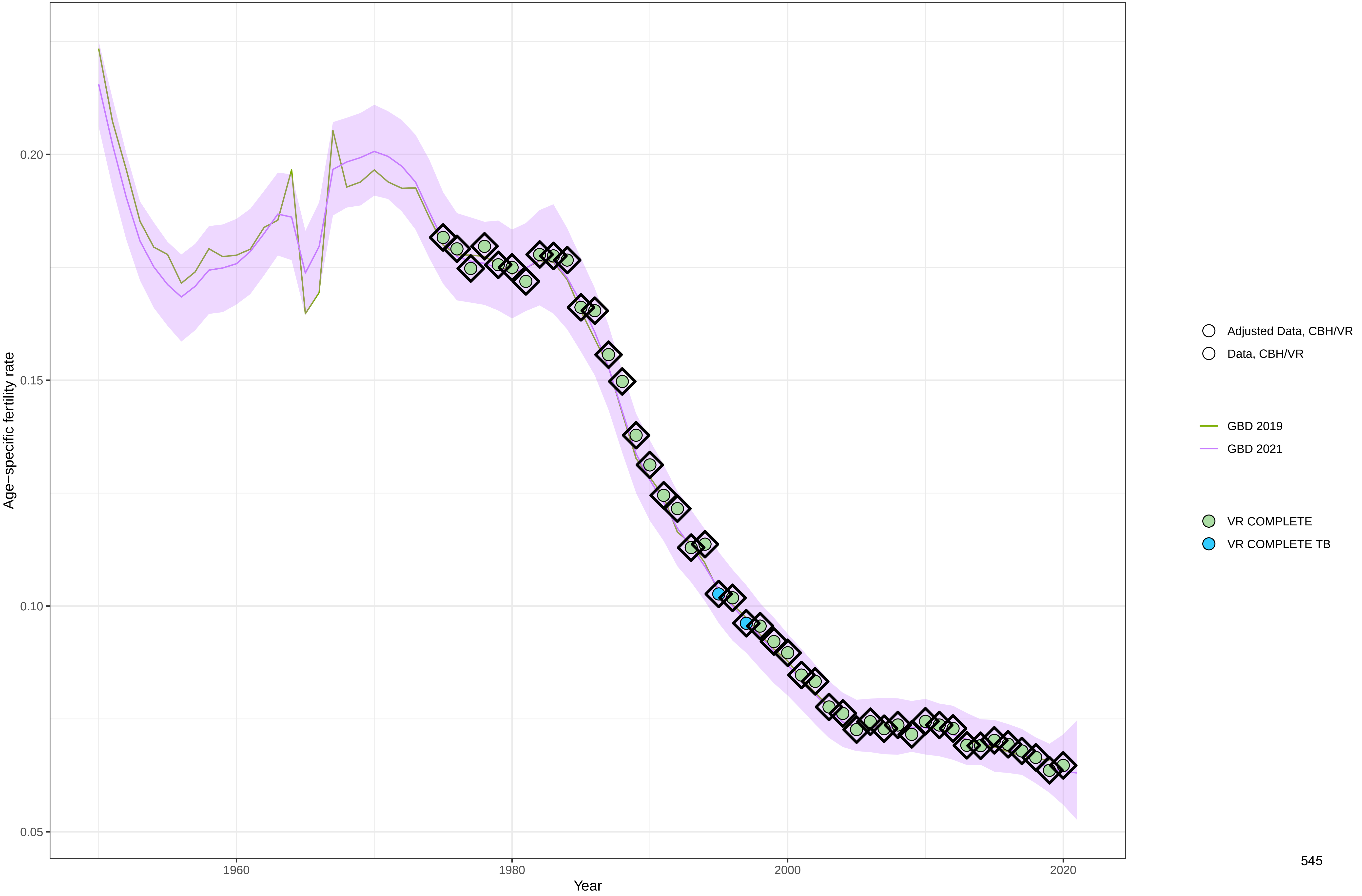

Age−specific fertility fitted trend lines, 1950−2021: Ky..to, Ages: 30−34
Data points enclosed with a diamond are reference sources

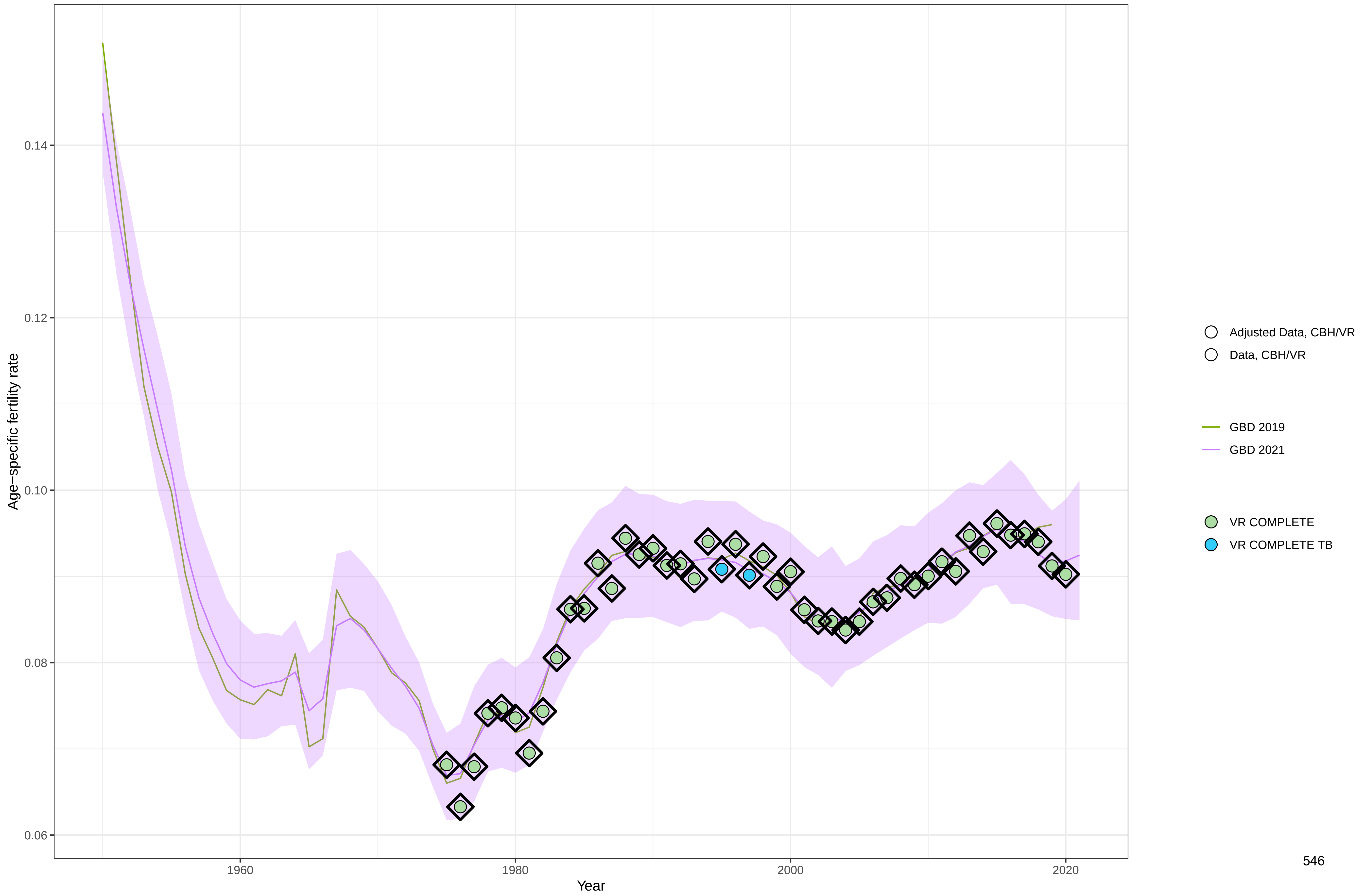

Age−specific fertility fitted trend lines, 1950−2021: Ky..to, Ages: 35−39
Data points enclosed with a diamond are reference sources

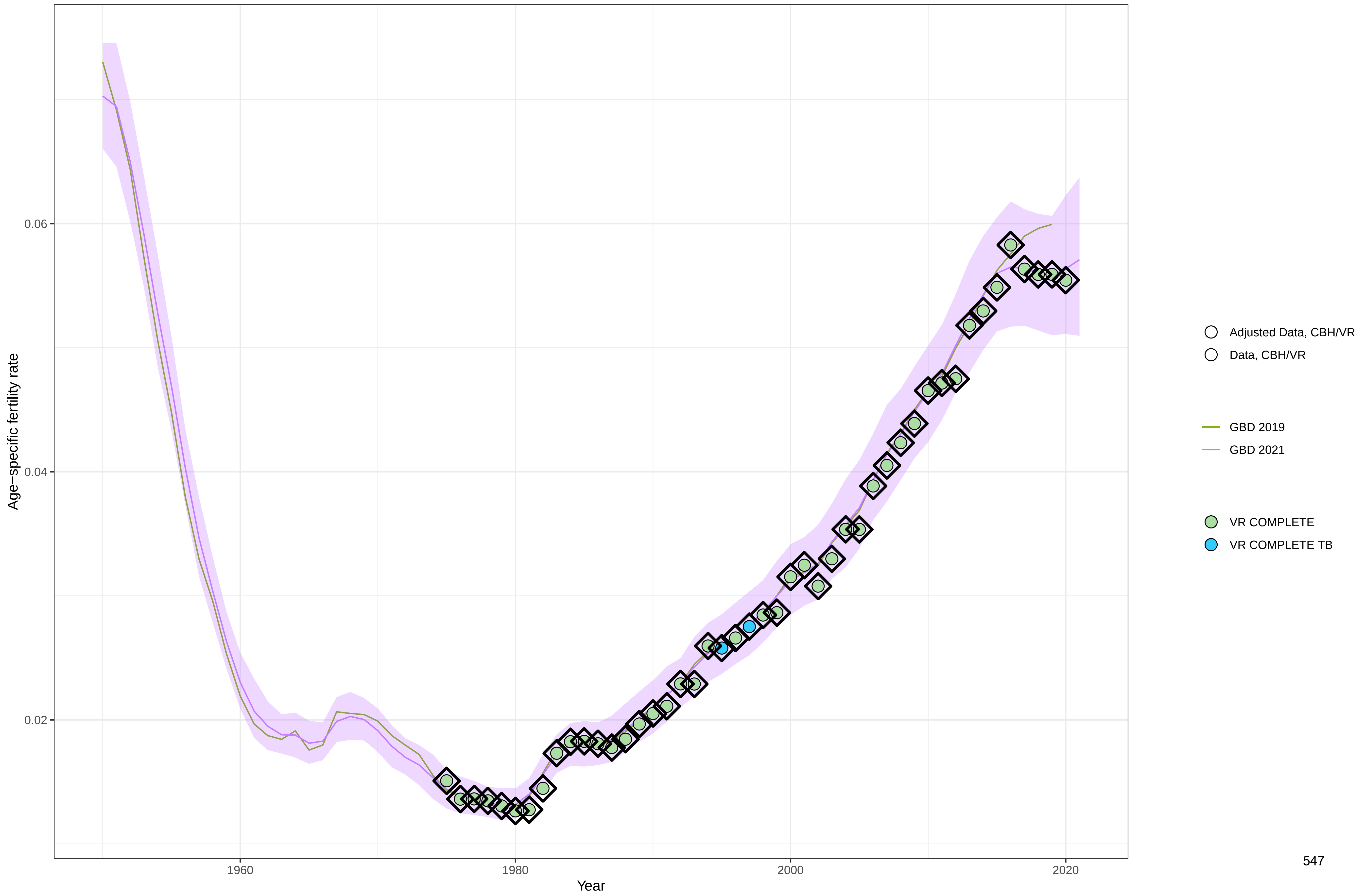

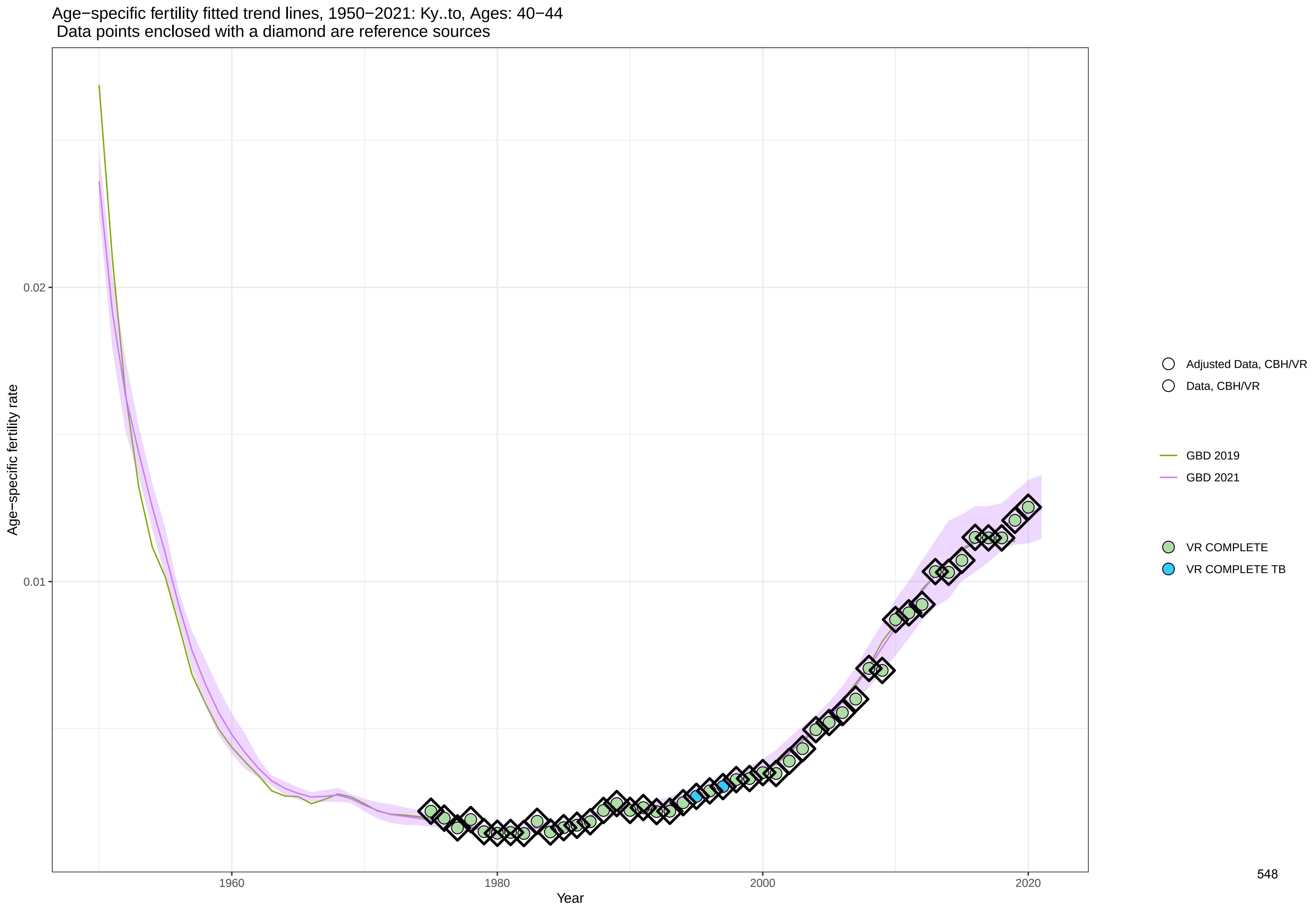

Age−specific fertility fitted trend lines, 1950−2021: Ky..to, Ages: 40−44
Data points enclosed with a diamond are reference sources
Age−specific fertility rate
0.02
0.01
1960
1980
2000
2020
Year
Adjusted Data, CBH/VR
Data, CBH/VR
GBD 2019
GBD 2021
VR COMPLETE
VR COMPLETE TB

Age−specific fertility fitted trend lines, 1950−2021: Ky..to, Ages: 45−49
Data points enclosed with a diamond are reference sources

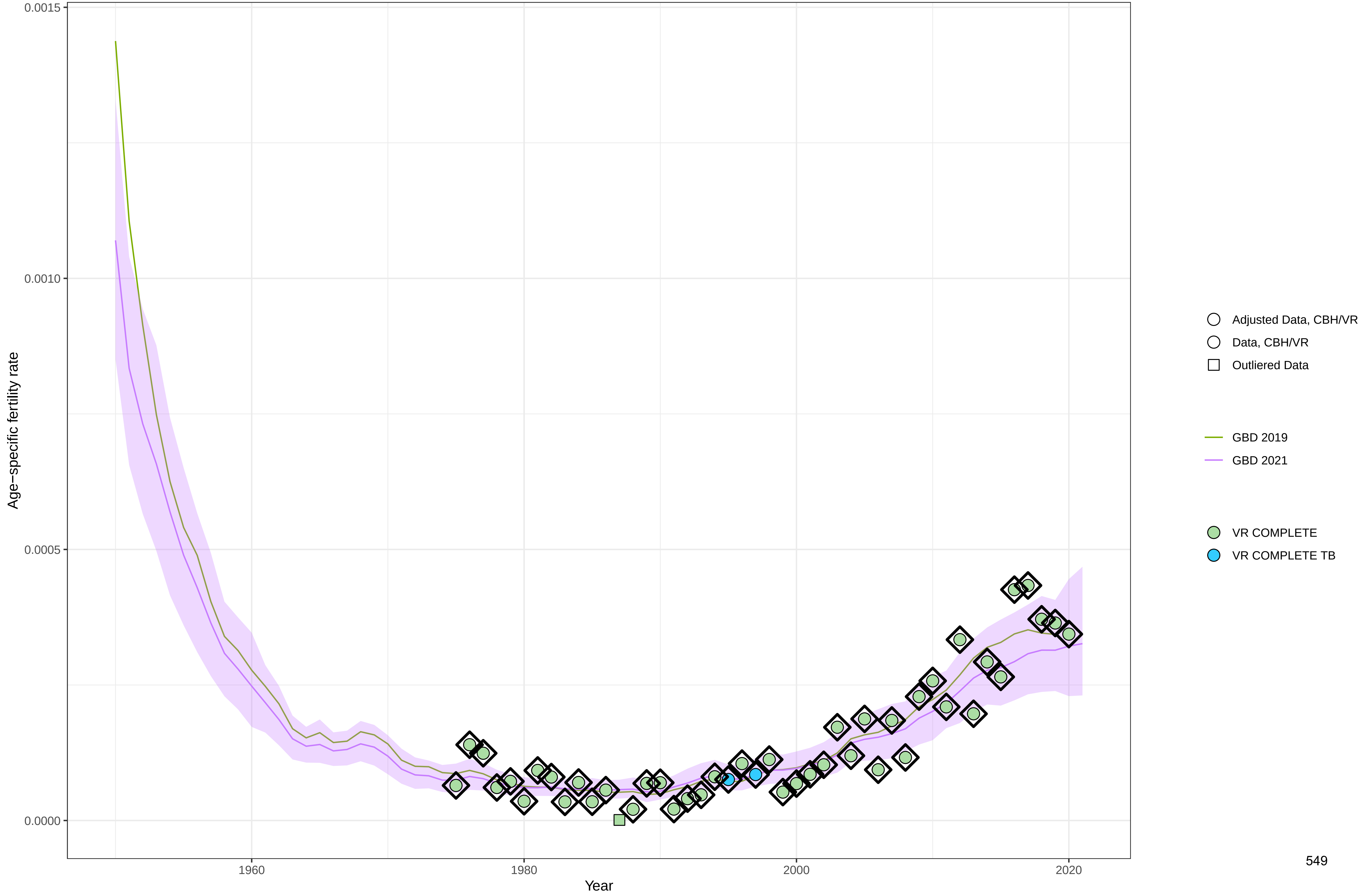

Age−specific fertility fitted trend lines, 1950−2021: Ky..to, Ages: 50−54
Data points enclosed with a diamond are reference sources

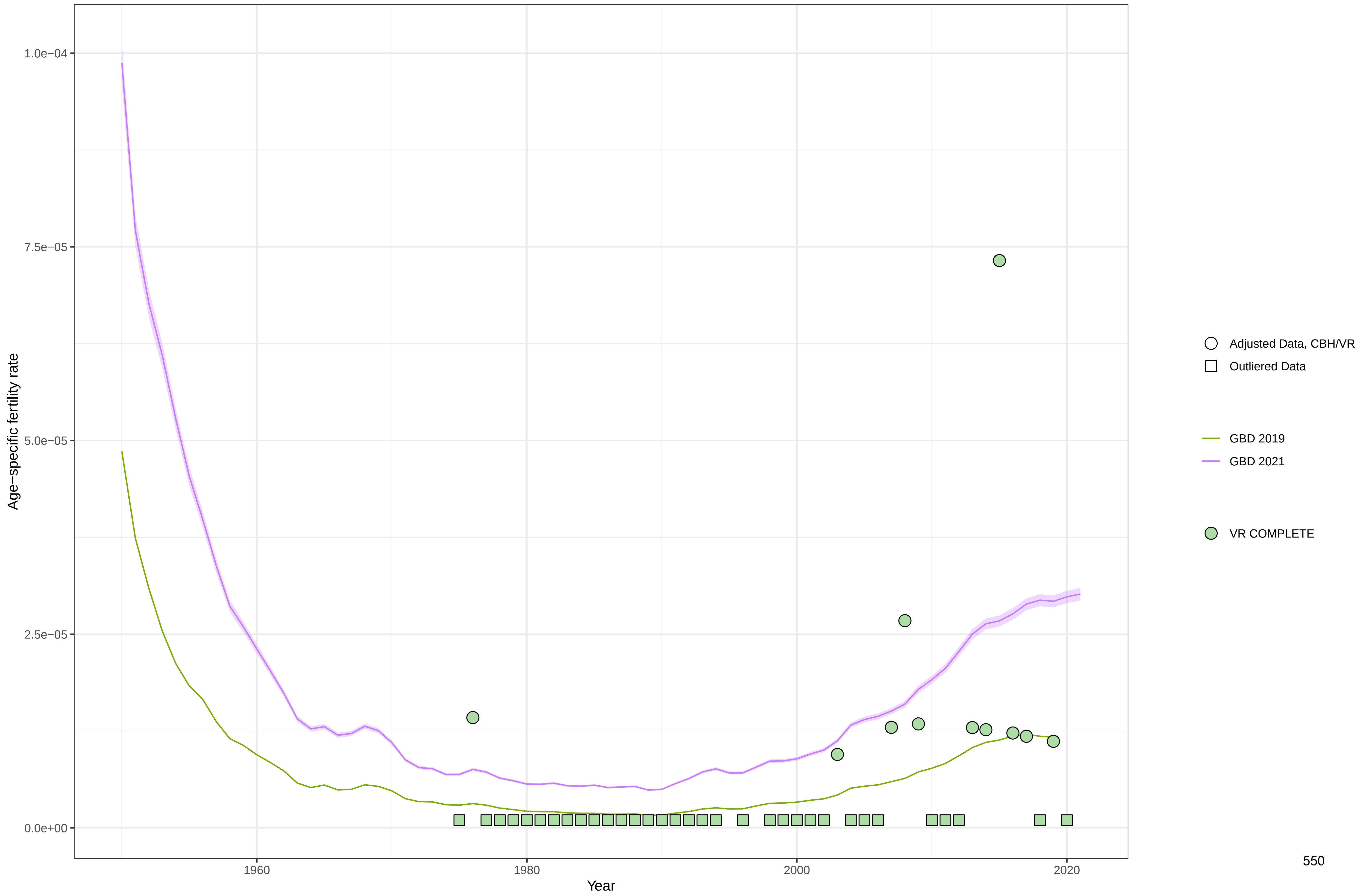

Total fertility rate fitted trend lines, 1950−2021: Ky..to TFR
Data points enclosed with a diamond are reference sources

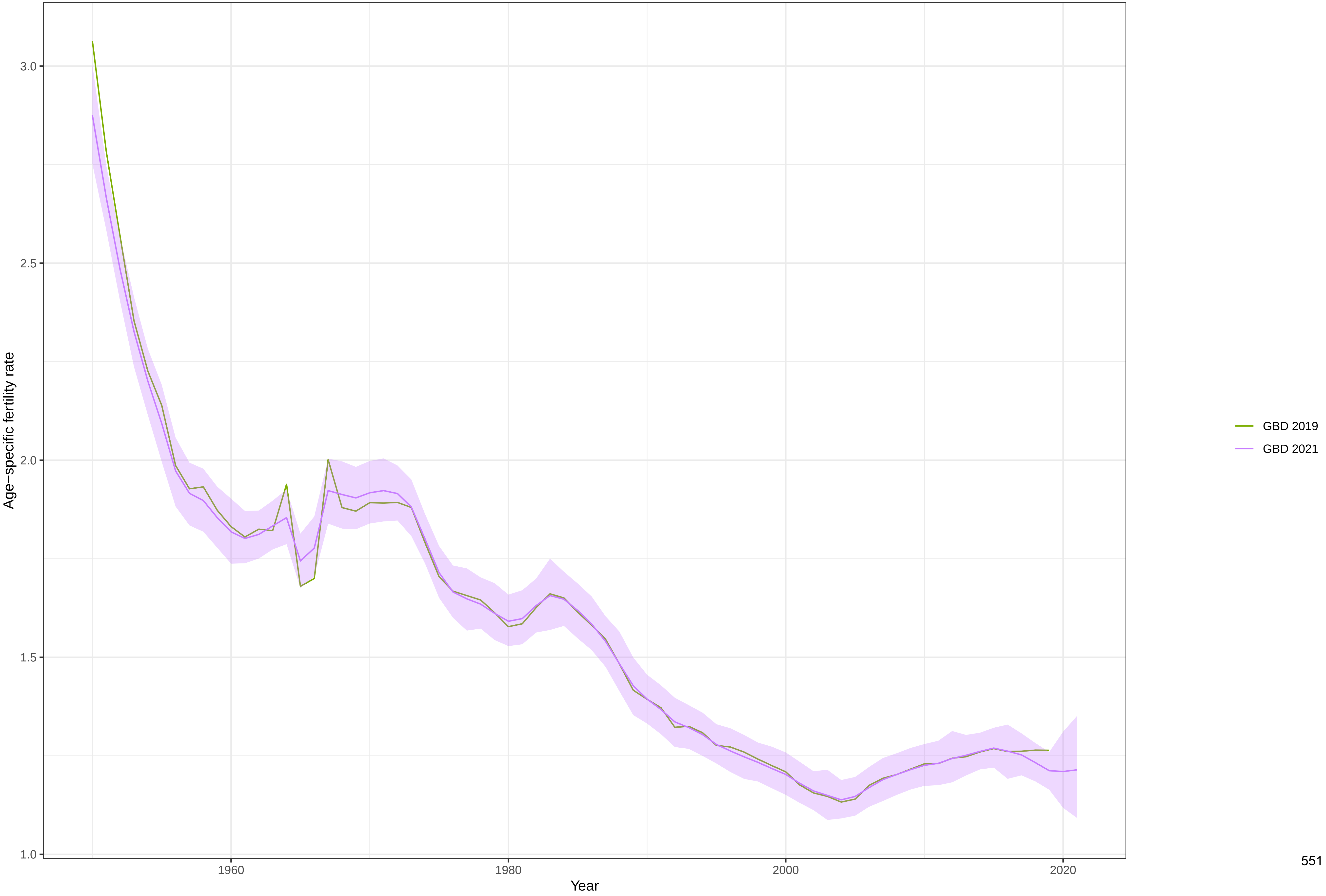

Age−specific fertility fitted trend lines, 1950−2021: Mie, Ages: 10−14
Data points enclosed with a diamond are reference sources

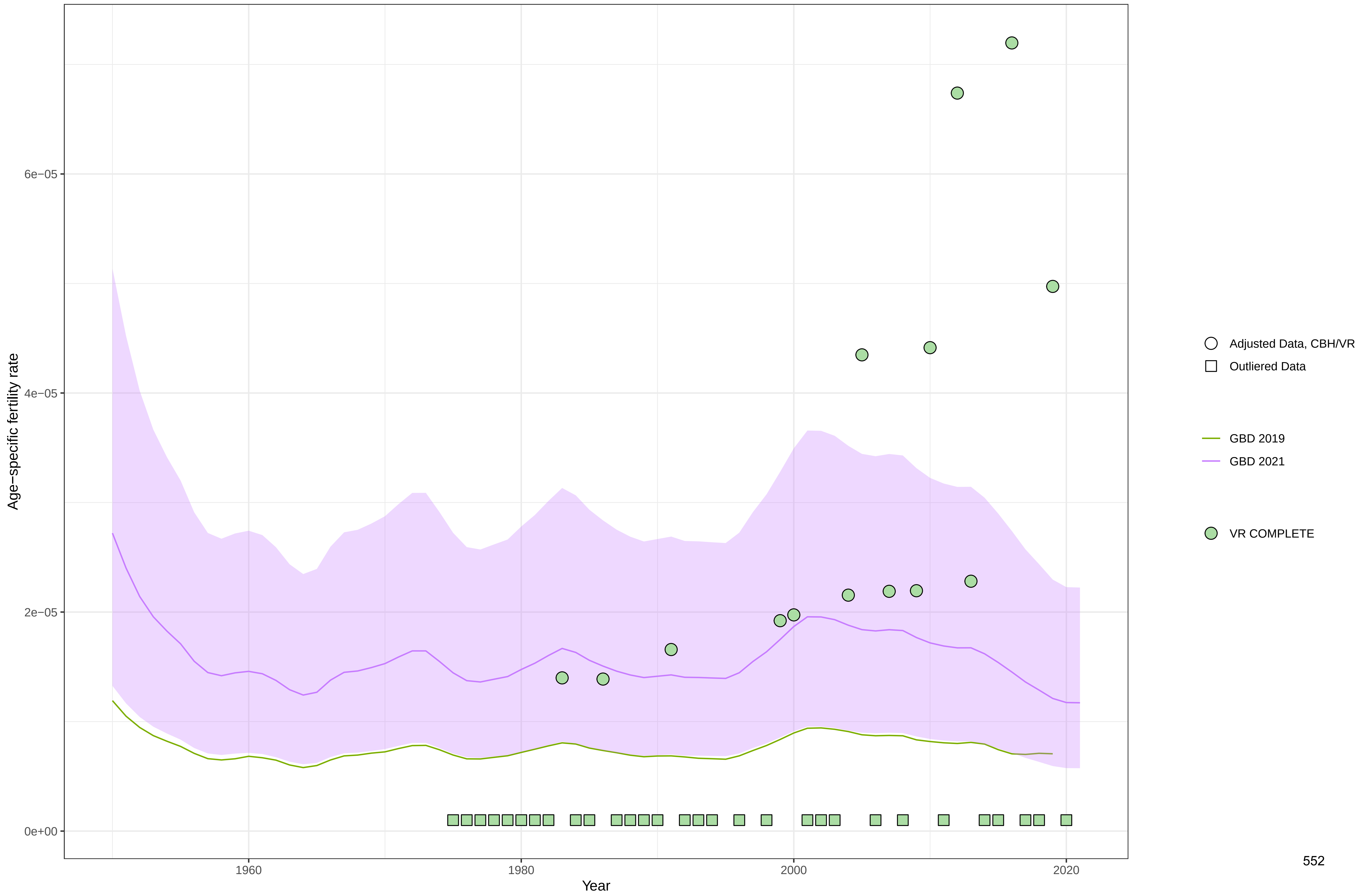

Age−specific fertility fitted trend lines, 1950−2021: Mie, Ages: 15−19
Data points enclosed with a diamond are reference sources

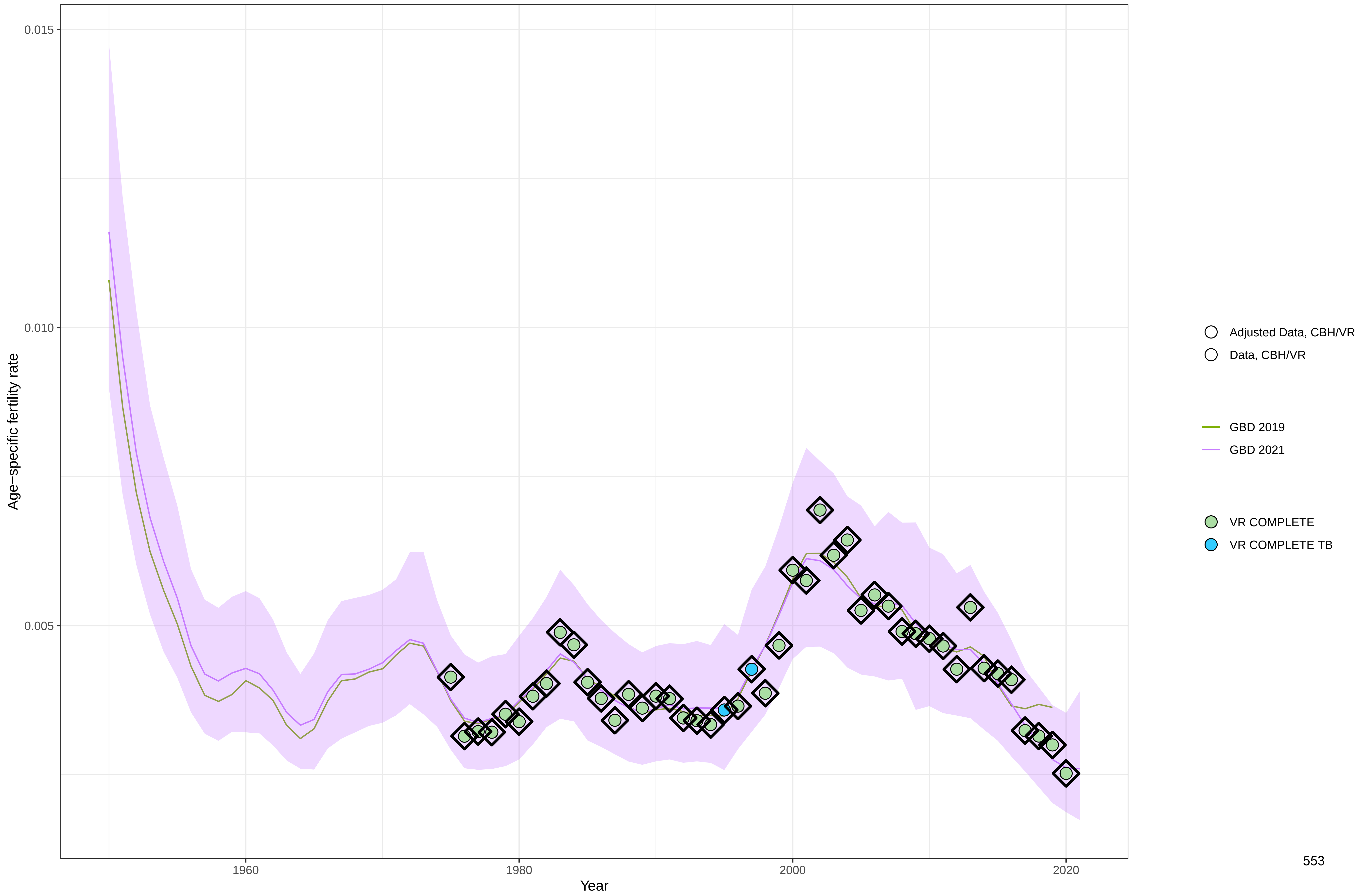

Age−specific fertility fitted trend lines, 1950−2021: Mie, Ages: 20−24
Data points enclosed with a diamond are reference sources

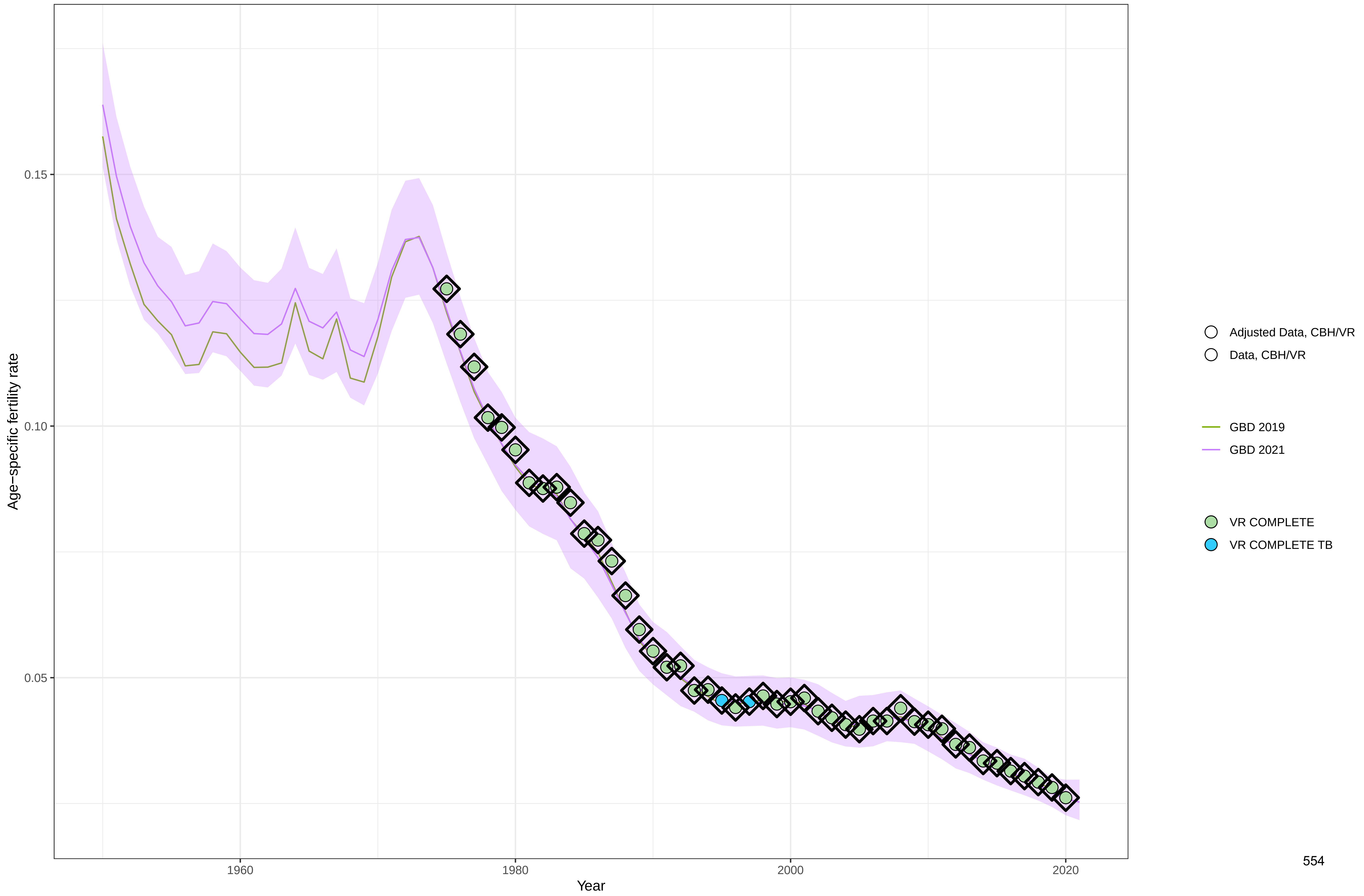

Age−specific fertility fitted trend lines, 1950−2021: Mie, Ages: 25−29
Data points enclosed with a diamond are reference sources

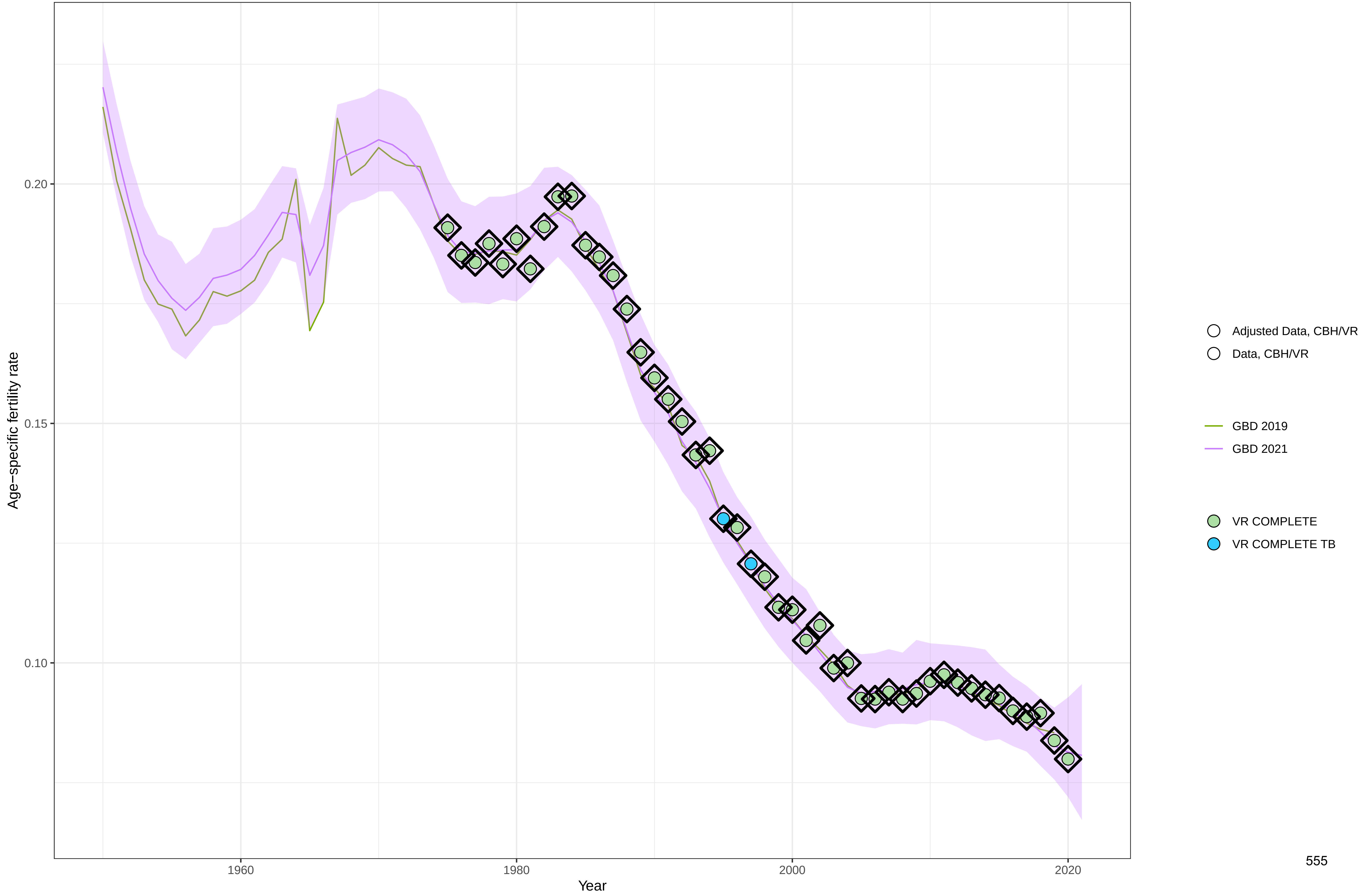

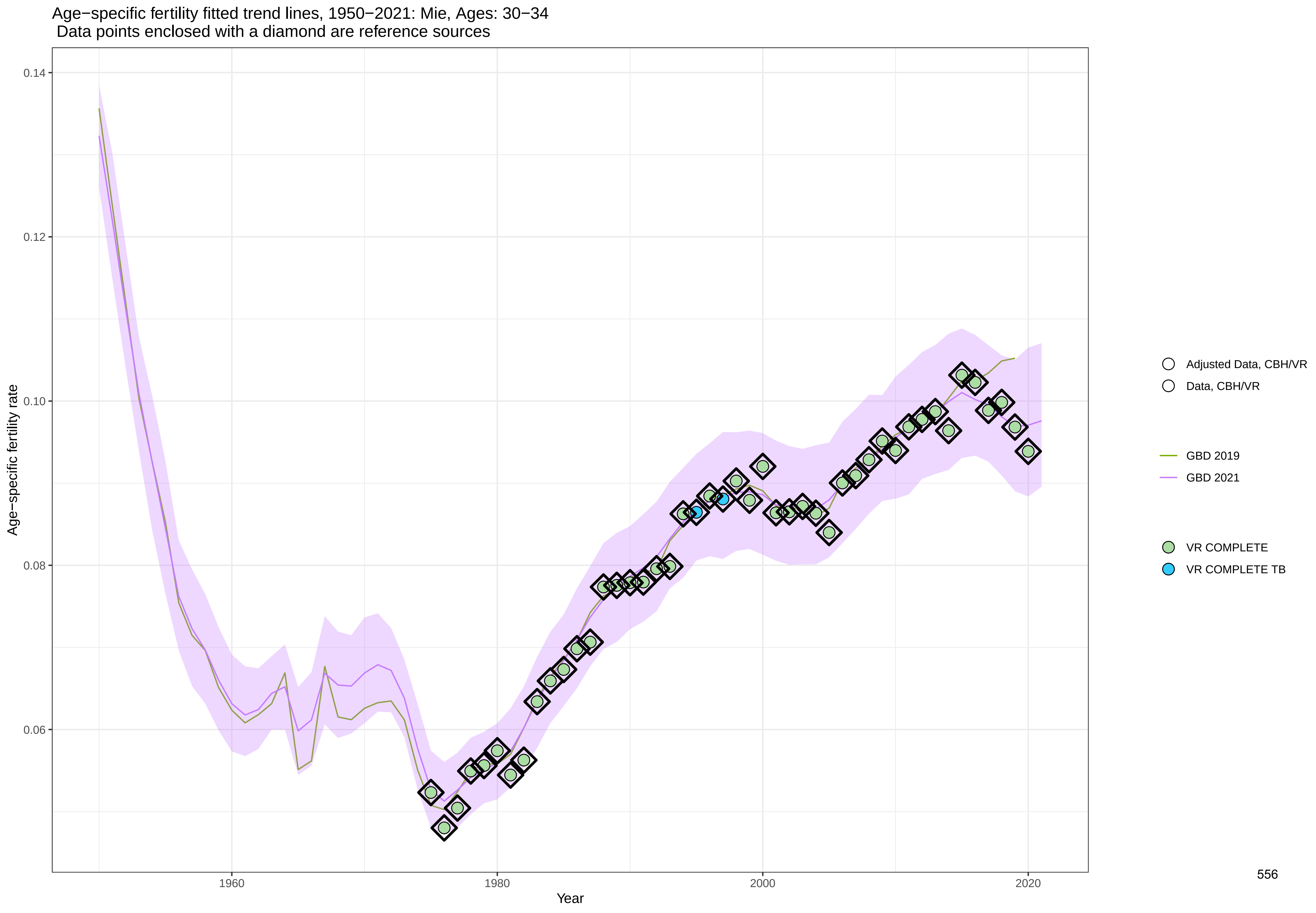

Age−specific fertility fitted trend lines, 1950−2021: Mie, Ages: 30−34
Data points enclosed with a diamond are reference sources
Age−specific fertility rate
0.14
0.12
0.10
0.08
0.06
1960
1980
2000
2020
Year
Adjusted Data, CBH/VR
Data, CBH/VR
GBD 2019
GBD 2021
VR COMPLETE
VR COMPLETE TB

Age−specific fertility fitted trend lines, 1950−2021: Mie, Ages: 35−39
Data points enclosed with a diamond are reference sources

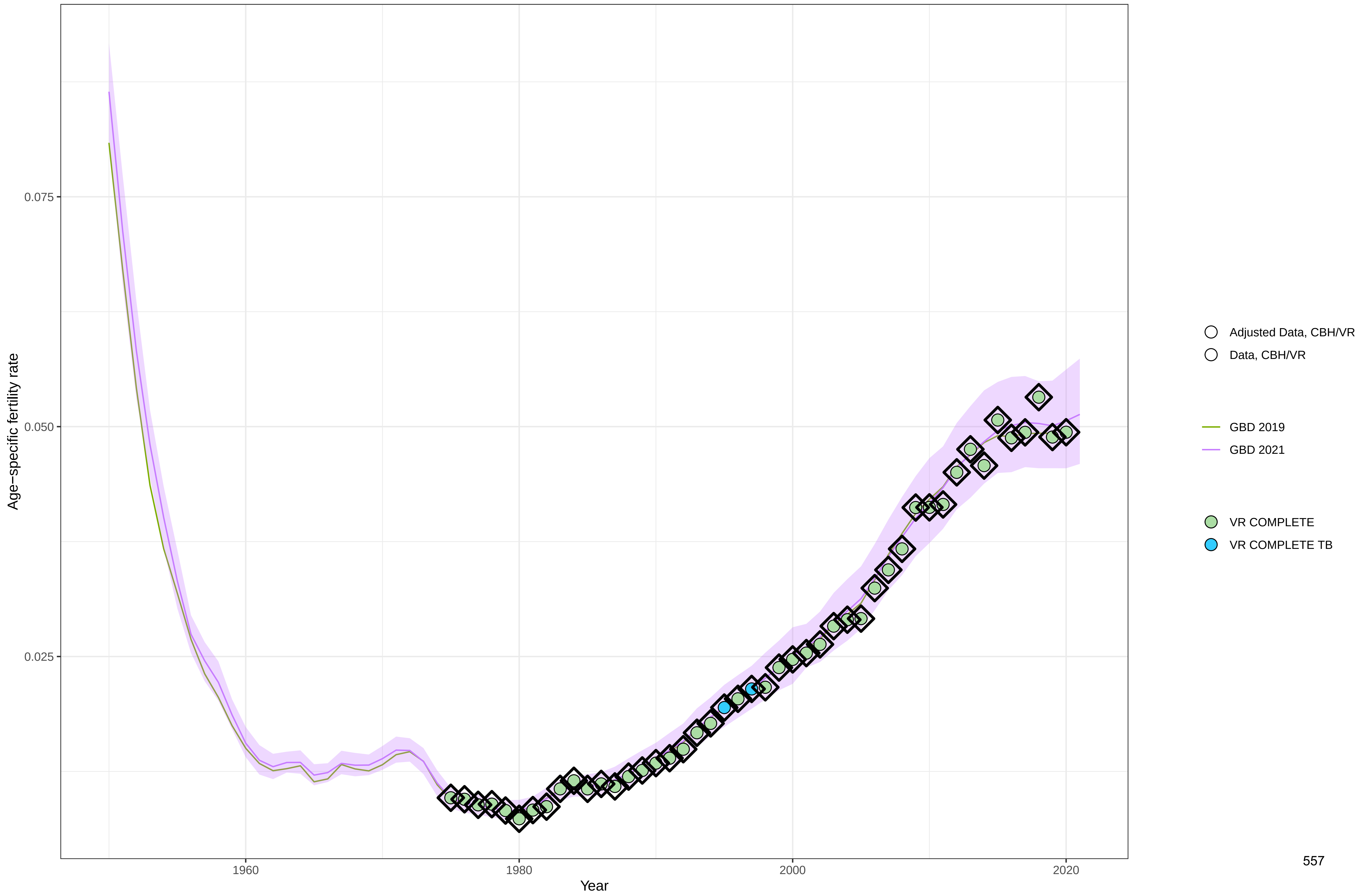

Age−specific fertility fitted trend lines, 1950−2021: Mie, Ages: 40−44
Data points enclosed with a diamond are reference sources

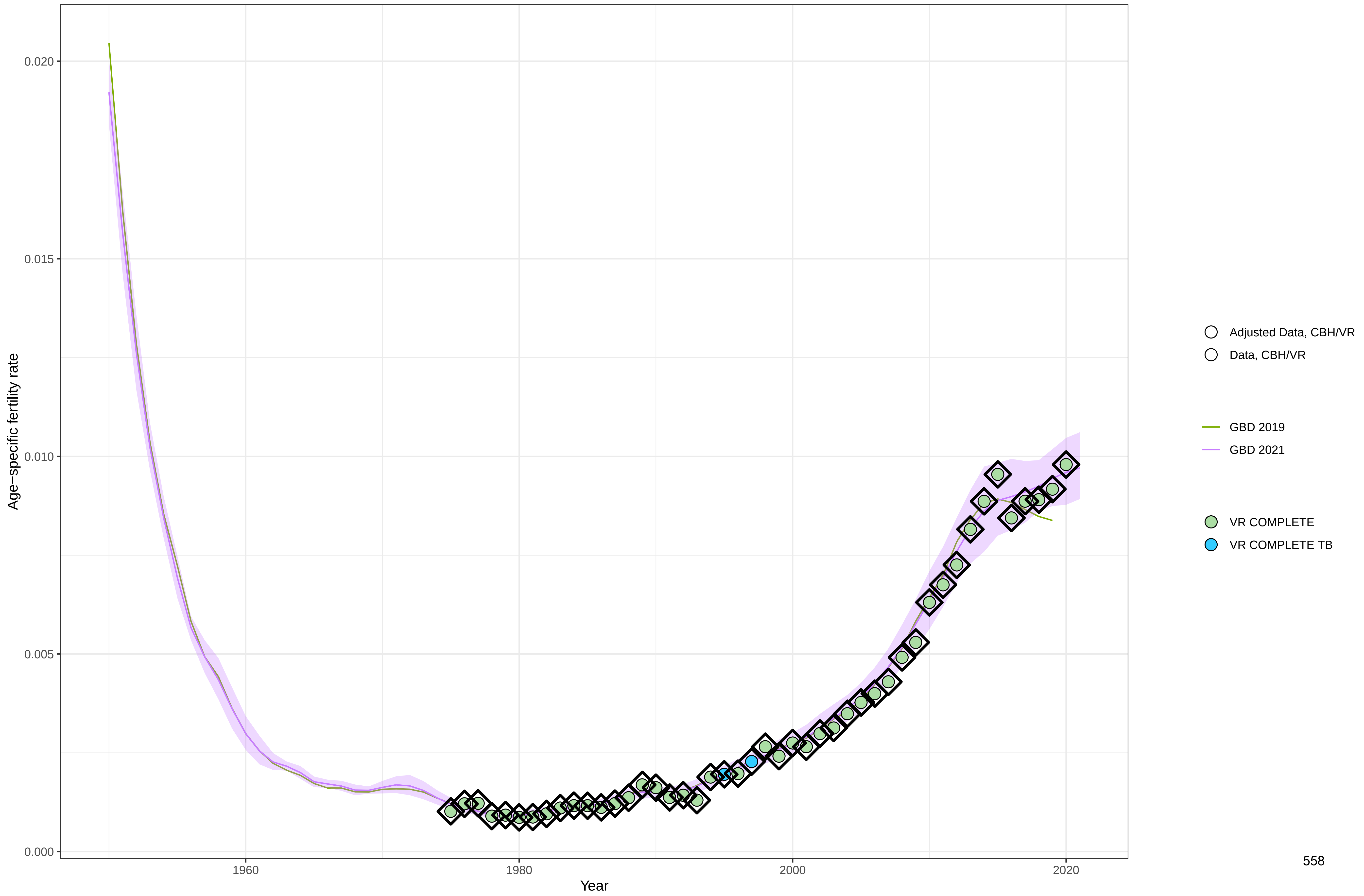

Age−specific fertility fitted trend lines, 1950−2021: Mie, Ages: 45−49
Data points enclosed with a diamond are reference sources

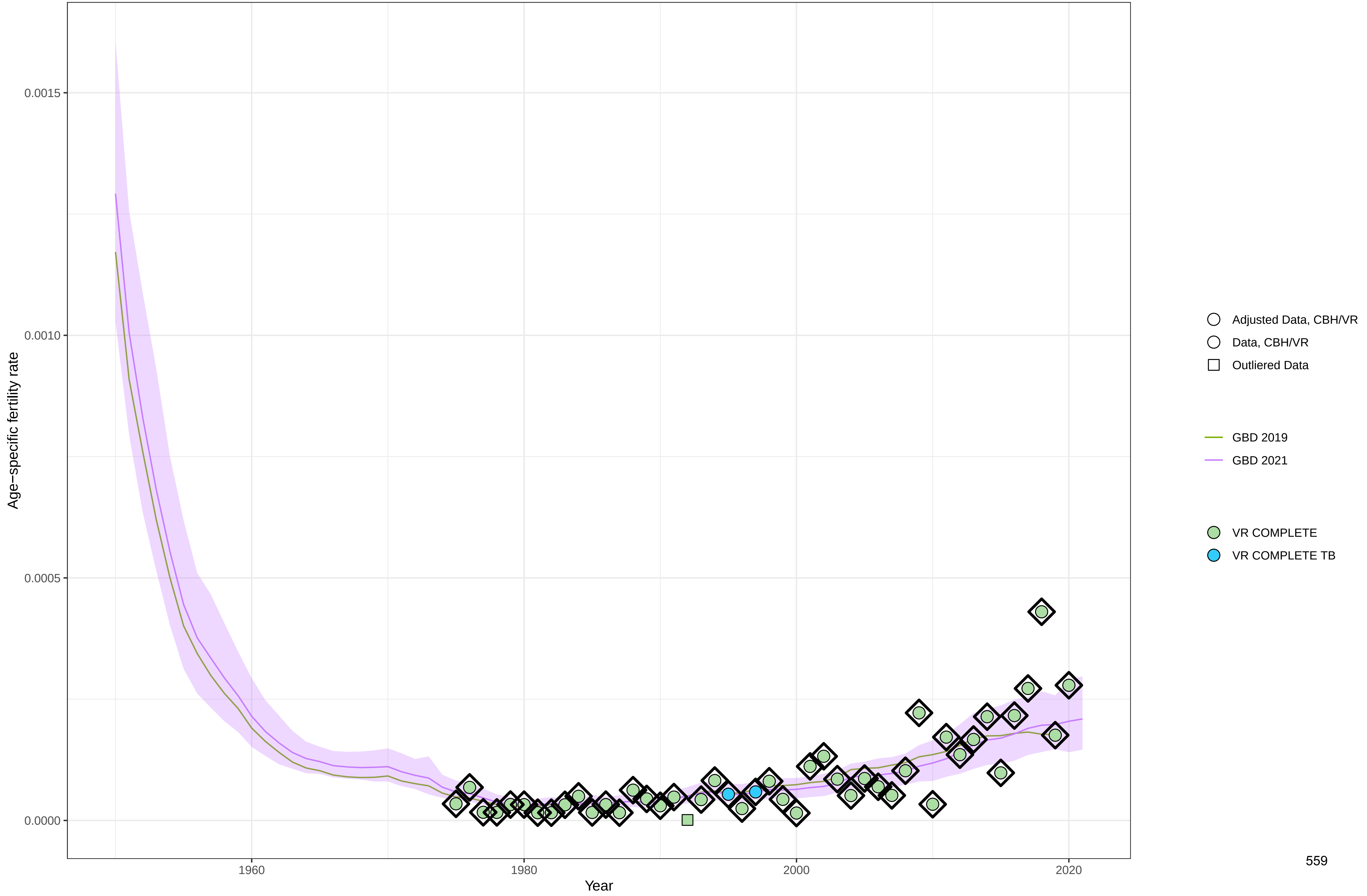

Age−specific fertility fitted trend lines, 1950−2021: Mie, Ages: 50−54
Data points enclosed with a diamond are reference sources

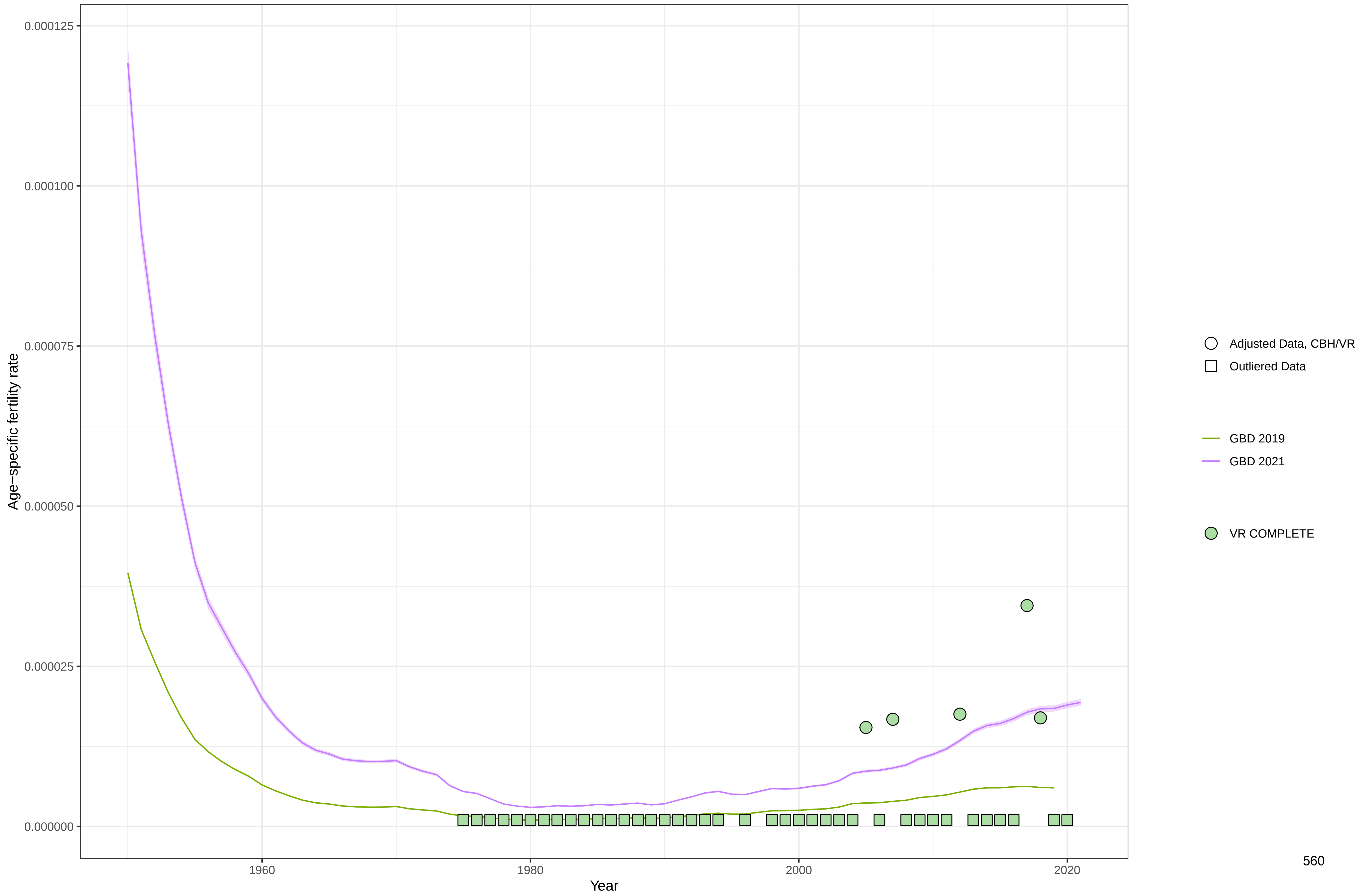

Total fertility rate fitted trend lines, 1950−2021: Mie TFR
Data points enclosed with a diamond are reference sources

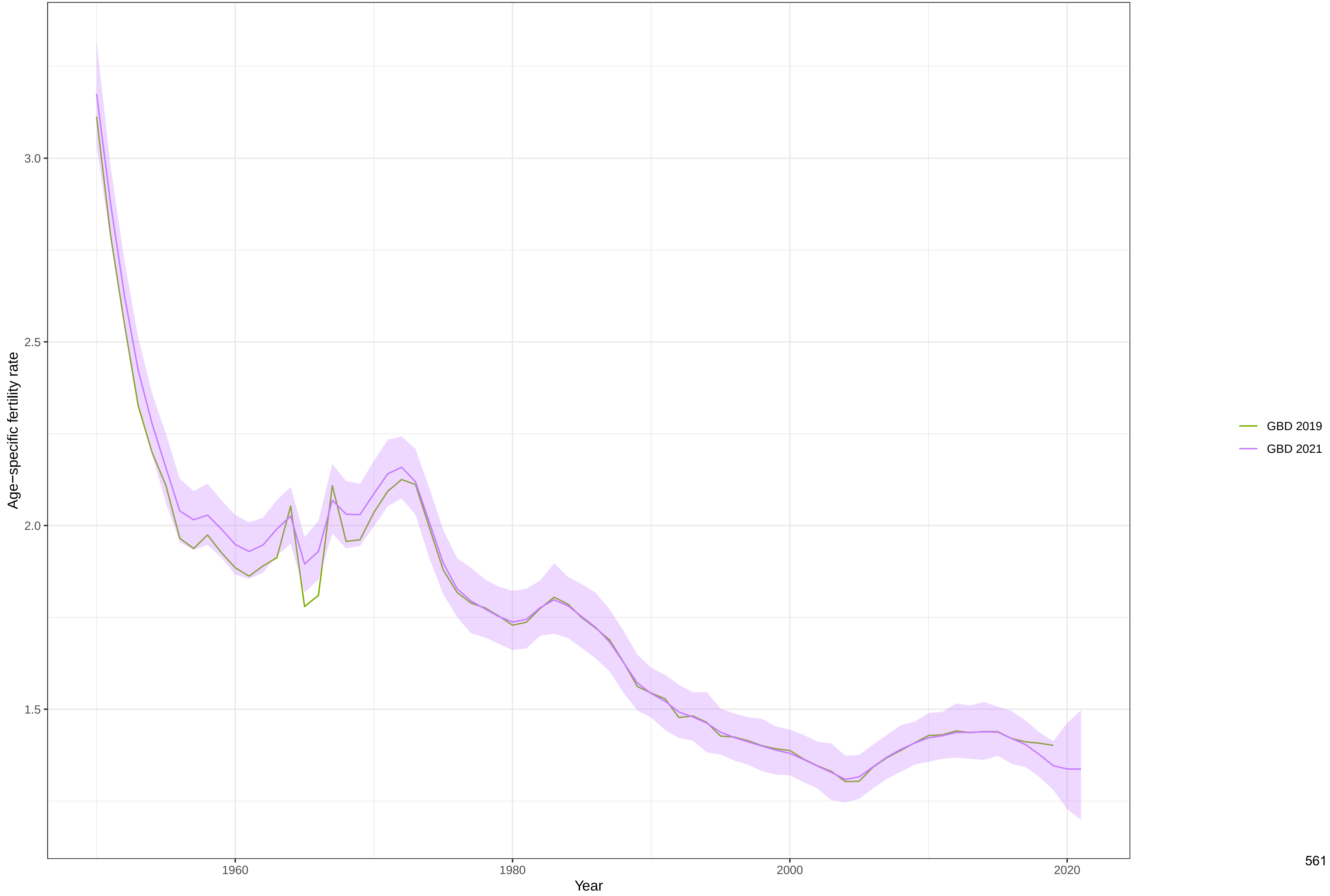

Age−specific fertility fitted trend lines, 1950−2021: Miyagi, Ages: 10−14
Data points enclosed with a diamond are reference sources

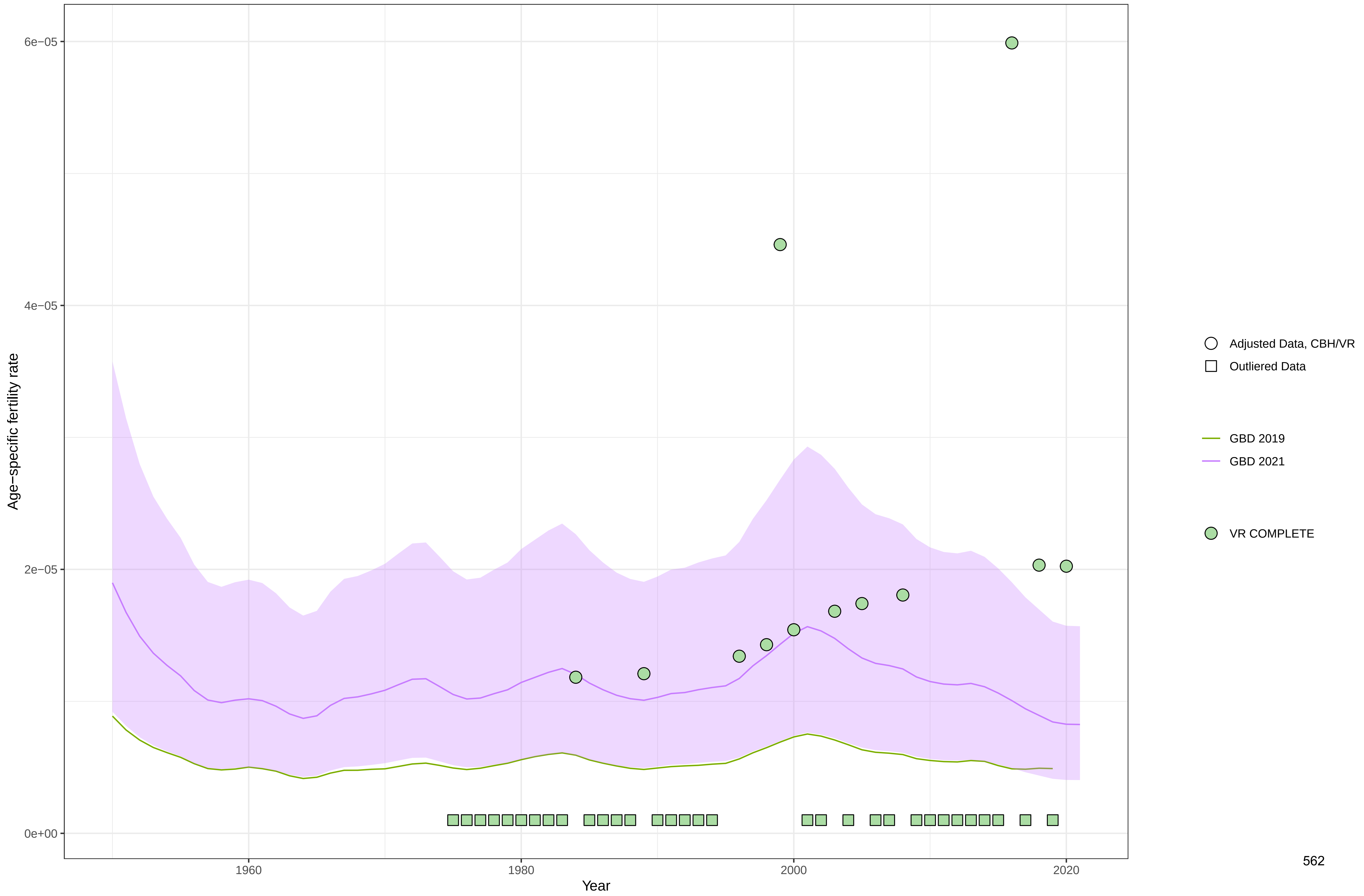

Age−specific fertility fitted trend lines, 1950−2021: Miyagi, Ages: 15−19
Data points enclosed with a diamond are reference sources

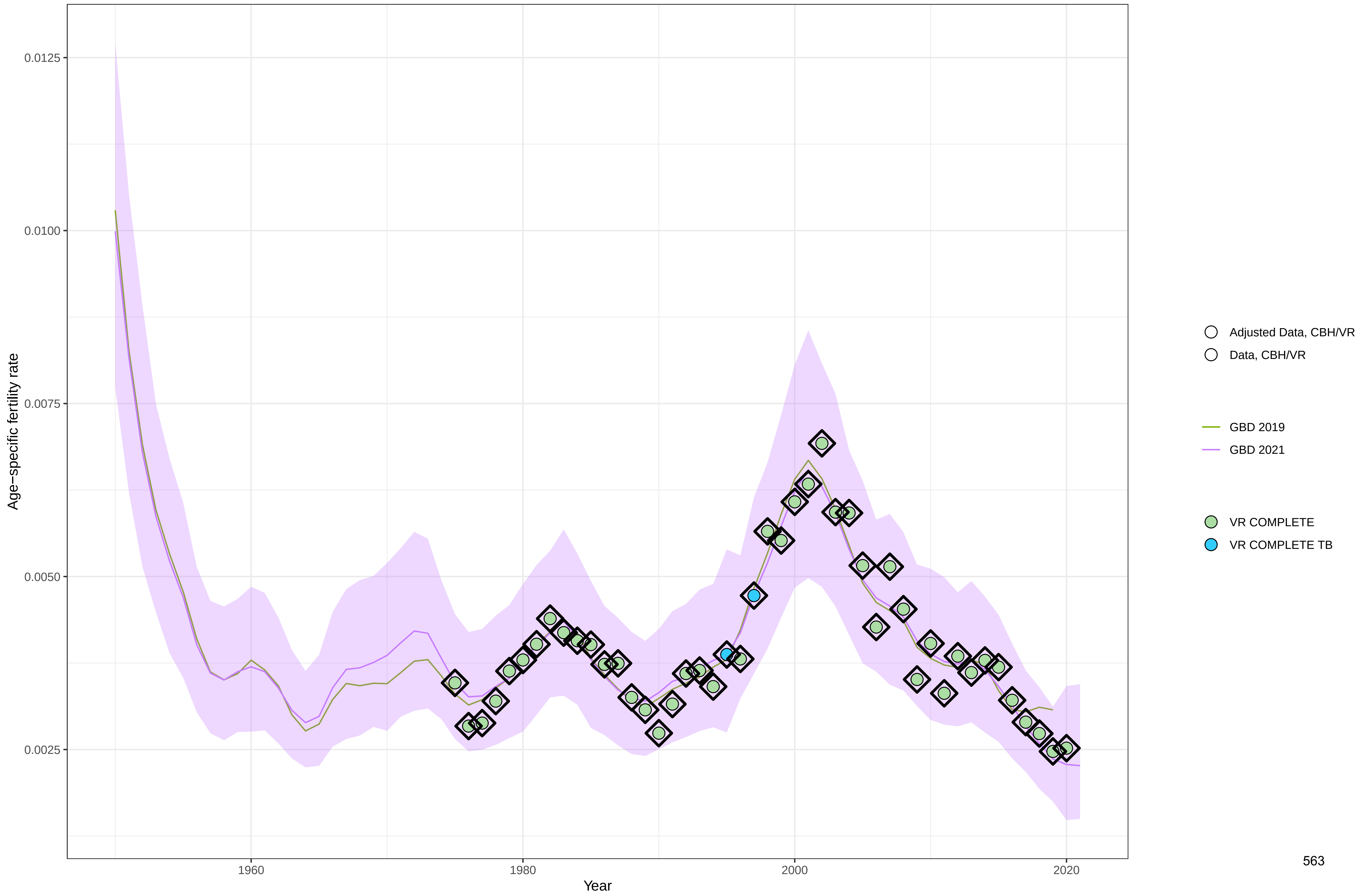

Age−specific fertility fitted trend lines, 1950−2021: Miyagi, Ages: 20−24
Data points enclosed with a diamond are reference sources

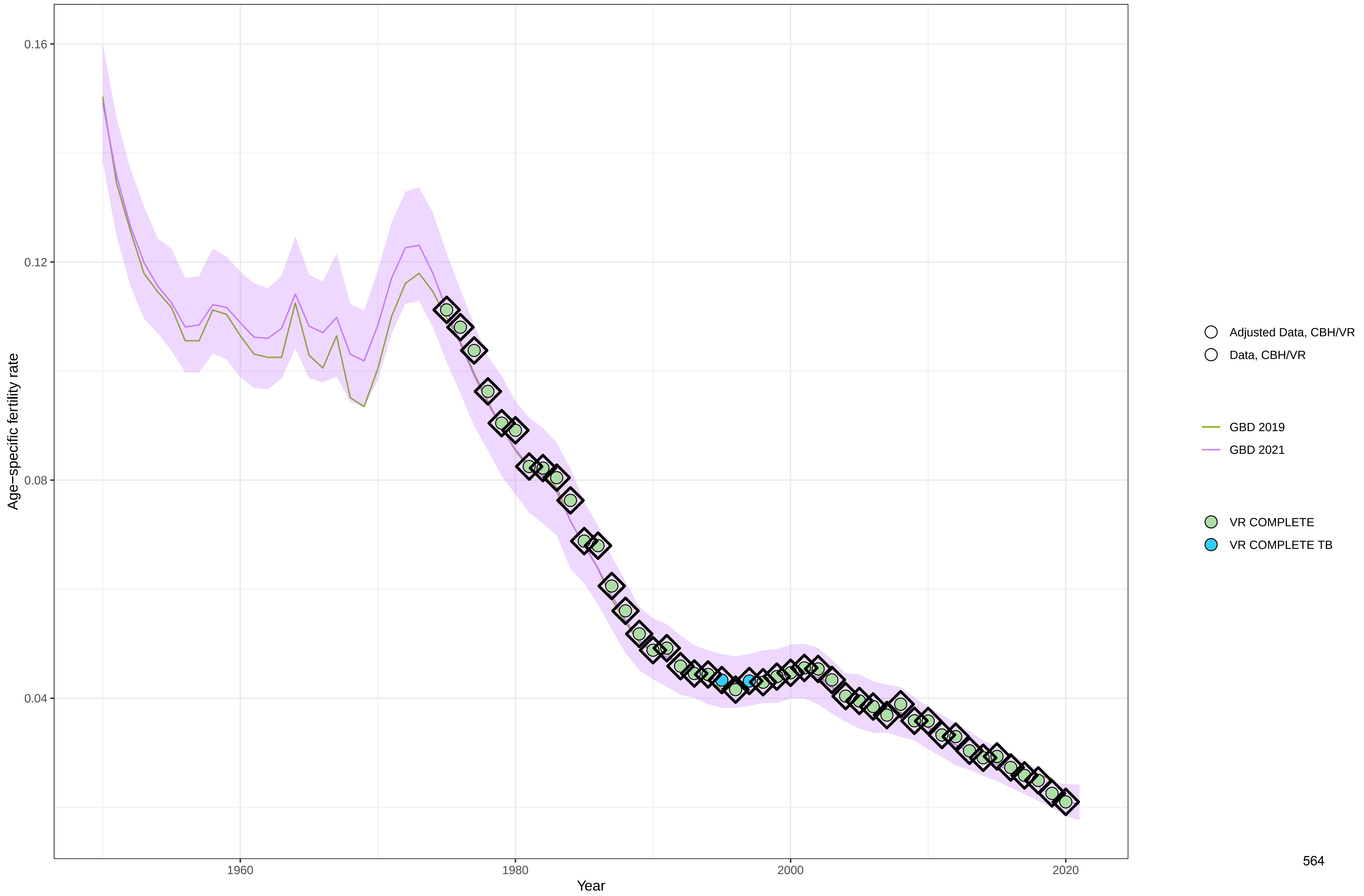

Age−specific fertility fitted trend lines, 1950−2021: Miyagi, Ages: 25−29
Data points enclosed with a diamond are reference sources

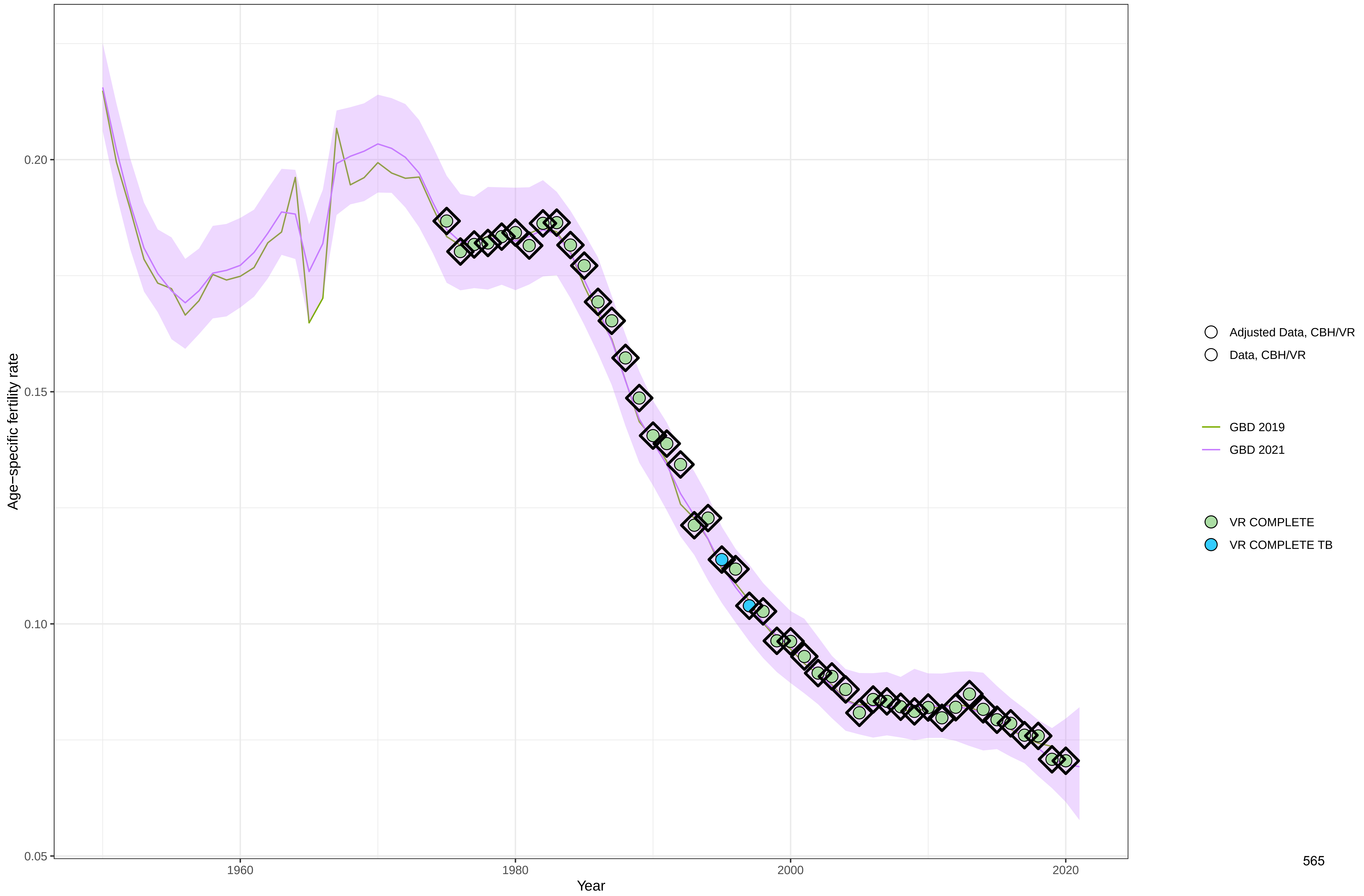

Age−specific fertility fitted trend lines, 1950−2021: Miyagi, Ages: 30−34
Data points enclosed with a diamond are reference sources

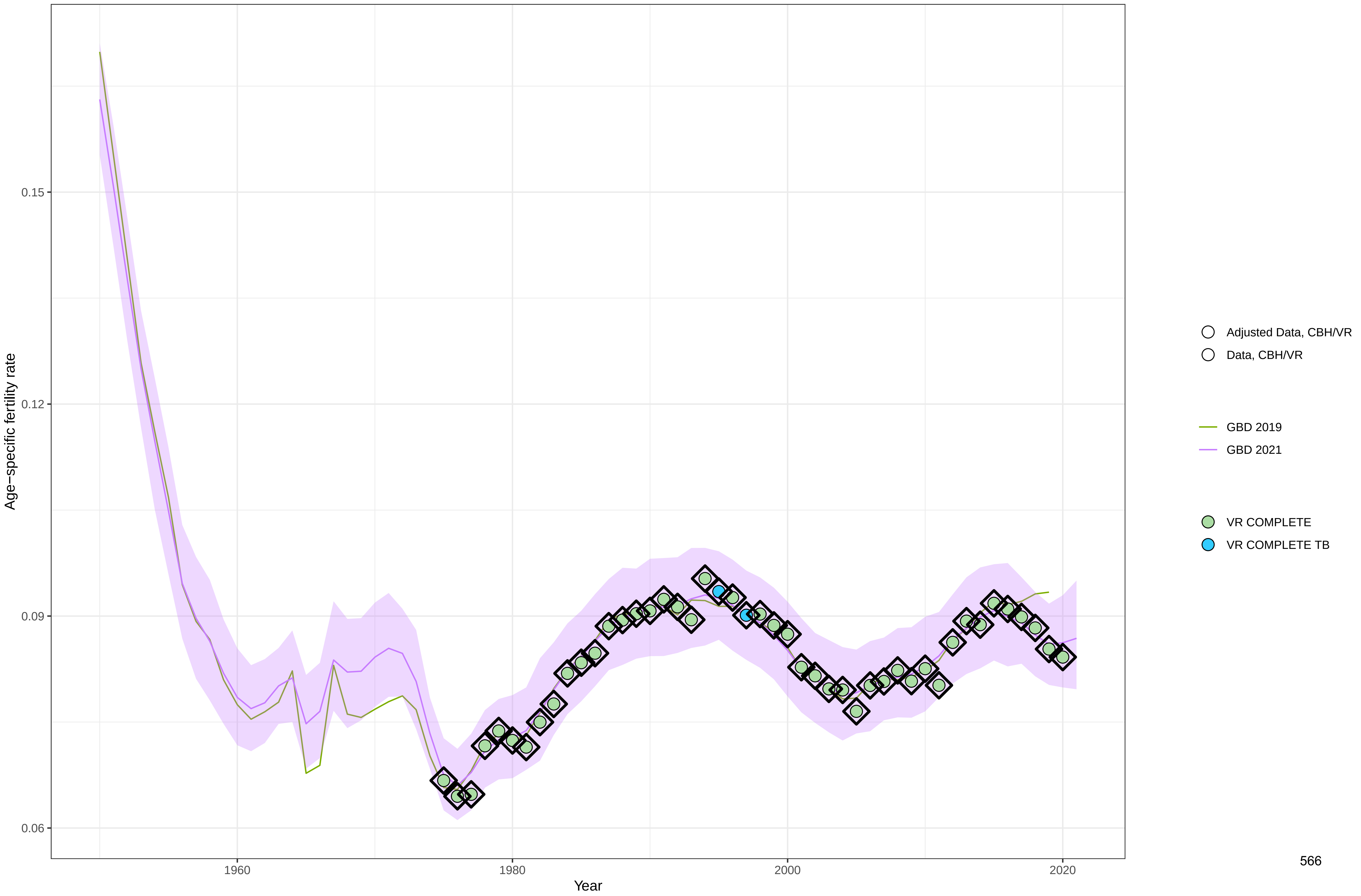

Age−specific fertility fitted trend lines, 1950−2021: Miyagi, Ages: 35−39
Data points enclosed with a diamond are reference sources

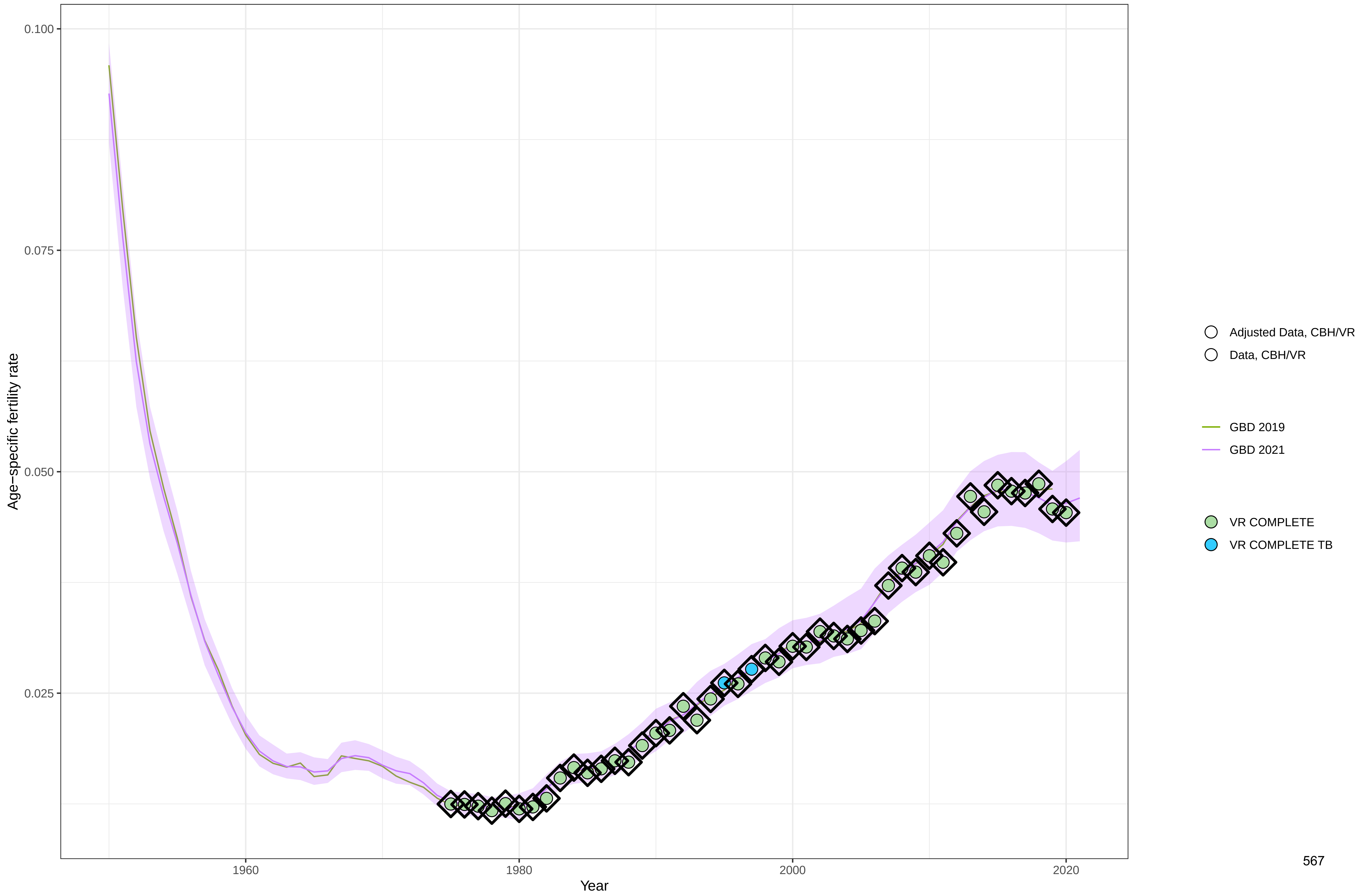

Age−specific fertility fitted trend lines, 1950−2021: Miyagi, Ages: 40−44
Data points enclosed with a diamond are reference sources

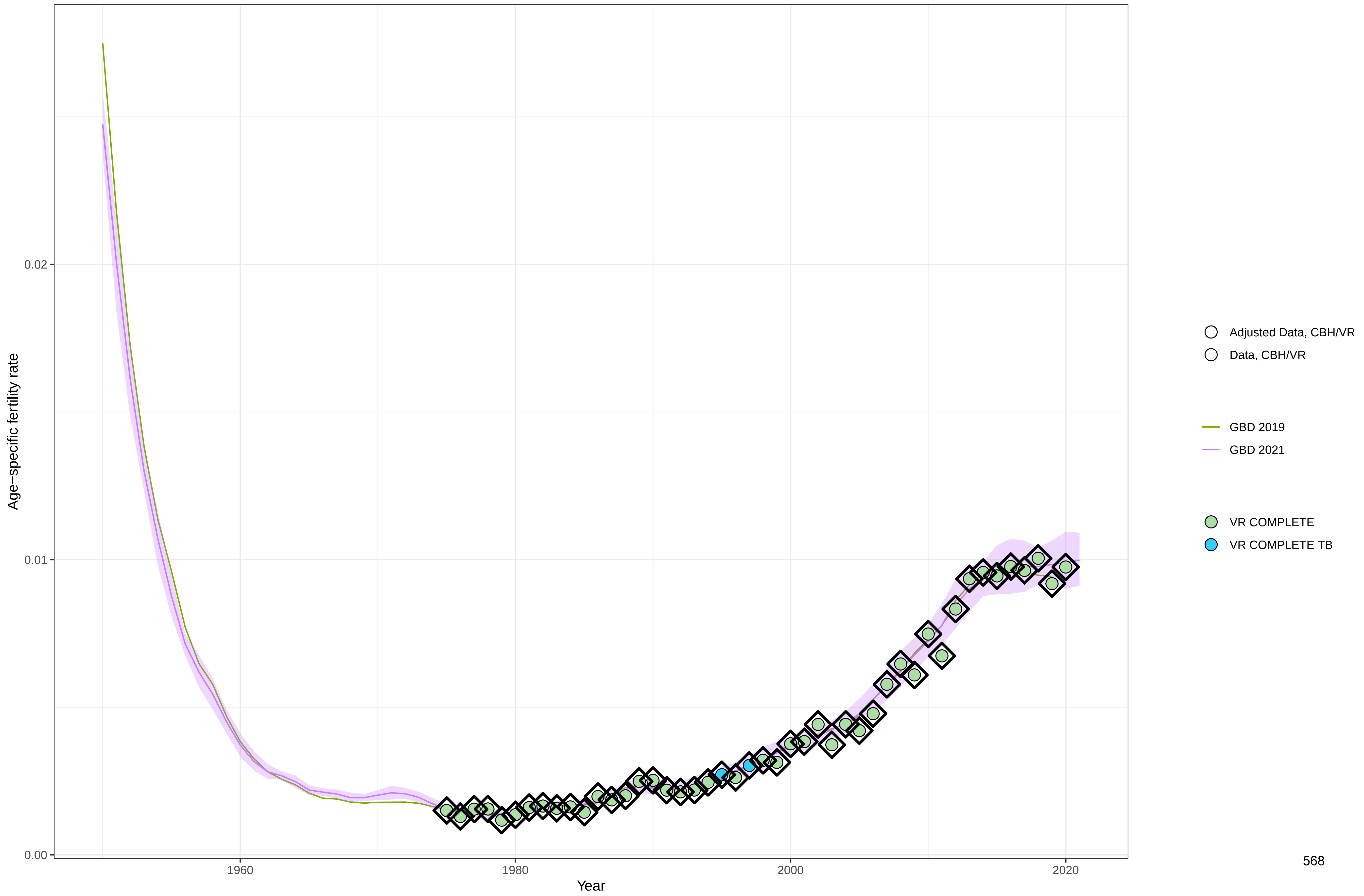

Age−specific fertility fitted trend lines, 1950−2021: Miyagi, Ages: 45−49
Data points enclosed with a diamond are reference sources

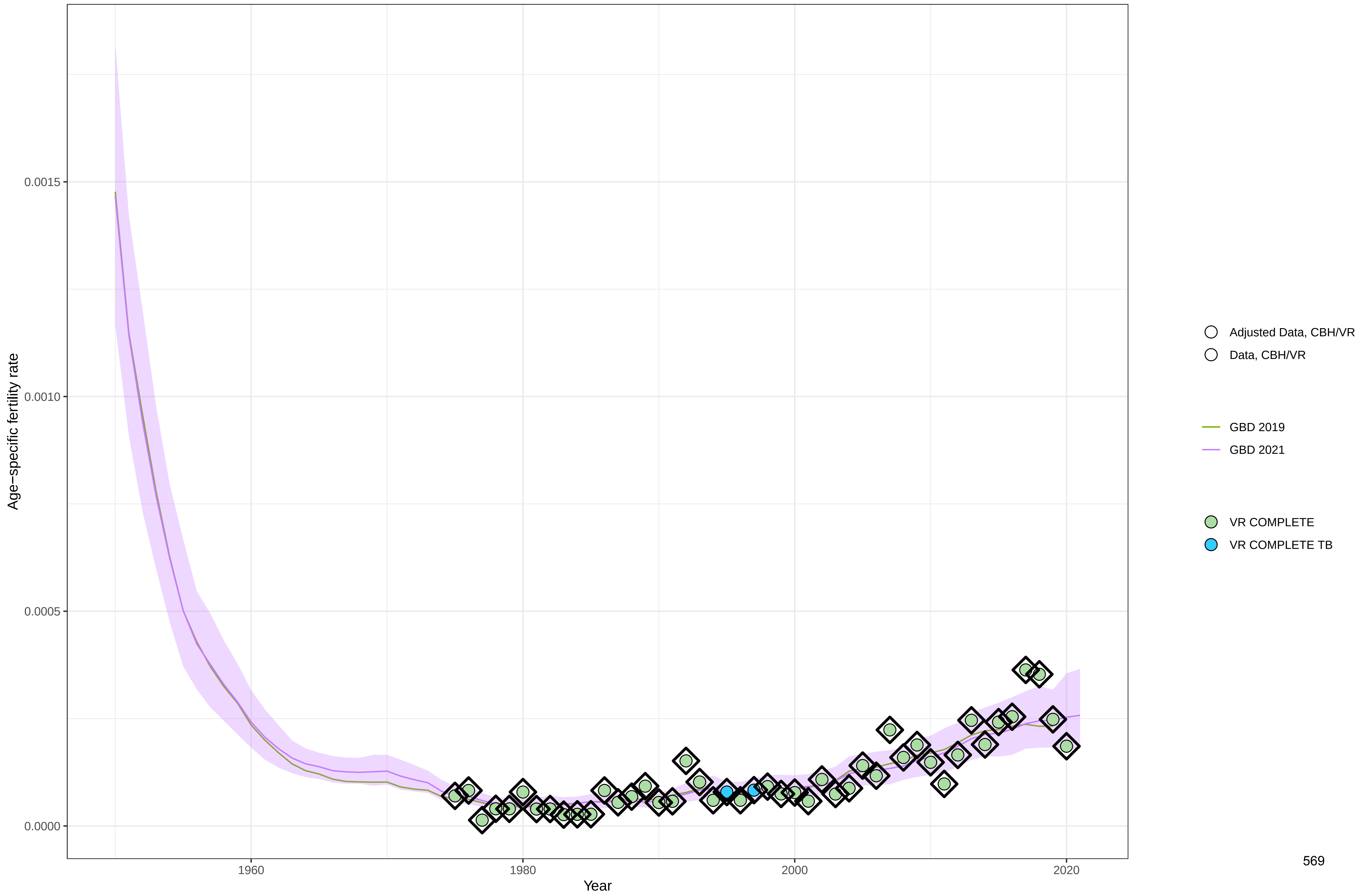

Age−specific fertility fitted trend lines, 1950−2021: Miyagi, Ages: 50−54
Data points enclosed with a diamond are reference sources

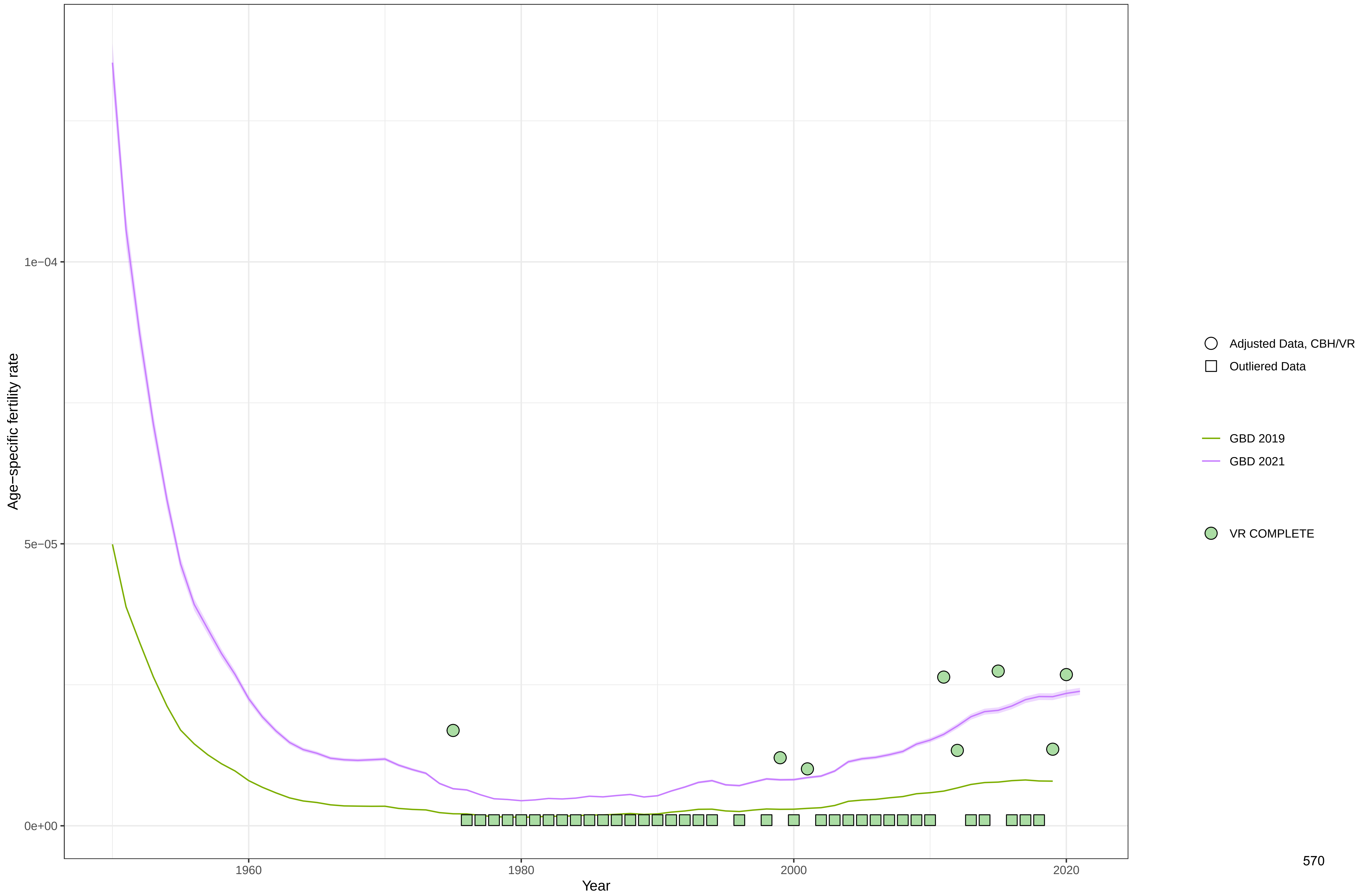

Total fertility rate fitted trend lines, 1950−2021: Miyagi TFR
Data points enclosed with a diamond are reference sources

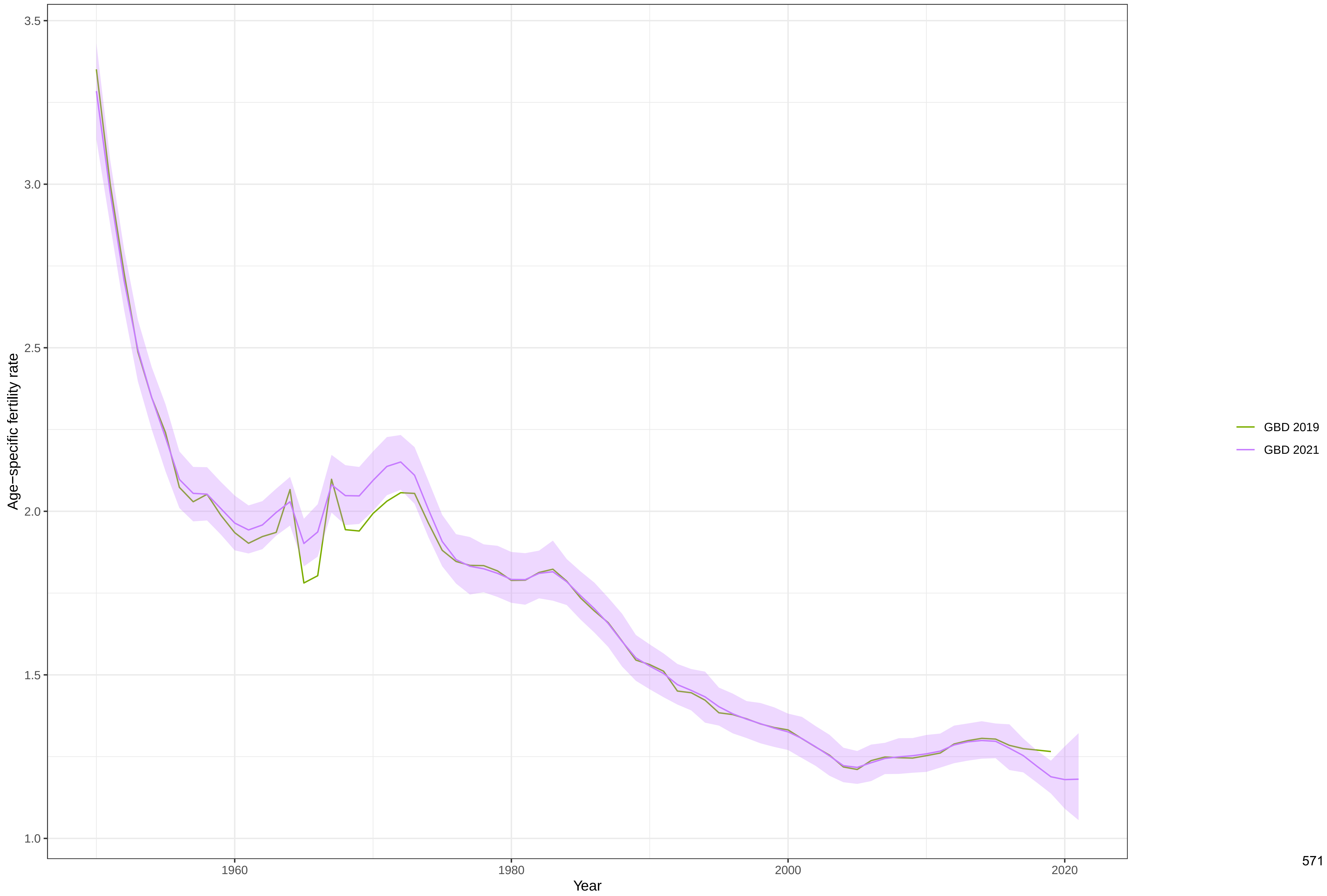

Age−specific fertility fitted trend lines, 1950−2021: Miyazaki, Ages: 10−14
Data points enclosed with a diamond are reference sources

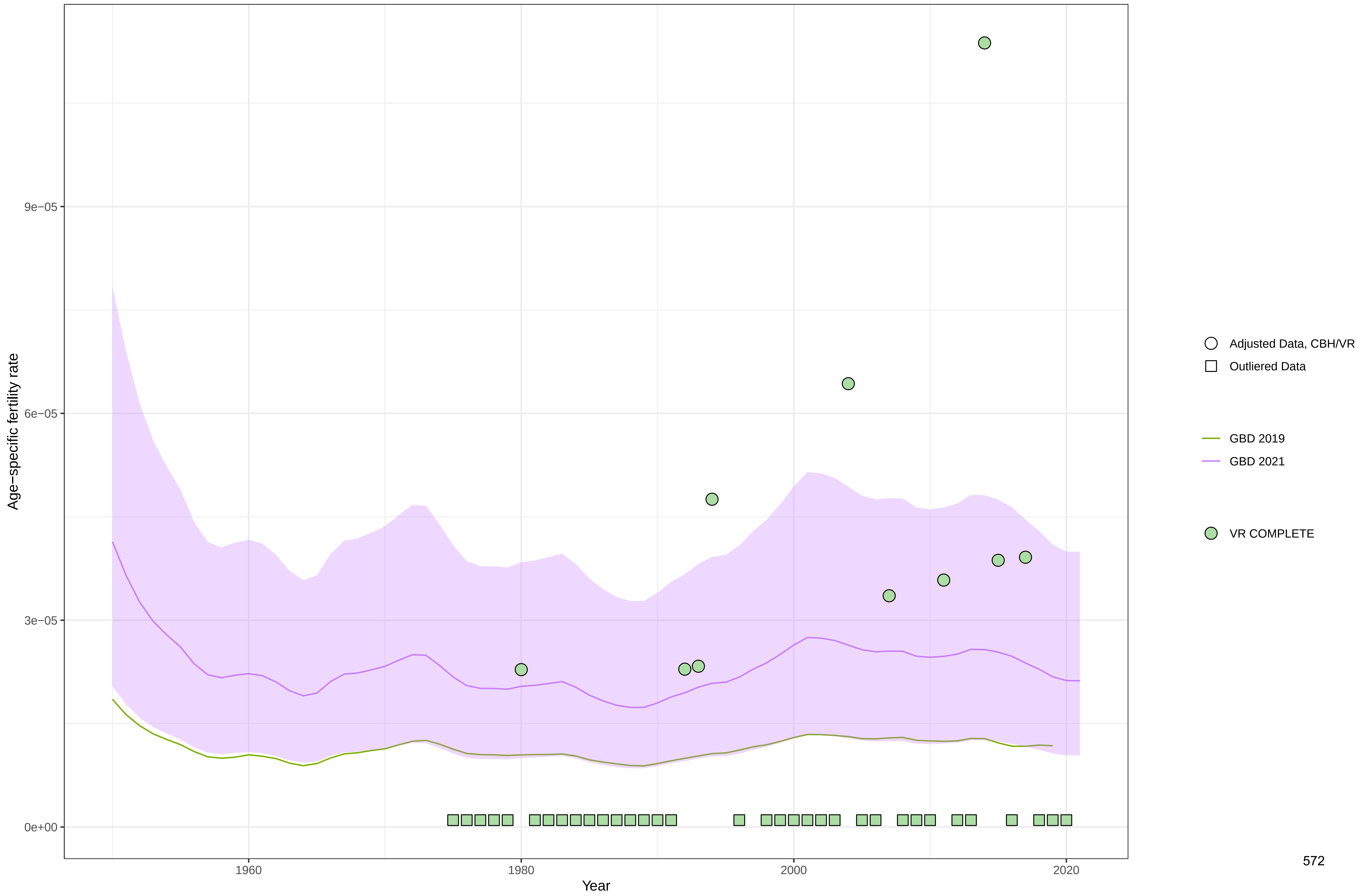

Age−specific fertility fitted trend lines, 1950−2021: Miyazaki, Ages: 15−19
Data points enclosed with a diamond are reference sources

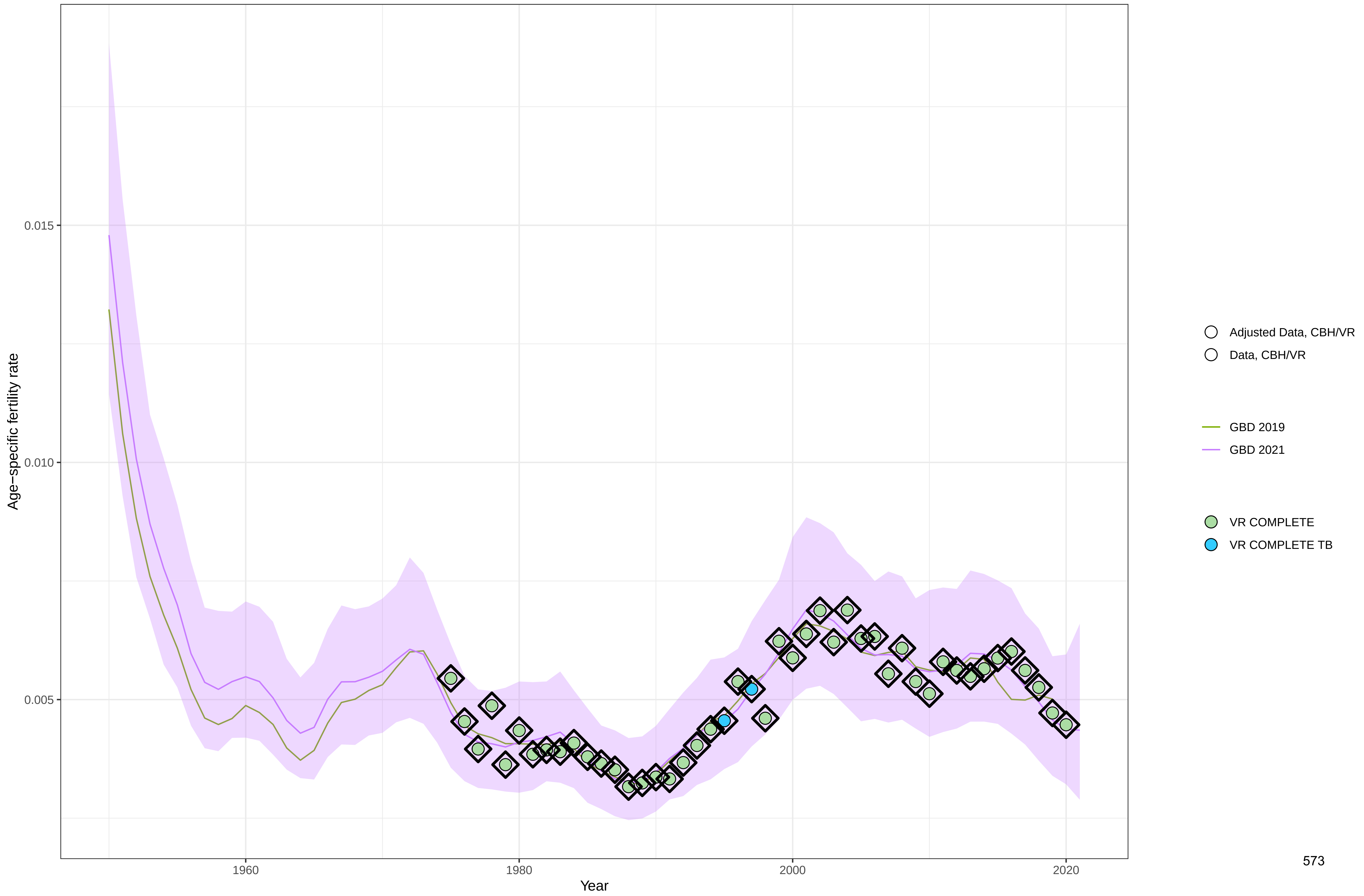

Age−specific fertility fitted trend lines, 1950−2021: Miyazaki, Ages: 20−24
Data points enclosed with a diamond are reference sources

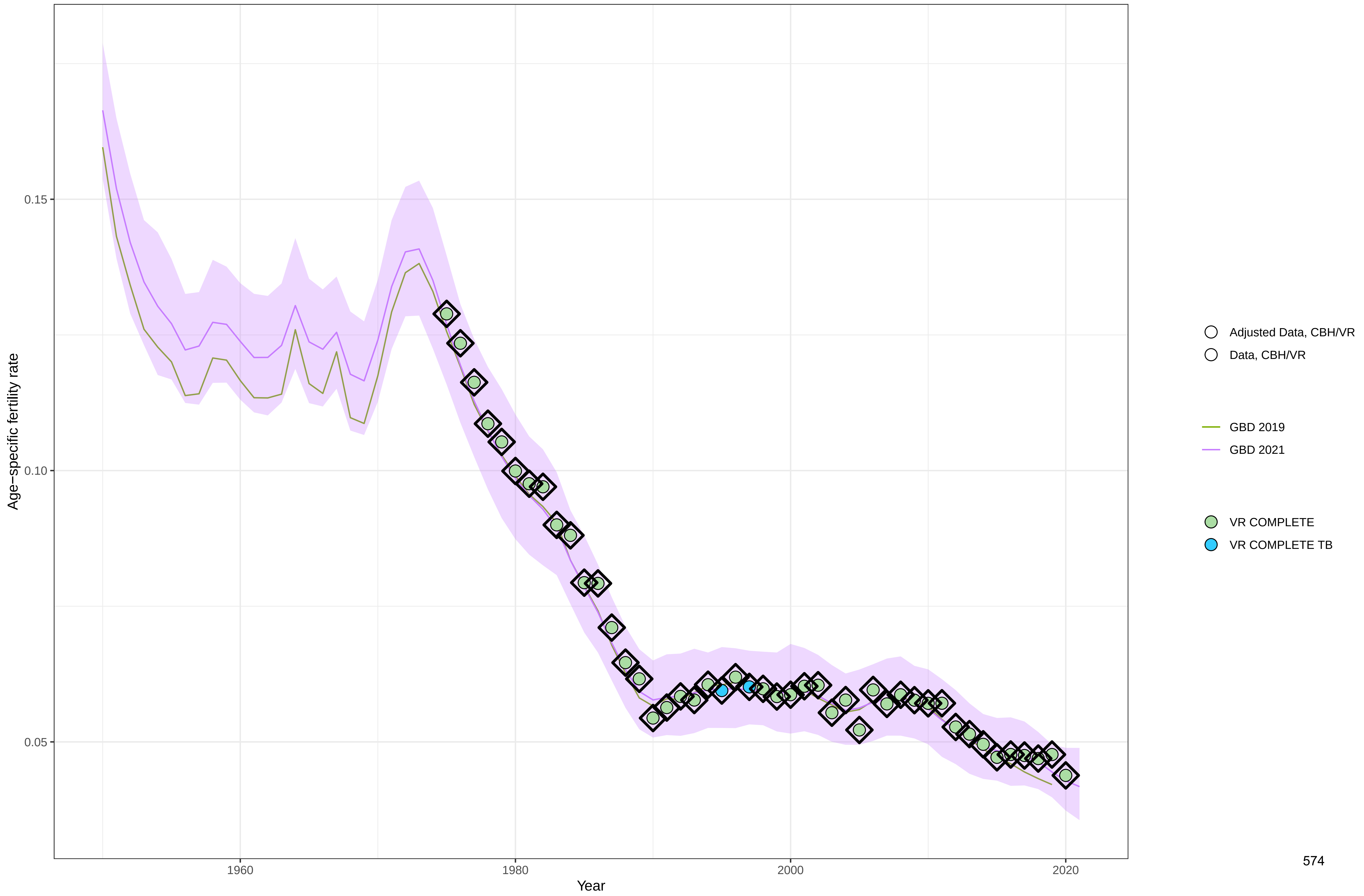

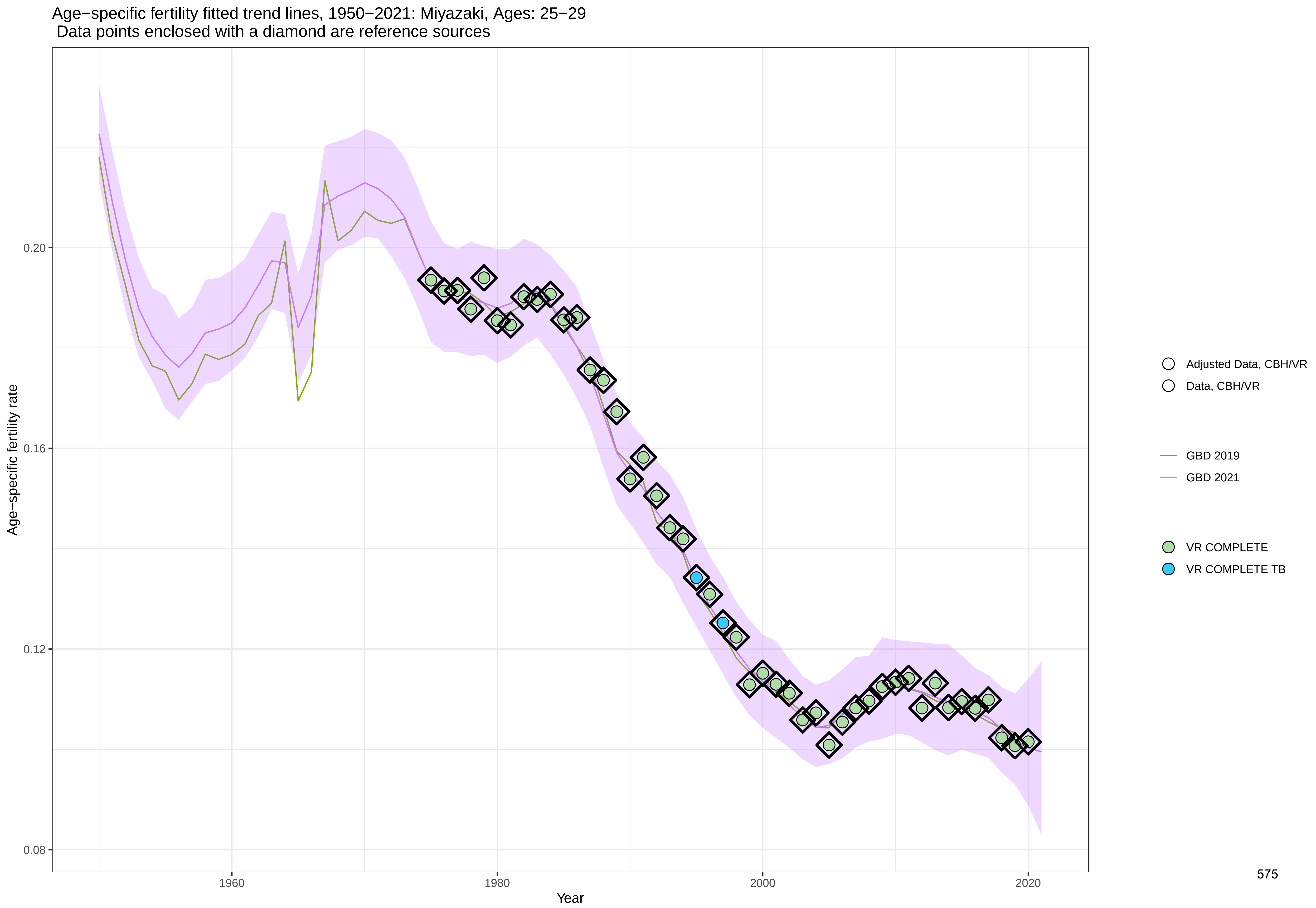

Age−specific fertility fitted trend lines, 1950−2021: Miyazaki, Ages: 25−29
Data points enclosed with a diamond are reference sources
Age−specific fertility rate
Year
0.20
0.16
0.12
0.08
1960
1980
2000
2020
Adjusted Data, CBH/VR
Data, CBH/VR
GBD 2019
GBD 2021
VR COMPLETE
VR COMPLETE TB

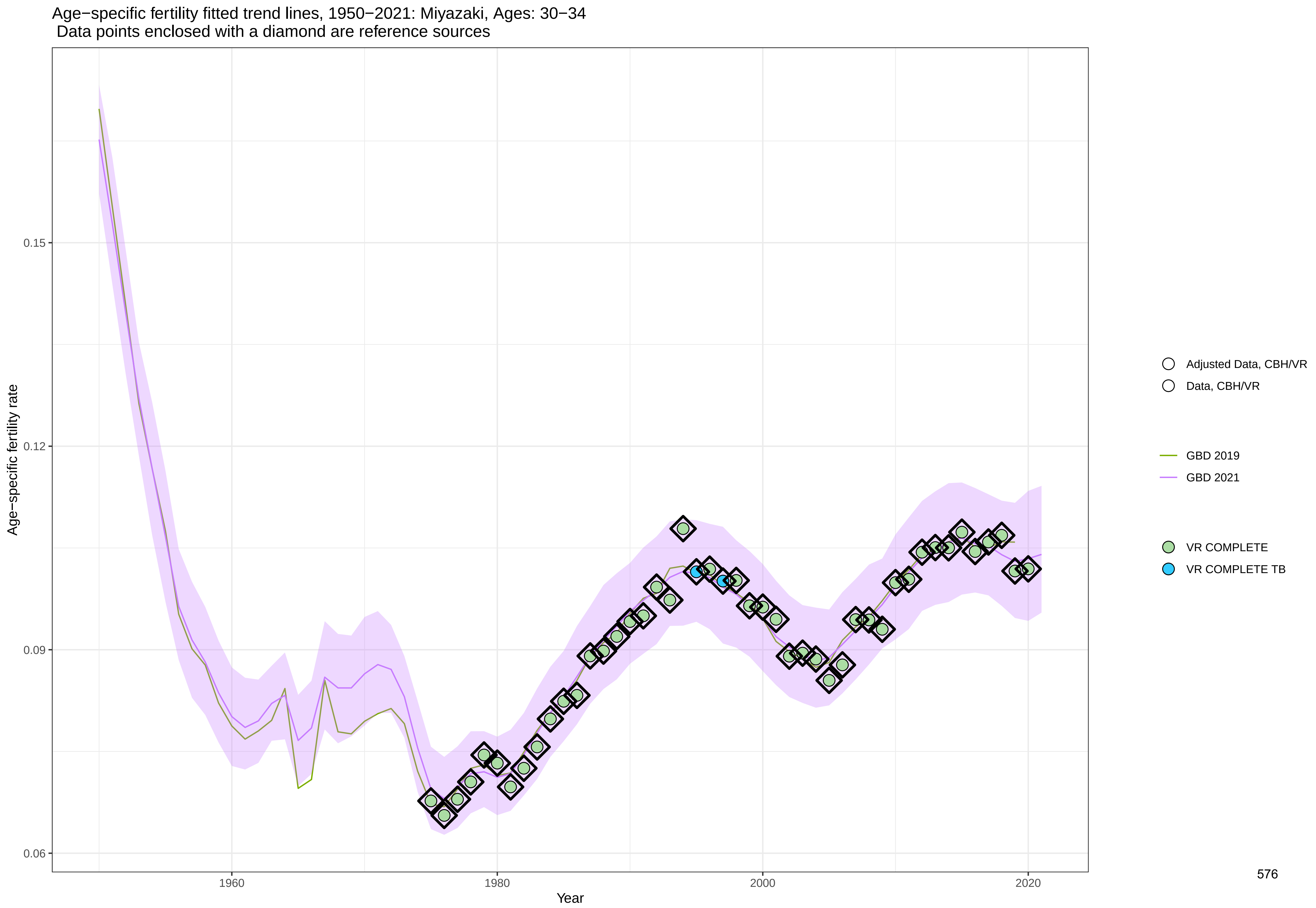
Age−specific fertility fitted trend lines, 1950−2021: Miyazaki, Ages: 30−34
Data points enclosed with a diamond are reference sources
Age−specific fertility rate
0.15
0.12
0.09
0.06
1960
1980
2000
2020
Year
Adjusted Data, CBH/VR
Data, CBH/VR
GBD 2019
GBD 2021
VR COMPLETE
VR COMPLETE TB

Age−specific fertility fitted trend lines, 1950−2021: Miyazaki, Ages: 35−39
Data points enclosed with a diamond are reference sources

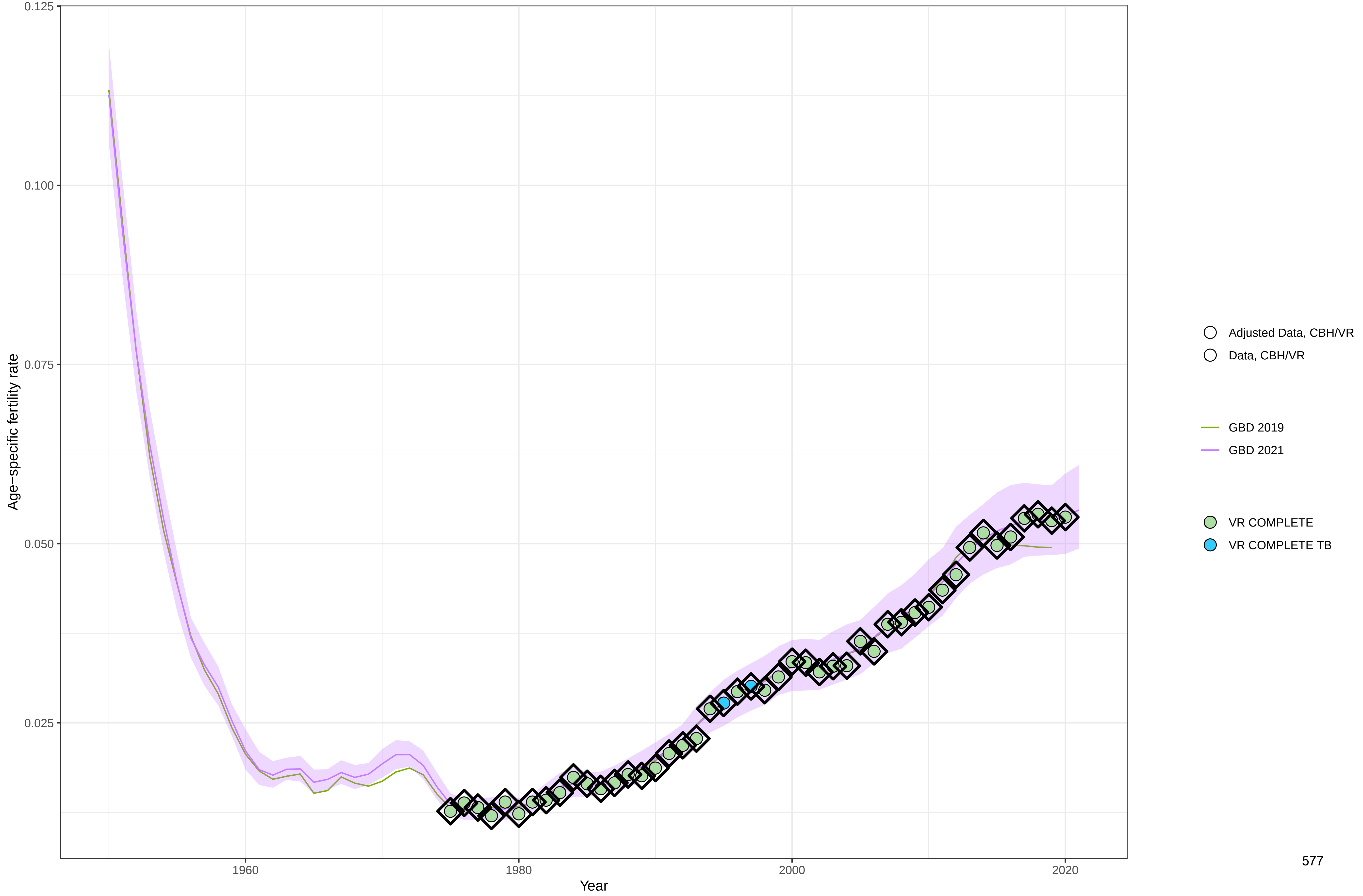

Age−specific fertility fitted trend lines, 1950−2021: Miyazaki, Ages: 40−44
Data points enclosed with a diamond are reference sources

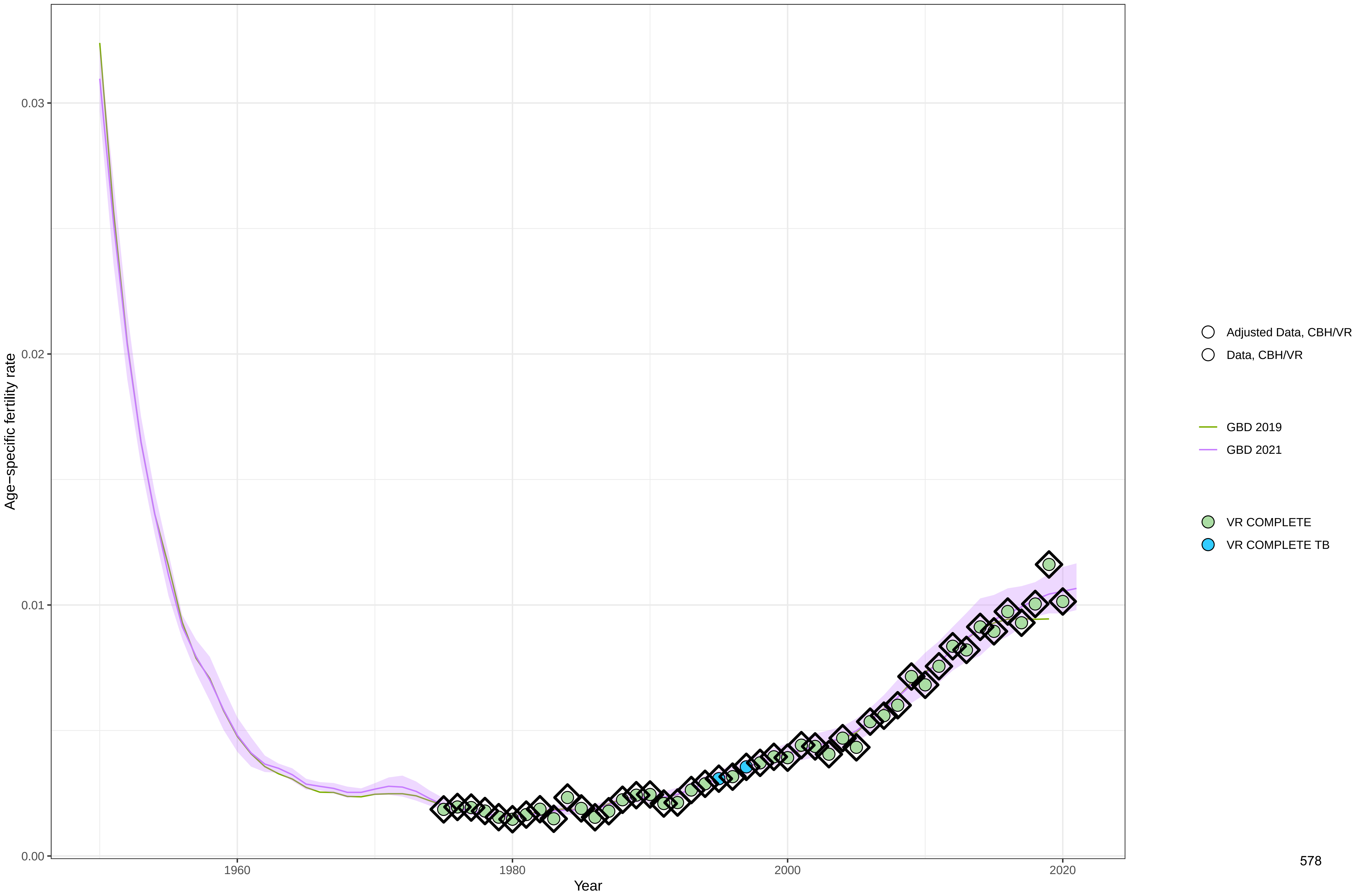

Age−specific fertility fitted trend lines, 1950−2021: Miyazaki, Ages: 45−49
Data points enclosed with a diamond are reference sources

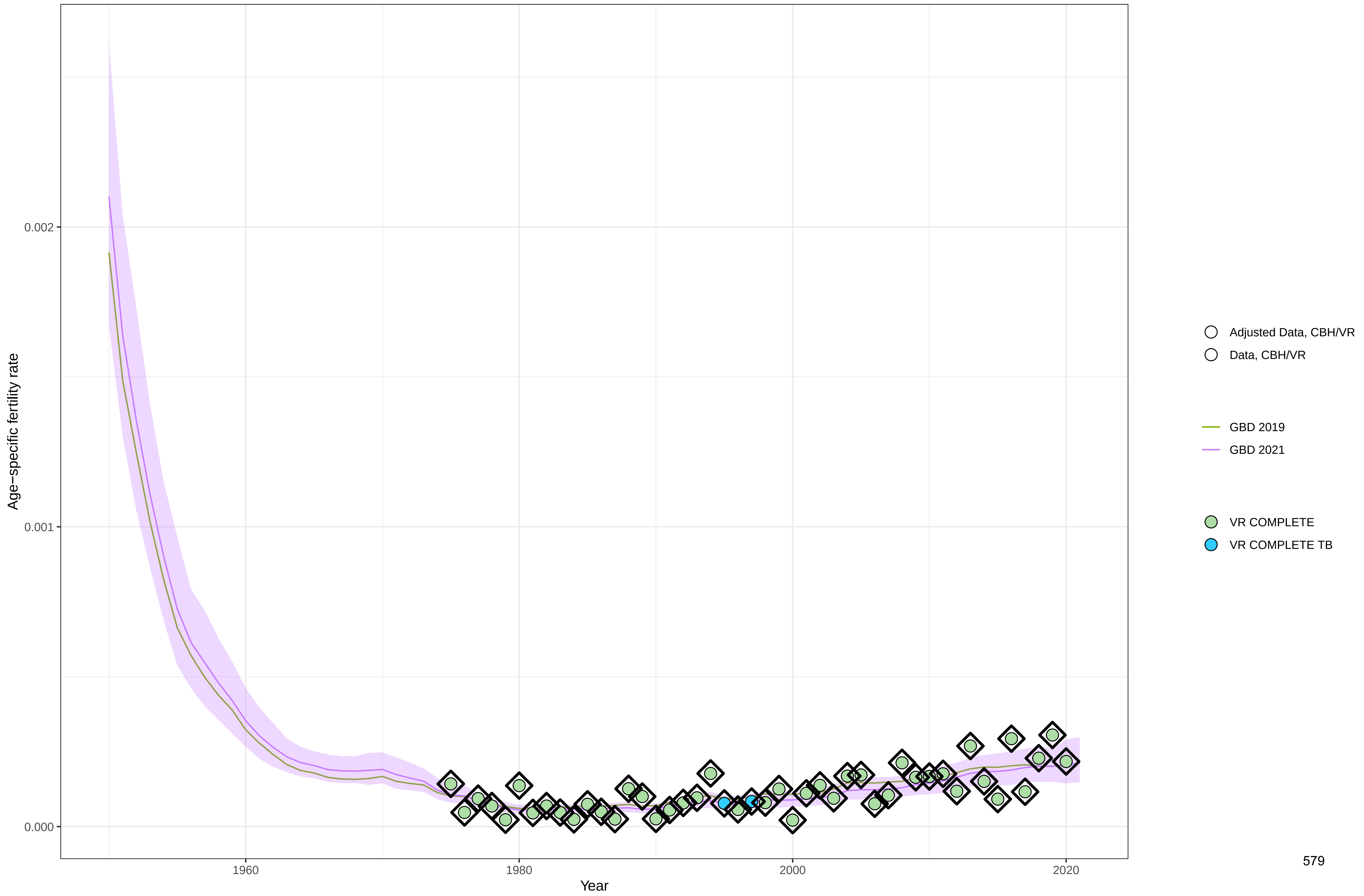

Age−specific fertility fitted trend lines, 1950−2021: Miyazaki, Ages: 50−54
Data points enclosed with a diamond are reference sources

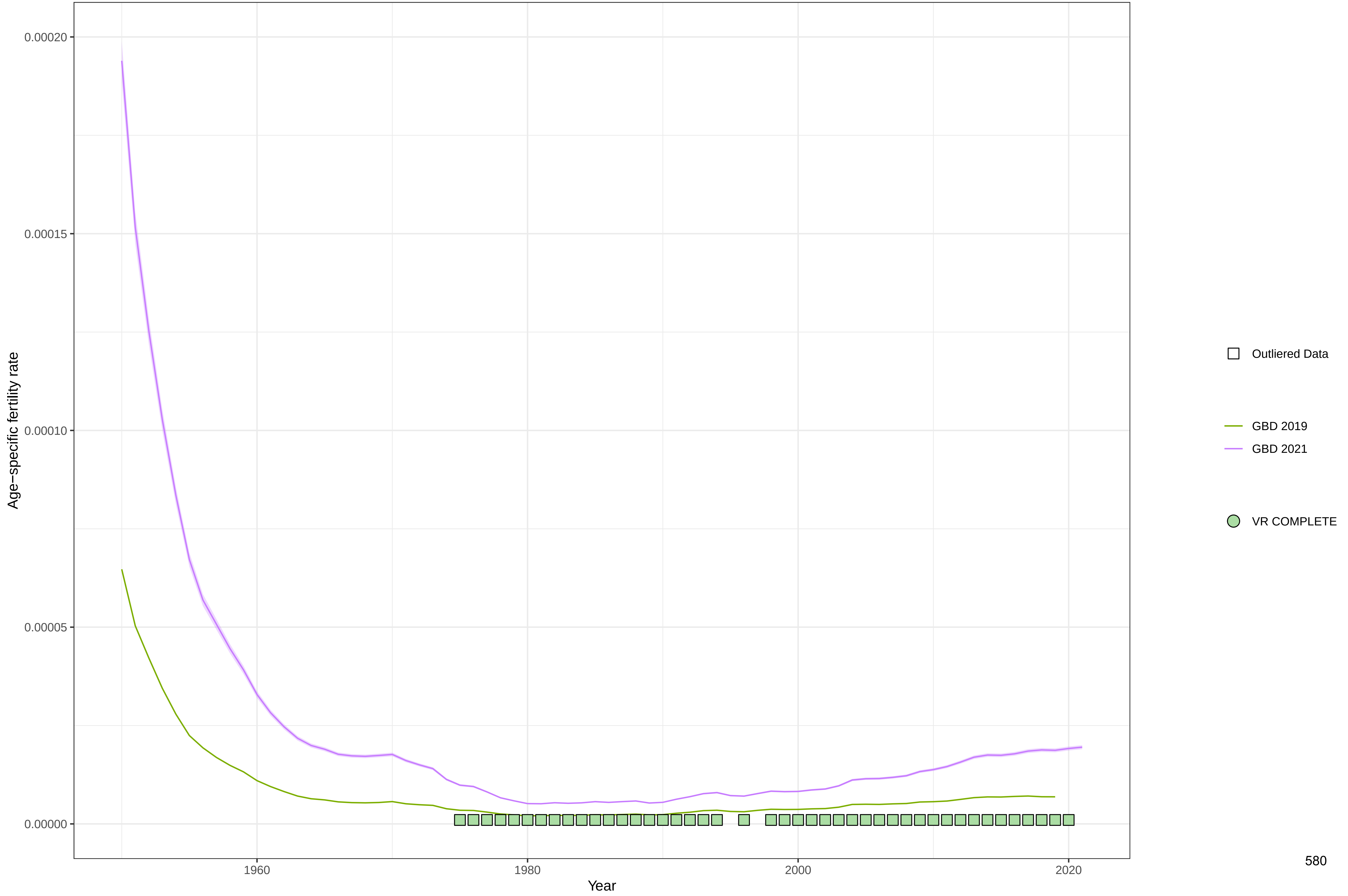

Total fertility rate fitted trend lines, 1950−2021: Miyazaki TFR
Data points enclosed with a diamond are reference sources

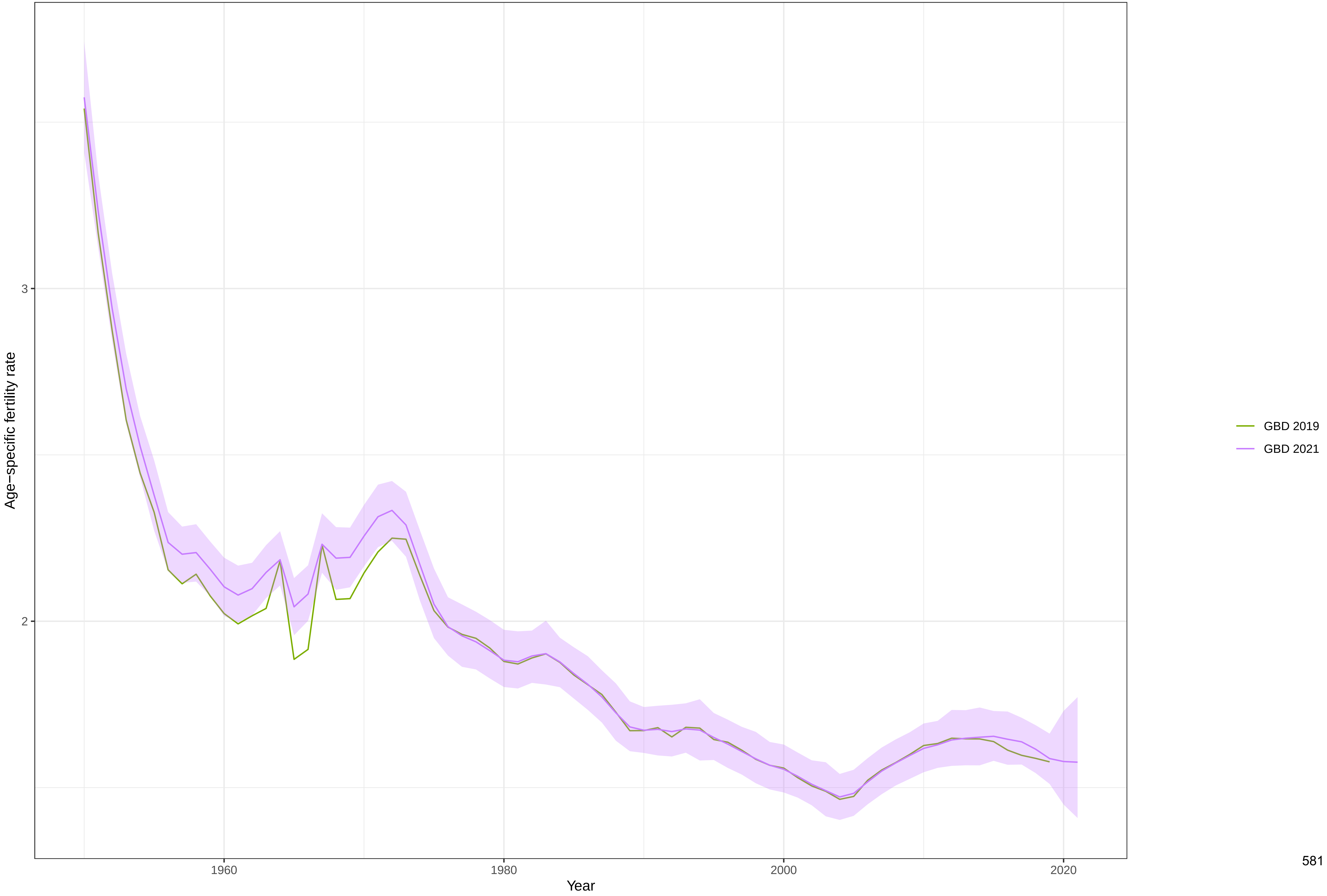

Age−specific fertility fitted trend lines, 1950−2021: Nagano, Ages: 10−14
Data points enclosed with a diamond are reference sources

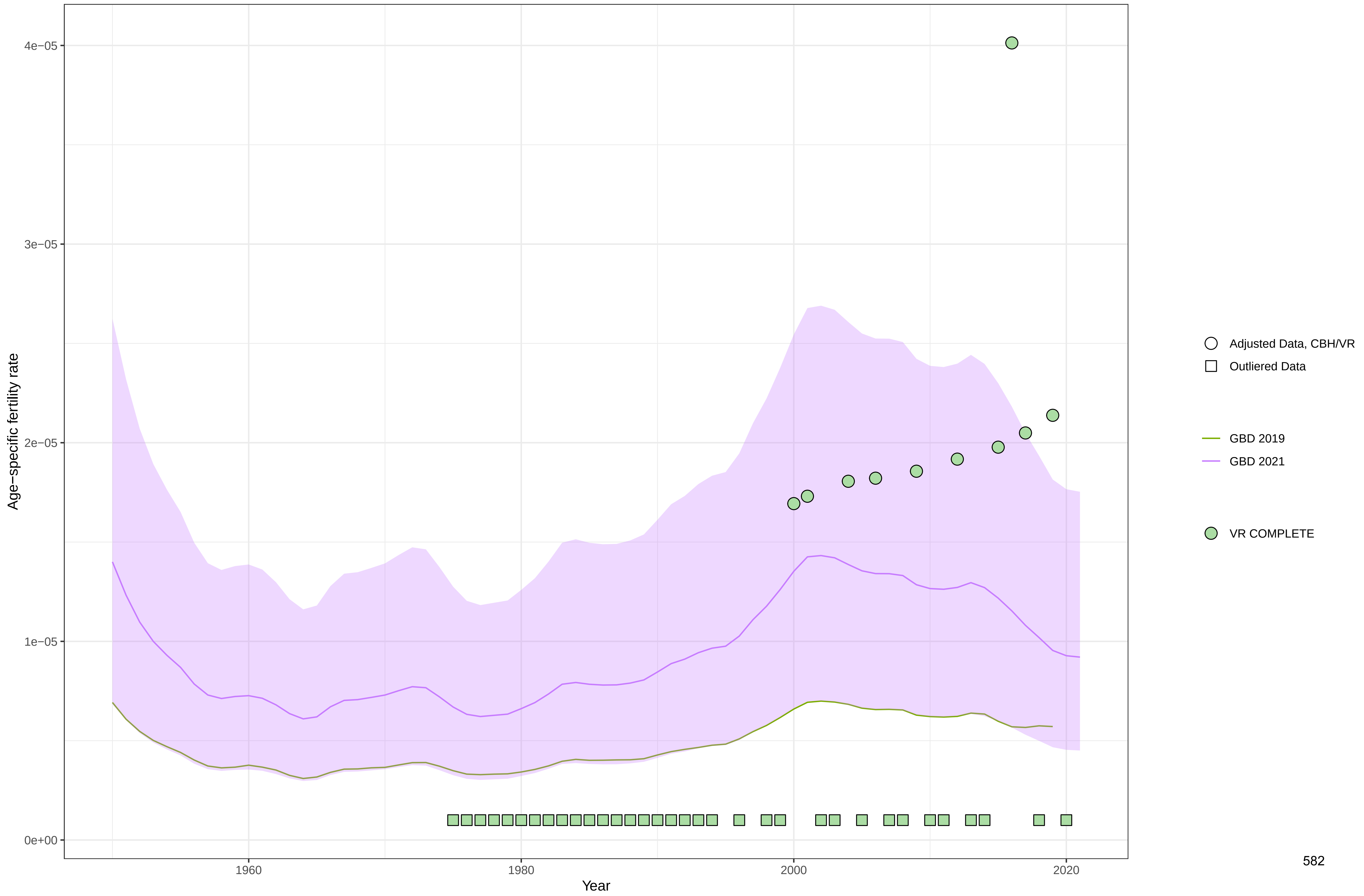

Age−specific fertility fitted trend lines, 1950−2021: Nagano, Ages: 15−19
Data points enclosed with a diamond are reference sources

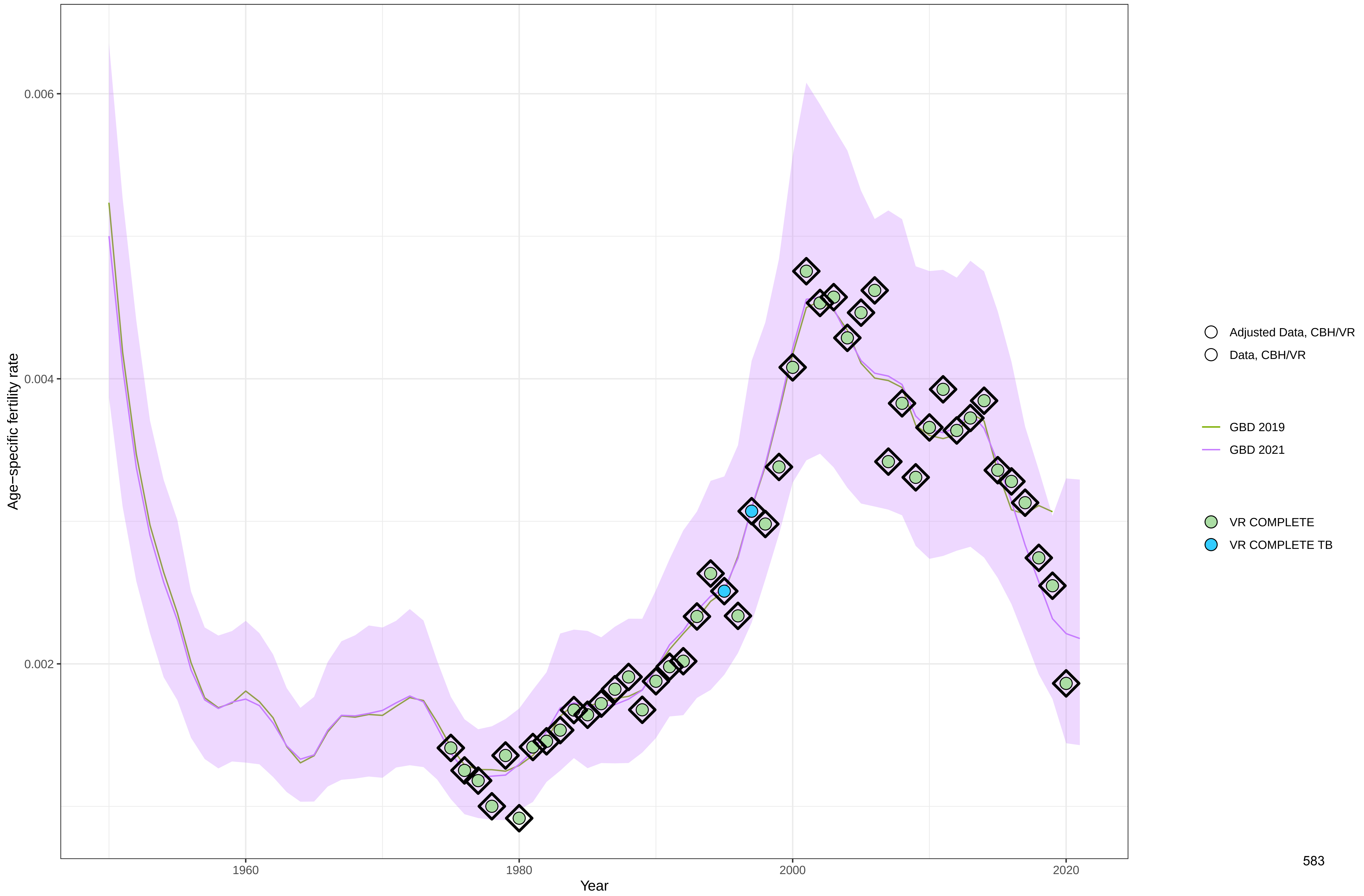

Age−specific fertility fitted trend lines, 1950−2021: Nagano, Ages: 20−24
Data points enclosed with a diamond are reference sources

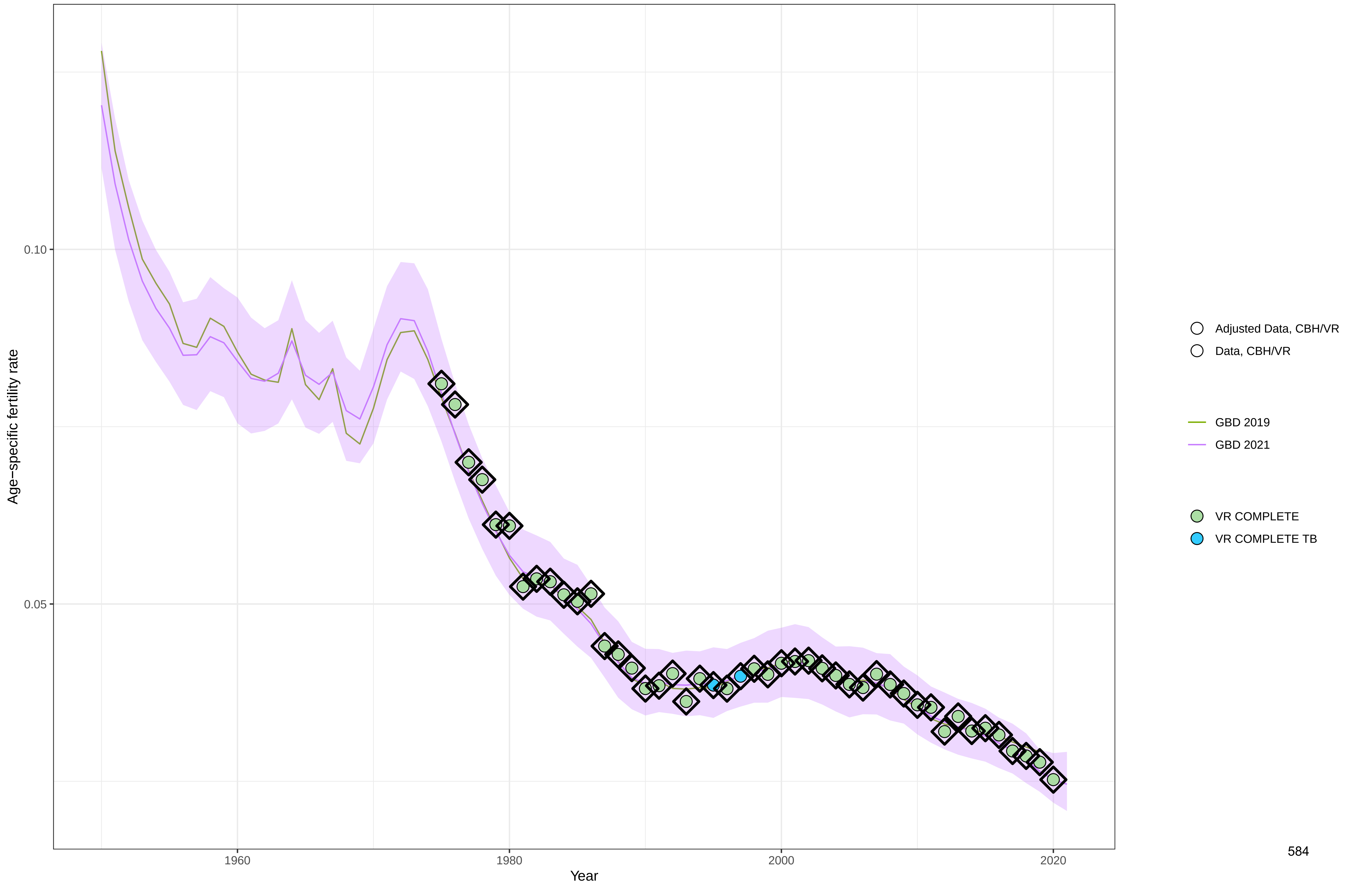

Age−specific fertility fitted trend lines, 1950−2021: Nagano, Ages: 25−29
Data points enclosed with a diamond are reference sources

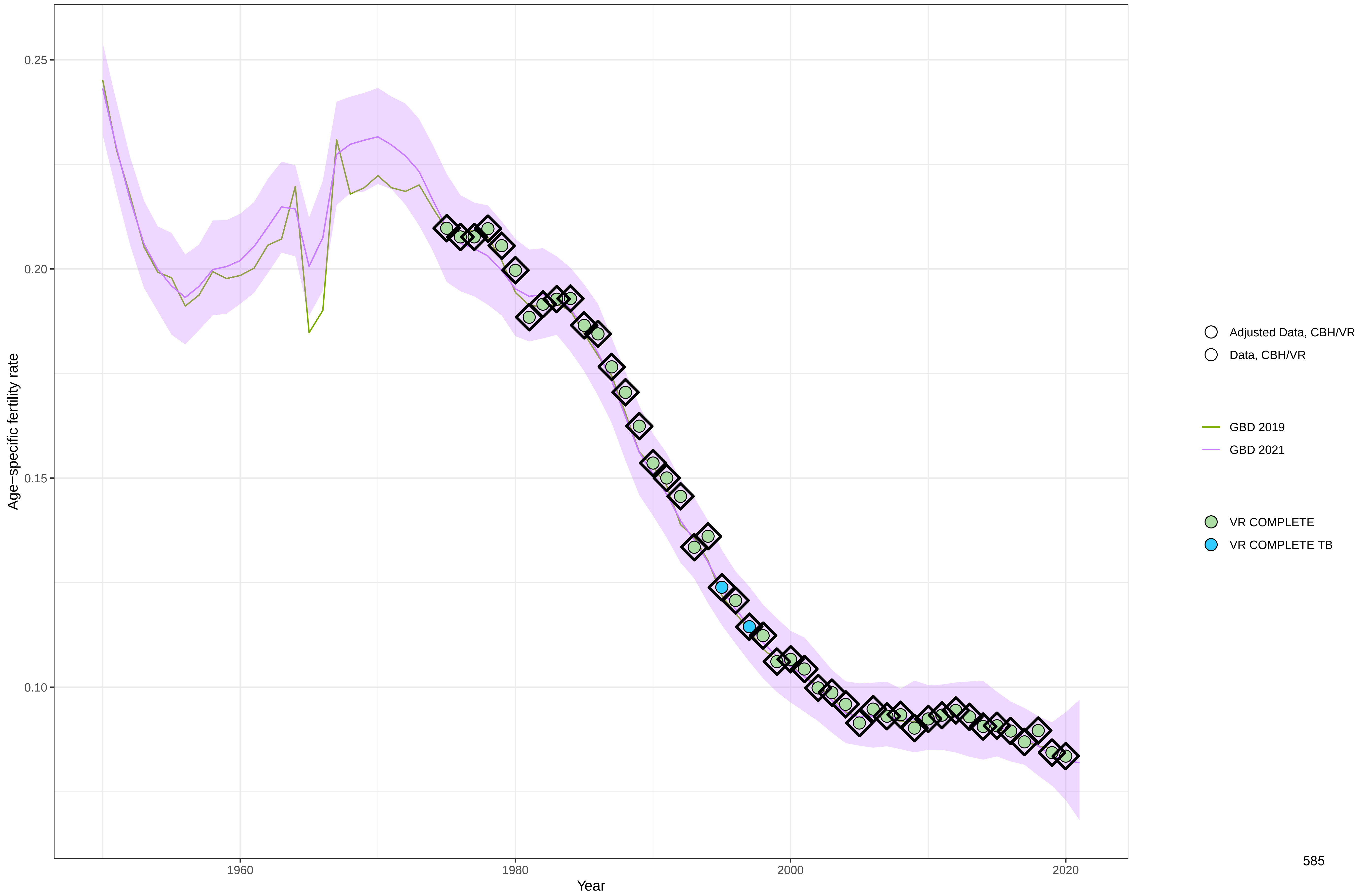

Age−specific fertility fitted trend lines, 1950−2021: Nagano, Ages: 30−34
Data points enclosed with a diamond are reference sources

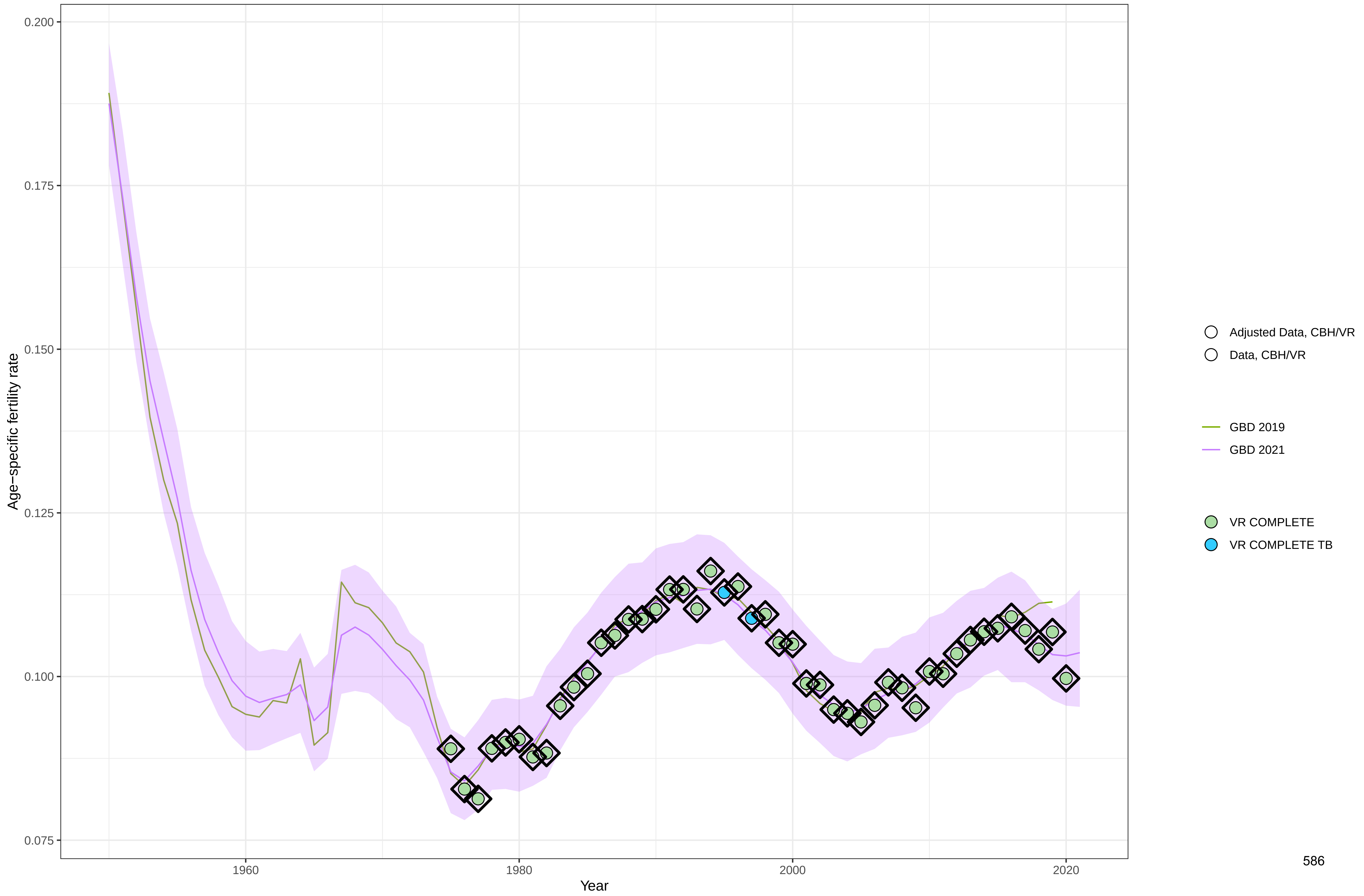

Age−specific fertility fitted trend lines, 1950−2021: Nagano, Ages: 35−39
Data points enclosed with a diamond are reference sources

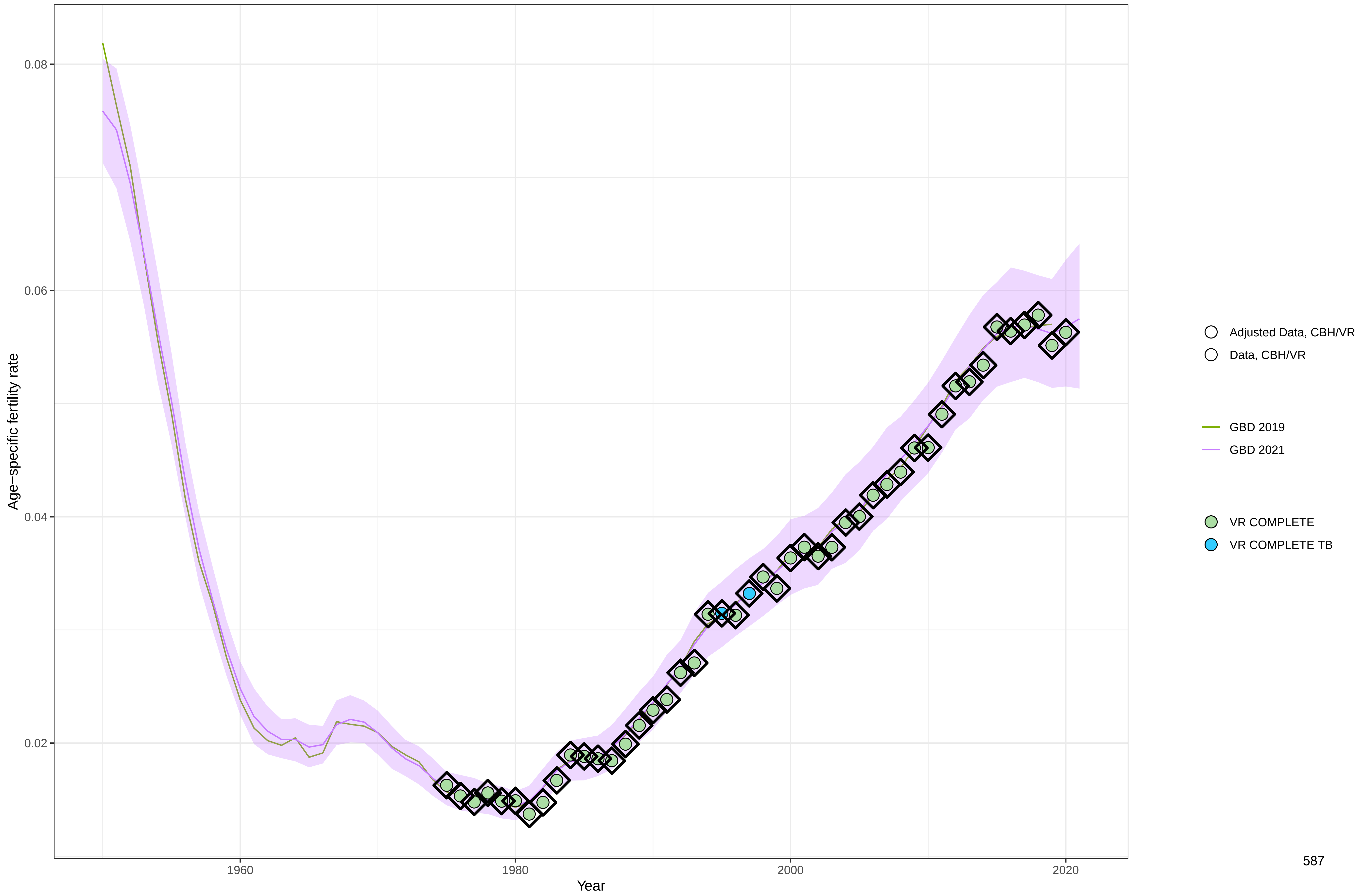

Age−specific fertility fitted trend lines, 1950−2021: Nagano, Ages: 40−44
Data points enclosed with a diamond are reference sources

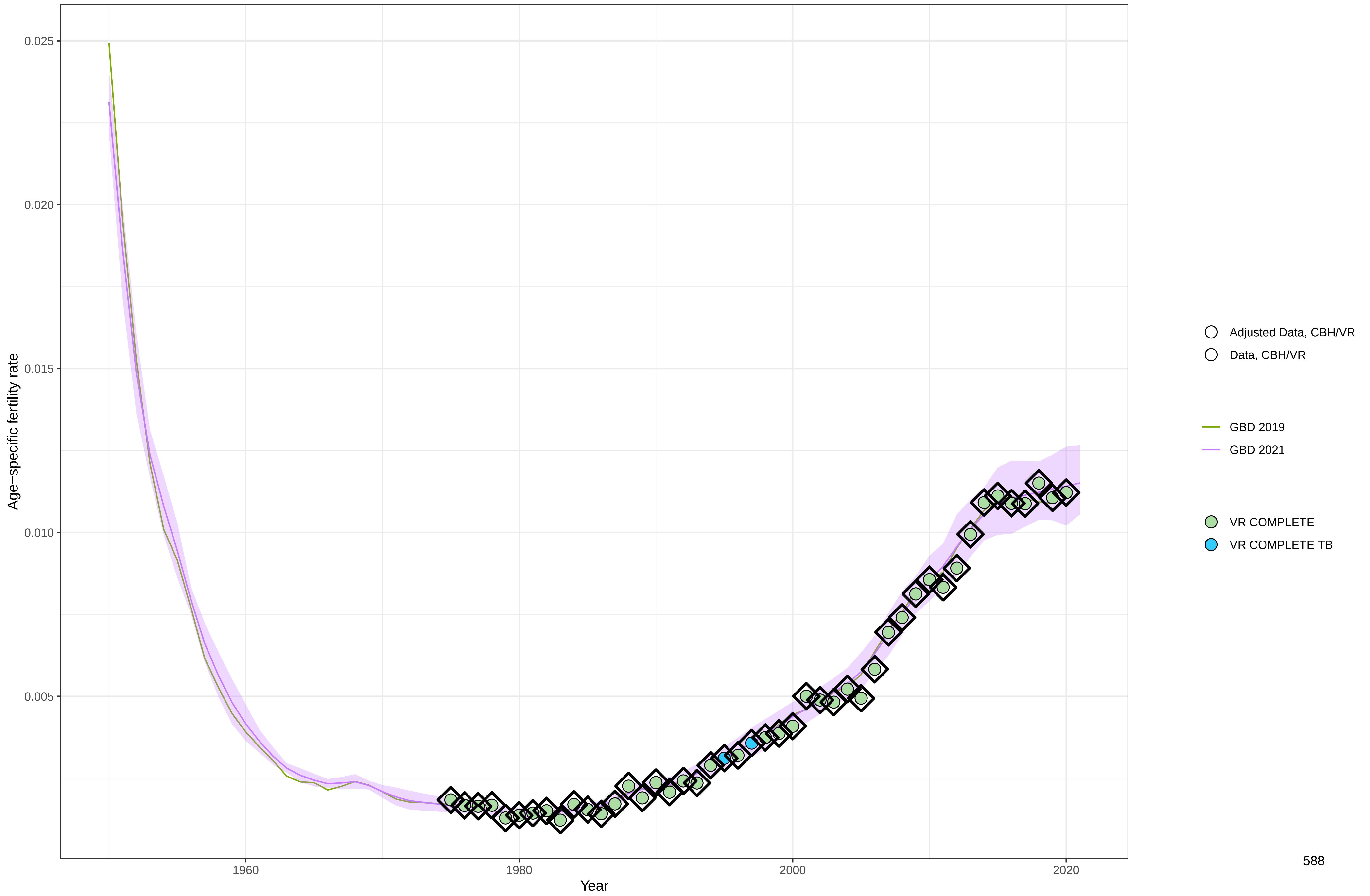

Age−specific fertility fitted trend lines, 1950−2021: Nagano, Ages: 45−49
Data points enclosed with a diamond are reference sources

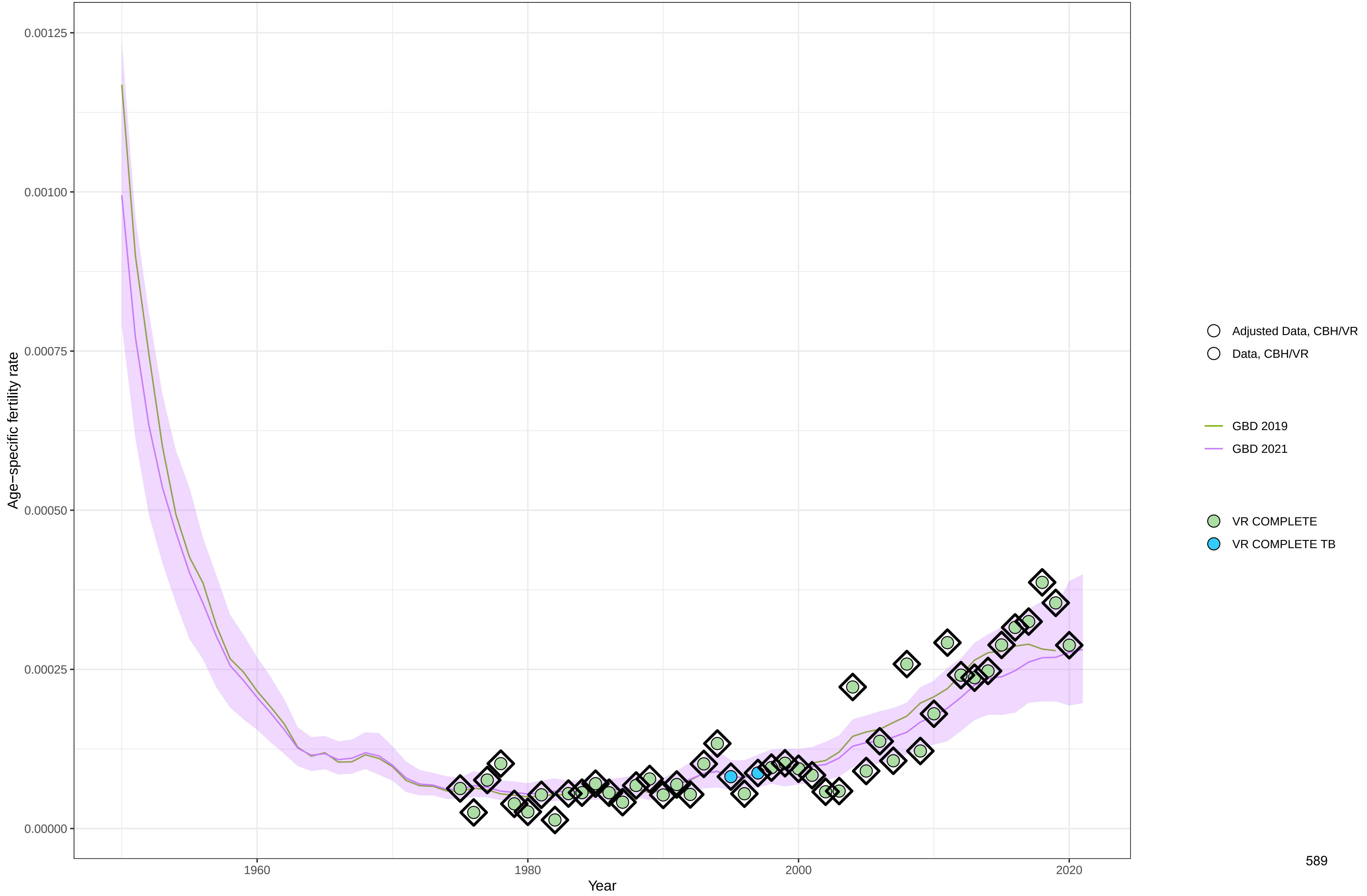

Age−specific fertility fitted trend lines, 1950−2021: Nagano, Ages: 50−54
Data points enclosed with a diamond are reference sources

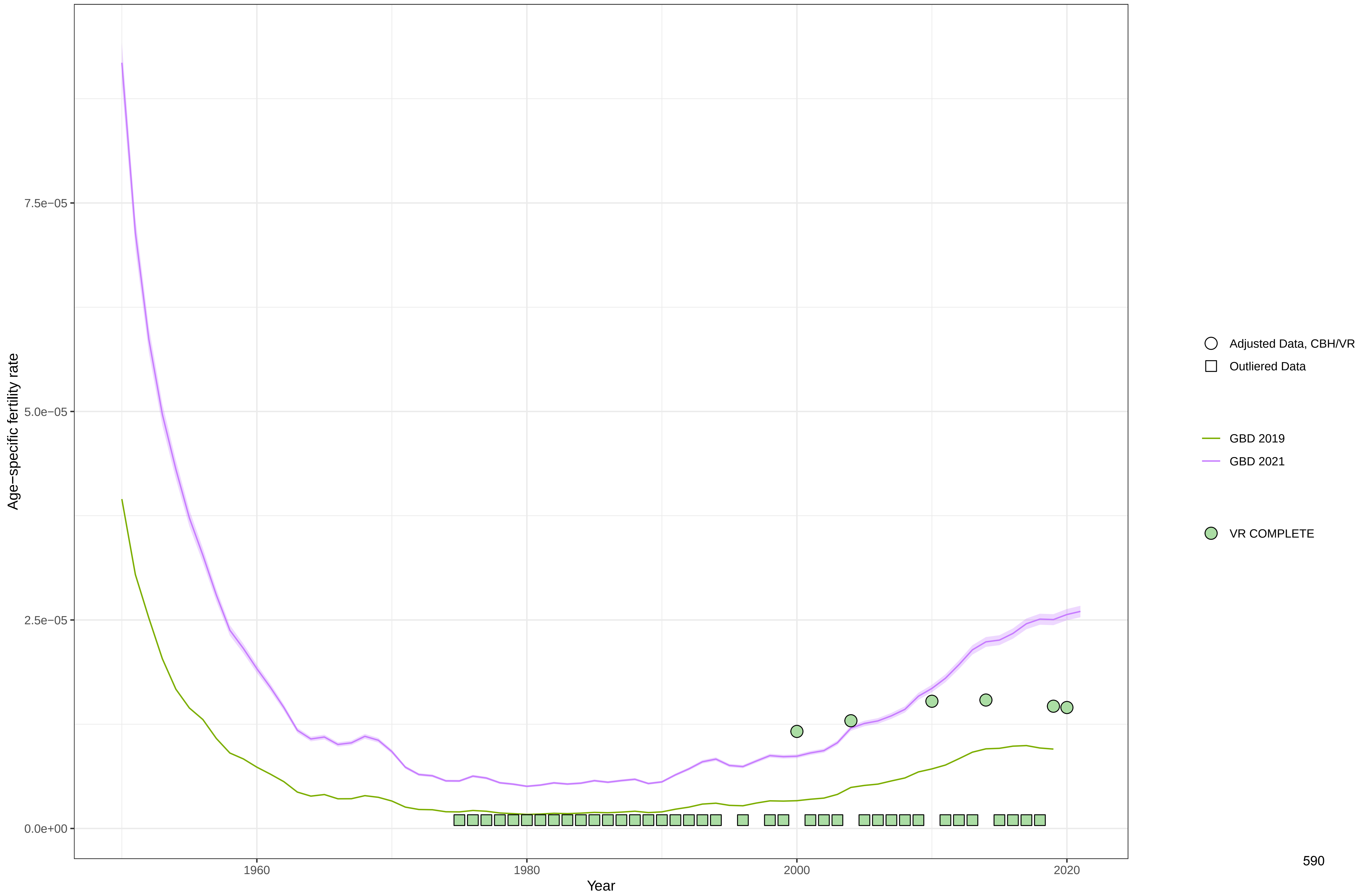

Total fertility rate fitted trend lines, 1950−2021: Nagano TFR
Data points enclosed with a diamond are reference sources

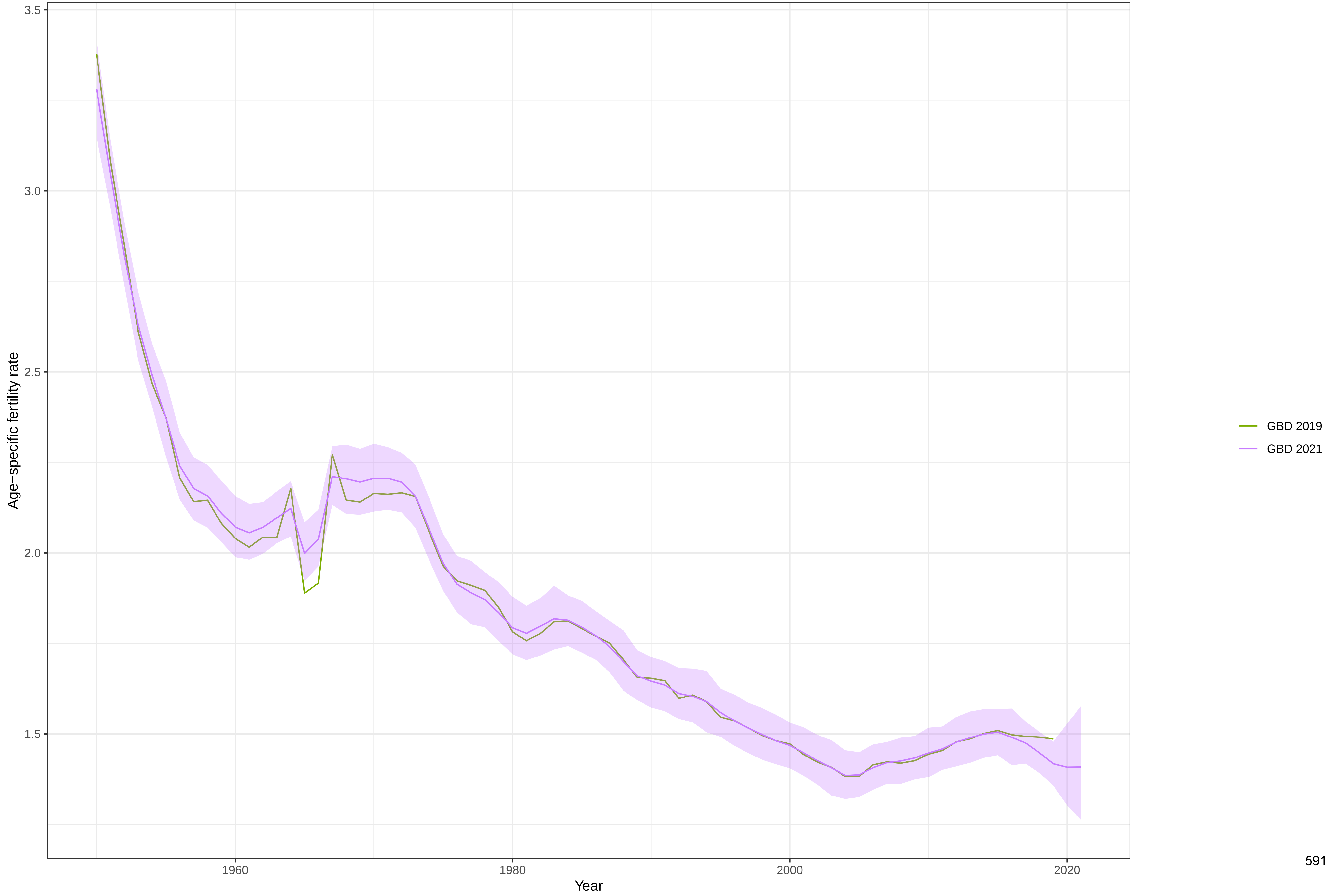

Age−specific fertility fitted trend lines, 1950−2021: Nagasaki, Ages: 10−14
Data points enclosed with a diamond are reference sources

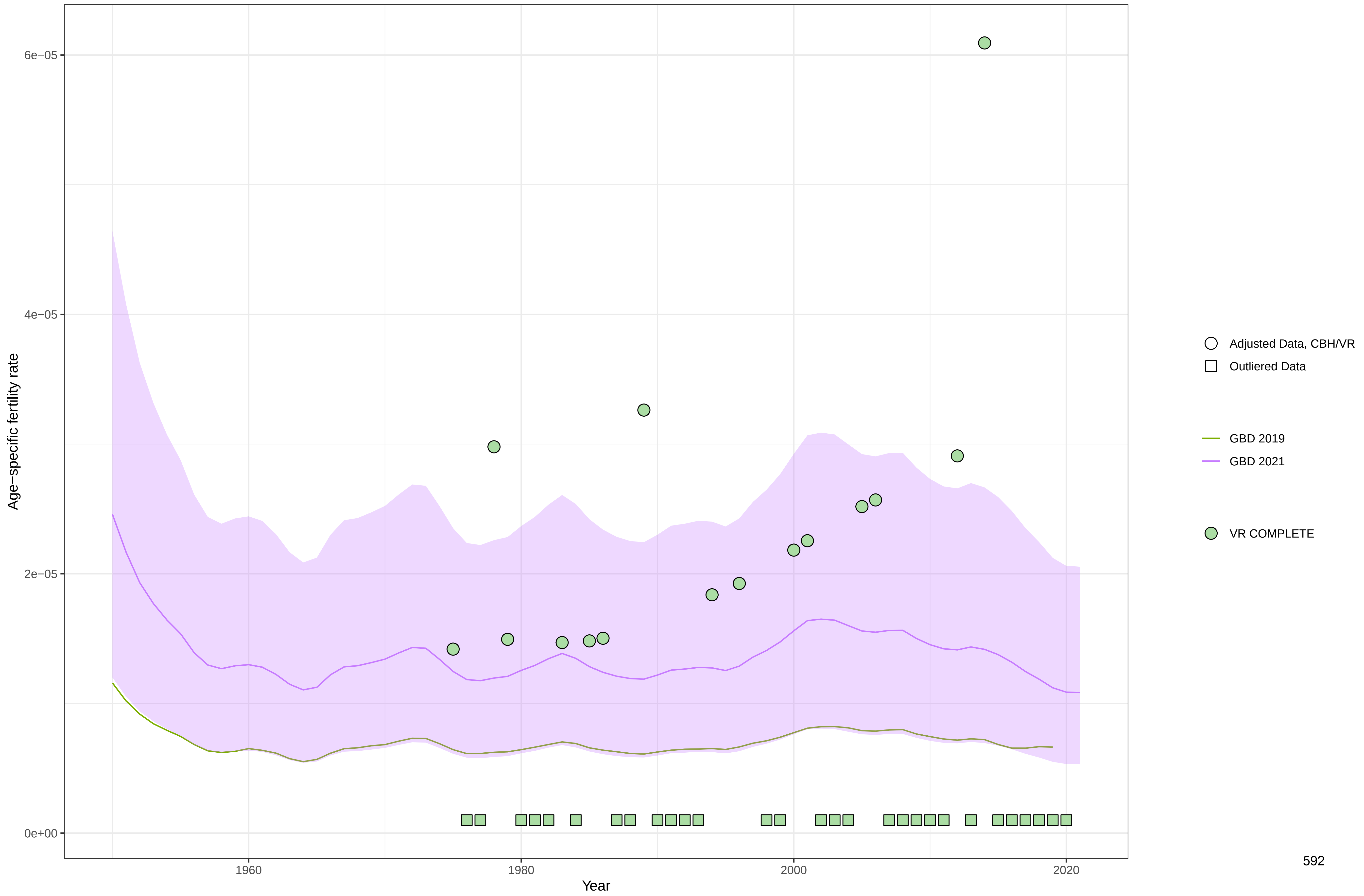

Age−specific fertility fitted trend lines, 1950−2021: Nagasaki, Ages: 15−19
Data points enclosed with a diamond are reference sources

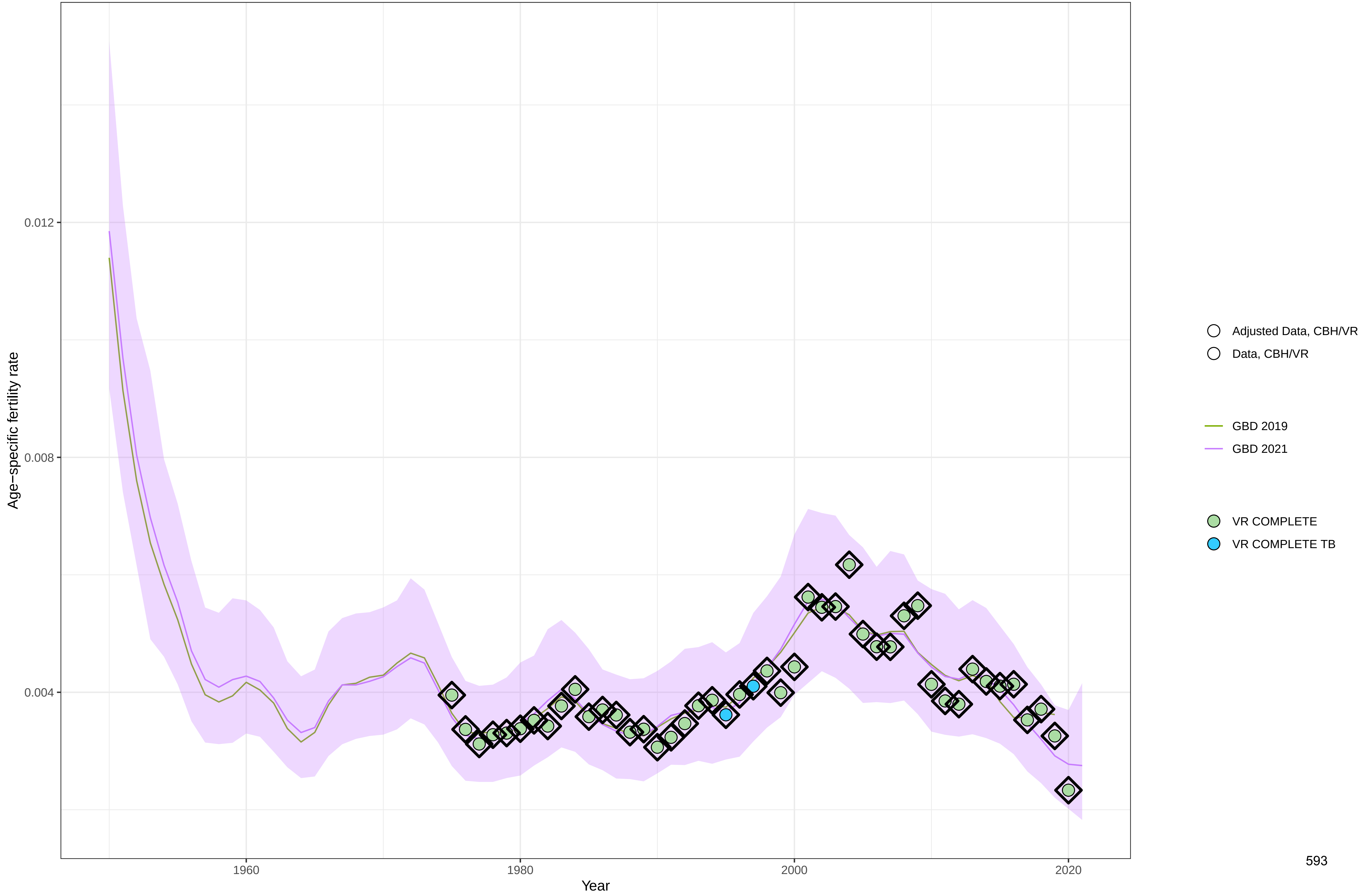

Age−specific fertility fitted trend lines, 1950−2021: Nagasaki, Ages: 20−24
Data points enclosed with a diamond are reference sources

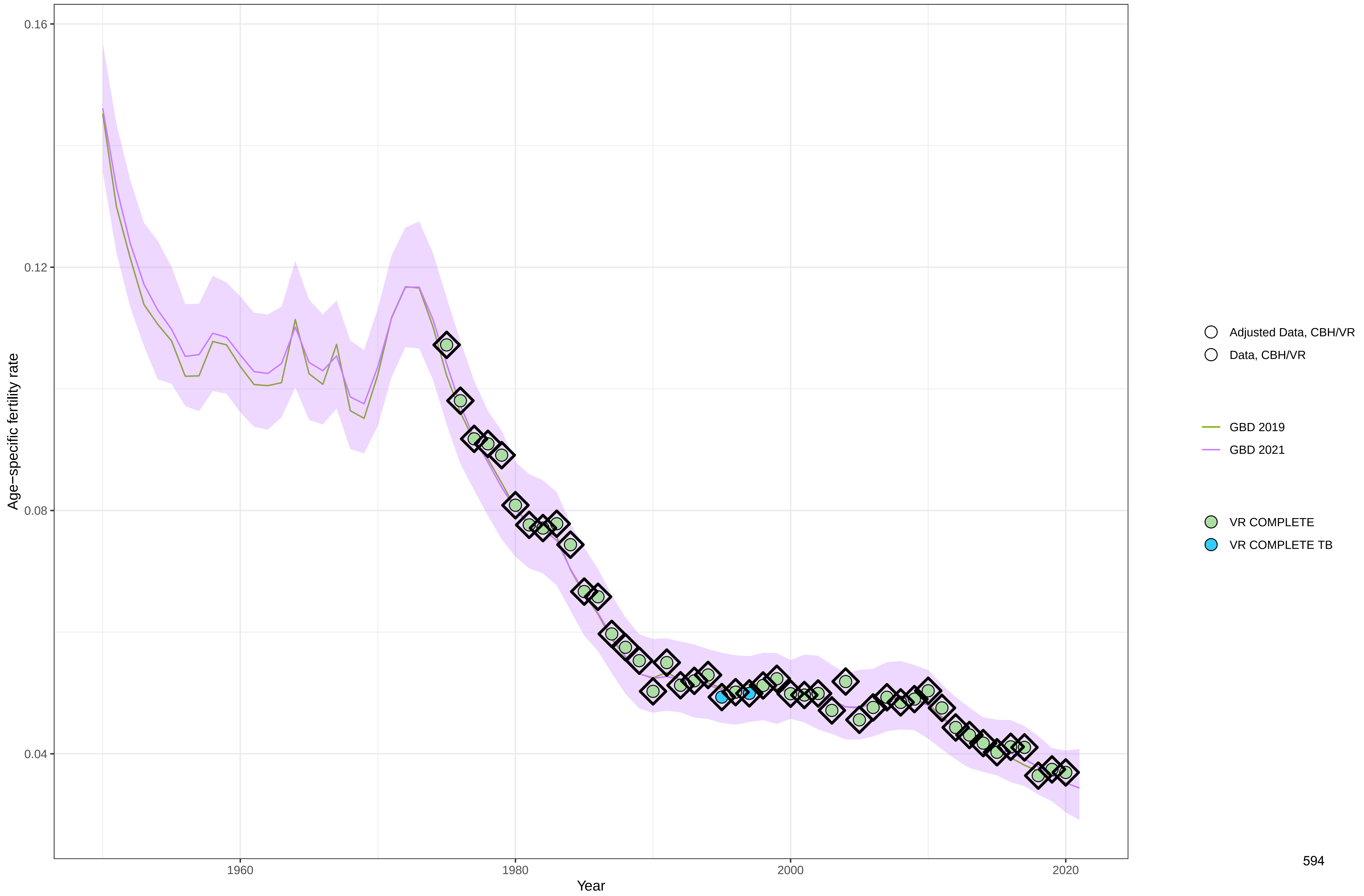

Age−specific fertility fitted trend lines, 1950−2021: Nagasaki, Ages: 25−29
Data points enclosed with a diamond are reference sources

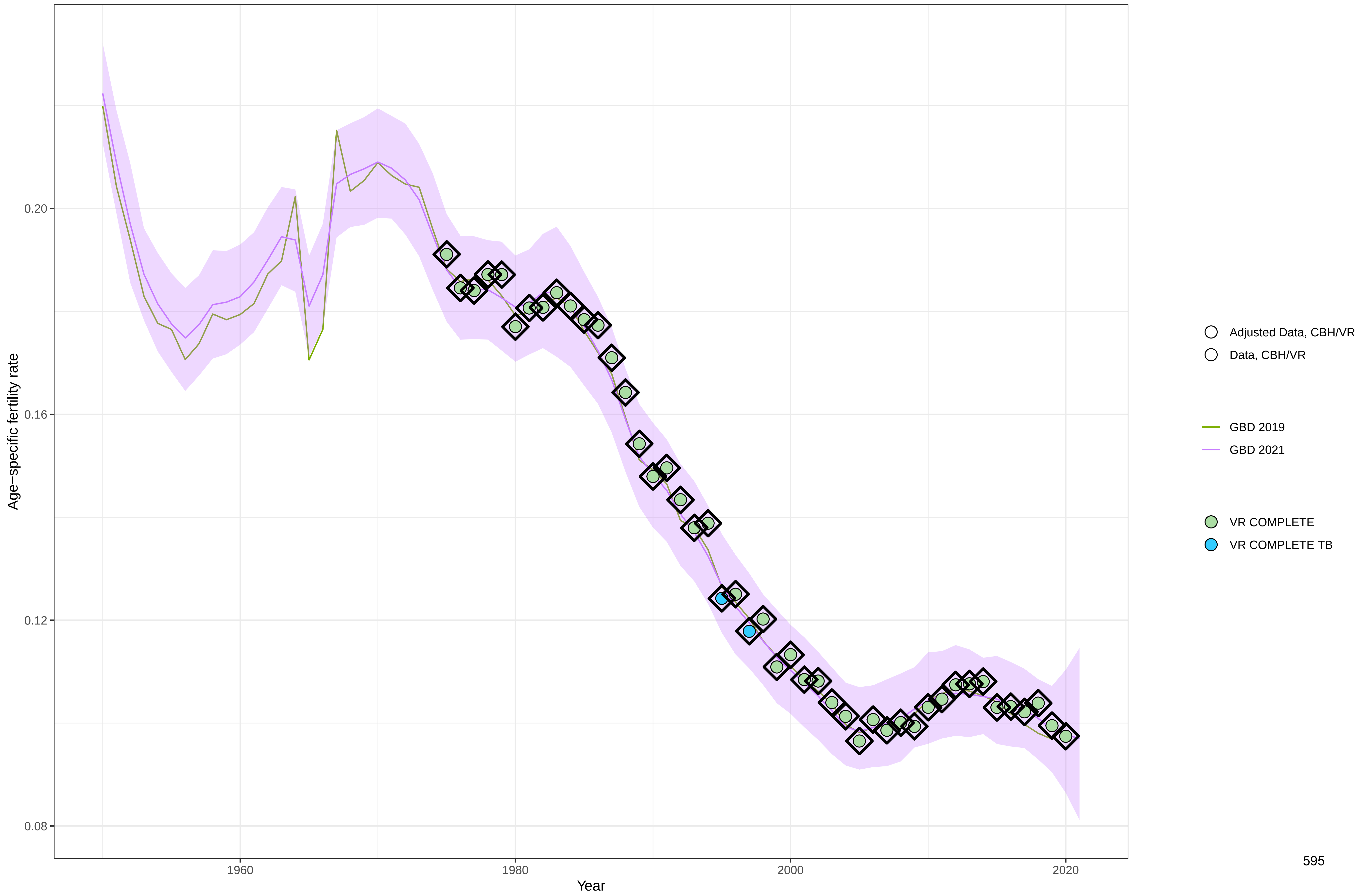

Age−specific fertility fitted trend lines, 1950−2021: Nagasaki, Ages: 30−34
Data points enclosed with a diamond are reference sources

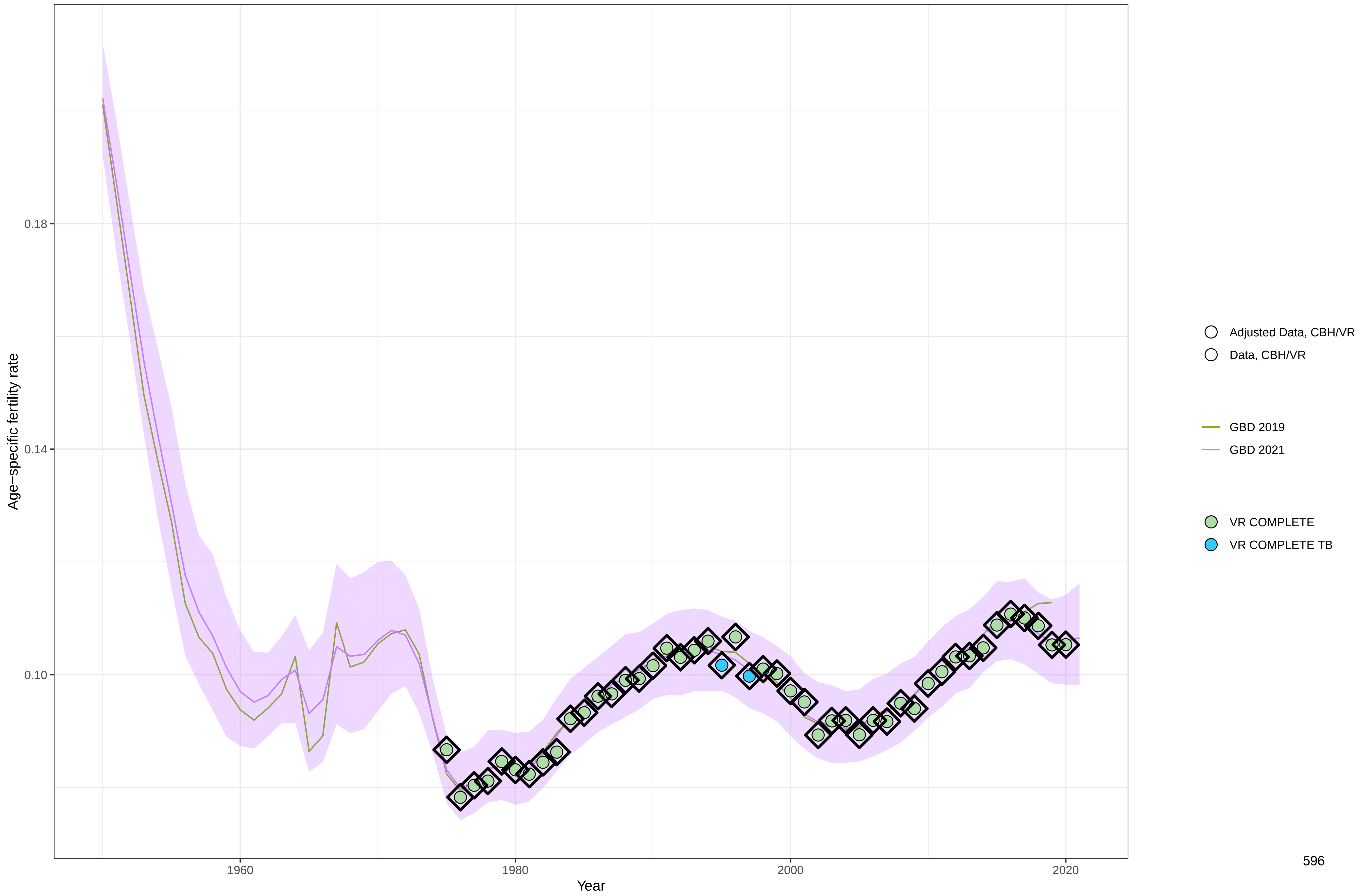

Age−specific fertility fitted trend lines, 1950−2021: Nagasaki, Ages: 35−39
Data points enclosed with a diamond are reference sources

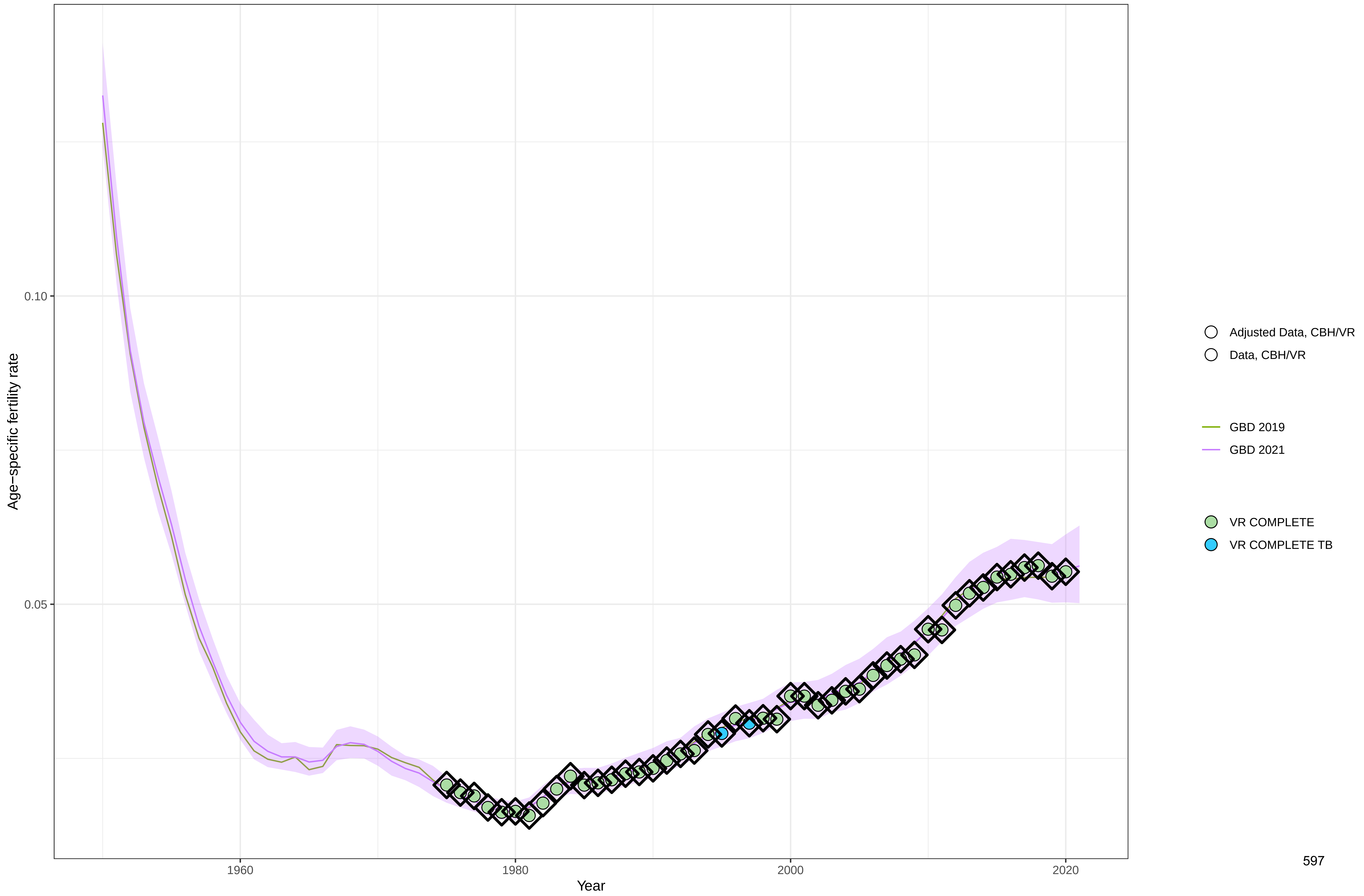

Age−specific fertility fitted trend lines, 1950−2021: Nagasaki, Ages: 40−44
Data points enclosed with a diamond are reference sources

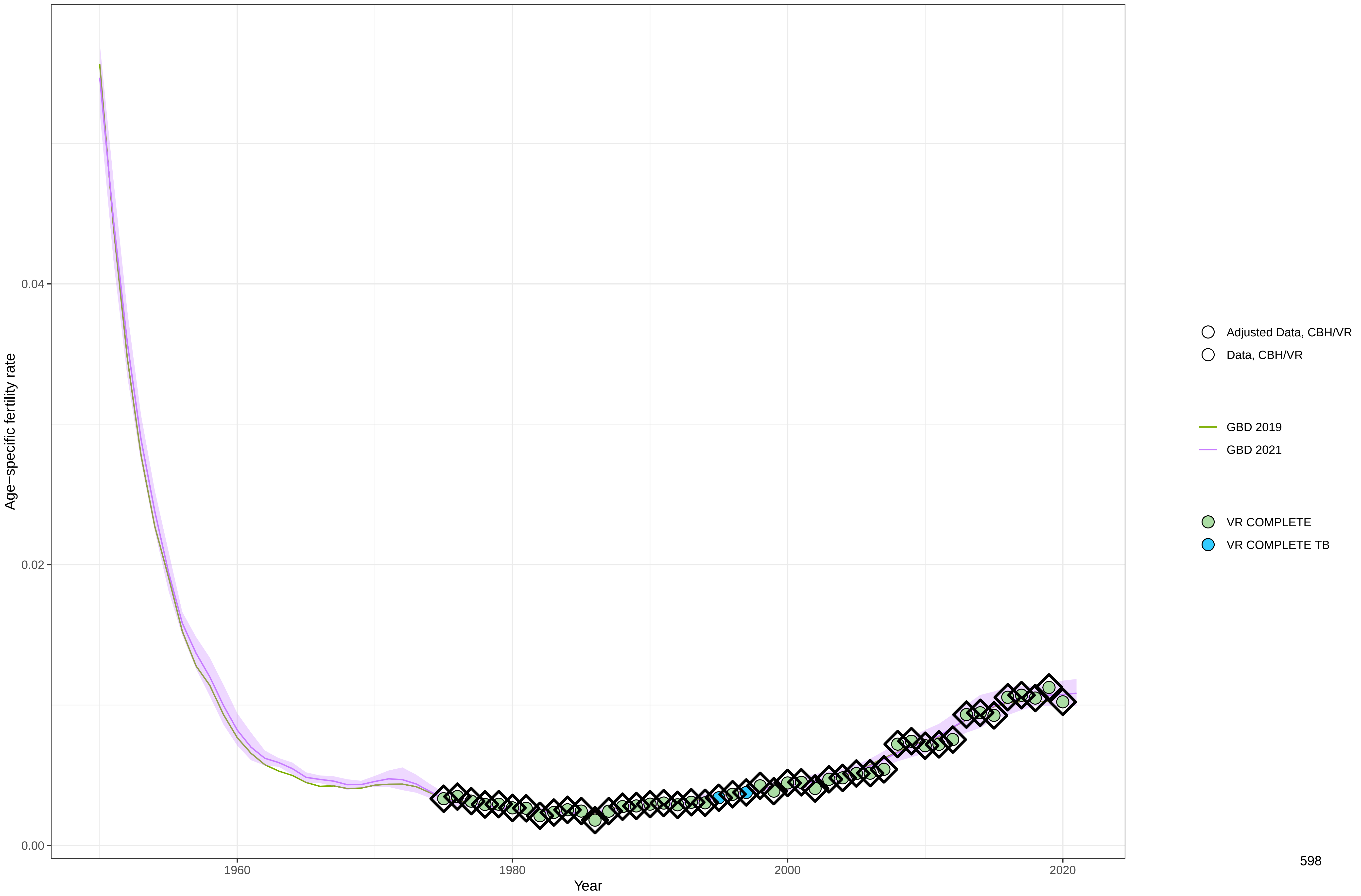

Age−specific fertility fitted trend lines, 1950−2021: Nagasaki, Ages: 45−49
Data points enclosed with a diamond are reference sources

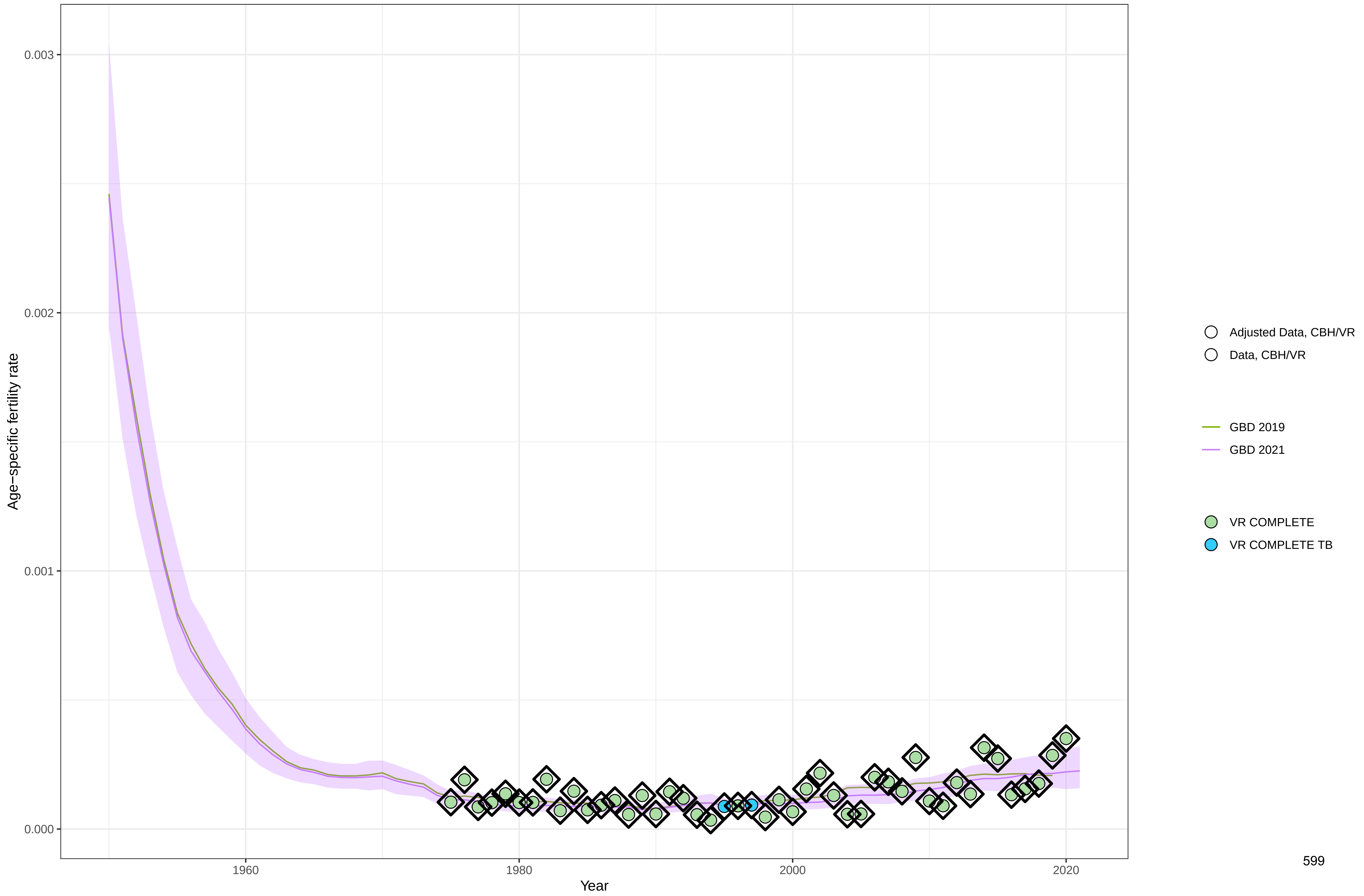

Age−specific fertility fitted trend lines, 1950−2021: Nagasaki, Ages: 50−54
Data points enclosed with a diamond are reference sources

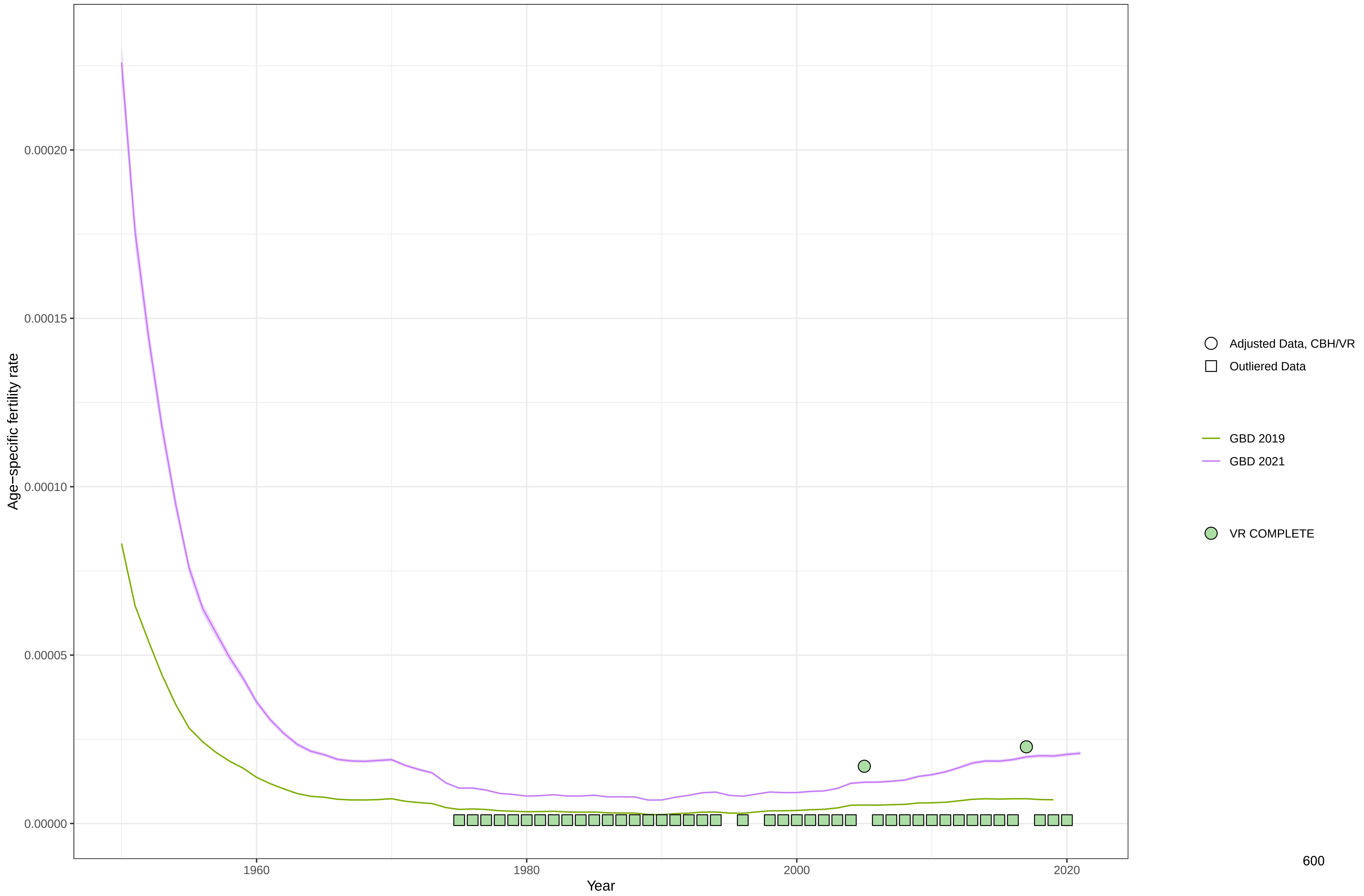

Total fertility rate fitted trend lines, 1950−2021: Nagasaki TFR
Data points enclosed with a diamond are reference sources

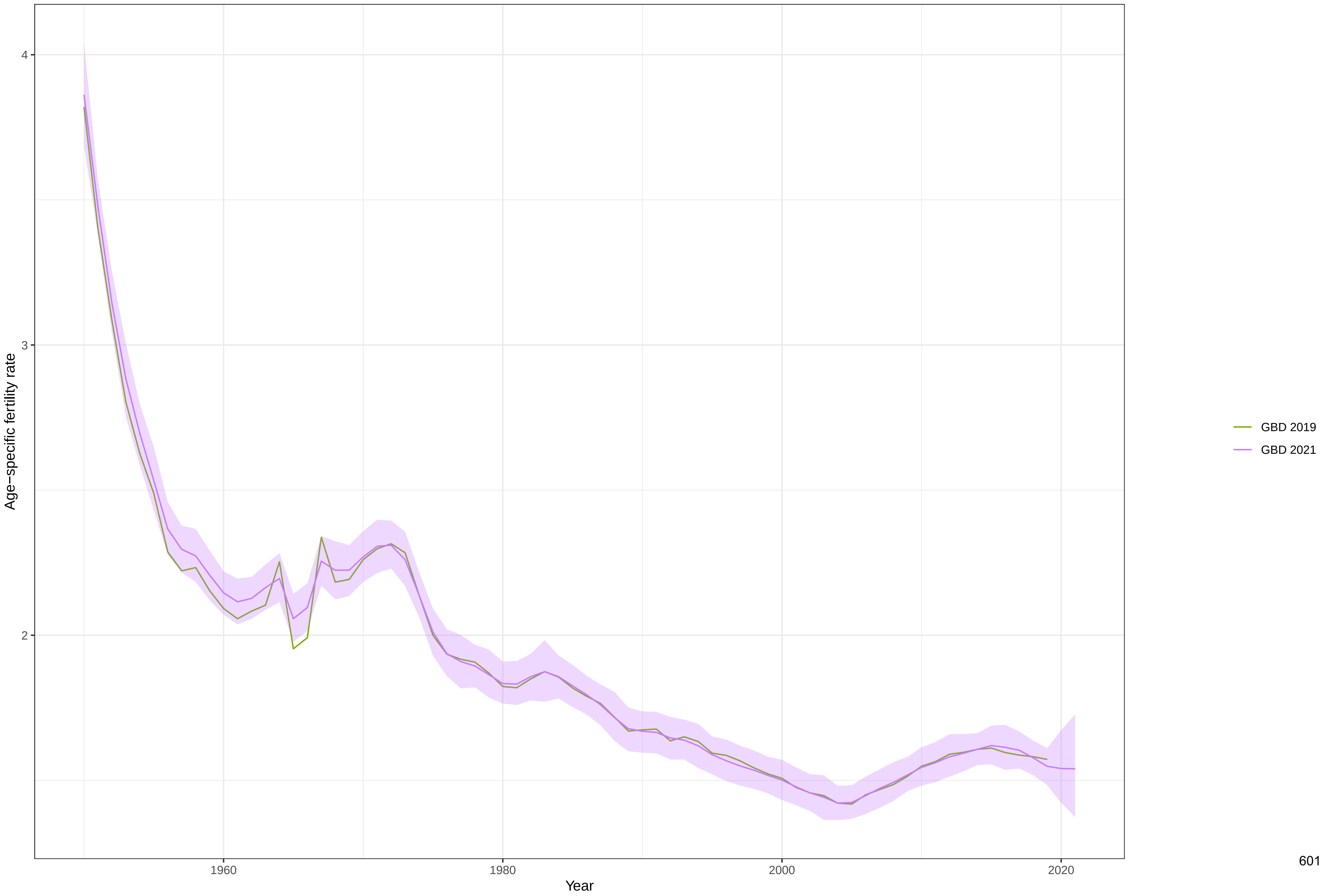

Age−specific fertility fitted trend lines, 1950−2021: Nara, Ages: 10−14
Data points enclosed with a diamond are reference sources

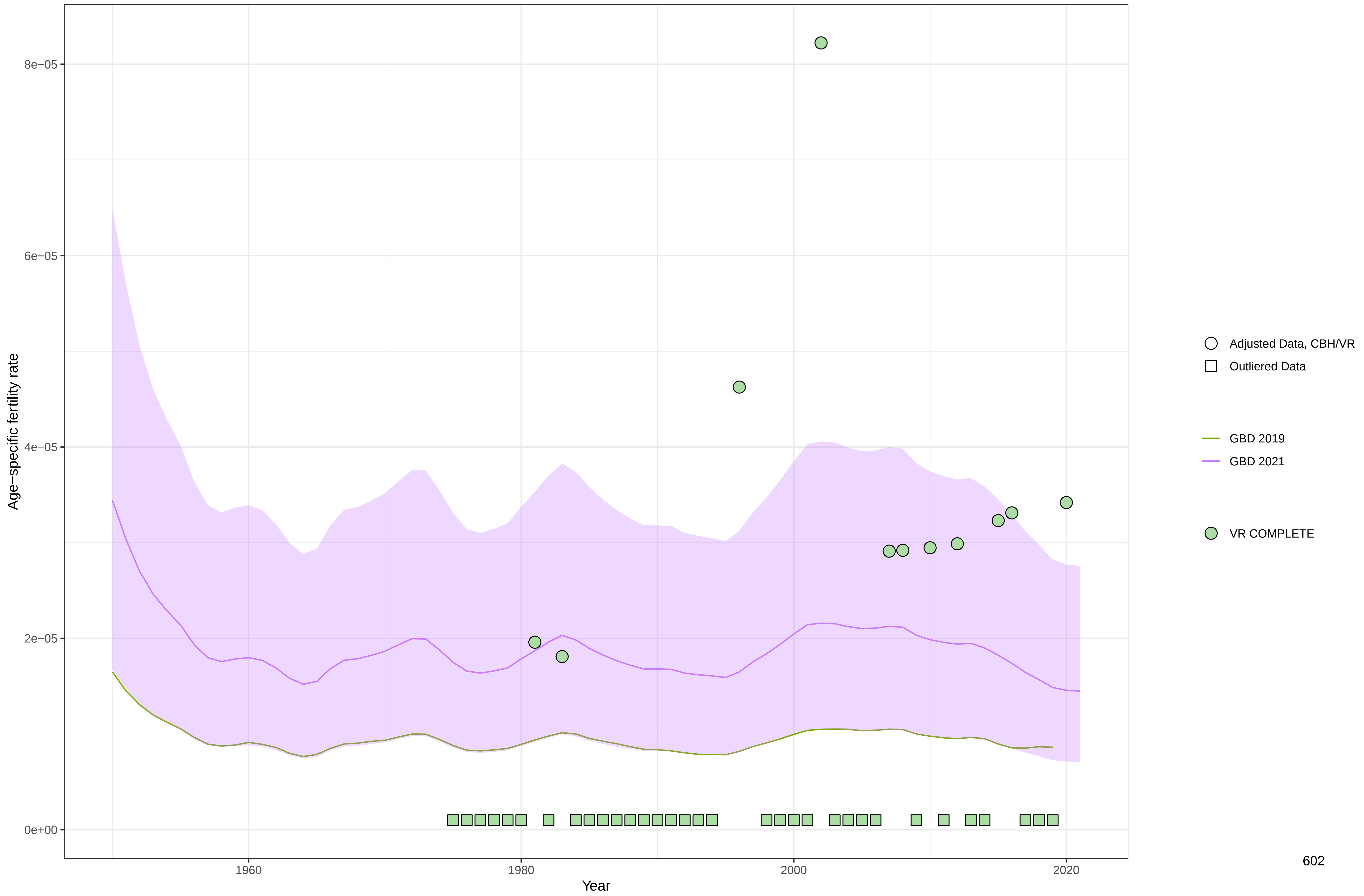

Age−specific fertility fitted trend lines, 1950−2021: Nara, Ages: 15−19
Data points enclosed with a diamond are reference sources

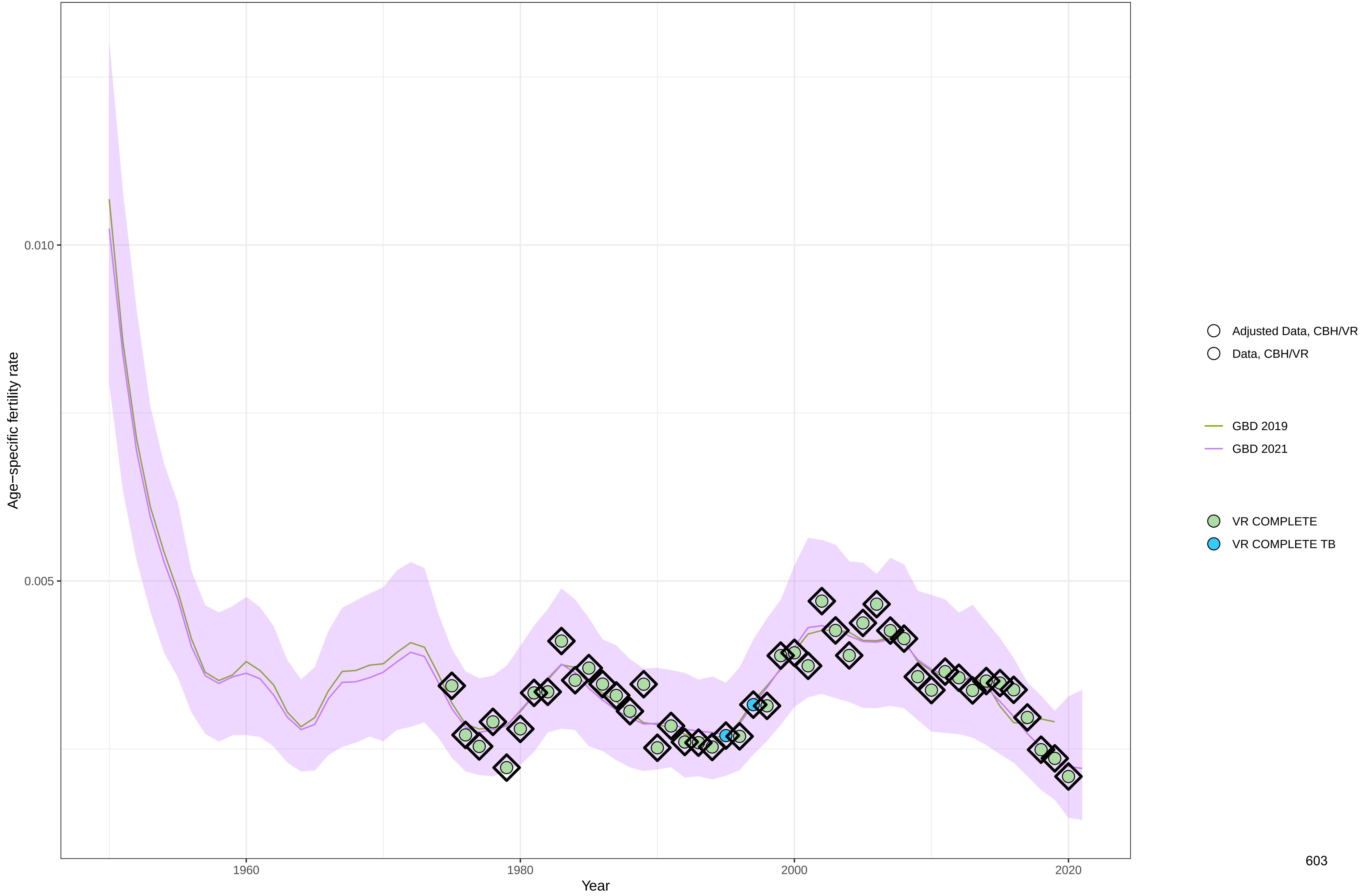

Age−specific fertility fitted trend lines, 1950−2021: Nara, Ages: 20−24
Data points enclosed with a diamond are reference sources

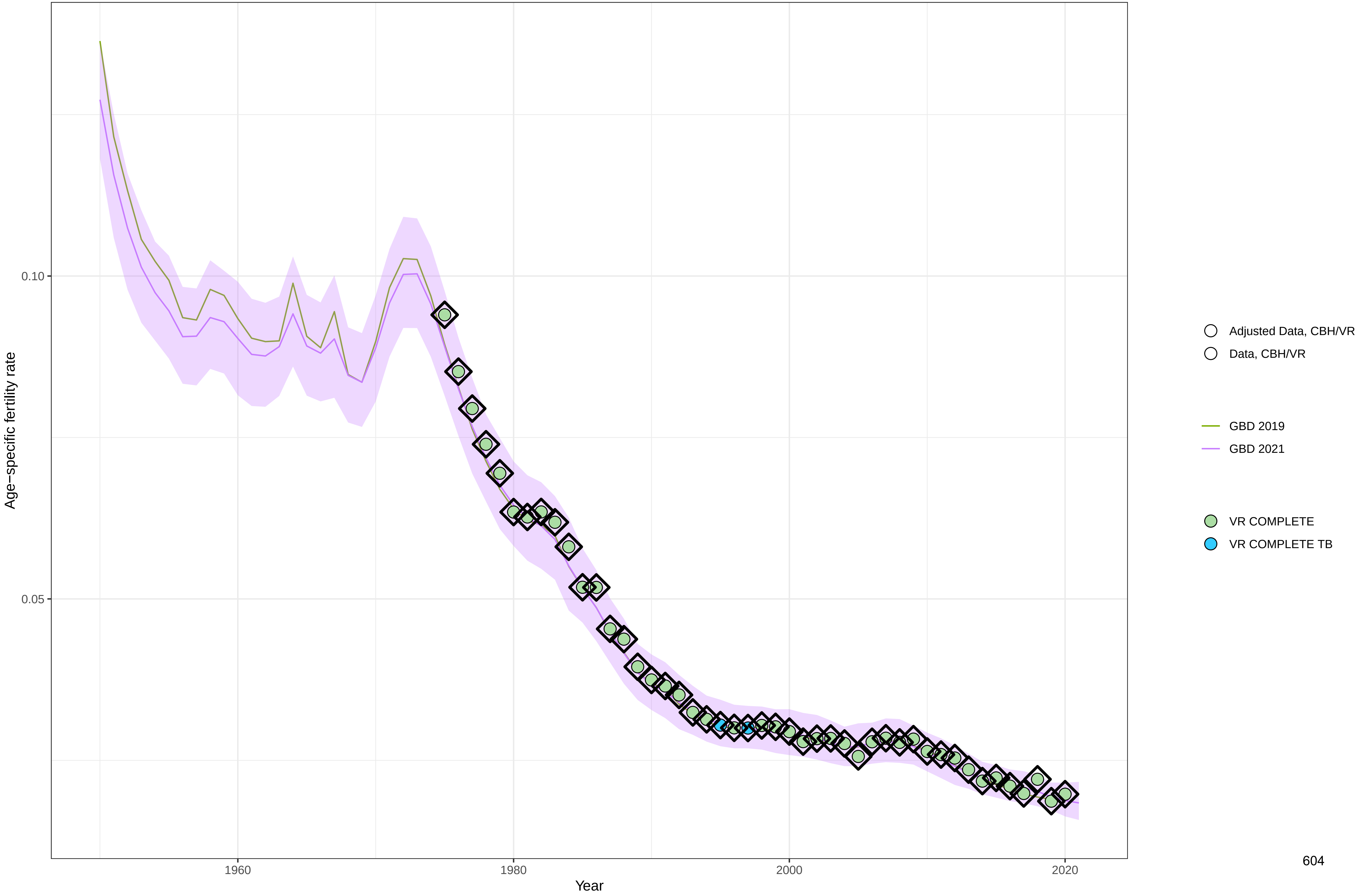

Age−specific fertility fitted trend lines, 1950−2021: Nara, Ages: 25−29
Data points enclosed with a diamond are reference sources

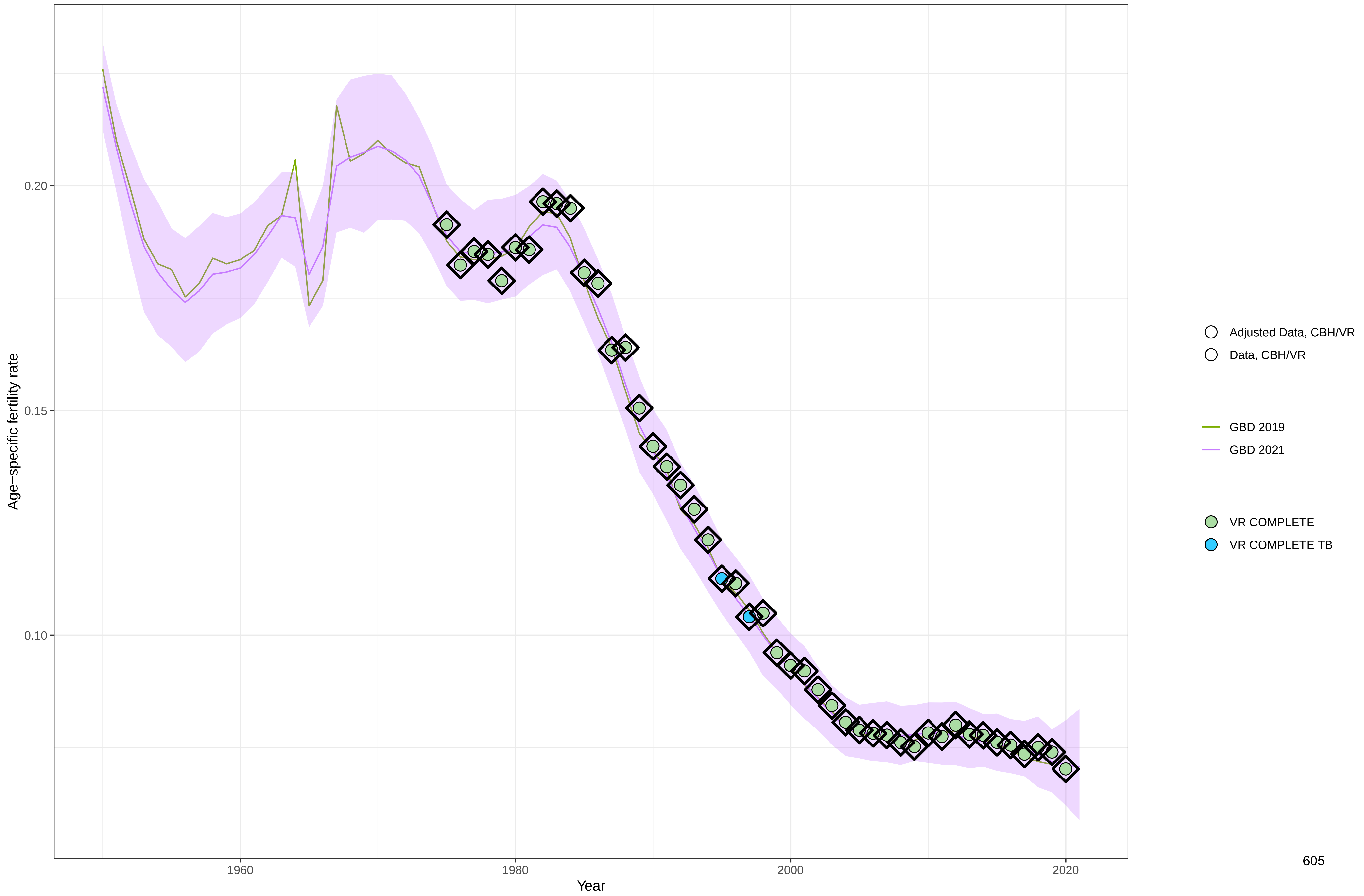

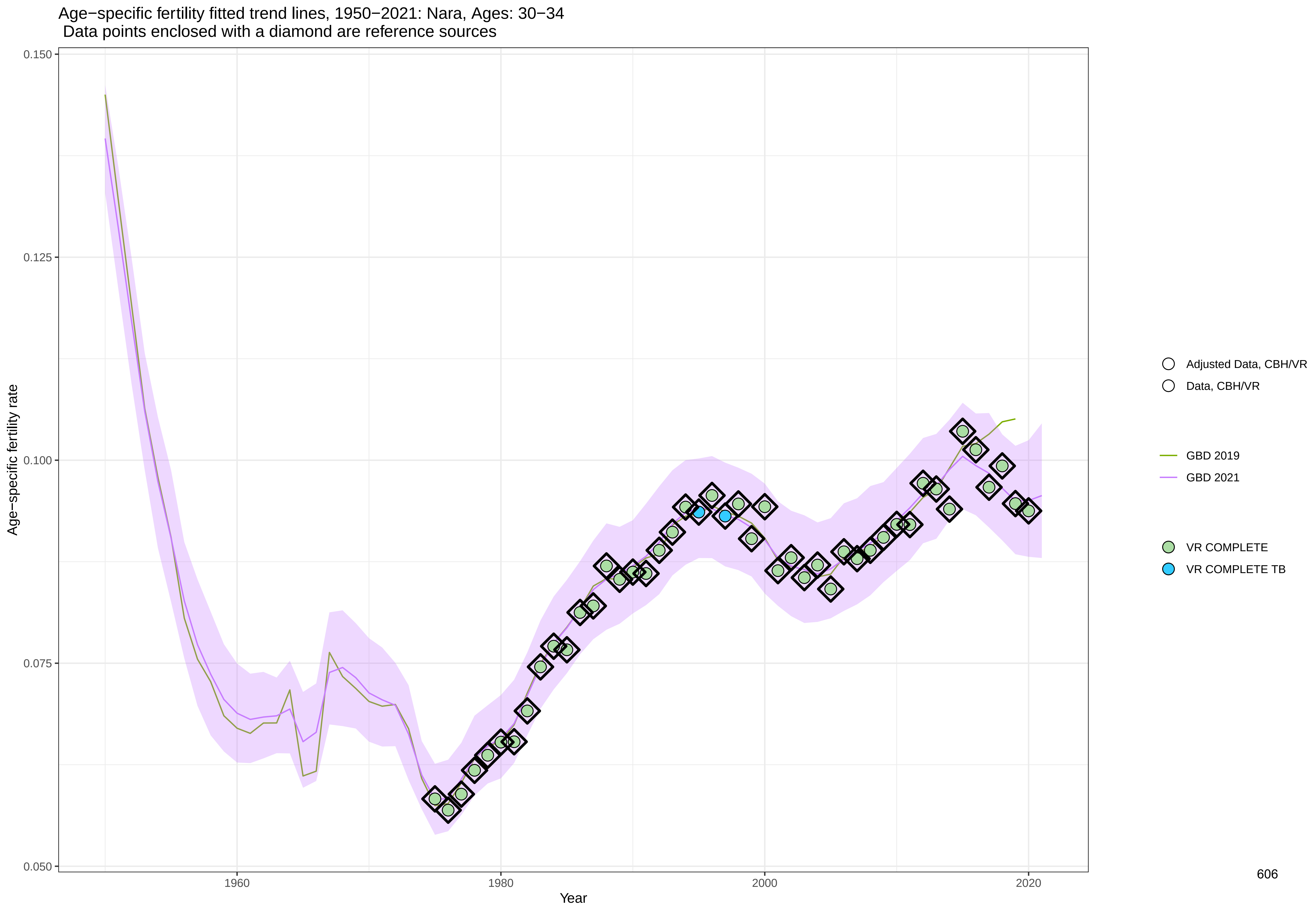
Age−specific fertility fitted trend lines, 1950−2021: Nara, Ages: 30−34
Data points enclosed with a diamond are reference sources
Age−specific fertility rate
Year
0.150
0.125
0.100
0.075
0.050
1960
1980
2000
2020
Adjusted Data, CBH/VR
Data, CBH/VR
GBD 2019
GBD 2021
VR COMPLETE
VR COMPLETE TB

Age−specific fertility fitted trend lines, 1950−2021: Nara, Ages: 35−39
Data points enclosed with a diamond are reference sources

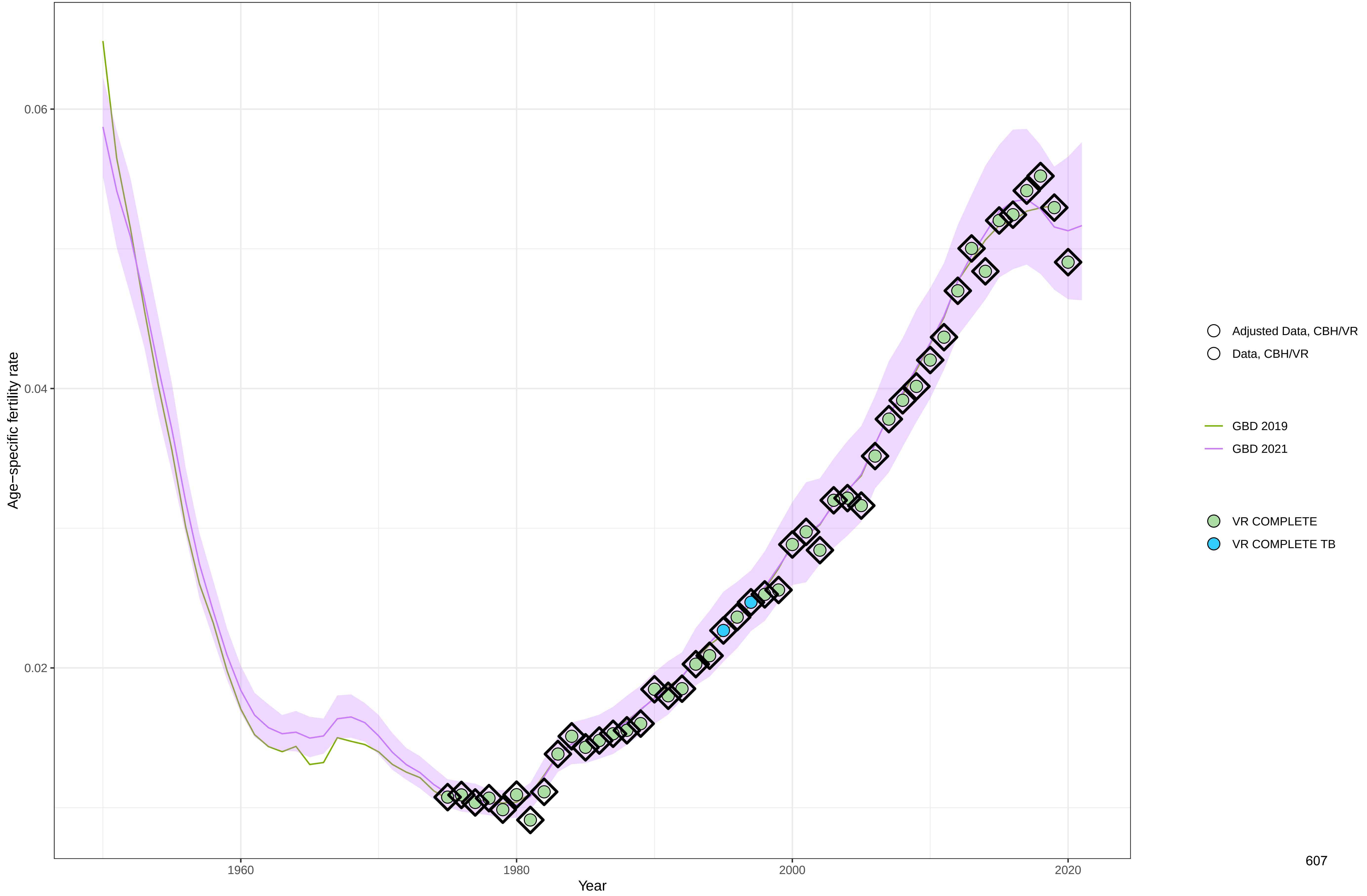

Age−specific fertility fitted trend lines, 1950−2021: Nara, Ages: 40−44
Data points enclosed with a diamond are reference sources

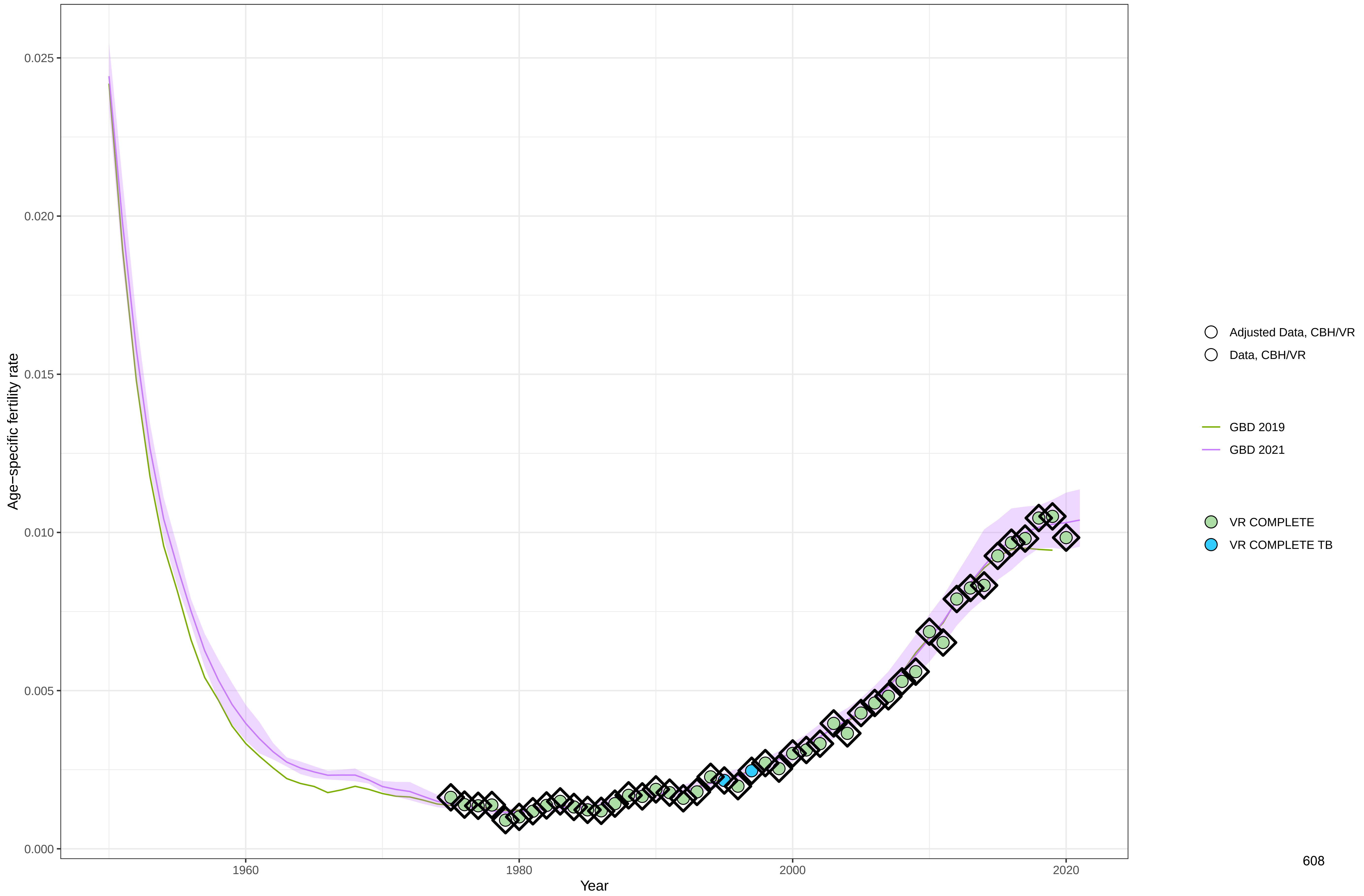

Age−specific fertility fitted trend lines, 1950−2021: Nara, Ages: 45−49
Data points enclosed with a diamond are reference sources

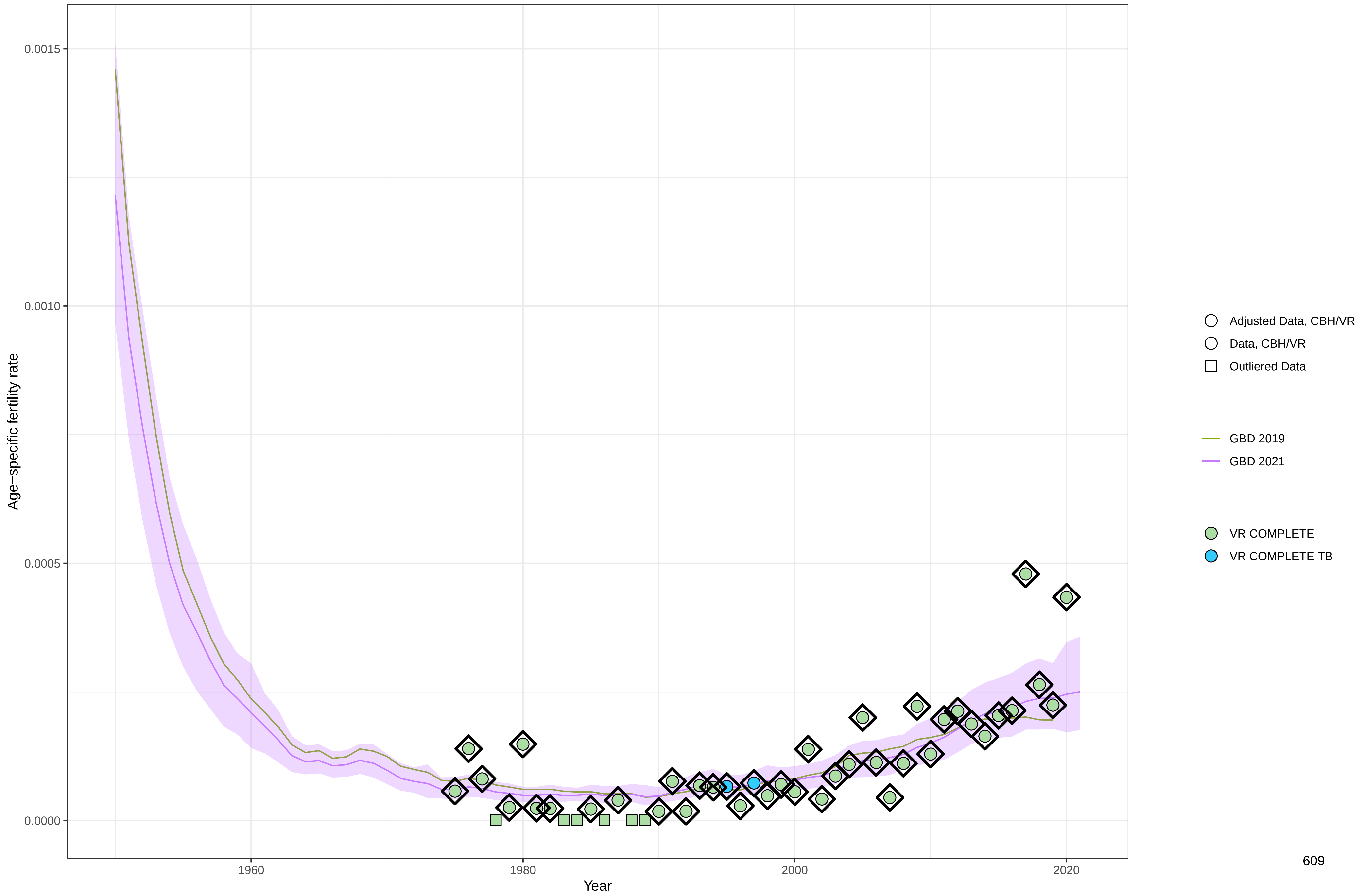

Age−specific fertility fitted trend lines, 1950−2021: Nara, Ages: 50−54
Data points enclosed with a diamond are reference sources

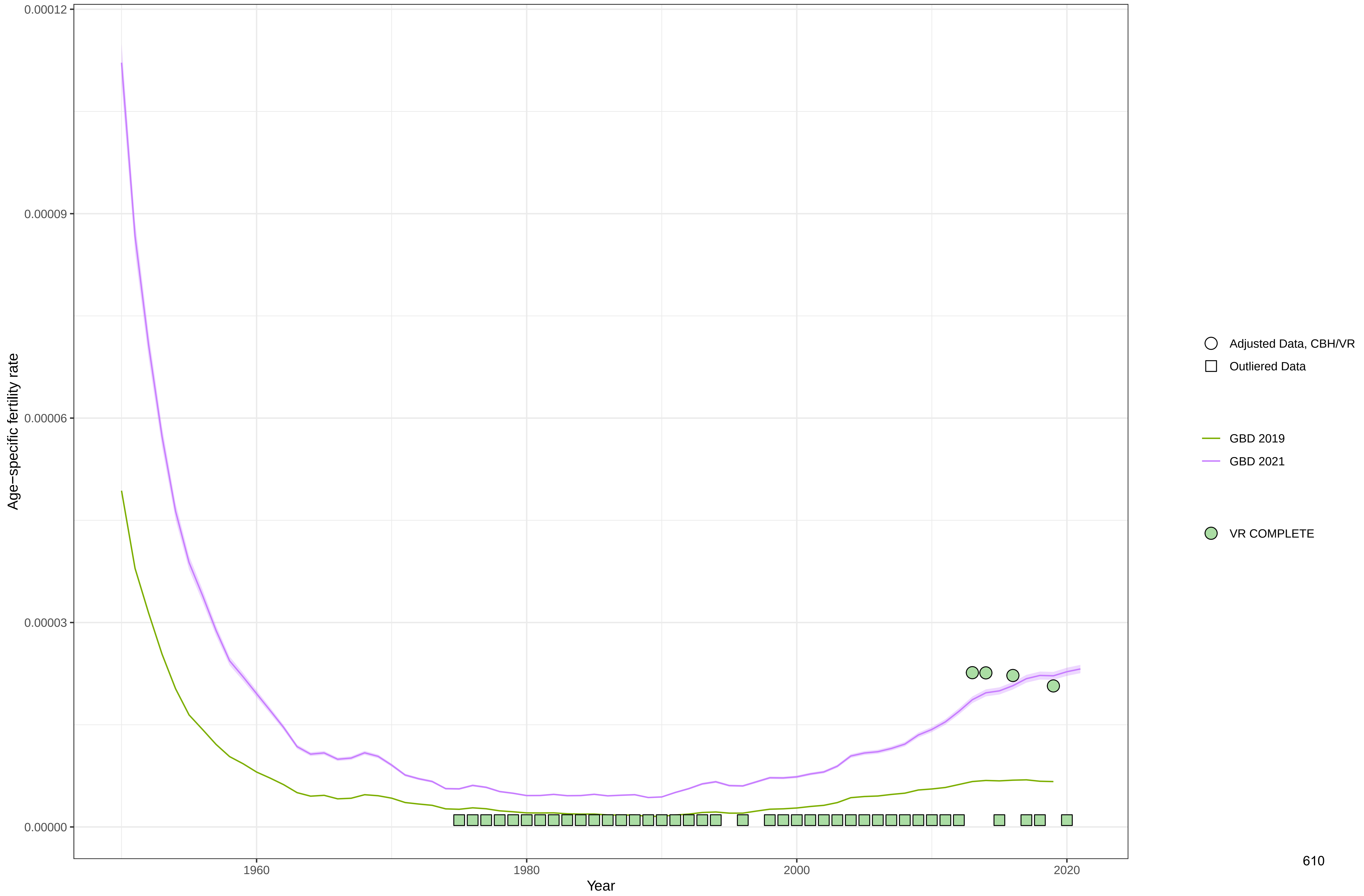

Total fertility rate fitted trend lines, 1950−2021: Nara TFR
Data points enclosed with a diamond are reference sources

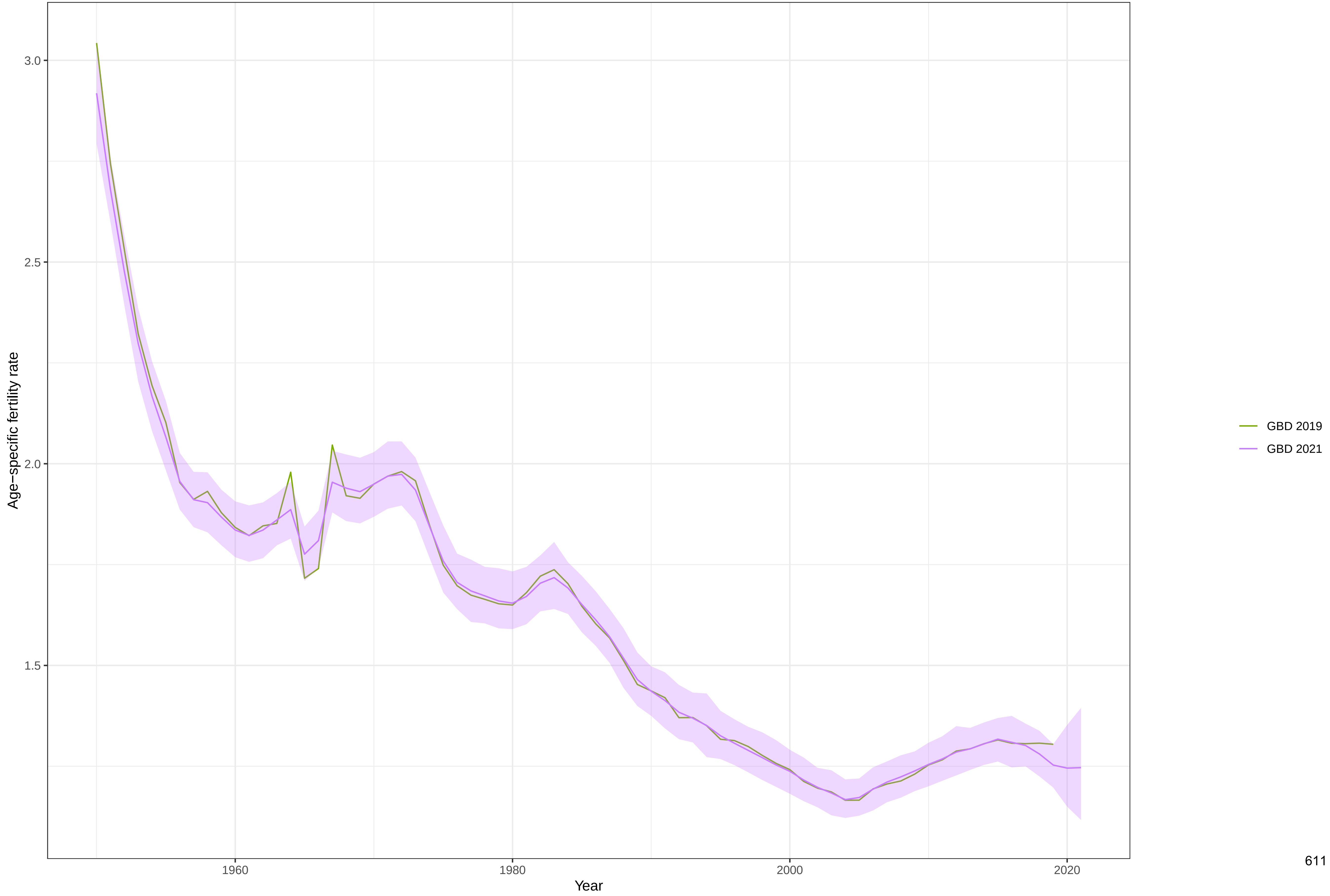

Age−specific fertility fitted trend lines, 1950−2021: Niigata, Ages: 10−14
Data points enclosed with a diamond are reference sources

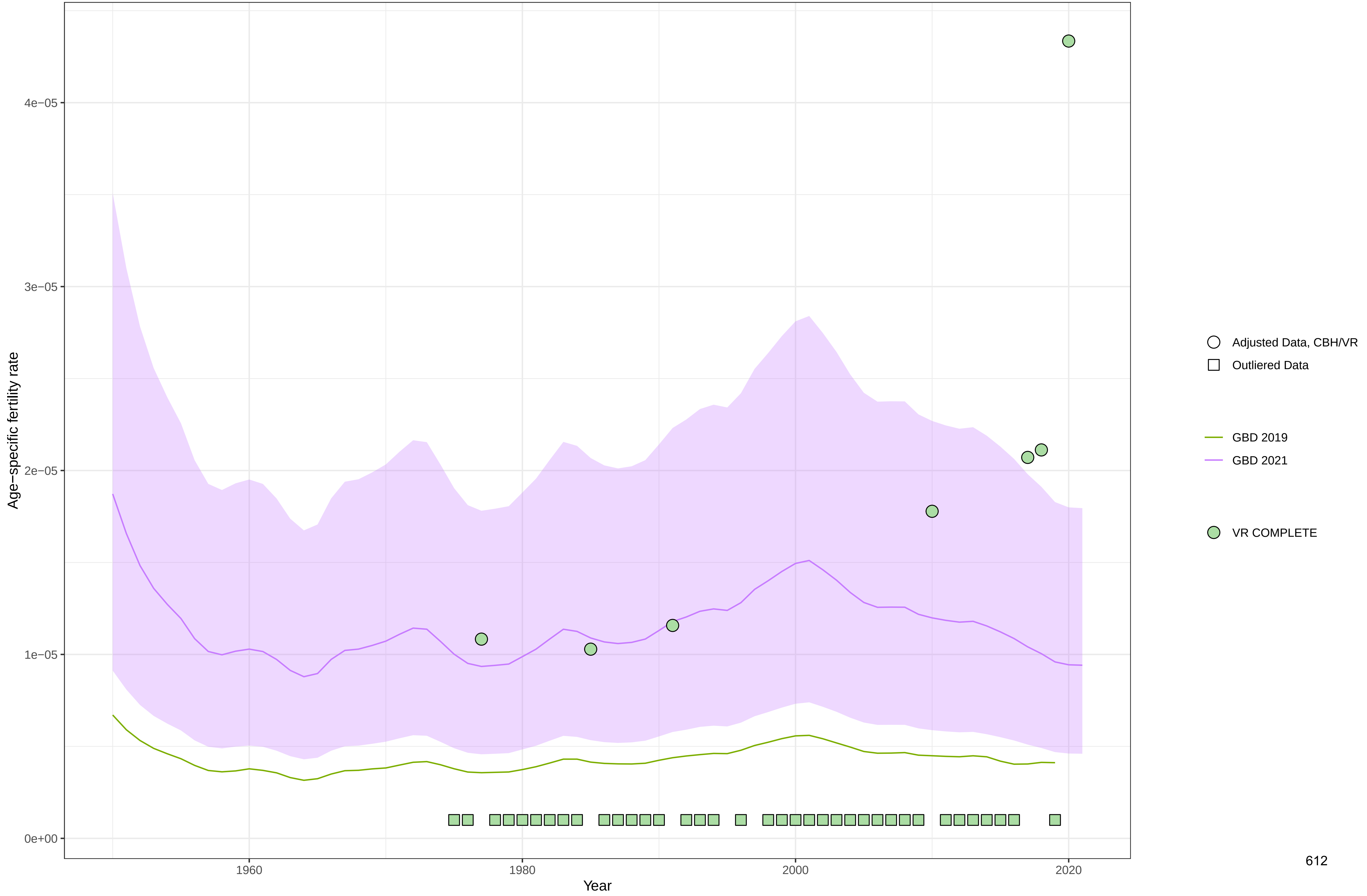

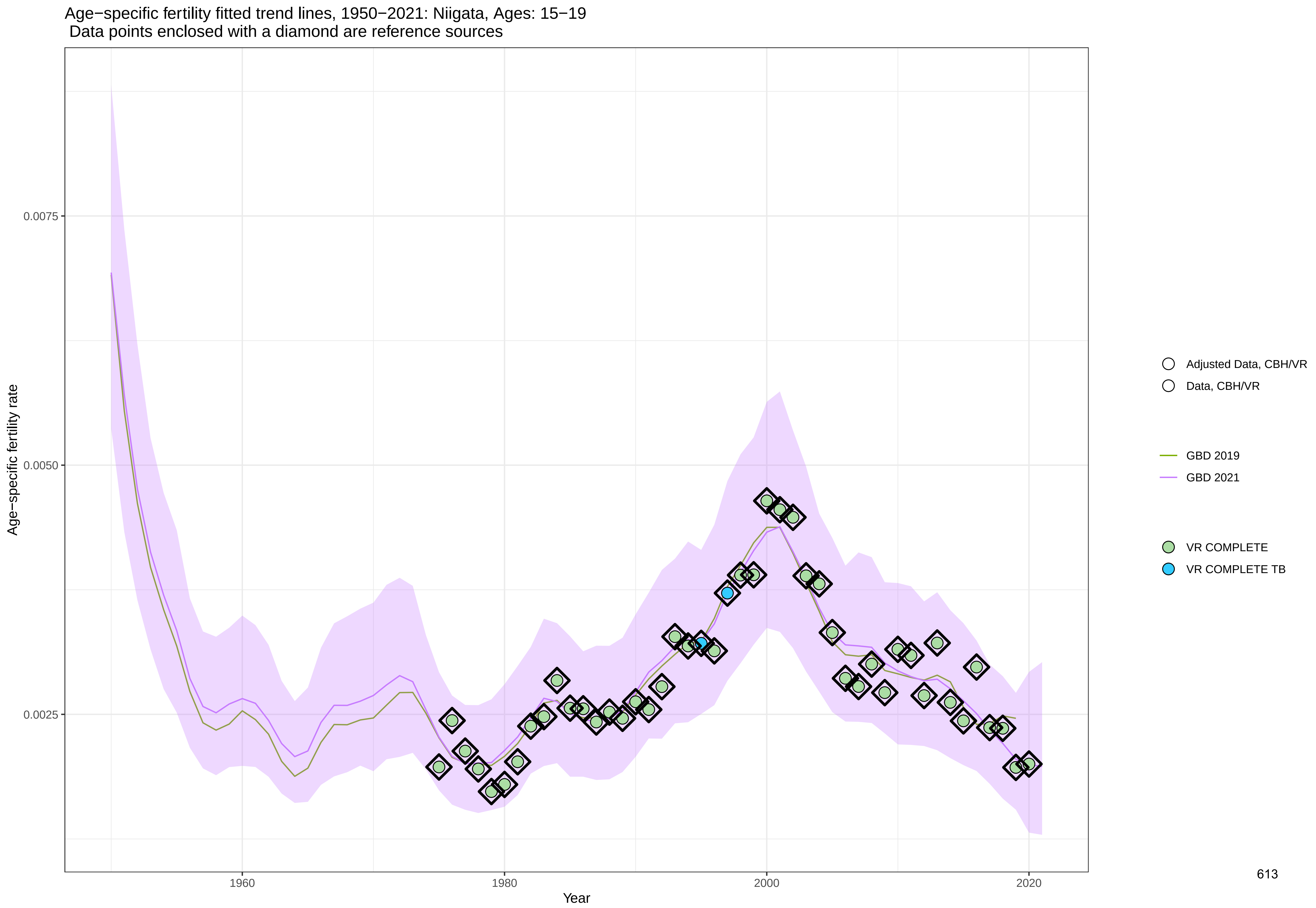
Age−specific fertility fitted trend lines, 1950−2021: Niigata, Ages: 15−19
Data points enclosed with a diamond are reference sources
Age−specific fertility rate
0.0075
0.0050
0.0025
1960
1980
2000
2020
Year
Adjusted Data, CBH/VR
Data, CBH/VR
GBD 2019
GBD 2021
VR COMPLETE
VR COMPLETE TB

Age−specific fertility fitted trend lines, 1950−2021: Niigata, Ages: 20−24
Data points enclosed with a diamond are reference sources

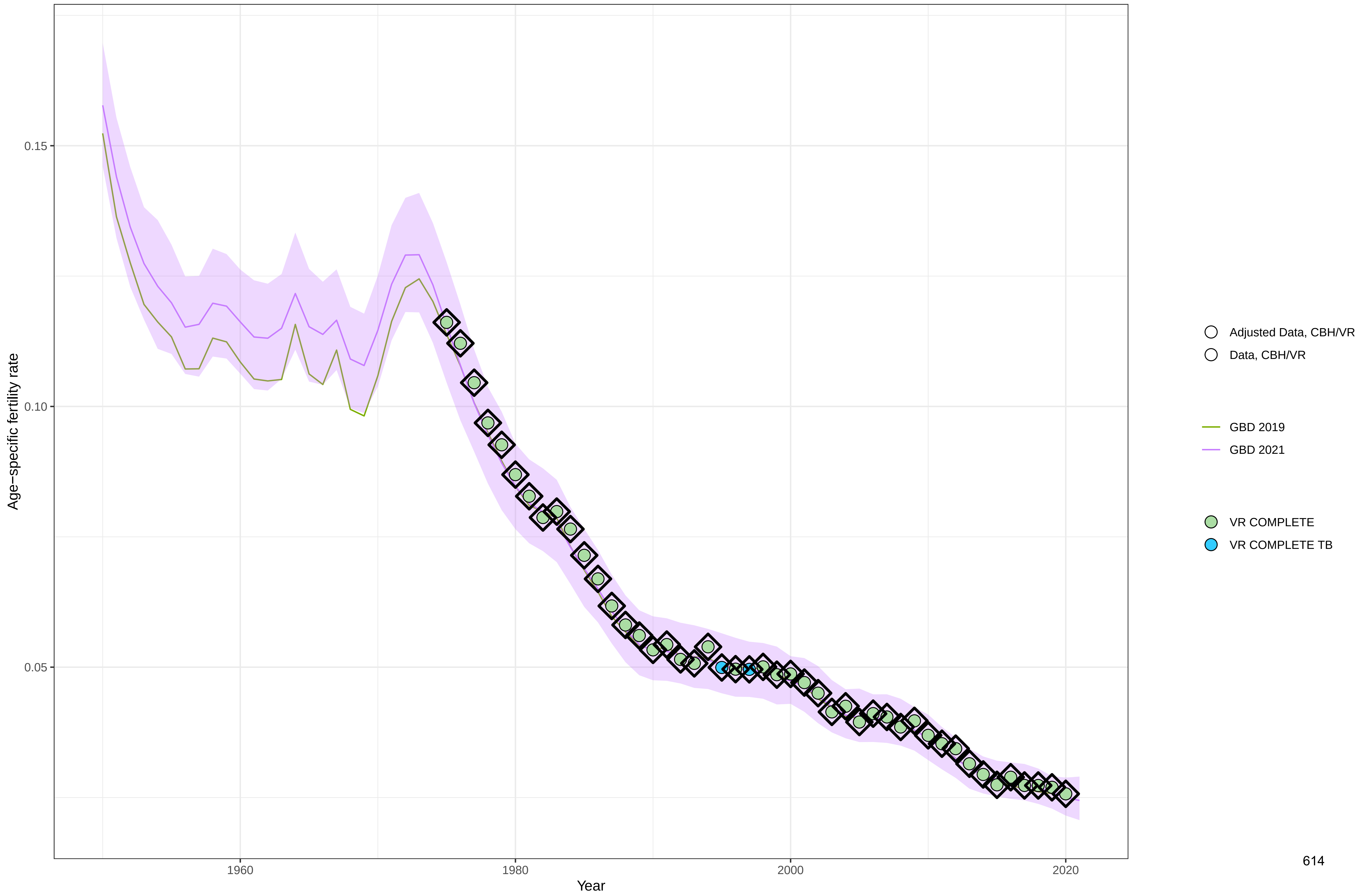

Age−specific fertility fitted trend lines, 1950−2021: Niigata, Ages: 25−29
Data points enclosed with a diamond are reference sources

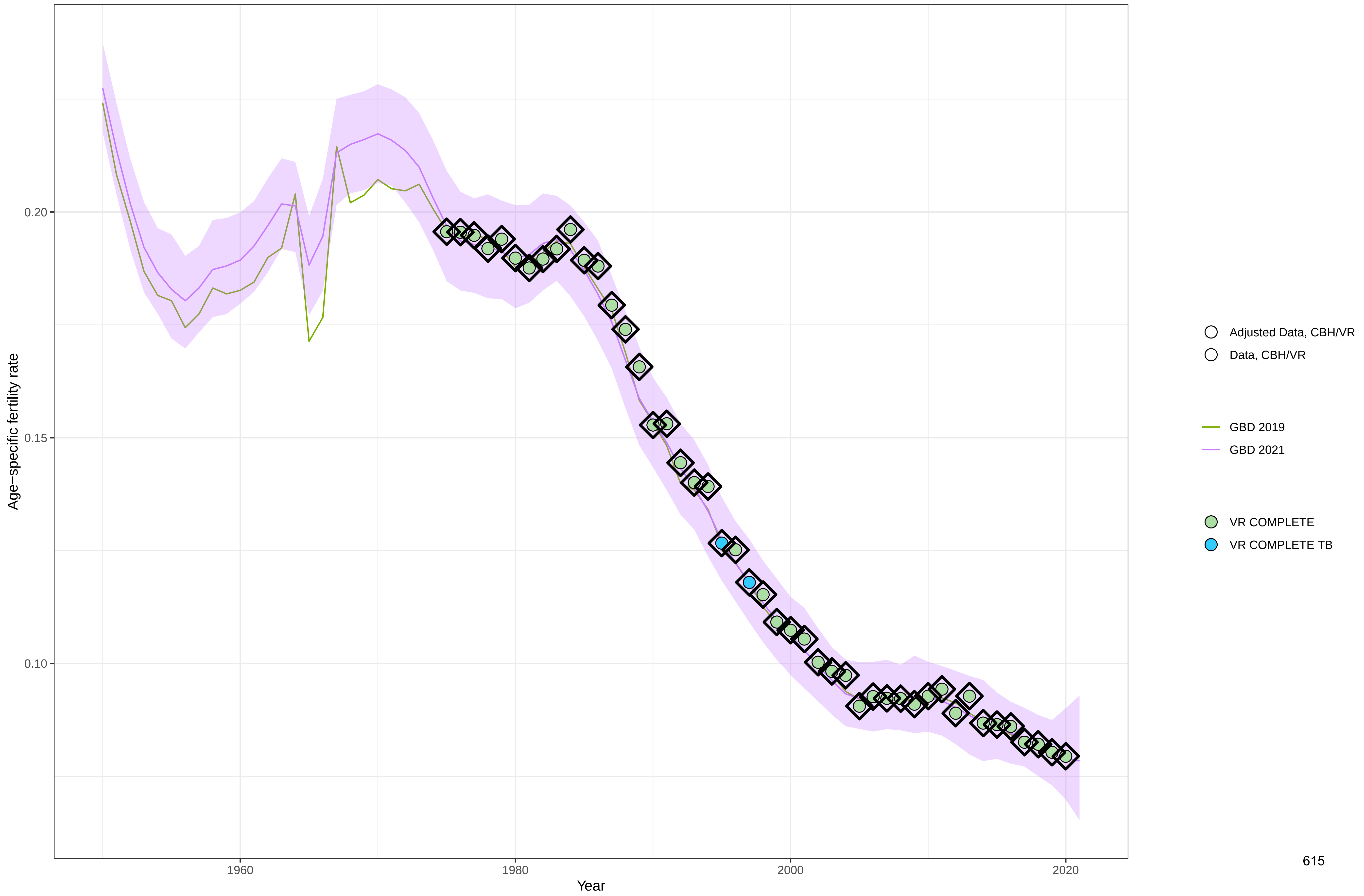

Age−specific fertility fitted trend lines, 1950−2021: Niigata, Ages: 30−34
Data points enclosed with a diamond are reference sources

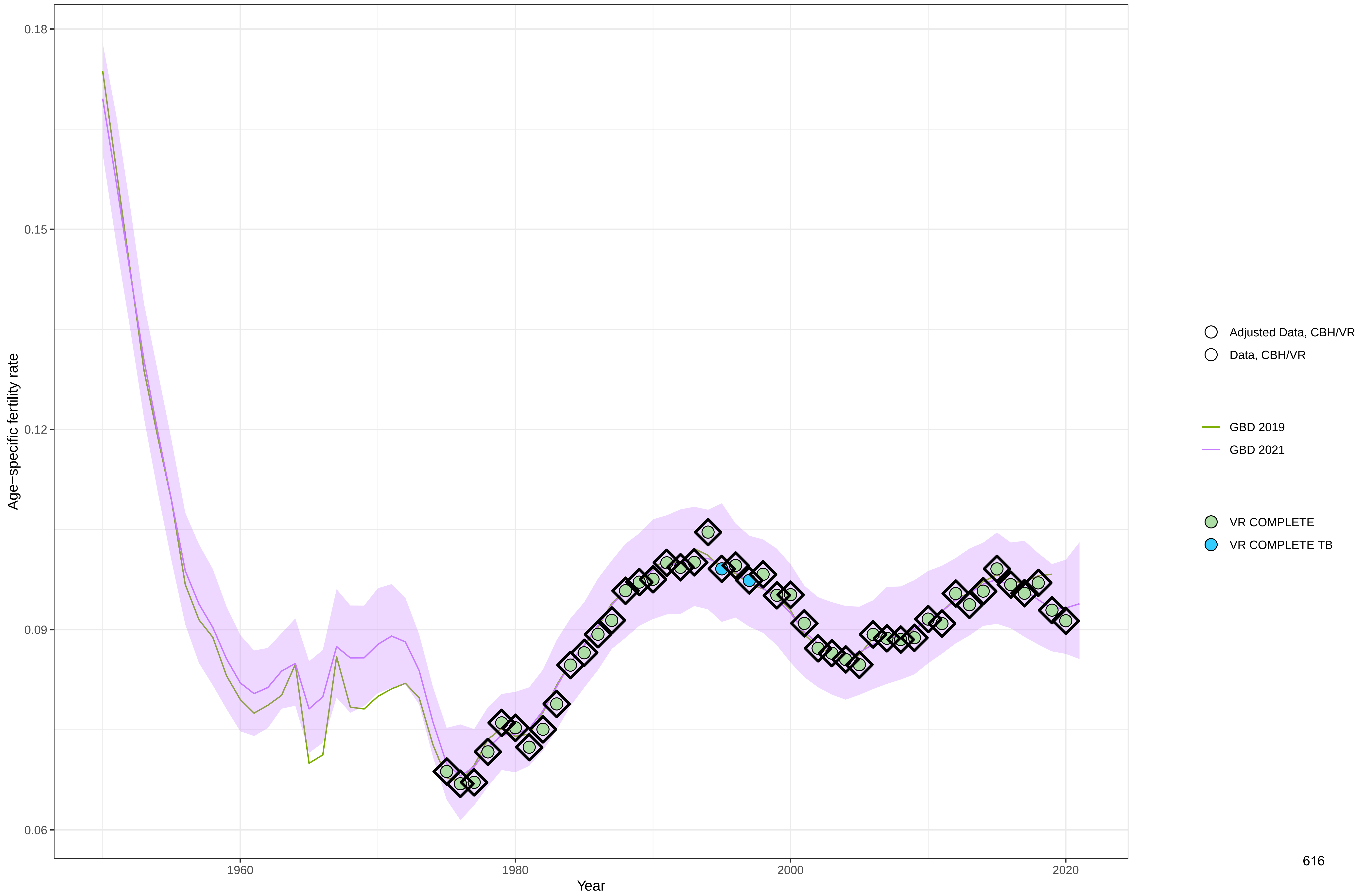

Age−specific fertility fitted trend lines, 1950−2021: Niigata, Ages: 35−39
Data points enclosed with a diamond are reference sources

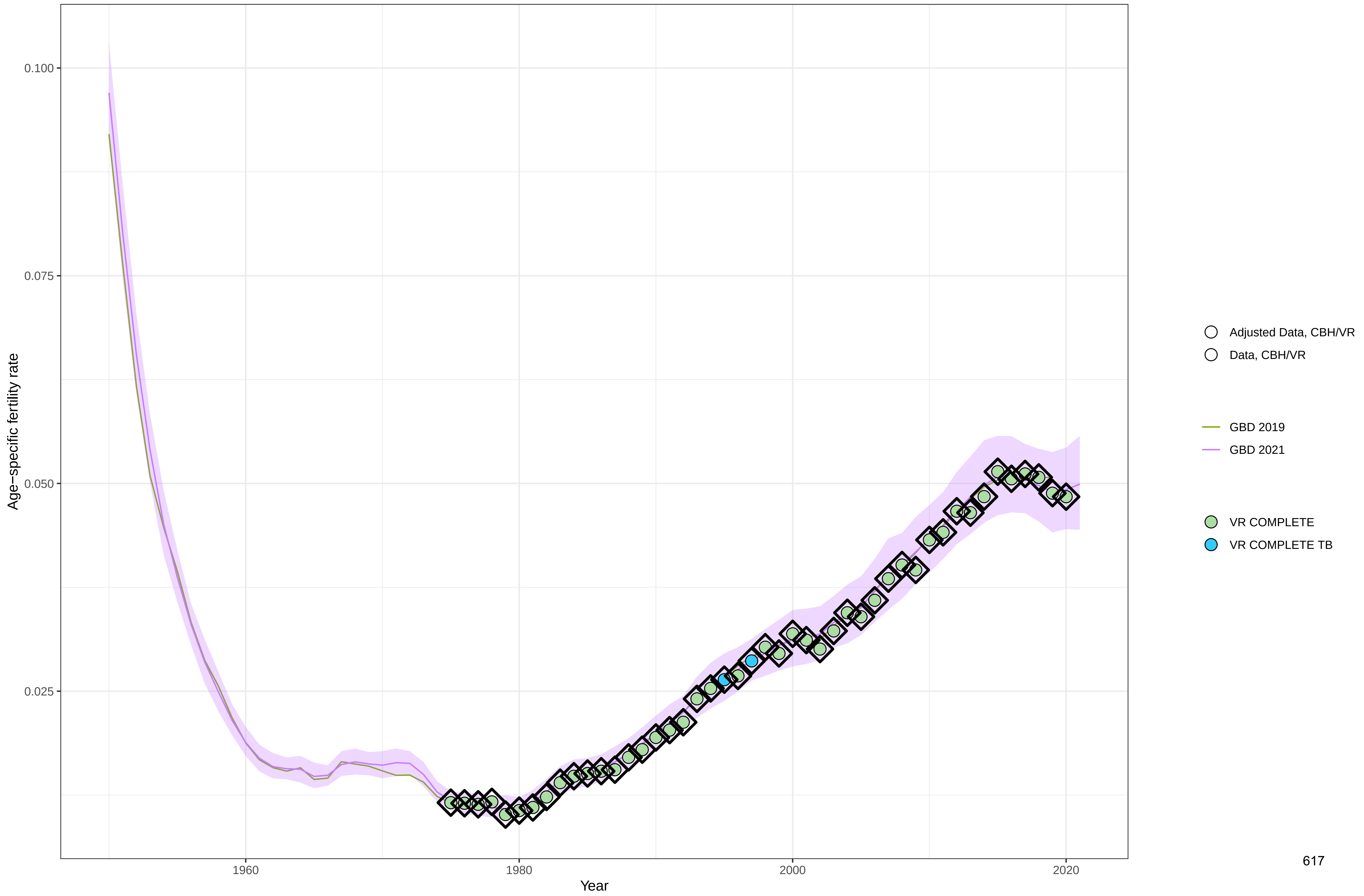

Age−specific fertility fitted trend lines, 1950−2021: Niigata, Ages: 40−44
Data points enclosed with a diamond are reference sources

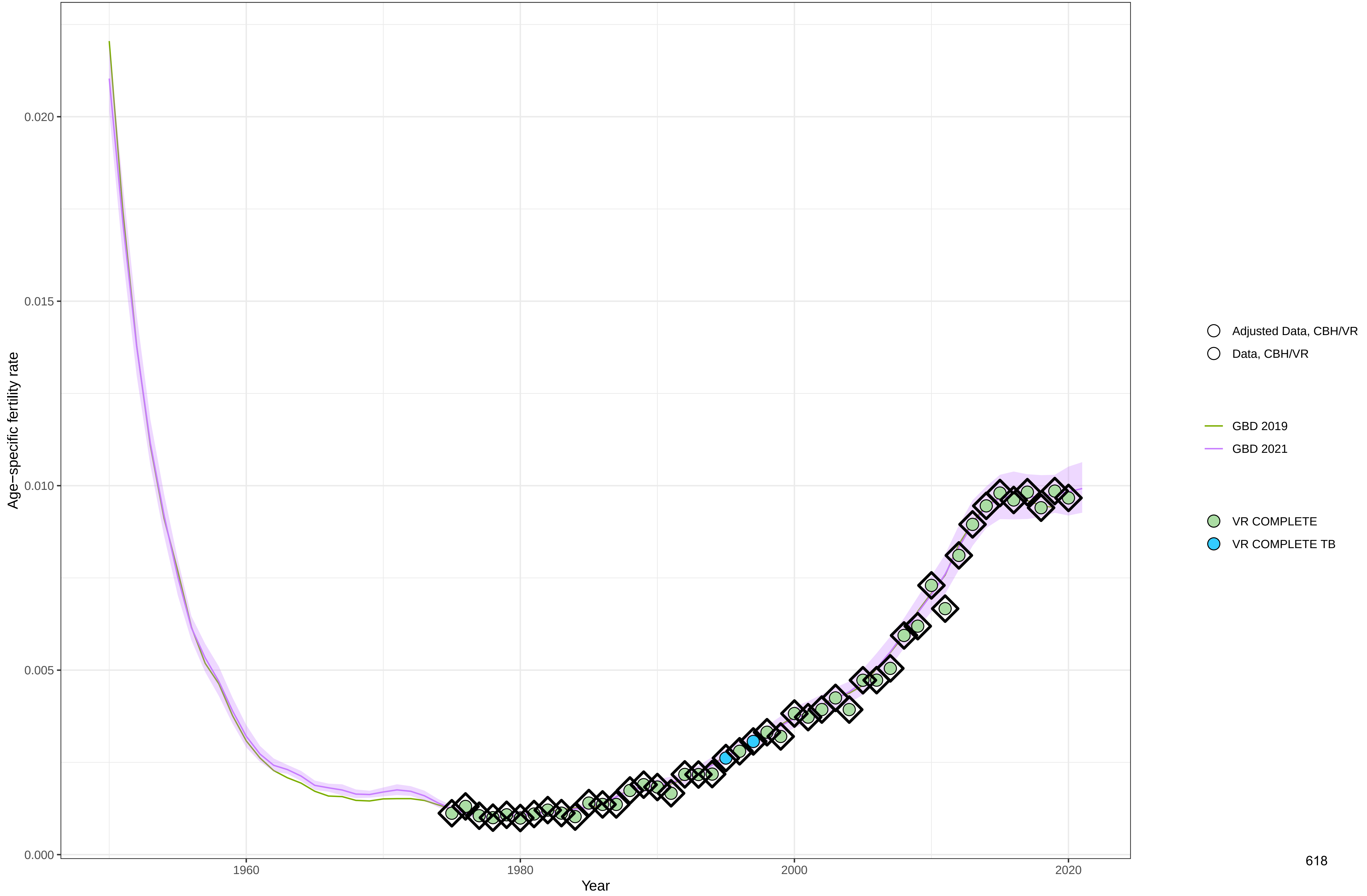

Age−specific fertility fitted trend lines, 1950−2021: Niigata, Ages: 45−49
Data points enclosed with a diamond are reference sources

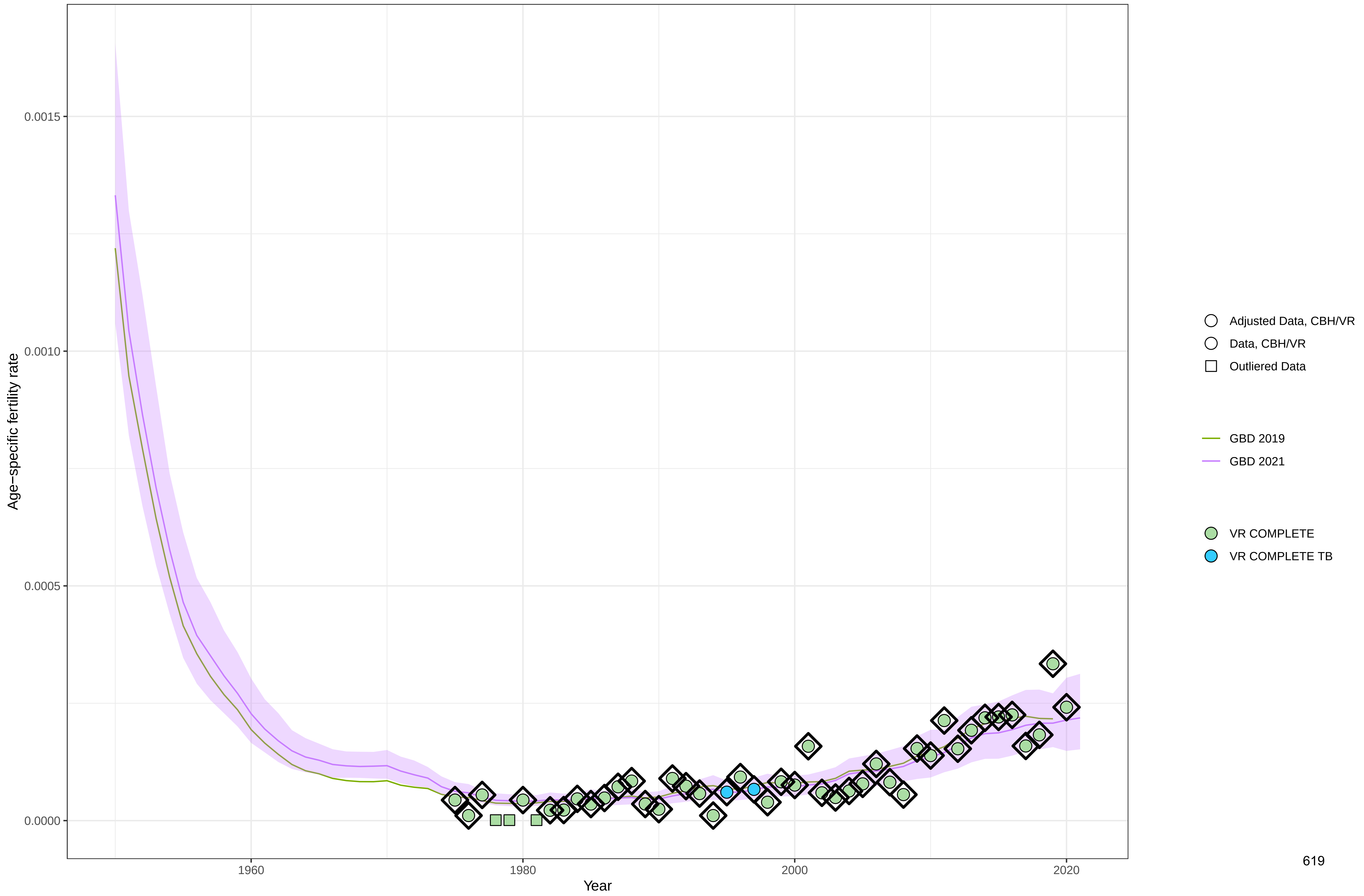

Age−specific fertility fitted trend lines, 1950−2021: Niigata, Ages: 50−54
Data points enclosed with a diamond are reference sources

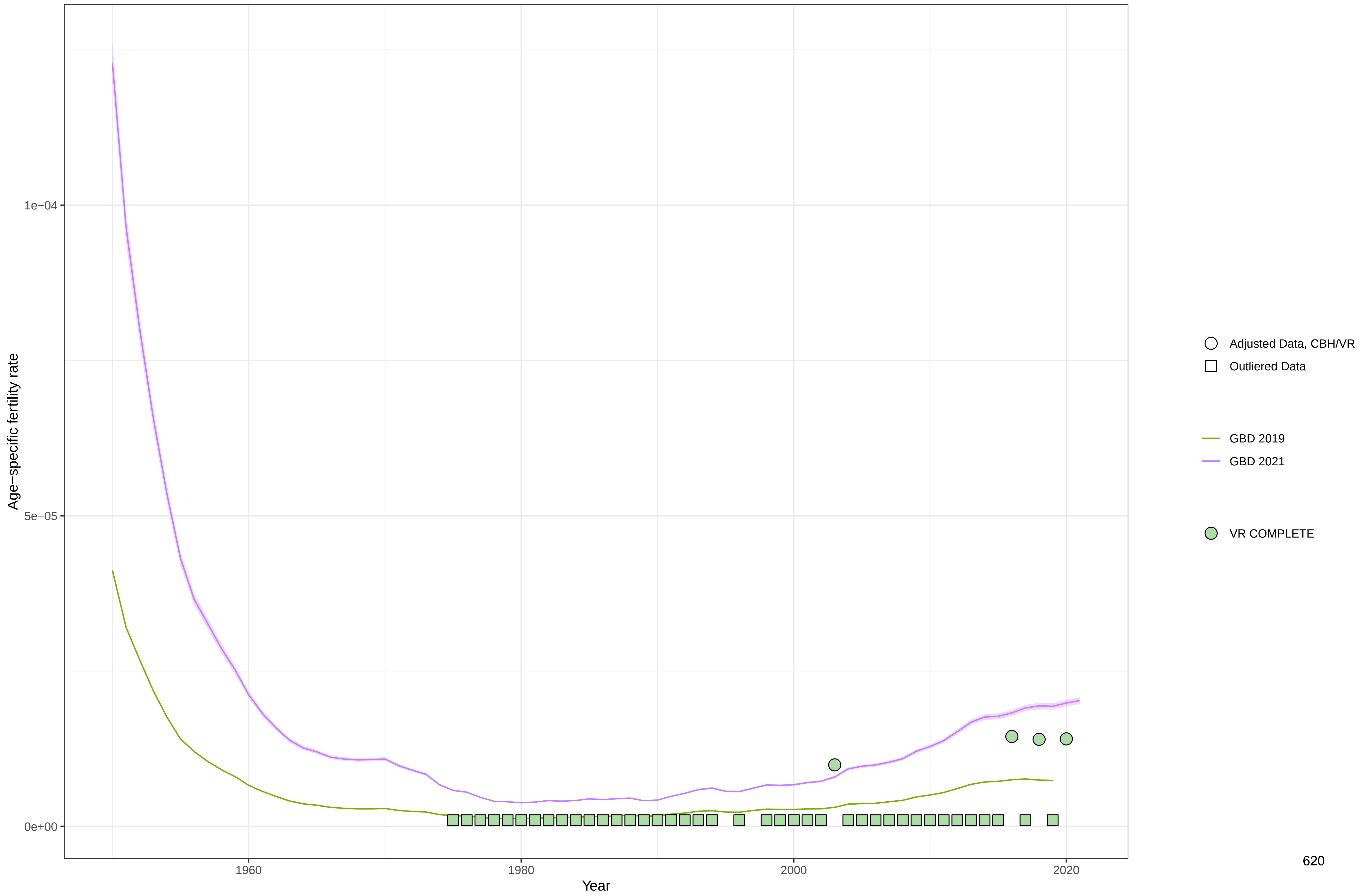

Total fertility rate fitted trend lines, 1950−2021: Niigata TFR
Data points enclosed with a diamond are reference sources

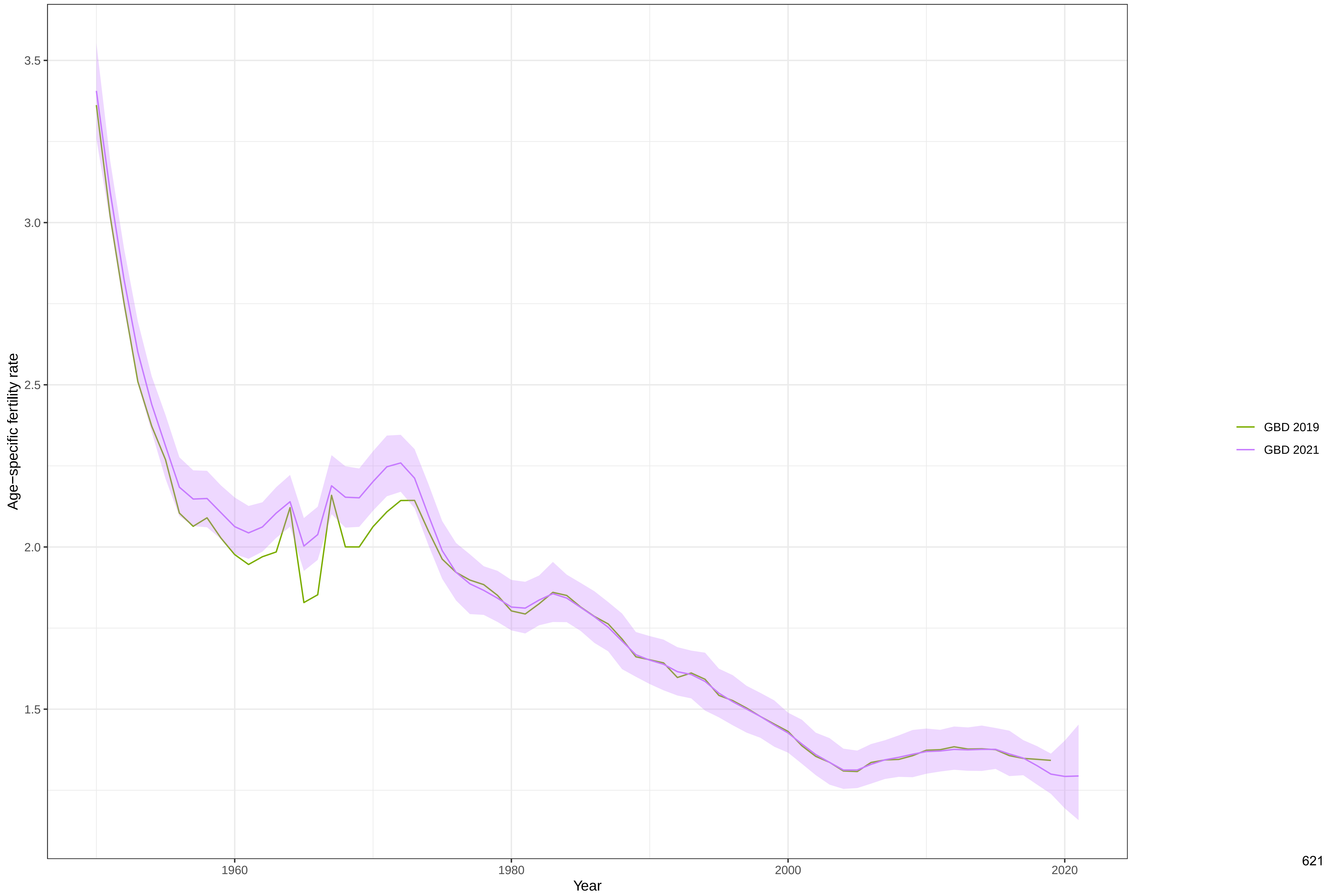

Age−specific fertility fitted trend lines, 1950−2021: ..ita, Ages: 10−14
Data points enclosed with a diamond are reference sources

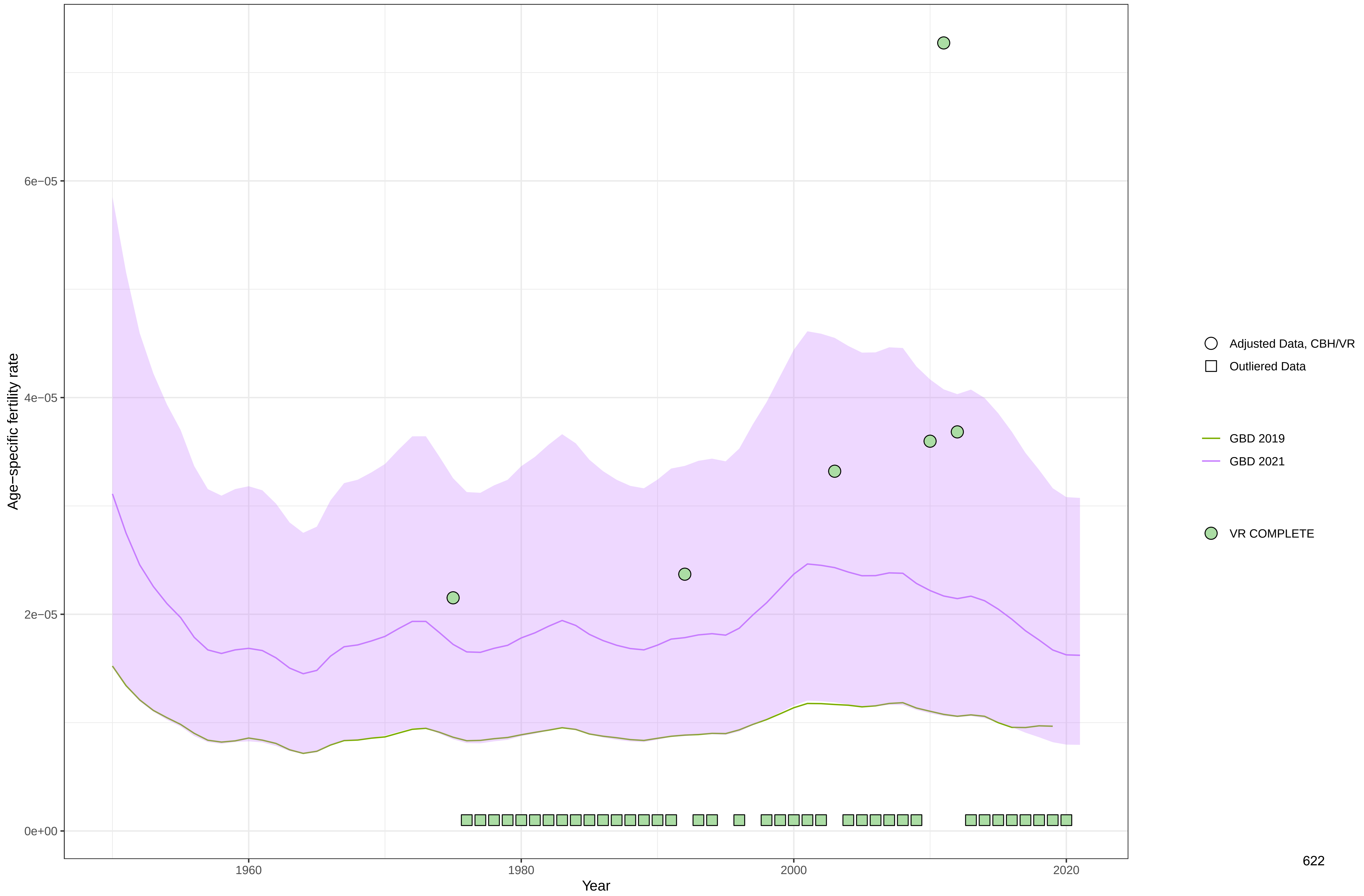

Age−specific fertility fitted trend lines, 1950−2021: ..ita, Ages: 15−19
Data points enclosed with a diamond are reference sources

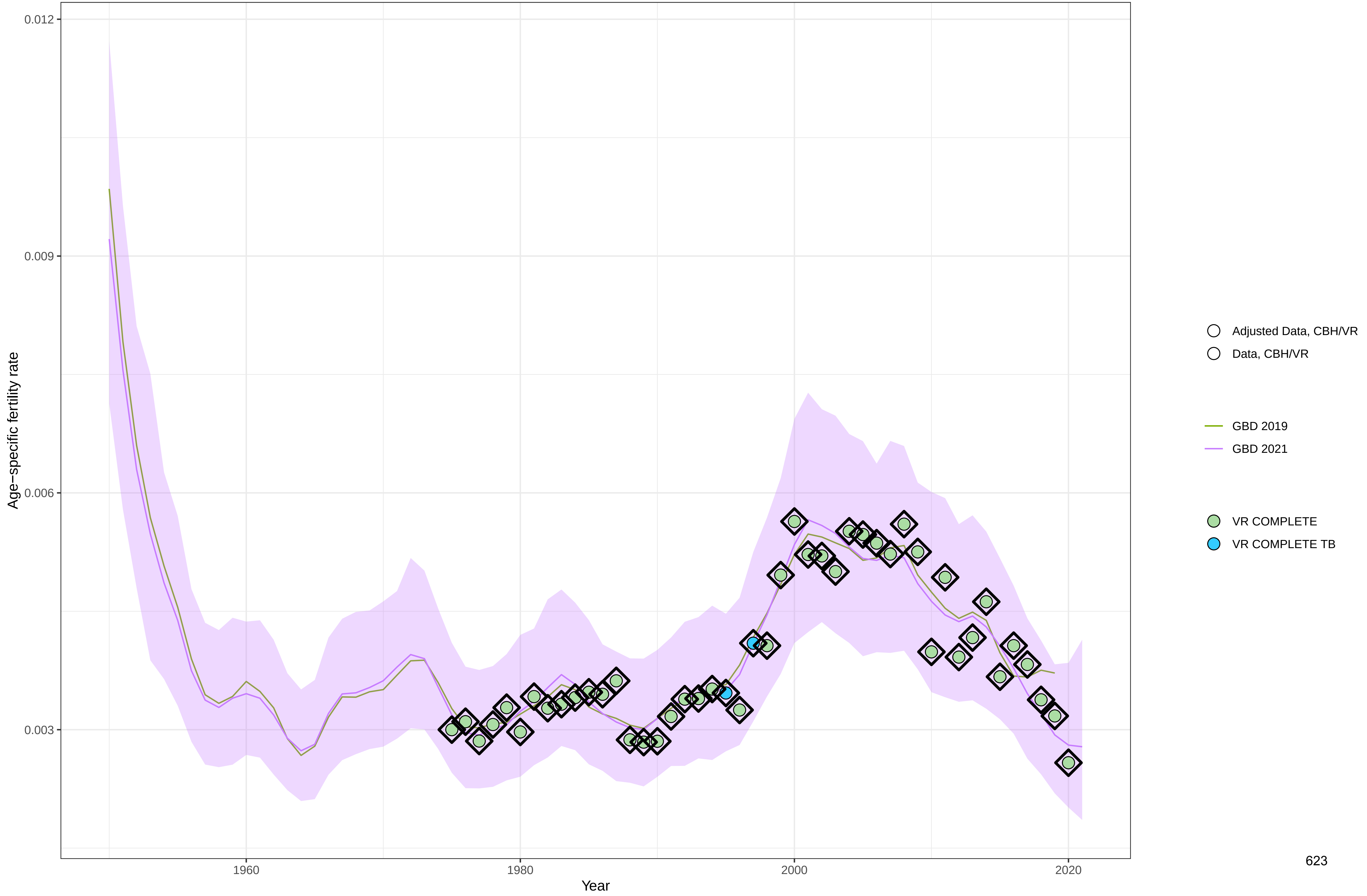

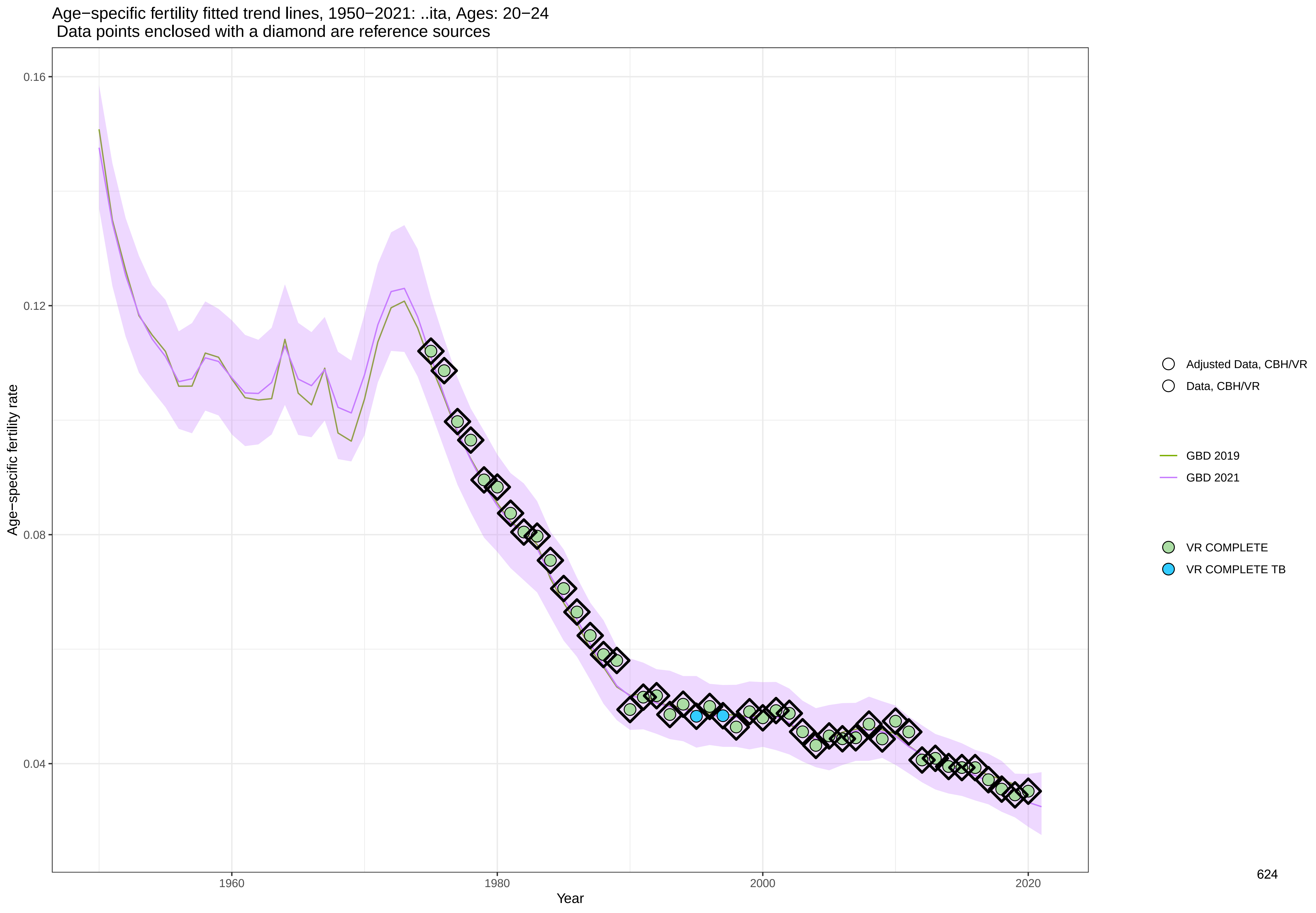

Age−specific fertility fitted trend lines, 1950−2021: ..ita, Ages: 20−24
Data points enclosed with a diamond are reference sources
Age−specific fertility rate
0.16
0.12
0.08
0.04
1960
1980
2000
2020
Year
Adjusted Data, CBH/VR
Data, CBH/VR
GBD 2019
GBD 2021
VR COMPLETE
VR COMPLETE TB

Age−specific fertility fitted trend lines, 1950−2021: ..ita, Ages: 25−29
Data points enclosed with a diamond are reference sources

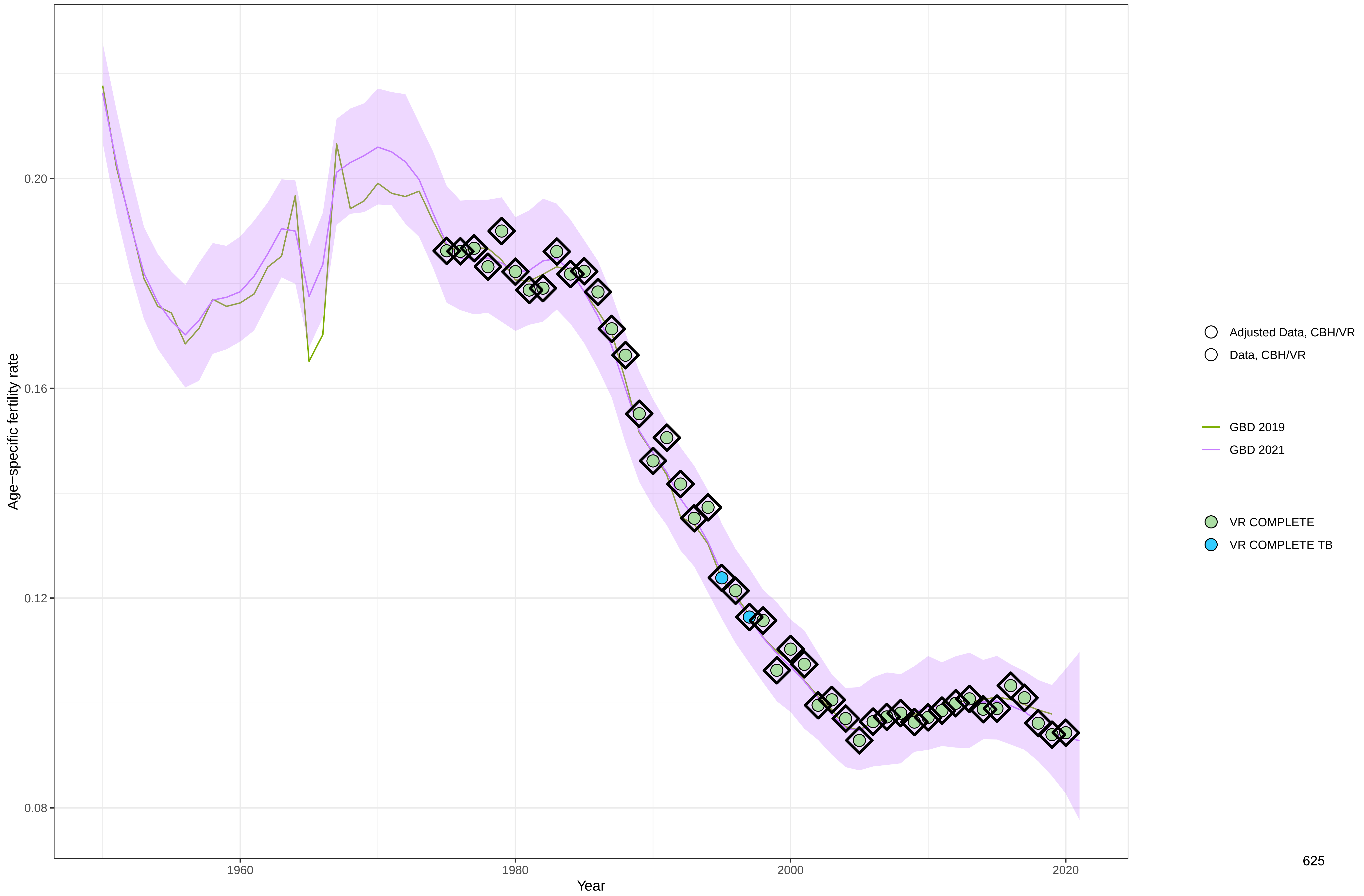

Age−specific fertility fitted trend lines, 1950−2021: ..ita, Ages: 30−34
Data points enclosed with a diamond are reference sources

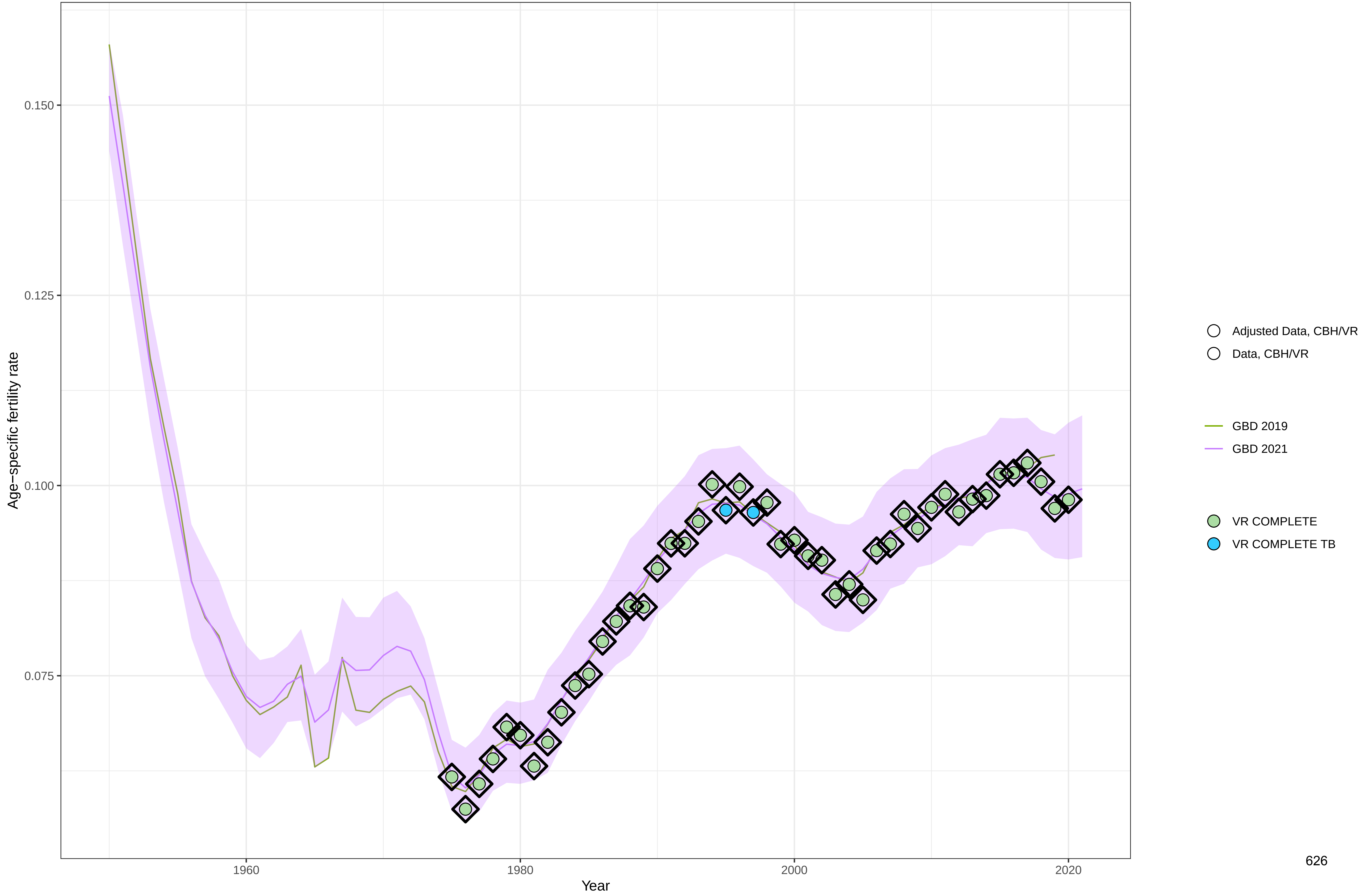

Age−specific fertility fitted trend lines, 1950−2021: ..ita, Ages: 35−39
Data points enclosed with a diamond are reference sources

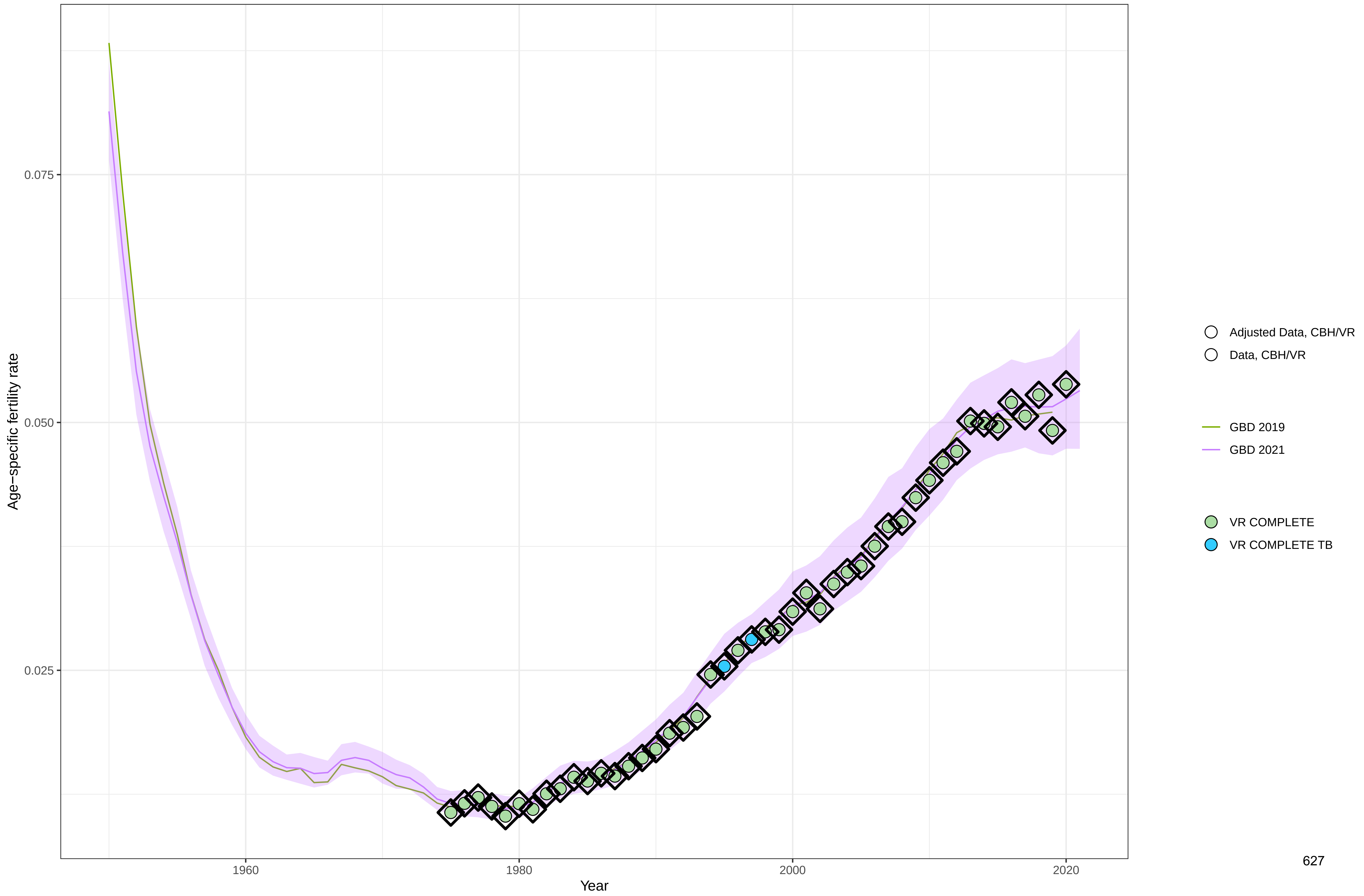

Age−specific fertility fitted trend lines, 1950−2021: ..ita, Ages: 40−44
Data points enclosed with a diamond are reference sources

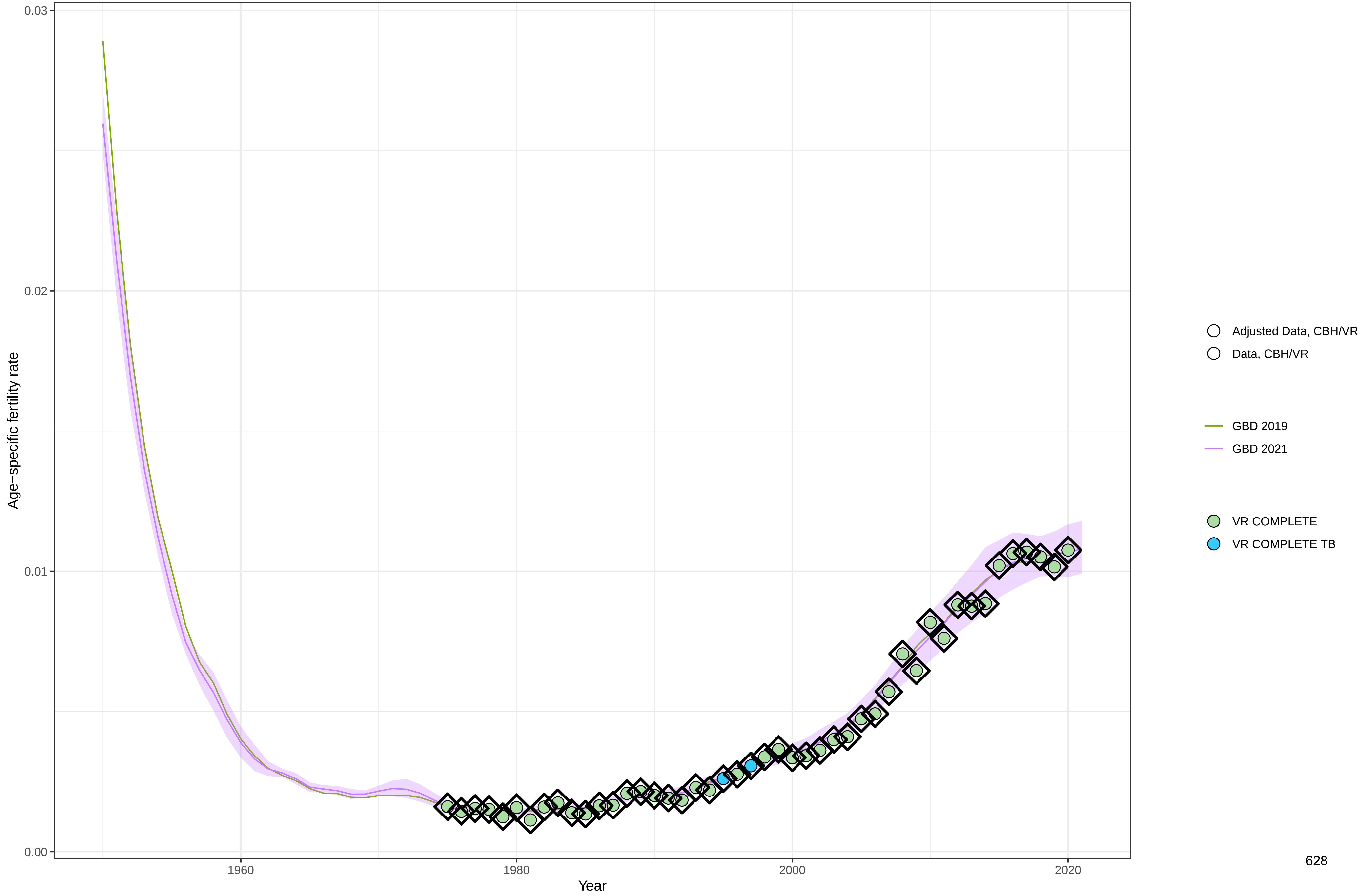

Age−specific fertility fitted trend lines, 1950−2021: ..ita, Ages: 45−49
Data points enclosed with a diamond are reference sources

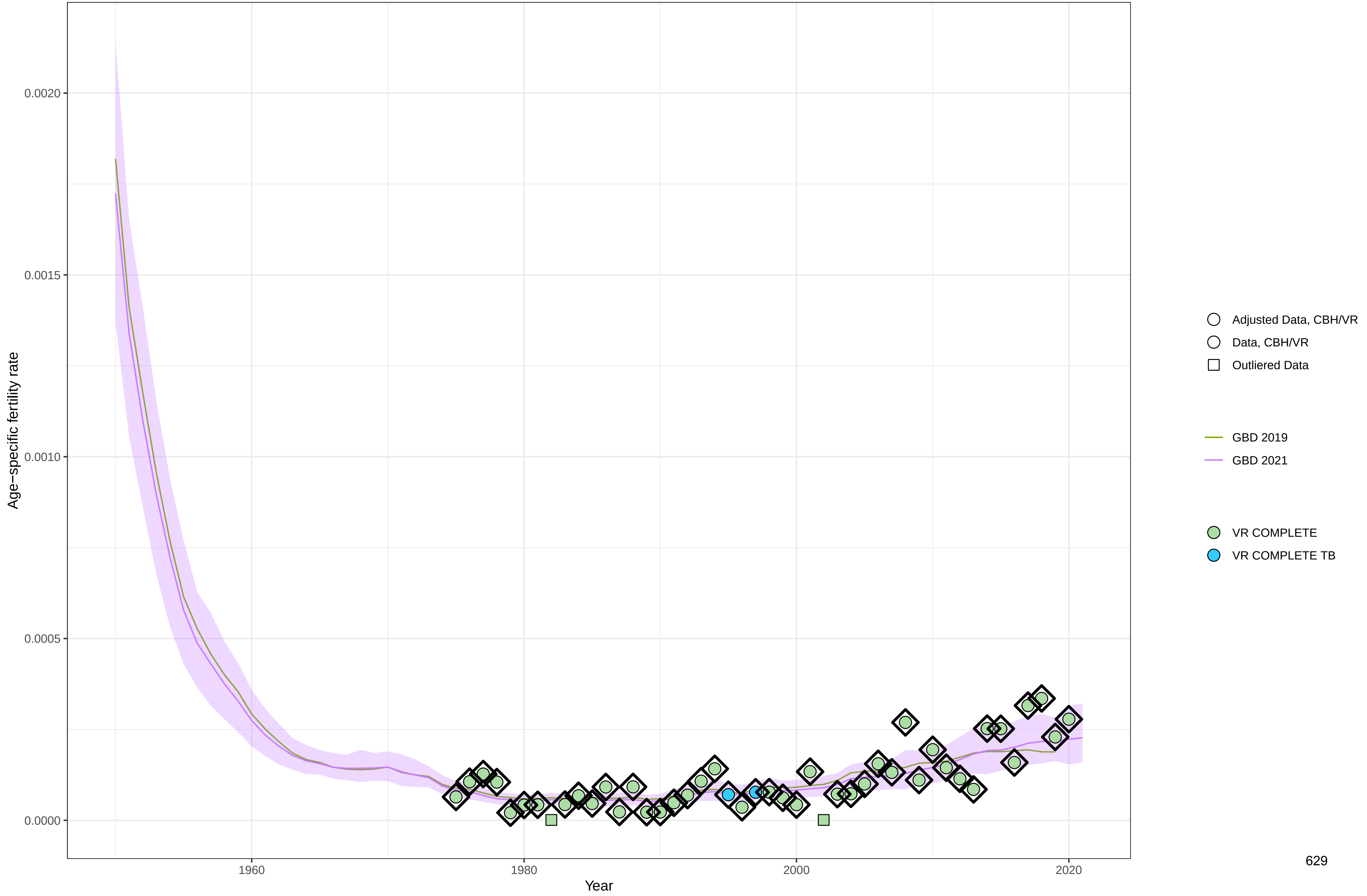

Age−specific fertility fitted trend lines, 1950−2021: ..ita, Ages: 50−54
Data points enclosed with a diamond are reference sources

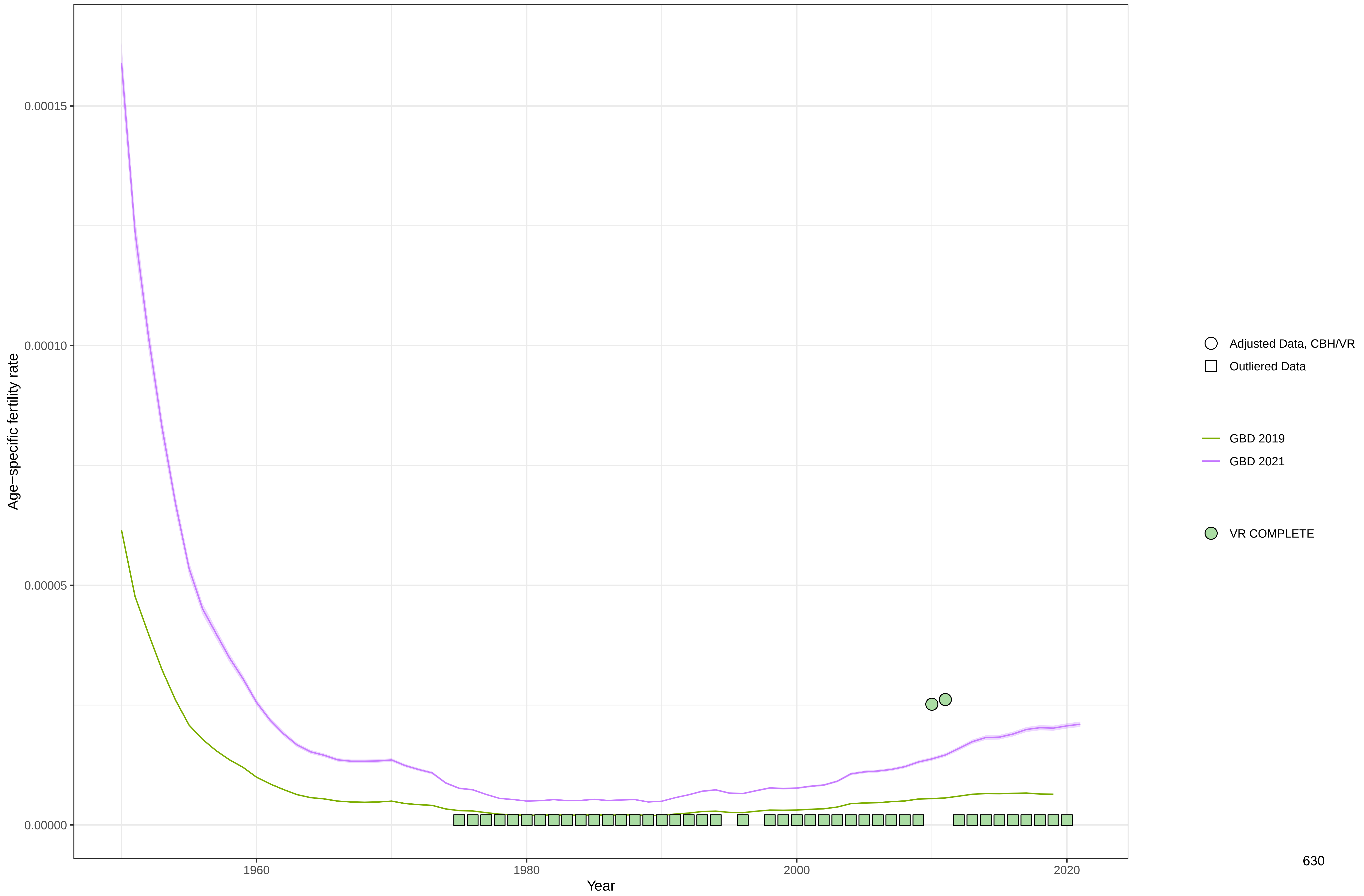

Total fertility rate fitted trend lines, 1950−2021: ..ita TFR
Data points enclosed with a diamond are reference sources

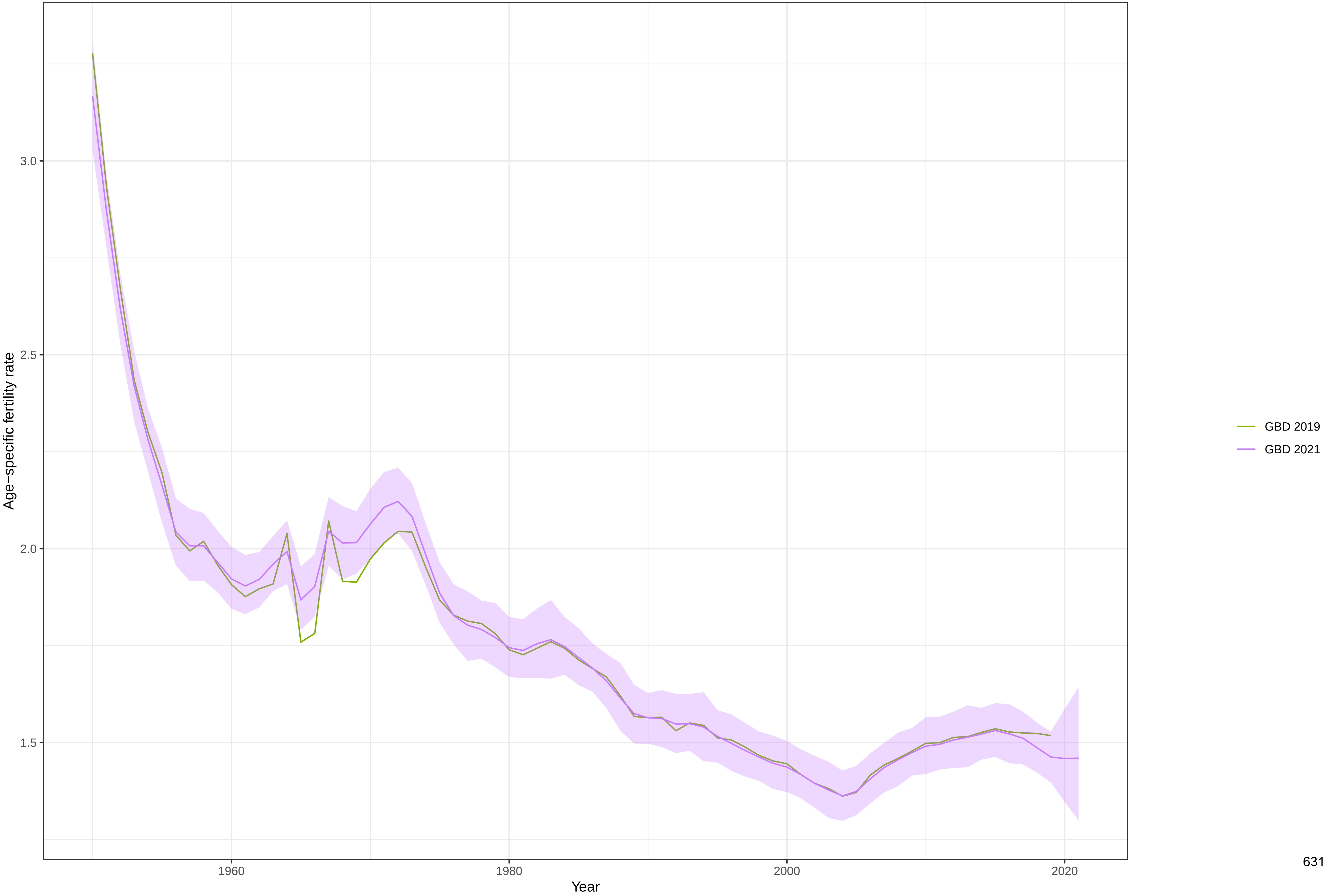

Age−specific fertility fitted trend lines, 1950−2021: Okayama, Ages: 10−14
Data points enclosed with a diamond are reference sources

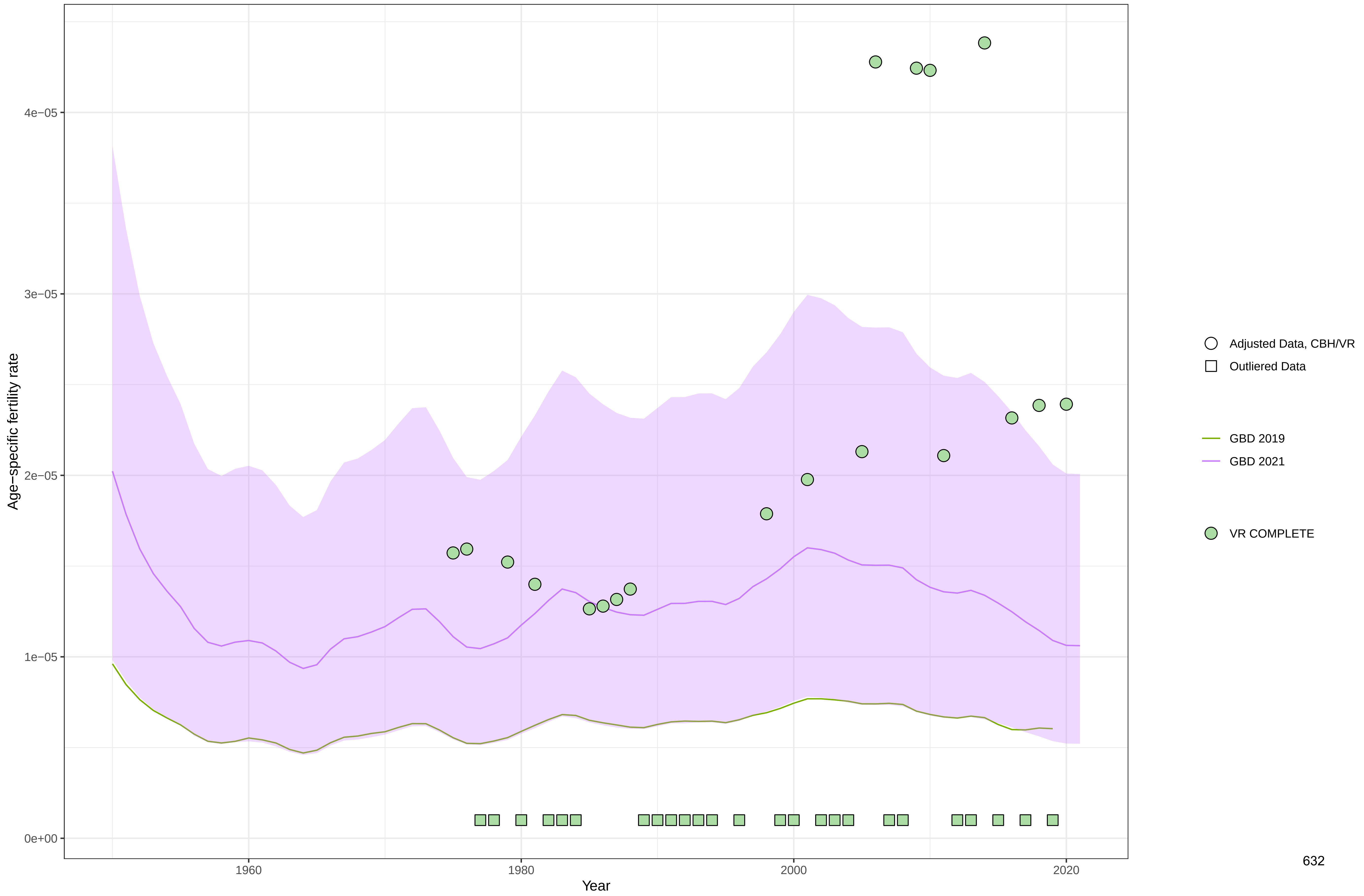

Age−specific fertility fitted trend lines, 1950−2021: Okayama, Ages: 15−19
Data points enclosed with a diamond are reference sources

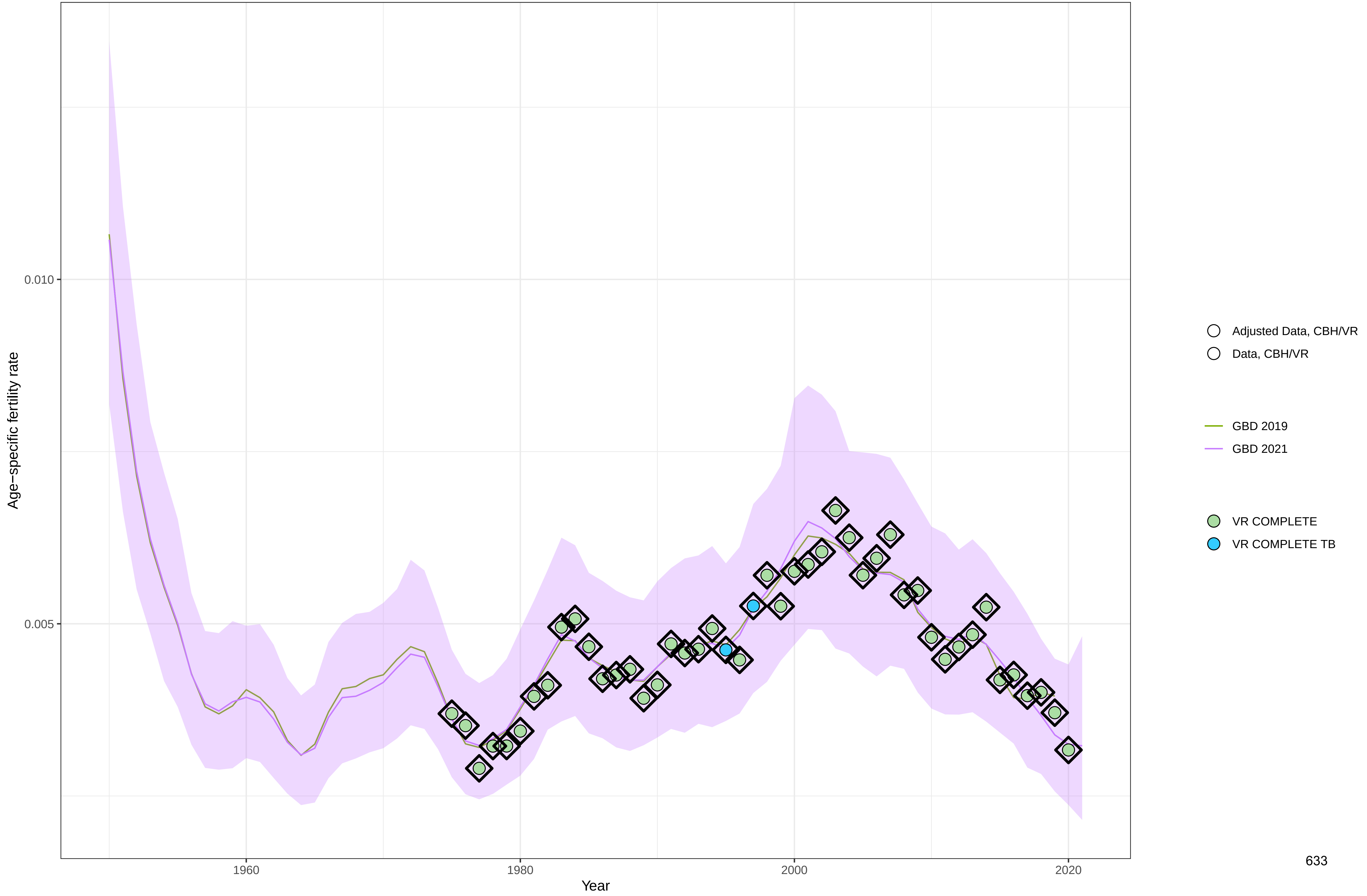

Age−specific fertility fitted trend lines, 1950−2021: Okayama, Ages: 20−24
Data points enclosed with a diamond are reference sources

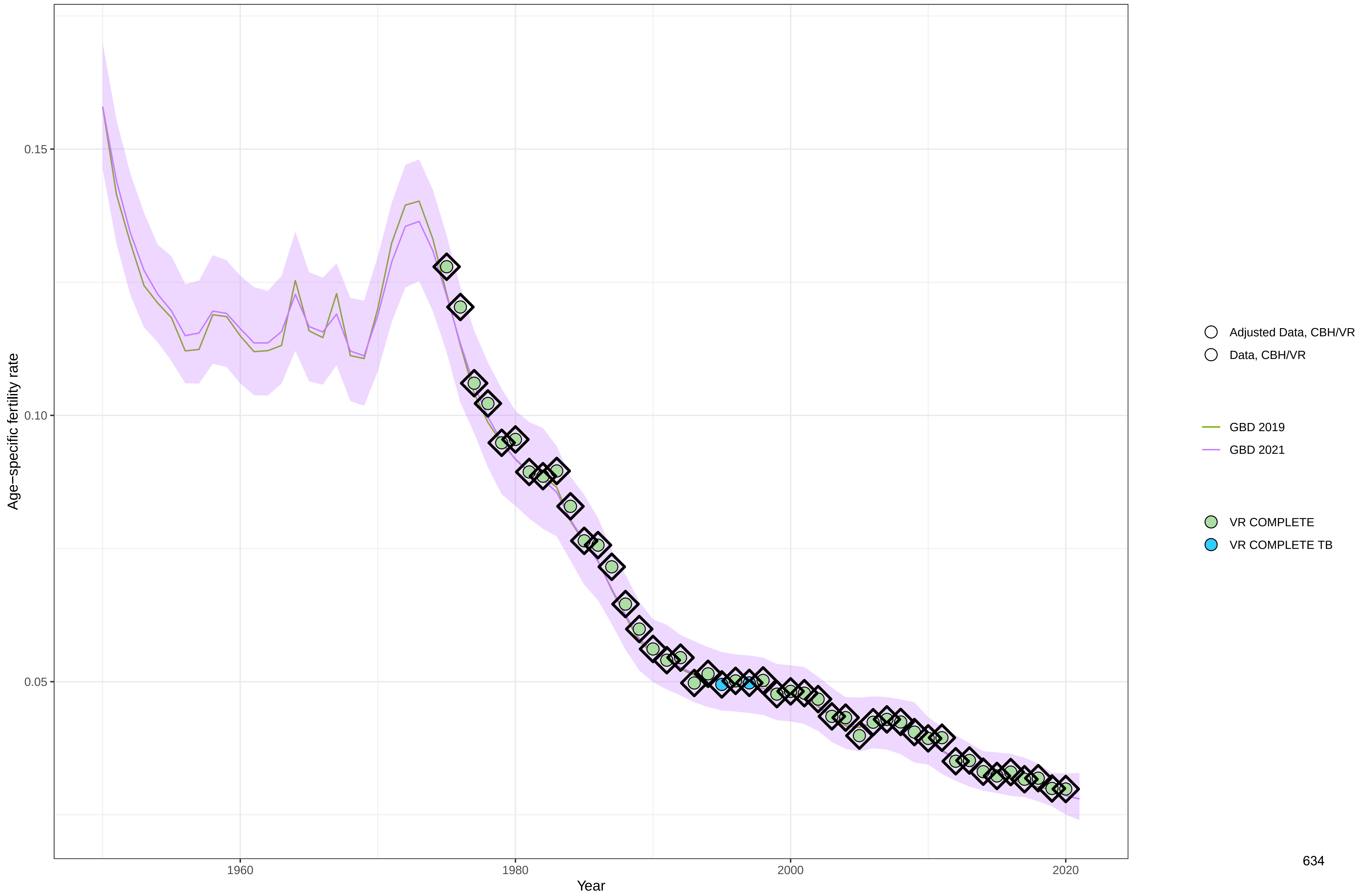

Age−specific fertility fitted trend lines, 1950−2021: Okayama, Ages: 25−29
Data points enclosed with a diamond are reference sources

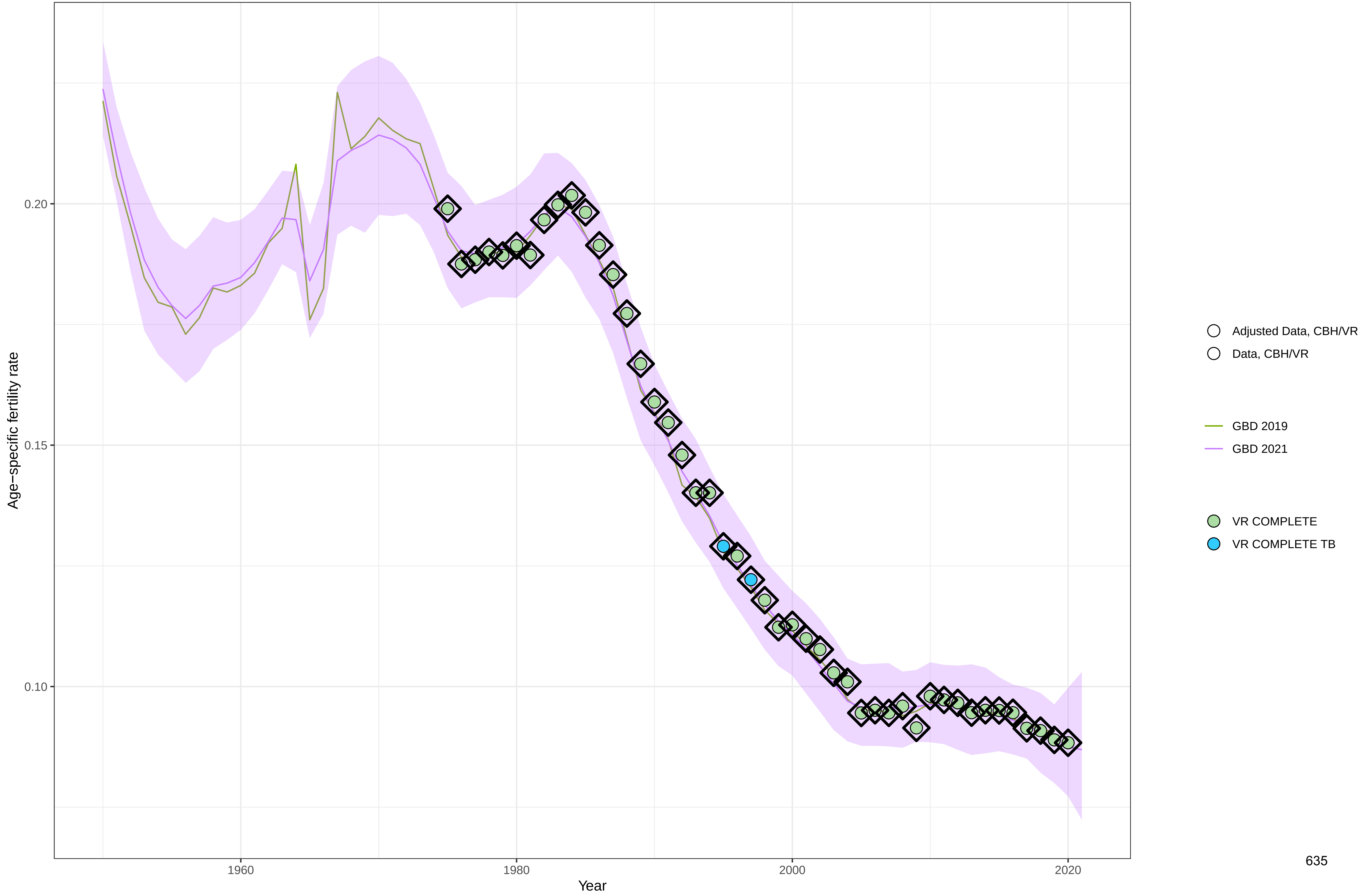

Age−specific fertility fitted trend lines, 1950−2021: Okayama, Ages: 30−34
Data points enclosed with a diamond are reference sources

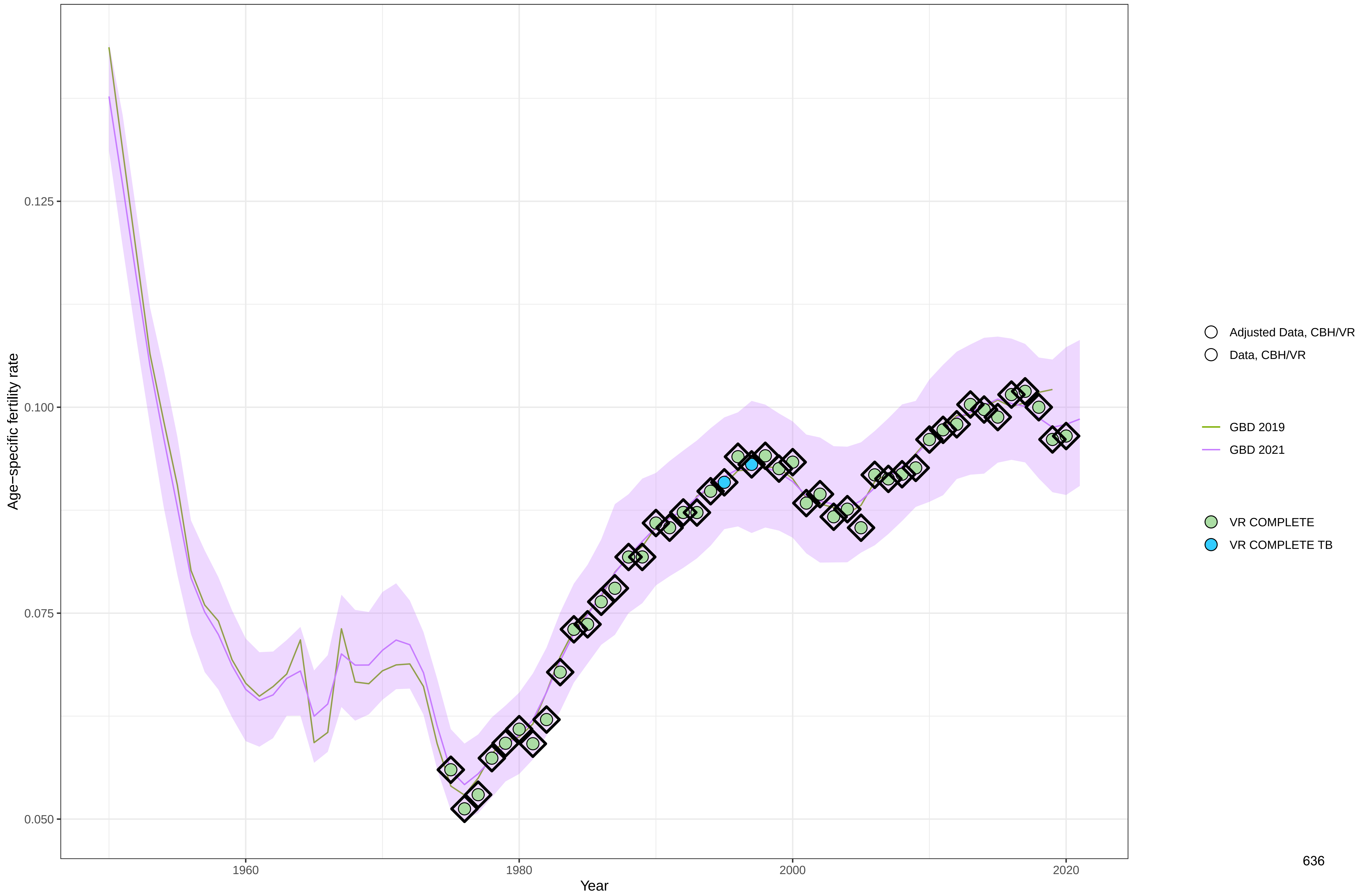

Age−specific fertility fitted trend lines, 1950−2021: Okayama, Ages: 35−39
Data points enclosed with a diamond are reference sources

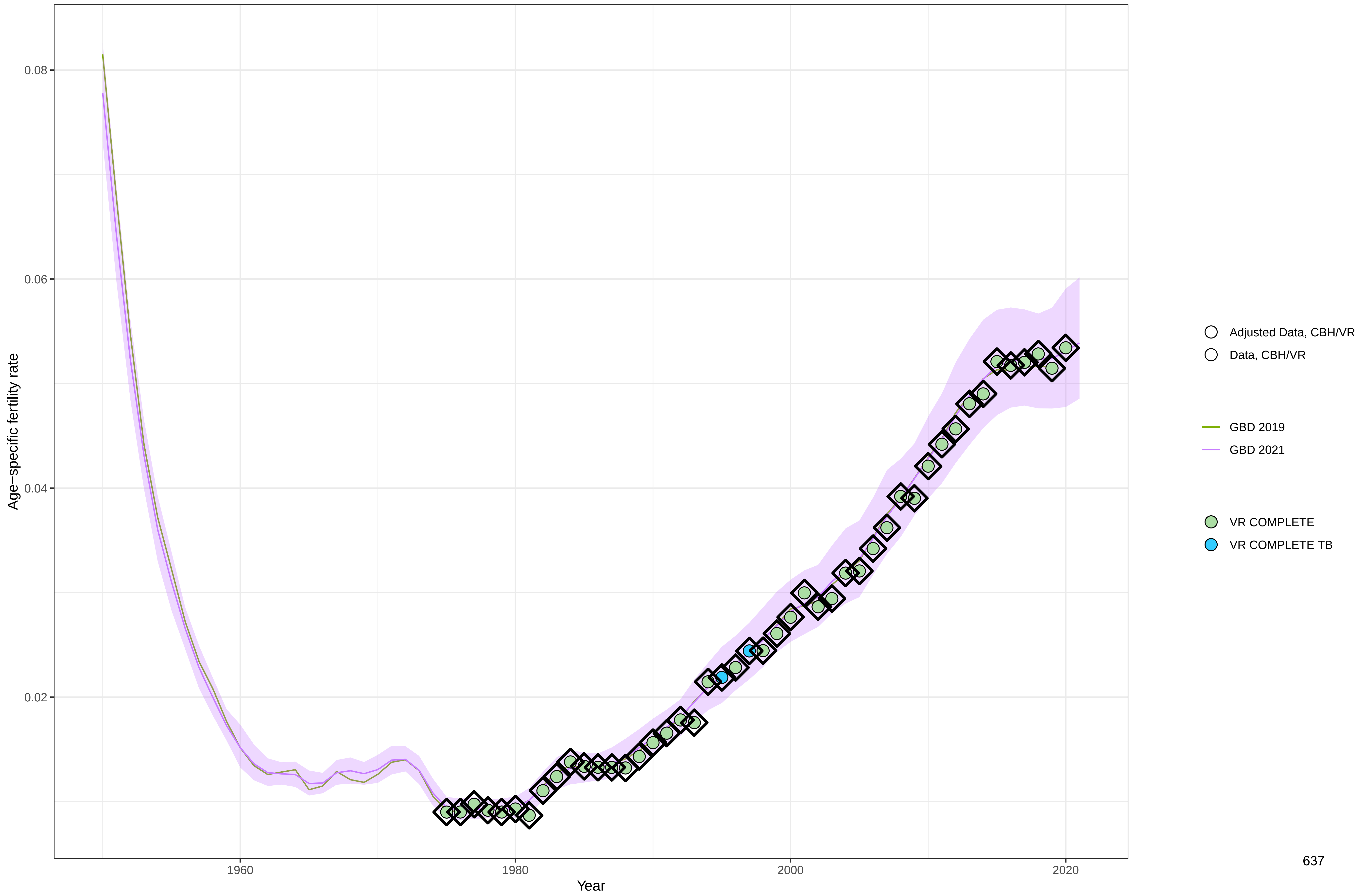

Age−specific fertility fitted trend lines, 1950−2021: Okayama, Ages: 40−44
Data points enclosed with a diamond are reference sources

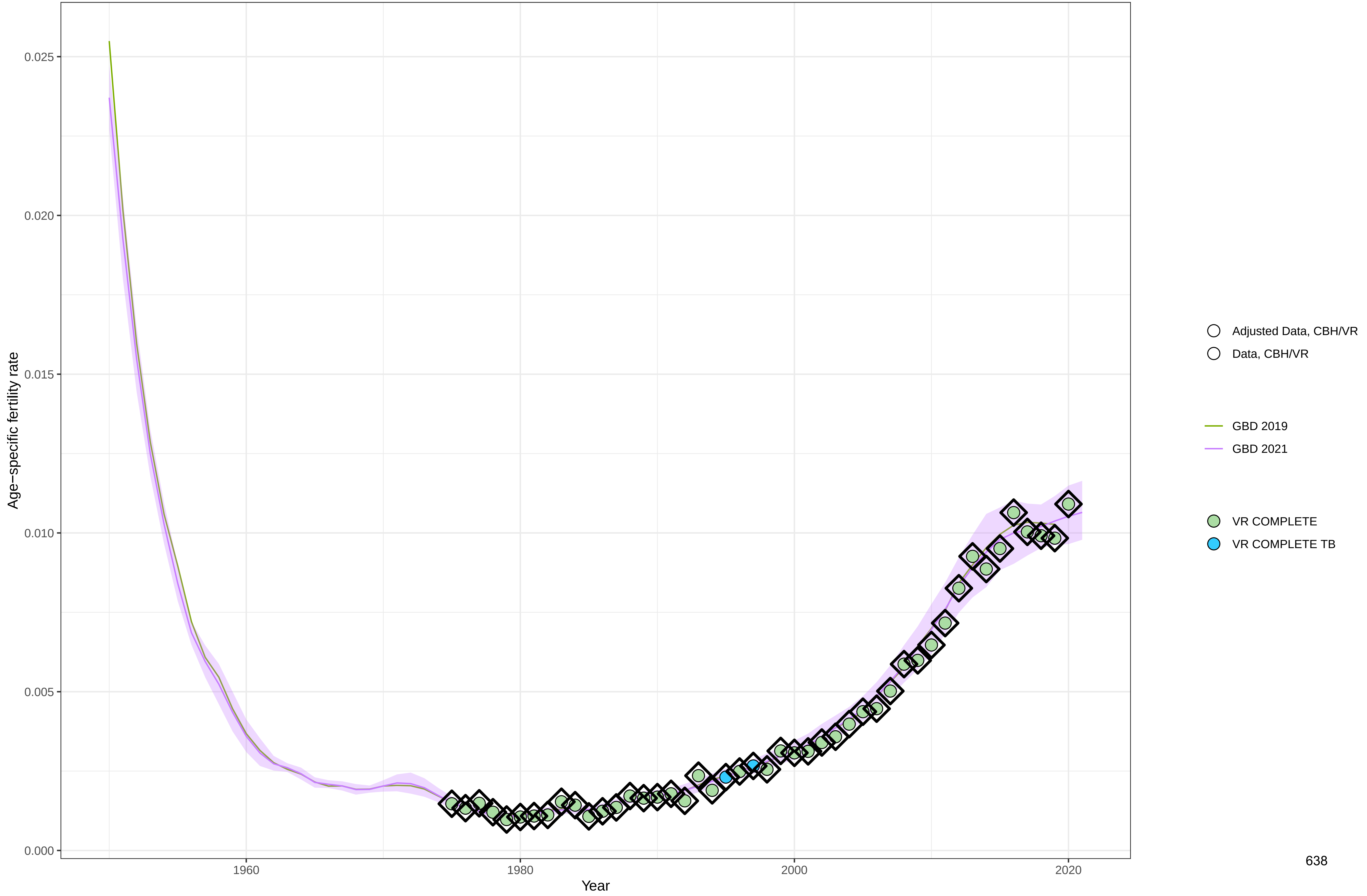

Age−specific fertility fitted trend lines, 1950−2021: Okayama, Ages: 45−49
Data points enclosed with a diamond are reference sources

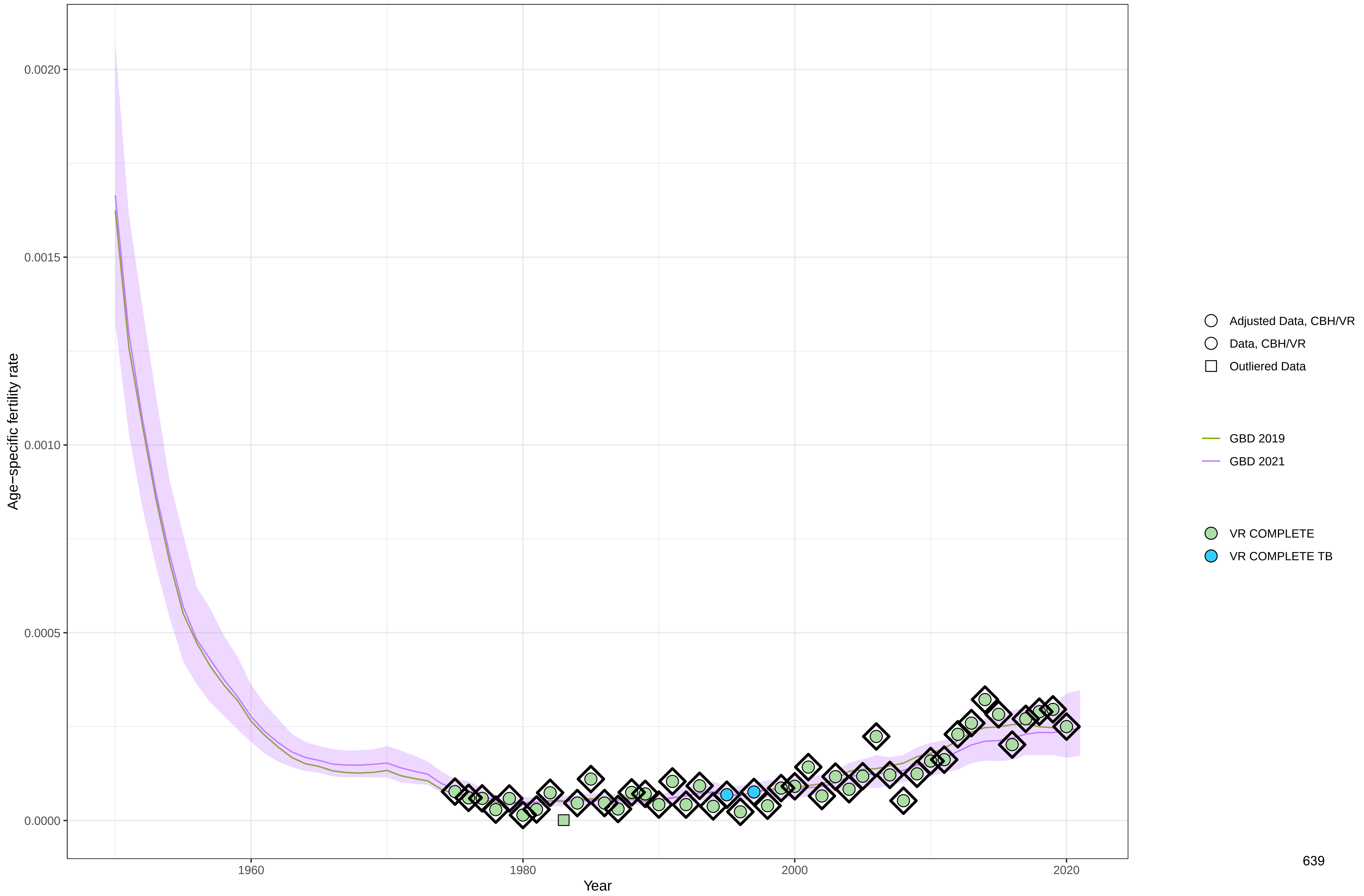

Age−specific fertility fitted trend lines, 1950−2021: Okayama, Ages: 50−54
Data points enclosed with a diamond are reference sources

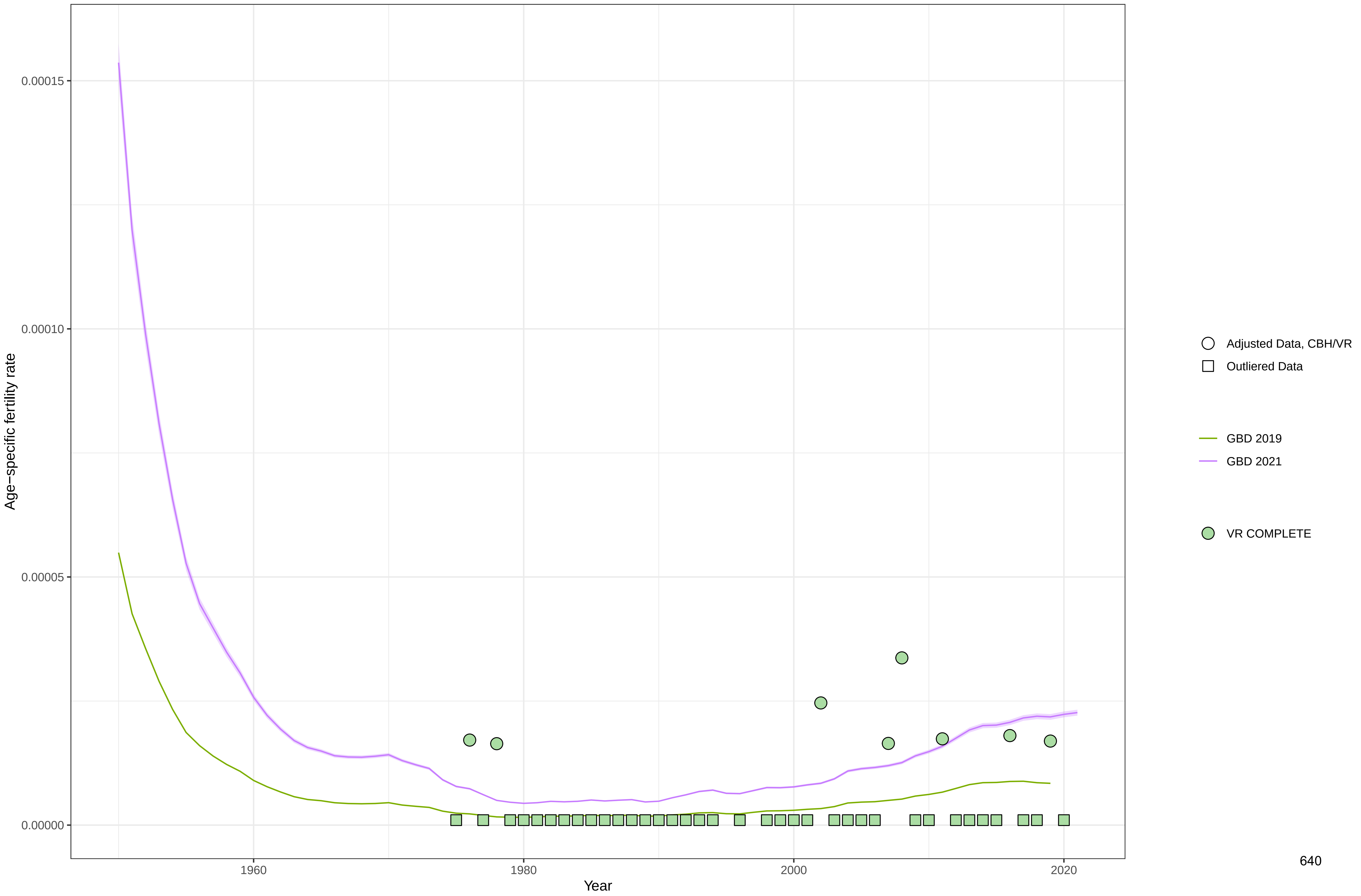

Total fertility rate fitted trend lines, 1950−2021: Okayama TFR
Data points enclosed with a diamond are reference sources

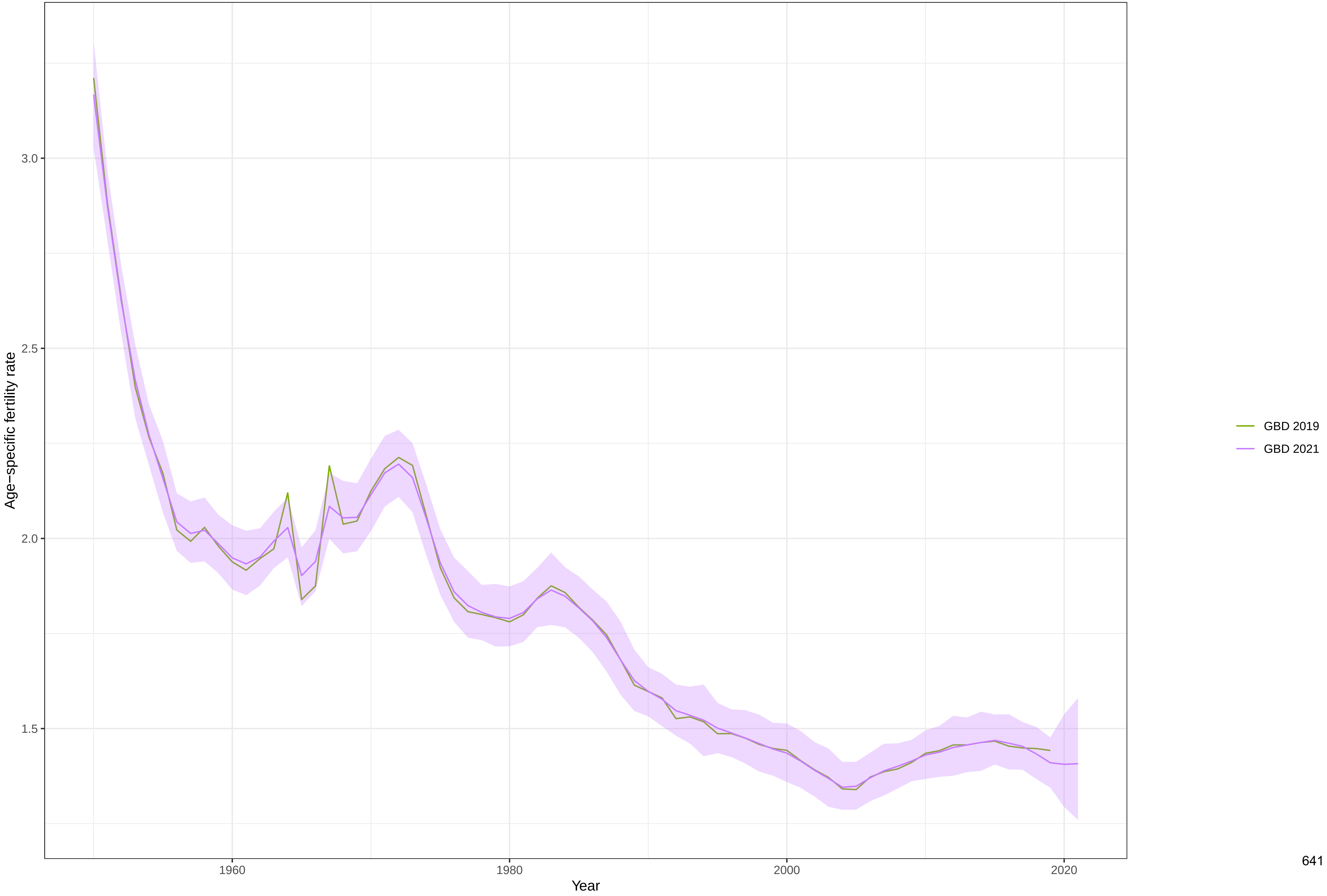

Age−specific fertility fitted trend lines, 1950−2021: Okinawa, Ages: 10−14
Data points enclosed with a diamond are reference sources

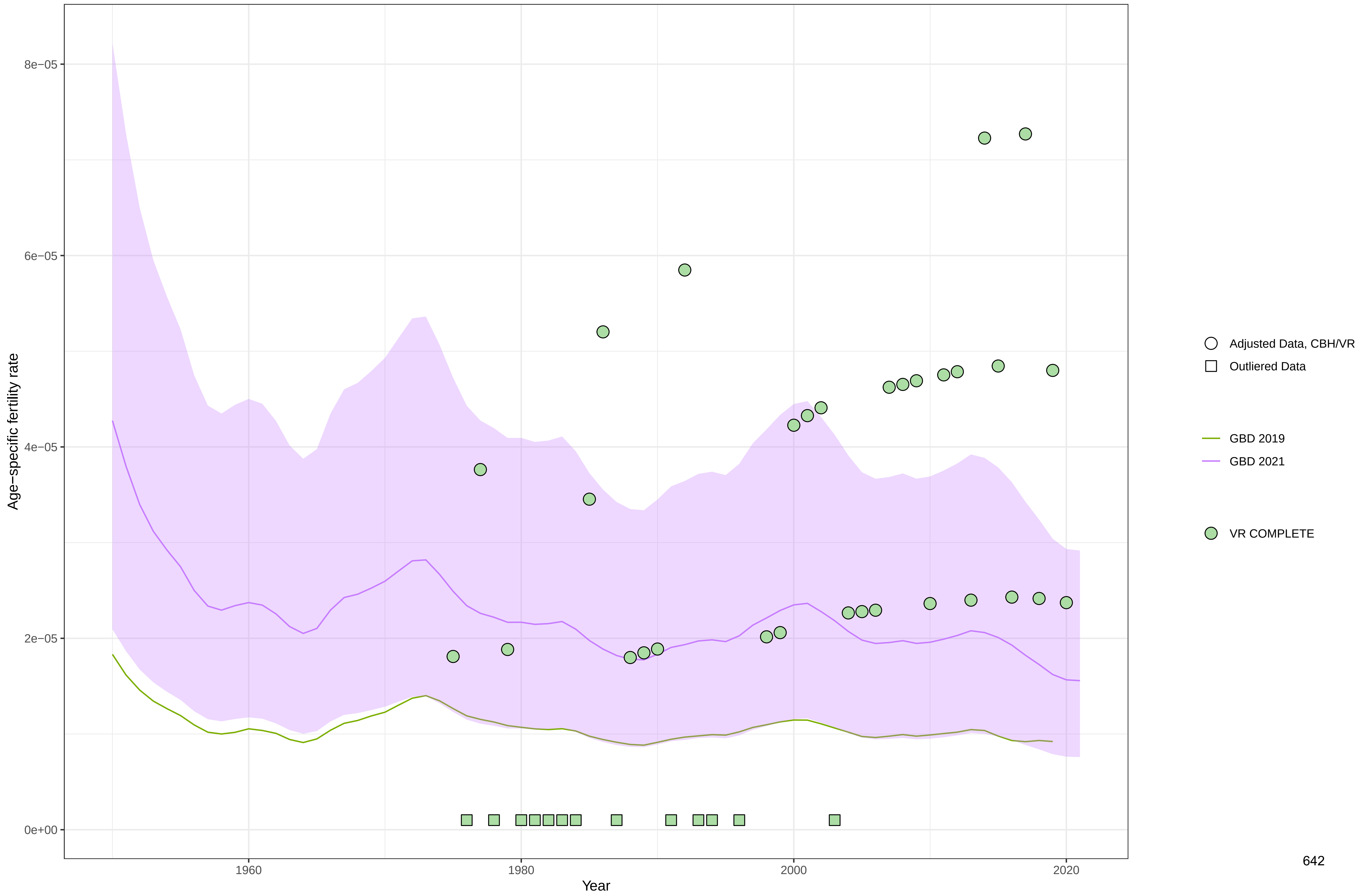

Age−specific fertility fitted trend lines, 1950−2021: Okinawa, Ages: 15−19
Data points enclosed with a diamond are reference sources

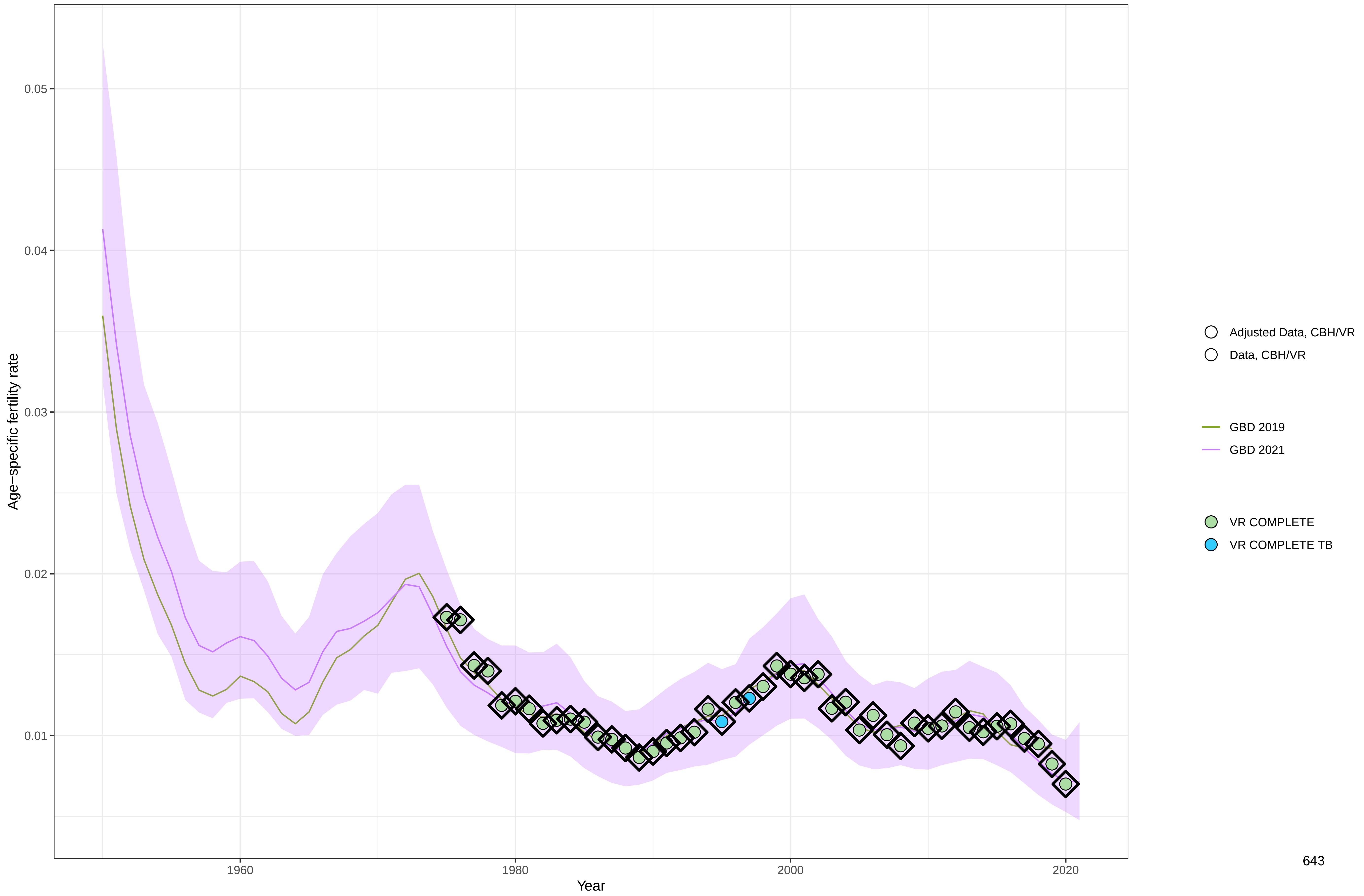

Age−specific fertility fitted trend lines, 1950−2021: Okinawa, Ages: 20−24
Data points enclosed with a diamond are reference sources

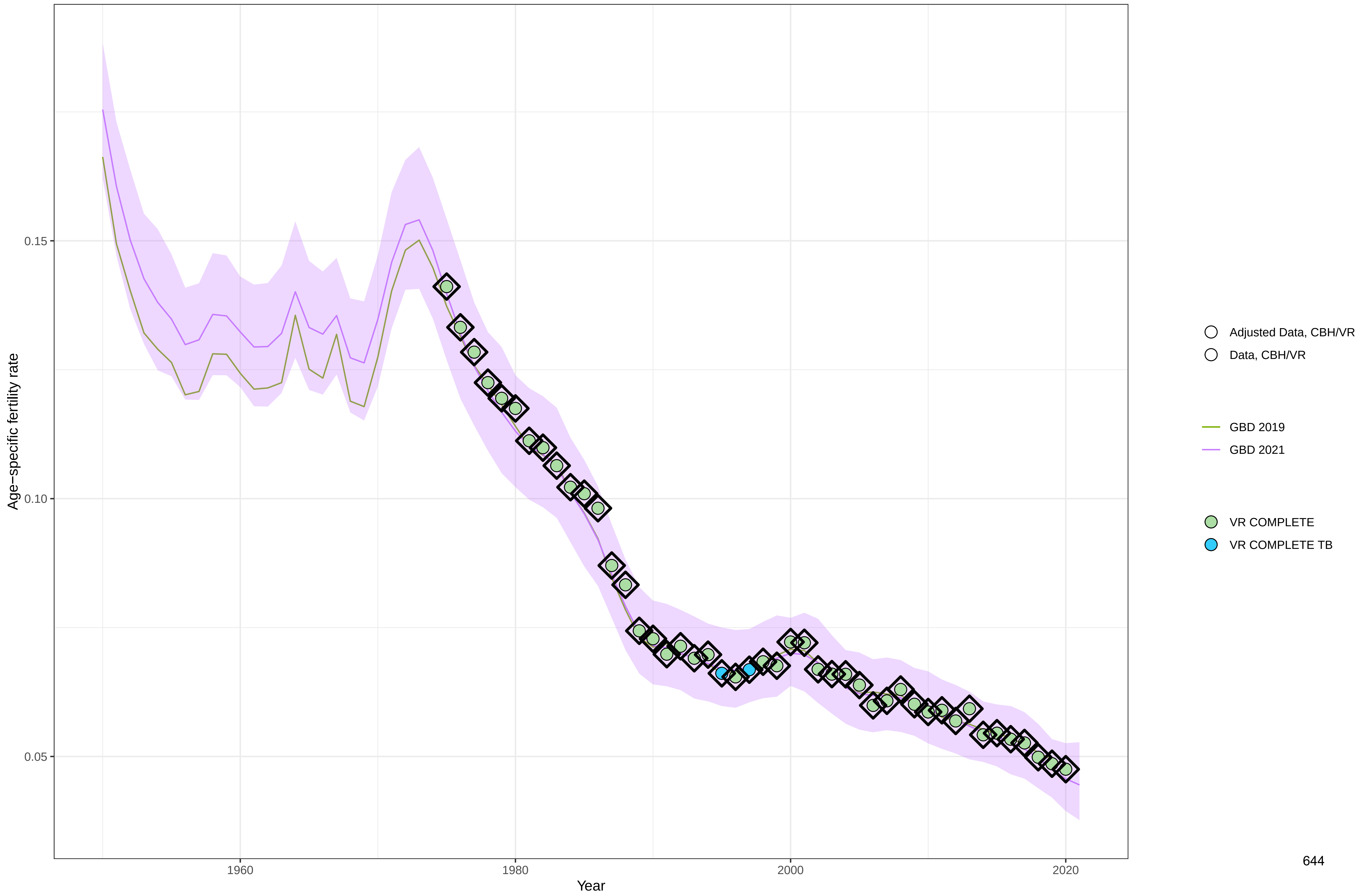

Age−specific fertility fitted trend lines, 1950−2021: Okinawa, Ages: 25−29
Data points enclosed with a diamond are reference sources

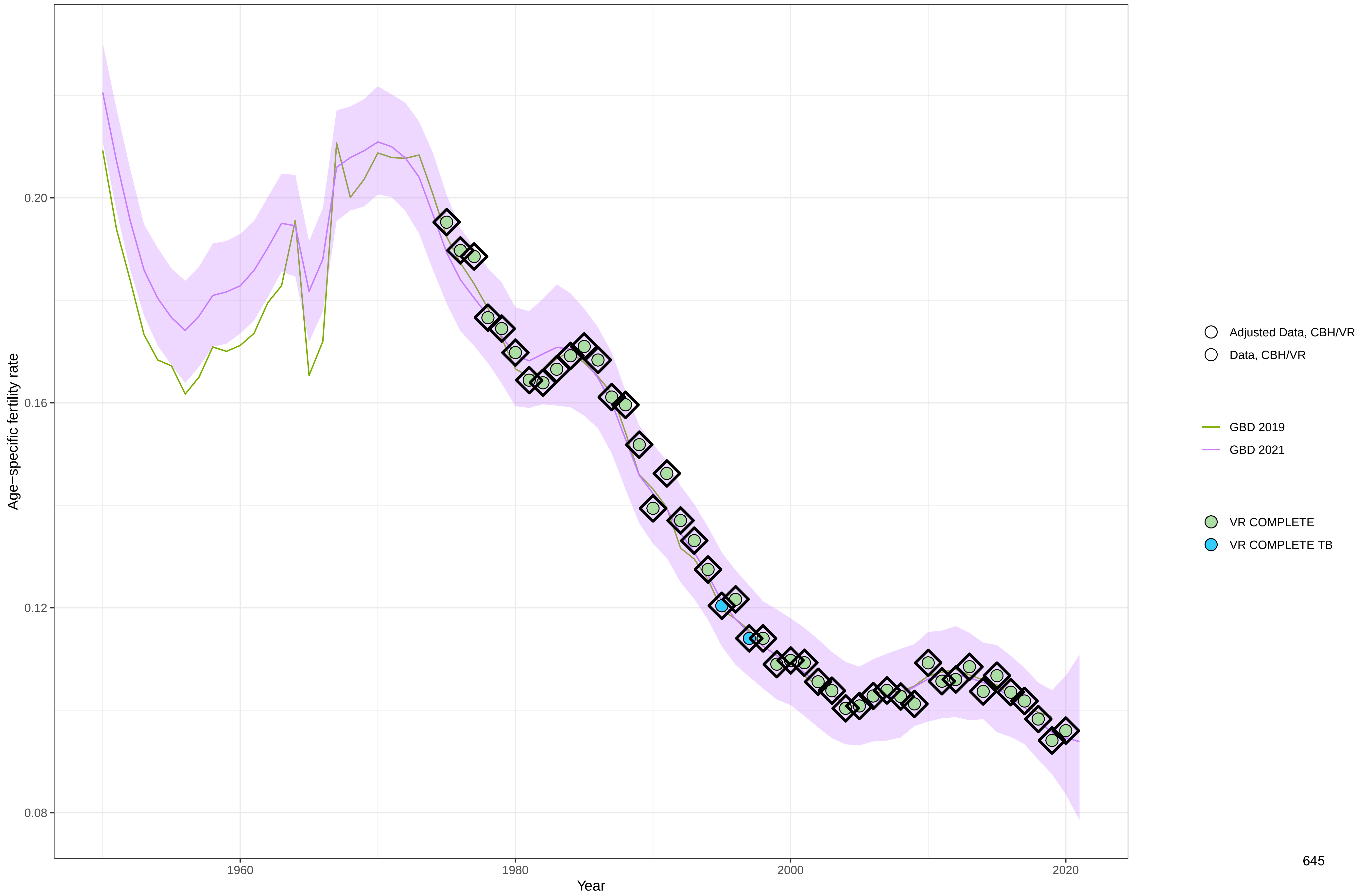

Age−specific fertility fitted trend lines, 1950−2021: Okinawa, Ages: 30−34
Data points enclosed with a diamond are reference sources

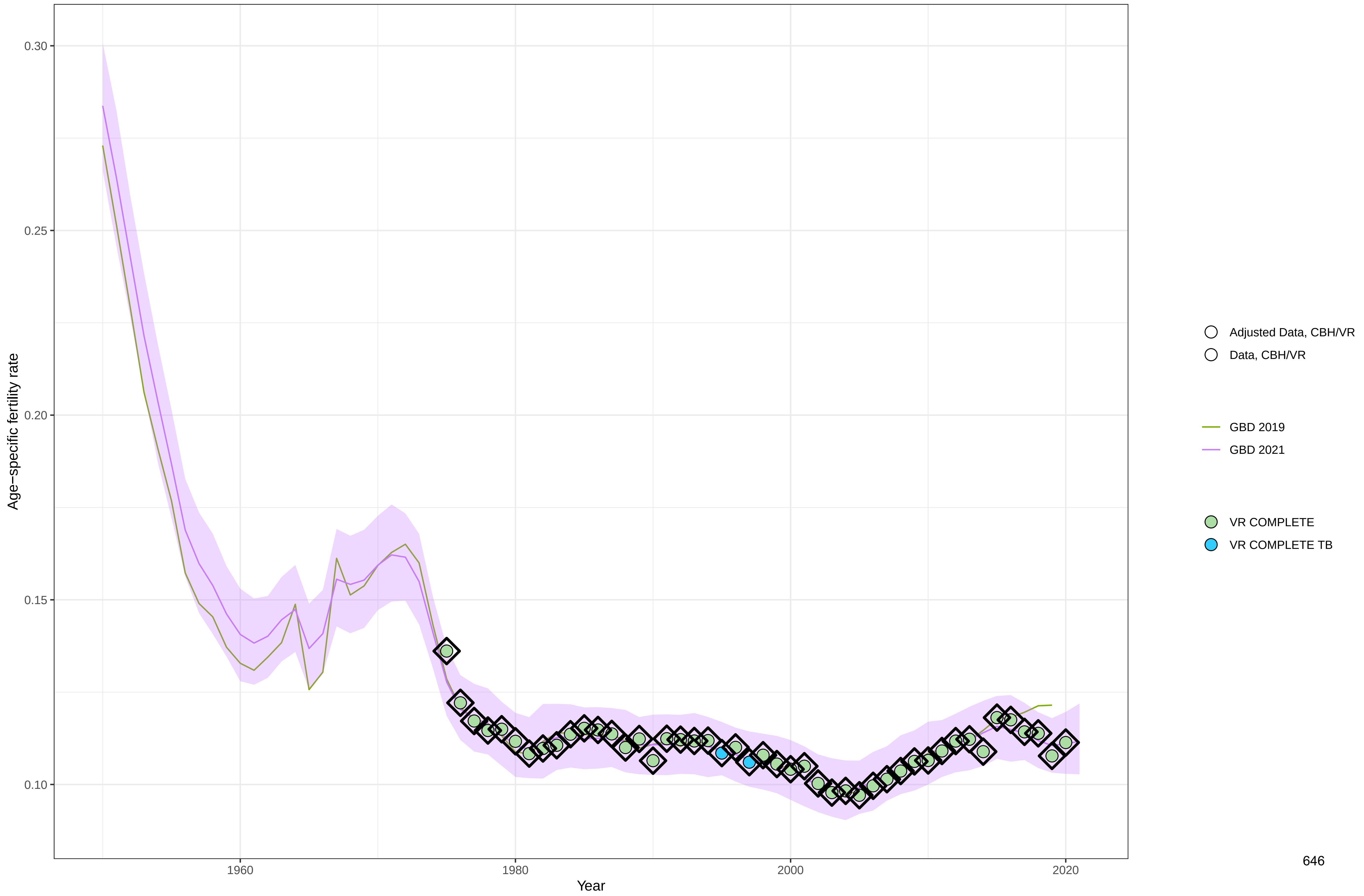

Age−specific fertility fitted trend lines, 1950−2021: Okinawa, Ages: 35−39
Data points enclosed with a diamond are reference sources

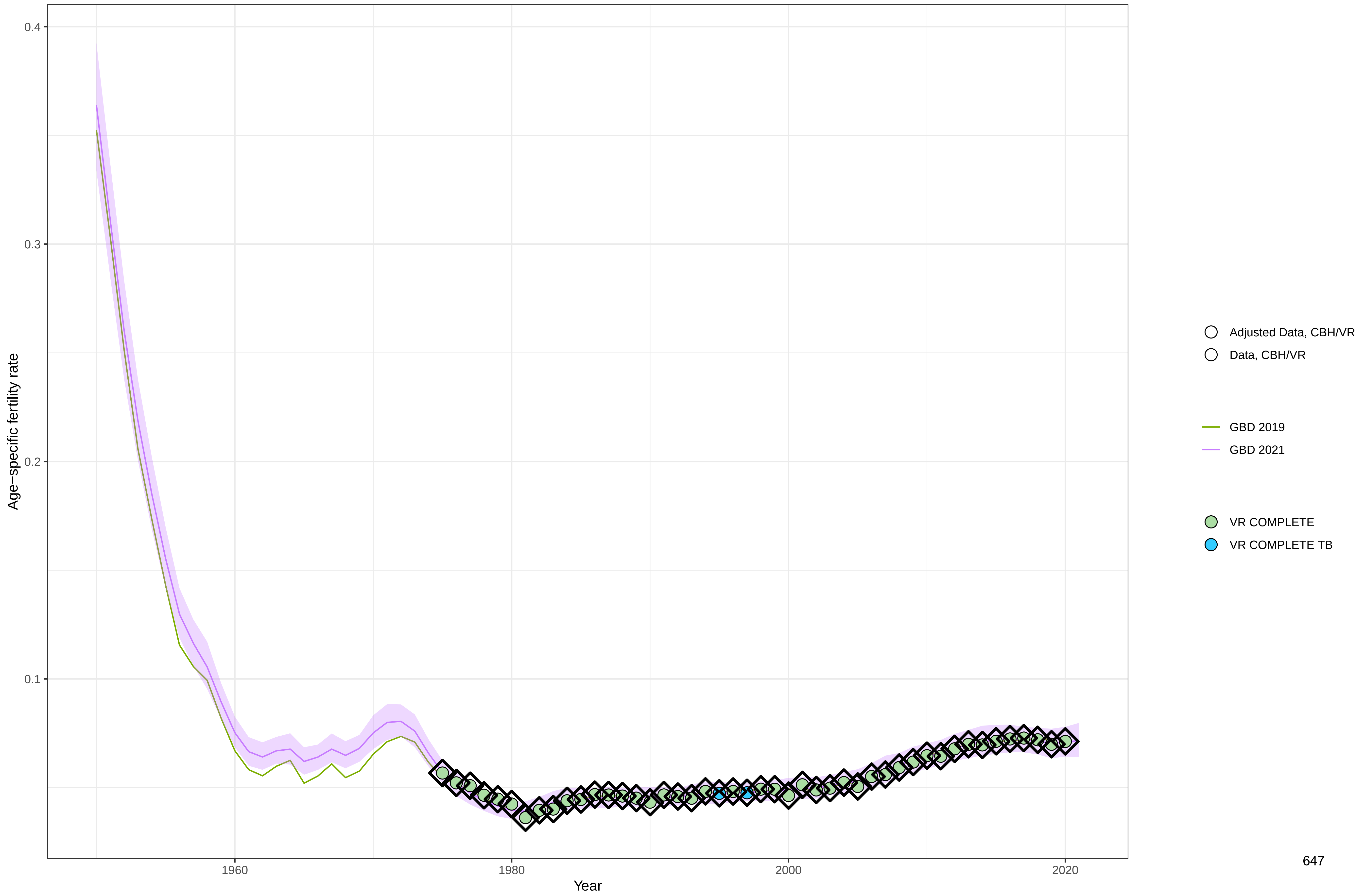

Age−specific fertility fitted trend lines, 1950−2021: Okinawa, Ages: 40−44
Data points enclosed with a diamond are reference sources

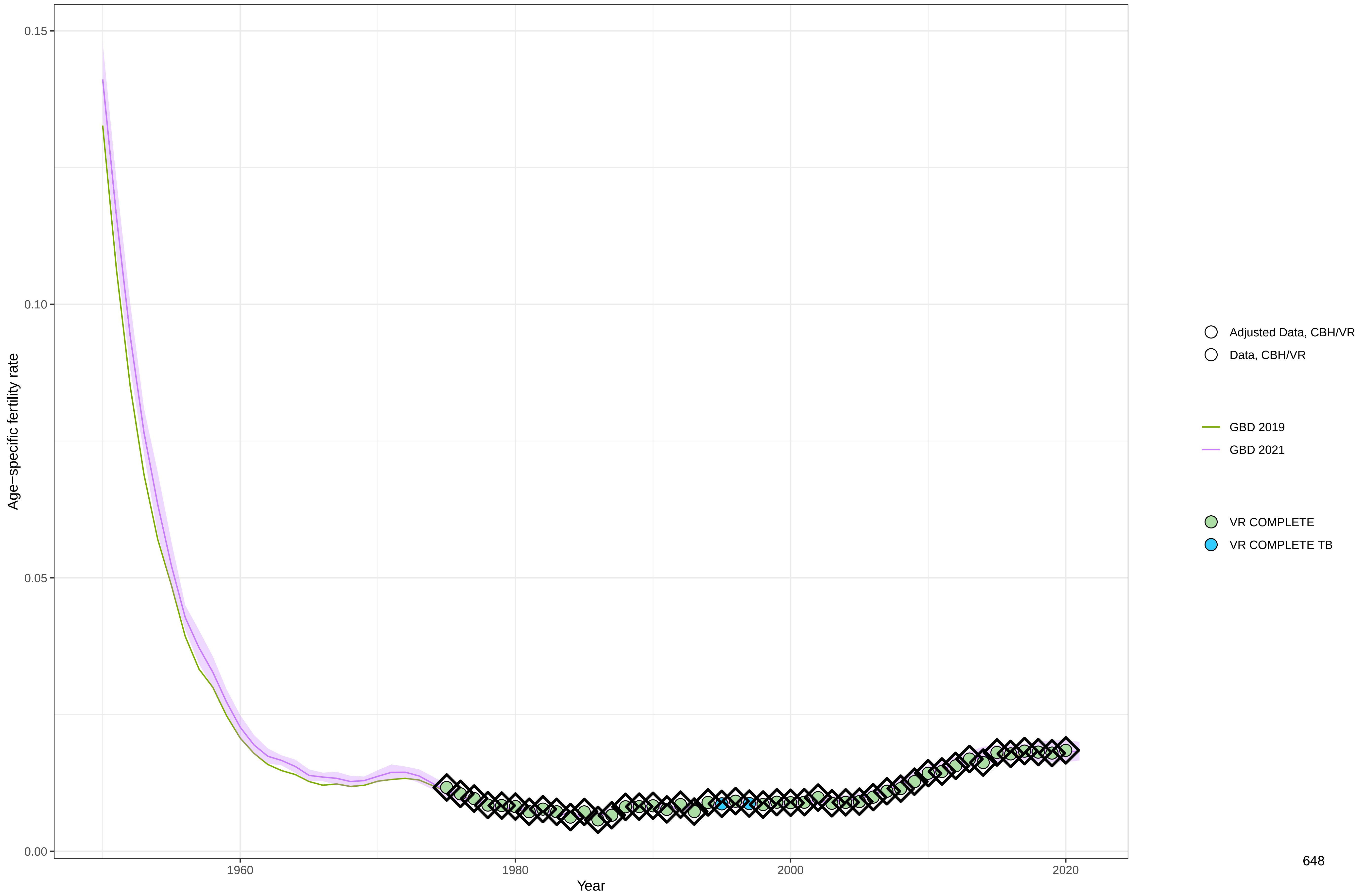

Age−specific fertility fitted trend lines, 1950−2021: Okinawa, Ages: 45−49
Data points enclosed with a diamond are reference sources

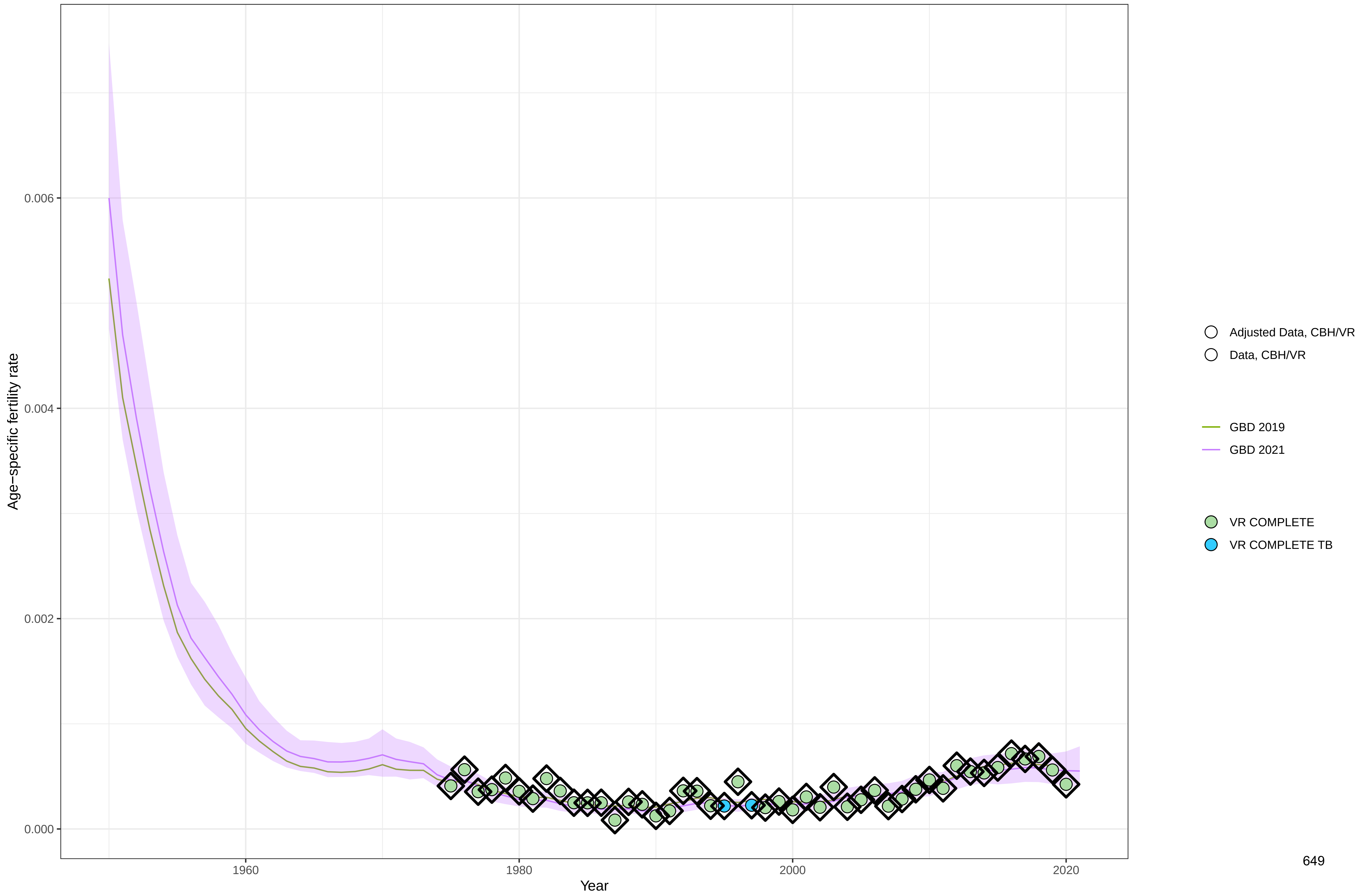

Age−specific fertility fitted trend lines, 1950−2021: Okinawa, Ages: 50−54
Data points enclosed with a diamond are reference sources

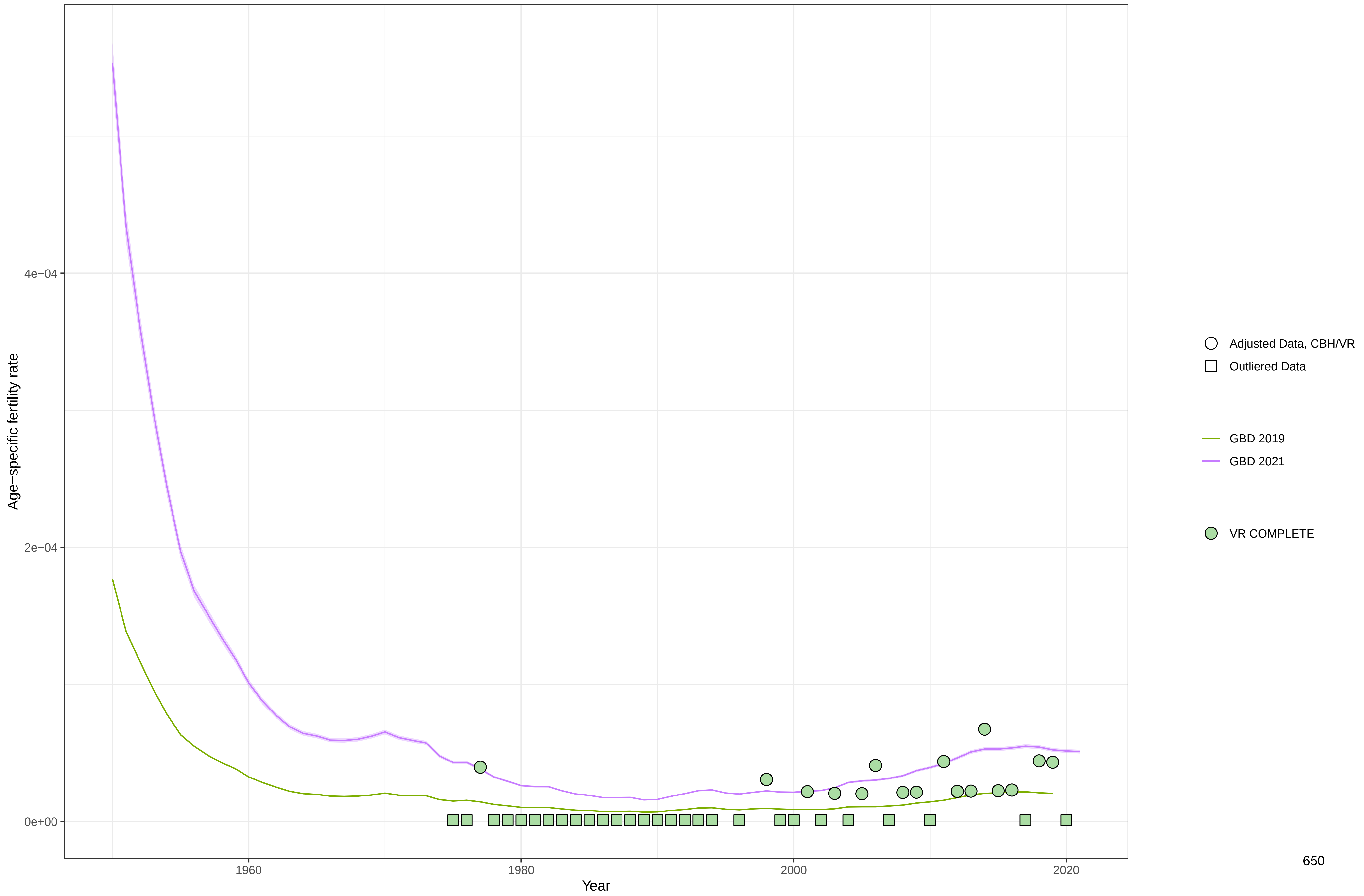

Total fertility rate fitted trend lines, 1950−2021: Okinawa TFR
Data points enclosed with a diamond are reference sources

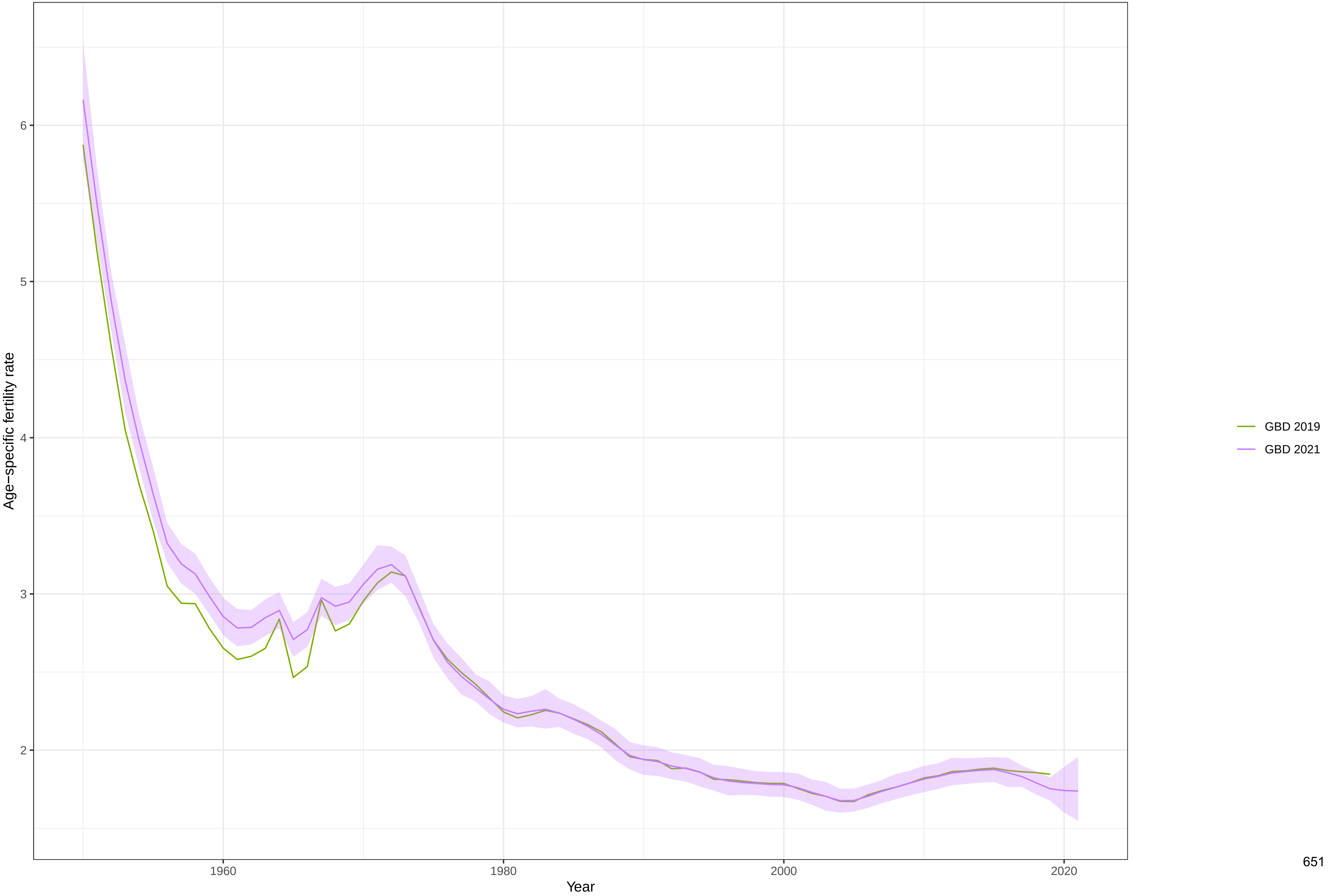

Age−specific fertility fitted trend lines, 1950−2021: ..saka, Ages: 10−14
Data points enclosed with a diamond are reference sources

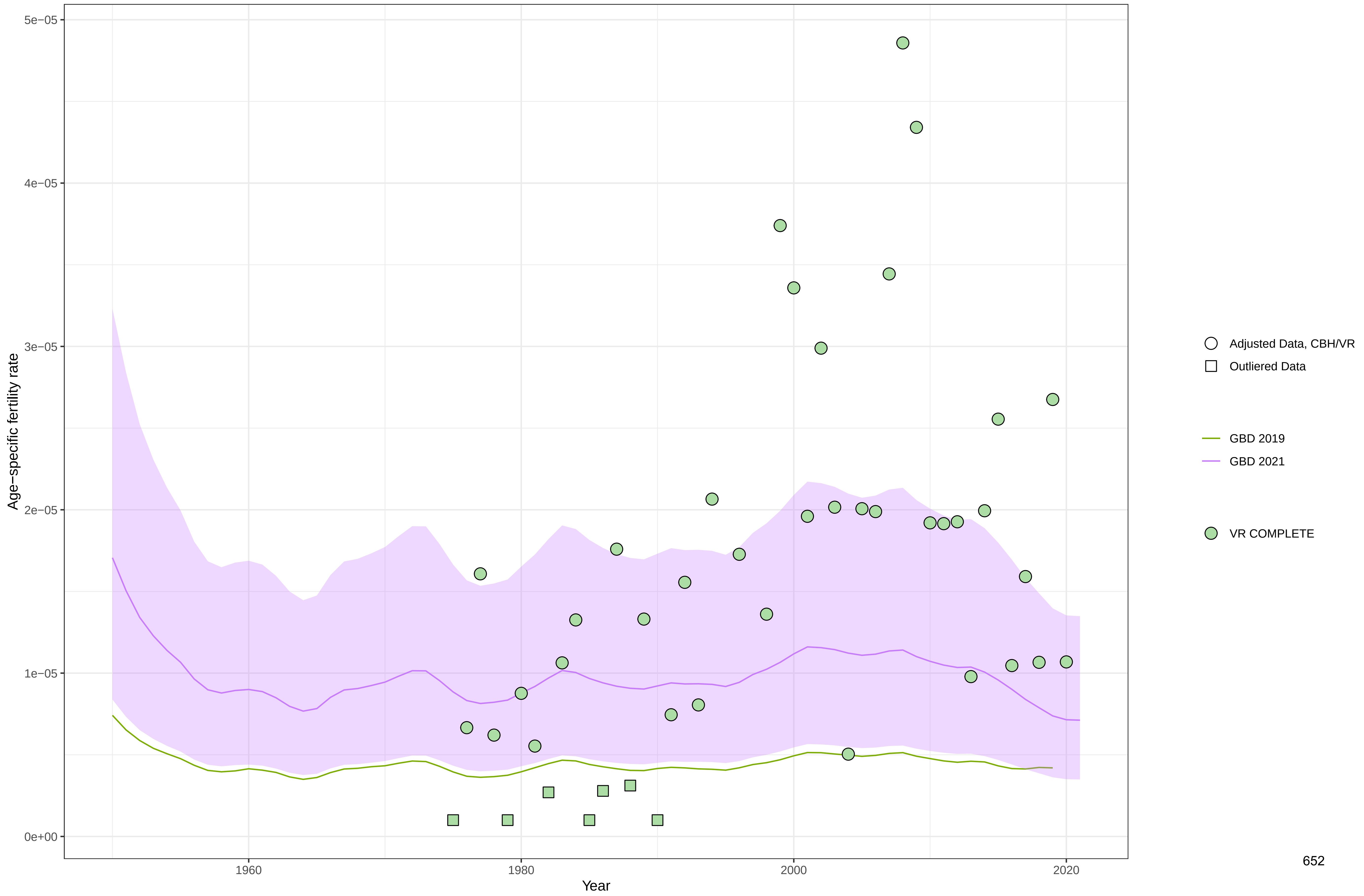

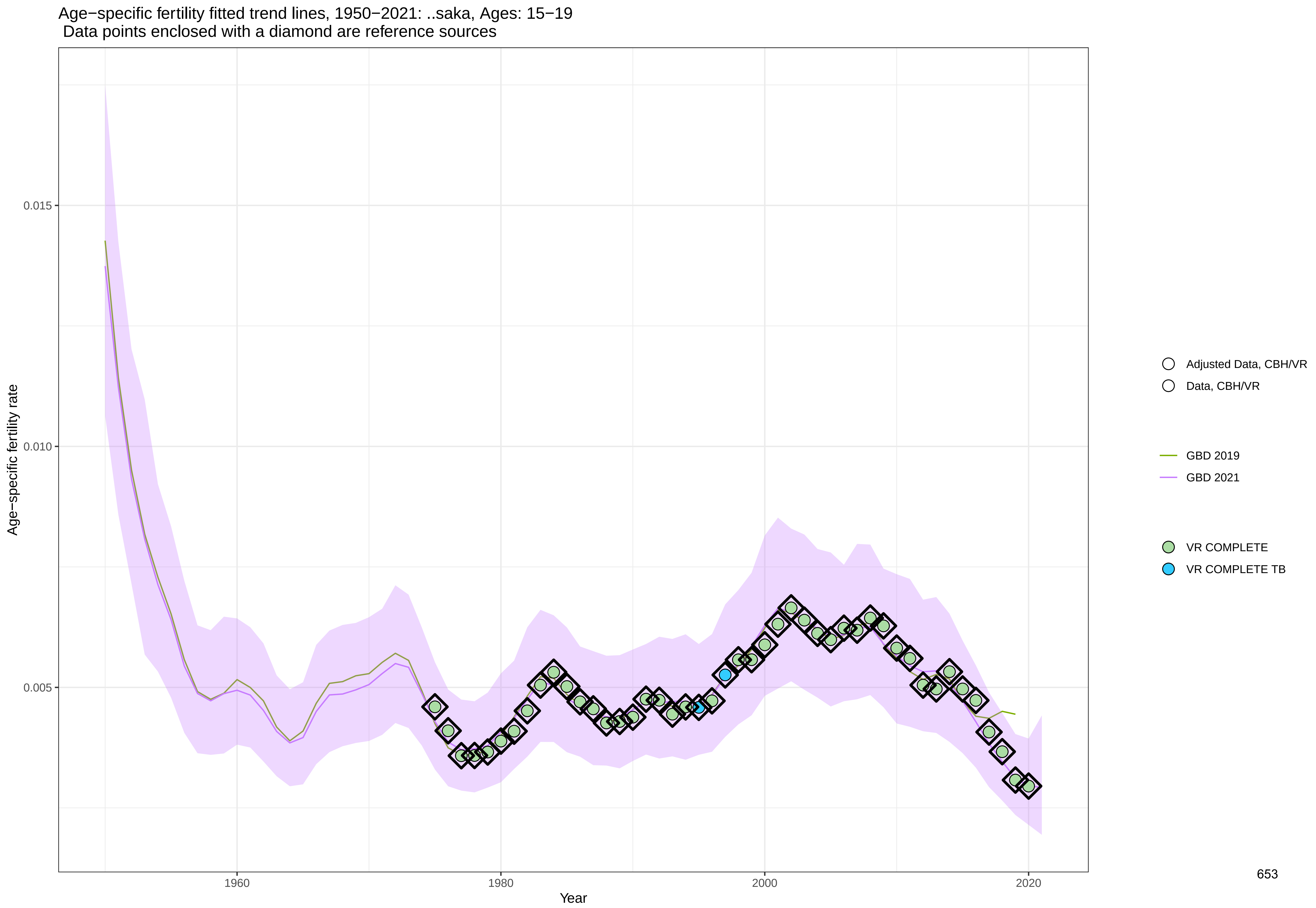
Age−specific fertility fitted trend lines, 1950−2021: ..saka, Ages: 15−19
Data points enclosed with a diamond are reference sources
Age−specific fertility rate
0.015
0.010
0.005
1960
1980
2000
2020
Year
Adjusted Data, CBH/VR
Data, CBH/VR
GBD 2019
GBD 2021
VR COMPLETE
VR COMPLETE TB

Age−specific fertility fitted trend lines, 1950−2021: ..saka, Ages: 20−24
Data points enclosed with a diamond are reference sources

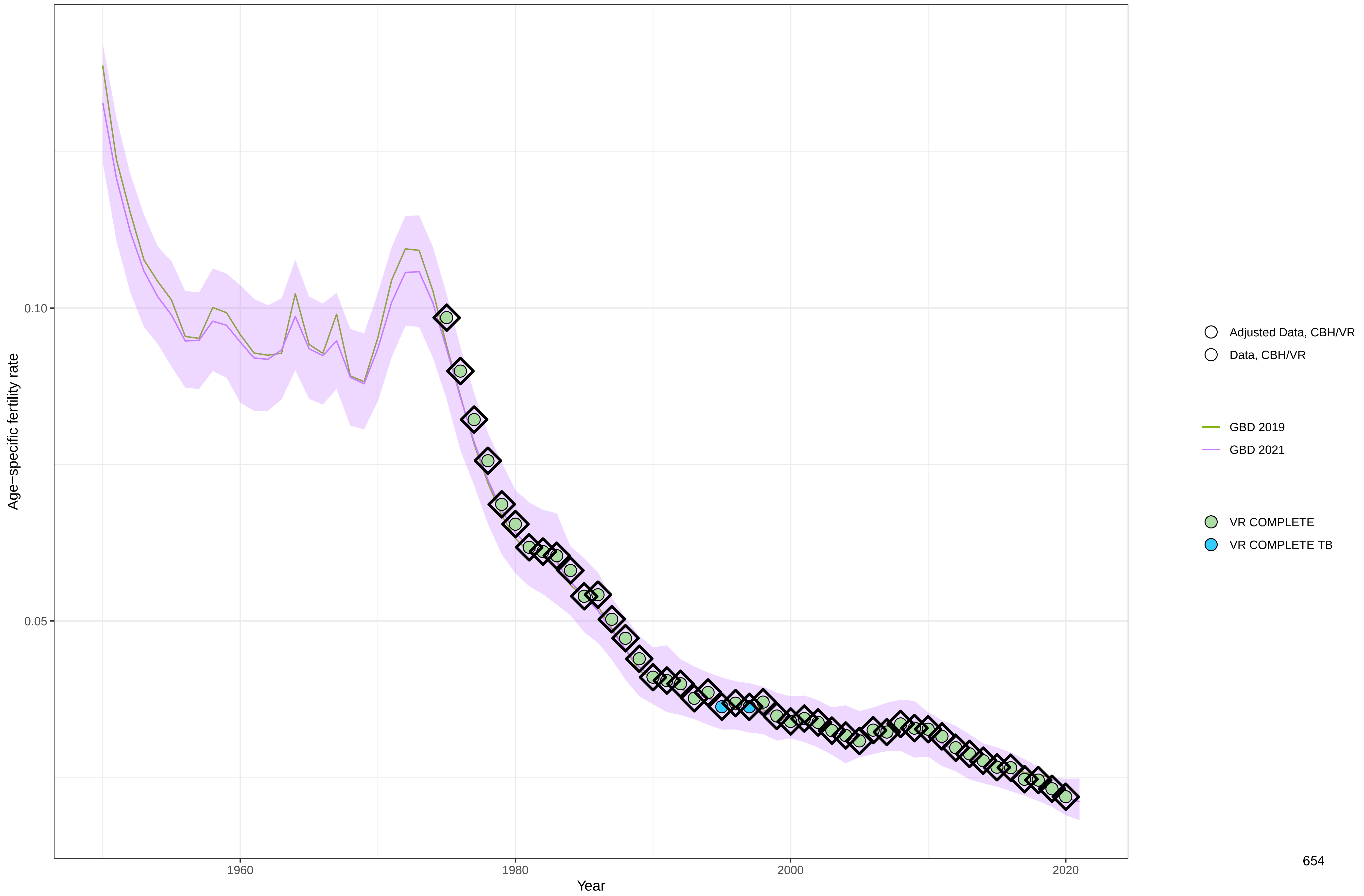

Age−specific fertility fitted trend lines, 1950−2021: ..saka, Ages: 25−29
Data points enclosed with a diamond are reference sources

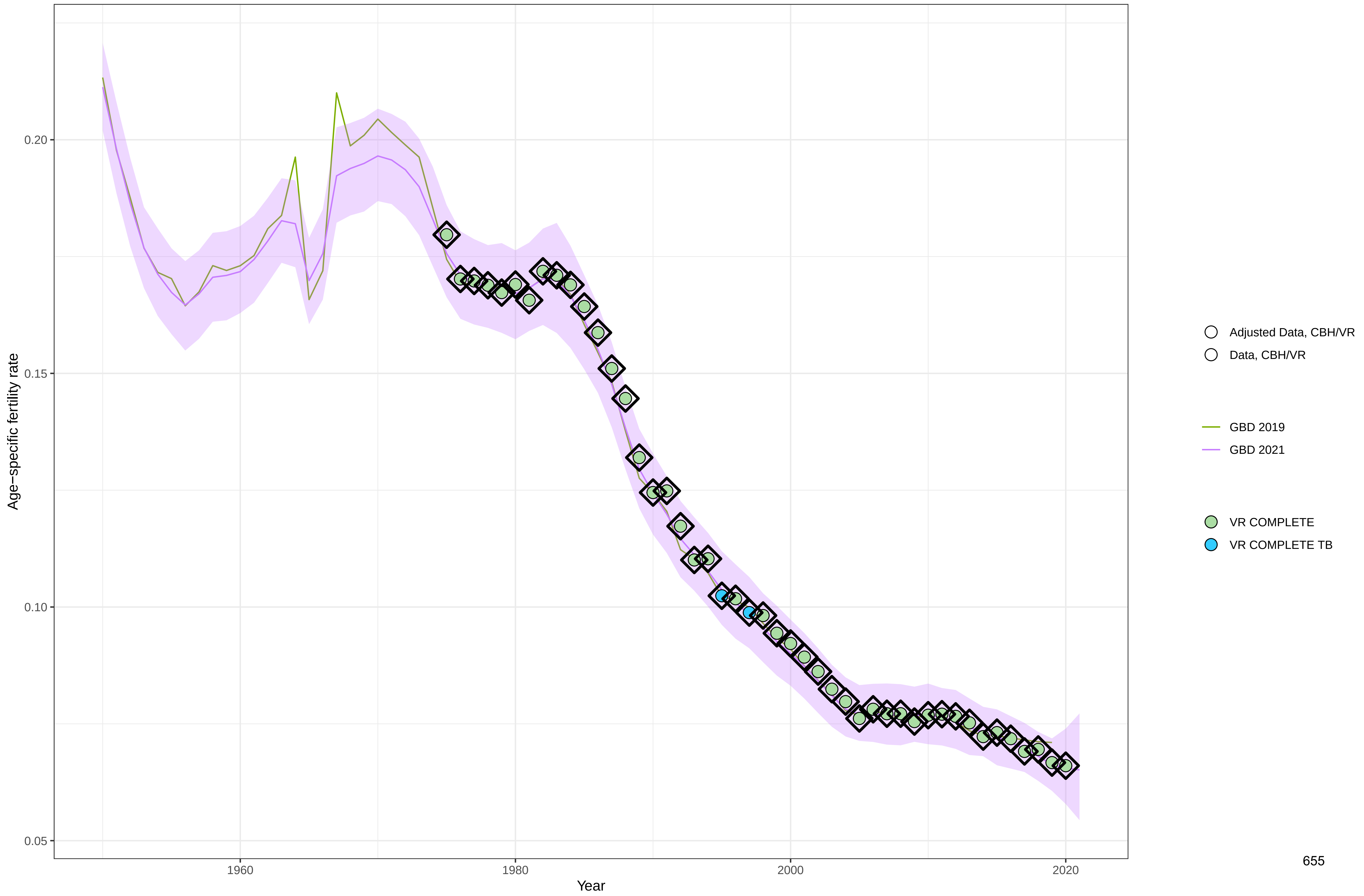

Age−specific fertility fitted trend lines, 1950−2021: ..saka, Ages: 30−34
Data points enclosed with a diamond are reference sources

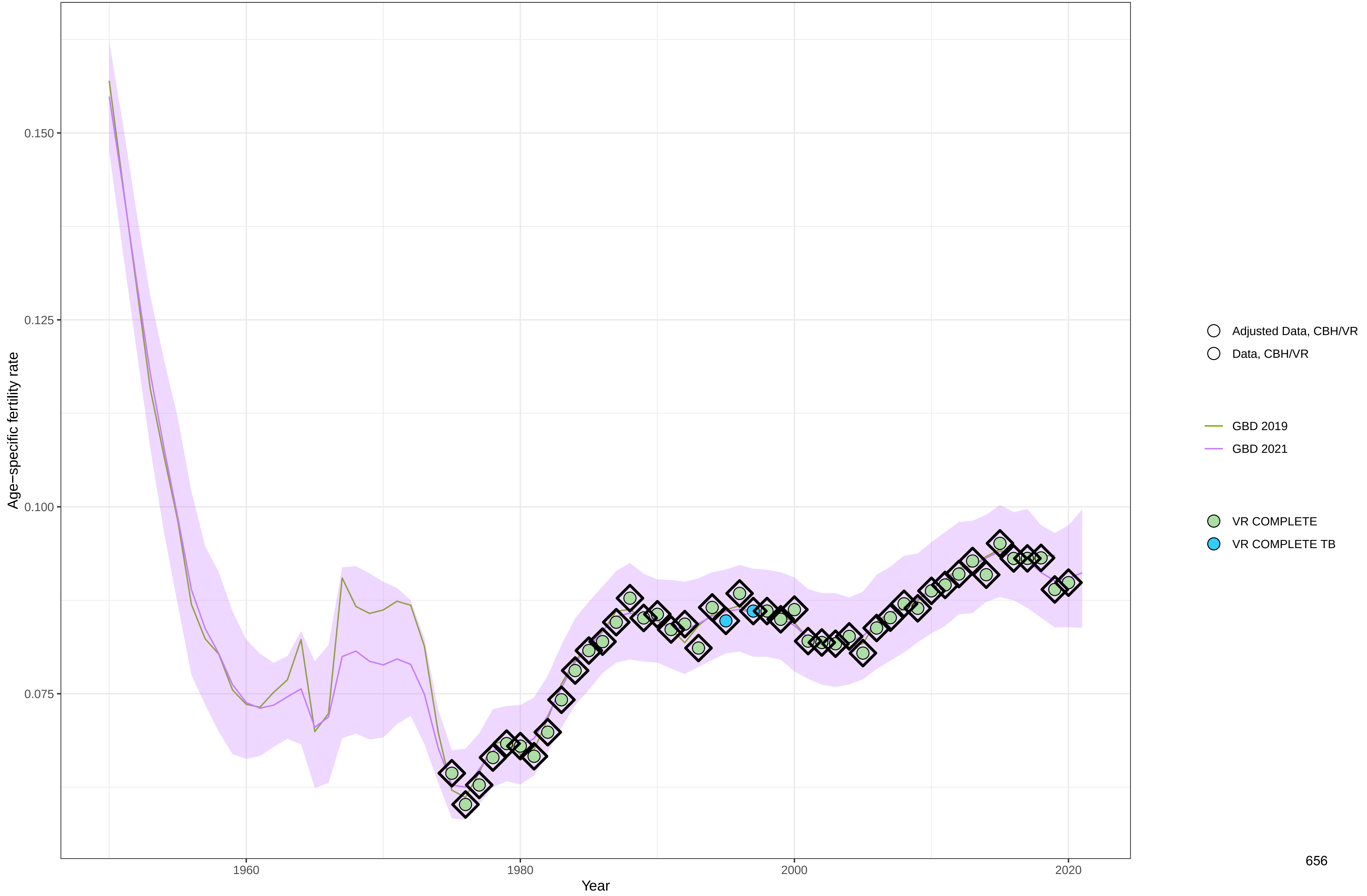

Age−specific fertility fitted trend lines, 1950−2021: ..saka, Ages: 35−39
Data points enclosed with a diamond are reference sources

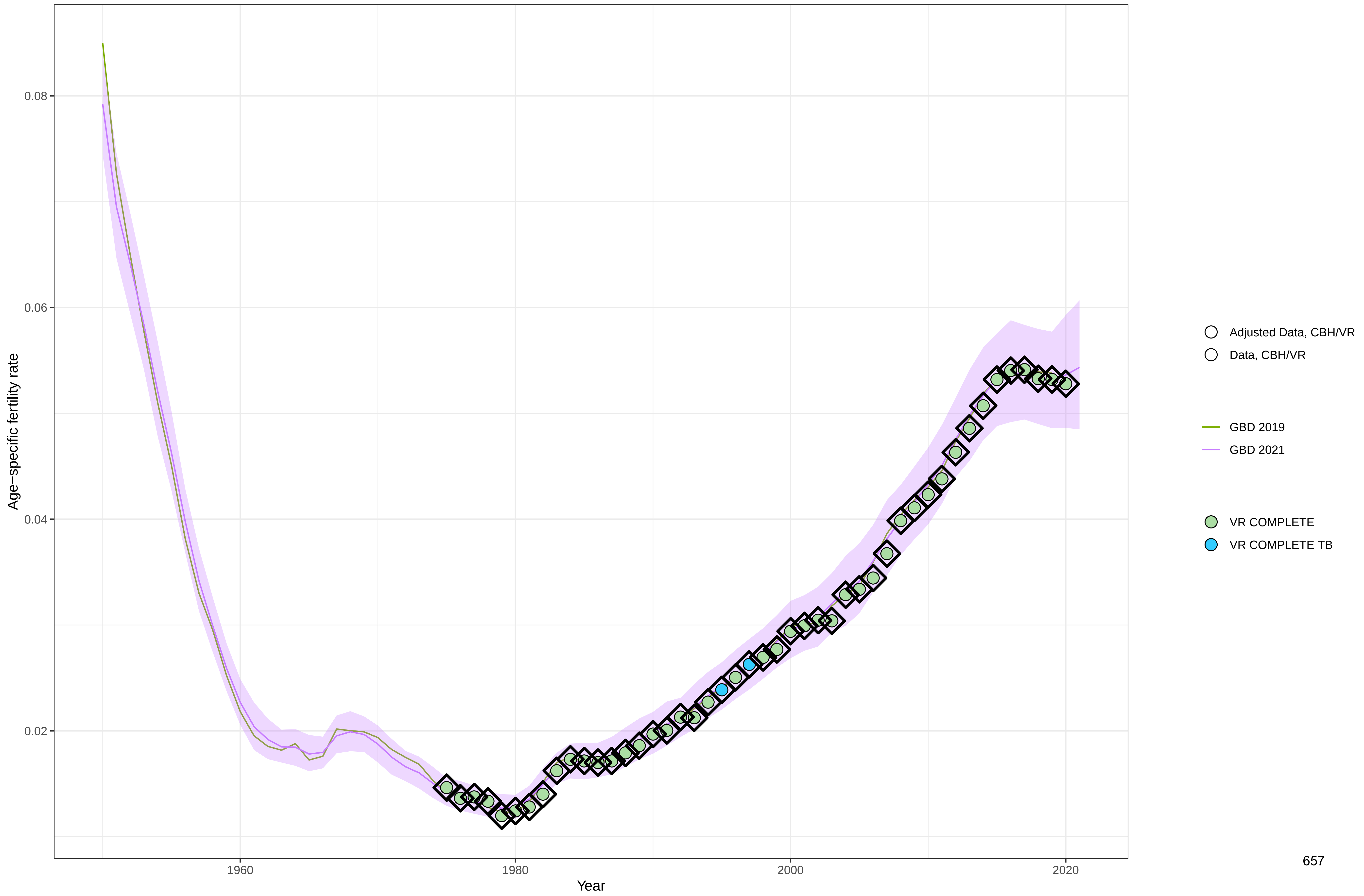

Age−specific fertility fitted trend lines, 1950−2021: ..saka, Ages: 40−44
Data points enclosed with a diamond are reference sources

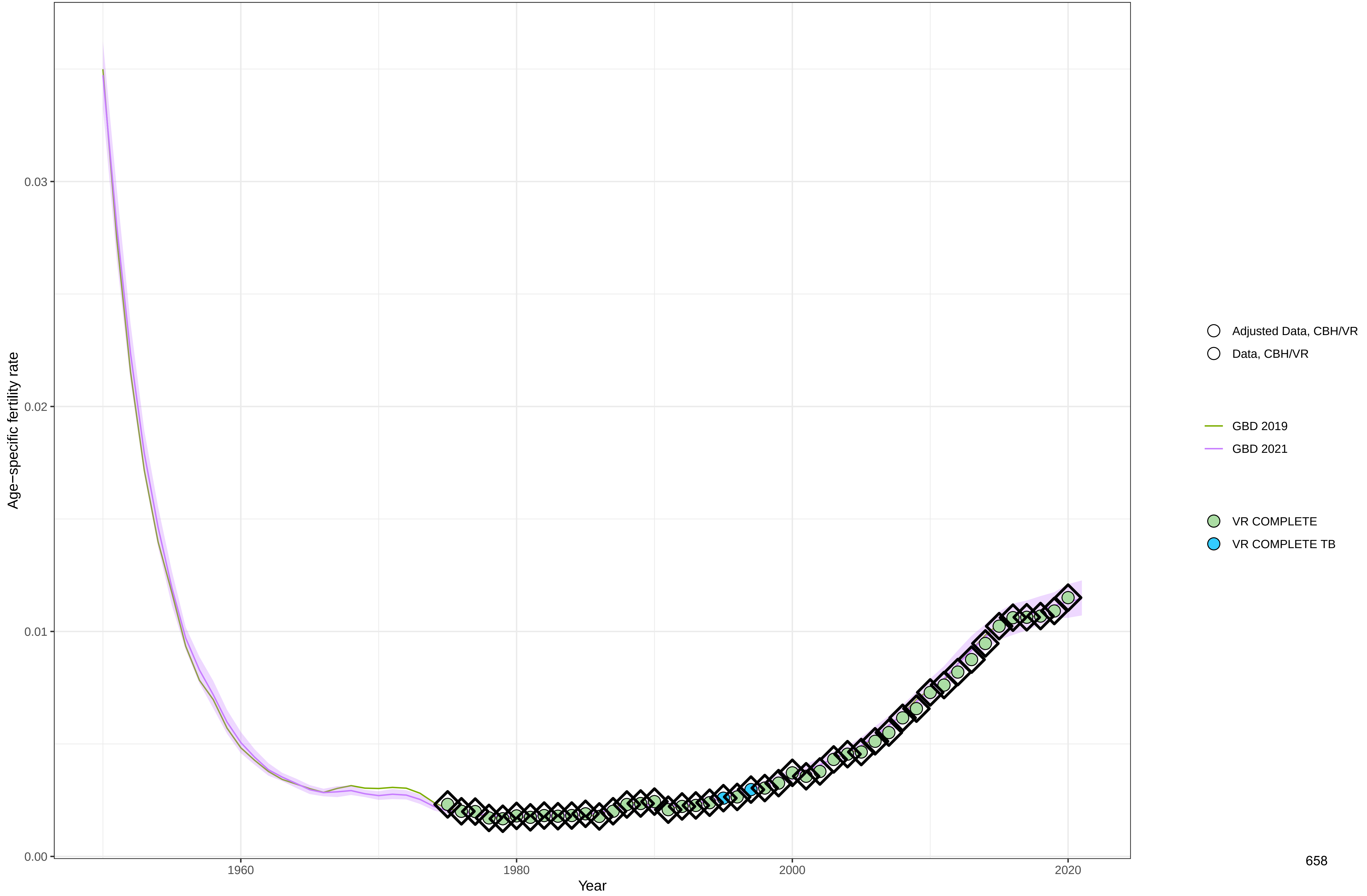

Age−specific fertility fitted trend lines, 1950−2021: ..saka, Ages: 45−49
Data points enclosed with a diamond are reference sources

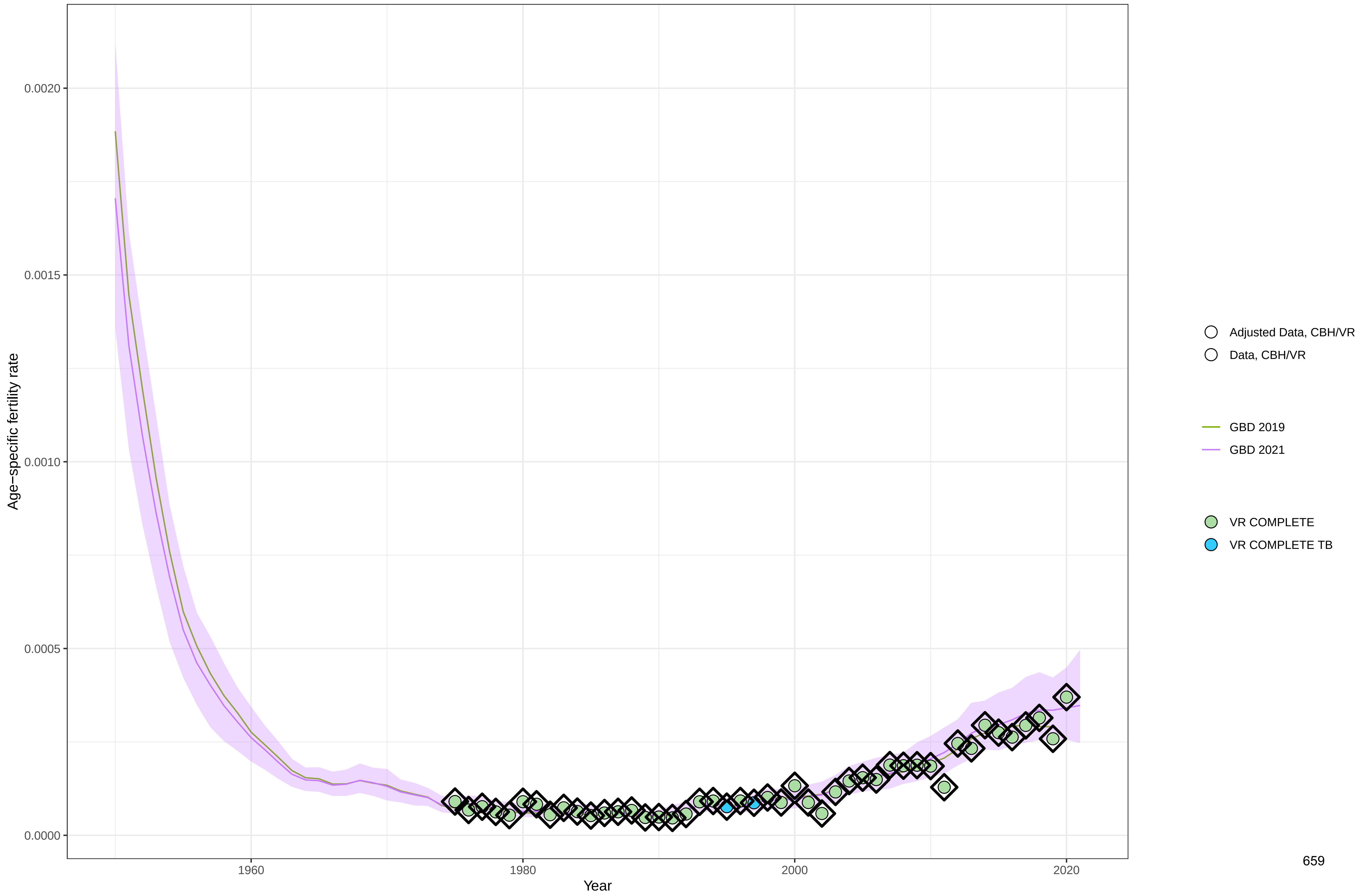

Age−specific fertility fitted trend lines, 1950−2021: ..saka, Ages: 50−54
Data points enclosed with a diamond are reference sources

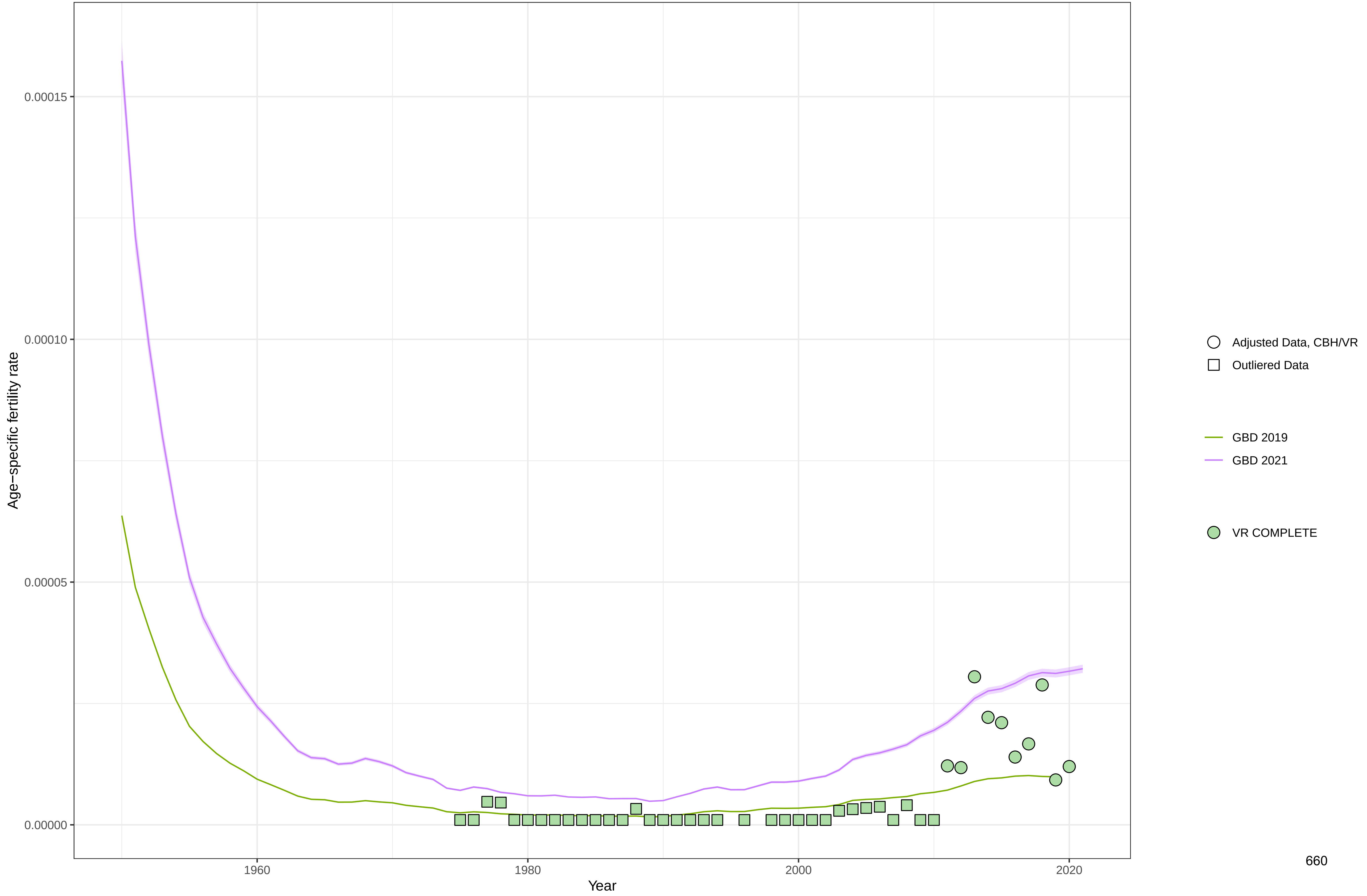

Total fertility rate fitted trend lines, 1950−2021: ..saka TFR
Data points enclosed with a diamond are reference sources

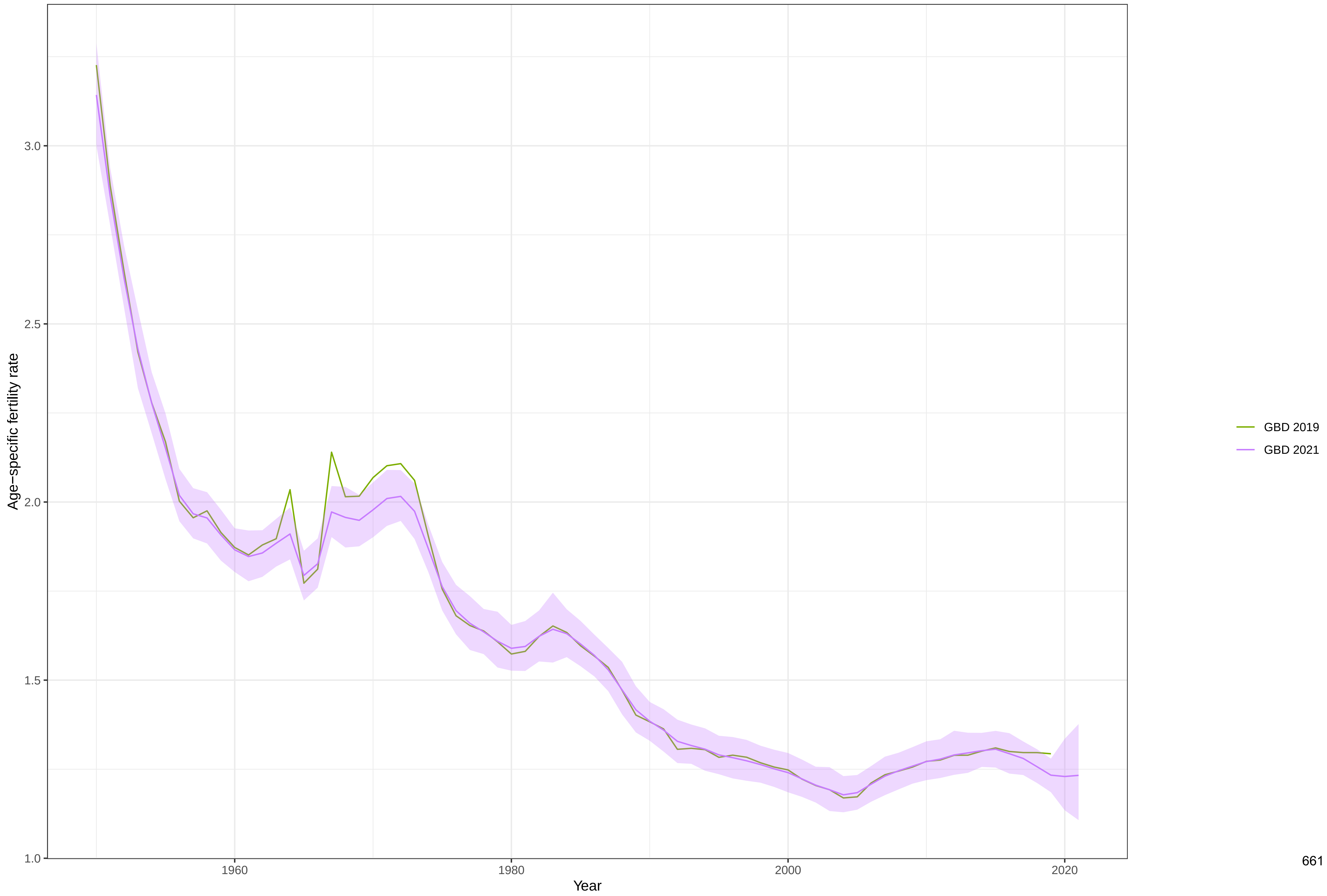

Age−specific fertility fitted trend lines, 1950−2021: Saga, Ages: 10−14
Data points enclosed with a diamond are reference sources

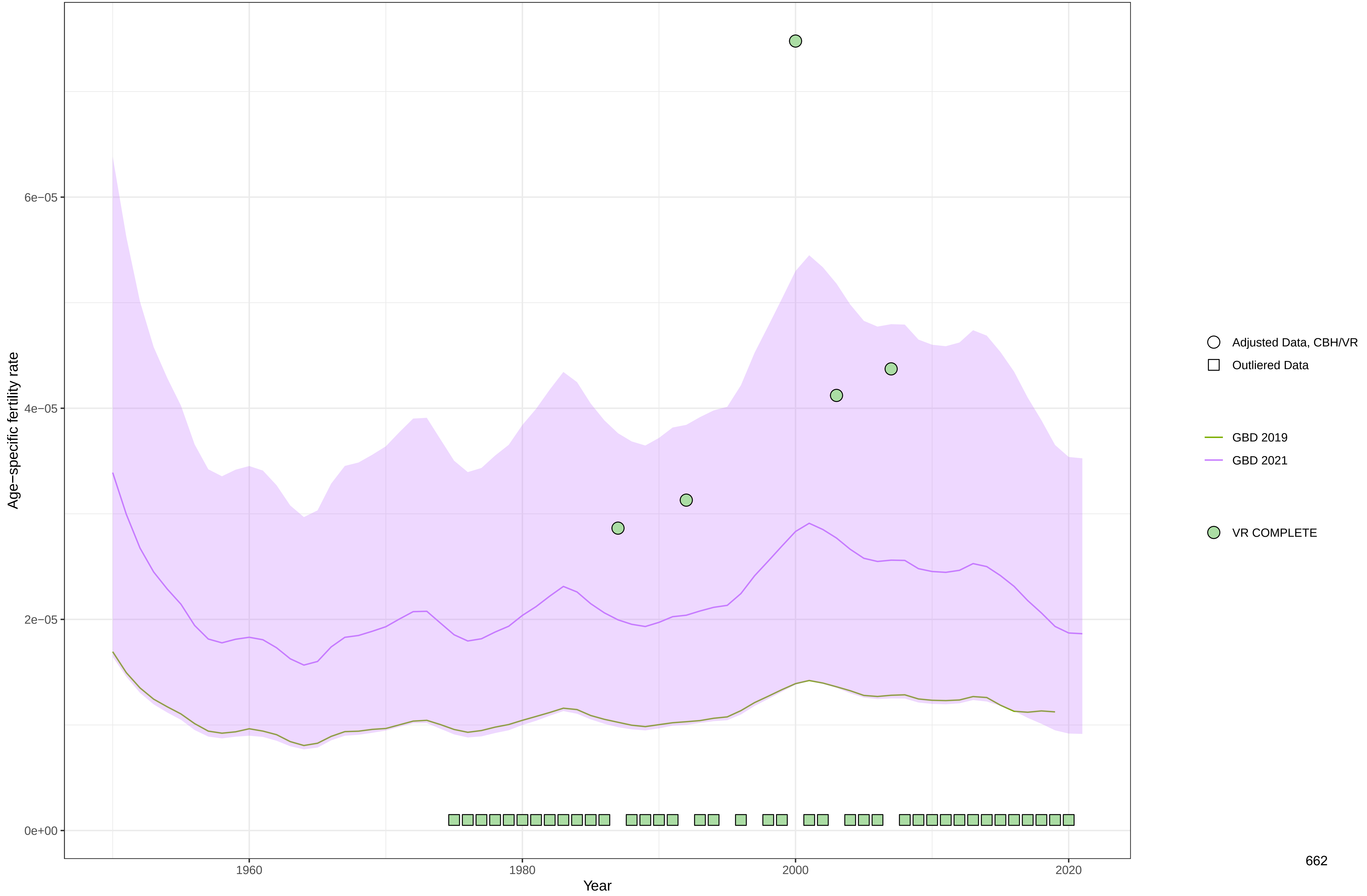

Age−specific fertility fitted trend lines, 1950−2021: Saga, Ages: 15−19
Data points enclosed with a diamond are reference sources

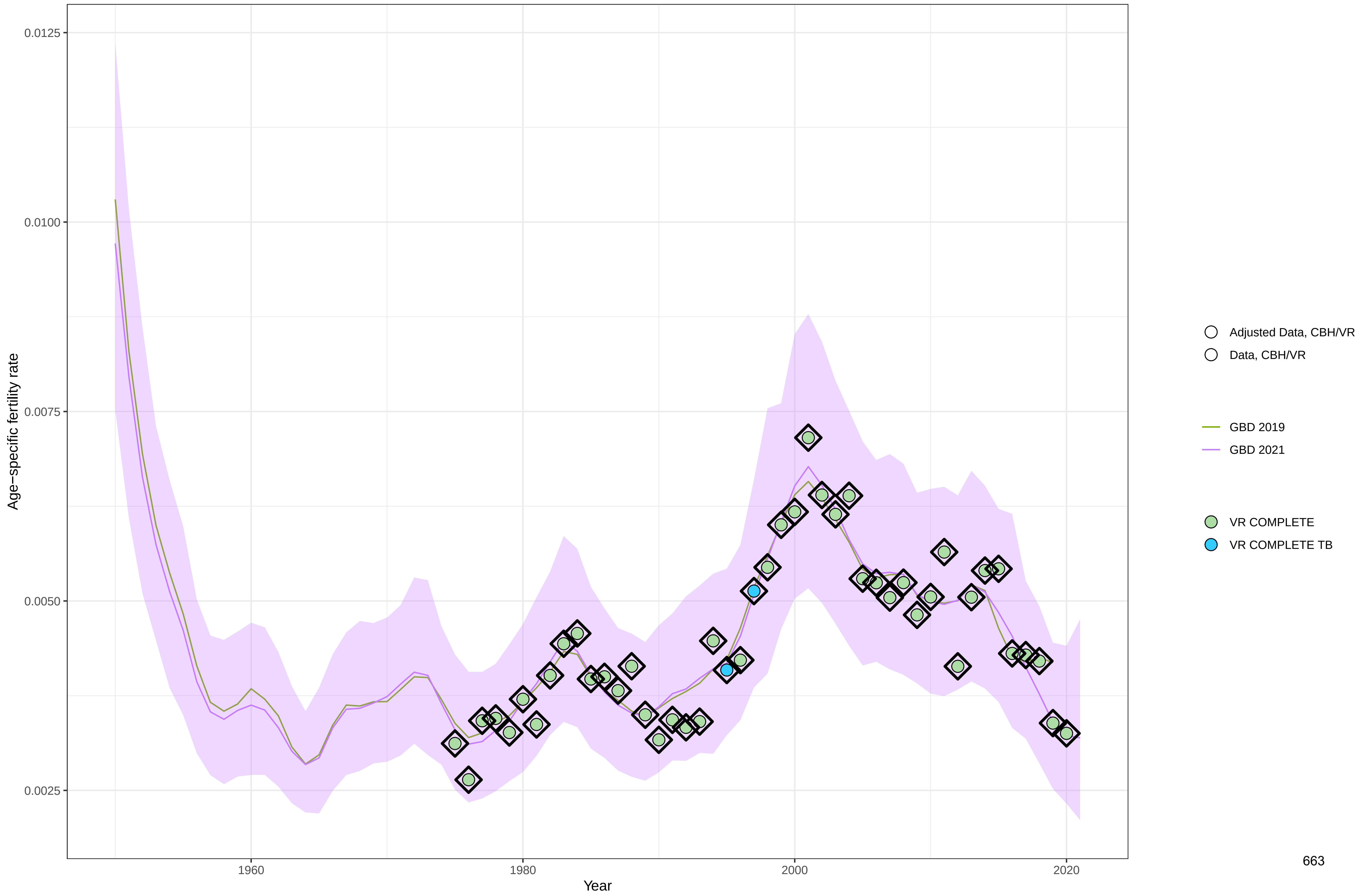

Age−specific fertility fitted trend lines, 1950−2021: Saga, Ages: 20−24
Data points enclosed with a diamond are reference sources

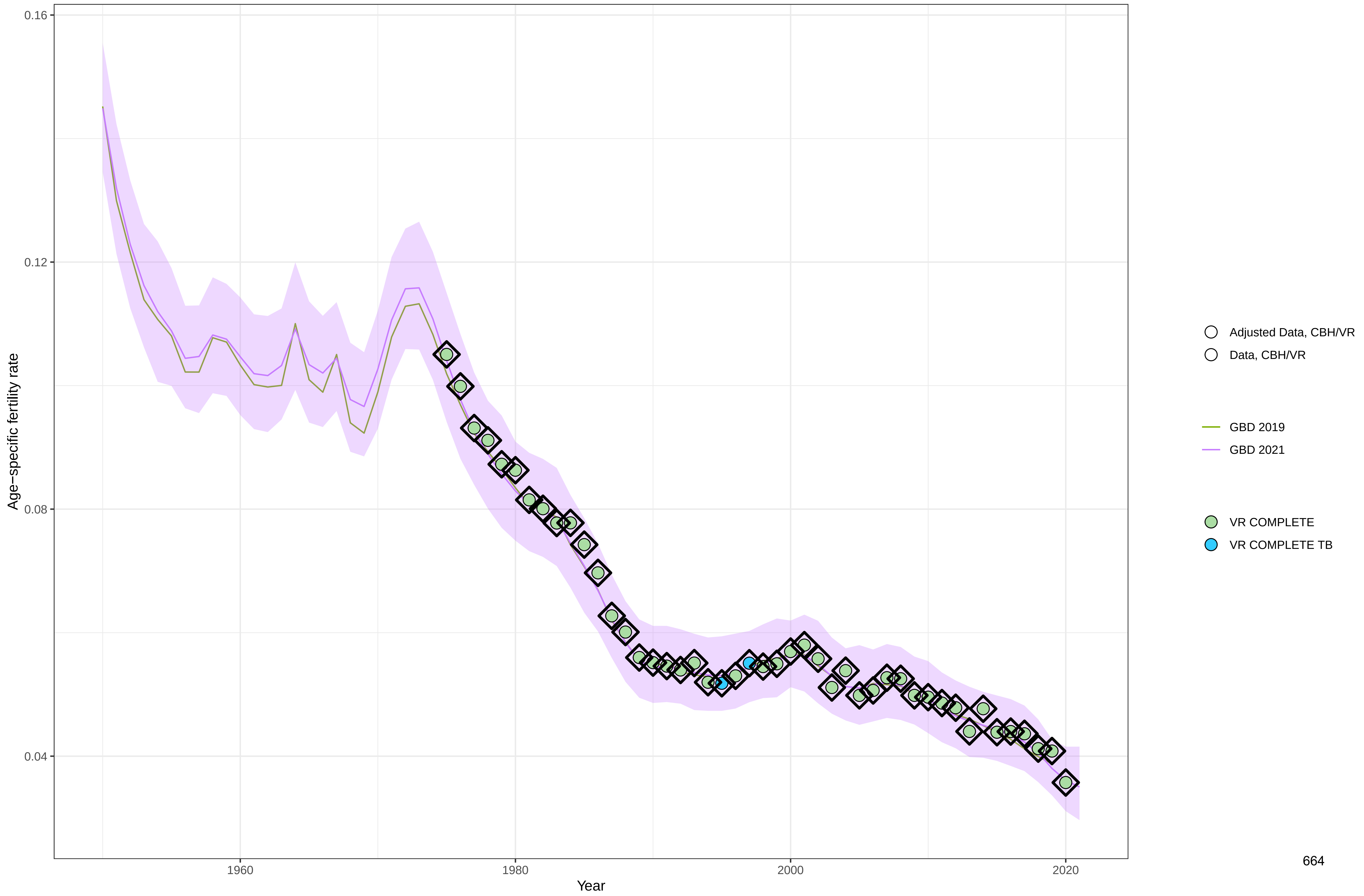

Age−specific fertility fitted trend lines, 1950−2021: Saga, Ages: 25−29
Data points enclosed with a diamond are reference sources

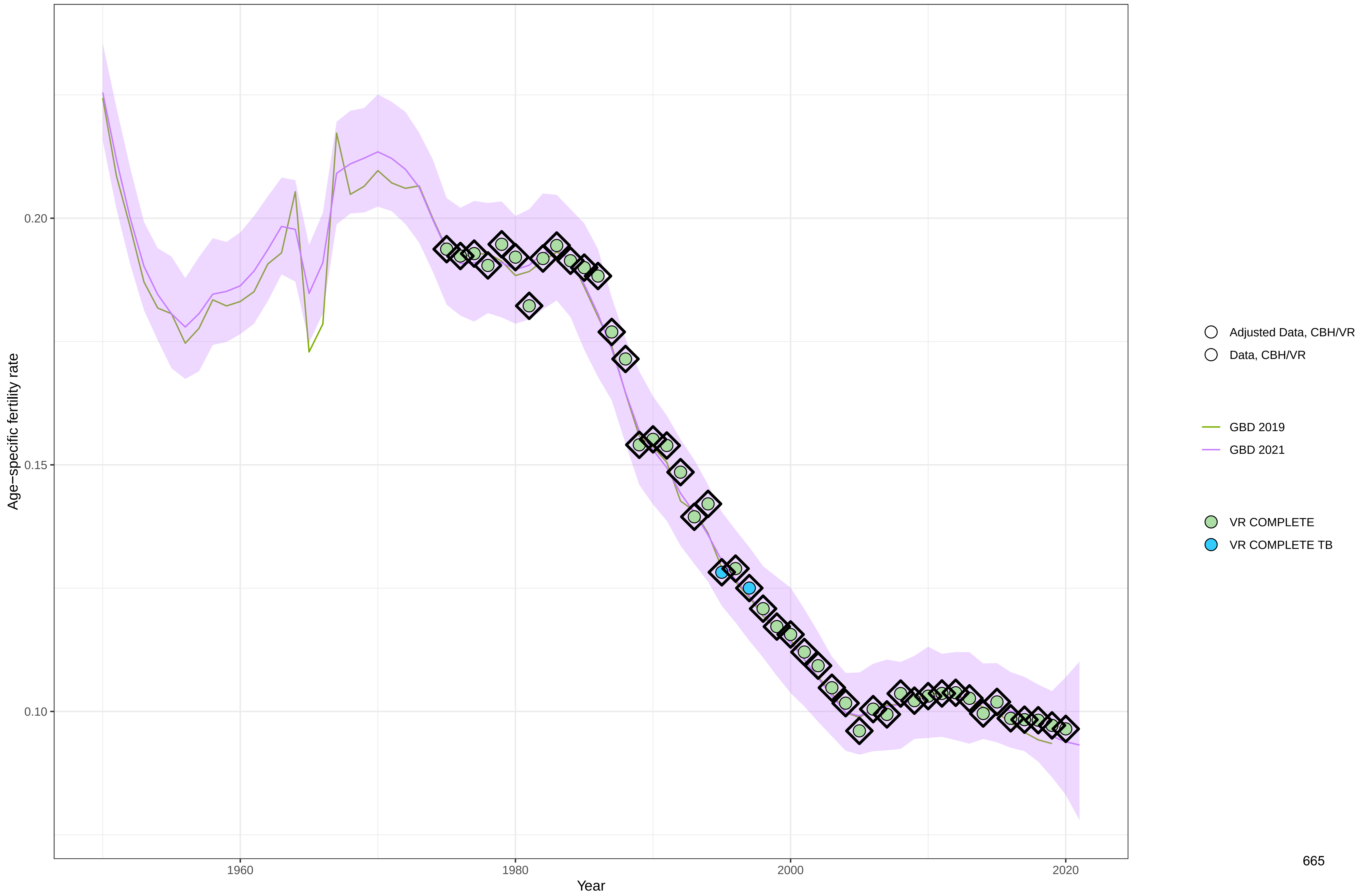

Age−specific fertility fitted trend lines, 1950−2021: Saga, Ages: 30−34
Data points enclosed with a diamond are reference sources

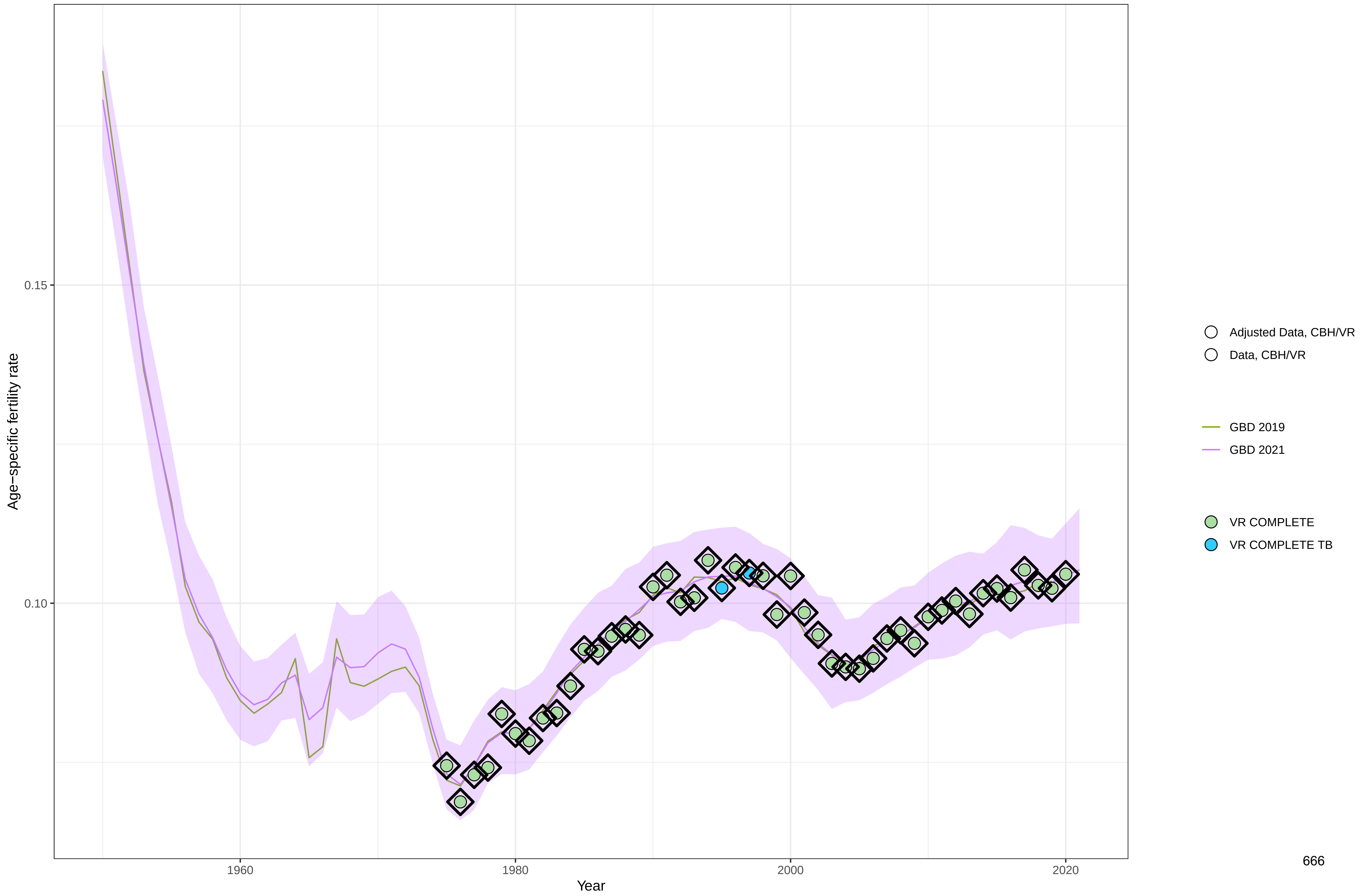

Age−specific fertility fitted trend lines, 1950−2021: Saga, Ages: 35−39
Data points enclosed with a diamond are reference sources

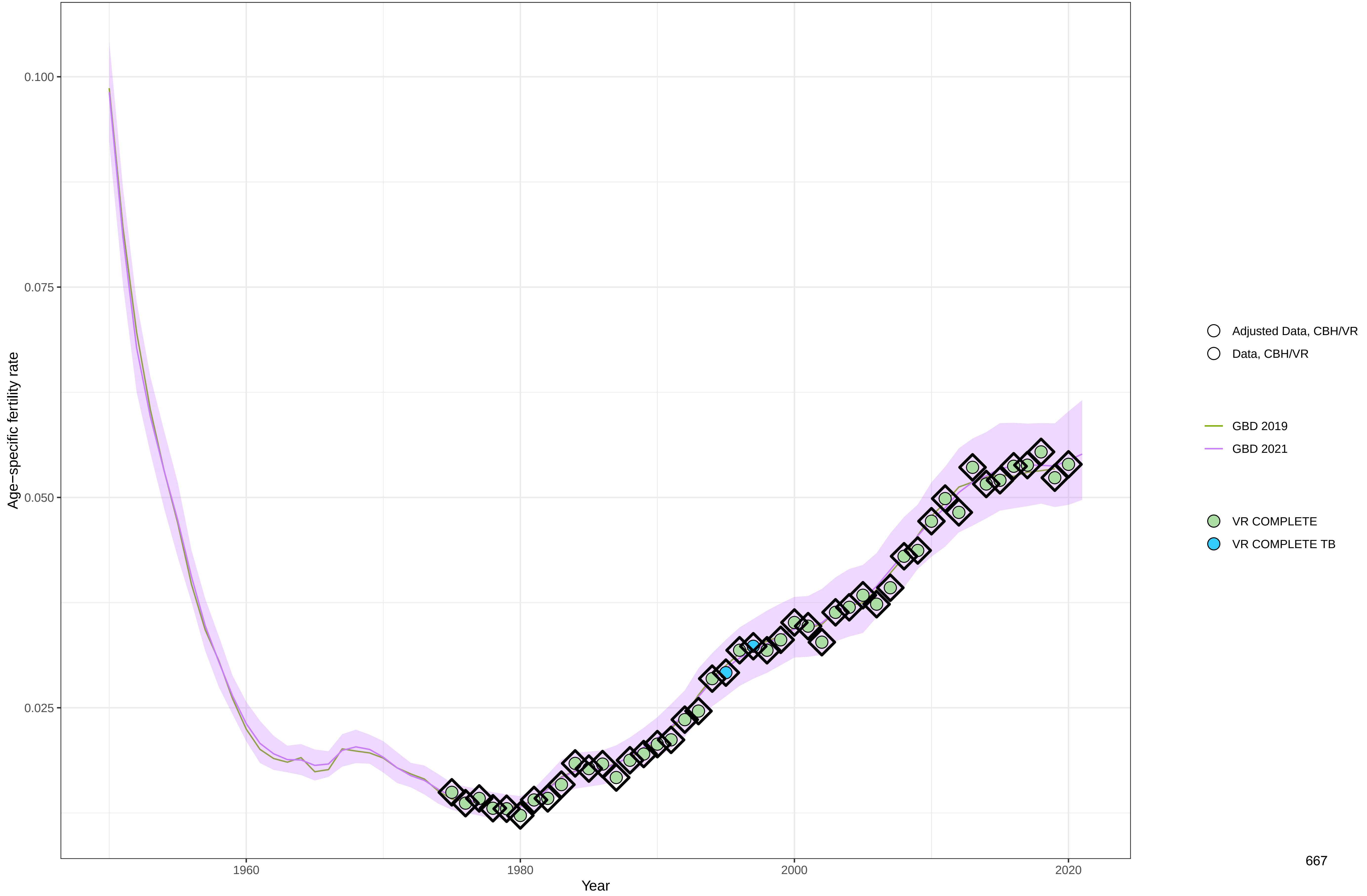

Age−specific fertility fitted trend lines, 1950−2021: Saga, Ages: 40−44
Data points enclosed with a diamond are reference sources

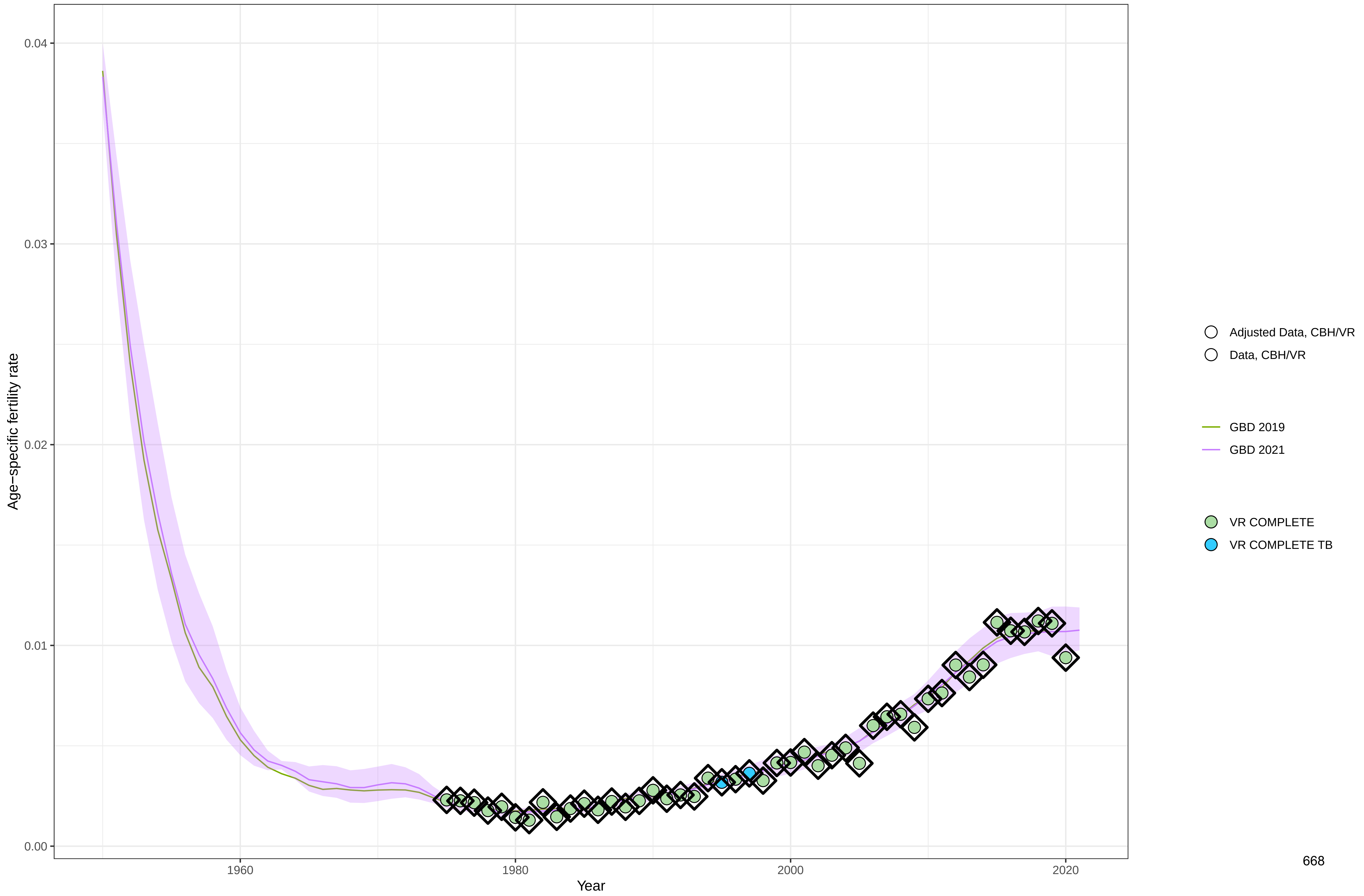

Age−specific fertility fitted trend lines, 1950−2021: Saga, Ages: 45−49
Data points enclosed with a diamond are reference sources

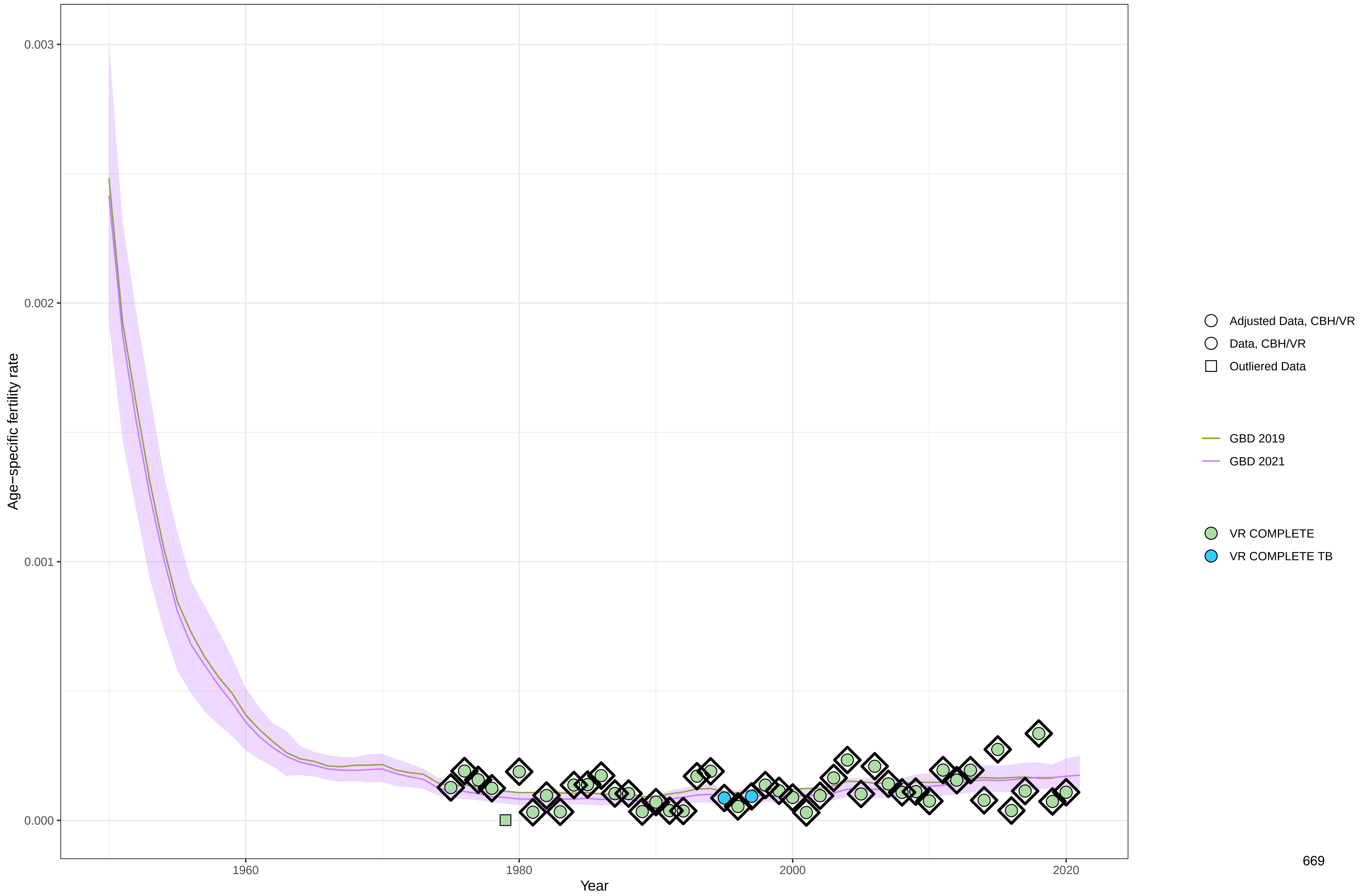

Age−specific fertility fitted trend lines, 1950−2021: Saga, Ages: 50−54
Data points enclosed with a diamond are reference sources

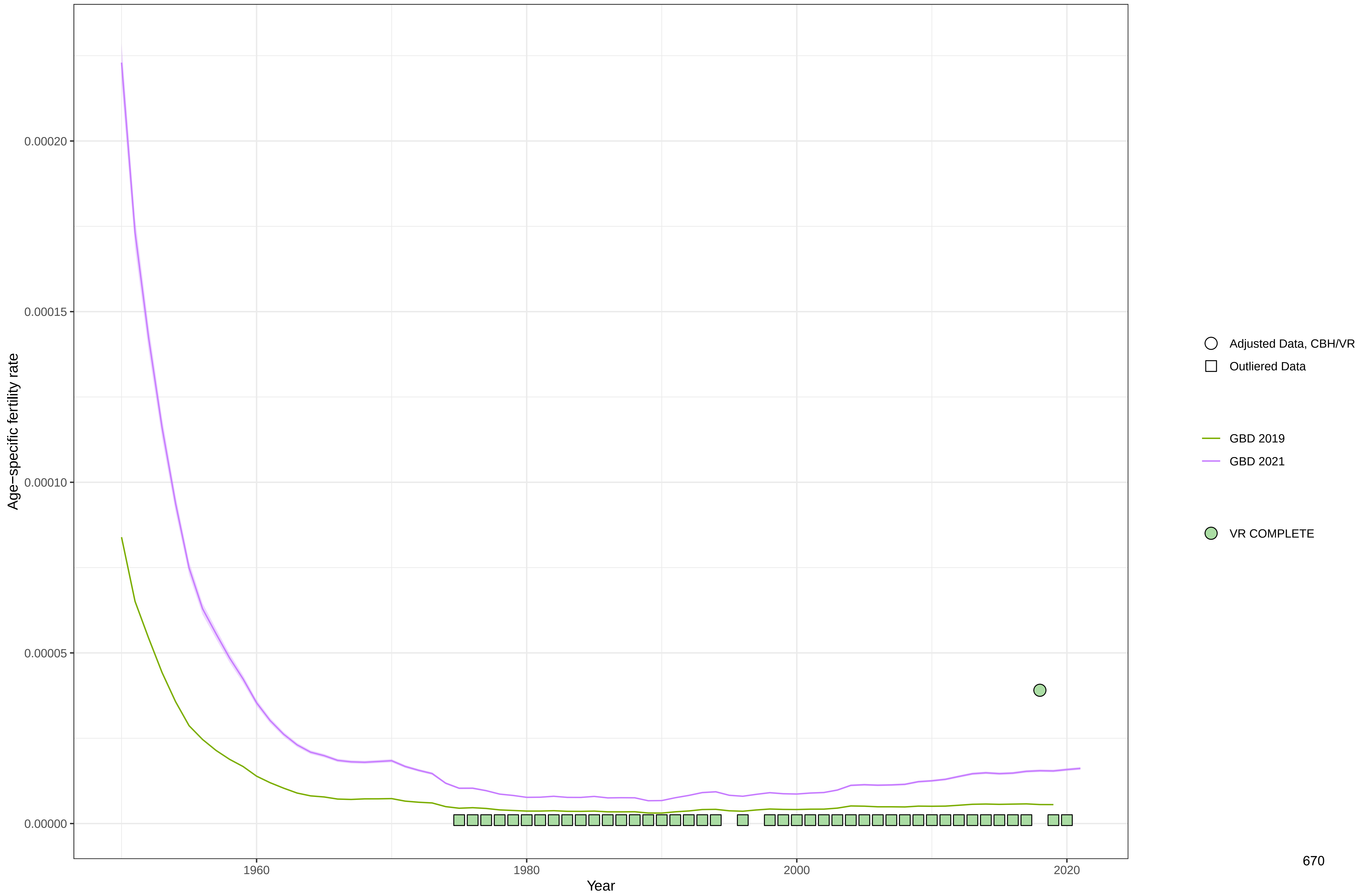

Total fertility rate fitted trend lines, 1950−2021: Saga TFR
Data points enclosed with a diamond are reference sources

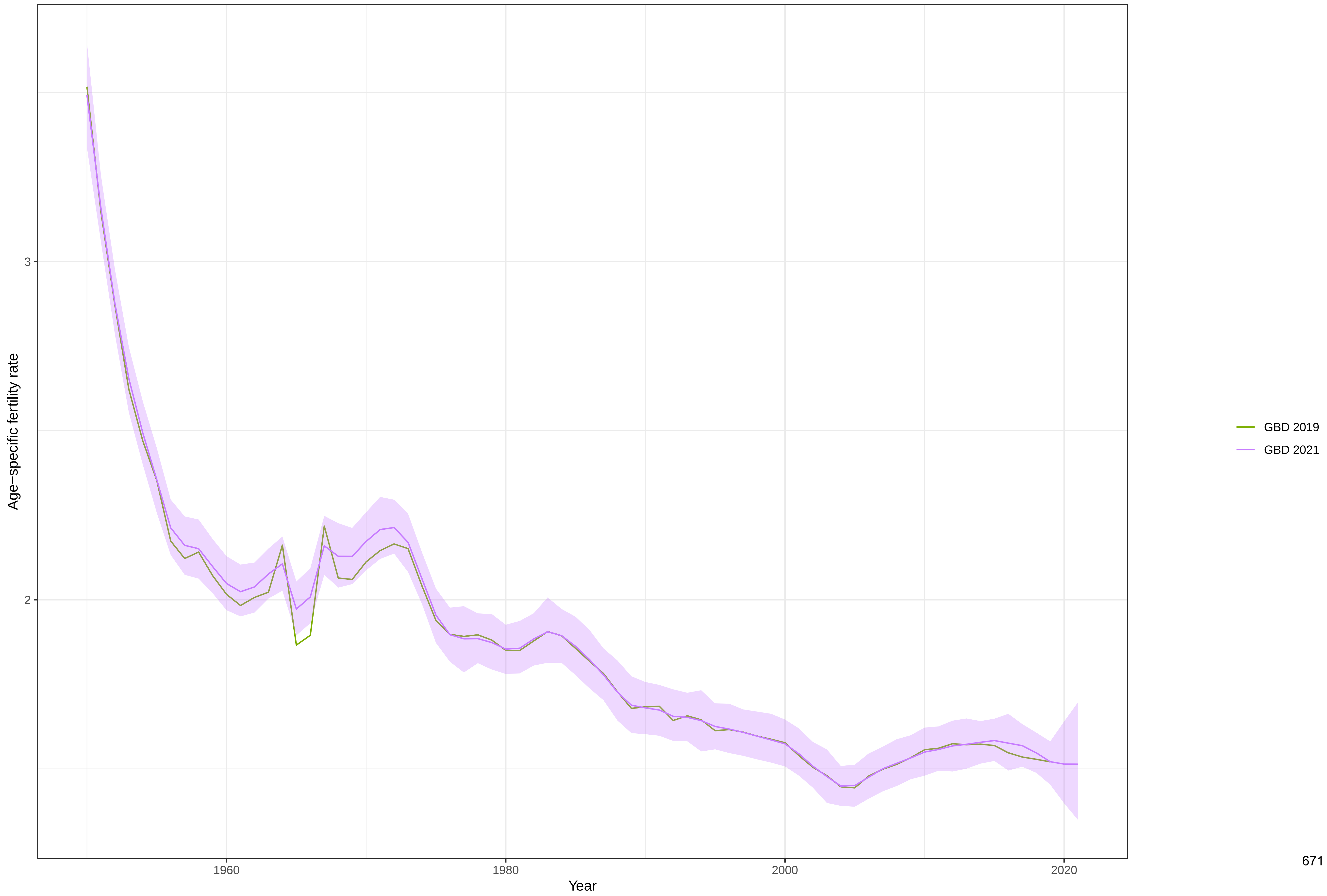

Age−specific fertility fitted trend lines, 1950−2021: Saitama, Ages: 10−14
Data points enclosed with a diamond are reference sources

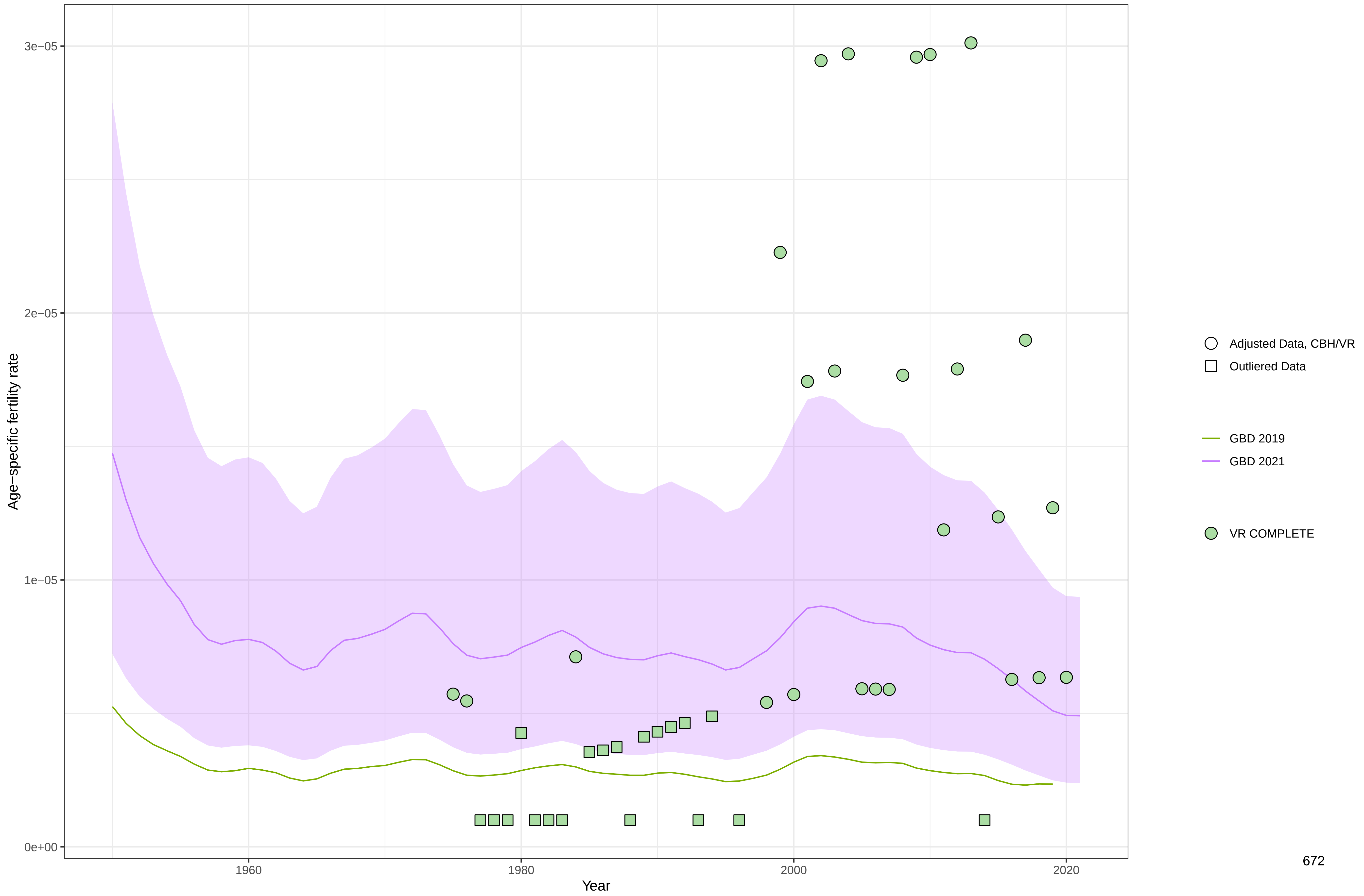

Age−specific fertility fitted trend lines, 1950−2021: Saitama, Ages: 15−19
Data points enclosed with a diamond are reference sources

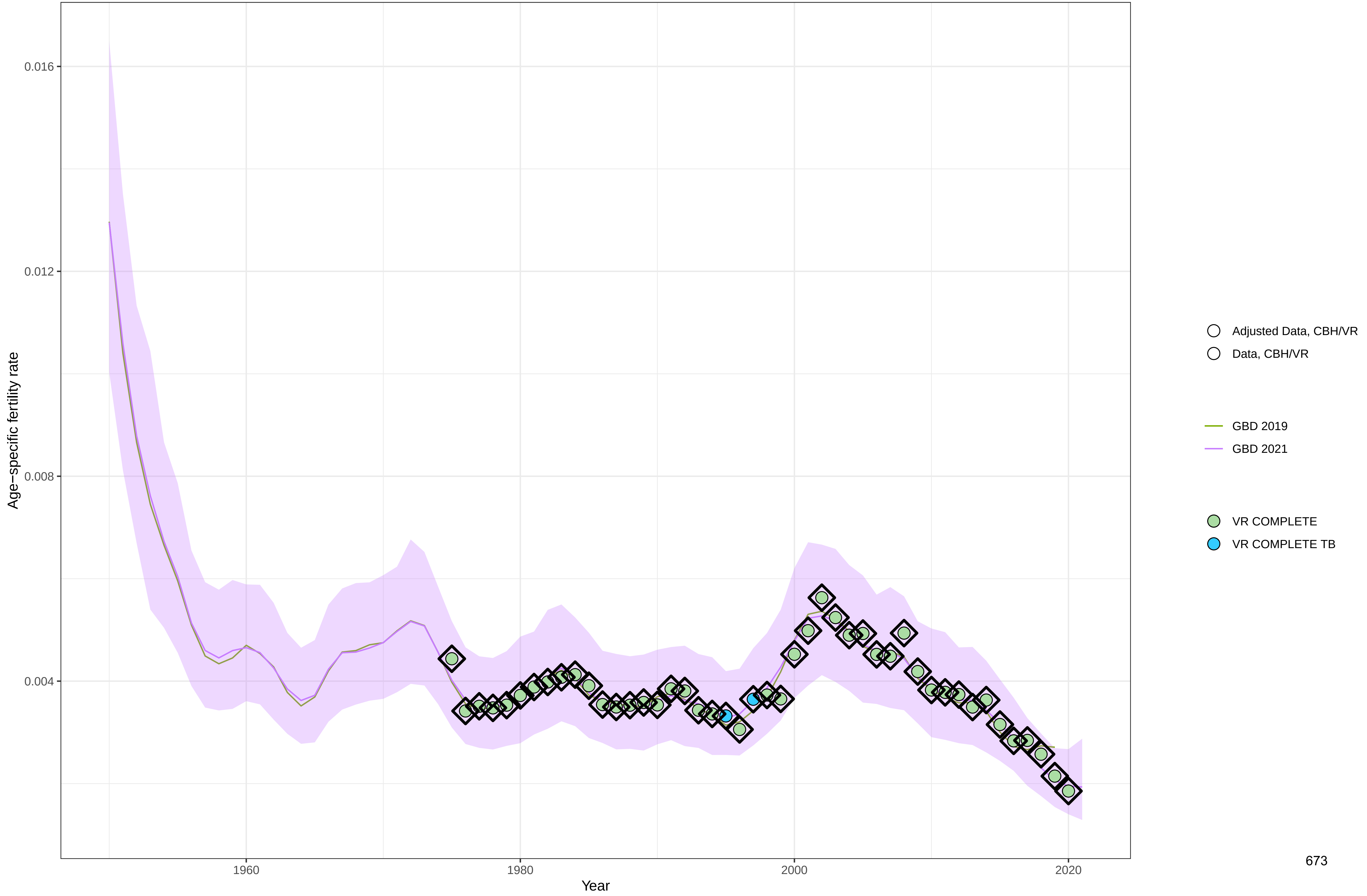

Age−specific fertility fitted trend lines, 1950−2021: Saitama, Ages: 20−24
Data points enclosed with a diamond are reference sources

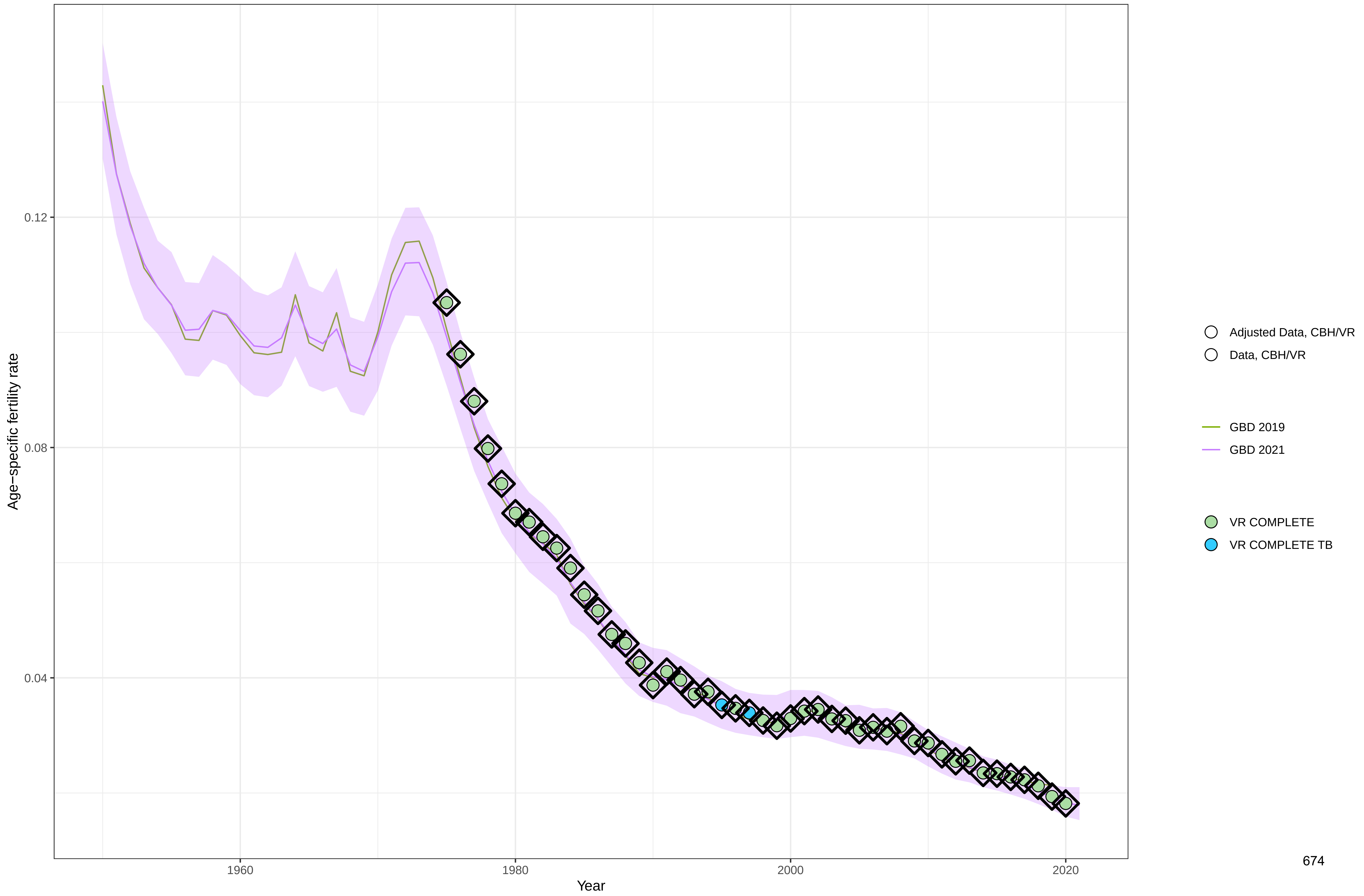

Age−specific fertility fitted trend lines, 1950−2021: Saitama, Ages: 25−29
Data points enclosed with a diamond are reference sources

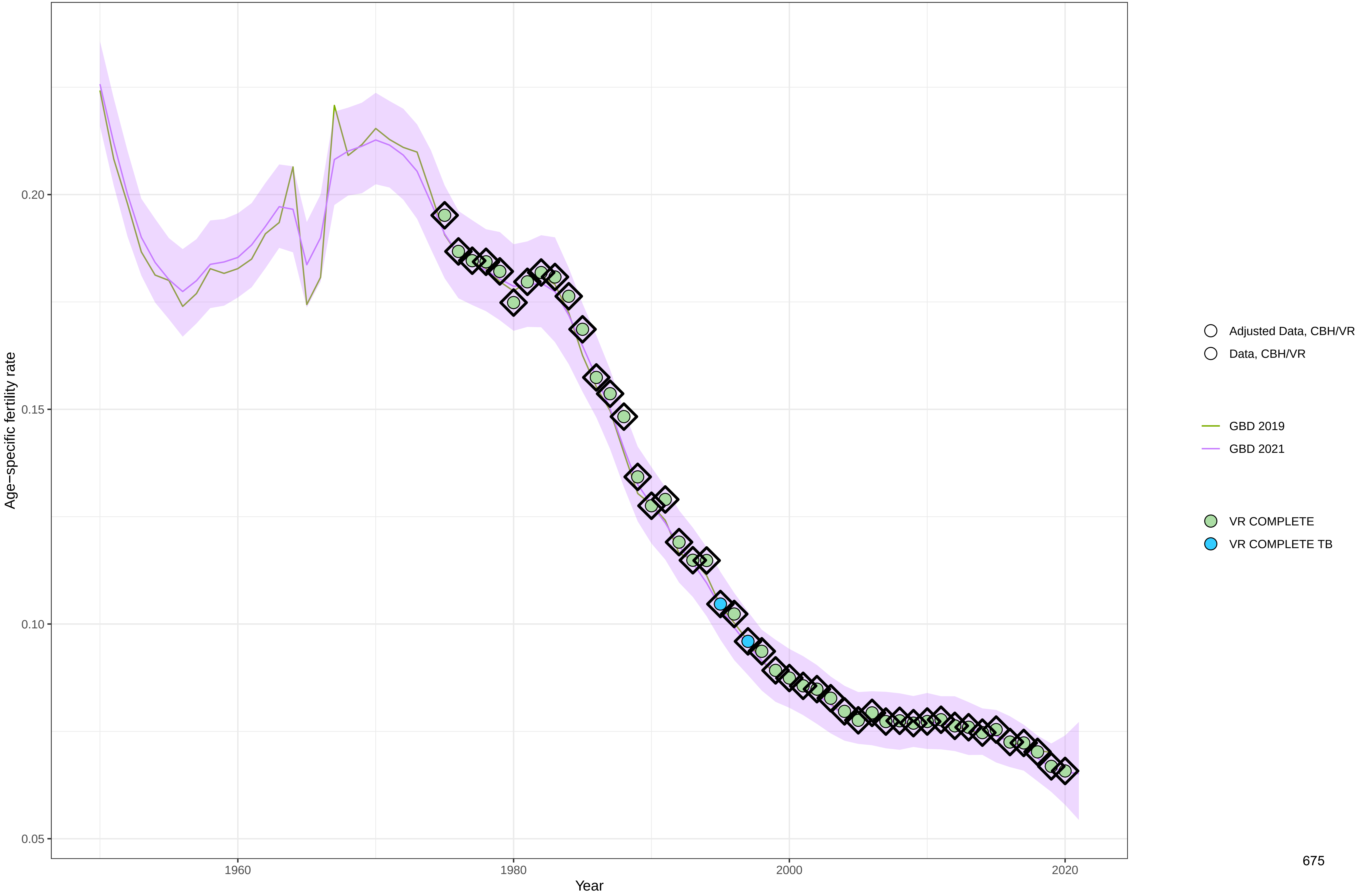

Age−specific fertility fitted trend lines, 1950−2021: Saitama, Ages: 30−34
Data points enclosed with a diamond are reference sources

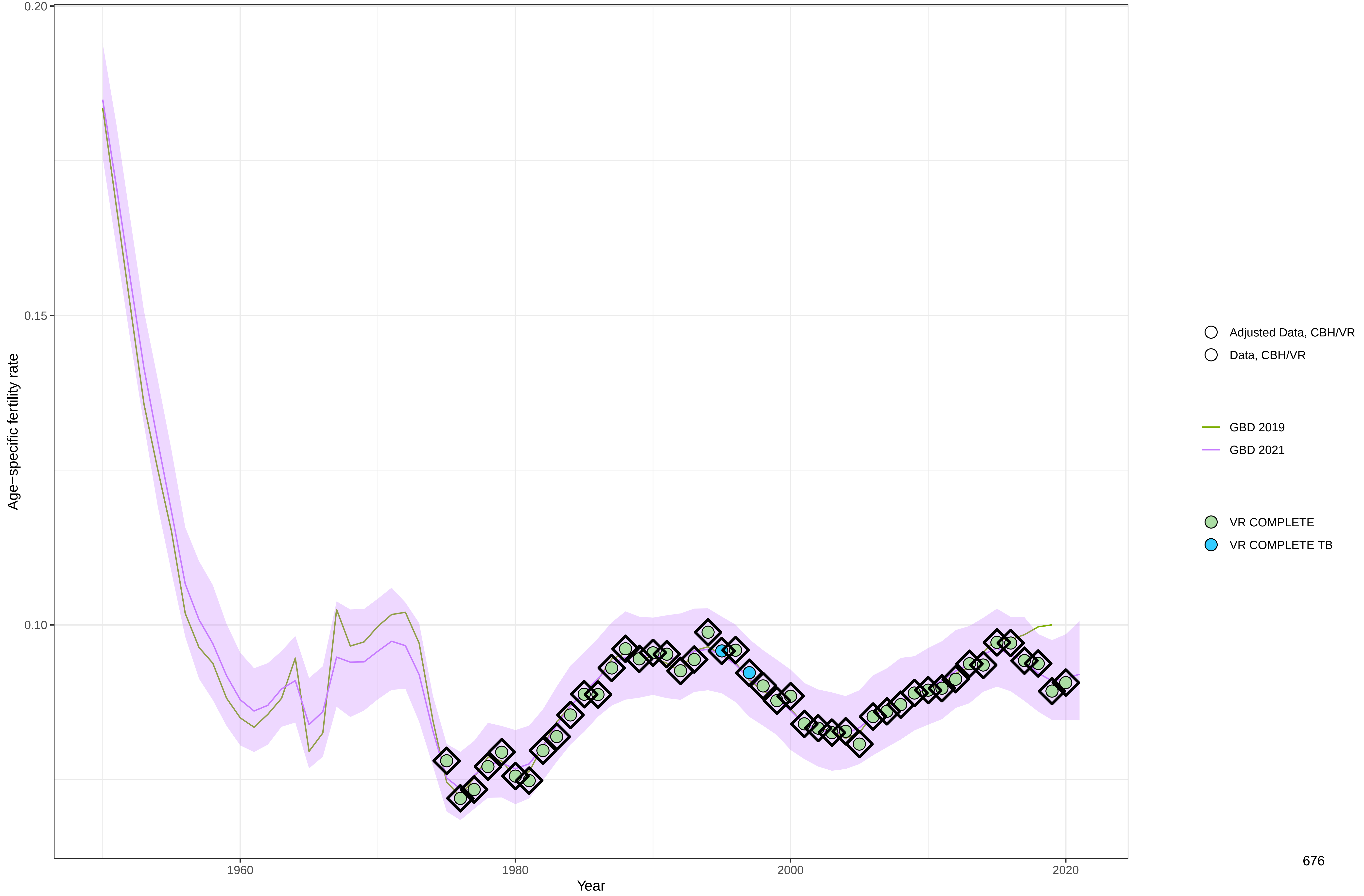

Age−specific fertility fitted trend lines, 1950−2021: Saitama, Ages: 35−39
Data points enclosed with a diamond are reference sources

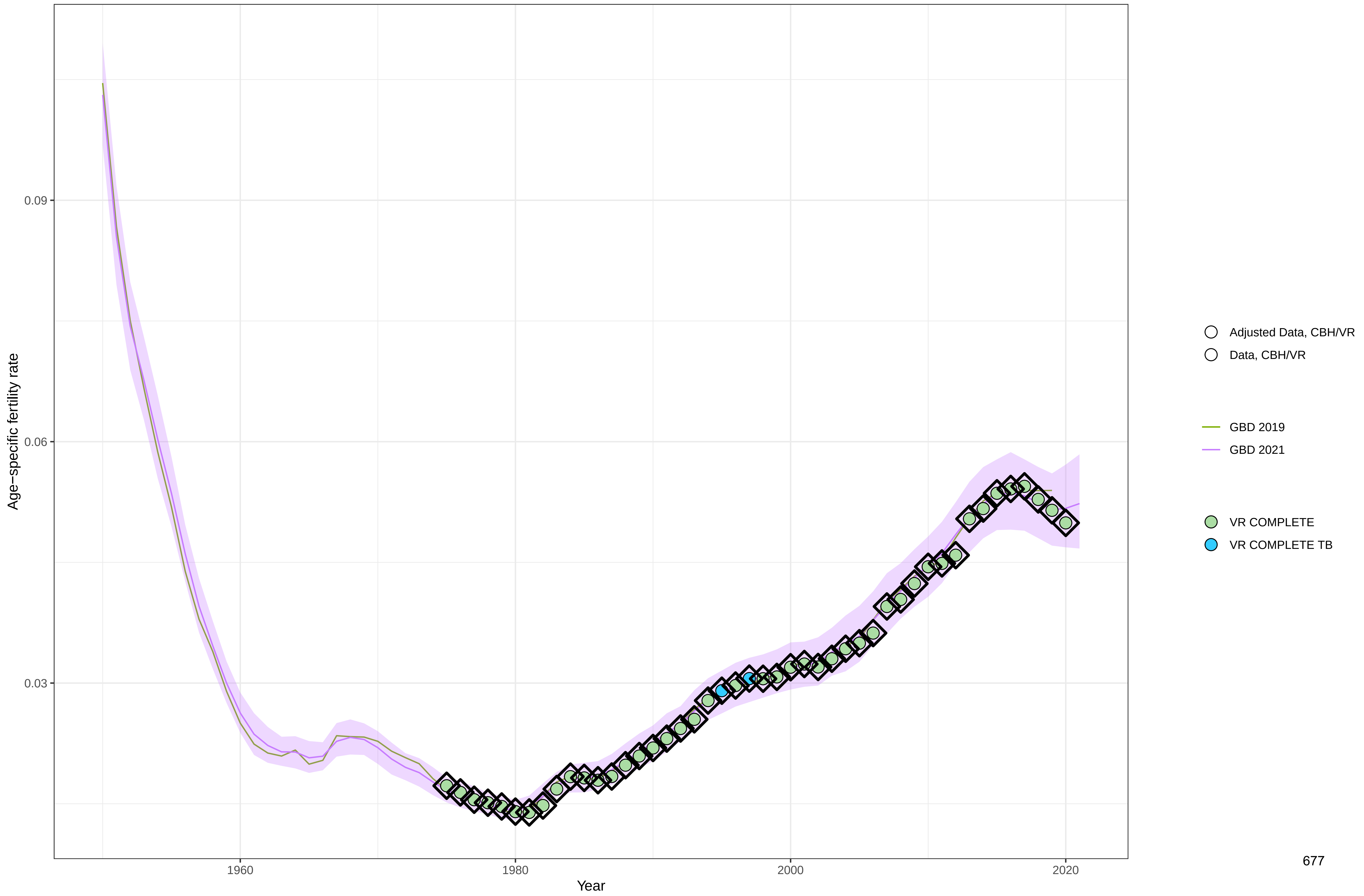

Age−specific fertility fitted trend lines, 1950−2021: Saitama, Ages: 40−44
Data points enclosed with a diamond are reference sources

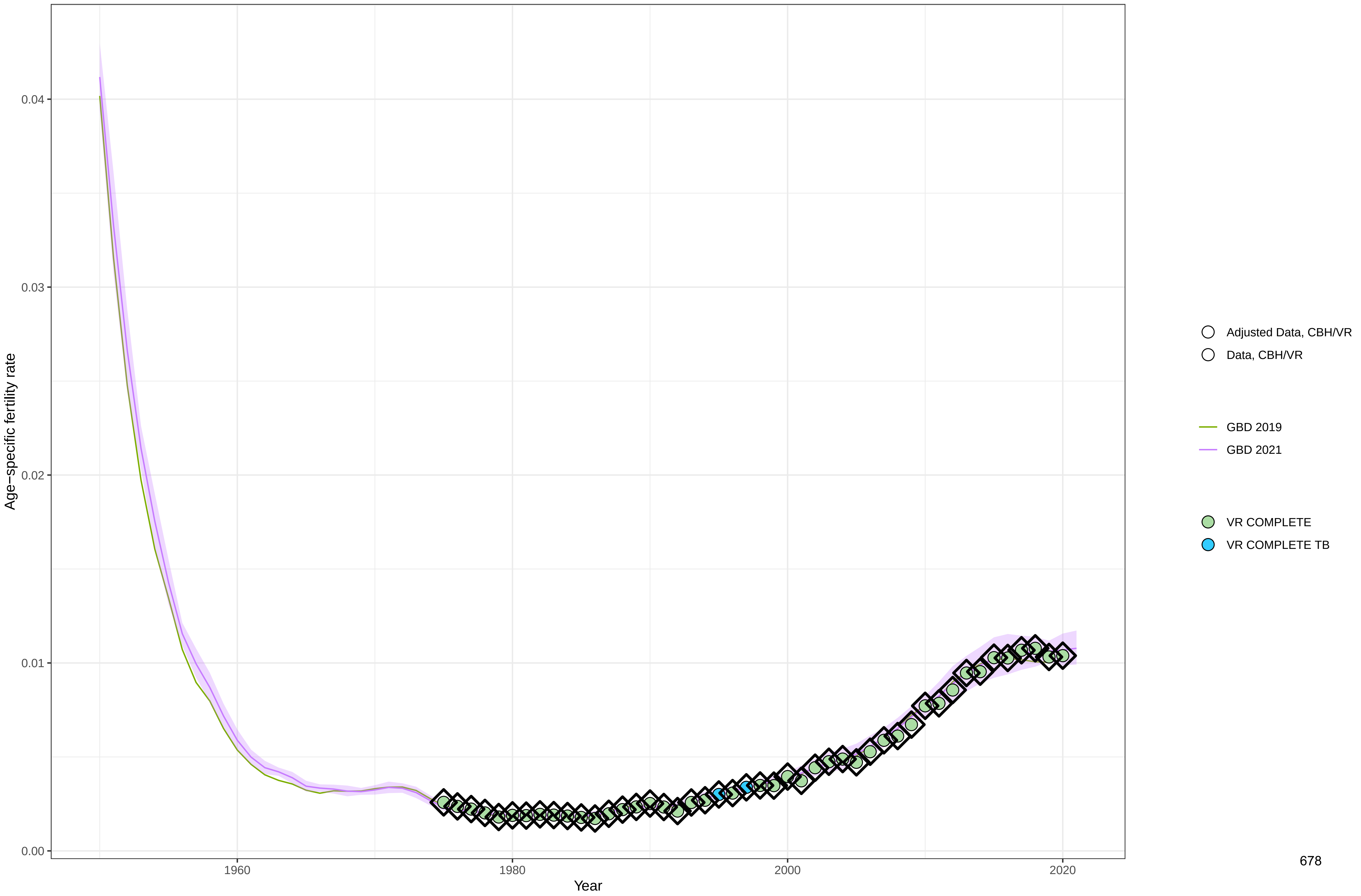

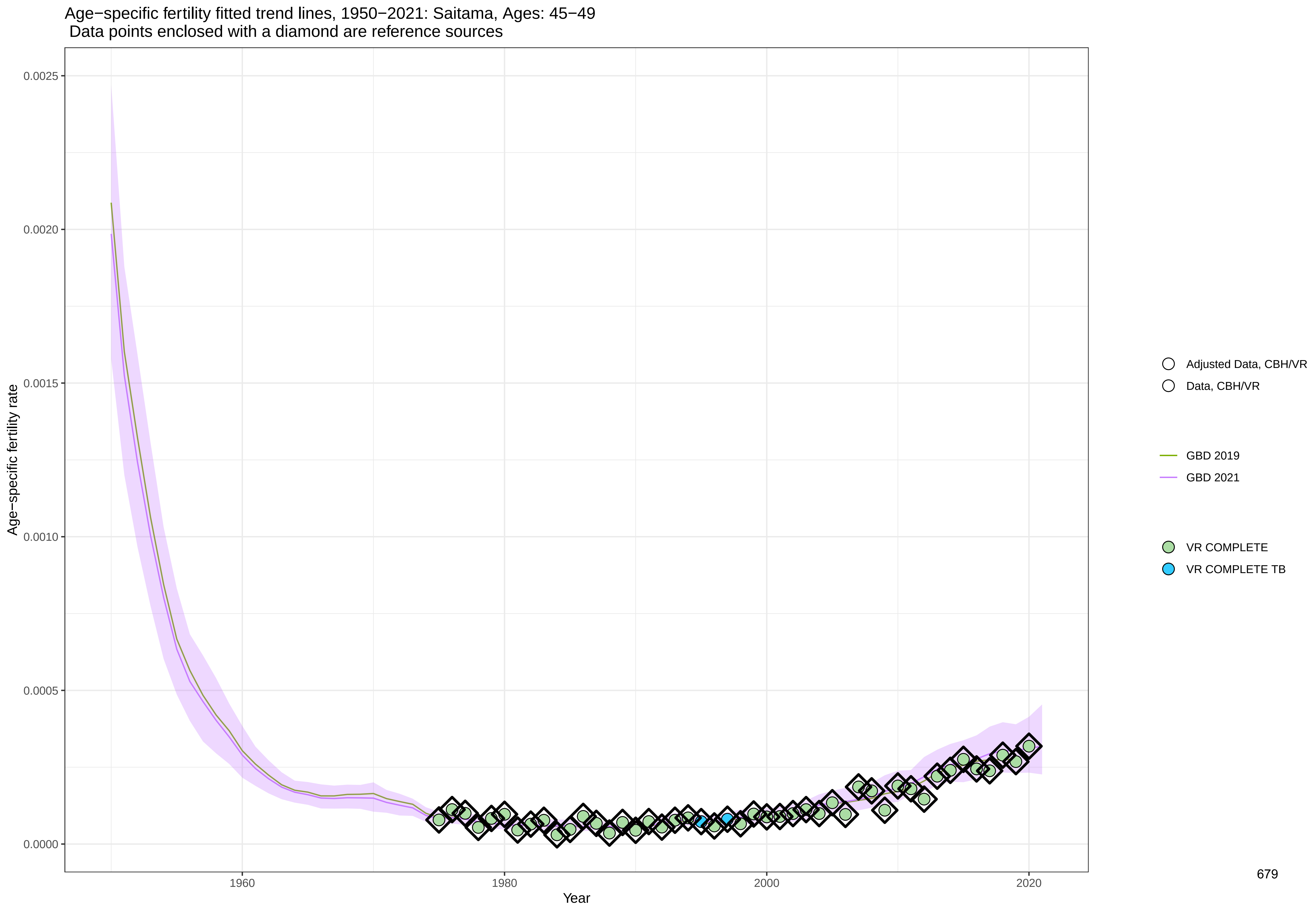

Age−specific fertility fitted trend lines, 1950−2021: Saitama, Ages: 45−49
Data points enclosed with a diamond are reference sources
Age−specific fertility rate
0.0025
0.0020
0.0015
0.0010
0.0005
0.0000
1960
1980
2000
2020
Year
Adjusted Data, CBH/VR
Data, CBH/VR
GBD 2019
GBD 2021
VR COMPLETE
VR COMPLETE TB

Age−specific fertility fitted trend lines, 1950−2021: Saitama, Ages: 50−54
Data points enclosed with a diamond are reference sources

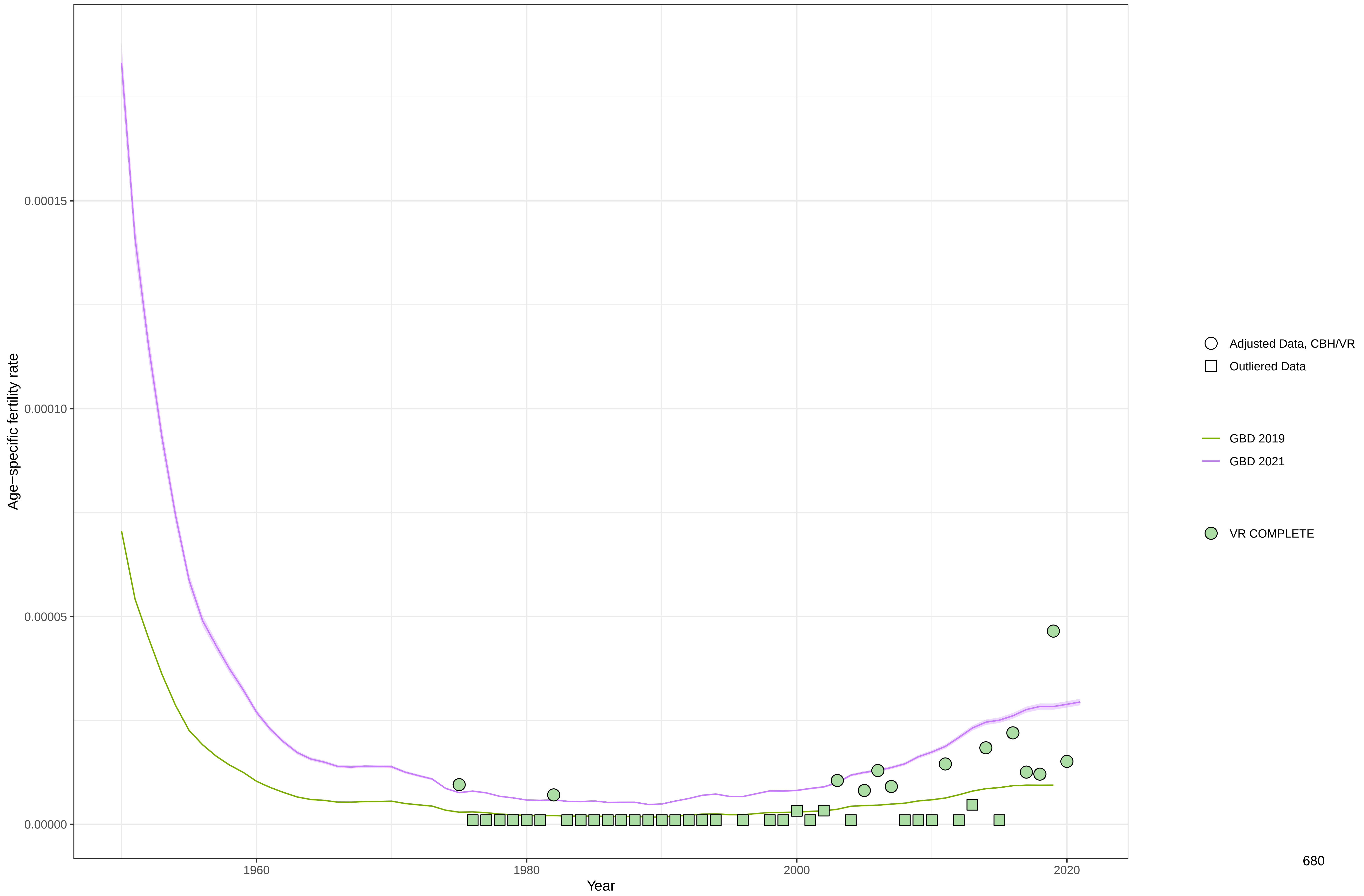

Total fertility rate fitted trend lines, 1950−2021: Saitama TFR
Data points enclosed with a diamond are reference sources

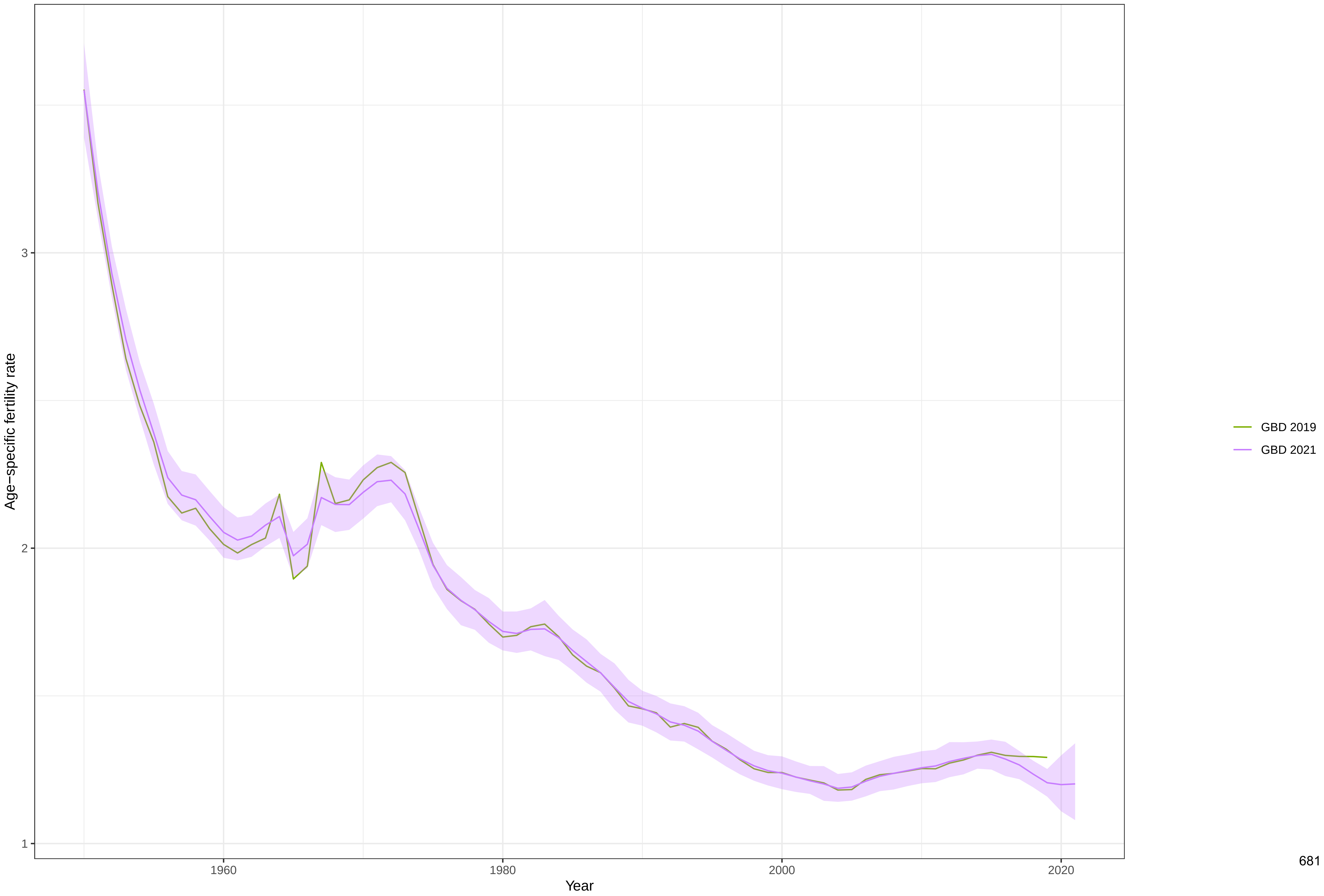

Age−specific fertility fitted trend lines, 1950−2021: Shiga, Ages: 10−14
Data points enclosed with a diamond are reference sources

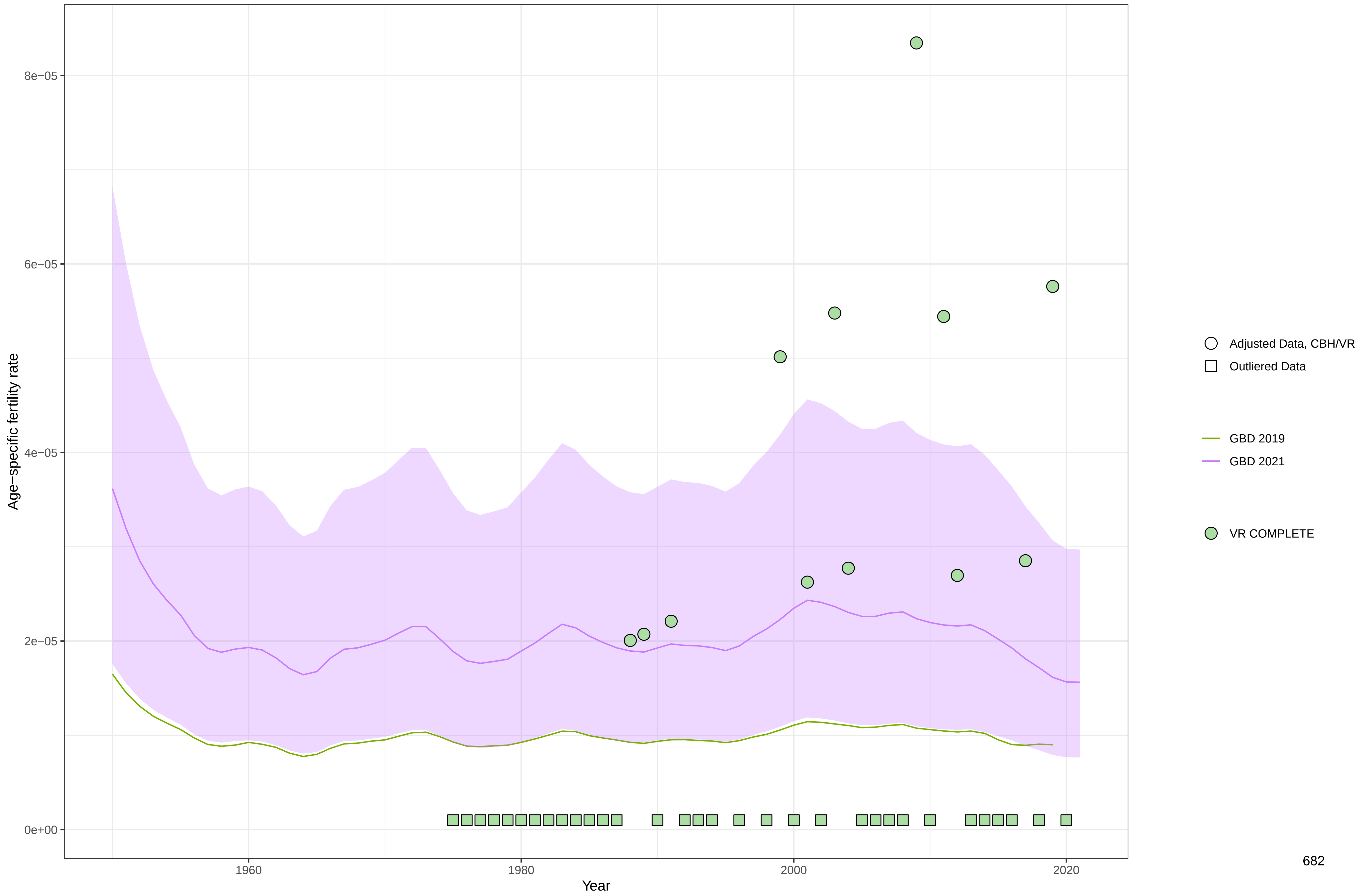

Age−specific fertility fitted trend lines, 1950−2021: Shiga, Ages: 15−19
Data points enclosed with a diamond are reference sources

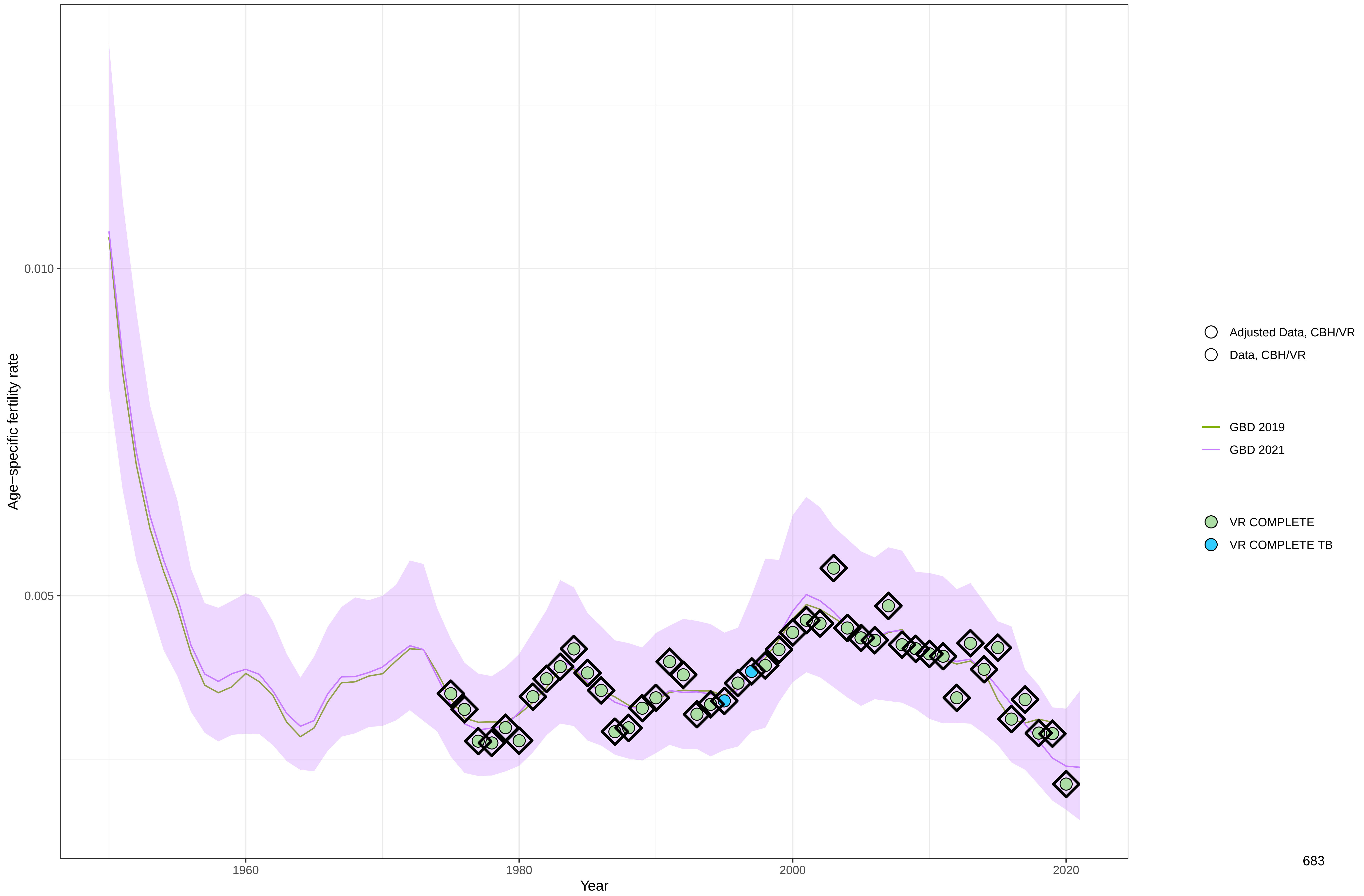

Age−specific fertility fitted trend lines, 1950−2021: Shiga, Ages: 20−24
Data points enclosed with a diamond are reference sources

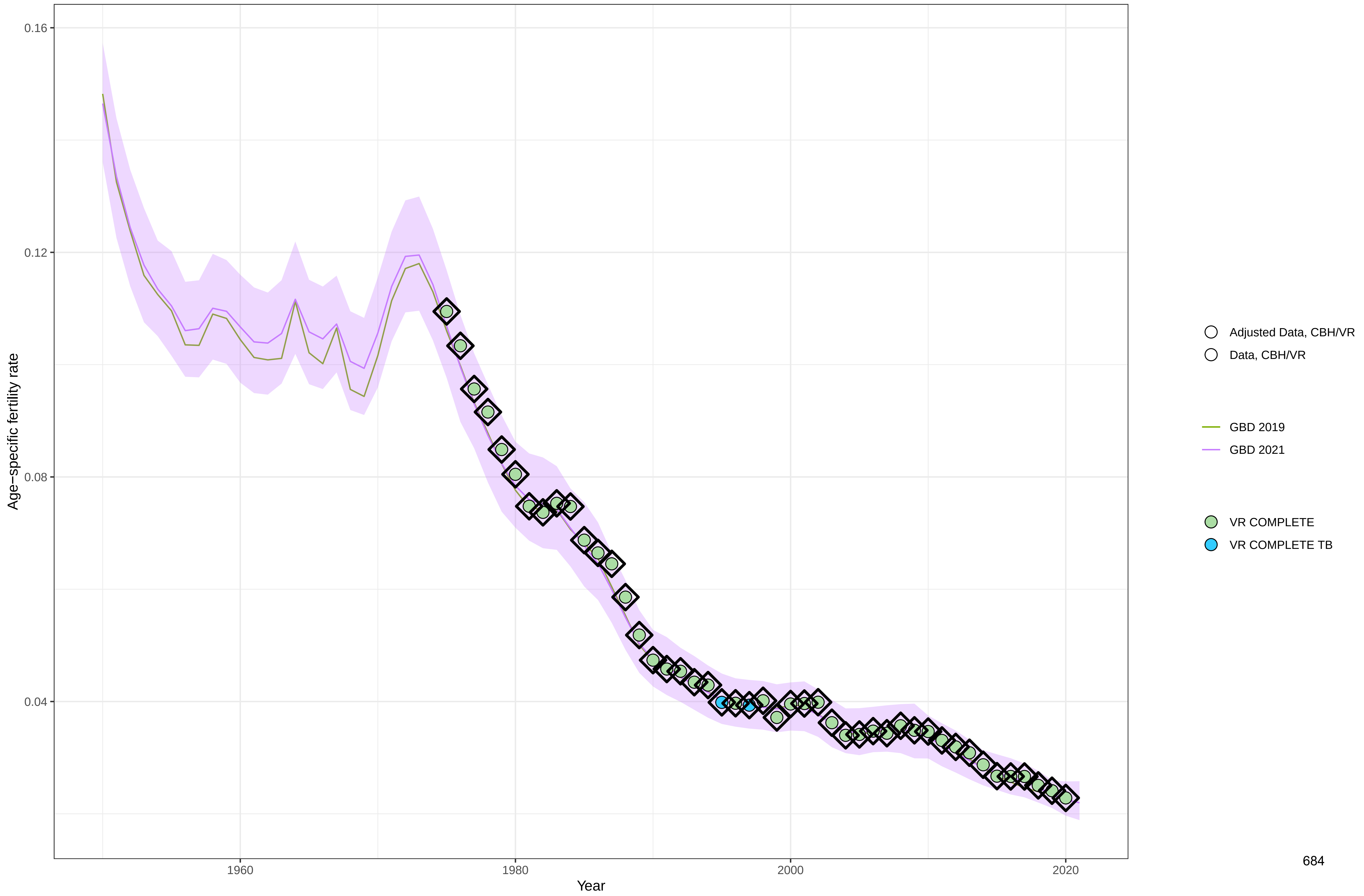

Age−specific fertility fitted trend lines, 1950−2021: Shiga, Ages: 25−29
Data points enclosed with a diamond are reference sources

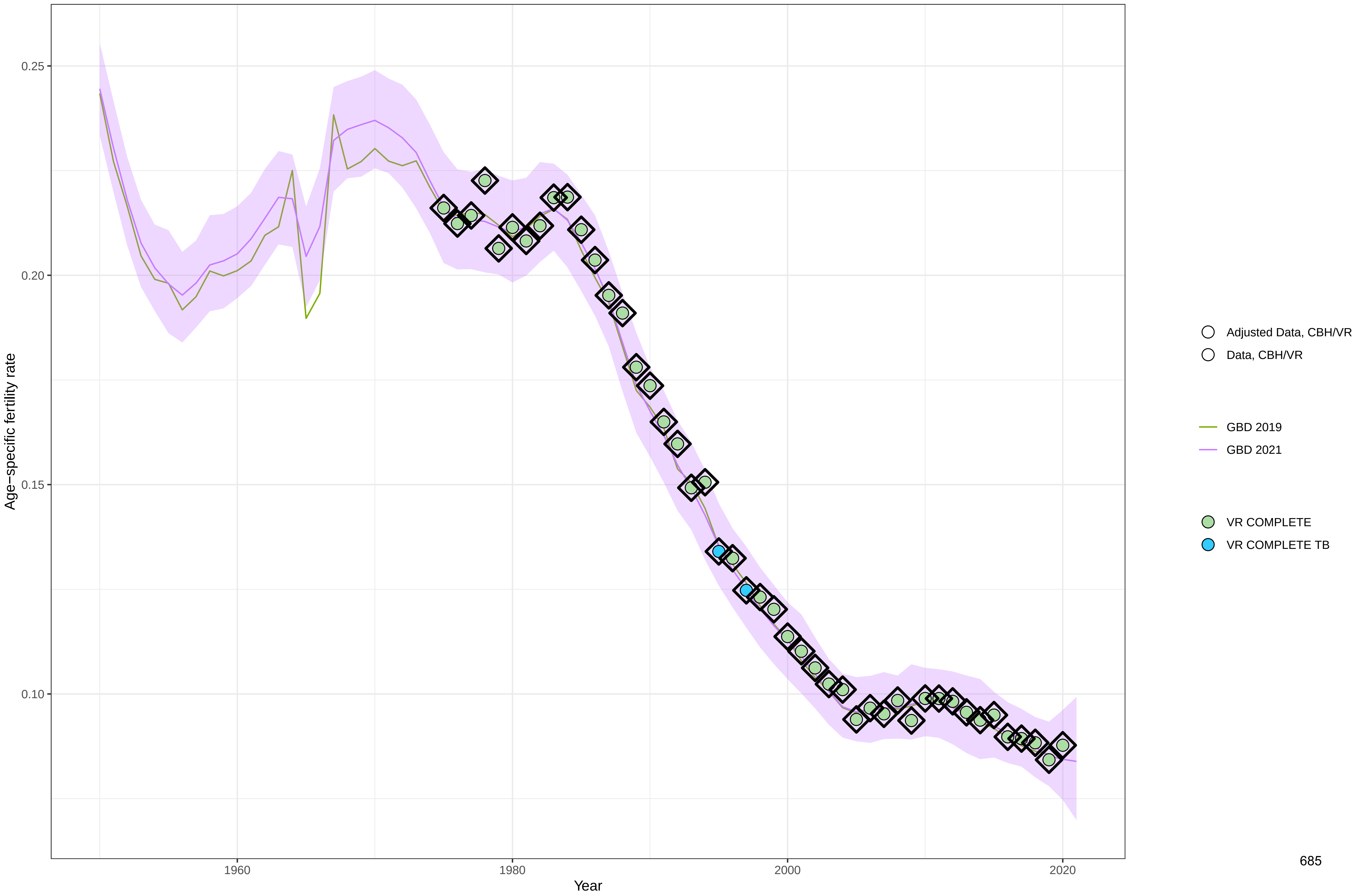

Age−specific fertility fitted trend lines, 1950−2021: Shiga, Ages: 30−34
Data points enclosed with a diamond are reference sources

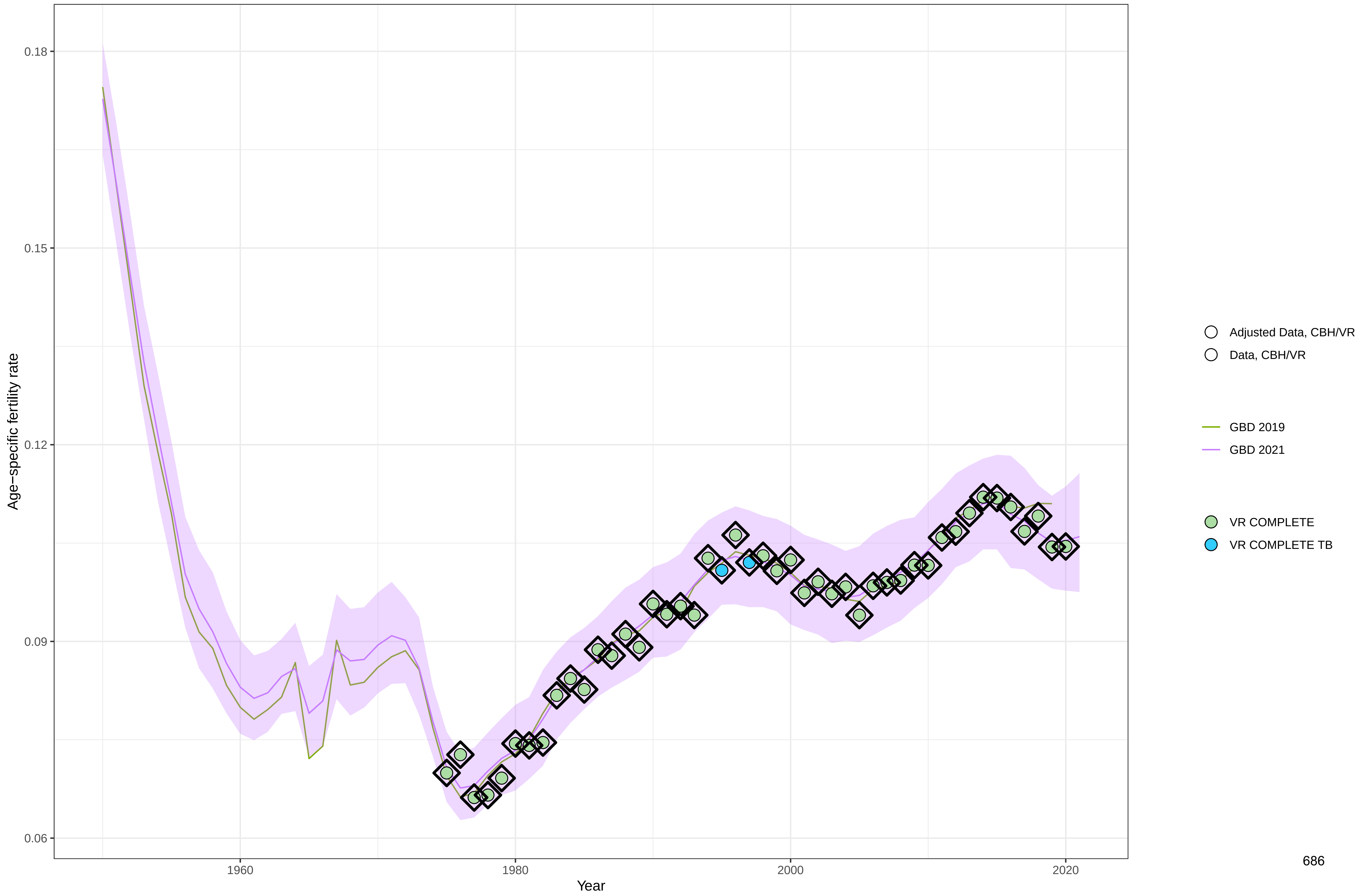

Age−specific fertility fitted trend lines, 1950−2021: Shiga, Ages: 35−39
Data points enclosed with a diamond are reference sources

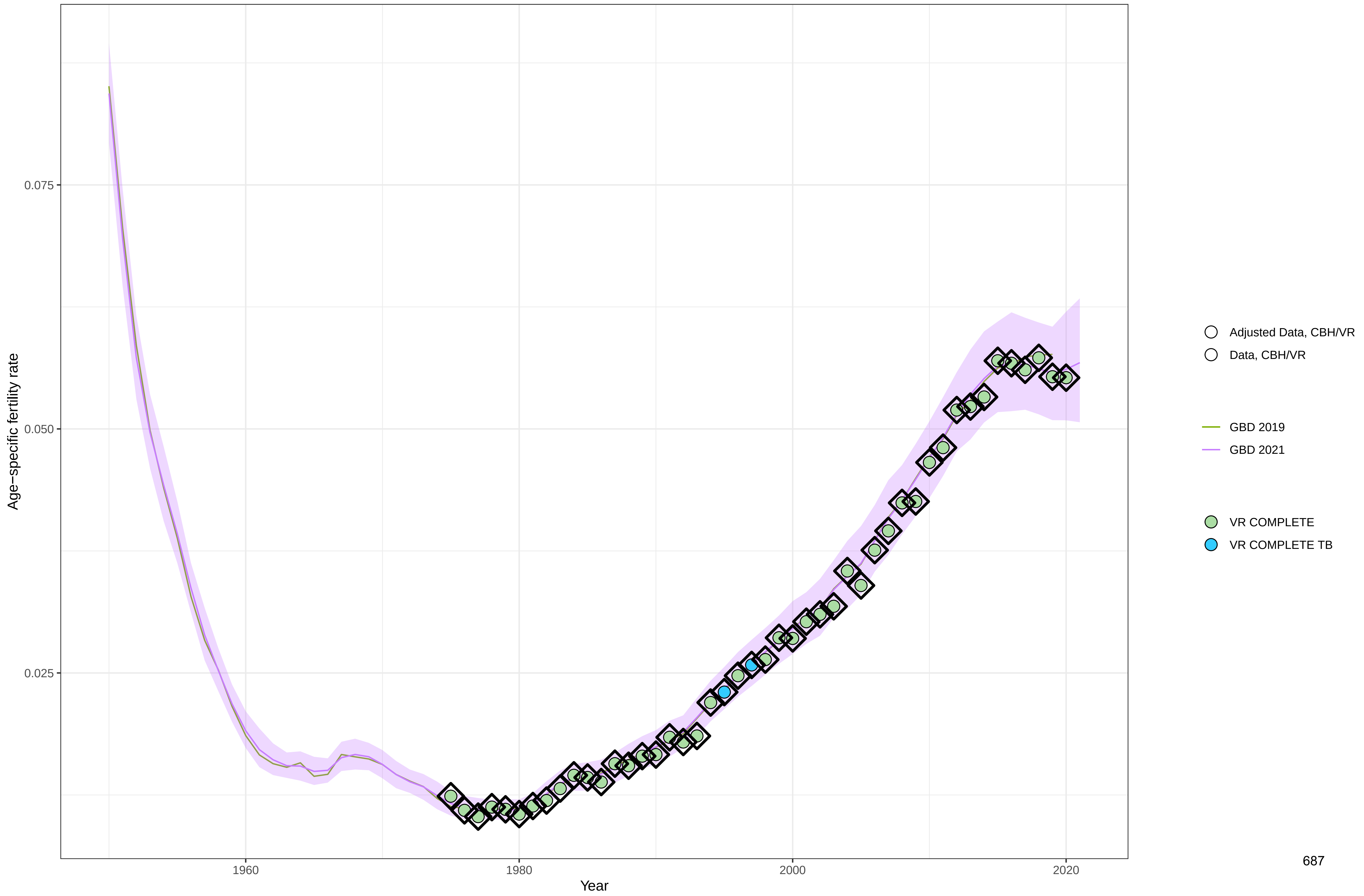

Age−specific fertility fitted trend lines, 1950−2021: Shiga, Ages: 40−44
Data points enclosed with a diamond are reference sources

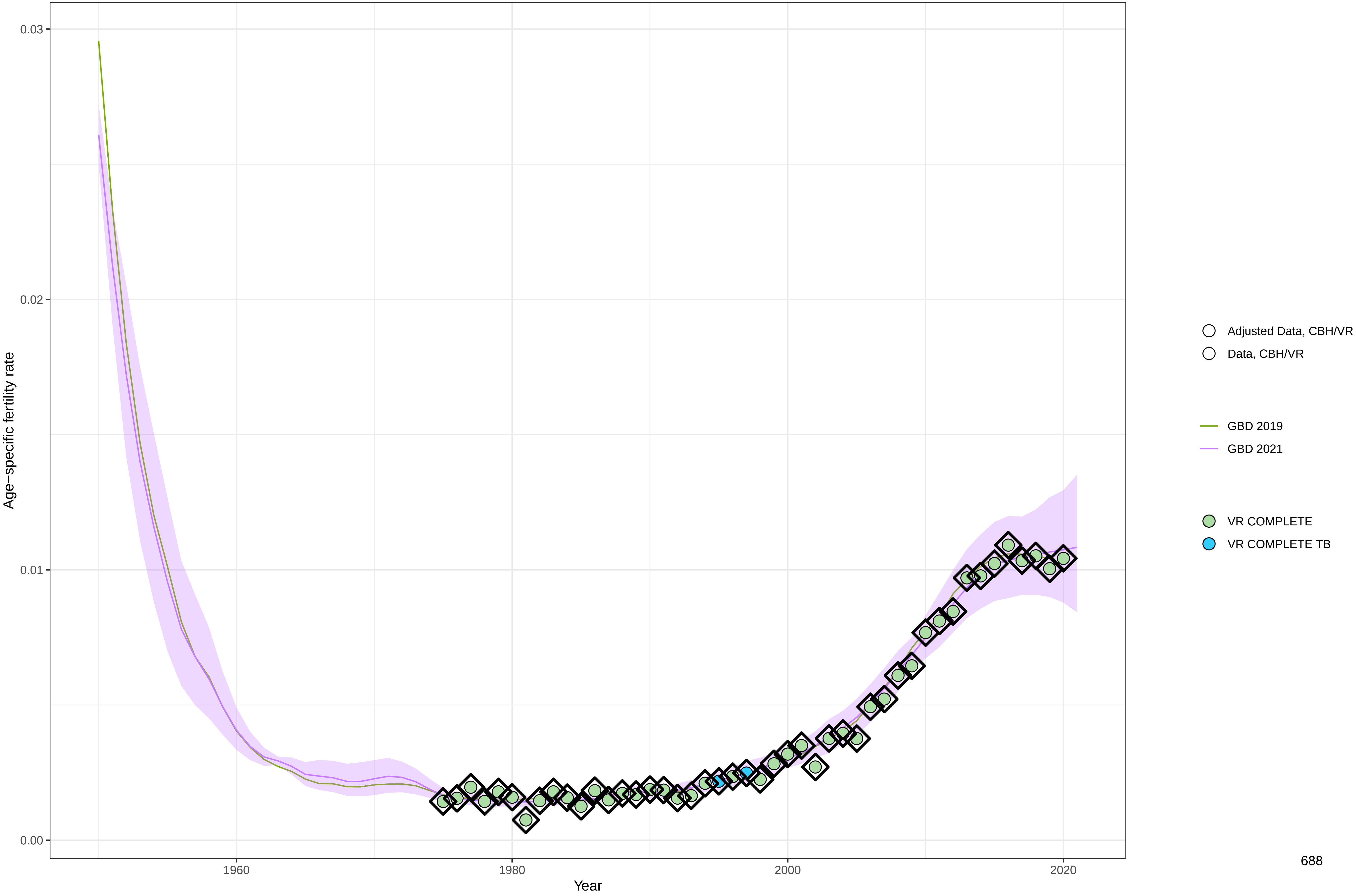

Age−specific fertility fitted trend lines, 1950−2021: Shiga, Ages: 45−49
Data points enclosed with a diamond are reference sources

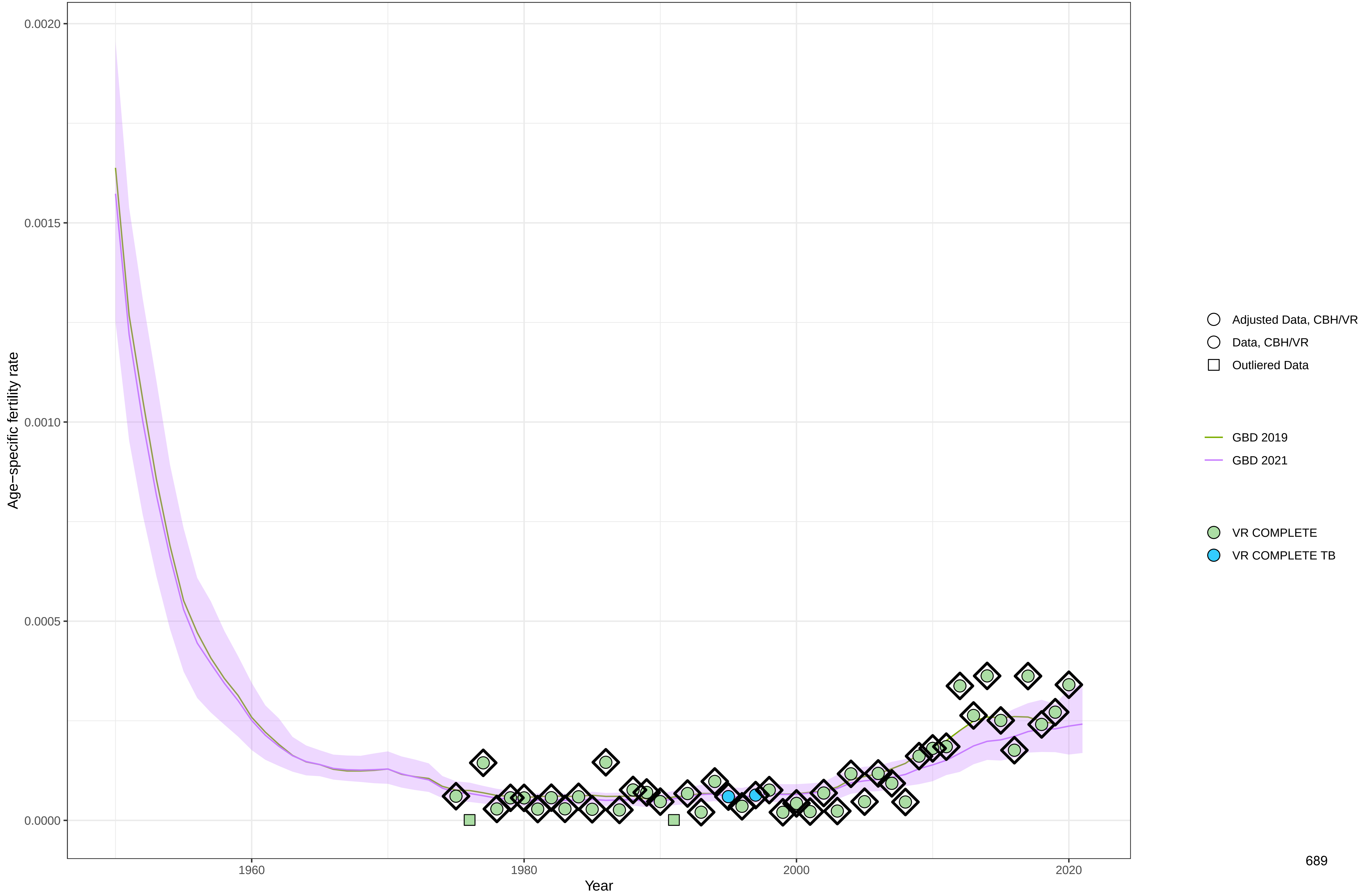

Age−specific fertility fitted trend lines, 1950−2021: Shiga, Ages: 50−54
Data points enclosed with a diamond are reference sources

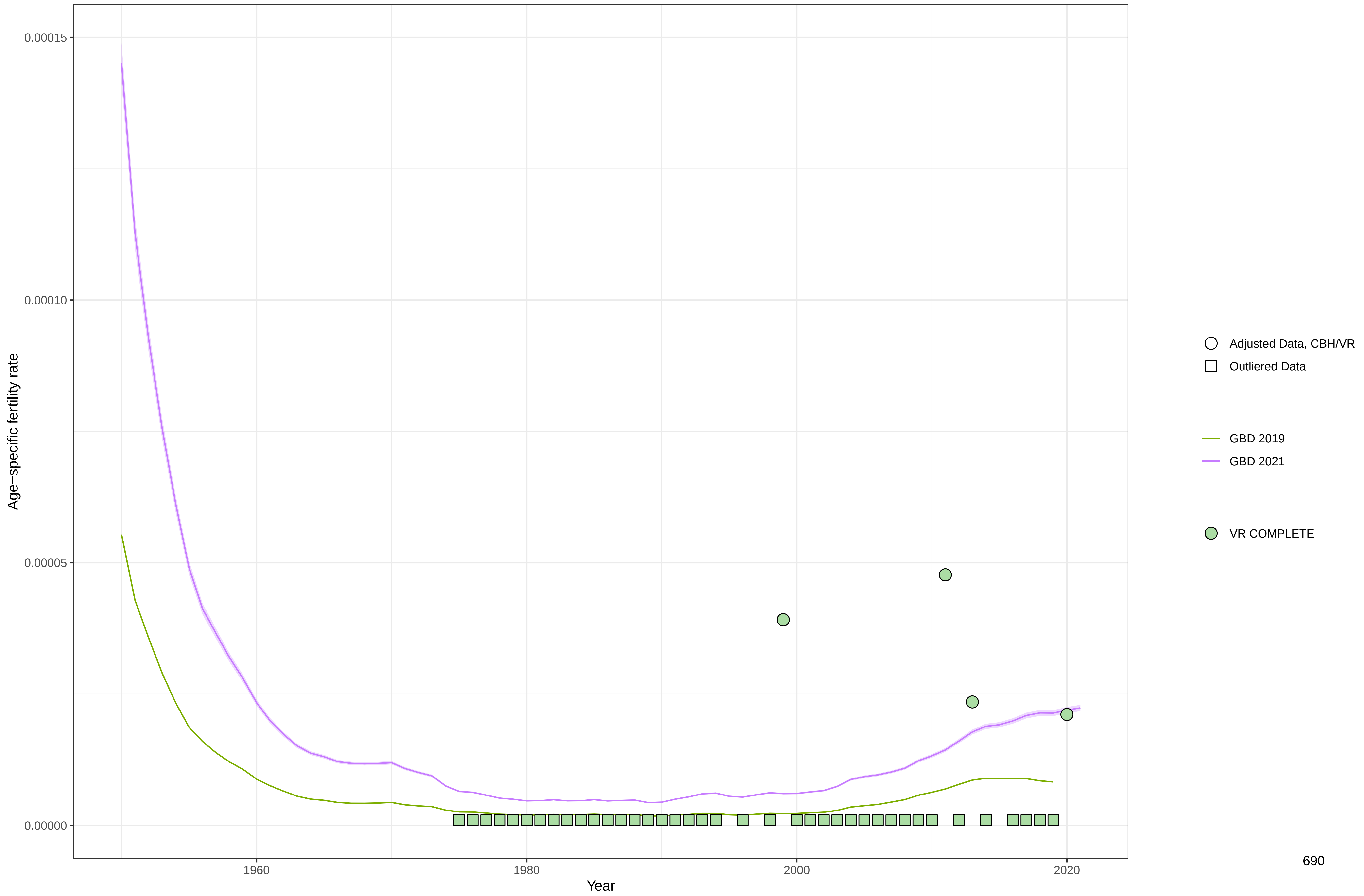

Total fertility rate fitted trend lines, 1950−2021: Shiga TFR
Data points enclosed with a diamond are reference sources

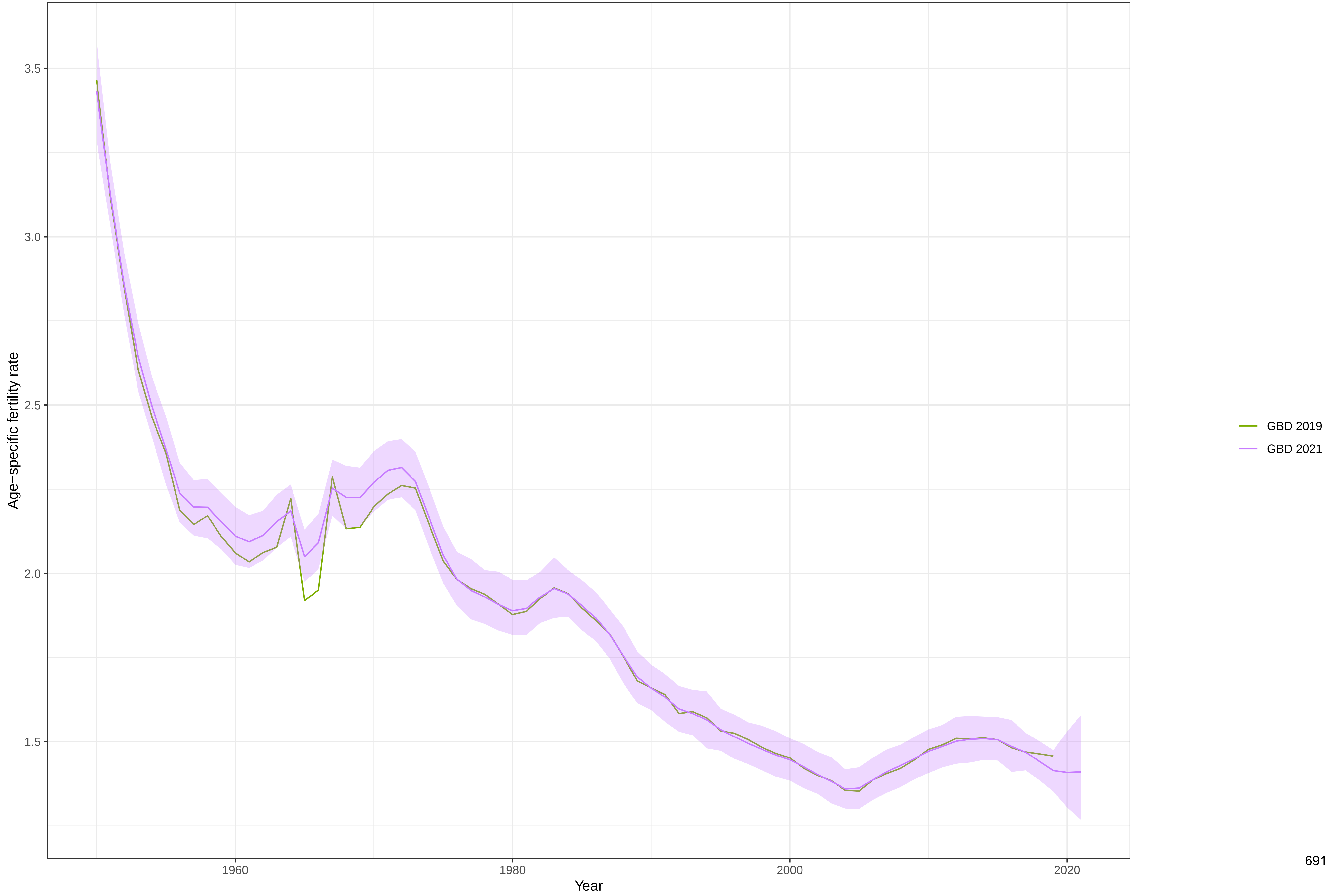

Age−specific fertility fitted trend lines, 1950−2021: Shimane, Ages: 10−14
Data points enclosed with a diamond are reference sources

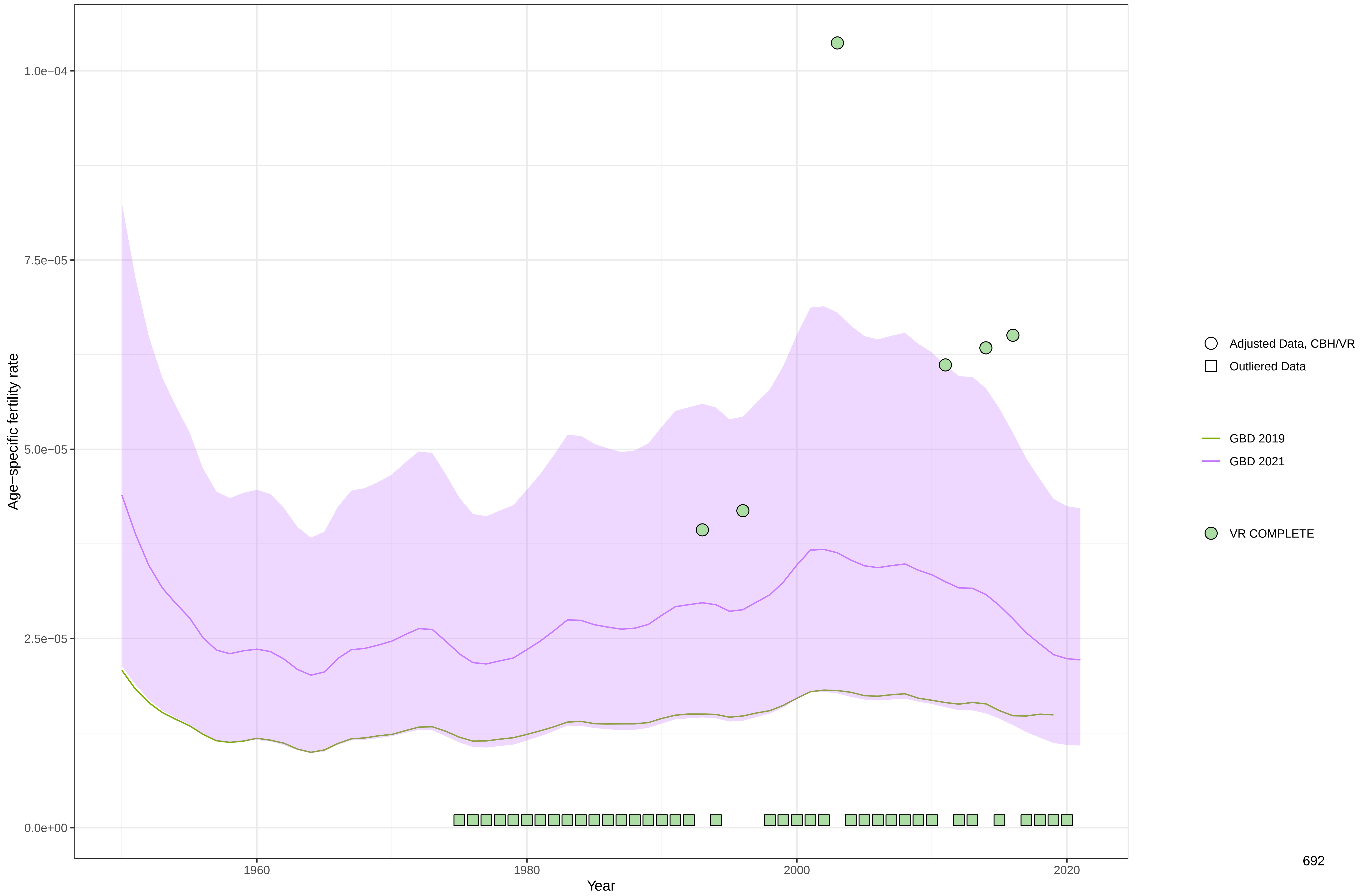

Age−specific fertility fitted trend lines, 1950−2021: Shimane, Ages: 15−19
Data points enclosed with a diamond are reference sources

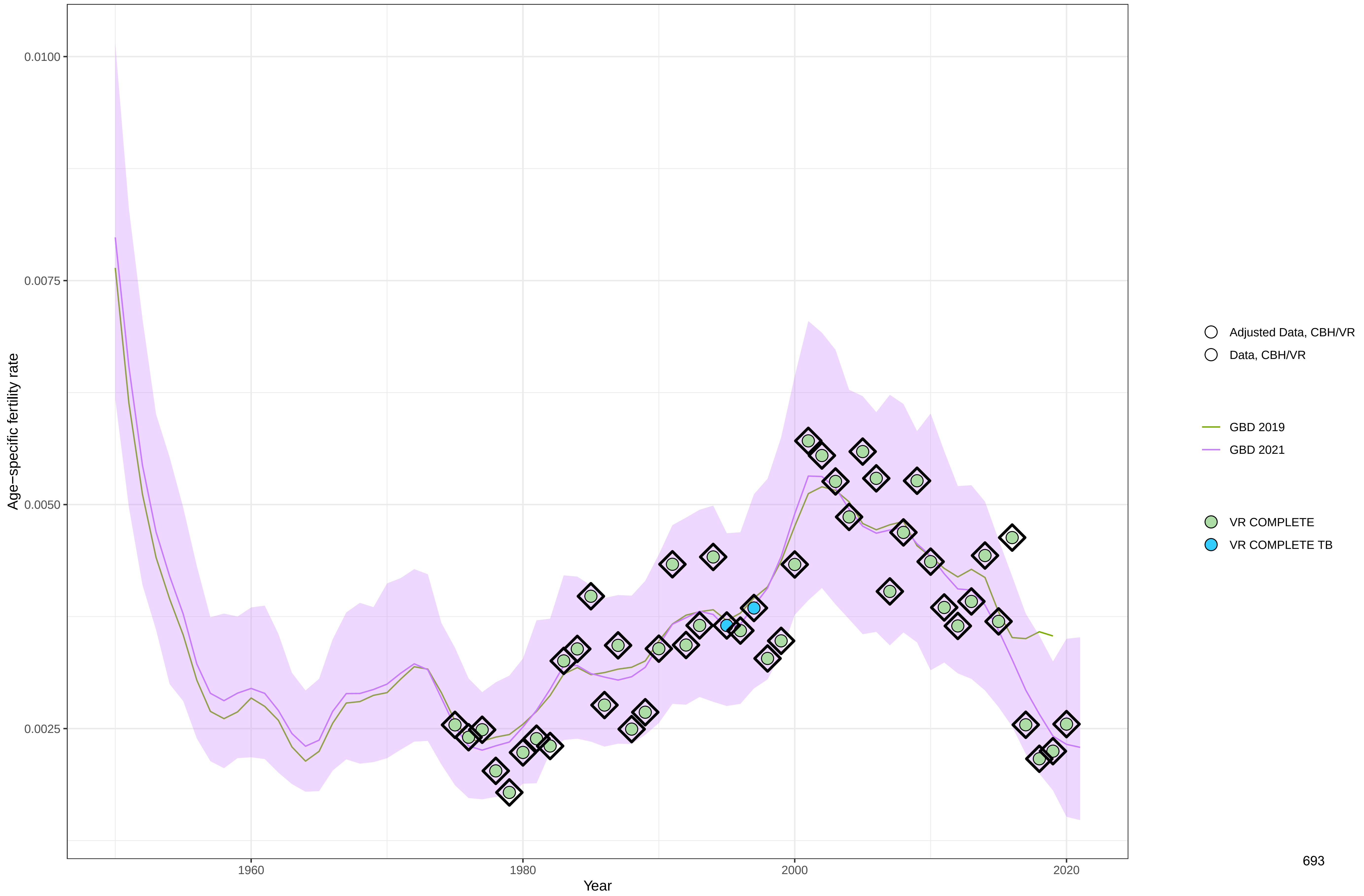

Age−specific fertility fitted trend lines, 1950−2021: Shimane, Ages: 20−24
Data points enclosed with a diamond are reference sources

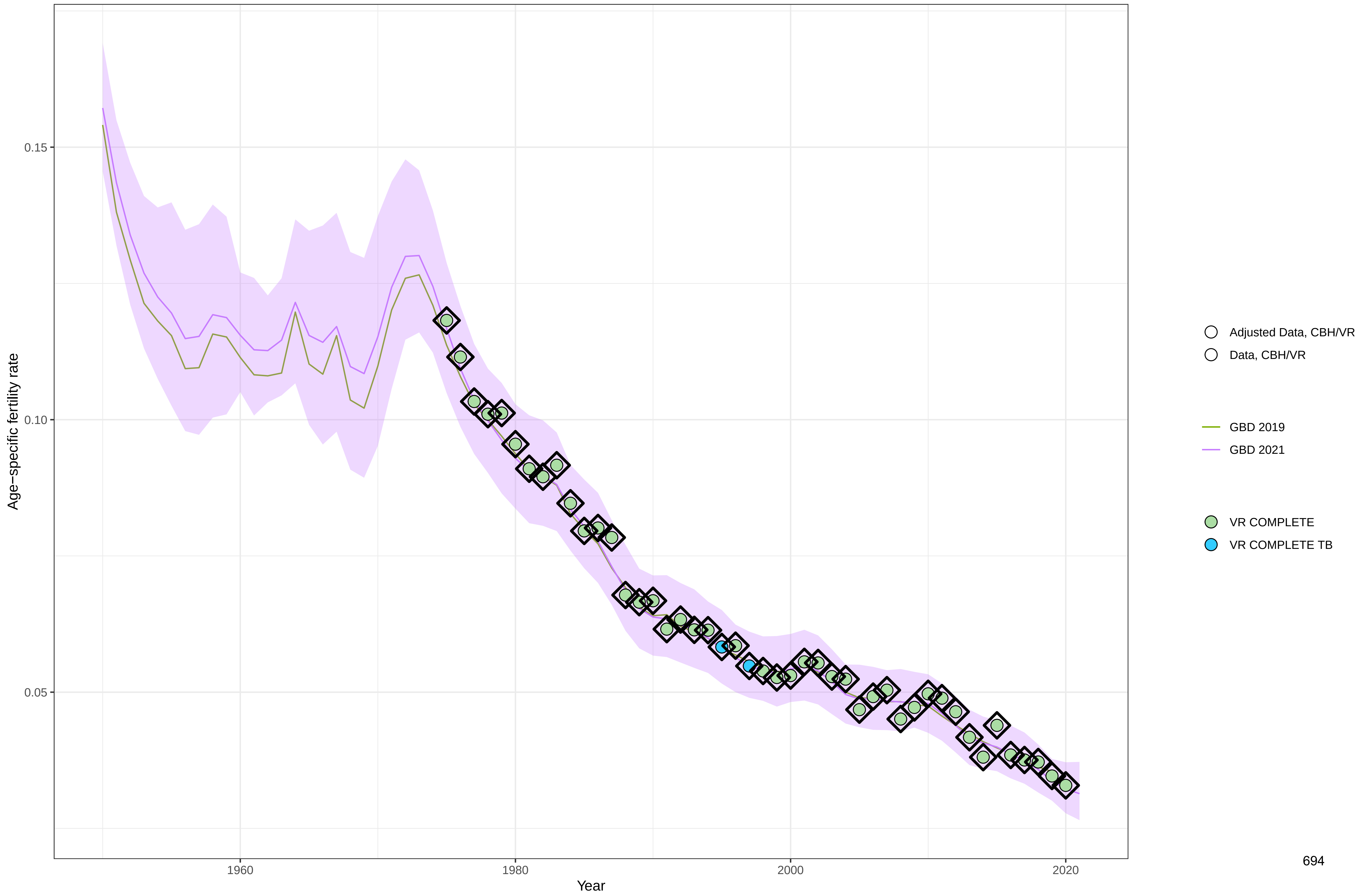

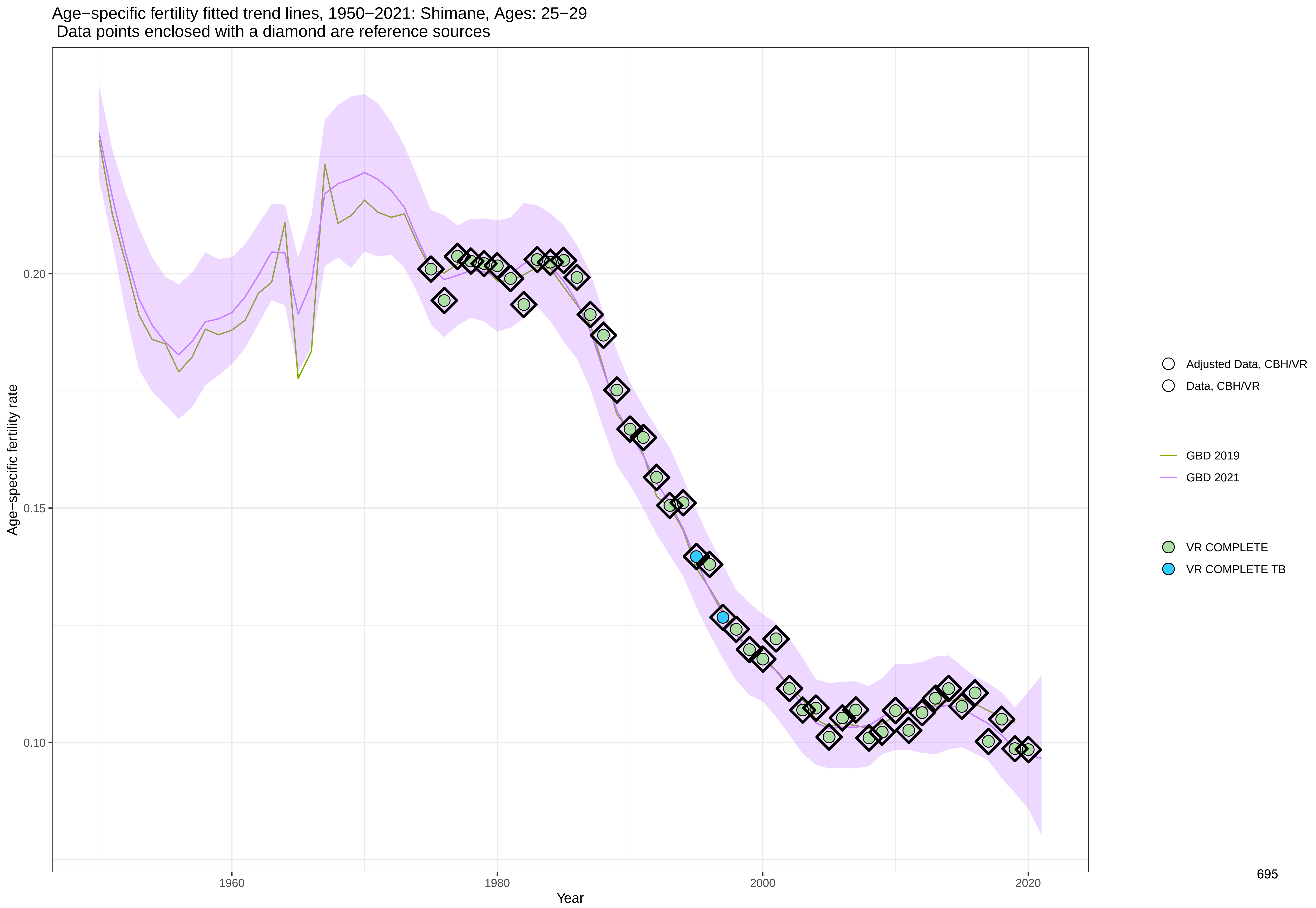
Age−specific fertility fitted trend lines, 1950−2021: Shimane, Ages: 25−29
Data points enclosed with a diamond are reference sources
Age−specific fertility rate
0.20
0.15
0.10
1960
1980
2000
2020
Year
Adjusted Data, CBH/VR
Data, CBH/VR
GBD 2019
GBD 2021
VR COMPLETE
VR COMPLETE TB

Age−specific fertility fitted trend lines, 1950−2021: Shimane, Ages: 30−34
Data points enclosed with a diamond are reference sources

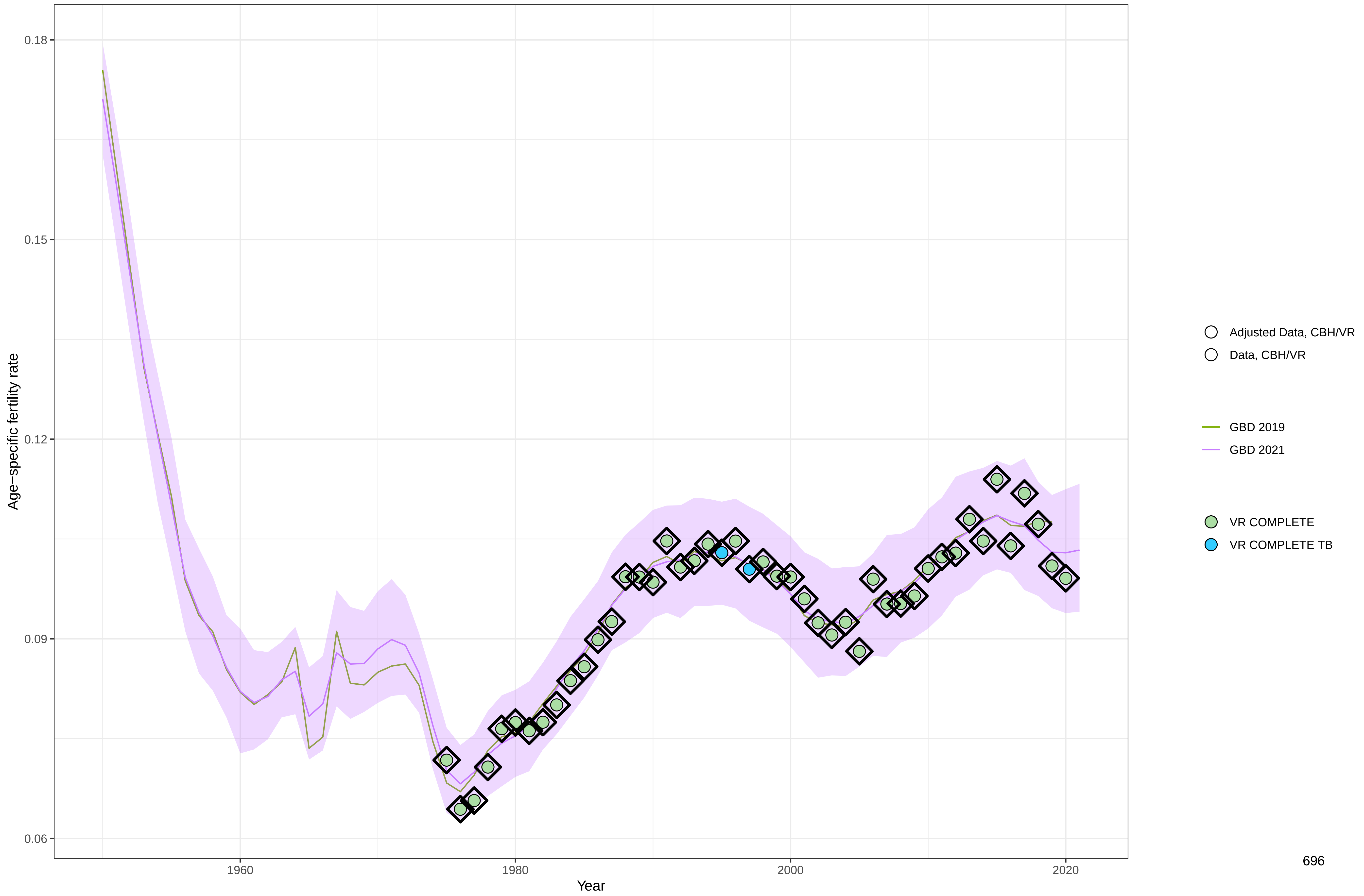

Age−specific fertility fitted trend lines, 1950−2021: Shimane, Ages: 35−39
Data points enclosed with a diamond are reference sources

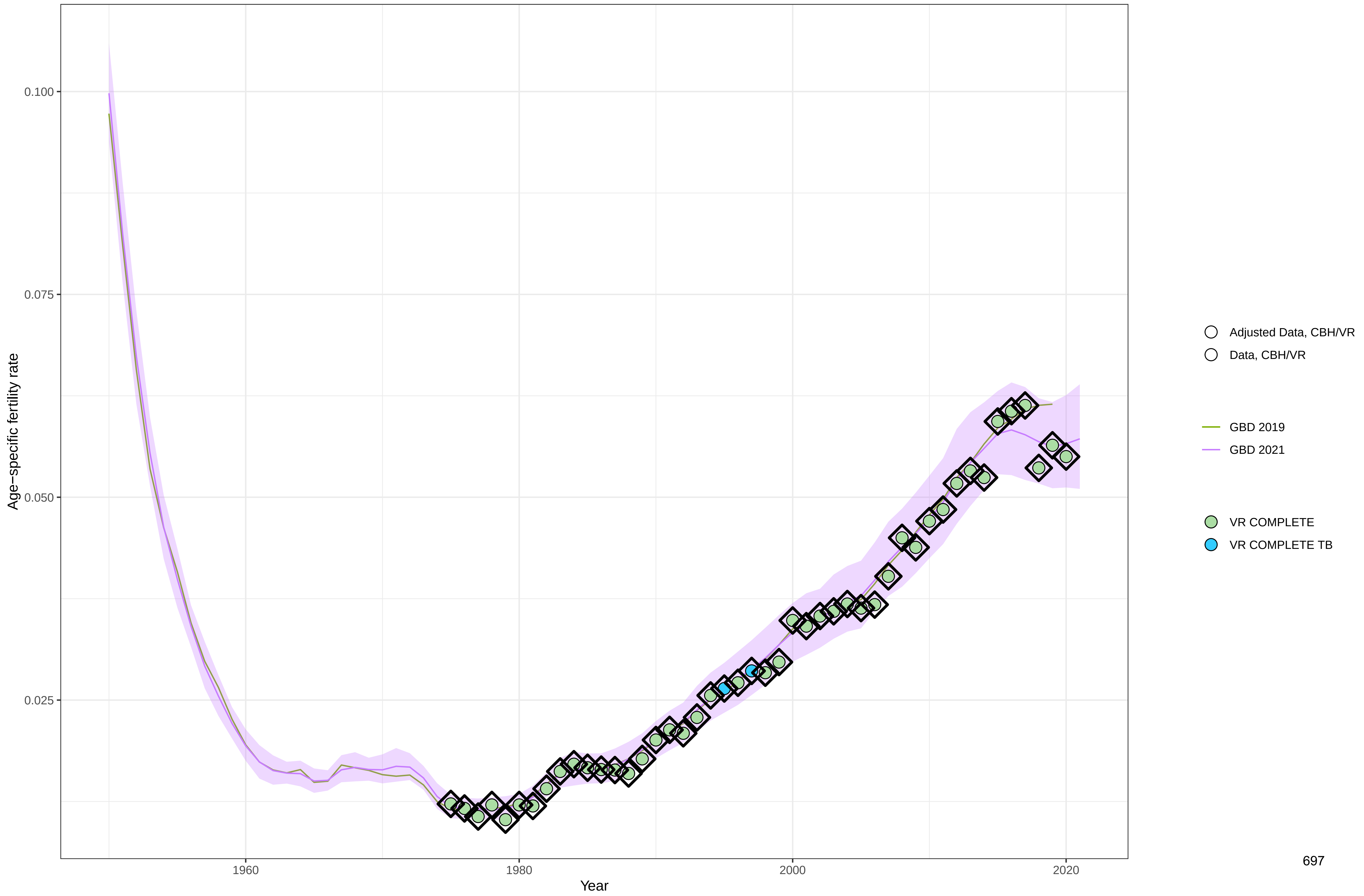

Age−specific fertility fitted trend lines, 1950−2021: Shimane, Ages: 40−44
Data points enclosed with a diamond are reference sources

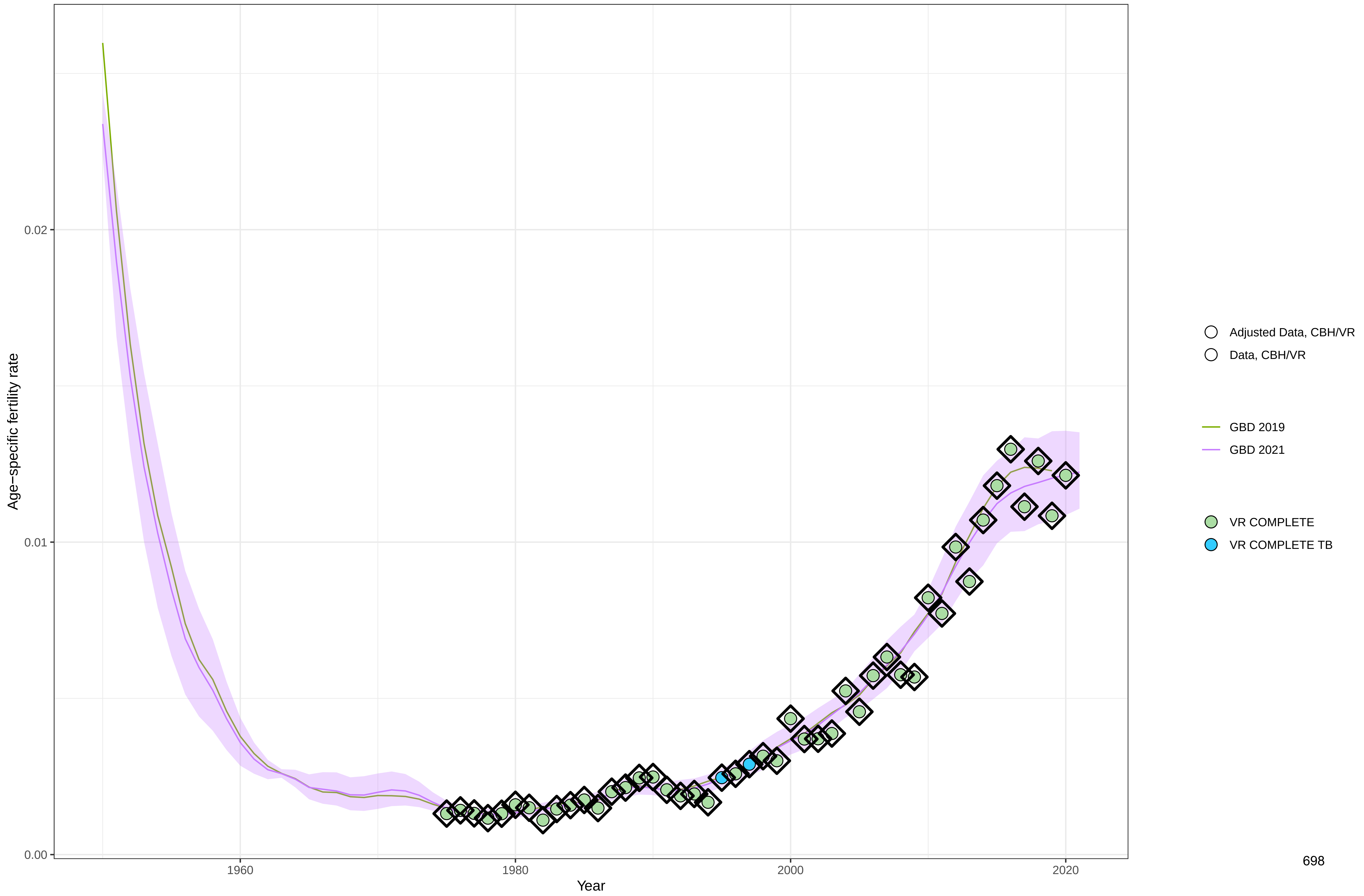

Age−specific fertility fitted trend lines, 1950−2021: Shimane, Ages: 45−49
Data points enclosed with a diamond are reference sources

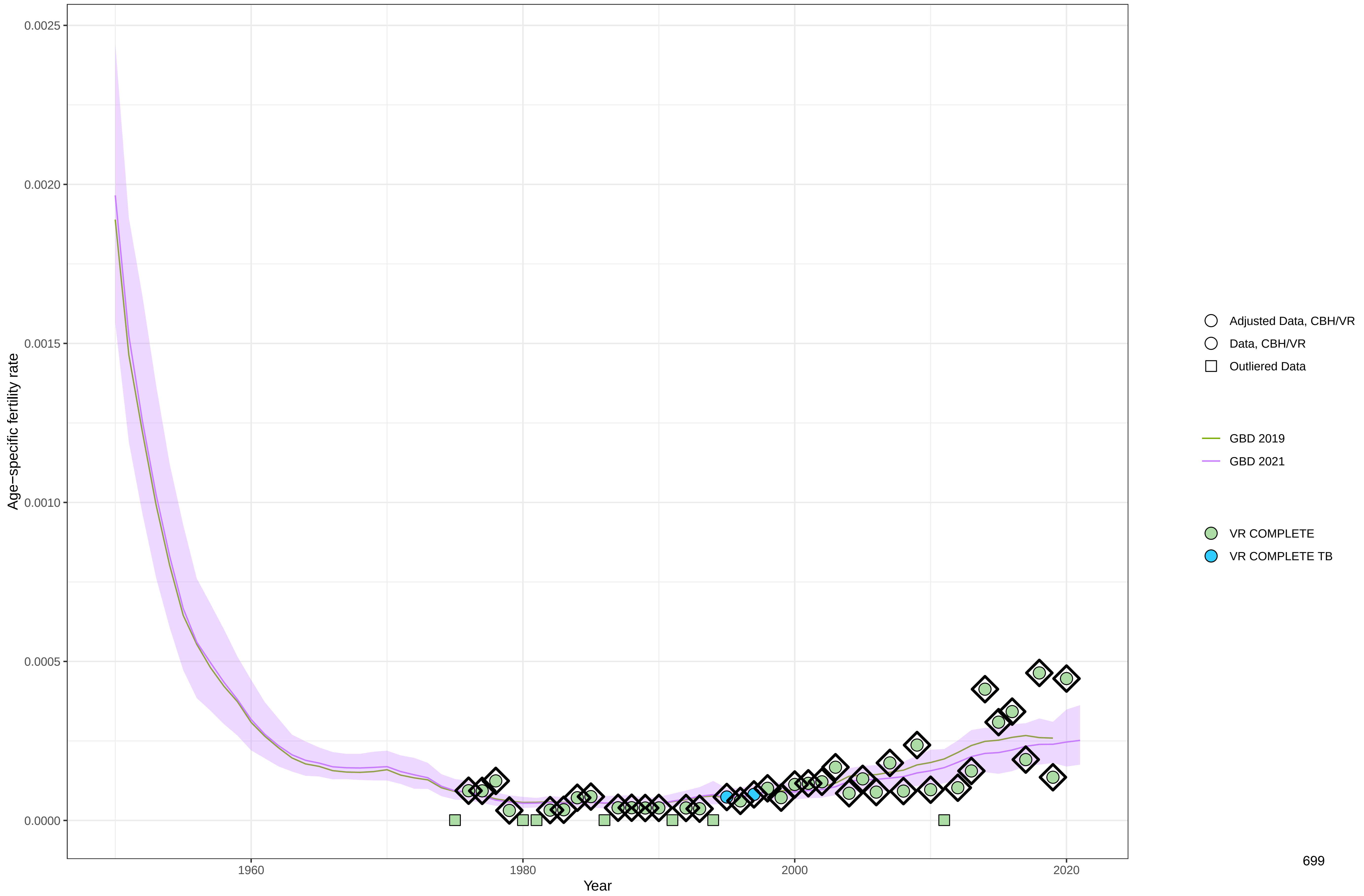

Age−specific fertility fitted trend lines, 1950−2021: Shimane, Ages: 50−54
Data points enclosed with a diamond are reference sources

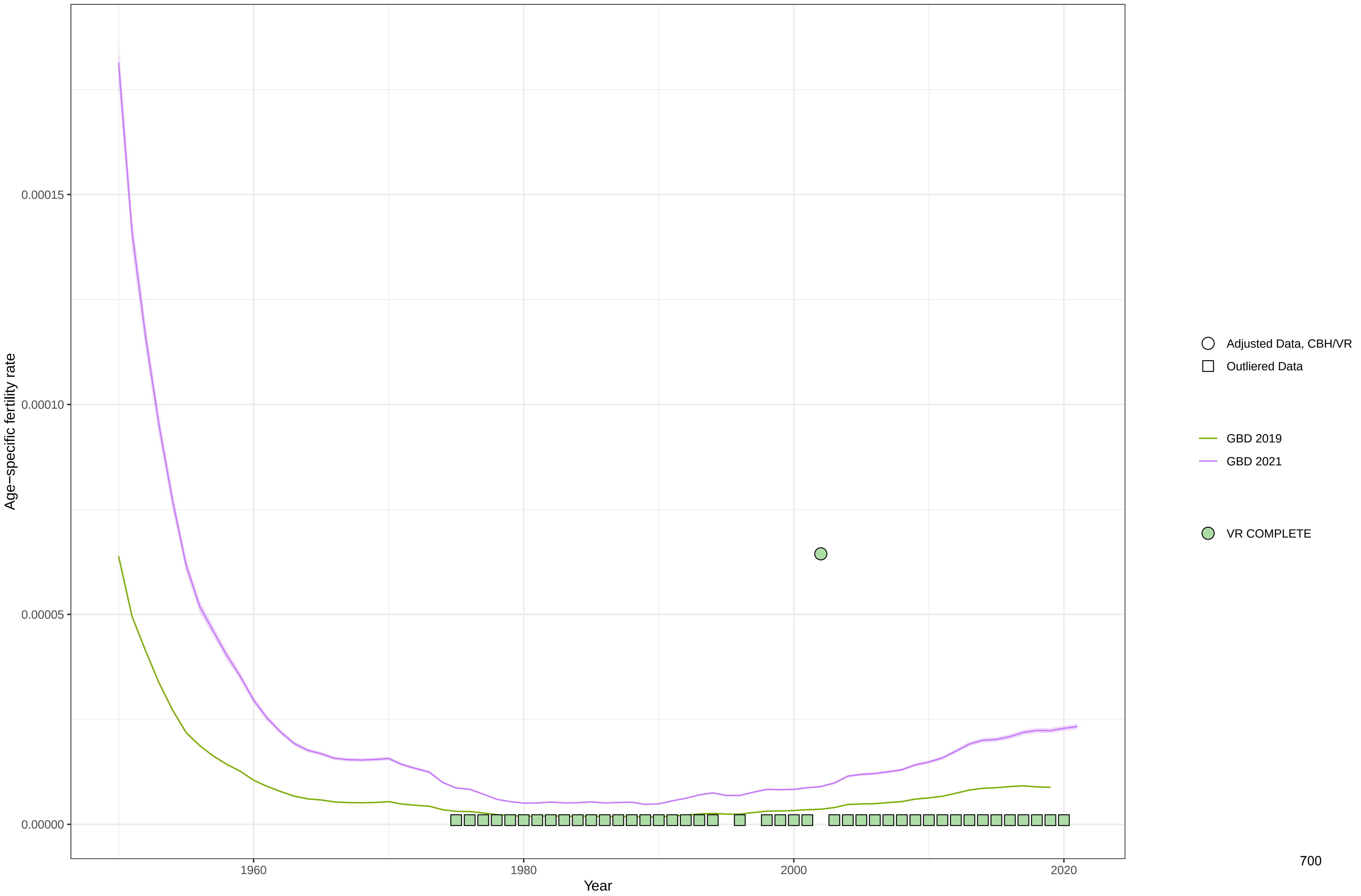

Total fertility rate fitted trend lines, 1950−2021: Shimane TFR
Data points enclosed with a diamond are reference sources

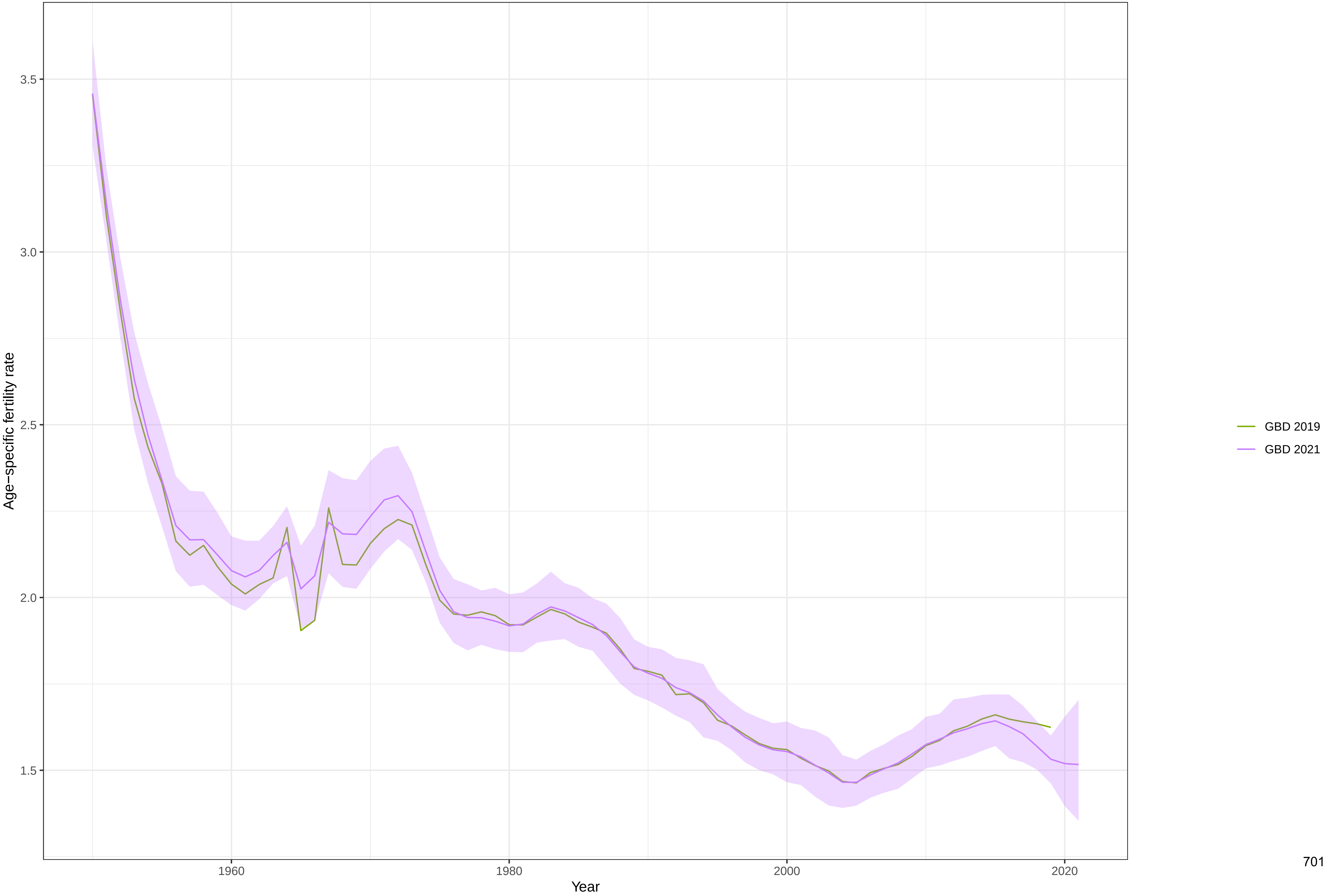

Age−specific fertility fitted trend lines, 1950−2021: Shizuoka, Ages: 10−14
Data points enclosed with a diamond are reference sources

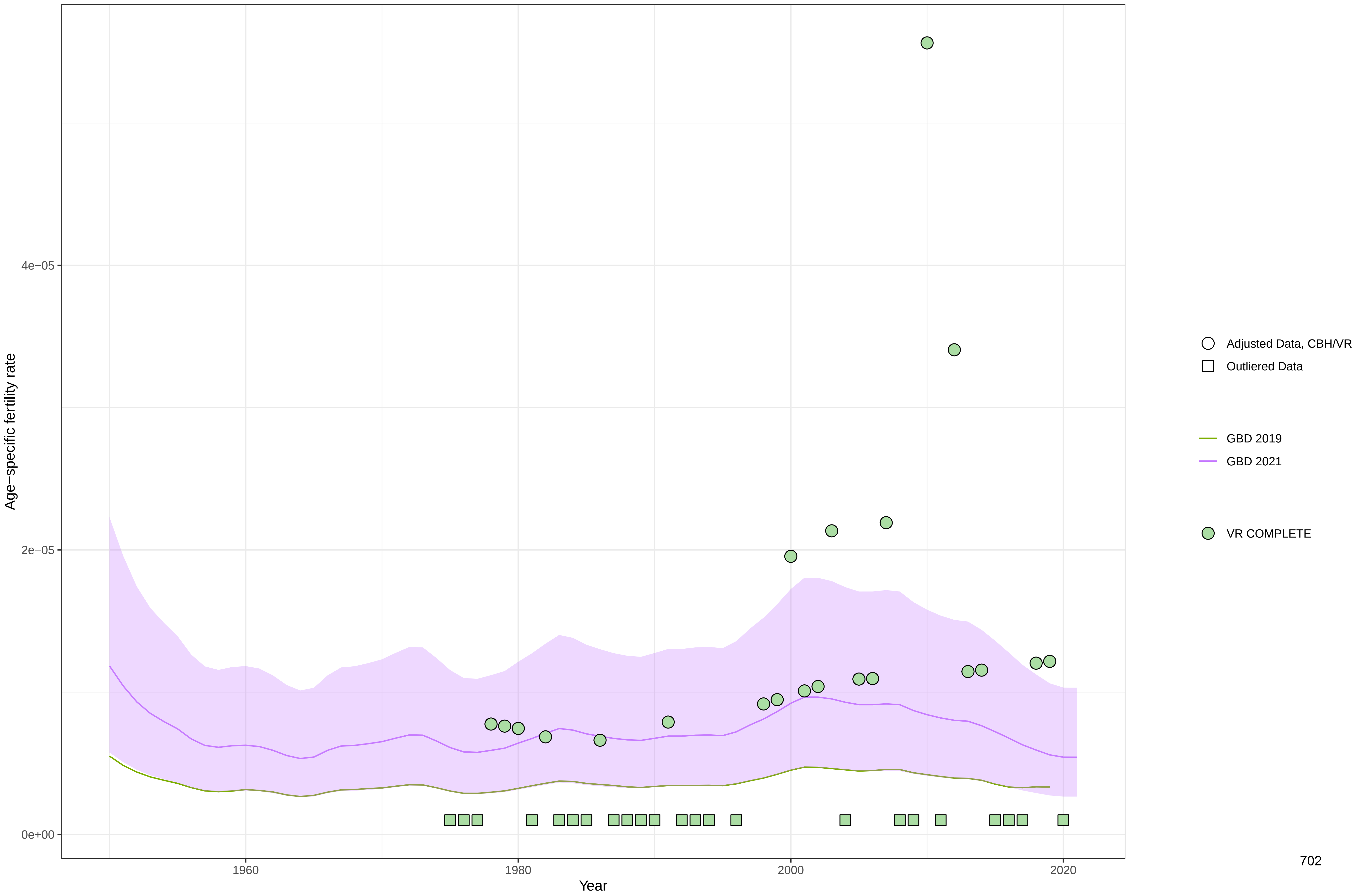

Age−specific fertility fitted trend lines, 1950−2021: Shizuoka, Ages: 15−19
Data points enclosed with a diamond are reference sources

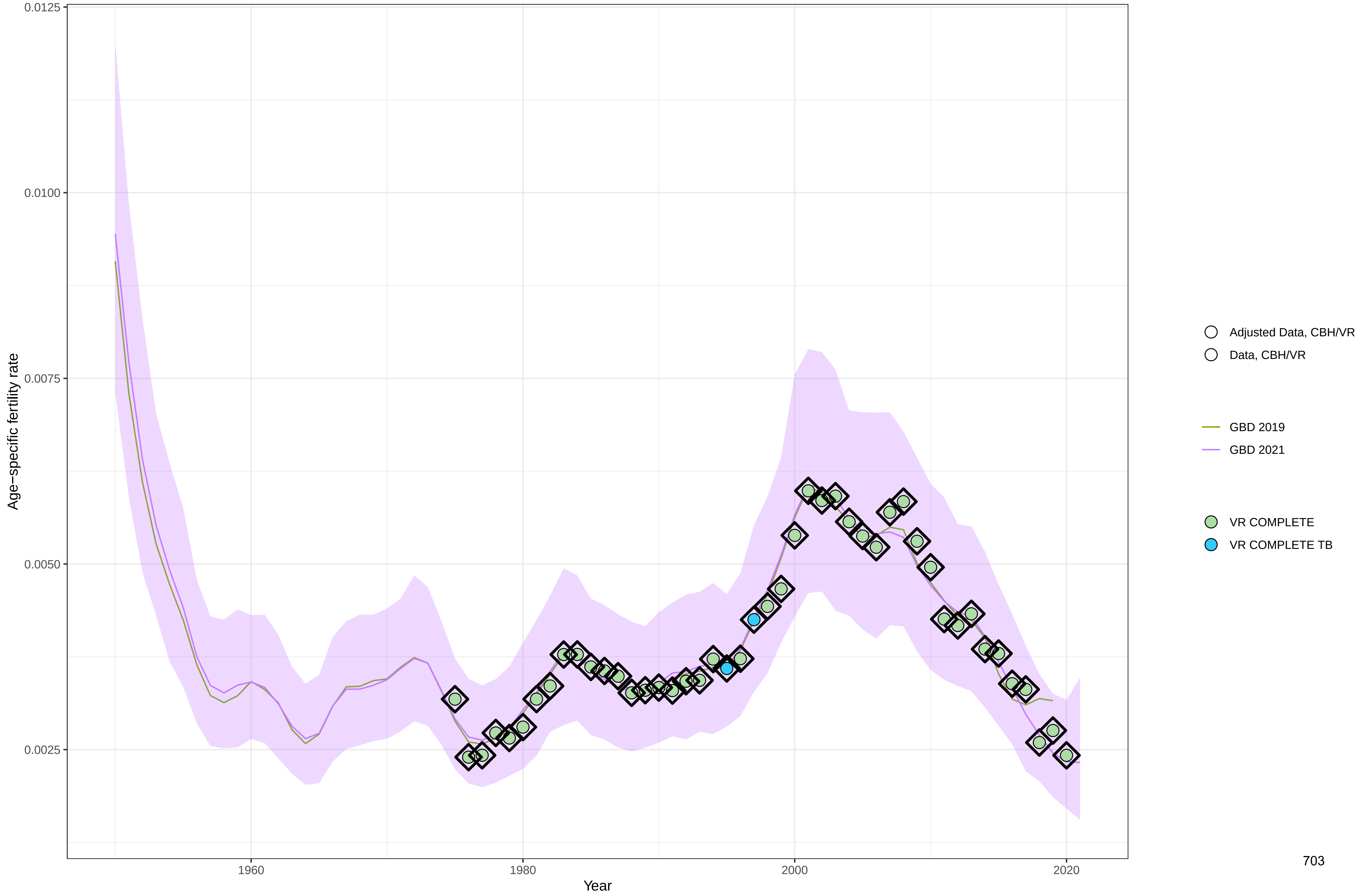

Age−specific fertility fitted trend lines, 1950−2021: Shizuoka, Ages: 20−24
Data points enclosed with a diamond are reference sources

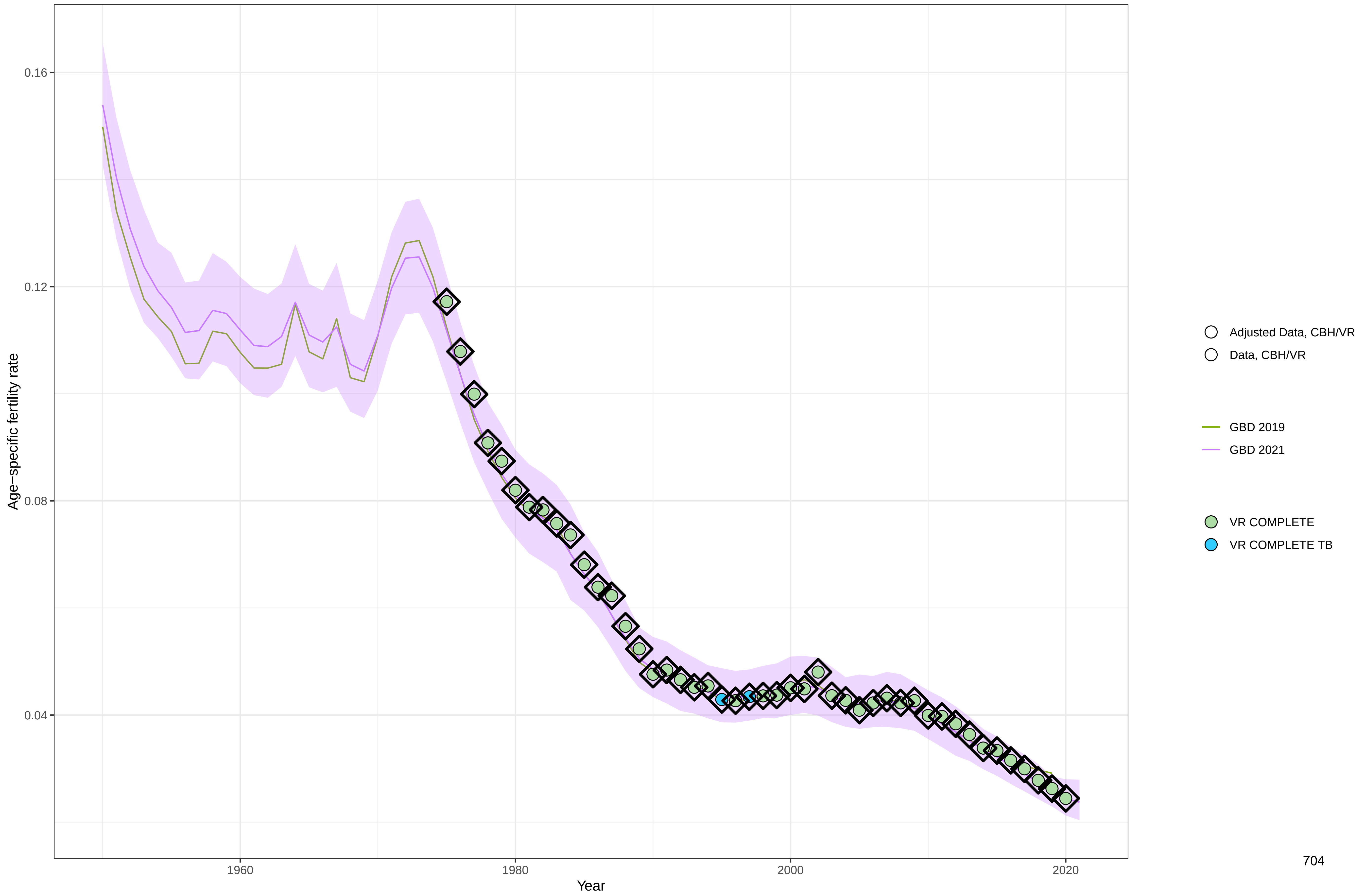

Age−specific fertility fitted trend lines, 1950−2021: Shizuoka, Ages: 25−29
Data points enclosed with a diamond are reference sources

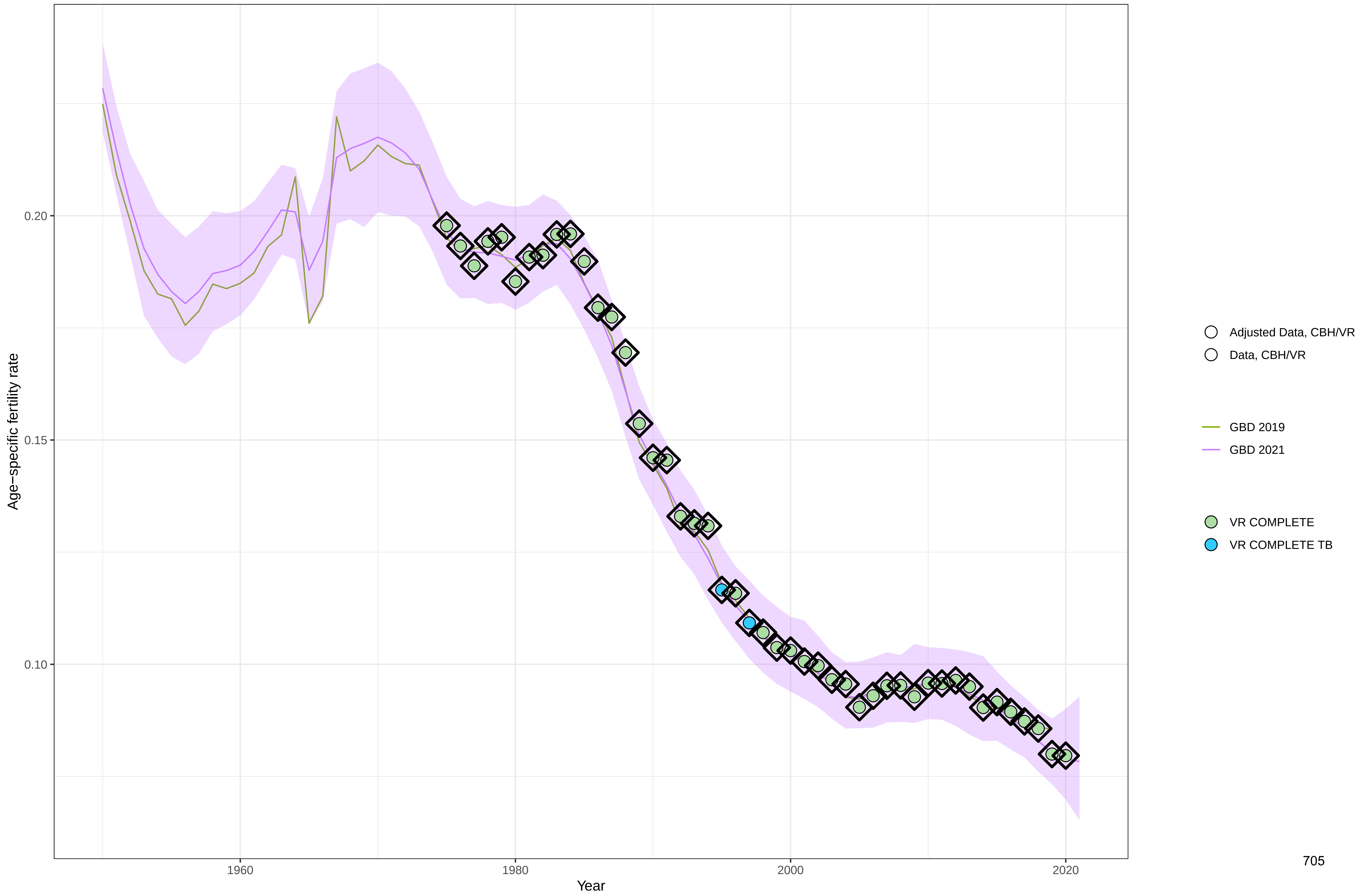

Age−specific fertility fitted trend lines, 1950−2021: Shizuoka, Ages: 30−34
Data points enclosed with a diamond are reference sources

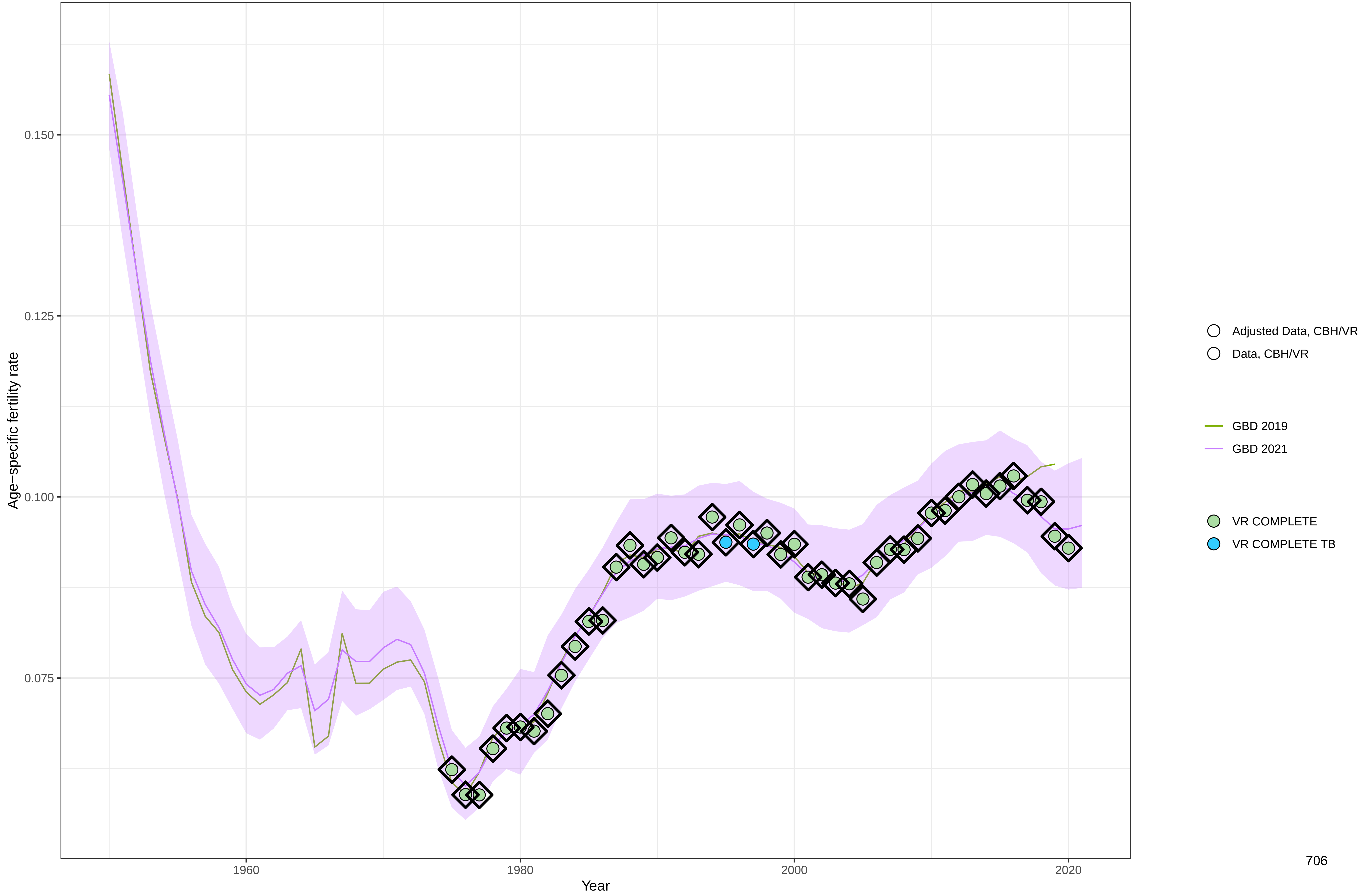

Age−specific fertility fitted trend lines, 1950−2021: Shizuoka, Ages: 35−39
Data points enclosed with a diamond are reference sources

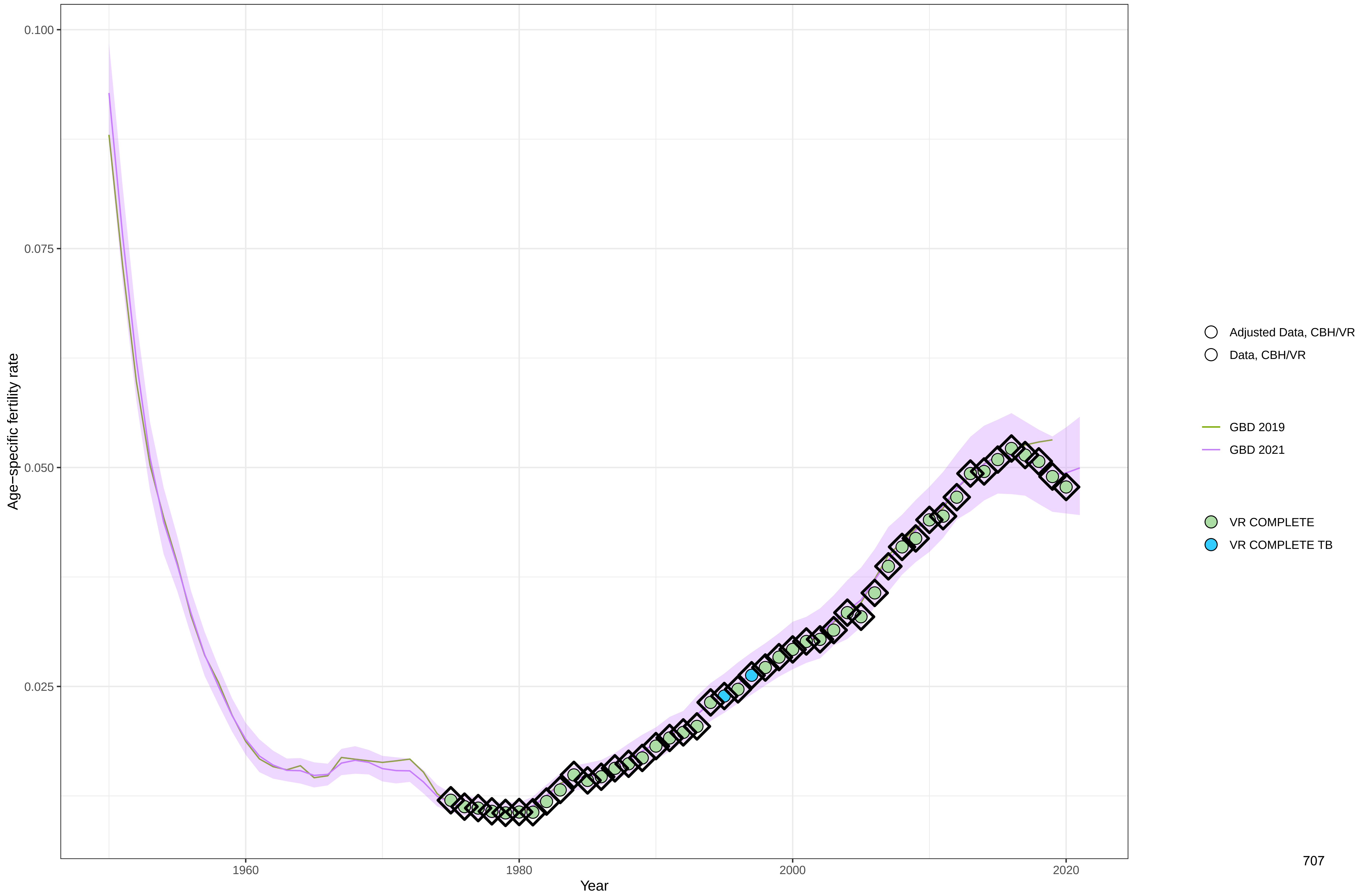

Age−specific fertility fitted trend lines, 1950−2021: Shizuoka, Ages: 40−44
Data points enclosed with a diamond are reference sources

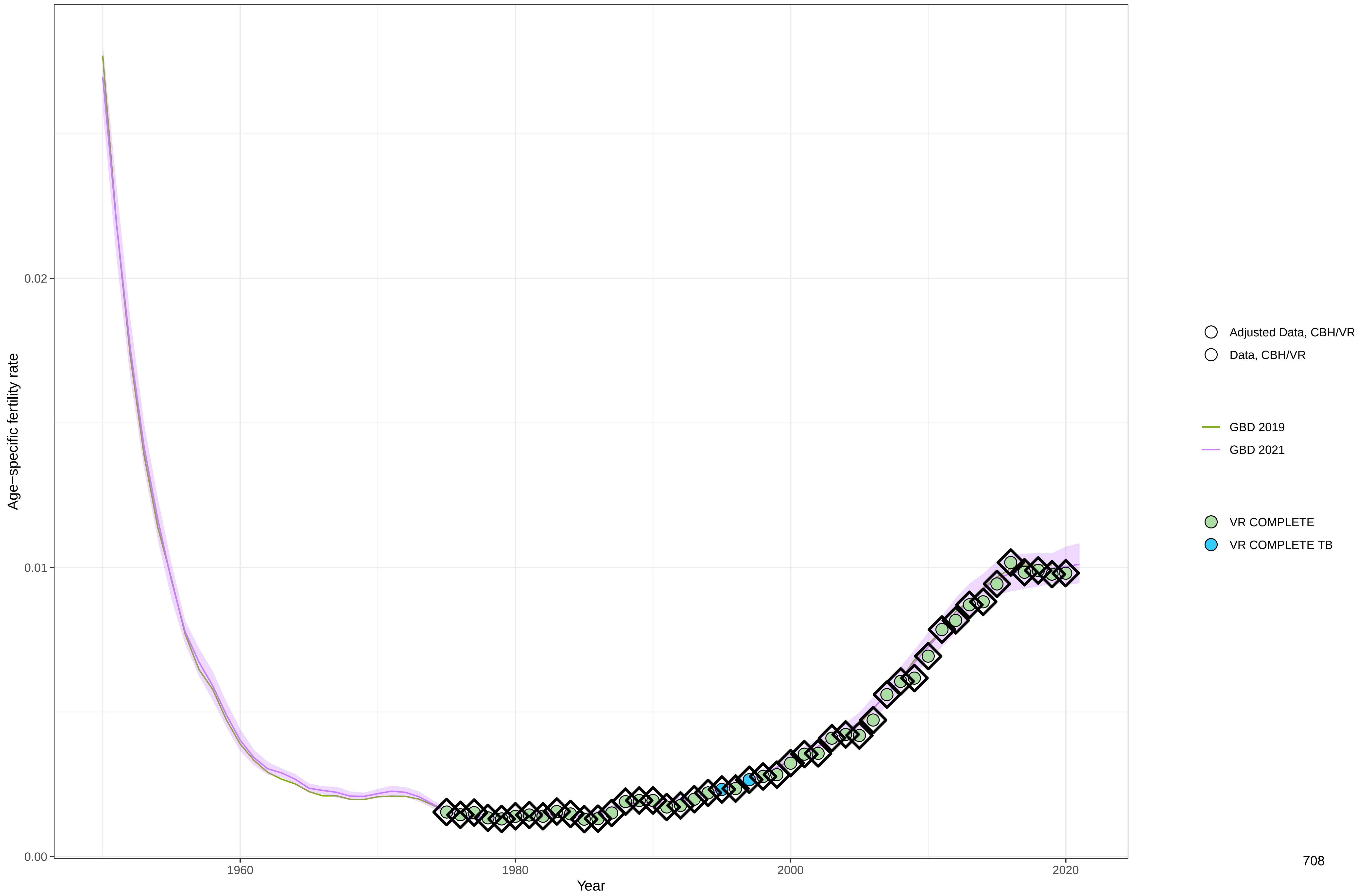

Age−specific fertility fitted trend lines, 1950−2021: Shizuoka, Ages: 45−49
Data points enclosed with a diamond are reference sources

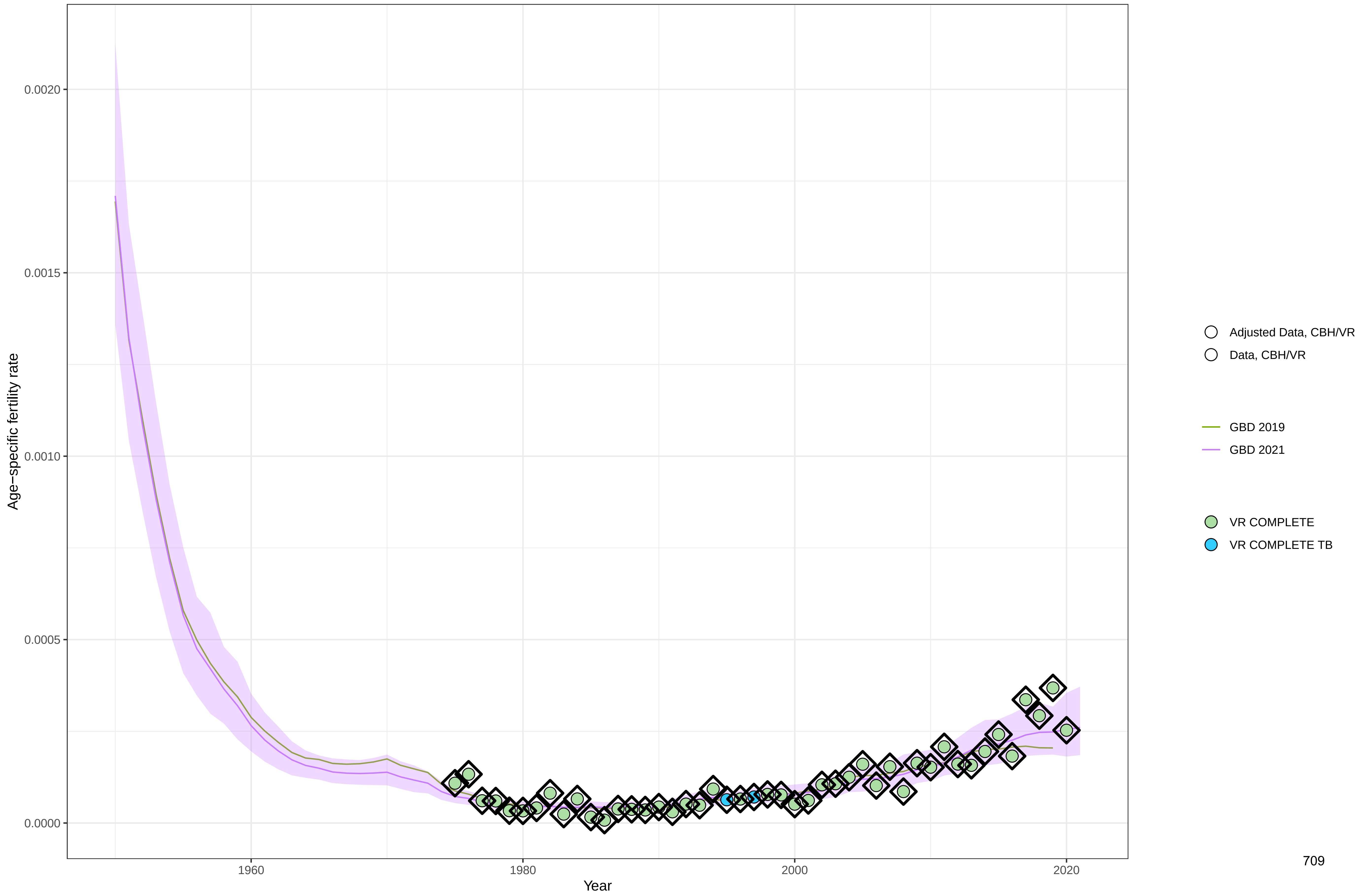

Age−specific fertility fitted trend lines, 1950−2021: Shizuoka, Ages: 50−54
Data points enclosed with a diamond are reference sources

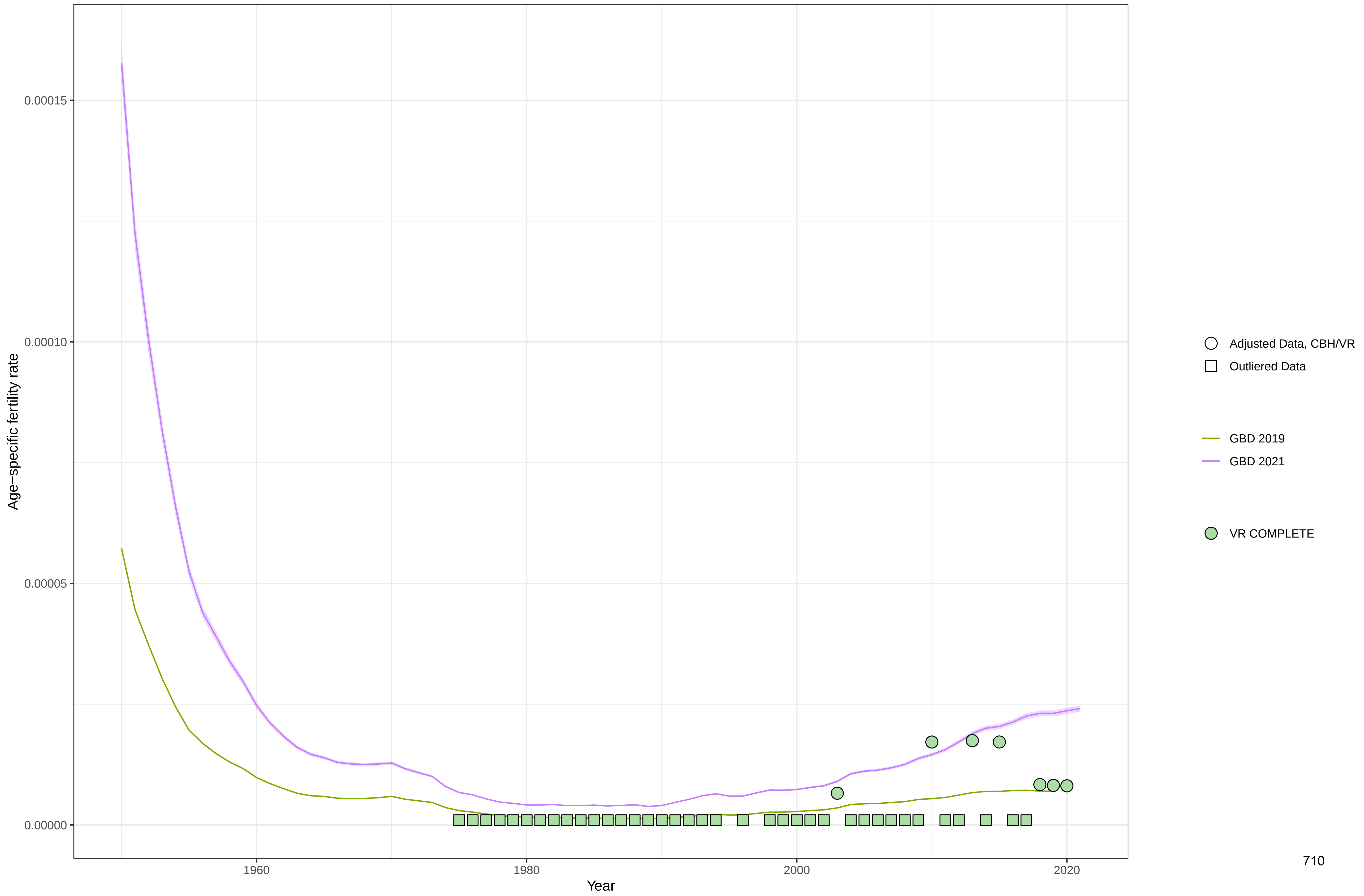

Total fertility rate fitted trend lines, 1950−2021: Shizuoka TFR
Data points enclosed with a diamond are reference sources

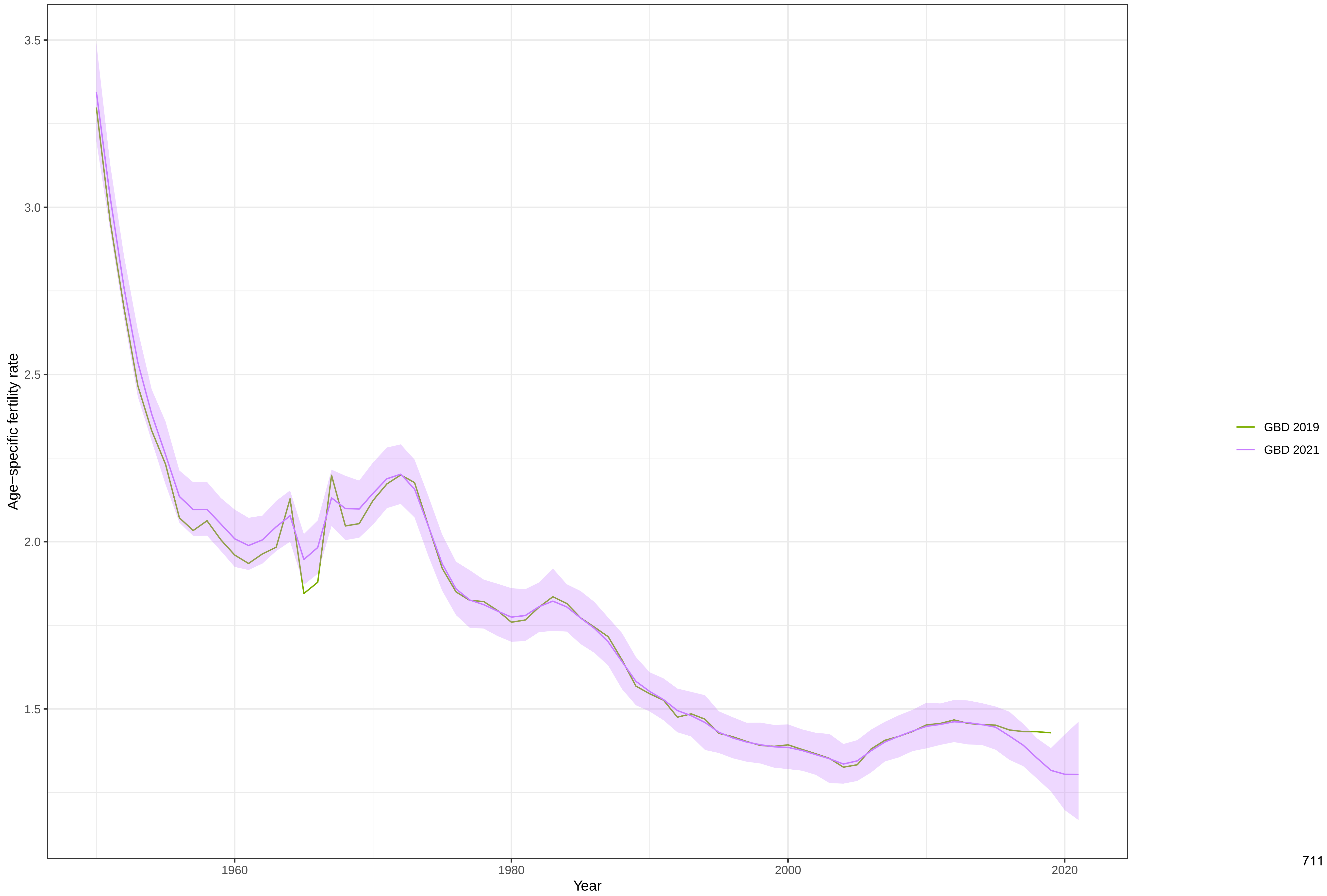

Age−specific fertility fitted trend lines, 1950−2021: Tochigi, Ages: 10−14
Data points enclosed with a diamond are reference sources

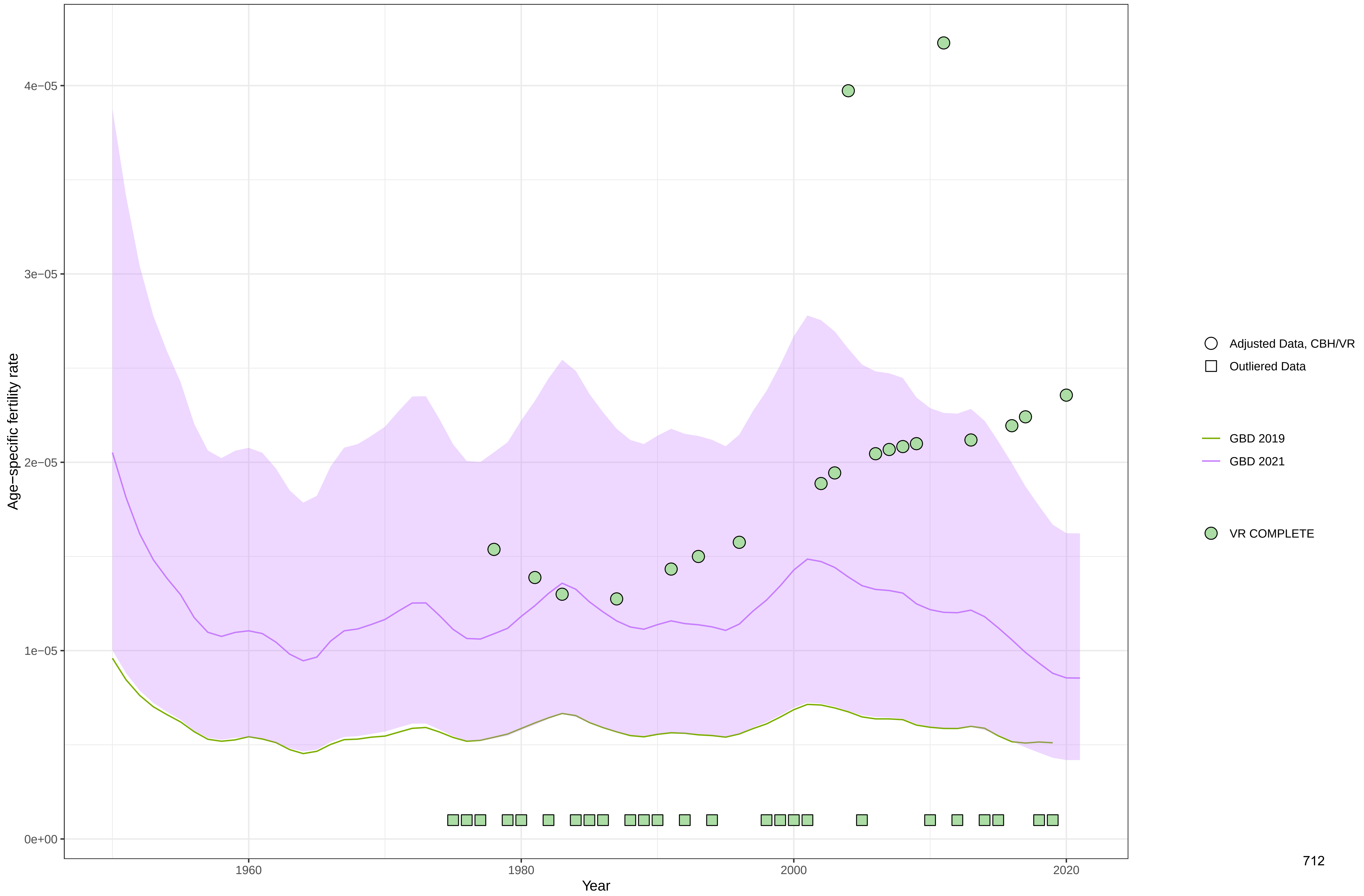

Age−specific fertility fitted trend lines, 1950−2021: Tochigi, Ages: 15−19
Data points enclosed with a diamond are reference sources

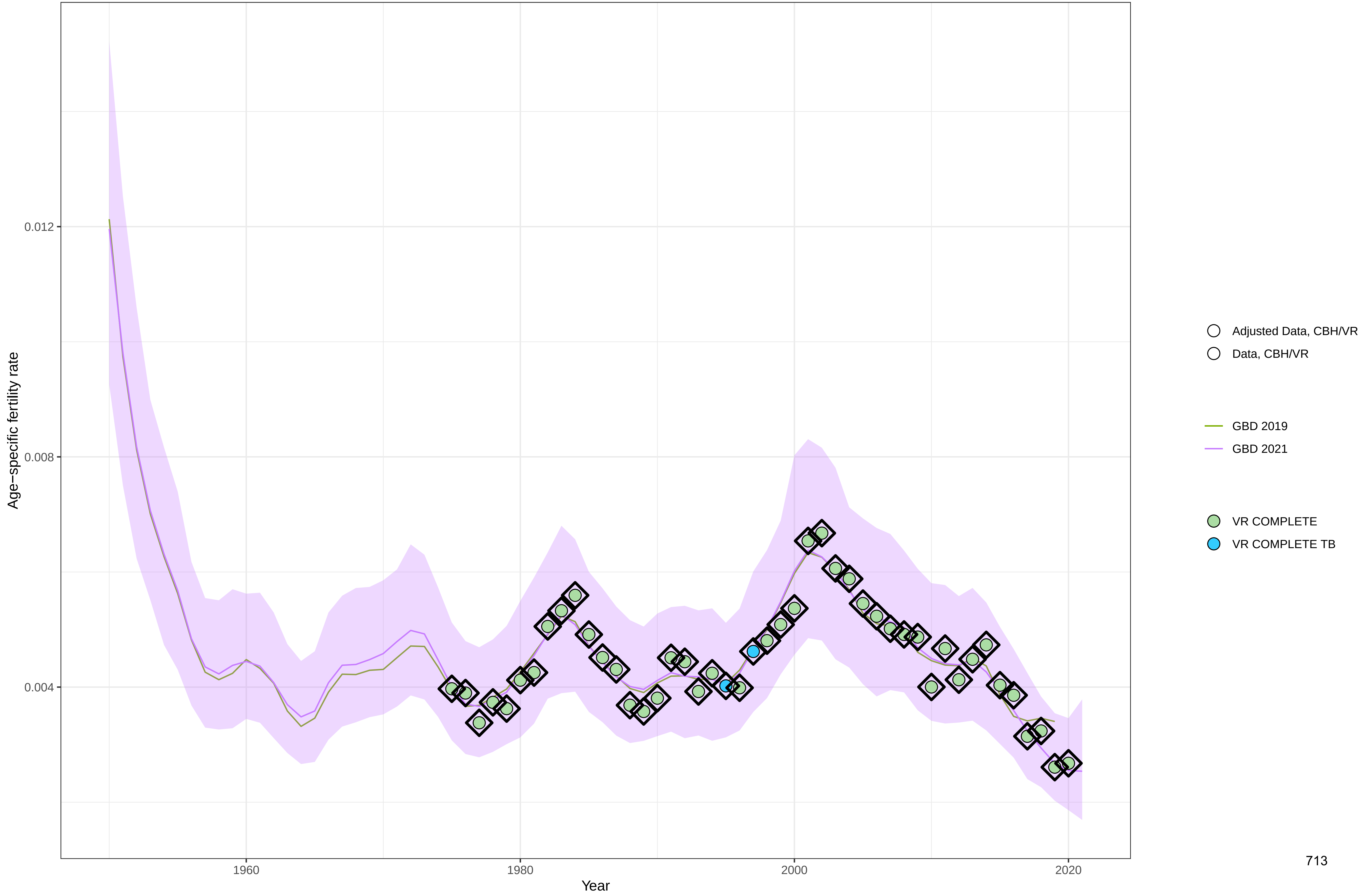

Age−specific fertility fitted trend lines, 1950−2021: Tochigi, Ages: 20−24
Data points enclosed with a diamond are reference sources

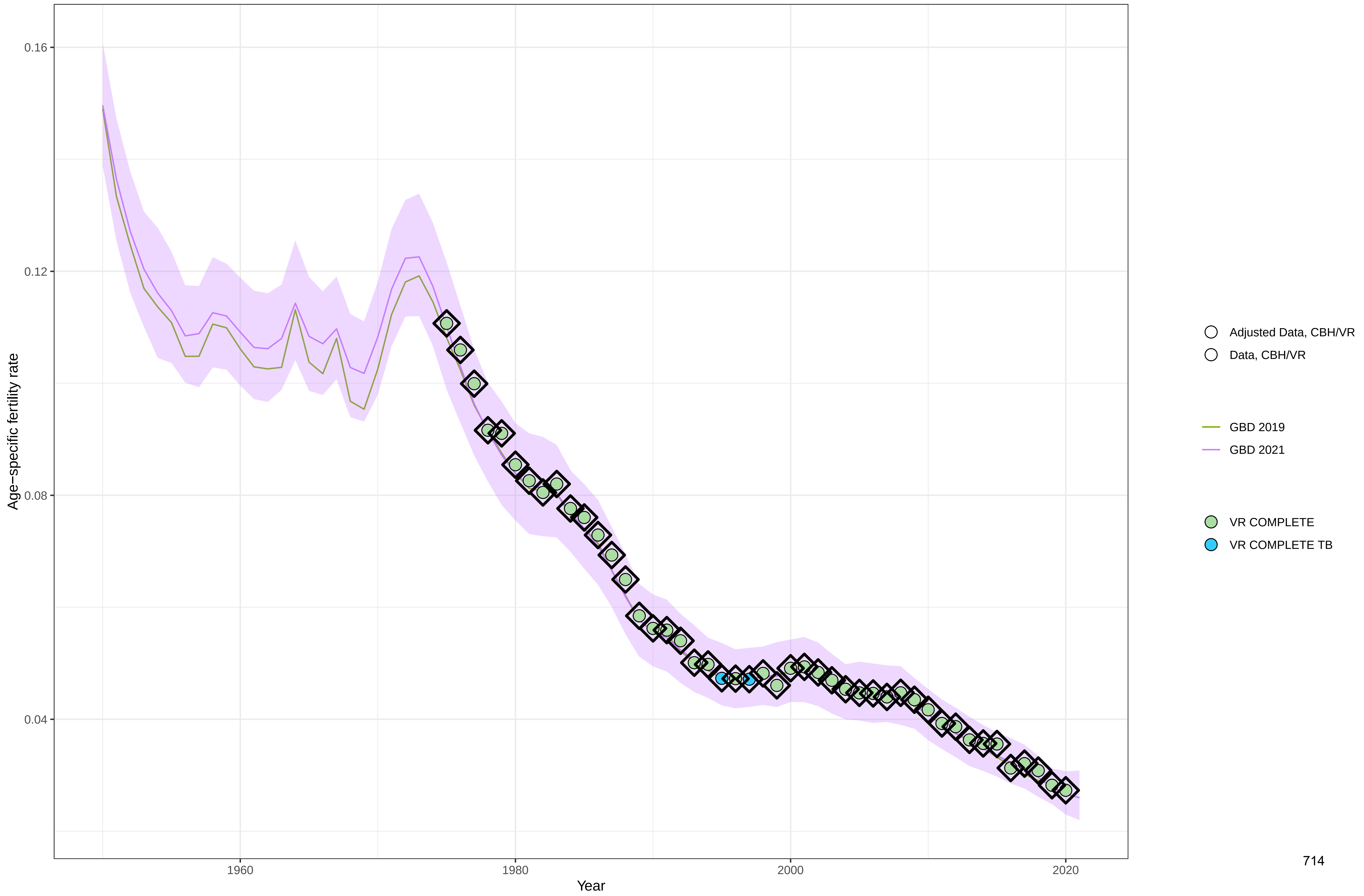

Age−specific fertility fitted trend lines, 1950−2021: Tochigi, Ages: 25−29
Data points enclosed with a diamond are reference sources

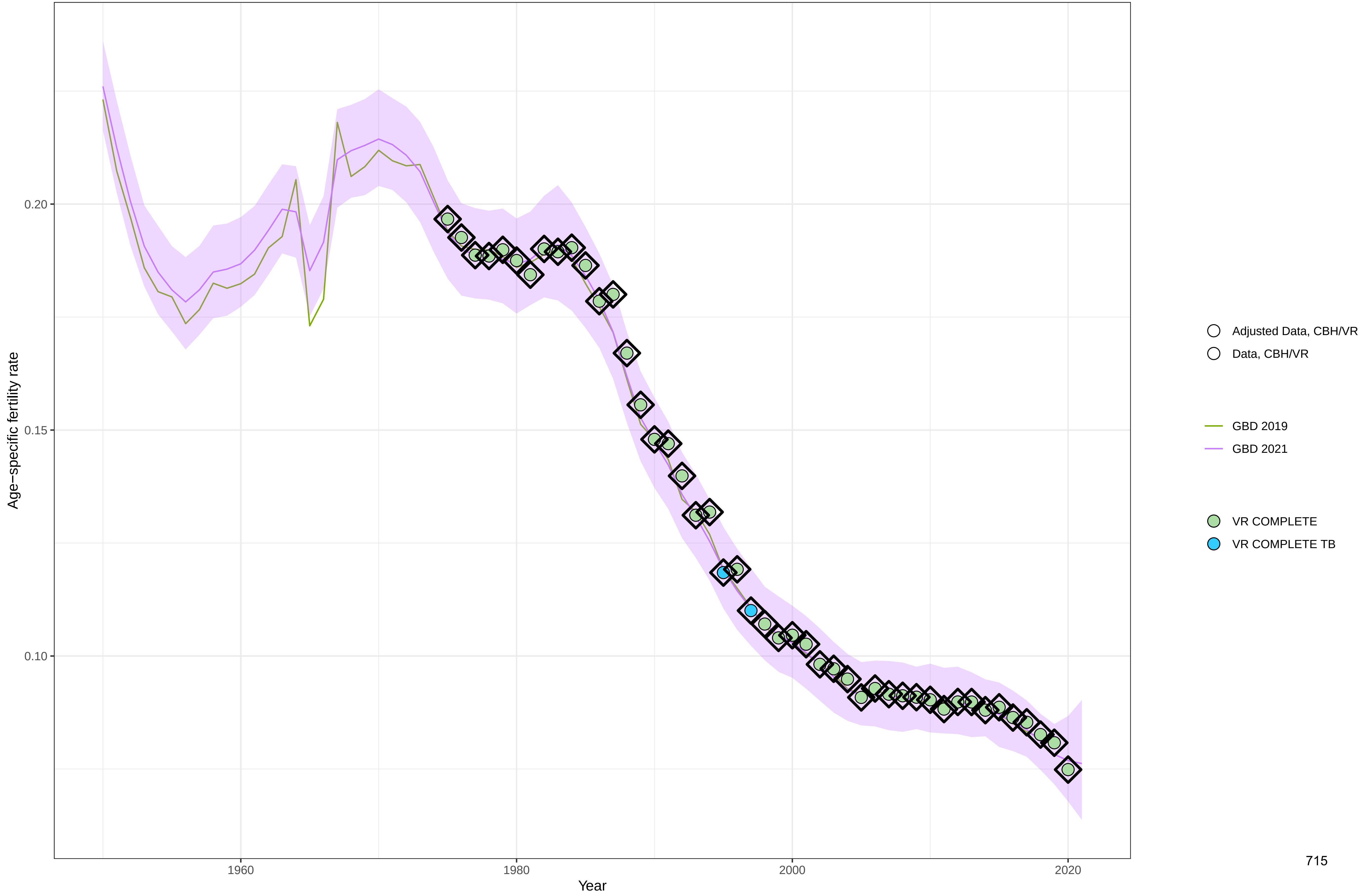

Age−specific fertility fitted trend lines, 1950−2021: Tochigi, Ages: 30−34
Data points enclosed with a diamond are reference sources

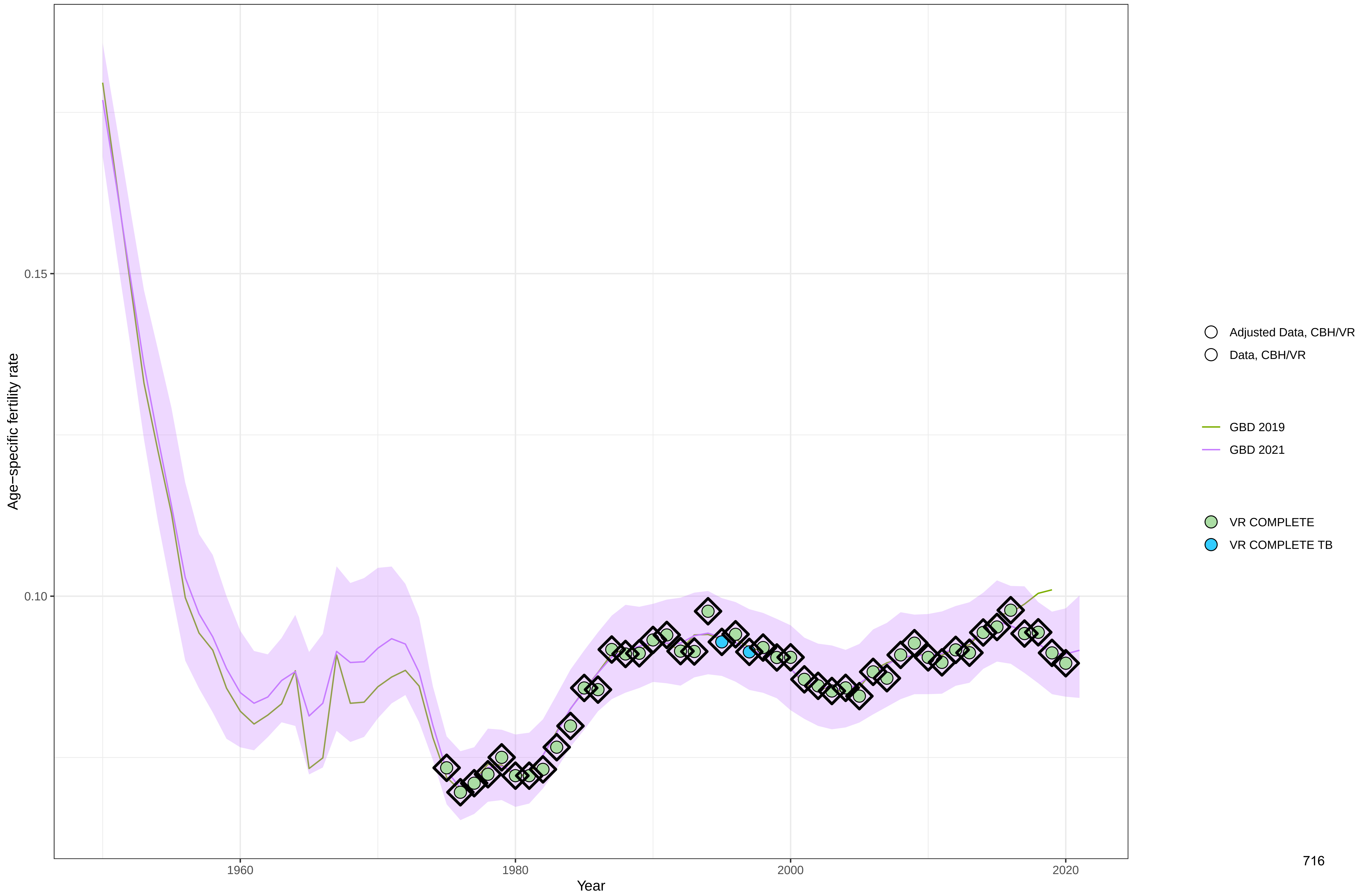

Age−specific fertility fitted trend lines, 1950−2021: Tochigi, Ages: 35−39
Data points enclosed with a diamond are reference sources

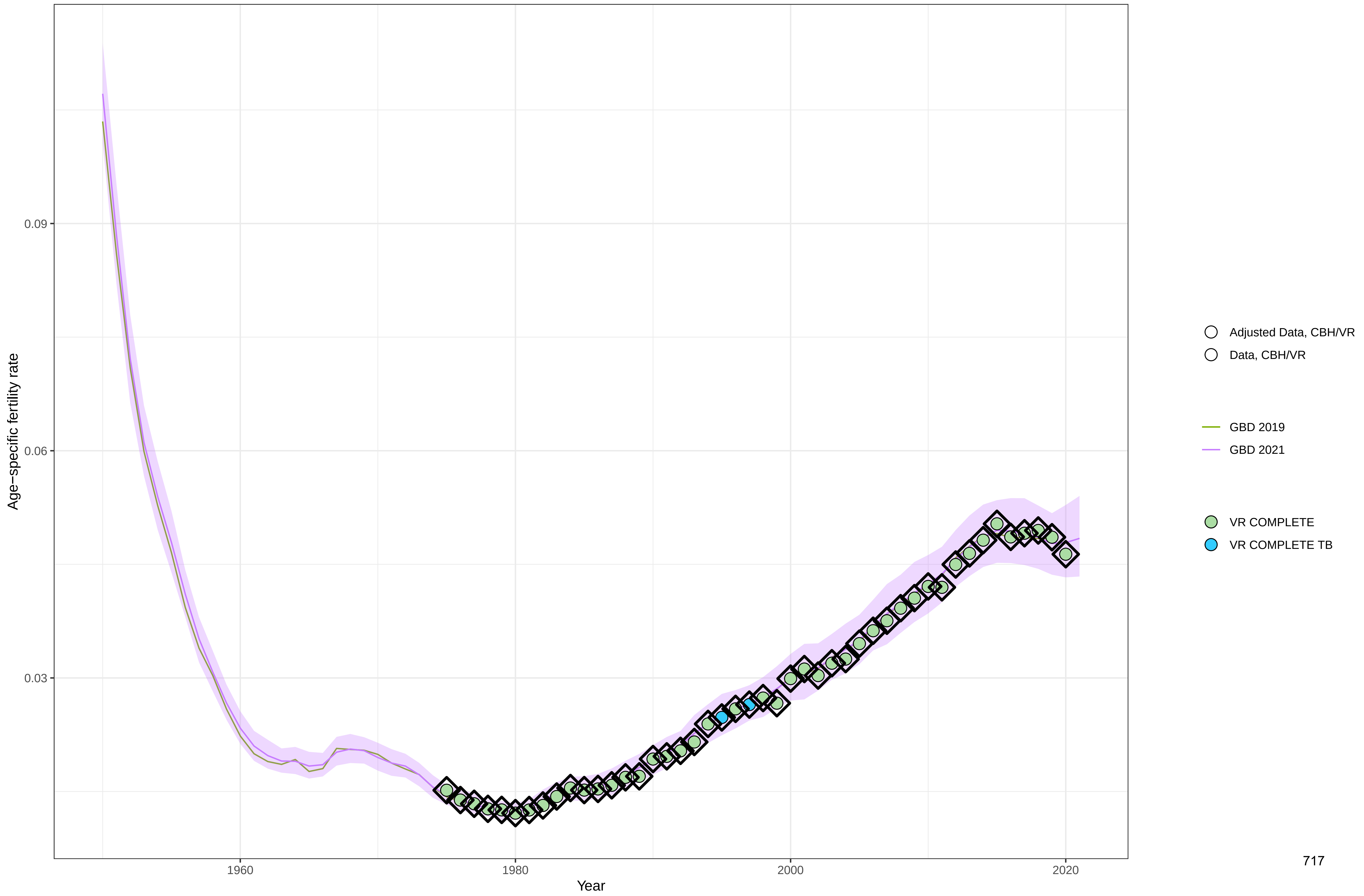

Age−specific fertility fitted trend lines, 1950−2021: Tochigi, Ages: 40−44
Data points enclosed with a diamond are reference sources

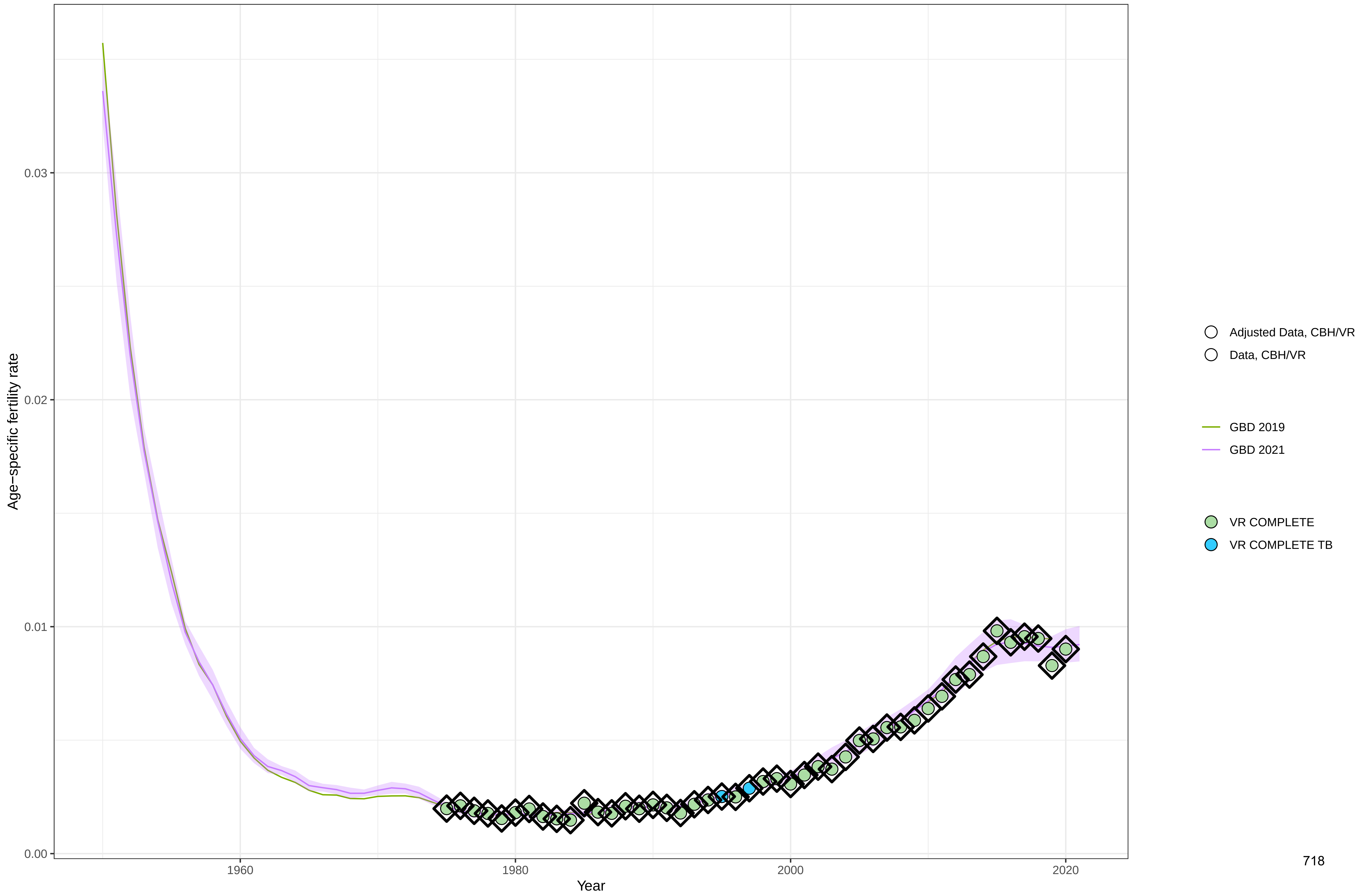

Age−specific fertility fitted trend lines, 1950−2021: Tochigi, Ages: 45−49
Data points enclosed with a diamond are reference sources

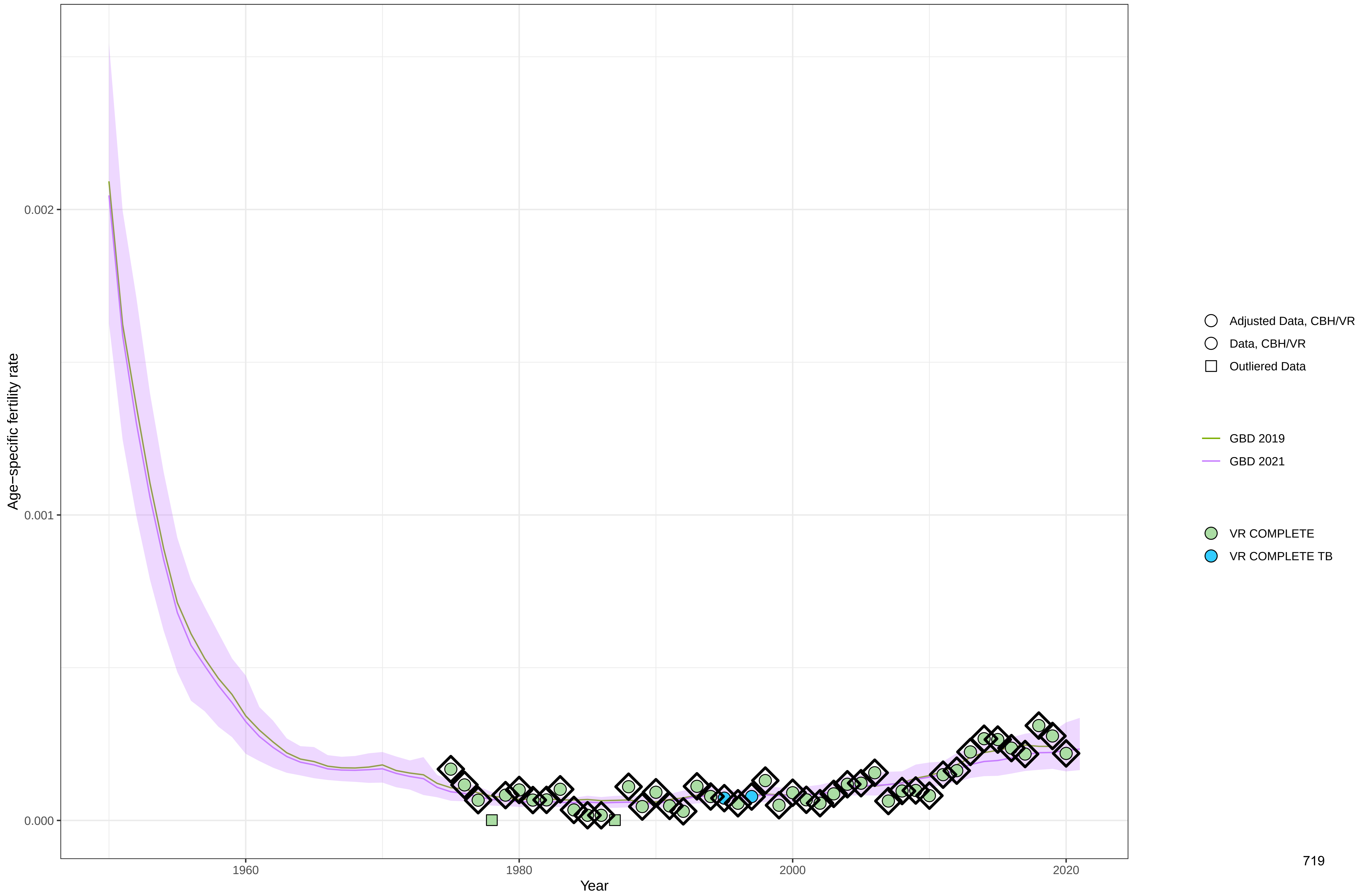

Age−specific fertility fitted trend lines, 1950−2021: Tochigi, Ages: 50−54
Data points enclosed with a diamond are reference sources

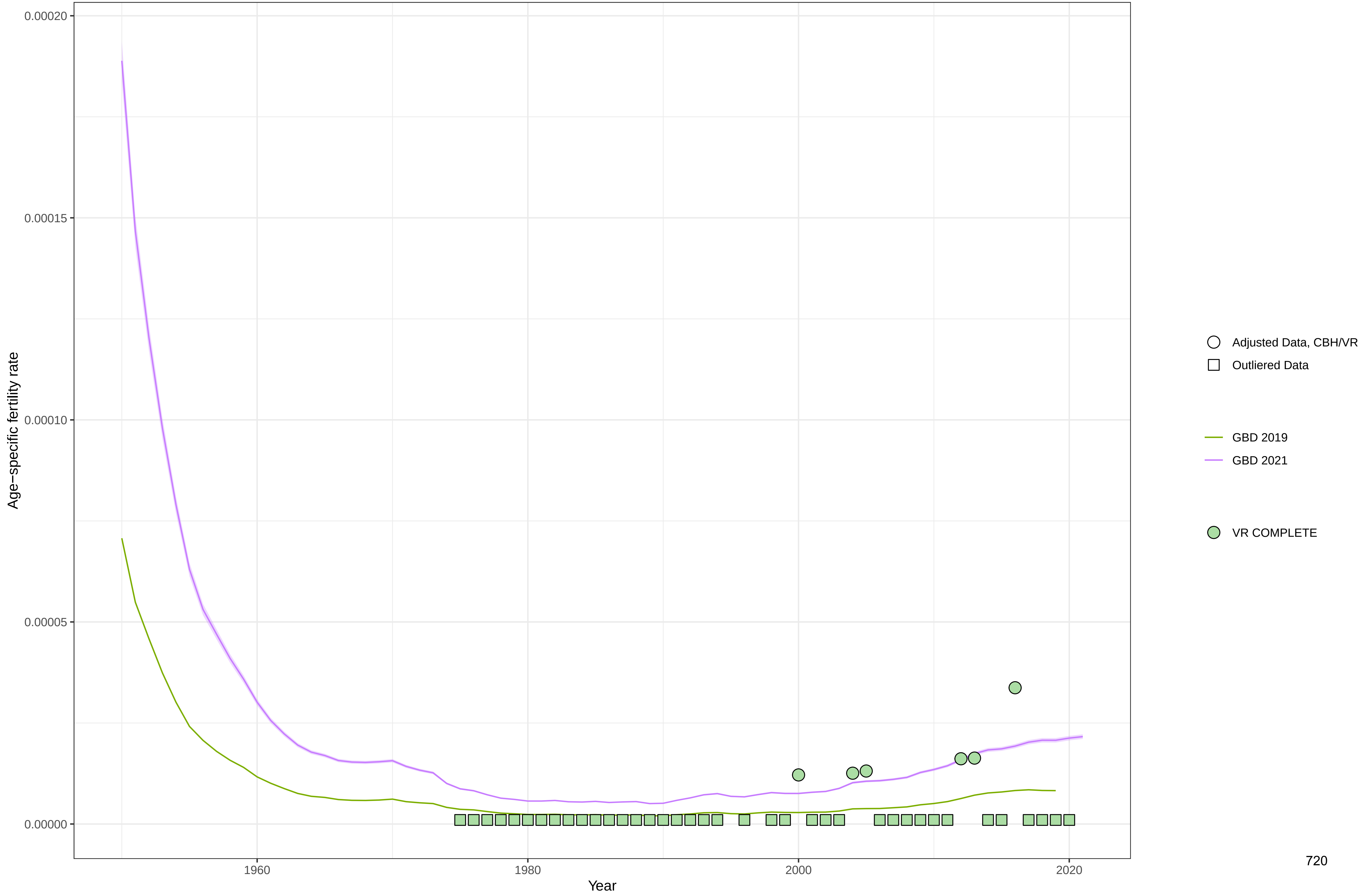

Total fertility rate fitted trend lines, 1950−2021: Tochigi TFR
Data points enclosed with a diamond are reference sources

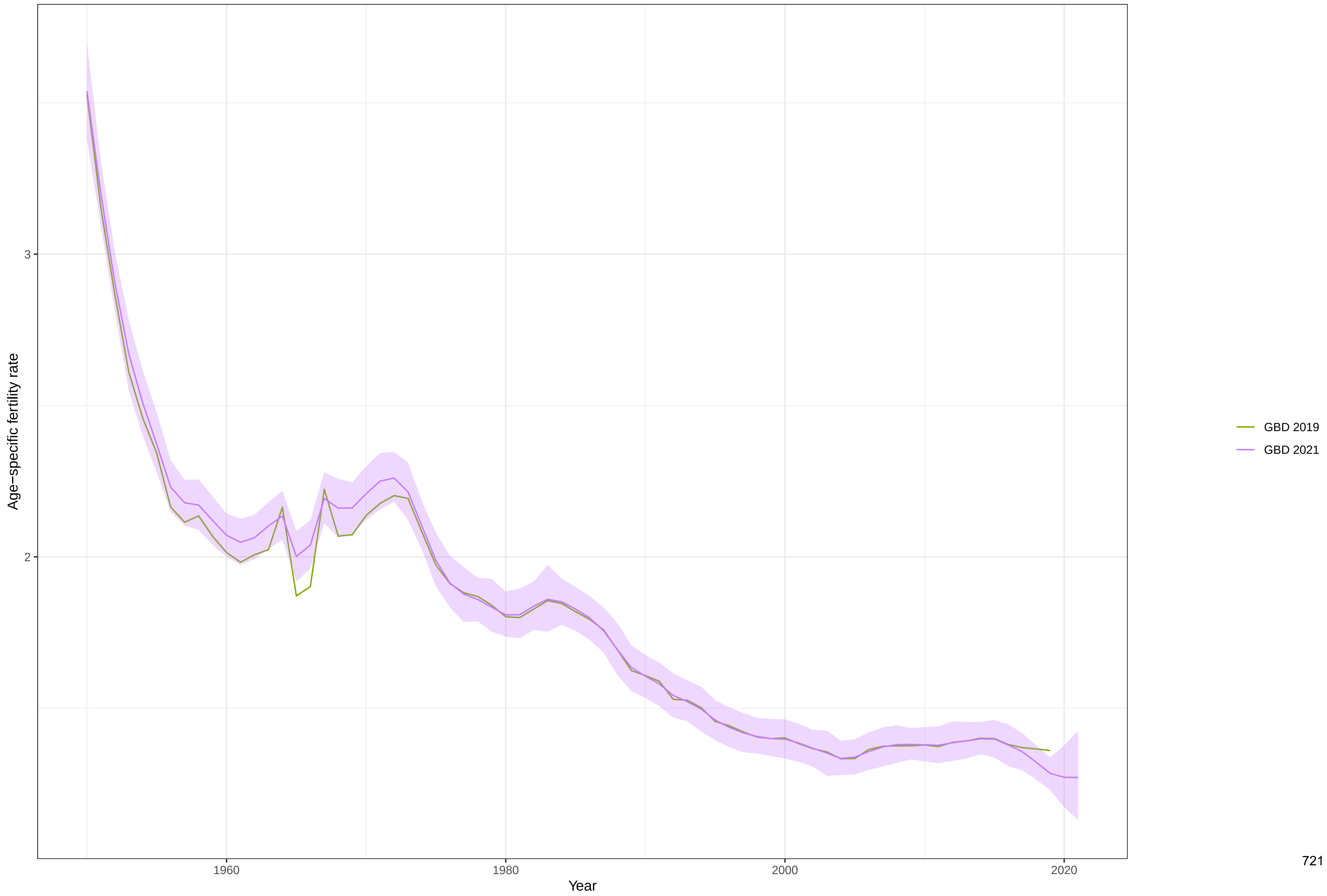

Age−specific fertility fitted trend lines, 1950−2021: Tokushima, Ages: 10−14
Data points enclosed with a diamond are reference sources

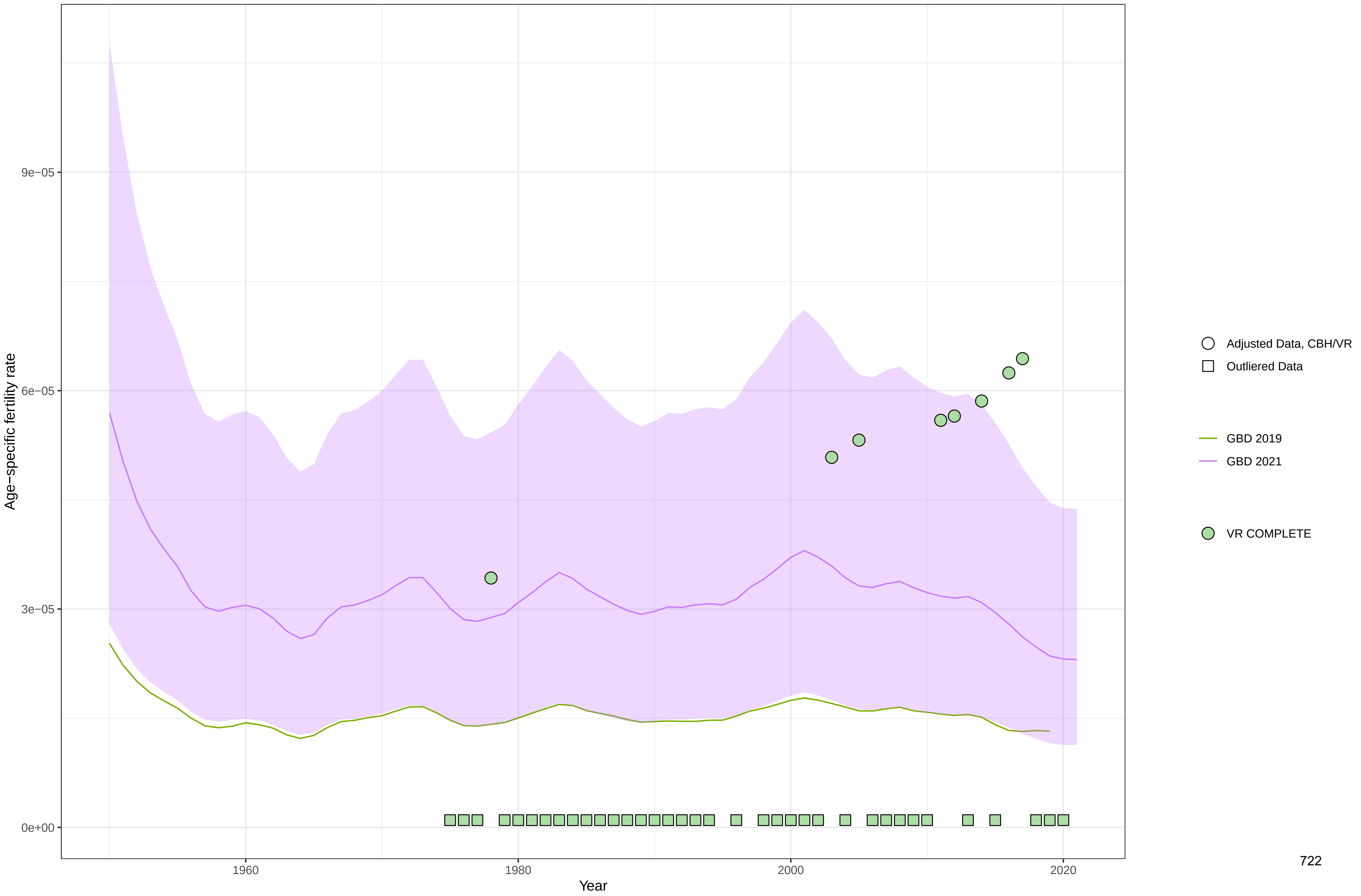

Age−specific fertility fitted trend lines, 1950−2021: Tokushima, Ages: 15−19
Data points enclosed with a diamond are reference sources

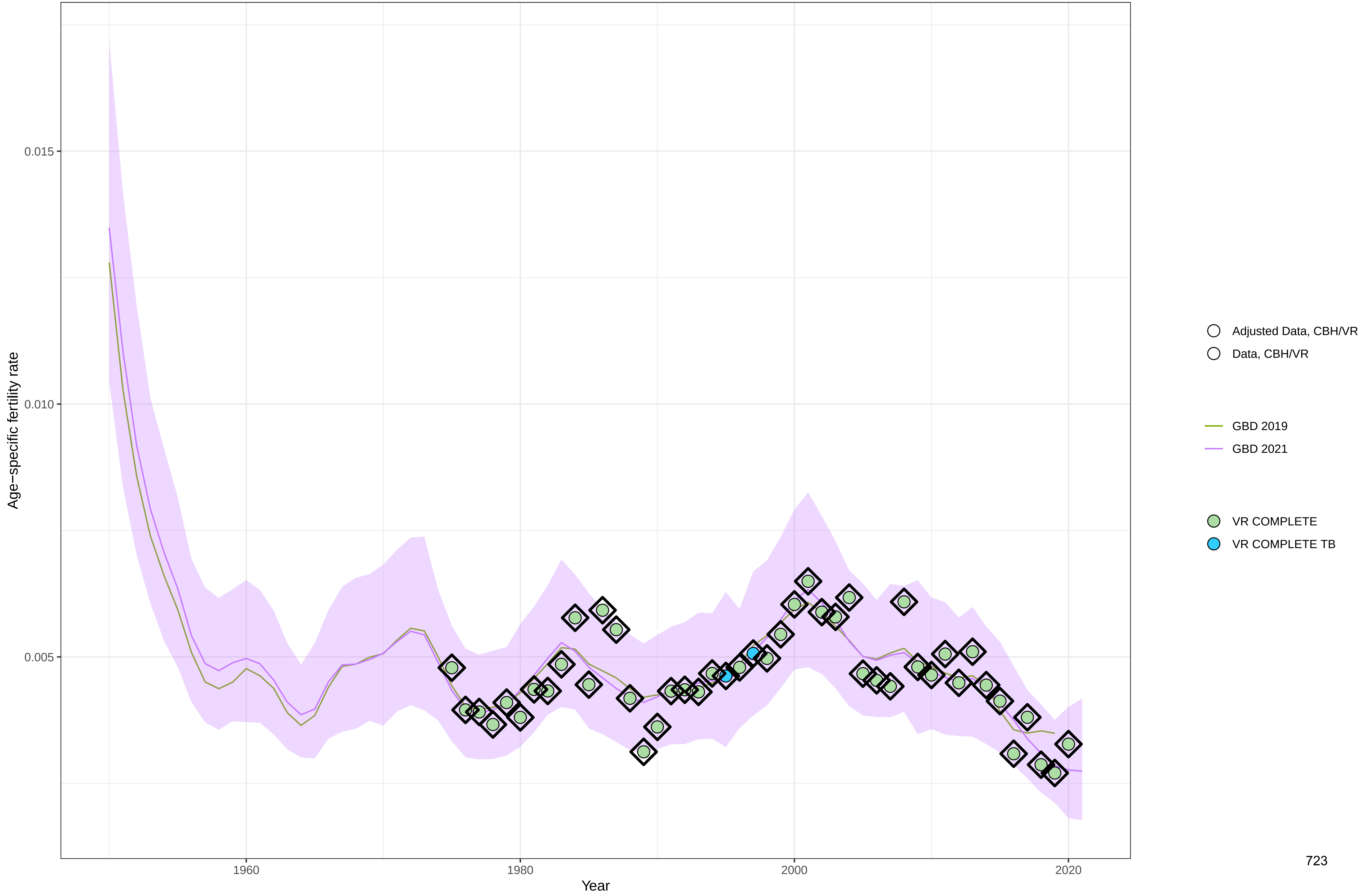

Age−specific fertility fitted trend lines, 1950−2021: Tokushima, Ages: 20−24
Data points enclosed with a diamond are reference sources

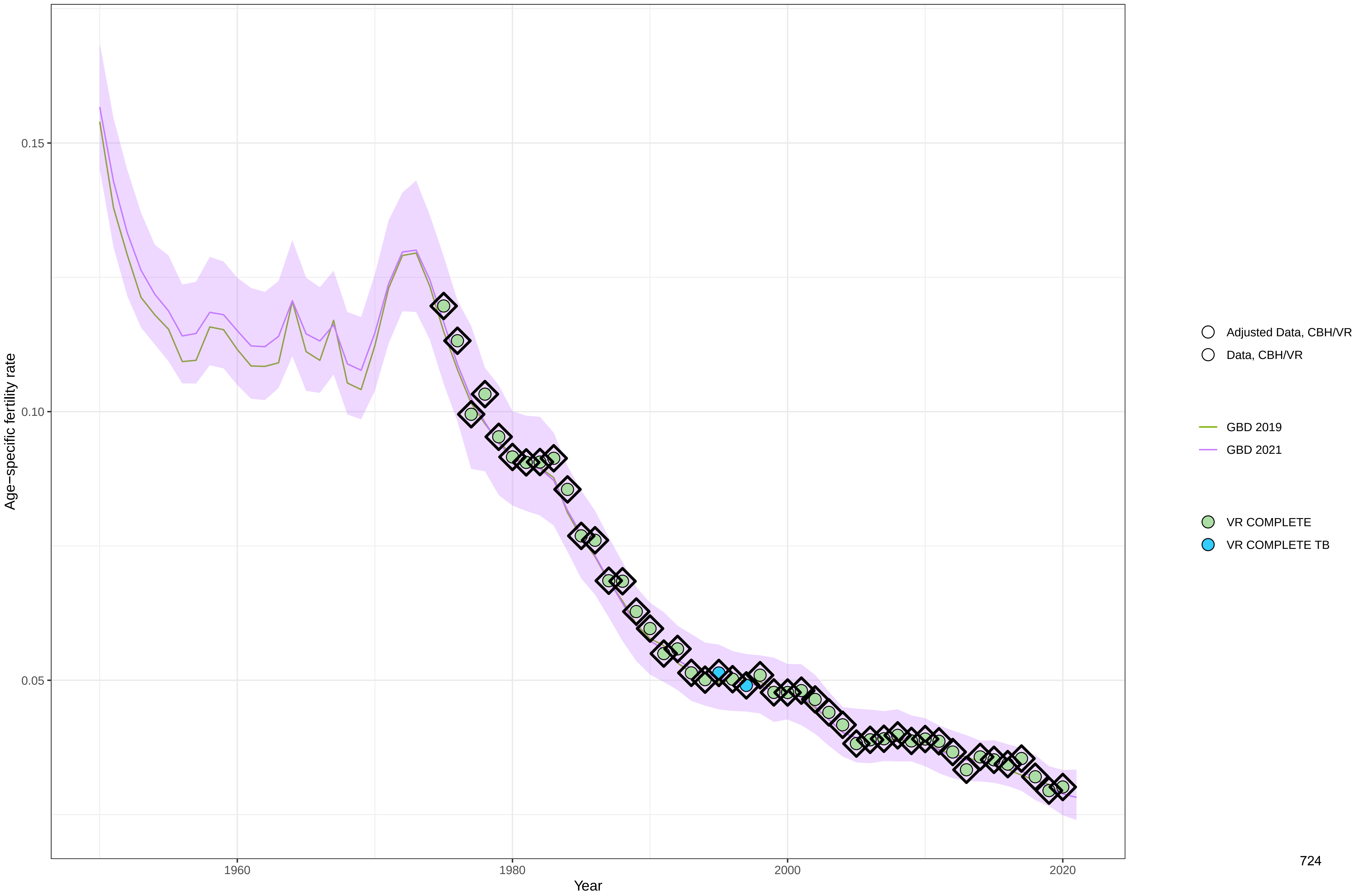

Age−specific fertility fitted trend lines, 1950−2021: Tokushima, Ages: 25−29
Data points enclosed with a diamond are reference sources

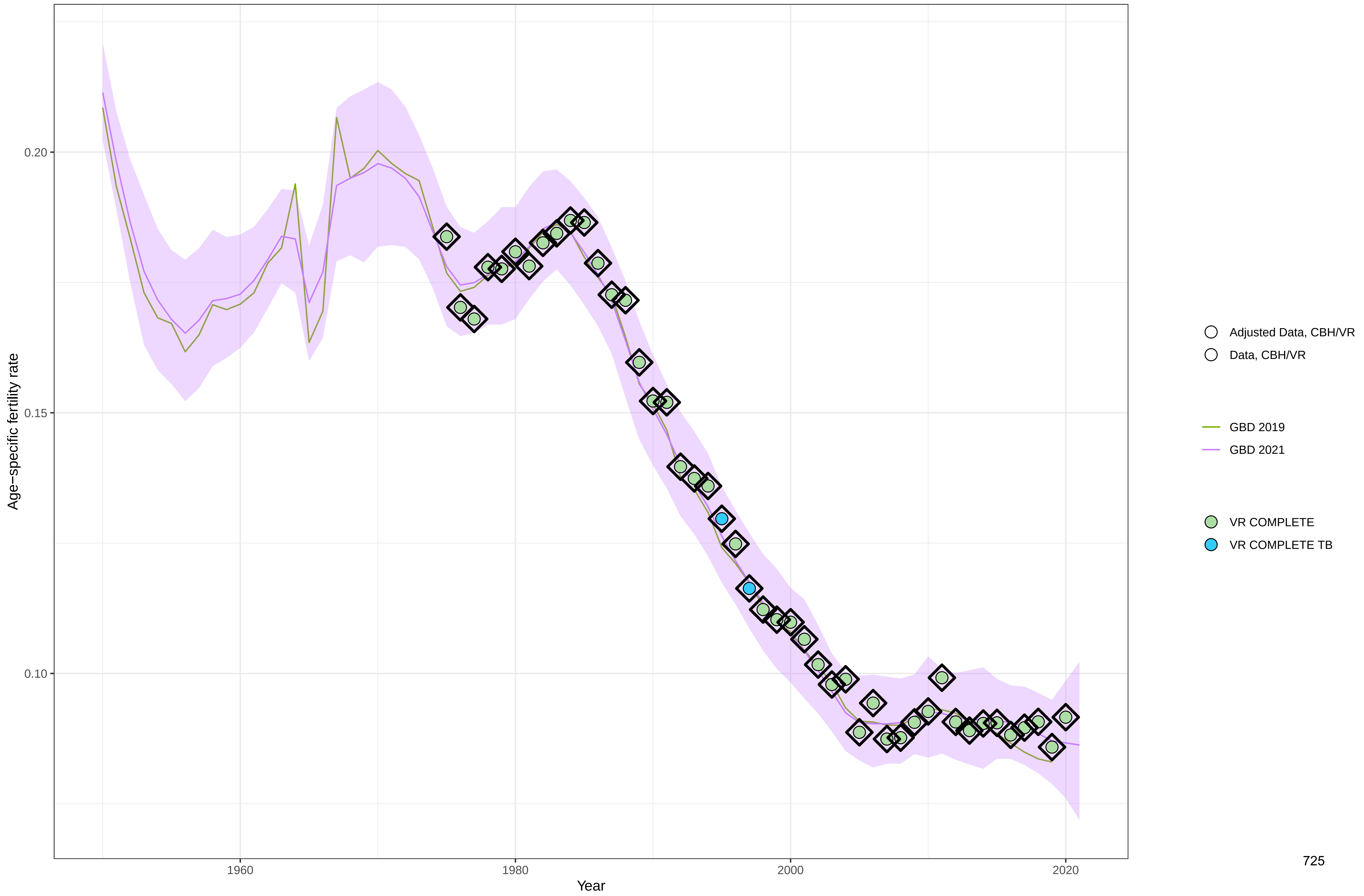

Age−specific fertility fitted trend lines, 1950−2021: Tokushima, Ages: 30−34
Data points enclosed with a diamond are reference sources

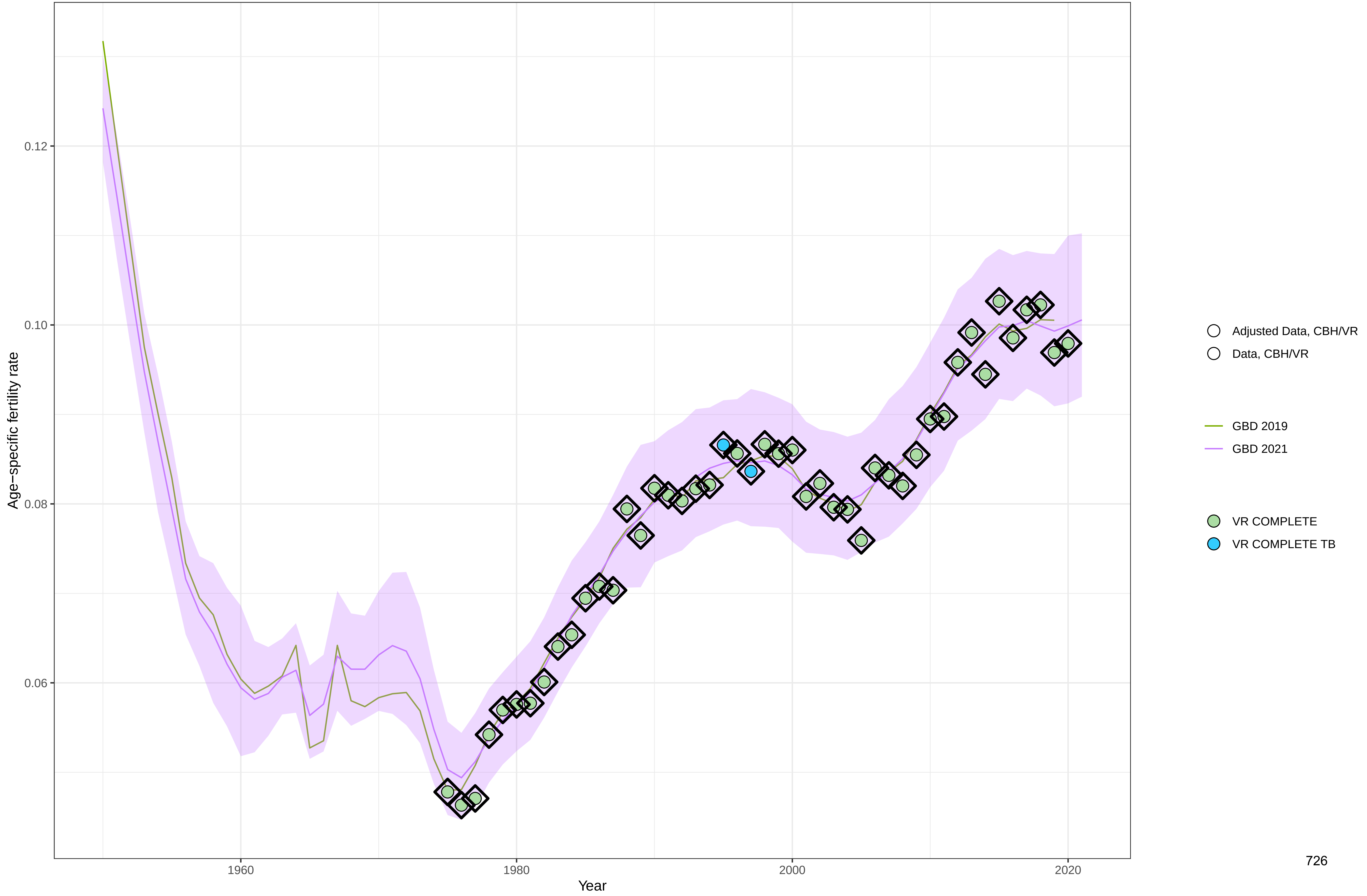

Age−specific fertility fitted trend lines, 1950−2021: Tokushima, Ages: 35−39
Data points enclosed with a diamond are reference sources

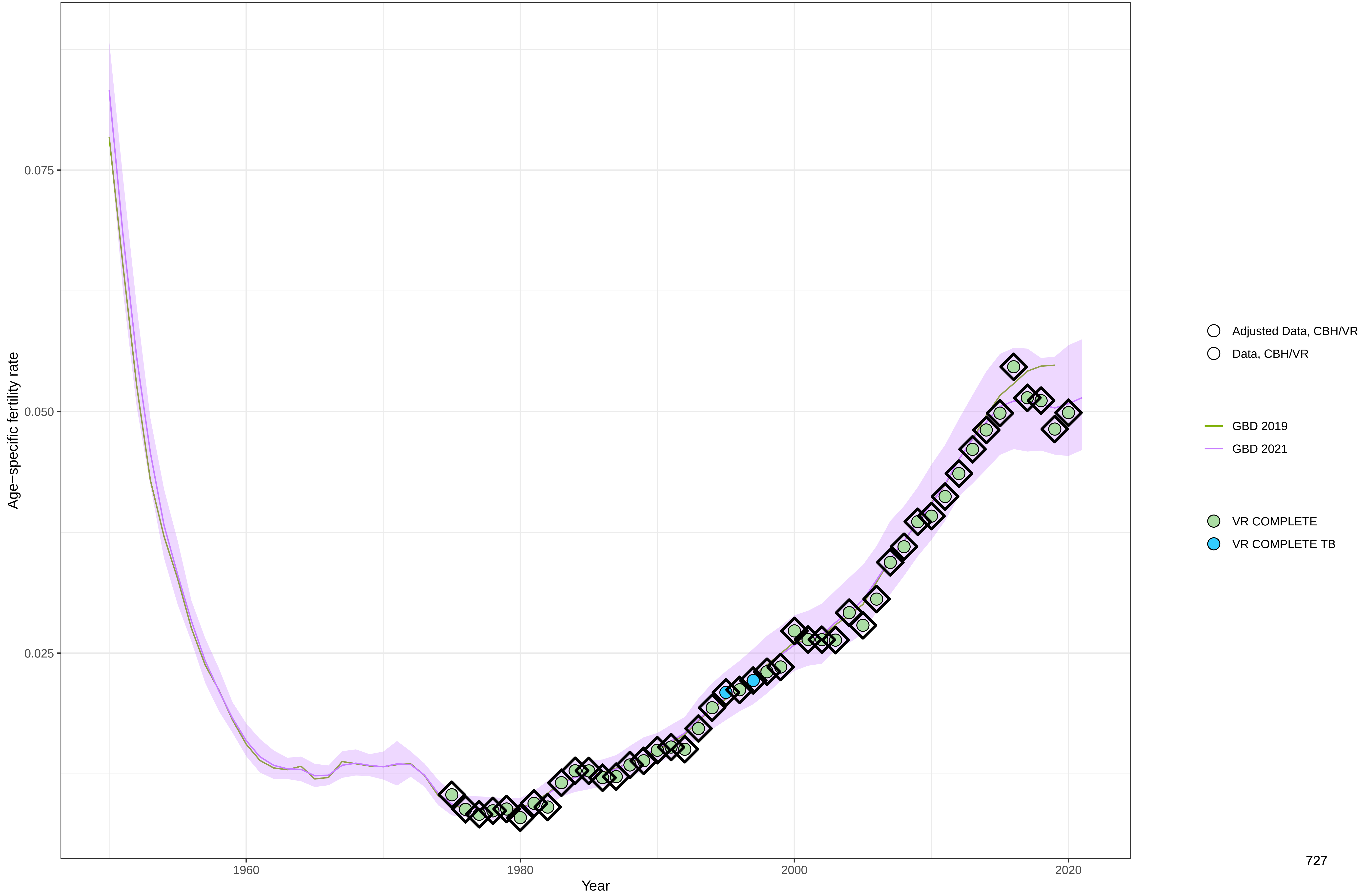

Age−specific fertility fitted trend lines, 1950−2021: Tokushima, Ages: 40−44
Data points enclosed with a diamond are reference sources

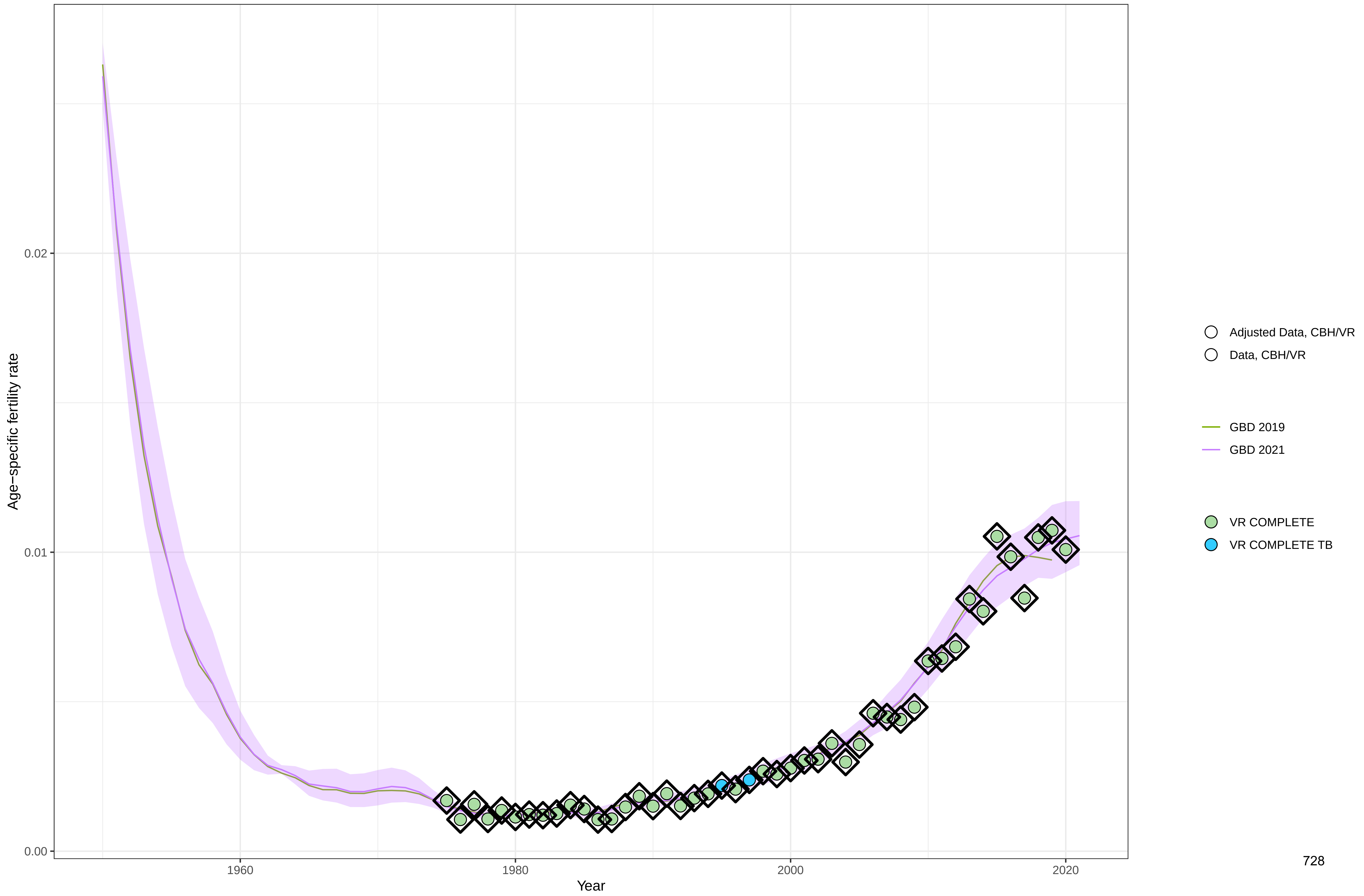

Age−specific fertility fitted trend lines, 1950−2021: Tokushima, Ages: 45−49
Data points enclosed with a diamond are reference sources

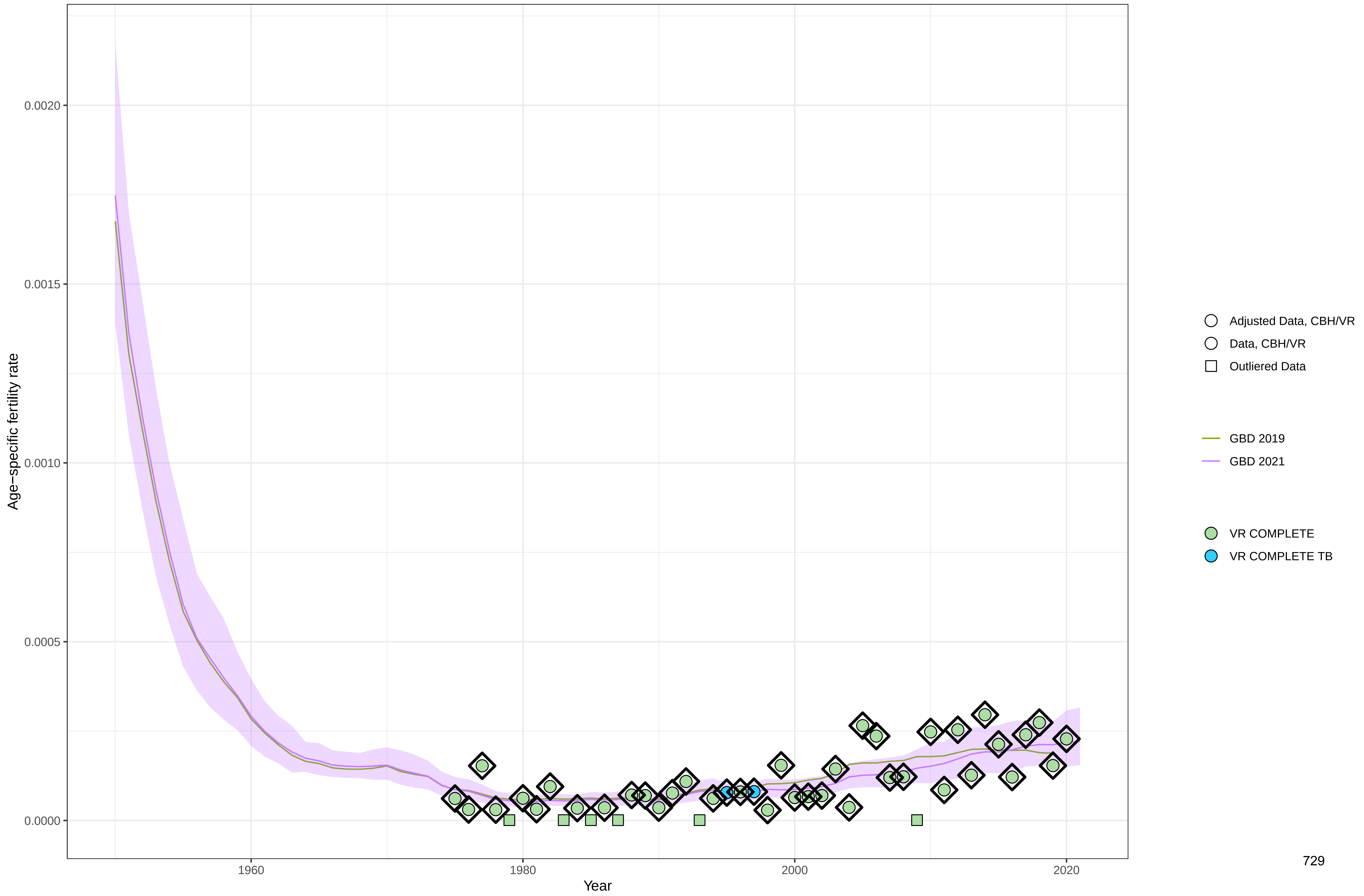

Age−specific fertility fitted trend lines, 1950−2021: Tokushima, Ages: 50−54
Data points enclosed with a diamond are reference sources

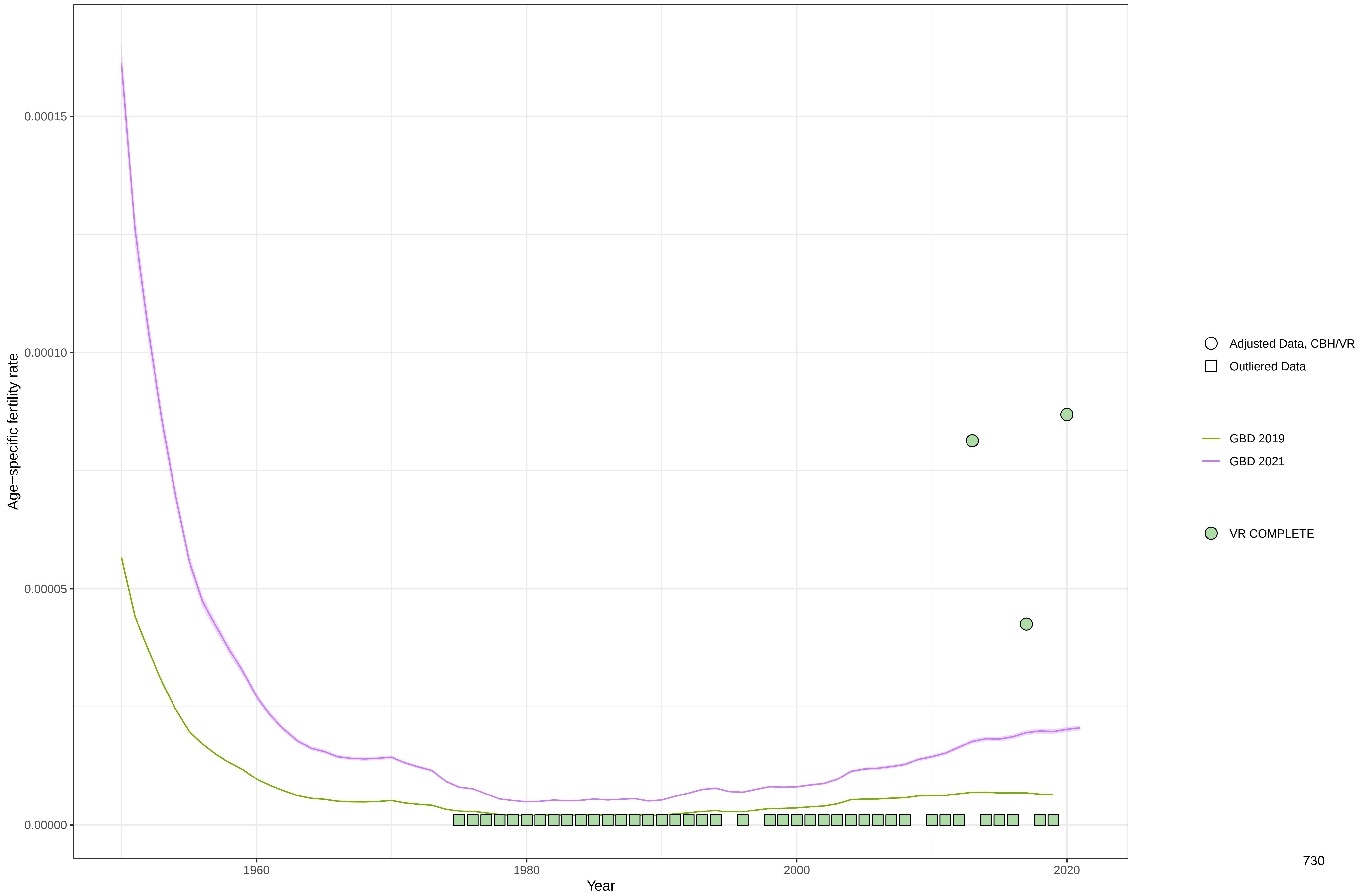

Total fertility rate fitted trend lines, 1950−2021: Tokushima TFR
Data points enclosed with a diamond are reference sources

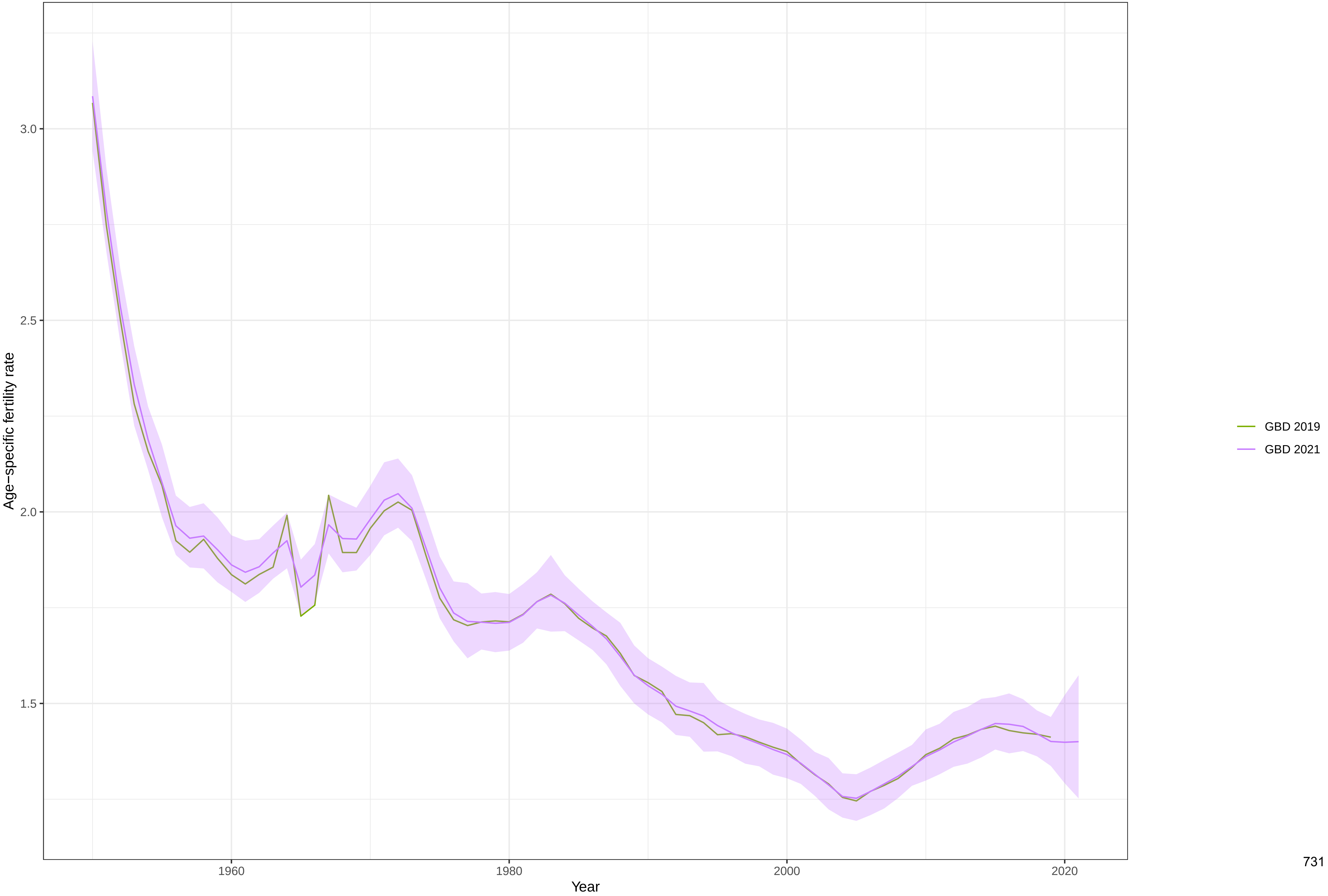

Age−specific fertility fitted trend lines, 1950−2021: T..ky.., Ages: 10−14
Data points enclosed with a diamond are reference sources

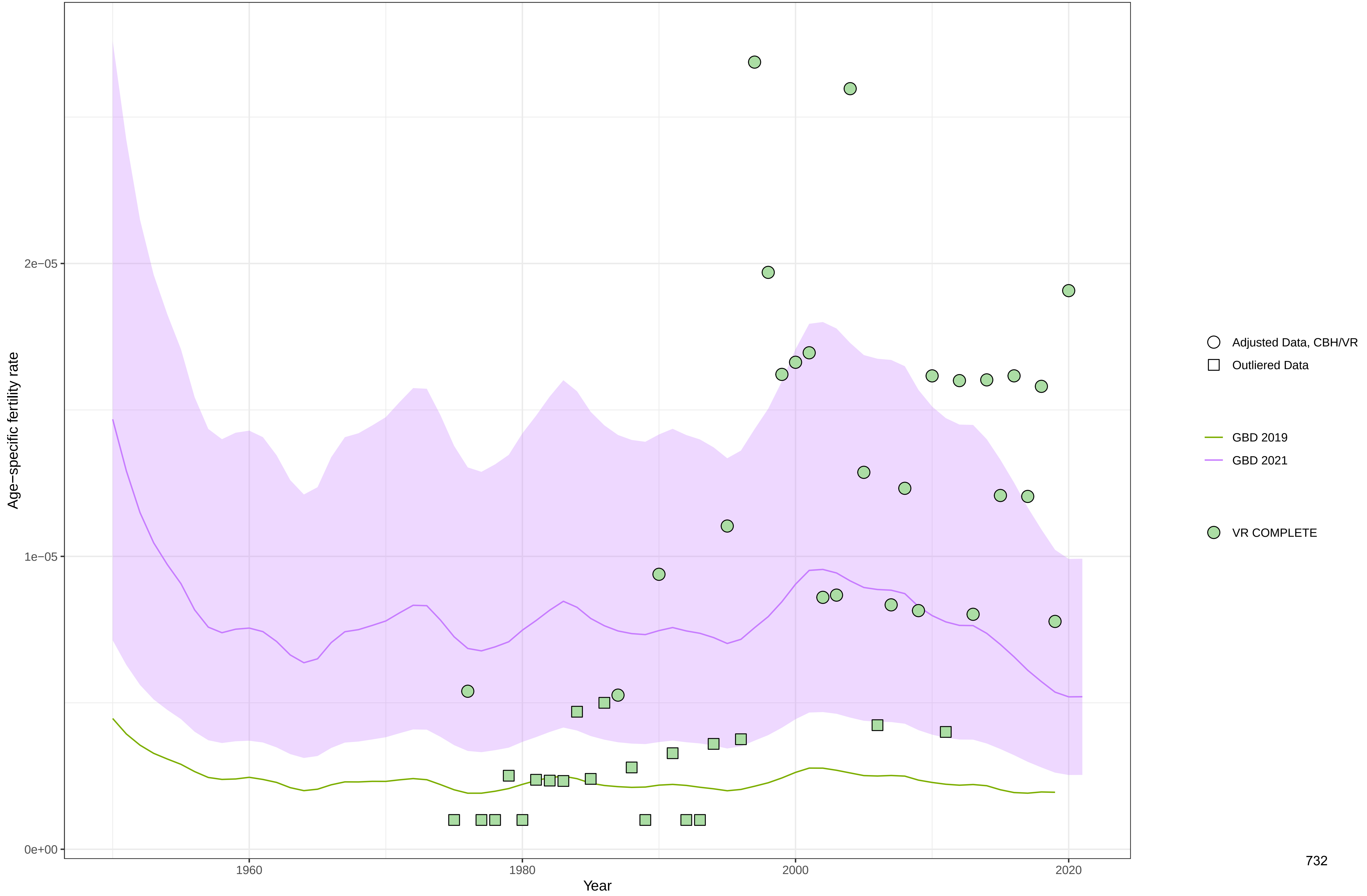

Age−specific fertility fitted trend lines, 1950−2021: T..ky.., Ages: 15−19
Data points enclosed with a diamond are reference sources

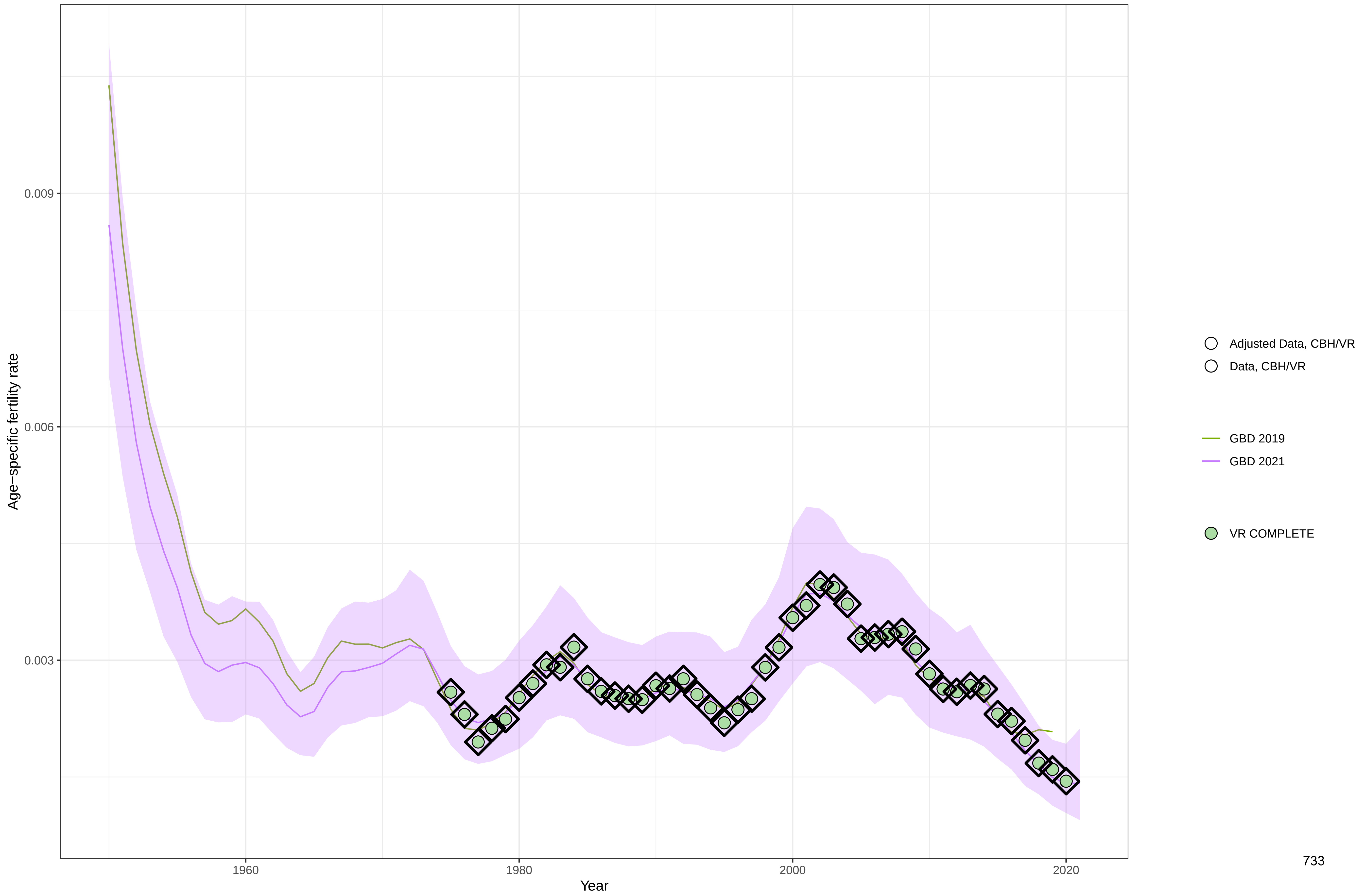

Age−specific fertility fitted trend lines, 1950−2021: T..ky.., Ages: 20−24
Data points enclosed with a diamond are reference sources

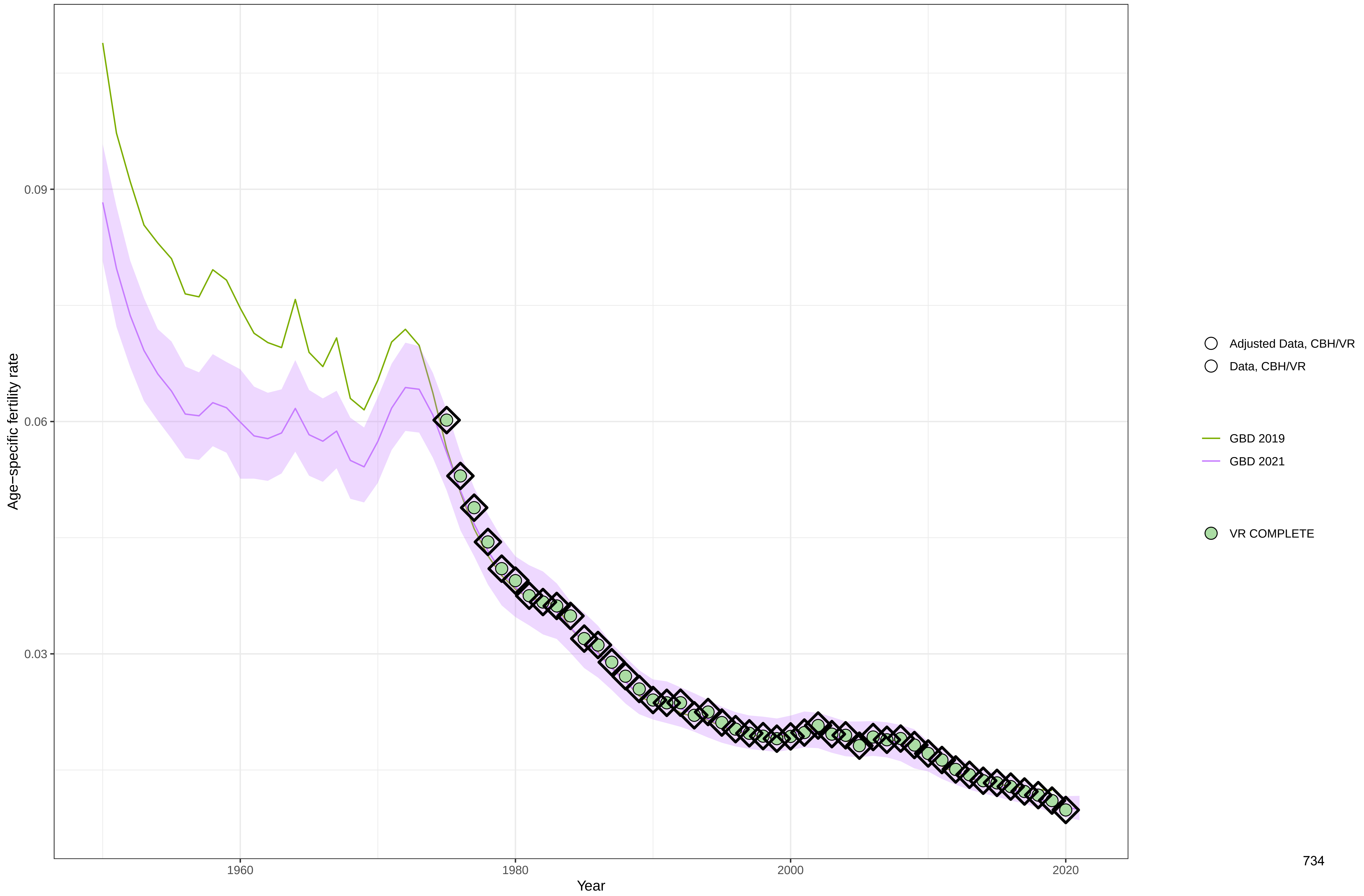

Age−specific fertility fitted trend lines, 1950−2021: T..ky.., Ages: 25−29
Data points enclosed with a diamond are reference sources

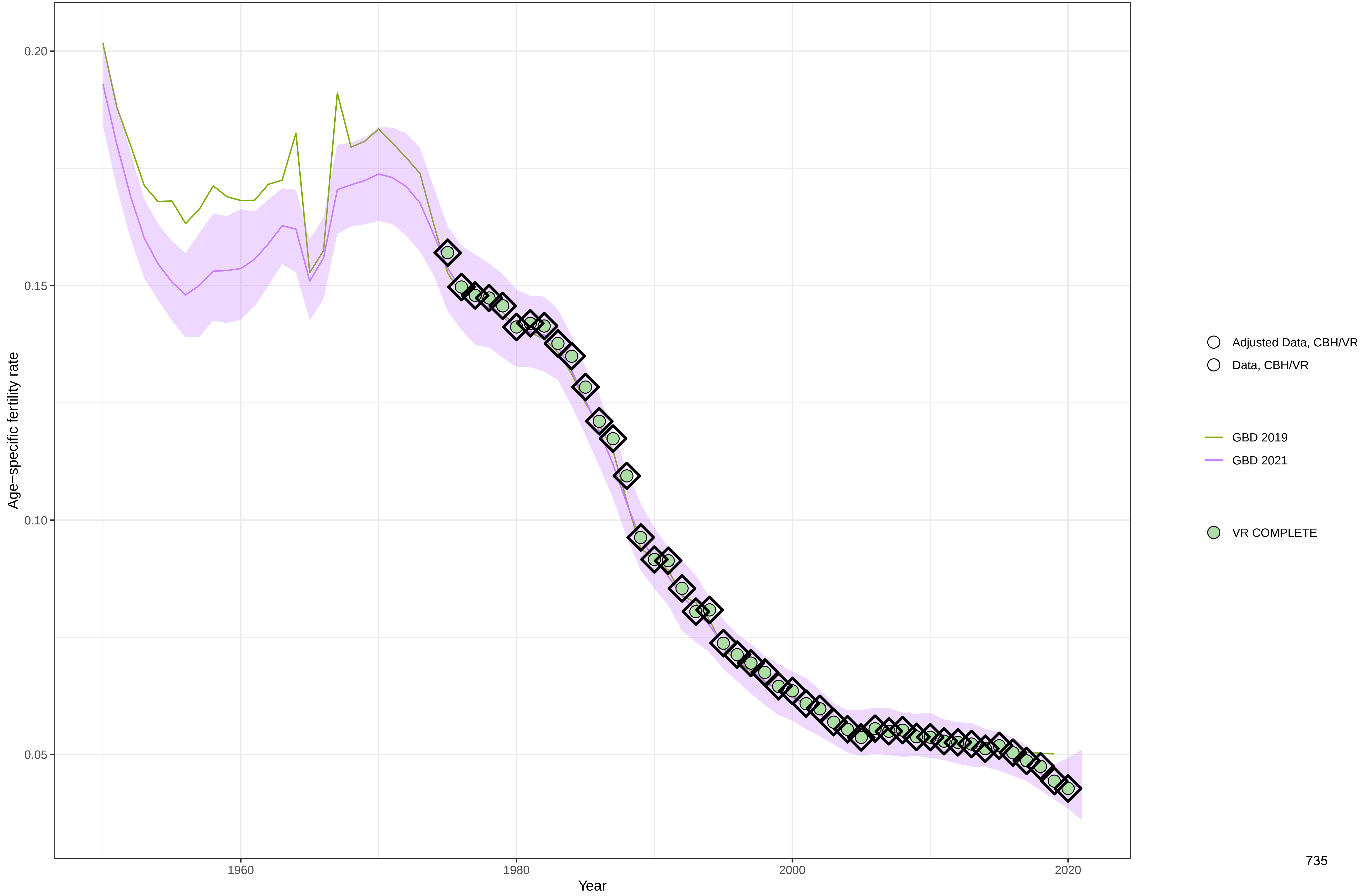

Age−specific fertility fitted trend lines, 1950−2021: T..ky.., Ages: 30−34
Data points enclosed with a diamond are reference sources

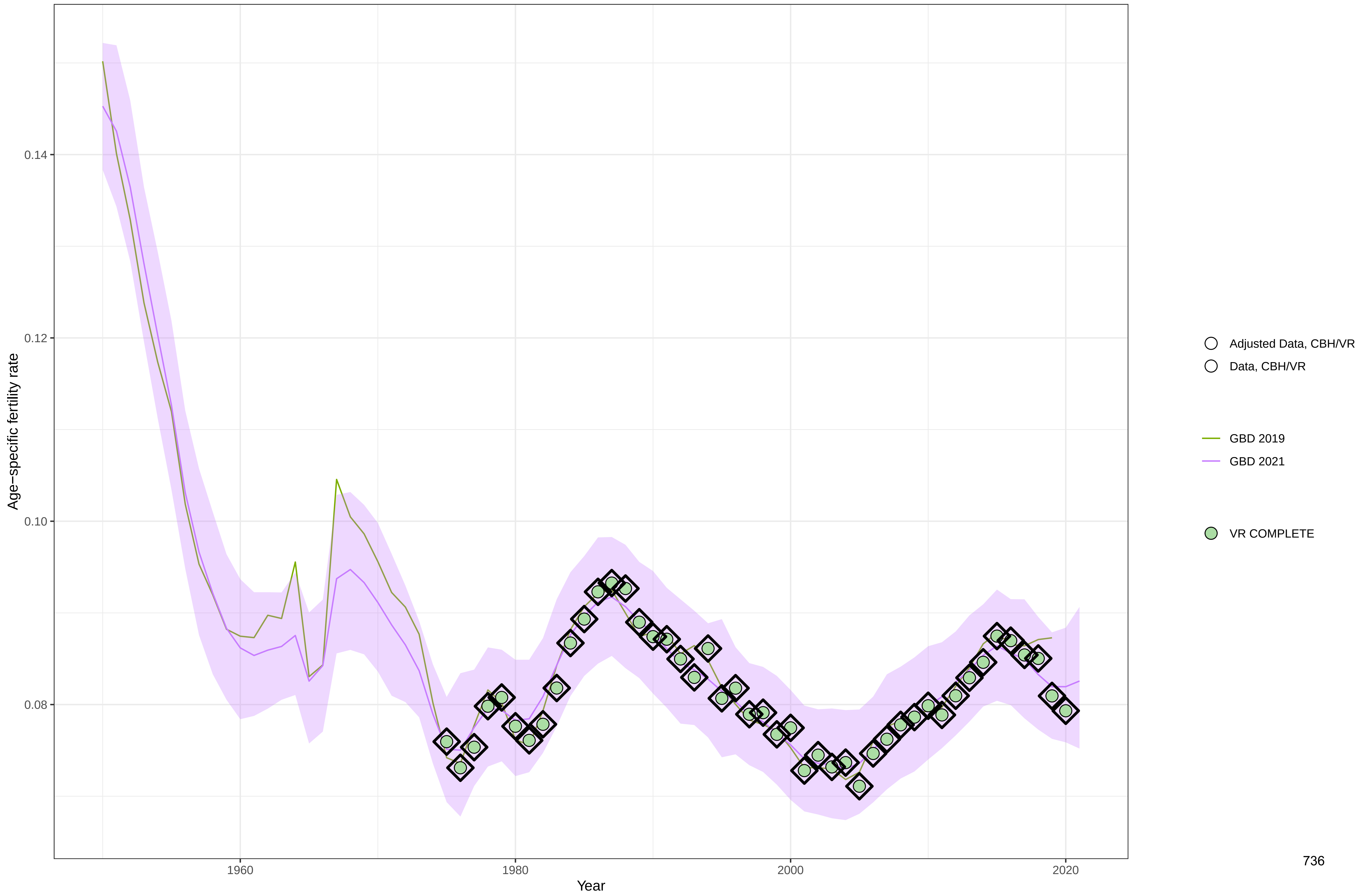

Age−specific fertility fitted trend lines, 1950−2021: T..ky.., Ages: 35−39
Data points enclosed with a diamond are reference sources

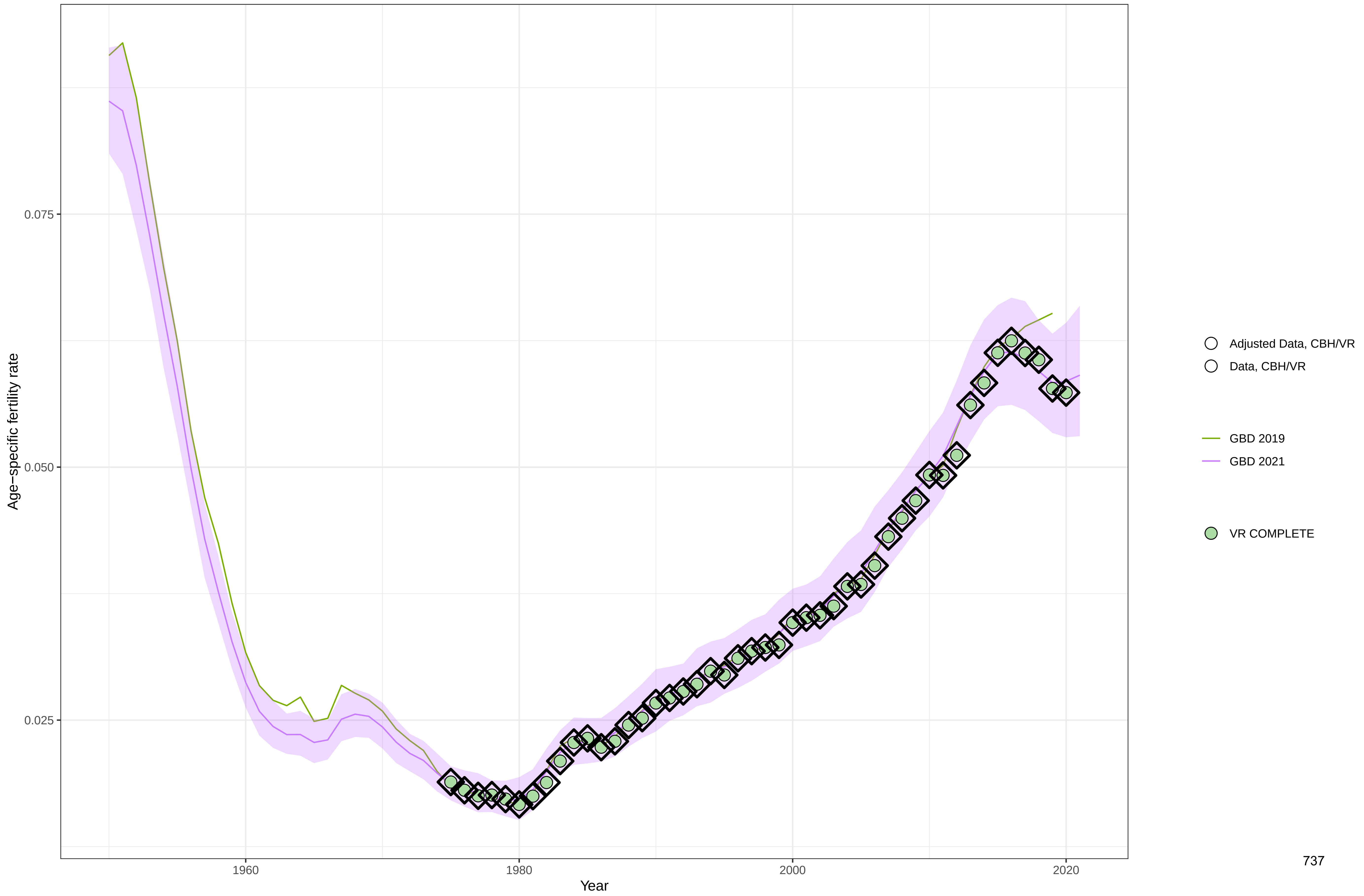

Age−specific fertility fitted trend lines, 1950−2021: T..ky.., Ages: 40−44
Data points enclosed with a diamond are reference sources

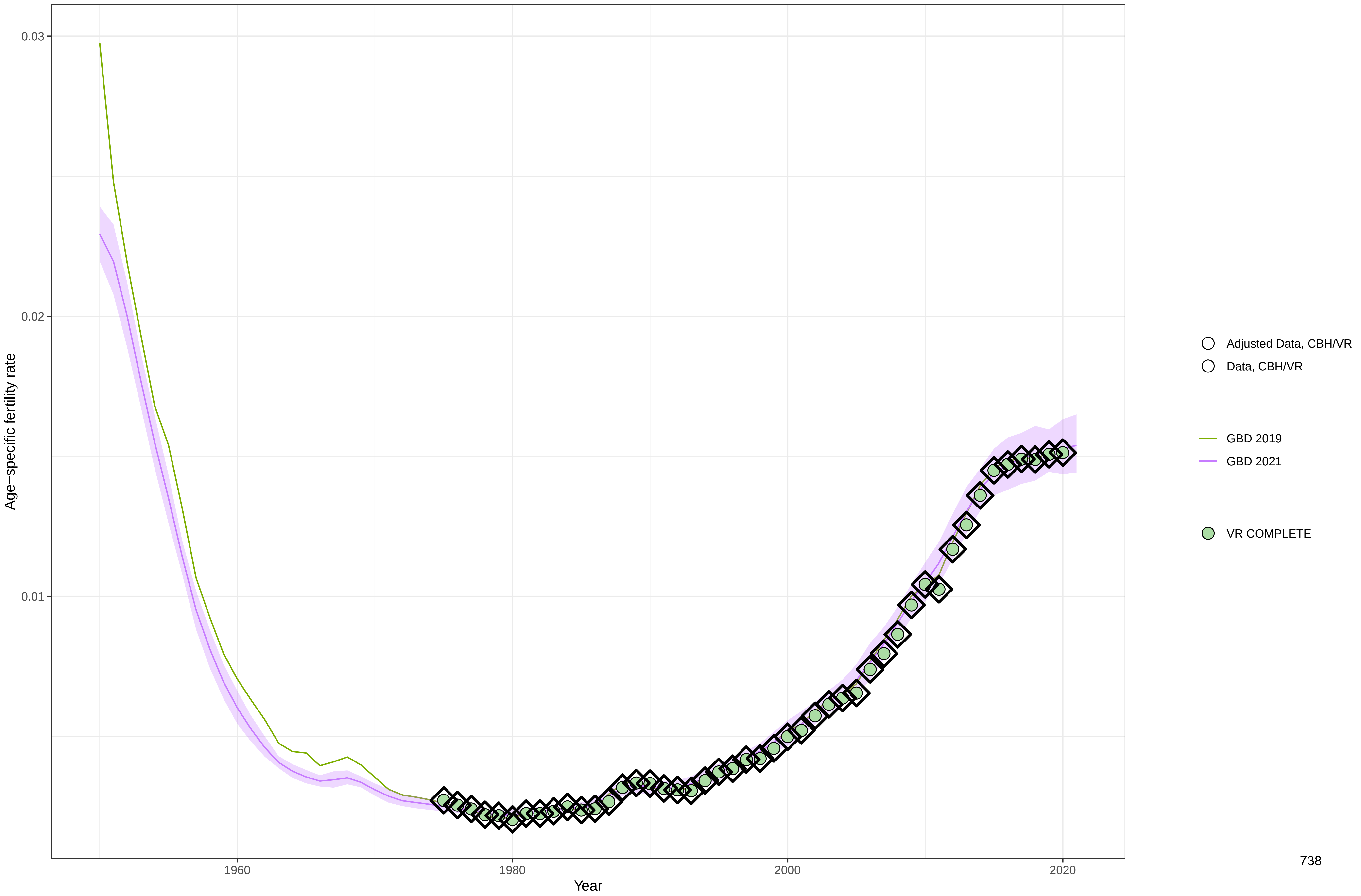

Age−specific fertility fitted trend lines, 1950−2021: T..ky.., Ages: 45−49
Data points enclosed with a diamond are reference sources

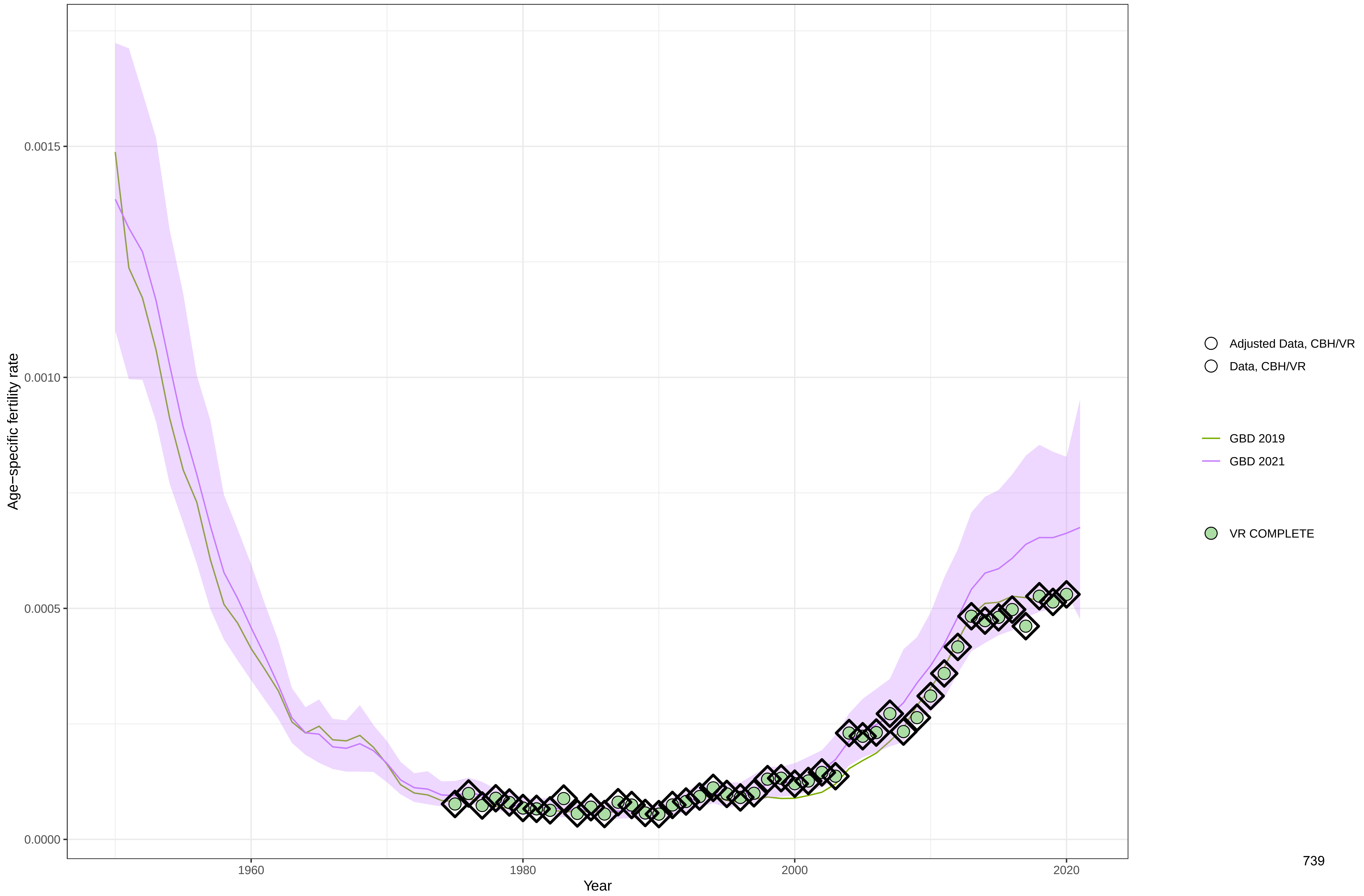

Age−specific fertility fitted trend lines, 1950−2021: T..ky.., Ages: 50−54
Data points enclosed with a diamond are reference sources

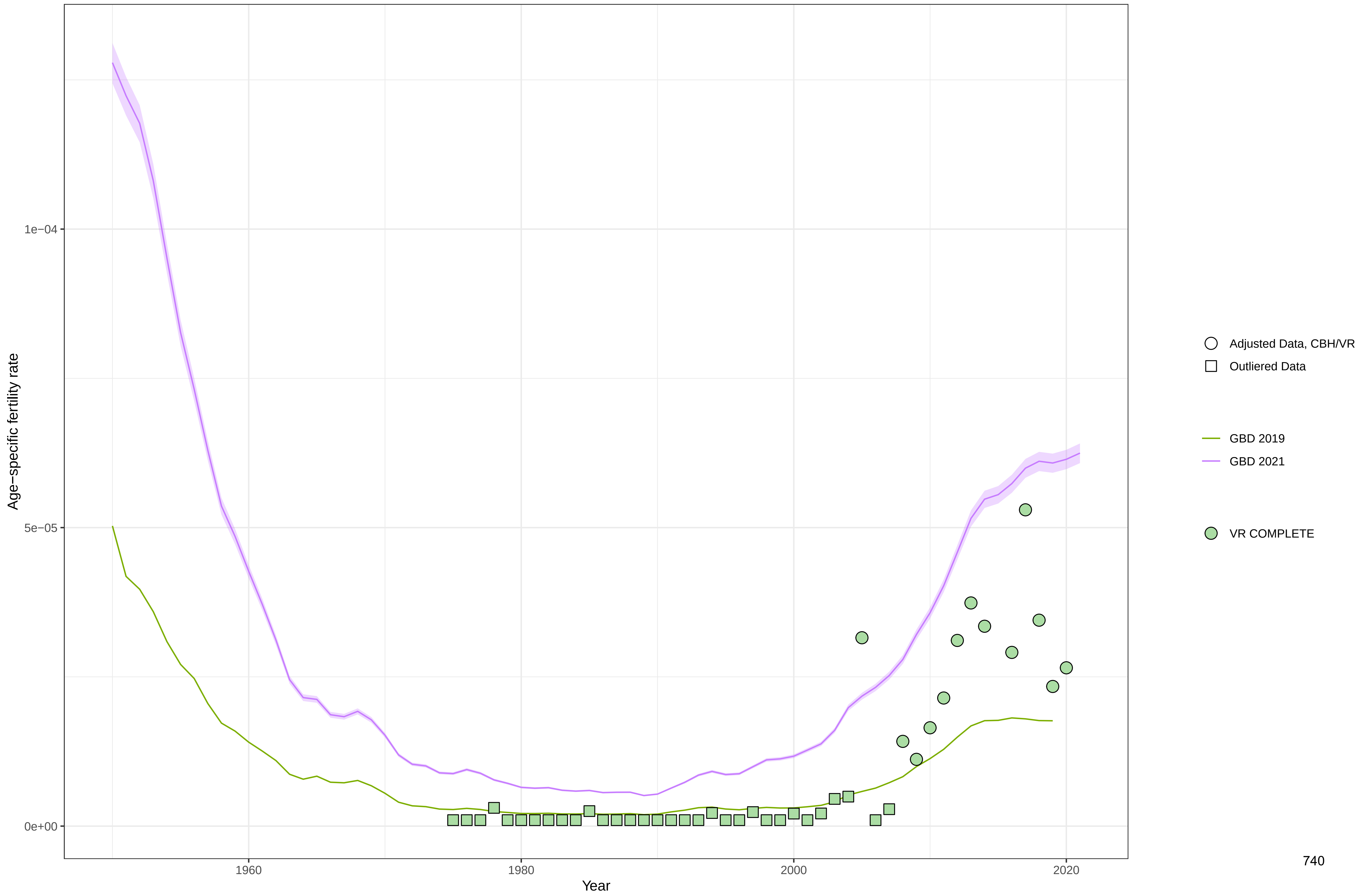

Total fertility rate fitted trend lines, 1950−2021: T..ky.. TFR
Data points enclosed with a diamond are reference sources

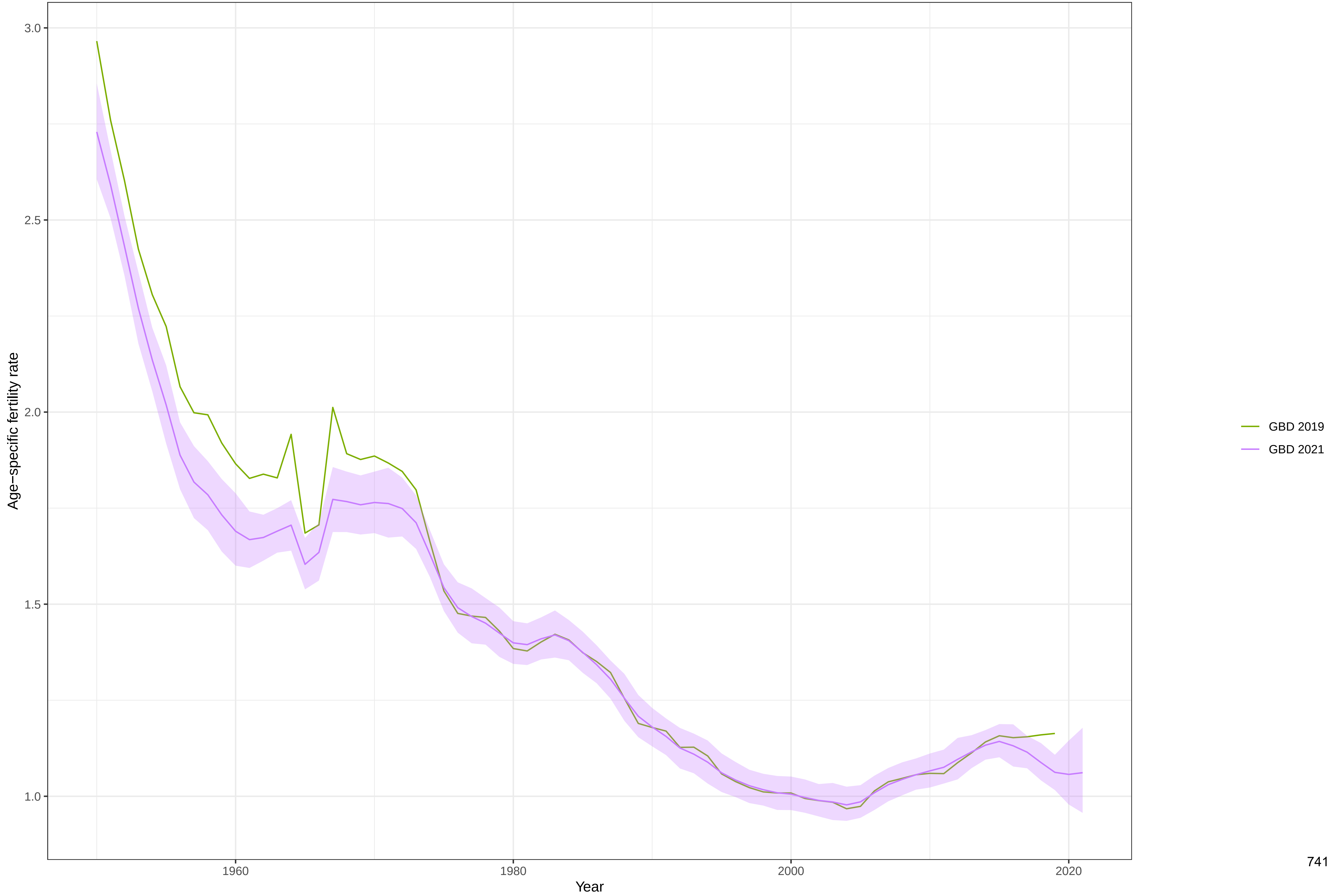

Age−specific fertility fitted trend lines, 1950−2021: Tottori, Ages: 10−14
Data points enclosed with a diamond are reference sources

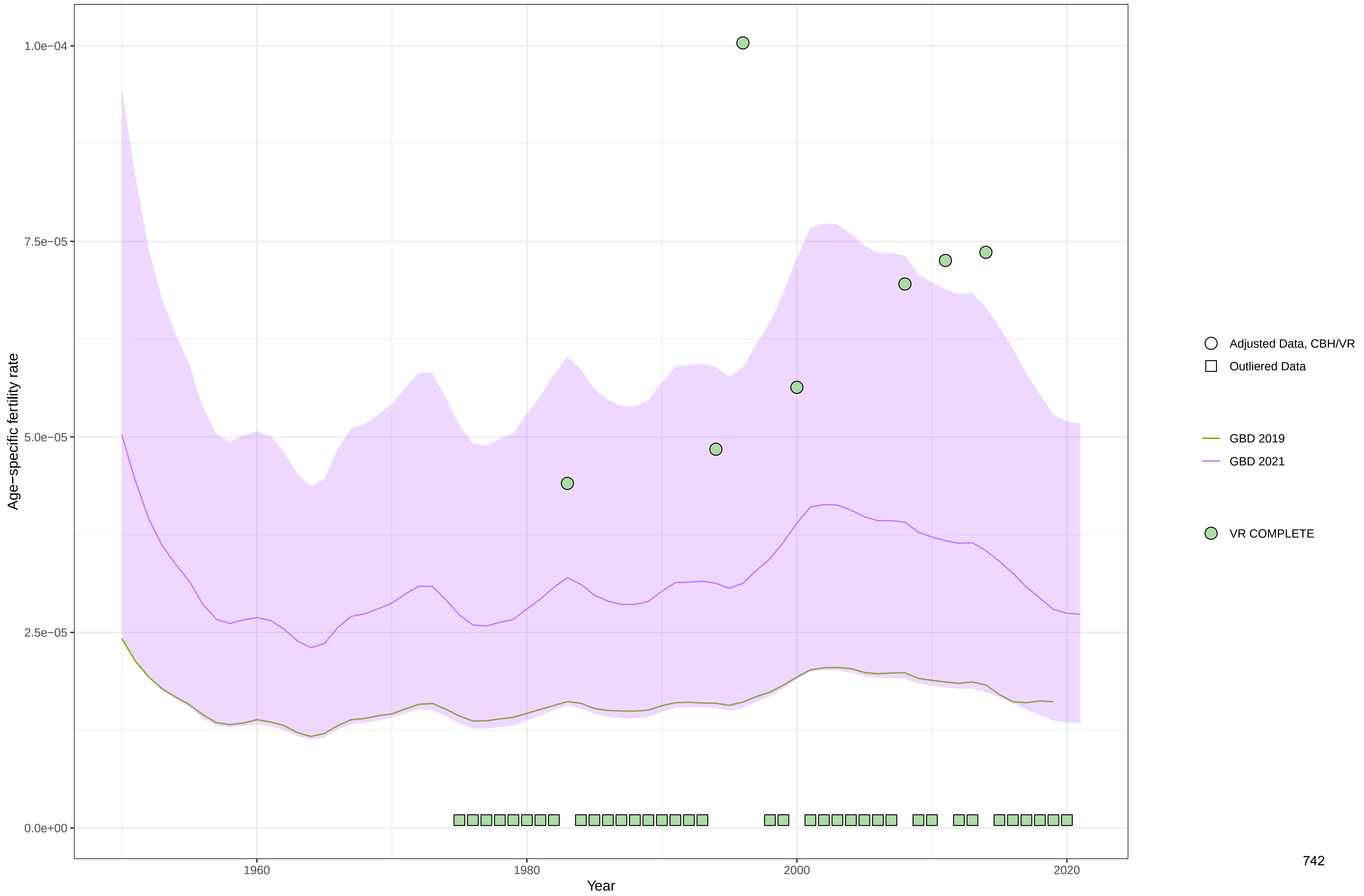

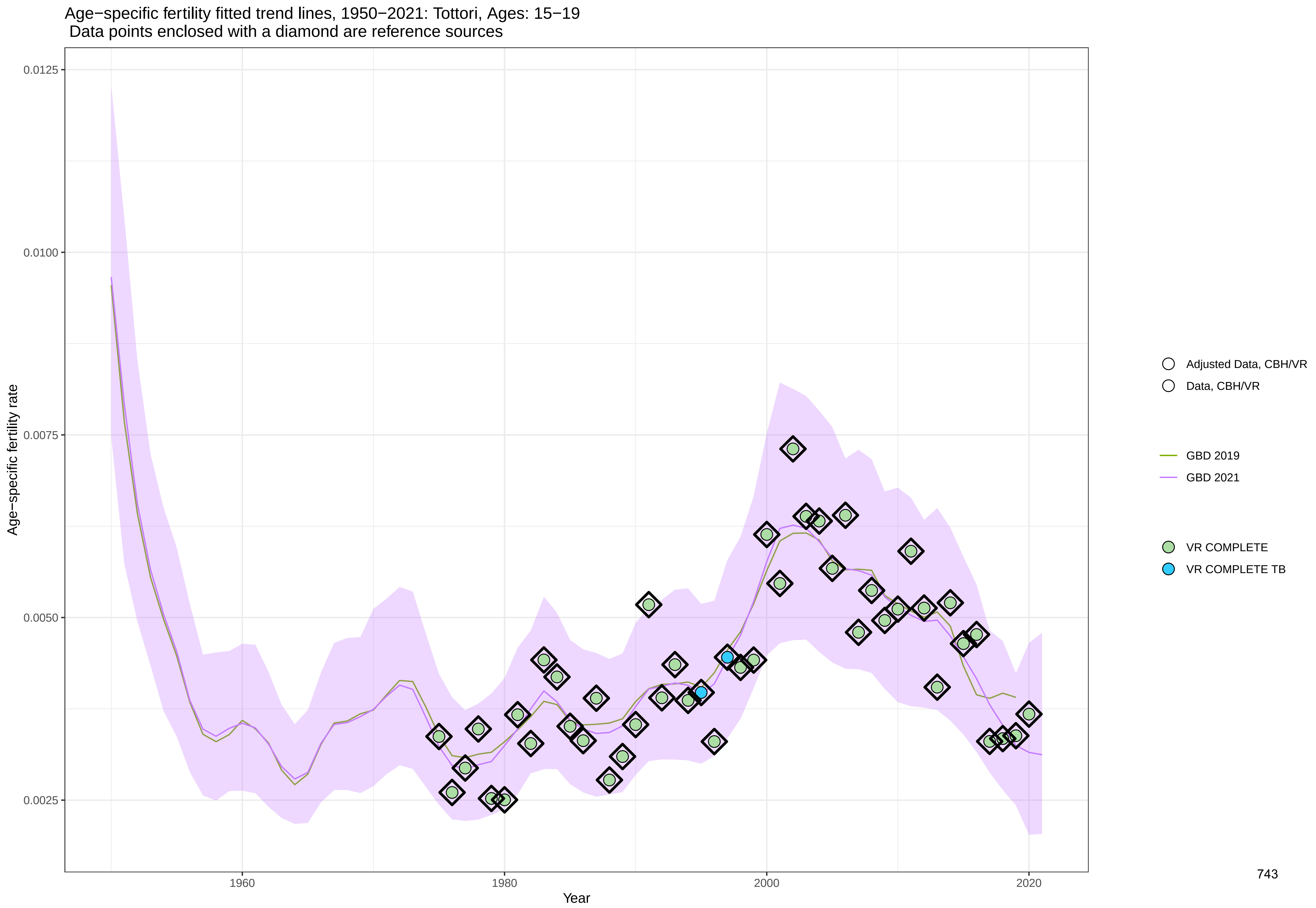
Age−specific fertility fitted trend lines, 1950−2021: Tottori, Ages: 15−19
Data points enclosed with a diamond are reference sources
Age−specific fertility rate
0.0125
0.0100
0.0075
0.0050
0.0025
1960
1980
2000
2020
Year
Adjusted Data, CBH/VR
Data, CBH/VR
GBD 2019
GBD 2021
VR COMPLETE
VR COMPLETE TB

Age−specific fertility fitted trend lines, 1950−2021: Tottori, Ages: 20−24
Data points enclosed with a diamond are reference sources

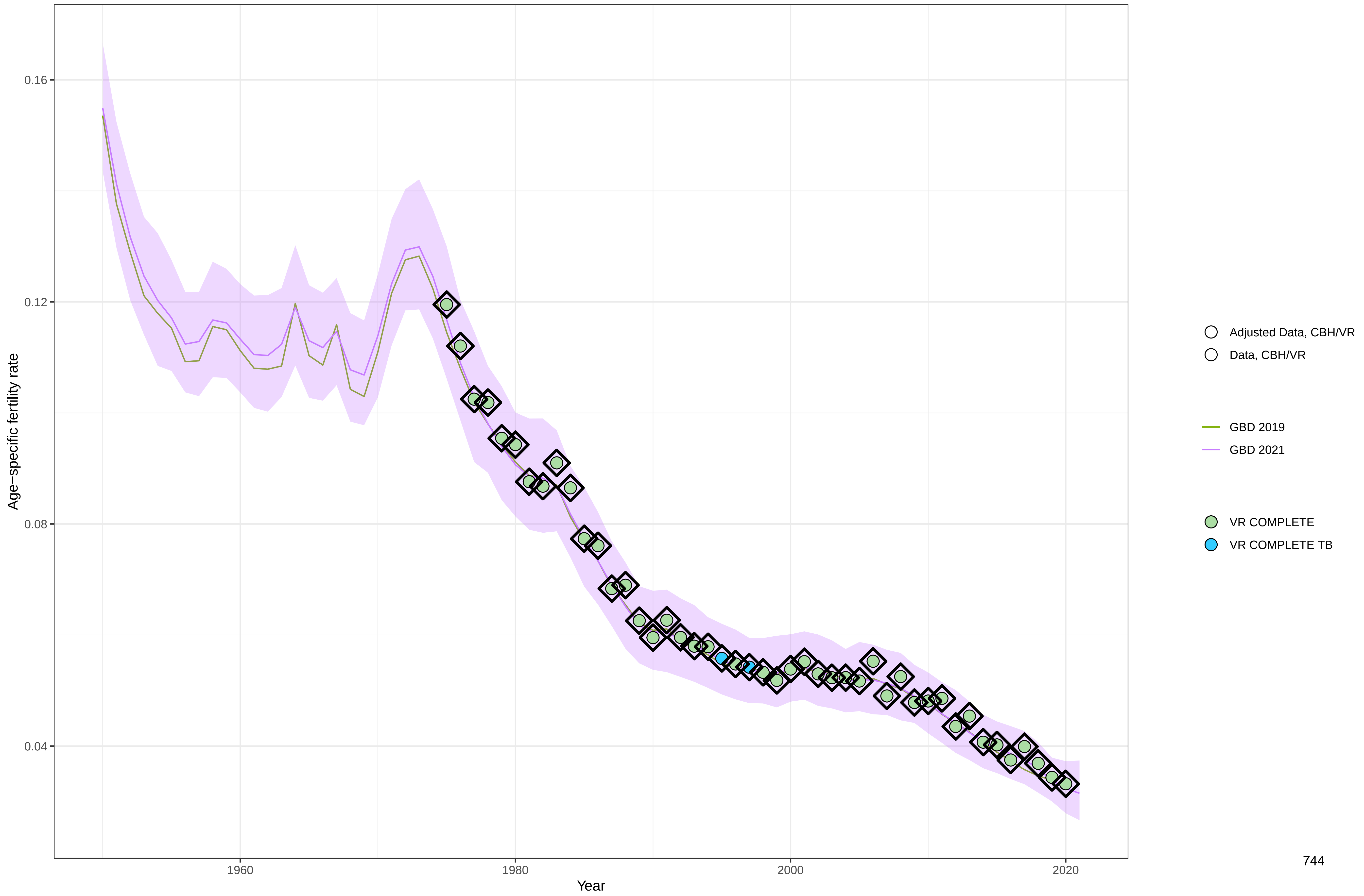

Age−specific fertility fitted trend lines, 1950−2021: Tottori, Ages: 25−29
Data points enclosed with a diamond are reference sources

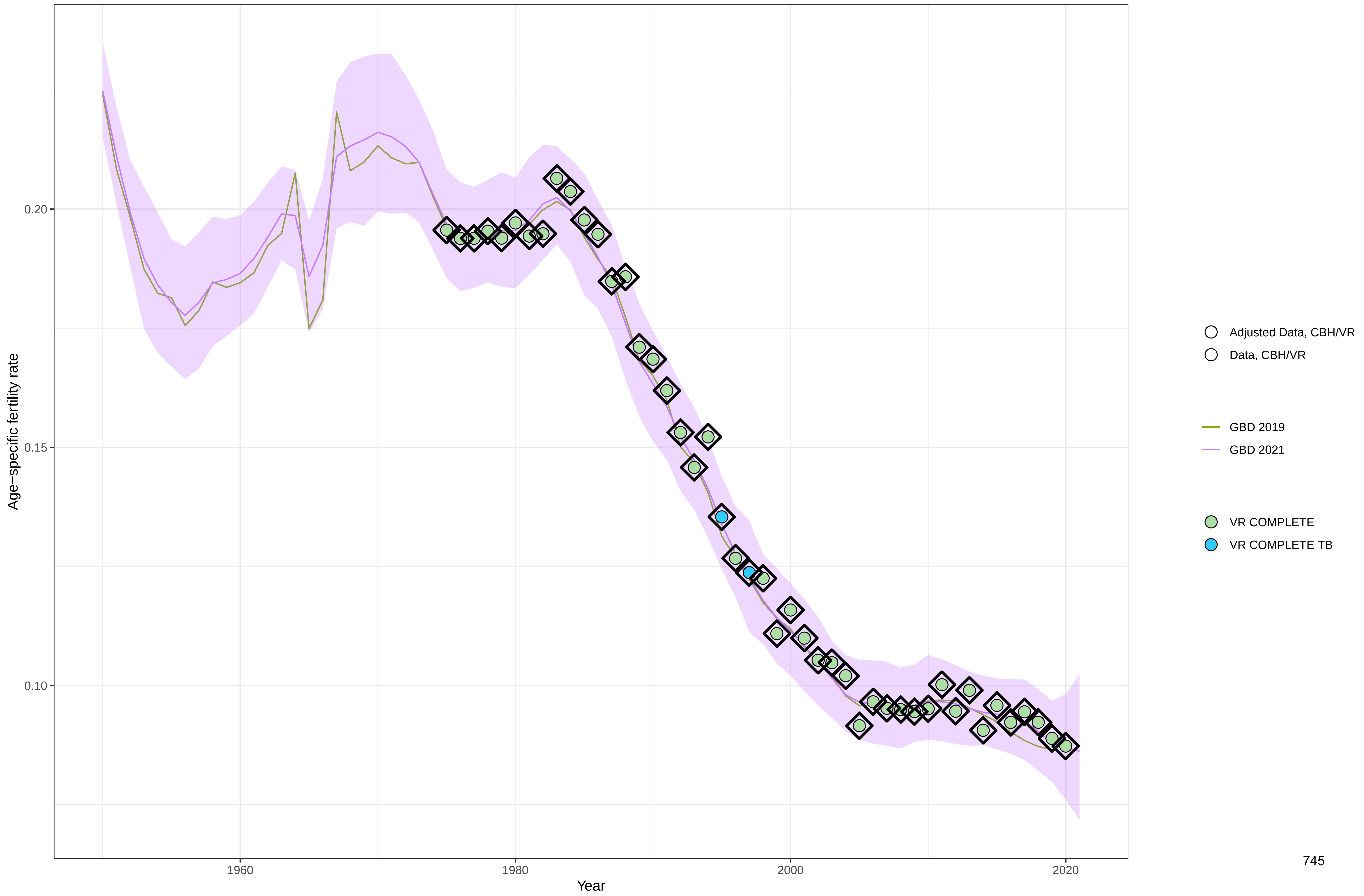

Age−specific fertility fitted trend lines, 1950−2021: Tottori, Ages: 30−34
Data points enclosed with a diamond are reference sources

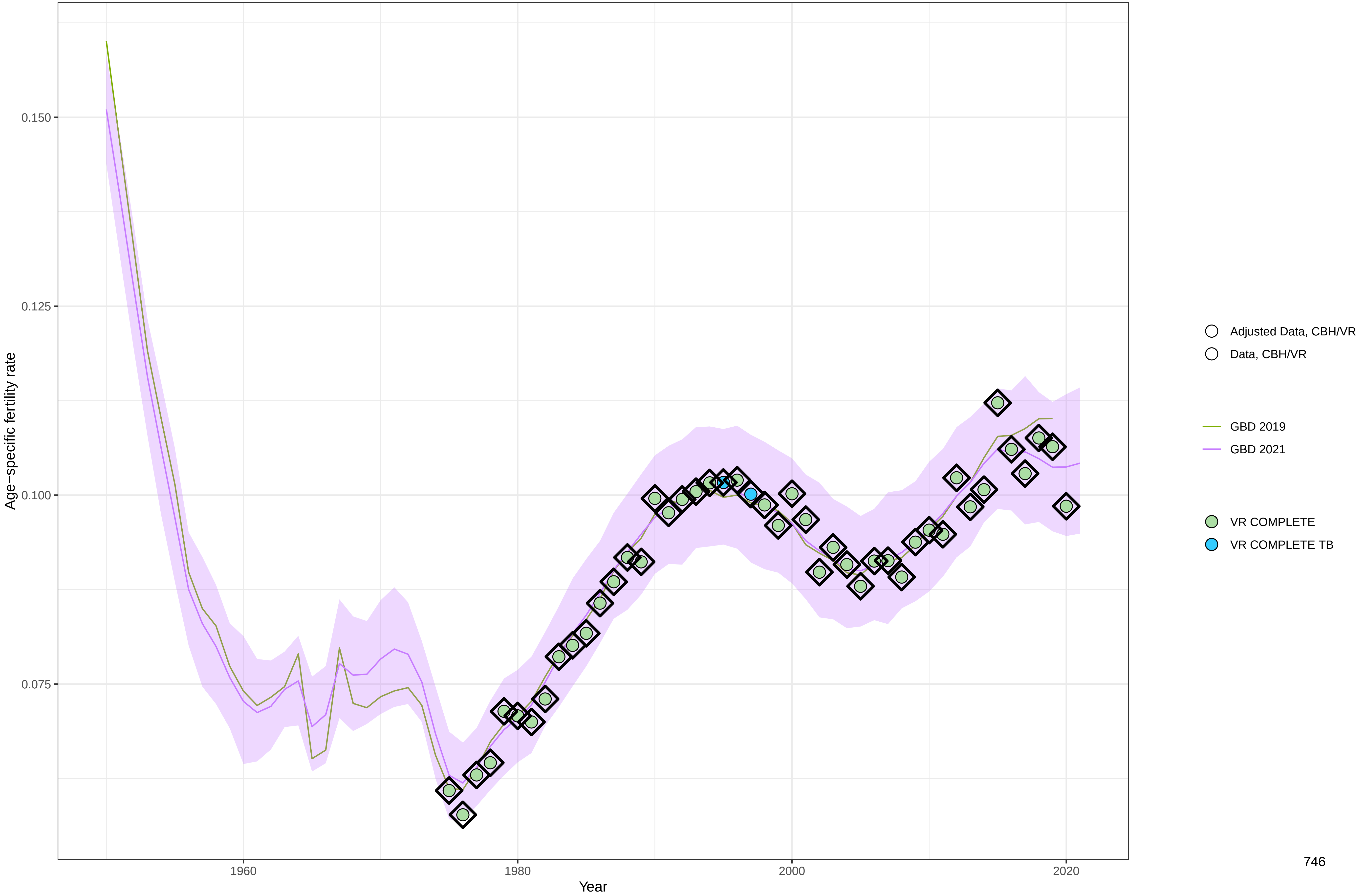

Age−specific fertility fitted trend lines, 1950−2021: Tottori, Ages: 35−39
Data points enclosed with a diamond are reference sources

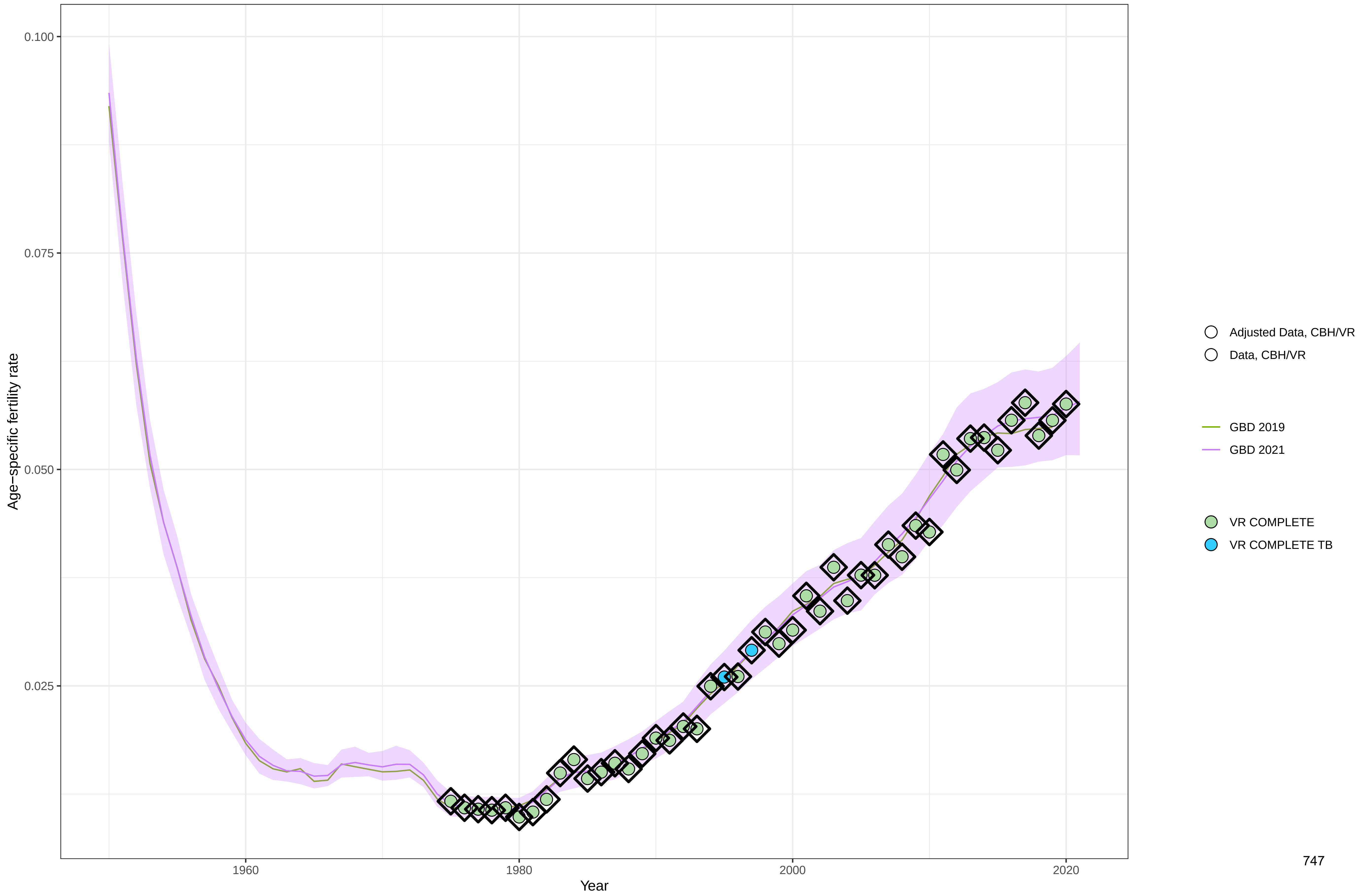

Age−specific fertility fitted trend lines, 1950−2021: Tottori, Ages: 40−44
Data points enclosed with a diamond are reference sources

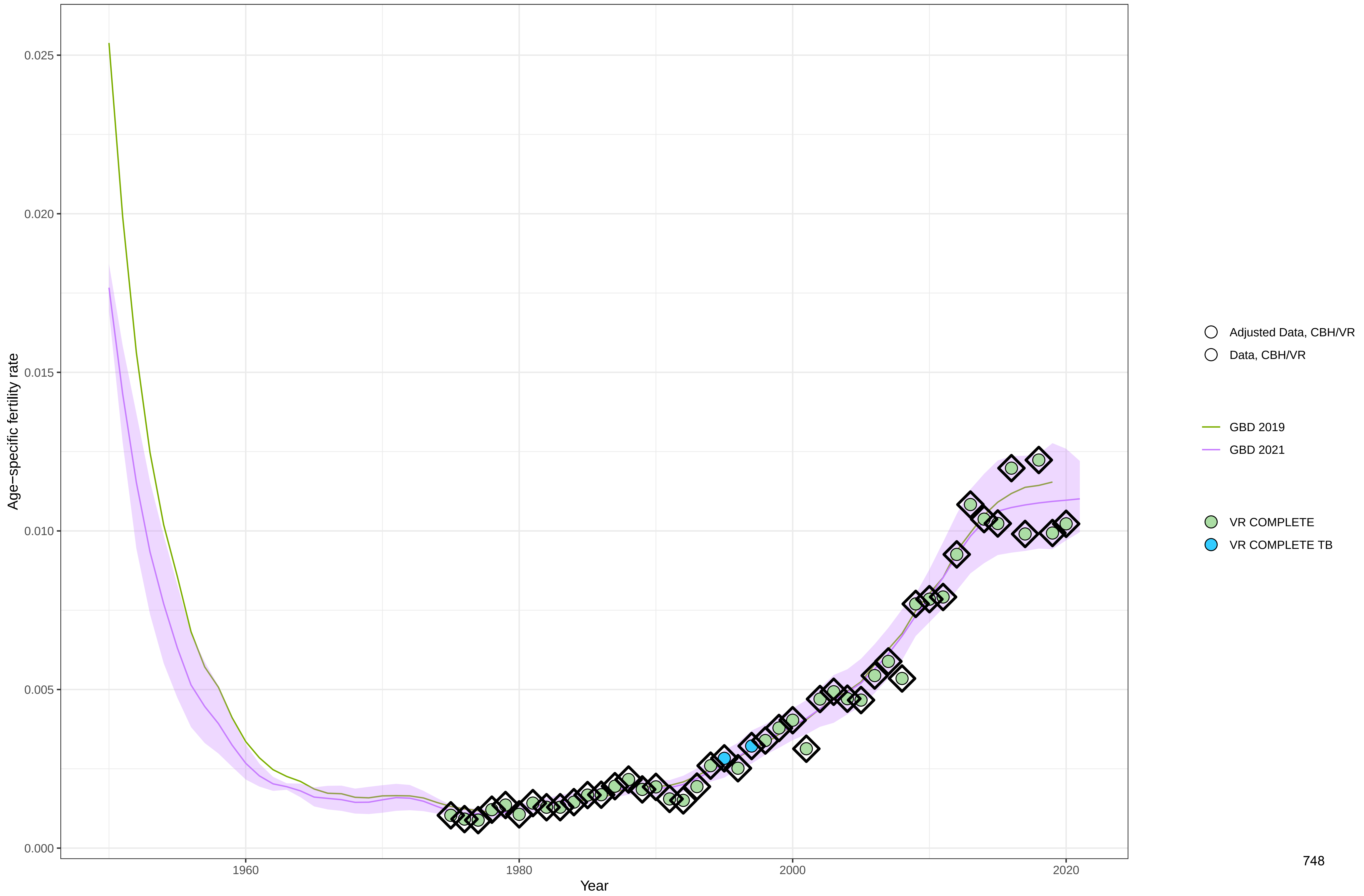

Age−specific fertility fitted trend lines, 1950−2021: Tottori, Ages: 45−49
Data points enclosed with a diamond are reference sources

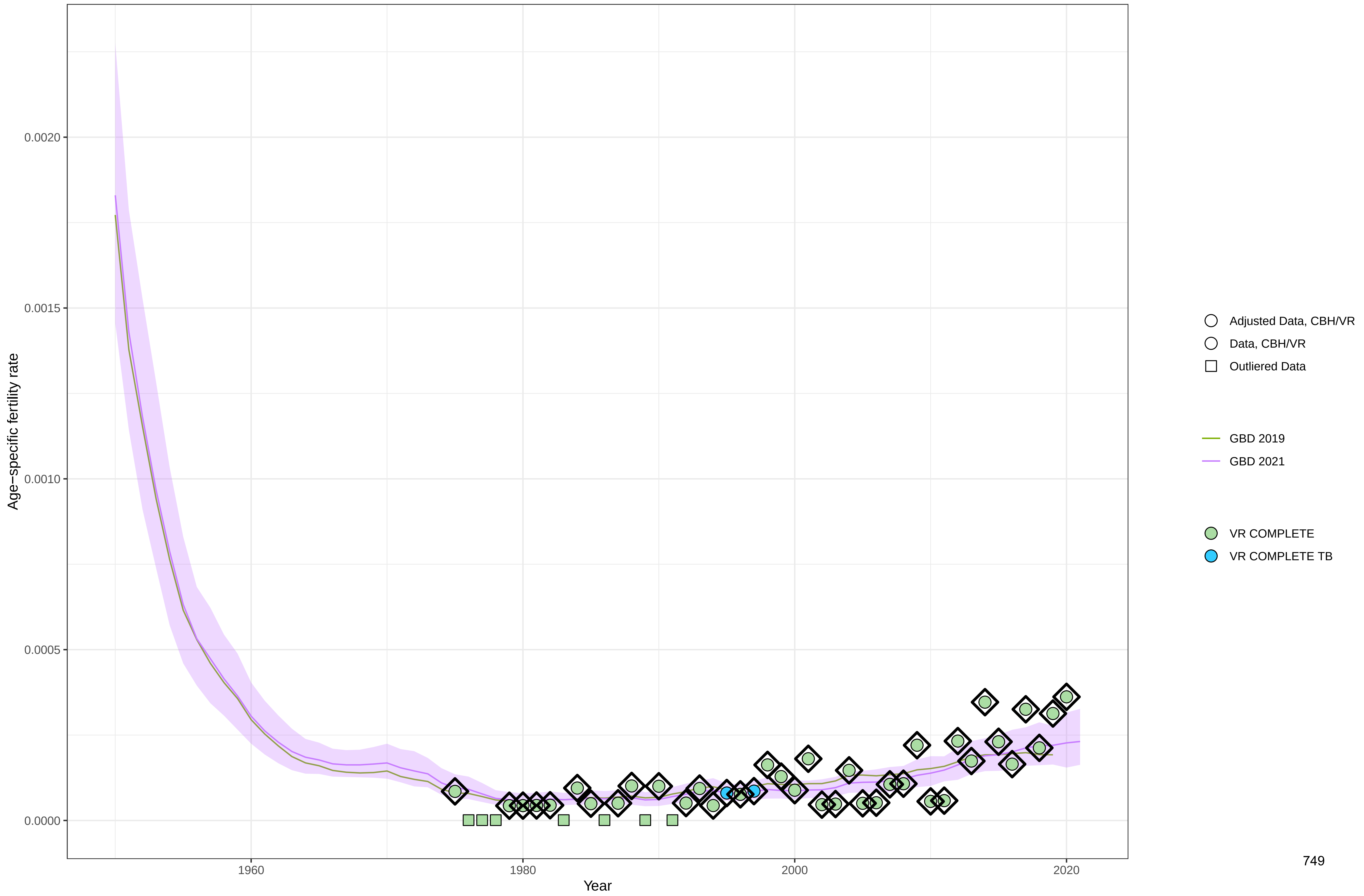

Age−specific fertility fitted trend lines, 1950−2021: Tottori, Ages: 50−54
Data points enclosed with a diamond are reference sources

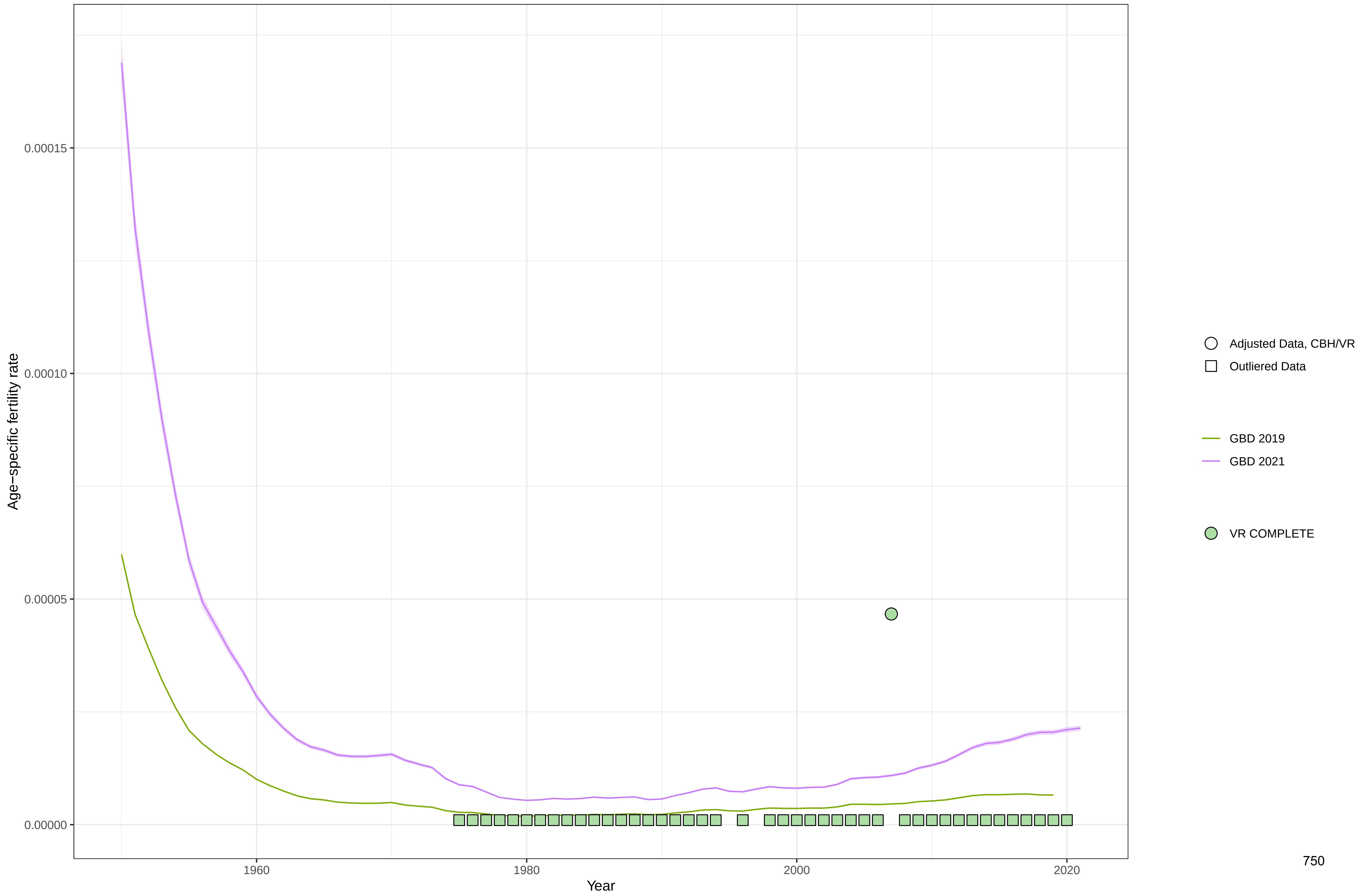

Total fertility rate fitted trend lines, 1950−2021: Tottori TFR
Data points enclosed with a diamond are reference sources

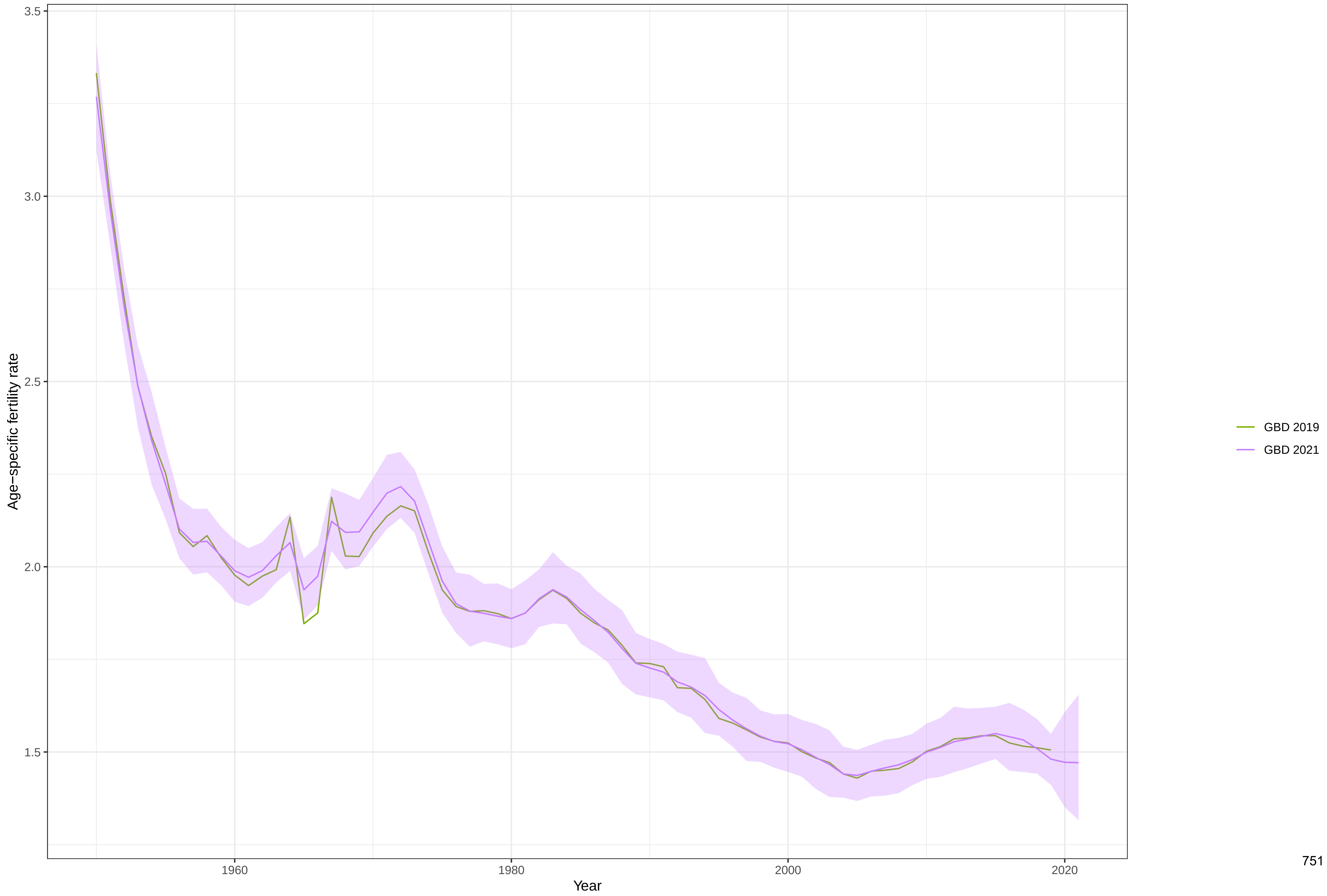

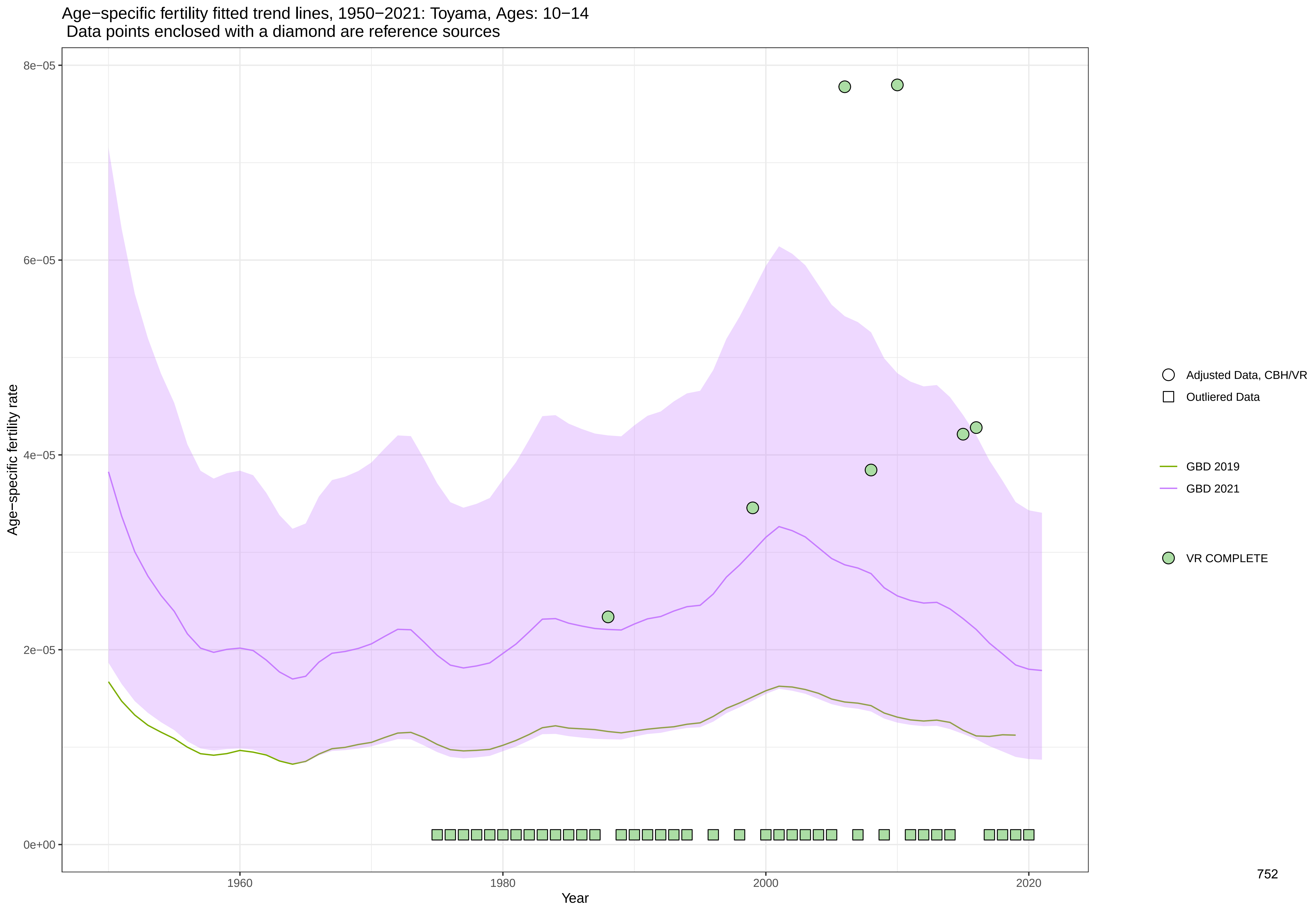

Age−specific fertility fitted trend lines, 1950−2021: Toyama, Ages: 10−14
Data points enclosed with a diamond are reference sources
8e−05
6e−05
4e−05
2e−05
0e+00
Age−specific fertility rate
1960
1980
2000
2020
Year
Adjusted Data, CBH/VR
Outliered Data
GBD 2019
GBD 2021
VR COMPLETE

Age−specific fertility fitted trend lines, 1950−2021: Toyama, Ages: 15−19
Data points enclosed with a diamond are reference sources

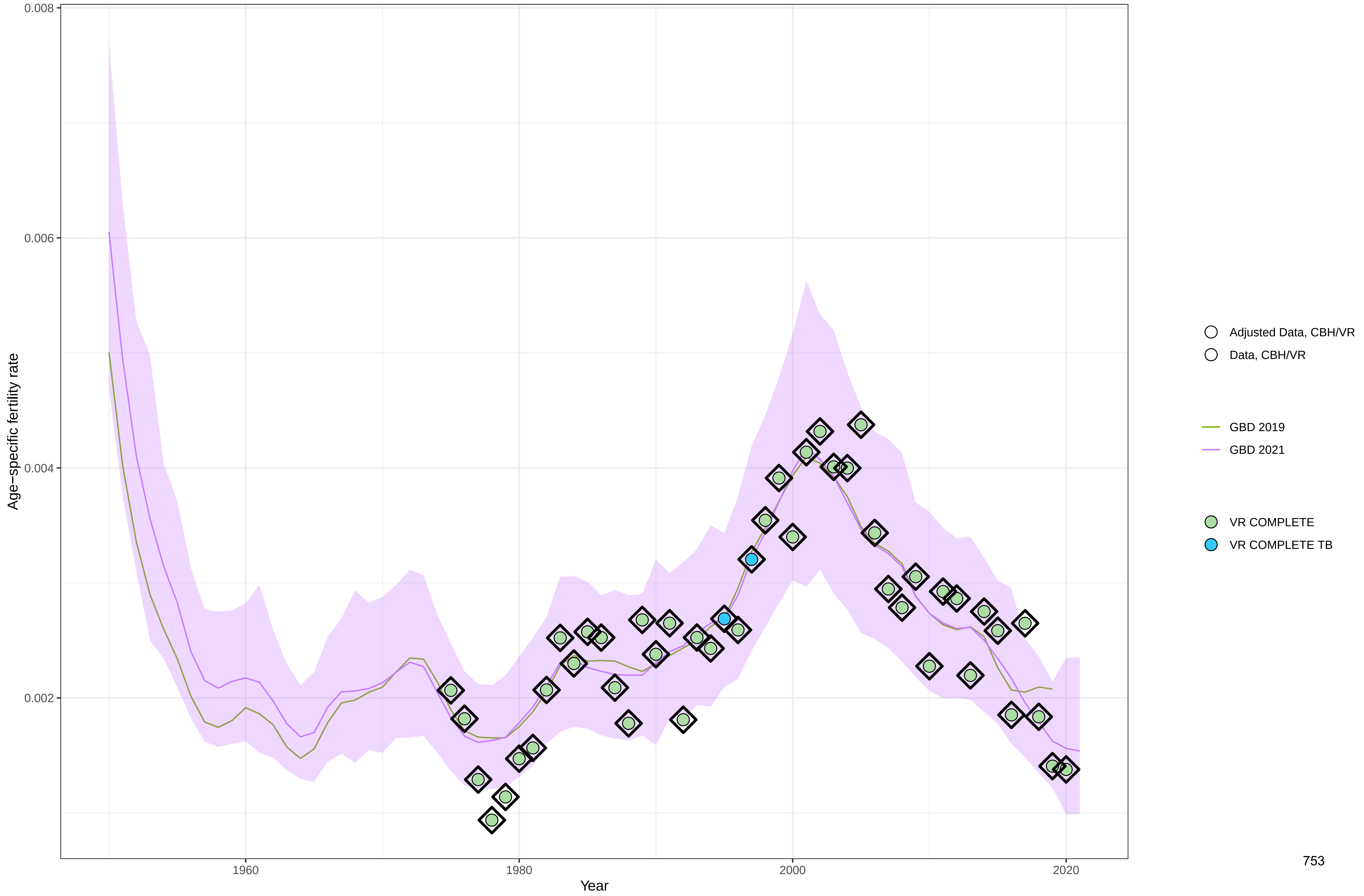

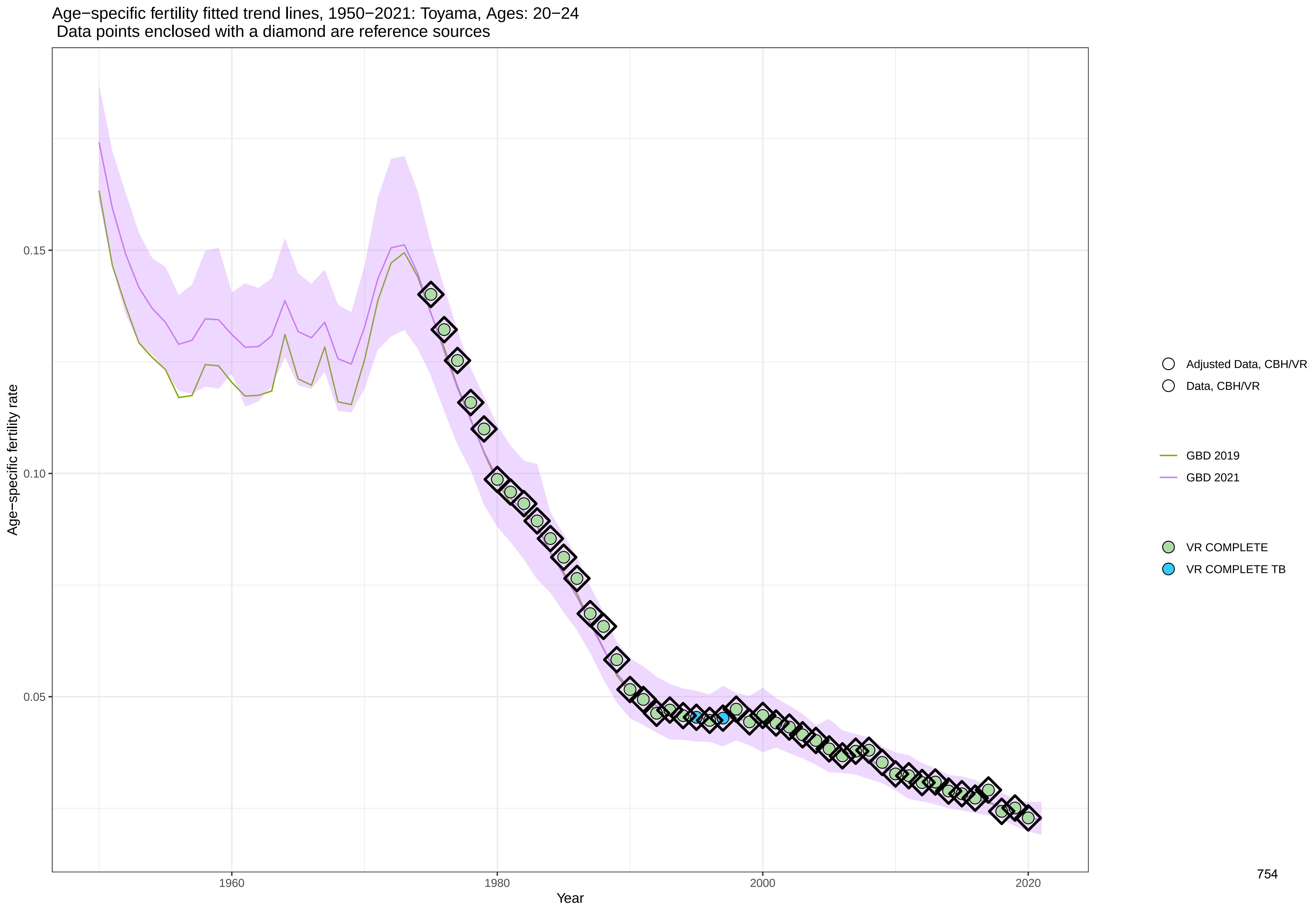
Age−specific fertility fitted trend lines, 1950−2021: Toyama, Ages: 20−24
Data points enclosed with a diamond are reference sources
Age−specific fertility rate
0.15
0.10
0.05
1960
1980
2000
2020
Year
Adjusted Data, CBH/VR
Data, CBH/VR
GBD 2019
GBD 2021
VR COMPLETE
VR COMPLETE TB

Age−specific fertility fitted trend lines, 1950−2021: Toyama, Ages: 25−29
Data points enclosed with a diamond are reference sources

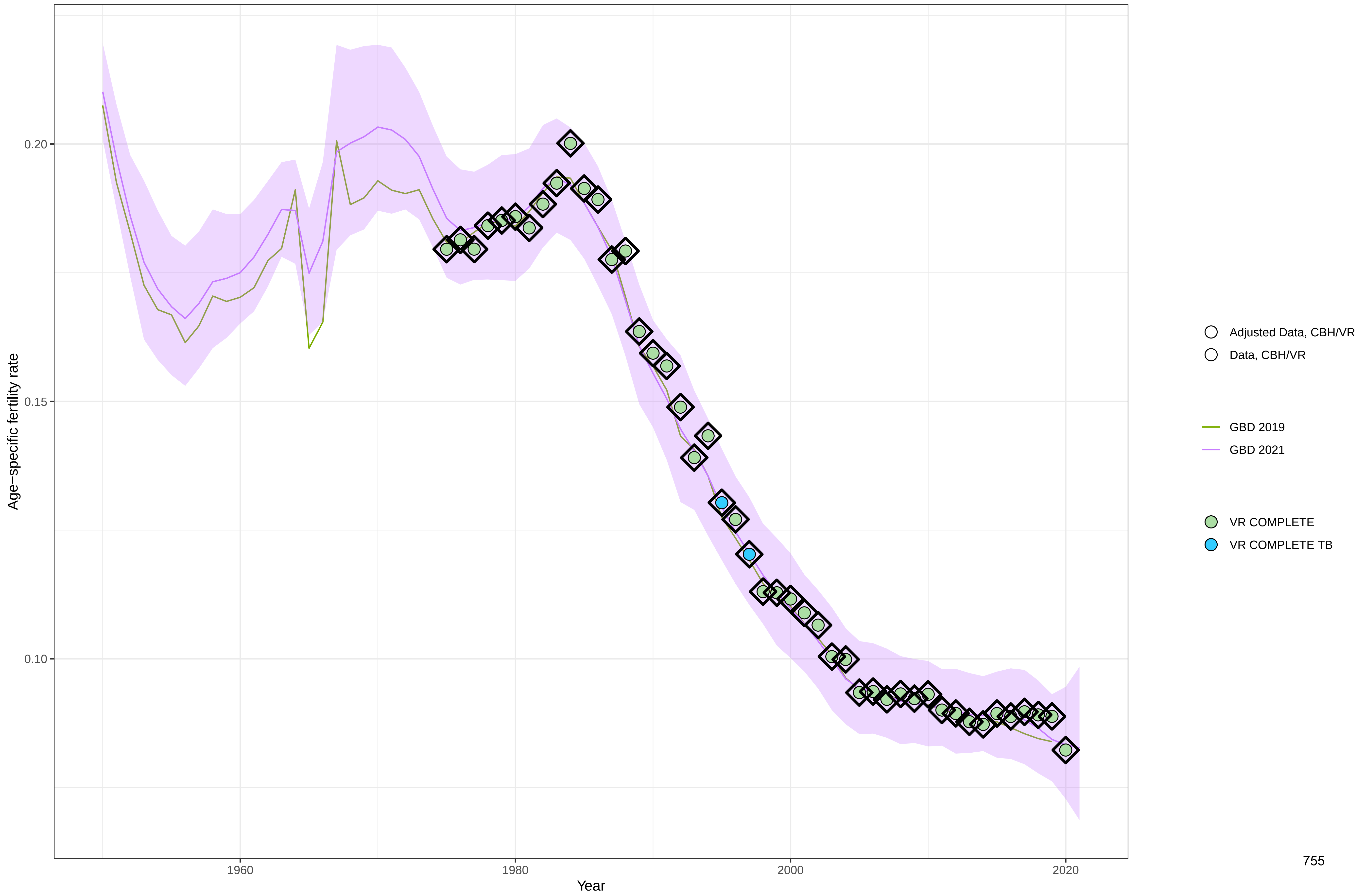

Age−specific fertility fitted trend lines, 1950−2021: Toyama, Ages: 30−34
Data points enclosed with a diamond are reference sources

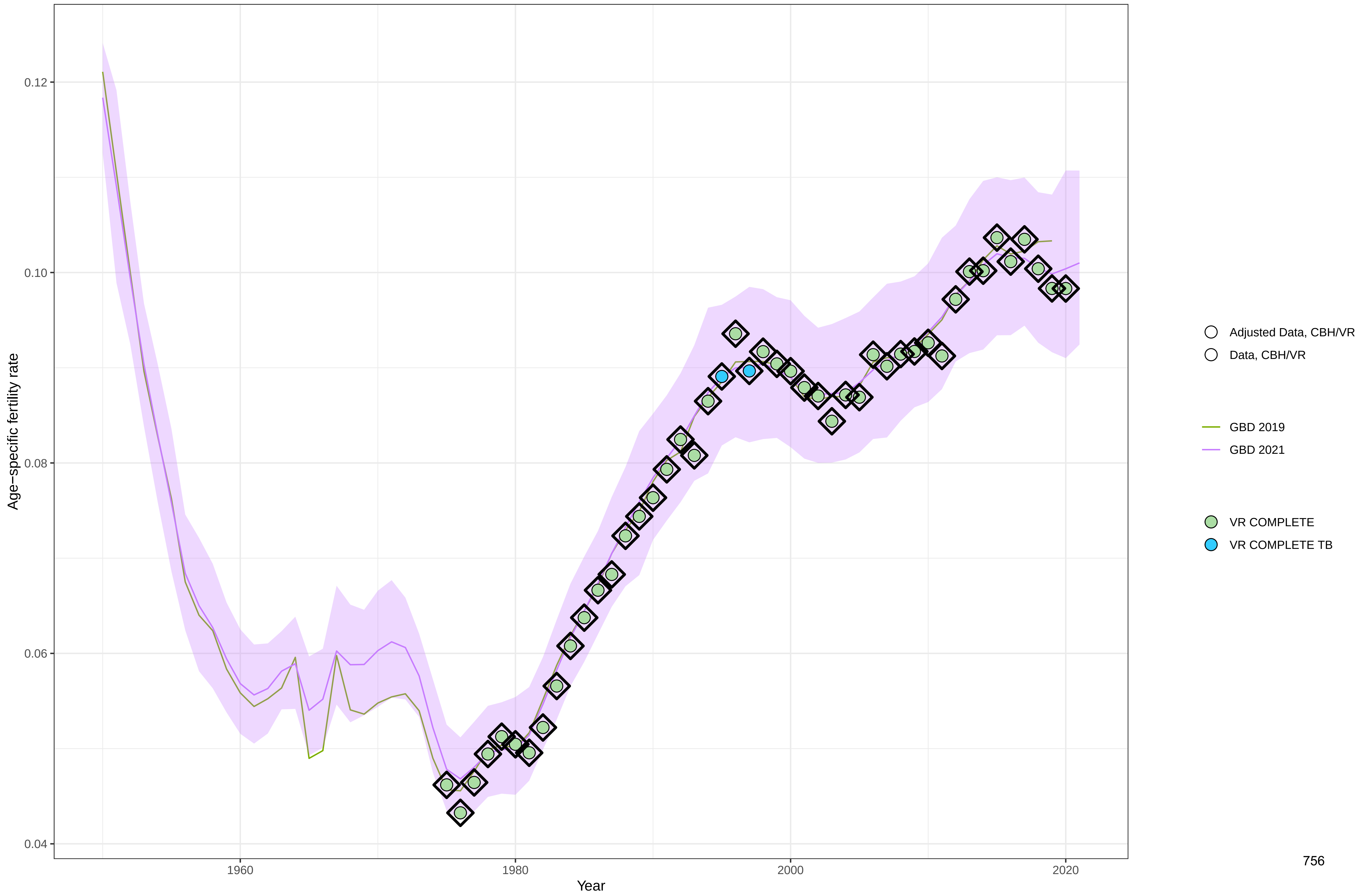

Age−specific fertility fitted trend lines, 1950−2021: Toyama, Ages: 35−39
Data points enclosed with a diamond are reference sources

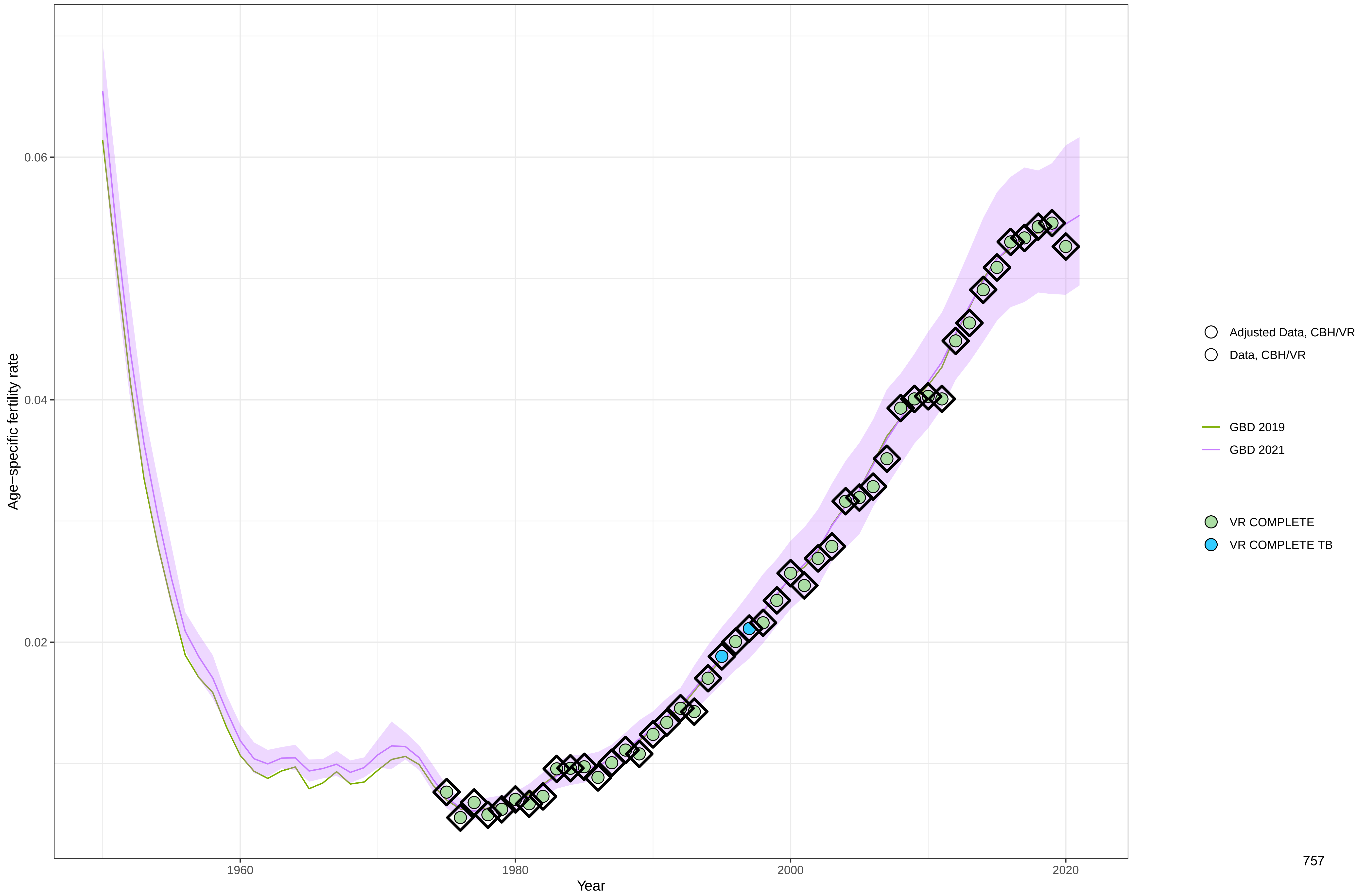

Age−specific fertility fitted trend lines, 1950−2021: Toyama, Ages: 40−44
Data points enclosed with a diamond are reference sources

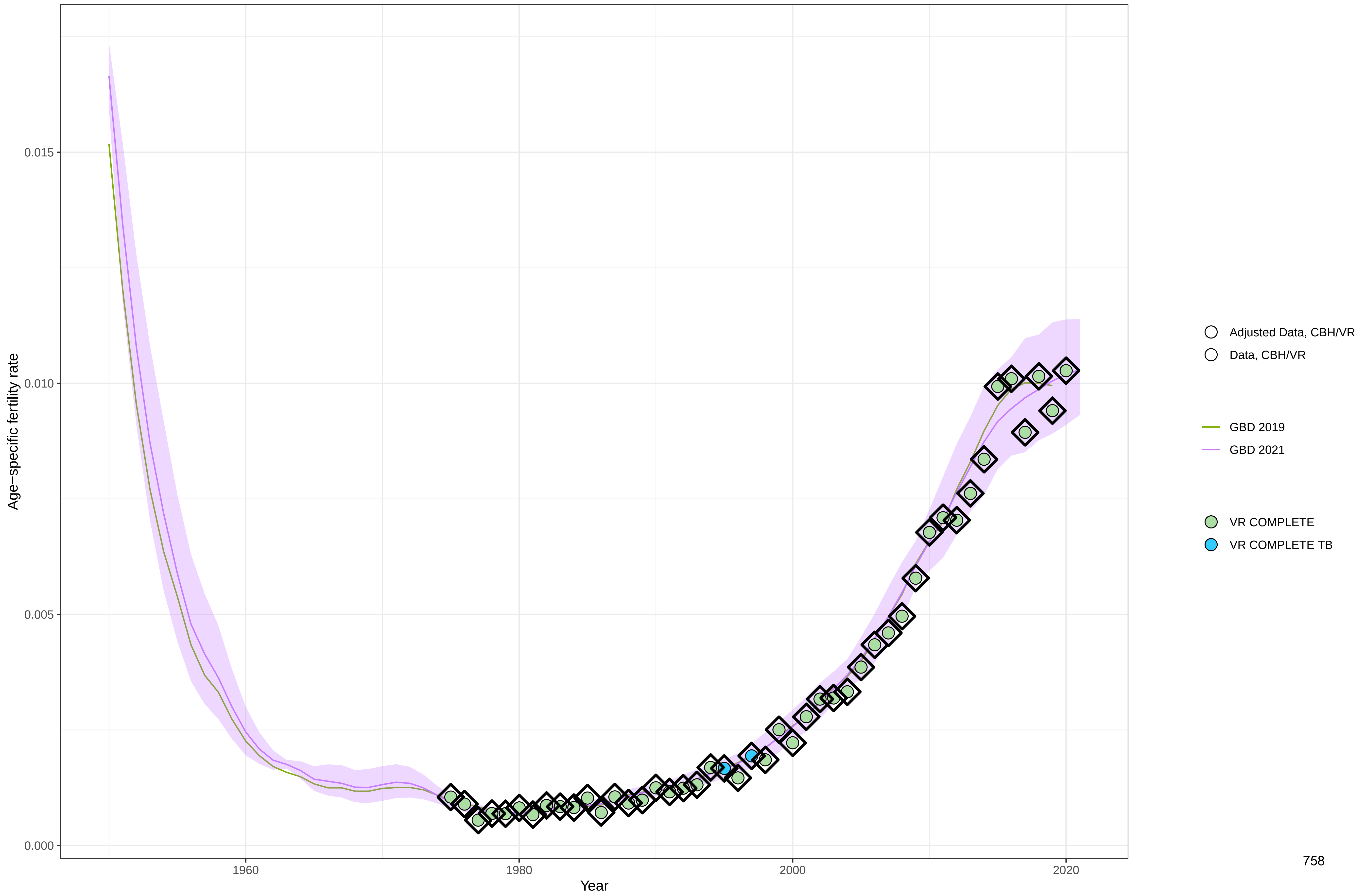

Age−specific fertility fitted trend lines, 1950−2021: Toyama, Ages: 45−49
Data points enclosed with a diamond are reference sources

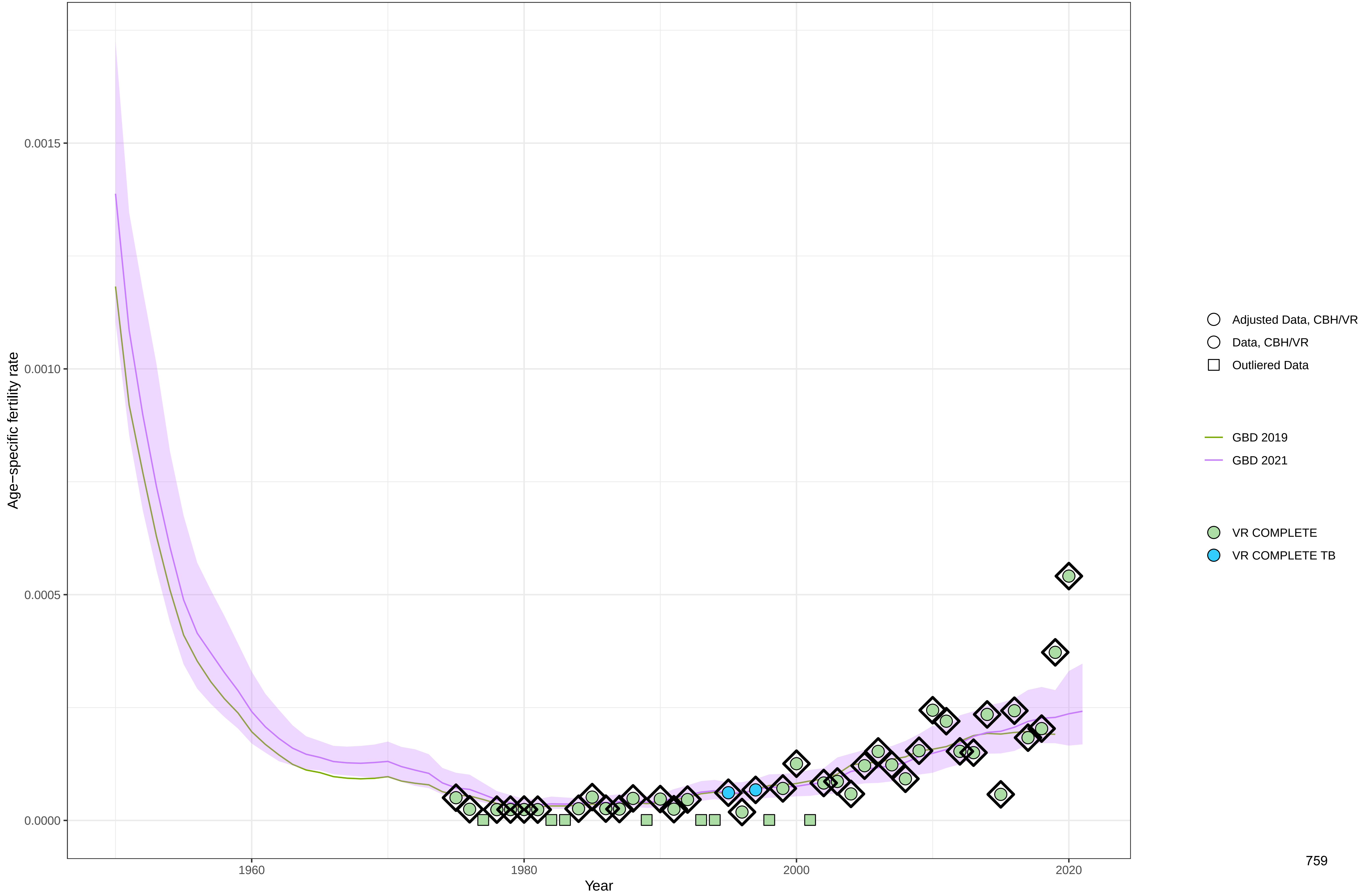

Age−specific fertility fitted trend lines, 1950−2021: Toyama, Ages: 50−54
Data points enclosed with a diamond are reference sources

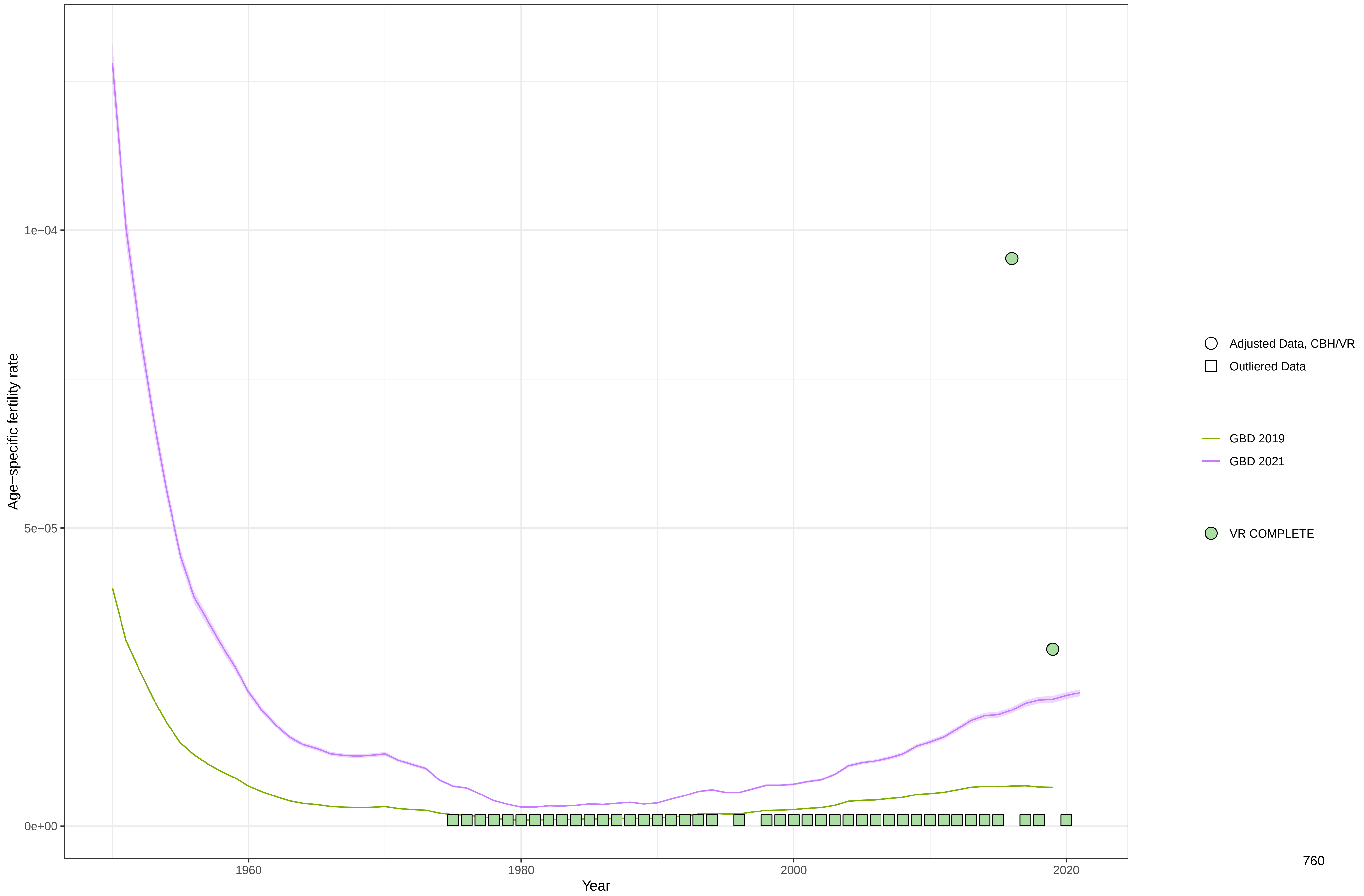

Total fertility rate fitted trend lines, 1950−2021: Toyama TFR
Data points enclosed with a diamond are reference sources

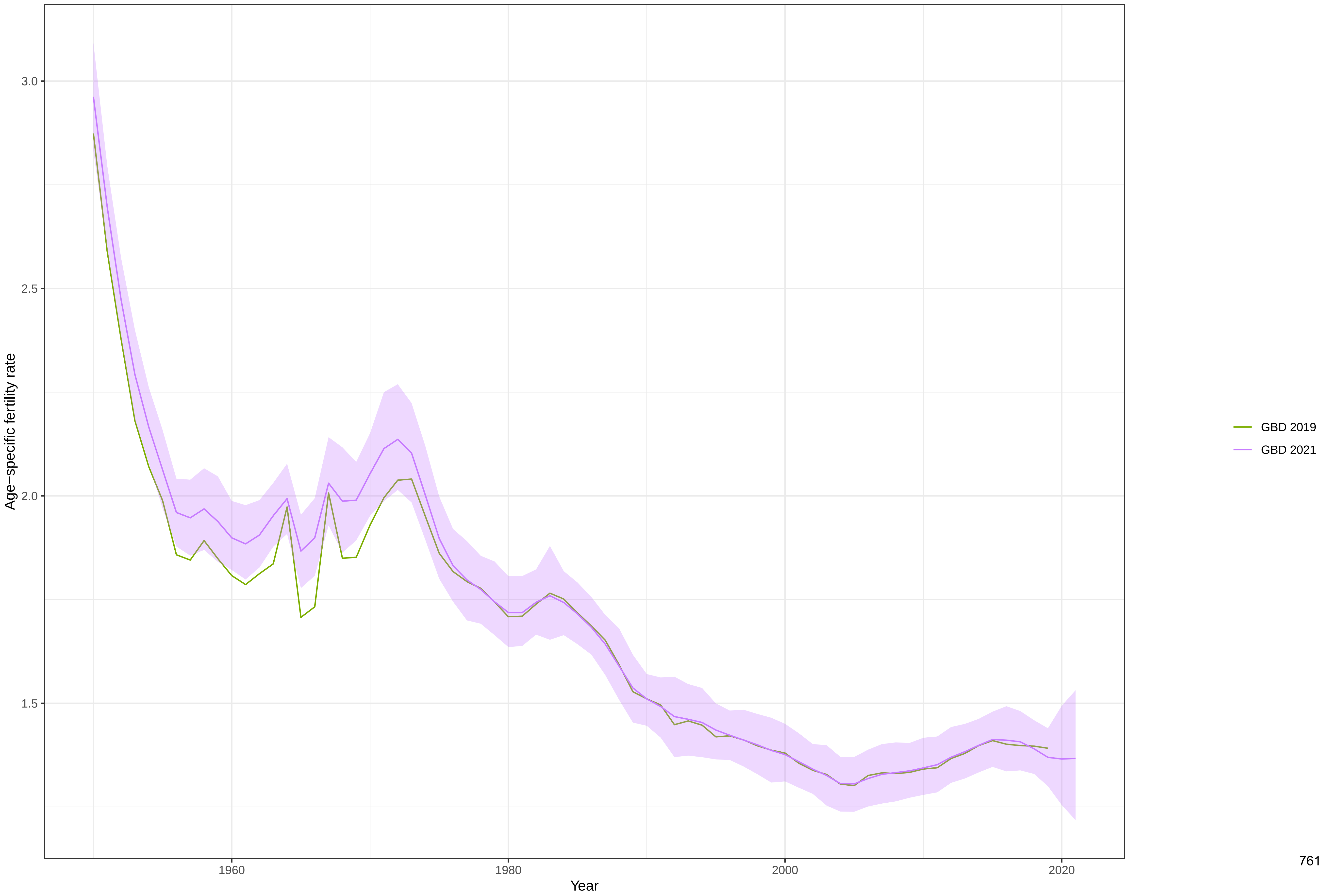

Age−specific fertility fitted trend lines, 1950−2021: Wakayama, Ages: 10−14
Data points enclosed with a diamond are reference sources

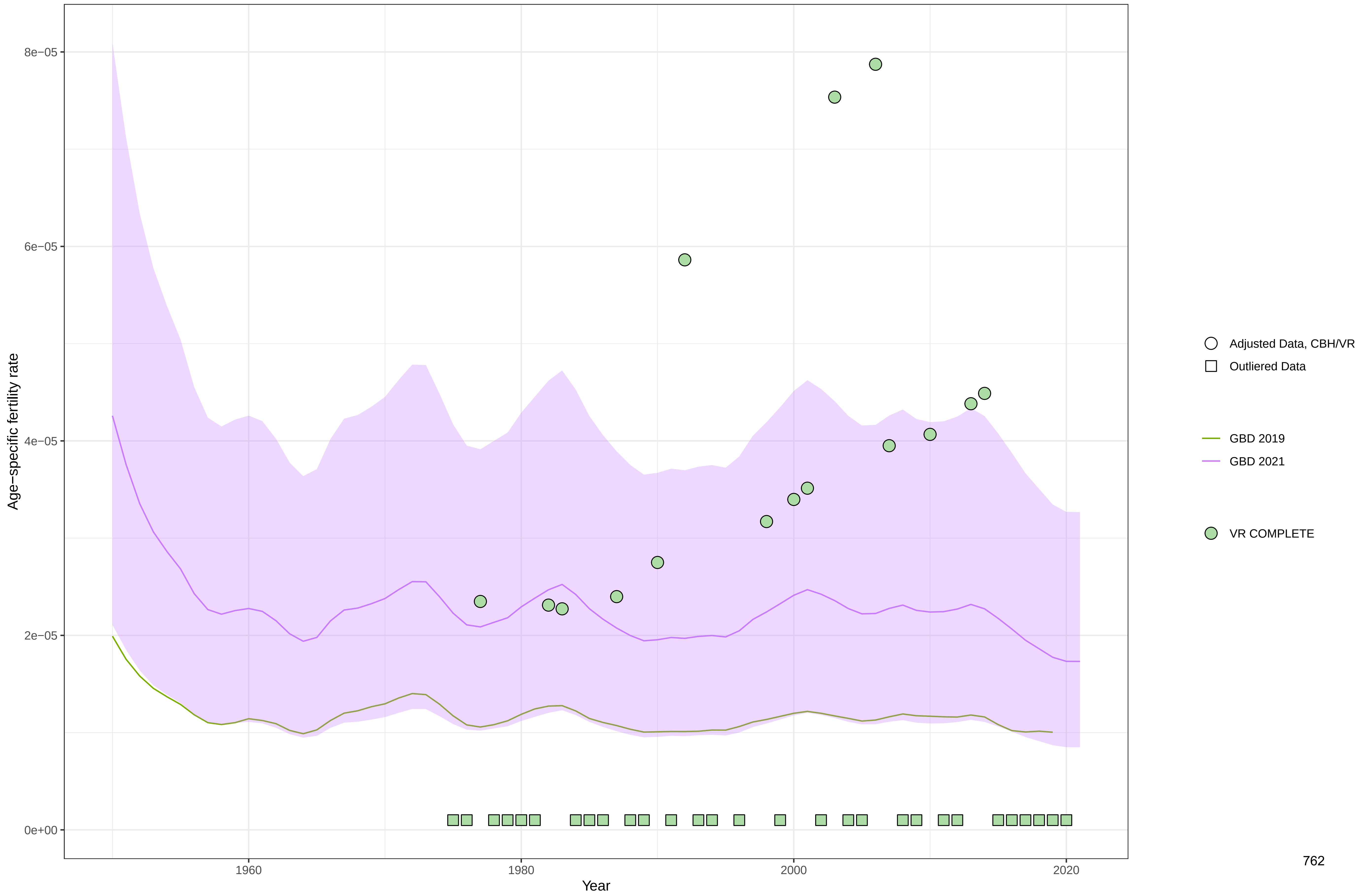

Age−specific fertility fitted trend lines, 1950−2021: Wakayama, Ages: 15−19
Data points enclosed with a diamond are reference sources

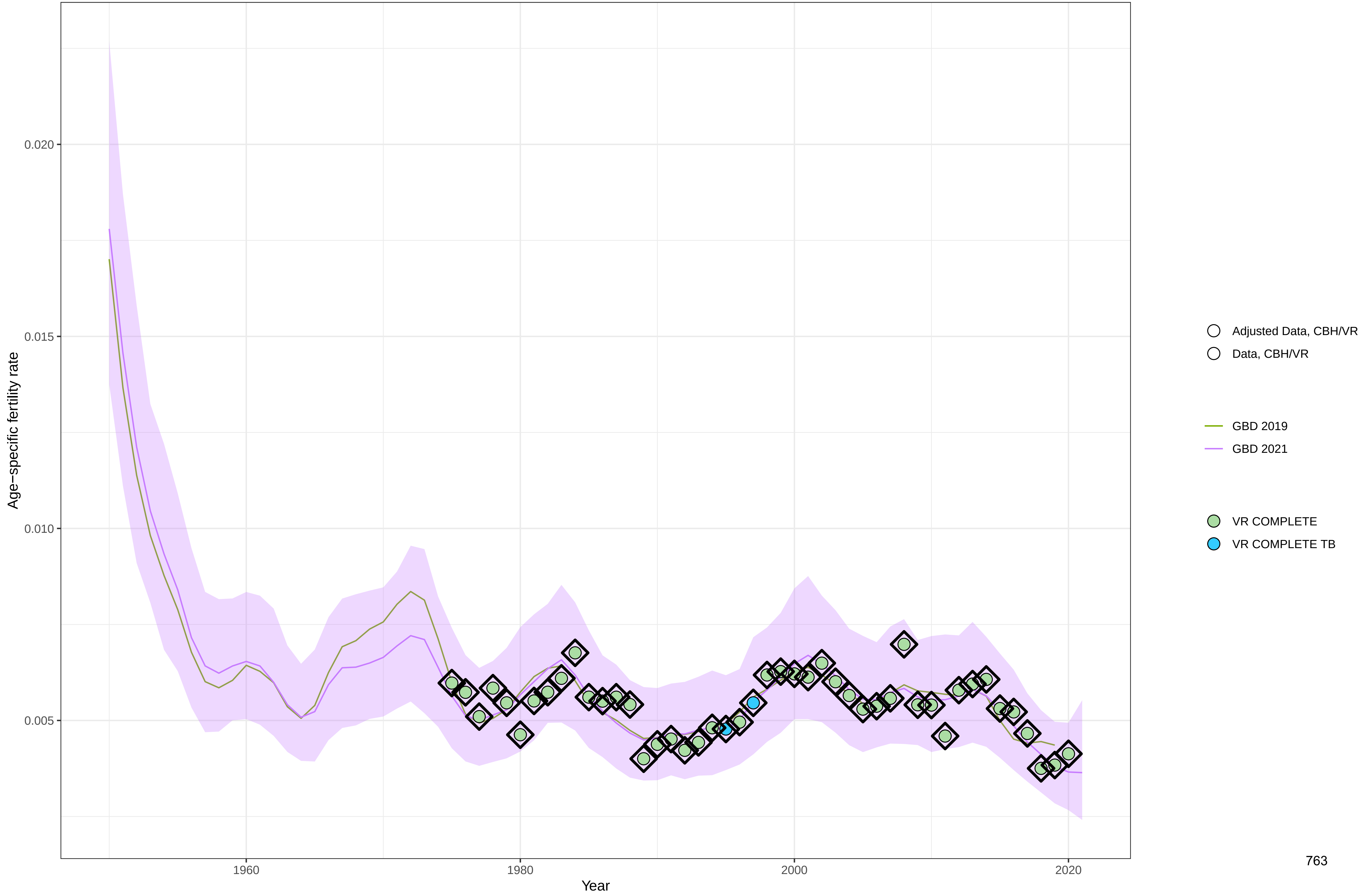

Age−specific fertility fitted trend lines, 1950−2021: Wakayama, Ages: 20−24
Data points enclosed with a diamond are reference sources

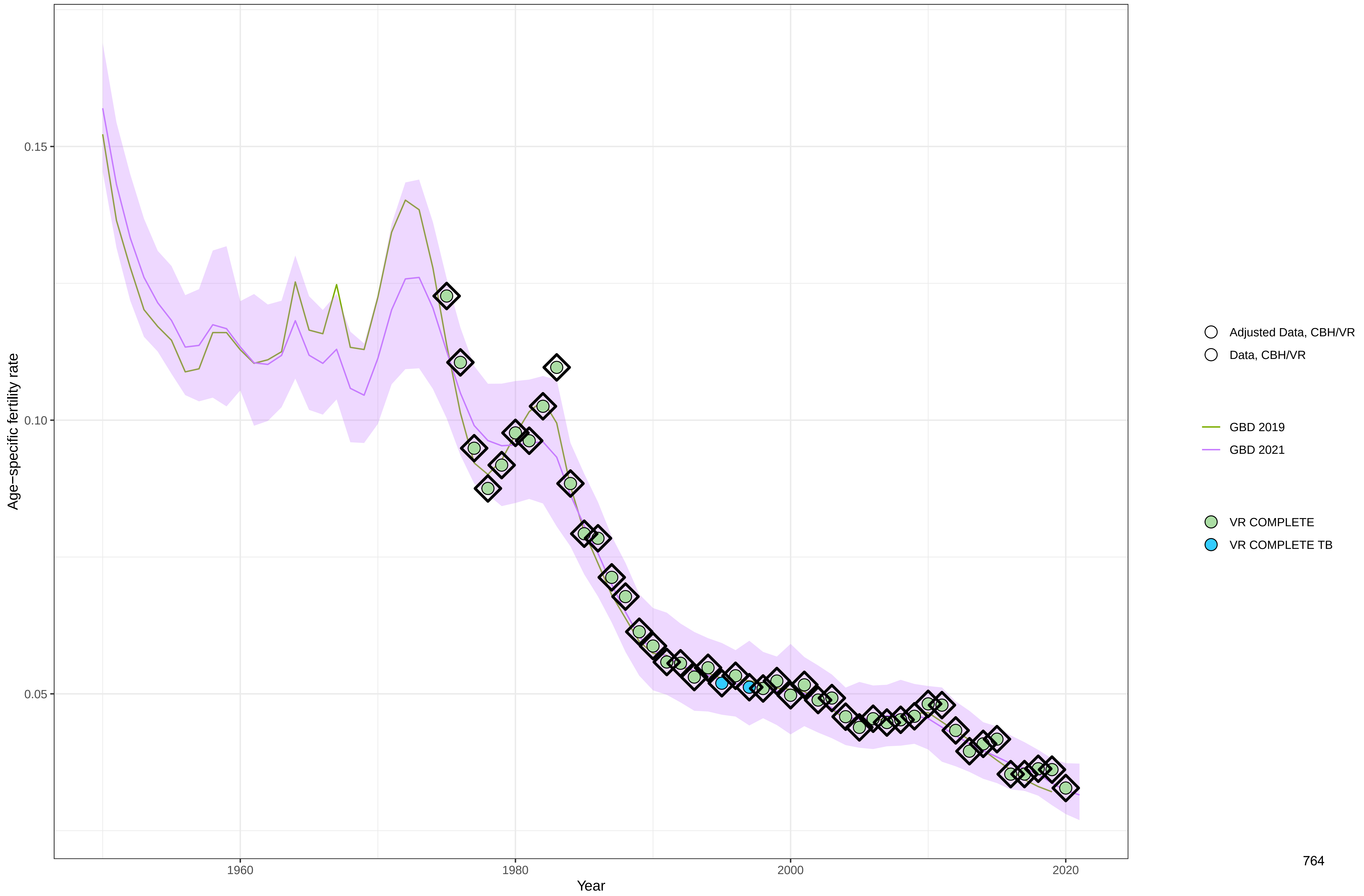

Age−specific fertility fitted trend lines, 1950−2021: Wakayama, Ages: 25−29
Data points enclosed with a diamond are reference sources

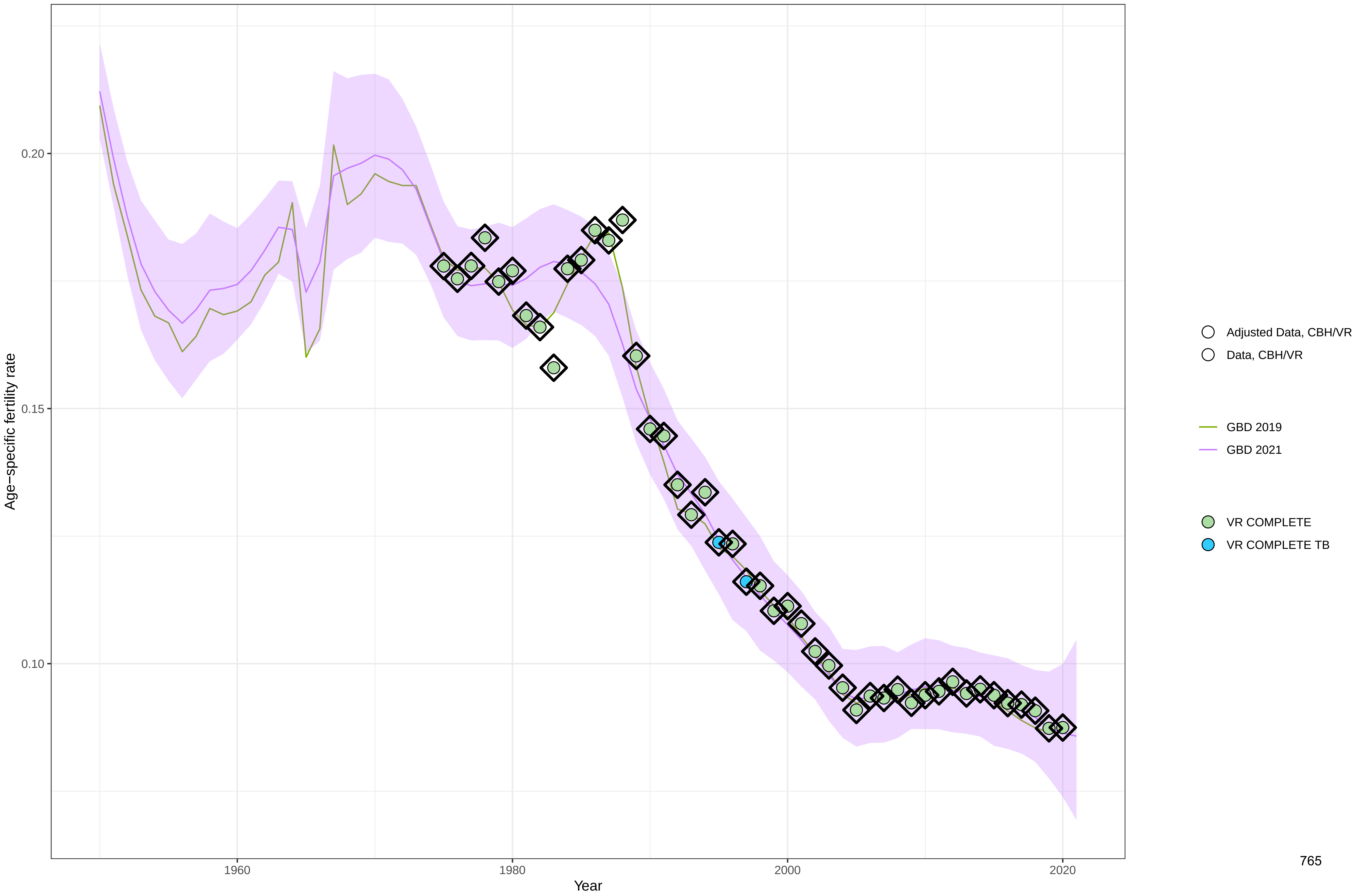

Age−specific fertility fitted trend lines, 1950−2021: Wakayama, Ages: 30−34
Data points enclosed with a diamond are reference sources

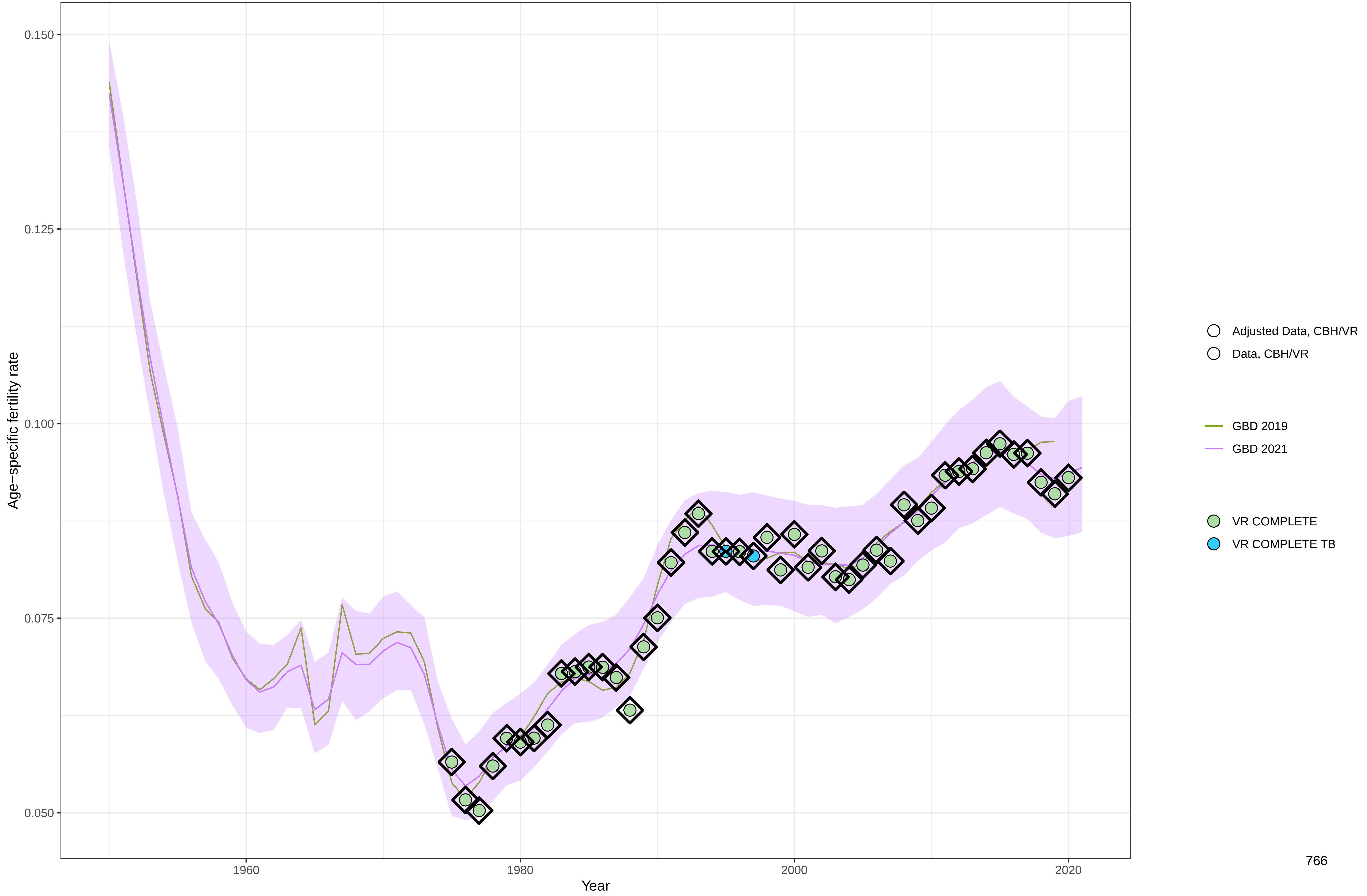

Age−specific fertility fitted trend lines, 1950−2021: Wakayama, Ages: 35−39
Data points enclosed with a diamond are reference sources

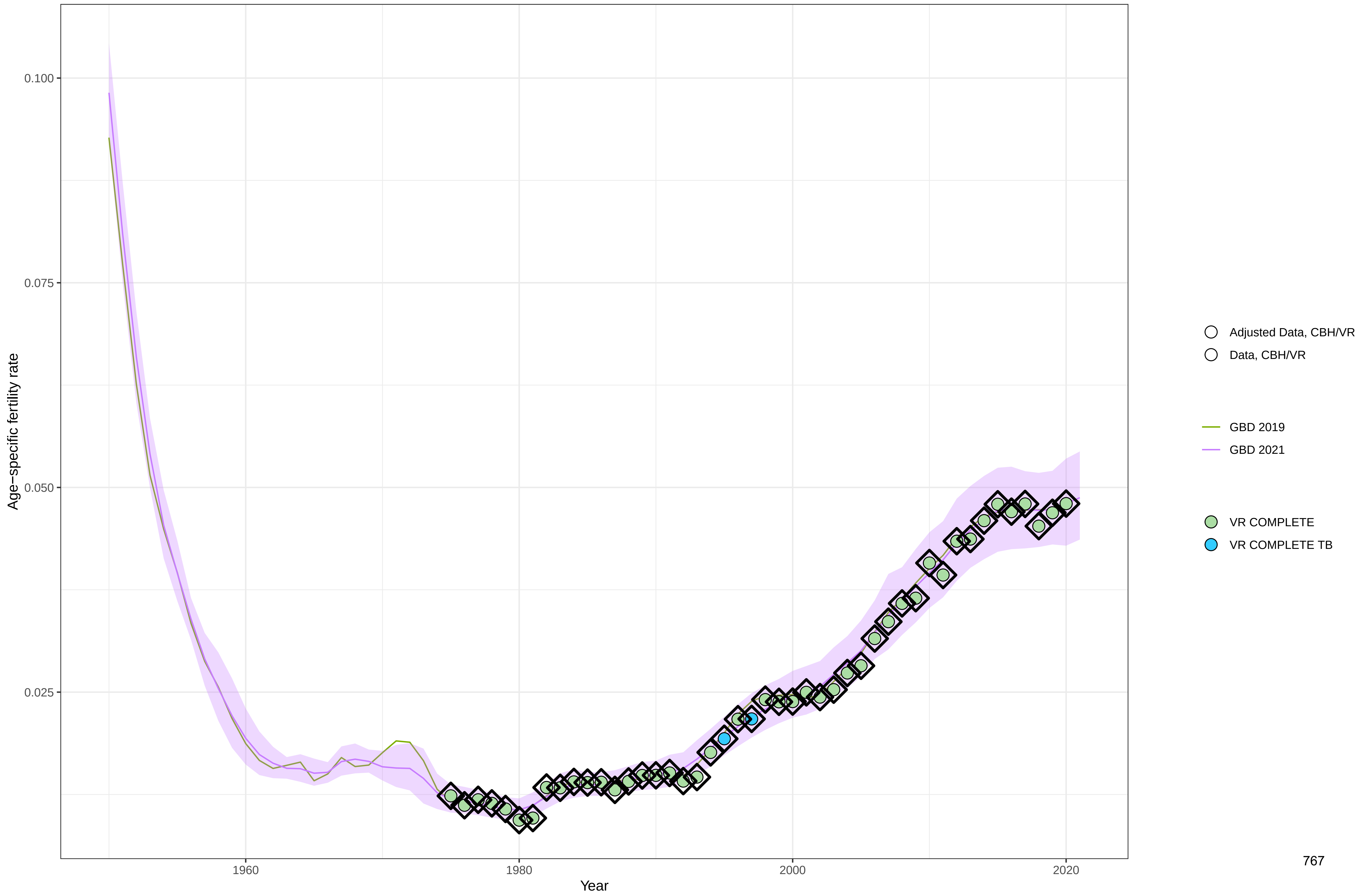

Age−specific fertility fitted trend lines, 1950−2021: Wakayama, Ages: 40−44
Data points enclosed with a diamond are reference sources

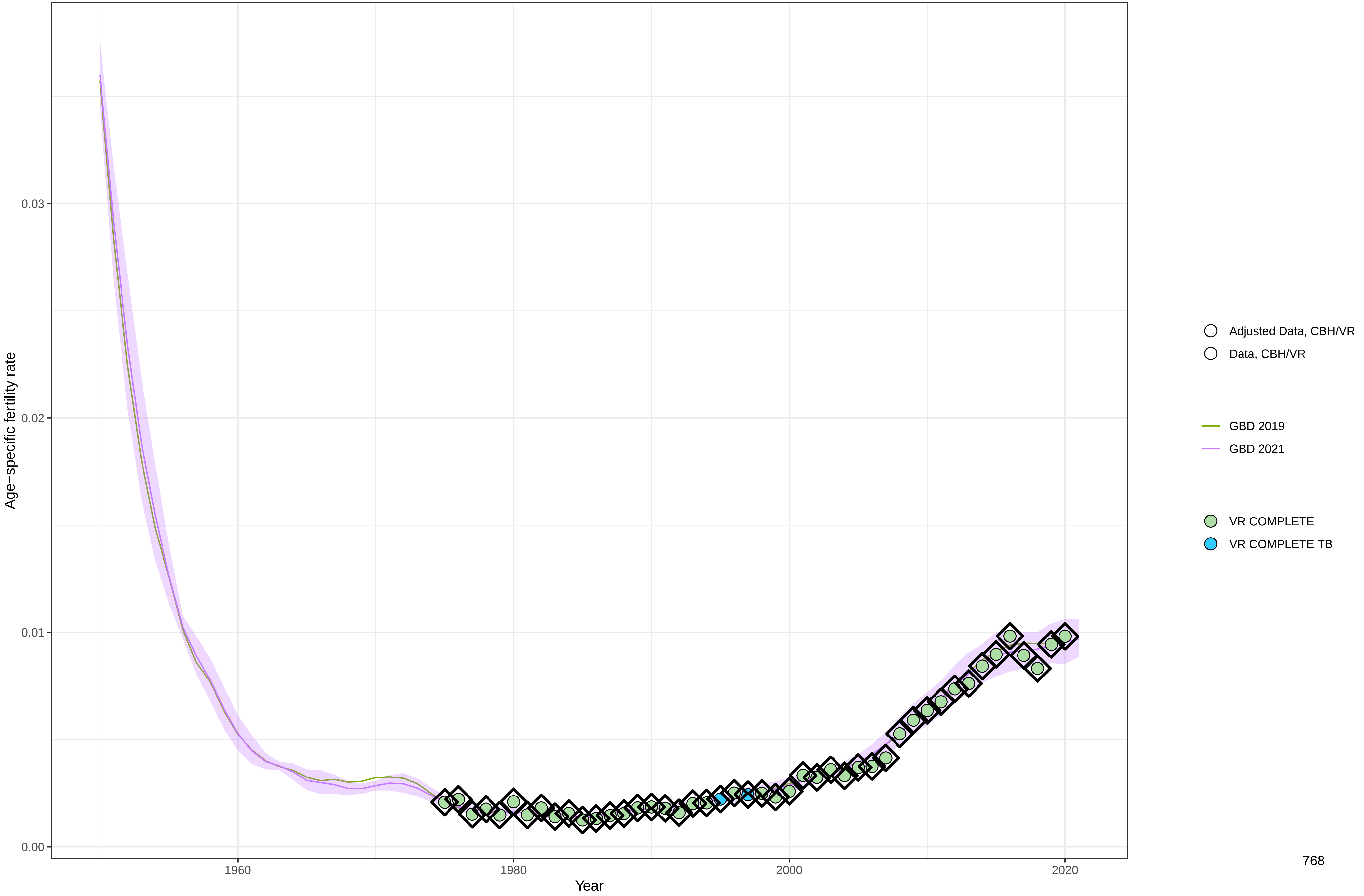

Age−specific fertility fitted trend lines, 1950−2021: Wakayama, Ages: 45−49
Data points enclosed with a diamond are reference sources

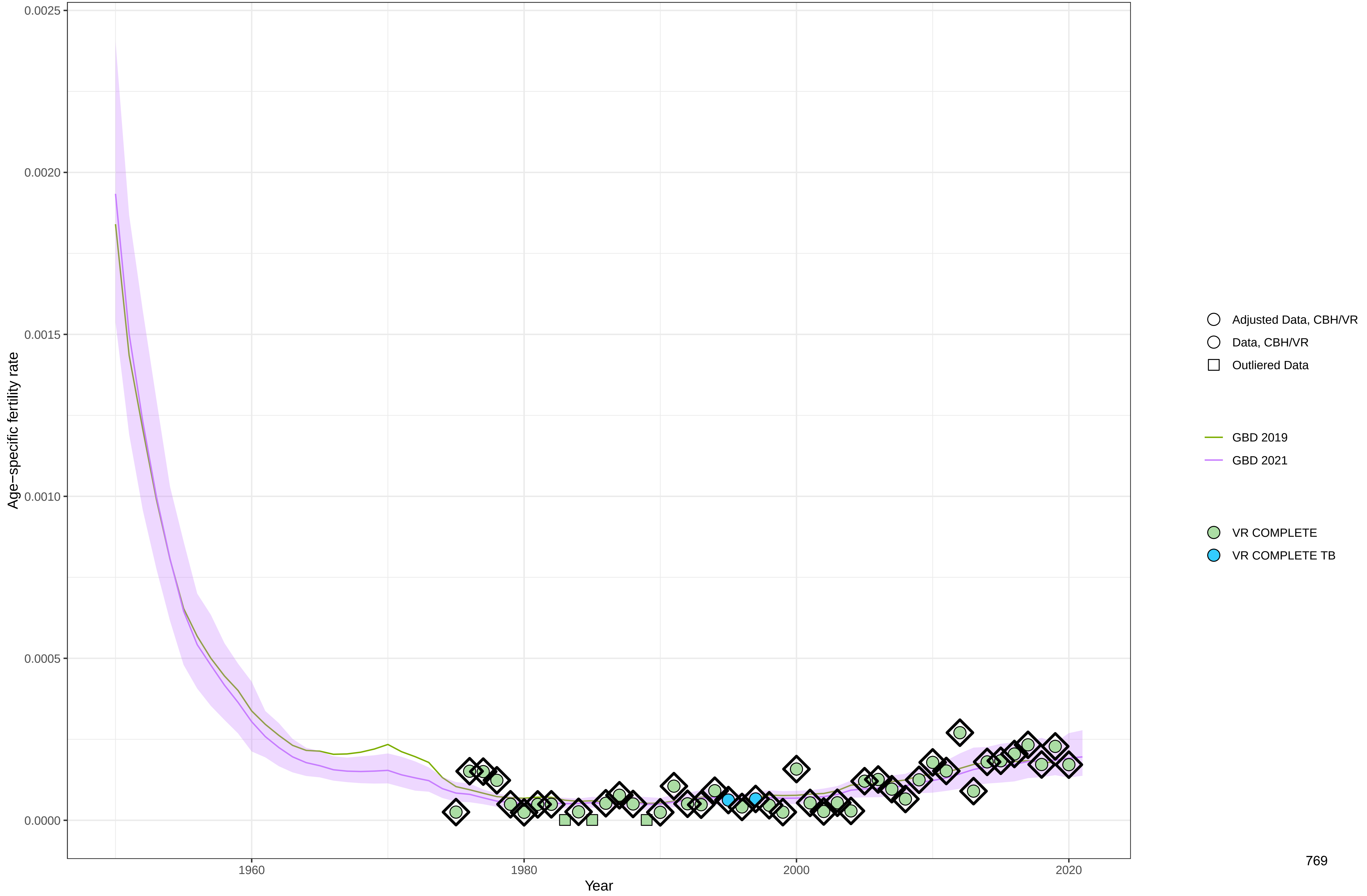

Age−specific fertility fitted trend lines, 1950−2021: Wakayama, Ages: 50−54
Data points enclosed with a diamond are reference sources

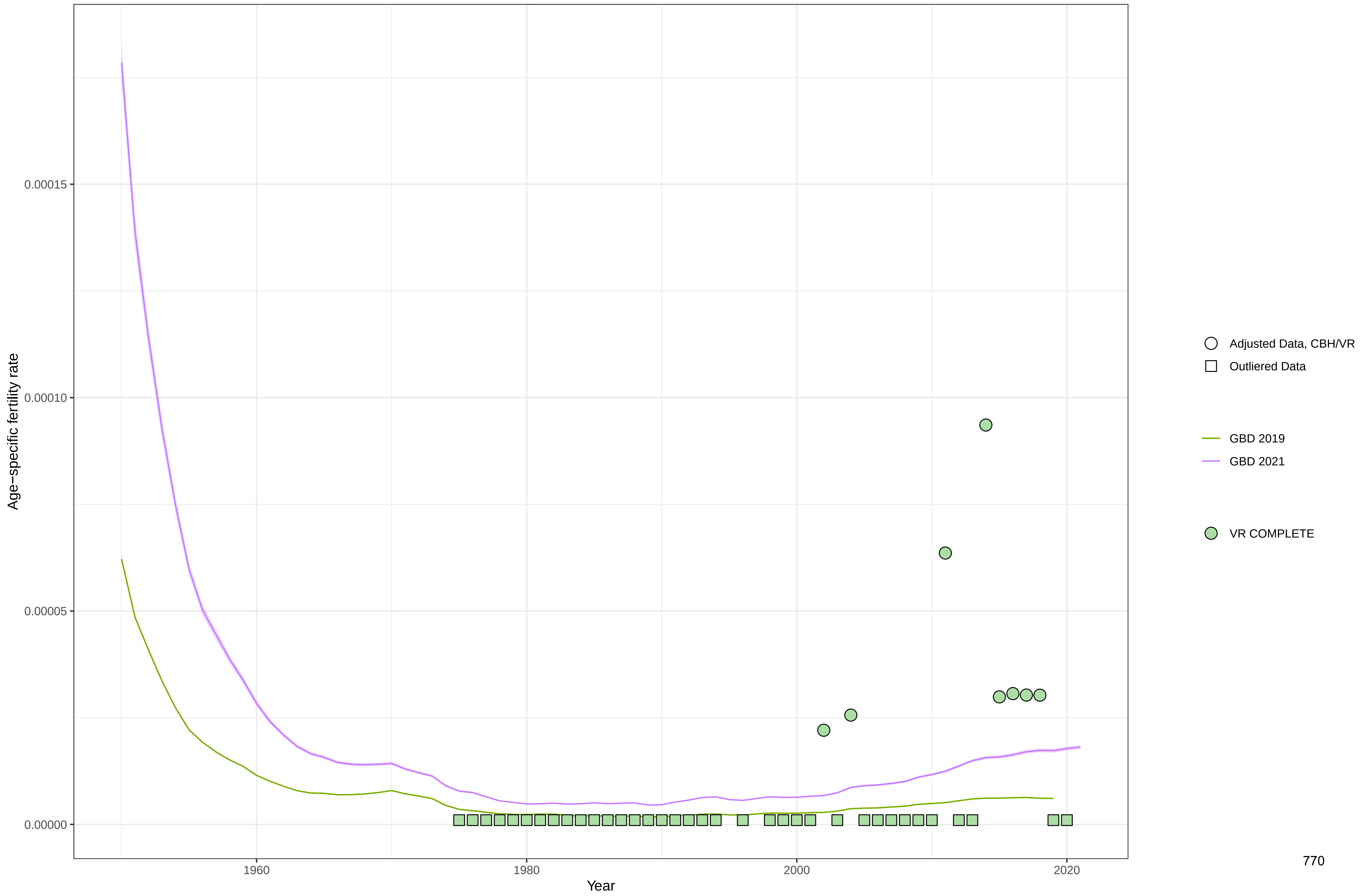

Total fertility rate fitted trend lines, 1950−2021: Wakayama TFR
Data points enclosed with a diamond are reference sources

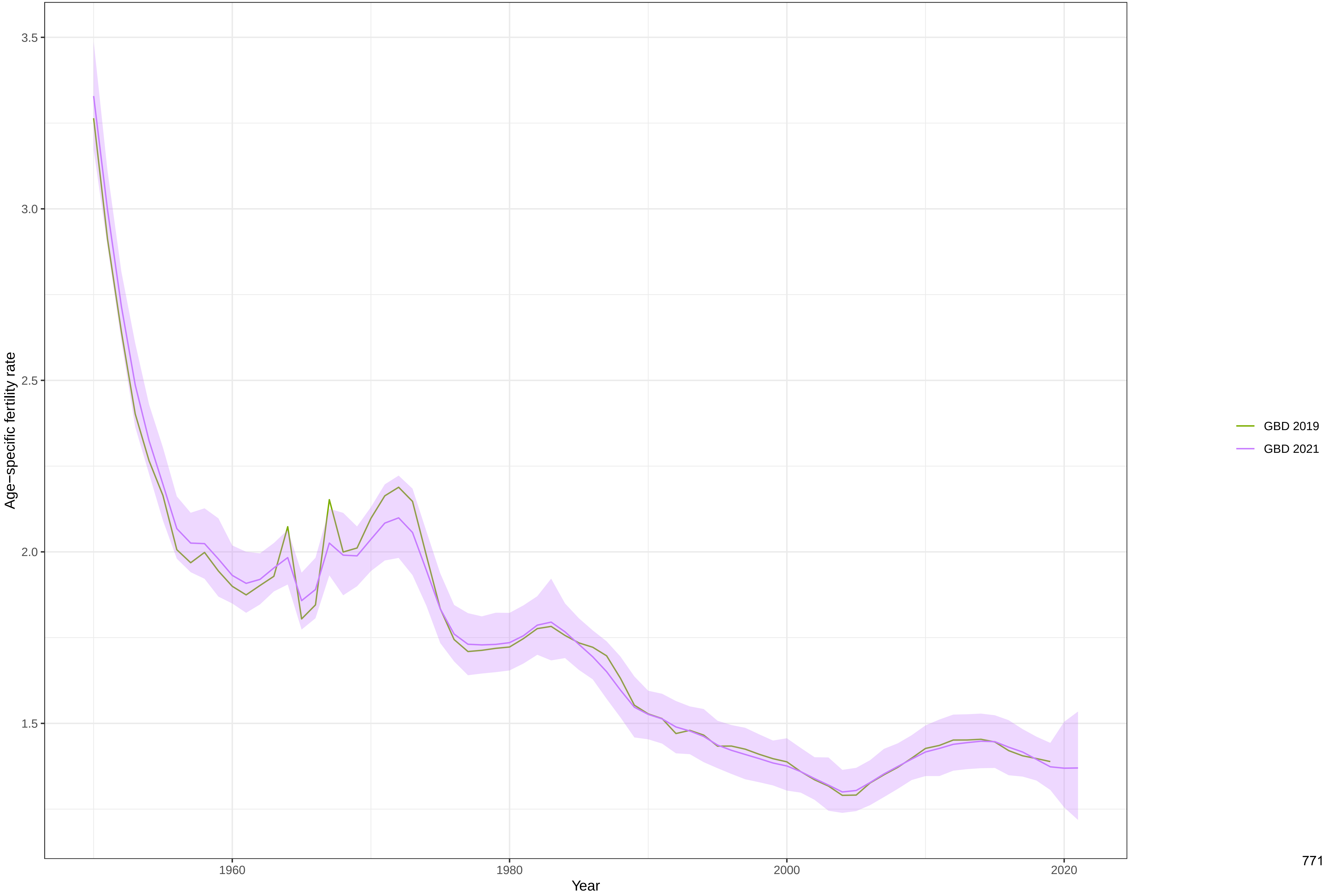

Age−specific fertility fitted trend lines, 1950−2021: Yamagata, Ages: 10−14
Data points enclosed with a diamond are reference sources

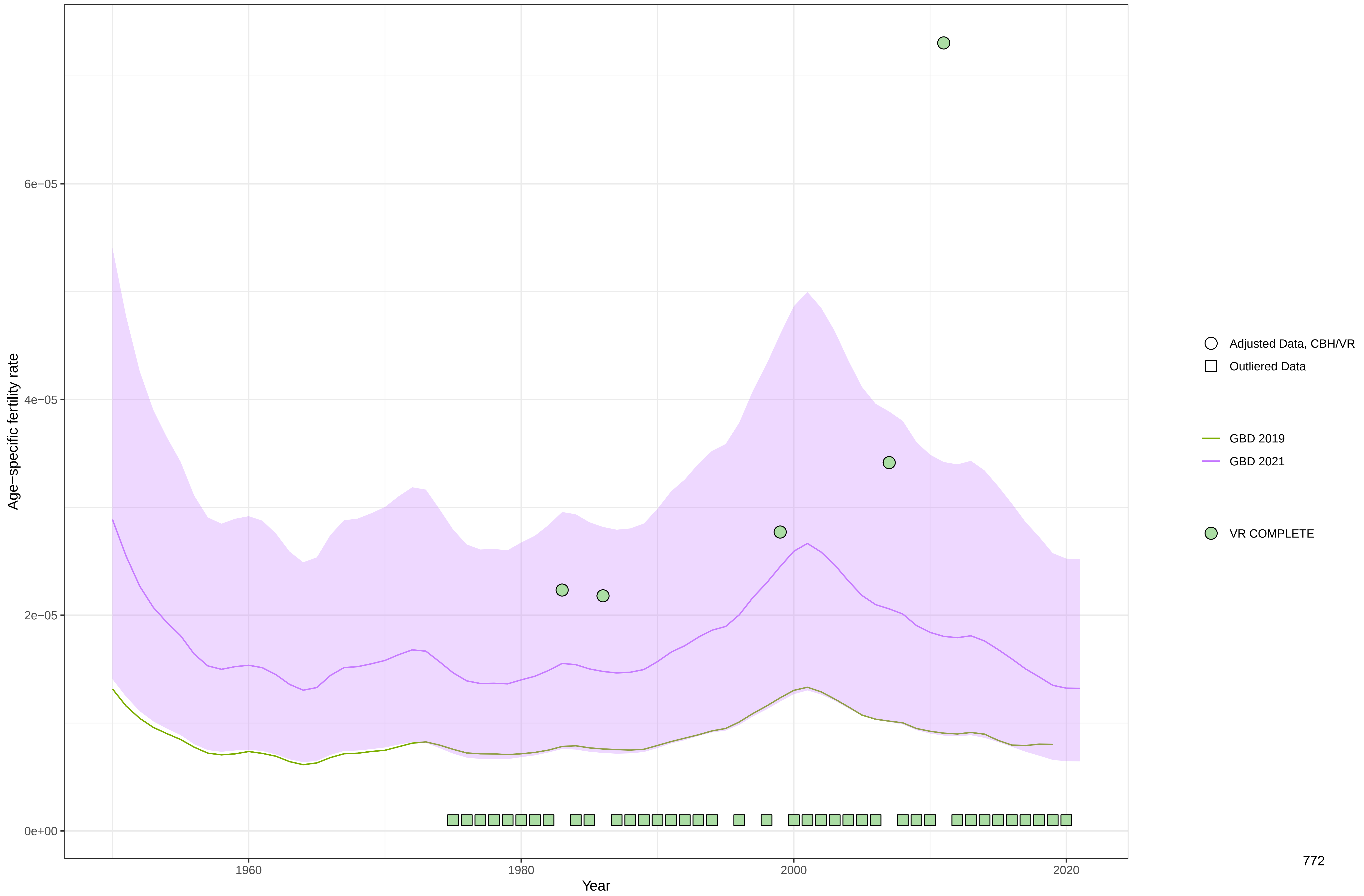

Age−specific fertility fitted trend lines, 1950−2021: Yamagata, Ages: 15−19
Data points enclosed with a diamond are reference sources

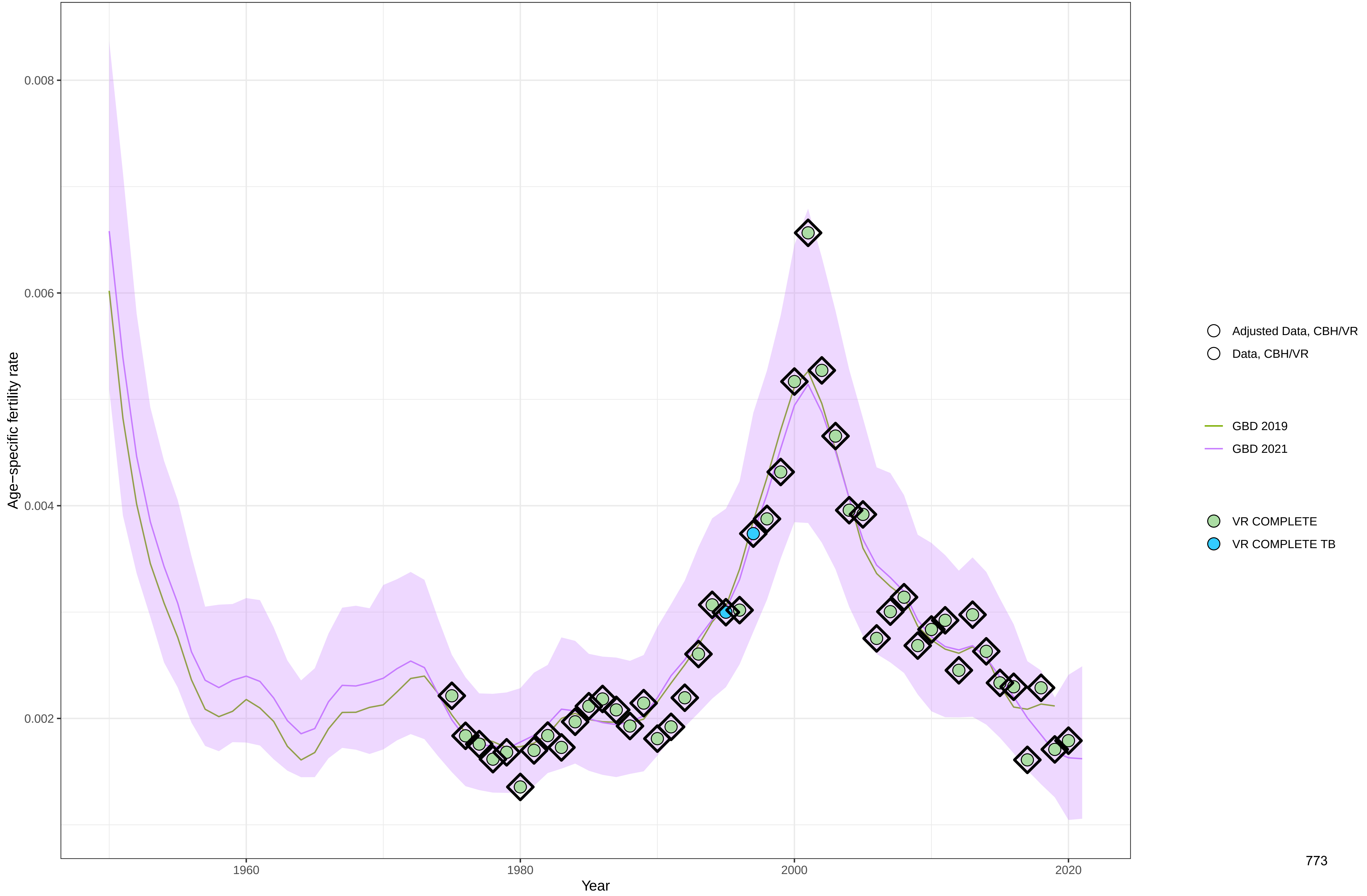

Age−specific fertility fitted trend lines, 1950−2021: Yamagata, Ages: 20−24
Data points enclosed with a diamond are reference sources

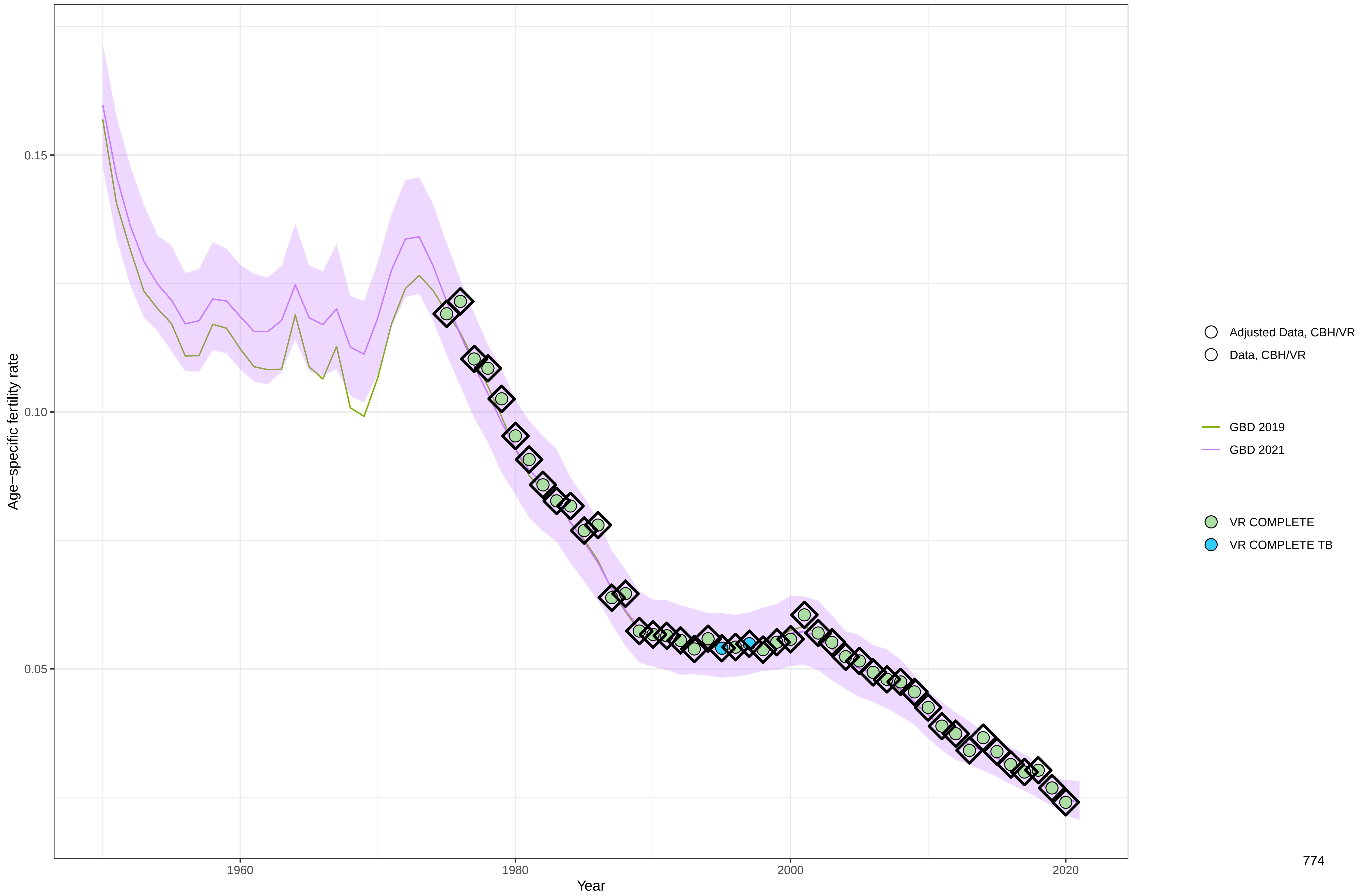

Age−specific fertility fitted trend lines, 1950−2021: Yamagata, Ages: 25−29
Data points enclosed with a diamond are reference sources

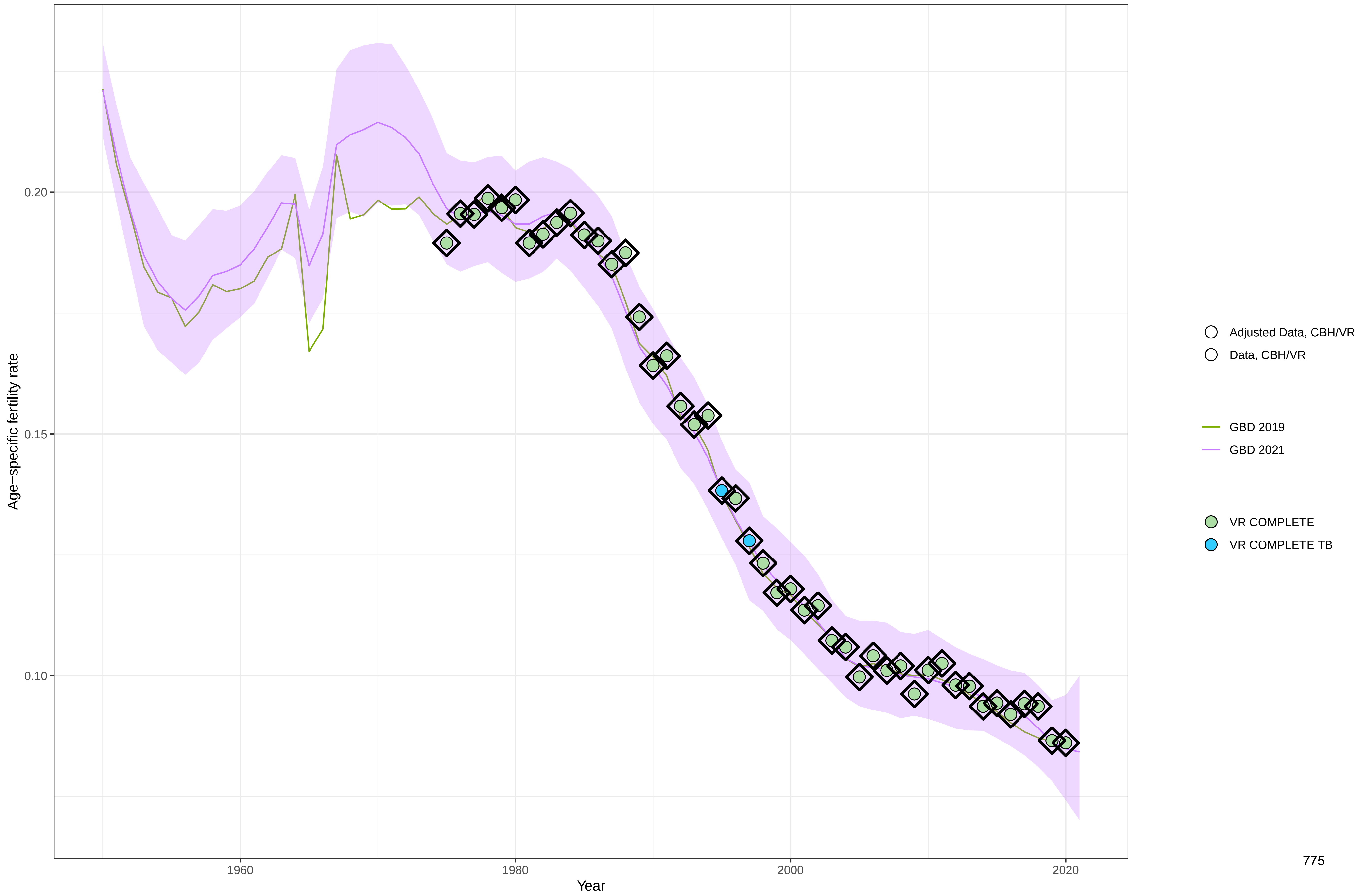

Age−specific fertility fitted trend lines, 1950−2021: Yamagata, Ages: 30−34
Data points enclosed with a diamond are reference sources

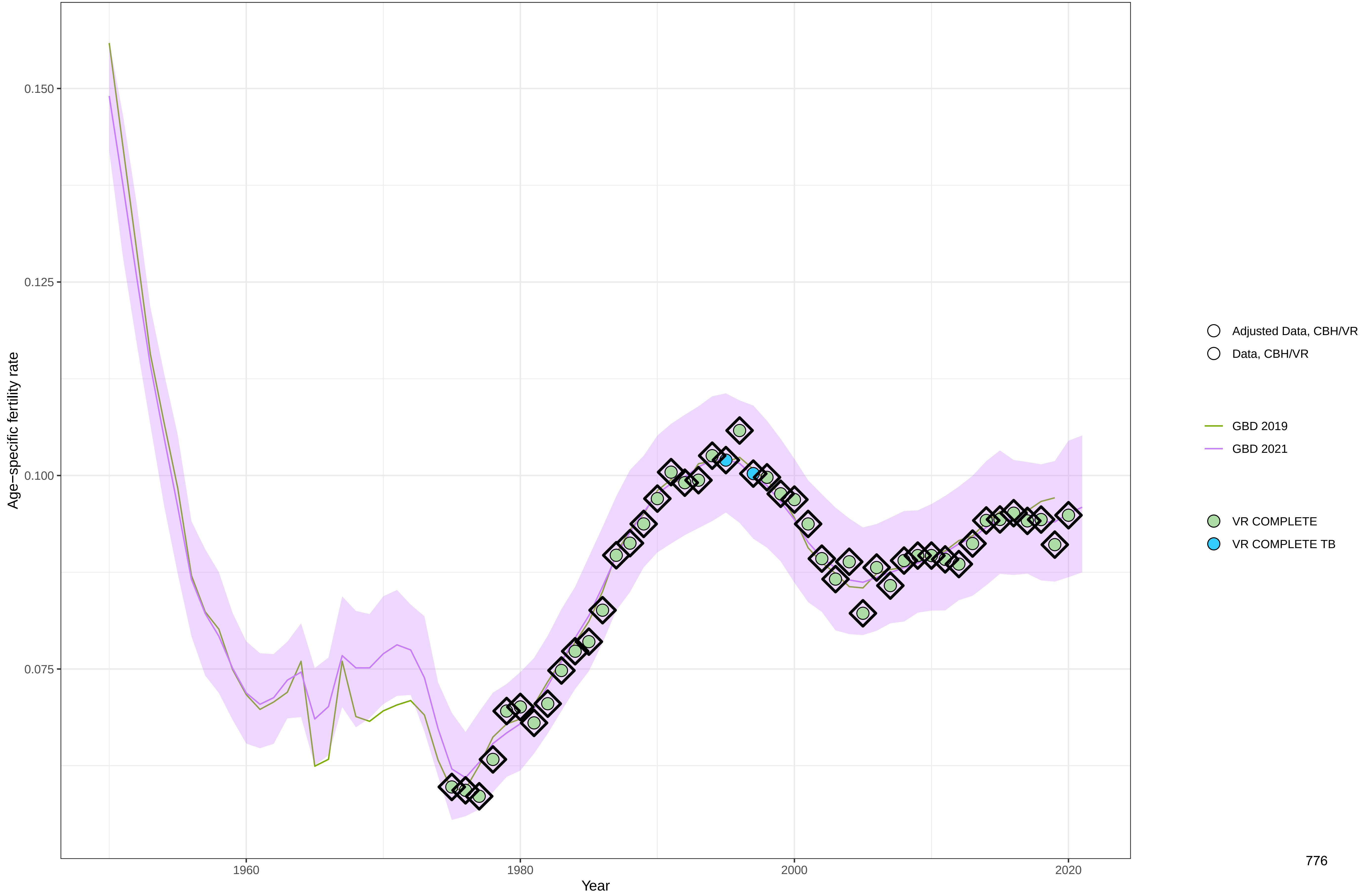

Age−specific fertility fitted trend lines, 1950−2021: Yamagata, Ages: 35−39
Data points enclosed with a diamond are reference sources

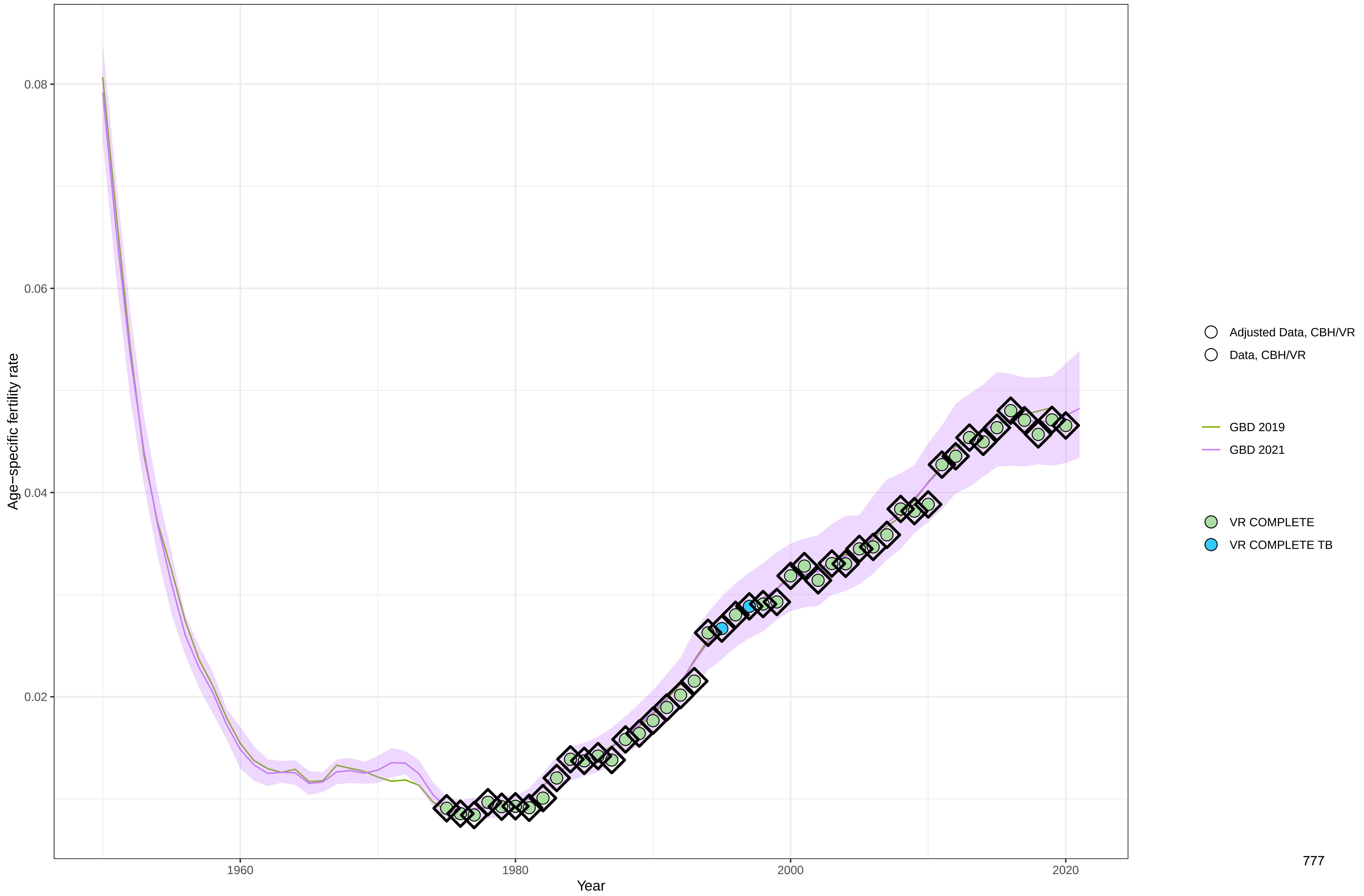

Age−specific fertility fitted trend lines, 1950−2021: Yamagata, Ages: 40−44
Data points enclosed with a diamond are reference sources

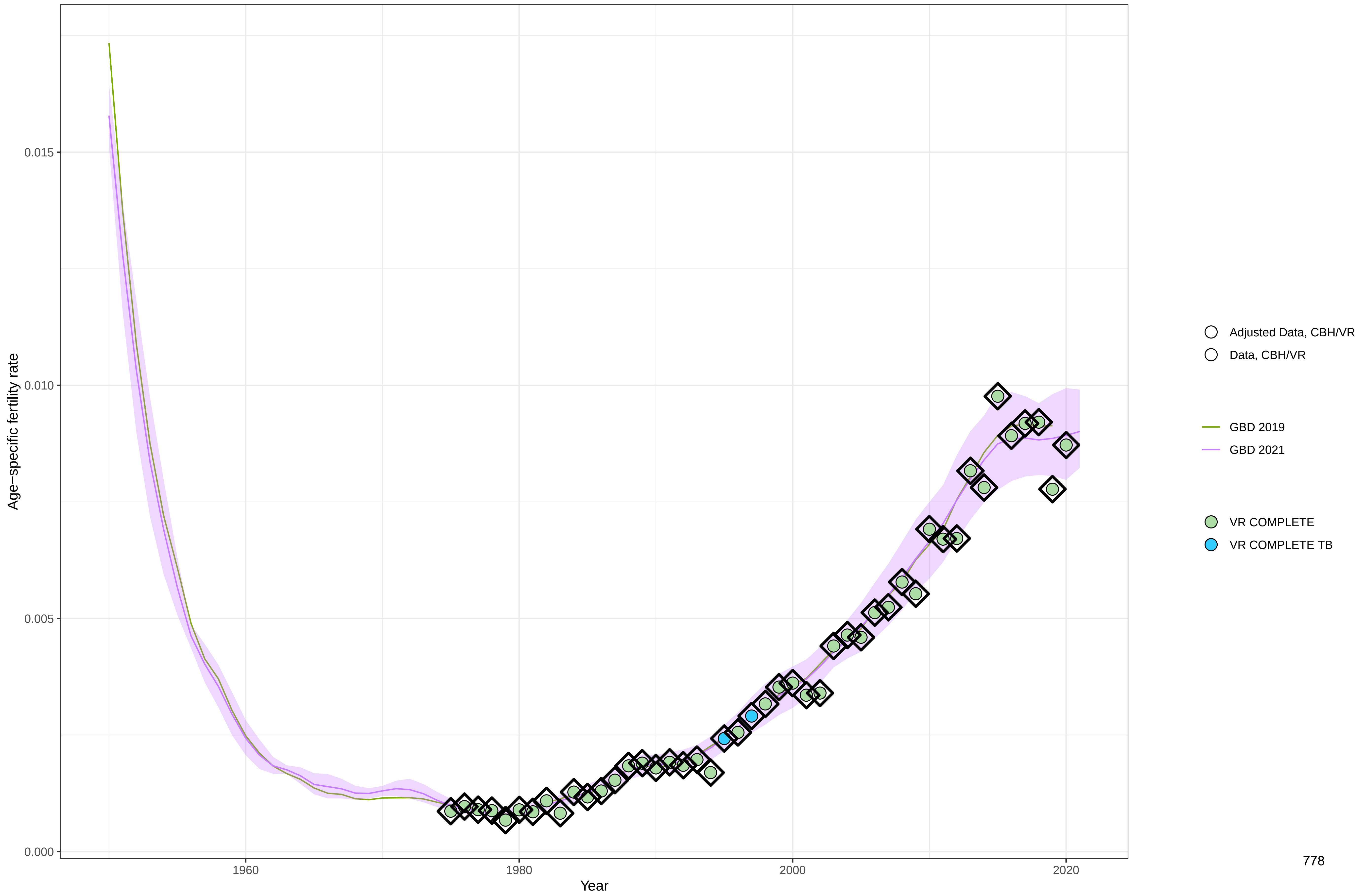

Age−specific fertility fitted trend lines, 1950−2021: Yamagata, Ages: 45−49
Data points enclosed with a diamond are reference sources

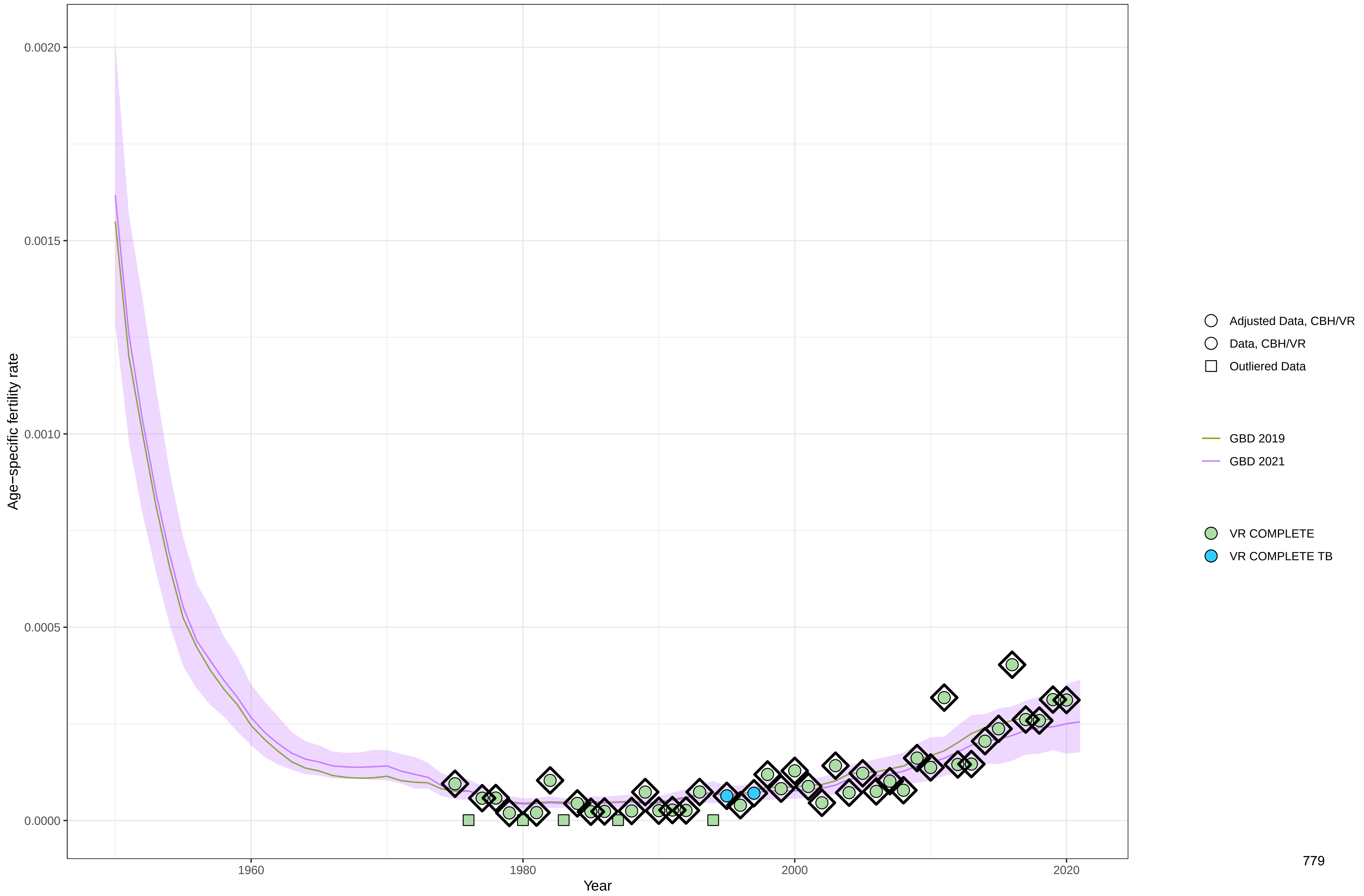

Age−specific fertility fitted trend lines, 1950−2021: Yamagata, Ages: 50−54
Data points enclosed with a diamond are reference sources

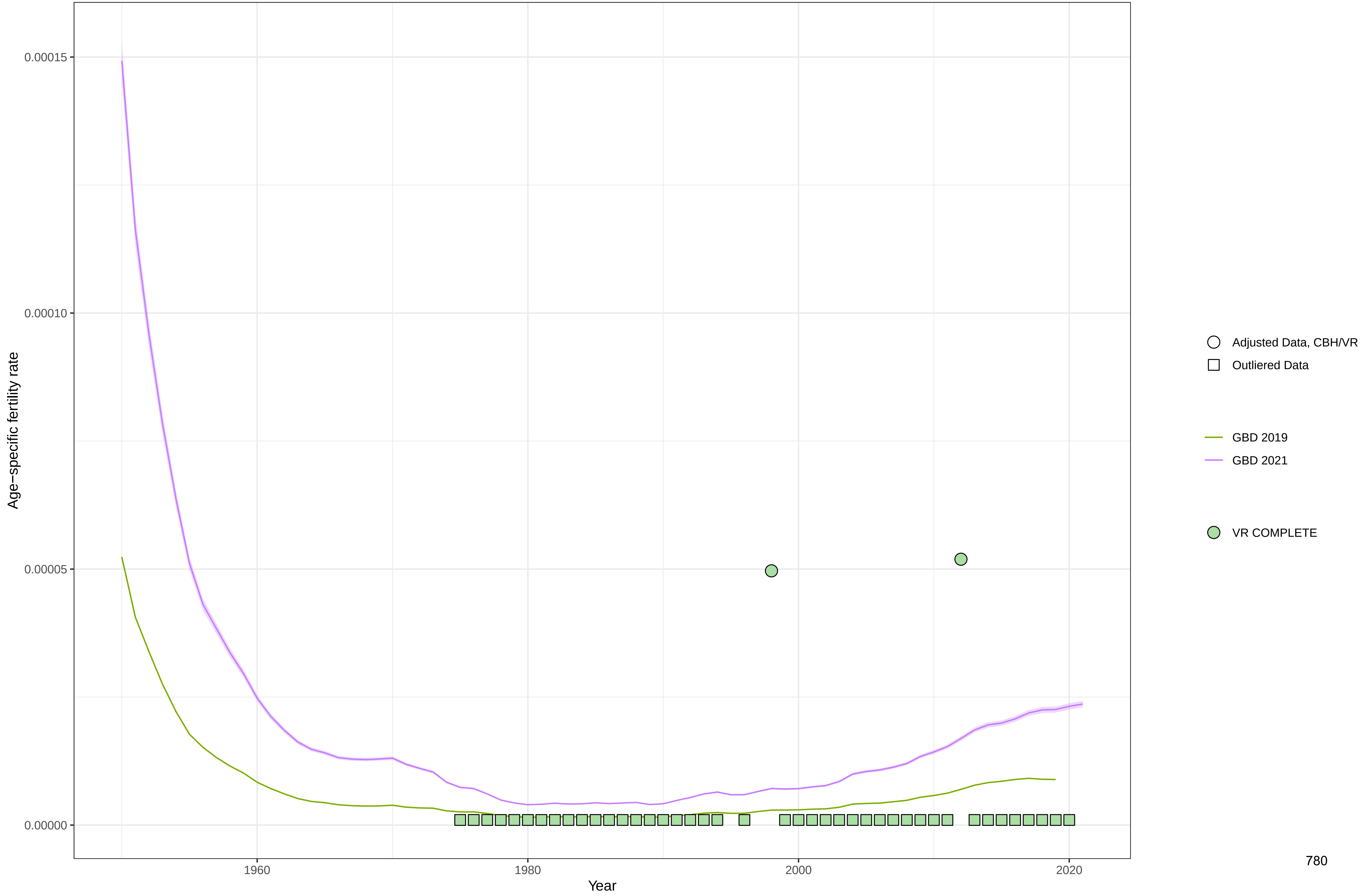

Total fertility rate fitted trend lines, 1950−2021: Yamagata TFR
Data points enclosed with a diamond are reference sources

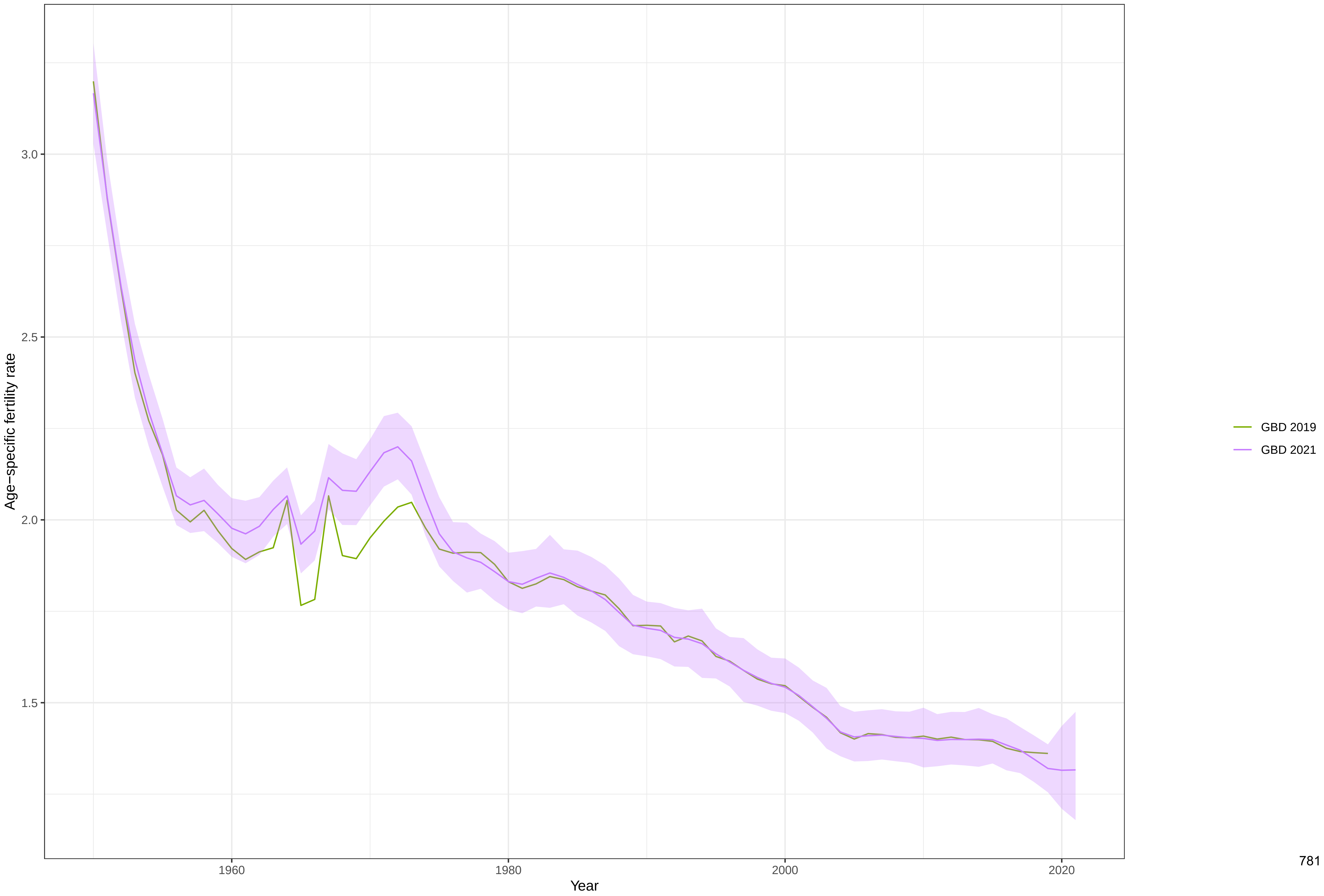

Age−specific fertility fitted trend lines, 1950−2021: Yamaguchi, Ages: 10−14
Data points enclosed with a diamond are reference sources

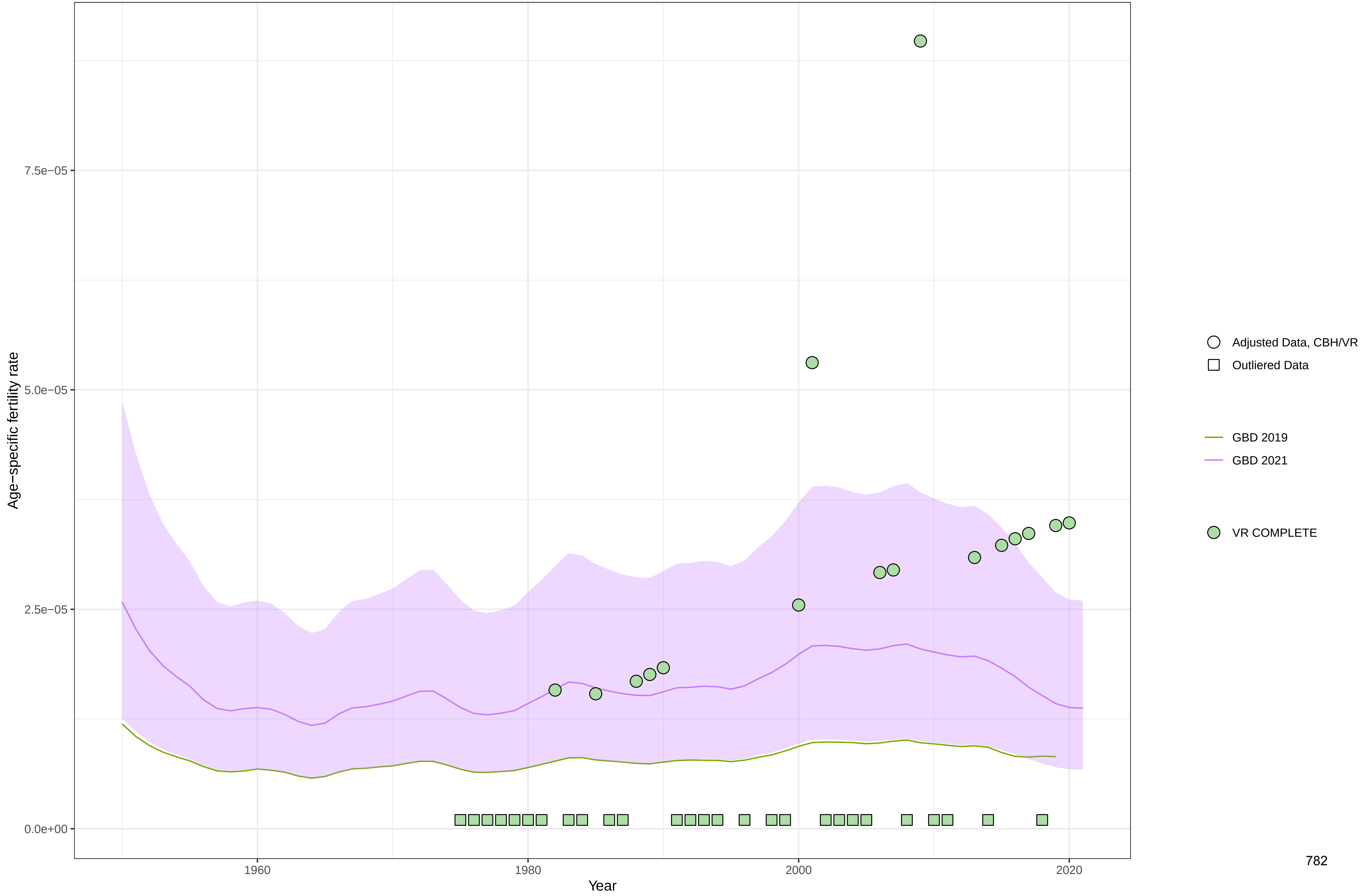

Age−specific fertility fitted trend lines, 1950−2021: Yamaguchi, Ages: 15−19
Data points enclosed with a diamond are reference sources

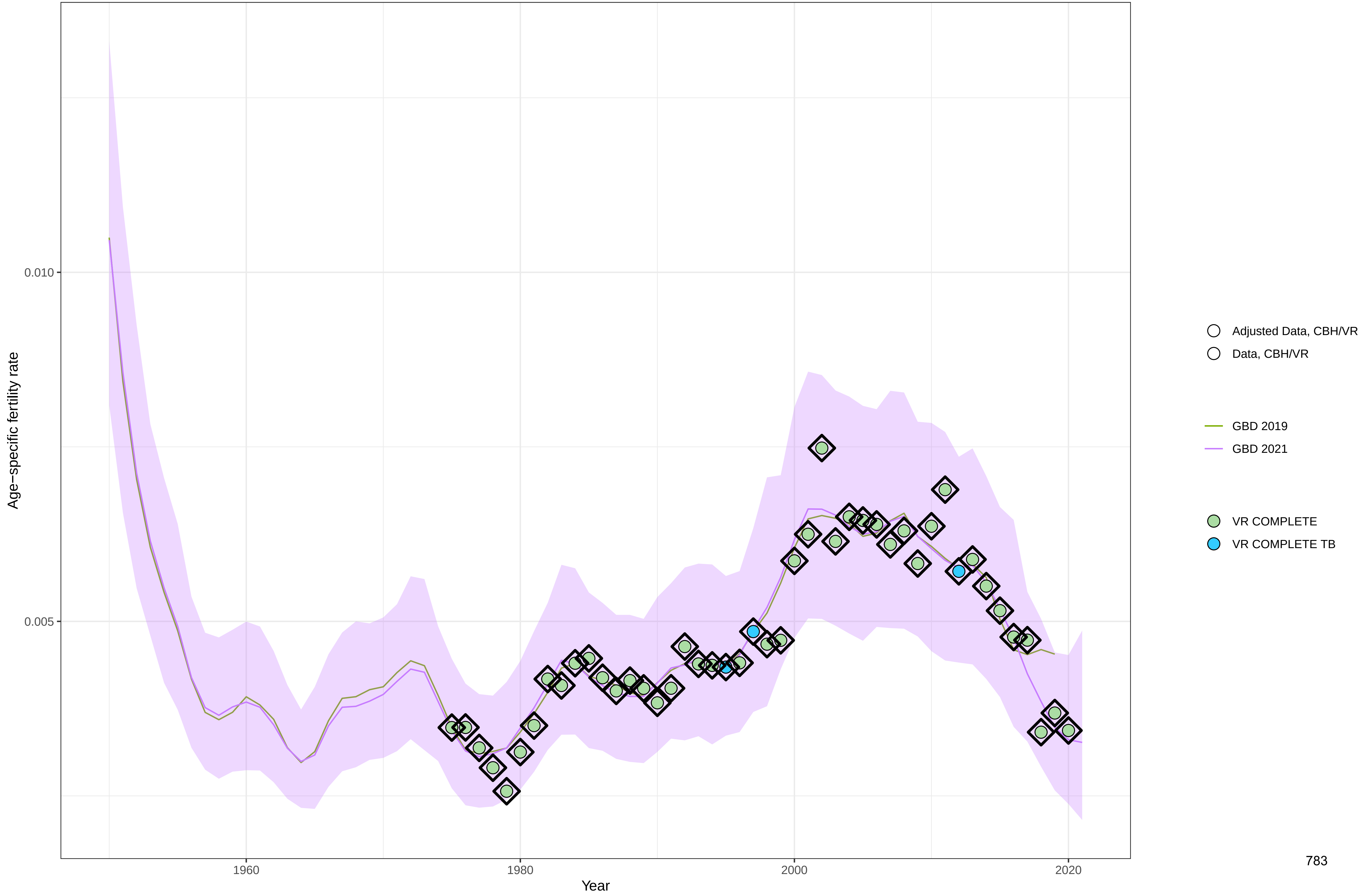

Age−specific fertility fitted trend lines, 1950−2021: Yamaguchi, Ages: 20−24
Data points enclosed with a diamond are reference sources

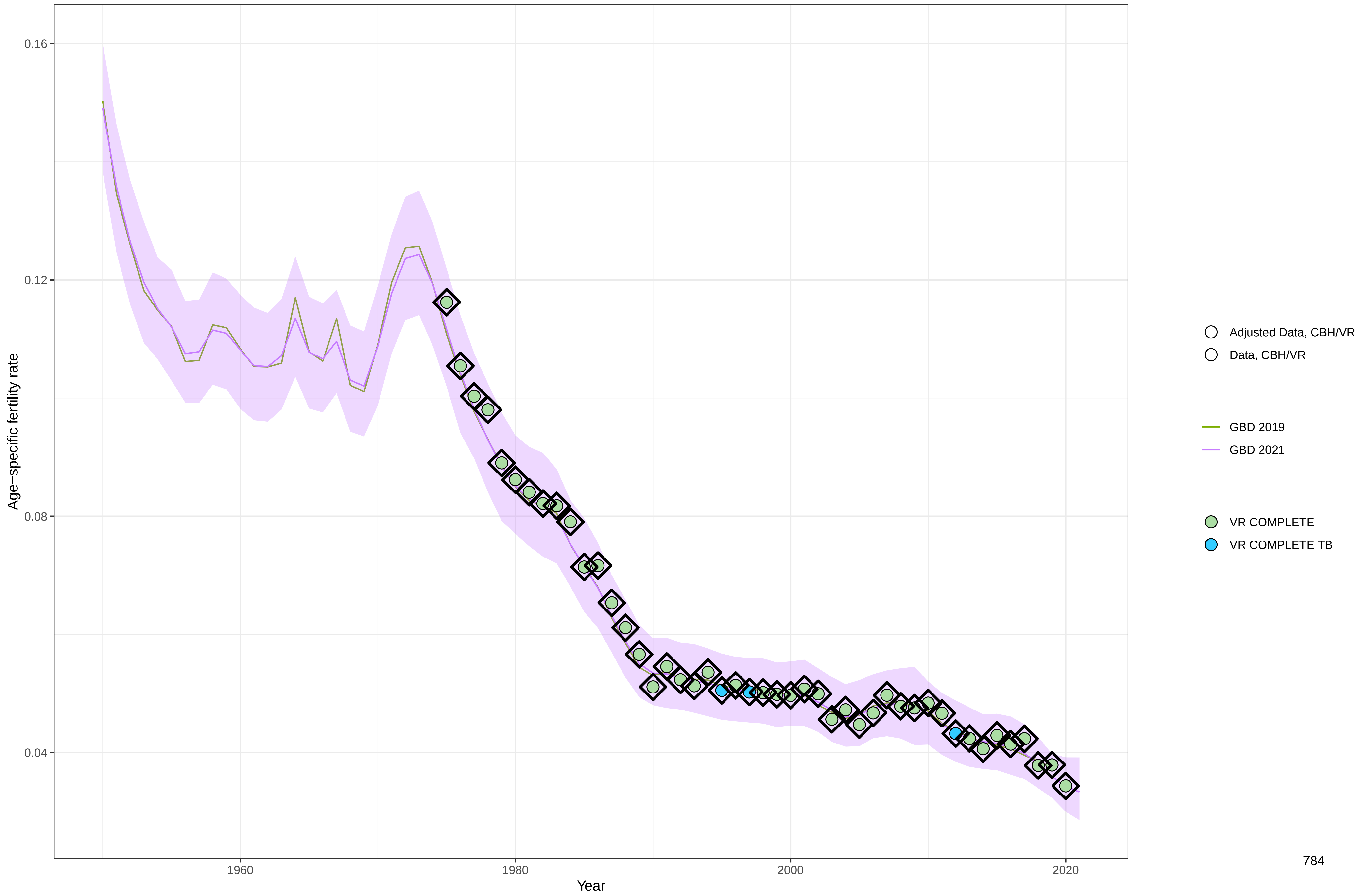

Age−specific fertility fitted trend lines, 1950−2021: Yamaguchi, Ages: 25−29
Data points enclosed with a diamond are reference sources

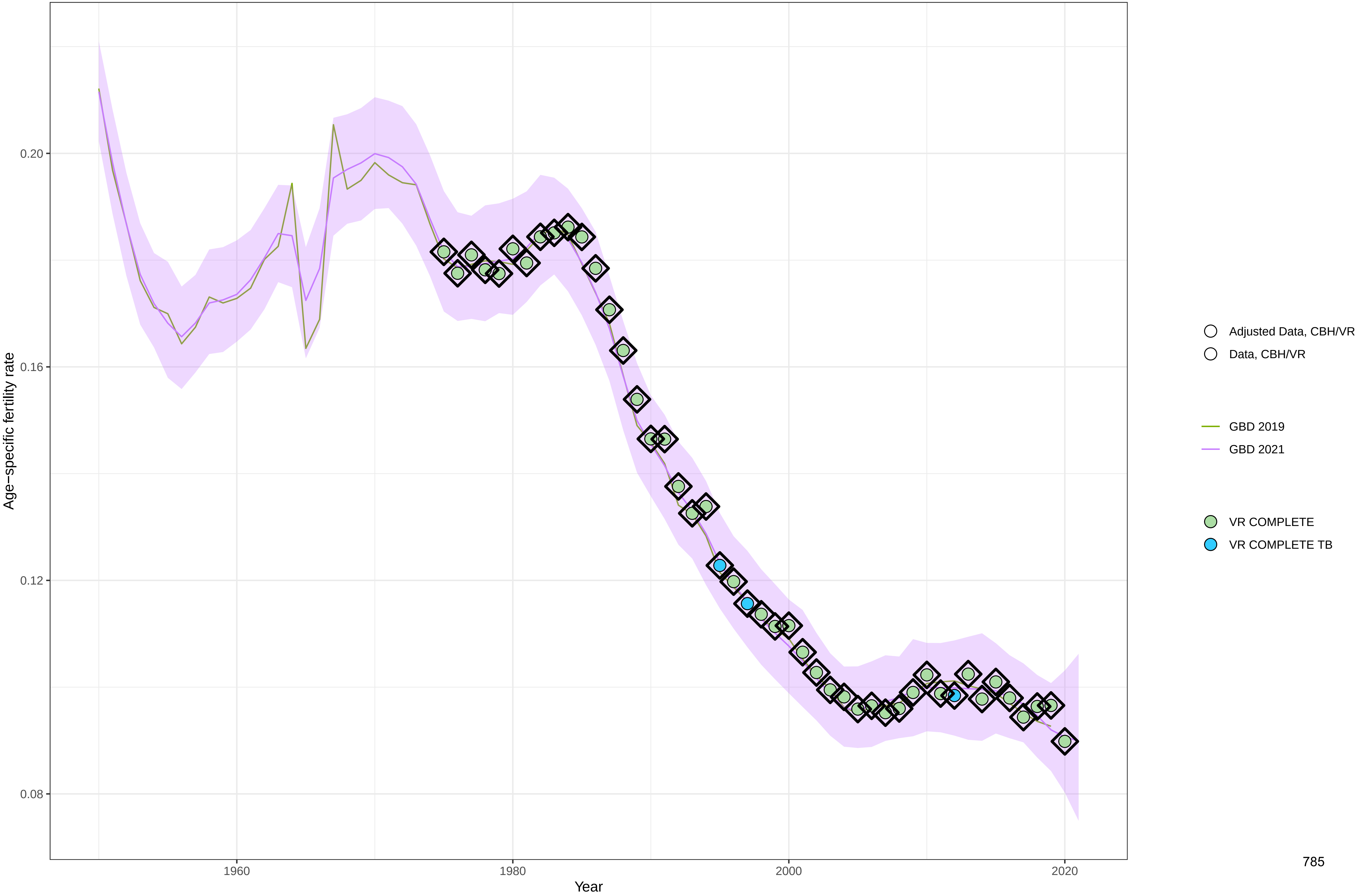

Age−specific fertility fitted trend lines, 1950−2021: Yamaguchi, Ages: 30−34
Data points enclosed with a diamond are reference sources

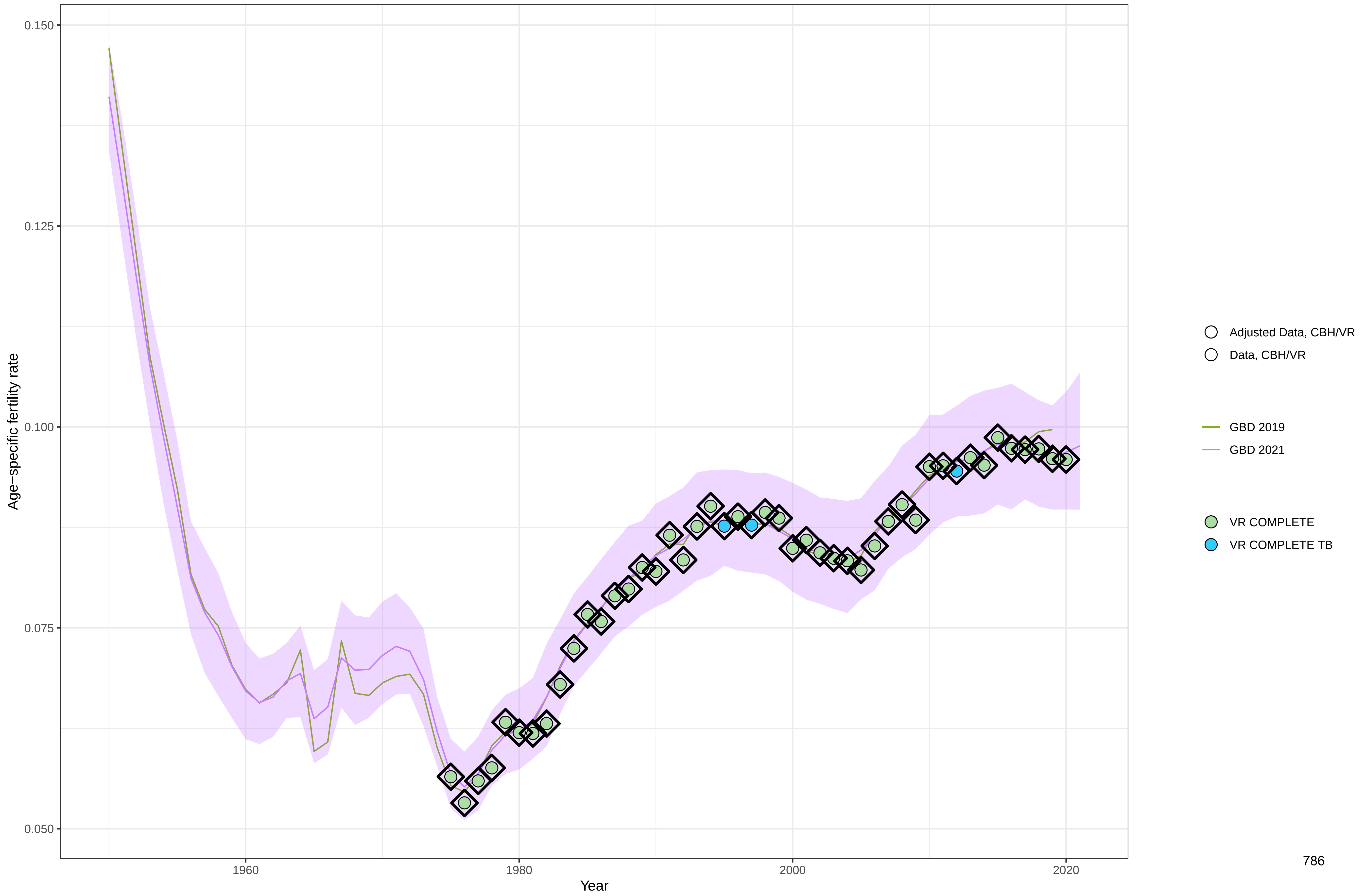

Age−specific fertility fitted trend lines, 1950−2021: Yamaguchi, Ages: 35−39
Data points enclosed with a diamond are reference sources

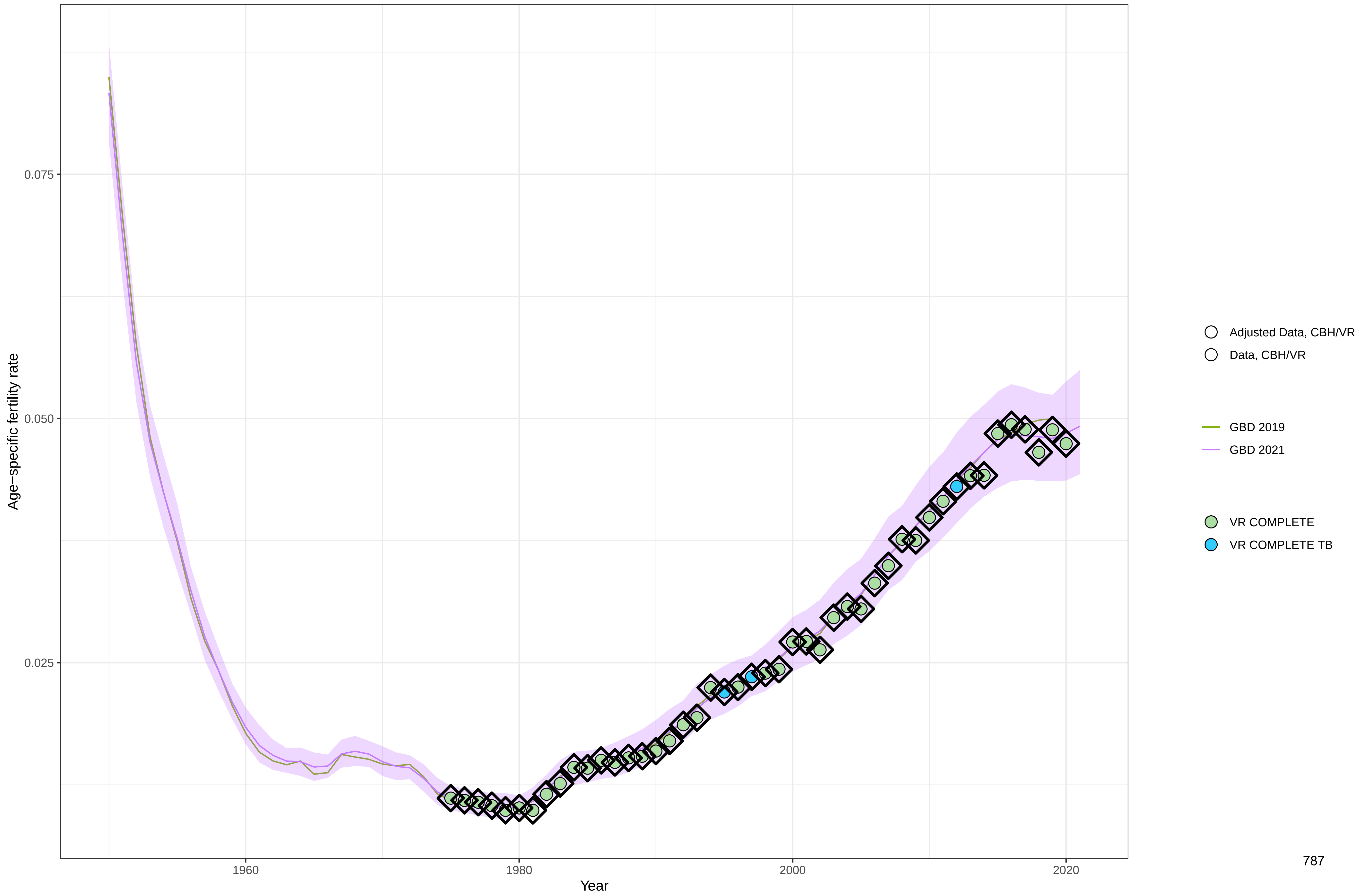

Age−specific fertility fitted trend lines, 1950−2021: Yamaguchi, Ages: 40−44
Data points enclosed with a diamond are reference sources

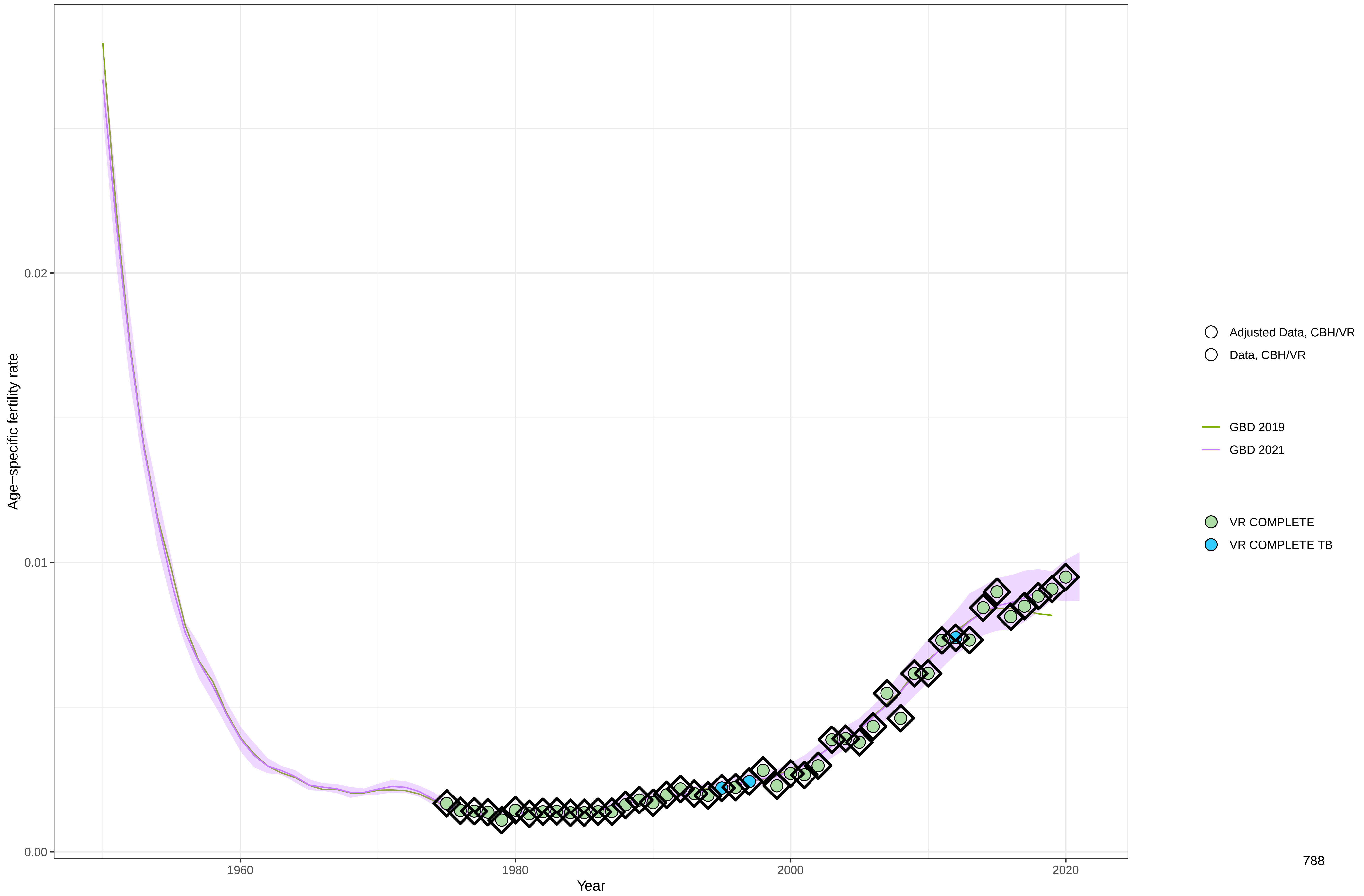

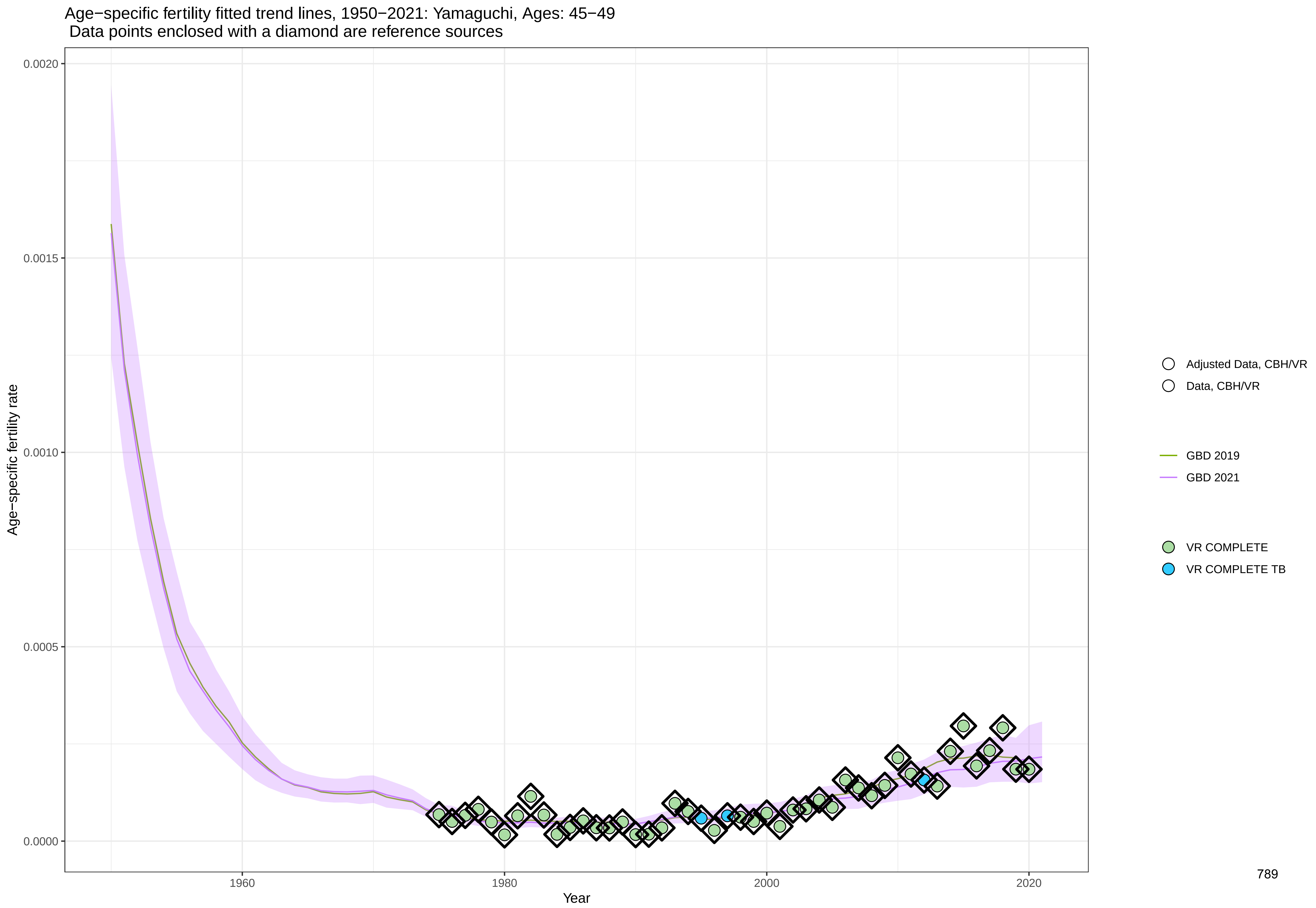

Age−specific fertility fitted trend lines, 1950−2021: Yamaguchi, Ages: 45−49
Data points enclosed with a diamond are reference sources
Age−specific fertility rate
0.0020
0.0015
0.0010
0.0005
0.0000
1960
1980
2000
2020
Year
Adjusted Data, CBH/VR
Data, CBH/VR
GBD 2019
GBD 2021
VR COMPLETE
VR COMPLETE TB

Age−specific fertility fitted trend lines, 1950−2021: Yamaguchi, Ages: 50−54
Data points enclosed with a diamond are reference sources

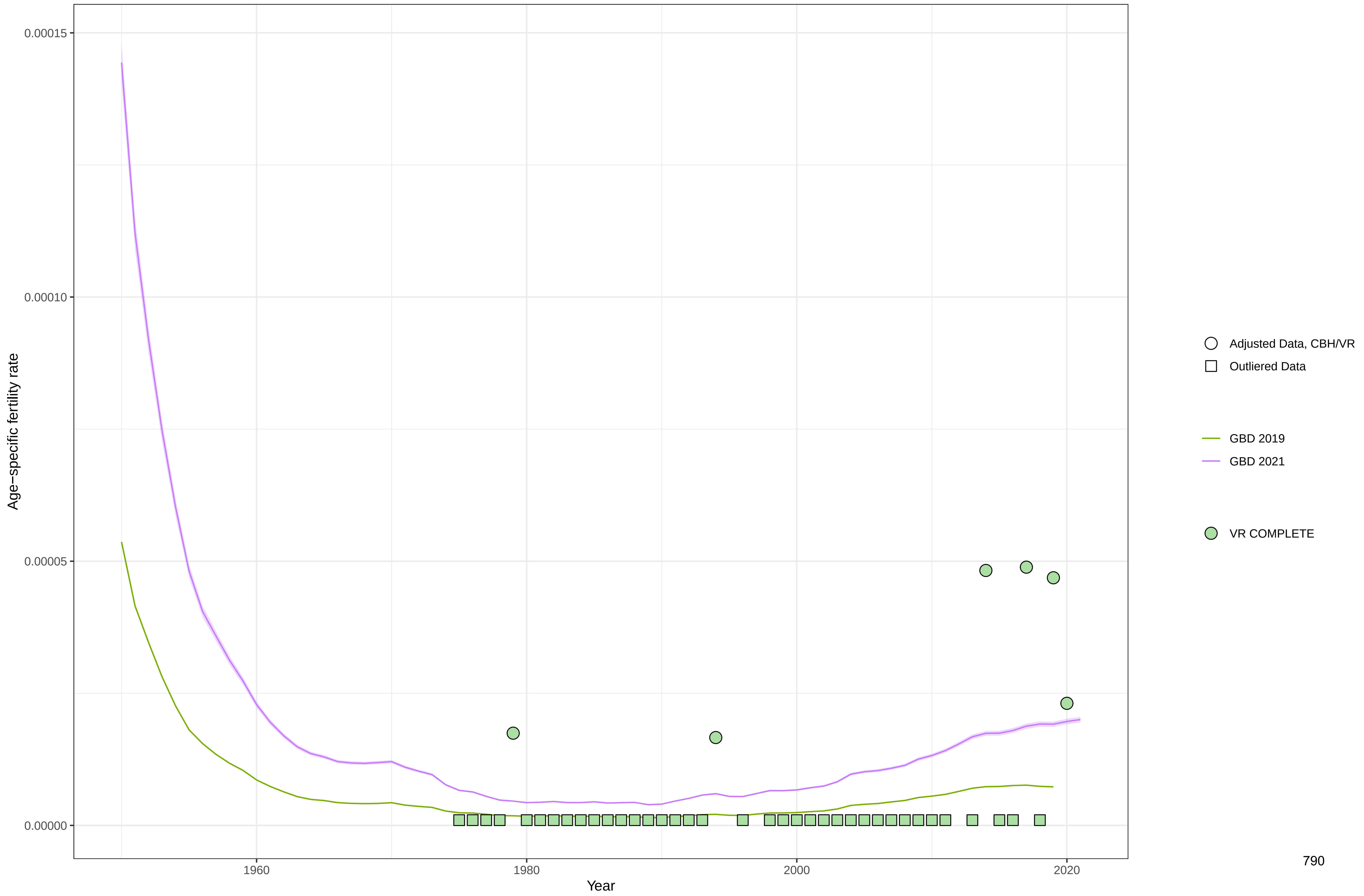

Total fertility rate fitted trend lines, 1950−2021: Yamaguchi TFR
Data points enclosed with a diamond are reference sources

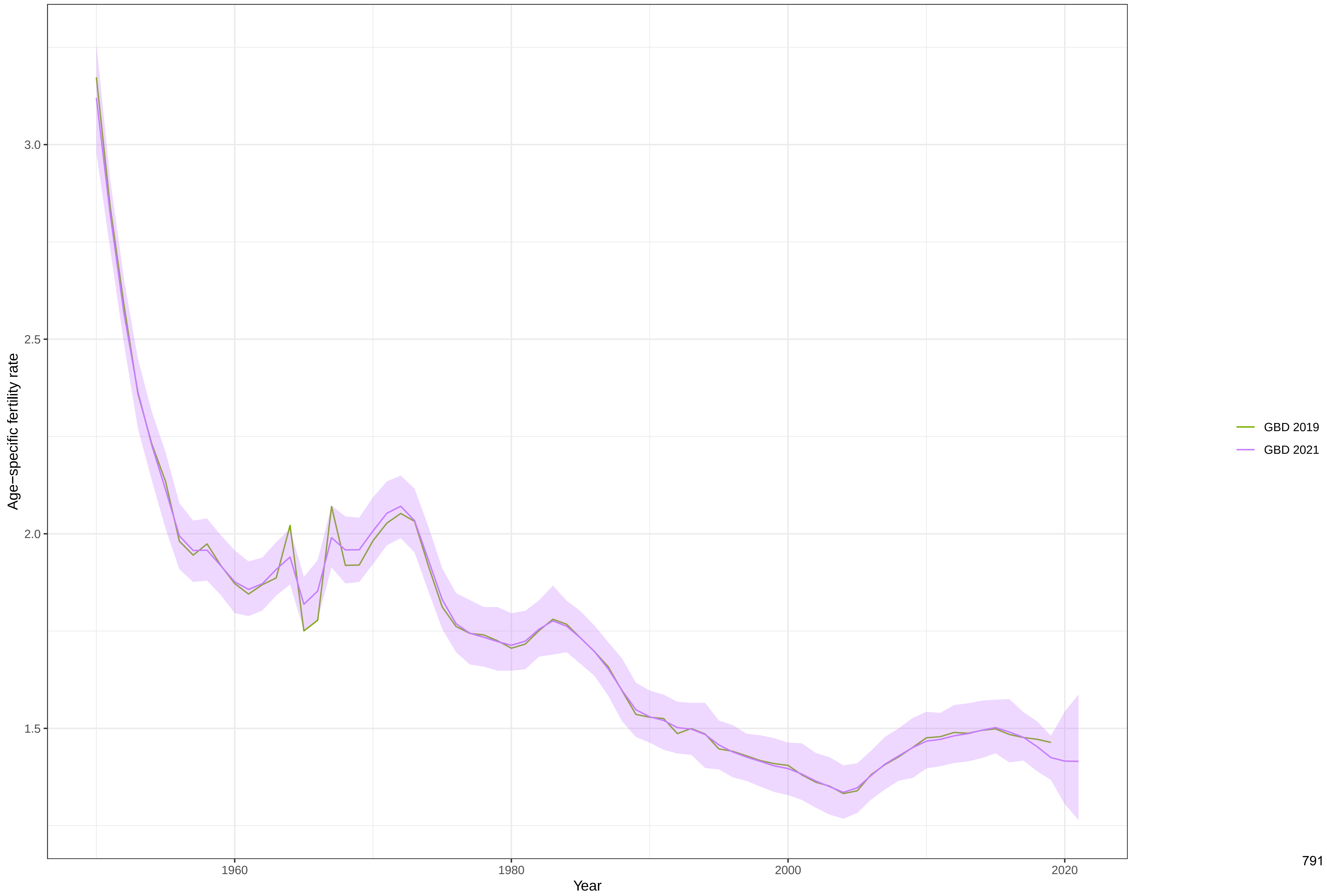

Age−specific fertility fitted trend lines, 1950−2021: Yamanashi, Ages: 10−14
Data points enclosed with a diamond are reference sources

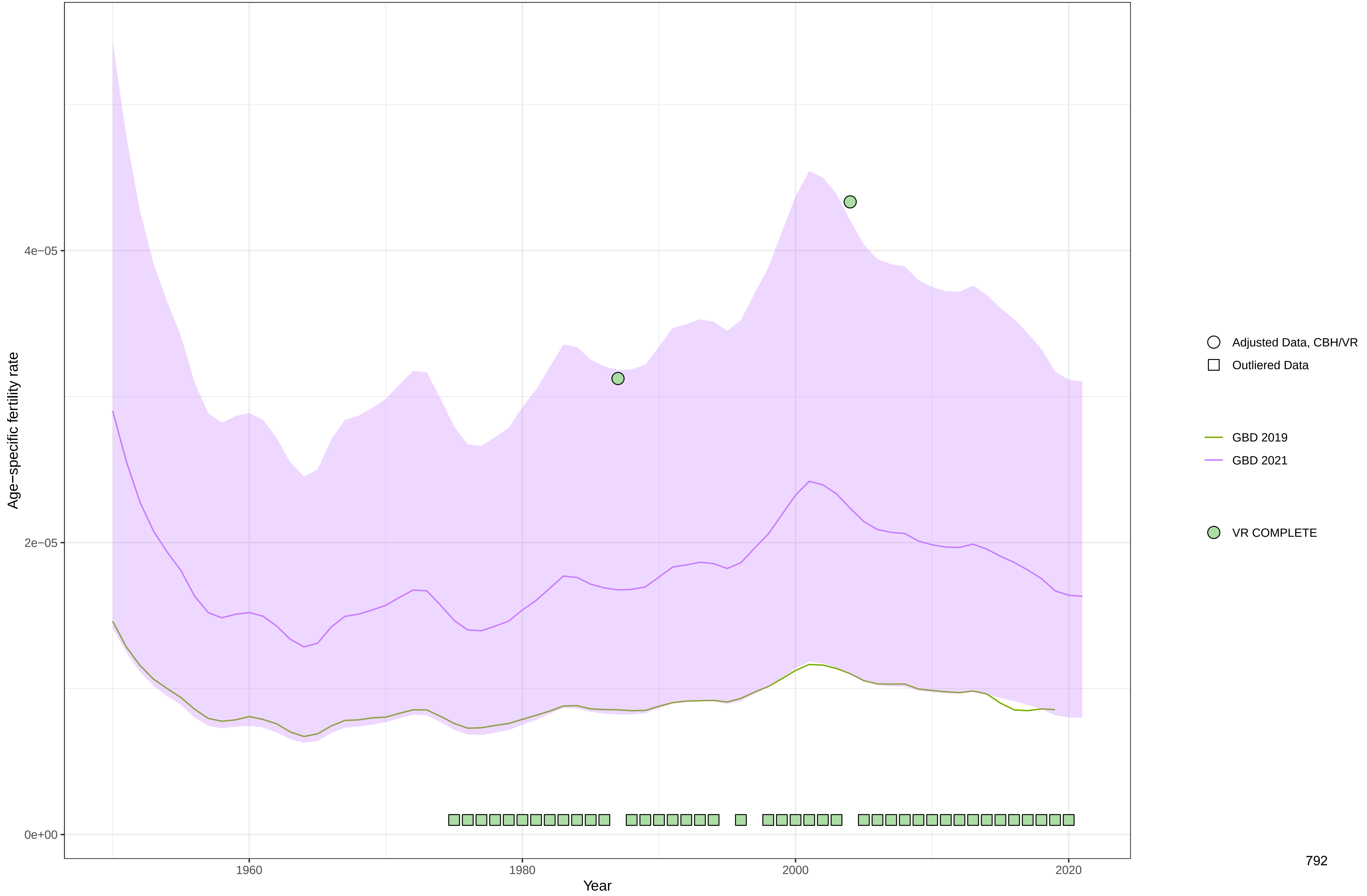

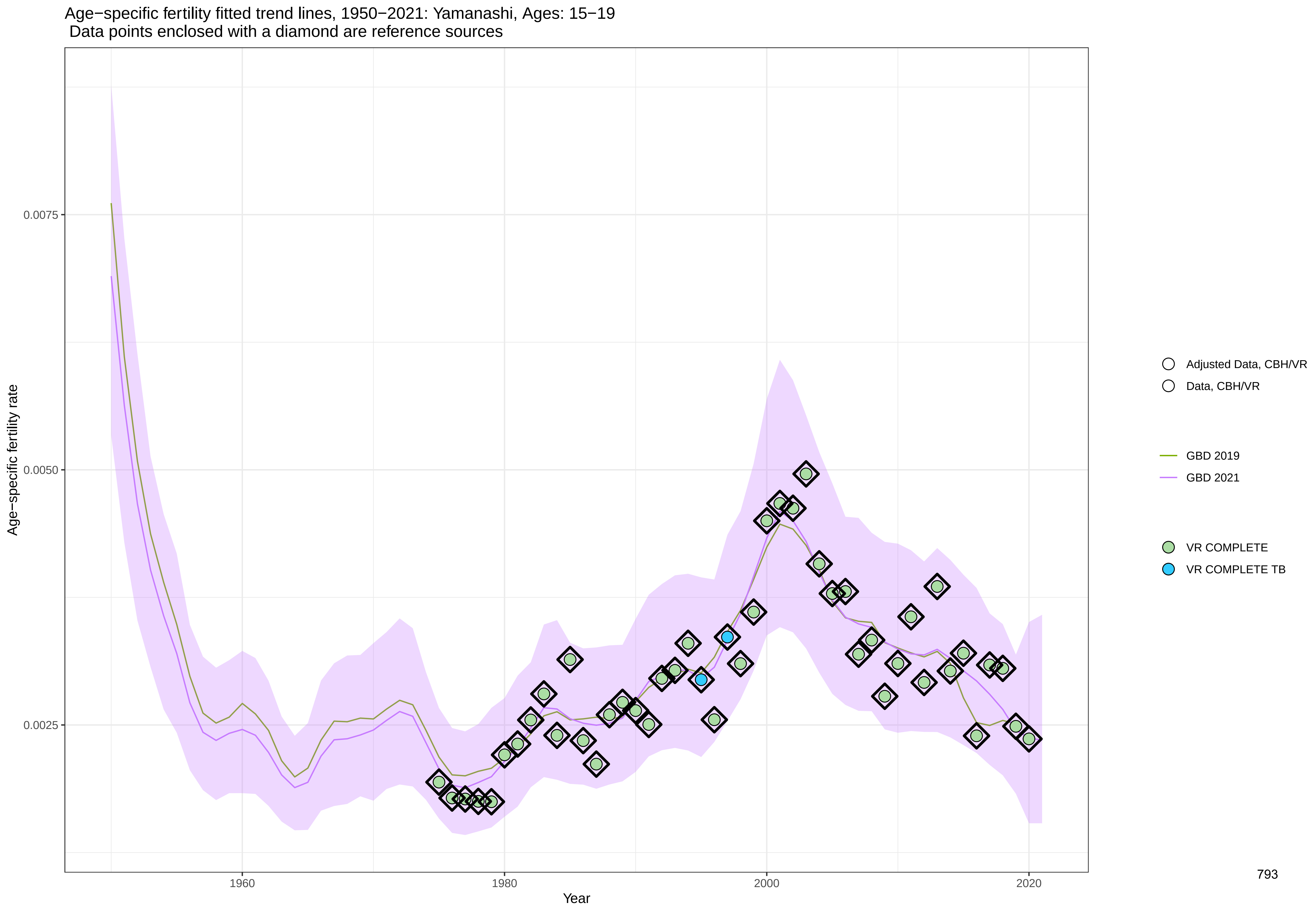

Age−specific fertility fitted trend lines, 1950−2021: Yamanashi, Ages: 15−19
Data points enclosed with a diamond are reference sources
Age−specific fertility rate
0.0075
0.0050
0.0025
1960
1980
2000
2020
Year
Adjusted Data, CBH/VR
Data, CBH/VR
GBD 2019
GBD 2021
VR COMPLETE
VR COMPLETE TB

Age−specific fertility fitted trend lines, 1950−2021: Yamanashi, Ages: 20−24
Data points enclosed with a diamond are reference sources

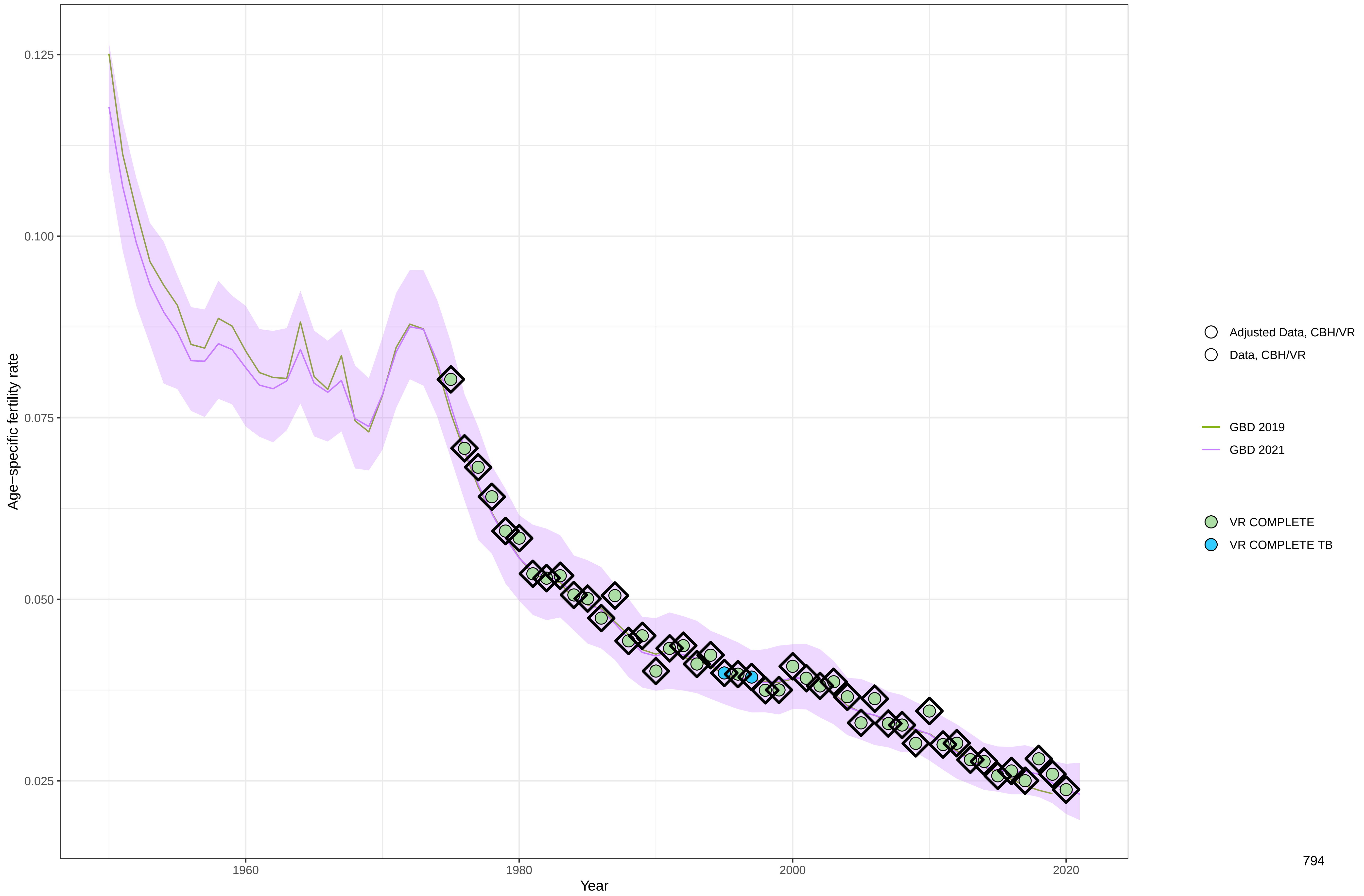

Age−specific fertility fitted trend lines, 1950−2021: Yamanashi, Ages: 25−29
Data points enclosed with a diamond are reference sources

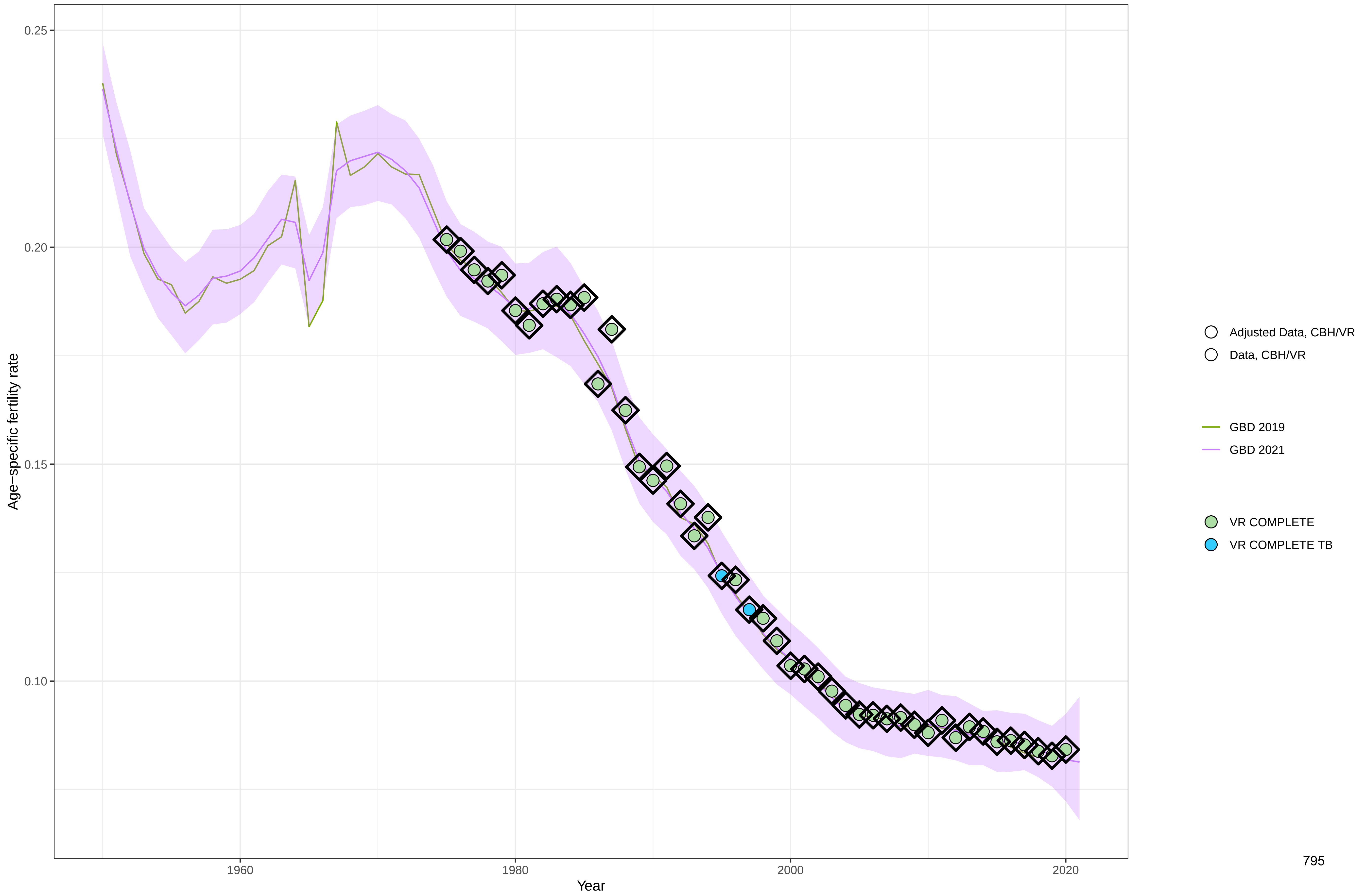

Age−specific fertility fitted trend lines, 1950−2021: Yamanashi, Ages: 30−34
Data points enclosed with a diamond are reference sources

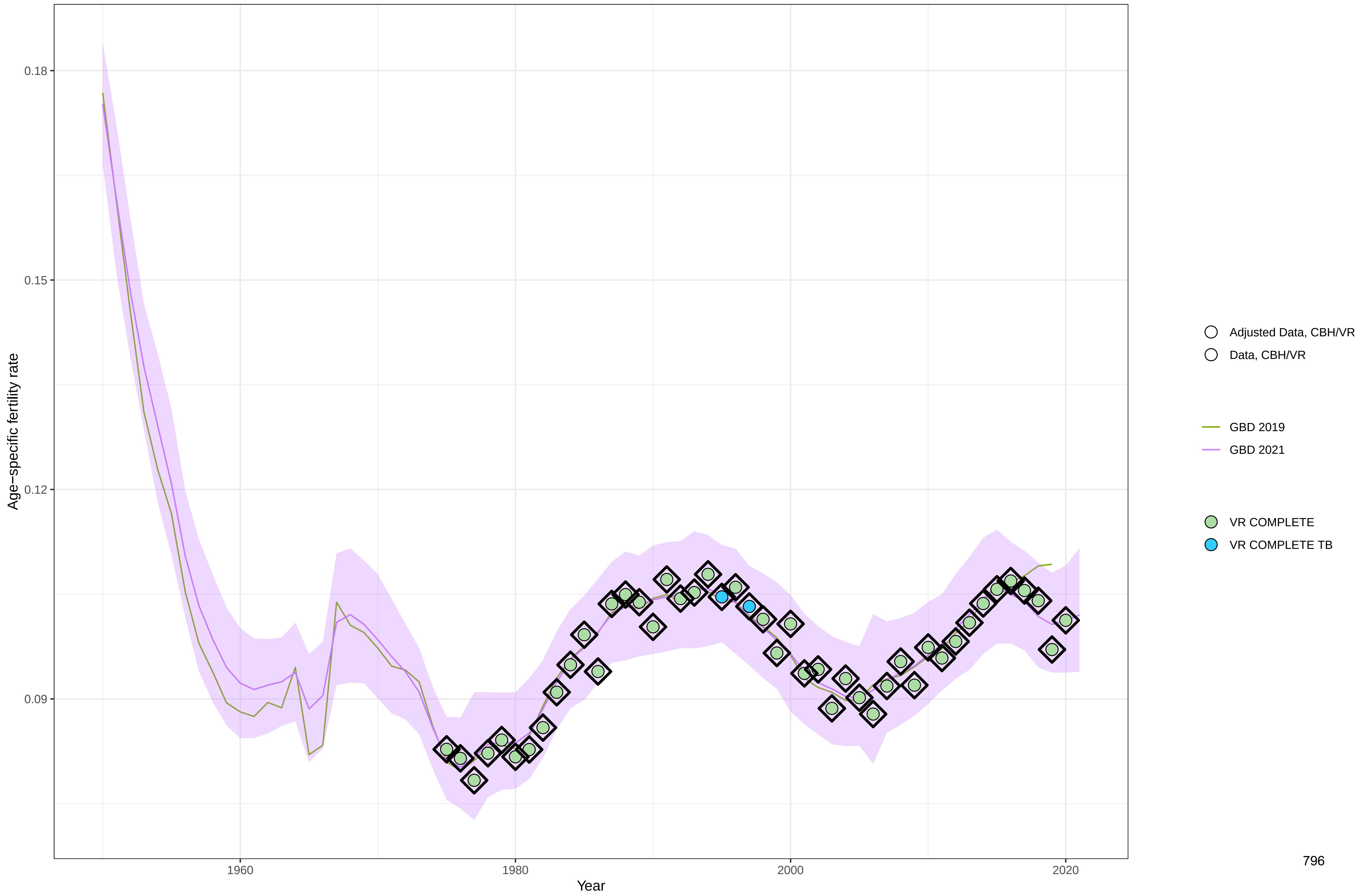

Age−specific fertility fitted trend lines, 1950−2021: Yamanashi, Ages: 35−39
Data points enclosed with a diamond are reference sources

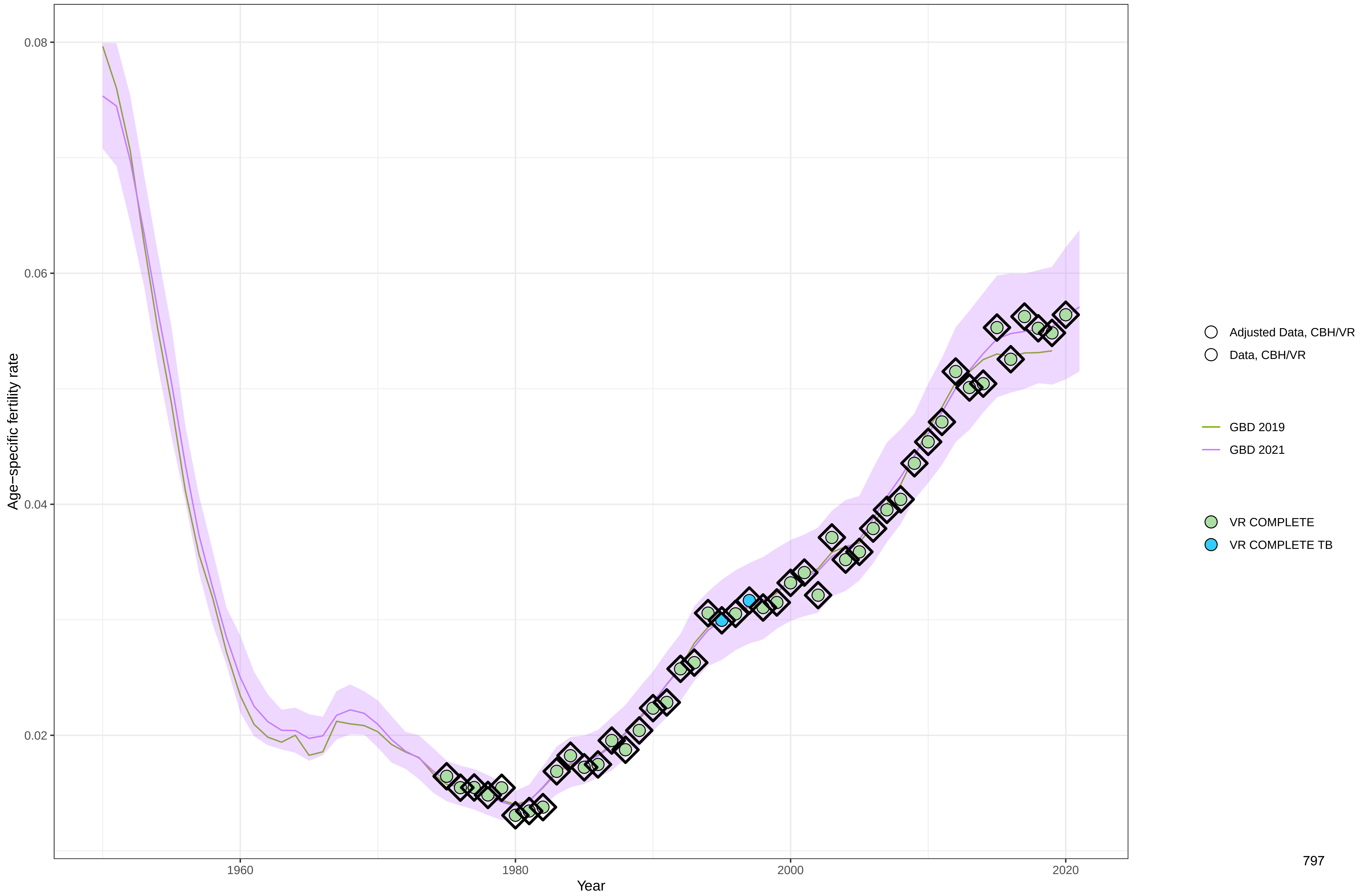

Age−specific fertility fitted trend lines, 1950−2021: Yamanashi, Ages: 40−44
Data points enclosed with a diamond are reference sources

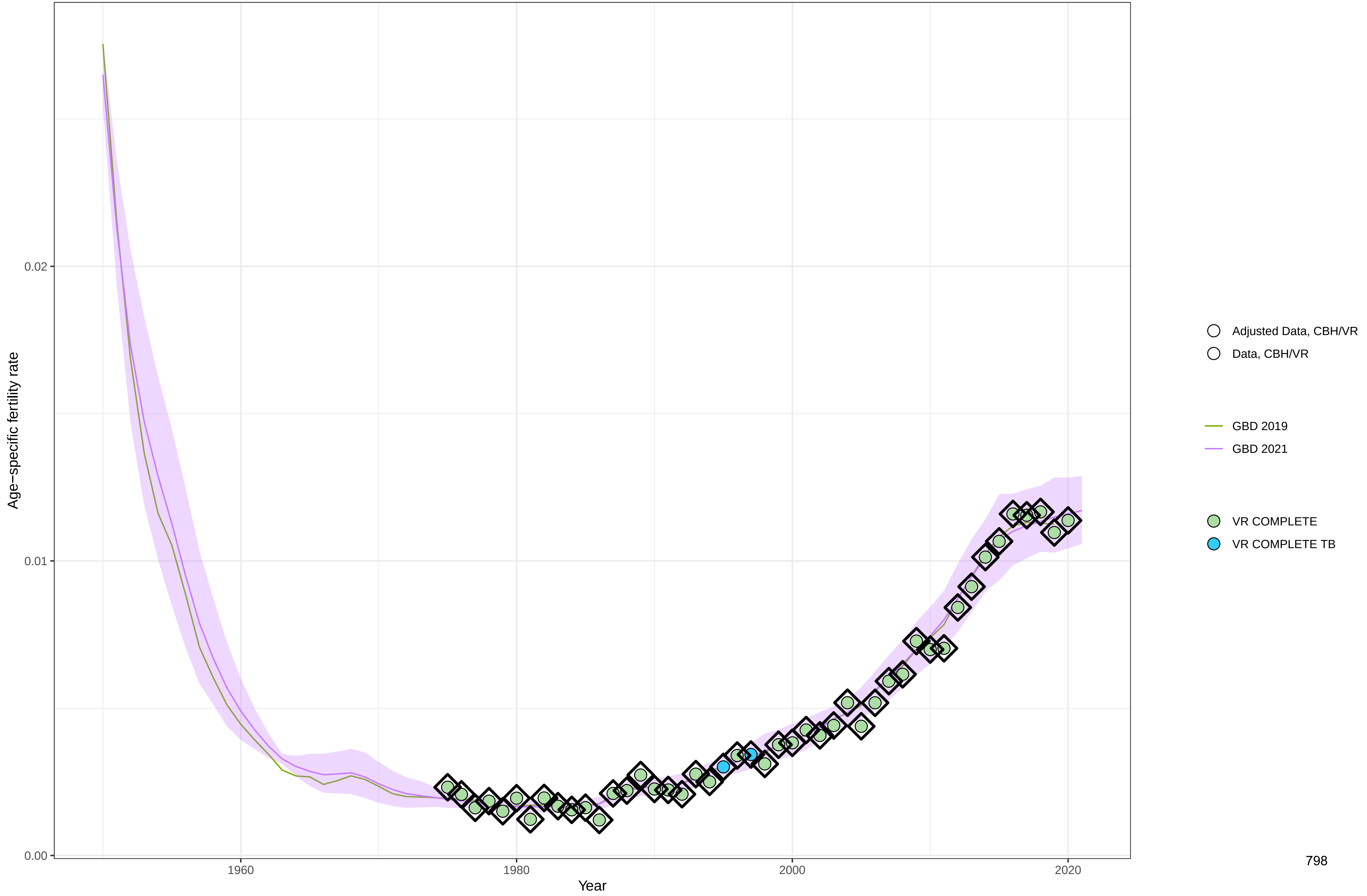

Age−specific fertility fitted trend lines, 1950−2021: Yamanashi, Ages: 45−49
Data points enclosed with a diamond are reference sources

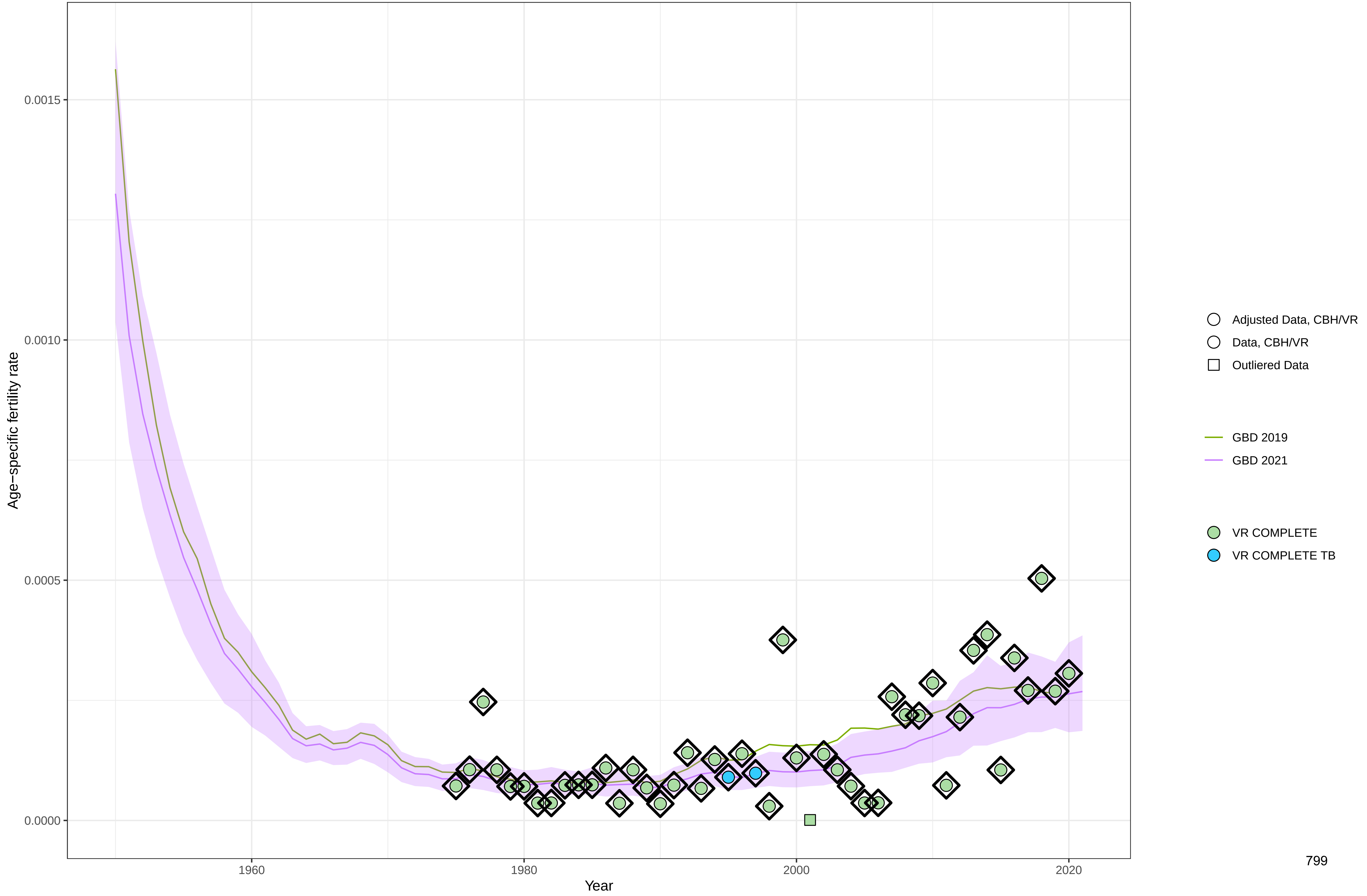

Age−specific fertility fitted trend lines, 1950−2021: Yamanashi, Ages: 50−54
Data points enclosed with a diamond are reference sources

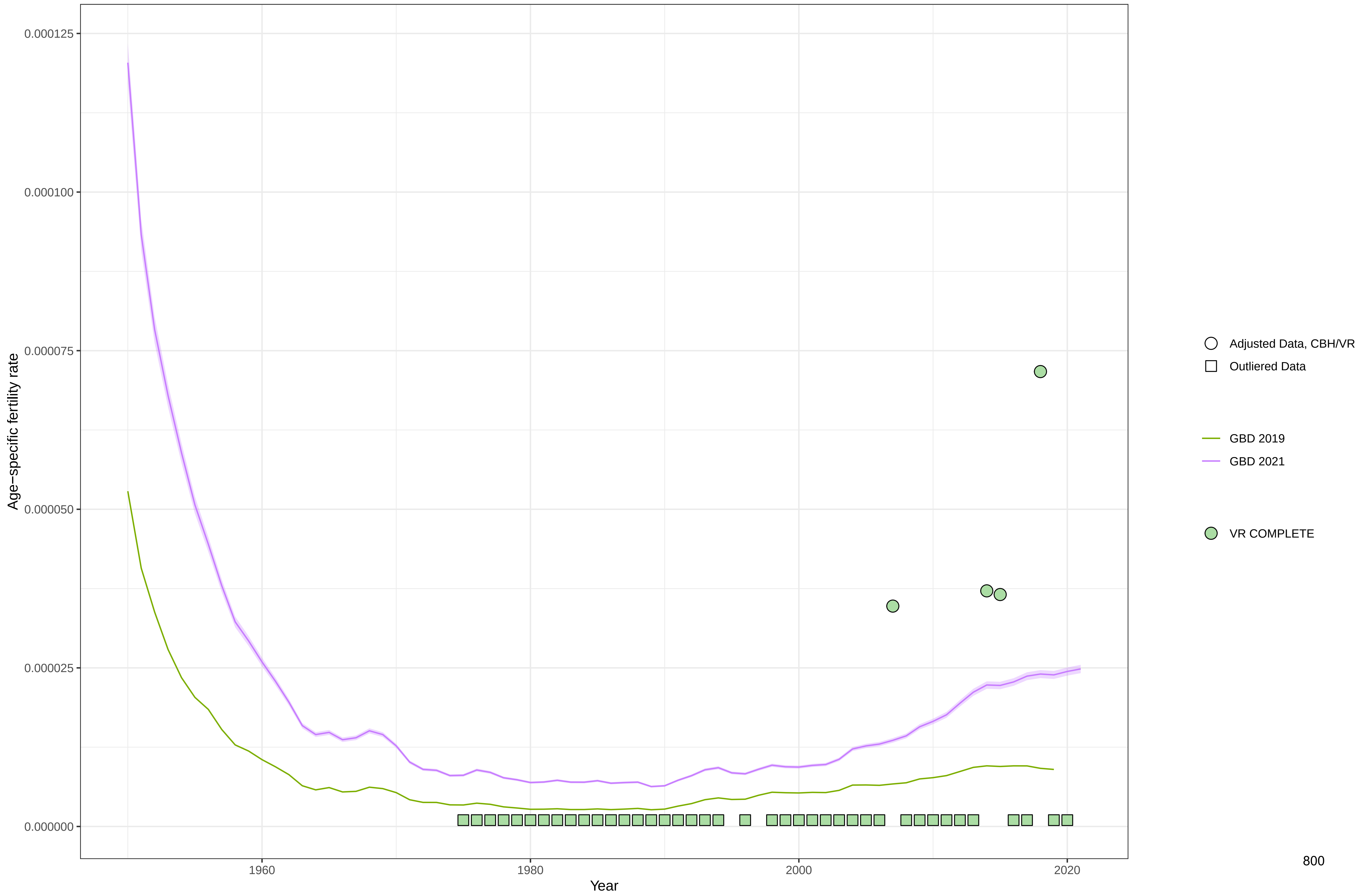

Total fertility rate fitted trend lines, 1950−2021: Yamanashi TFR
Data points enclosed with a diamond are reference sources

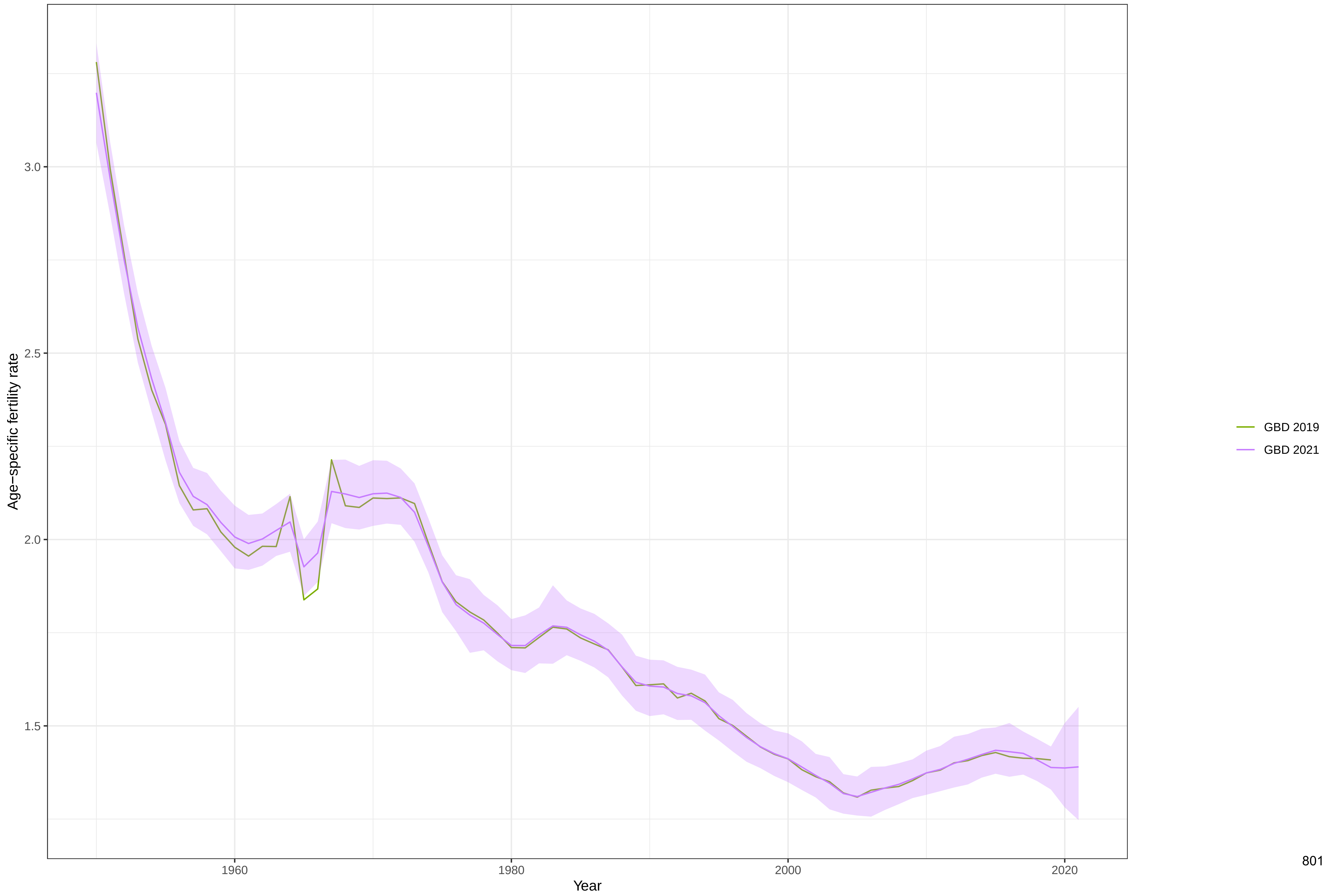

Age−specific fertility fitted trend lines, 1950−2021: South Korea, Ages: 10−14
Data points enclosed with a diamond are reference sources

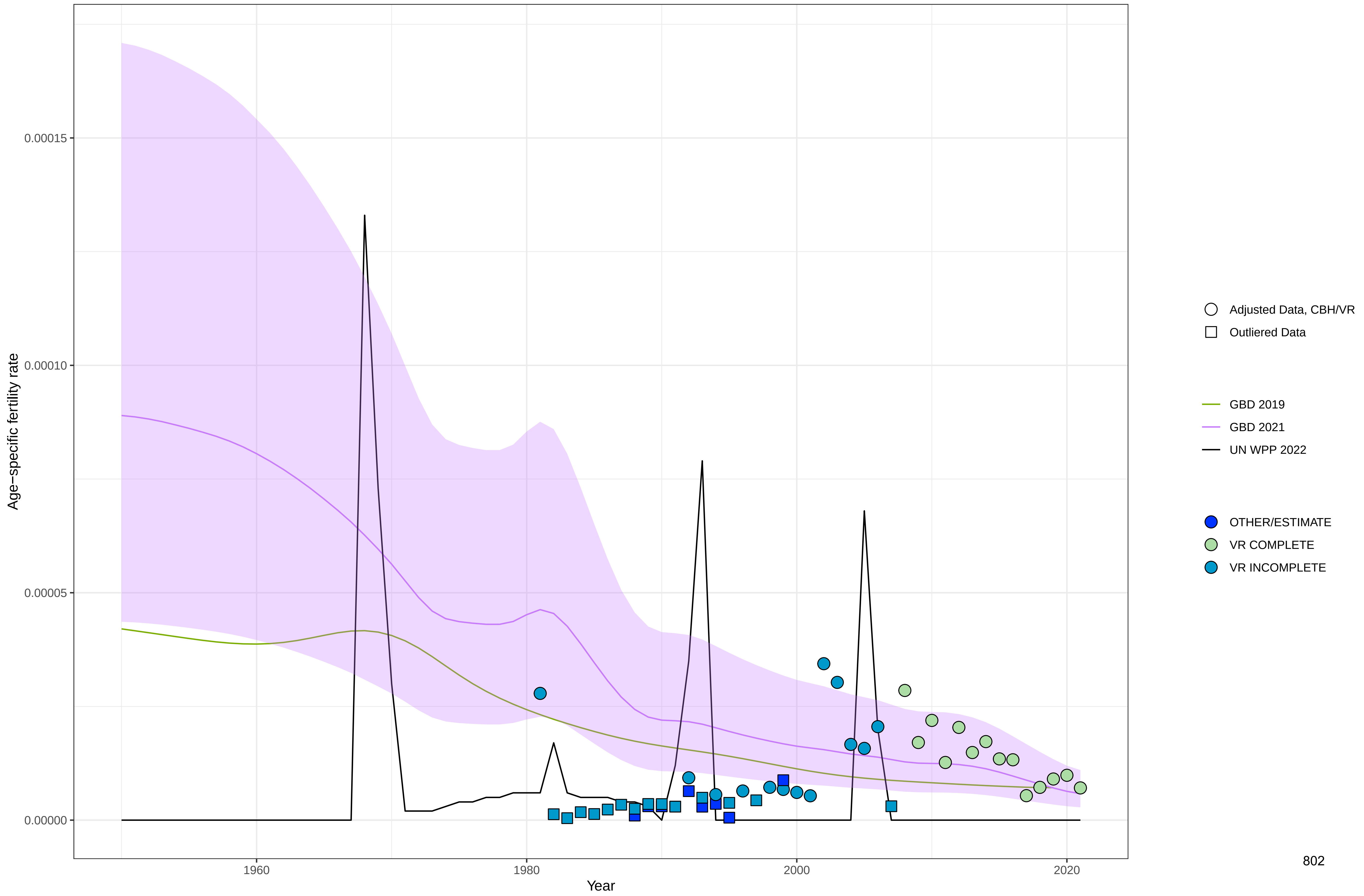

Age−specific fertility fitted trend lines, 1950−2021: South Korea, Ages: 15−19
Data points enclosed with a diamond are reference sources

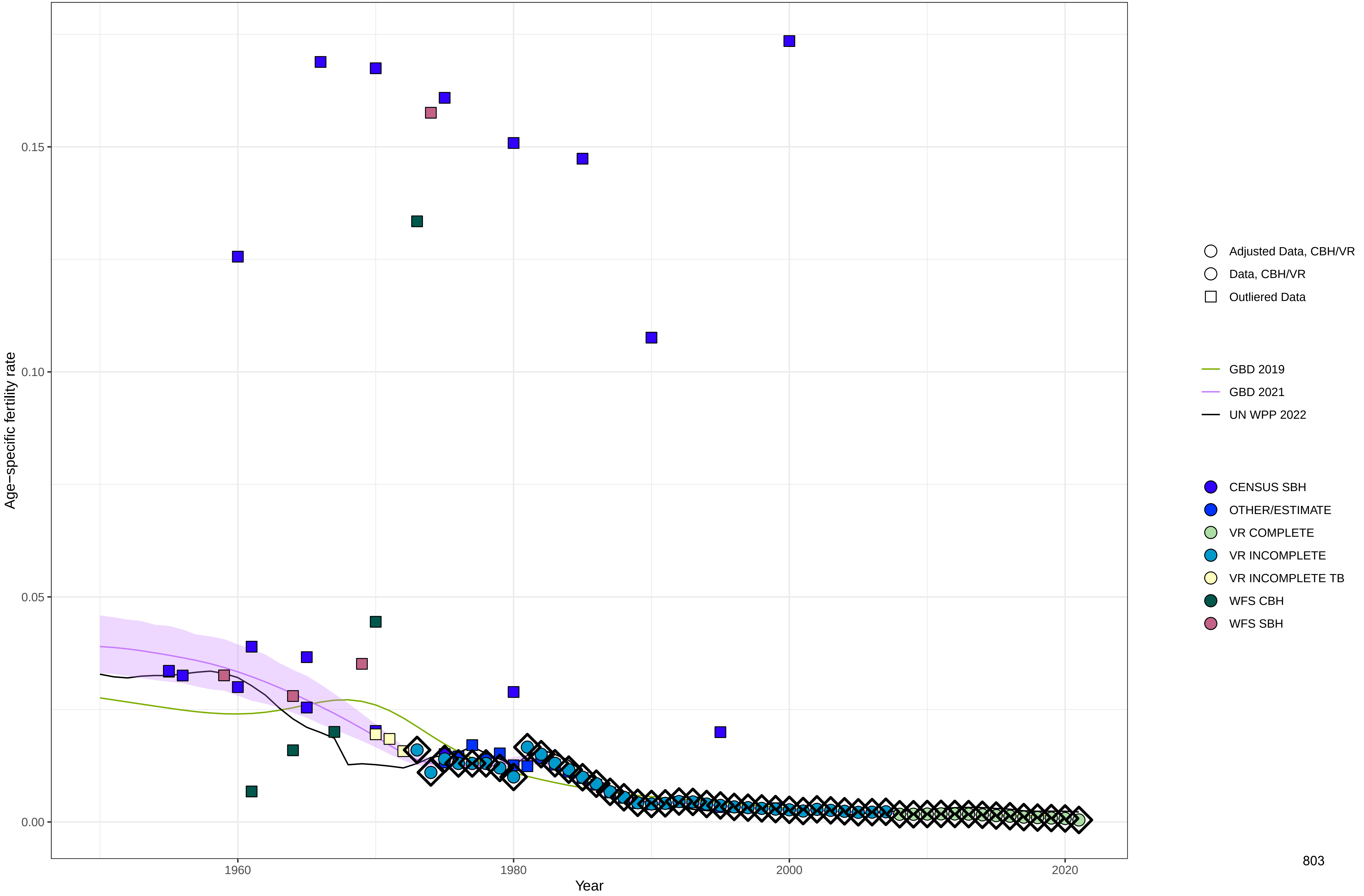

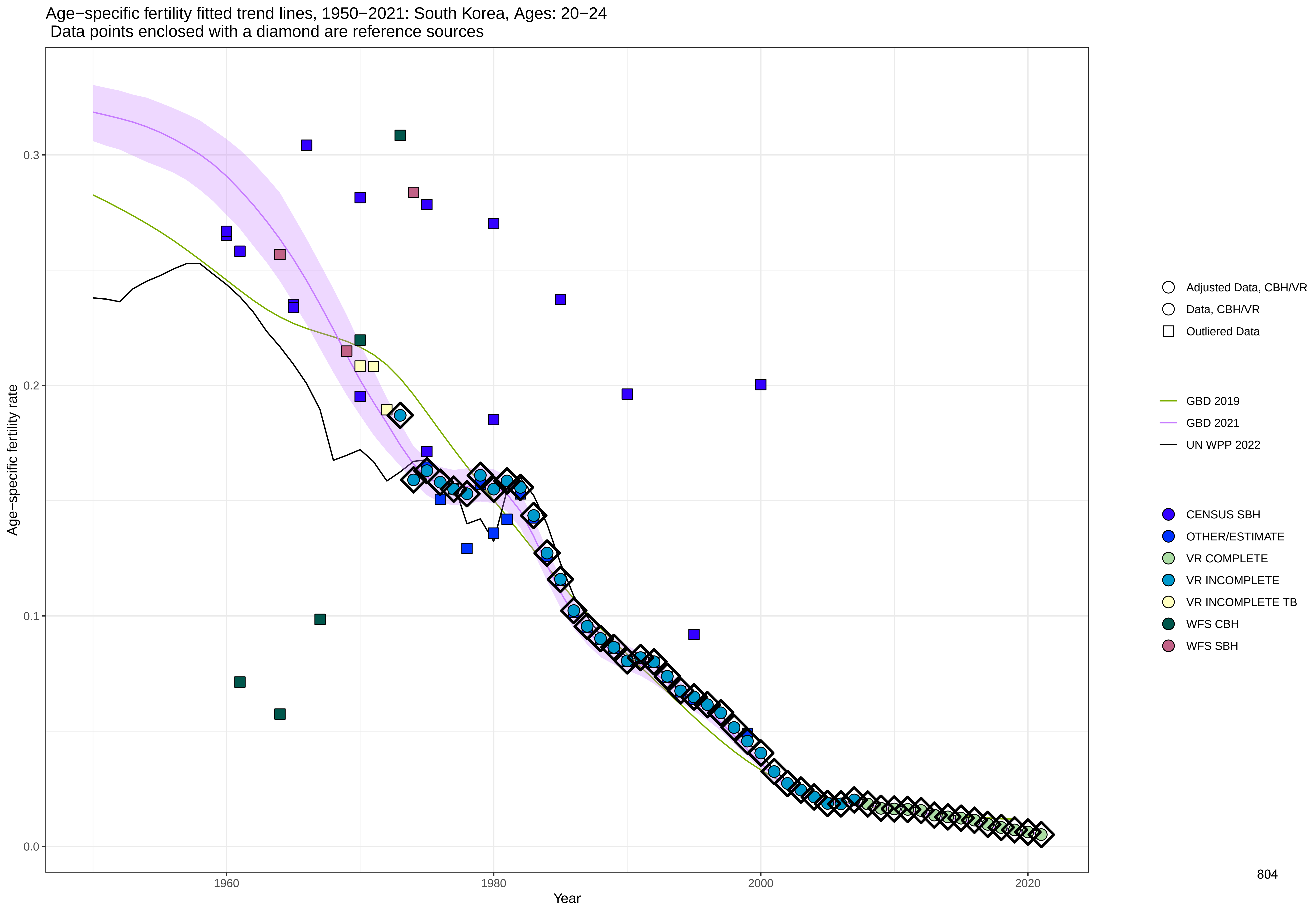
Age−specific fertility fitted trend lines, 1950−2021: South Korea, Ages: 20−24
Data points enclosed with a diamond are reference sources
Age−specific fertility rate
Year
0.0
0.1
0.2
0.3
1960
1980
2000
2020
Adjusted Data, CBH/VR
Data, CBH/VR
Outliered Data
GBD 2019
GBD 2021
UN WPP 2022
CENSUS SBH
OTHER/ESTIMATE
VR COMPLETE
VR INCOMPLETE
VR INCOMPLETE TB
WFS CBH
WFS SBH

Age−specific fertility fitted trend lines, 1950−2021: South Korea, Ages: 25−29
Data points enclosed with a diamond are reference sources

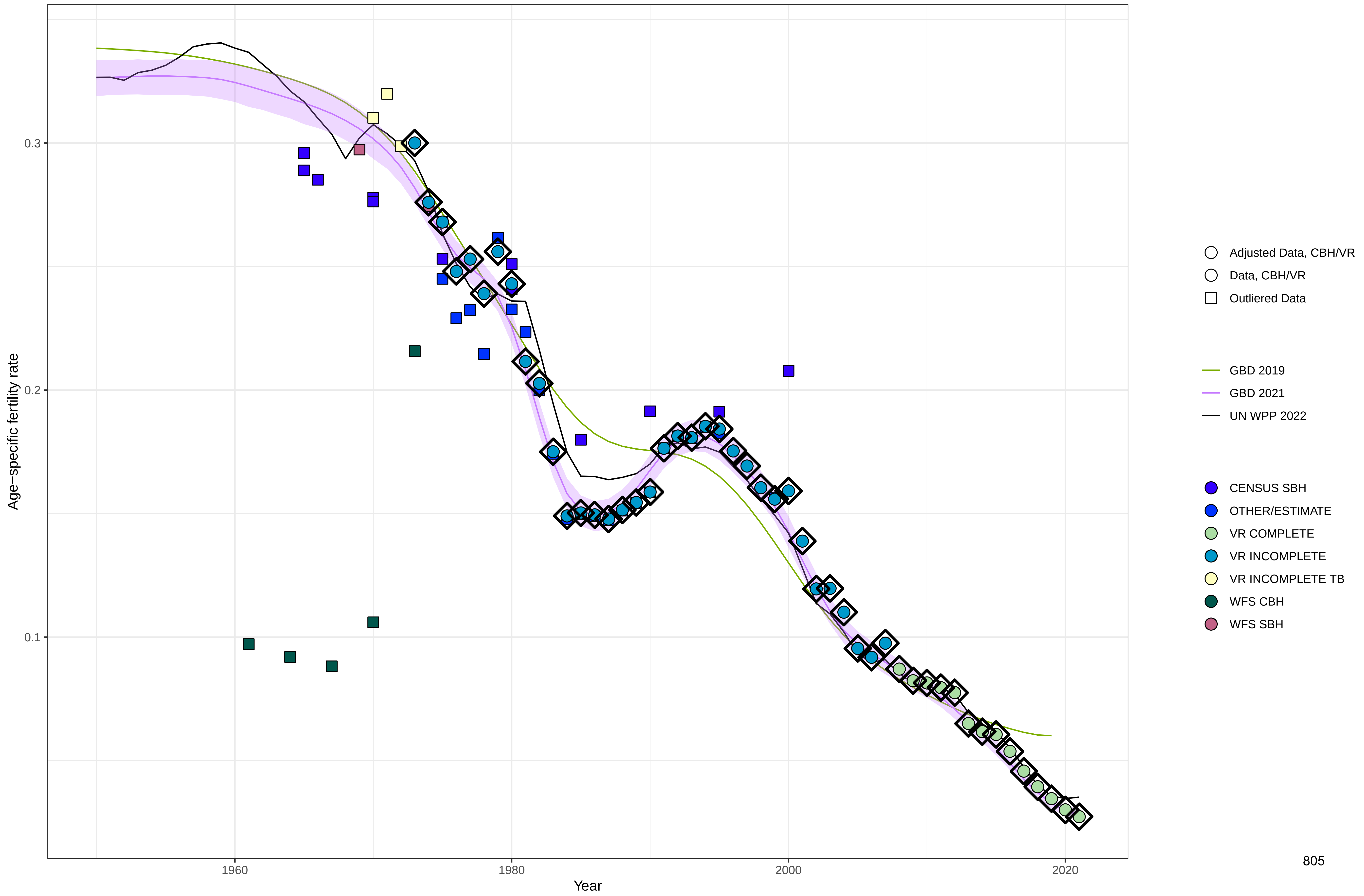

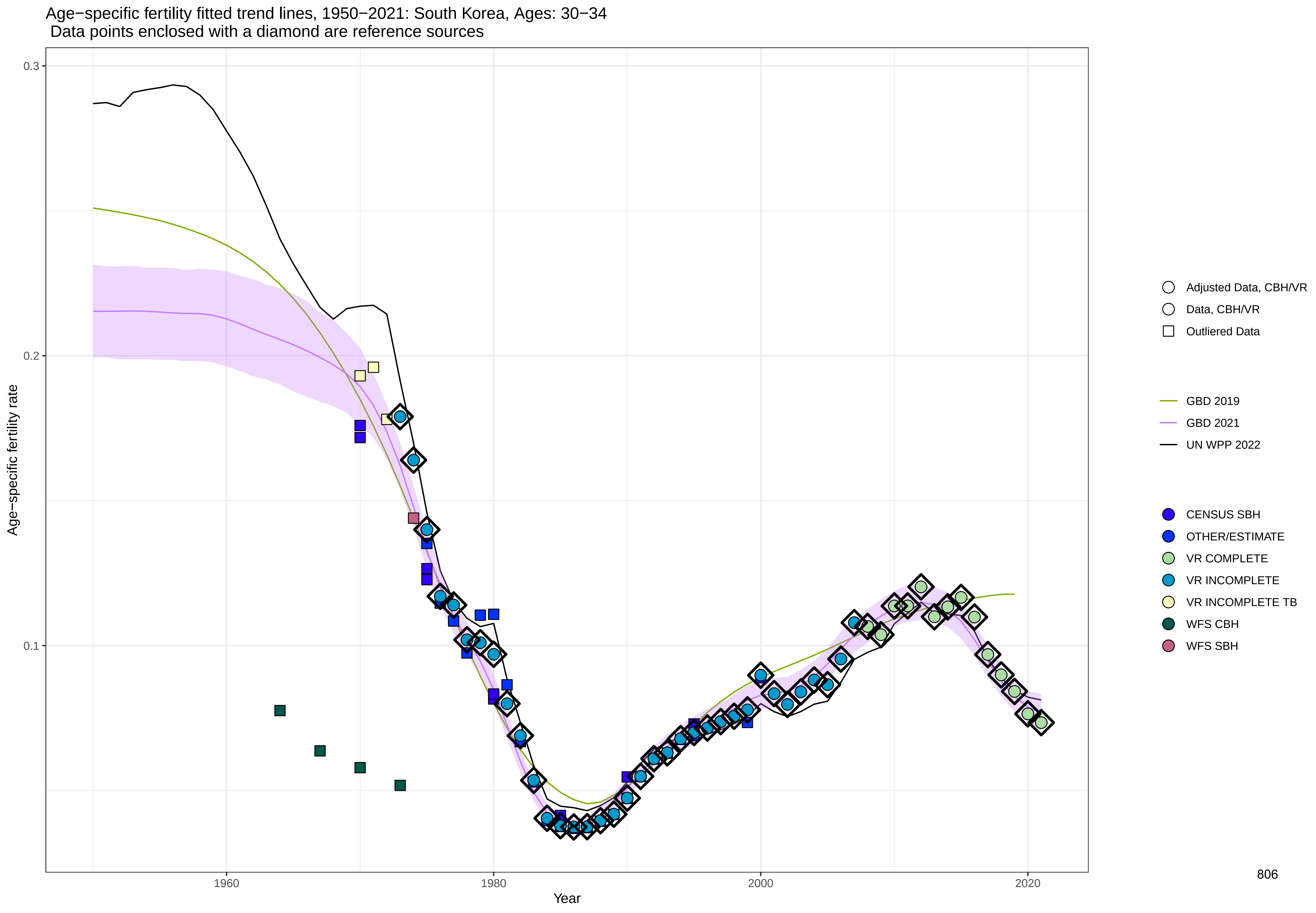
Age−specific fertility fitted trend lines, 1950−2021: South Korea, Ages: 30−34
Data points enclosed with a diamond are reference sources
Age−specific fertility rate
0.3
0.2
0.1
1960
1980
2000
2020
Year
Adjusted Data, CBH/VR
Data, CBH/VR
Outliered Data
GBD 2019
GBD 2021
UN WPP 2022
CENSUS SBH
OTHER/ESTIMATE
VR COMPLETE
VR INCOMPLETE
VR INCOMPLETE TB
WFS CBH
WFS SBH

Age−specific fertility fitted trend lines, 1950−2021: South Korea, Ages: 35−39
Data points enclosed with a diamond are reference sources

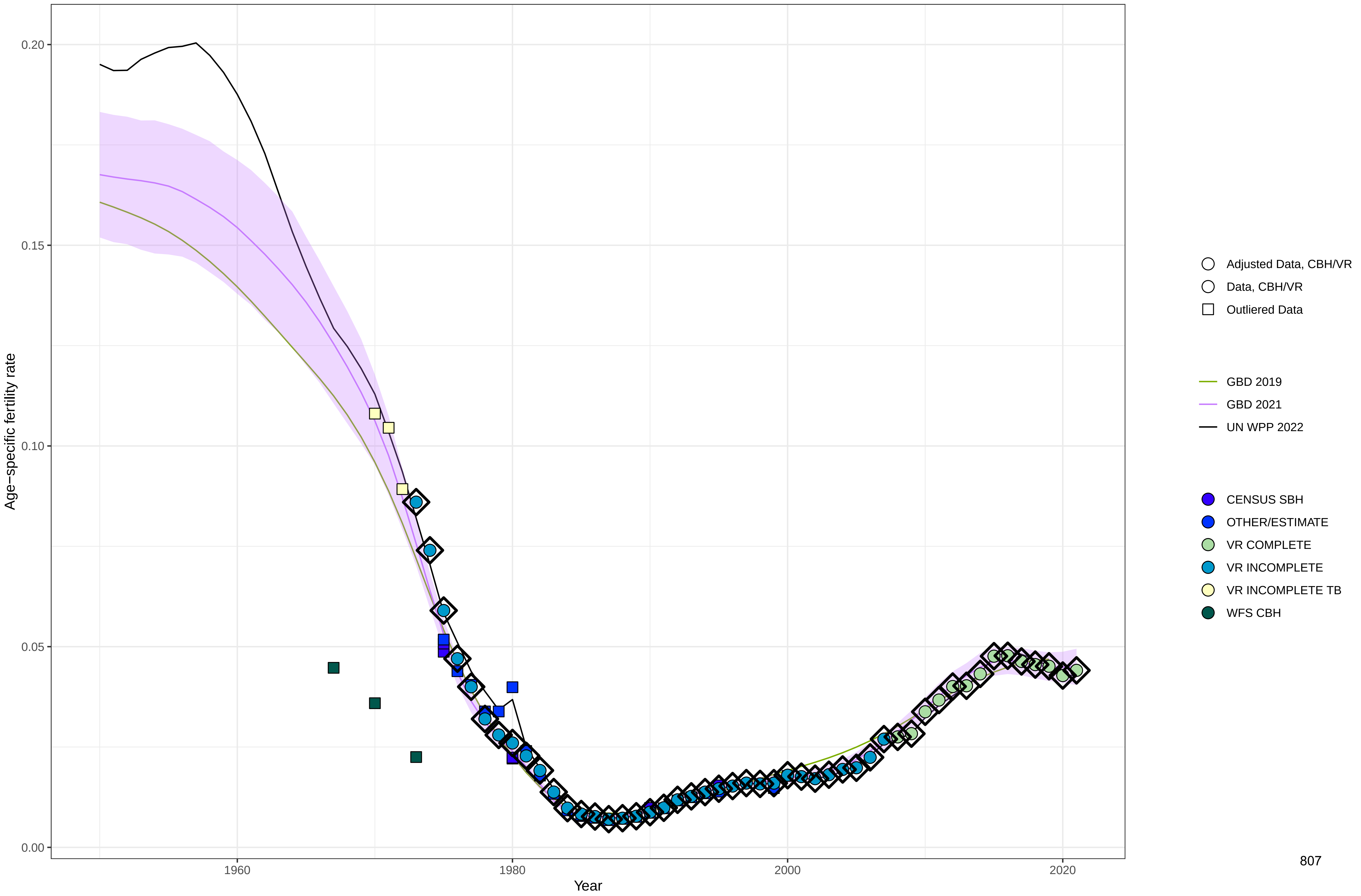

Age−specific fertility fitted trend lines, 1950−2021: South Korea, Ages: 40−44
Data points enclosed with a diamond are reference sources

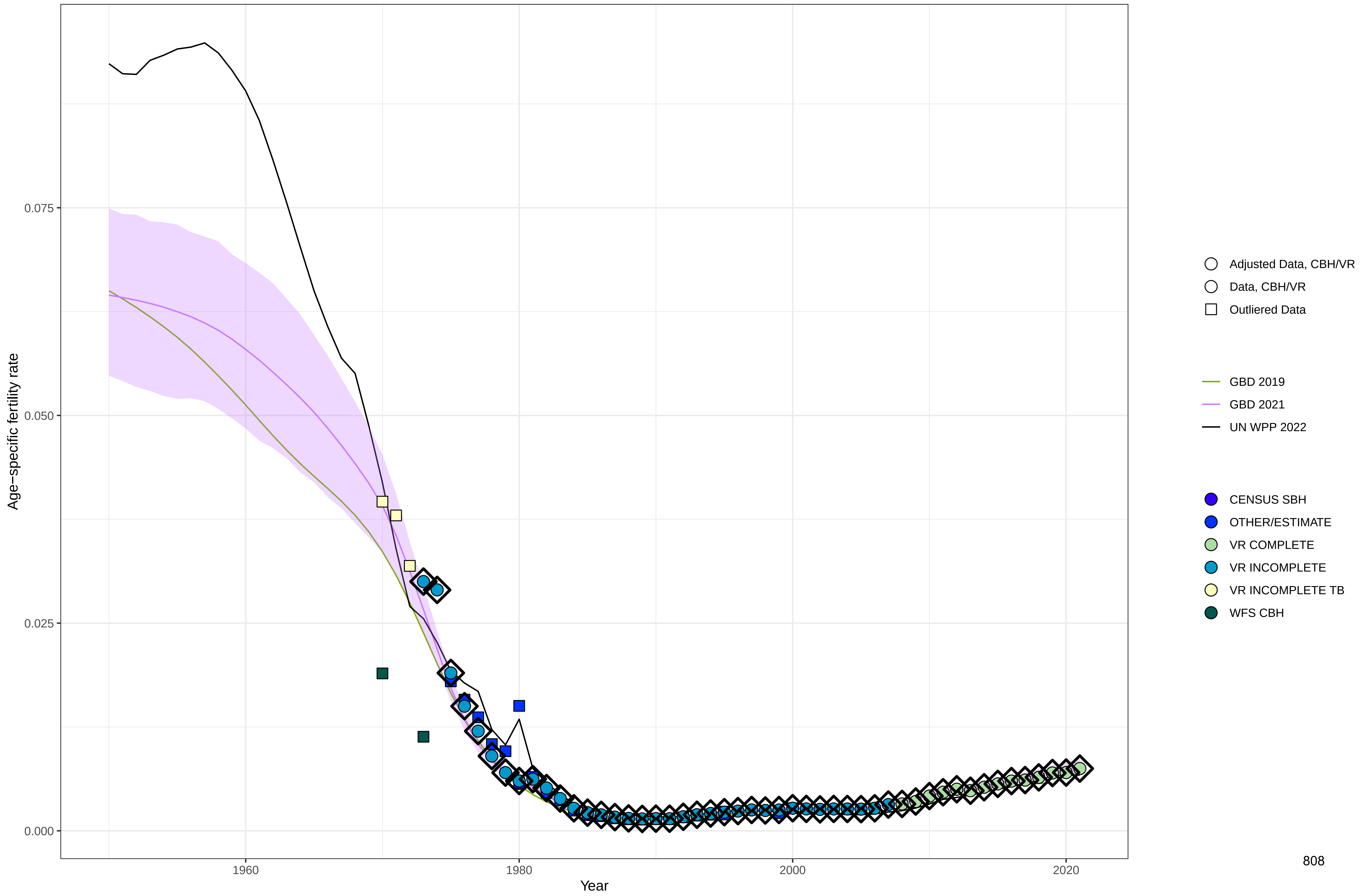

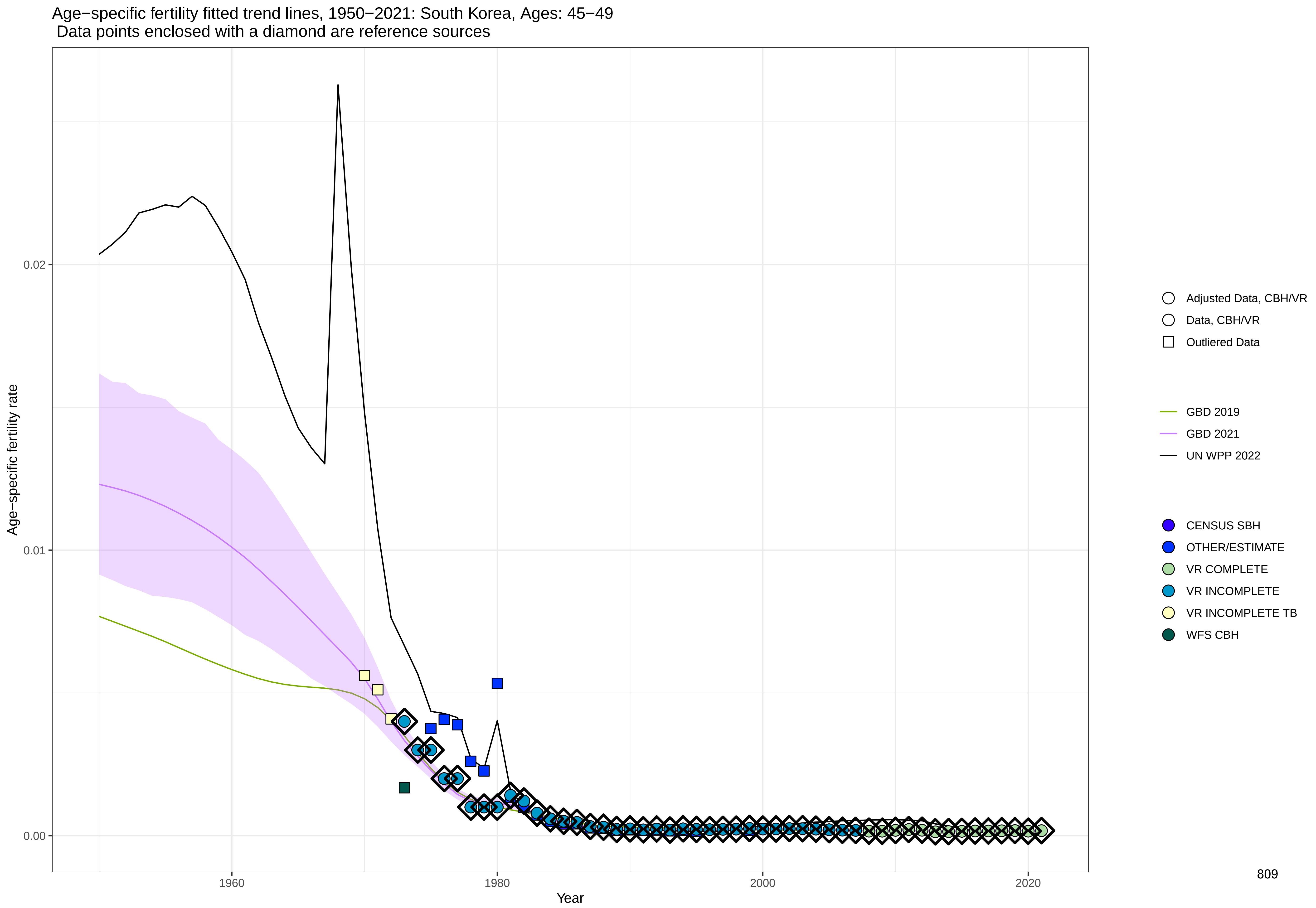

Age−specific fertility fitted trend lines, 1950−2021: South Korea, Ages: 45−49
Data points enclosed with a diamond are reference sources
Age−specific fertility rate
0.00
0.01
0.02
Year
1960
1980
2000
2020
Adjusted Data, CBH/VR
Data, CBH/VR
Outliered Data
GBD 2019
GBD 2021
UN WPP 2022
CENSUS SBH
OTHER/ESTIMATE
VR COMPLETE
VR INCOMPLETE
VR INCOMPLETE TB
WFS CBH

Age−specific fertility fitted trend lines, 1950−2021: South Korea, Ages: 50−54
Data points enclosed with a diamond are reference sources

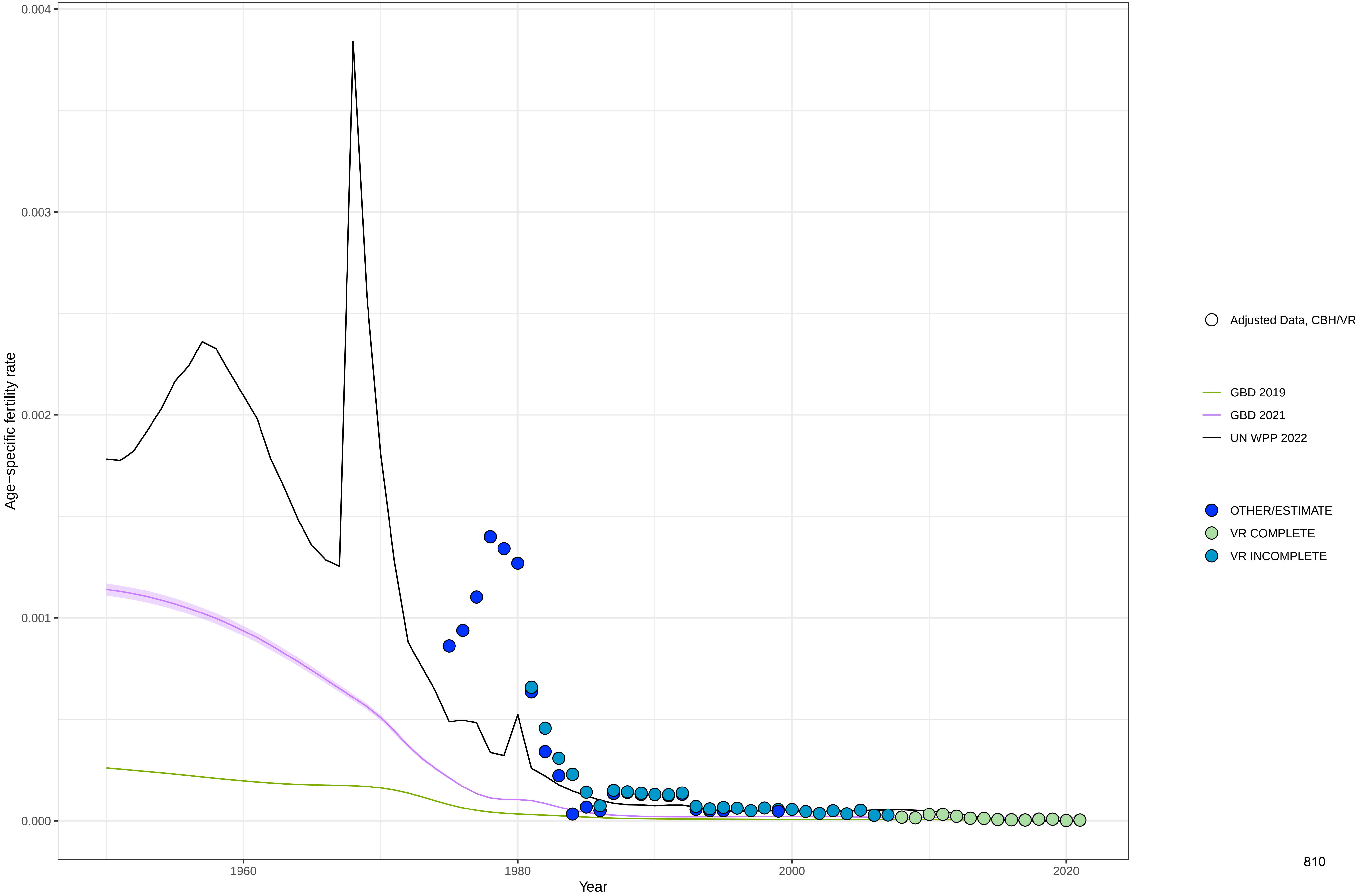

Total fertility rate fitted trend lines, 1950−2021: South Korea TFR
Data points enclosed with a diamond are reference sources

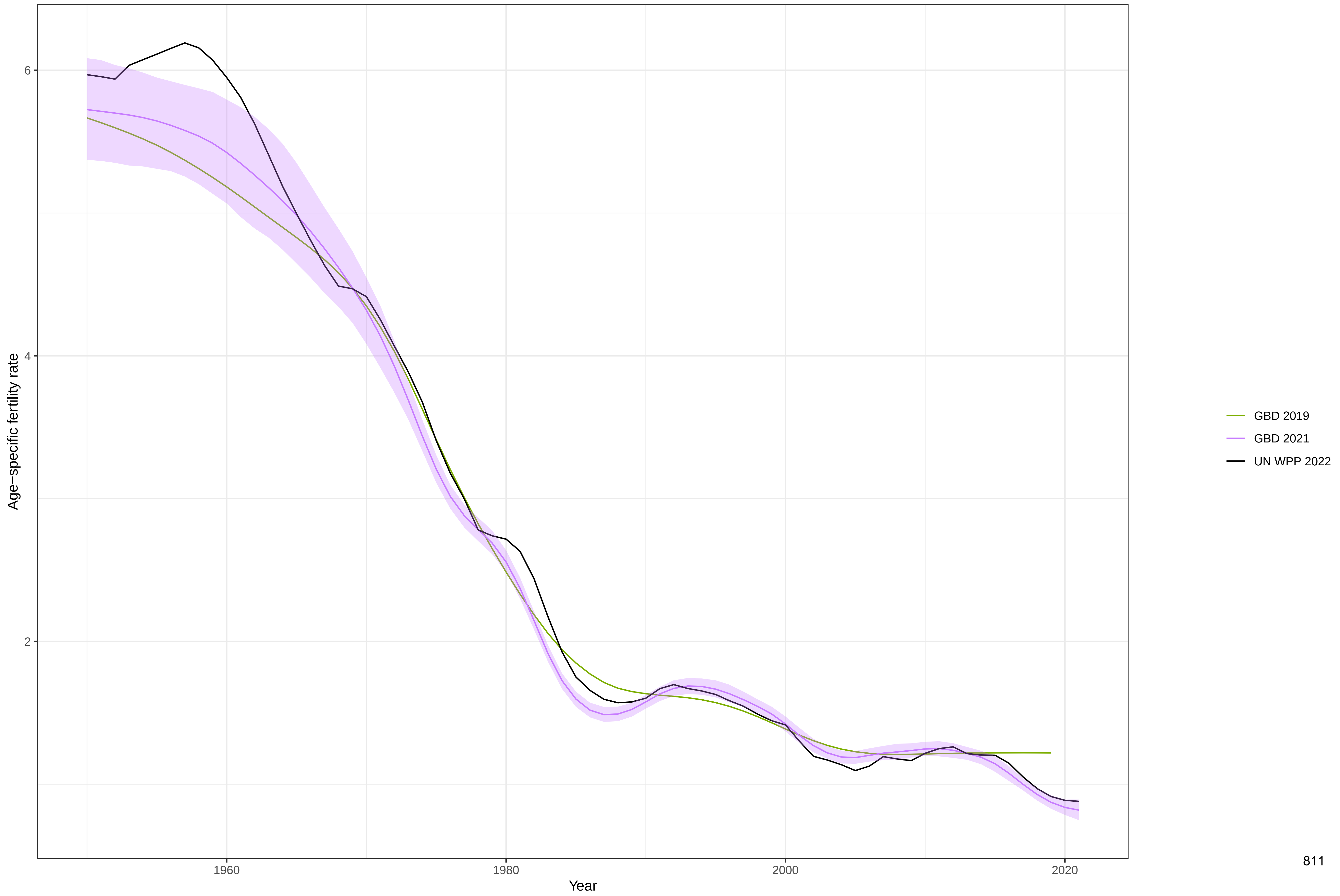

Age−specific fertility fitted trend lines, 1950−2021: Singapore, Ages: 10−14
Data points enclosed with a diamond are reference sources

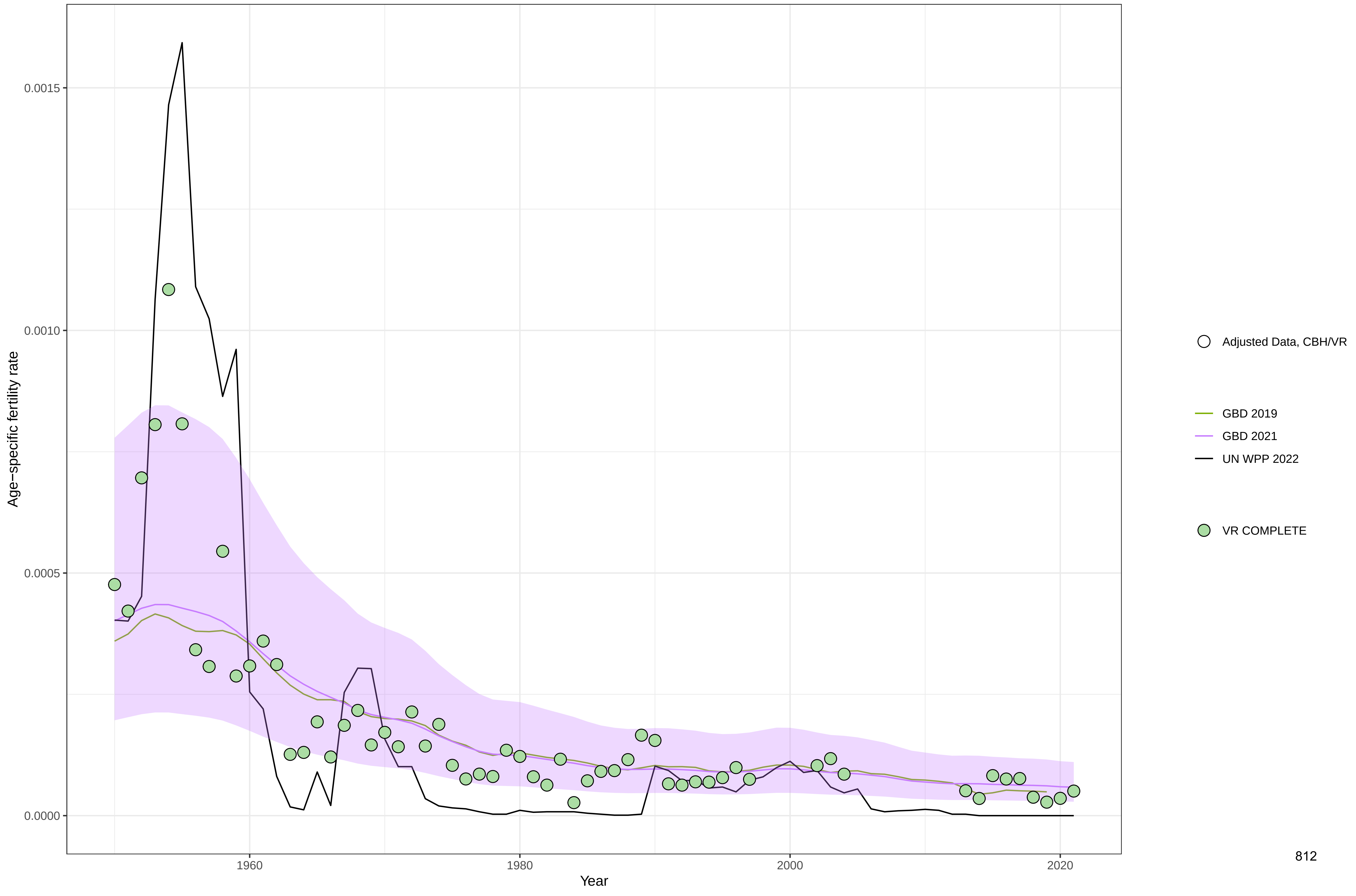

Age−specific fertility fitted trend lines, 1950−2021: Singapore, Ages: 15−19
Data points enclosed with a diamond are reference sources

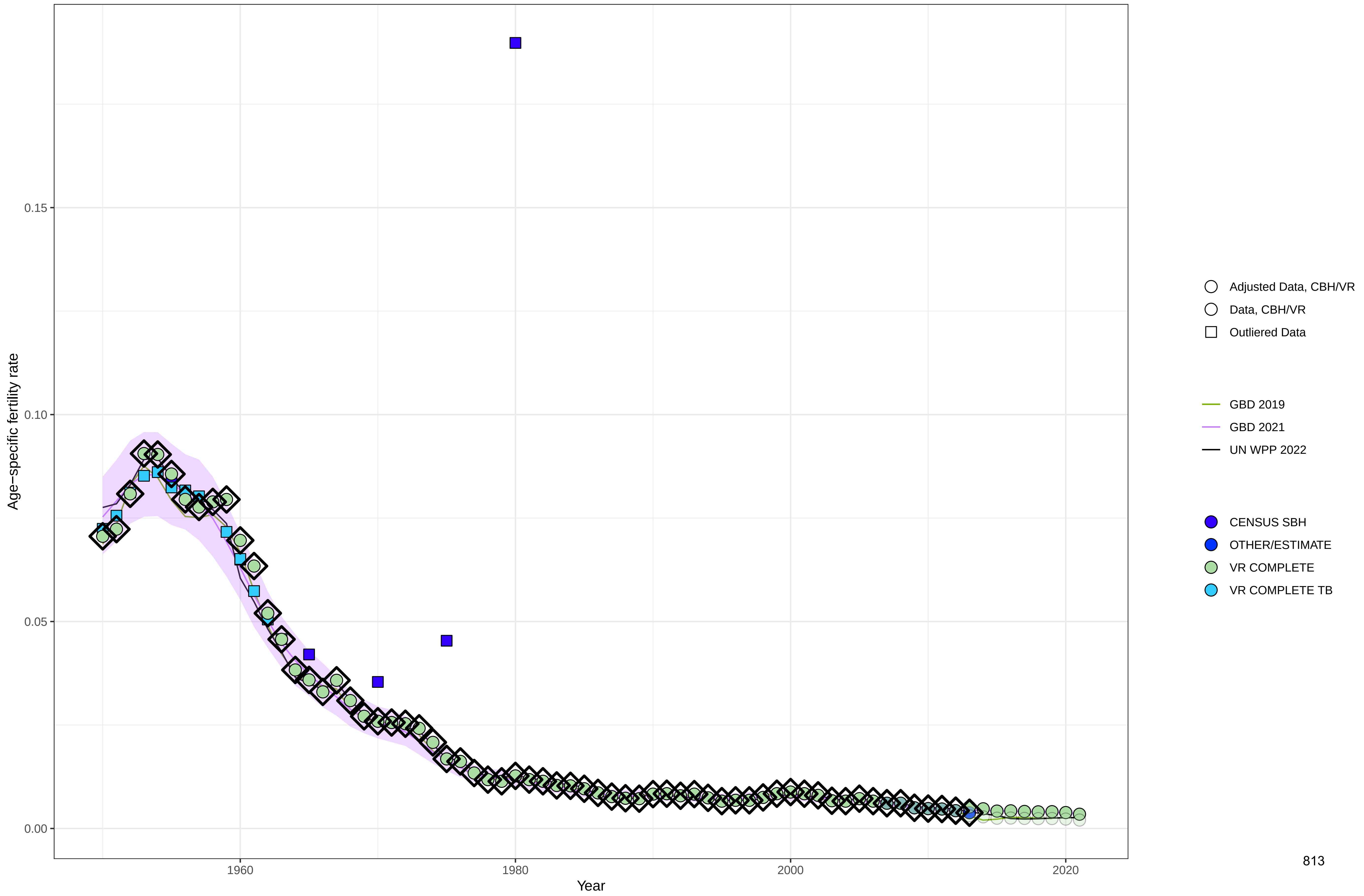

Age−specific fertility fitted trend lines, 1950−2021: Singapore, Ages: 20−24
Data points enclosed with a diamond are reference sources

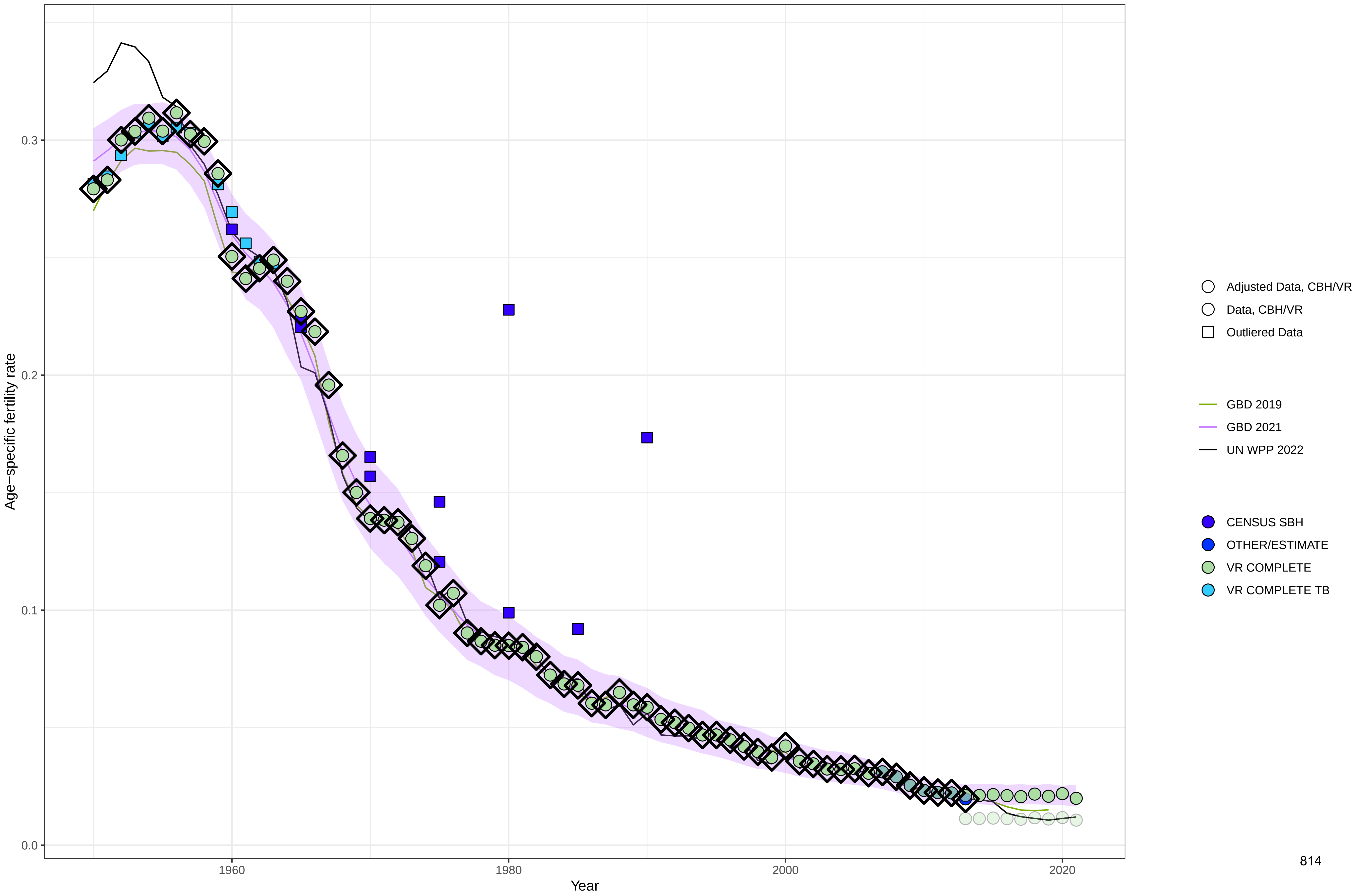

Age−specific fertility fitted trend lines, 1950−2021: Singapore, Ages: 25−29
Data points enclosed with a diamond are reference sources

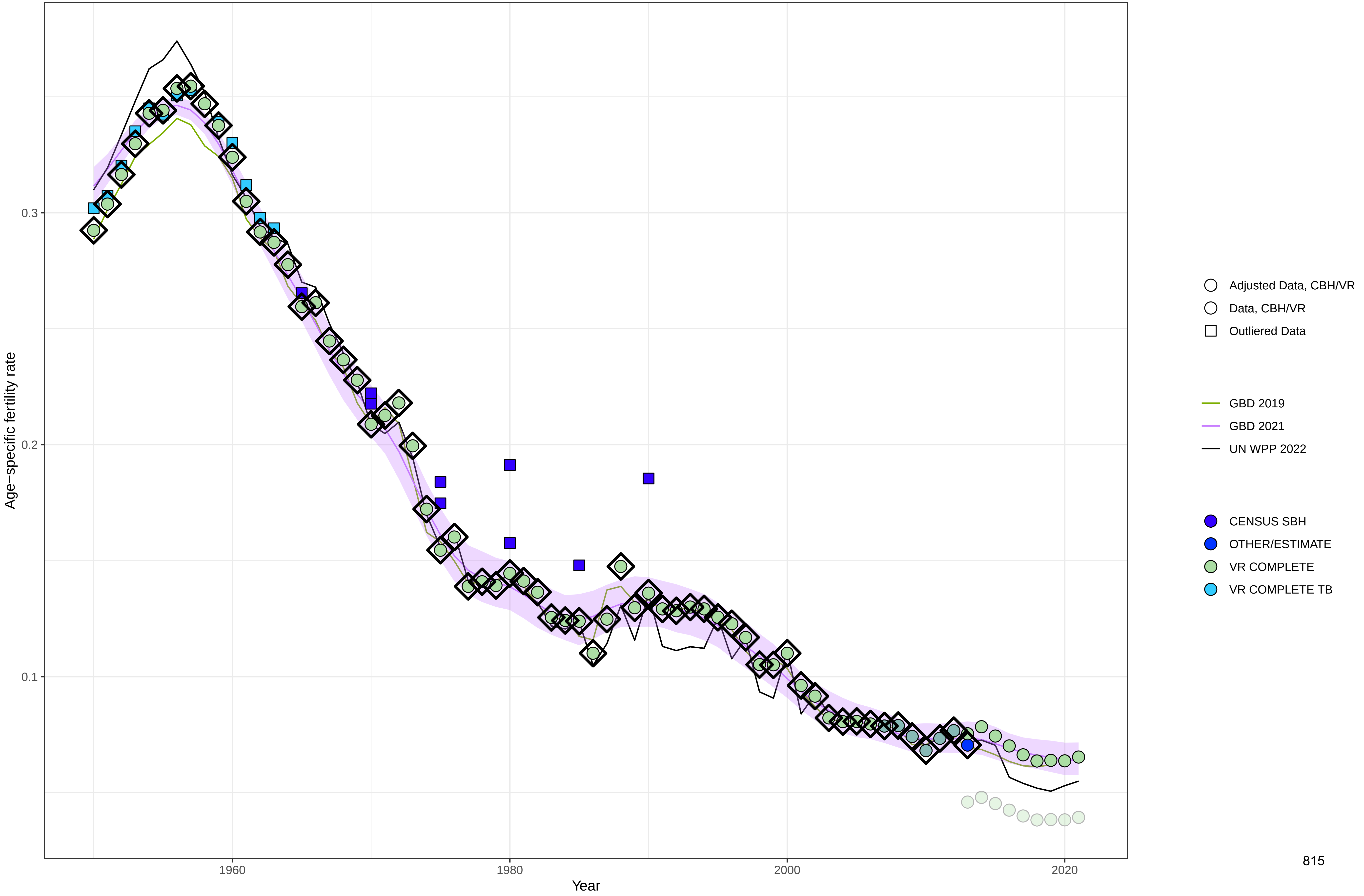

Age−specific fertility fitted trend lines, 1950−2021: Singapore, Ages: 30−34
Data points enclosed with a diamond are reference sources

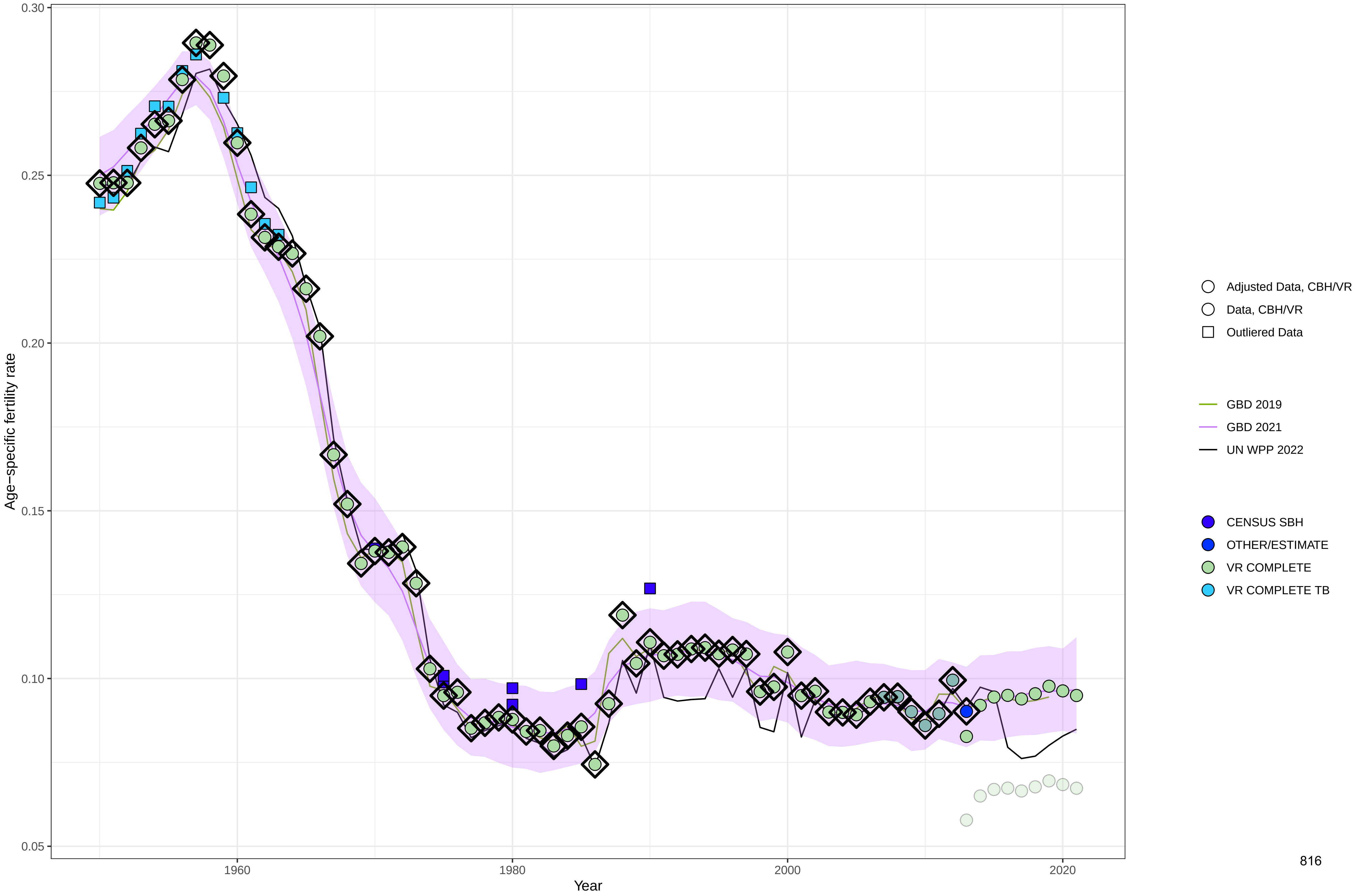

Age−specific fertility fitted trend lines, 1950−2021: Singapore, Ages: 35−39
Data points enclosed with a diamond are reference sources

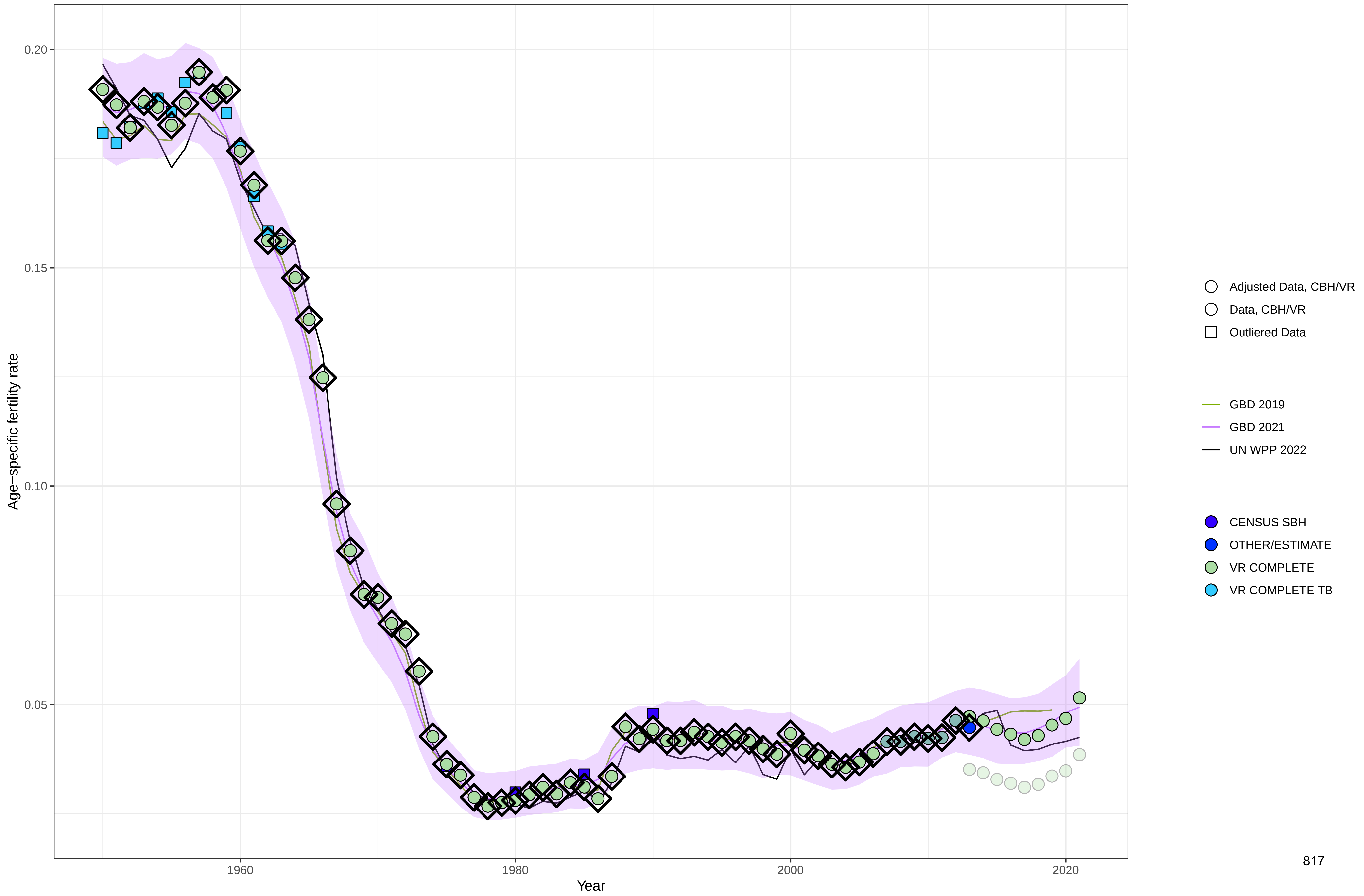

Age−specific fertility fitted trend lines, 1950−2021: Singapore, Ages: 40−44
Data points enclosed with a diamond are reference sources

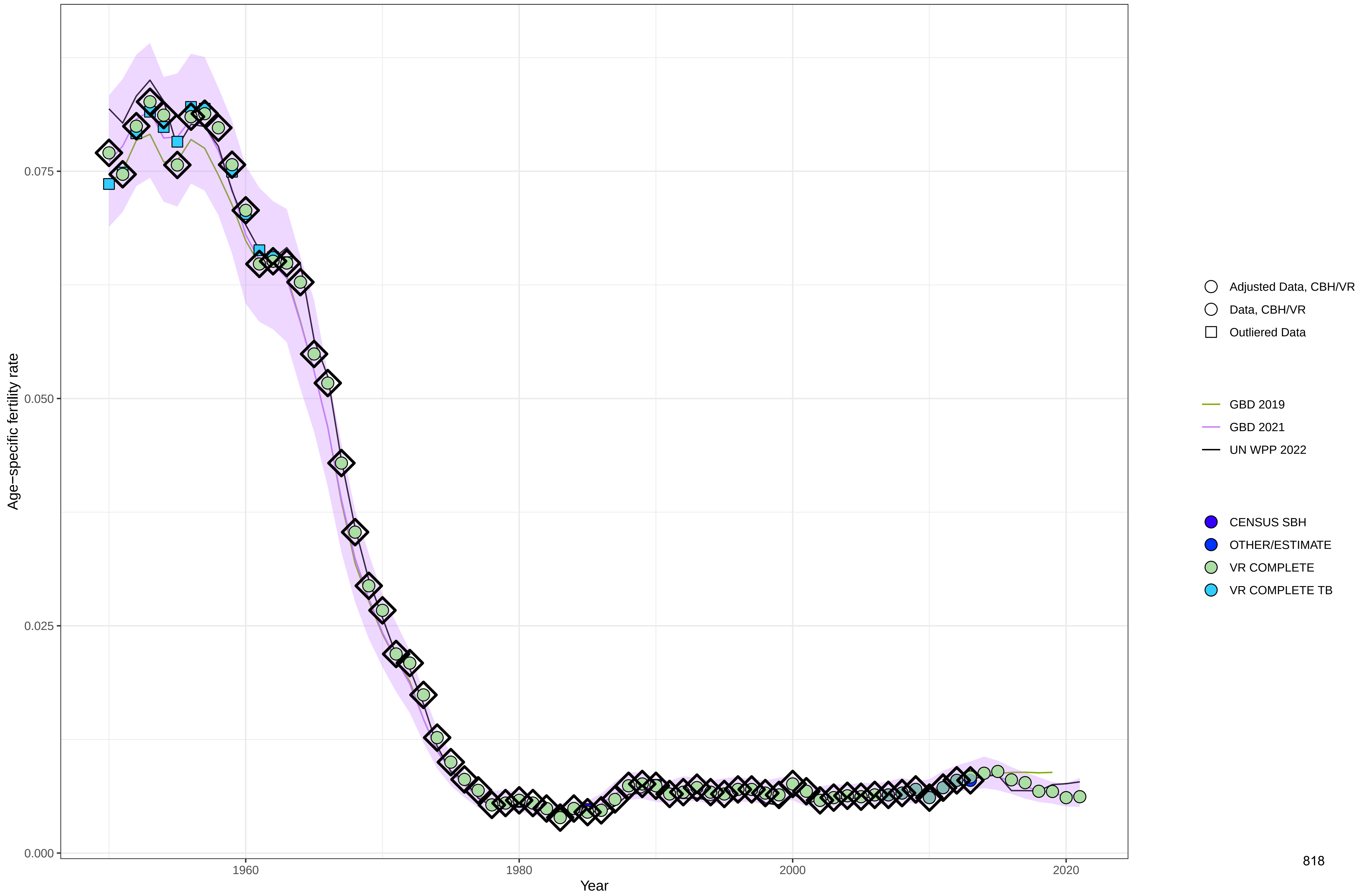

Age−specific fertility fitted trend lines, 1950−2021: Singapore, Ages: 45−49
Data points enclosed with a diamond are reference sources

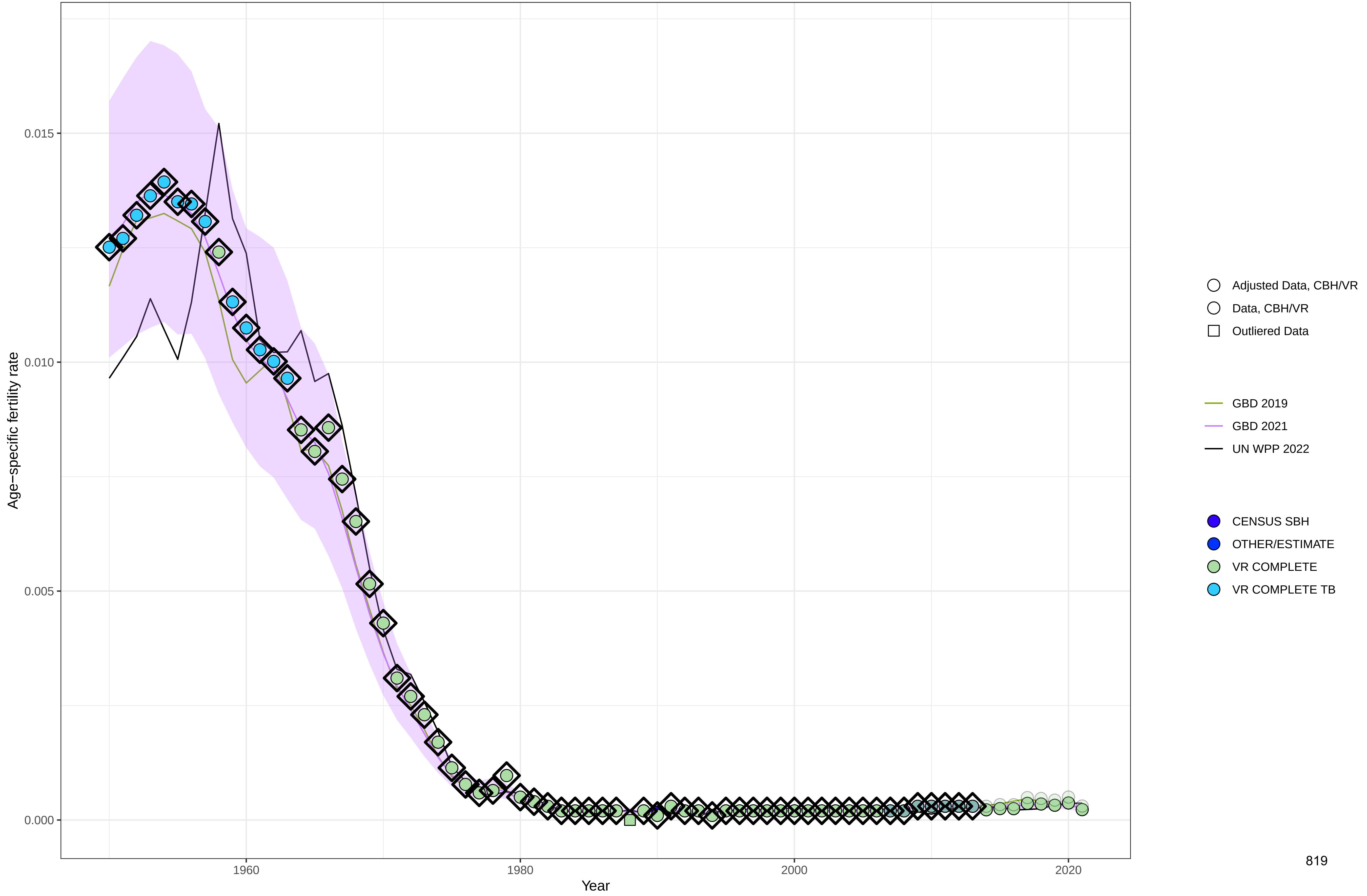

Age−specific fertility fitted trend lines, 1950−2021: Singapore, Ages: 50−54
Data points enclosed with a diamond are reference sources

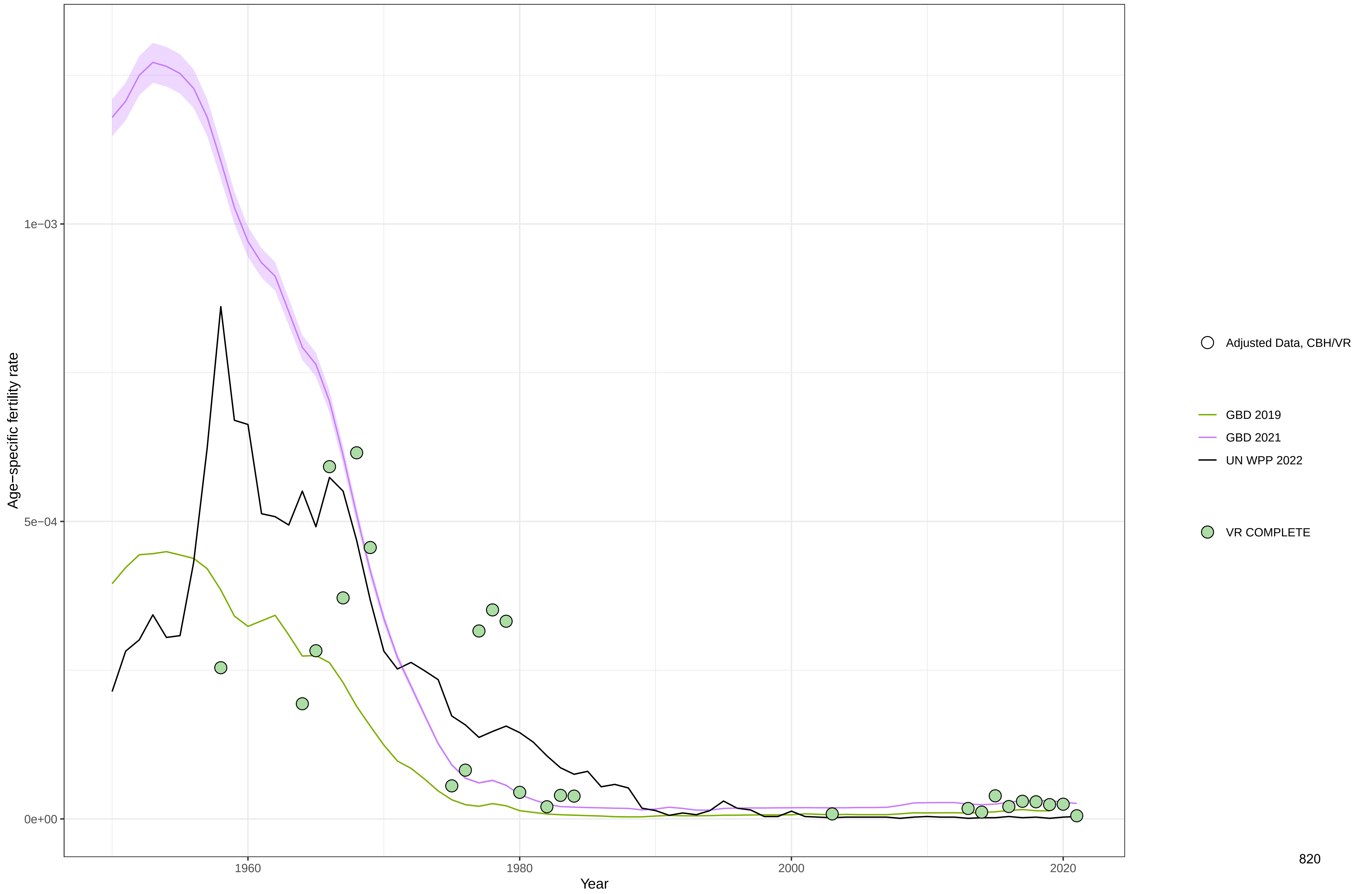

Total fertility rate fitted trend lines, 1950−2021: Singapore TFR
Data points enclosed with a diamond are reference sources

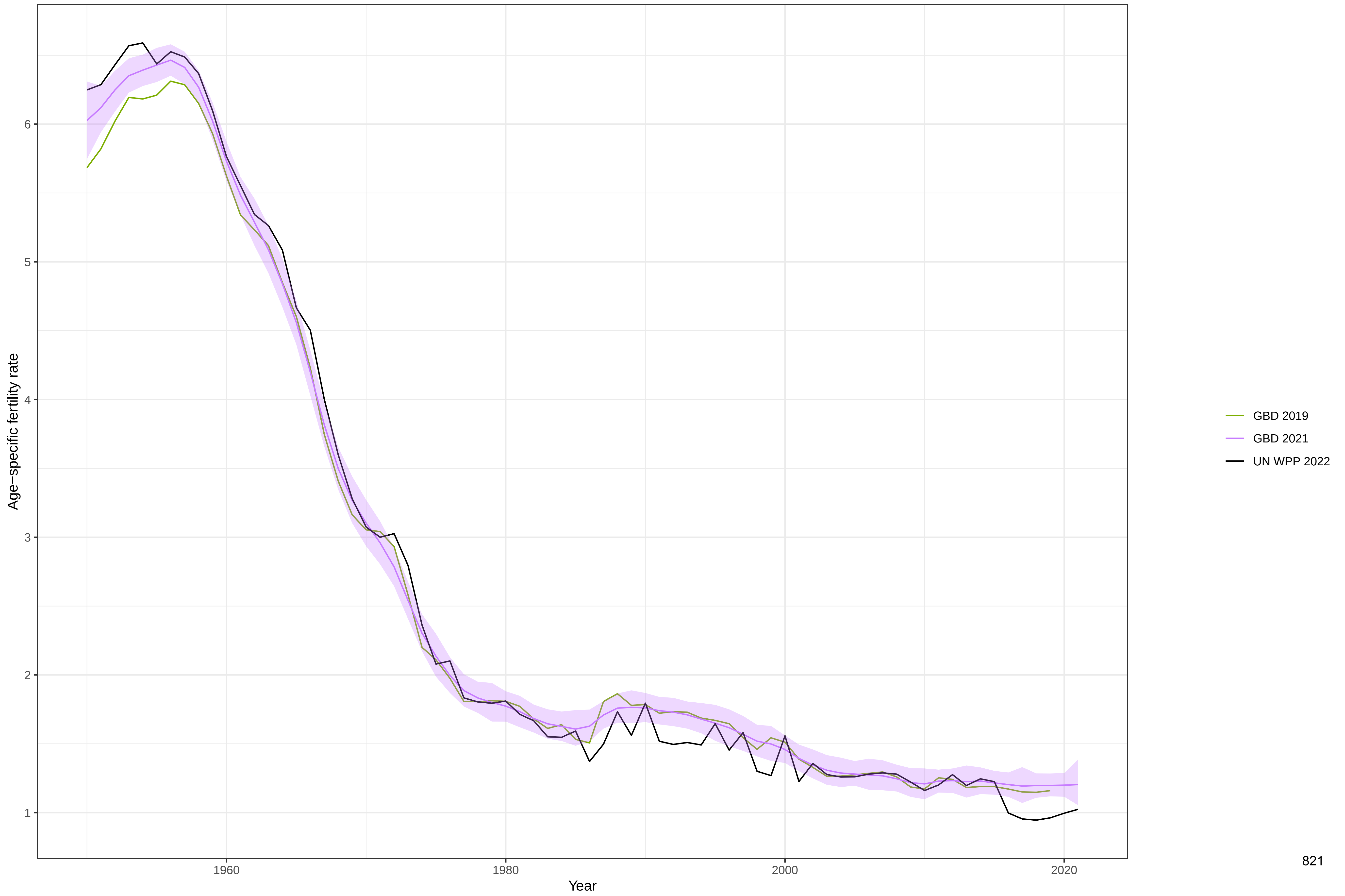

Age−specific fertility fitted trend lines, 1950−2021: Canada, Ages: 10−14
Data points enclosed with a diamond are reference sources

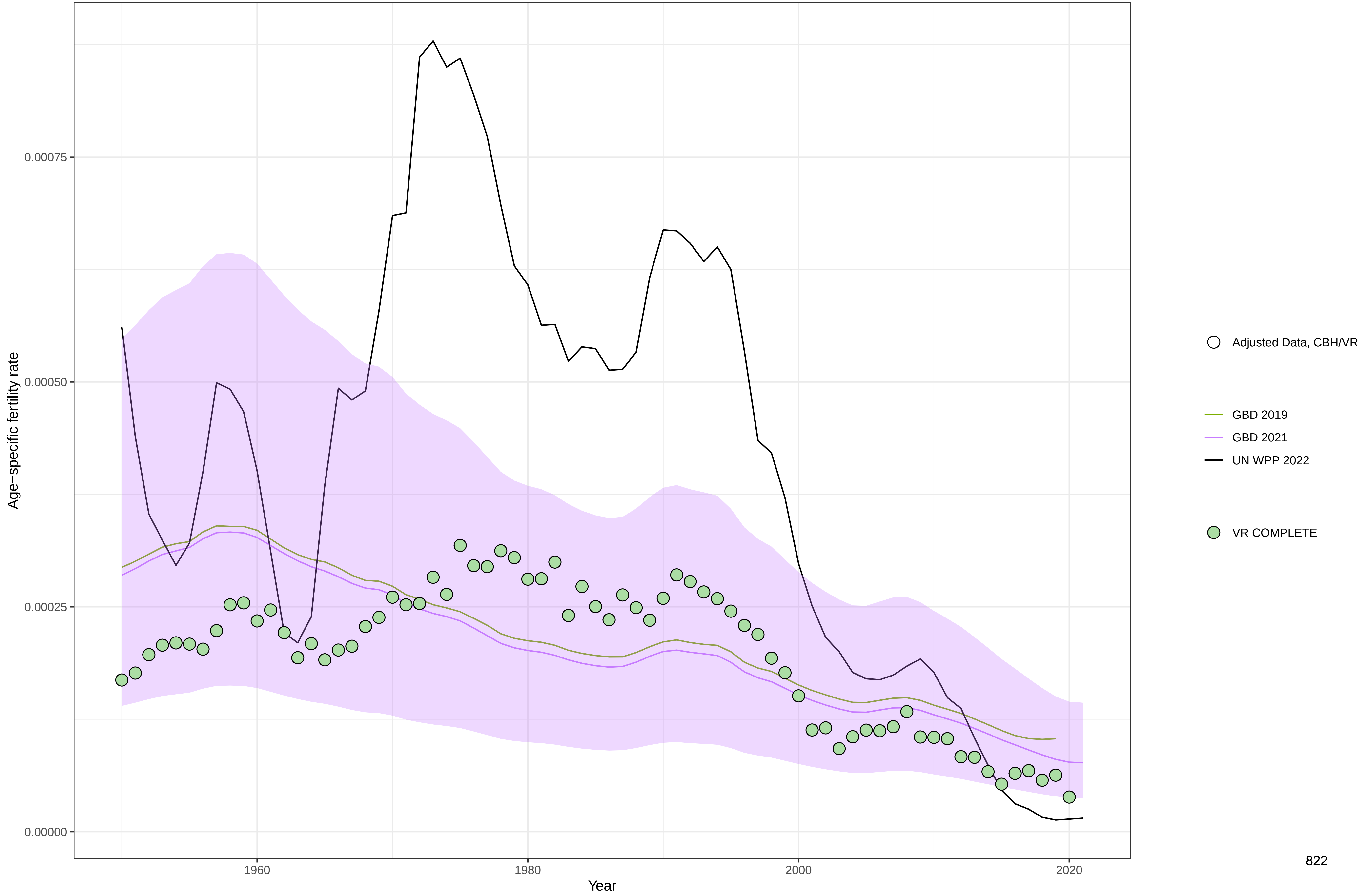

Age−specific fertility fitted trend lines, 1950−2021: Canada, Ages: 15−19
Data points enclosed with a diamond are reference sources

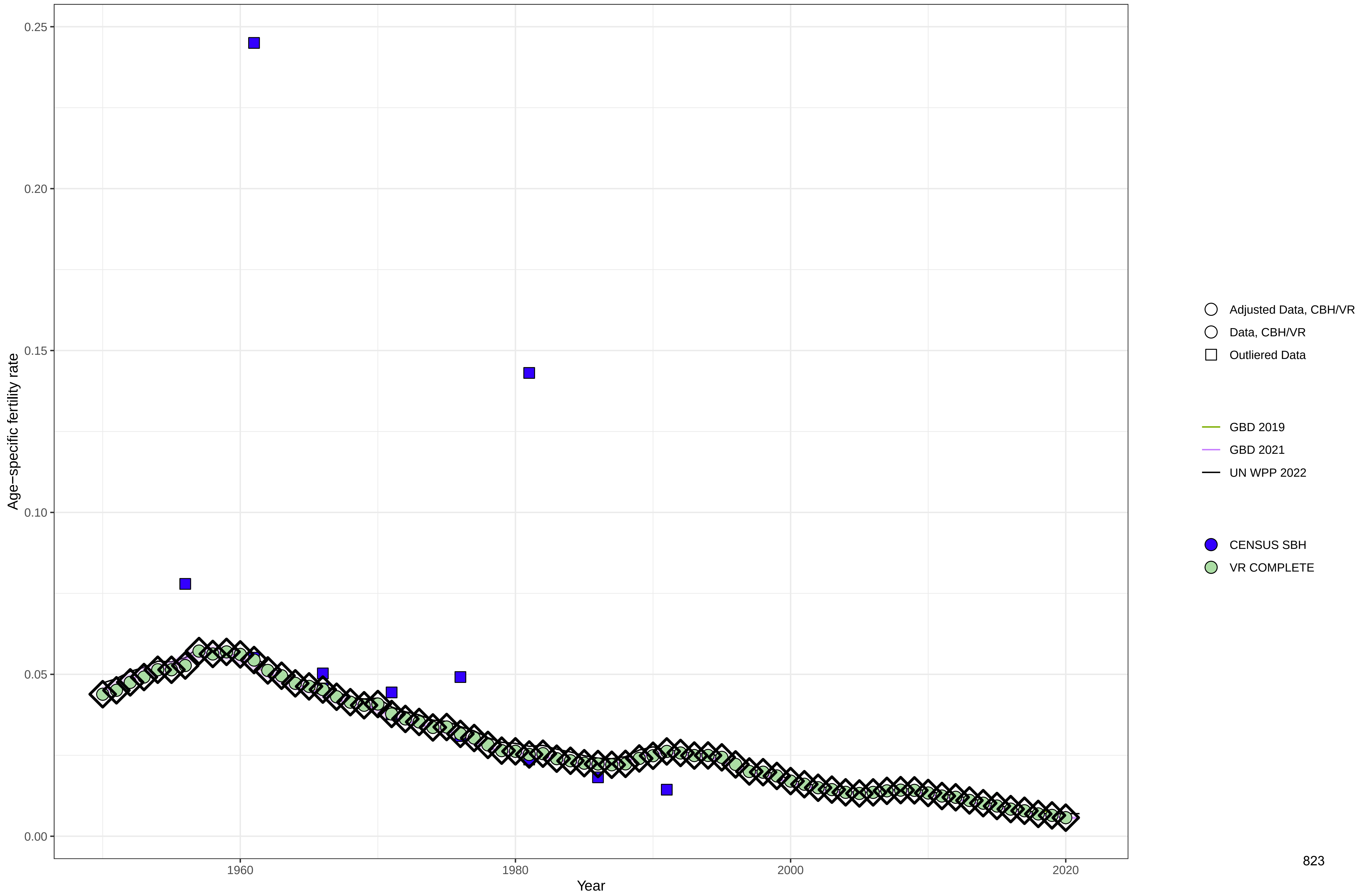

Age−specific fertility fitted trend lines, 1950−2021: Canada, Ages: 20−24
Data points enclosed with a diamond are reference sources

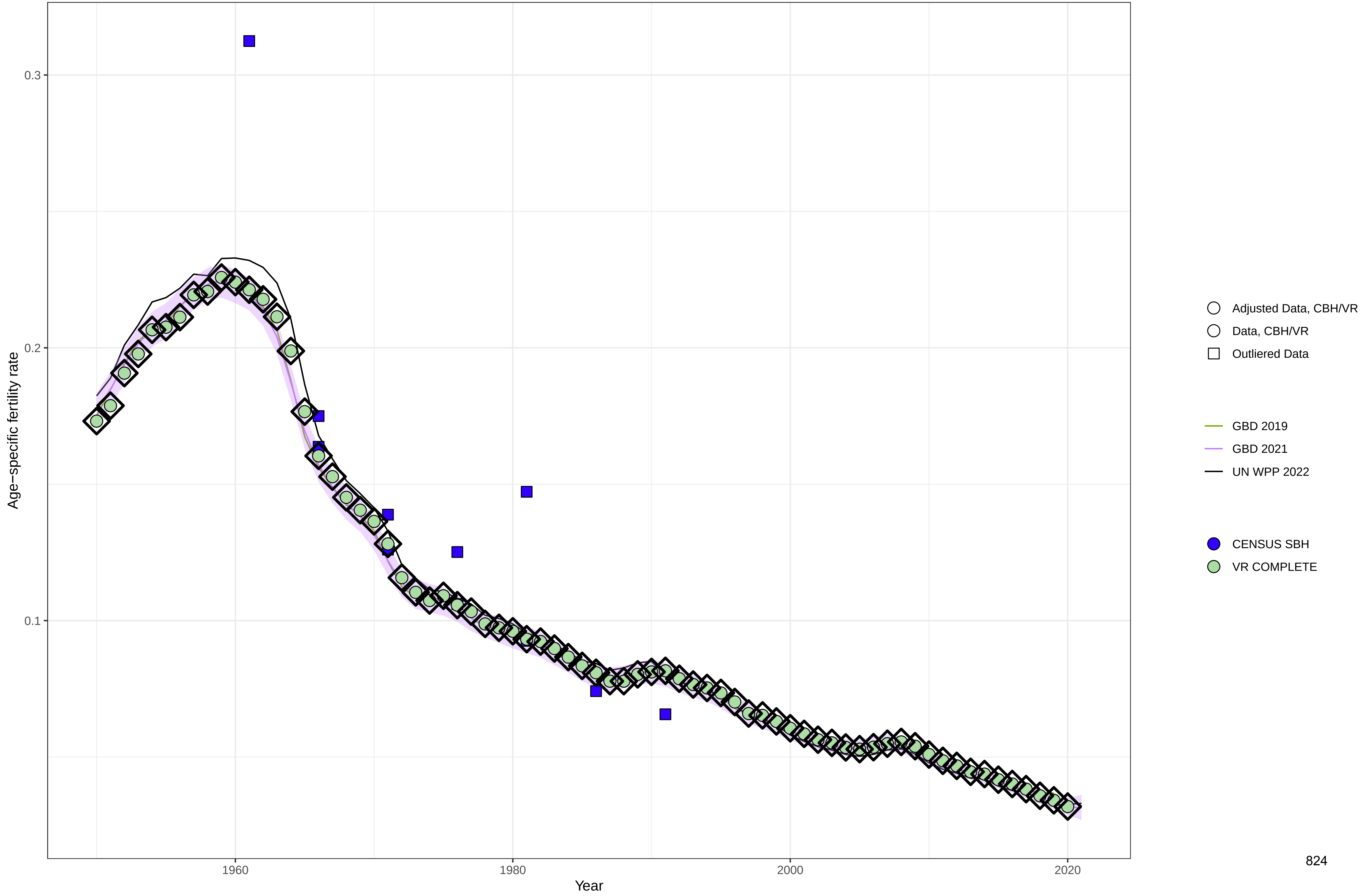

Age−specific fertility fitted trend lines, 1950−2021: Canada, Ages: 25−29
Data points enclosed with a diamond are reference sources

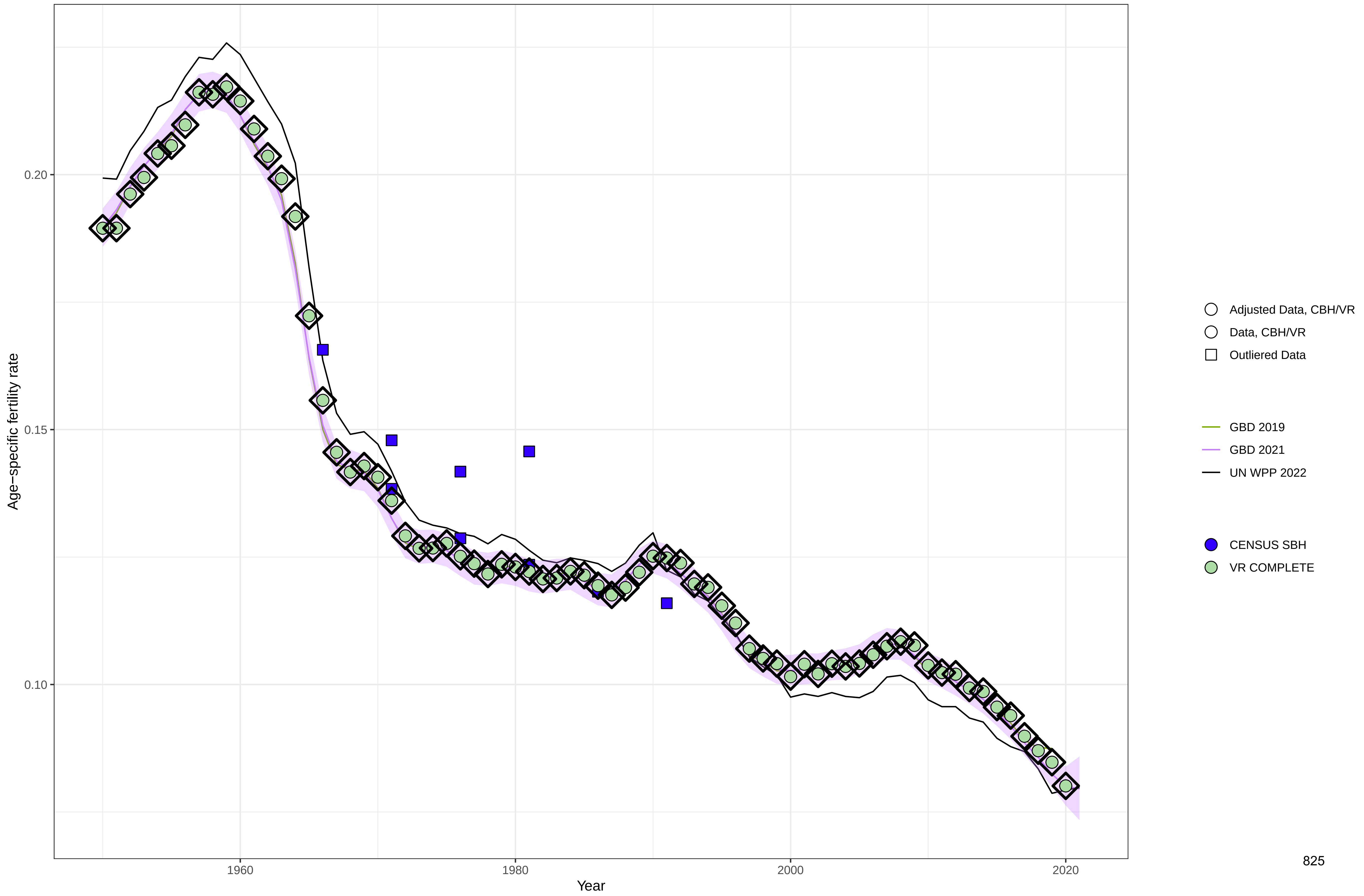

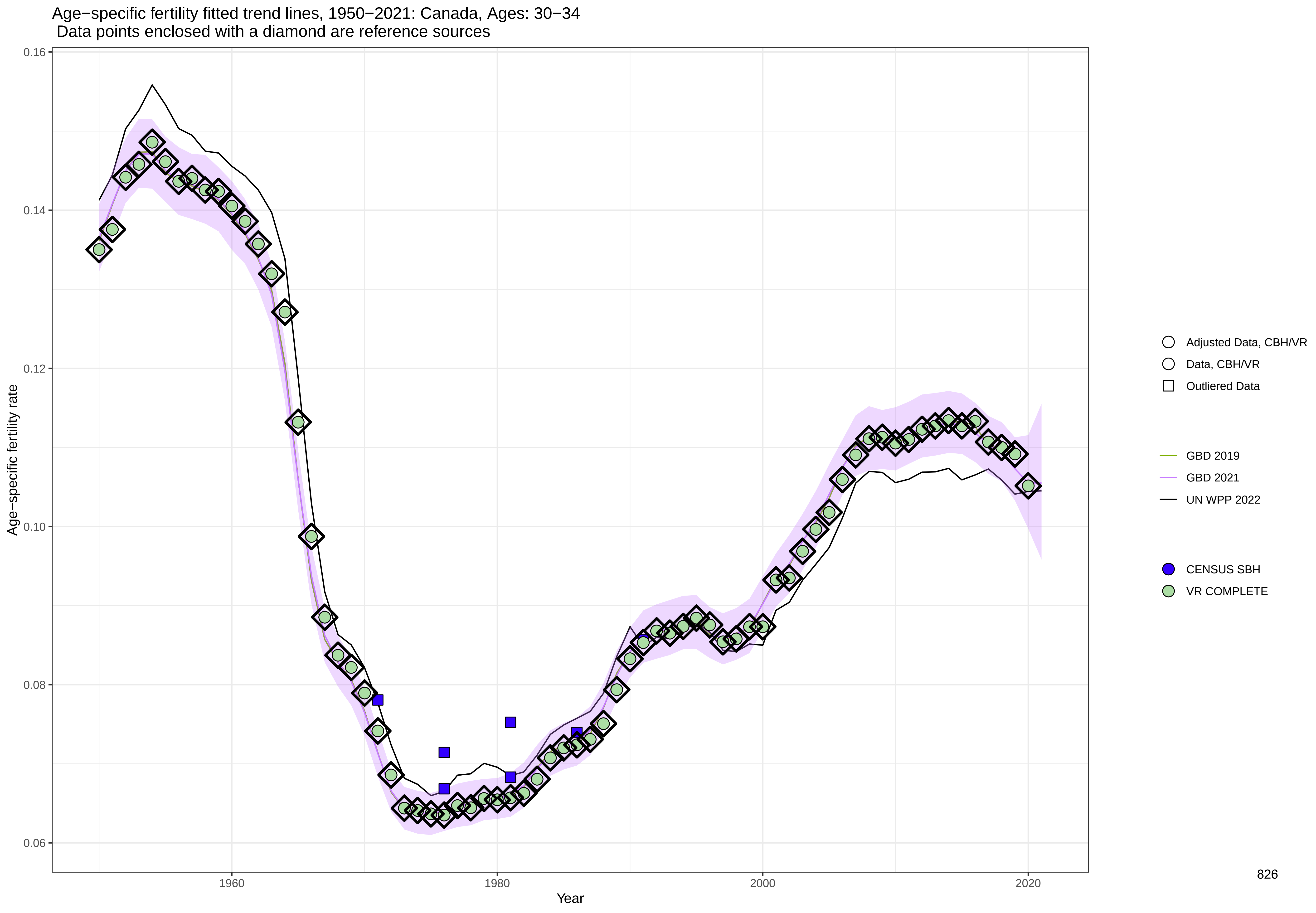

Age−specific fertility fitted trend lines, 1950−2021: Canada, Ages: 30−34
Data points enclosed with a diamond are reference sources
Age−specific fertility rate
0.16
0.14
0.12
0.10
0.08
0.06
1960
1980
2000
2020
Year
Adjusted Data, CBH/VR
Data, CBH/VR
Outliered Data
GBD 2019
GBD 2021
UN WPP 2022
CENSUS SBH
VR COMPLETE

Age−specific fertility fitted trend lines, 1950−2021: Canada, Ages: 35−39
Data points enclosed with a diamond are reference sources

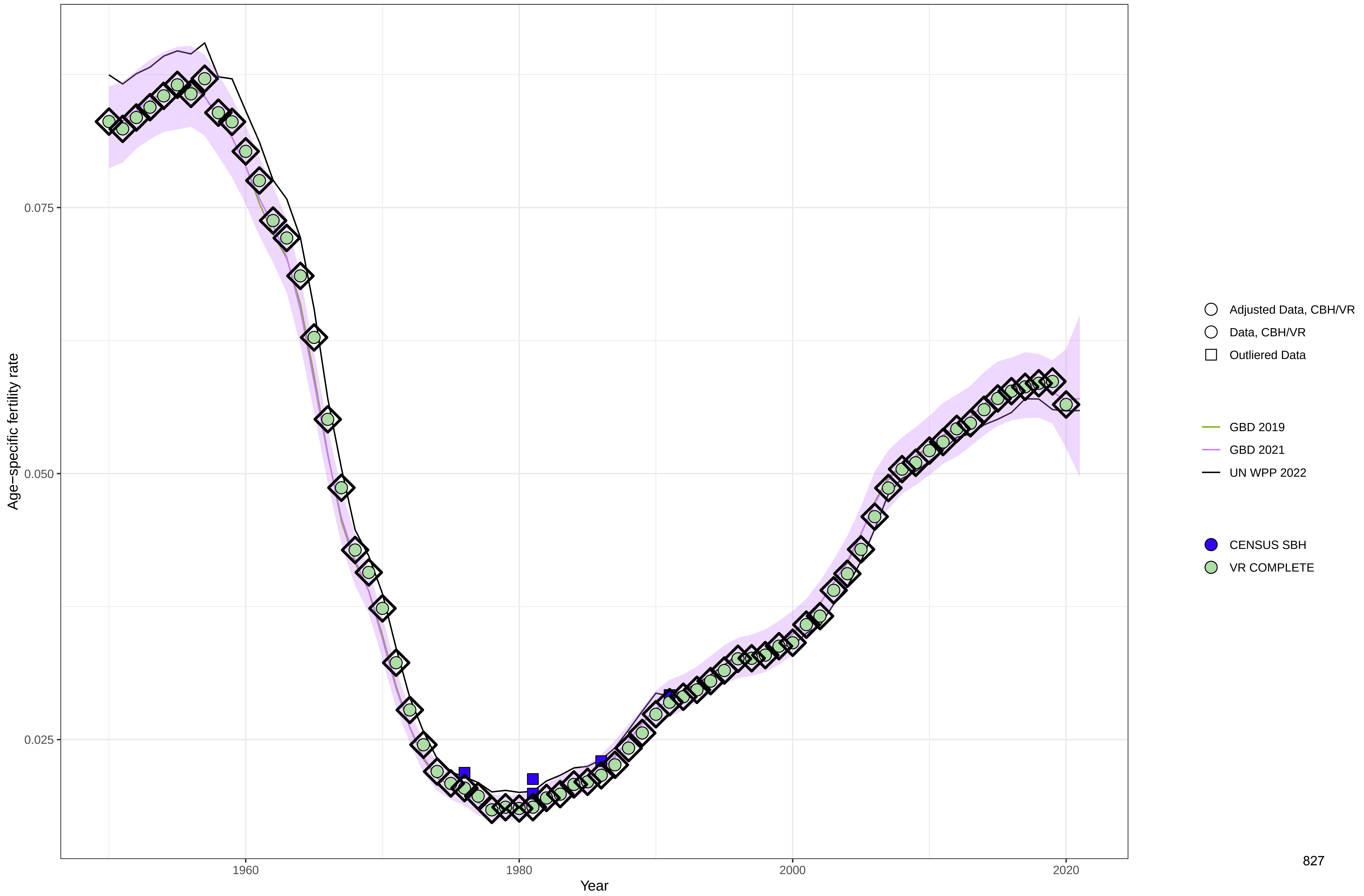

Age−specific fertility fitted trend lines, 1950−2021: Canada, Ages: 40−44
Data points enclosed with a diamond are reference sources

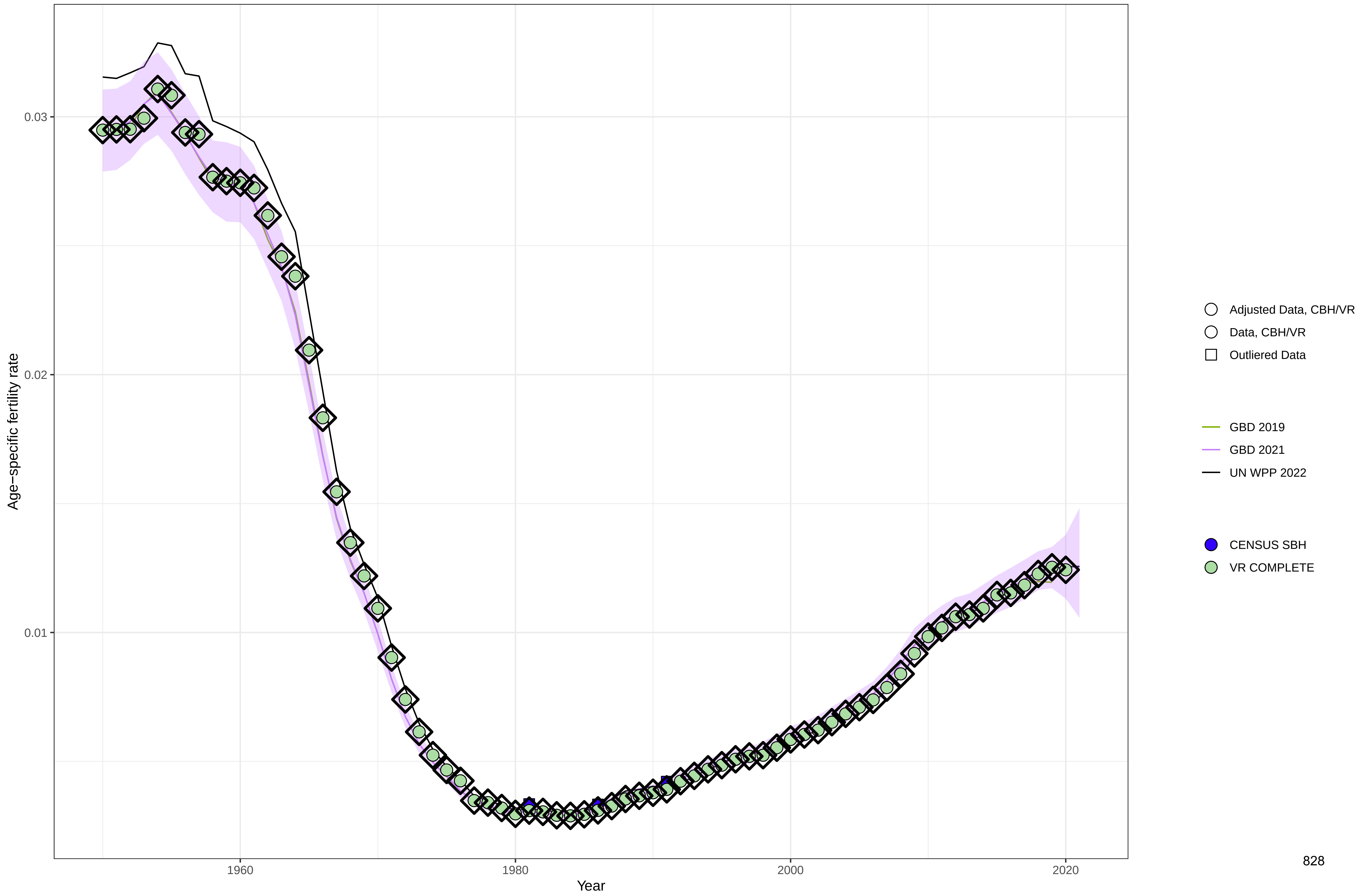

Age−specific fertility fitted trend lines, 1950−2021: Canada, Ages: 45−49
Data points enclosed with a diamond are reference sources

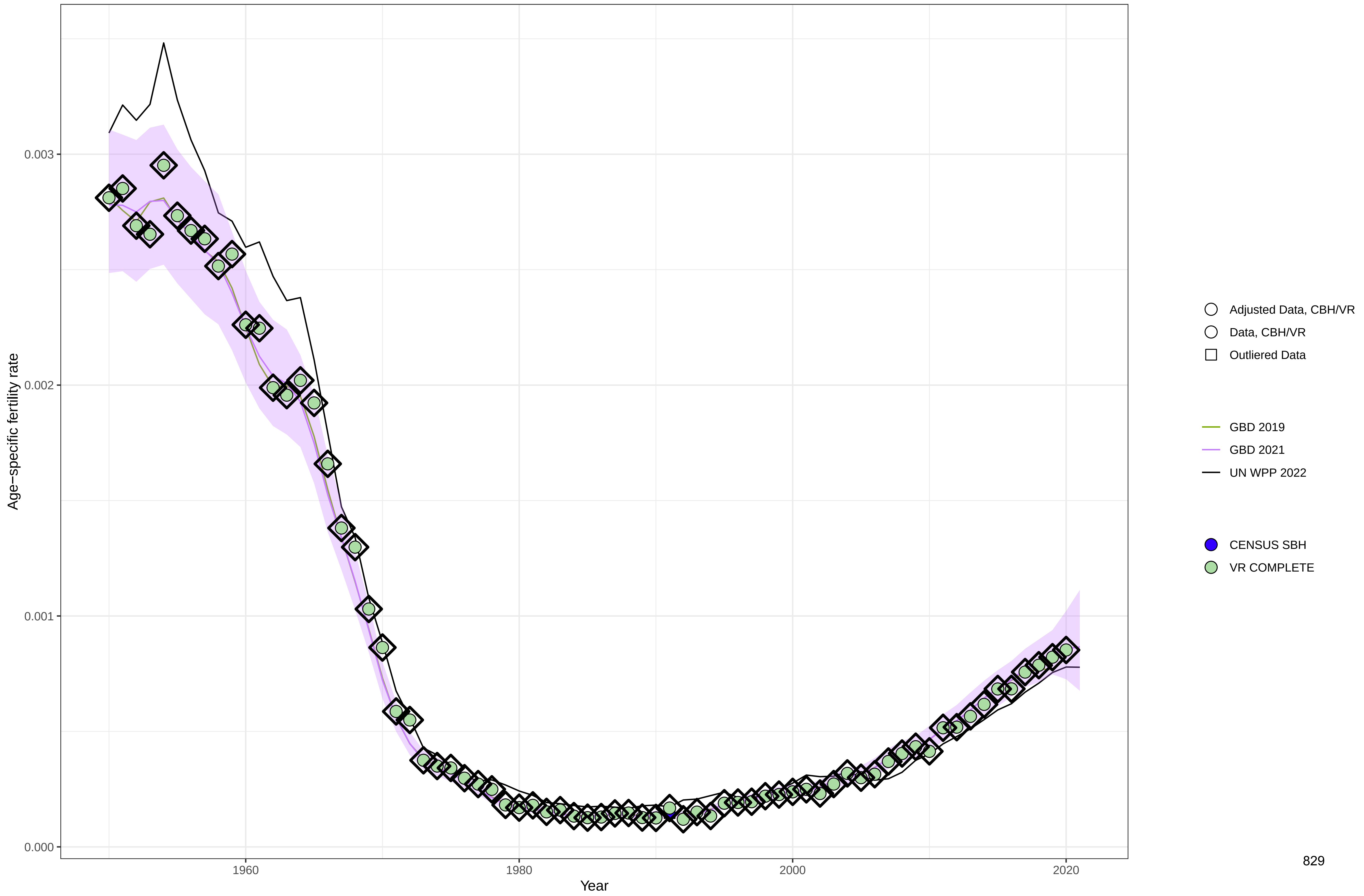

Age−specific fertility fitted trend lines, 1950−2021: Canada, Ages: 50−54
Data points enclosed with a diamond are reference sources

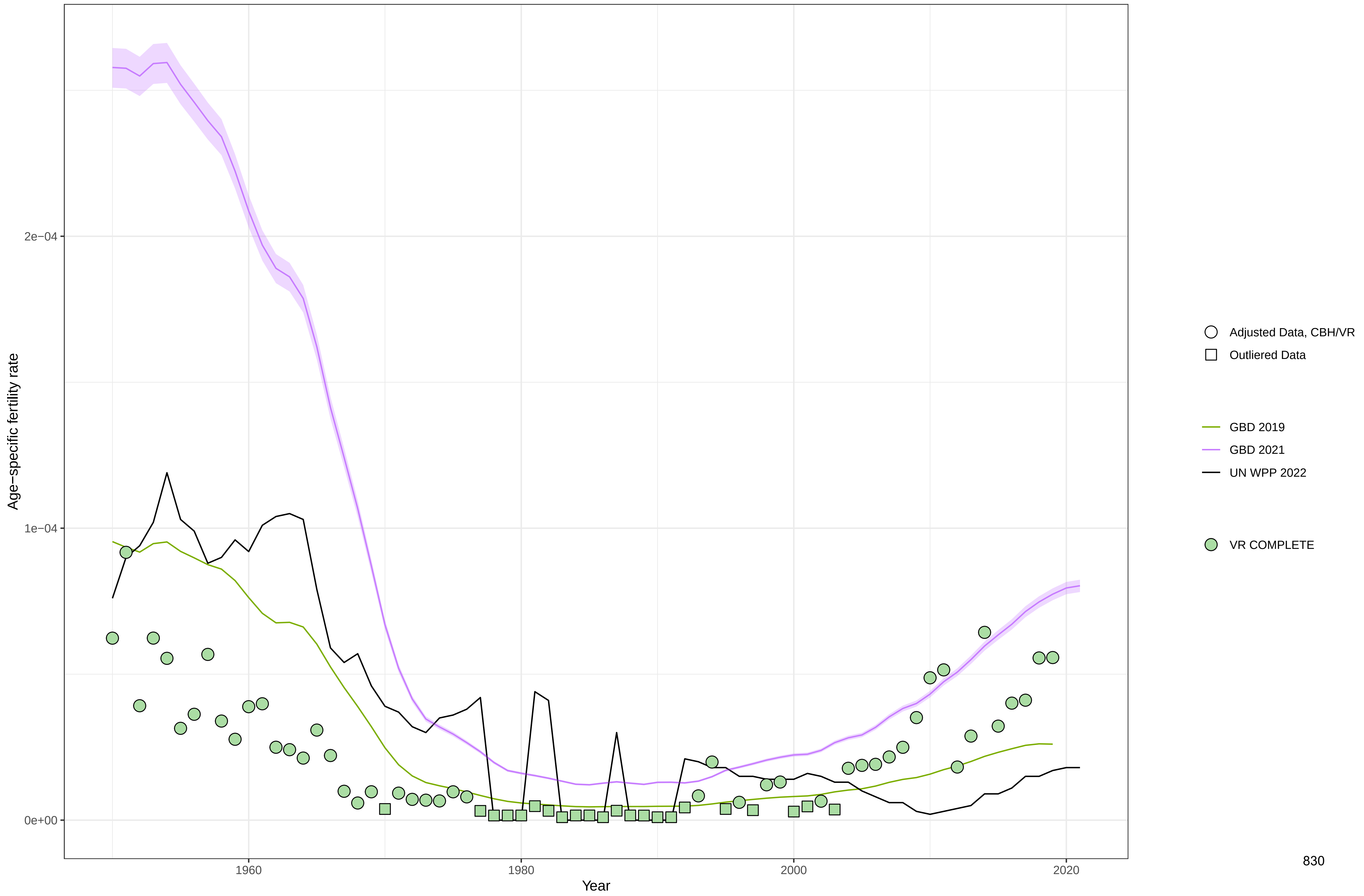

Total fertility rate fitted trend lines, 1950−2021: Canada TFR
Data points enclosed with a diamond are reference sources

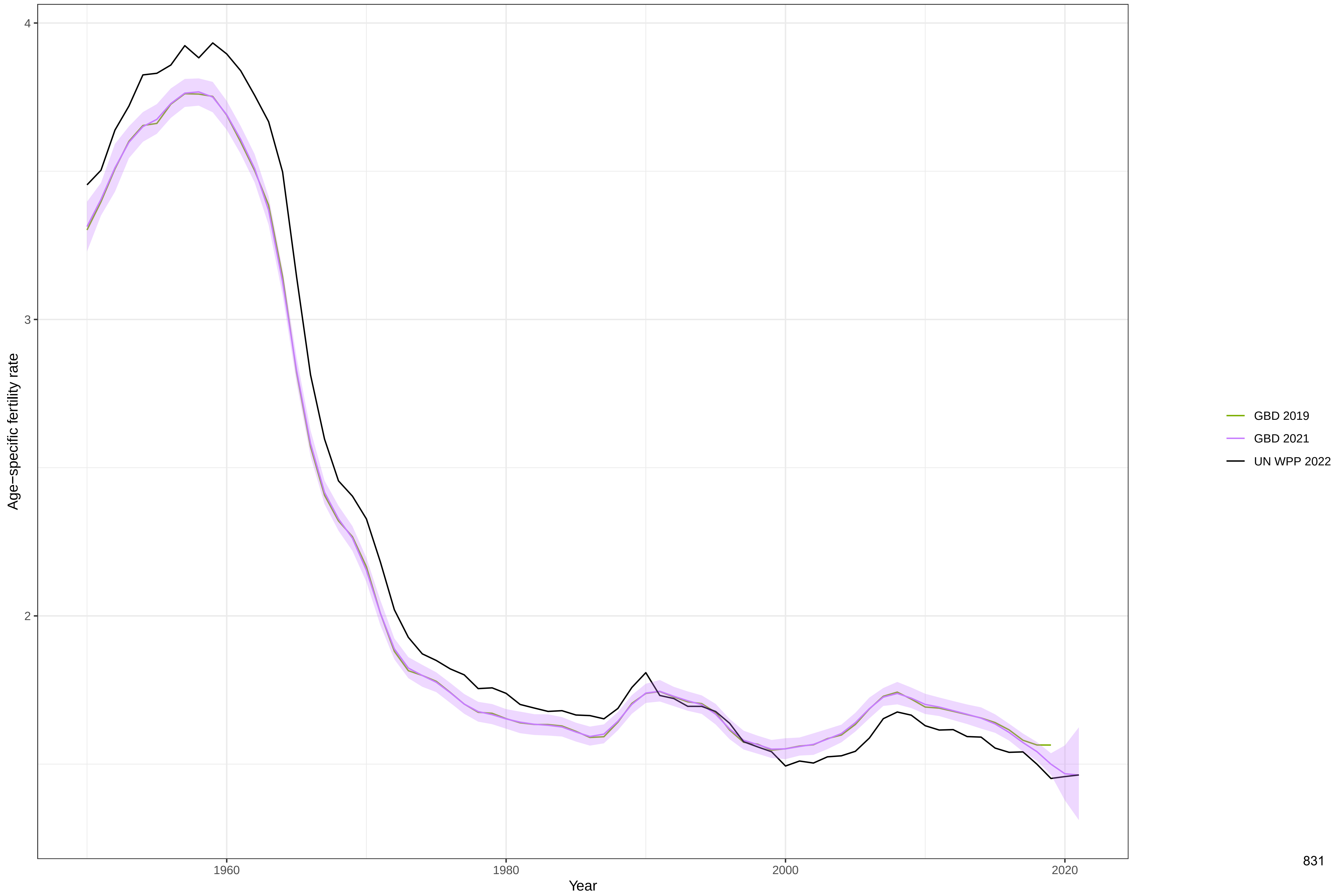

Age−specific fertility fitted trend lines, 1950−2021: Greenland, Ages: 10−14
Data points enclosed with a diamond are reference sources

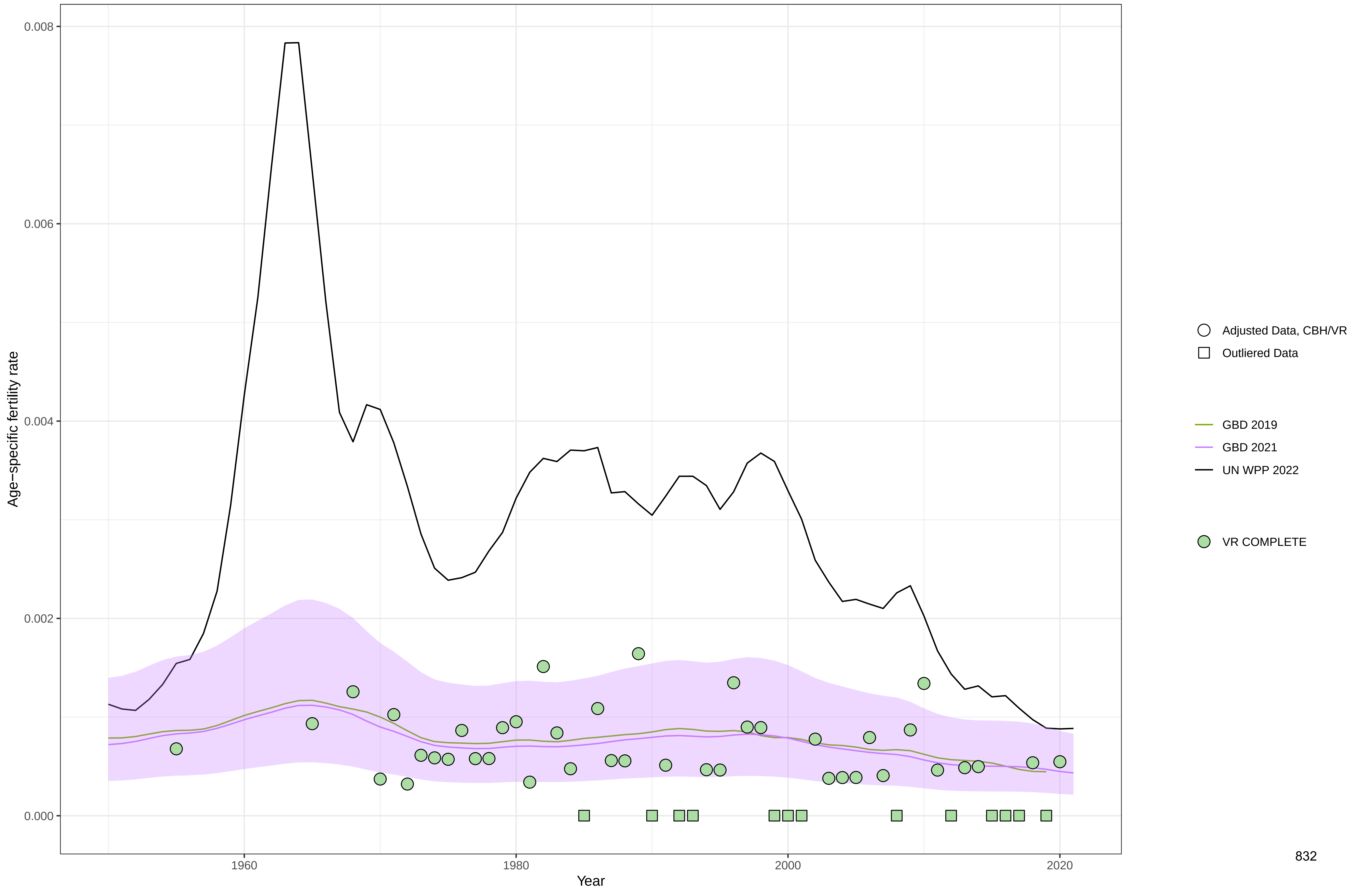

Age−specific fertility fitted trend lines, 1950−2021: Greenland, Ages: 15−19
Data points enclosed with a diamond are reference sources

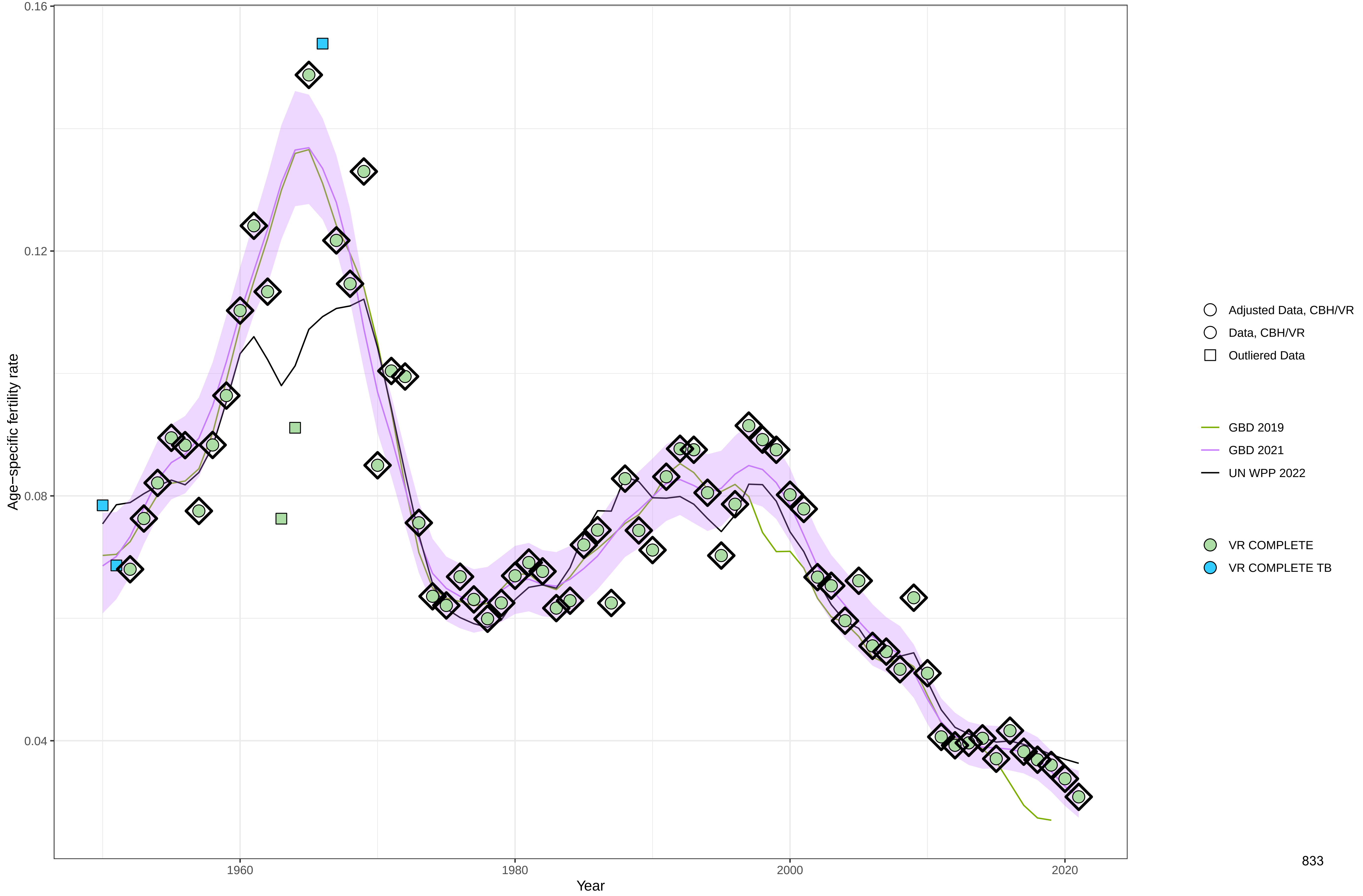

Age−specific fertility fitted trend lines, 1950−2021: Greenland, Ages: 20−24
Data points enclosed with a diamond are reference sources

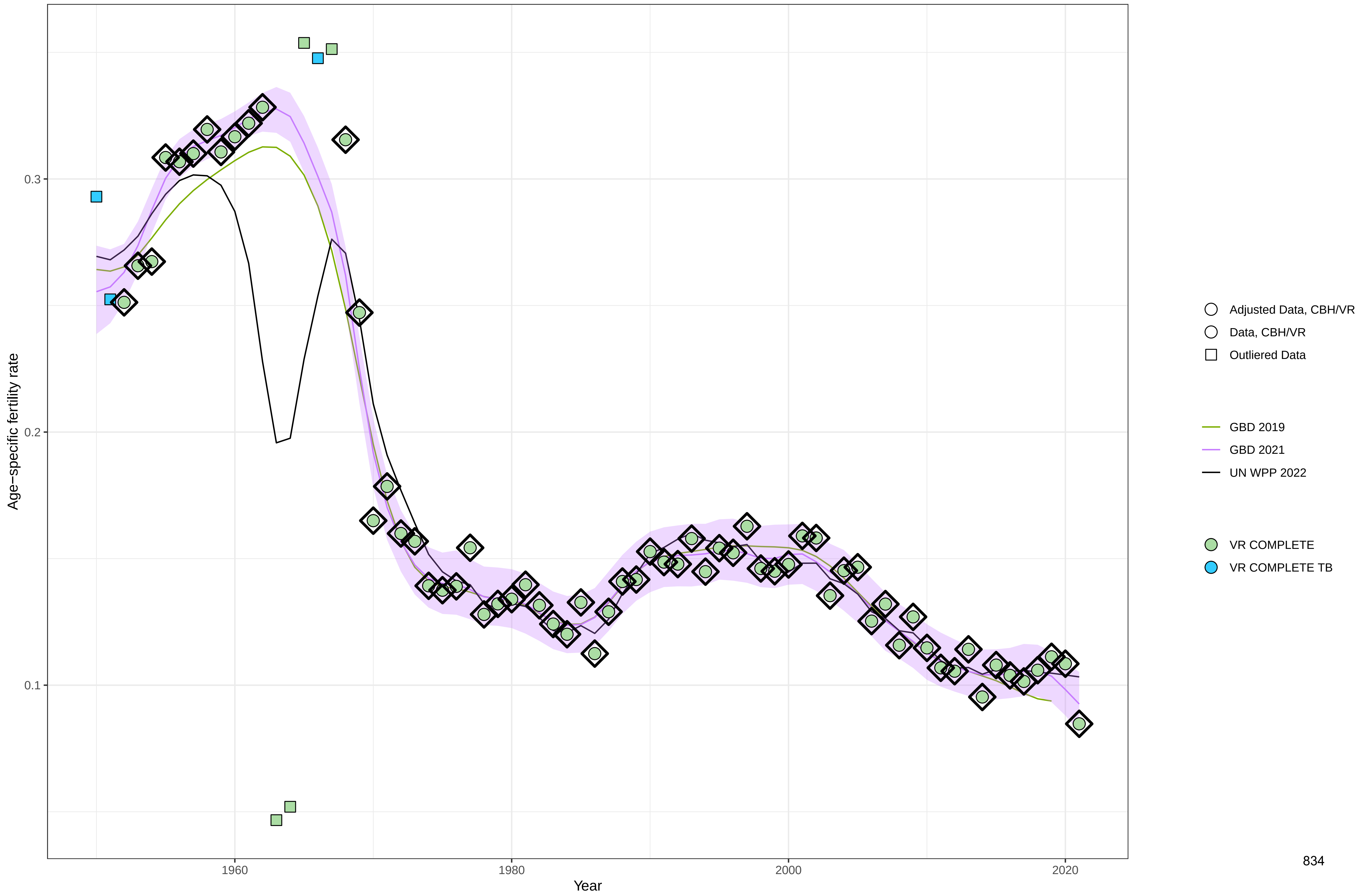

Age−specific fertility fitted trend lines, 1950−2021: Greenland, Ages: 25−29
Data points enclosed with a diamond are reference sources

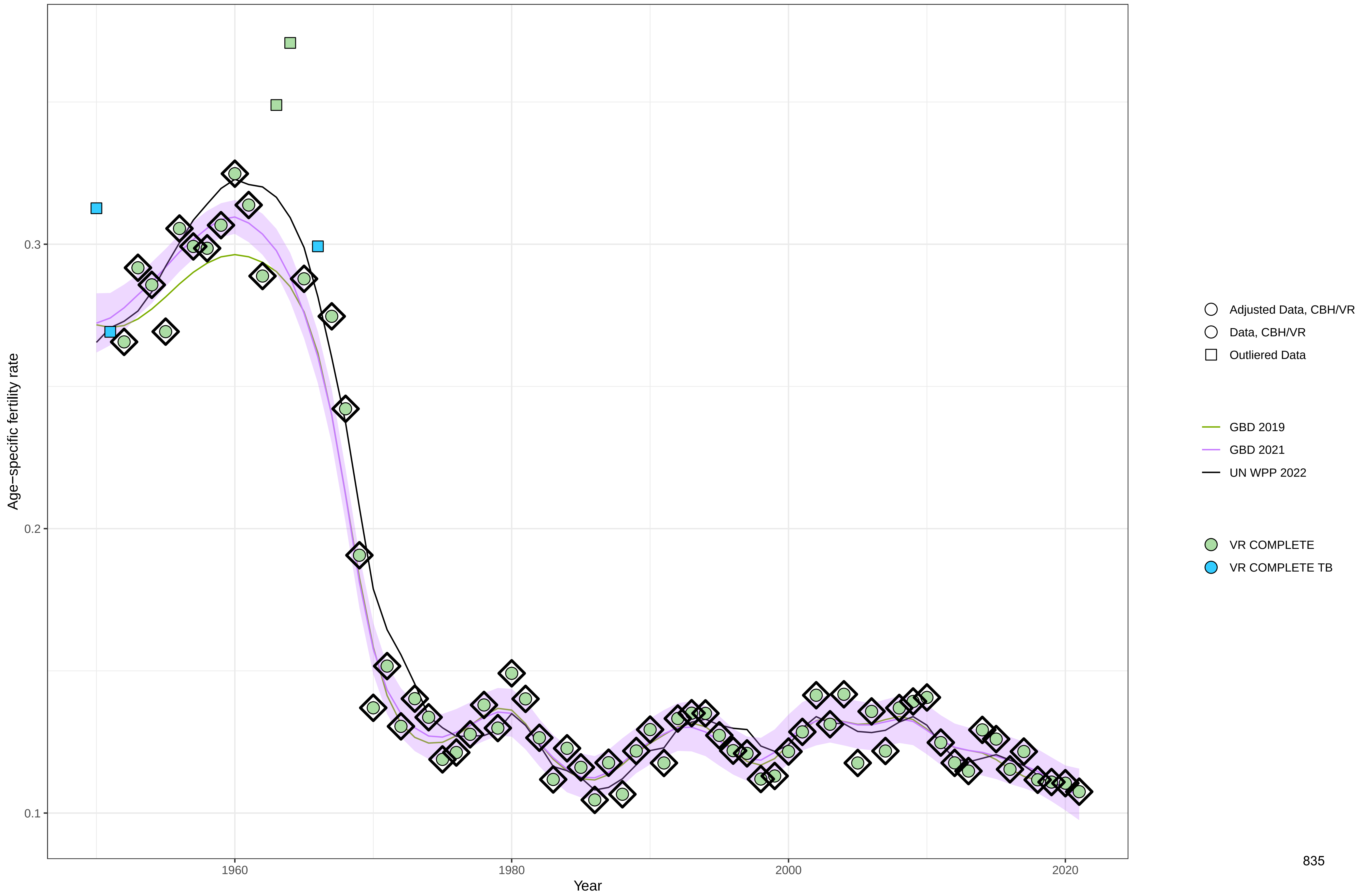

Age−specific fertility fitted trend lines, 1950−2021: Greenland, Ages: 30−34
Data points enclosed with a diamond are reference sources

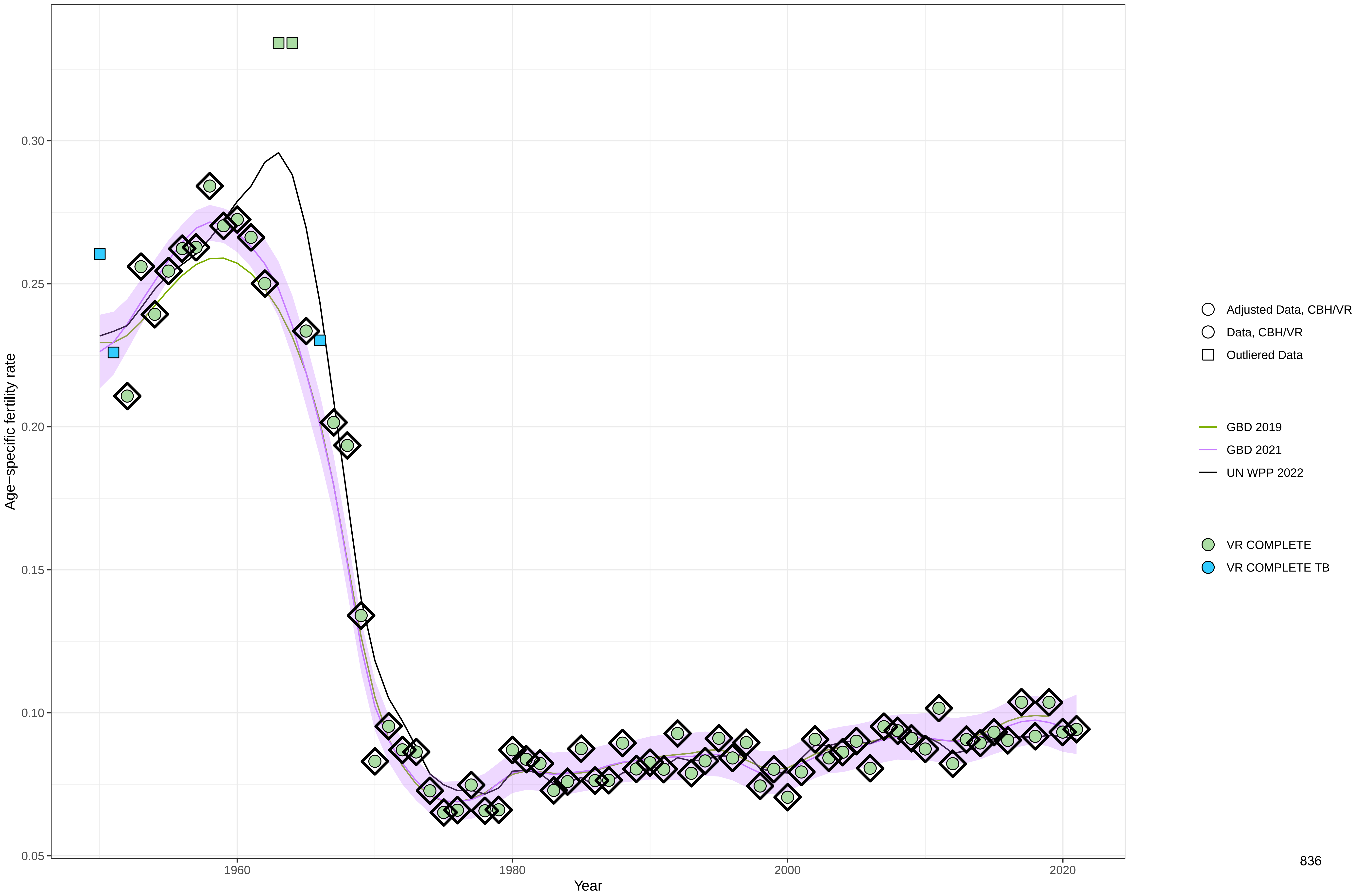

Age−specific fertility fitted trend lines, 1950−2021: Greenland, Ages: 35−39
Data points enclosed with a diamond are reference sources

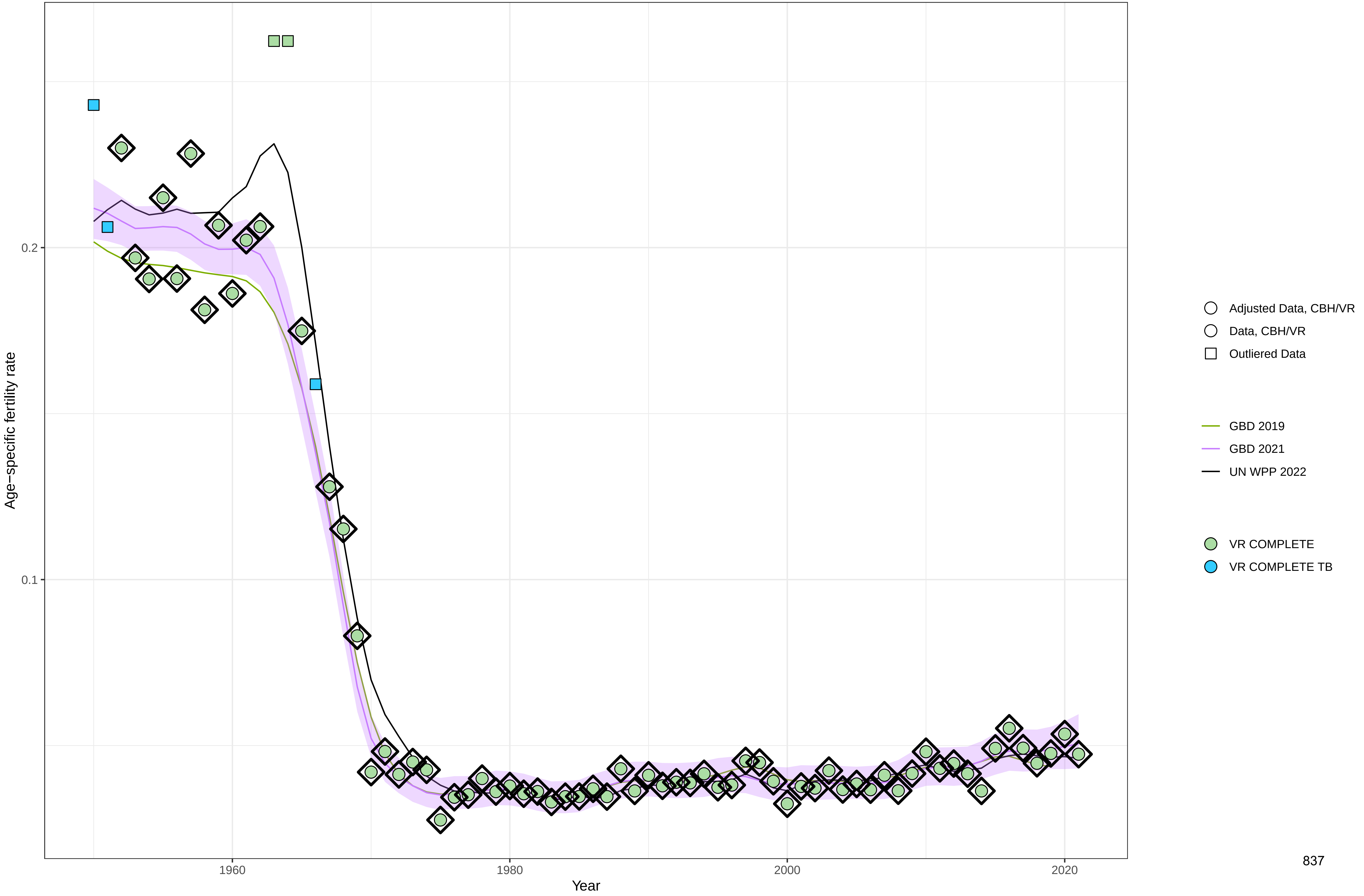

Age−specific fertility fitted trend lines, 1950−2021: Greenland, Ages: 40−44
Data points enclosed with a diamond are reference sources

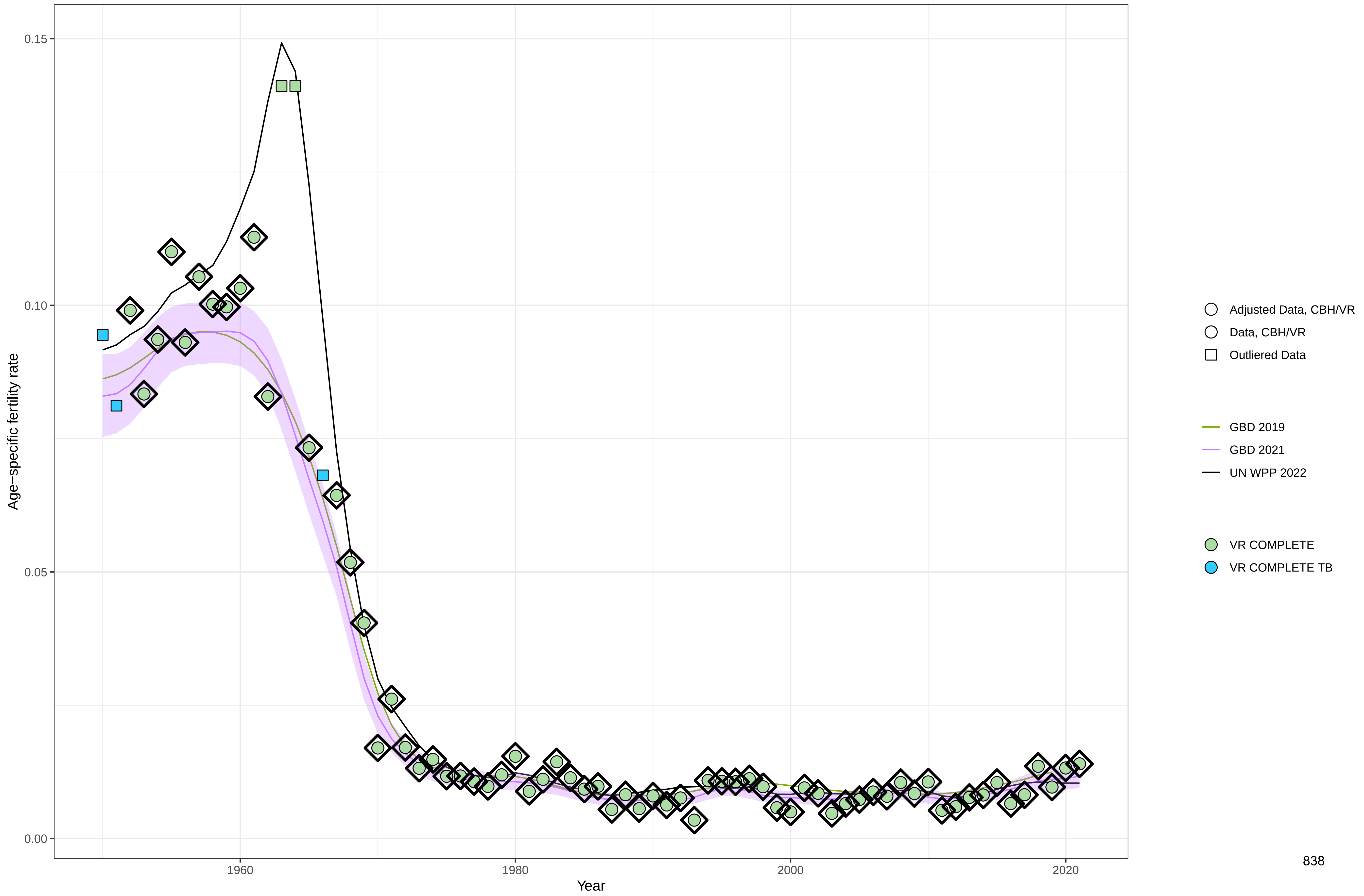

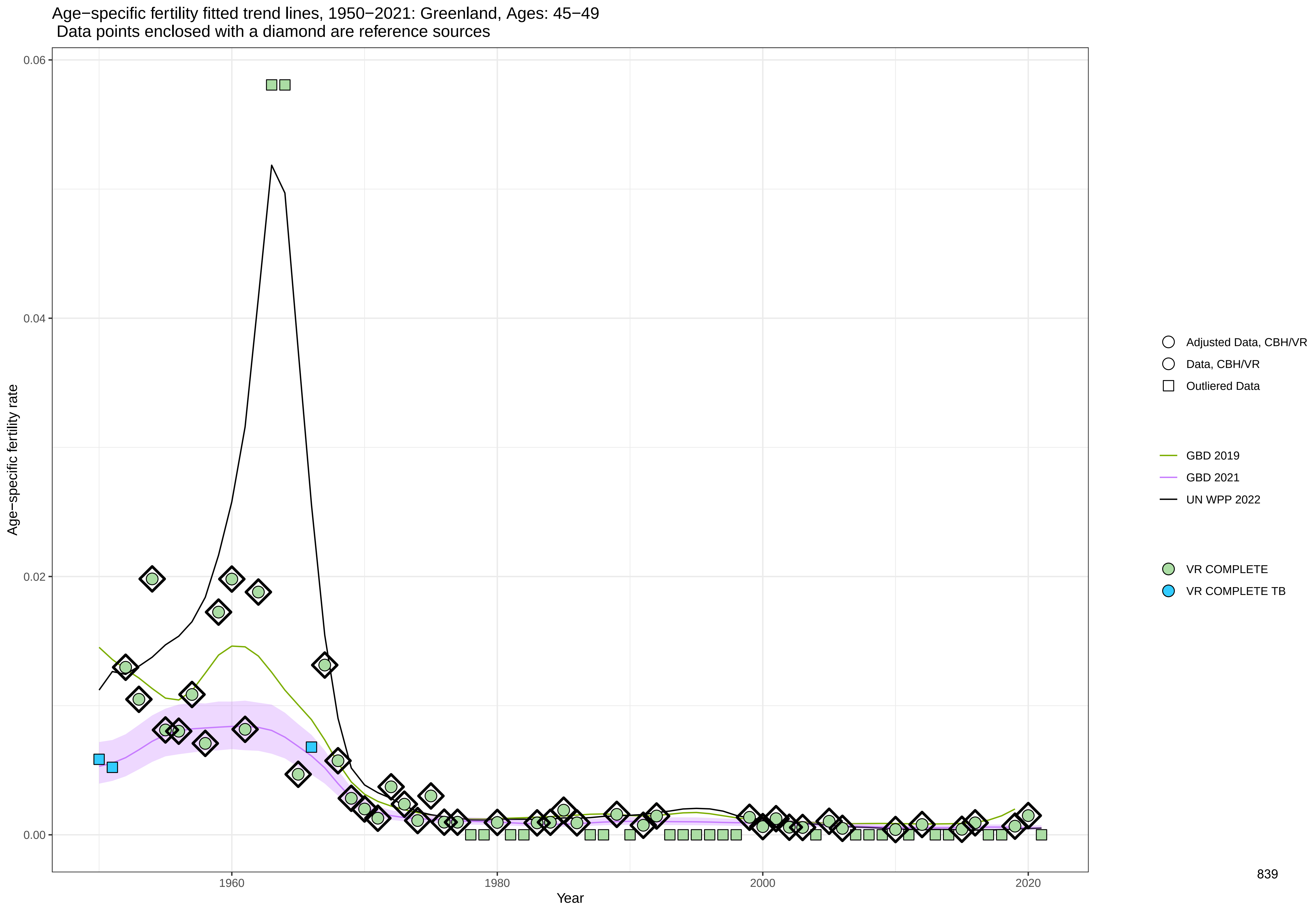

Age−specific fertility fitted trend lines, 1950−2021: Greenland, Ages: 45−49
Data points enclosed with a diamond are reference sources
0.06
0.04
0.02
0.00
Age−specific fertility rate
1960
1980
2000
2020
Year
Adjusted Data, CBH/VR
Data, CBH/VR
Outliered Data
GBD 2019
GBD 2021
UN WPP 2022
VR COMPLETE
VR COMPLETE TB

Age−specific fertility fitted trend lines, 1950−2021: Greenland, Ages: 50−54
Data points enclosed with a diamond are reference sources

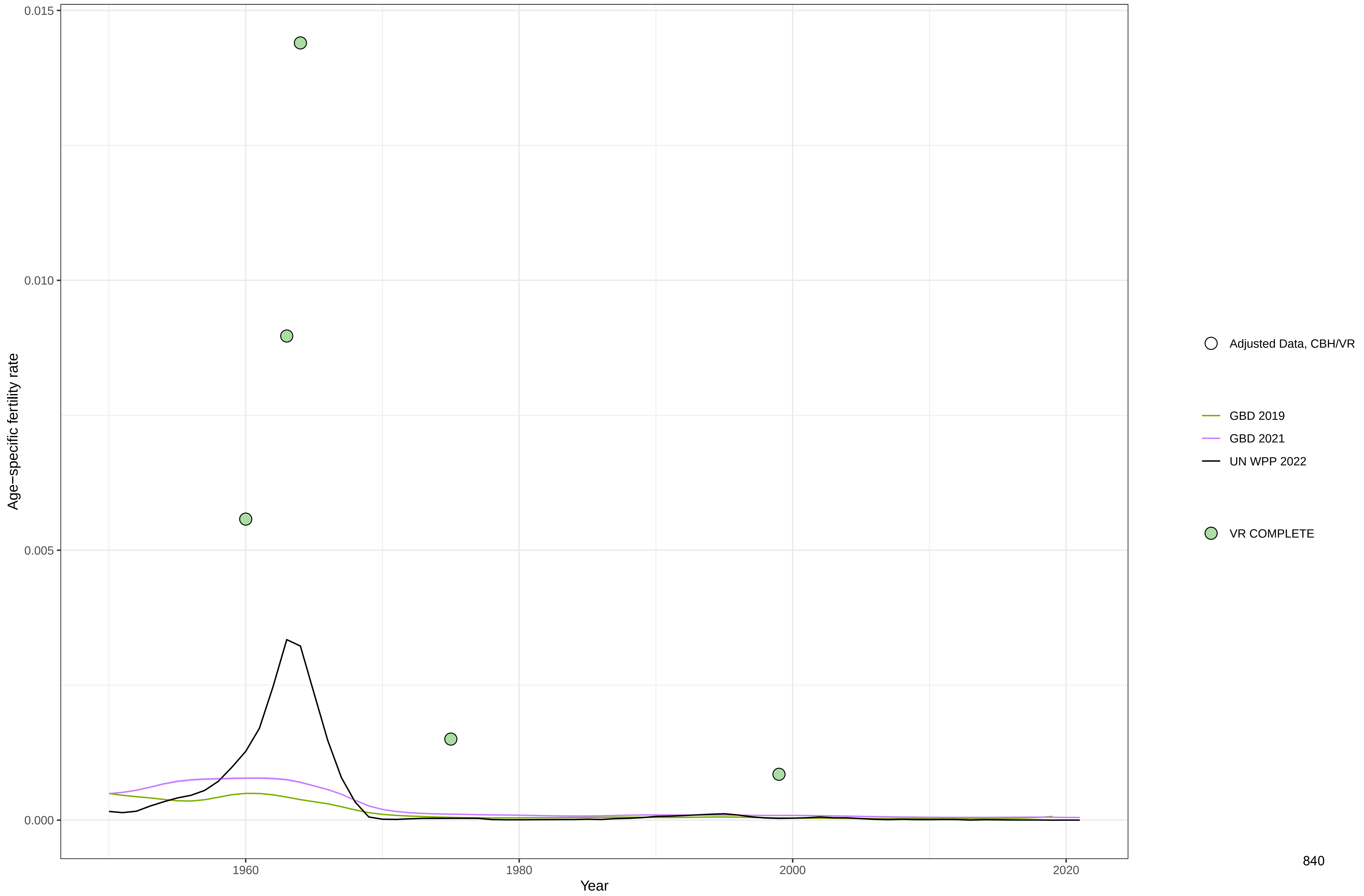

Total fertility rate fitted trend lines, 1950−2021: Greenland TFR
Data points enclosed with a diamond are reference sources

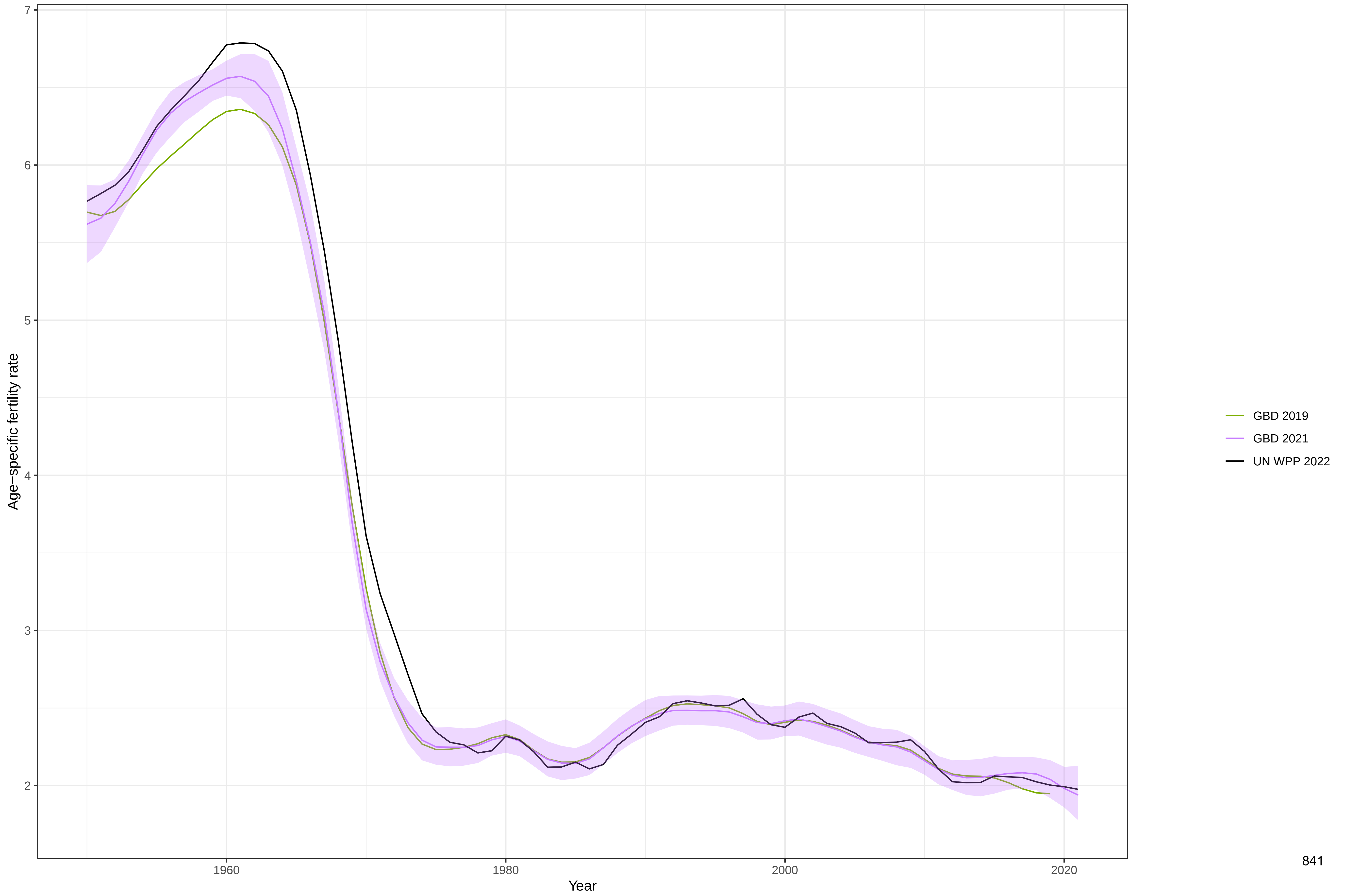

Age−specific fertility fitted trend lines, 1950−2021: USA, Ages: 10−14
Data points enclosed with a diamond are reference sources

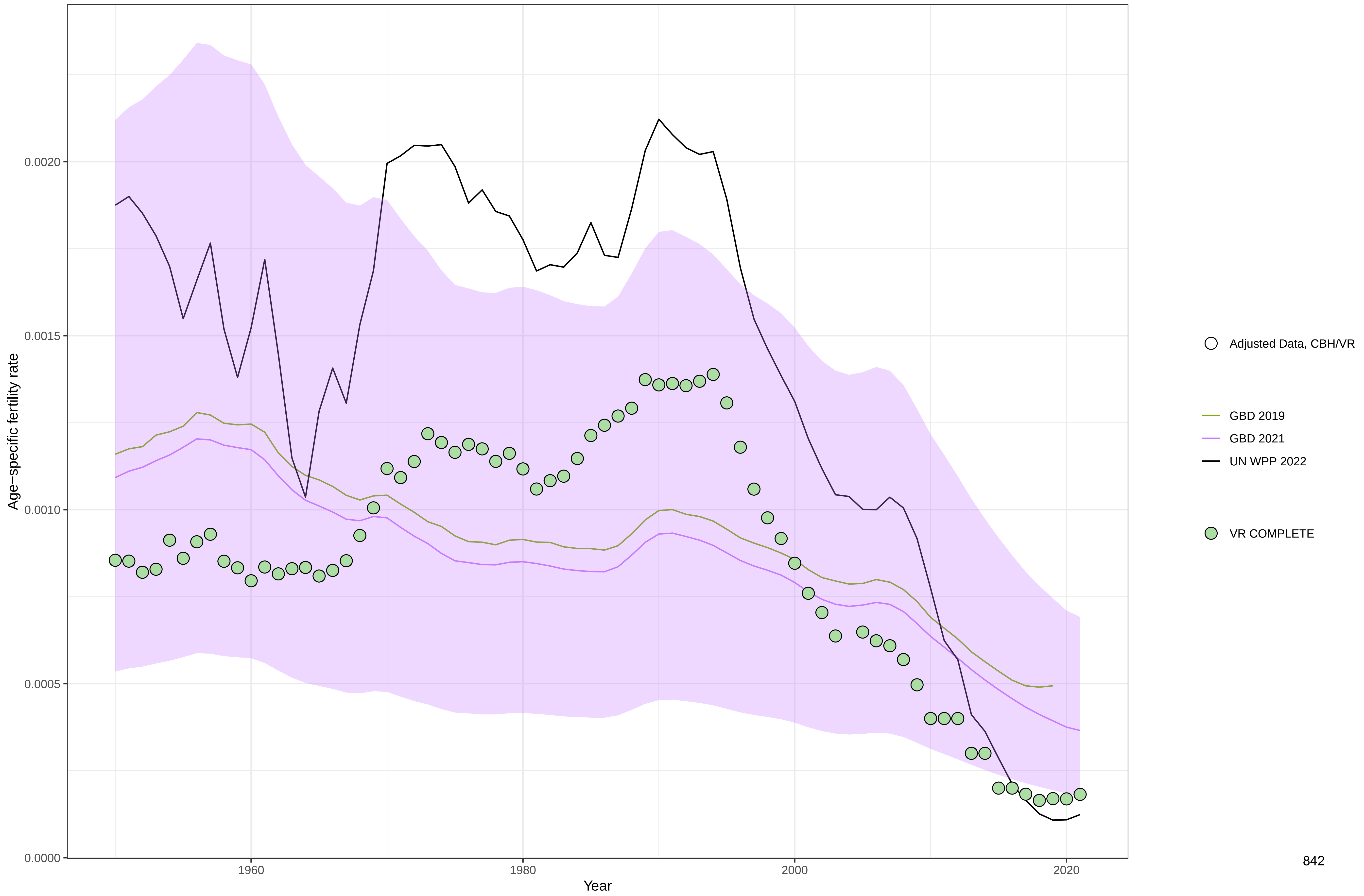

Age−specific fertility fitted trend lines, 1950−2021: USA, Ages: 15−19
Data points enclosed with a diamond are reference sources

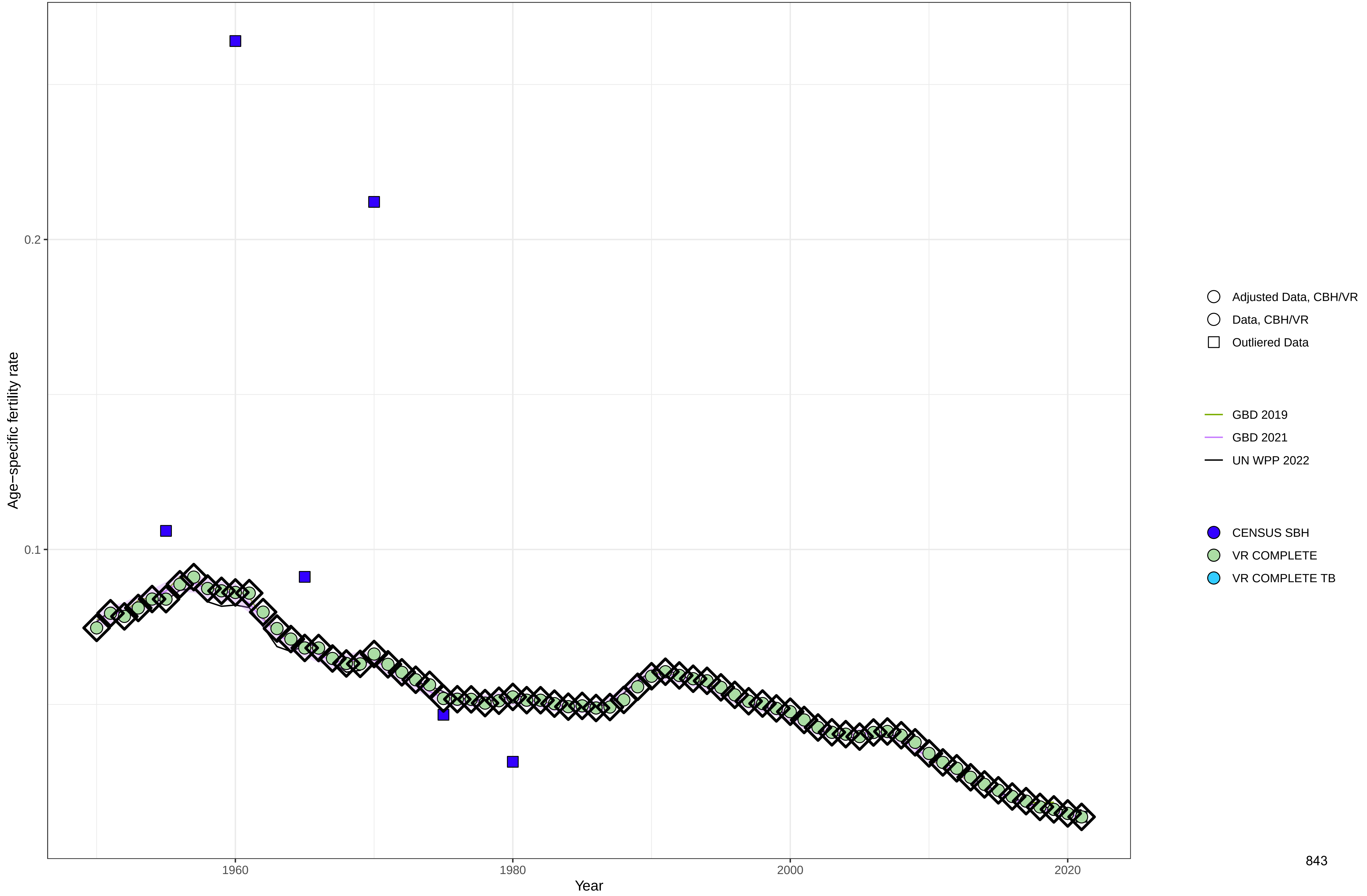

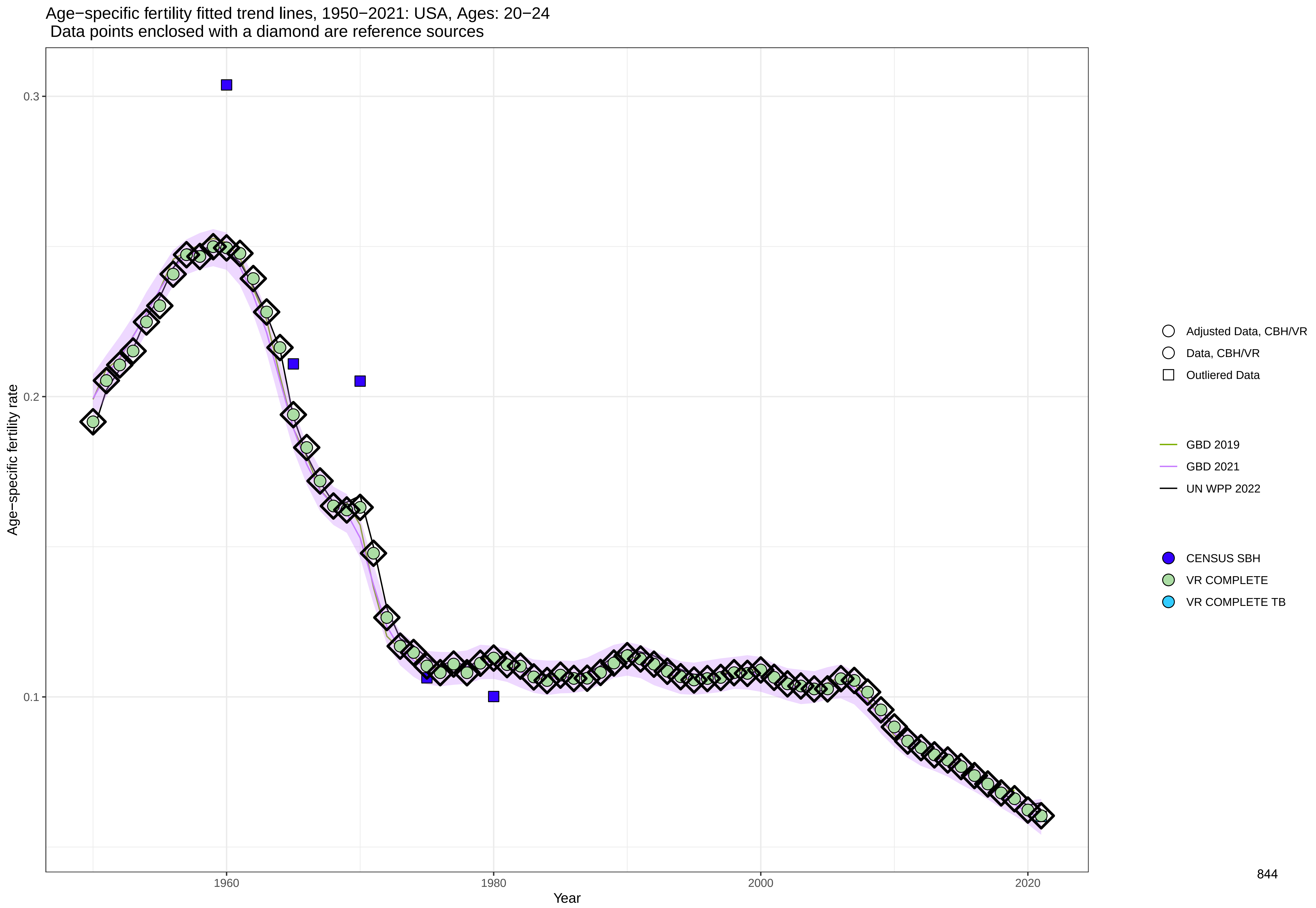
Age−specific fertility fitted trend lines, 1950−2021: USA, Ages: 20−24
Data points enclosed with a diamond are reference sources
0.3
0.2
0.1
Age−specific fertility rate
1960
1980
2000
2020
Year
Adjusted Data, CBH/VR
Data, CBH/VR
Outliered Data
GBD 2019
GBD 2021
UN WPP 2022
CENSUS SBH
VR COMPLETE
VR COMPLETE TB

Age−specific fertility fitted trend lines, 1950−2021: USA, Ages: 25−29
Data points enclosed with a diamond are reference sources

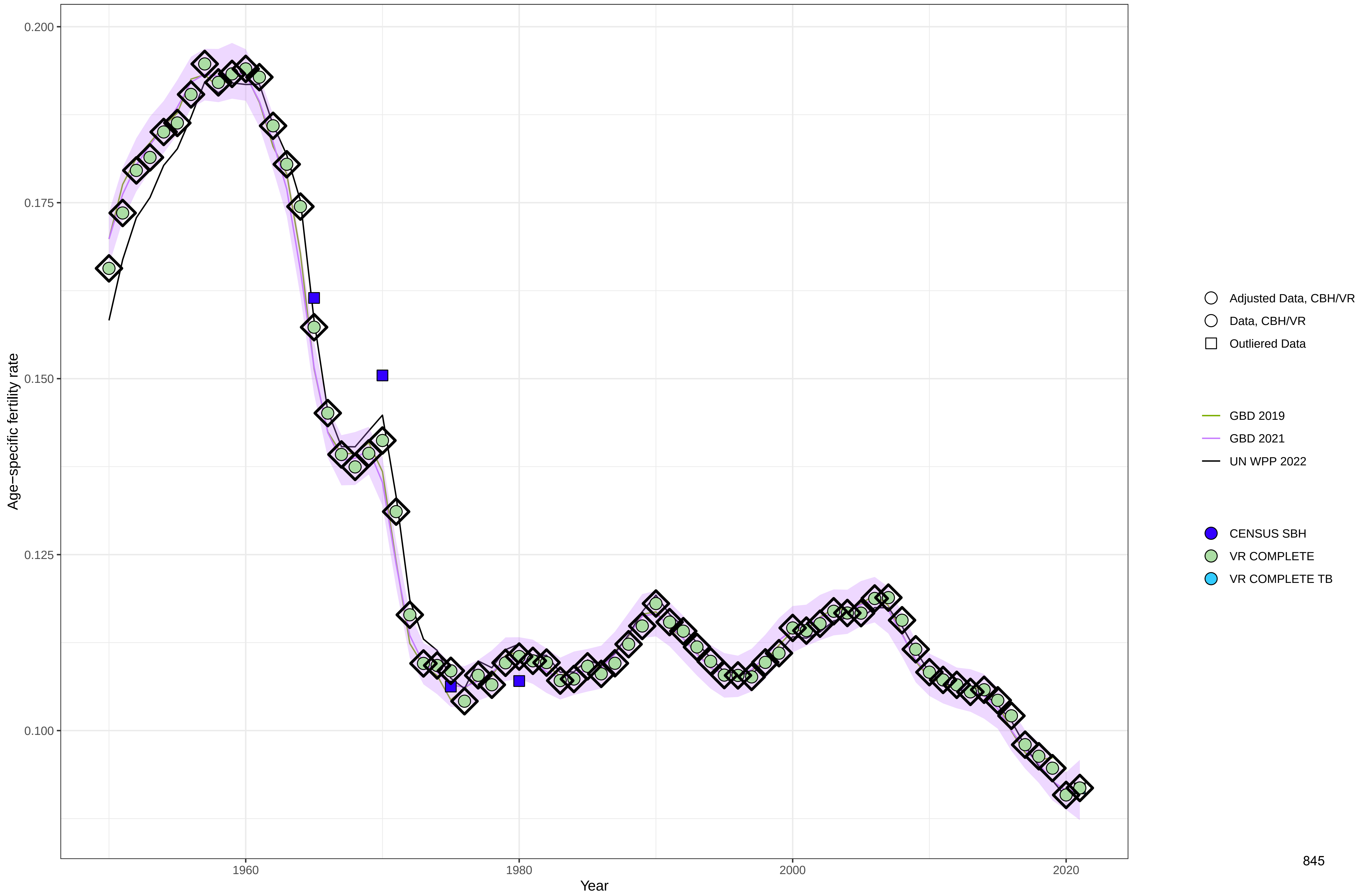

Age−specific fertility fitted trend lines, 1950−2021: USA, Ages: 30−34
Data points enclosed with a diamond are reference sources

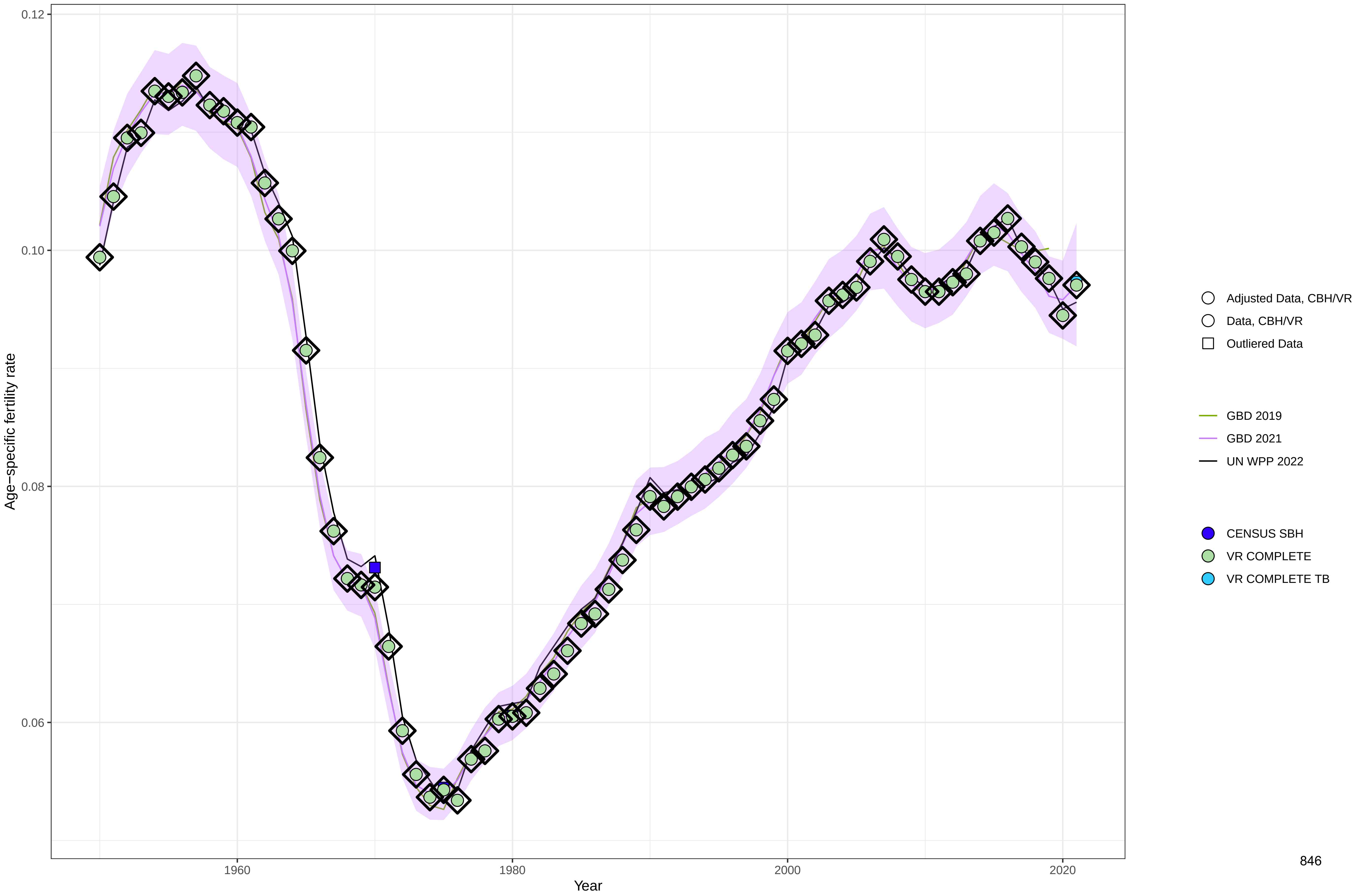

Age−specific fertility fitted trend lines, 1950−2021: USA, Ages: 35−39
Data points enclosed with a diamond are reference sources

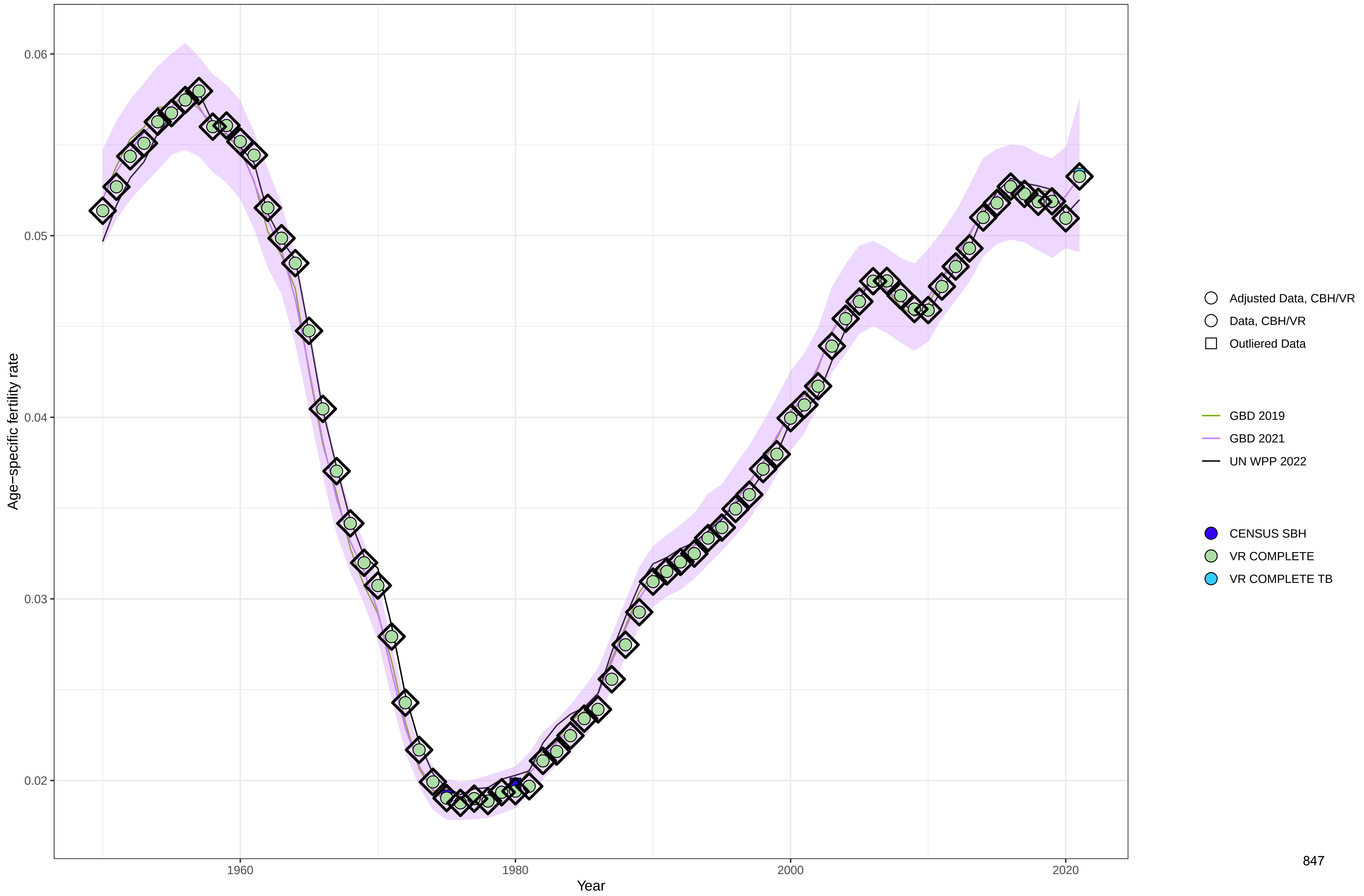

Age−specific fertility fitted trend lines, 1950−2021: USA, Ages: 40−44
Data points enclosed with a diamond are reference sources

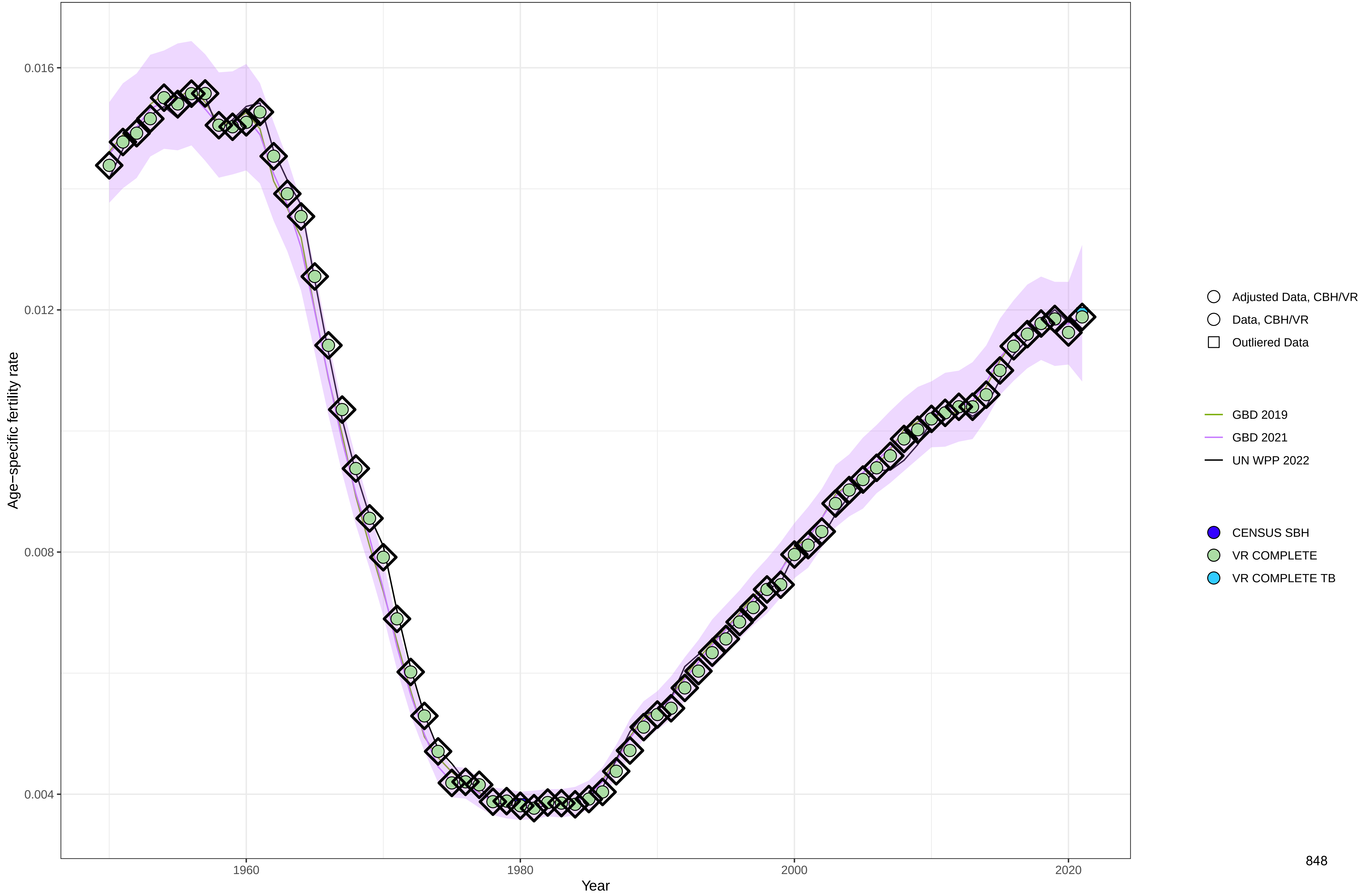

Age−specific fertility fitted trend lines, 1950−2021: USA, Ages: 45−49
Data points enclosed with a diamond are reference sources

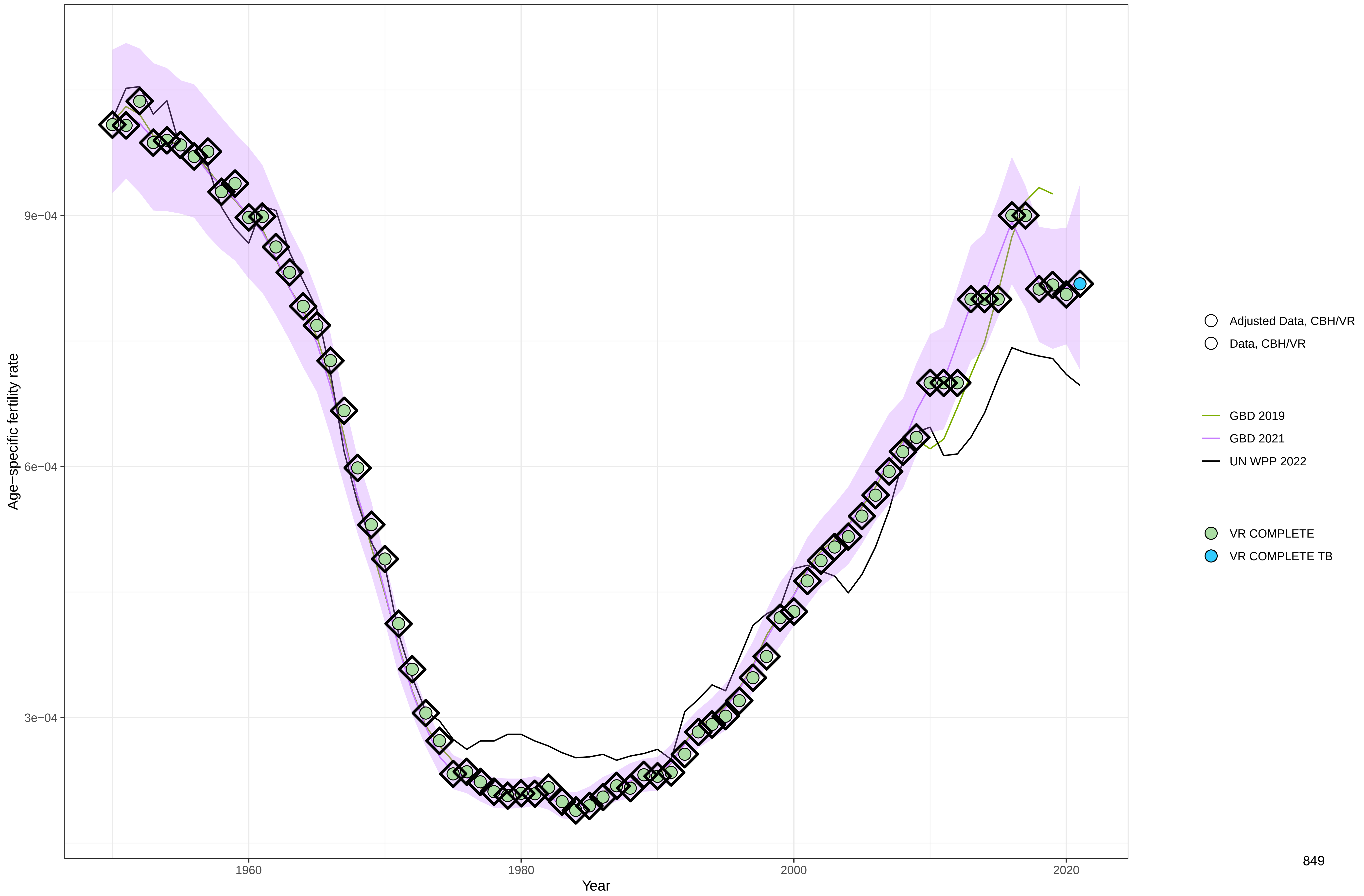

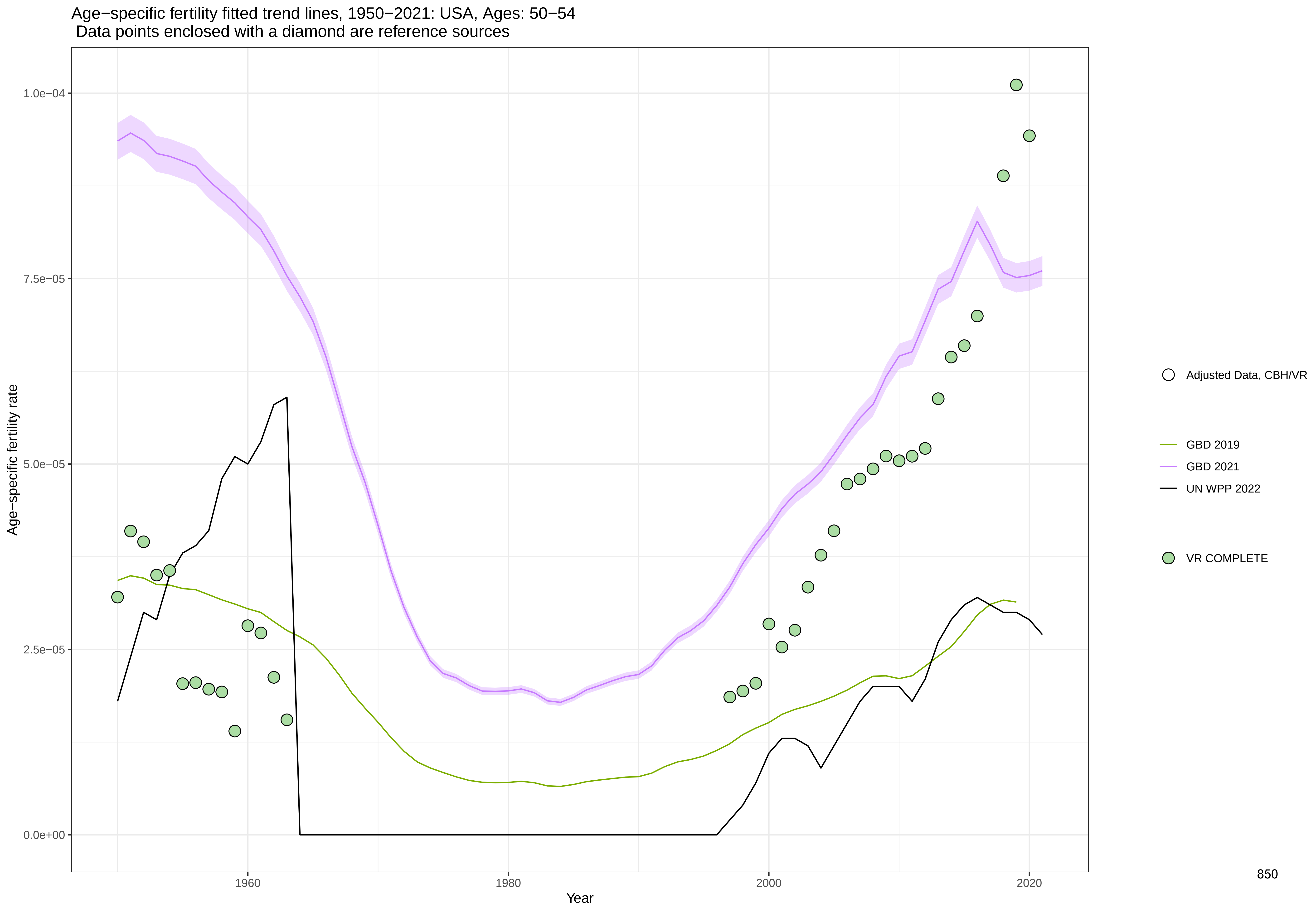

Age−specific fertility fitted trend lines, 1950−2021: USA, Ages: 50−54
Data points enclosed with a diamond are reference sources
Age−specific fertility rate
Year
1.0e−04
7.5e−05
5.0e−05
2.5e−05
0.0e+00
1960
1980
2000
2020
Adjusted Data, CBH/VR
GBD 2019
GBD 2021
UN WPP 2022
VR COMPLETE

Total fertility rate fitted trend lines, 1950−2021: USA TFR
Data points enclosed with a diamond are reference sources

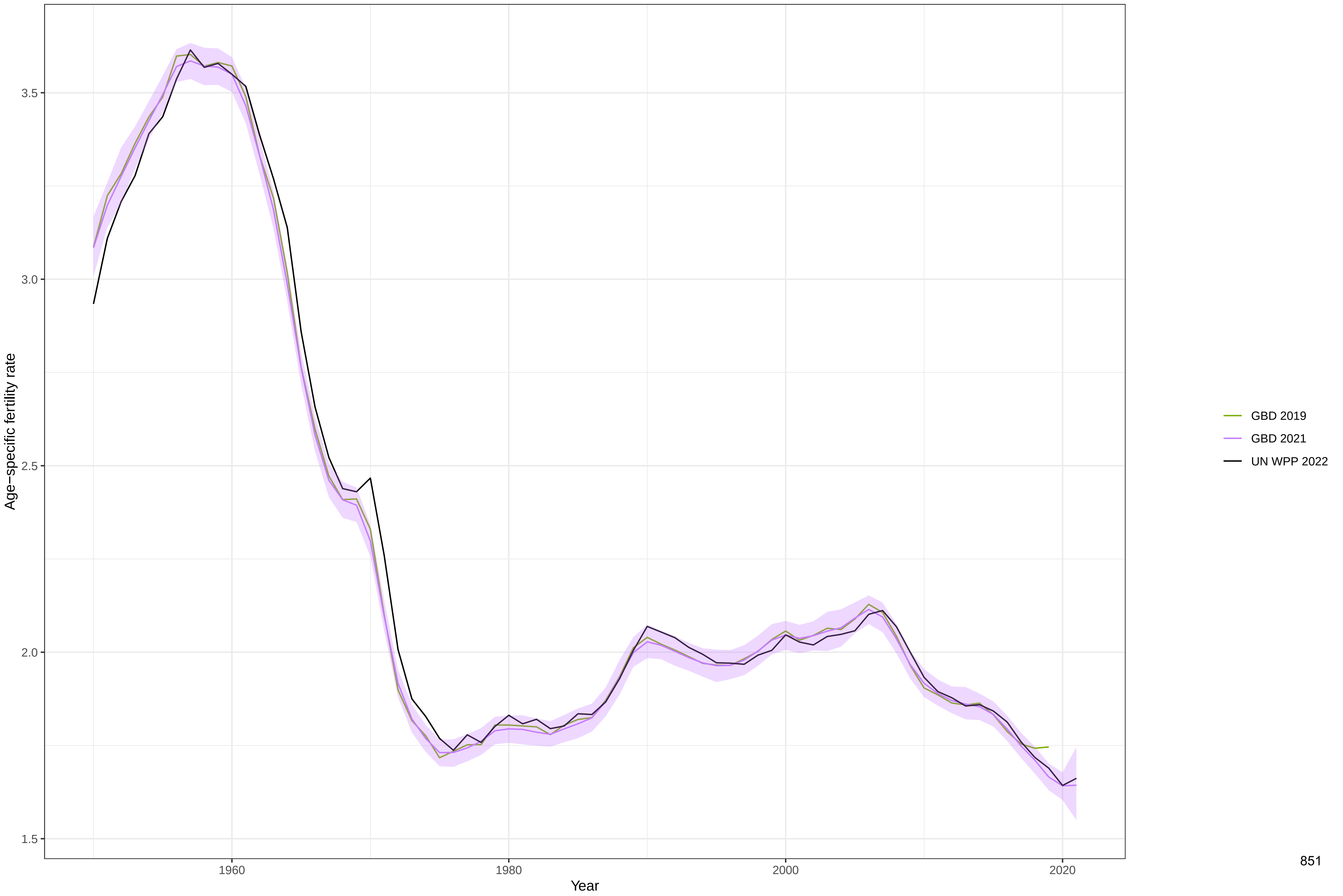

Age−specific fertility fitted trend lines, 1950−2021: Alabama, Ages: 10−14
Data points enclosed with a diamond are reference sources

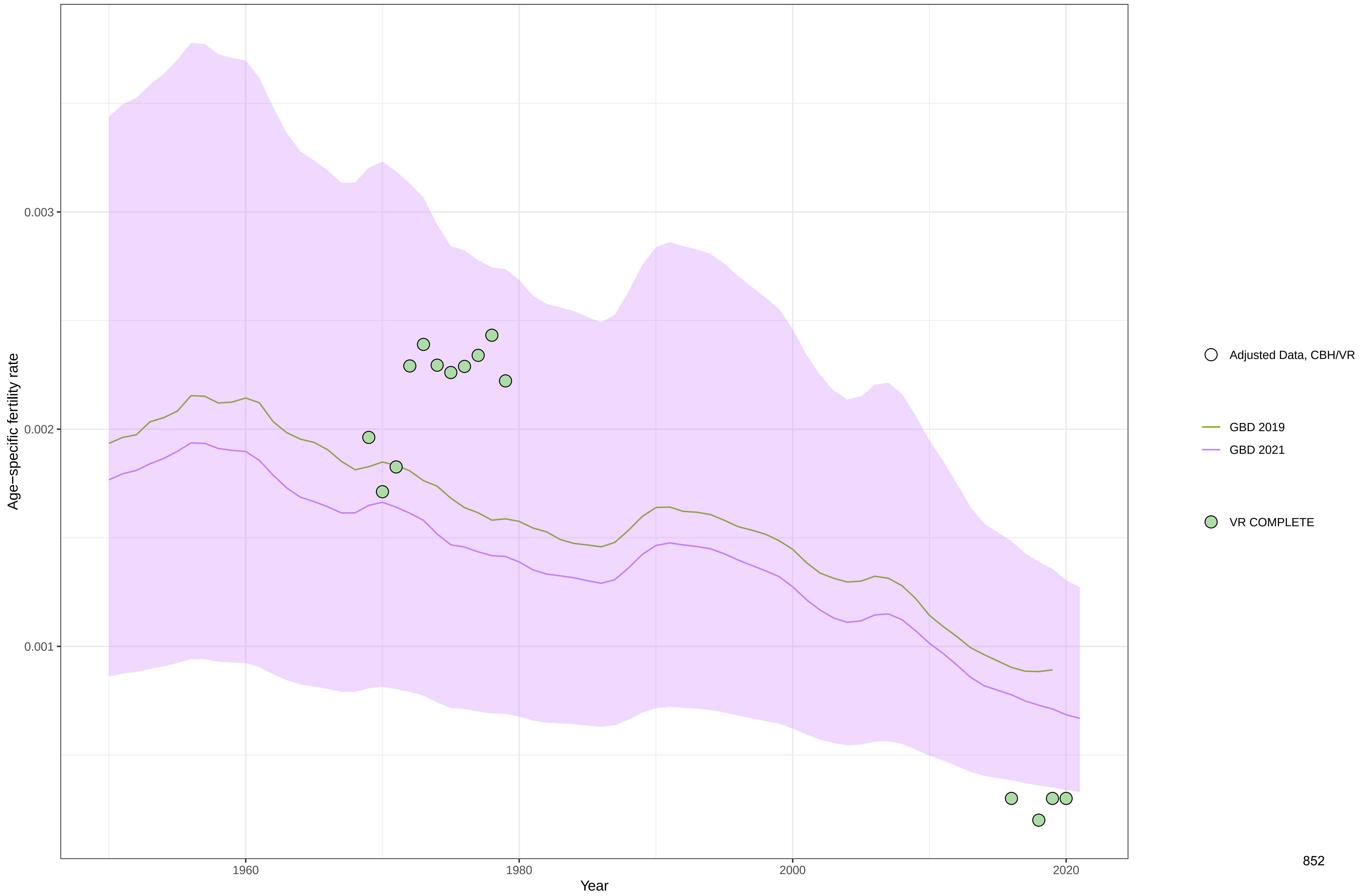

Age−specific fertility fitted trend lines, 1950−2021: Alabama, Ages: 15−19
Data points enclosed with a diamond are reference sources

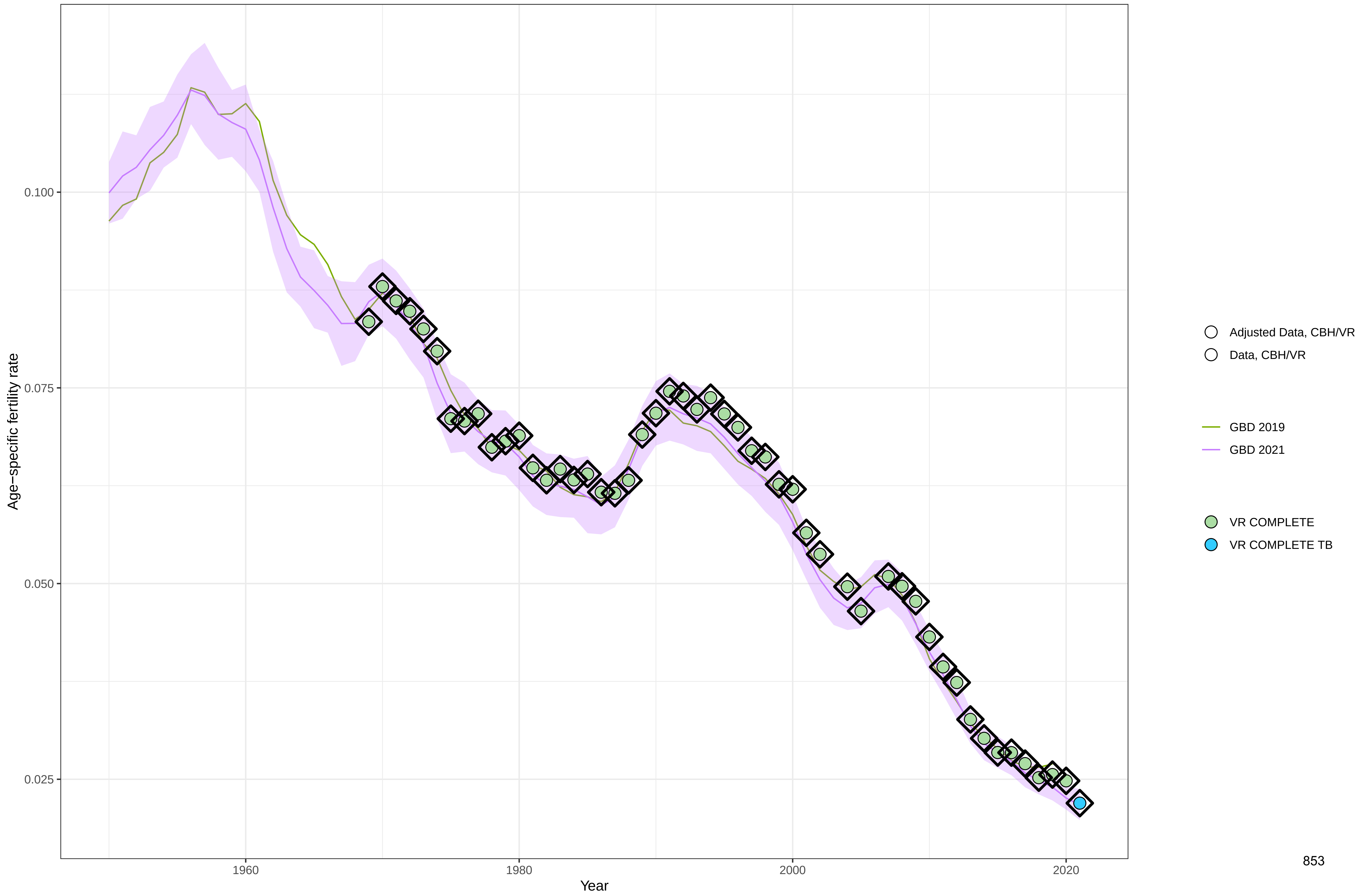

Age−specific fertility fitted trend lines, 1950−2021: Alabama, Ages: 20−24
Data points enclosed with a diamond are reference sources

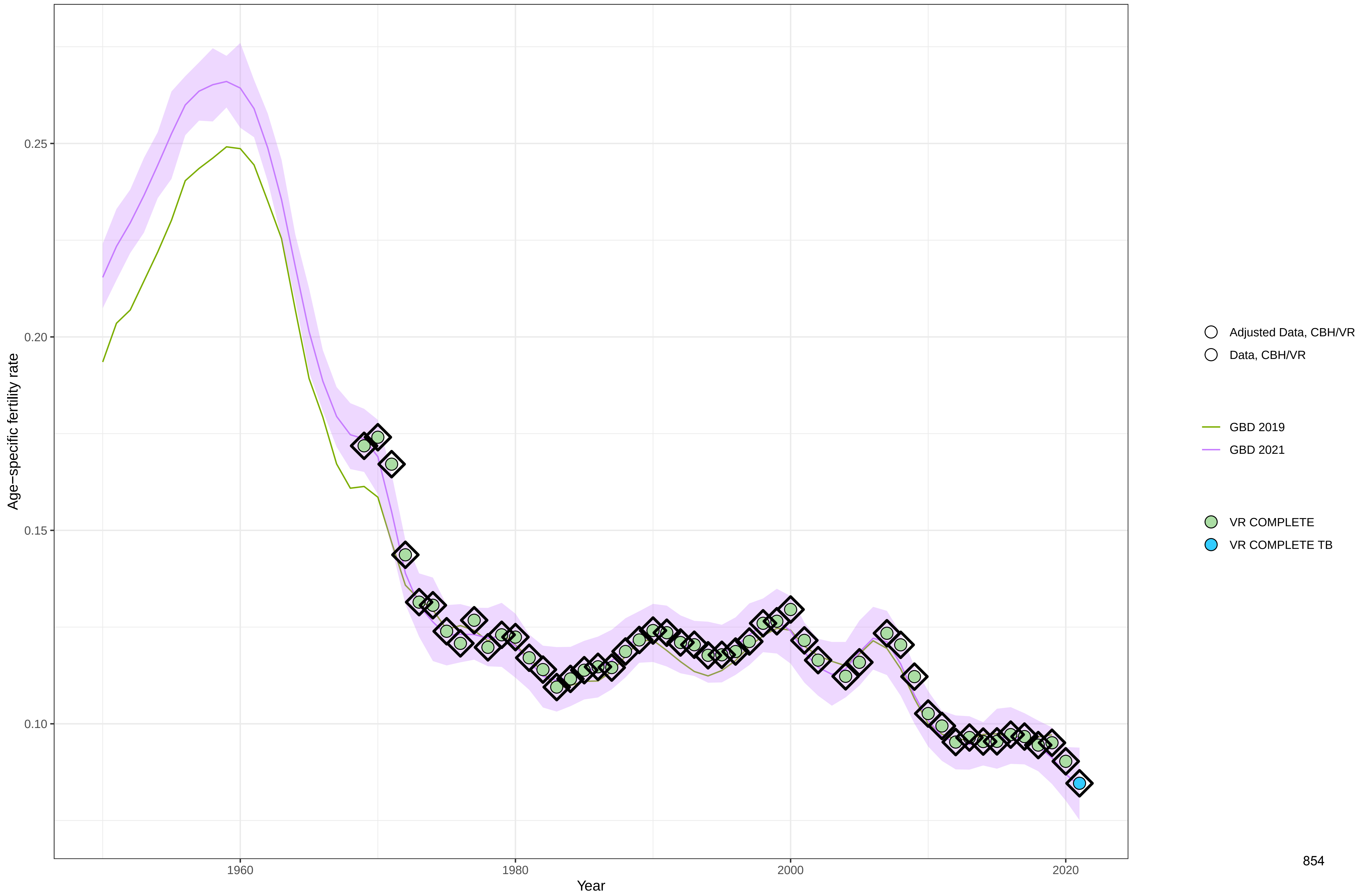

Age−specific fertility fitted trend lines, 1950−2021: Alabama, Ages: 25−29
Data points enclosed with a diamond are reference sources

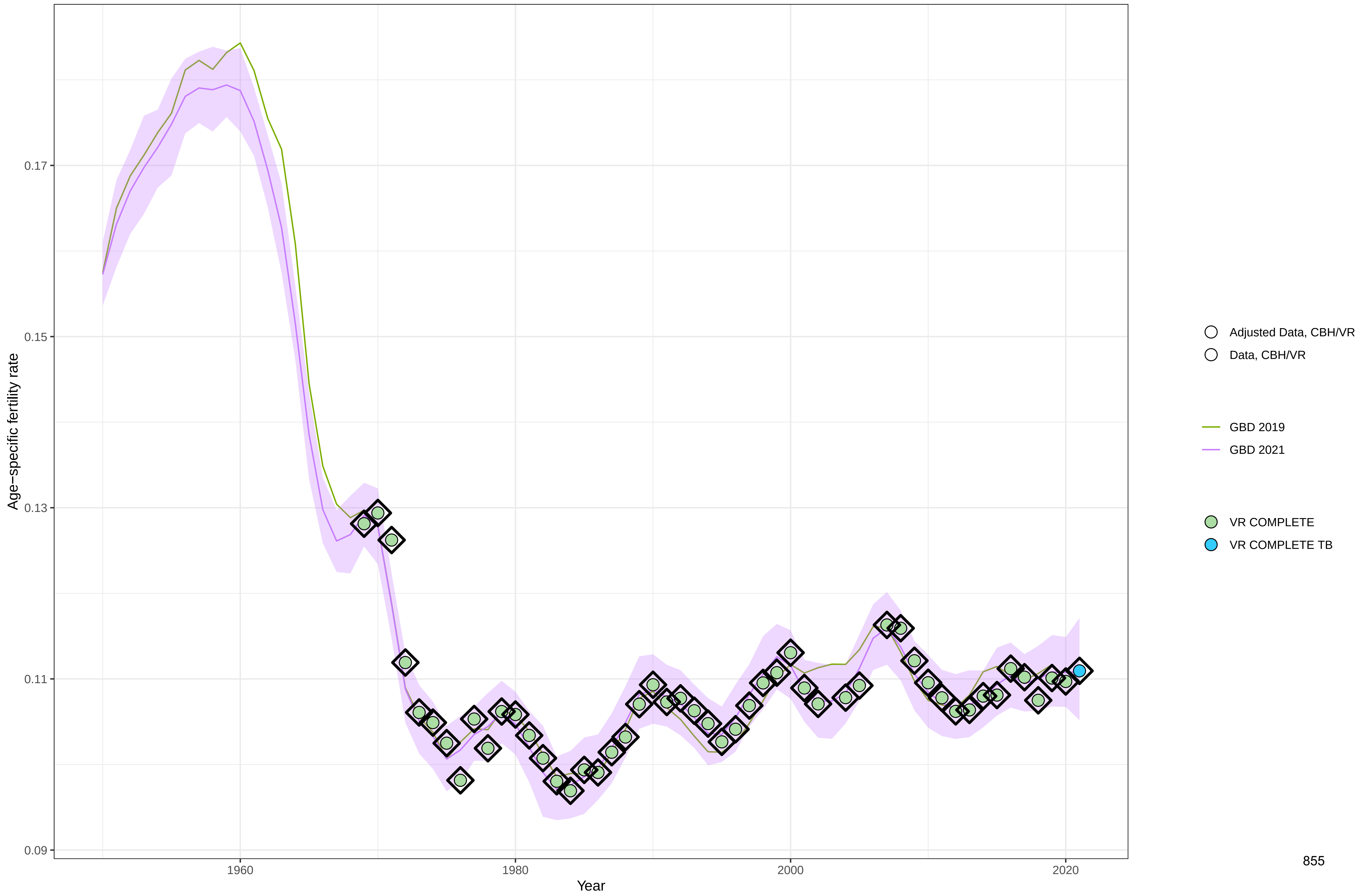

Age−specific fertility fitted trend lines, 1950−2021: Alabama, Ages: 30−34
Data points enclosed with a diamond are reference sources

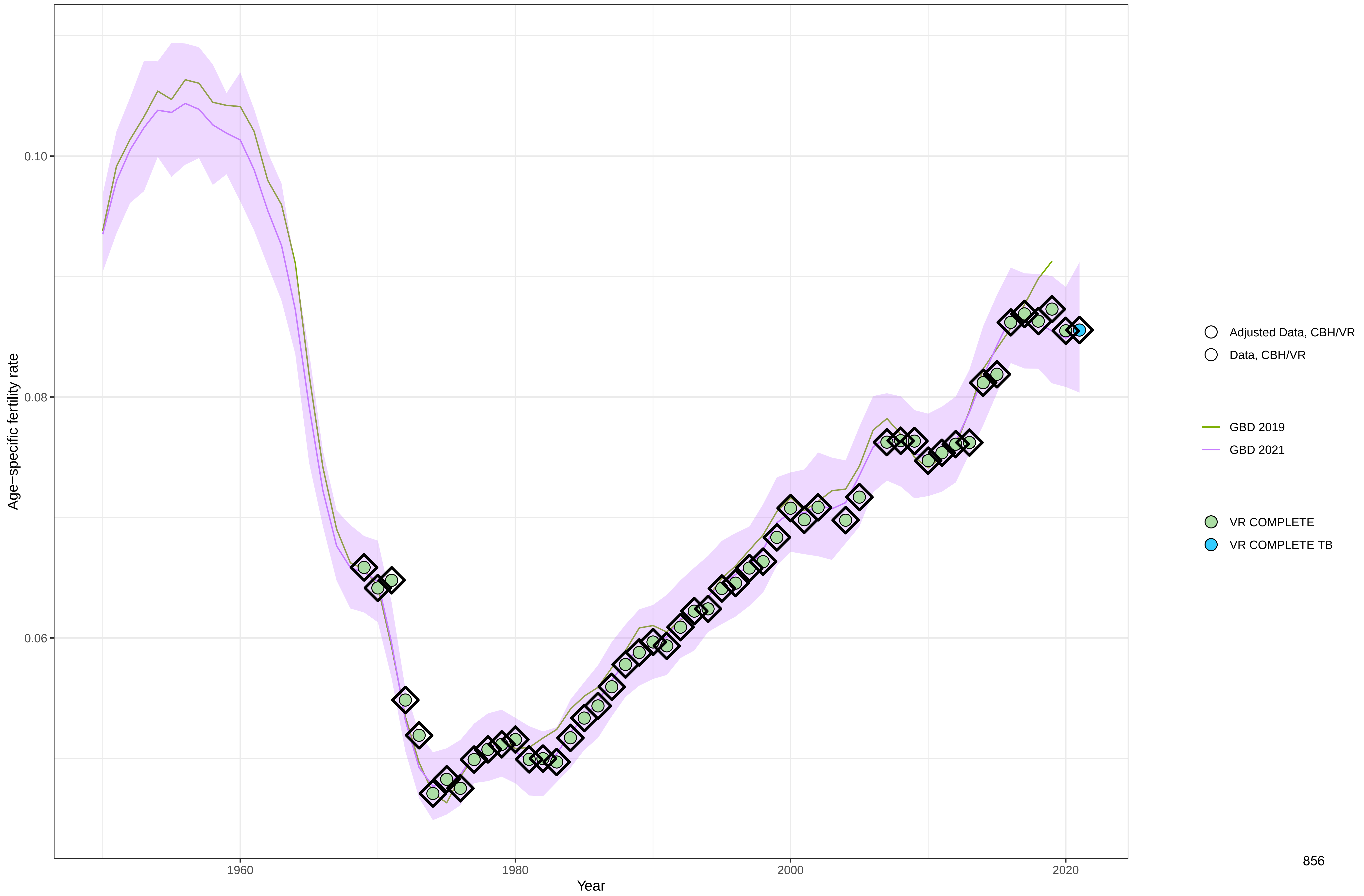

Age−specific fertility fitted trend lines, 1950−2021: Alabama, Ages: 35−39
Data points enclosed with a diamond are reference sources

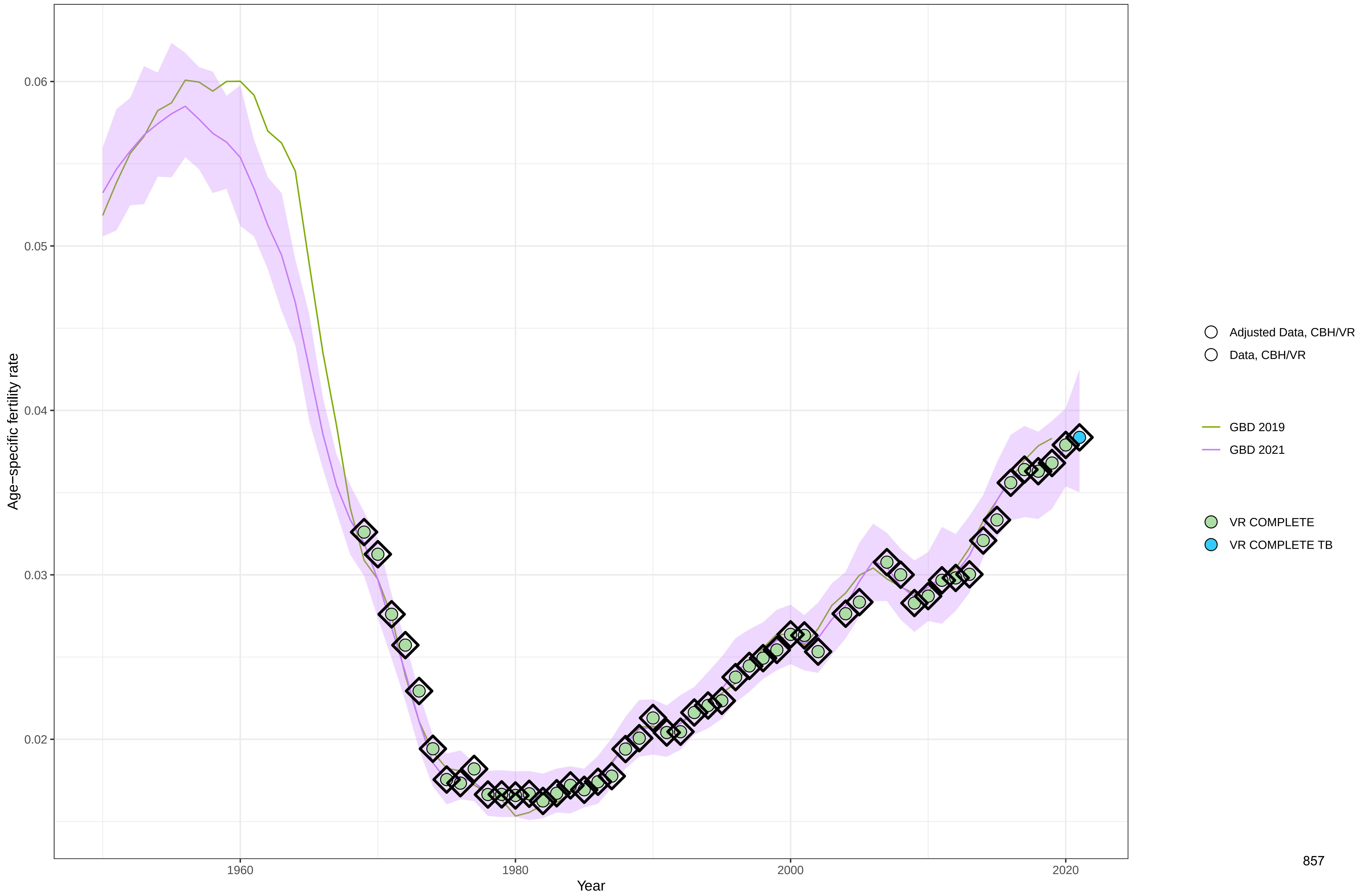

Age−specific fertility fitted trend lines, 1950−2021: Alabama, Ages: 40−44
Data points enclosed with a diamond are reference sources

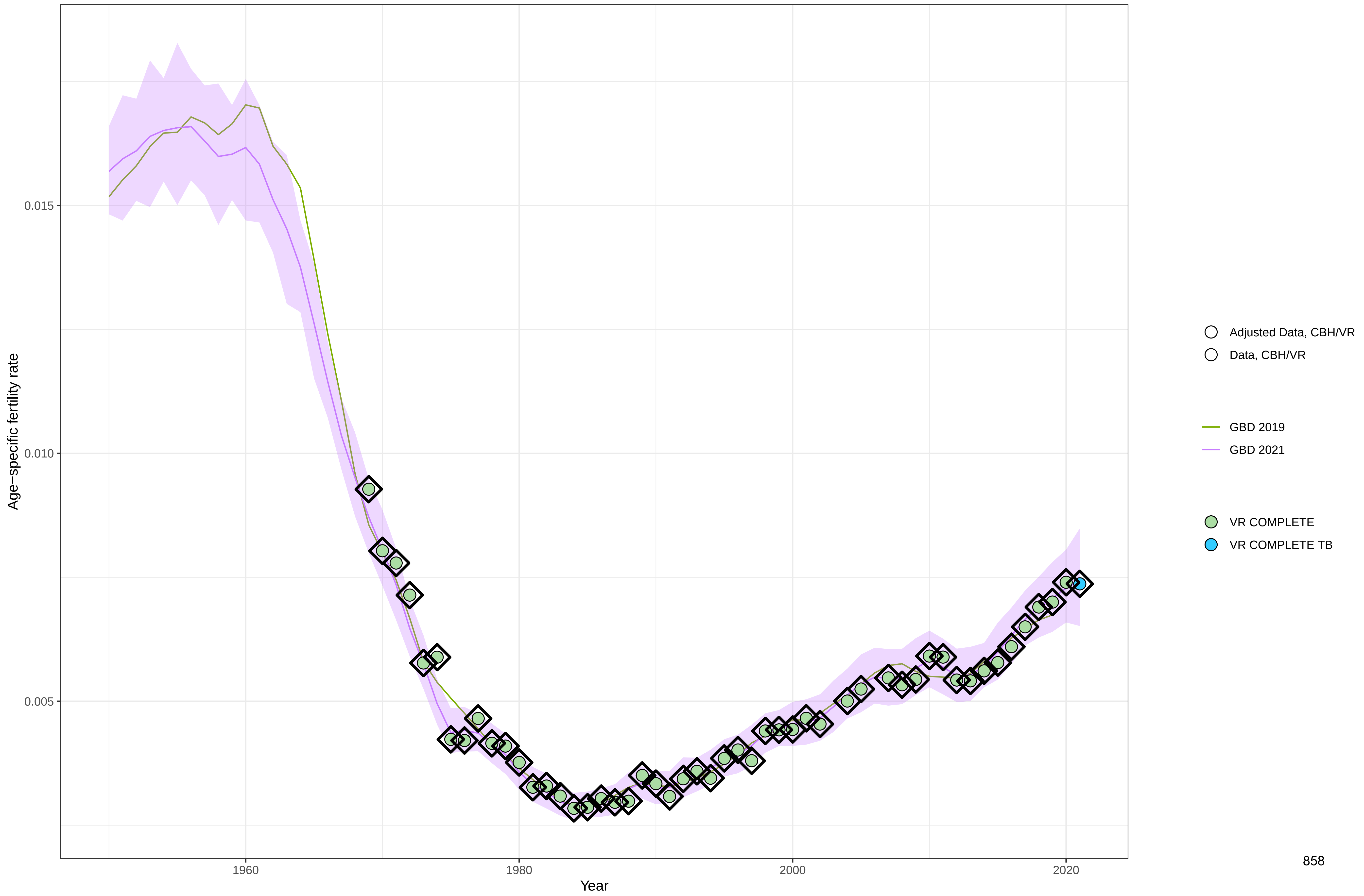

Age−specific fertility fitted trend lines, 1950−2021: Alabama, Ages: 45−49
Data points enclosed with a diamond are reference sources

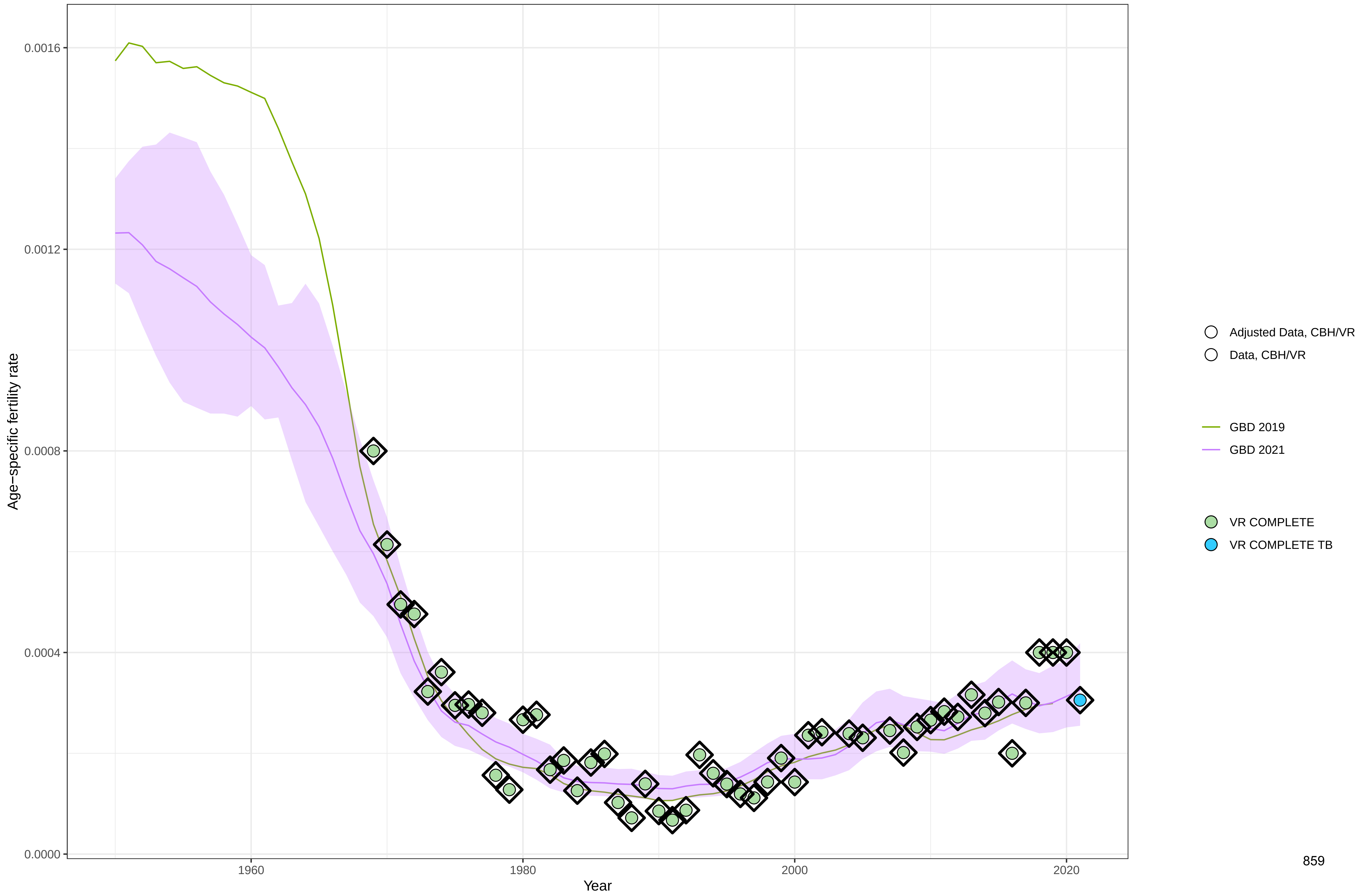

Age−specific fertility fitted trend lines, 1950−2021: Alabama, Ages: 50−54
Data points enclosed with a diamond are reference sources

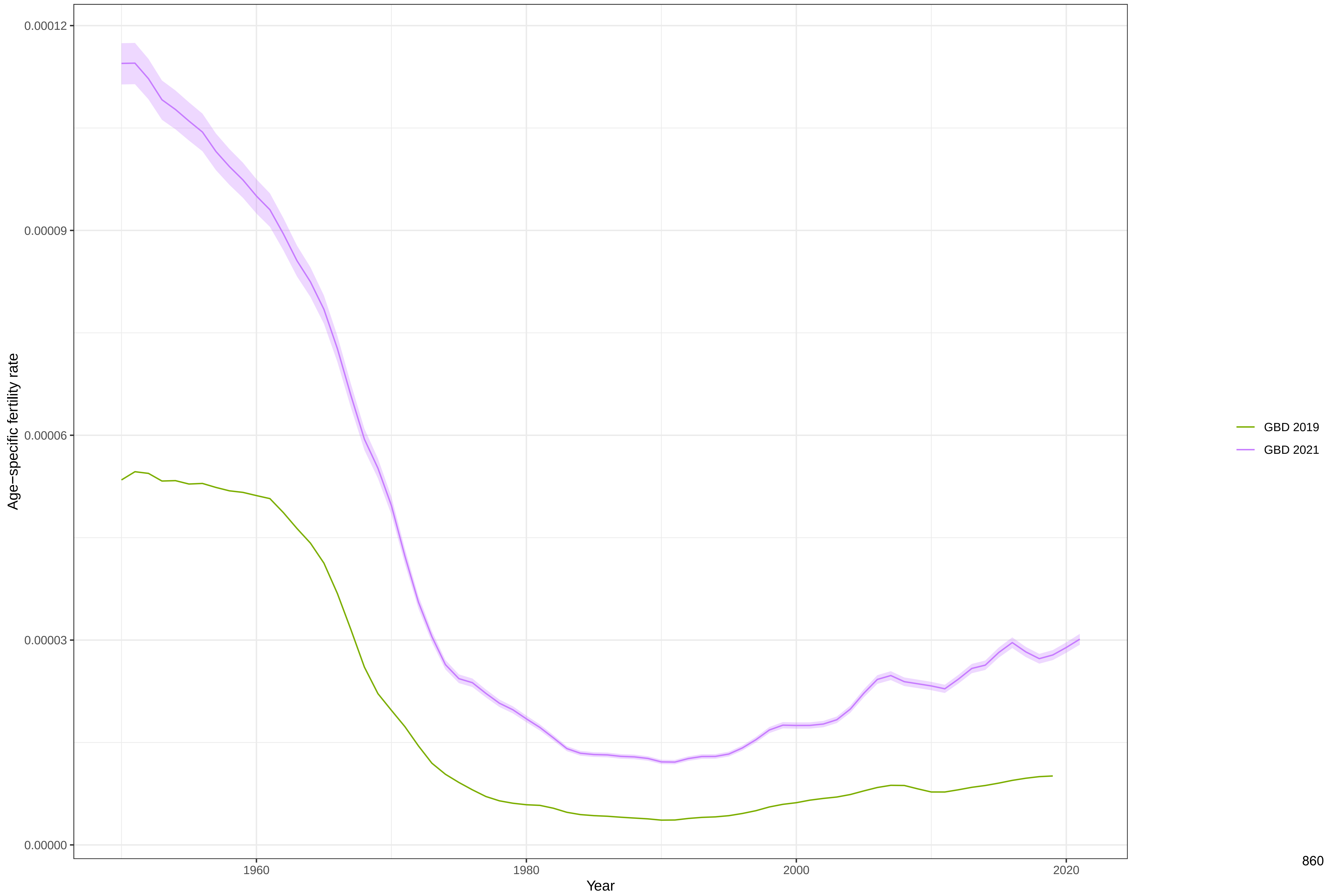

Total fertility rate fitted trend lines, 1950−2021: Alabama TFR
Data points enclosed with a diamond are reference sources

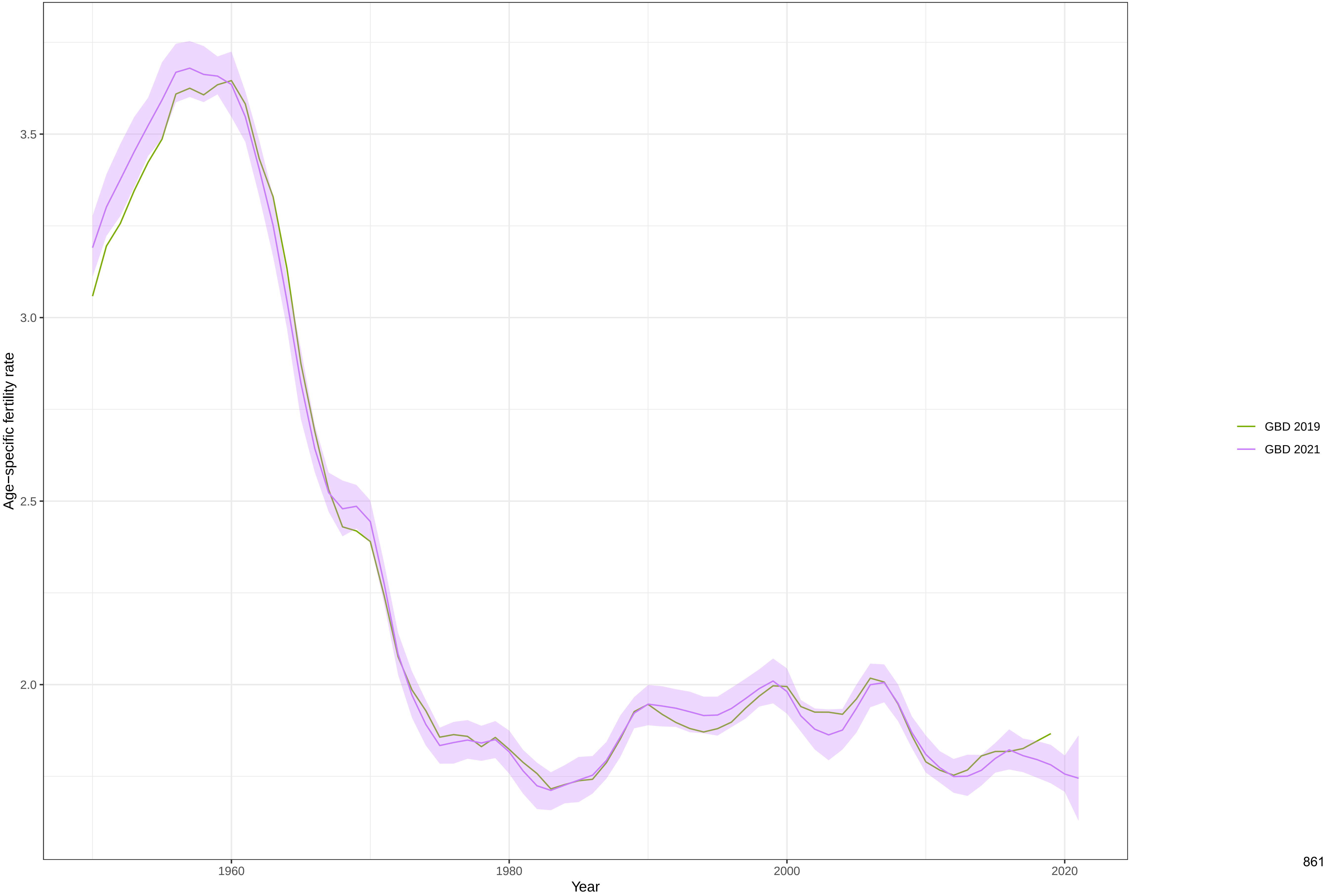

Age−specific fertility fitted trend lines, 1950−2021: Alaska, Ages: 10−14
Data points enclosed with a diamond are reference sources

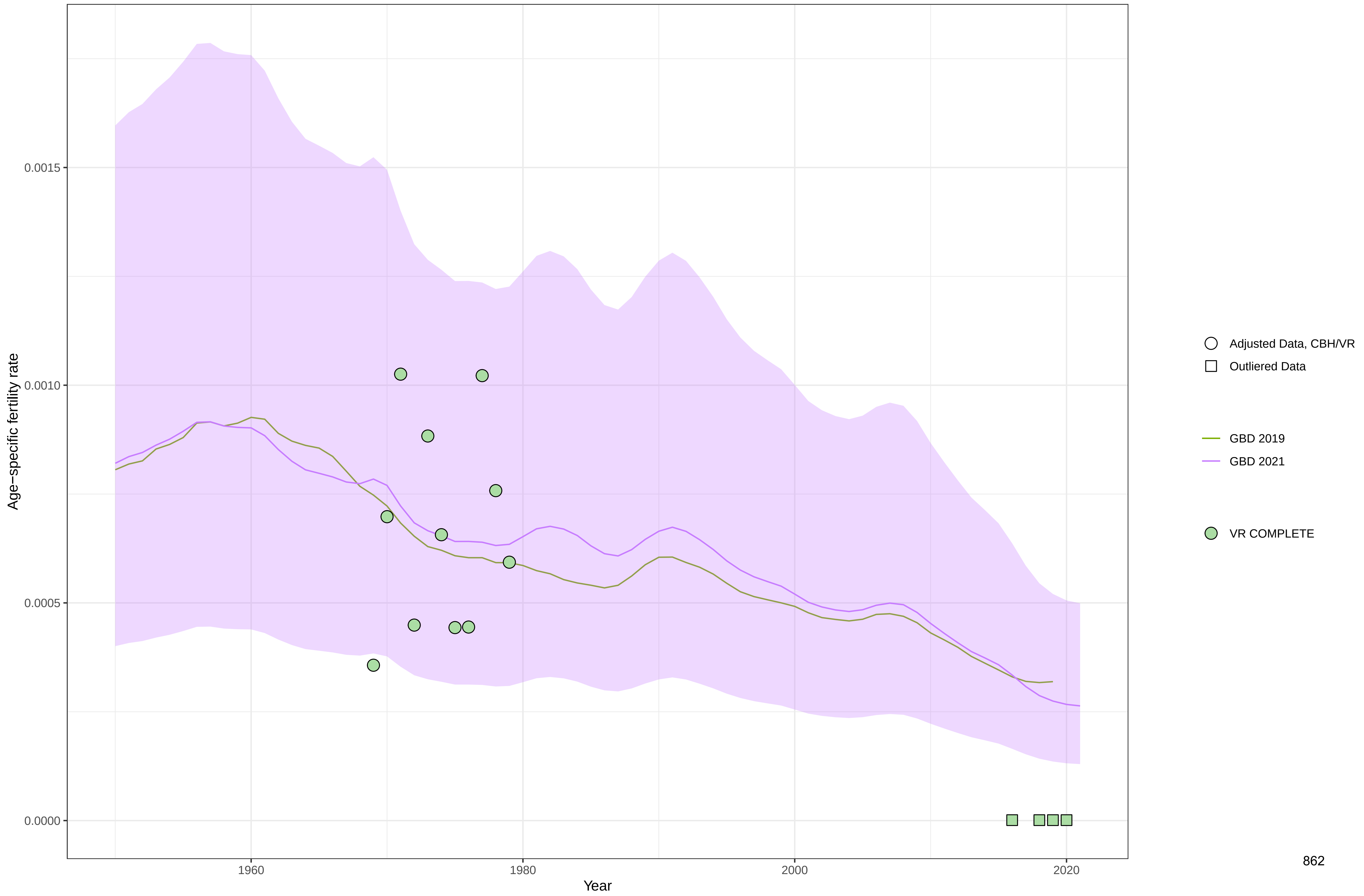

Age−specific fertility fitted trend lines, 1950−2021: Alaska, Ages: 15−19
Data points enclosed with a diamond are reference sources

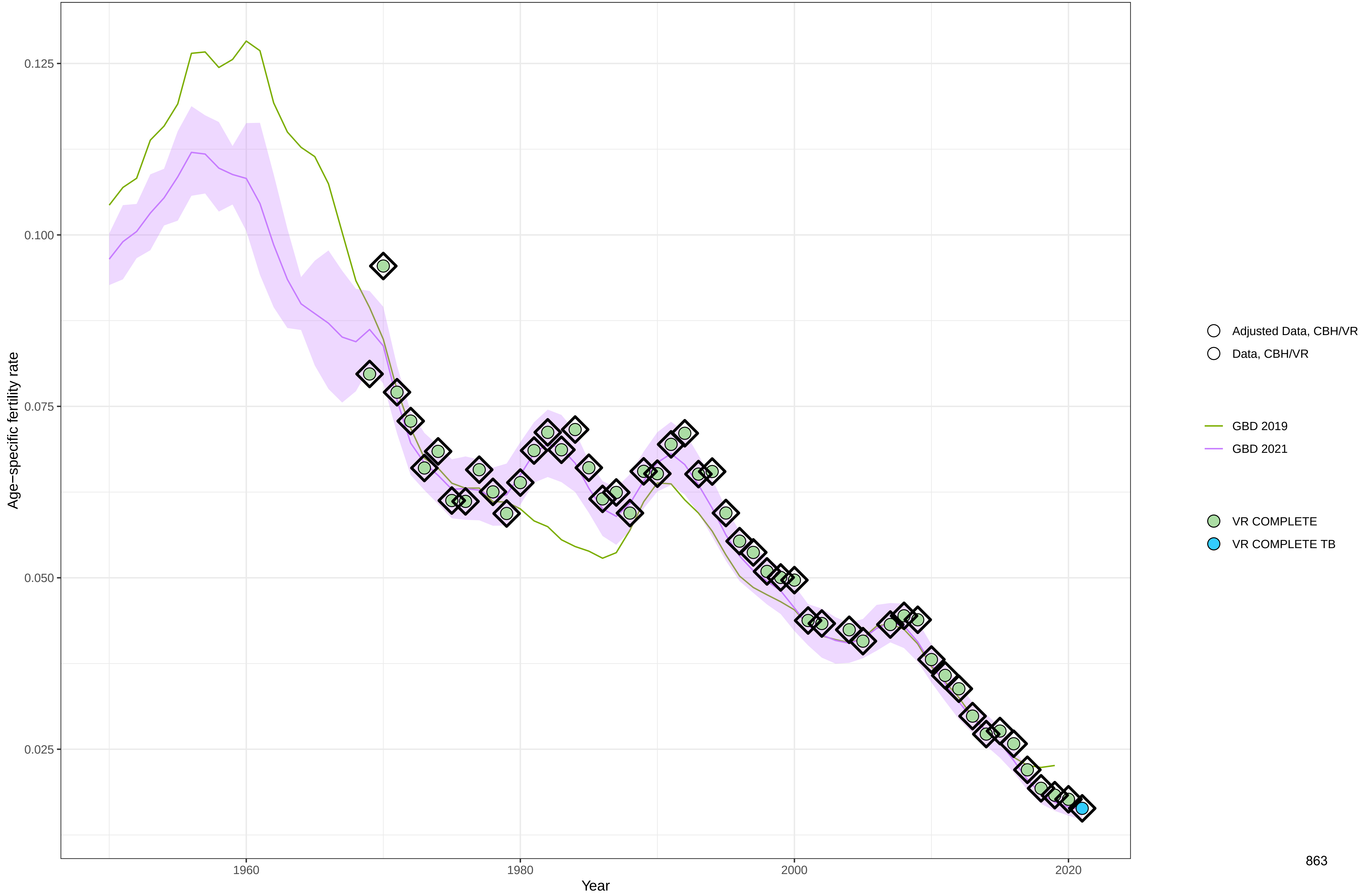

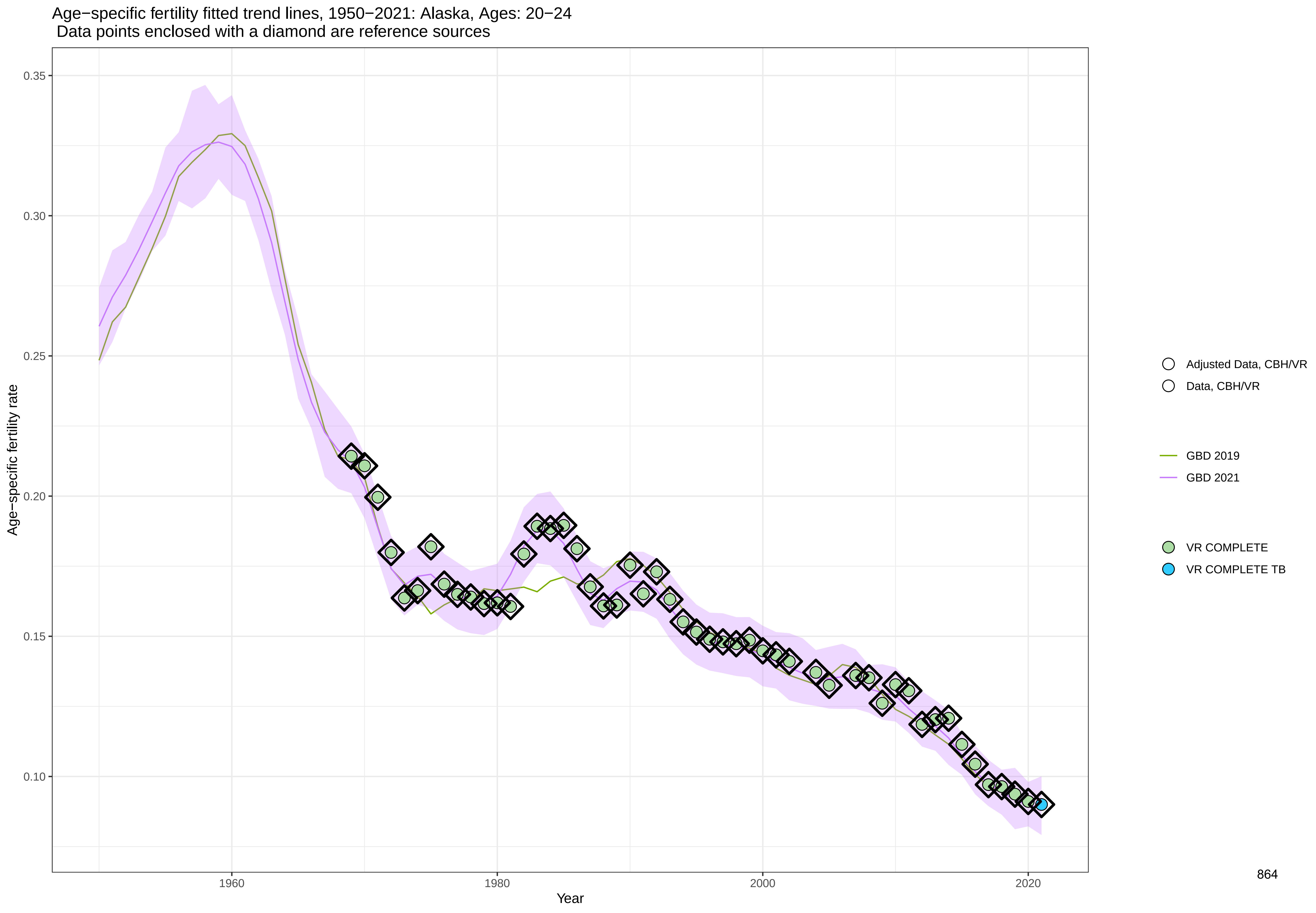

Age−specific fertility fitted trend lines, 1950−2021: Alaska, Ages: 20−24
Data points enclosed with a diamond are reference sources
Age−specific fertility rate
Year
0.35
0.30
0.25
0.20
0.15
0.10
1960
1980
2000
2020
Adjusted Data, CBH/VR
Data, CBH/VR
GBD 2019
GBD 2021
VR COMPLETE
VR COMPLETE TB

Age−specific fertility fitted trend lines, 1950−2021: Alaska, Ages: 25−29
Data points enclosed with a diamond are reference sources

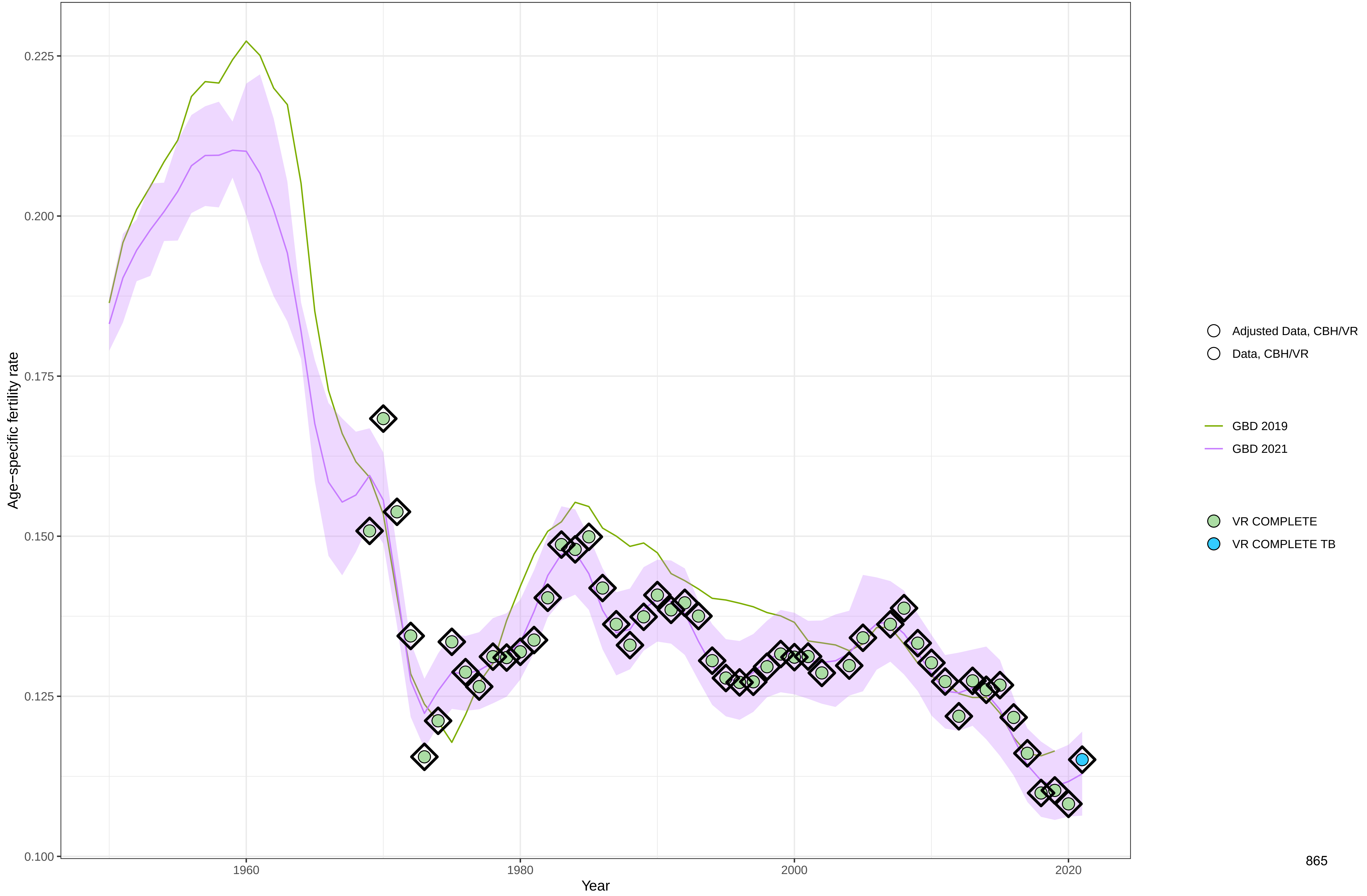

Age−specific fertility fitted trend lines, 1950−2021: Alaska, Ages: 30−34
Data points enclosed with a diamond are reference sources

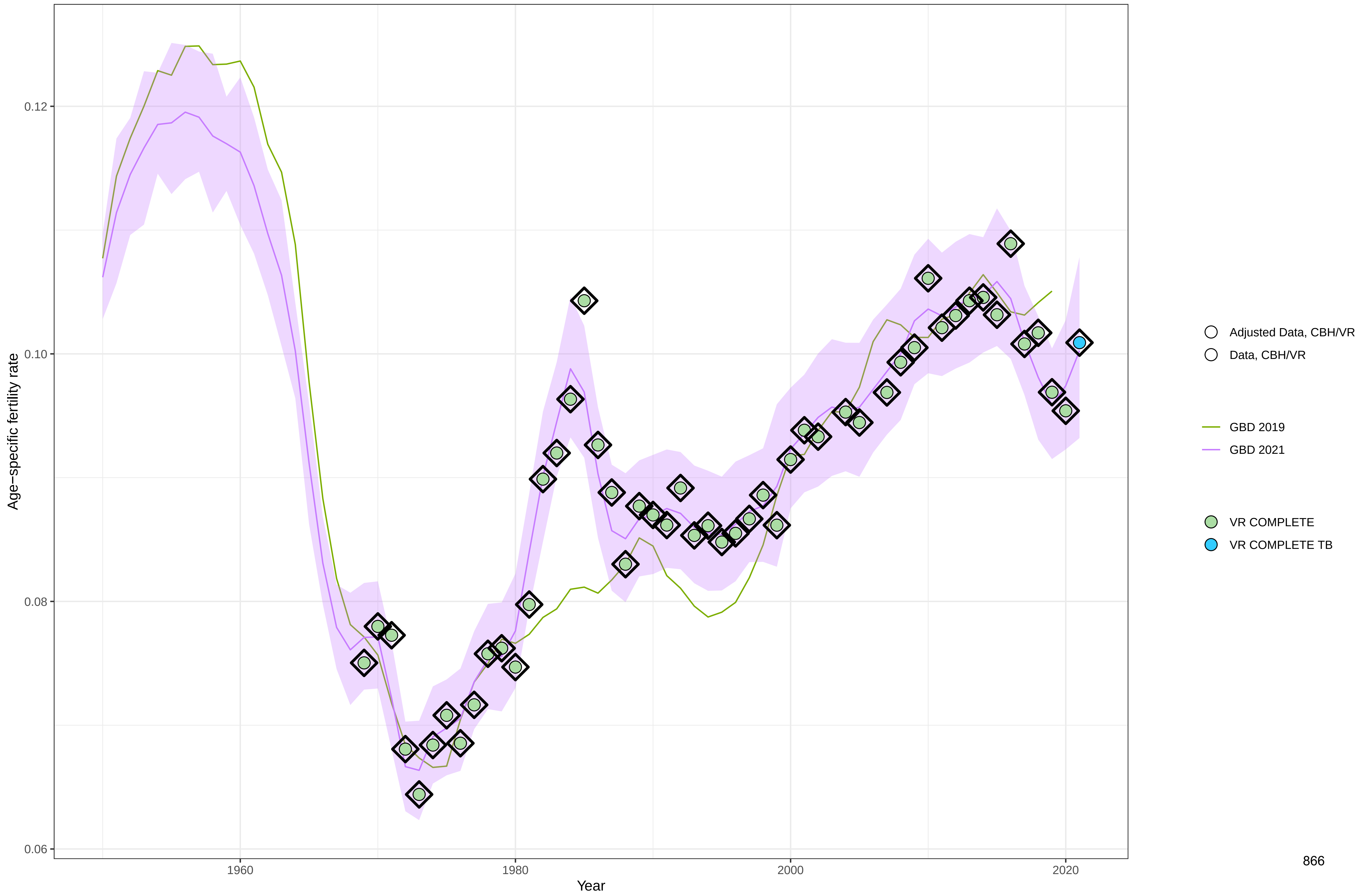

Age−specific fertility fitted trend lines, 1950−2021: Alaska, Ages: 35−39
Data points enclosed with a diamond are reference sources

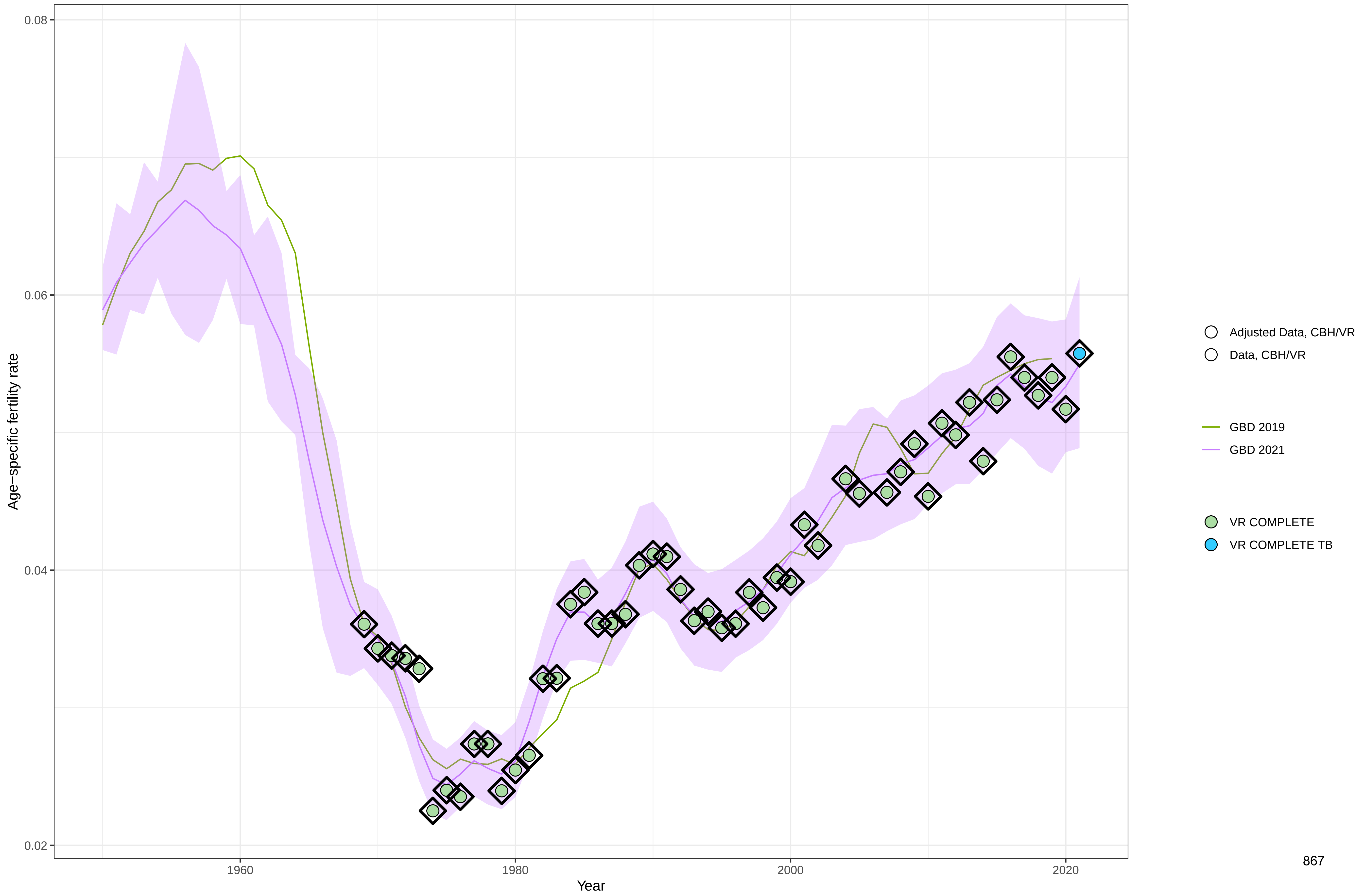

Age−specific fertility fitted trend lines, 1950−2021: Alaska, Ages: 40−44
Data points enclosed with a diamond are reference sources

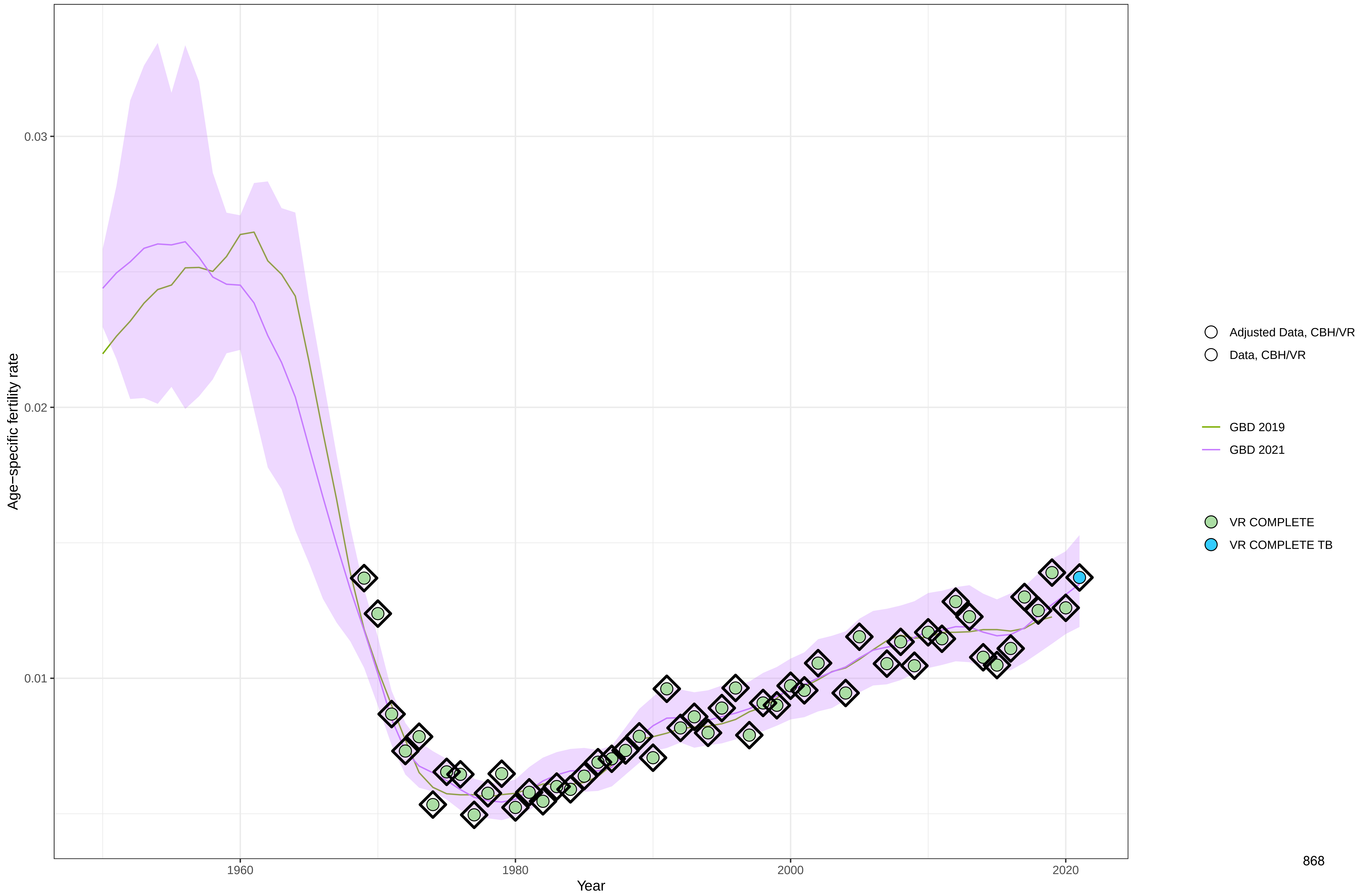

Age−specific fertility fitted trend lines, 1950−2021: Alaska, Ages: 45−49
Data points enclosed with a diamond are reference sources

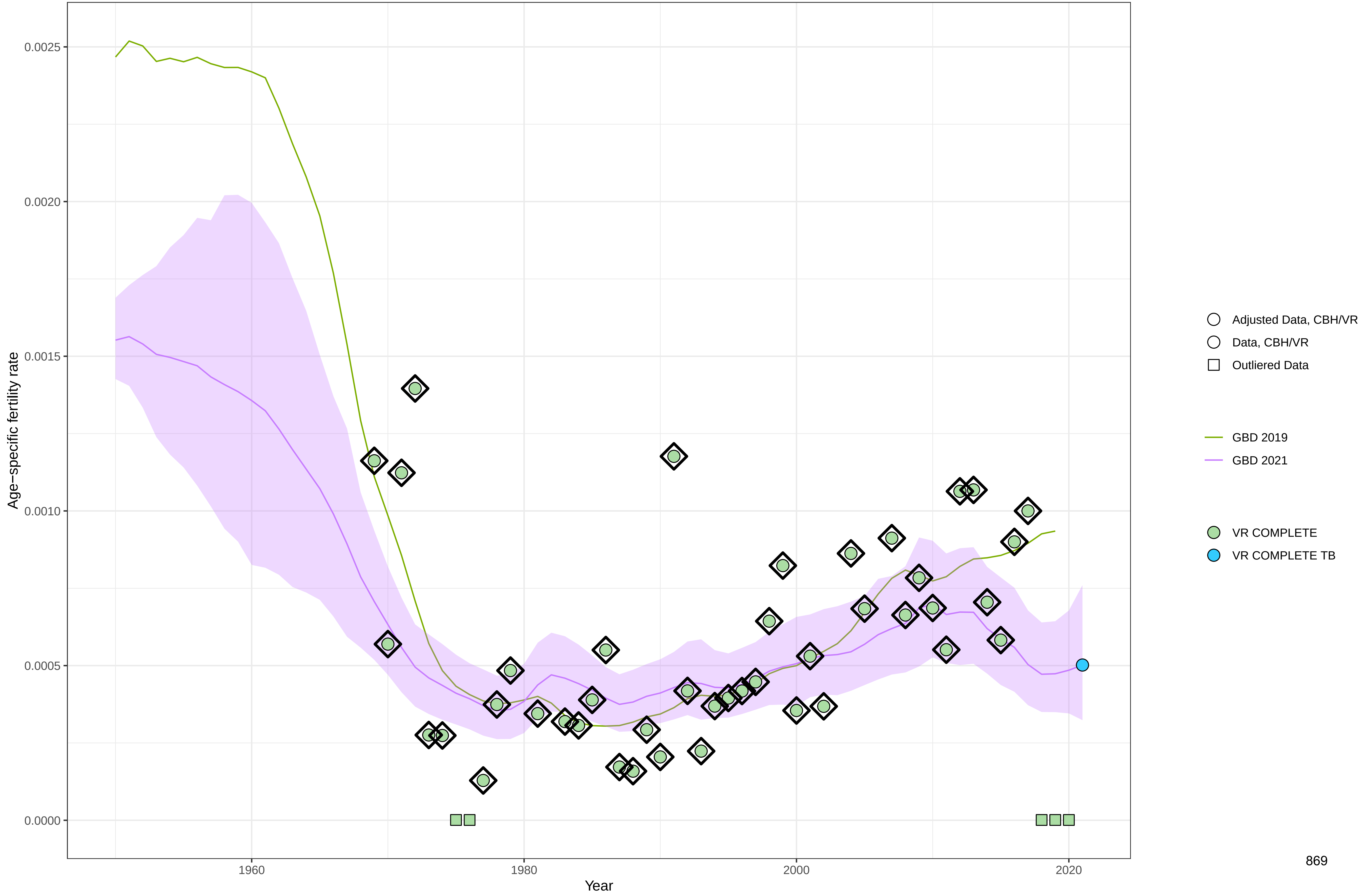

Age−specific fertility fitted trend lines, 1950−2021: Alaska, Ages: 50−54
Data points enclosed with a diamond are reference sources

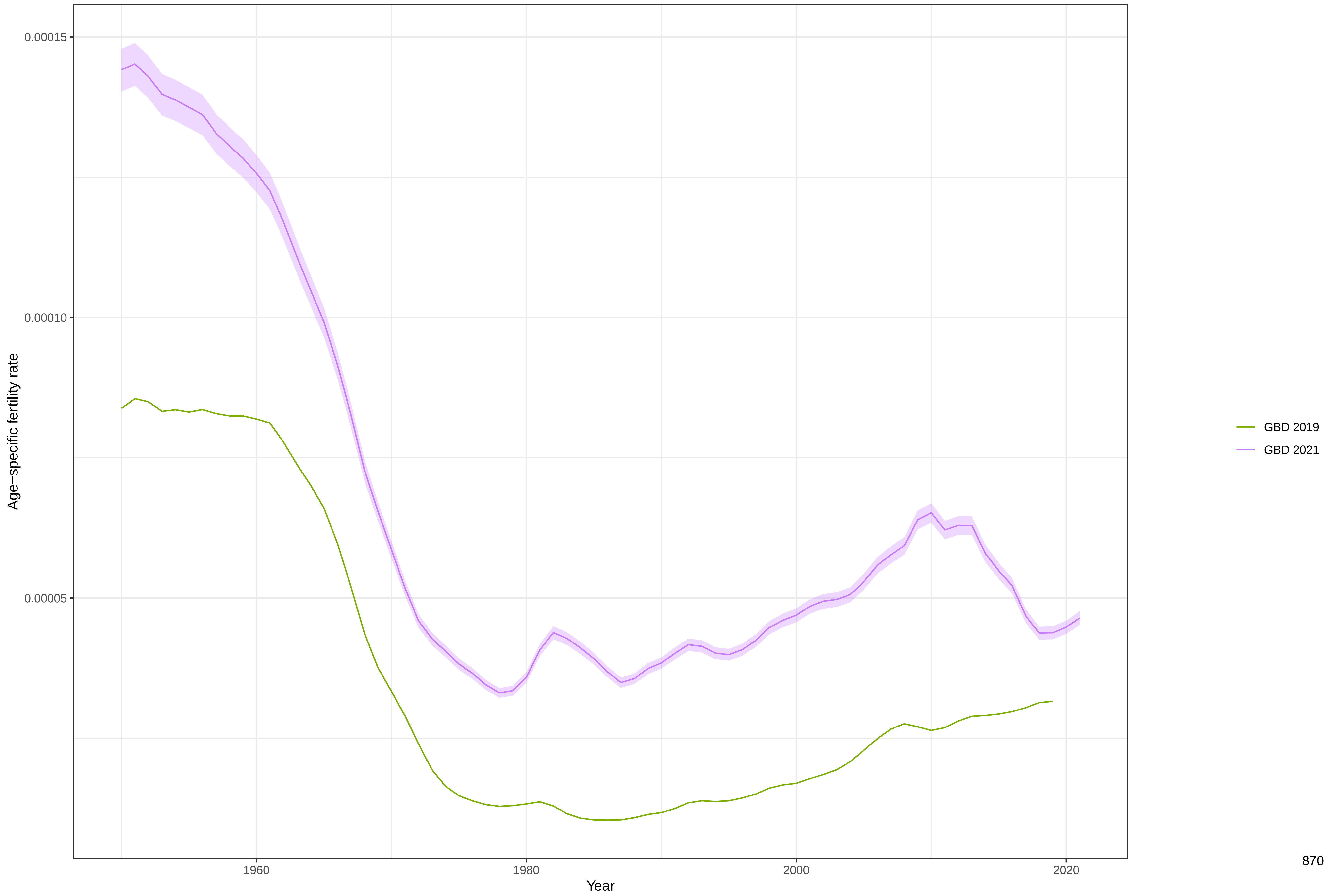

Total fertility rate fitted trend lines, 1950−2021: Alaska TFR
Data points enclosed with a diamond are reference sources

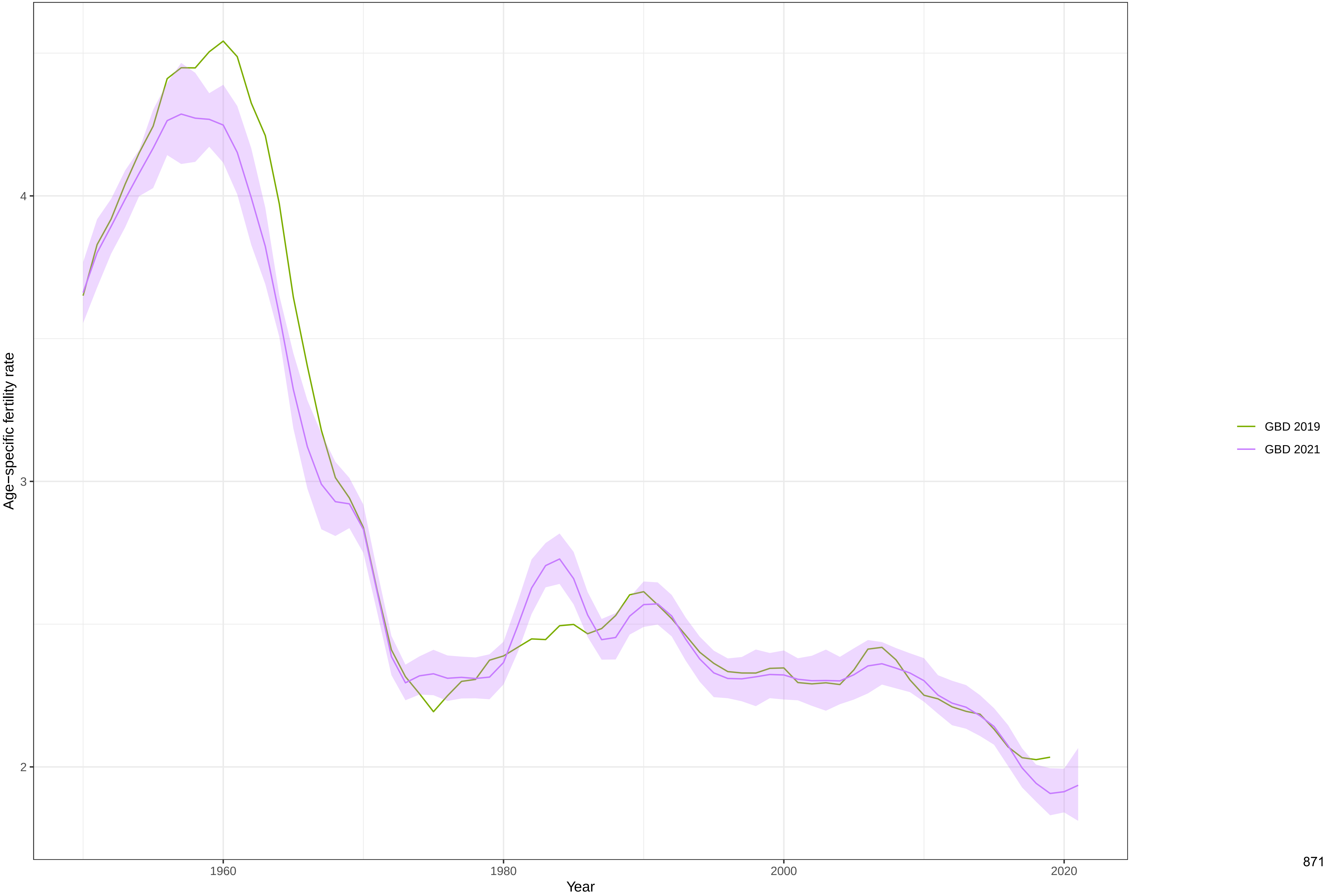

Age−specific fertility fitted trend lines, 1950−2021: Arizona, Ages: 10−14
Data points enclosed with a diamond are reference sources

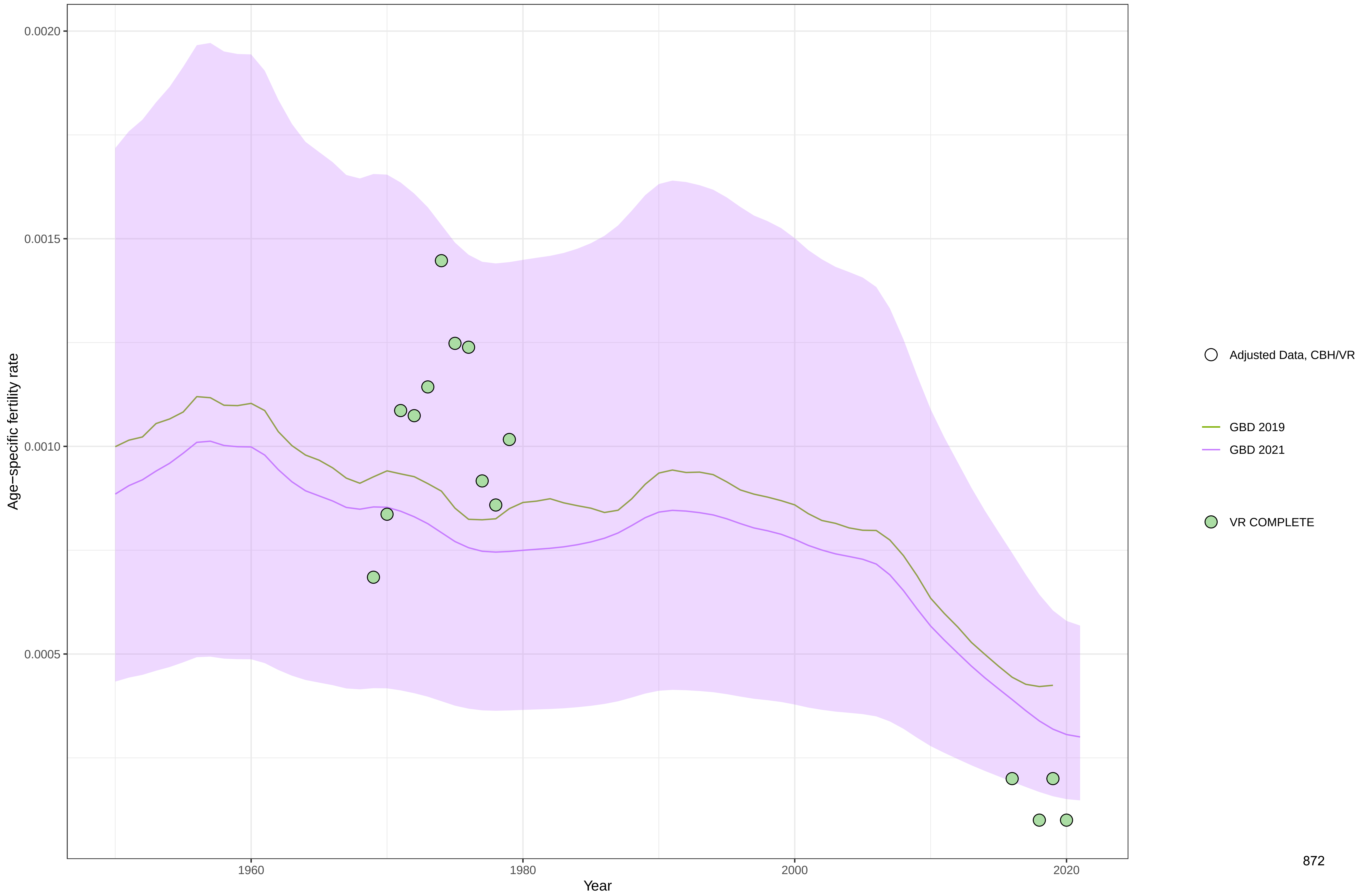

Age−specific fertility fitted trend lines, 1950−2021: Arizona, Ages: 15−19
Data points enclosed with a diamond are reference sources

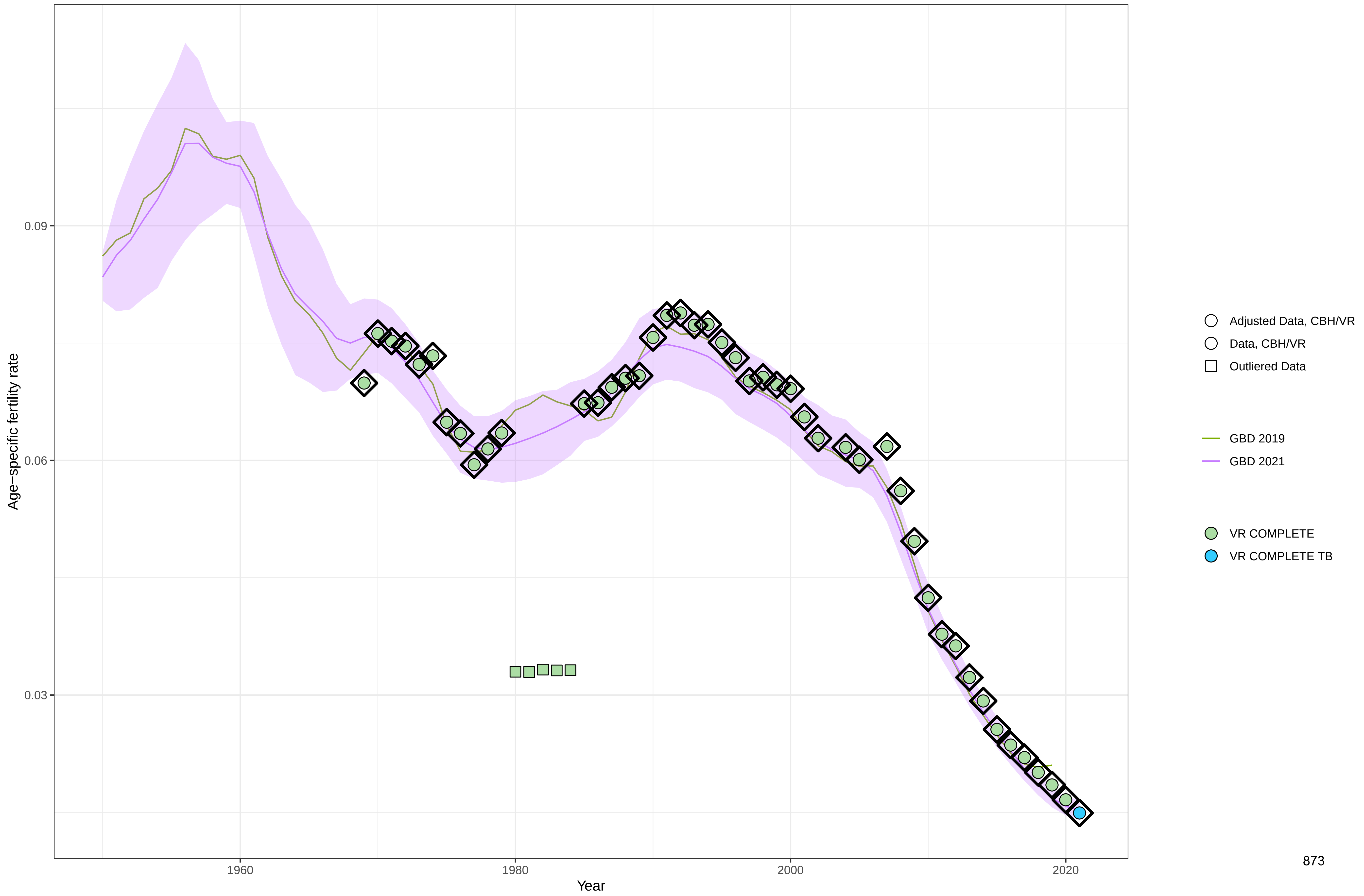

Age−specific fertility fitted trend lines, 1950−2021: Arizona, Ages: 20−24
Data points enclosed with a diamond are reference sources

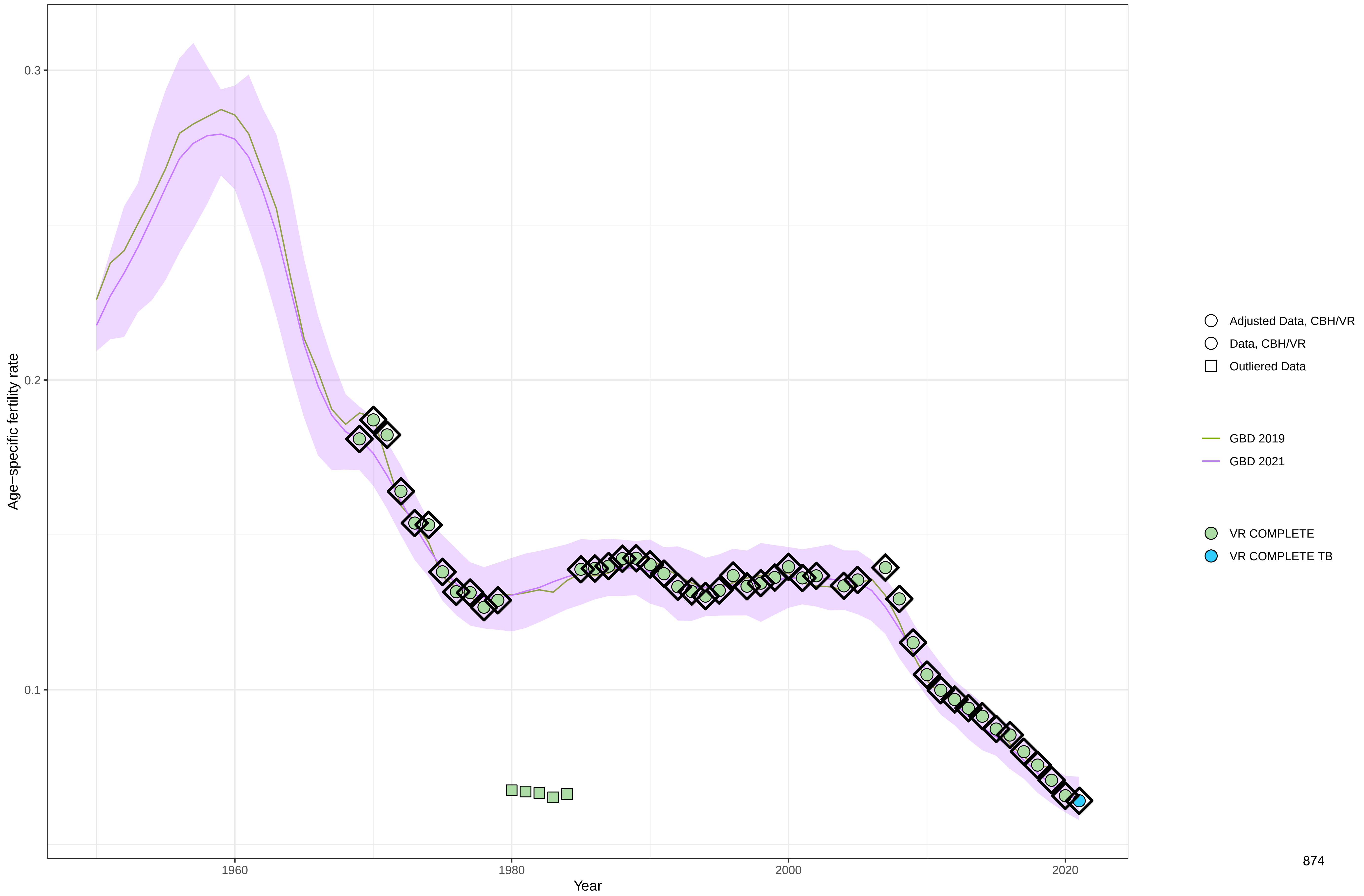

Age−specific fertility fitted trend lines, 1950−2021: Arizona, Ages: 25−29
Data points enclosed with a diamond are reference sources

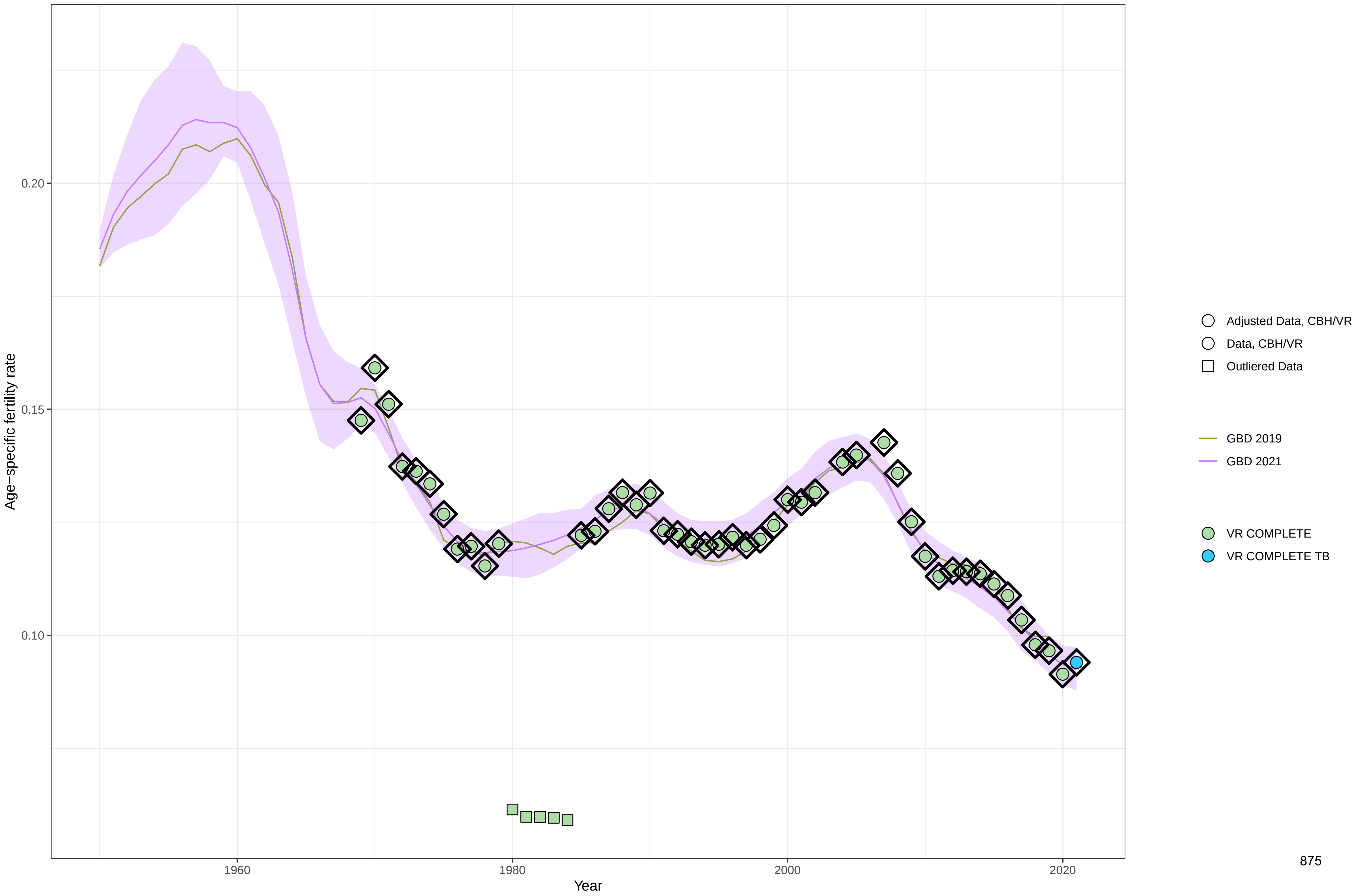

Age−specific fertility fitted trend lines, 1950−2021: Arizona, Ages: 30−34
Data points enclosed with a diamond are reference sources

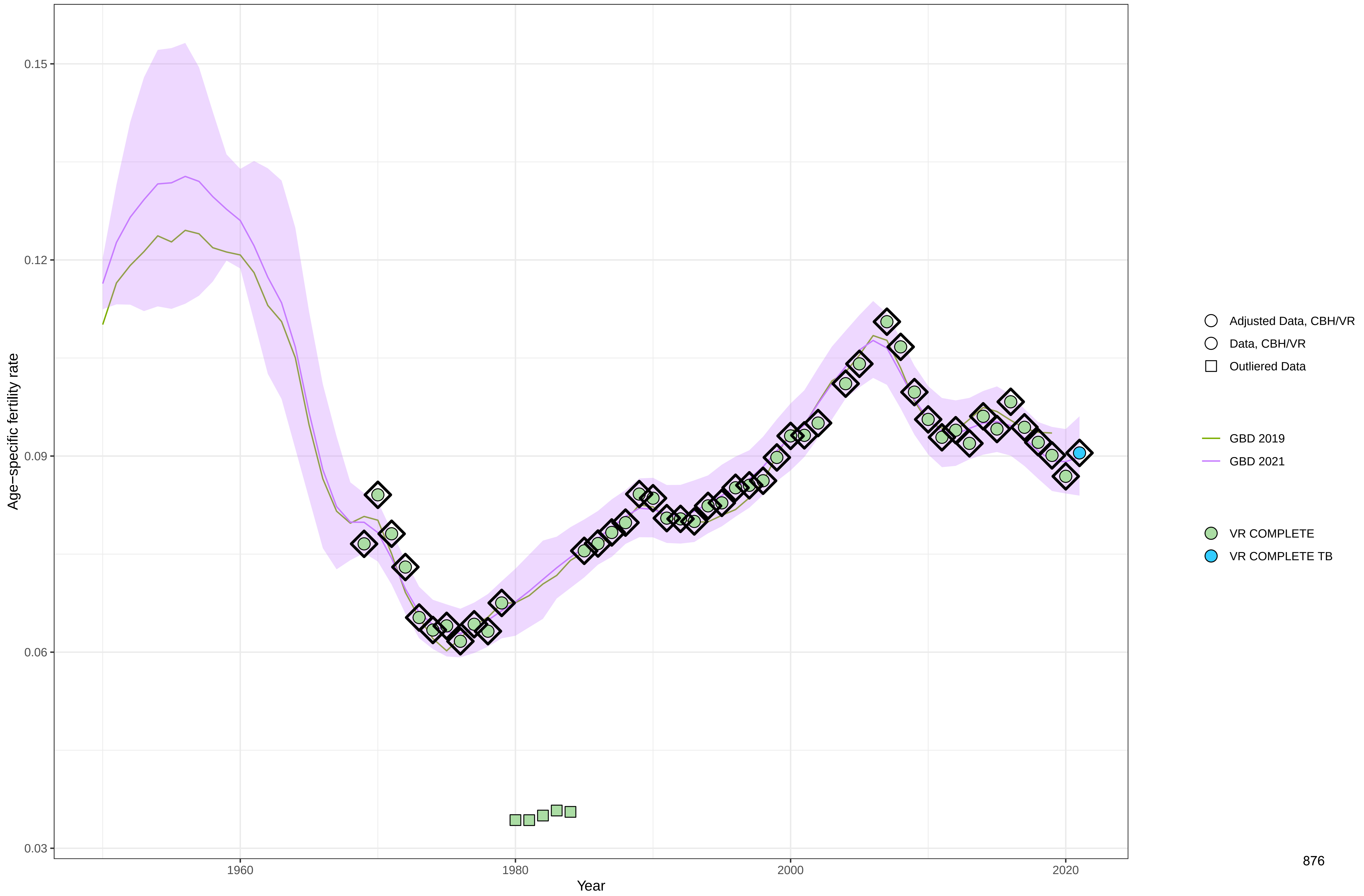

Age−specific fertility fitted trend lines, 1950−2021: Arizona, Ages: 35−39
Data points enclosed with a diamond are reference sources

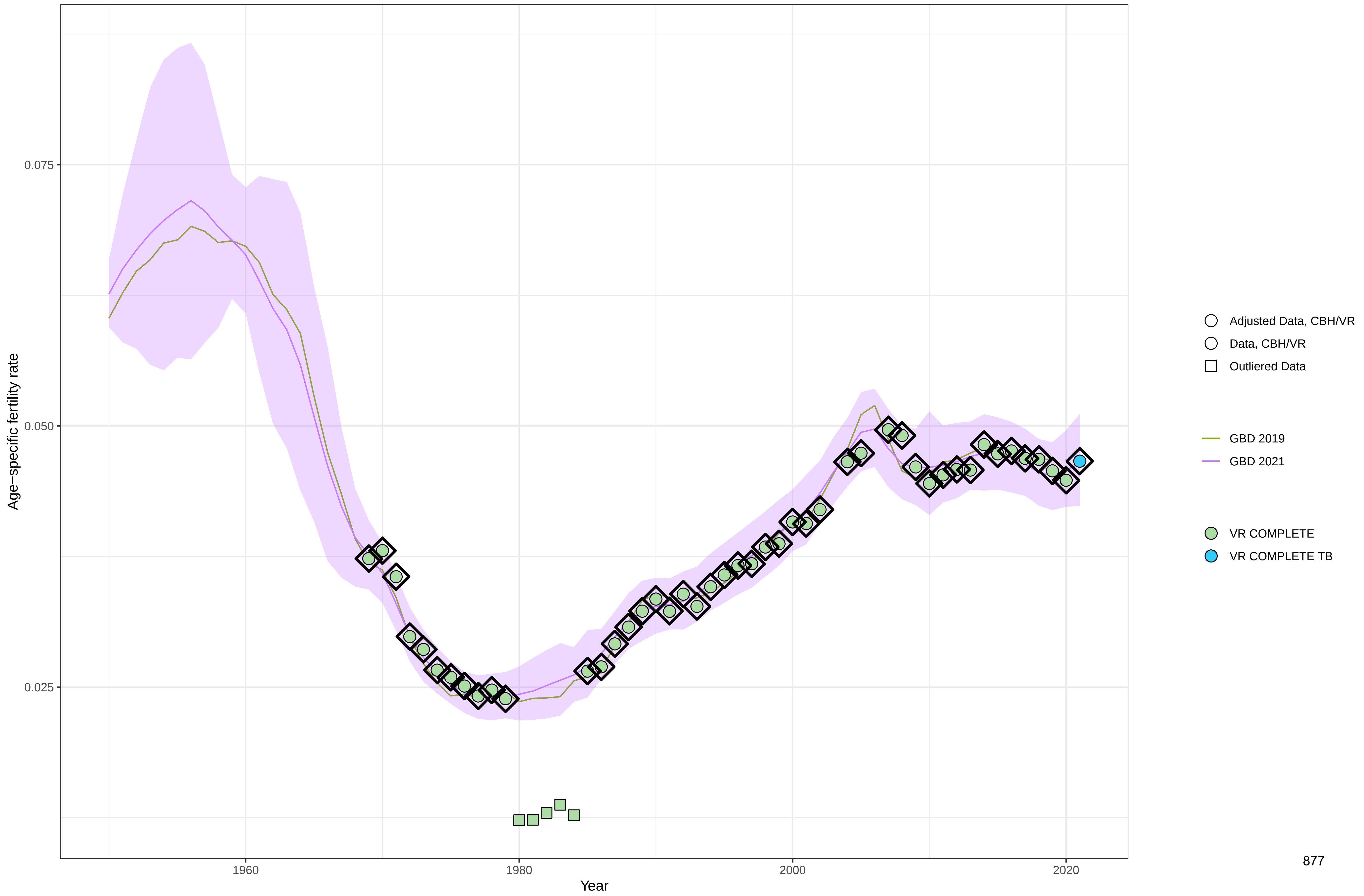

Age−specific fertility fitted trend lines, 1950−2021: Arizona, Ages: 40−44
Data points enclosed with a diamond are reference sources

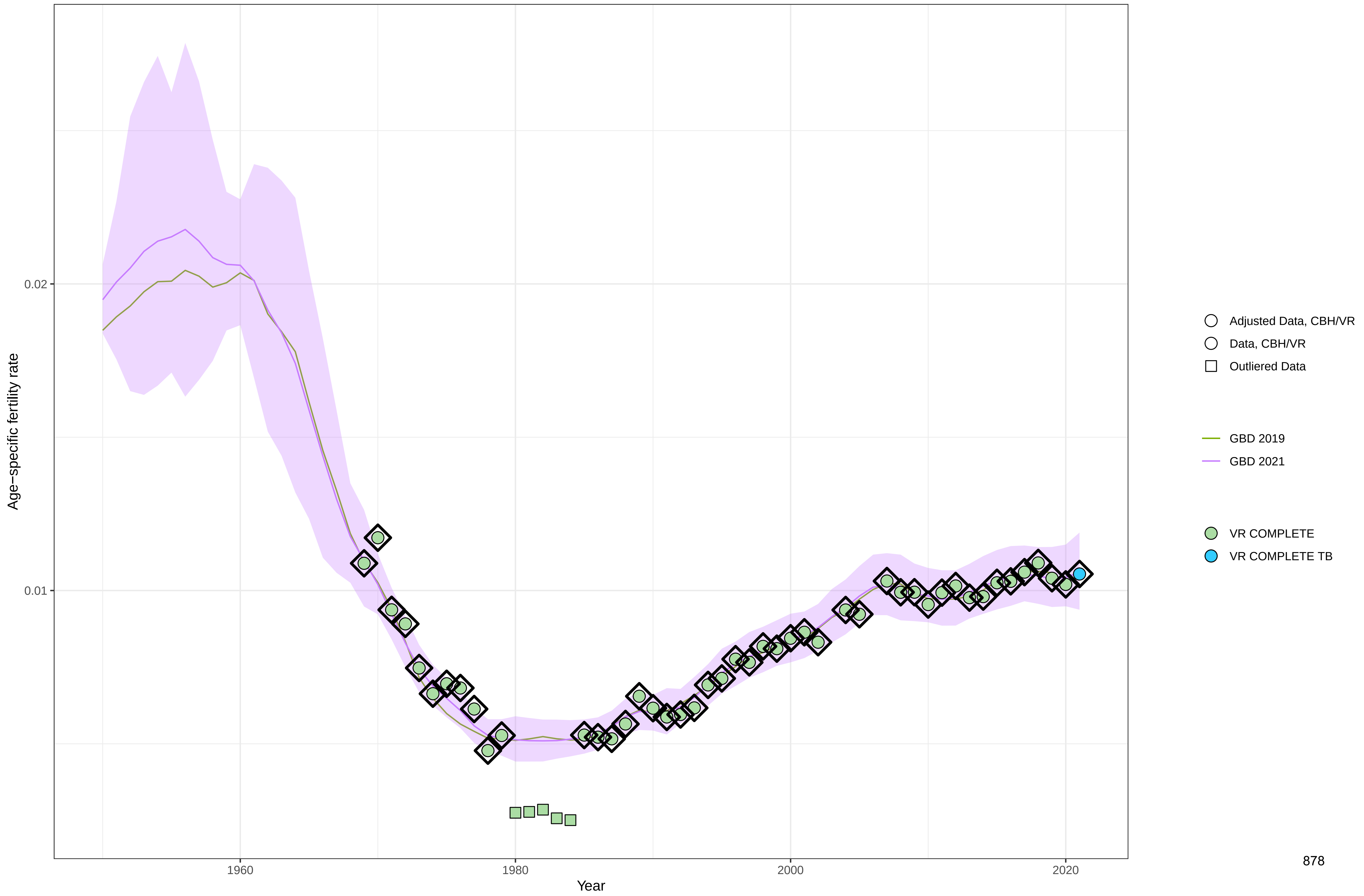

Age−specific fertility fitted trend lines, 1950−2021: Arizona, Ages: 45−49
Data points enclosed with a diamond are reference sources

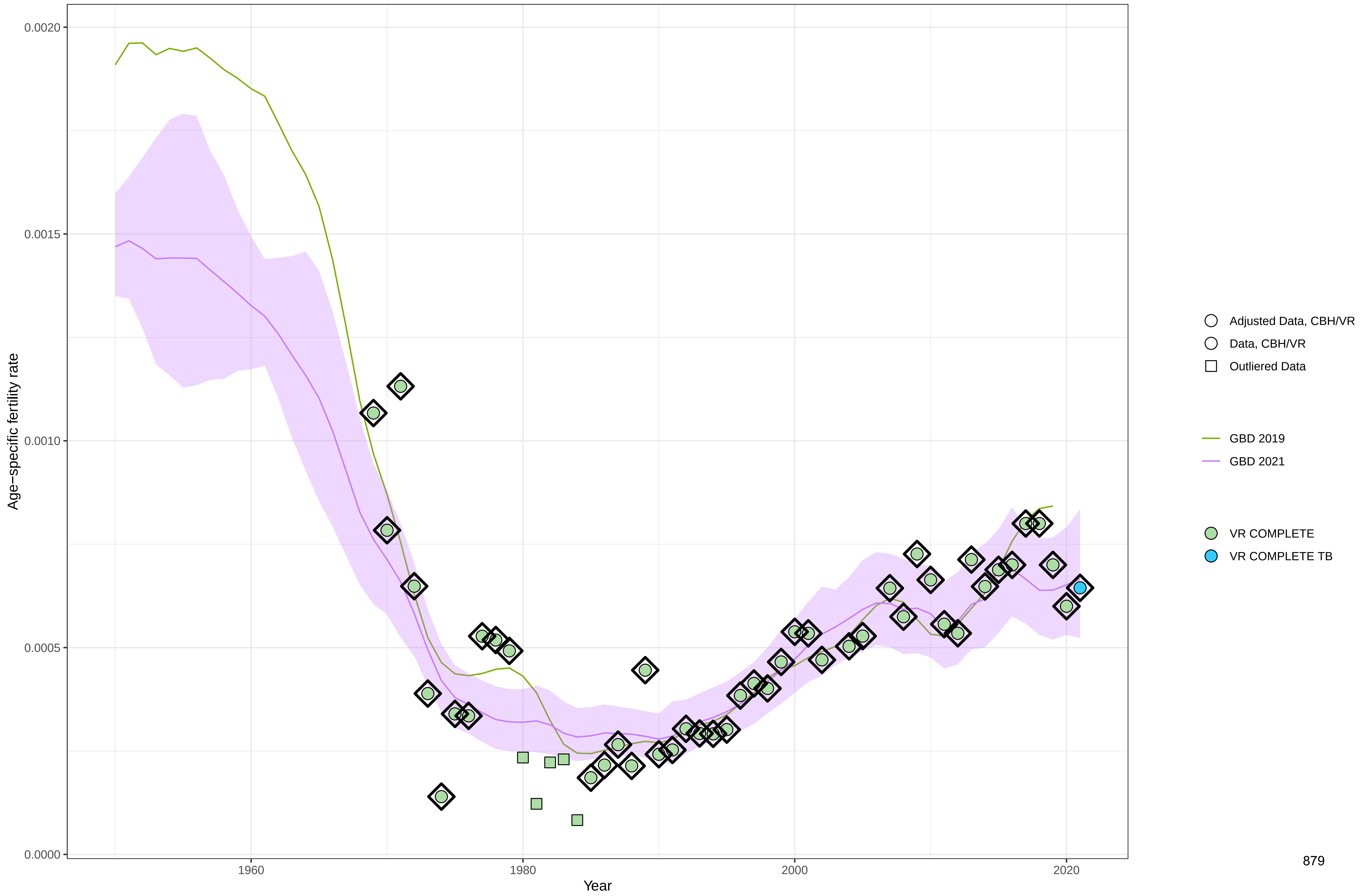

Age−specific fertility fitted trend lines, 1950−2021: Arizona, Ages: 50−54
Data points enclosed with a diamond are reference sources

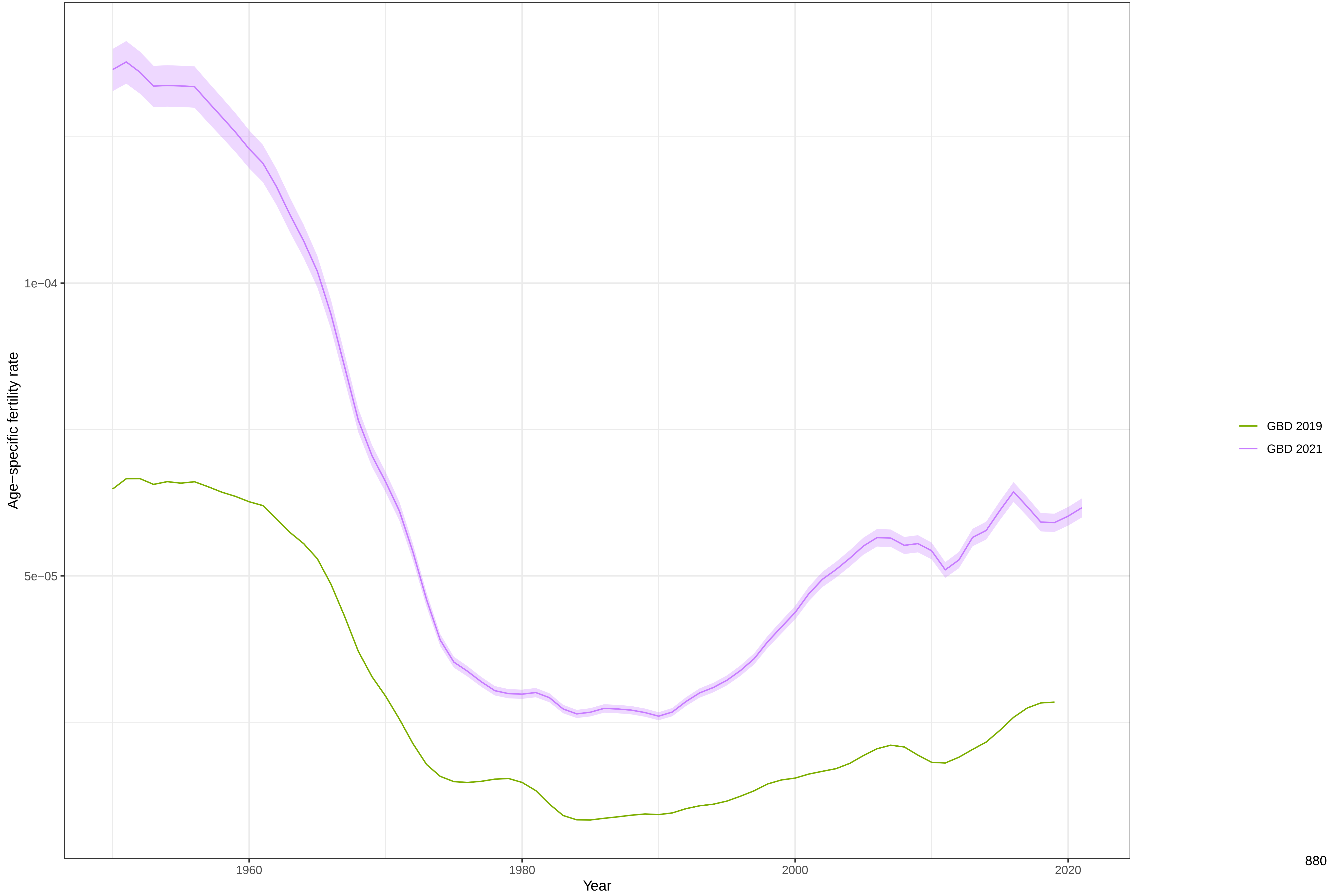

Total fertility rate fitted trend lines, 1950−2021: Arizona TFR
Data points enclosed with a diamond are reference sources

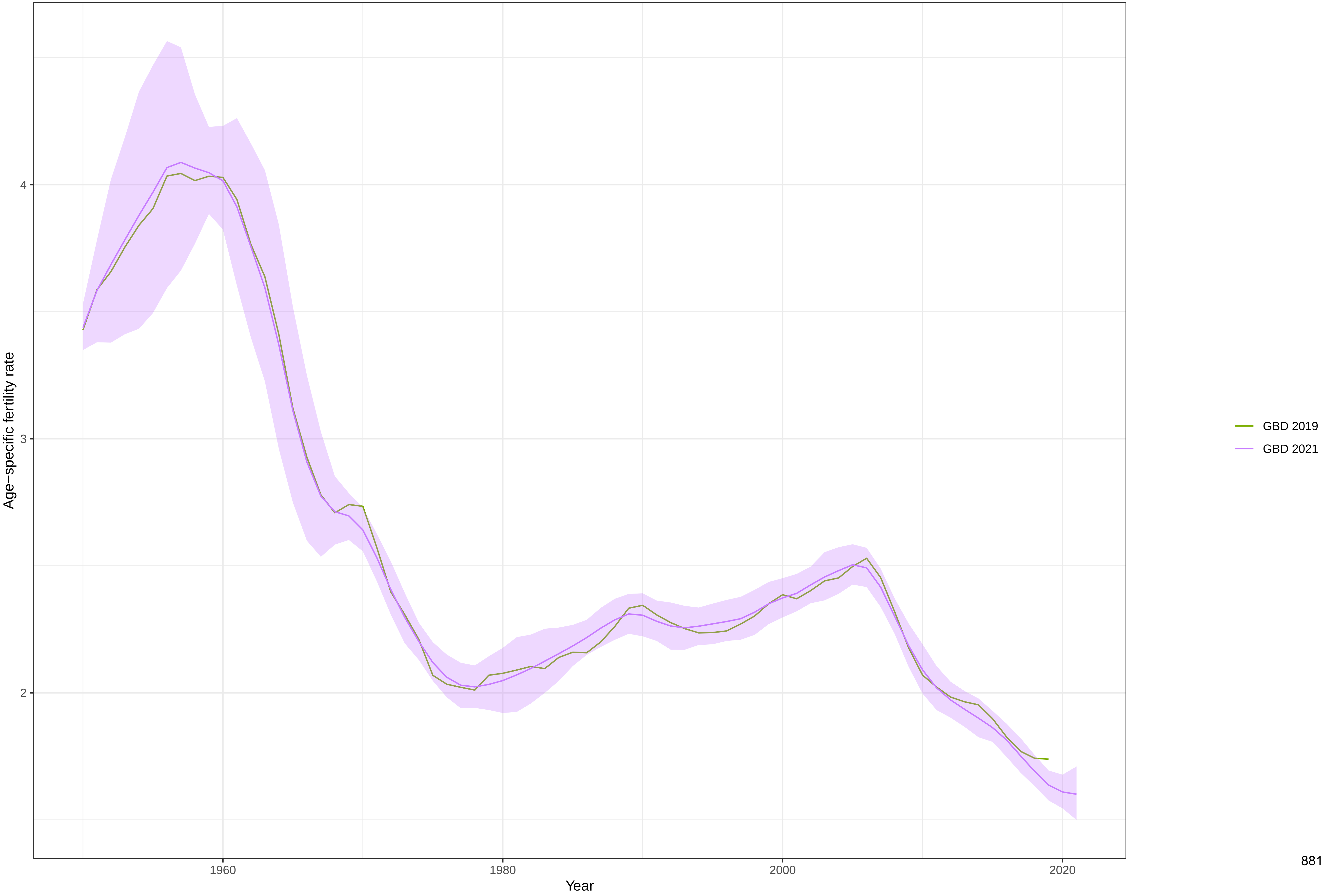

Age−specific fertility fitted trend lines, 1950−2021: Arkansas, Ages: 10−14
Data points enclosed with a diamond are reference sources

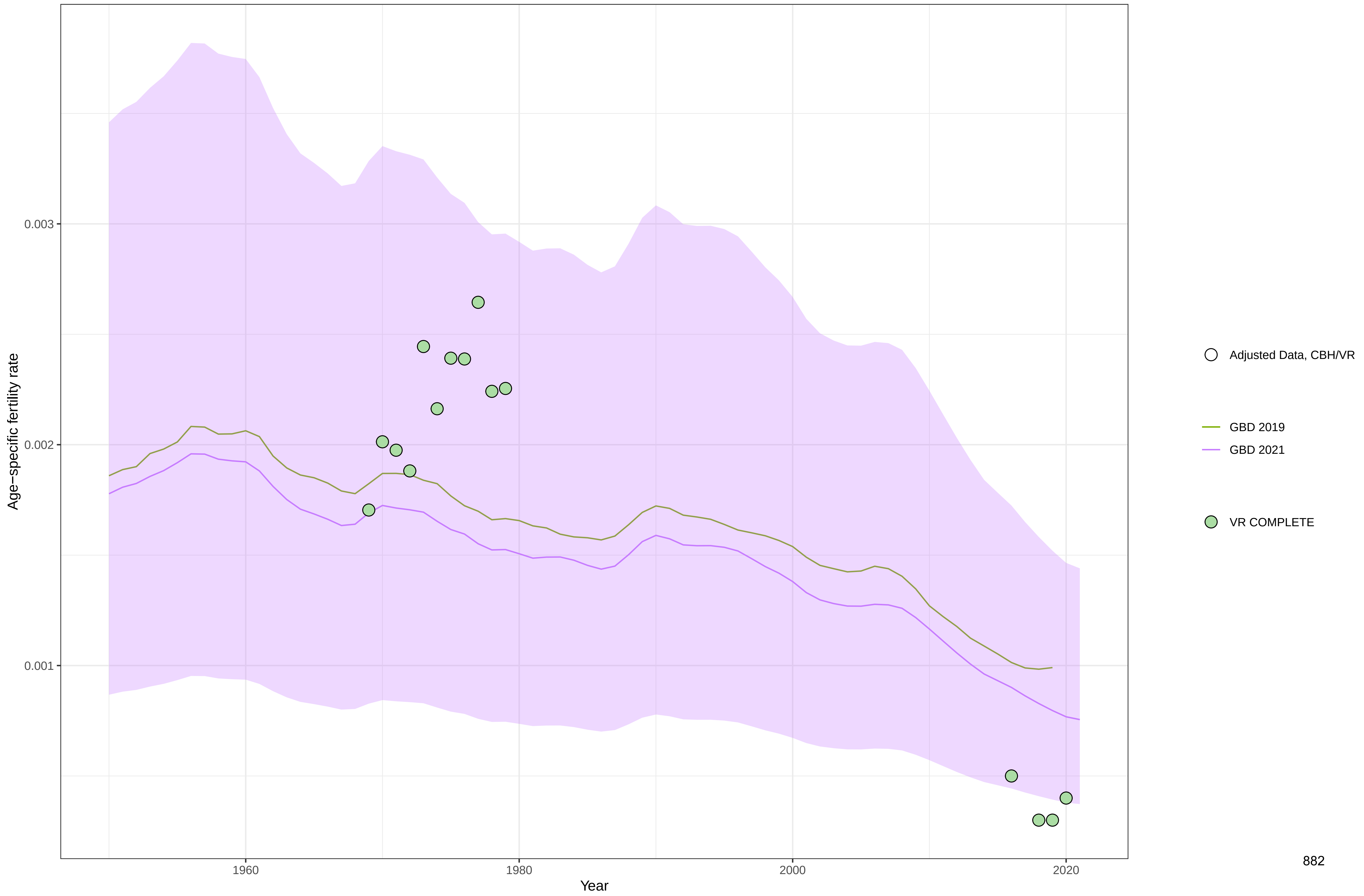

Age−specific fertility fitted trend lines, 1950−2021: Arkansas, Ages: 15−19
Data points enclosed with a diamond are reference sources

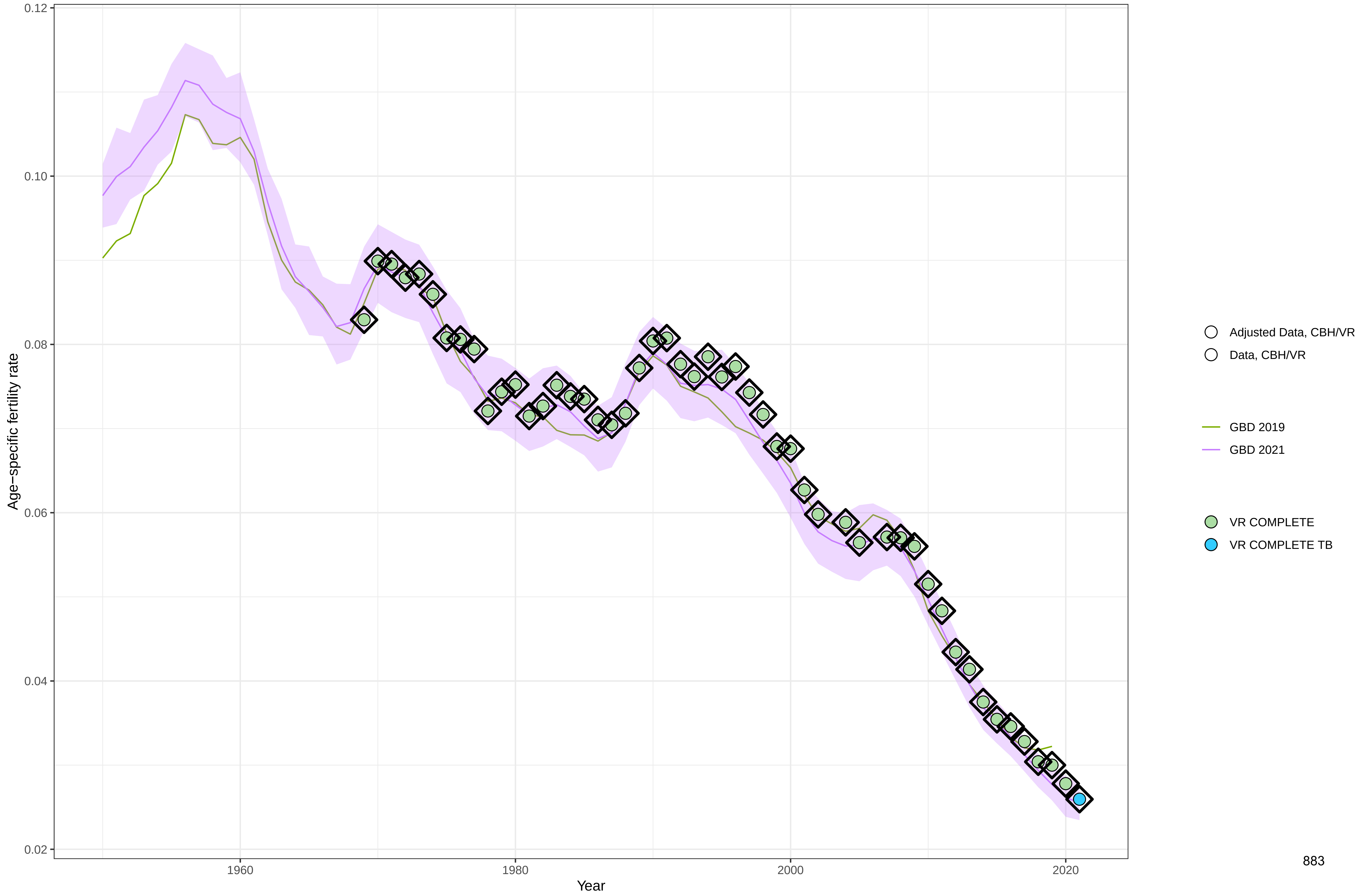

Age−specific fertility fitted trend lines, 1950−2021: Arkansas, Ages: 20−24
Data points enclosed with a diamond are reference sources

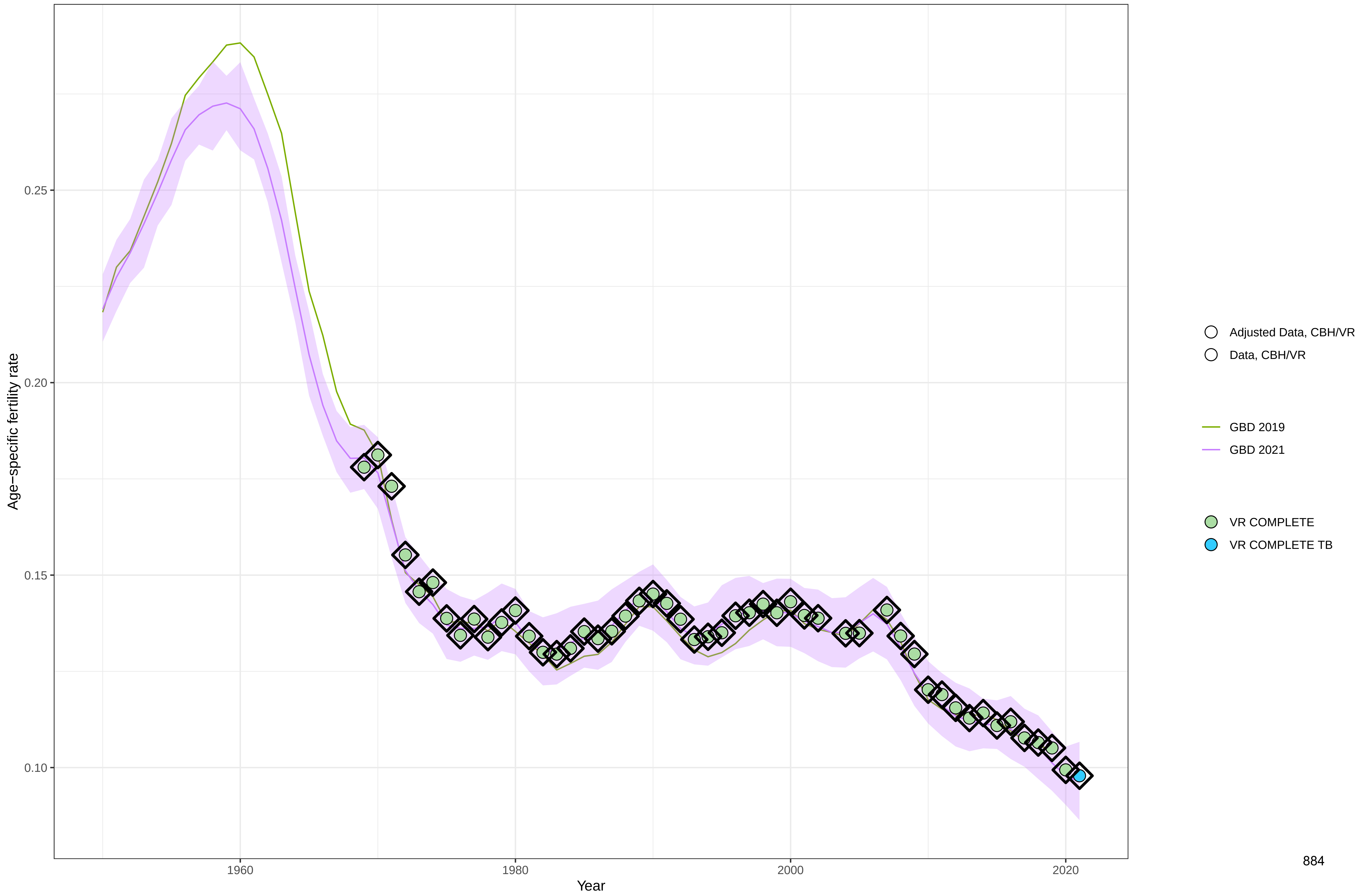

Age−specific fertility fitted trend lines, 1950−2021: Arkansas, Ages: 25−29
Data points enclosed with a diamond are reference sources

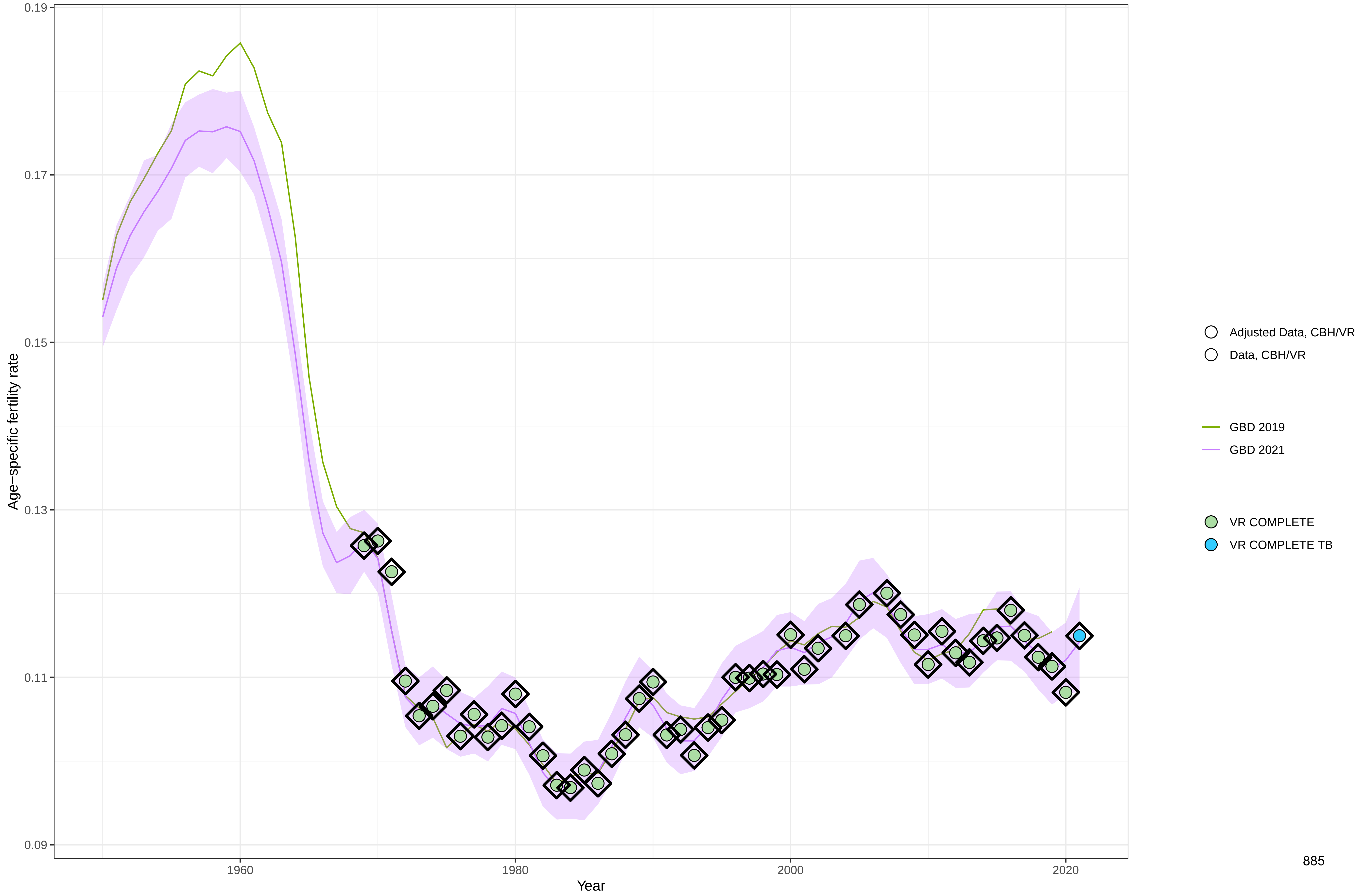

Age−specific fertility fitted trend lines, 1950−2021: Arkansas, Ages: 30−34
Data points enclosed with a diamond are reference sources

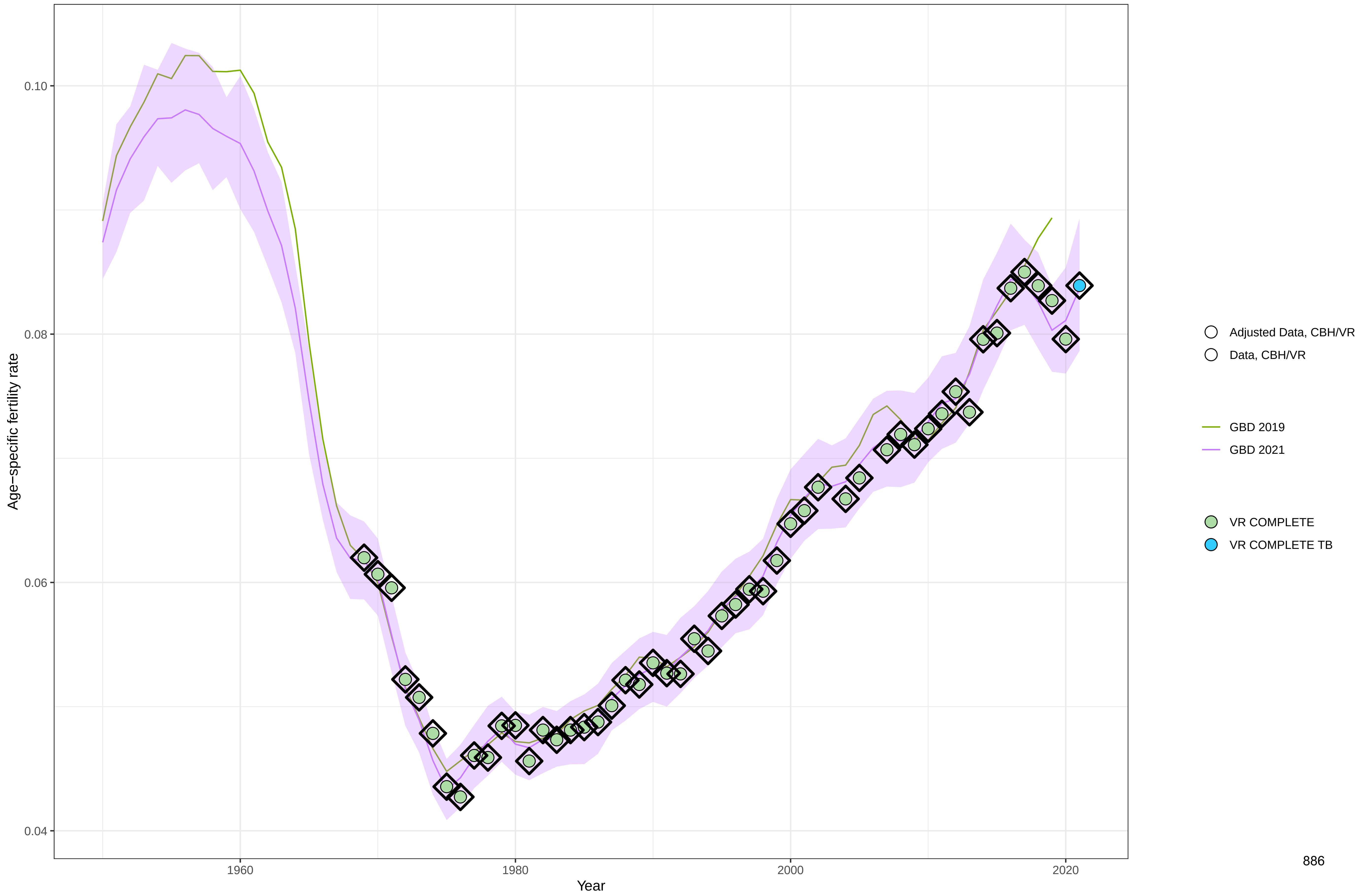

Age−specific fertility fitted trend lines, 1950−2021: Arkansas, Ages: 35−39
Data points enclosed with a diamond are reference sources

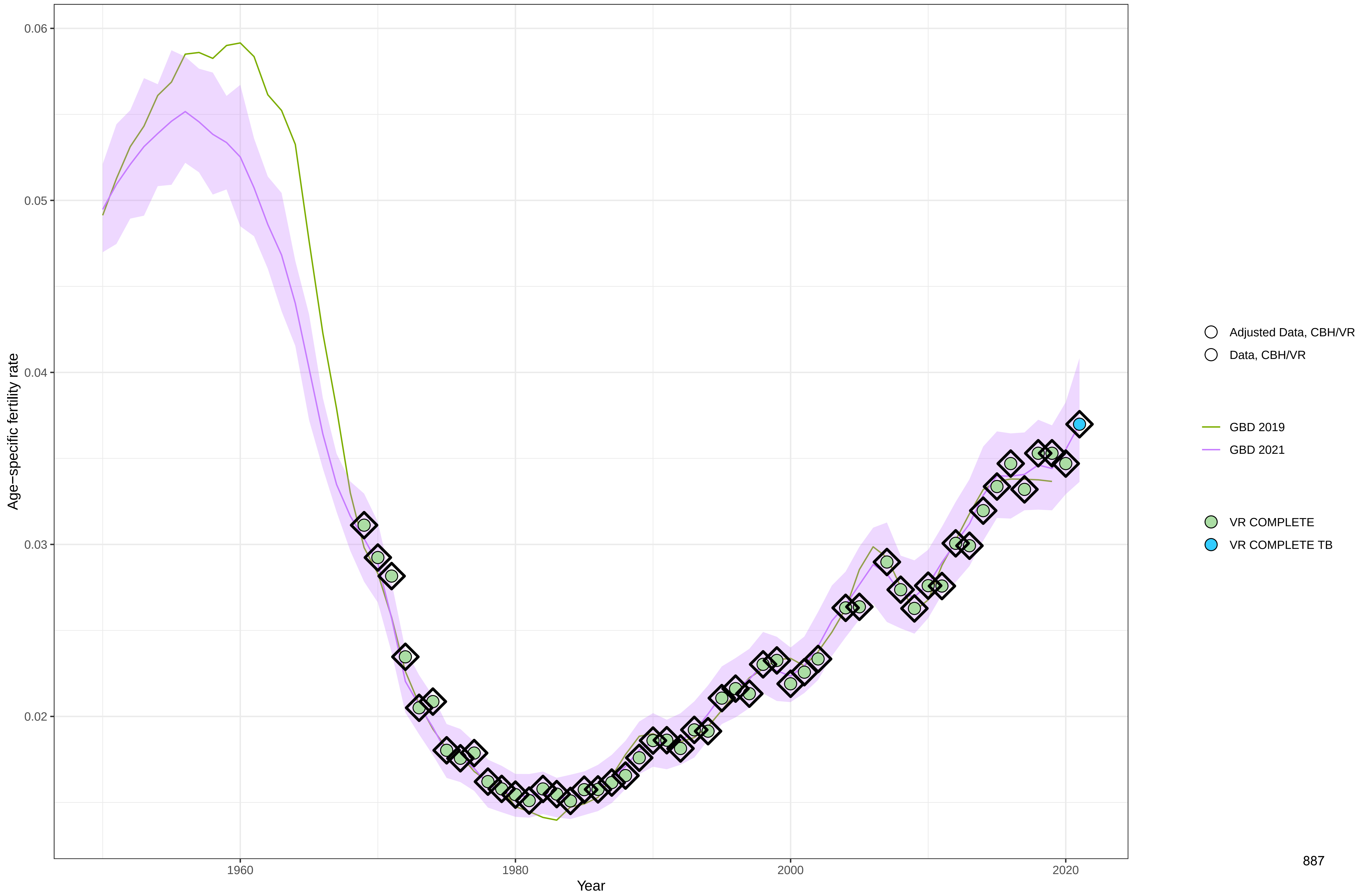

Age−specific fertility fitted trend lines, 1950−2021: Arkansas, Ages: 40−44
Data points enclosed with a diamond are reference sources

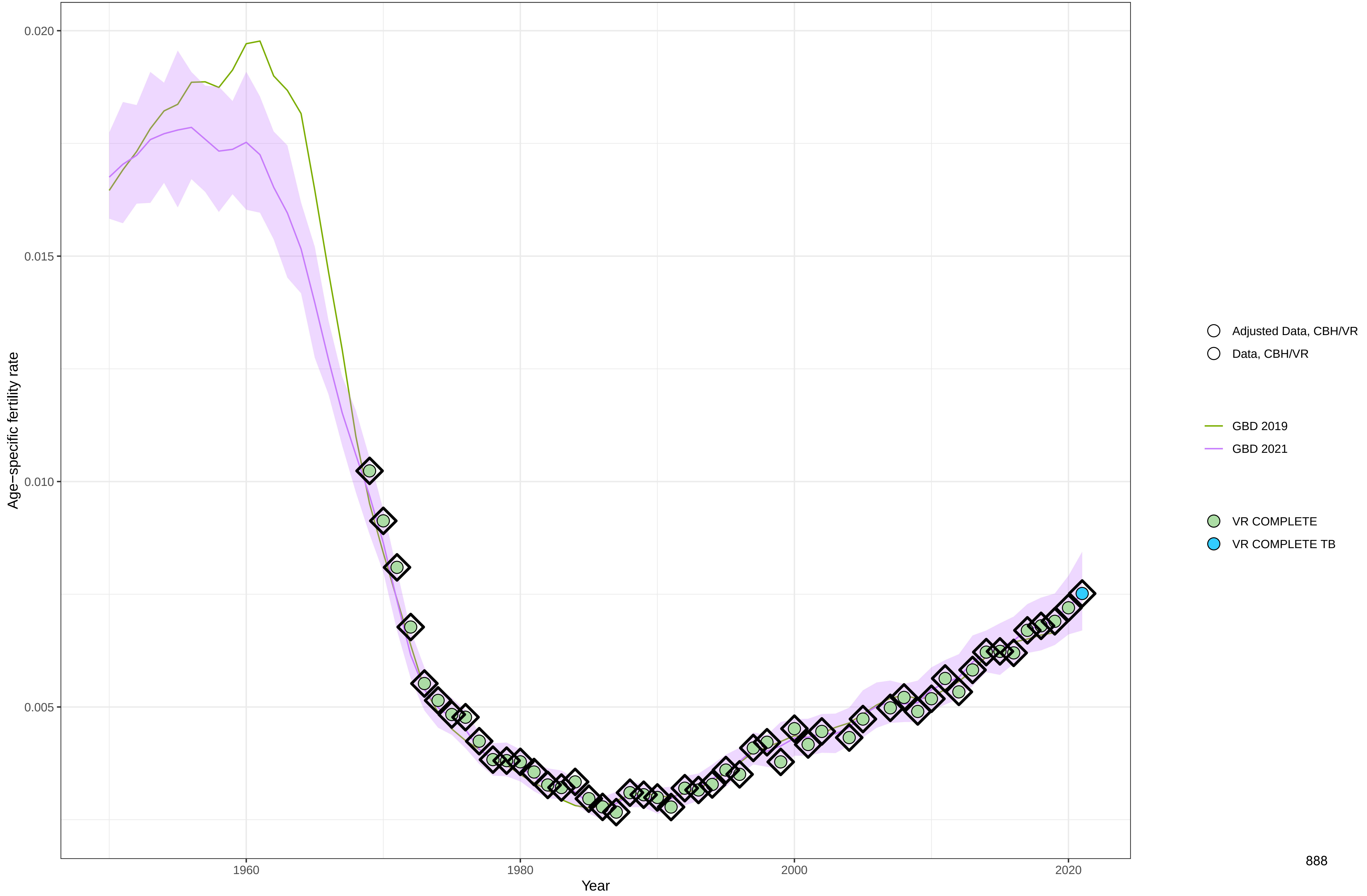

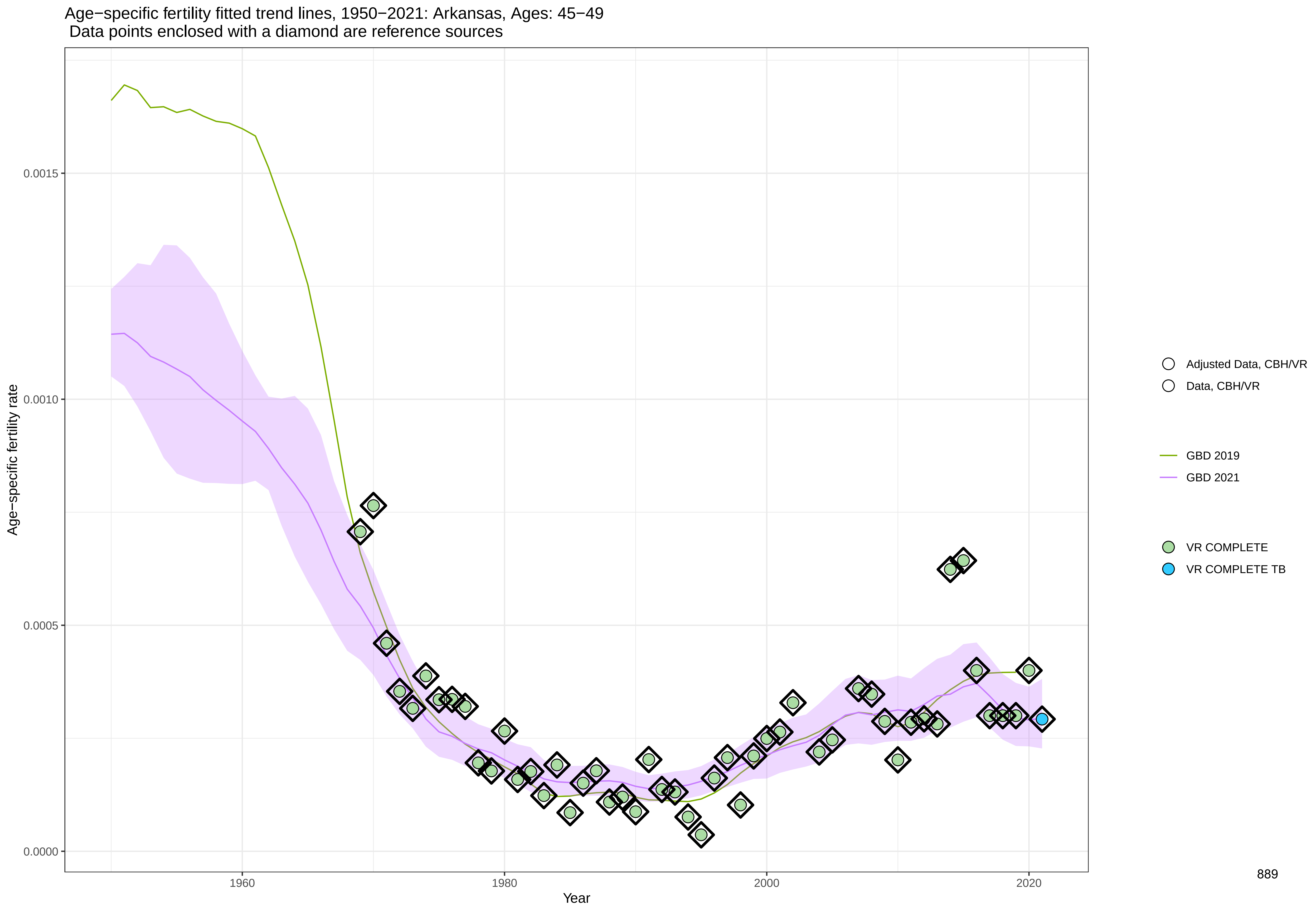
Age−specific fertility fitted trend lines, 1950−2021: Arkansas, Ages: 45−49
Data points enclosed with a diamond are reference sources
Age−specific fertility rate
0.0000
0.0005
0.0010
0.0015
1960
1980
2000
2020
Year
Adjusted Data, CBH/VR
Data, CBH/VR
GBD 2019
GBD 2021
VR COMPLETE
VR COMPLETE TB

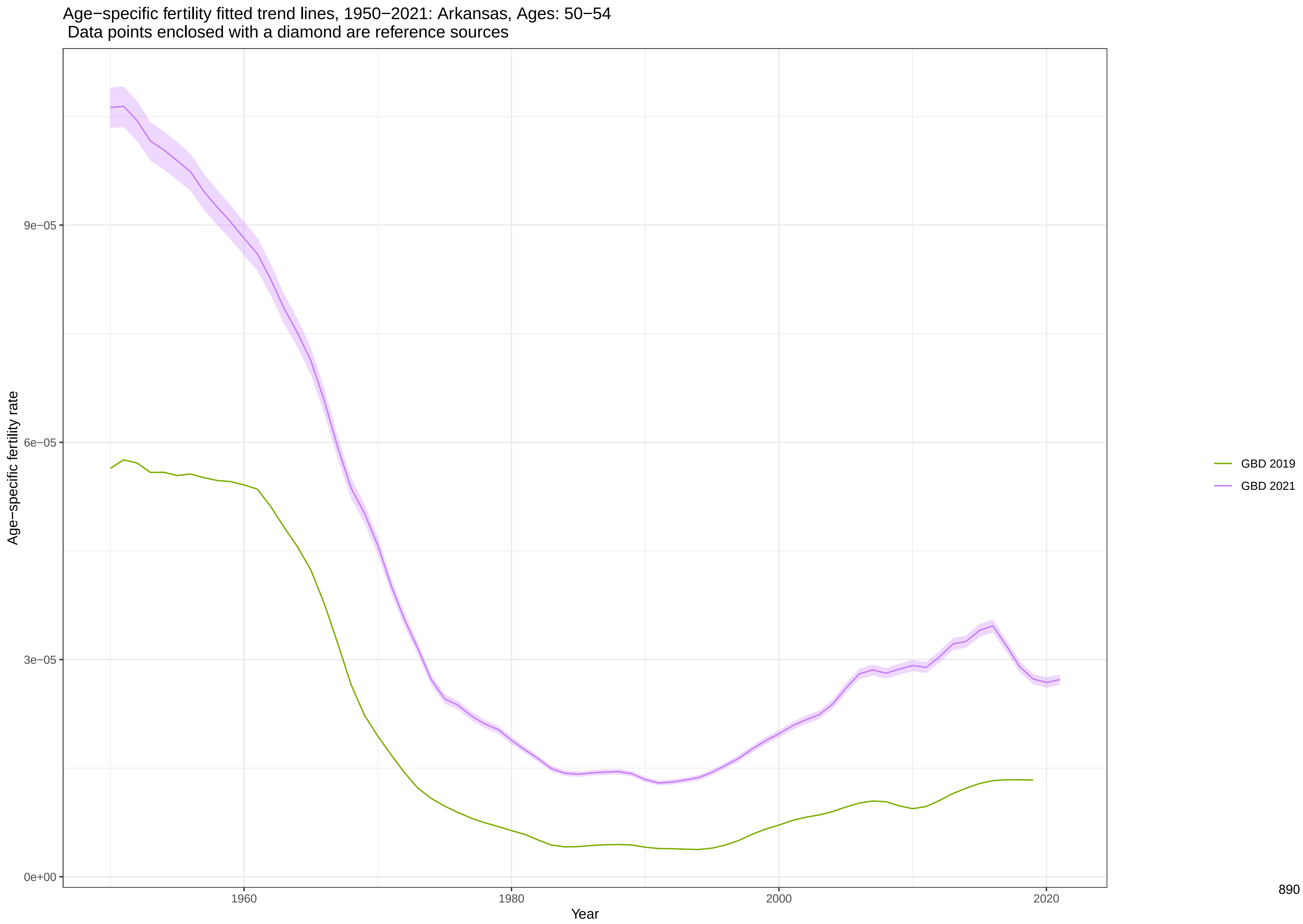
Age−specific fertility fitted trend lines, 1950−2021: Arkansas, Ages: 50−54
Data points enclosed with a diamond are reference sources
Age−specific fertility rate
9e−05
6e−05
3e−05
0e+00
1960
1980
2000
2020
Year
GBD 2019
GBD 2021

Total fertility rate fitted trend lines, 1950−2021: Arkansas TFR
Data points enclosed with a diamond are reference sources

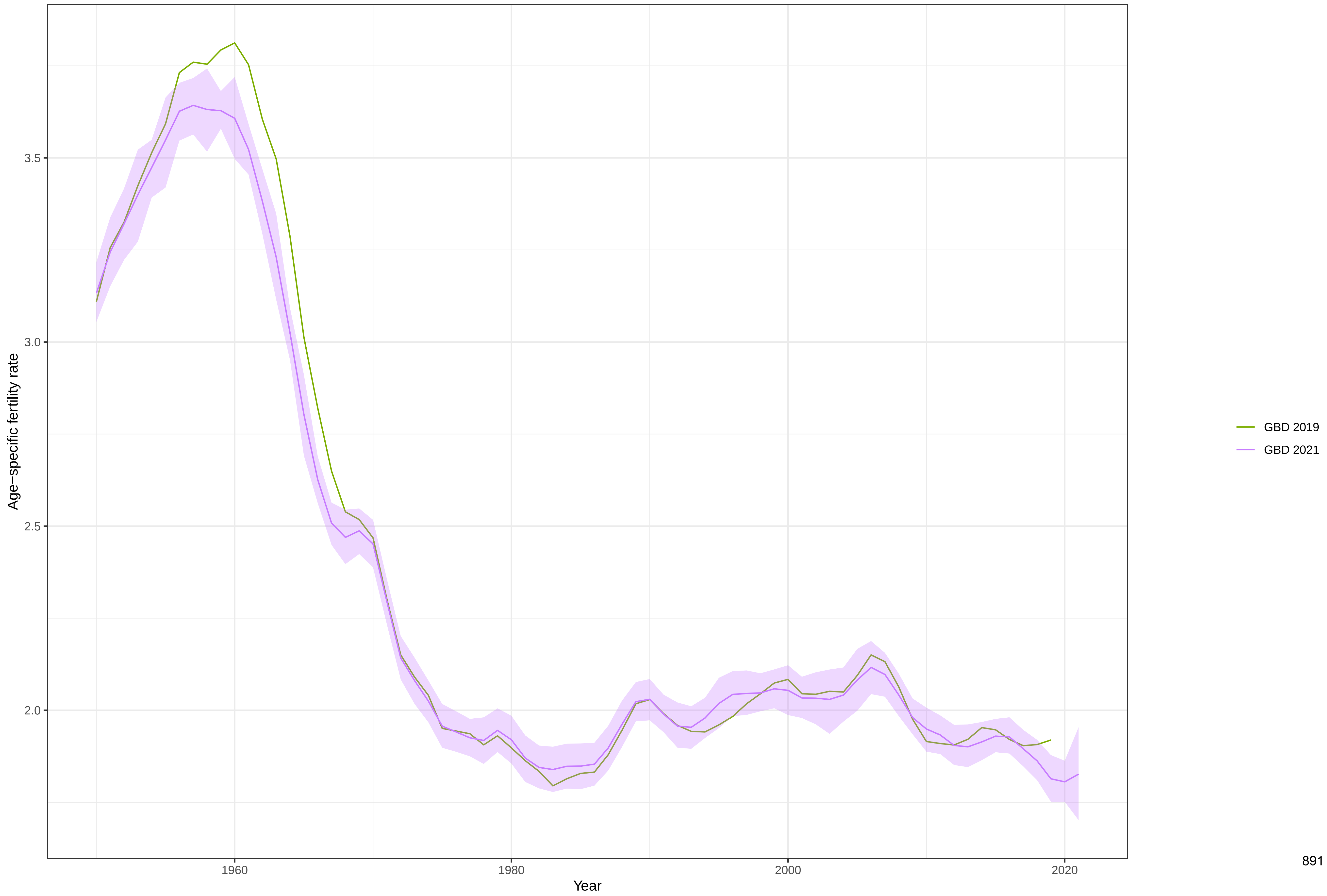

Age−specific fertility fitted trend lines, 1950−2021: California, Ages: 10−14
Data points enclosed with a diamond are reference sources

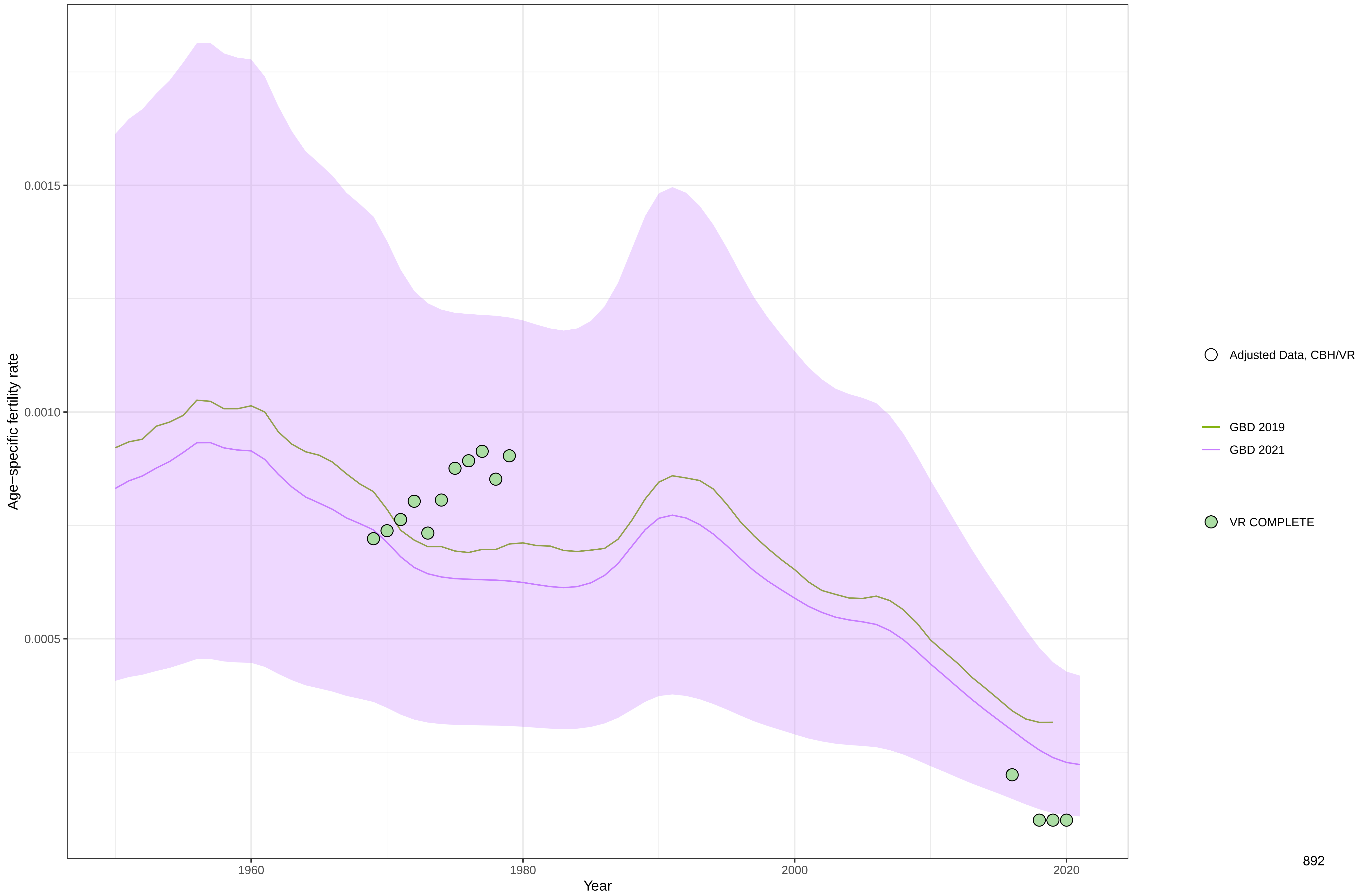

Age−specific fertility fitted trend lines, 1950−2021: California, Ages: 15−19
Data points enclosed with a diamond are reference sources

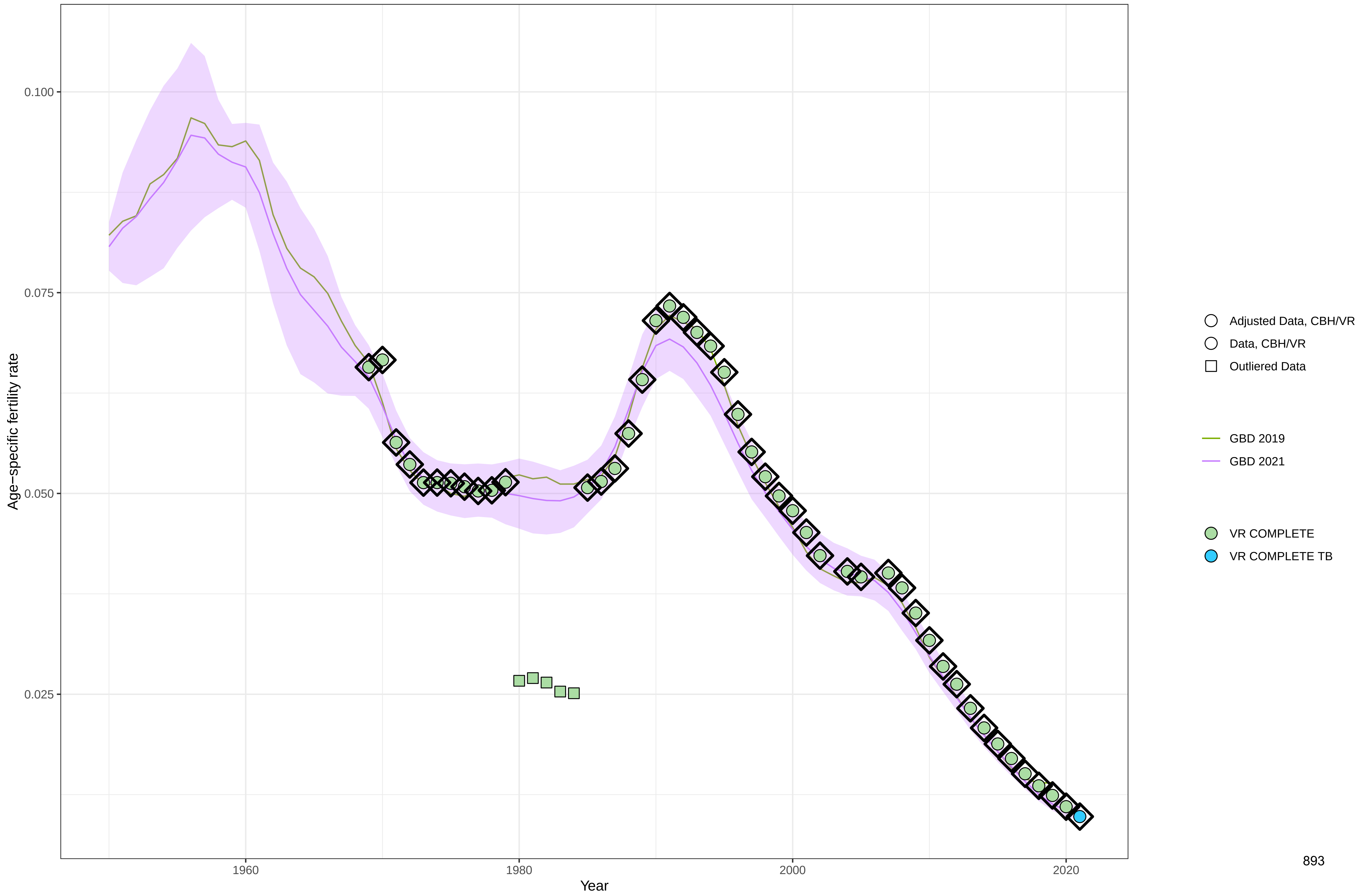

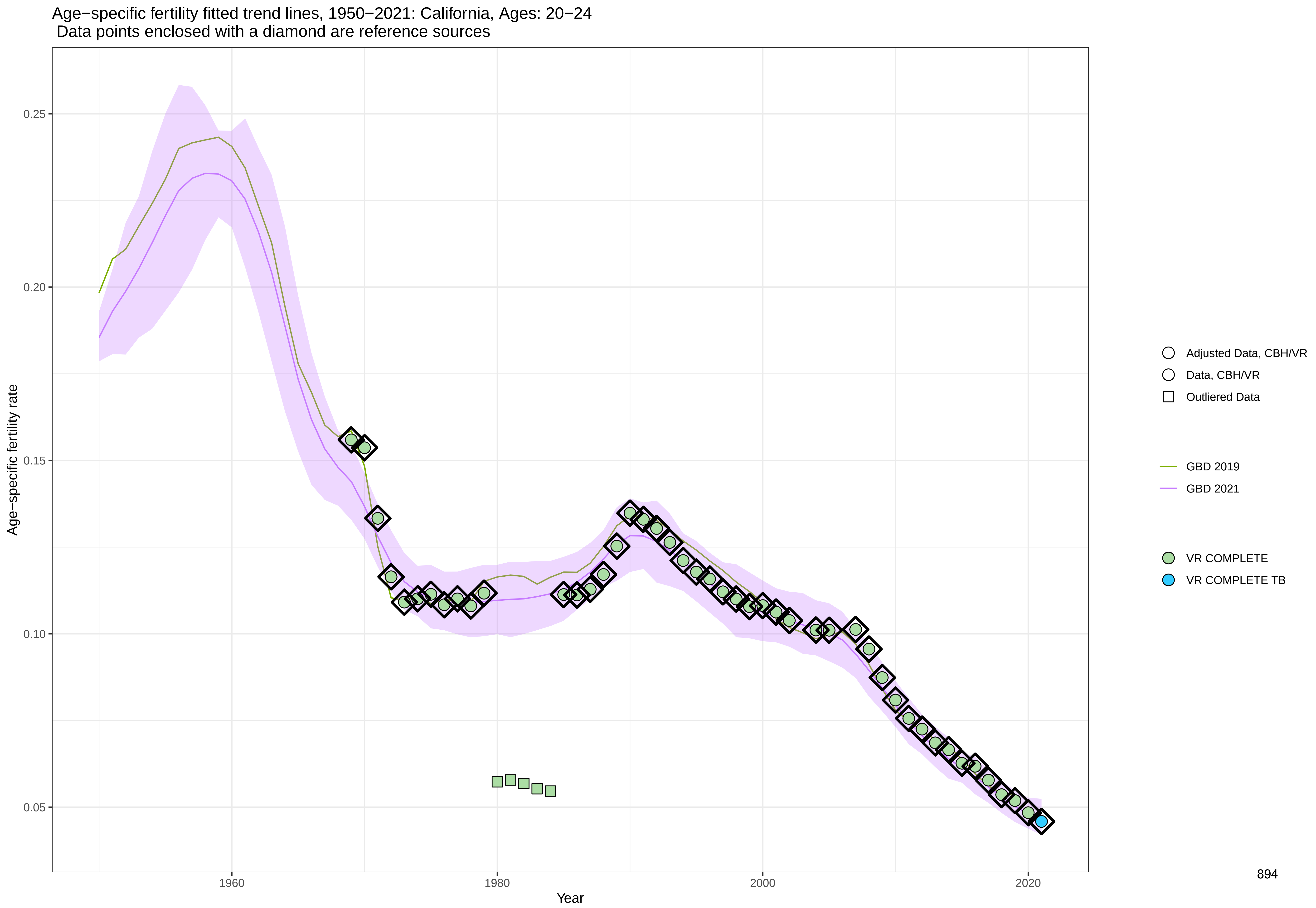
Age−specific fertility fitted trend lines, 1950−2021: California, Ages: 20−24
Data points enclosed with a diamond are reference sources
0.25
0.20
0.15
0.10
0.05
Age−specific fertility rate
1960
1980
2000
2020
Year
Adjusted Data, CBH/VR
Data, CBH/VR
Outliered Data
GBD 2019
GBD 2021
VR COMPLETE
VR COMPLETE TB

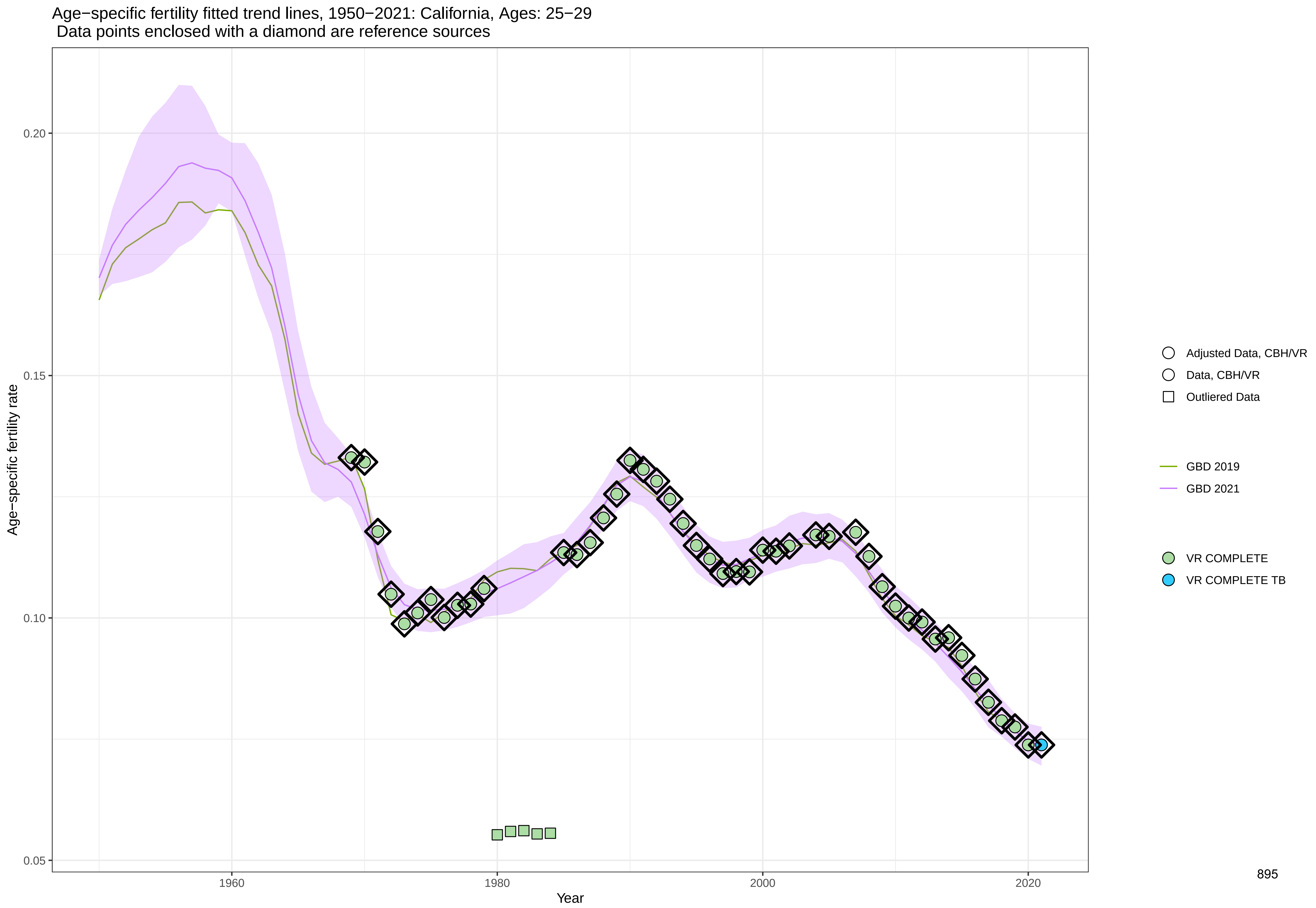
Age−specific fertility fitted trend lines, 1950−2021: California, Ages: 25−29
Data points enclosed with a diamond are reference sources
Age−specific fertility rate
0.20
0.15
0.10
0.05
1960
1980
2000
2020
Year
Adjusted Data, CBH/VR
Data, CBH/VR
Outliered Data
GBD 2019
GBD 2021
VR COMPLETE
VR COMPLETE TB

Age−specific fertility fitted trend lines, 1950−2021: California, Ages: 30−34
Data points enclosed with a diamond are reference sources

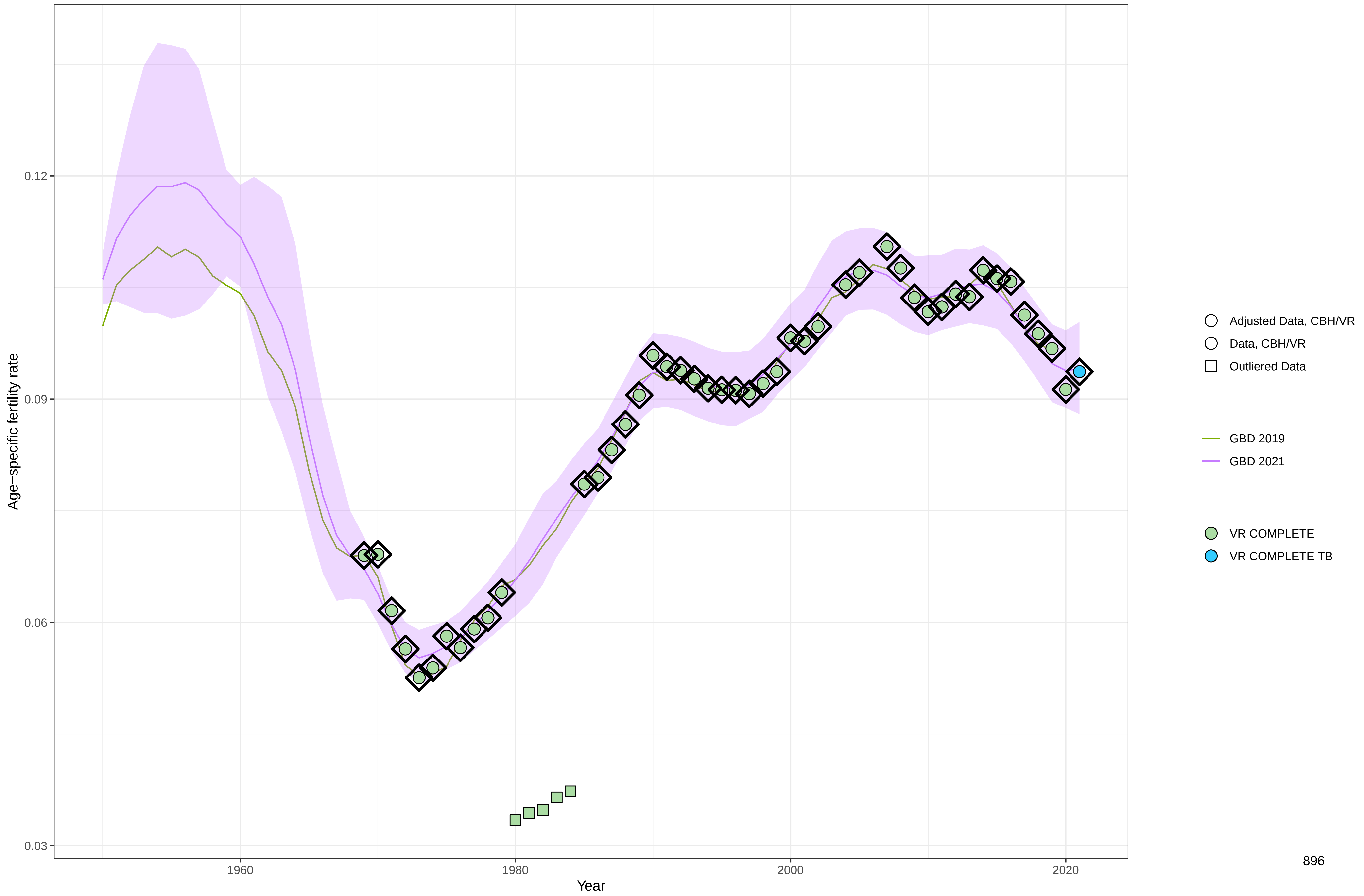

Age−specific fertility fitted trend lines, 1950−2021: California, Ages: 35−39
Data points enclosed with a diamond are reference sources

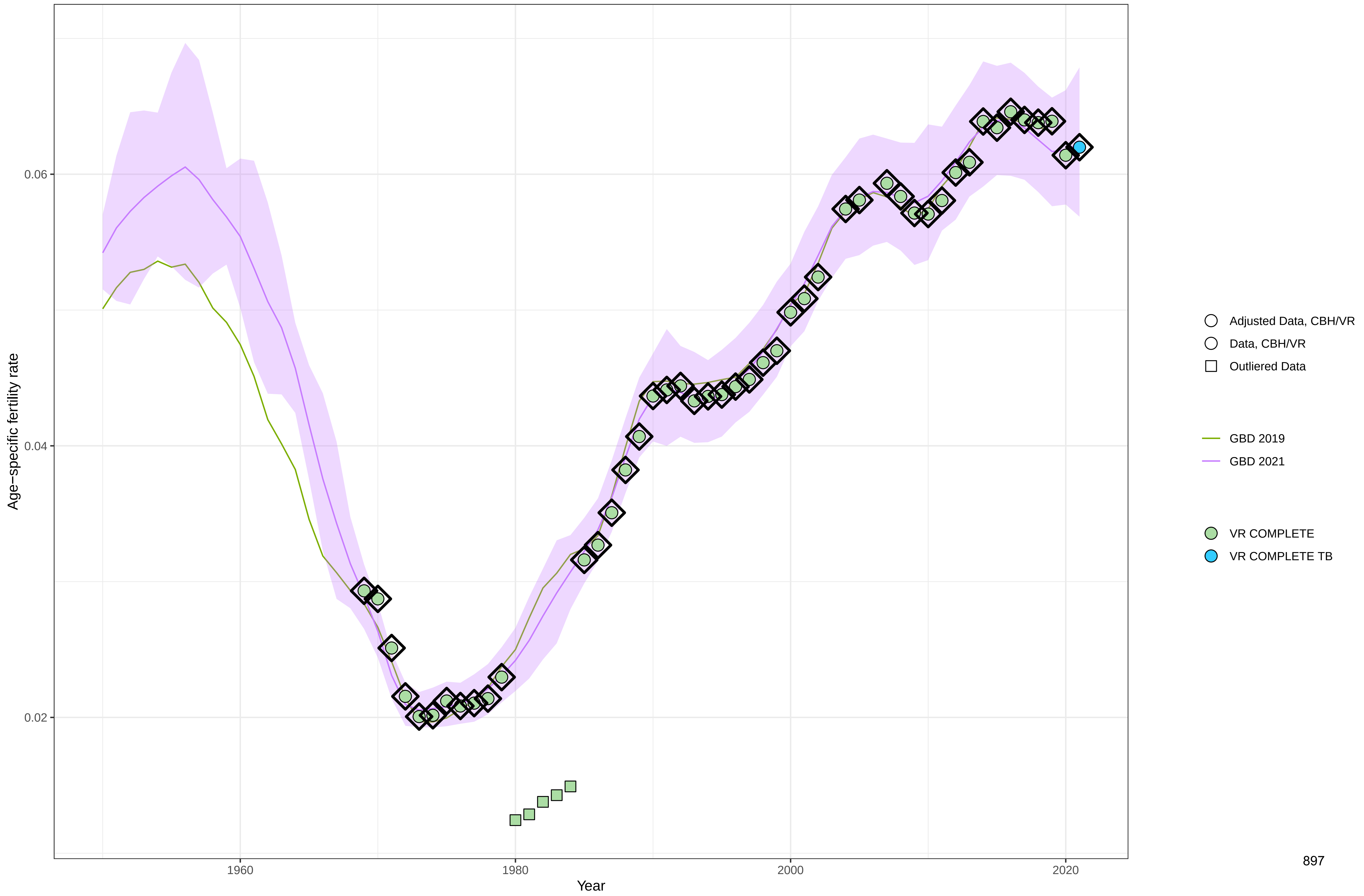

Age−specific fertility fitted trend lines, 1950−2021: California, Ages: 40−44
Data points enclosed with a diamond are reference sources

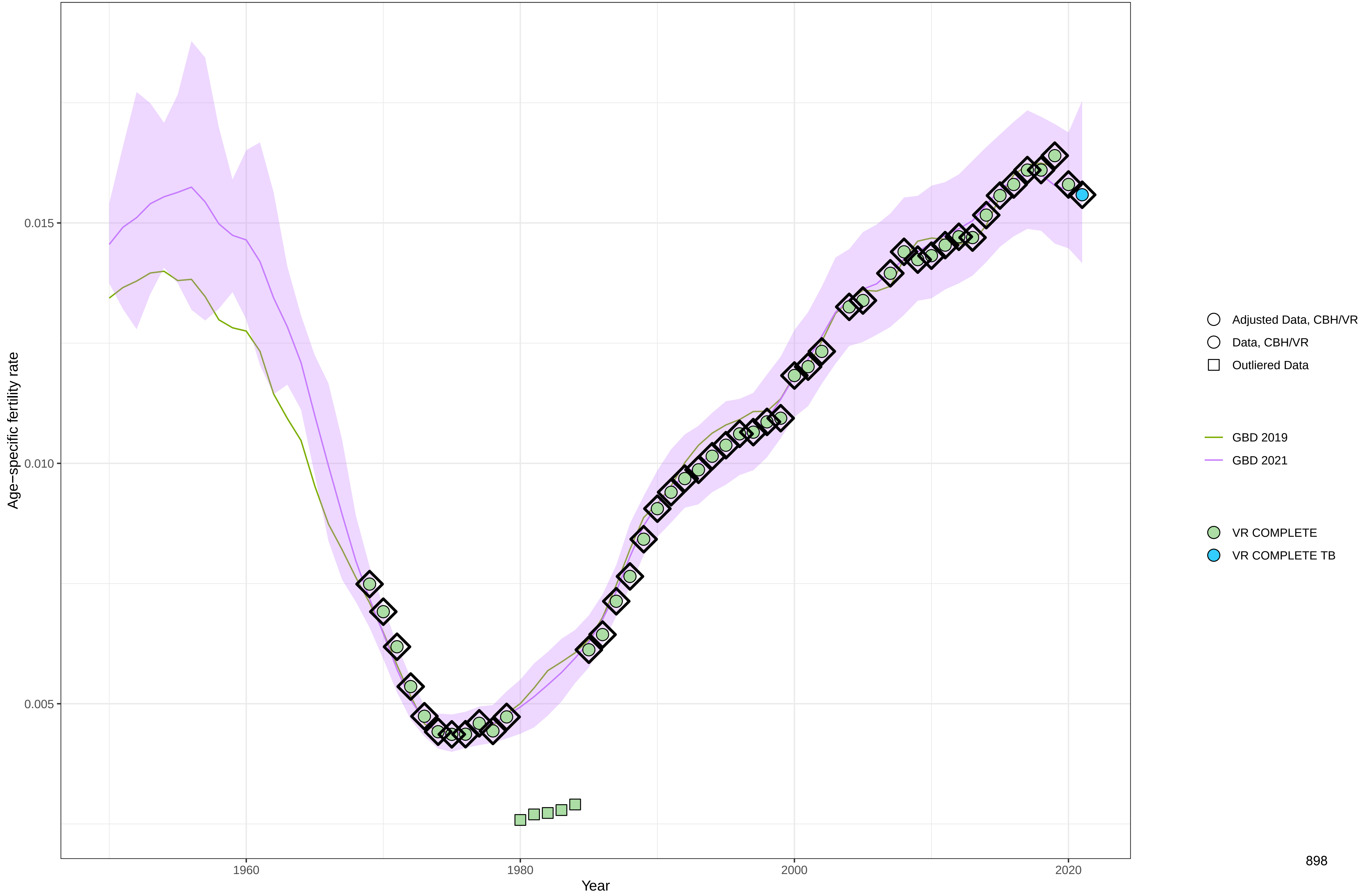

Age−specific fertility fitted trend lines, 1950−2021: California, Ages: 45−49
Data points enclosed with a diamond are reference sources

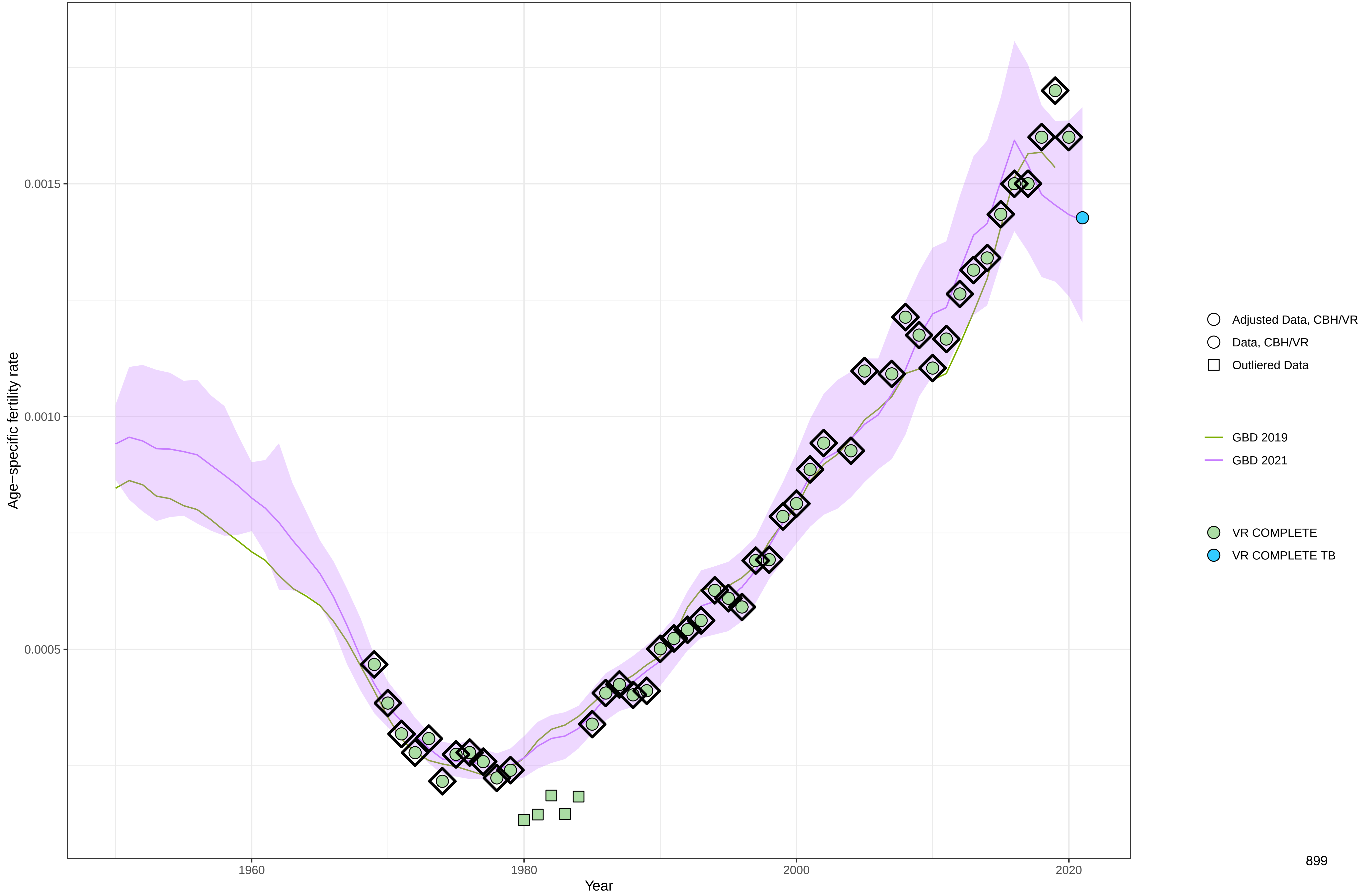

Age−specific fertility fitted trend lines, 1950−2021: California, Ages: 50−54
Data points enclosed with a diamond are reference sources

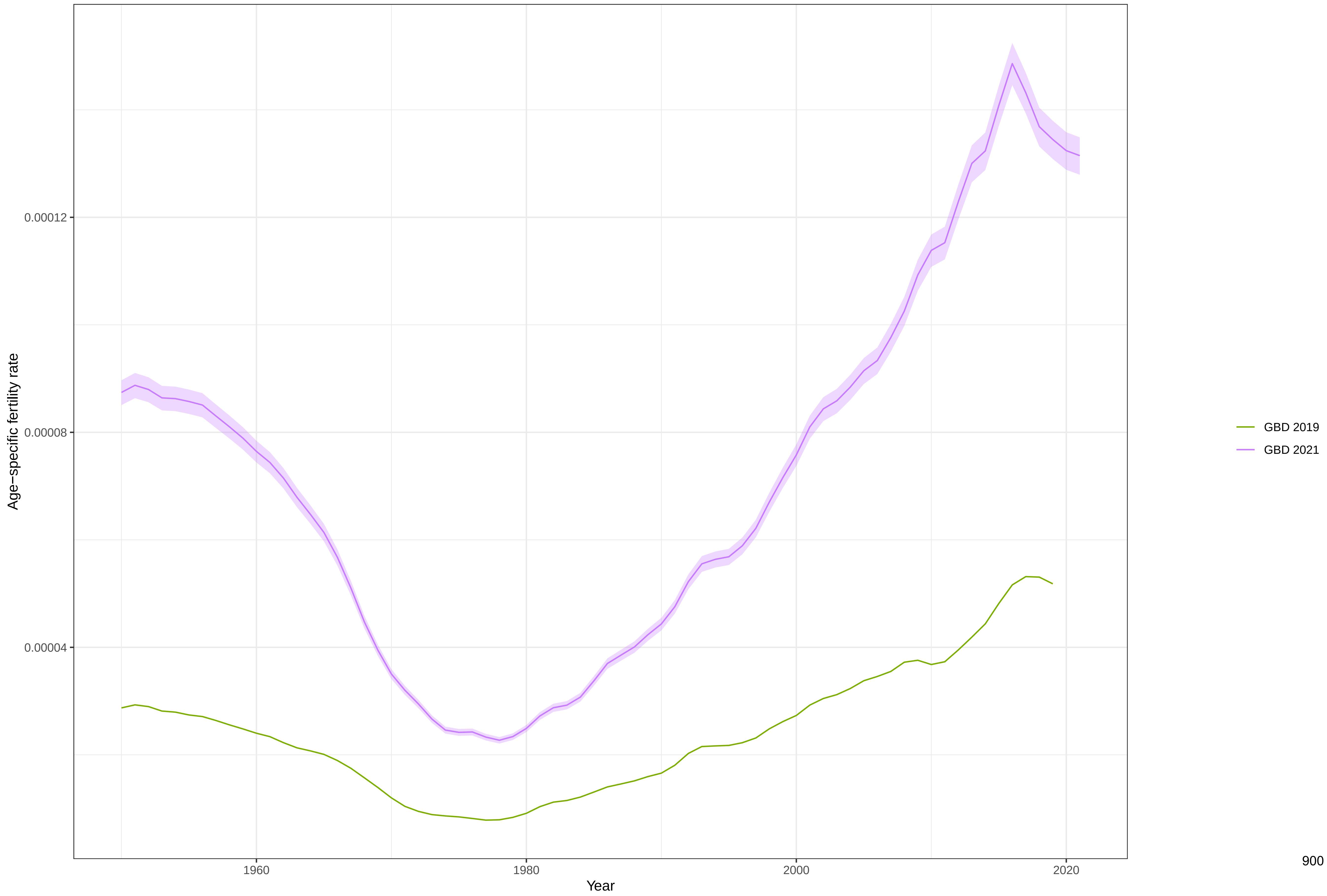

Total fertility rate fitted trend lines, 1950−2021: California TFR
Data points enclosed with a diamond are reference sources

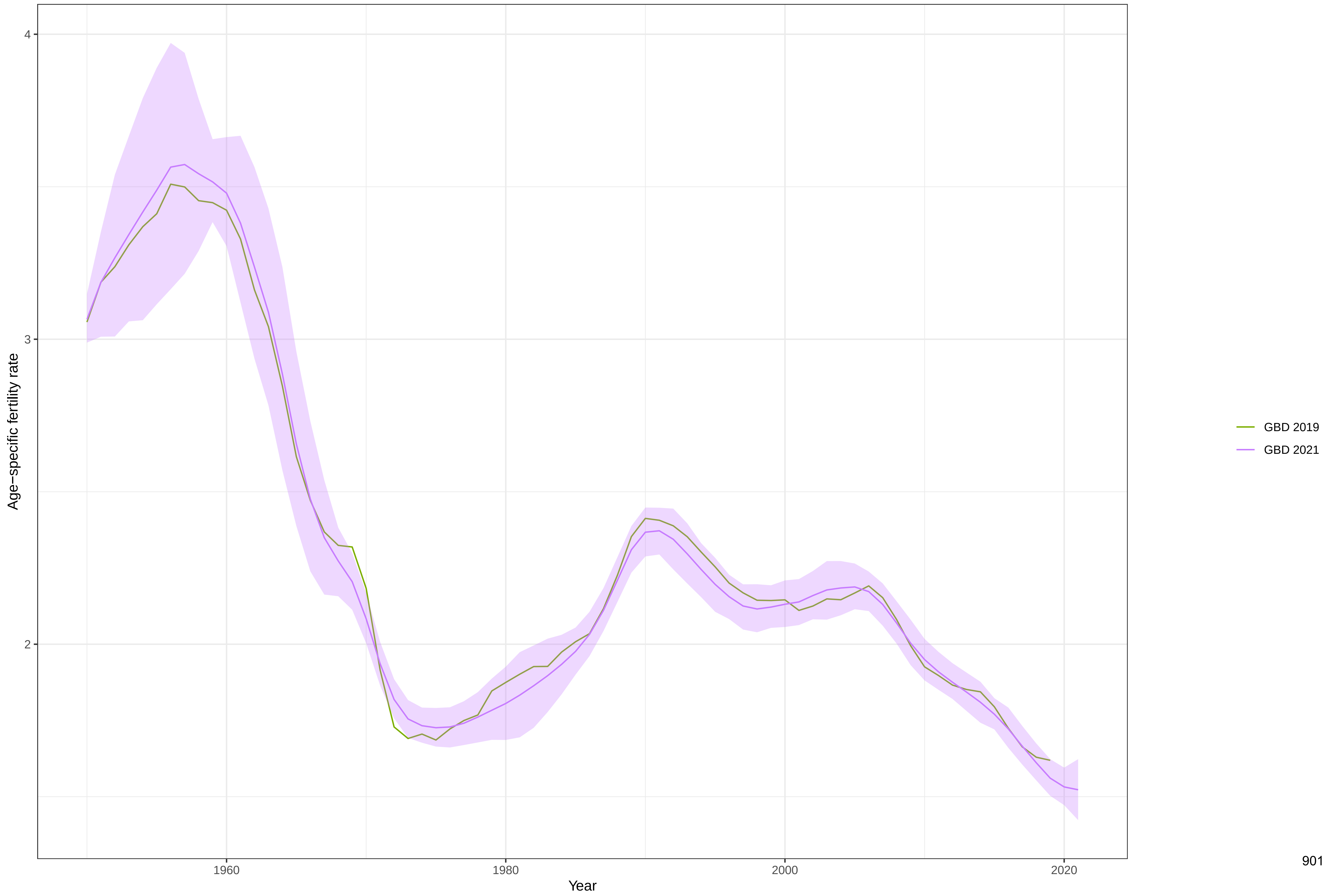

Age−specific fertility fitted trend lines, 1950−2021: Colorado, Ages: 10−14
Data points enclosed with a diamond are reference sources

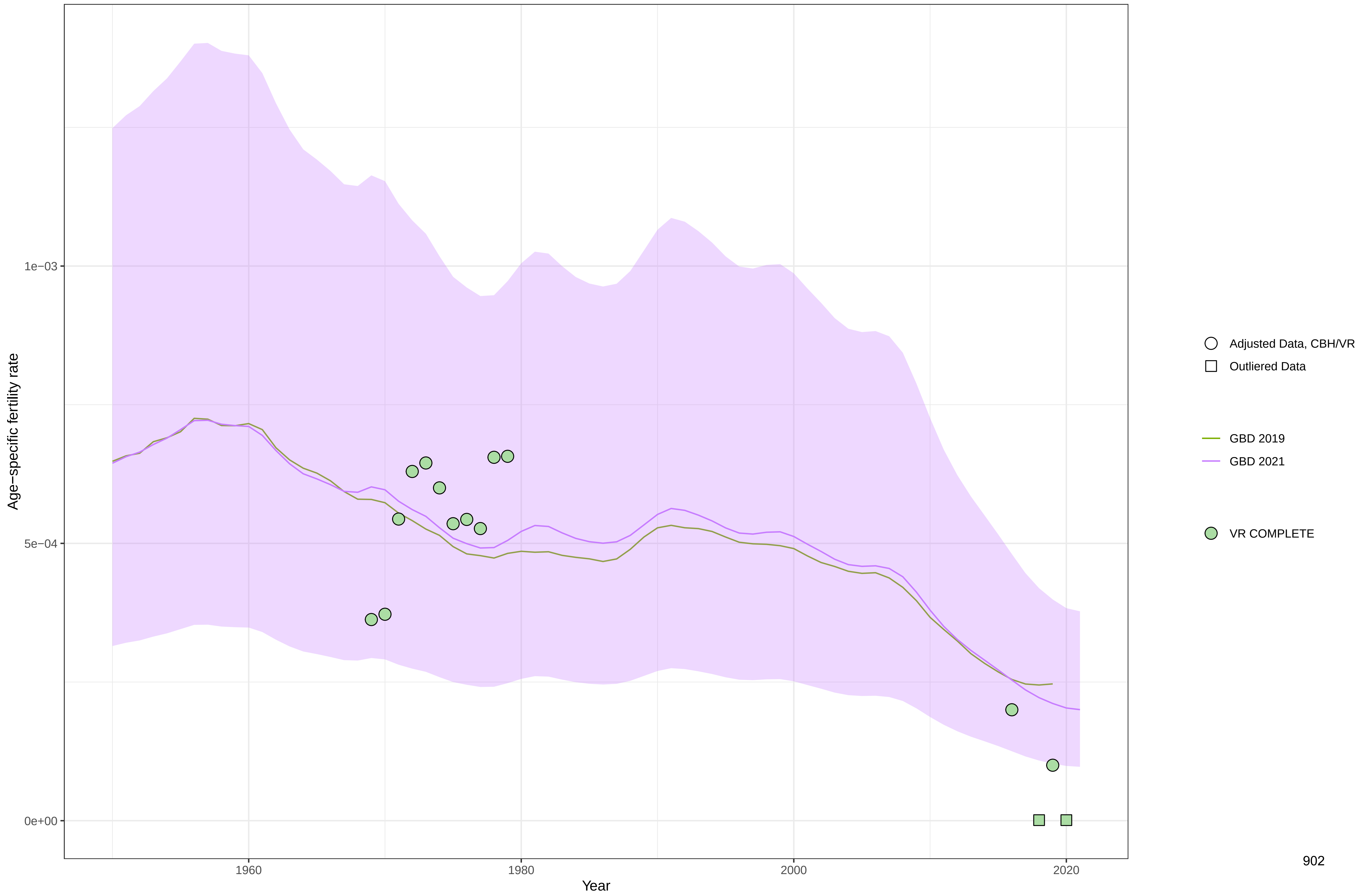

Age−specific fertility fitted trend lines, 1950−2021: Colorado, Ages: 15−19
Data points enclosed with a diamond are reference sources

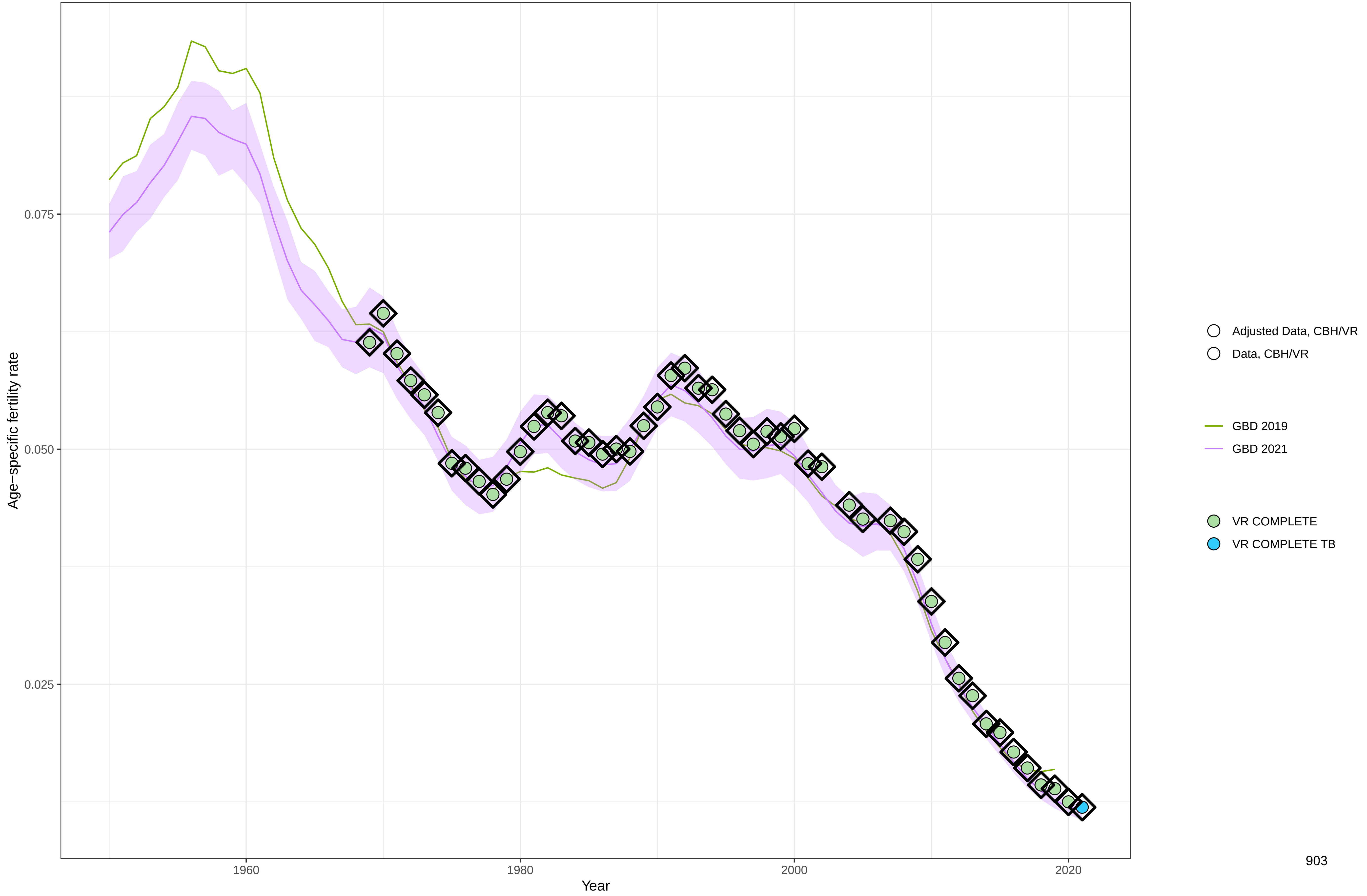

Age−specific fertility fitted trend lines, 1950−2021: Colorado, Ages: 20−24
Data points enclosed with a diamond are reference sources

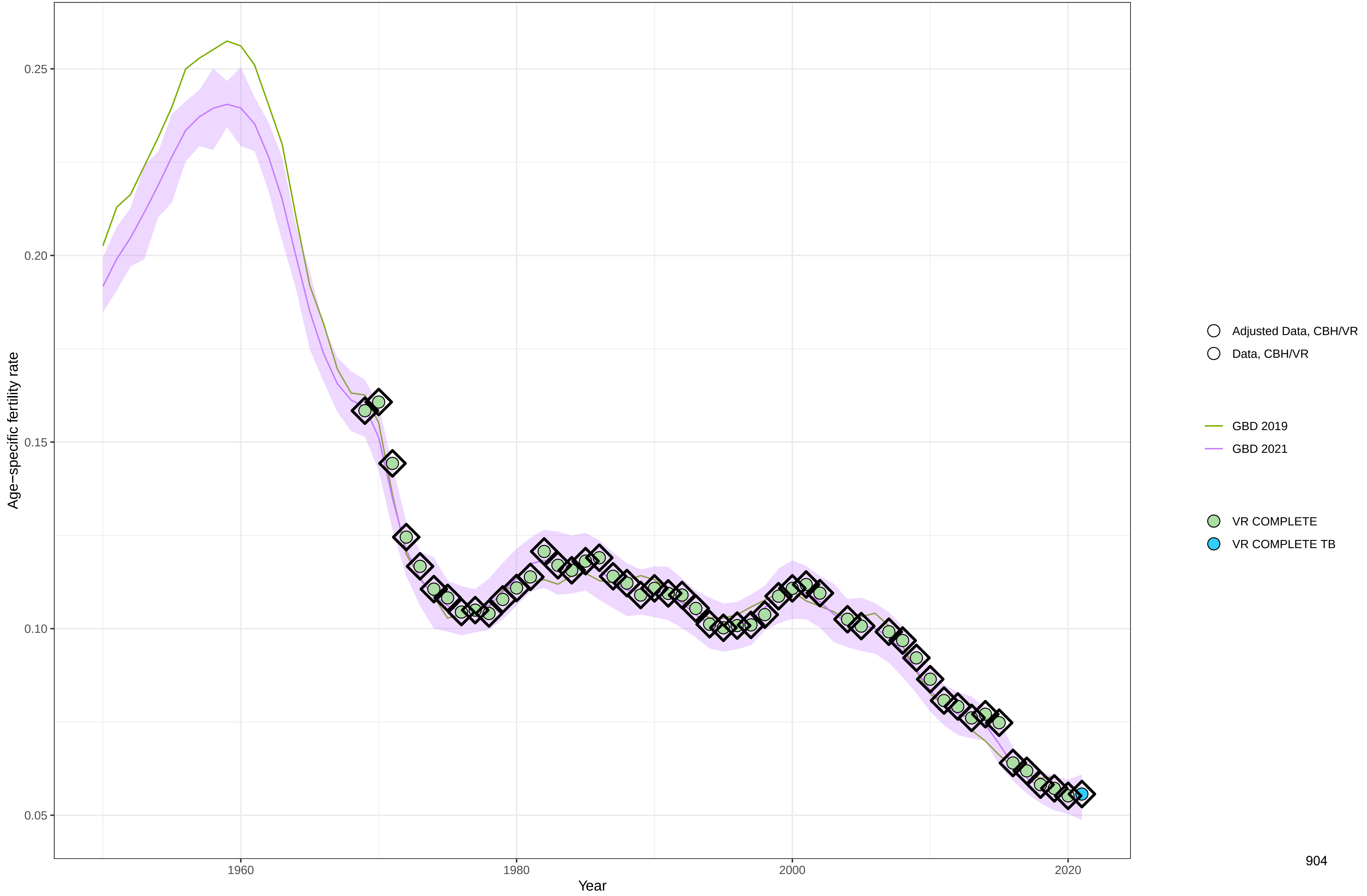

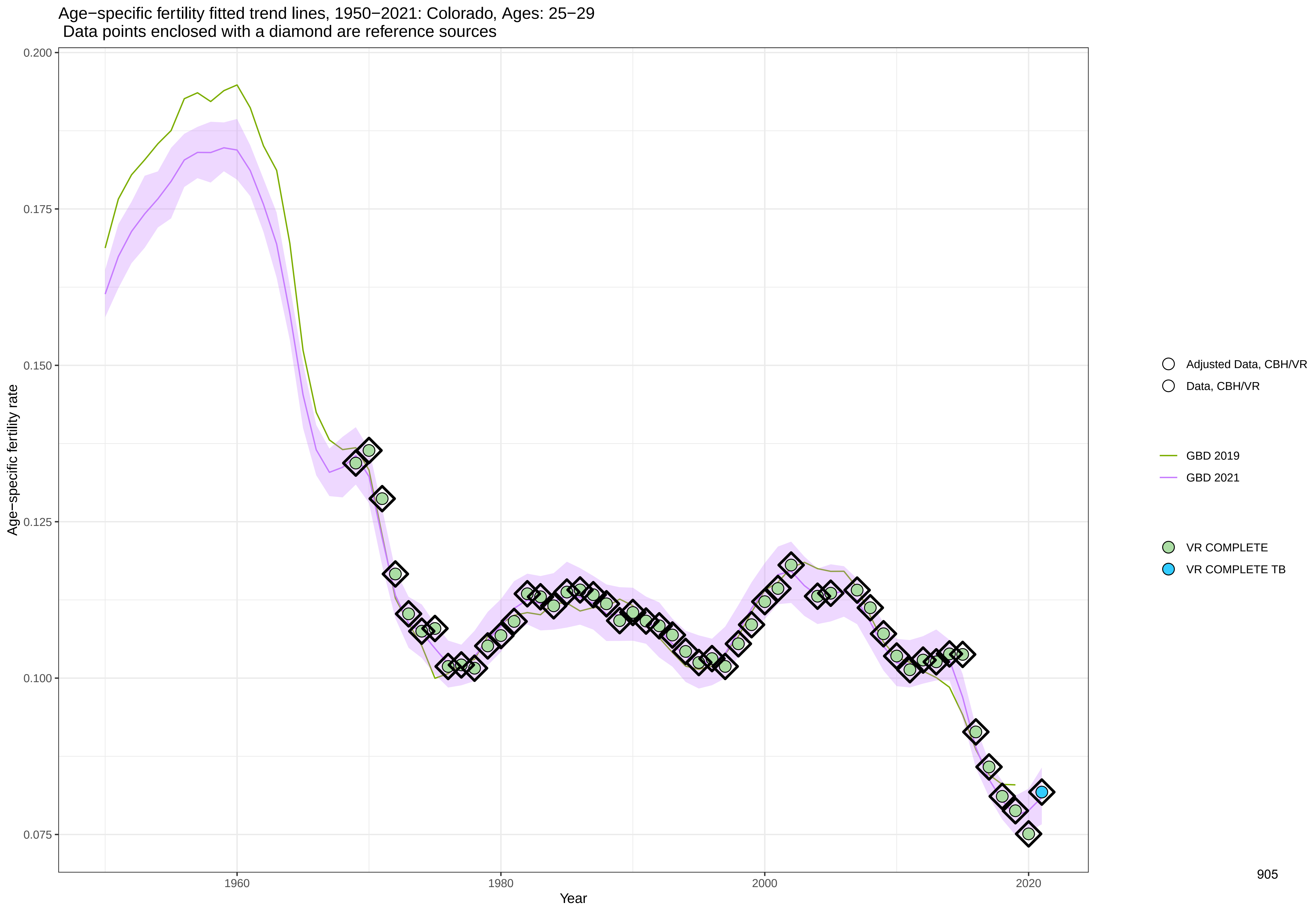

Age−specific fertility fitted trend lines, 1950−2021: Colorado, Ages: 25−29
Data points enclosed with a diamond are reference sources
Age−specific fertility rate
Year
0.200
0.175
0.150
0.125
0.100
0.075
1960
1980
2000
2020
Adjusted Data, CBH/VR
Data, CBH/VR
GBD 2019
GBD 2021
VR COMPLETE
VR COMPLETE TB

Age−specific fertility fitted trend lines, 1950−2021: Colorado, Ages: 30−34
Data points enclosed with a diamond are reference sources

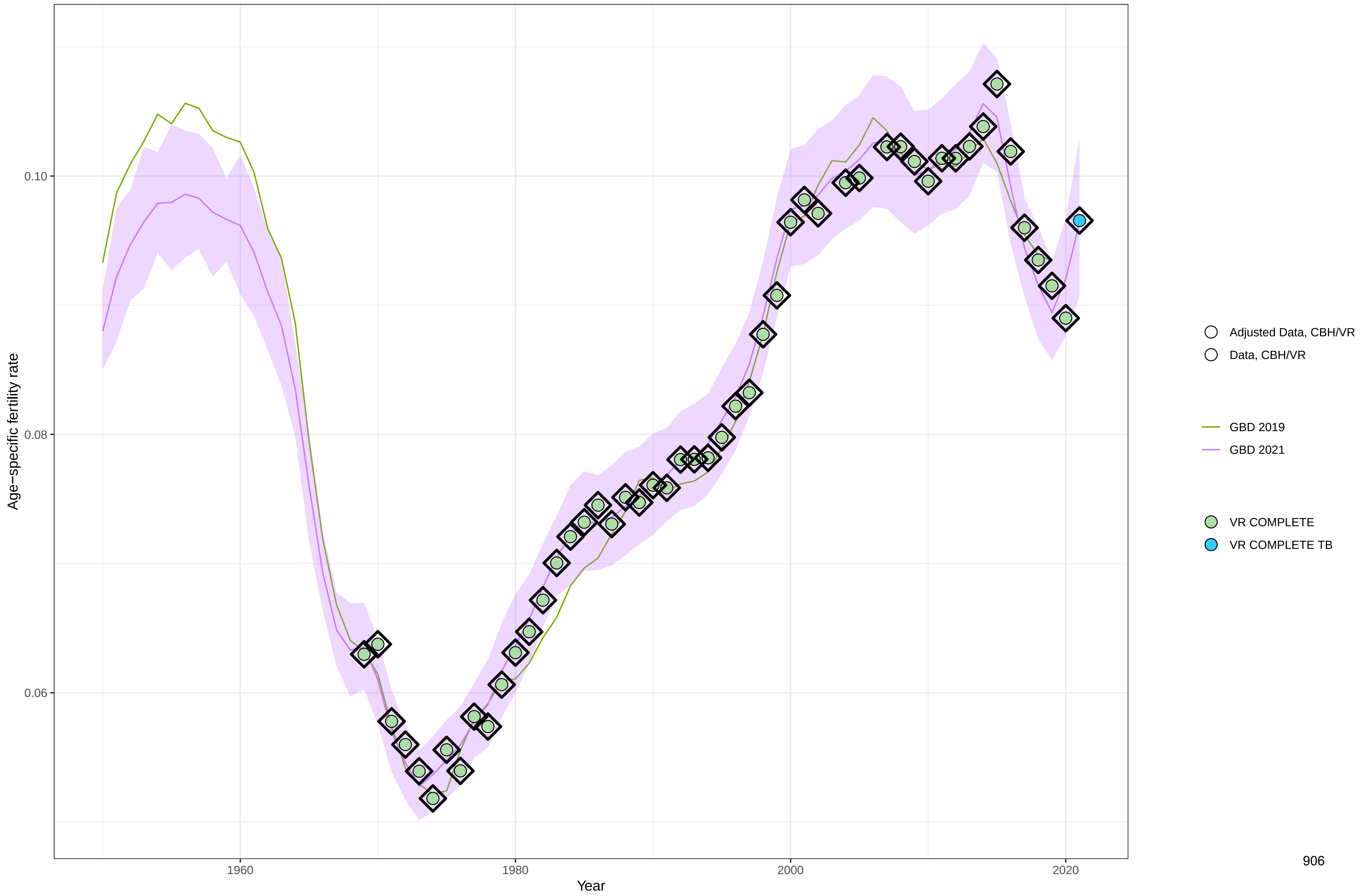

Age−specific fertility fitted trend lines, 1950−2021: Colorado, Ages: 35−39
Data points enclosed with a diamond are reference sources

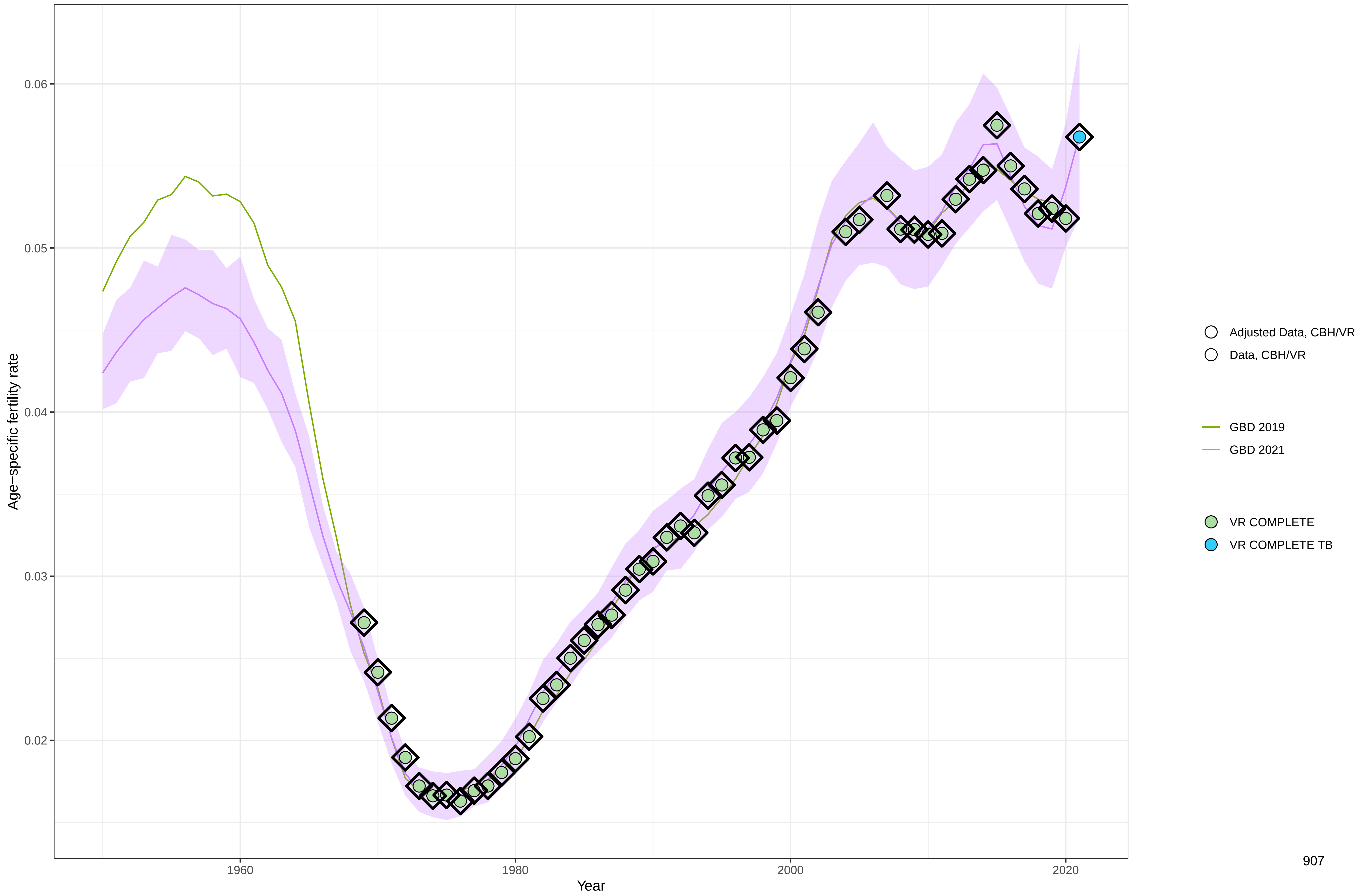

Age−specific fertility fitted trend lines, 1950−2021: Colorado, Ages: 40−44
Data points enclosed with a diamond are reference sources

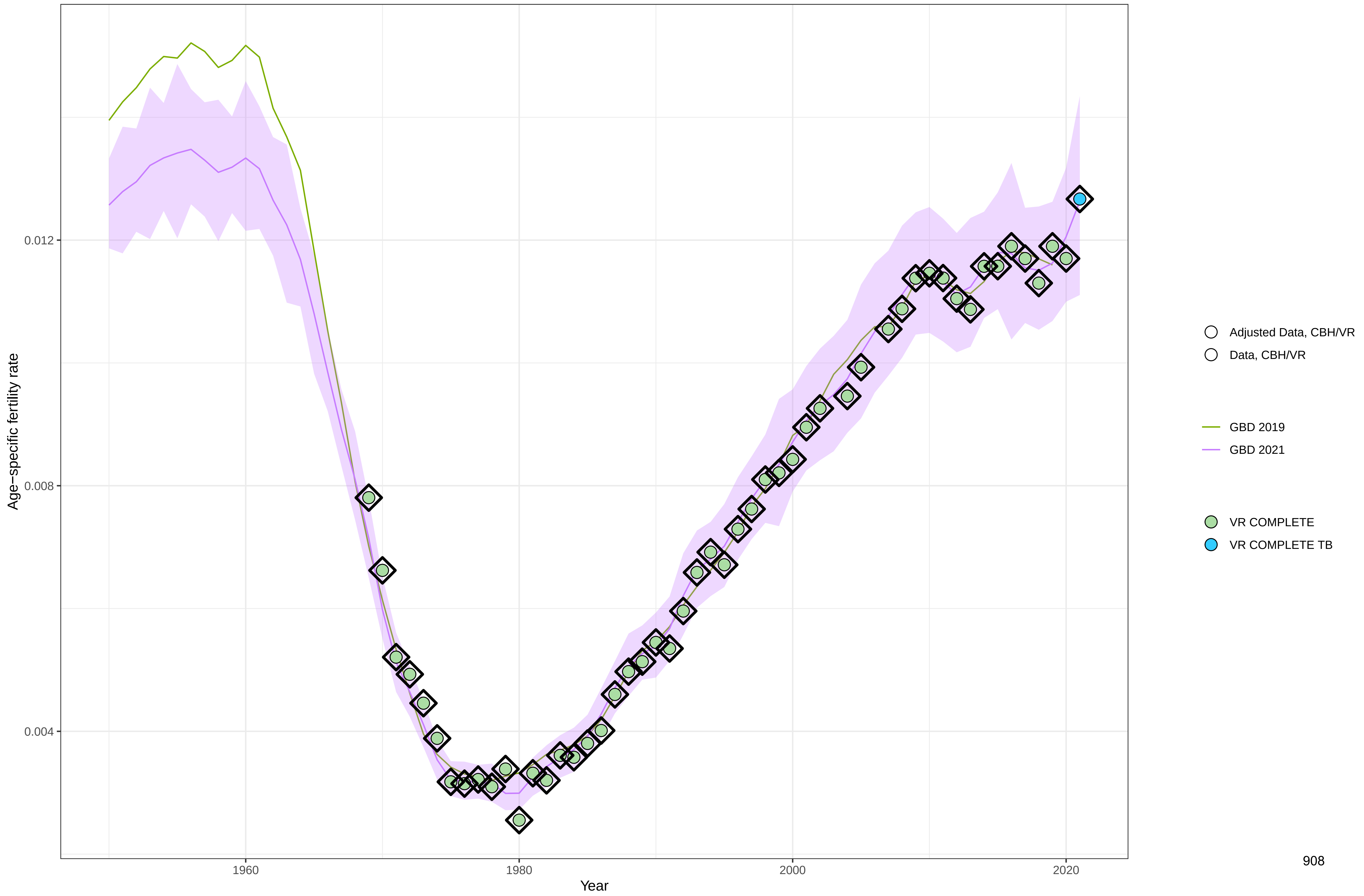

Age−specific fertility fitted trend lines, 1950−2021: Colorado, Ages: 45−49
Data points enclosed with a diamond are reference sources

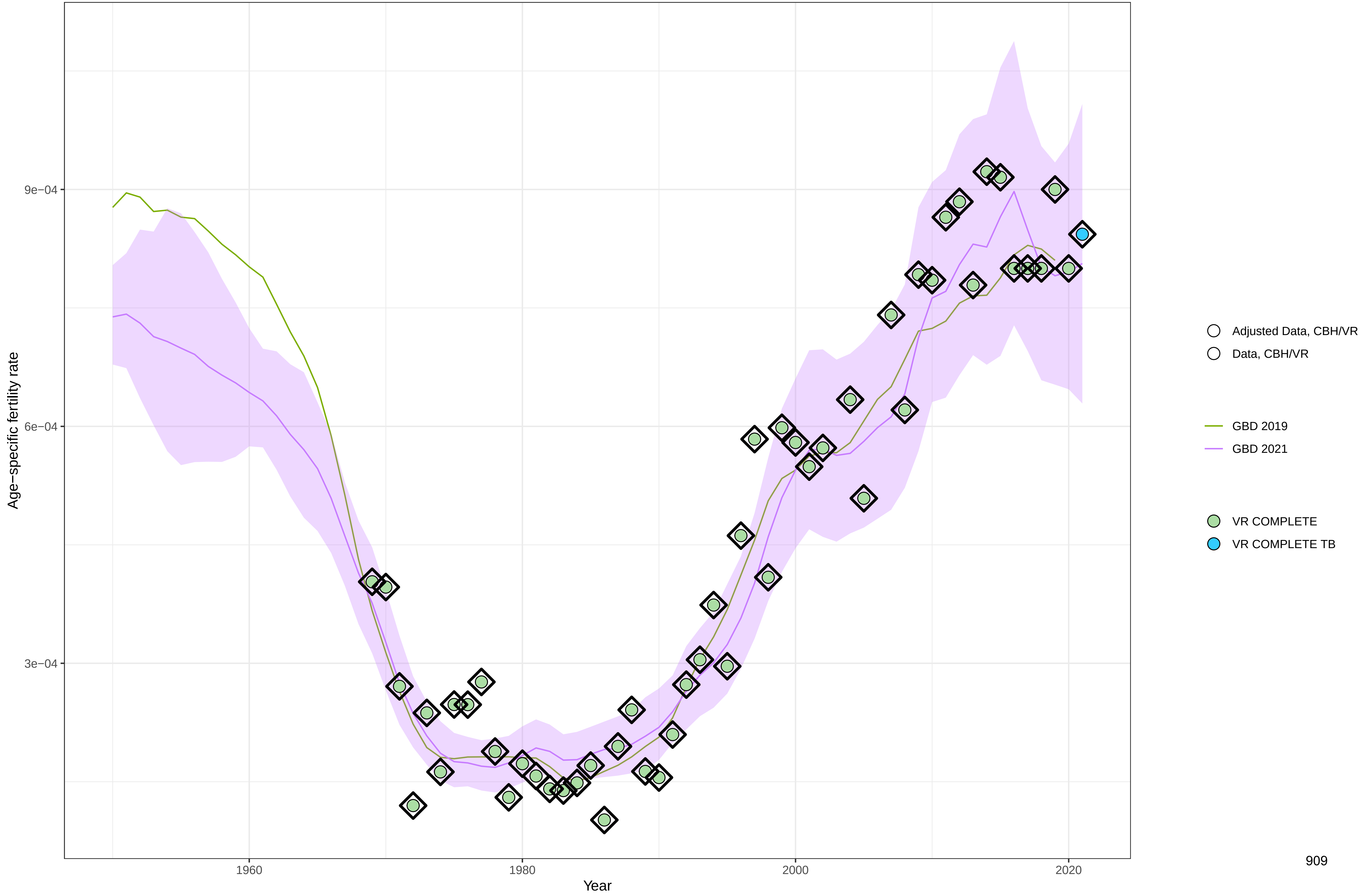

Age−specific fertility fitted trend lines, 1950−2021: Colorado, Ages: 50−54
Data points enclosed with a diamond are reference sources

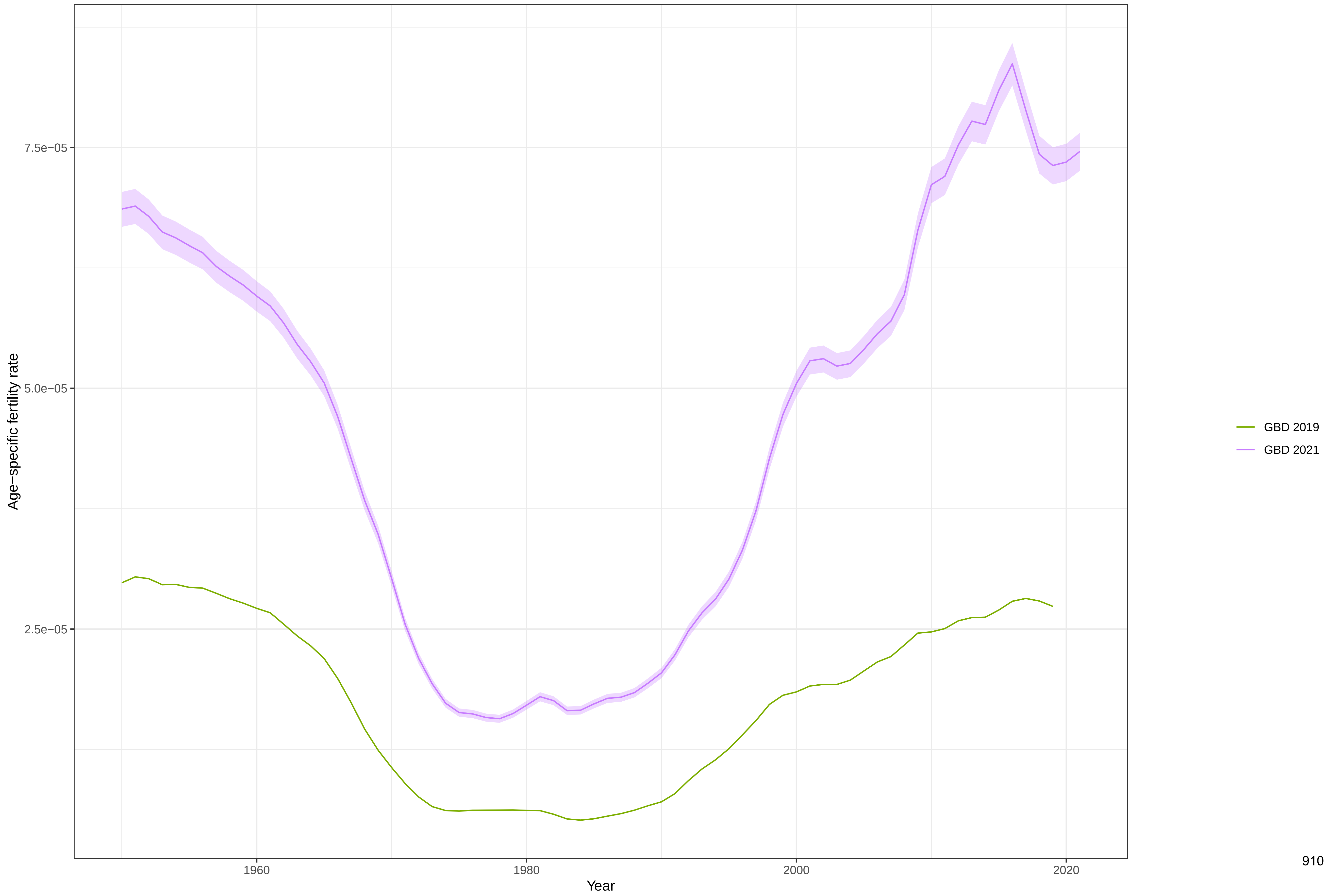

Total fertility rate fitted trend lines, 1950−2021: Colorado TFR
Data points enclosed with a diamond are reference sources

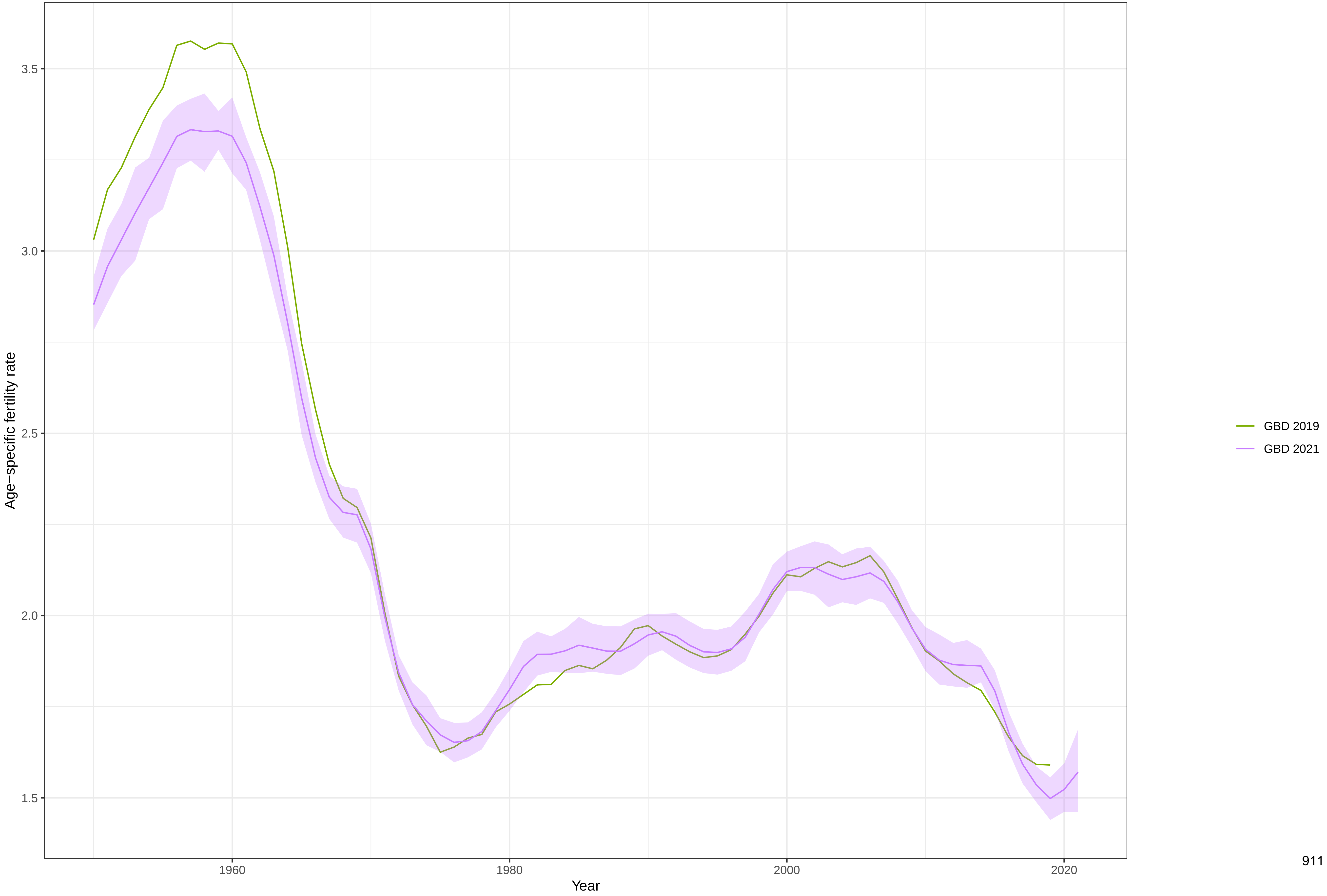

Age−specific fertility fitted trend lines, 1950−2021: Connecticut, Ages: 10−14
Data points enclosed with a diamond are reference sources

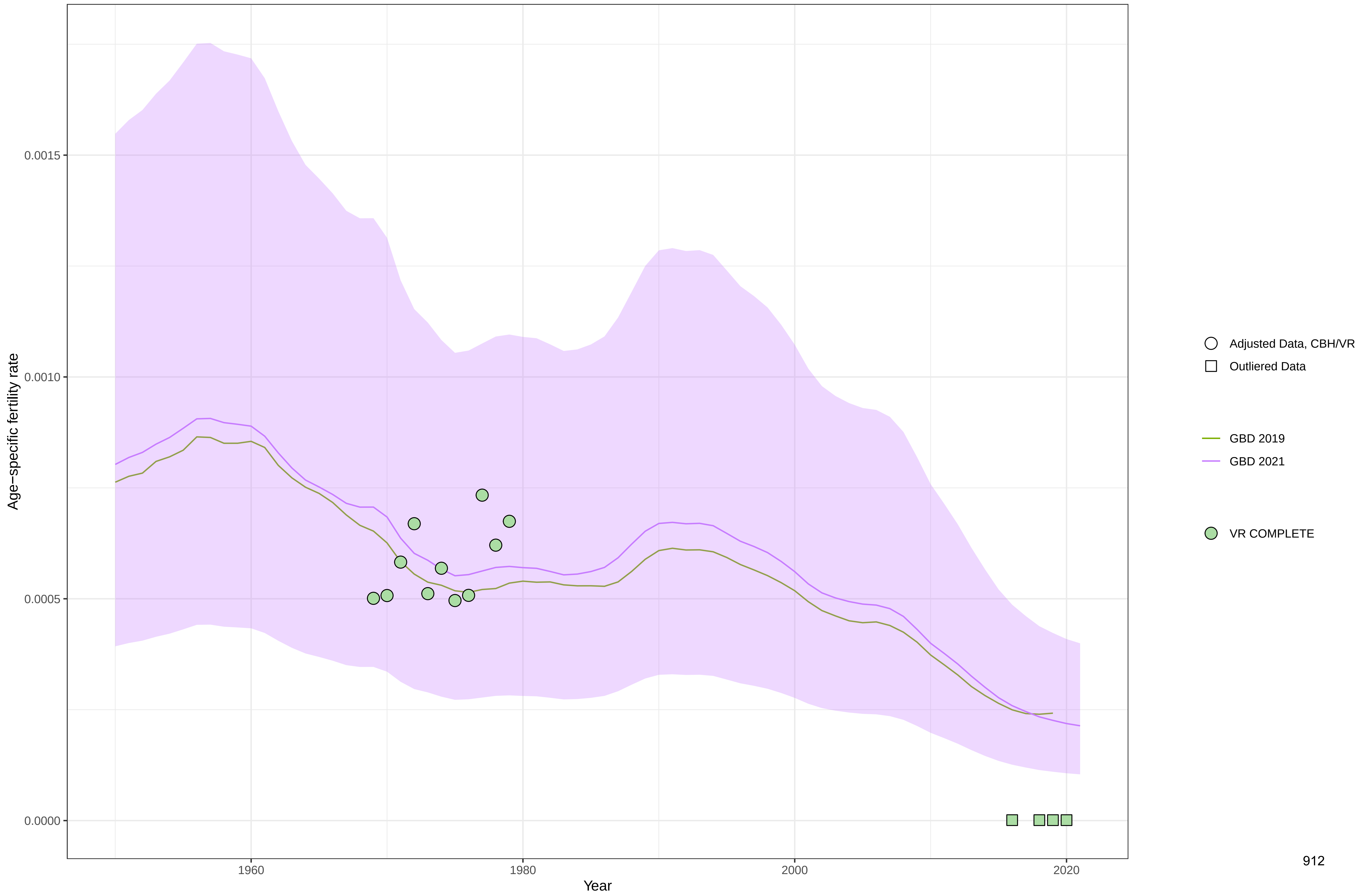

Age−specific fertility fitted trend lines, 1950−2021: Connecticut, Ages: 15−19
Data points enclosed with a diamond are reference sources

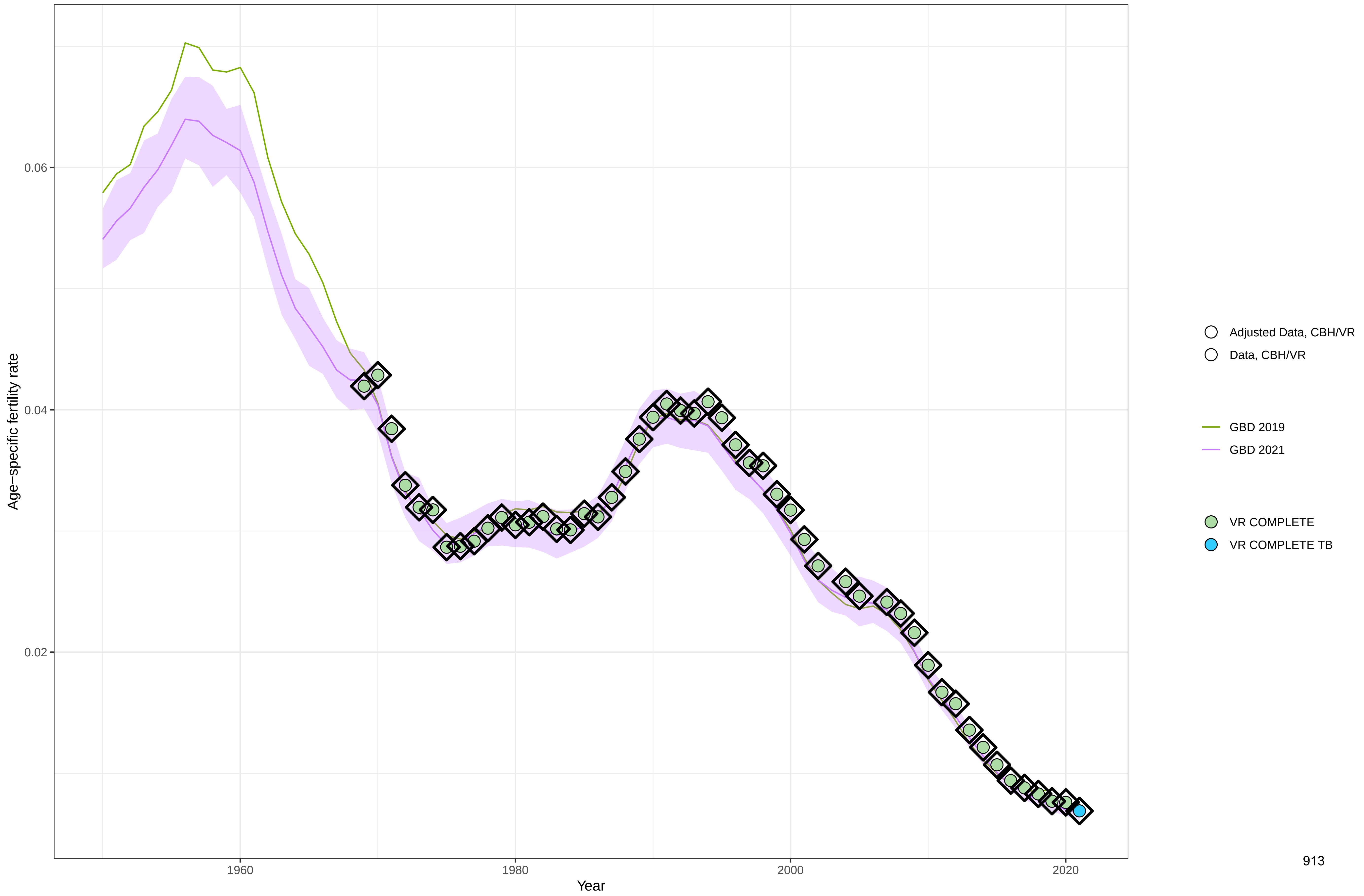

Age−specific fertility fitted trend lines, 1950−2021: Connecticut, Ages: 20−24
Data points enclosed with a diamond are reference sources

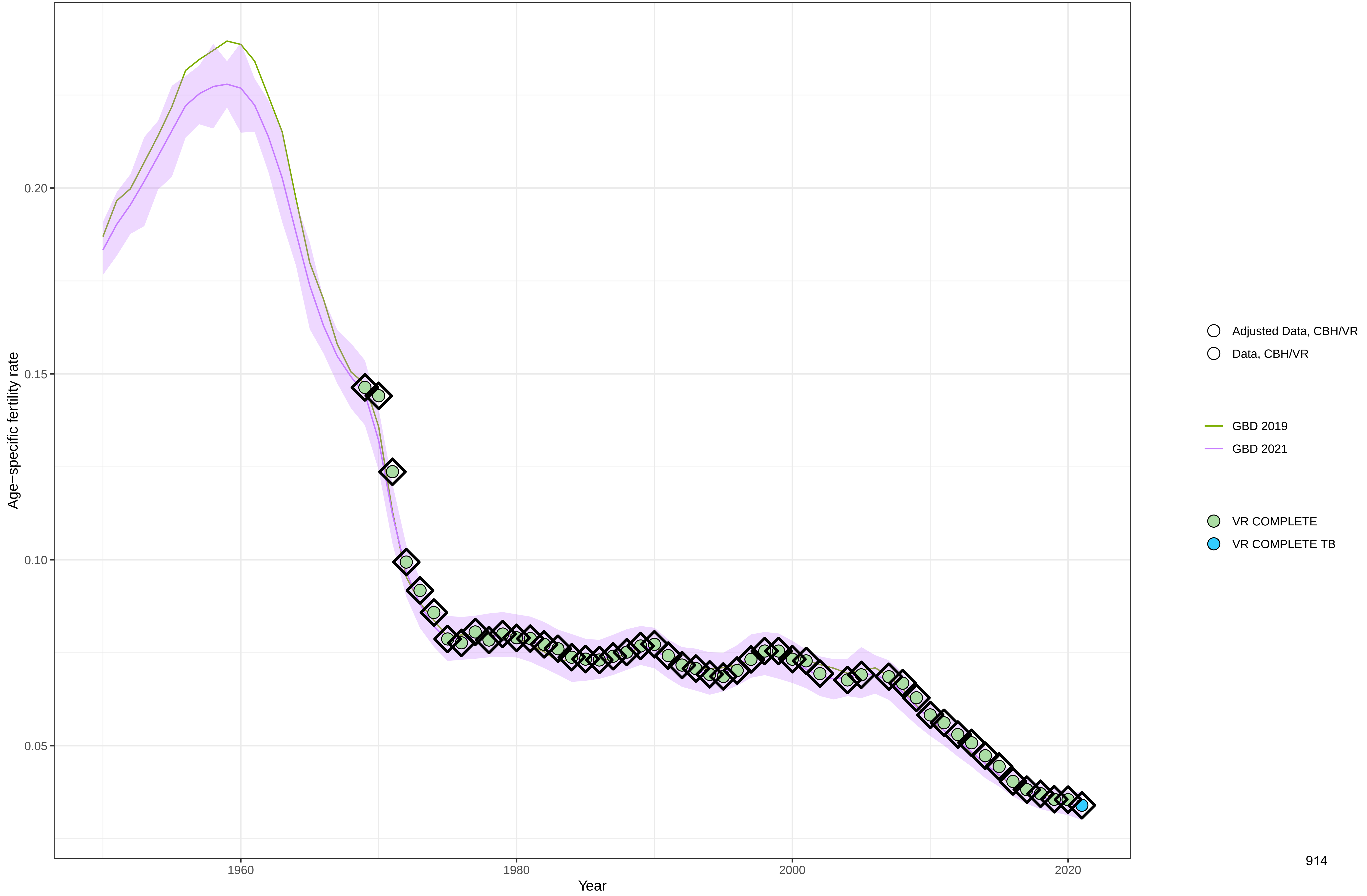

Age−specific fertility fitted trend lines, 1950−2021: Connecticut, Ages: 25−29
Data points enclosed with a diamond are reference sources

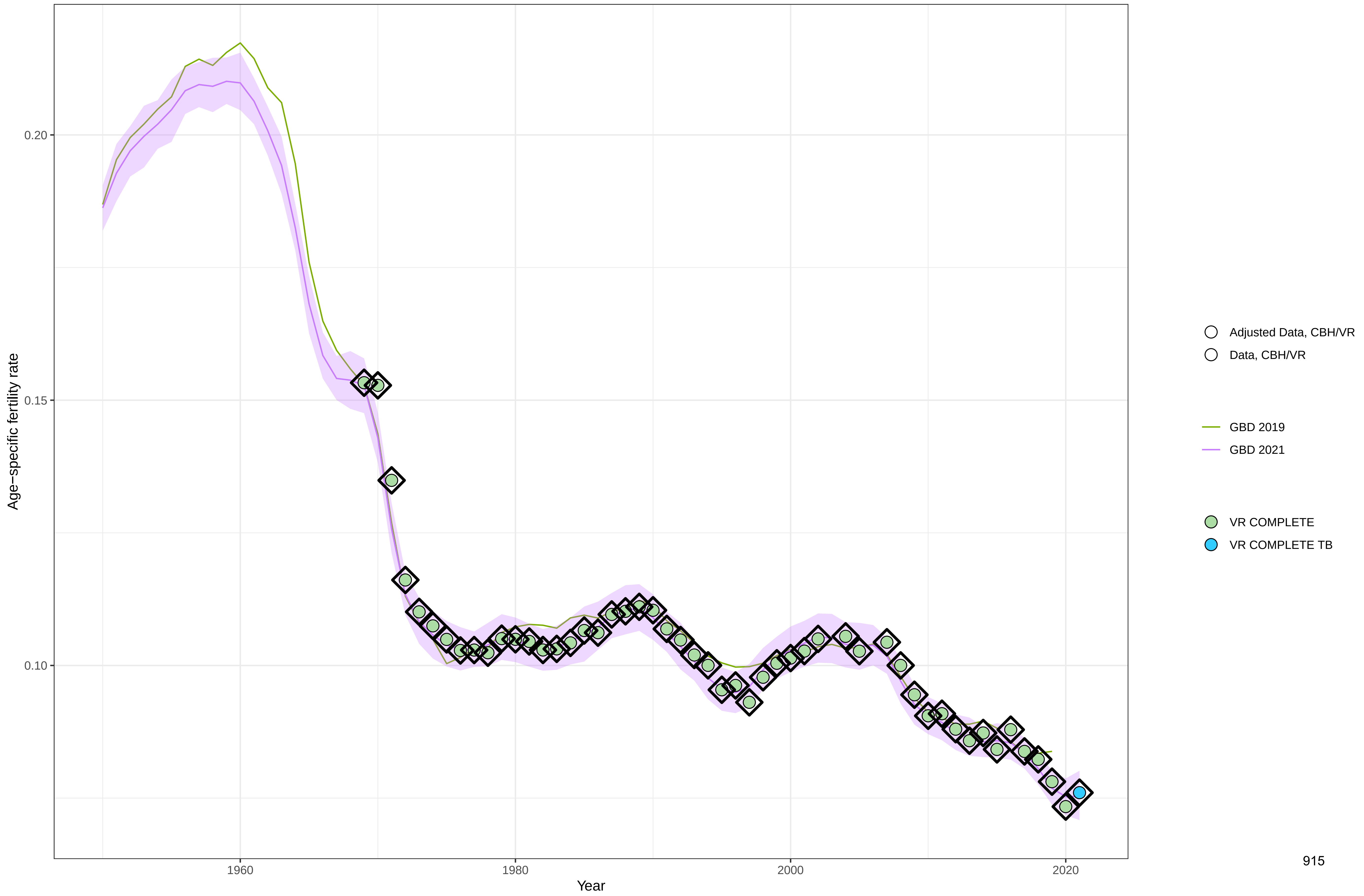

Age−specific fertility fitted trend lines, 1950−2021: Connecticut, Ages: 30−34
Data points enclosed with a diamond are reference sources

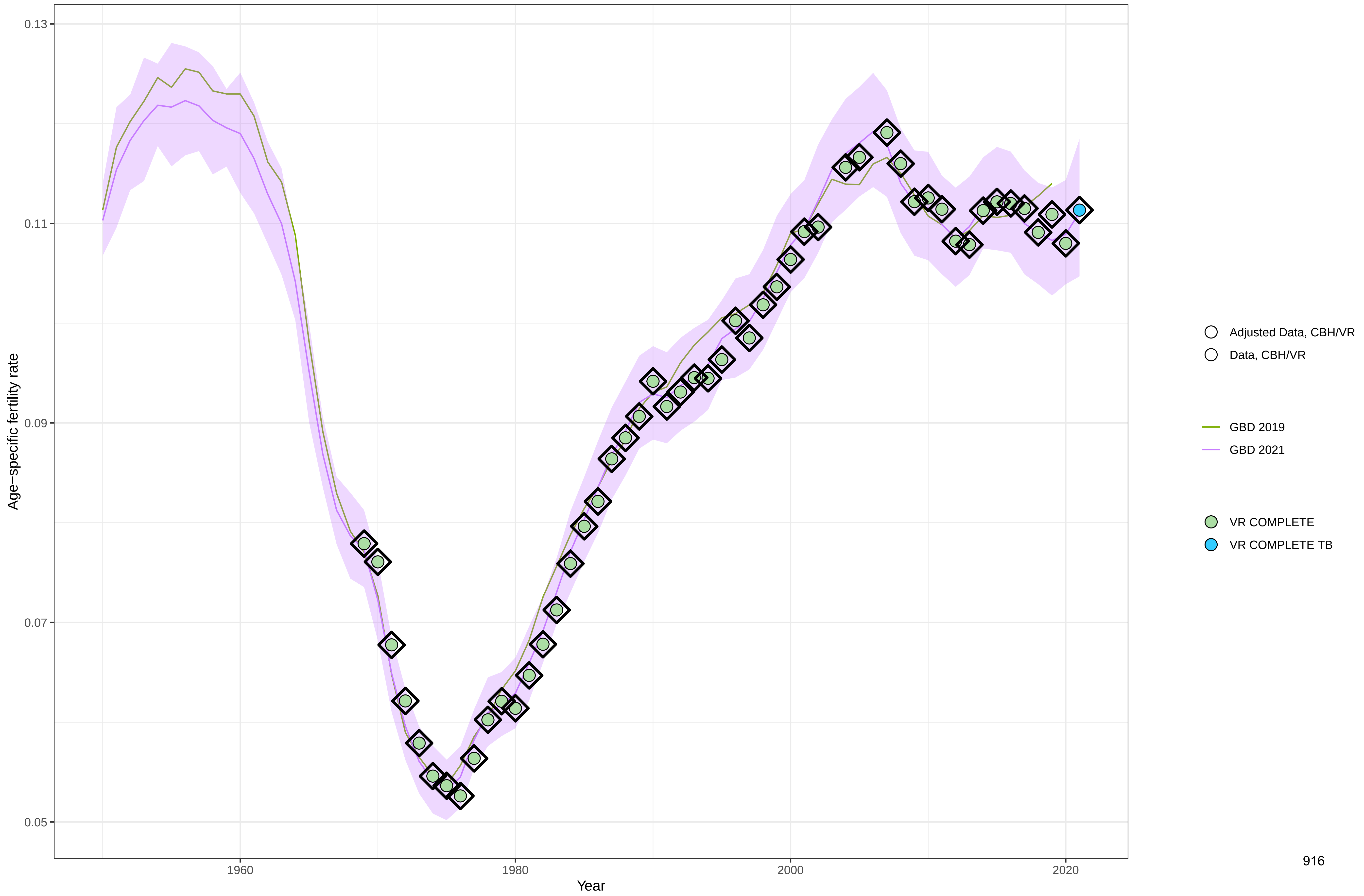

Age−specific fertility fitted trend lines, 1950−2021: Connecticut, Ages: 35−39
Data points enclosed with a diamond are reference sources

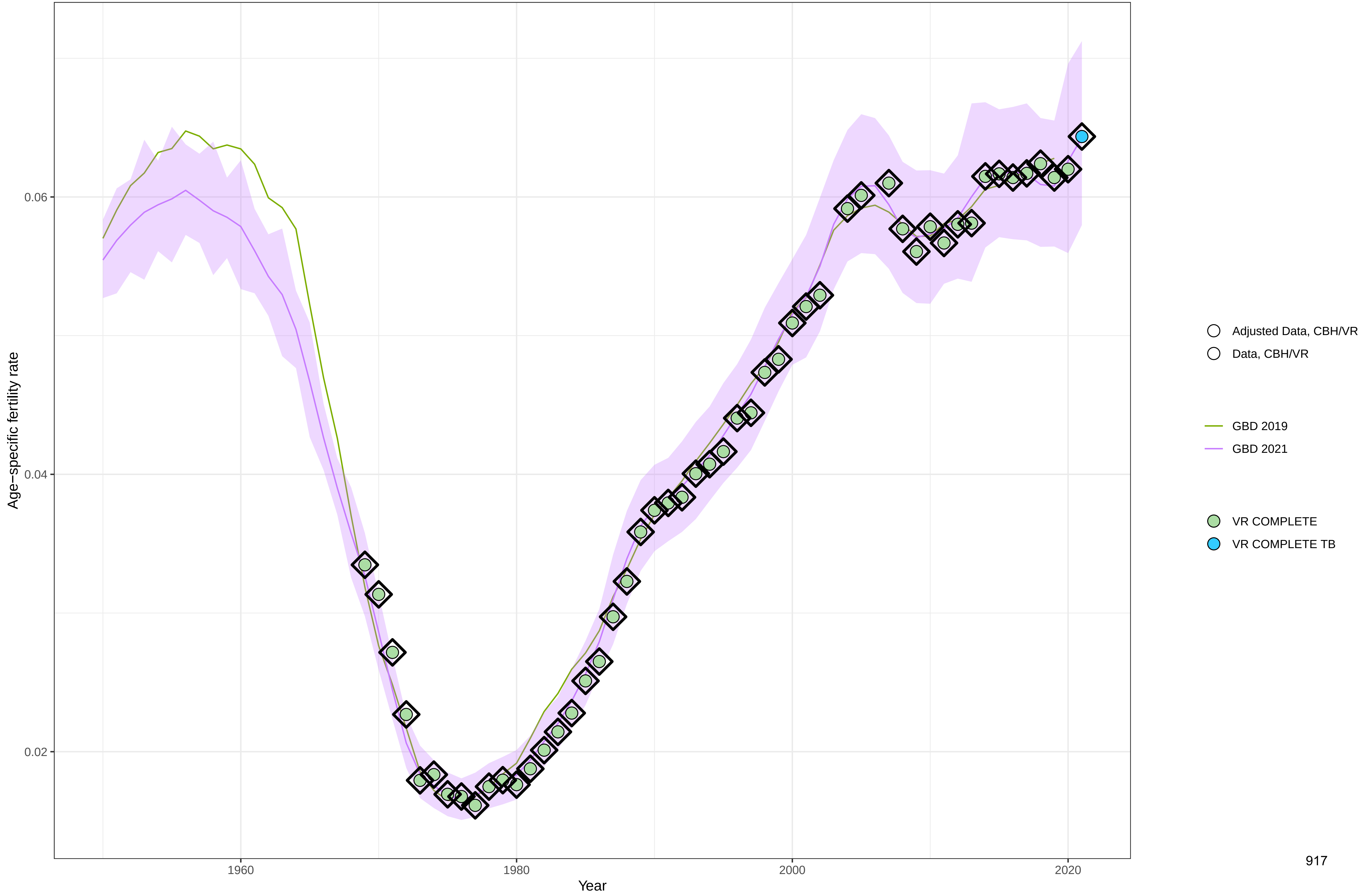

Age−specific fertility fitted trend lines, 1950−2021: Connecticut, Ages: 40−44
Data points enclosed with a diamond are reference sources

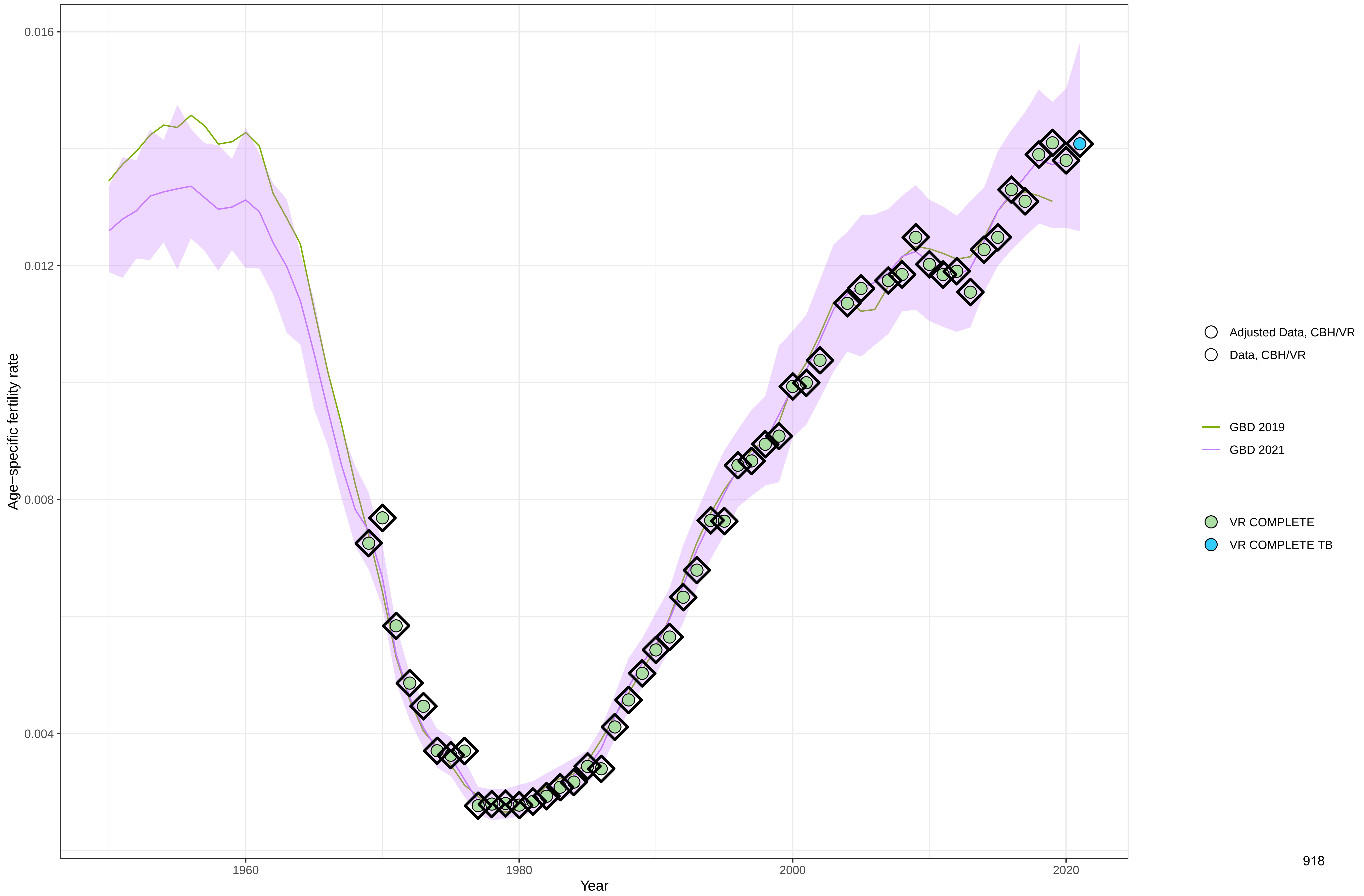

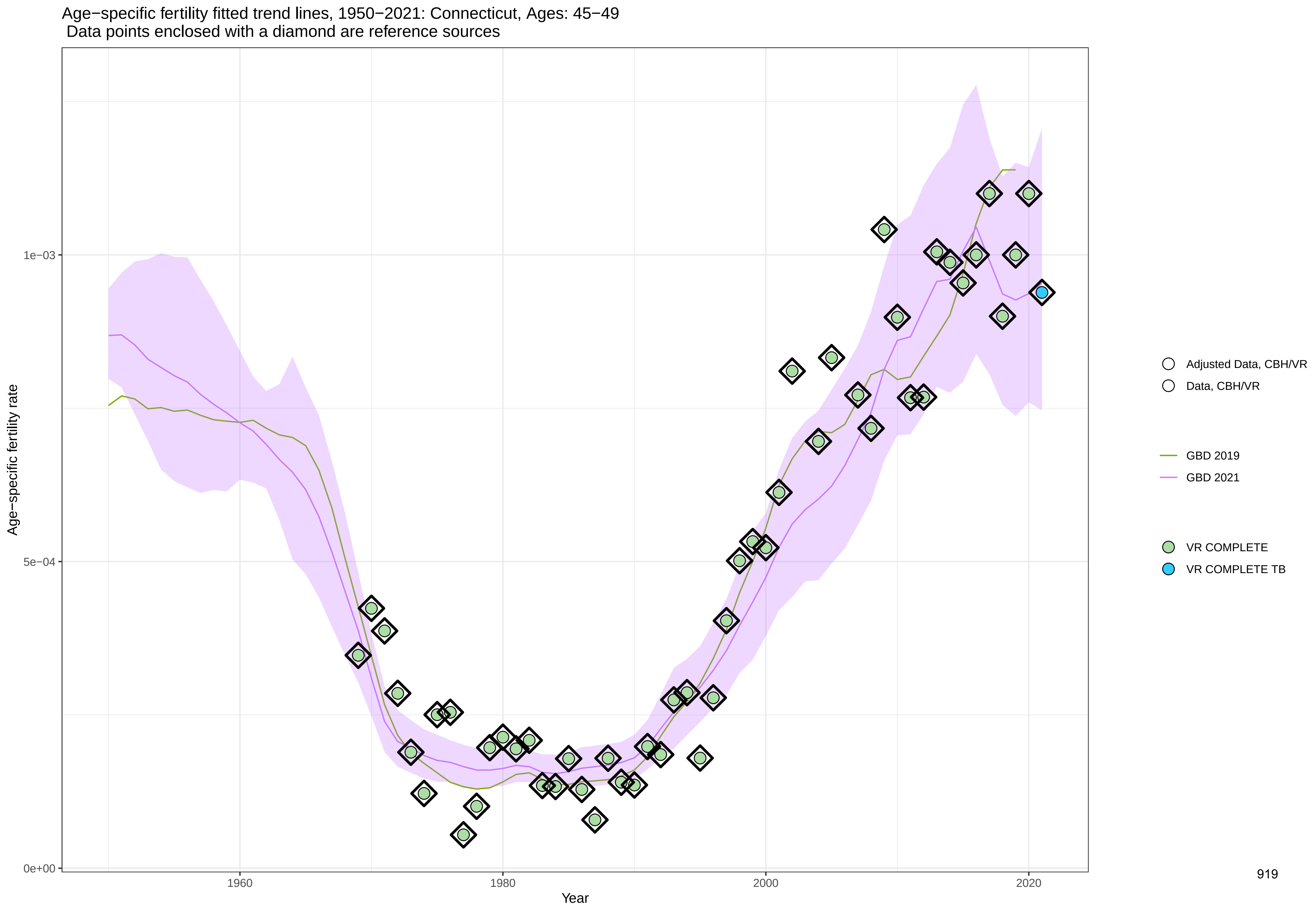

Age−specific fertility fitted trend lines, 1950−2021: Connecticut, Ages: 45−49
Data points enclosed with a diamond are reference sources
Age−specific fertility rate
1e−03
5e−04
0e+00
1960
1980
2000
2020
Year
Adjusted Data, CBH/VR
Data, CBH/VR
GBD 2019
GBD 2021
VR COMPLETE
VR COMPLETE TB

Age−specific fertility fitted trend lines, 1950−2021: Connecticut, Ages: 50−54
Data points enclosed with a diamond are reference sources

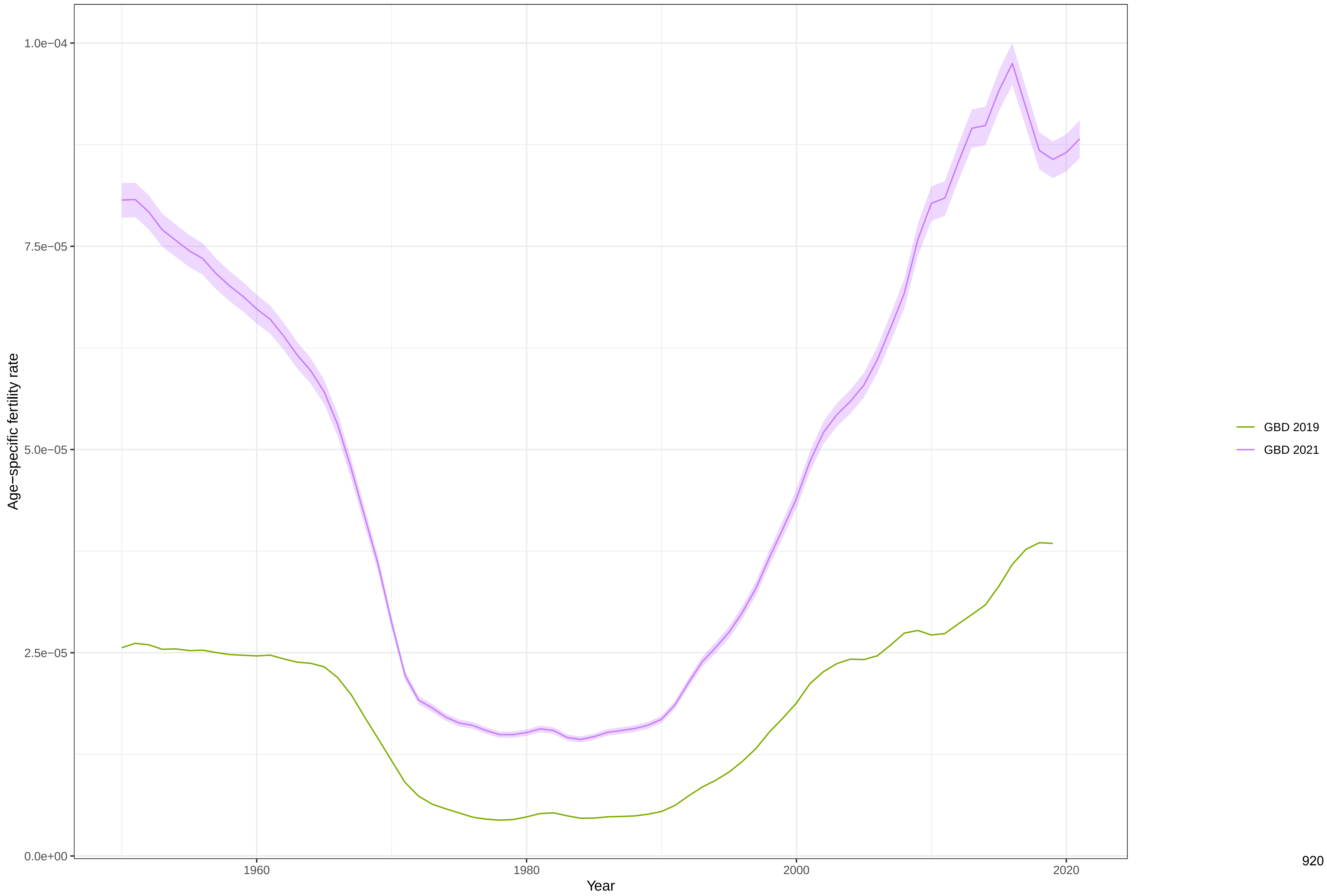

Total fertility rate fitted trend lines, 1950−2021: Connecticut TFR
Data points enclosed with a diamond are reference sources

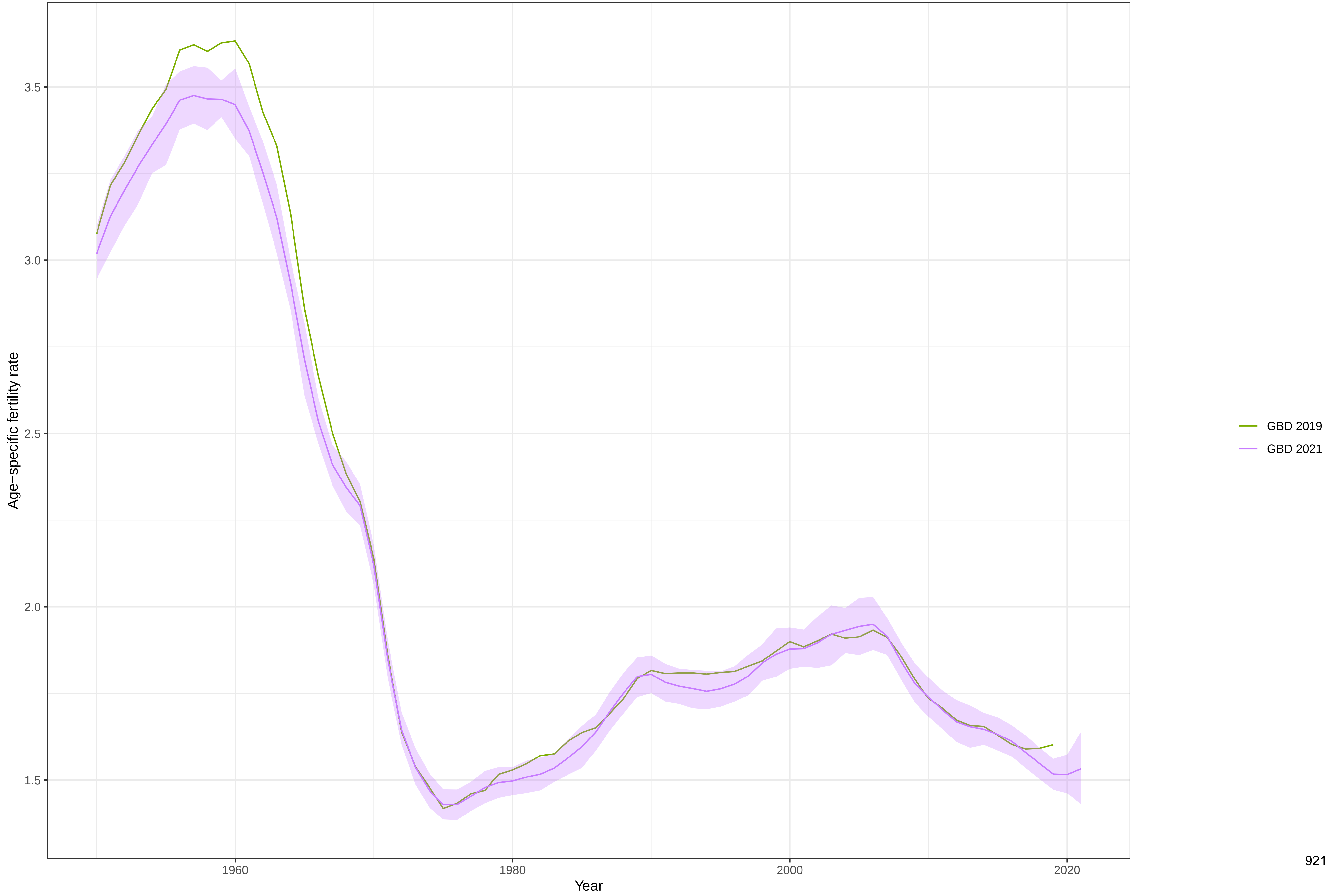

Age−specific fertility fitted trend lines, 1950−2021: Delaware, Ages: 10−14
Data points enclosed with a diamond are reference sources

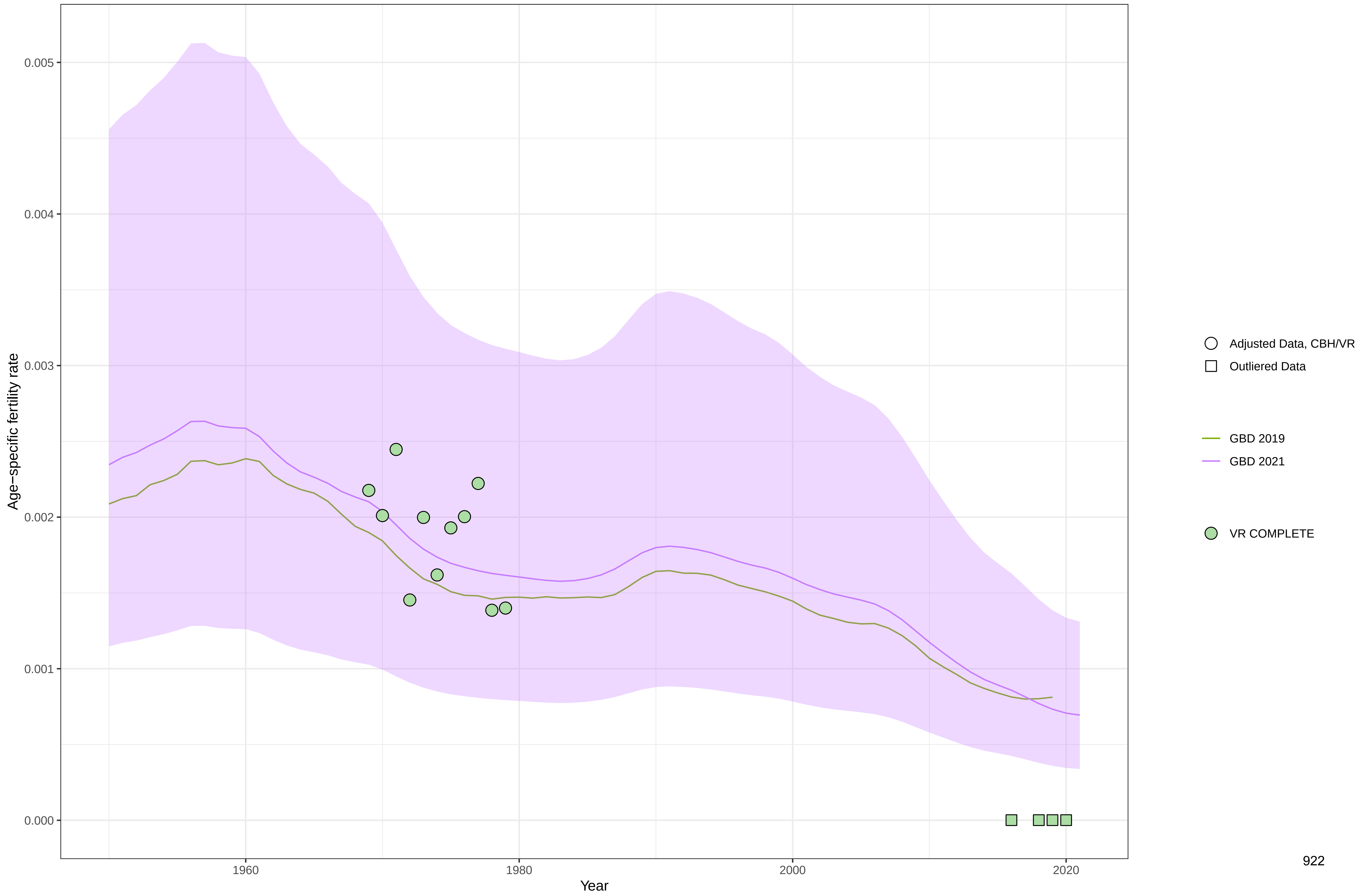

Age−specific fertility fitted trend lines, 1950−2021: Delaware, Ages: 15−19
Data points enclosed with a diamond are reference sources

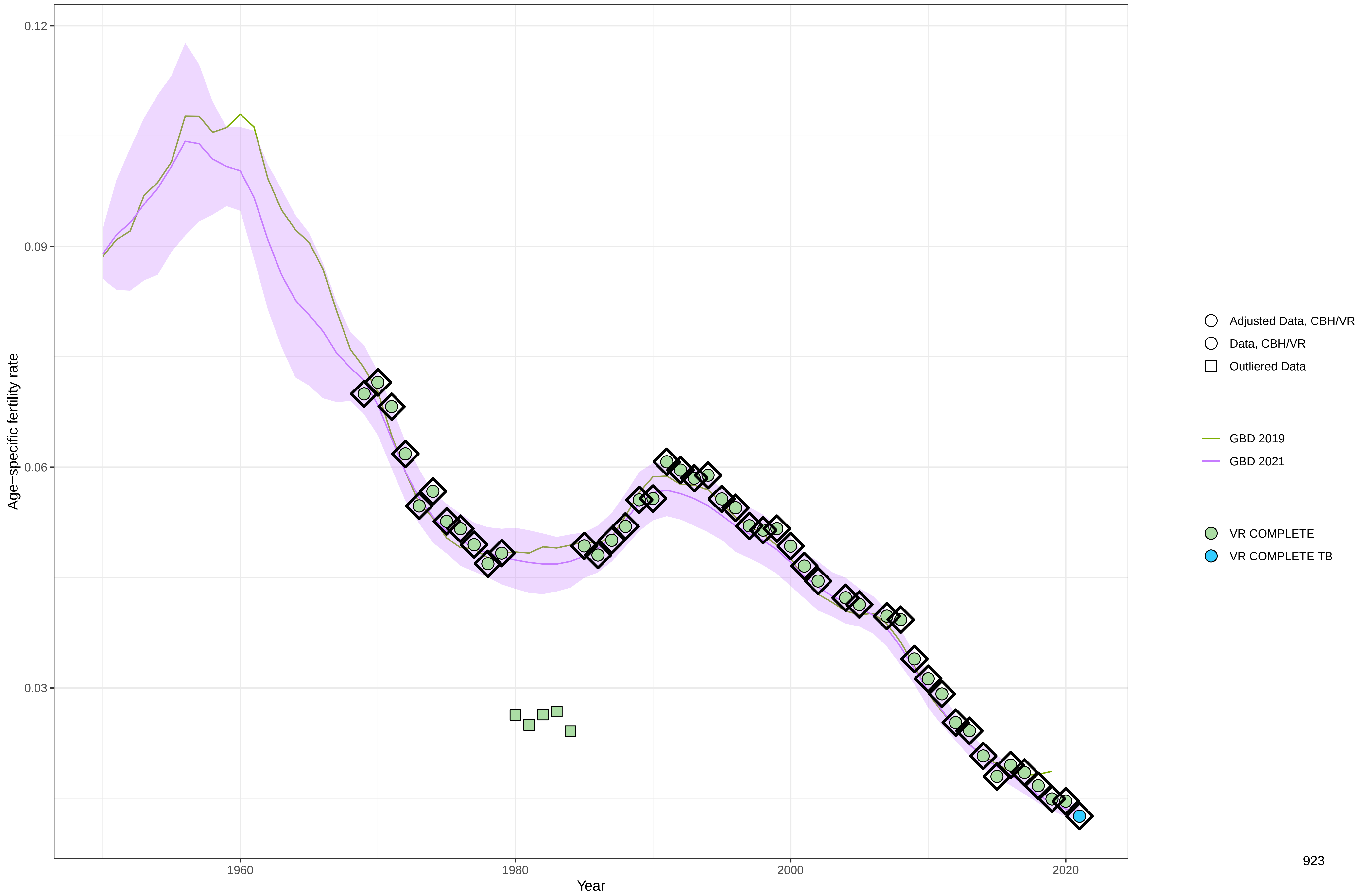

Age−specific fertility fitted trend lines, 1950−2021: Delaware, Ages: 20−24
Data points enclosed with a diamond are reference sources

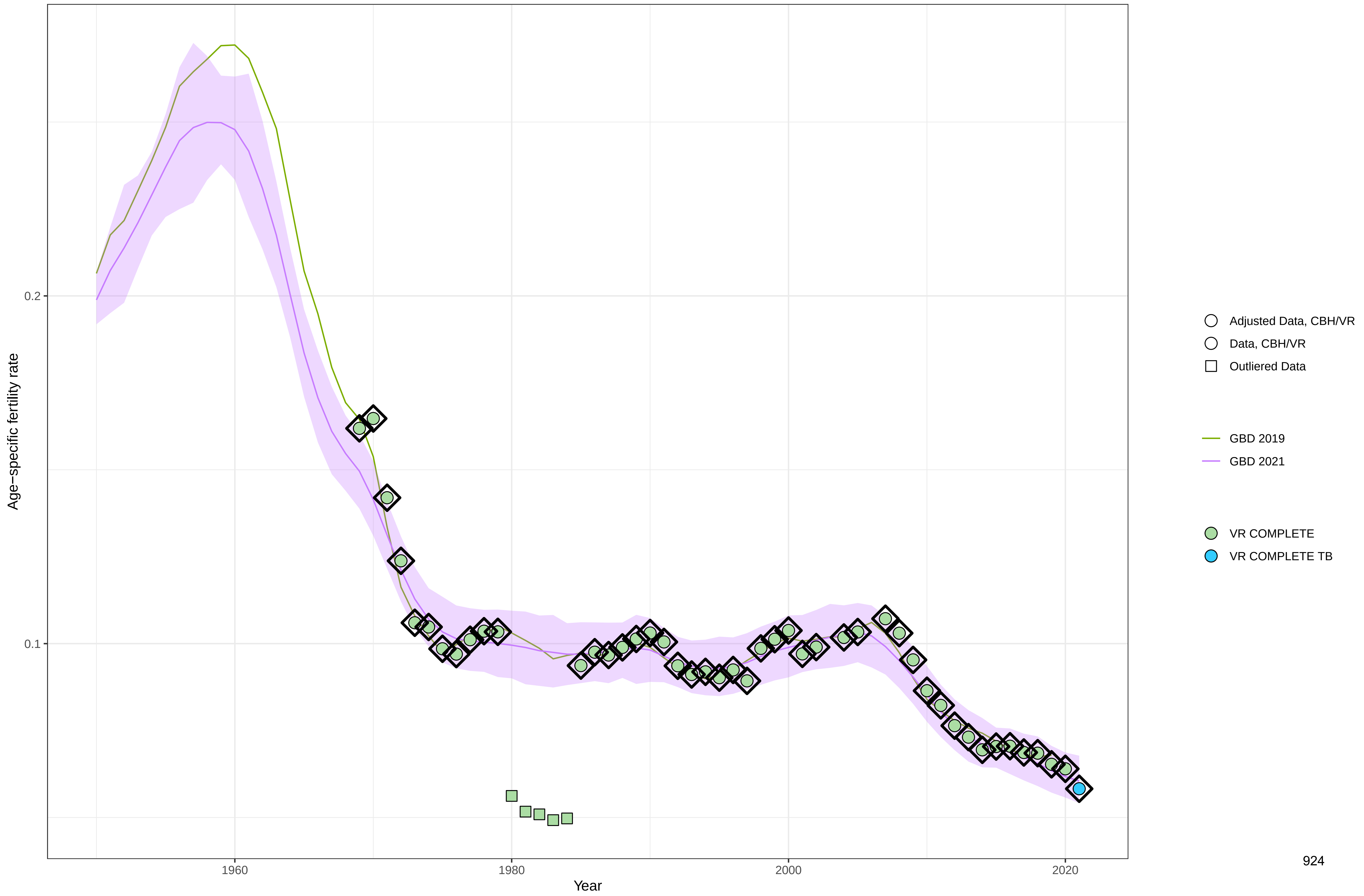

Age−specific fertility fitted trend lines, 1950−2021: Delaware, Ages: 25−29
Data points enclosed with a diamond are reference sources

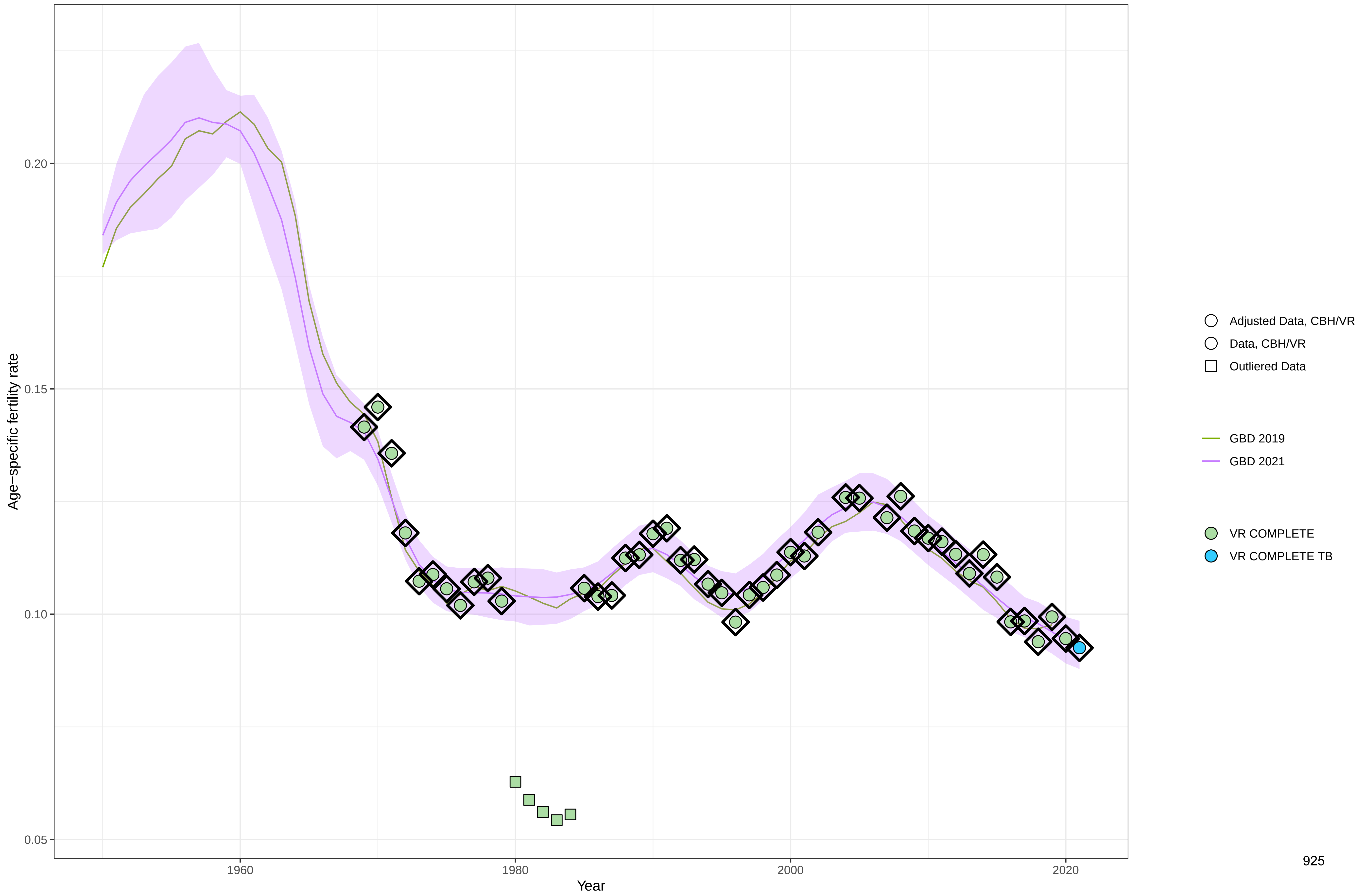

Age−specific fertility fitted trend lines, 1950−2021: Delaware, Ages: 30−34
Data points enclosed with a diamond are reference sources

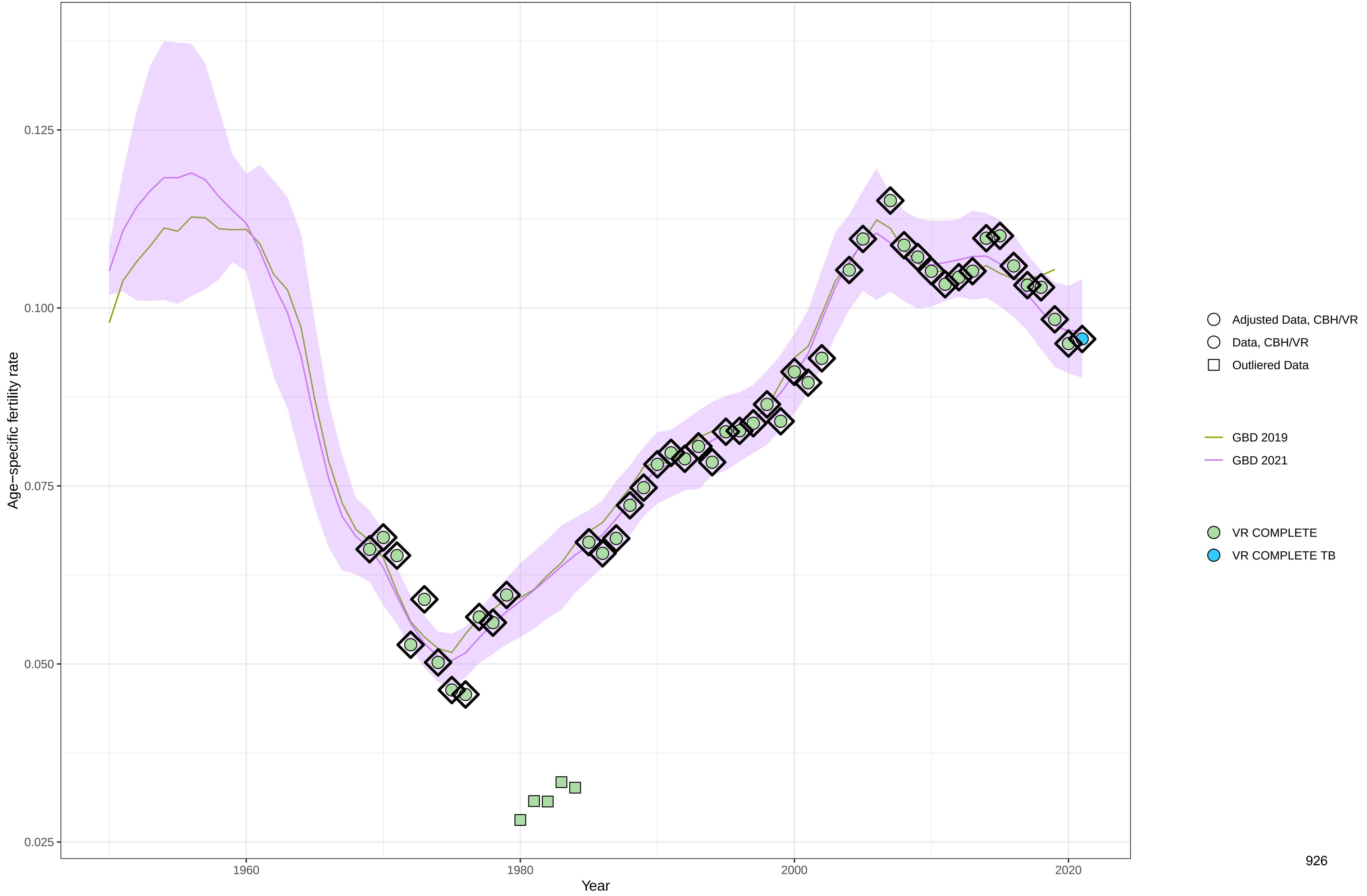

Age−specific fertility fitted trend lines, 1950−2021: Delaware, Ages: 35−39
Data points enclosed with a diamond are reference sources

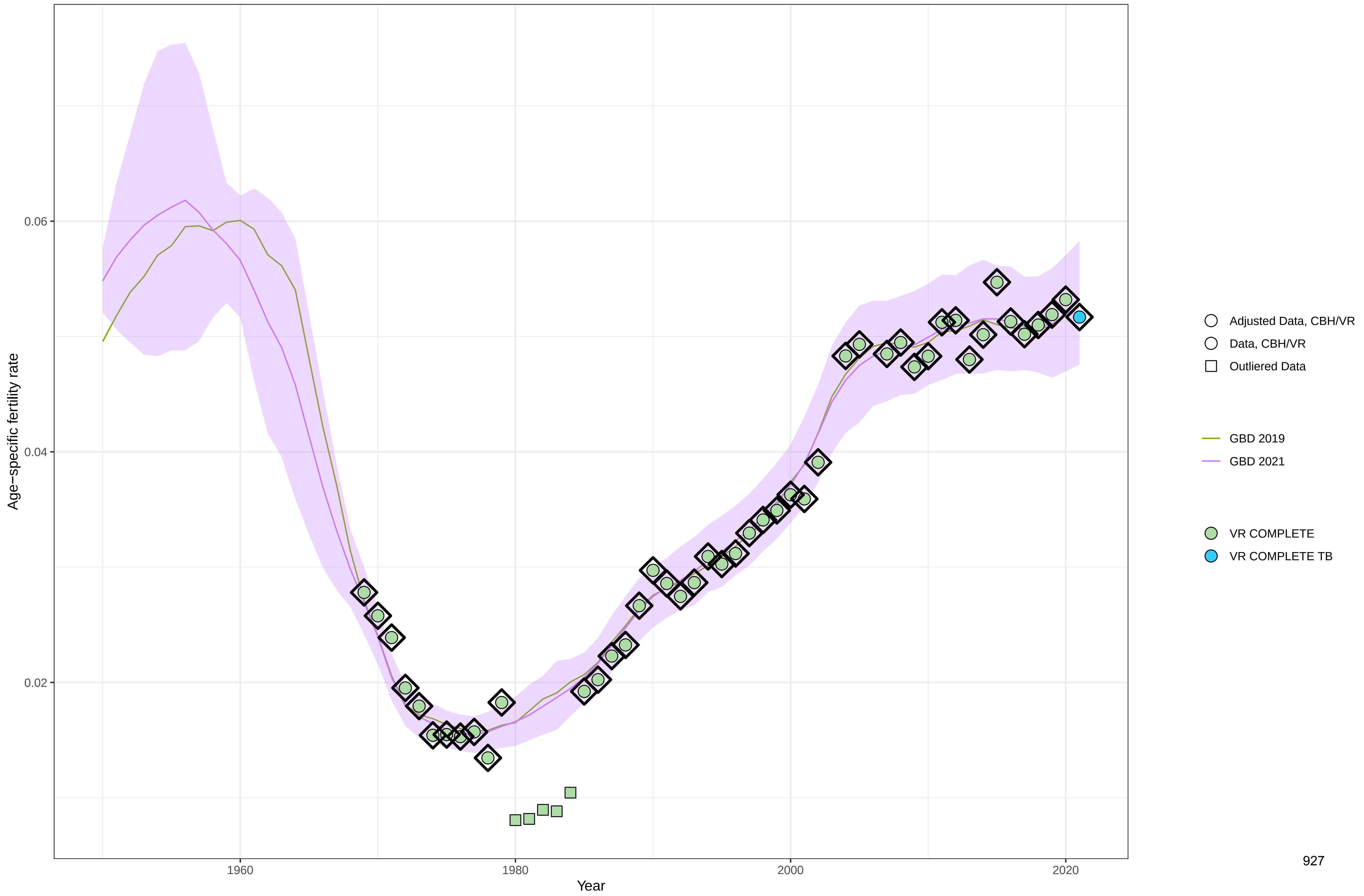

Age−specific fertility fitted trend lines, 1950−2021: Delaware, Ages: 40−44
Data points enclosed with a diamond are reference sources

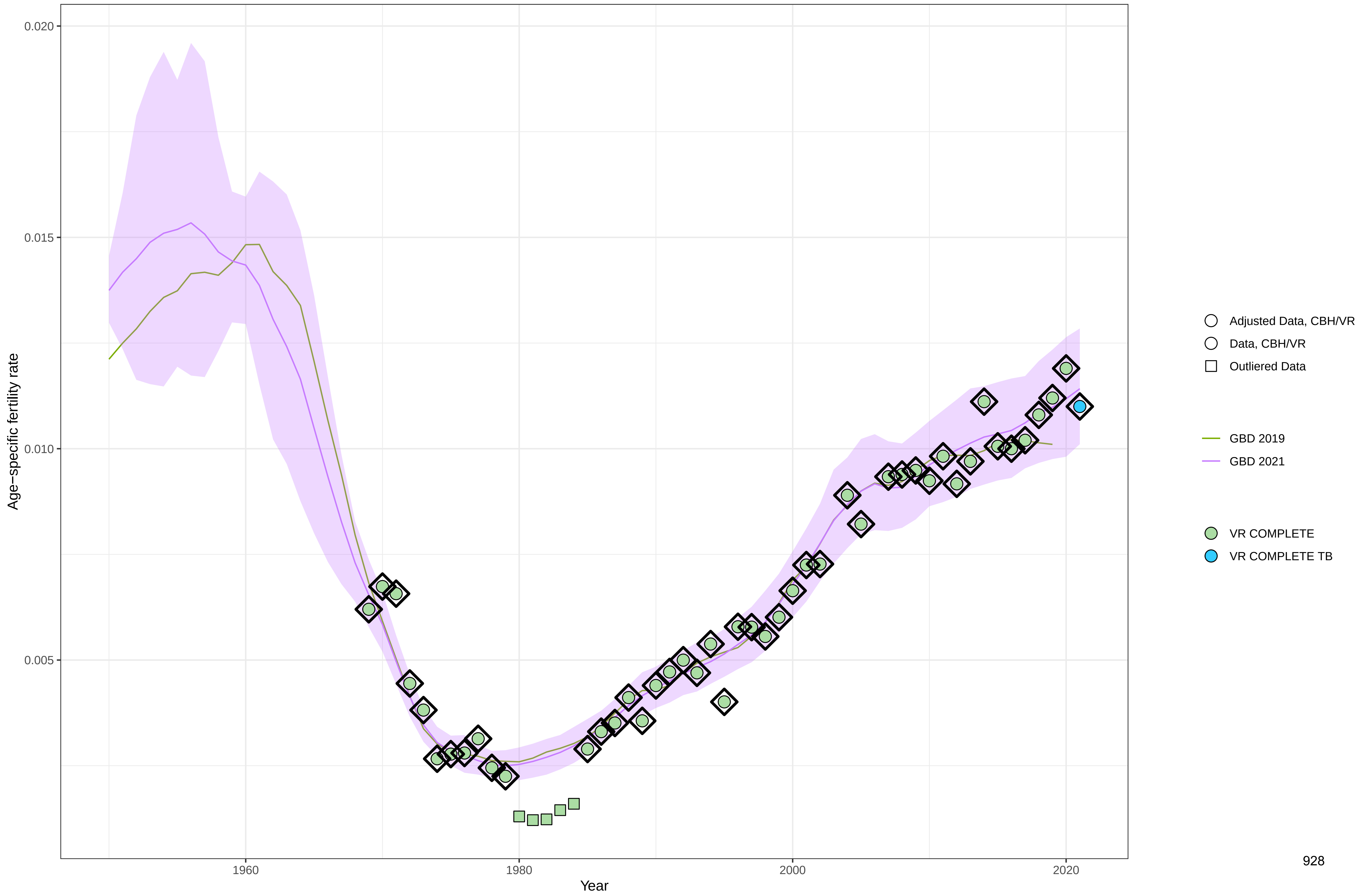

Age−specific fertility fitted trend lines, 1950−2021: Delaware, Ages: 45−49
Data points enclosed with a diamond are reference sources

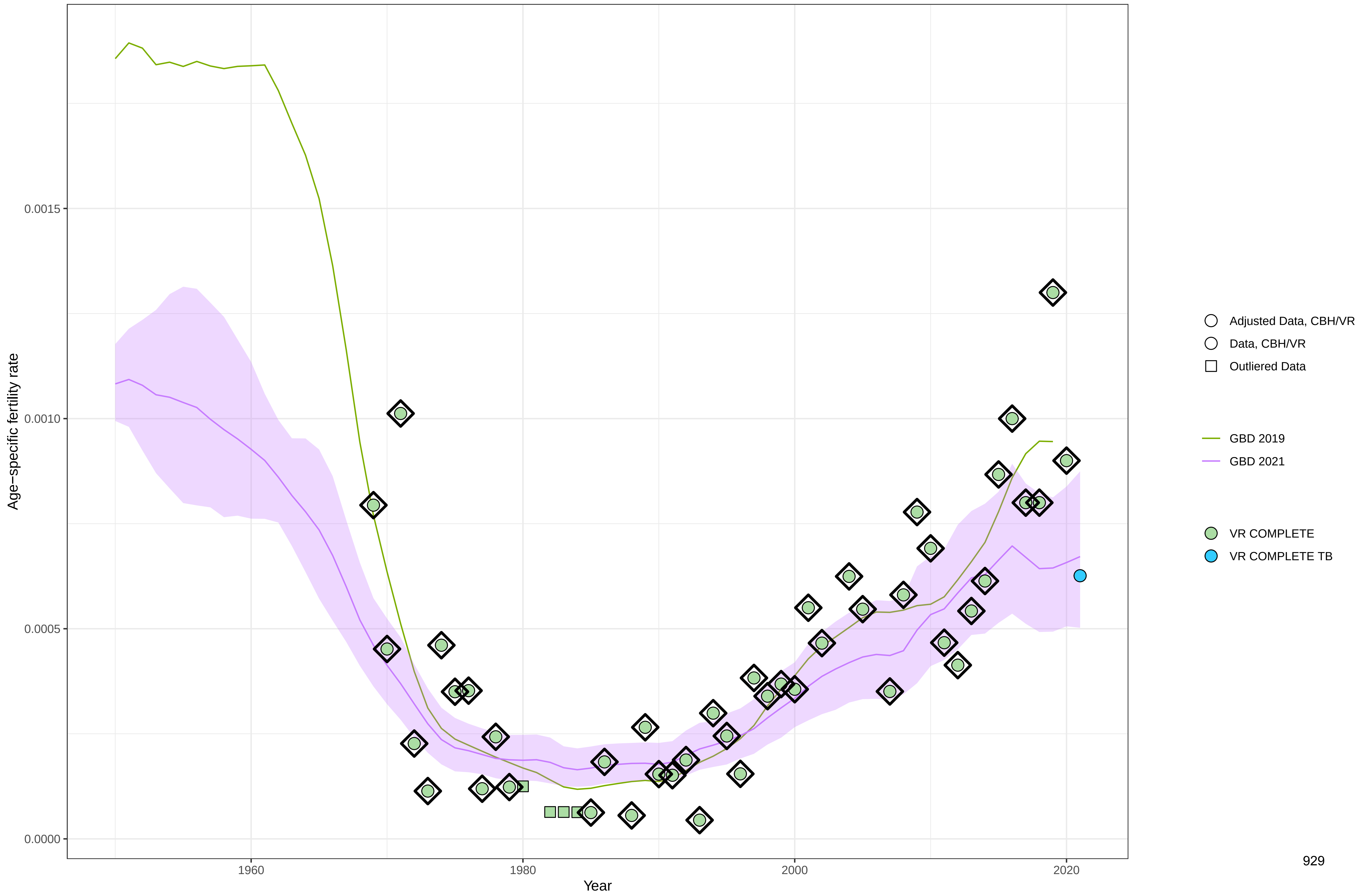

Age−specific fertility fitted trend lines, 1950−2021: Delaware, Ages: 50−54
Data points enclosed with a diamond are reference sources

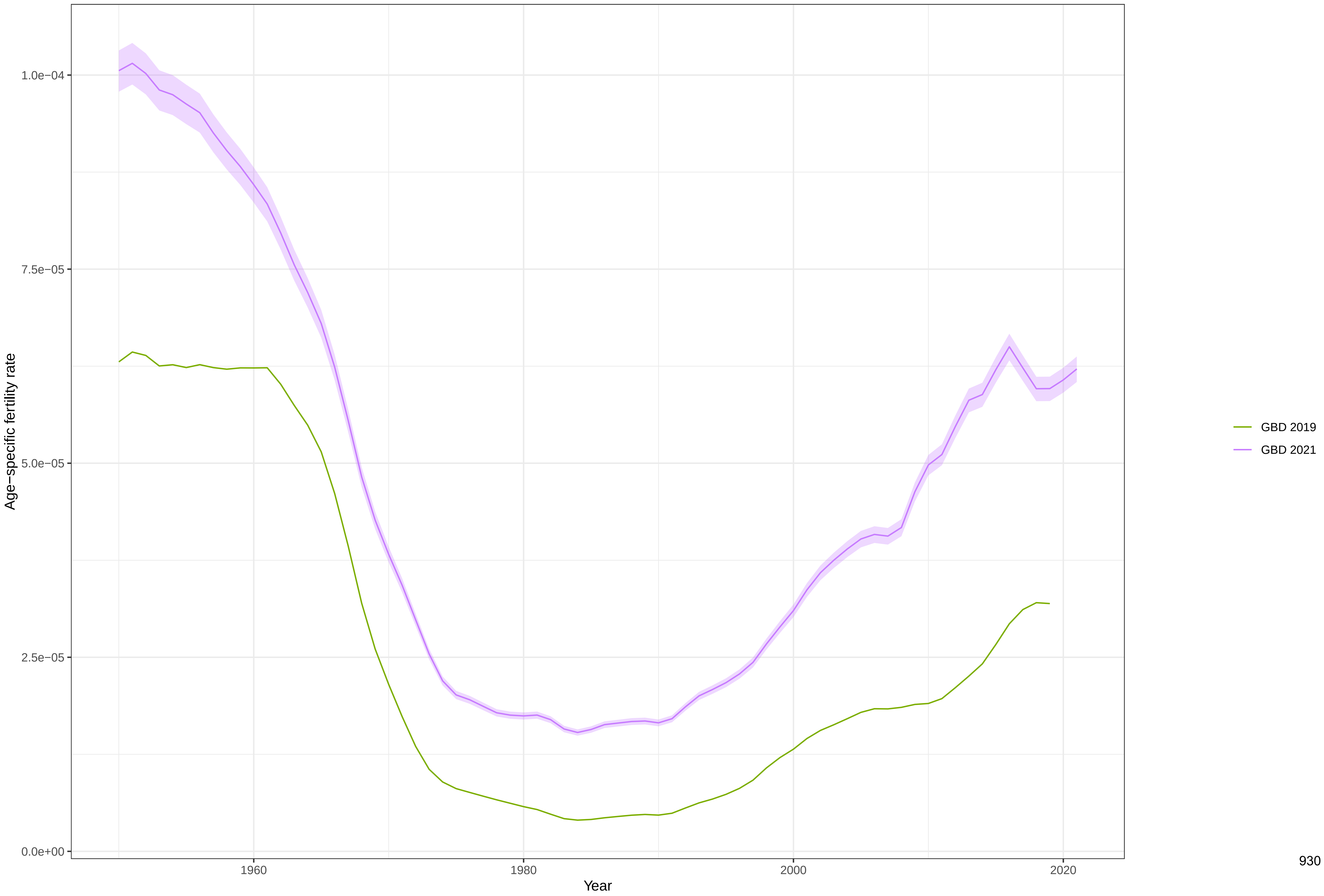

Total fertility rate fitted trend lines, 1950−2021: Delaware TFR
Data points enclosed with a diamond are reference sources

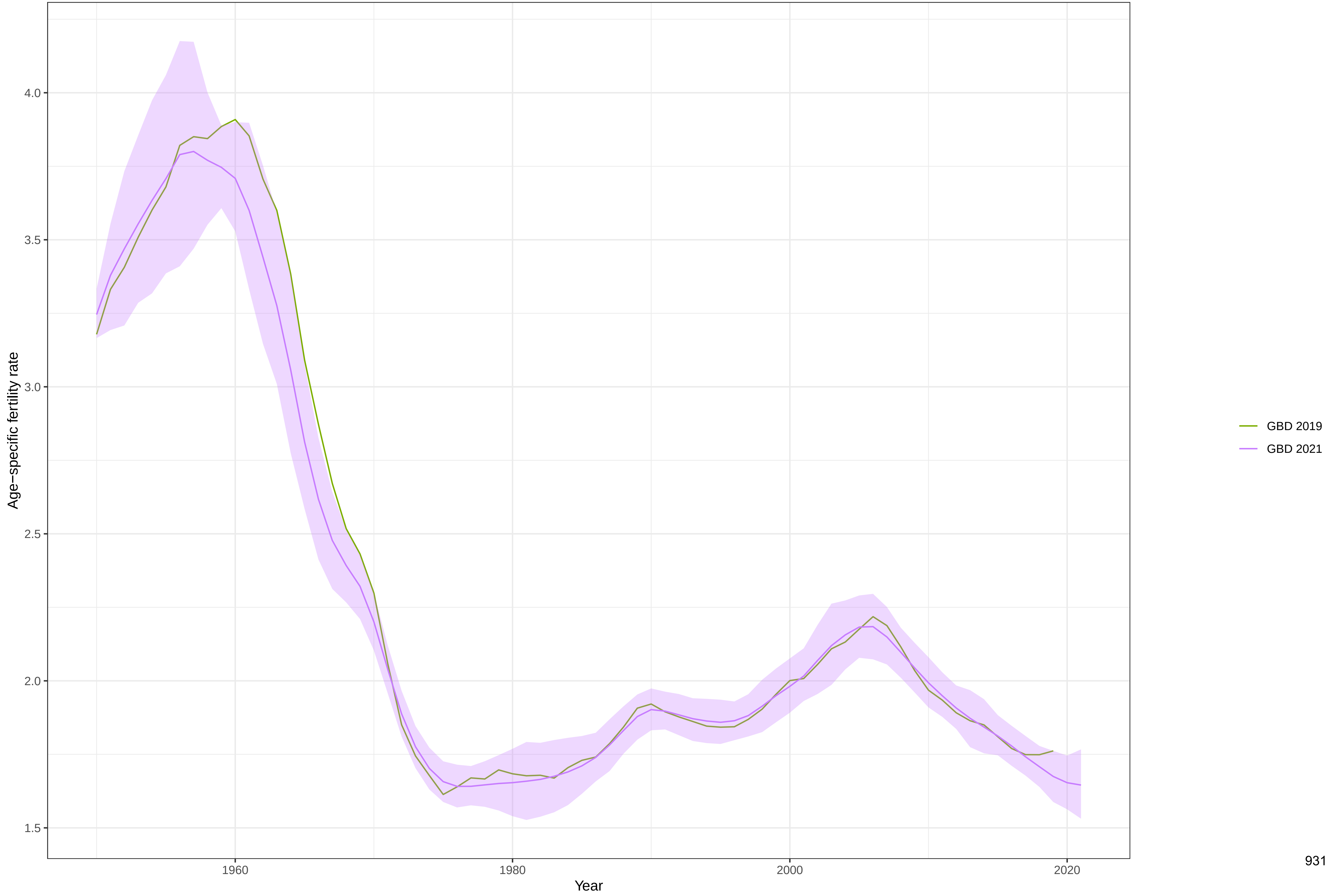

Age−specific fertility fitted trend lines, 1950−2021: Washington, DC, Ages: 10−14
Data points enclosed with a diamond are reference sources

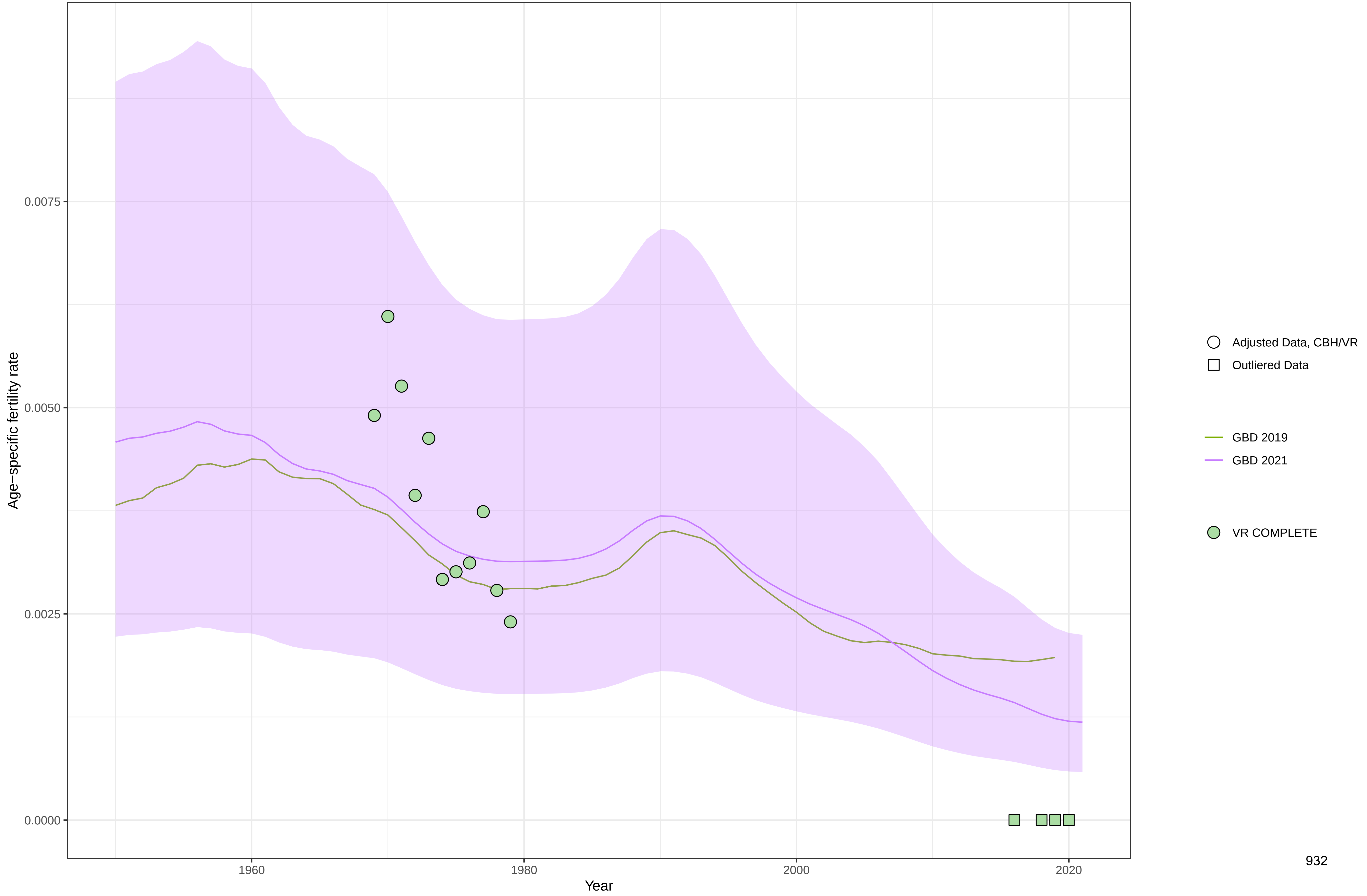

Age−specific fertility fitted trend lines, 1950−2021: Washington, DC, Ages: 15−19
Data points enclosed with a diamond are reference sources

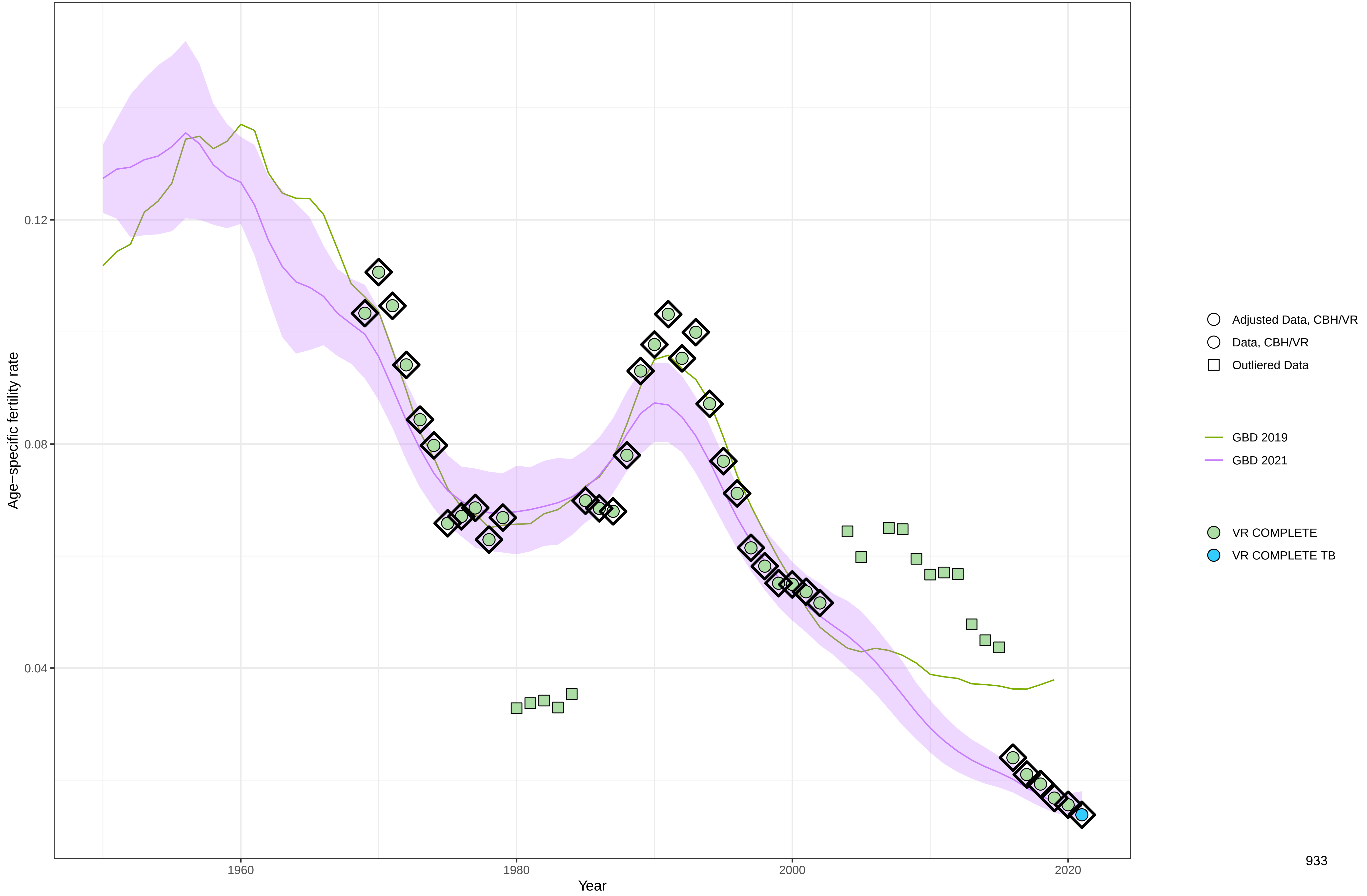

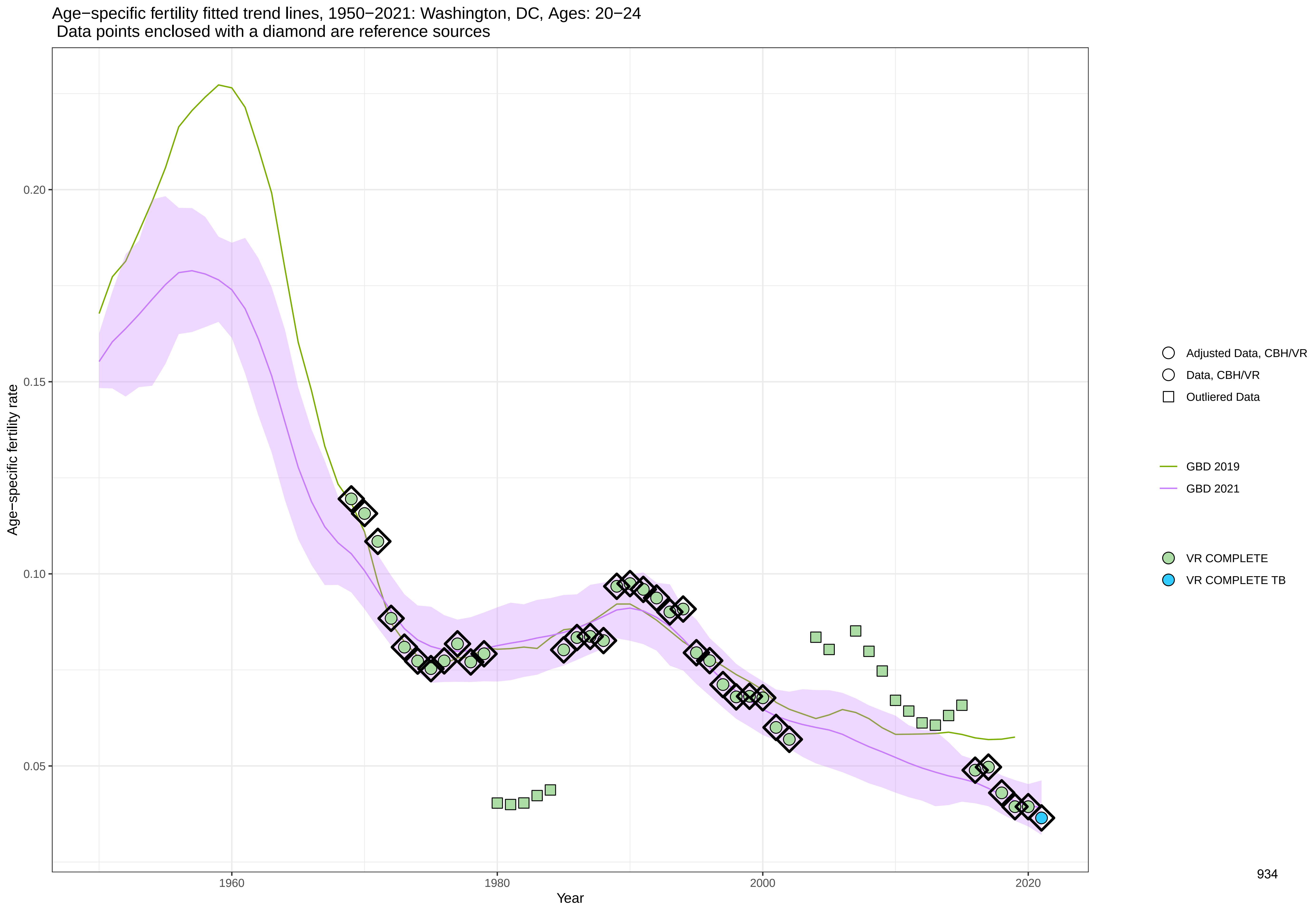
Age−specific fertility fitted trend lines, 1950−2021: Washington, DC, Ages: 20−24
Data points enclosed with a diamond are reference sources
Age−specific fertility rate
0.20
0.15
0.10
0.05
1960
1980
2000
2020
Year
Adjusted Data, CBH/VR
Data, CBH/VR
Outliered Data
GBD 2019
GBD 2021
VR COMPLETE
VR COMPLETE TB

Age−specific fertility fitted trend lines, 1950−2021: Washington, DC, Ages: 25−29
Data points enclosed with a diamond are reference sources

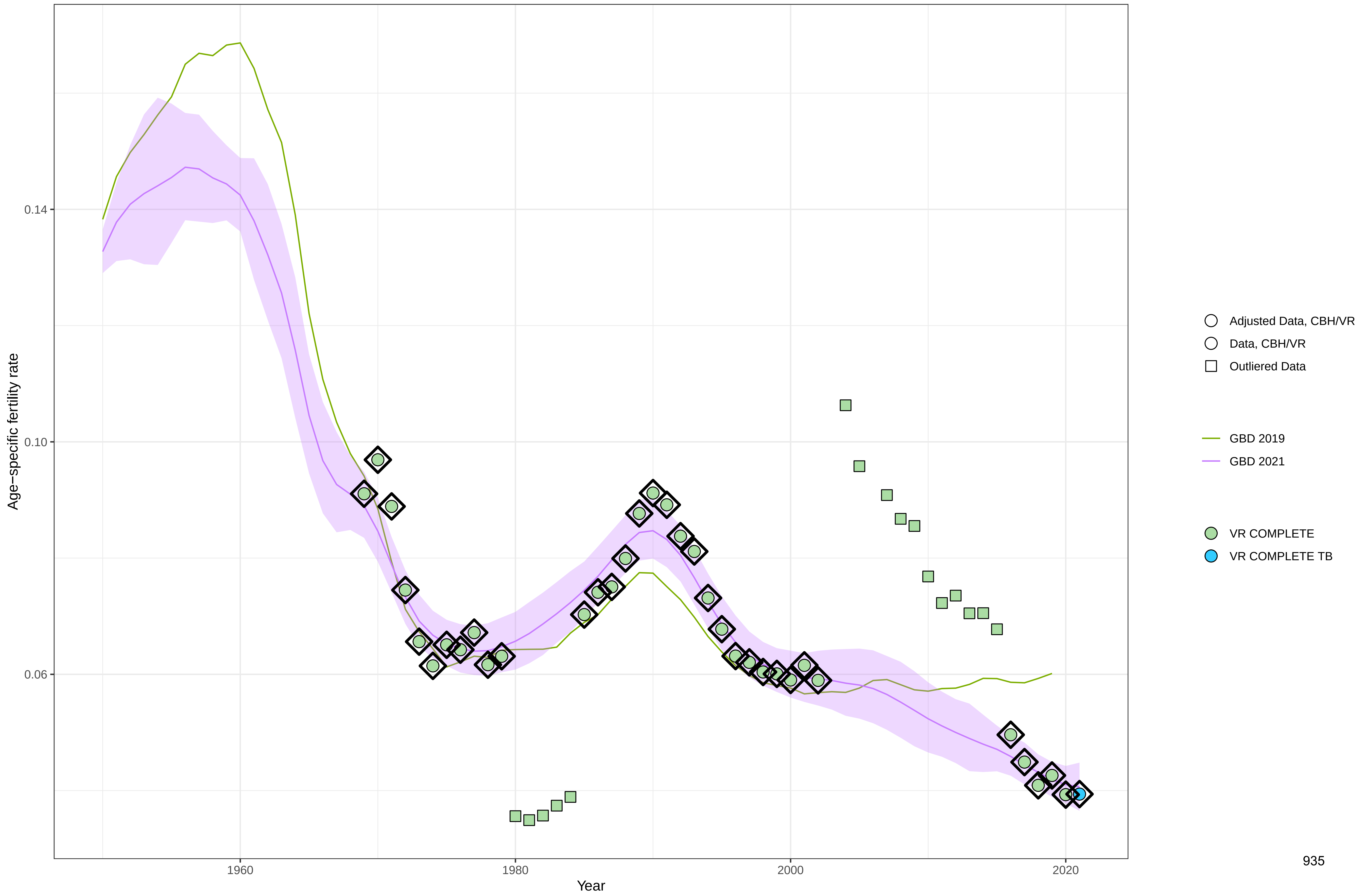

Age−specific fertility fitted trend lines, 1950−2021: Washington, DC, Ages: 30−34
Data points enclosed with a diamond are reference sources

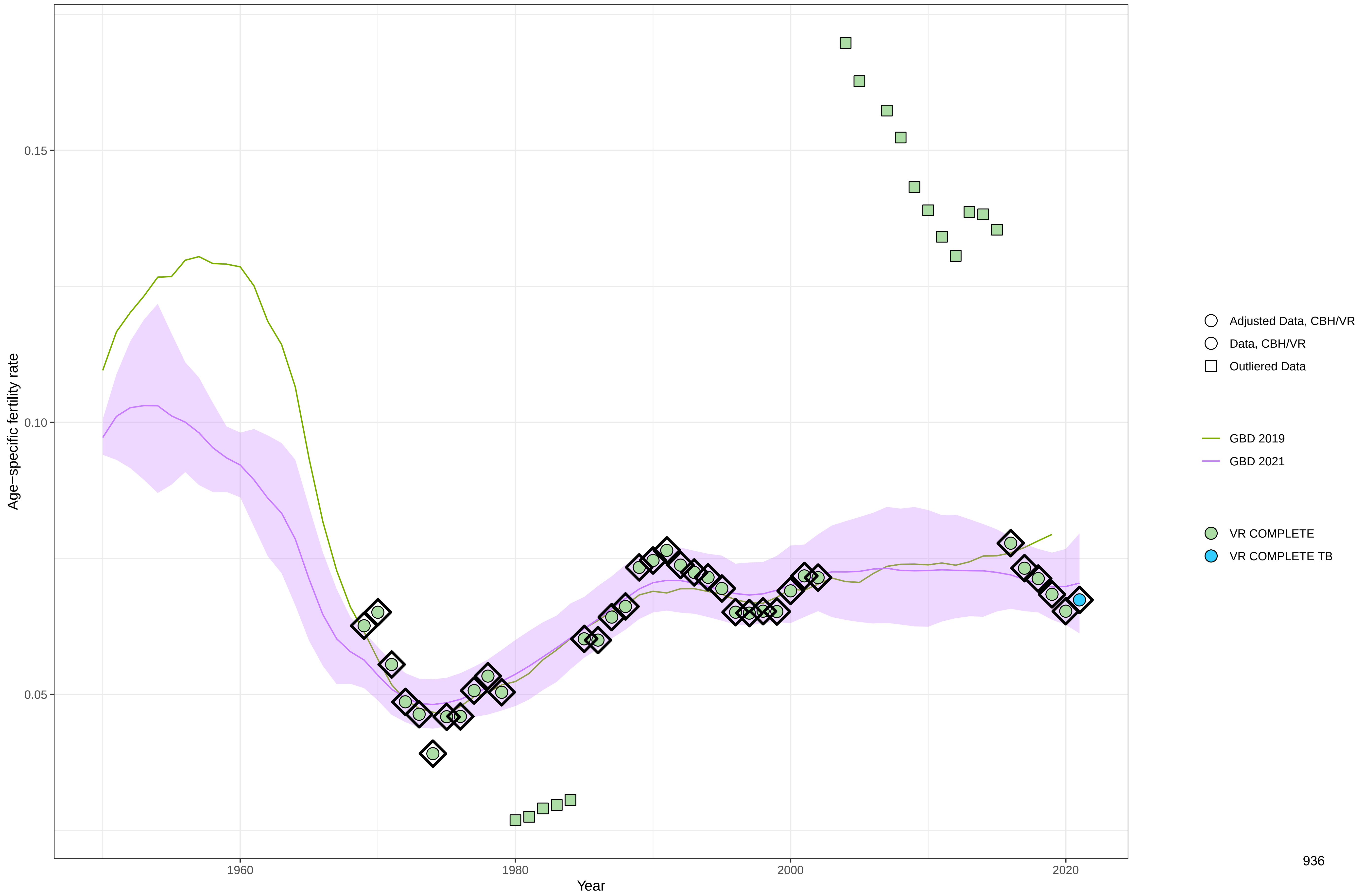

Age−specific fertility fitted trend lines, 1950−2021: Washington, DC, Ages: 35−39
Data points enclosed with a diamond are reference sources

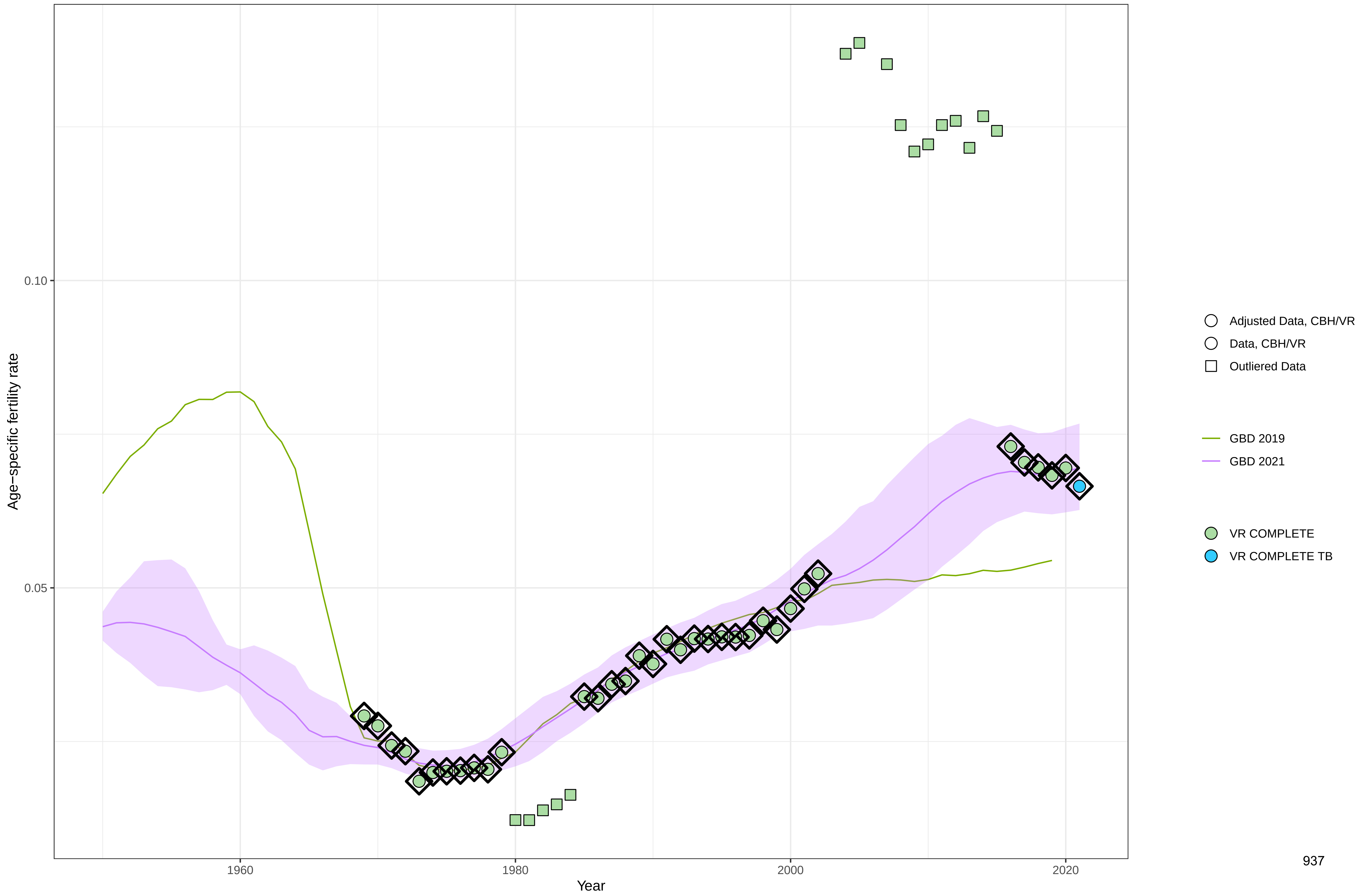

Age−specific fertility fitted trend lines, 1950−2021: Washington, DC, Ages: 40−44
Data points enclosed with a diamond are reference sources

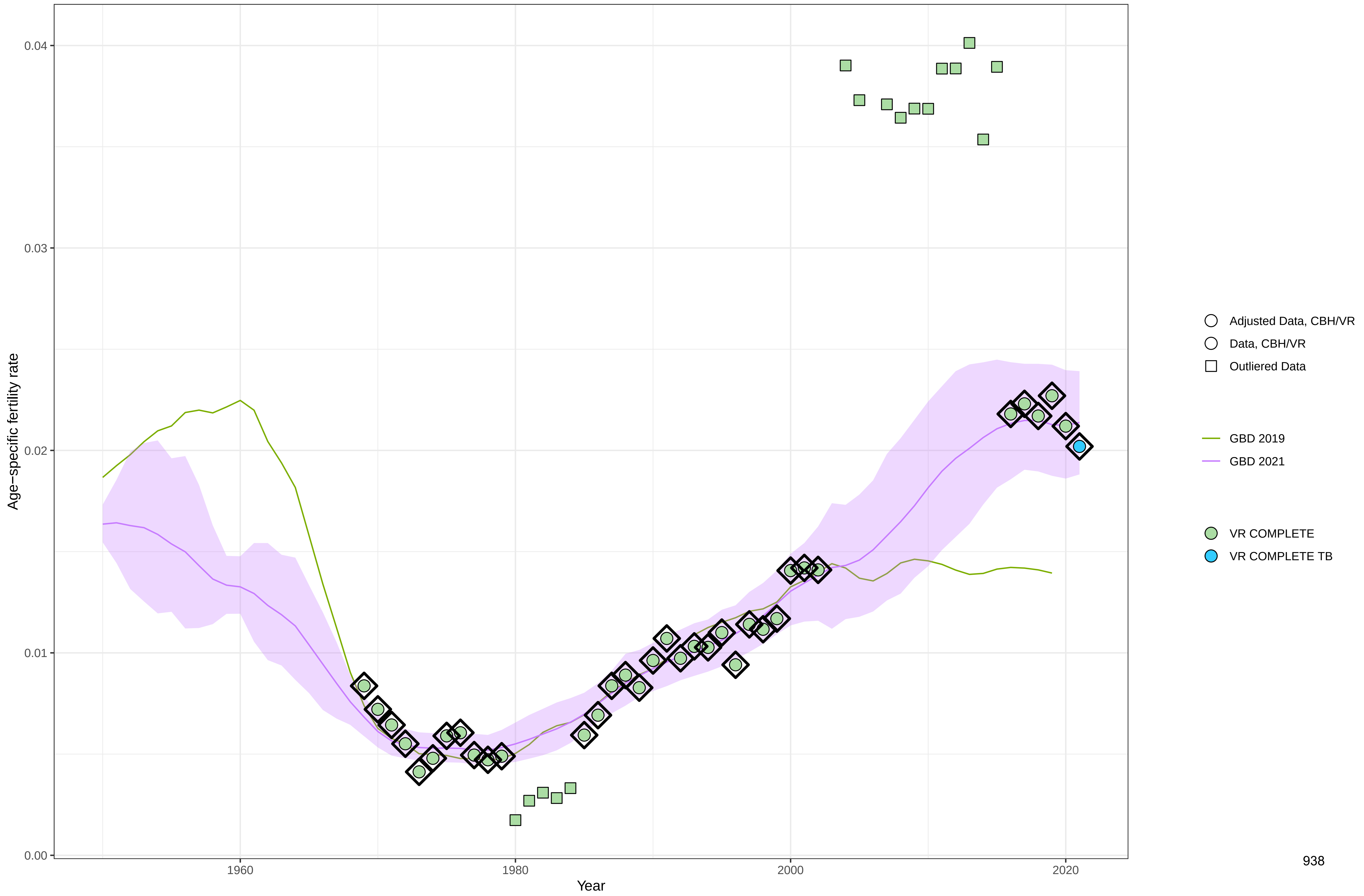

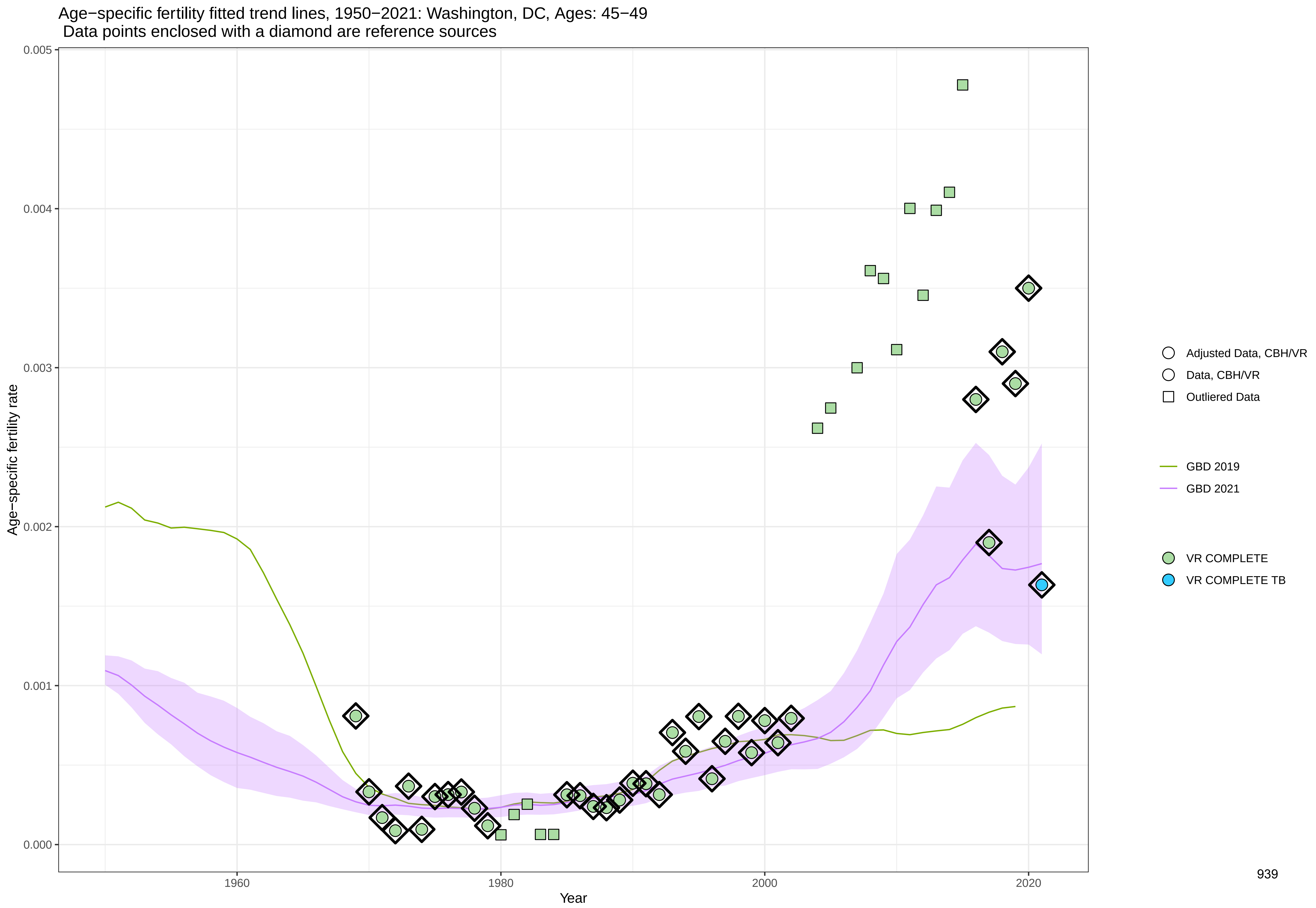
Age−specific fertility fitted trend lines, 1950−2021: Washington, DC, Ages: 45−49
Data points enclosed with a diamond are reference sources
Age−specific fertility rate
0.000
0.001
0.002
0.003
0.004
0.005
Year
1960
1980
2000
2020
Adjusted Data, CBH/VR
Data, CBH/VR
Outliered Data
GBD 2019
GBD 2021
VR COMPLETE
VR COMPLETE TB

Age−specific fertility fitted trend lines, 1950−2021: Washington, DC, Ages: 50−54
Data points enclosed with a diamond are reference sources

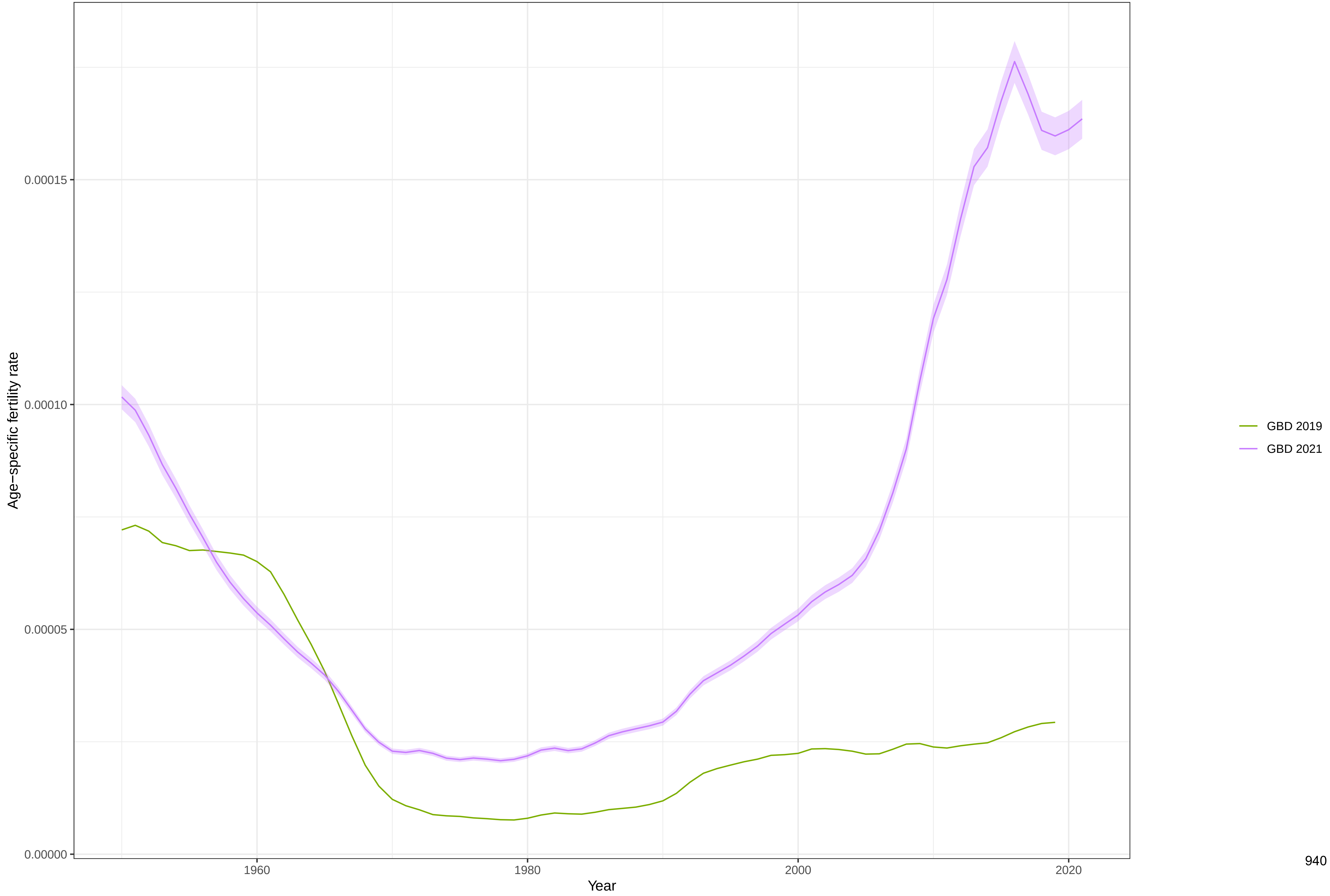

Total fertility rate fitted trend lines, 1950−2021: Washington, DC TFR
Data points enclosed with a diamond are reference sources

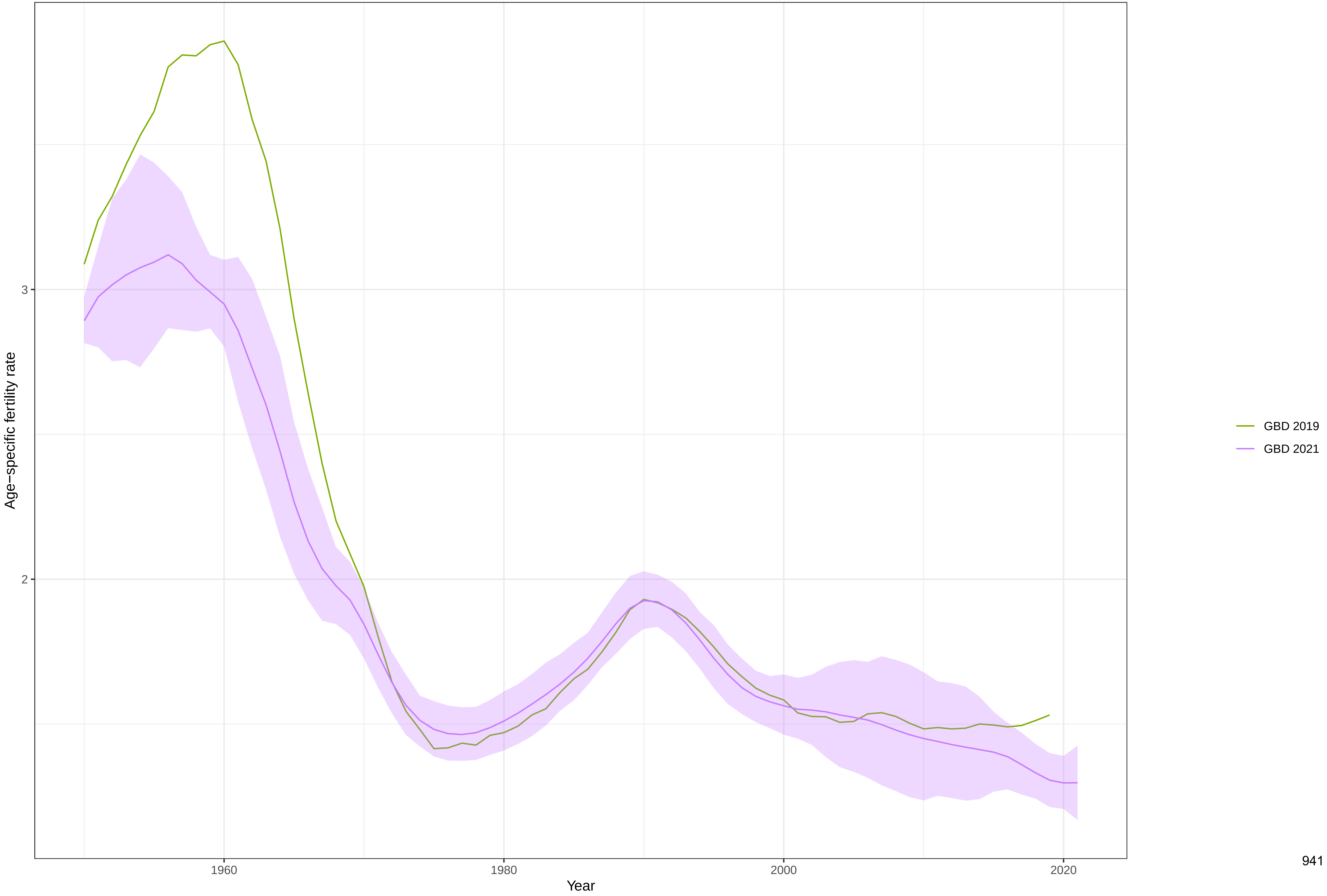

Age−specific fertility fitted trend lines, 1950−2021: Florida, Ages: 10−14
Data points enclosed with a diamond are reference sources

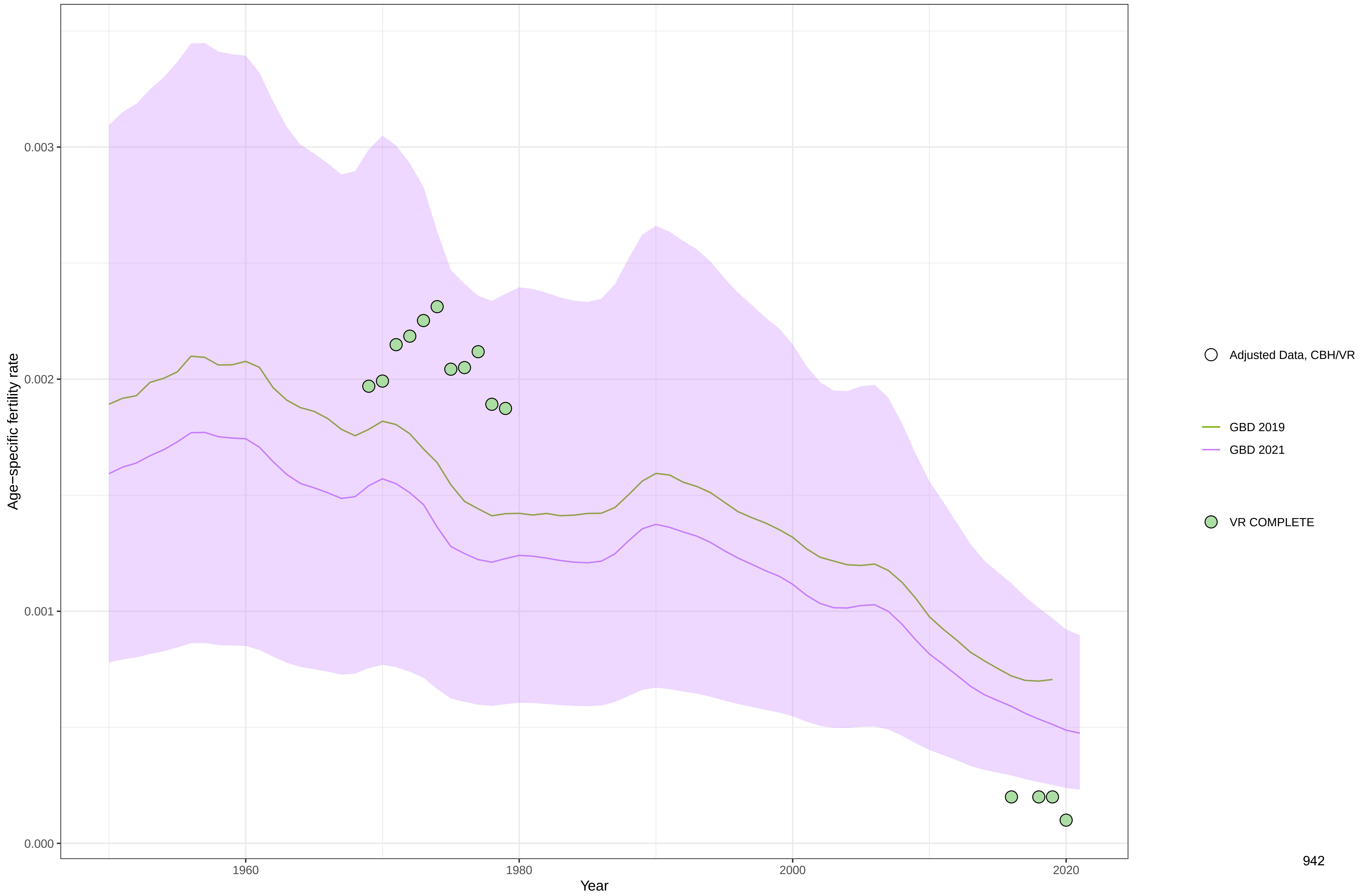

Age−specific fertility fitted trend lines, 1950−2021: Florida, Ages: 15−19
Data points enclosed with a diamond are reference sources

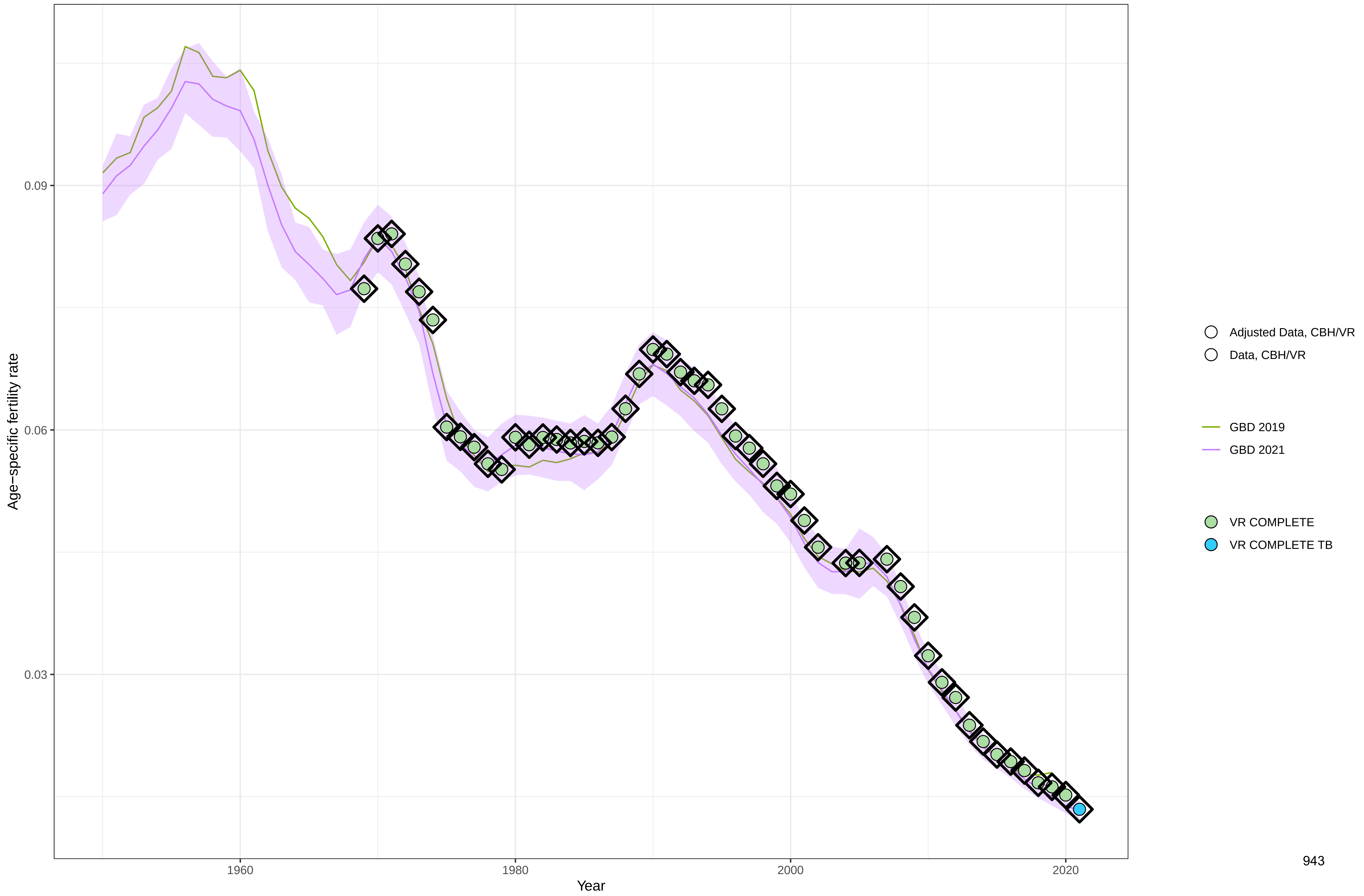

Age−specific fertility fitted trend lines, 1950−2021: Florida, Ages: 20−24
Data points enclosed with a diamond are reference sources

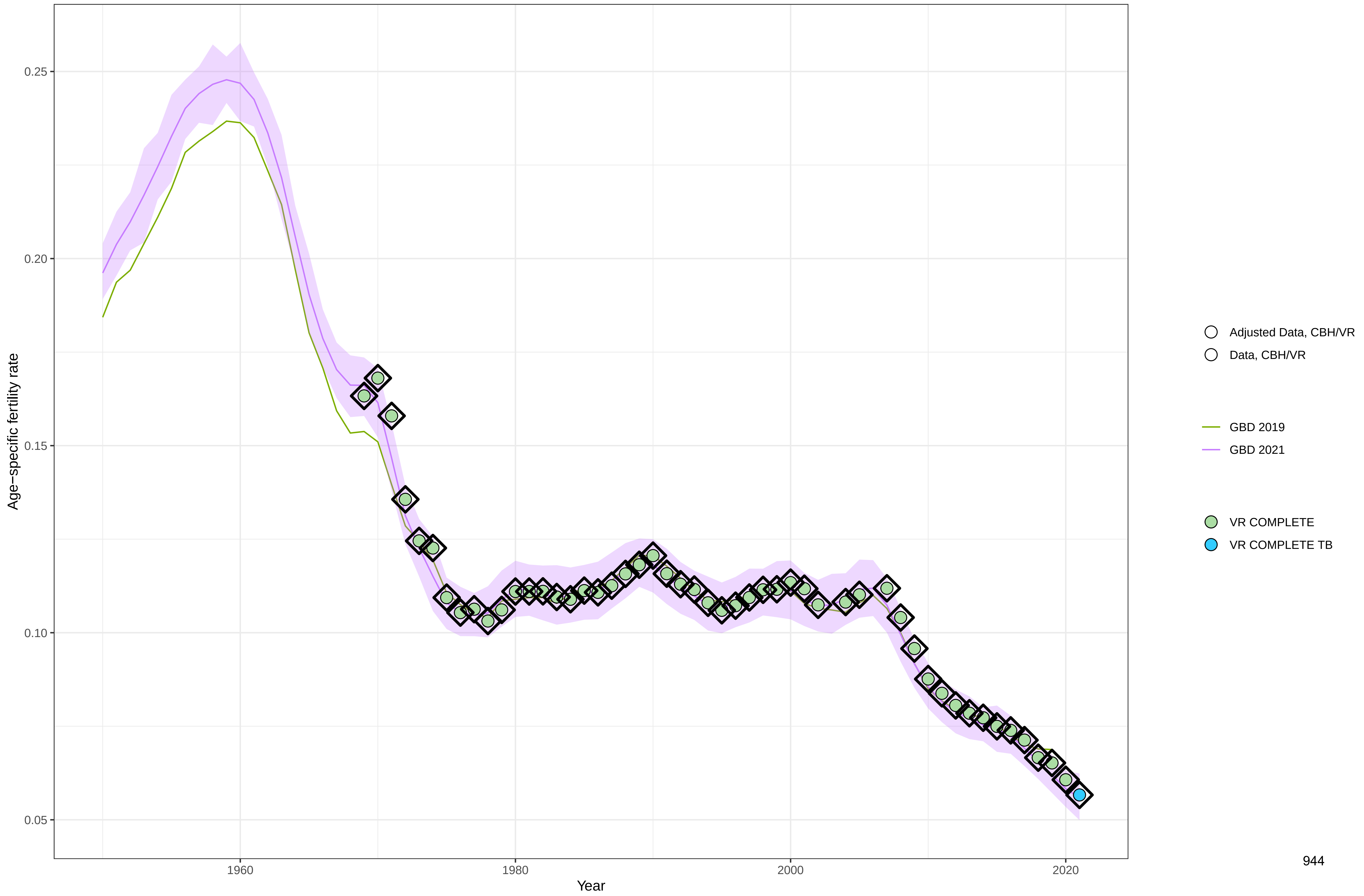

Age−specific fertility fitted trend lines, 1950−2021: Florida, Ages: 25−29
Data points enclosed with a diamond are reference sources

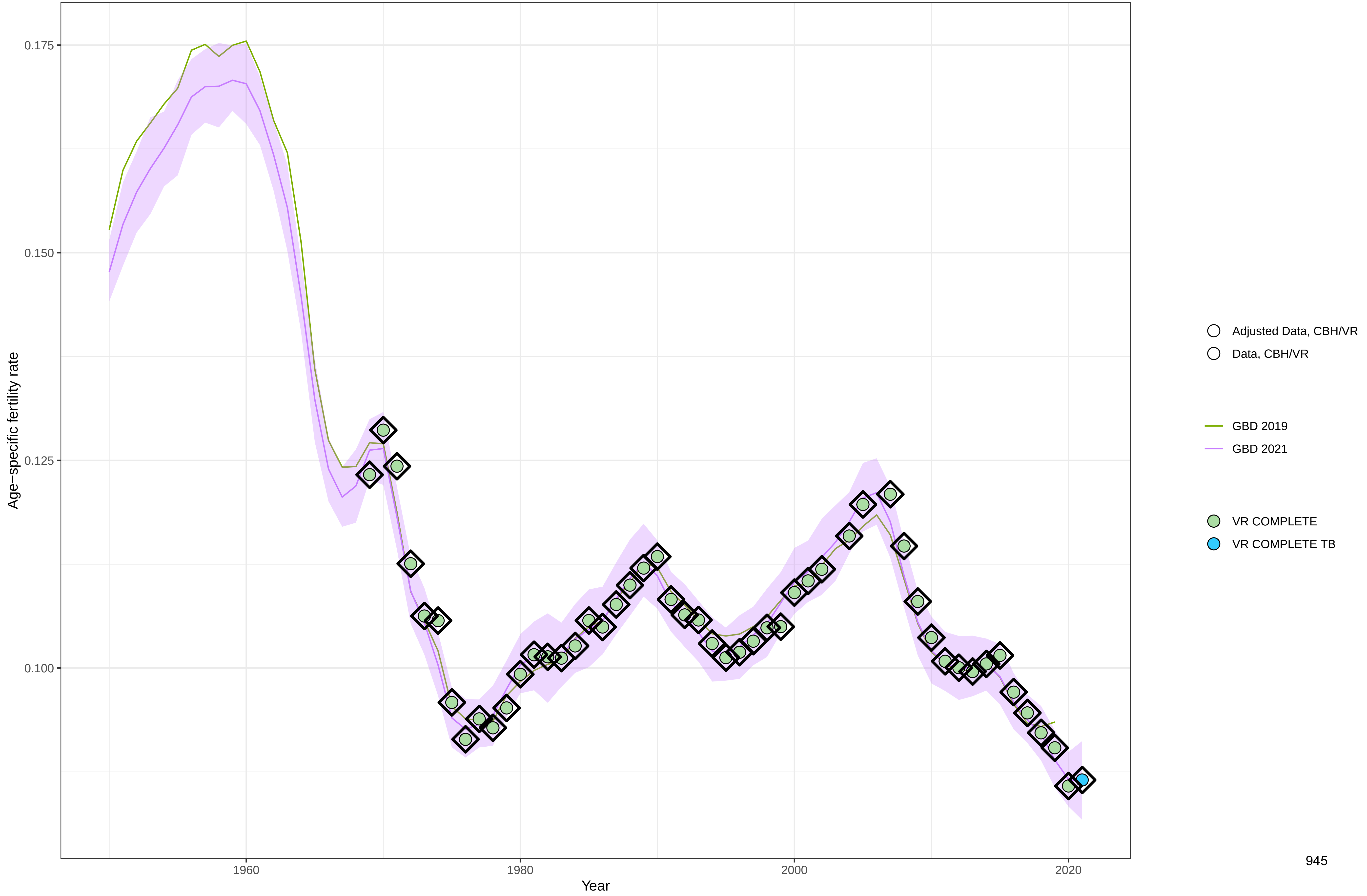

Age−specific fertility fitted trend lines, 1950−2021: Florida, Ages: 30−34
Data points enclosed with a diamond are reference sources

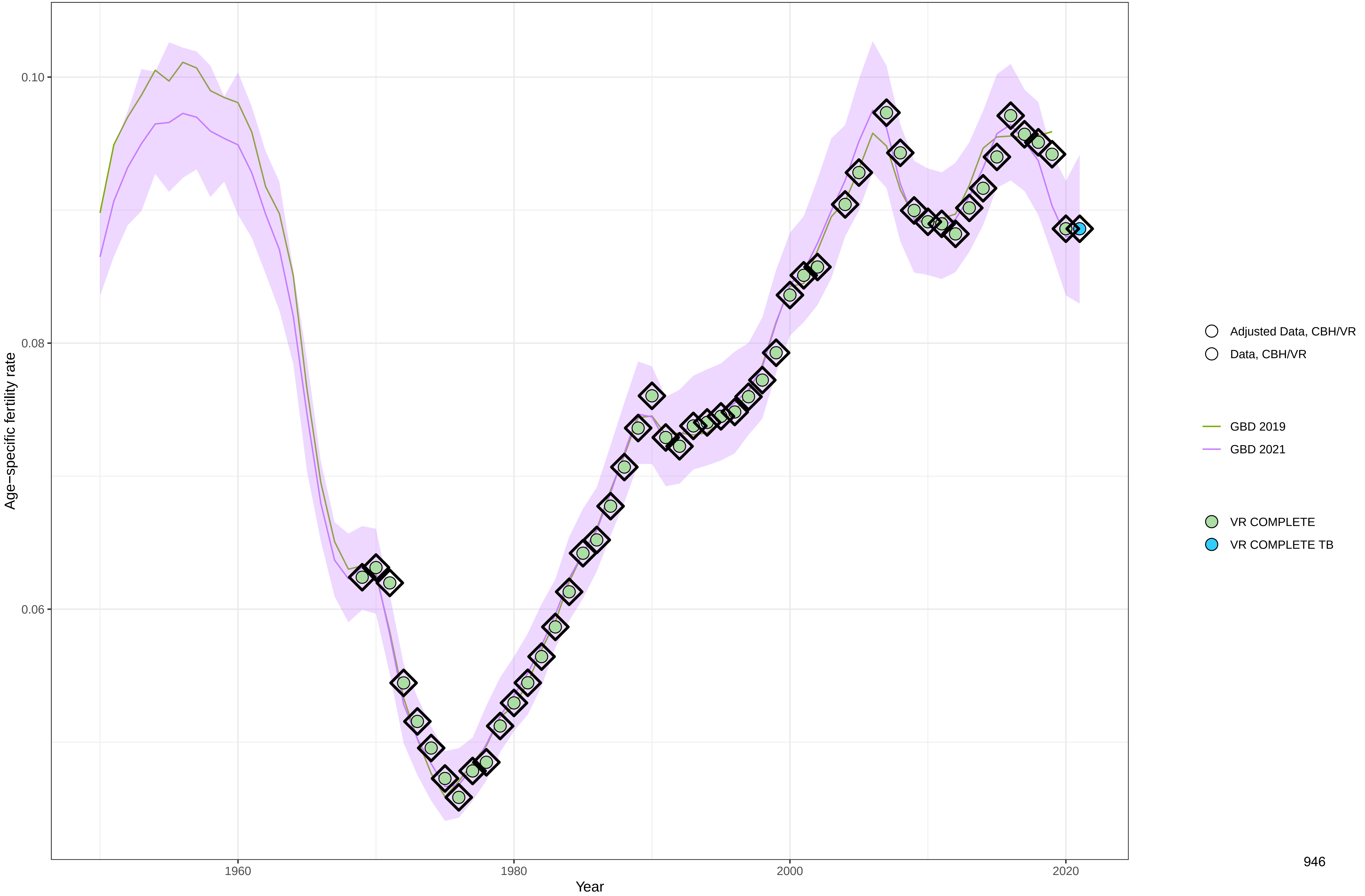

Age−specific fertility fitted trend lines, 1950−2021: Florida, Ages: 35−39
Data points enclosed with a diamond are reference sources

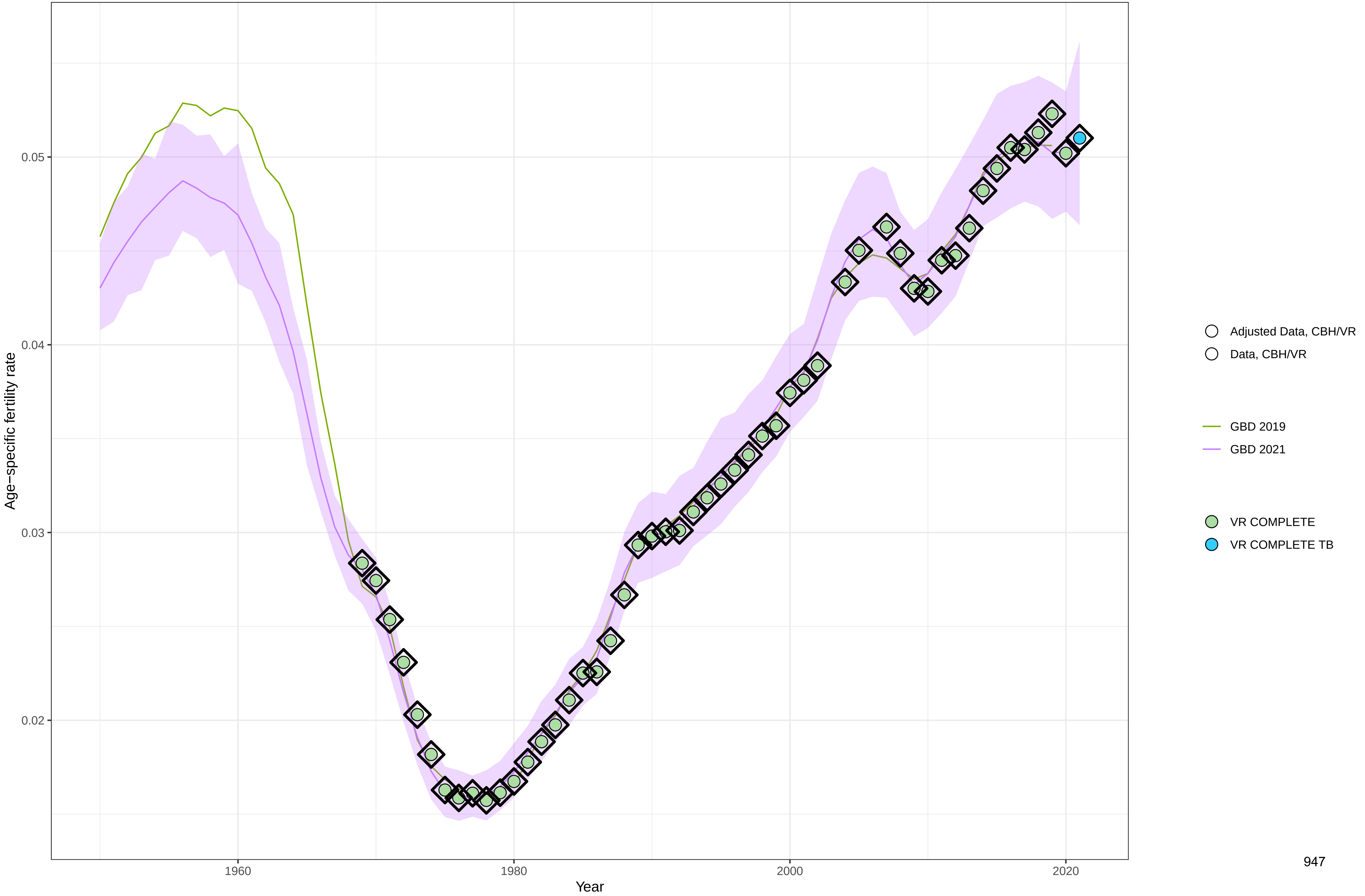

Age−specific fertility fitted trend lines, 1950−2021: Florida, Ages: 40−44
Data points enclosed with a diamond are reference sources

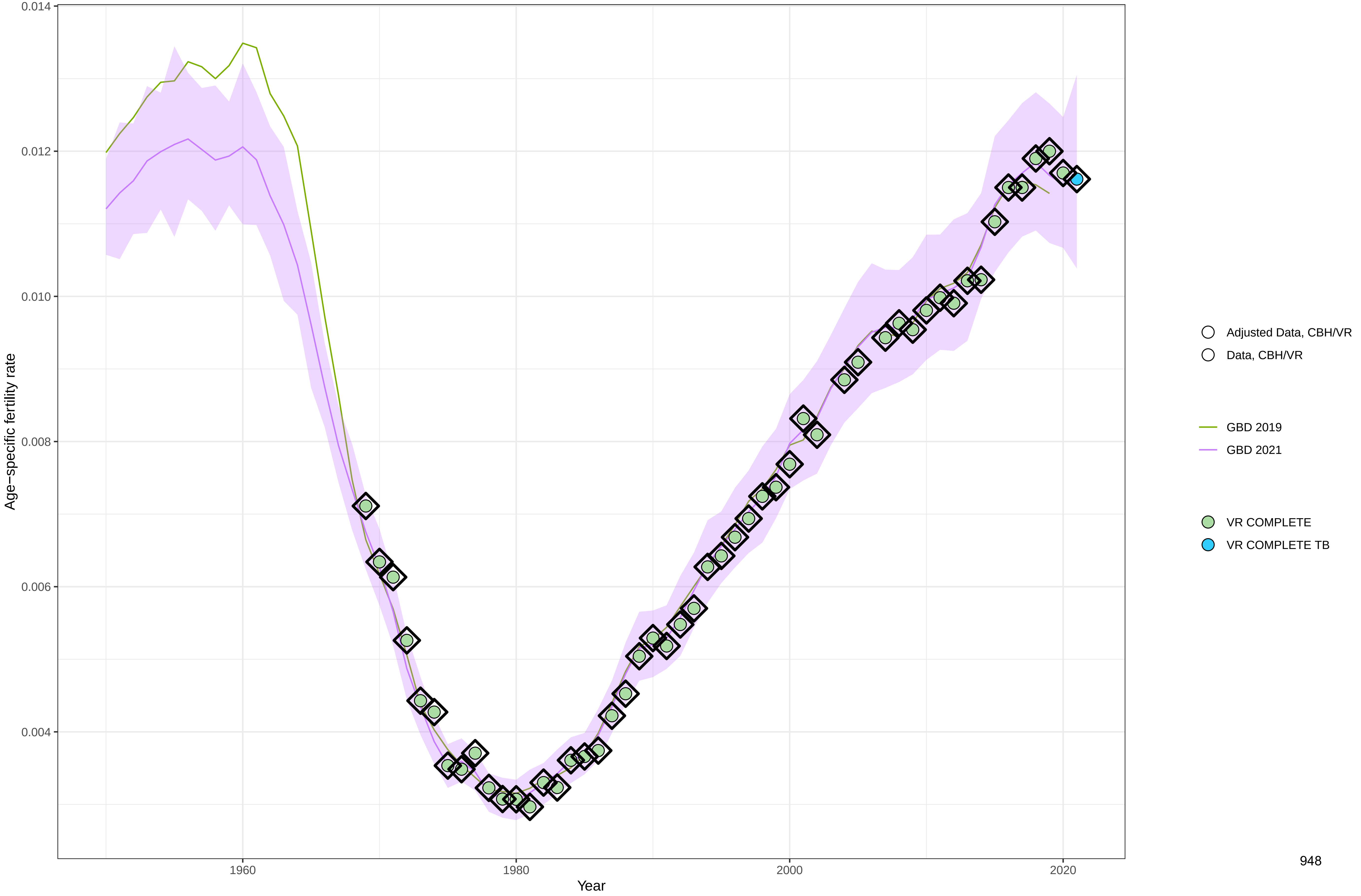

Age−specific fertility fitted trend lines, 1950−2021: Florida, Ages: 45−49
Data points enclosed with a diamond are reference sources

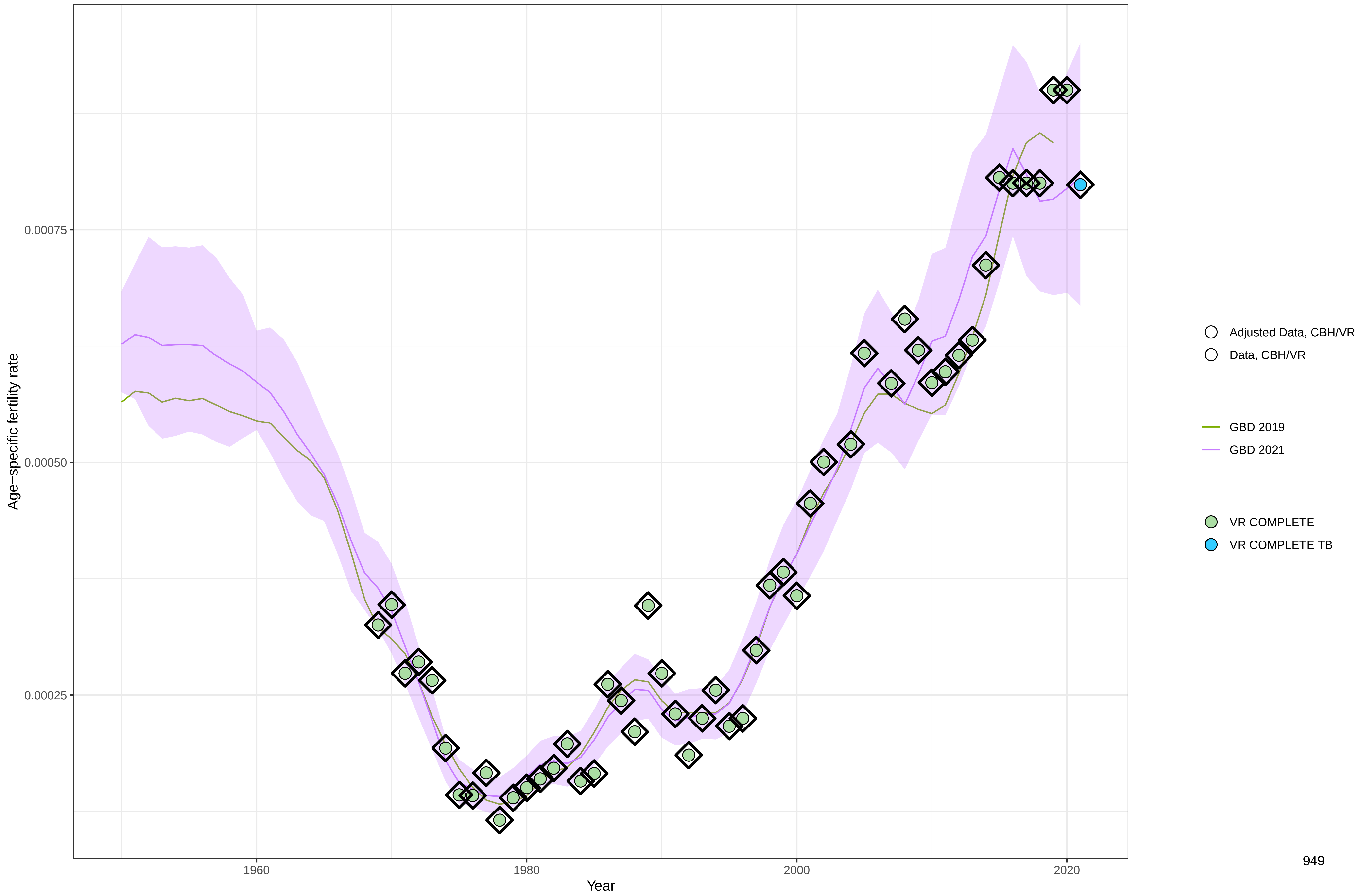

Age−specific fertility fitted trend lines, 1950−2021: Florida, Ages: 50−54
Data points enclosed with a diamond are reference sources

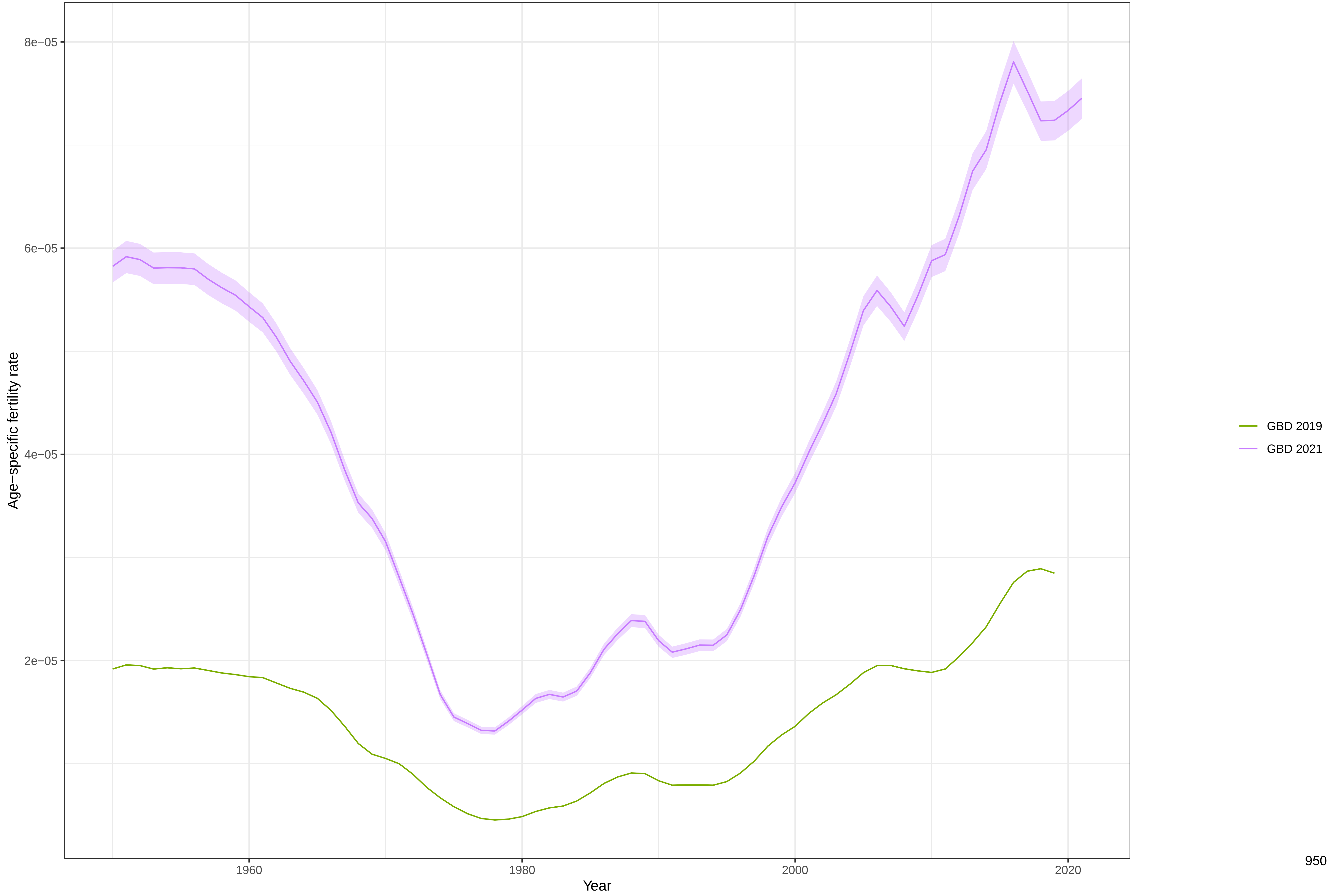

Total fertility rate fitted trend lines, 1950−2021: Florida TFR
Data points enclosed with a diamond are reference sources

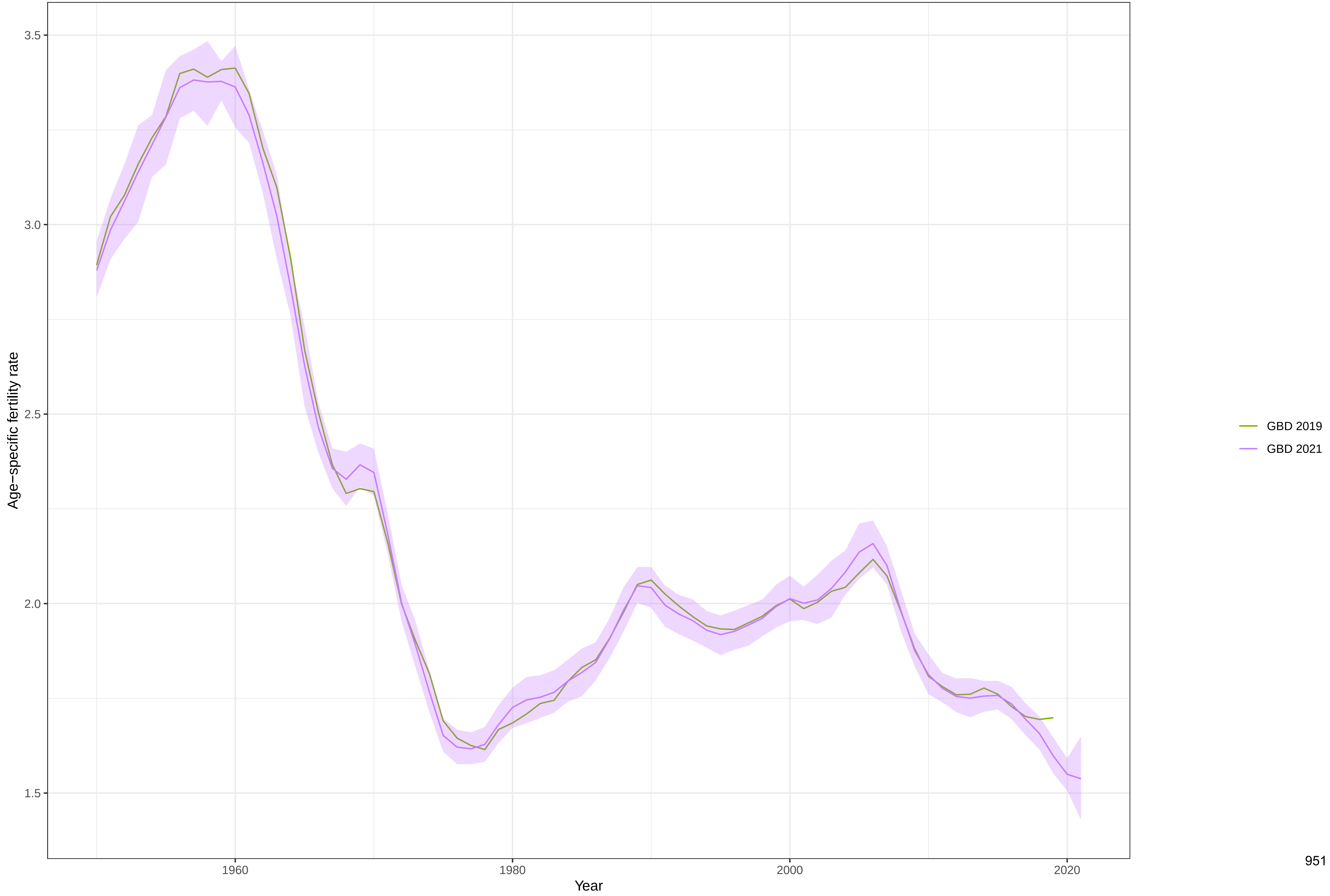

Age−specific fertility fitted trend lines, 1950−2021: Georgia, Ages: 10−14
Data points enclosed with a diamond are reference sources

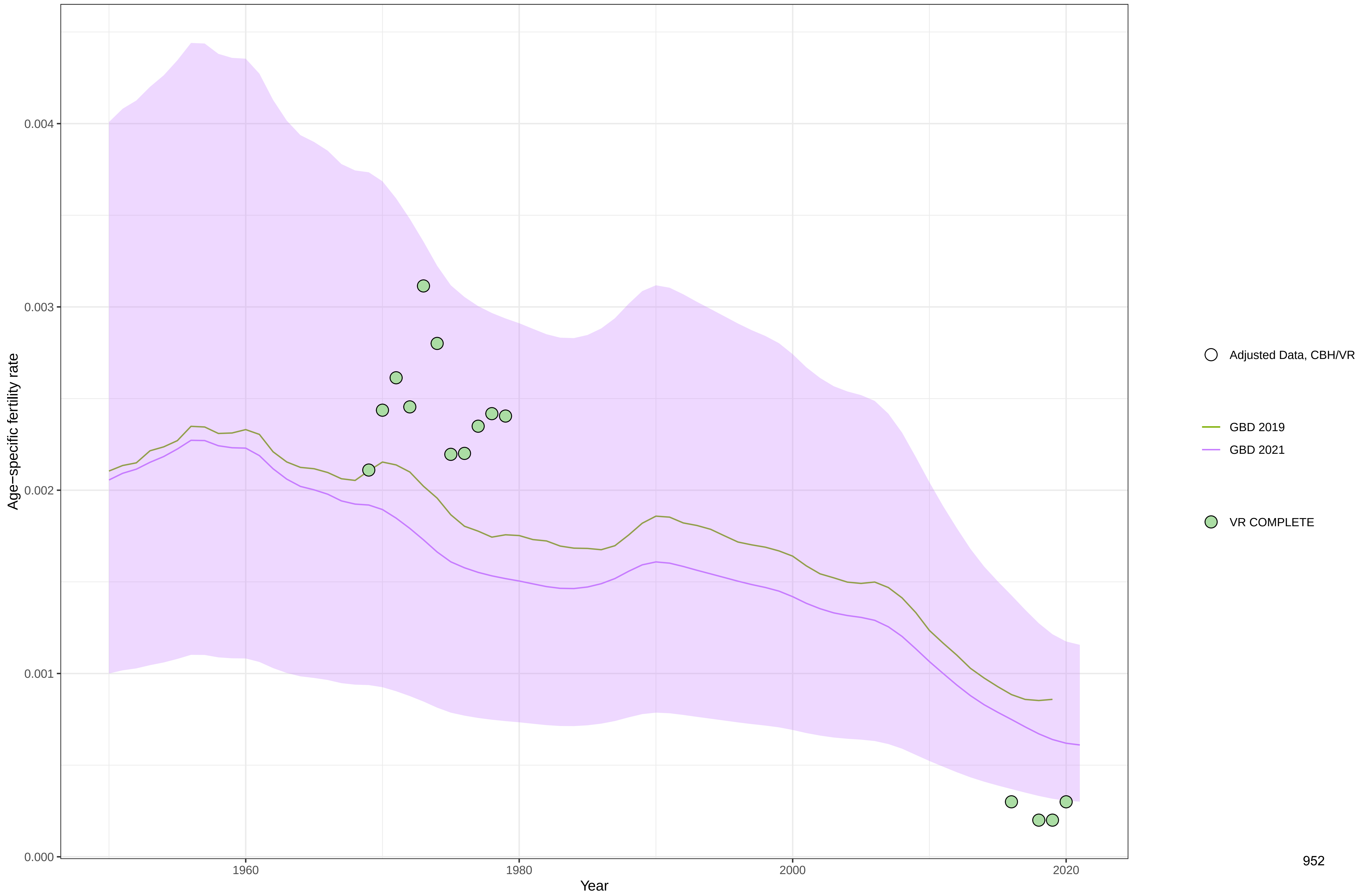

Age−specific fertility fitted trend lines, 1950−2021: Georgia, Ages: 15−19
Data points enclosed with a diamond are reference sources

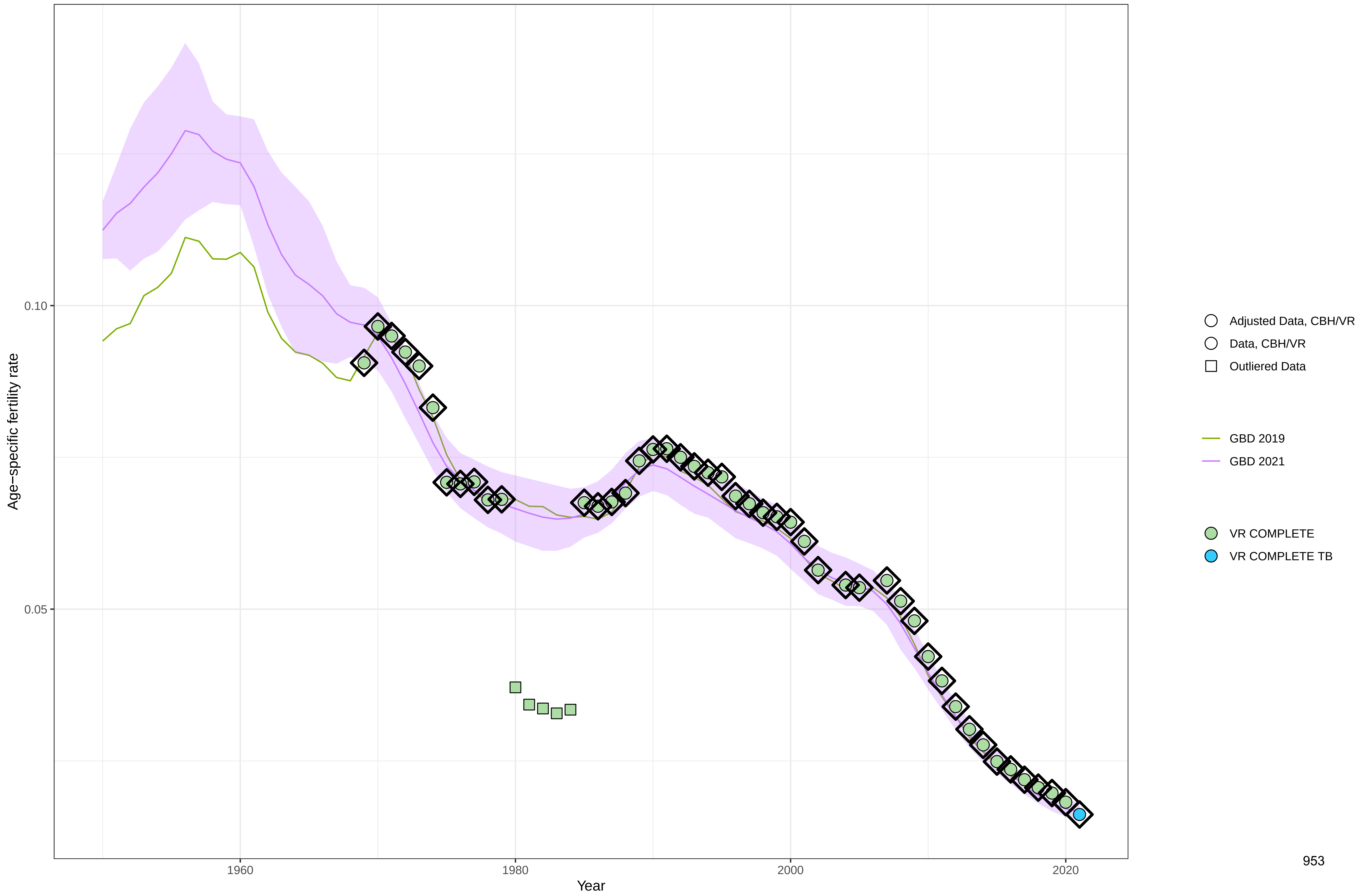

Age−specific fertility fitted trend lines, 1950−2021: Georgia, Ages: 20−24
Data points enclosed with a diamond are reference sources

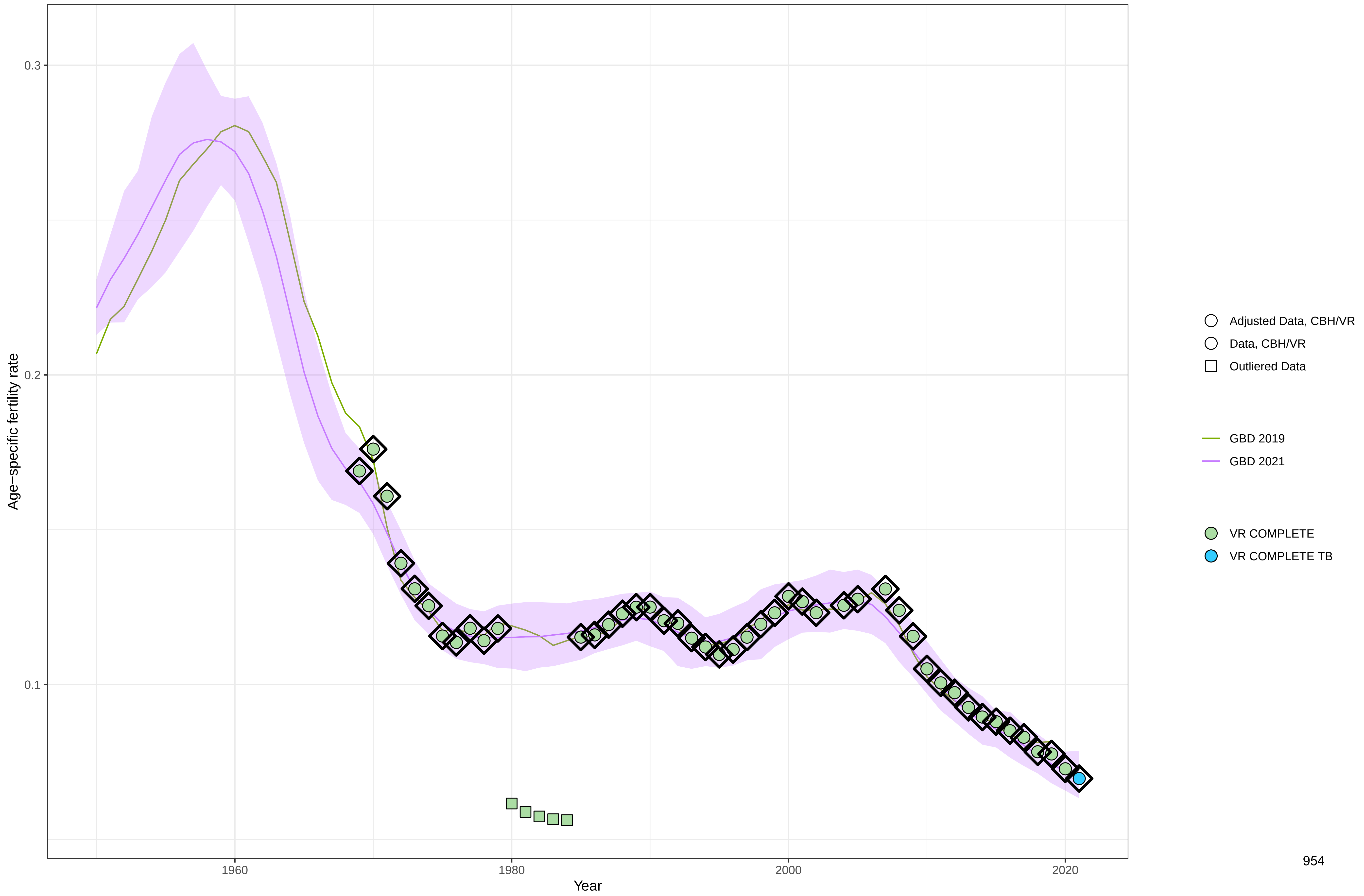

Age−specific fertility fitted trend lines, 1950−2021: Georgia, Ages: 25−29
Data points enclosed with a diamond are reference sources

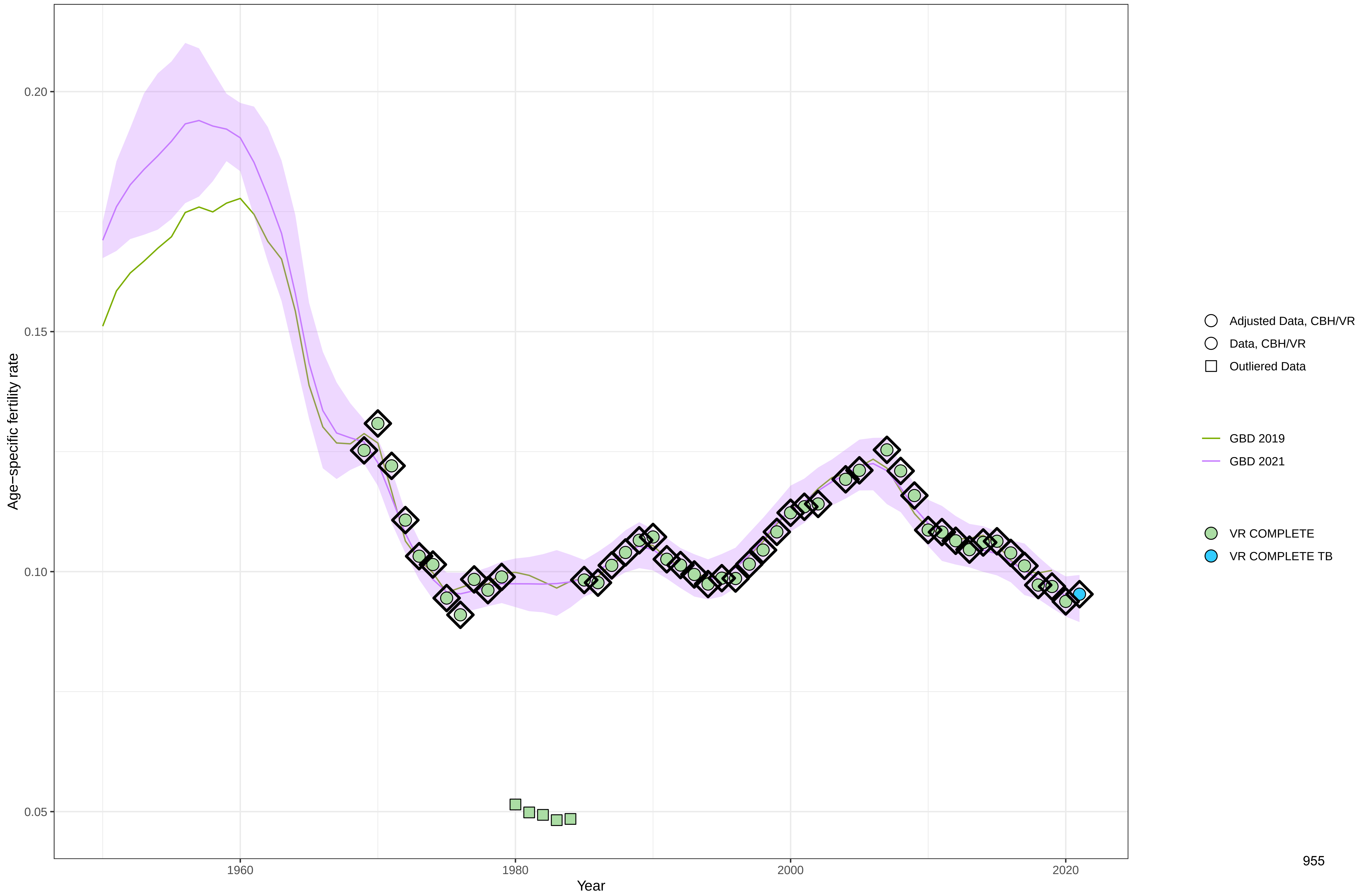

Age−specific fertility fitted trend lines, 1950−2021: Georgia, Ages: 30−34
Data points enclosed with a diamond are reference sources

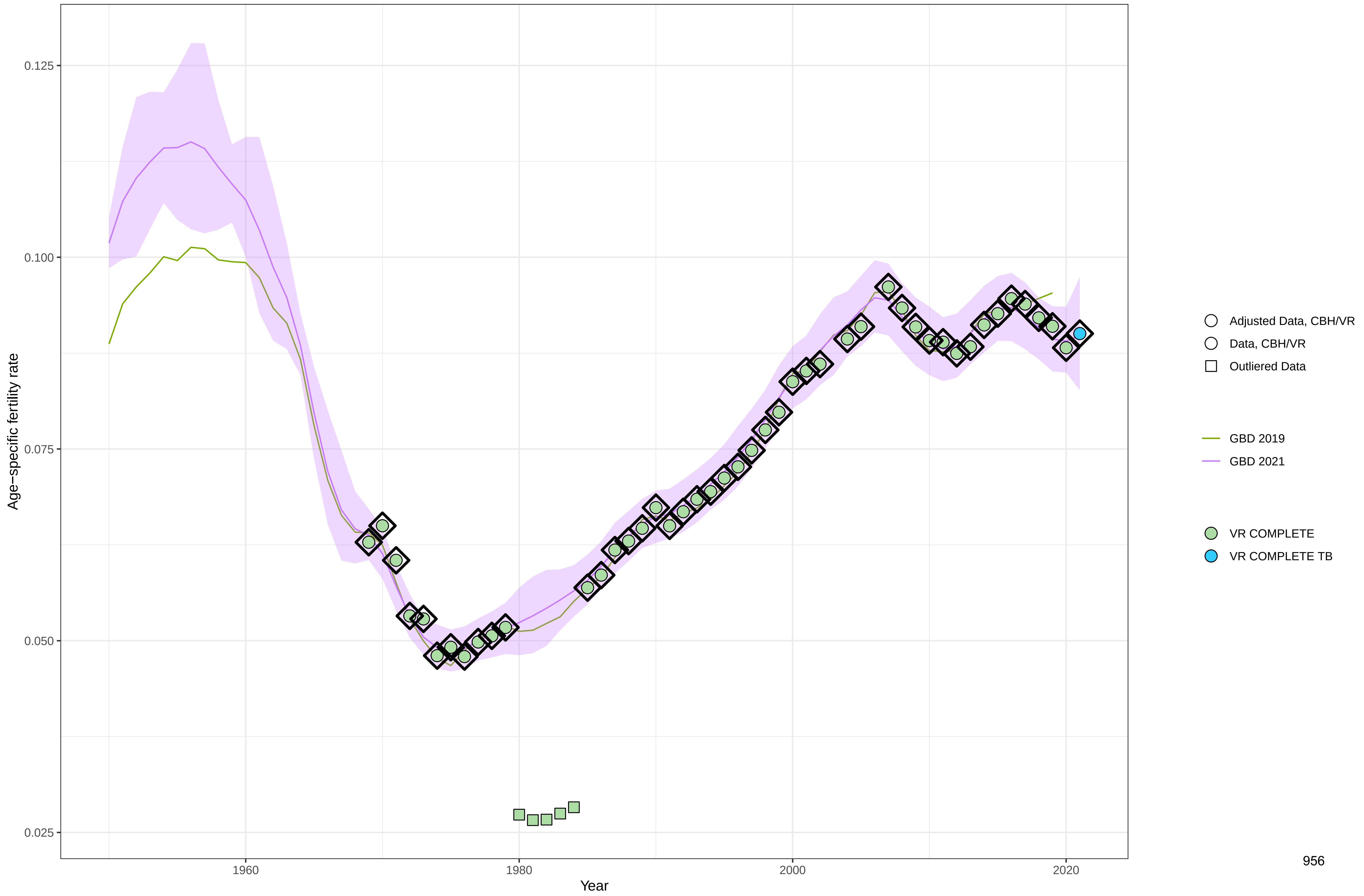

Age−specific fertility fitted trend lines, 1950−2021: Georgia, Ages: 35−39
Data points enclosed with a diamond are reference sources

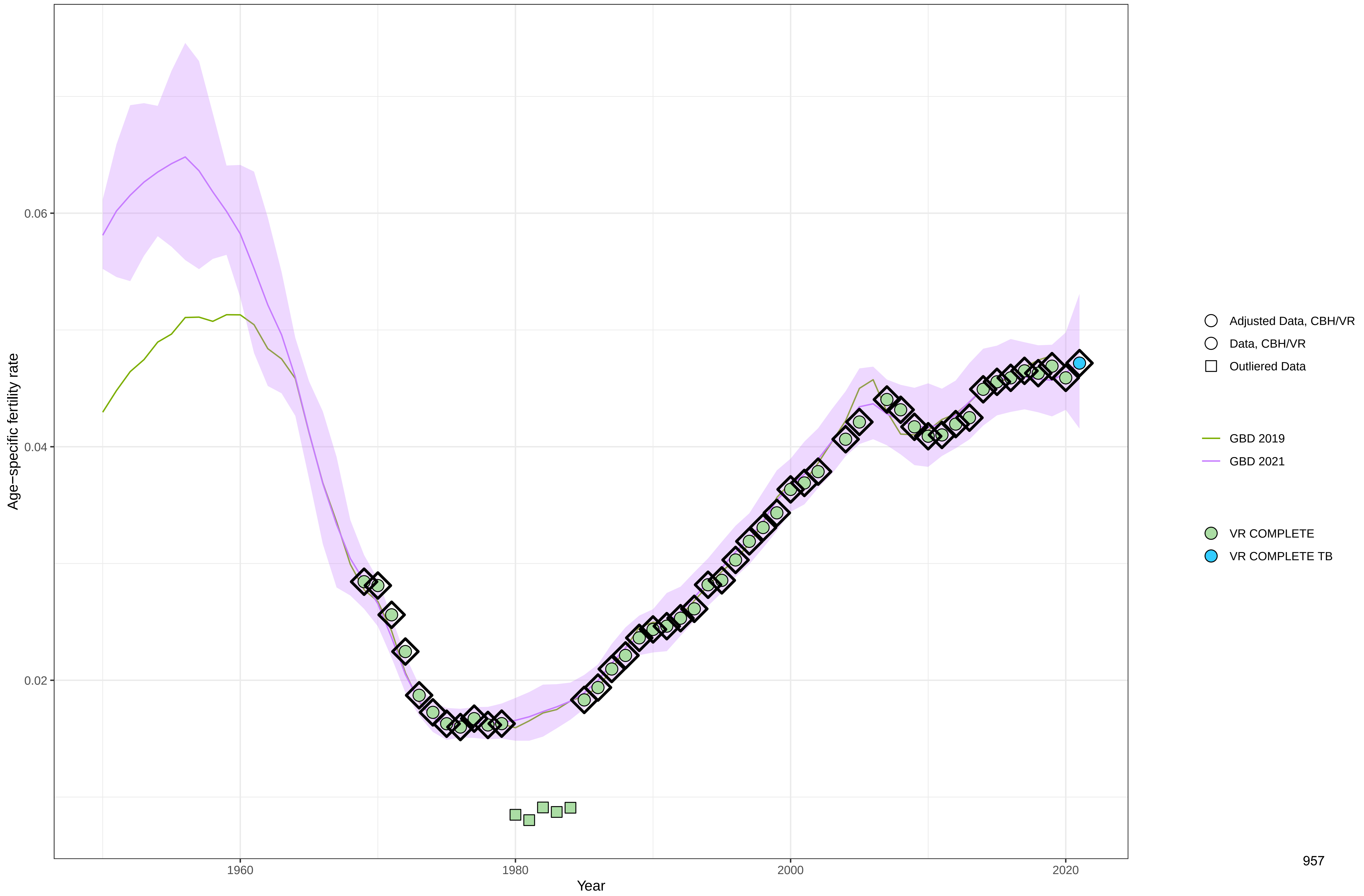

Age−specific fertility fitted trend lines, 1950−2021: Georgia, Ages: 40−44
Data points enclosed with a diamond are reference sources

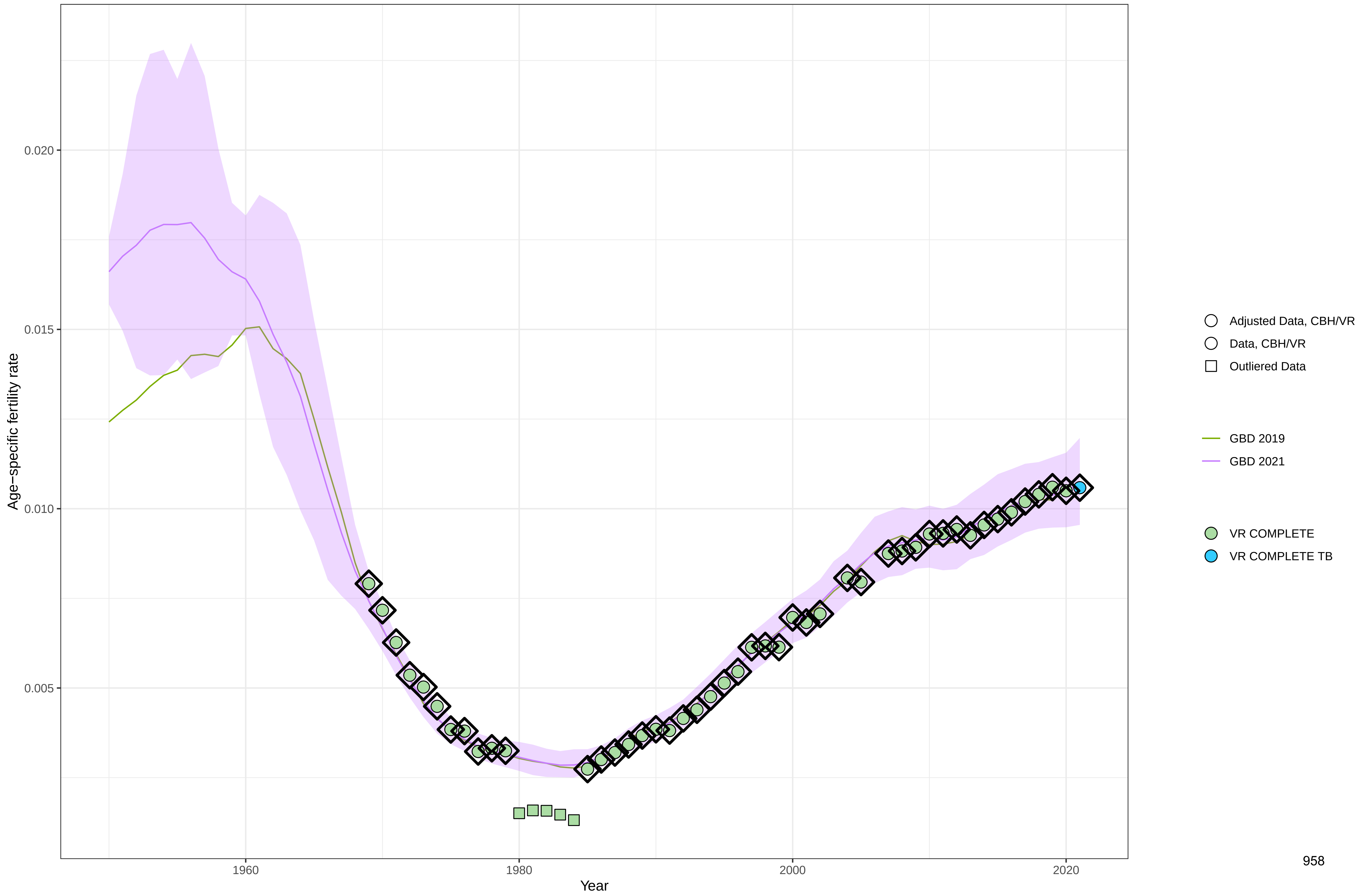

Age−specific fertility fitted trend lines, 1950−2021: Georgia, Ages: 45−49
Data points enclosed with a diamond are reference sources

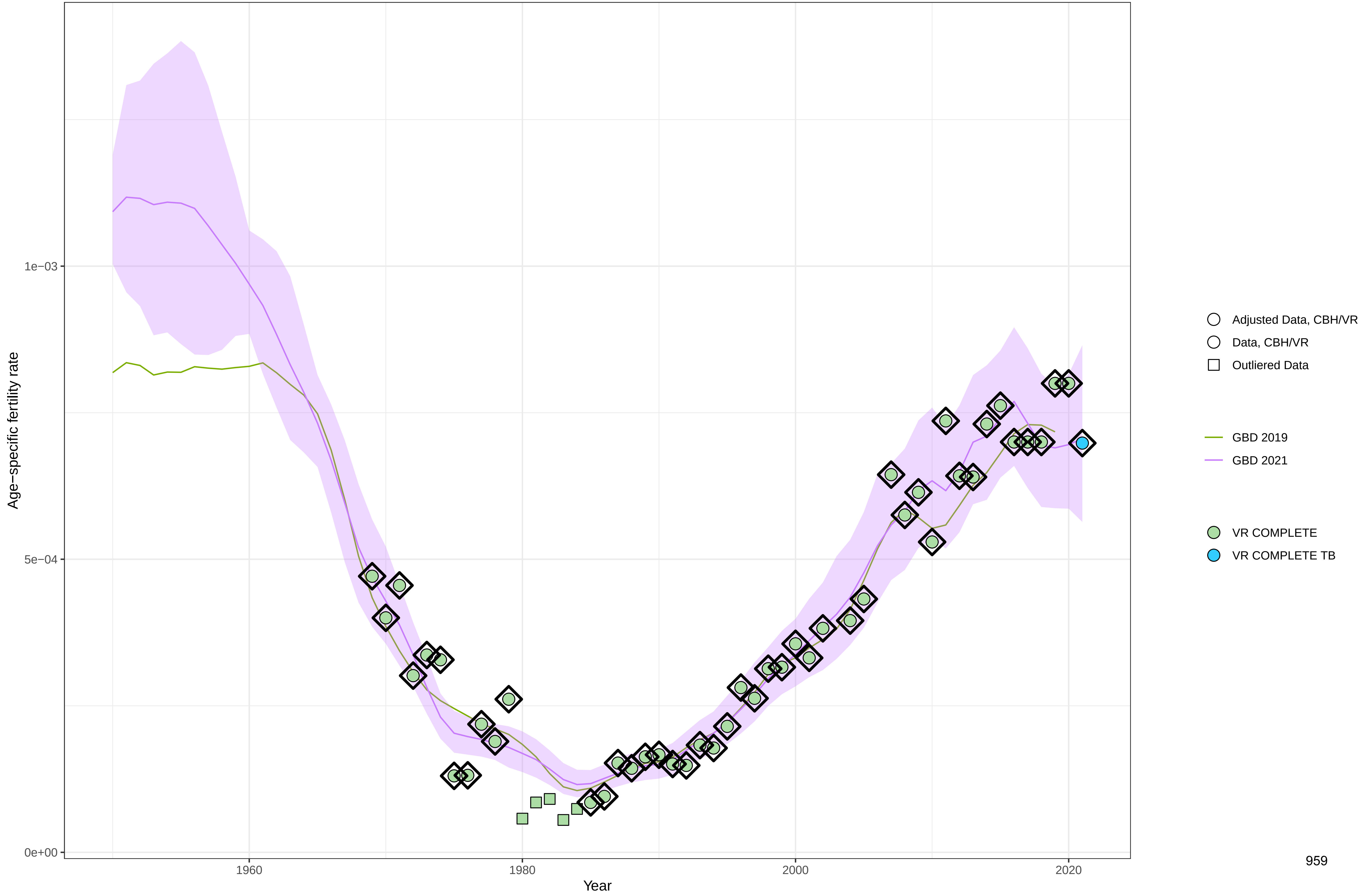

Age−specific fertility fitted trend lines, 1950−2021: Georgia, Ages: 50−54
Data points enclosed with a diamond are reference sources

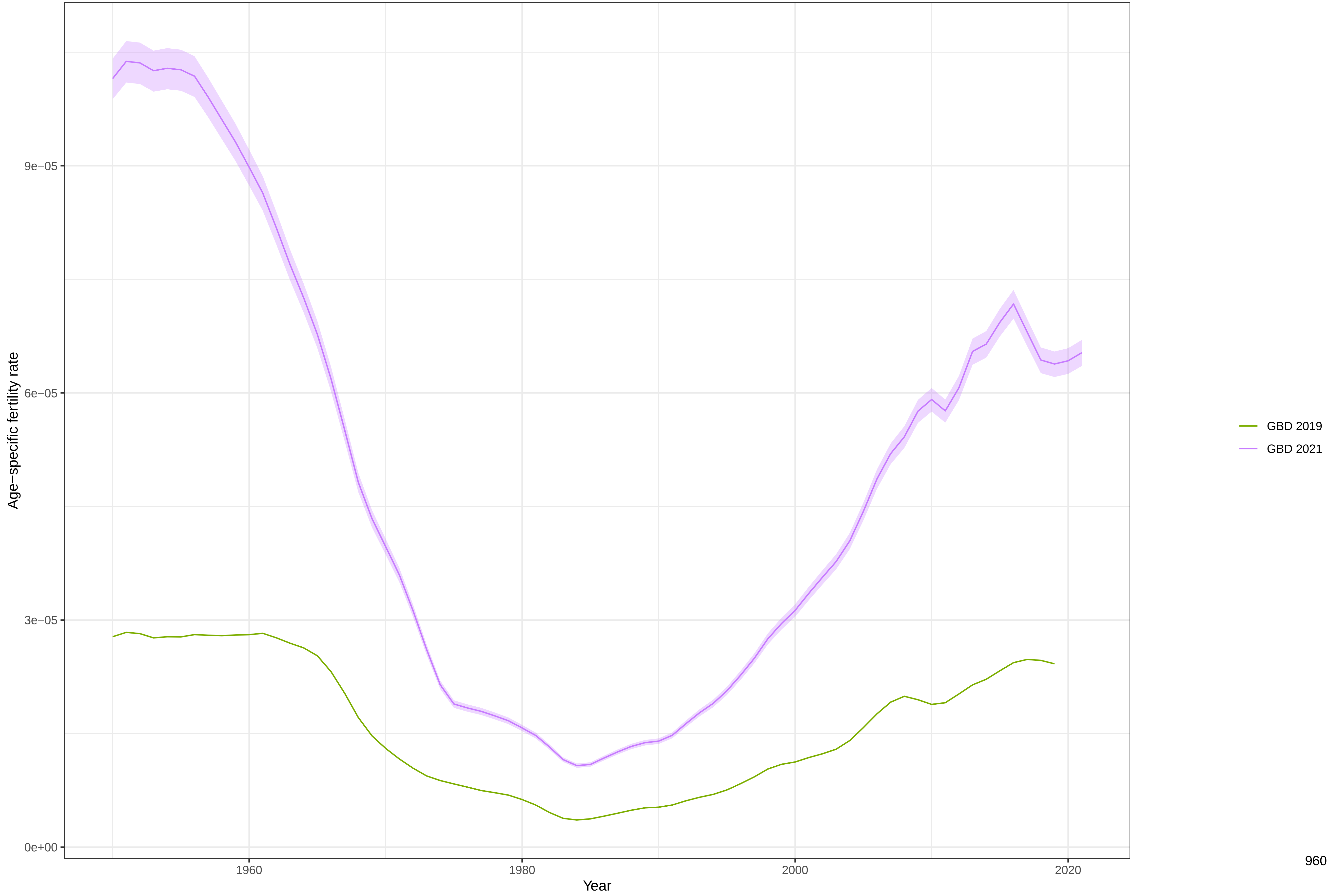

Total fertility rate fitted trend lines, 1950−2021: Georgia TFR
Data points enclosed with a diamond are reference sources

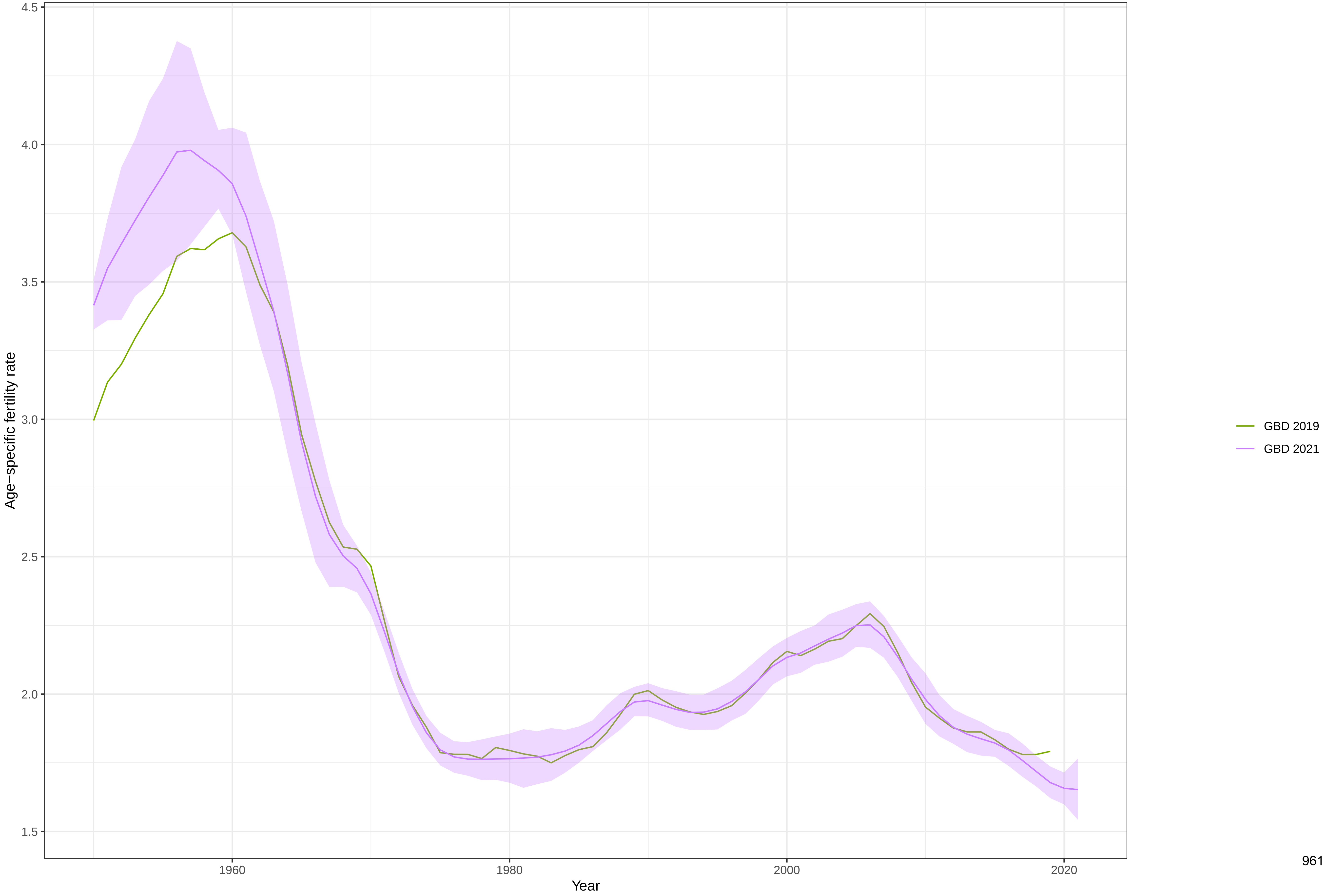

Age−specific fertility fitted trend lines, 1950−2021: Hawaii, Ages: 10−14
Data points enclosed with a diamond are reference sources

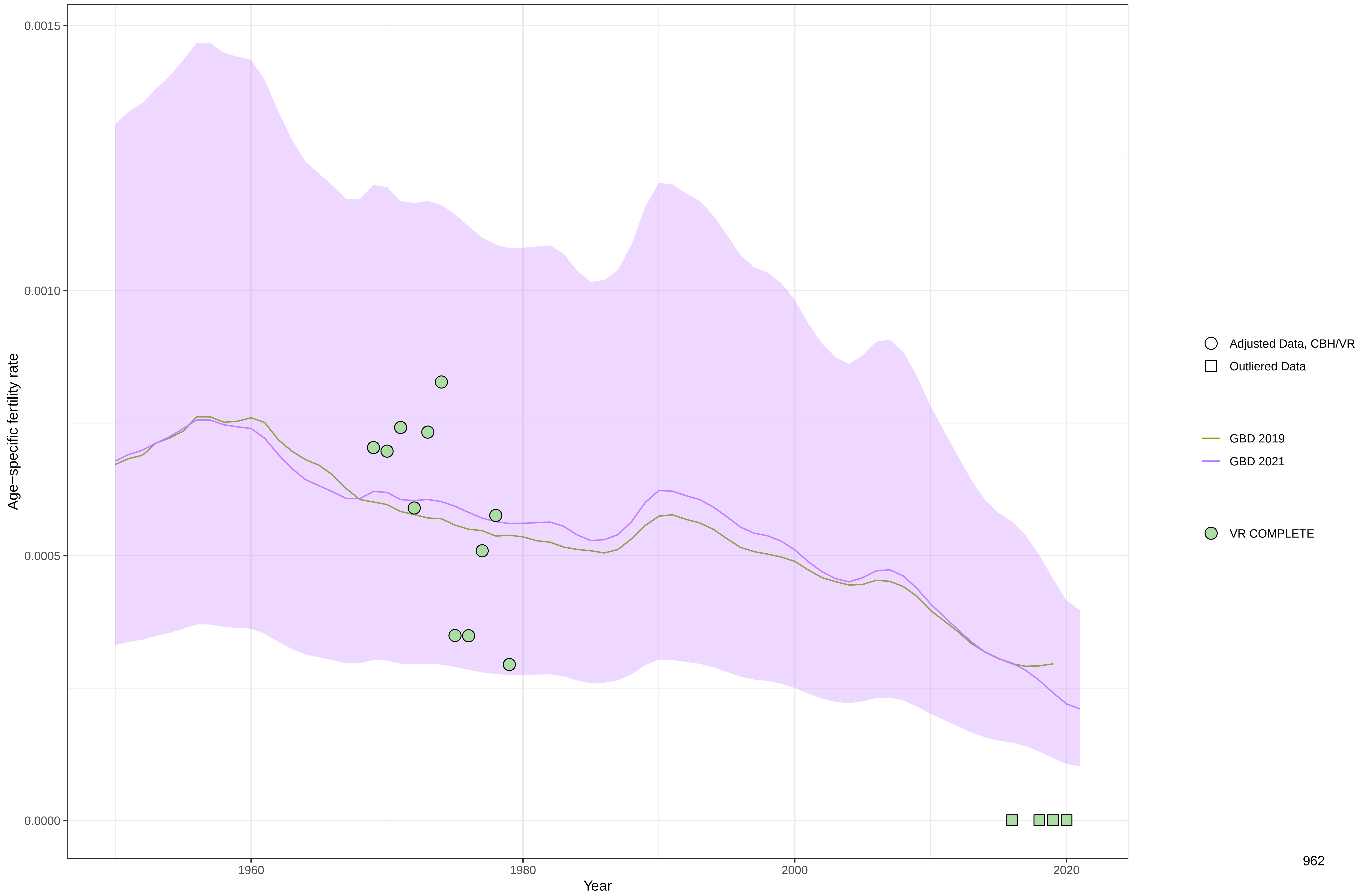

Age−specific fertility fitted trend lines, 1950−2021: Hawaii, Ages: 15−19
Data points enclosed with a diamond are reference sources

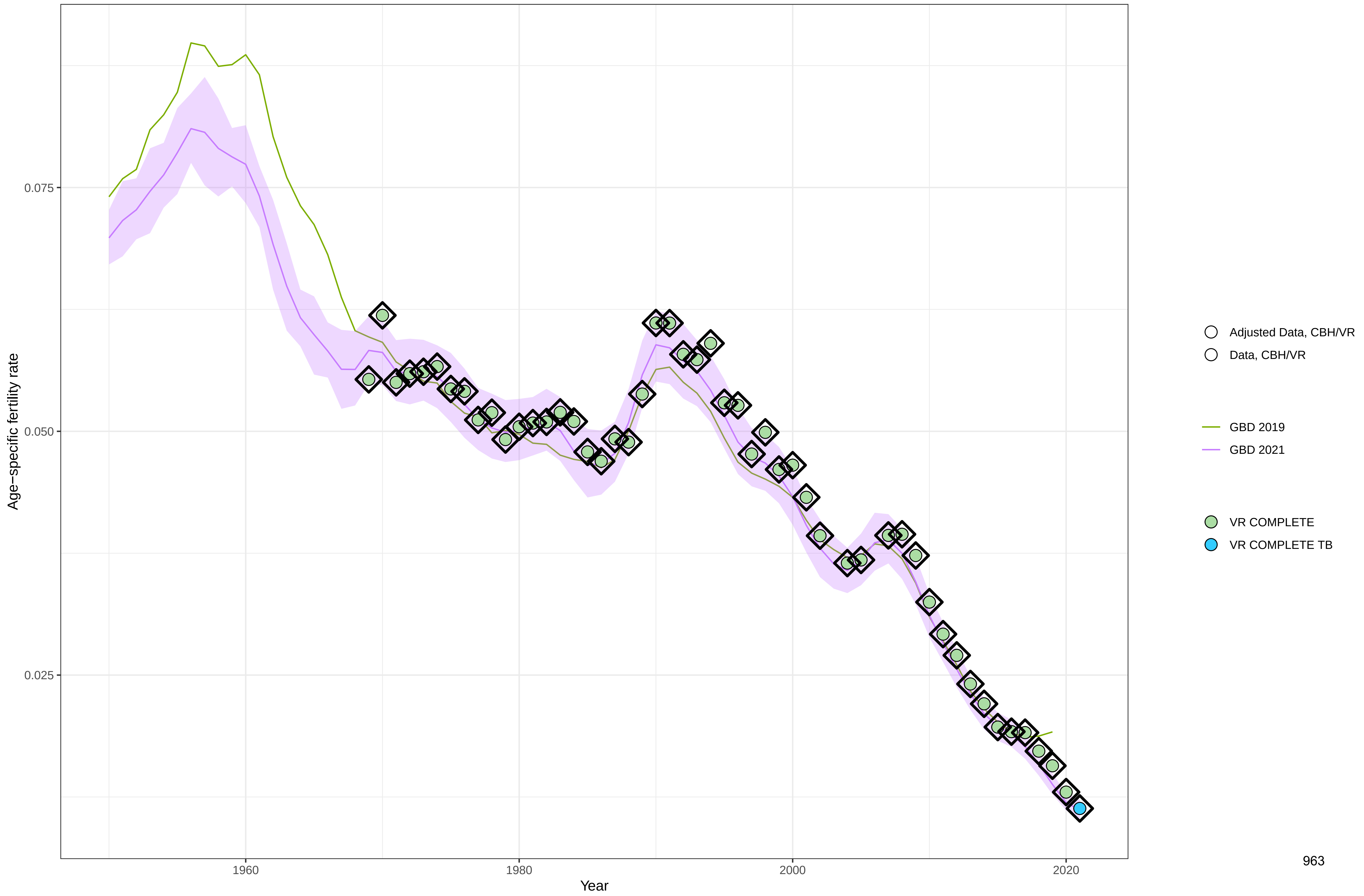

Age−specific fertility fitted trend lines, 1950−2021: Hawaii, Ages: 20−24
Data points enclosed with a diamond are reference sources

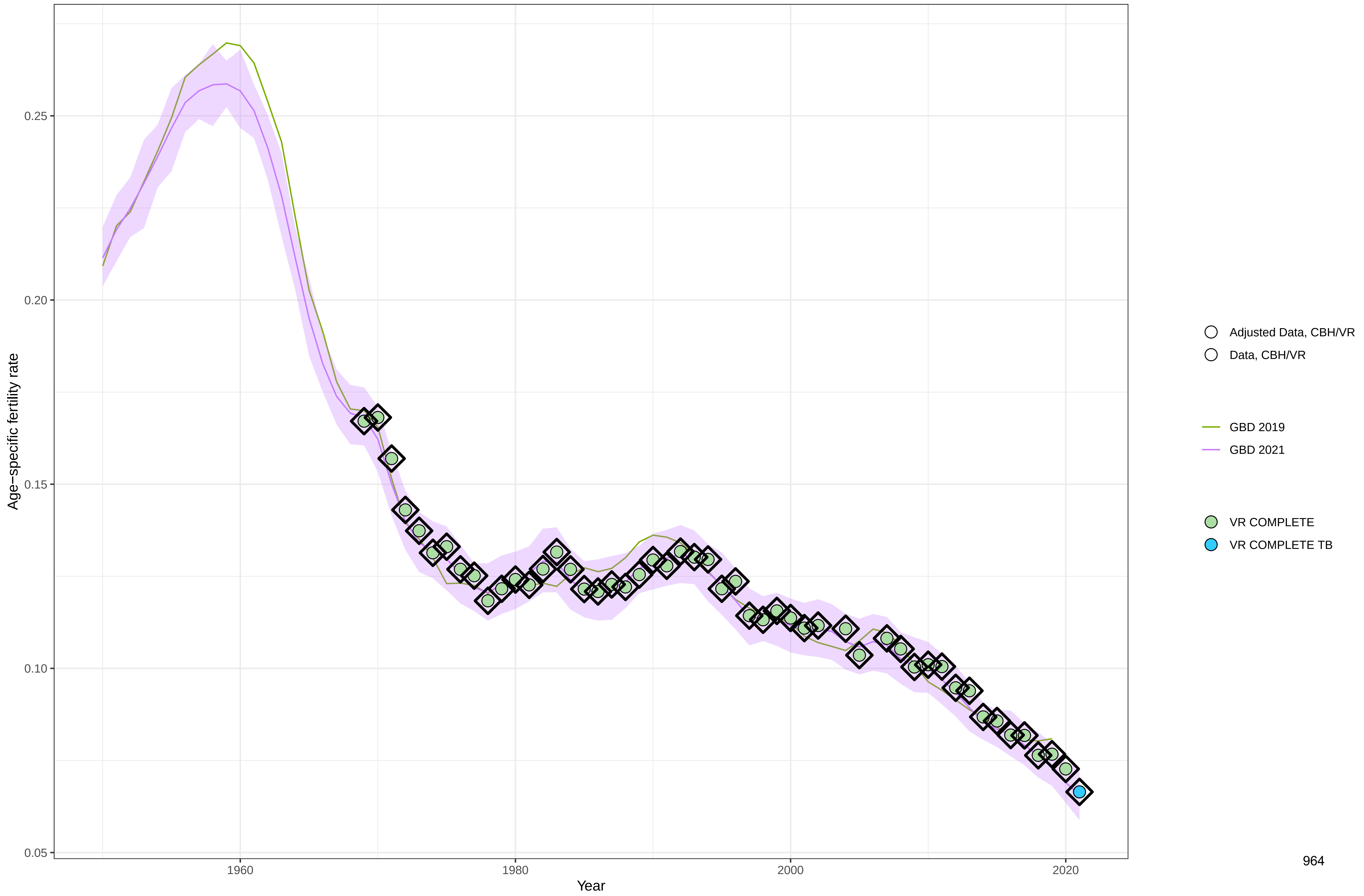

Age−specific fertility fitted trend lines, 1950−2021: Hawaii, Ages: 25−29
Data points enclosed with a diamond are reference sources

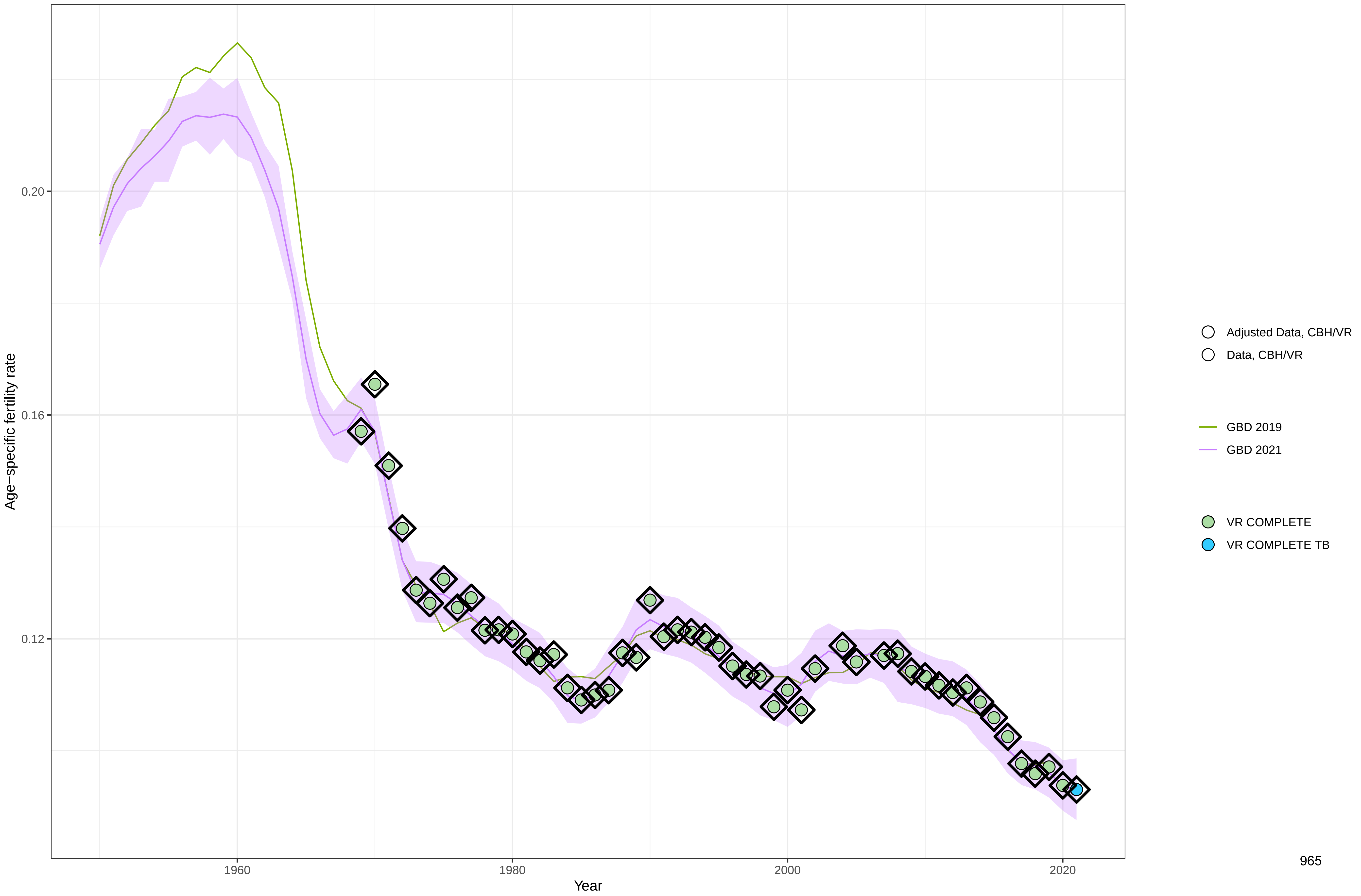

Age−specific fertility fitted trend lines, 1950−2021: Hawaii, Ages: 30−34
Data points enclosed with a diamond are reference sources

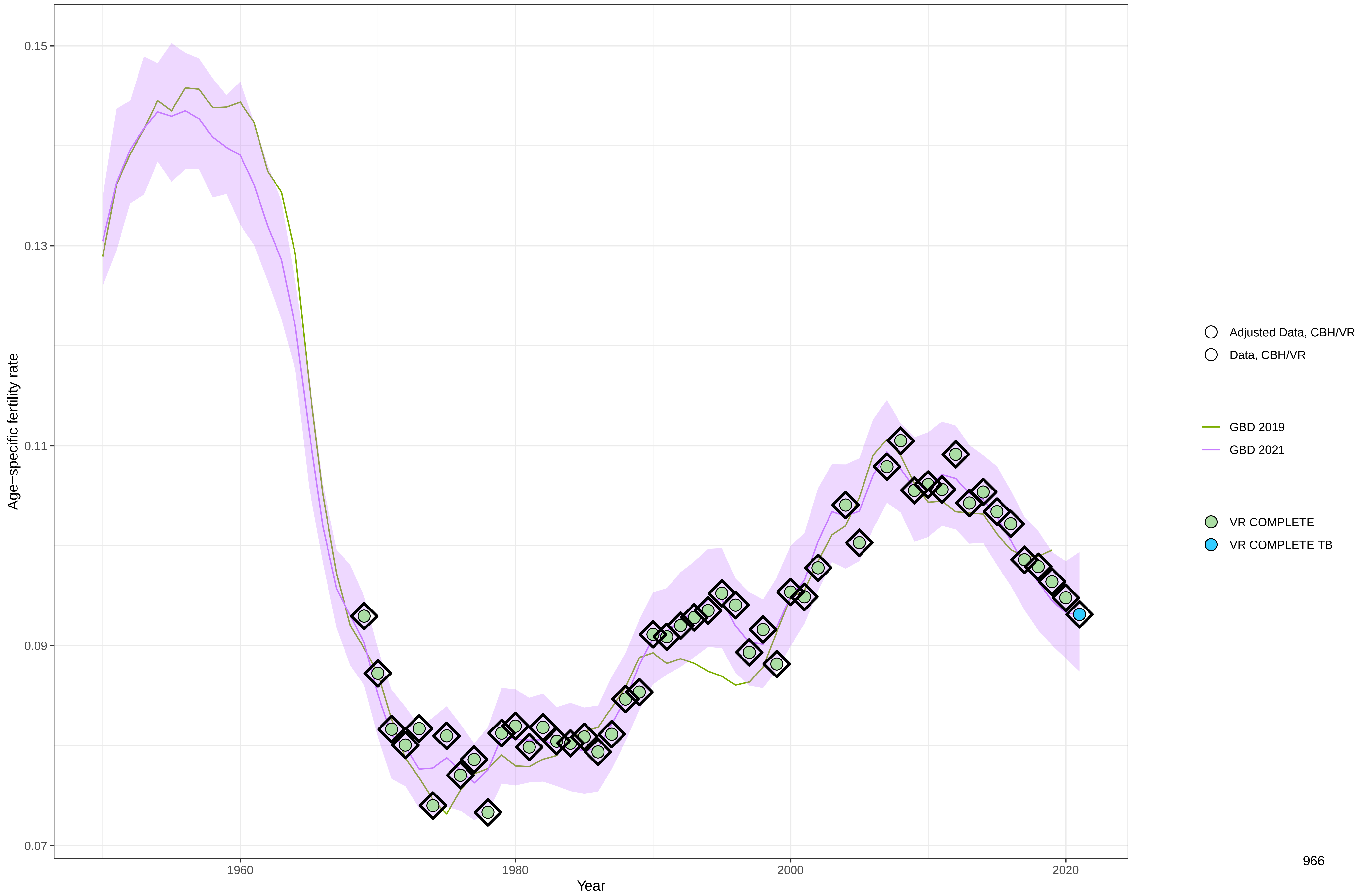

Age−specific fertility fitted trend lines, 1950−2021: Hawaii, Ages: 35−39
Data points enclosed with a diamond are reference sources

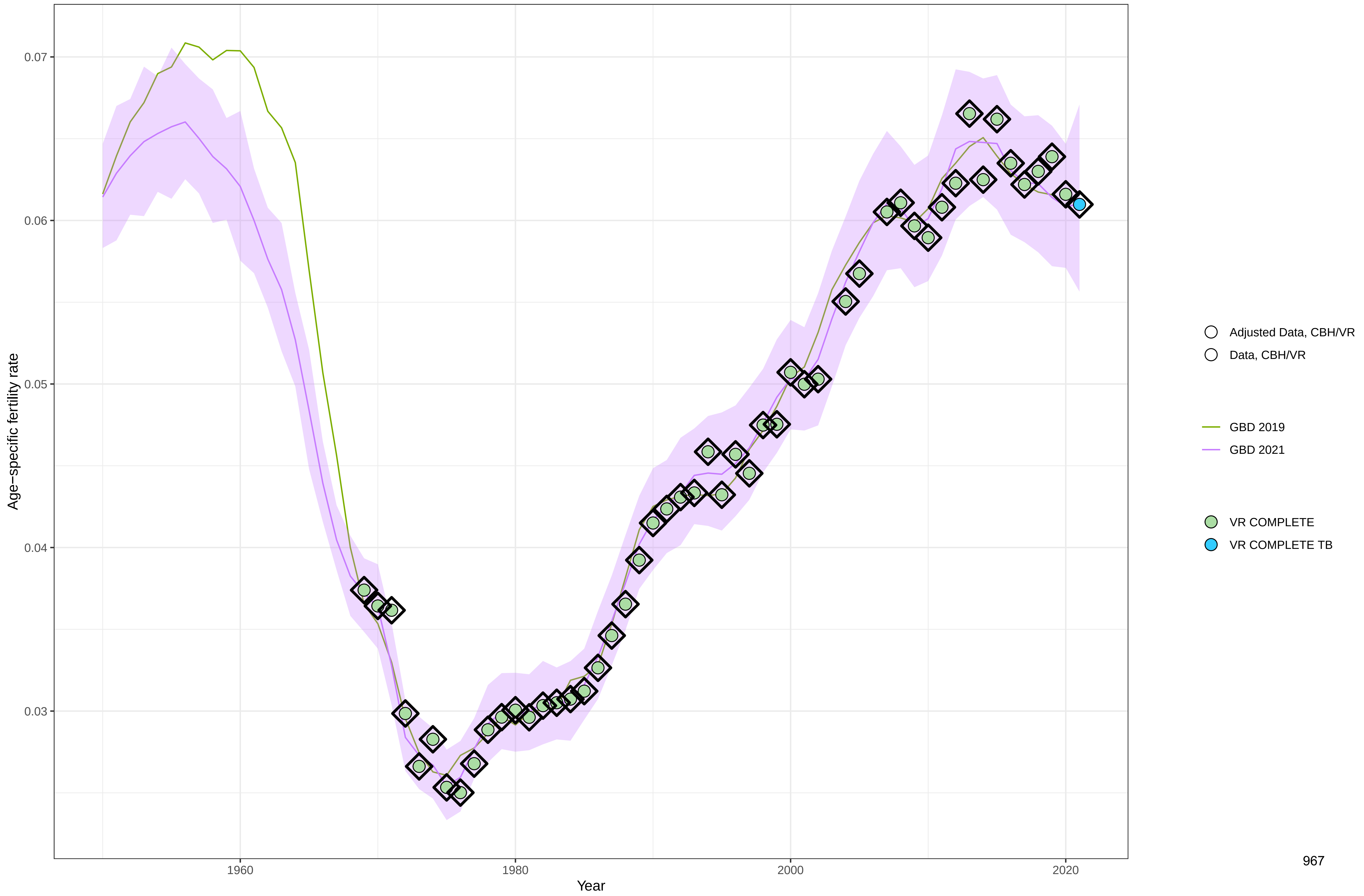

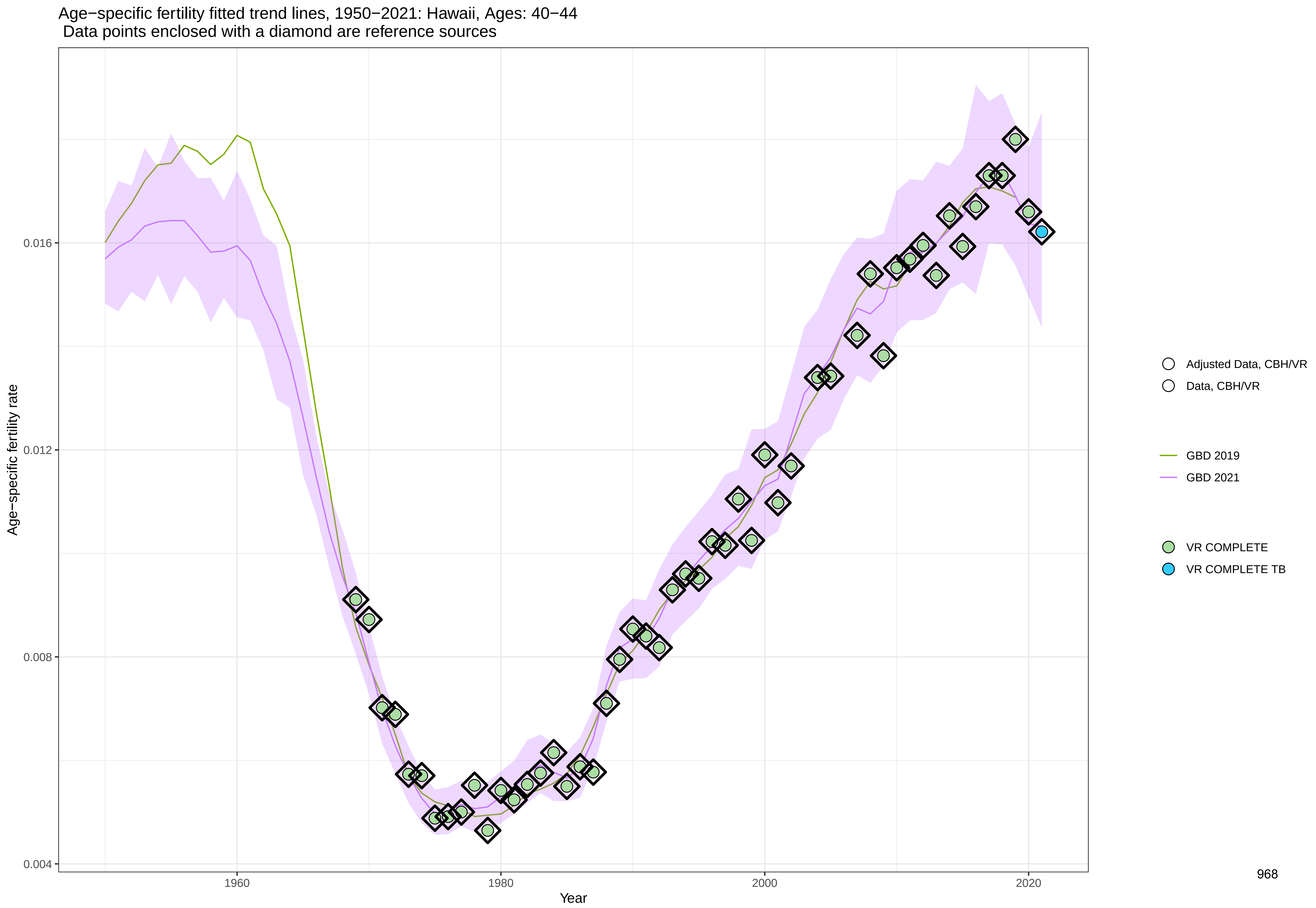
Age−specific fertility fitted trend lines, 1950−2021: Hawaii, Ages: 40−44
Data points enclosed with a diamond are reference sources
Age−specific fertility rate
0.016
0.012
0.008
0.004
1960
1980
2000
2020
Year
Adjusted Data, CBH/VR
Data, CBH/VR
GBD 2019
GBD 2021
VR COMPLETE
VR COMPLETE TB

Age−specific fertility fitted trend lines, 1950−2021: Hawaii, Ages: 45−49
Data points enclosed with a diamond are reference sources

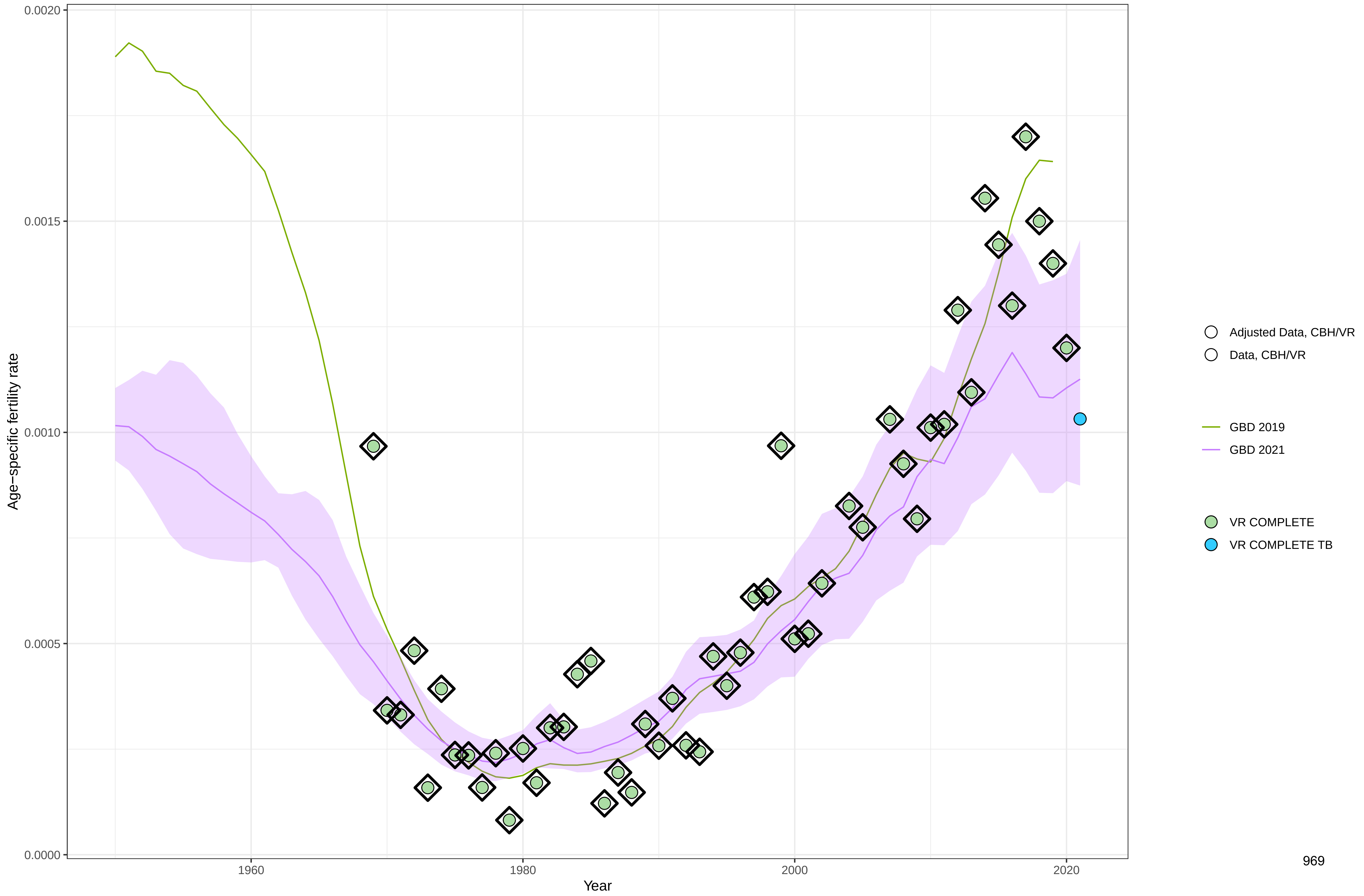

Age−specific fertility fitted trend lines, 1950−2021: Hawaii, Ages: 50−54
Data points enclosed with a diamond are reference sources

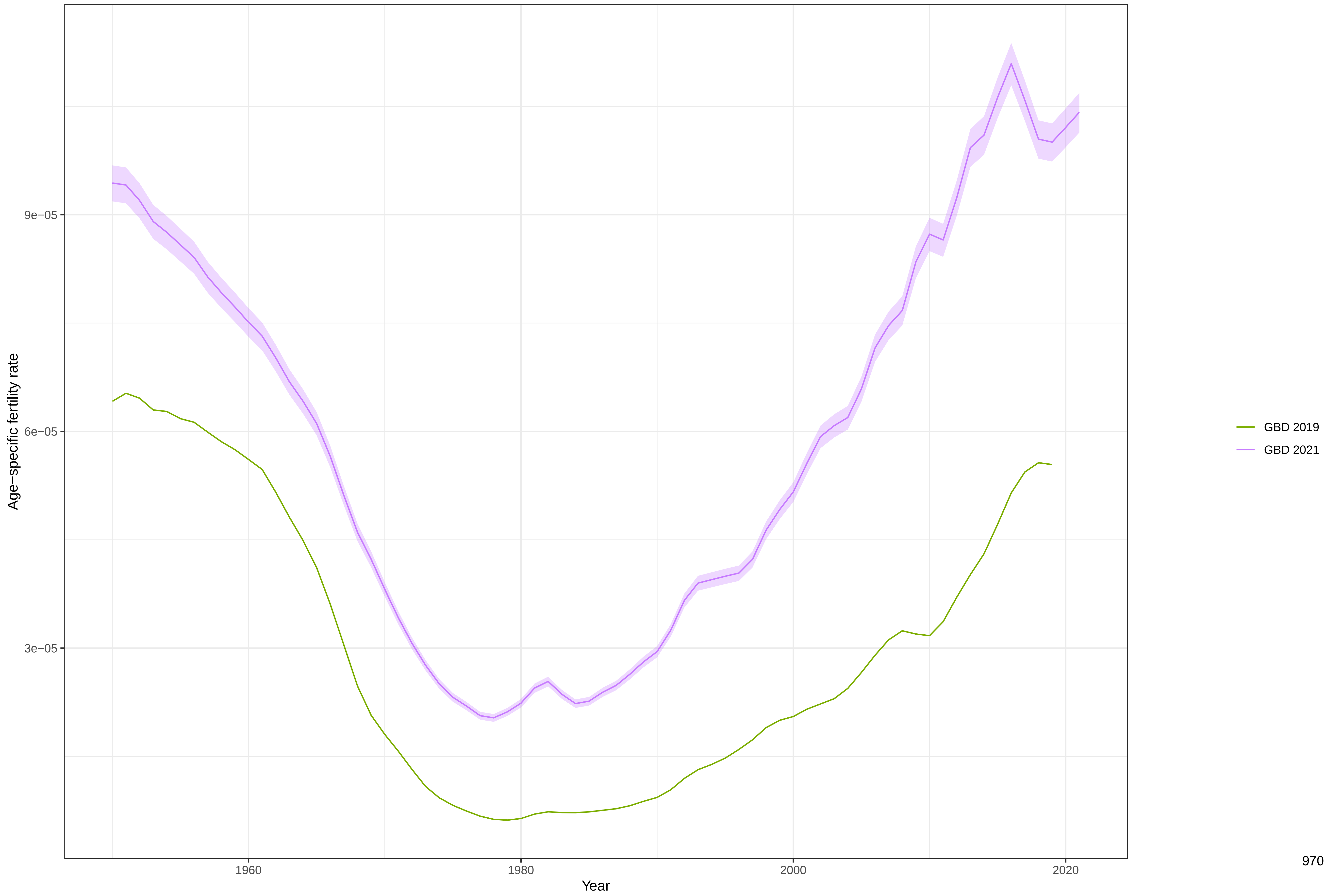

Total fertility rate fitted trend lines, 1950−2021: Hawaii TFR
Data points enclosed with a diamond are reference sources

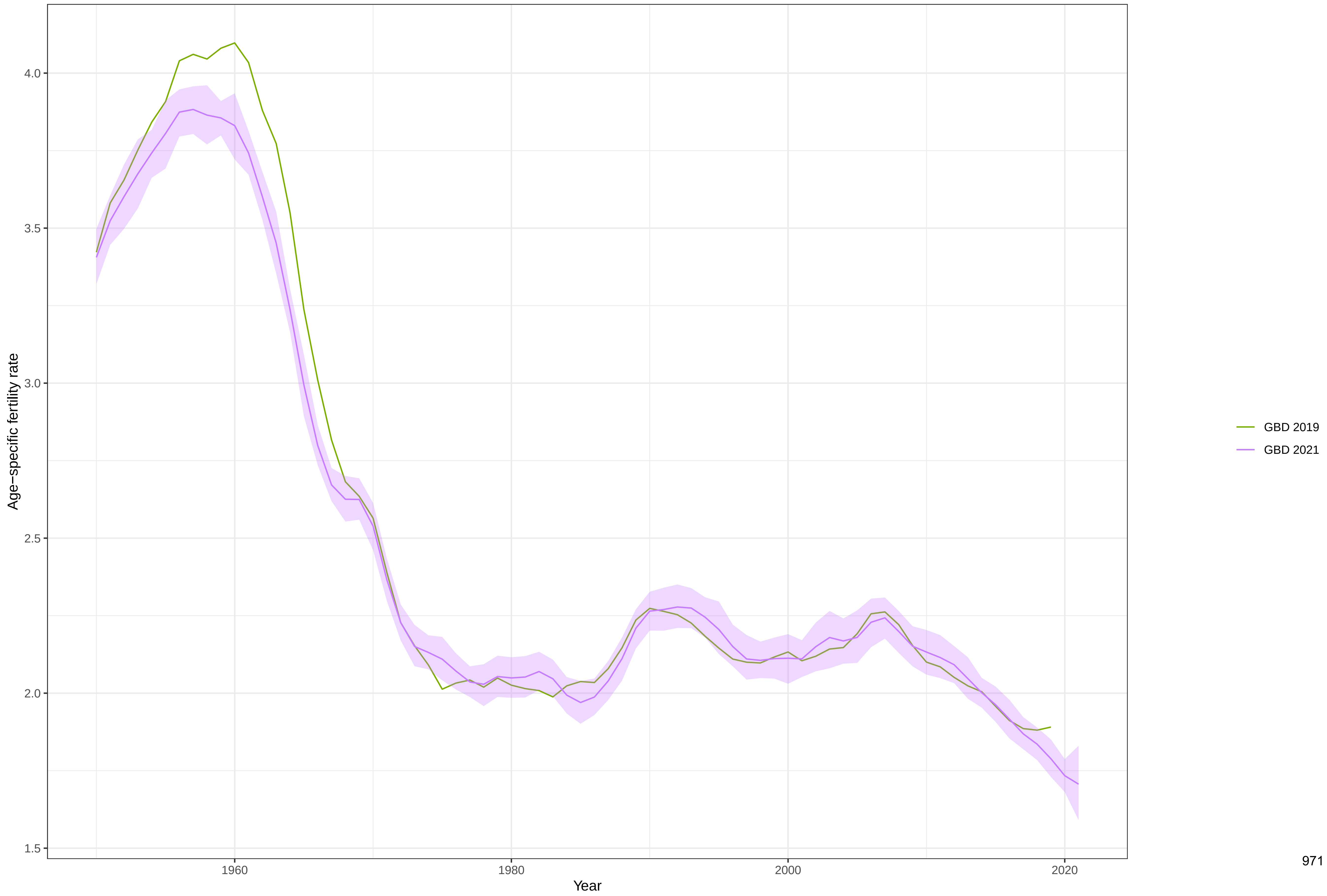

Age−specific fertility fitted trend lines, 1950−2021: Idaho, Ages: 10−14
Data points enclosed with a diamond are reference sources

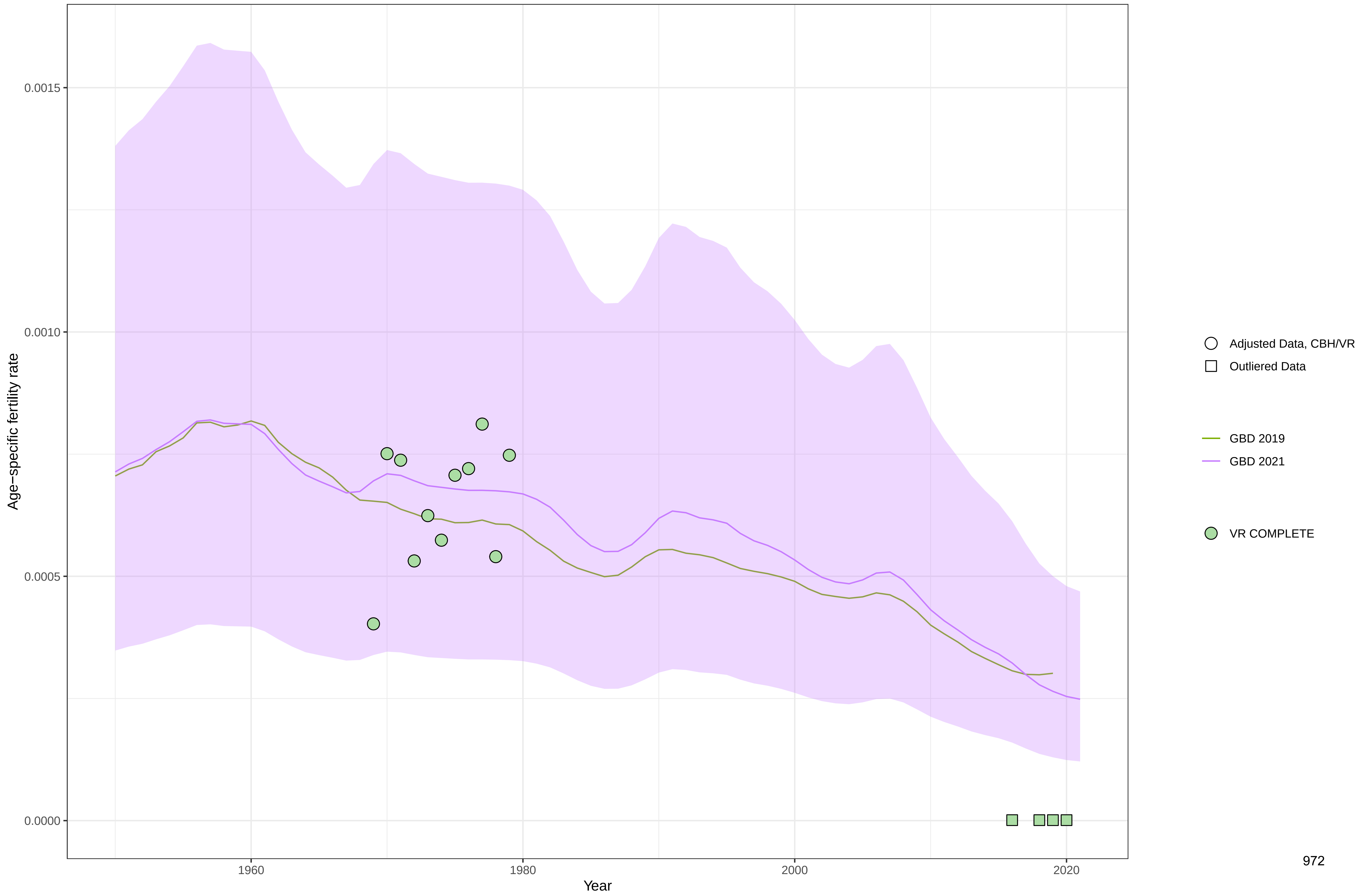

Age−specific fertility fitted trend lines, 1950−2021: Idaho, Ages: 15−19
Data points enclosed with a diamond are reference sources

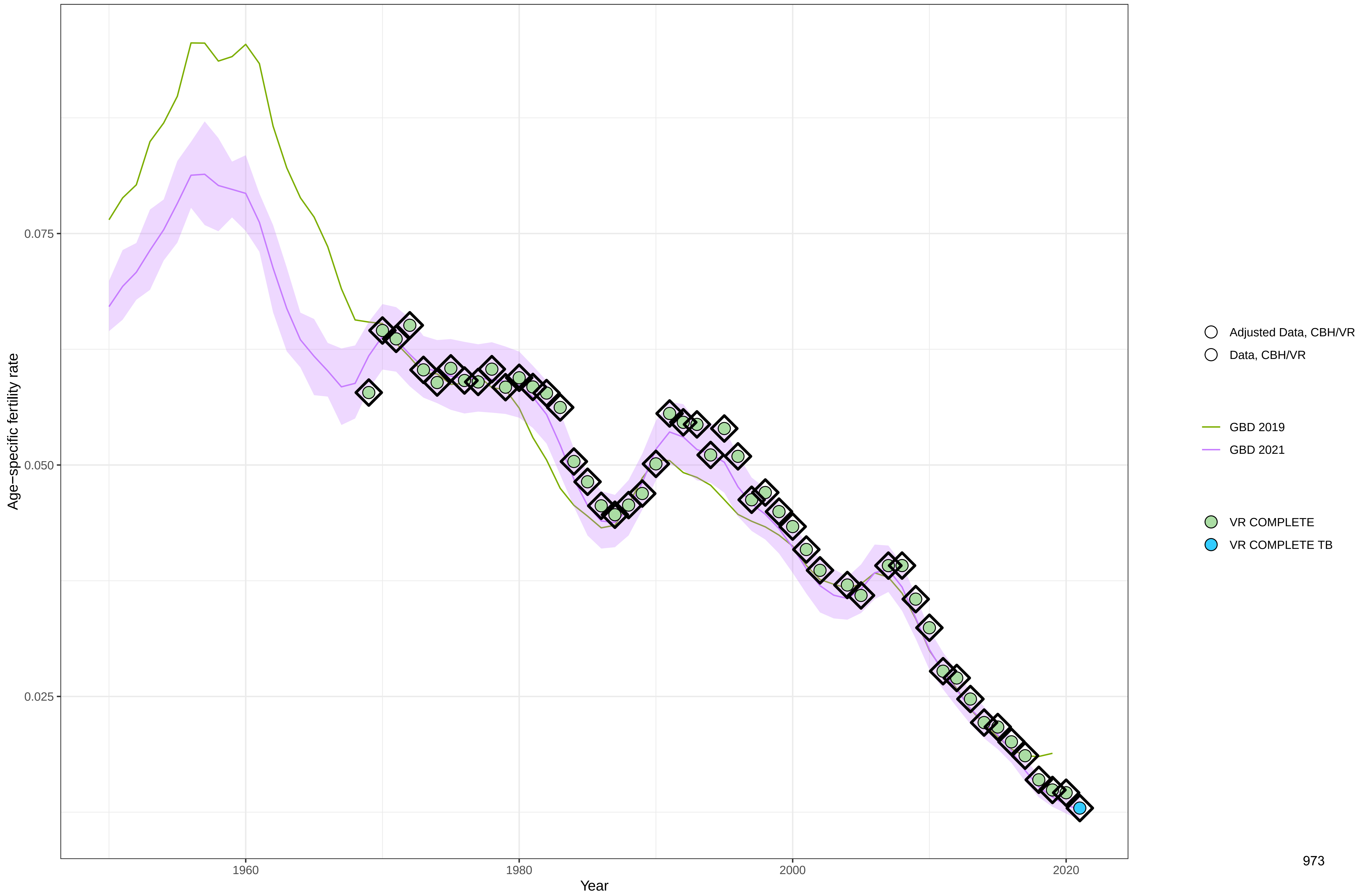

Age−specific fertility fitted trend lines, 1950−2021: Idaho, Ages: 20−24
Data points enclosed with a diamond are reference sources

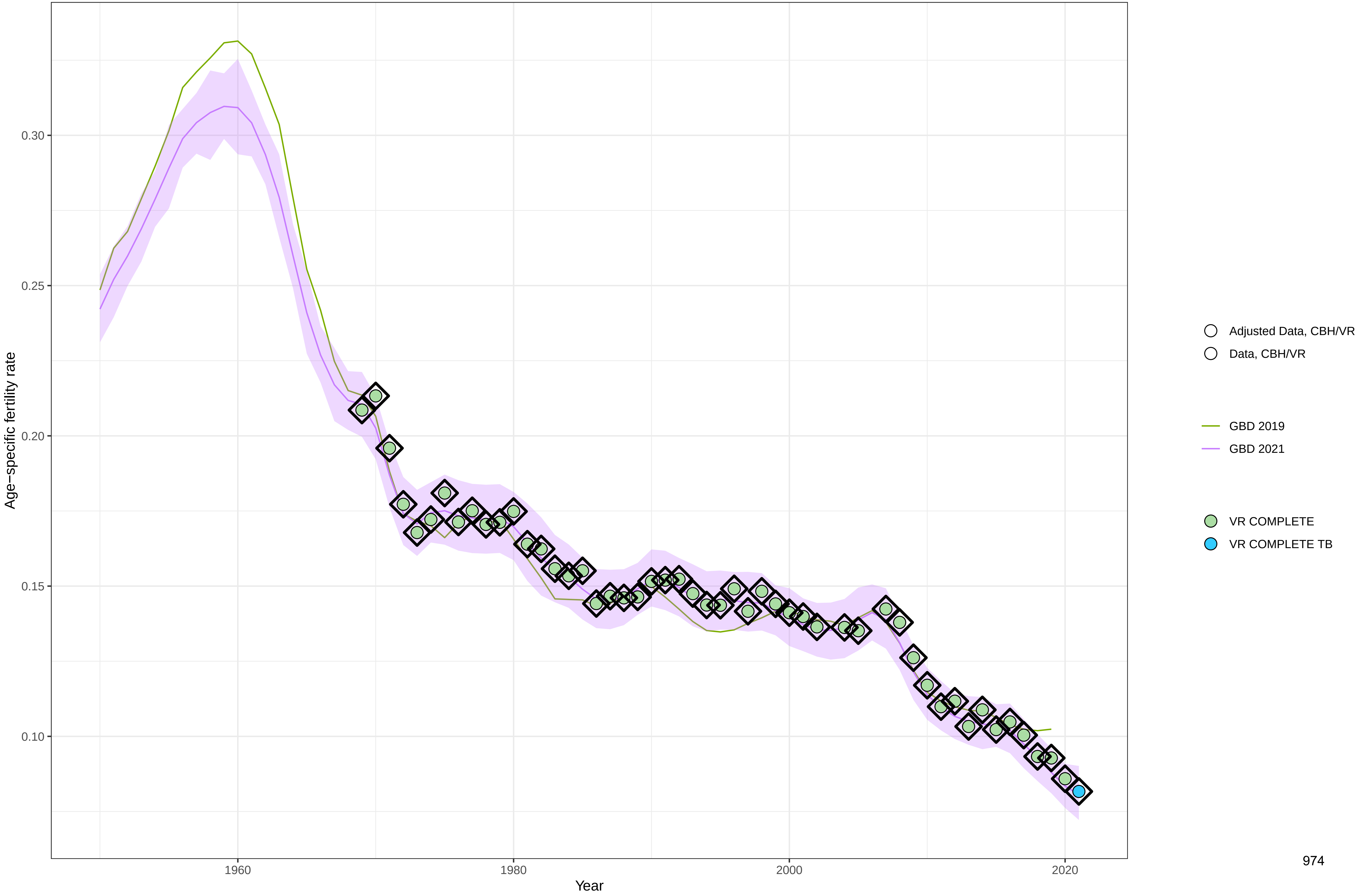

Age−specific fertility fitted trend lines, 1950−2021: Idaho, Ages: 25−29
Data points enclosed with a diamond are reference sources

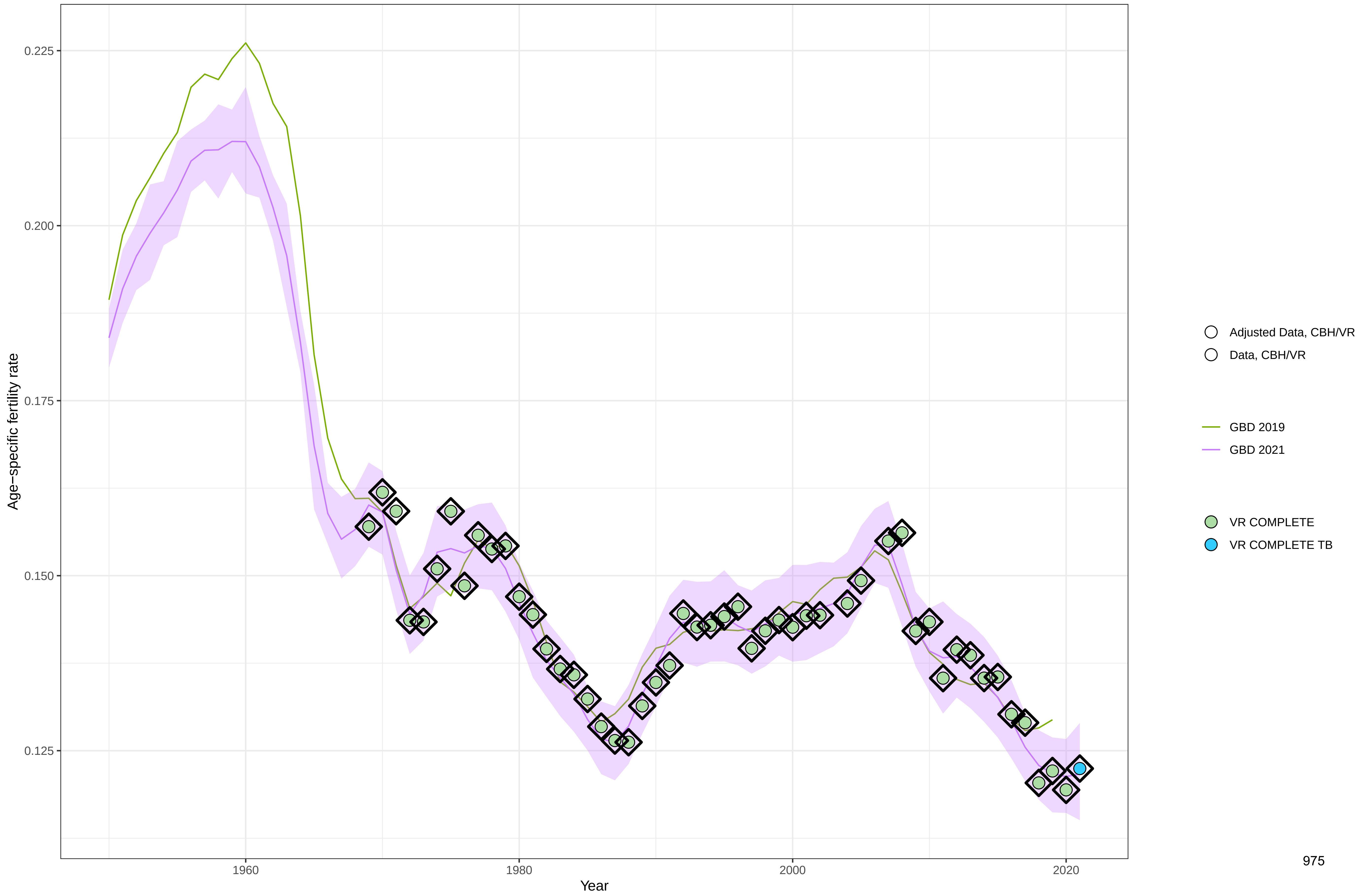

Age−specific fertility fitted trend lines, 1950−2021: Idaho, Ages: 30−34
Data points enclosed with a diamond are reference sources

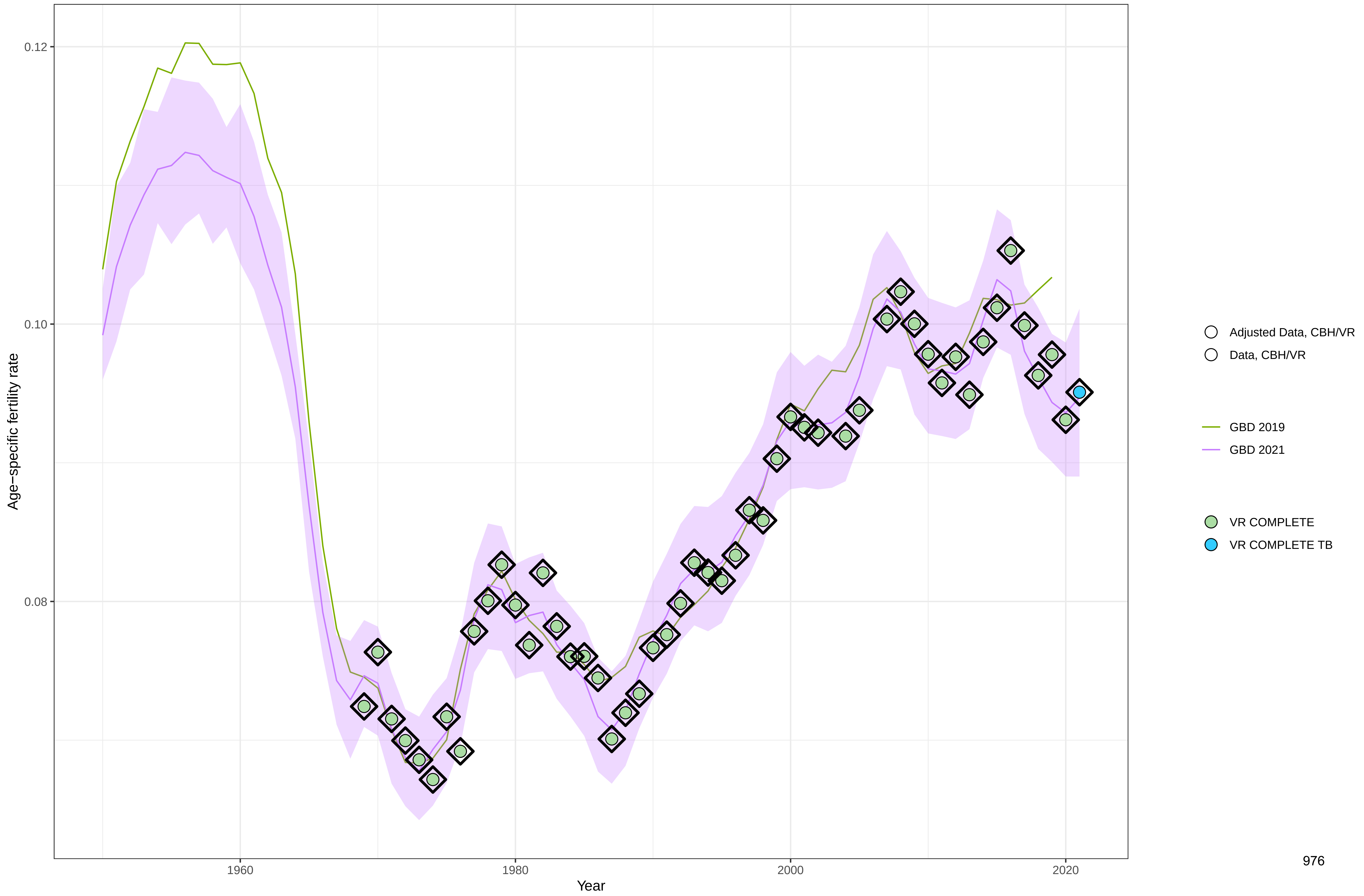

Age−specific fertility fitted trend lines, 1950−2021: Idaho, Ages: 35−39
Data points enclosed with a diamond are reference sources

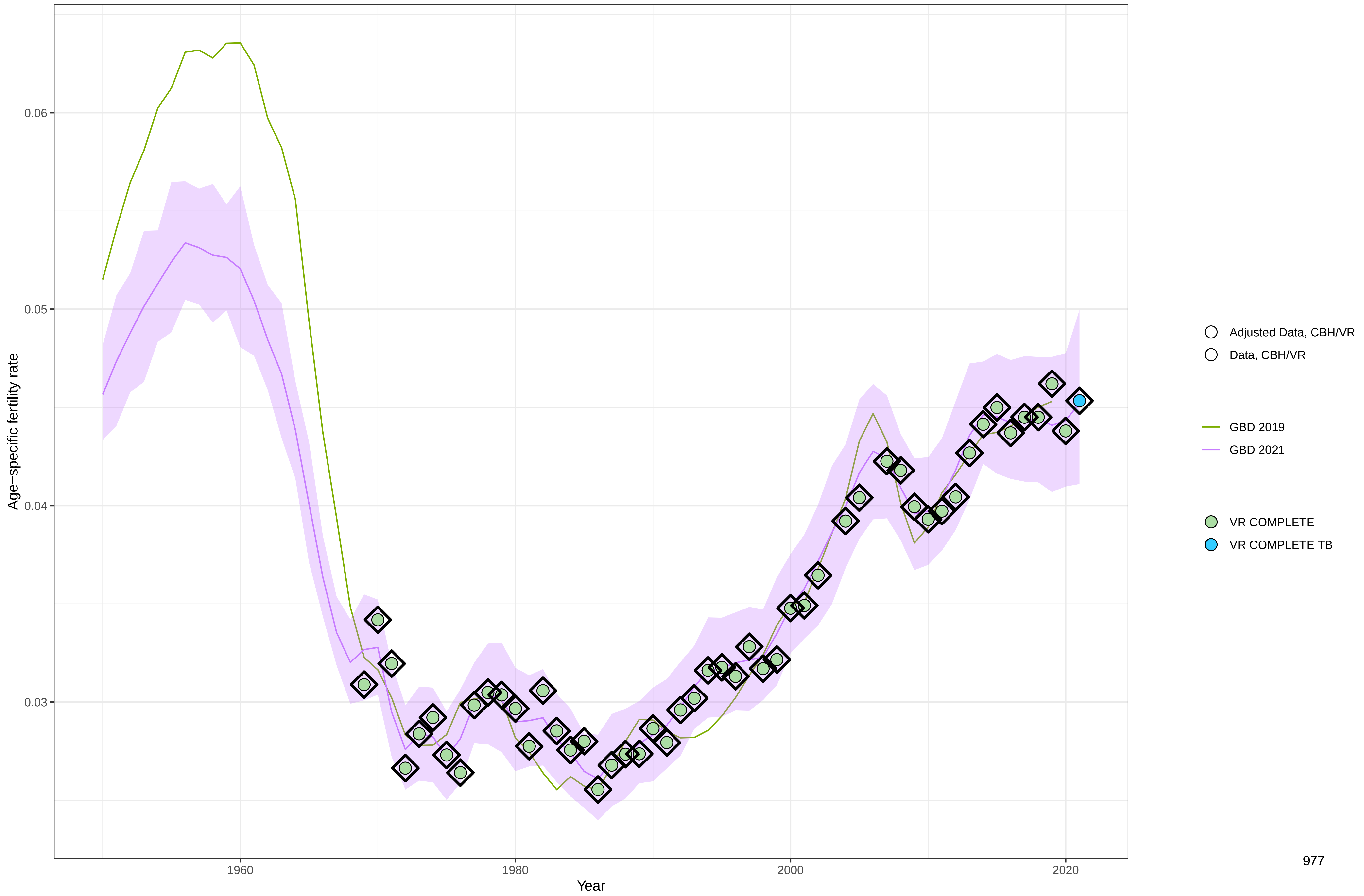

Age−specific fertility fitted trend lines, 1950−2021: Idaho, Ages: 40−44
Data points enclosed with a diamond are reference sources

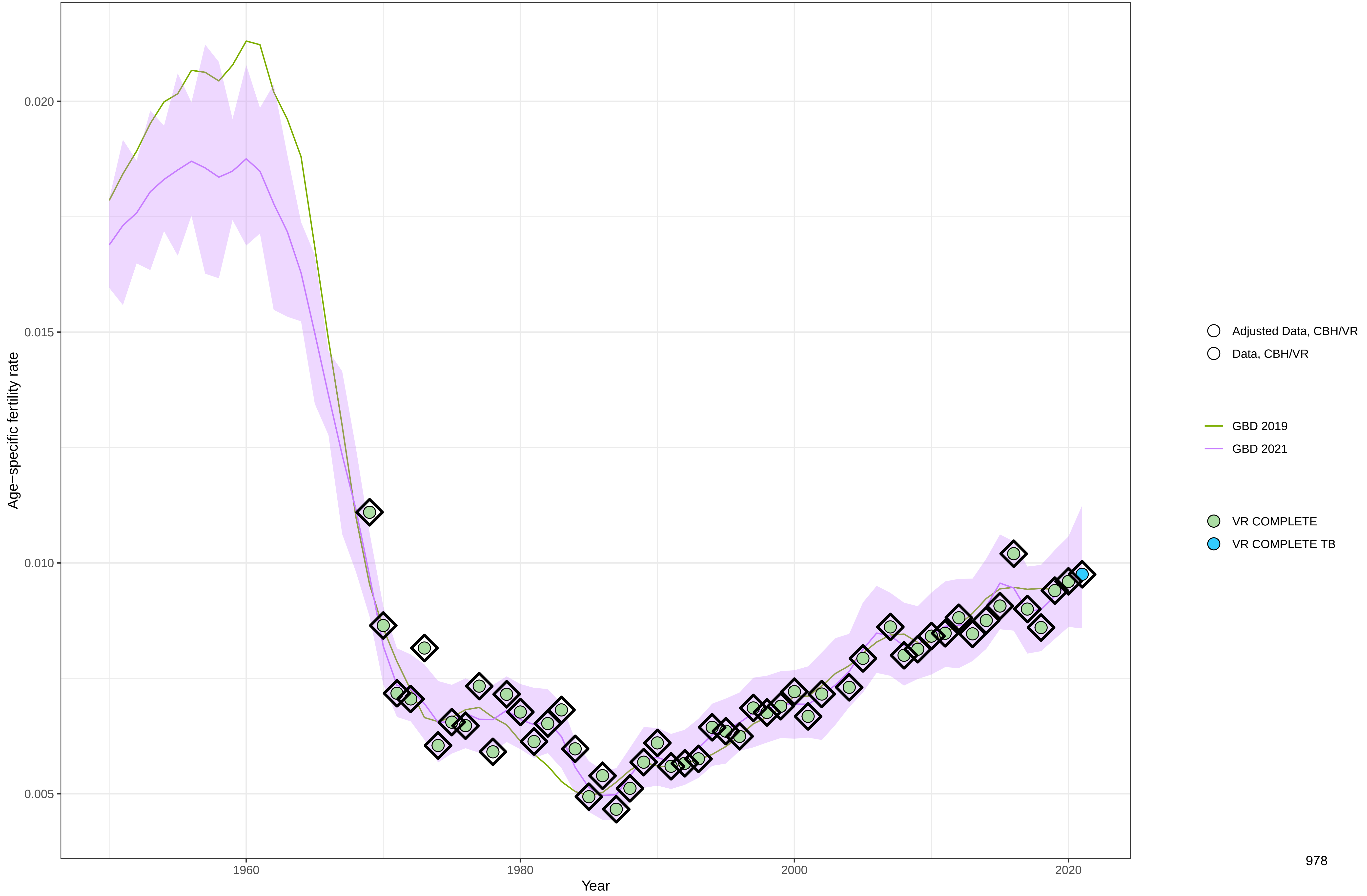

Age−specific fertility fitted trend lines, 1950−2021: Idaho, Ages: 45−49
Data points enclosed with a diamond are reference sources

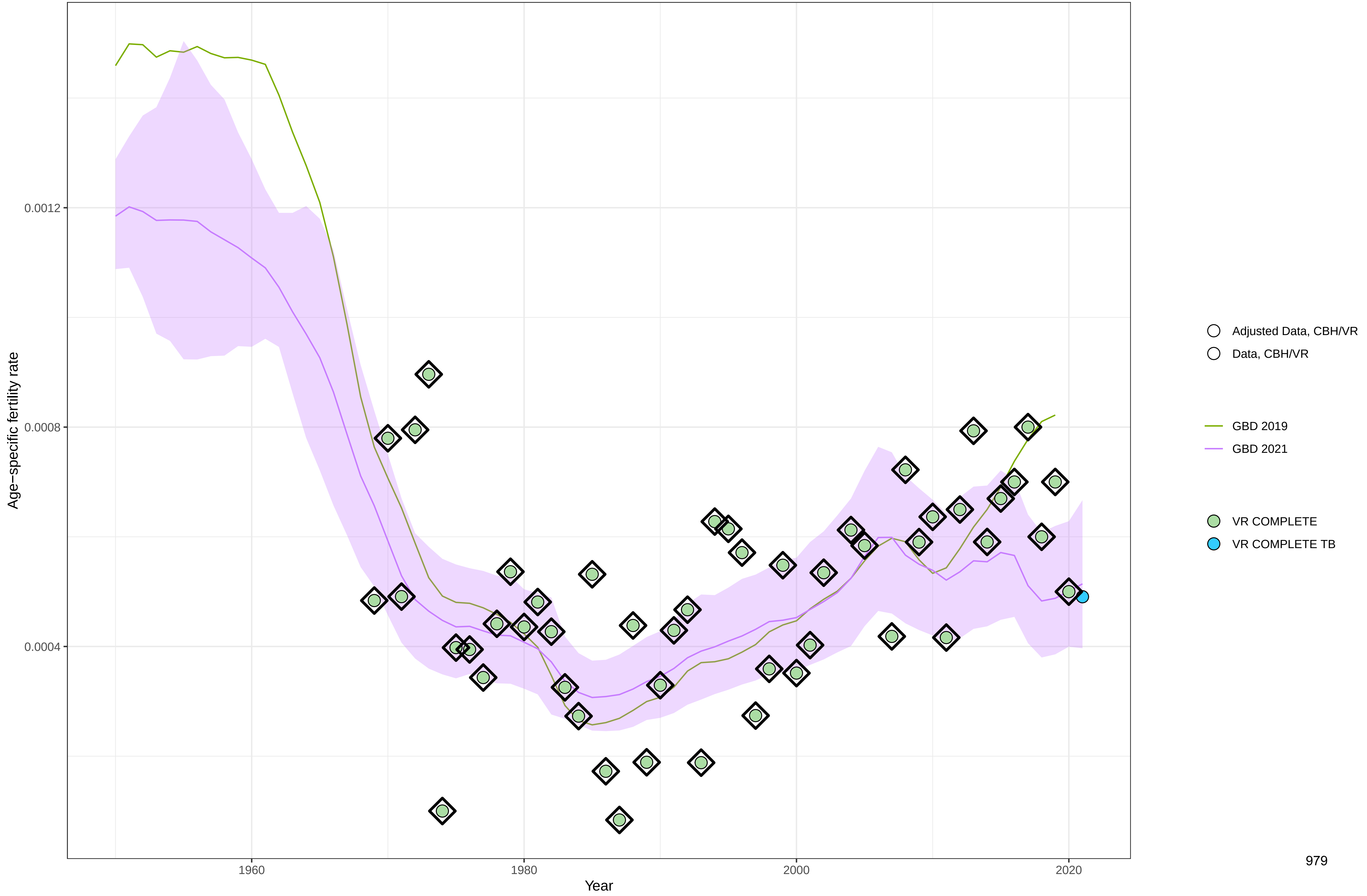

Age−specific fertility fitted trend lines, 1950−2021: Idaho, Ages: 50−54
Data points enclosed with a diamond are reference sources

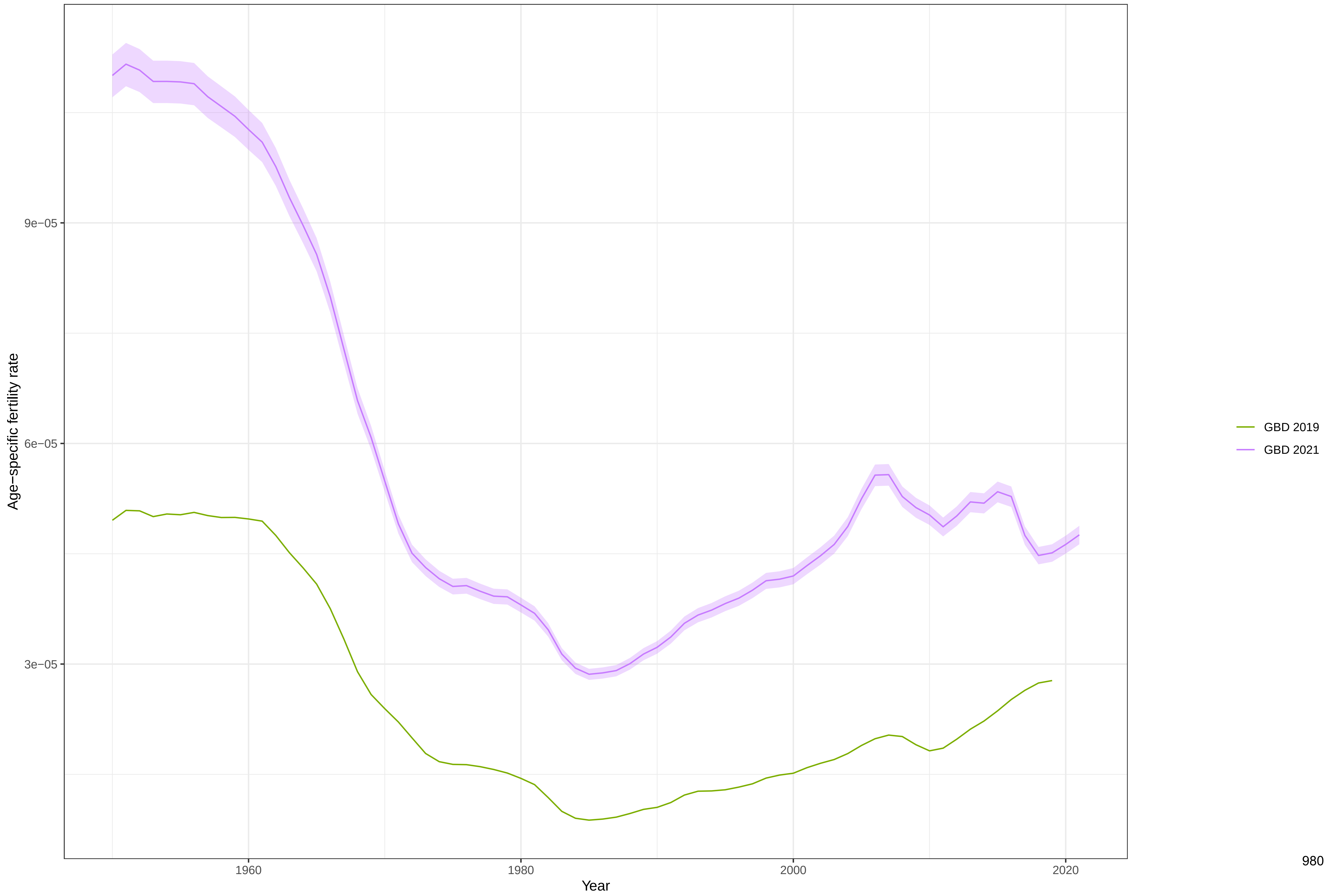

Total fertility rate fitted trend lines, 1950−2021: Idaho TFR
Data points enclosed with a diamond are reference sources

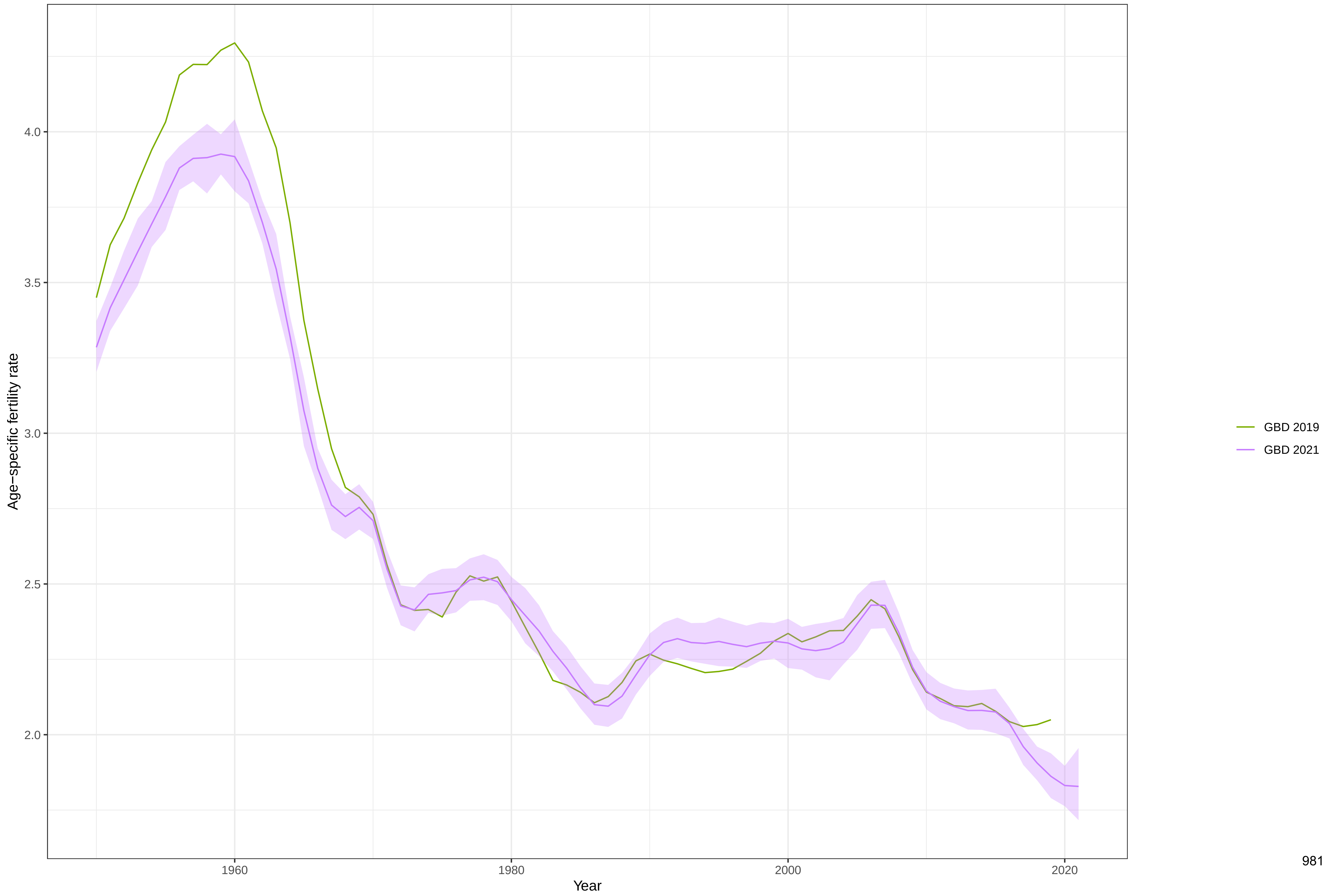

Age−specific fertility fitted trend lines, 1950−2021: Illinois, Ages: 10−14
Data points enclosed with a diamond are reference sources

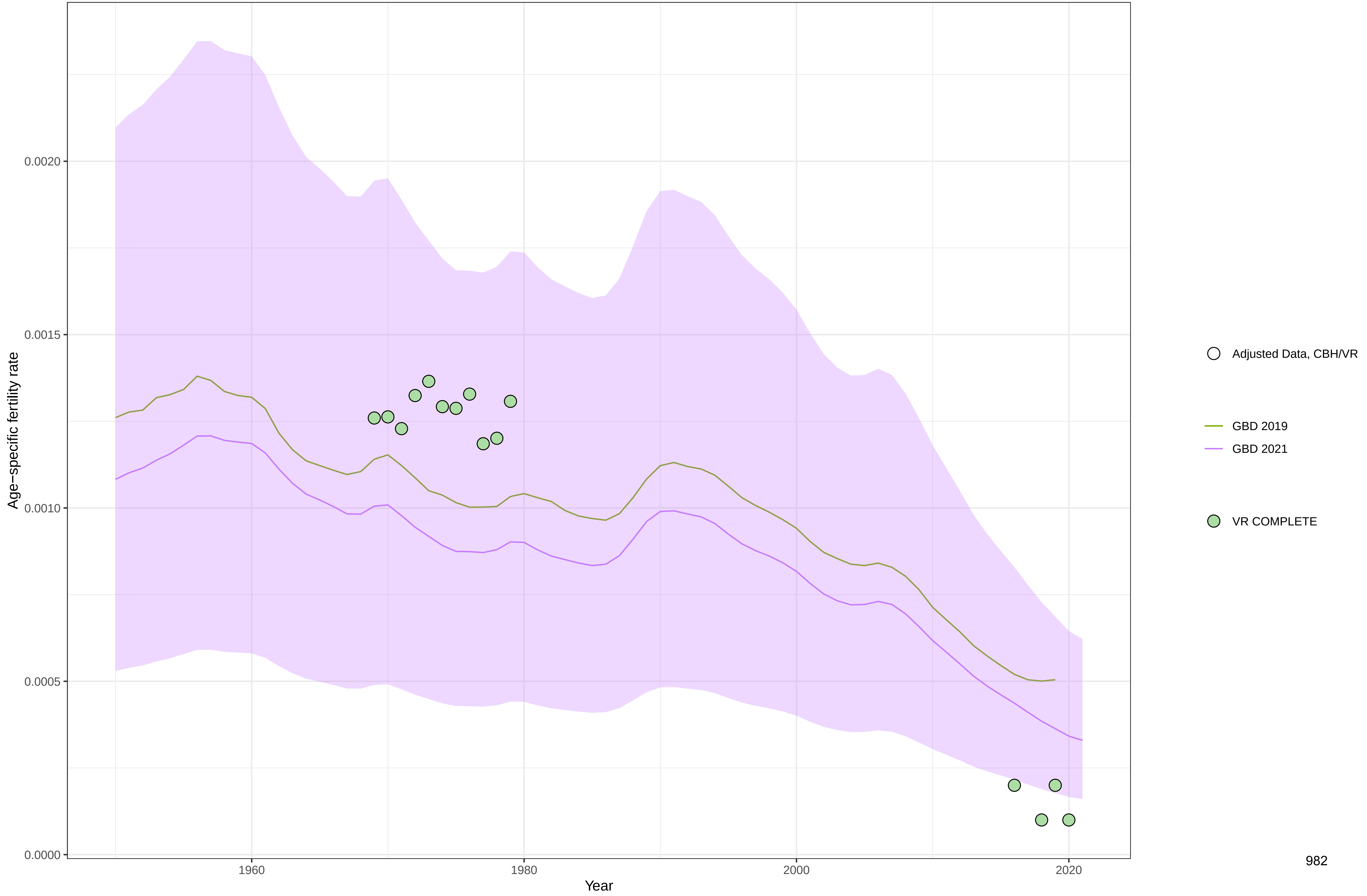

Age−specific fertility fitted trend lines, 1950−2021: Illinois, Ages: 15−19
Data points enclosed with a diamond are reference sources

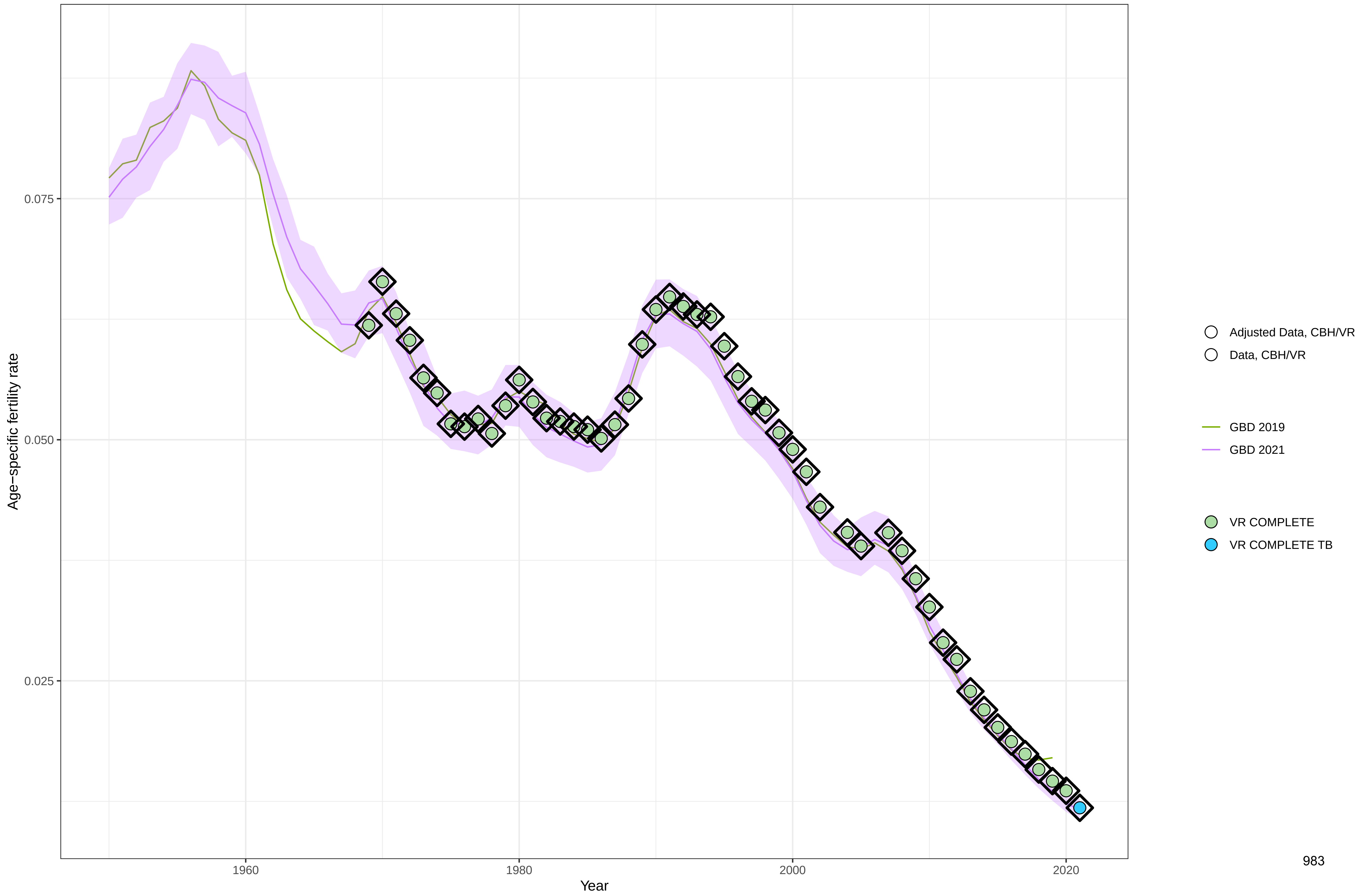

Age−specific fertility fitted trend lines, 1950−2021: Illinois, Ages: 20−24
Data points enclosed with a diamond are reference sources

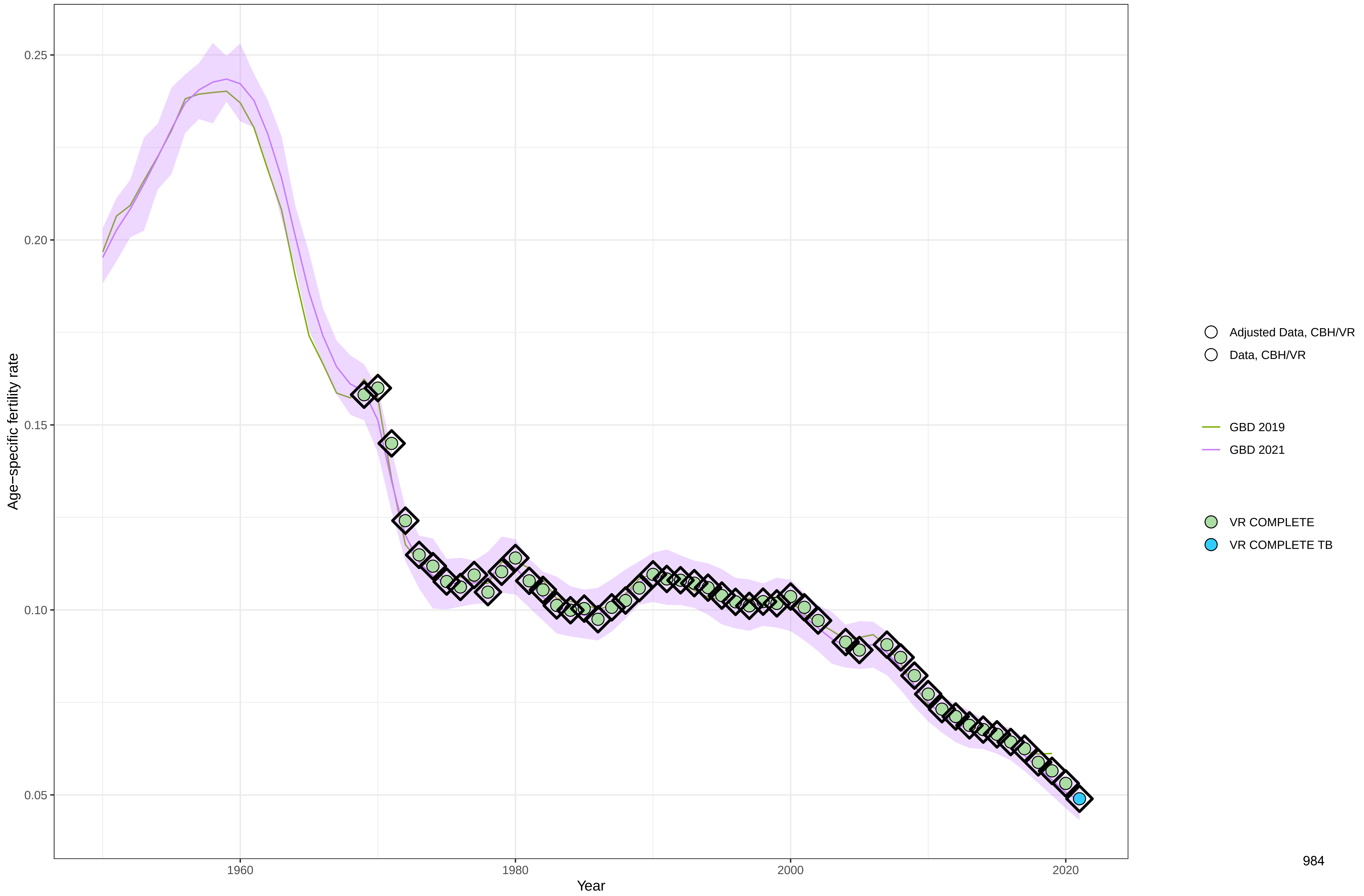

Age−specific fertility fitted trend lines, 1950−2021: Illinois, Ages: 25−29
Data points enclosed with a diamond are reference sources

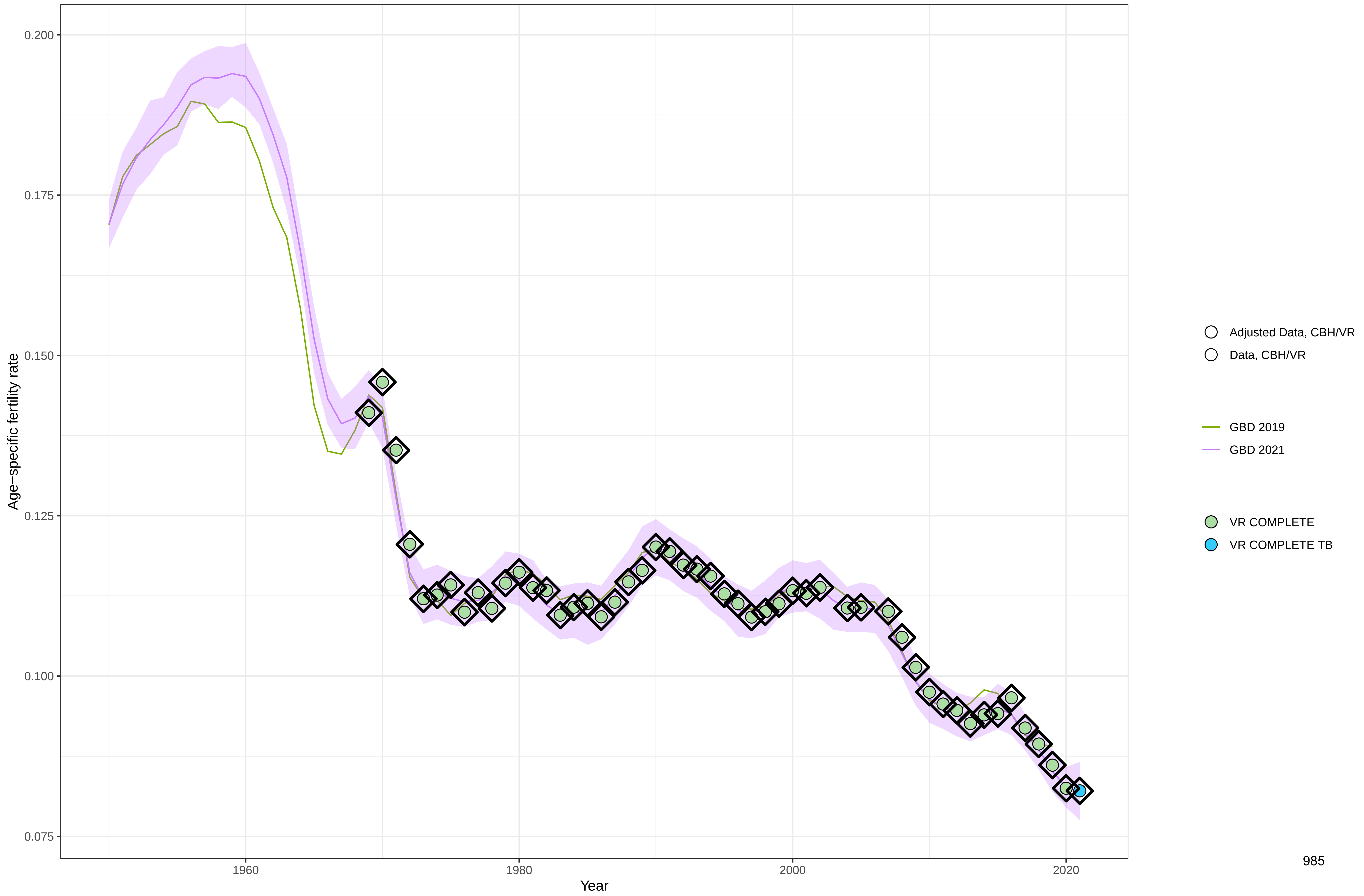

Age−specific fertility fitted trend lines, 1950−2021: Illinois, Ages: 30−34
Data points enclosed with a diamond are reference sources

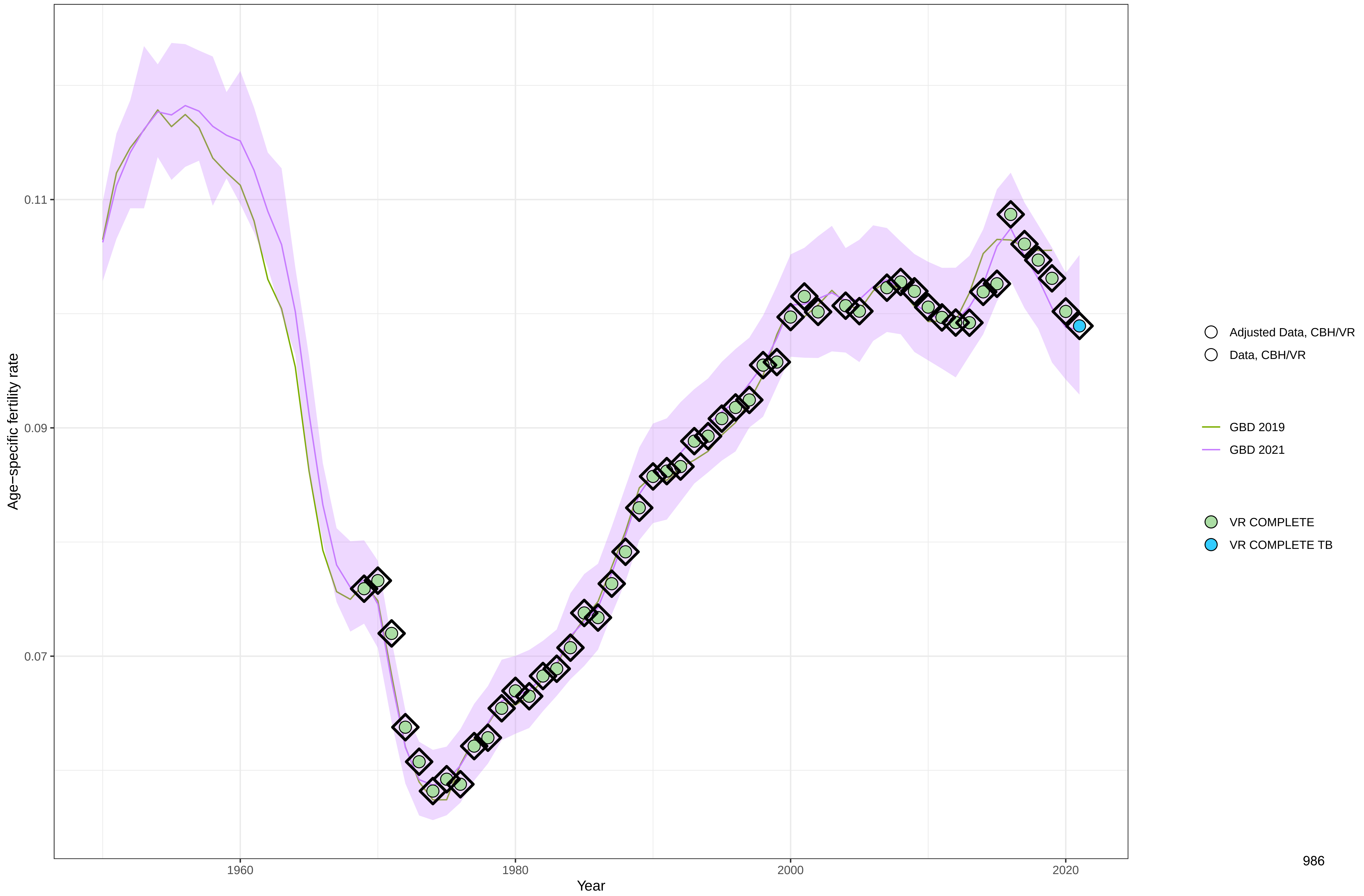

Age−specific fertility fitted trend lines, 1950−2021: Illinois, Ages: 35−39
Data points enclosed with a diamond are reference sources

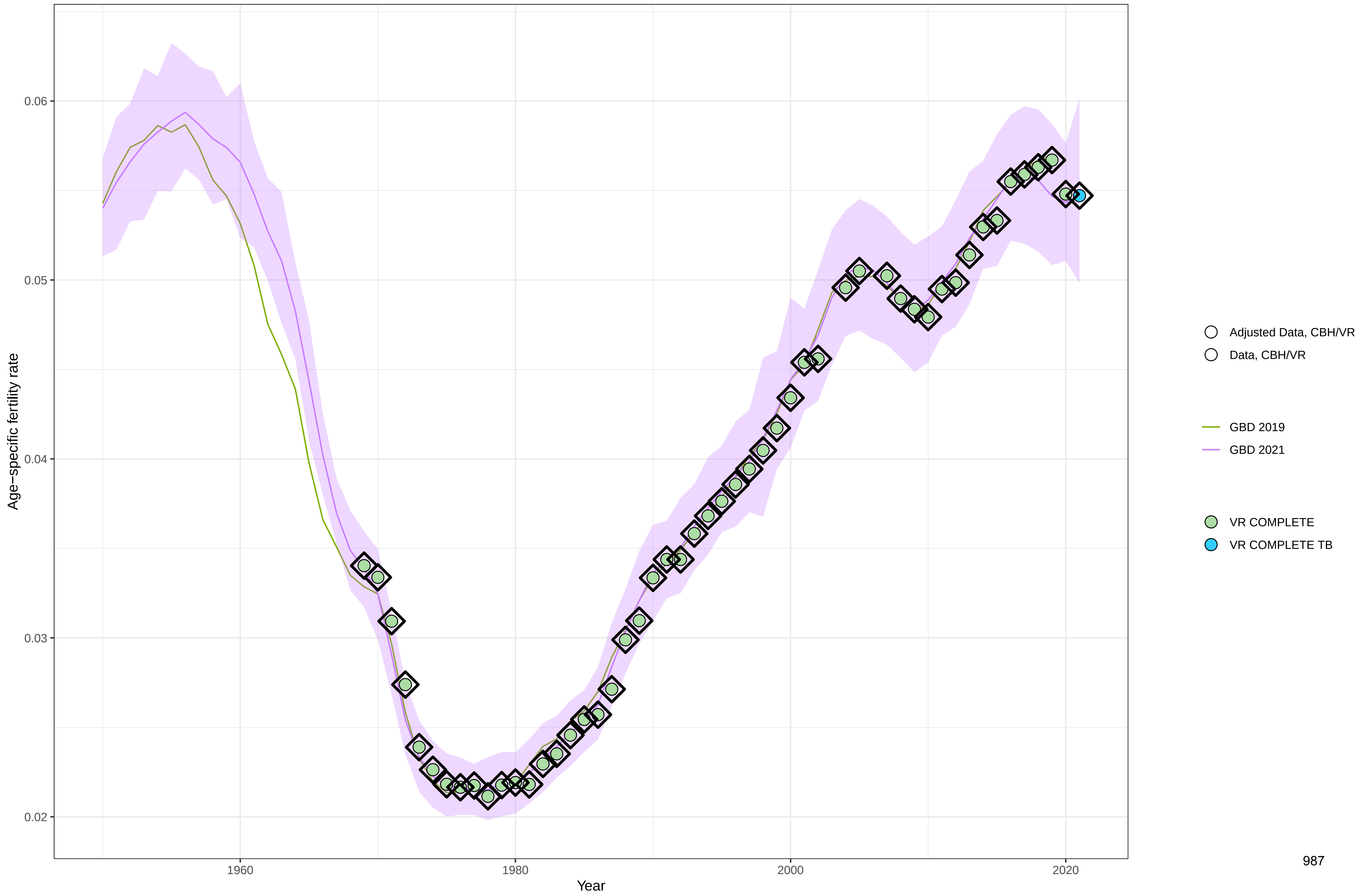

Age−specific fertility fitted trend lines, 1950−2021: Illinois, Ages: 40−44
Data points enclosed with a diamond are reference sources

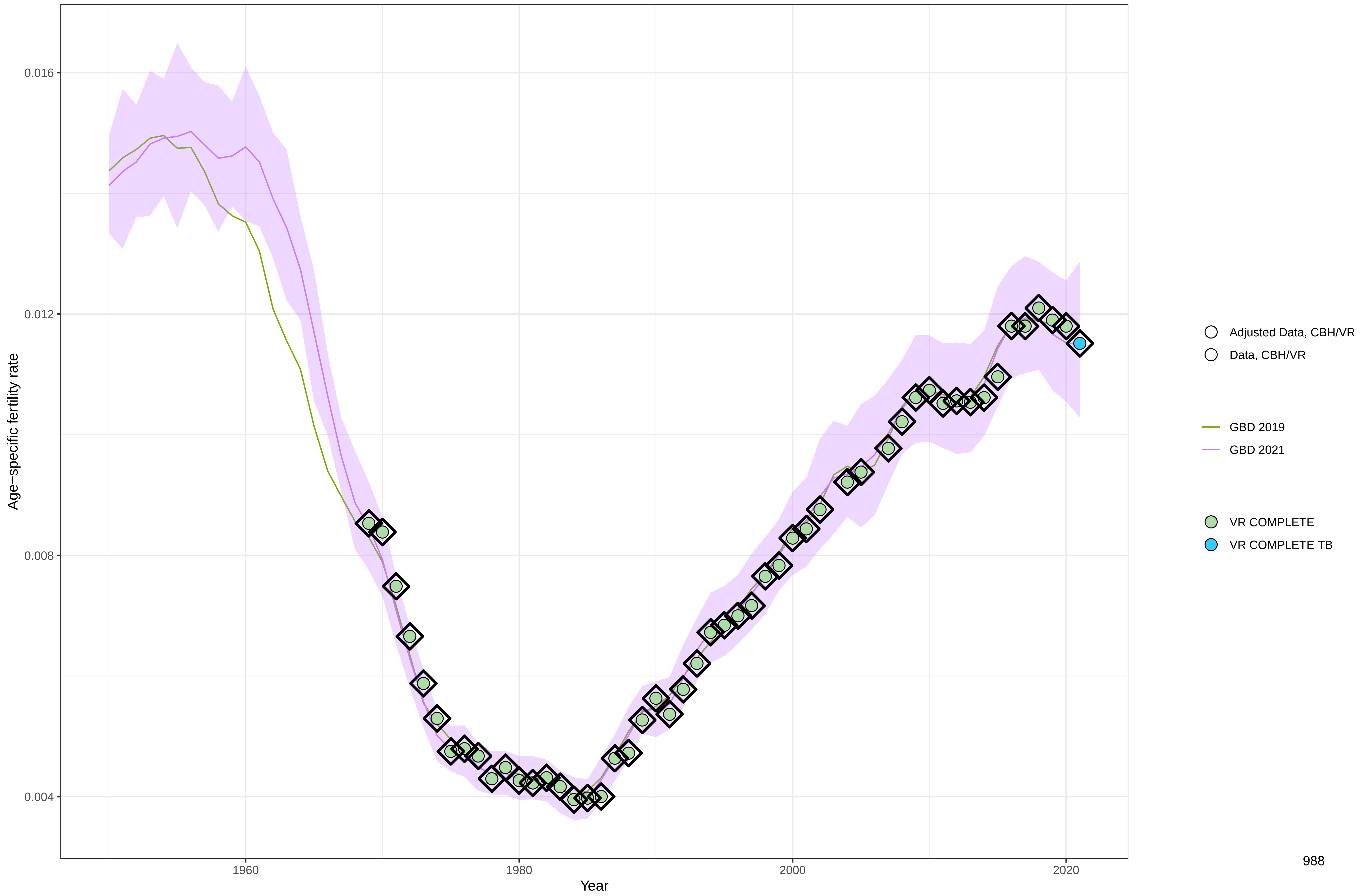

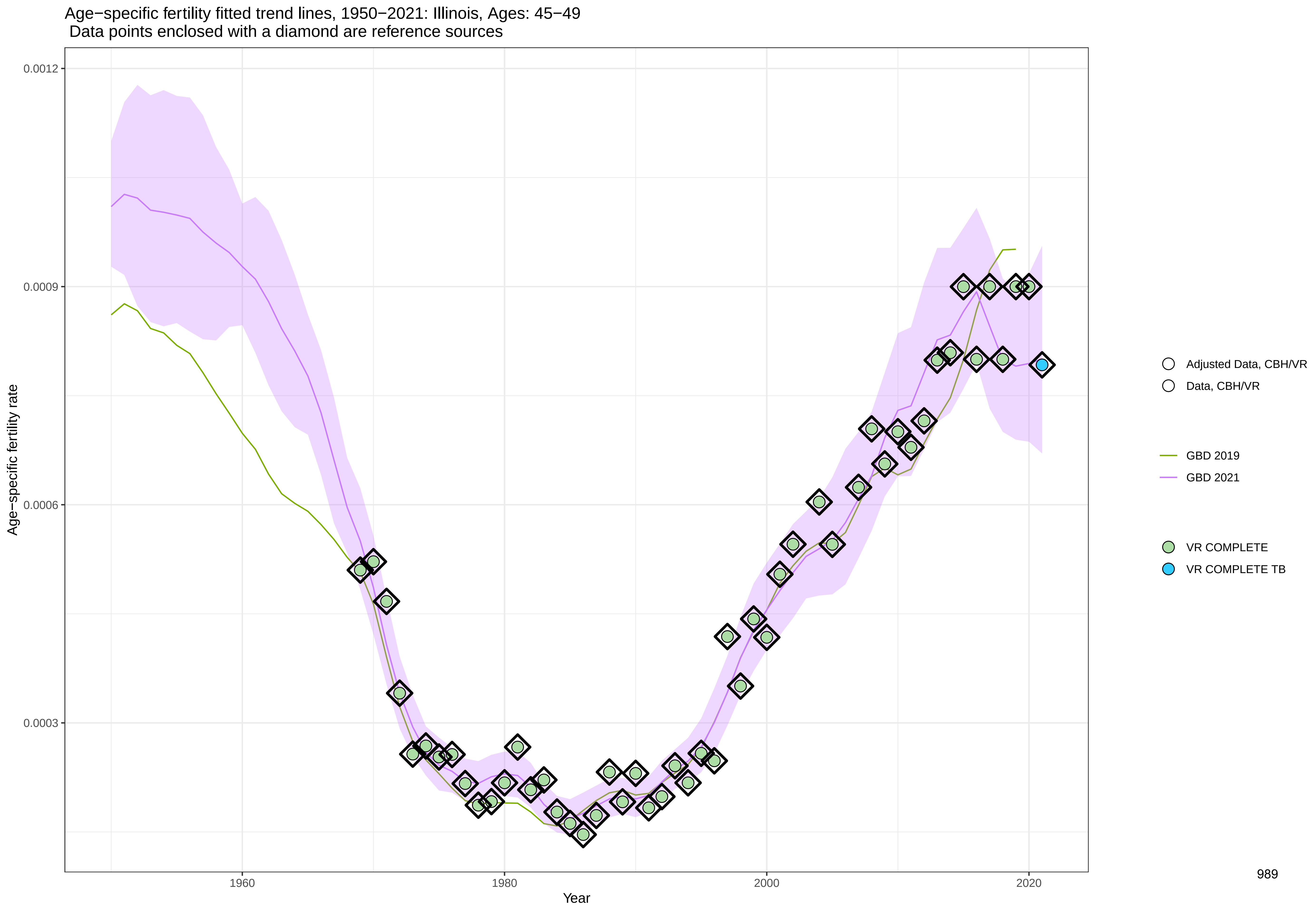
Age−specific fertility fitted trend lines, 1950−2021: Illinois, Ages: 45−49
Data points enclosed with a diamond are reference sources
0.0012
0.0009
0.0006
0.0003
Age−specific fertility rate
1960
1980
2000
2020
Year
Adjusted Data, CBH/VR
Data, CBH/VR
GBD 2019
GBD 2021
VR COMPLETE
VR COMPLETE TB

Age−specific fertility fitted trend lines, 1950−2021: Illinois, Ages: 50−54
Data points enclosed with a diamond are reference sources

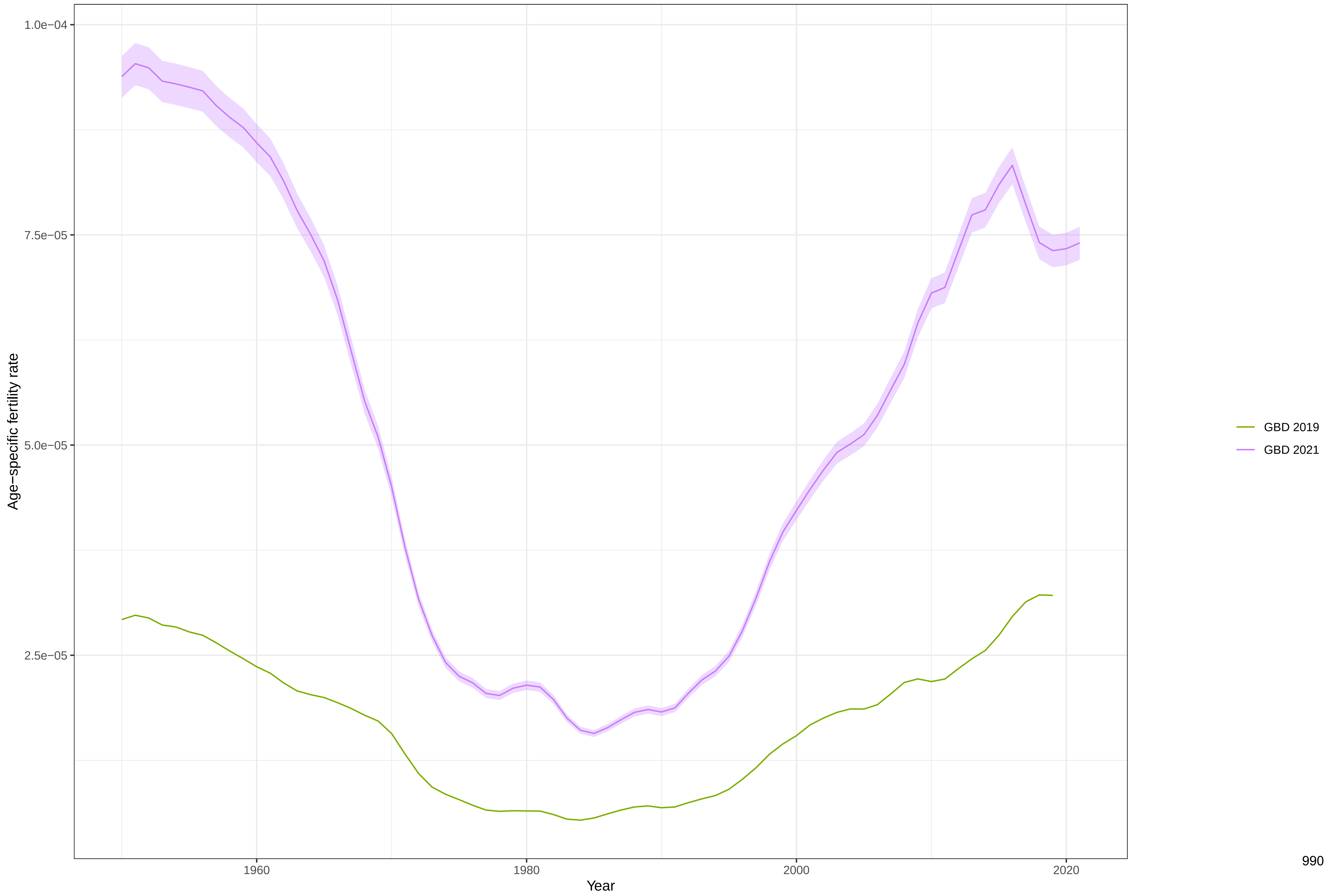

Total fertility rate fitted trend lines, 1950−2021: Illinois TFR
Data points enclosed with a diamond are reference sources

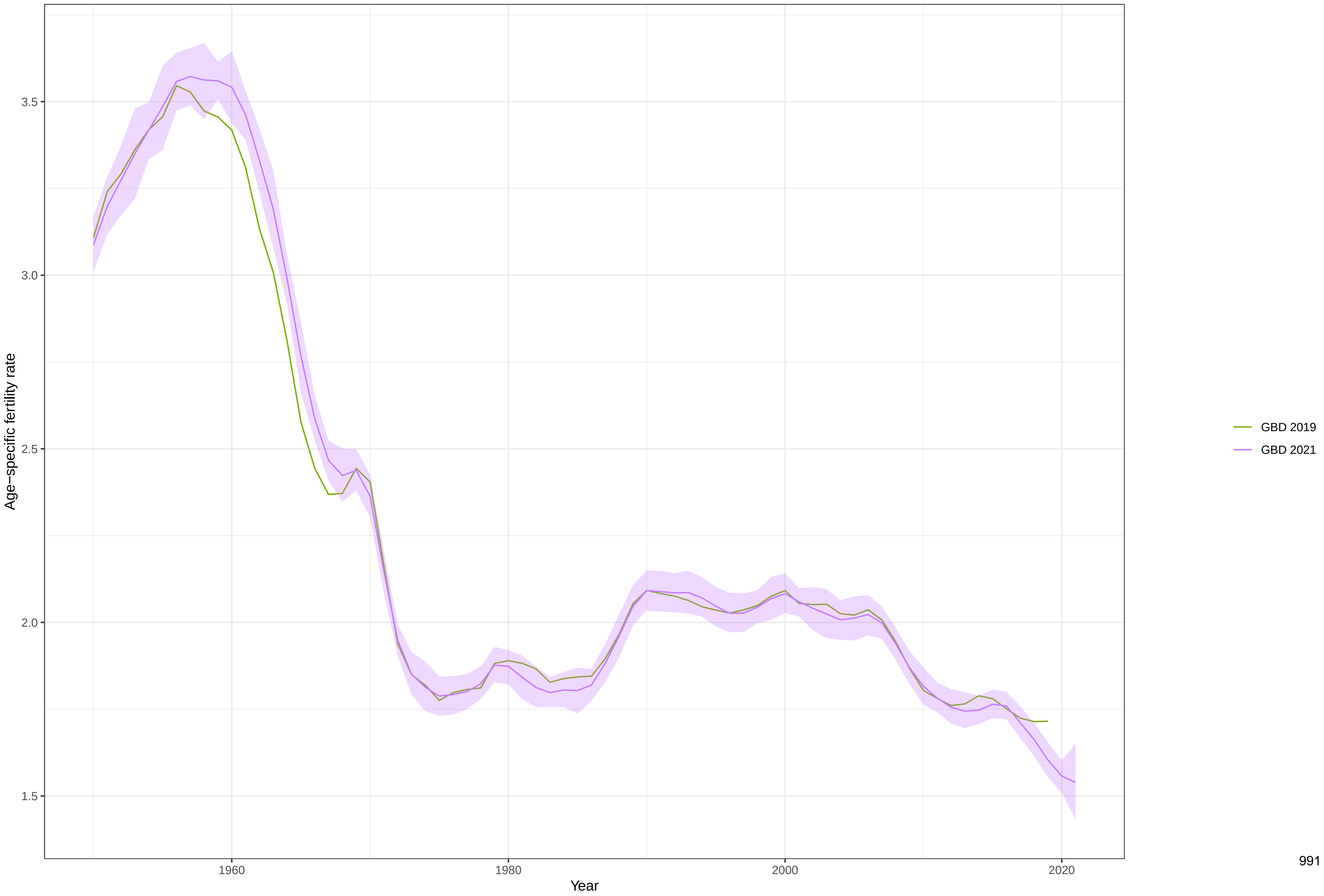

Age−specific fertility fitted trend lines, 1950−2021: Indiana, Ages: 10−14
Data points enclosed with a diamond are reference sources

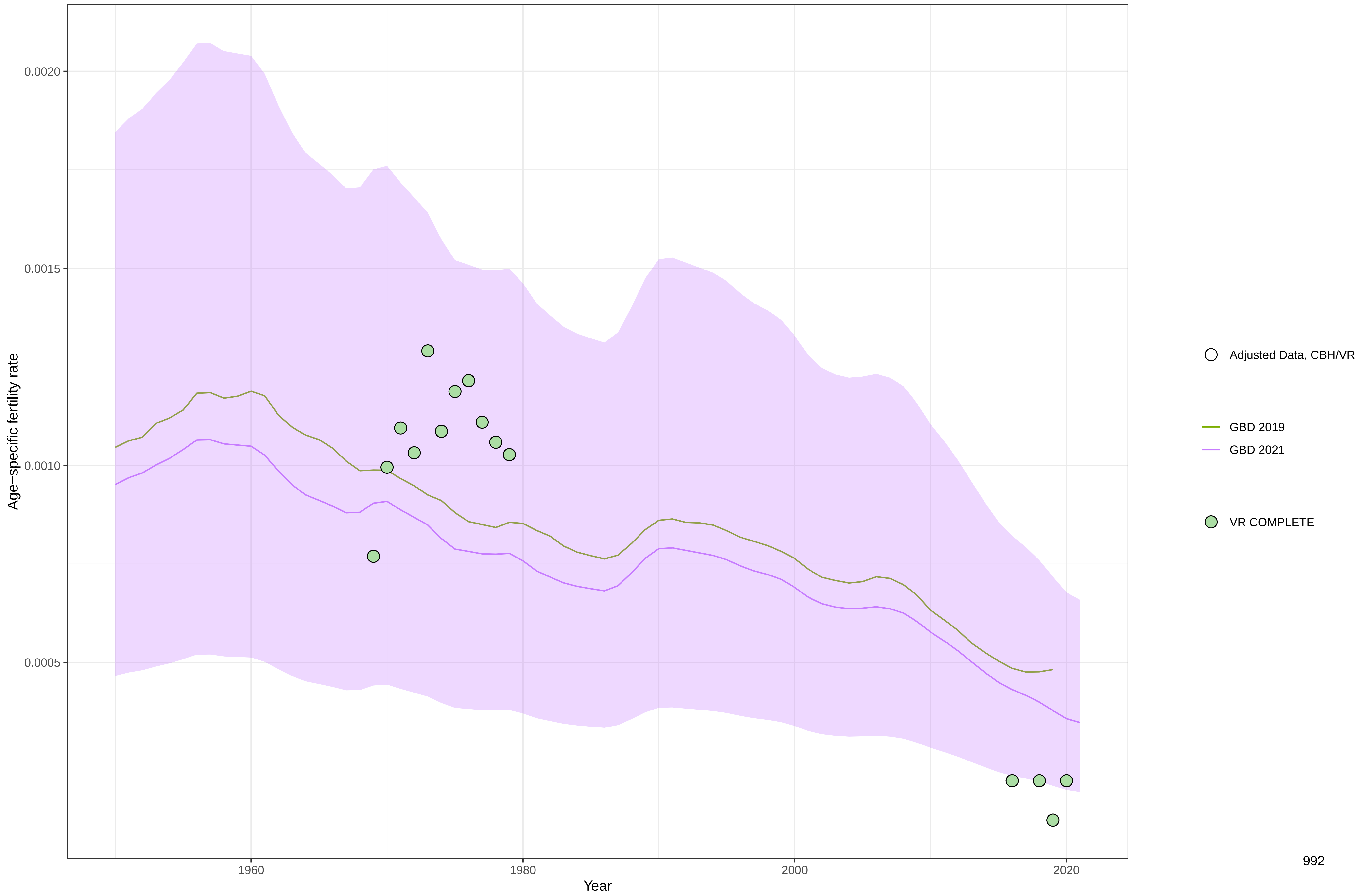

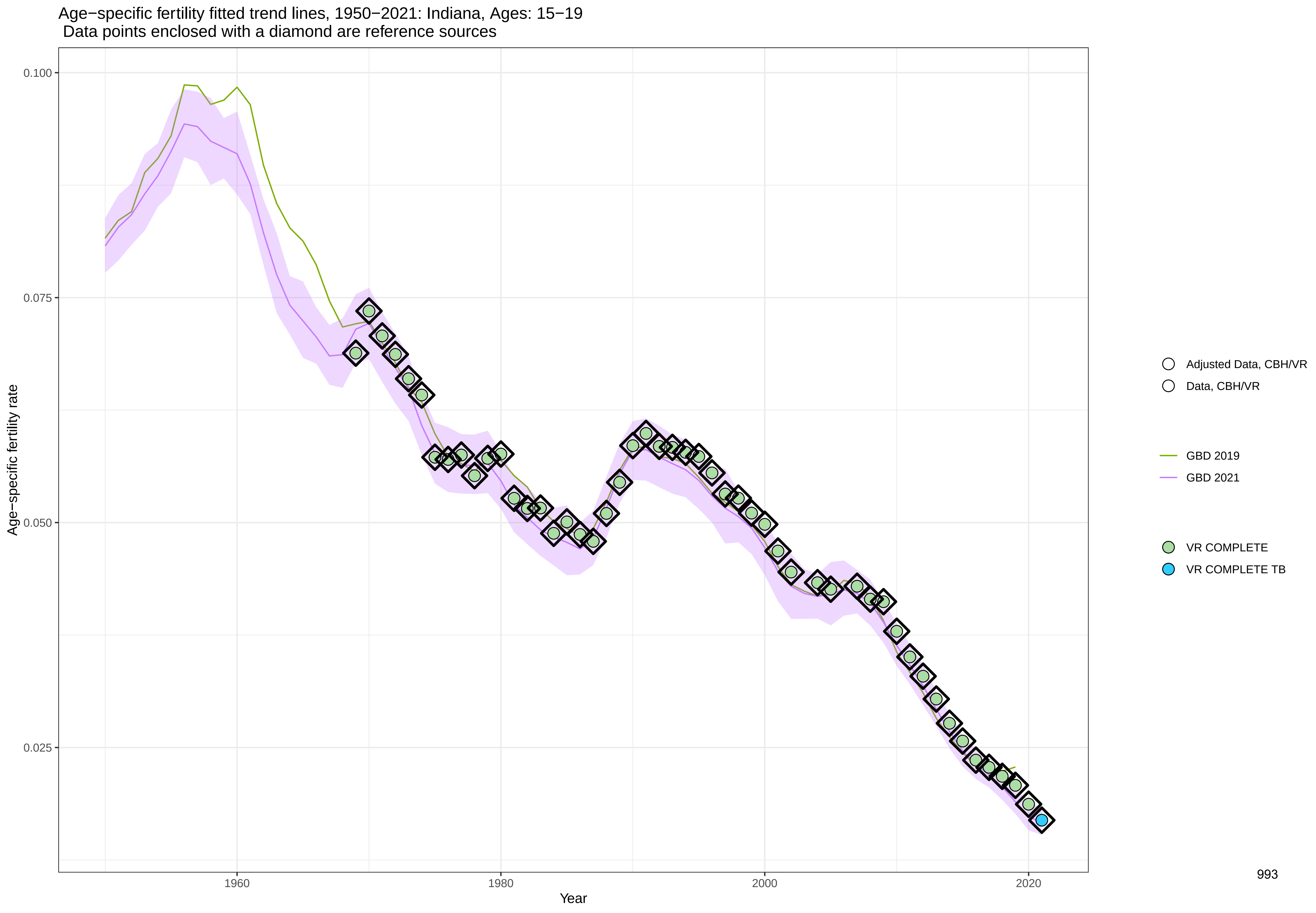
Age−specific fertility fitted trend lines, 1950−2021: Indiana, Ages: 15−19
Data points enclosed with a diamond are reference sources
Age−specific fertility rate
0.100
0.075
0.050
0.025
1960
1980
2000
2020
Year
Adjusted Data, CBH/VR
Data, CBH/VR
GBD 2019
GBD 2021
VR COMPLETE
VR COMPLETE TB

Age−specific fertility fitted trend lines, 1950−2021: Indiana, Ages: 20−24
Data points enclosed with a diamond are reference sources

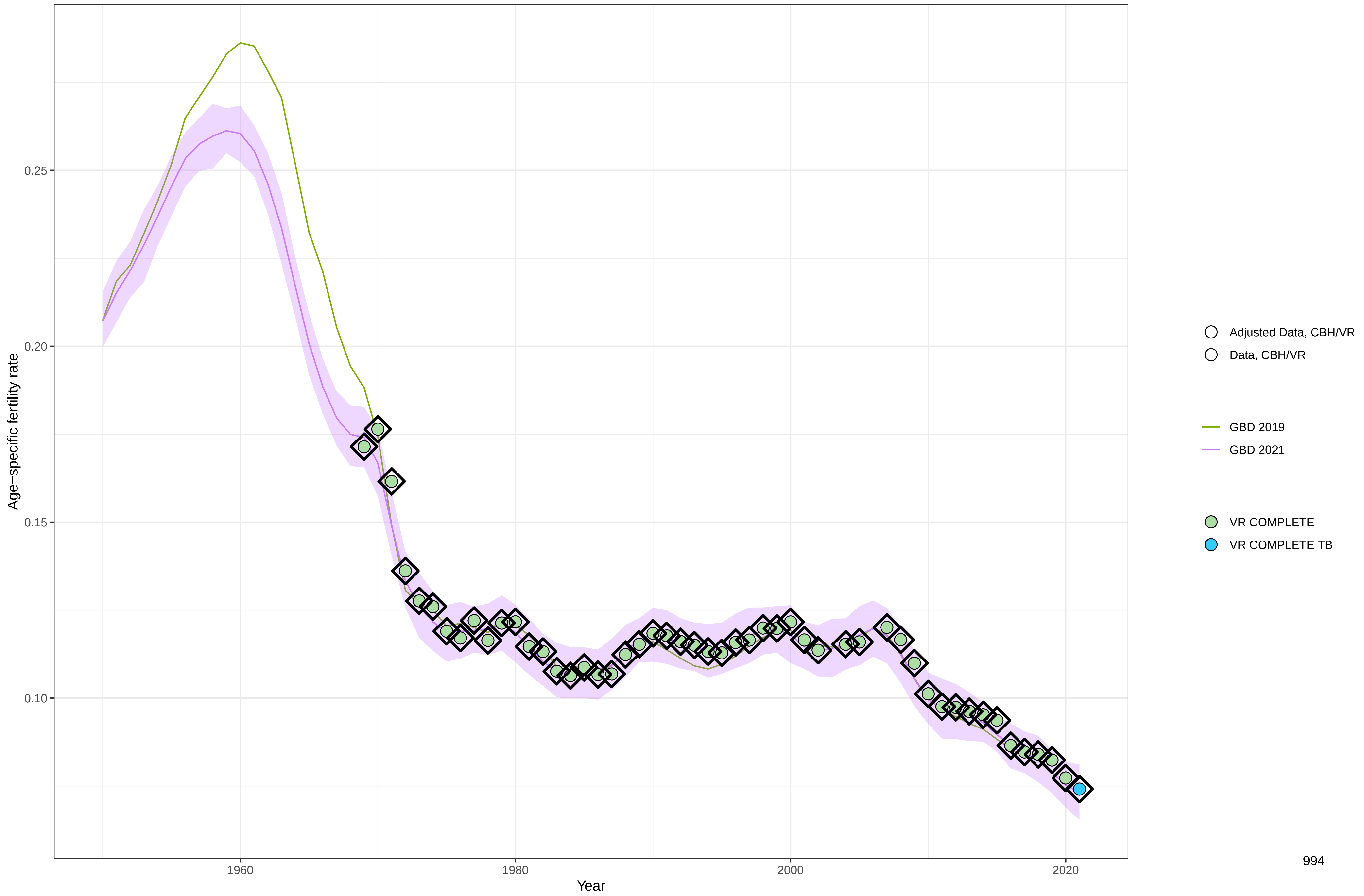

Age−specific fertility fitted trend lines, 1950−2021: Indiana, Ages: 25−29
Data points enclosed with a diamond are reference sources

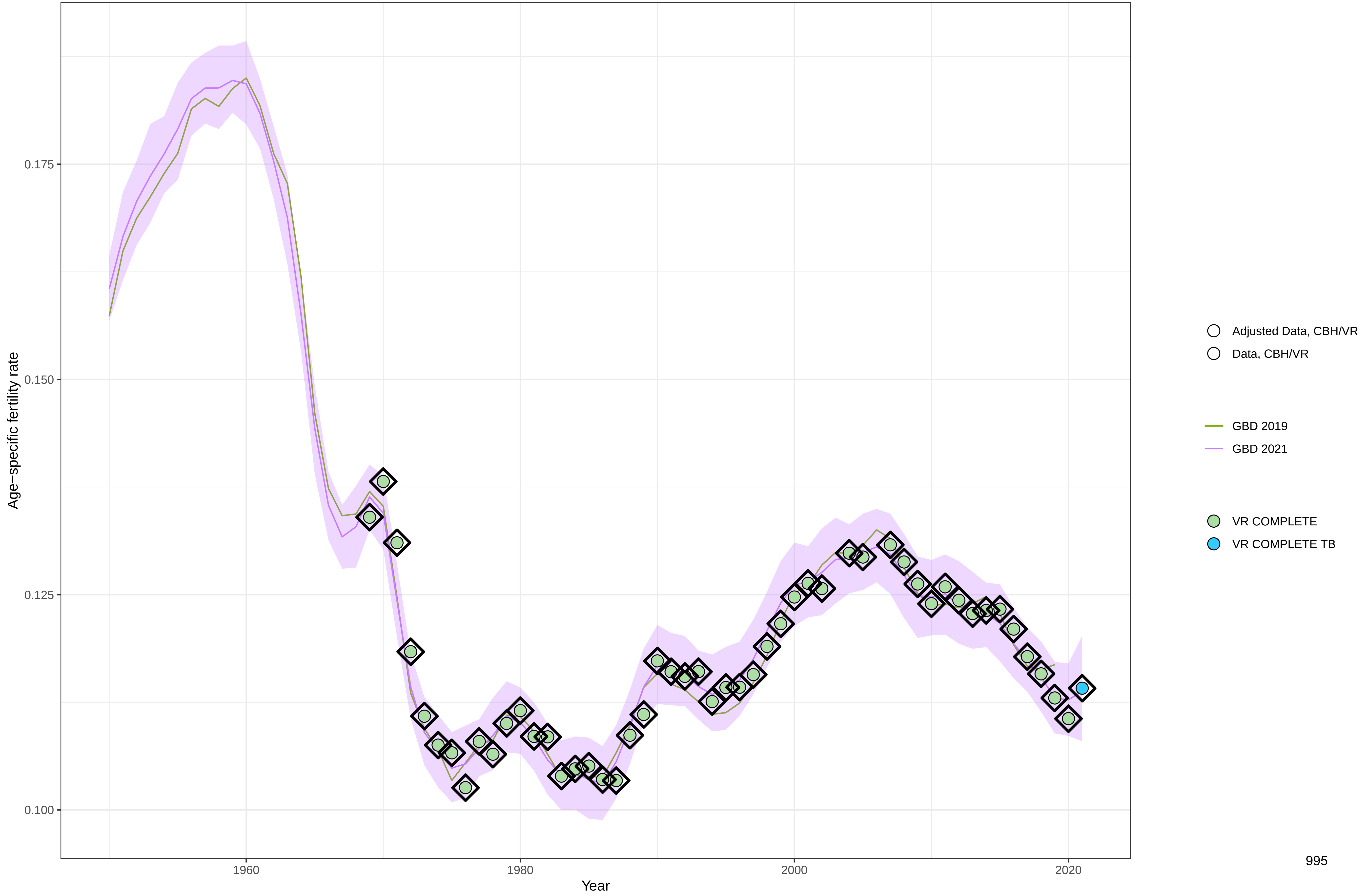

Age−specific fertility fitted trend lines, 1950−2021: Indiana, Ages: 30−34
Data points enclosed with a diamond are reference sources

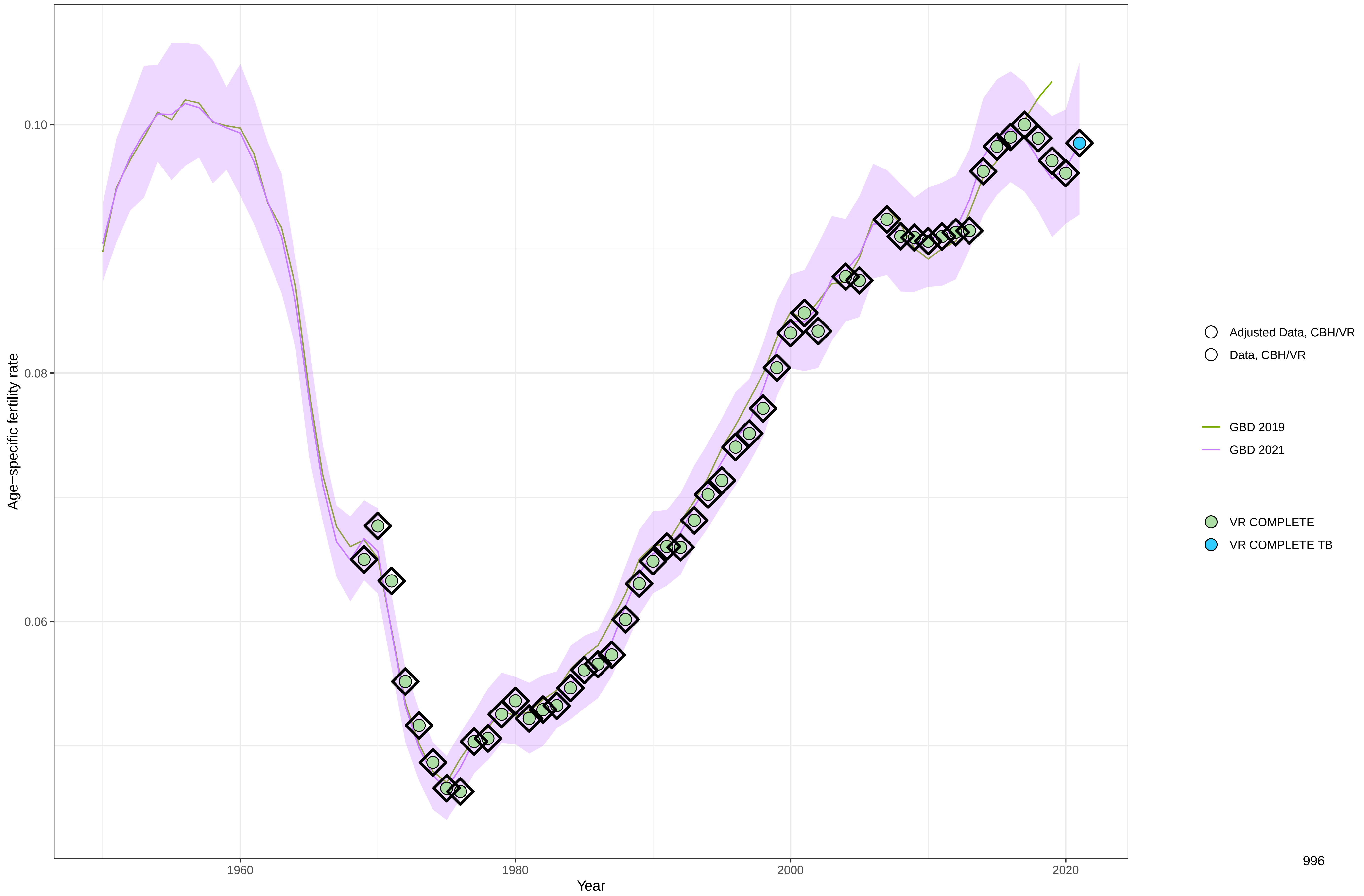

Age−specific fertility fitted trend lines, 1950−2021: Indiana, Ages: 35−39
Data points enclosed with a diamond are reference sources

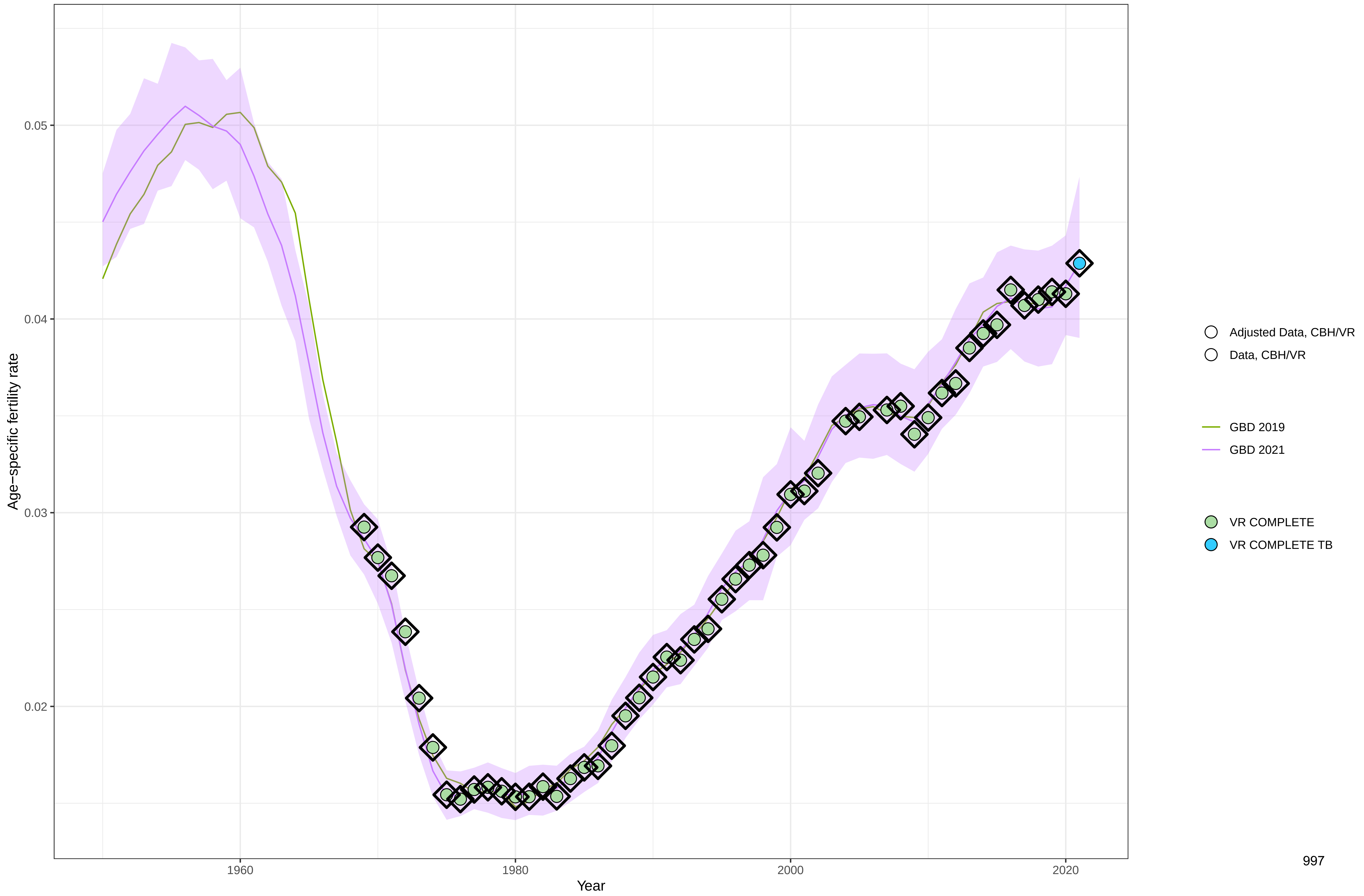

Age−specific fertility fitted trend lines, 1950−2021: Indiana, Ages: 40−44
Data points enclosed with a diamond are reference sources

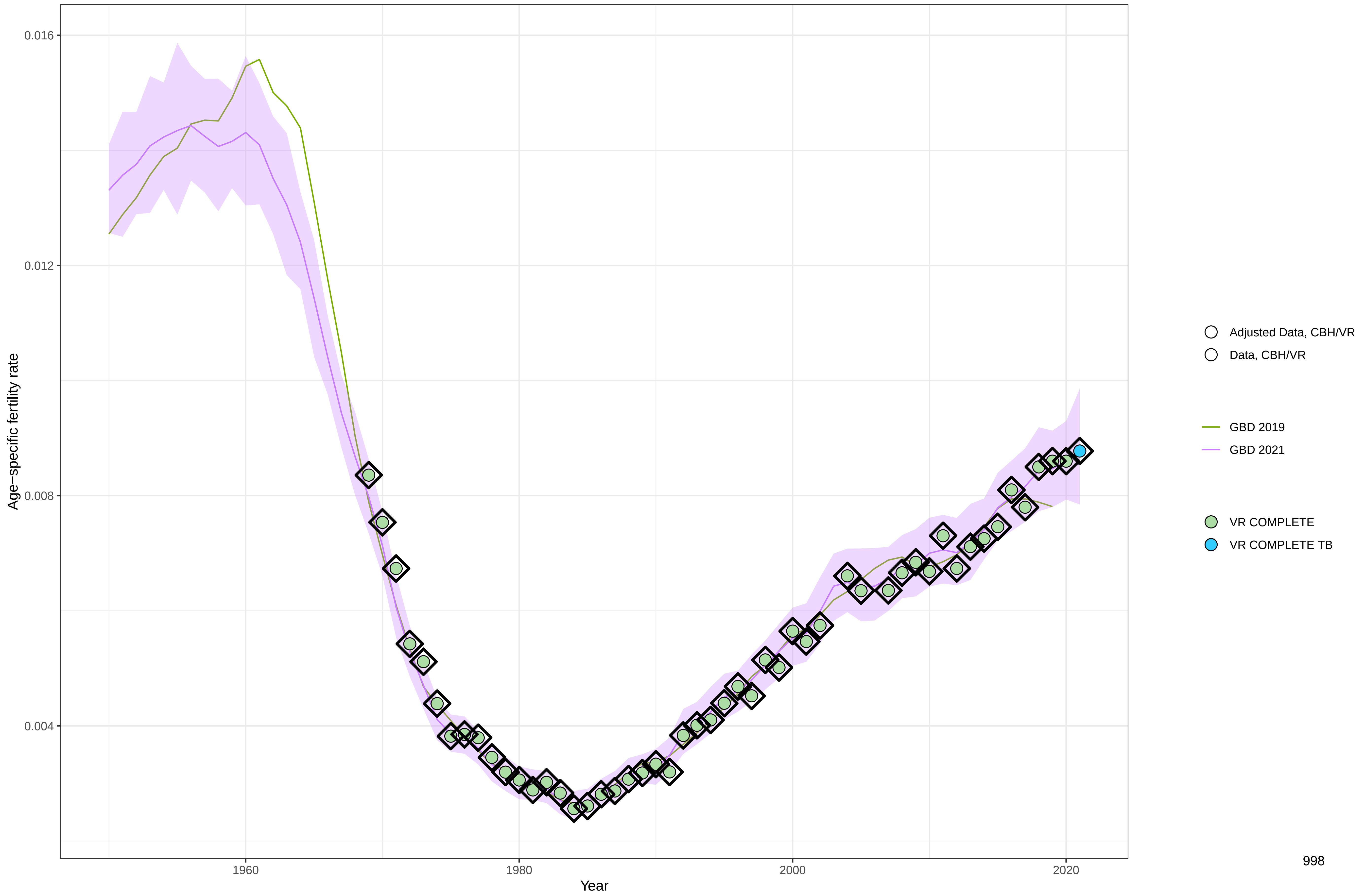

Age−specific fertility fitted trend lines, 1950−2021: Indiana, Ages: 45−49
Data points enclosed with a diamond are reference sources

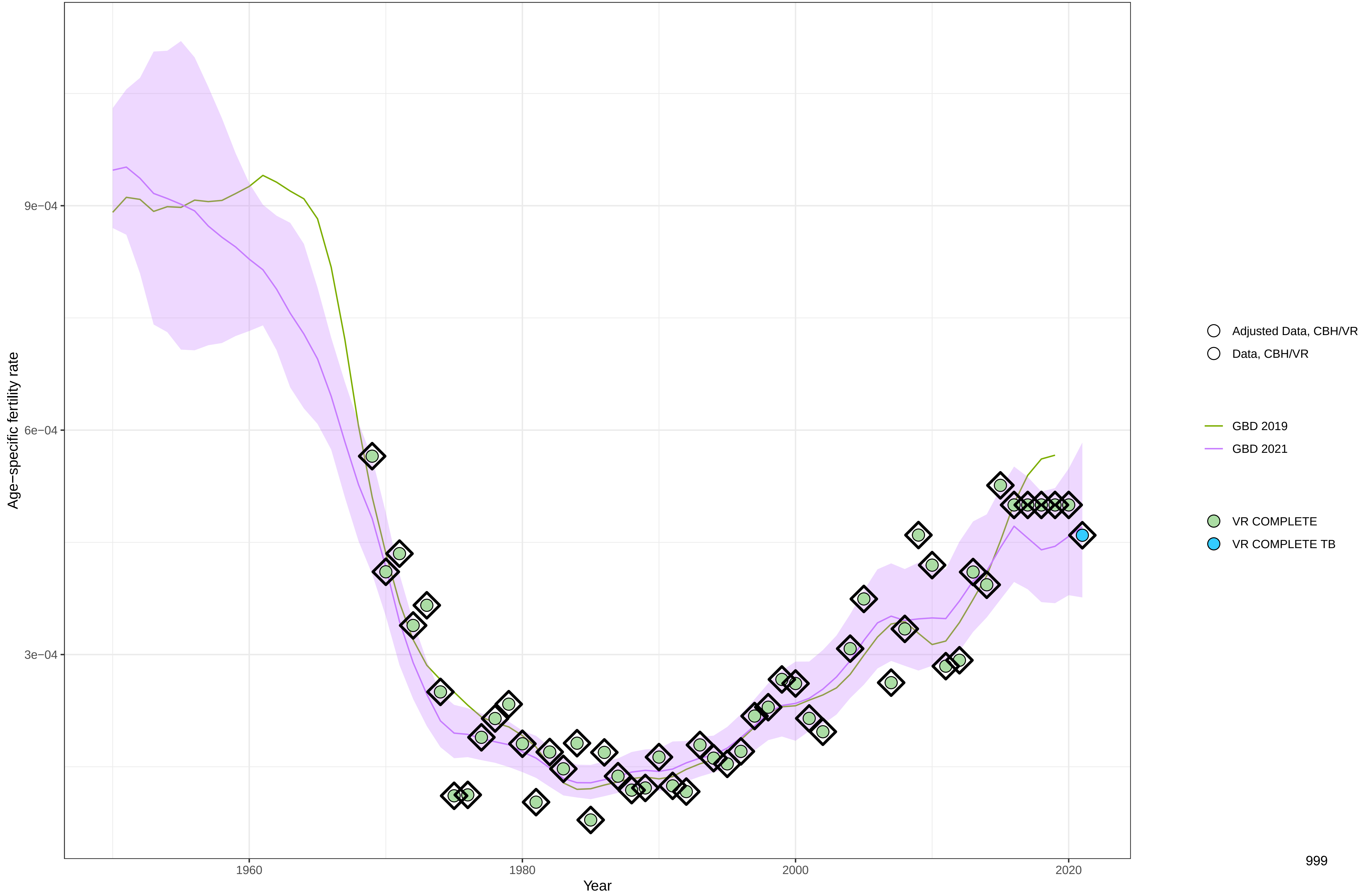

Age−specific fertility fitted trend lines, 1950−2021: Indiana, Ages: 50−54
Data points enclosed with a diamond are reference sources

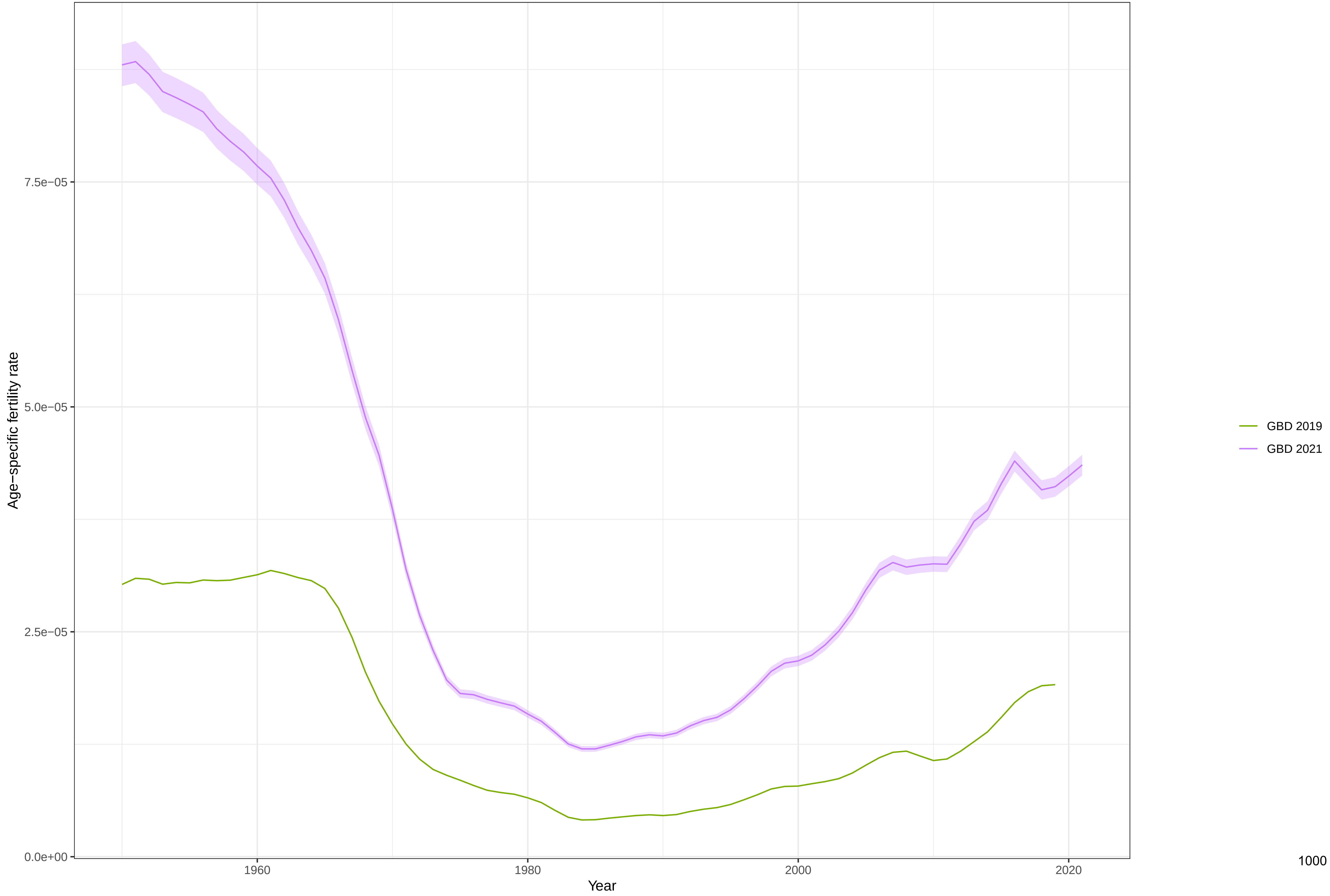

1000

Total fertility rate fitted trend lines, 1950−2021: Indiana TFR
Data points enclosed with a diamond are reference sources

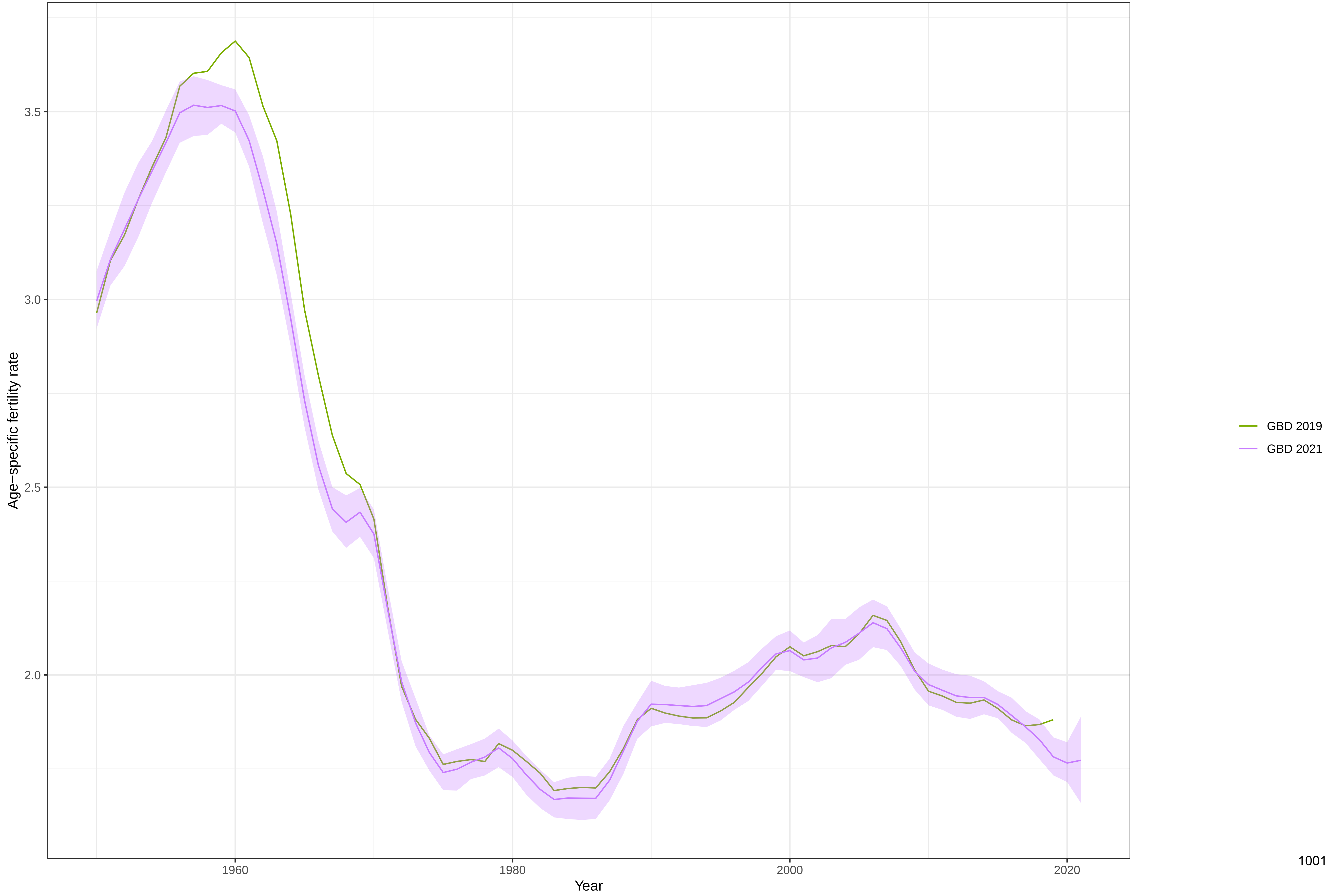

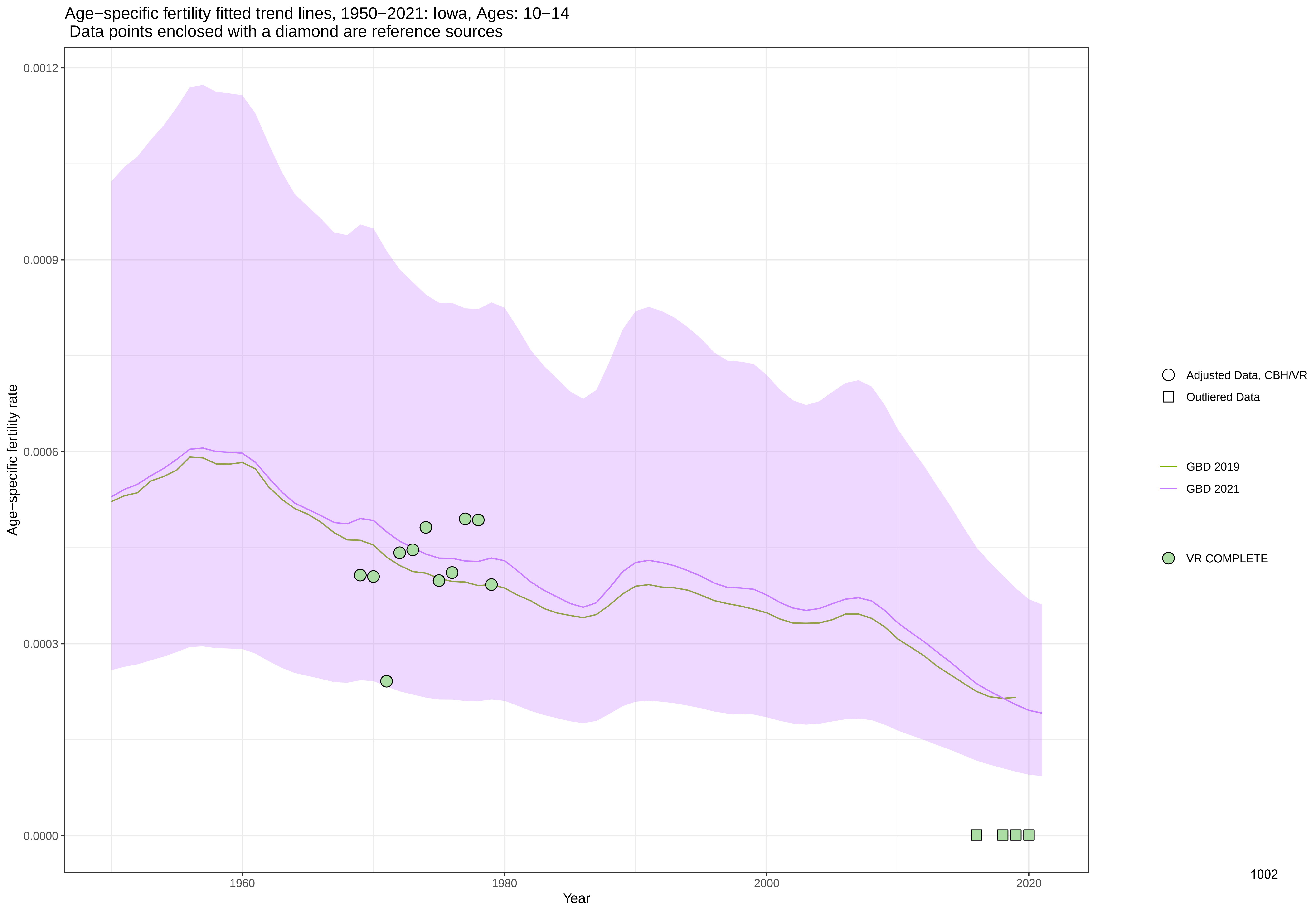

Age−specific fertility fitted trend lines, 1950−2021: Iowa, Ages: 10−14
Data points enclosed with a diamond are reference sources
0.0012
0.0009
0.0006
0.0003
0.0000
Age−specific fertility rate
1960
1980
2000
2020
Year
Adjusted Data, CBH/VR
Outliered Data
GBD 2019
GBD 2021
VR COMPLETE

Age−specific fertility fitted trend lines, 1950−2021: Iowa, Ages: 15−19
Data points enclosed with a diamond are reference sources

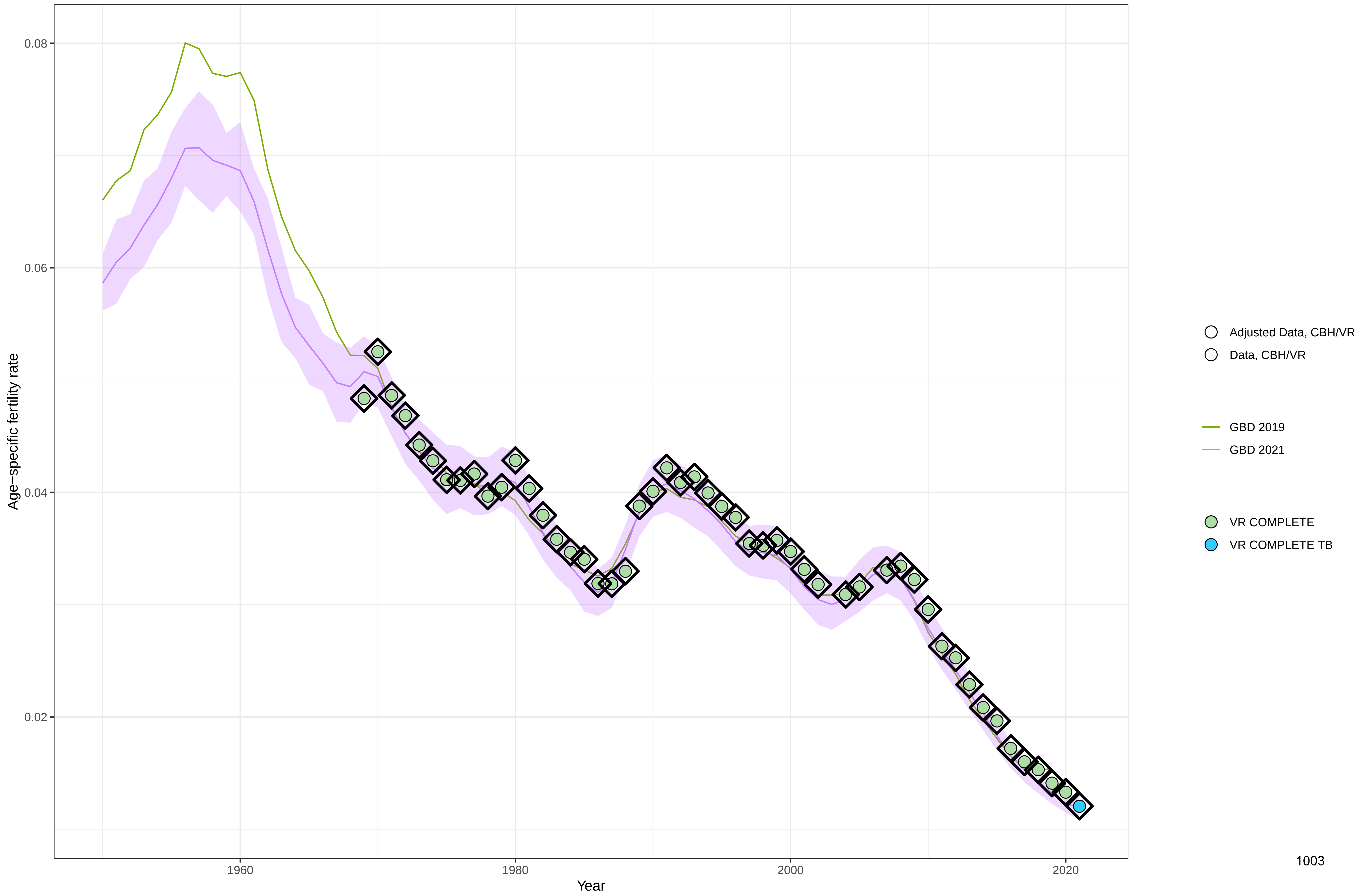

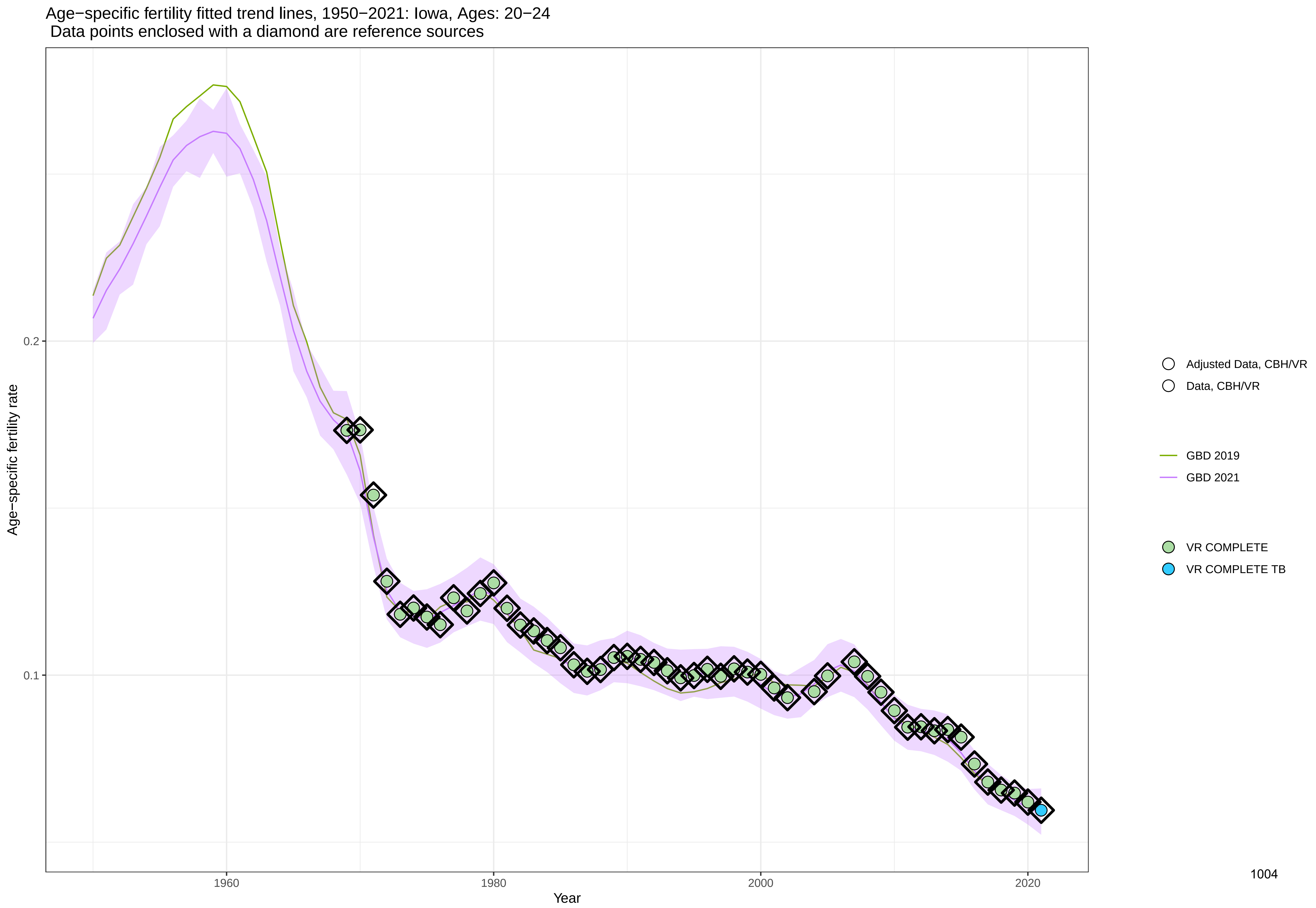

Age−specific fertility fitted trend lines, 1950−2021: Iowa, Ages: 20−24
Data points enclosed with a diamond are reference sources
Age−specific fertility rate
0.2
0.1
1960
1980
2000
2020
Year
Adjusted Data, CBH/VR
Data, CBH/VR
GBD 2019
GBD 2021
VR COMPLETE
VR COMPLETE TB

Age−specific fertility fitted trend lines, 1950−2021: Iowa, Ages: 25−29
Data points enclosed with a diamond are reference sources

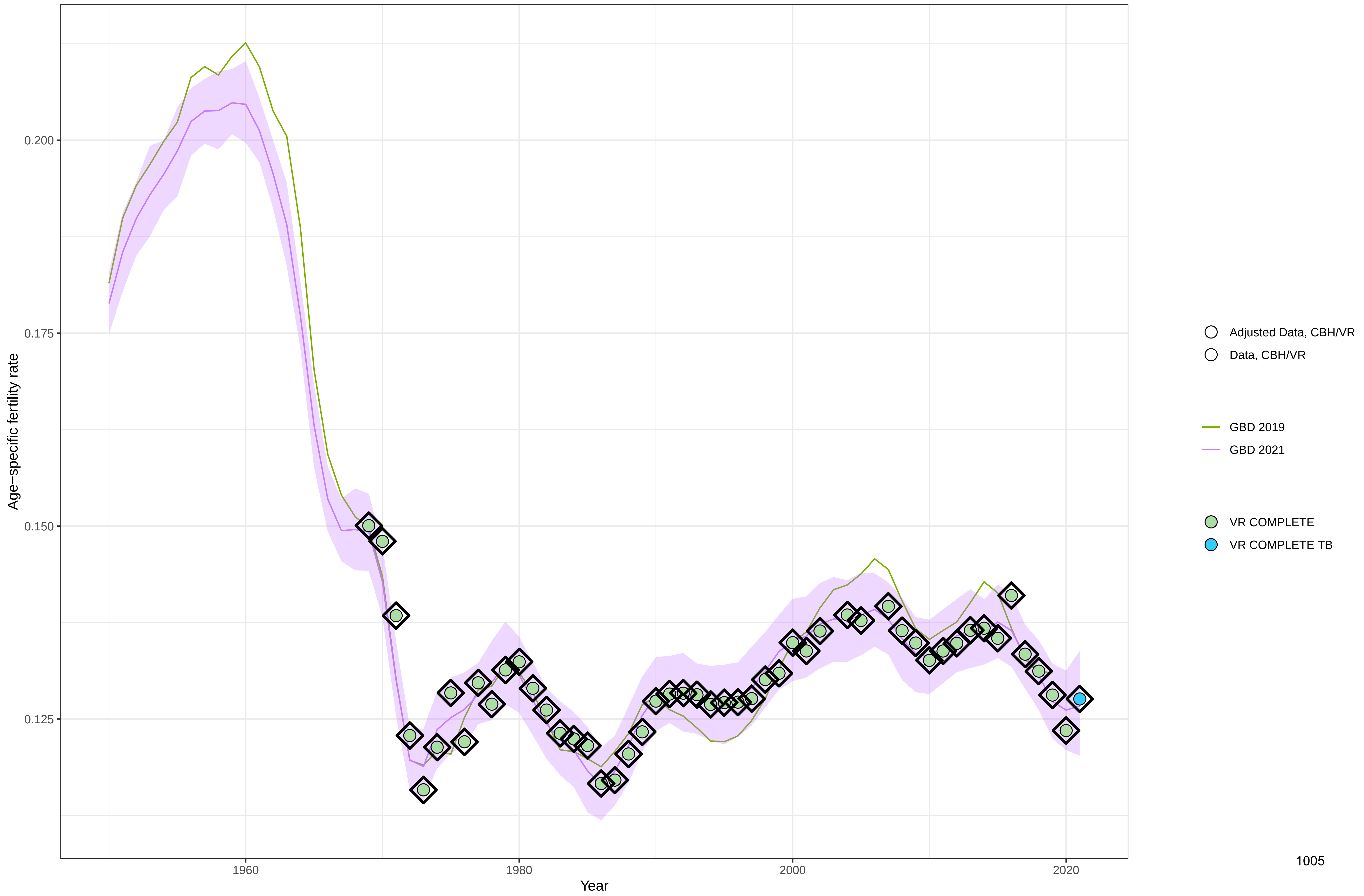

Age−specific fertility fitted trend lines, 1950−2021: Iowa, Ages: 30−34
Data points enclosed with a diamond are reference sources

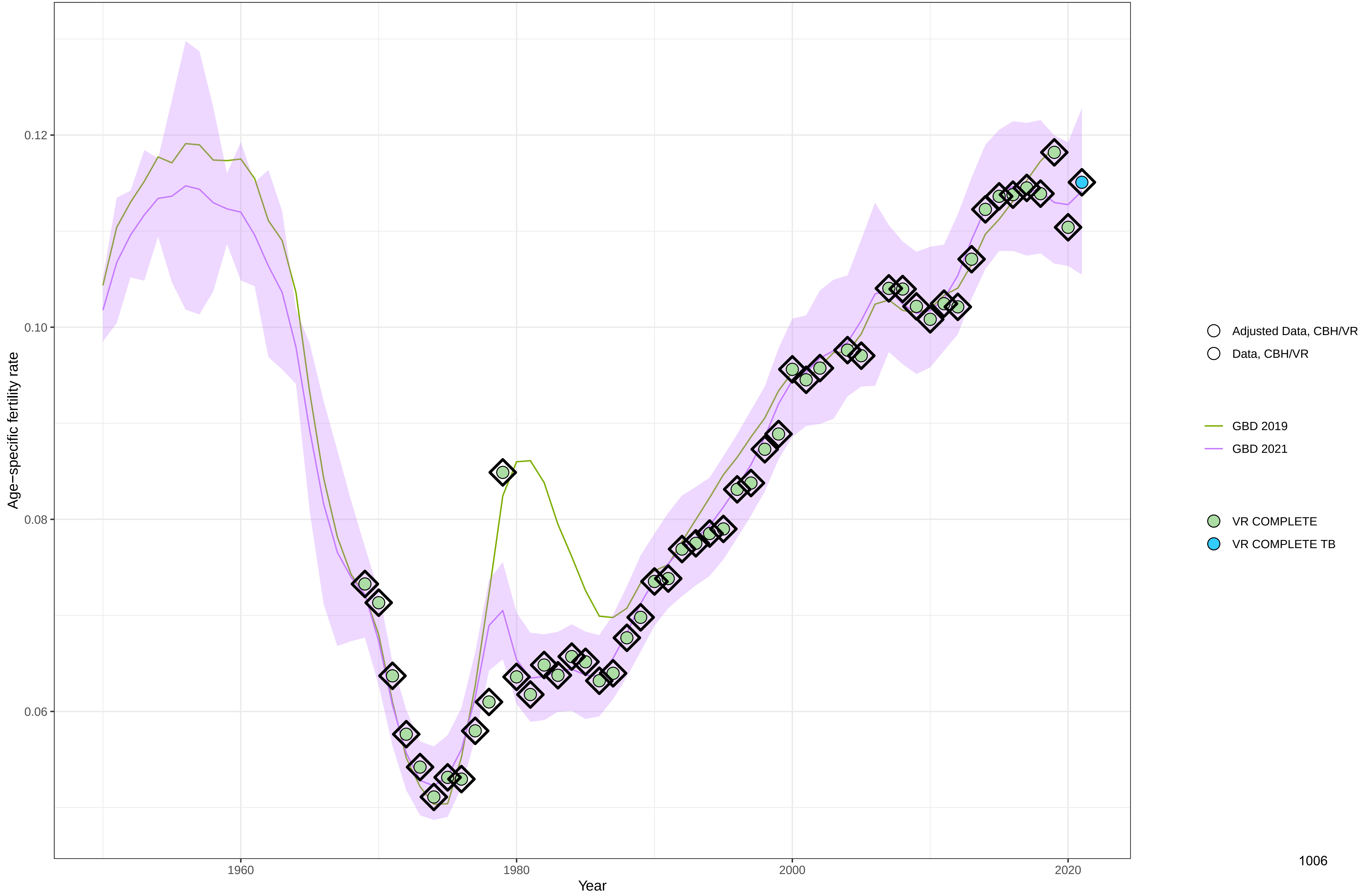

Age−specific fertility fitted trend lines, 1950−2021: Iowa, Ages: 35−39
Data points enclosed with a diamond are reference sources

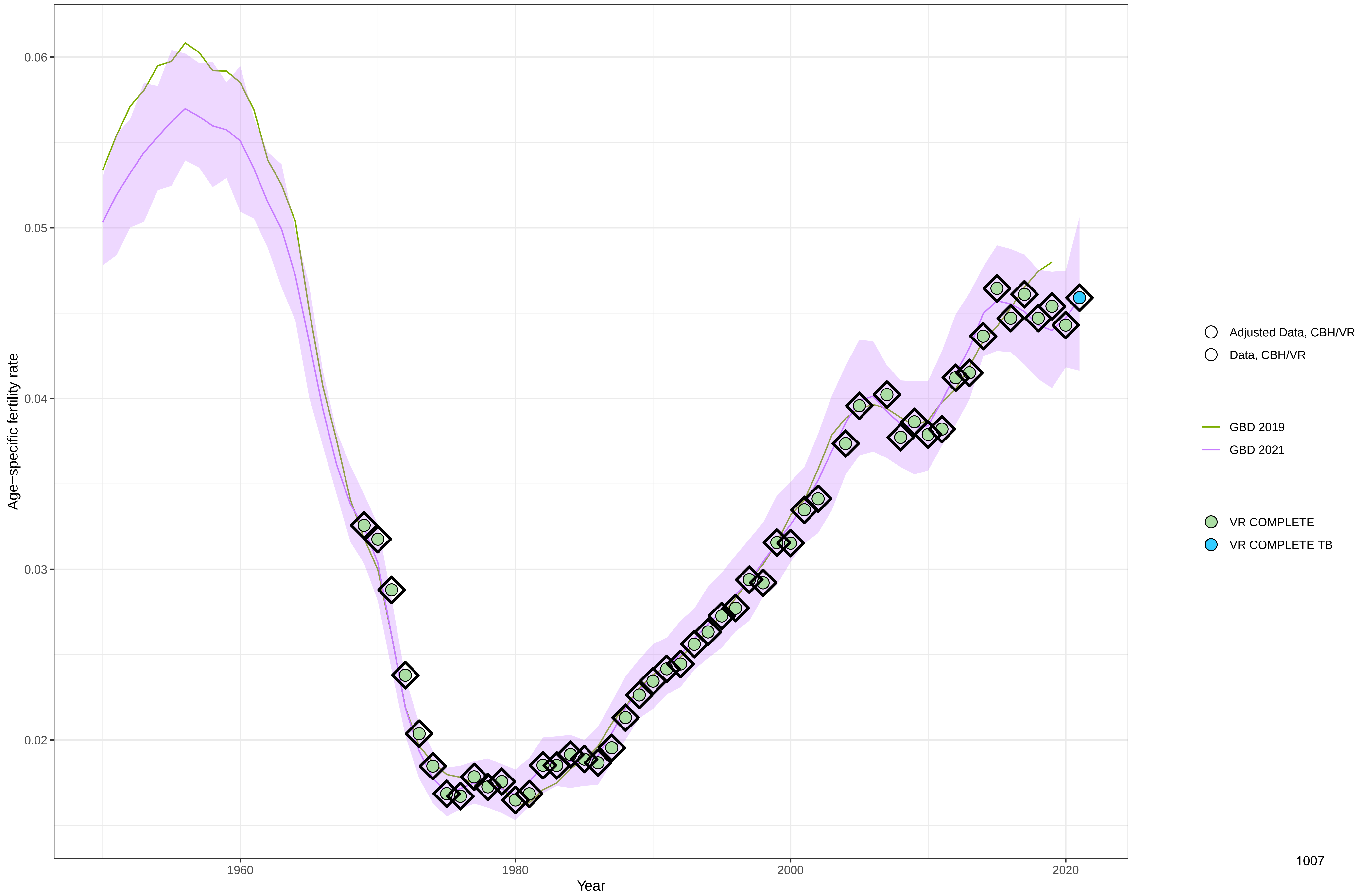

Age−specific fertility fitted trend lines, 1950−2021: Iowa, Ages: 40−44
Data points enclosed with a diamond are reference sources

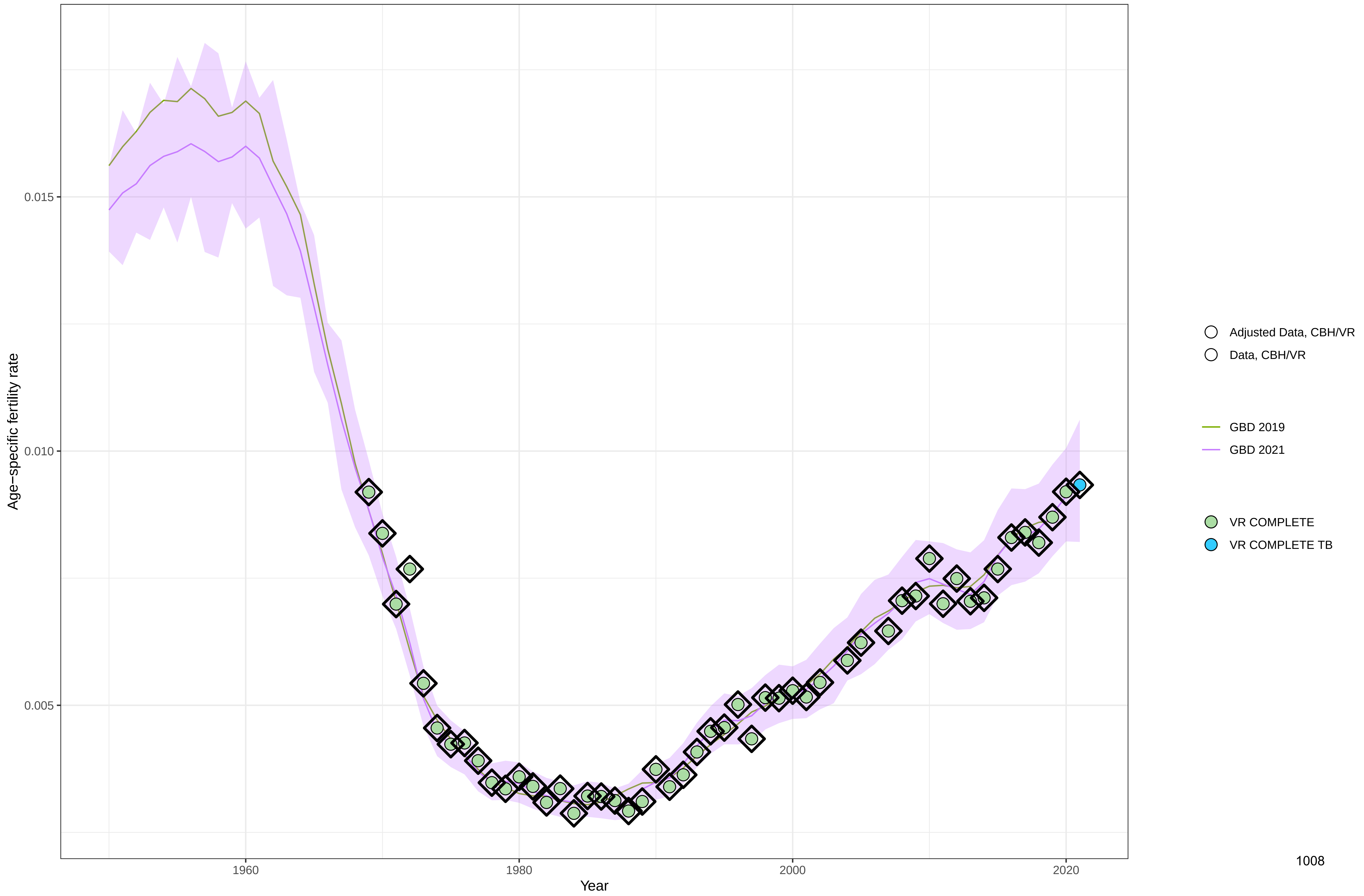

Age−specific fertility fitted trend lines, 1950−2021: Iowa, Ages: 45−49
Data points enclosed with a diamond are reference sources

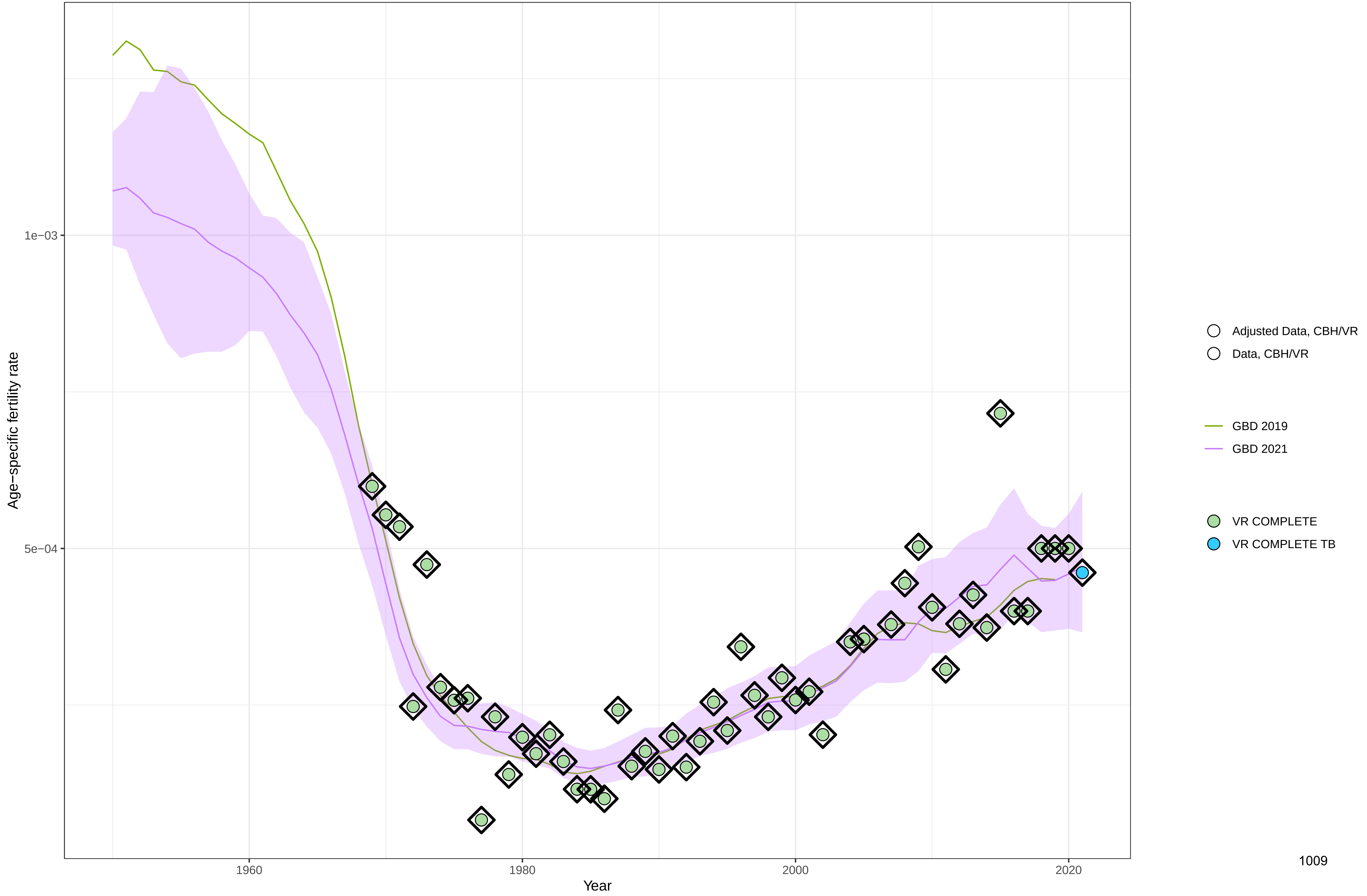

Age−specific fertility fitted trend lines, 1950−2021: Iowa, Ages: 50−54
Data points enclosed with a diamond are reference sources

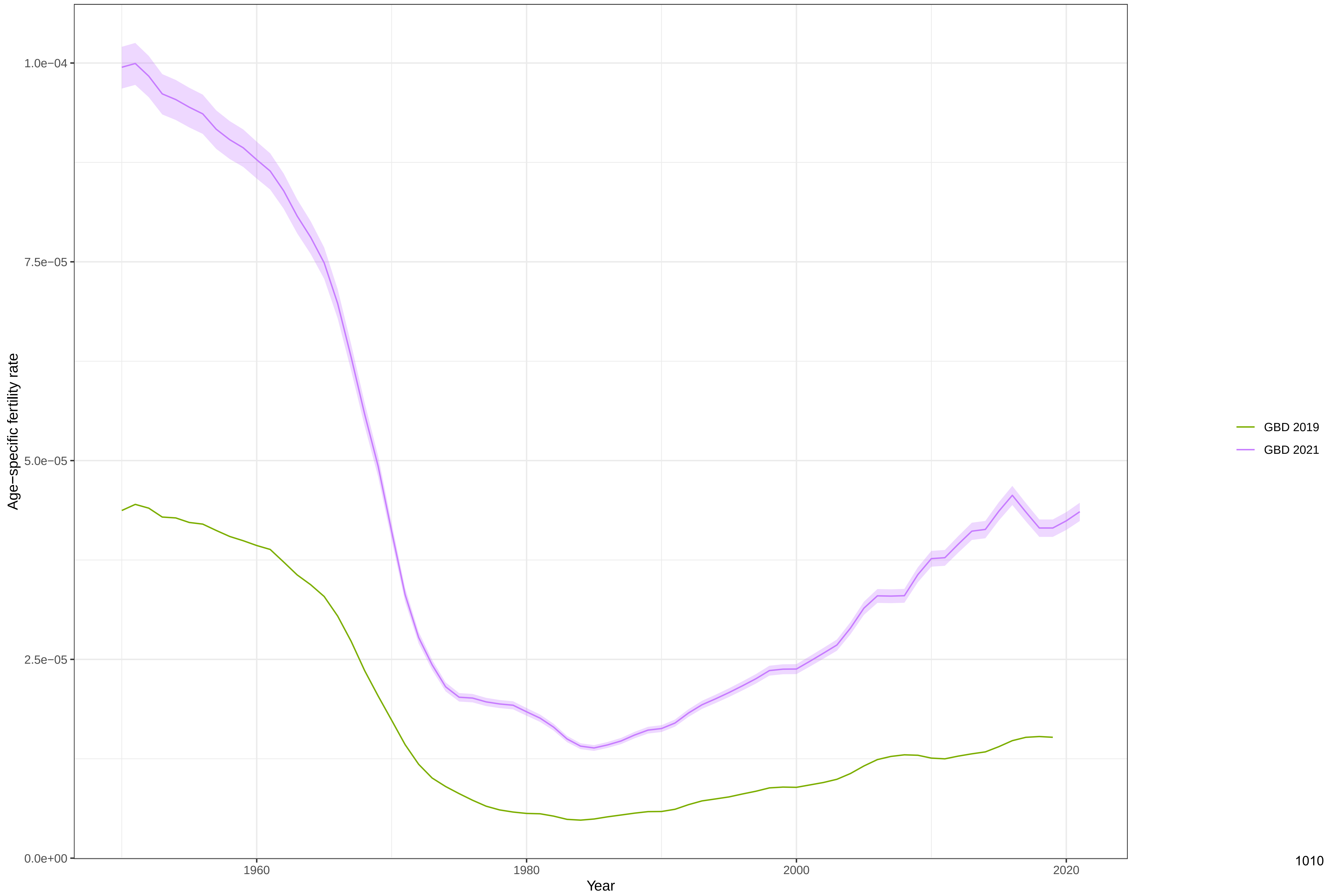

Total fertility rate fitted trend lines, 1950−2021: Iowa TFR
Data points enclosed with a diamond are reference sources

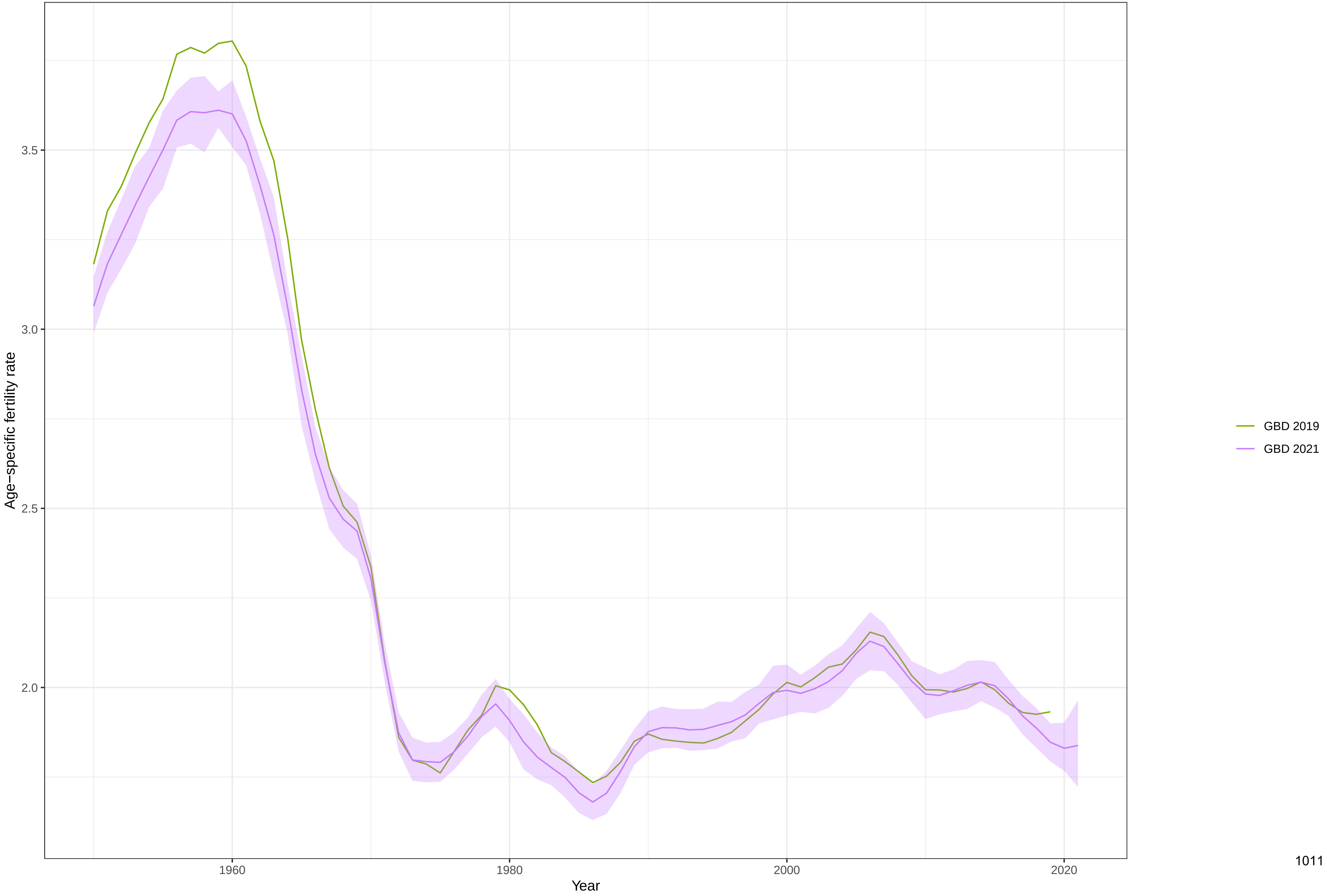

Age−specific fertility fitted trend lines, 1950−2021: Kansas, Ages: 10−14
Data points enclosed with a diamond are reference sources

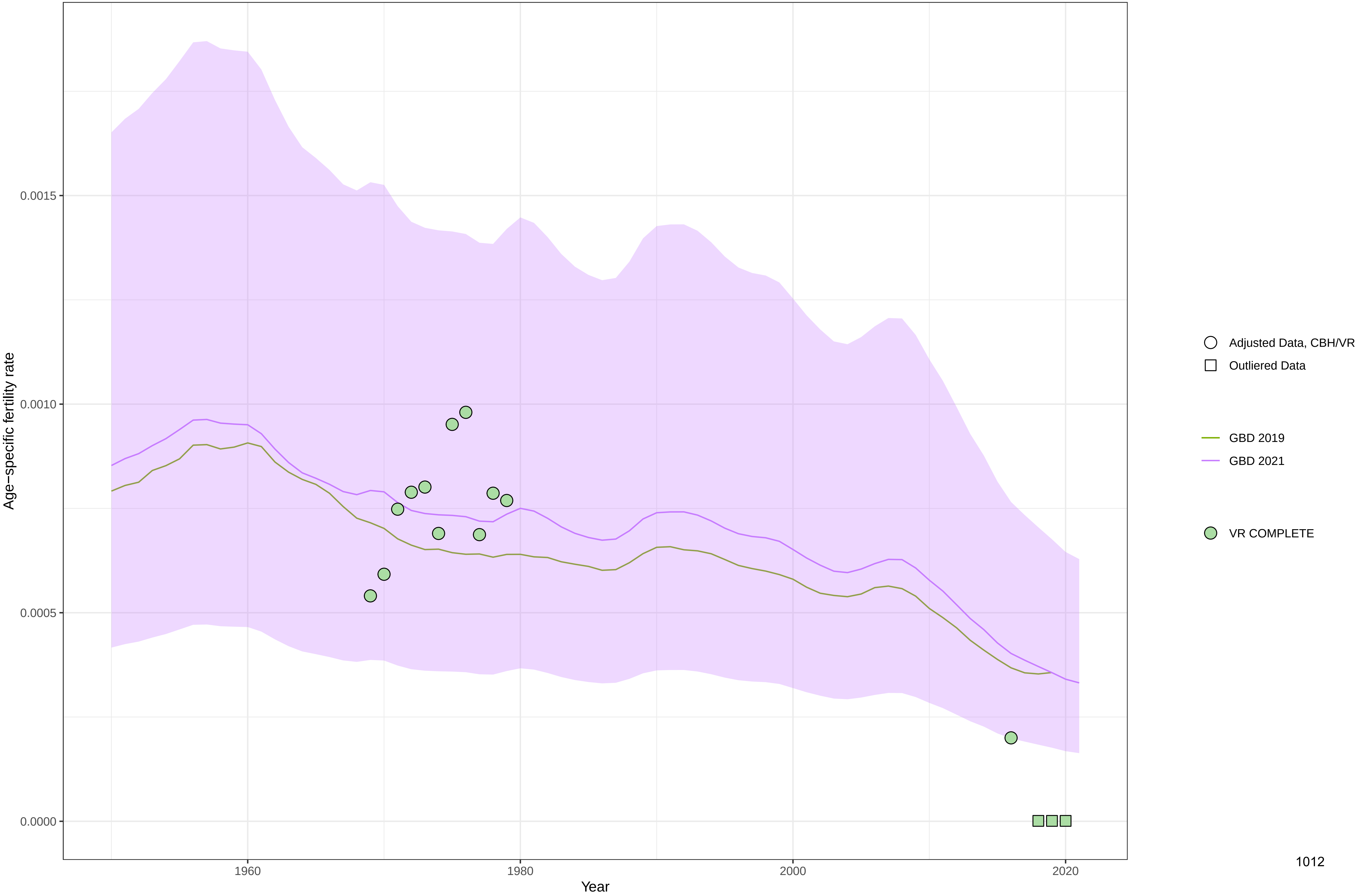

Age−specific fertility fitted trend lines, 1950−2021: Kansas, Ages: 15−19
Data points enclosed with a diamond are reference sources

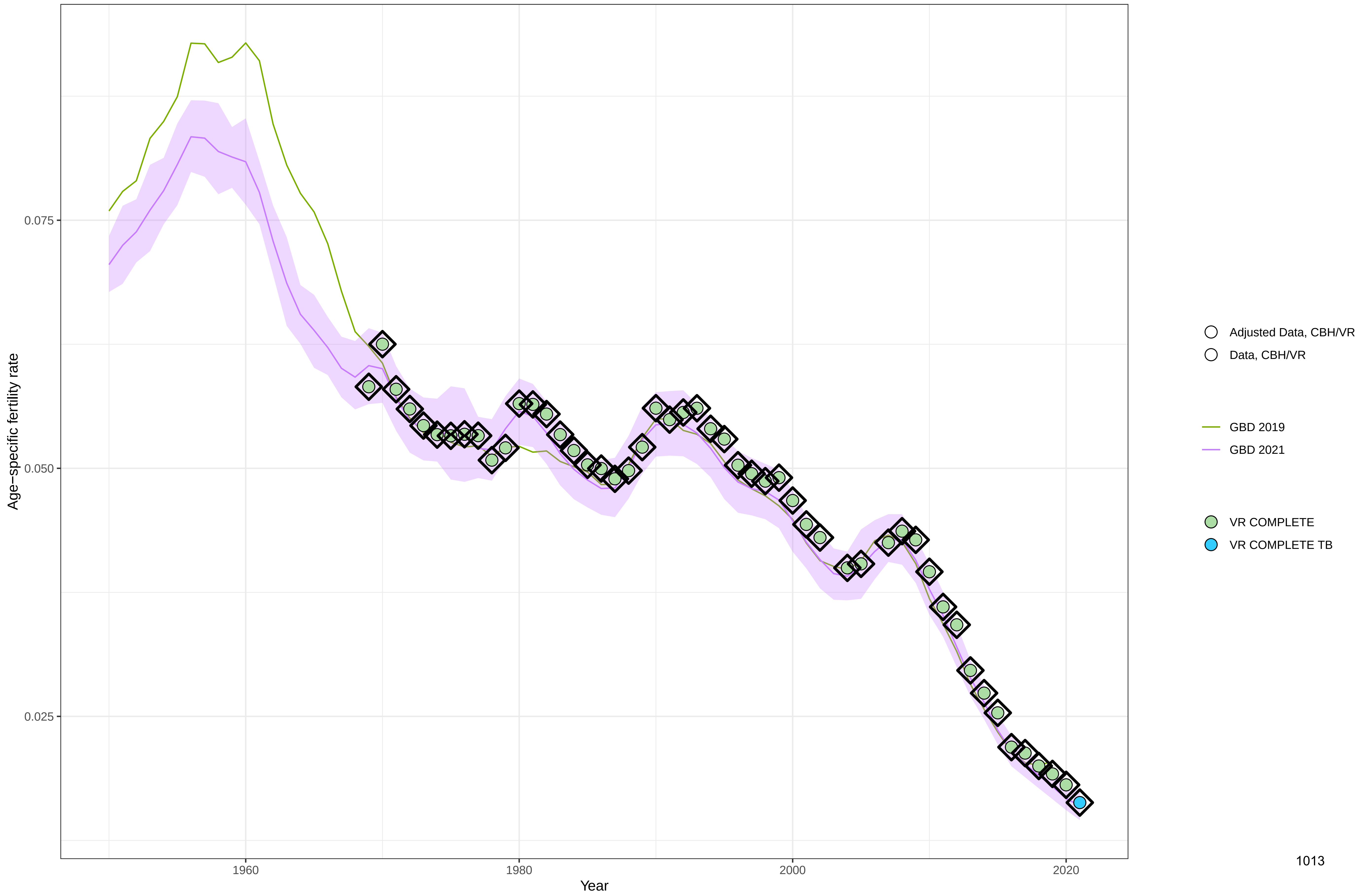

Age−specific fertility fitted trend lines, 1950−2021: Kansas, Ages: 20−24
Data points enclosed with a diamond are reference sources

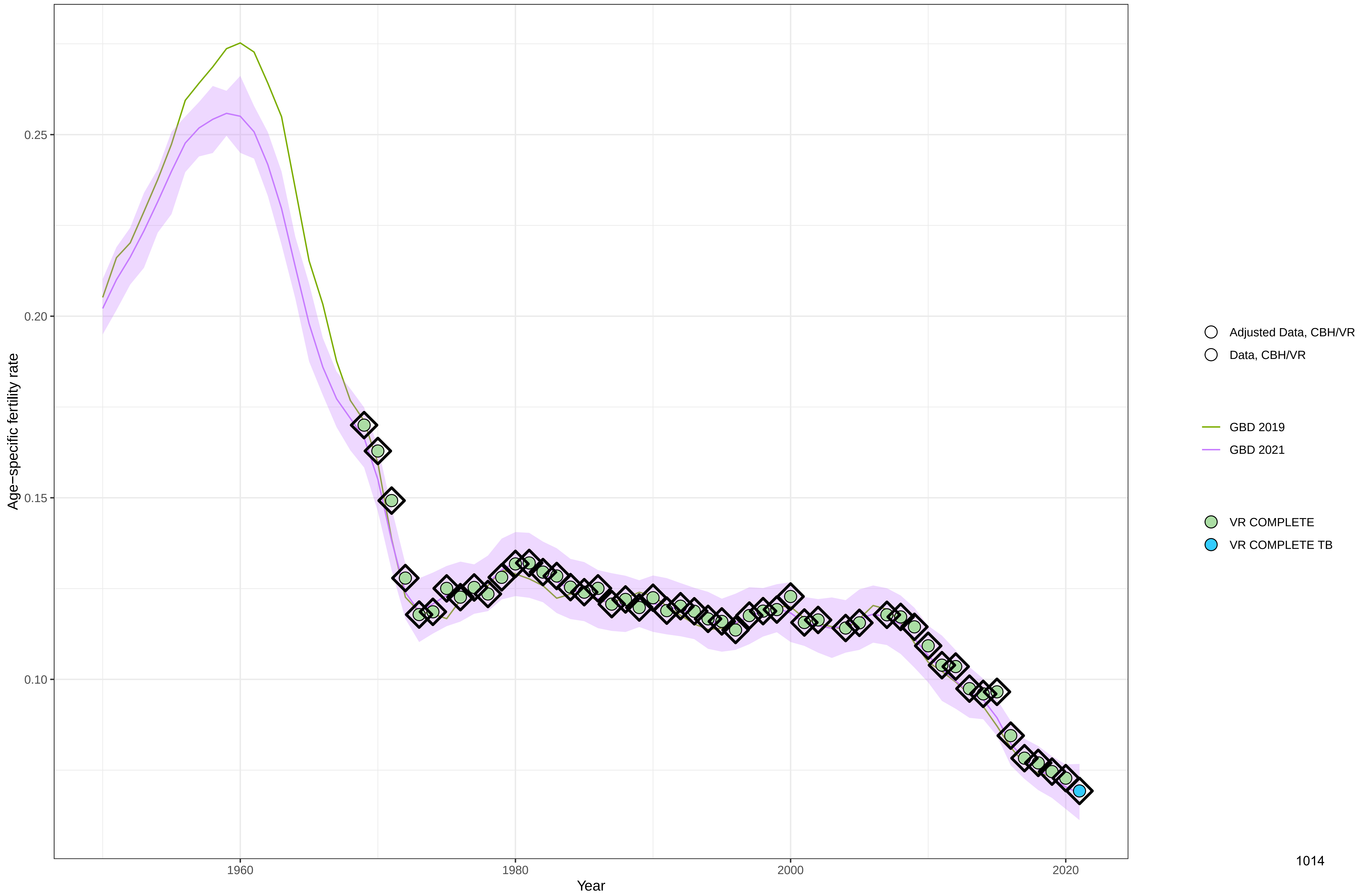

Age−specific fertility fitted trend lines, 1950−2021: Kansas, Ages: 25−29
Data points enclosed with a diamond are reference sources

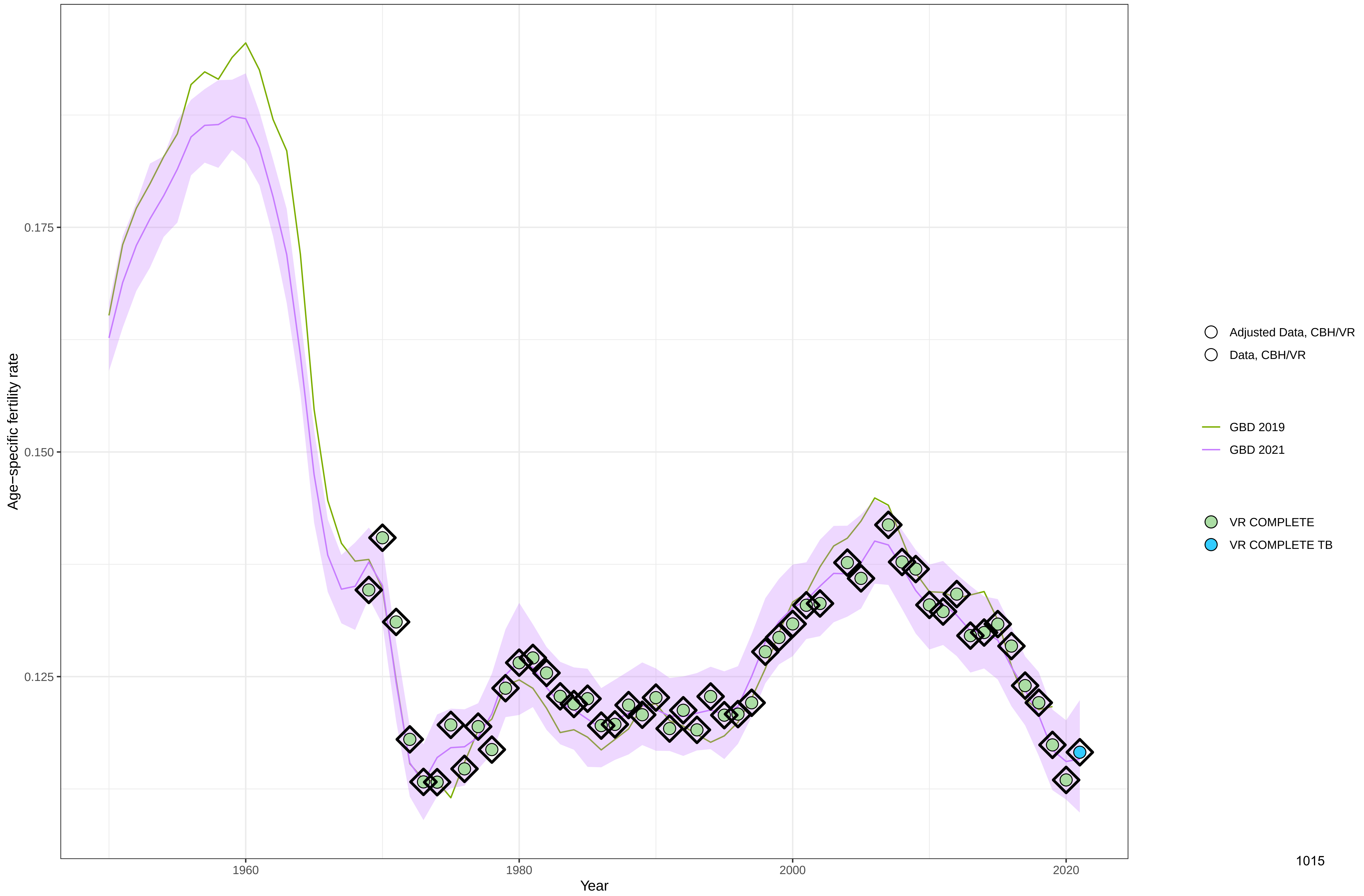

Age−specific fertility fitted trend lines, 1950−2021: Kansas, Ages: 30−34
Data points enclosed with a diamond are reference sources

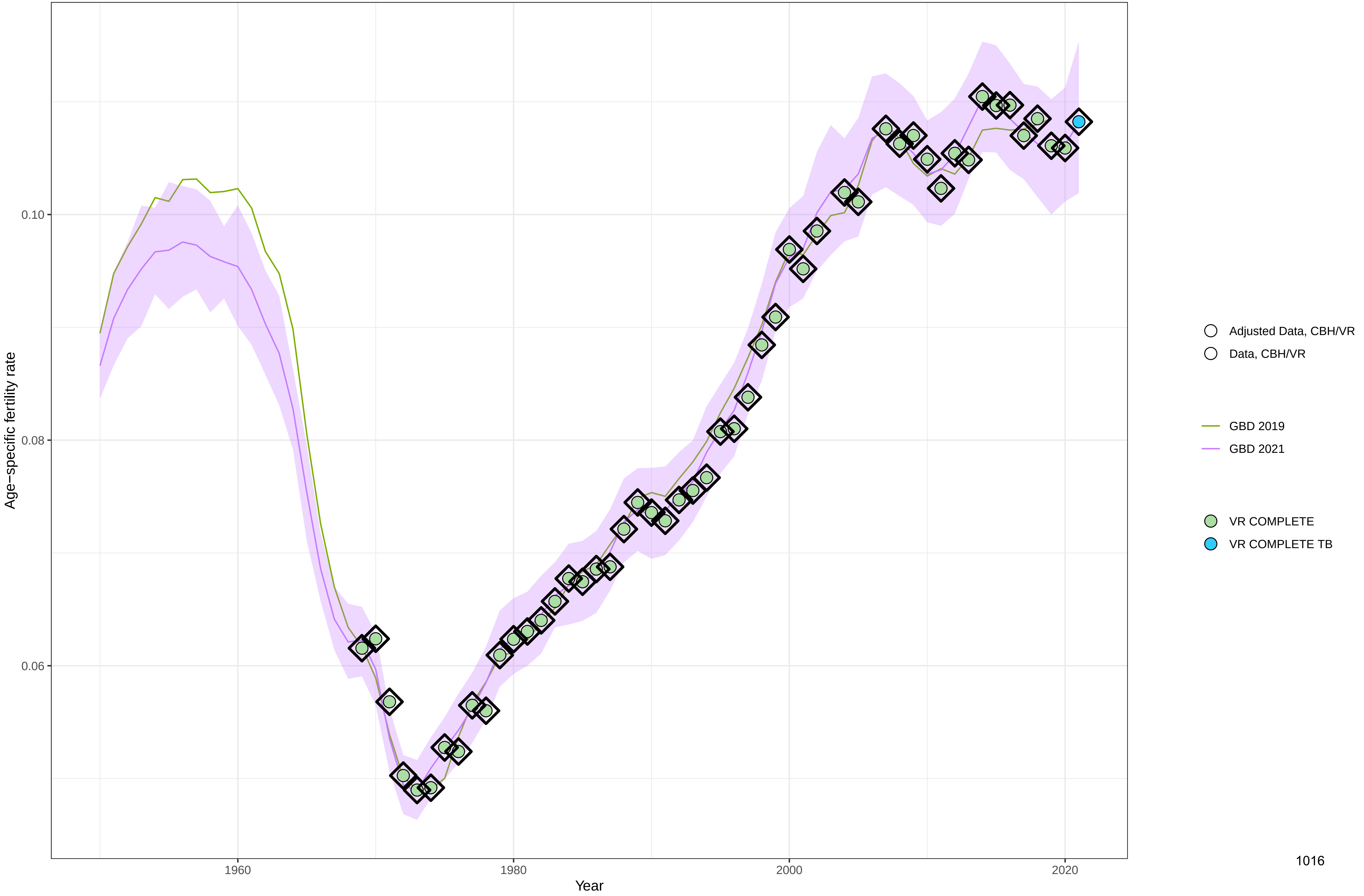

Age−specific fertility fitted trend lines, 1950−2021: Kansas, Ages: 35−39
Data points enclosed with a diamond are reference sources

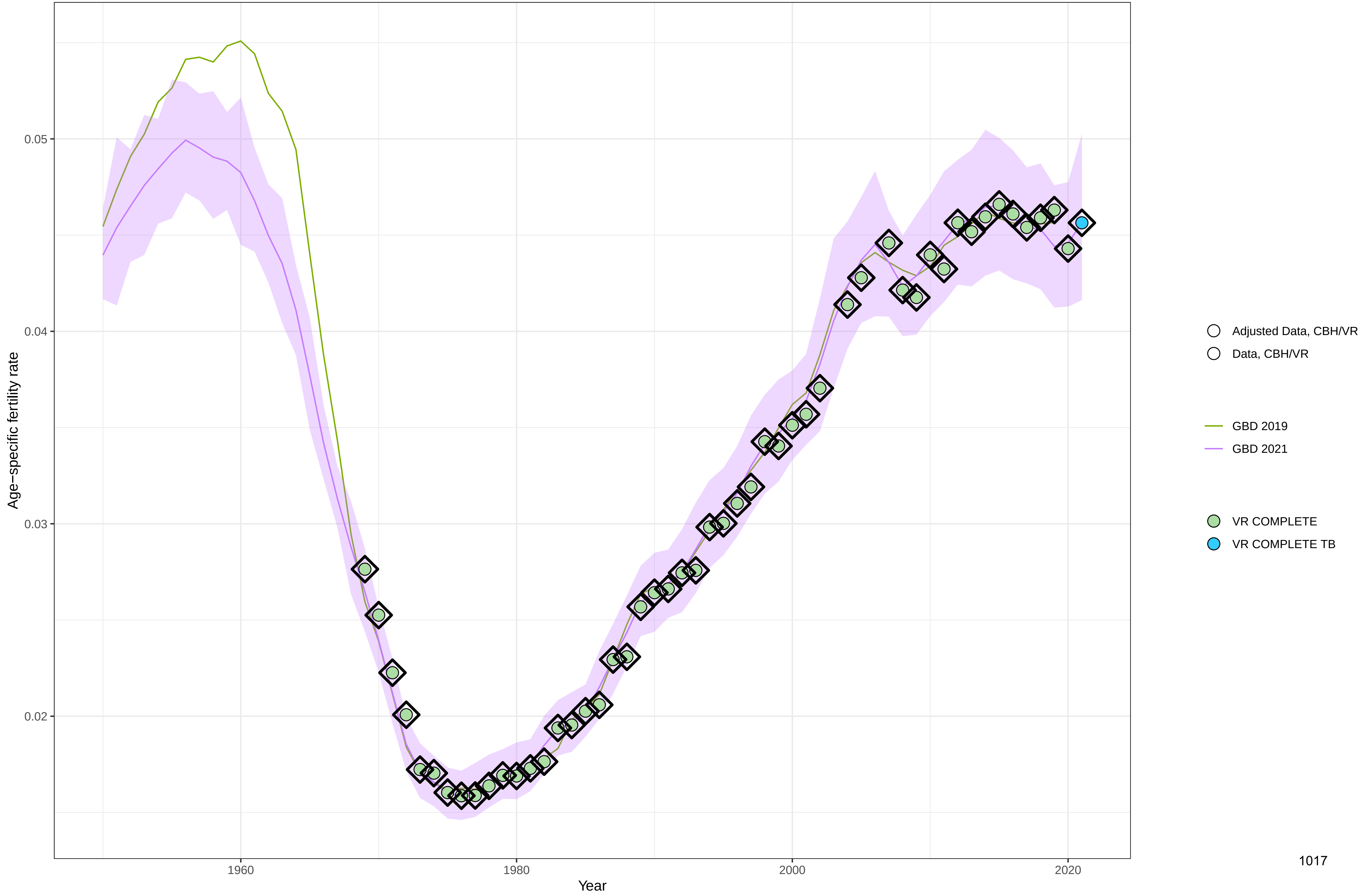

Age−specific fertility fitted trend lines, 1950−2021: Kansas, Ages: 40−44
Data points enclosed with a diamond are reference sources

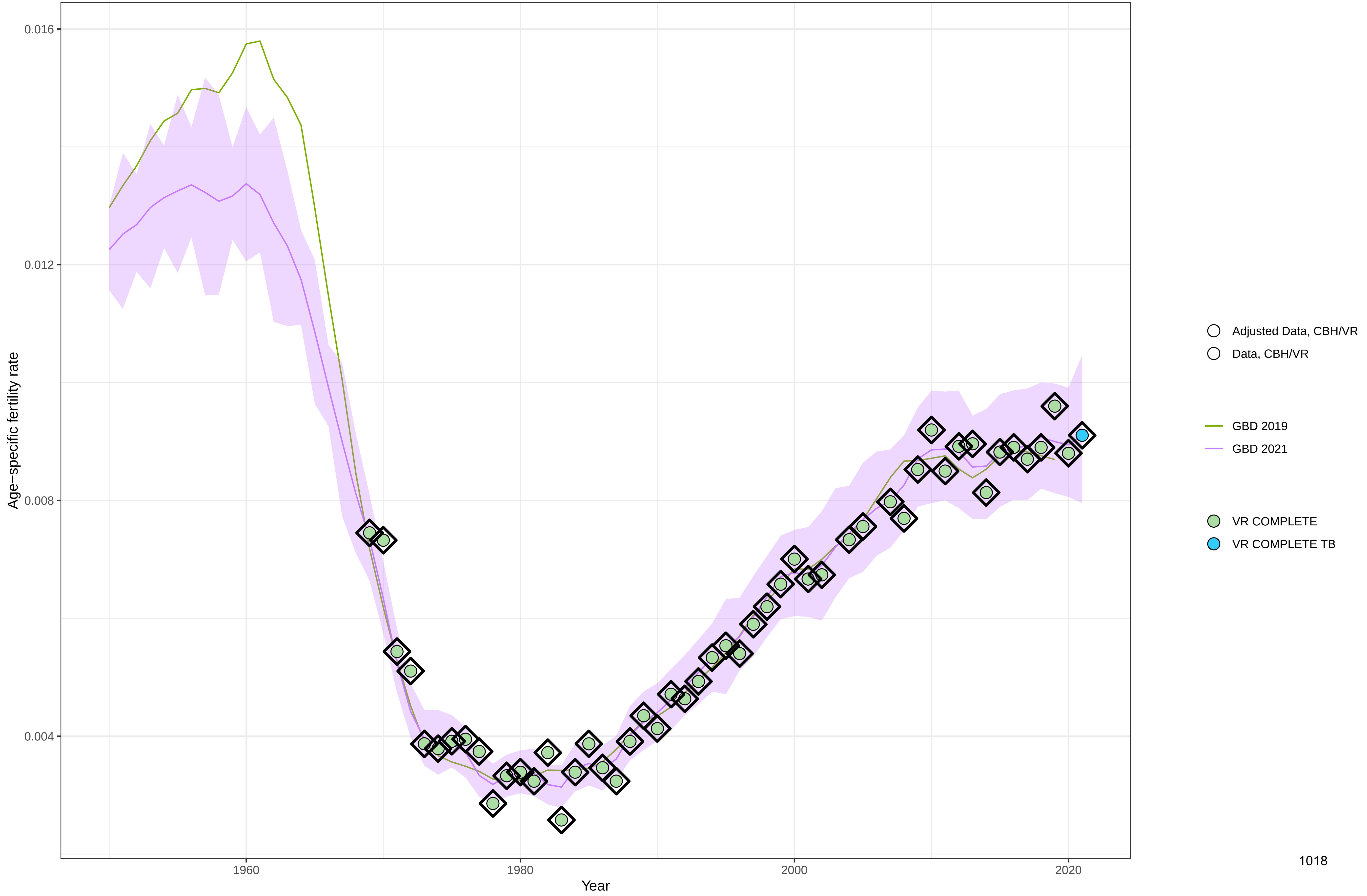

Age−specific fertility fitted trend lines, 1950−2021: Kansas, Ages: 45−49
Data points enclosed with a diamond are reference sources

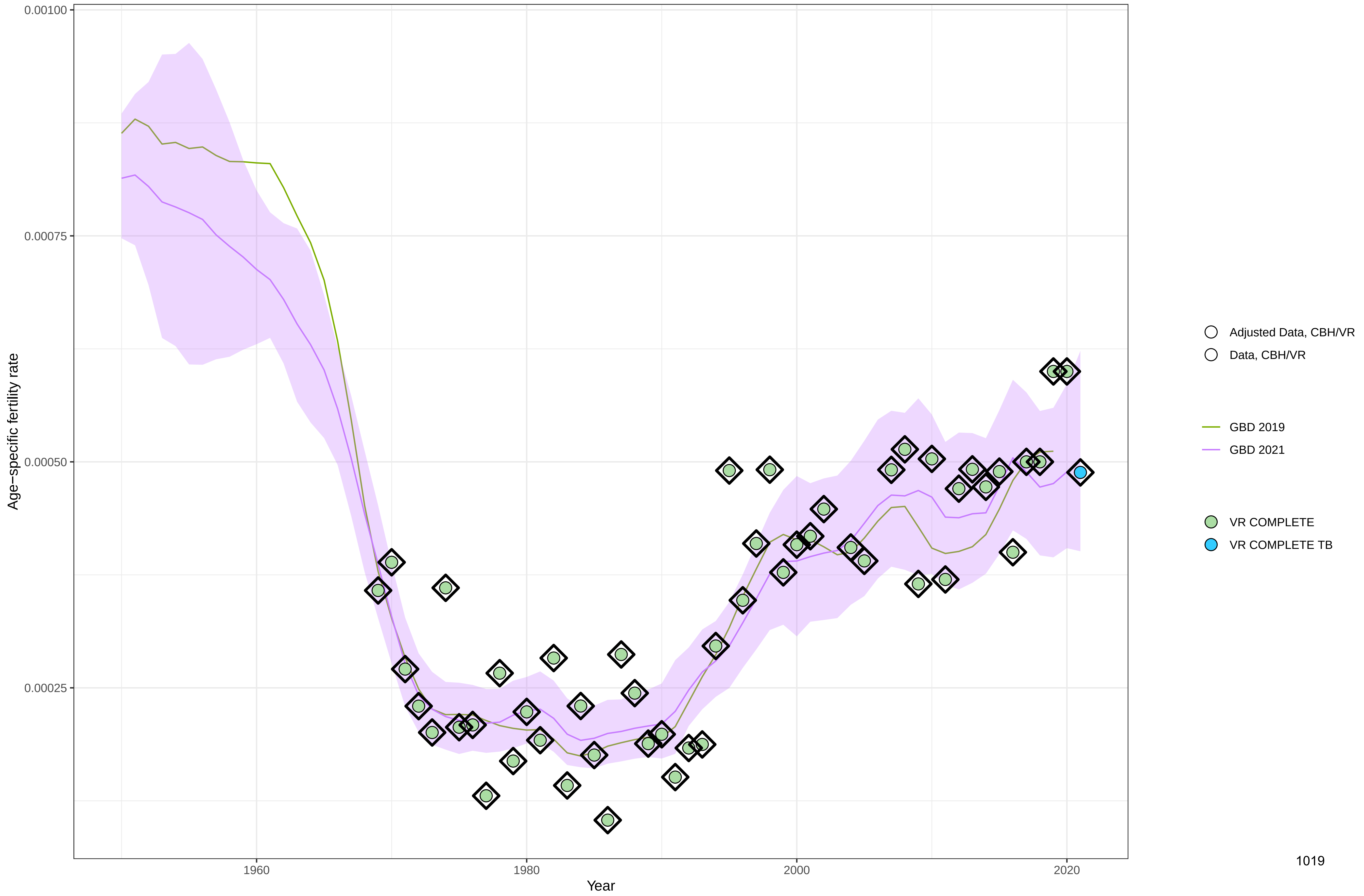

Age−specific fertility fitted trend lines, 1950−2021: Kansas, Ages: 50−54
Data points enclosed with a diamond are reference sources

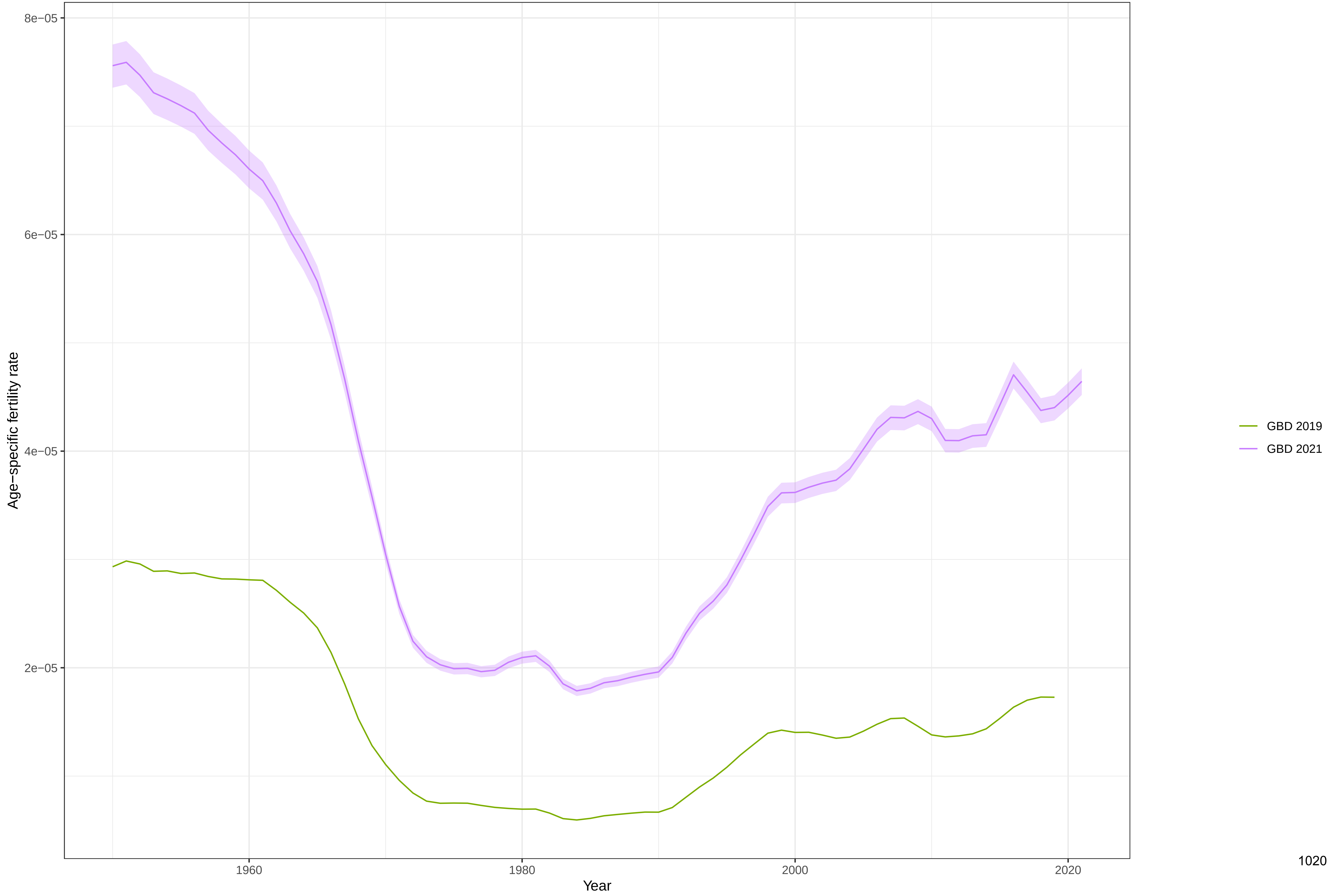

Total fertility rate fitted trend lines, 1950−2021: Kansas TFR
Data points enclosed with a diamond are reference sources

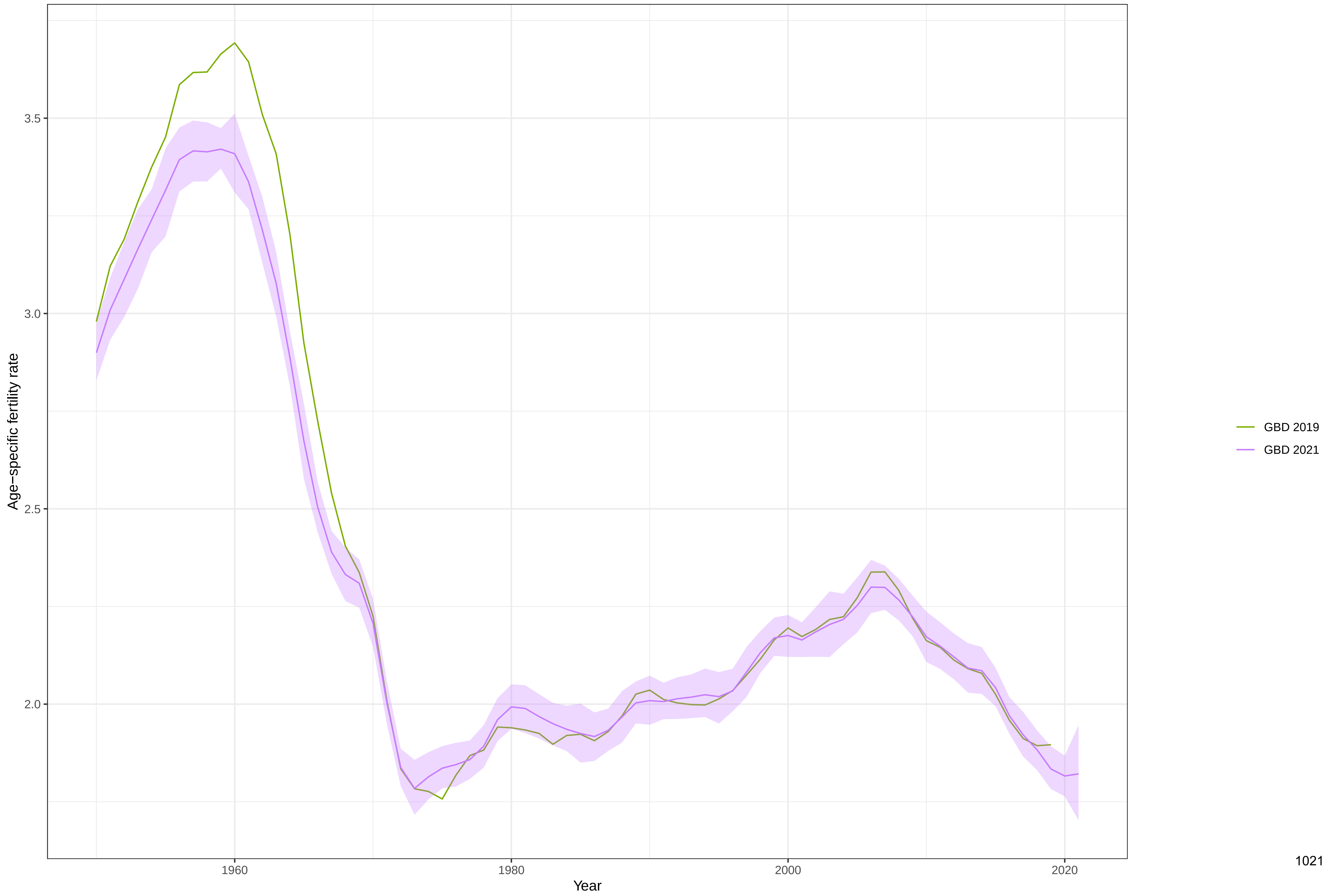

Age−specific fertility fitted trend lines, 1950−2021: Kentucky, Ages: 10−14
Data points enclosed with a diamond are reference sources

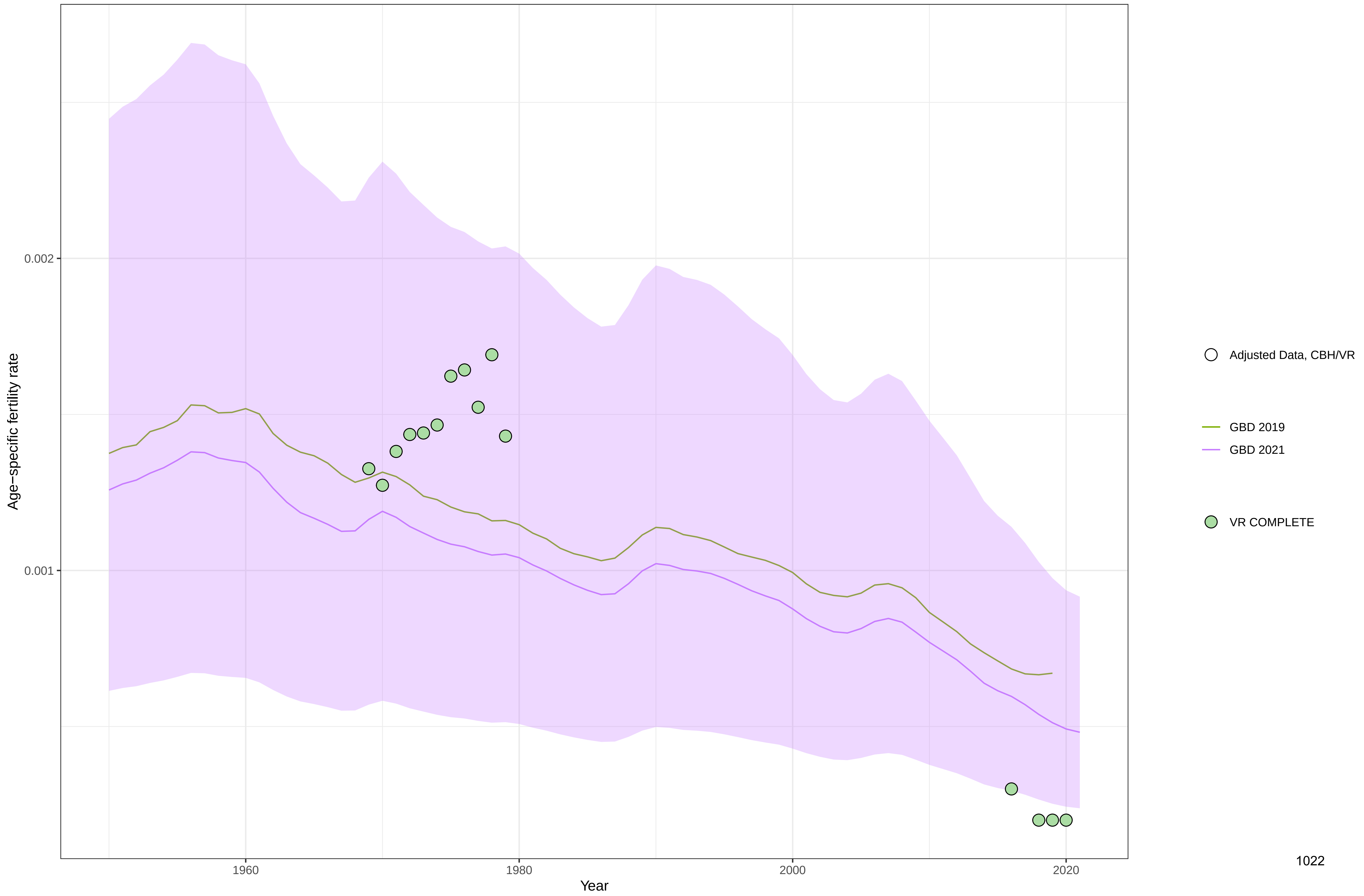

Age−specific fertility fitted trend lines, 1950−2021: Kentucky, Ages: 15−19
Data points enclosed with a diamond are reference sources

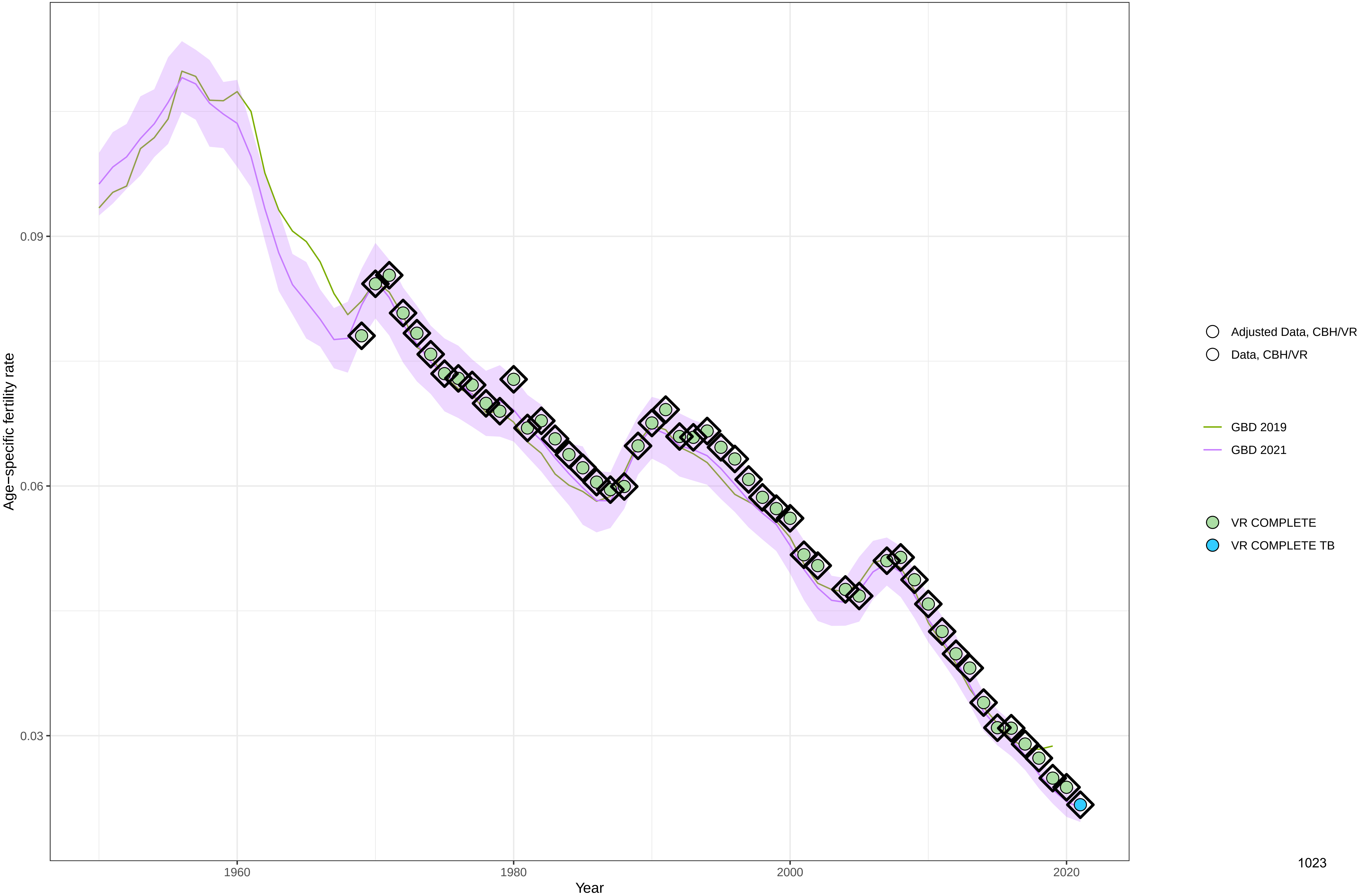

Age−specific fertility fitted trend lines, 1950−2021: Kentucky, Ages: 20−24
Data points enclosed with a diamond are reference sources

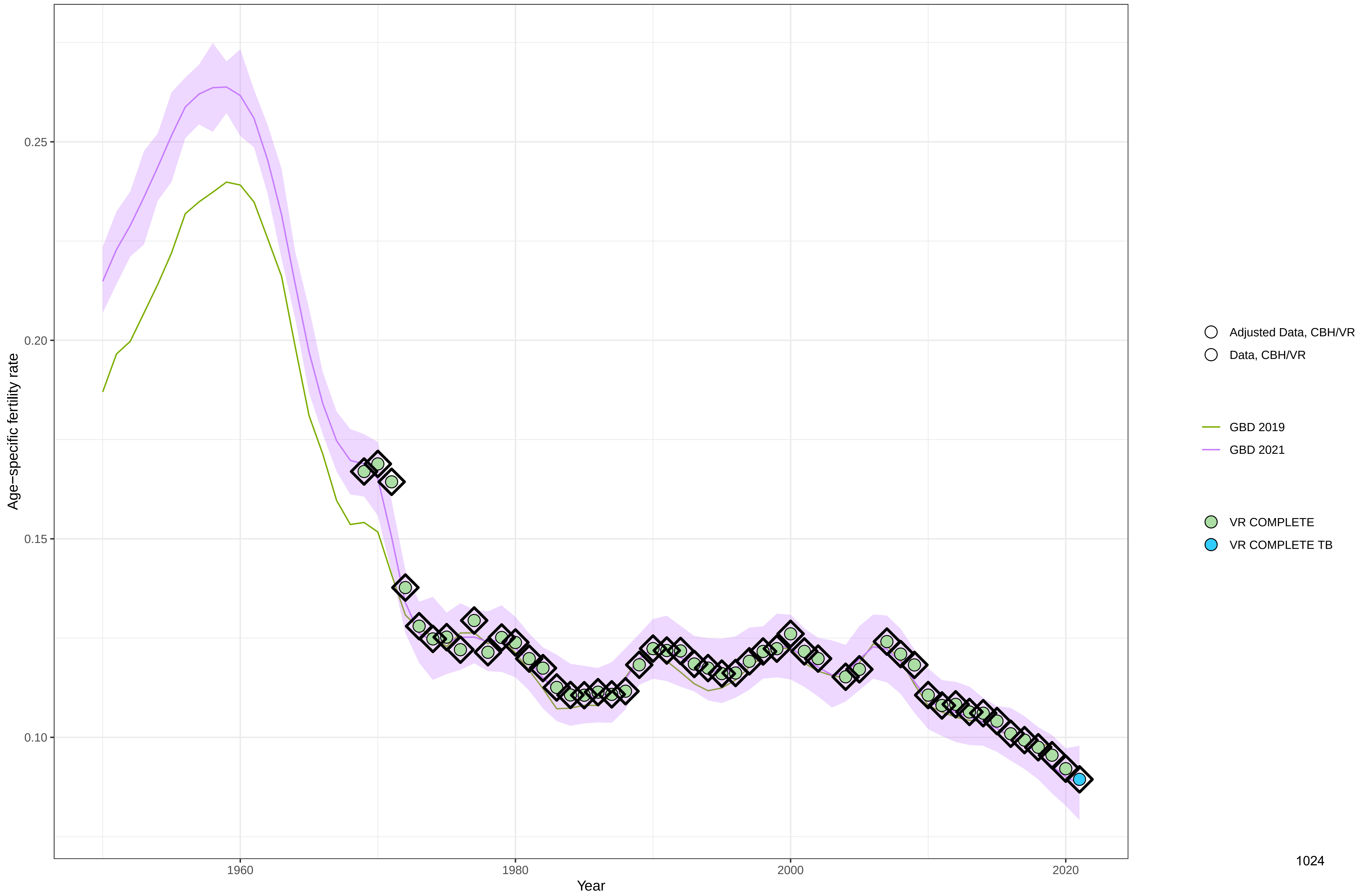

Age−specific fertility fitted trend lines, 1950−2021: Kentucky, Ages: 25−29
Data points enclosed with a diamond are reference sources

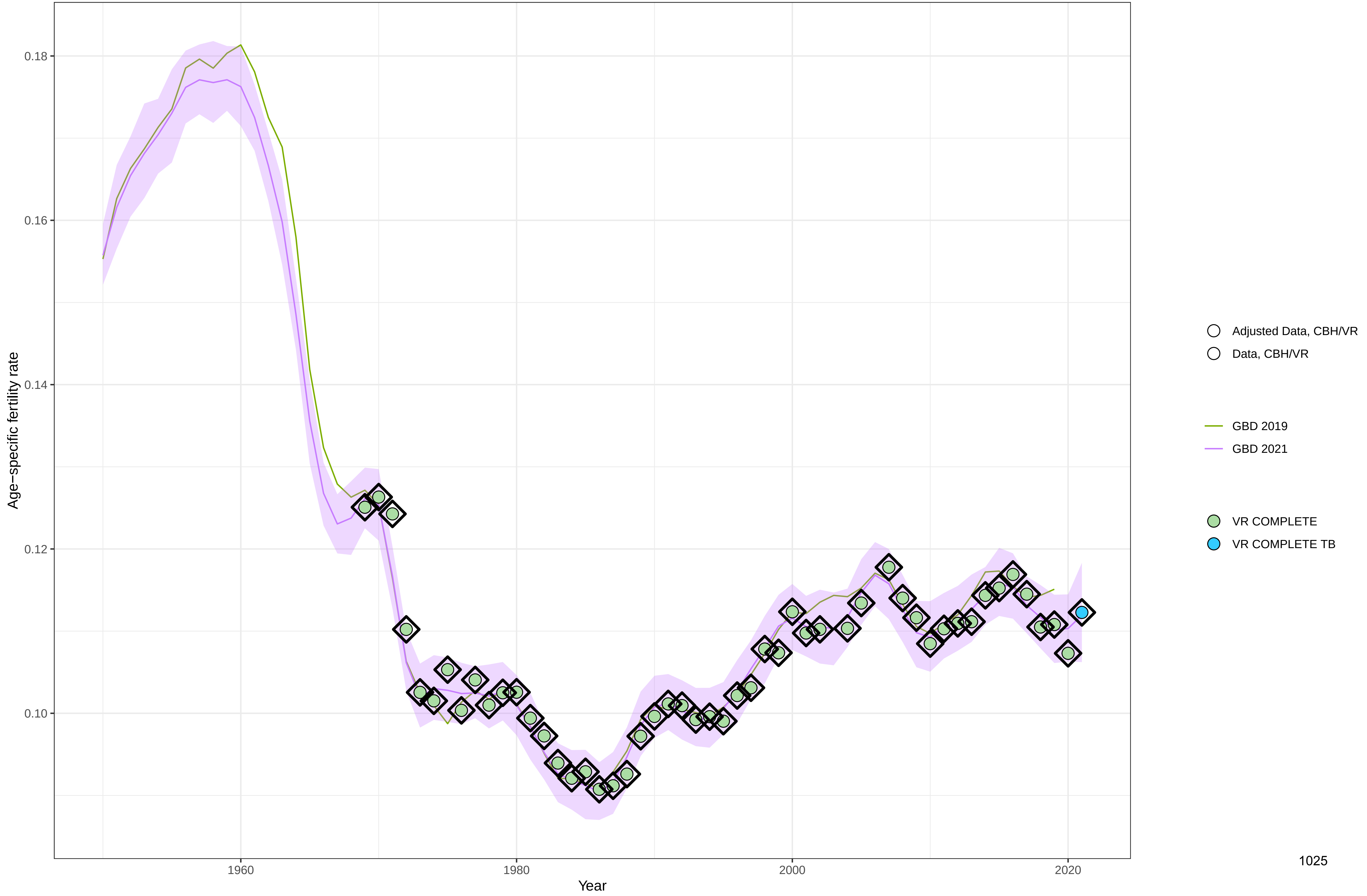

Age−specific fertility fitted trend lines, 1950−2021: Kentucky, Ages: 30−34
Data points enclosed with a diamond are reference sources

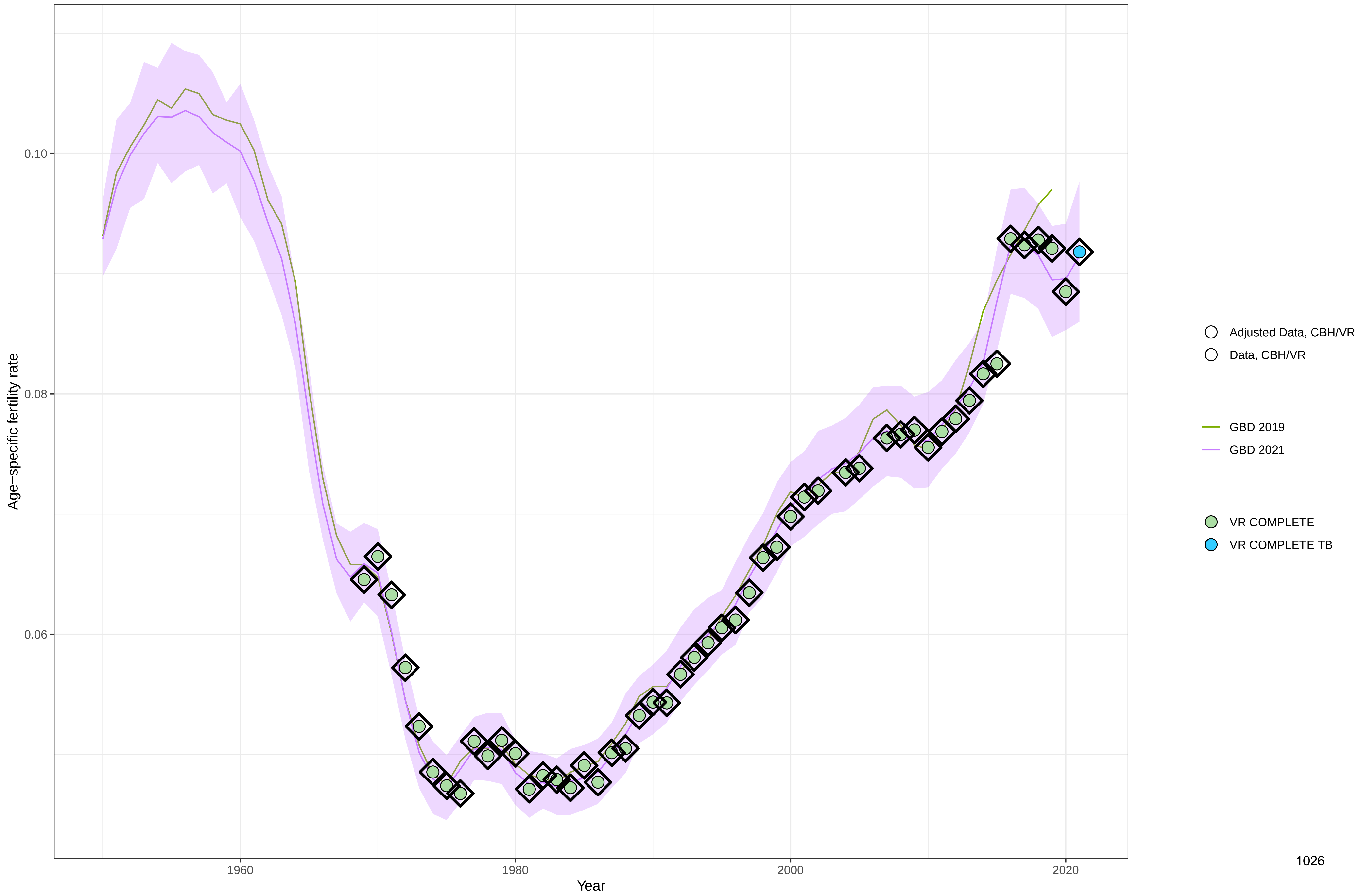

Age−specific fertility fitted trend lines, 1950−2021: Kentucky, Ages: 35−39
Data points enclosed with a diamond are reference sources

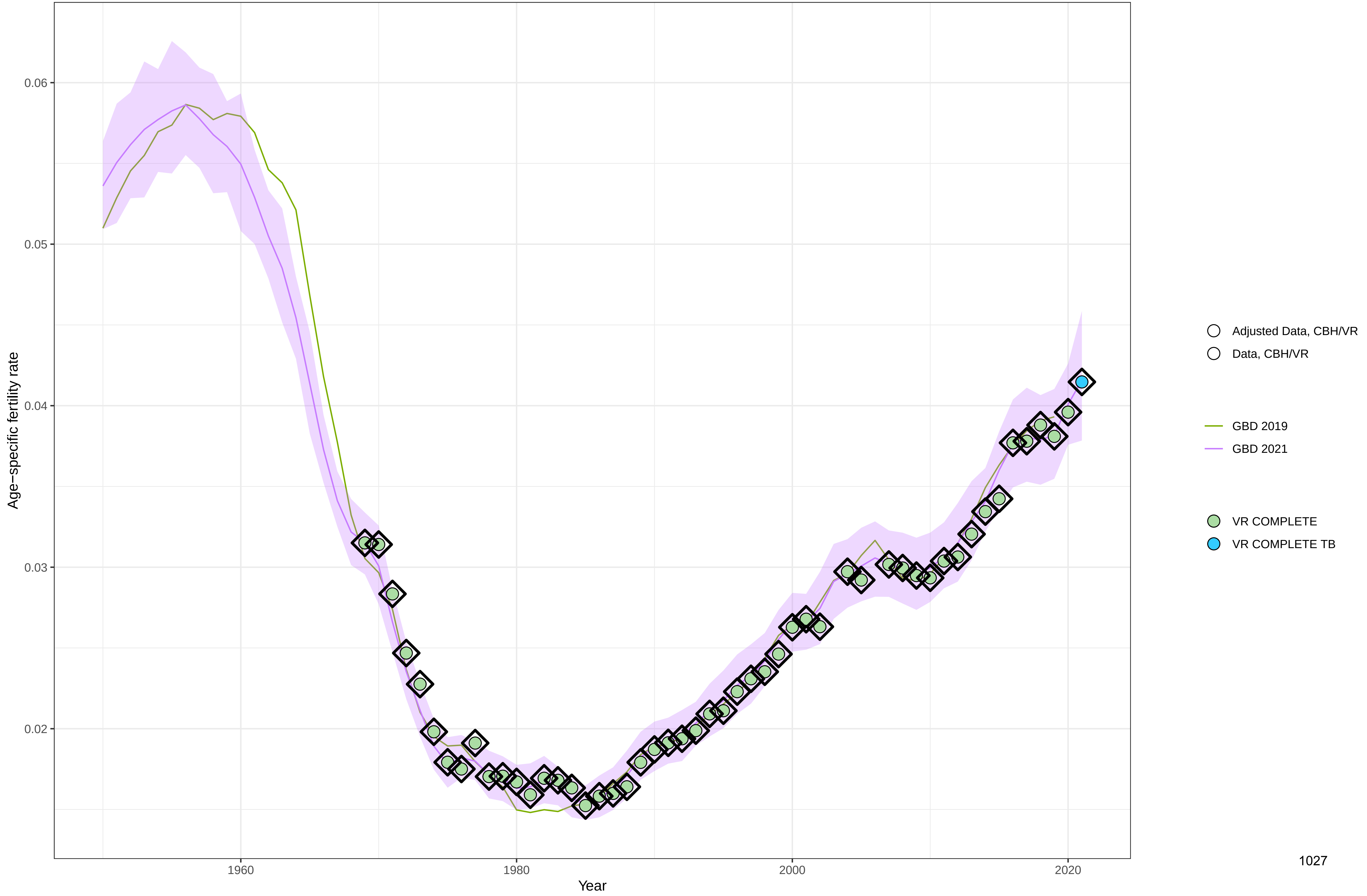

Age−specific fertility fitted trend lines, 1950−2021: Kentucky, Ages: 40−44
Data points enclosed with a diamond are reference sources

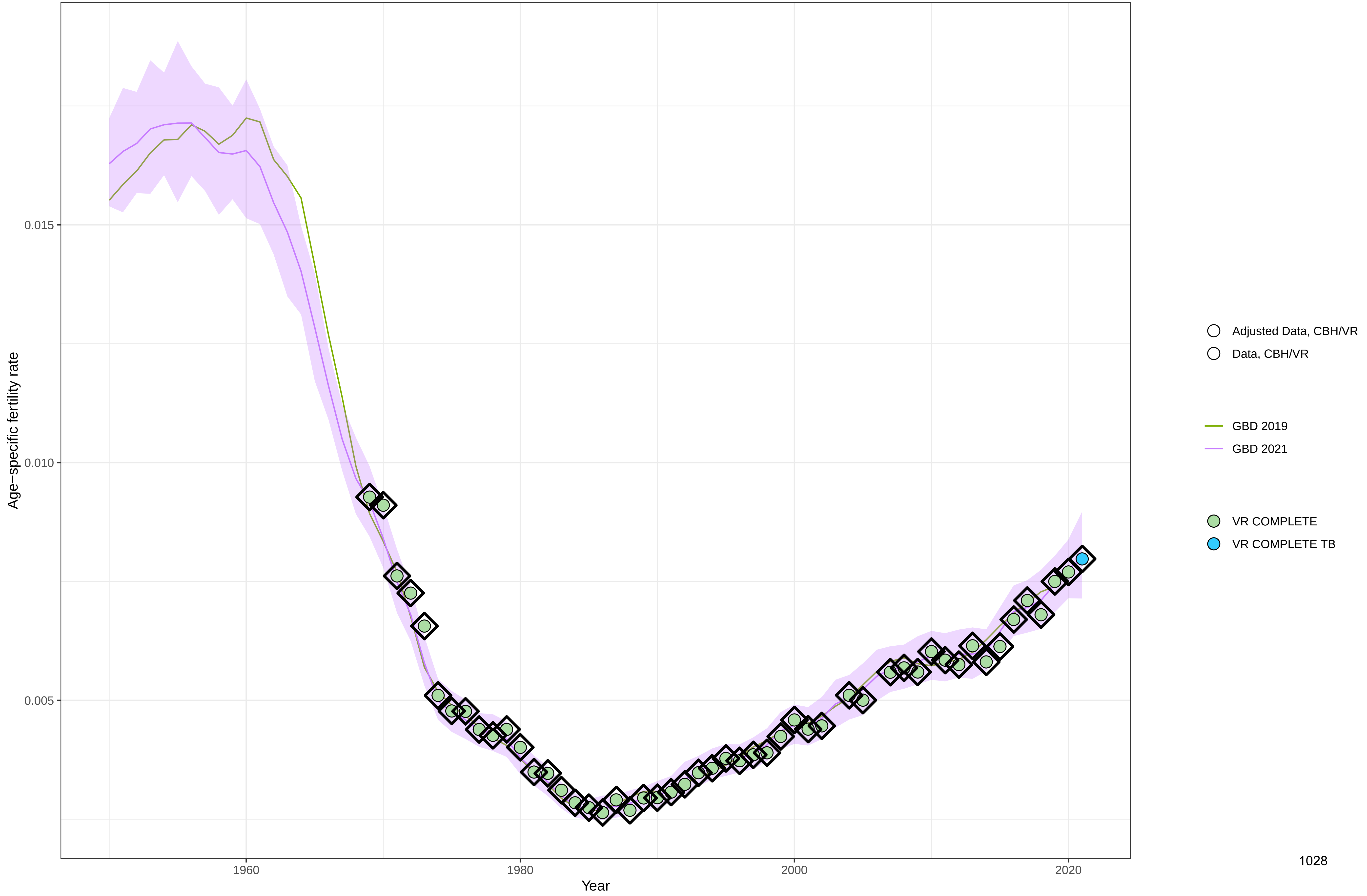

Age−specific fertility fitted trend lines, 1950−2021: Kentucky, Ages: 45−49
Data points enclosed with a diamond are reference sources

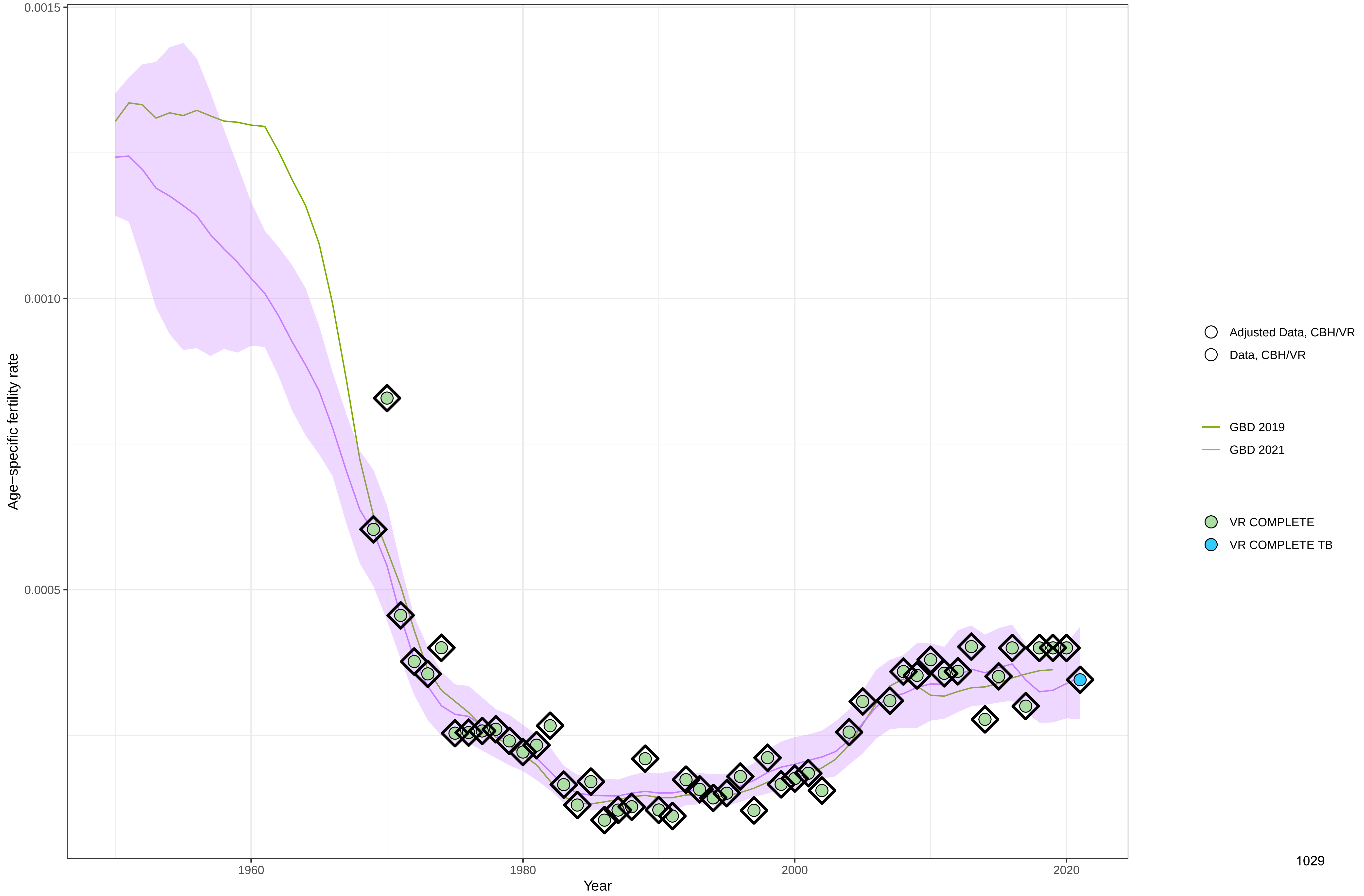

Age−specific fertility fitted trend lines, 1950−2021: Kentucky, Ages: 50−54
Data points enclosed with a diamond are reference sources

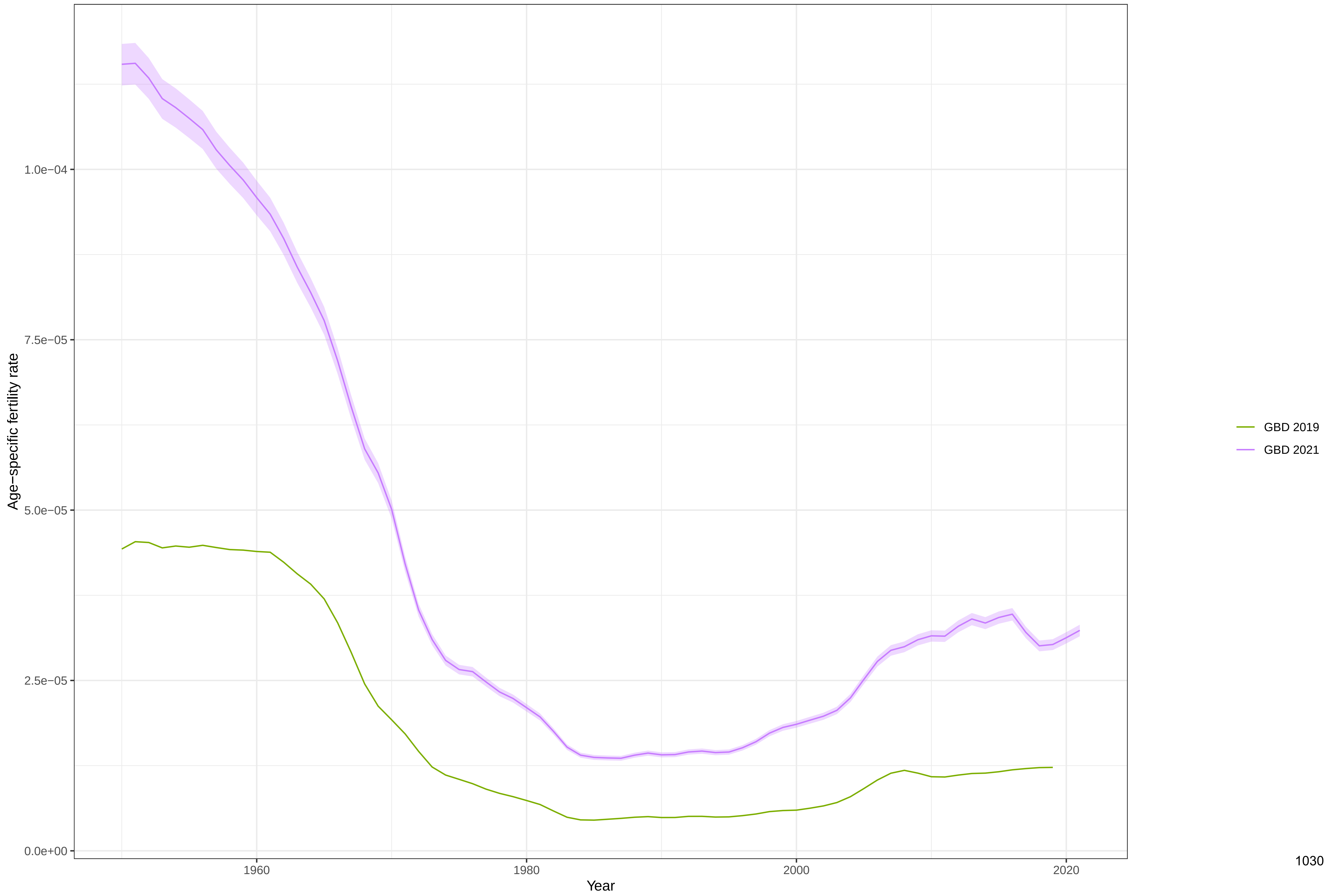

Total fertility rate fitted trend lines, 1950−2021: Kentucky TFR
Data points enclosed with a diamond are reference sources

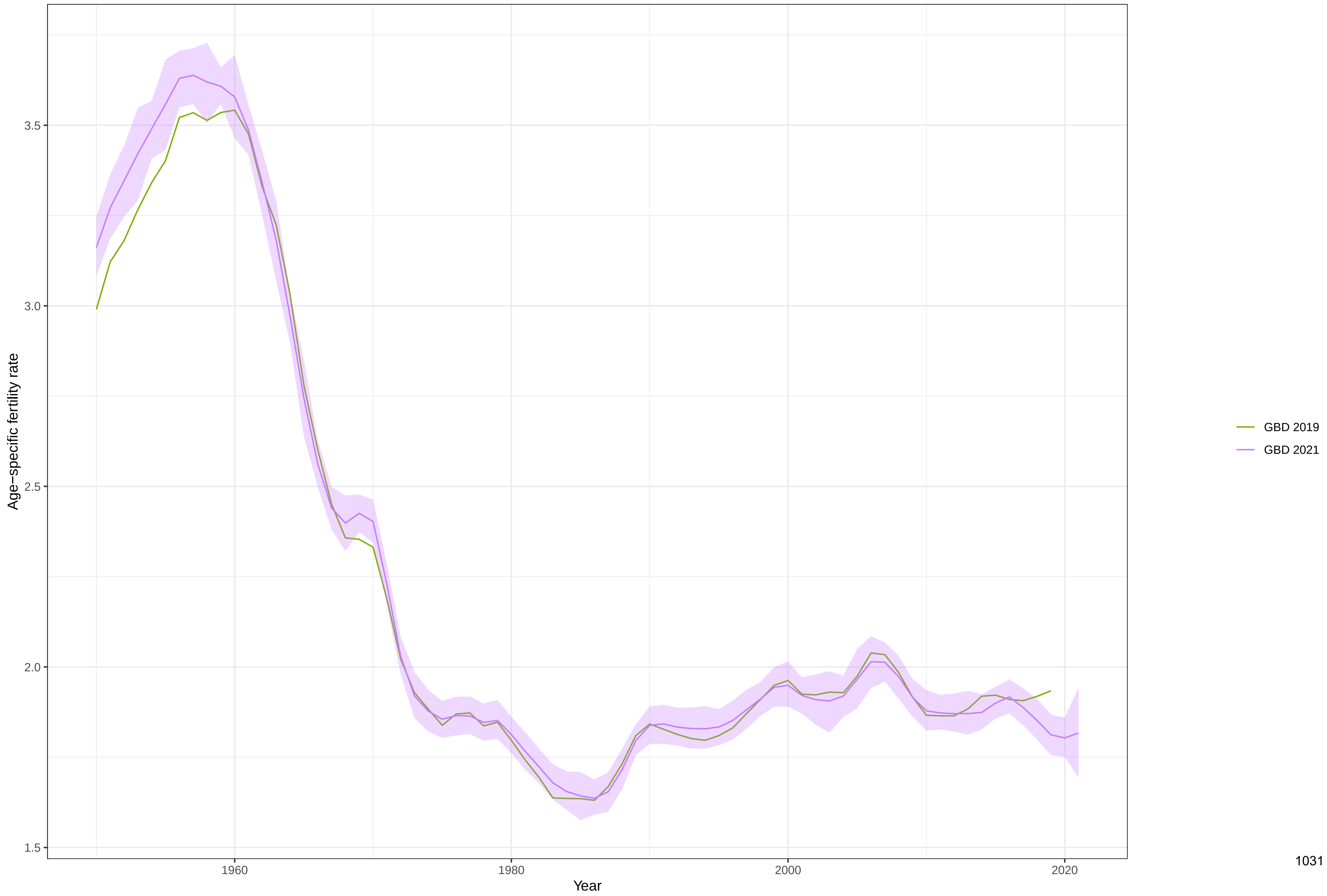

Age−specific fertility fitted trend lines, 1950−2021: Louisiana, Ages: 10−14
Data points enclosed with a diamond are reference sources

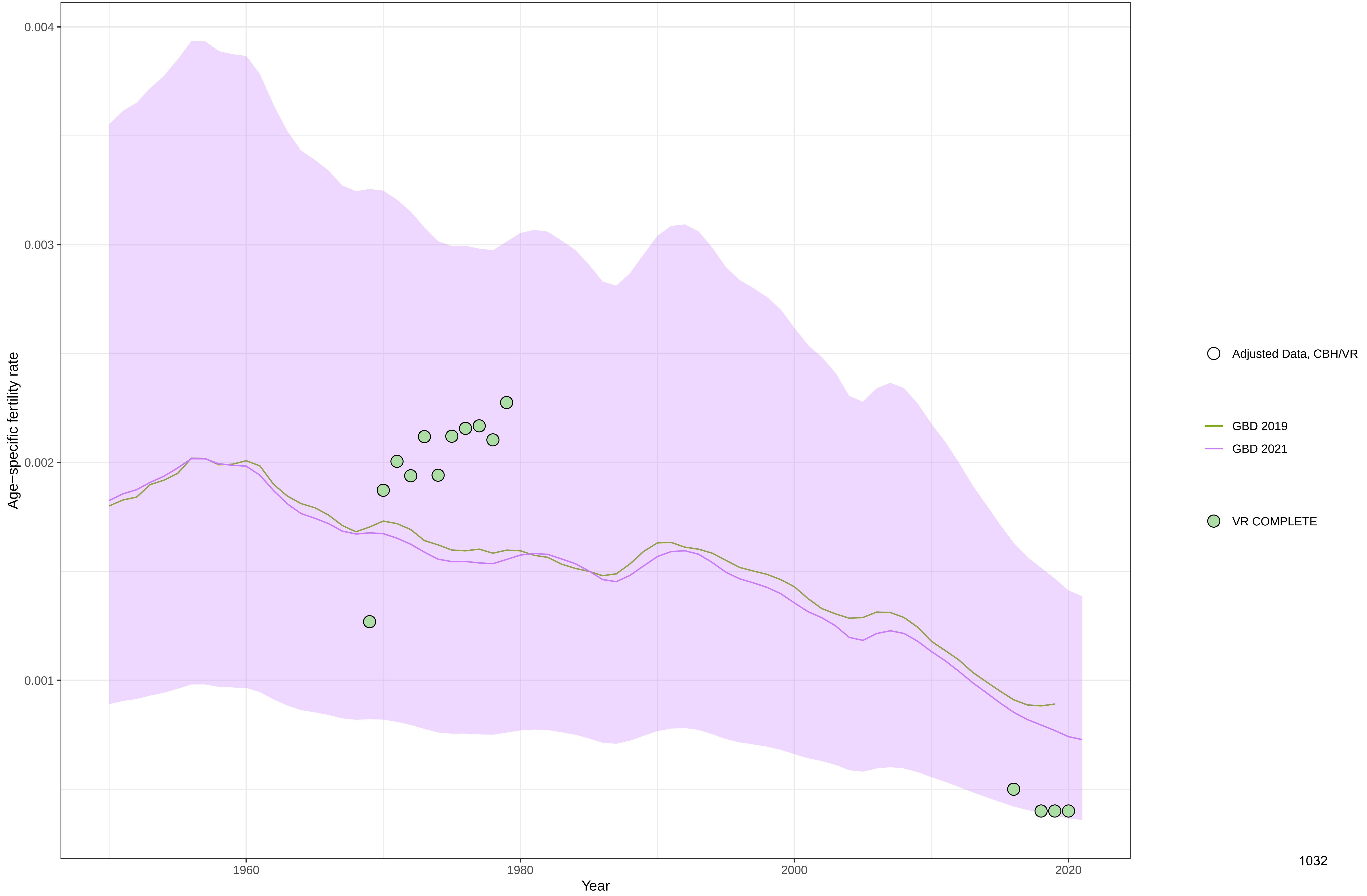

Age−specific fertility fitted trend lines, 1950−2021: Louisiana, Ages: 15−19
Data points enclosed with a diamond are reference sources

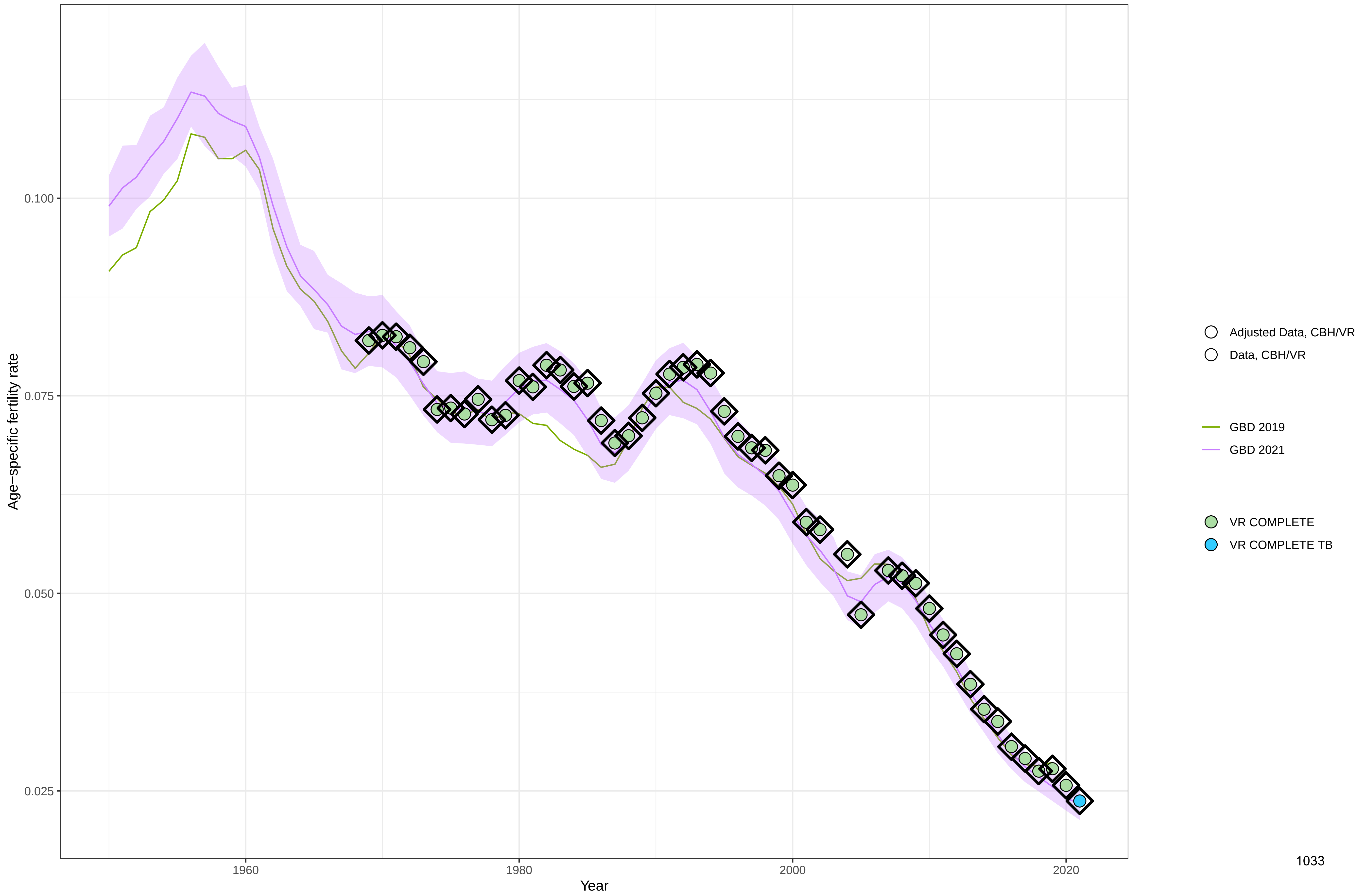

Age−specific fertility fitted trend lines, 1950−2021: Louisiana, Ages: 20−24
Data points enclosed with a diamond are reference sources

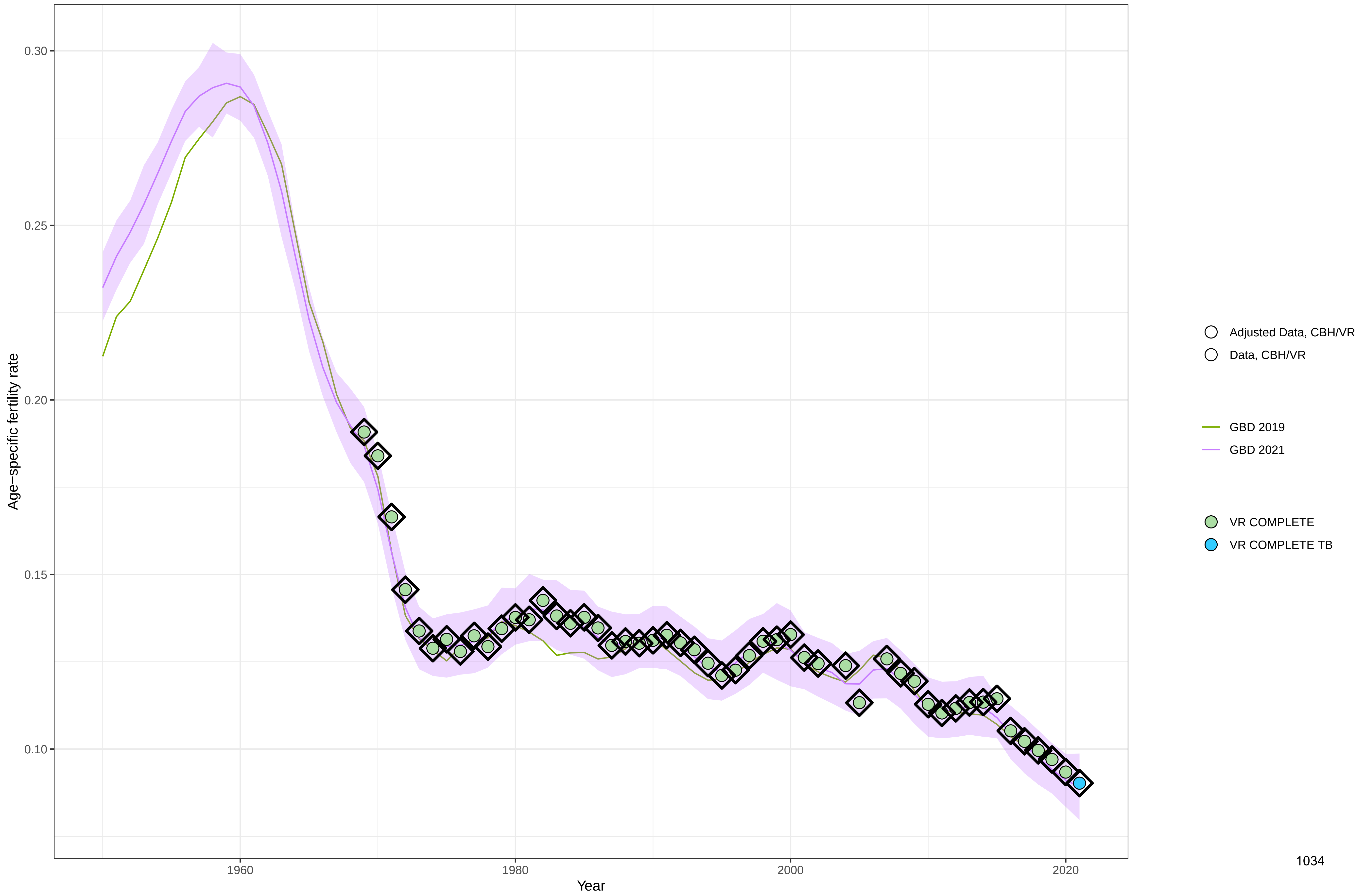

Age−specific fertility fitted trend lines, 1950−2021: Louisiana, Ages: 25−29
Data points enclosed with a diamond are reference sources

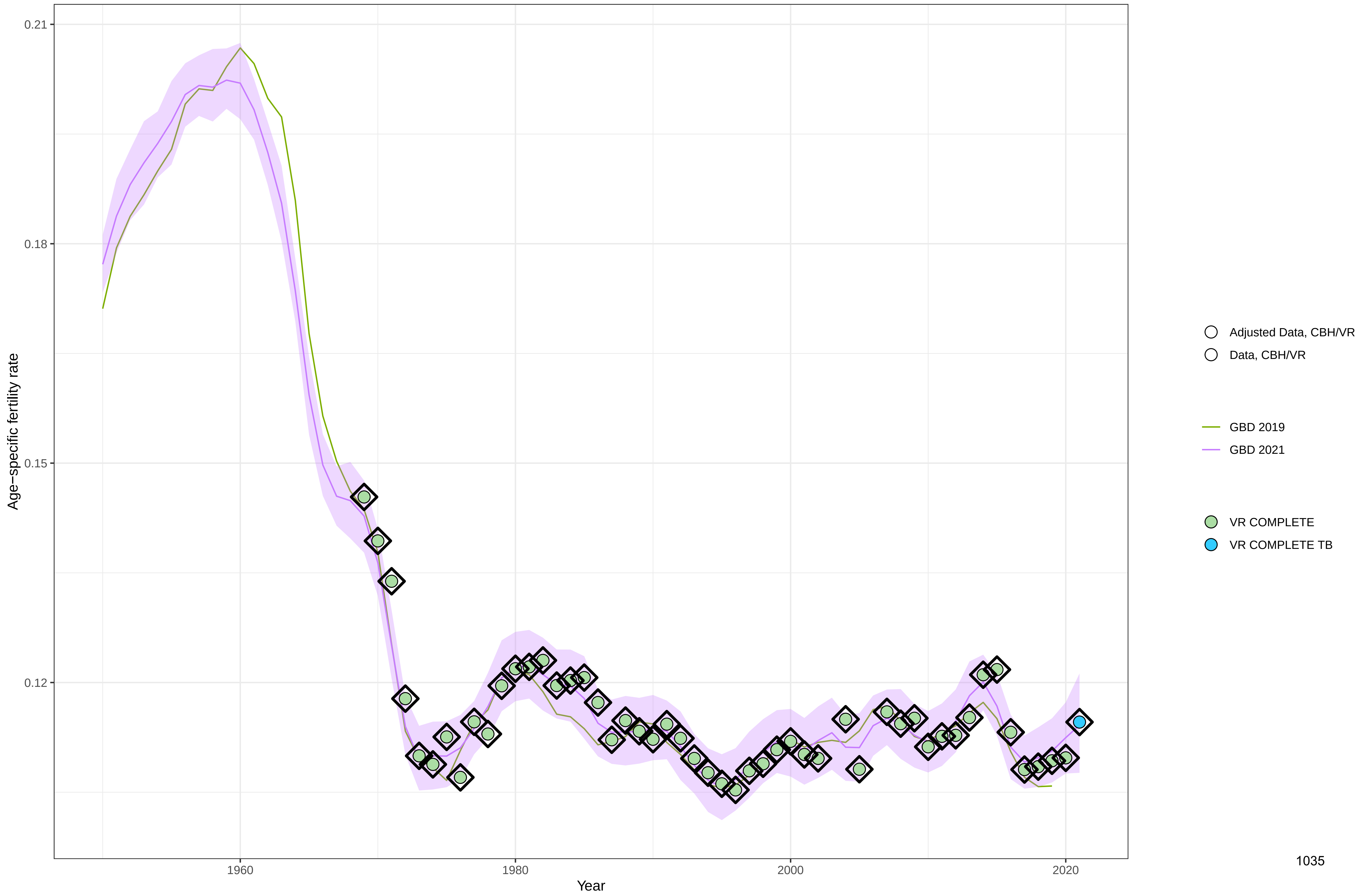

Age−specific fertility fitted trend lines, 1950−2021: Louisiana, Ages: 30−34
Data points enclosed with a diamond are reference sources

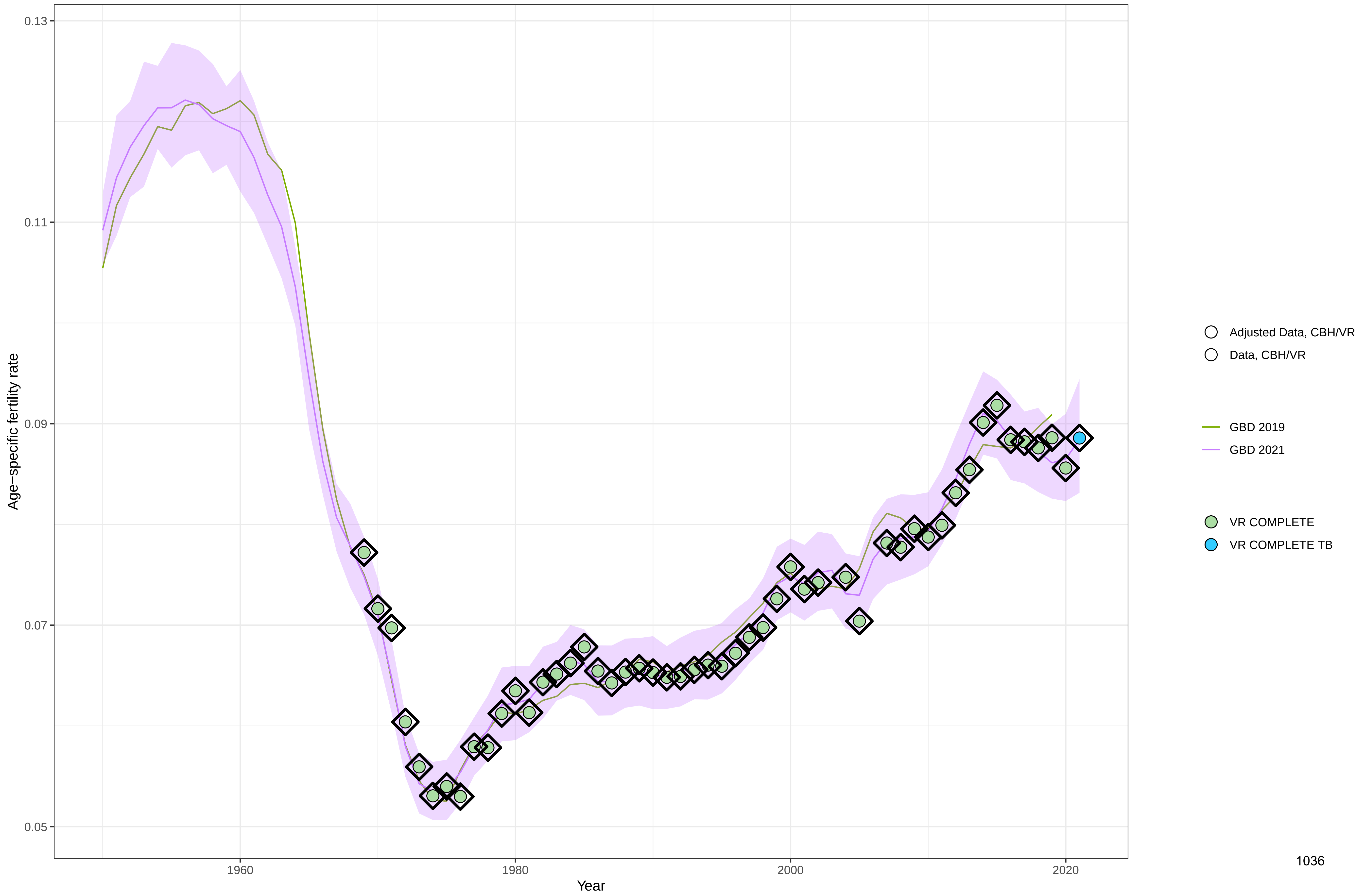

Age−specific fertility fitted trend lines, 1950−2021: Louisiana, Ages: 35−39
Data points enclosed with a diamond are reference sources

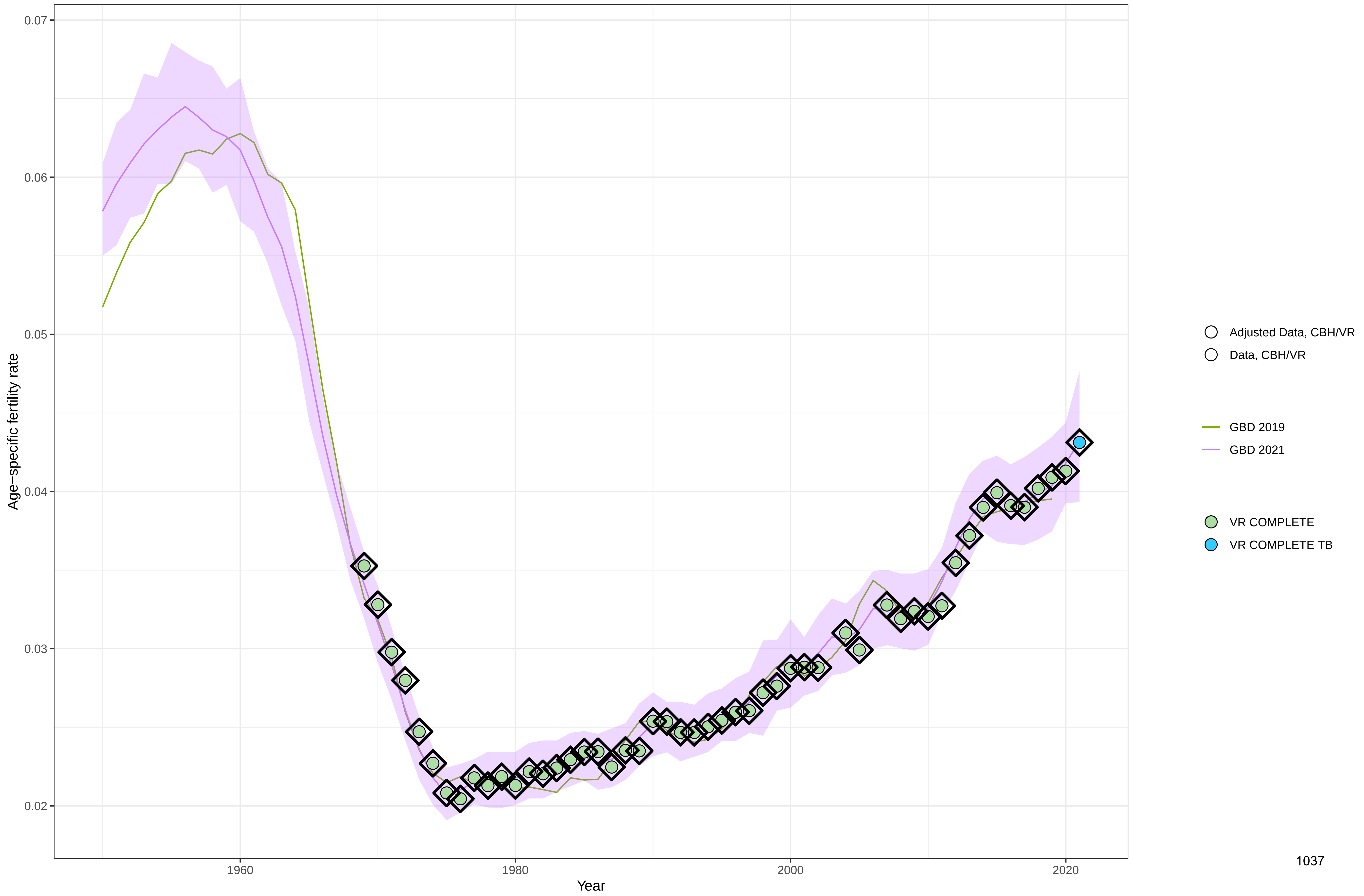

Age−specific fertility fitted trend lines, 1950−2021: Louisiana, Ages: 40−44
Data points enclosed with a diamond are reference sources

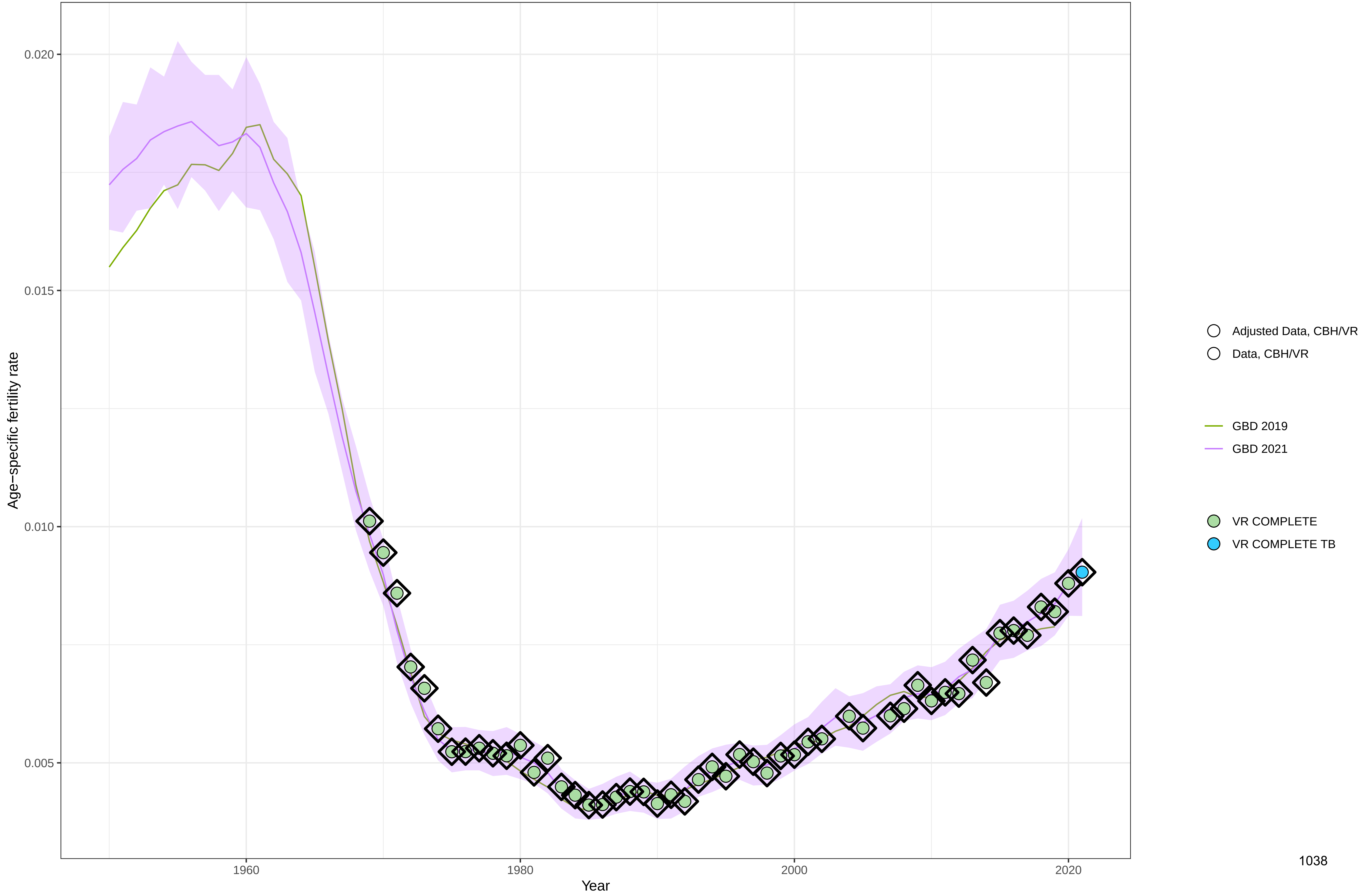

Age−specific fertility fitted trend lines, 1950−2021: Louisiana, Ages: 45−49
Data points enclosed with a diamond are reference sources

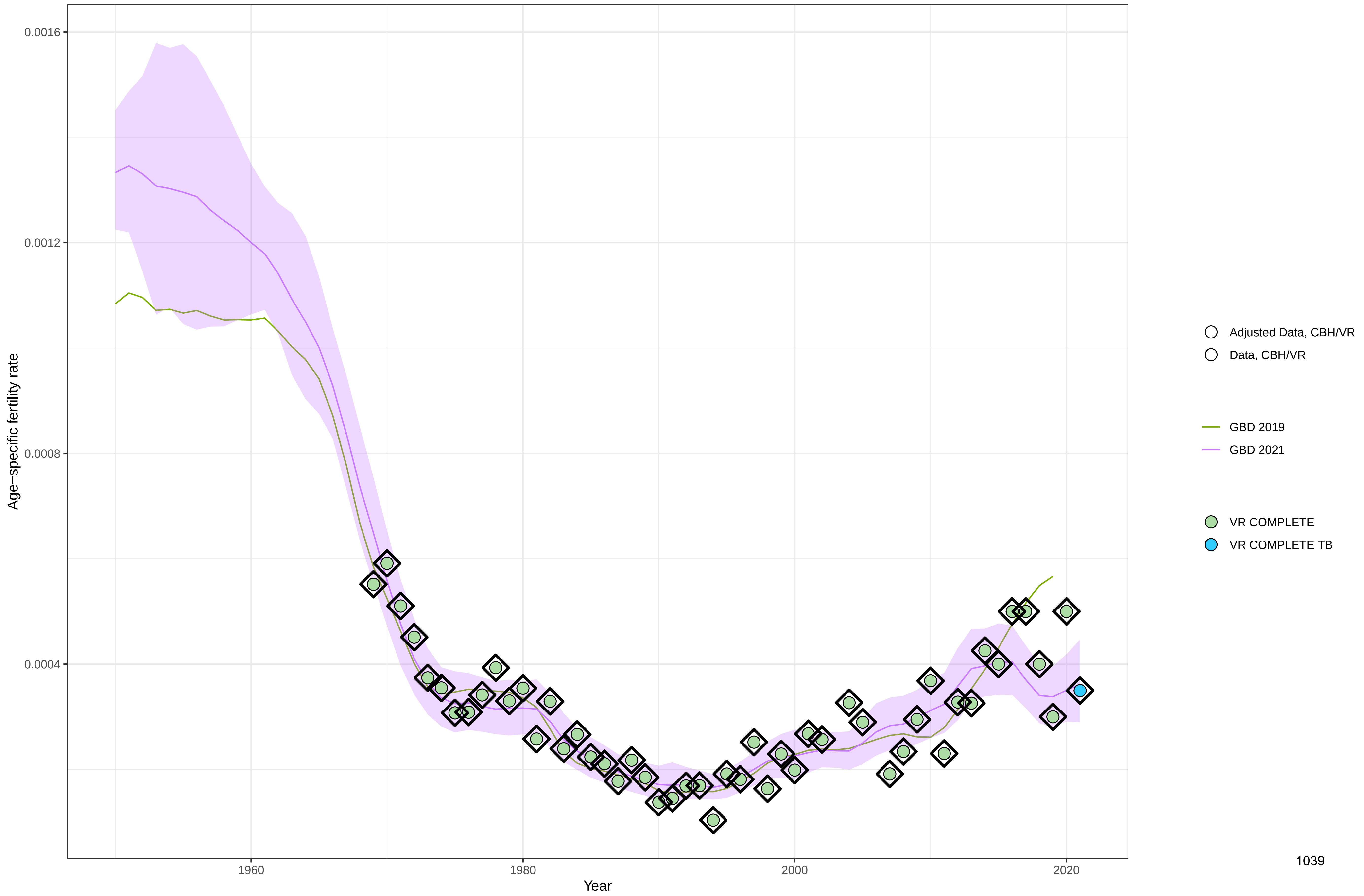

Age−specific fertility fitted trend lines, 1950−2021: Louisiana, Ages: 50−54
Data points enclosed with a diamond are reference sources

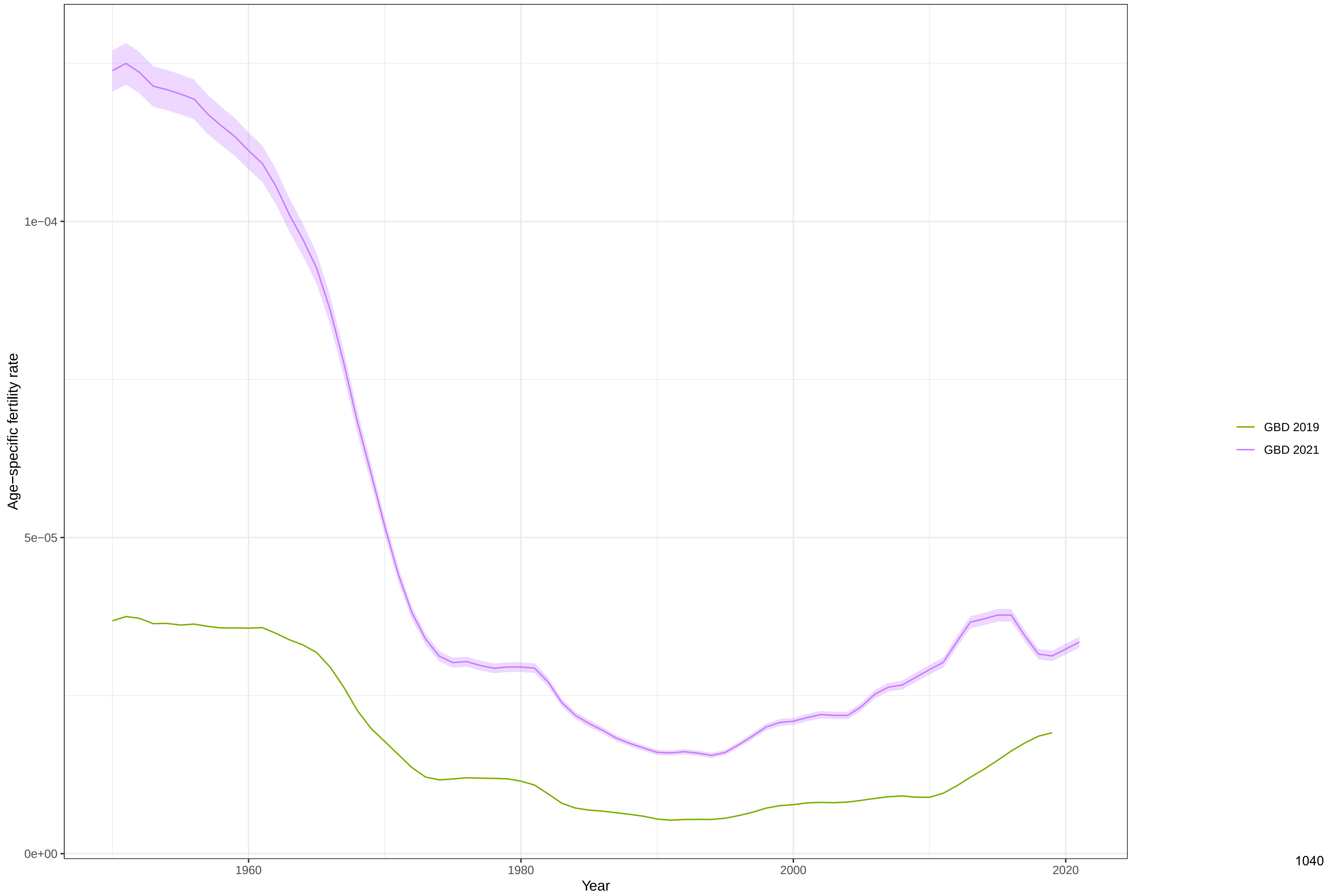

Total fertility rate fitted trend lines, 1950−2021: Louisiana TFR
Data points enclosed with a diamond are reference sources

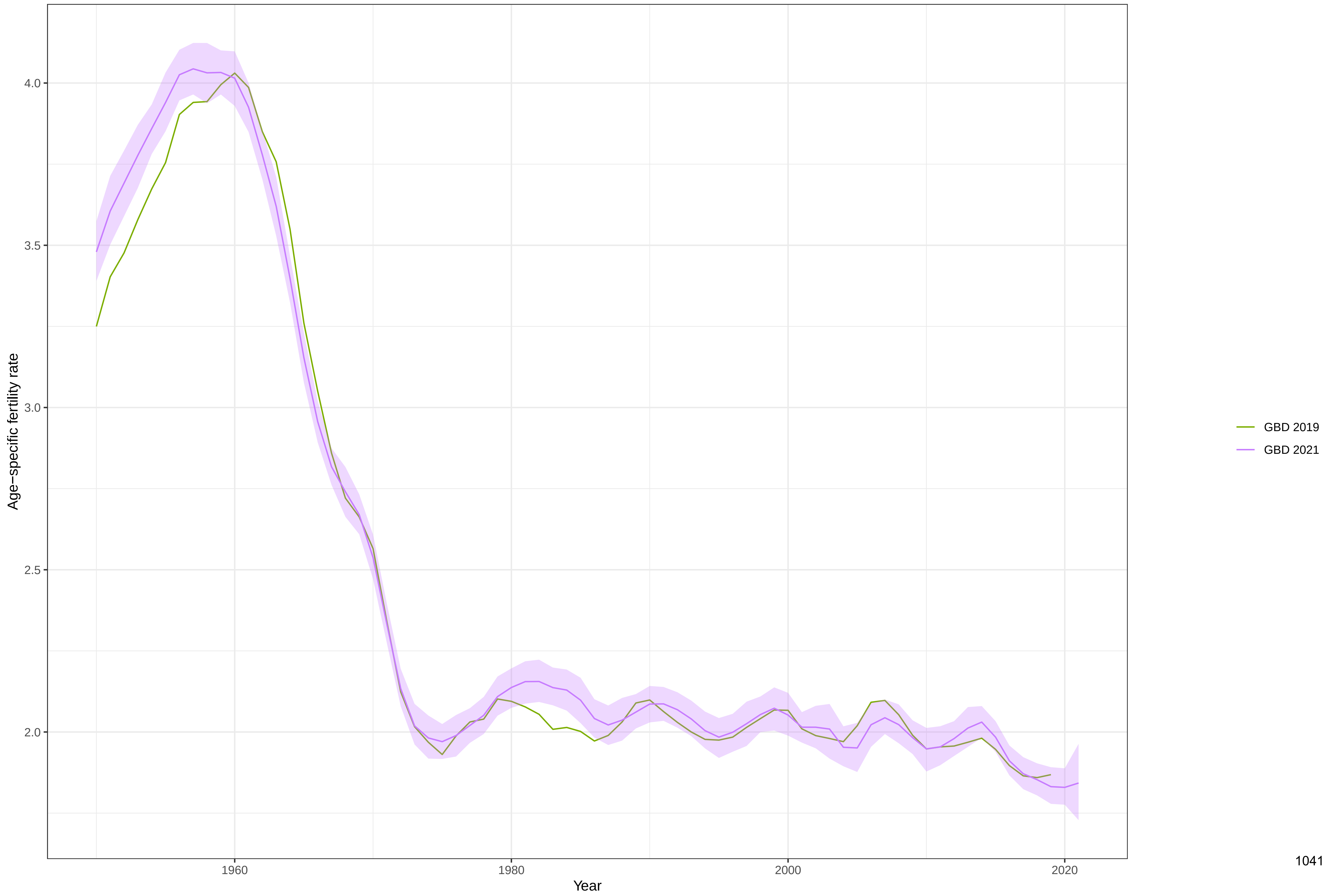

Age−specific fertility fitted trend lines, 1950−2021: Maine, Ages: 10−14
Data points enclosed with a diamond are reference sources

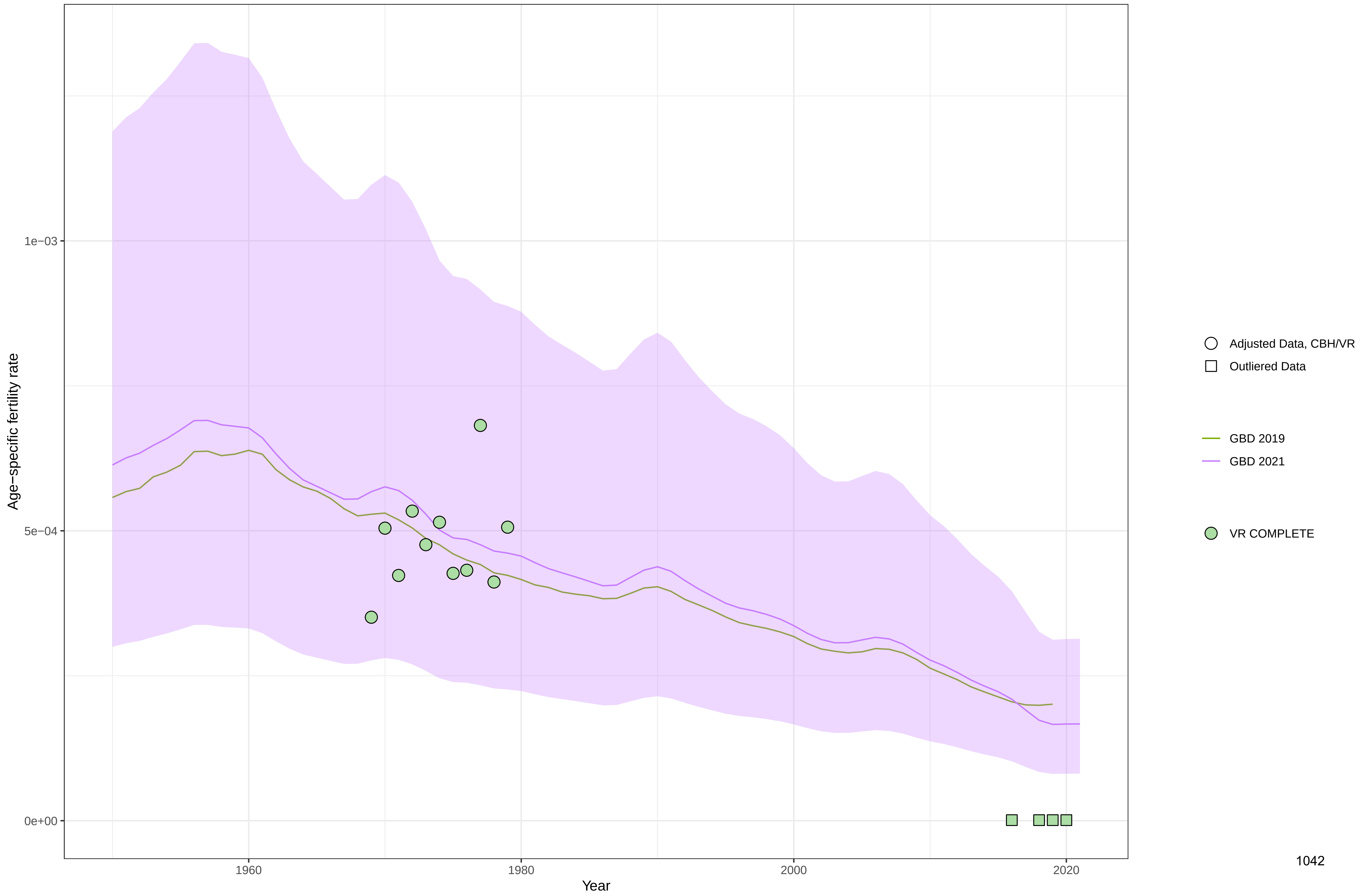

Age−specific fertility fitted trend lines, 1950−2021: Maine, Ages: 15−19
Data points enclosed with a diamond are reference sources

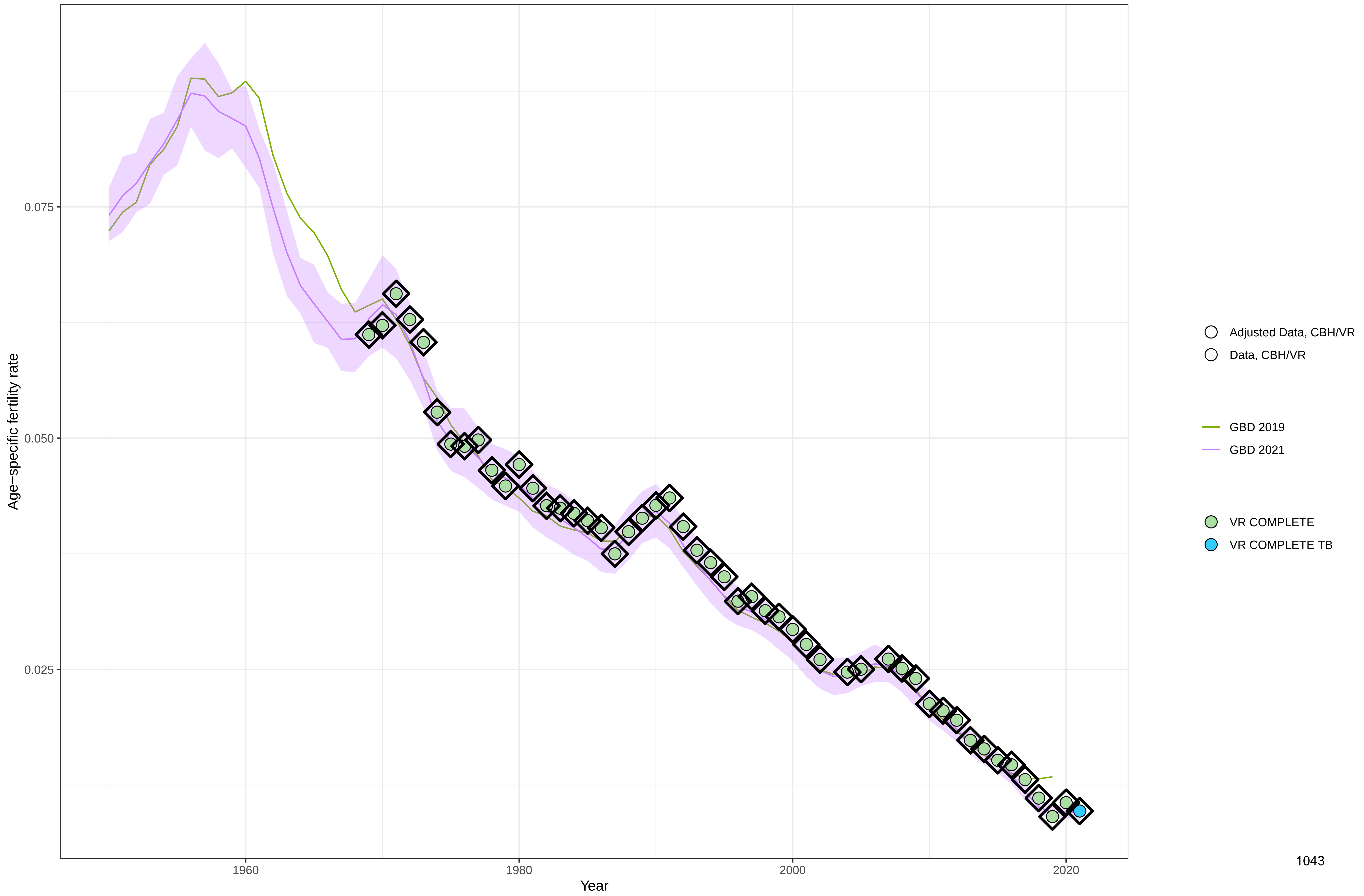

Age−specific fertility fitted trend lines, 1950−2021: Maine, Ages: 20−24
Data points enclosed with a diamond are reference sources

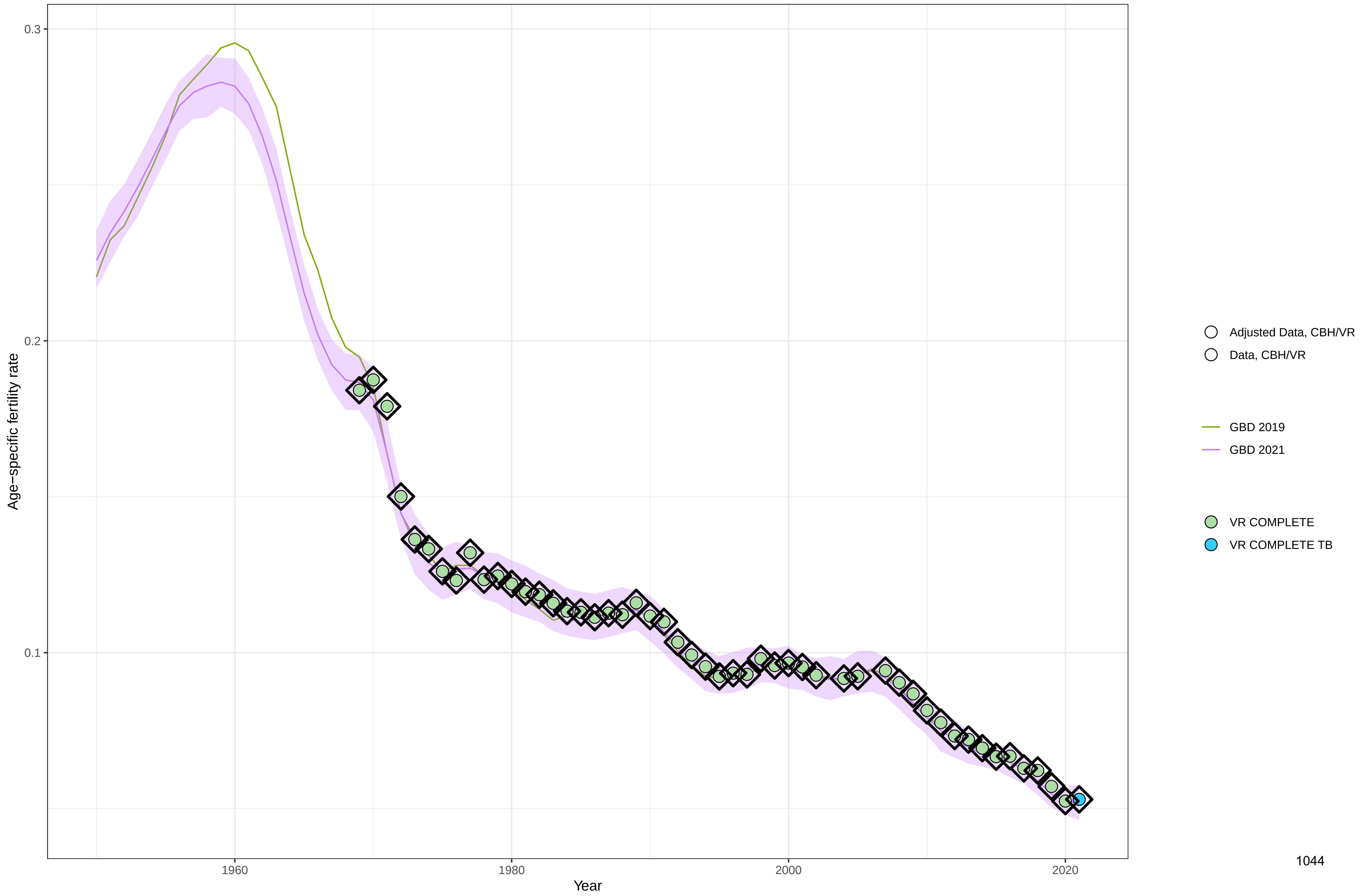

Age−specific fertility fitted trend lines, 1950−2021: Maine, Ages: 25−29
Data points enclosed with a diamond are reference sources

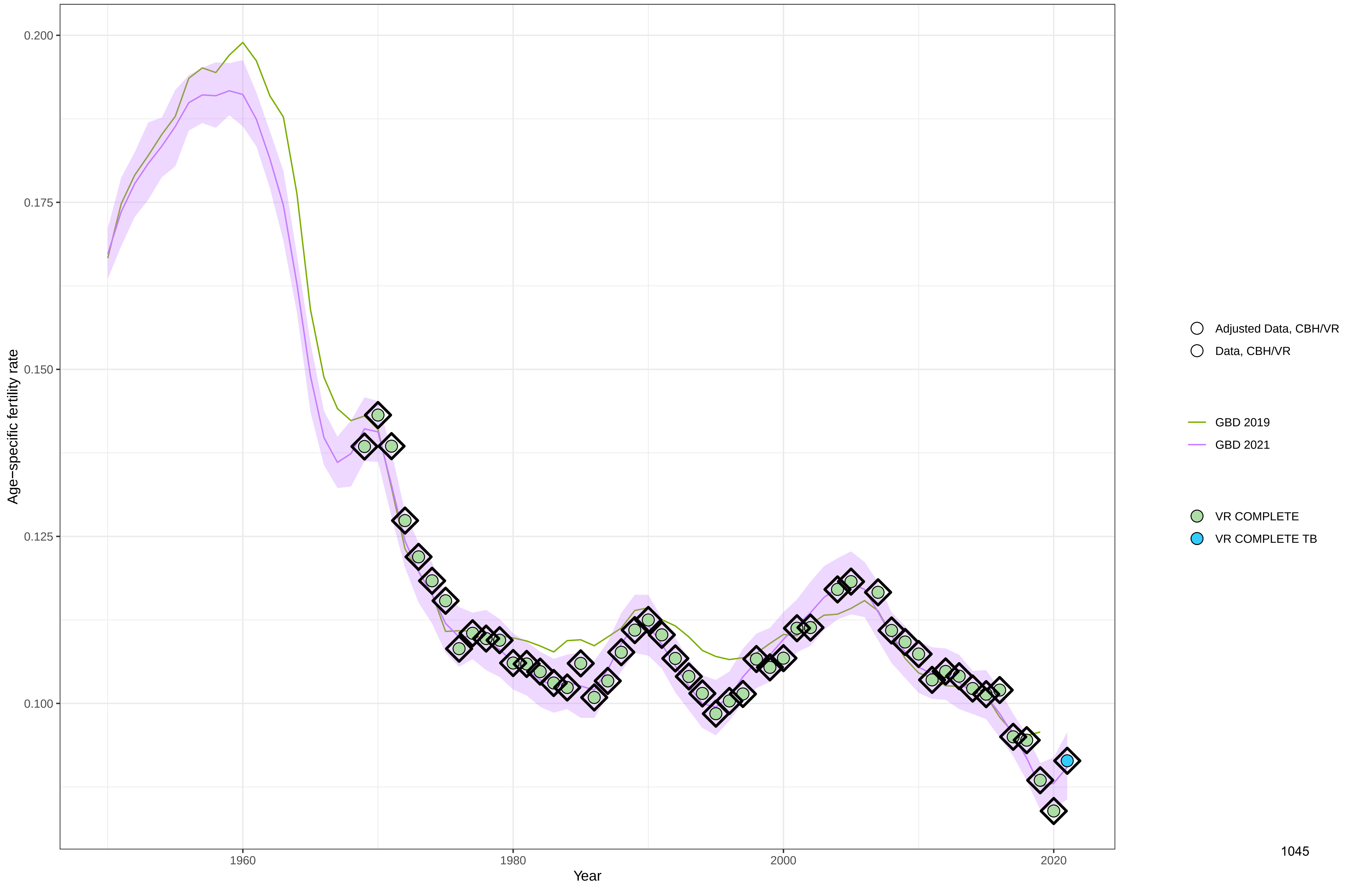

Age−specific fertility fitted trend lines, 1950−2021: Maine, Ages: 30−34
Data points enclosed with a diamond are reference sources

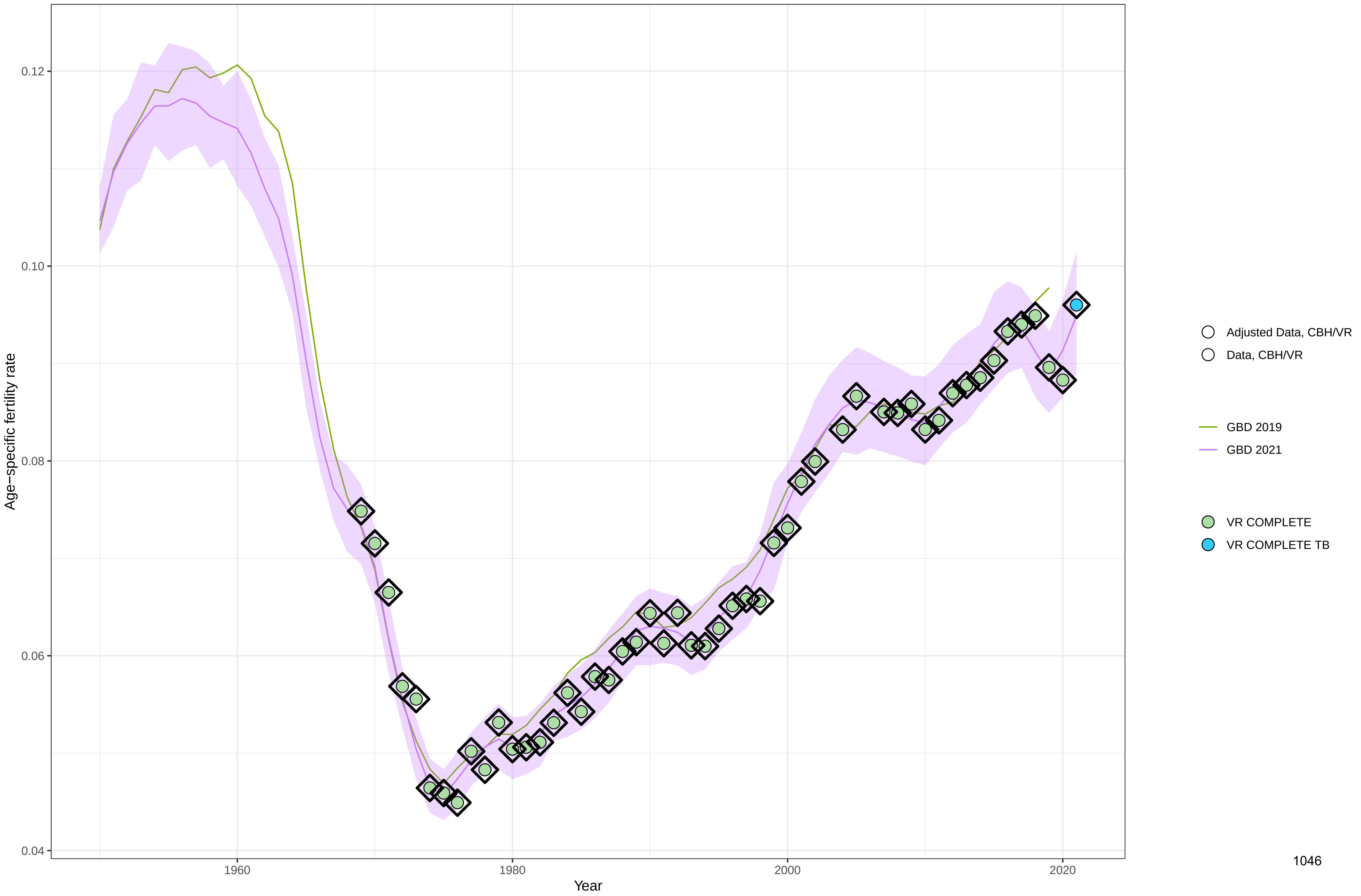

Age−specific fertility fitted trend lines, 1950−2021: Maine, Ages: 35−39
Data points enclosed with a diamond are reference sources

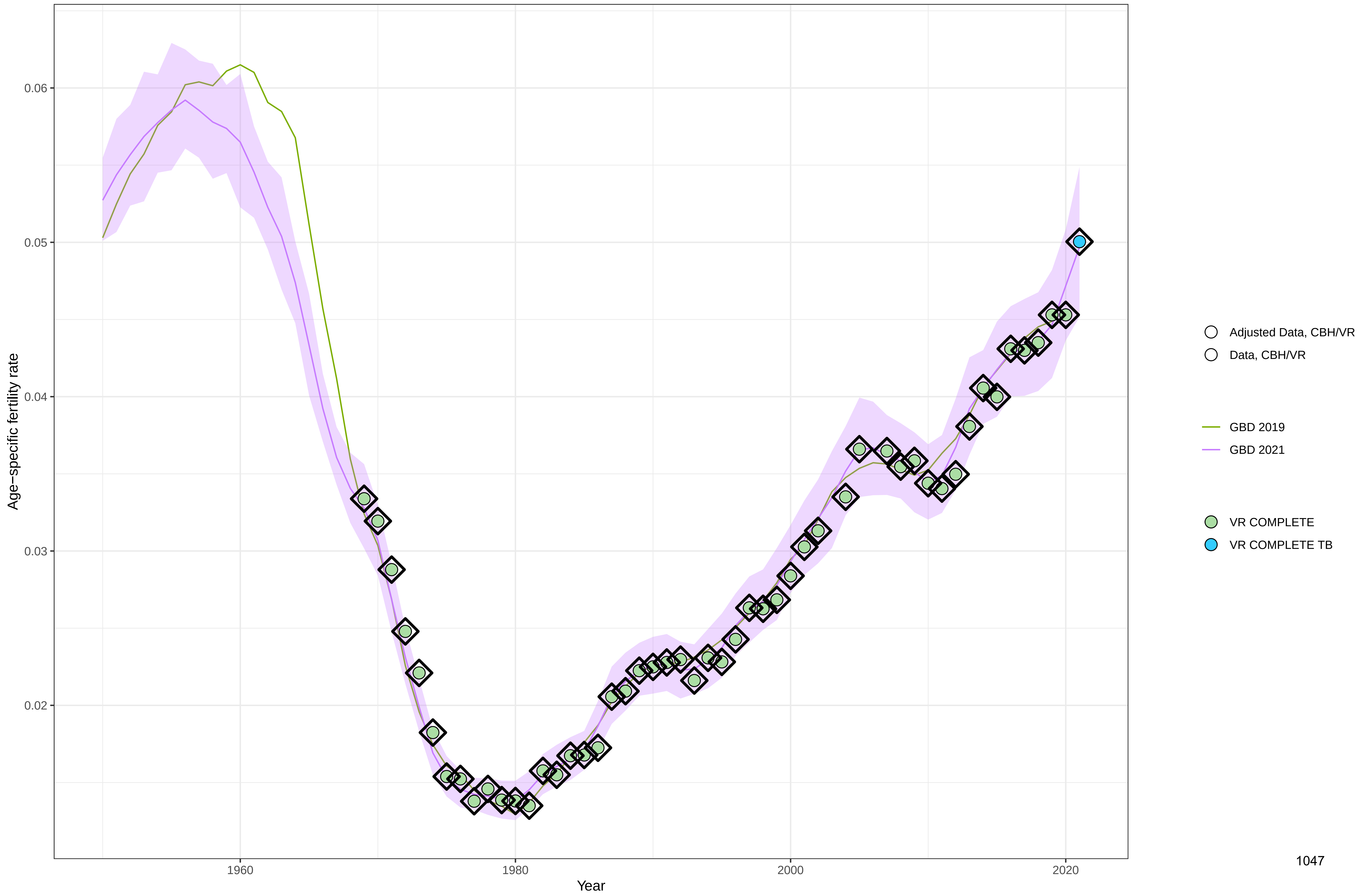

1047

Age−specific fertility fitted trend lines, 1950−2021: Maine, Ages: 40−44
Data points enclosed with a diamond are reference sources

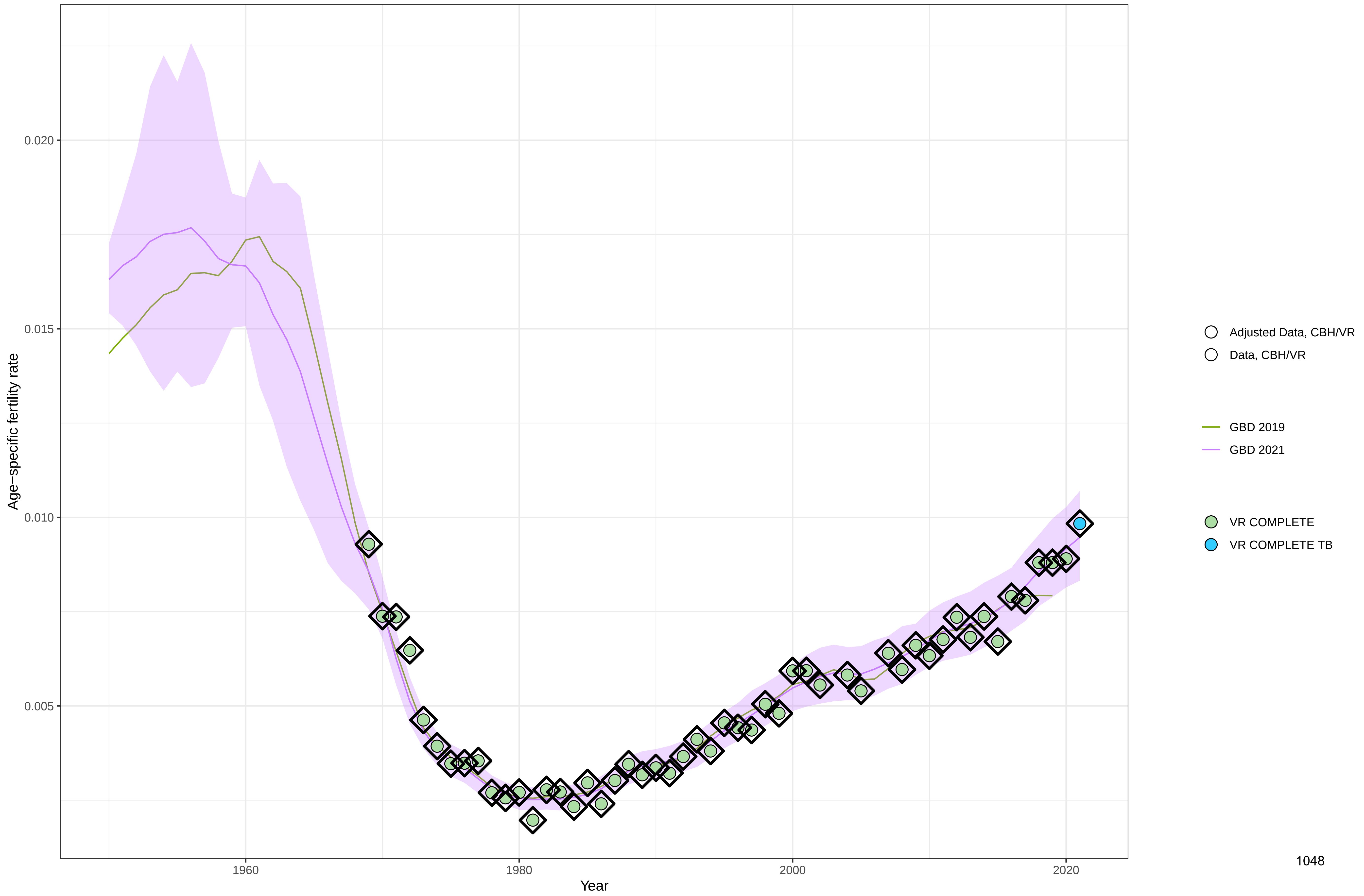

Age−specific fertility fitted trend lines, 1950−2021: Maine, Ages: 45−49
Data points enclosed with a diamond are reference sources

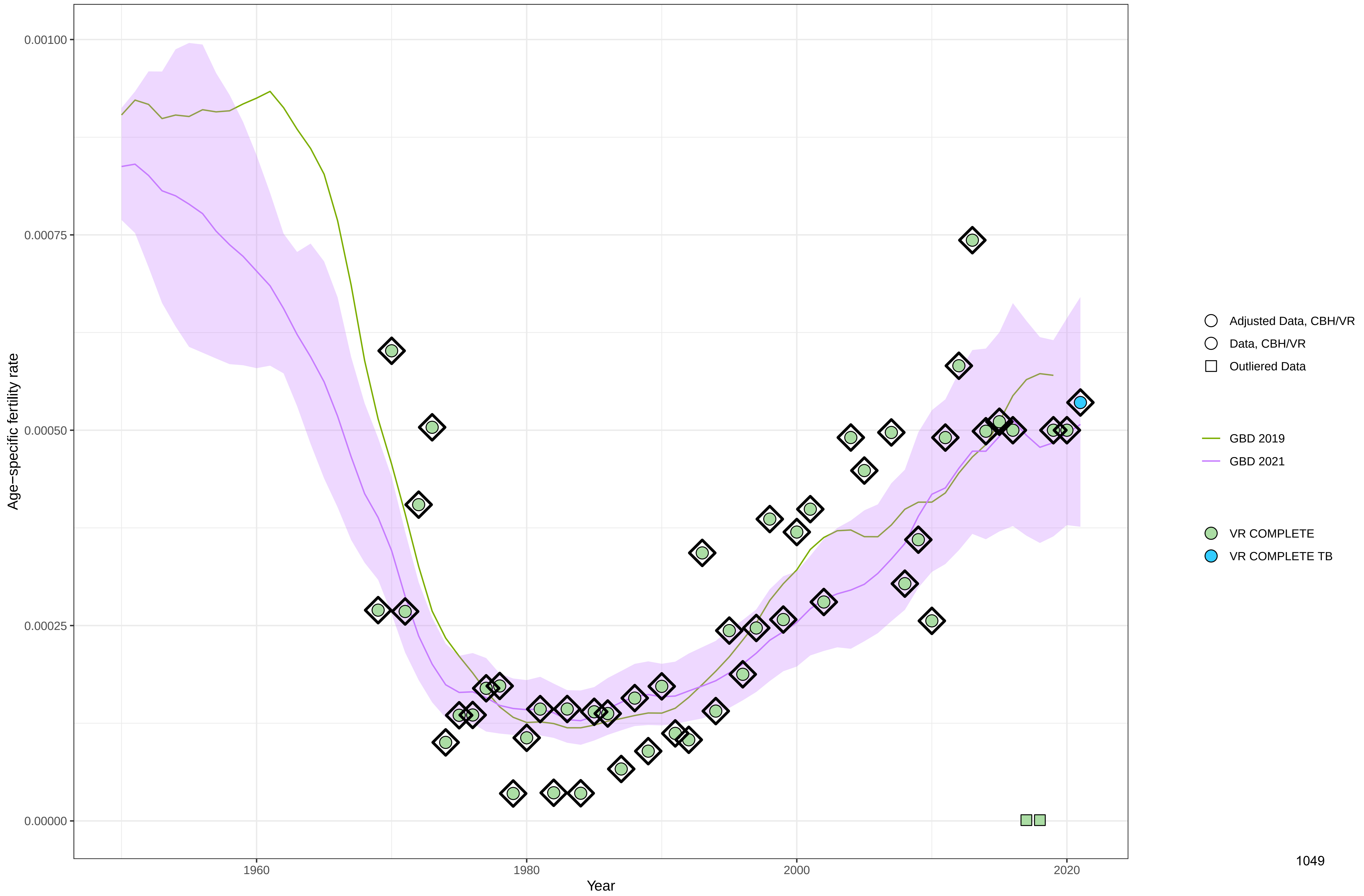

Age−specific fertility fitted trend lines, 1950−2021: Maine, Ages: 50−54
Data points enclosed with a diamond are reference sources

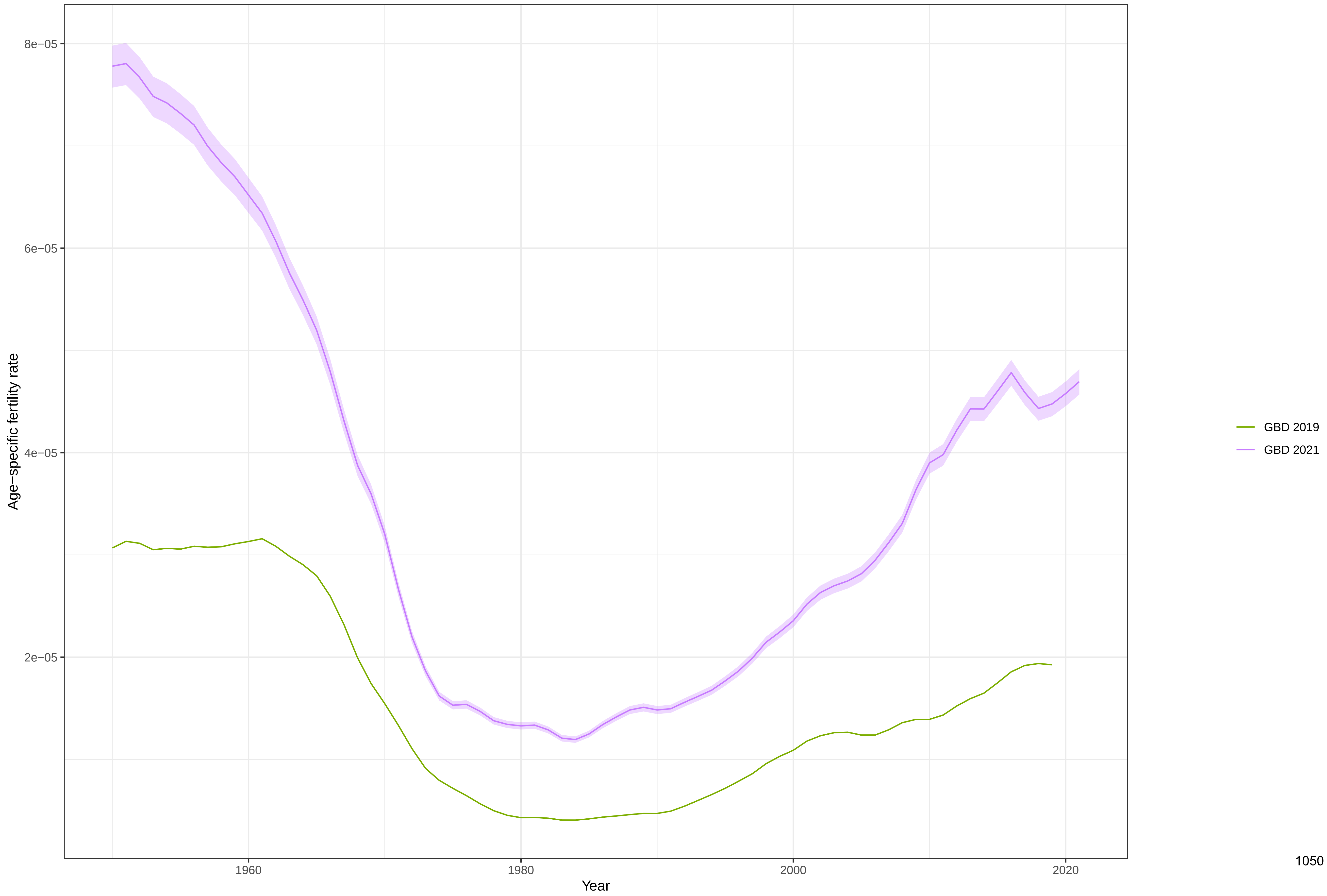

Total fertility rate fitted trend lines, 1950−2021: Maine TFR
Data points enclosed with a diamond are reference sources

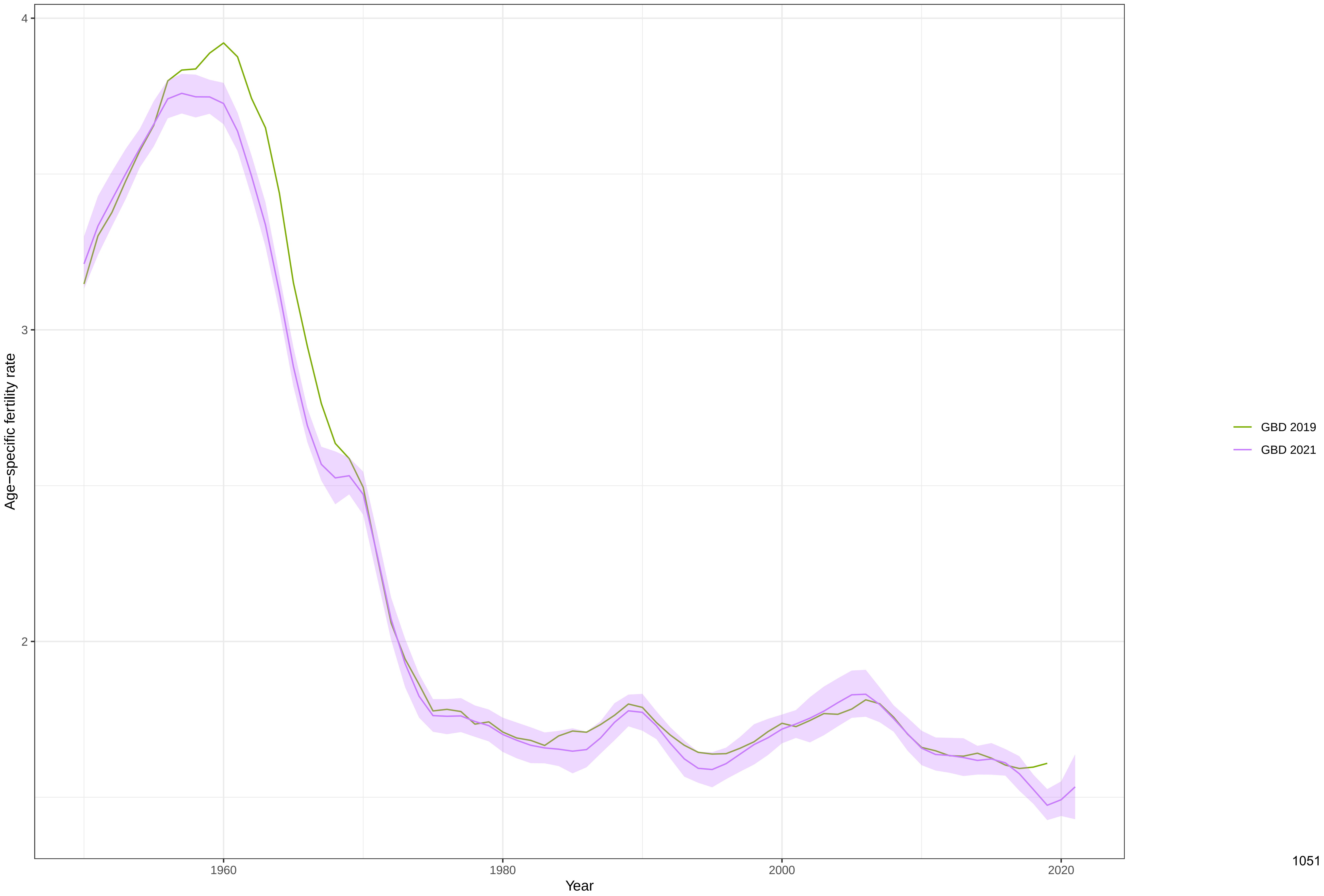

Age−specific fertility fitted trend lines, 1950−2021: Maryland, Ages: 10−14
Data points enclosed with a diamond are reference sources

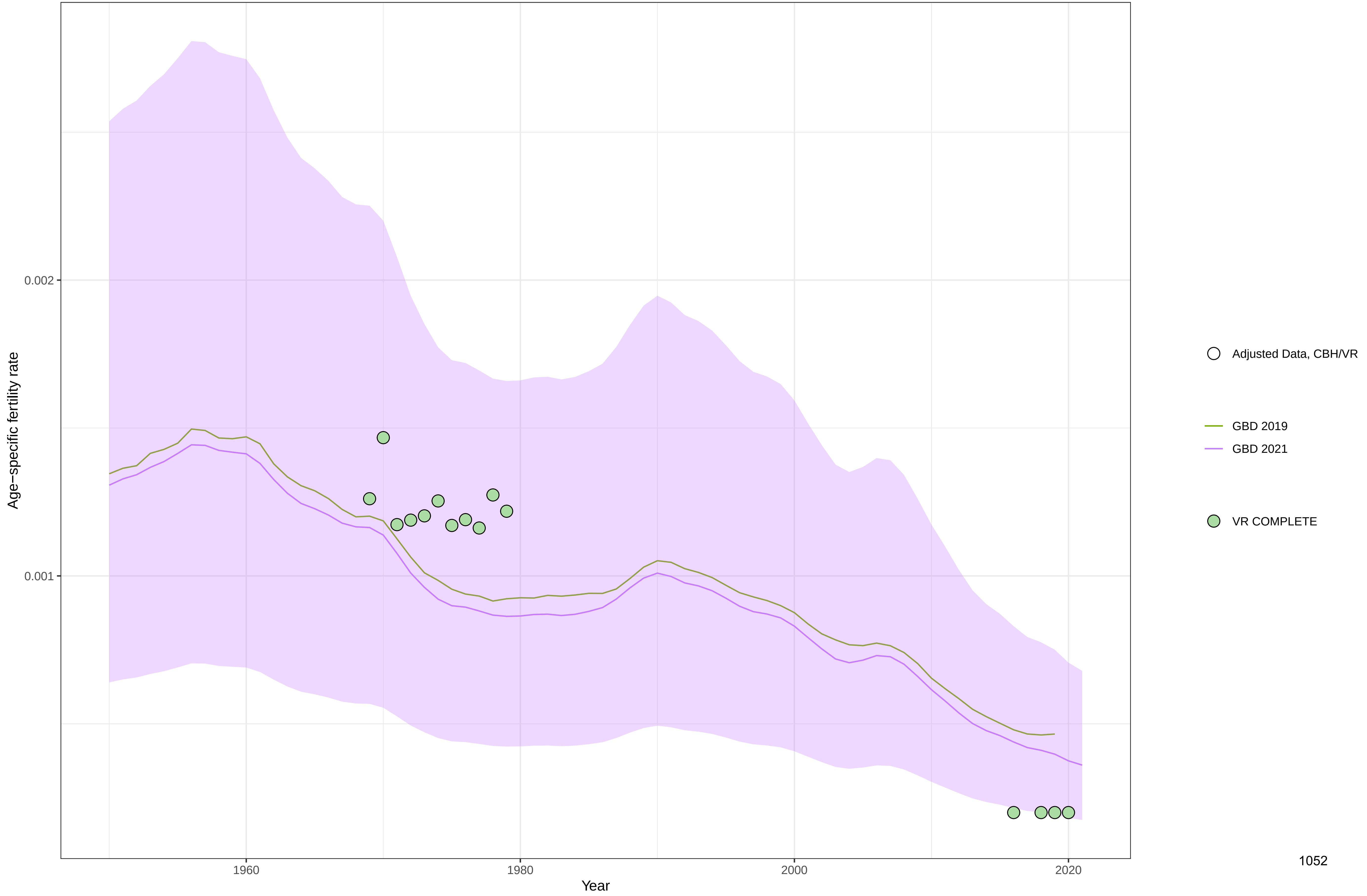

Age−specific fertility fitted trend lines, 1950−2021: Maryland, Ages: 15−19
Data points enclosed with a diamond are reference sources

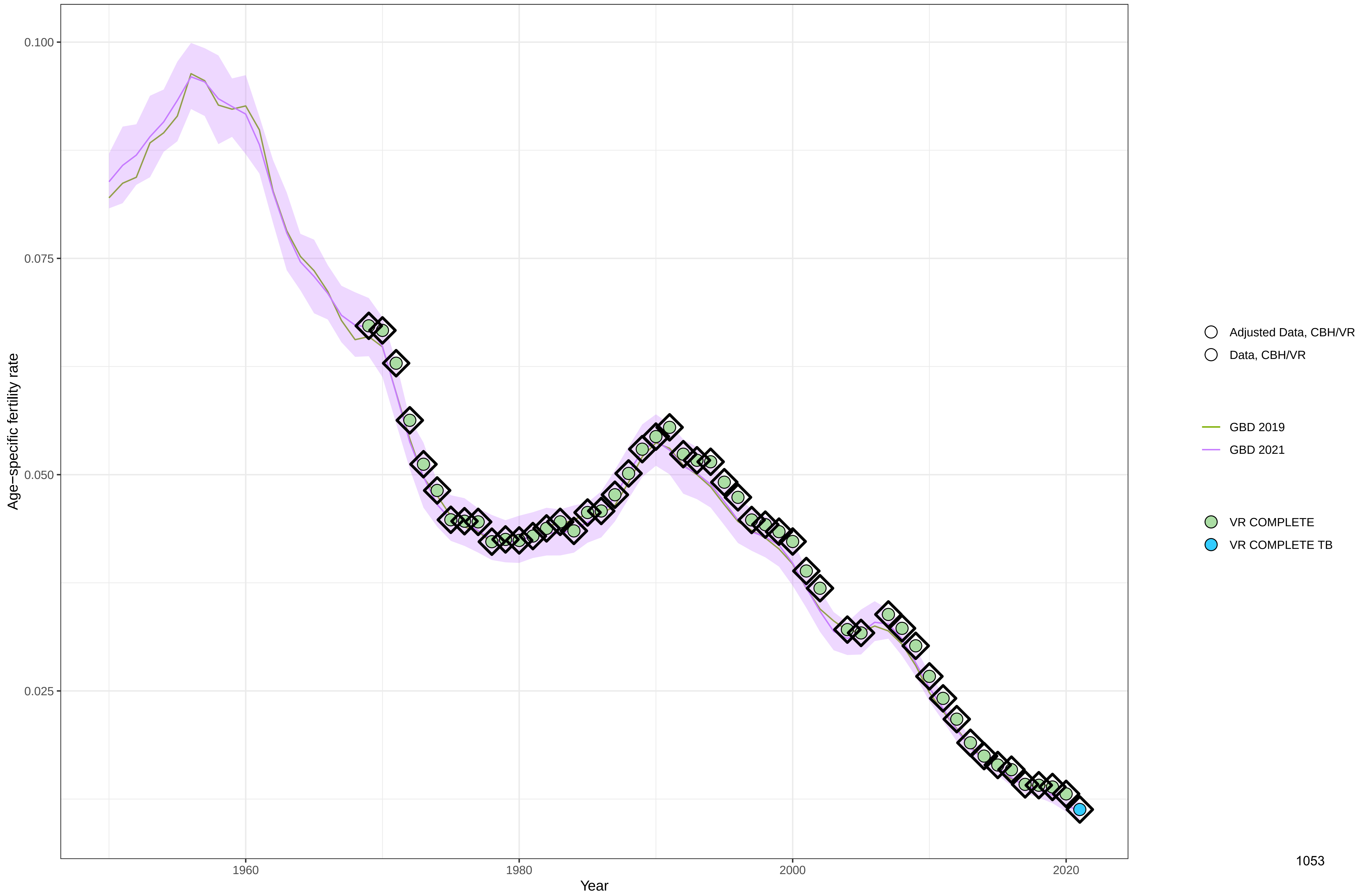

Age−specific fertility fitted trend lines, 1950−2021: Maryland, Ages: 20−24
Data points enclosed with a diamond are reference sources

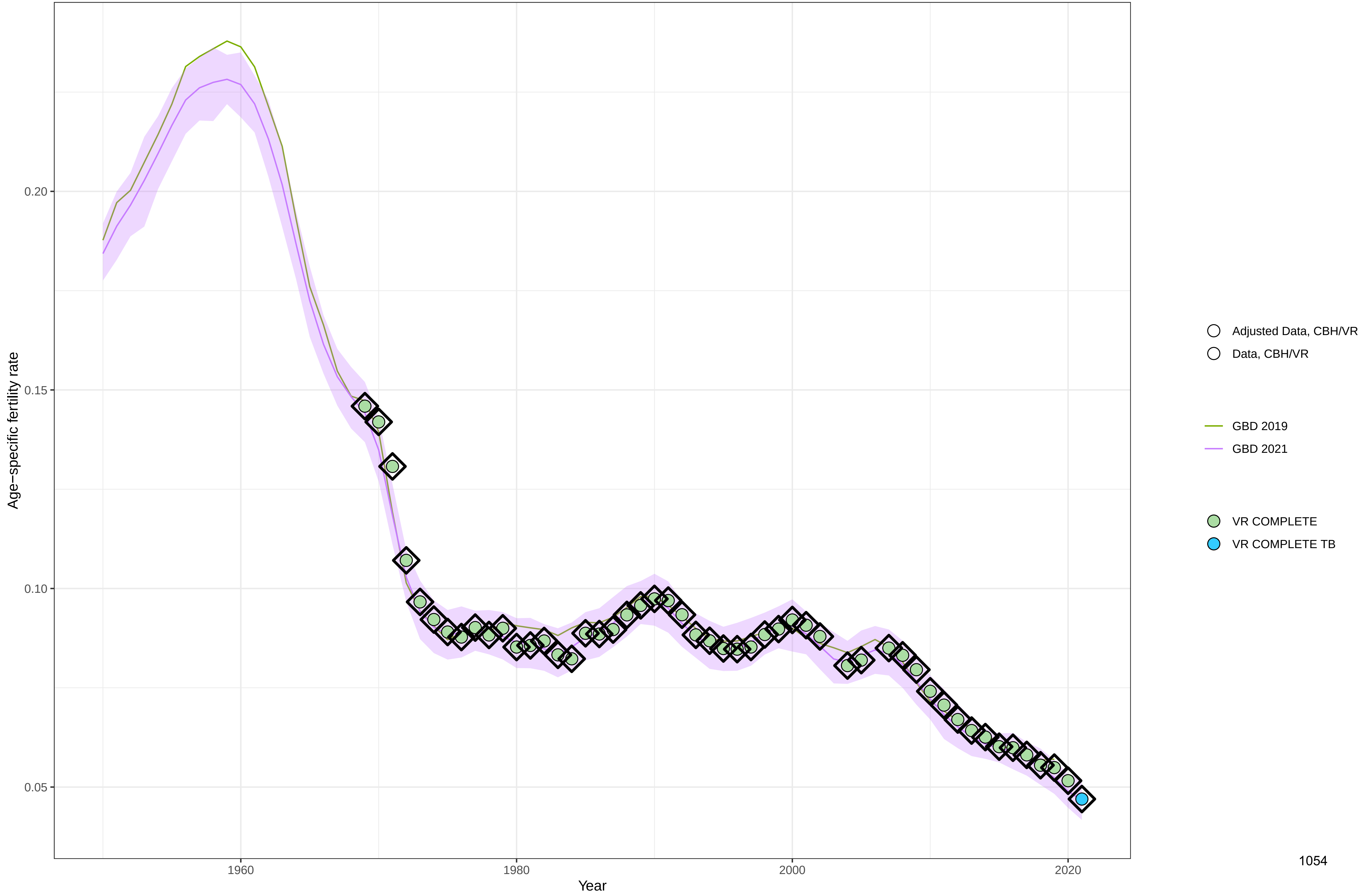

Age−specific fertility fitted trend lines, 1950−2021: Maryland, Ages: 25−29
Data points enclosed with a diamond are reference sources

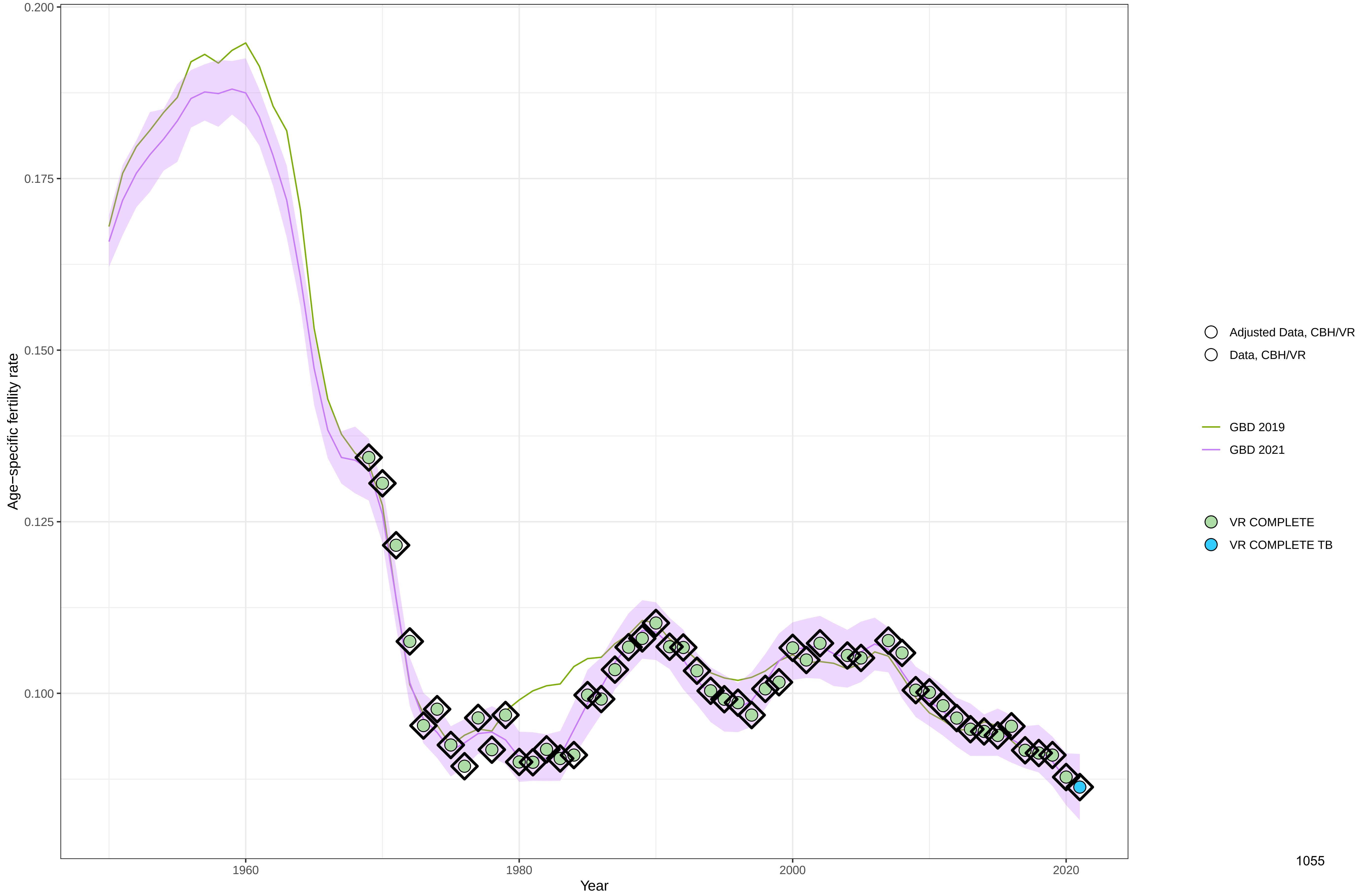

Age−specific fertility fitted trend lines, 1950−2021: Maryland, Ages: 30−34
Data points enclosed with a diamond are reference sources

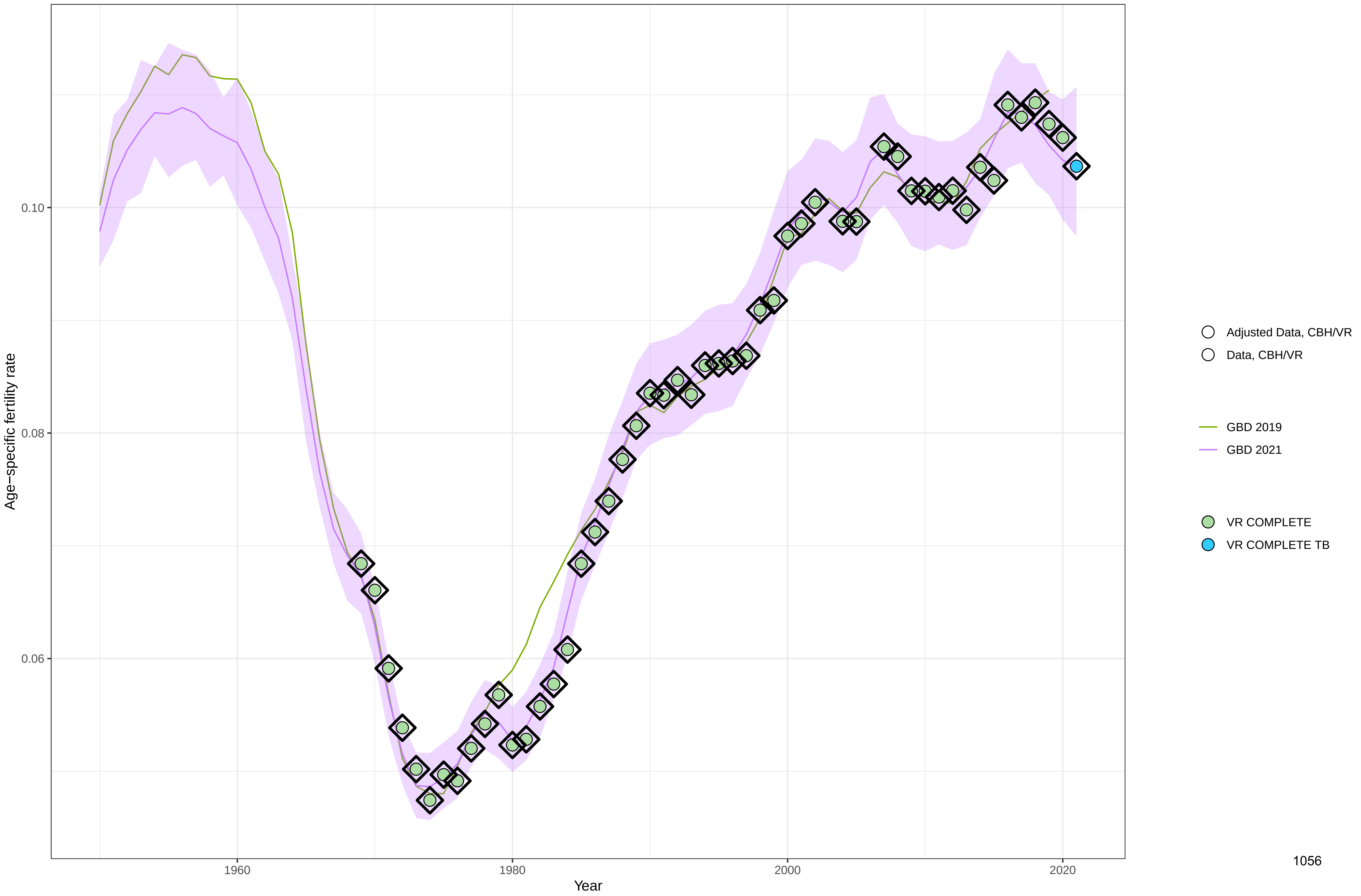

Age−specific fertility fitted trend lines, 1950−2021: Maryland, Ages: 35−39
Data points enclosed with a diamond are reference sources

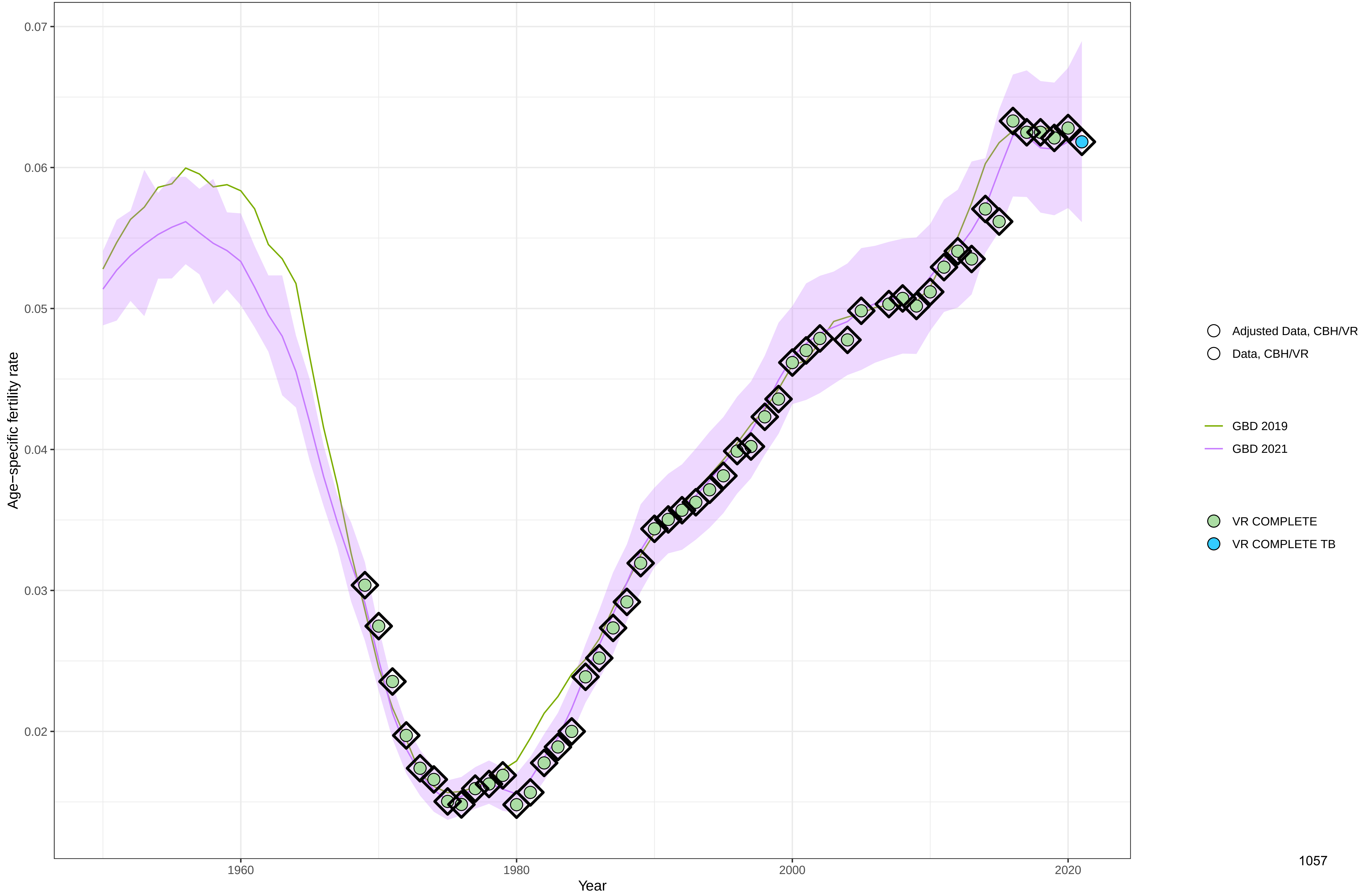

Age−specific fertility fitted trend lines, 1950−2021: Maryland, Ages: 40−44
Data points enclosed with a diamond are reference sources

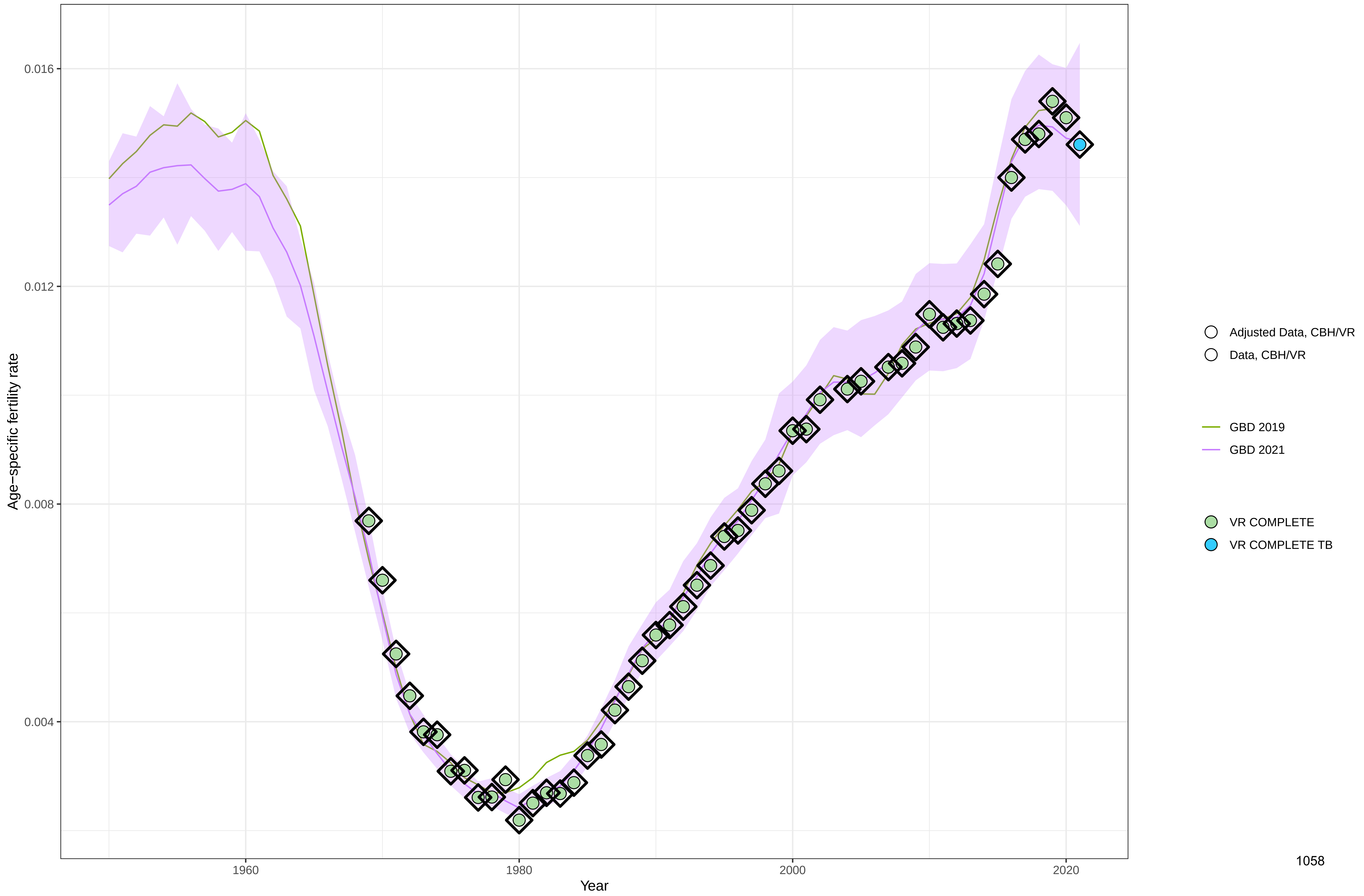

Age−specific fertility fitted trend lines, 1950−2021: Maryland, Ages: 45−49
Data points enclosed with a diamond are reference sources

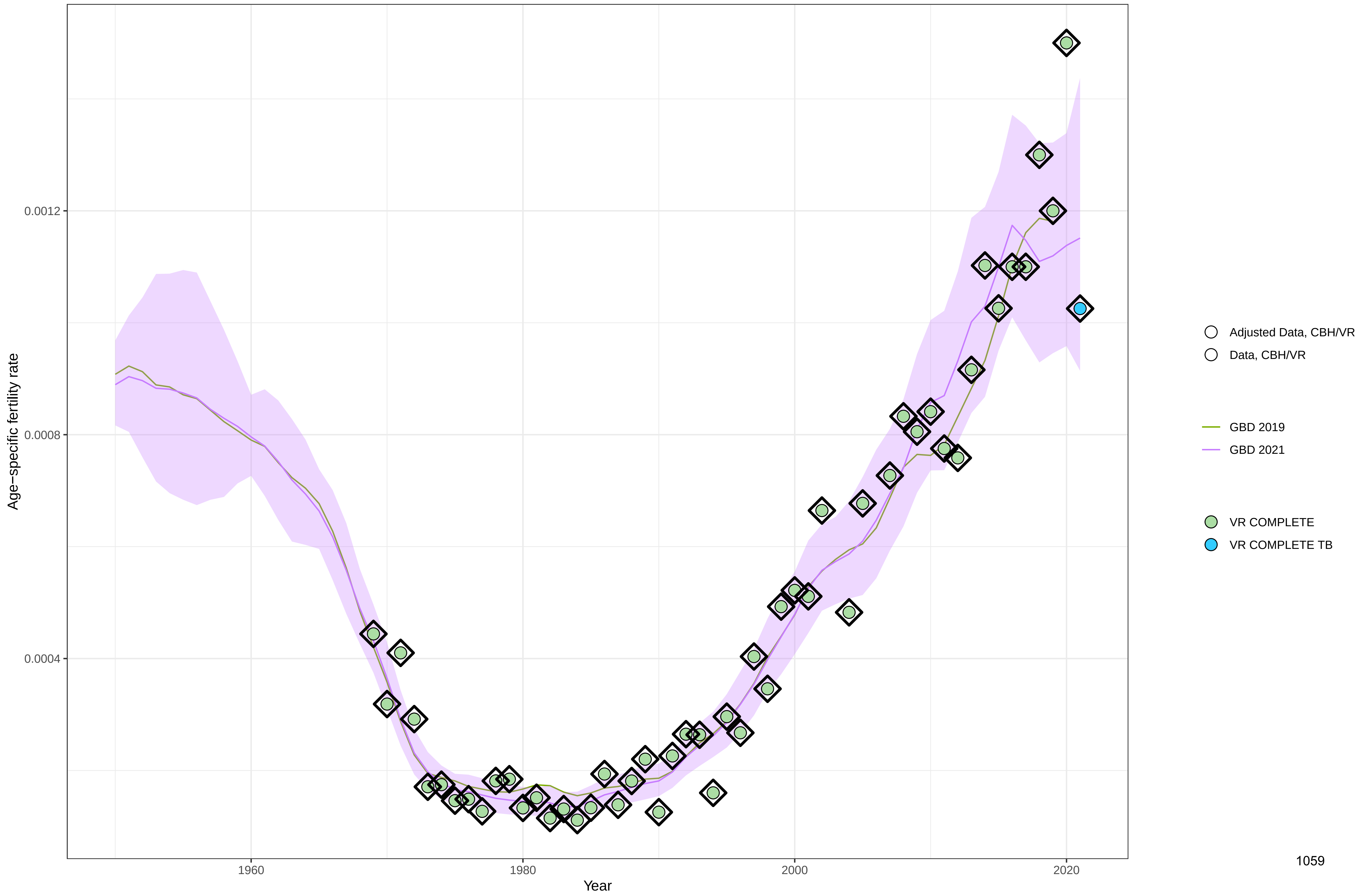

Age−specific fertility fitted trend lines, 1950−2021: Maryland, Ages: 50−54
Data points enclosed with a diamond are reference sources

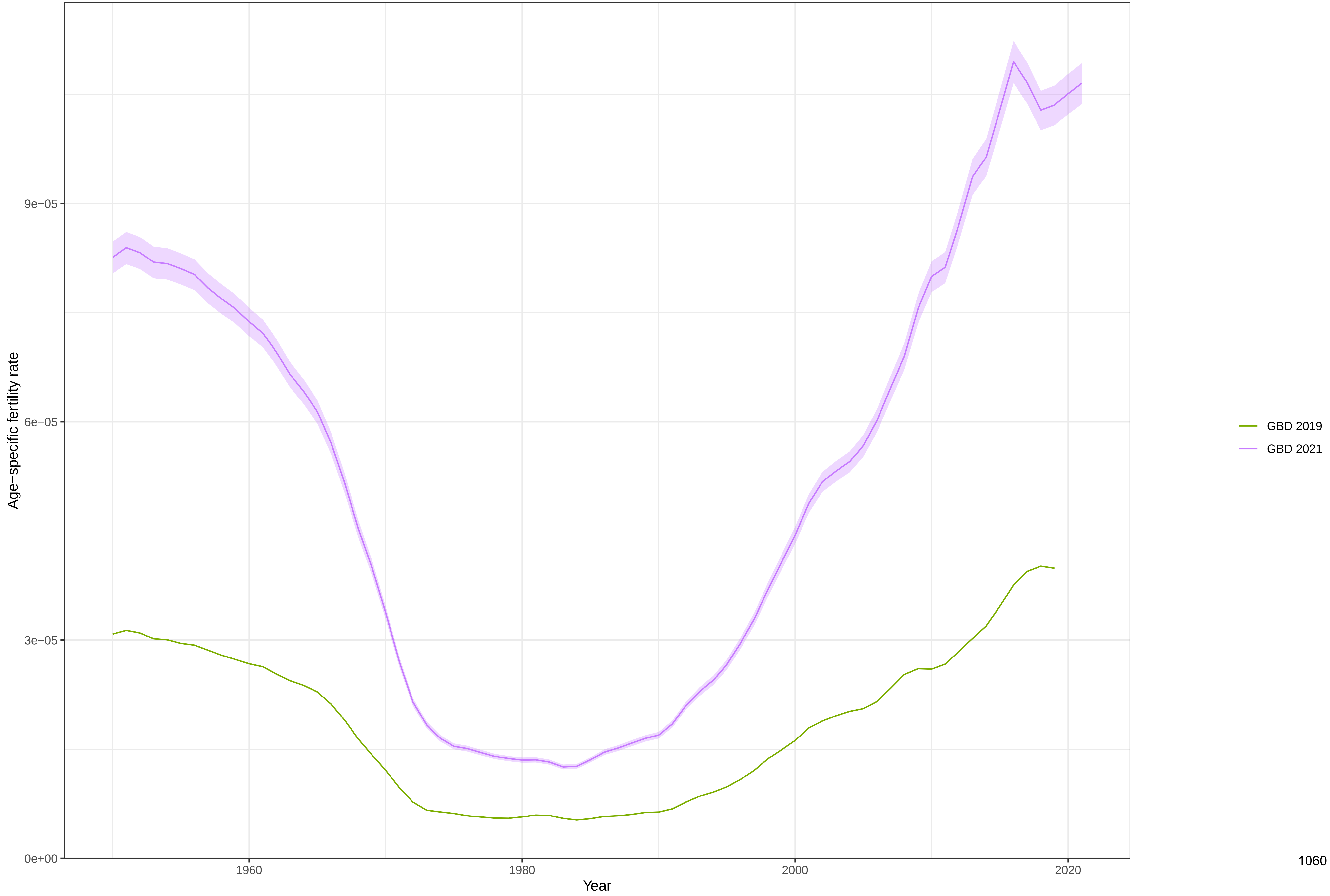

Total fertility rate fitted trend lines, 1950−2021: Maryland TFR
Data points enclosed with a diamond are reference sources

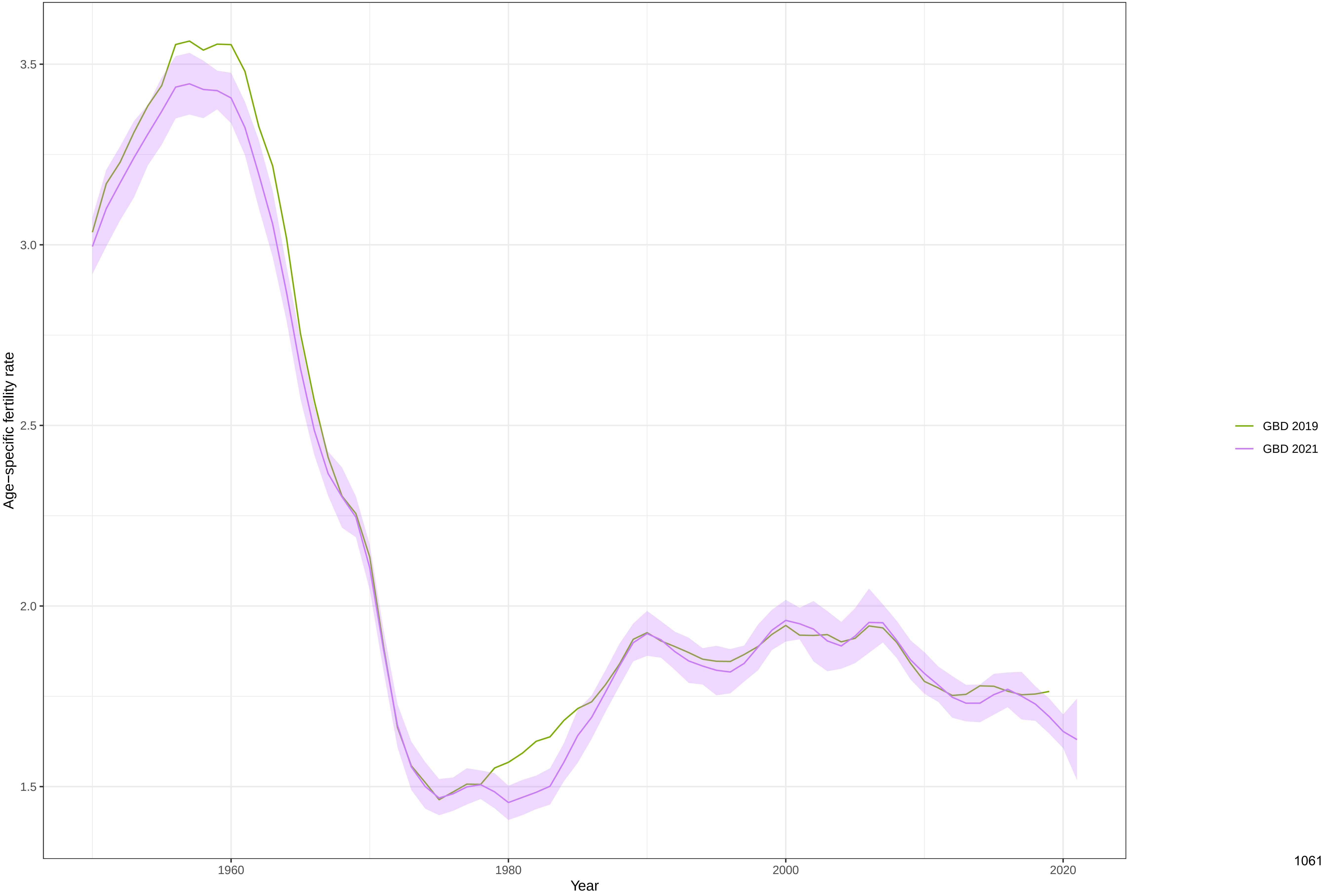

Age−specific fertility fitted trend lines, 1950−2021: Massachusetts, Ages: 10−14
Data points enclosed with a diamond are reference sources

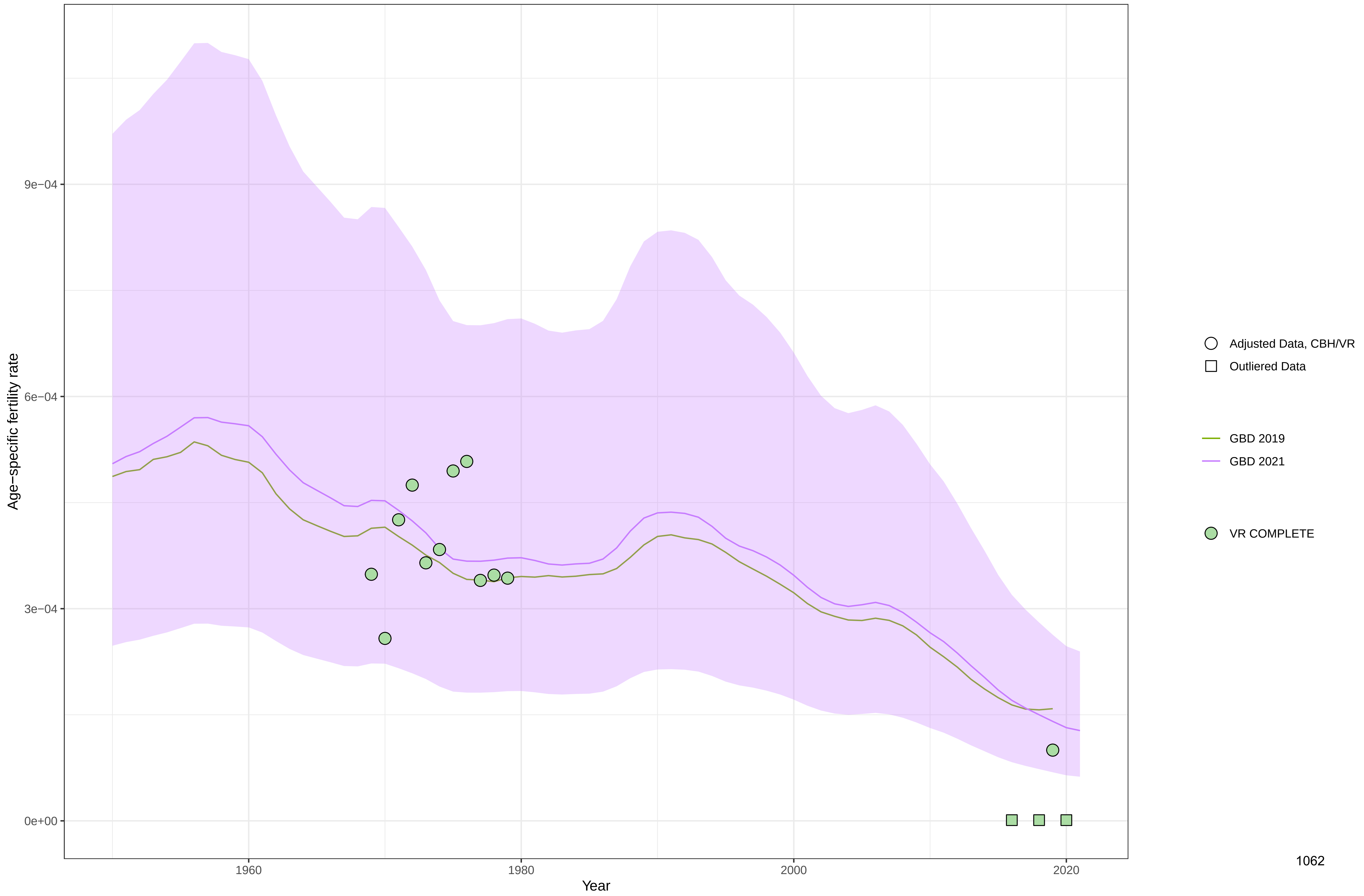

Age−specific fertility fitted trend lines, 1950−2021: Massachusetts, Ages: 15−19
Data points enclosed with a diamond are reference sources

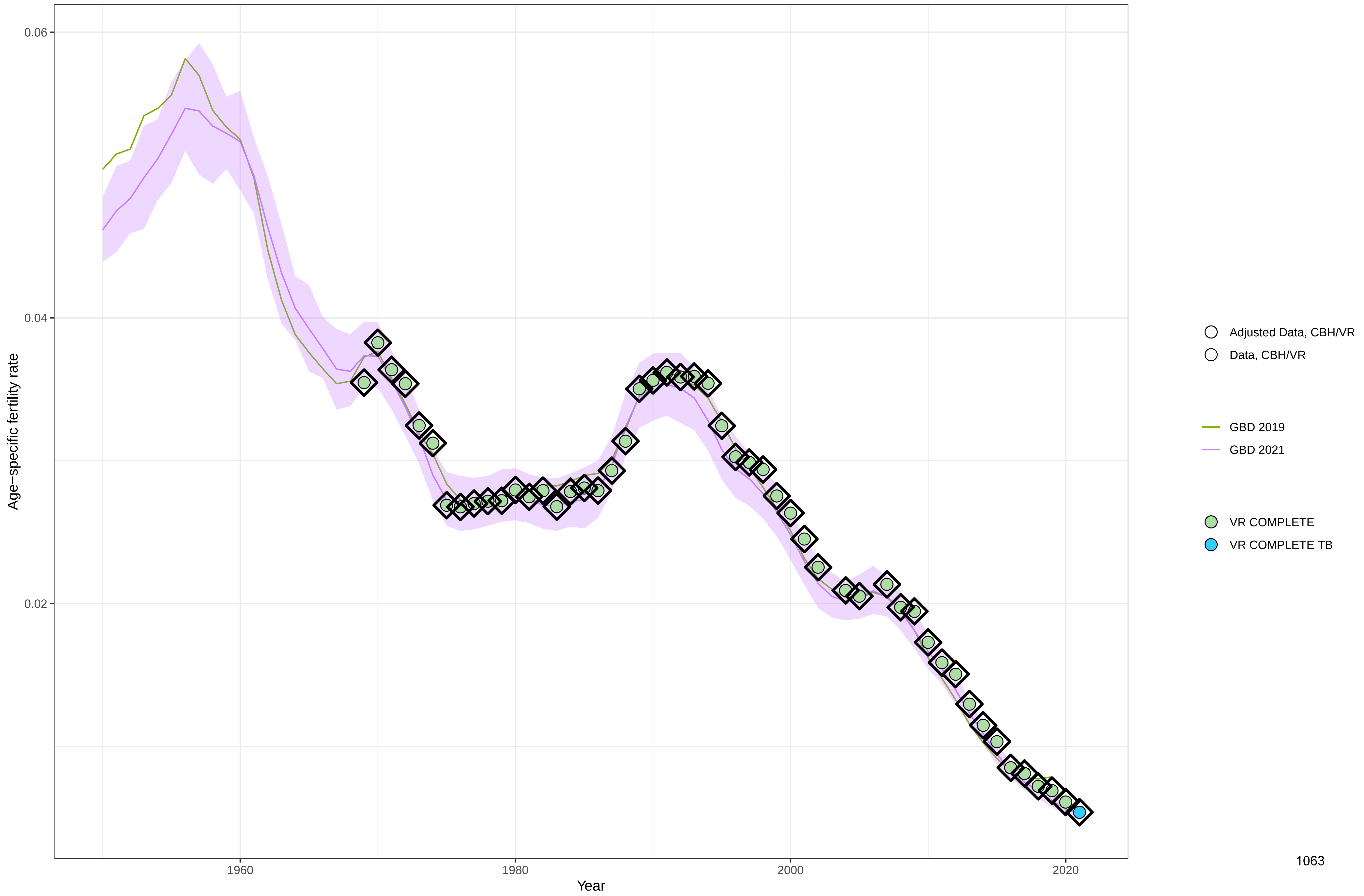

Age−specific fertility fitted trend lines, 1950−2021: Massachusetts, Ages: 20−24
Data points enclosed with a diamond are reference sources

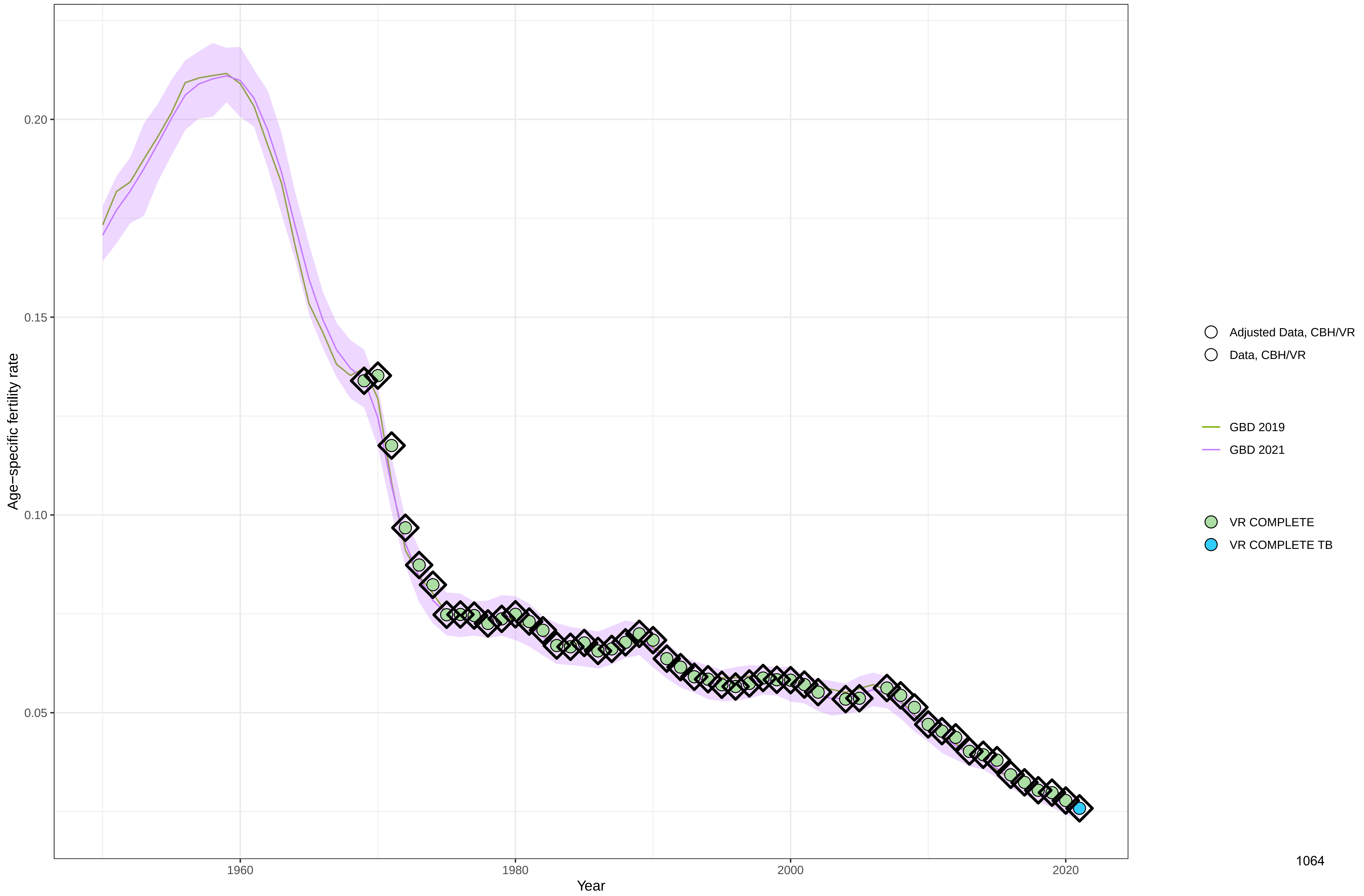

Age−specific fertility fitted trend lines, 1950−2021: Massachusetts, Ages: 25−29
Data points enclosed with a diamond are reference sources

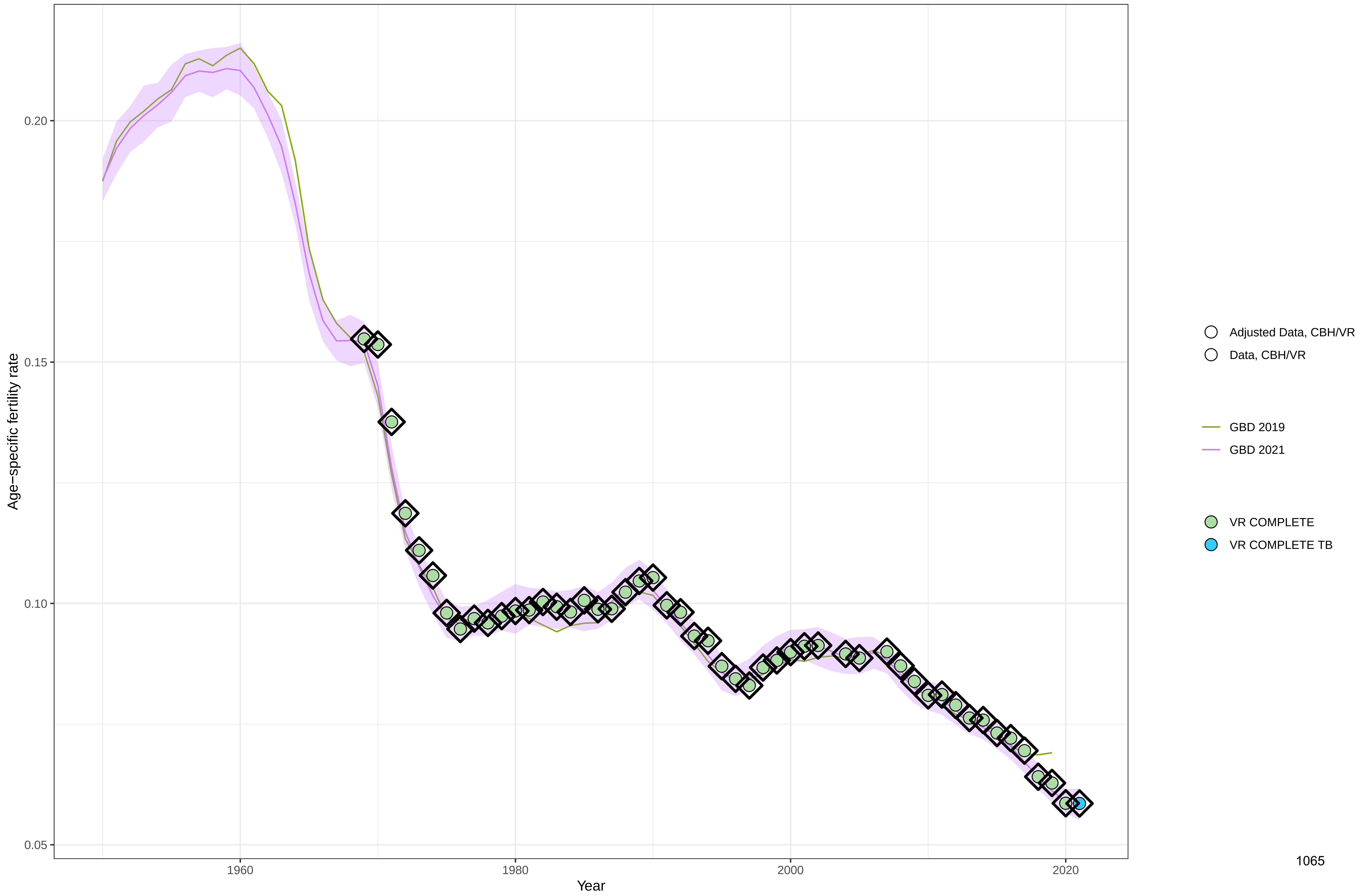

Age−specific fertility fitted trend lines, 1950−2021: Massachusetts, Ages: 30−34
Data points enclosed with a diamond are reference sources

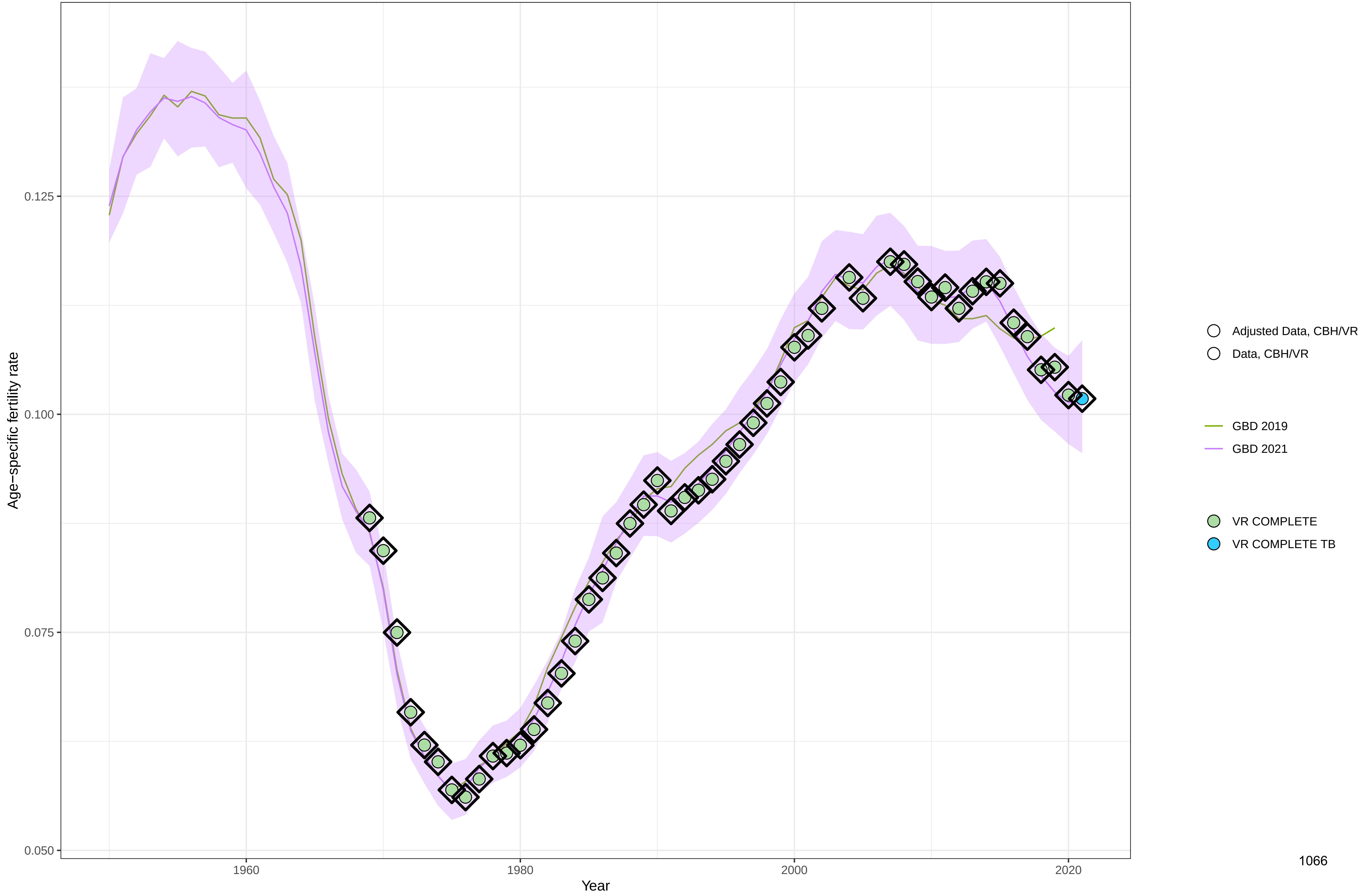

Age−specific fertility fitted trend lines, 1950−2021: Massachusetts, Ages: 35−39
Data points enclosed with a diamond are reference sources

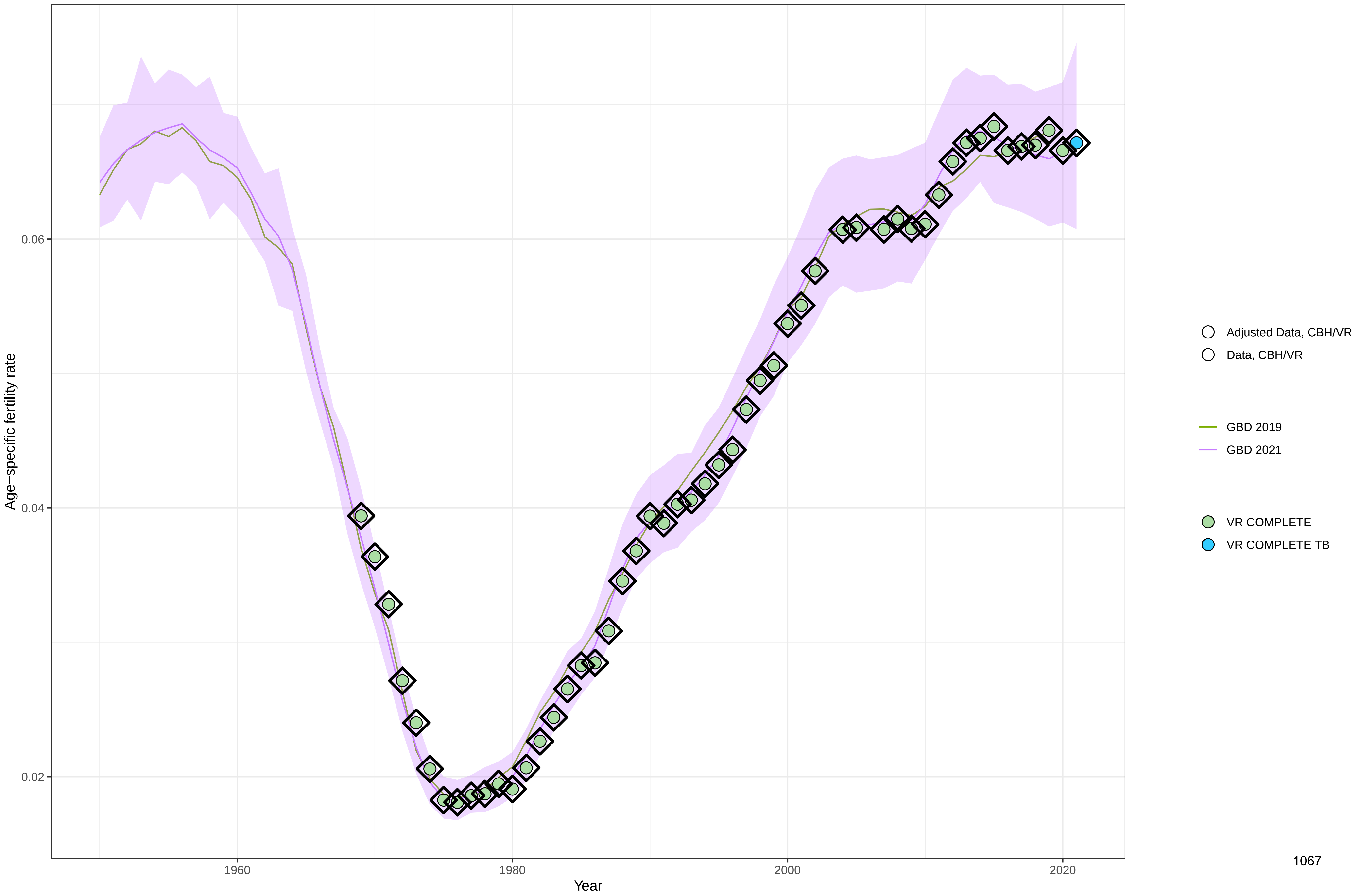

Age−specific fertility fitted trend lines, 1950−2021: Massachusetts, Ages: 40−44
Data points enclosed with a diamond are reference sources

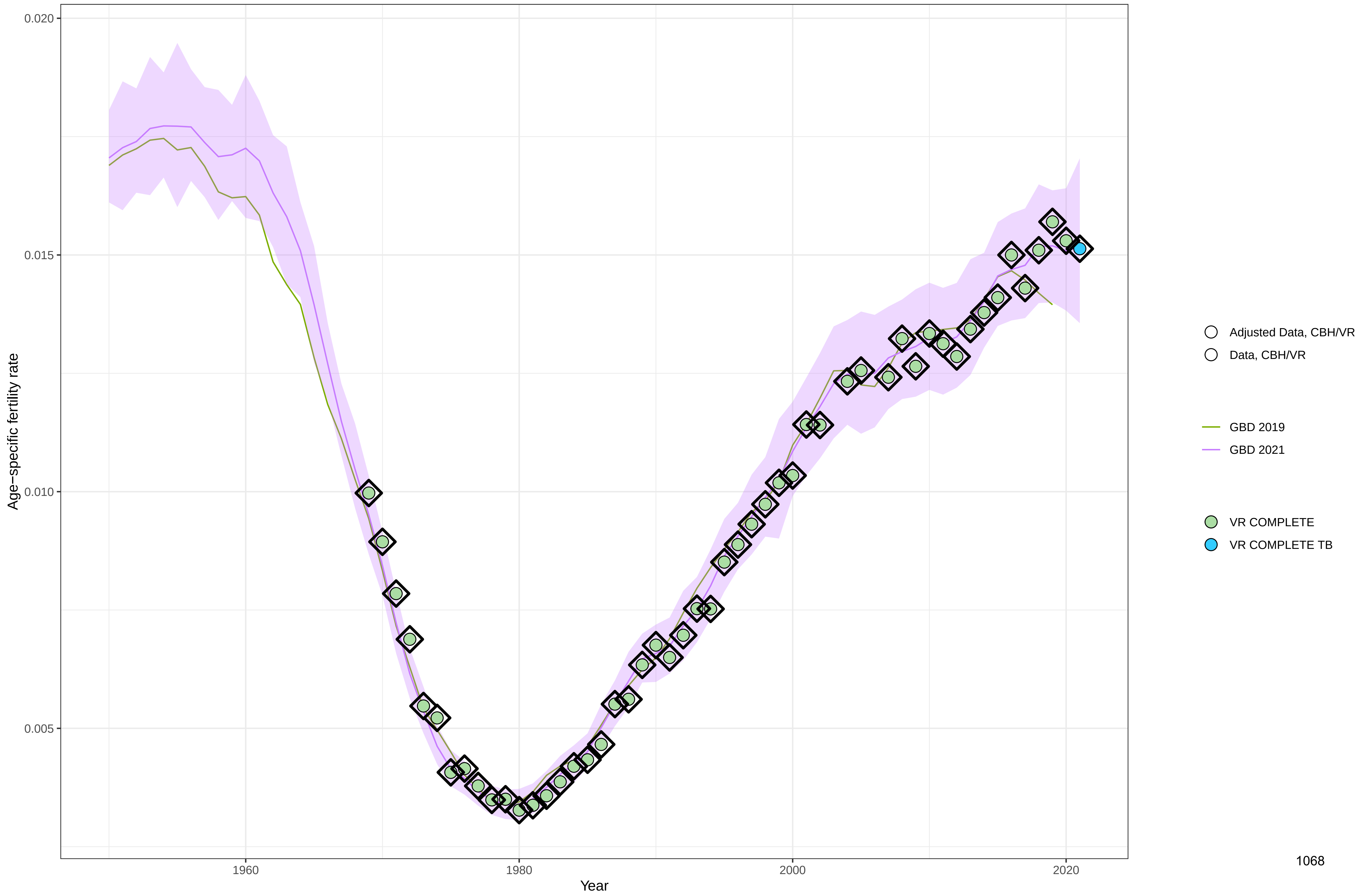

Age−specific fertility fitted trend lines, 1950−2021: Massachusetts, Ages: 45−49
Data points enclosed with a diamond are reference sources

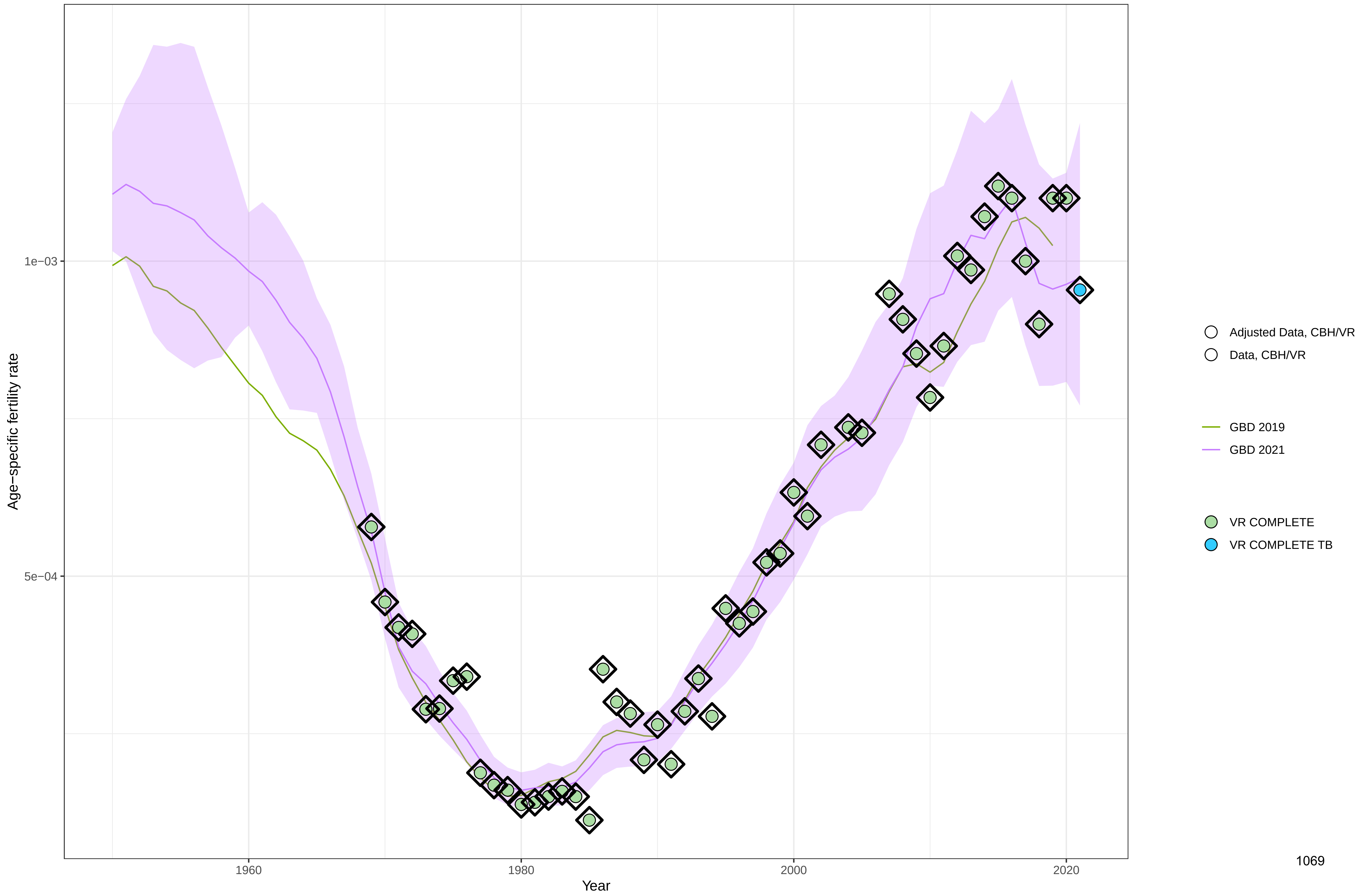

Age−specific fertility fitted trend lines, 1950−2021: Massachusetts, Ages: 50−54
Data points enclosed with a diamond are reference sources

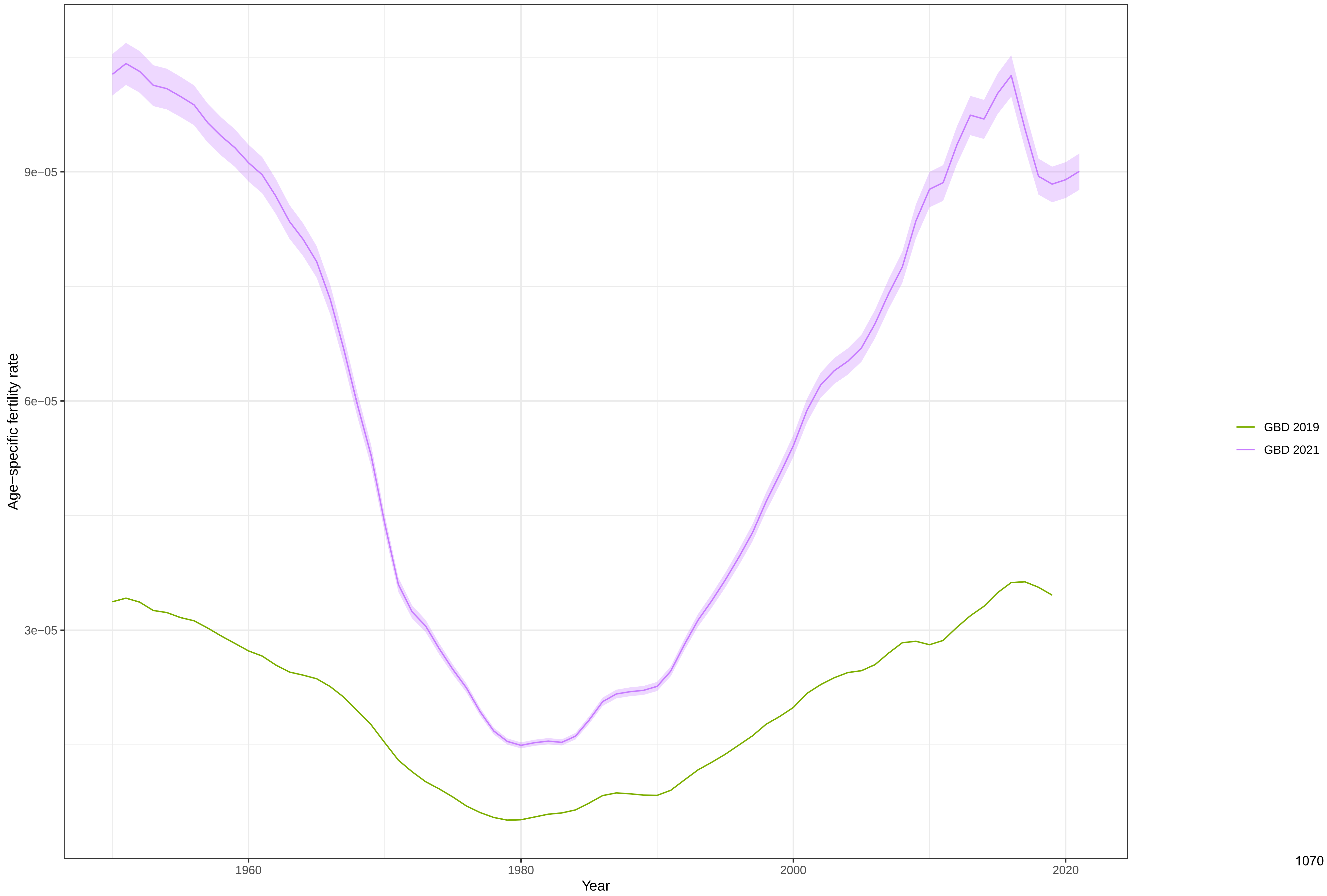

Total fertility rate fitted trend lines, 1950−2021: Massachusetts TFR
Data points enclosed with a diamond are reference sources

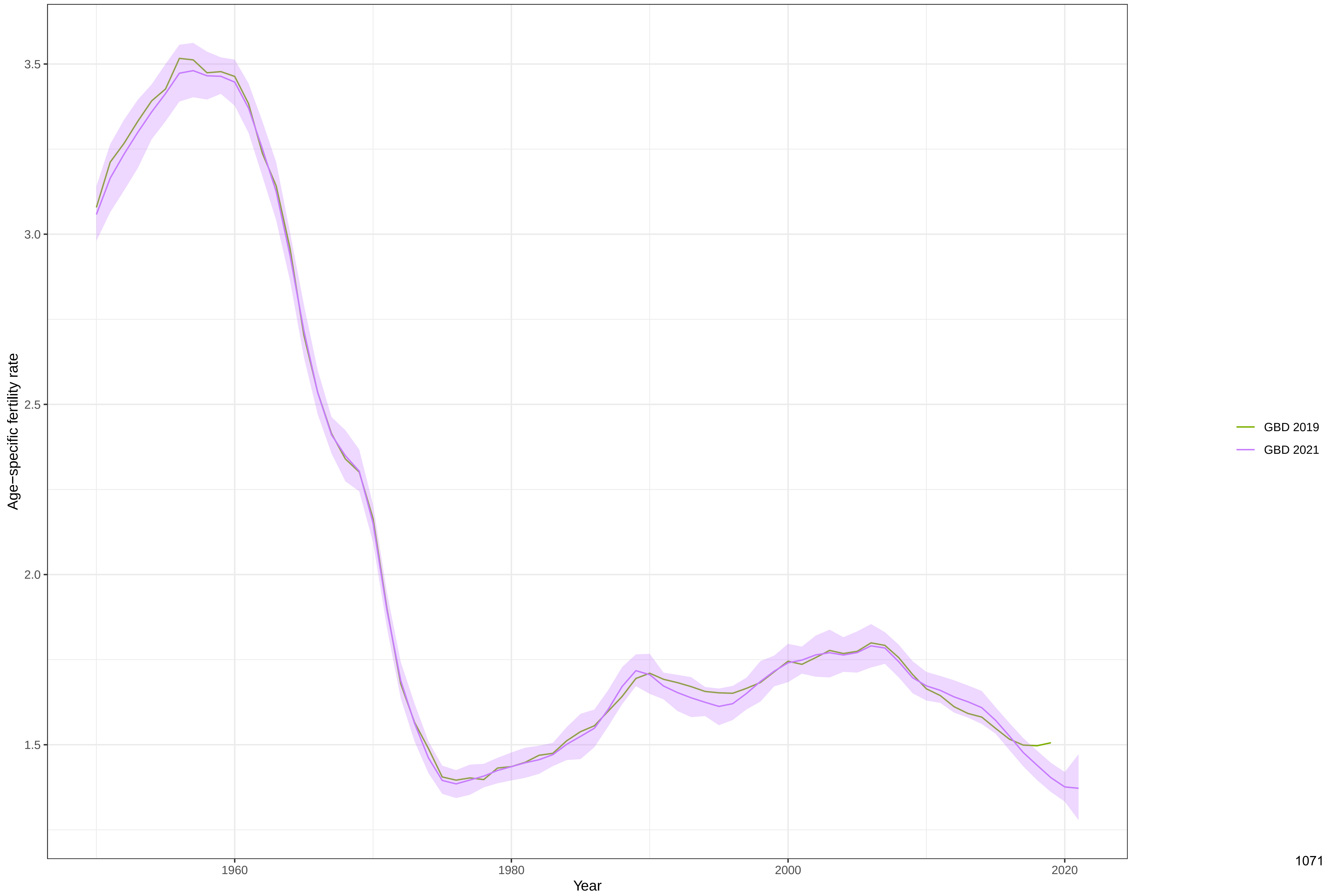

Age−specific fertility fitted trend lines, 1950−2021: Michigan, Ages: 10−14
Data points enclosed with a diamond are reference sources

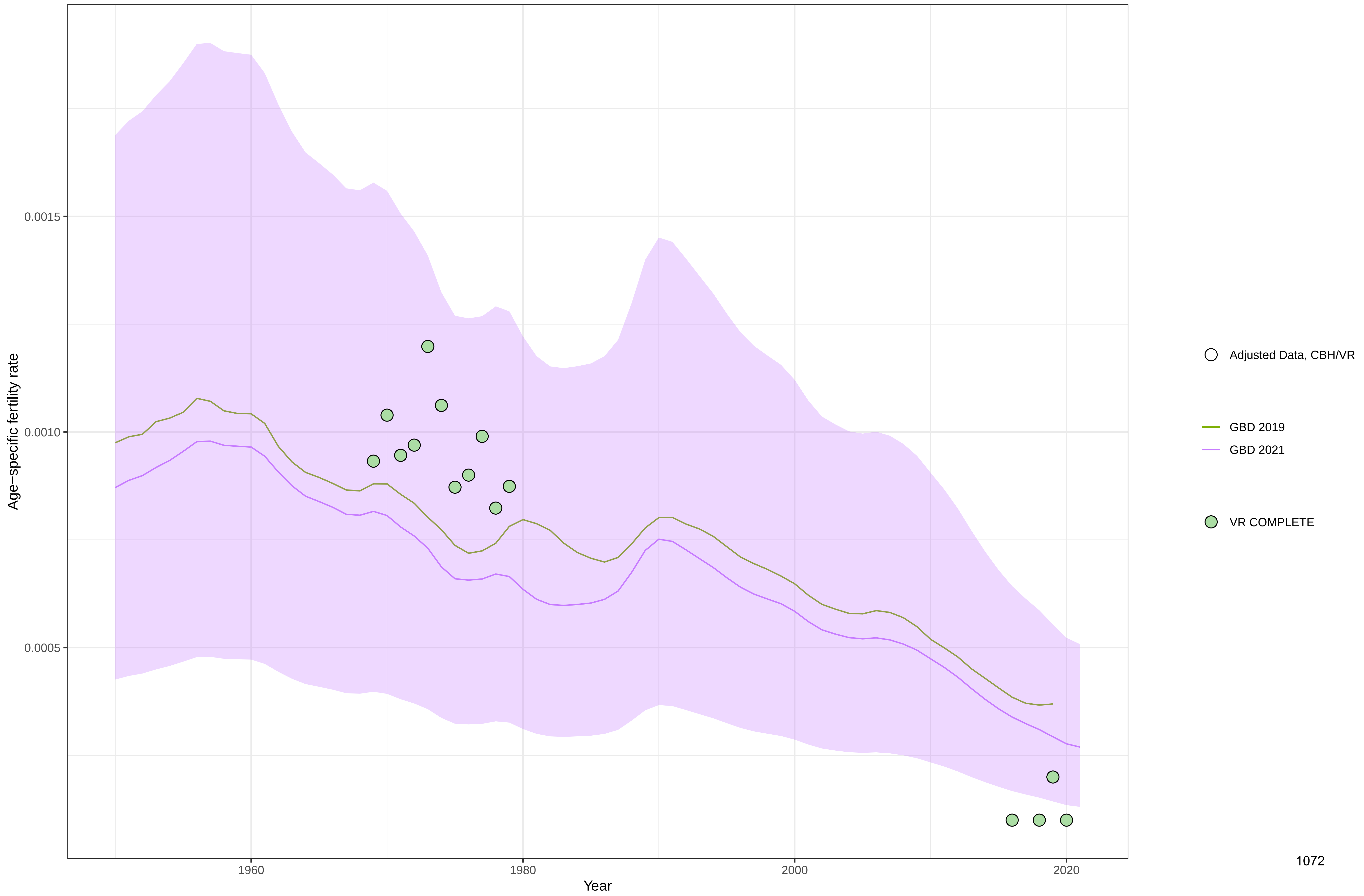

Age−specific fertility fitted trend lines, 1950−2021: Michigan, Ages: 15−19
Data points enclosed with a diamond are reference sources

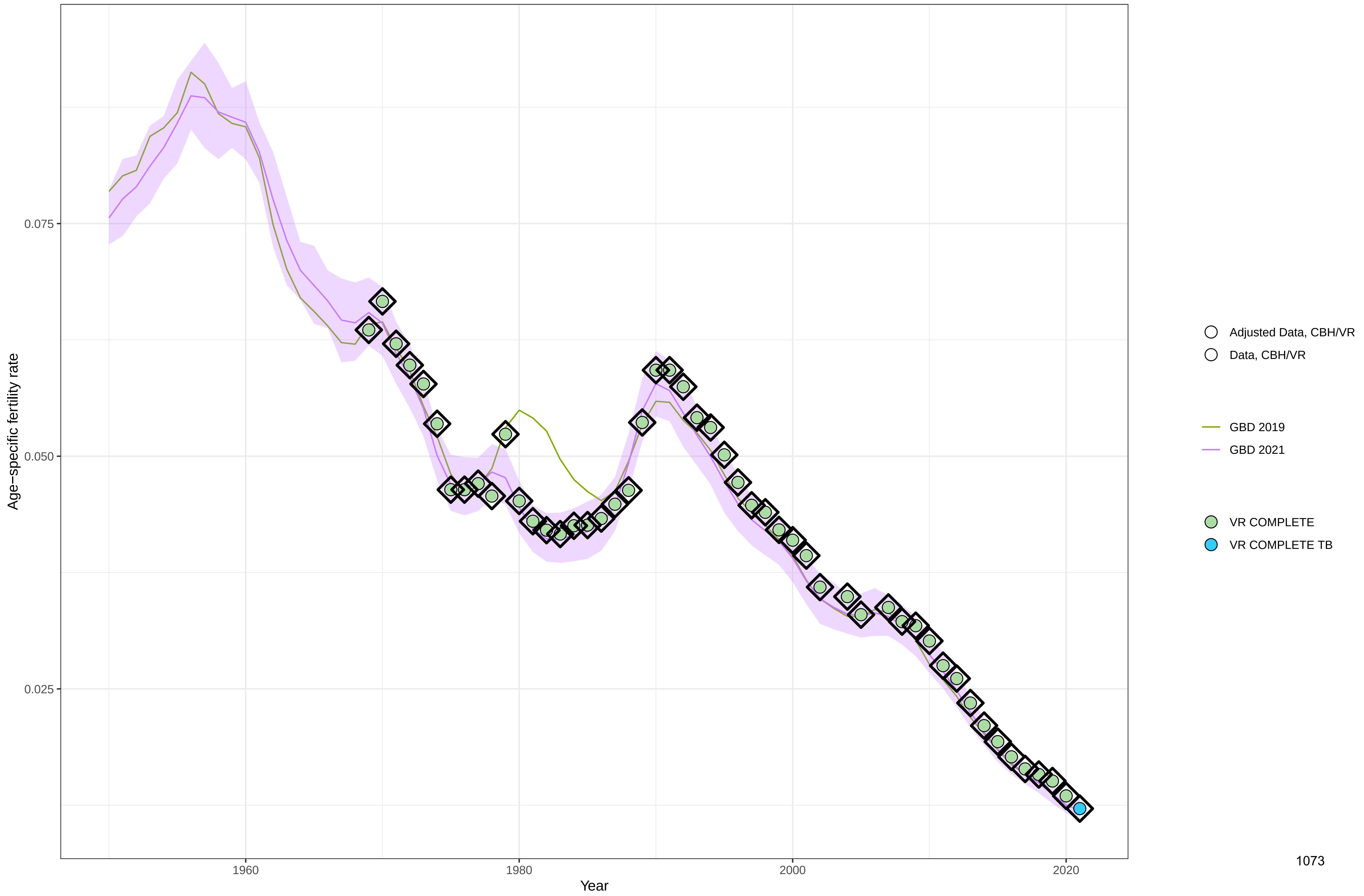

Age−specific fertility fitted trend lines, 1950−2021: Michigan, Ages: 20−24
Data points enclosed with a diamond are reference sources

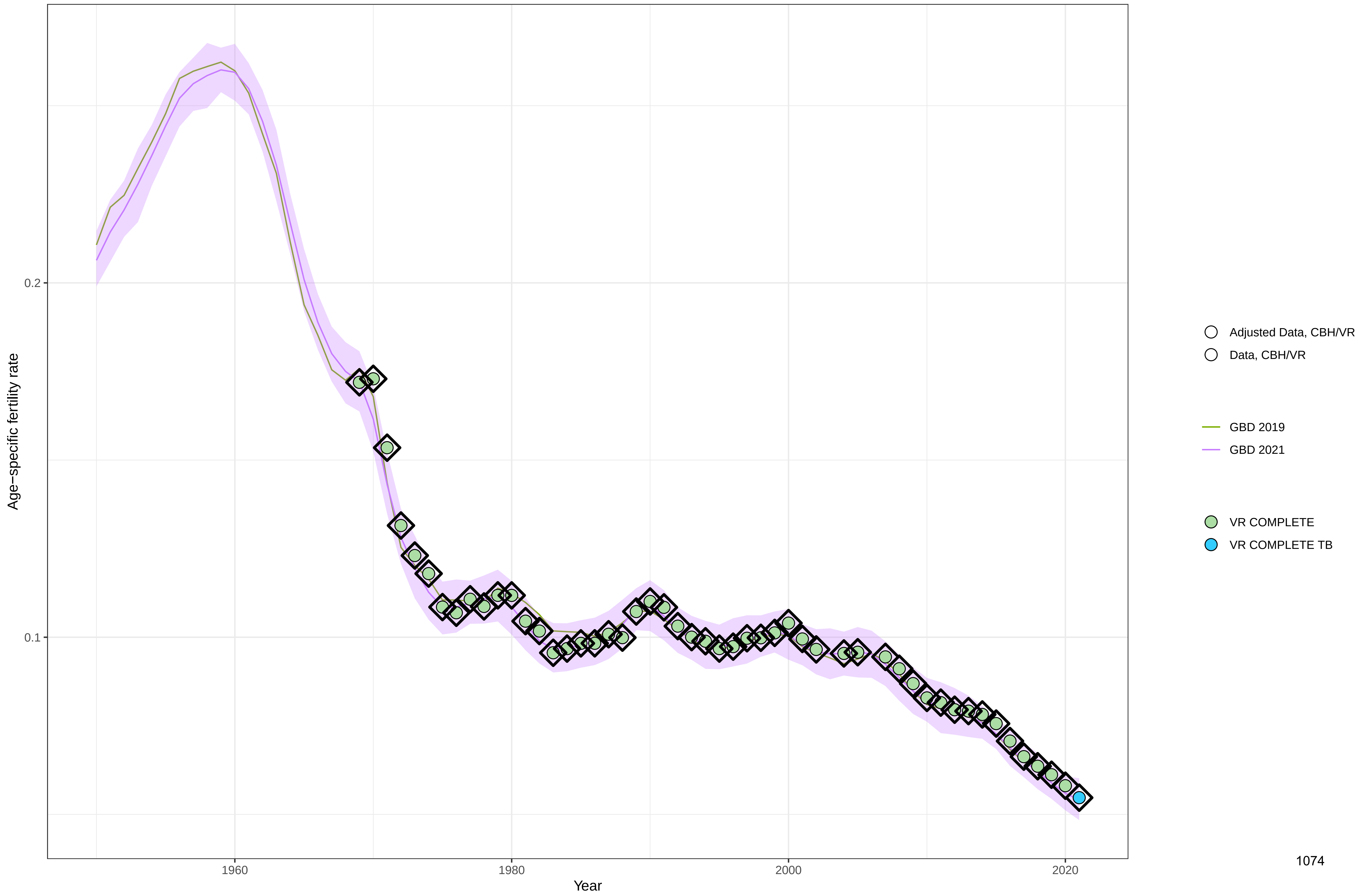

Age−specific fertility fitted trend lines, 1950−2021: Michigan, Ages: 25−29
Data points enclosed with a diamond are reference sources

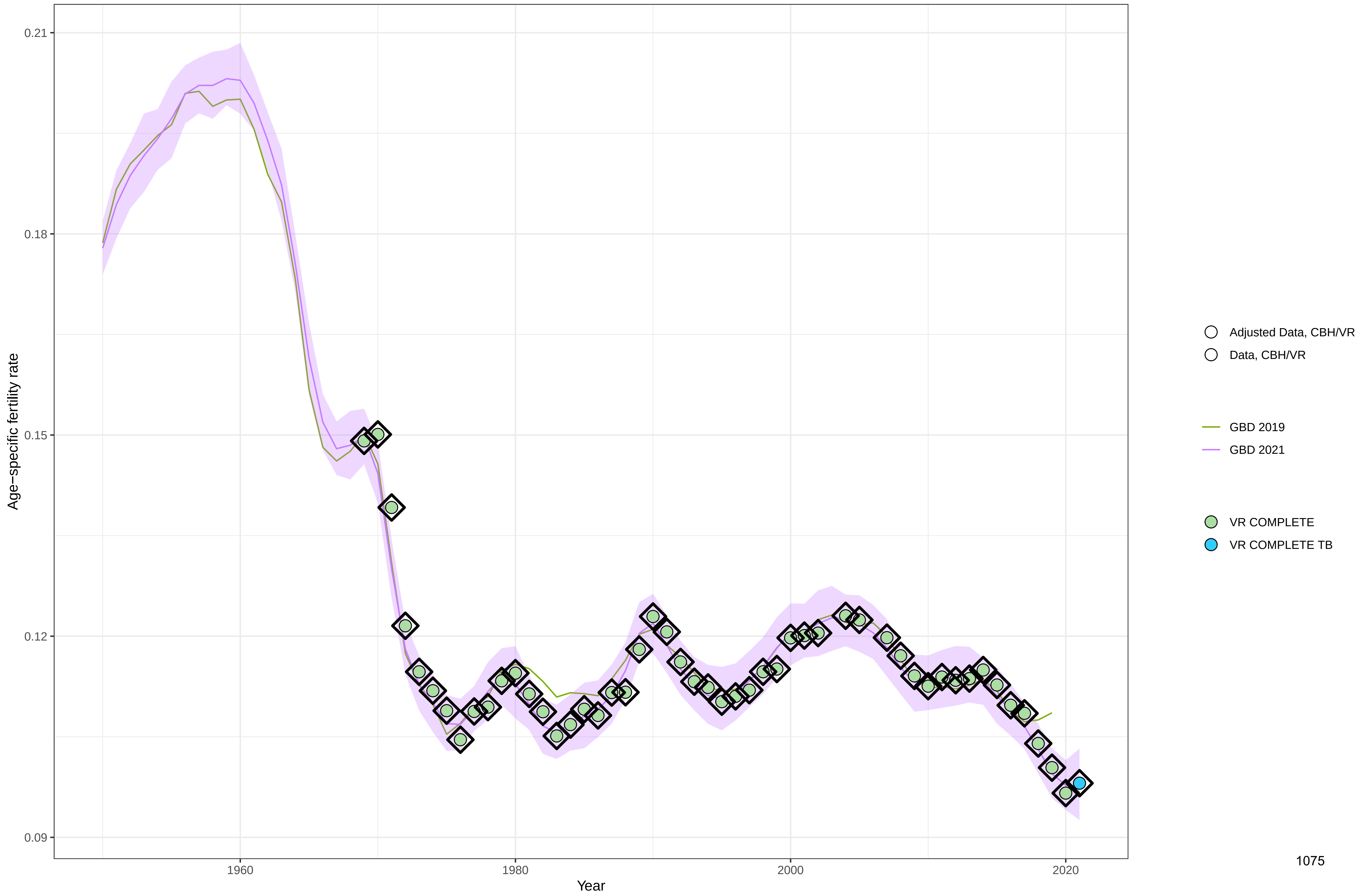

Age−specific fertility fitted trend lines, 1950−2021: Michigan, Ages: 30−34
Data points enclosed with a diamond are reference sources

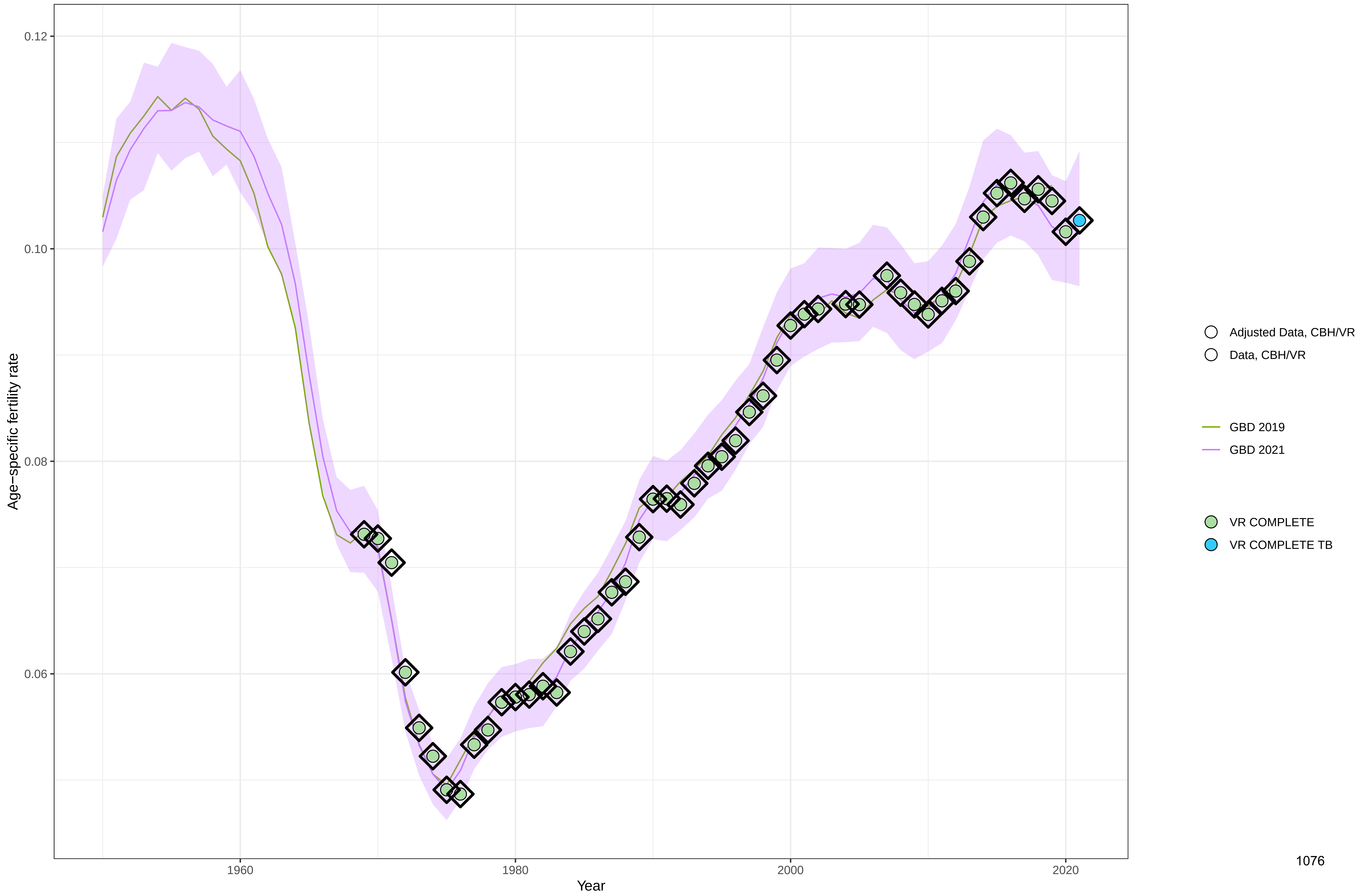

Age−specific fertility fitted trend lines, 1950−2021: Michigan, Ages: 35−39
Data points enclosed with a diamond are reference sources

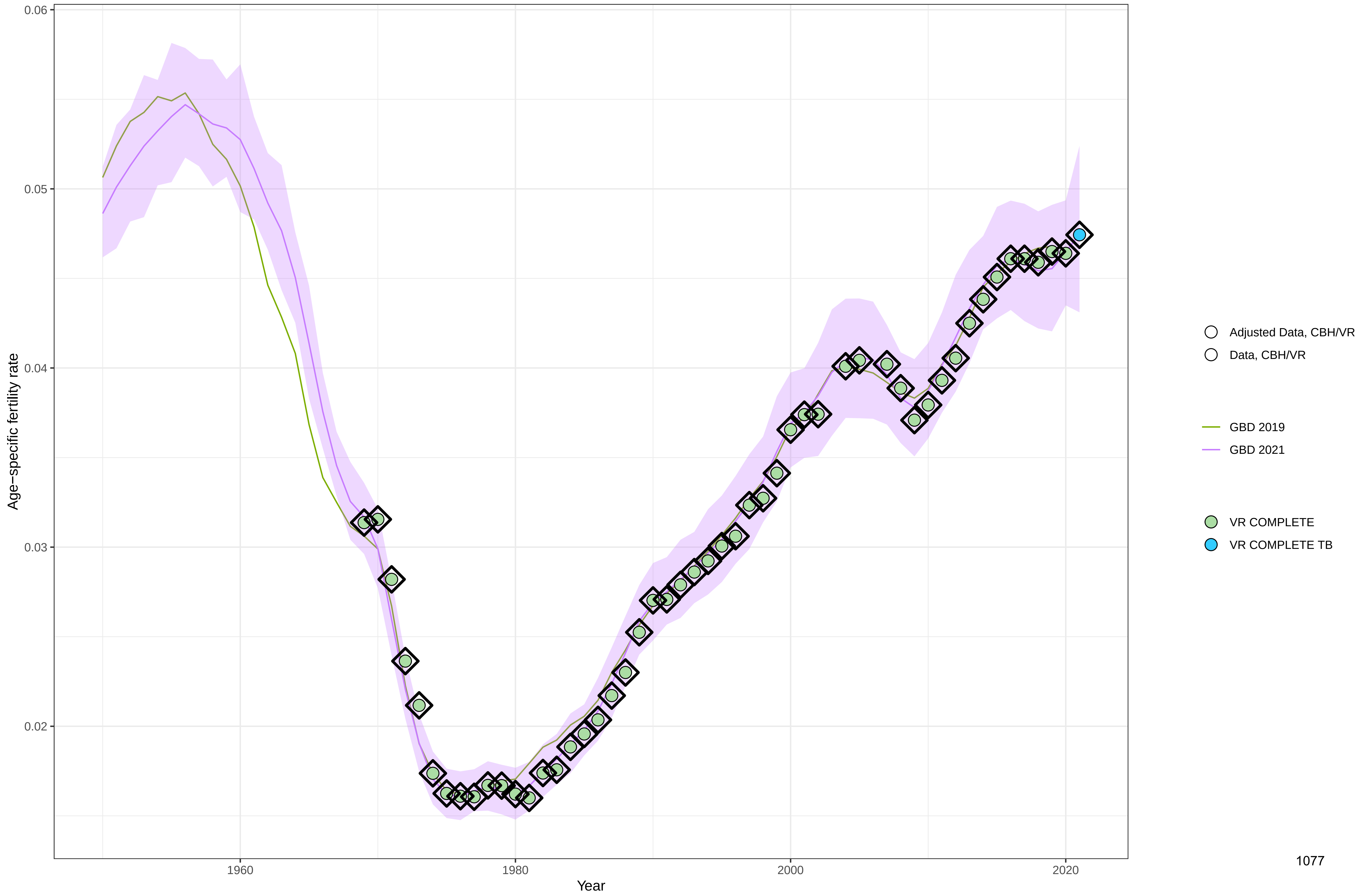

Age−specific fertility fitted trend lines, 1950−2021: Michigan, Ages: 40−44
Data points enclosed with a diamond are reference sources

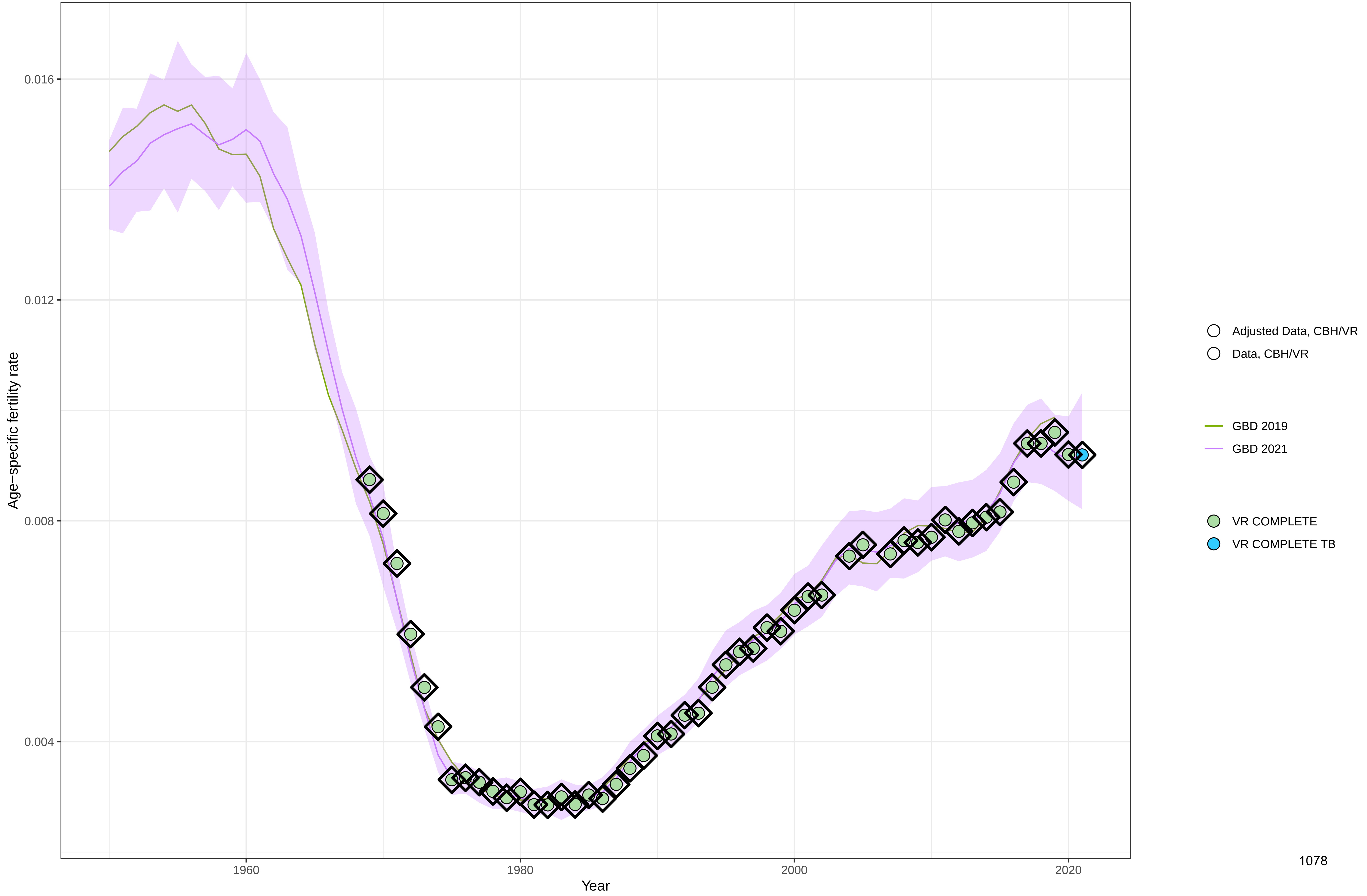

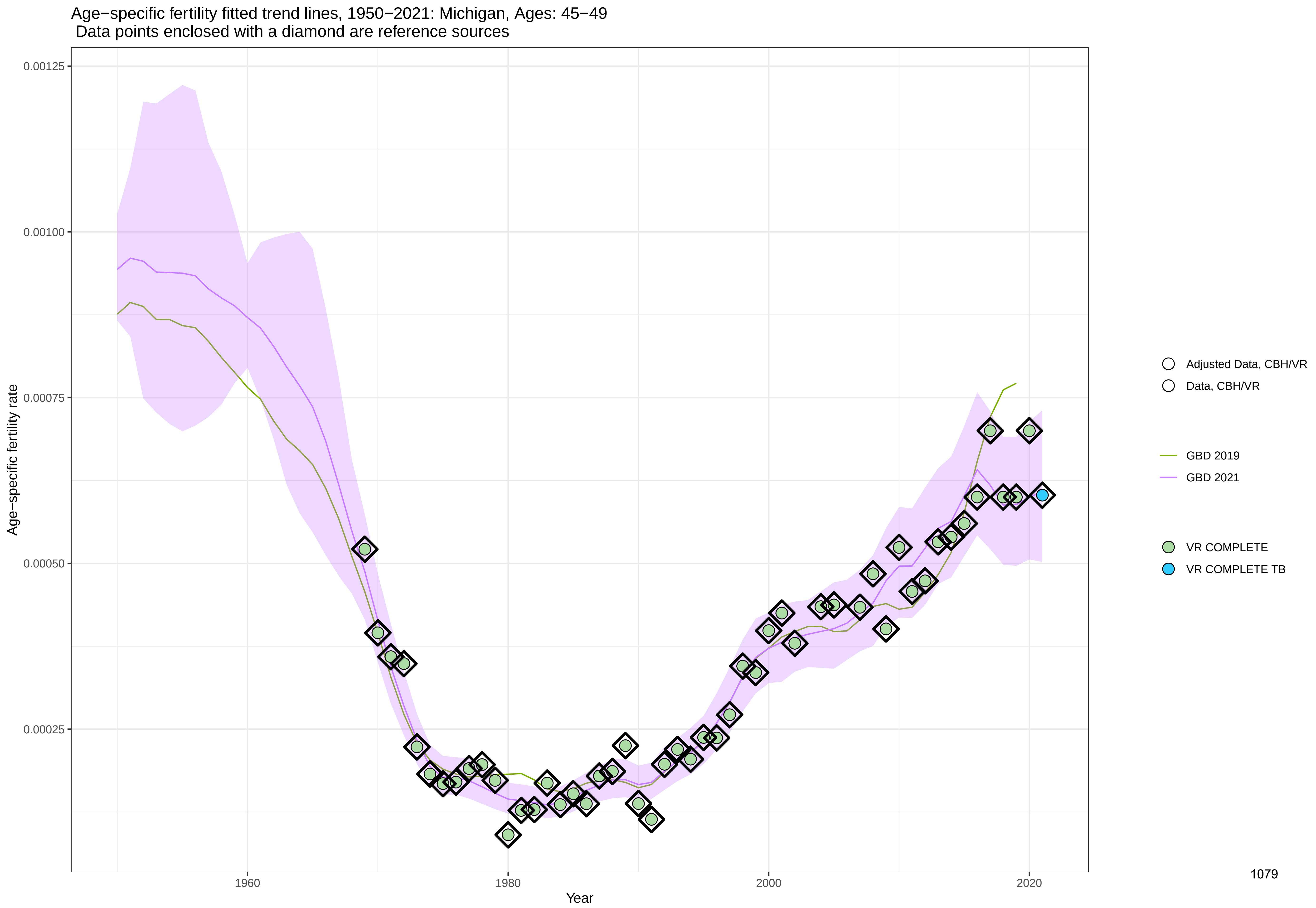

Age−specific fertility fitted trend lines, 1950−2021: Michigan, Ages: 45−49
Data points enclosed with a diamond are reference sources
Age−specific fertility rate
0.00125
0.00100
0.00075
0.00050
0.00025
1960
1980
2000
2020
Year
Adjusted Data, CBH/VR
Data, CBH/VR
GBD 2019
GBD 2021
VR COMPLETE
VR COMPLETE TB

Age−specific fertility fitted trend lines, 1950−2021: Michigan, Ages: 50−54
Data points enclosed with a diamond are reference sources

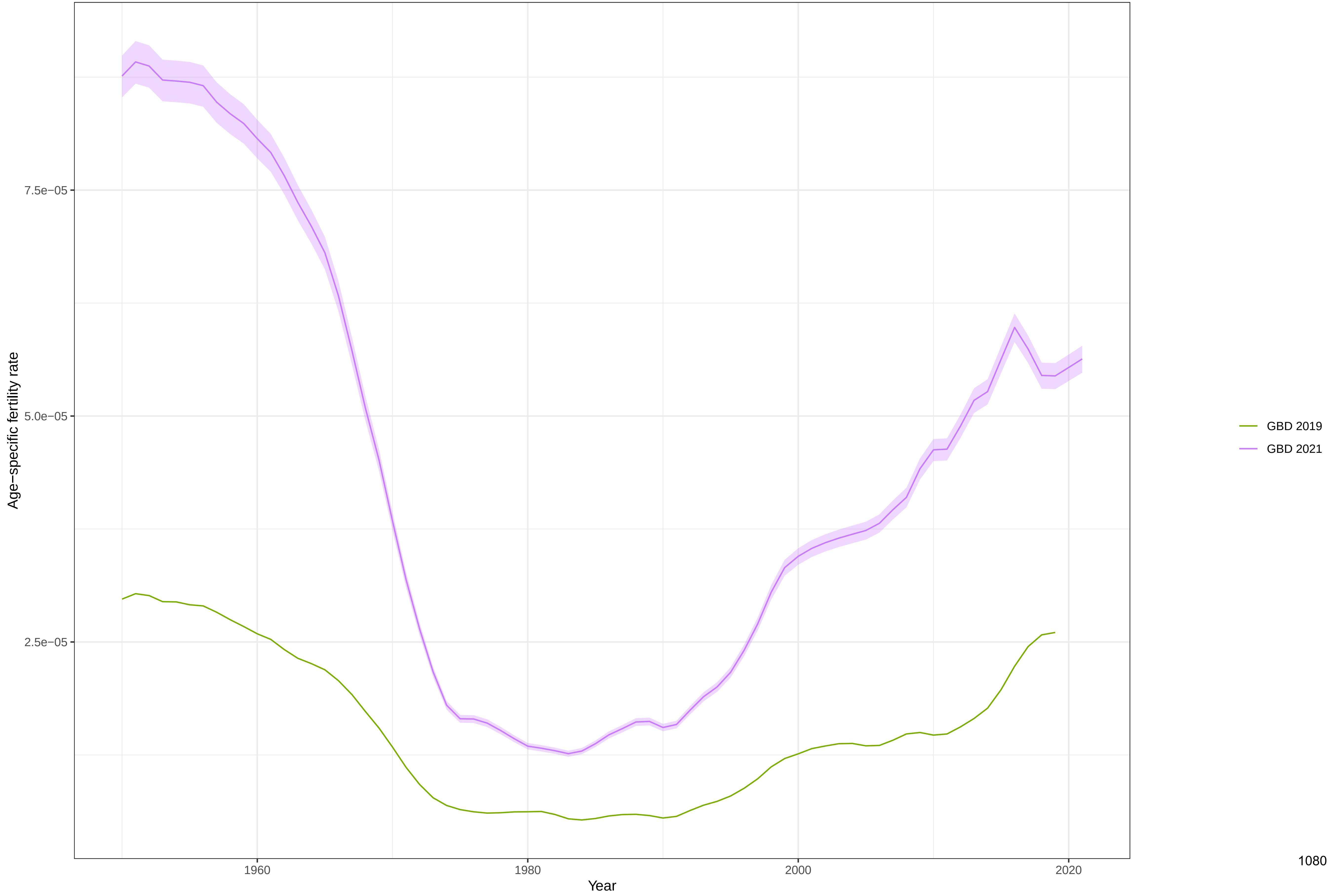

Total fertility rate fitted trend lines, 1950−2021: Michigan TFR
Data points enclosed with a diamond are reference sources

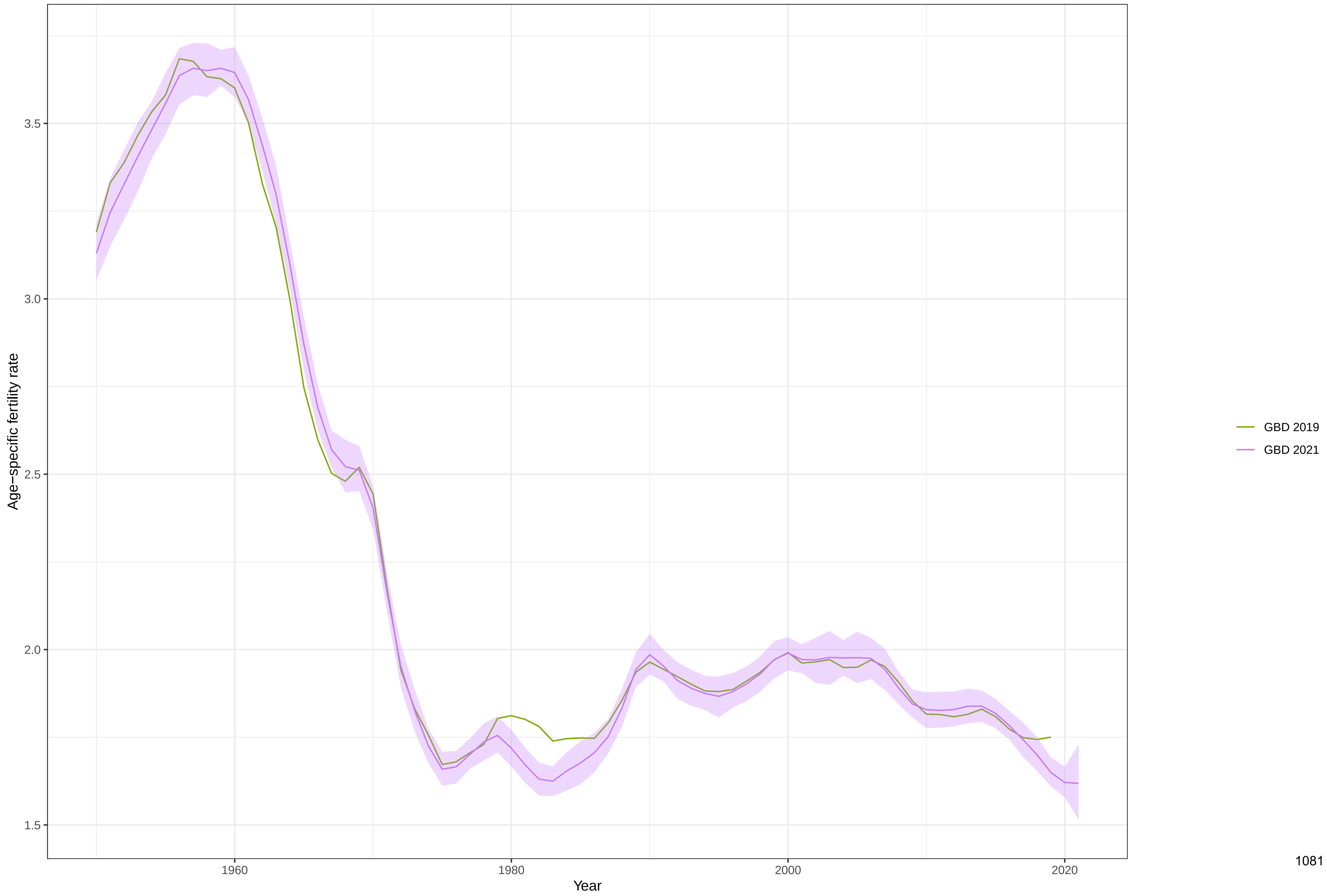

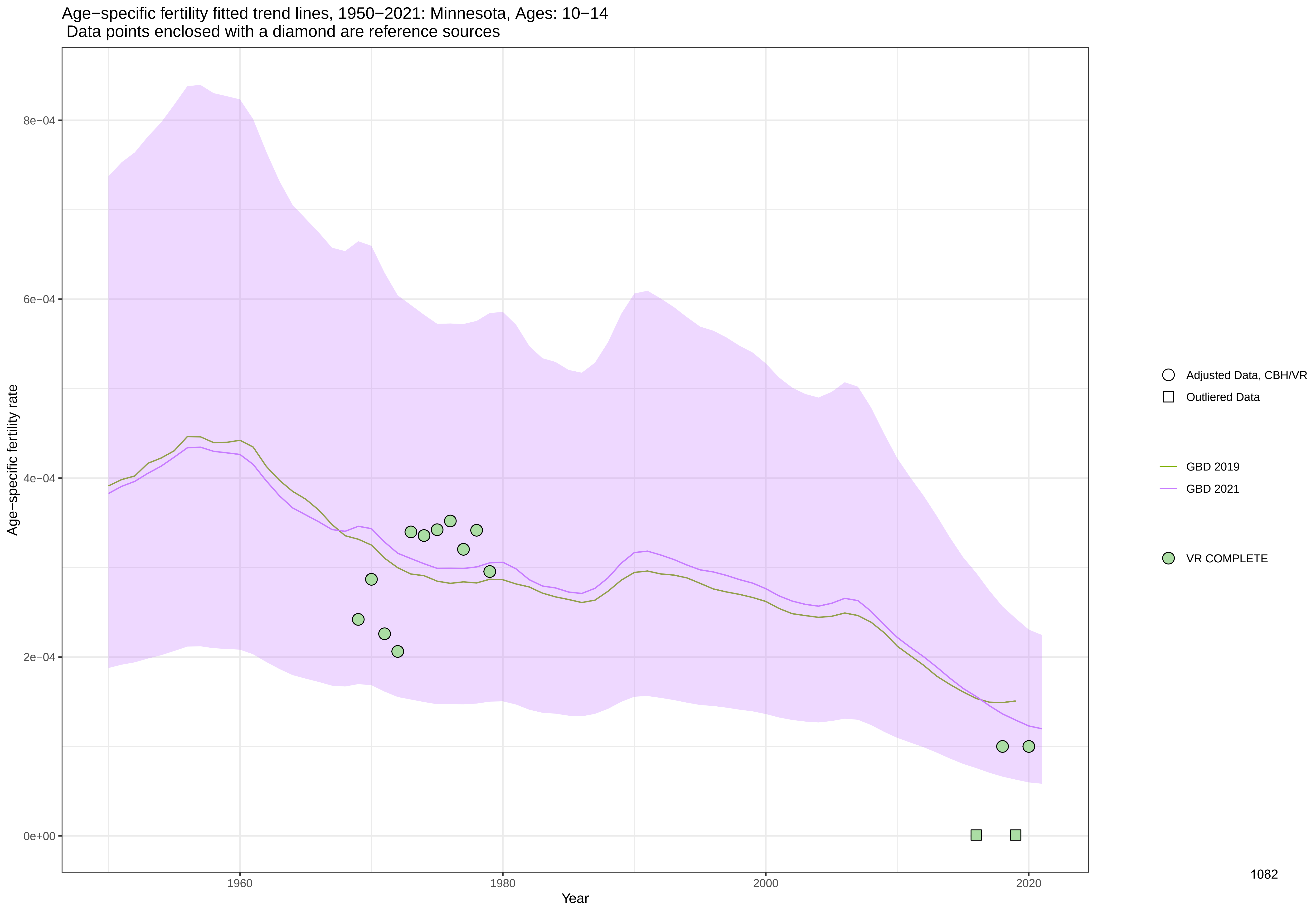

Age−specific fertility fitted trend lines, 1950−2021: Minnesota, Ages: 10−14
Data points enclosed with a diamond are reference sources
Age−specific fertility rate
8e−04
6e−04
4e−04
2e−04
0e+00
1960
1980
2000
2020
Year
Adjusted Data, CBH/VR
Outliered Data
GBD 2019
GBD 2021
VR COMPLETE

1082

Age−specific fertility fitted trend lines, 1950−2021: Minnesota, Ages: 15−19
Data points enclosed with a diamond are reference sources

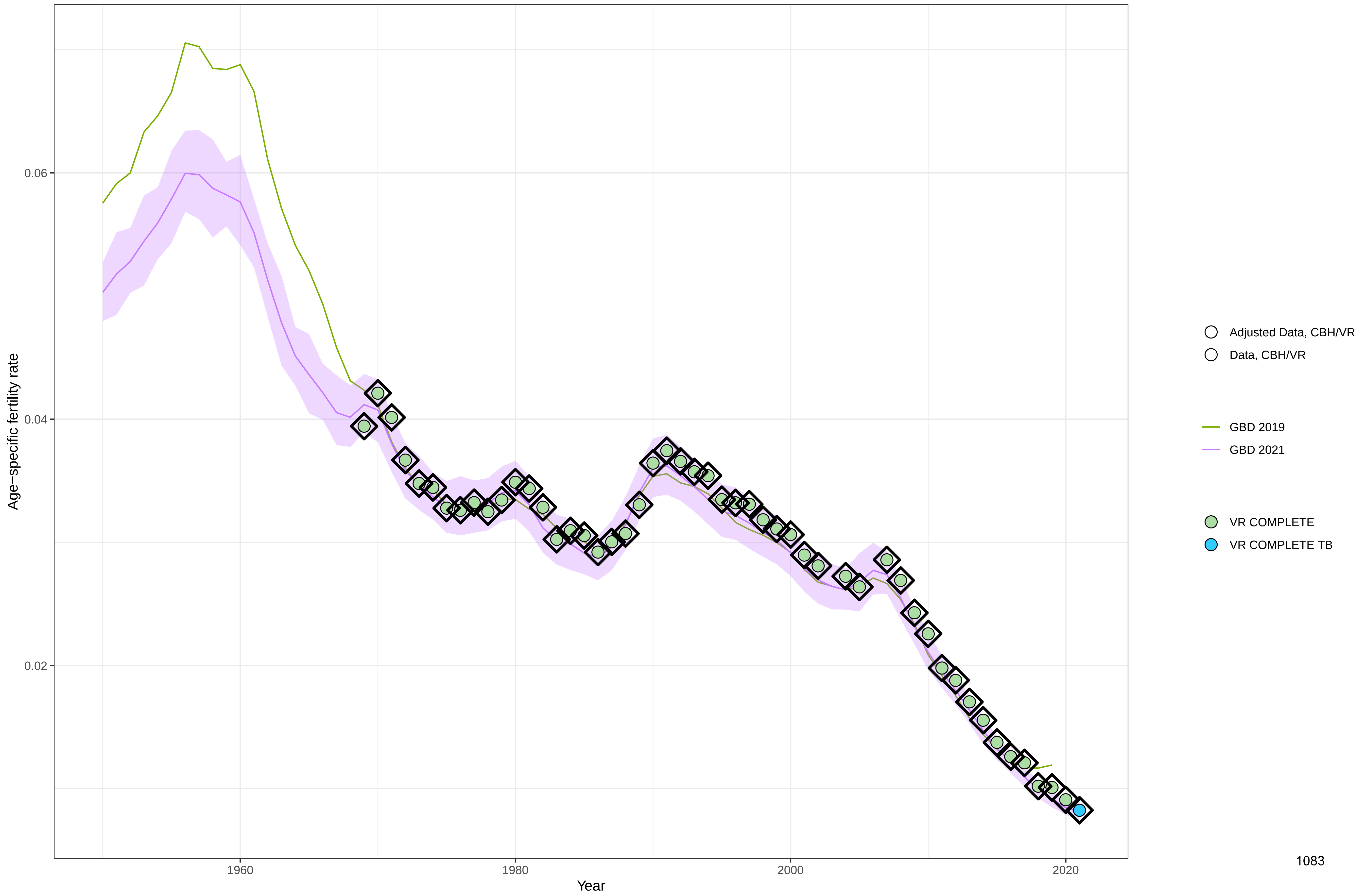

Age−specific fertility fitted trend lines, 1950−2021: Minnesota, Ages: 20−24
Data points enclosed with a diamond are reference sources

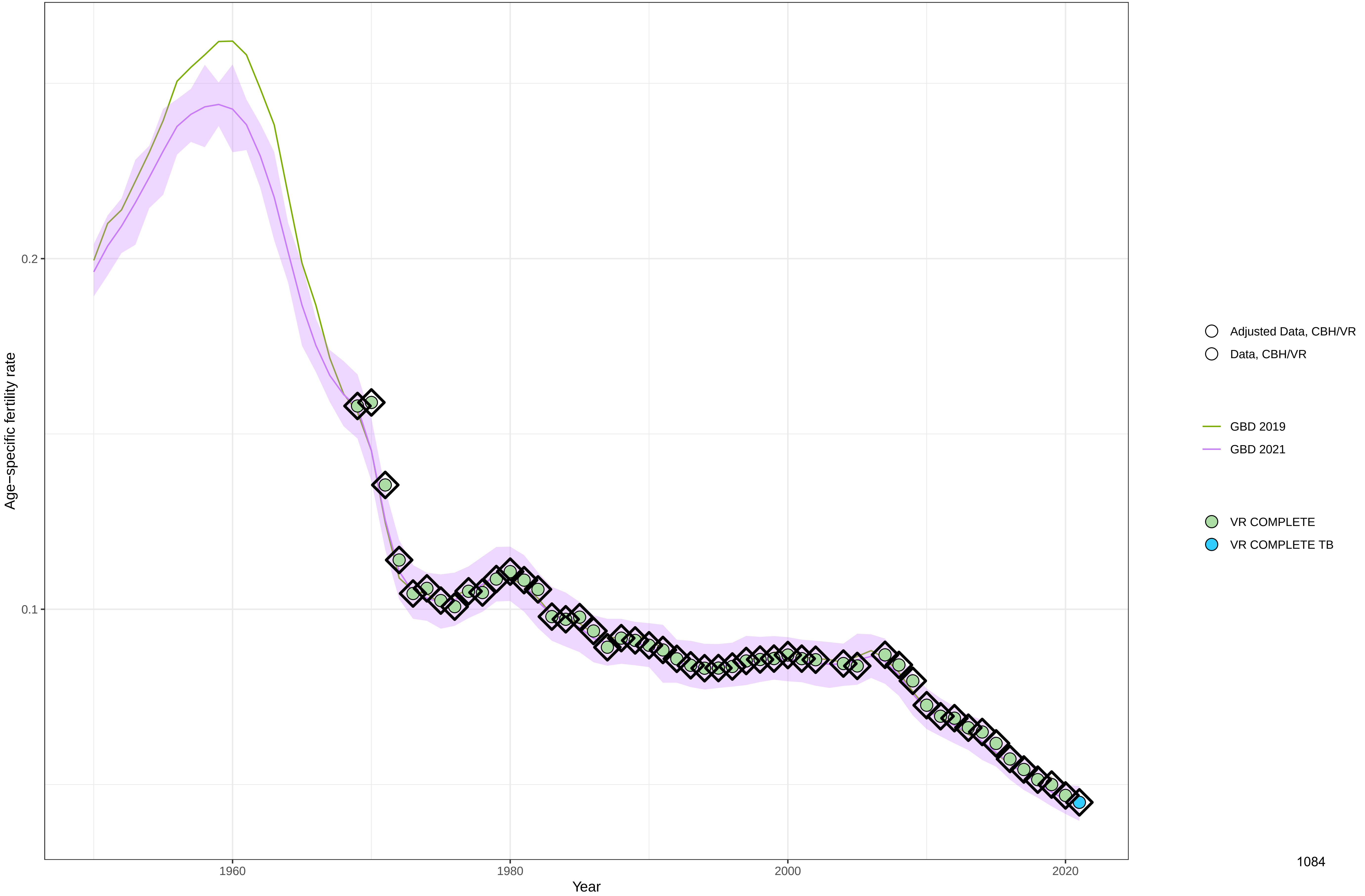

Age−specific fertility fitted trend lines, 1950−2021: Minnesota, Ages: 25−29
Data points enclosed with a diamond are reference sources

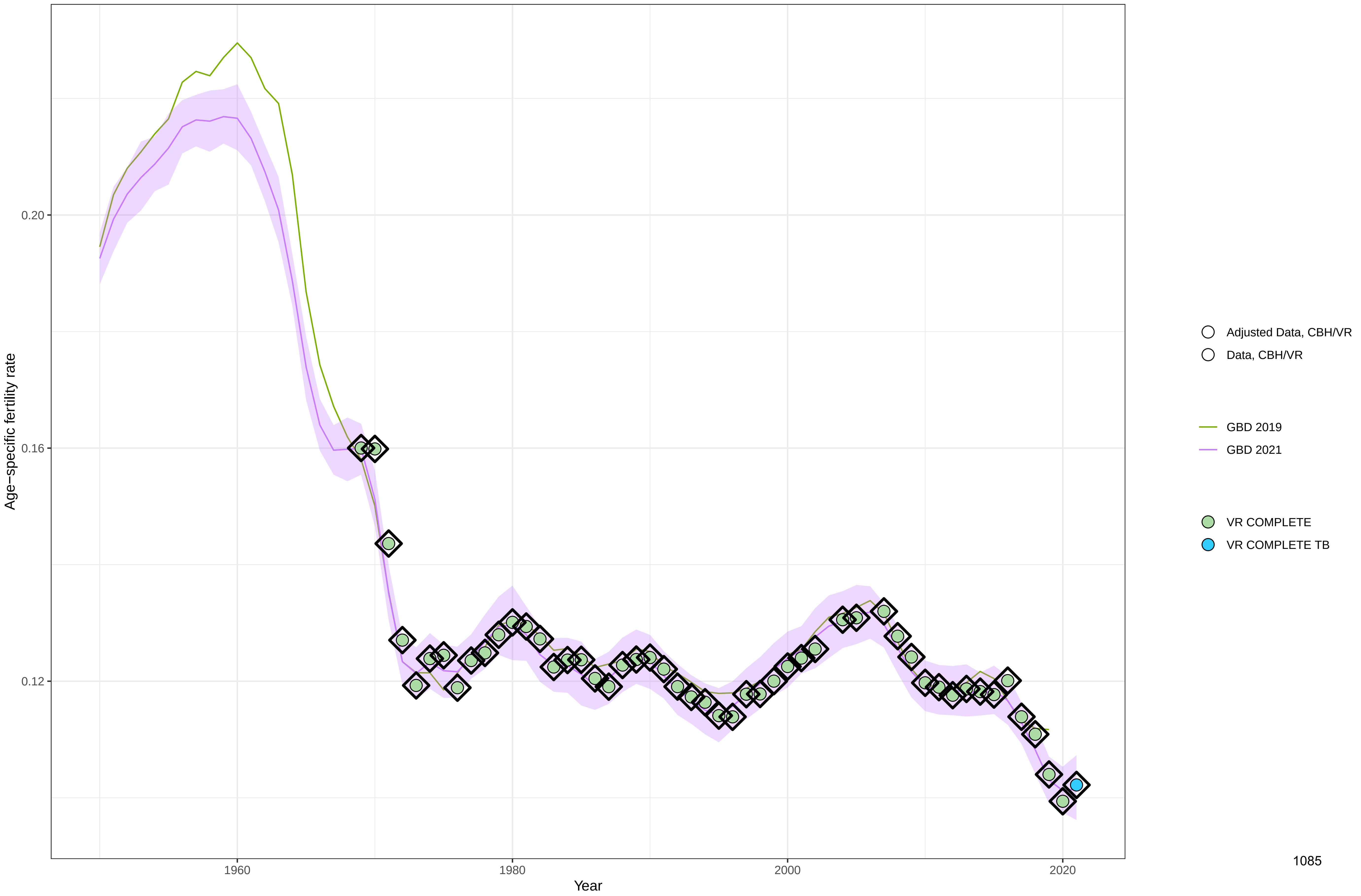

Age−specific fertility fitted trend lines, 1950−2021: Minnesota, Ages: 30−34
Data points enclosed with a diamond are reference sources

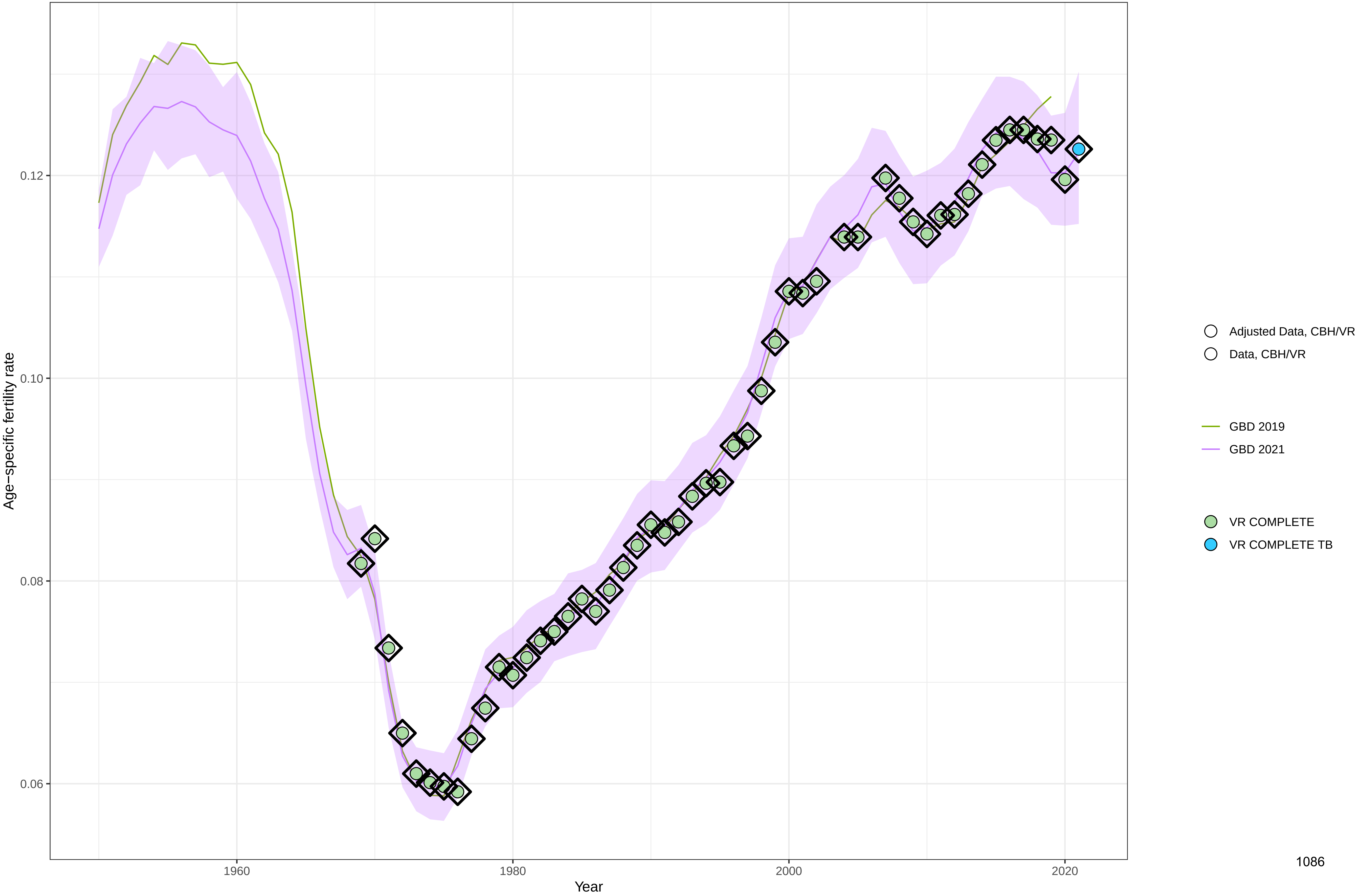

Age−specific fertility fitted trend lines, 1950−2021: Minnesota, Ages: 35−39
Data points enclosed with a diamond are reference sources

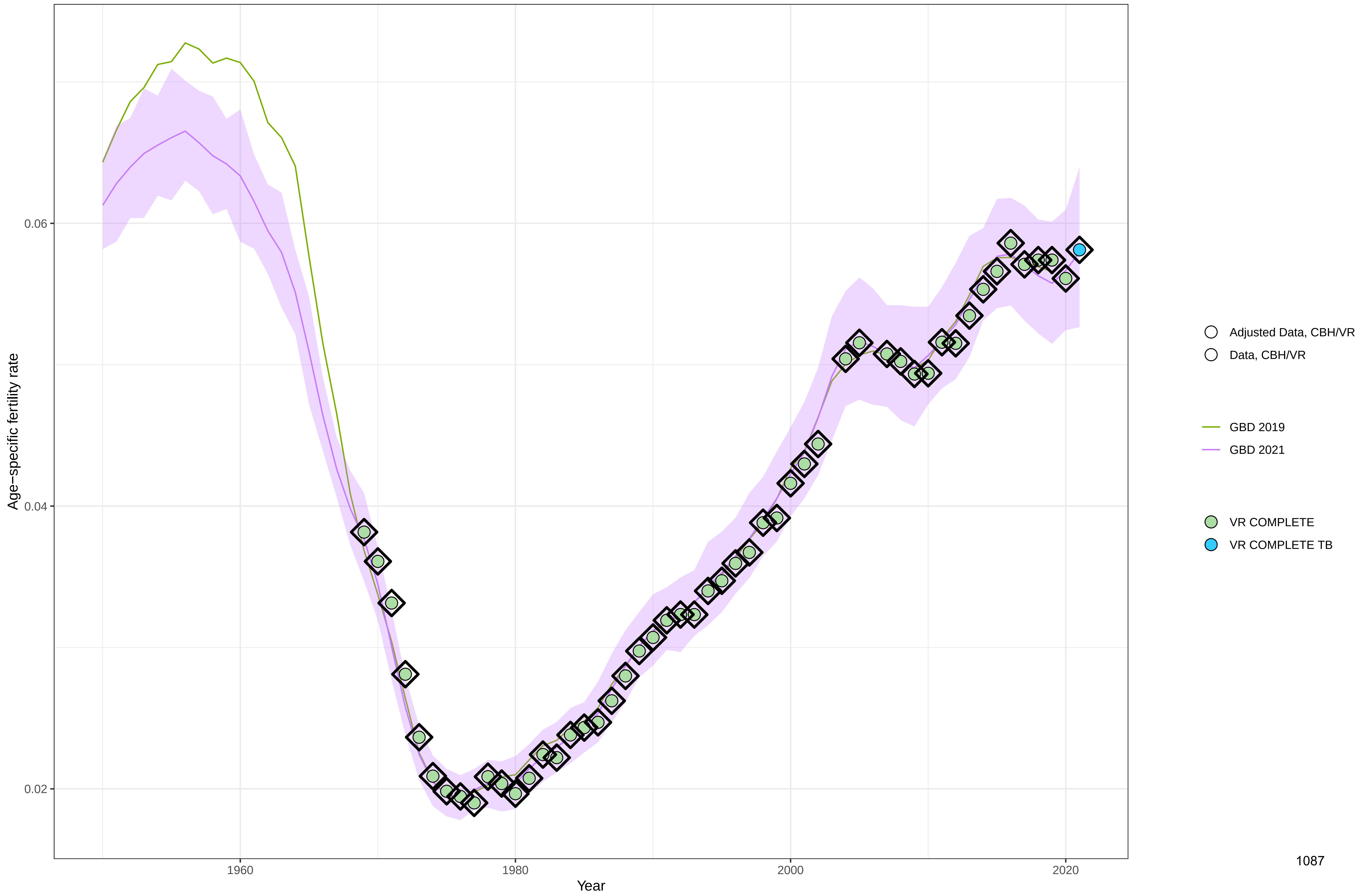

Age−specific fertility fitted trend lines, 1950−2021: Minnesota, Ages: 40−44
Data points enclosed with a diamond are reference sources

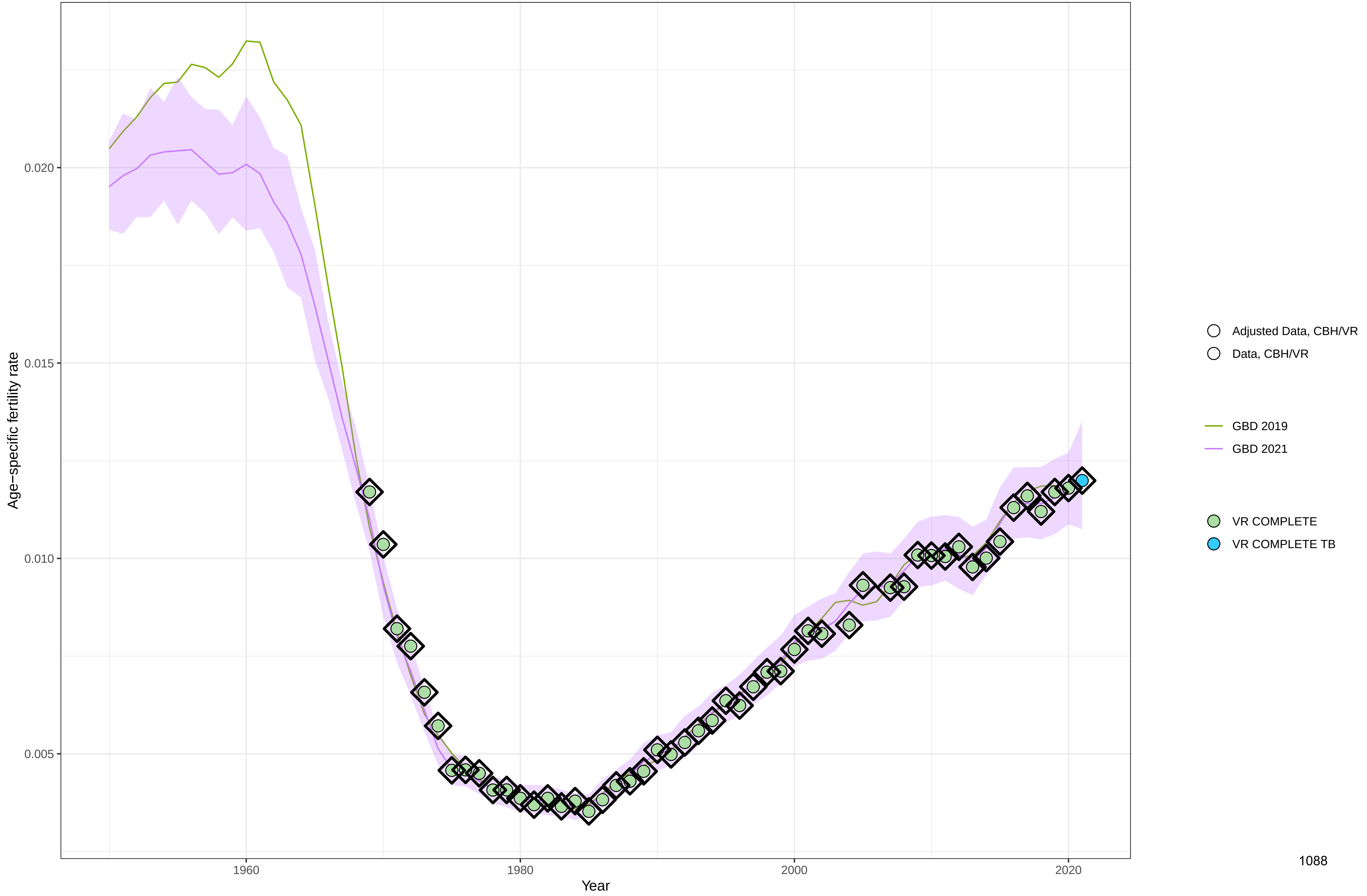

Age−specific fertility fitted trend lines, 1950−2021: Minnesota, Ages: 45−49
Data points enclosed with a diamond are reference sources

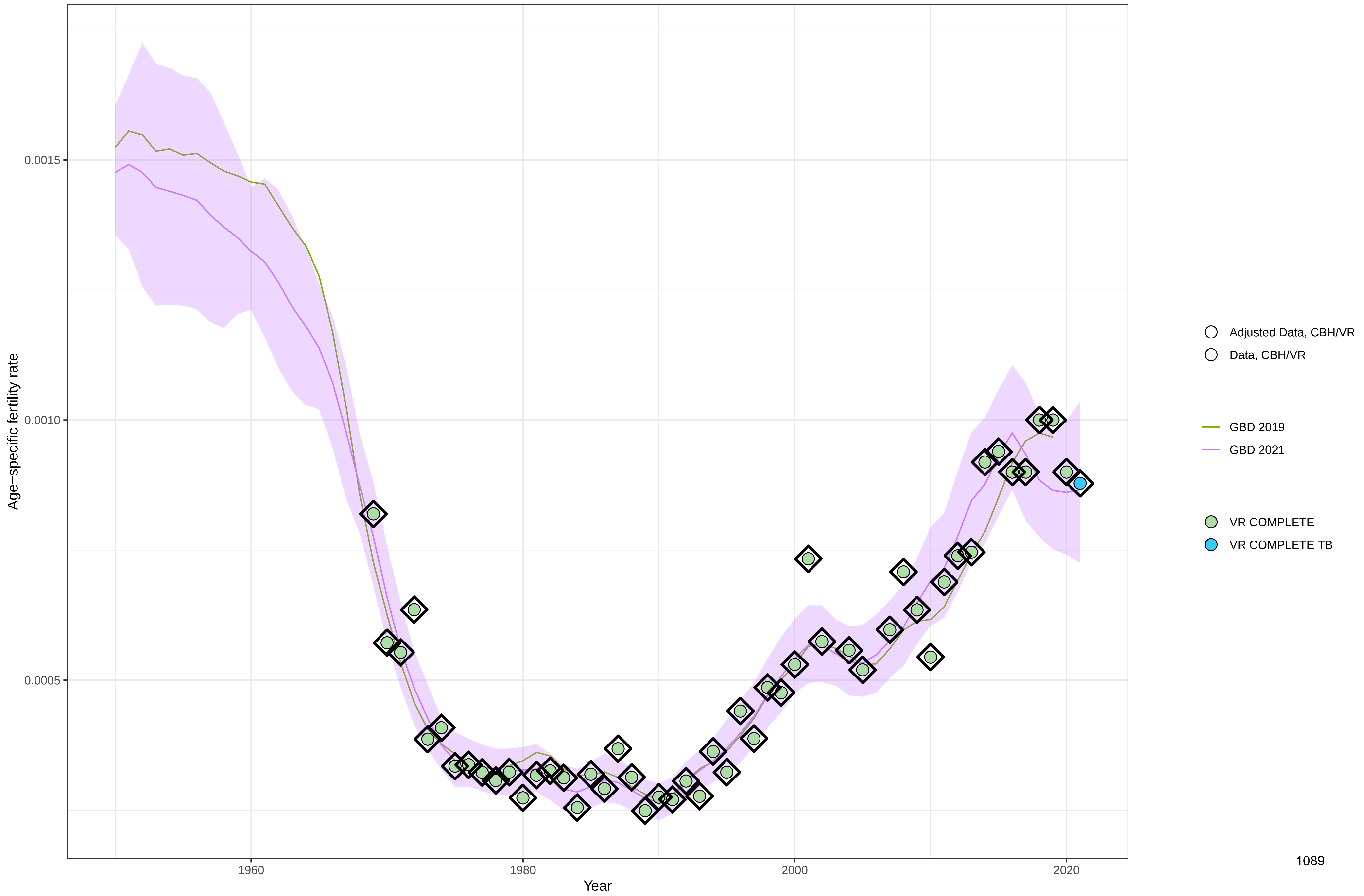

Age−specific fertility fitted trend lines, 1950−2021: Minnesota, Ages: 50−54
Data points enclosed with a diamond are reference sources

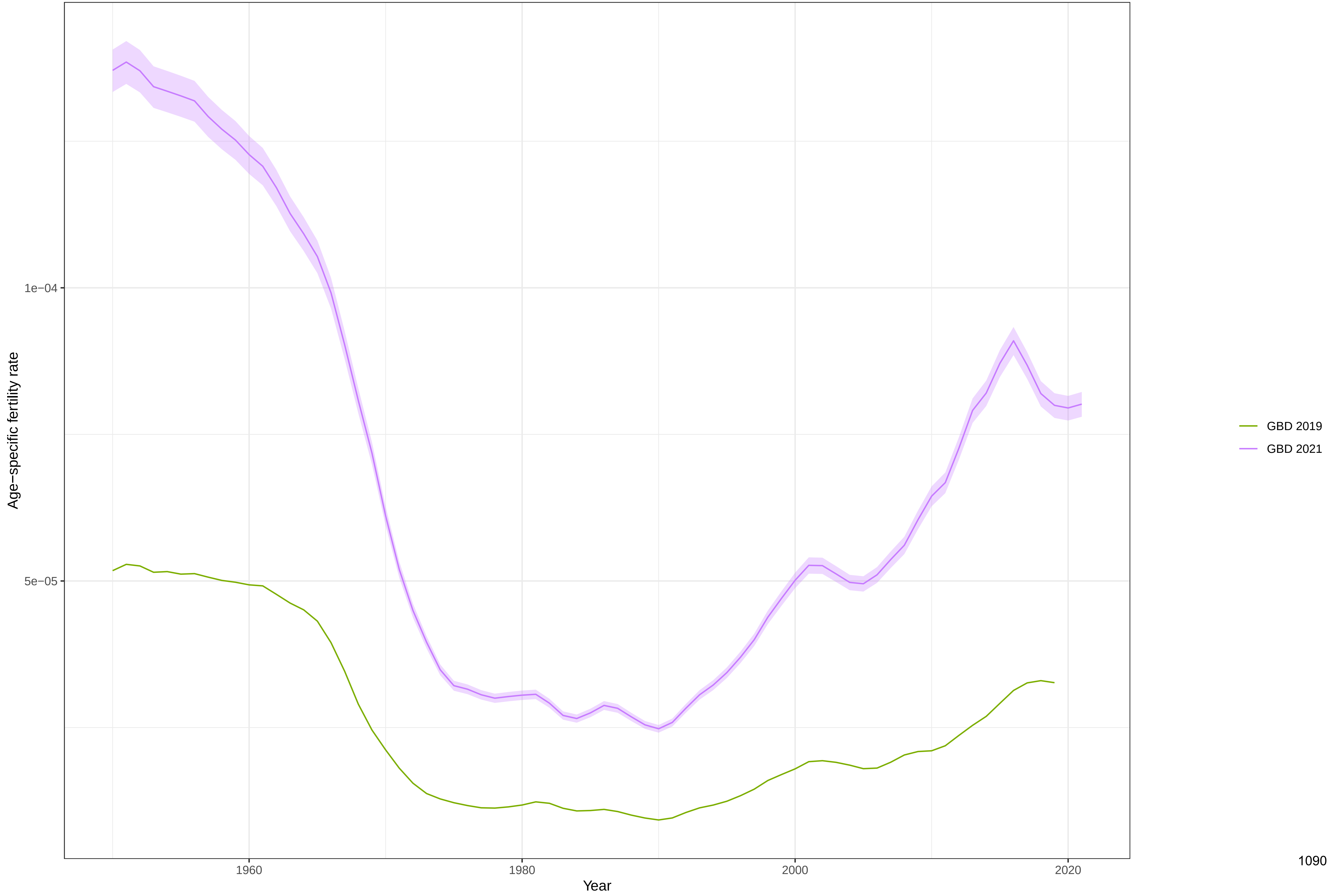

Total fertility rate fitted trend lines, 1950−2021: Minnesota TFR
Data points enclosed with a diamond are reference sources

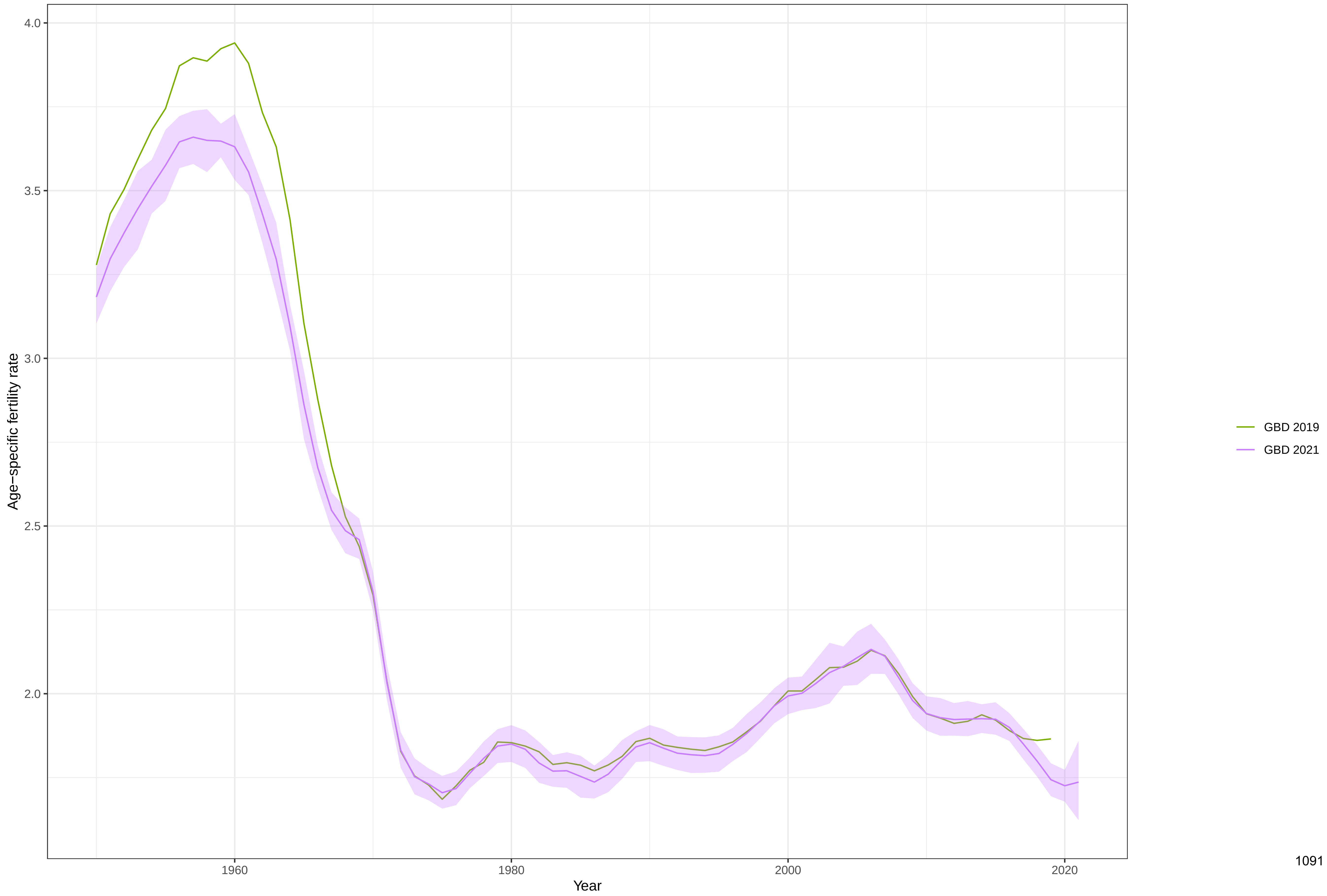

Age−specific fertility fitted trend lines, 1950−2021: Mississippi, Ages: 10−14
Data points enclosed with a diamond are reference sources

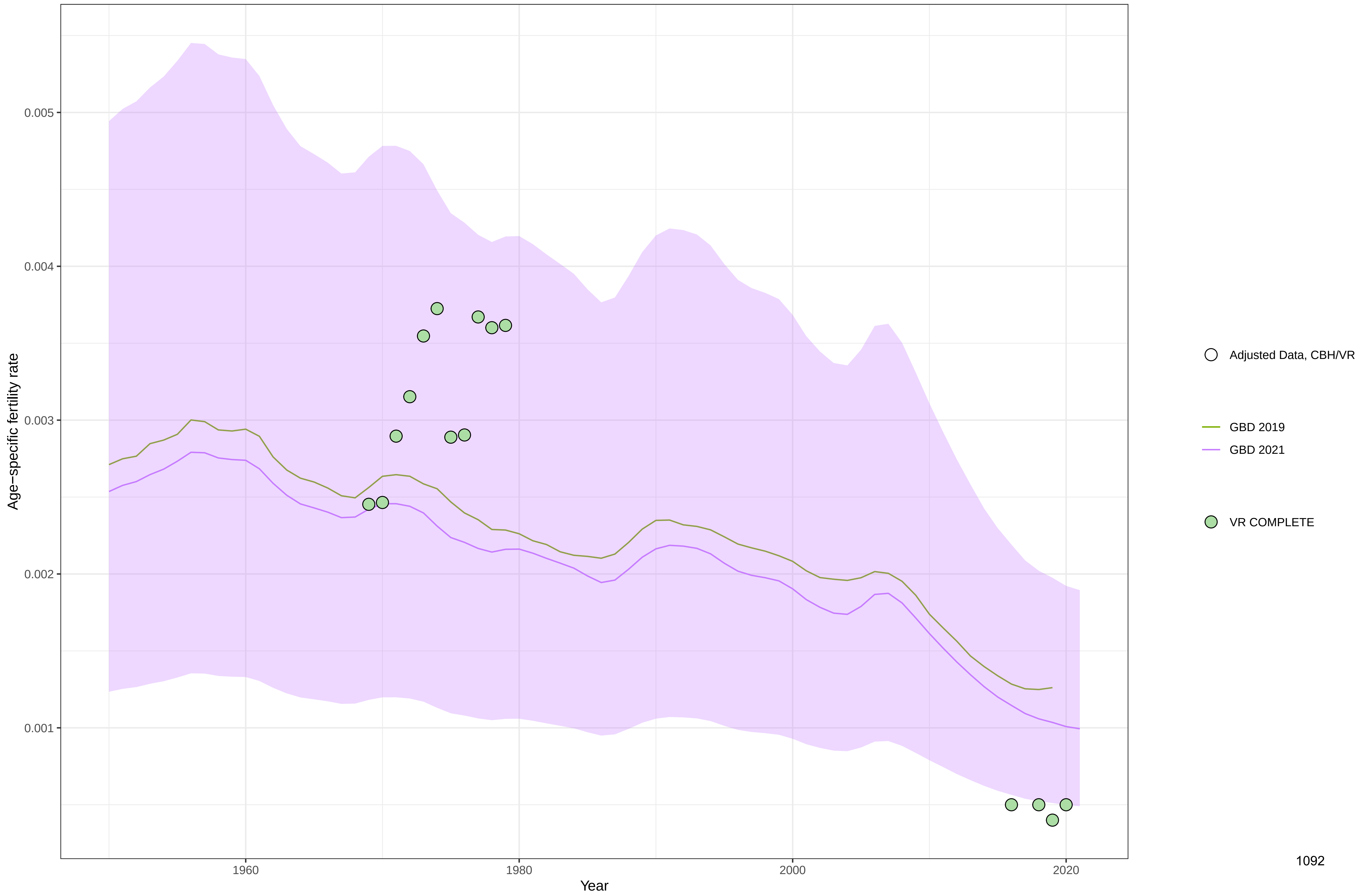

Age−specific fertility fitted trend lines, 1950−2021: Mississippi, Ages: 15−19
Data points enclosed with a diamond are reference sources

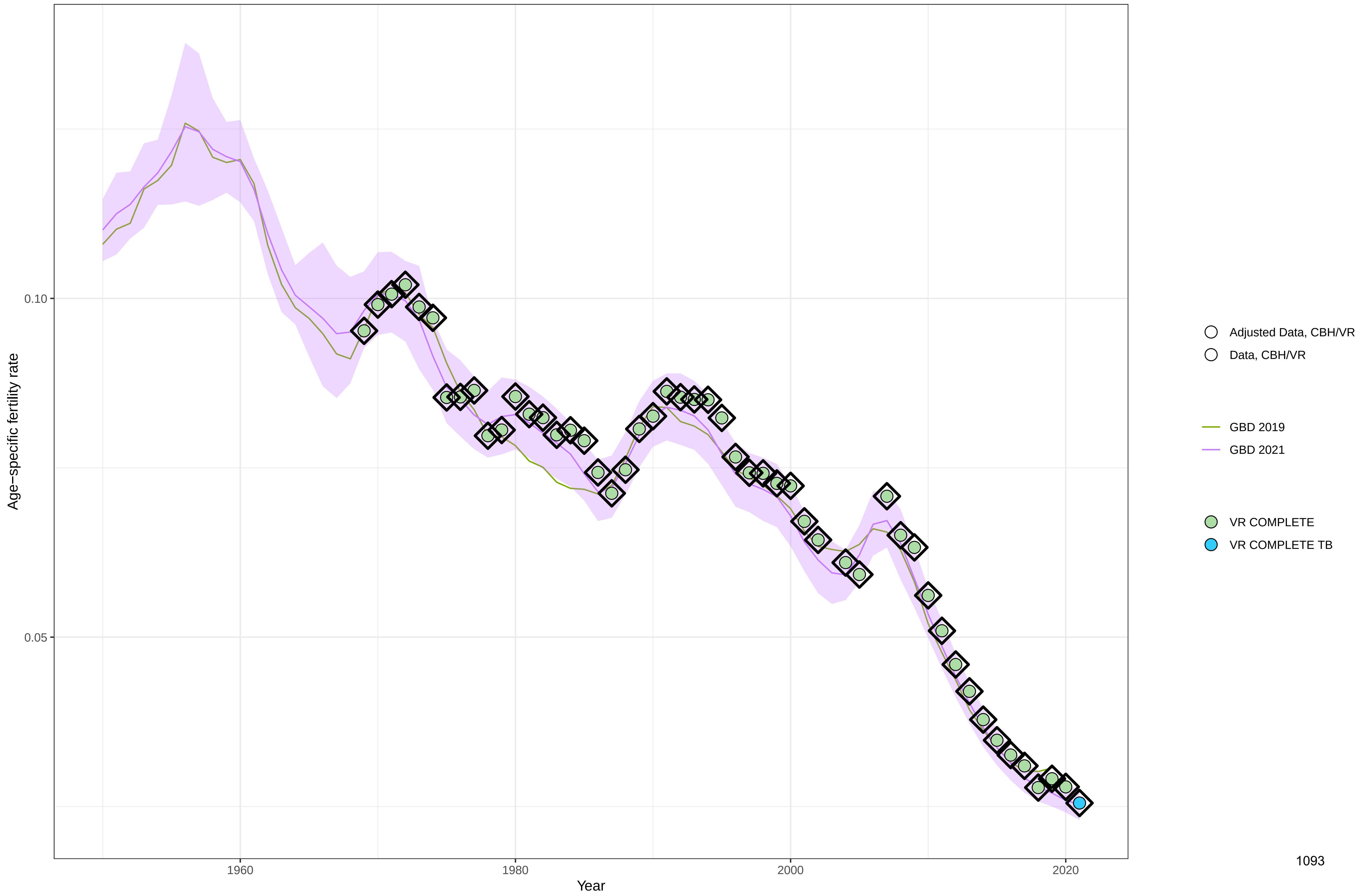

Age−specific fertility fitted trend lines, 1950−2021: Mississippi, Ages: 20−24
Data points enclosed with a diamond are reference sources

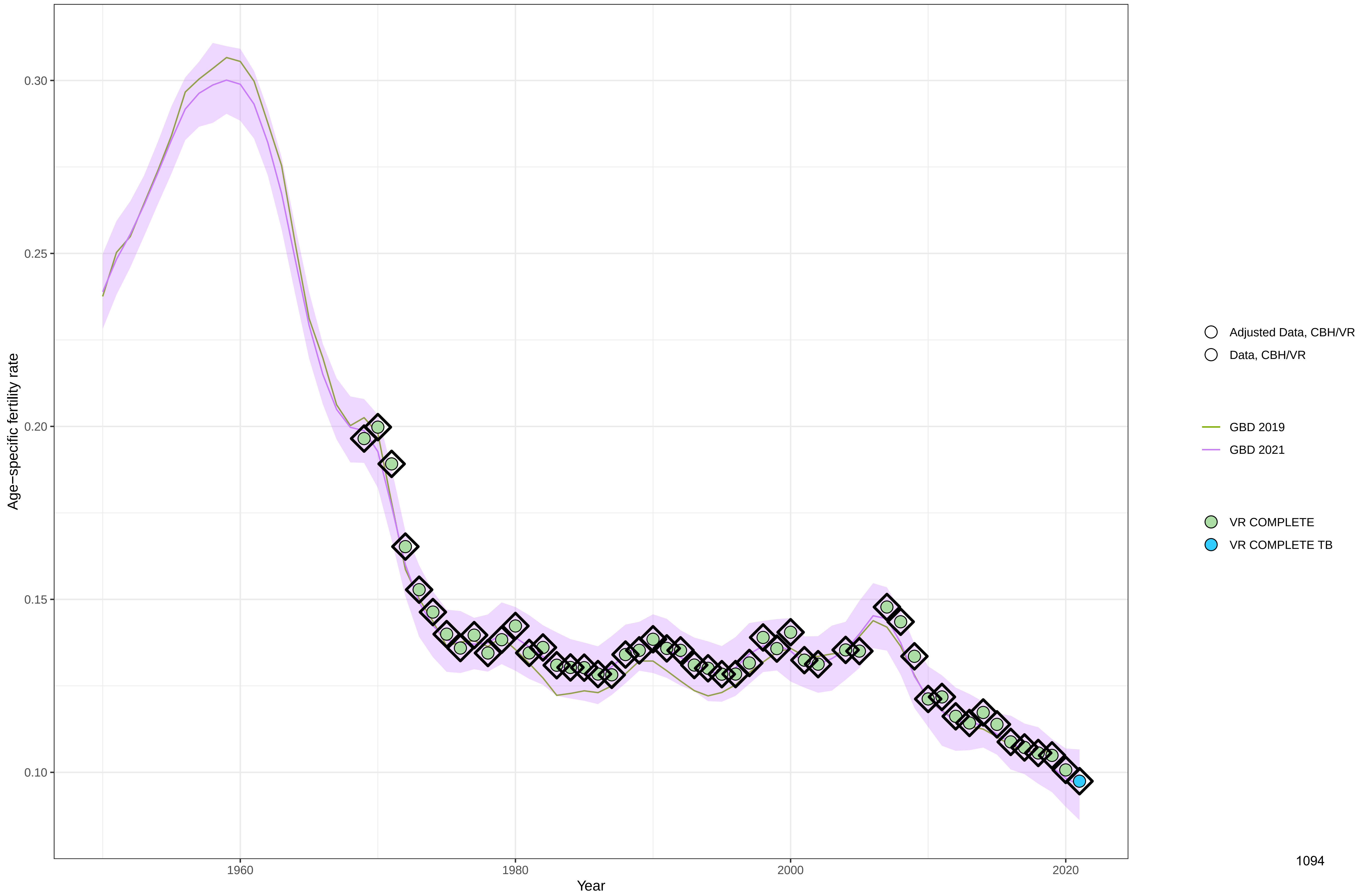

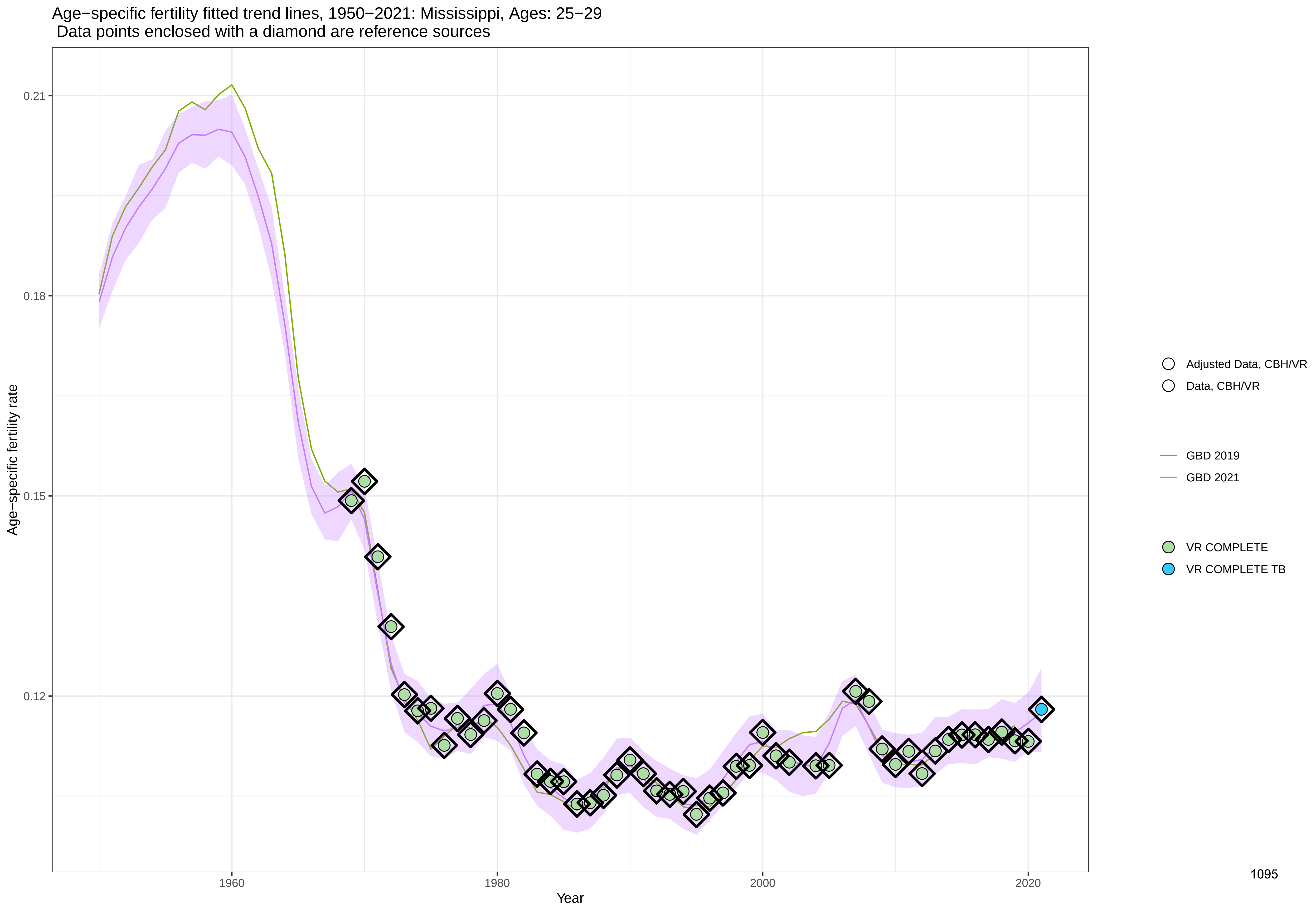

Age−specific fertility fitted trend lines, 1950−2021: Mississippi, Ages: 25−29
Data points enclosed with a diamond are reference sources
Age−specific fertility rate
0.21
0.18
0.15
0.12
1960
1980
2000
2020
Year
Adjusted Data, CBH/VR
Data, CBH/VR
GBD 2019
GBD 2021
VR COMPLETE
VR COMPLETE TB

Age−specific fertility fitted trend lines, 1950−2021: Mississippi, Ages: 30−34
Data points enclosed with a diamond are reference sources

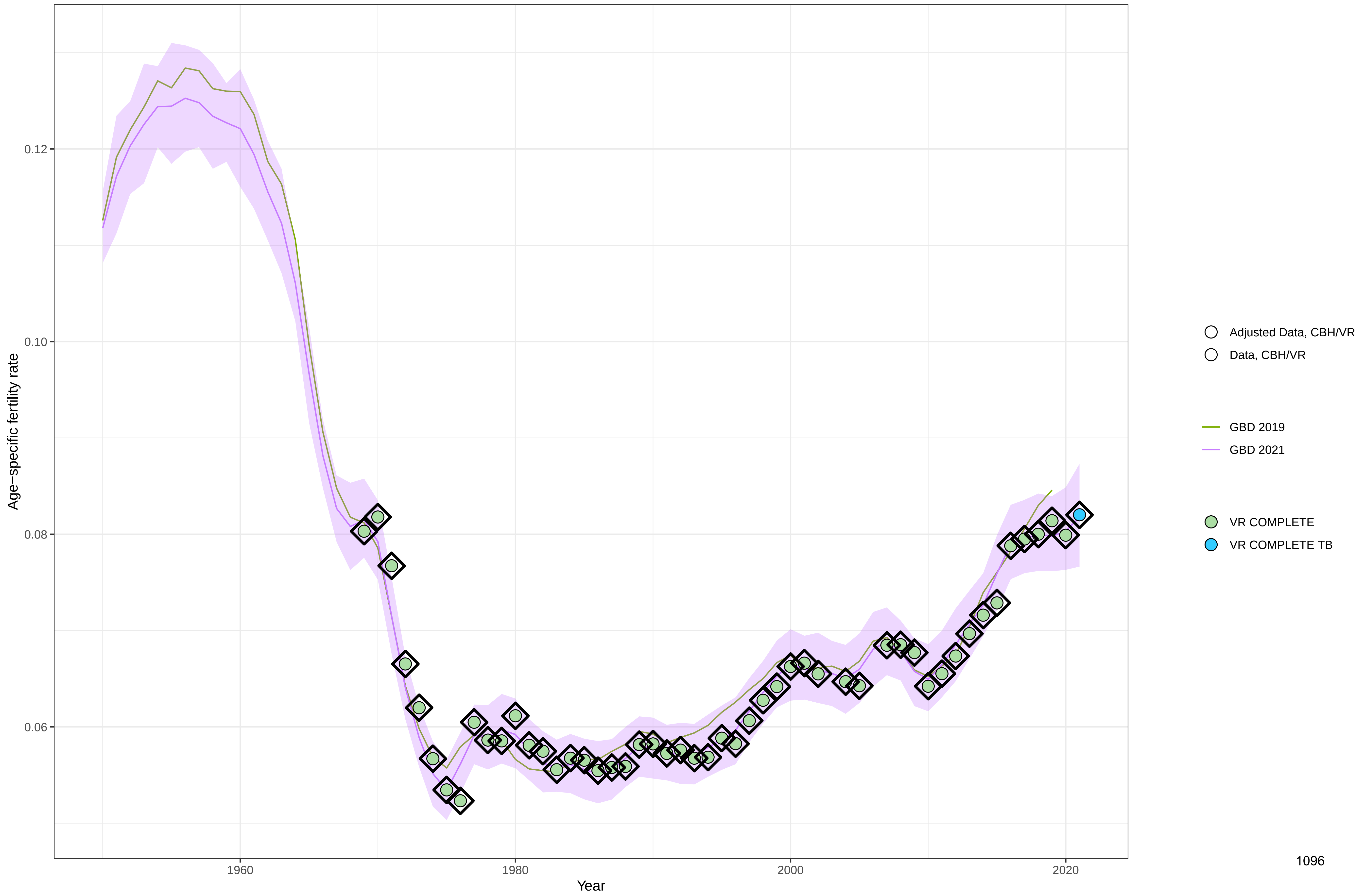

Age−specific fertility fitted trend lines, 1950−2021: Mississippi, Ages: 35−39
Data points enclosed with a diamond are reference sources

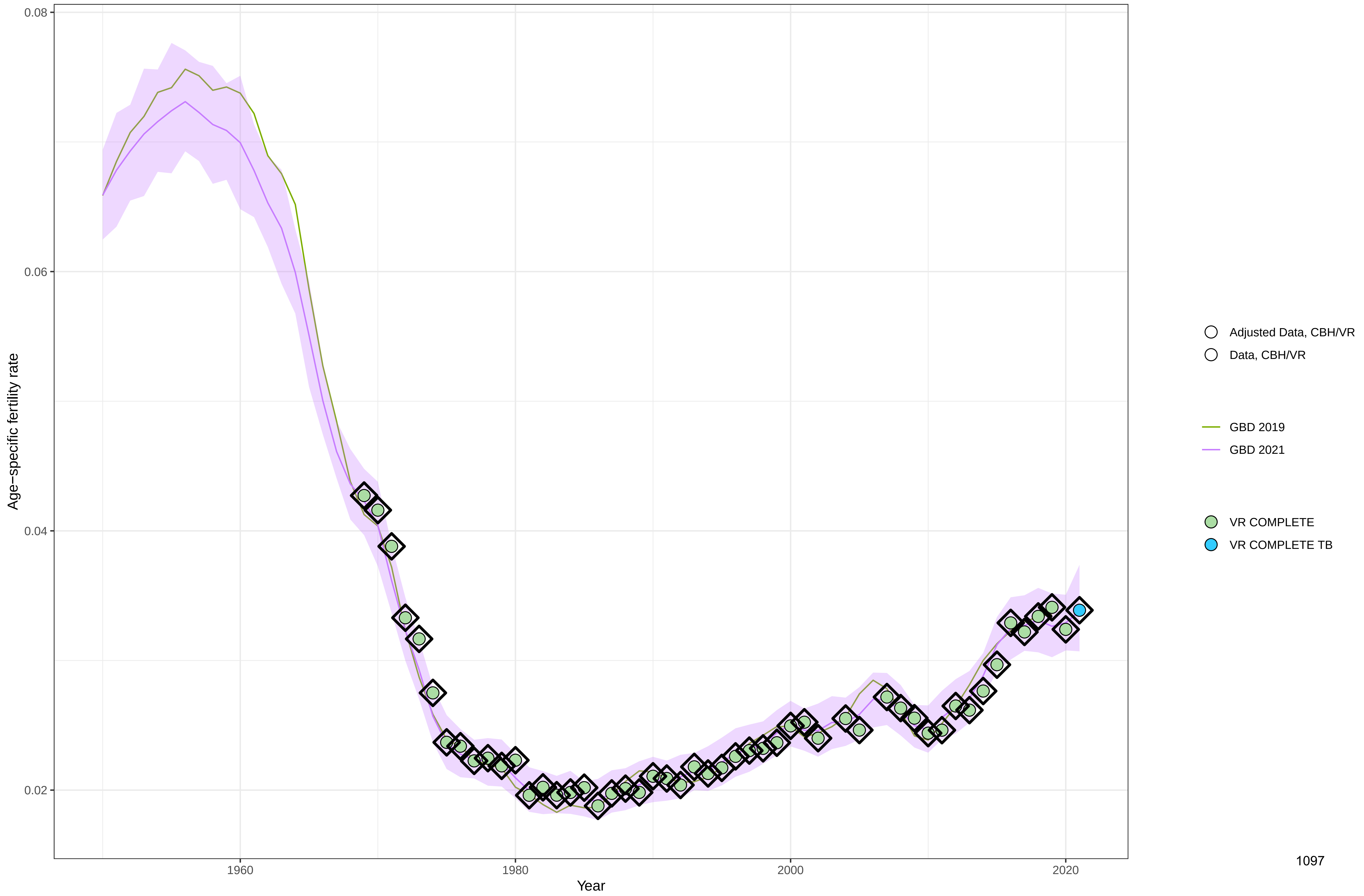

Age−specific fertility fitted trend lines, 1950−2021: Mississippi, Ages: 40−44
Data points enclosed with a diamond are reference sources

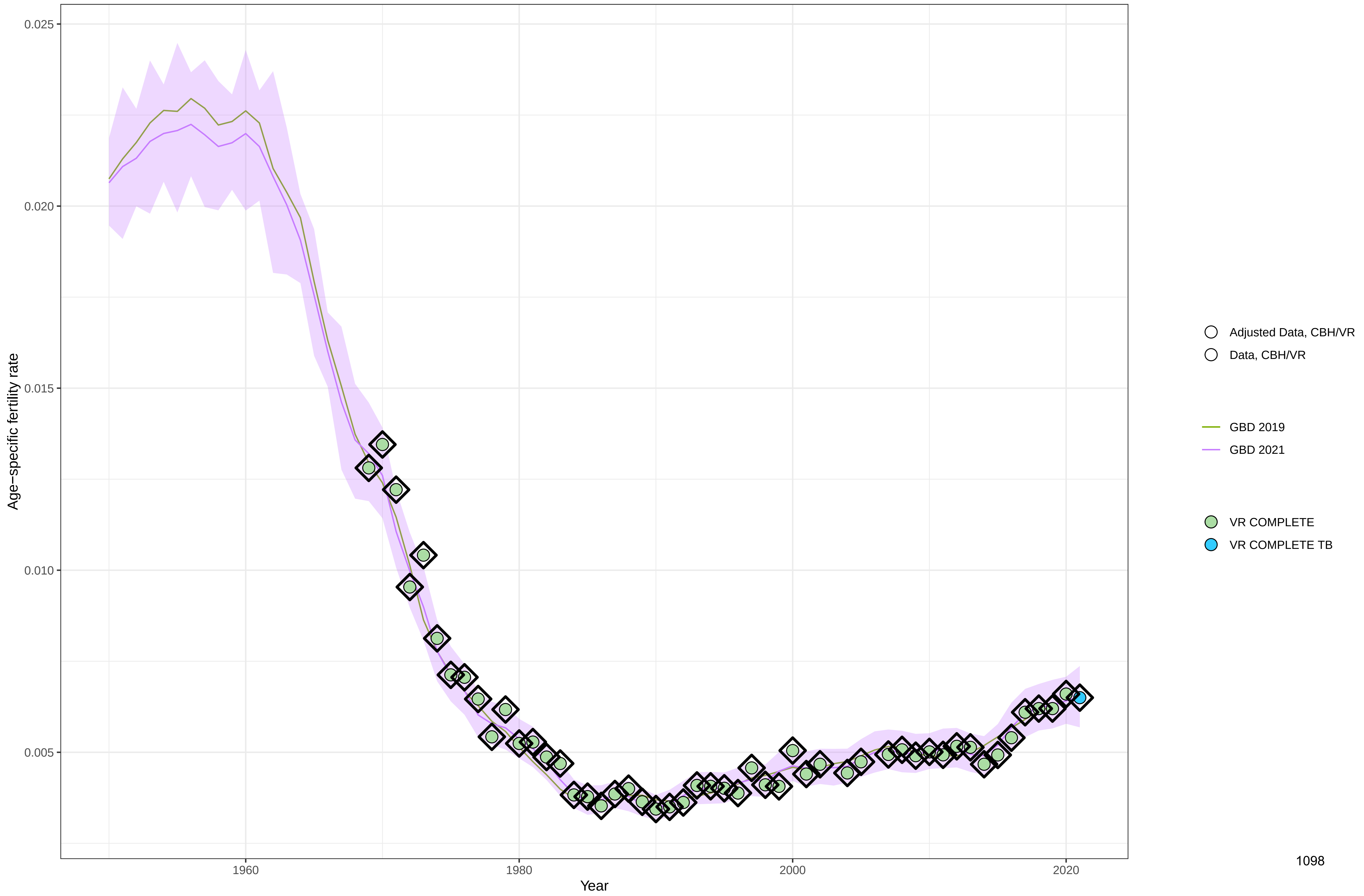

Age−specific fertility fitted trend lines, 1950−2021: Mississippi, Ages: 45−49
Data points enclosed with a diamond are reference sources

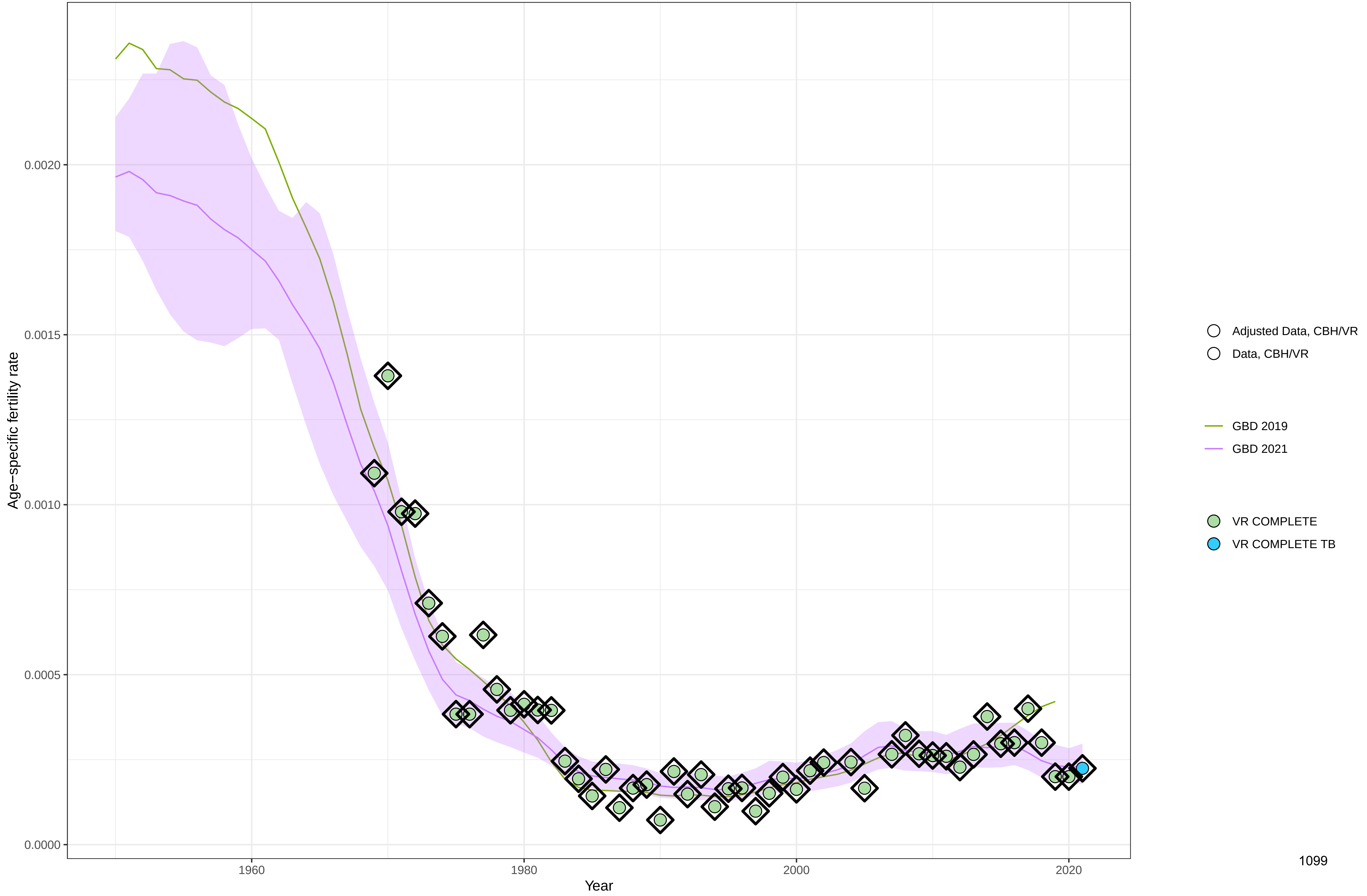

Age−specific fertility fitted trend lines, 1950−2021: Mississippi, Ages: 50−54
Data points enclosed with a diamond are reference sources

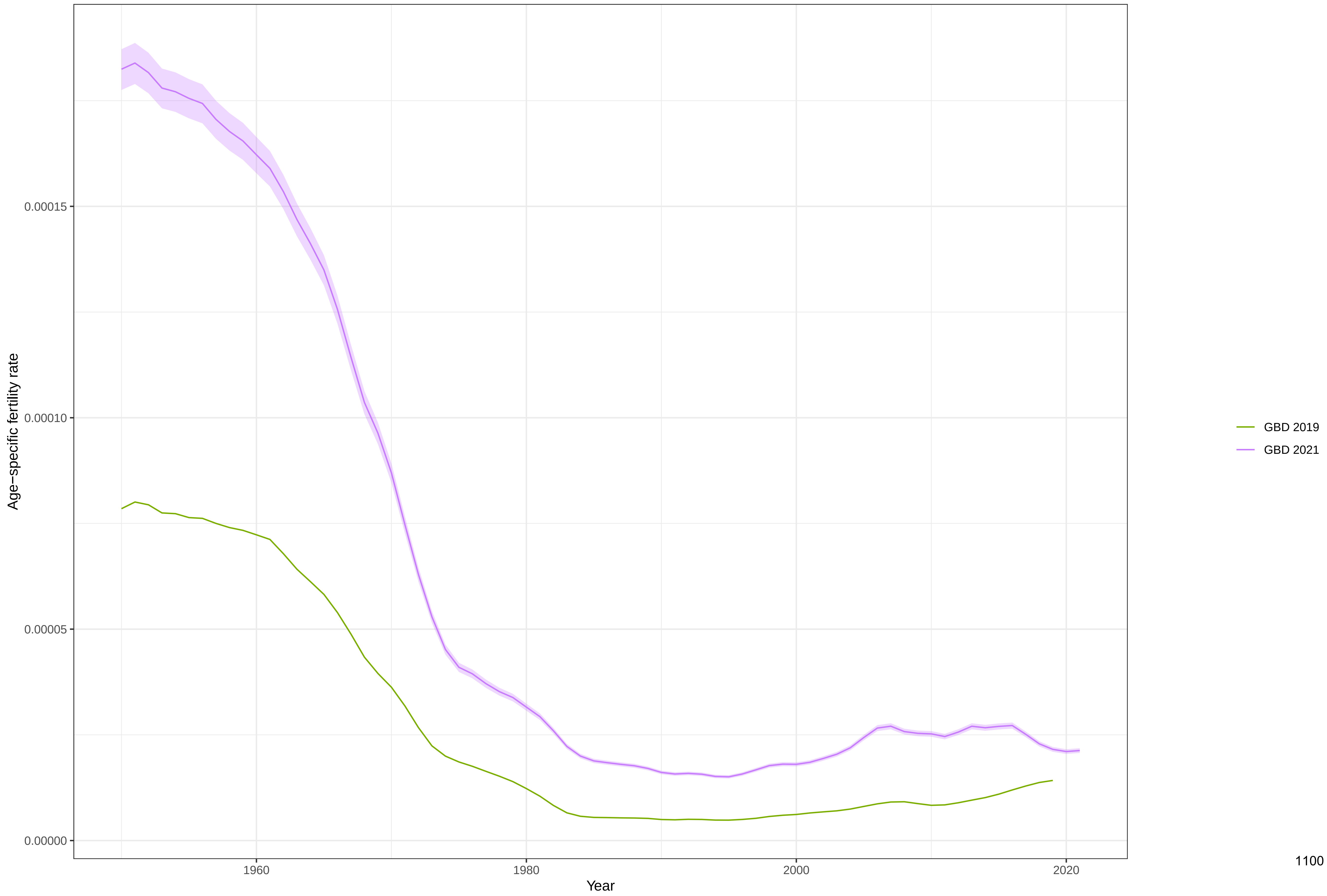

Total fertility rate fitted trend lines, 1950−2021: Mississippi TFR
Data points enclosed with a diamond are reference sources

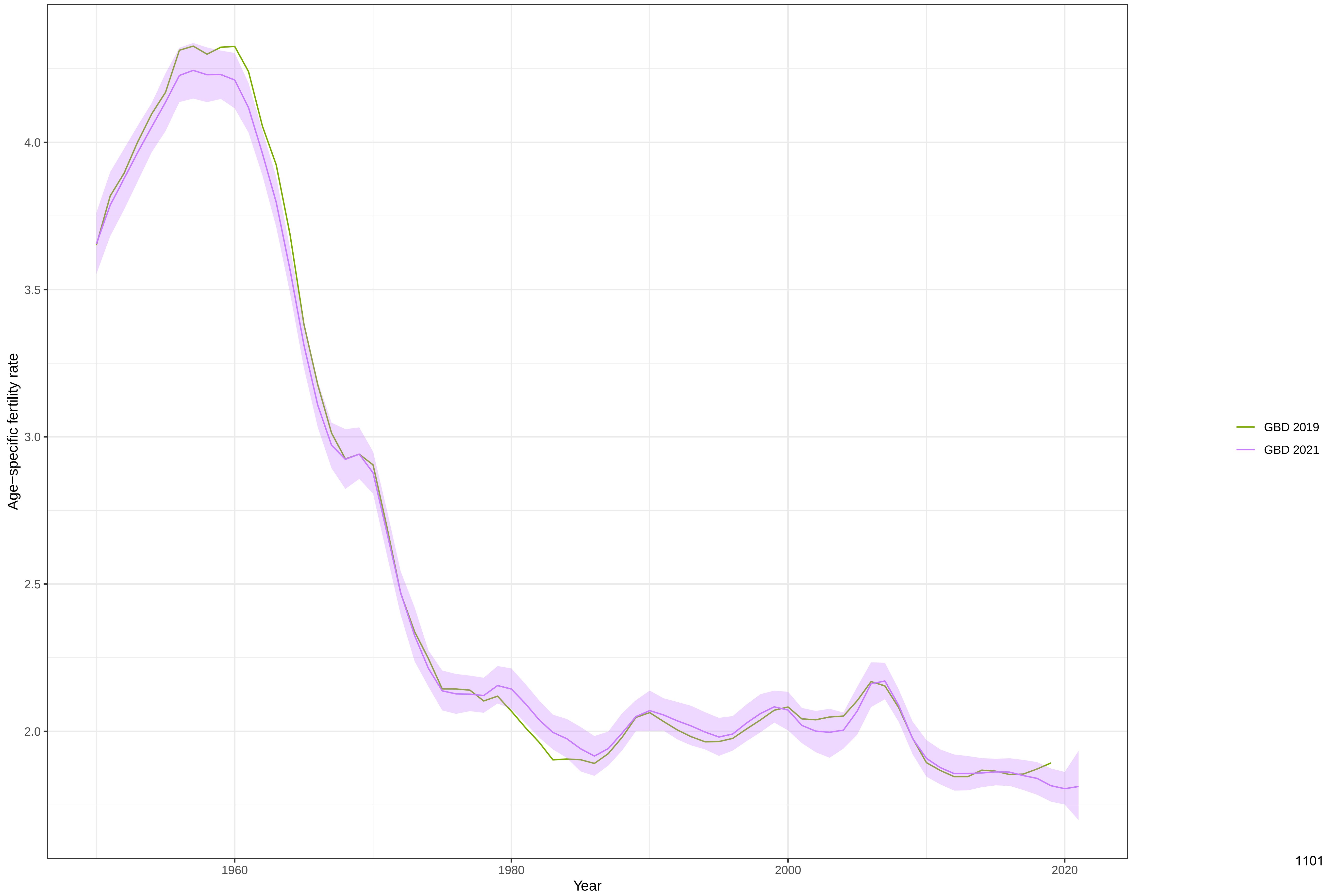

Age−specific fertility fitted trend lines, 1950−2021: Missouri, Ages: 10−14
Data points enclosed with a diamond are reference sources

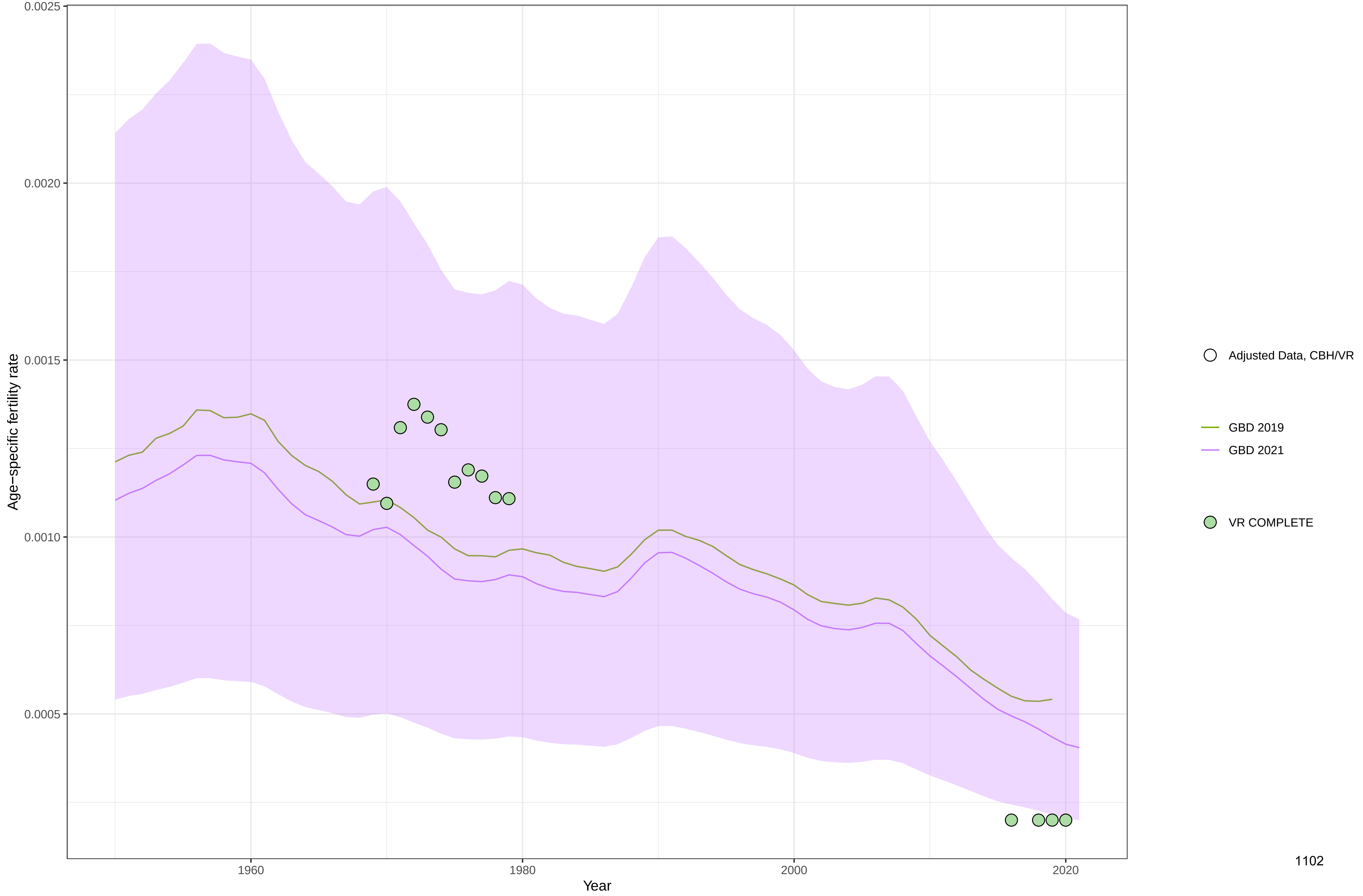

Age−specific fertility fitted trend lines, 1950−2021: Missouri, Ages: 15−19
Data points enclosed with a diamond are reference sources

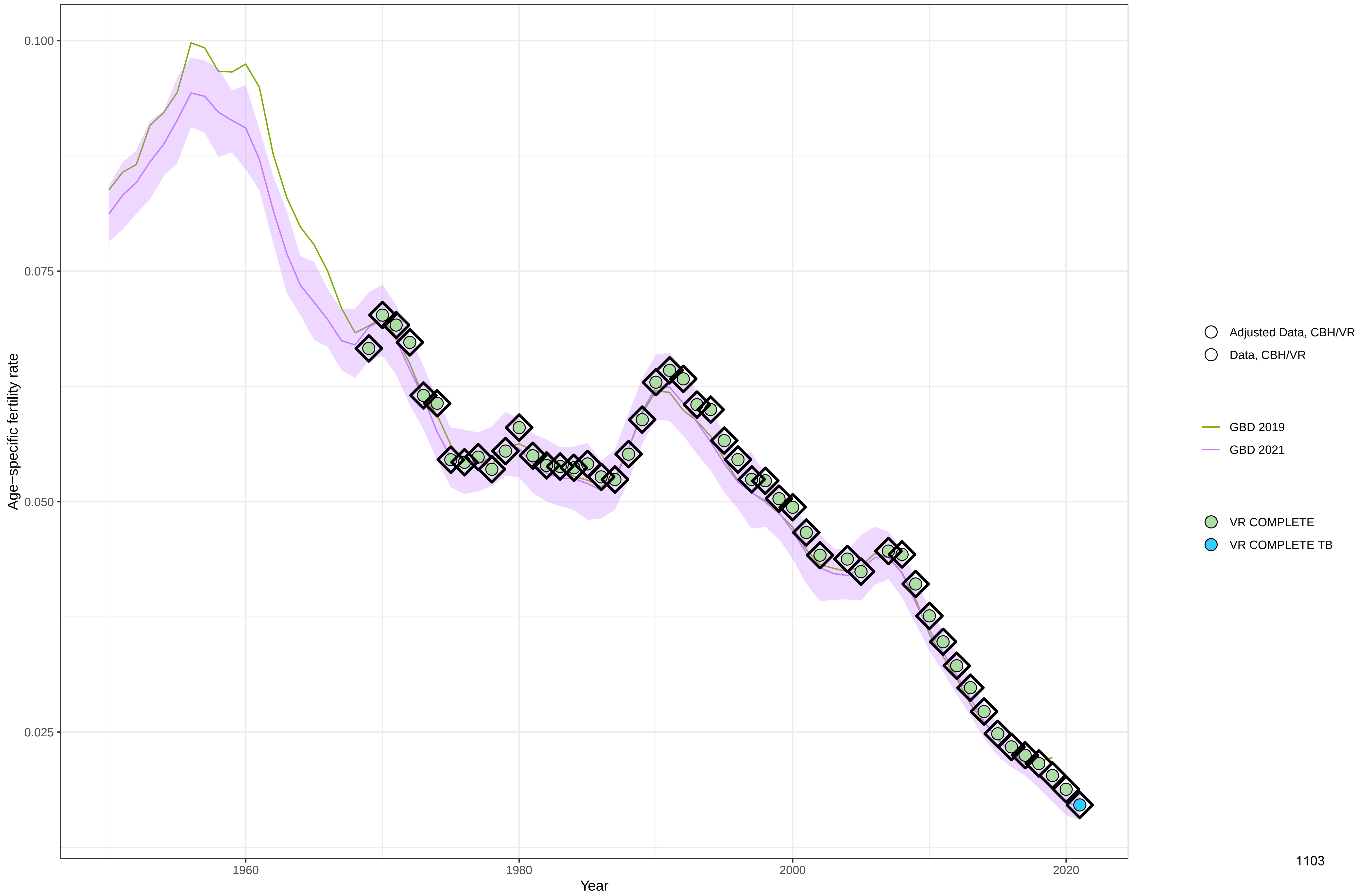

Age−specific fertility fitted trend lines, 1950−2021: Missouri, Ages: 20−24
Data points enclosed with a diamond are reference sources

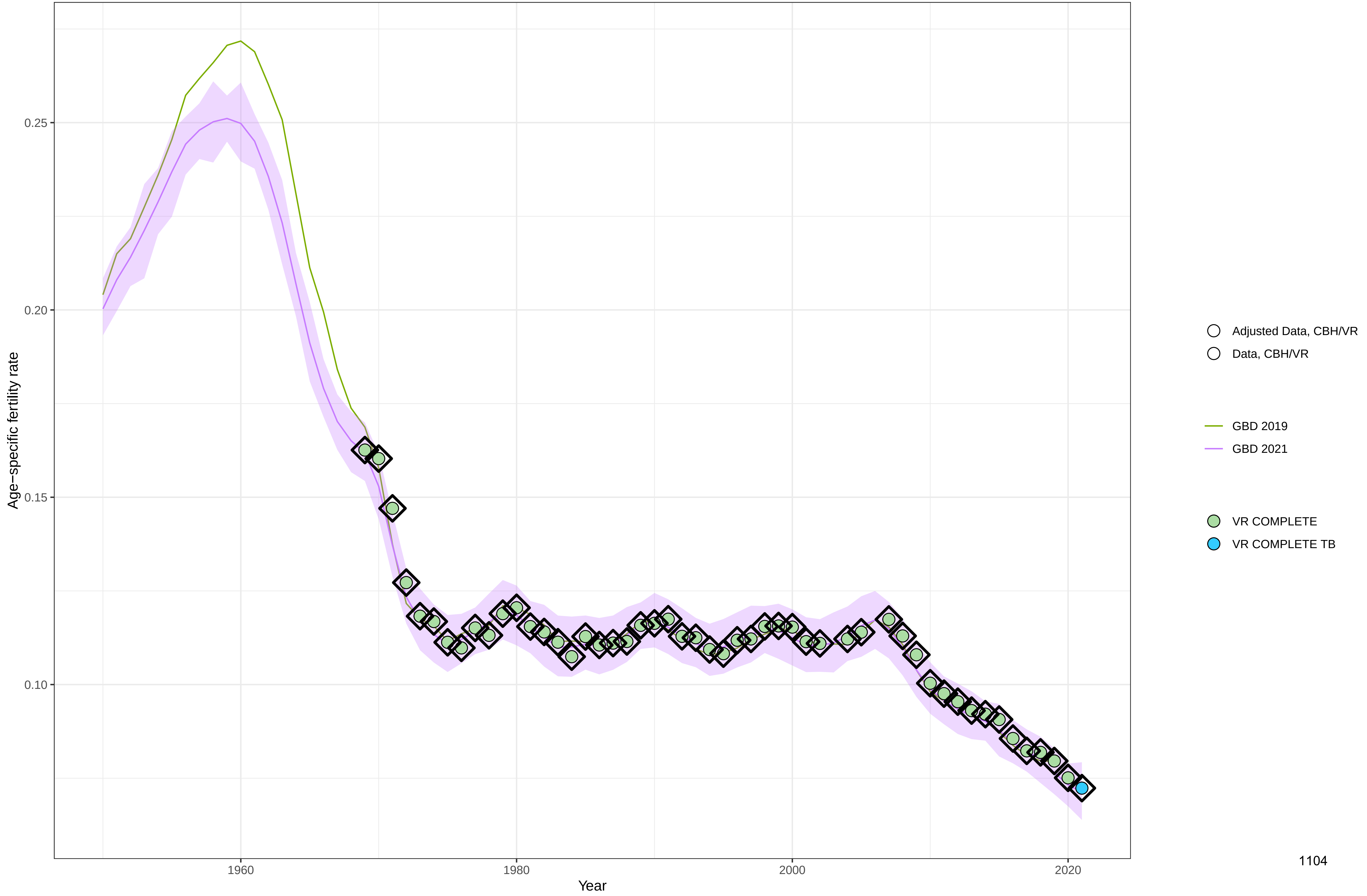

Age−specific fertility fitted trend lines, 1950−2021: Missouri, Ages: 25−29
Data points enclosed with a diamond are reference sources

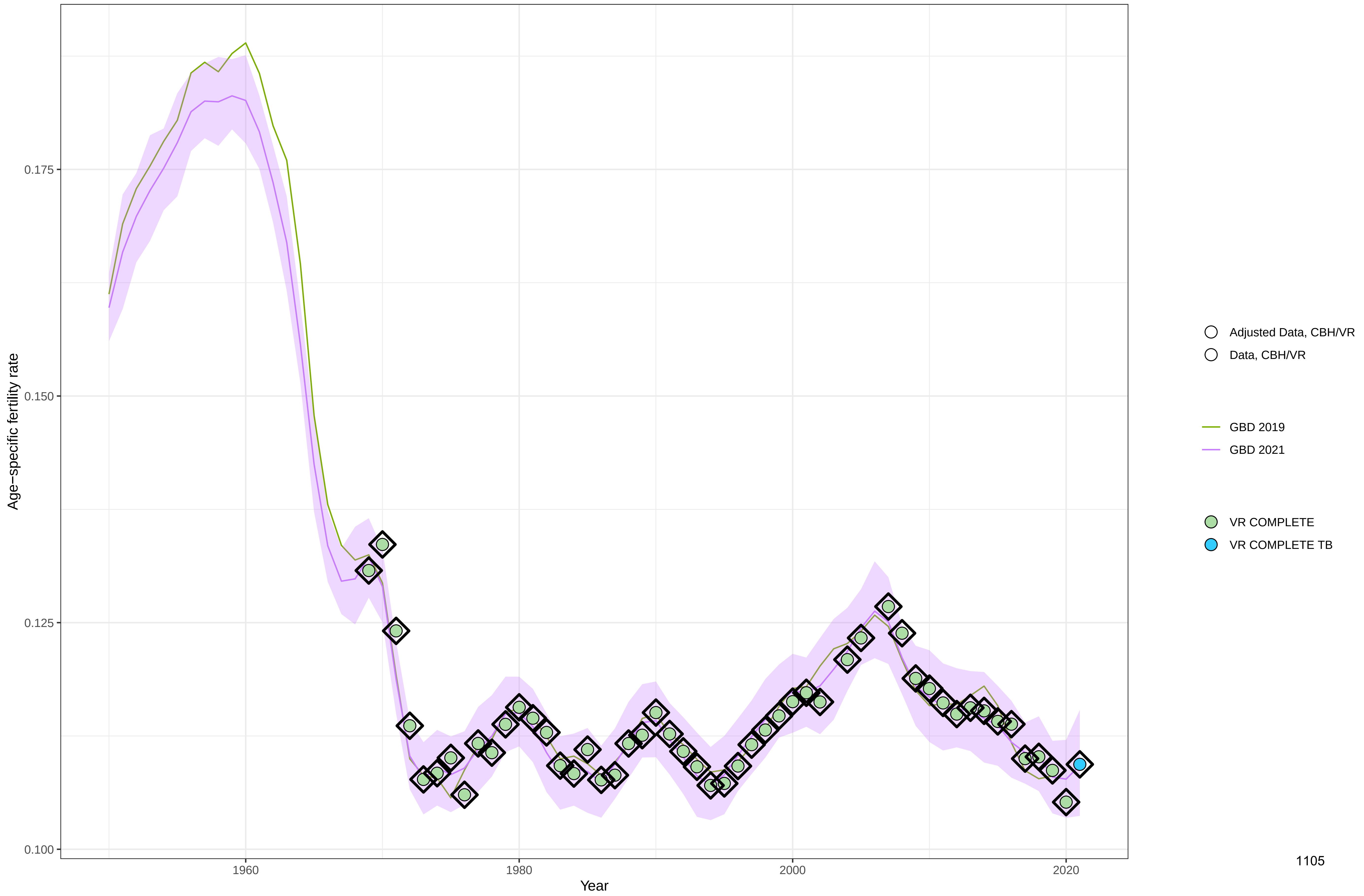

Age−specific fertility fitted trend lines, 1950−2021: Missouri, Ages: 30−34
Data points enclosed with a diamond are reference sources

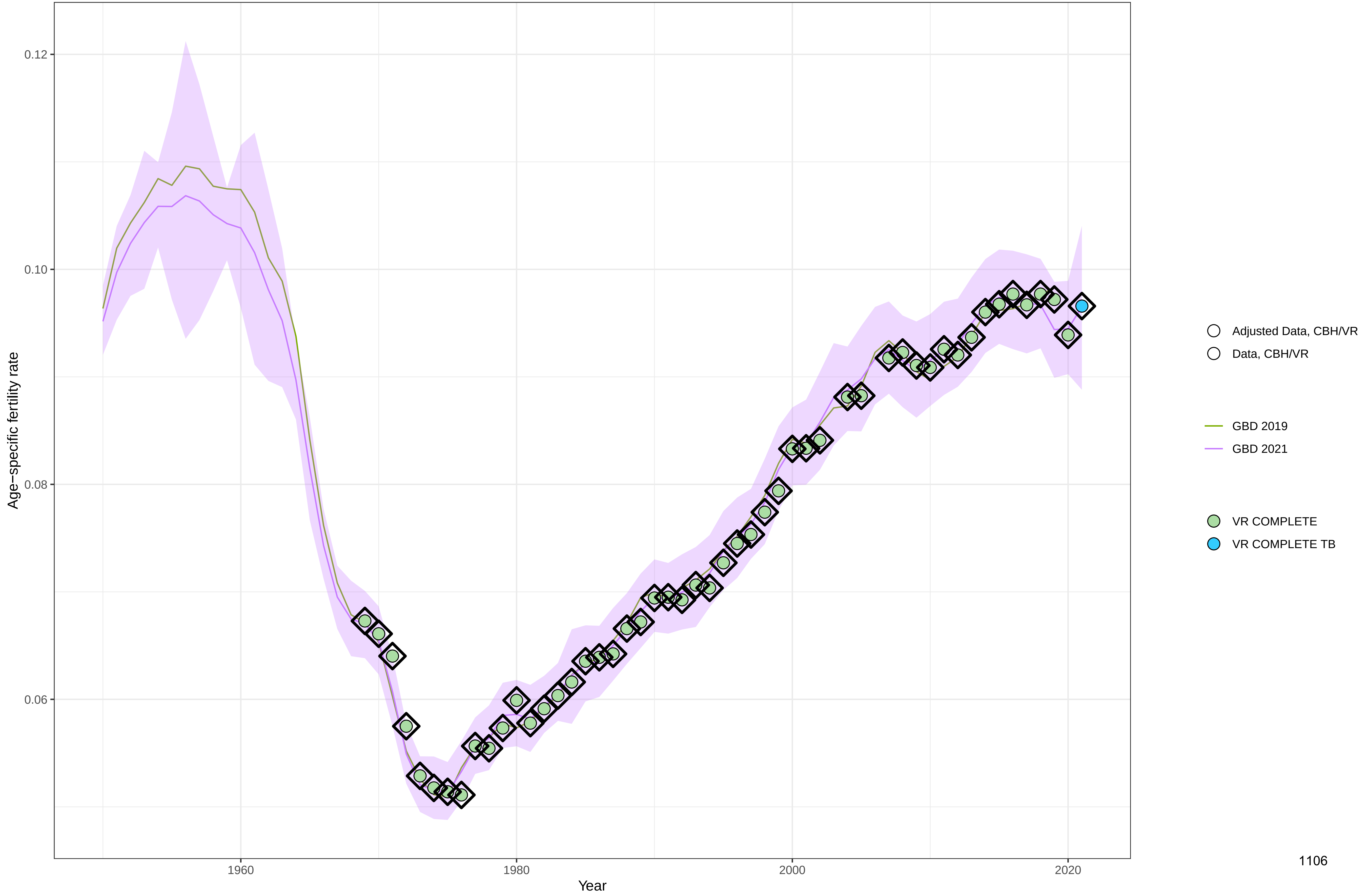

Age−specific fertility fitted trend lines, 1950−2021: Missouri, Ages: 35−39
Data points enclosed with a diamond are reference sources

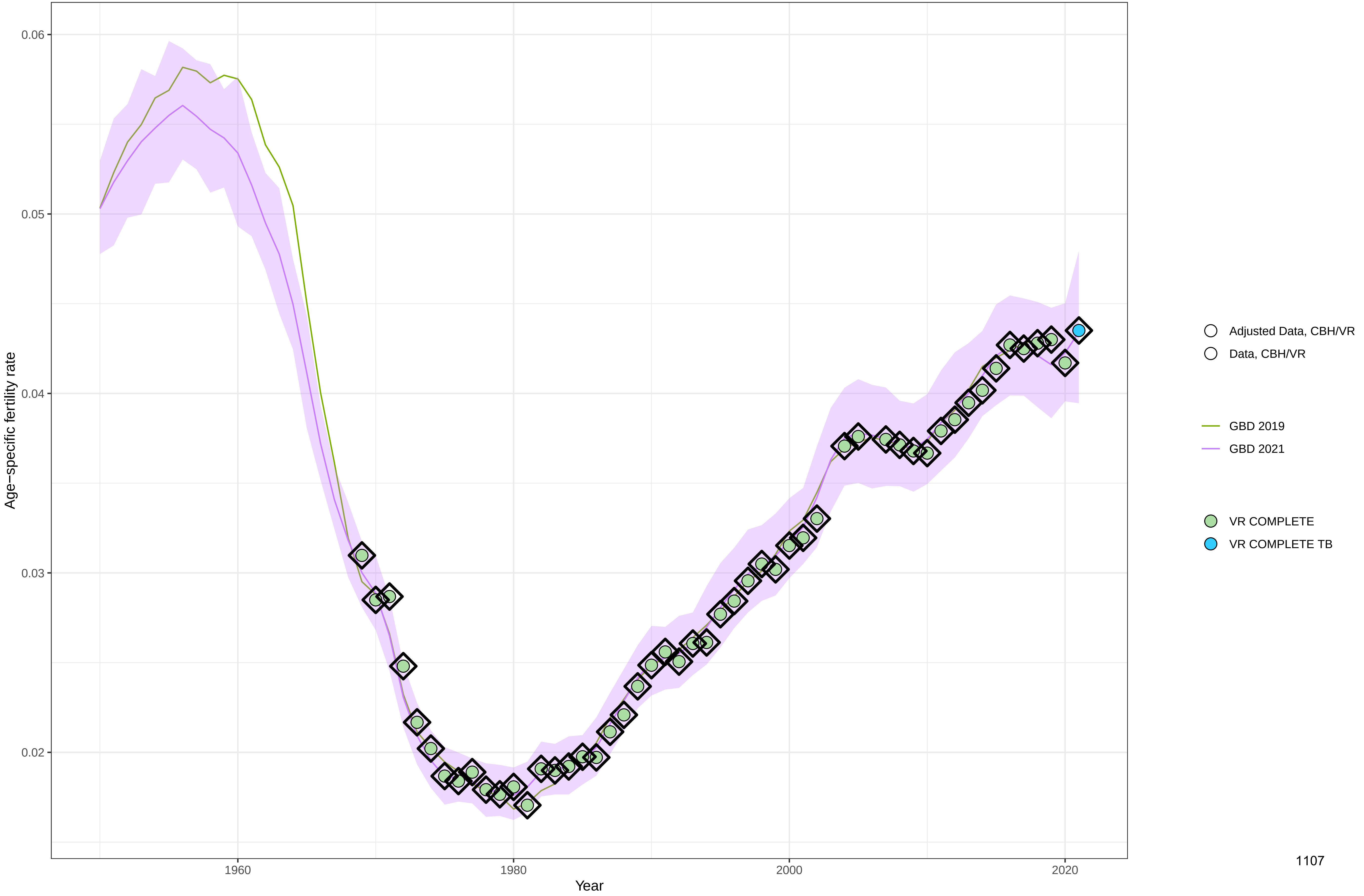

Age−specific fertility fitted trend lines, 1950−2021: Missouri, Ages: 40−44
Data points enclosed with a diamond are reference sources

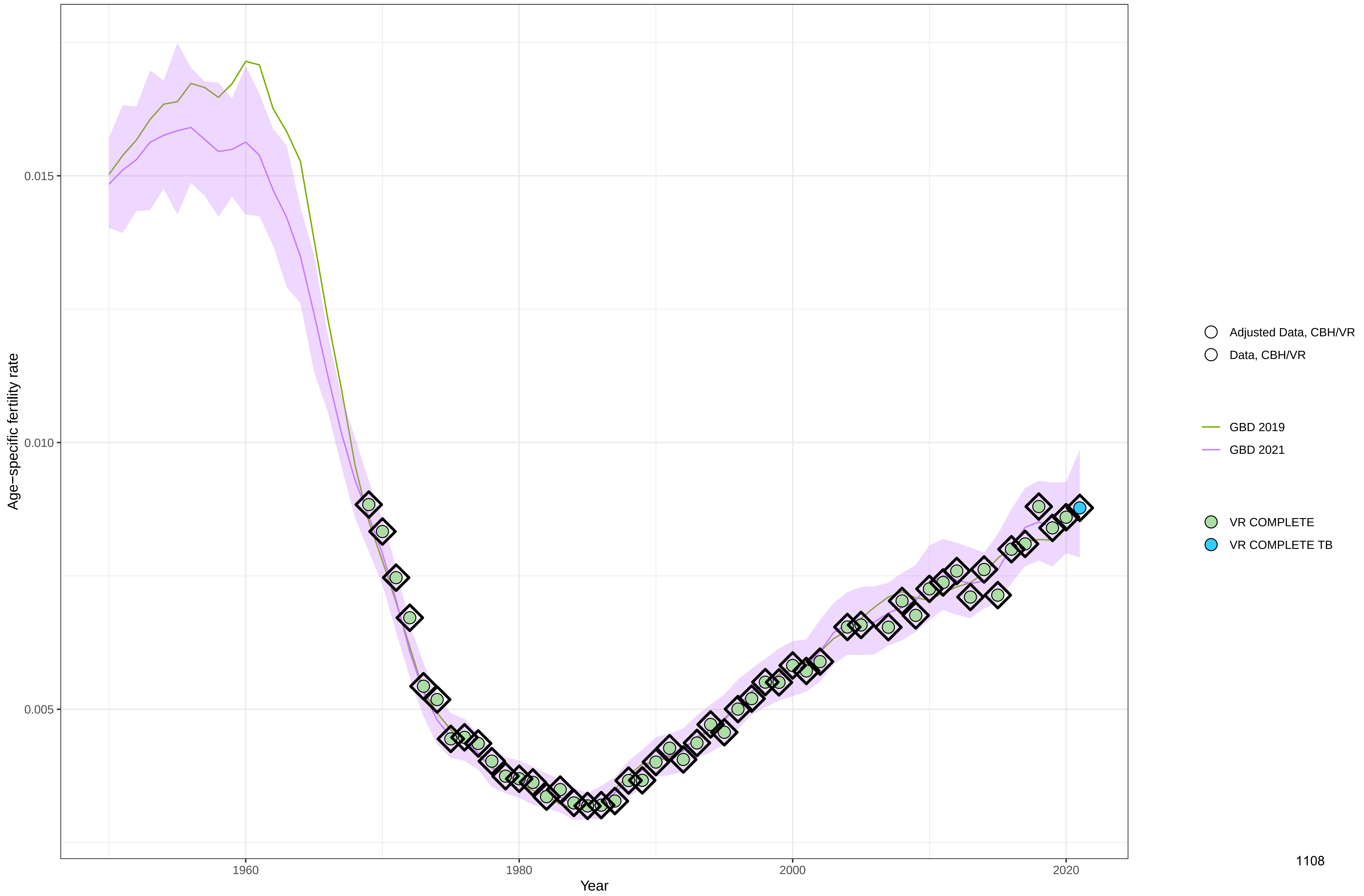

Age−specific fertility fitted trend lines, 1950−2021: Missouri, Ages: 45−49
Data points enclosed with a diamond are reference sources

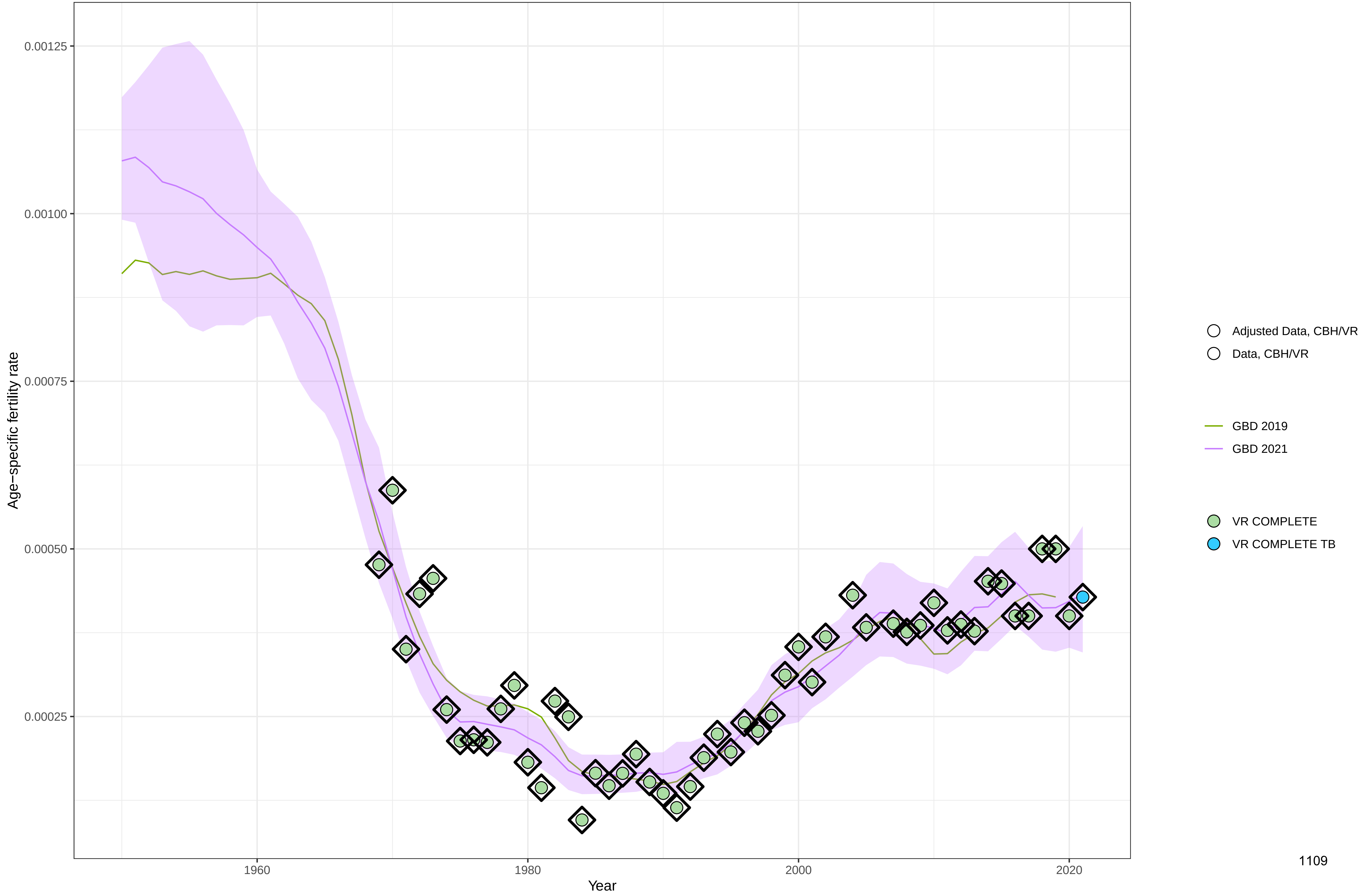

Age−specific fertility fitted trend lines, 1950−2021: Missouri, Ages: 50−54
Data points enclosed with a diamond are reference sources

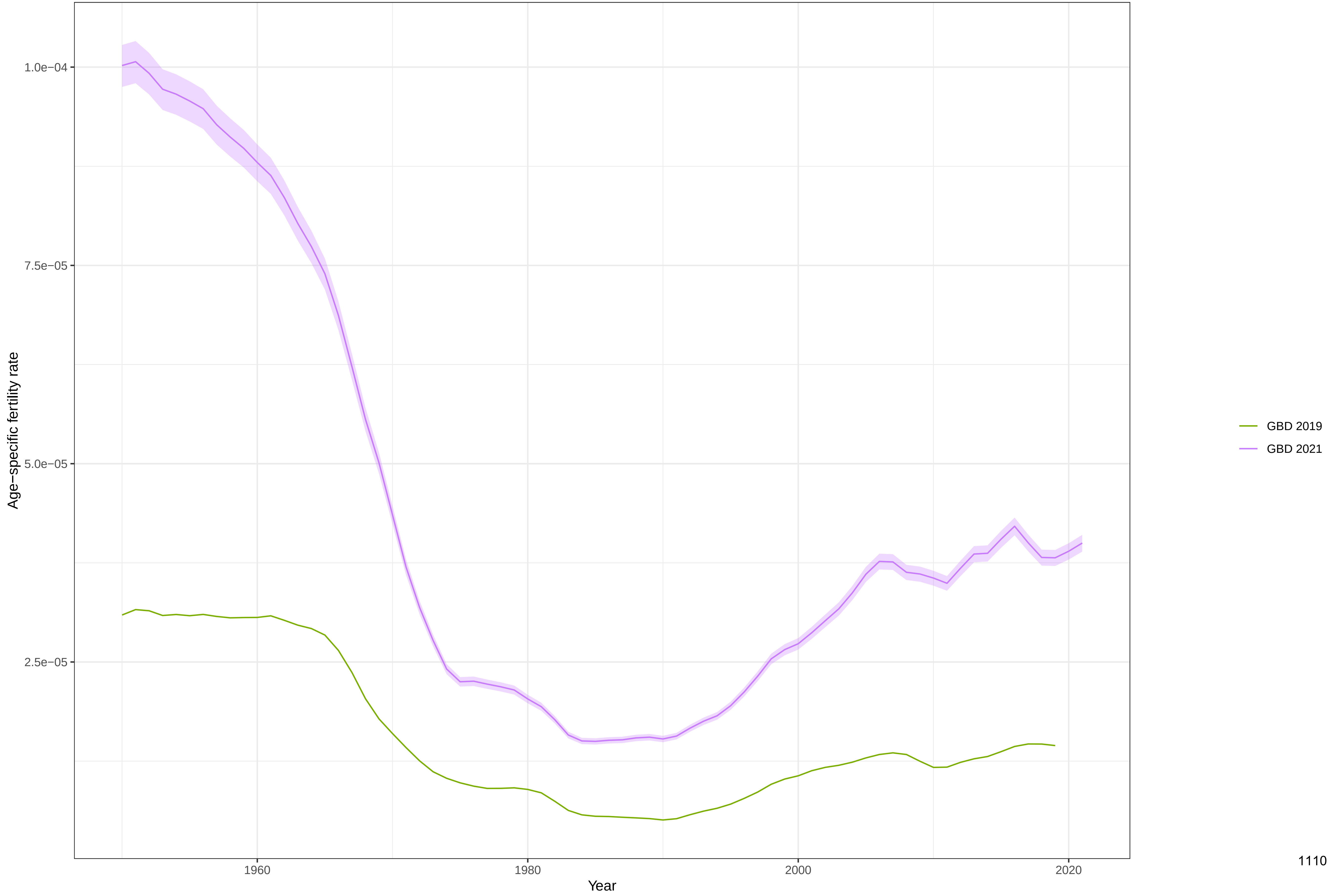

Total fertility rate fitted trend lines, 1950−2021: Missouri TFR
Data points enclosed with a diamond are reference sources

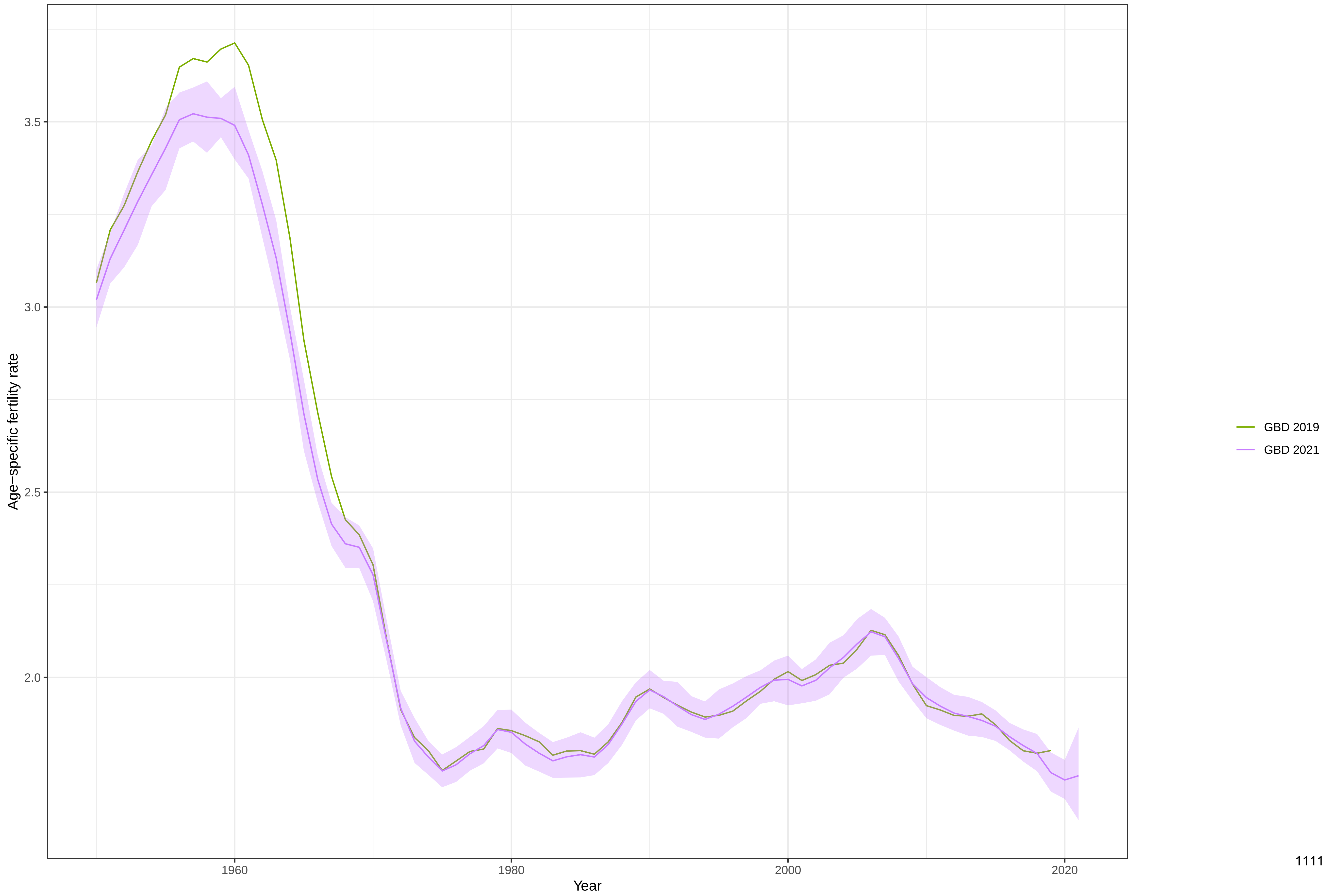

Age−specific fertility fitted trend lines, 1950−2021: Montana, Ages: 10−14
Data points enclosed with a diamond are reference sources

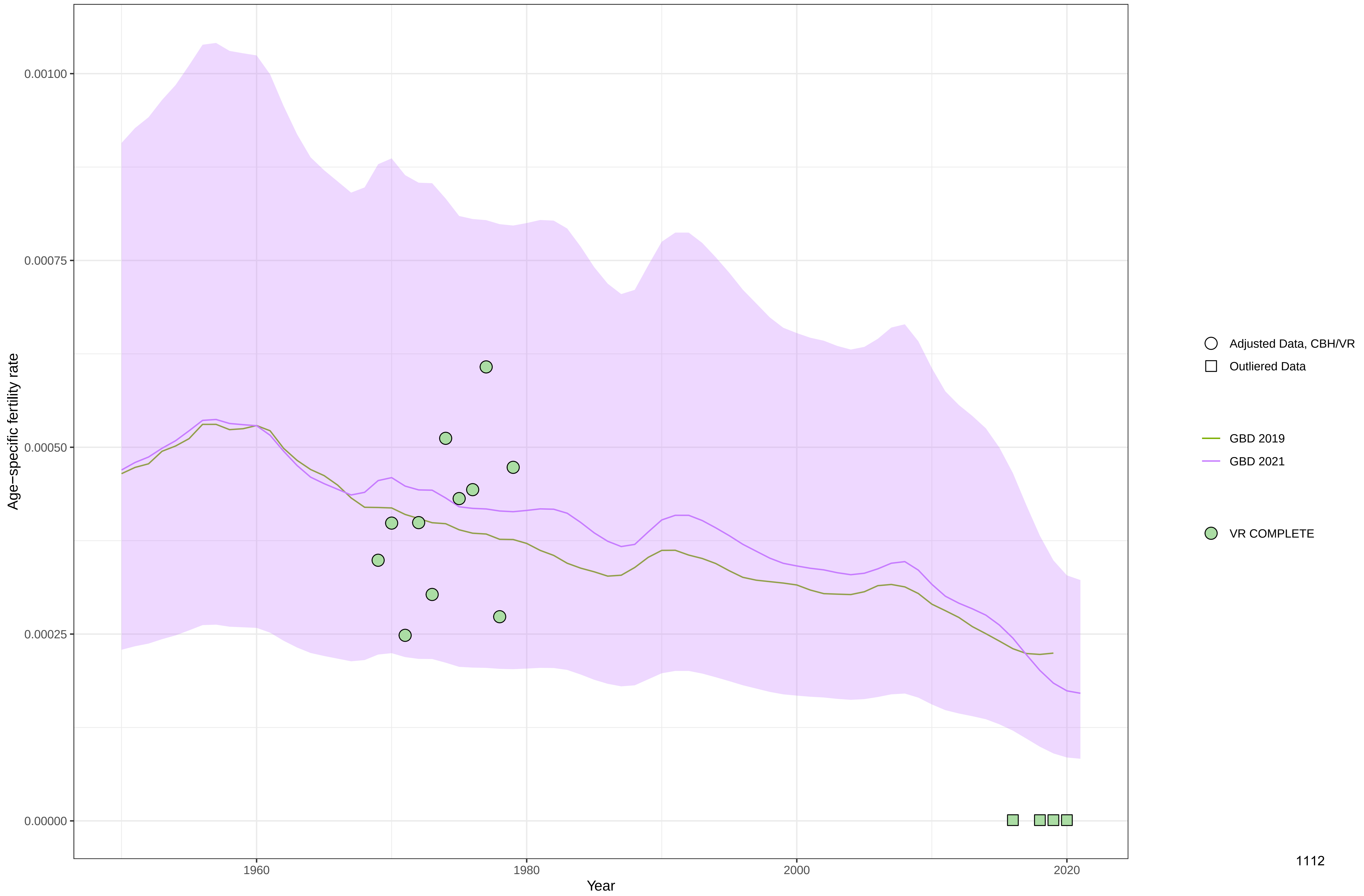

Age−specific fertility fitted trend lines, 1950−2021: Montana, Ages: 15−19
Data points enclosed with a diamond are reference sources

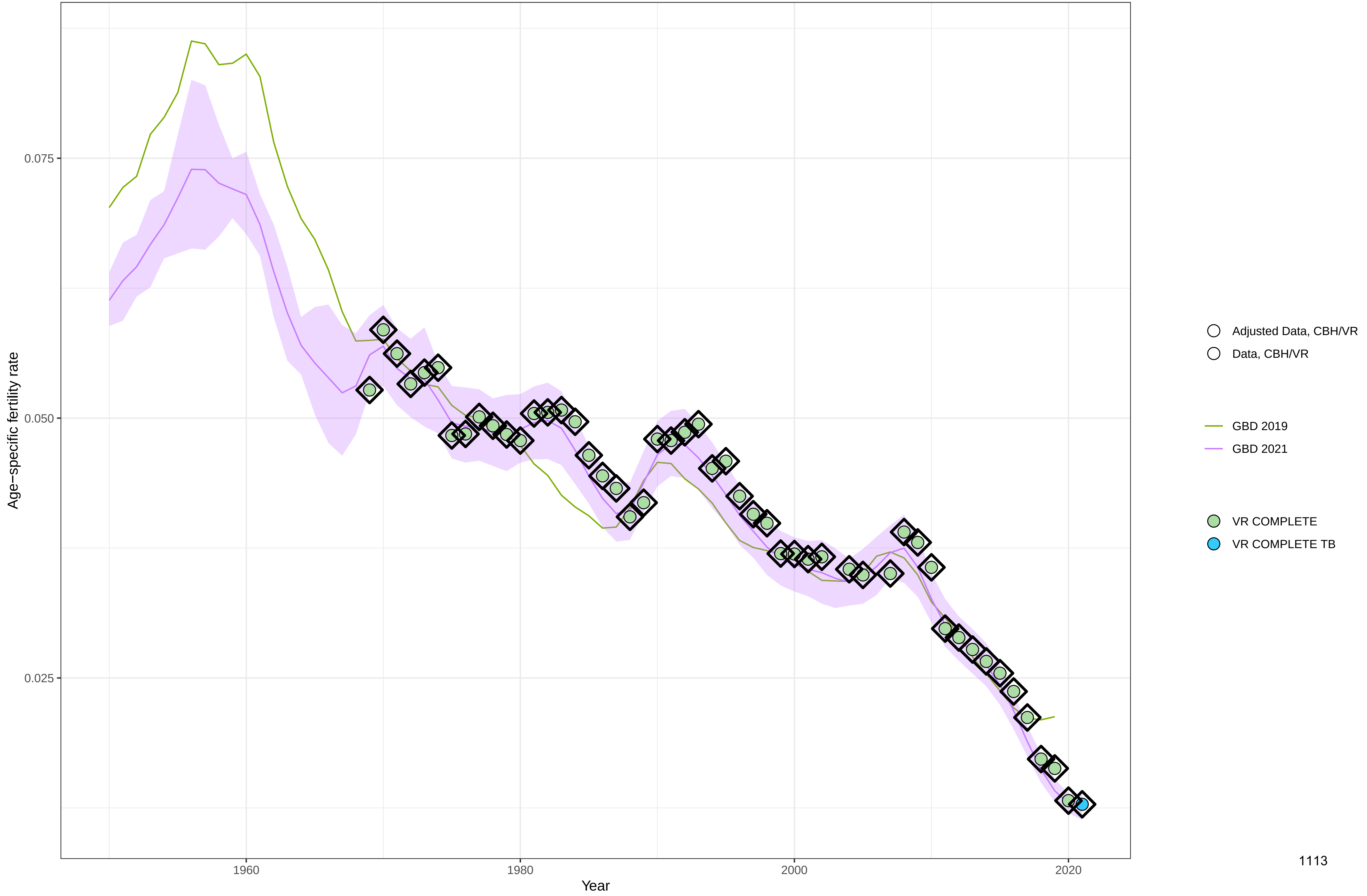

Age−specific fertility fitted trend lines, 1950−2021: Montana, Ages: 20−24
Data points enclosed with a diamond are reference sources

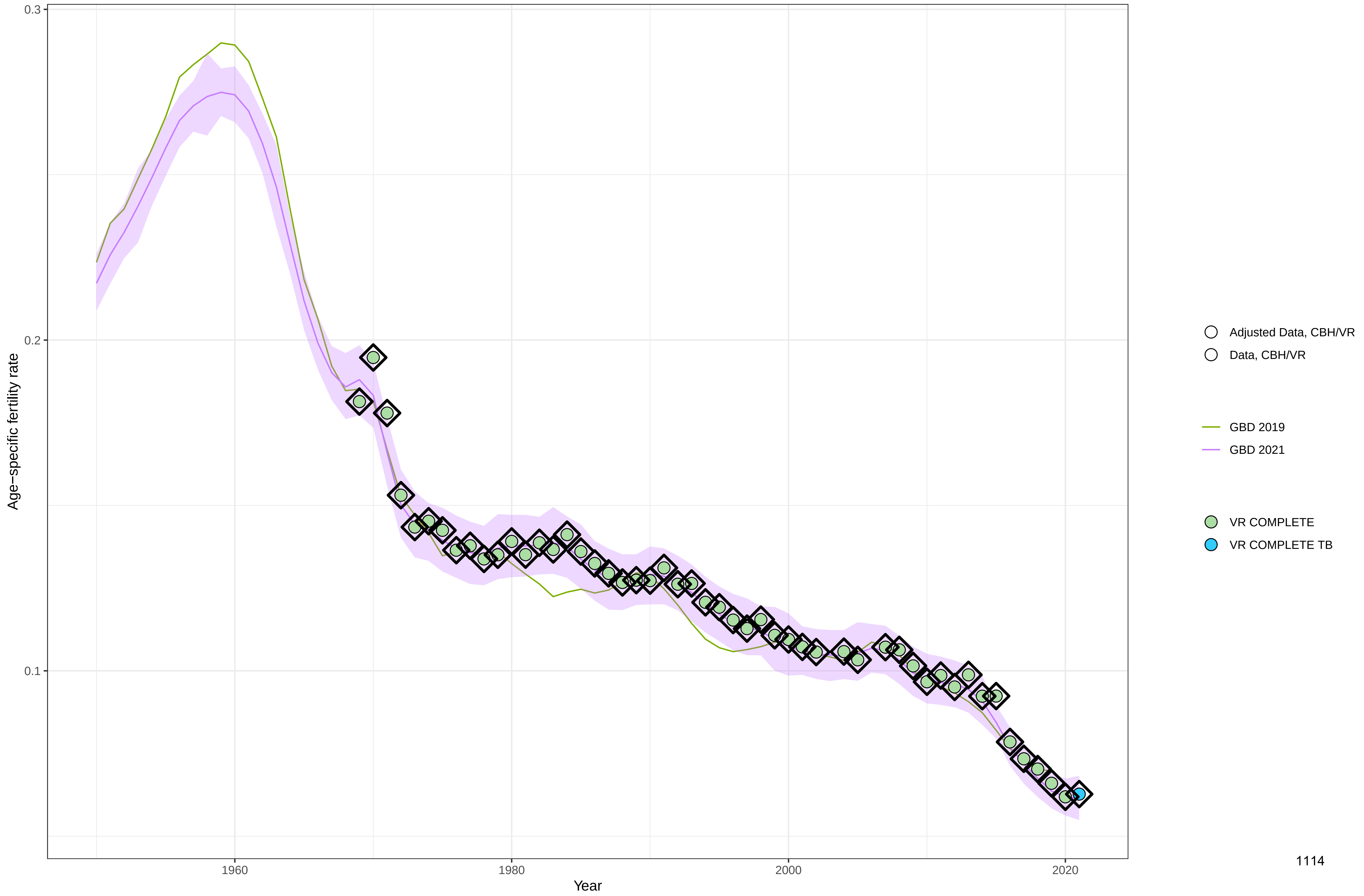

Age−specific fertility fitted trend lines, 1950−2021: Montana, Ages: 25−29
Data points enclosed with a diamond are reference sources

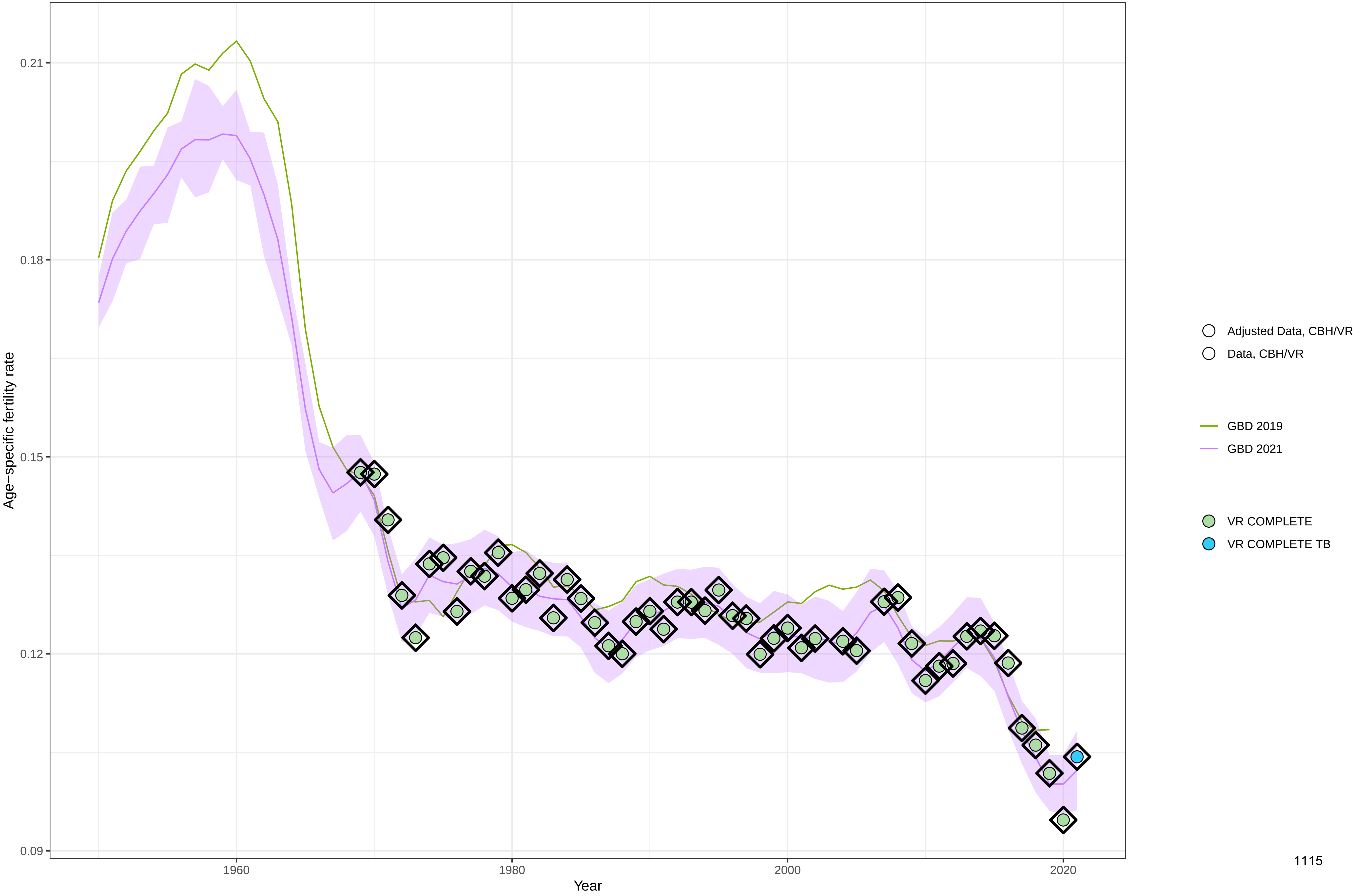

Age−specific fertility fitted trend lines, 1950−2021: Montana, Ages: 30−34
Data points enclosed with a diamond are reference sources

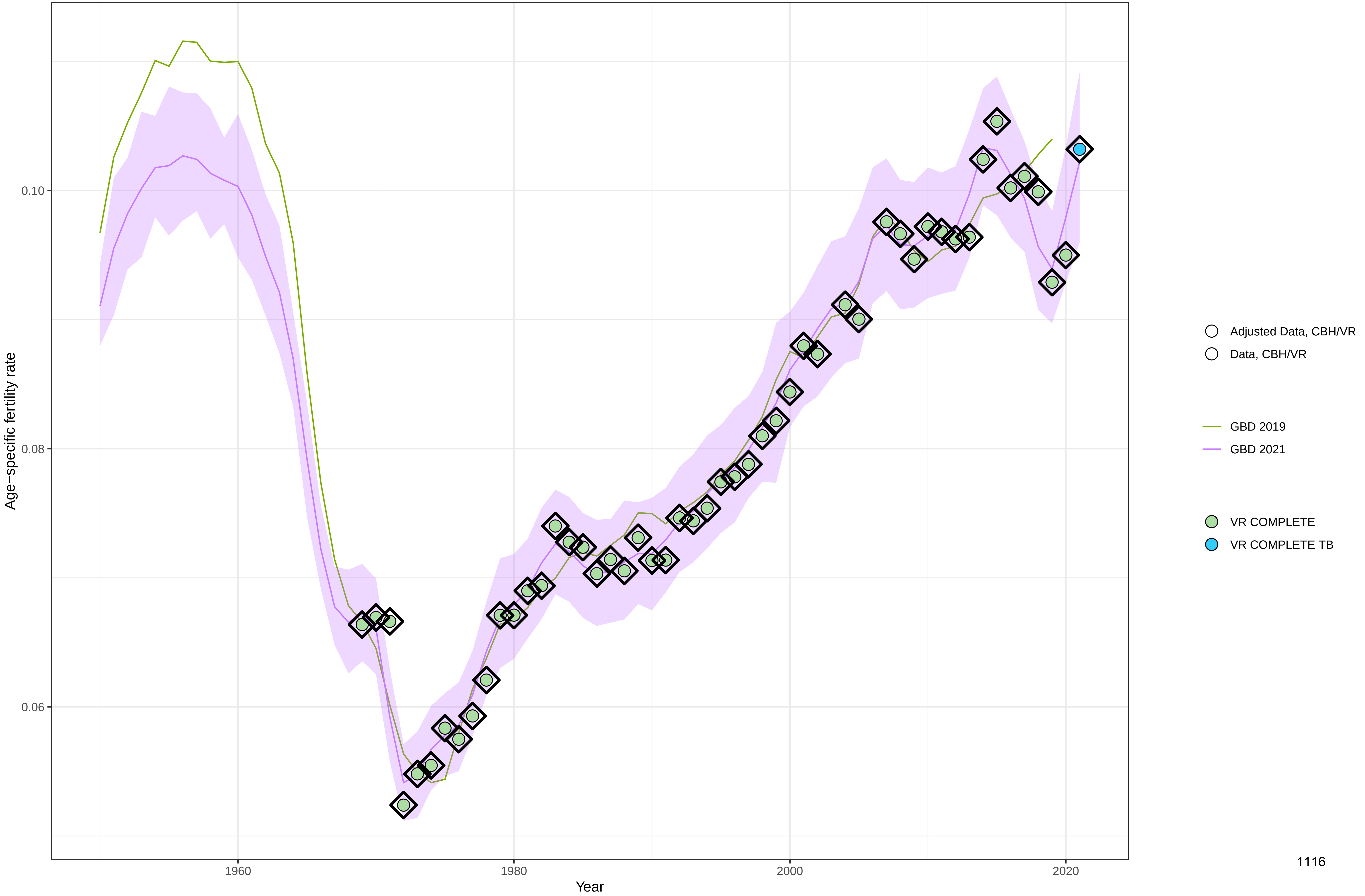

Age−specific fertility fitted trend lines, 1950−2021: Montana, Ages: 35−39
Data points enclosed with a diamond are reference sources

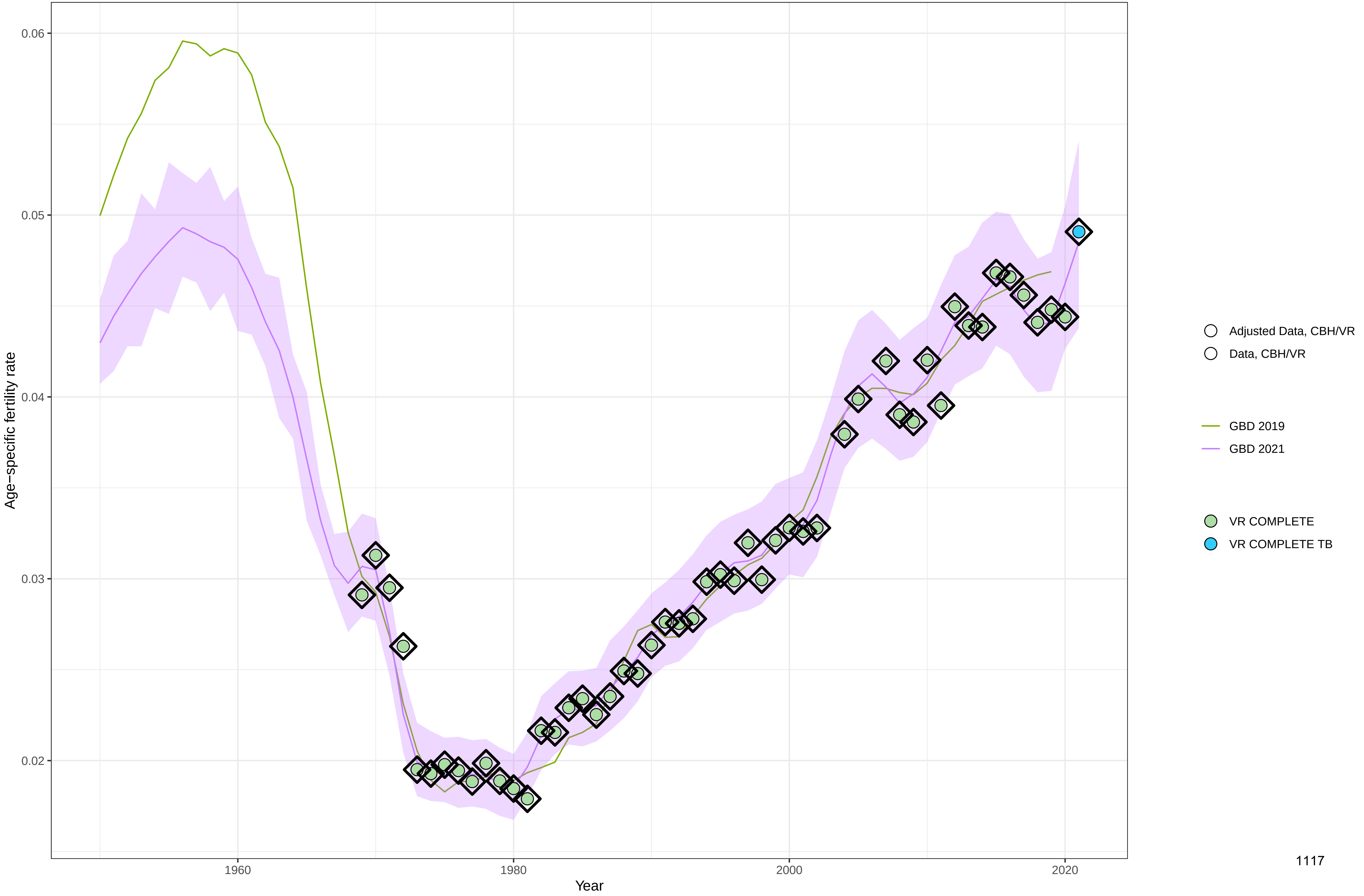

Age−specific fertility fitted trend lines, 1950−2021: Montana, Ages: 40−44
Data points enclosed with a diamond are reference sources

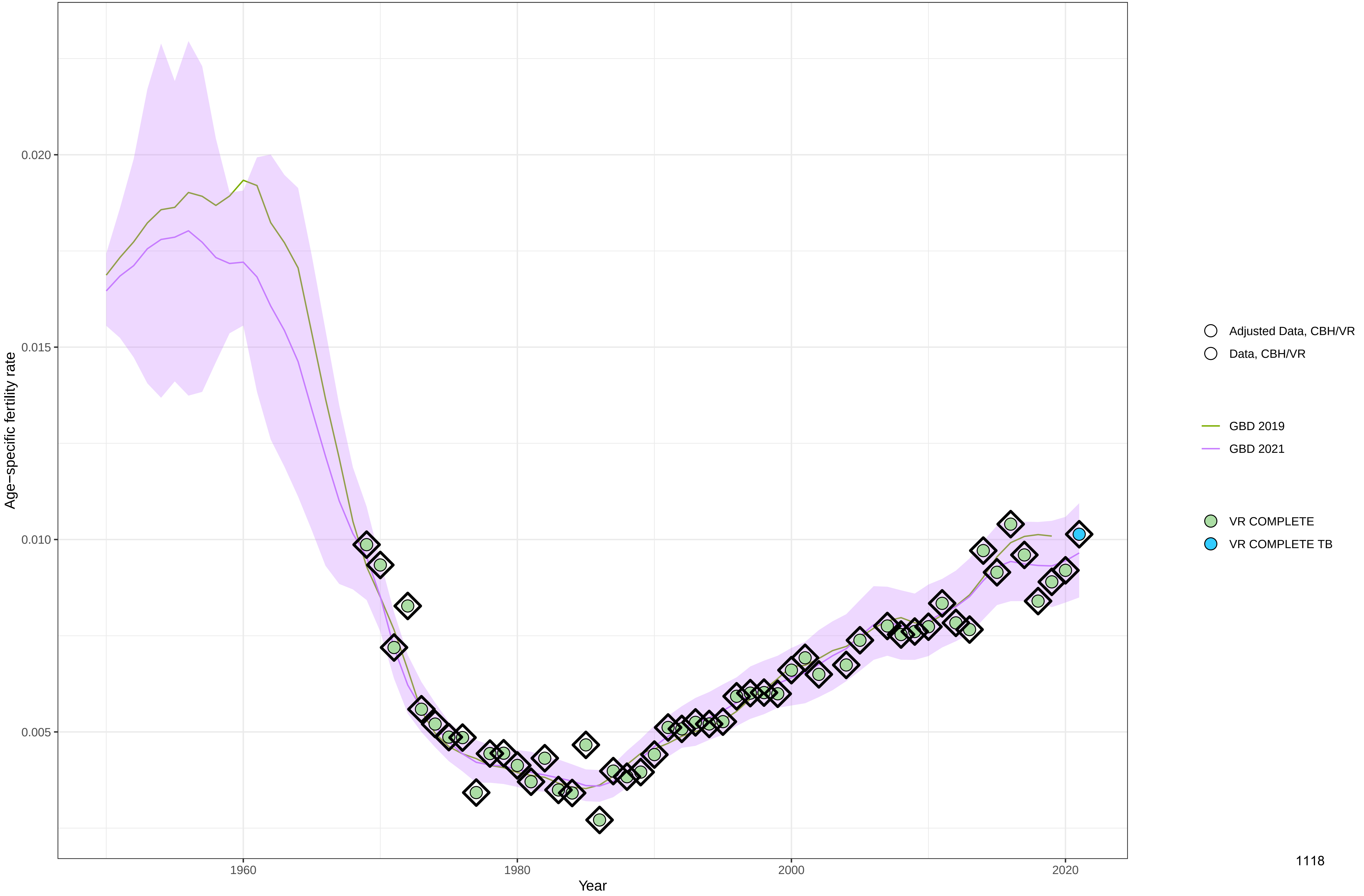

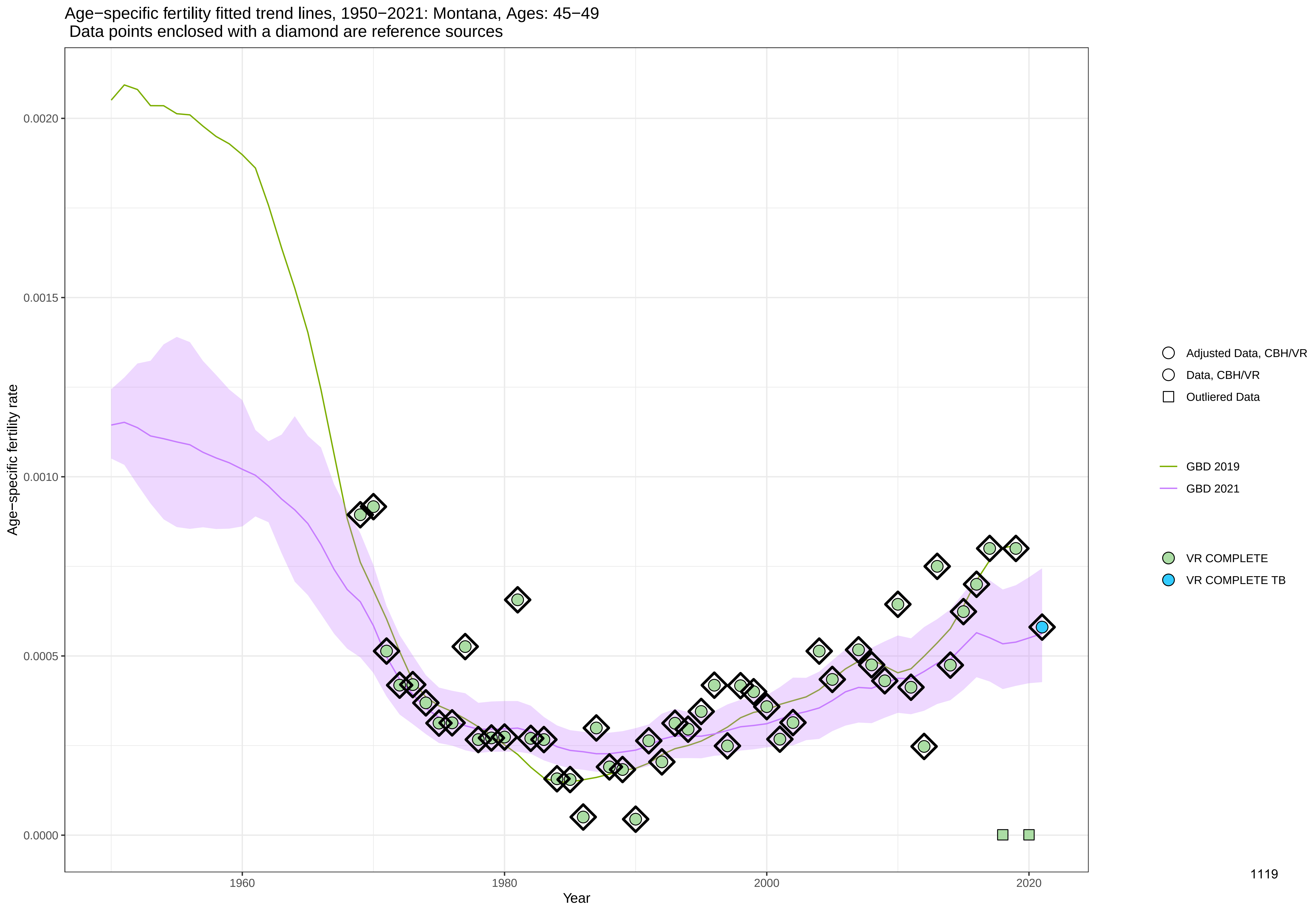
Age−specific fertility fitted trend lines, 1950−2021: Montana, Ages: 45−49
Data points enclosed with a diamond are reference sources
Age−specific fertility rate
0.0020
0.0015
0.0010
0.0005
0.0000
1960
1980
2000
2020
Year
Adjusted Data, CBH/VR
Data, CBH/VR
Outliered Data
GBD 2019
GBD 2021
VR COMPLETE
VR COMPLETE TB

Age−specific fertility fitted trend lines, 1950−2021: Montana, Ages: 50−54
Data points enclosed with a diamond are reference sources

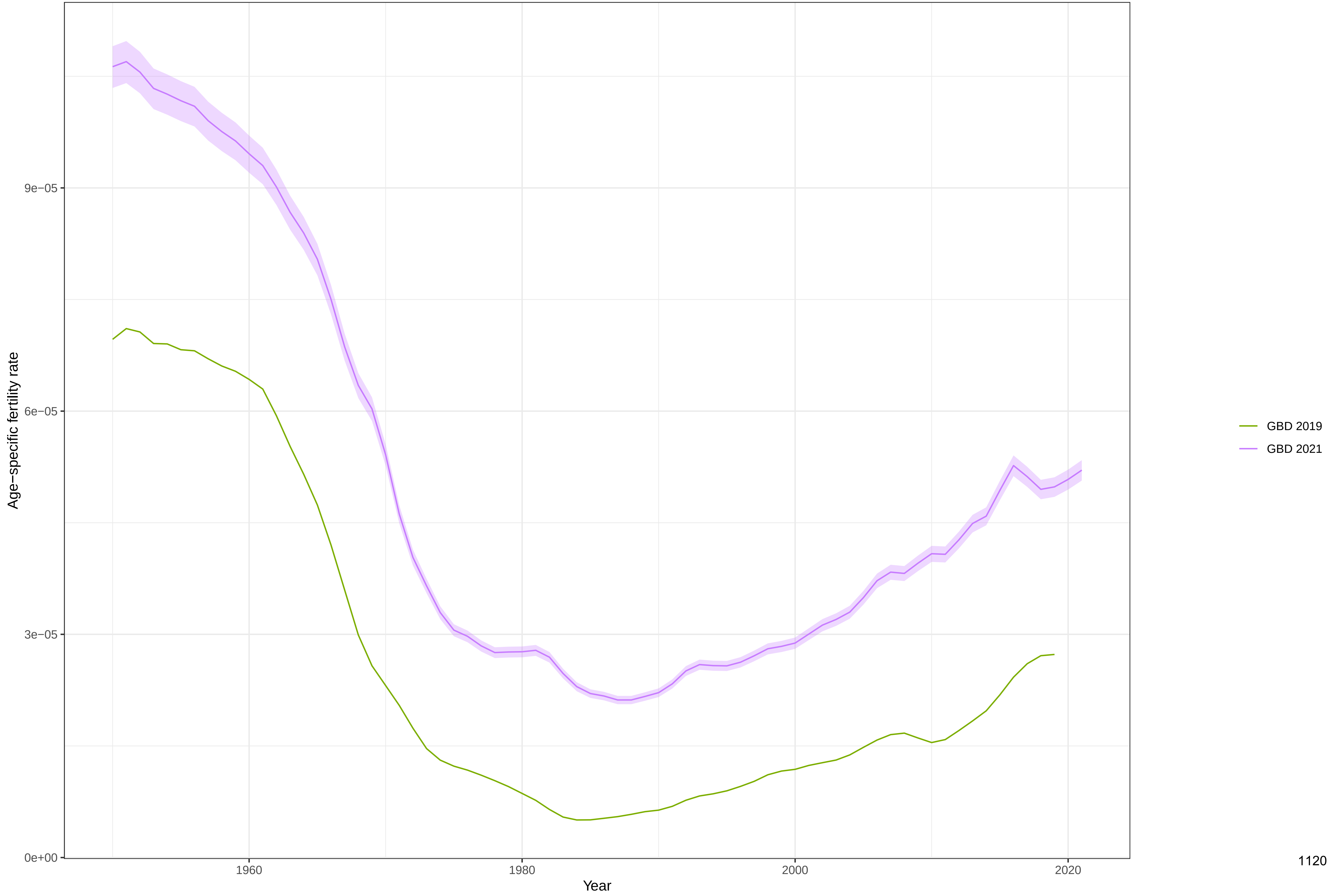

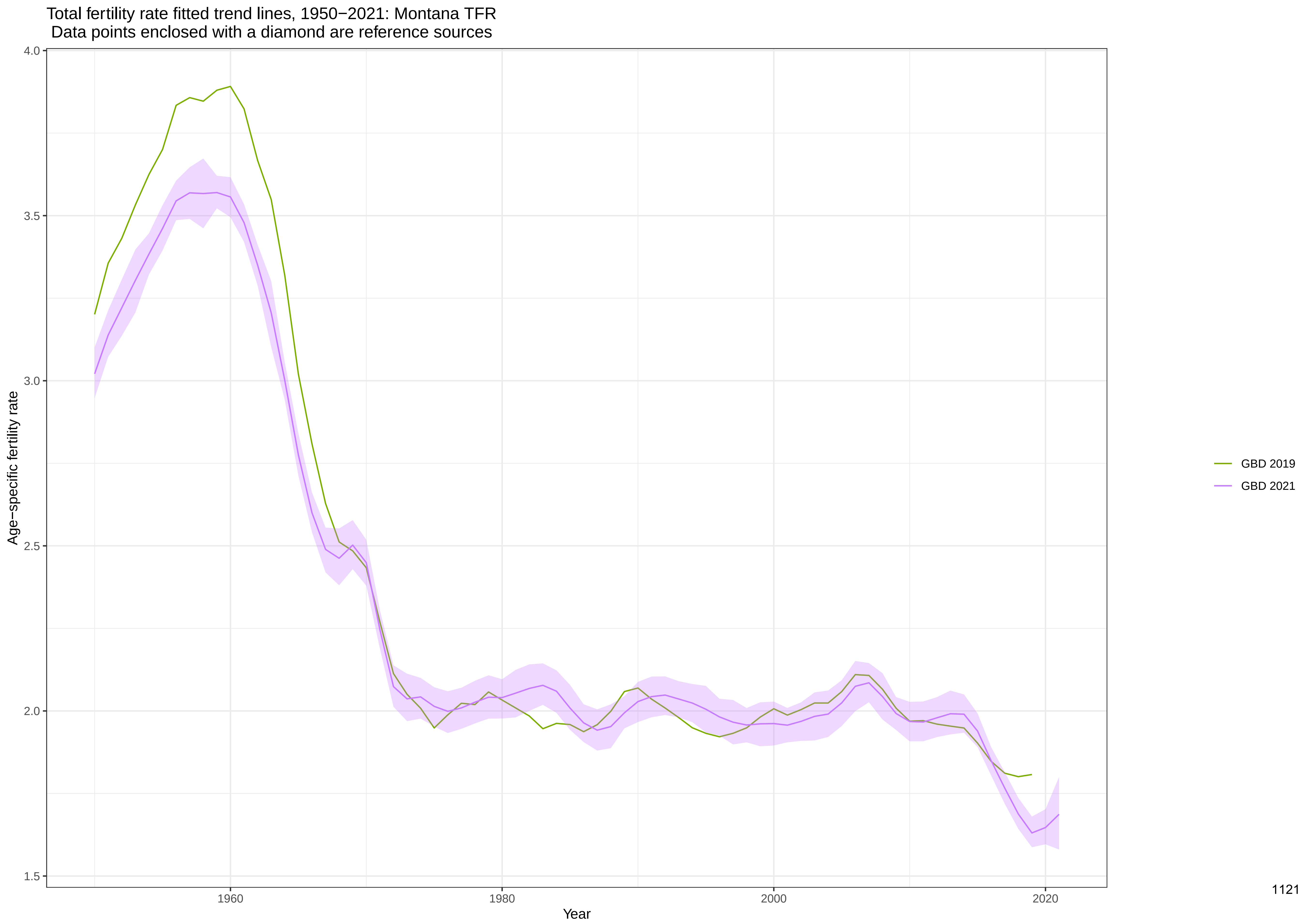
Total fertility rate fitted trend lines, 1950−2021: Montana TFR
Data points enclosed with a diamond are reference sources
Age−specific fertility rate
Year
4.0
3.5
3.0
2.5
2.0
1.5
1960
1980
2000
2020
GBD 2019
GBD 2021

Age−specific fertility fitted trend lines, 1950−2021: Nebraska, Ages: 10−14
Data points enclosed with a diamond are reference sources

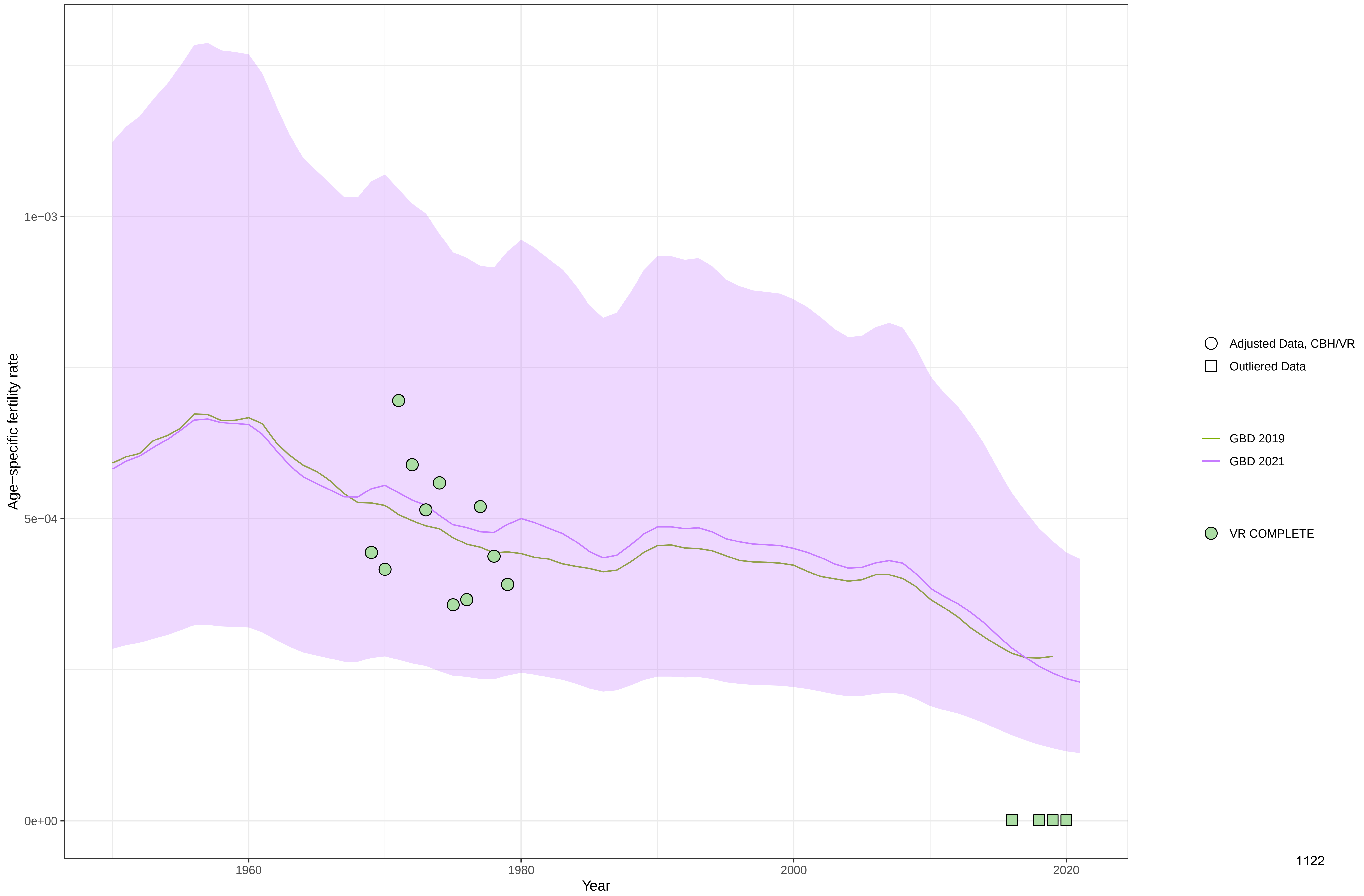

Age−specific fertility fitted trend lines, 1950−2021: Nebraska, Ages: 15−19
Data points enclosed with a diamond are reference sources

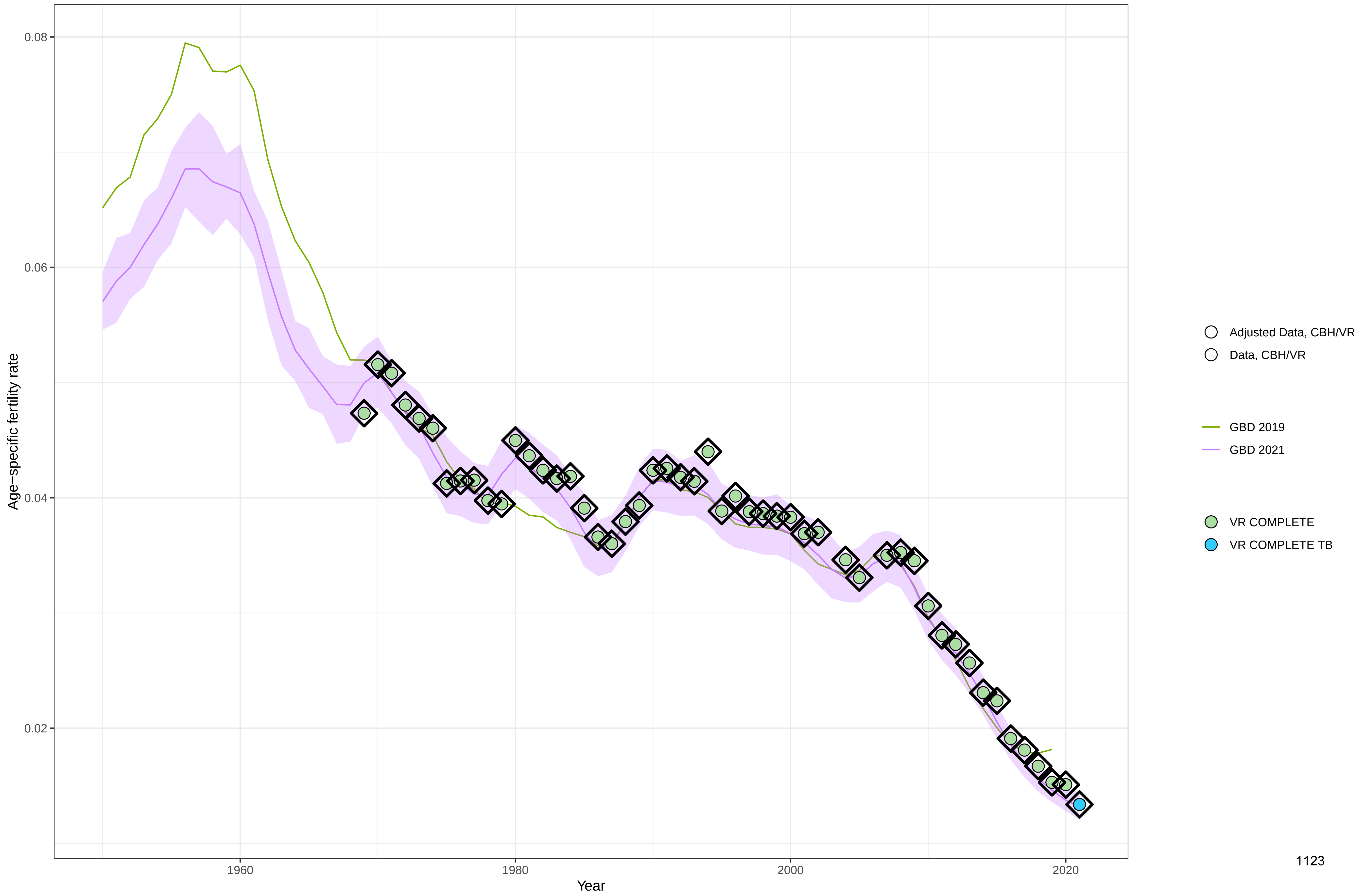

Age−specific fertility fitted trend lines, 1950−2021: Nebraska, Ages: 20−24
Data points enclosed with a diamond are reference sources

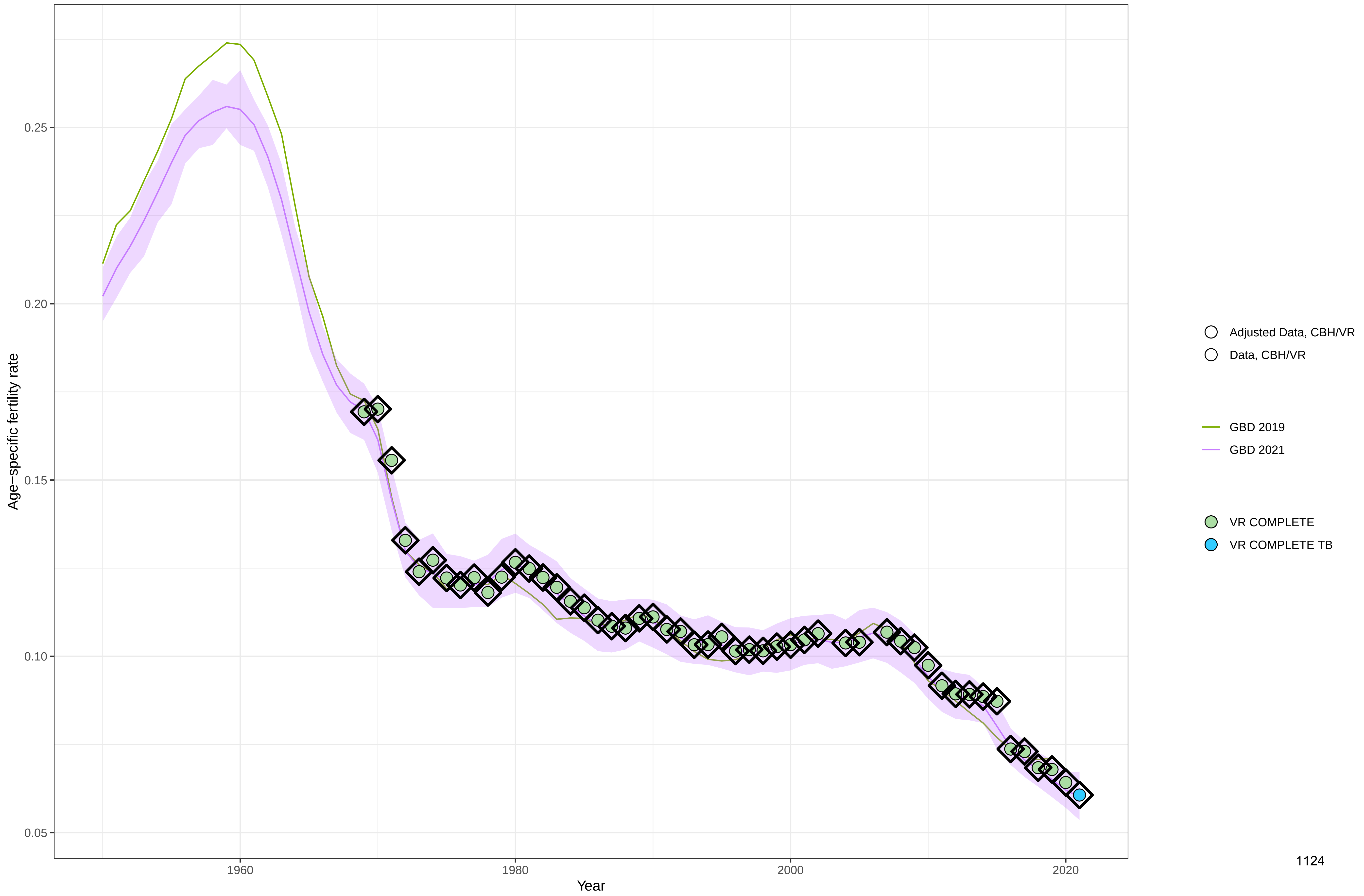

Age−specific fertility fitted trend lines, 1950−2021: Nebraska, Ages: 25−29
Data points enclosed with a diamond are reference sources

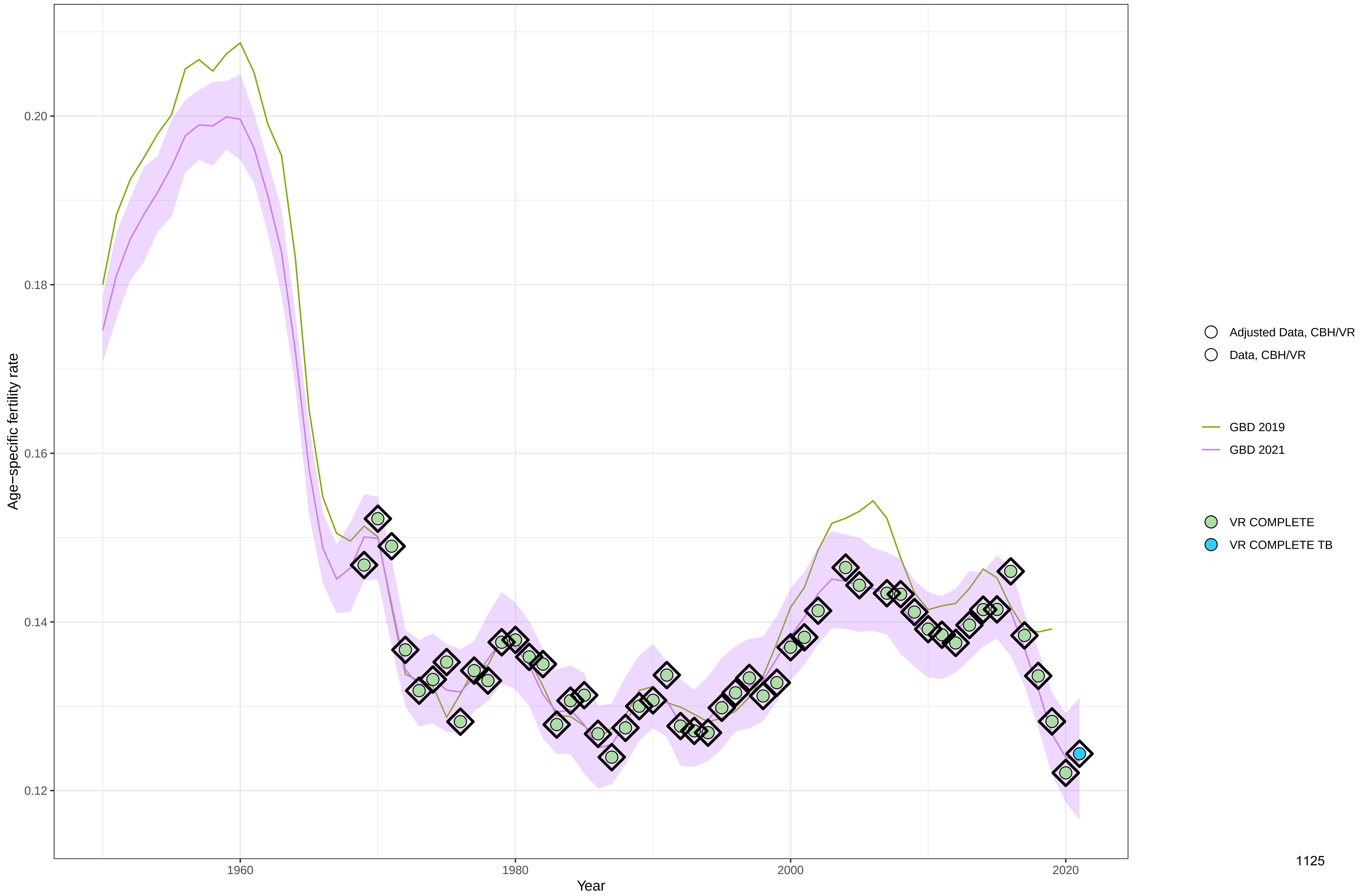

Age−specific fertility fitted trend lines, 1950−2021: Nebraska, Ages: 30−34
Data points enclosed with a diamond are reference sources

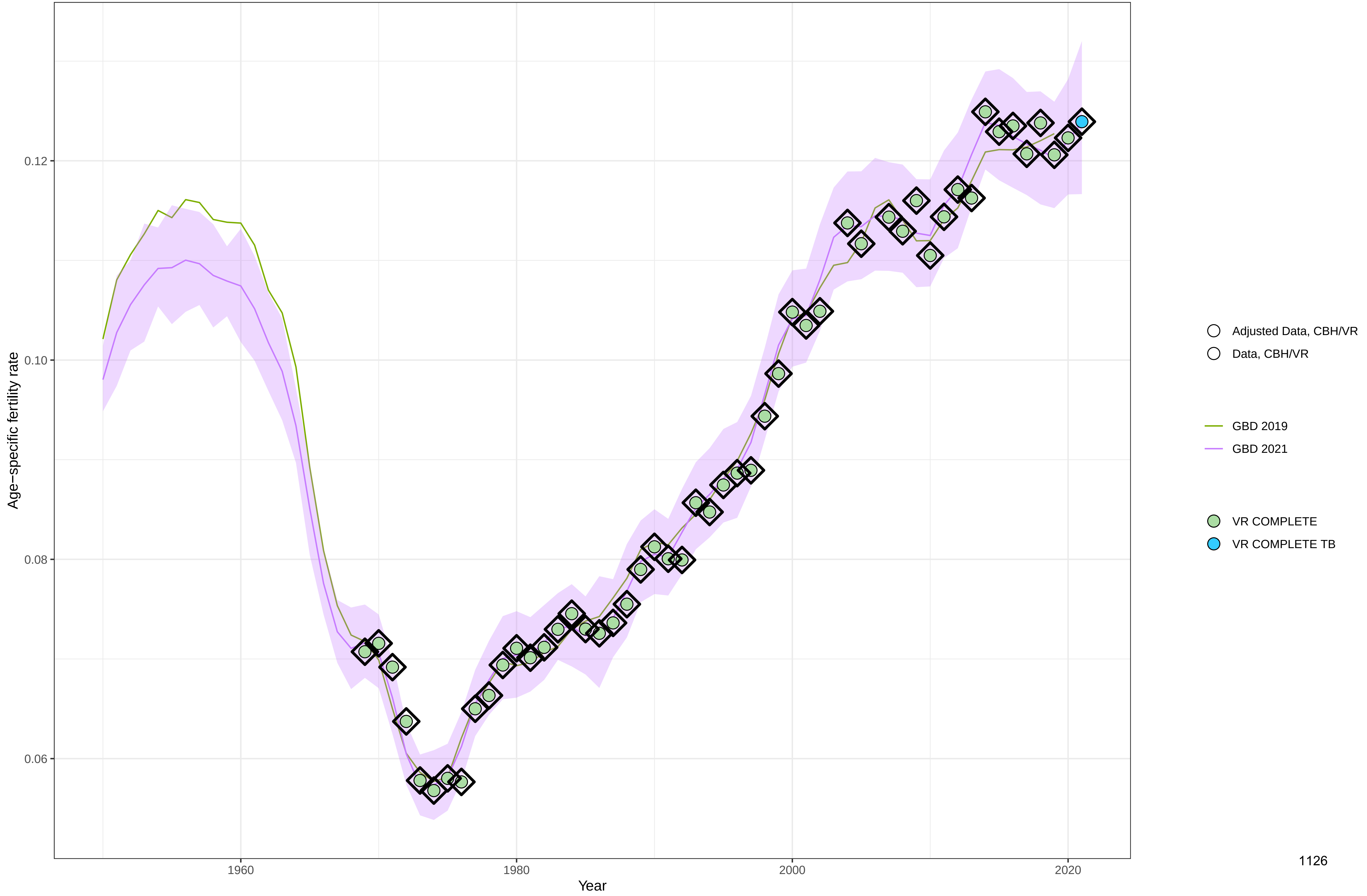

Age−specific fertility fitted trend lines, 1950−2021: Nebraska, Ages: 35−39
Data points enclosed with a diamond are reference sources

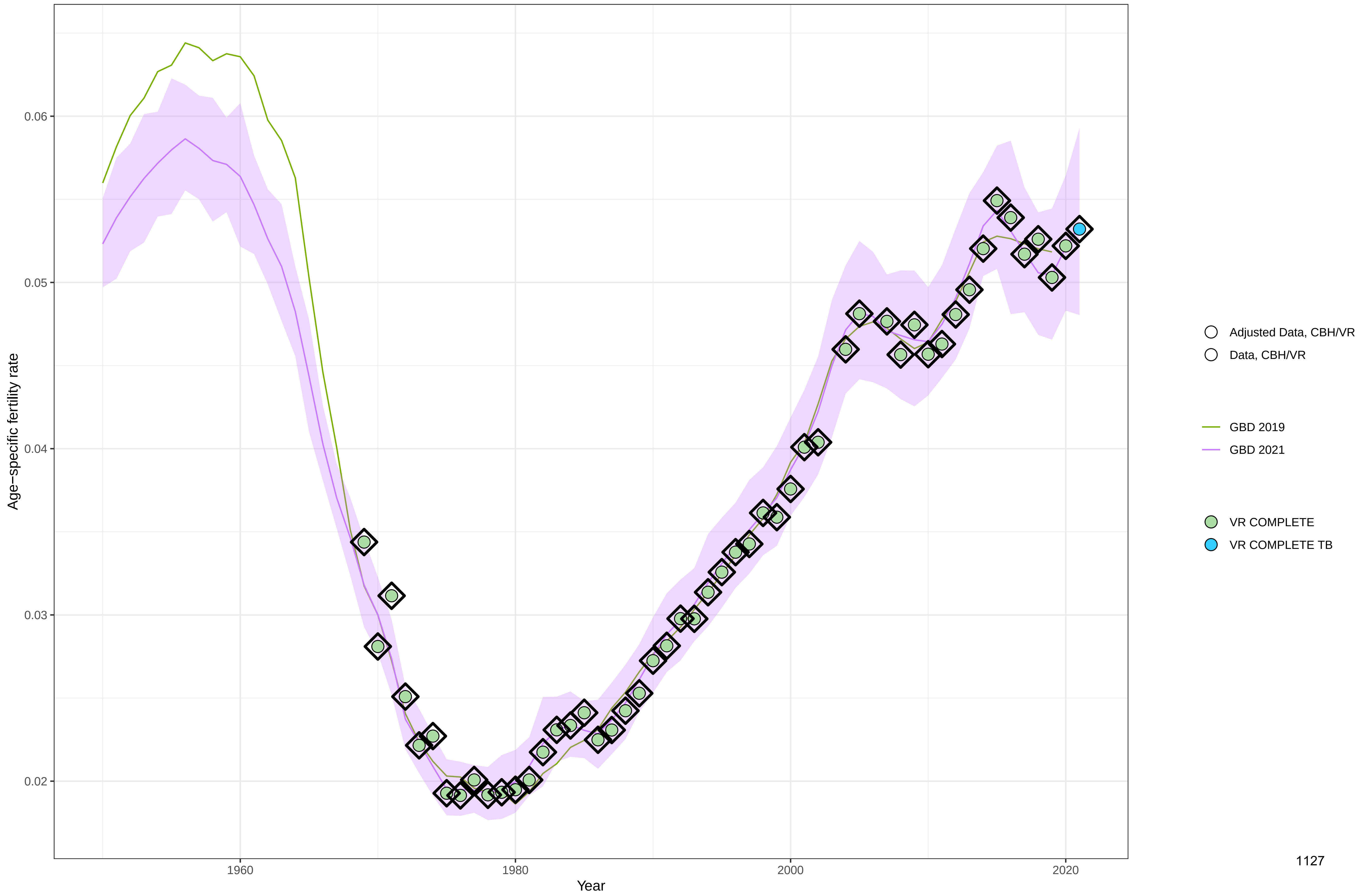

Age−specific fertility fitted trend lines, 1950−2021: Nebraska, Ages: 40−44
Data points enclosed with a diamond are reference sources

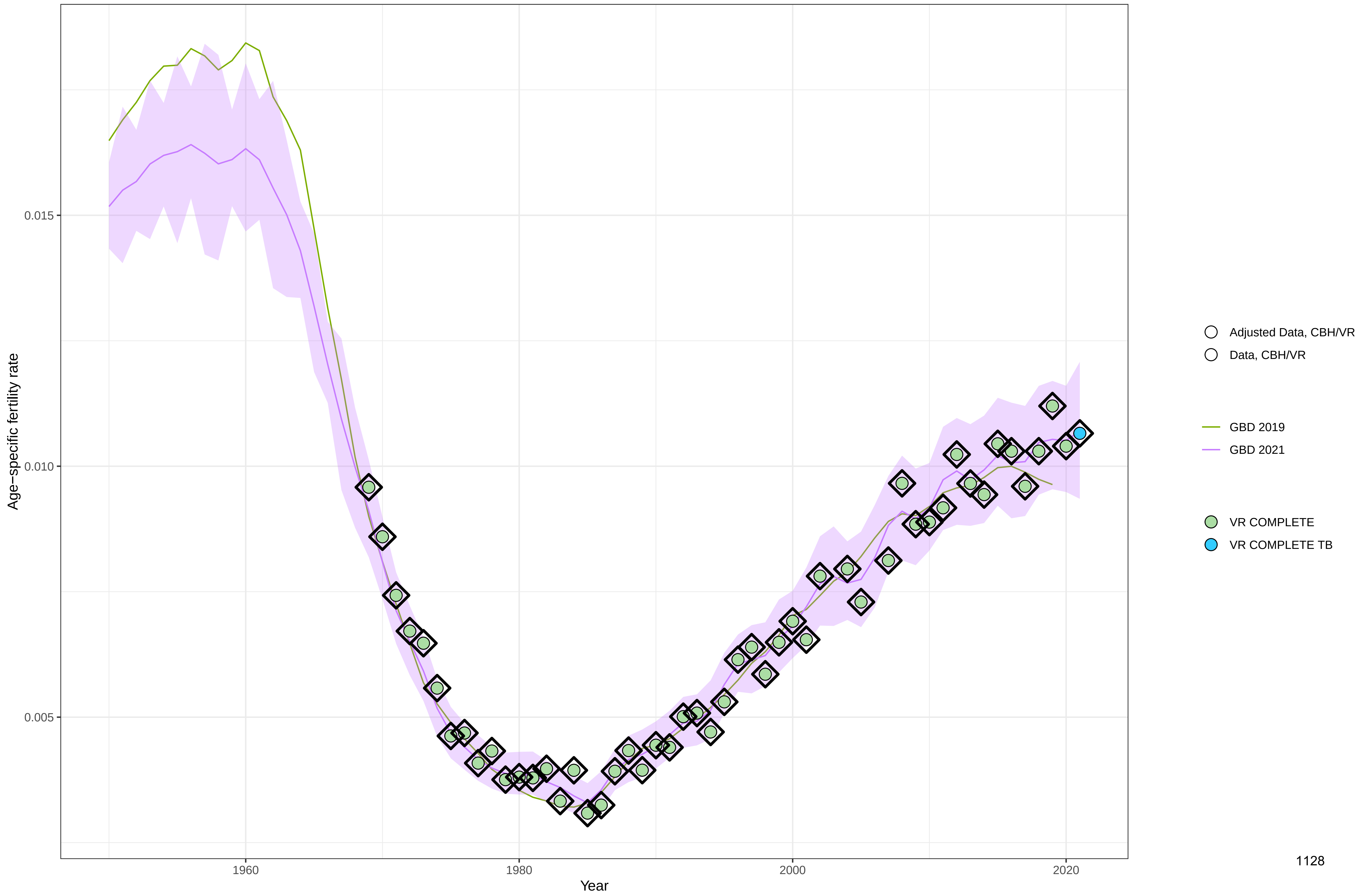

Age−specific fertility fitted trend lines, 1950−2021: Nebraska, Ages: 45−49
Data points enclosed with a diamond are reference sources

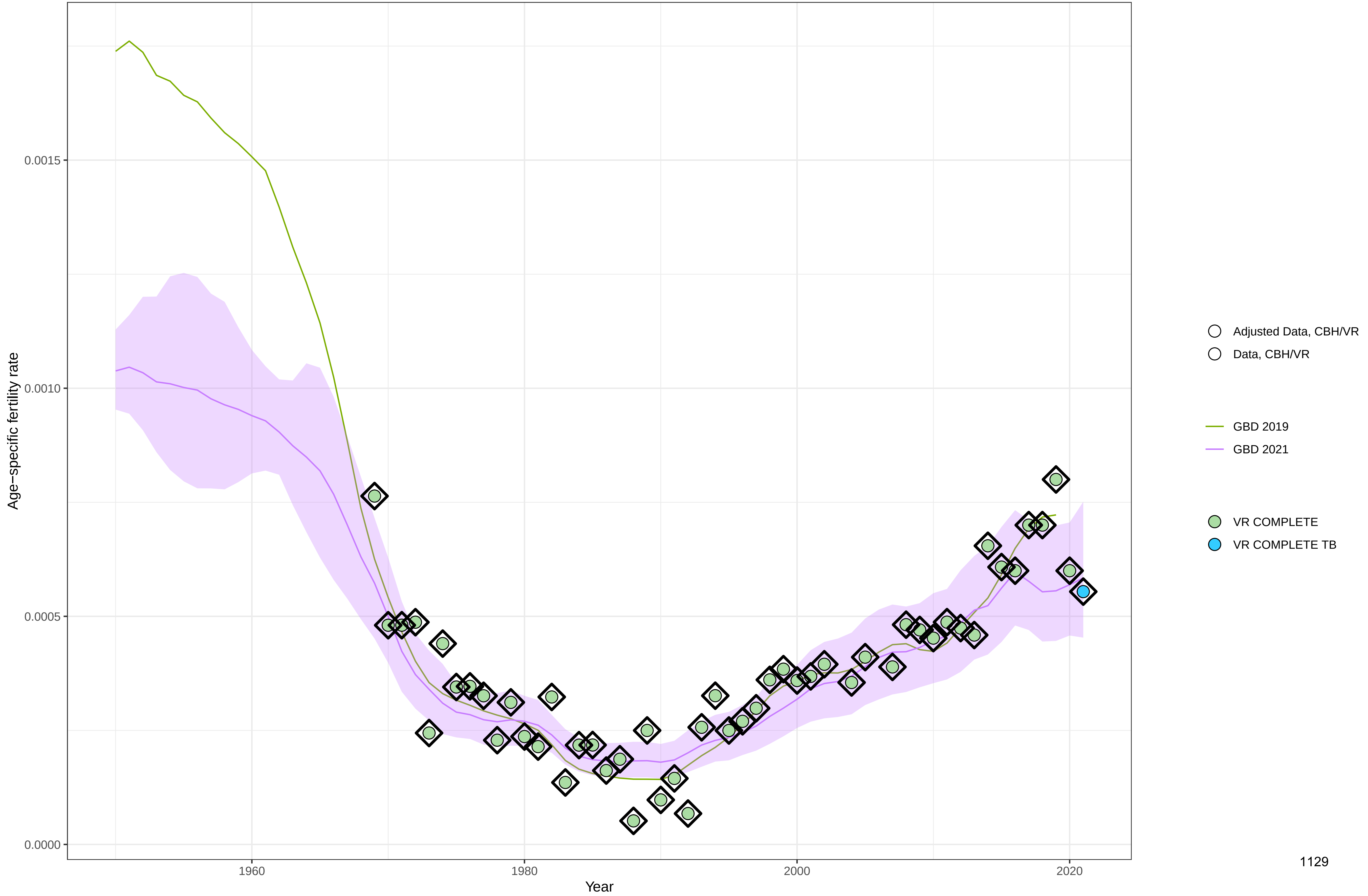

Age−specific fertility fitted trend lines, 1950−2021: Nebraska, Ages: 50−54
Data points enclosed with a diamond are reference sources

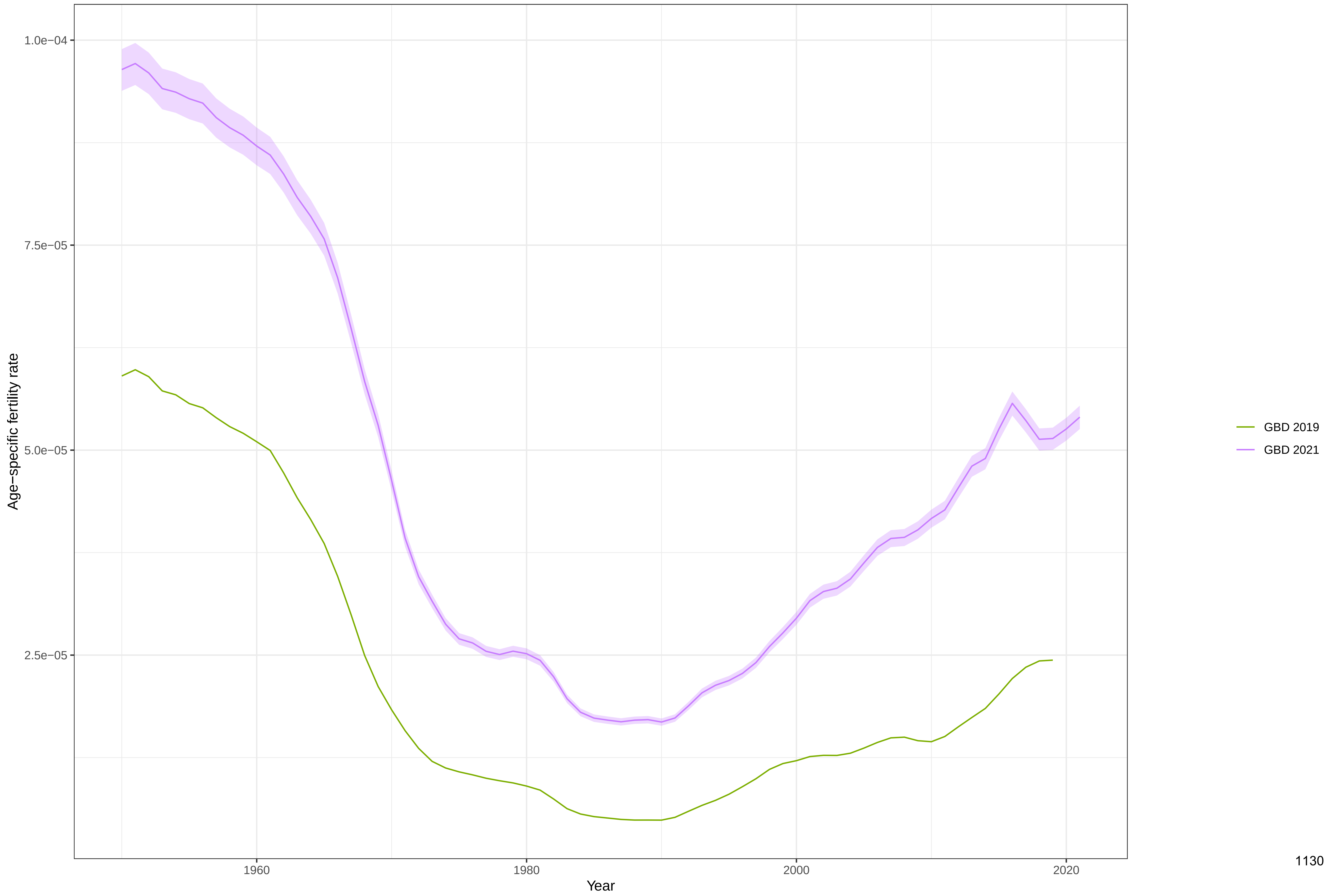

Total fertility rate fitted trend lines, 1950−2021: Nebraska TFR
Data points enclosed with a diamond are reference sources

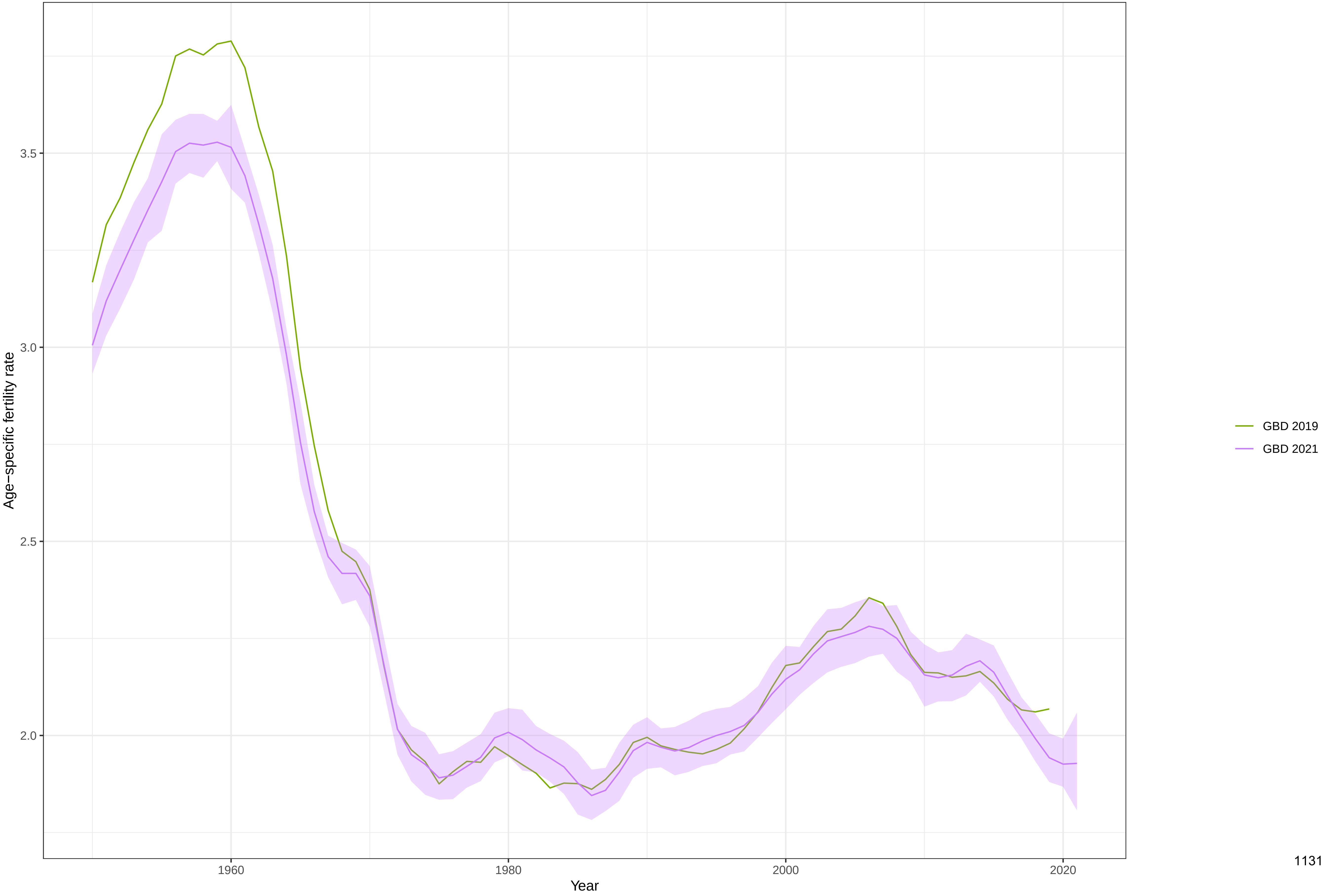

Age−specific fertility fitted trend lines, 1950−2021: Nevada, Ages: 10−14
Data points enclosed with a diamond are reference sources

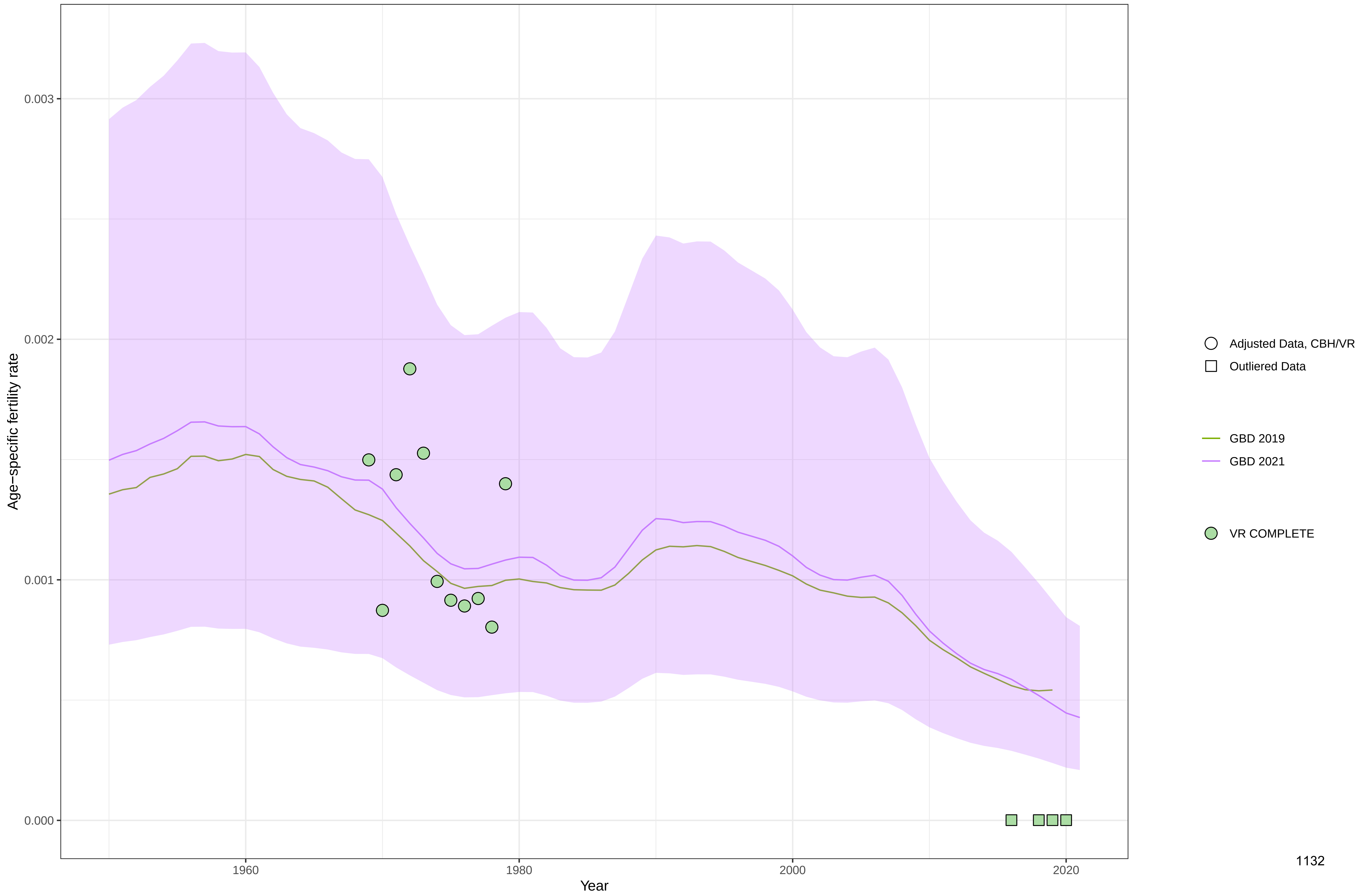

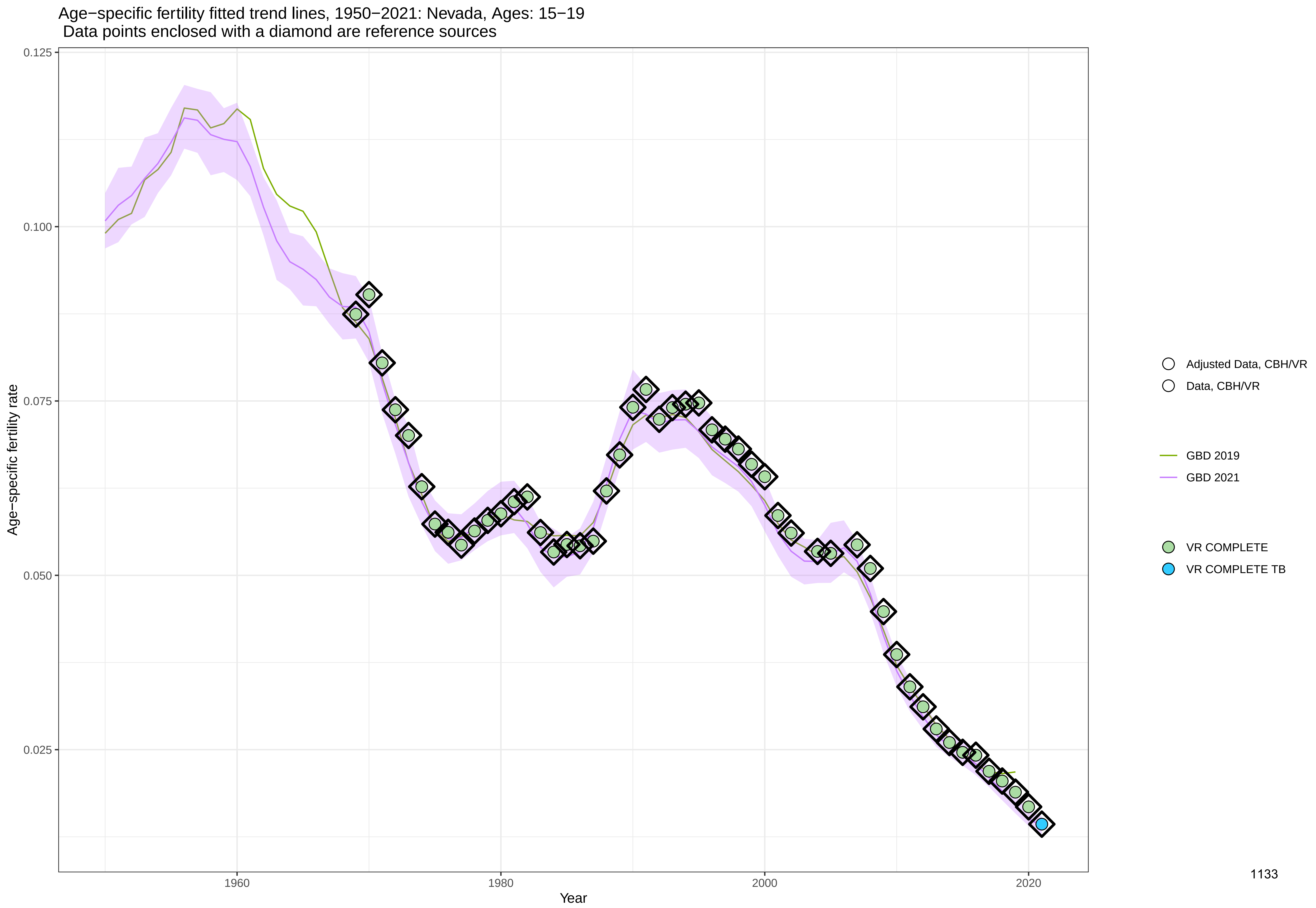
Age−specific fertility fitted trend lines, 1950−2021: Nevada, Ages: 15−19
Data points enclosed with a diamond are reference sources
Age−specific fertility rate
Year
0.125
0.100
0.075
0.050
0.025
1960
1980
2000
2020
Adjusted Data, CBH/VR
Data, CBH/VR
GBD 2019
GBD 2021
VR COMPLETE
VR COMPLETE TB

Age−specific fertility fitted trend lines, 1950−2021: Nevada, Ages: 20−24
Data points enclosed with a diamond are reference sources

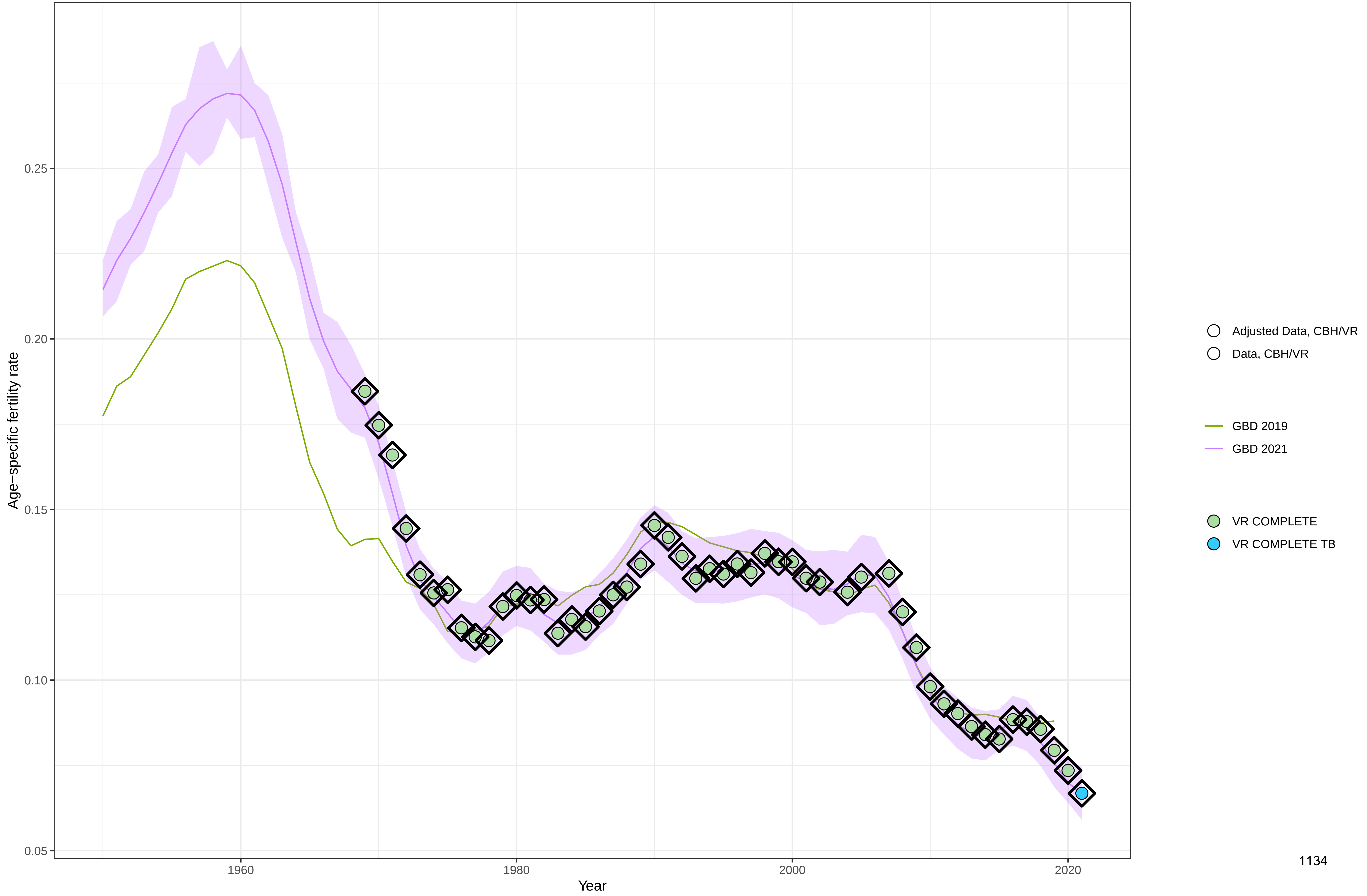

Age−specific fertility fitted trend lines, 1950−2021: Nevada, Ages: 25−29
Data points enclosed with a diamond are reference sources

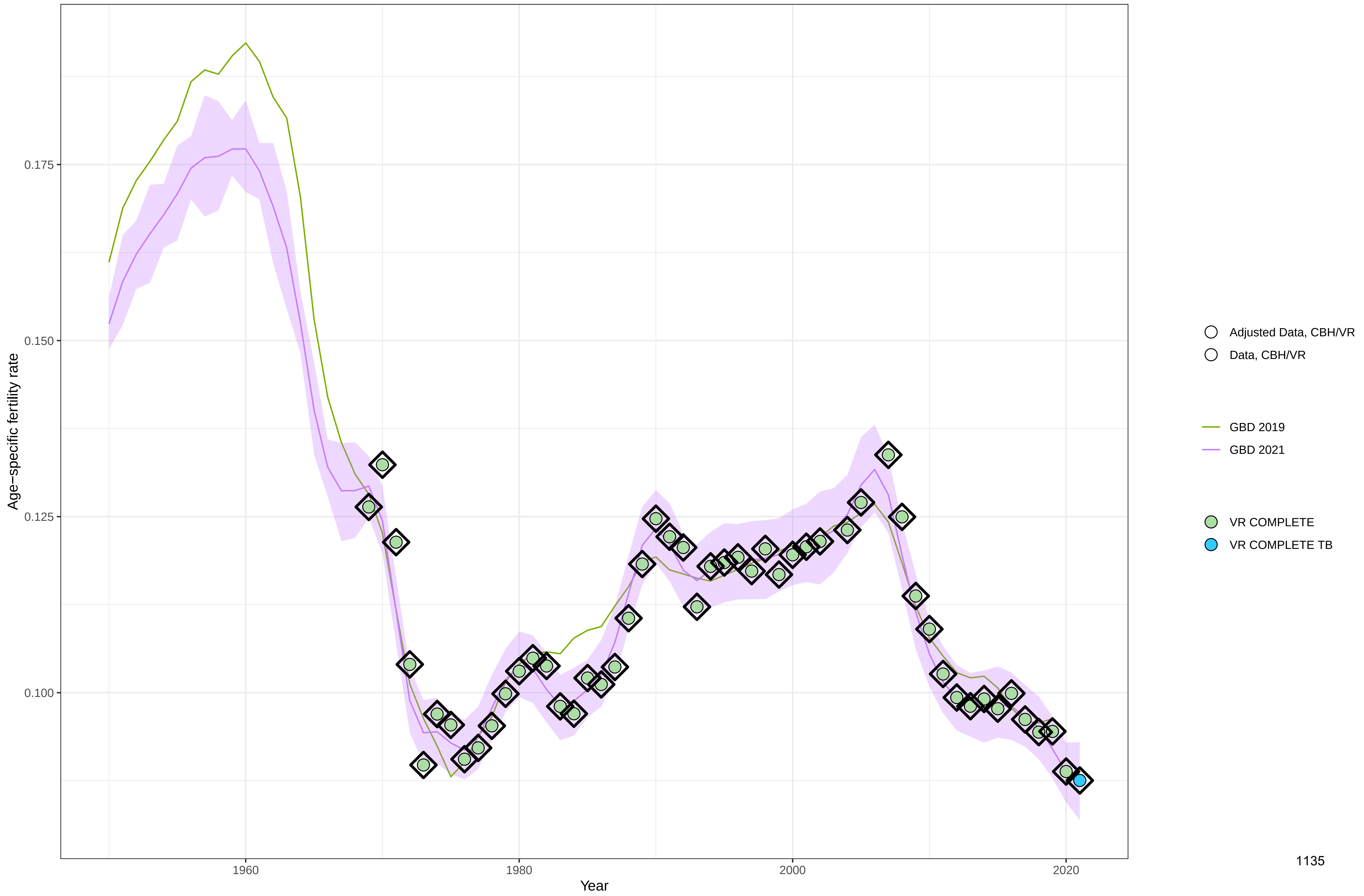

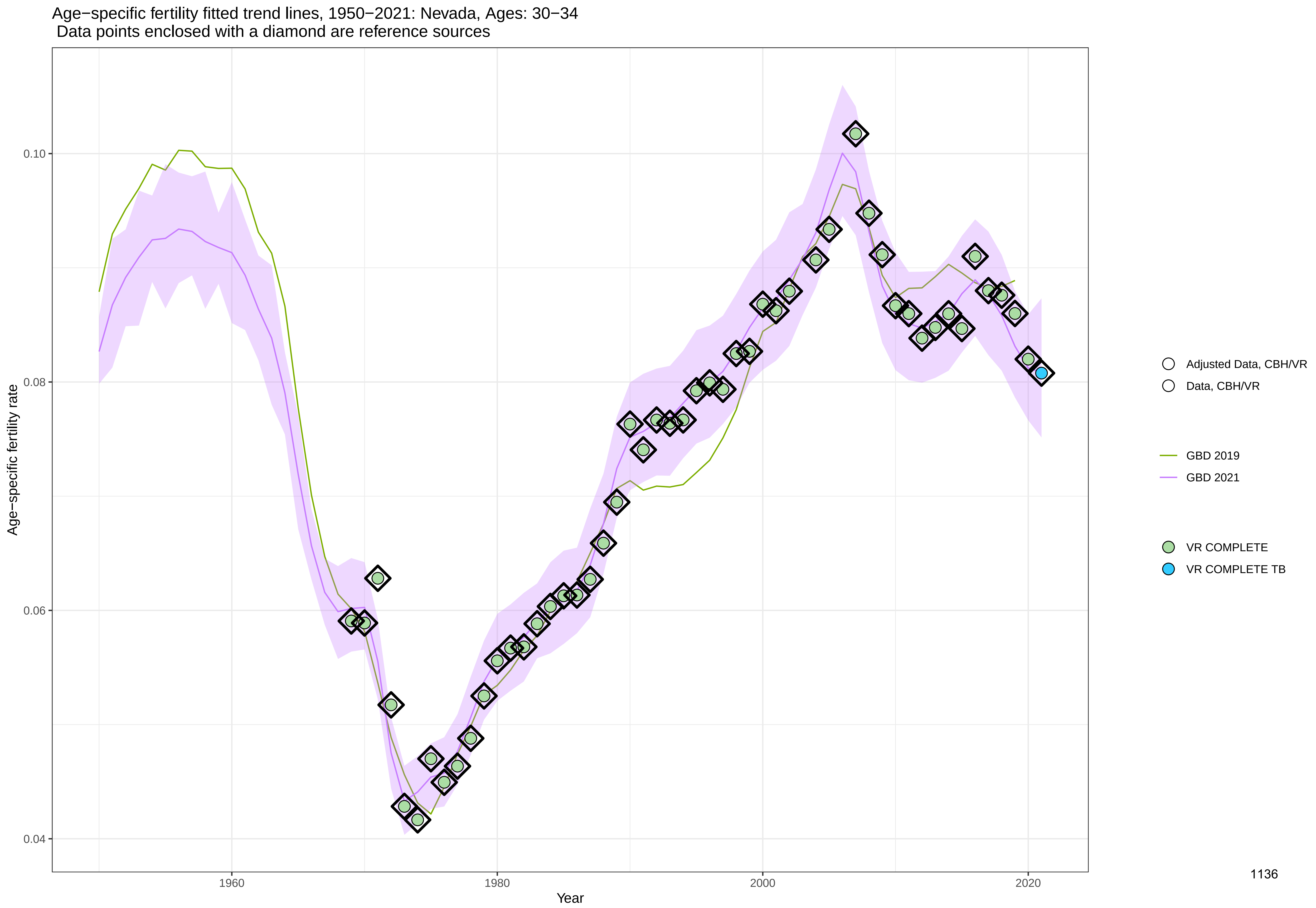

Age−specific fertility fitted trend lines, 1950−2021: Nevada, Ages: 30−34
Data points enclosed with a diamond are reference sources
Age−specific fertility rate
0.10
0.08
0.06
0.04
1960
1980
2000
2020
Year
Adjusted Data, CBH/VR
Data, CBH/VR
GBD 2019
GBD 2021
VR COMPLETE
VR COMPLETE TB

Age−specific fertility fitted trend lines, 1950−2021: Nevada, Ages: 35−39
Data points enclosed with a diamond are reference sources

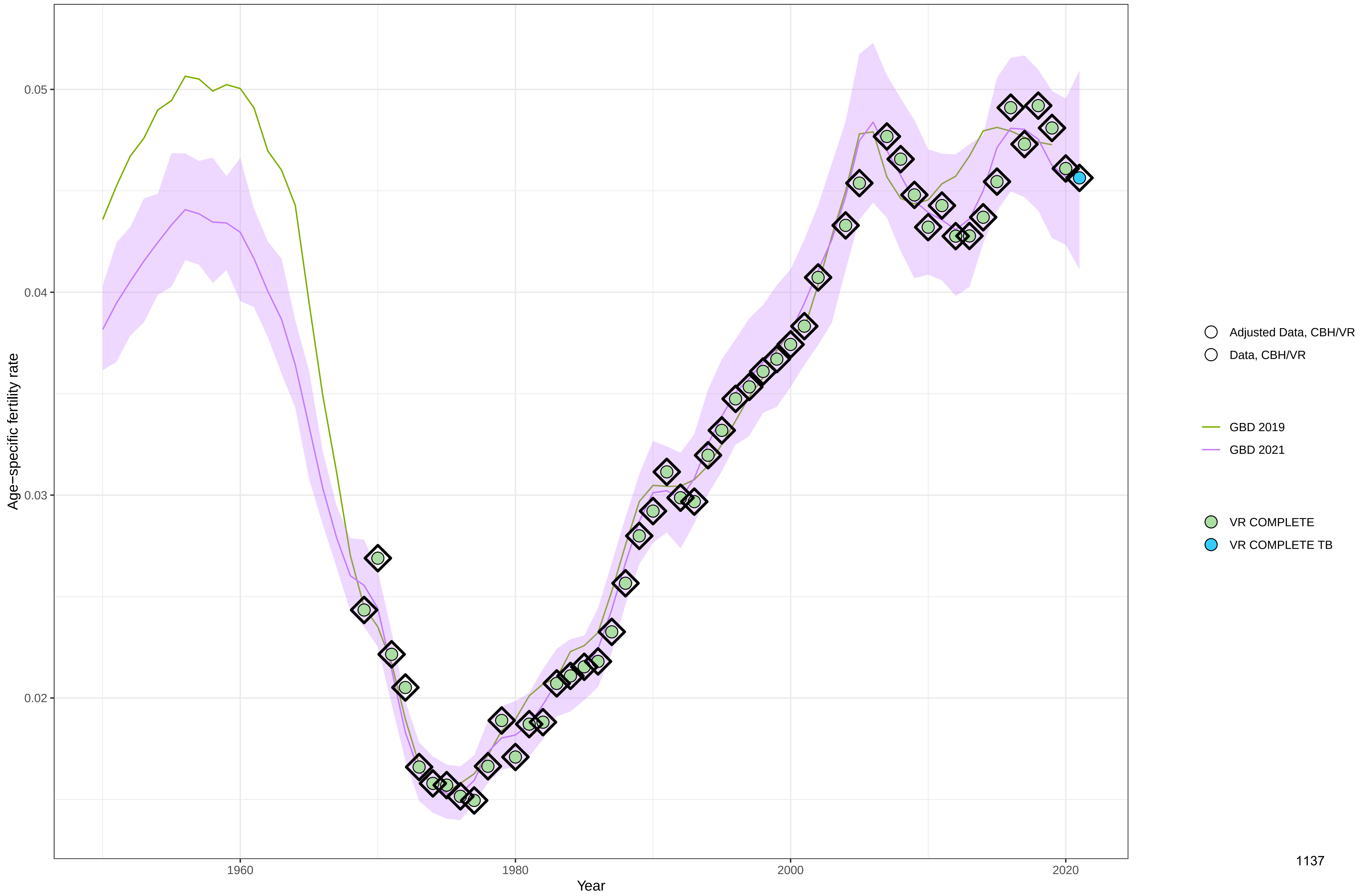

Age−specific fertility fitted trend lines, 1950−2021: Nevada, Ages: 40−44
Data points enclosed with a diamond are reference sources

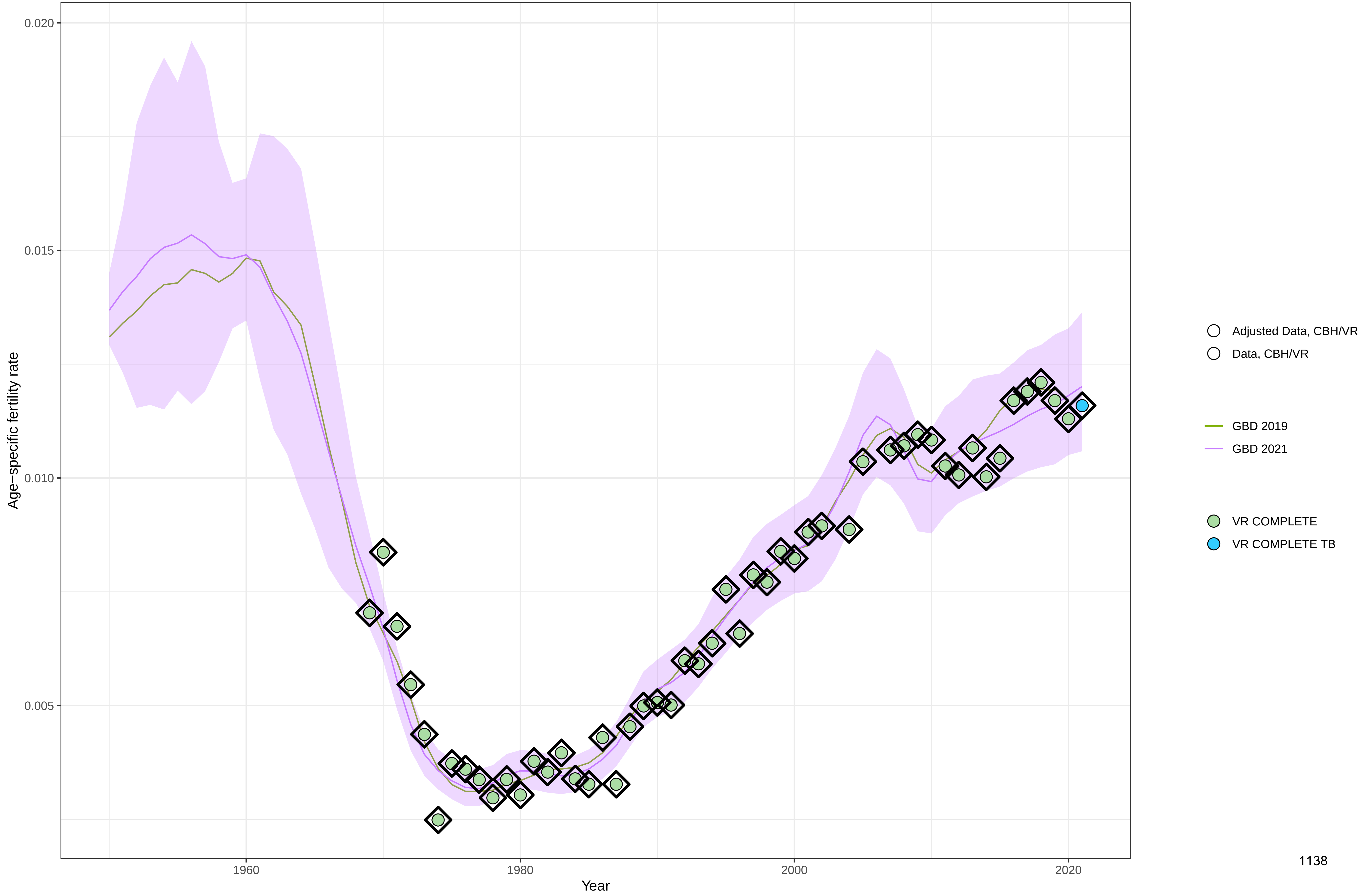

Age−specific fertility fitted trend lines, 1950−2021: Nevada, Ages: 45−49
Data points enclosed with a diamond are reference sources

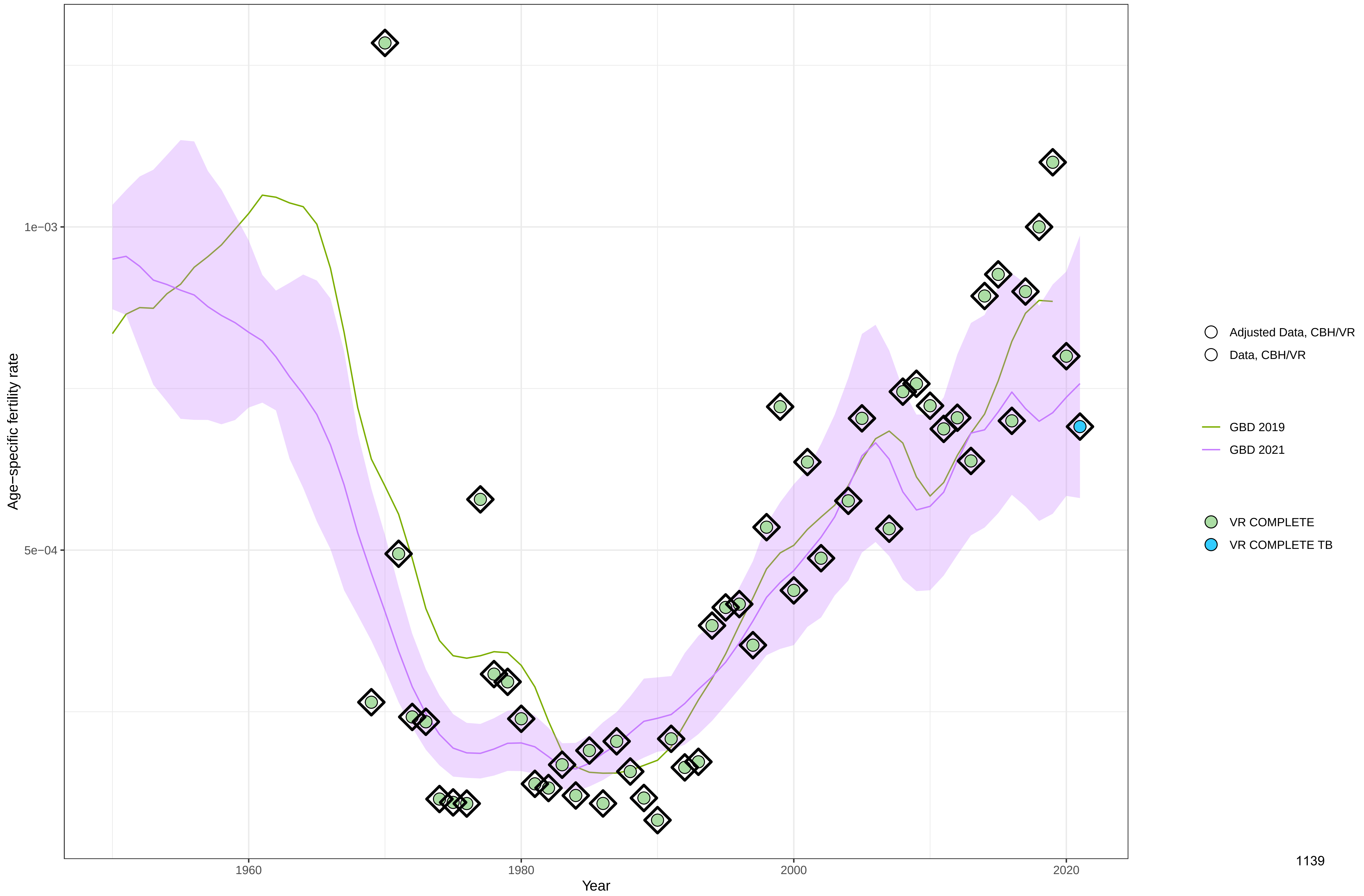

Age−specific fertility fitted trend lines, 1950−2021: Nevada, Ages: 50−54
Data points enclosed with a diamond are reference sources

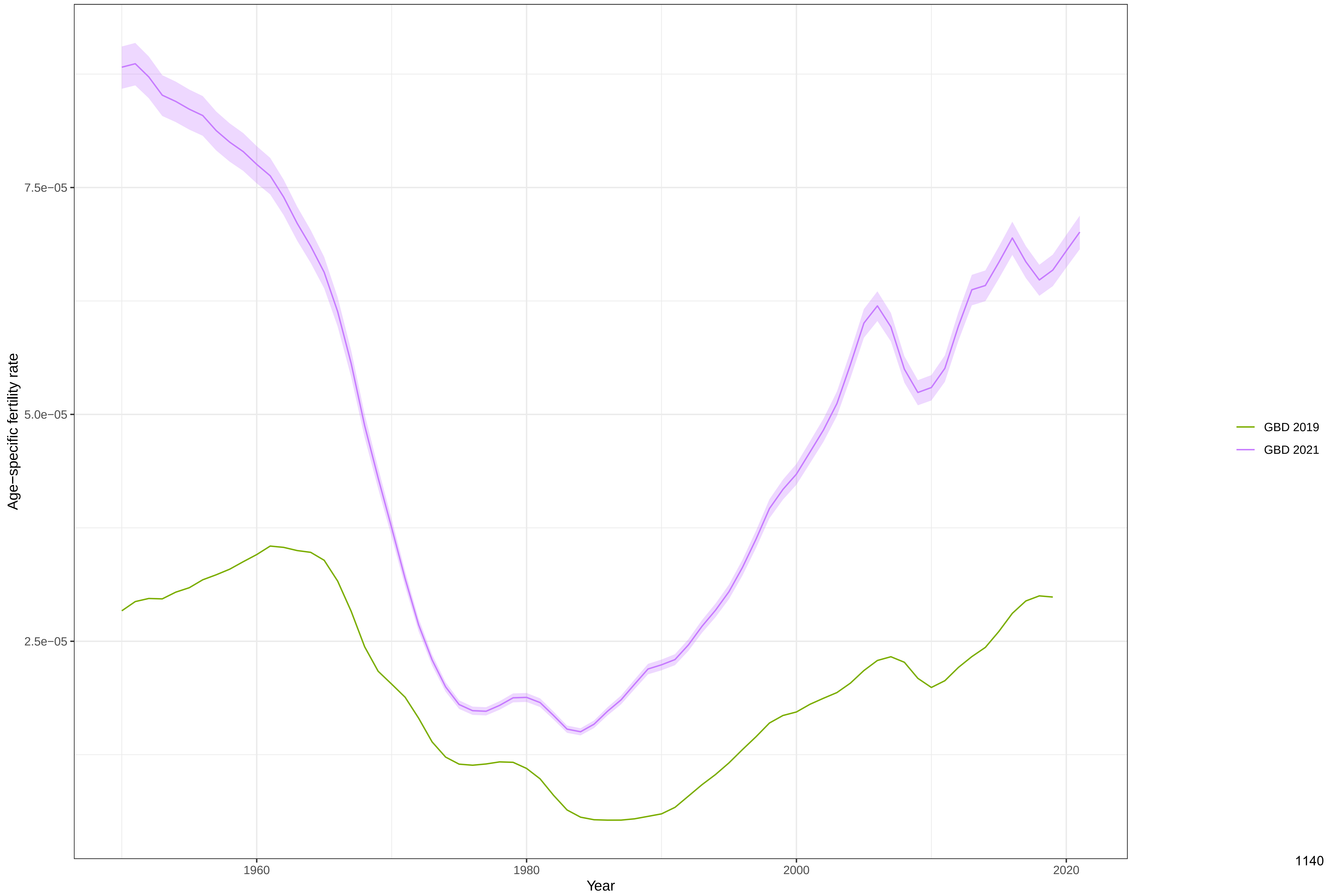

Total fertility rate fitted trend lines, 1950−2021: Nevada TFR
Data points enclosed with a diamond are reference sources

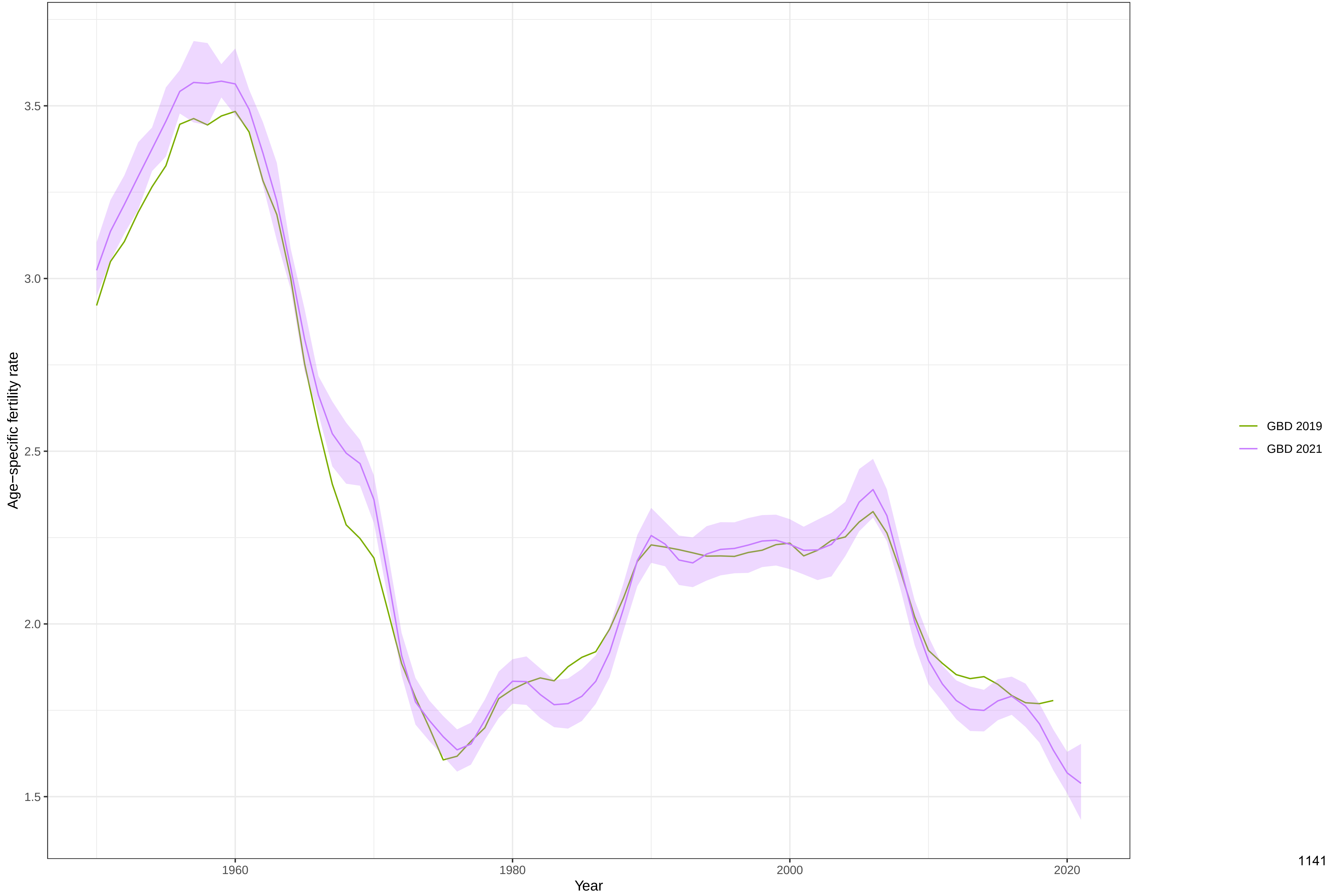

Age−specific fertility fitted trend lines, 1950−2021: New Hampshire, Ages: 10−14
Data points enclosed with a diamond are reference sources

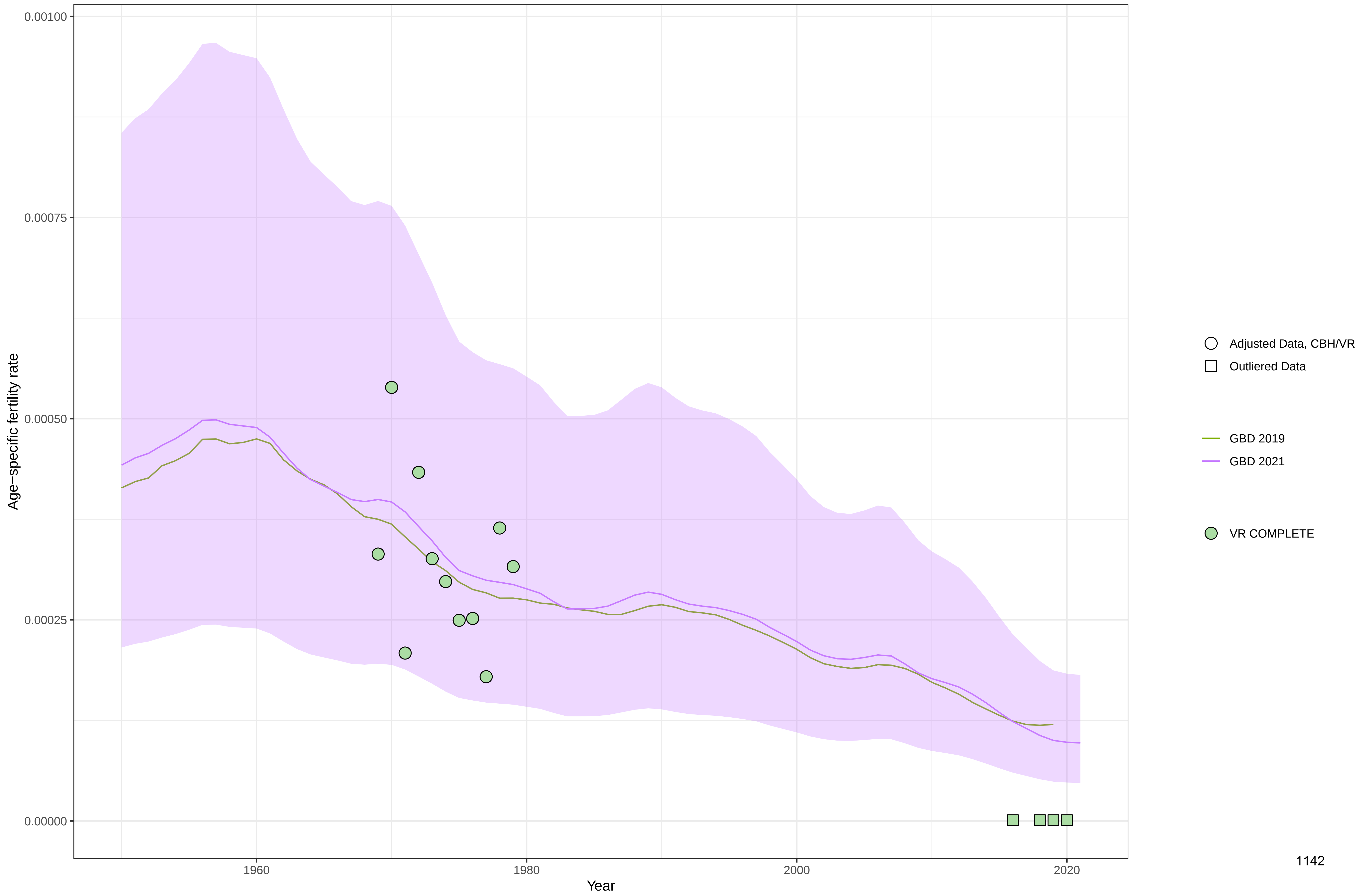

Age−specific fertility fitted trend lines, 1950−2021: New Hampshire, Ages: 15−19
Data points enclosed with a diamond are reference sources

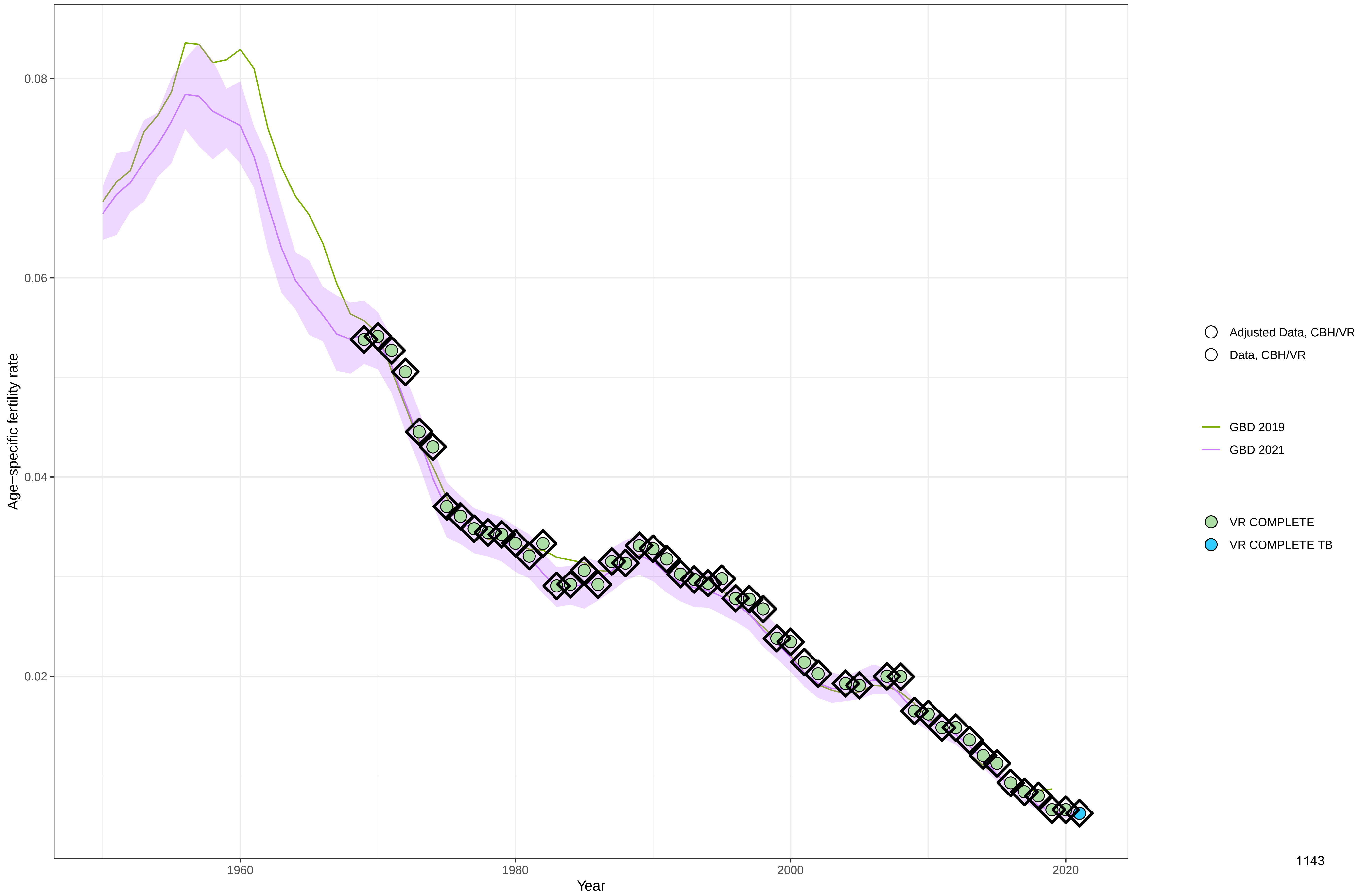

Age−specific fertility fitted trend lines, 1950−2021: New Hampshire, Ages: 20−24
Data points enclosed with a diamond are reference sources

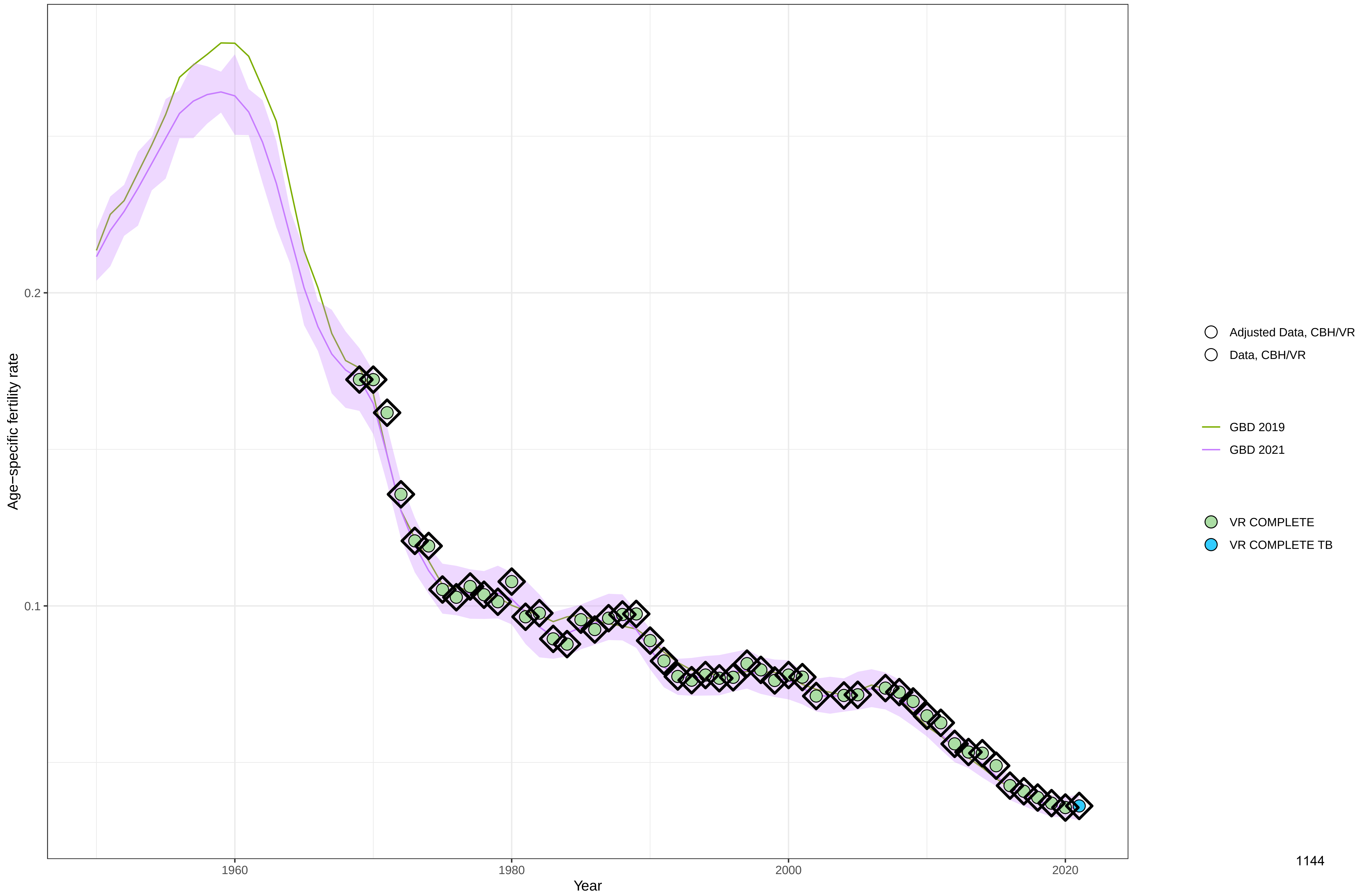

Age−specific fertility fitted trend lines, 1950−2021: New Hampshire, Ages: 25−29
Data points enclosed with a diamond are reference sources

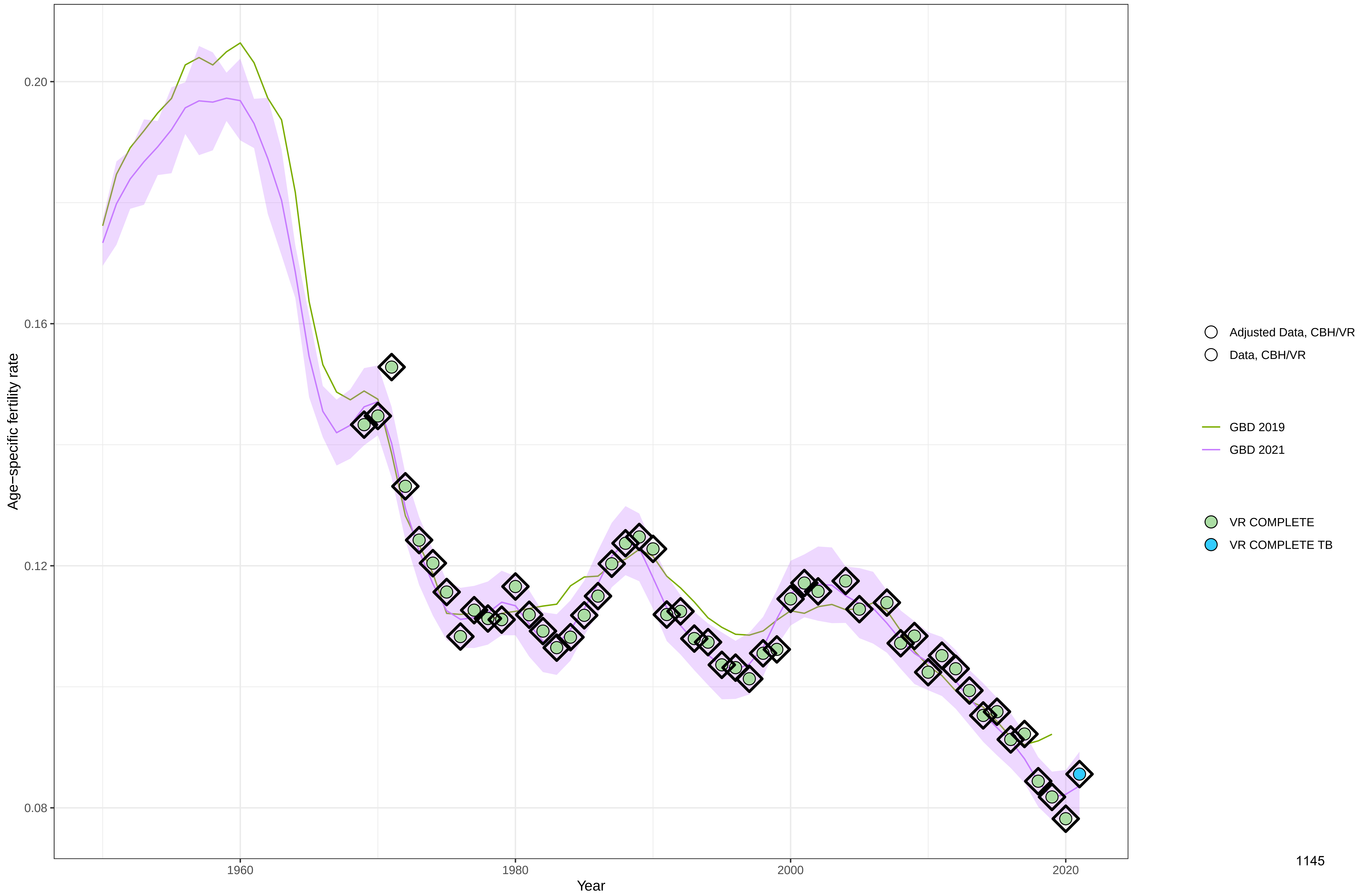

Age−specific fertility fitted trend lines, 1950−2021: New Hampshire, Ages: 30−34
Data points enclosed with a diamond are reference sources

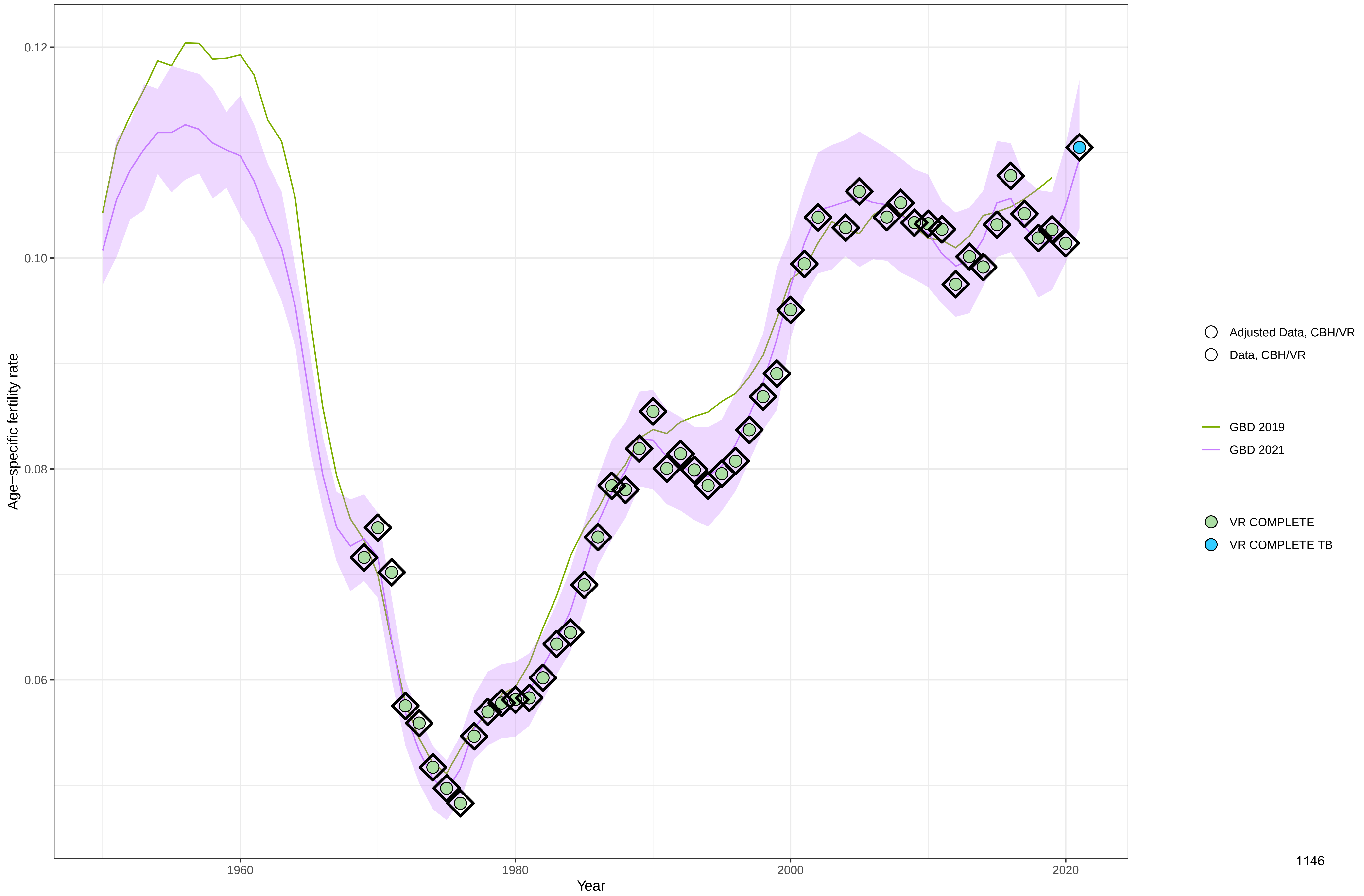

Age−specific fertility fitted trend lines, 1950−2021: New Hampshire, Ages: 35−39
Data points enclosed with a diamond are reference sources

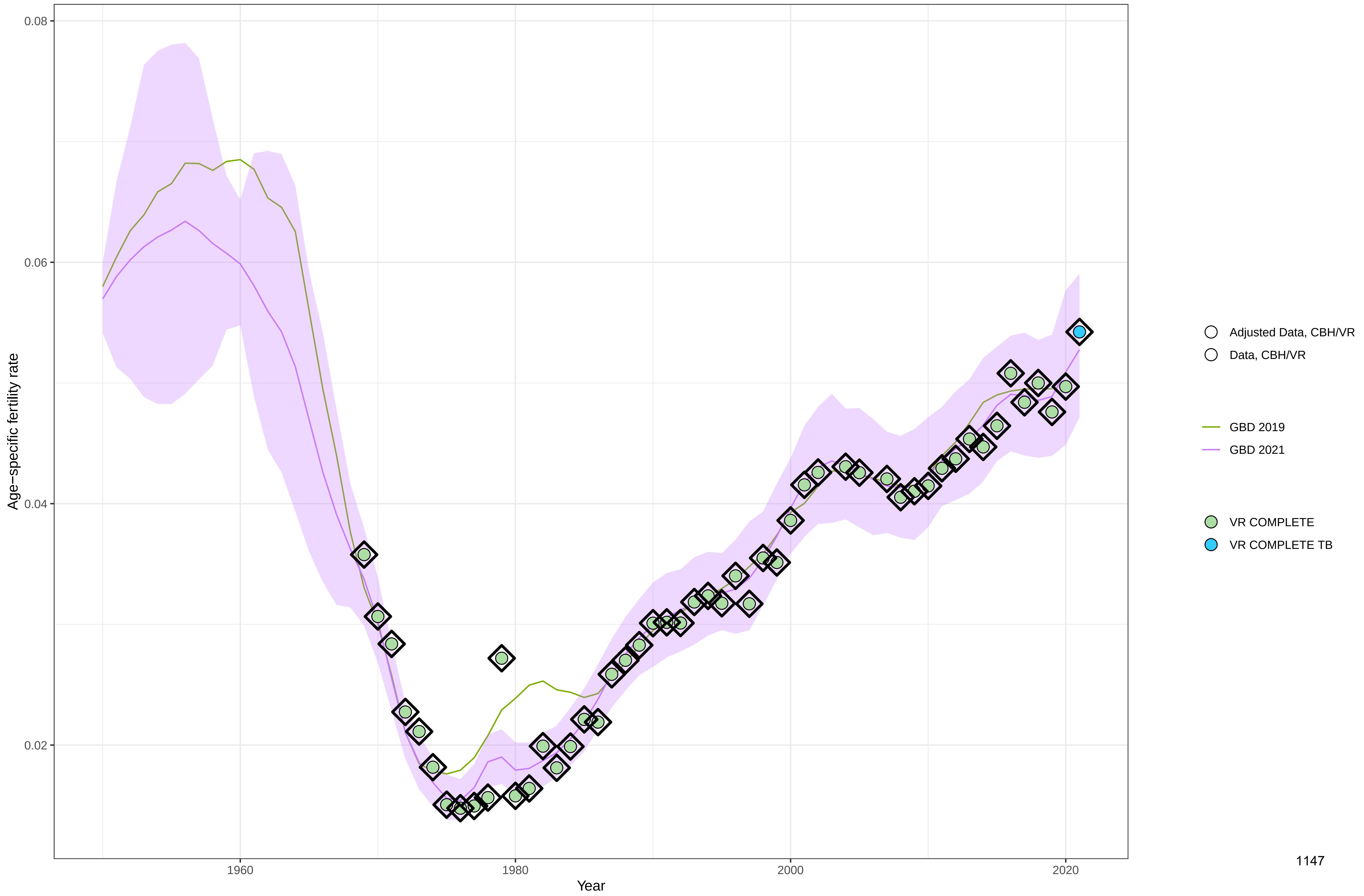

Age−specific fertility fitted trend lines, 1950−2021: New Hampshire, Ages: 40−44
Data points enclosed with a diamond are reference sources

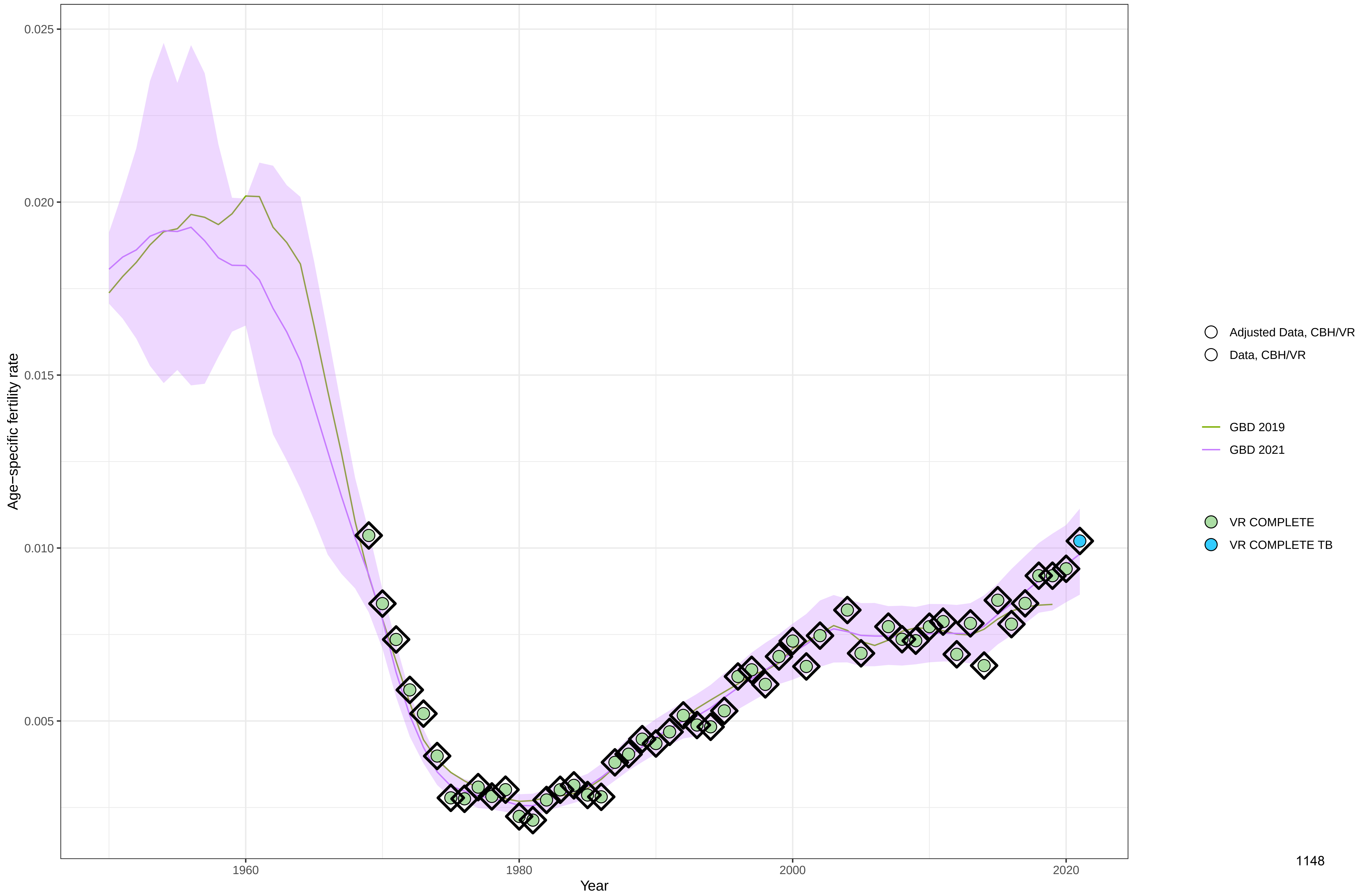

Age−specific fertility fitted trend lines, 1950−2021: New Hampshire, Ages: 45−49
Data points enclosed with a diamond are reference sources

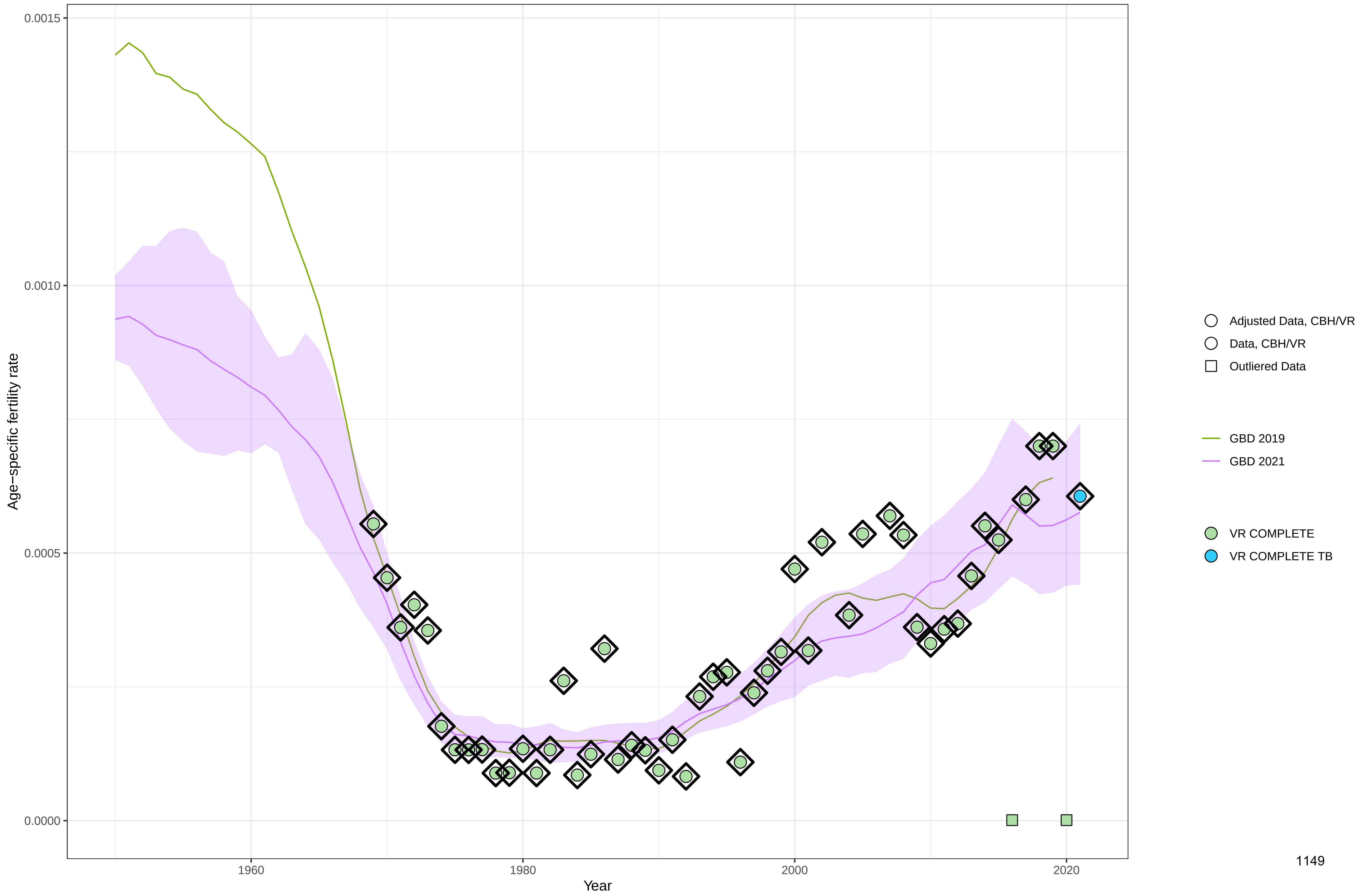

Age−specific fertility fitted trend lines, 1950−2021: New Hampshire, Ages: 50−54
Data points enclosed with a diamond are reference sources

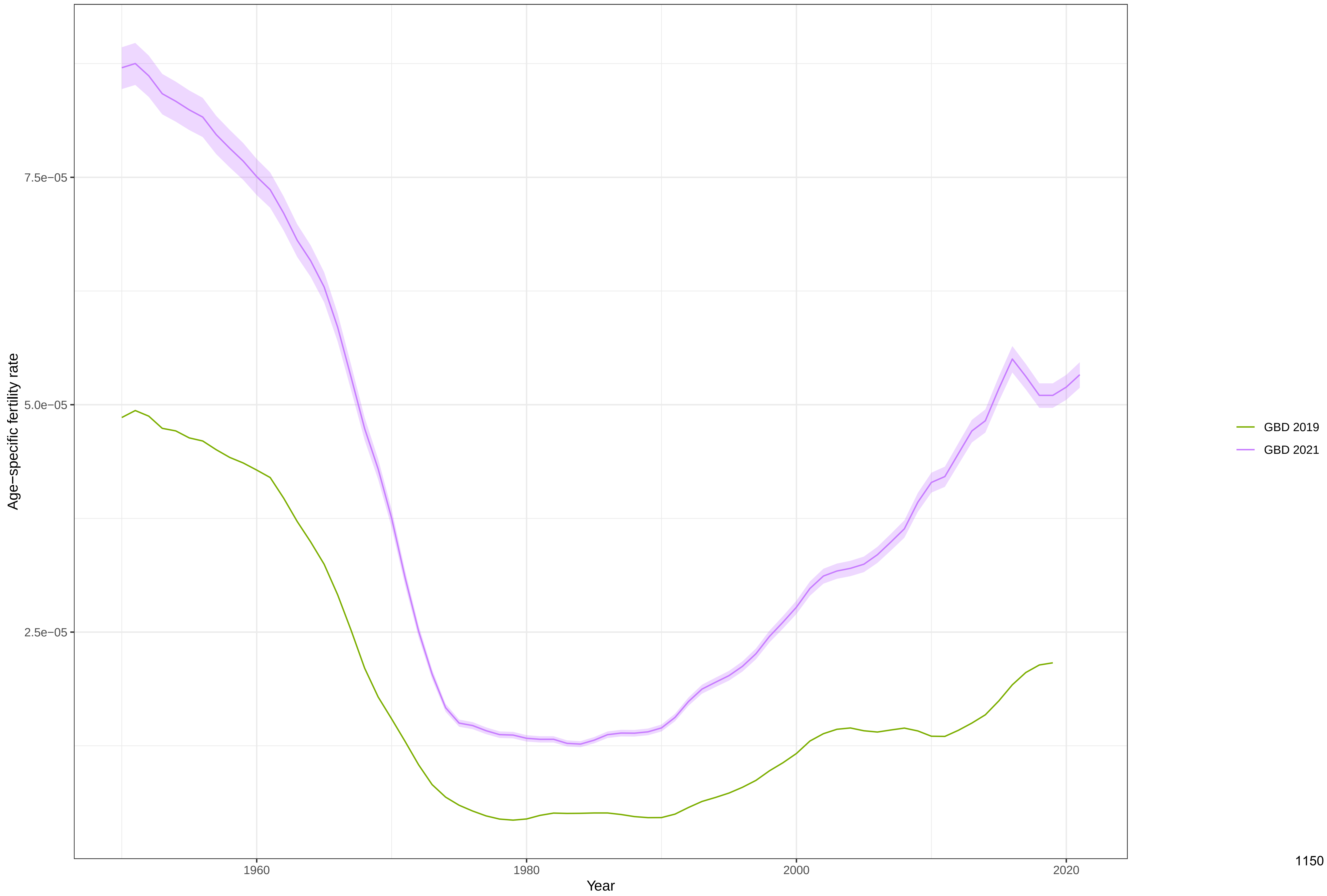

Total fertility rate fitted trend lines, 1950−2021: New Hampshire TFR
Data points enclosed with a diamond are reference sources

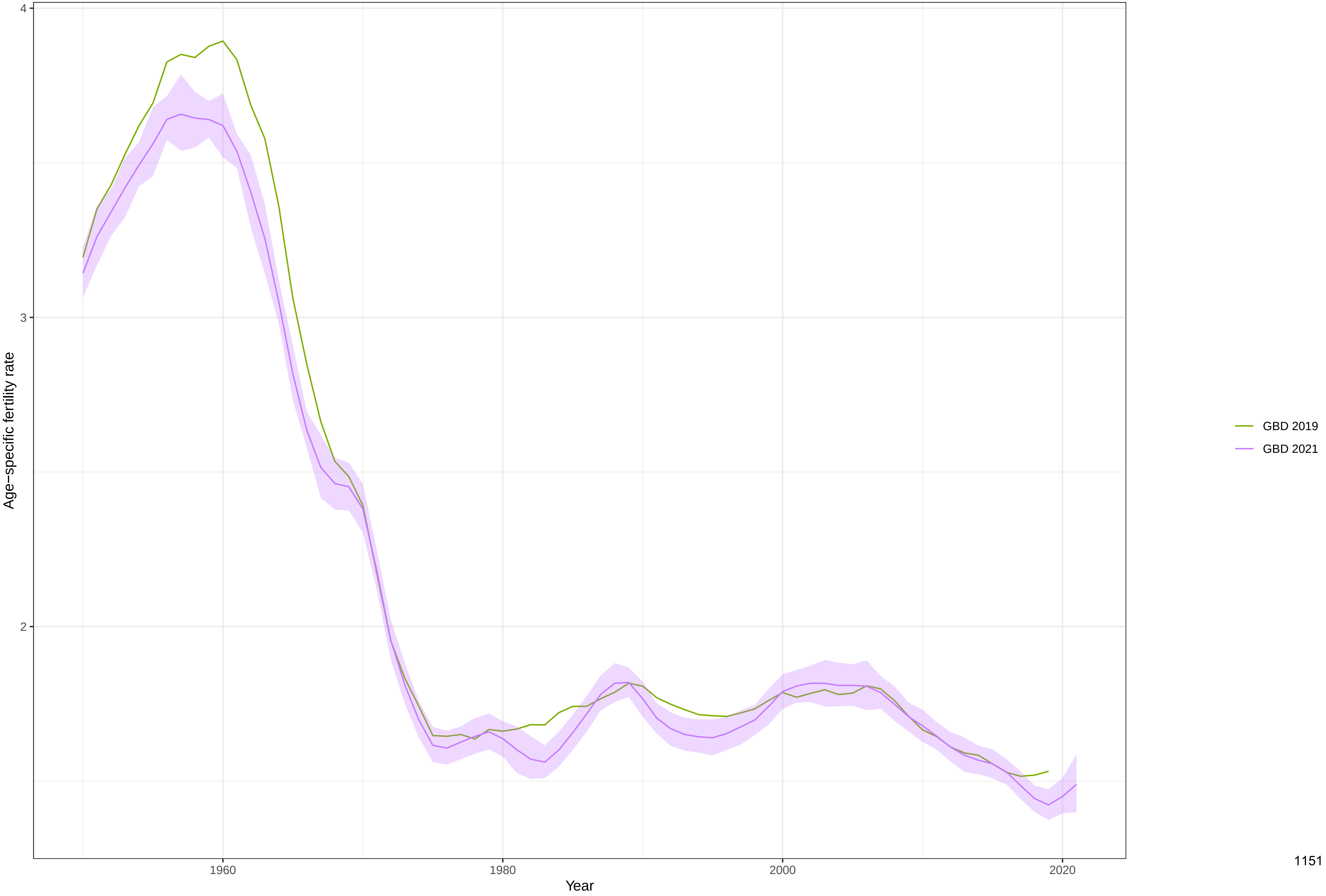

Age−specific fertility fitted trend lines, 1950−2021: New Jersey, Ages: 10−14
Data points enclosed with a diamond are reference sources

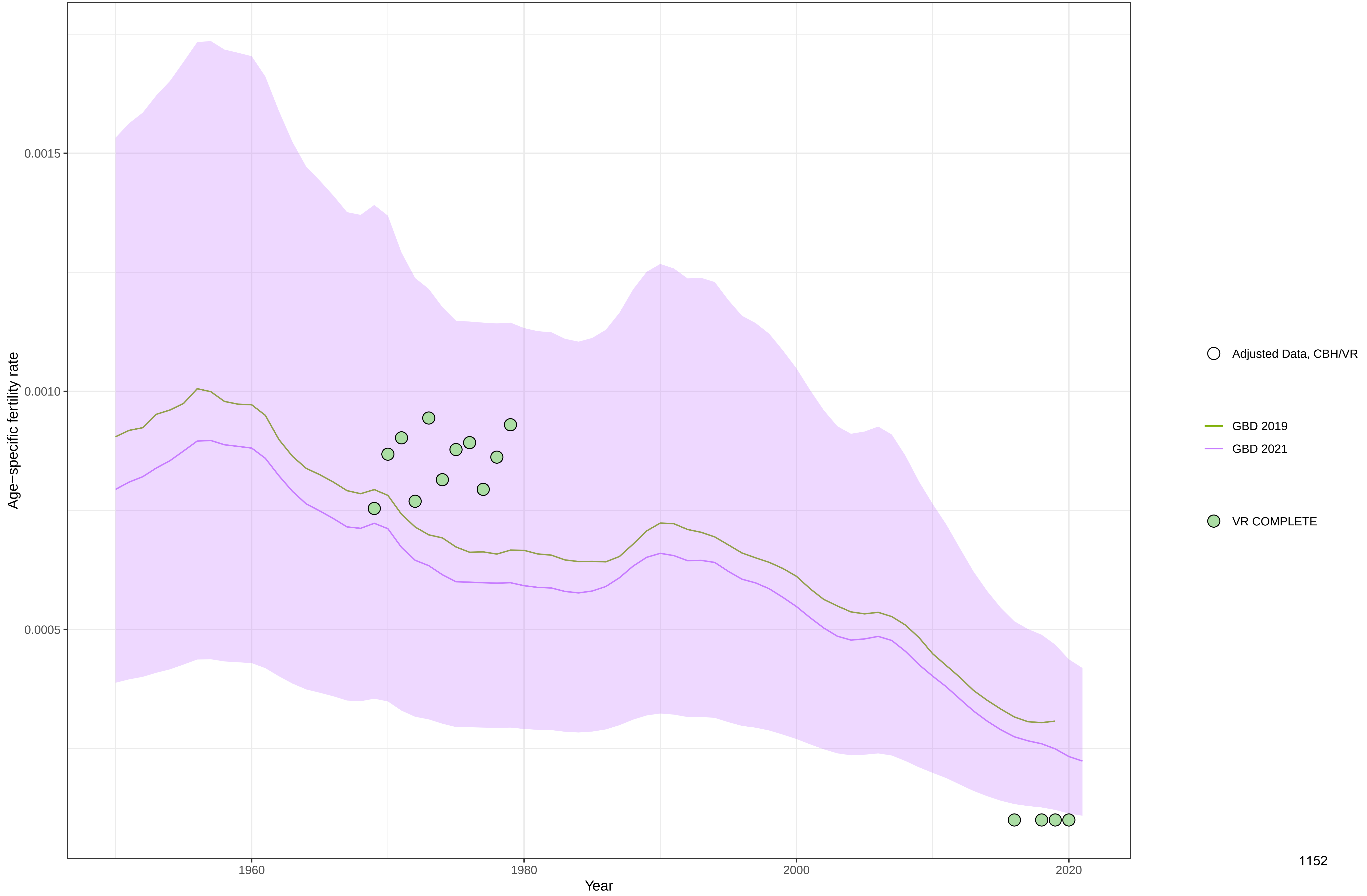

Age−specific fertility fitted trend lines, 1950−2021: New Jersey, Ages: 15−19
Data points enclosed with a diamond are reference sources

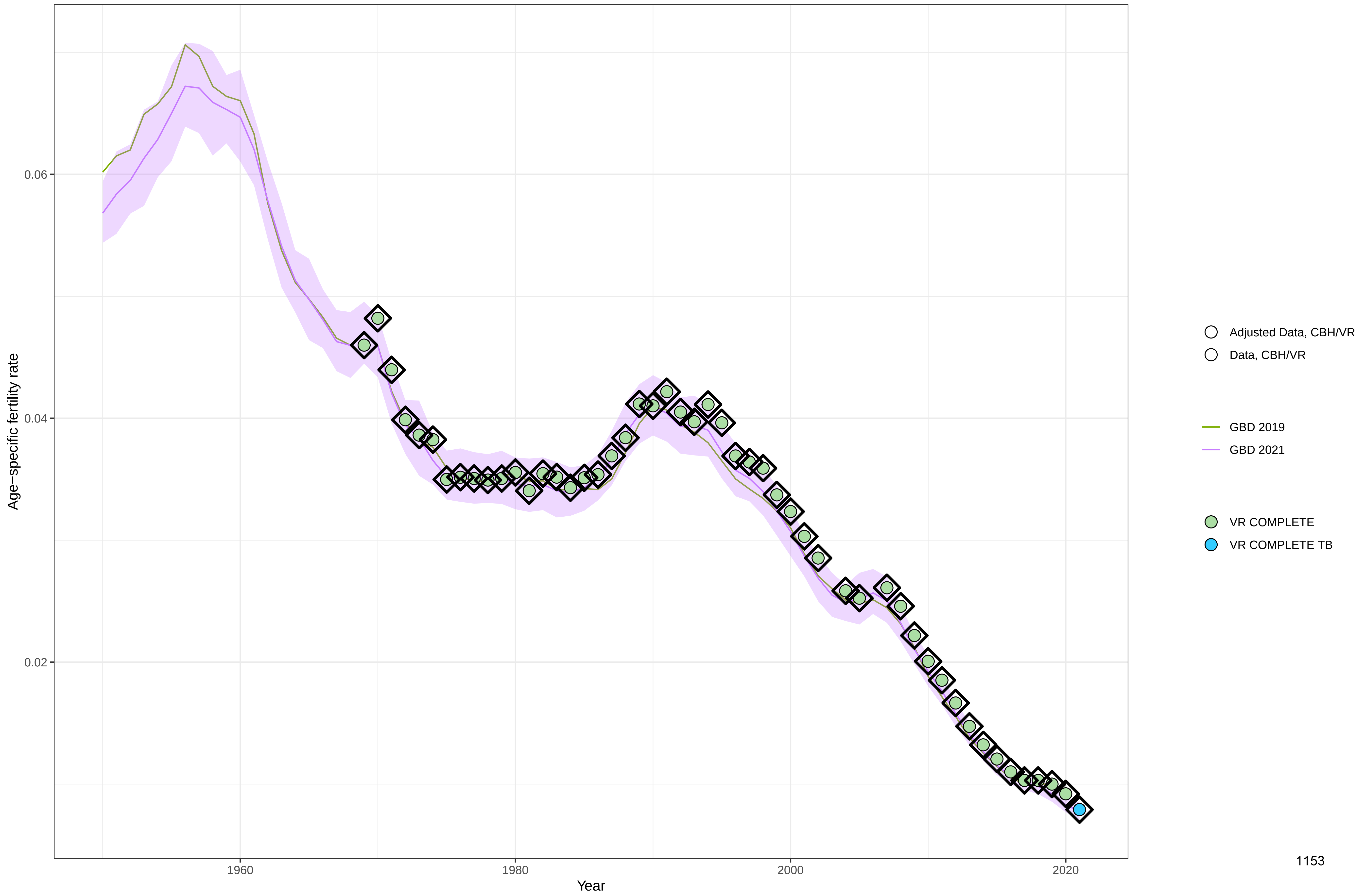

Age−specific fertility fitted trend lines, 1950−2021: New Jersey, Ages: 20−24
Data points enclosed with a diamond are reference sources

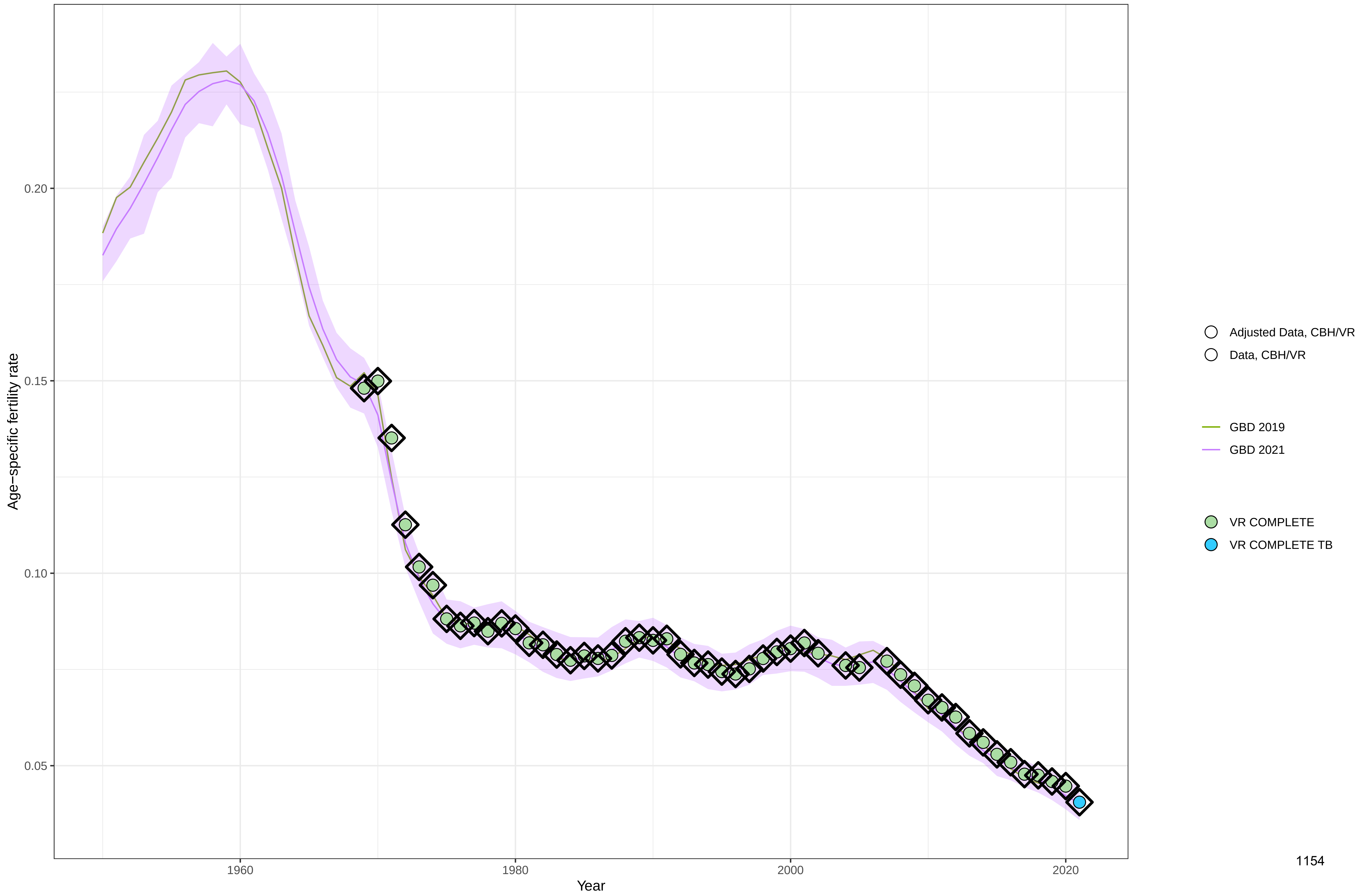

Age−specific fertility fitted trend lines, 1950−2021: New Jersey, Ages: 25−29
Data points enclosed with a diamond are reference sources

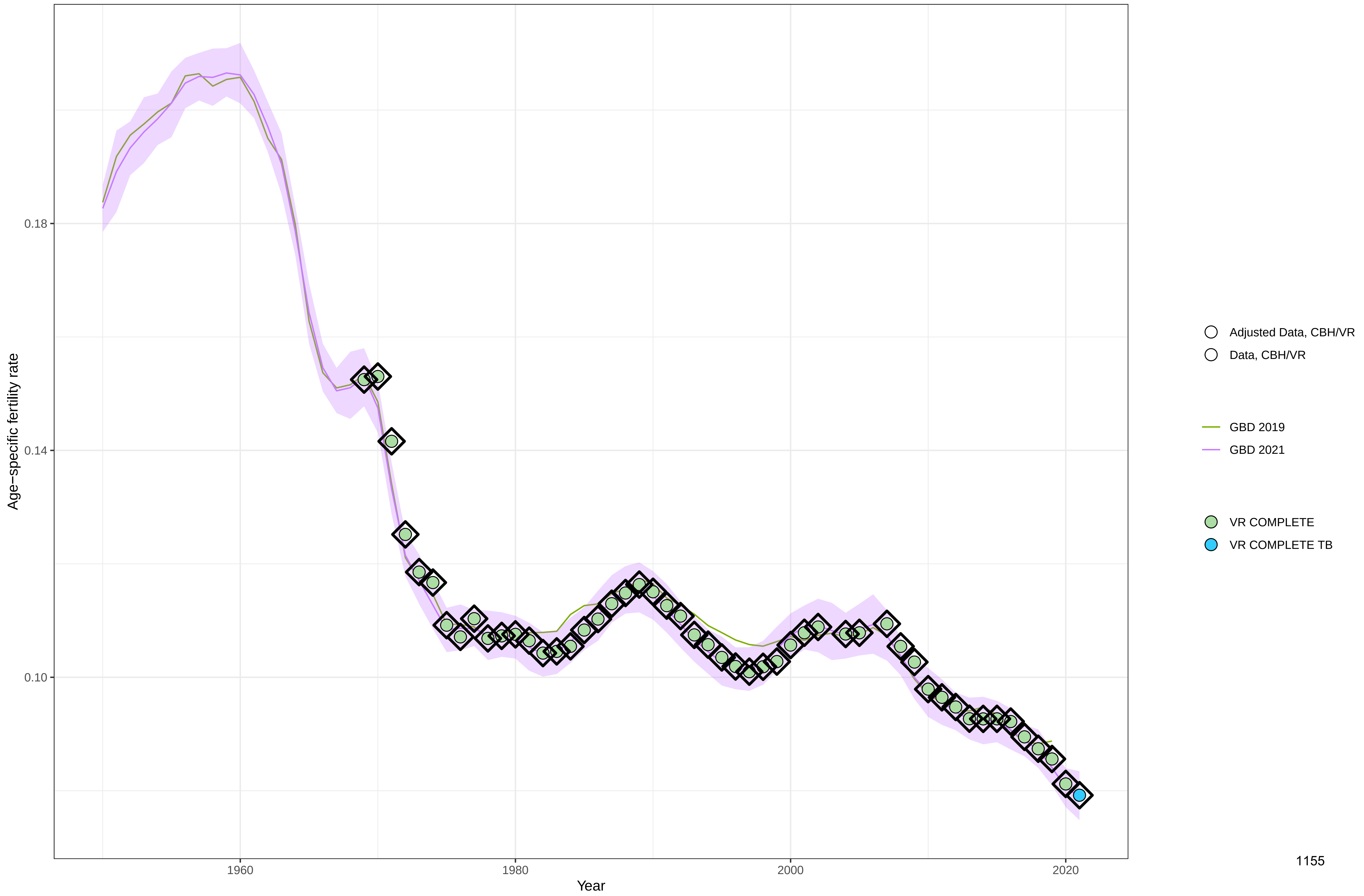

Age−specific fertility fitted trend lines, 1950−2021: New Jersey, Ages: 30−34
Data points enclosed with a diamond are reference sources

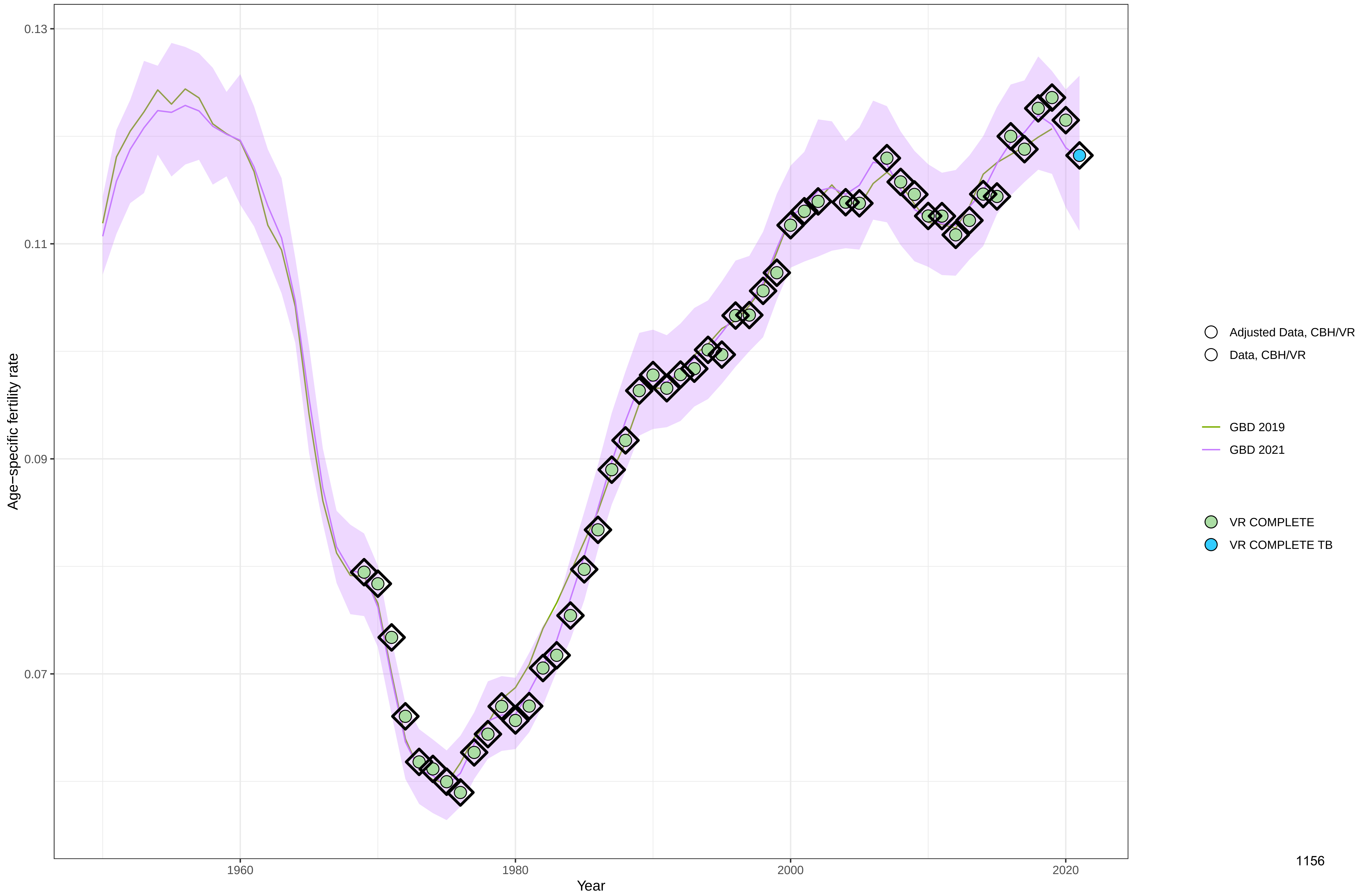

Age−specific fertility fitted trend lines, 1950−2021: New Jersey, Ages: 35−39
Data points enclosed with a diamond are reference sources

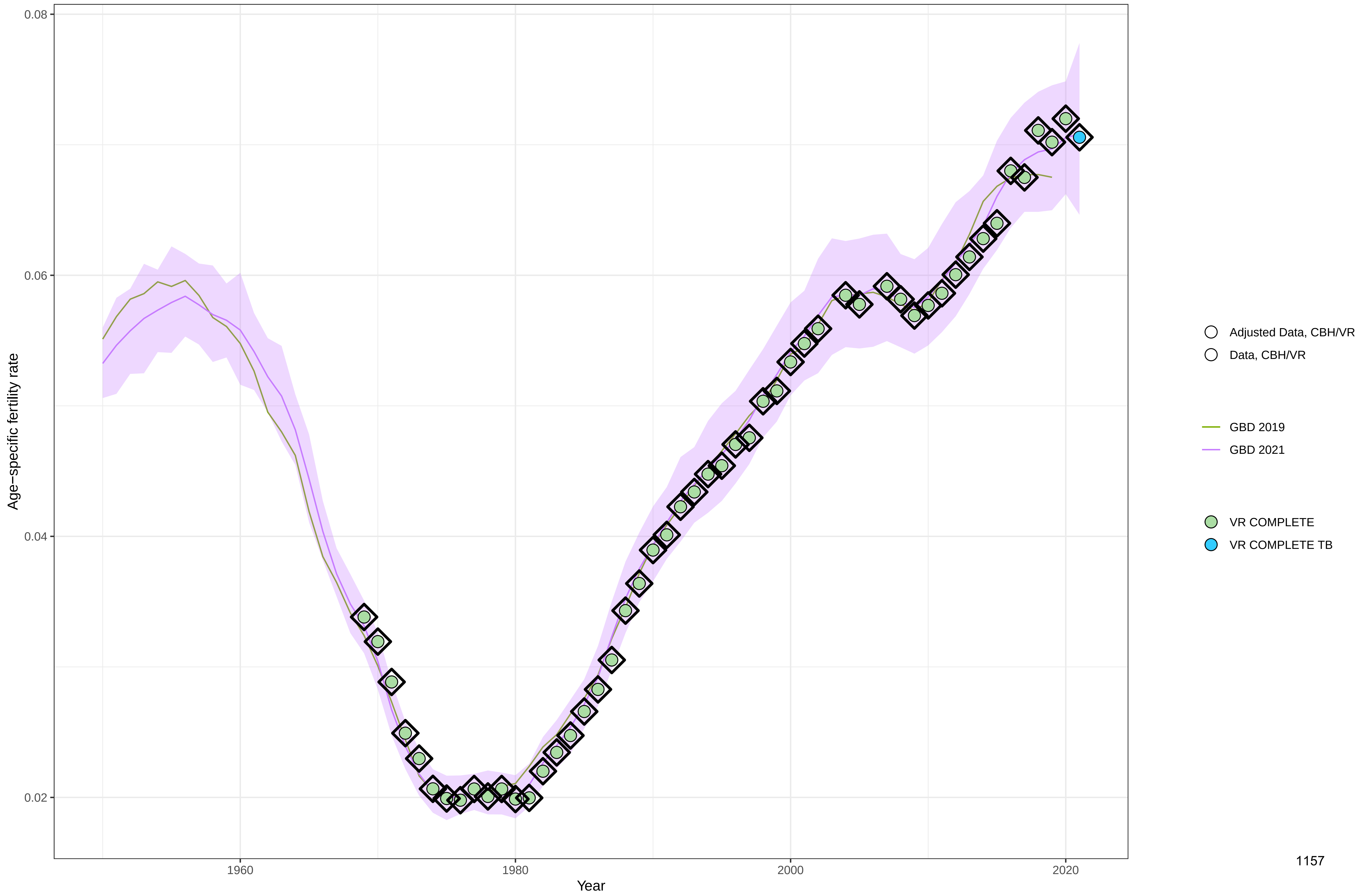

Age−specific fertility fitted trend lines, 1950−2021: New Jersey, Ages: 40−44
Data points enclosed with a diamond are reference sources

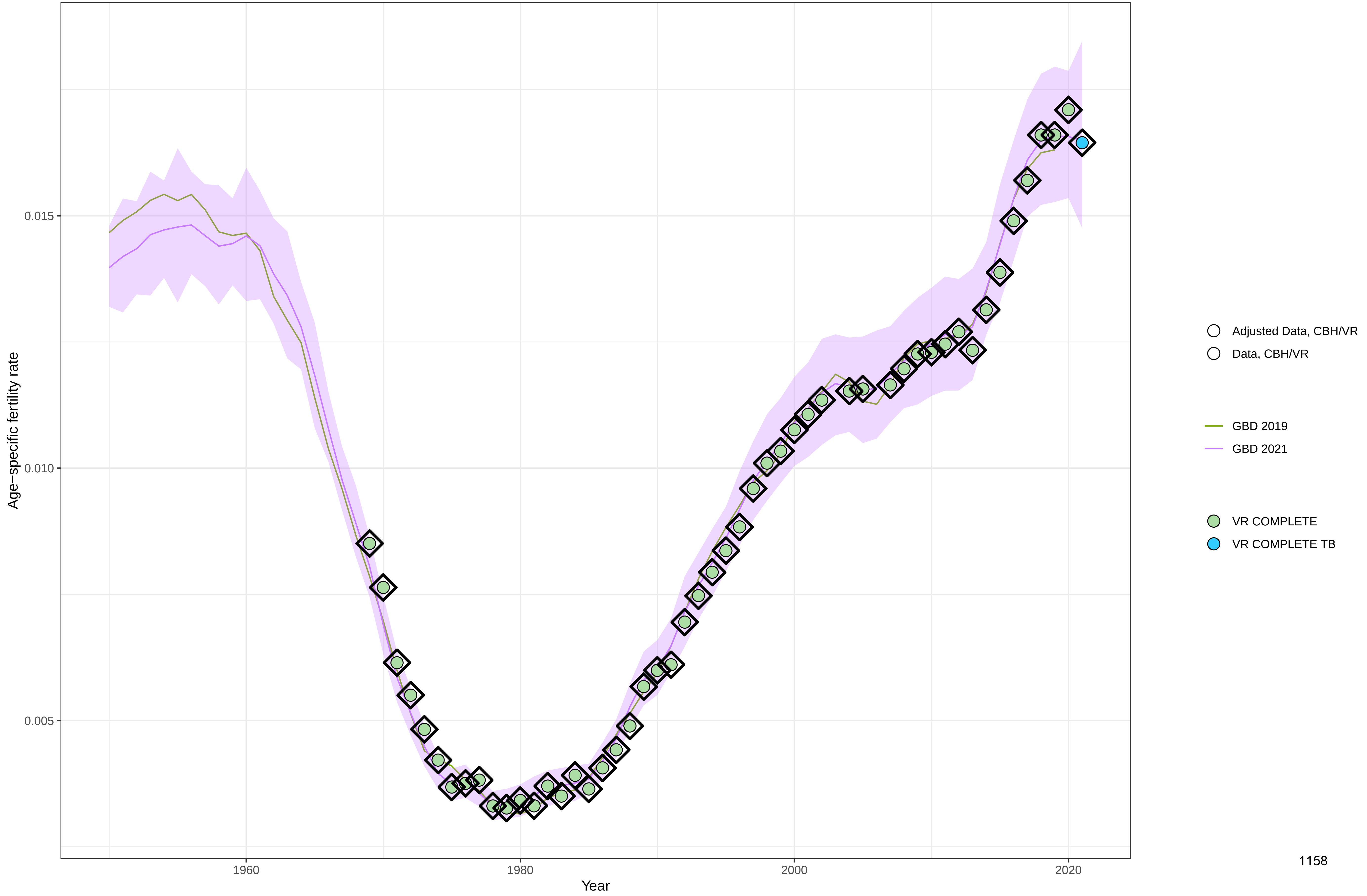

Age−specific fertility fitted trend lines, 1950−2021: New Jersey, Ages: 45−49
Data points enclosed with a diamond are reference sources

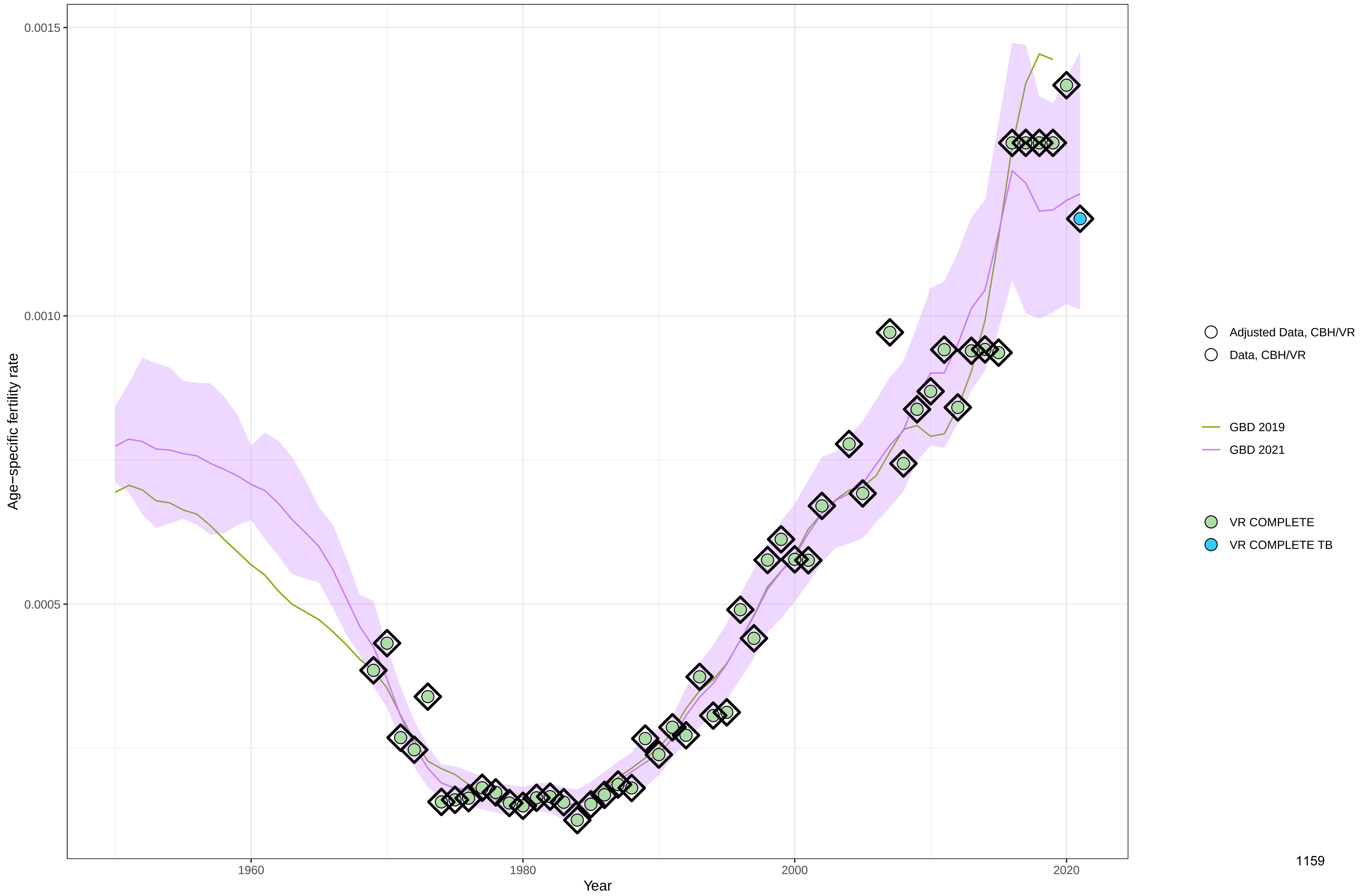

Age−specific fertility fitted trend lines, 1950−2021: New Jersey, Ages: 50−54
Data points enclosed with a diamond are reference sources

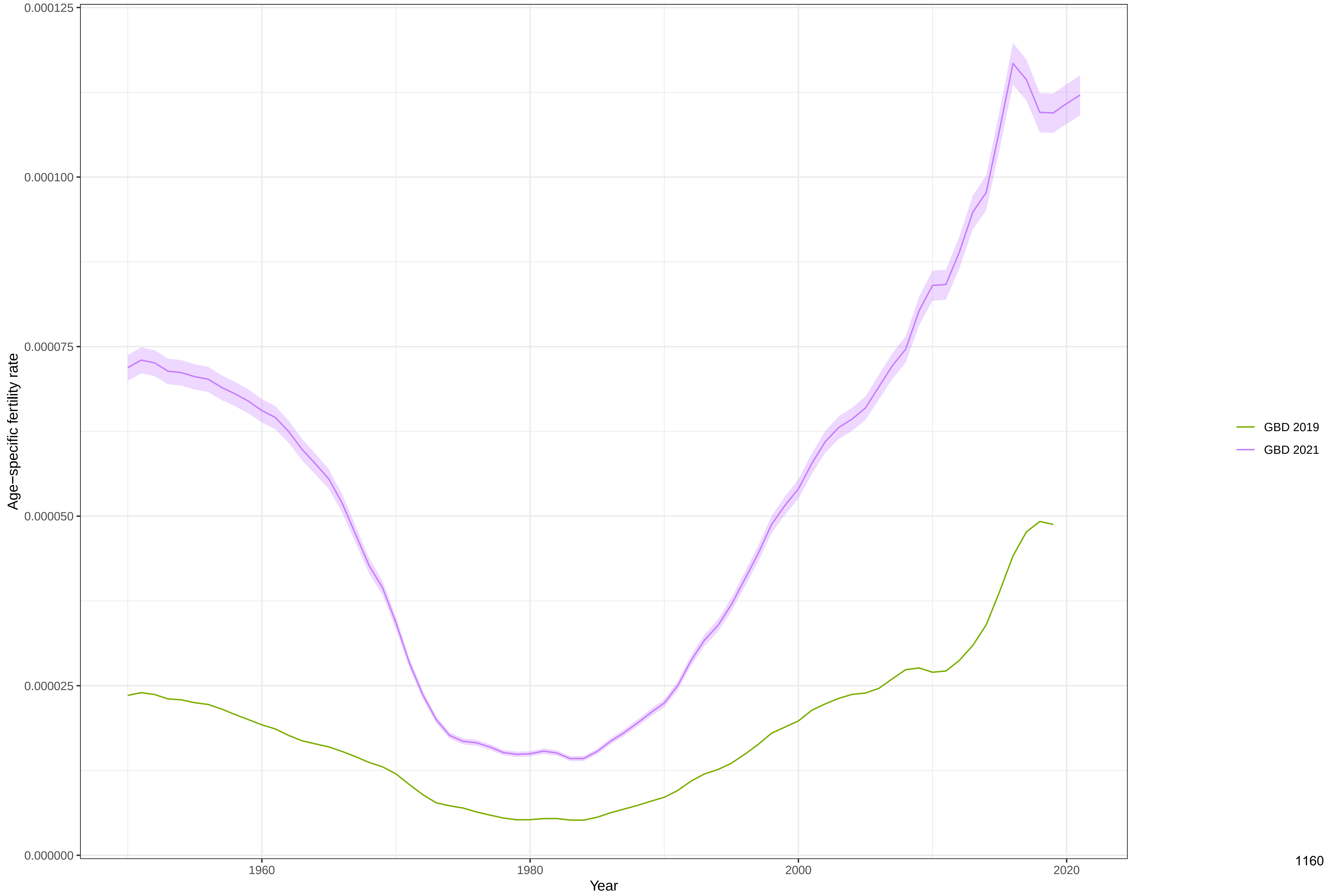

Total fertility rate fitted trend lines, 1950−2021: New Jersey TFR
Data points enclosed with a diamond are reference sources

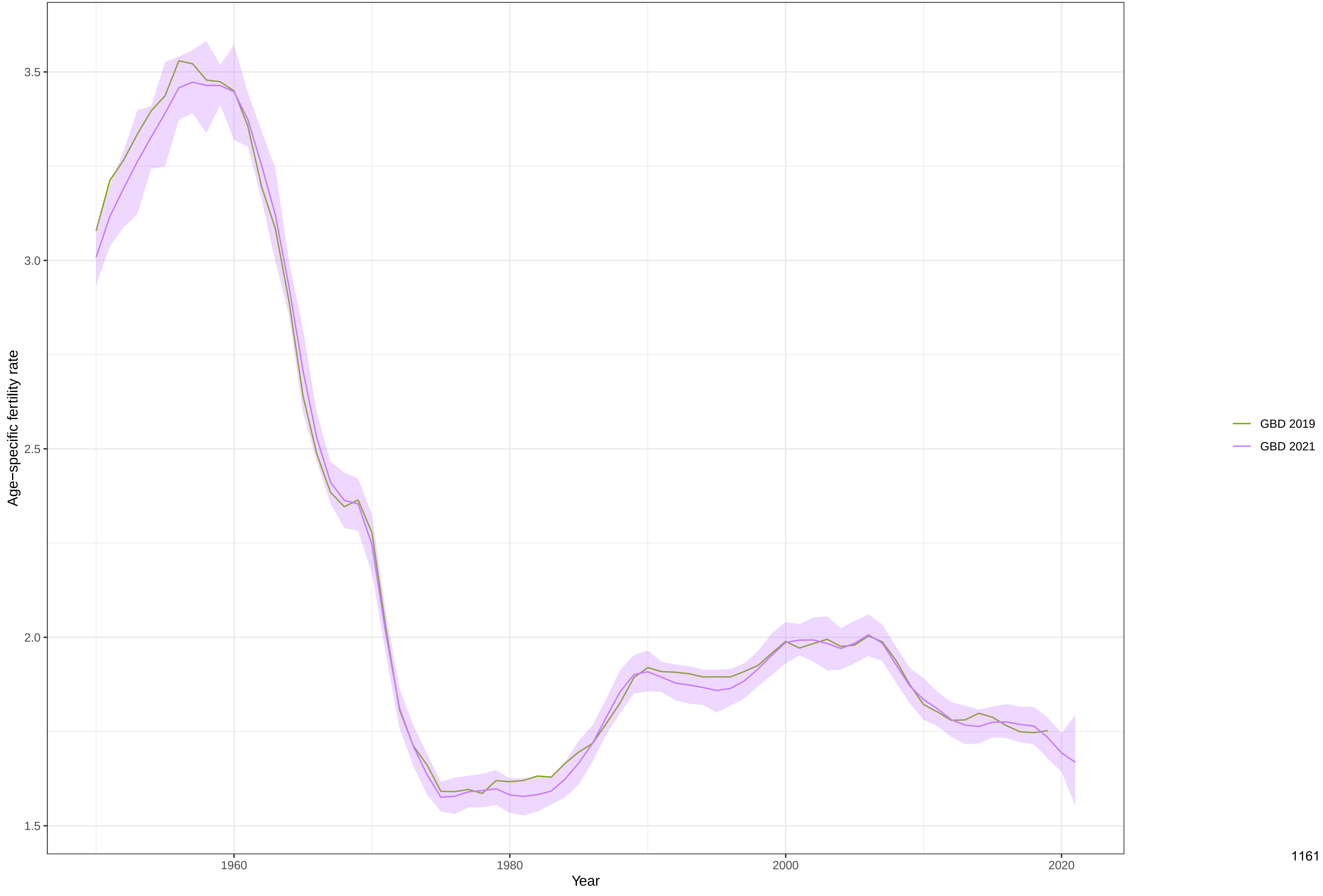

Age−specific fertility fitted trend lines, 1950−2021: New Mexico, Ages: 10−14
Data points enclosed with a diamond are reference sources

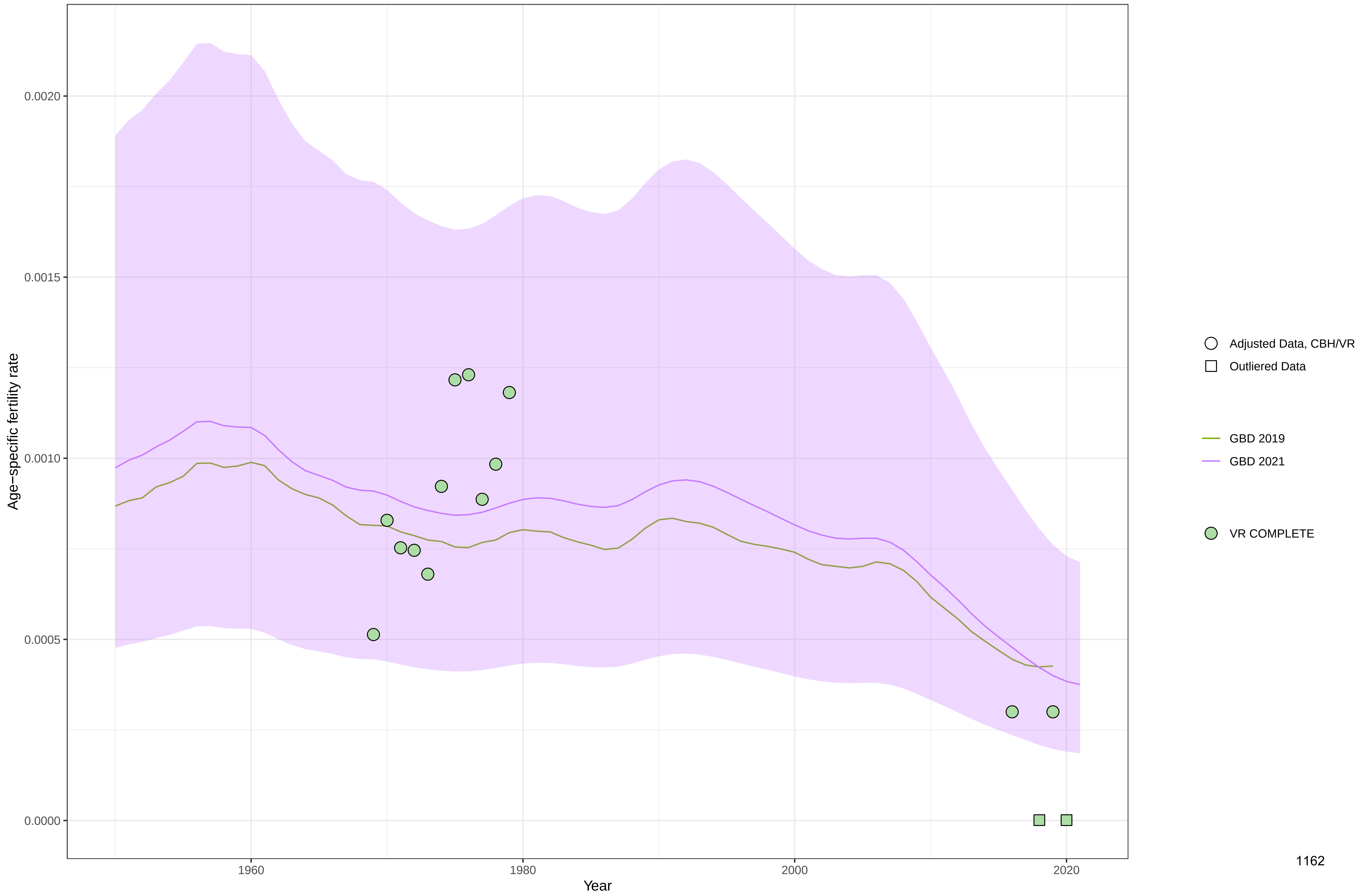

Age−specific fertility fitted trend lines, 1950−2021: New Mexico, Ages: 15−19
Data points enclosed with a diamond are reference sources

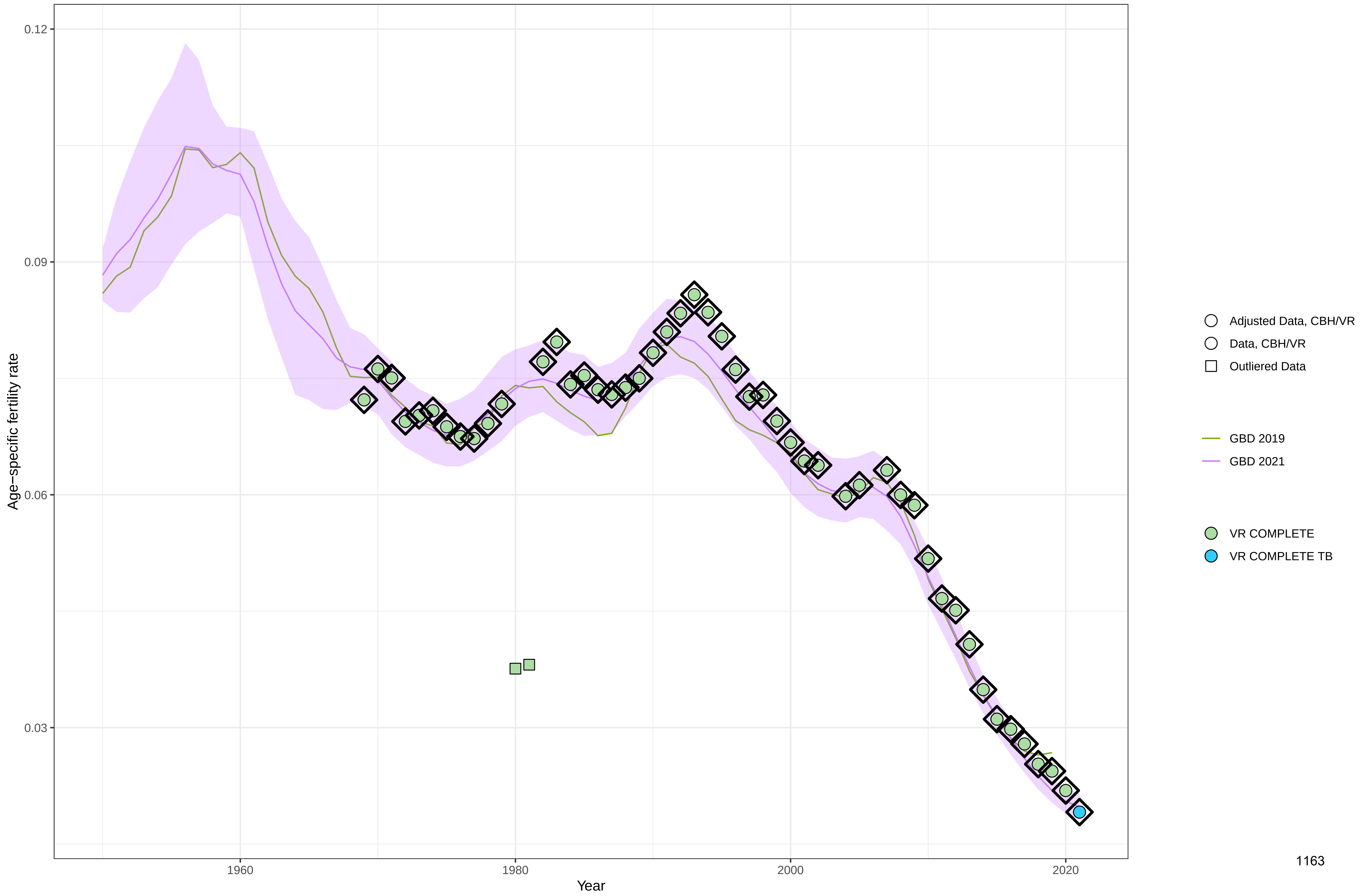

Age−specific fertility fitted trend lines, 1950−2021: New Mexico, Ages: 20−24
Data points enclosed with a diamond are reference sources

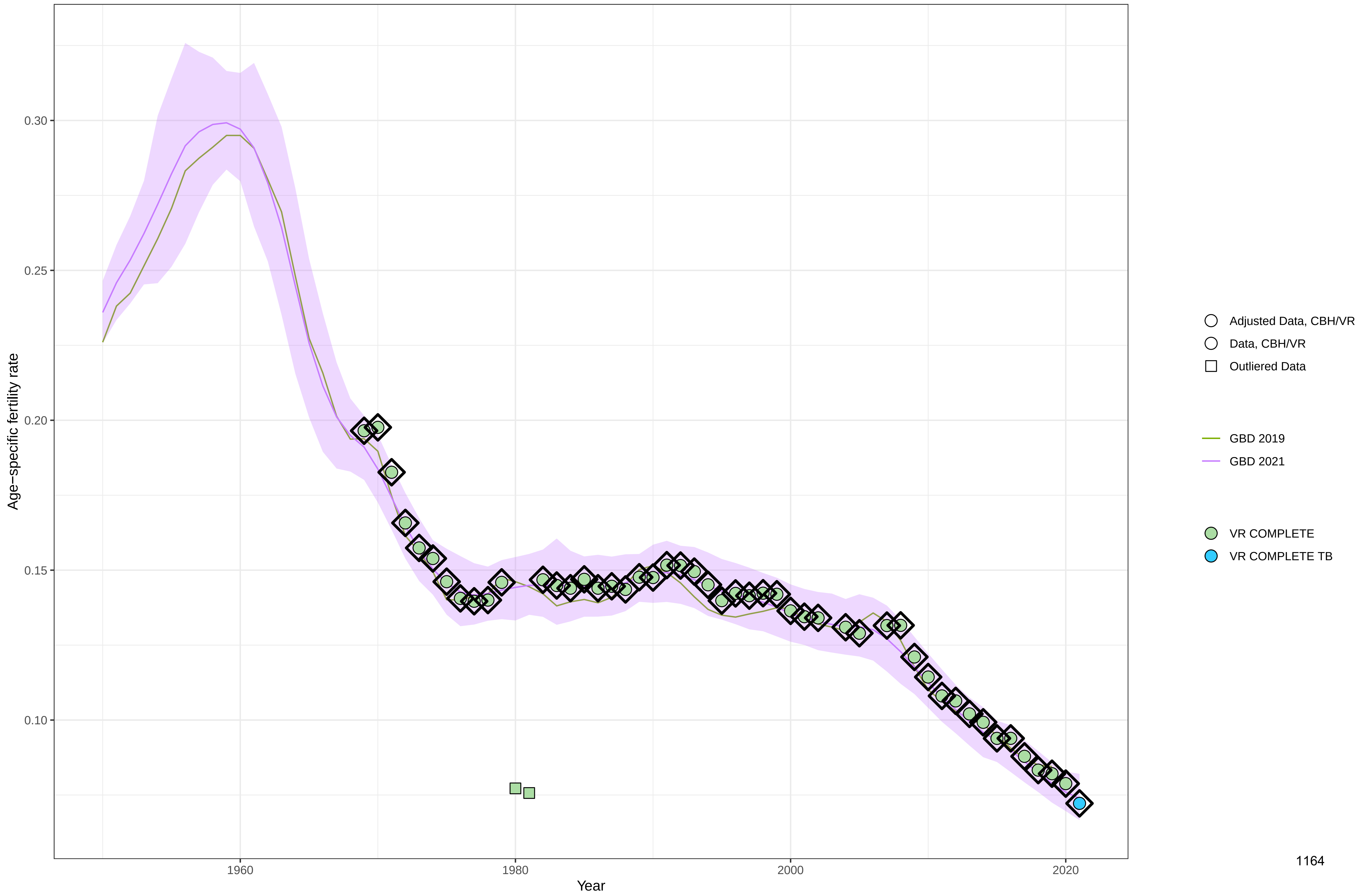

Age−specific fertility fitted trend lines, 1950−2021: New Mexico, Ages: 25−29
Data points enclosed with a diamond are reference sources

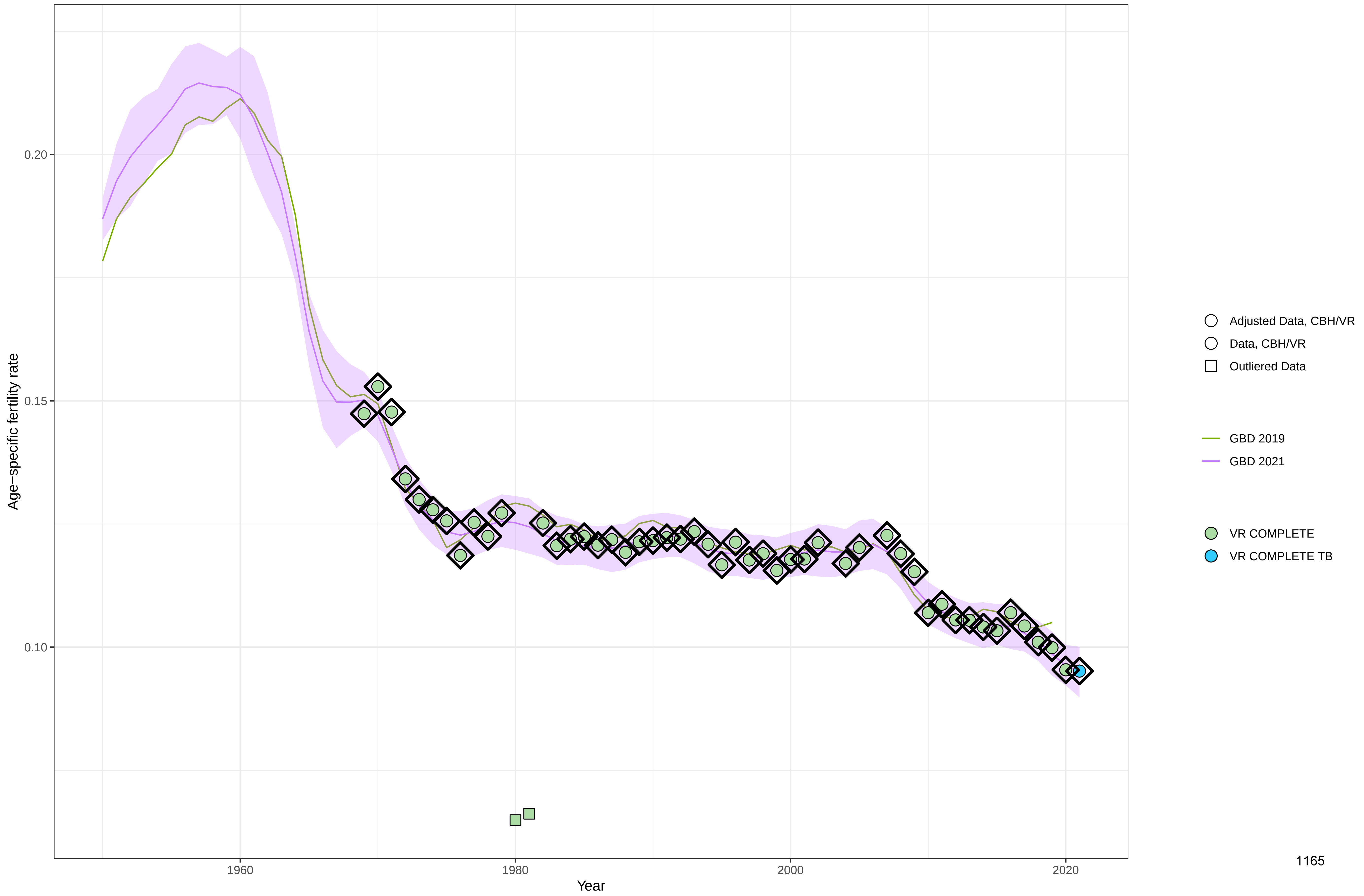

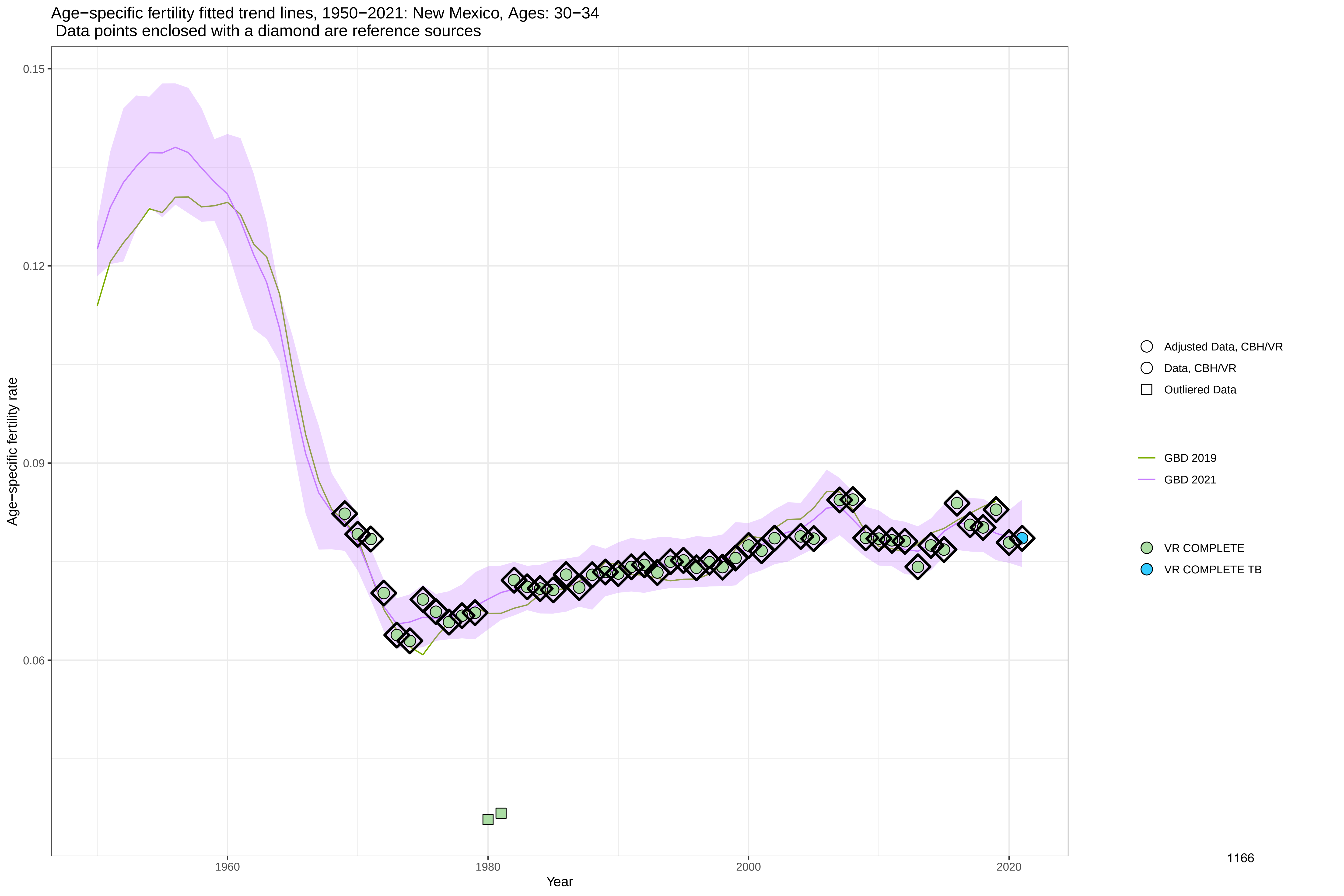

Age−specific fertility fitted trend lines, 1950−2021: New Mexico, Ages: 30−34
Data points enclosed with a diamond are reference sources
Age−specific fertility rate
0.15
0.12
0.09
0.06
1960
1980
2000
2020
Year
Adjusted Data, CBH/VR
Data, CBH/VR
Outliered Data
GBD 2019
GBD 2021
VR COMPLETE
VR COMPLETE TB
1166

Age−specific fertility fitted trend lines, 1950−2021: New Mexico, Ages: 35−39
Data points enclosed with a diamond are reference sources

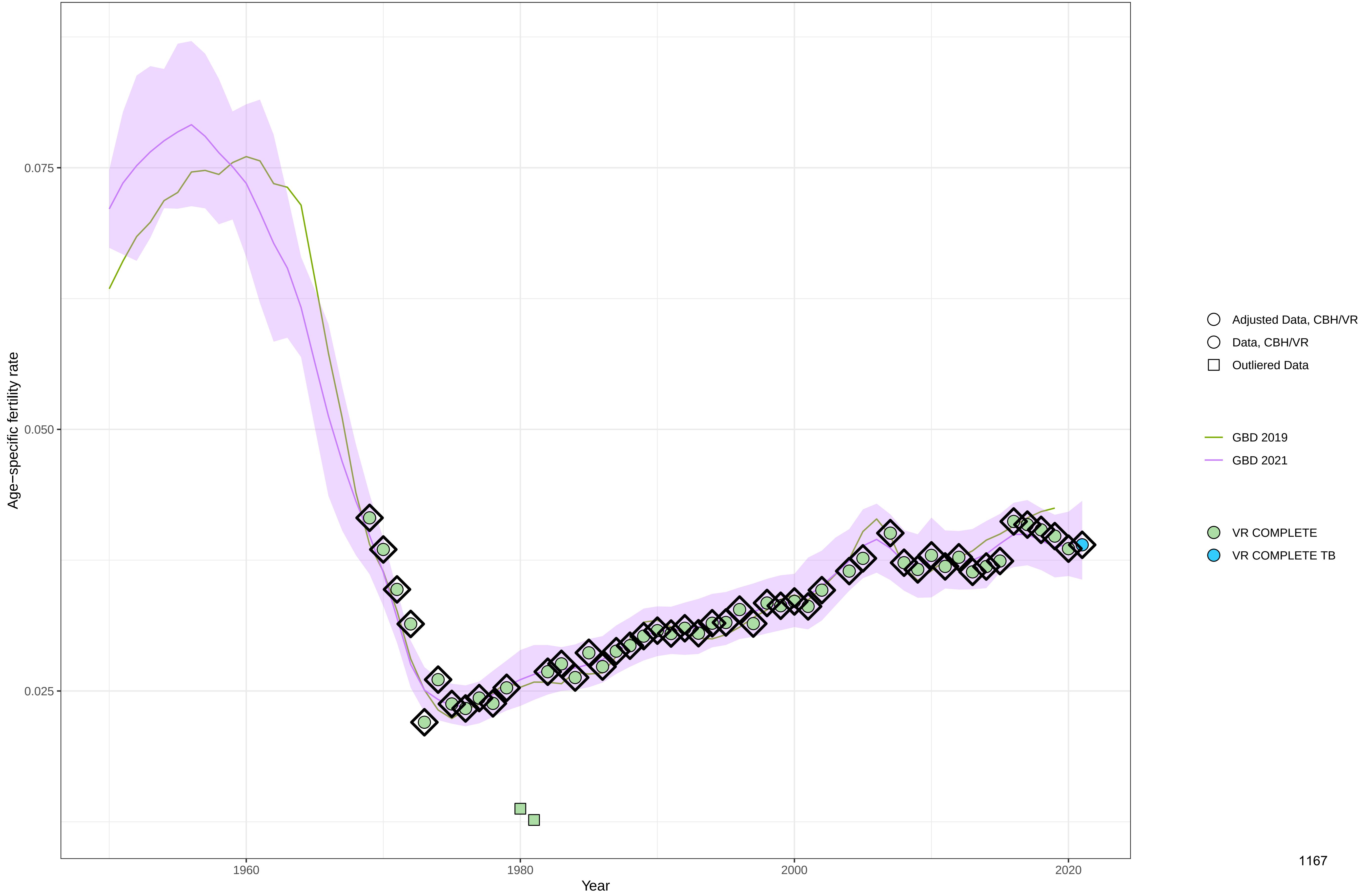

Age−specific fertility fitted trend lines, 1950−2021: New Mexico, Ages: 40−44
Data points enclosed with a diamond are reference sources

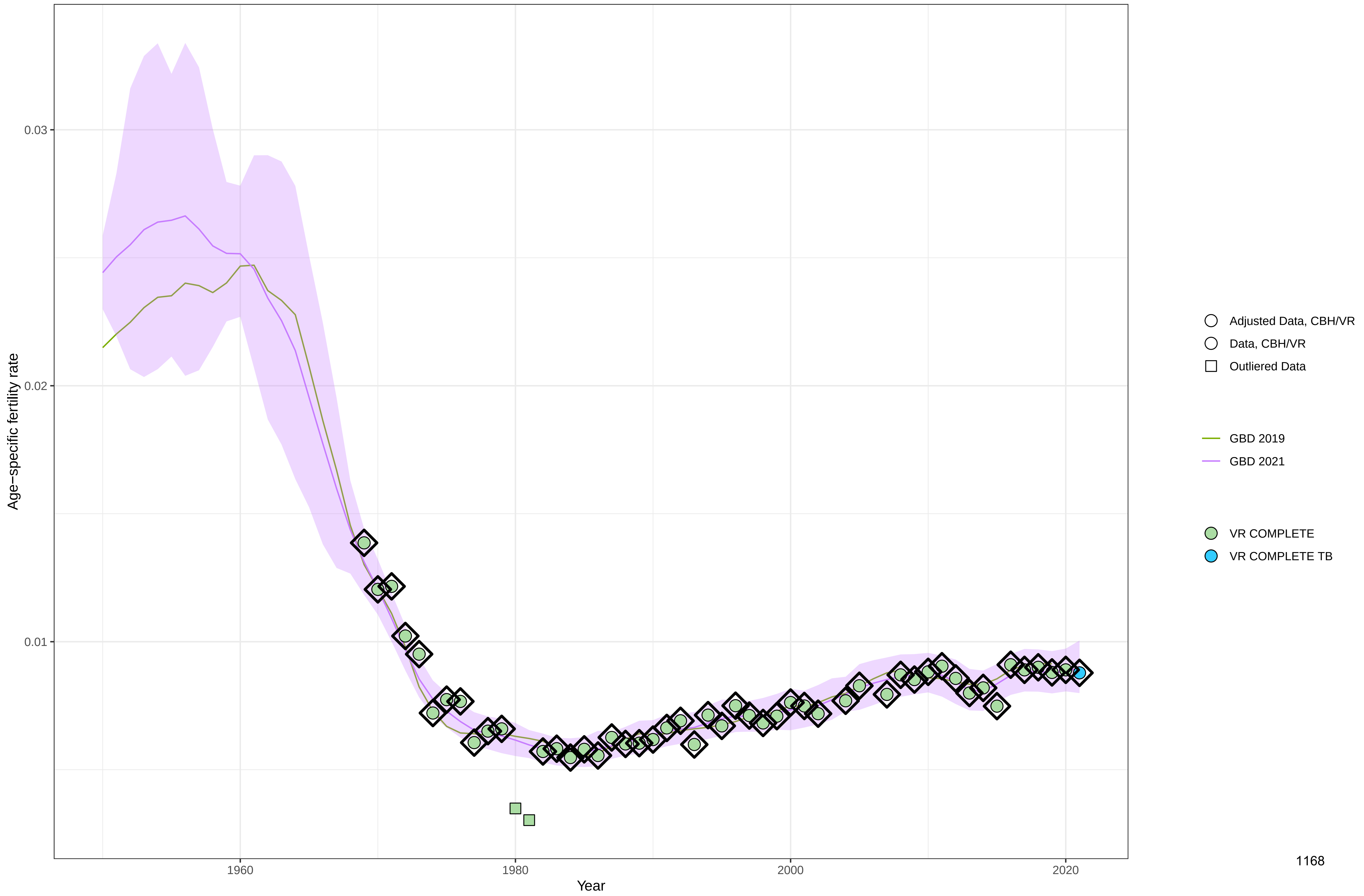

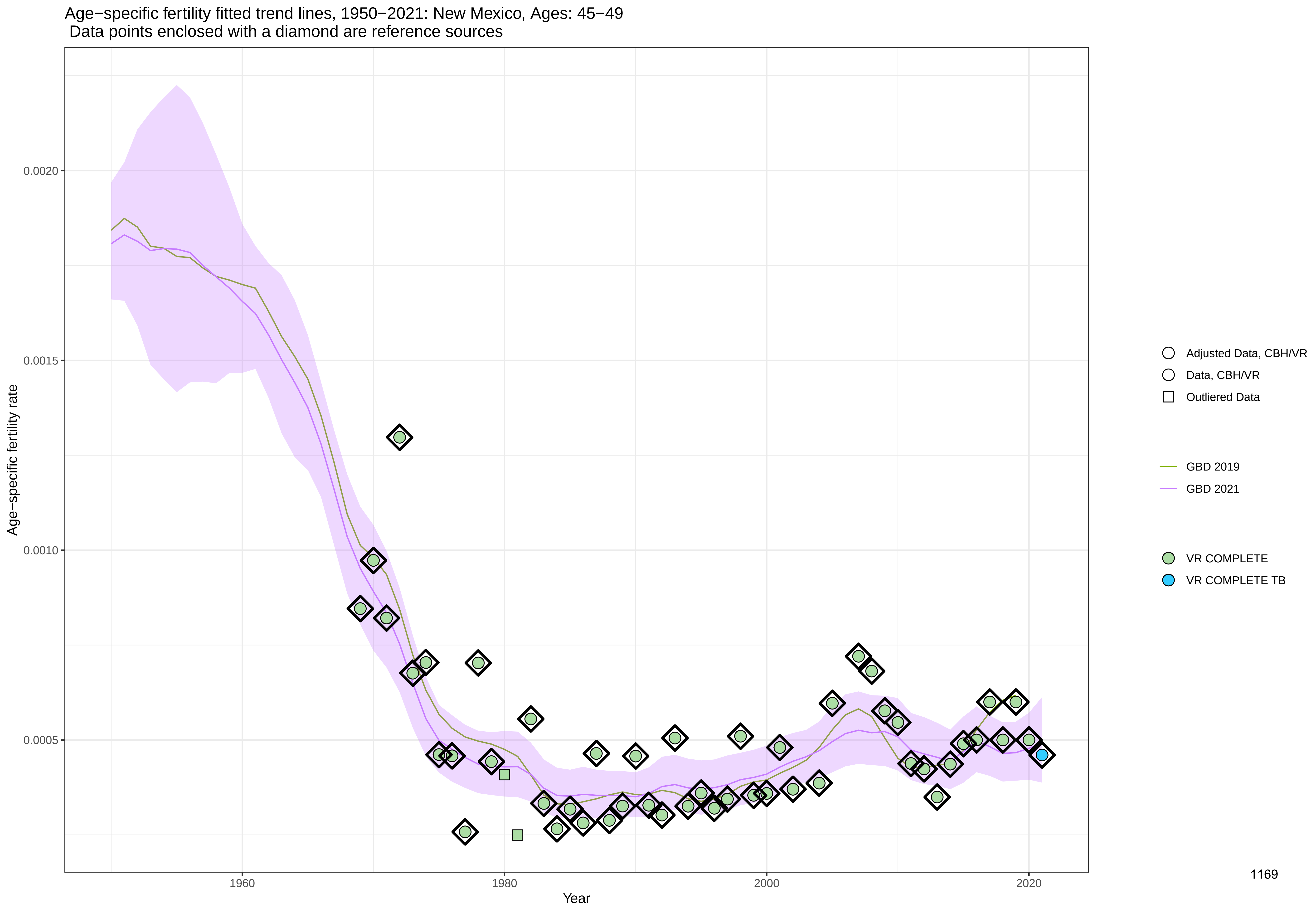

Age−specific fertility fitted trend lines, 1950−2021: New Mexico, Ages: 45−49
Data points enclosed with a diamond are reference sources
Age−specific fertility rate
0.0020
0.0015
0.0010
0.0005
Year
1960
1980
2000
2020
Adjusted Data, CBH/VR
Data, CBH/VR
Outliered Data
GBD 2019
GBD 2021
VR COMPLETE
VR COMPLETE TB

Age−specific fertility fitted trend lines, 1950−2021: New Mexico, Ages: 50−54
Data points enclosed with a diamond are reference sources

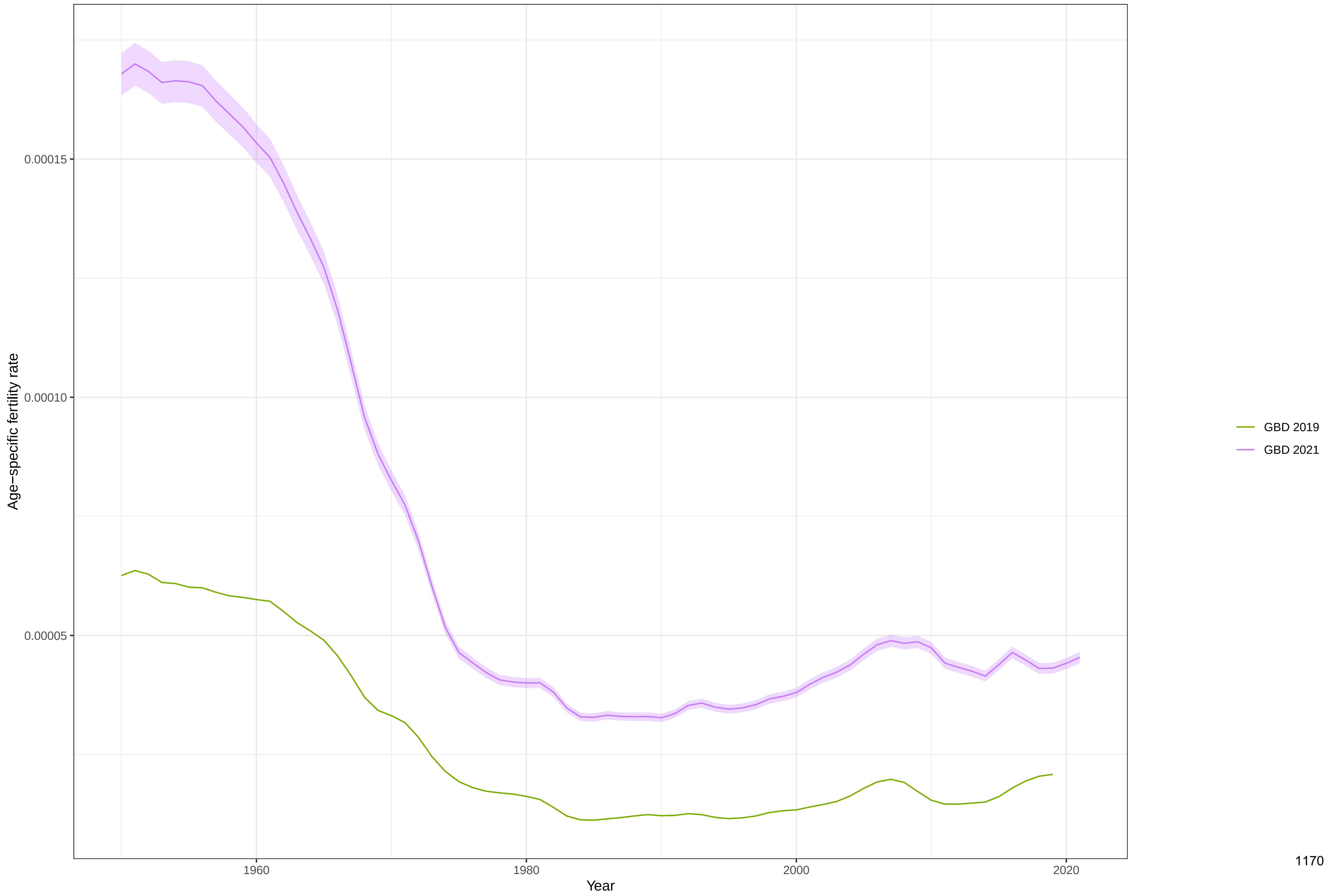

Total fertility rate fitted trend lines, 1950−2021: New Mexico TFR
Data points enclosed with a diamond are reference sources

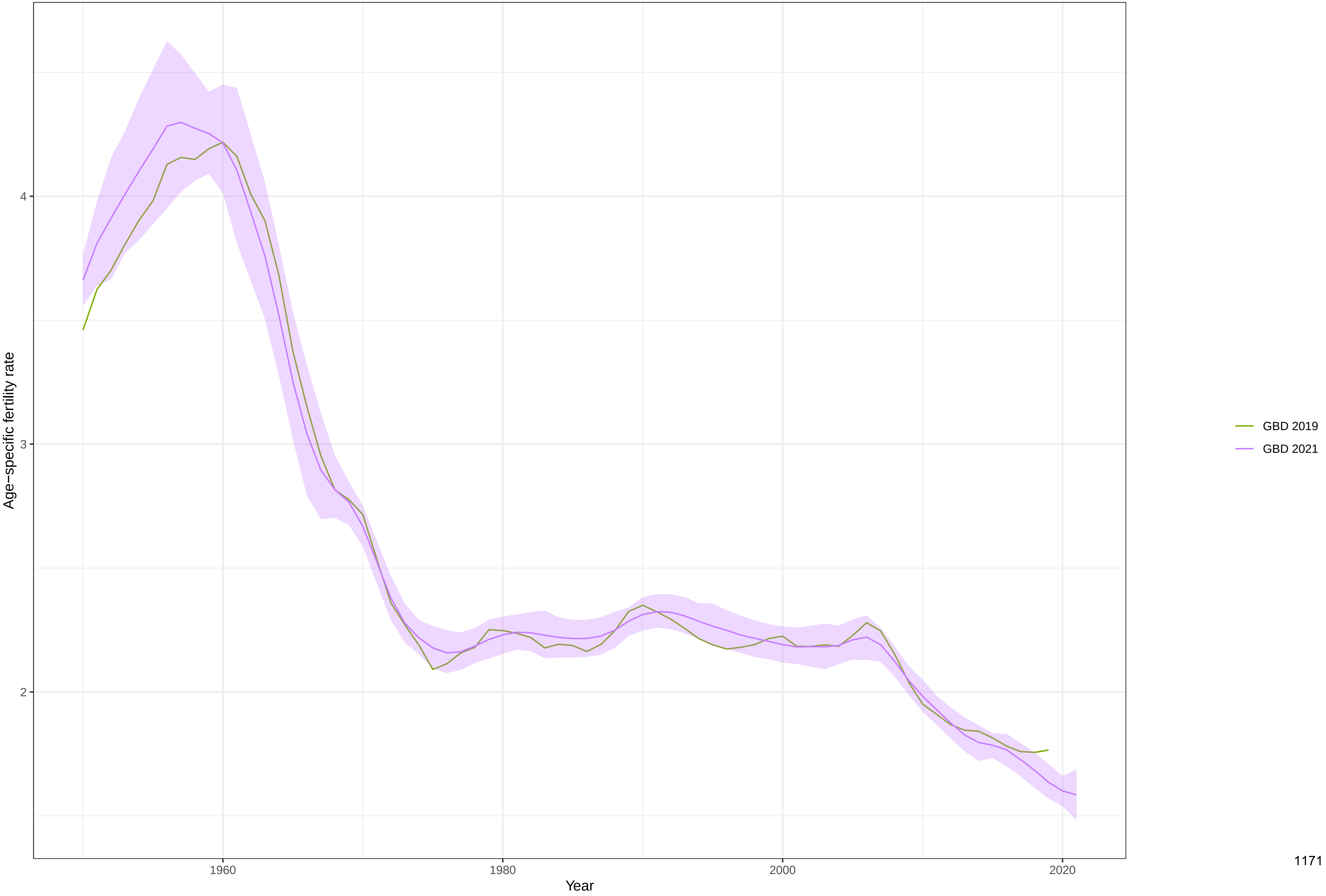

Age−specific fertility fitted trend lines, 1950−2021: New York, Ages: 10−14
Data points enclosed with a diamond are reference sources

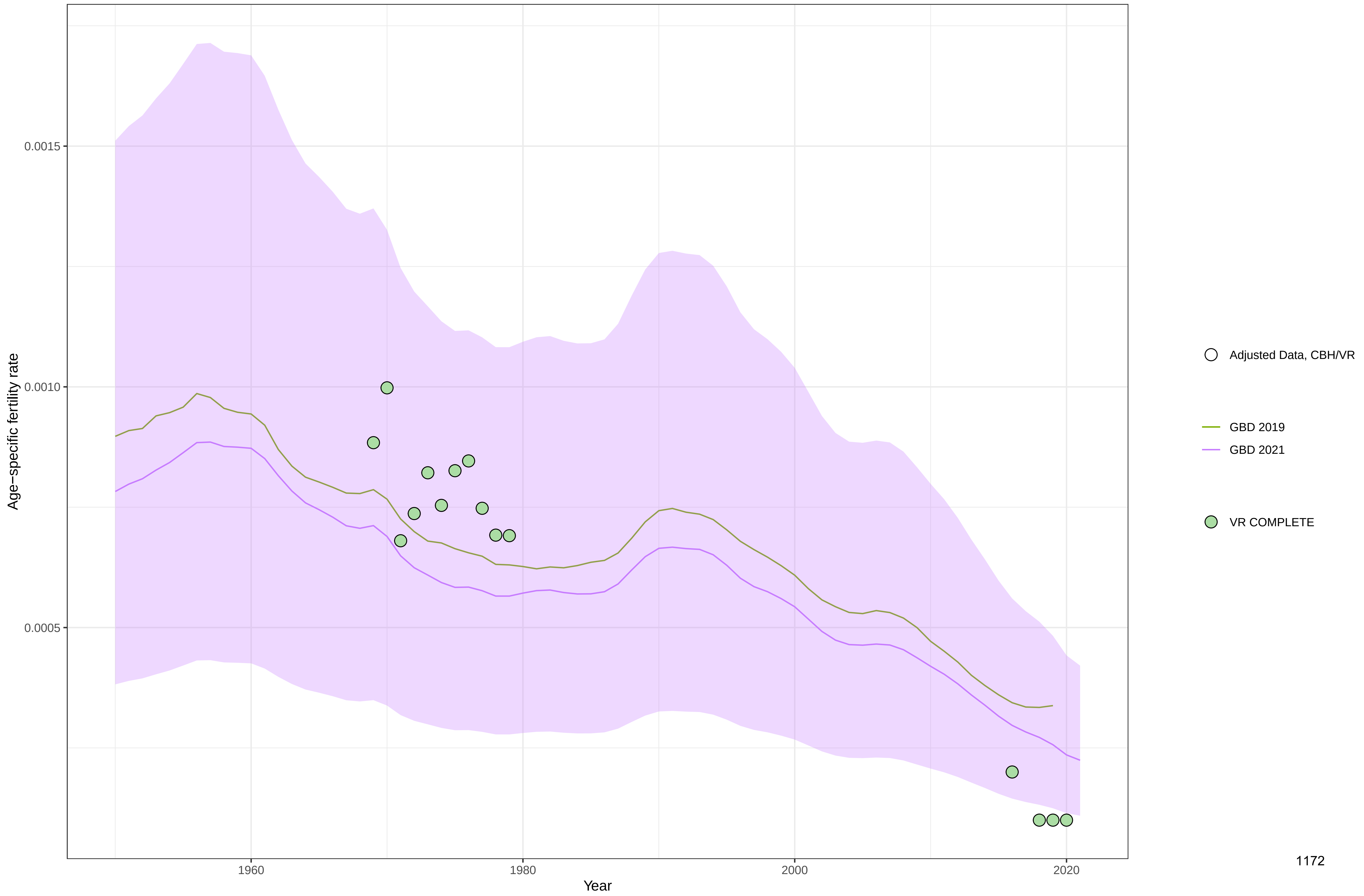

Age−specific fertility fitted trend lines, 1950−2021: New York, Ages: 15−19
Data points enclosed with a diamond are reference sources

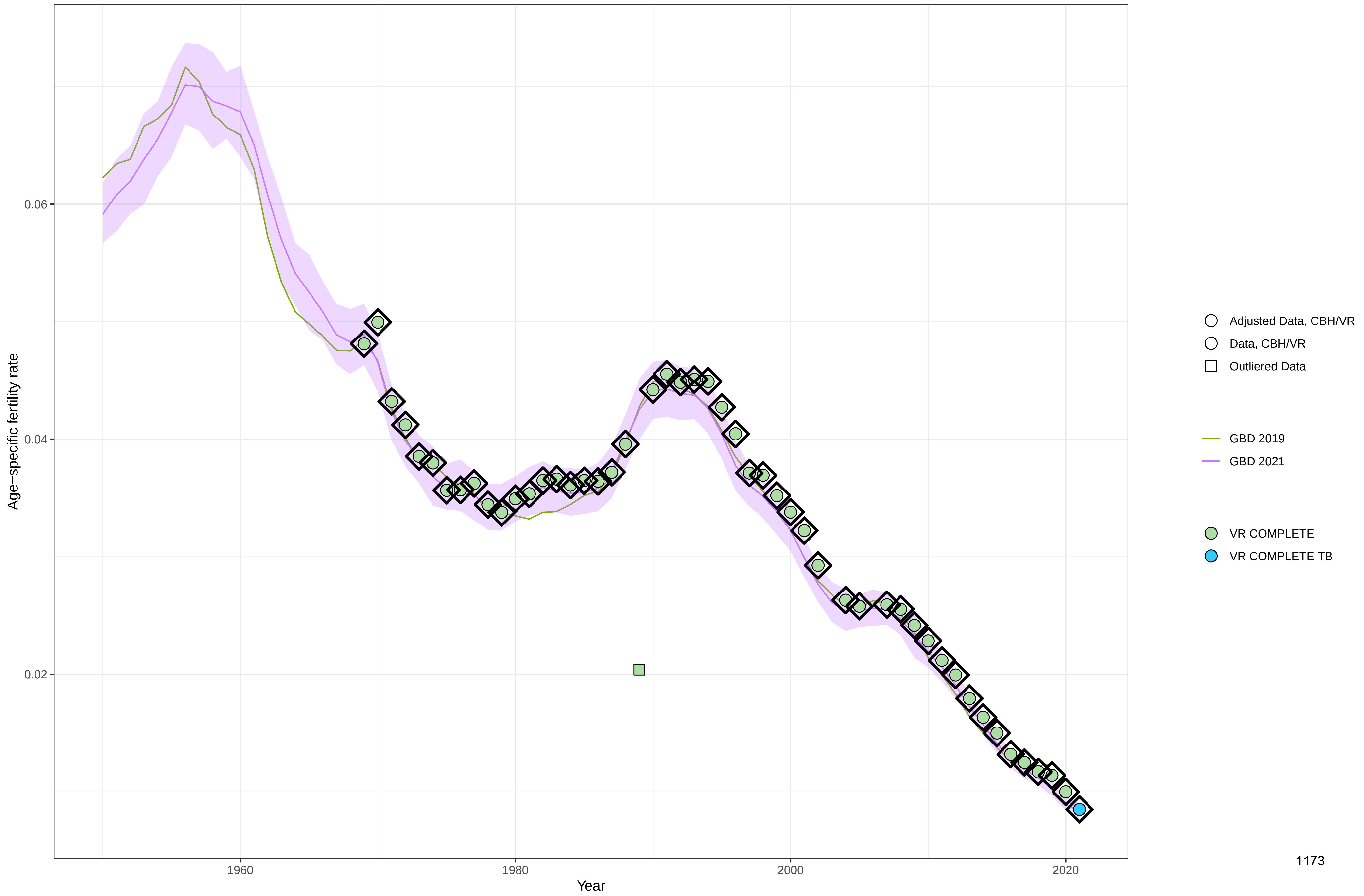

Age−specific fertility fitted trend lines, 1950−2021: New York, Ages: 20−24
Data points enclosed with a diamond are reference sources

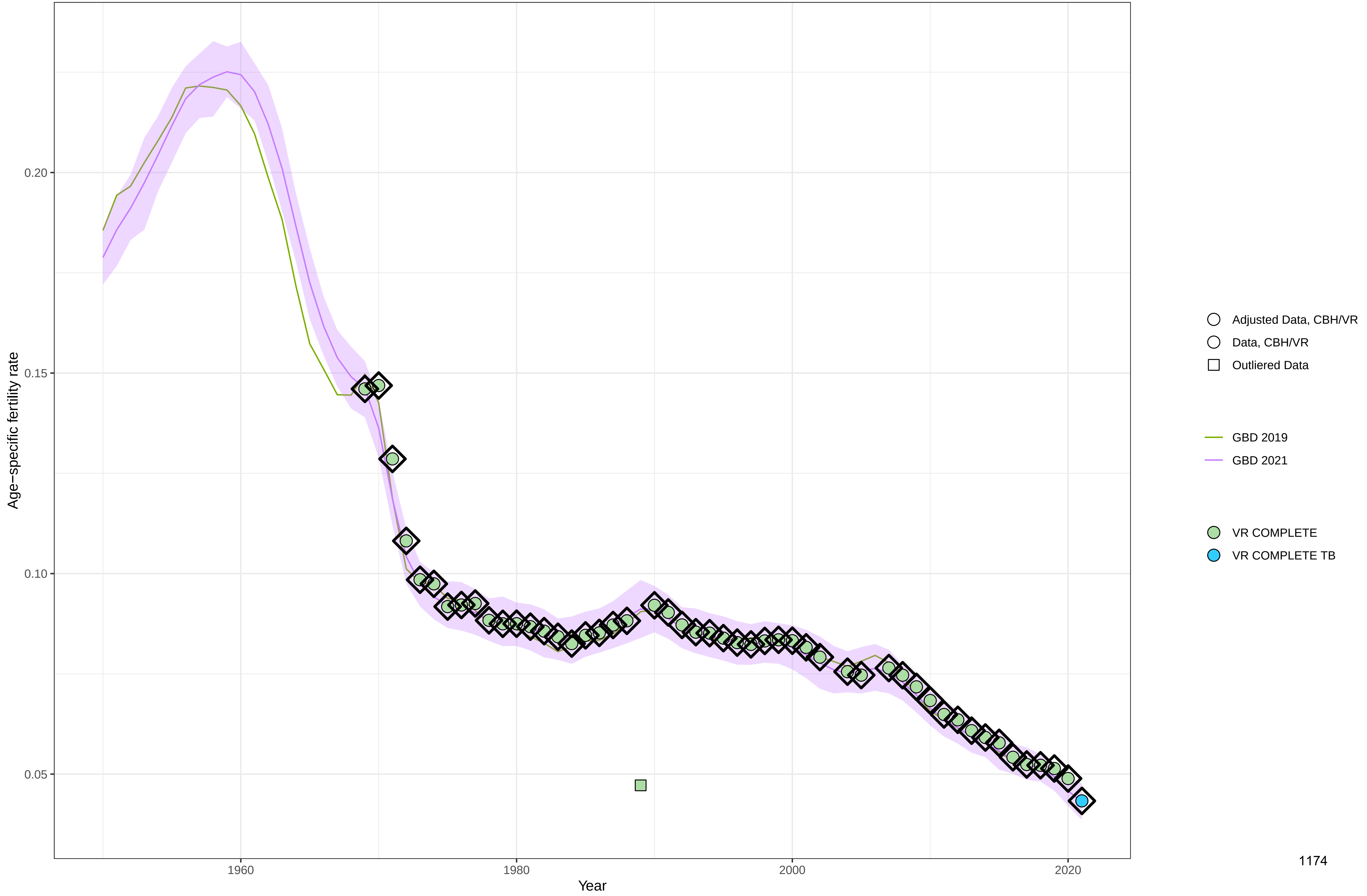

Age−specific fertility fitted trend lines, 1950−2021: New York, Ages: 25−29
Data points enclosed with a diamond are reference sources

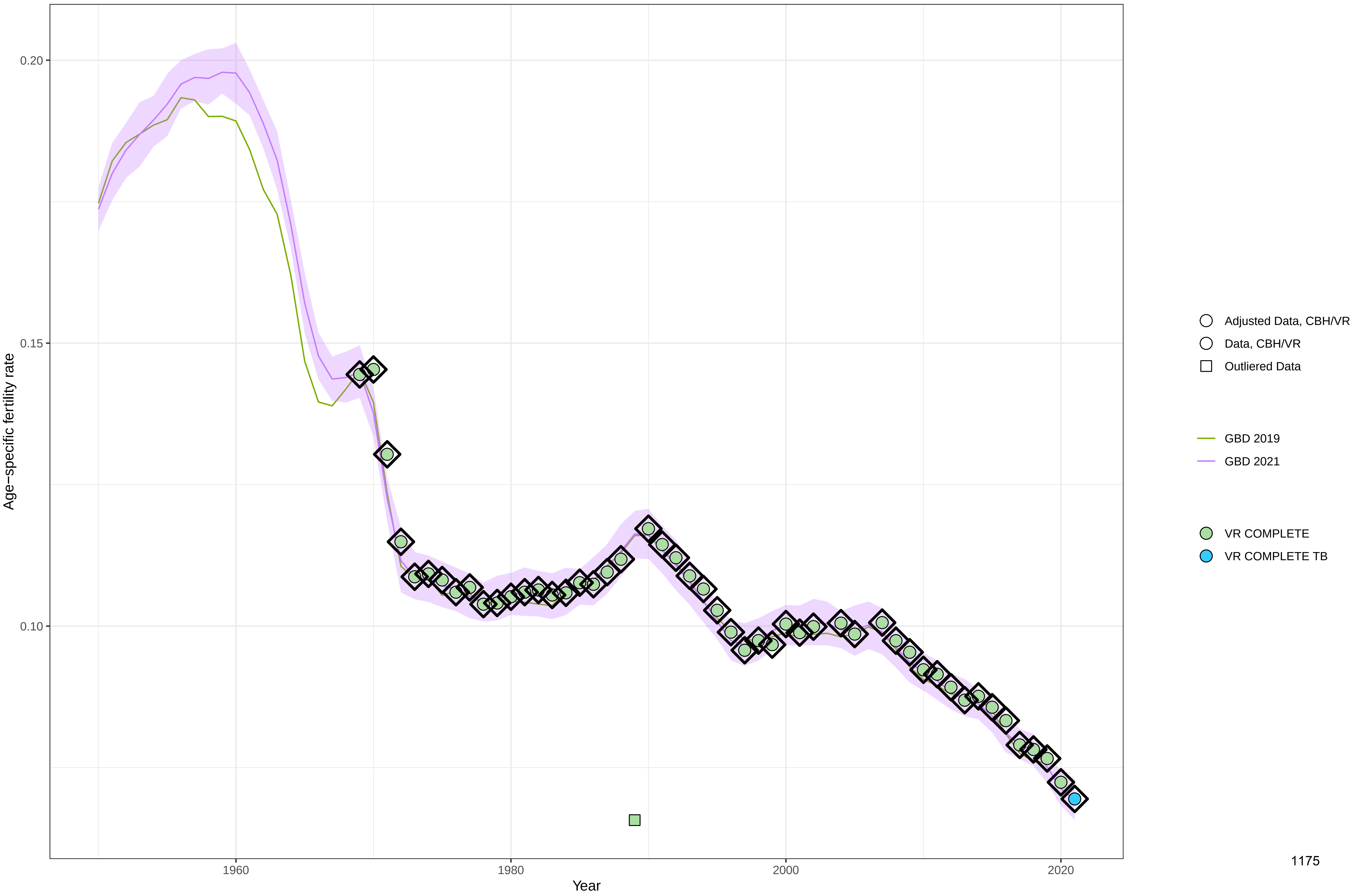

Age−specific fertility fitted trend lines, 1950−2021: New York, Ages: 30−34
Data points enclosed with a diamond are reference sources

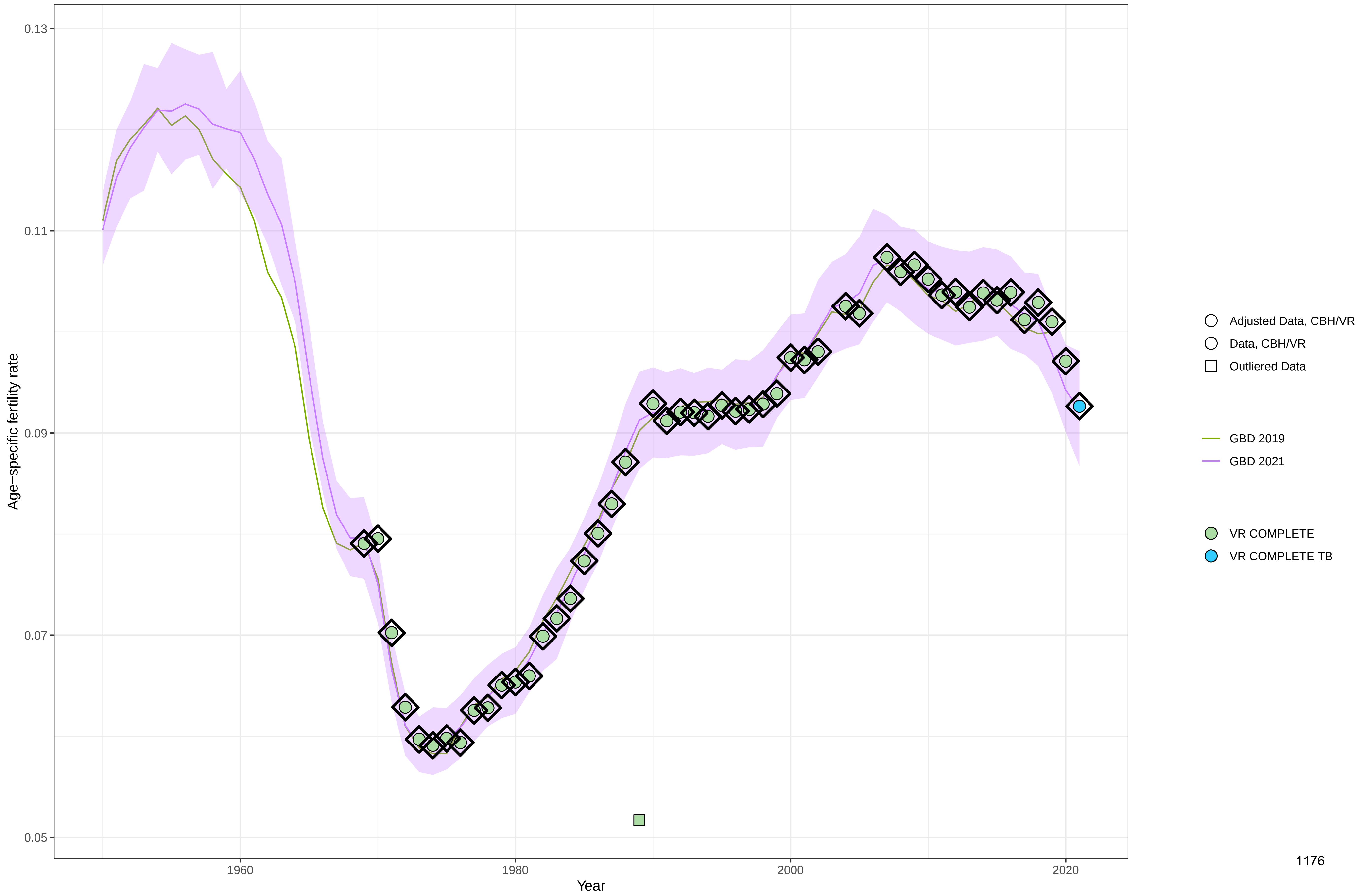

Age−specific fertility fitted trend lines, 1950−2021: New York, Ages: 35−39
Data points enclosed with a diamond are reference sources

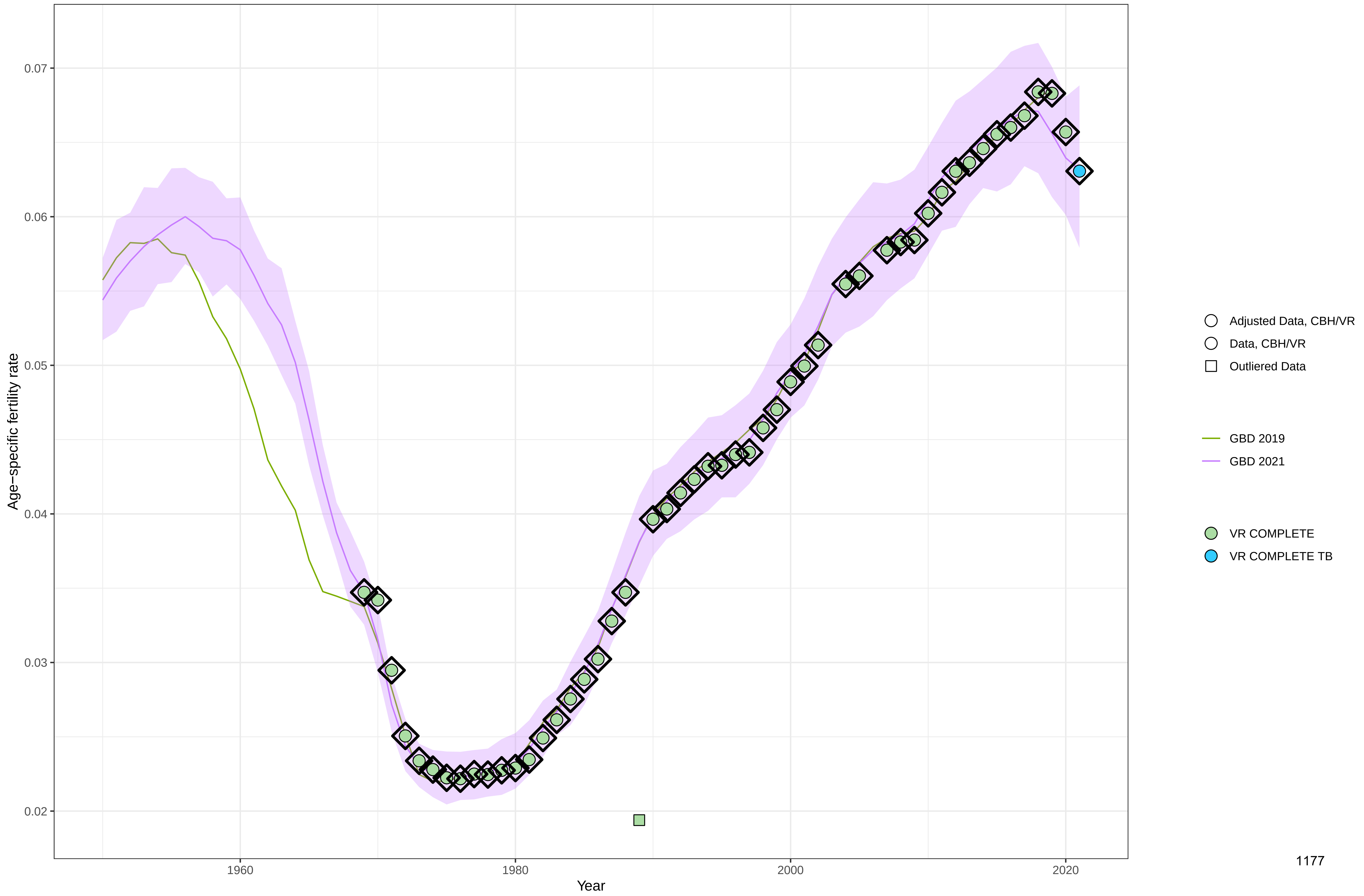

Age−specific fertility fitted trend lines, 1950−2021: New York, Ages: 40−44
Data points enclosed with a diamond are reference sources

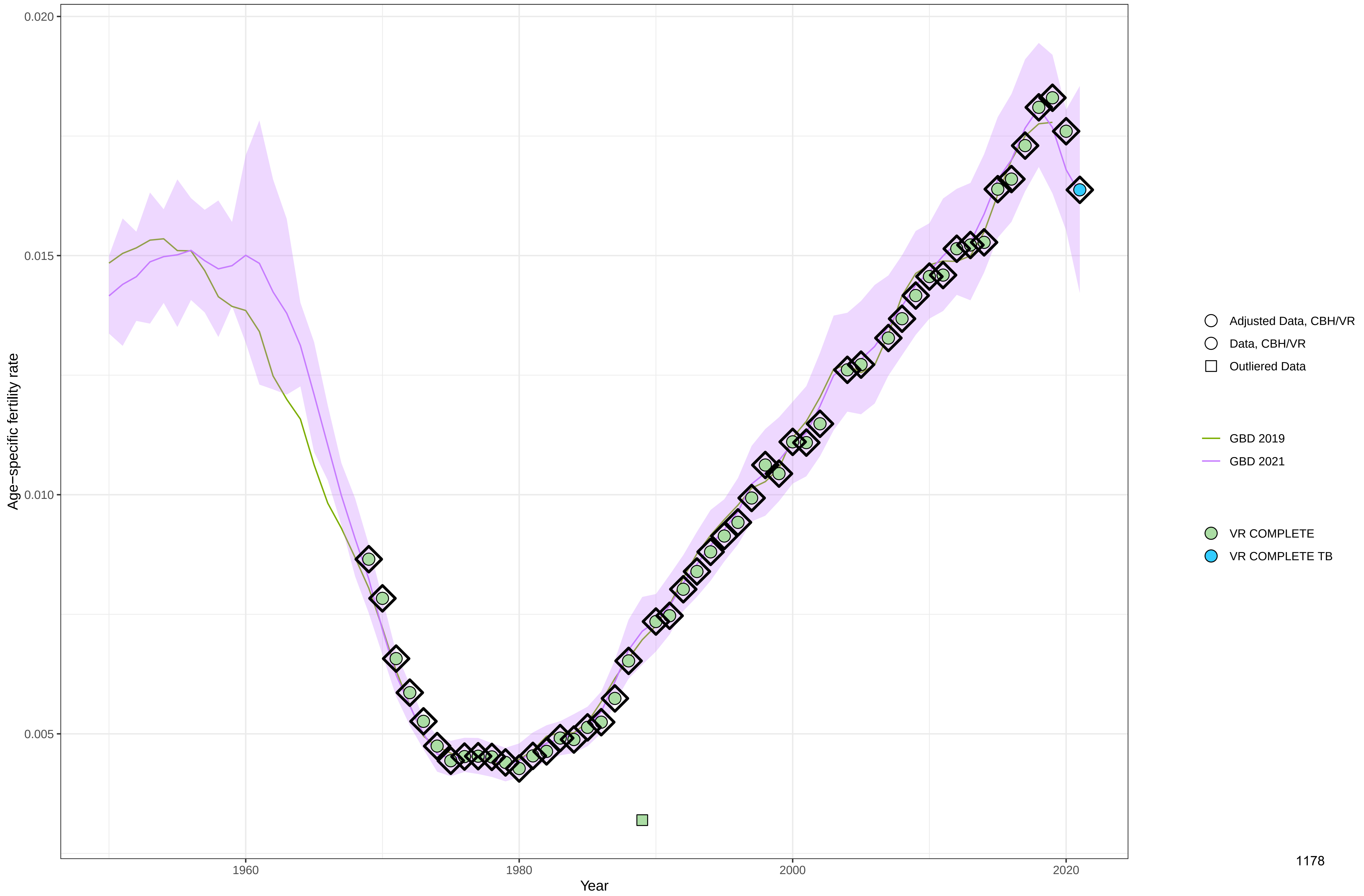

Age−specific fertility fitted trend lines, 1950−2021: New York, Ages: 45−49
Data points enclosed with a diamond are reference sources

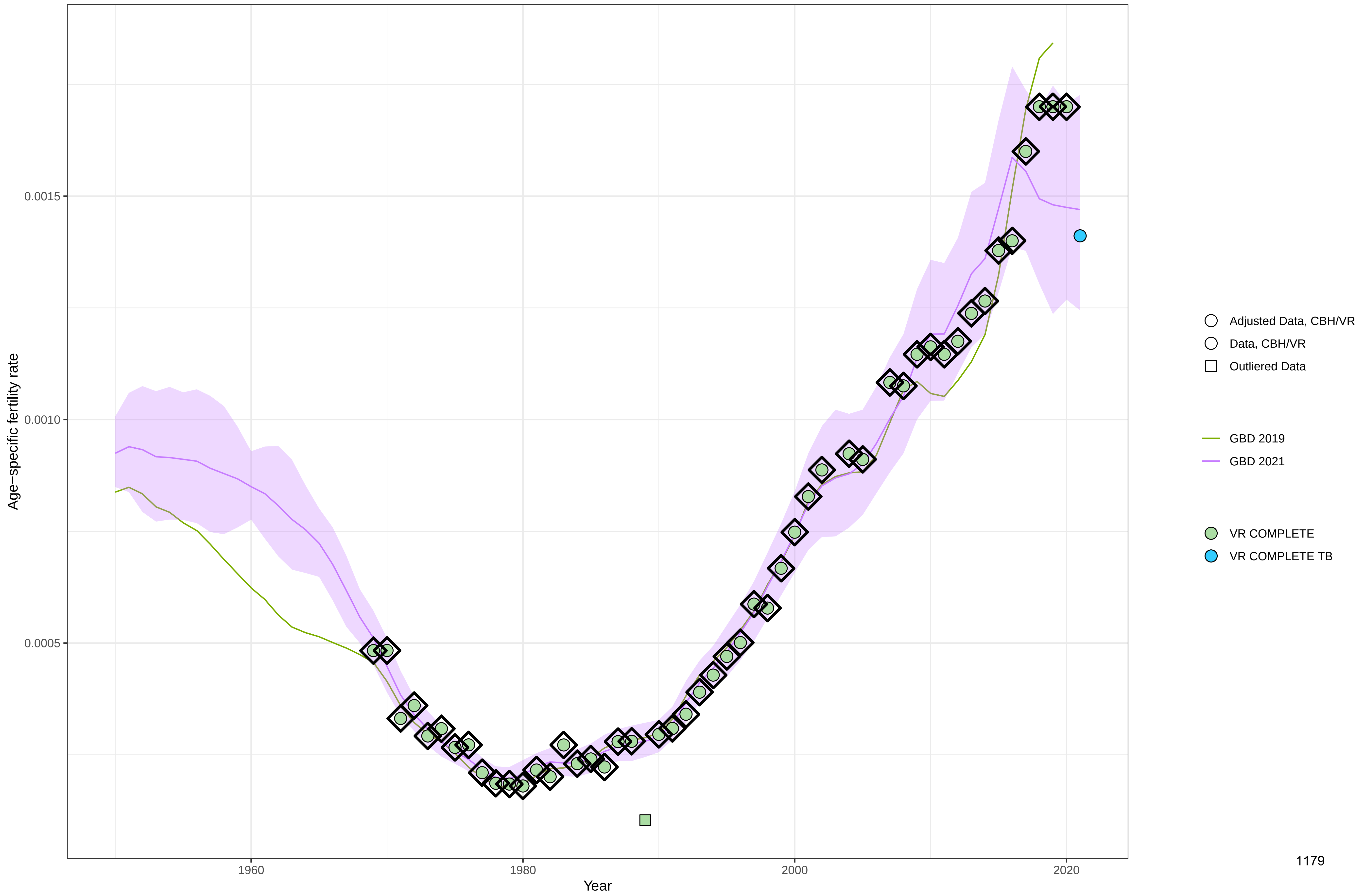

Age−specific fertility fitted trend lines, 1950−2021: New York, Ages: 50−54
Data points enclosed with a diamond are reference sources

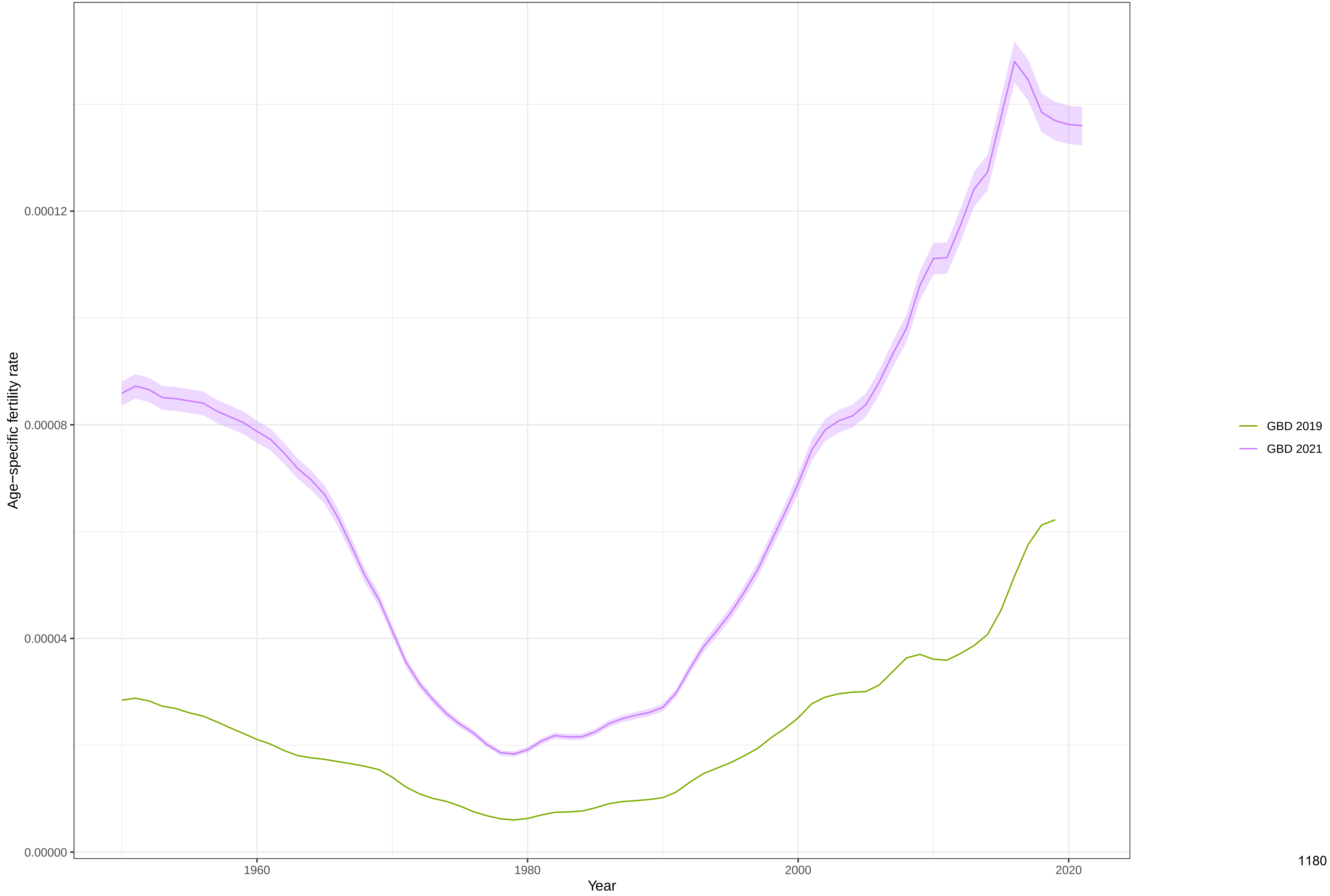

Total fertility rate fitted trend lines, 1950−2021: New York TFR
Data points enclosed with a diamond are reference sources

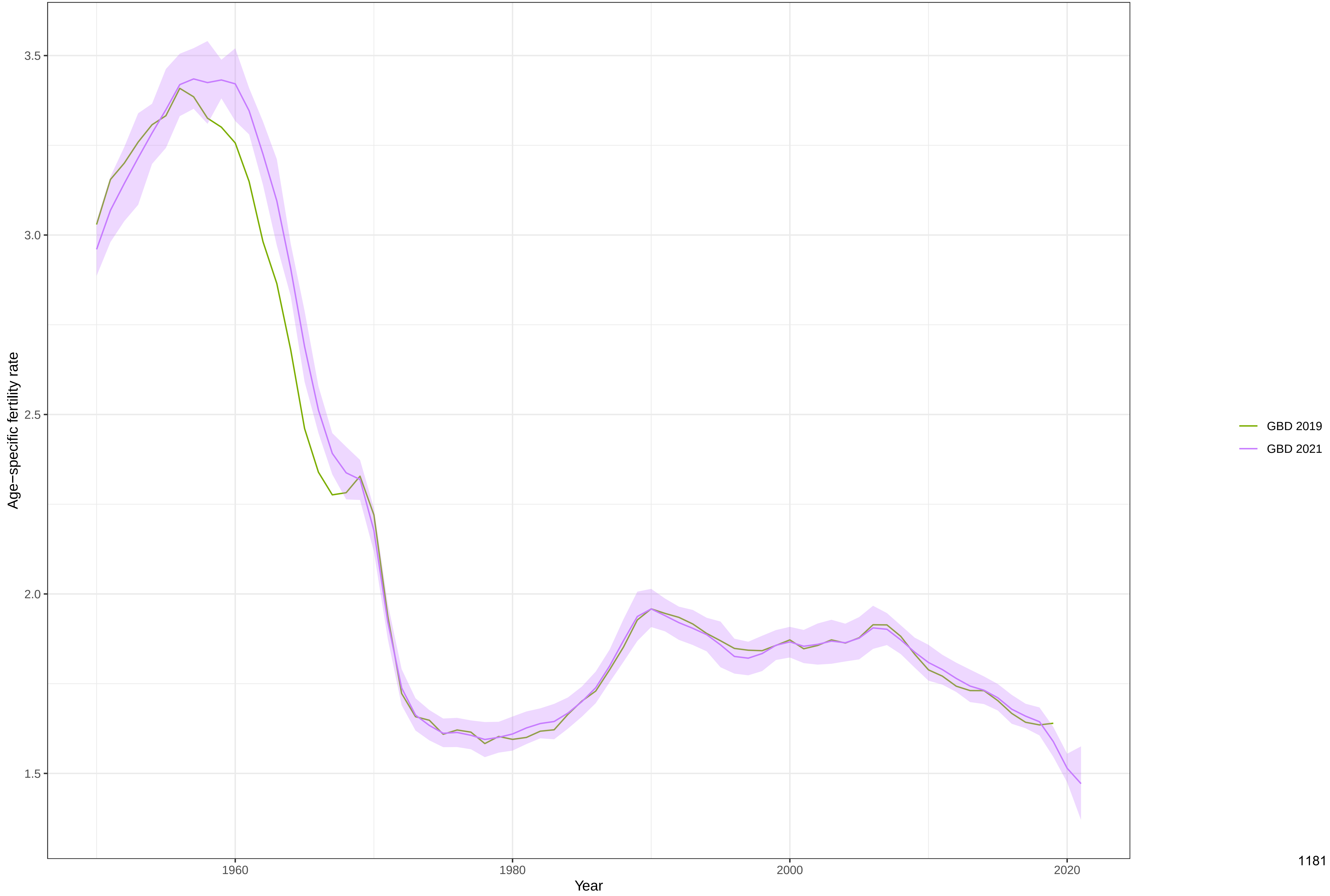

Age−specific fertility fitted trend lines, 1950−2021: North Carolina, Ages: 10−14
Data points enclosed with a diamond are reference sources

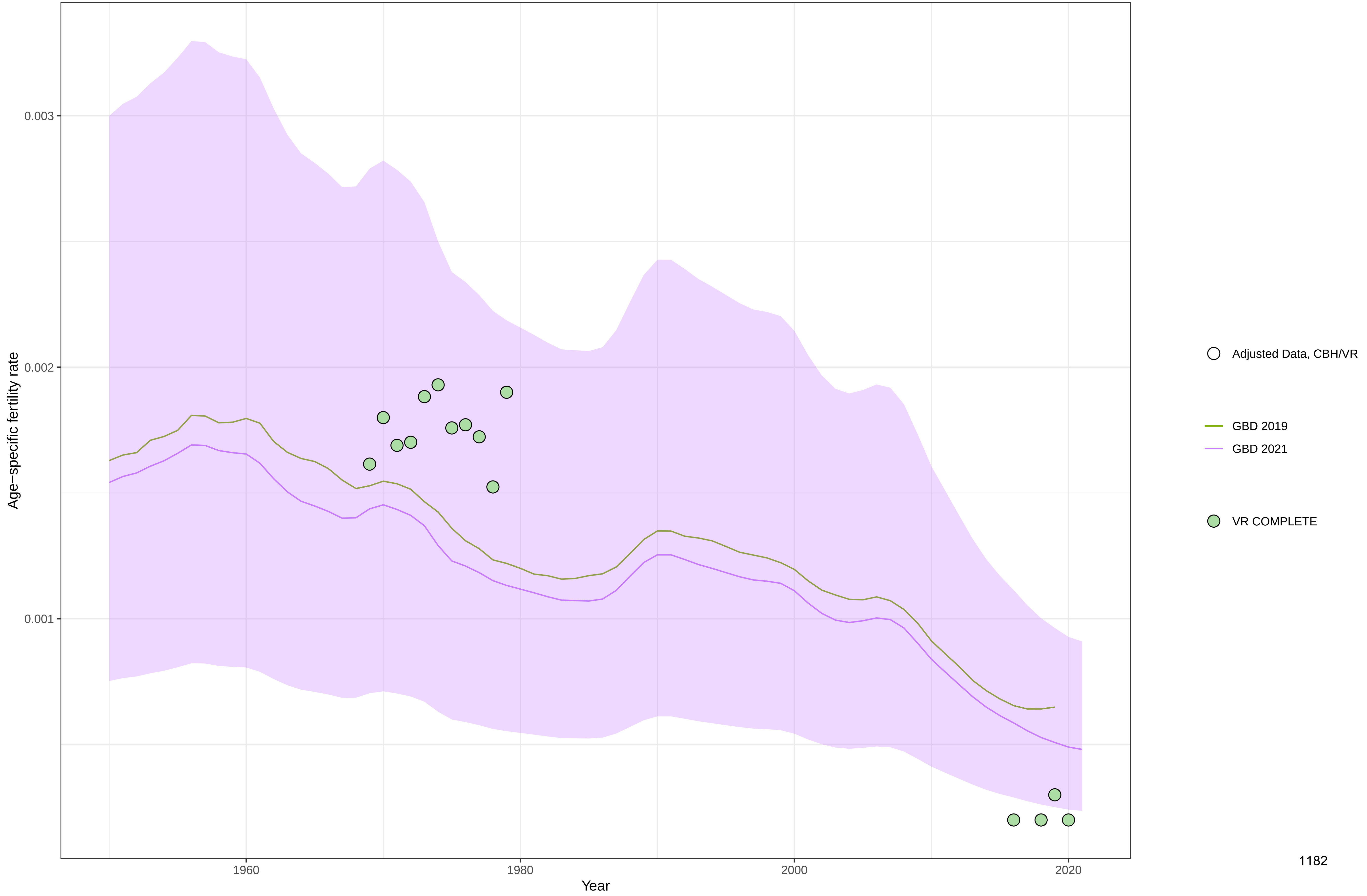

Age−specific fertility fitted trend lines, 1950−2021: North Carolina, Ages: 15−19
Data points enclosed with a diamond are reference sources

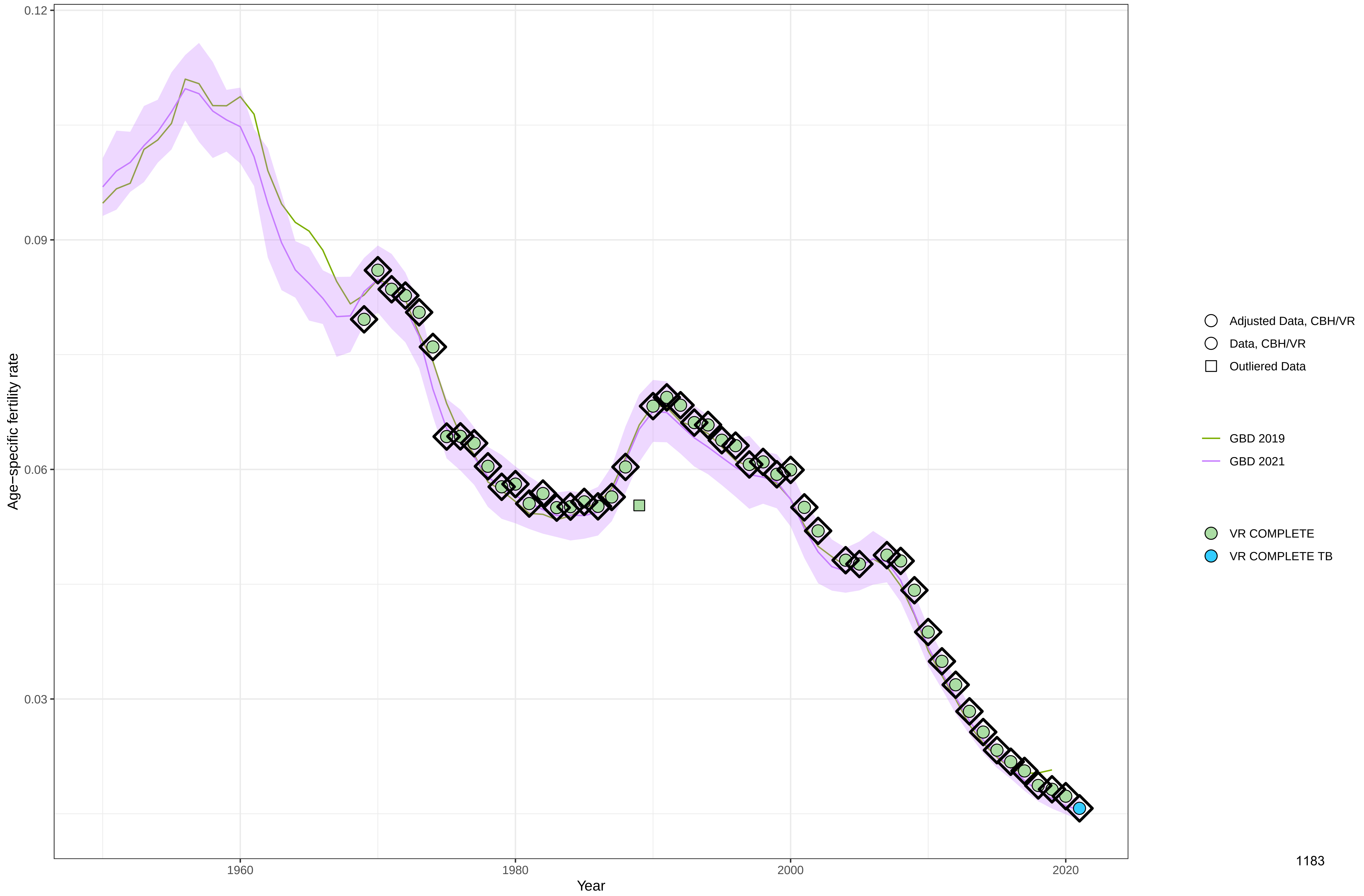

Age−specific fertility fitted trend lines, 1950−2021: North Carolina, Ages: 20−24
Data points enclosed with a diamond are reference sources

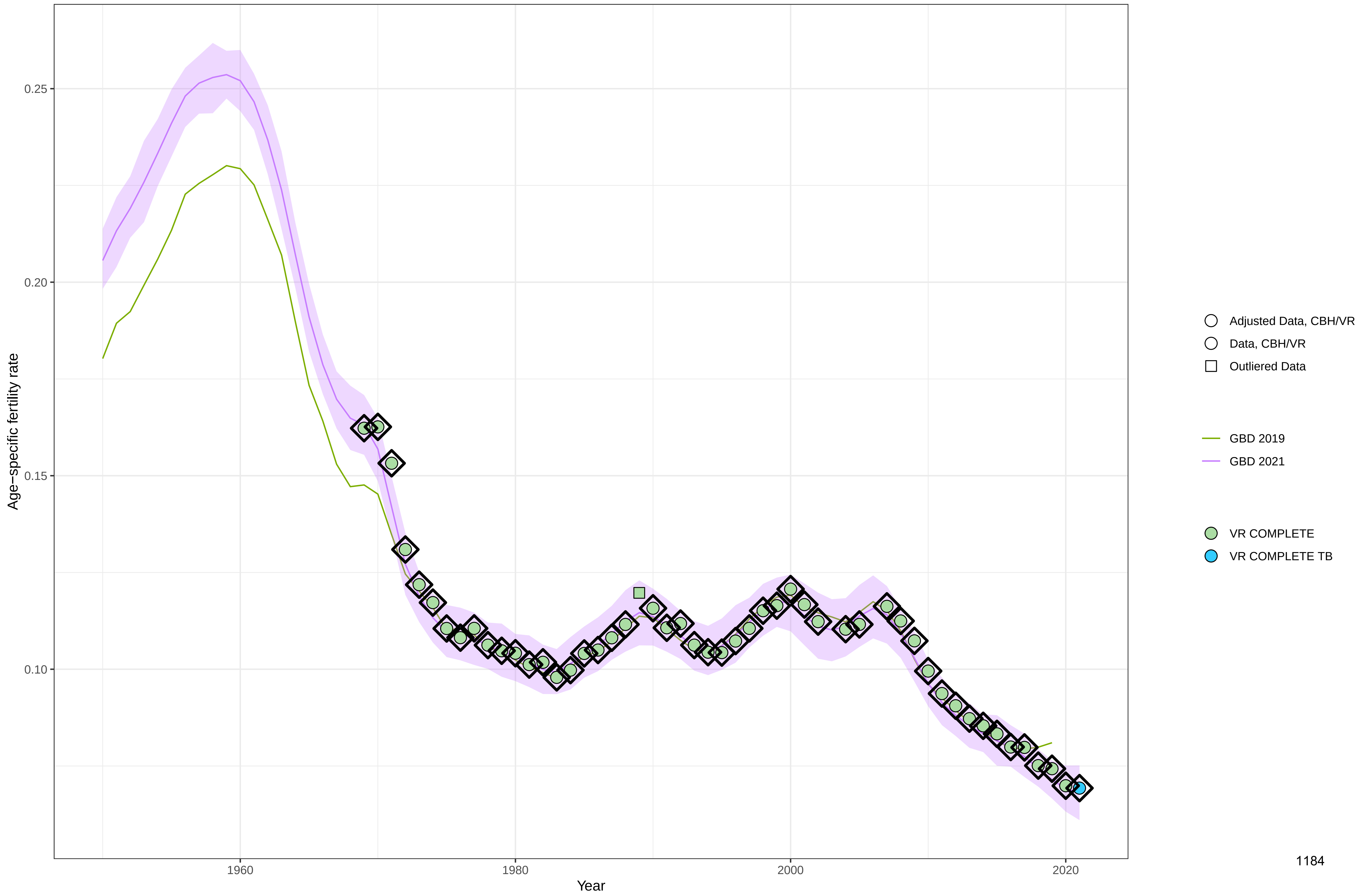

Age−specific fertility fitted trend lines, 1950−2021: North Carolina, Ages: 25−29
Data points enclosed with a diamond are reference sources

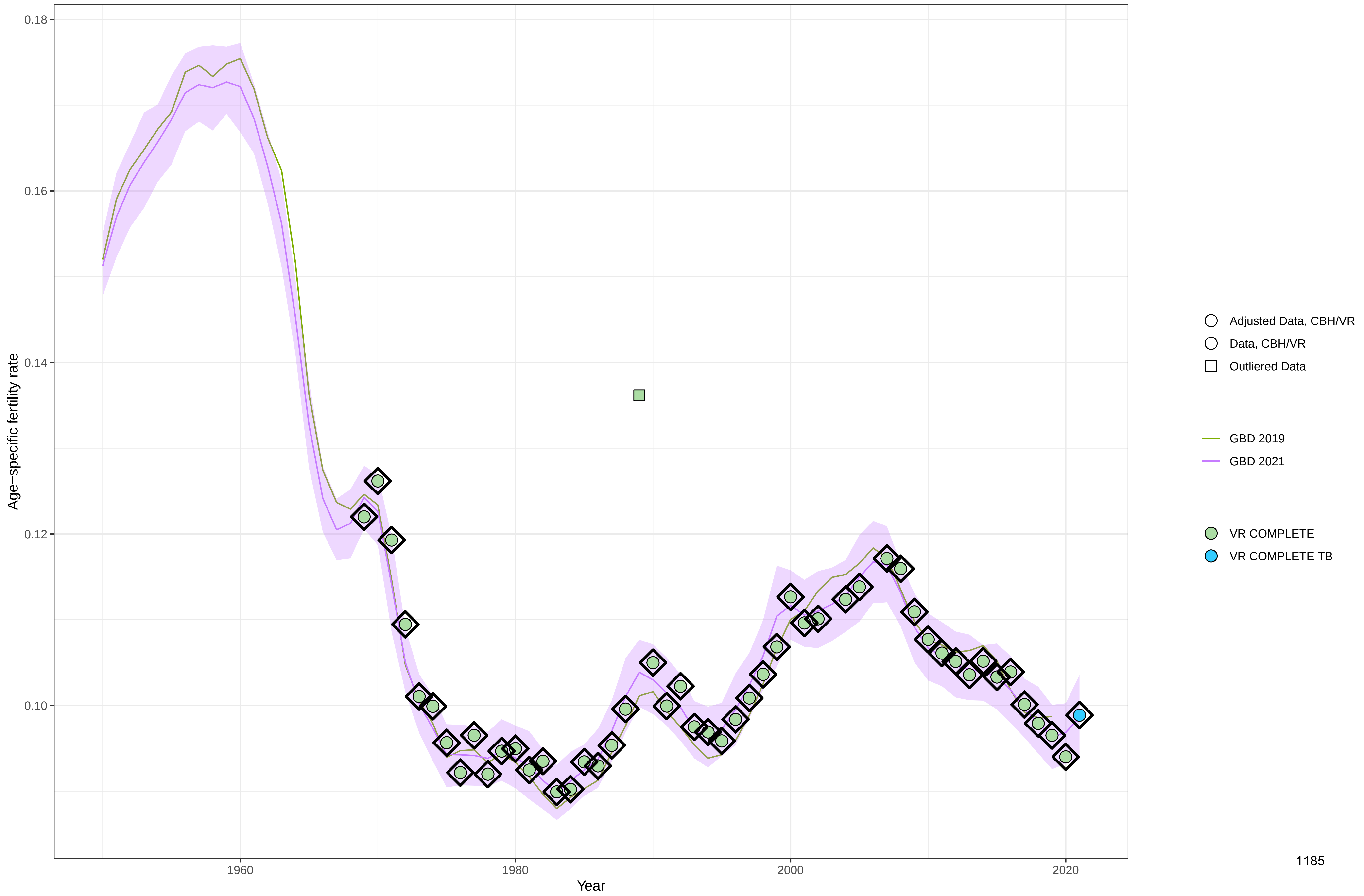

Age−specific fertility fitted trend lines, 1950−2021: North Carolina, Ages: 30−34
Data points enclosed with a diamond are reference sources

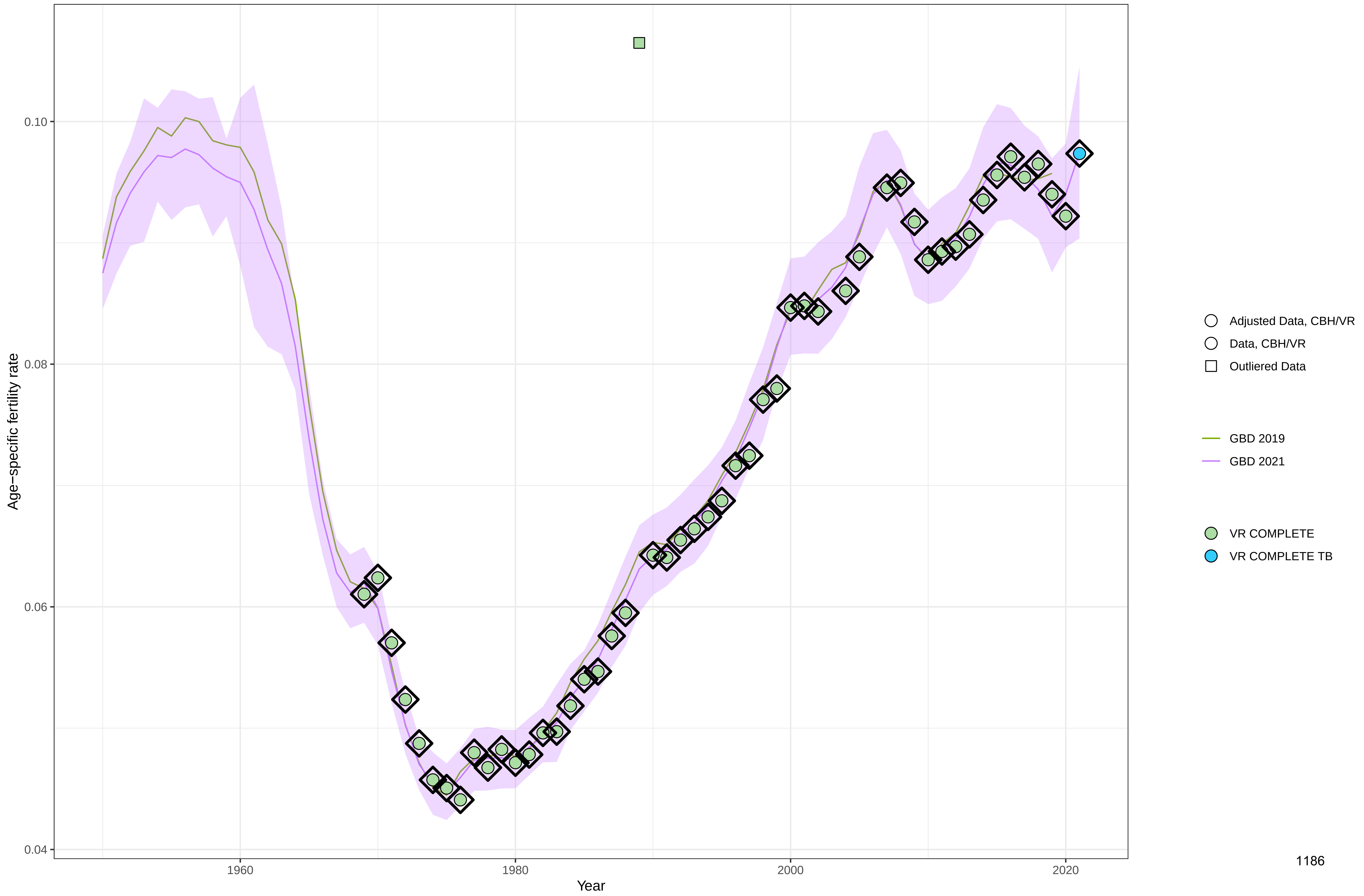

Age−specific fertility fitted trend lines, 1950−2021: North Carolina, Ages: 35−39
Data points enclosed with a diamond are reference sources

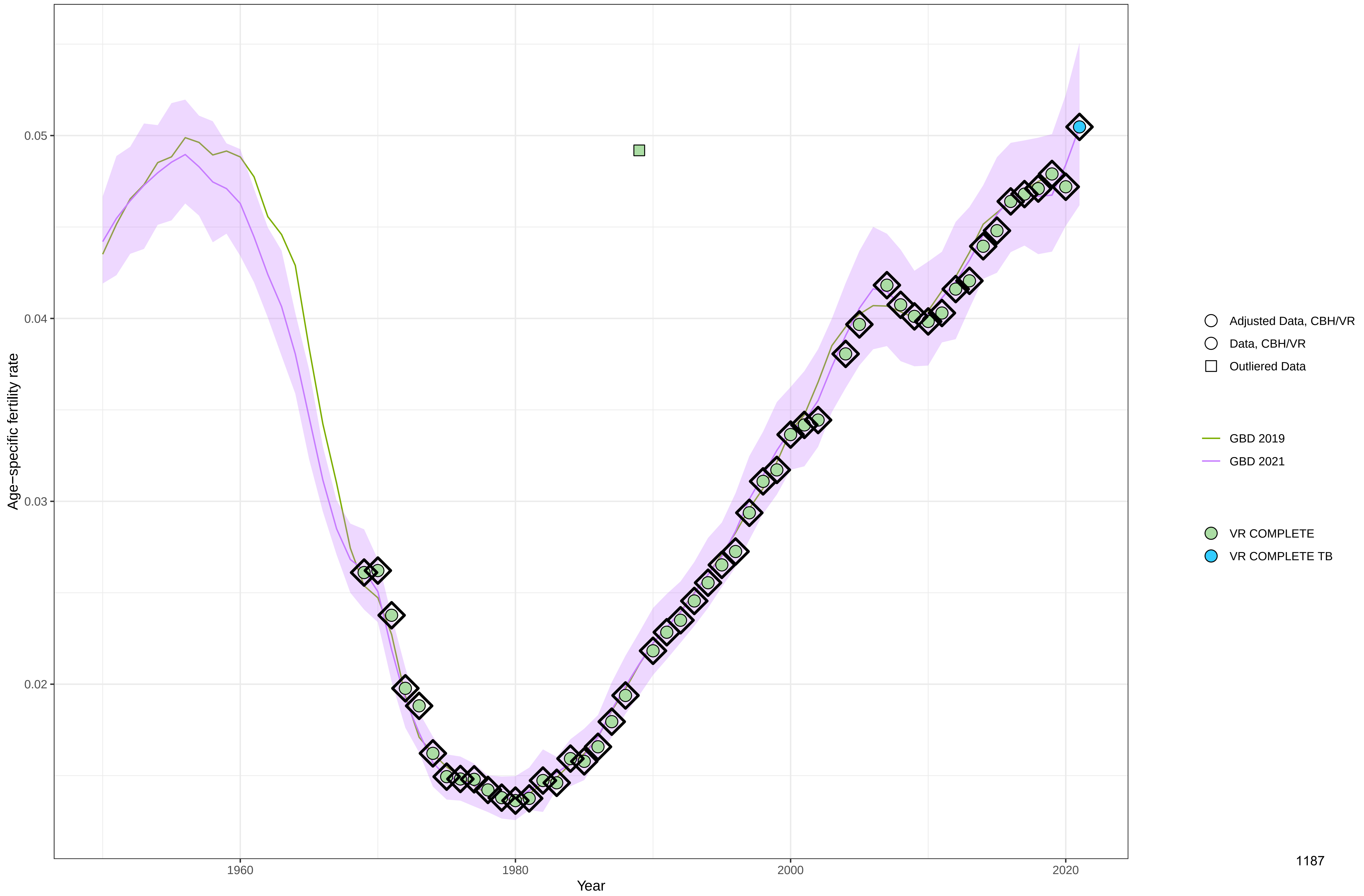

Age−specific fertility fitted trend lines, 1950−2021: North Carolina, Ages: 40−44
Data points enclosed with a diamond are reference sources

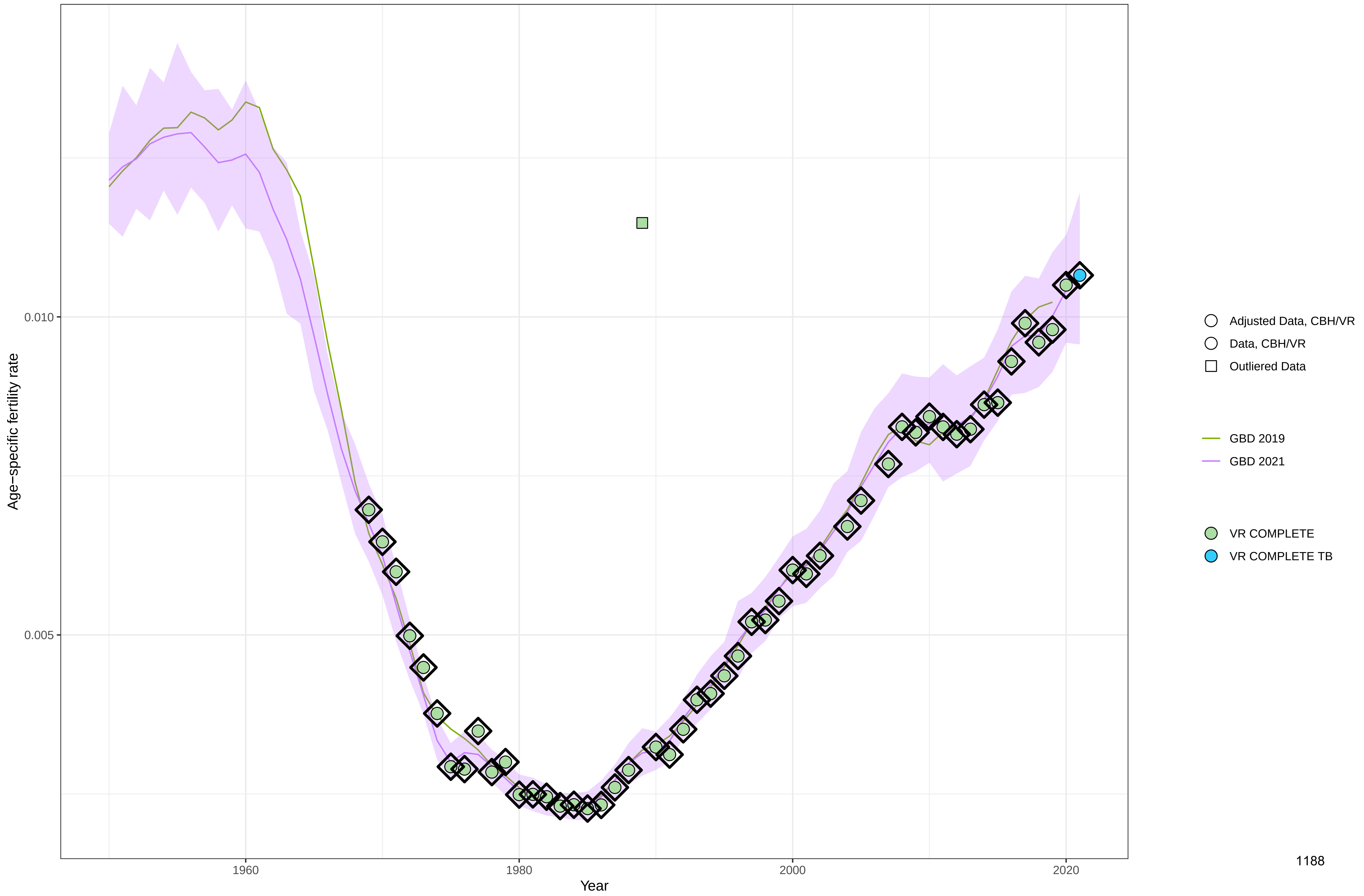

Age−specific fertility fitted trend lines, 1950−2021: North Carolina, Ages: 45−49
Data points enclosed with a diamond are reference sources

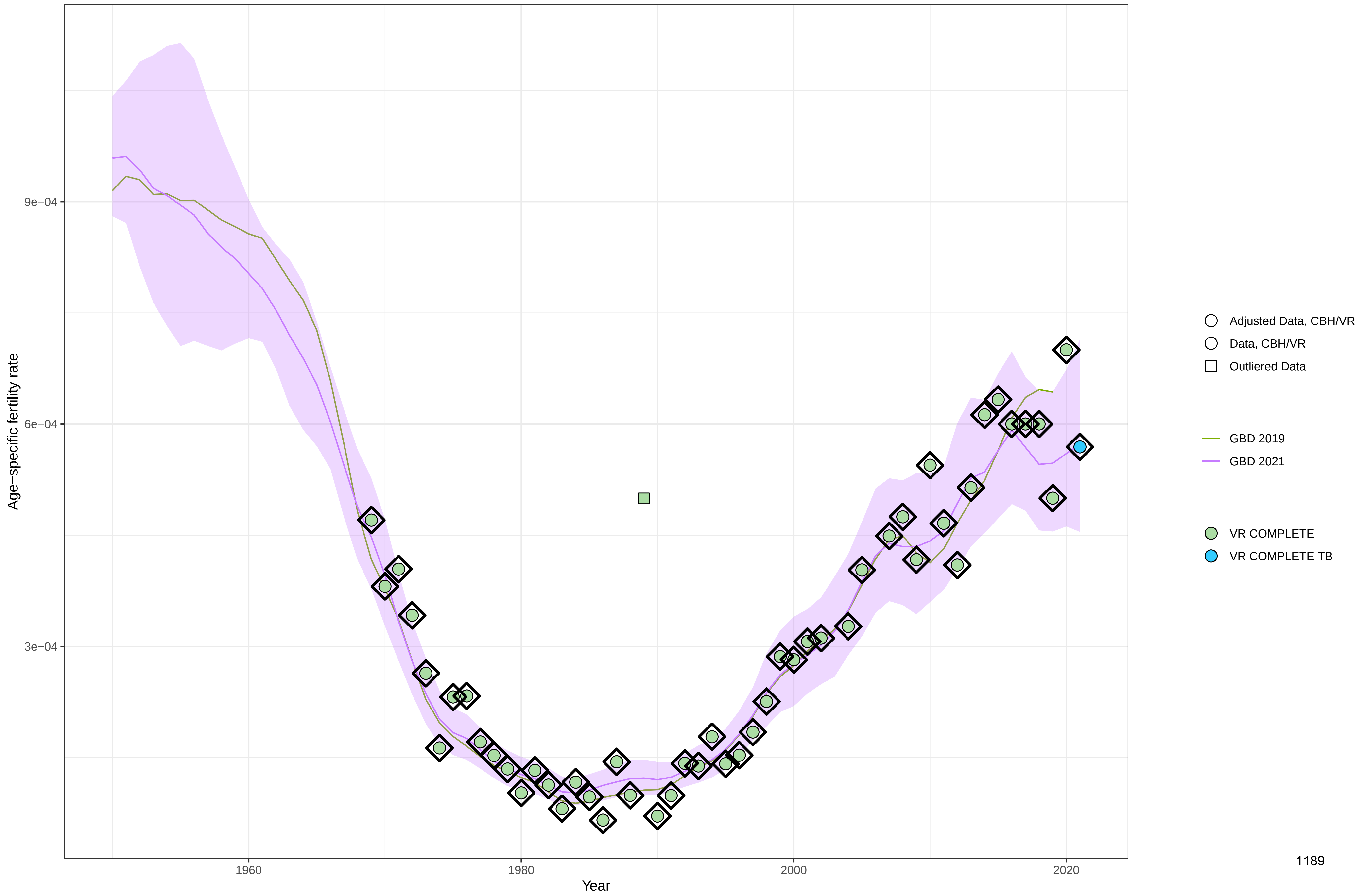

Age−specific fertility fitted trend lines, 1950−2021: North Carolina, Ages: 50−54
Data points enclosed with a diamond are reference sources

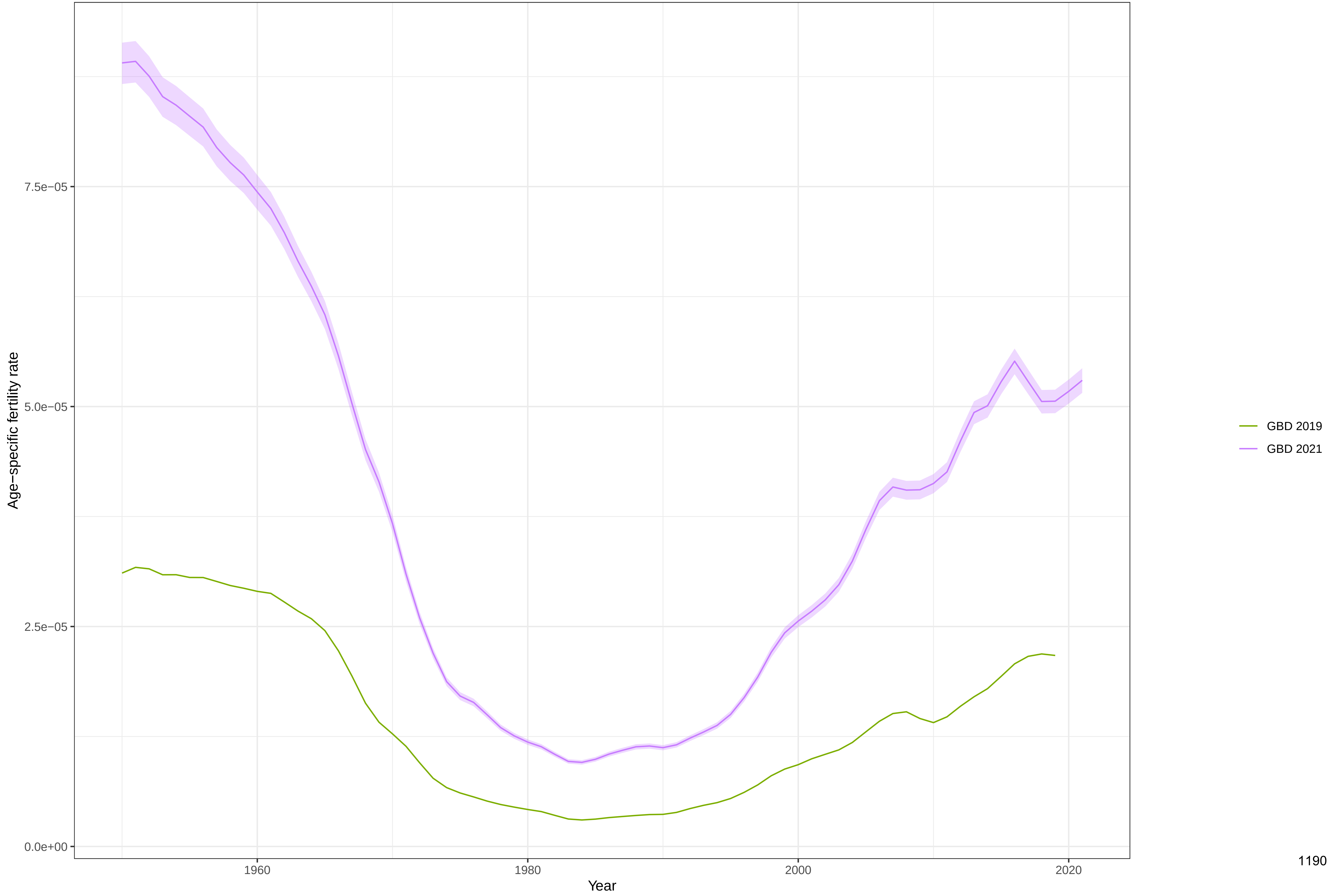

Total fertility rate fitted trend lines, 1950−2021: North Carolina TFR
Data points enclosed with a diamond are reference sources

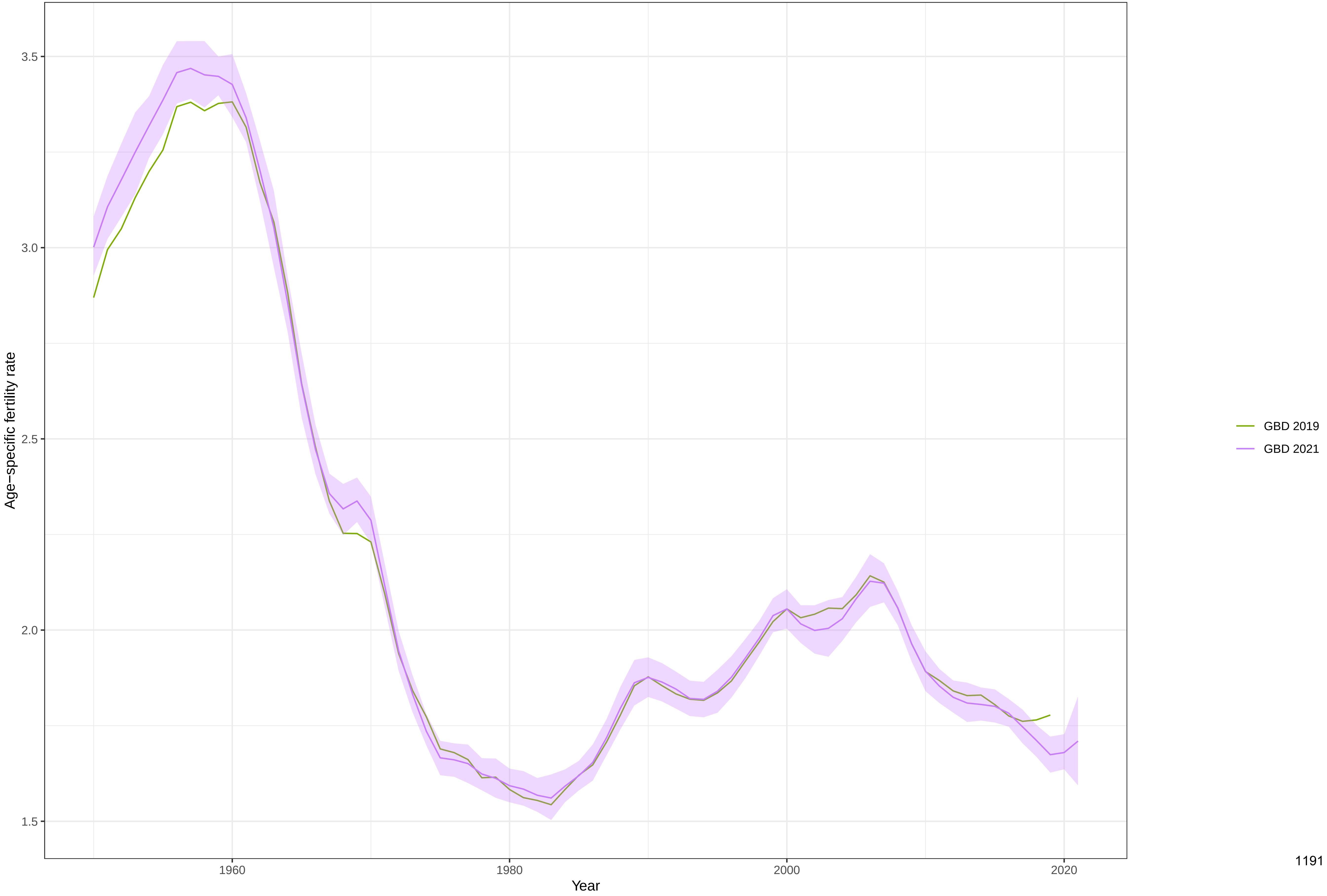

Age−specific fertility fitted trend lines, 1950−2021: North Dakota, Ages: 10−14
Data points enclosed with a diamond are reference sources

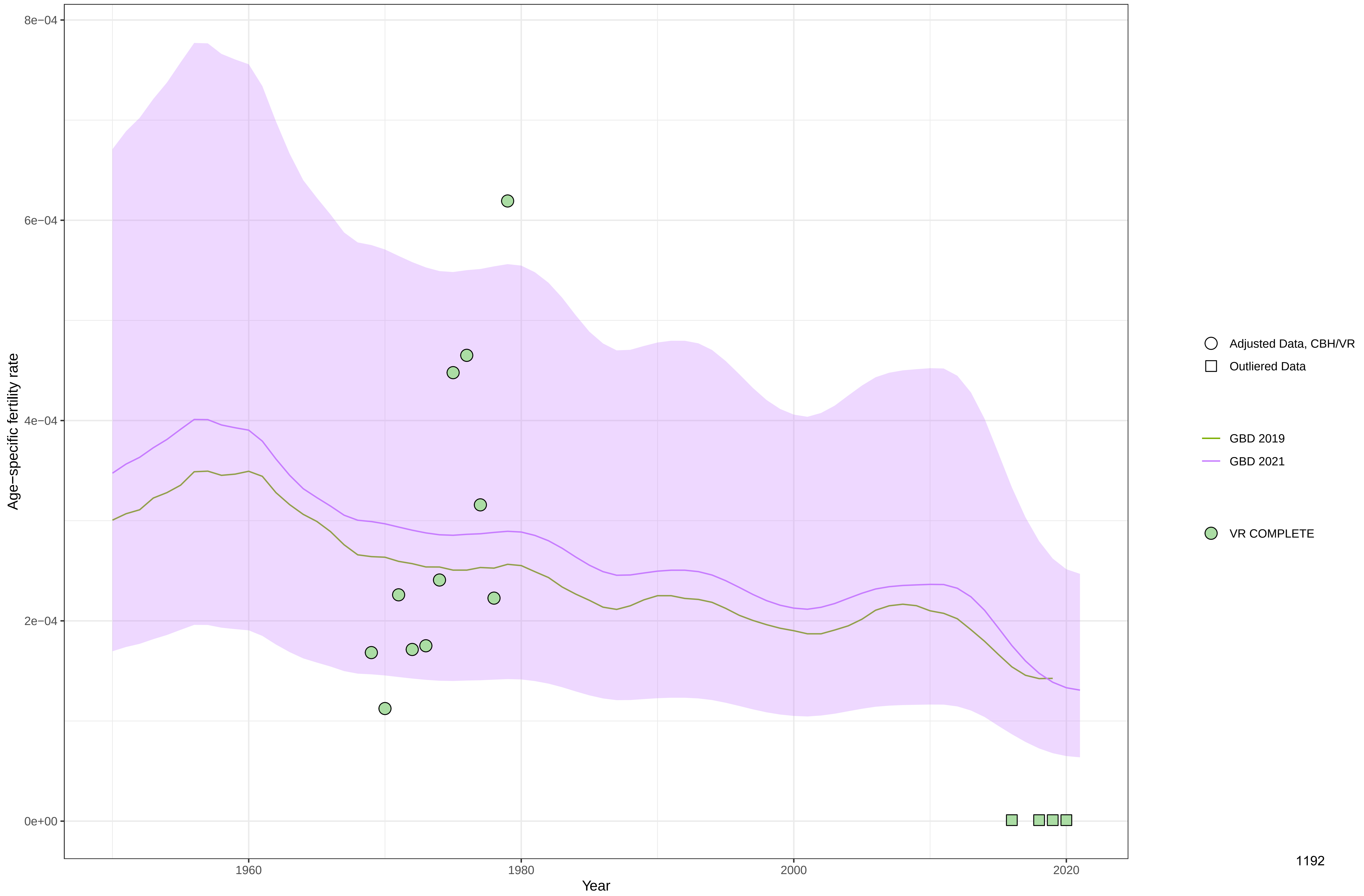

Age−specific fertility fitted trend lines, 1950−2021: North Dakota, Ages: 15−19
Data points enclosed with a diamond are reference sources

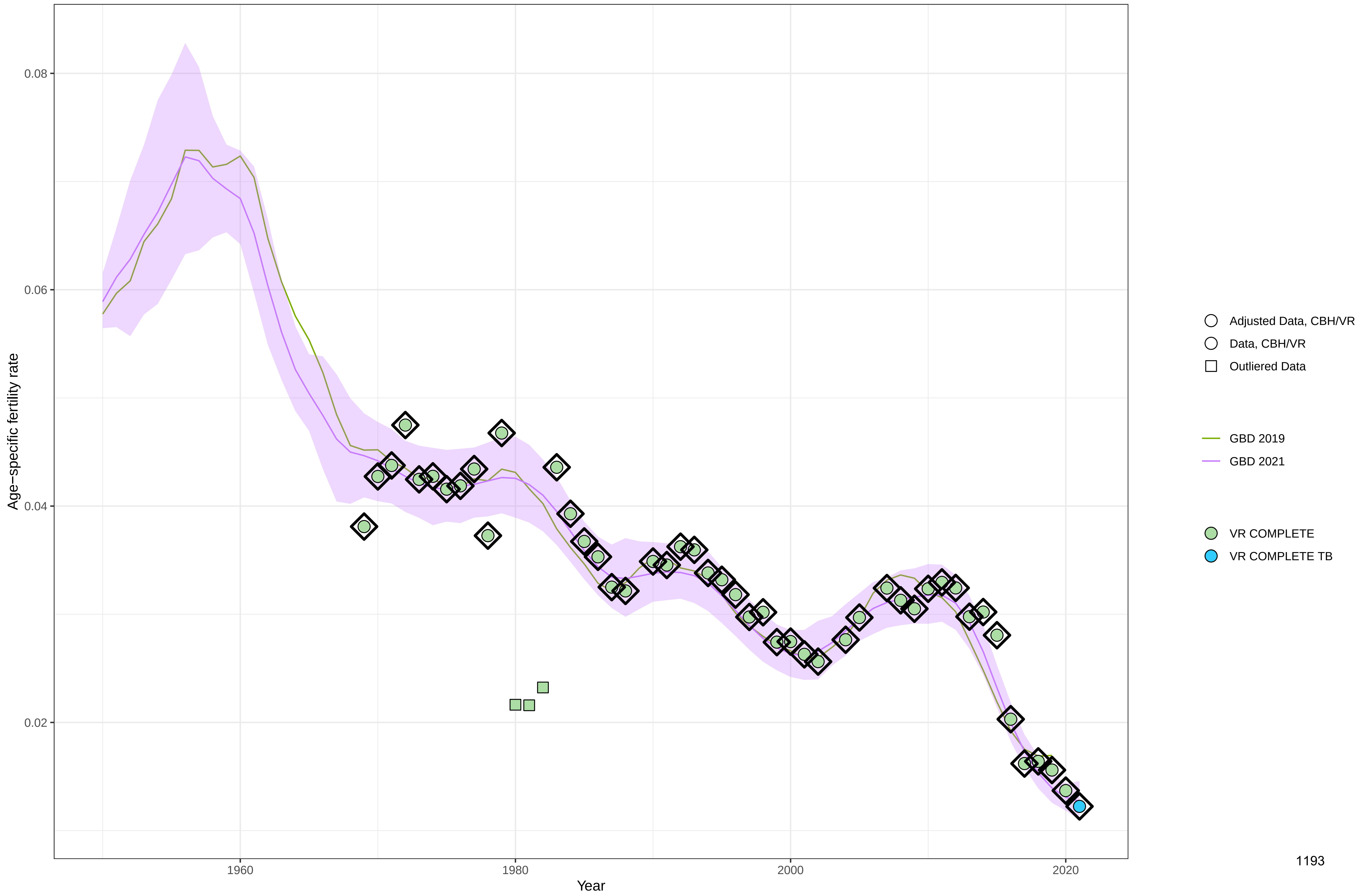

Age−specific fertility fitted trend lines, 1950−2021: North Dakota, Ages: 20−24
Data points enclosed with a diamond are reference sources

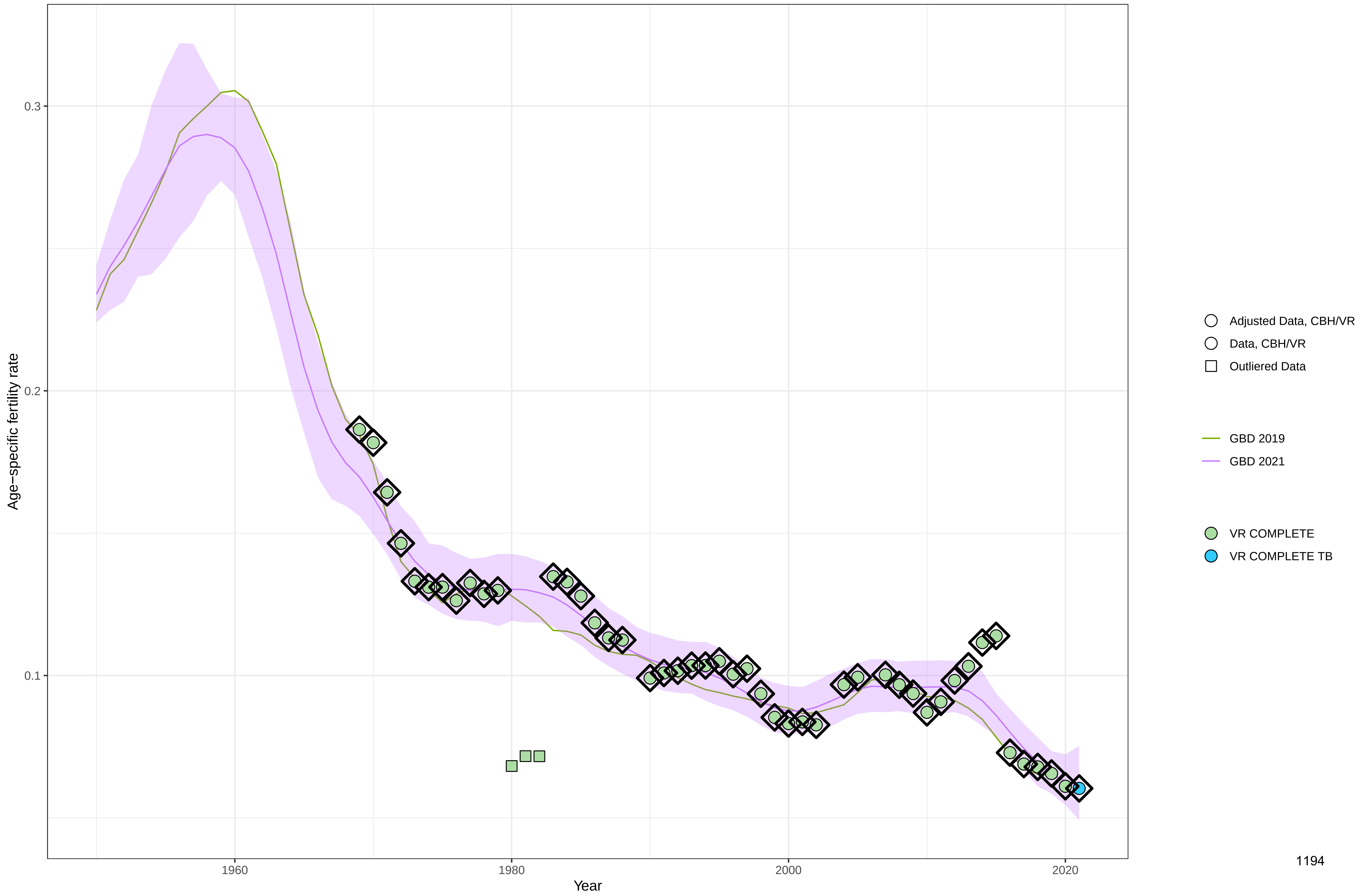

Age−specific fertility fitted trend lines, 1950−2021: North Dakota, Ages: 25−29
Data points enclosed with a diamond are reference sources

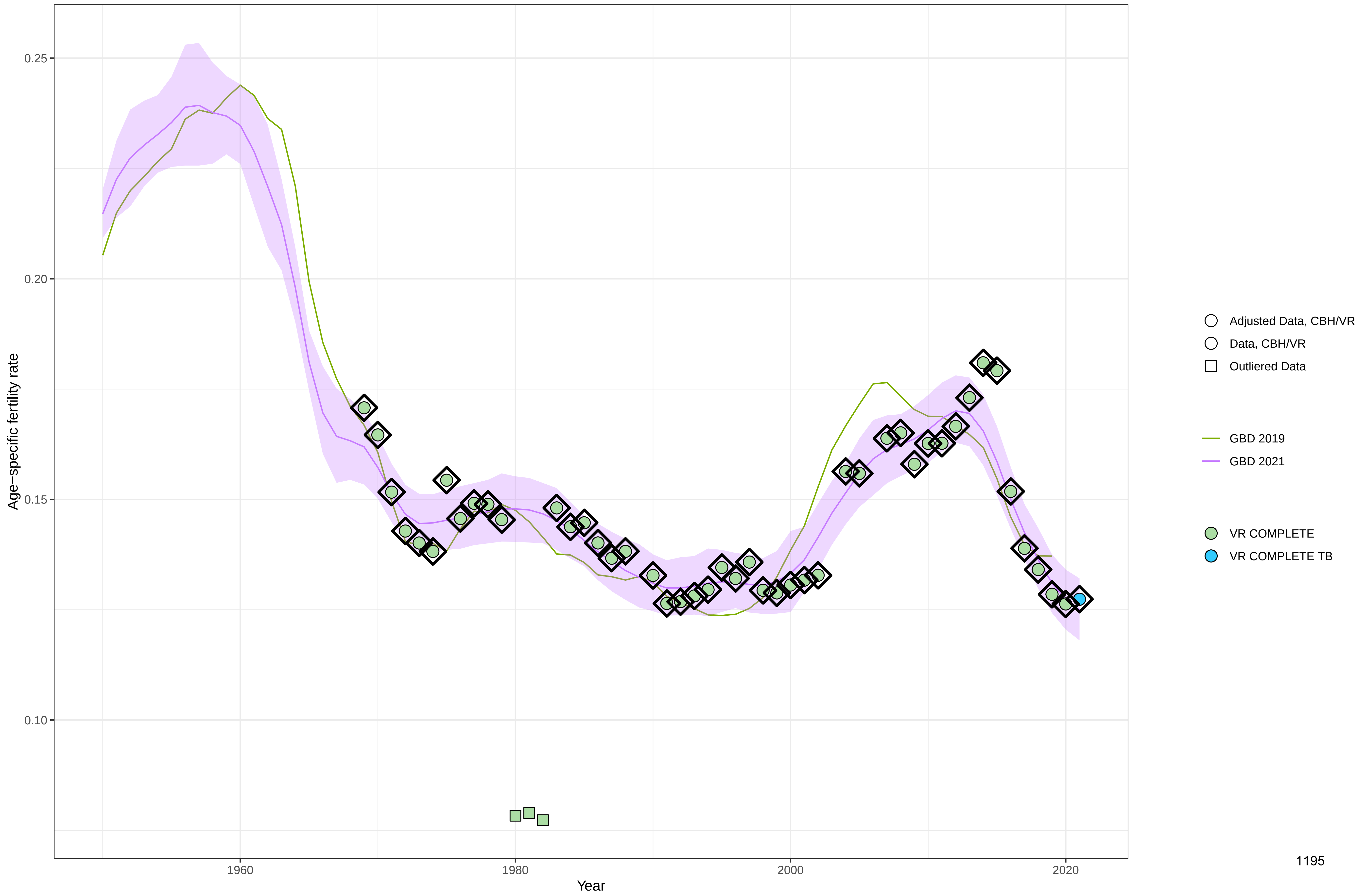

Age−specific fertility fitted trend lines, 1950−2021: North Dakota, Ages: 30−34
Data points enclosed with a diamond are reference sources

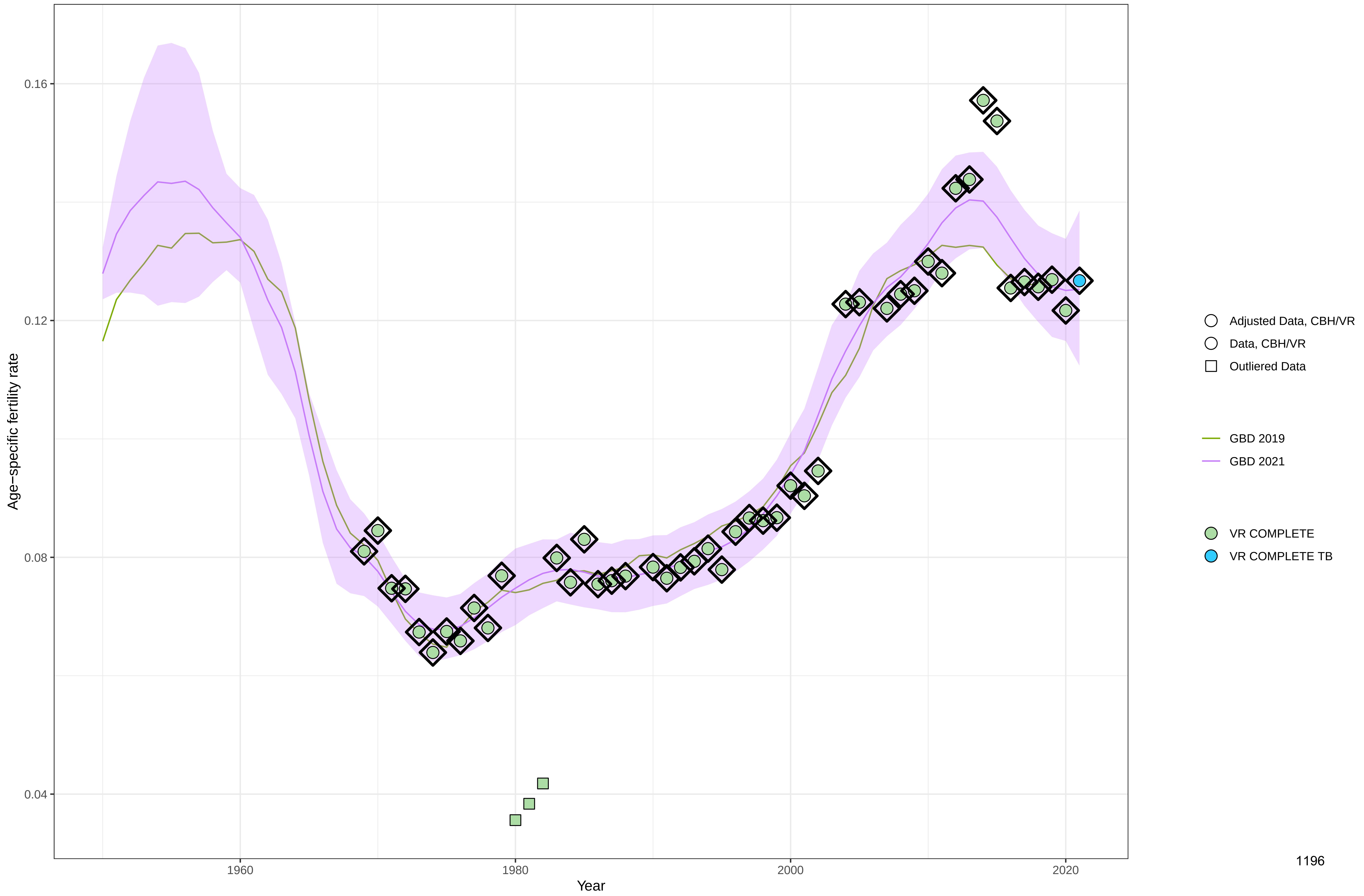

Age−specific fertility fitted trend lines, 1950−2021: North Dakota, Ages: 35−39
Data points enclosed with a diamond are reference sources

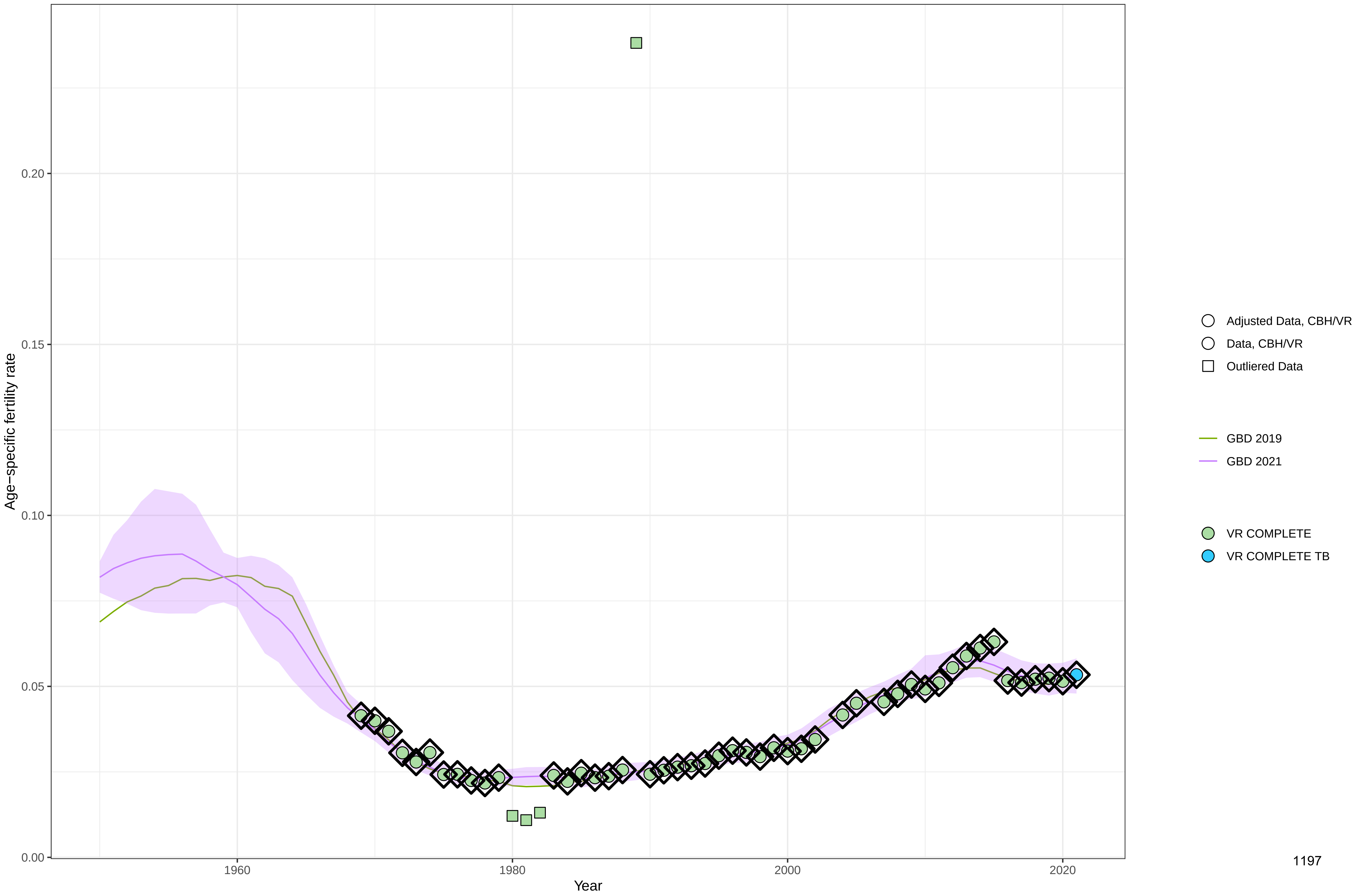

Age−specific fertility fitted trend lines, 1950−2021: North Dakota, Ages: 40−44
Data points enclosed with a diamond are reference sources

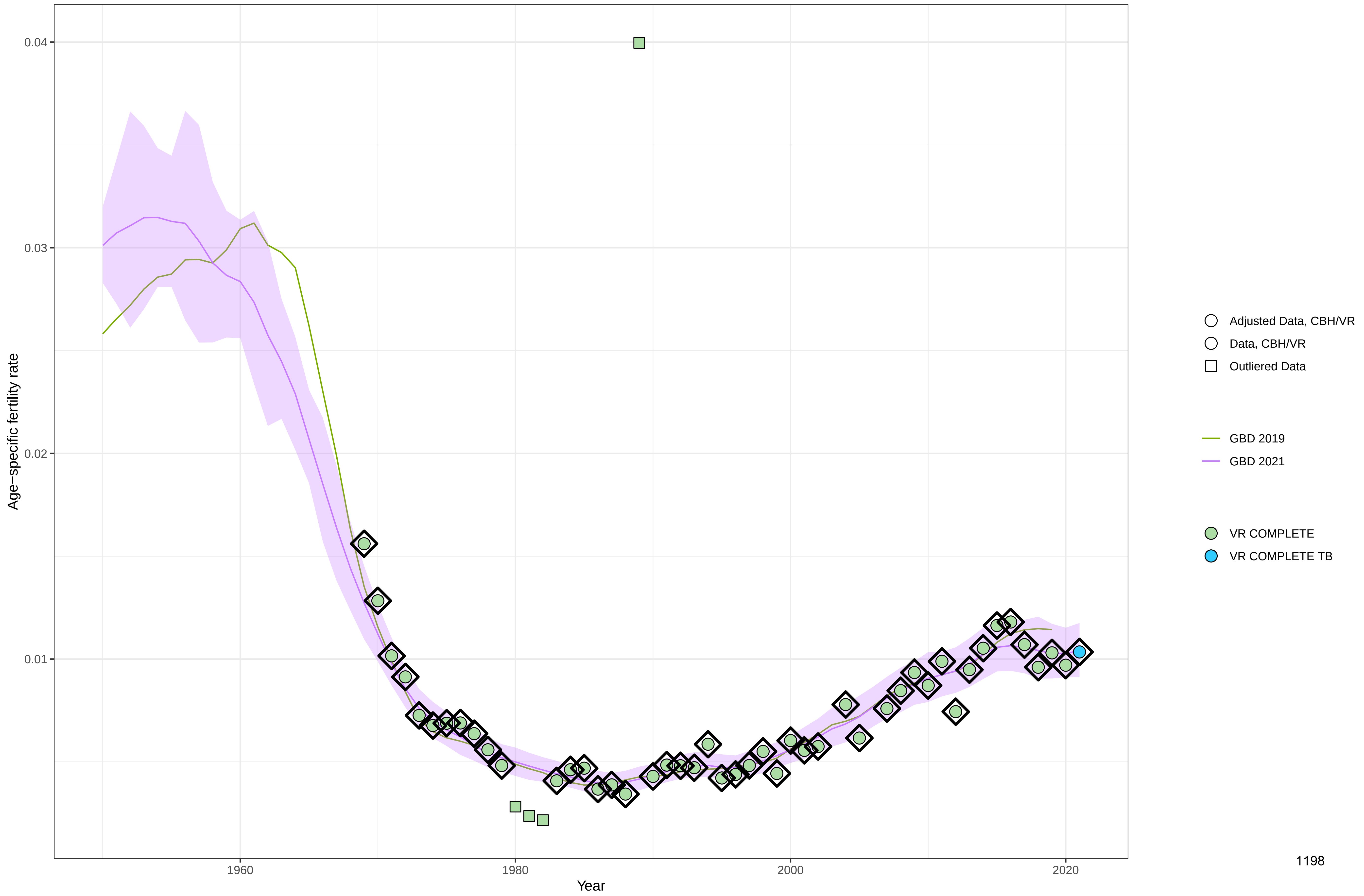

Age−specific fertility fitted trend lines, 1950−2021: North Dakota, Ages: 45−49
Data points enclosed with a diamond are reference sources

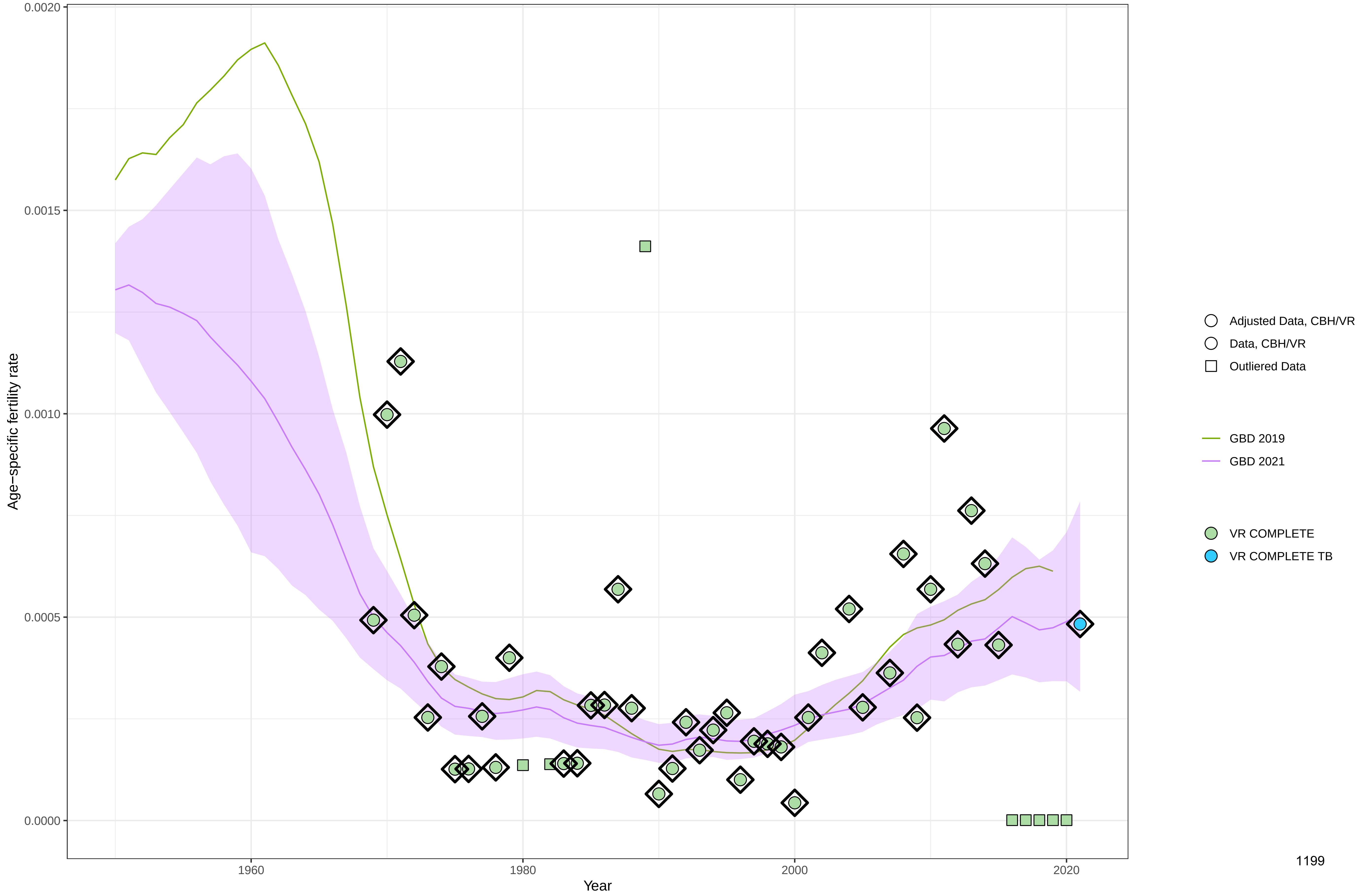

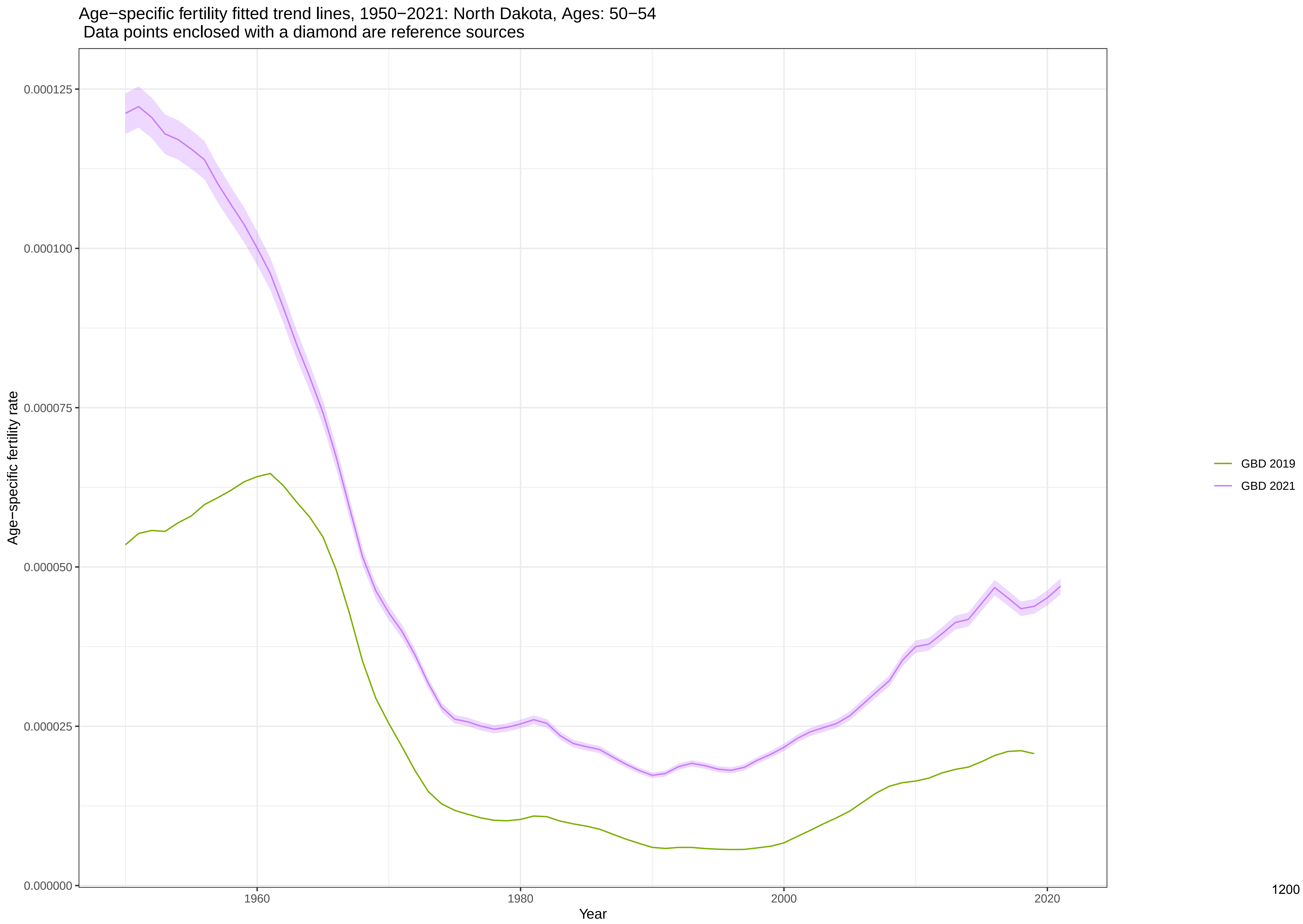

Age−specific fertility fitted trend lines, 1950−2021: North Dakota, Ages: 50−54
Data points enclosed with a diamond are reference sources
0.000125
0.000100
0.000075
0.000050
0.000025
0.000000
Age−specific fertility rate
1960
1980
2000
2020
Year
GBD 2019
GBD 2021

Total fertility rate fitted trend lines, 1950−2021: North Dakota TFR
Data points enclosed with a diamond are reference sources

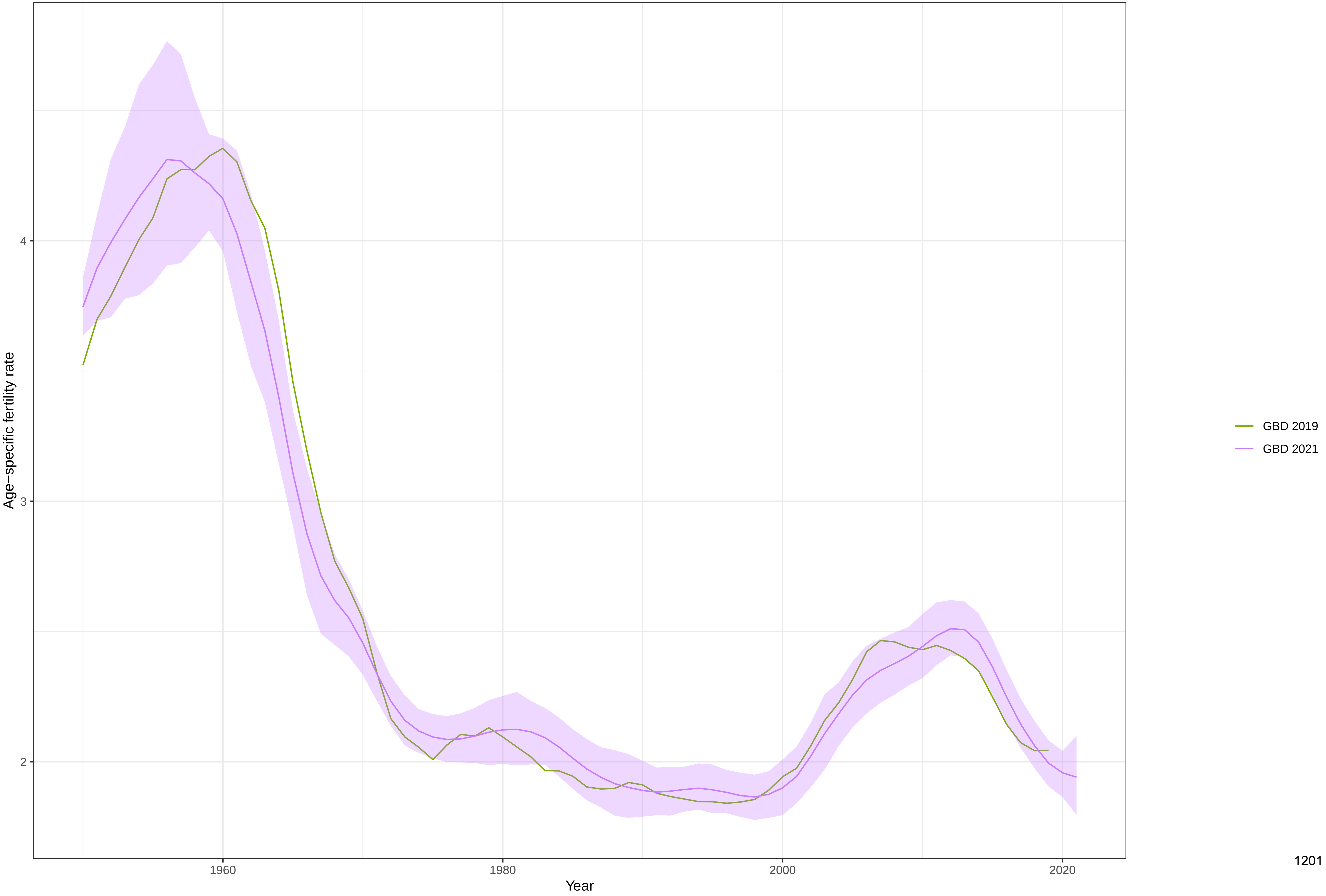

Age−specific fertility fitted trend lines, 1950−2021: Ohio, Ages: 10−14
Data points enclosed with a diamond are reference sources

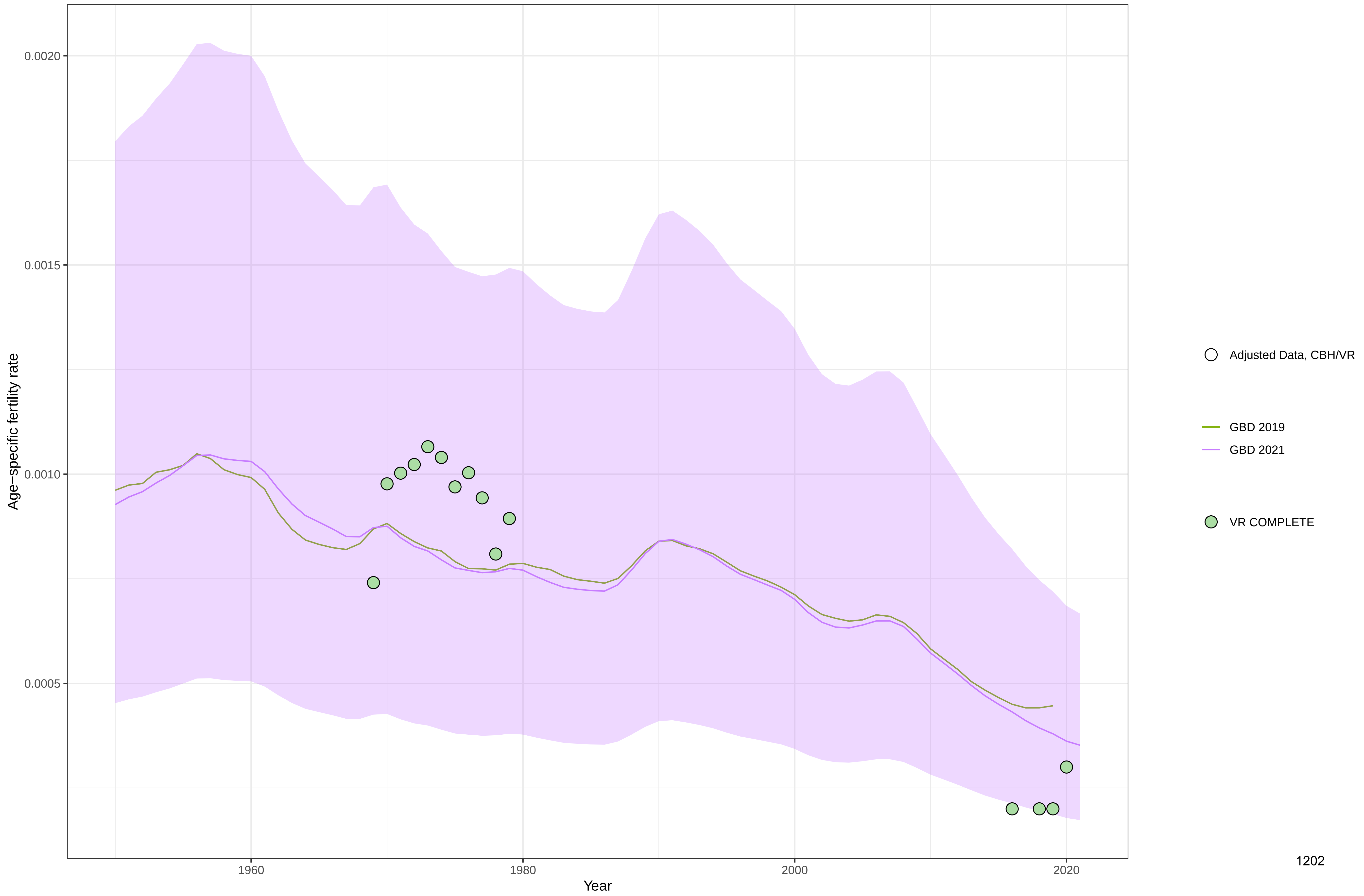

Age−specific fertility fitted trend lines, 1950−2021: Ohio, Ages: 15−19
Data points enclosed with a diamond are reference sources

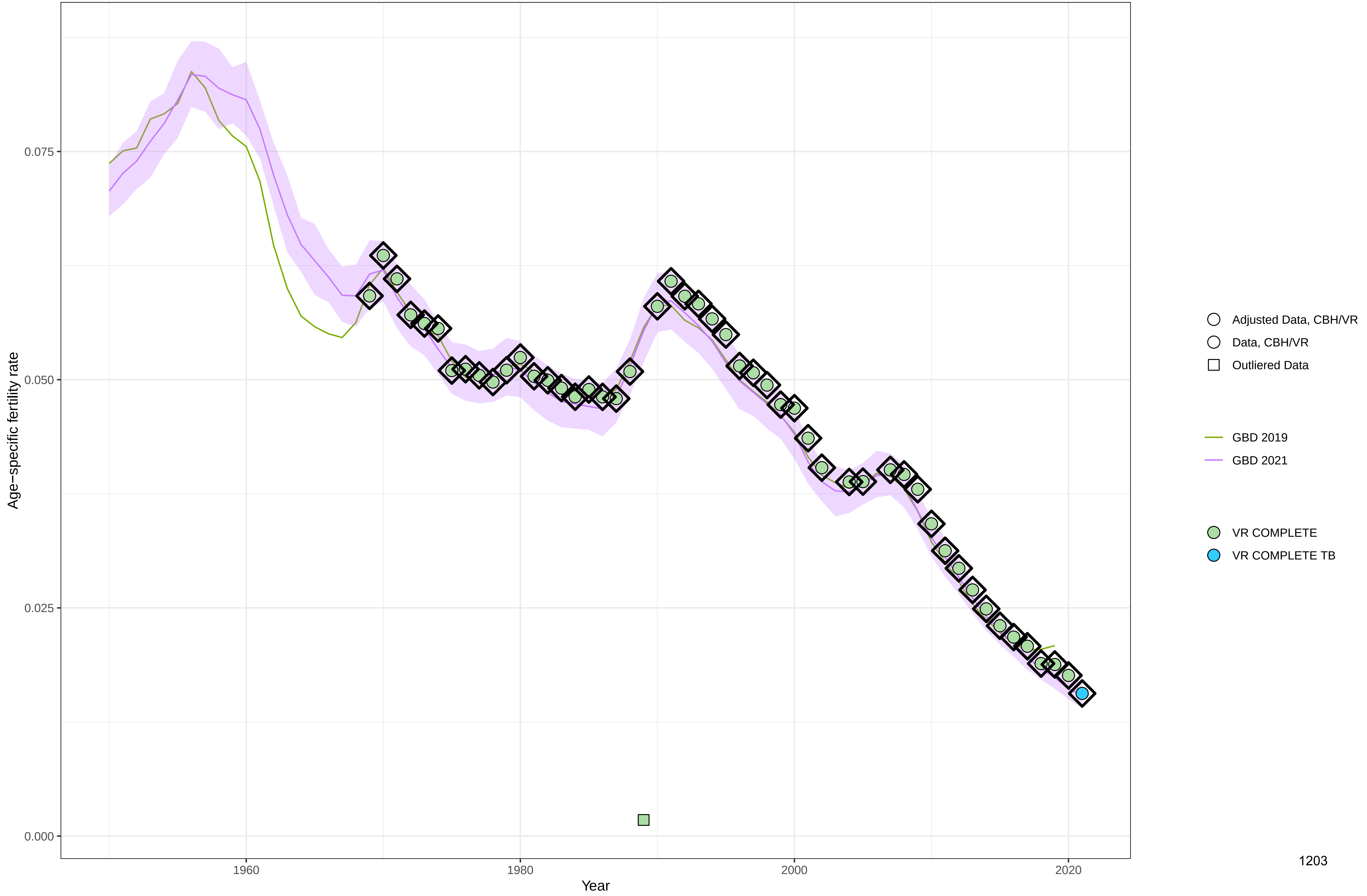

Age−specific fertility fitted trend lines, 1950−2021: Ohio, Ages: 20−24
Data points enclosed with a diamond are reference sources

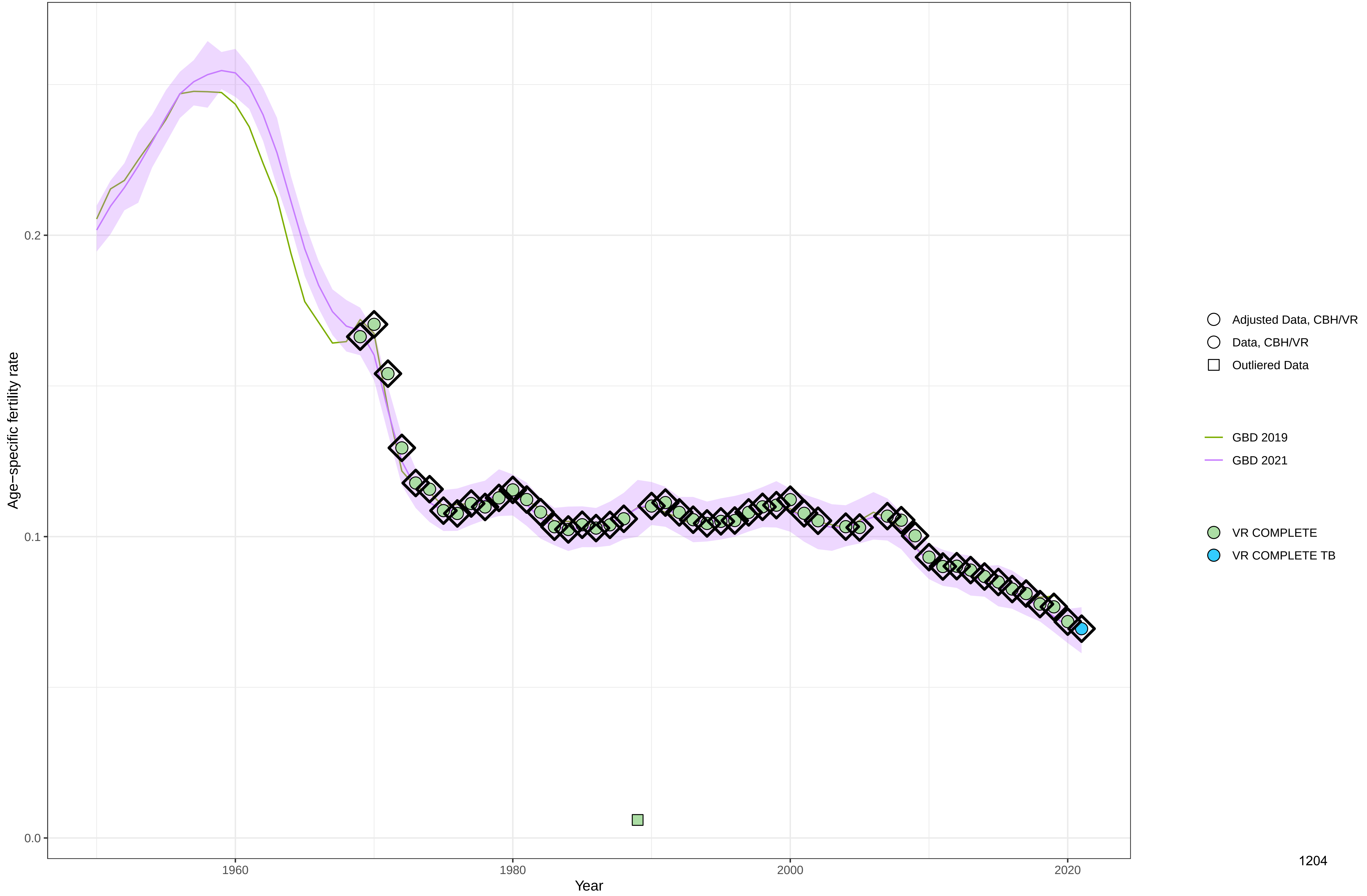

Age−specific fertility fitted trend lines, 1950−2021: Ohio, Ages: 25−29
Data points enclosed with a diamond are reference sources

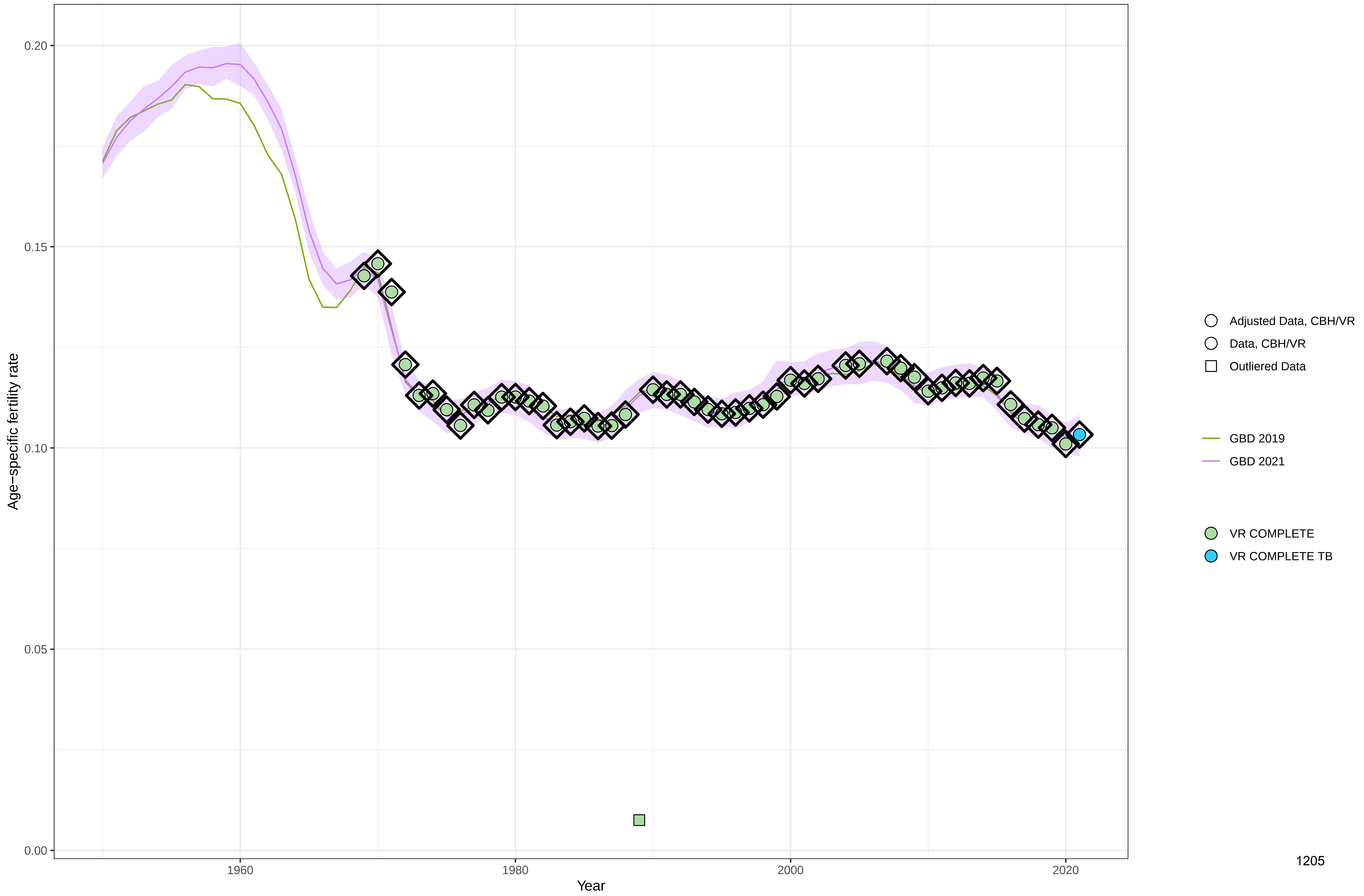

Age−specific fertility fitted trend lines, 1950−2021: Ohio, Ages: 30−34
Data points enclosed with a diamond are reference sources

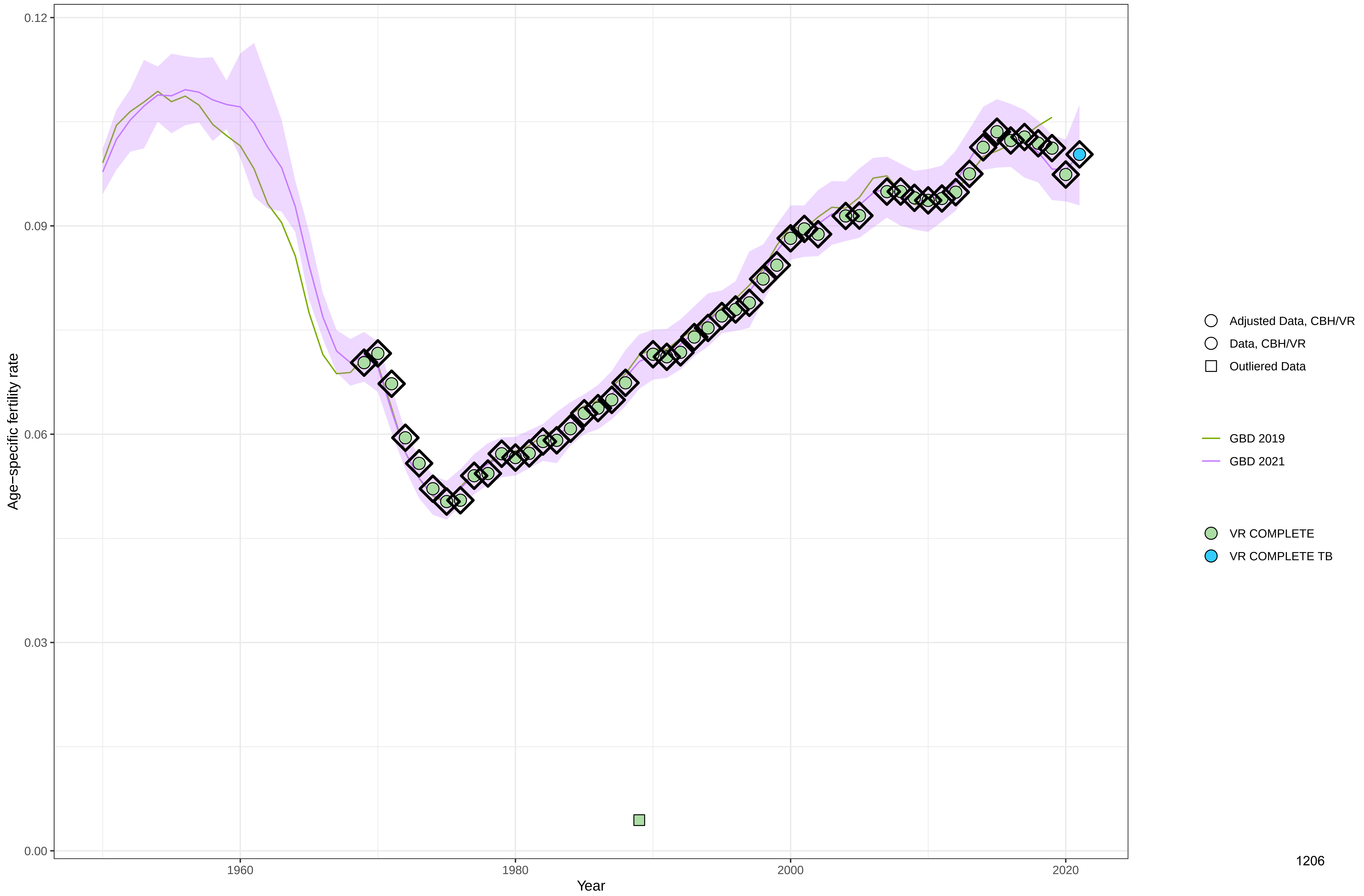

Age−specific fertility fitted trend lines, 1950−2021: Ohio, Ages: 35−39
Data points enclosed with a diamond are reference sources

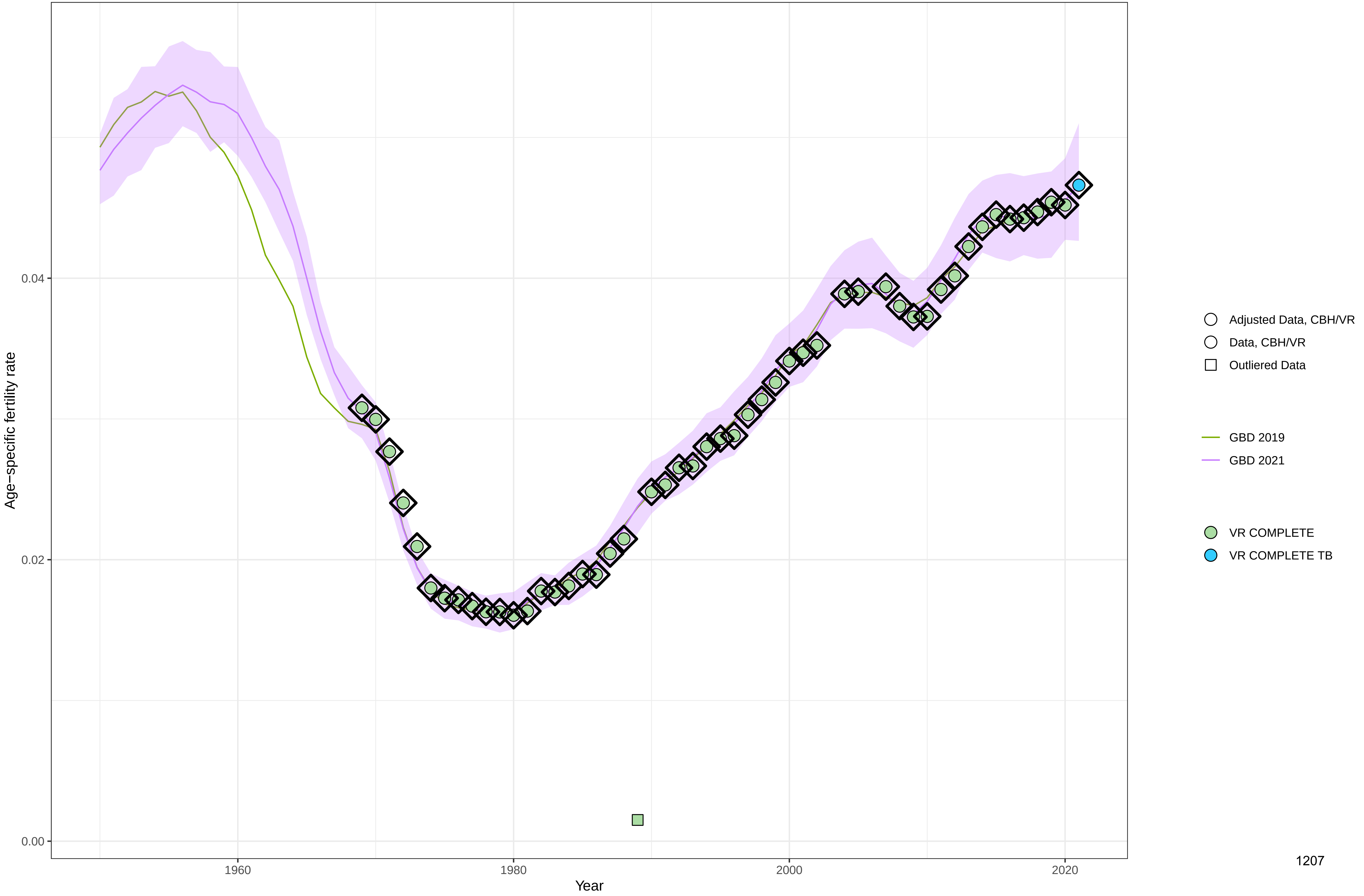

Age−specific fertility fitted trend lines, 1950−2021: Ohio, Ages: 40−44
Data points enclosed with a diamond are reference sources

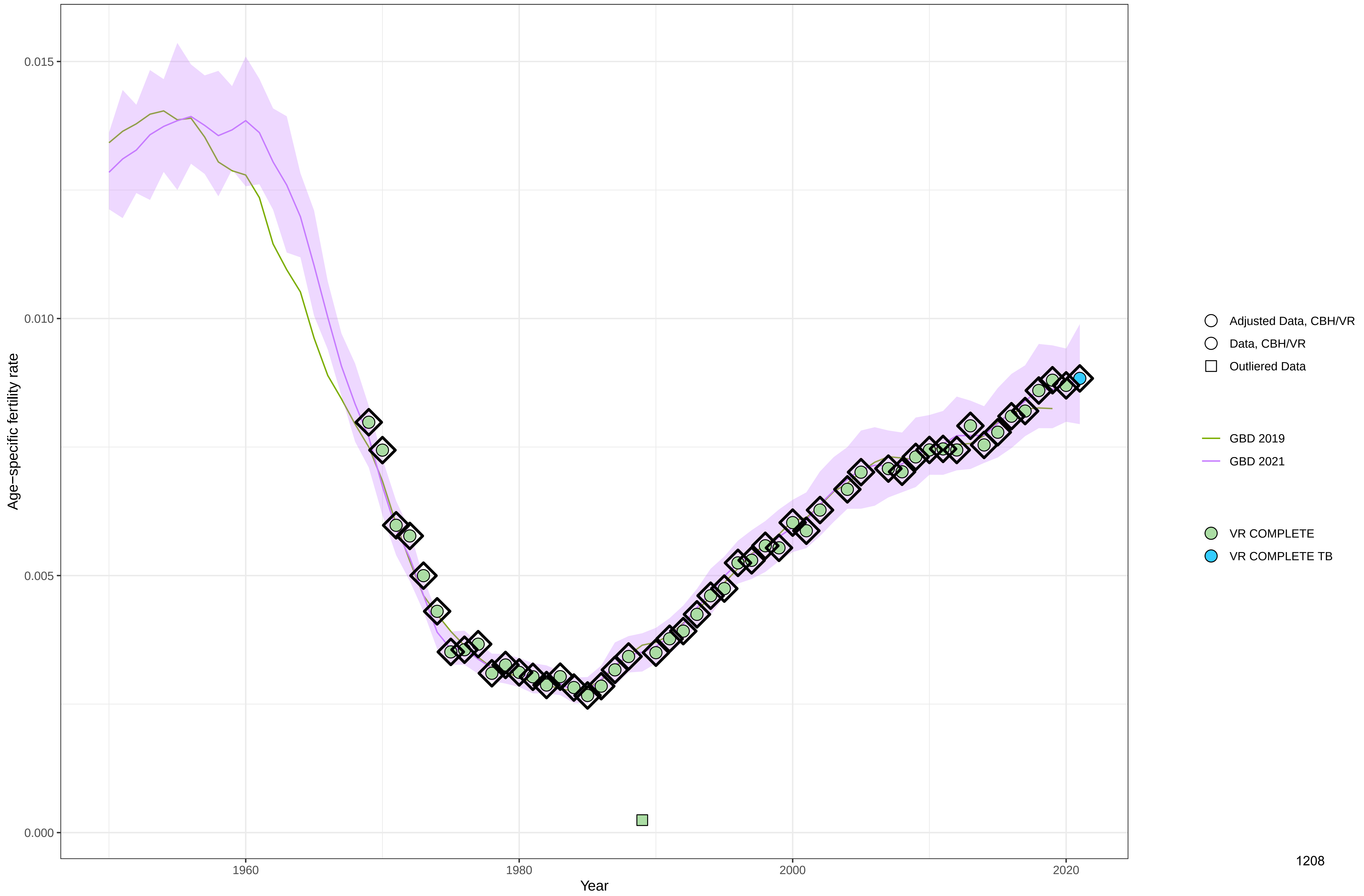

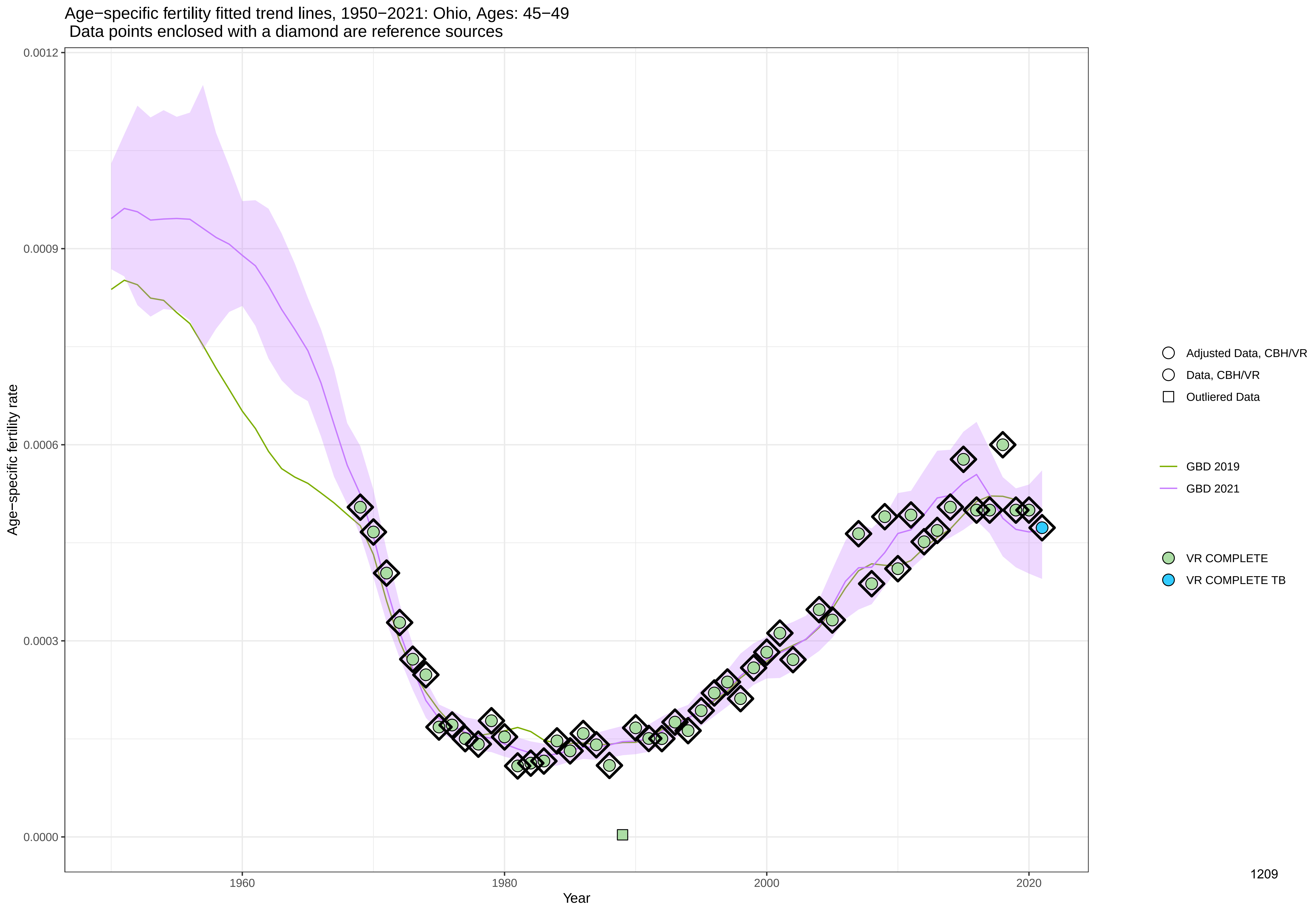

Age−specific fertility fitted trend lines, 1950−2021: Ohio, Ages: 45−49
Data points enclosed with a diamond are reference sources
Age−specific fertility rate
Year
0.0000
0.0003
0.0006
0.0009
0.0012
1960
1980
2000
2020
Adjusted Data, CBH/VR
Data, CBH/VR
Outliered Data
GBD 2019
GBD 2021
VR COMPLETE
VR COMPLETE TB

Age−specific fertility fitted trend lines, 1950−2021: Ohio, Ages: 50−54
Data points enclosed with a diamond are reference sources

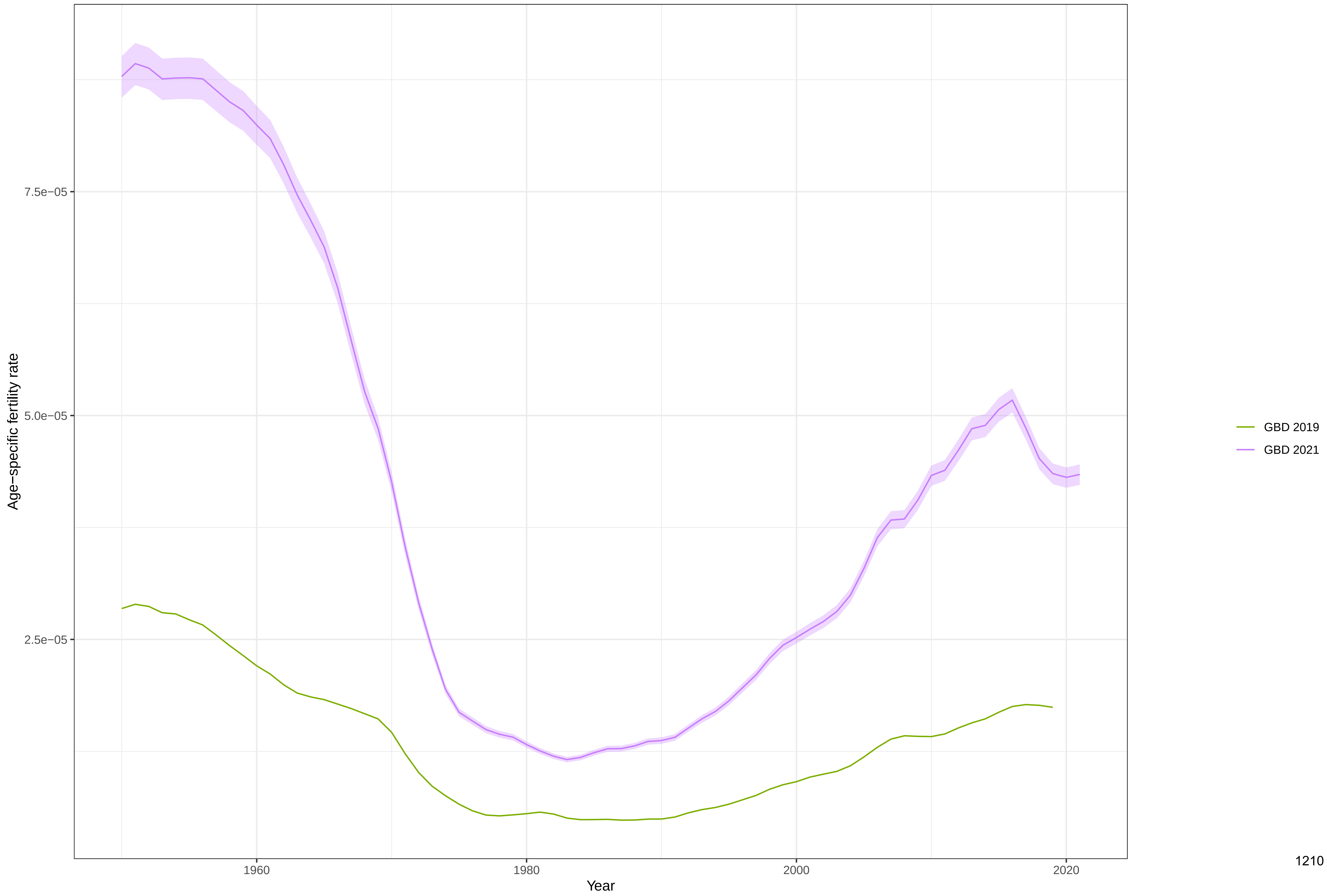

Total fertility rate fitted trend lines, 1950−2021: Ohio TFR
Data points enclosed with a diamond are reference sources

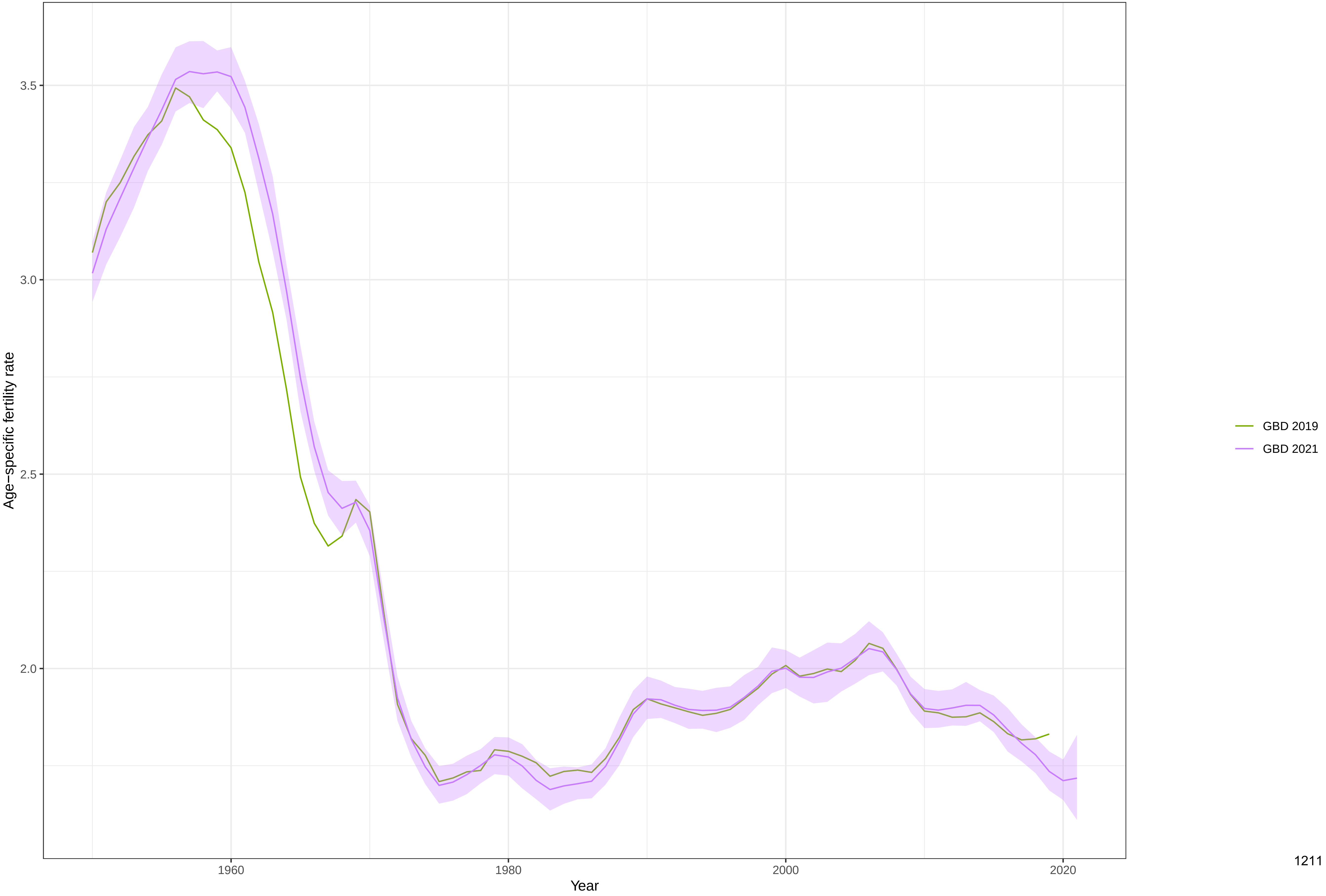

Age−specific fertility fitted trend lines, 1950−2021: Oklahoma, Ages: 10−14
Data points enclosed with a diamond are reference sources

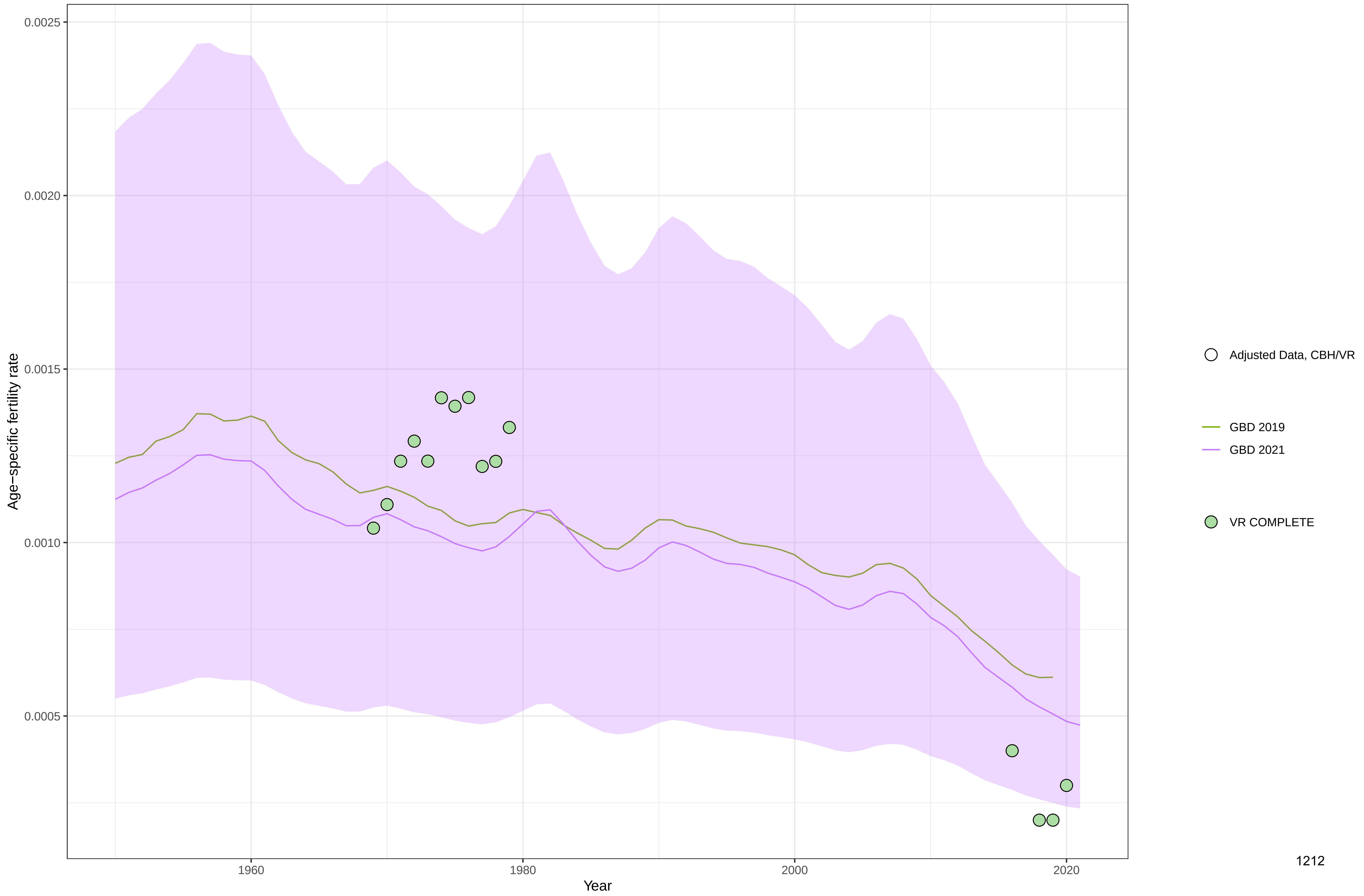

Age−specific fertility fitted trend lines, 1950−2021: Oklahoma, Ages: 15−19
Data points enclosed with a diamond are reference sources

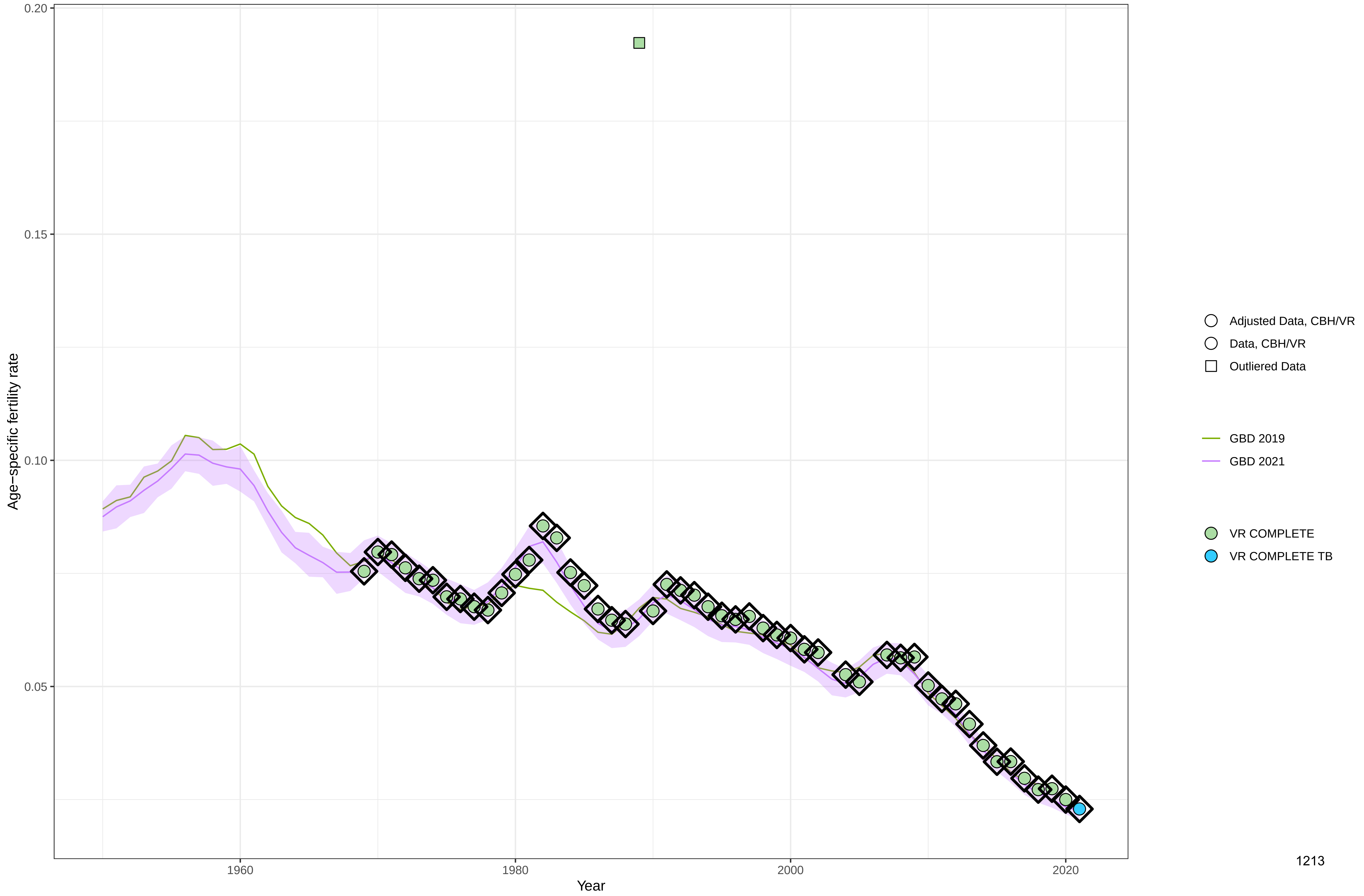

Age−specific fertility fitted trend lines, 1950−2021: Oklahoma, Ages: 20−24
Data points enclosed with a diamond are reference sources

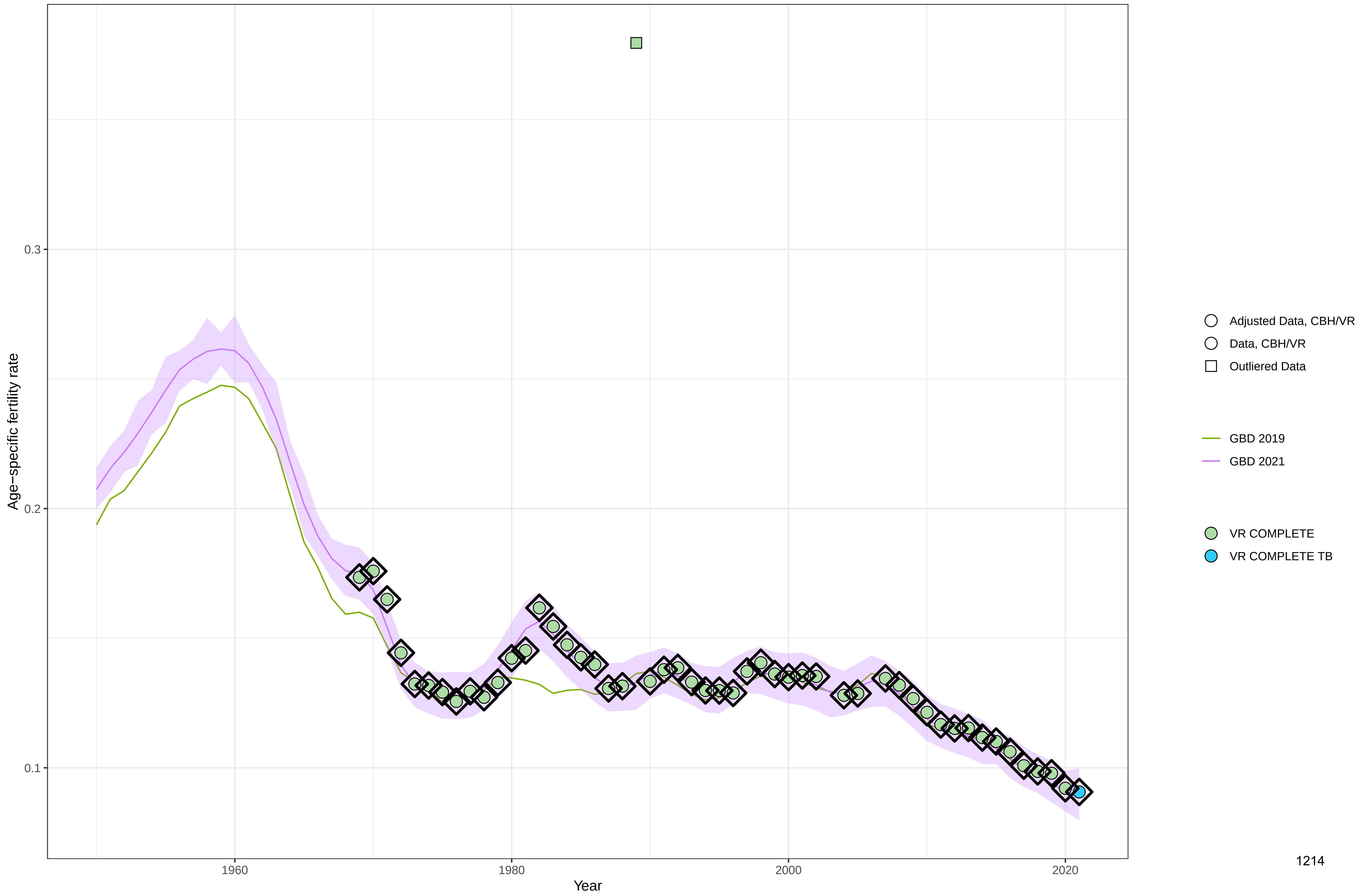

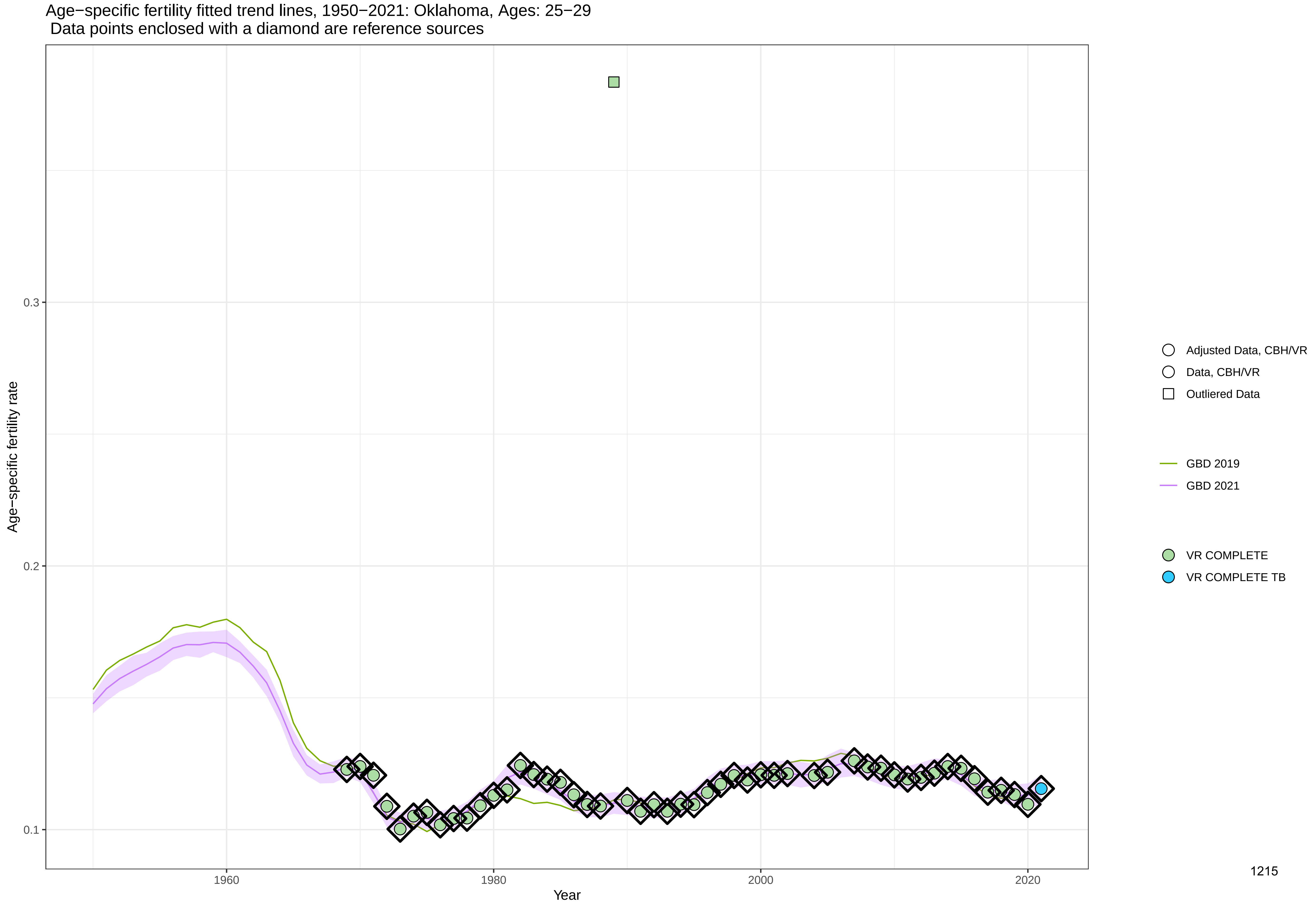
Age−specific fertility fitted trend lines, 1950−2021: Oklahoma, Ages: 25−29
Data points enclosed with a diamond are reference sources
Age−specific fertility rate
0.3
0.2
0.1
1960
1980
2000
2020
Year
Adjusted Data, CBH/VR
Data, CBH/VR
Outliered Data
GBD 2019
GBD 2021
VR COMPLETE
VR COMPLETE TB

Age−specific fertility fitted trend lines, 1950−2021: Oklahoma, Ages: 30−34
Data points enclosed with a diamond are reference sources

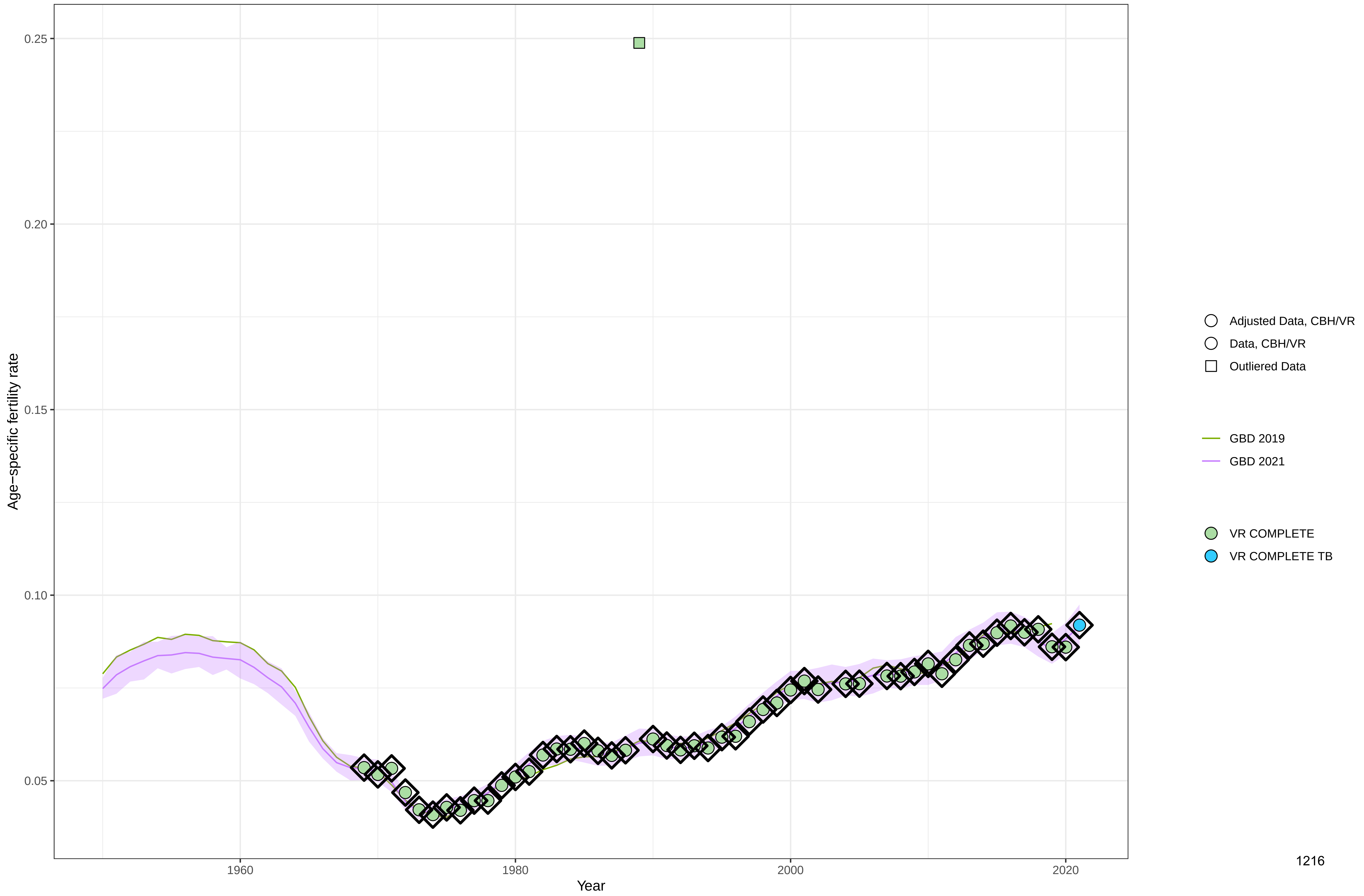

Age−specific fertility fitted trend lines, 1950−2021: Oklahoma, Ages: 35−39
Data points enclosed with a diamond are reference sources

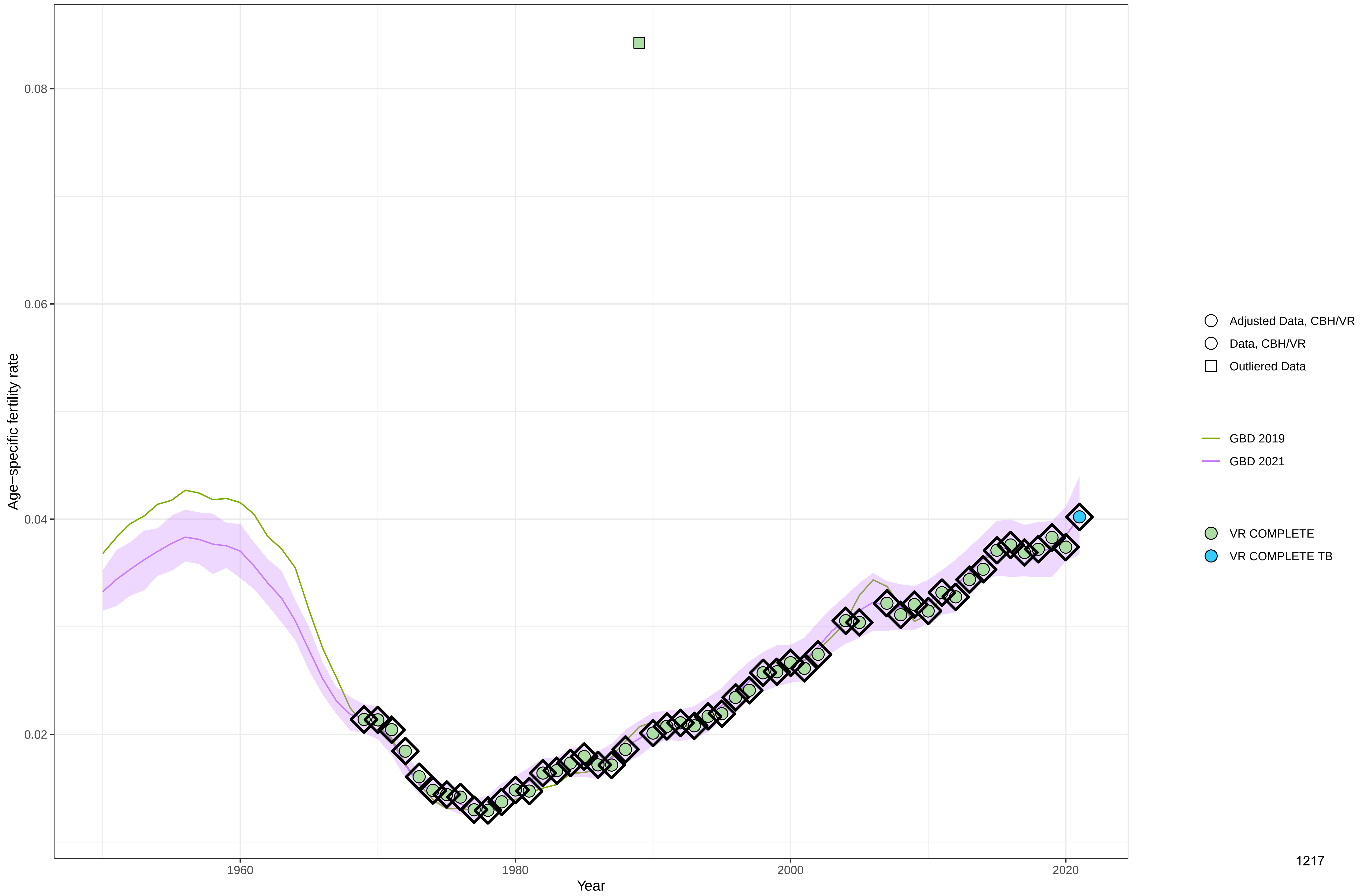

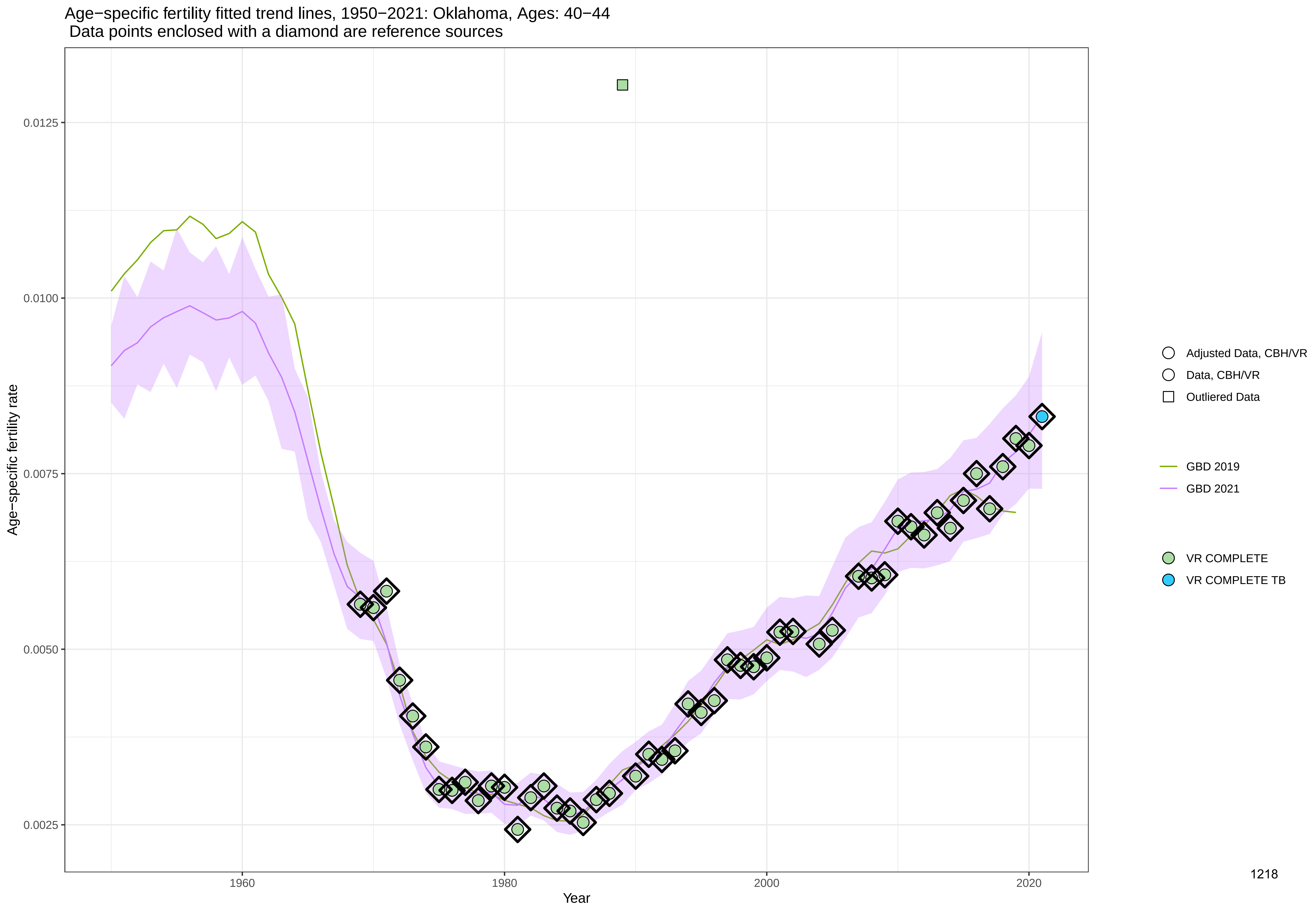
Age−specific fertility fitted trend lines, 1950−2021: Oklahoma, Ages: 40−44
Data points enclosed with a diamond are reference sources
Age−specific fertility rate
0.0125
0.0100
0.0075
0.0050
0.0025
1960
1980
2000
2020
Year
Adjusted Data, CBH/VR
Data, CBH/VR
Outliered Data
GBD 2019
GBD 2021
VR COMPLETE
VR COMPLETE TB

Age−specific fertility fitted trend lines, 1950−2021: Oklahoma, Ages: 45−49
Data points enclosed with a diamond are reference sources

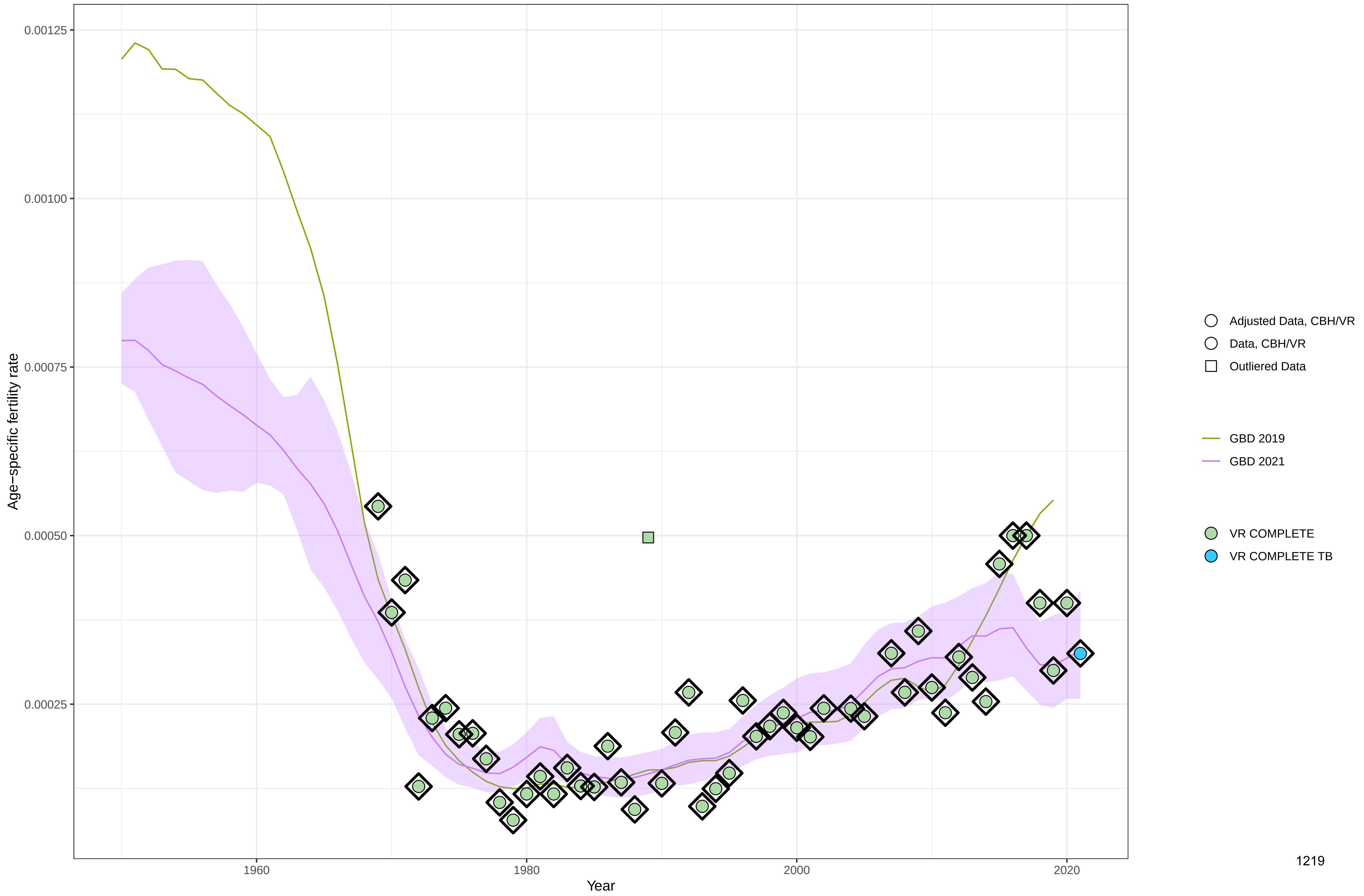

Age−specific fertility fitted trend lines, 1950−2021: Oklahoma, Ages: 50−54
Data points enclosed with a diamond are reference sources

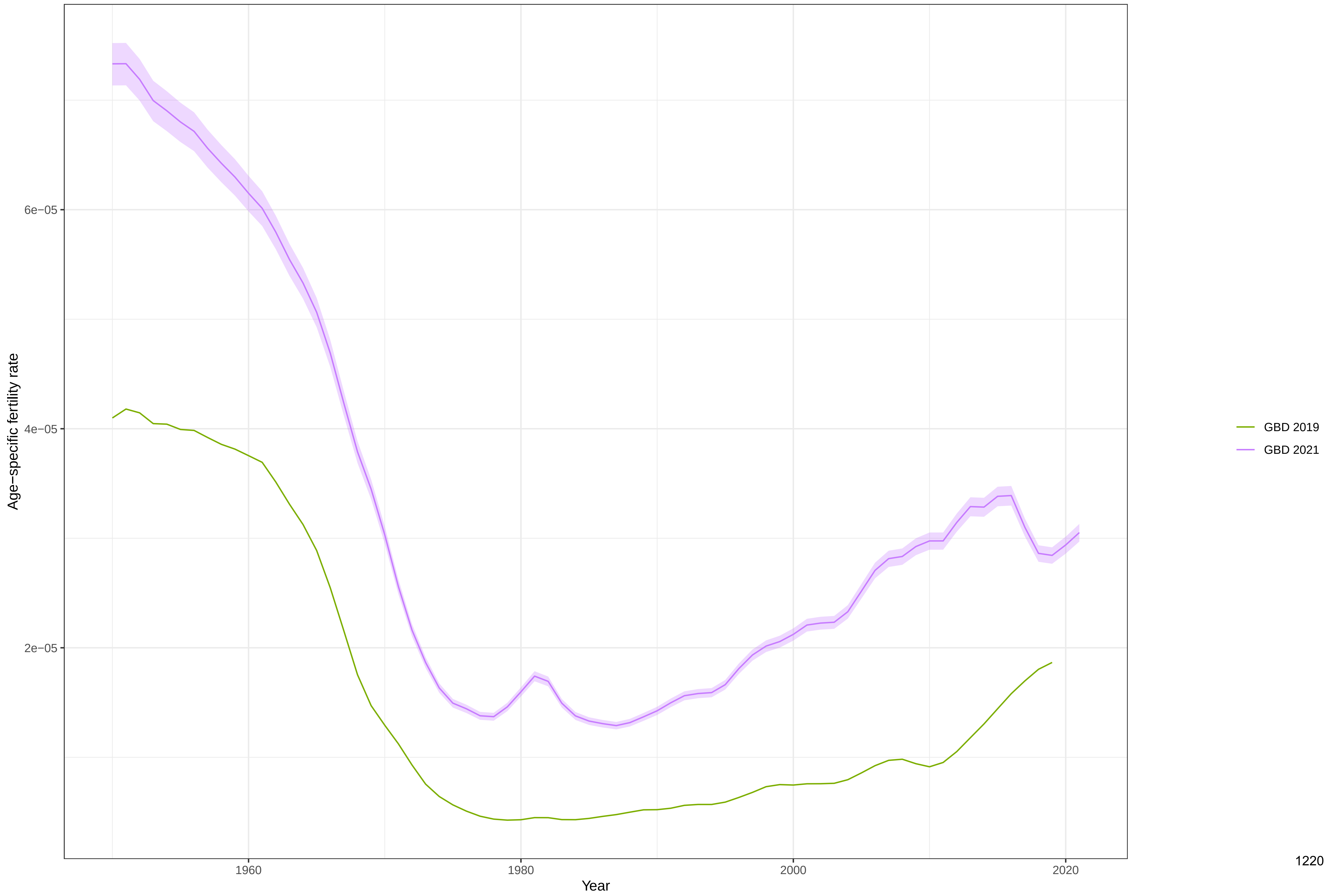

Total fertility rate fitted trend lines, 1950−2021: Oklahoma TFR
Data points enclosed with a diamond are reference sources

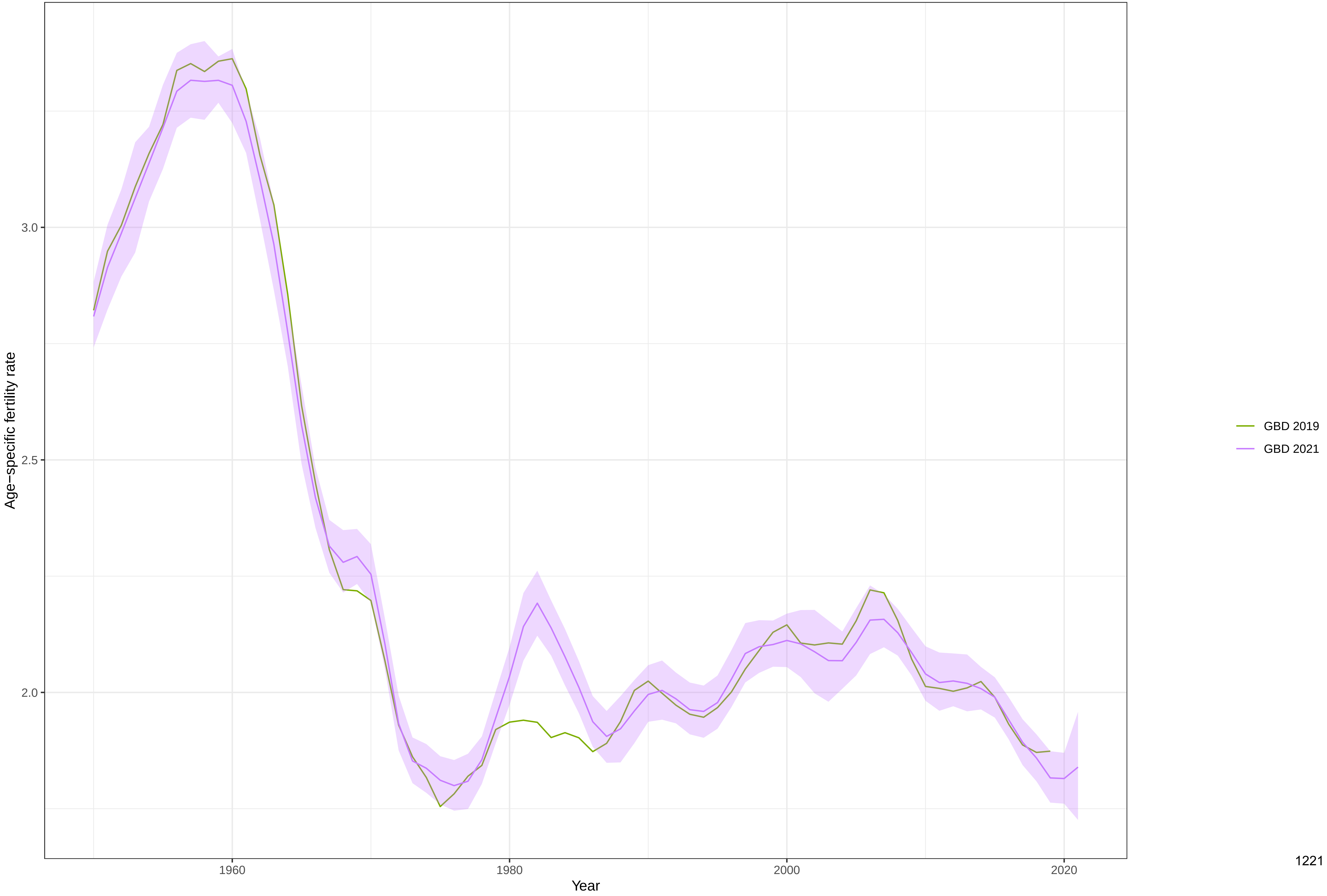

Age−specific fertility fitted trend lines, 1950−2021: Oregon, Ages: 10−14
Data points enclosed with a diamond are reference sources

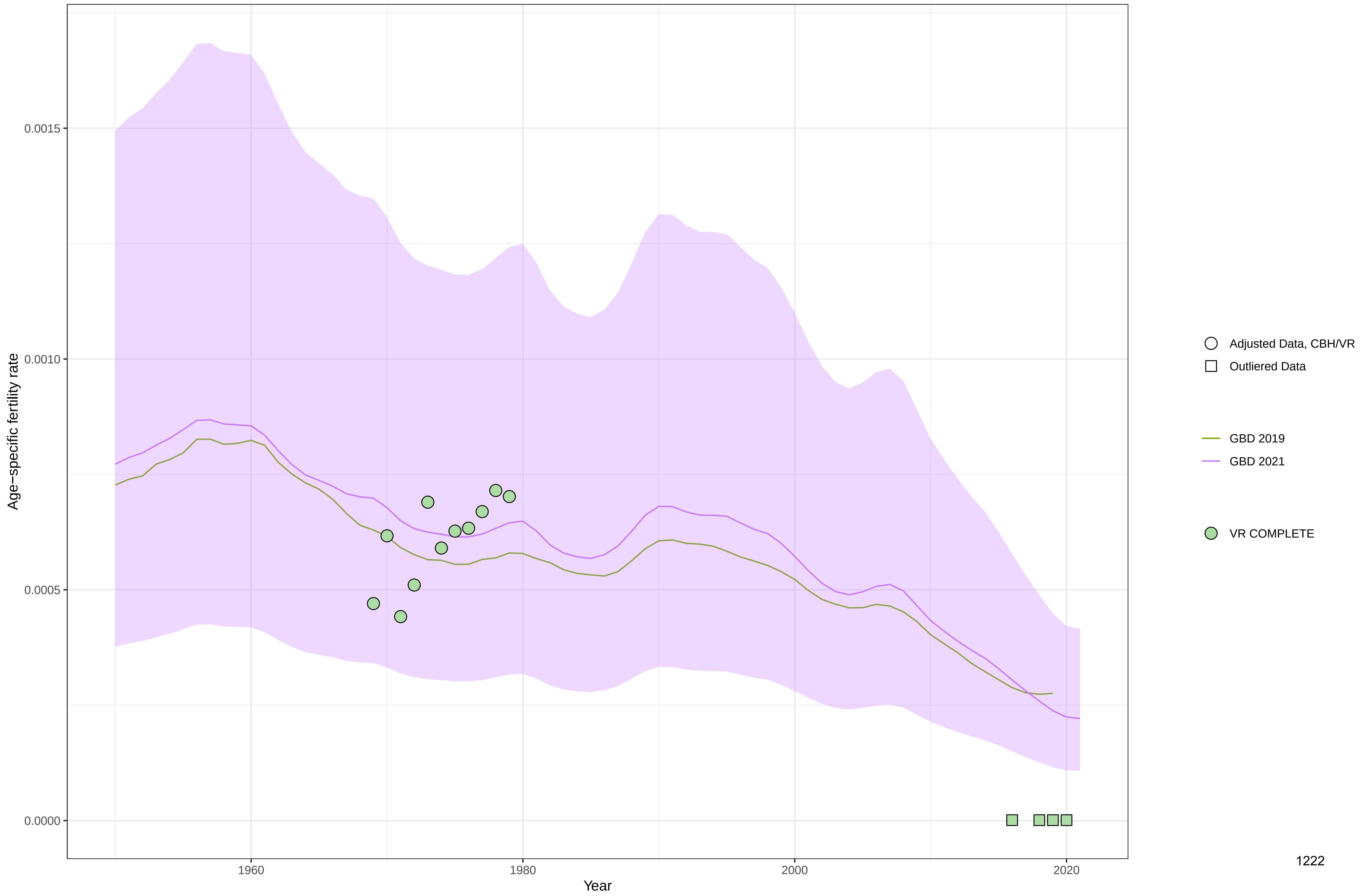

Age−specific fertility fitted trend lines, 1950−2021: Oregon, Ages: 15−19
Data points enclosed with a diamond are reference sources

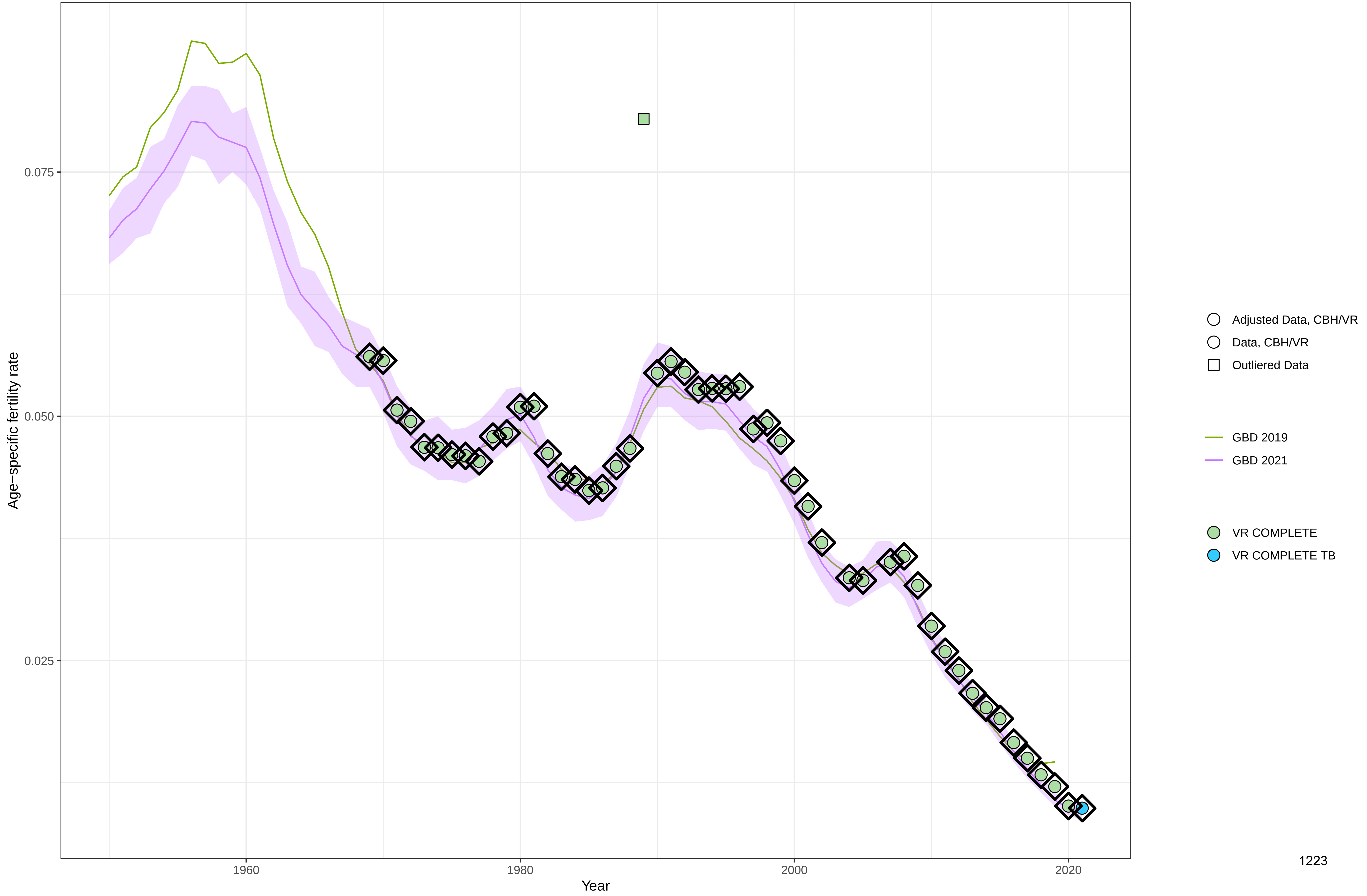

Age−specific fertility fitted trend lines, 1950−2021: Oregon, Ages: 20−24
Data points enclosed with a diamond are reference sources

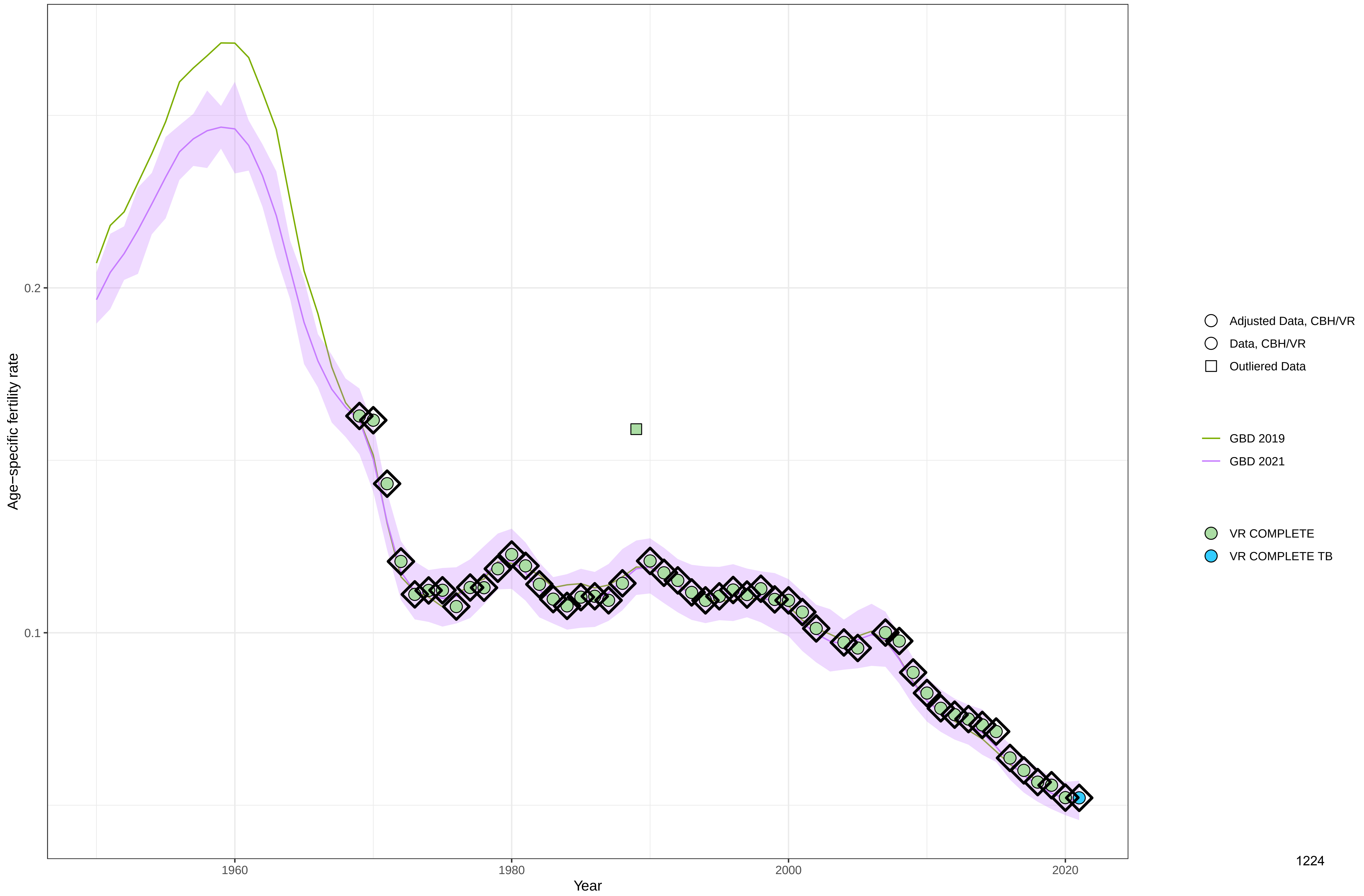

Age−specific fertility fitted trend lines, 1950−2021: Oregon, Ages: 25−29
Data points enclosed with a diamond are reference sources

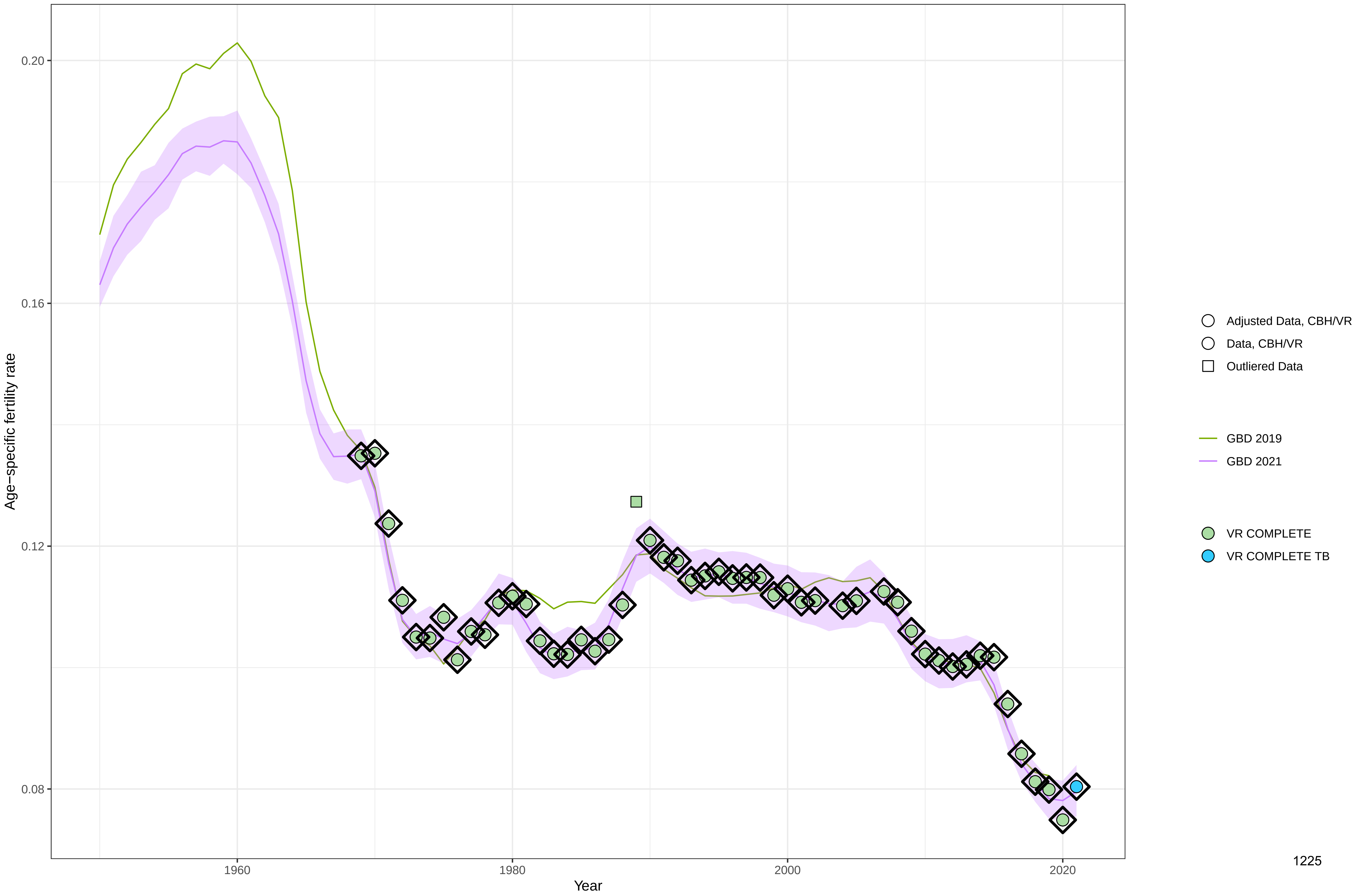

Age−specific fertility fitted trend lines, 1950−2021: Oregon, Ages: 30−34
Data points enclosed with a diamond are reference sources

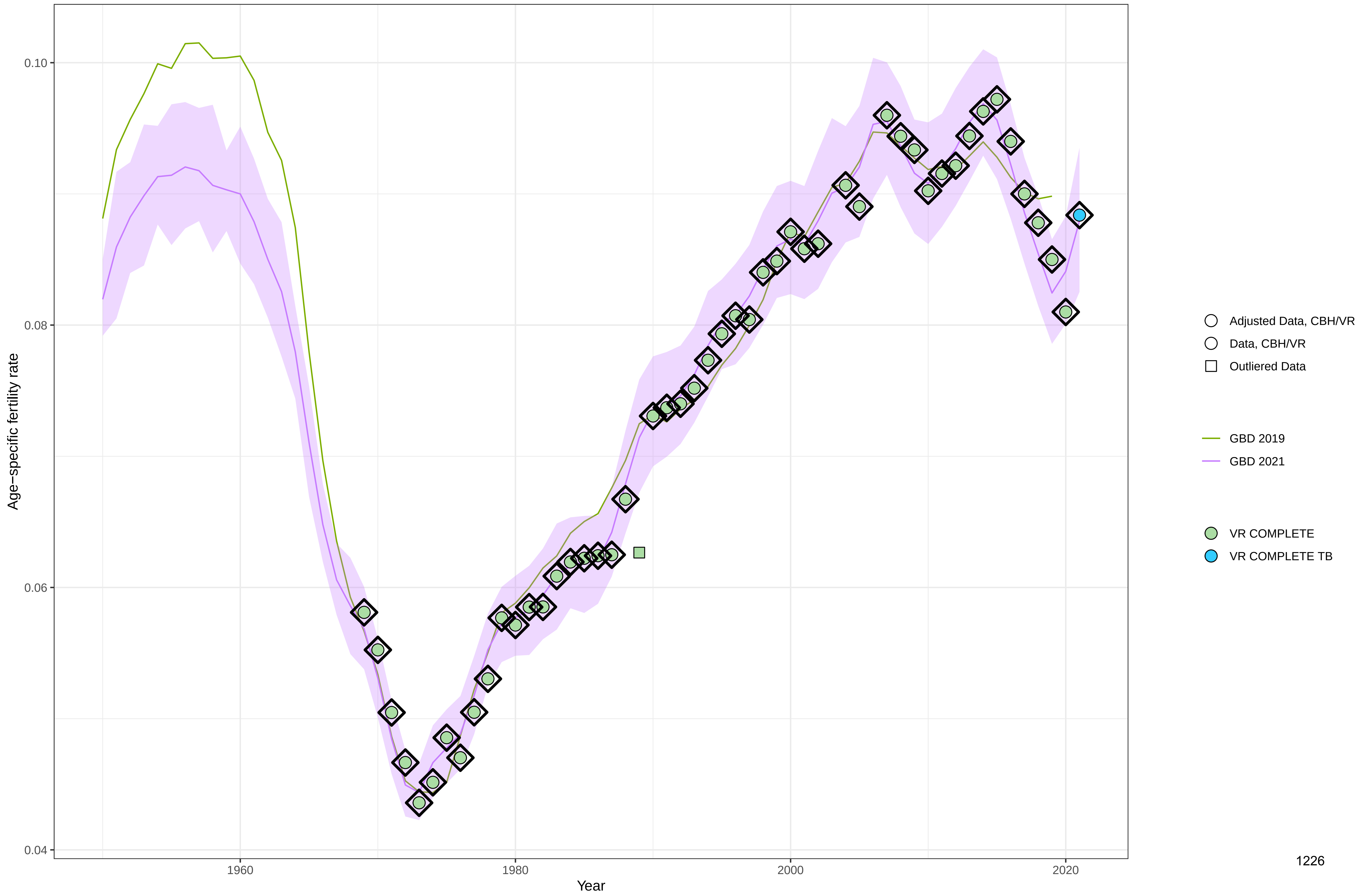

Age−specific fertility fitted trend lines, 1950−2021: Oregon, Ages: 35−39
Data points enclosed with a diamond are reference sources

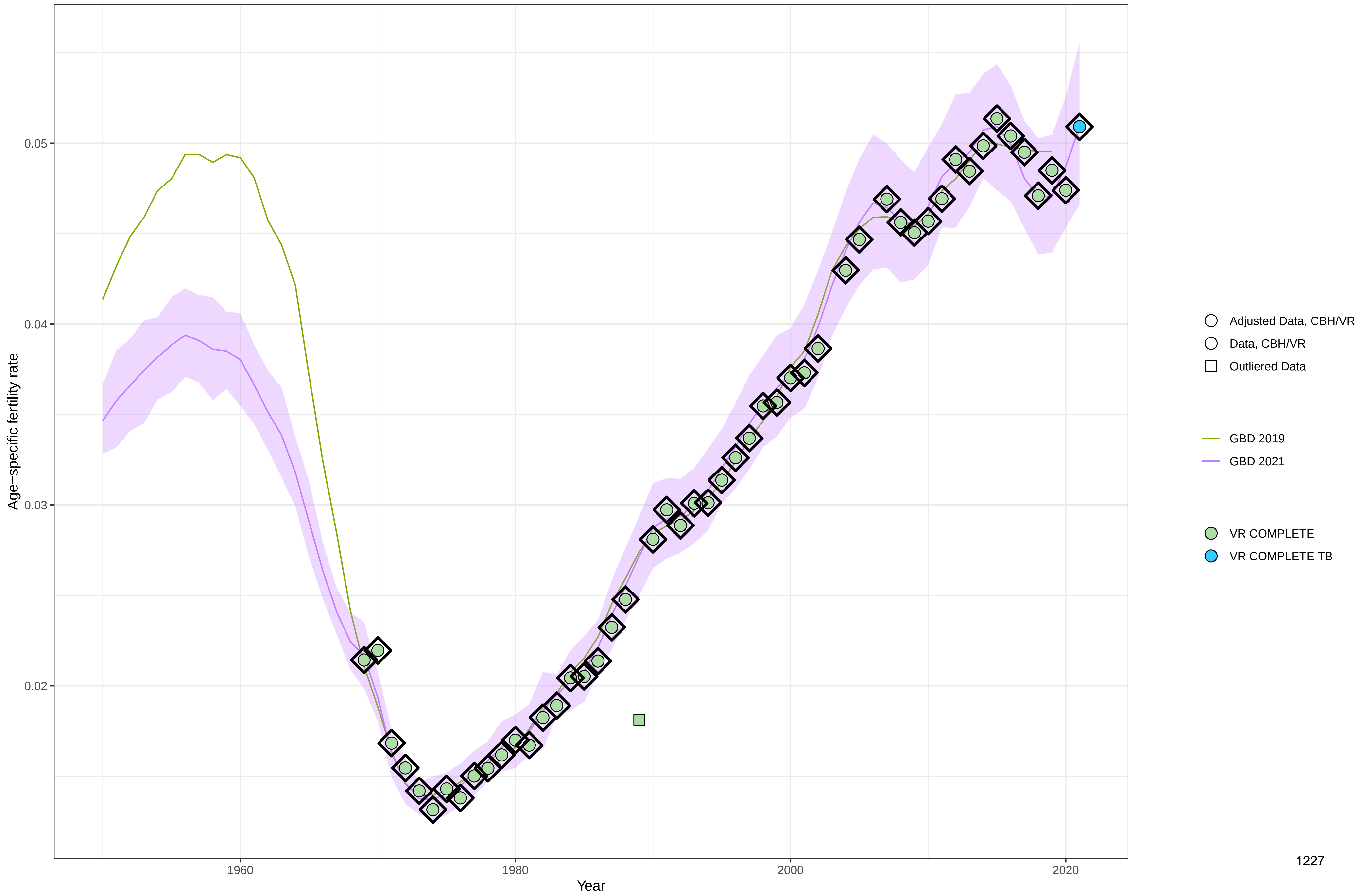

Age−specific fertility fitted trend lines, 1950−2021: Oregon, Ages: 40−44
Data points enclosed with a diamond are reference sources

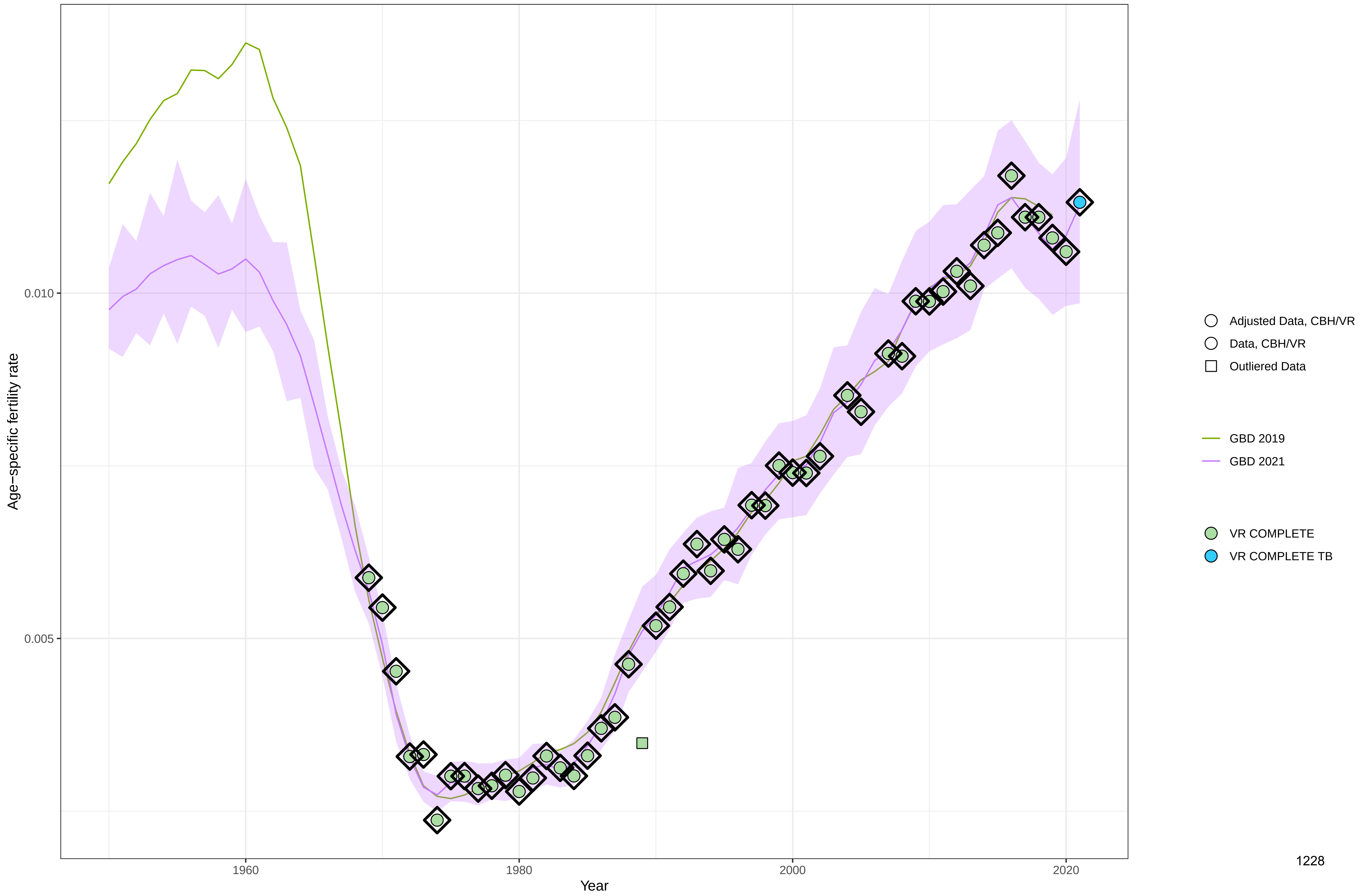

Age−specific fertility fitted trend lines, 1950−2021: Oregon, Ages: 45−49
Data points enclosed with a diamond are reference sources

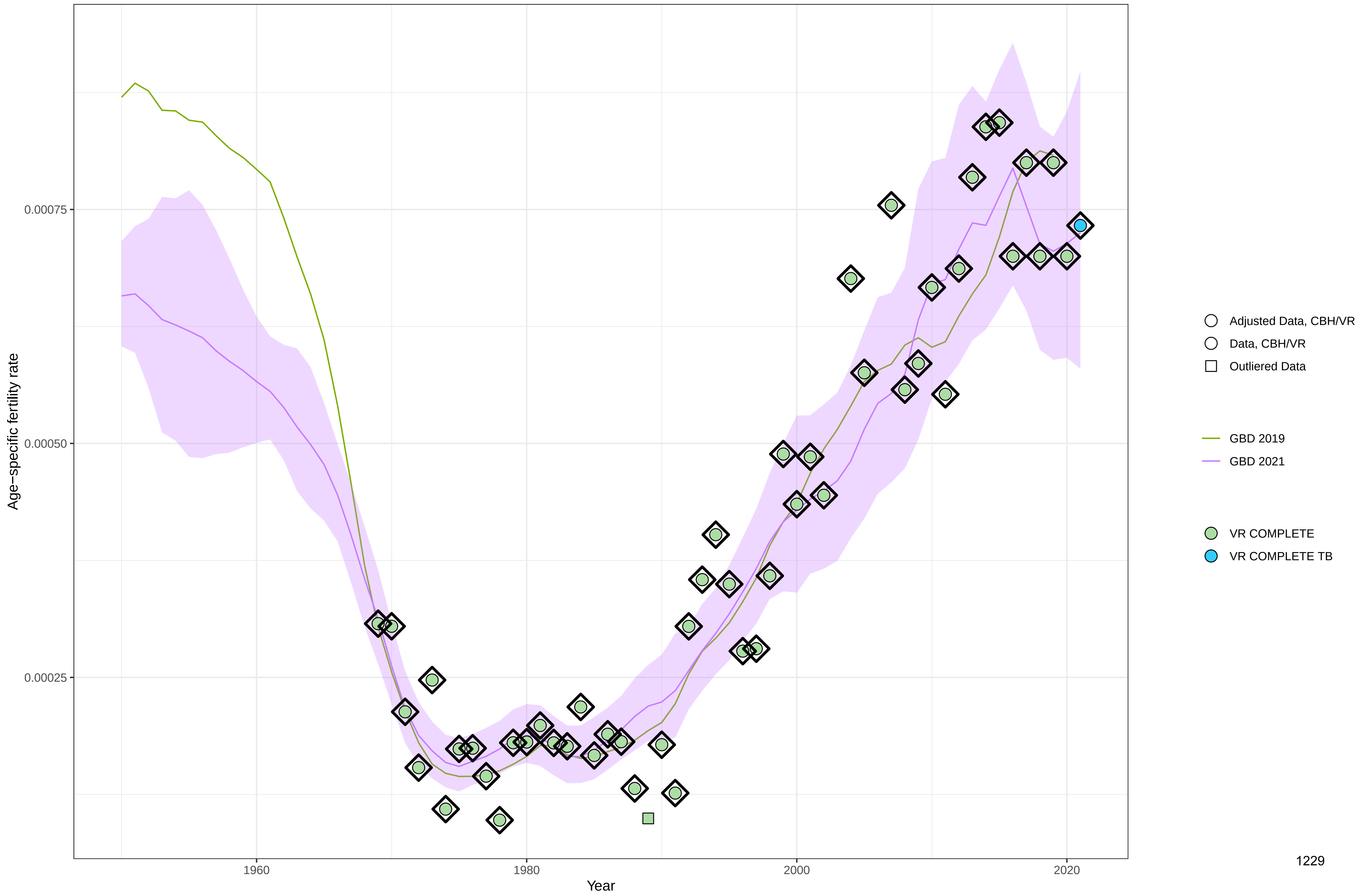

Age−specific fertility fitted trend lines, 1950−2021: Oregon, Ages: 50−54
Data points enclosed with a diamond are reference sources

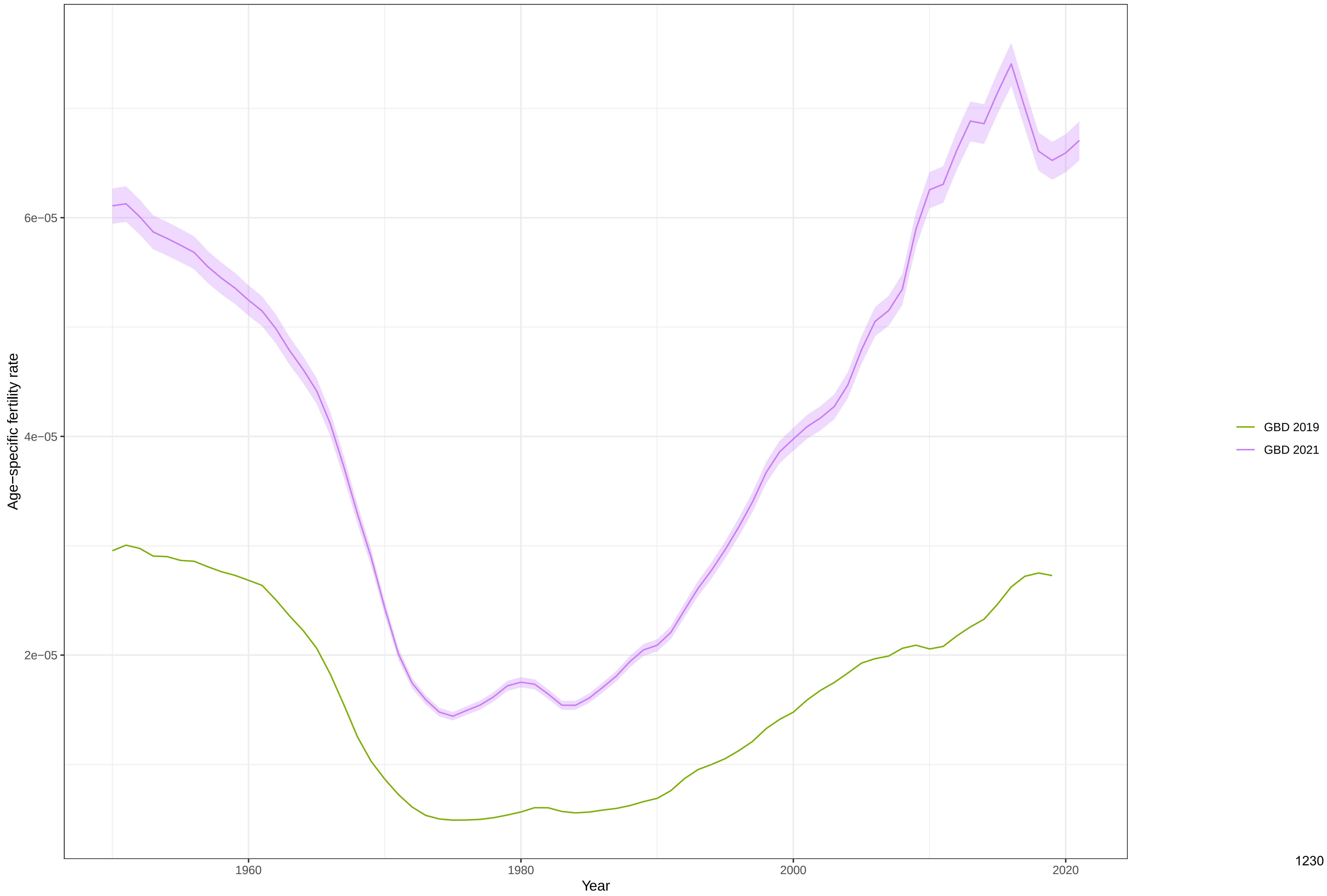

Total fertility rate fitted trend lines, 1950−2021: Oregon TFR
Data points enclosed with a diamond are reference sources

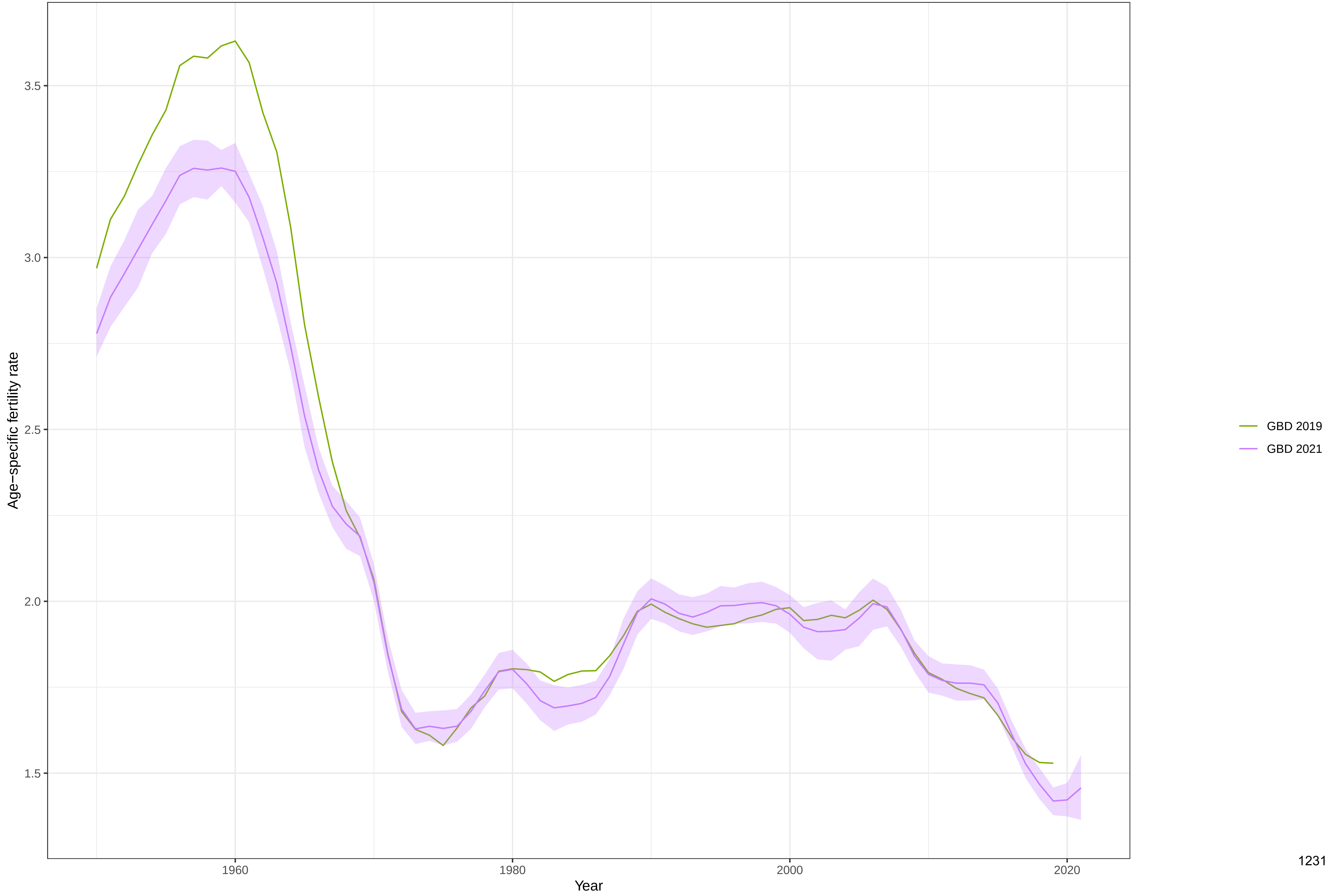

Age−specific fertility fitted trend lines, 1950−2021: Pennsylvania, Ages: 10−14
Data points enclosed with a diamond are reference sources

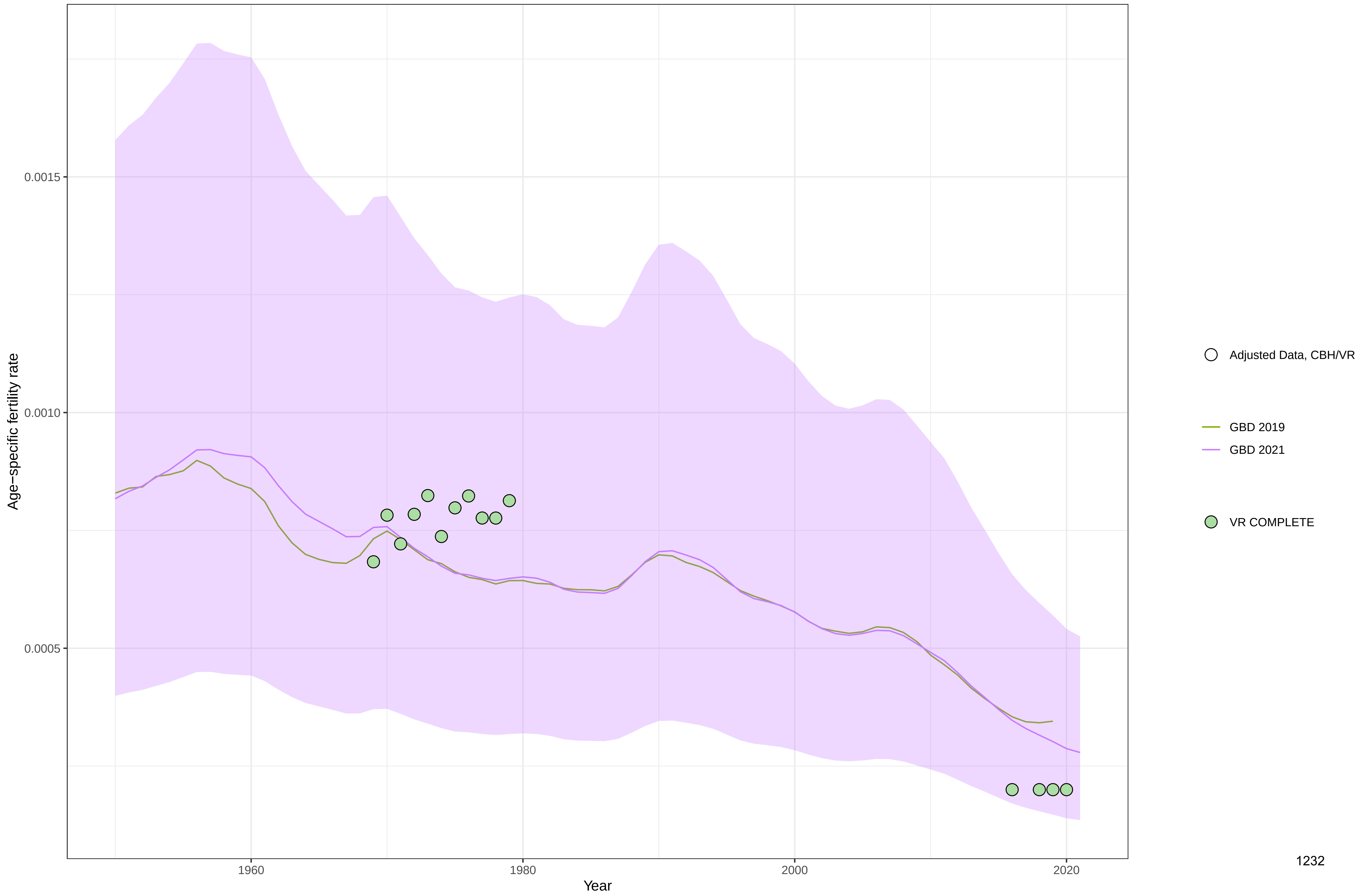

Age−specific fertility fitted trend lines, 1950−2021: Pennsylvania, Ages: 15−19
Data points enclosed with a diamond are reference sources

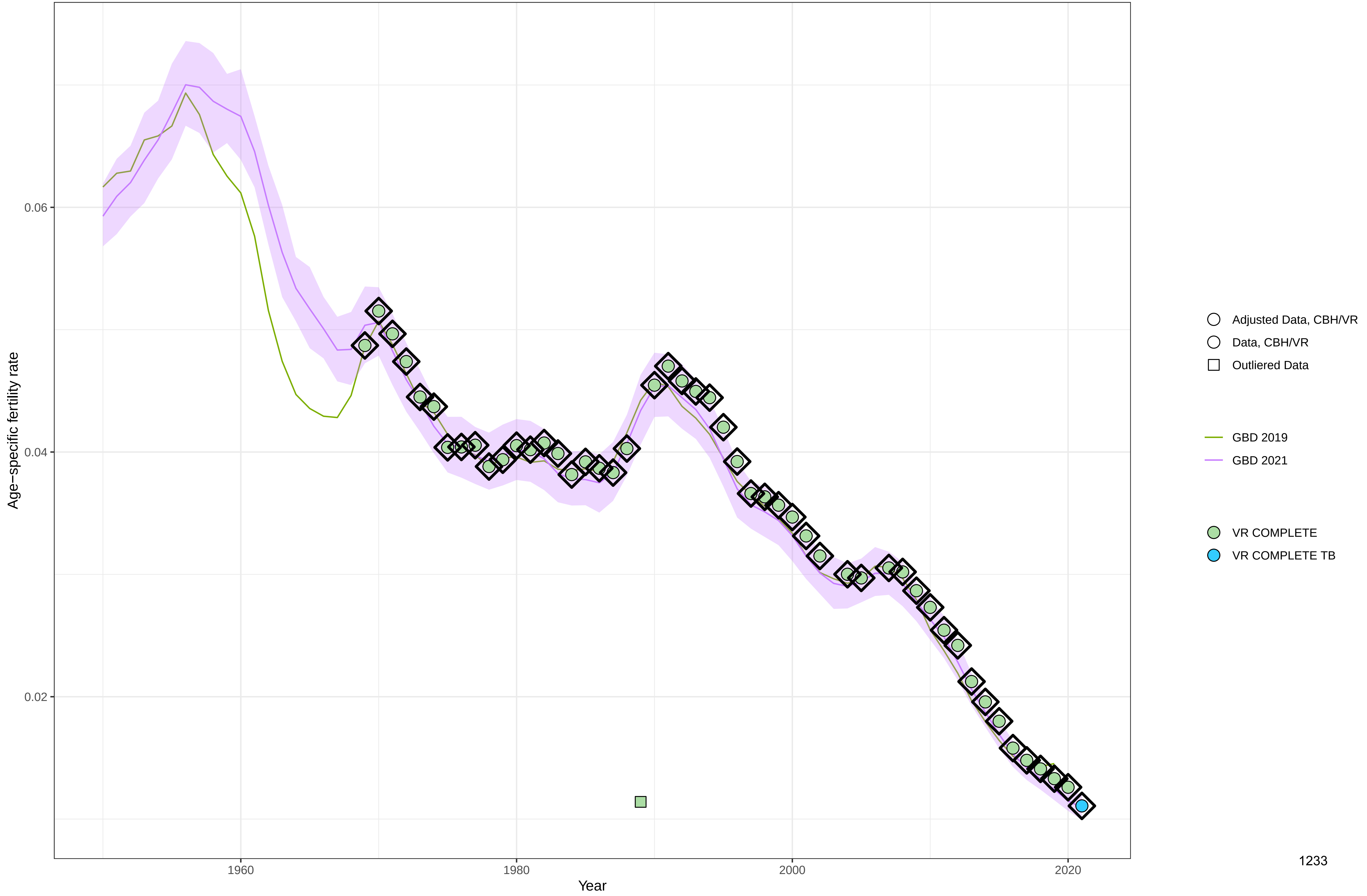

Age−specific fertility fitted trend lines, 1950−2021: Pennsylvania, Ages: 20−24
Data points enclosed with a diamond are reference sources

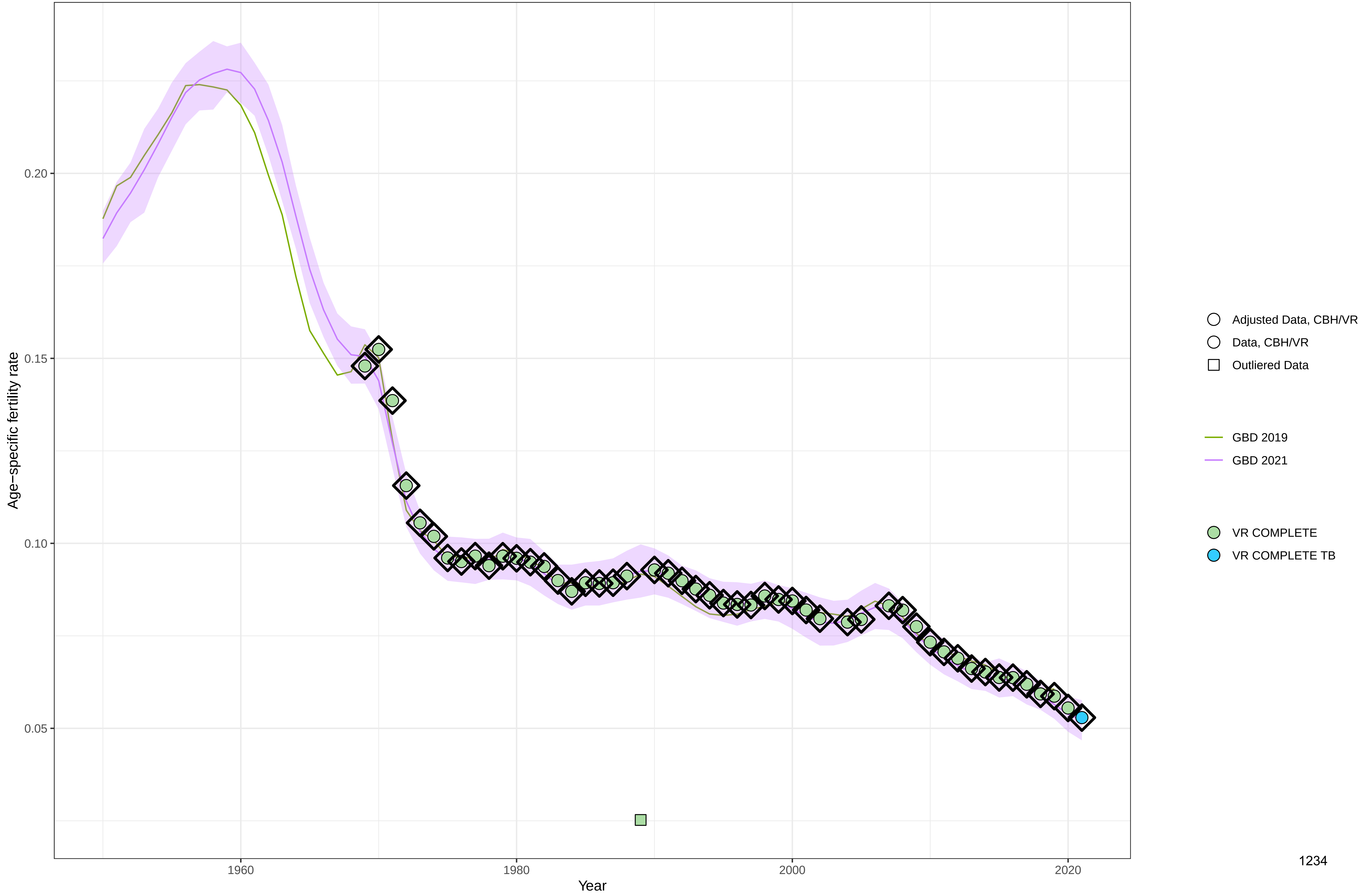

Age−specific fertility fitted trend lines, 1950−2021: Pennsylvania, Ages: 25−29
Data points enclosed with a diamond are reference sources

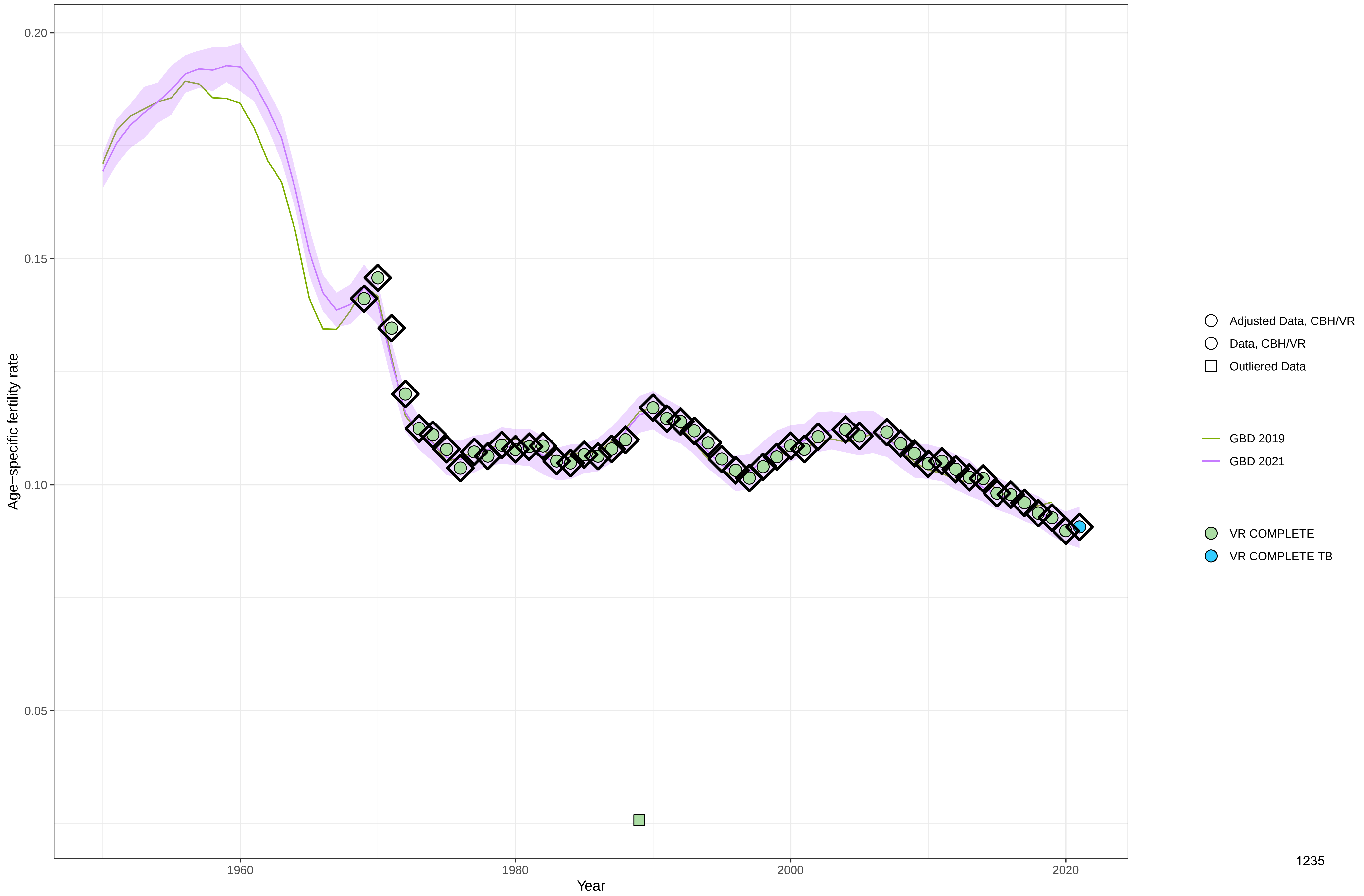

Age−specific fertility fitted trend lines, 1950−2021: Pennsylvania, Ages: 30−34
Data points enclosed with a diamond are reference sources

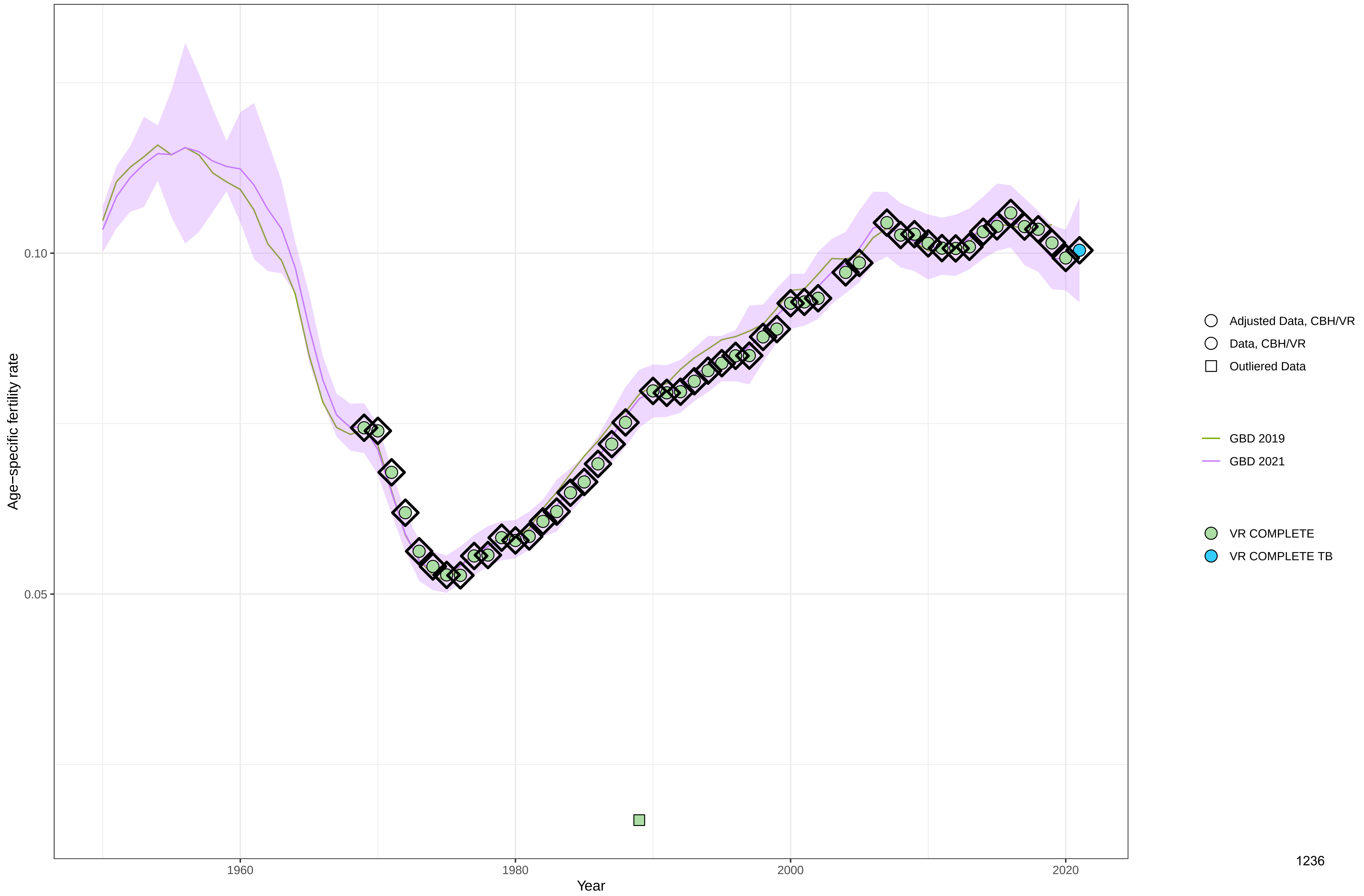

Age−specific fertility fitted trend lines, 1950−2021: Pennsylvania, Ages: 35−39
Data points enclosed with a diamond are reference sources

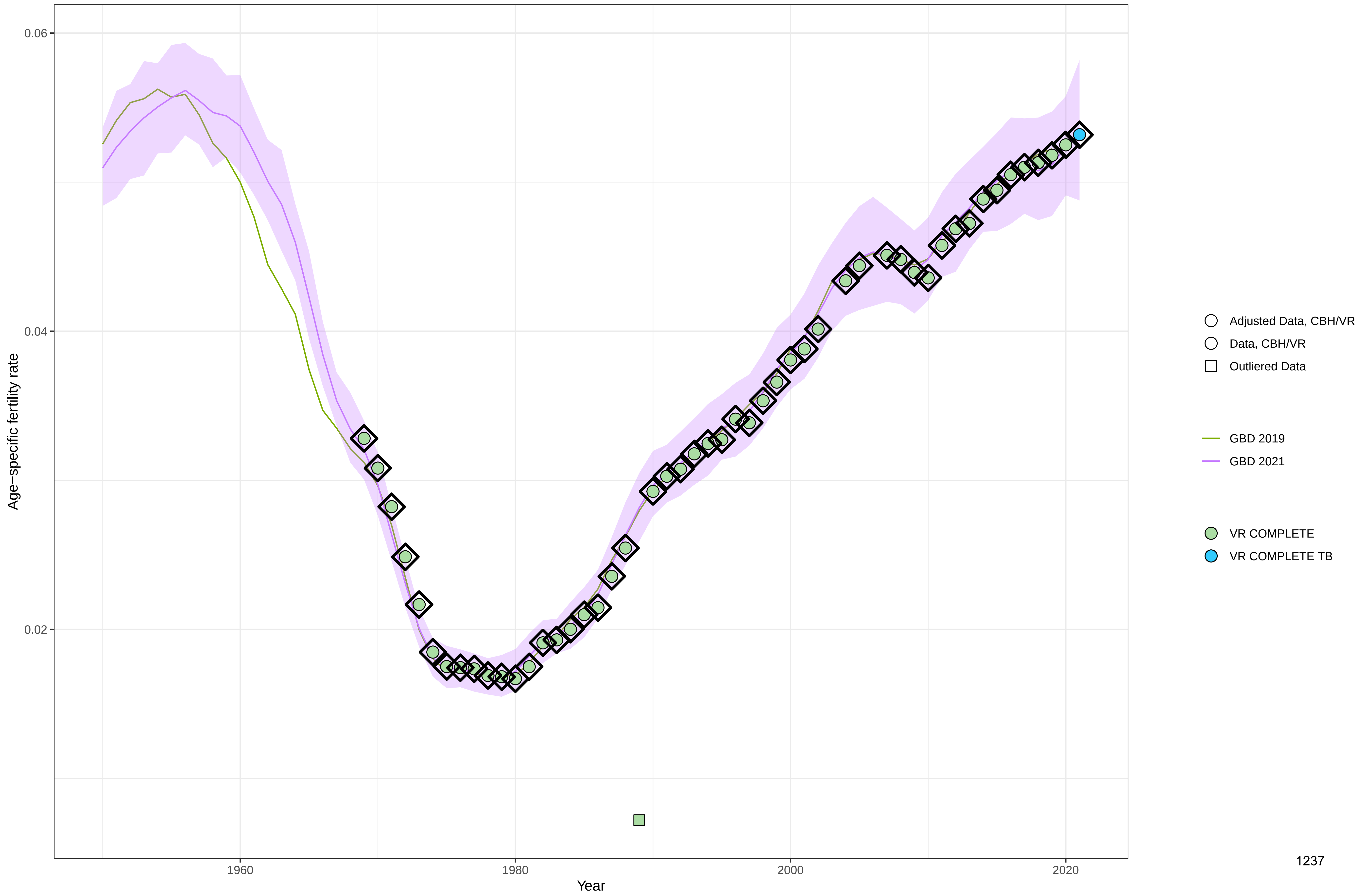

Age−specific fertility fitted trend lines, 1950−2021: Pennsylvania, Ages: 40−44
Data points enclosed with a diamond are reference sources

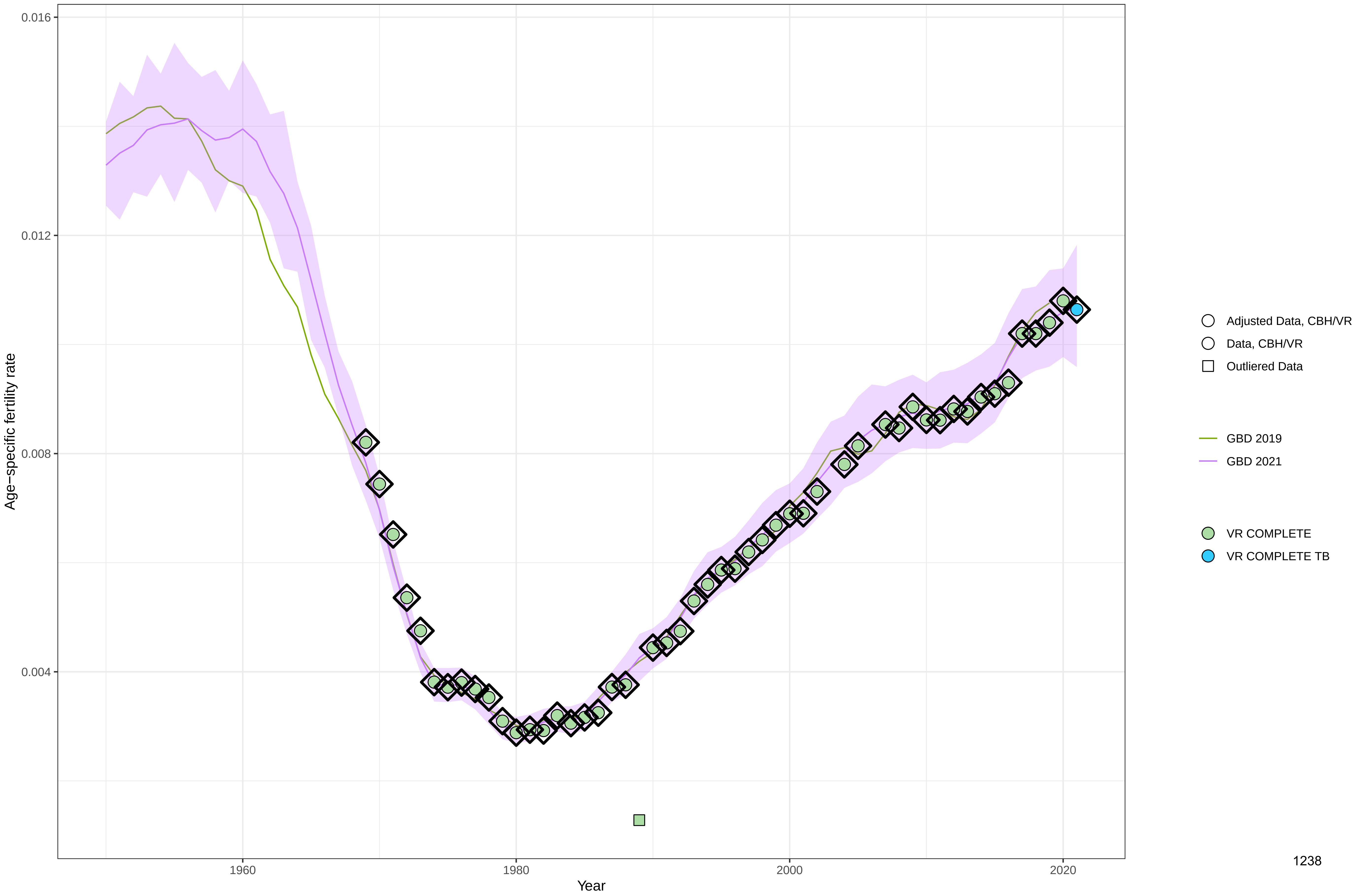

Age−specific fertility fitted trend lines, 1950−2021: Pennsylvania, Ages: 45−49
Data points enclosed with a diamond are reference sources

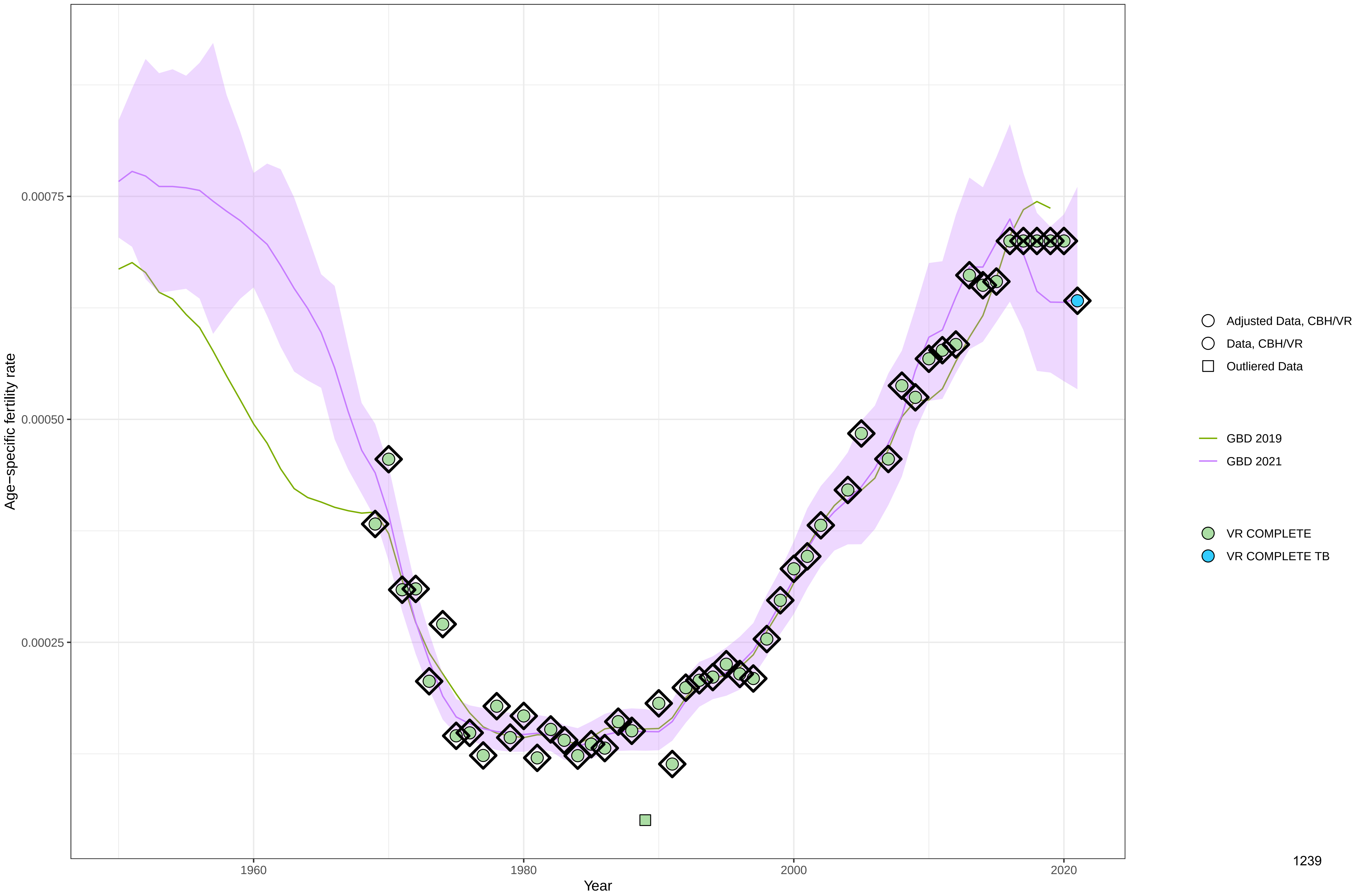

Age−specific fertility fitted trend lines, 1950−2021: Pennsylvania, Ages: 50−54
Data points enclosed with a diamond are reference sources

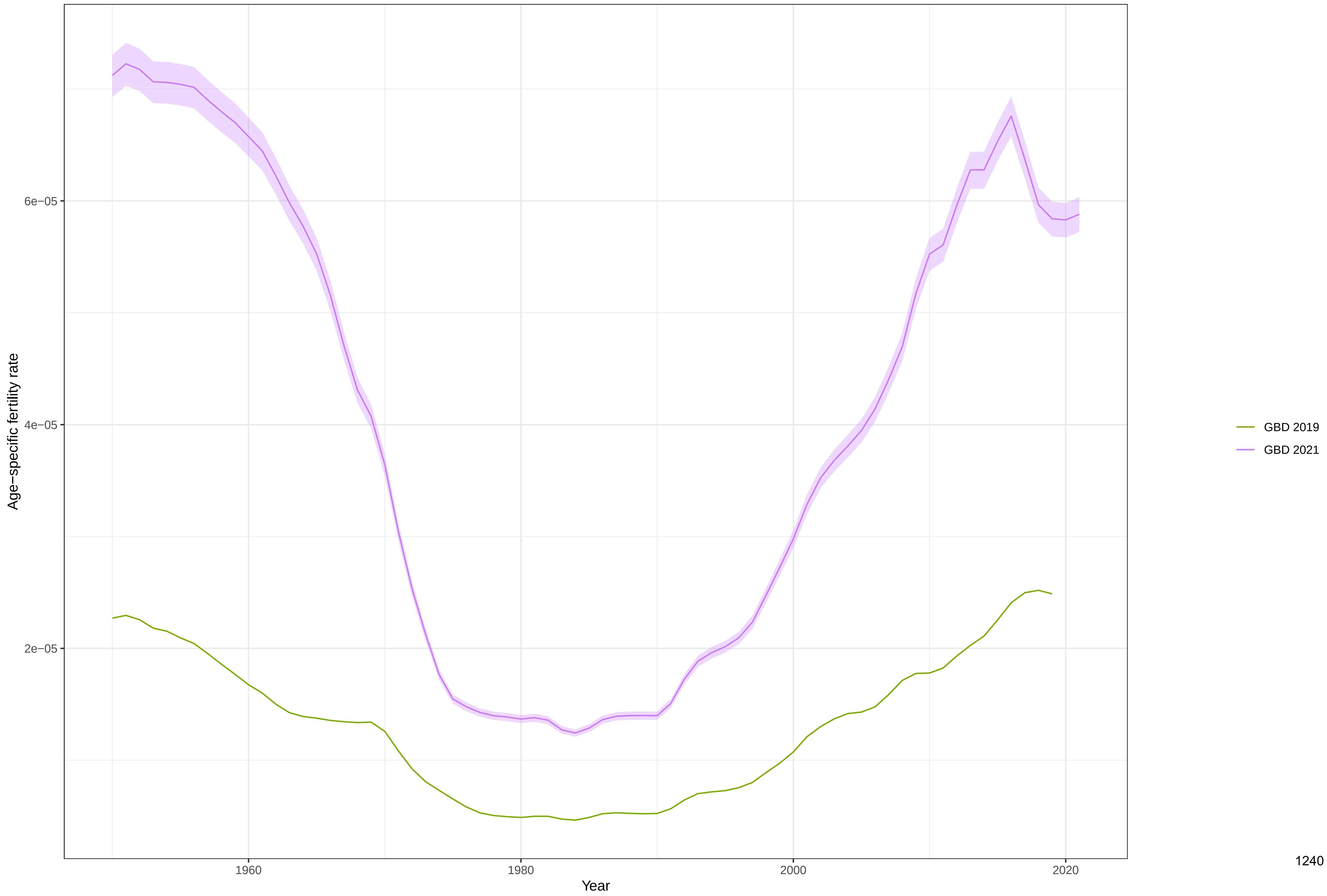

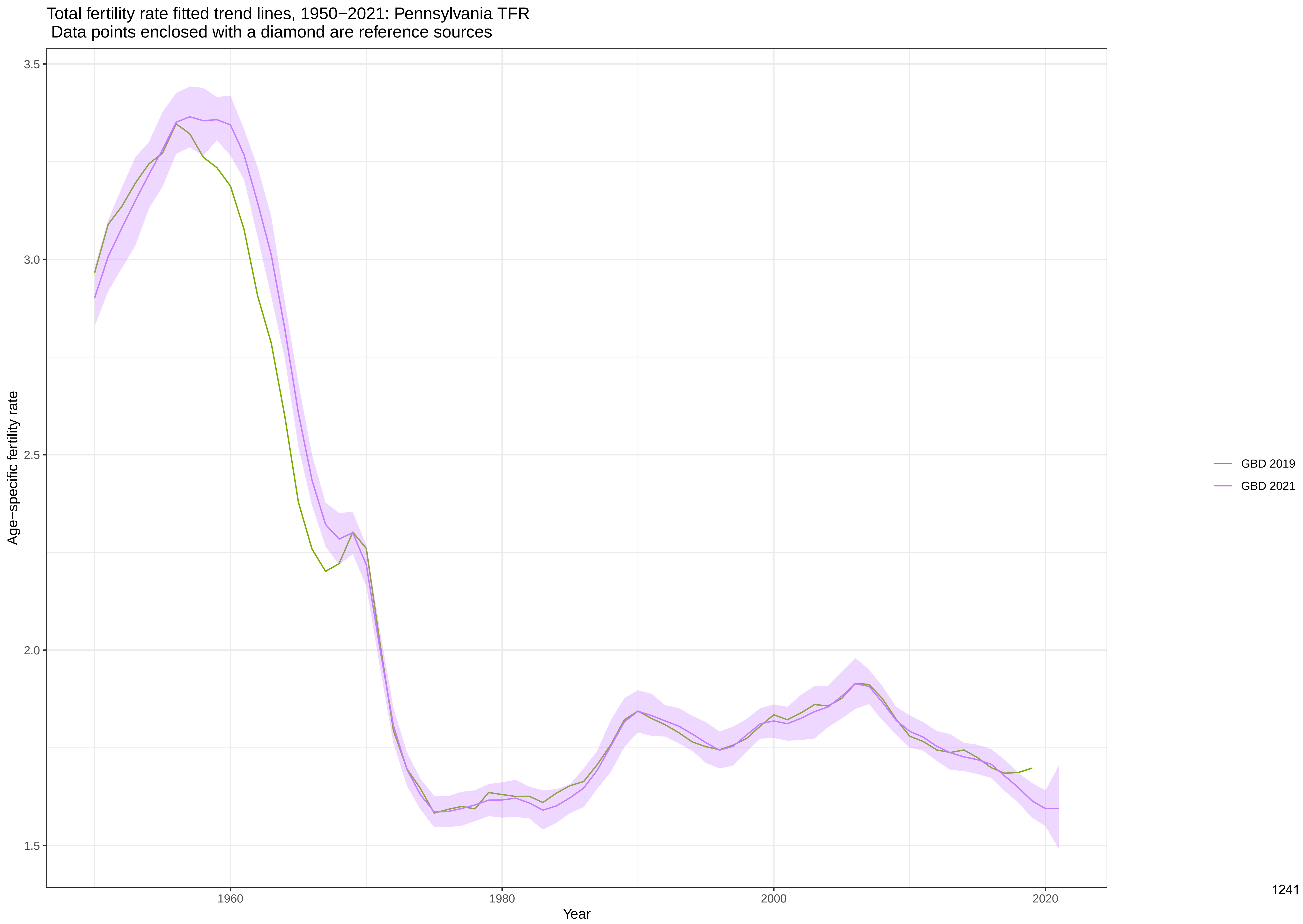
Total fertility rate fitted trend lines, 1950−2021: Pennsylvania TFR
Data points enclosed with a diamond are reference sources
Age−specific fertility rate
3.5
3.0
2.5
2.0
1.5
1960
1980
2000
2020
Year
GBD 2019
GBD 2021

Age−specific fertility fitted trend lines, 1950−2021: Rhode Island, Ages: 10−14
Data points enclosed with a diamond are reference sources

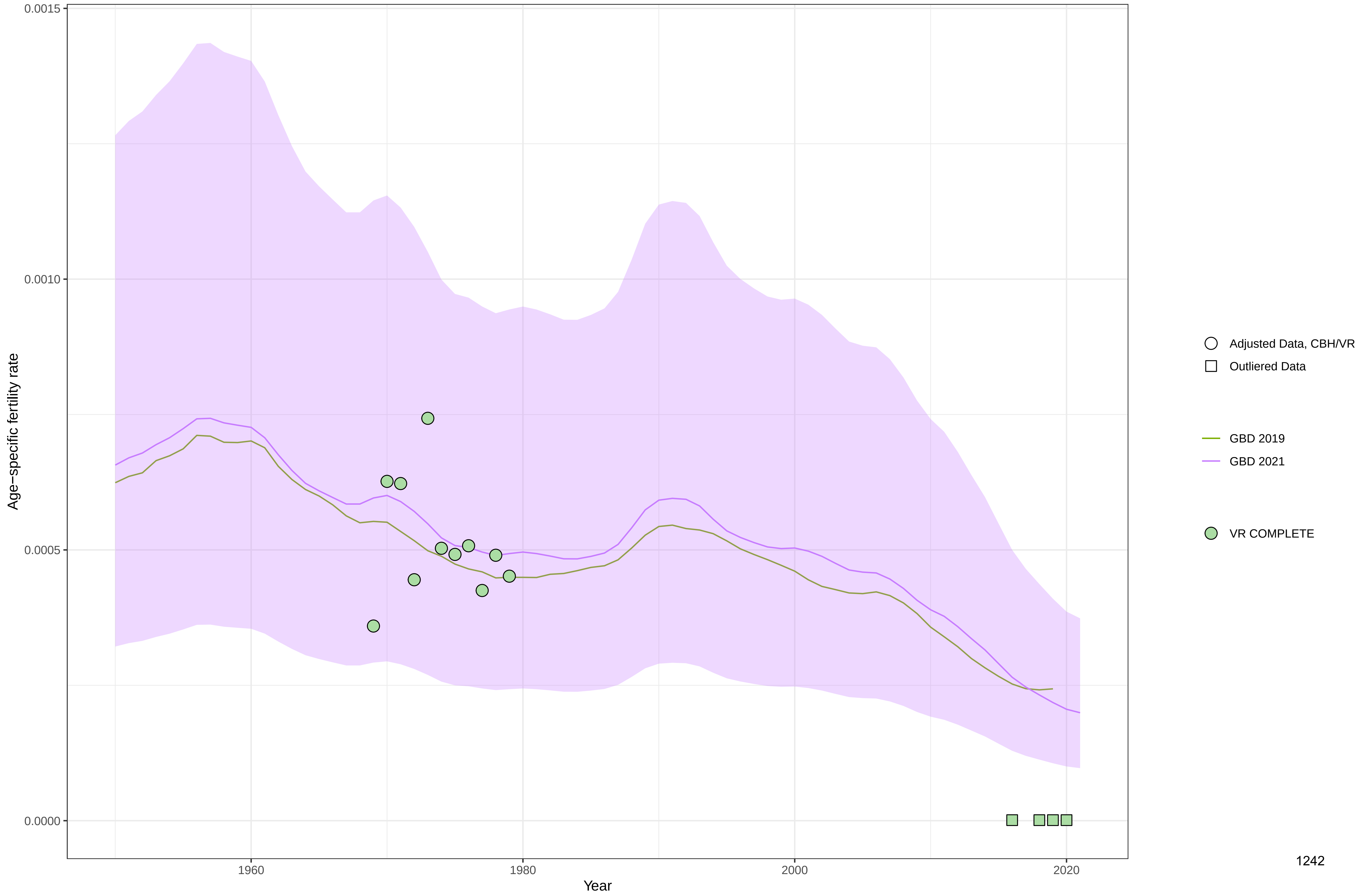

Age−specific fertility fitted trend lines, 1950−2021: Rhode Island, Ages: 15−19
Data points enclosed with a diamond are reference sources

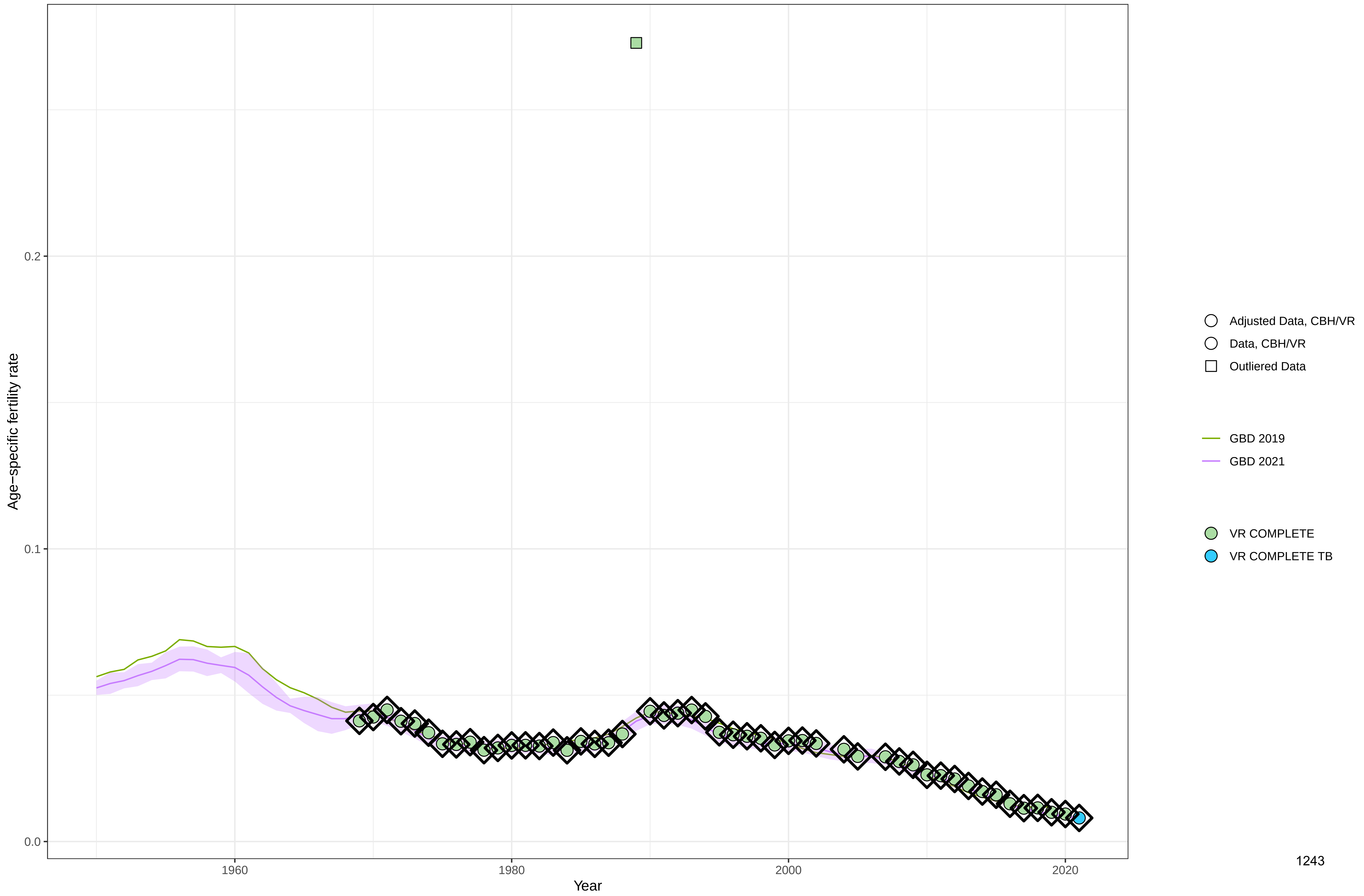

Age−specific fertility fitted trend lines, 1950−2021: Rhode Island, Ages: 20−24
Data points enclosed with a diamond are reference sources

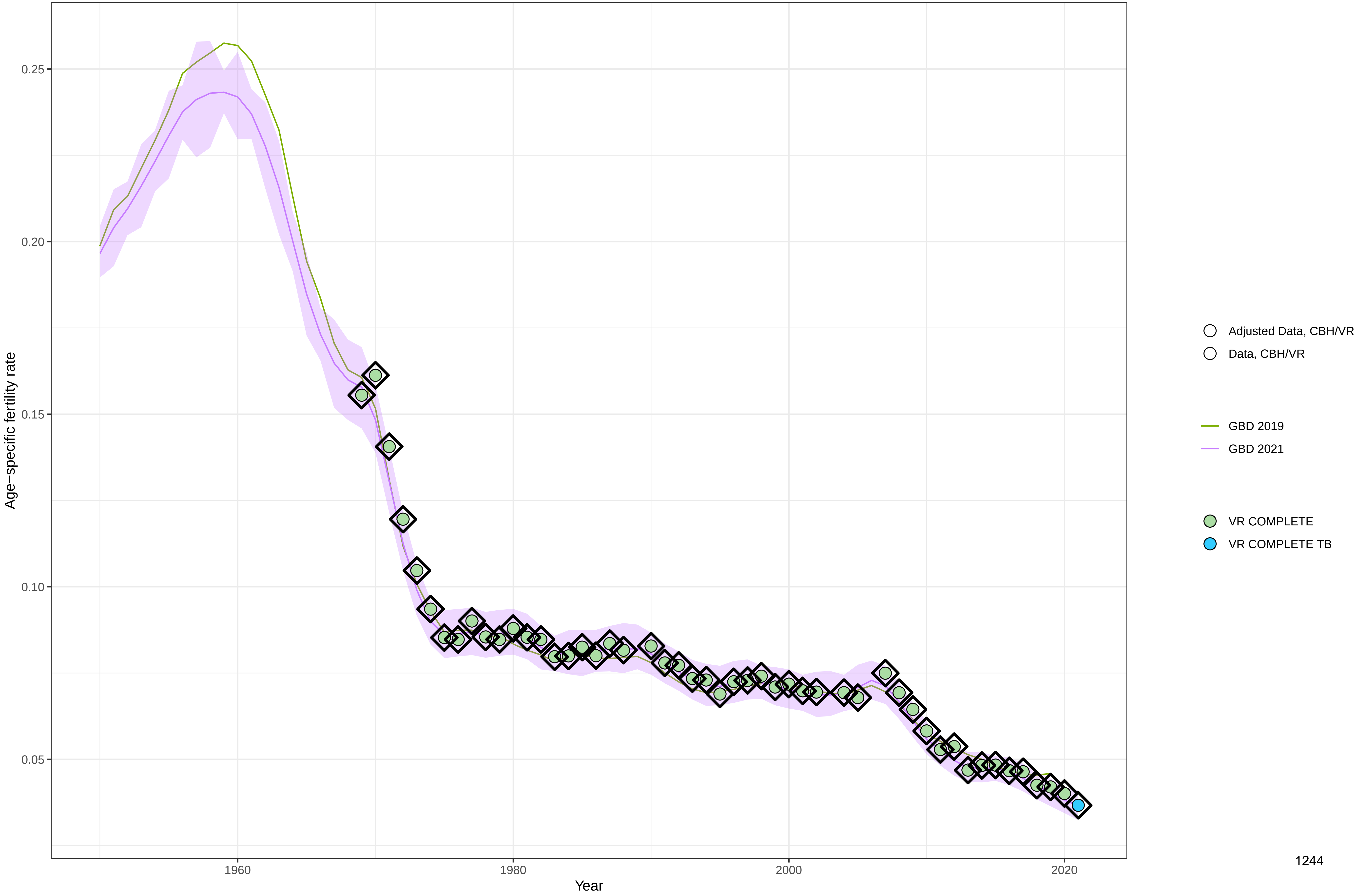

Age−specific fertility fitted trend lines, 1950−2021: Rhode Island, Ages: 25−29
Data points enclosed with a diamond are reference sources

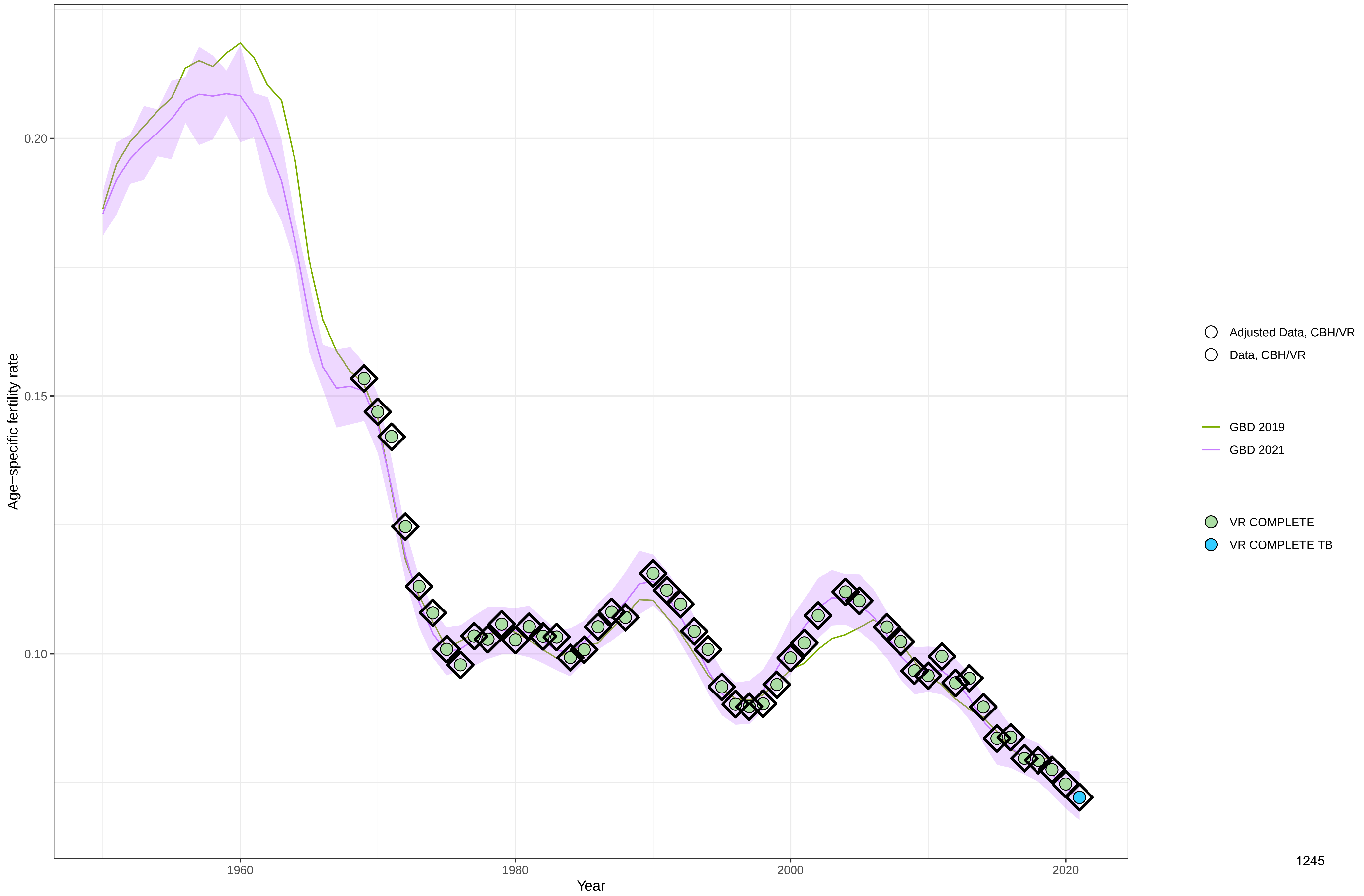

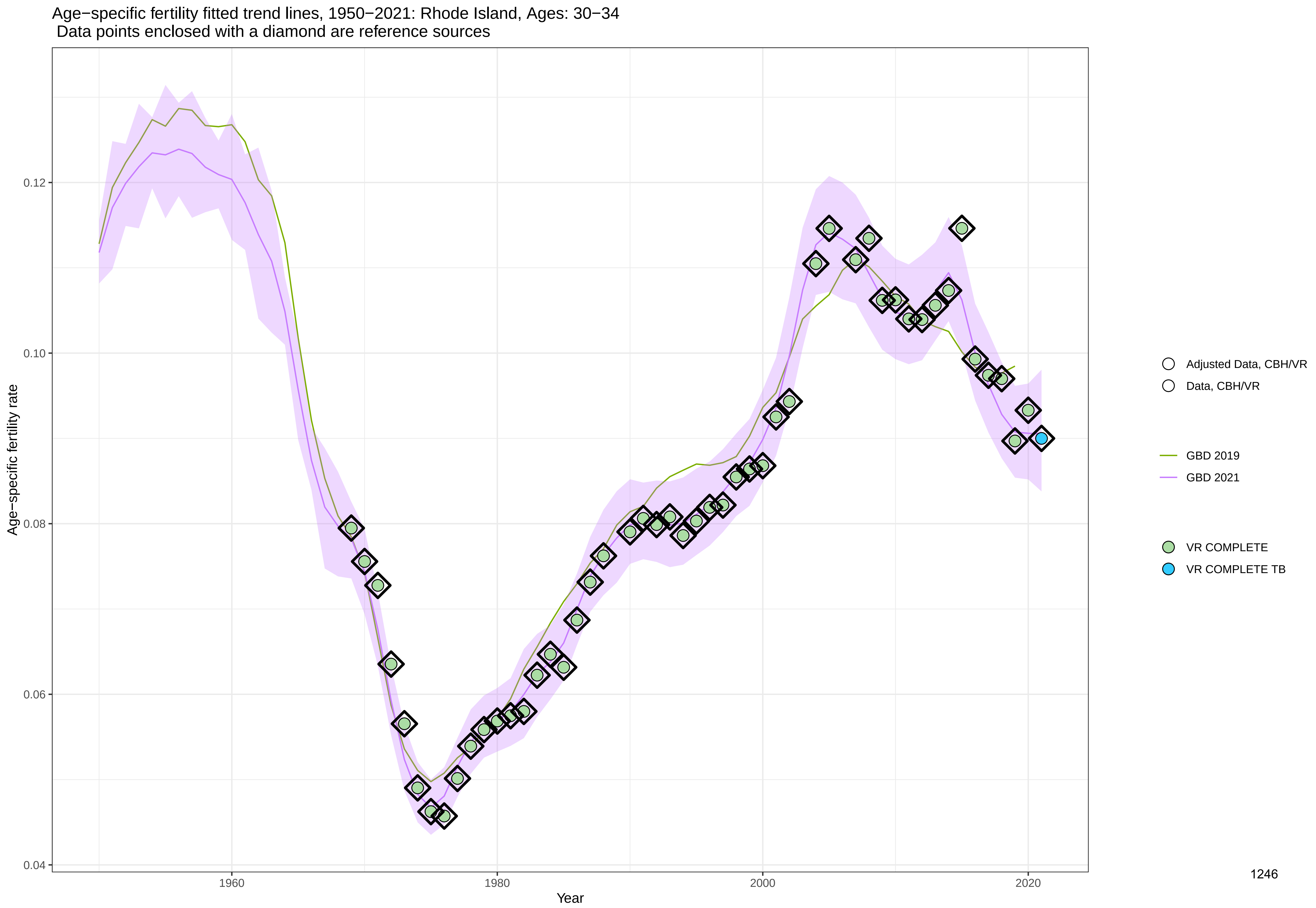

Age−specific fertility fitted trend lines, 1950−2021: Rhode Island, Ages: 30−34
Data points enclosed with a diamond are reference sources
Age−specific fertility rate
0.12
0.10
0.08
0.06
0.04
1960
1980
2000
2020
Year
Adjusted Data, CBH/VR
Data, CBH/VR
GBD 2019
GBD 2021
VR COMPLETE
VR COMPLETE TB

Age−specific fertility fitted trend lines, 1950−2021: Rhode Island, Ages: 35−39
Data points enclosed with a diamond are reference sources

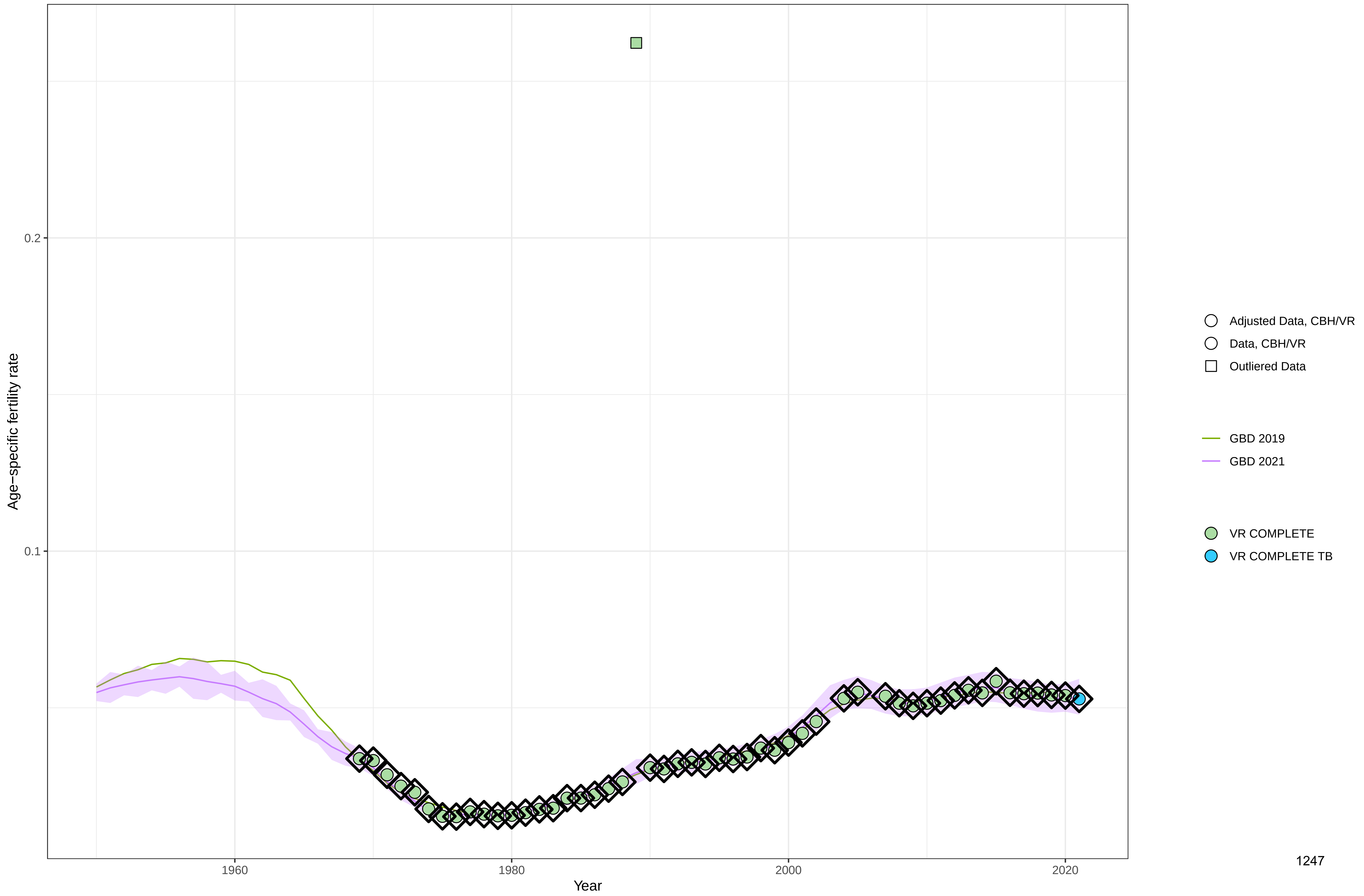

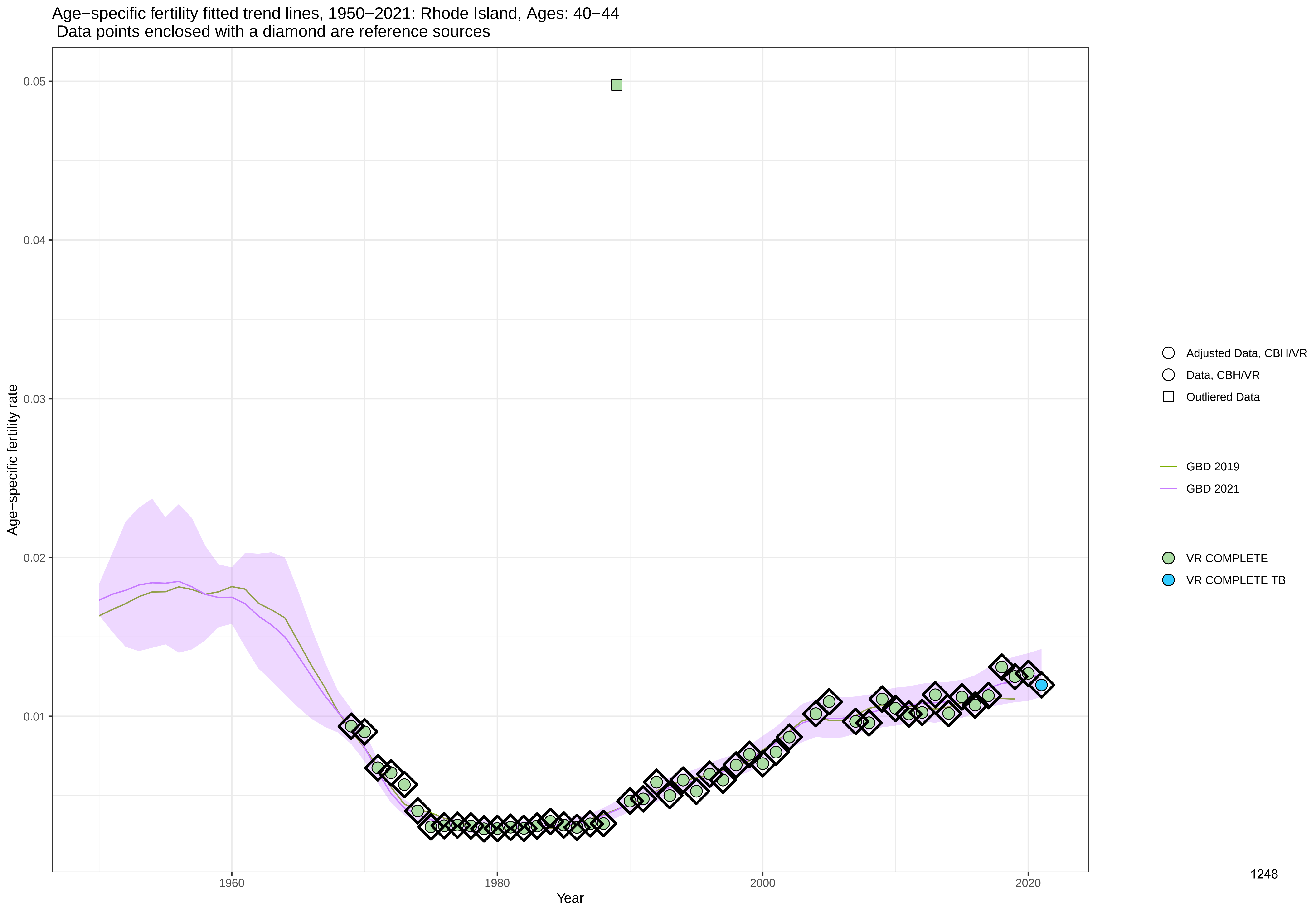

Age−specific fertility fitted trend lines, 1950−2021: Rhode Island, Ages: 40−44
Data points enclosed with a diamond are reference sources
Age−specific fertility rate
0.05
0.04
0.03
0.02
0.01
1960
1980
2000
2020
Year
Adjusted Data, CBH/VR
Data, CBH/VR
Outliered Data
GBD 2019
GBD 2021
VR COMPLETE
VR COMPLETE TB

Age−specific fertility fitted trend lines, 1950−2021: Rhode Island, Ages: 45−49
Data points enclosed with a diamond are reference sources

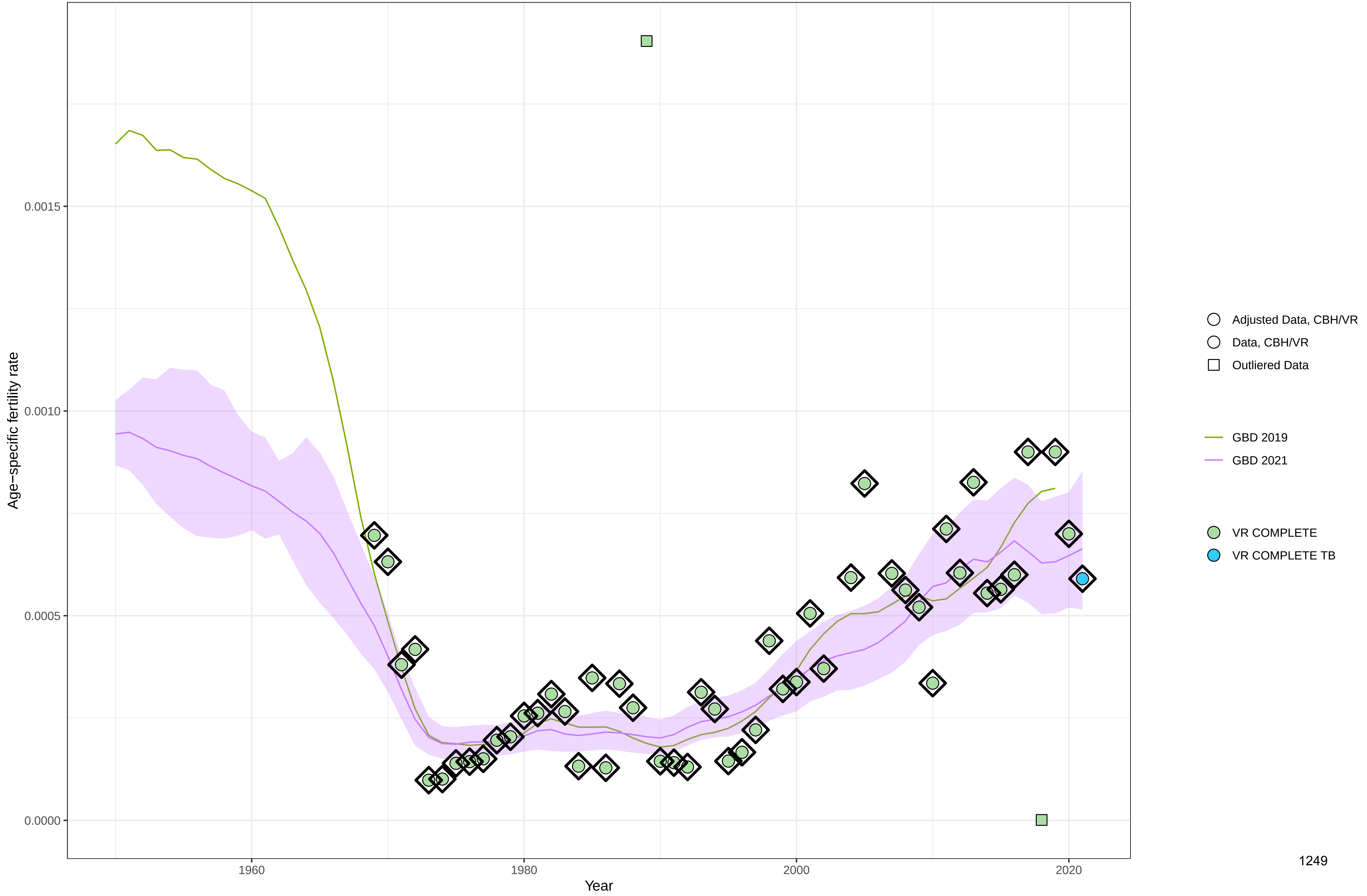

Age−specific fertility fitted trend lines, 1950−2021: Rhode Island, Ages: 50−54
Data points enclosed with a diamond are reference sources

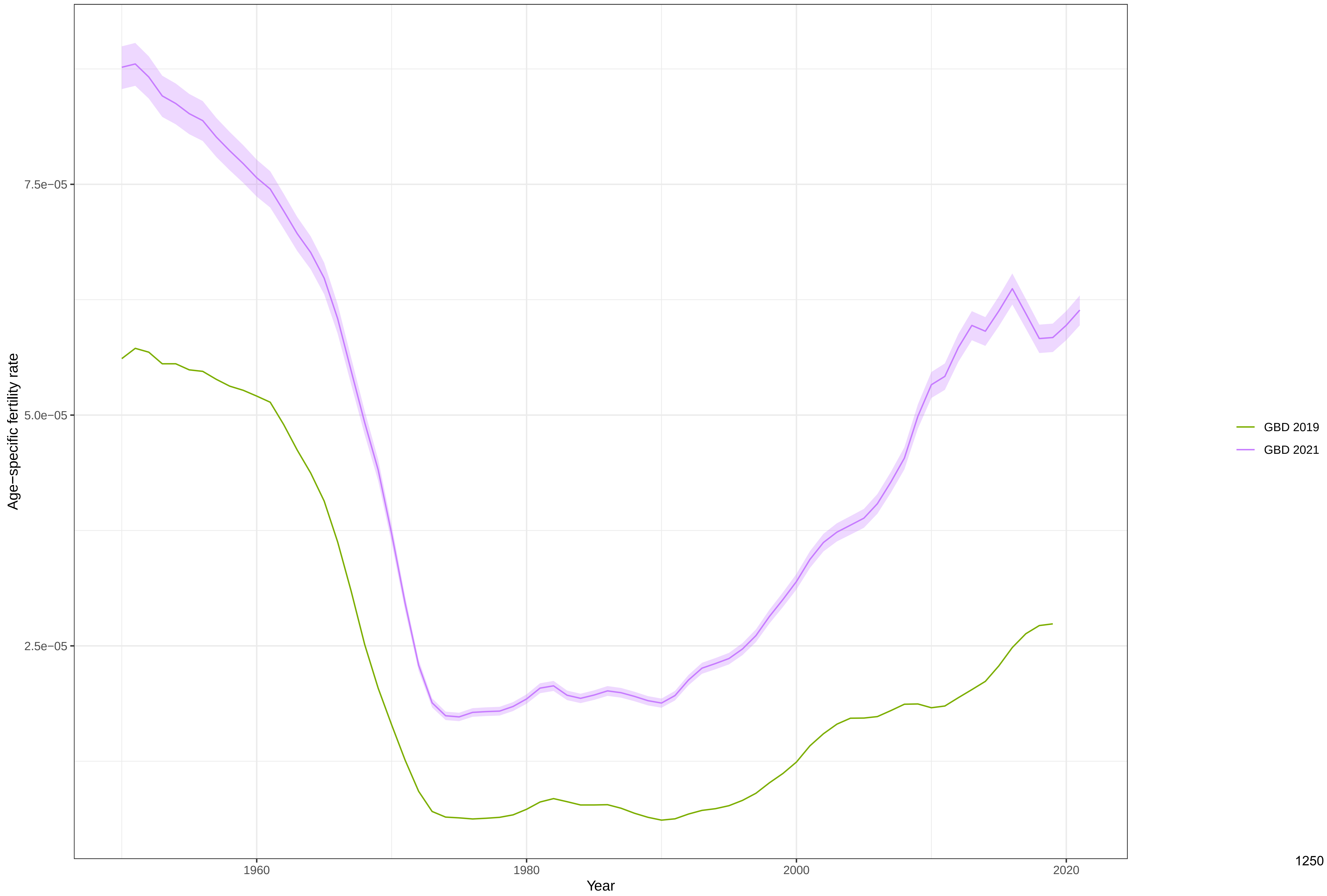

Total fertility rate fitted trend lines, 1950−2021: Rhode Island TFR
Data points enclosed with a diamond are reference sources

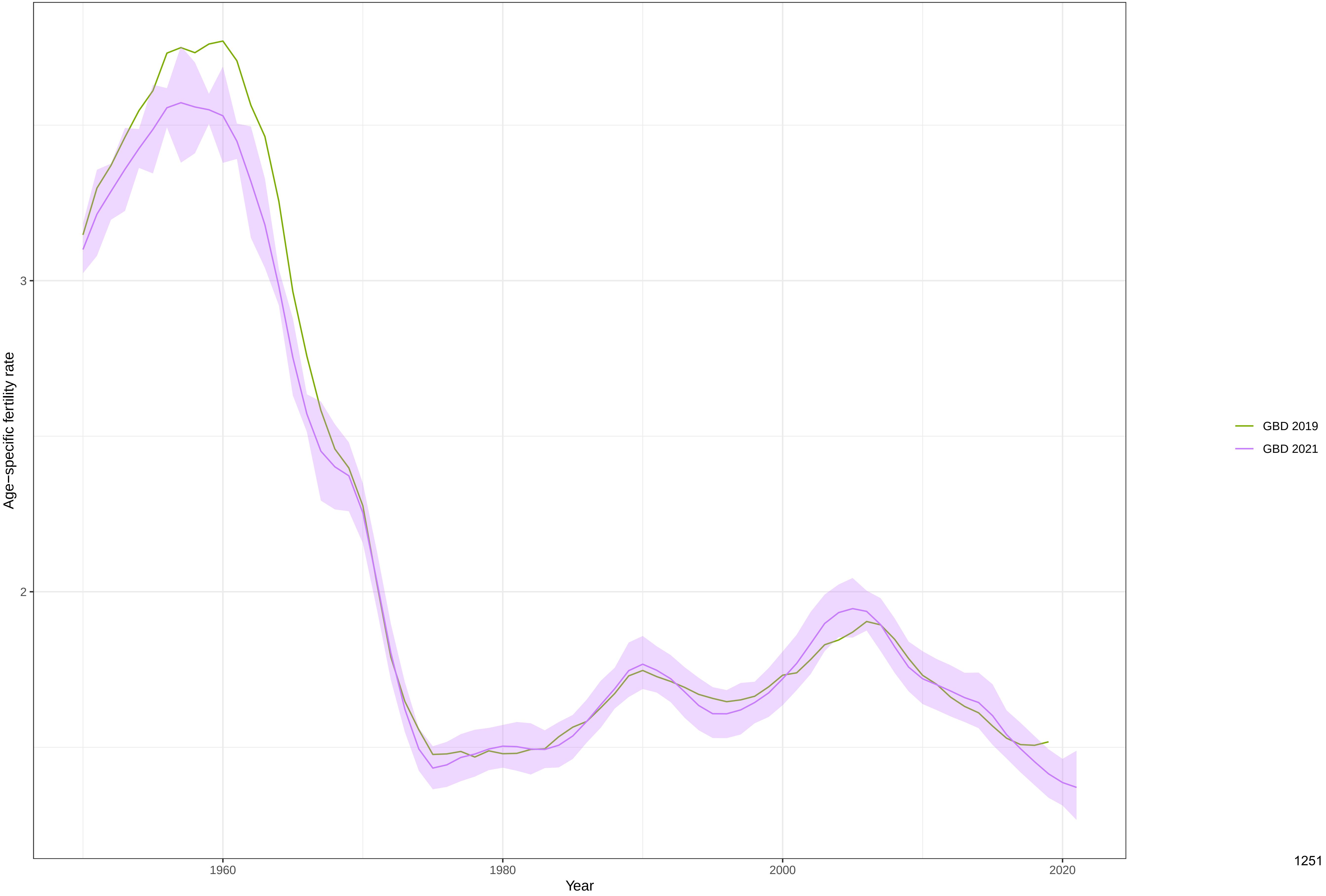

Age−specific fertility fitted trend lines, 1950−2021: South Carolina, Ages: 10−14
Data points enclosed with a diamond are reference sources

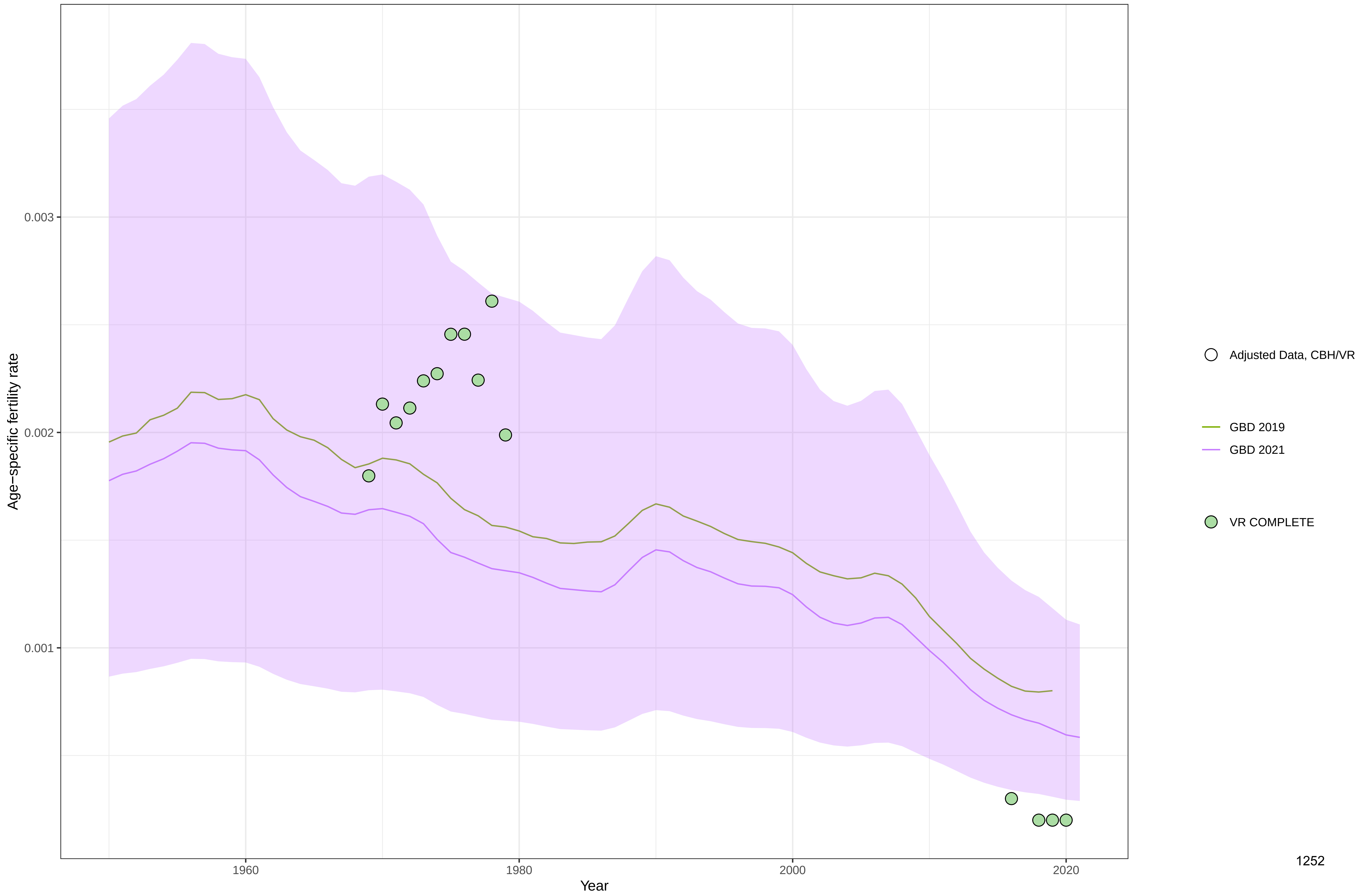

Age−specific fertility fitted trend lines, 1950−2021: South Carolina, Ages: 15−19
Data points enclosed with a diamond are reference sources

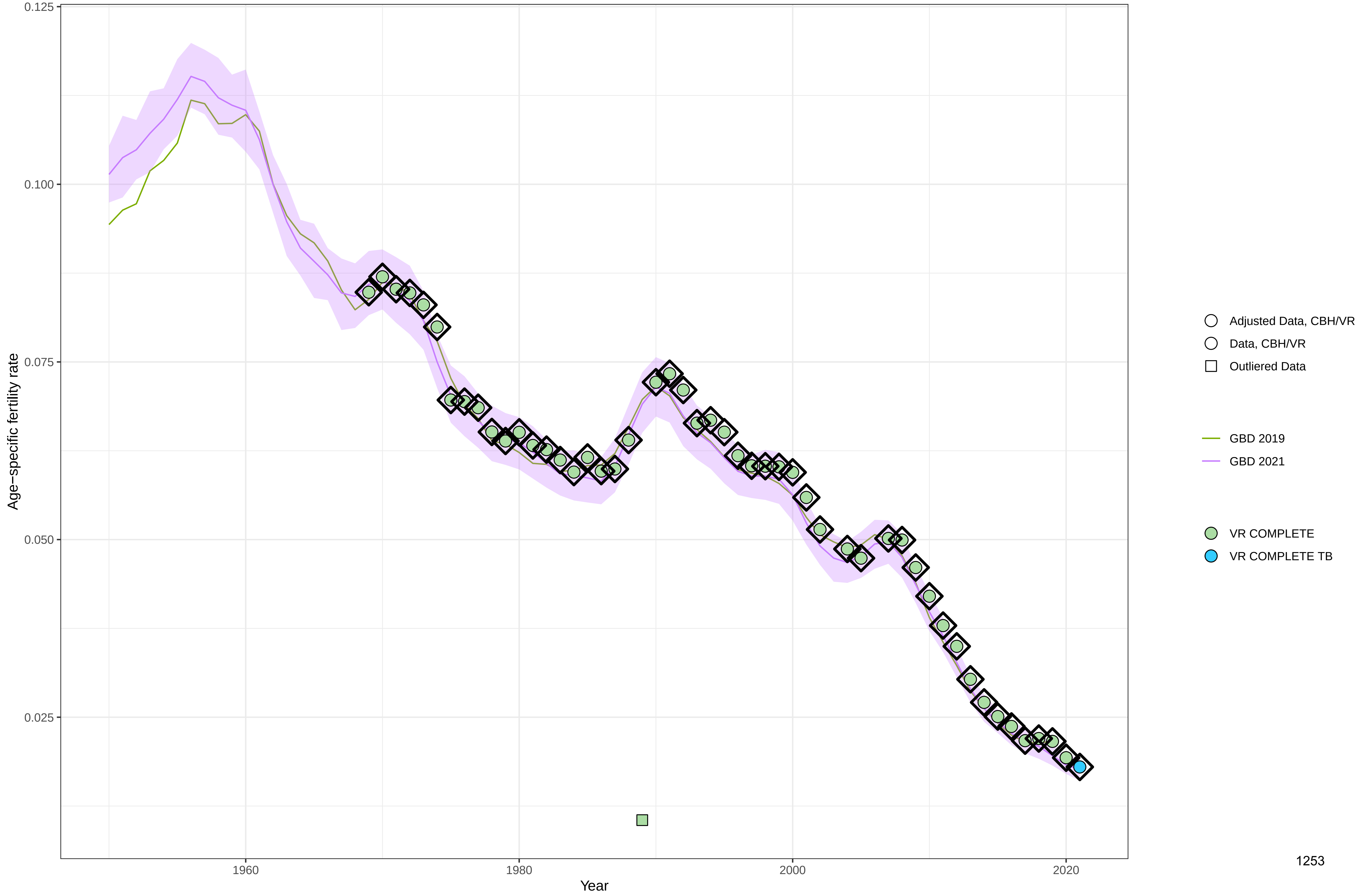

Age−specific fertility fitted trend lines, 1950−2021: South Carolina, Ages: 20−24
Data points enclosed with a diamond are reference sources

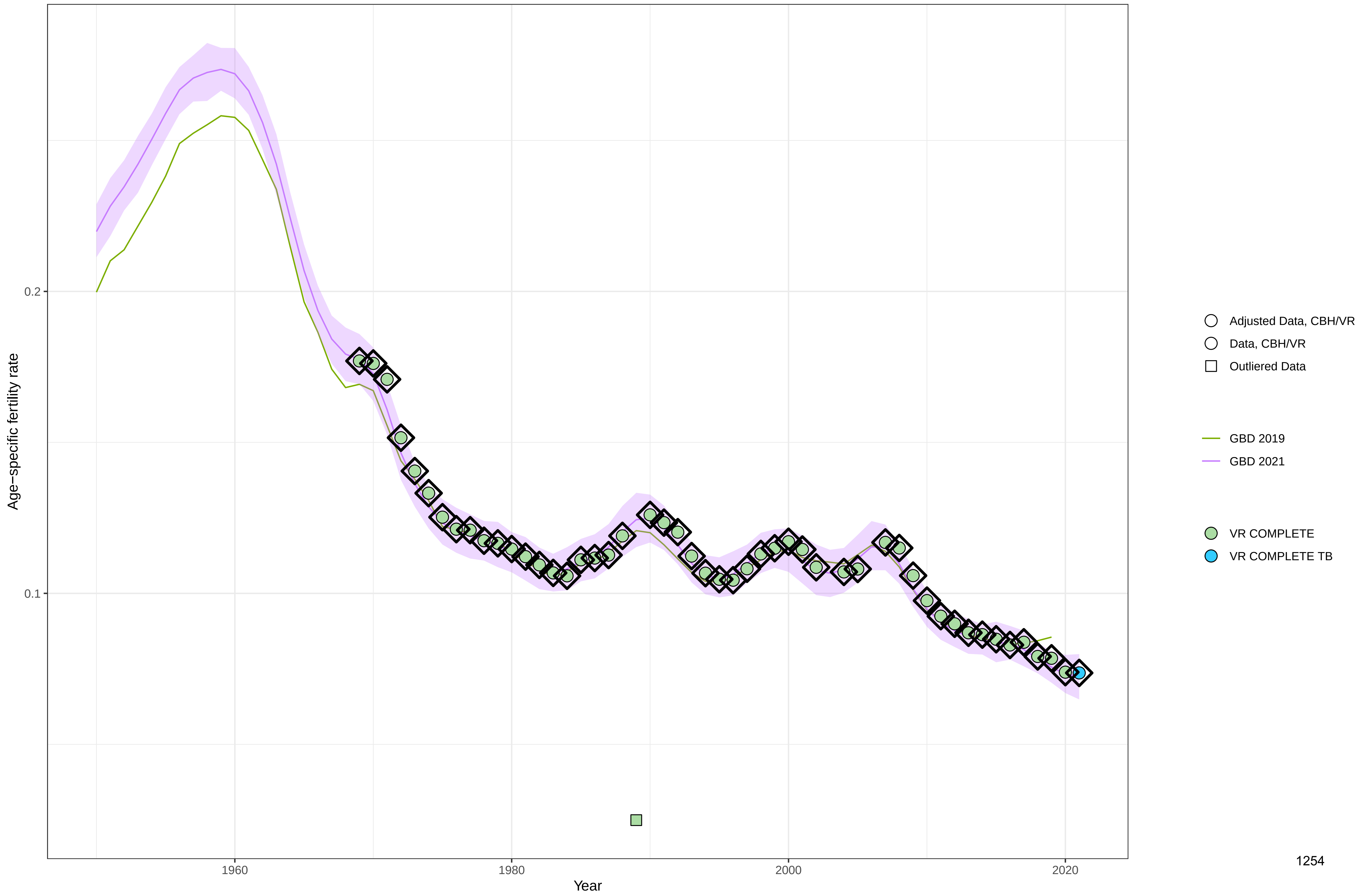

Age−specific fertility fitted trend lines, 1950−2021: South Carolina, Ages: 25−29
Data points enclosed with a diamond are reference sources

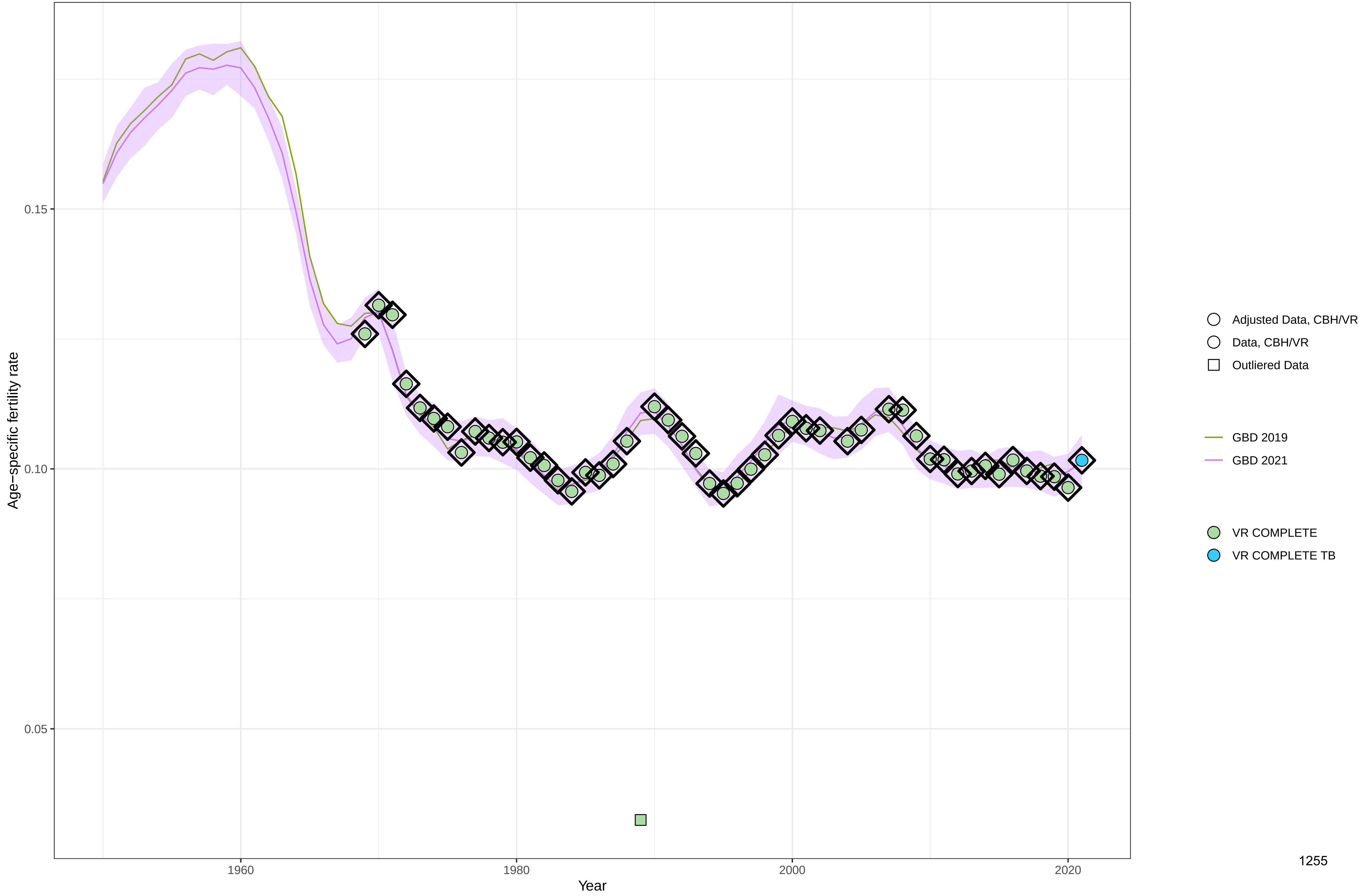

Age−specific fertility fitted trend lines, 1950−2021: South Carolina, Ages: 30−34
Data points enclosed with a diamond are reference sources

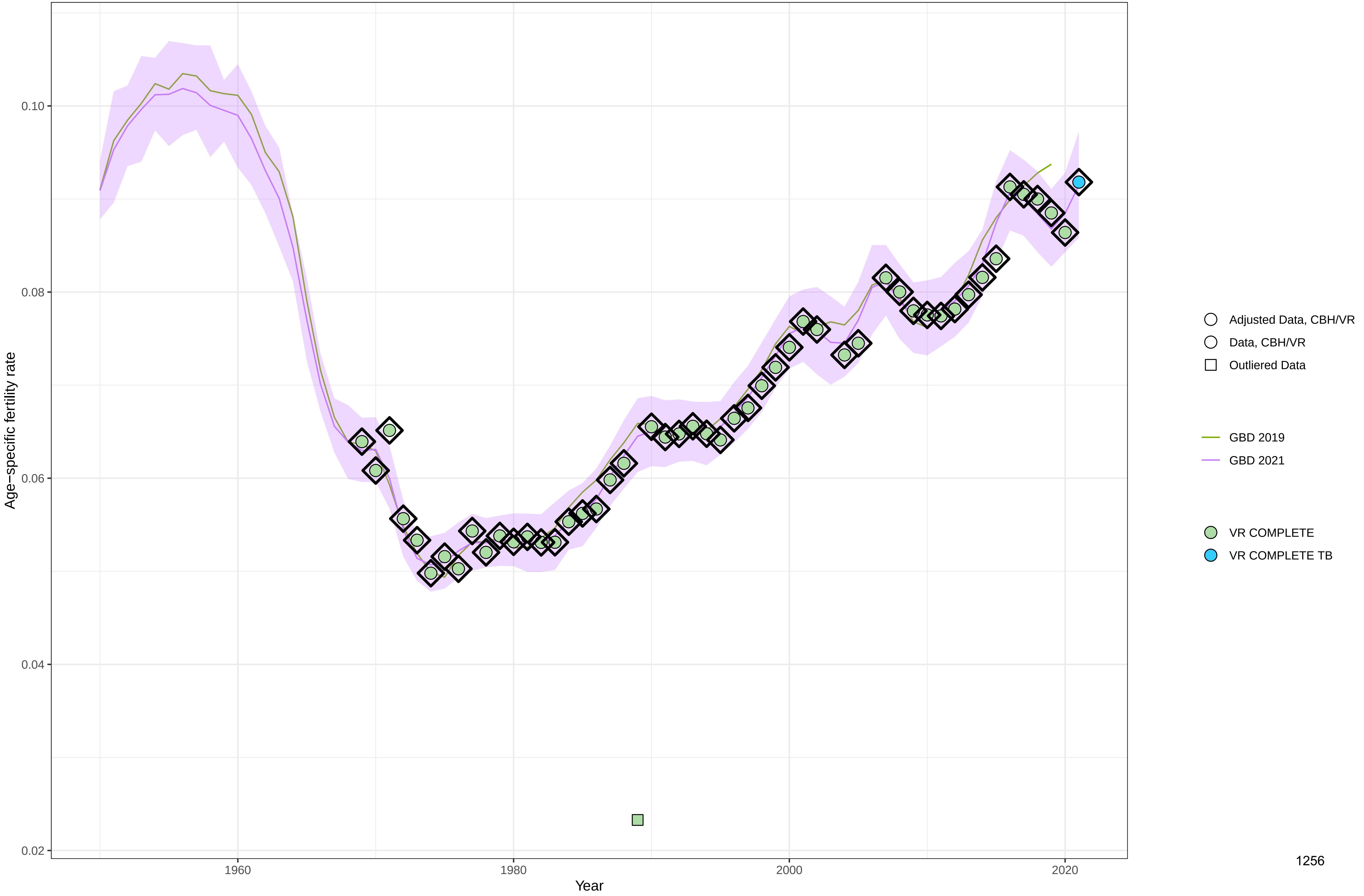

Age−specific fertility fitted trend lines, 1950−2021: South Carolina, Ages: 35−39
Data points enclosed with a diamond are reference sources

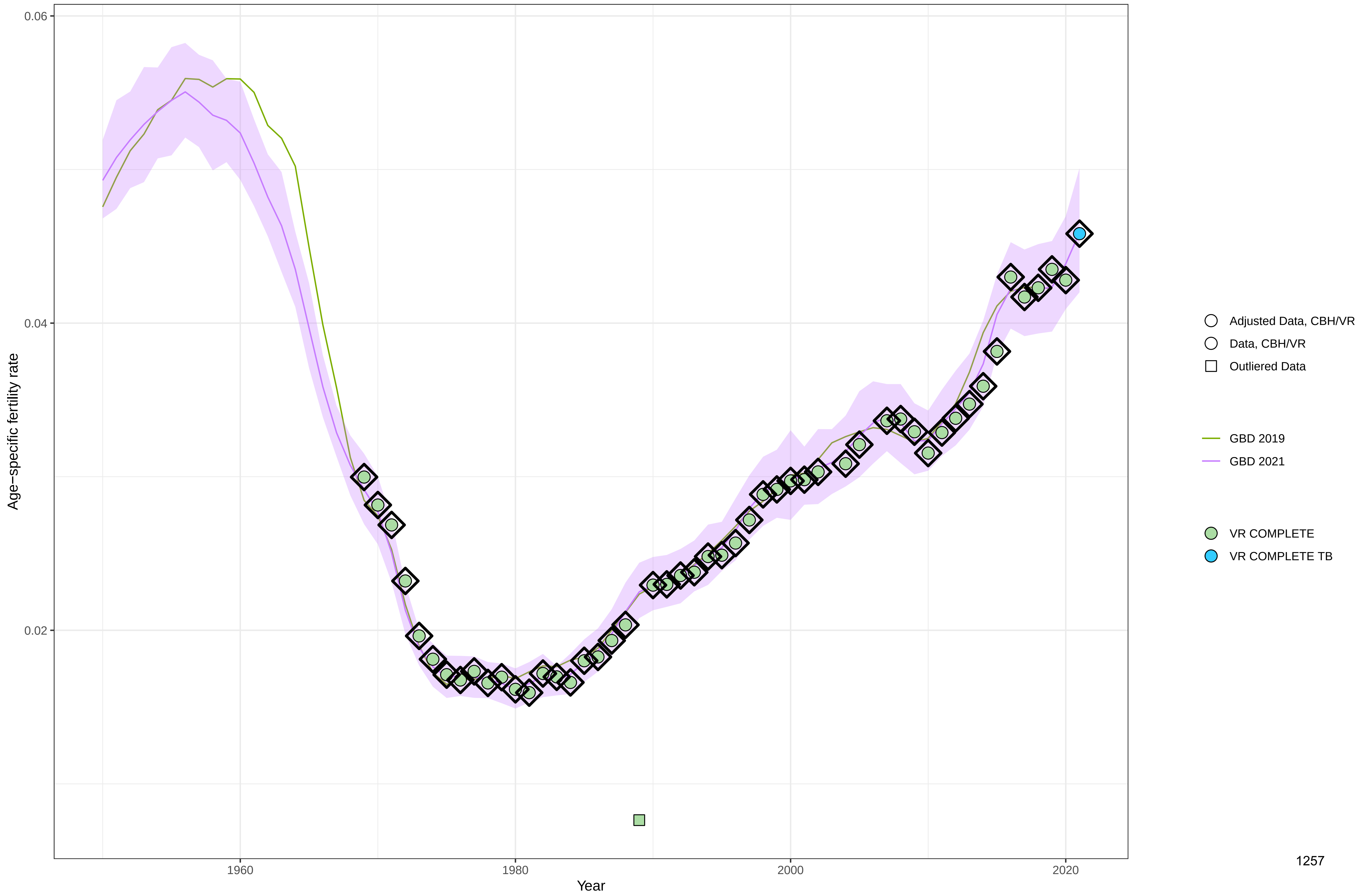

Age−specific fertility fitted trend lines, 1950−2021: South Carolina, Ages: 40−44
Data points enclosed with a diamond are reference sources

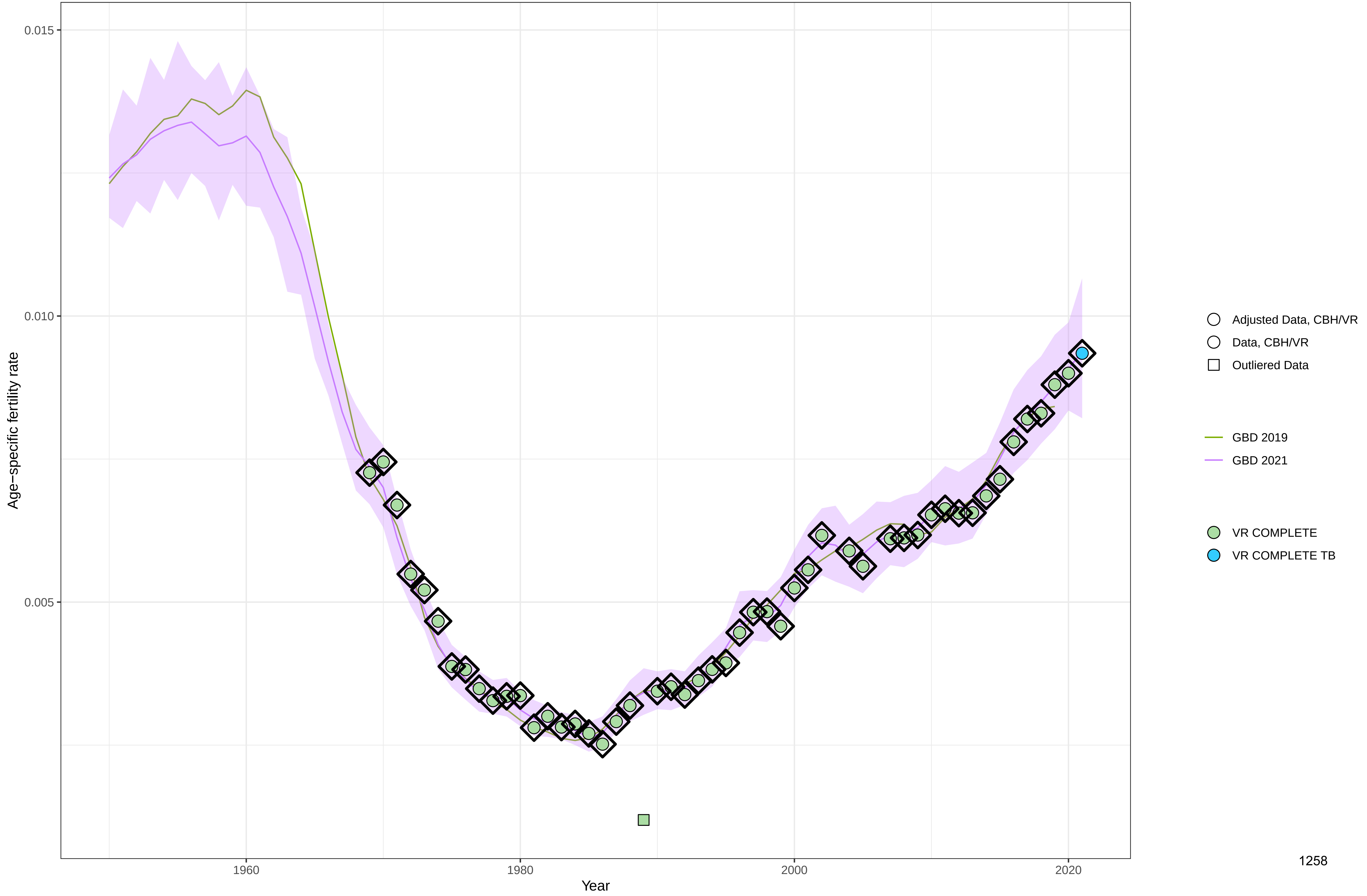

Age−specific fertility fitted trend lines, 1950−2021: South Carolina, Ages: 45−49
Data points enclosed with a diamond are reference sources

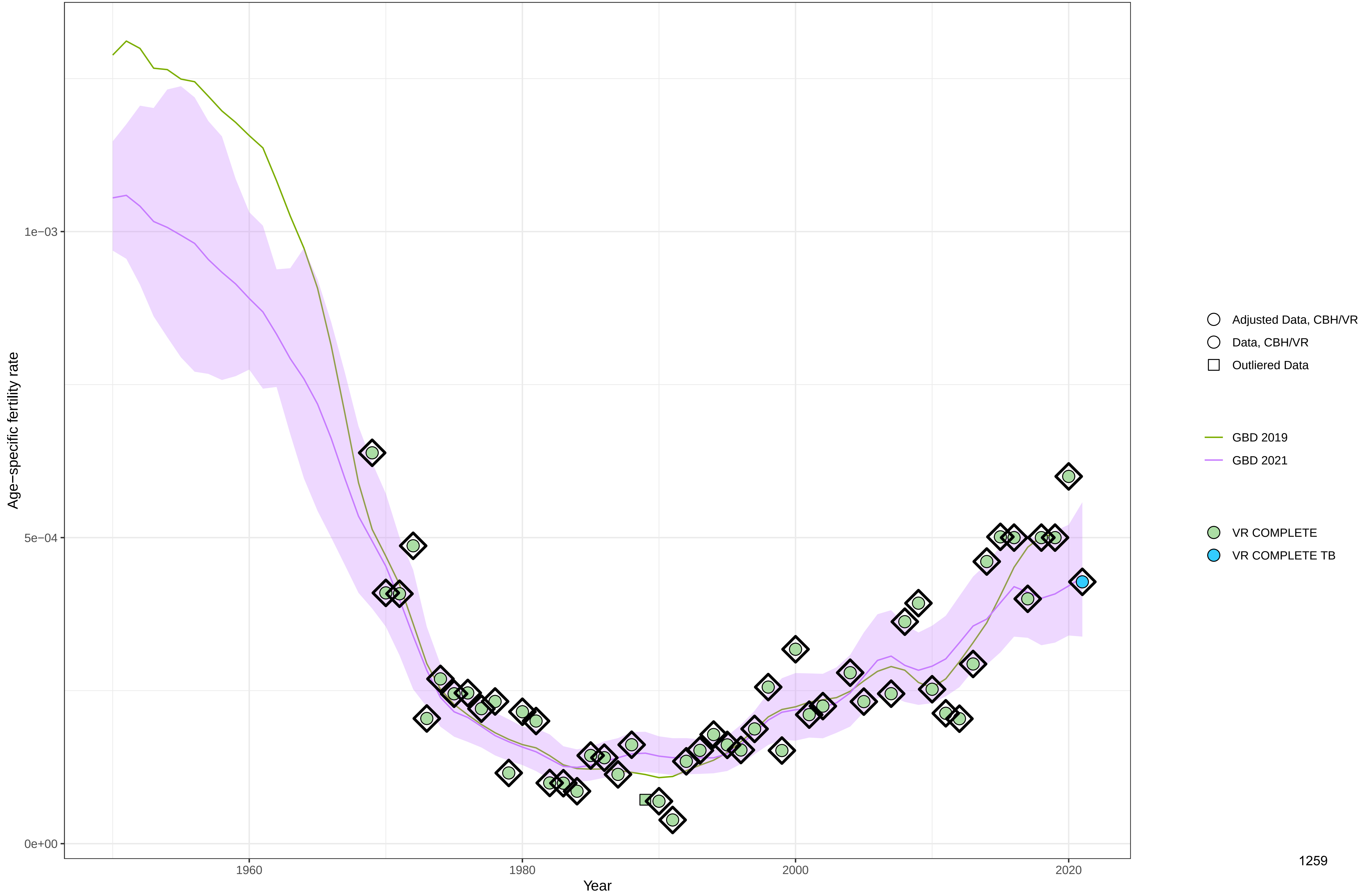

Age−specific fertility fitted trend lines, 1950−2021: South Carolina, Ages: 50−54
Data points enclosed with a diamond are reference sources

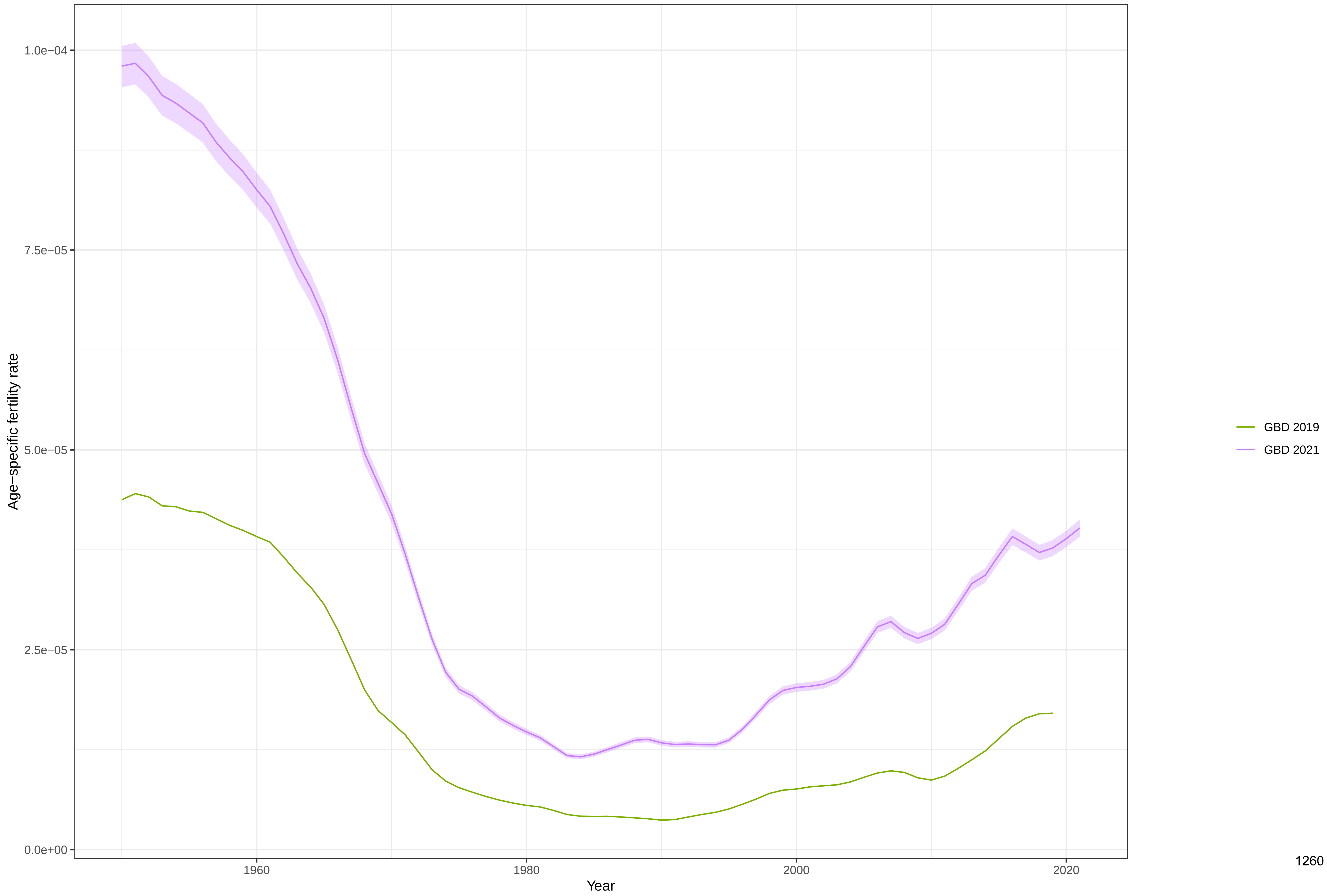

1260

Total fertility rate fitted trend lines, 1950−2021: South Carolina TFR
Data points enclosed with a diamond are reference sources

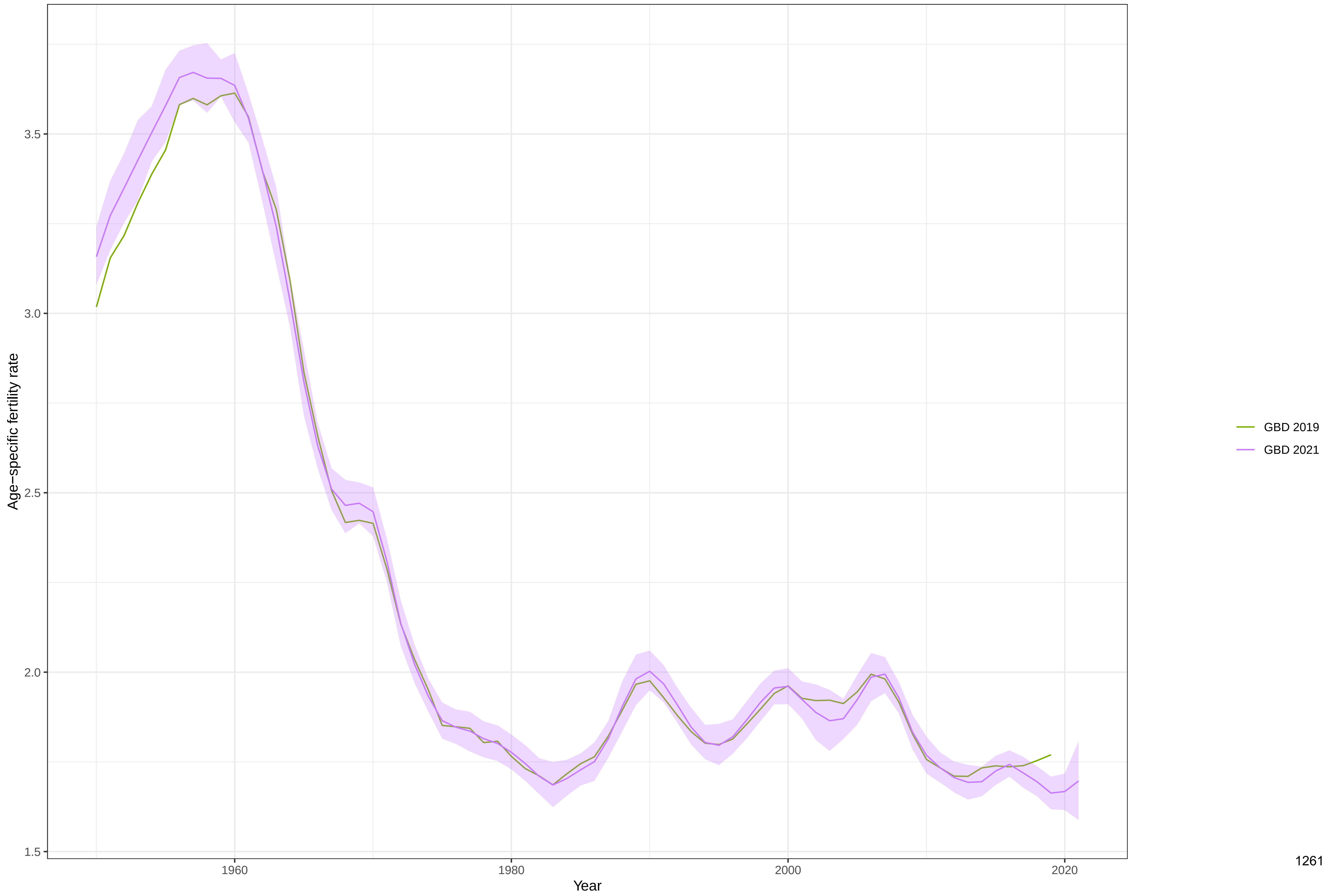

Age−specific fertility fitted trend lines, 1950−2021: South Dakota, Ages: 10−14
Data points enclosed with a diamond are reference sources

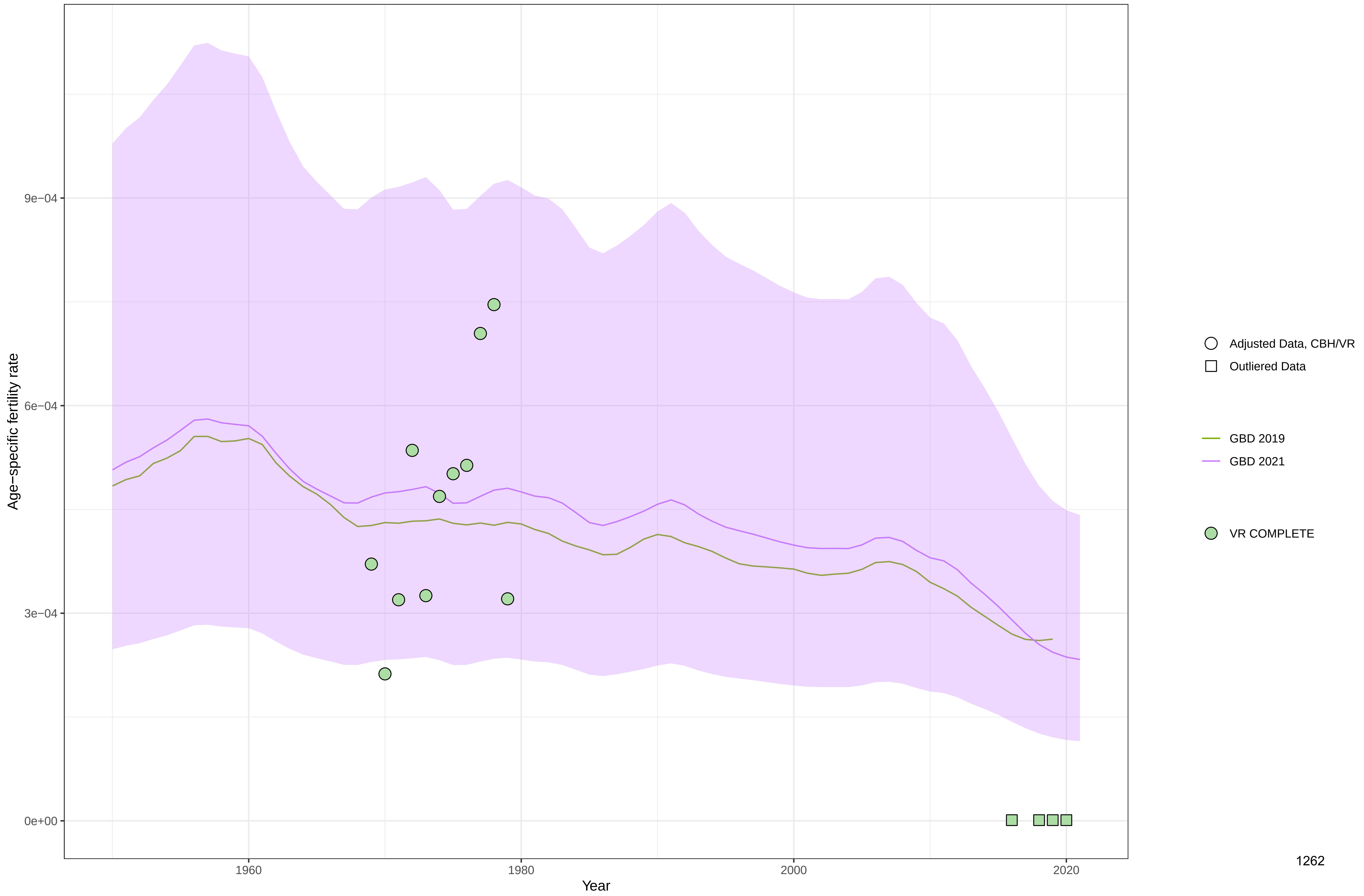

Age−specific fertility fitted trend lines, 1950−2021: South Dakota, Ages: 15−19
Data points enclosed with a diamond are reference sources

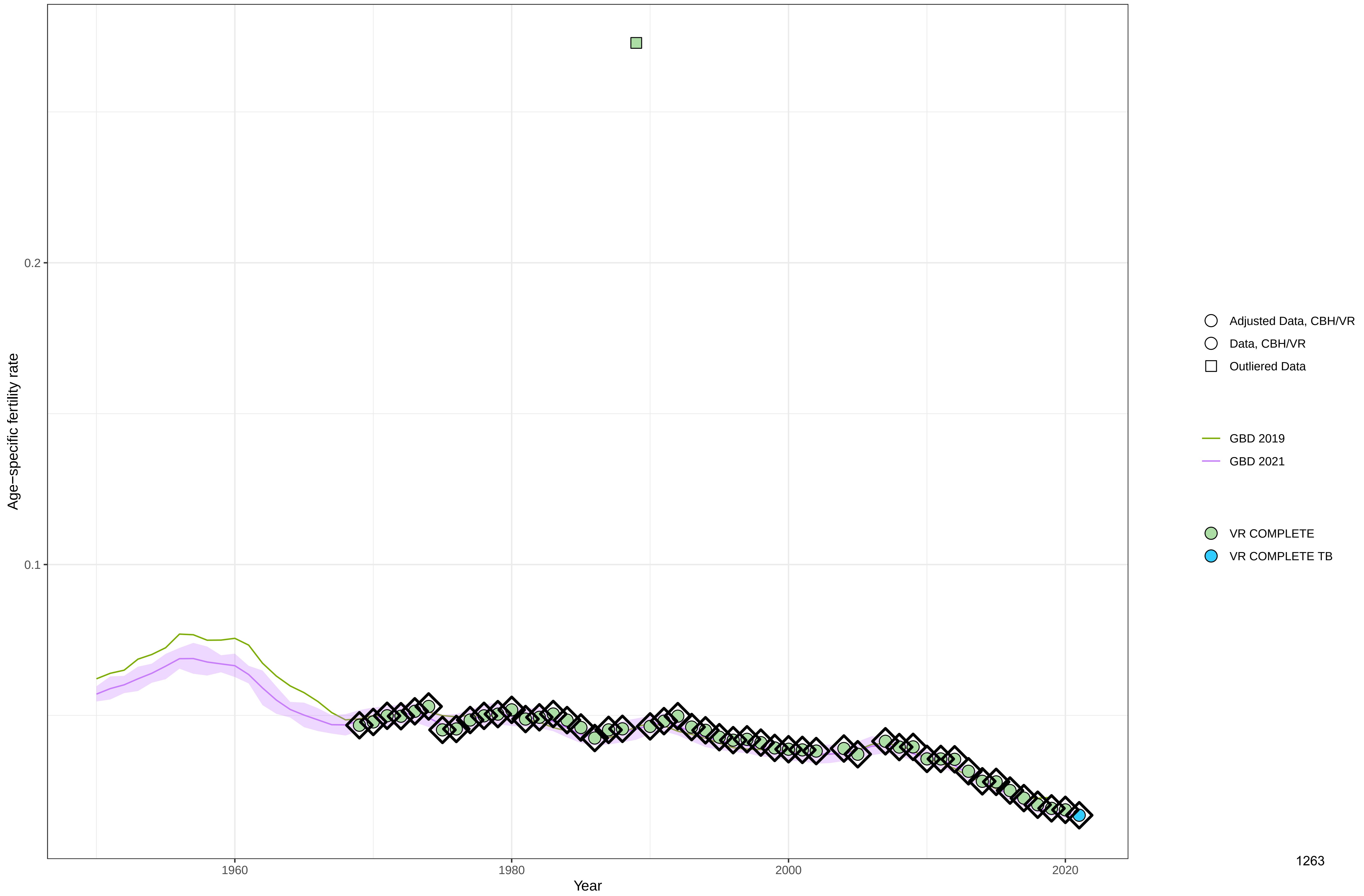

Age−specific fertility fitted trend lines, 1950−2021: South Dakota, Ages: 20−24
Data points enclosed with a diamond are reference sources

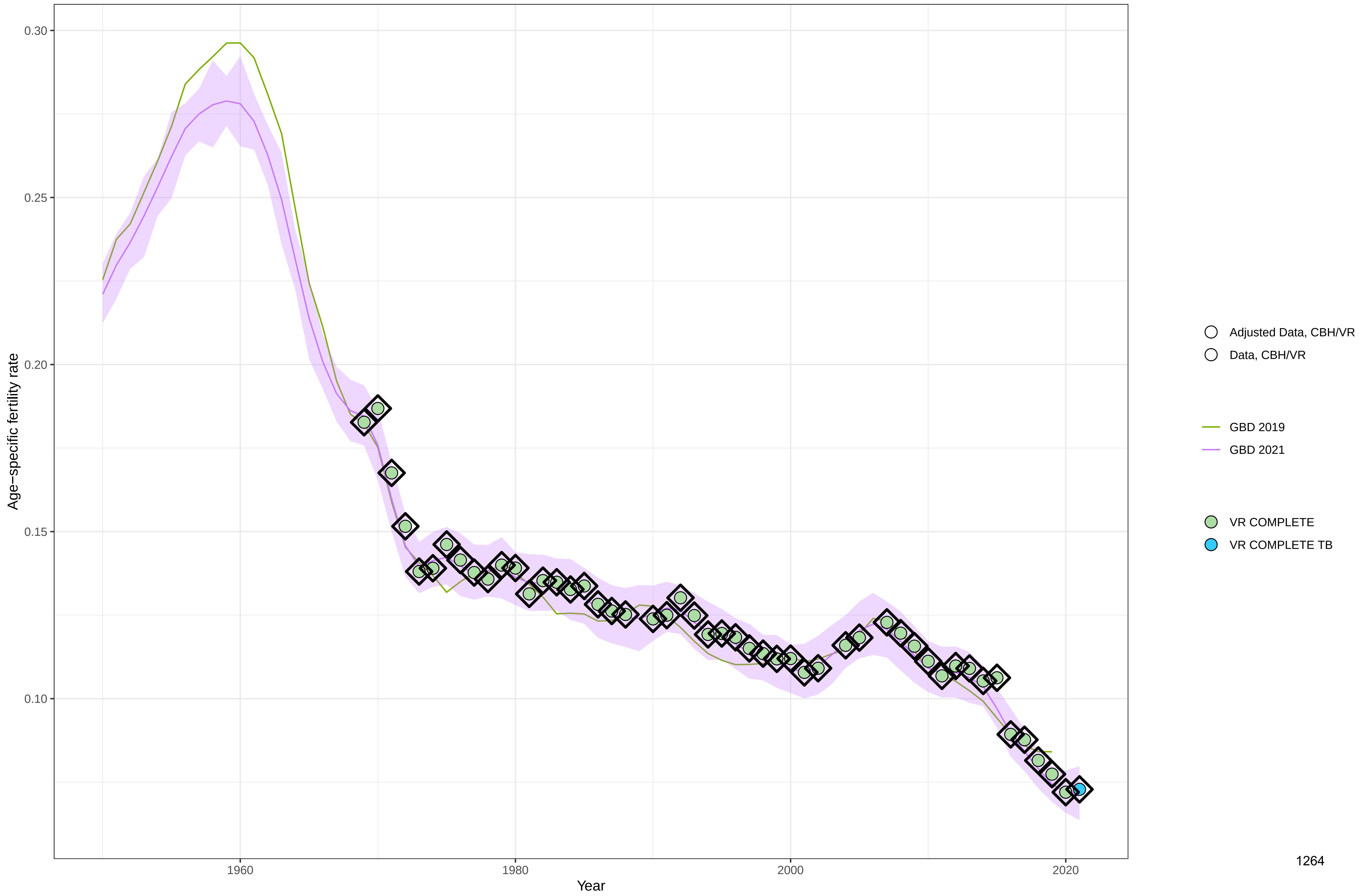

Age−specific fertility fitted trend lines, 1950−2021: South Dakota, Ages: 25−29
Data points enclosed with a diamond are reference sources

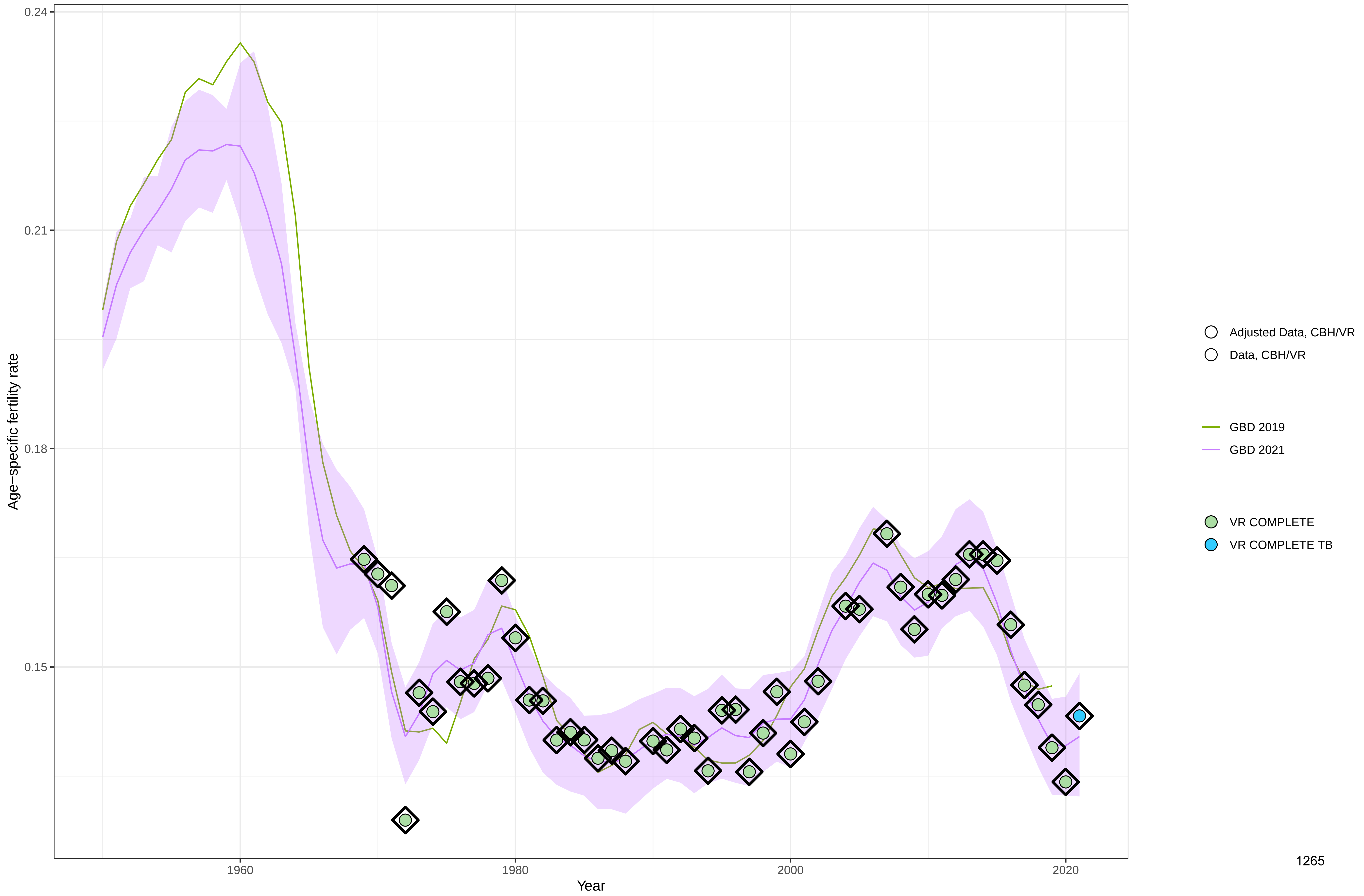

Age−specific fertility fitted trend lines, 1950−2021: South Dakota, Ages: 30−34
Data points enclosed with a diamond are reference sources

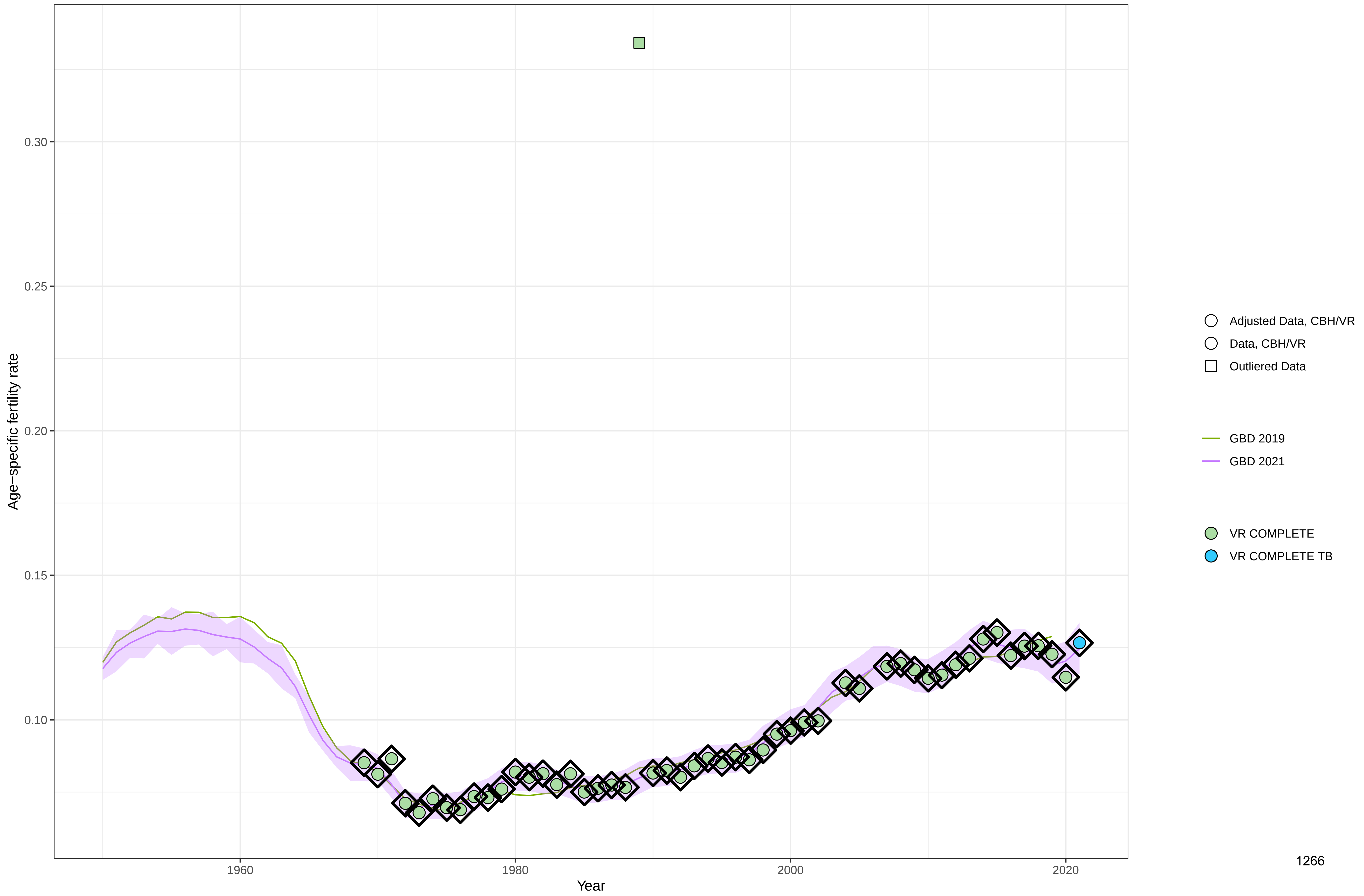

Age−specific fertility fitted trend lines, 1950−2021: South Dakota, Ages: 35−39
Data points enclosed with a diamond are reference sources

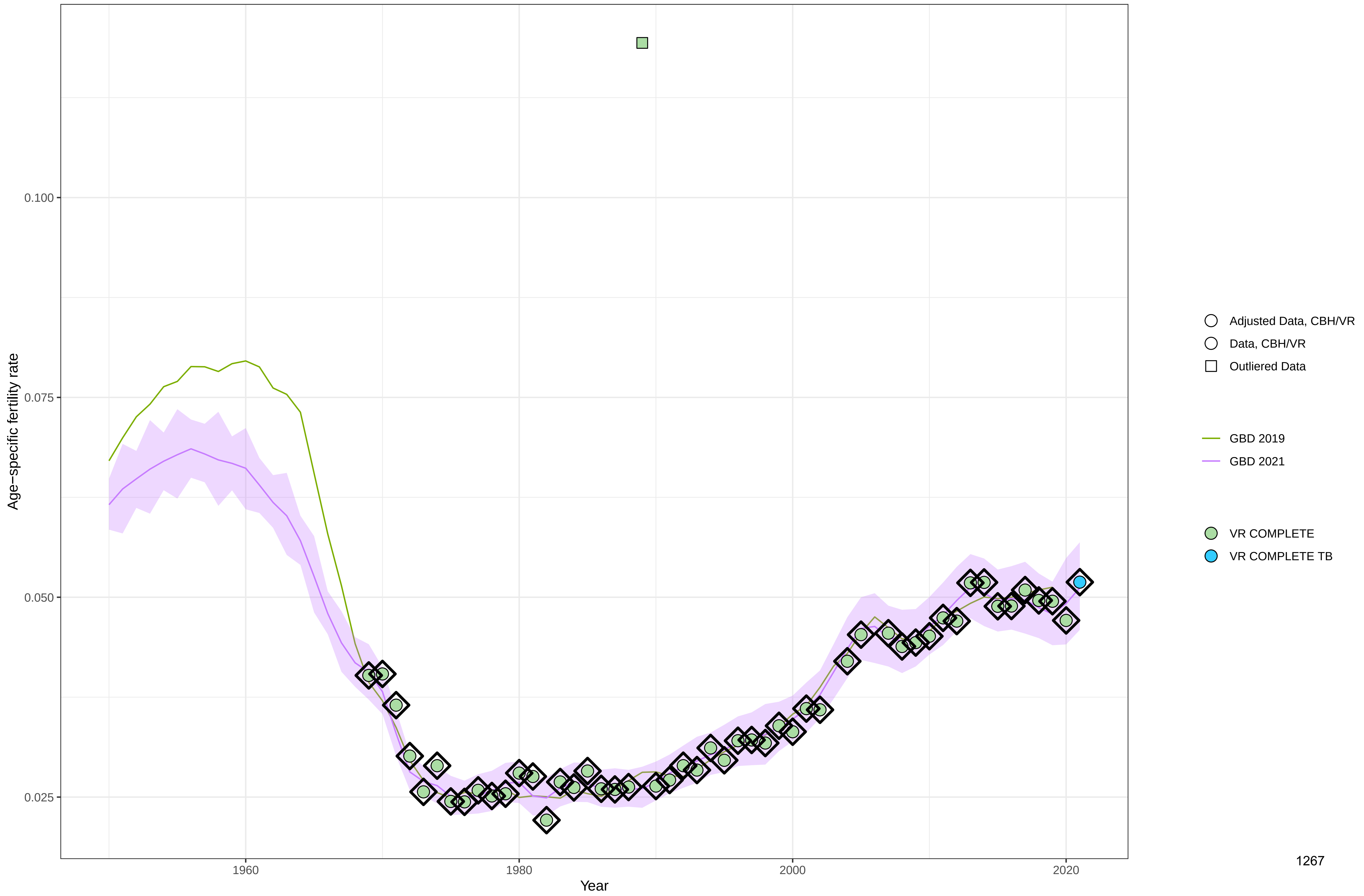

Age−specific fertility fitted trend lines, 1950−2021: South Dakota, Ages: 40−44
Data points enclosed with a diamond are reference sources

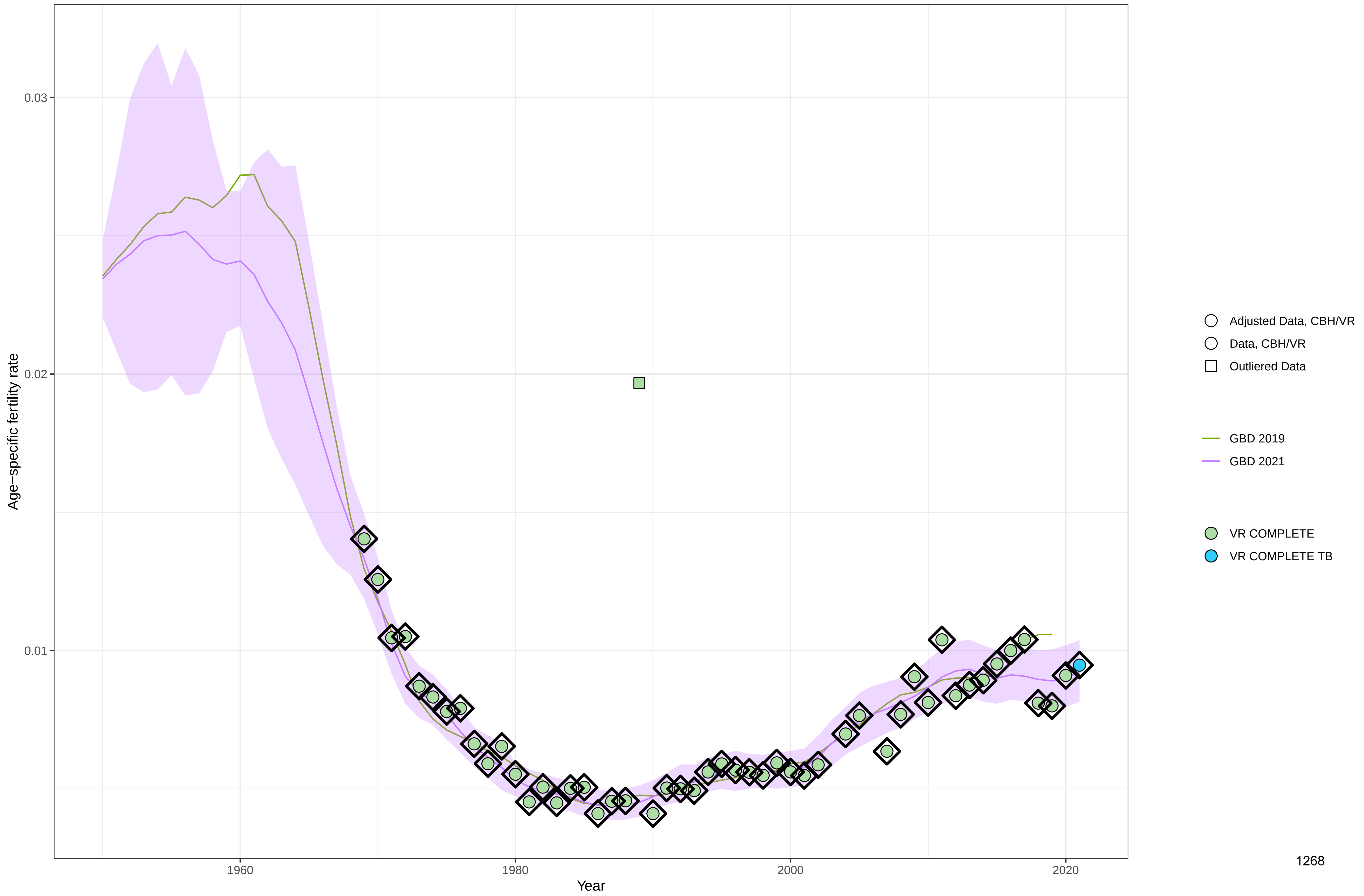

Age−specific fertility fitted trend lines, 1950−2021: South Dakota, Ages: 45−49
Data points enclosed with a diamond are reference sources

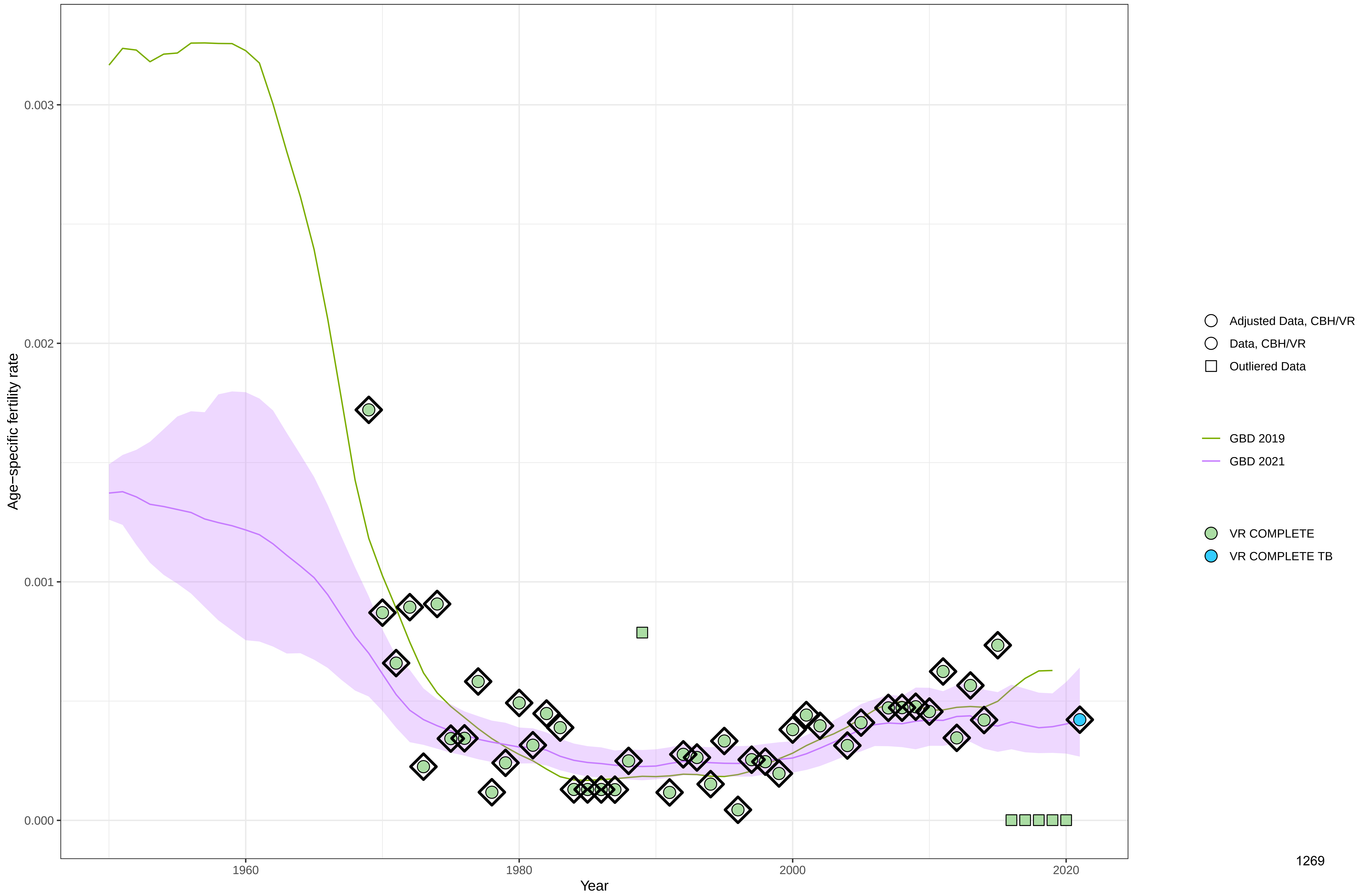

Age−specific fertility fitted trend lines, 1950−2021: South Dakota, Ages: 50−54
Data points enclosed with a diamond are reference sources

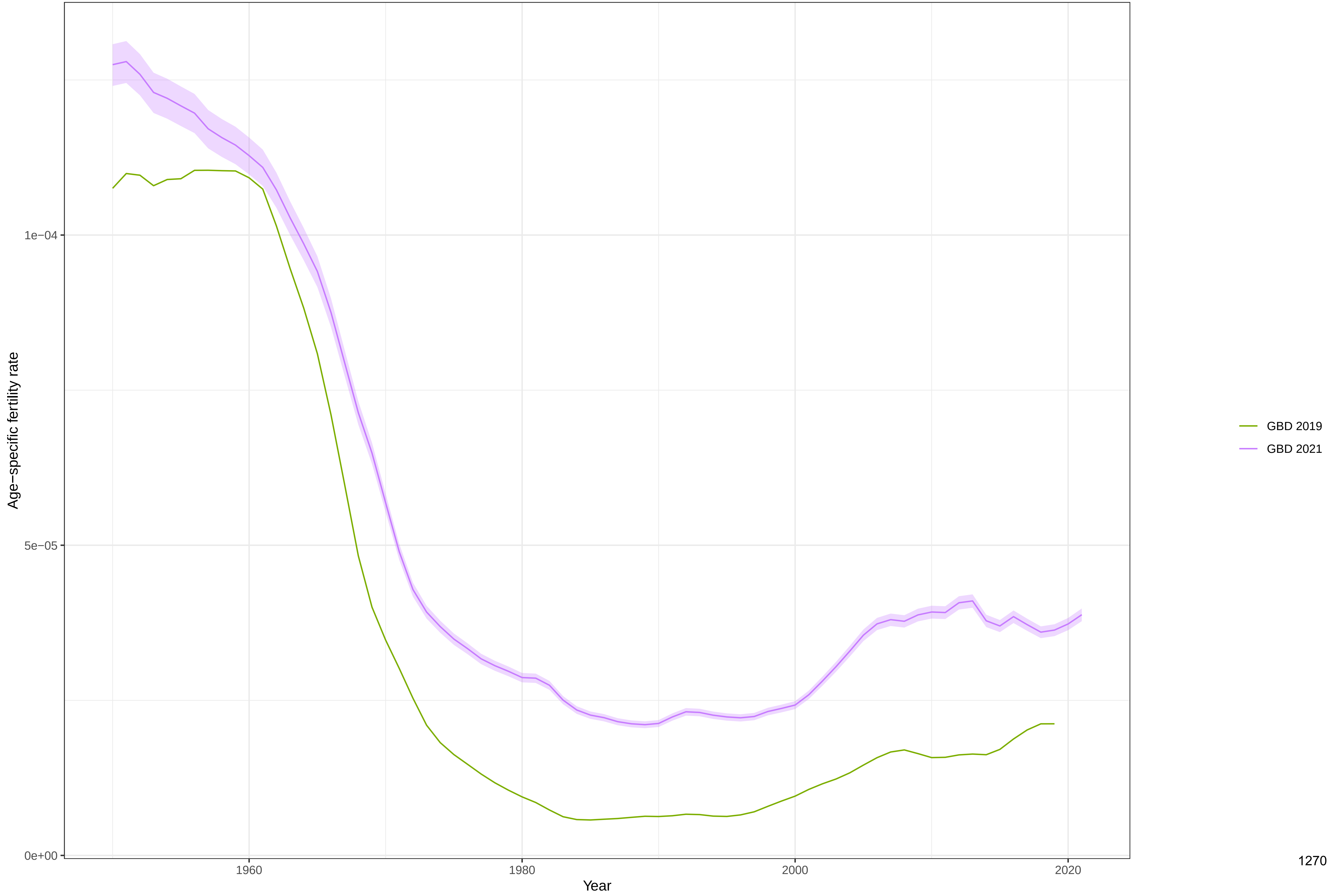

Total fertility rate fitted trend lines, 1950−2021: South Dakota TFR
Data points enclosed with a diamond are reference sources

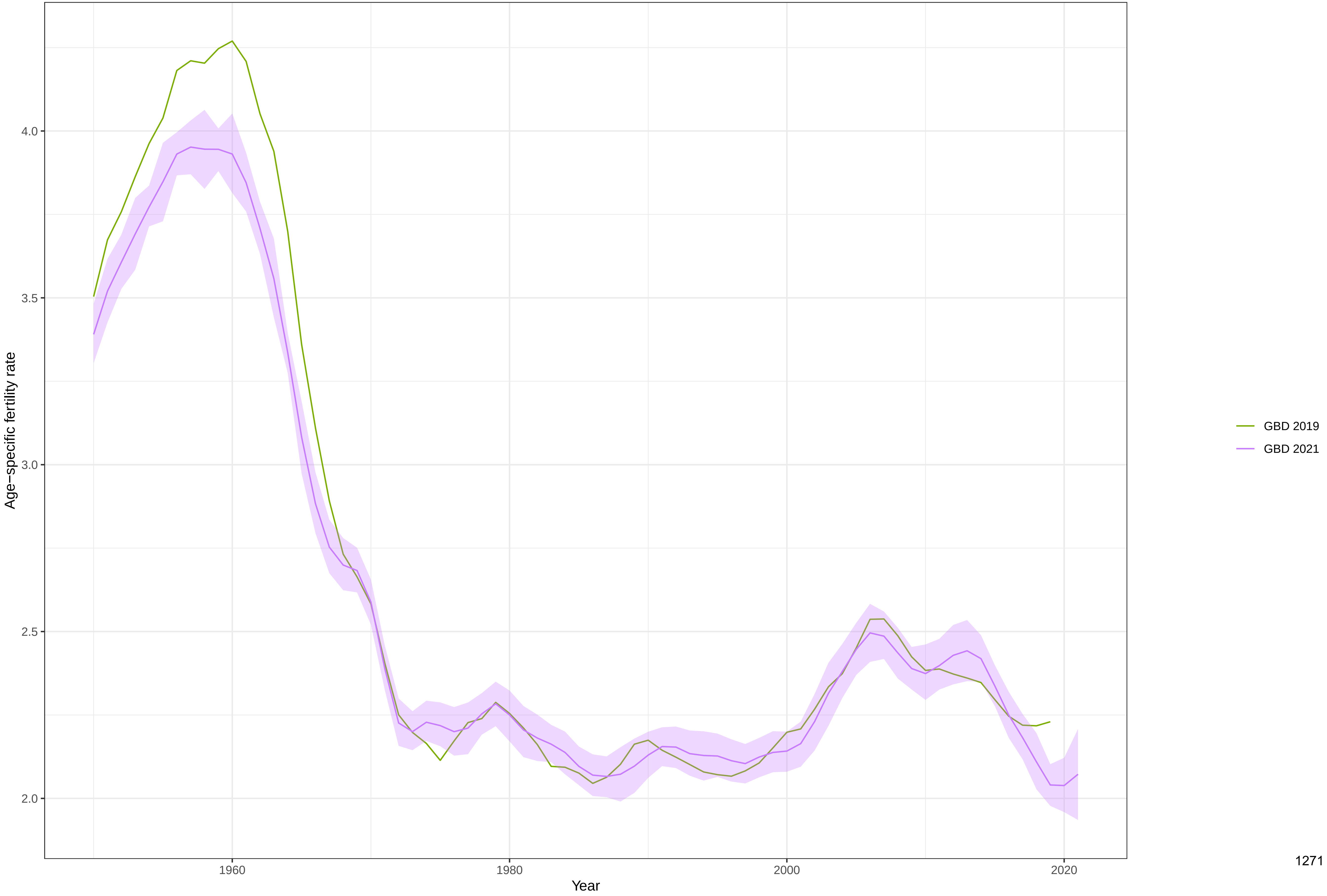

Age−specific fertility fitted trend lines, 1950−2021: Tennessee, Ages: 10−14
Data points enclosed with a diamond are reference sources

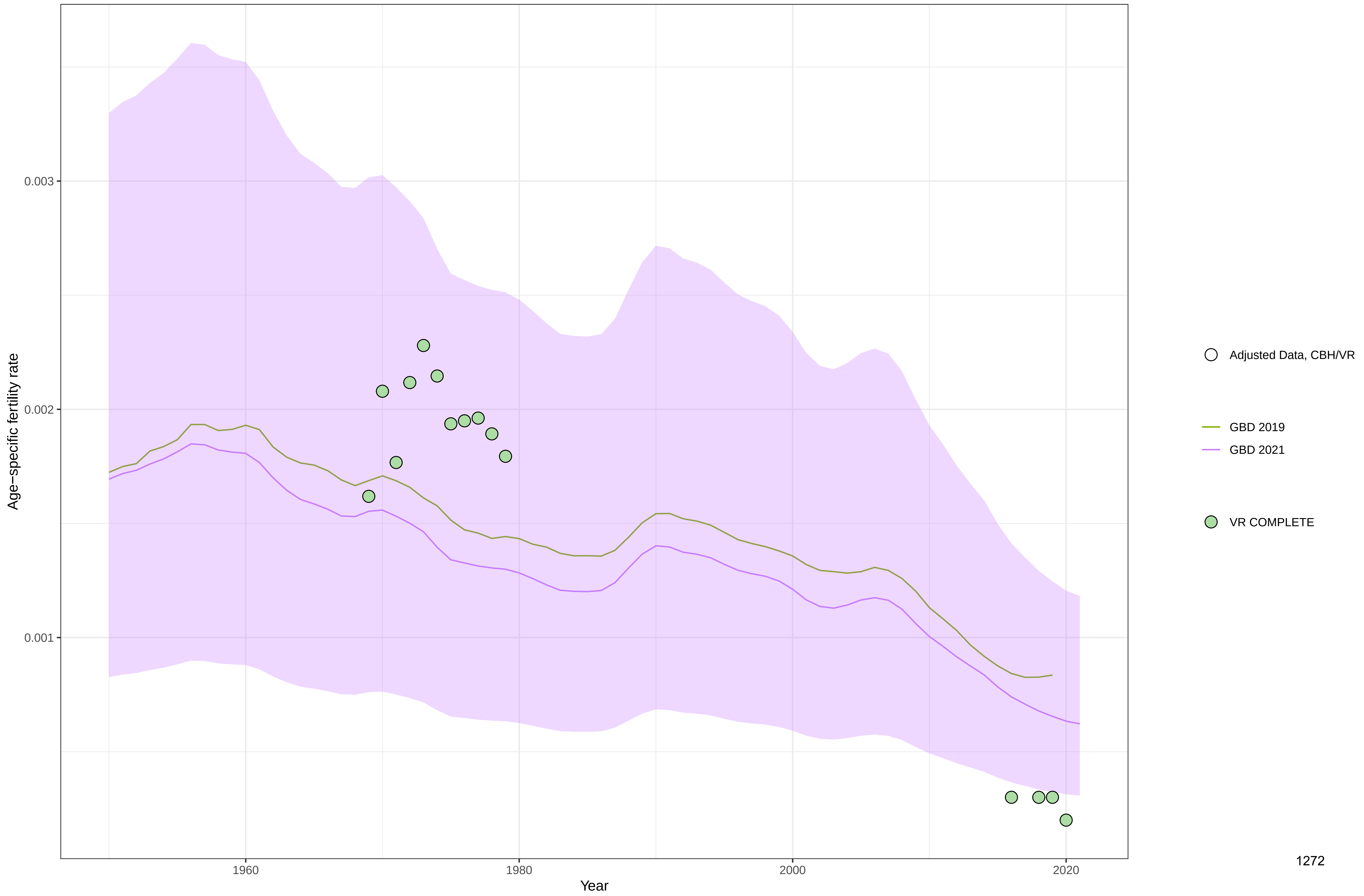

Age−specific fertility fitted trend lines, 1950−2021: Tennessee, Ages: 15−19
Data points enclosed with a diamond are reference sources

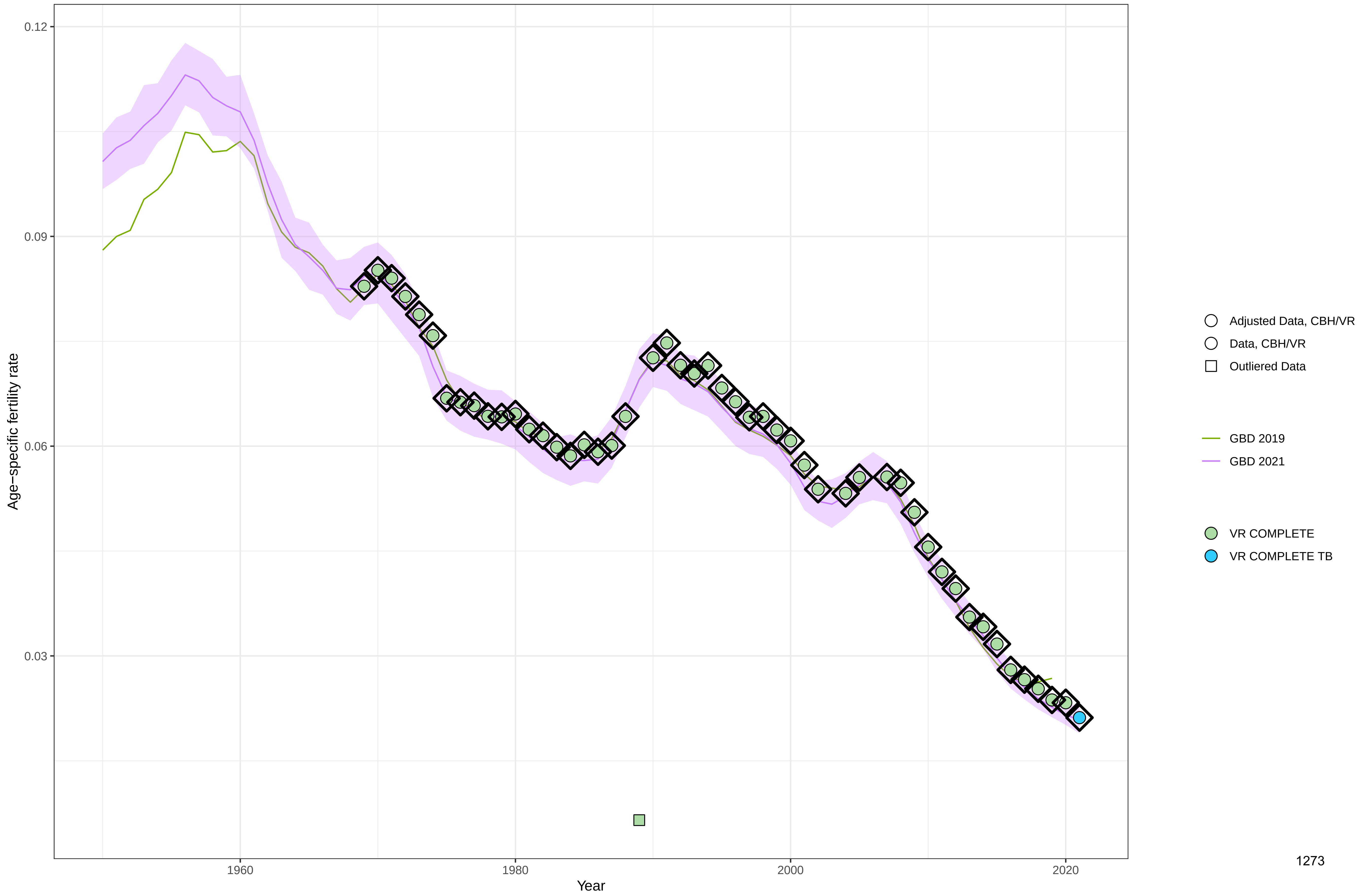

Age−specific fertility fitted trend lines, 1950−2021: Tennessee, Ages: 20−24
Data points enclosed with a diamond are reference sources

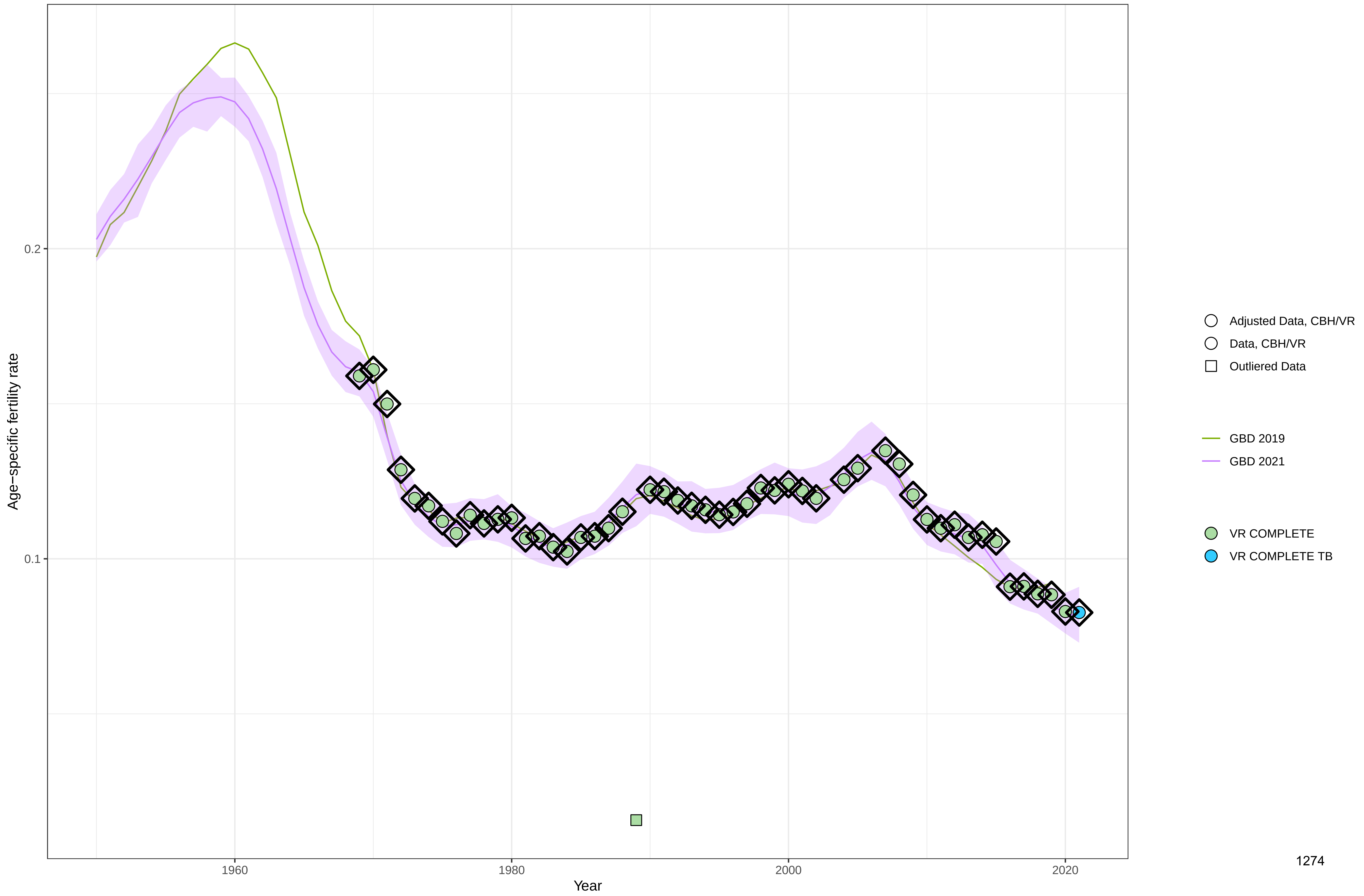

Age−specific fertility fitted trend lines, 1950−2021: Tennessee, Ages: 25−29
Data points enclosed with a diamond are reference sources

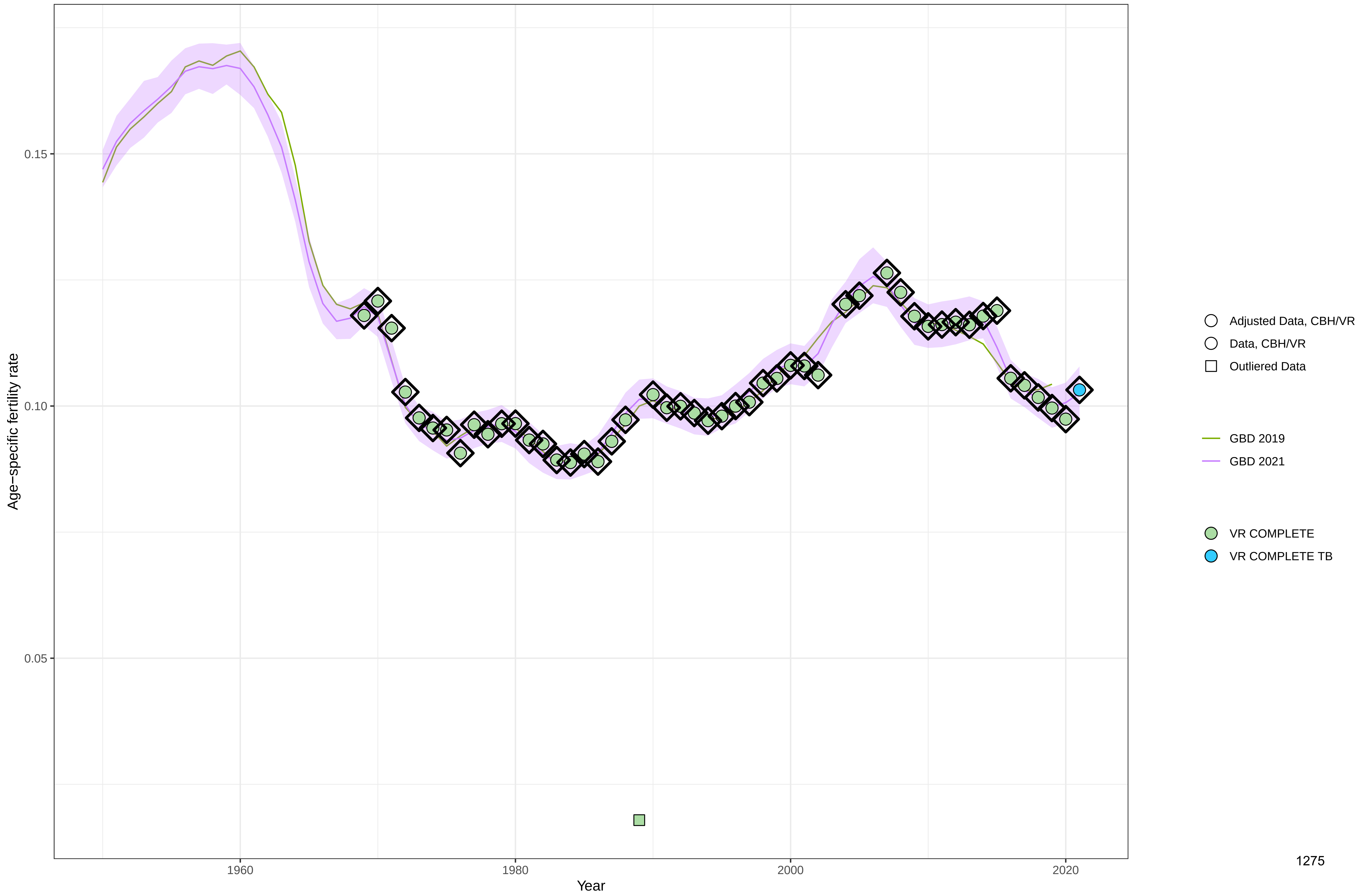

Age−specific fertility fitted trend lines, 1950−2021: Tennessee, Ages: 30−34
Data points enclosed with a diamond are reference sources

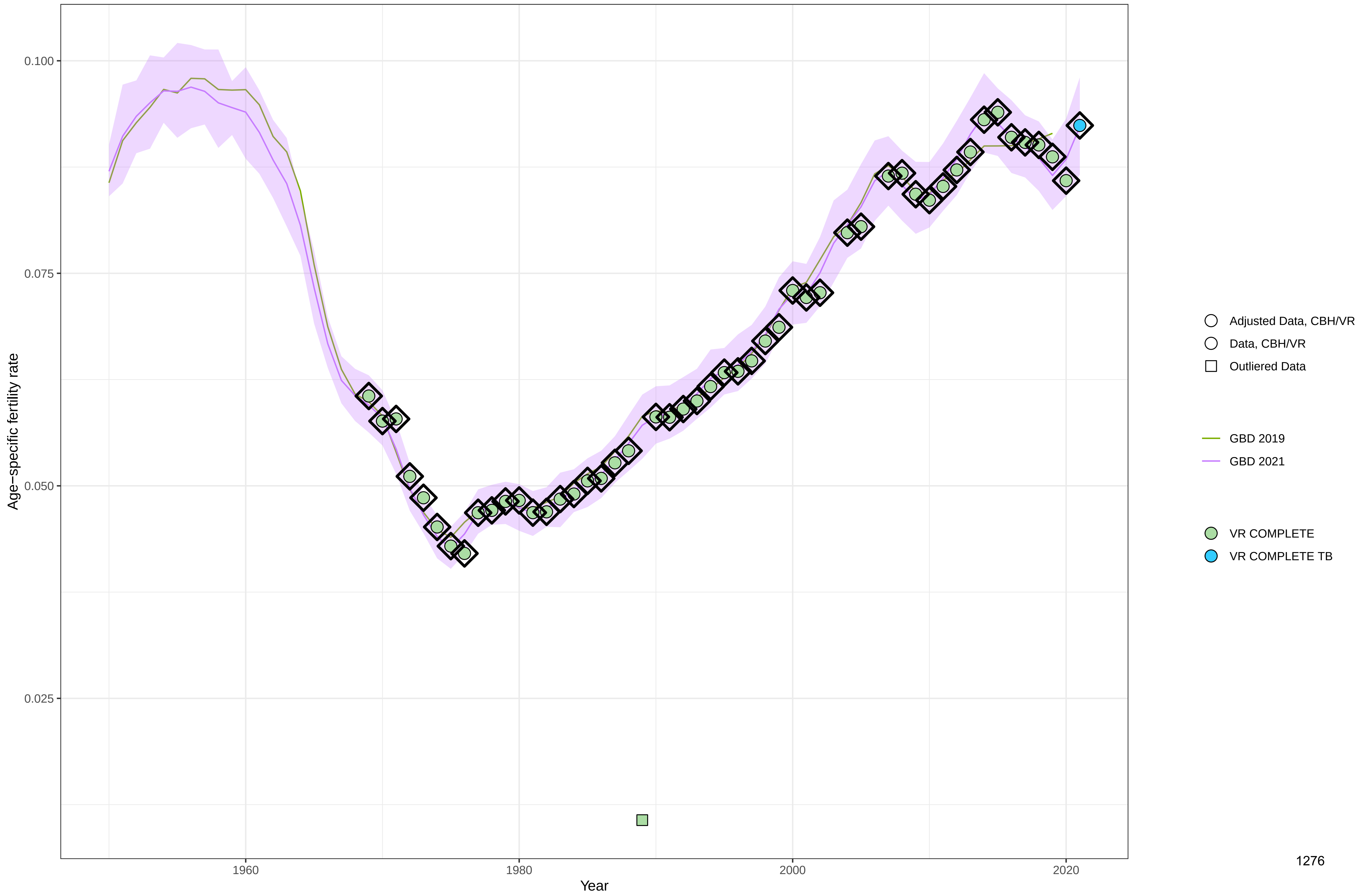

Age−specific fertility fitted trend lines, 1950−2021: Tennessee, Ages: 35−39
Data points enclosed with a diamond are reference sources

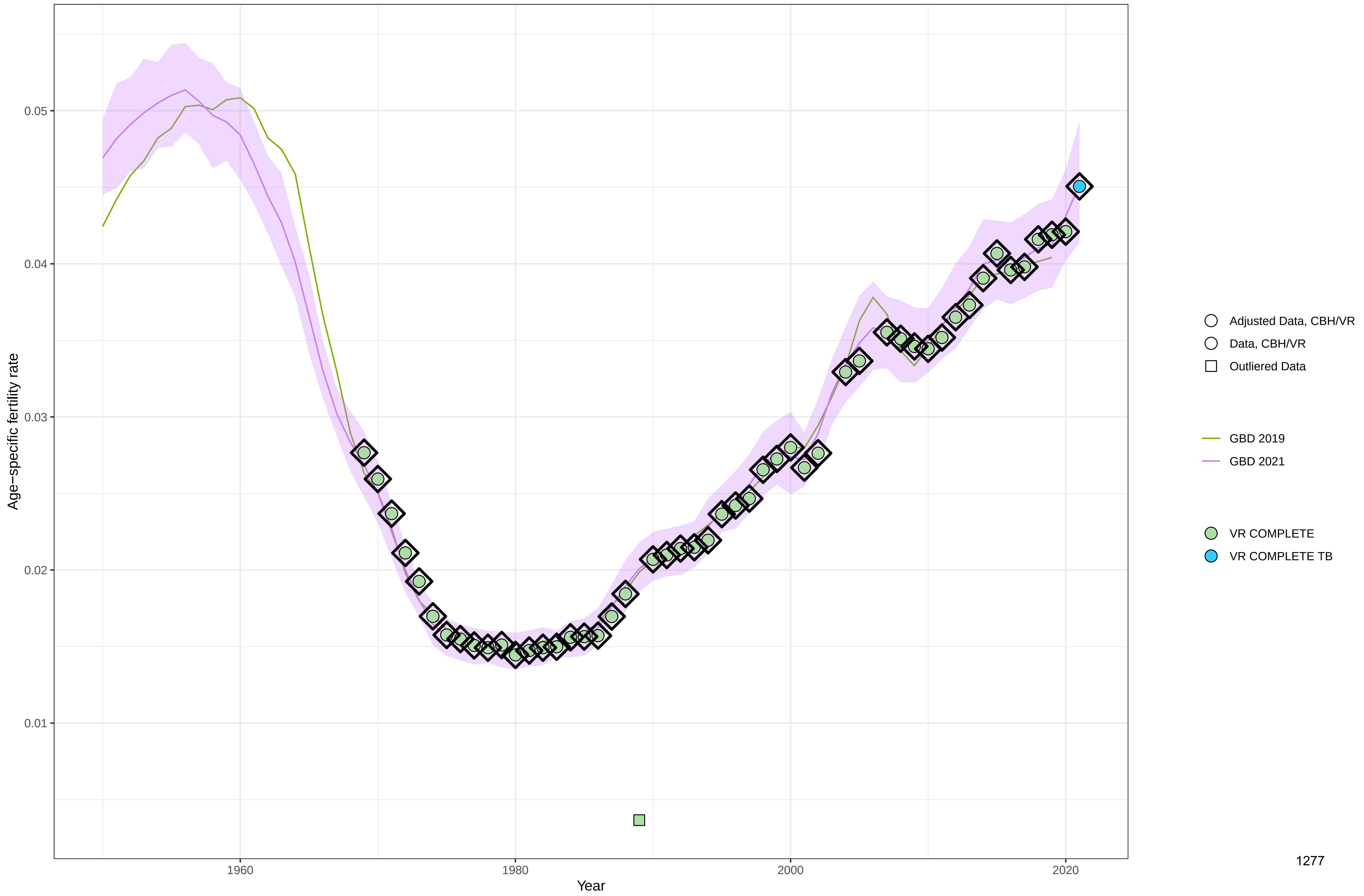

Age−specific fertility fitted trend lines, 1950−2021: Tennessee, Ages: 40−44
Data points enclosed with a diamond are reference sources

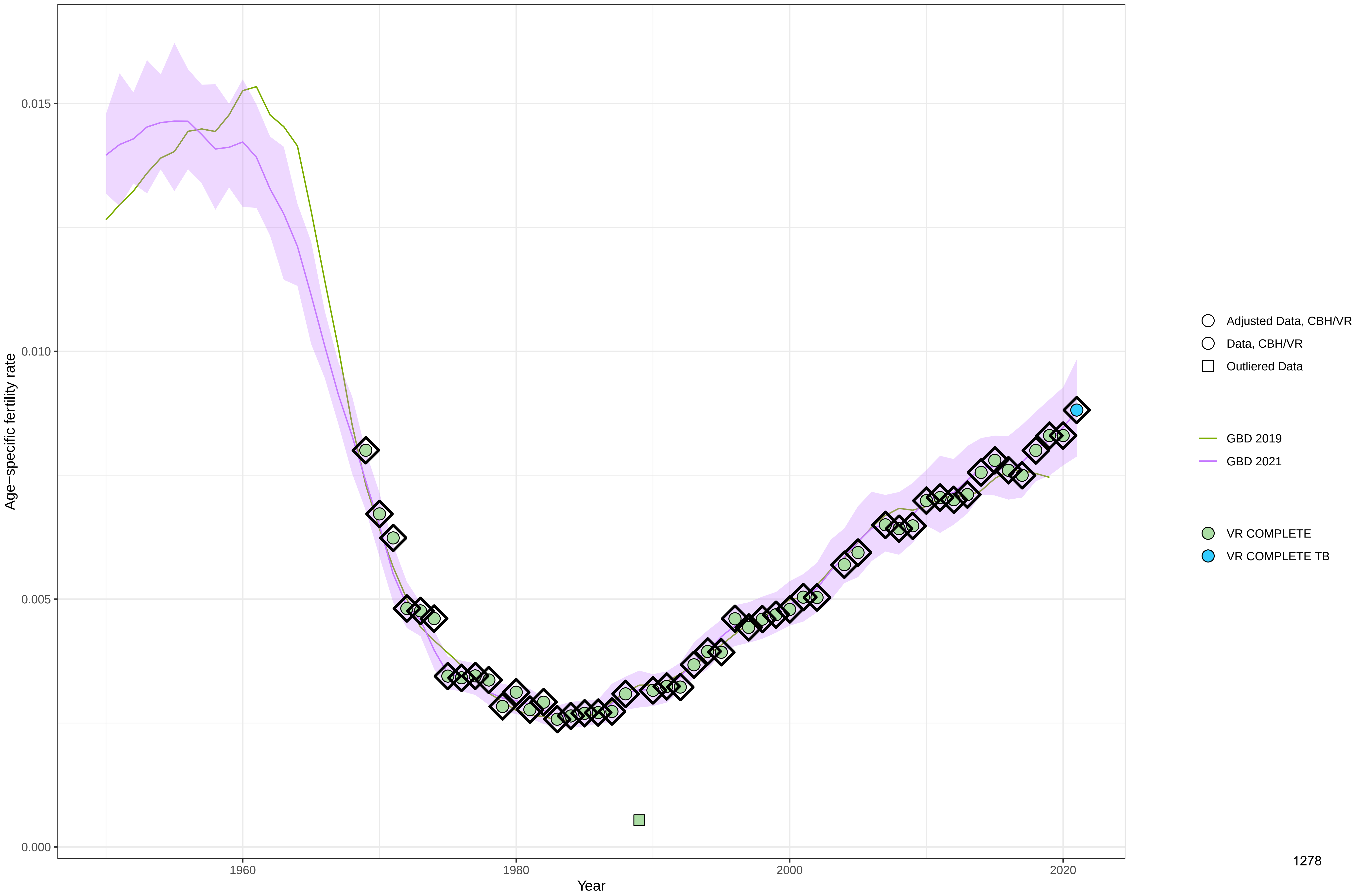

Age−specific fertility fitted trend lines, 1950−2021: Tennessee, Ages: 45−49
Data points enclosed with a diamond are reference sources

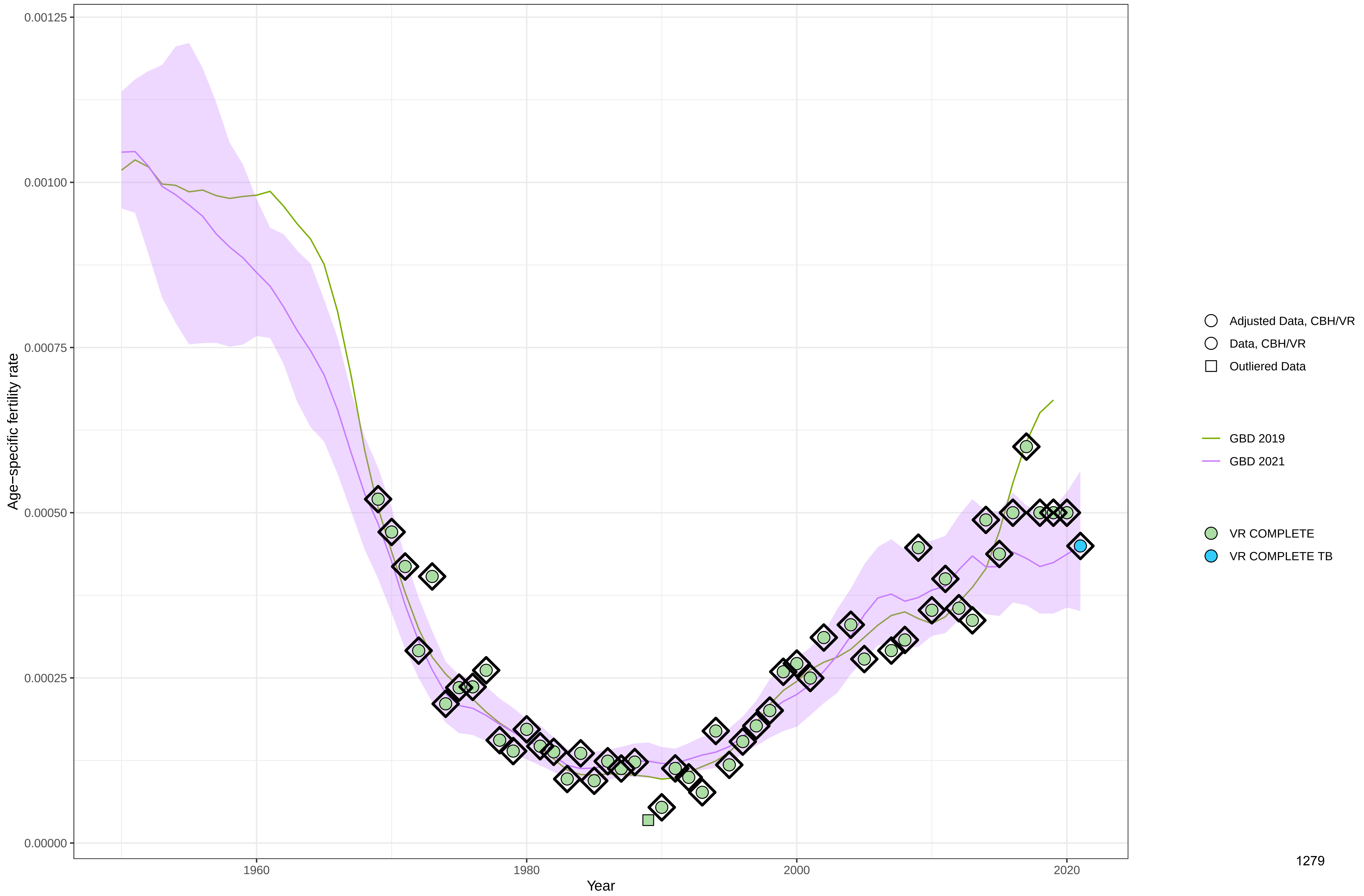

Age−specific fertility fitted trend lines, 1950−2021: Tennessee, Ages: 50−54
Data points enclosed with a diamond are reference sources

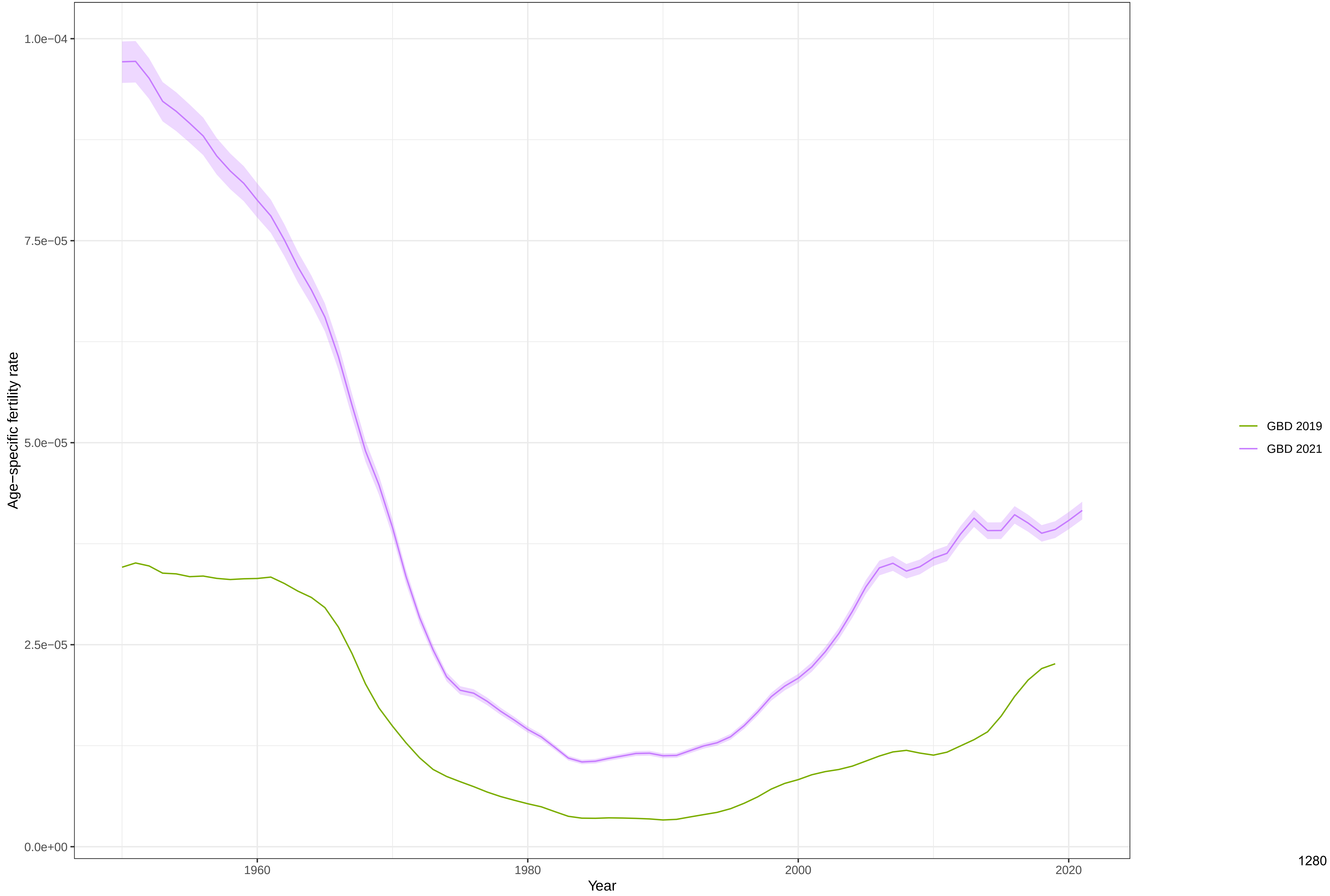

Total fertility rate fitted trend lines, 1950−2021: Tennessee TFR
Data points enclosed with a diamond are reference sources

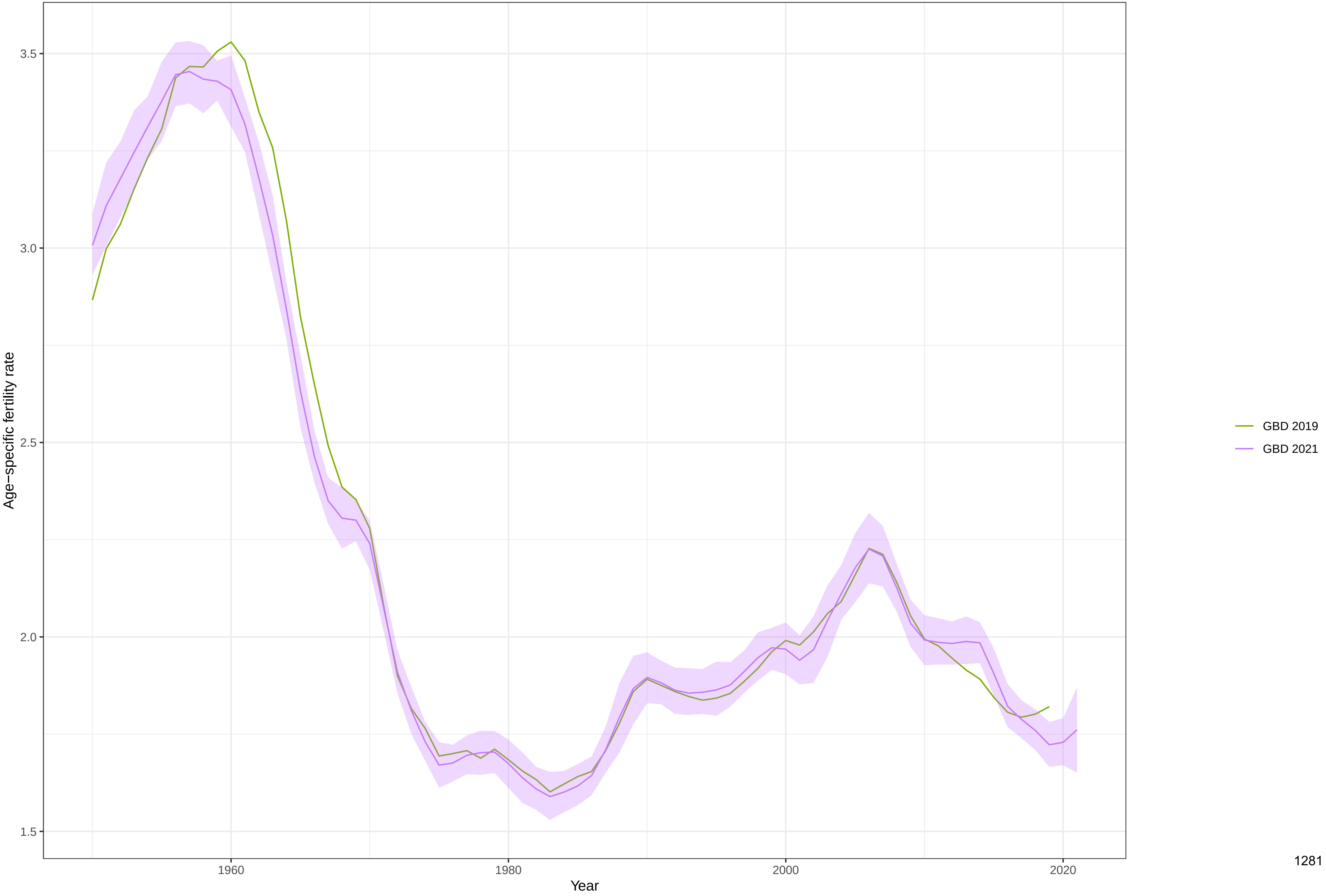

Age−specific fertility fitted trend lines, 1950−2021: Texas, Ages: 10−14
Data points enclosed with a diamond are reference sources

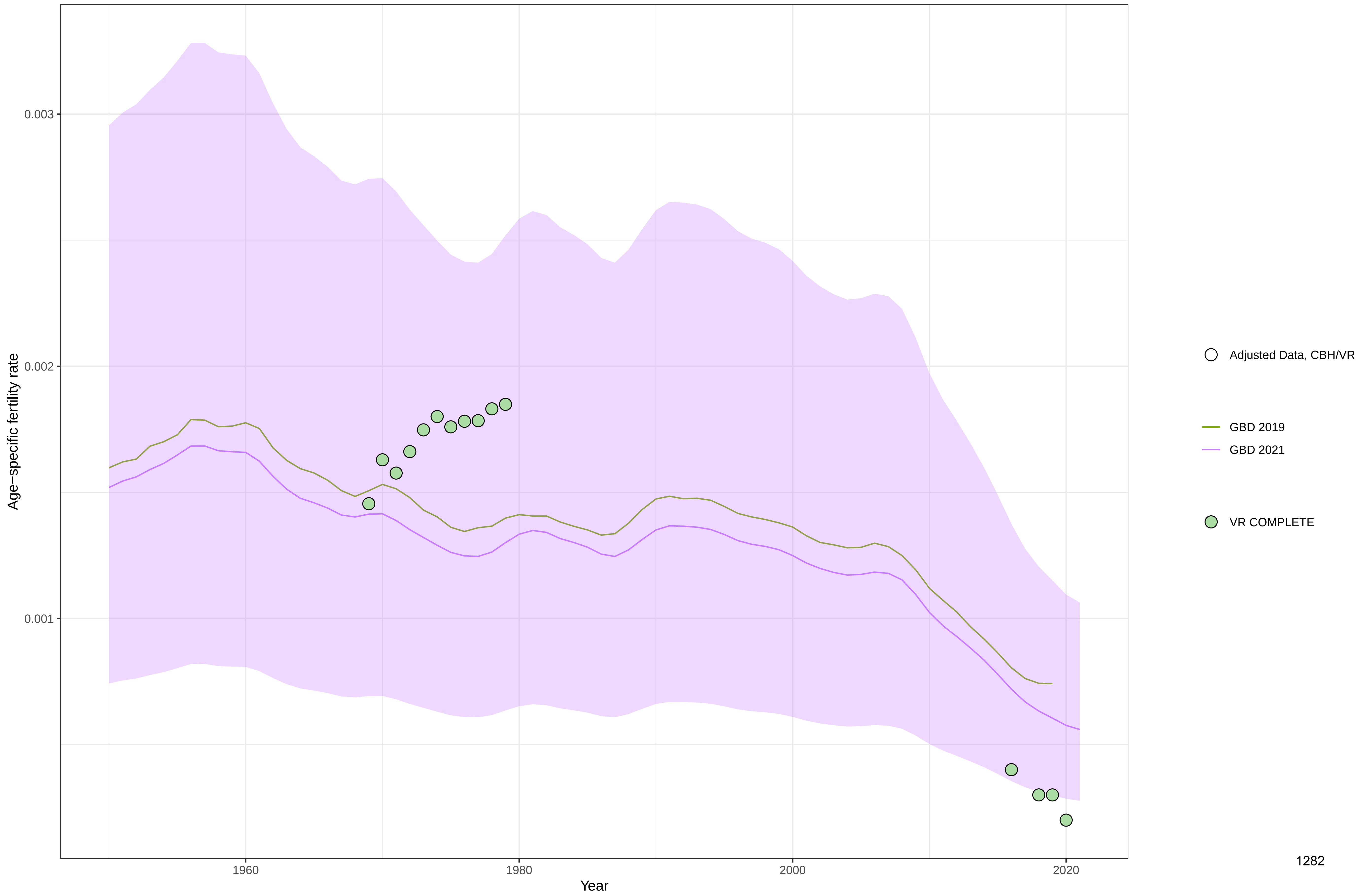

Age−specific fertility fitted trend lines, 1950−2021: Texas, Ages: 15−19
Data points enclosed with a diamond are reference sources

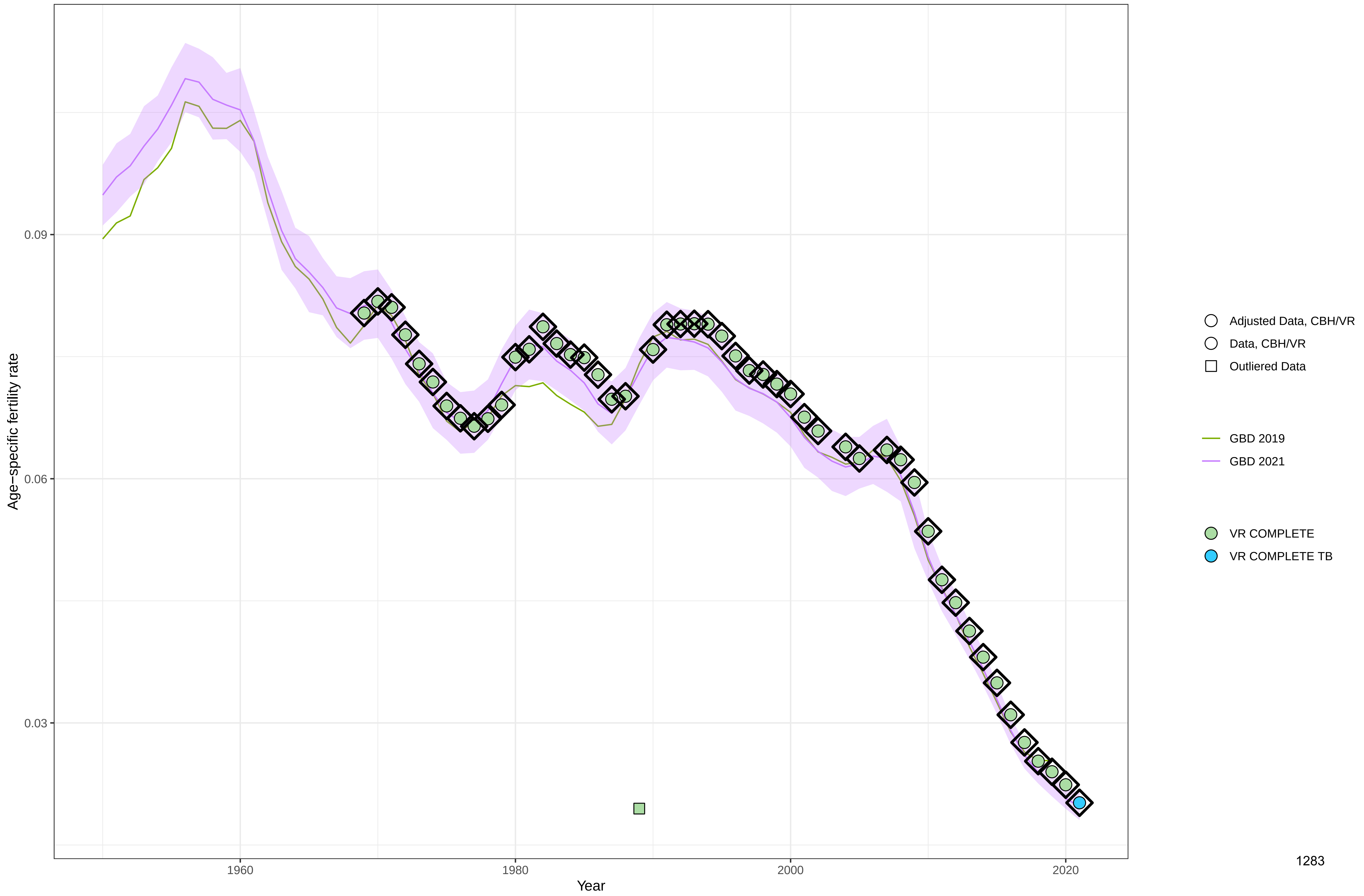

Age−specific fertility fitted trend lines, 1950−2021: Texas, Ages: 20−24
Data points enclosed with a diamond are reference sources

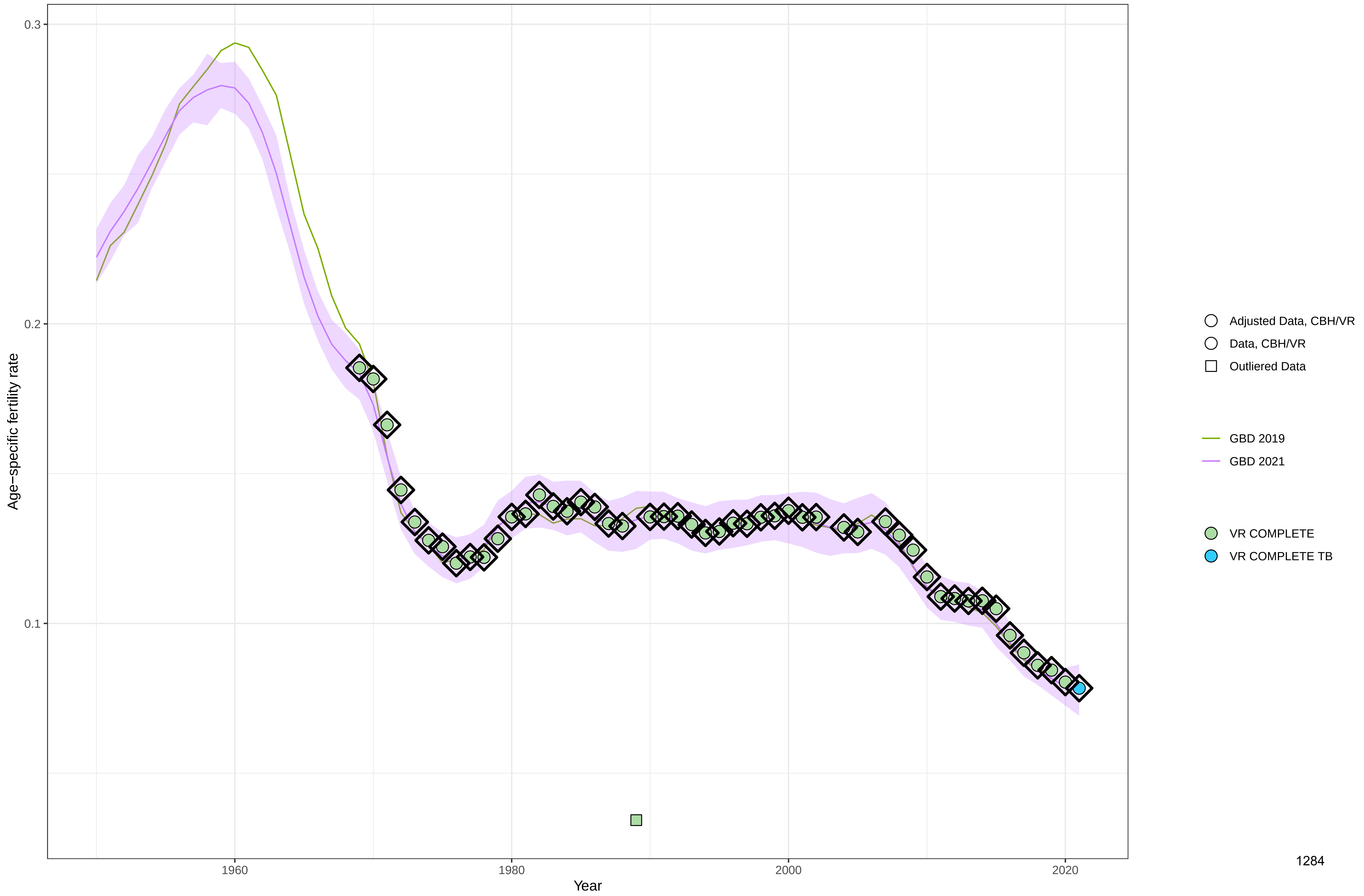

Age−specific fertility fitted trend lines, 1950−2021: Texas, Ages: 25−29
Data points enclosed with a diamond are reference sources

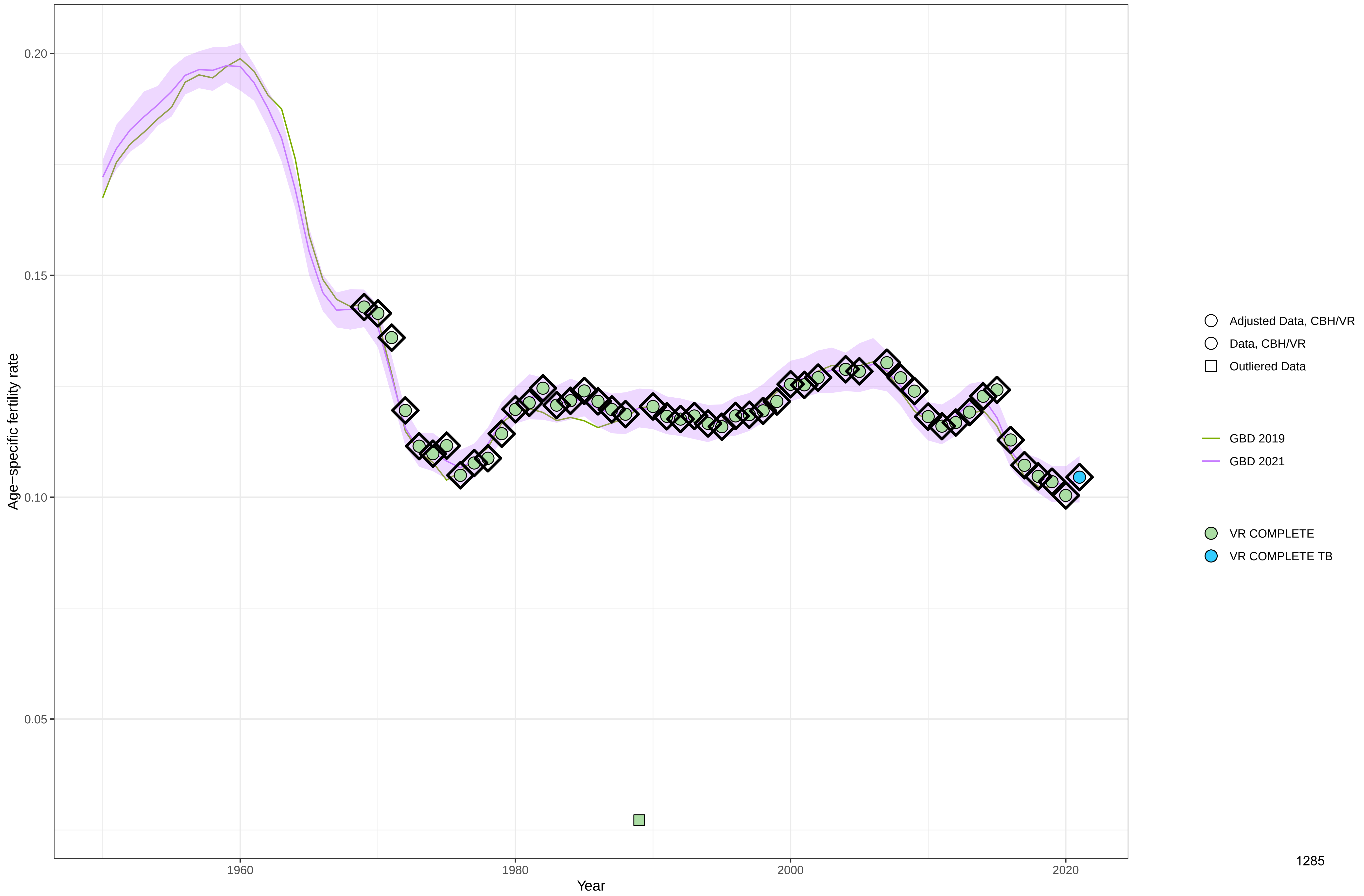

Age−specific fertility fitted trend lines, 1950−2021: Texas, Ages: 30−34
Data points enclosed with a diamond are reference sources

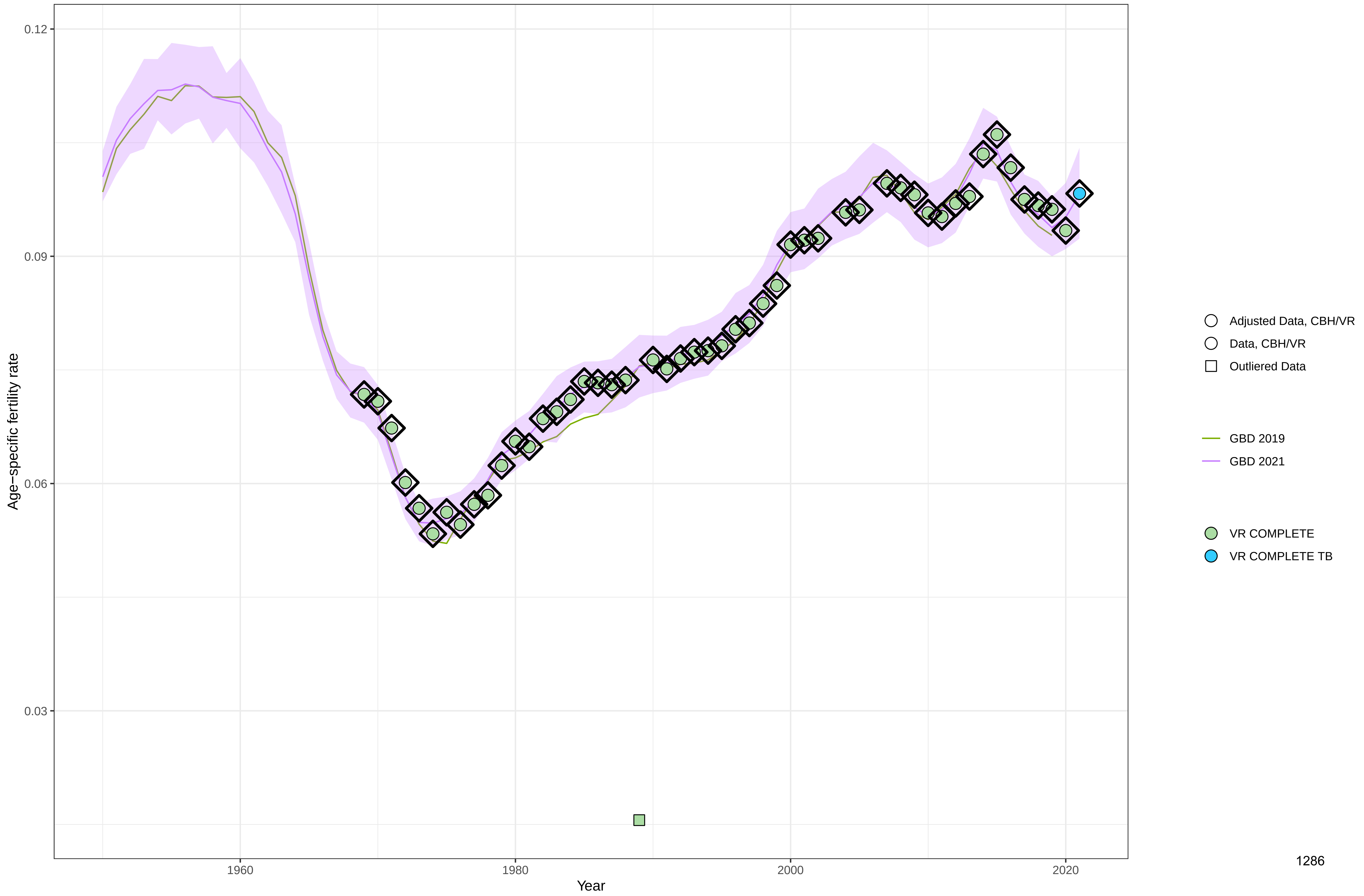

Age−specific fertility fitted trend lines, 1950−2021: Texas, Ages: 35−39
Data points enclosed with a diamond are reference sources

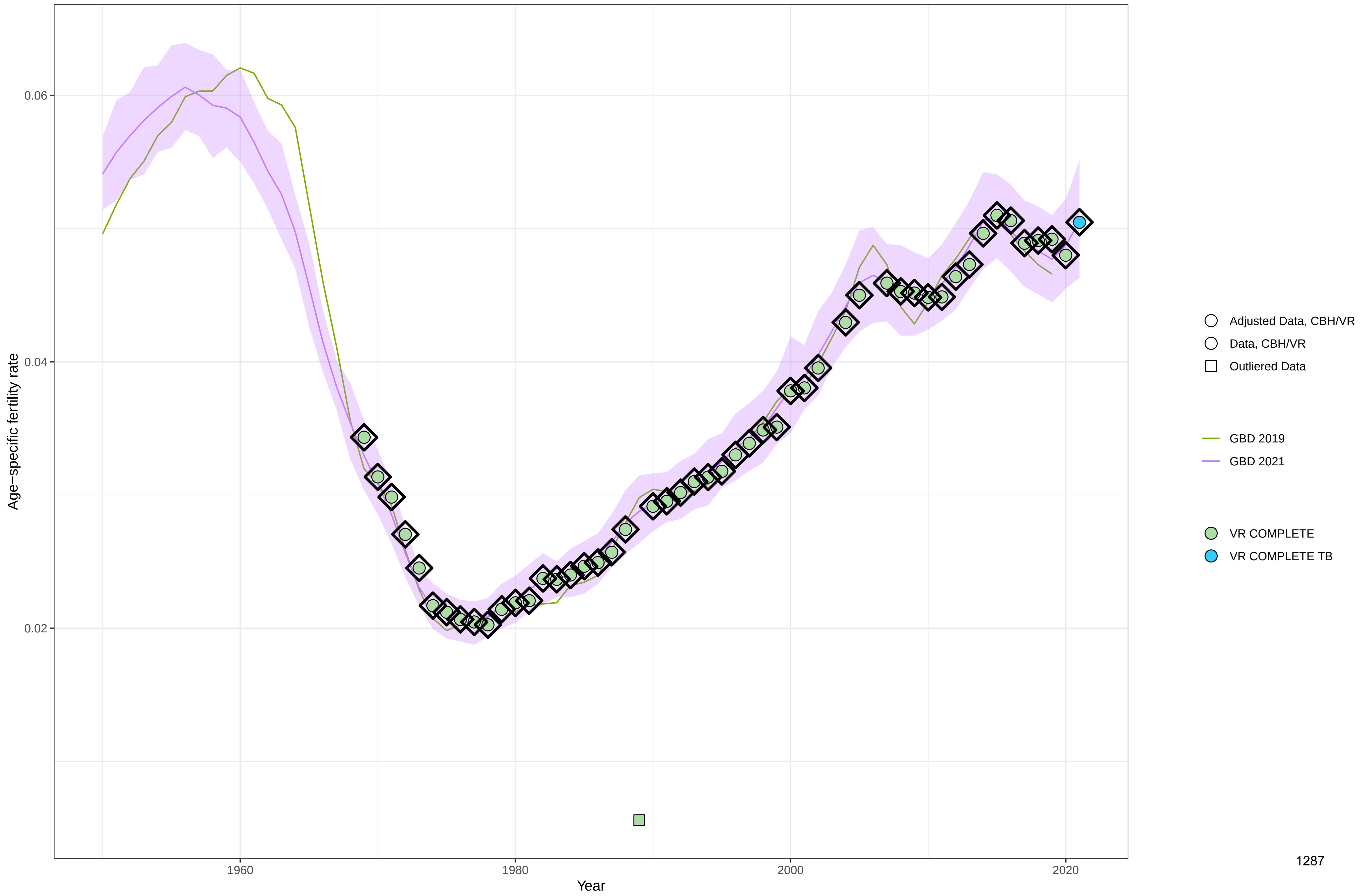

Age−specific fertility fitted trend lines, 1950−2021: Texas, Ages: 40−44
Data points enclosed with a diamond are reference sources

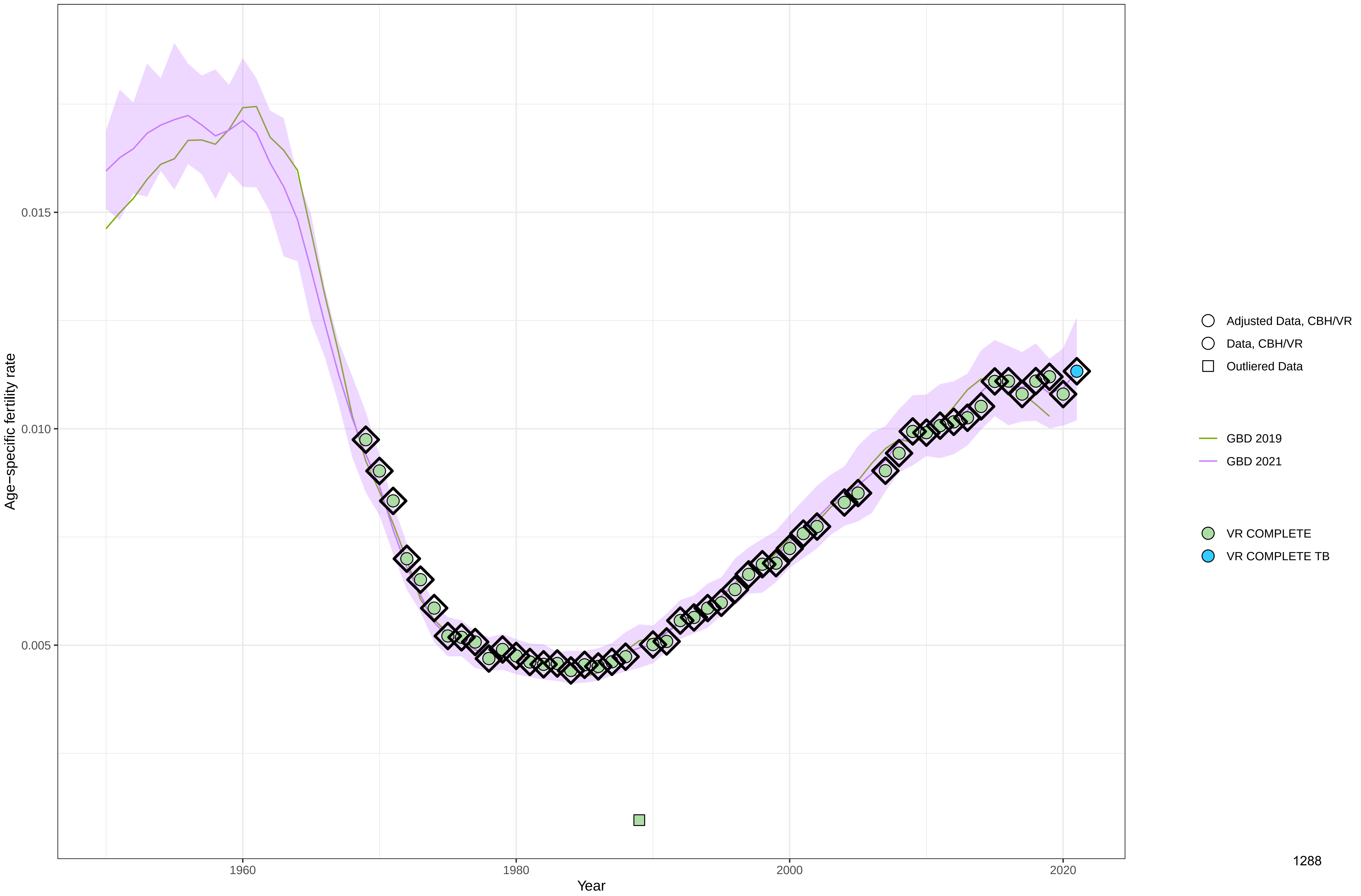

Age−specific fertility fitted trend lines, 1950−2021: Texas, Ages: 45−49
Data points enclosed with a diamond are reference sources

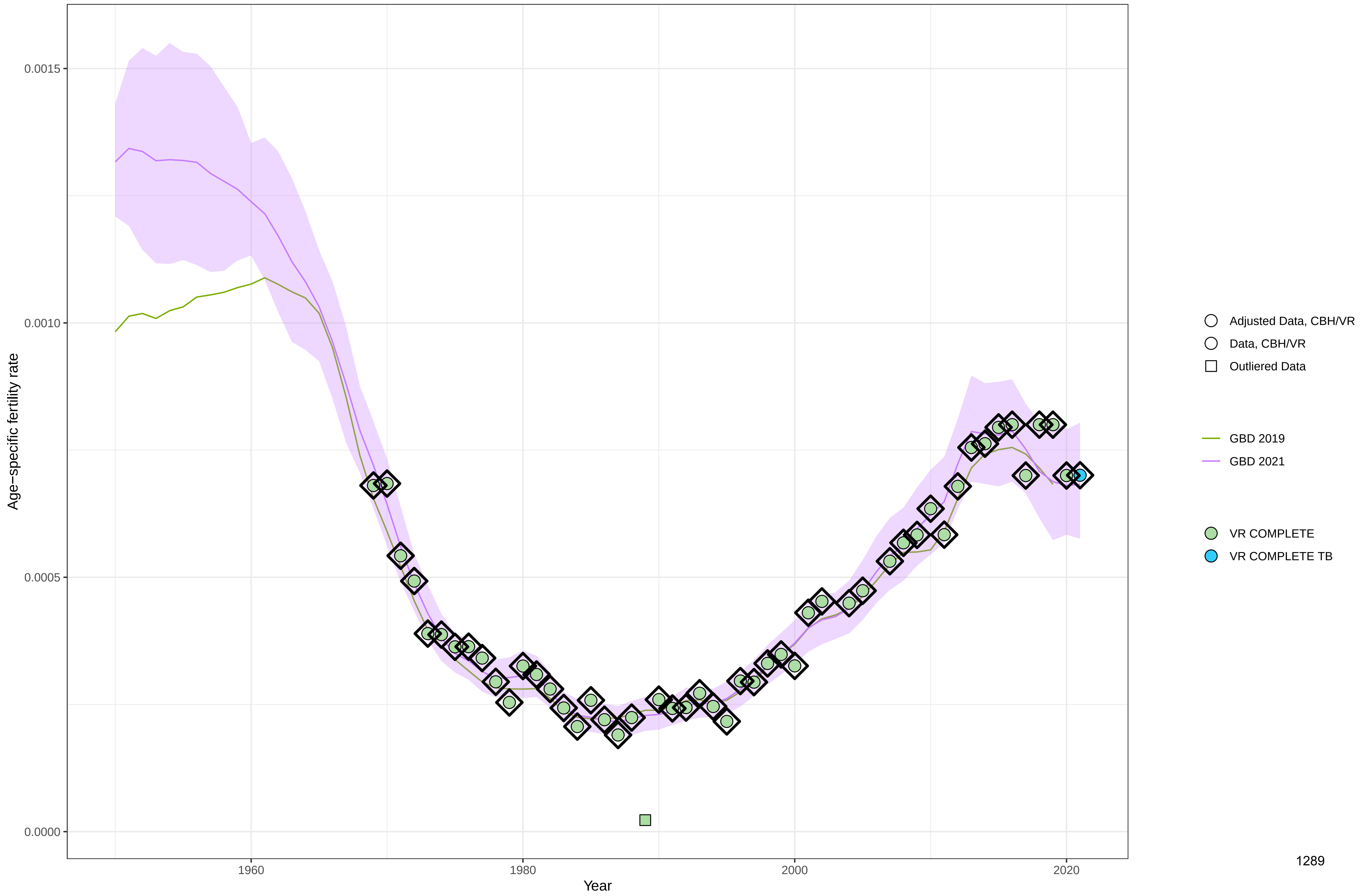

Age−specific fertility fitted trend lines, 1950−2021: Texas, Ages: 50−54
Data points enclosed with a diamond are reference sources

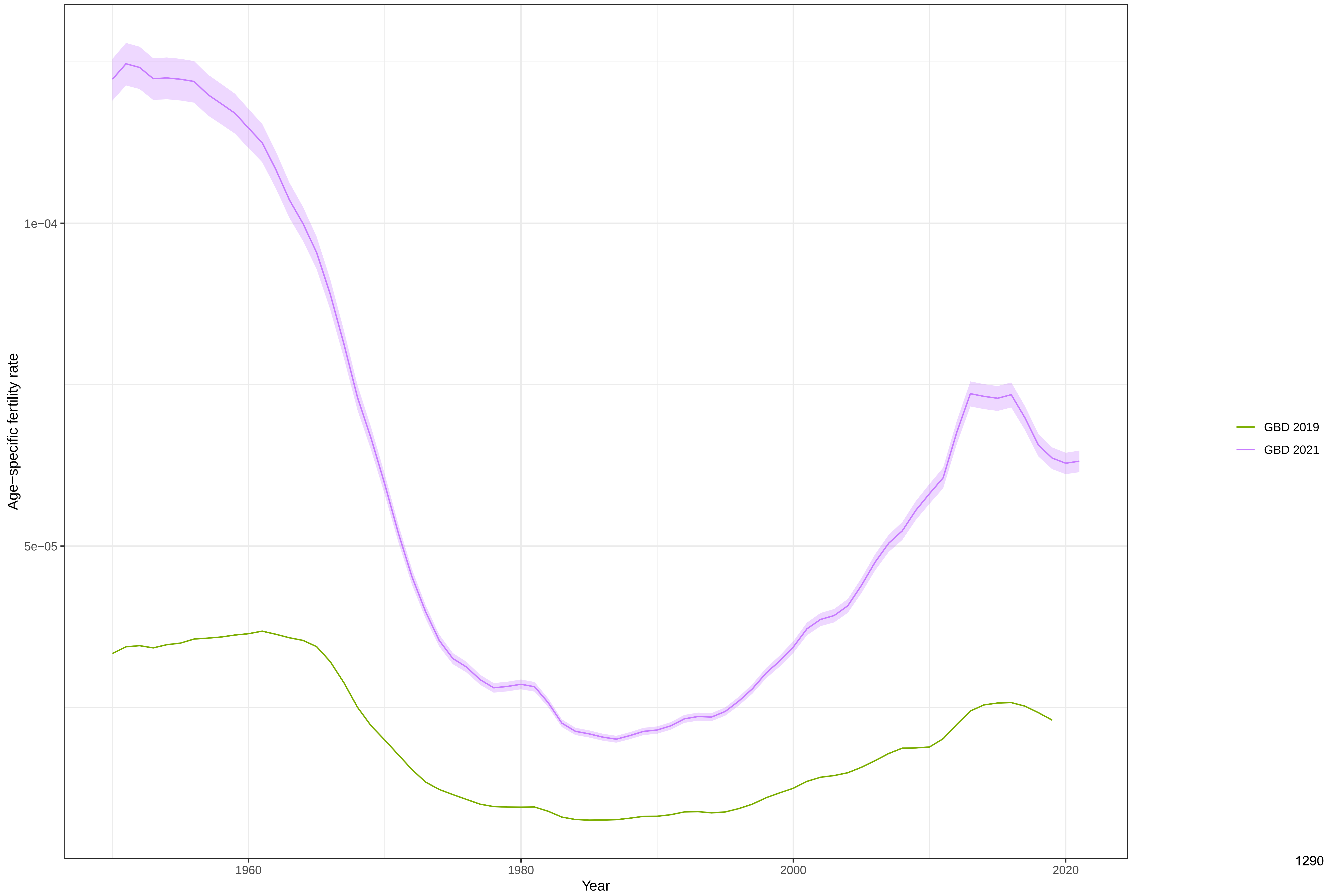

Total fertility rate fitted trend lines, 1950−2021: Texas TFR
Data points enclosed with a diamond are reference sources

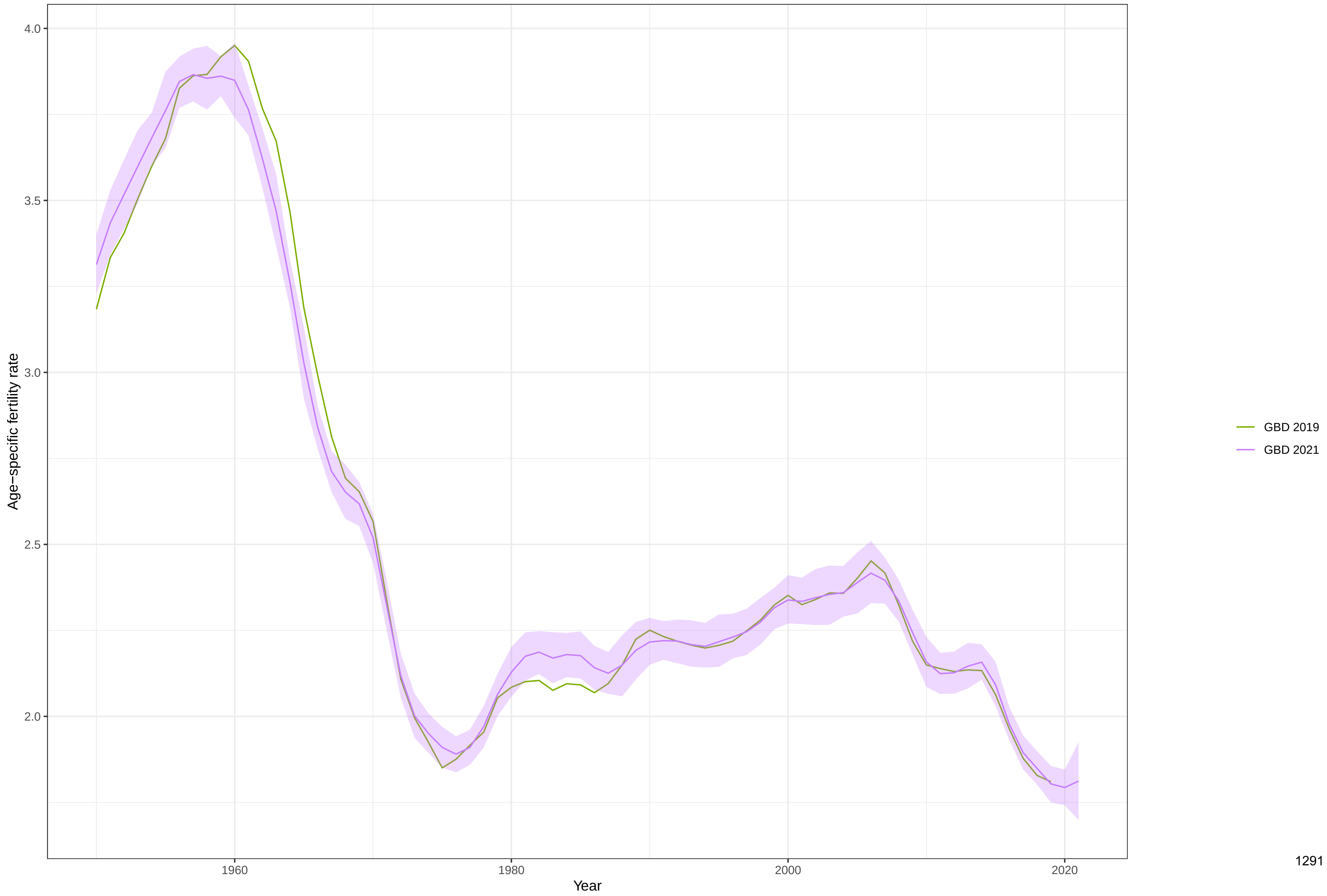

Age−specific fertility fitted trend lines, 1950−2021: Utah, Ages: 10−14
Data points enclosed with a diamond are reference sources

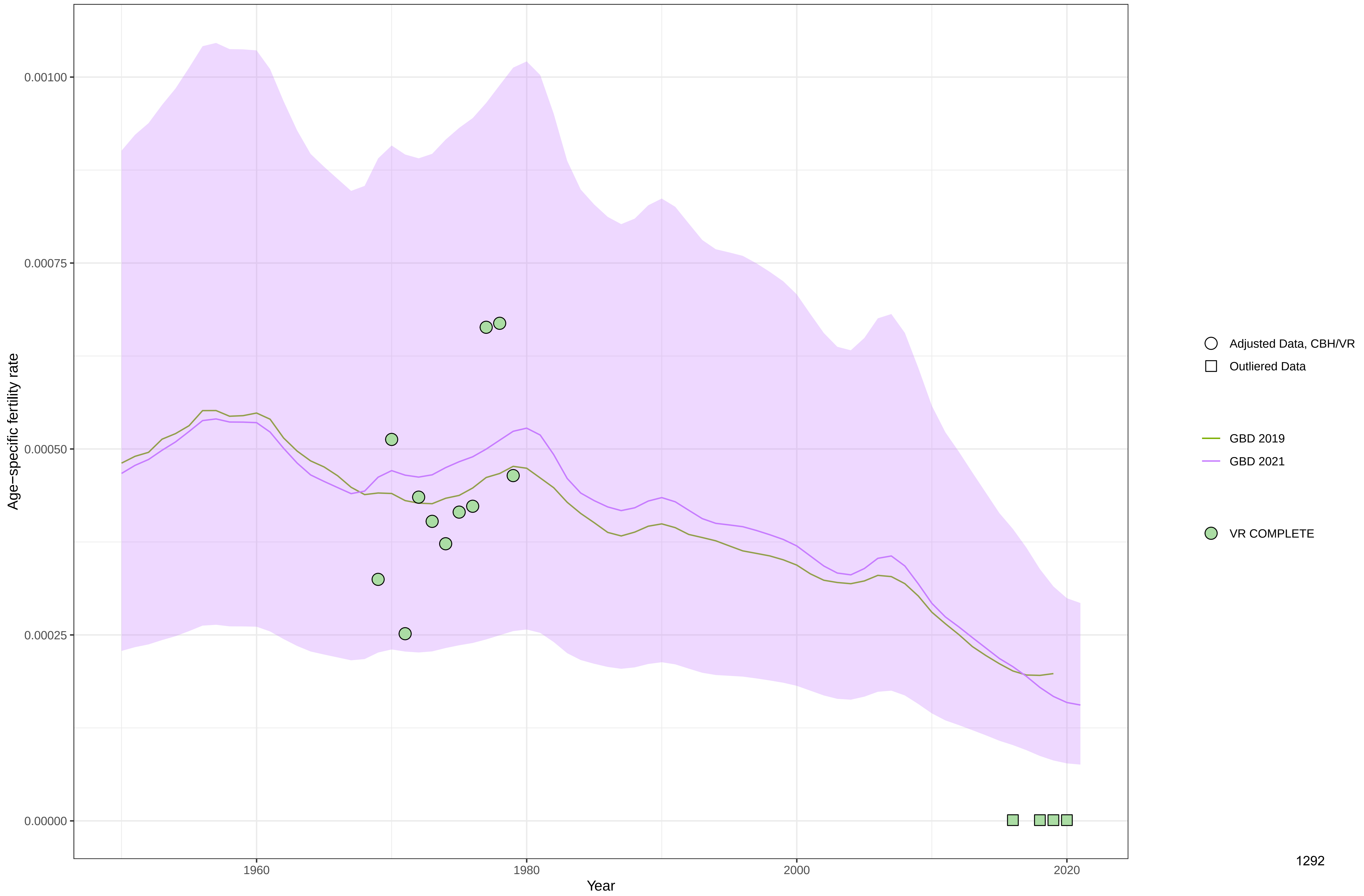

Age−specific fertility fitted trend lines, 1950−2021: Utah, Ages: 15−19
Data points enclosed with a diamond are reference sources

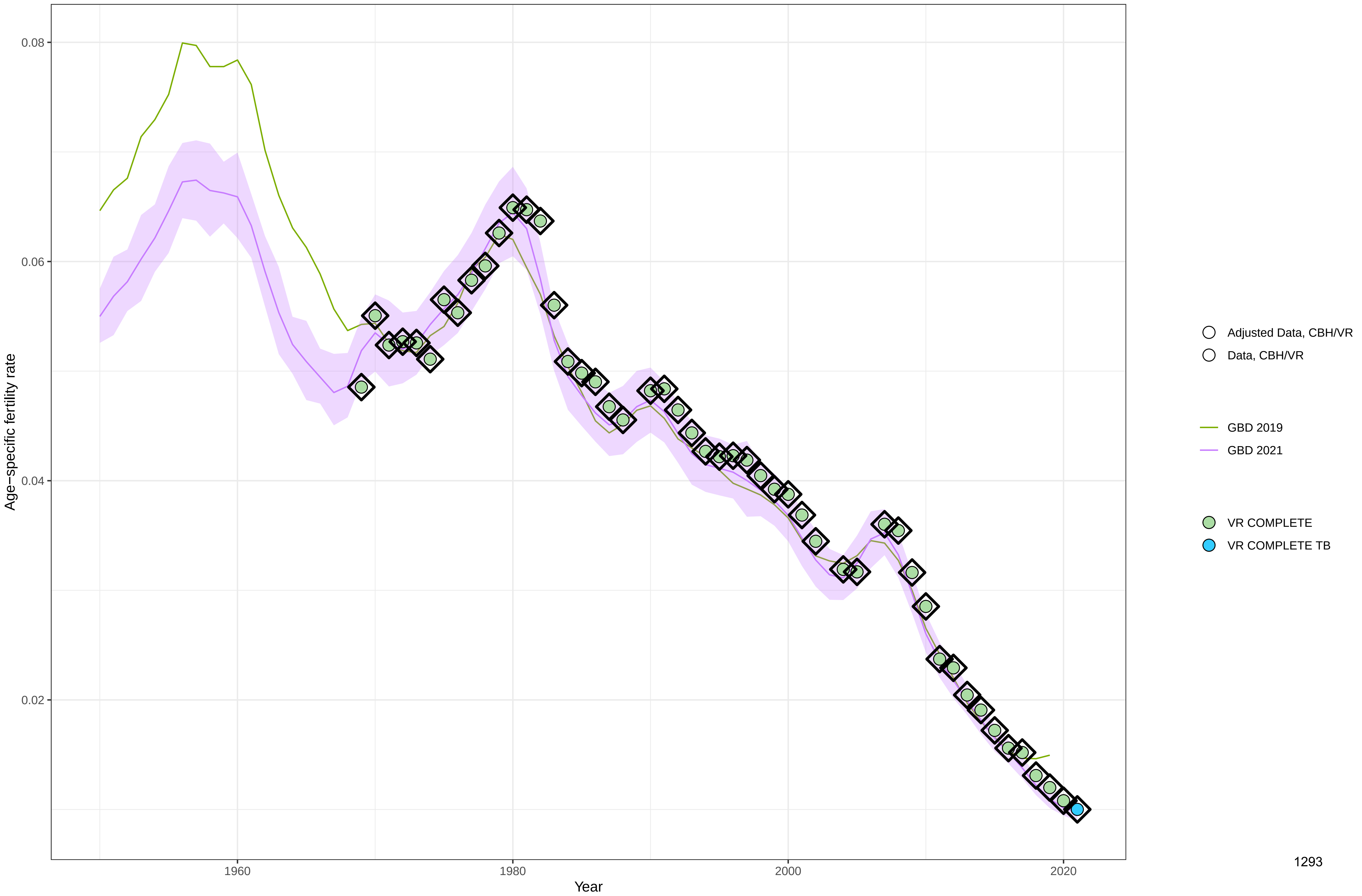

Age−specific fertility fitted trend lines, 1950−2021: Utah, Ages: 20−24
Data points enclosed with a diamond are reference sources

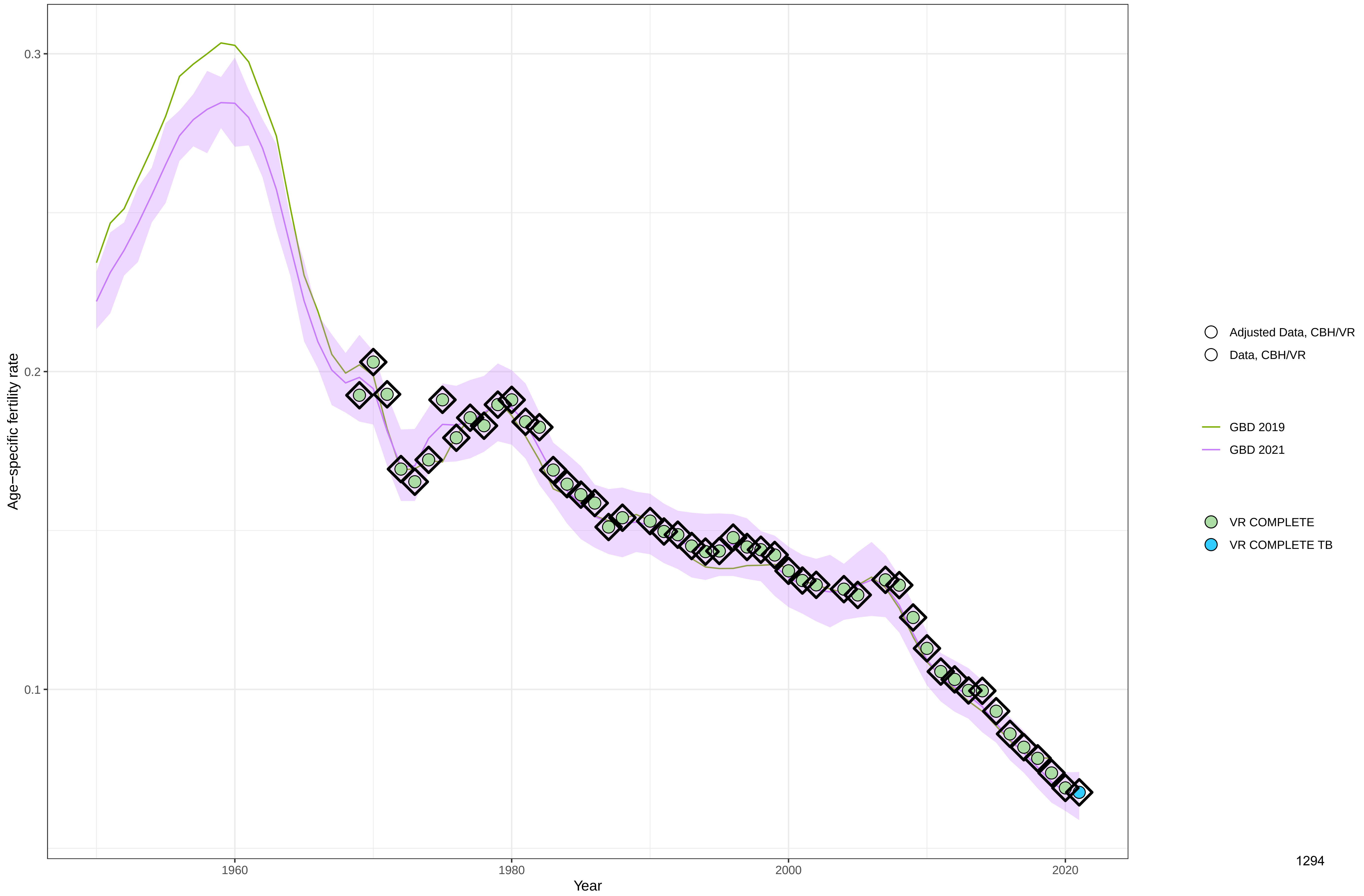

Age−specific fertility fitted trend lines, 1950−2021: Utah, Ages: 25−29
Data points enclosed with a diamond are reference sources

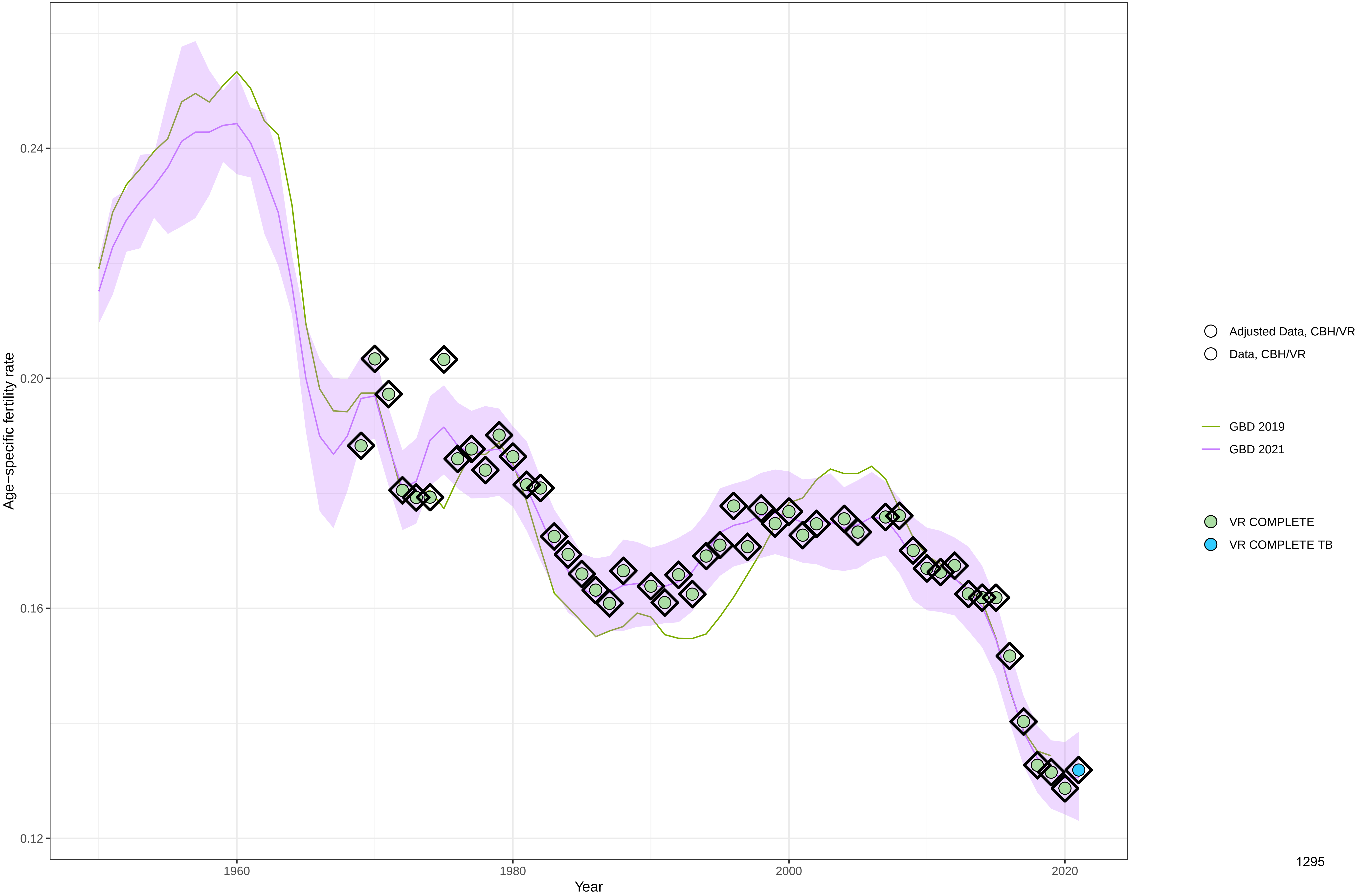

Age−specific fertility fitted trend lines, 1950−2021: Utah, Ages: 30−34
Data points enclosed with a diamond are reference sources

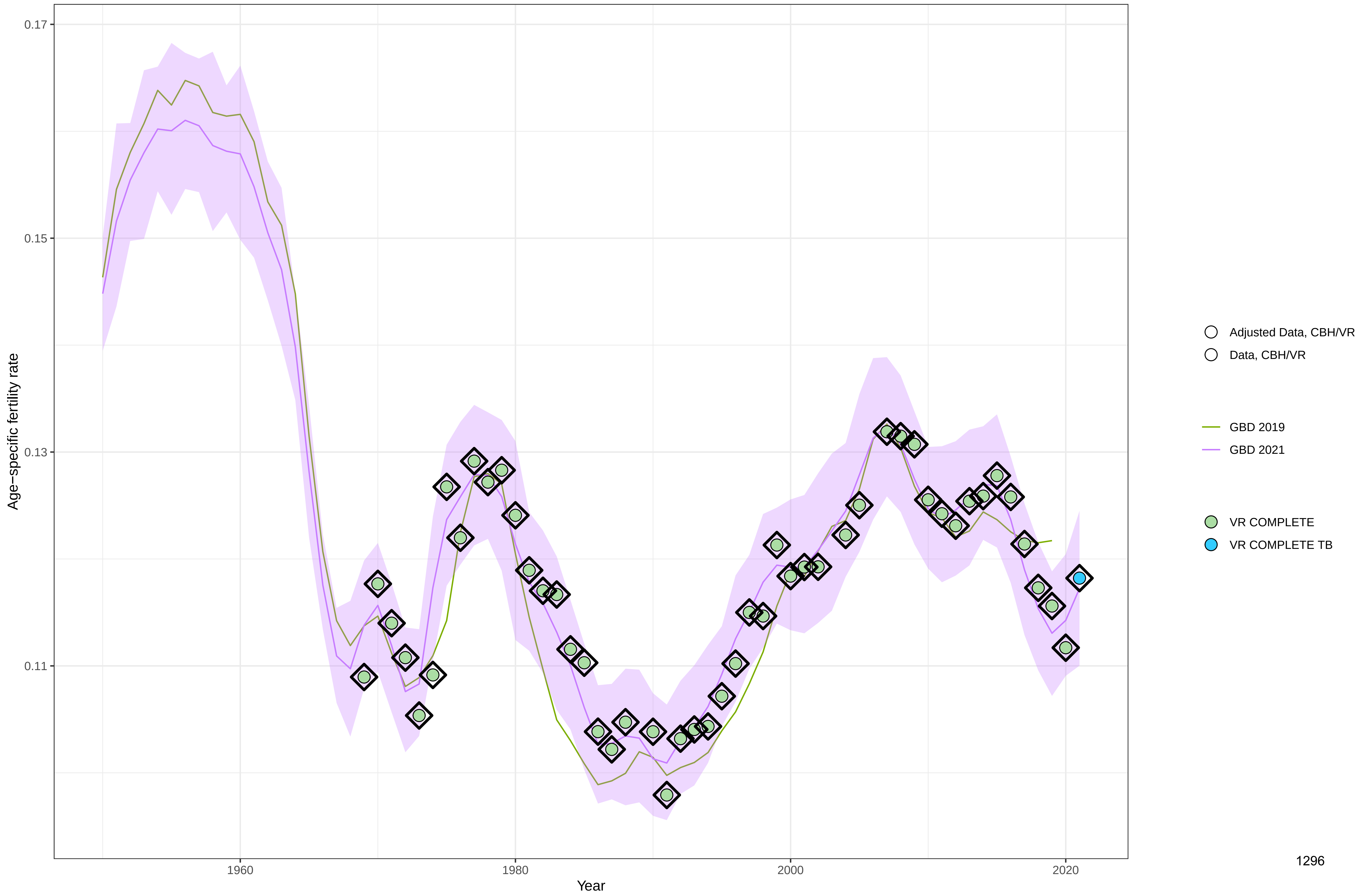

Age−specific fertility fitted trend lines, 1950−2021: Utah, Ages: 35−39
Data points enclosed with a diamond are reference sources

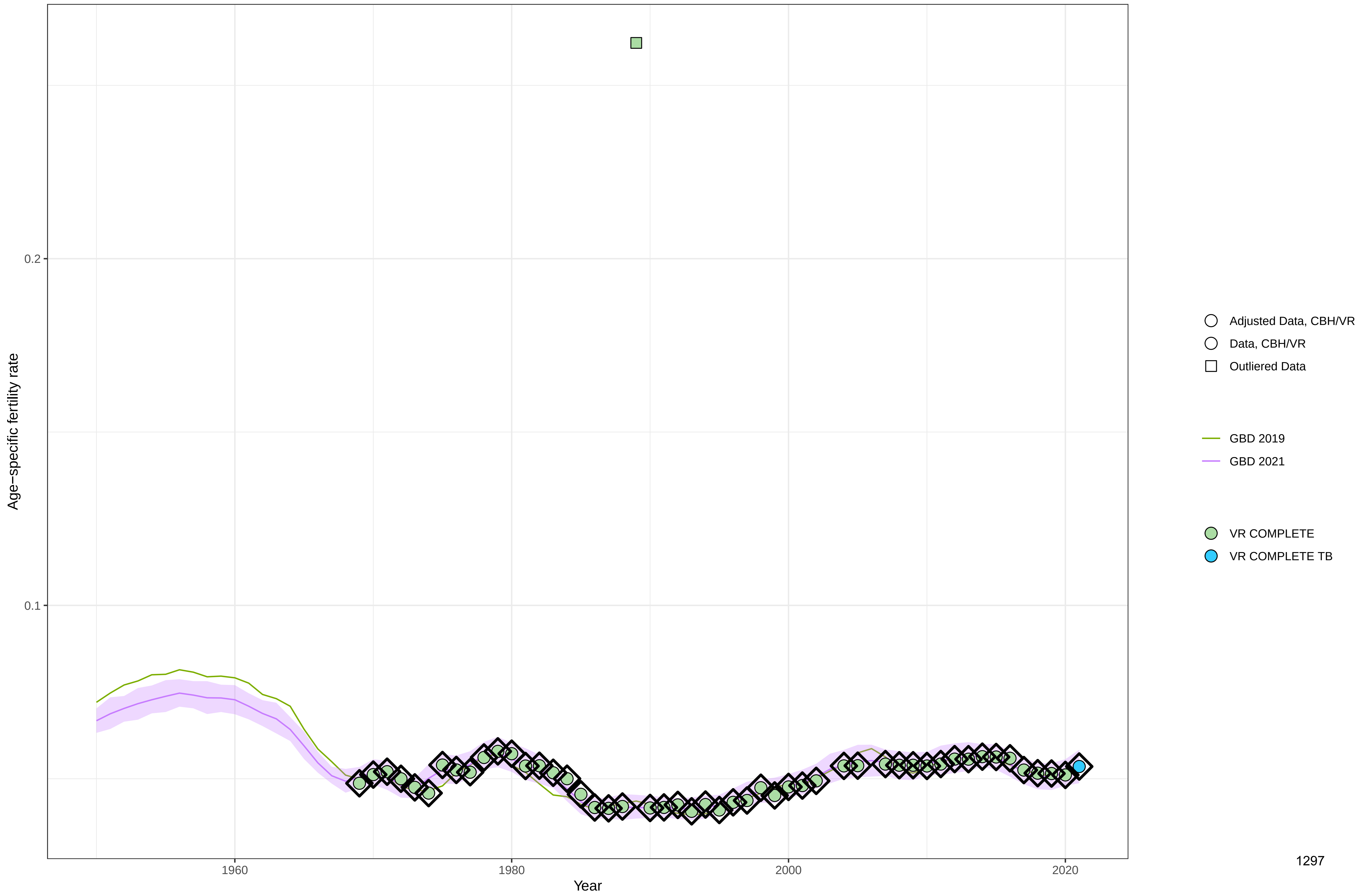

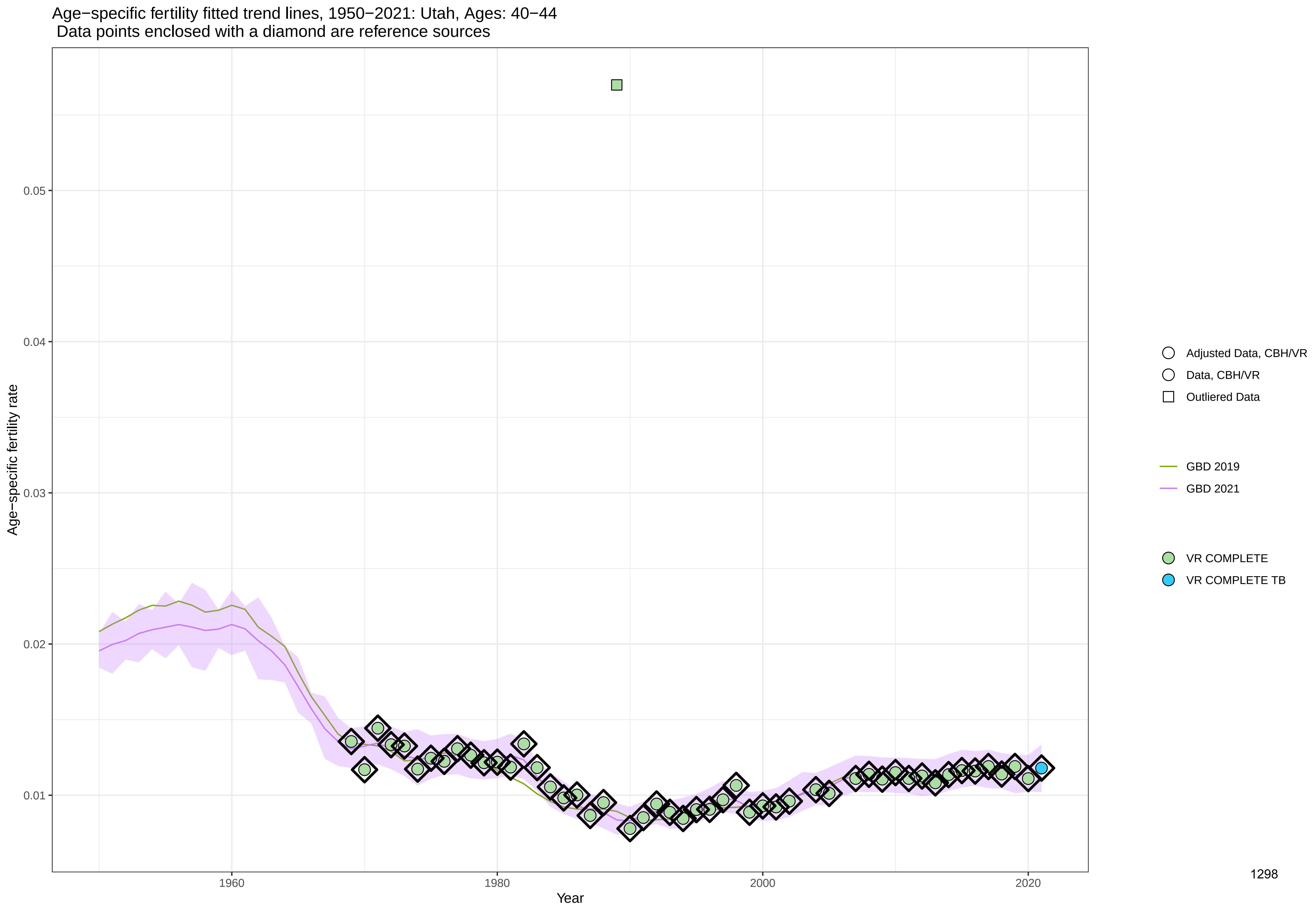
Age−specific fertility fitted trend lines, 1950−2021: Utah, Ages: 40−44
Data points enclosed with a diamond are reference sources
Age−specific fertility rate
0.05
0.04
0.03
0.02
0.01
1960
1980
2000
2020
Year
Adjusted Data, CBH/VR
Data, CBH/VR
Outliered Data
GBD 2019
GBD 2021
VR COMPLETE
VR COMPLETE TB

Age−specific fertility fitted trend lines, 1950−2021: Utah, Ages: 45−49
Data points enclosed with a diamond are reference sources

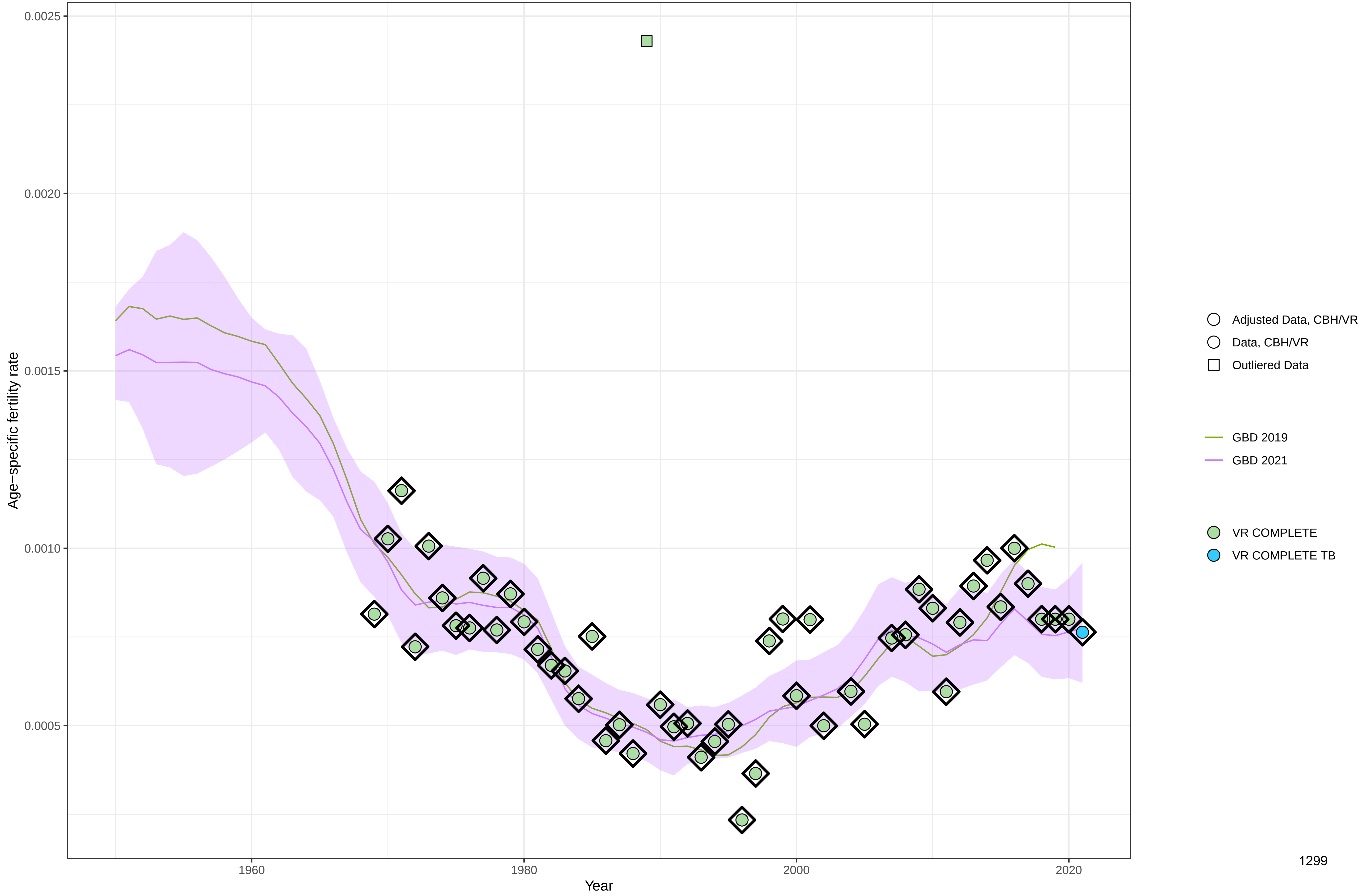

Age−specific fertility fitted trend lines, 1950−2021: Utah, Ages: 50−54
Data points enclosed with a diamond are reference sources

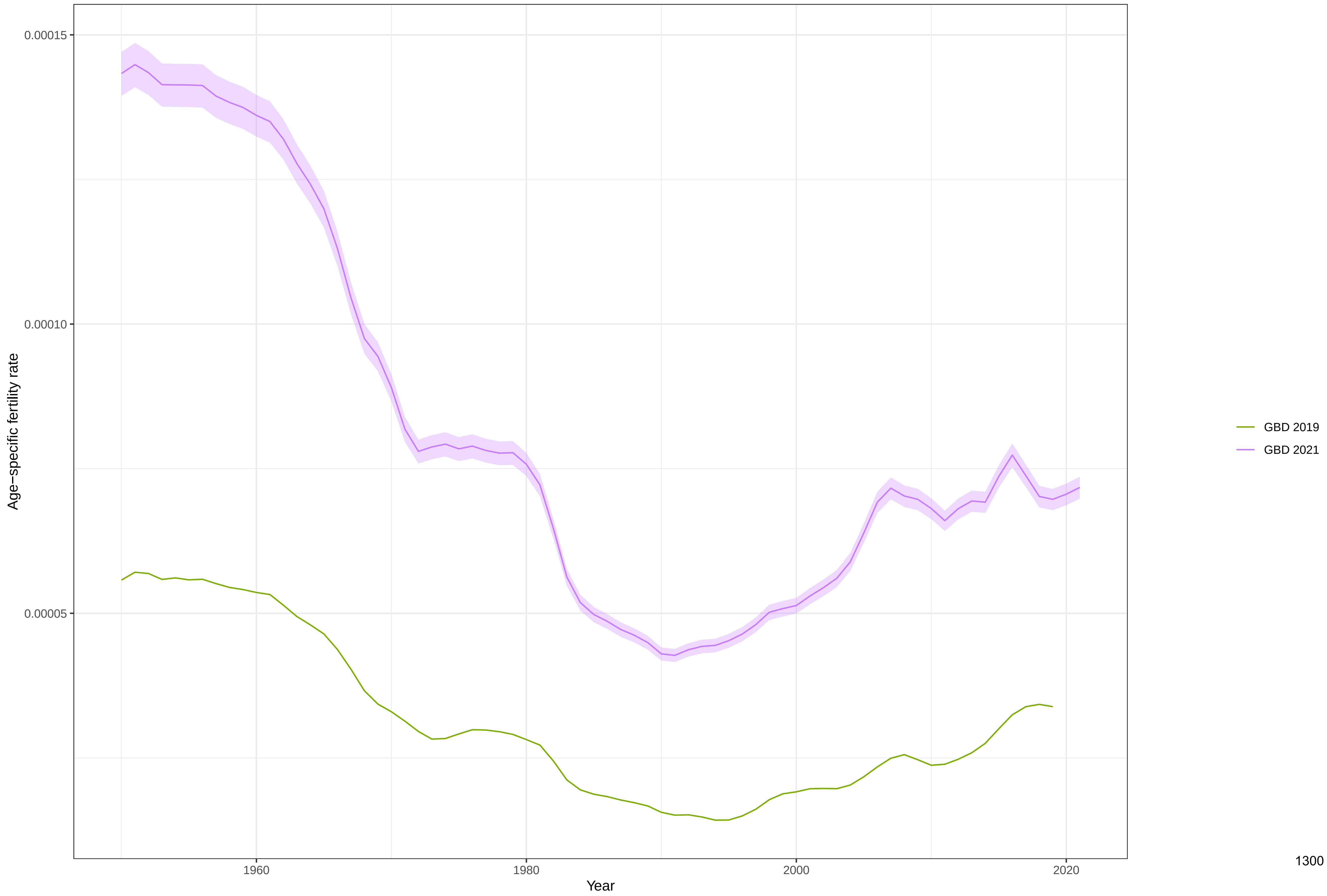

Total fertility rate fitted trend lines, 1950−2021: Utah TFR
Data points enclosed with a diamond are reference sources

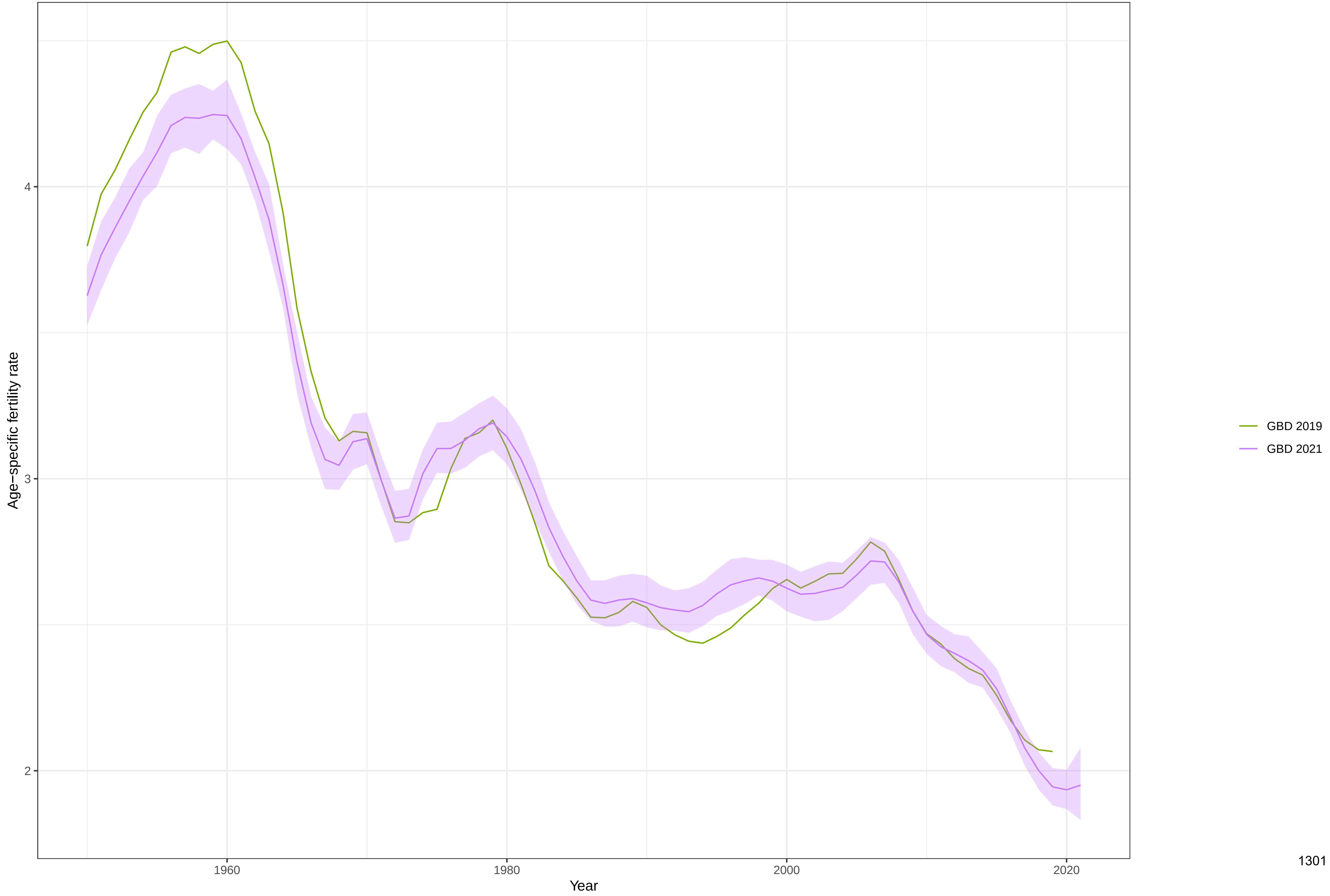

Age−specific fertility fitted trend lines, 1950−2021: Vermont, Ages: 10−14
Data points enclosed with a diamond are reference sources

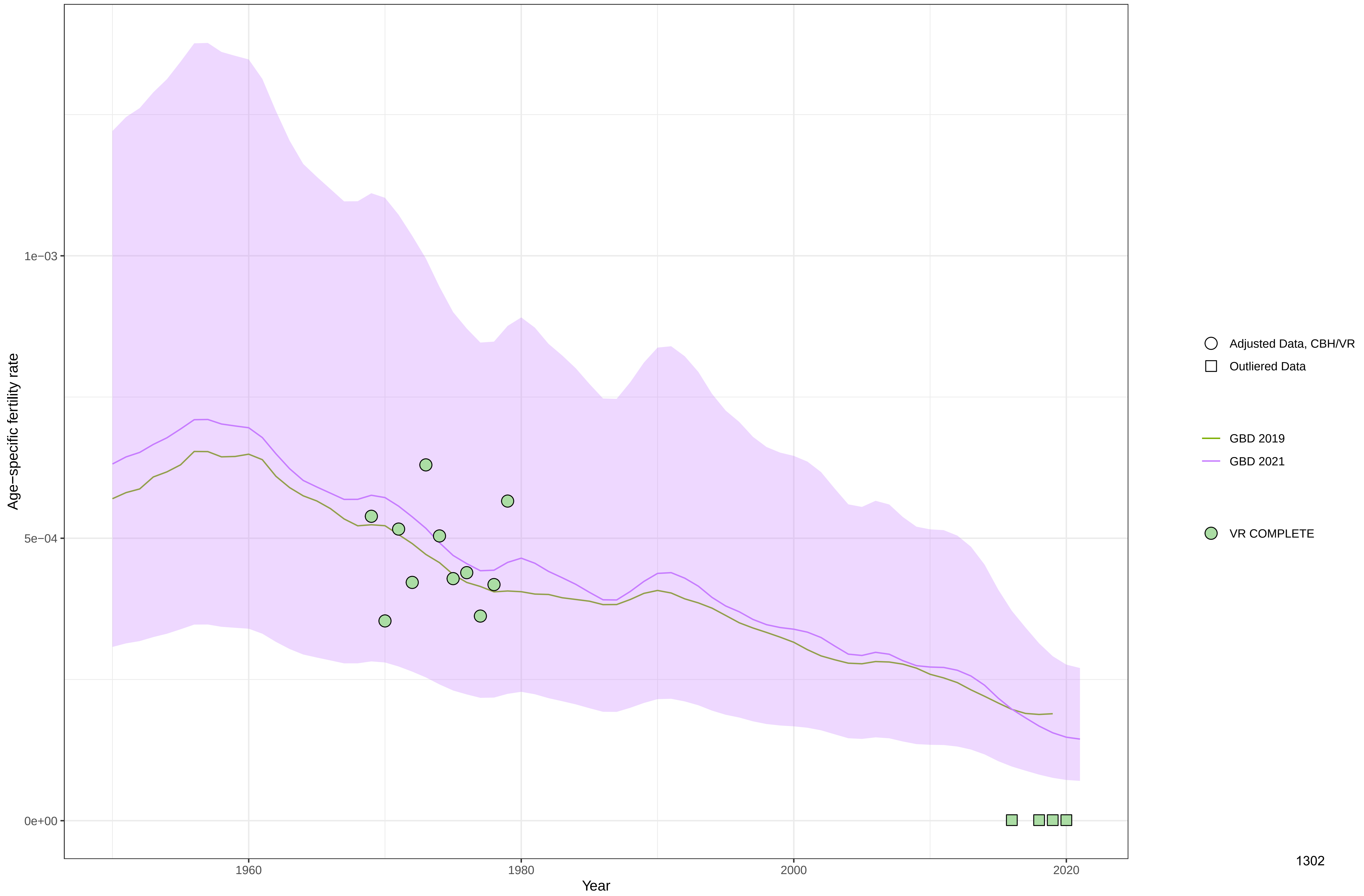

Age−specific fertility fitted trend lines, 1950−2021: Vermont, Ages: 15−19
Data points enclosed with a diamond are reference sources

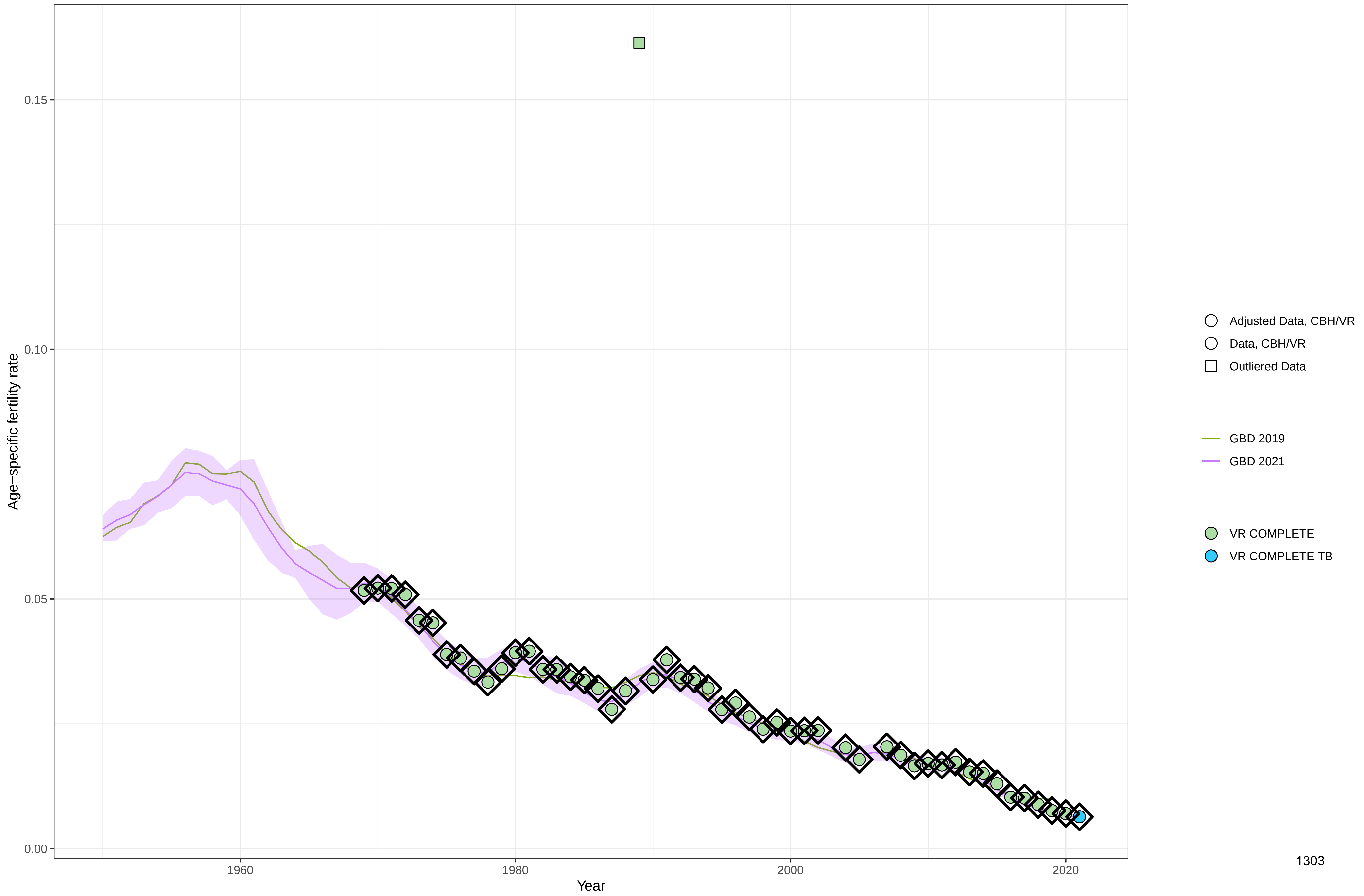

Age−specific fertility fitted trend lines, 1950−2021: Vermont, Ages: 20−24
Data points enclosed with a diamond are reference sources

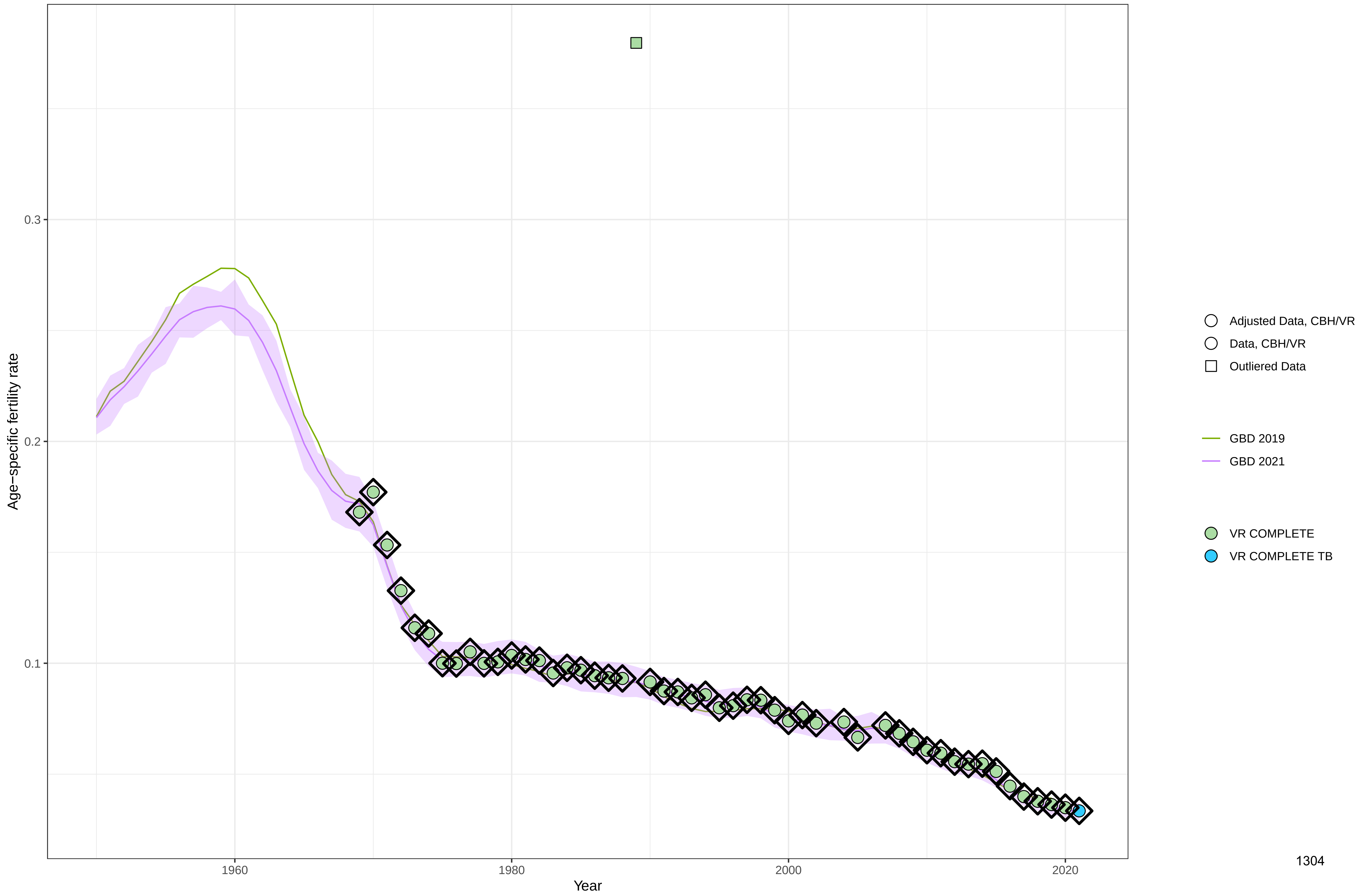

Age−specific fertility fitted trend lines, 1950−2021: Vermont, Ages: 25−29
Data points enclosed with a diamond are reference sources

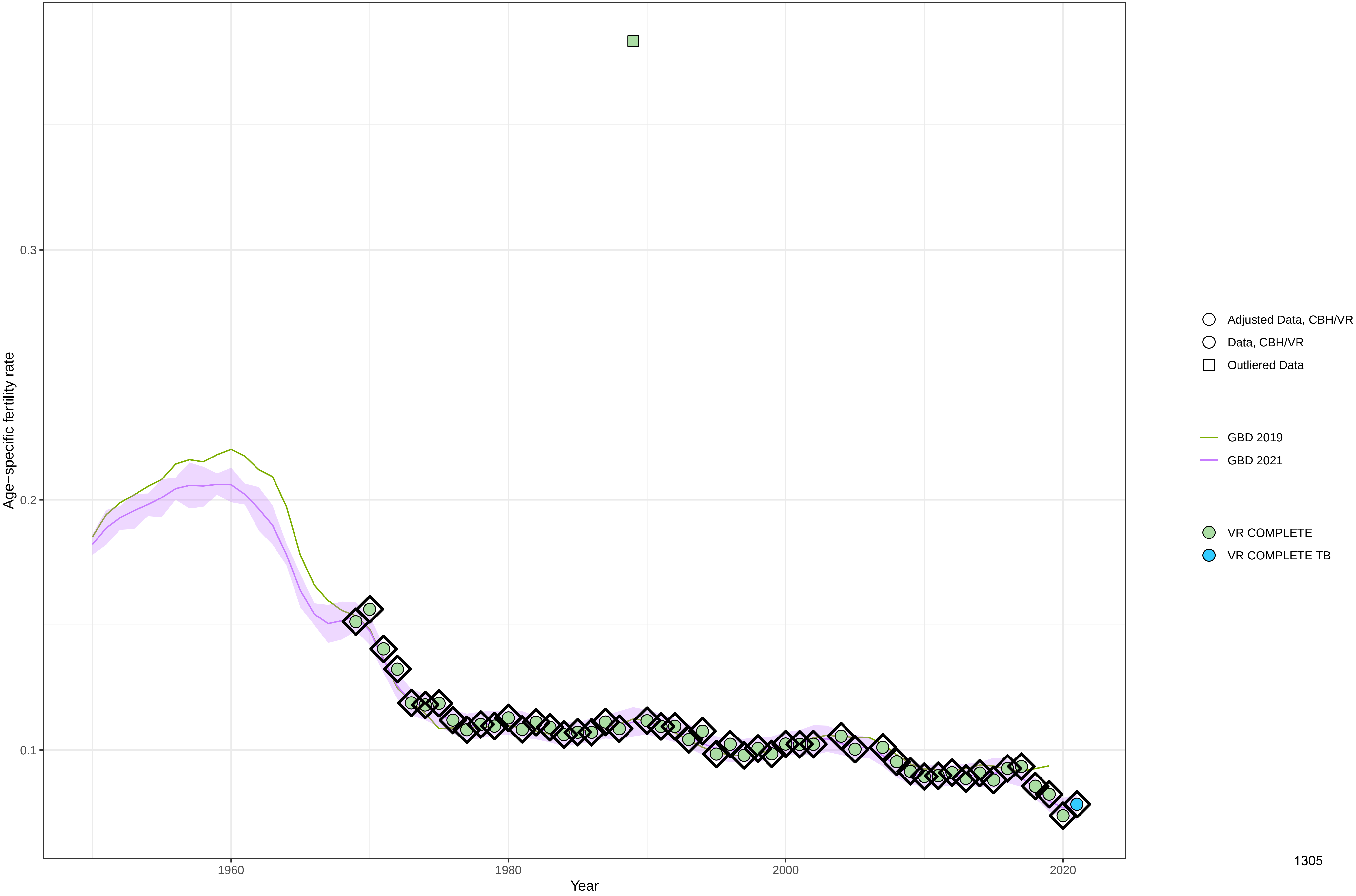

Age−specific fertility fitted trend lines, 1950−2021: Vermont, Ages: 30−34
Data points enclosed with a diamond are reference sources

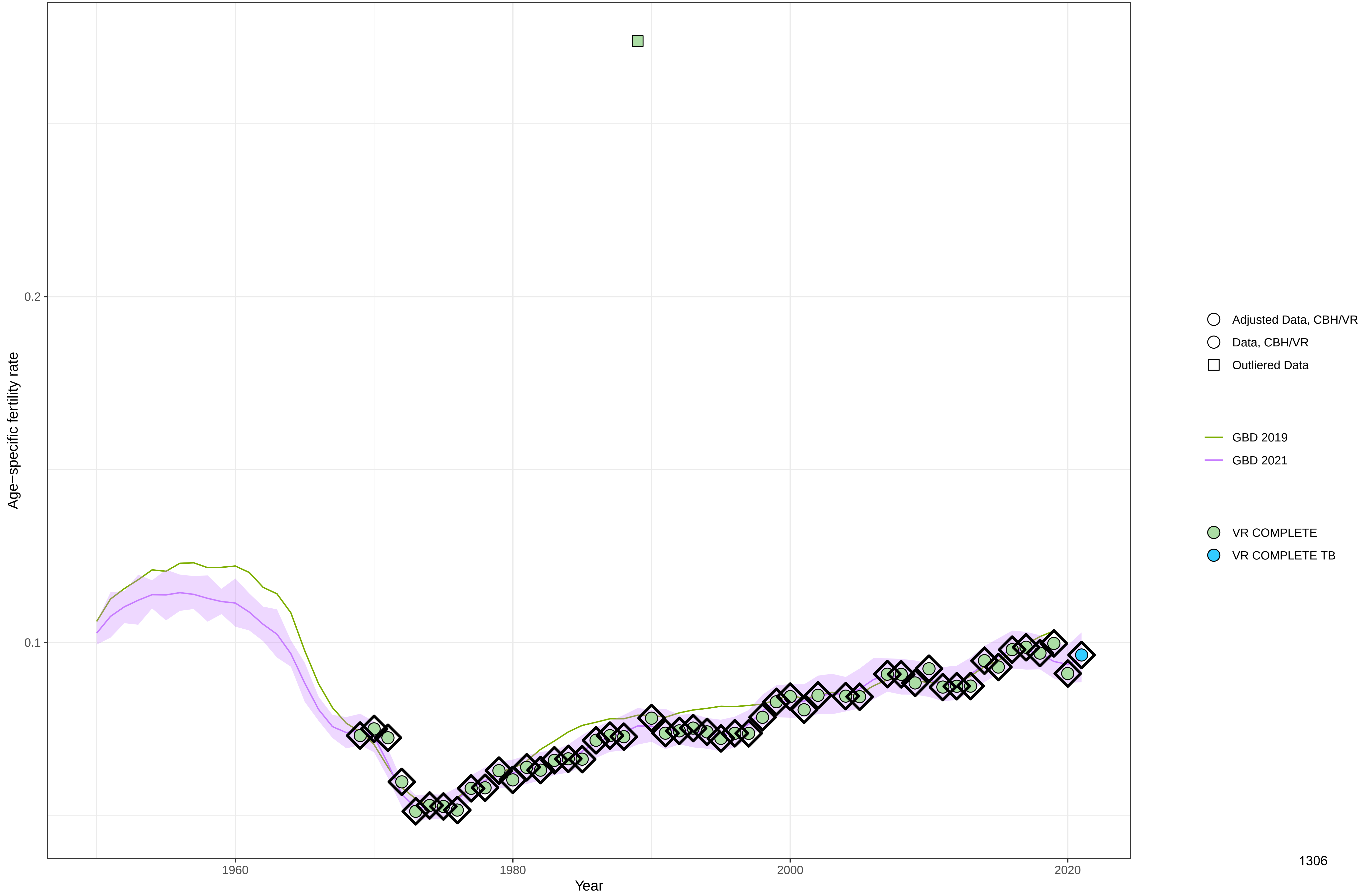

Age−specific fertility fitted trend lines, 1950−2021: Vermont, Ages: 35−39
Data points enclosed with a diamond are reference sources

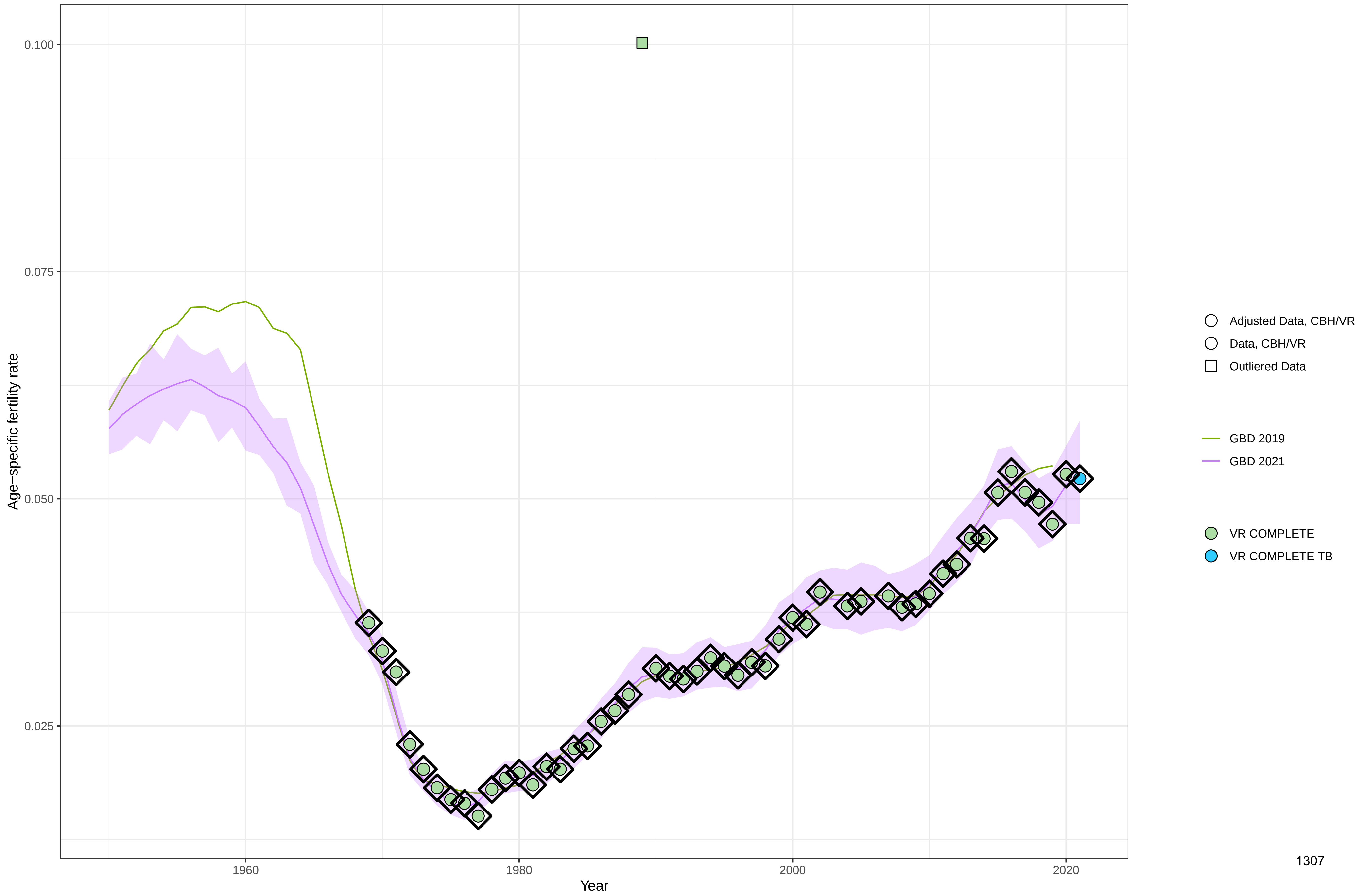

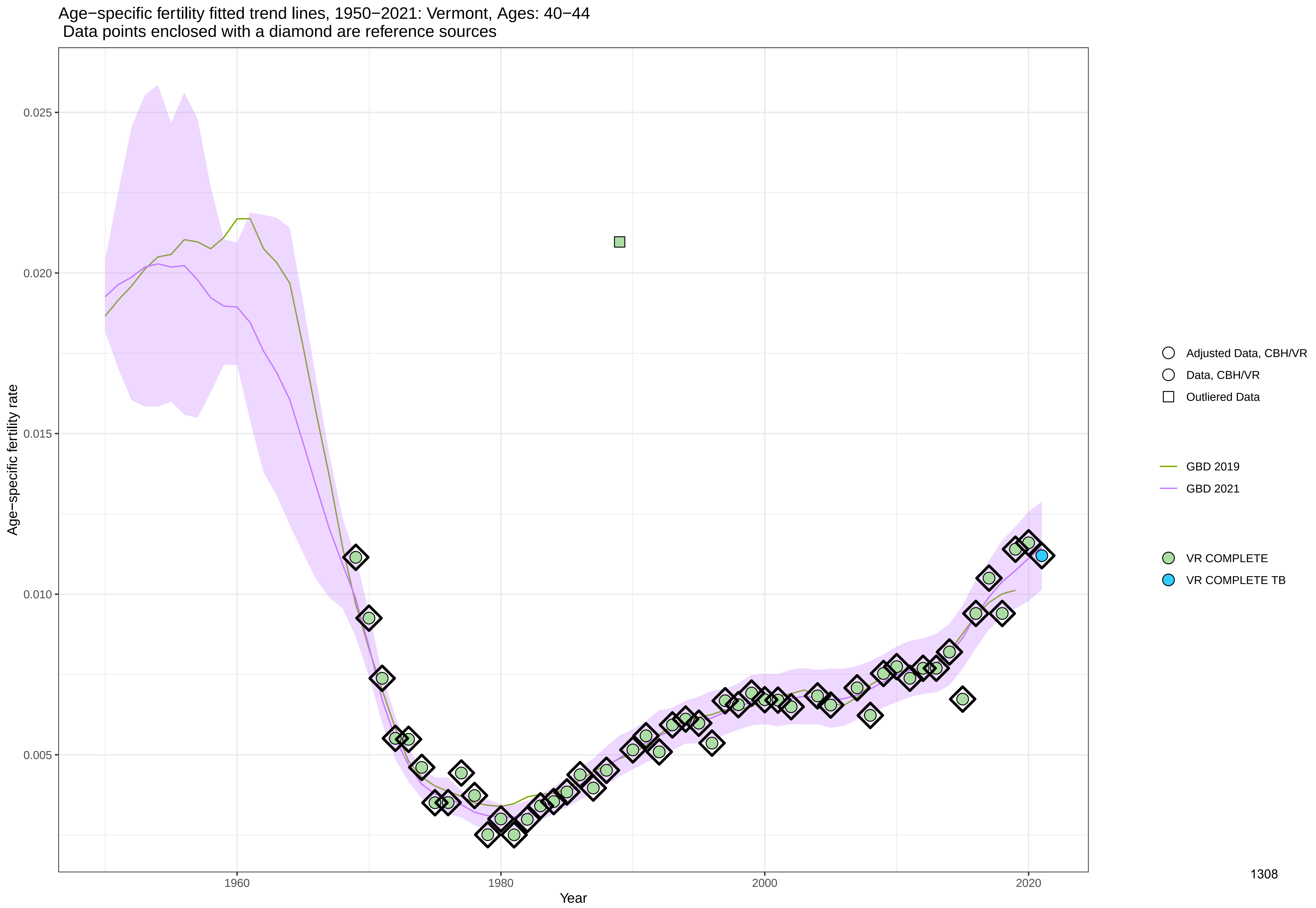
Age−specific fertility fitted trend lines, 1950−2021: Vermont, Ages: 40−44
Data points enclosed with a diamond are reference sources
Age−specific fertility rate
0.025
0.020
0.015
0.010
0.005
1960
1980
2000
2020
Year
Adjusted Data, CBH/VR
Data, CBH/VR
Outliered Data
GBD 2019
GBD 2021
VR COMPLETE
VR COMPLETE TB

Age−specific fertility fitted trend lines, 1950−2021: Vermont, Ages: 45−49
Data points enclosed with a diamond are reference sources

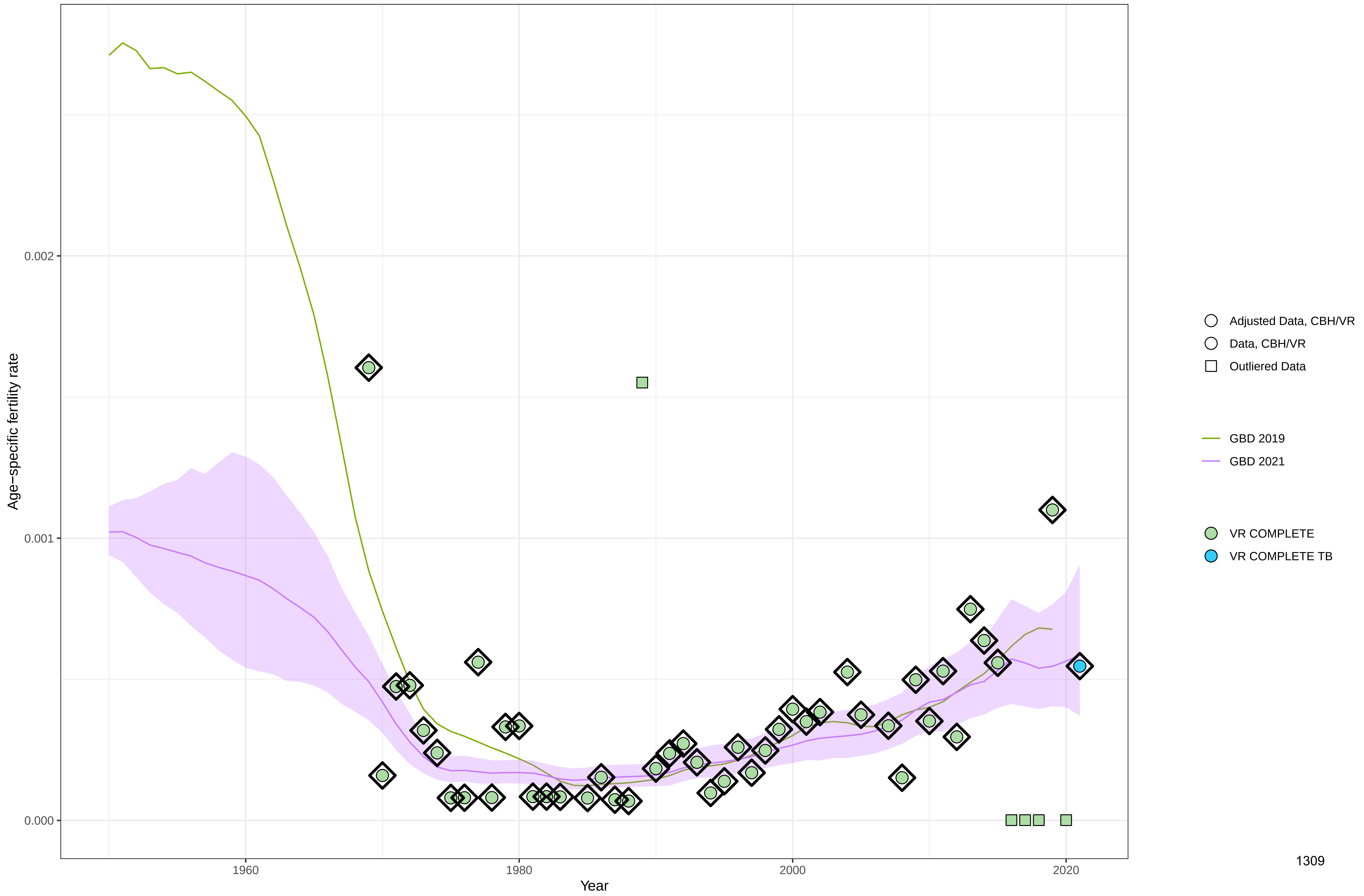

Age−specific fertility fitted trend lines, 1950−2021: Vermont, Ages: 50−54
Data points enclosed with a diamond are reference sources

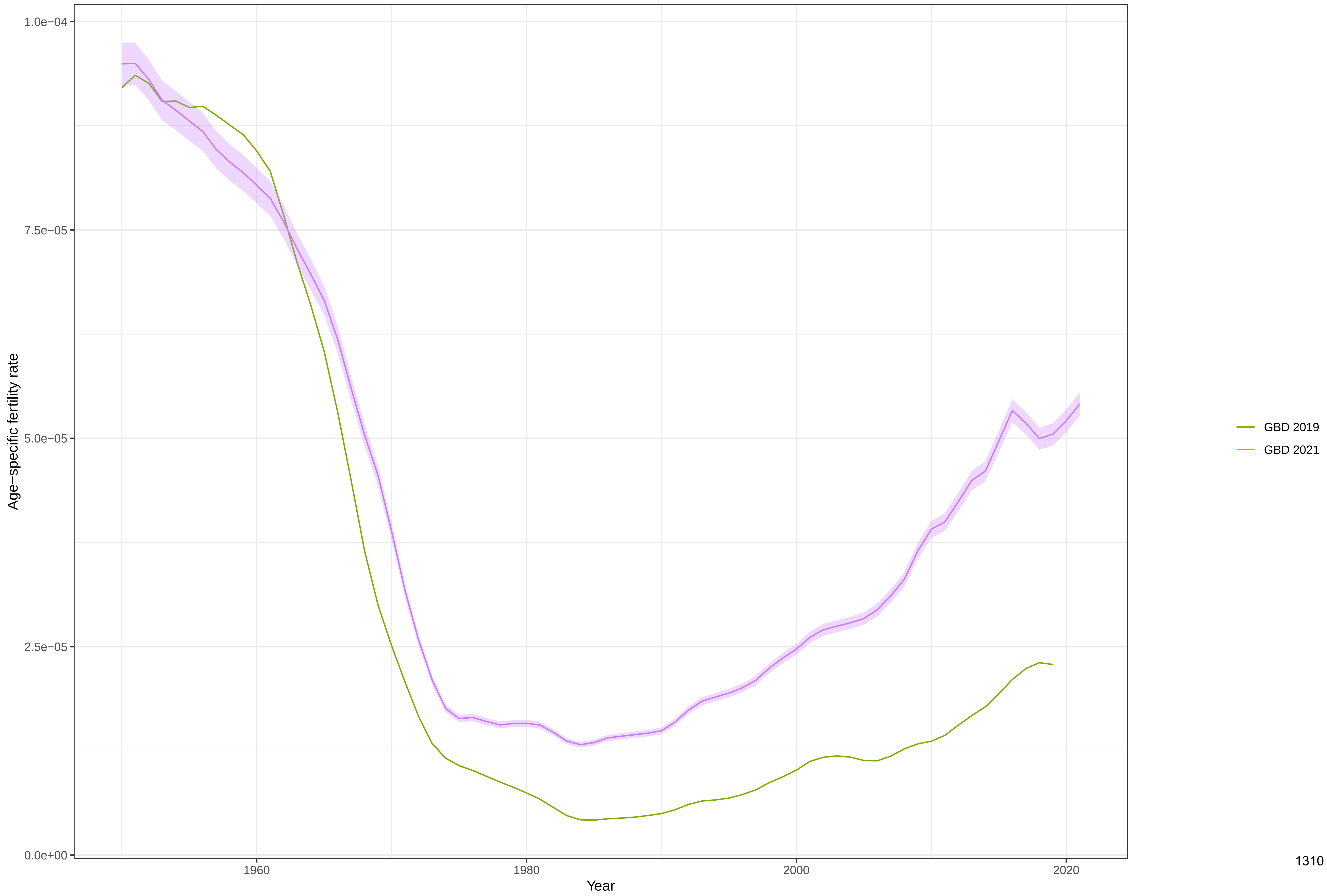

Total fertility rate fitted trend lines, 1950−2021: Vermont TFR
Data points enclosed with a diamond are reference sources

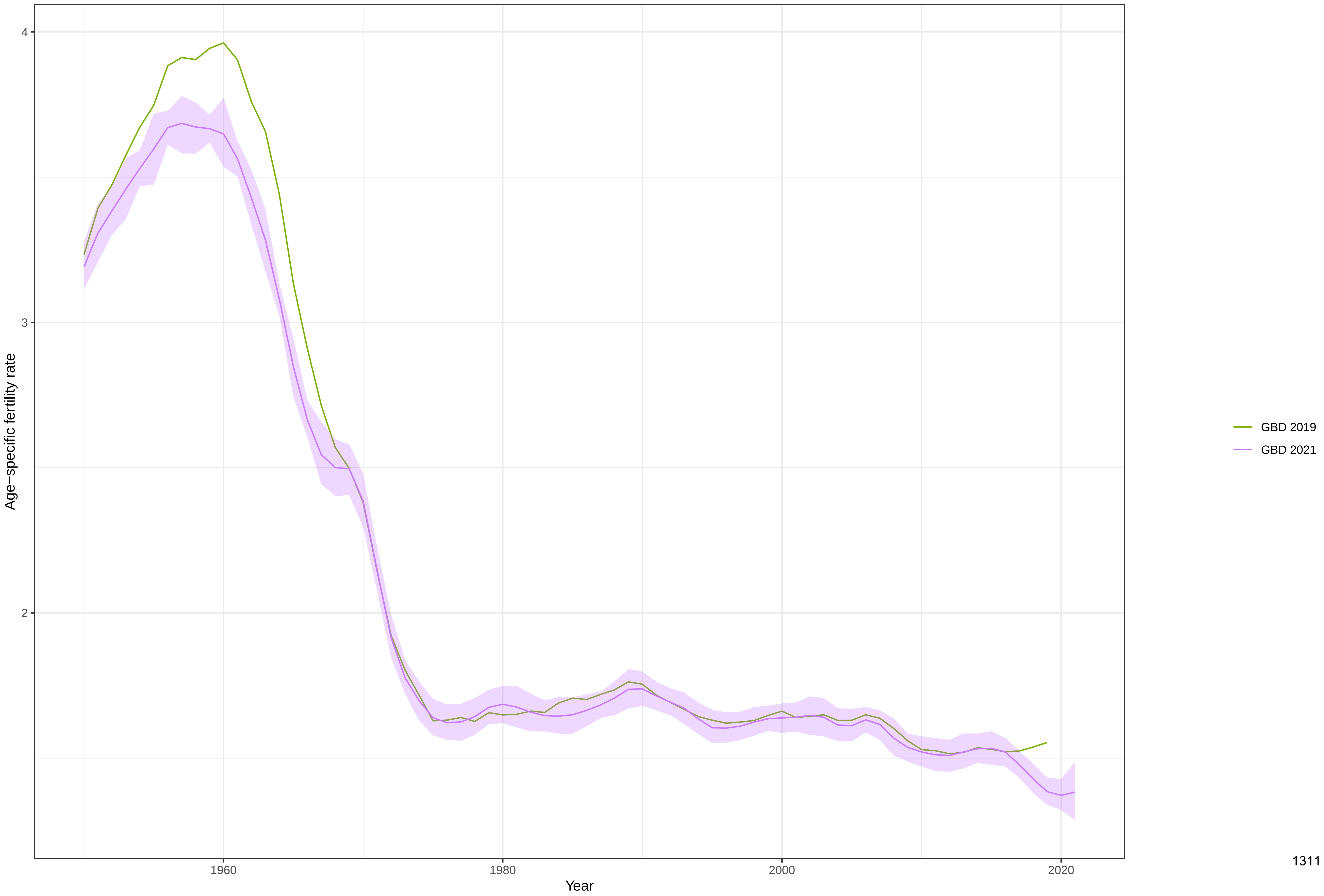

Age−specific fertility fitted trend lines, 1950−2021: Virginia, Ages: 10−14
Data points enclosed with a diamond are reference sources

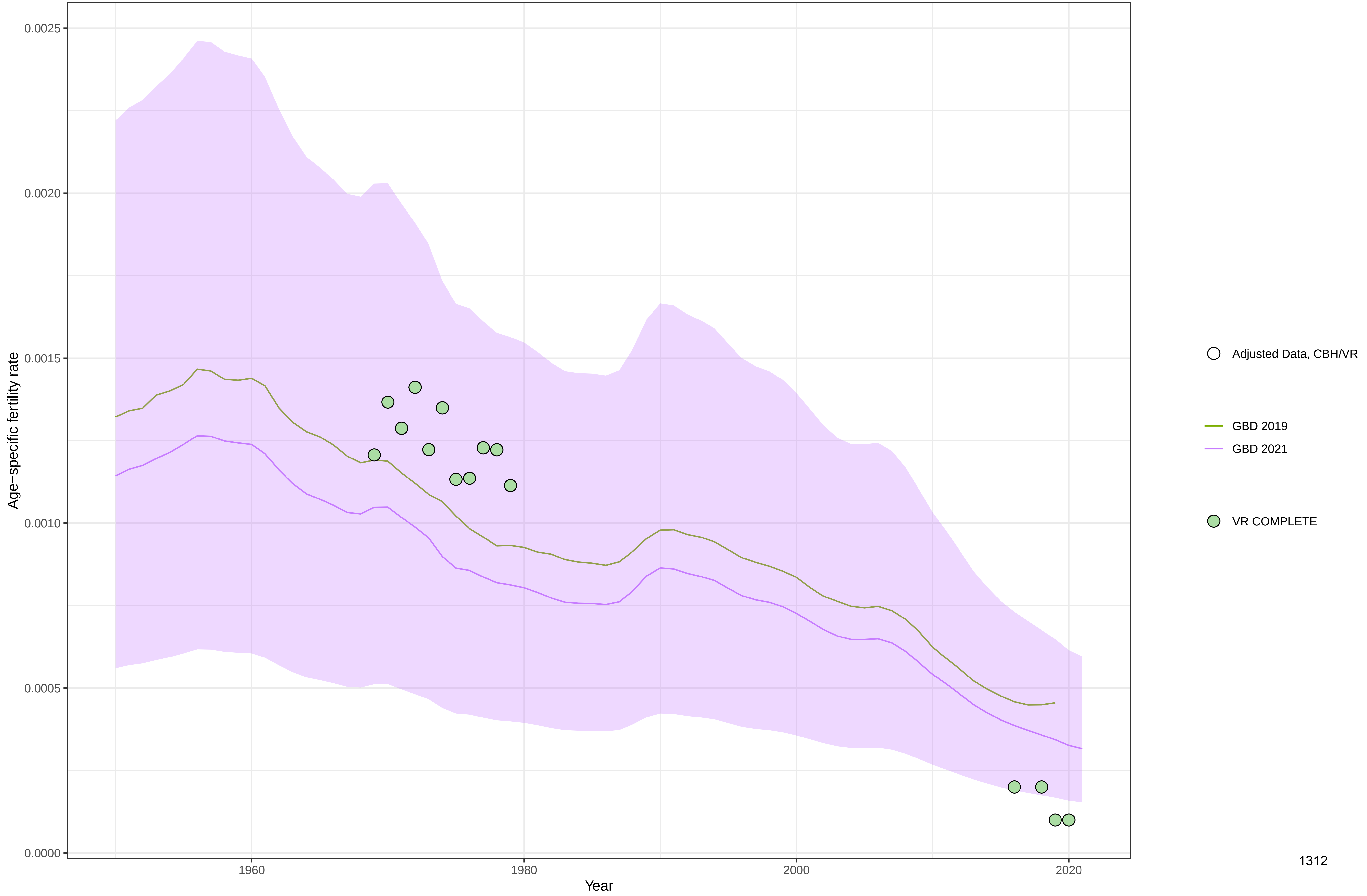

Age−specific fertility fitted trend lines, 1950−2021: Virginia, Ages: 15−19
Data points enclosed with a diamond are reference sources

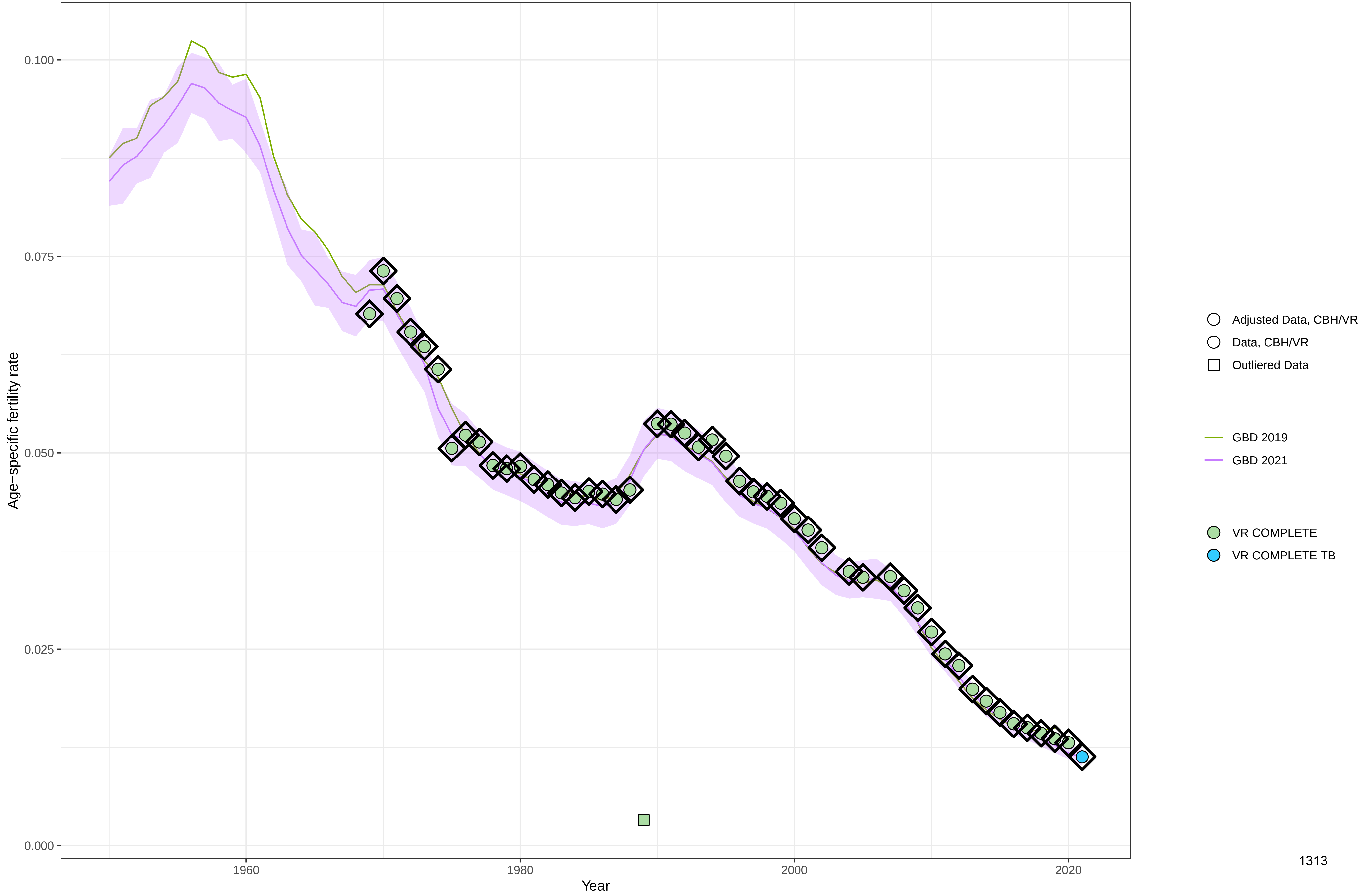

Age−specific fertility fitted trend lines, 1950−2021: Virginia, Ages: 20−24
Data points enclosed with a diamond are reference sources

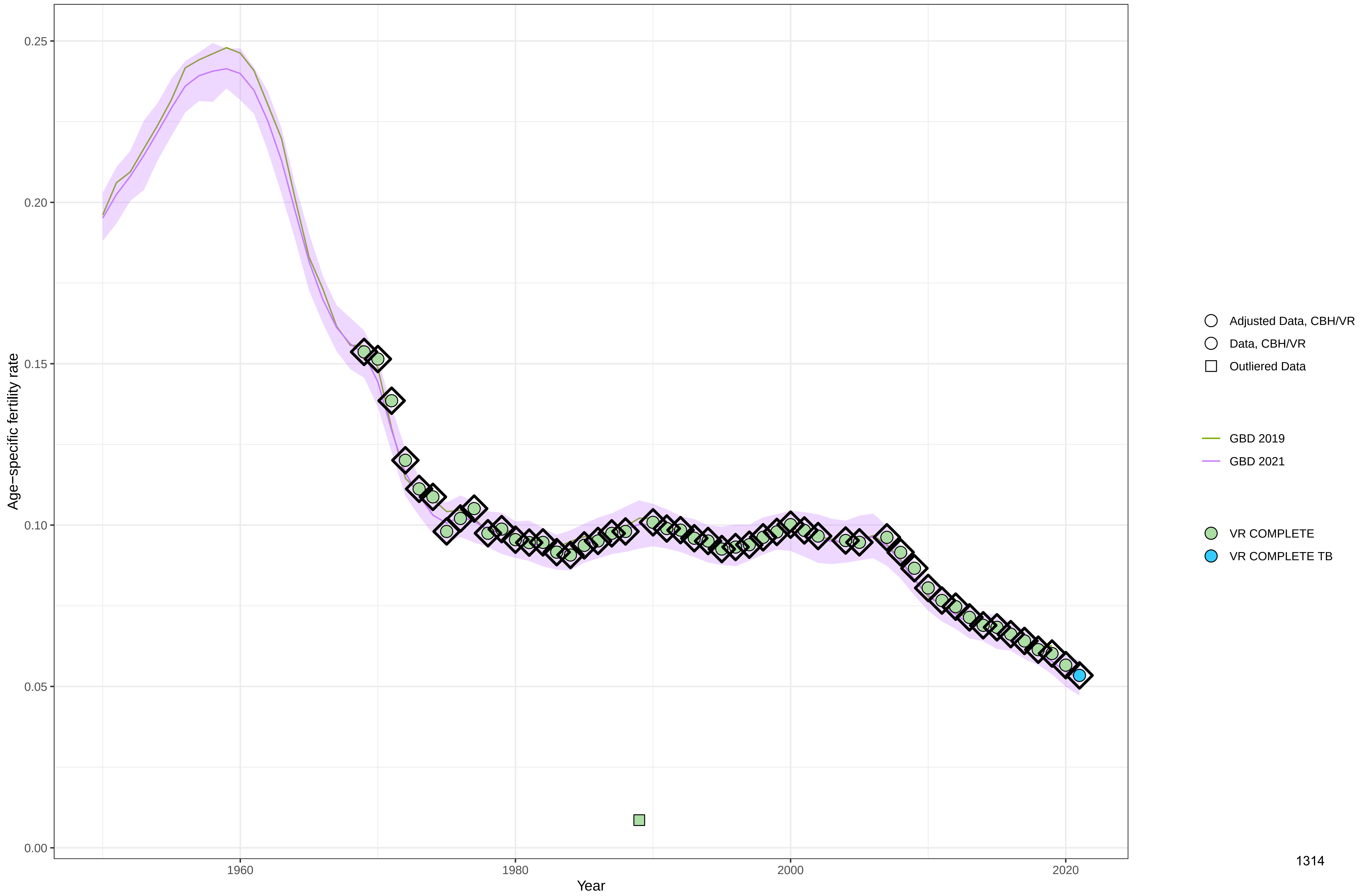

Age−specific fertility fitted trend lines, 1950−2021: Virginia, Ages: 25−29
Data points enclosed with a diamond are reference sources

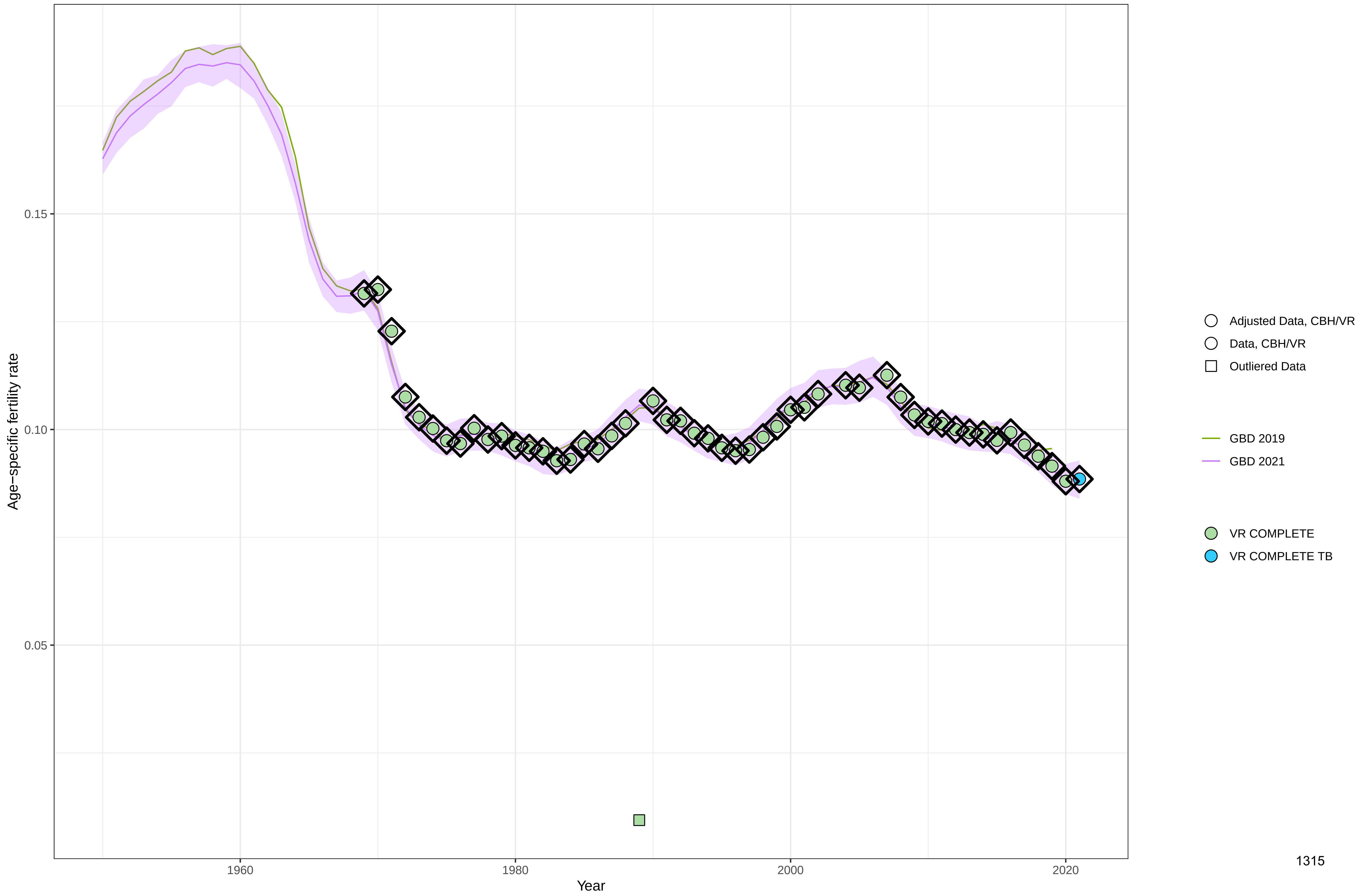

Age−specific fertility fitted trend lines, 1950−2021: Virginia, Ages: 30−34
Data points enclosed with a diamond are reference sources

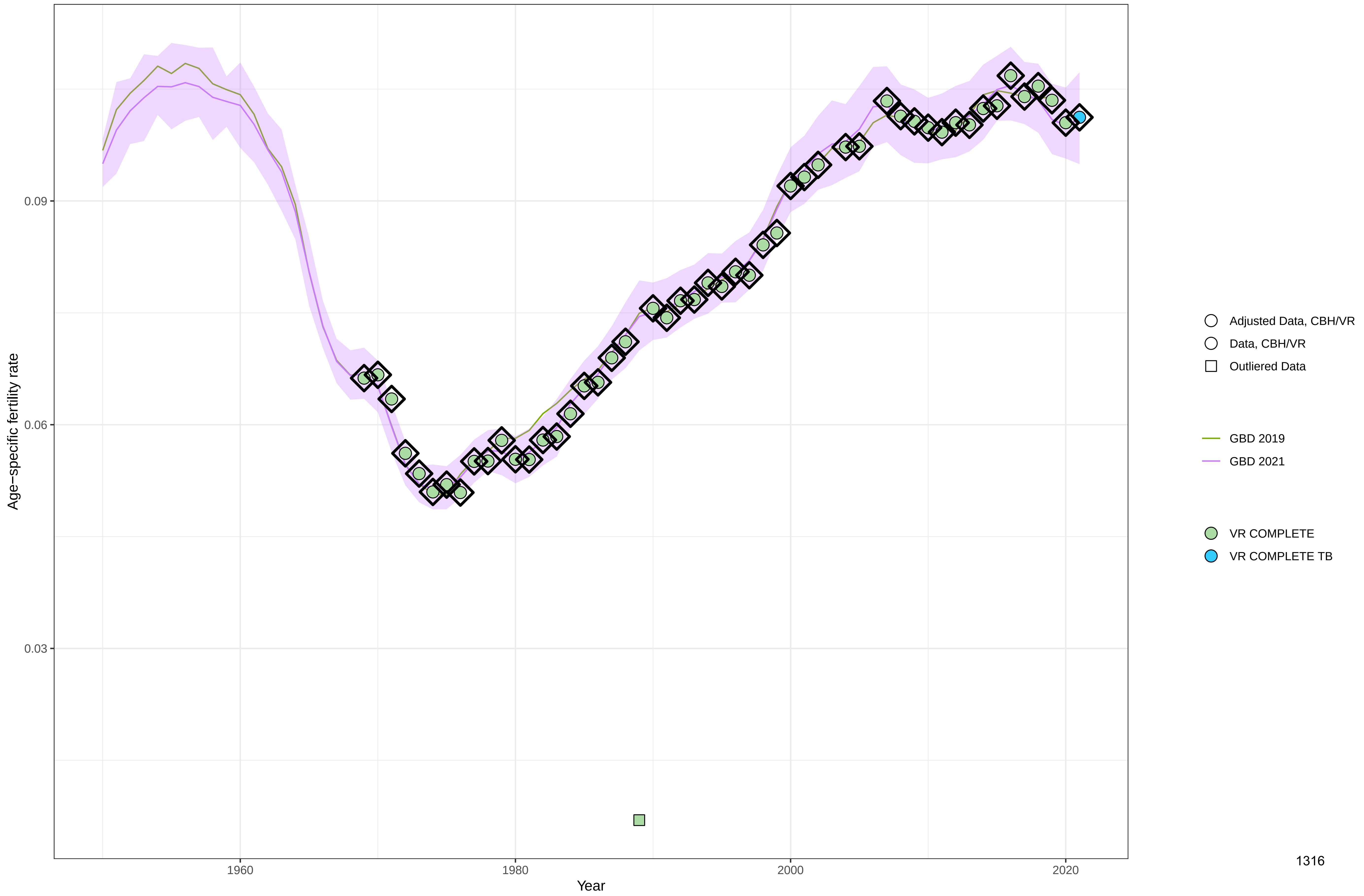

Age−specific fertility fitted trend lines, 1950−2021: Virginia, Ages: 35−39
Data points enclosed with a diamond are reference sources

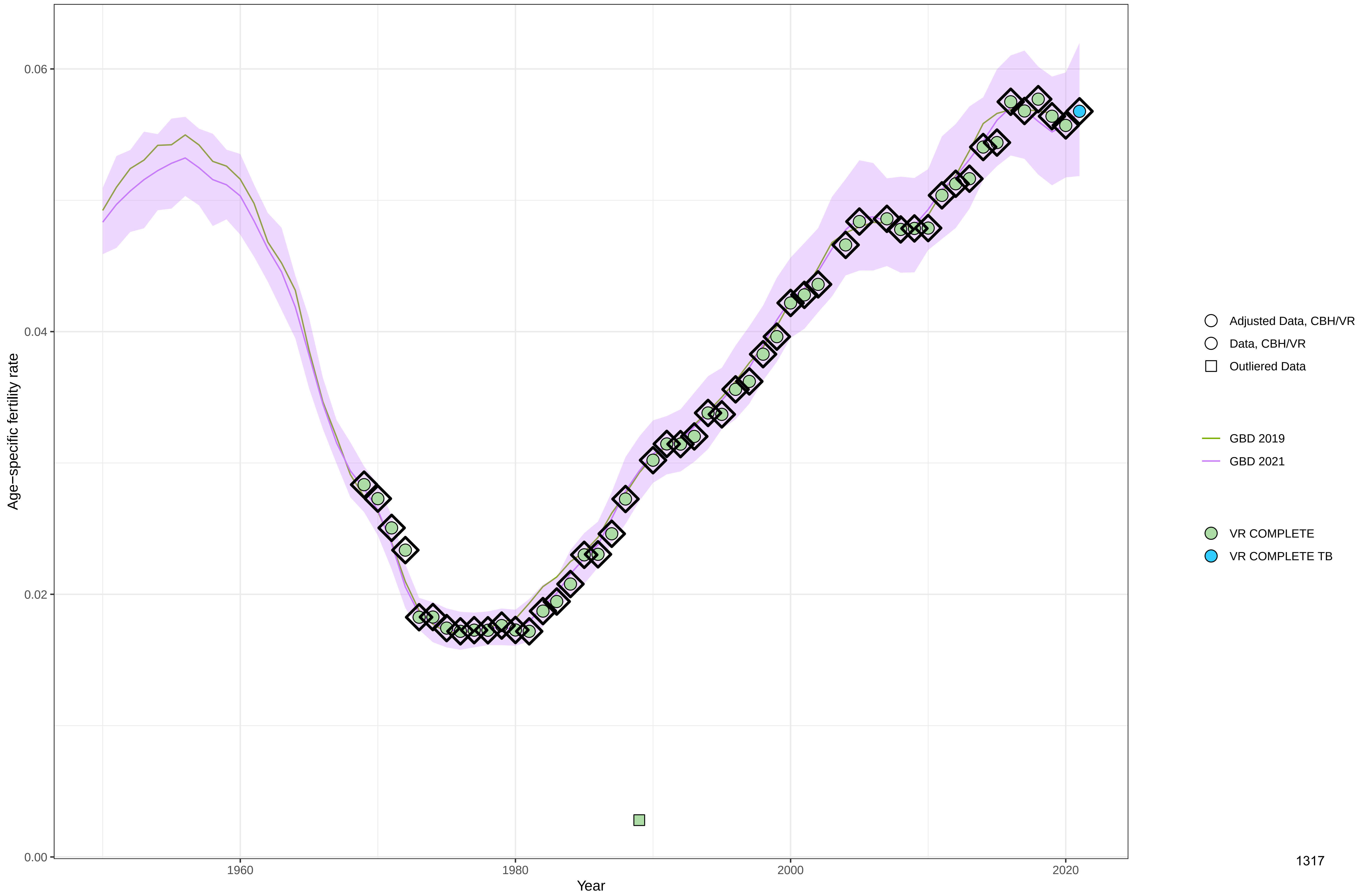

Age−specific fertility fitted trend lines, 1950−2021: Virginia, Ages: 40−44
Data points enclosed with a diamond are reference sources

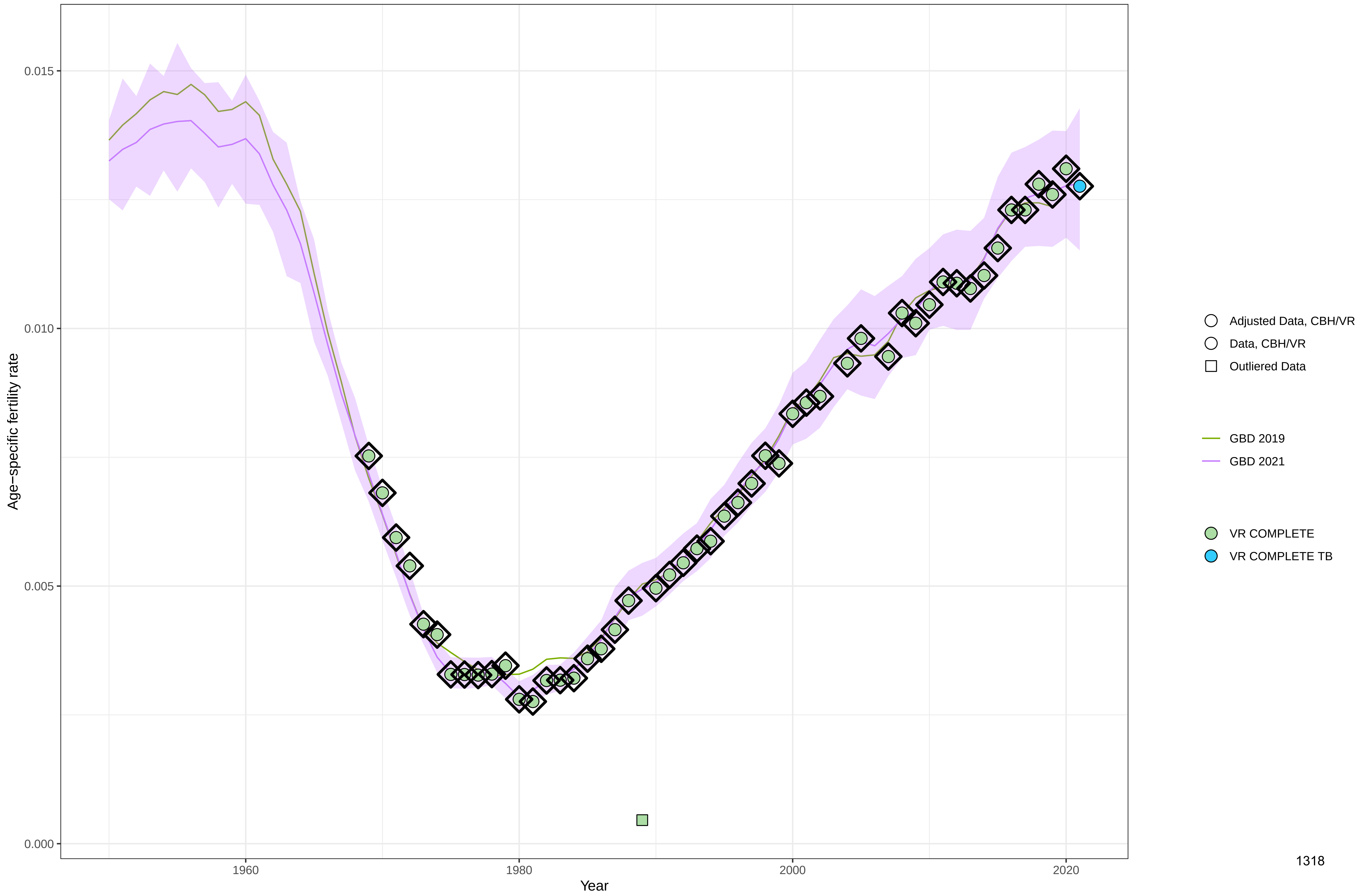

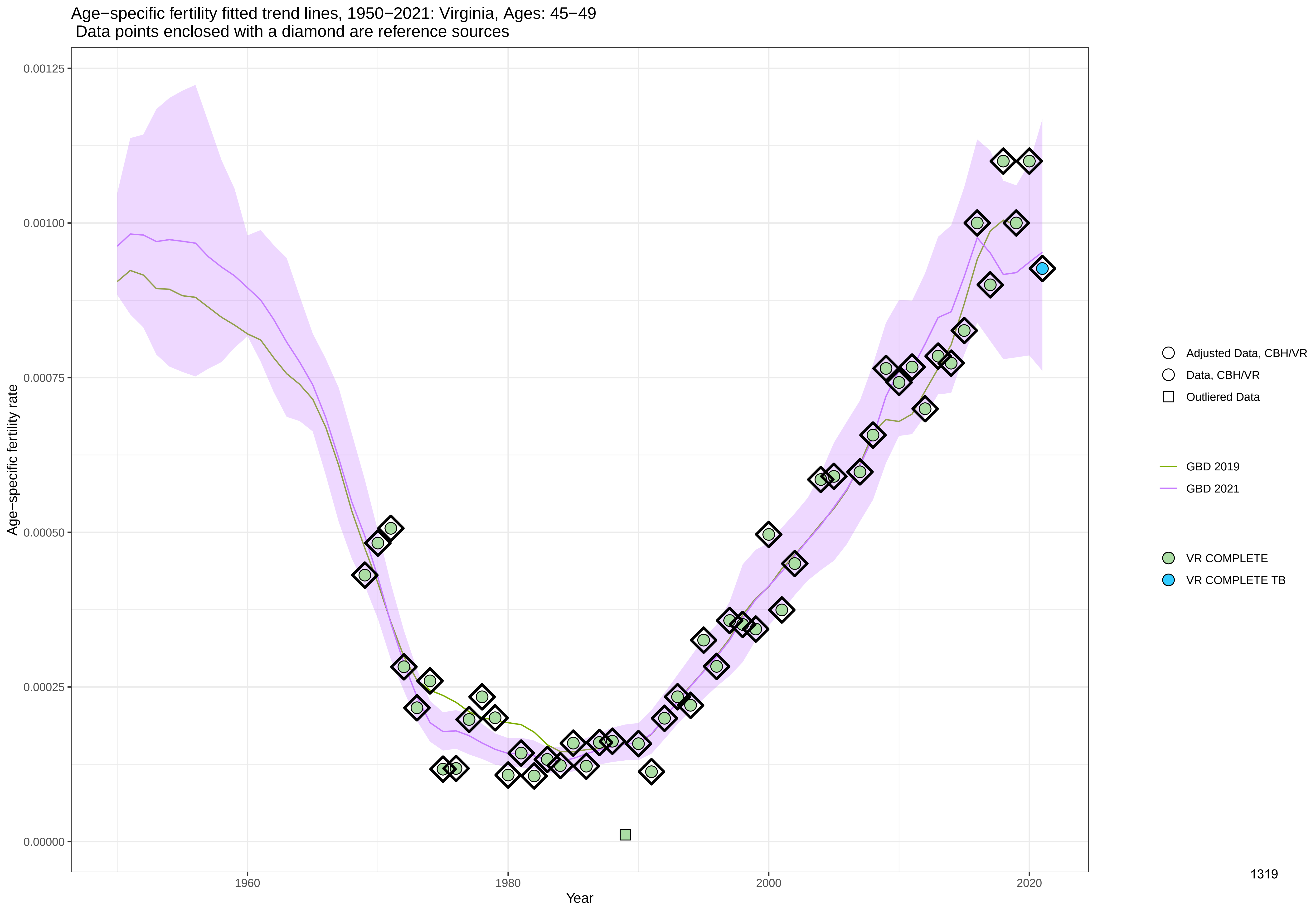

Age−specific fertility fitted trend lines, 1950−2021: Virginia, Ages: 45−49
Data points enclosed with a diamond are reference sources
Age−specific fertility rate
0.00000
0.00025
0.00050
0.00075
0.00100
0.00125
1960
1980
2000
2020
Year
Adjusted Data, CBH/VR
Data, CBH/VR
Outliered Data
GBD 2019
GBD 2021
VR COMPLETE
VR COMPLETE TB

Age−specific fertility fitted trend lines, 1950−2021: Virginia, Ages: 50−54
Data points enclosed with a diamond are reference sources

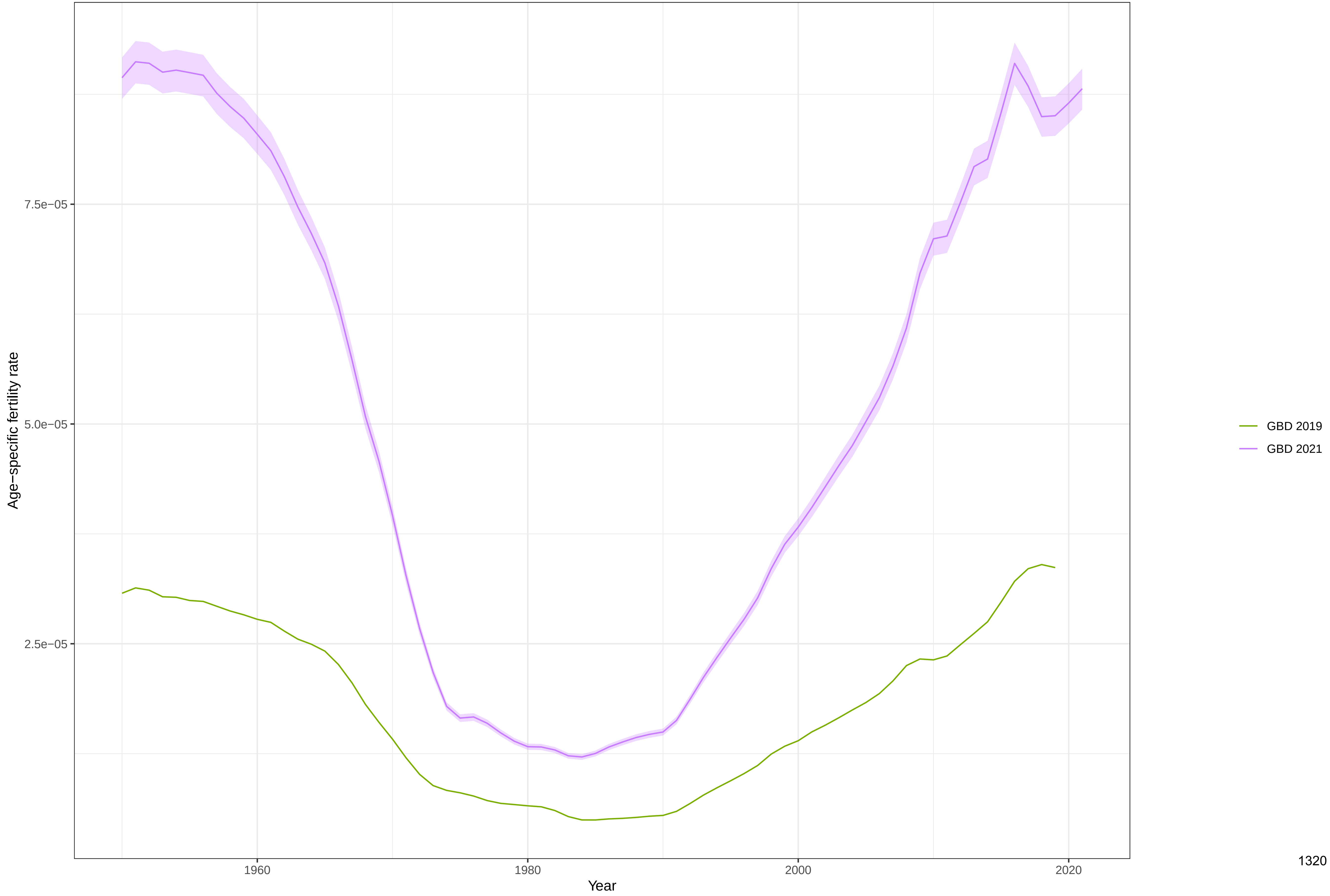

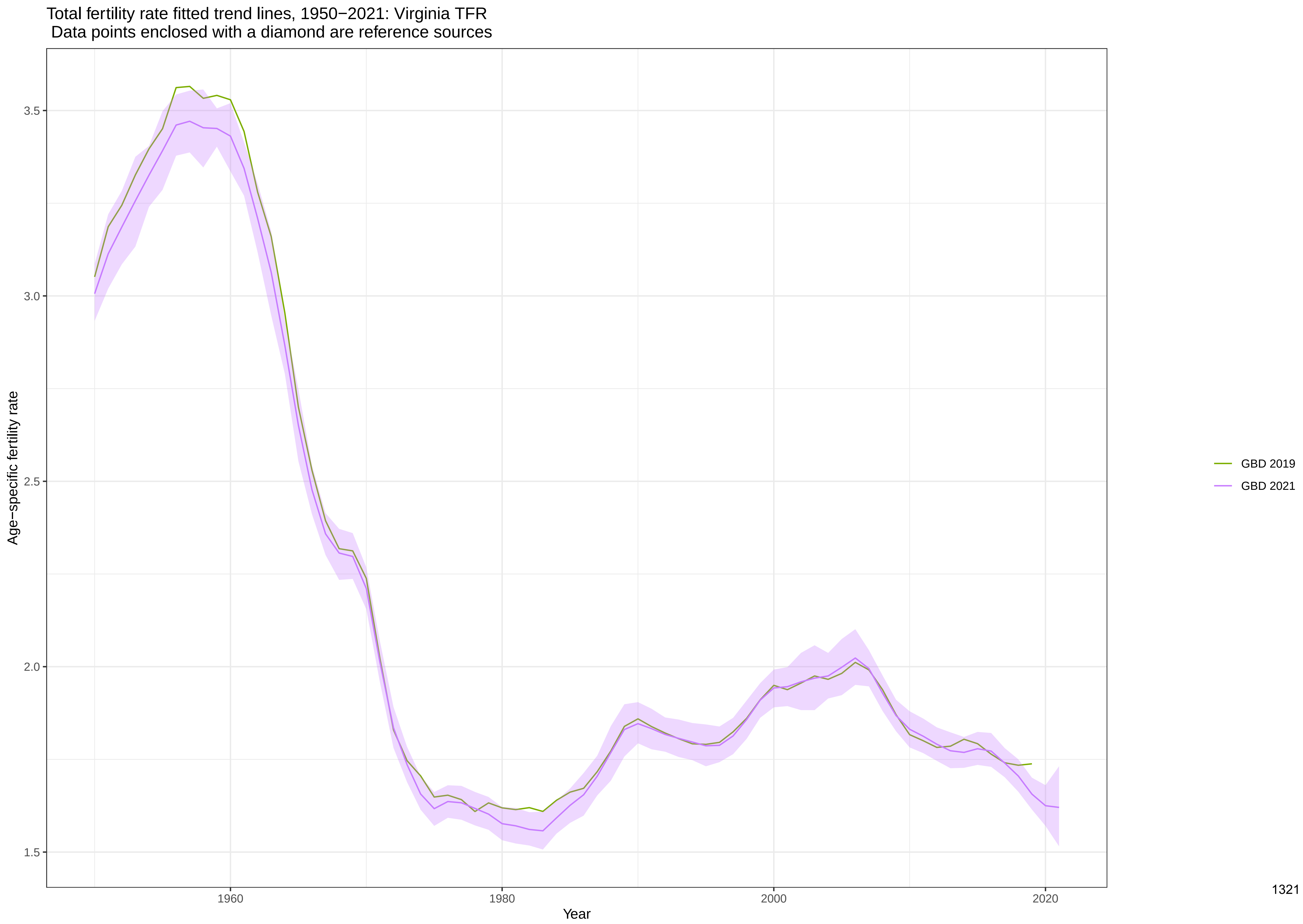

Total fertility rate fitted trend lines, 1950−2021: Virginia TFR
Data points enclosed with a diamond are reference sources
Age−specific fertility rate
Year
3.5
3.0
2.5
2.0
1.5
1960
1980
2000
2020
GBD 2019
GBD 2021

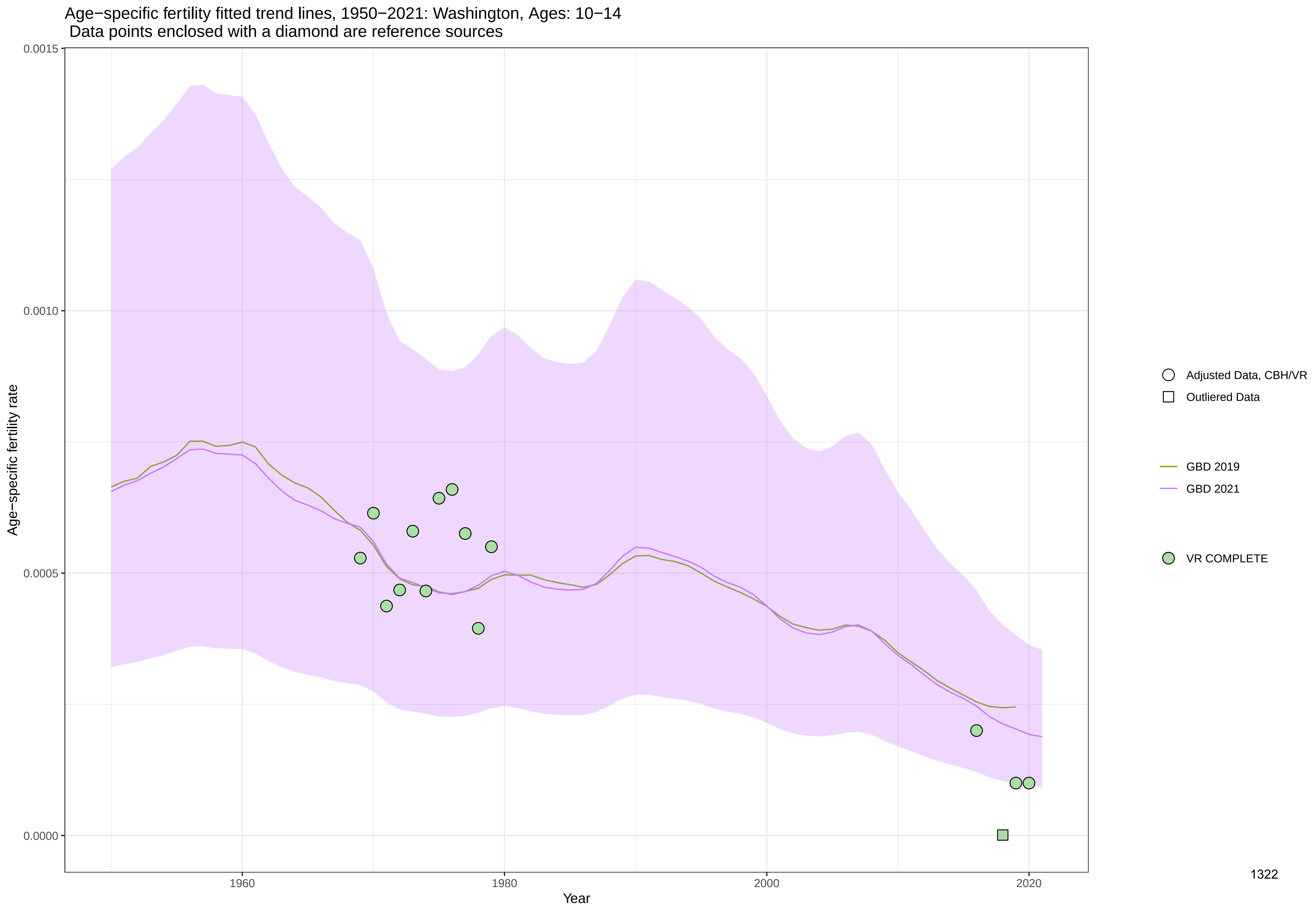

Age−specific fertility fitted trend lines, 1950−2021: Washington, Ages: 10−14
Data points enclosed with a diamond are reference sources
0.0015
0.0010
0.0005
0.0000
Age−specific fertility rate
1960
1980
2000
2020
Year
Adjusted Data, CBH/VR
Outliered Data
GBD 2019
GBD 2021
VR COMPLETE

Age−specific fertility fitted trend lines, 1950−2021: Washington, Ages: 15−19
Data points enclosed with a diamond are reference sources

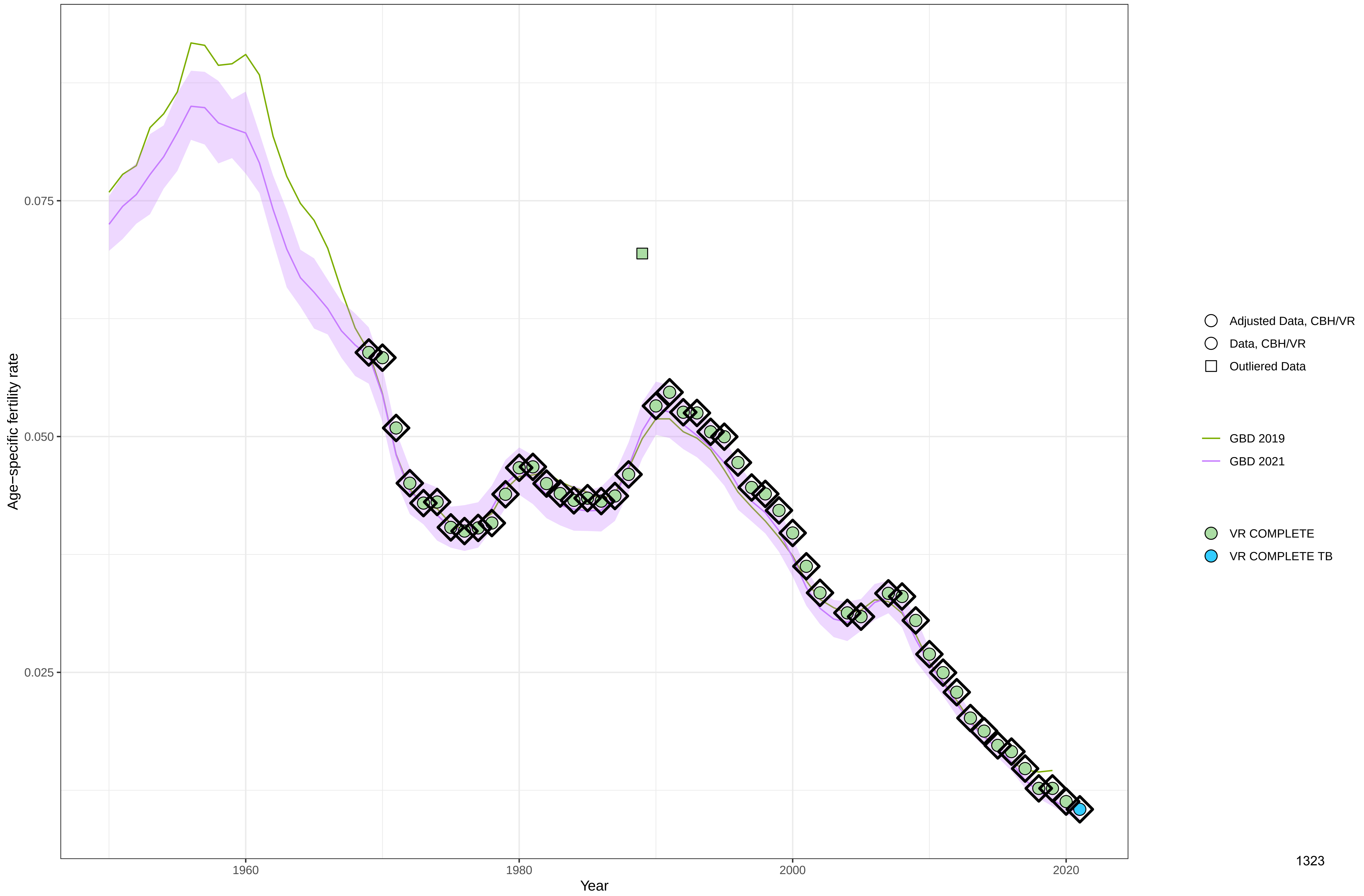

Age−specific fertility fitted trend lines, 1950−2021: Washington, Ages: 20−24
Data points enclosed with a diamond are reference sources

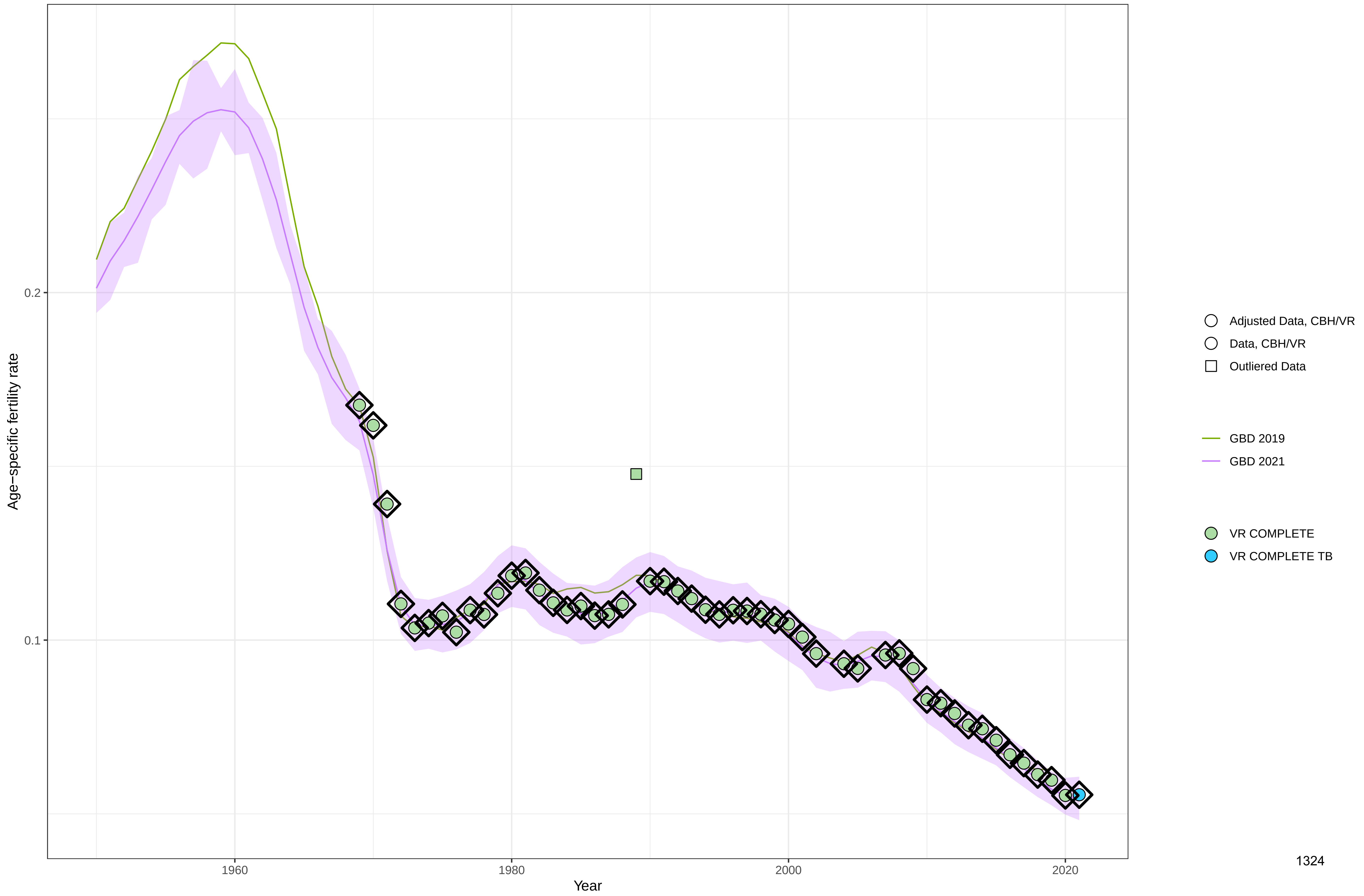

Age−specific fertility fitted trend lines, 1950−2021: Washington, Ages: 25−29
Data points enclosed with a diamond are reference sources

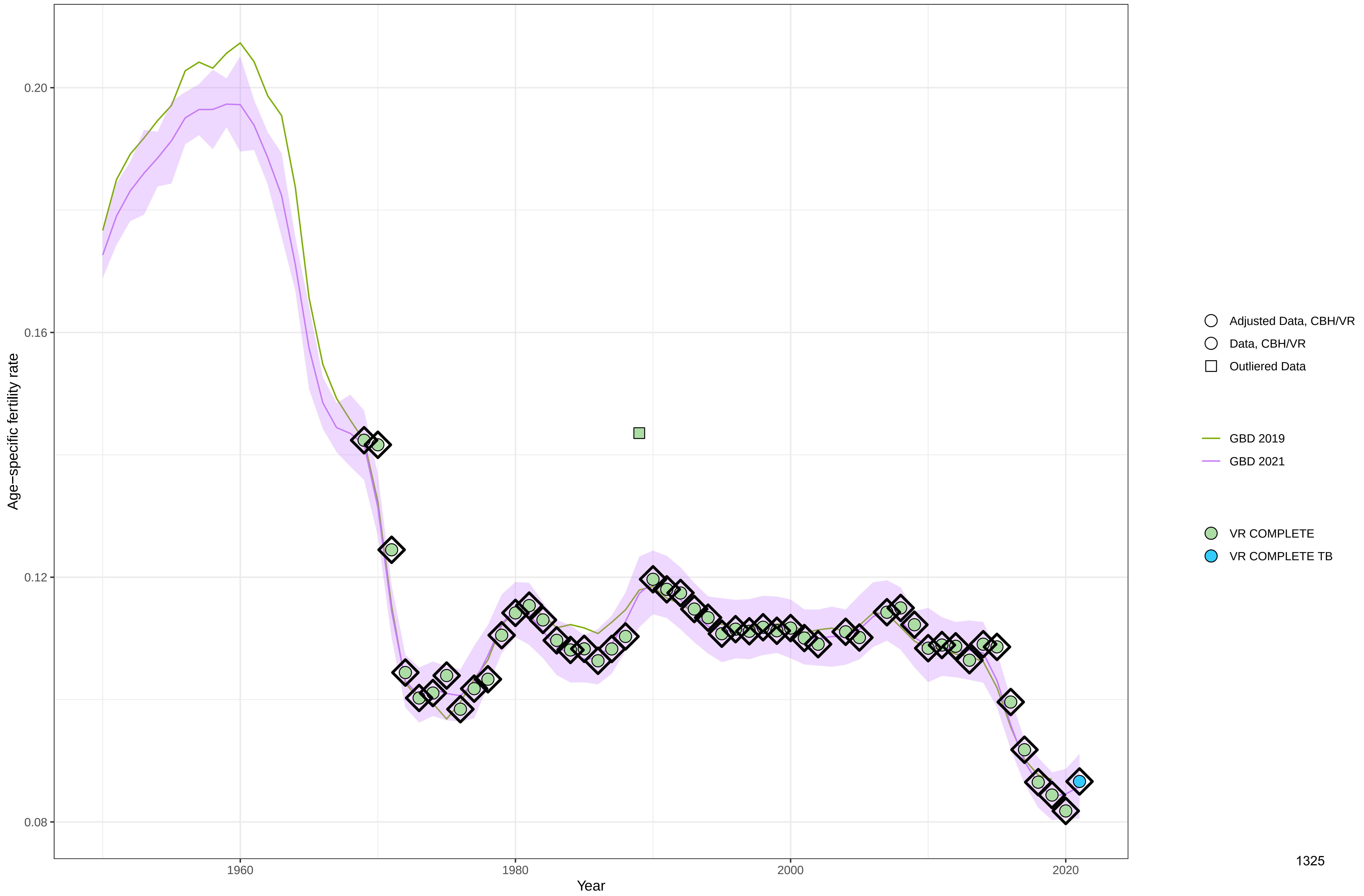

Age−specific fertility fitted trend lines, 1950−2021: Washington, Ages: 30−34
Data points enclosed with a diamond are reference sources

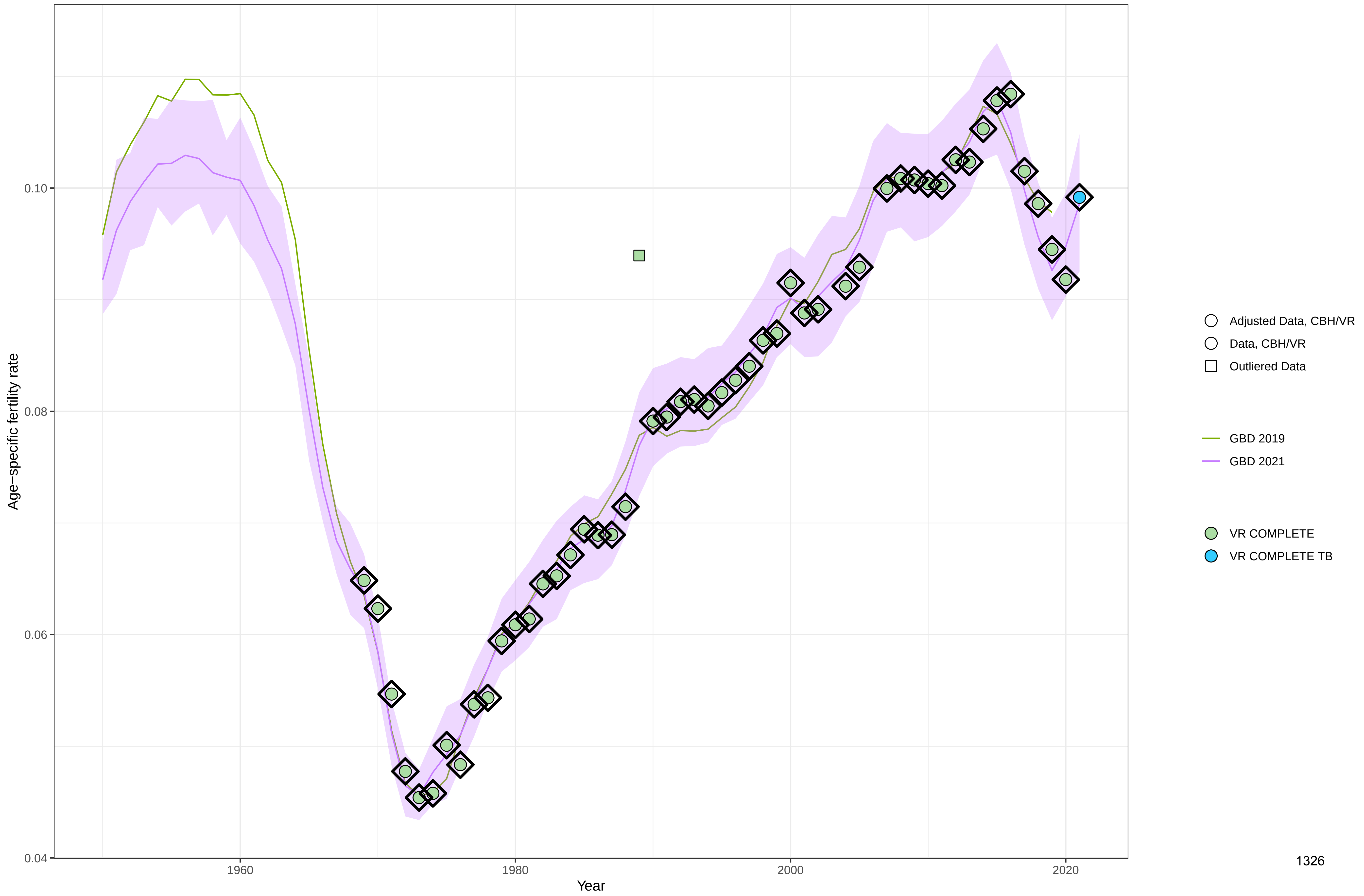

Age−specific fertility fitted trend lines, 1950−2021: Washington, Ages: 35−39
Data points enclosed with a diamond are reference sources

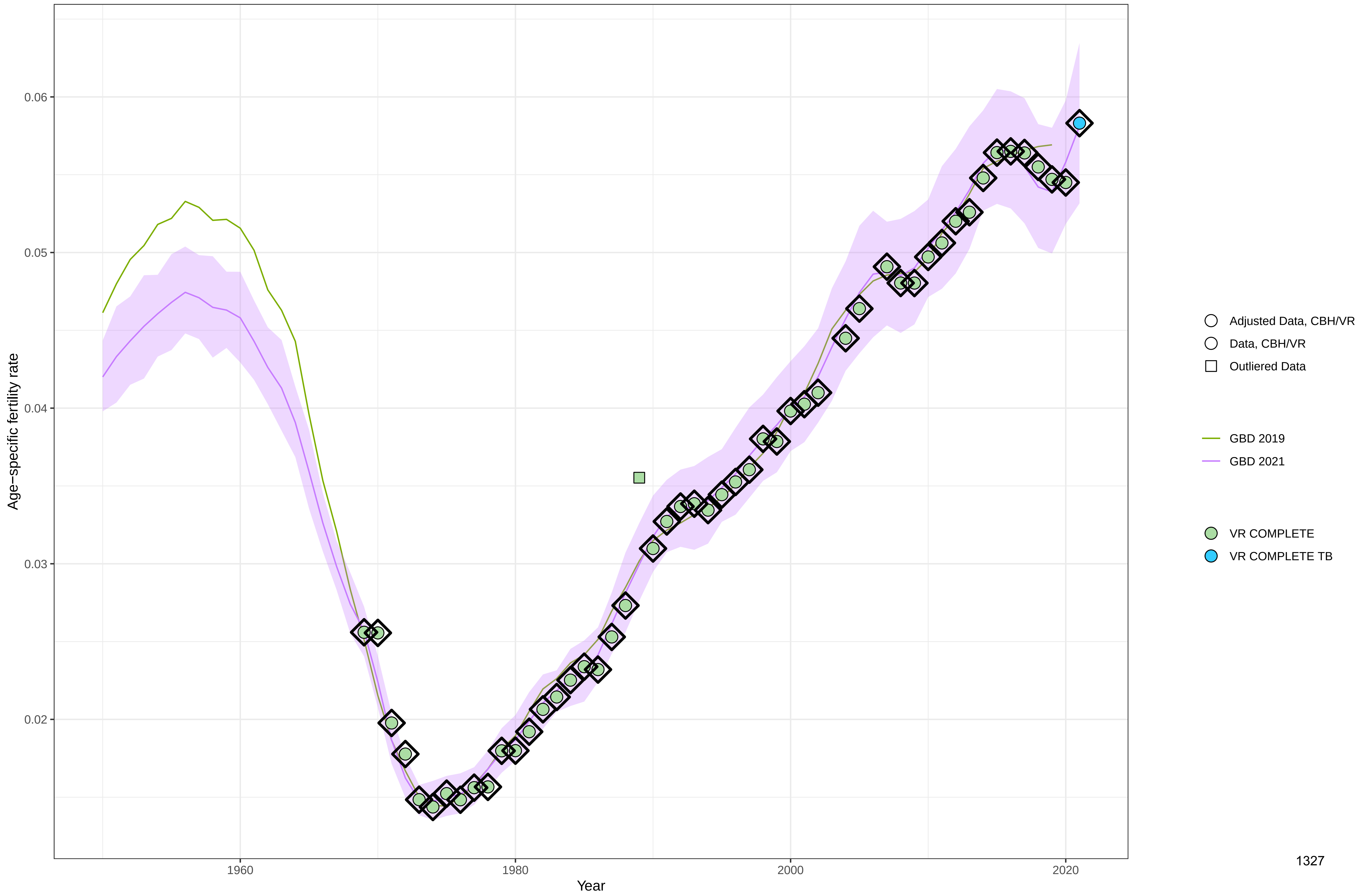

Age−specific fertility fitted trend lines, 1950−2021: Washington, Ages: 40−44
Data points enclosed with a diamond are reference sources

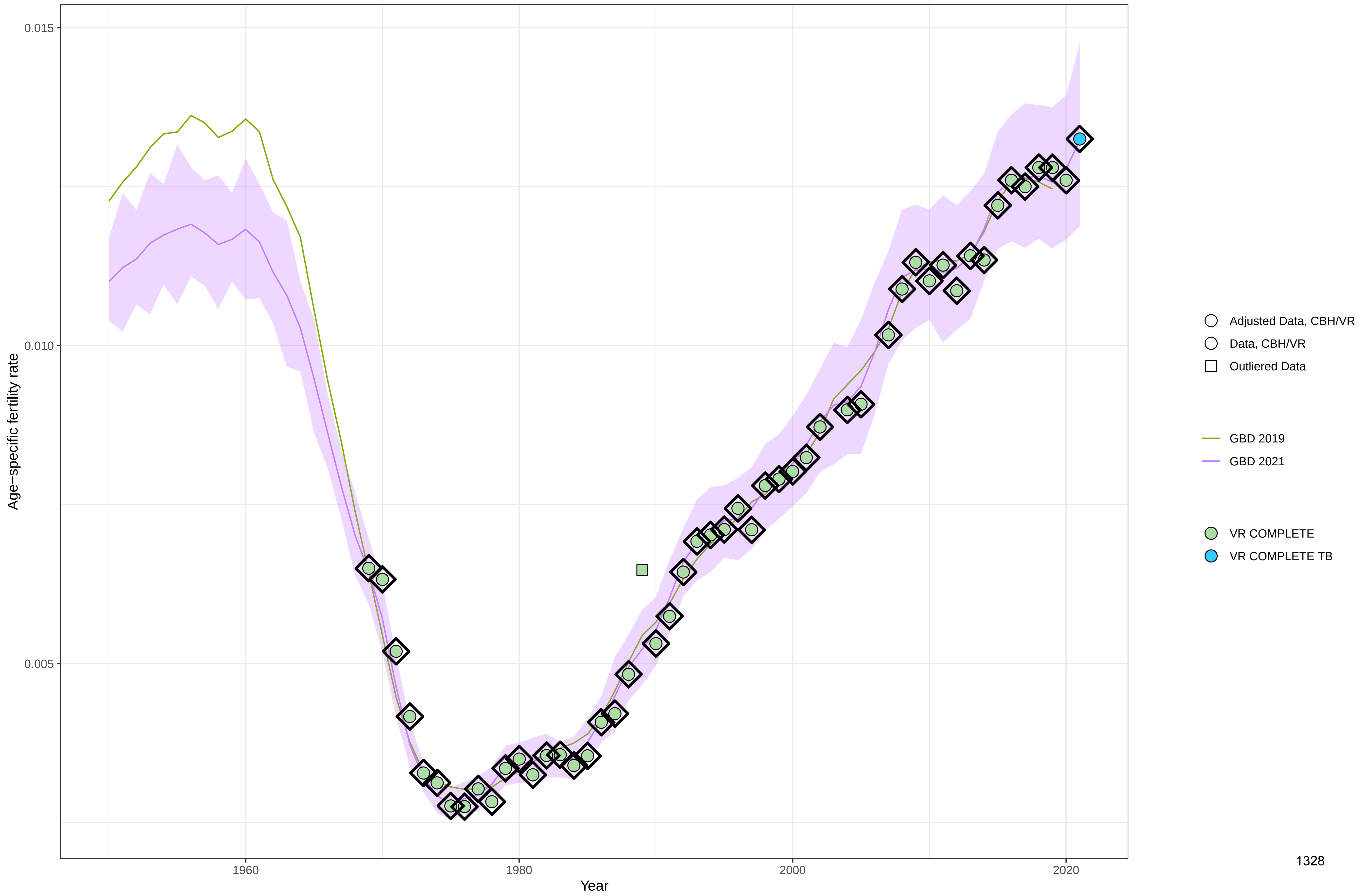

Age−specific fertility fitted trend lines, 1950−2021: Washington, Ages: 45−49
Data points enclosed with a diamond are reference sources

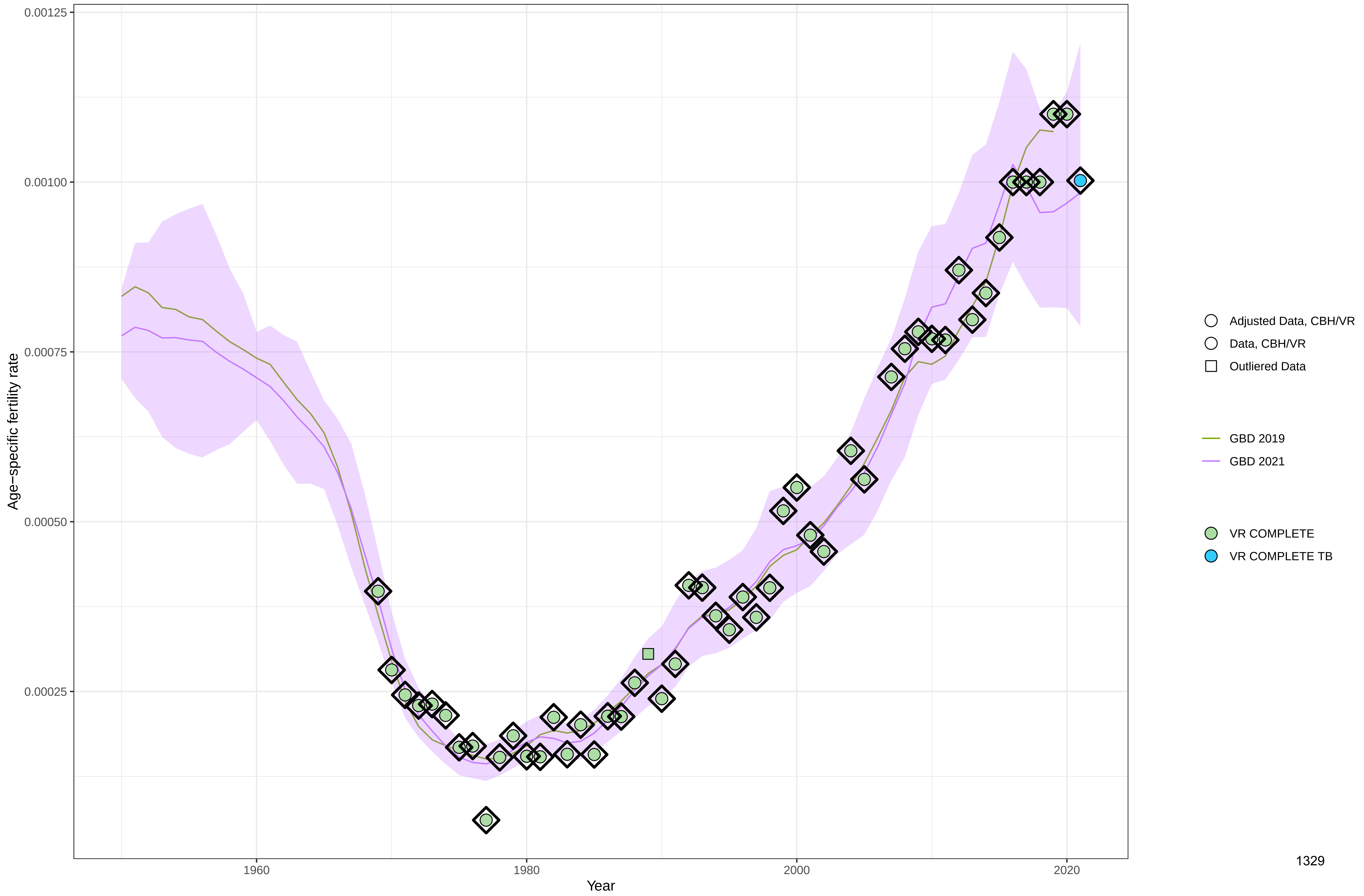

Age−specific fertility fitted trend lines, 1950−2021: Washington, Ages: 50−54
Data points enclosed with a diamond are reference sources

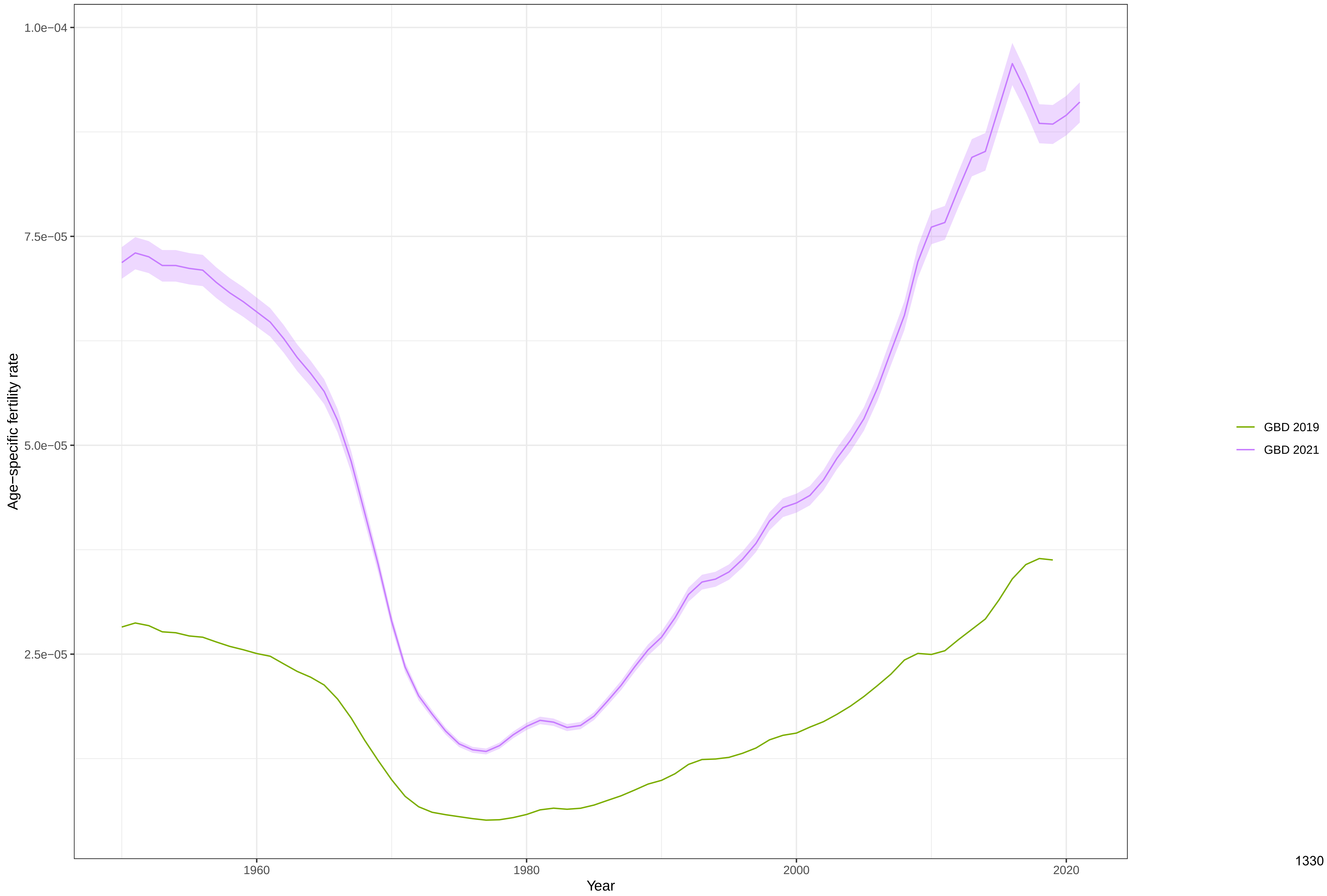

Total fertility rate fitted trend lines, 1950−2021: Washington TFR
Data points enclosed with a diamond are reference sources

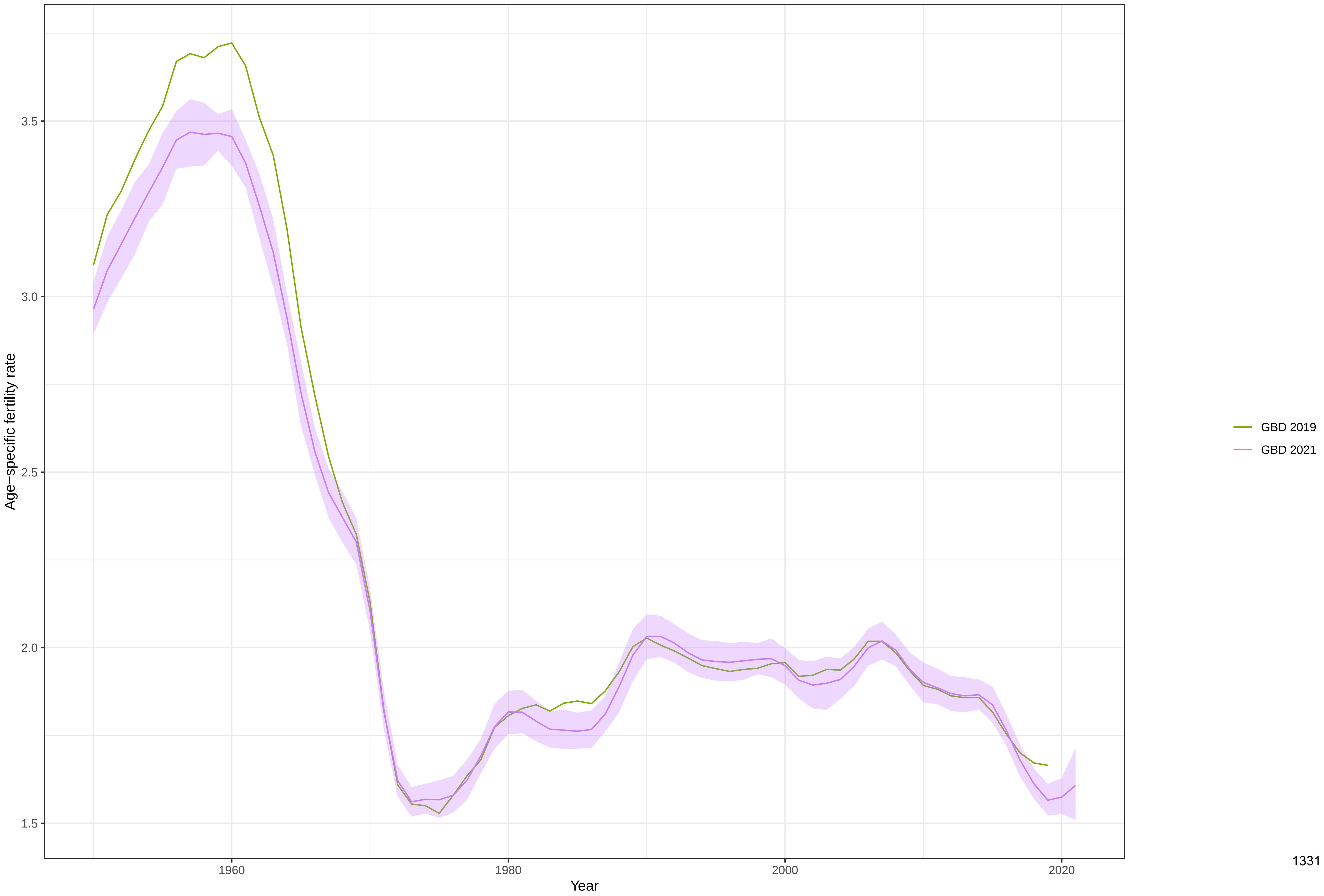

Age−specific fertility fitted trend lines, 1950−2021: West Virginia, Ages: 10−14
Data points enclosed with a diamond are reference sources

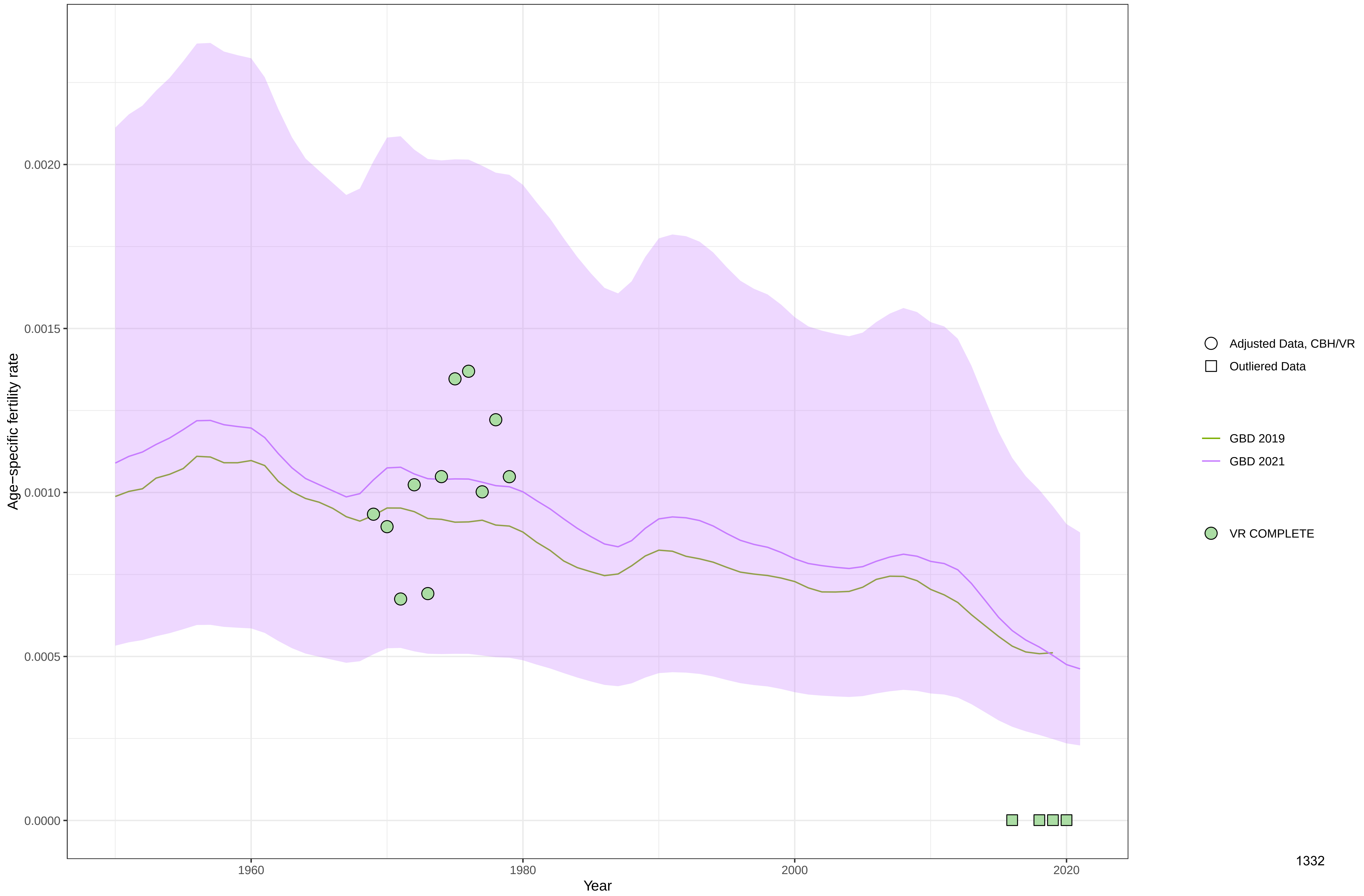

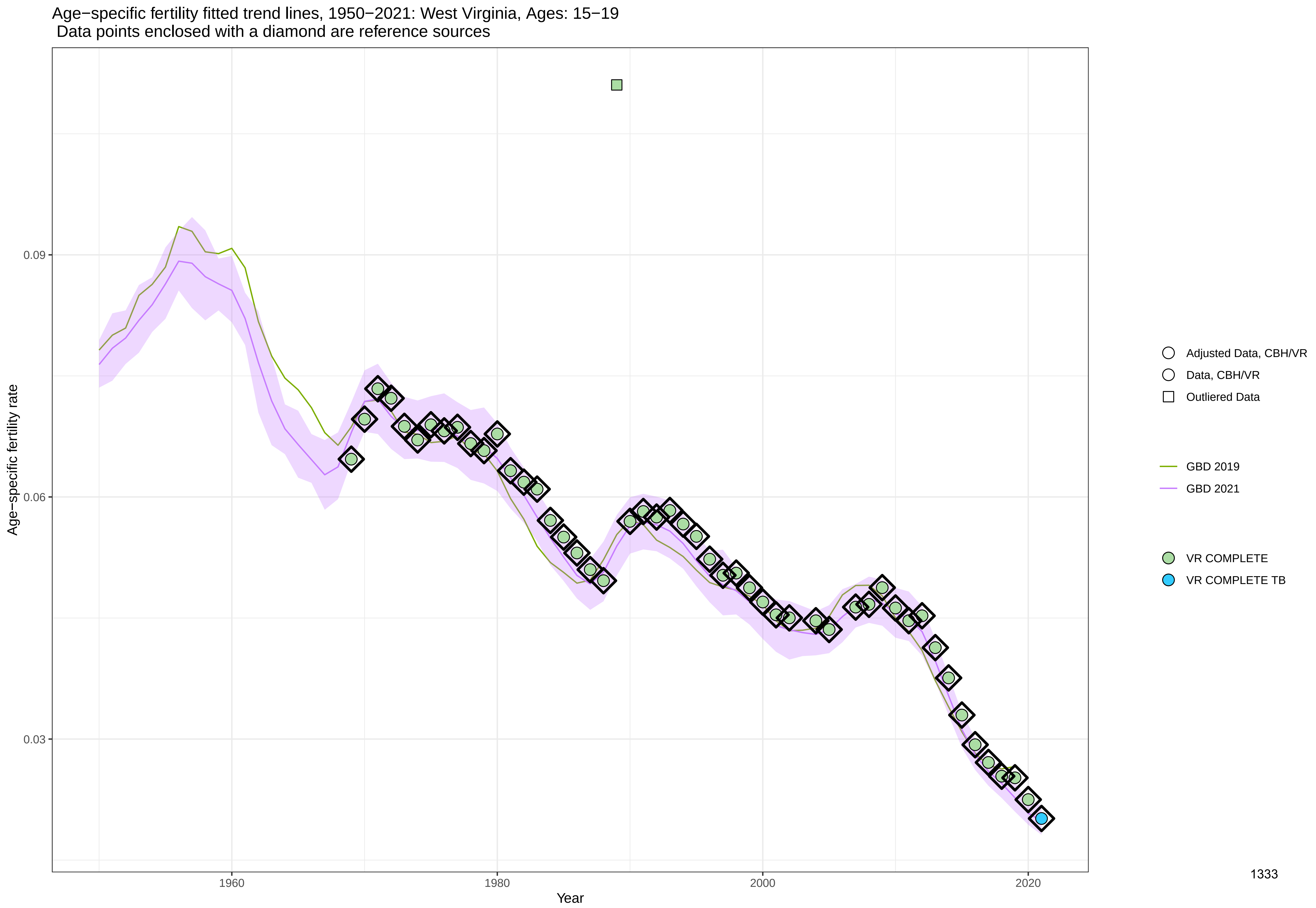

Age−specific fertility fitted trend lines, 1950−2021: West Virginia, Ages: 15−19
Data points enclosed with a diamond are reference sources
0.09
0.06
0.03
Age−specific fertility rate
1960
1980
2000
2020
Year
Adjusted Data, CBH/VR
Data, CBH/VR
Outliered Data
GBD 2019
GBD 2021
VR COMPLETE
VR COMPLETE TB

Age−specific fertility fitted trend lines, 1950−2021: West Virginia, Ages: 20−24
Data points enclosed with a diamond are reference sources

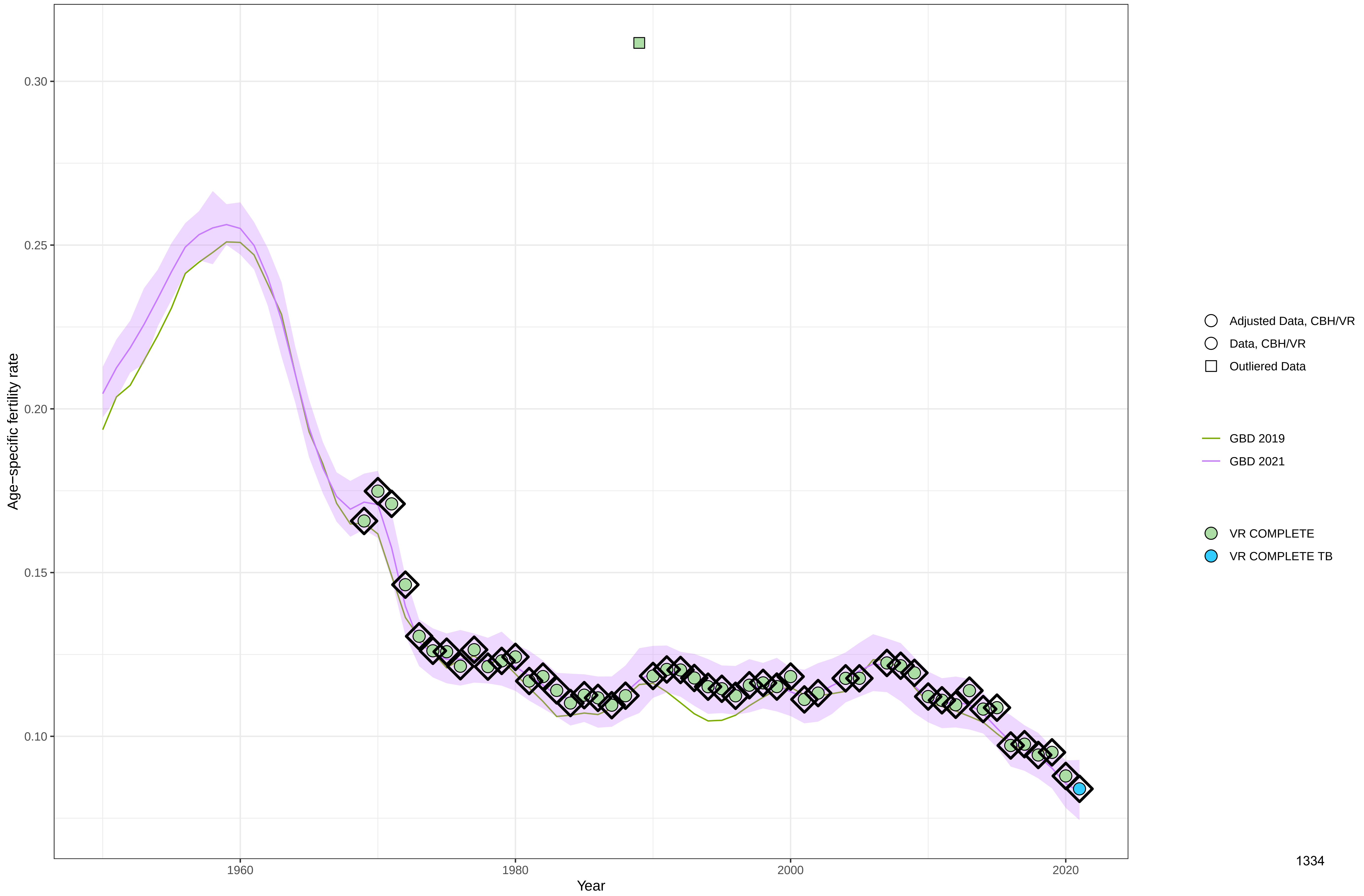

Age−specific fertility fitted trend lines, 1950−2021: West Virginia, Ages: 25−29
Data points enclosed with a diamond are reference sources

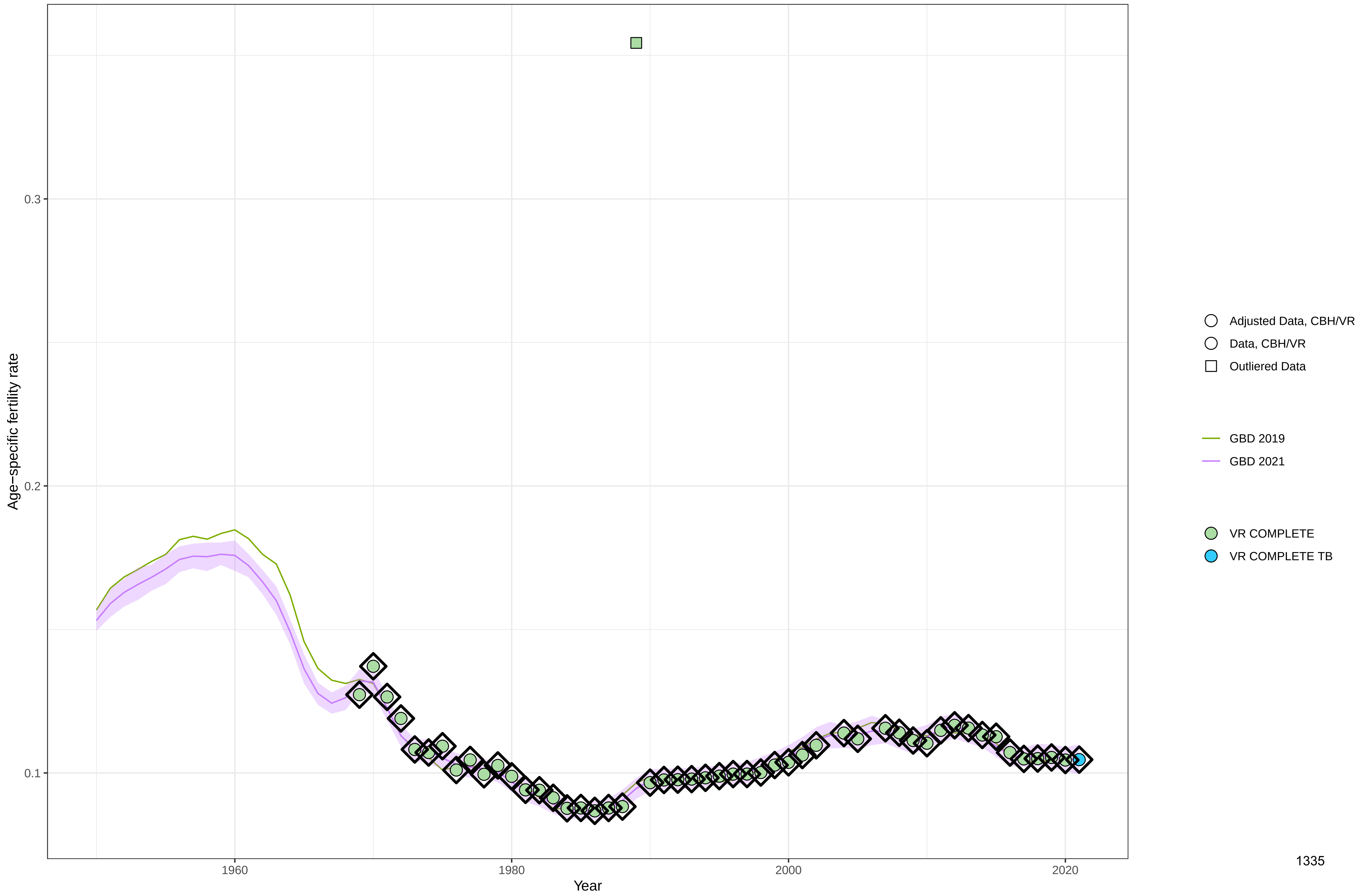

Age−specific fertility fitted trend lines, 1950−2021: West Virginia, Ages: 30−34
Data points enclosed with a diamond are reference sources

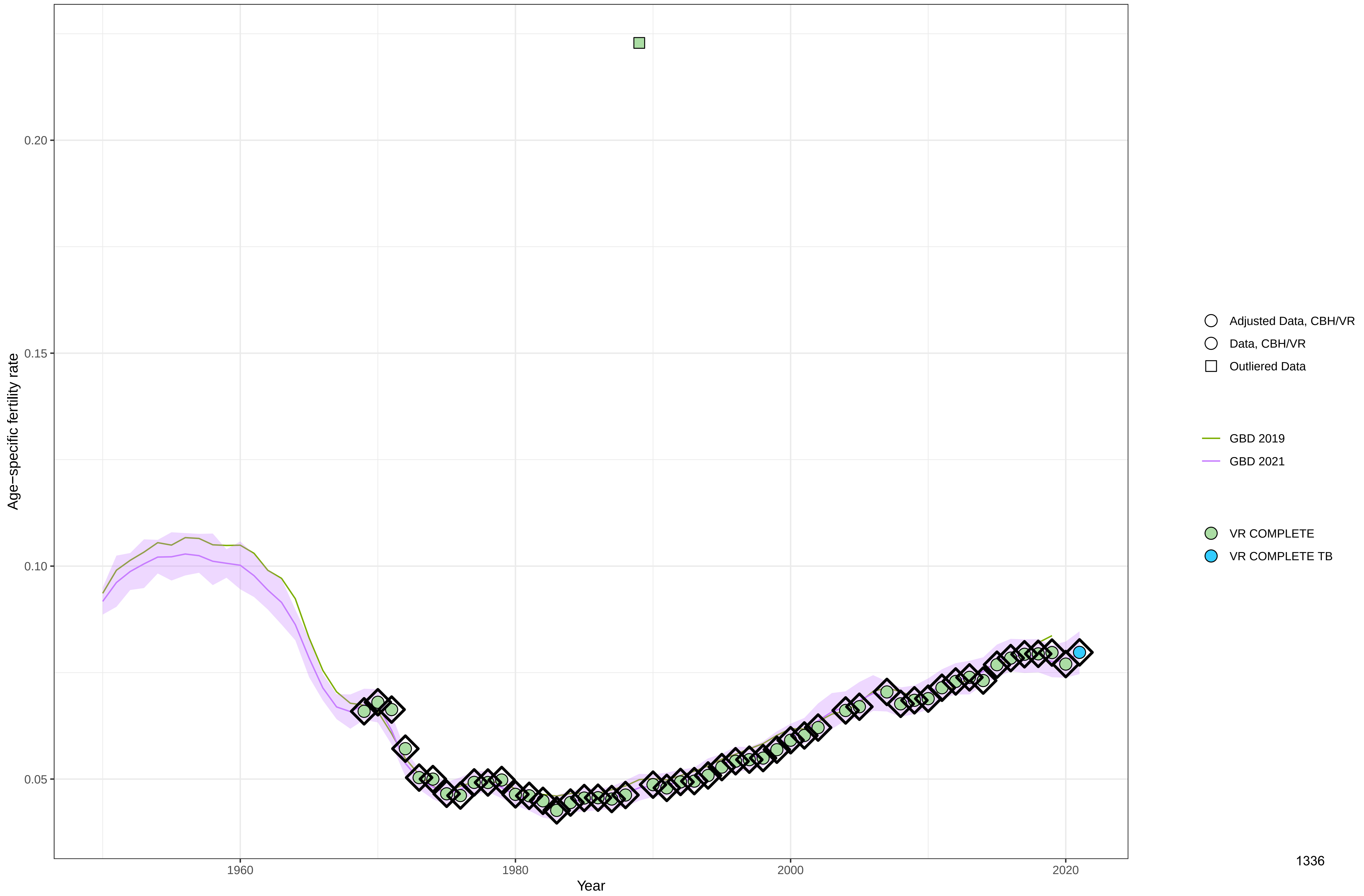

Age−specific fertility fitted trend lines, 1950−2021: West Virginia, Ages: 35−39
Data points enclosed with a diamond are reference sources

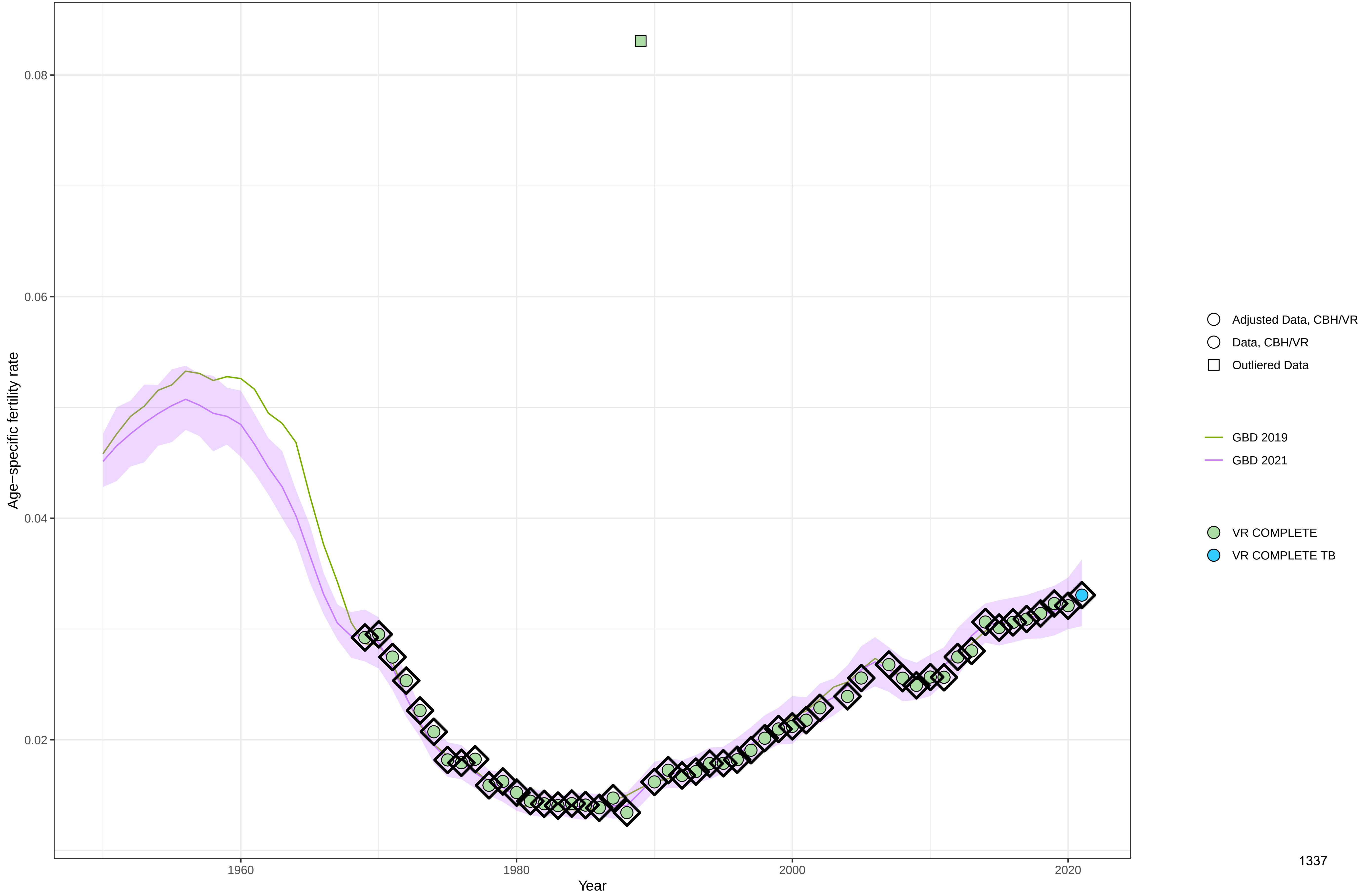

Age−specific fertility fitted trend lines, 1950−2021: West Virginia, Ages: 40−44
Data points enclosed with a diamond are reference sources

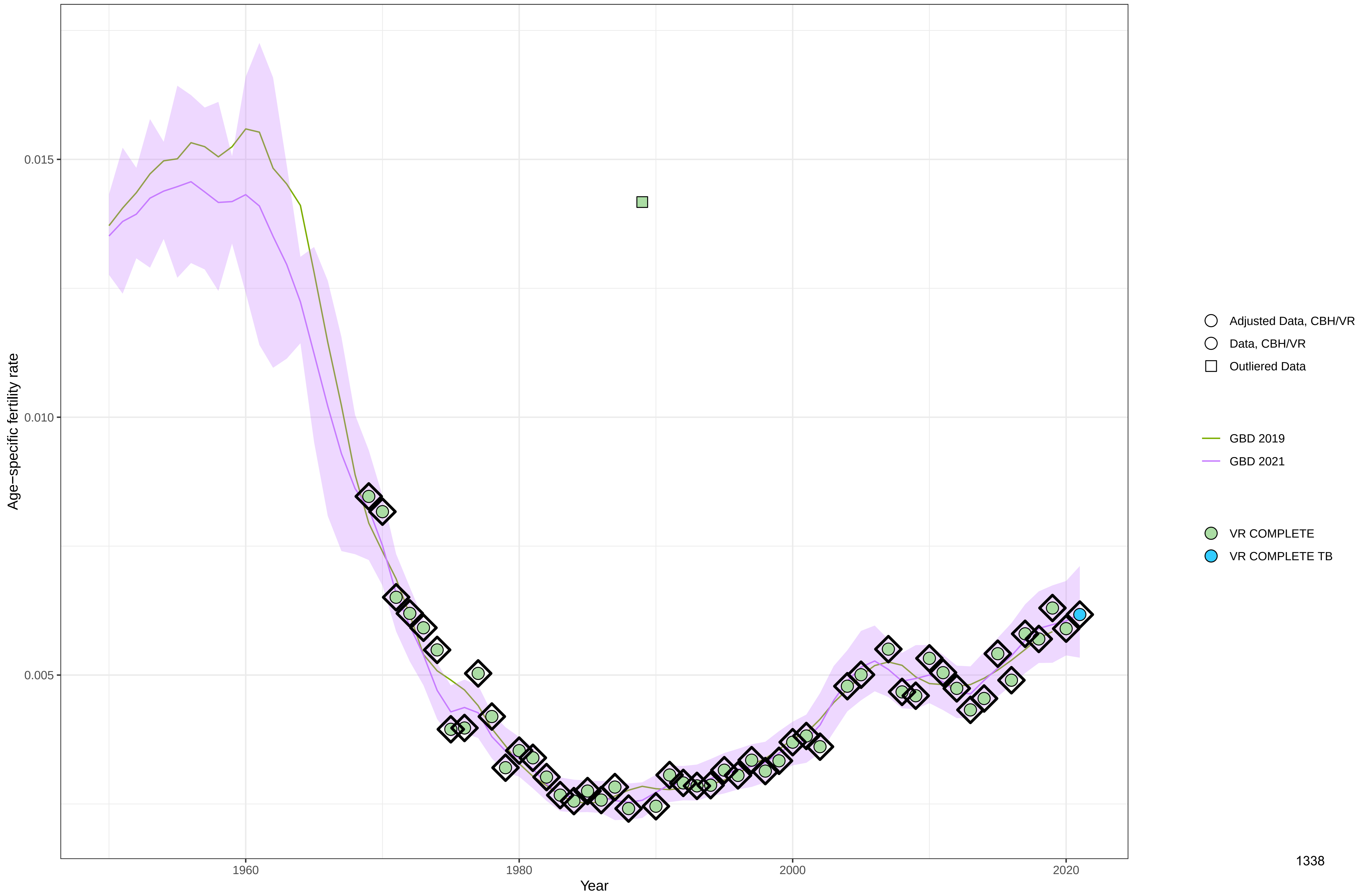

Age−specific fertility fitted trend lines, 1950−2021: West Virginia, Ages: 45−49
Data points enclosed with a diamond are reference sources

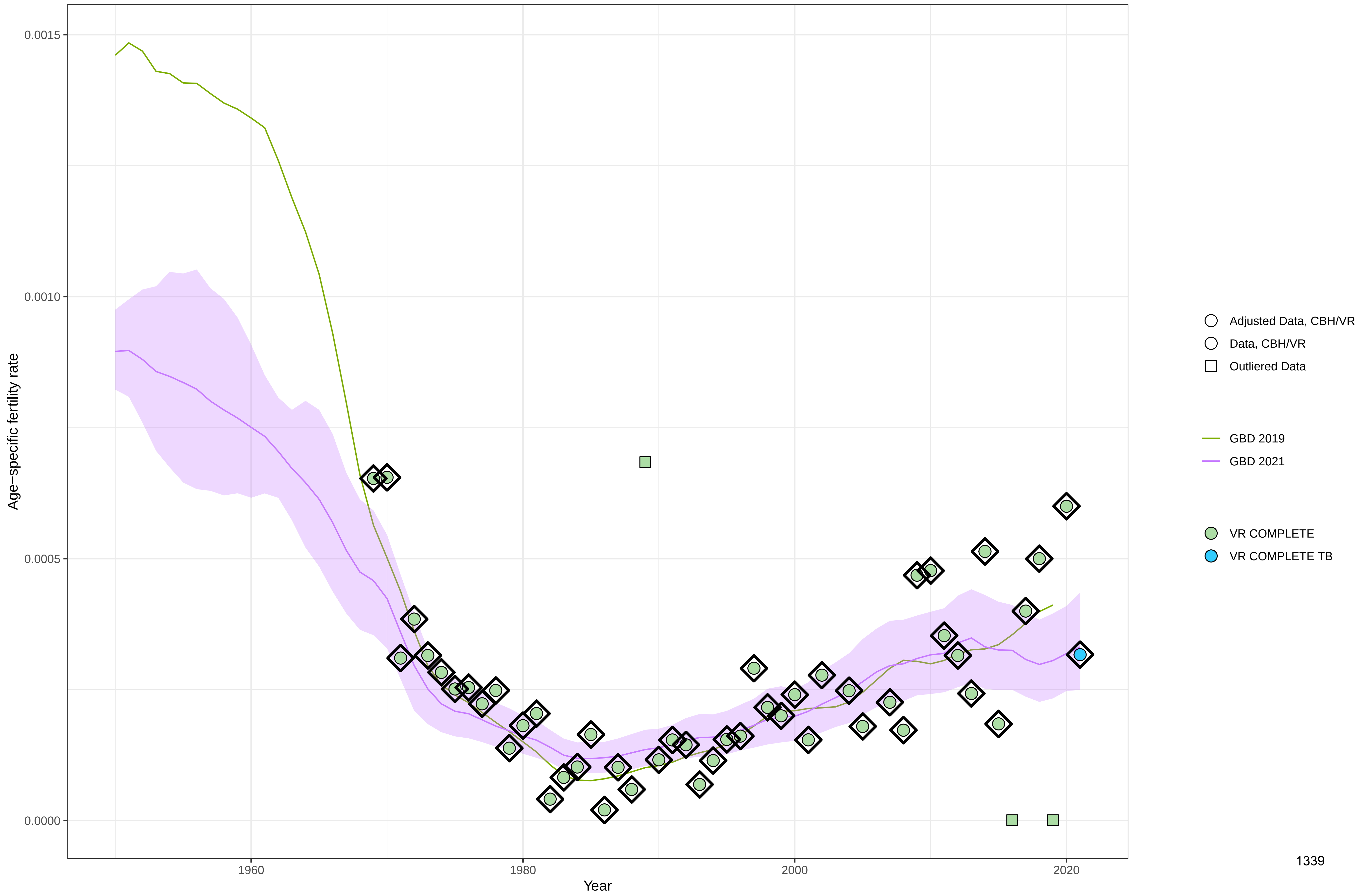

Age−specific fertility fitted trend lines, 1950−2021: West Virginia, Ages: 50−54
Data points enclosed with a diamond are reference sources

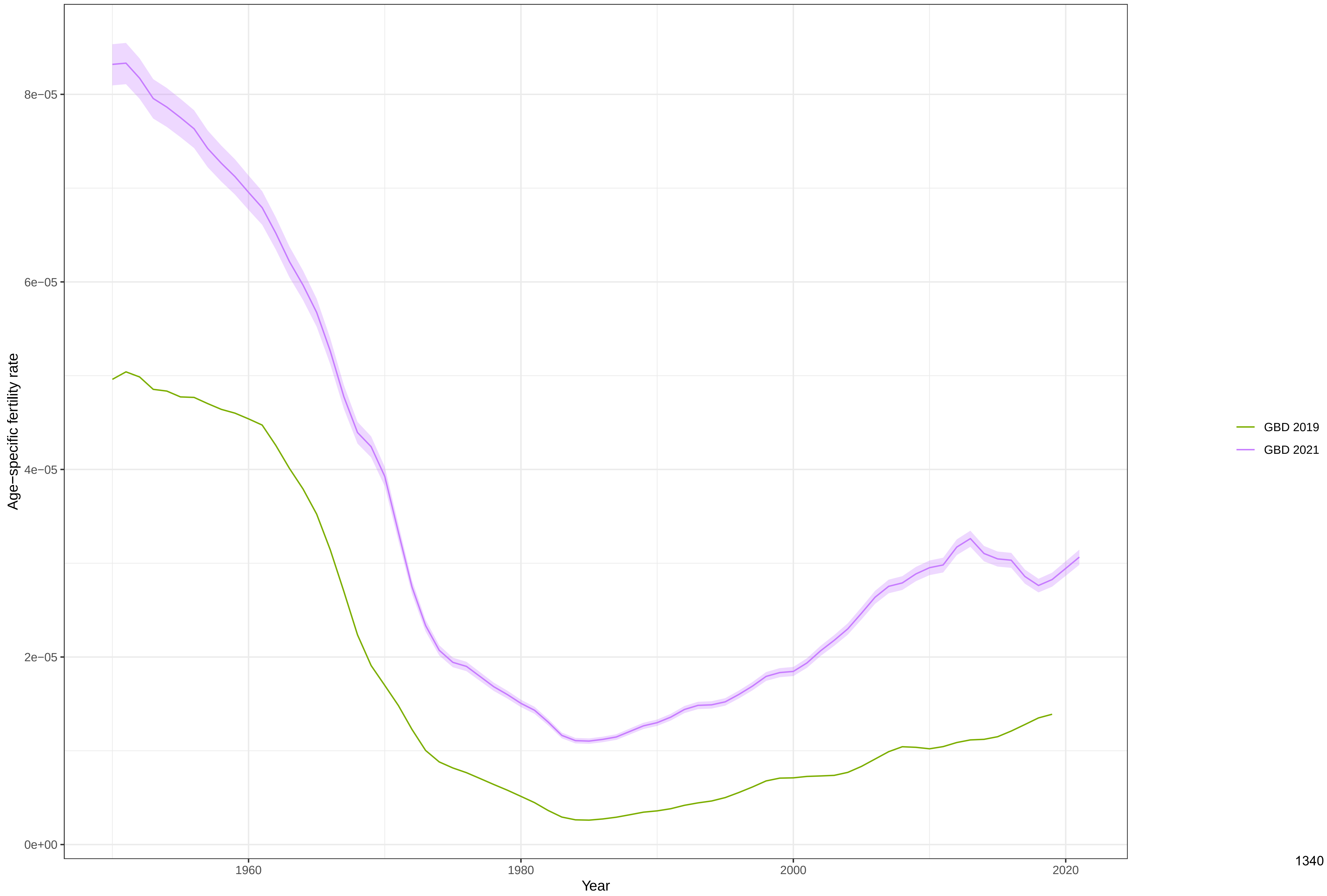

Total fertility rate fitted trend lines, 1950−2021: West Virginia TFR
Data points enclosed with a diamond are reference sources

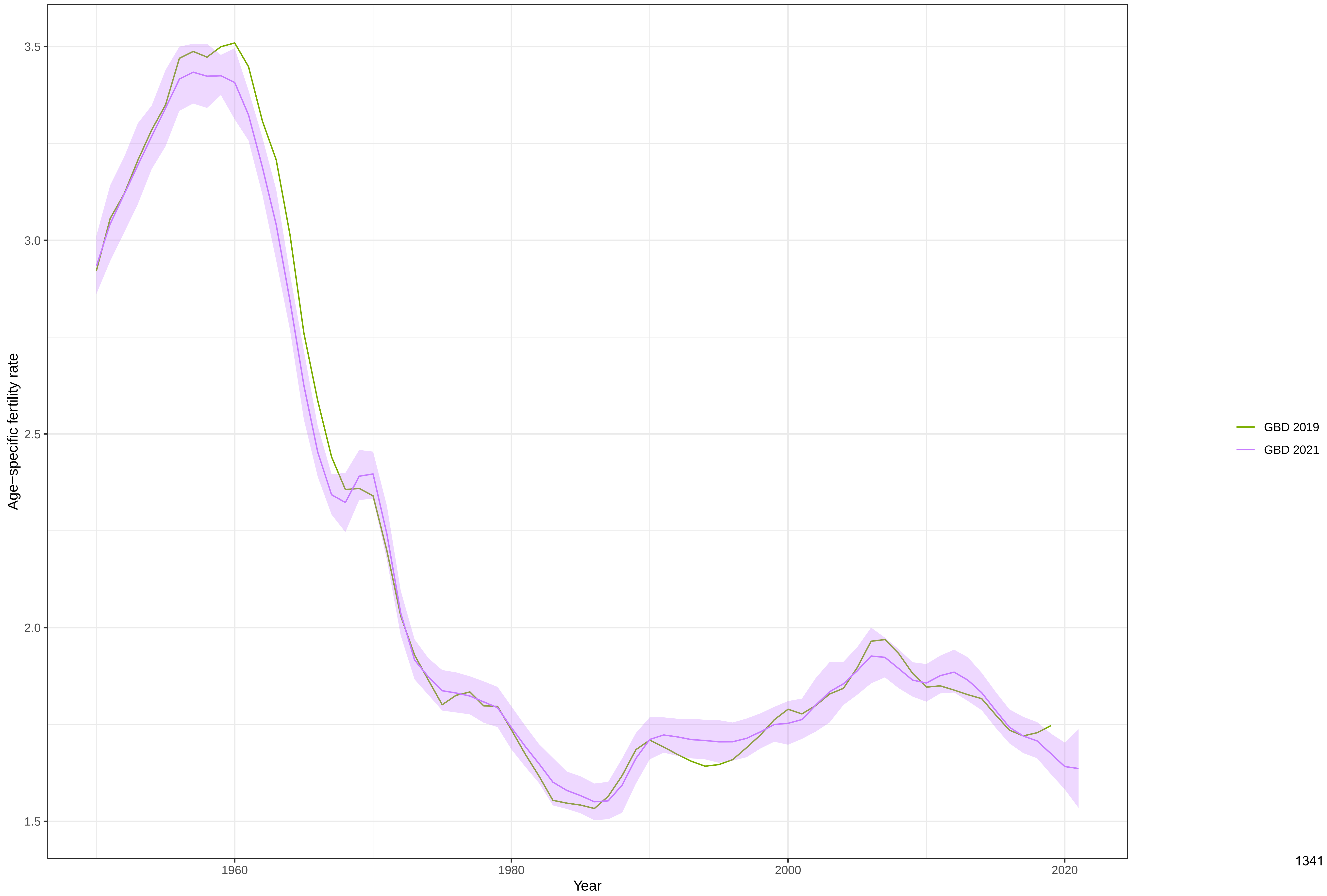

Age−specific fertility fitted trend lines, 1950−2021: Wisconsin, Ages: 10−14
Data points enclosed with a diamond are reference sources

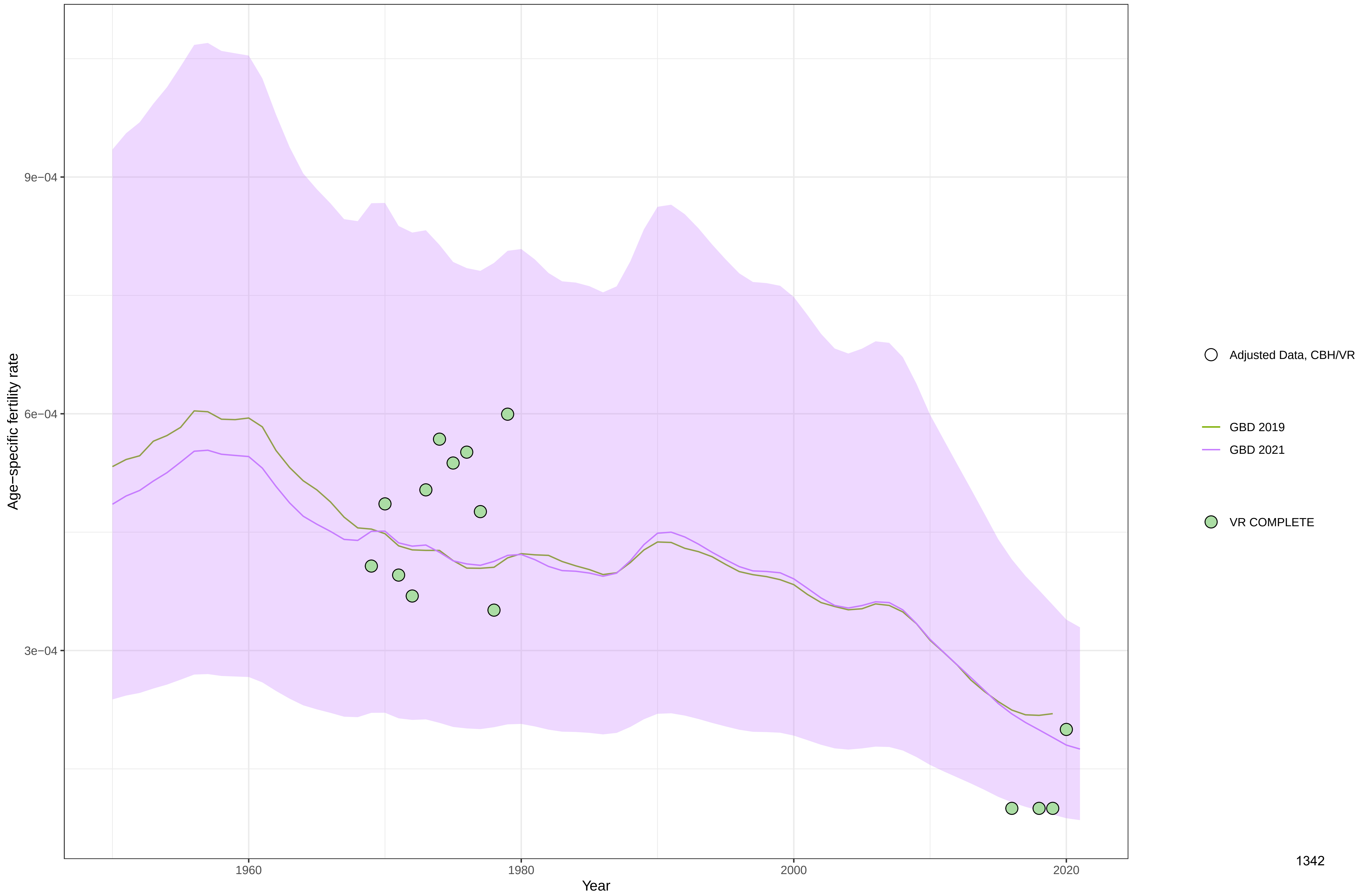

Age−specific fertility fitted trend lines, 1950−2021: Wisconsin, Ages: 15−19
Data points enclosed with a diamond are reference sources

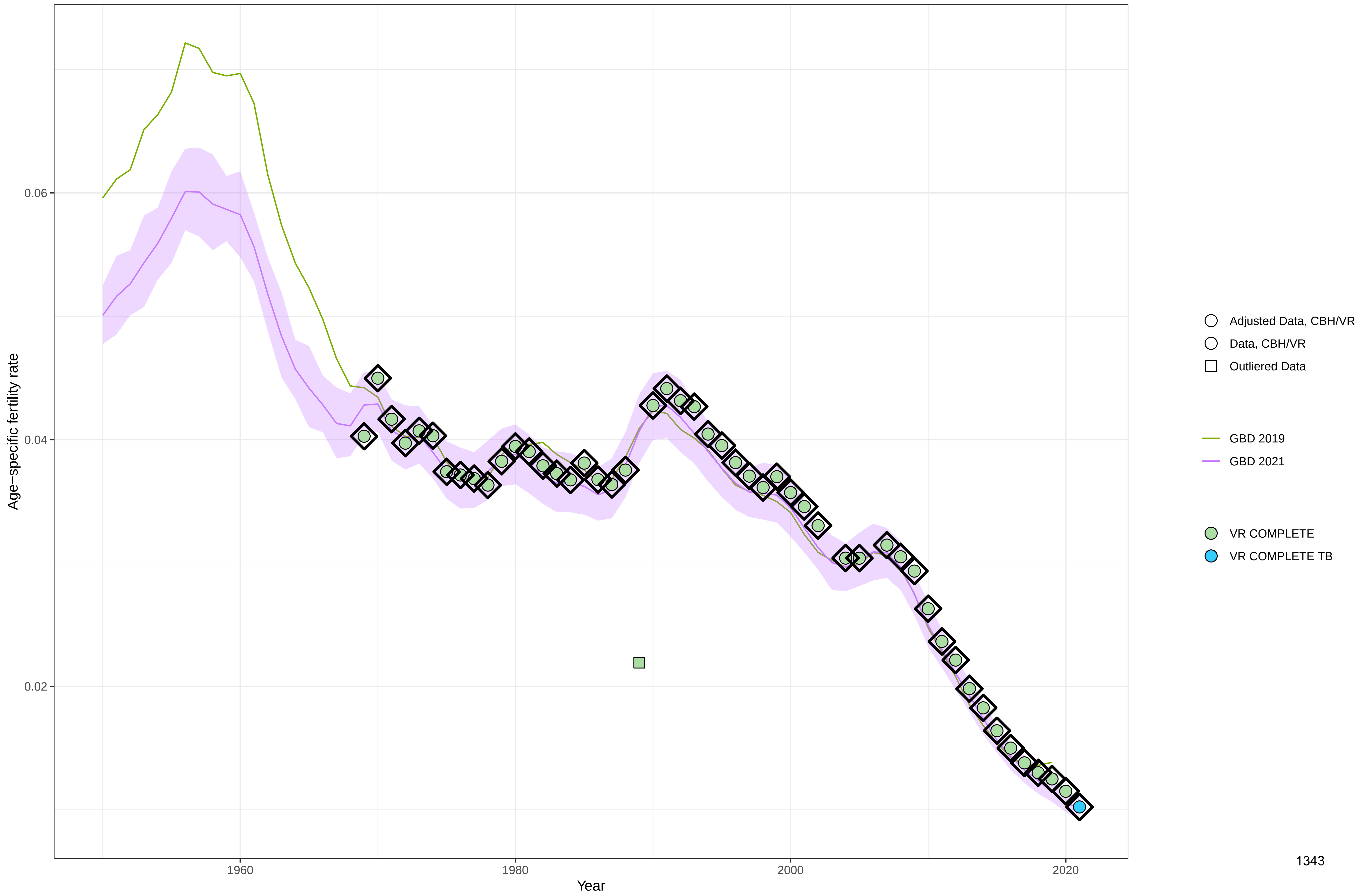

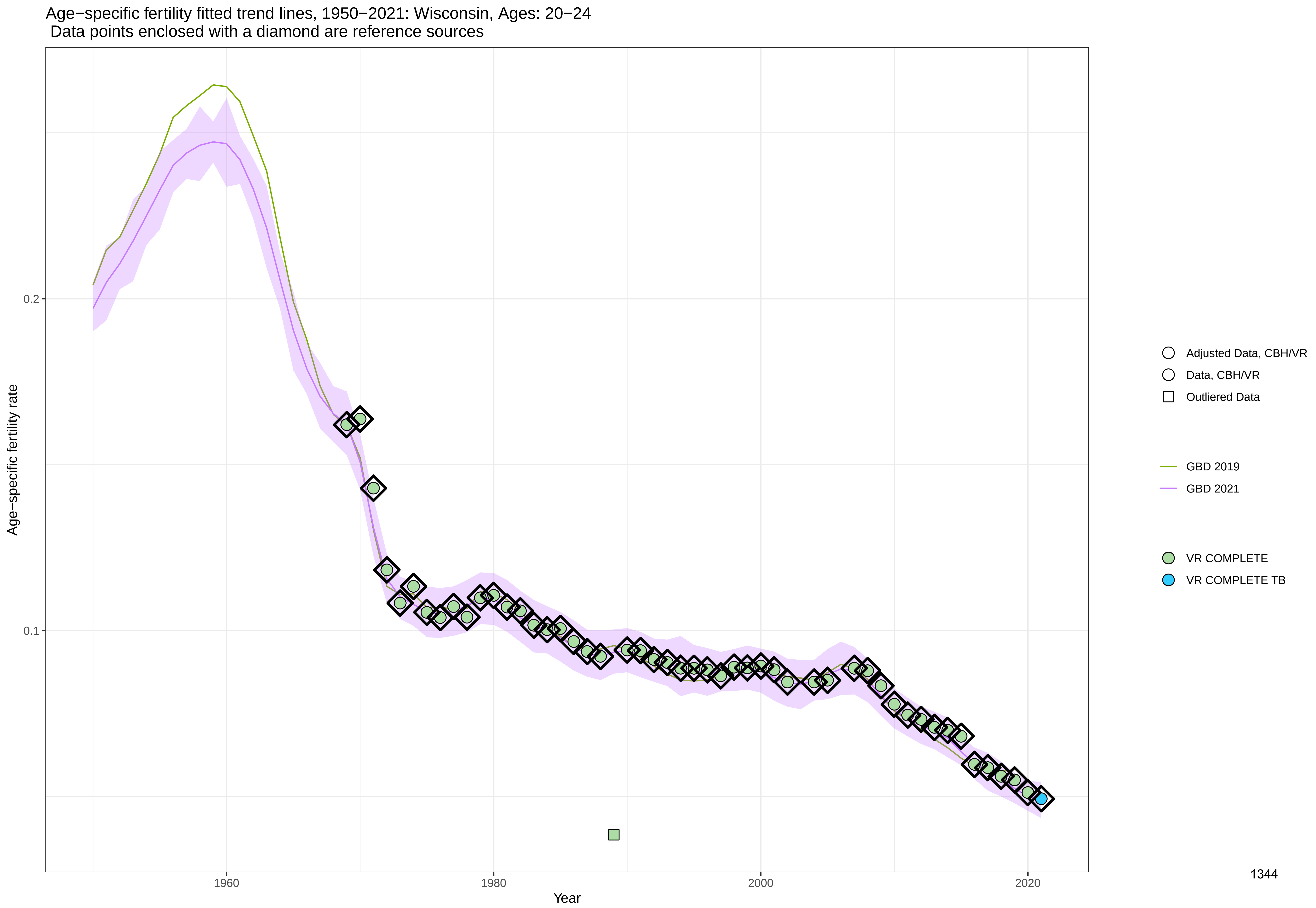

Age−specific fertility fitted trend lines, 1950−2021: Wisconsin, Ages: 20−24
Data points enclosed with a diamond are reference sources
Age−specific fertility rate
0.2
0.1
1960
1980
2000
2020
Year
Adjusted Data, CBH/VR
Data, CBH/VR
Outliered Data
GBD 2019
GBD 2021
VR COMPLETE
VR COMPLETE TB

Age−specific fertility fitted trend lines, 1950−2021: Wisconsin, Ages: 25−29
Data points enclosed with a diamond are reference sources

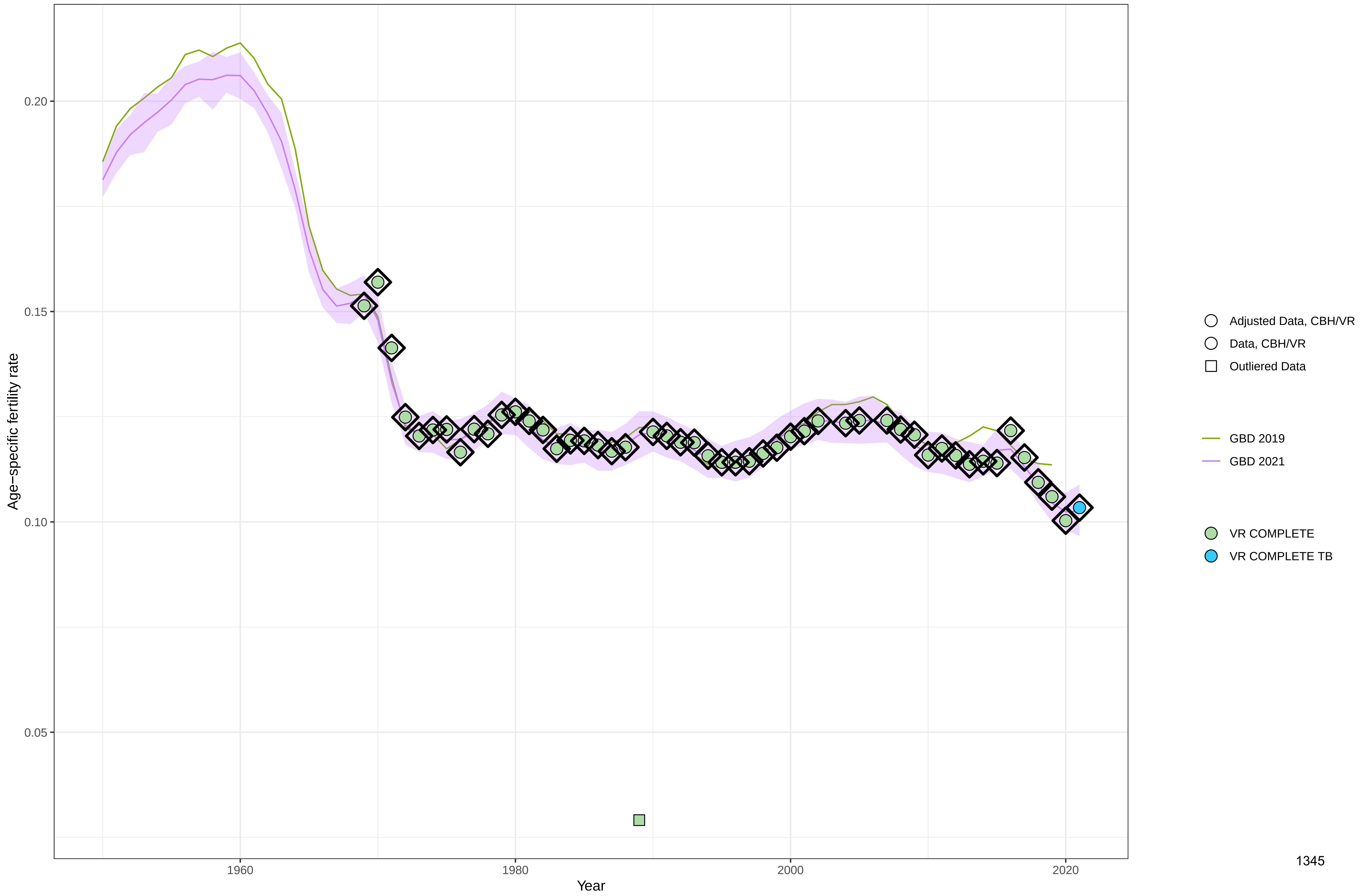

Age−specific fertility fitted trend lines, 1950−2021: Wisconsin, Ages: 30−34
Data points enclosed with a diamond are reference sources

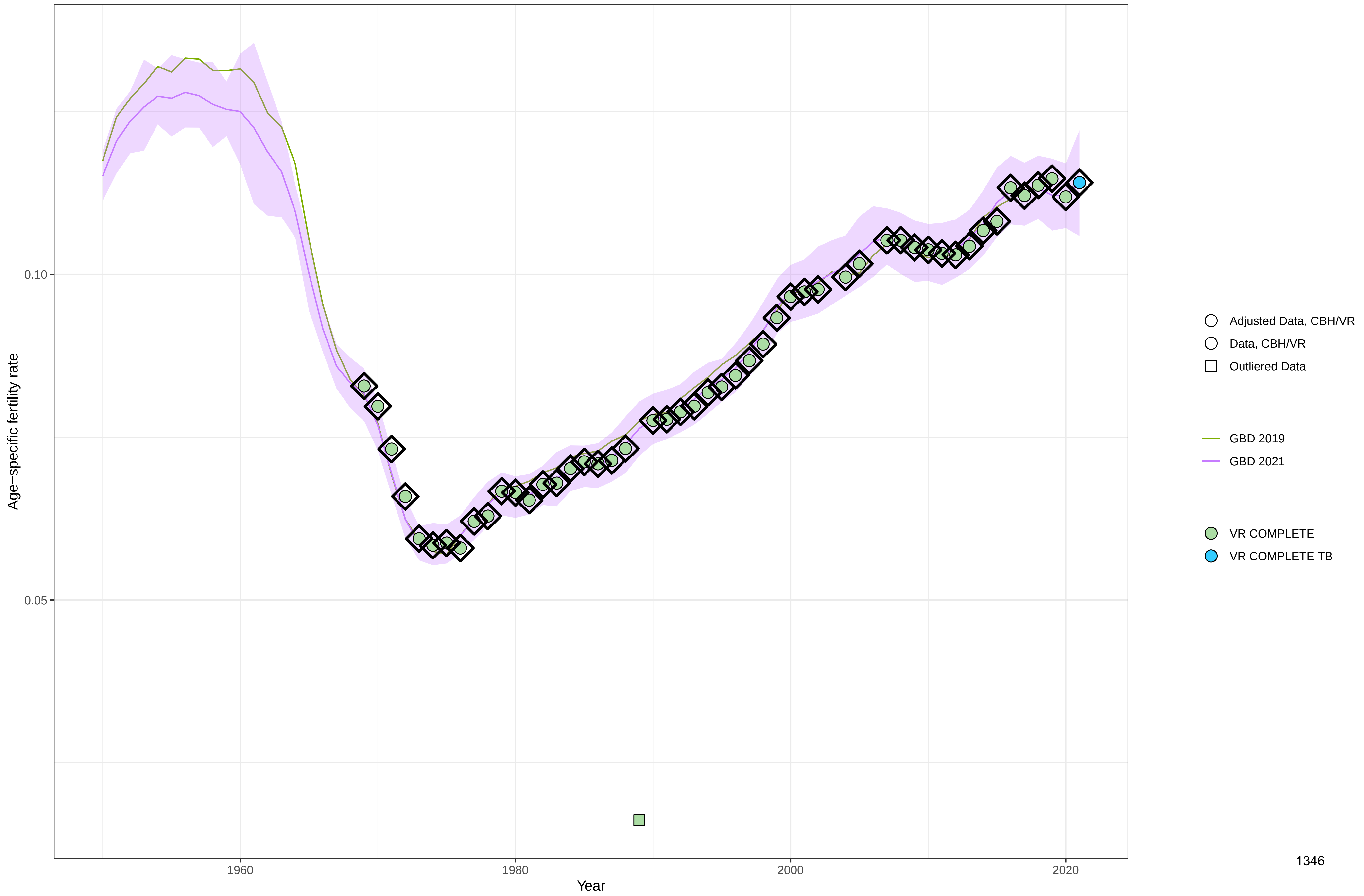

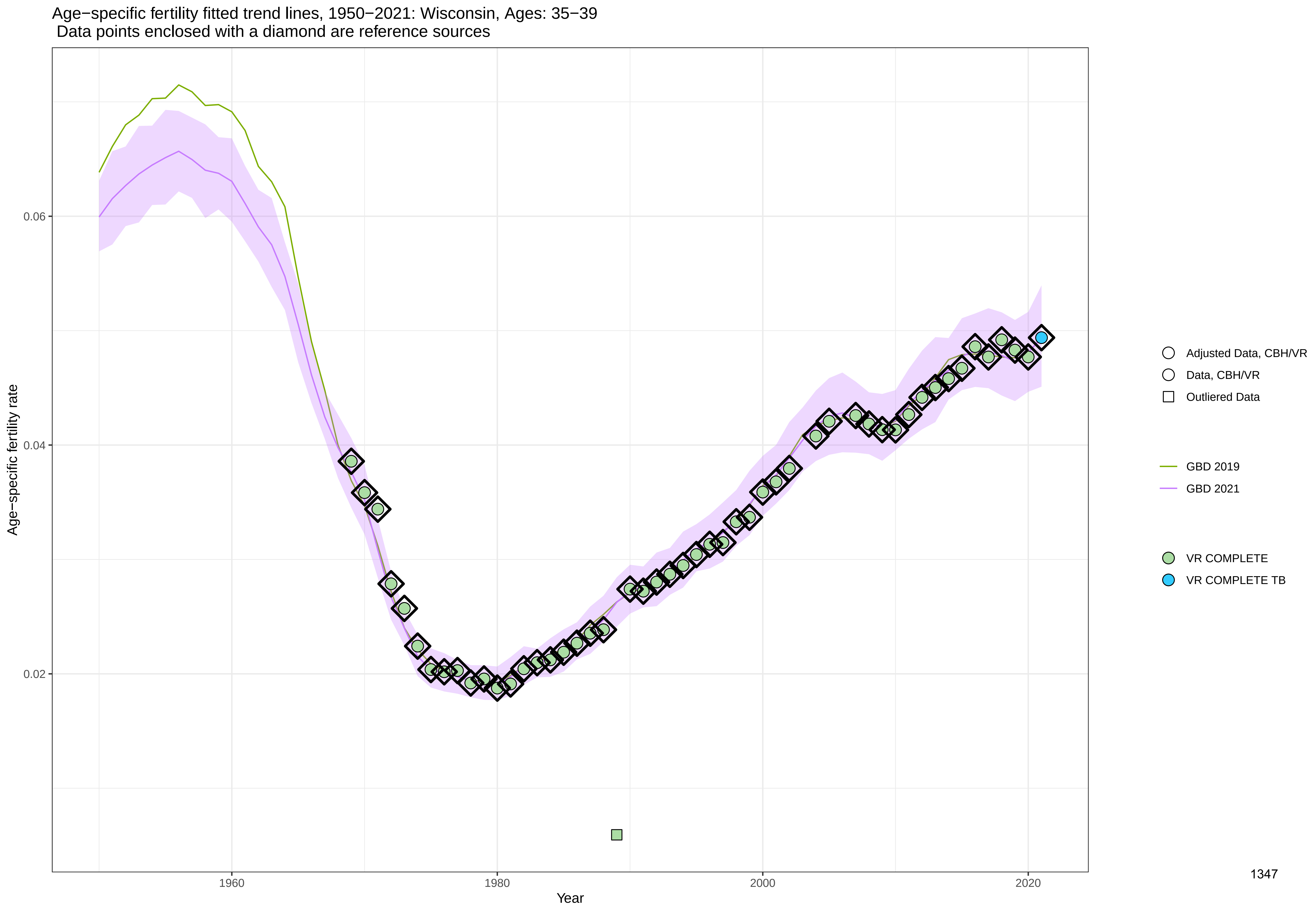

Age−specific fertility fitted trend lines, 1950−2021: Wisconsin, Ages: 35−39
Data points enclosed with a diamond are reference sources
Age−specific fertility rate
0.06
0.04
0.02
1960
1980
2000
2020
Year
Adjusted Data, CBH/VR
Data, CBH/VR
Outliered Data
GBD 2019
GBD 2021
VR COMPLETE
VR COMPLETE TB

Age−specific fertility fitted trend lines, 1950−2021: Wisconsin, Ages: 40−44
Data points enclosed with a diamond are reference sources

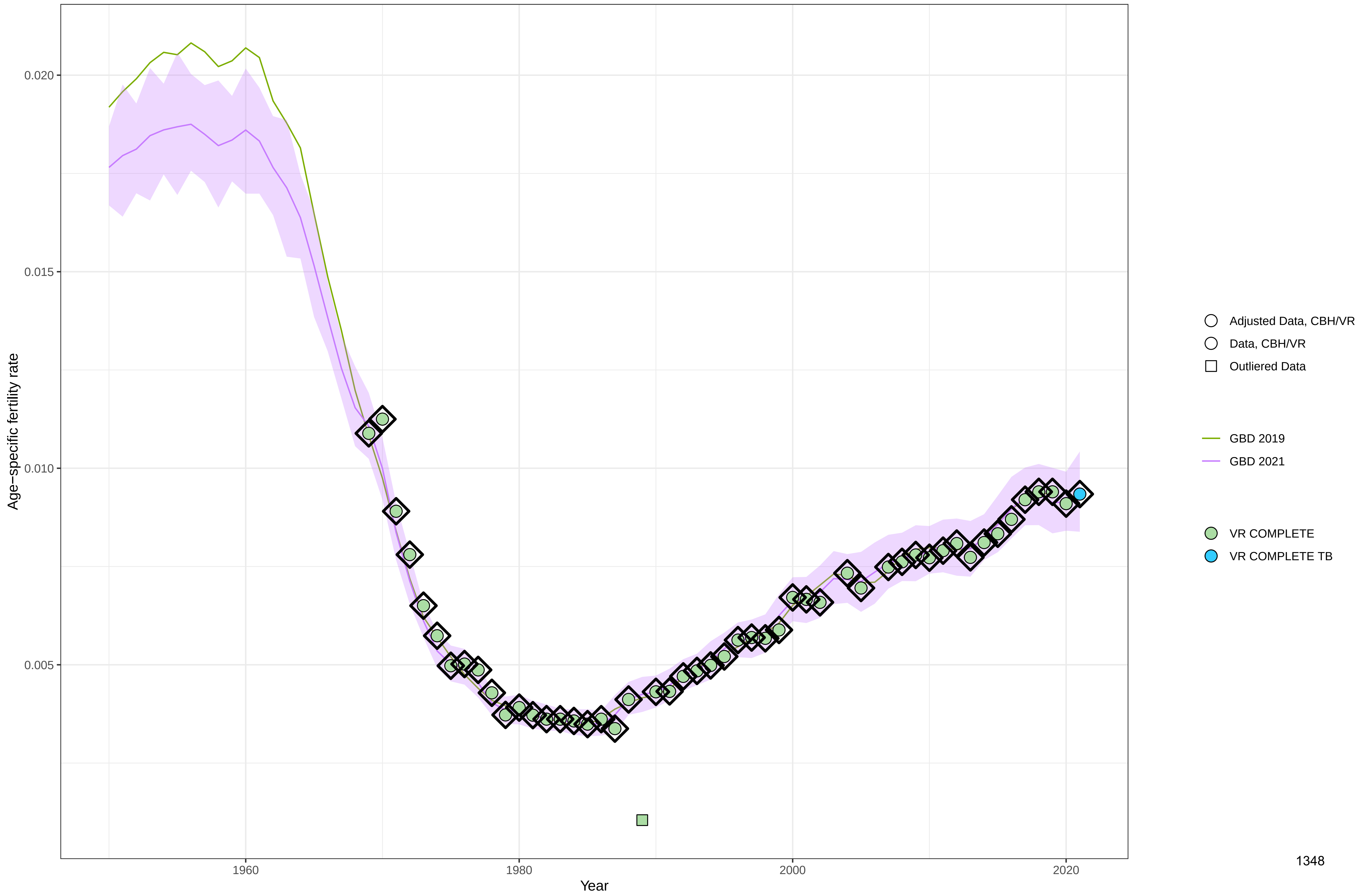

Age−specific fertility fitted trend lines, 1950−2021: Wisconsin, Ages: 45−49
Data points enclosed with a diamond are reference sources

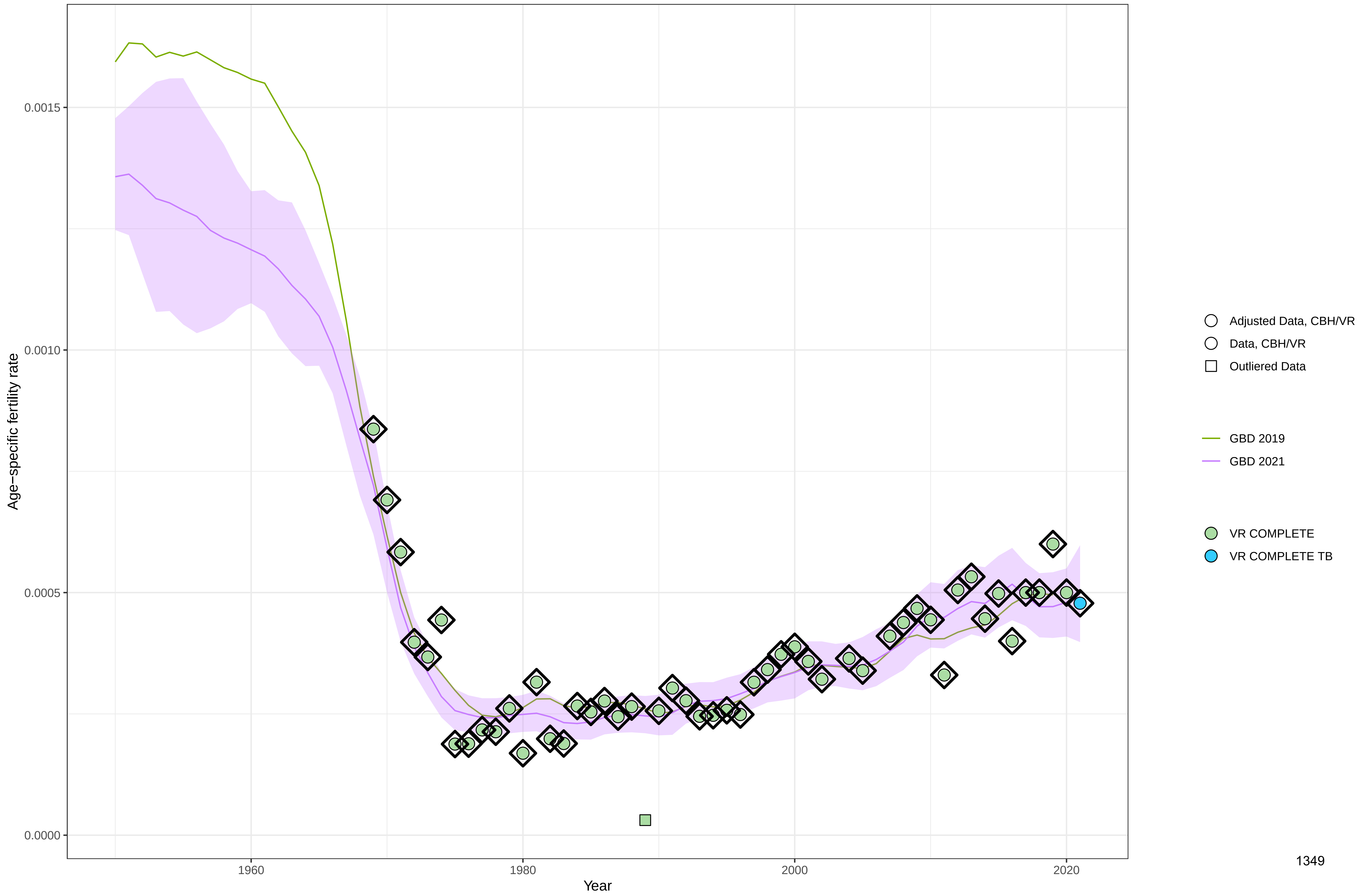

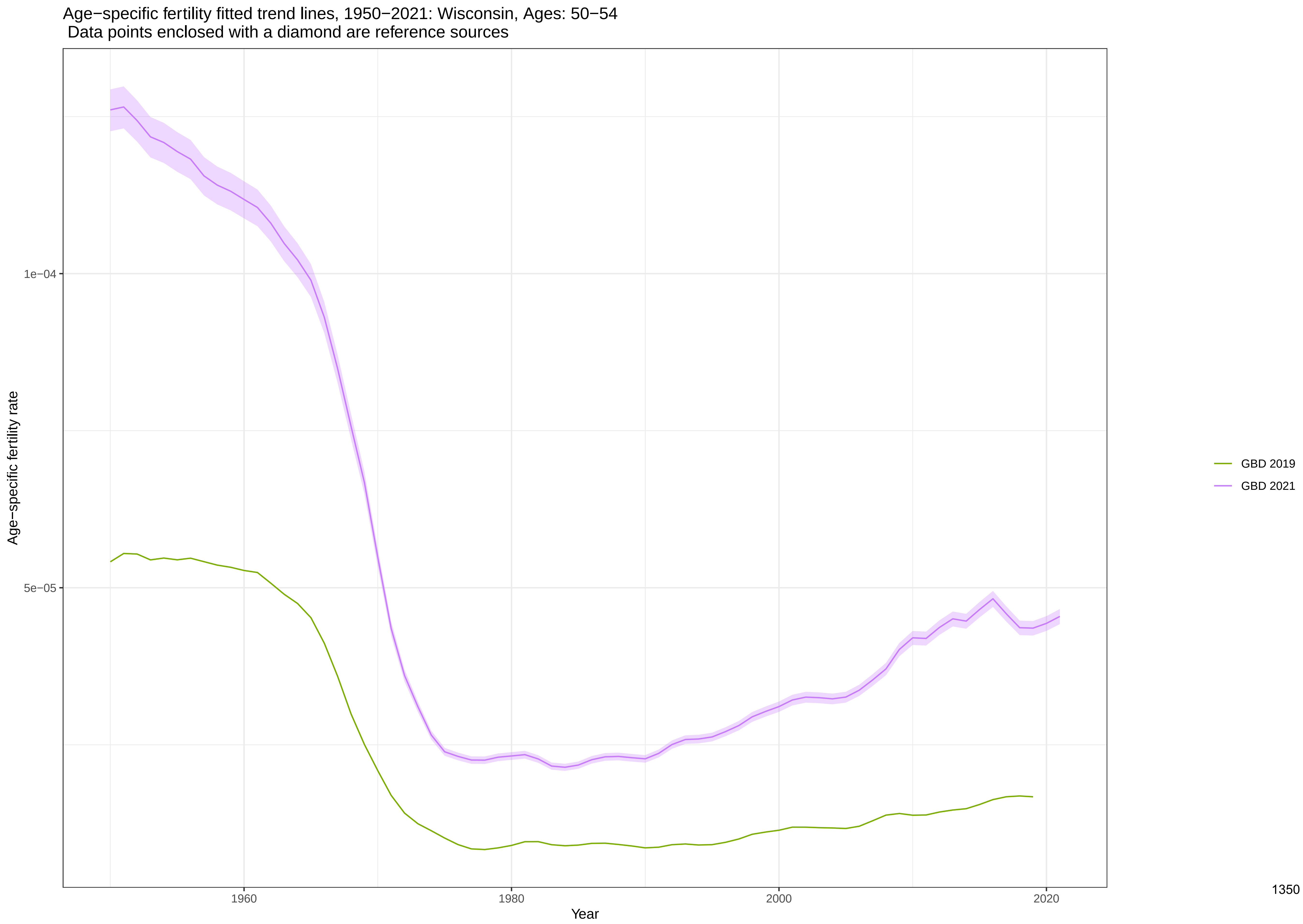
Age−specific fertility fitted trend lines, 1950−2021: Wisconsin, Ages: 50−54
Data points enclosed with a diamond are reference sources
Age−specific fertility rate
1e−04
5e−05
1960
1980
2000
2020
Year
GBD 2019
GBD 2021

Total fertility rate fitted trend lines, 1950−2021: Wisconsin TFR
Data points enclosed with a diamond are reference sources

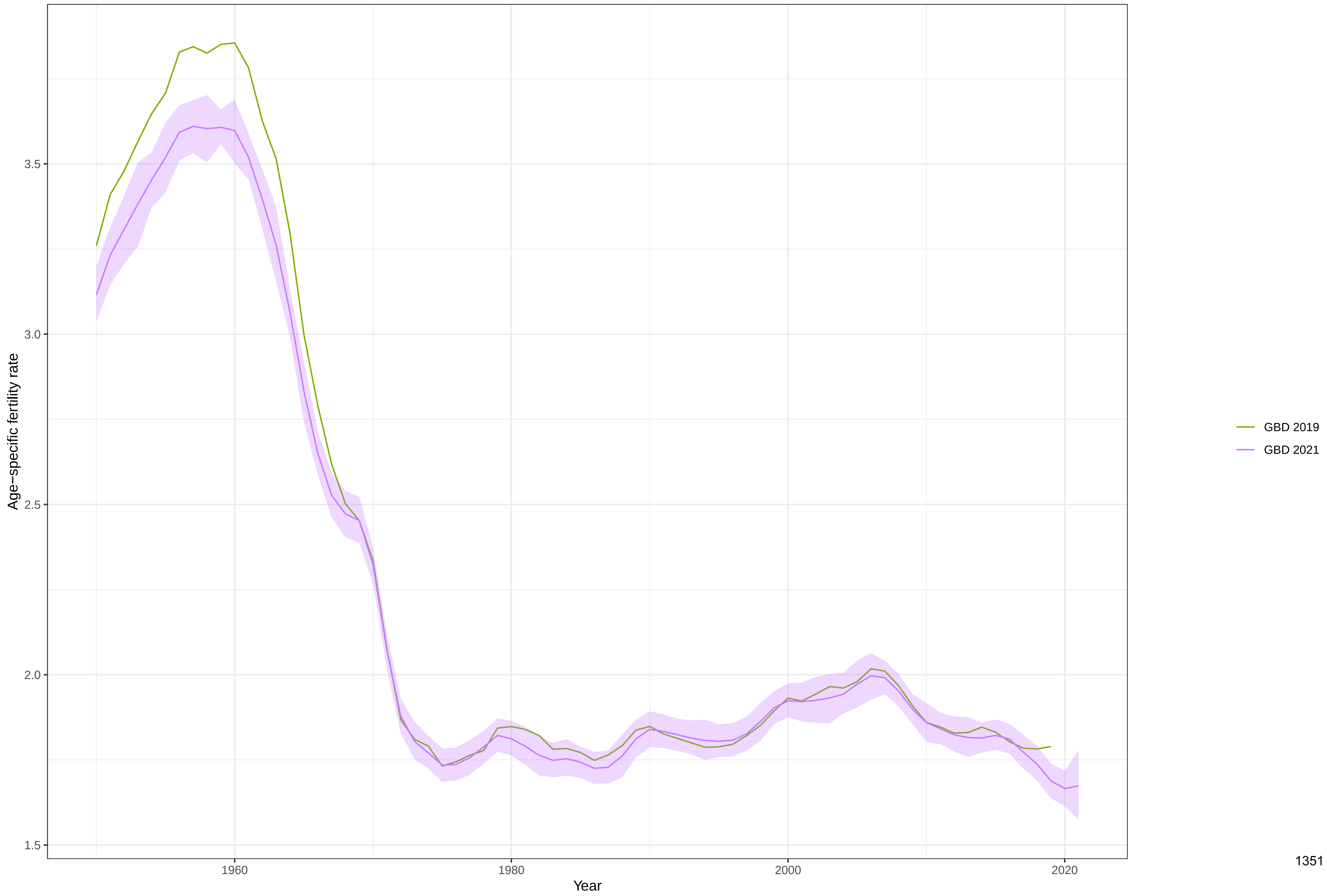

Age−specific fertility fitted trend lines, 1950−2021: Wyoming, Ages: 10−14
Data points enclosed with a diamond are reference sources

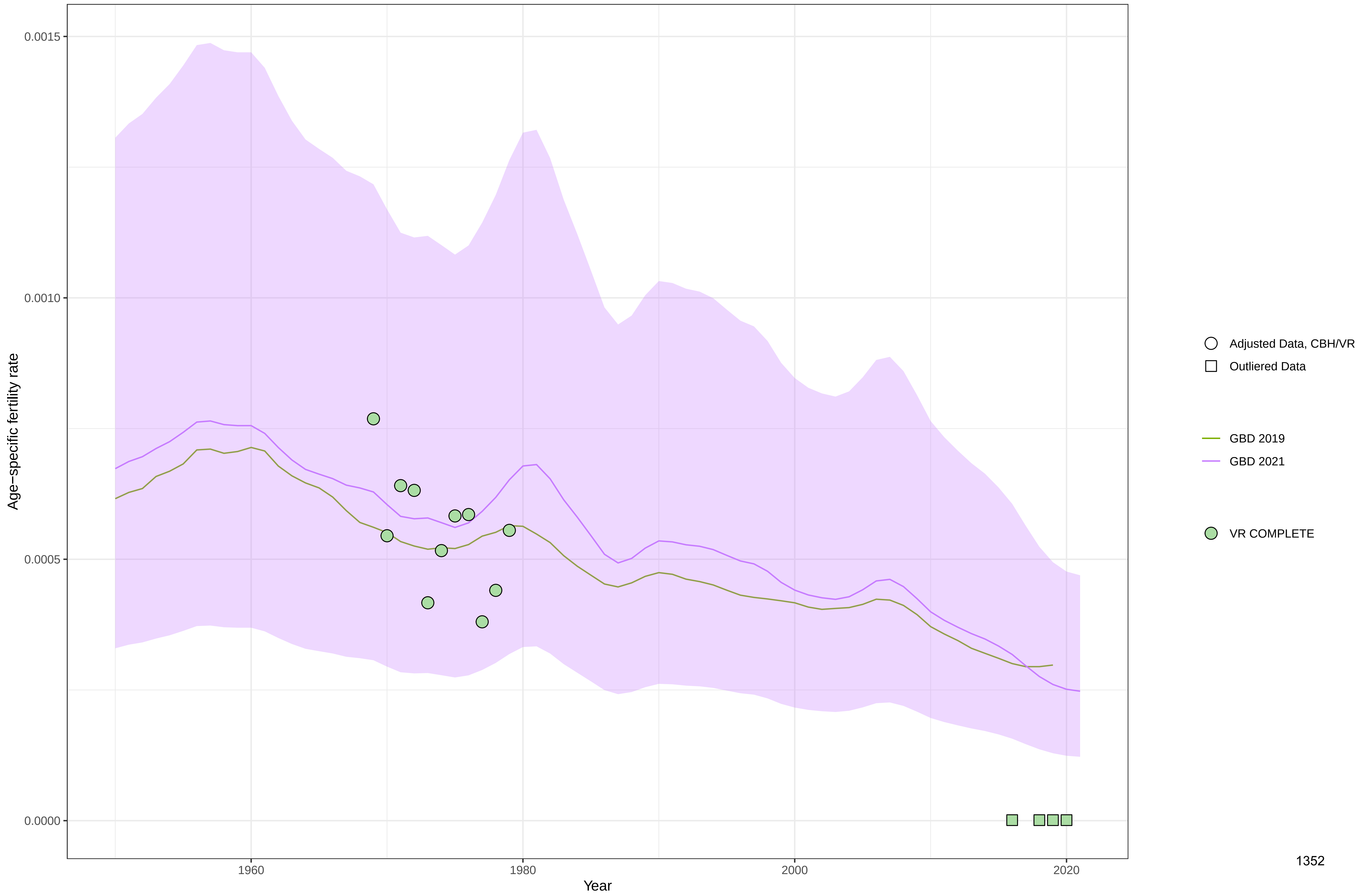

Age−specific fertility fitted trend lines, 1950−2021: Wyoming, Ages: 15−19
Data points enclosed with a diamond are reference sources

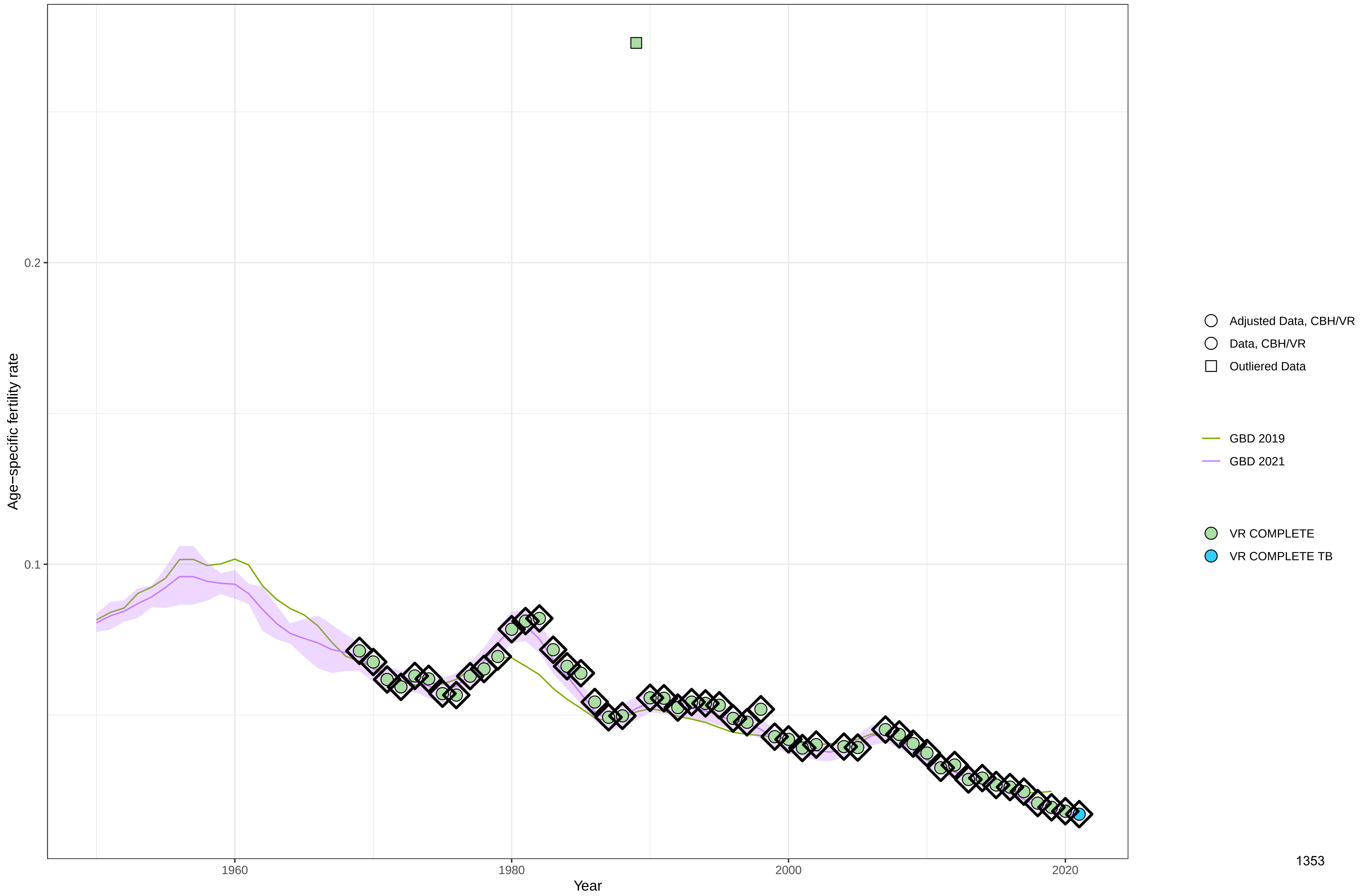

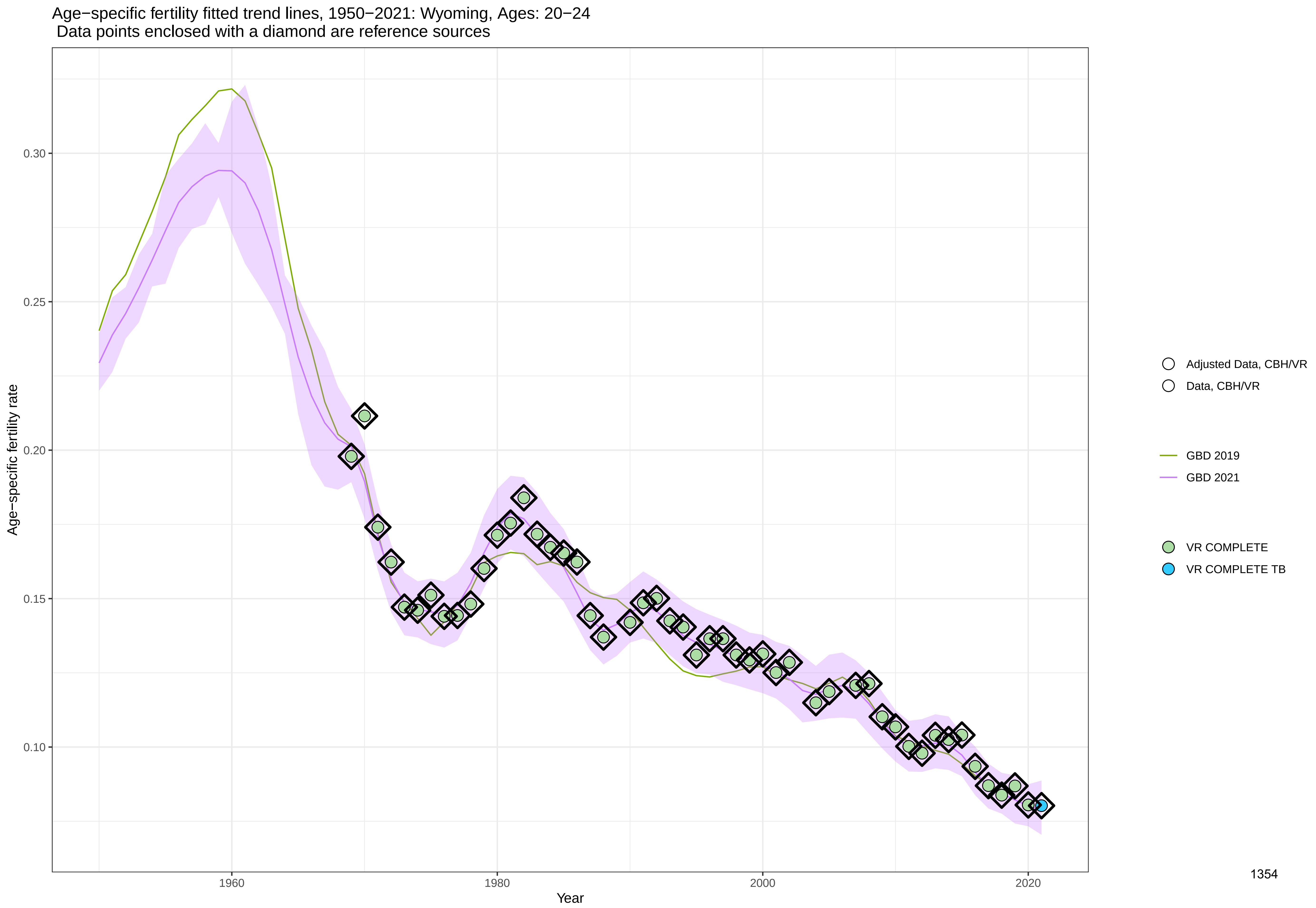

Age−specific fertility fitted trend lines, 1950−2021: Wyoming, Ages: 20−24
Data points enclosed with a diamond are reference sources
Age−specific fertility rate
0.30
0.25
0.20
0.15
0.10
1960
1980
2000
2020
Year
Adjusted Data, CBH/VR
Data, CBH/VR
GBD 2019
GBD 2021
VR COMPLETE
VR COMPLETE TB

Age−specific fertility fitted trend lines, 1950−2021: Wyoming, Ages: 25−29
Data points enclosed with a diamond are reference sources

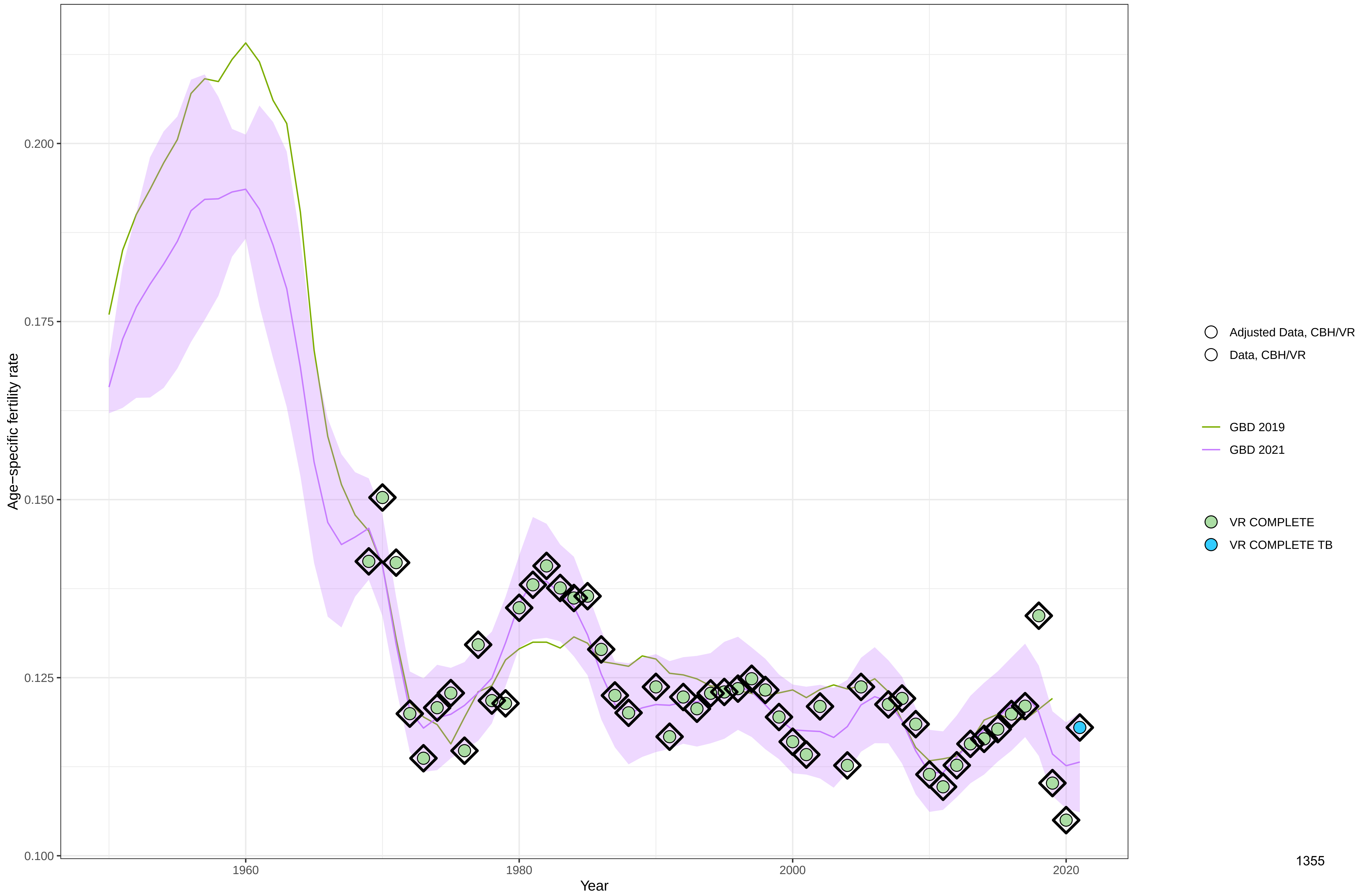

Age−specific fertility fitted trend lines, 1950−2021: Wyoming, Ages: 30−34
Data points enclosed with a diamond are reference sources

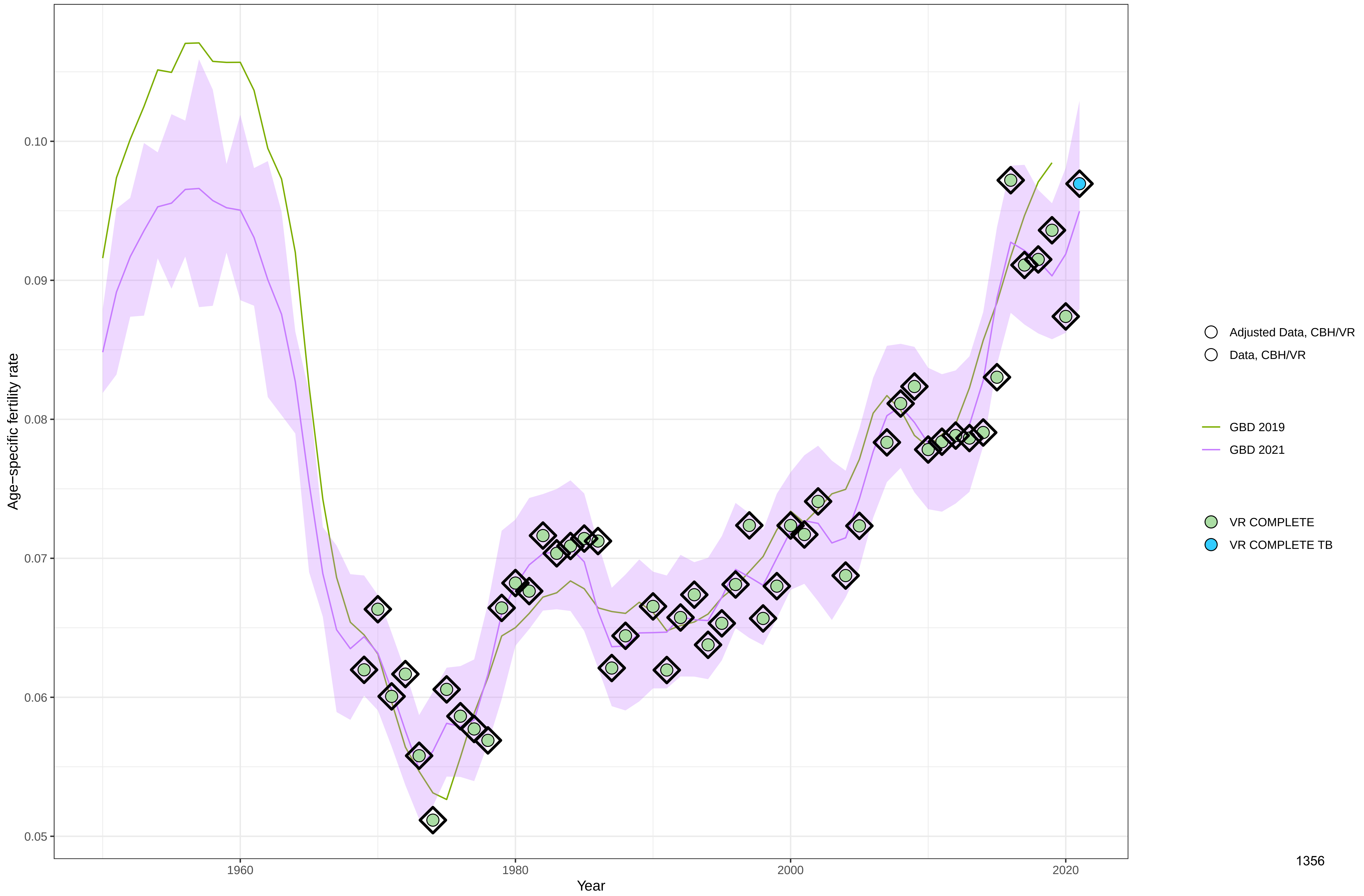

Age−specific fertility fitted trend lines, 1950−2021: Wyoming, Ages: 35−39
Data points enclosed with a diamond are reference sources

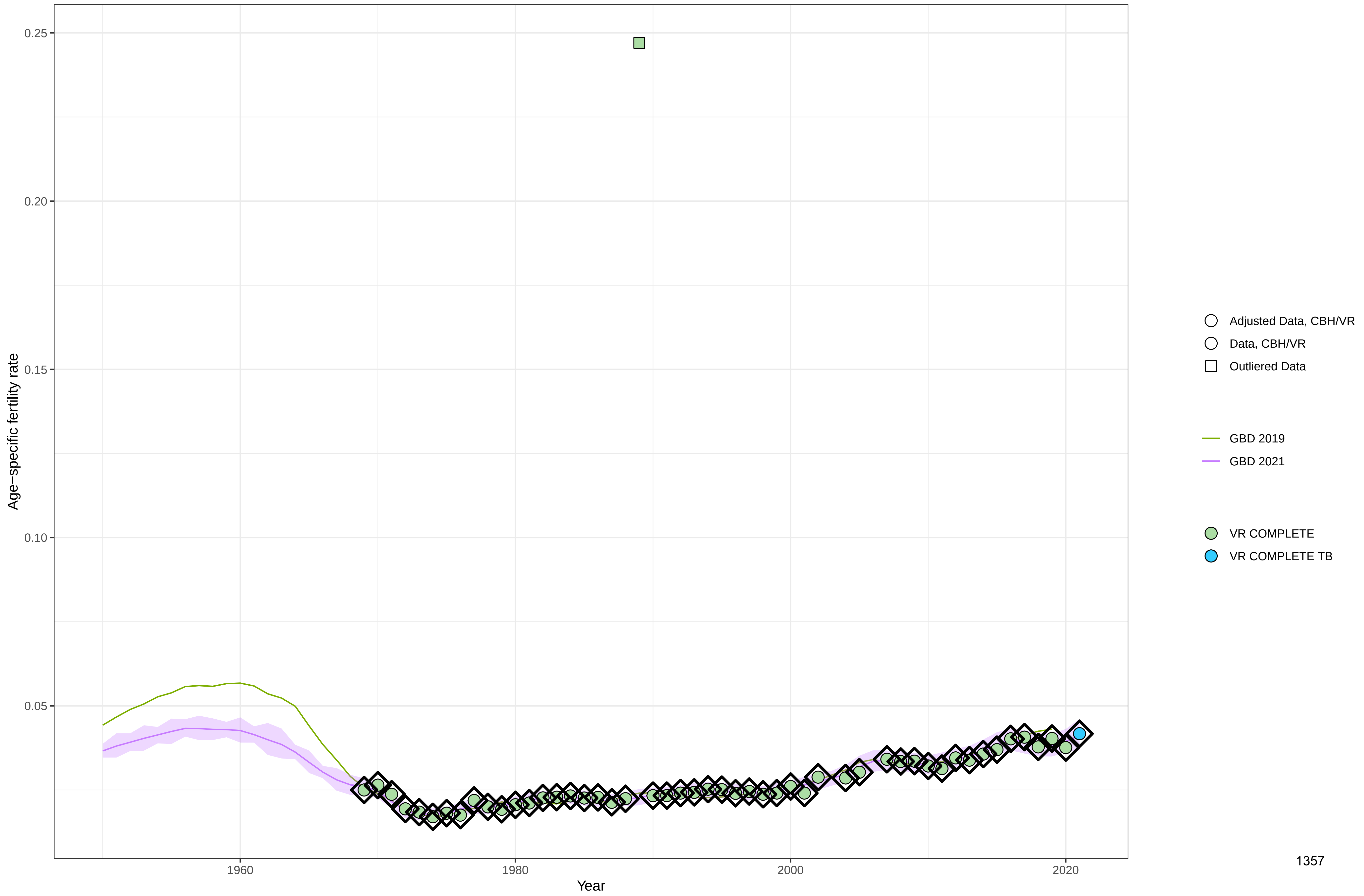

Age−specific fertility fitted trend lines, 1950−2021: Wyoming, Ages: 40−44
Data points enclosed with a diamond are reference sources

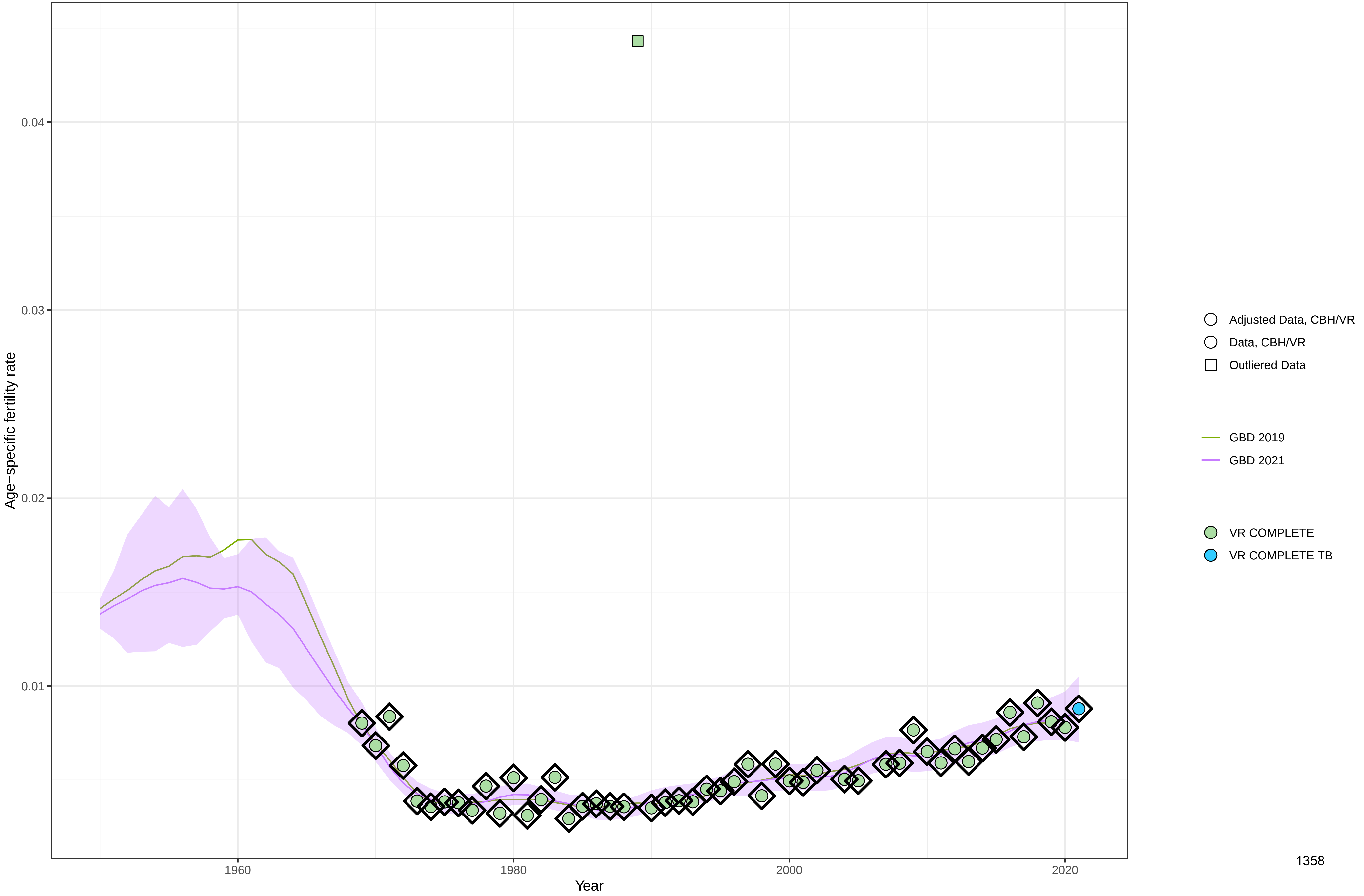

Age−specific fertility fitted trend lines, 1950−2021: Wyoming, Ages: 45−49
Data points enclosed with a diamond are reference sources

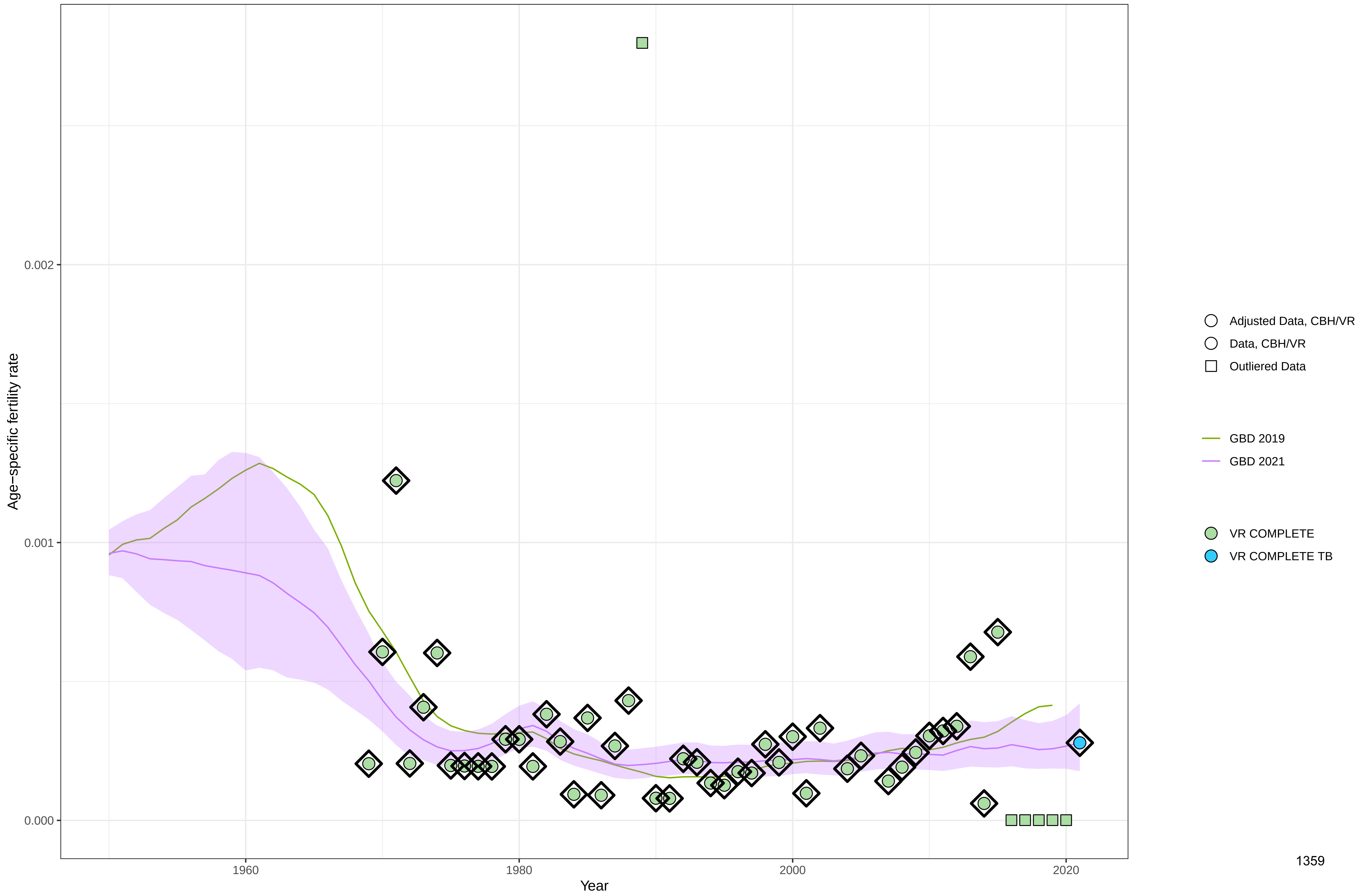

Age−specific fertility fitted trend lines, 1950−2021: Wyoming, Ages: 50−54
Data points enclosed with a diamond are reference sources

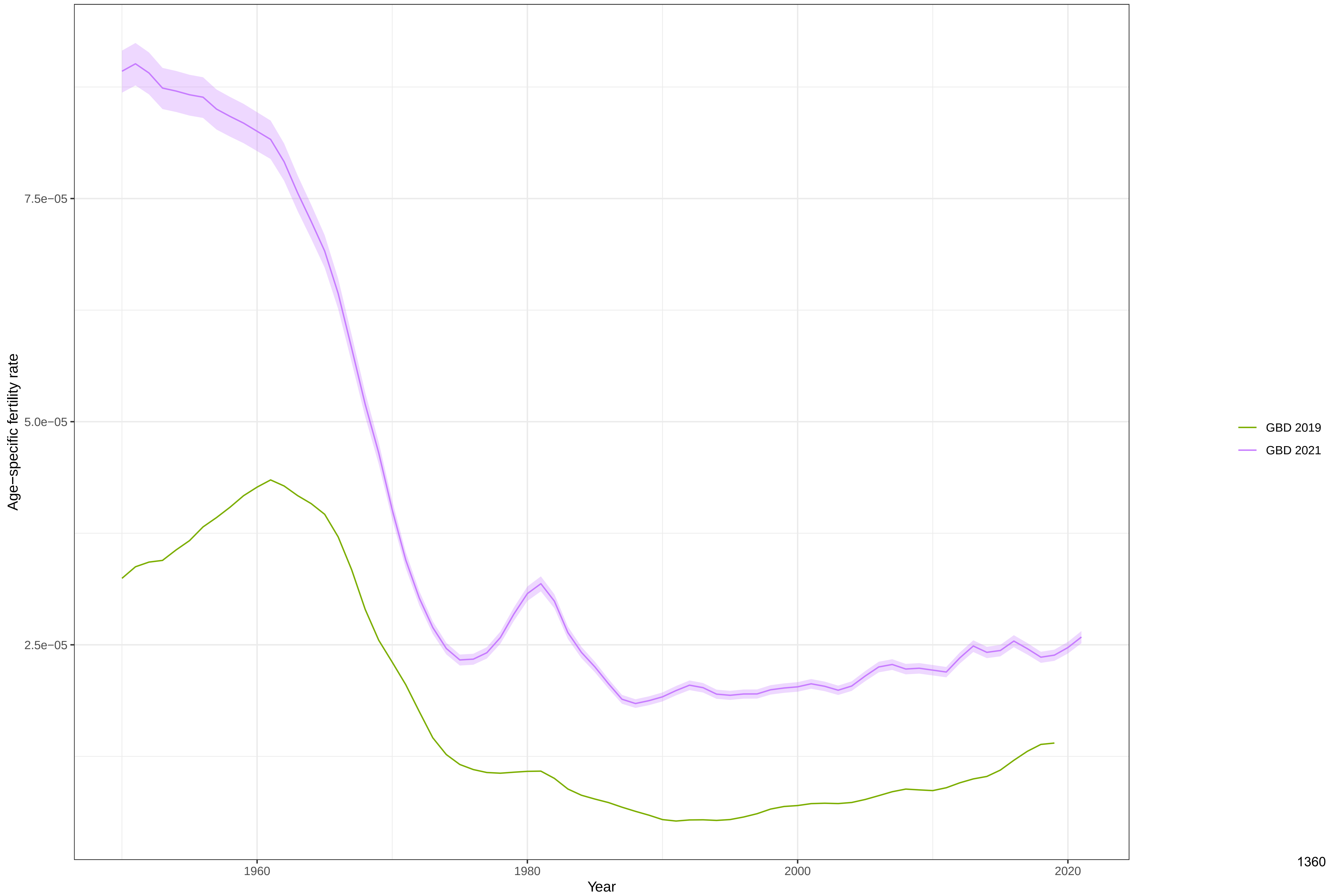

Total fertility rate fitted trend lines, 1950−2021: Wyoming TFR
Data points enclosed with a diamond are reference sources

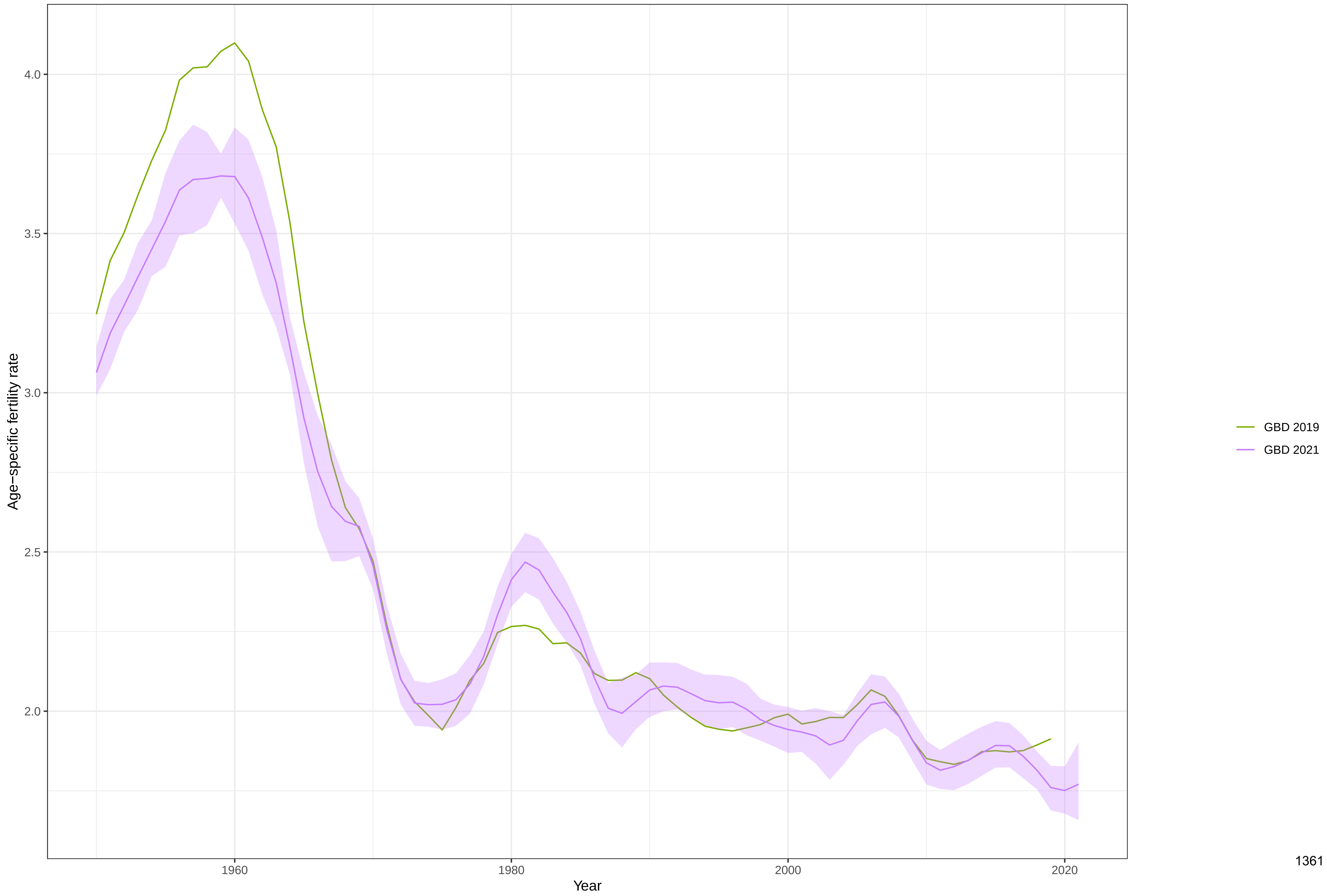

Age−specific fertility fitted trend lines, 1950−2021: Argentina, Ages: 10−14
Data points enclosed with a diamond are reference sources

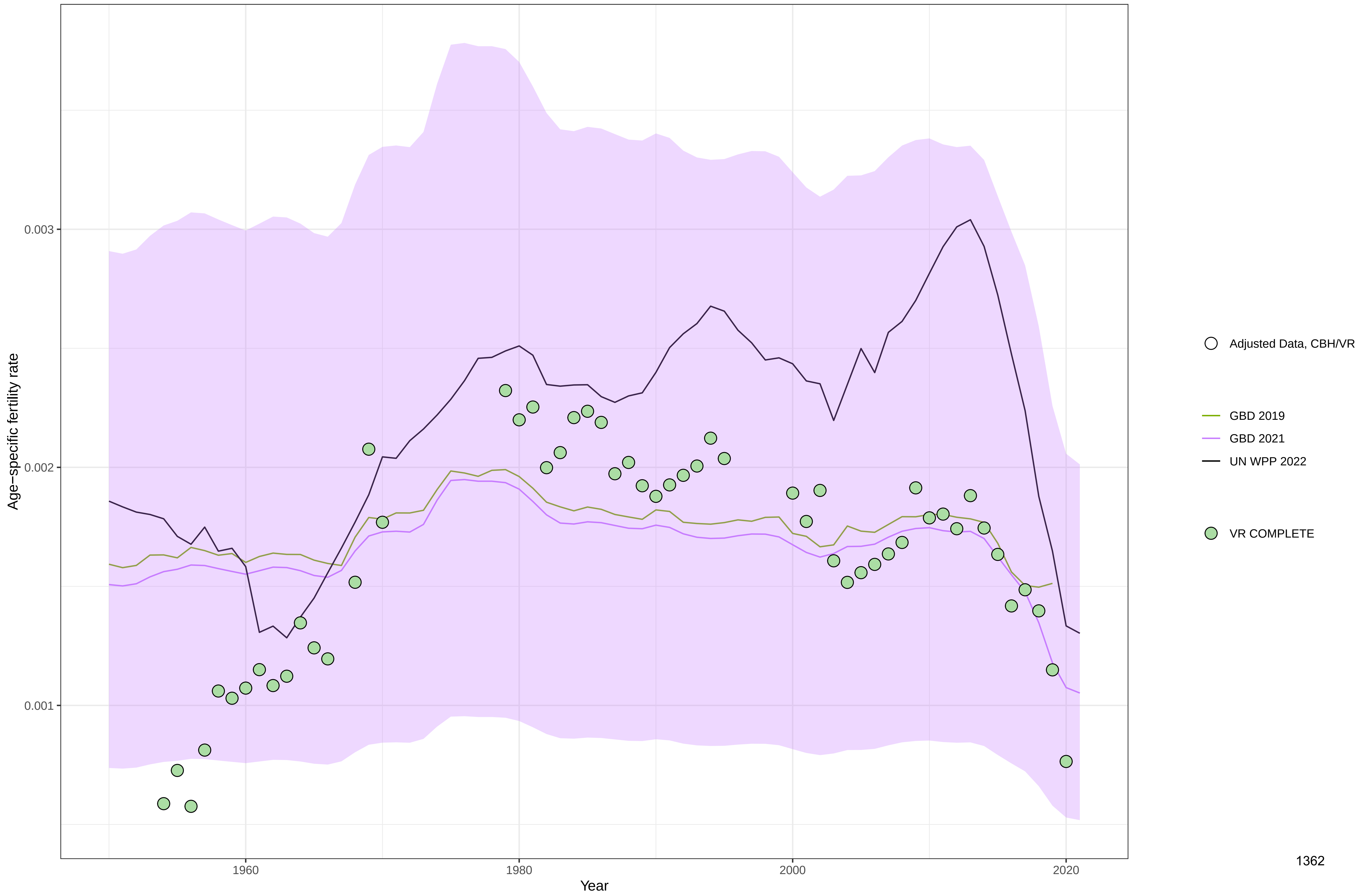

Age−specific fertility fitted trend lines, 1950−2021: Argentina, Ages: 15−19
Data points enclosed with a diamond are reference sources

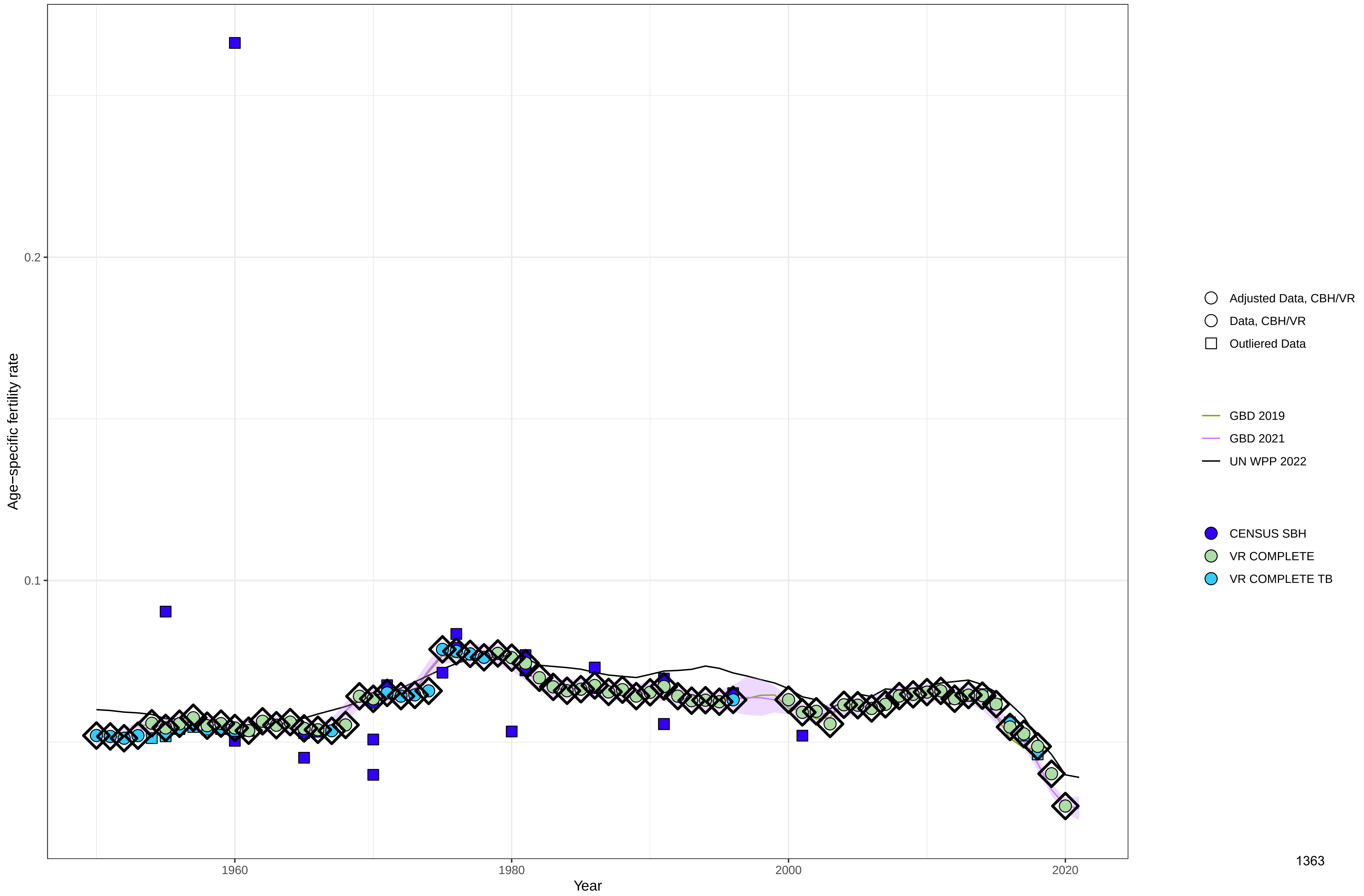

Age−specific fertility fitted trend lines, 1950−2021: Argentina, Ages: 20−24
Data points enclosed with a diamond are reference sources

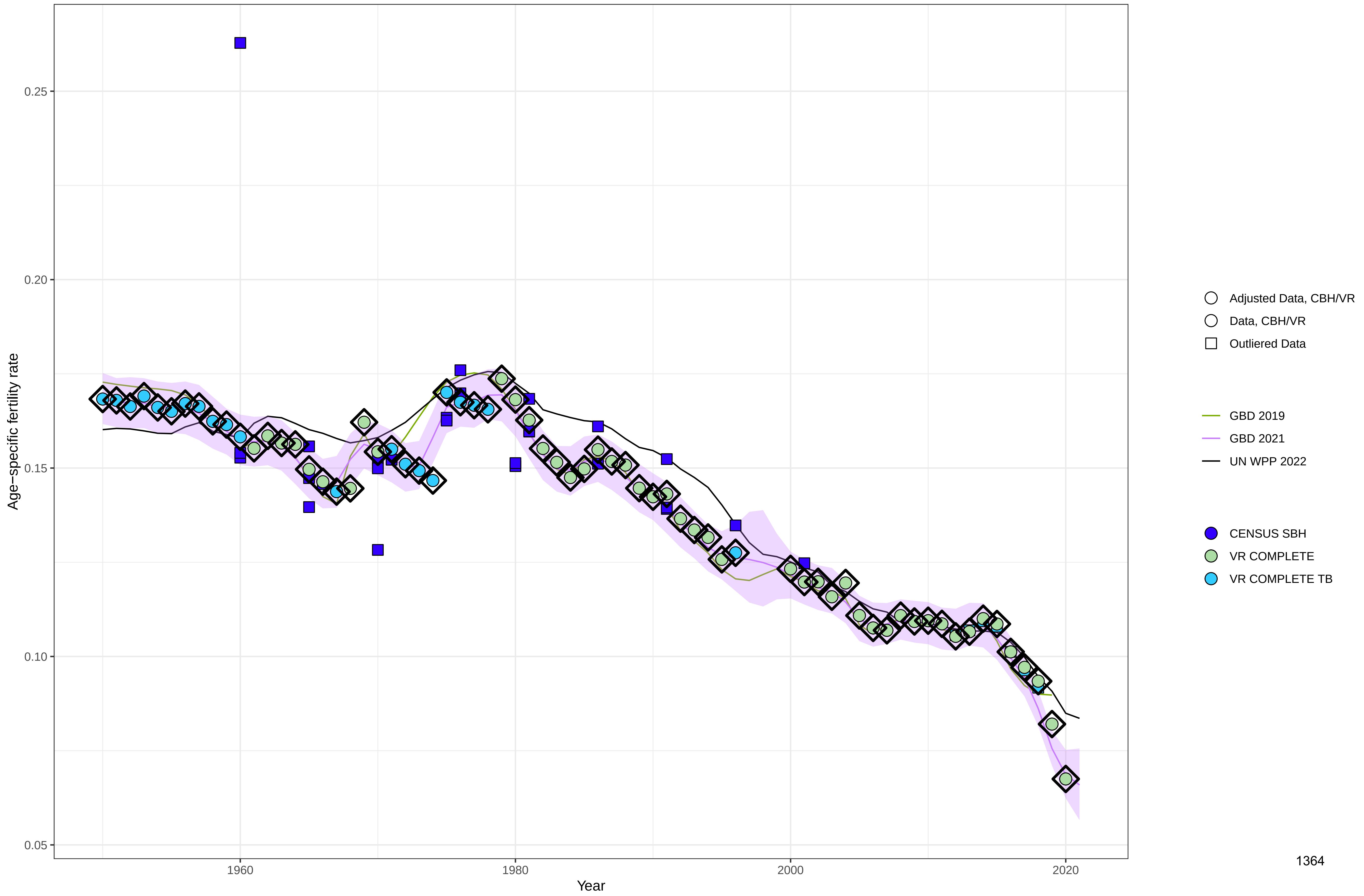

Age−specific fertility fitted trend lines, 1950−2021: Argentina, Ages: 25−29
Data points enclosed with a diamond are reference sources

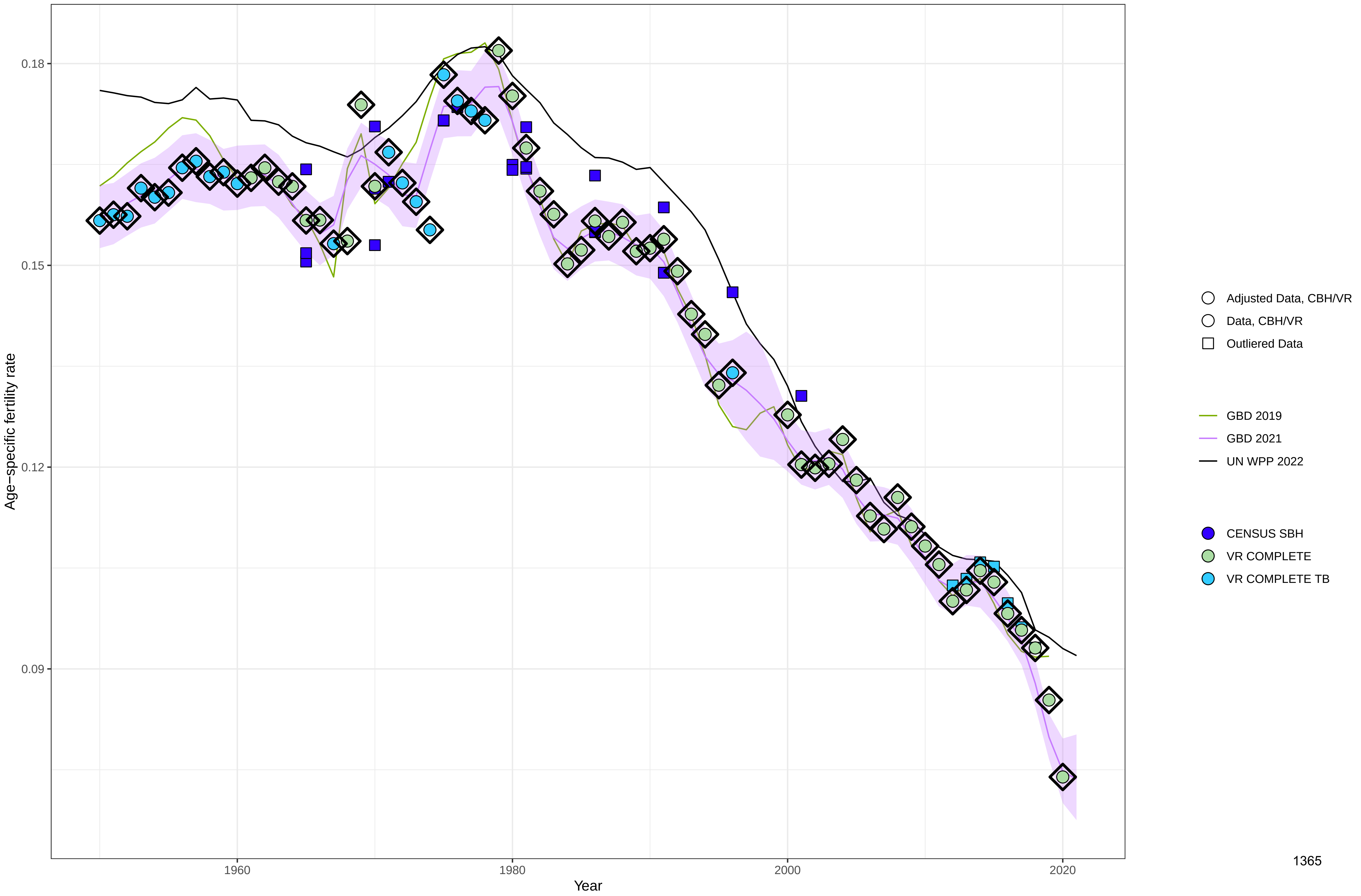

Age−specific fertility fitted trend lines, 1950−2021: Argentina, Ages: 30−34
Data points enclosed with a diamond are reference sources

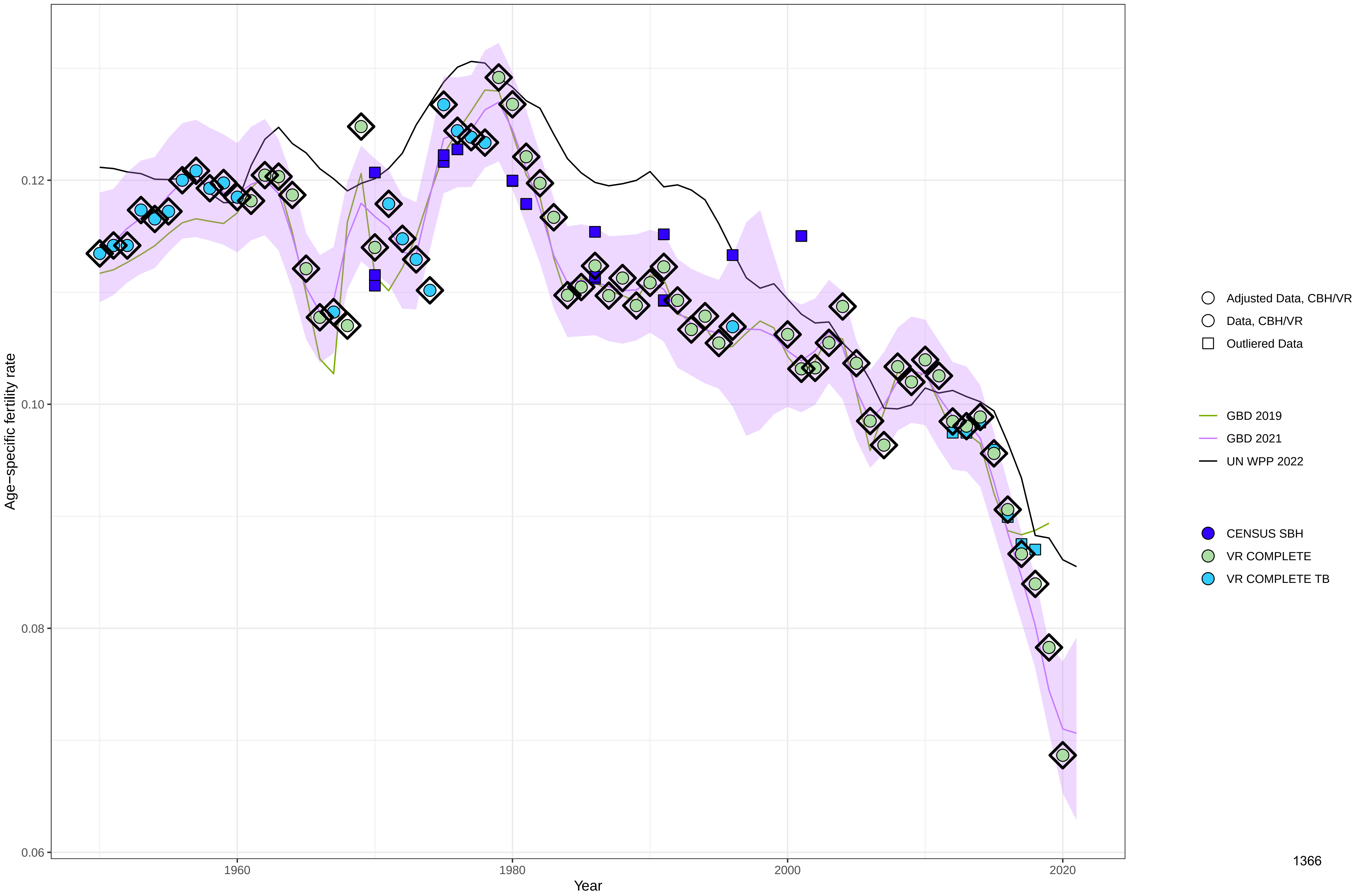

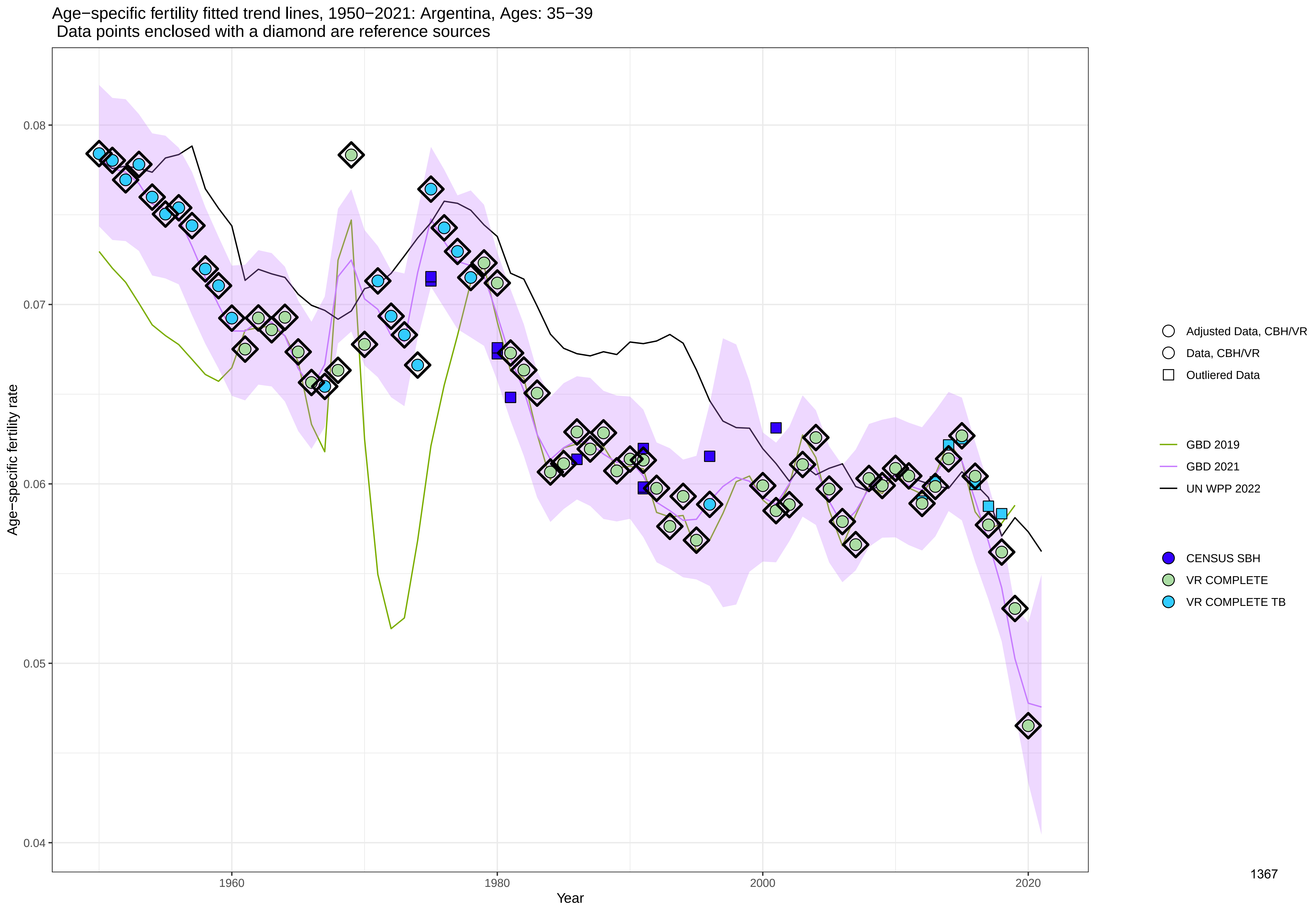

Age−specific fertility fitted trend lines, 1950−2021: Argentina, Ages: 35−39
Data points enclosed with a diamond are reference sources
Age−specific fertility rate
0.08
0.07
0.06
0.05
0.04
1960
1980
2000
2020
Year
Adjusted Data, CBH/VR
Data, CBH/VR
Outliered Data
GBD 2019
GBD 2021
UN WPP 2022
CENSUS SBH
VR COMPLETE
VR COMPLETE TB

Age−specific fertility fitted trend lines, 1950−2021: Argentina, Ages: 40−44
Data points enclosed with a diamond are reference sources

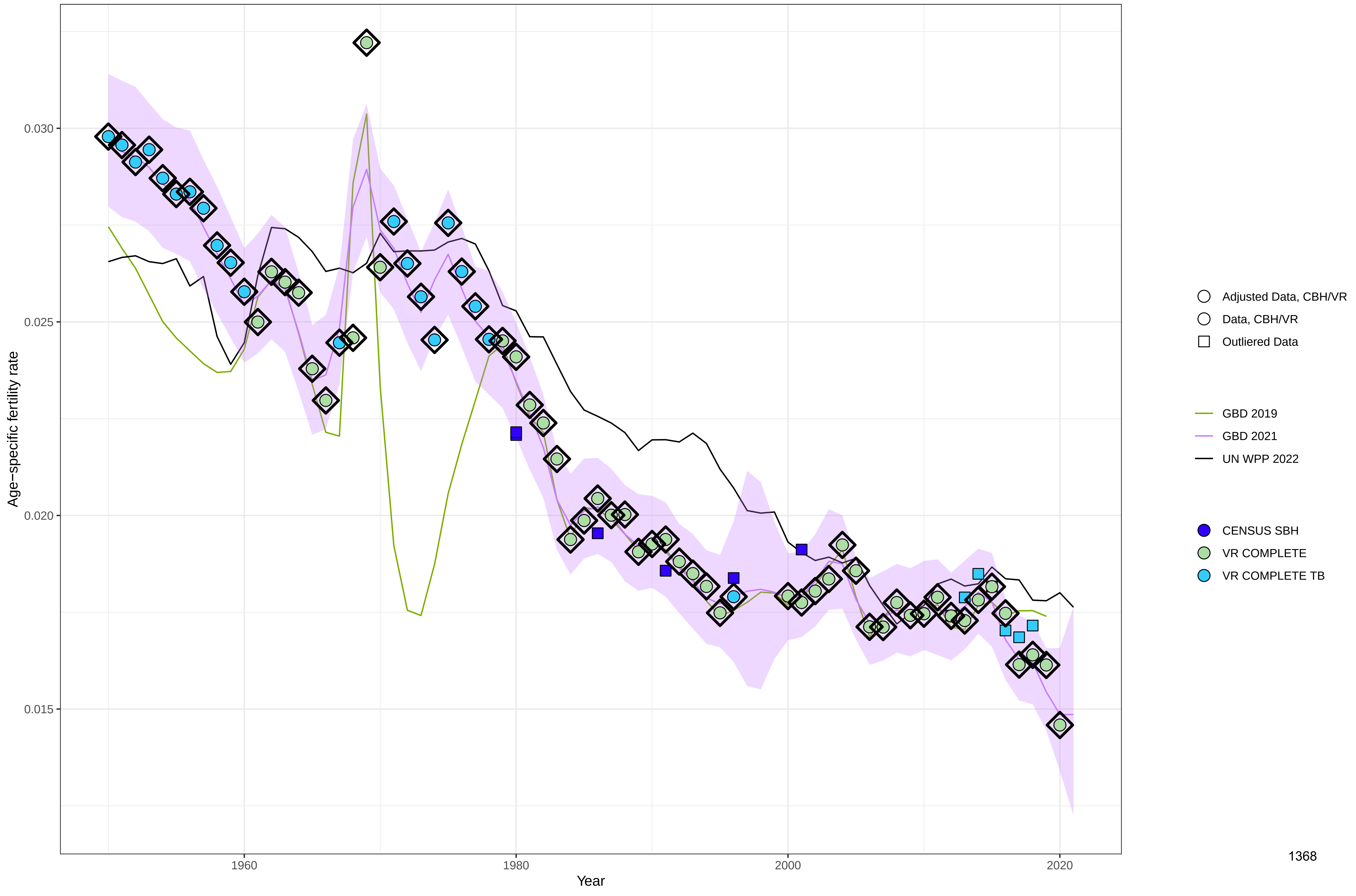

Age−specific fertility fitted trend lines, 1950−2021: Argentina, Ages: 45−49
Data points enclosed with a diamond are reference sources

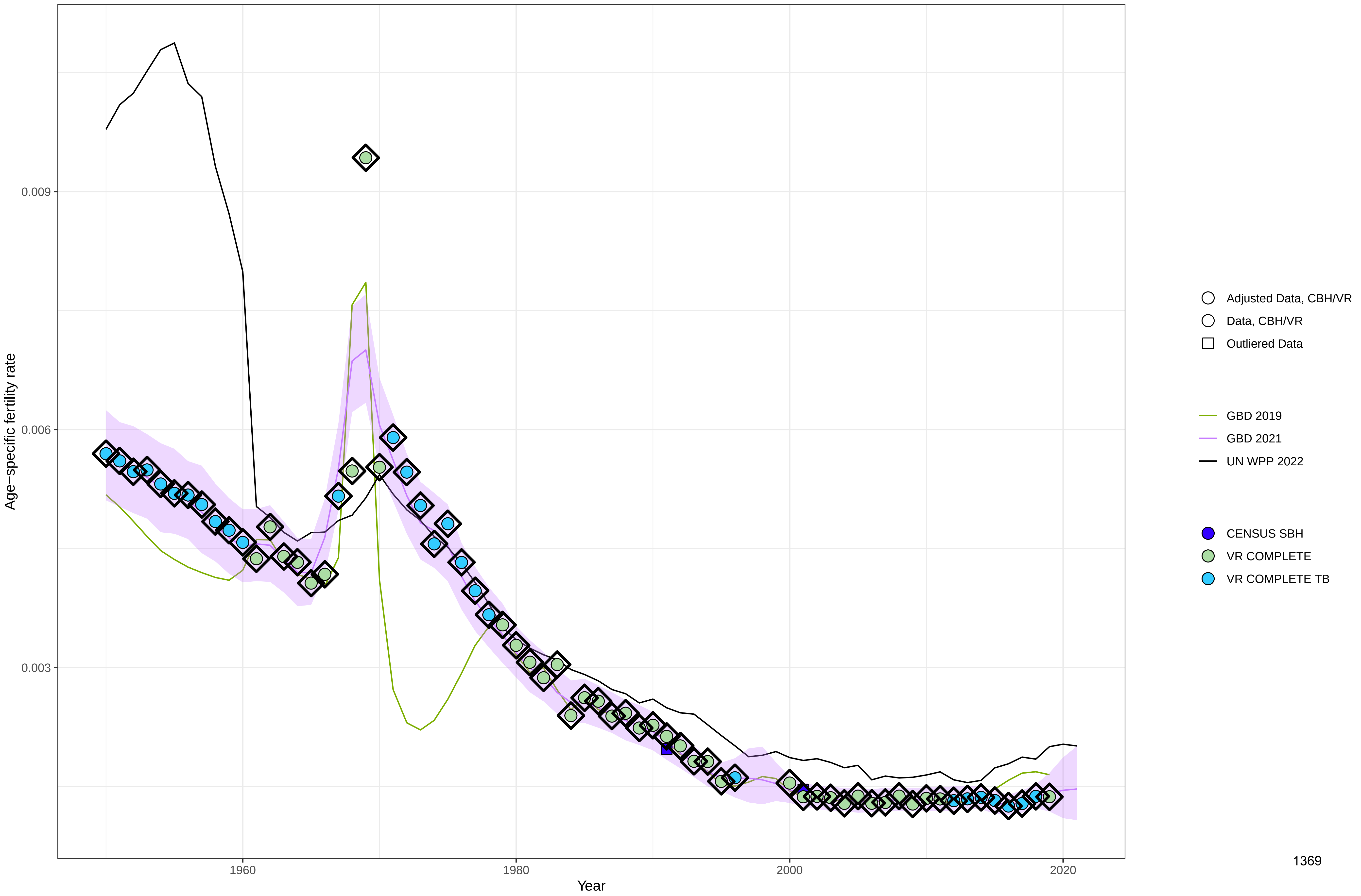

Age−specific fertility fitted trend lines, 1950−2021: Argentina, Ages: 50−54
Data points enclosed with a diamond are reference sources

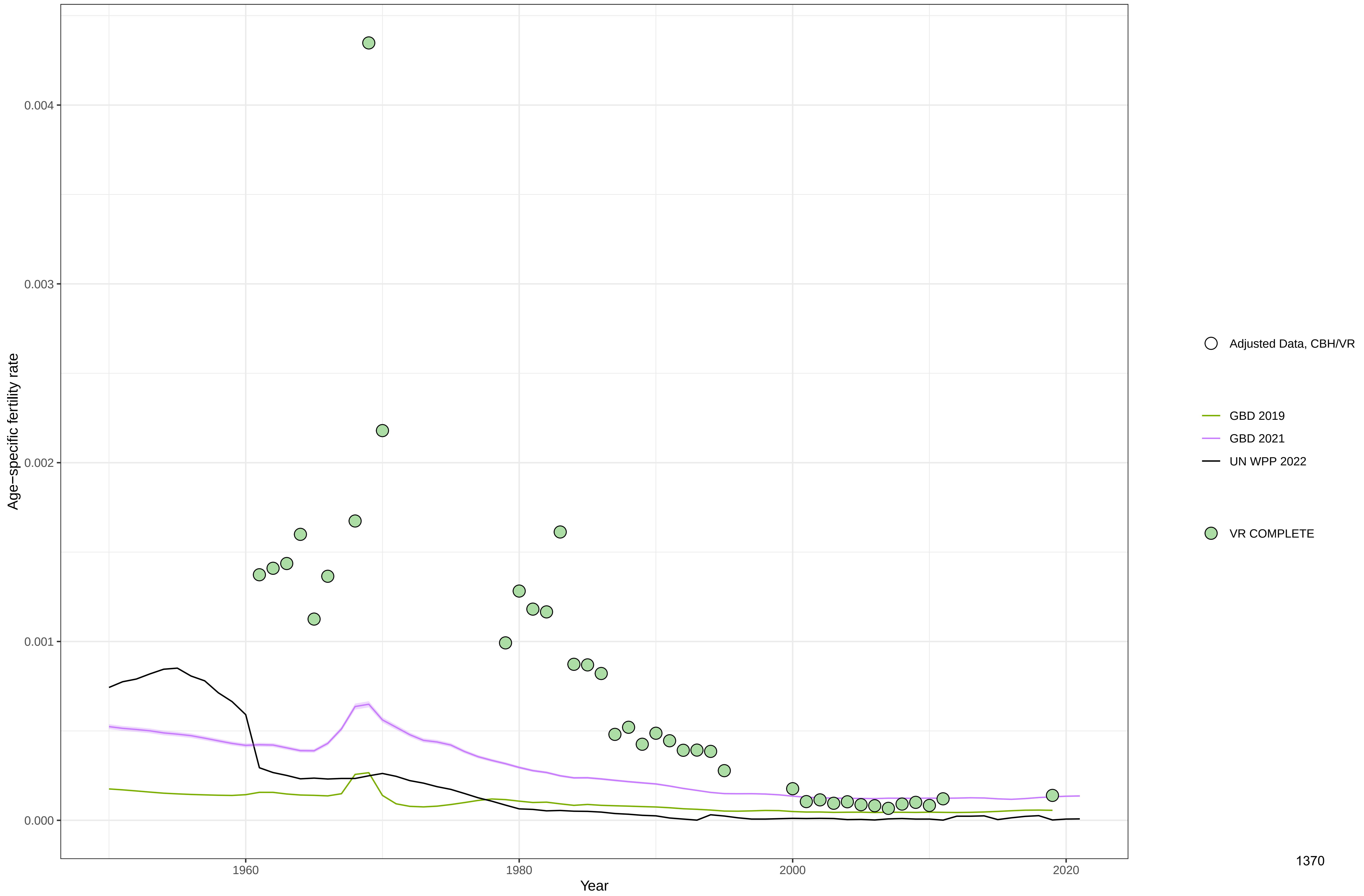

Total fertility rate fitted trend lines, 1950−2021: Argentina TFR
Data points enclosed with a diamond are reference sources

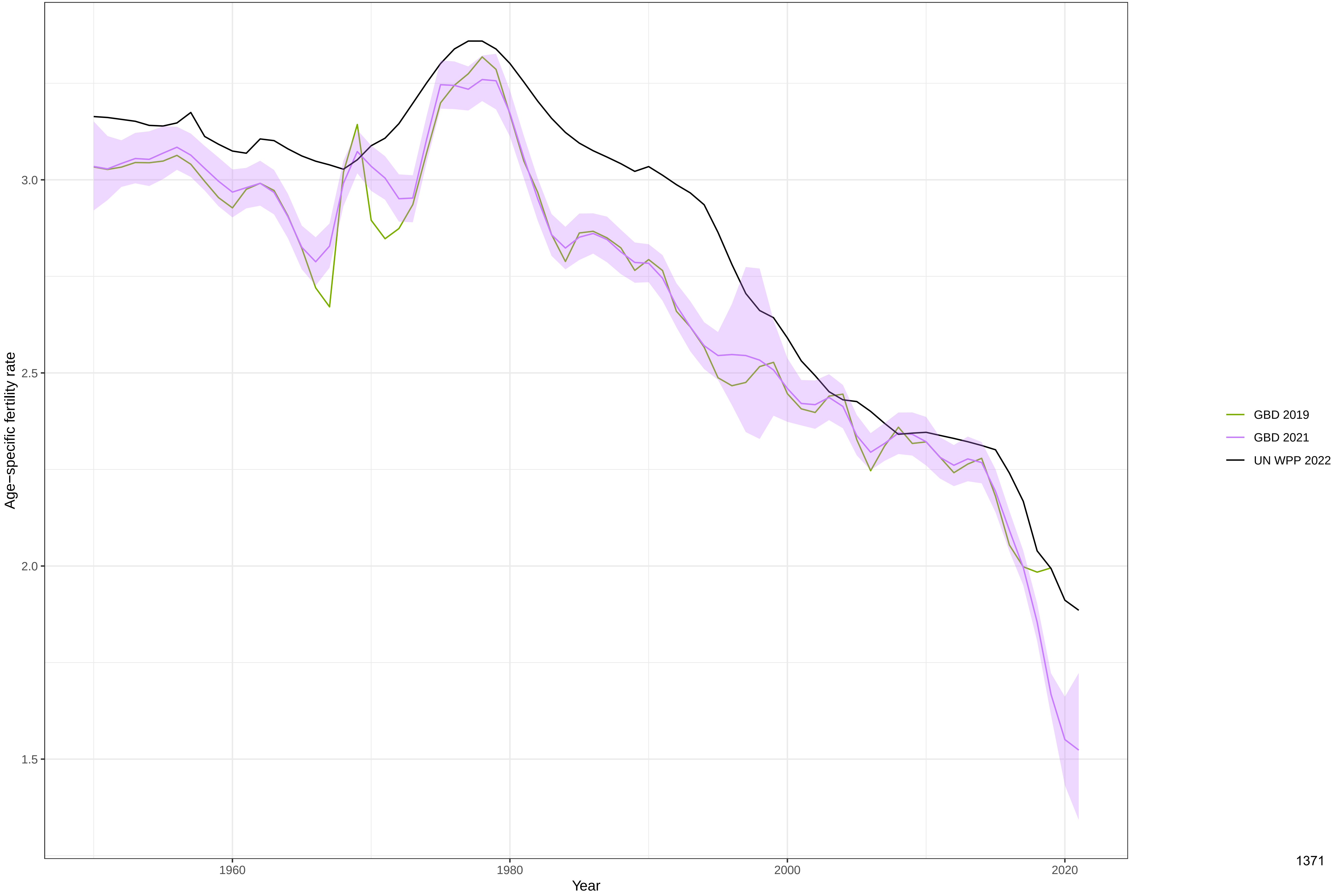

Age−specific fertility fitted trend lines, 1950−2021: Chile, Ages: 10−14
Data points enclosed with a diamond are reference sources

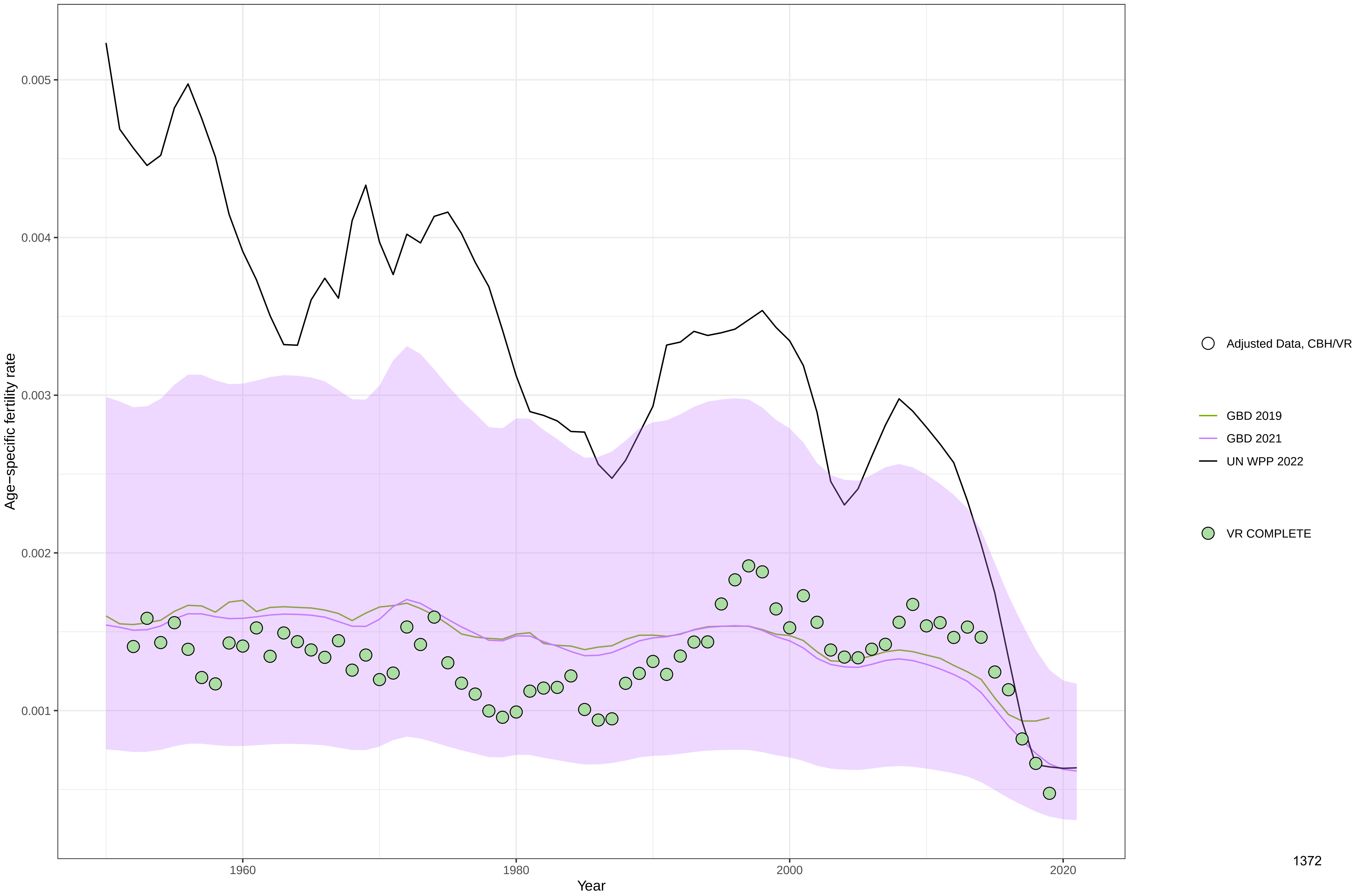

Age−specific fertility fitted trend lines, 1950−2021: Chile, Ages: 15−19
Data points enclosed with a diamond are reference sources

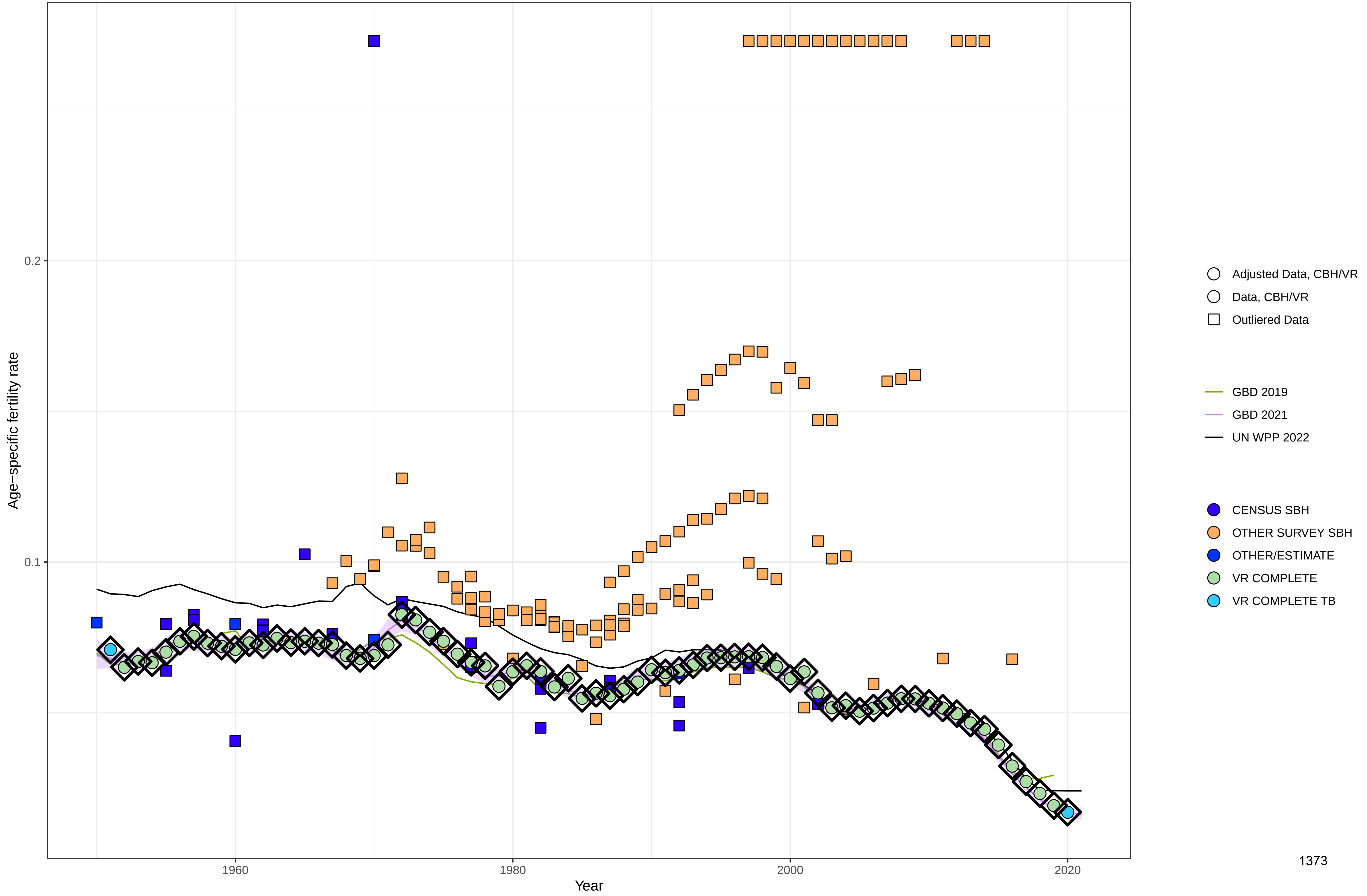

Age−specific fertility fitted trend lines, 1950−2021: Chile, Ages: 20−24
Data points enclosed with a diamond are reference sources

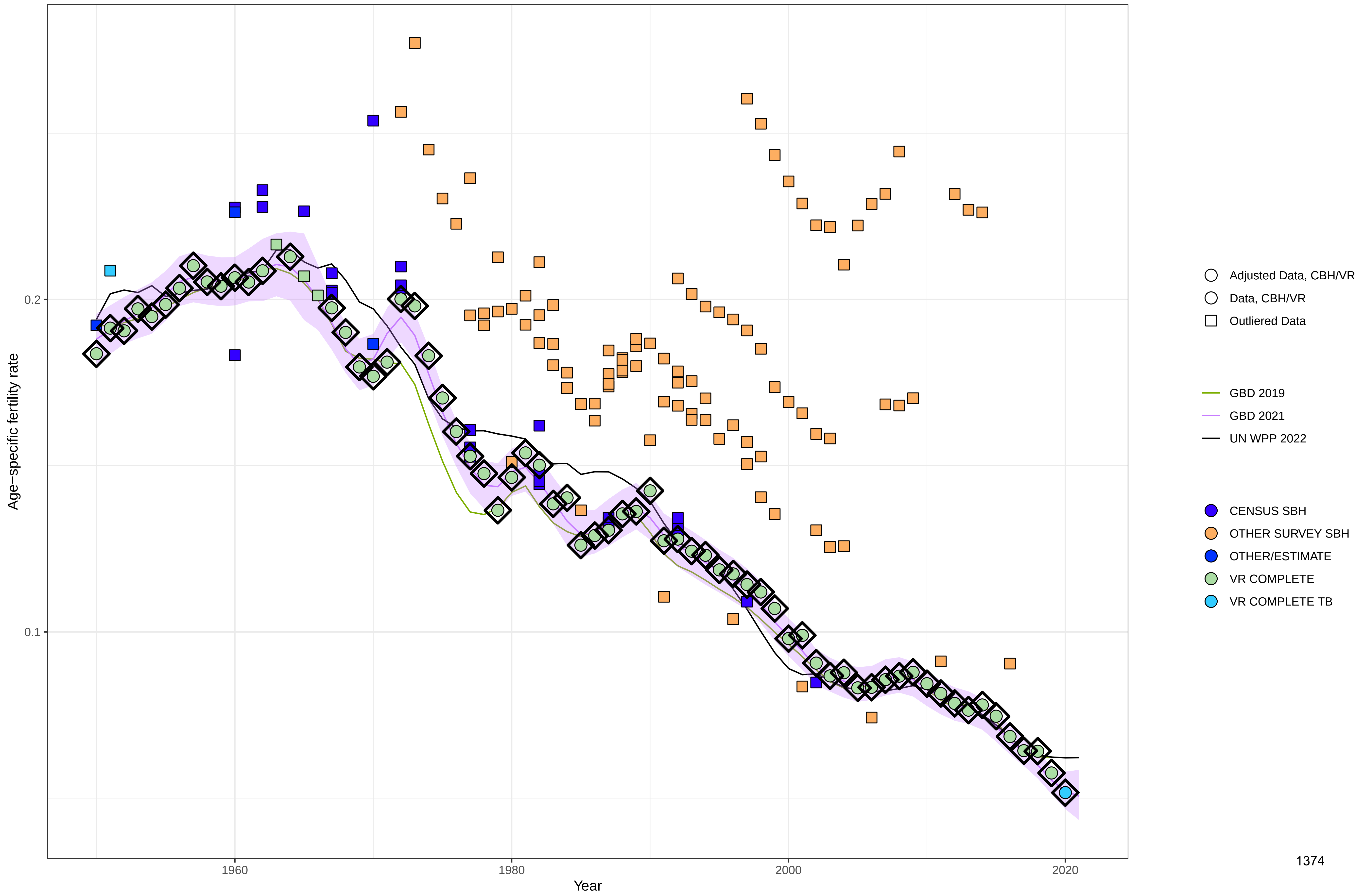

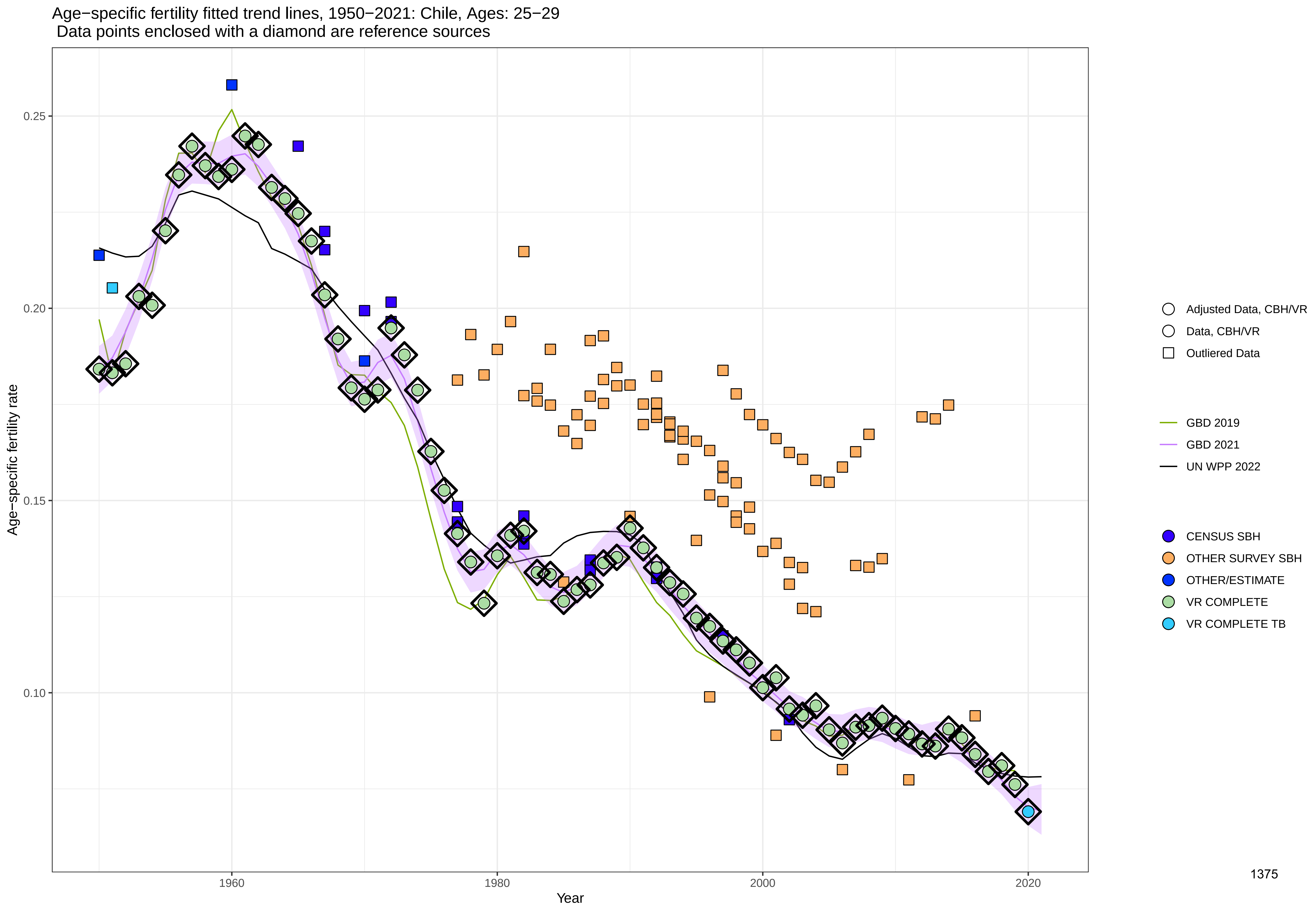

Age−specific fertility fitted trend lines, 1950−2021: Chile, Ages: 25−29
Data points enclosed with a diamond are reference sources
Age−specific fertility rate
0.25
0.20
0.15
0.10
1960
1980
2000
2020
Year
Adjusted Data, CBH/VR
Data, CBH/VR
Outliered Data
GBD 2019
GBD 2021
UN WPP 2022
CENSUS SBH
OTHER SURVEY SBH
OTHER/ESTIMATE
VR COMPLETE
VR COMPLETE TB

Age−specific fertility fitted trend lines, 1950−2021: Chile, Ages: 30−34
Data points enclosed with a diamond are reference sources

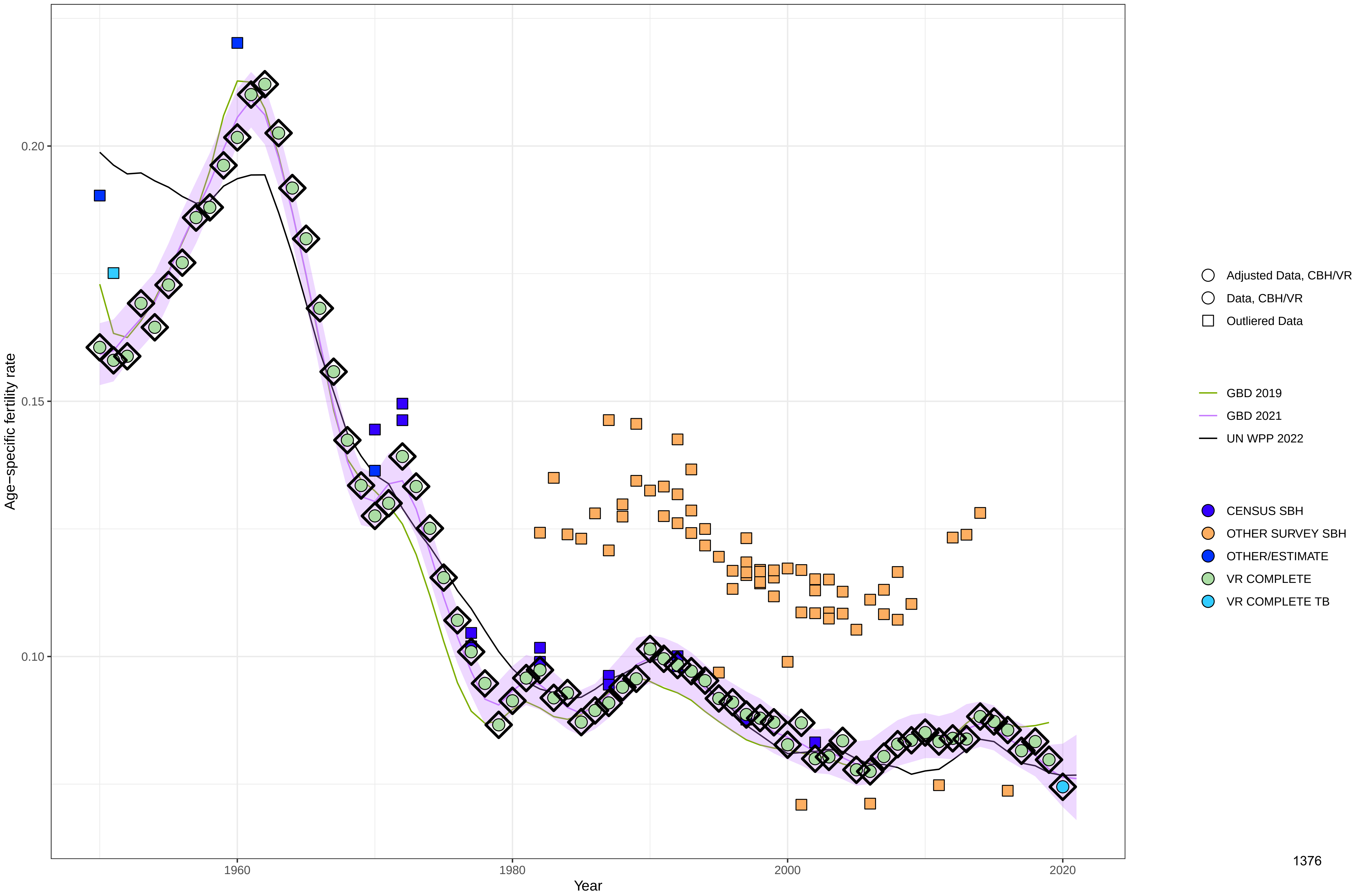

Age−specific fertility fitted trend lines, 1950−2021: Chile, Ages: 35−39
Data points enclosed with a diamond are reference sources

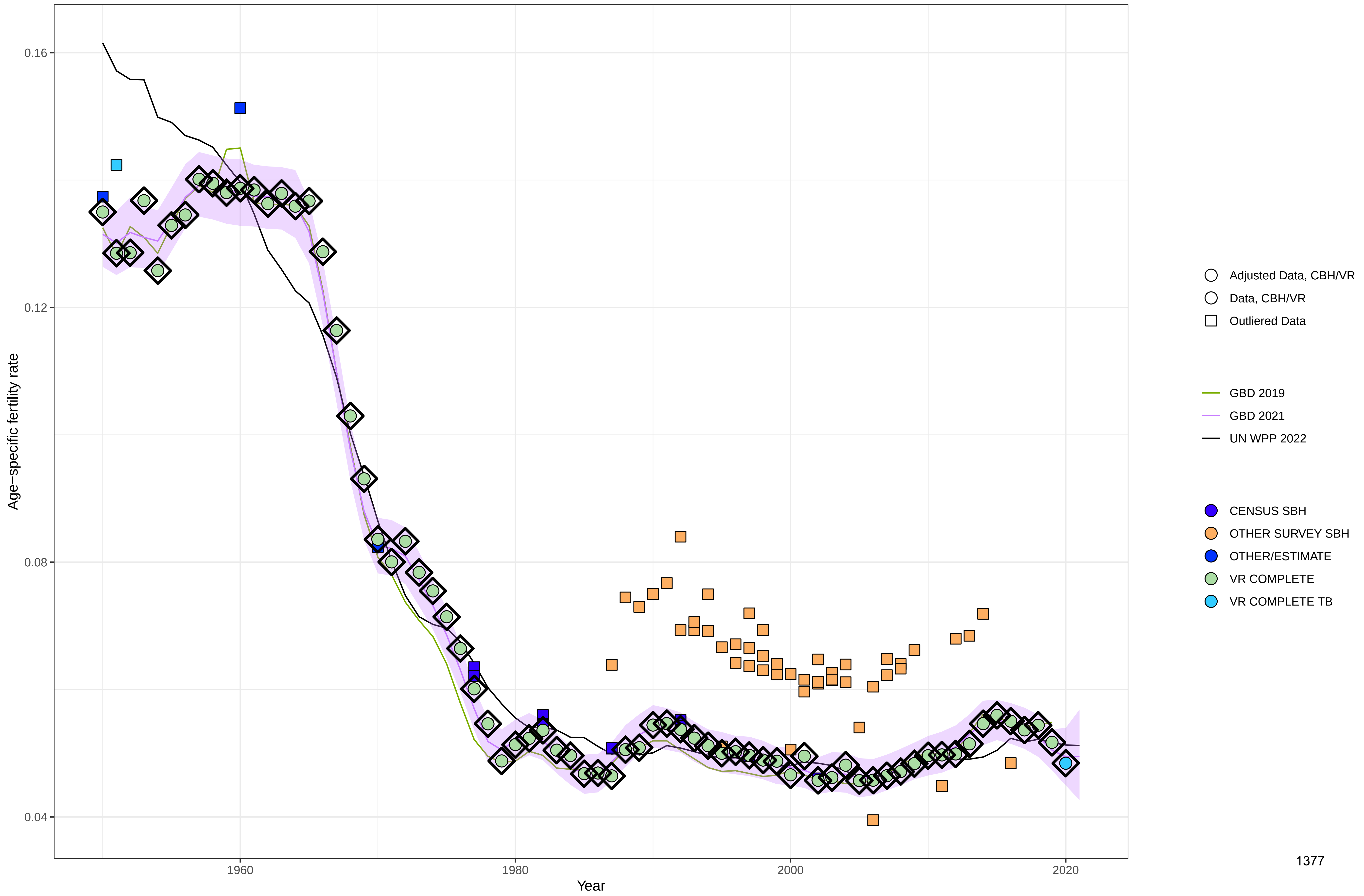

Age−specific fertility fitted trend lines, 1950−2021: Chile, Ages: 40−44
Data points enclosed with a diamond are reference sources

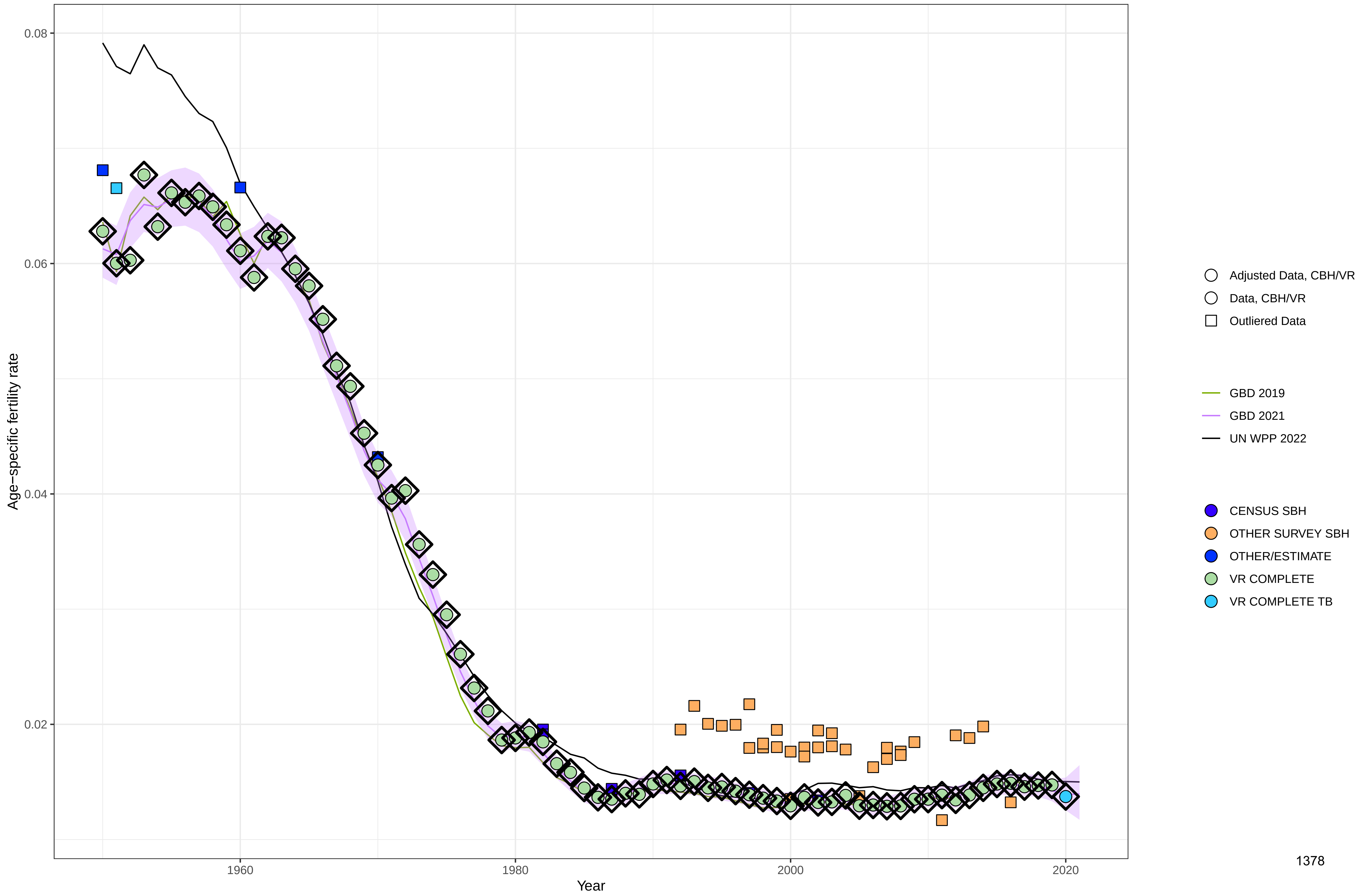

Age−specific fertility fitted trend lines, 1950−2021: Chile, Ages: 45−49
Data points enclosed with a diamond are reference sources

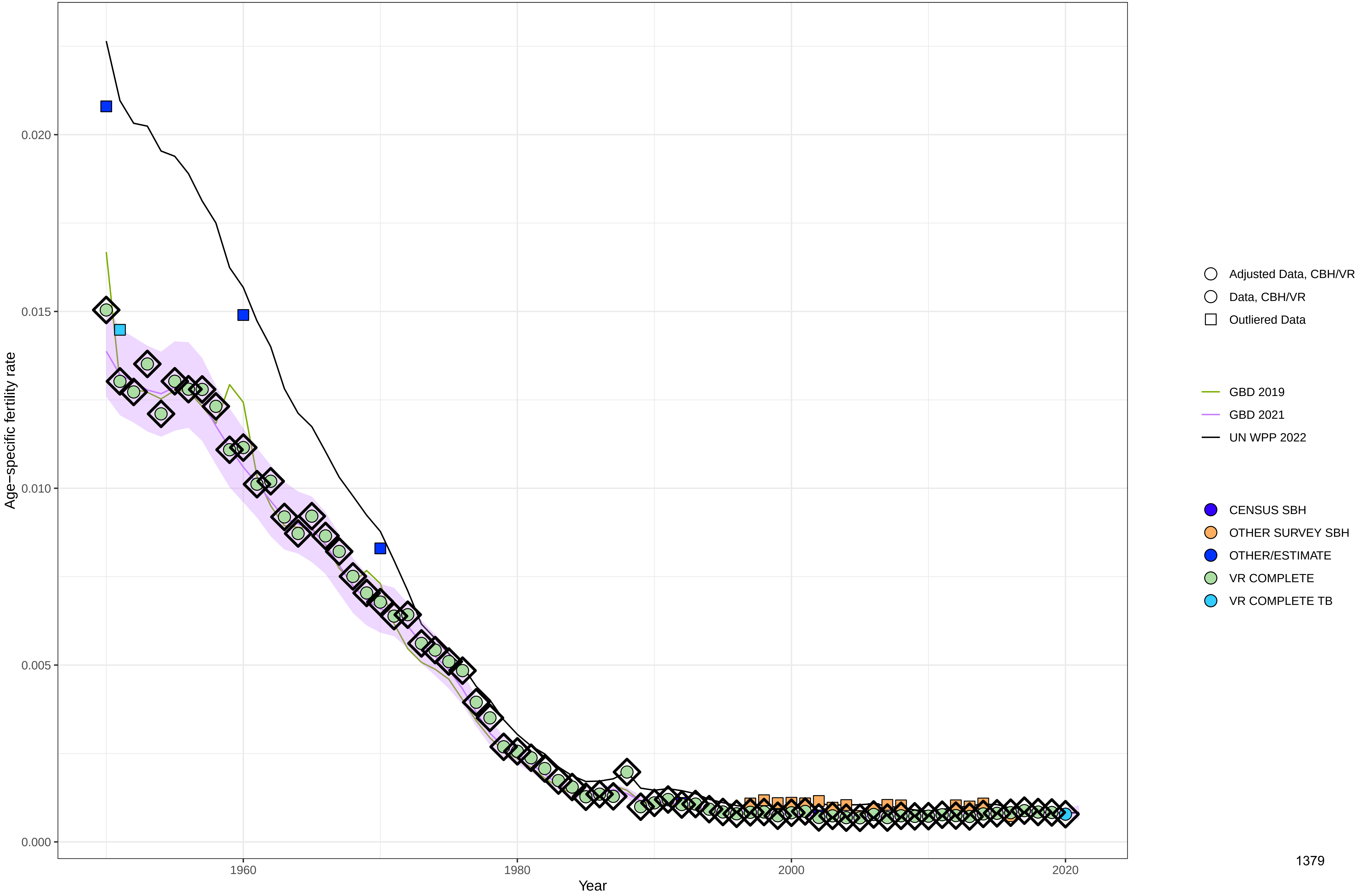

Age−specific fertility fitted trend lines, 1950−2021: Chile, Ages: 50−54
Data points enclosed with a diamond are reference sources

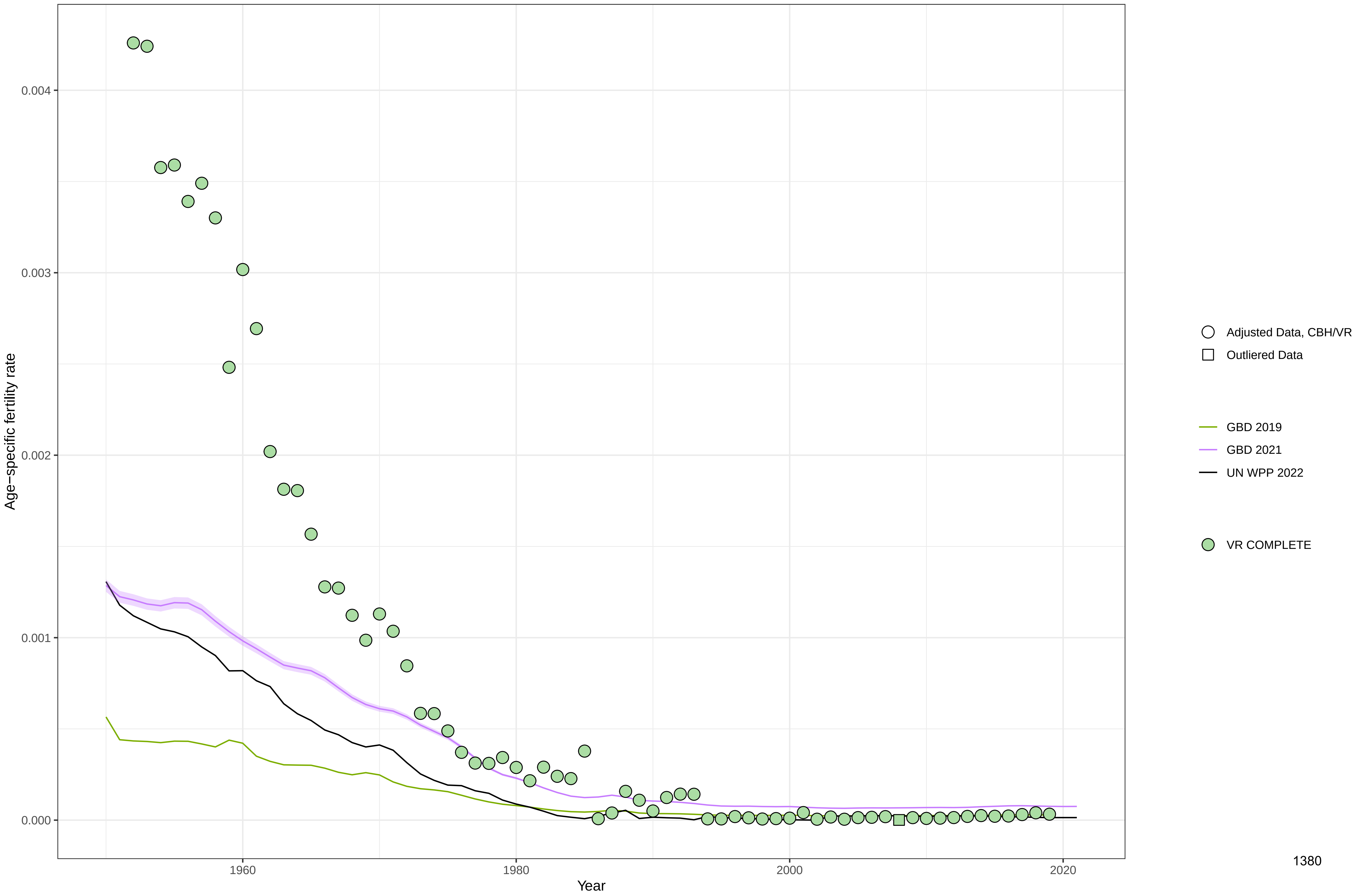

Total fertility rate fitted trend lines, 1950−2021: Chile TFR
Data points enclosed with a diamond are reference sources

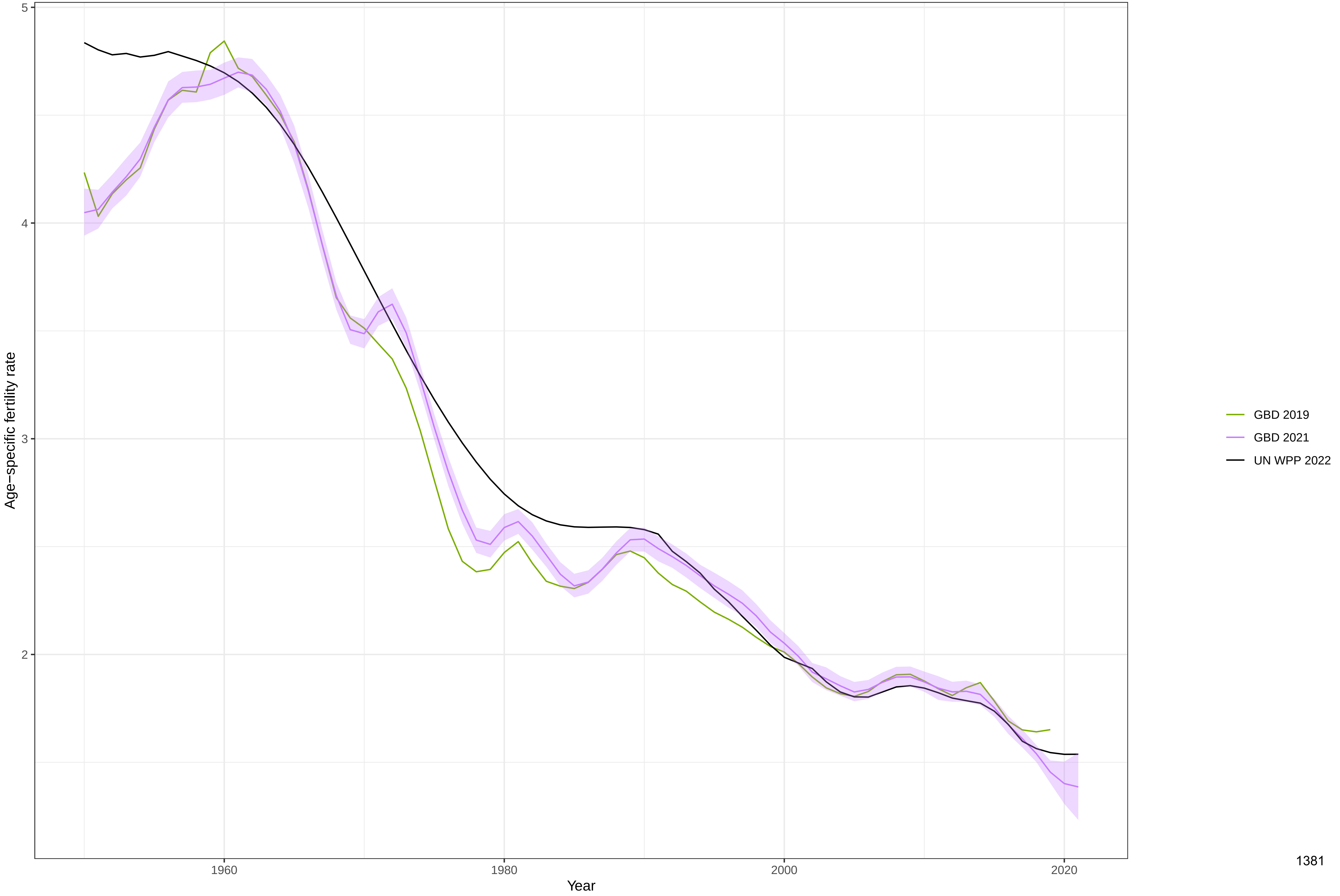

Age−specific fertility fitted trend lines, 1950−2021: Uruguay, Ages: 10−14
Data points enclosed with a diamond are reference sources

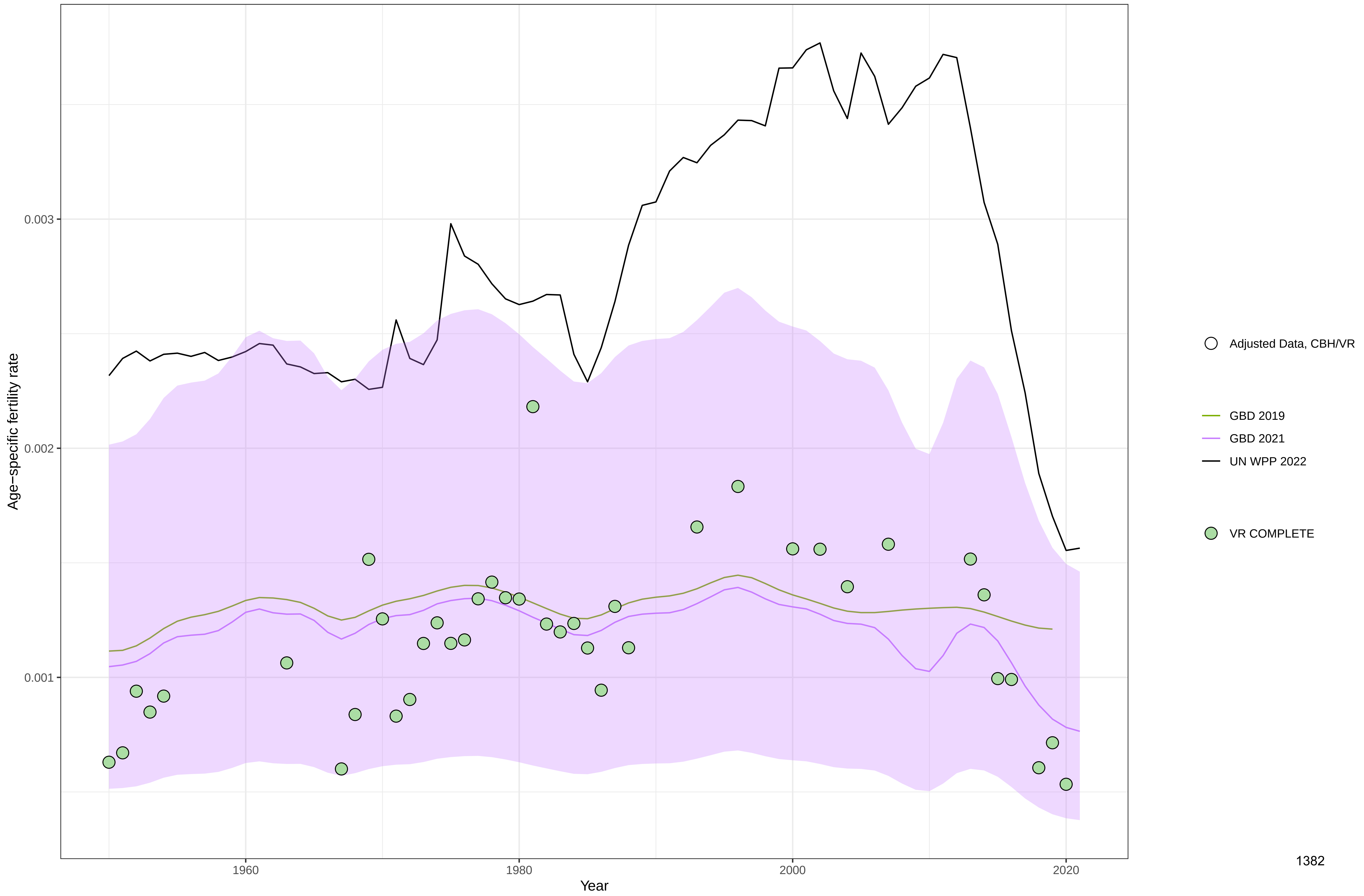

Age−specific fertility fitted trend lines, 1950−2021: Uruguay, Ages: 15−19
Data points enclosed with a diamond are reference sources

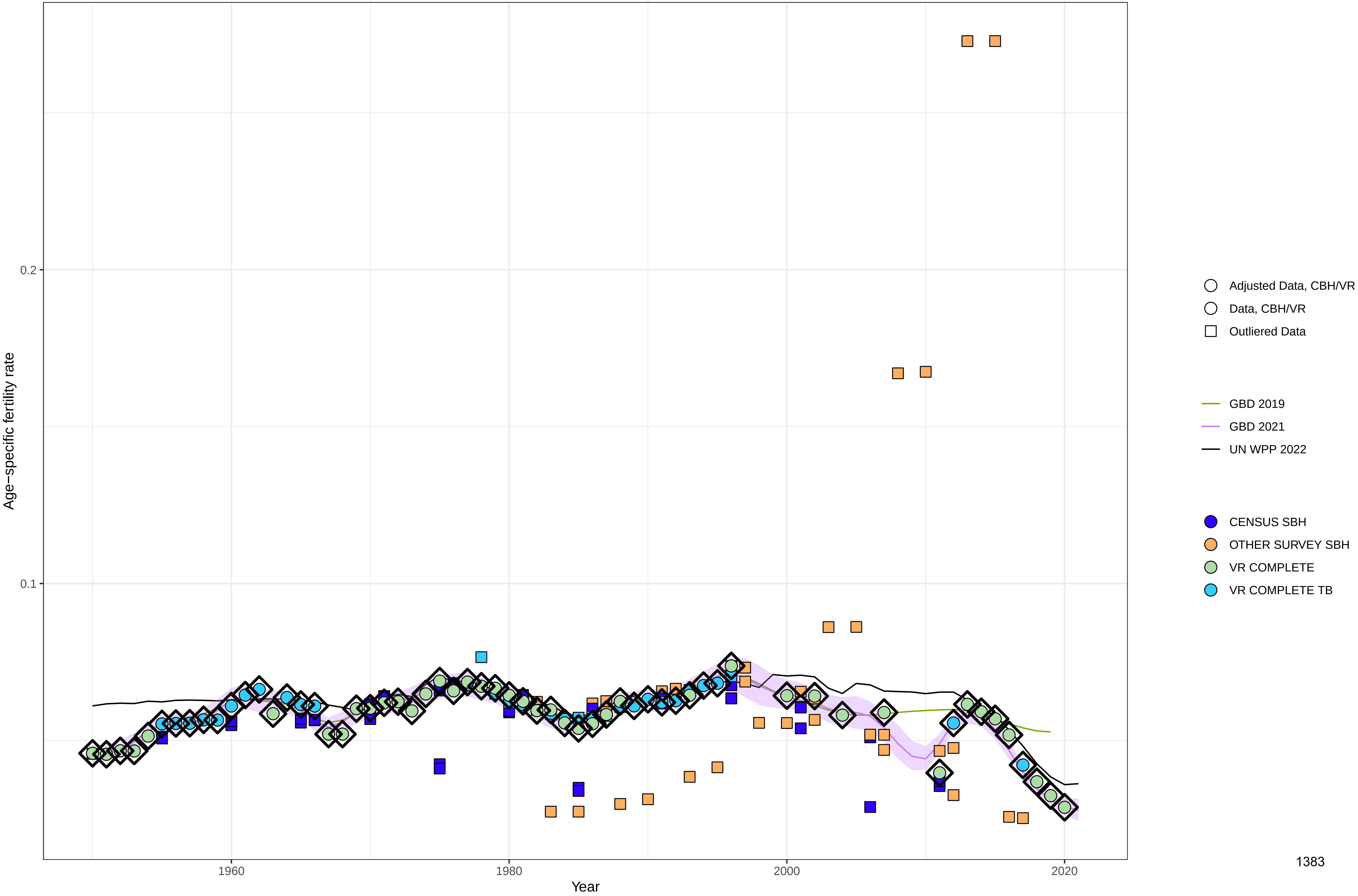

Age−specific fertility fitted trend lines, 1950−2021: Uruguay, Ages: 20−24
Data points enclosed with a diamond are reference sources

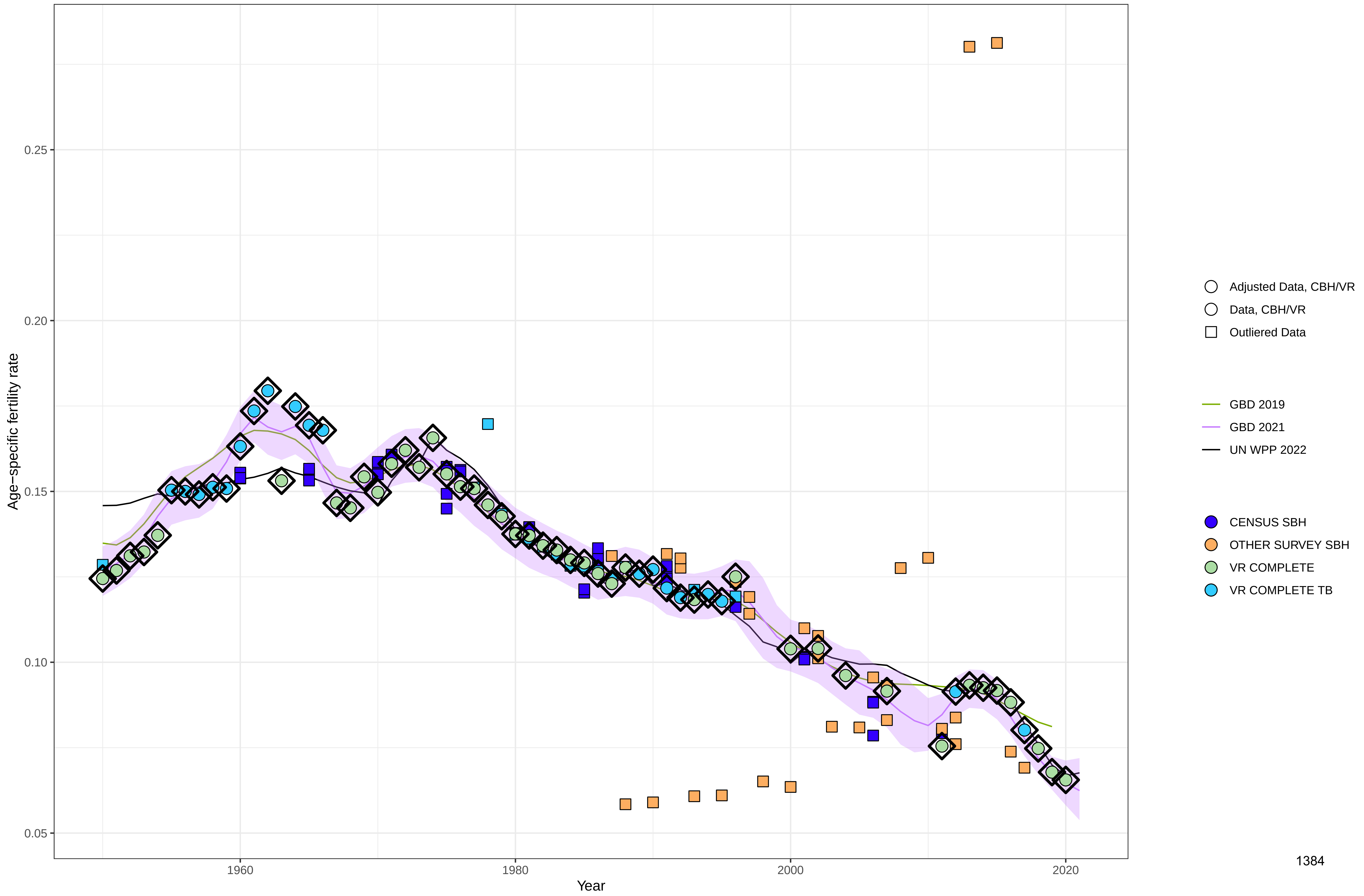

Age−specific fertility fitted trend lines, 1950−2021: Uruguay, Ages: 25−29
Data points enclosed with a diamond are reference sources

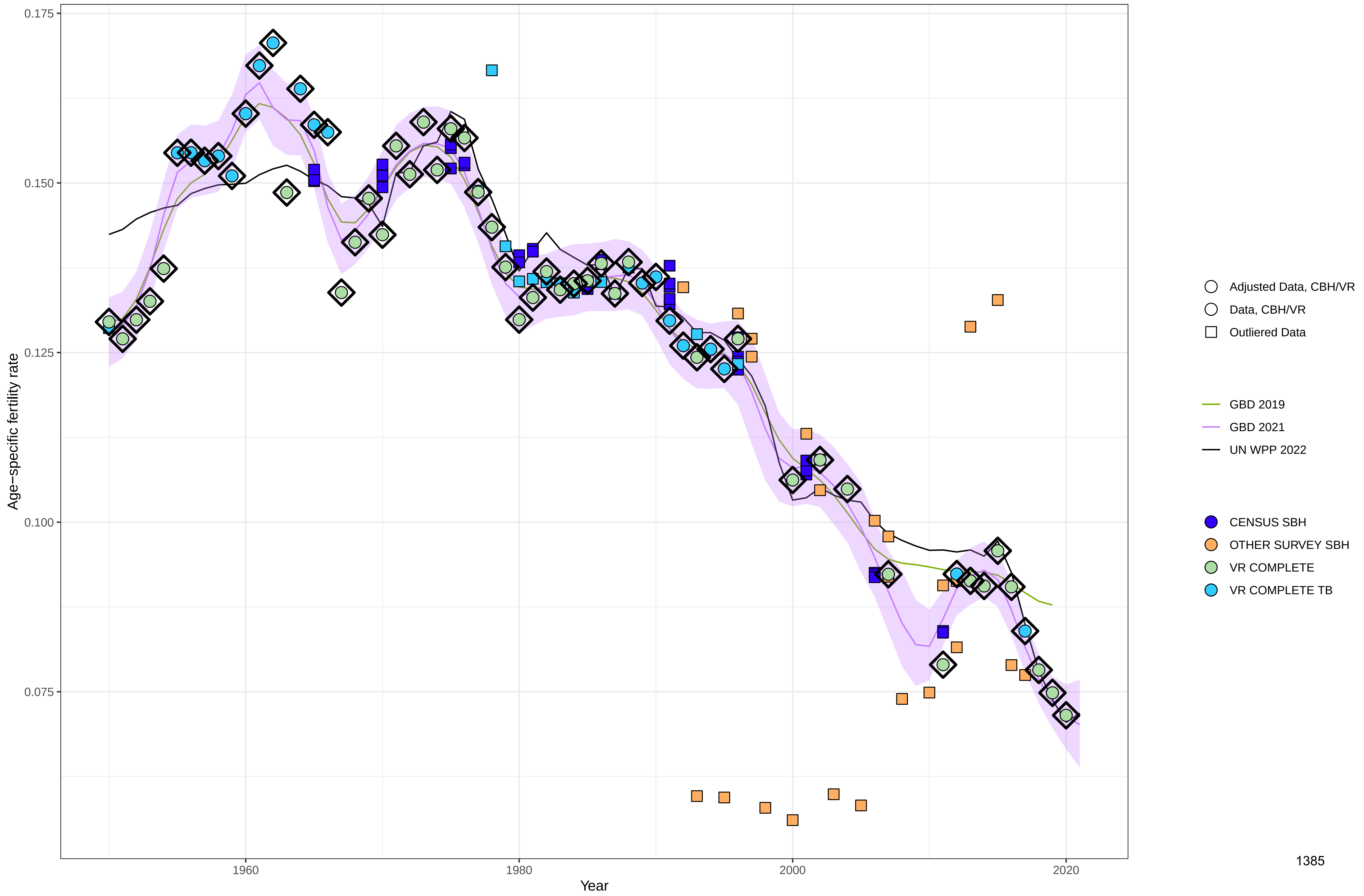

Age−specific fertility fitted trend lines, 1950−2021: Uruguay, Ages: 30−34
Data points enclosed with a diamond are reference sources

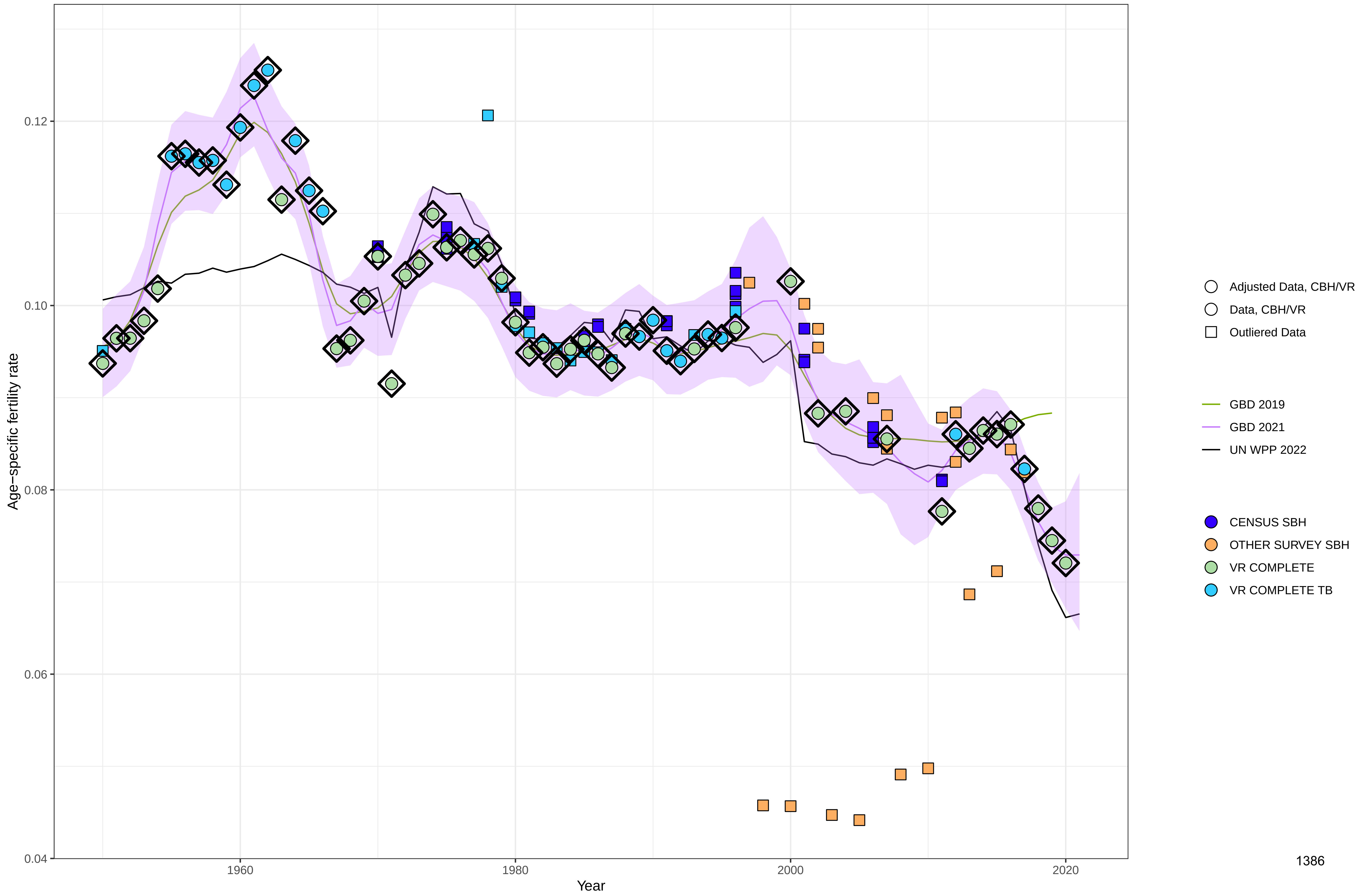

Age−specific fertility fitted trend lines, 1950−2021: Uruguay, Ages: 35−39
Data points enclosed with a diamond are reference sources

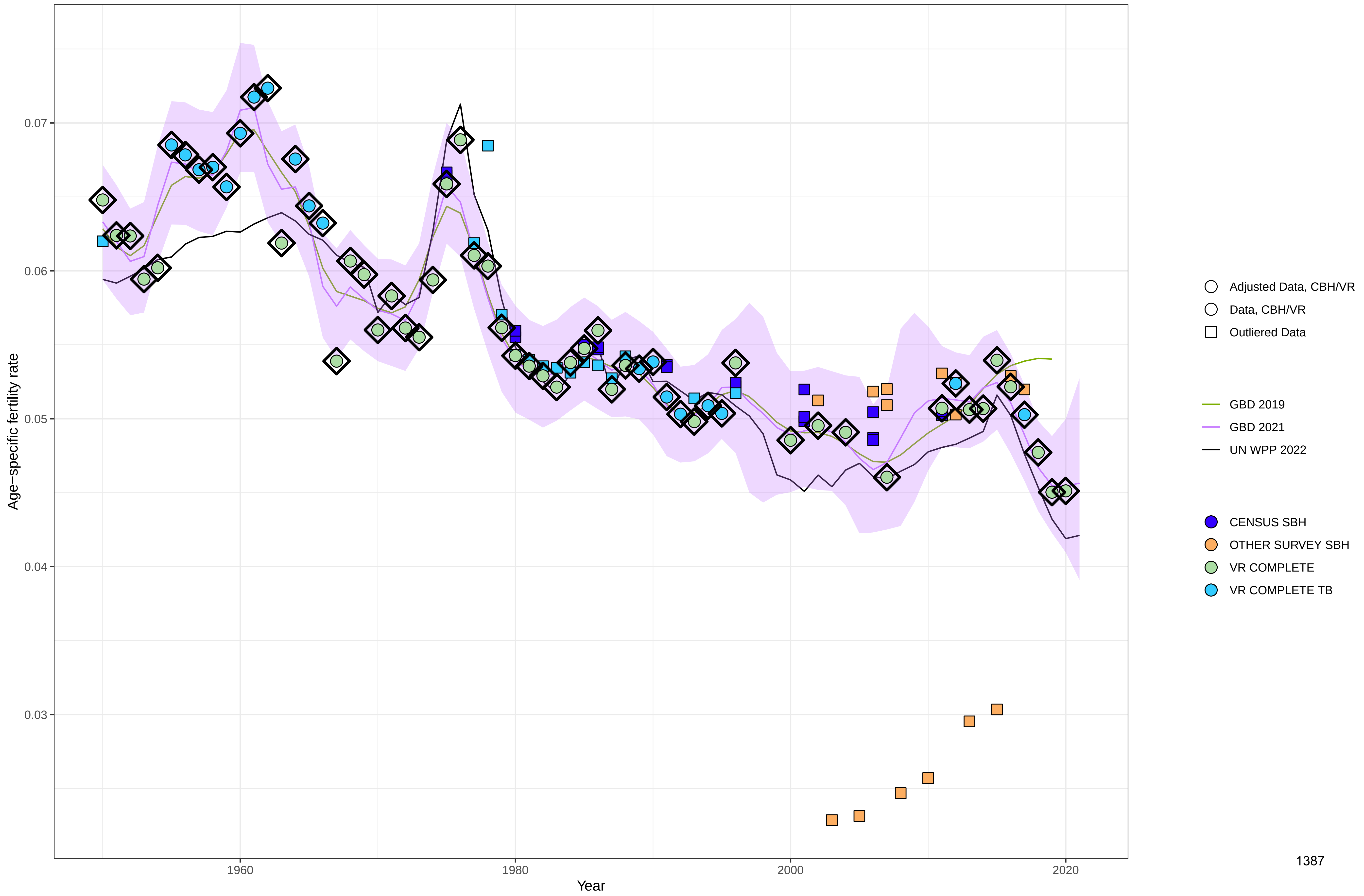

Age−specific fertility fitted trend lines, 1950−2021: Uruguay, Ages: 40−44
Data points enclosed with a diamond are reference sources

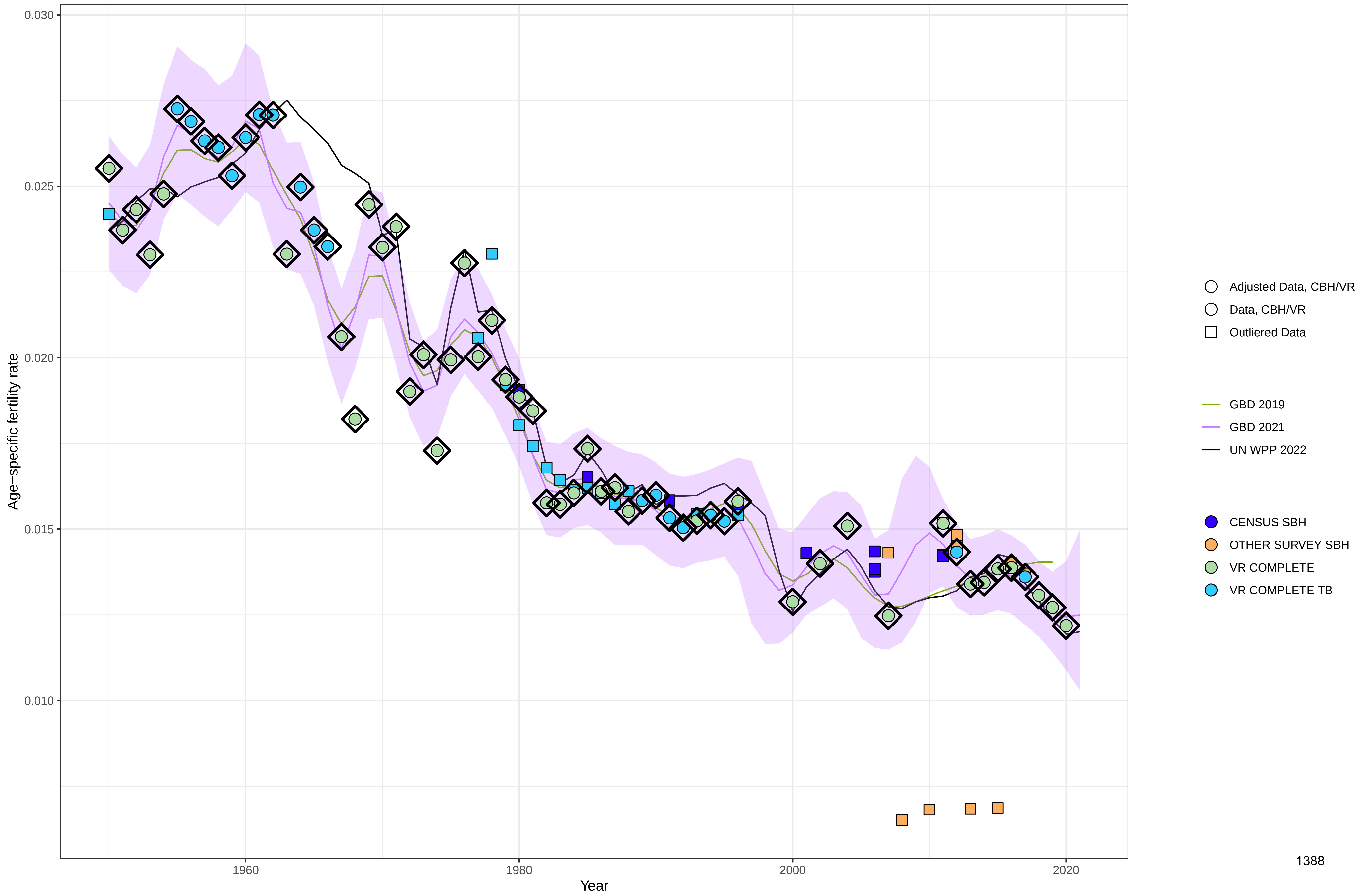

Age−specific fertility fitted trend lines, 1950−2021: Uruguay, Ages: 45−49
Data points enclosed with a diamond are reference sources

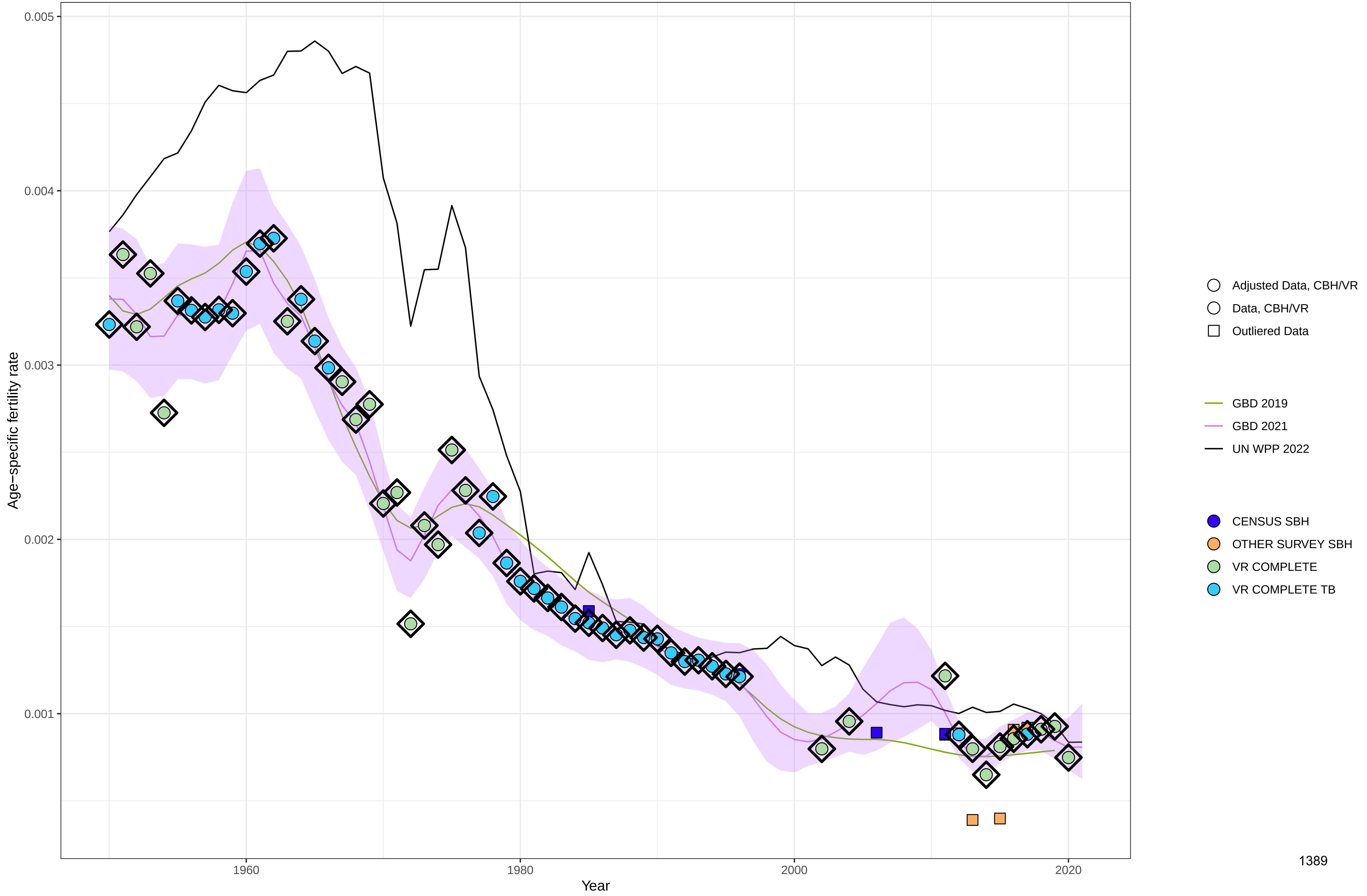

Age−specific fertility fitted trend lines, 1950−2021: Uruguay, Ages: 50−54
Data points enclosed with a diamond are reference sources

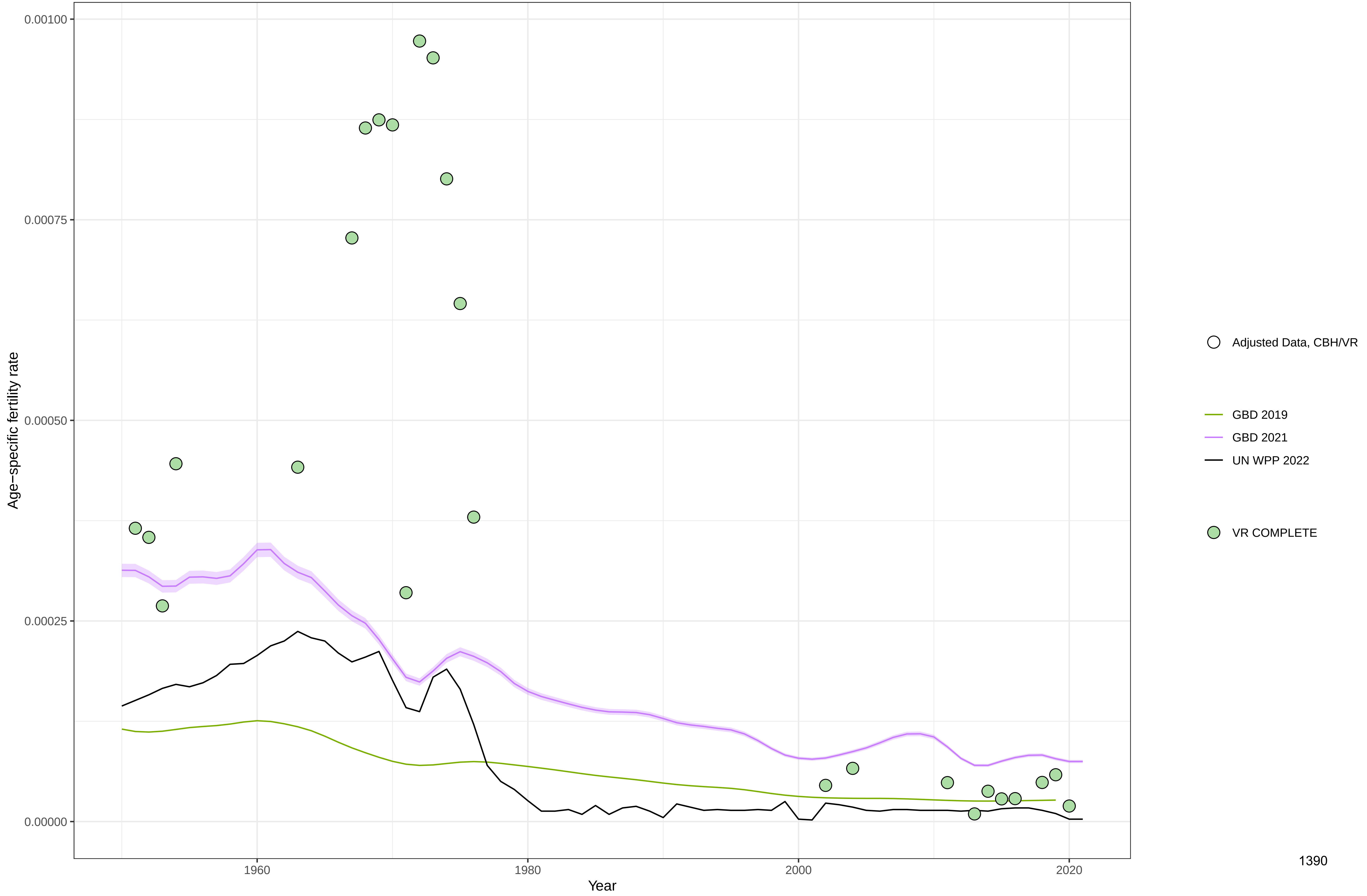

Total fertility rate fitted trend lines, 1950−2021: Uruguay TFR
Data points enclosed with a diamond are reference sources

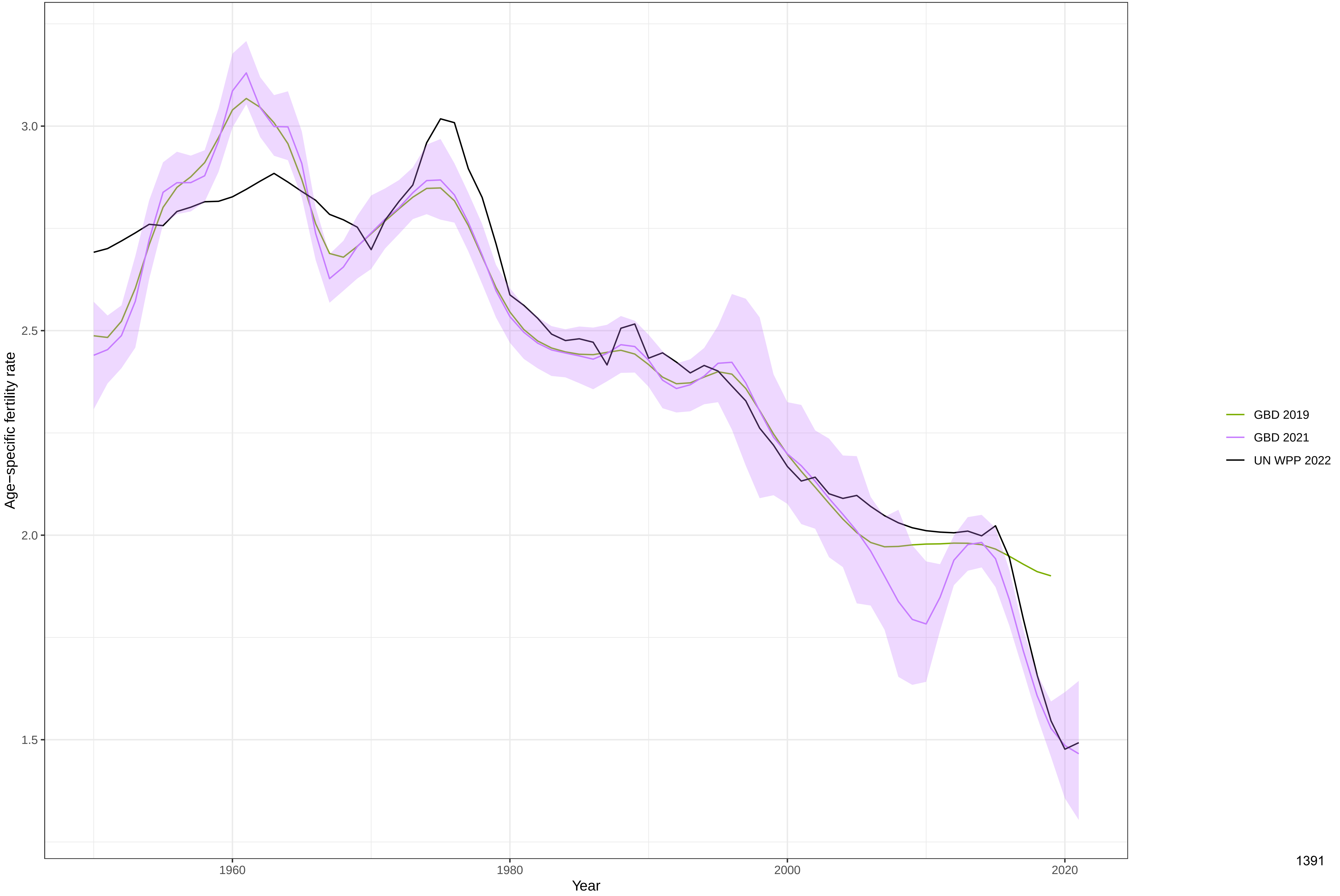

Age−specific fertility fitted trend lines, 1950−2021: Andorra, Ages: 10−14
Data points enclosed with a diamond are reference sources

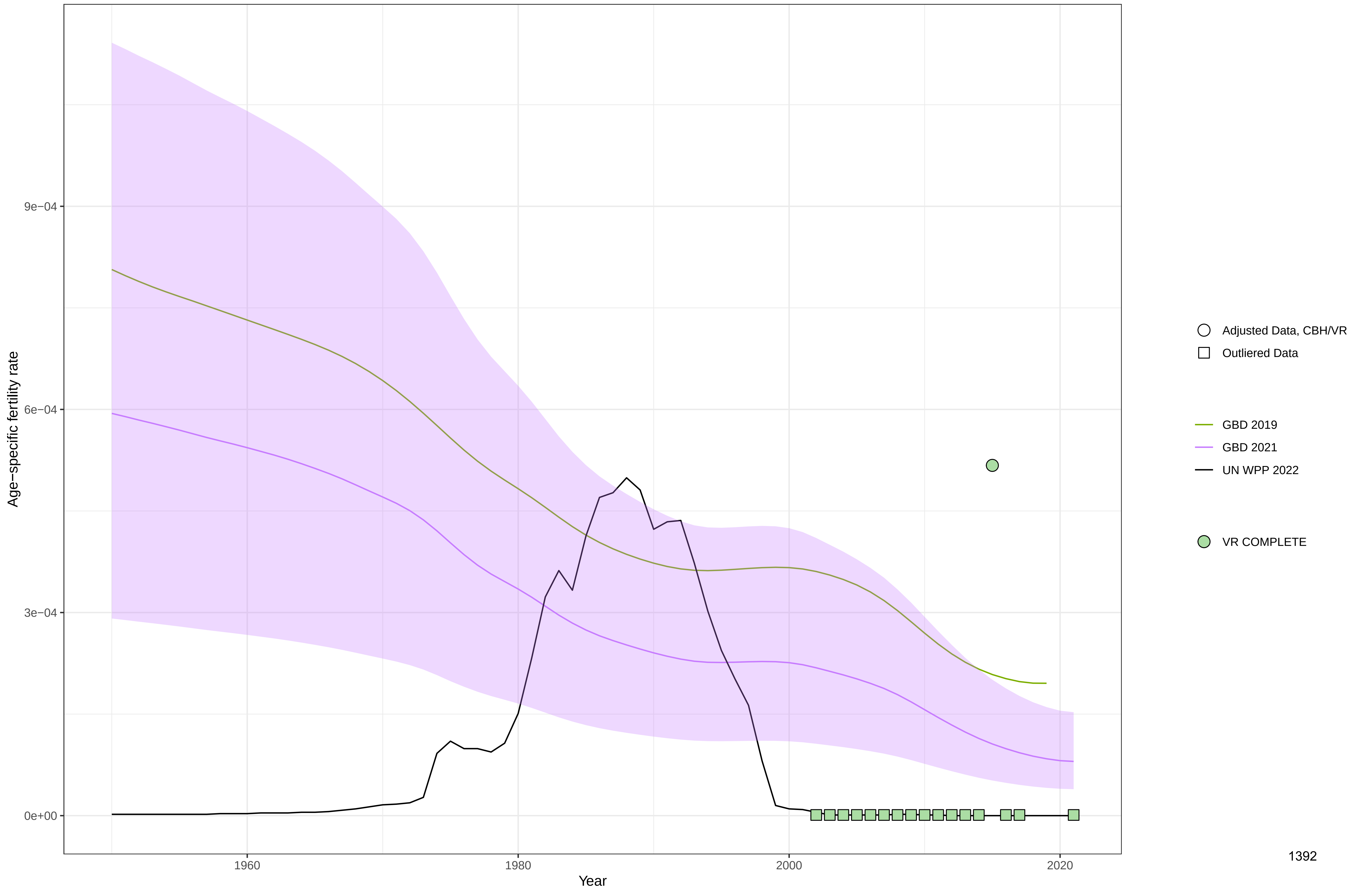

Age−specific fertility fitted trend lines, 1950−2021: Andorra, Ages: 15−19
Data points enclosed with a diamond are reference sources

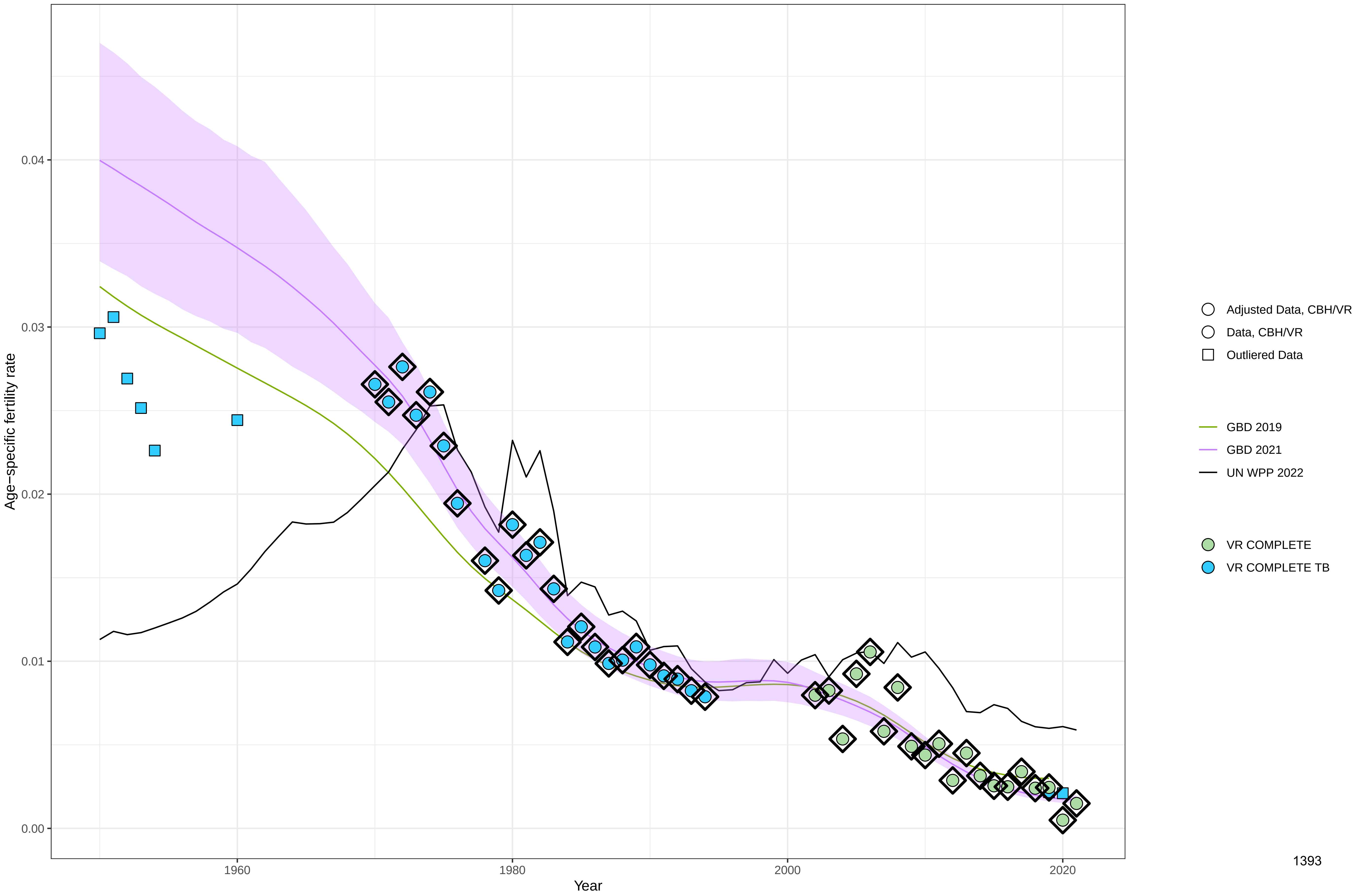

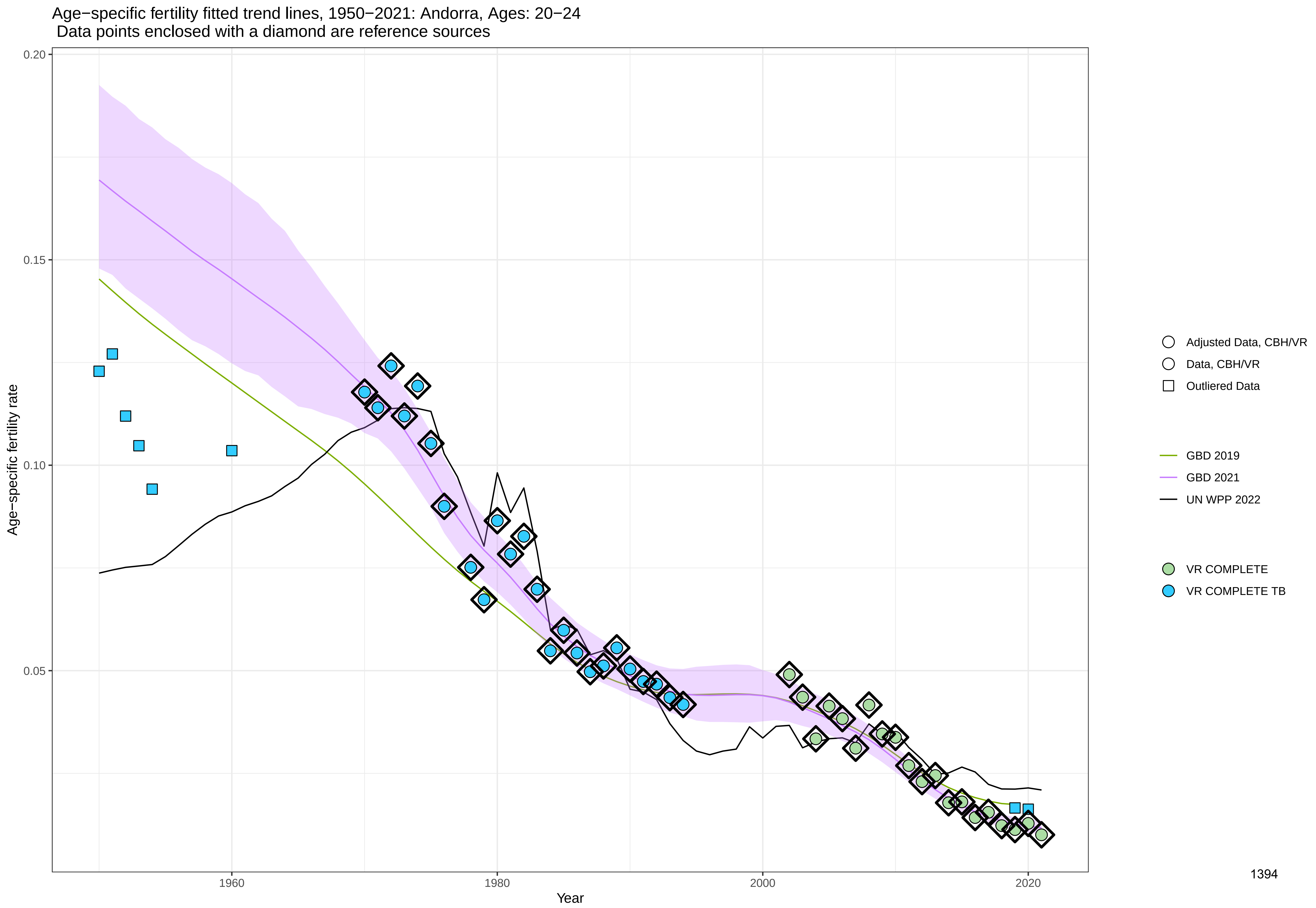
Age−specific fertility fitted trend lines, 1950−2021: Andorra, Ages: 20−24
Data points enclosed with a diamond are reference sources
Age−specific fertility rate
0.20
0.15
0.10
0.05
1960
1980
2000
2020
Year
Adjusted Data, CBH/VR
Data, CBH/VR
Outliered Data
GBD 2019
GBD 2021
UN WPP 2022
VR COMPLETE
VR COMPLETE TB

Age−specific fertility fitted trend lines, 1950−2021: Andorra, Ages: 25−29
Data points enclosed with a diamond are reference sources

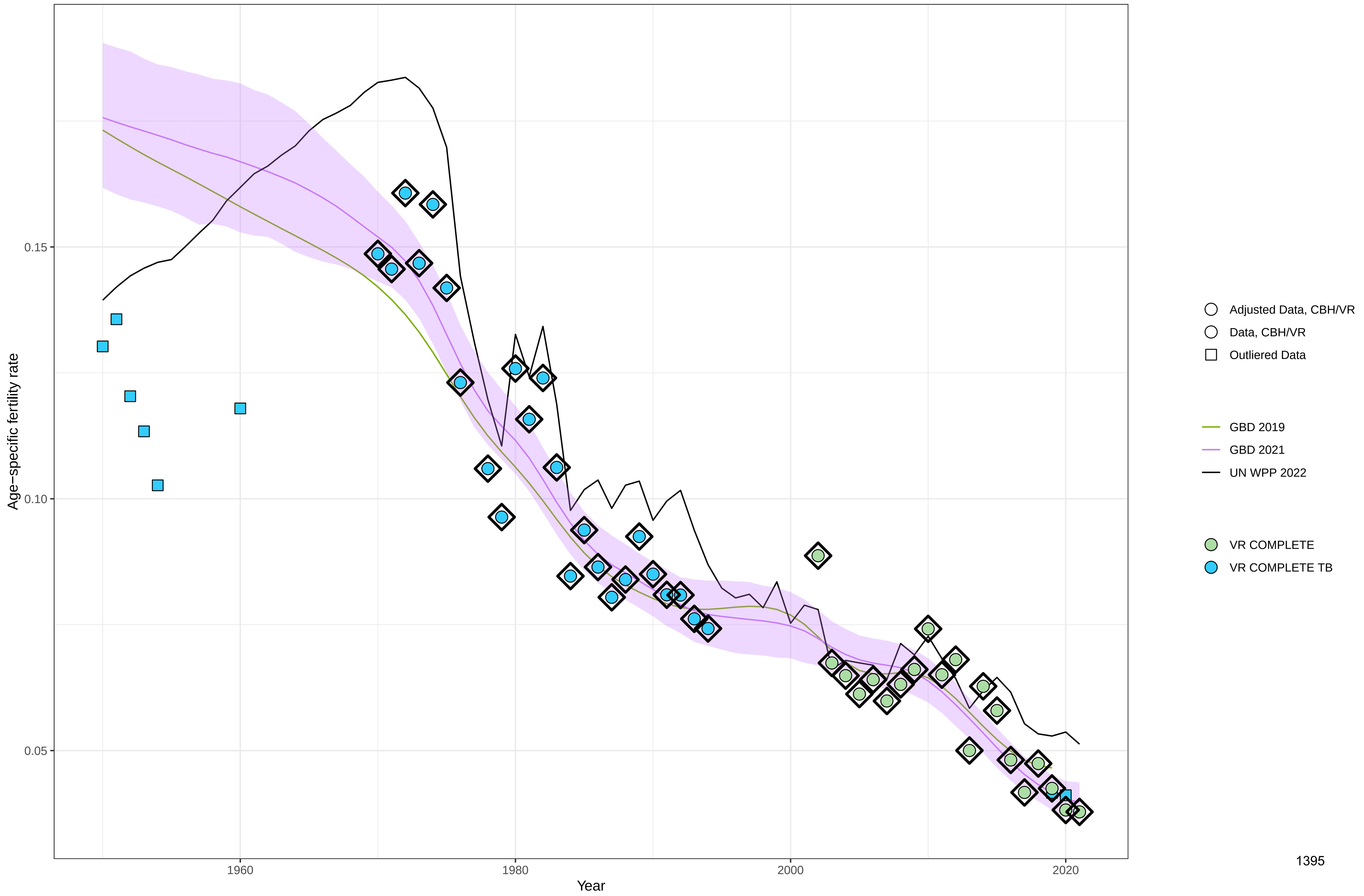

Age−specific fertility fitted trend lines, 1950−2021: Andorra, Ages: 30−34
Data points enclosed with a diamond are reference sources

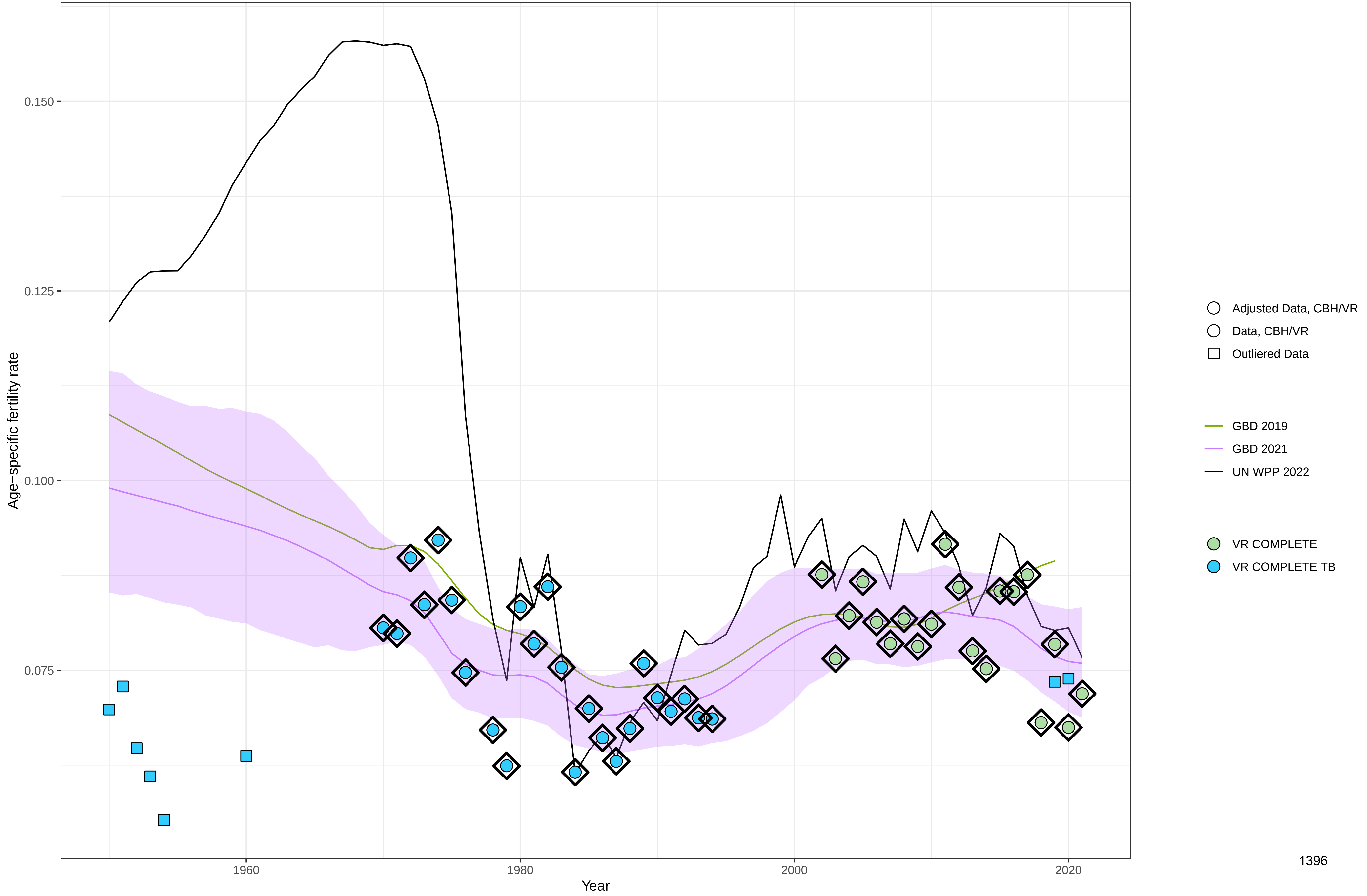

Age−specific fertility fitted trend lines, 1950−2021: Andorra, Ages: 35−39
Data points enclosed with a diamond are reference sources

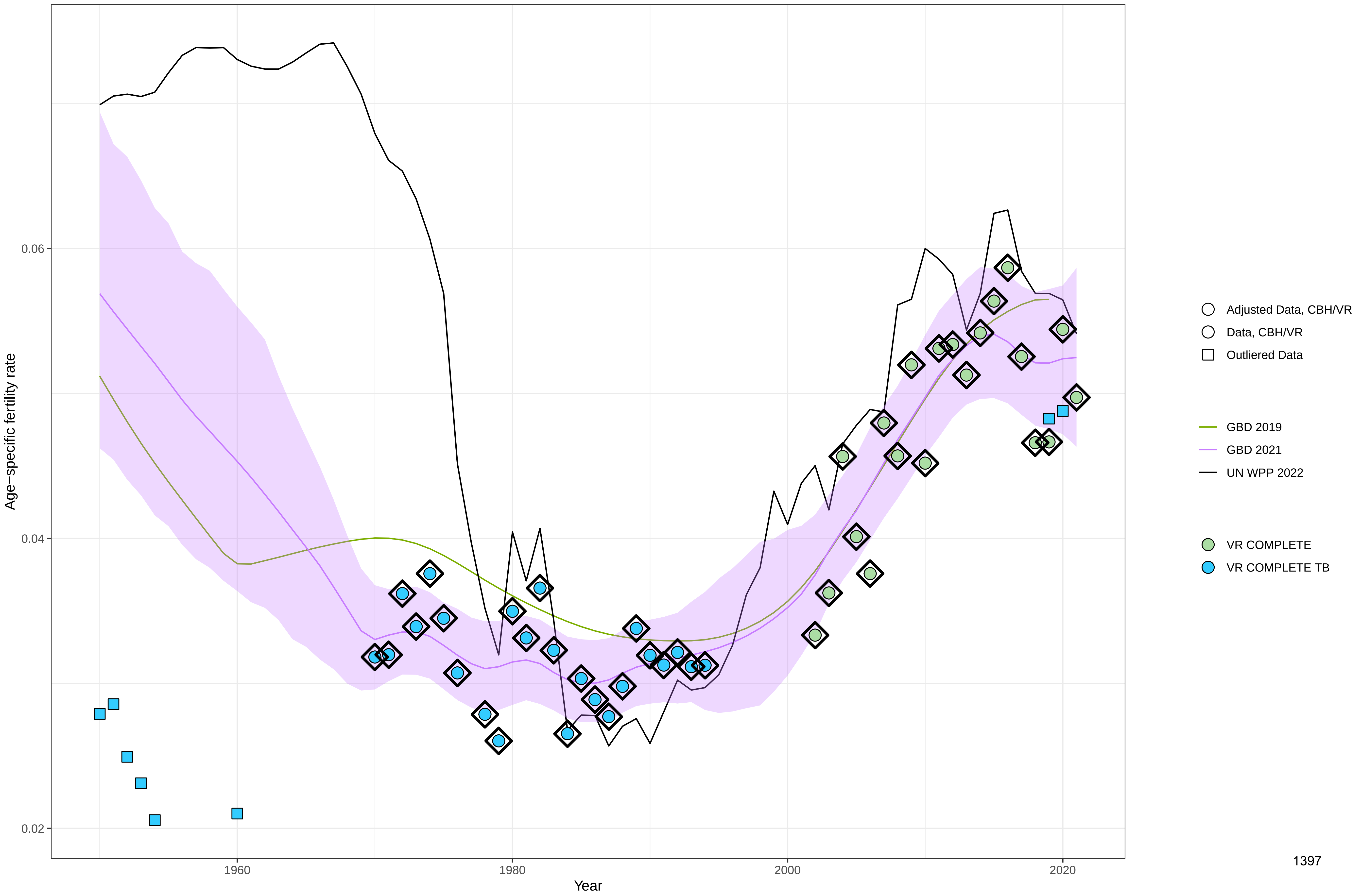

Age−specific fertility fitted trend lines, 1950−2021: Andorra, Ages: 40−44
Data points enclosed with a diamond are reference sources

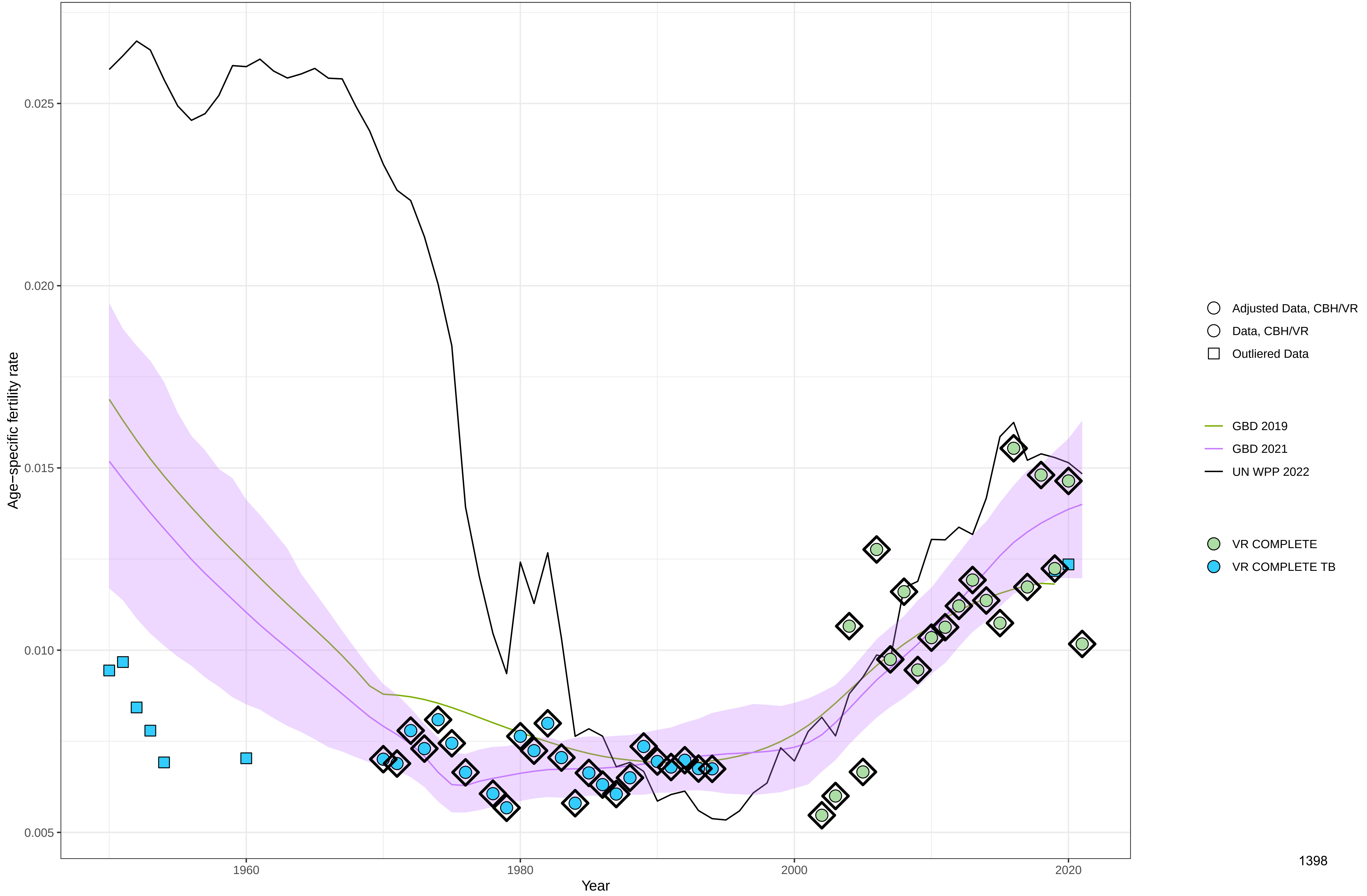

Age−specific fertility fitted trend lines, 1950−2021: Andorra, Ages: 45−49
Data points enclosed with a diamond are reference sources

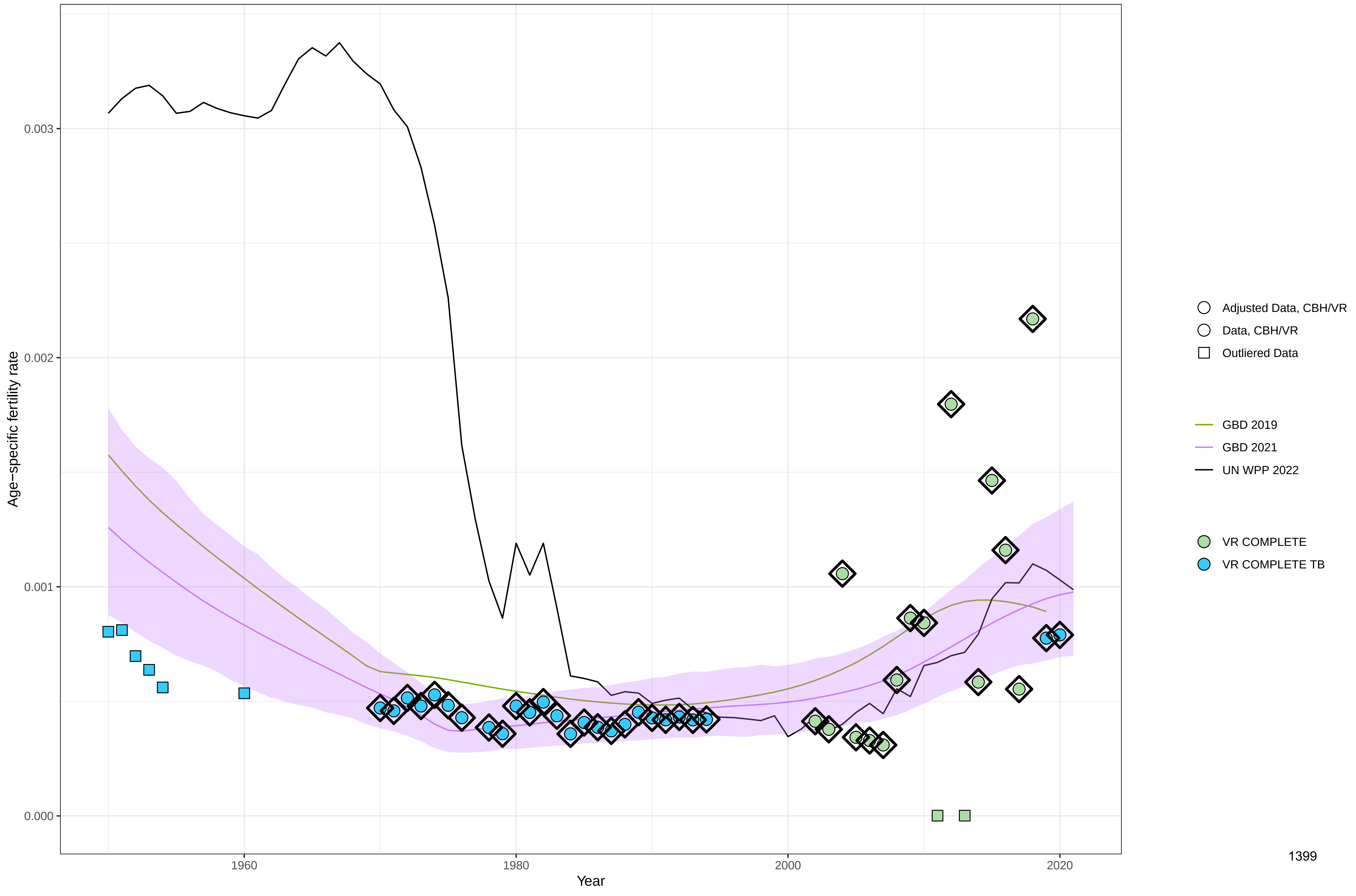

Age−specific fertility fitted trend lines, 1950−2021: Andorra, Ages: 50−54
Data points enclosed with a diamond are reference sources

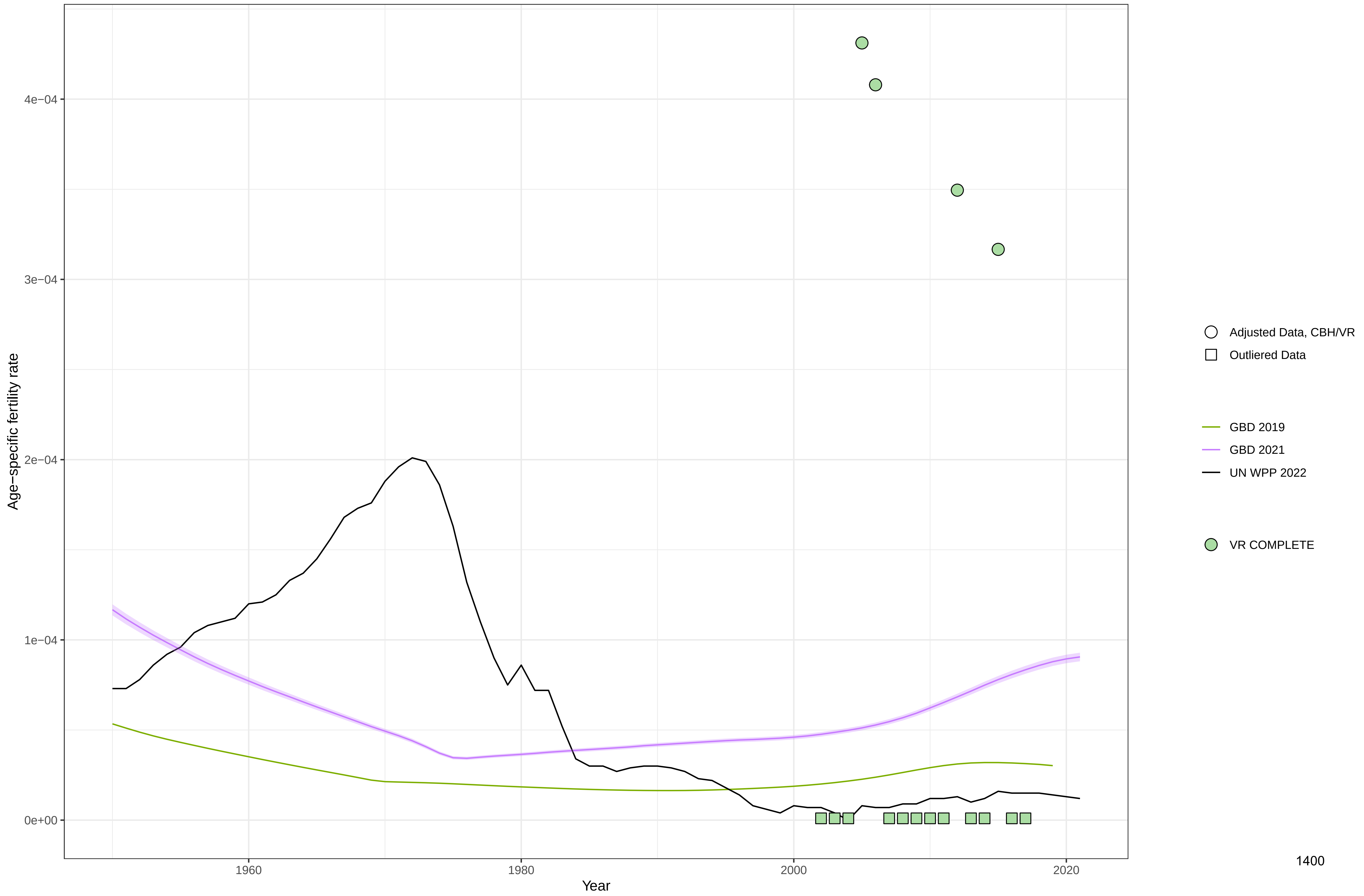

Total fertility rate fitted trend lines, 1950−2021: Andorra TFR
Data points enclosed with a diamond are reference sources

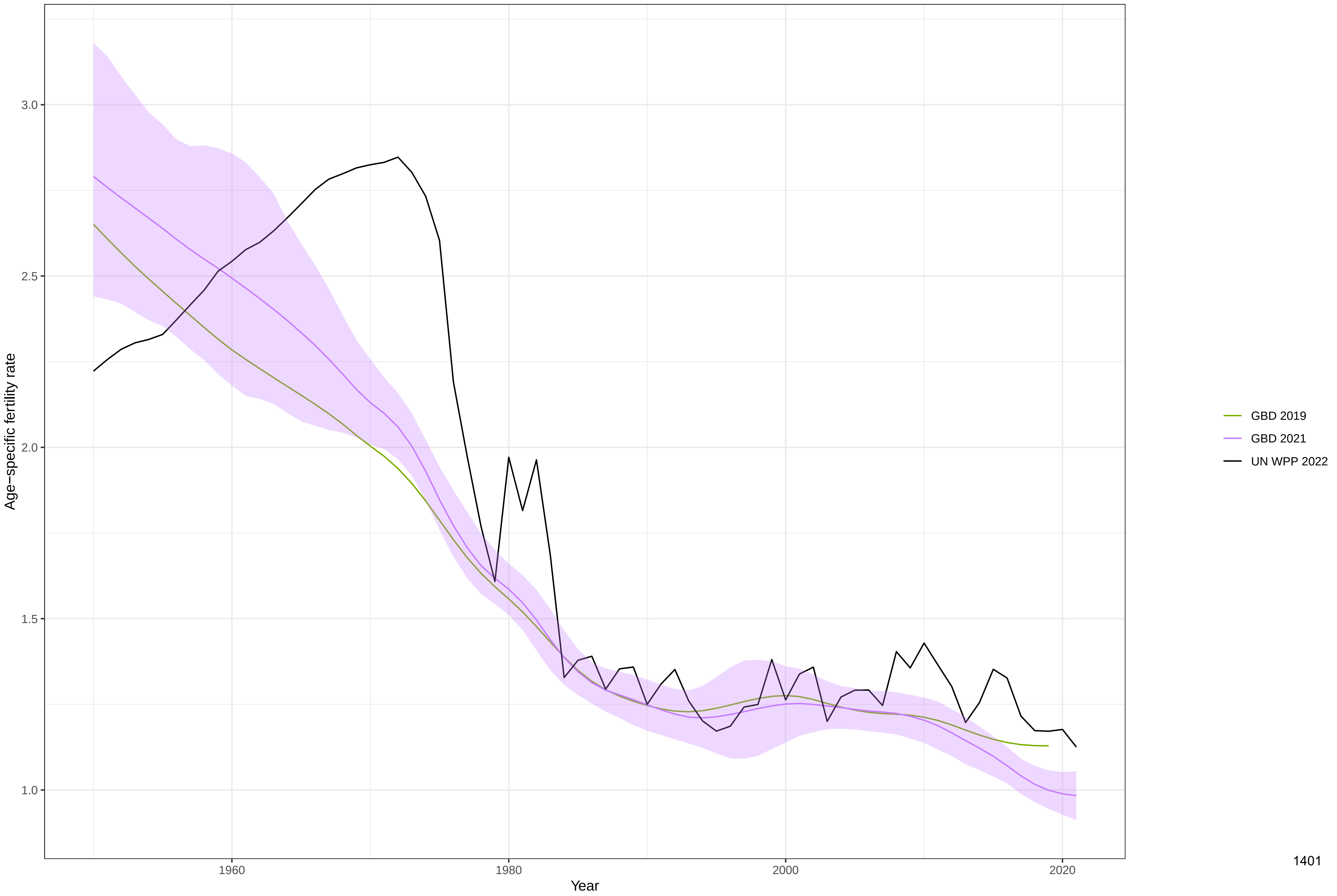

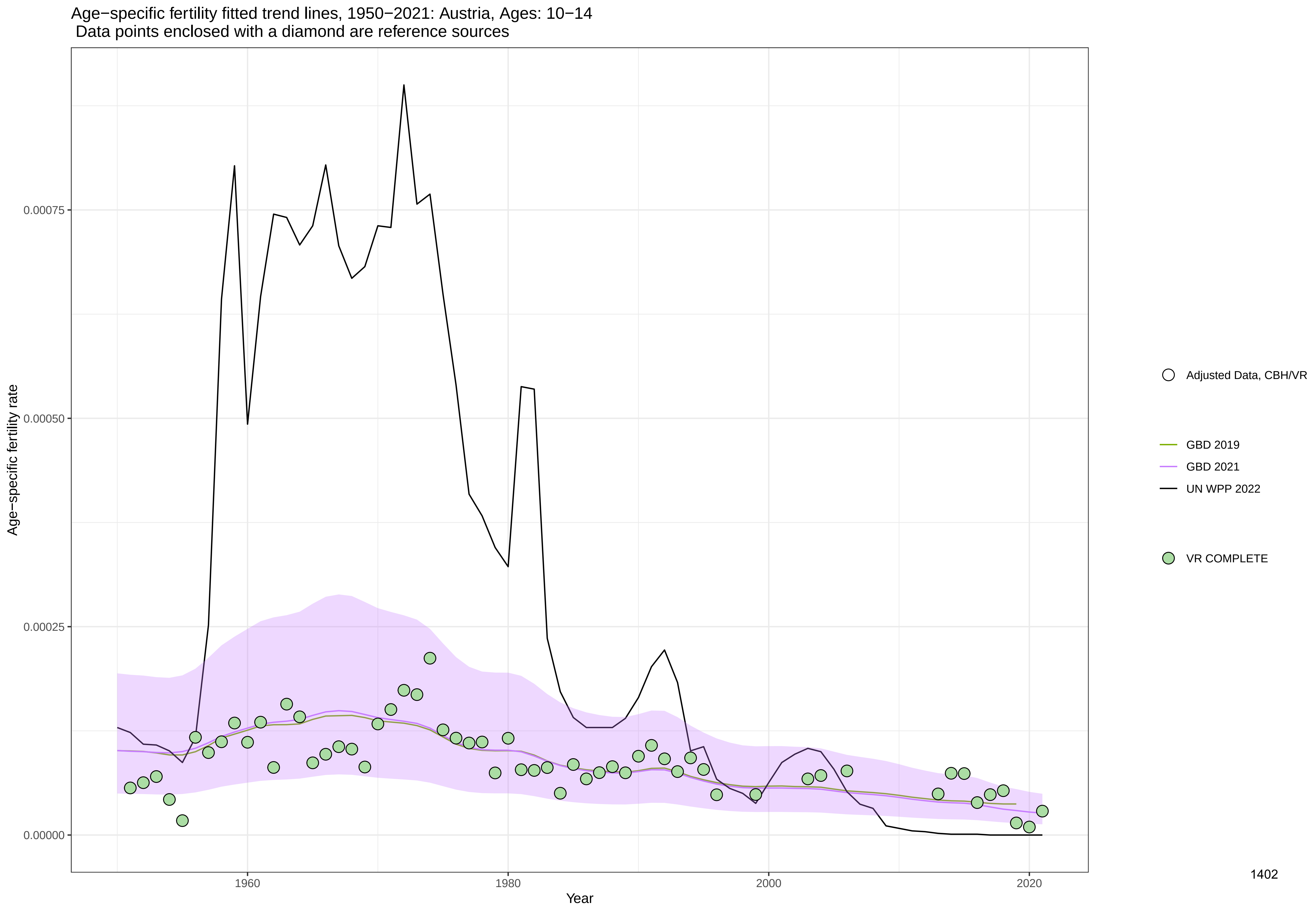

Age−specific fertility fitted trend lines, 1950−2021: Austria, Ages: 10−14
Data points enclosed with a diamond are reference sources
Age−specific fertility rate
0.00000
0.00025
0.00050
0.00075
1960
1980
2000
2020
Year
Adjusted Data, CBH/VR
GBD 2019
GBD 2021
UN WPP 2022
VR COMPLETE

Age−specific fertility fitted trend lines, 1950−2021: Austria, Ages: 15−19
Data points enclosed with a diamond are reference sources

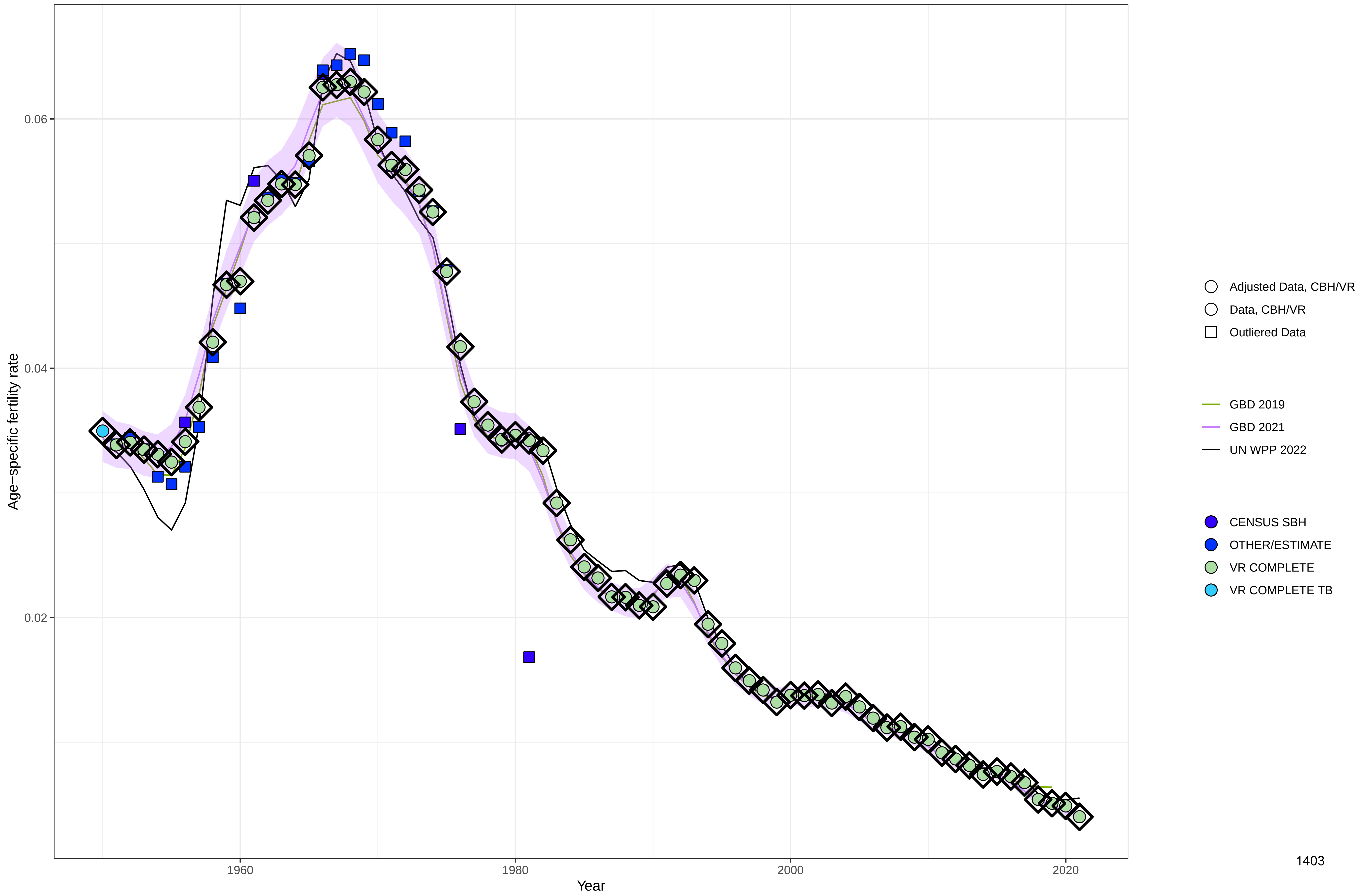

Age−specific fertility fitted trend lines, 1950−2021: Austria, Ages: 20−24
Data points enclosed with a diamond are reference sources

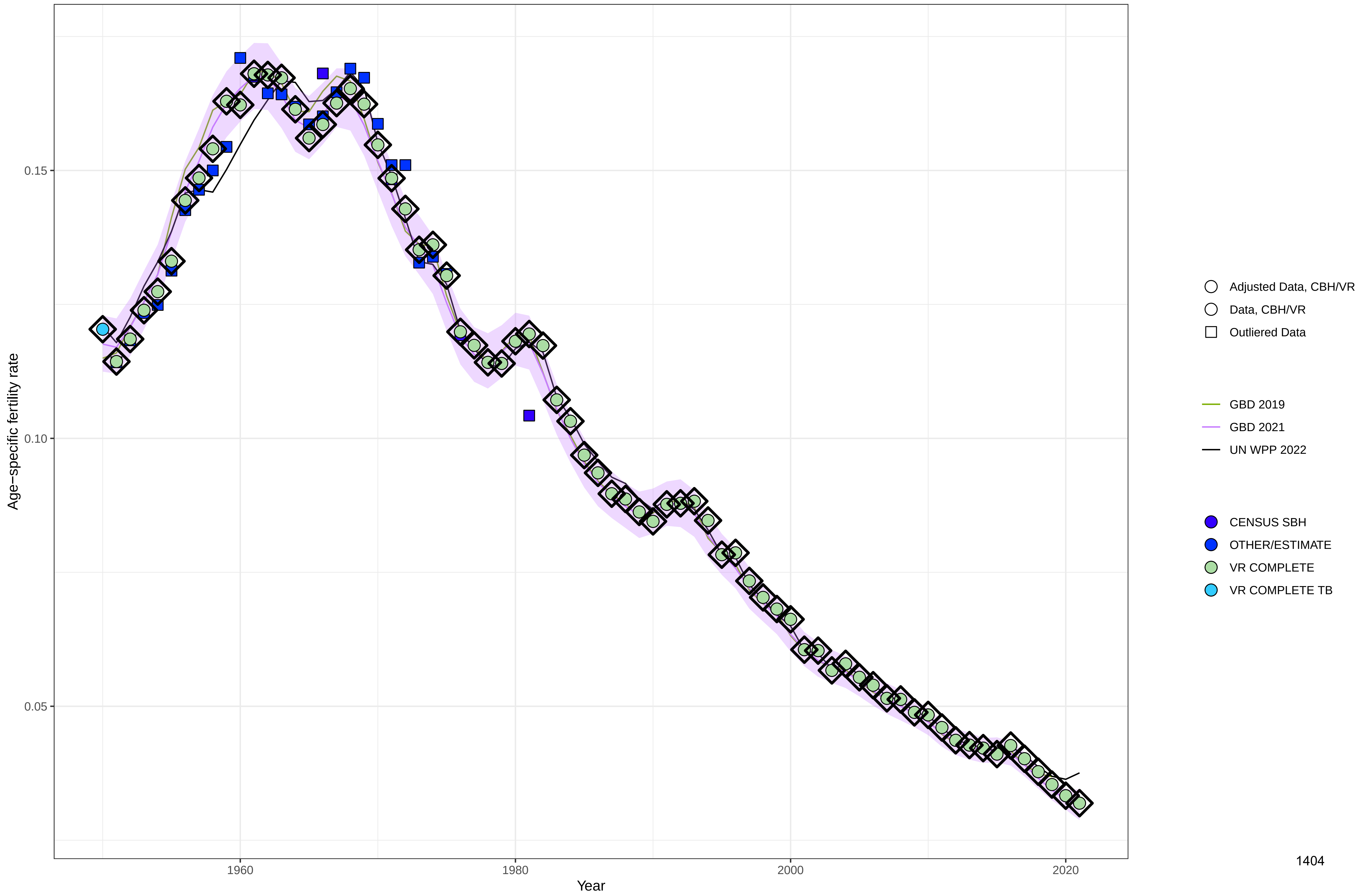

Age−specific fertility fitted trend lines, 1950−2021: Austria, Ages: 25−29
Data points enclosed with a diamond are reference sources

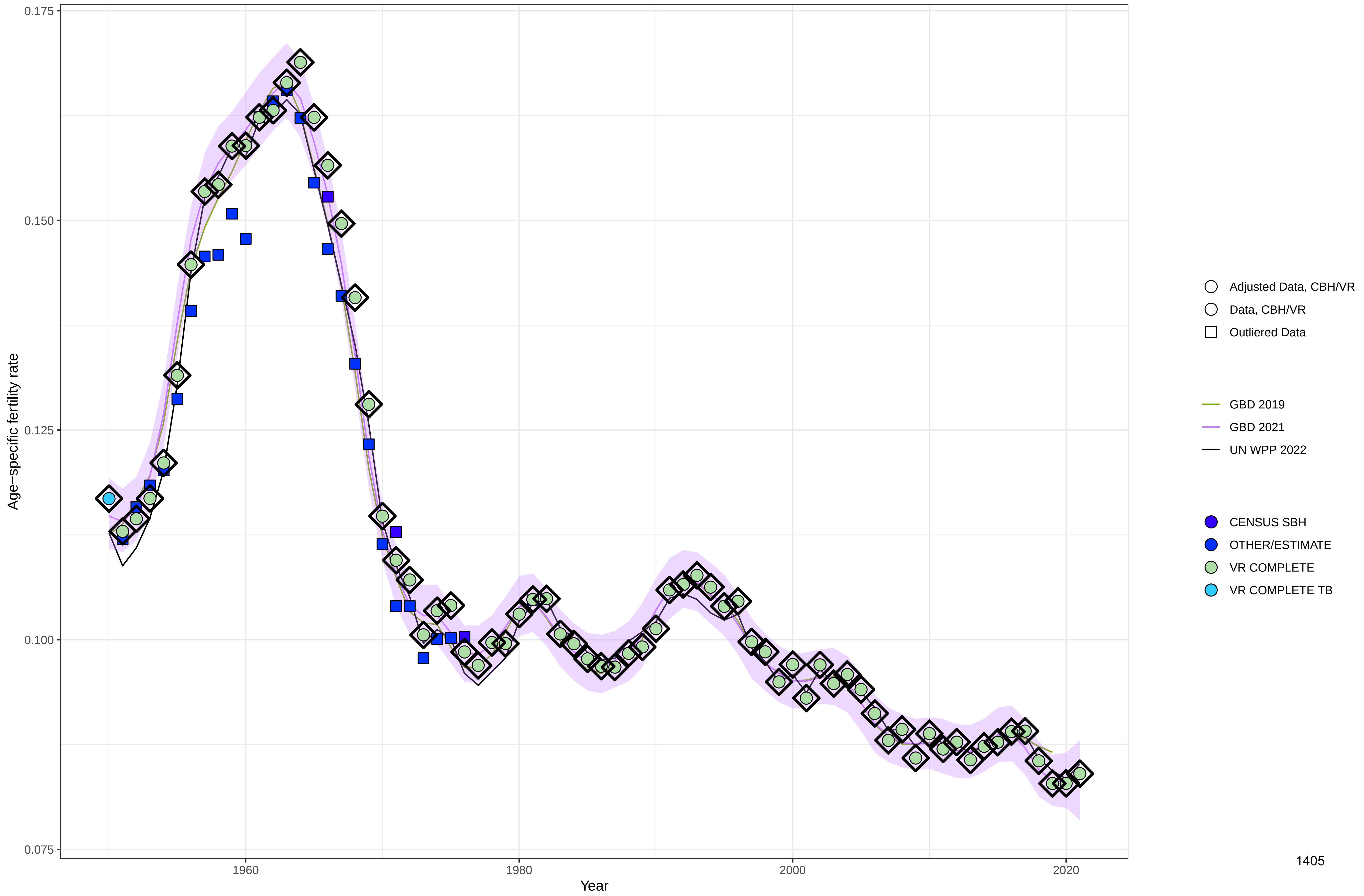

Age−specific fertility fitted trend lines, 1950−2021: Austria, Ages: 30−34
Data points enclosed with a diamond are reference sources

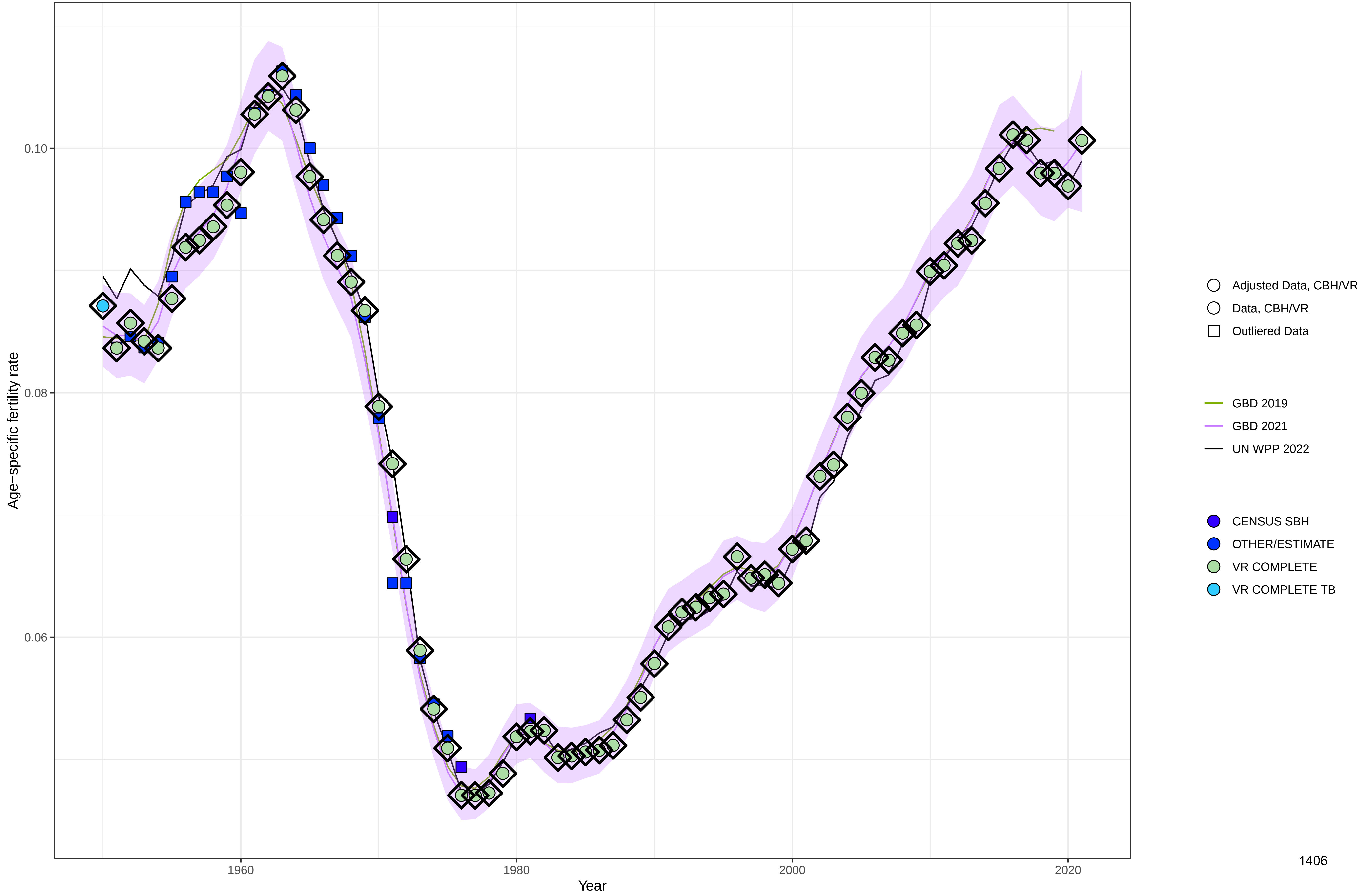

Age−specific fertility fitted trend lines, 1950−2021: Austria, Ages: 35−39
Data points enclosed with a diamond are reference sources

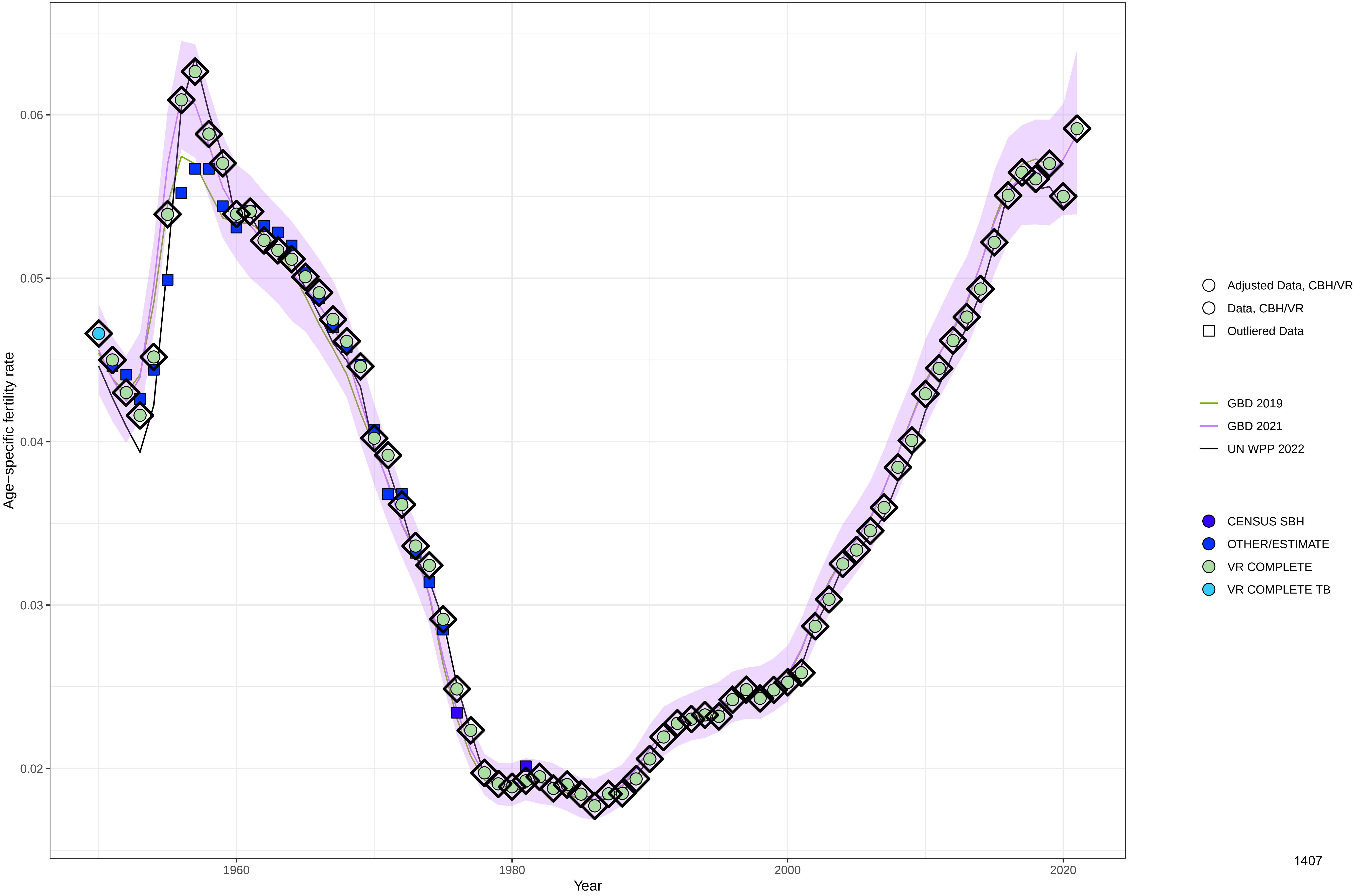

Age−specific fertility fitted trend lines, 1950−2021: Austria, Ages: 40−44
Data points enclosed with a diamond are reference sources

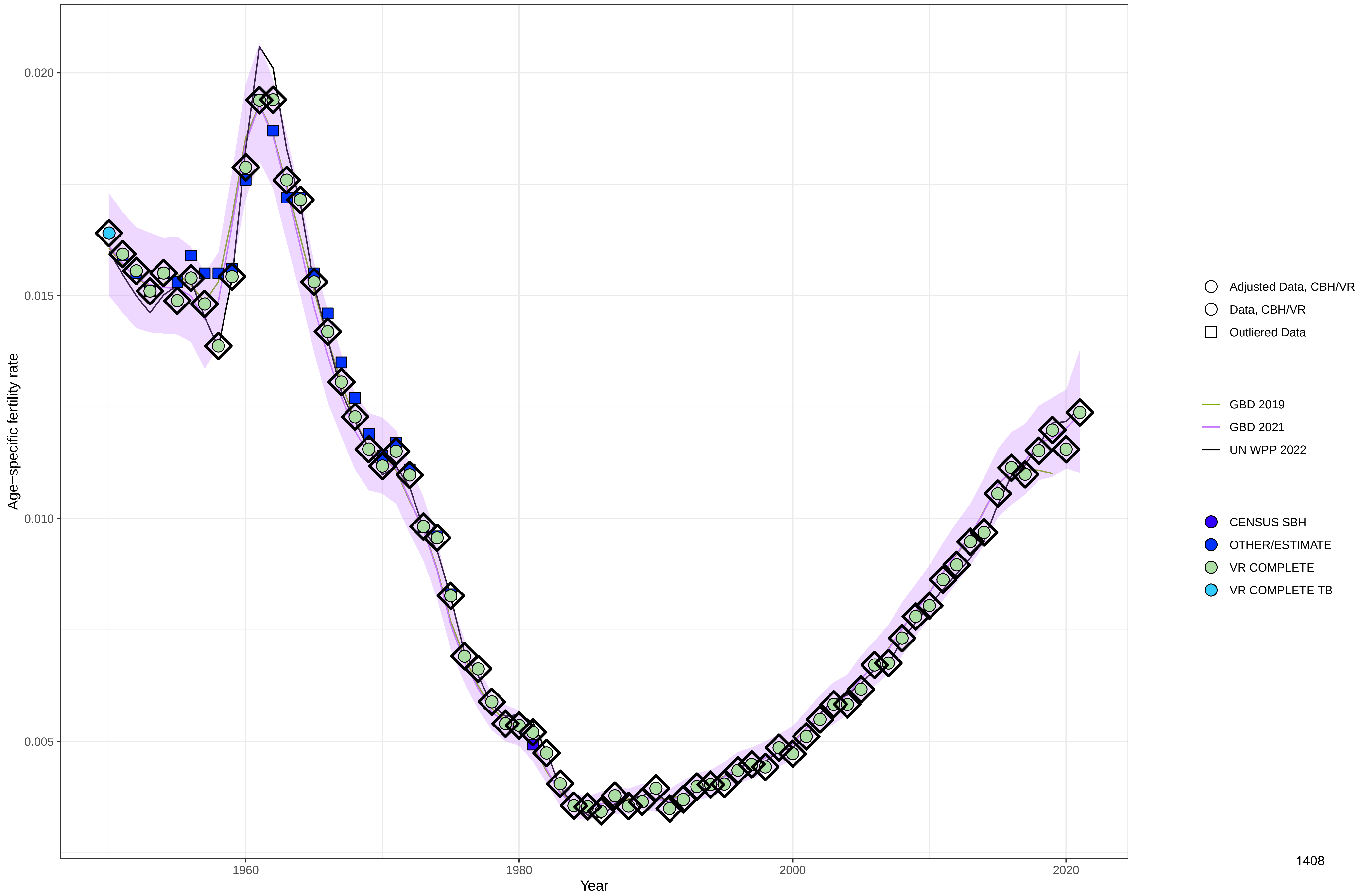

Age−specific fertility fitted trend lines, 1950−2021: Austria, Ages: 45−49
Data points enclosed with a diamond are reference sources

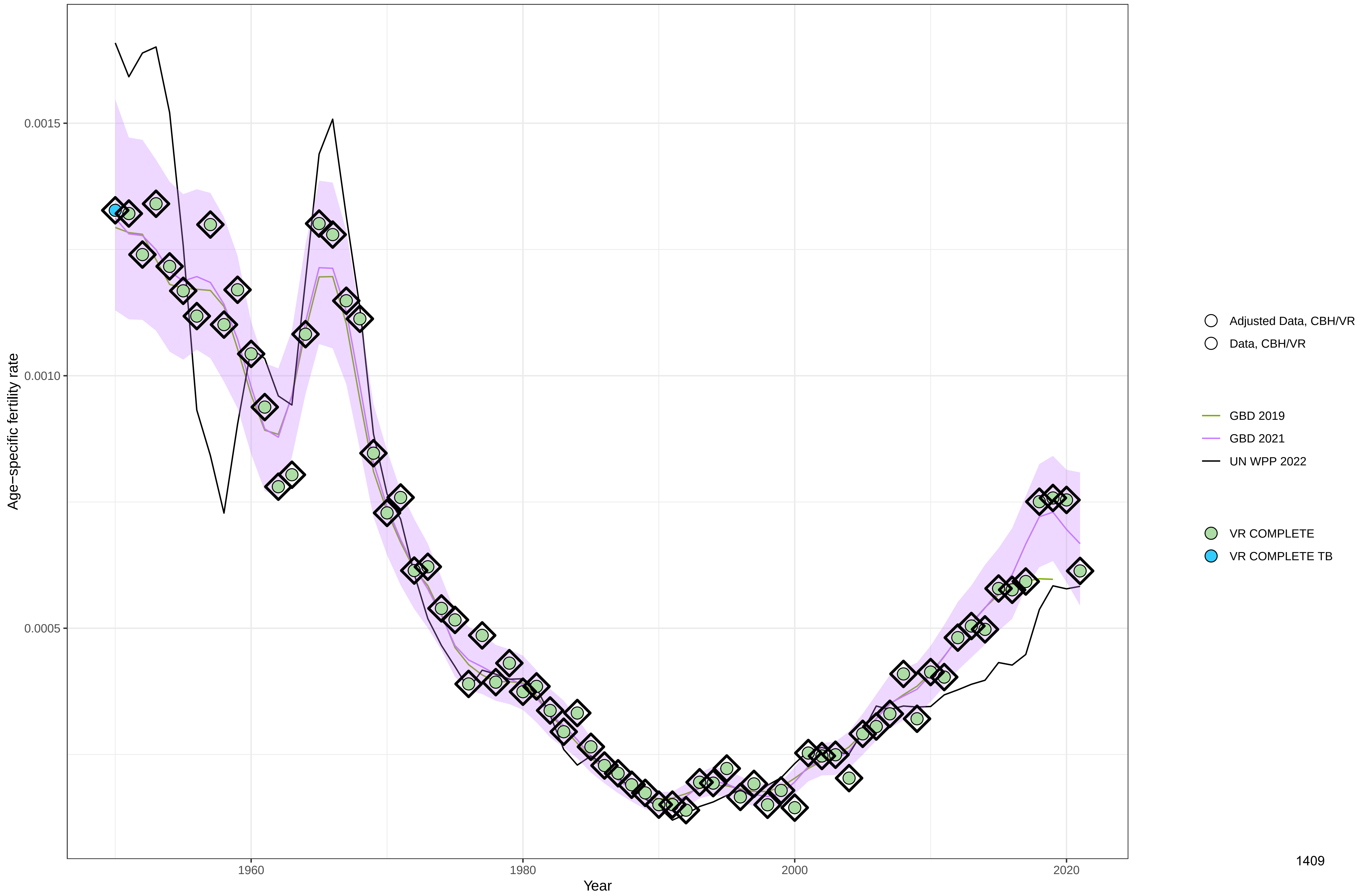

Age−specific fertility fitted trend lines, 1950−2021: Austria, Ages: 50−54
Data points enclosed with a diamond are reference sources

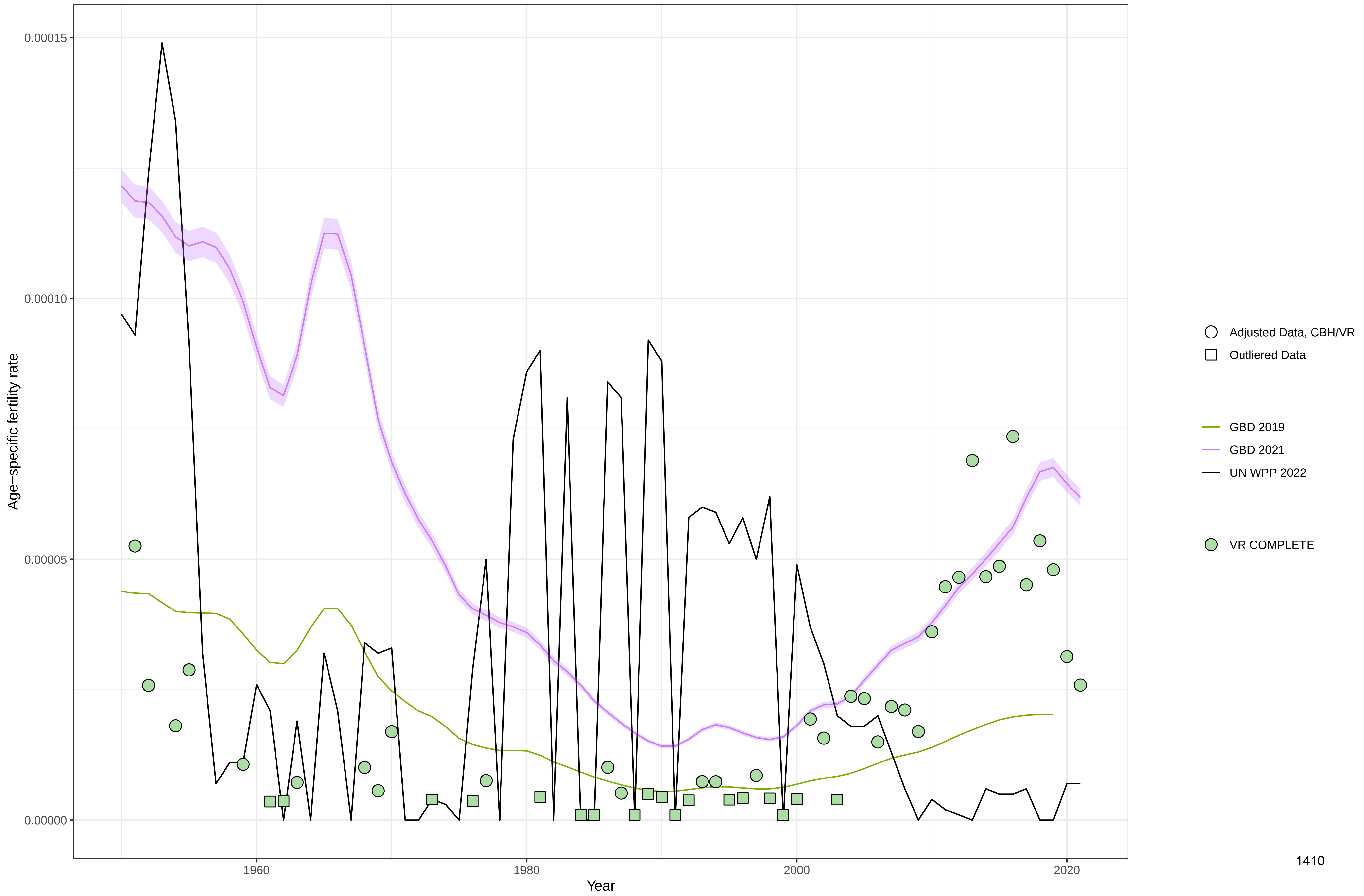

Total fertility rate fitted trend lines, 1950−2021: Austria TFR
Data points enclosed with a diamond are reference sources

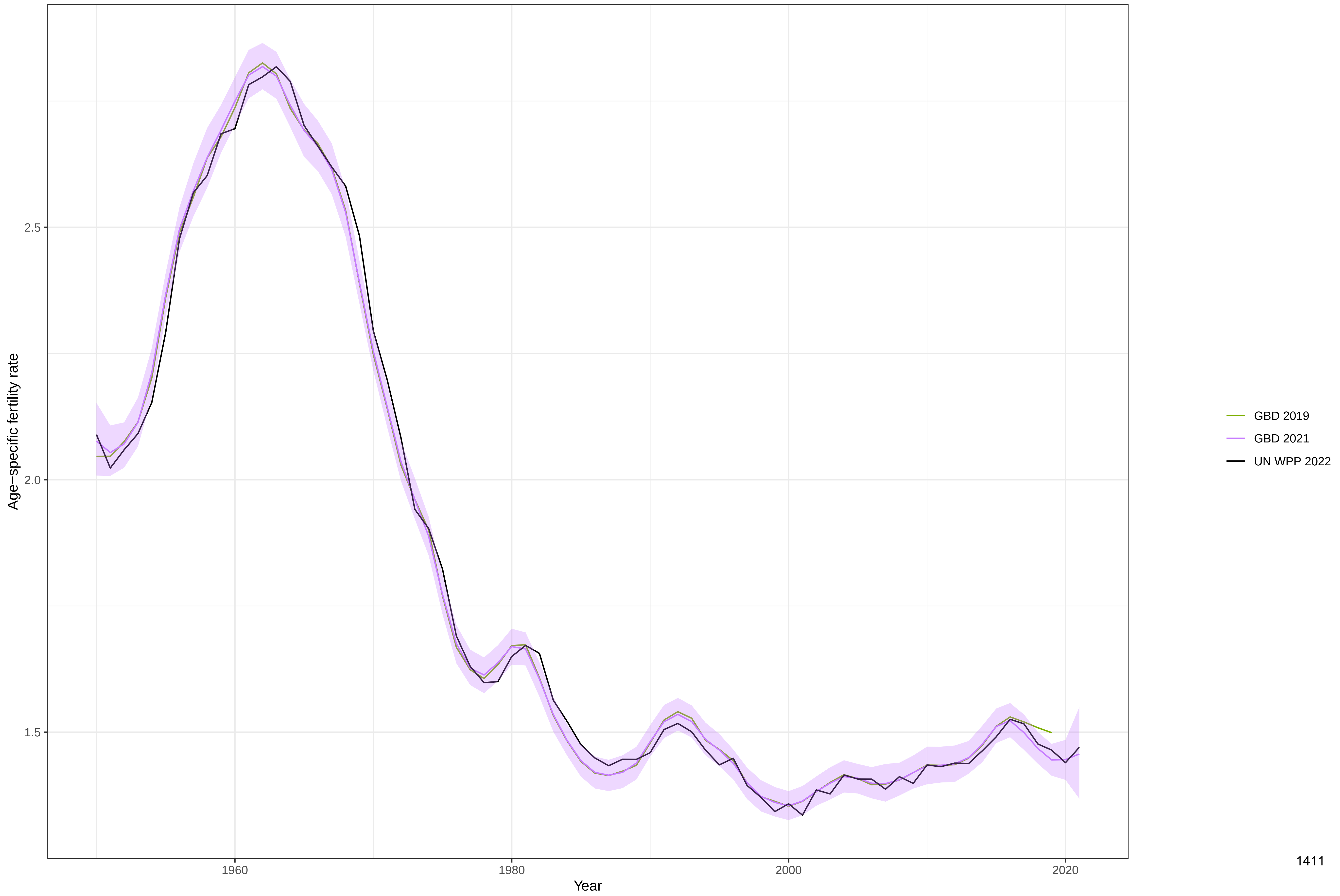

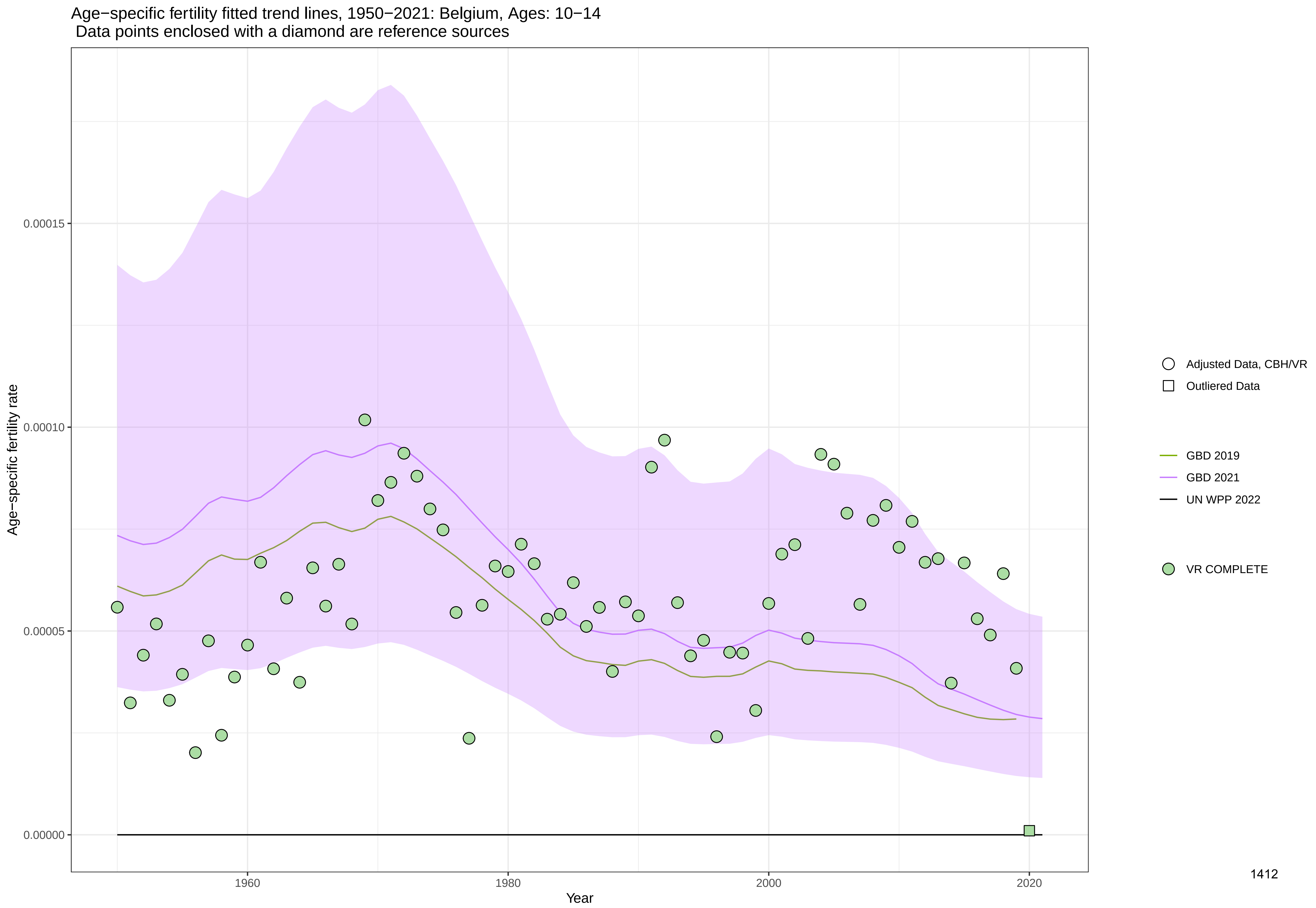

Age−specific fertility fitted trend lines, 1950−2021: Belgium, Ages: 10−14
Data points enclosed with a diamond are reference sources
Age−specific fertility rate
0.00015
0.00010
0.00005
0.00000
1960
1980
2000
2020
Year
Adjusted Data, CBH/VR
Outliered Data
GBD 2019
GBD 2021
UN WPP 2022
VR COMPLETE

Age−specific fertility fitted trend lines, 1950−2021: Belgium, Ages: 15−19
Data points enclosed with a diamond are reference sources

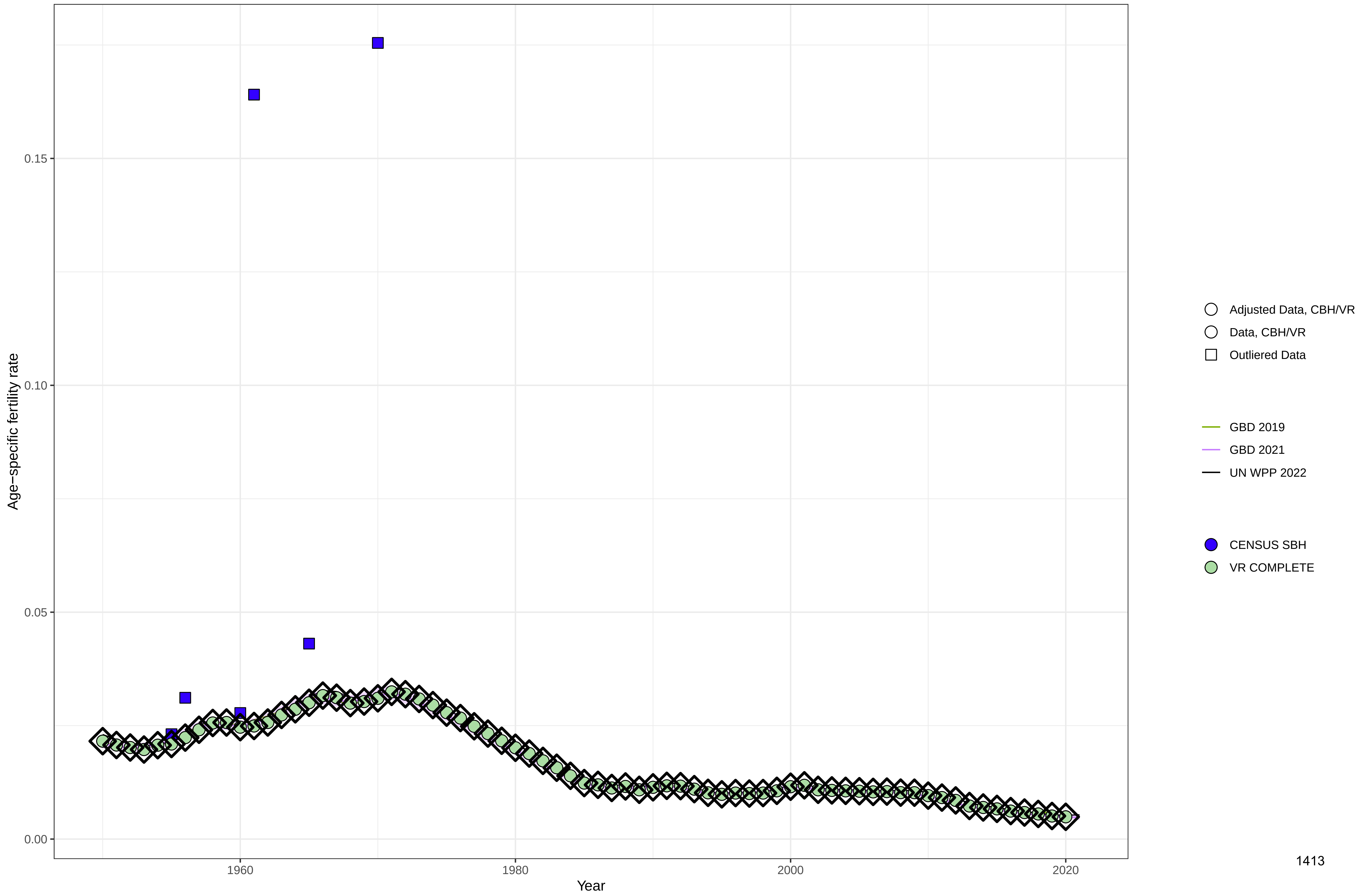

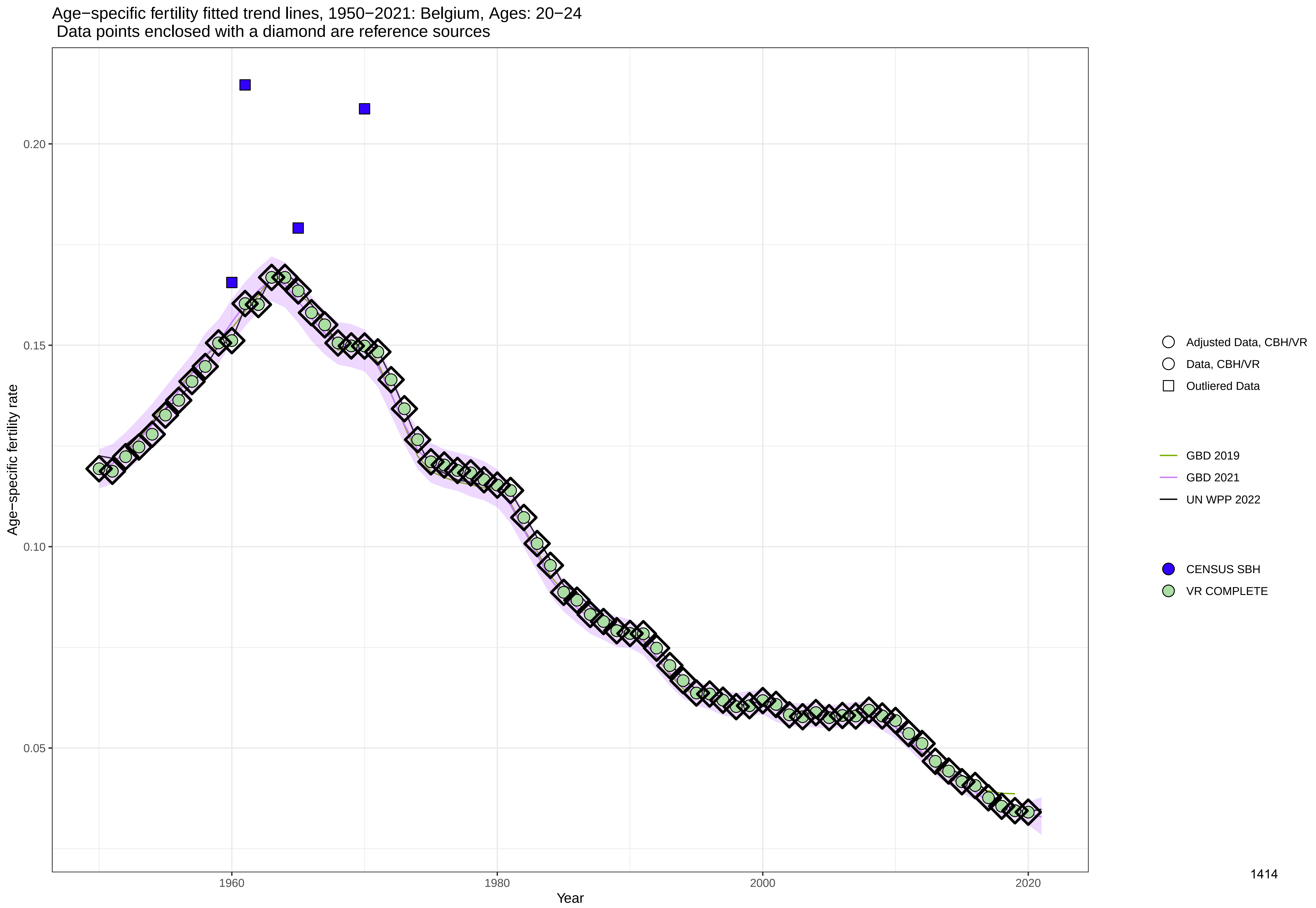

Age−specific fertility fitted trend lines, 1950−2021: Belgium, Ages: 20−24
Data points enclosed with a diamond are reference sources
Age−specific fertility rate
Year
0.20
0.15
0.10
0.05
1960
1980
2000
2020
Adjusted Data, CBH/VR
Data, CBH/VR
Outliered Data
GBD 2019
GBD 2021
UN WPP 2022
CENSUS SBH
VR COMPLETE

Age−specific fertility fitted trend lines, 1950−2021: Belgium, Ages: 25−29
Data points enclosed with a diamond are reference sources

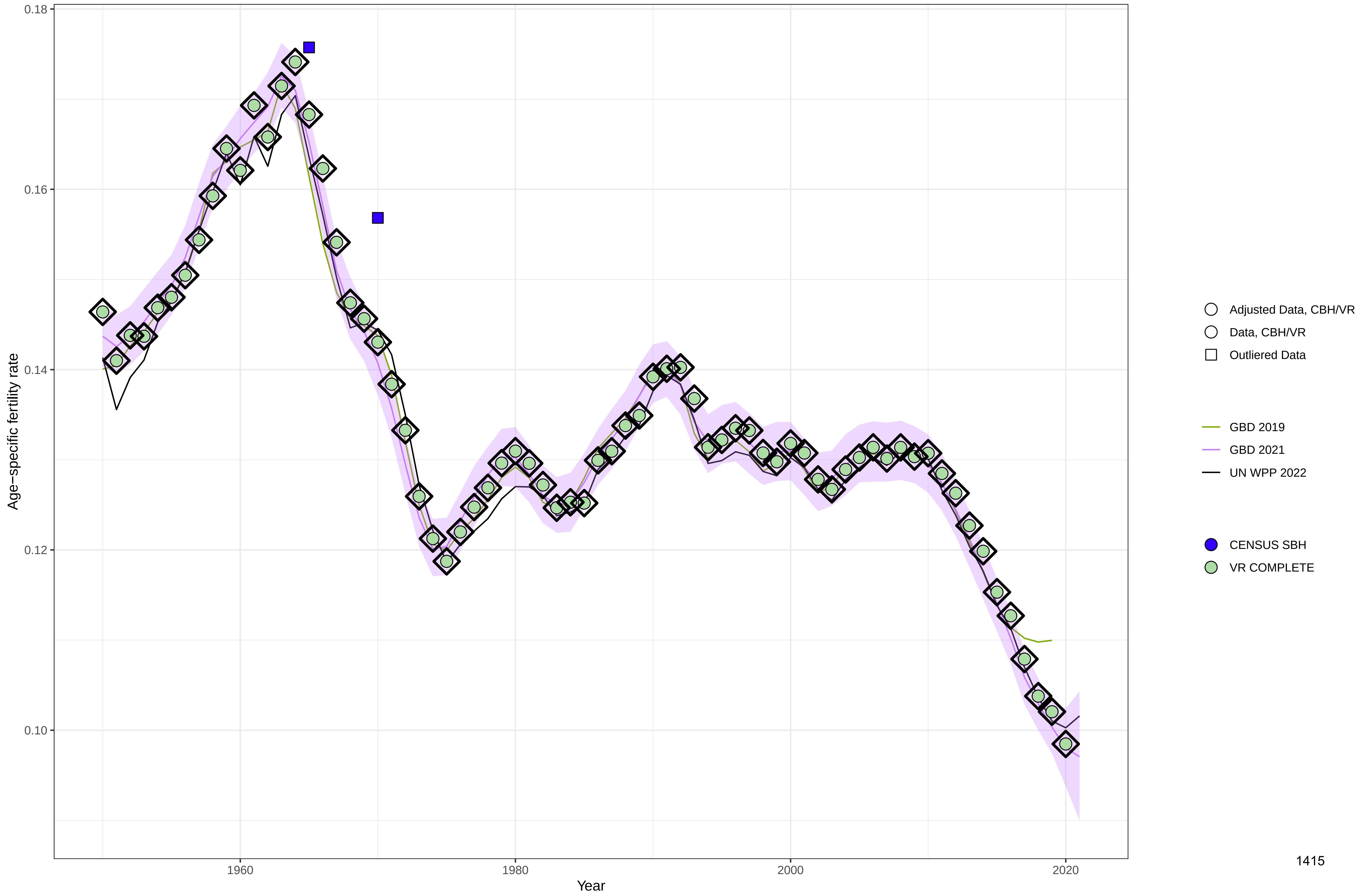

Age−specific fertility fitted trend lines, 1950−2021: Belgium, Ages: 30−34
Data points enclosed with a diamond are reference sources

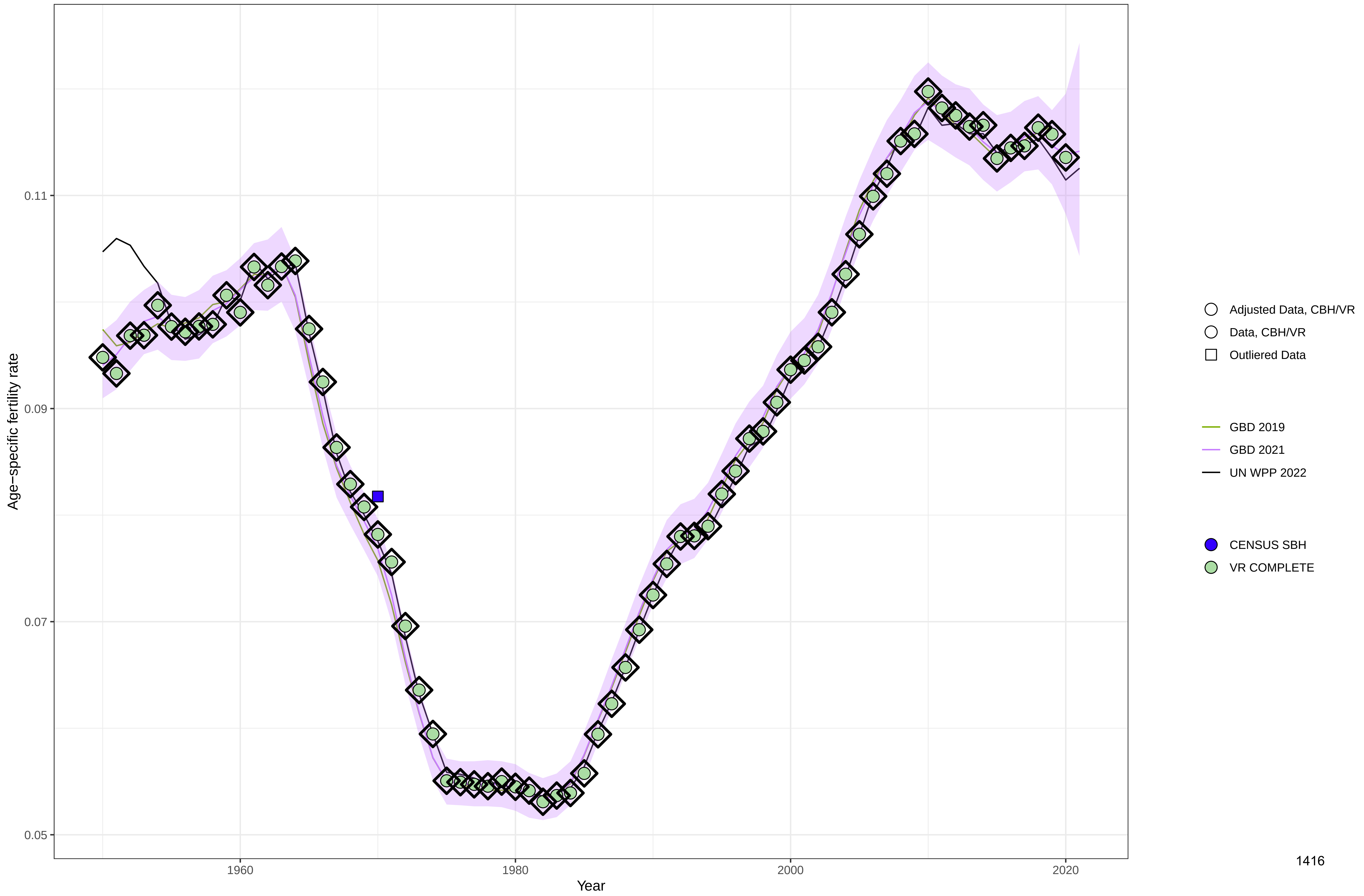

Age−specific fertility fitted trend lines, 1950−2021: Belgium, Ages: 35−39
Data points enclosed with a diamond are reference sources

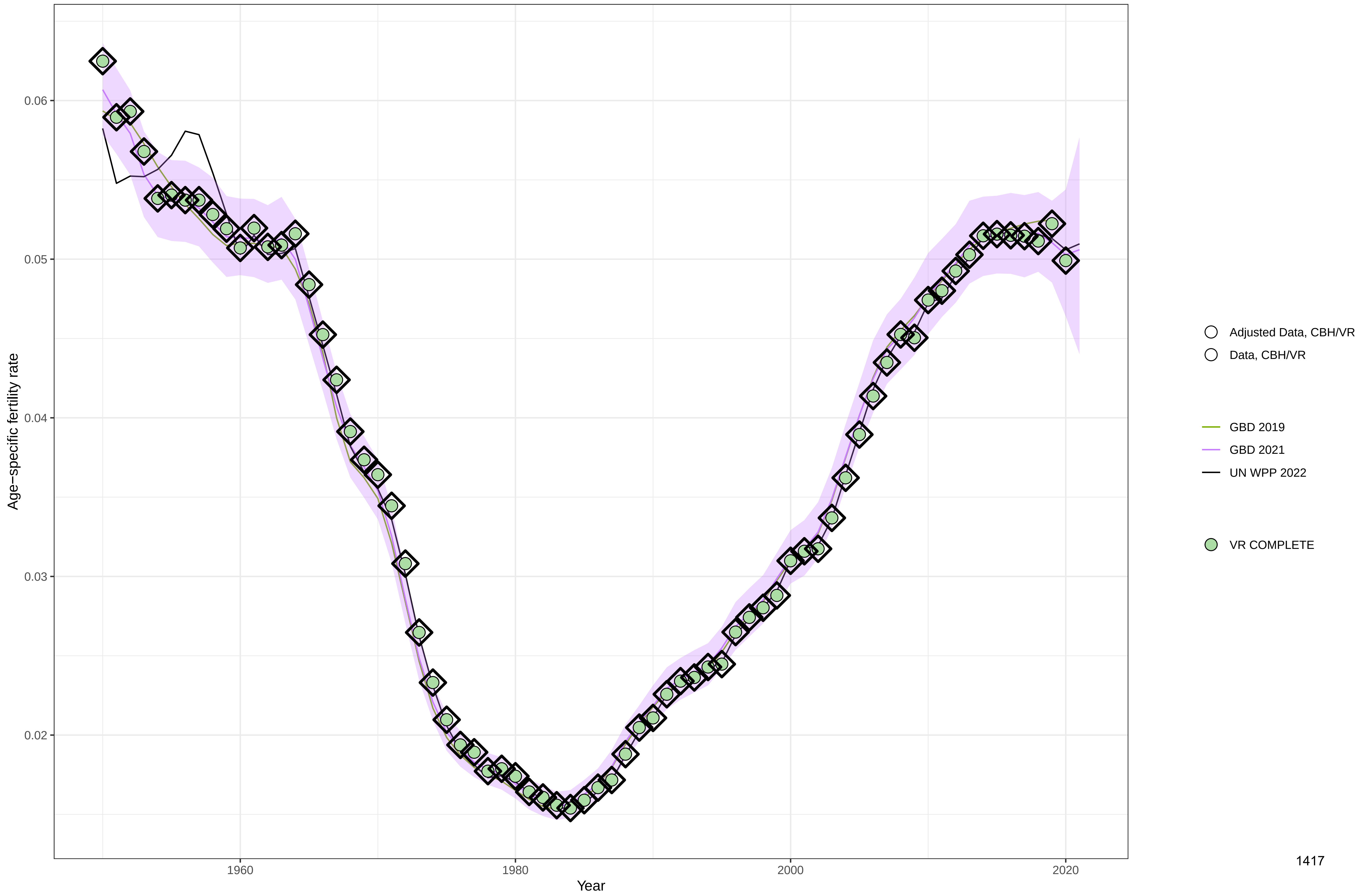

Age−specific fertility fitted trend lines, 1950−2021: Belgium, Ages: 40−44
Data points enclosed with a diamond are reference sources

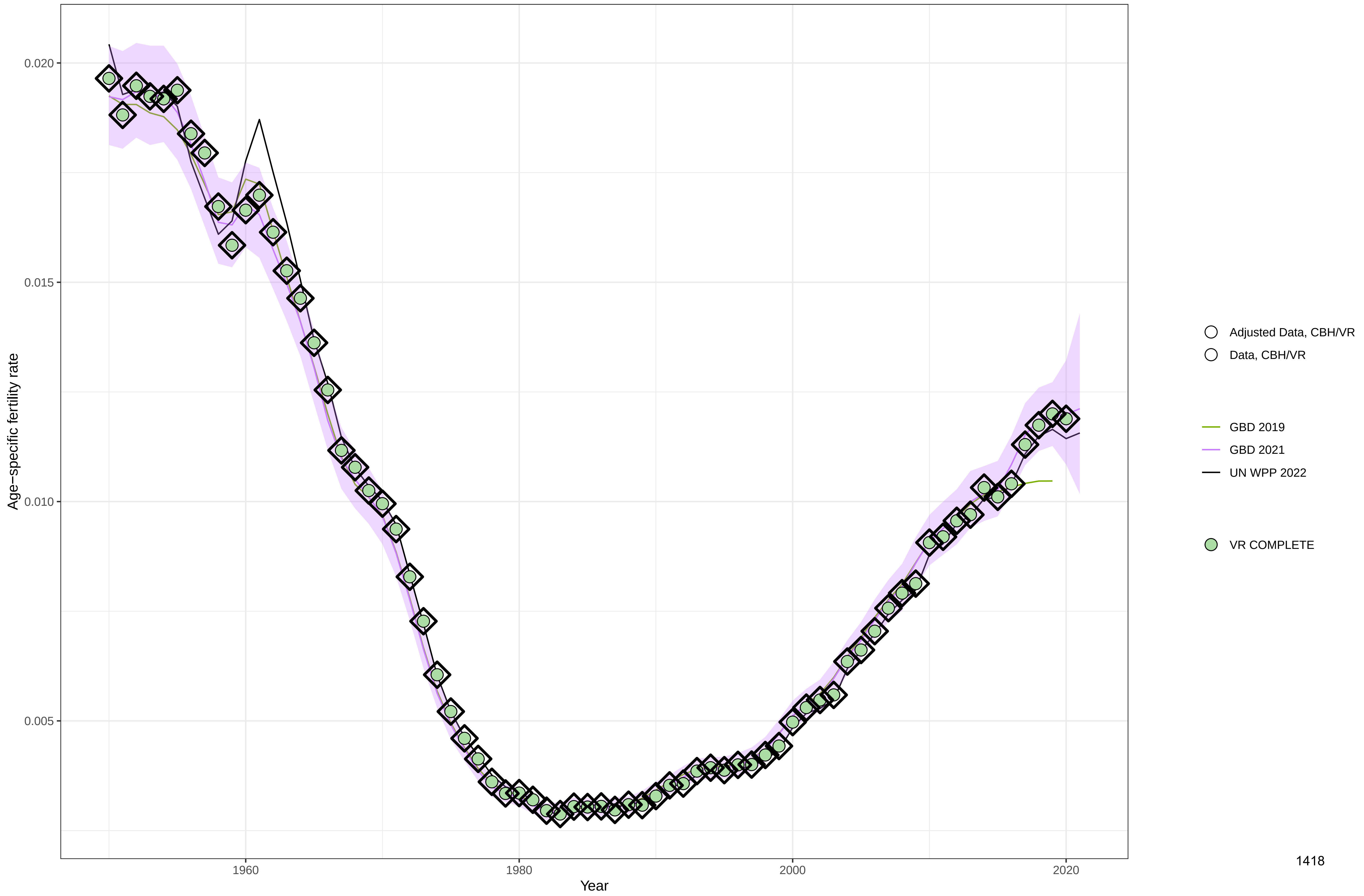

Age−specific fertility fitted trend lines, 1950−2021: Belgium, Ages: 45−49
Data points enclosed with a diamond are reference sources

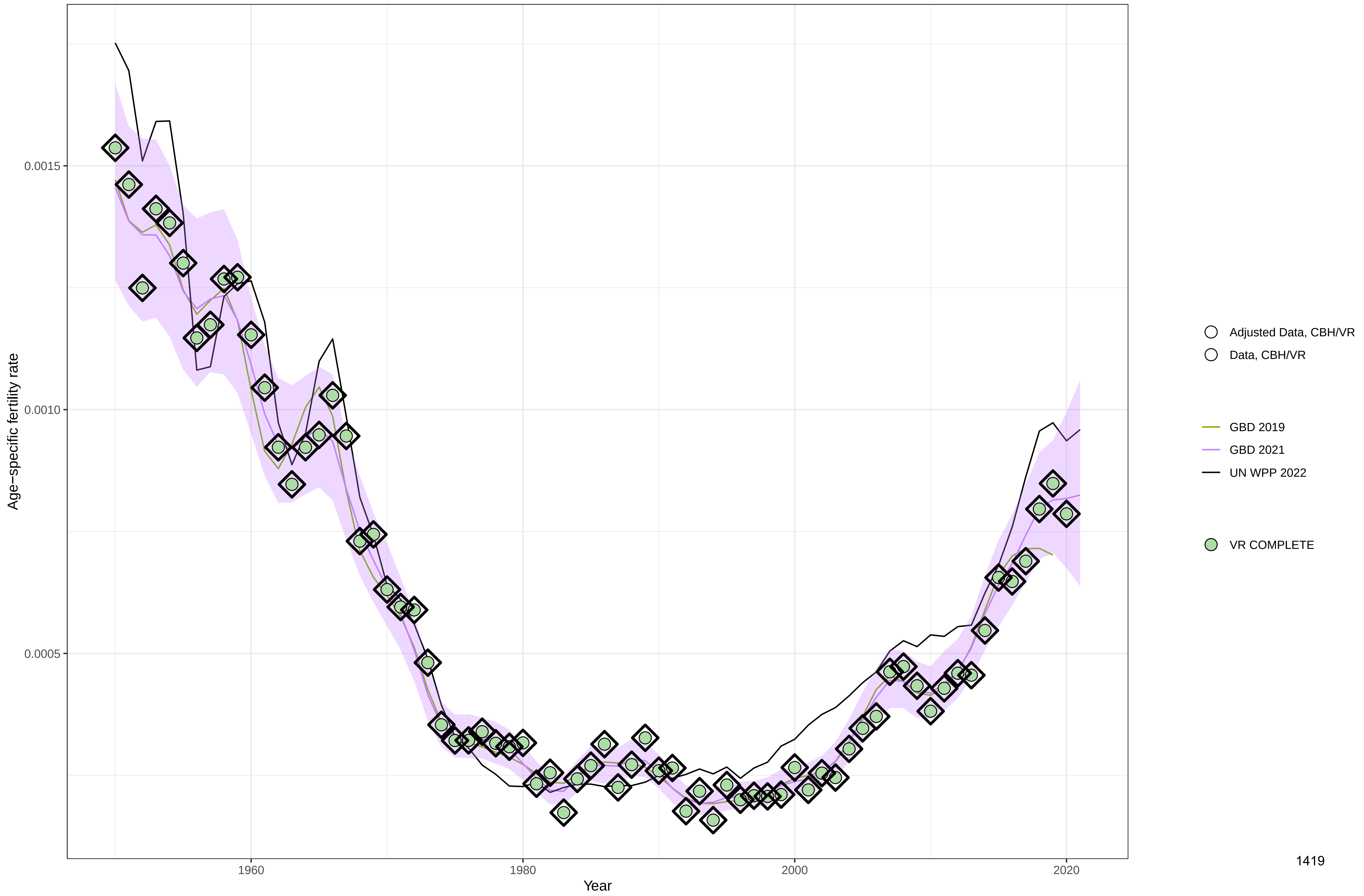

Age−specific fertility fitted trend lines, 1950−2021: Belgium, Ages: 50−54
Data points enclosed with a diamond are reference sources

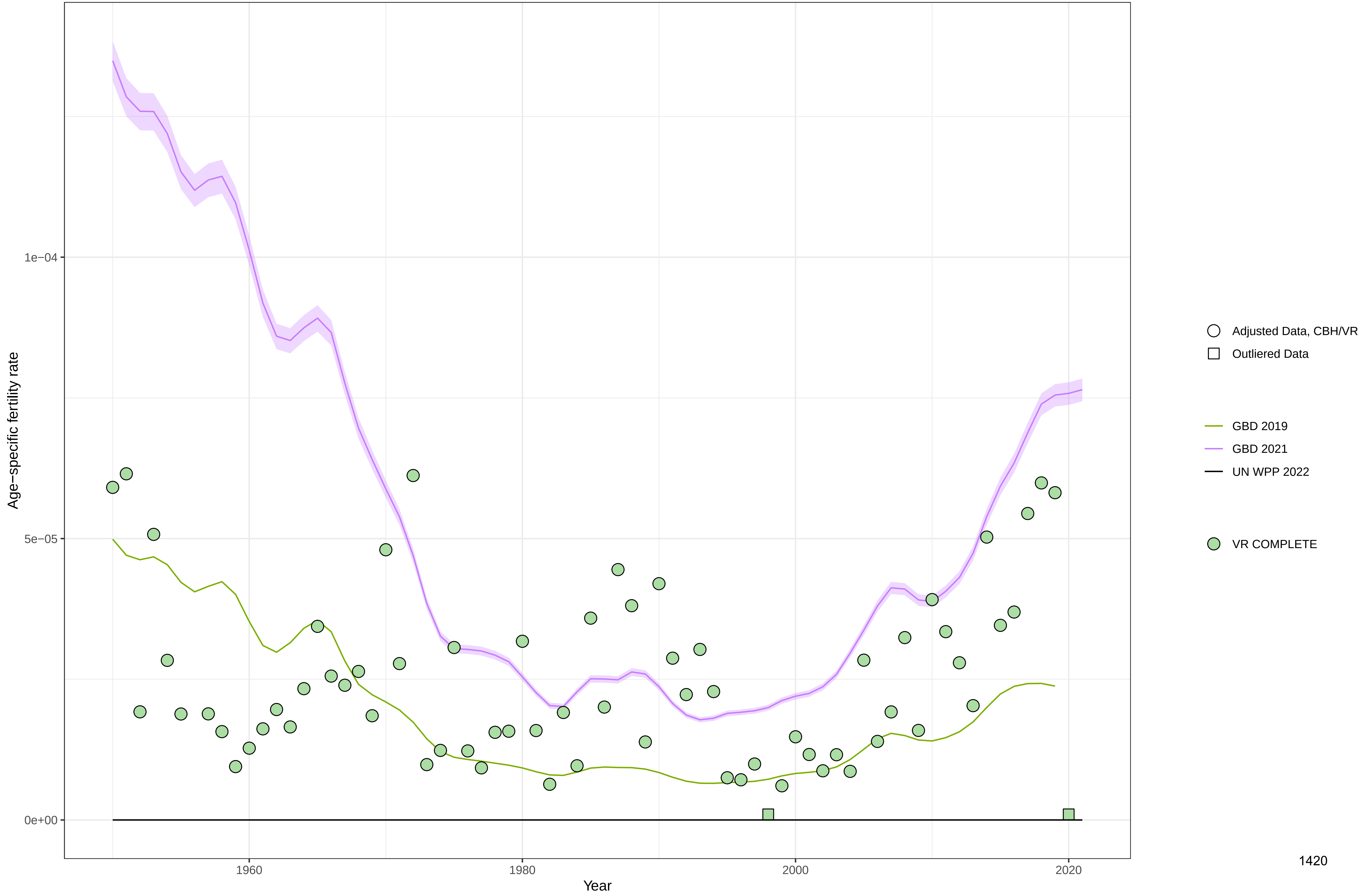

Total fertility rate fitted trend lines, 1950−2021: Belgium TFR
Data points enclosed with a diamond are reference sources

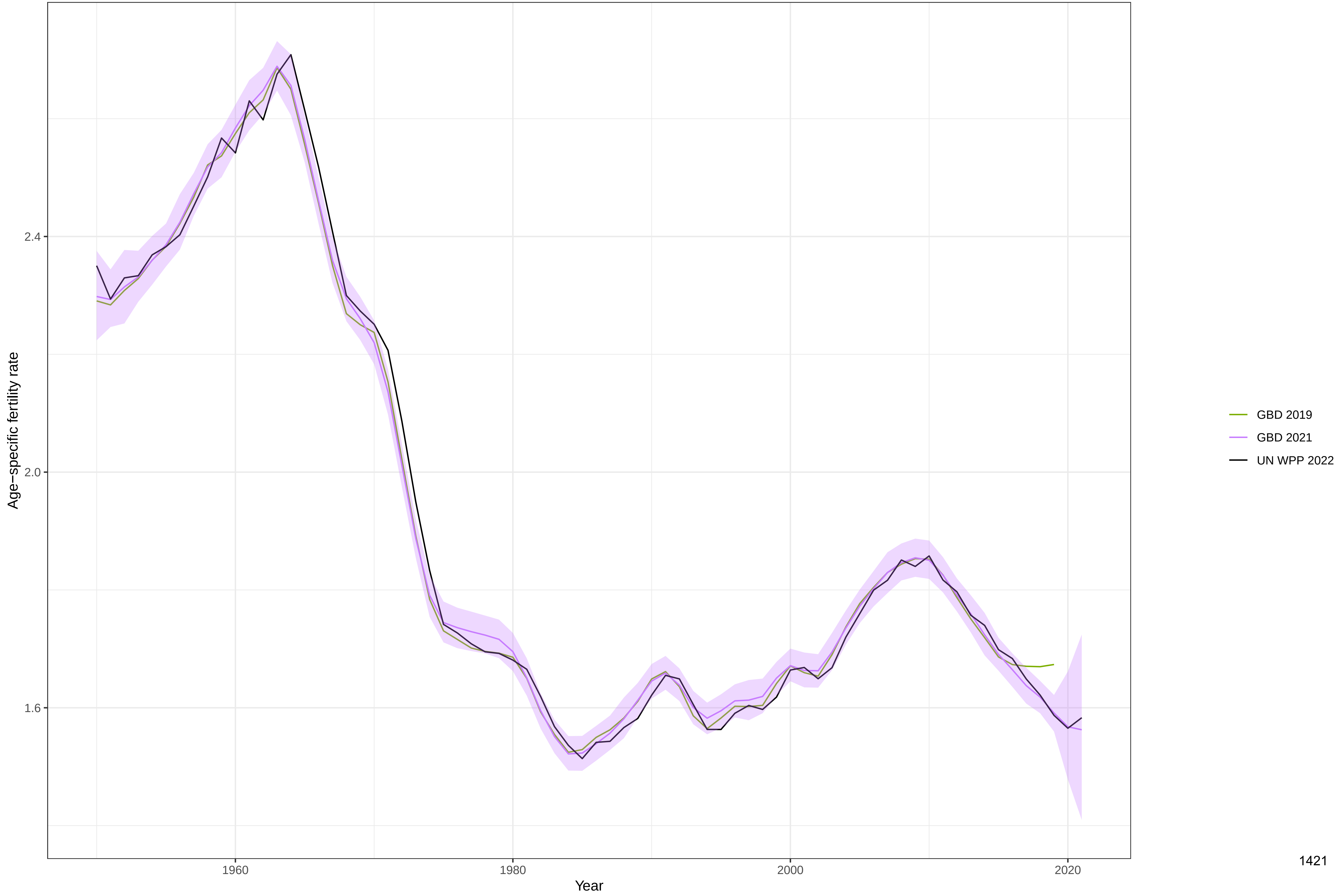

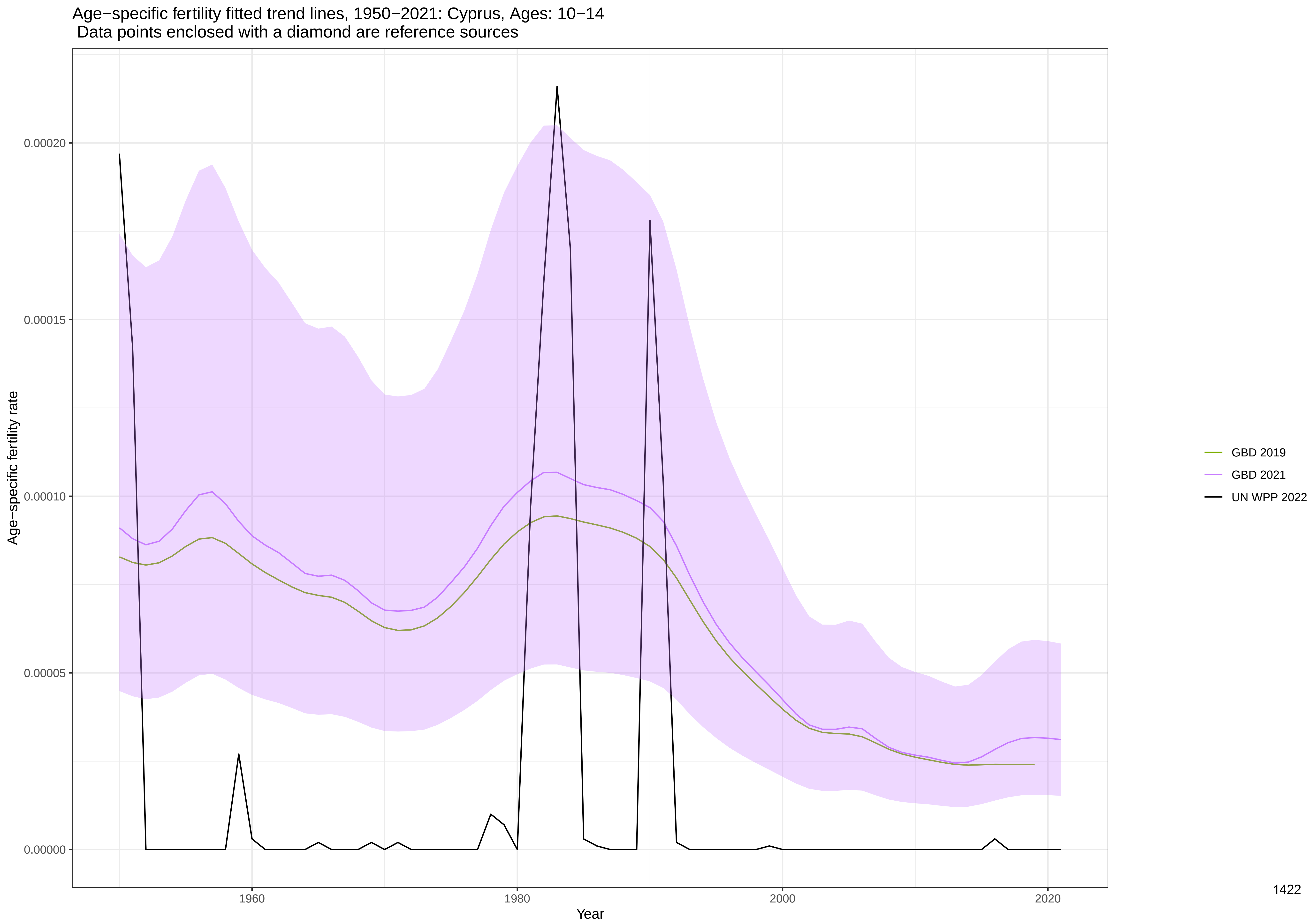

Age−specific fertility fitted trend lines, 1950−2021: Cyprus, Ages: 10−14
Data points enclosed with a diamond are reference sources
Age−specific fertility rate
0.00000
0.00005
0.00010
0.00015
0.00020
1960
1980
2000
2020
Year
GBD 2019
GBD 2021
UN WPP 2022

Age−specific fertility fitted trend lines, 1950−2021: Cyprus, Ages: 15−19
Data points enclosed with a diamond are reference sources

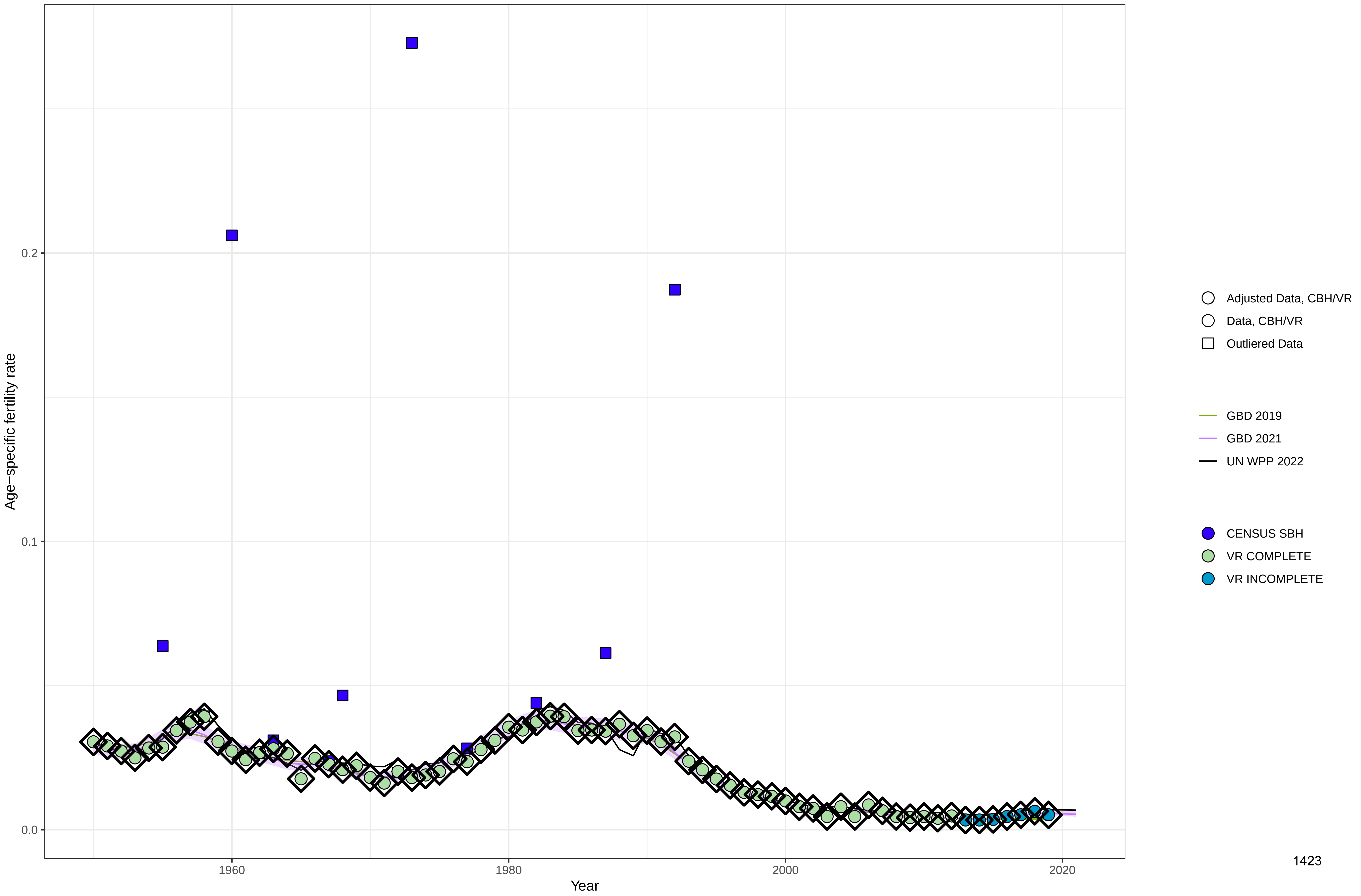

Age−specific fertility fitted trend lines, 1950−2021: Cyprus, Ages: 20−24
Data points enclosed with a diamond are reference sources

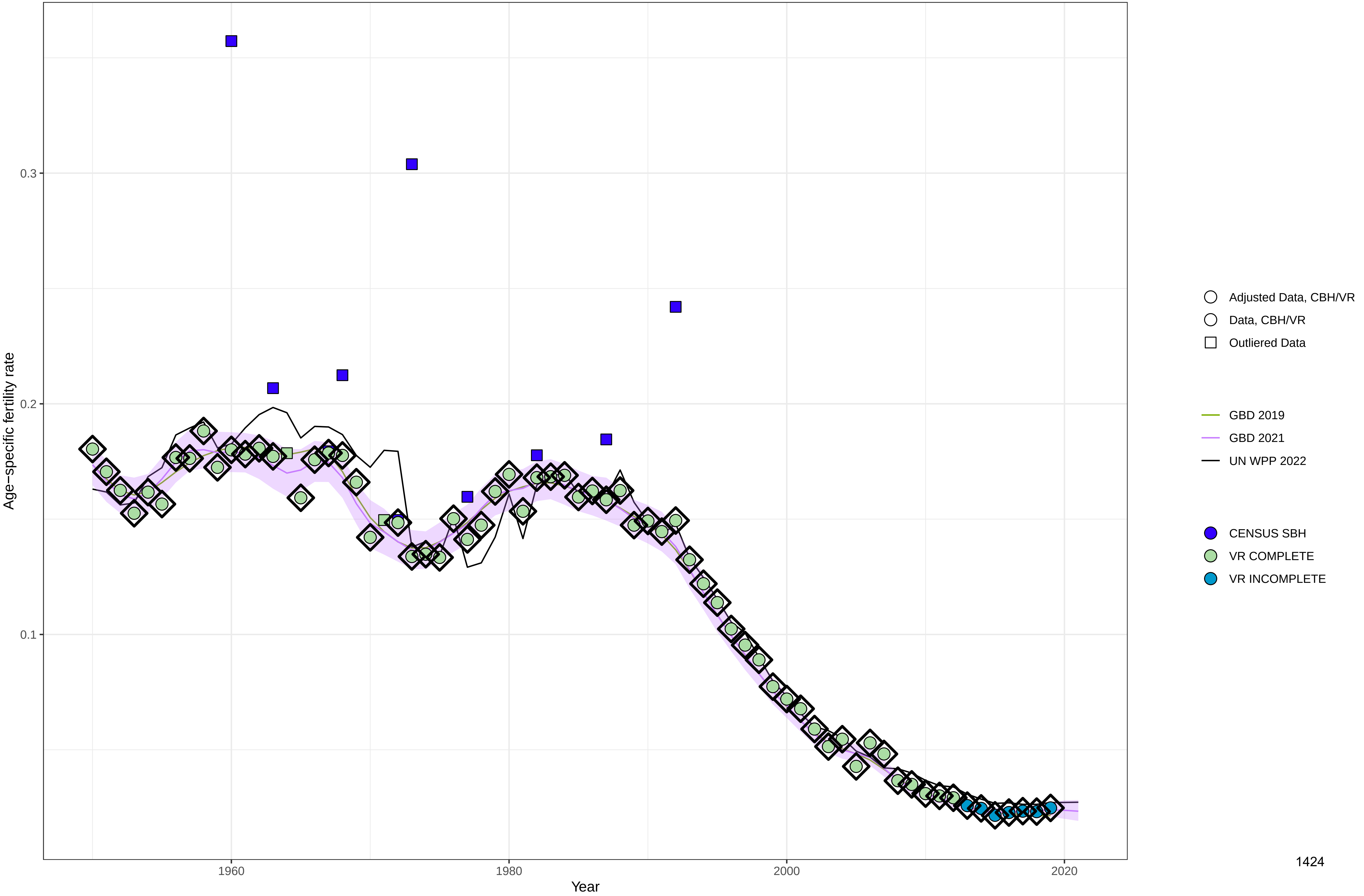

Age−specific fertility fitted trend lines, 1950−2021: Cyprus, Ages: 25−29
Data points enclosed with a diamond are reference sources

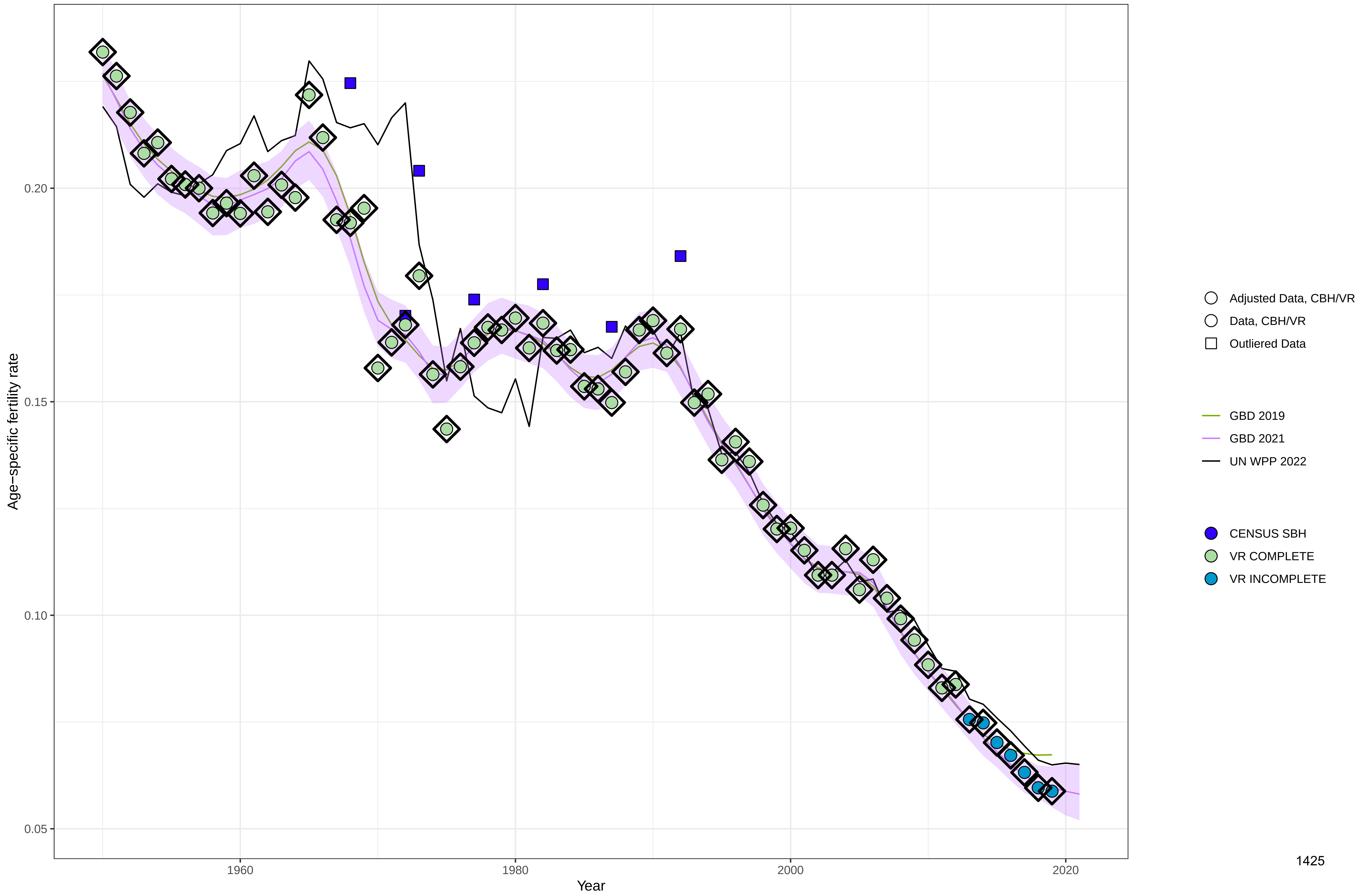

Age−specific fertility fitted trend lines, 1950−2021: Cyprus, Ages: 30−34
Data points enclosed with a diamond are reference sources

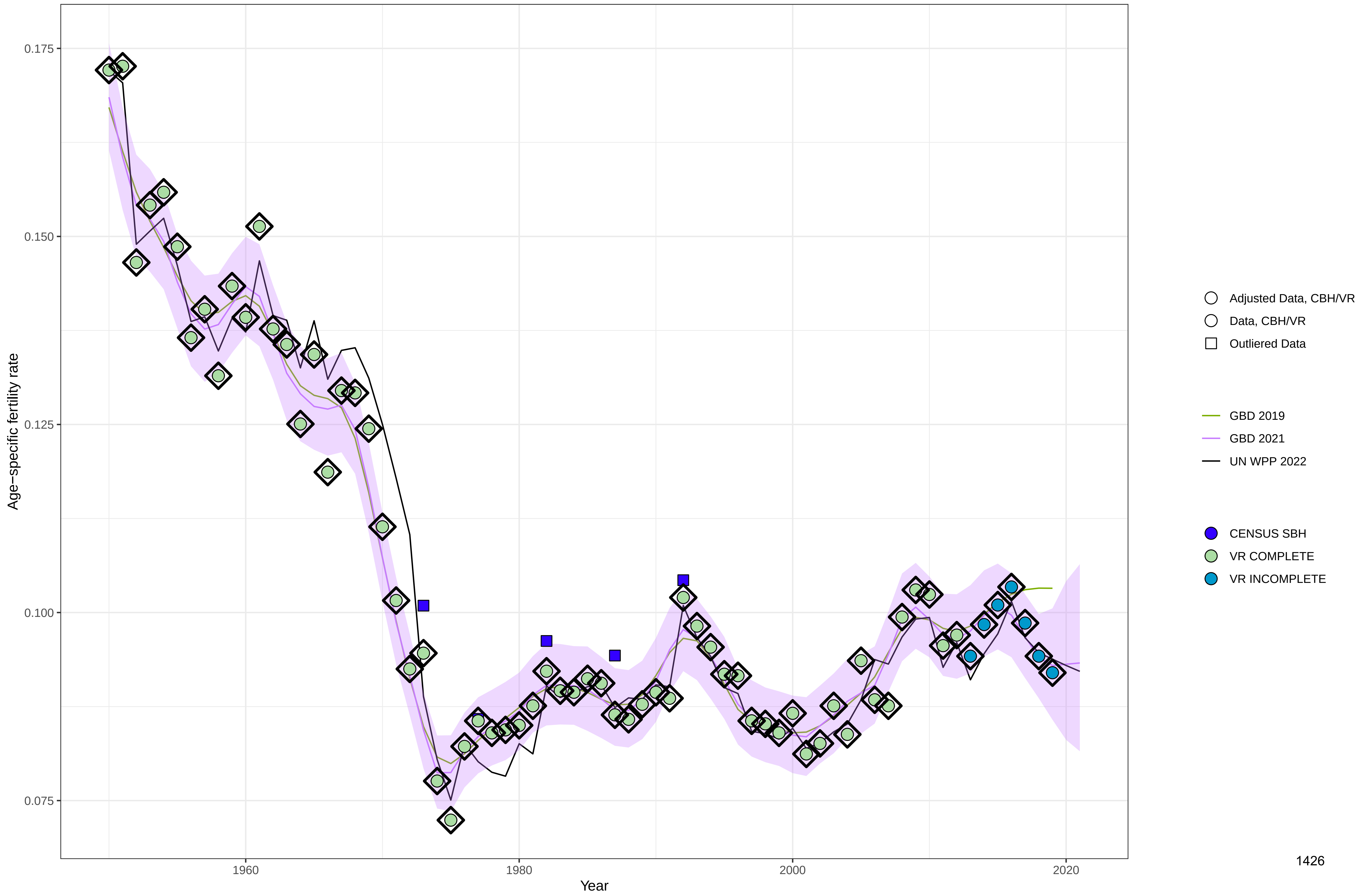

Age−specific fertility fitted trend lines, 1950−2021: Cyprus, Ages: 35−39
Data points enclosed with a diamond are reference sources

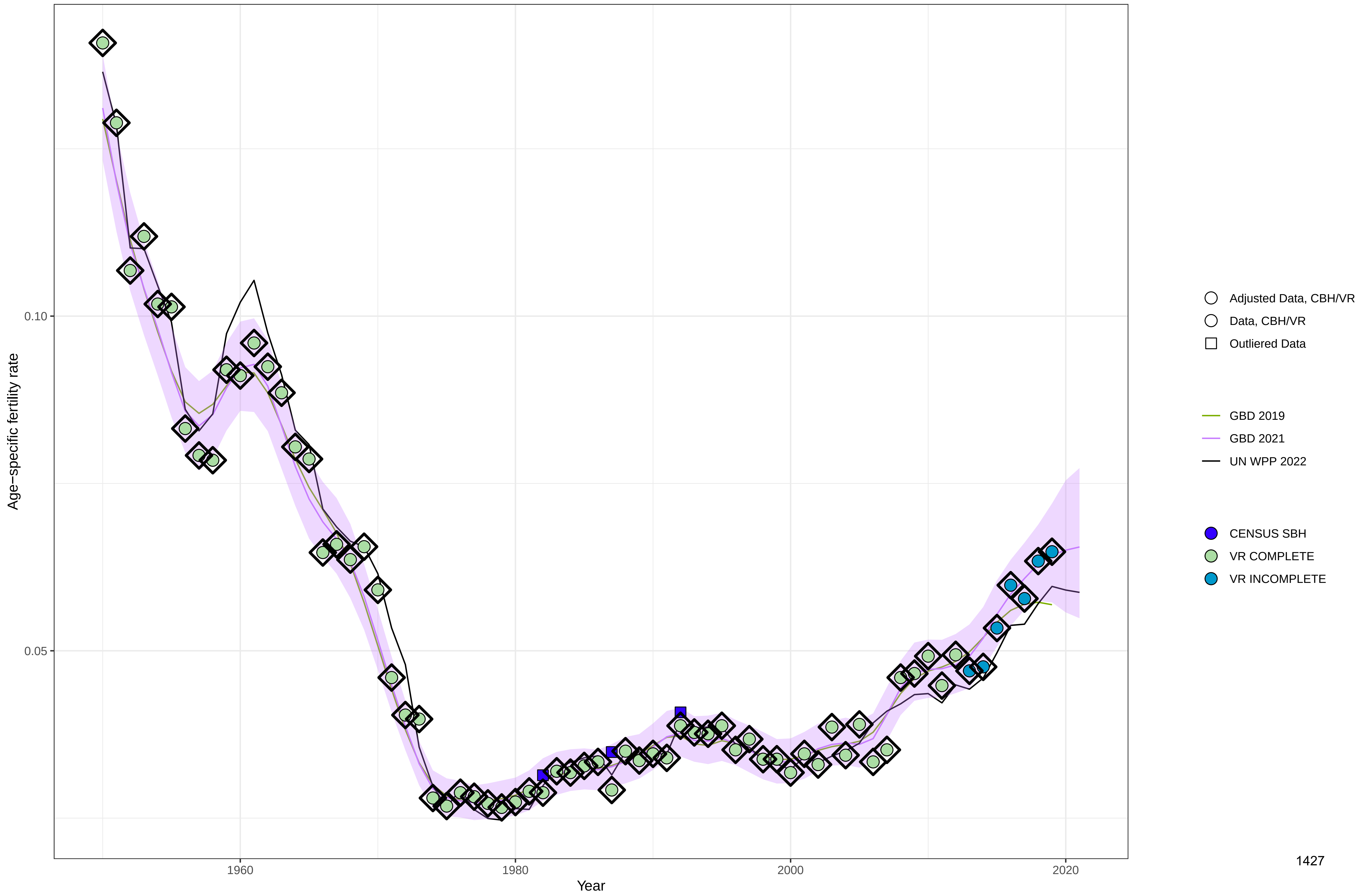

Age−specific fertility fitted trend lines, 1950−2021: Cyprus, Ages: 40−44
Data points enclosed with a diamond are reference sources

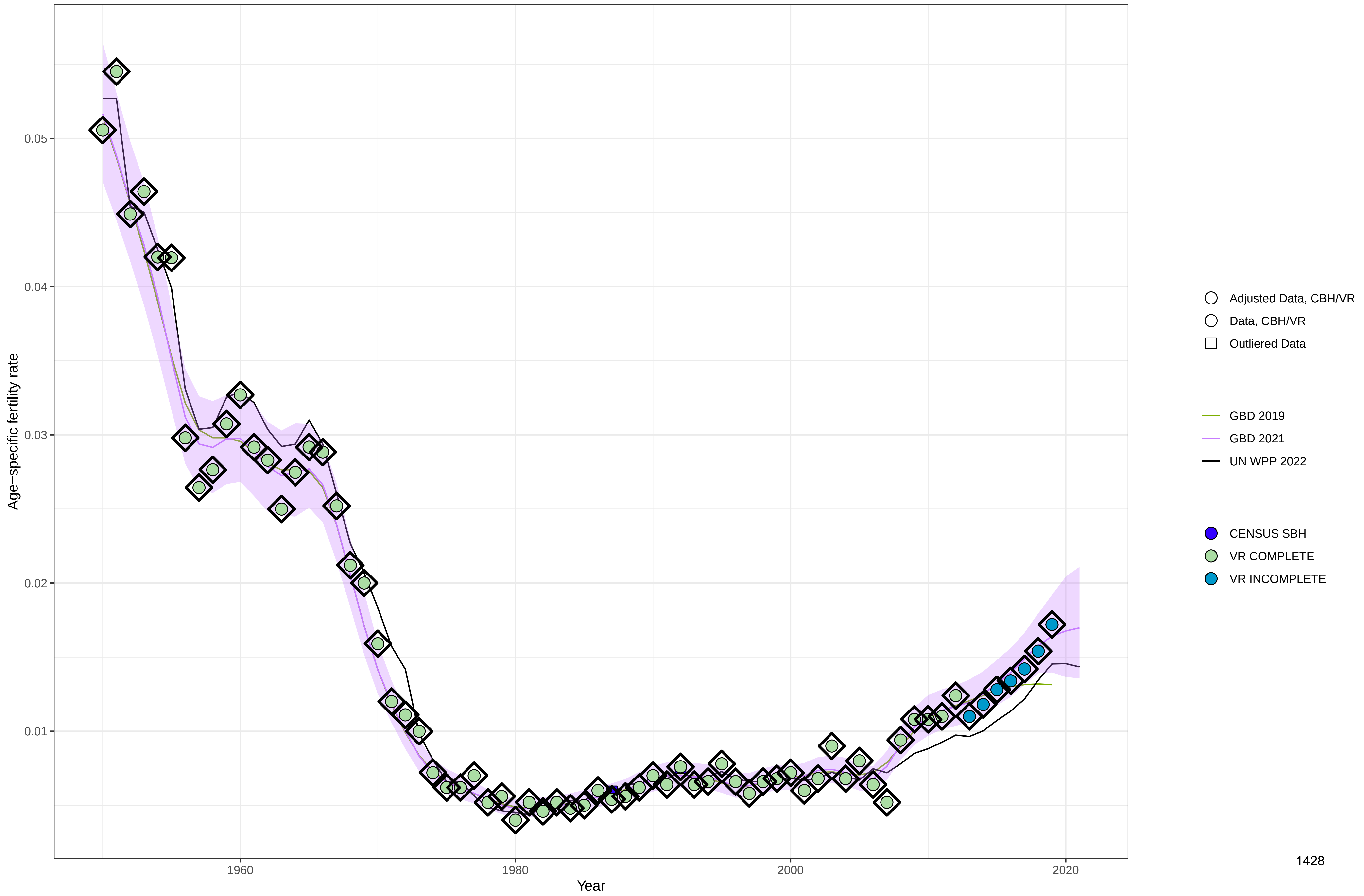

Age−specific fertility fitted trend lines, 1950−2021: Cyprus, Ages: 45−49
Data points enclosed with a diamond are reference sources

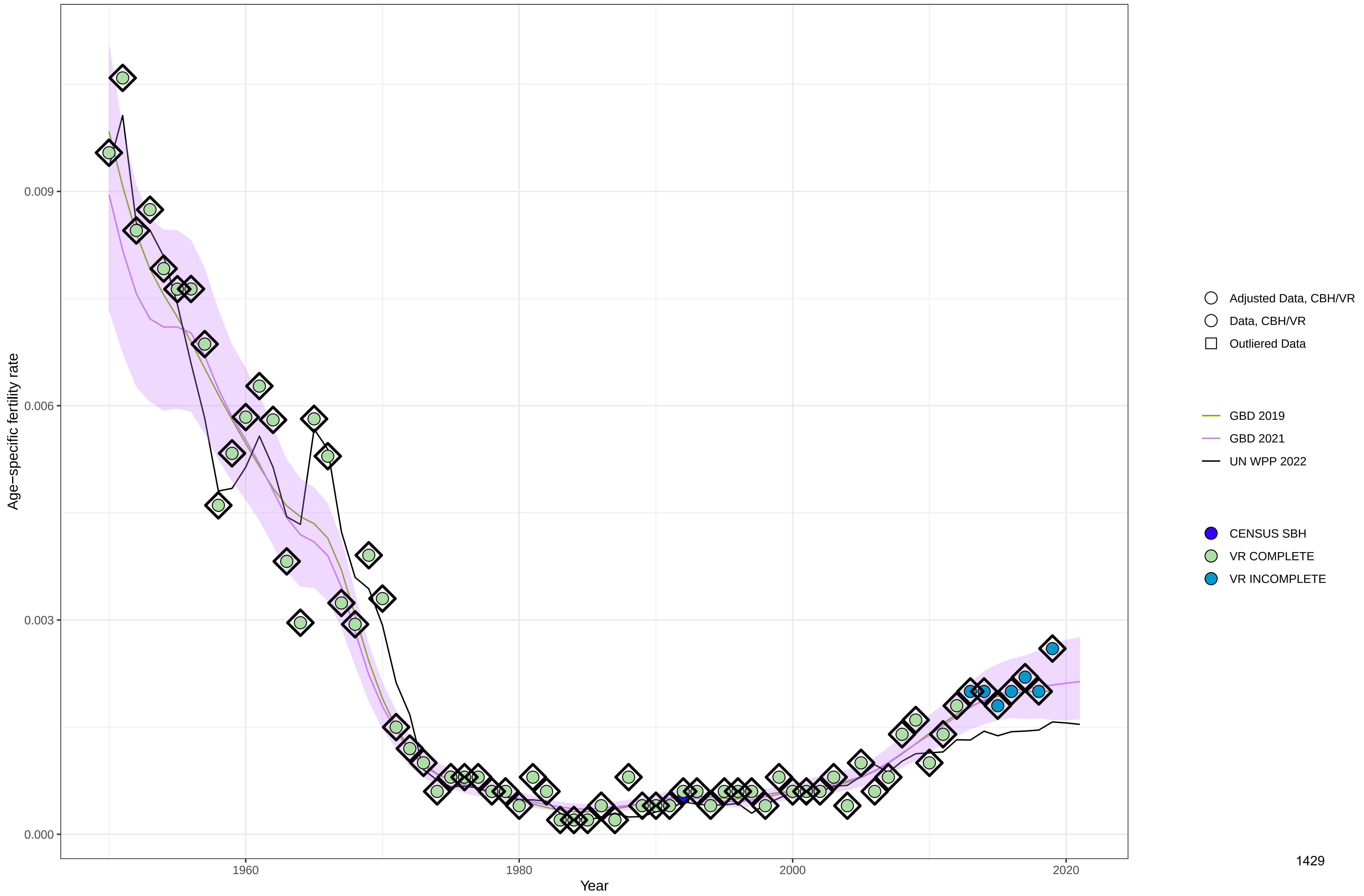

Age−specific fertility fitted trend lines, 1950−2021: Cyprus, Ages: 50−54
Data points enclosed with a diamond are reference sources

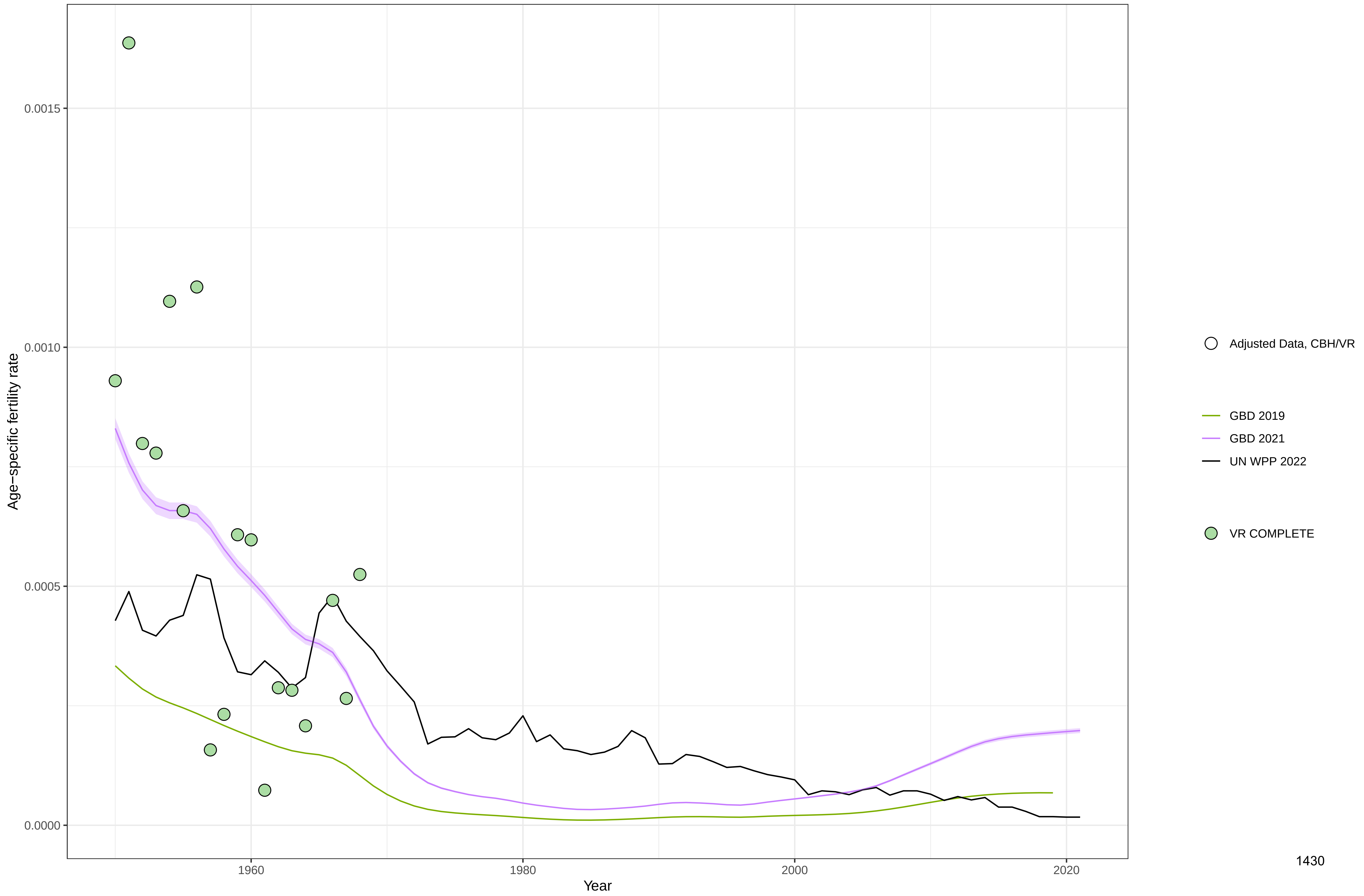

Total fertility rate fitted trend lines, 1950−2021: Cyprus TFR
Data points enclosed with a diamond are reference sources

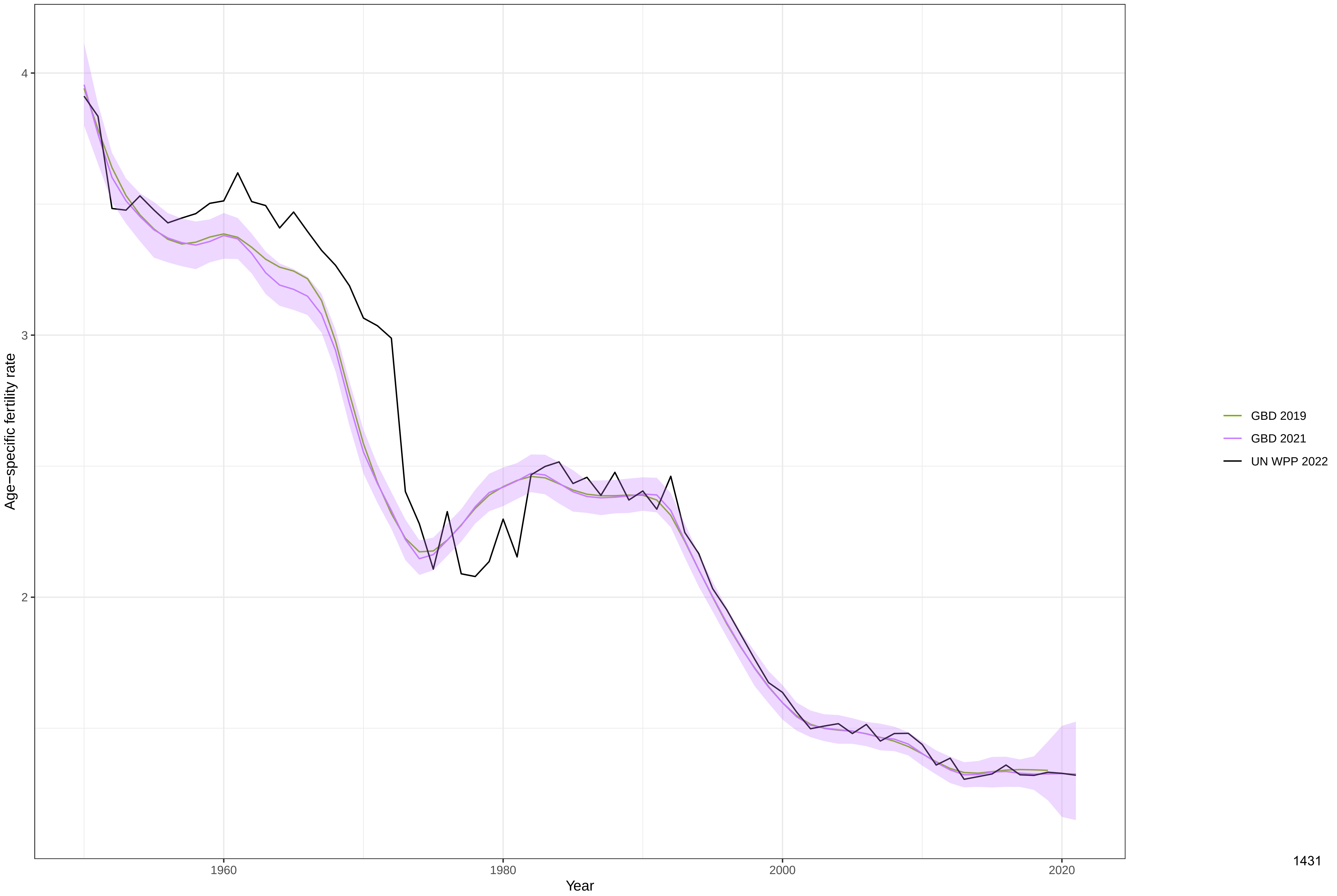

Age−specific fertility fitted trend lines, 1950−2021: Denmark, Ages: 10−14
Data points enclosed with a diamond are reference sources

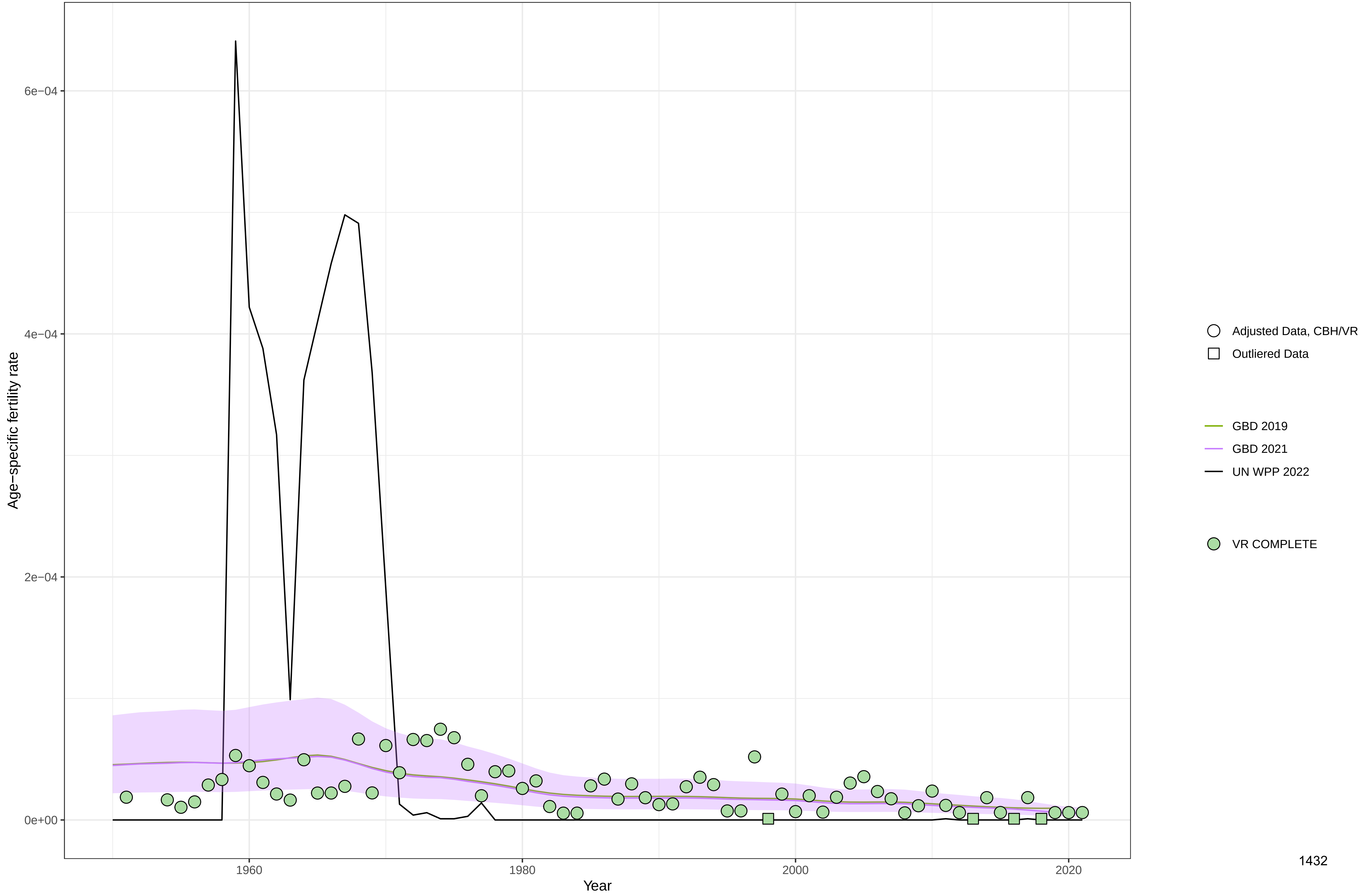

Age−specific fertility fitted trend lines, 1950−2021: Denmark, Ages: 15−19
Data points enclosed with a diamond are reference sources

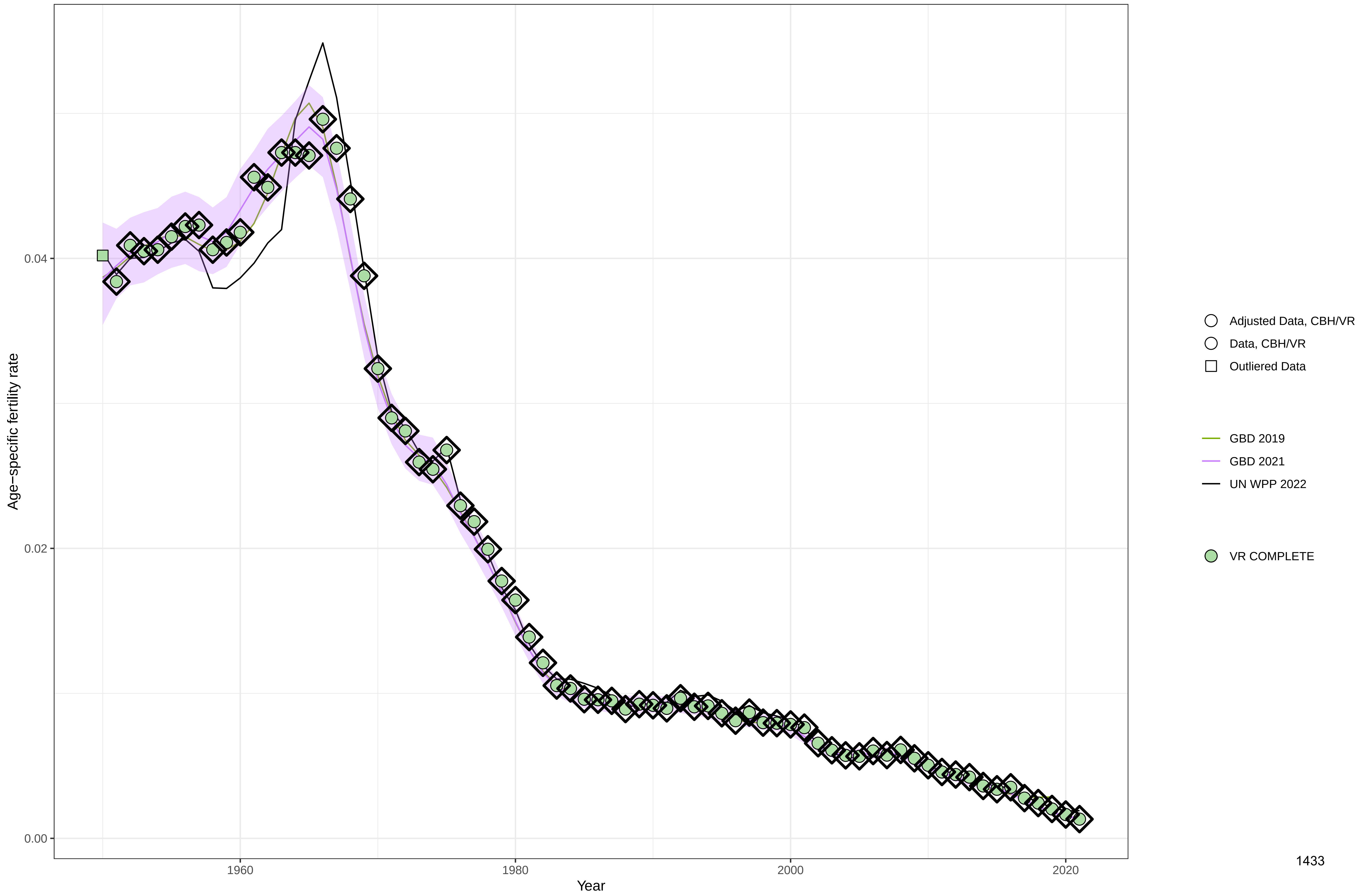

Age−specific fertility fitted trend lines, 1950−2021: Denmark, Ages: 20−24
Data points enclosed with a diamond are reference sources

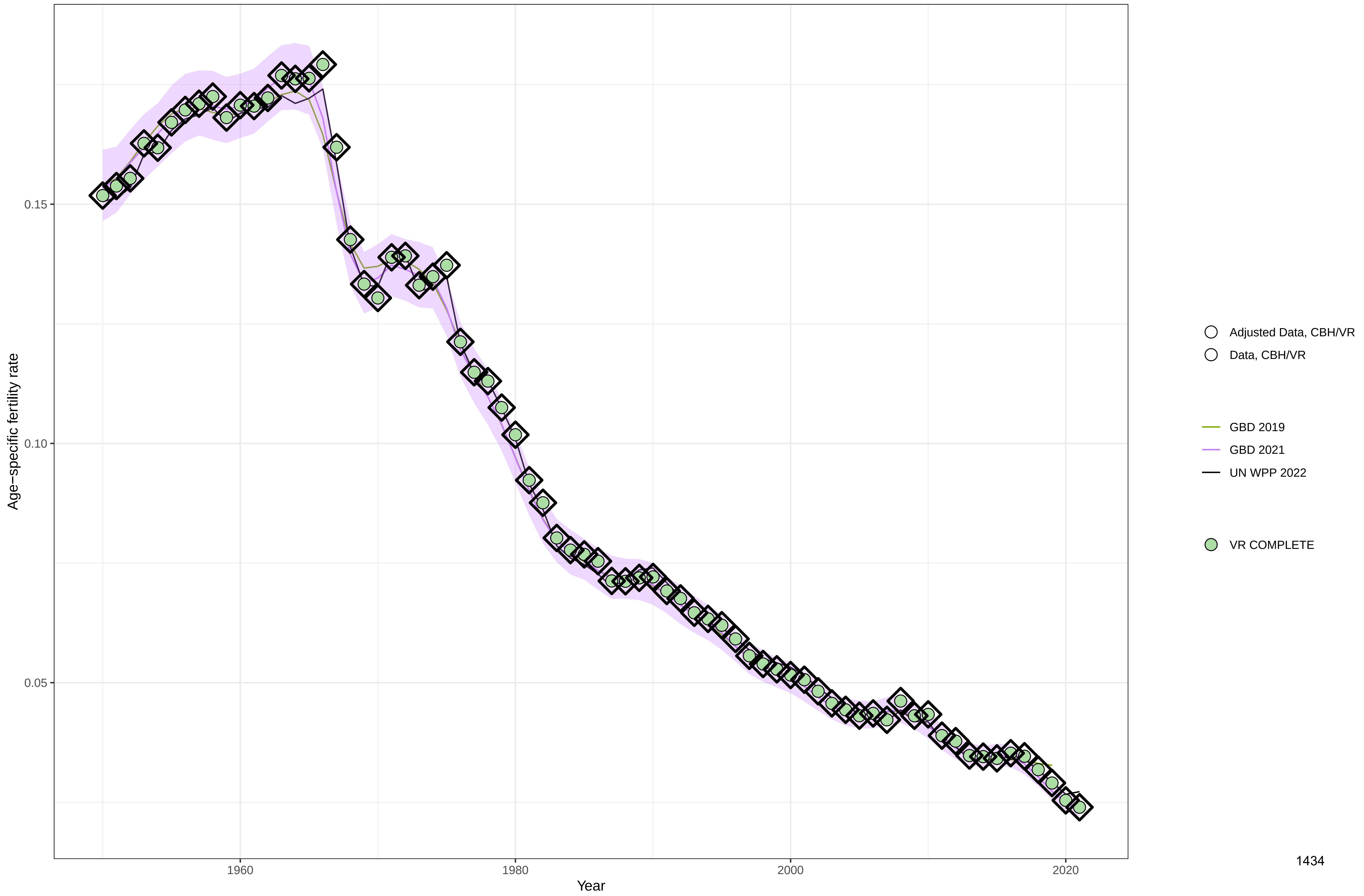

Age−specific fertility fitted trend lines, 1950−2021: Denmark, Ages: 25−29
Data points enclosed with a diamond are reference sources

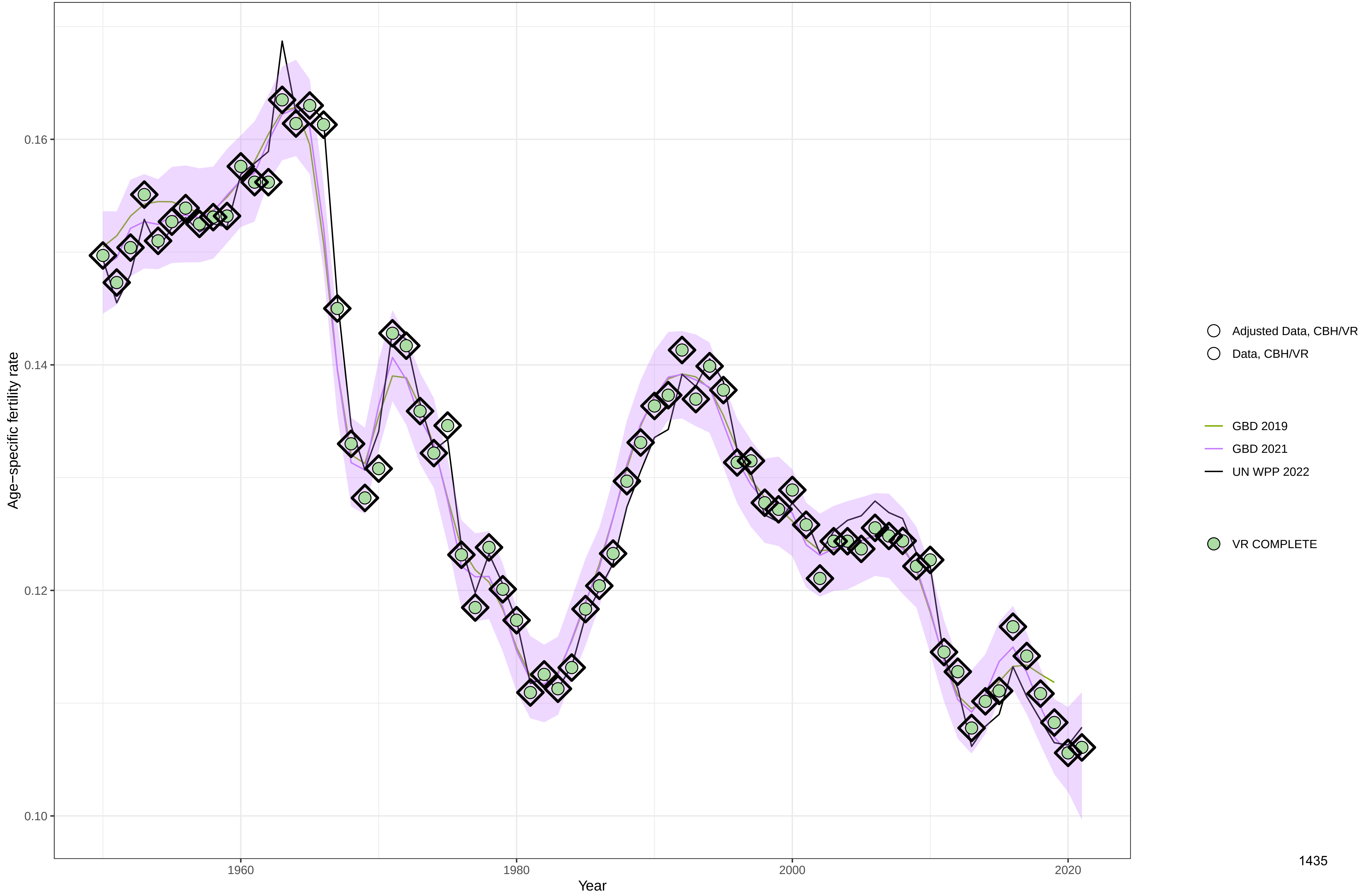

Age−specific fertility fitted trend lines, 1950−2021: Denmark, Ages: 30−34
Data points enclosed with a diamond are reference sources

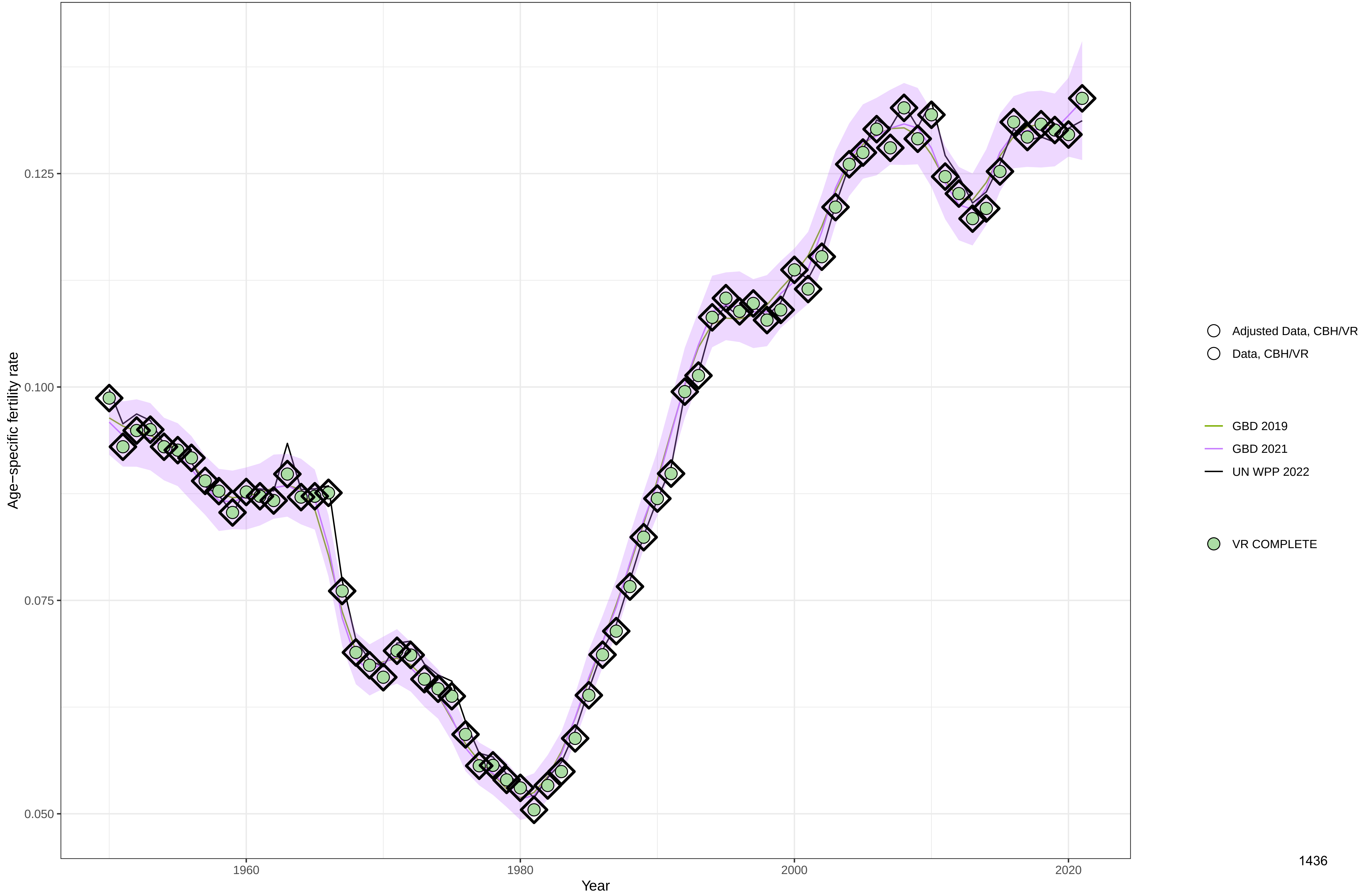

Age−specific fertility fitted trend lines, 1950−2021: Denmark, Ages: 35−39
Data points enclosed with a diamond are reference sources

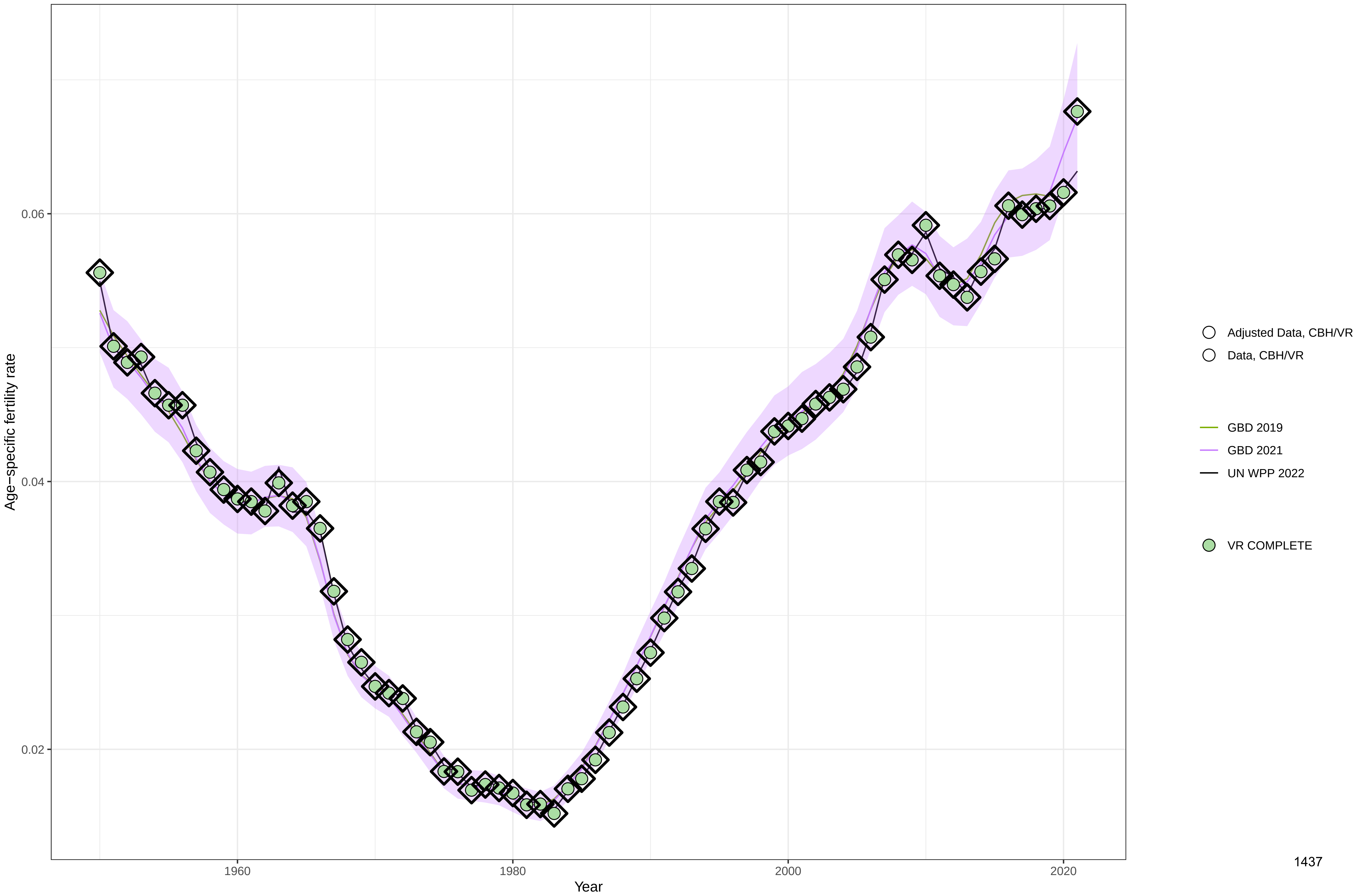

Age−specific fertility fitted trend lines, 1950−2021: Denmark, Ages: 40−44
Data points enclosed with a diamond are reference sources

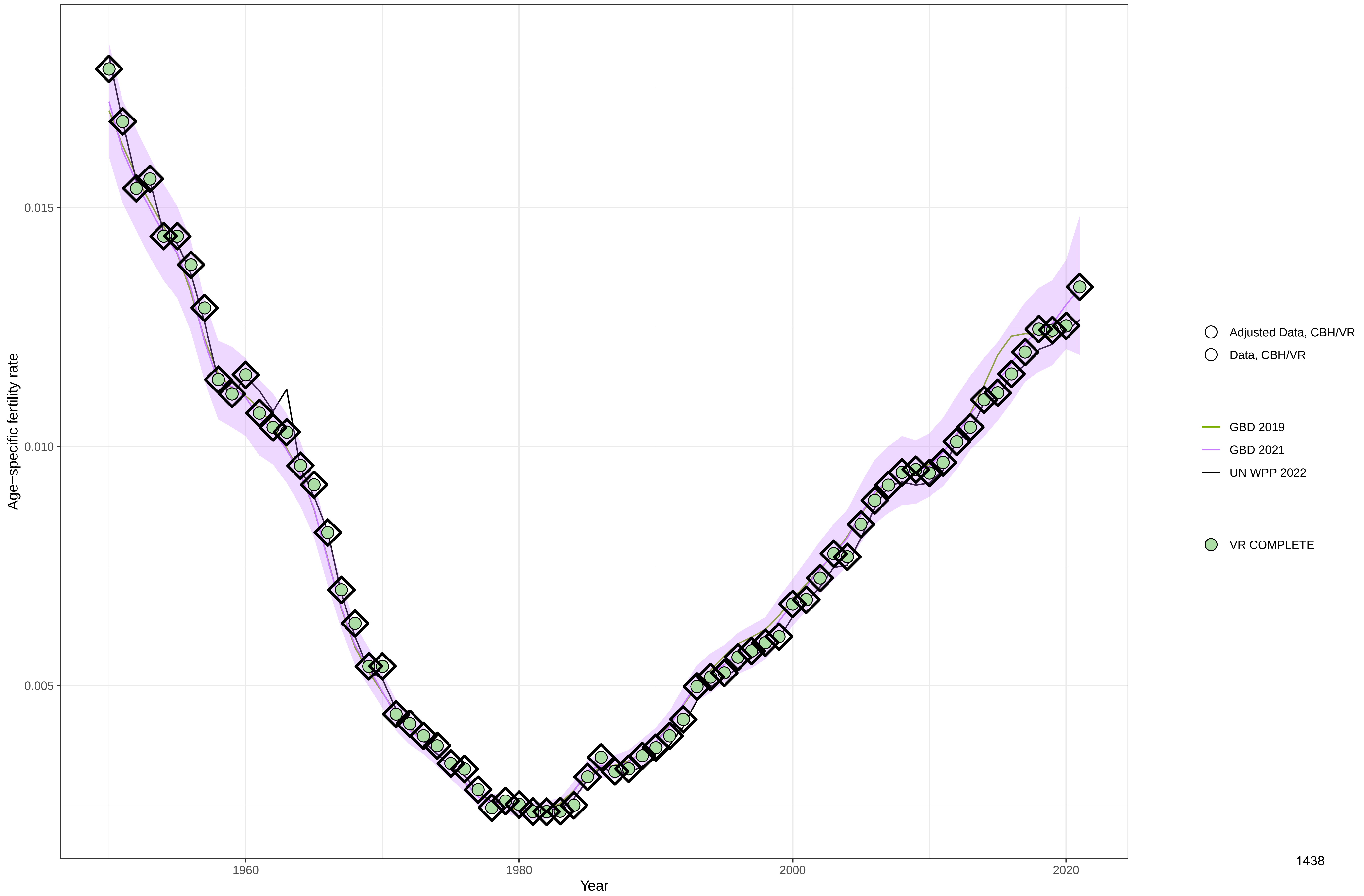

Age−specific fertility fitted trend lines, 1950−2021: Denmark, Ages: 45−49
Data points enclosed with a diamond are reference sources

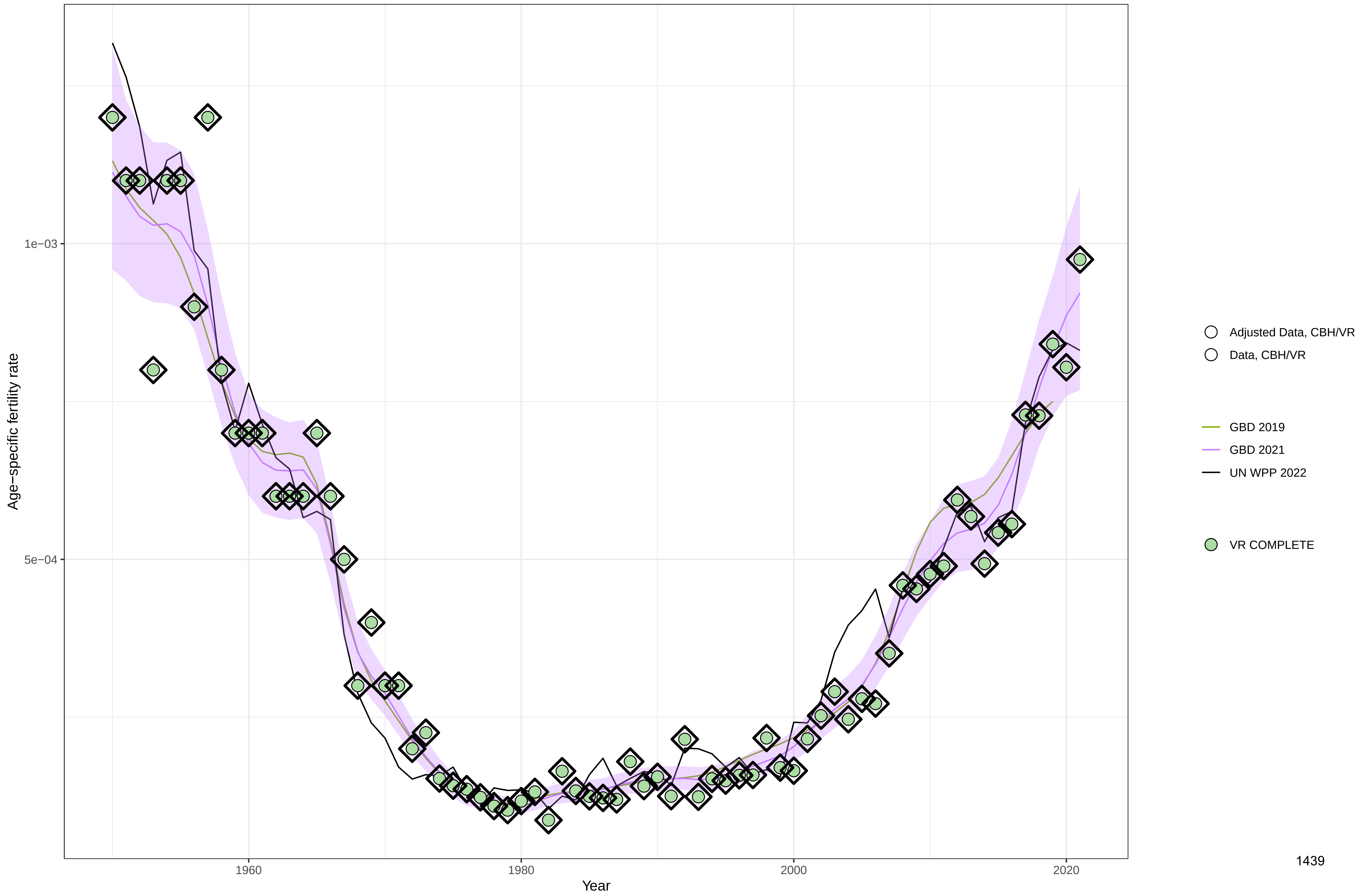

Age−specific fertility fitted trend lines, 1950−2021: Denmark, Ages: 50−54
Data points enclosed with a diamond are reference sources

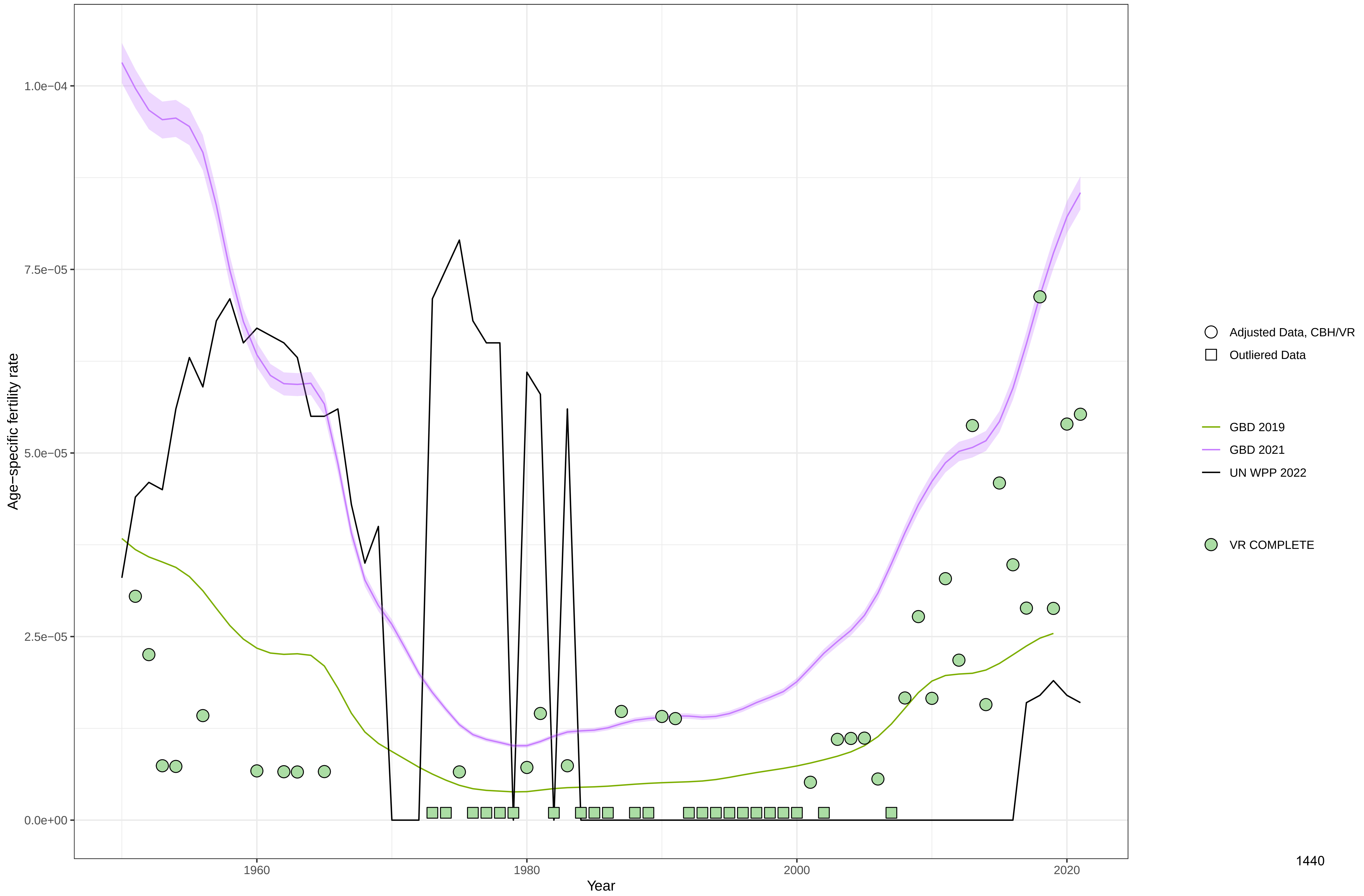

Total fertility rate fitted trend lines, 1950−2021: Denmark TFR
Data points enclosed with a diamond are reference sources

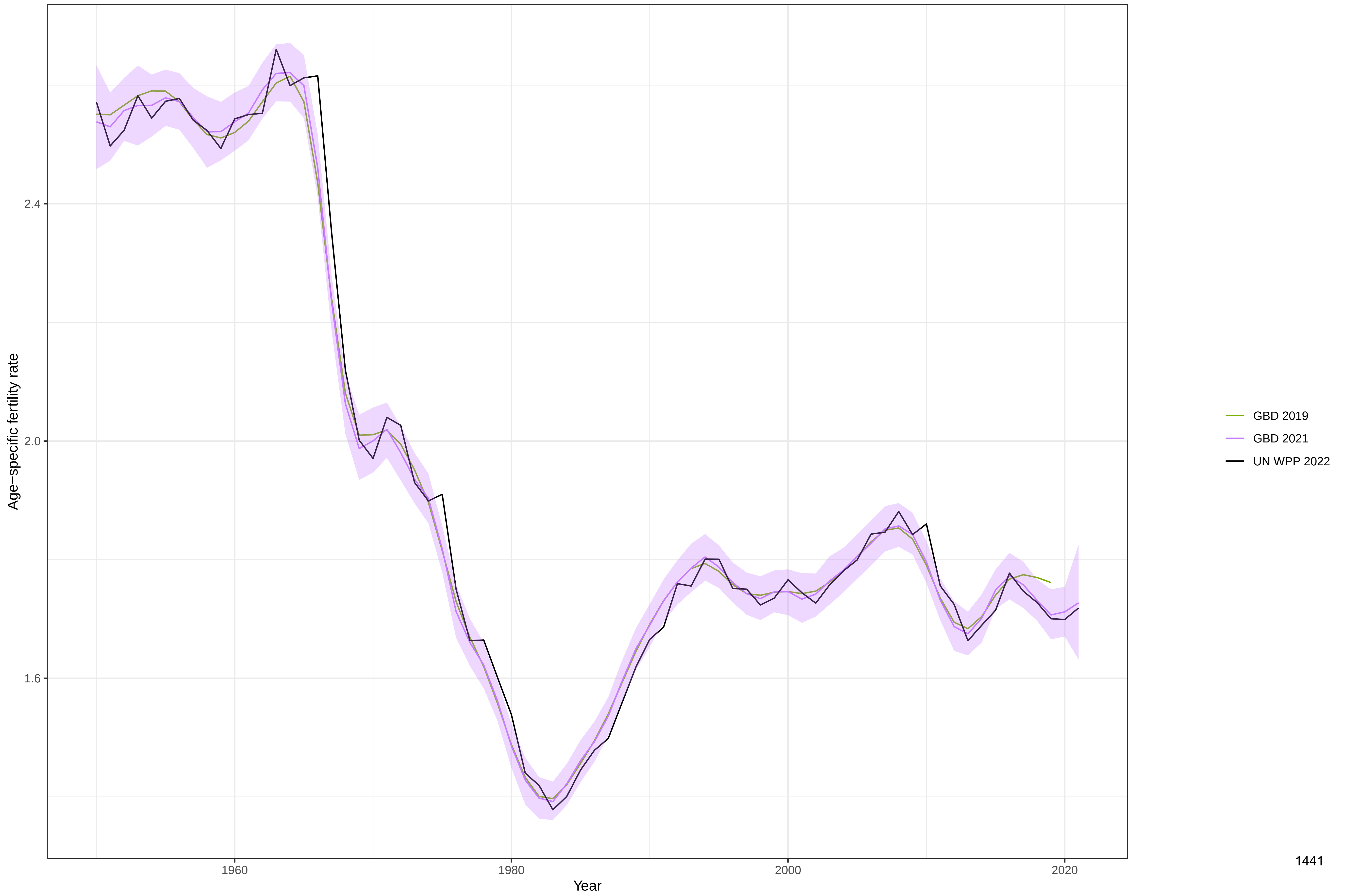

Age−specific fertility fitted trend lines, 1950−2021: Finland, Ages: 10−14
Data points enclosed with a diamond are reference sources

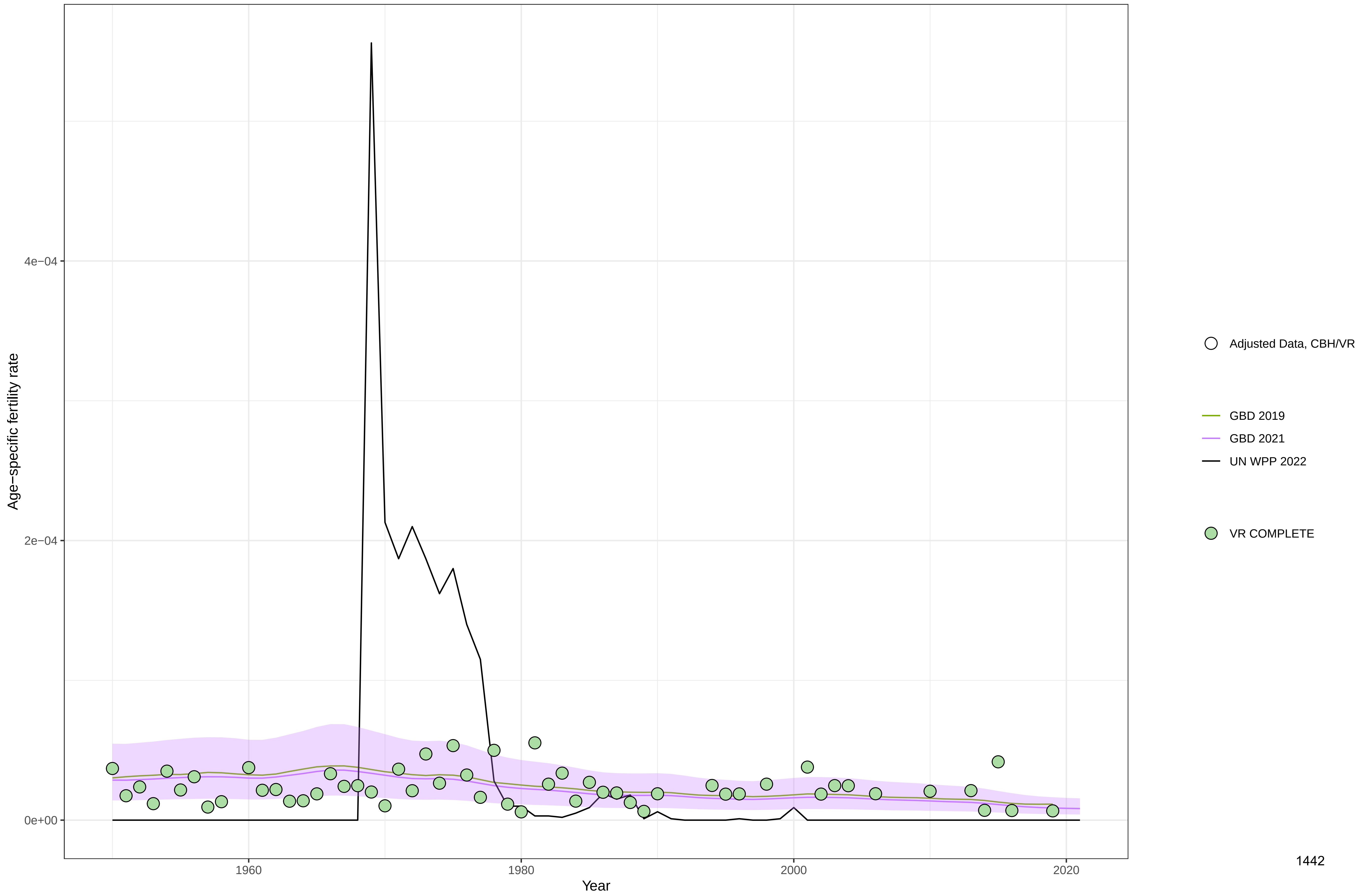

Age−specific fertility fitted trend lines, 1950−2021: Finland, Ages: 15−19
Data points enclosed with a diamond are reference sources

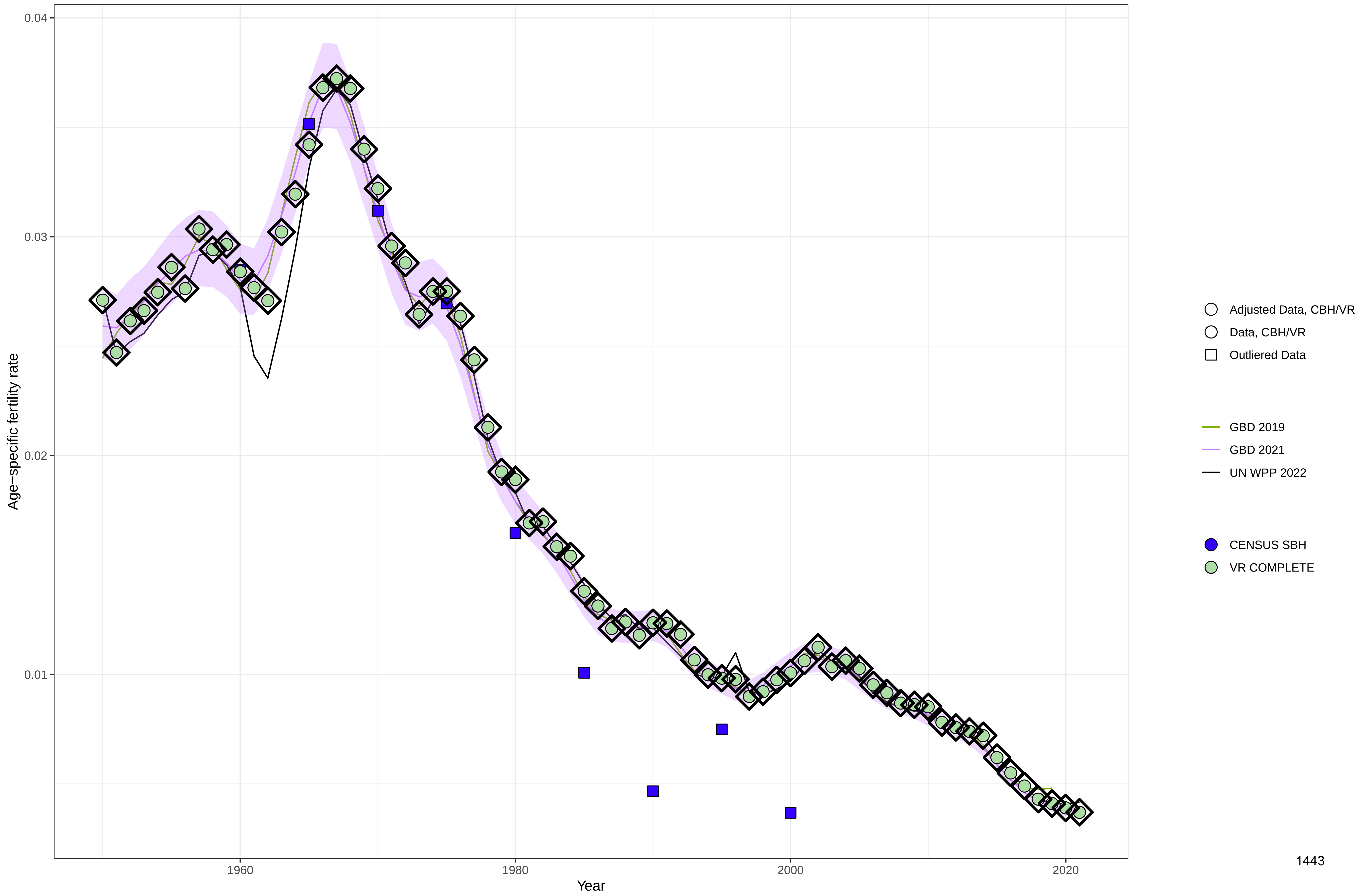

Age−specific fertility fitted trend lines, 1950−2021: Finland, Ages: 20−24
Data points enclosed with a diamond are reference sources

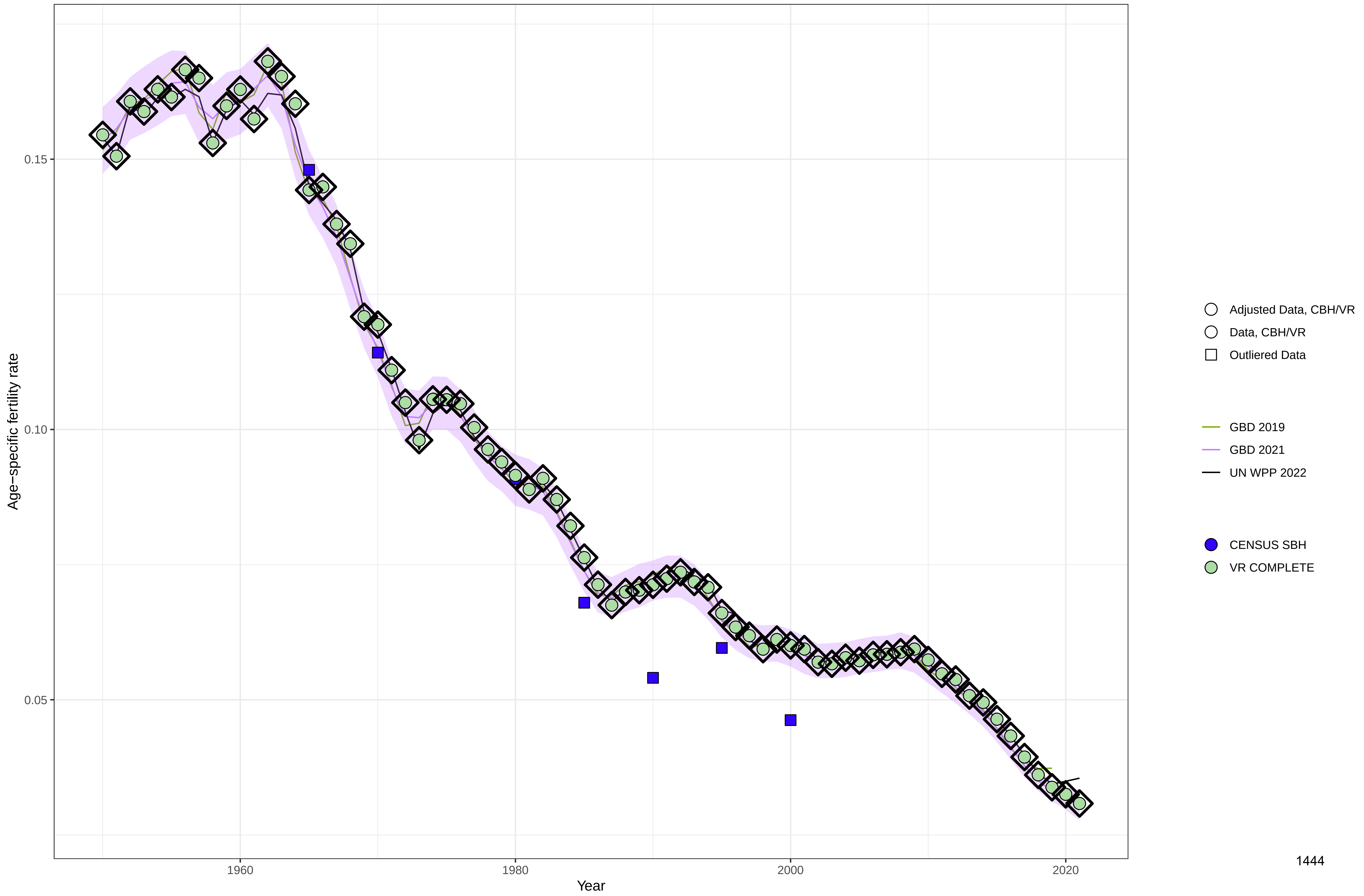

Age−specific fertility fitted trend lines, 1950−2021: Finland, Ages: 25−29
Data points enclosed with a diamond are reference sources

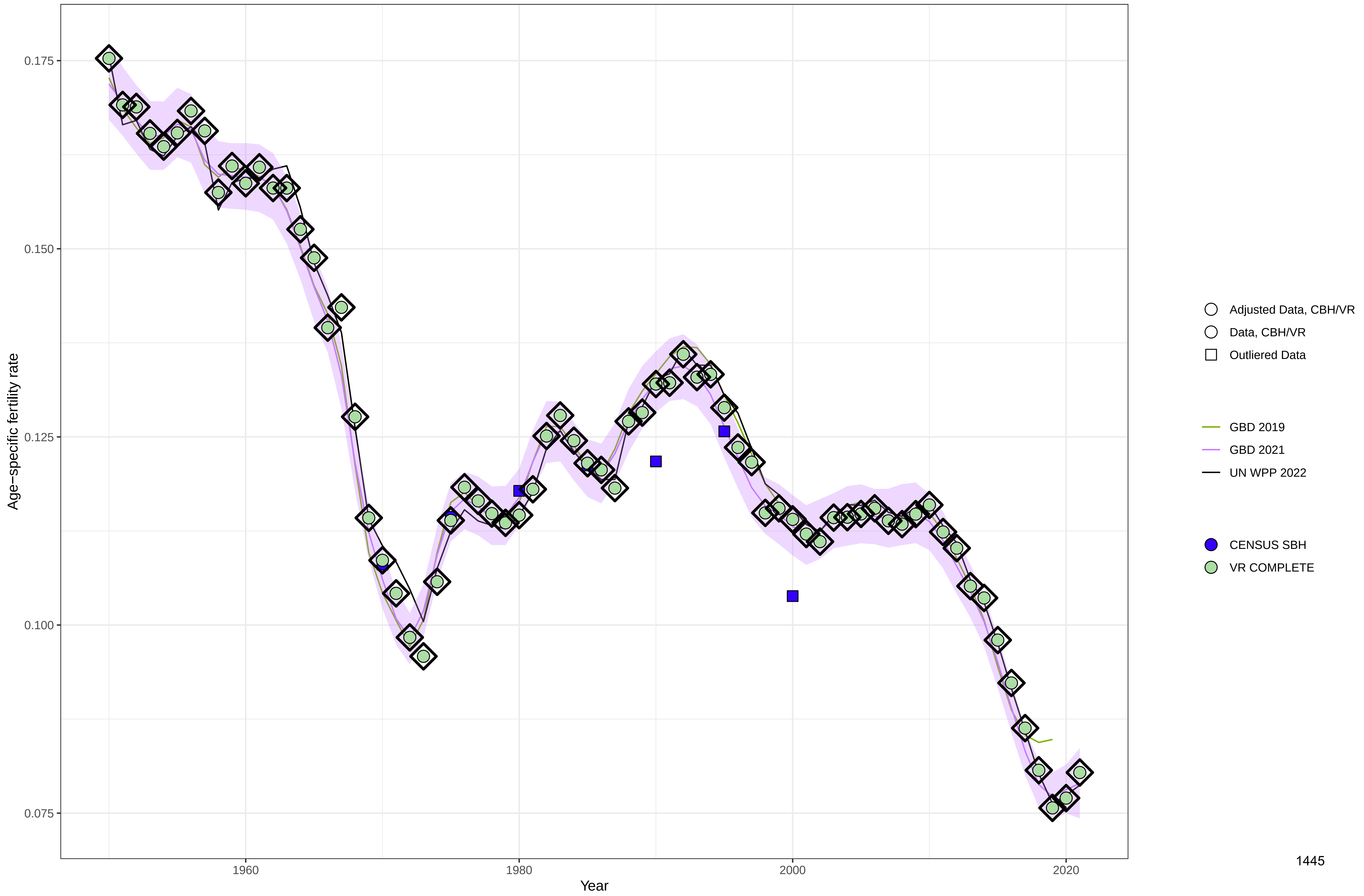

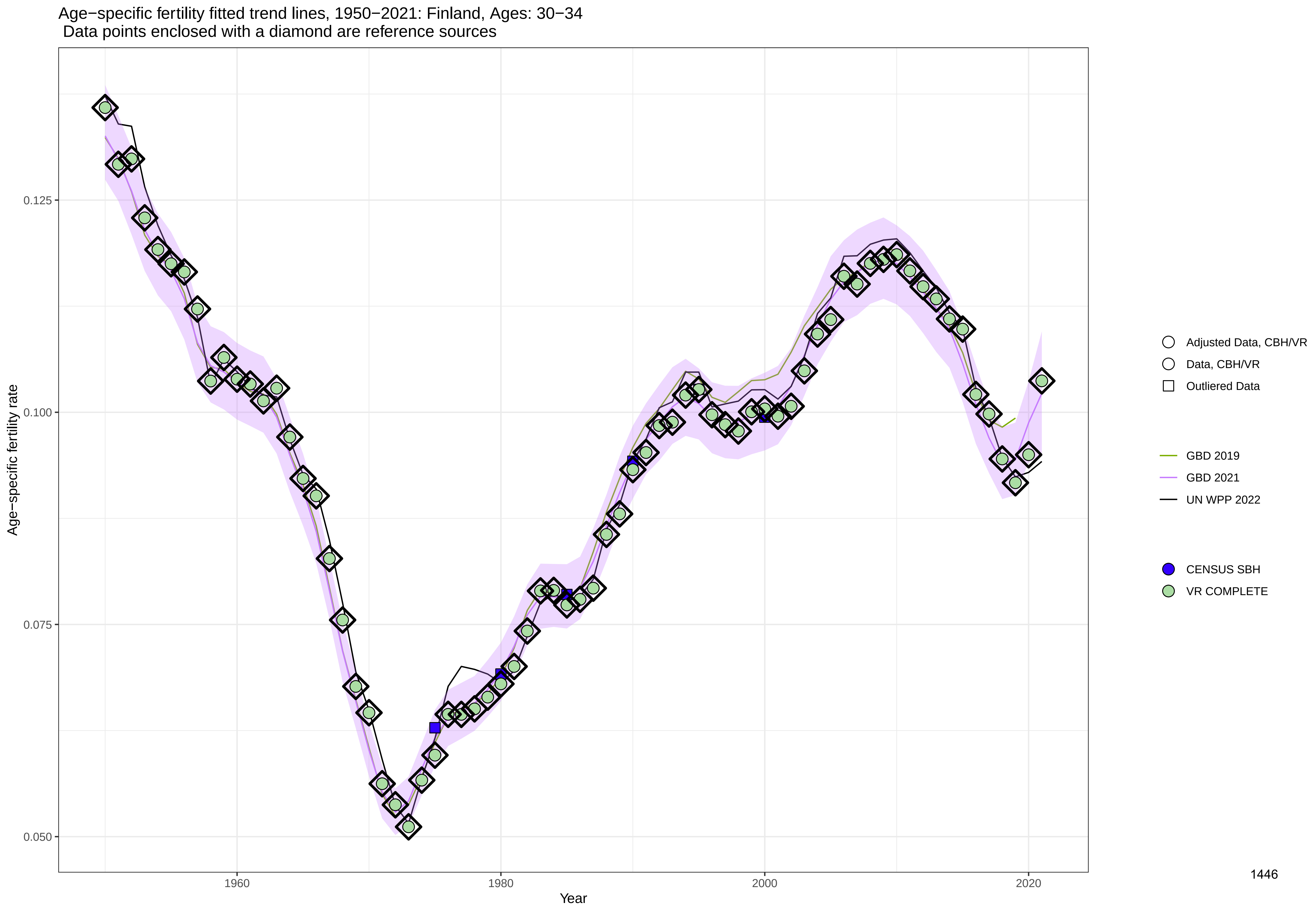
Age−specific fertility fitted trend lines, 1950−2021: Finland, Ages: 30−34
Data points enclosed with a diamond are reference sources
Age−specific fertility rate
0.125
0.100
0.075
0.050
1960
1980
2000
2020
Year
Adjusted Data, CBH/VR
Data, CBH/VR
Outliered Data
GBD 2019
GBD 2021
UN WPP 2022
CENSUS SBH
VR COMPLETE

Age−specific fertility fitted trend lines, 1950−2021: Finland, Ages: 35−39
Data points enclosed with a diamond are reference sources

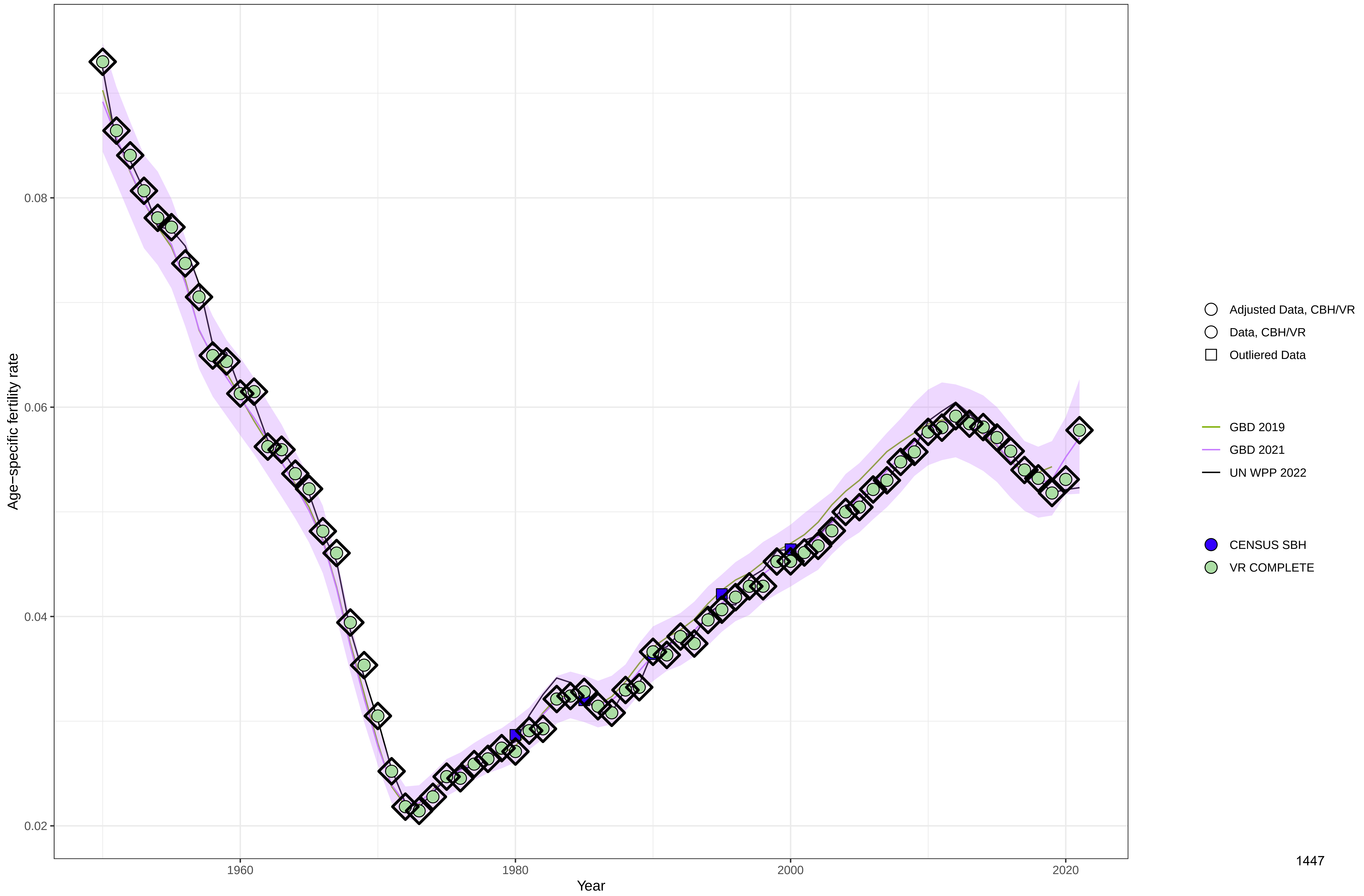

Age−specific fertility fitted trend lines, 1950−2021: Finland, Ages: 40−44
Data points enclosed with a diamond are reference sources

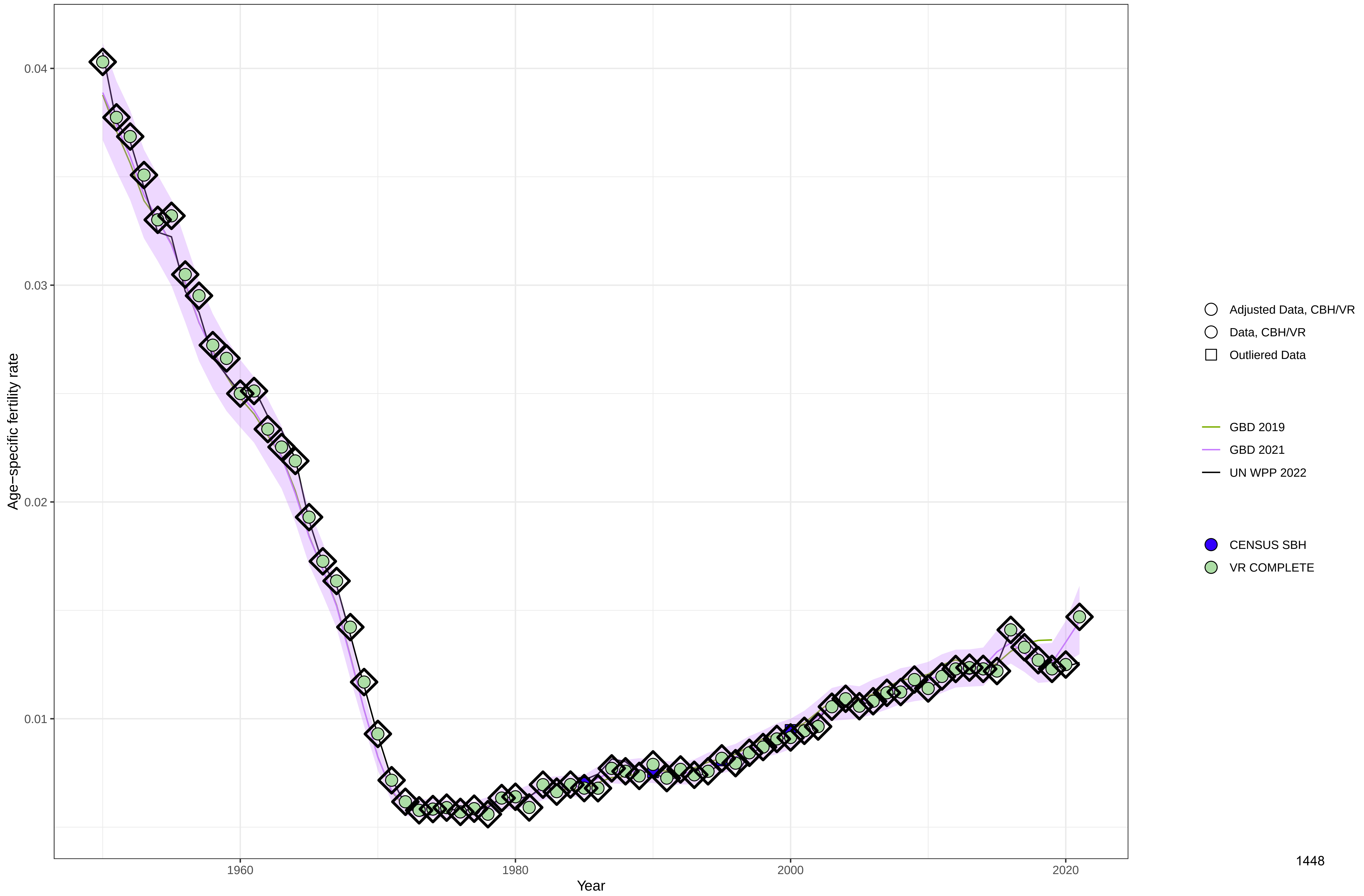

Age−specific fertility fitted trend lines, 1950−2021: Finland, Ages: 45−49
Data points enclosed with a diamond are reference sources

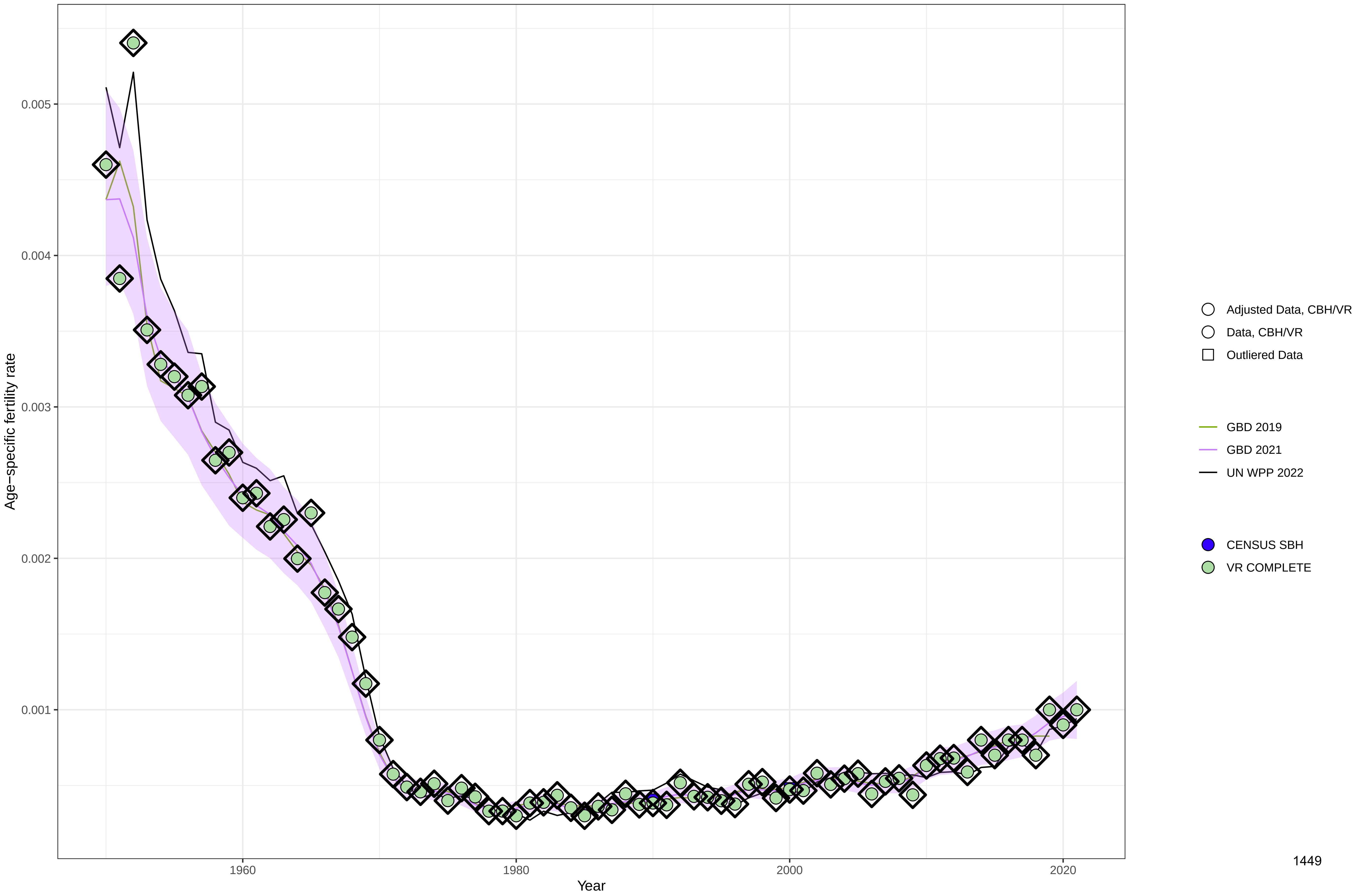

Age−specific fertility fitted trend lines, 1950−2021: Finland, Ages: 50−54
Data points enclosed with a diamond are reference sources

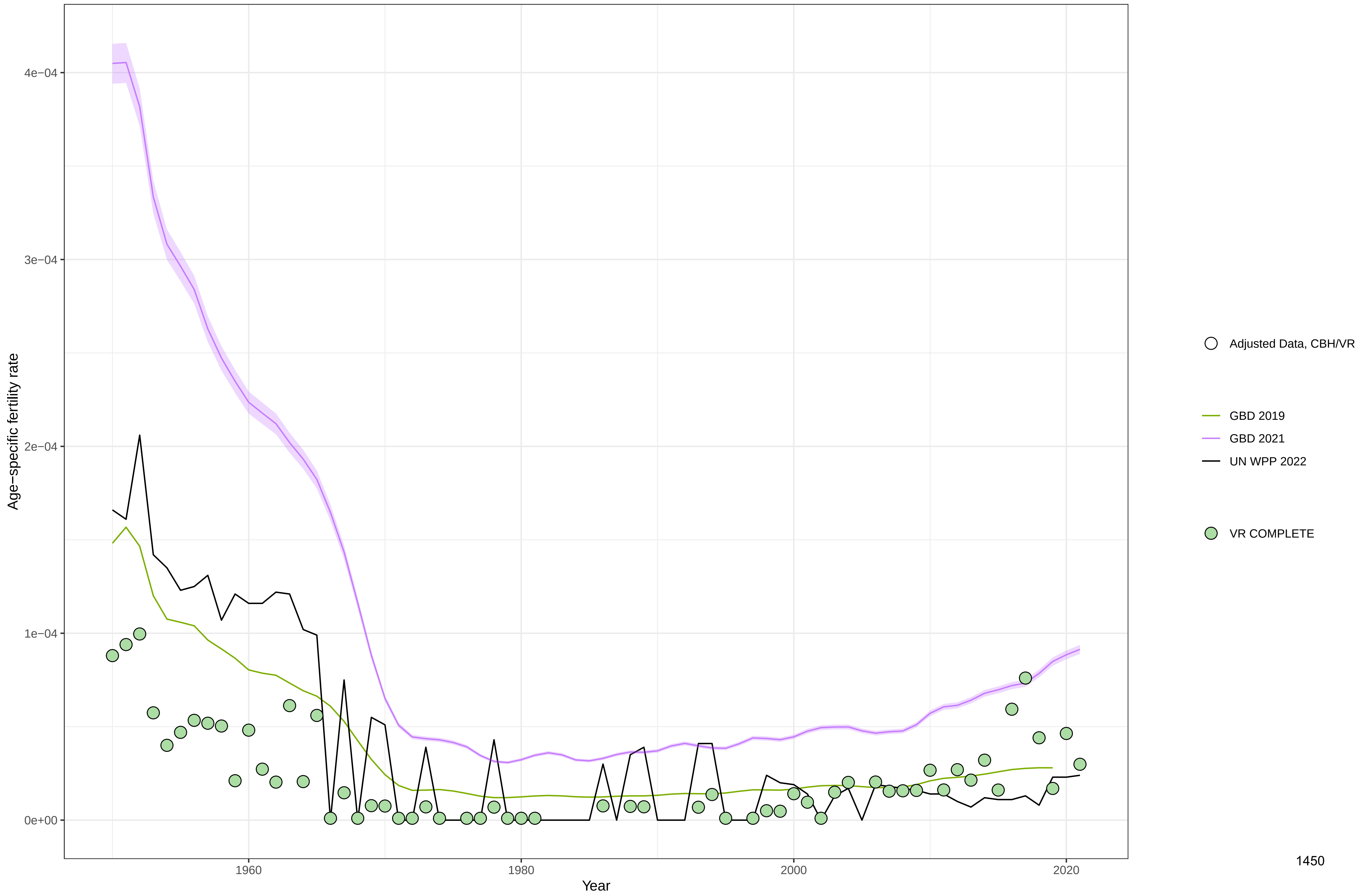

Total fertility rate fitted trend lines, 1950−2021: Finland TFR
Data points enclosed with a diamond are reference sources

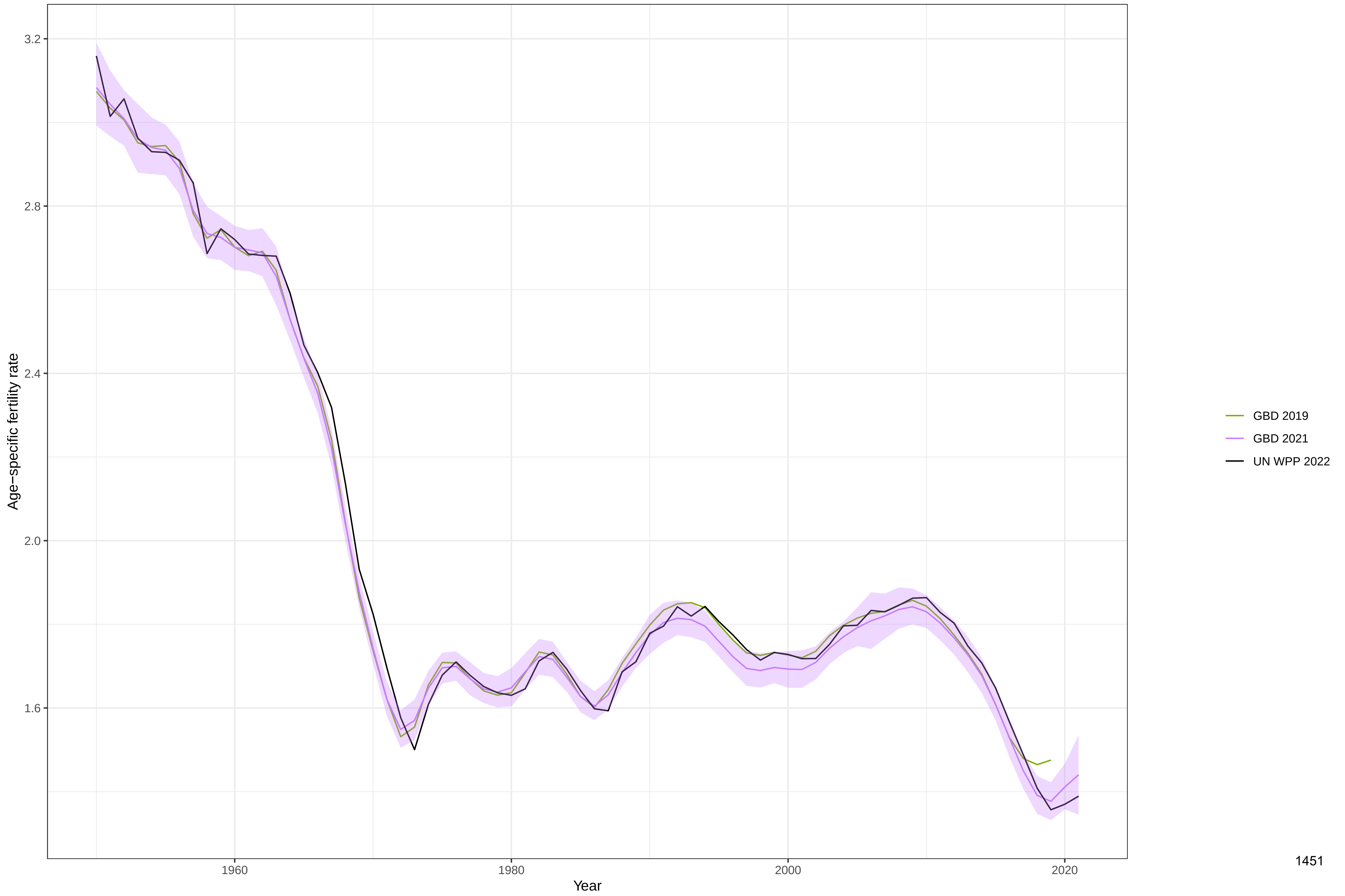

Age−specific fertility fitted trend lines, 1950−2021: France, Ages: 10−14
Data points enclosed with a diamond are reference sources

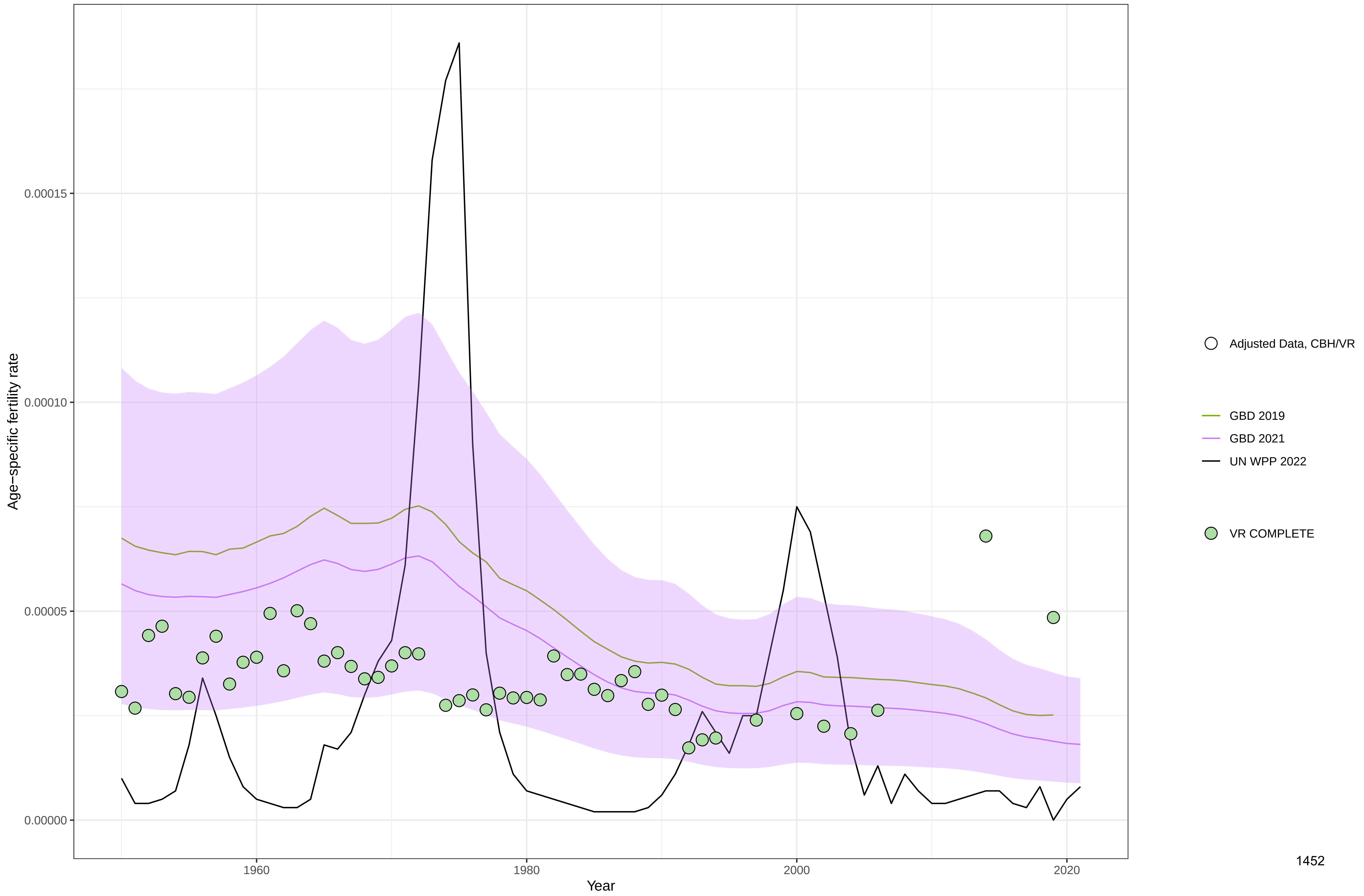

Age−specific fertility fitted trend lines, 1950−2021: France, Ages: 15−19
Data points enclosed with a diamond are reference sources

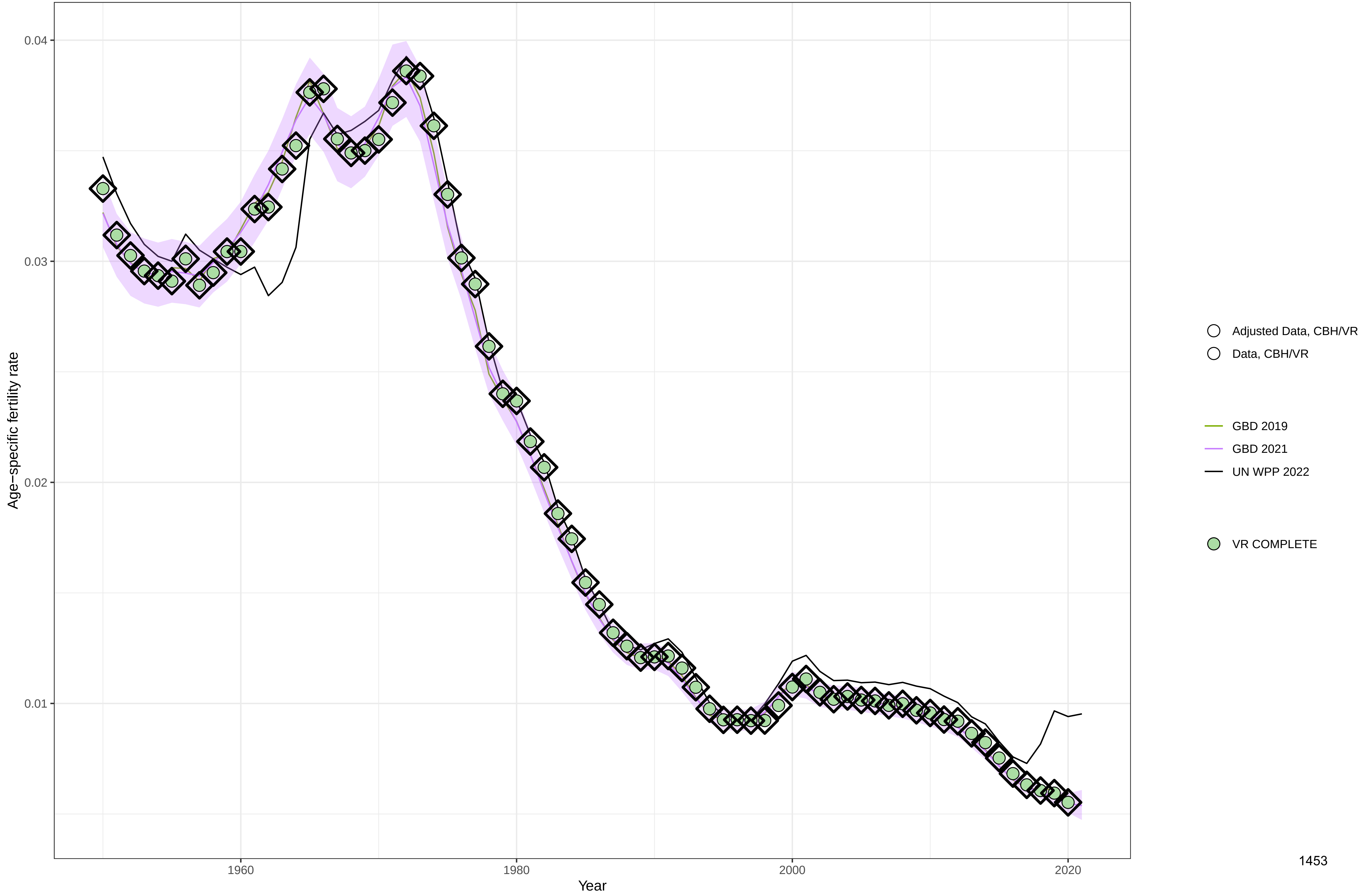

Age−specific fertility fitted trend lines, 1950−2021: France, Ages: 20−24
Data points enclosed with a diamond are reference sources

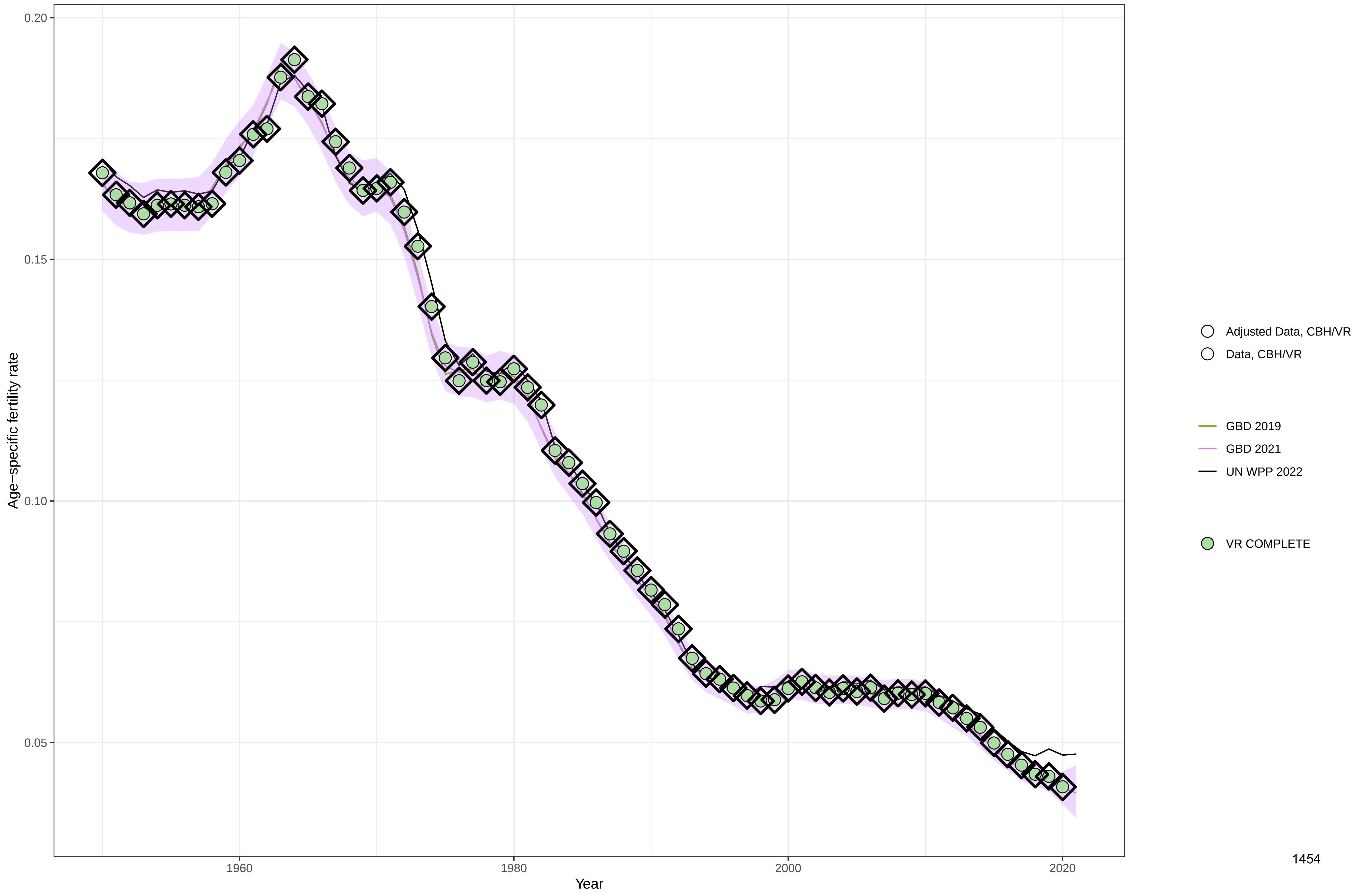

Age−specific fertility fitted trend lines, 1950−2021: France, Ages: 25−29
Data points enclosed with a diamond are reference sources

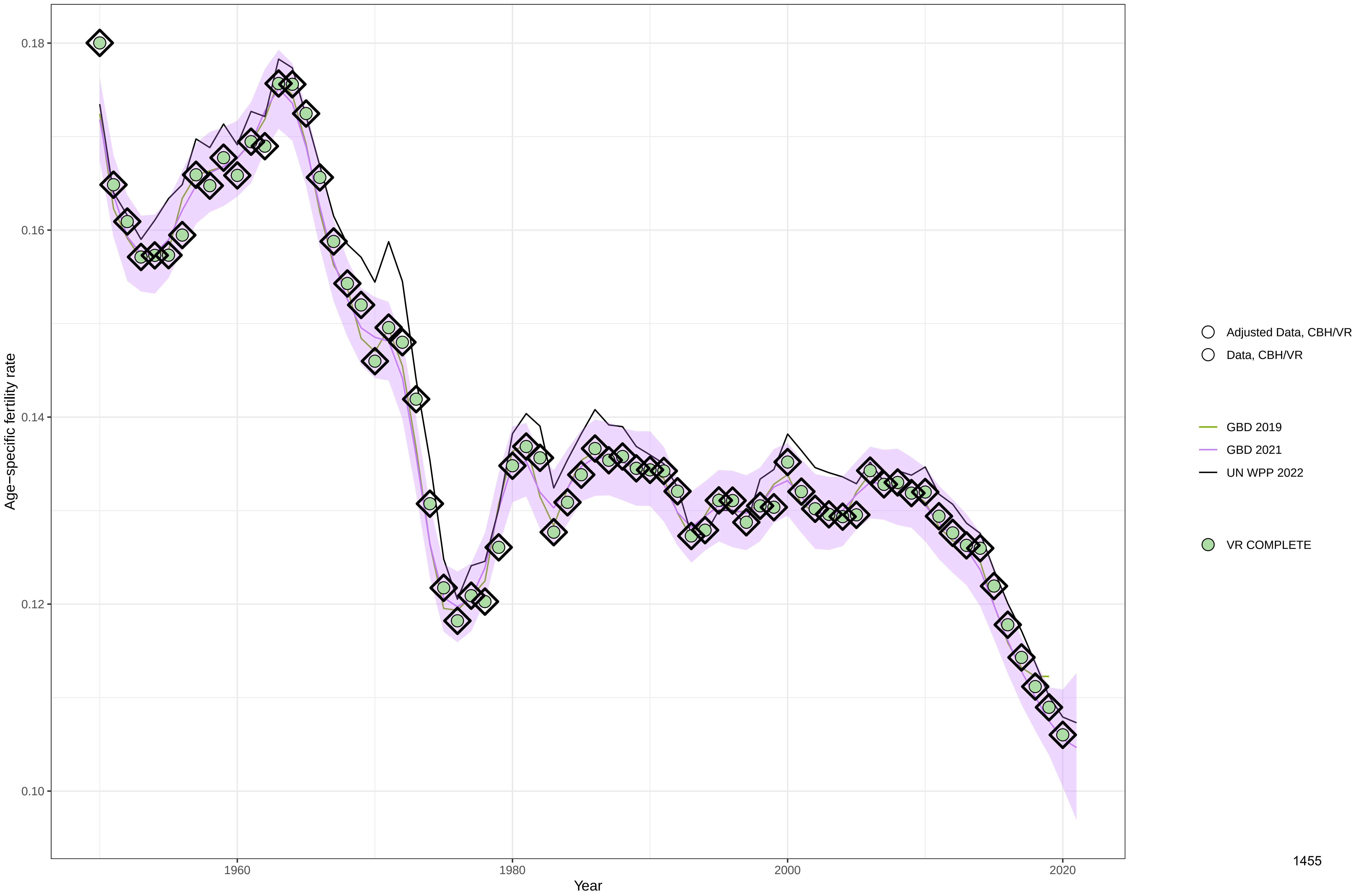

Age−specific fertility fitted trend lines, 1950−2021: France, Ages: 30−34
Data points enclosed with a diamond are reference sources

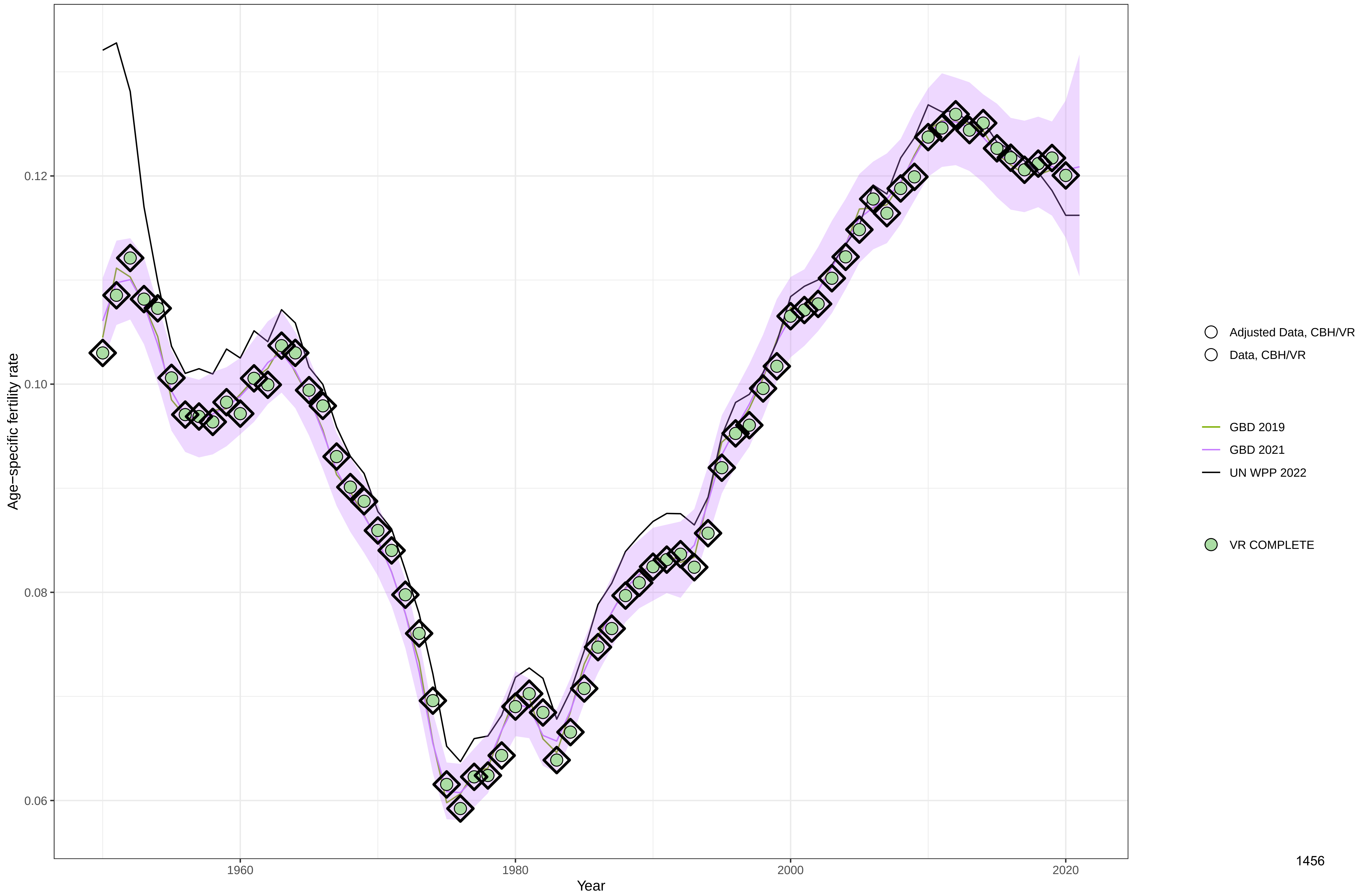

Age−specific fertility fitted trend lines, 1950−2021: France, Ages: 35−39
Data points enclosed with a diamond are reference sources

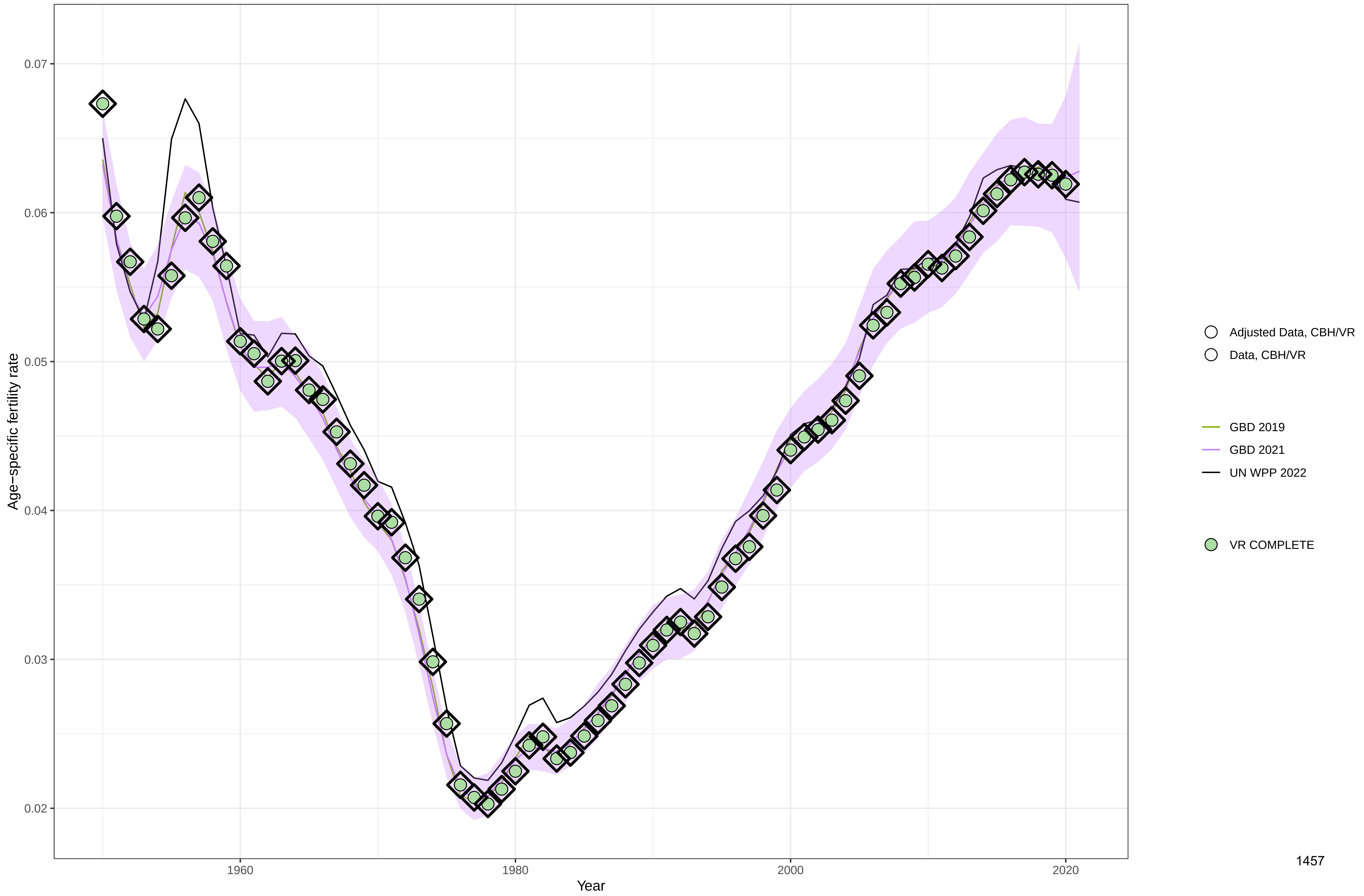

Age−specific fertility fitted trend lines, 1950−2021: France, Ages: 40−44
Data points enclosed with a diamond are reference sources

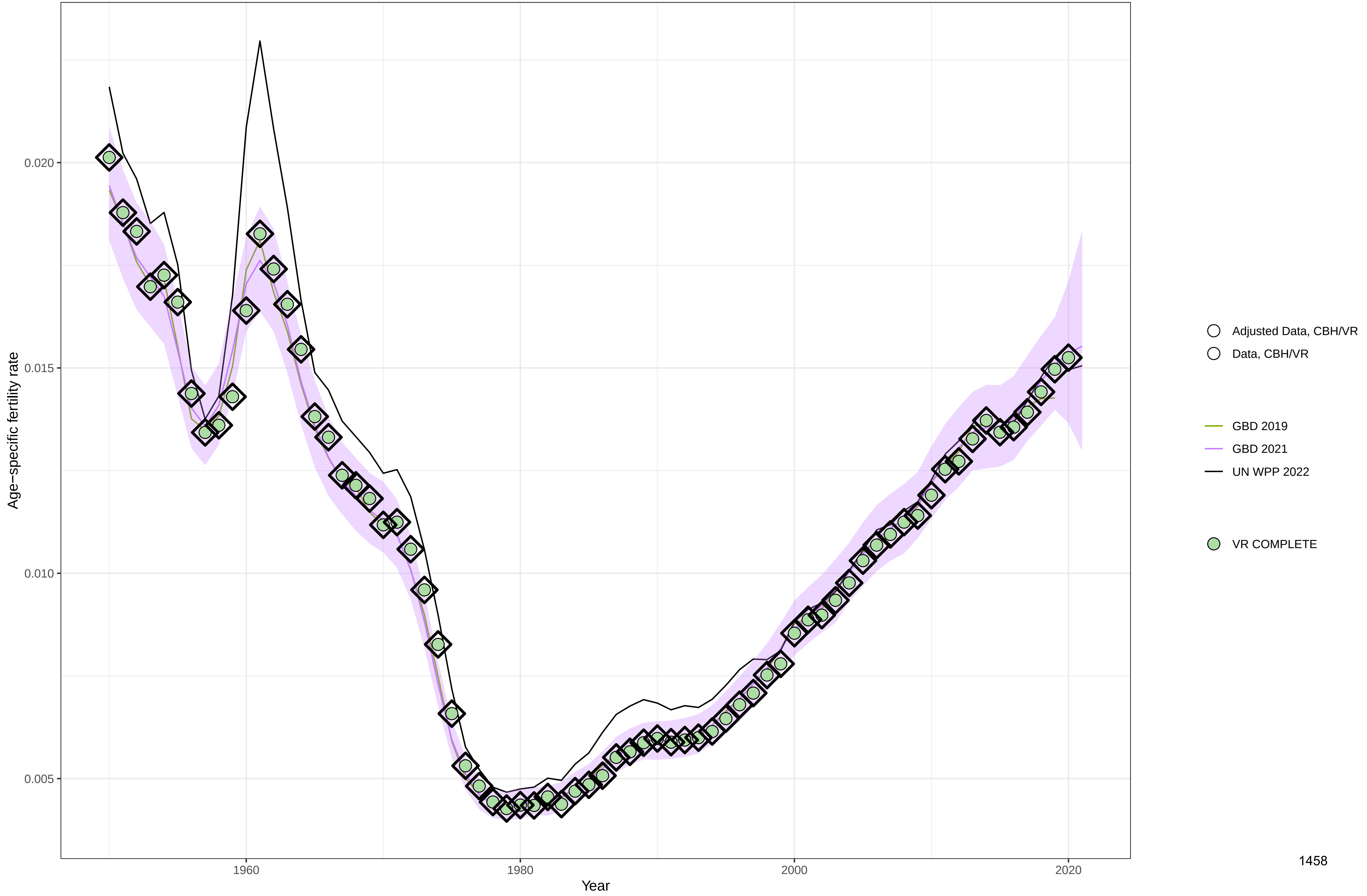

Age−specific fertility fitted trend lines, 1950−2021: France, Ages: 45−49
Data points enclosed with a diamond are reference sources

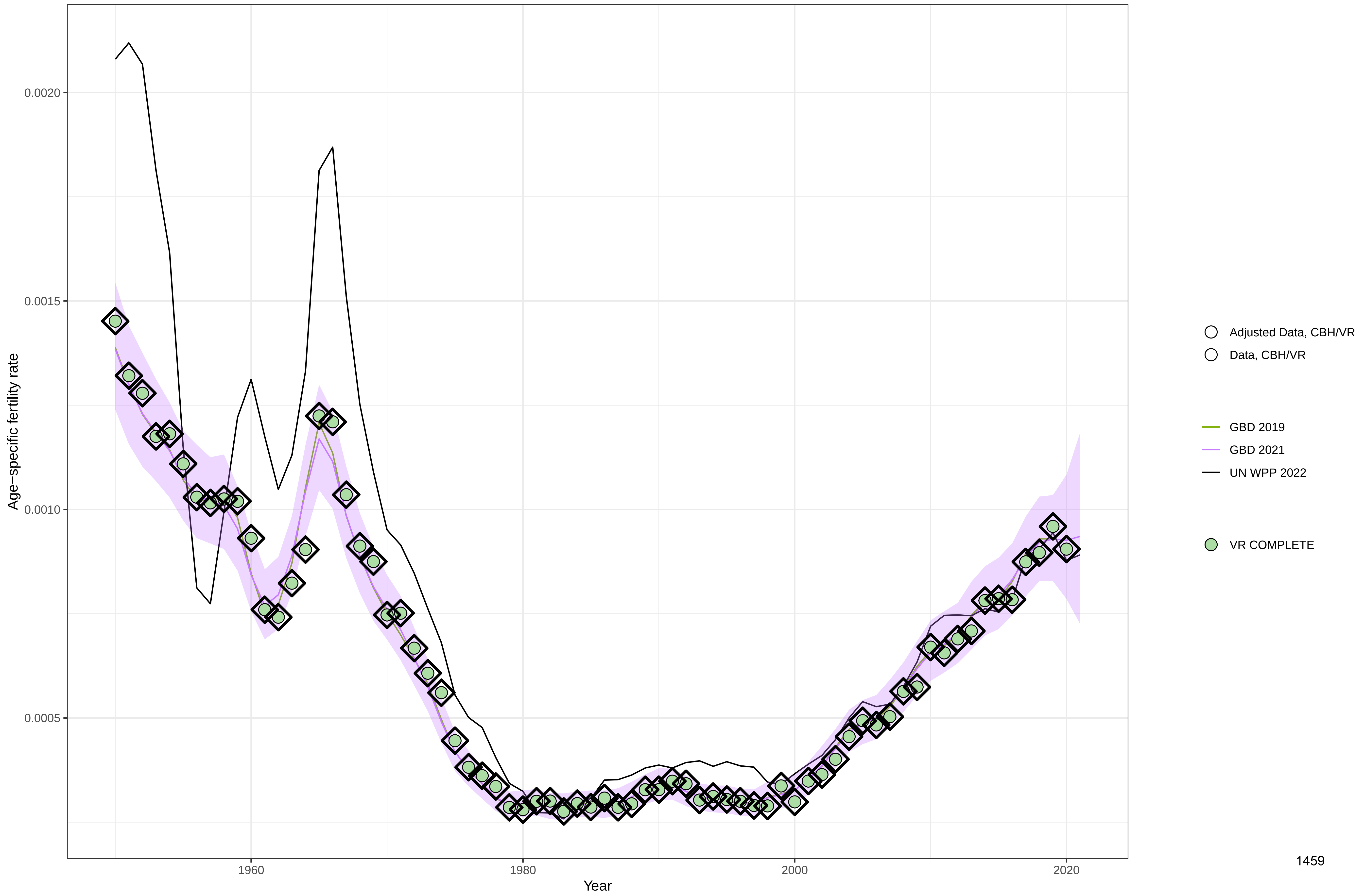

Age−specific fertility fitted trend lines, 1950−2021: France, Ages: 50−54
Data points enclosed with a diamond are reference sources

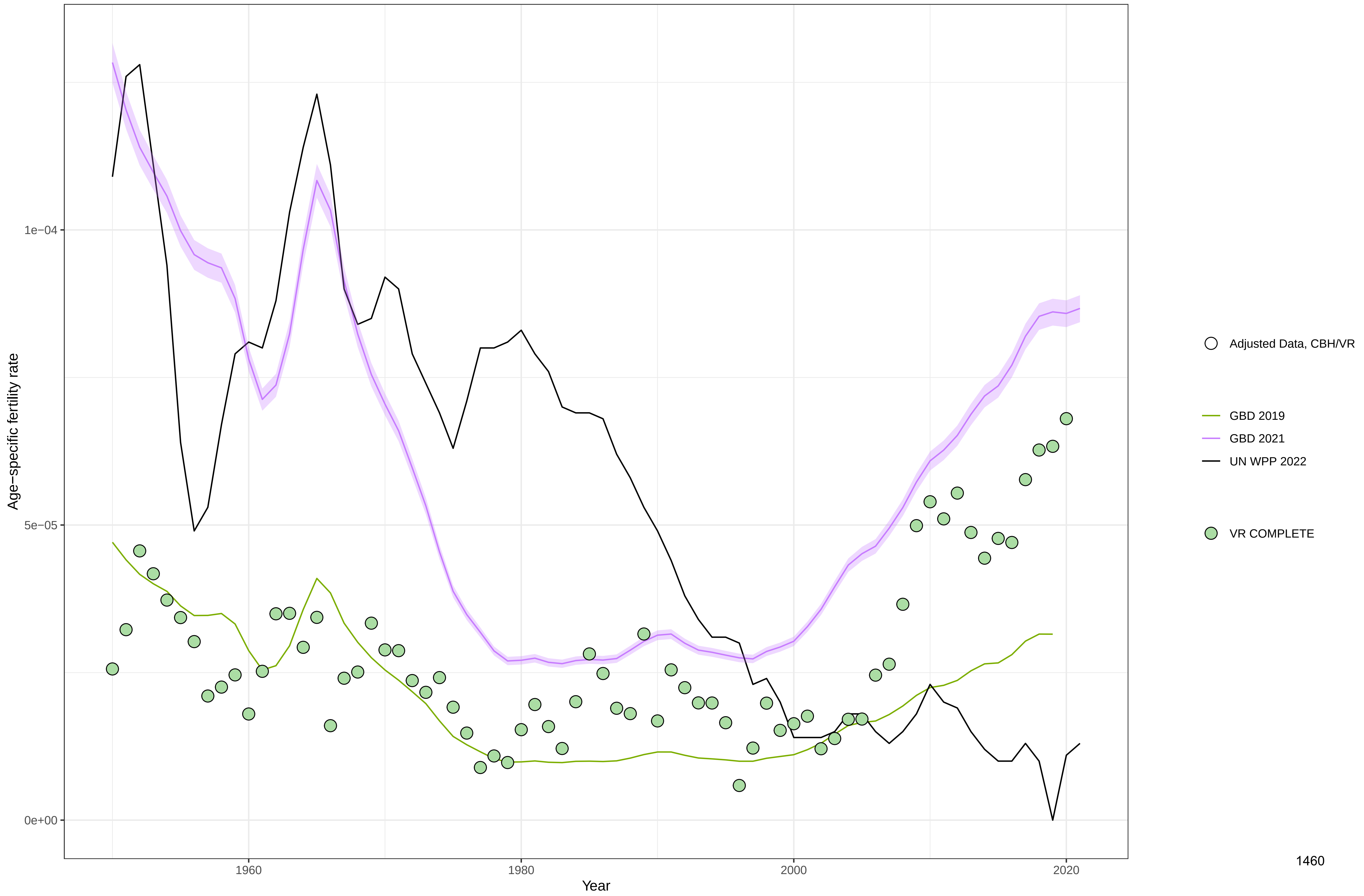

Total fertility rate fitted trend lines, 1950−2021: France TFR
Data points enclosed with a diamond are reference sources

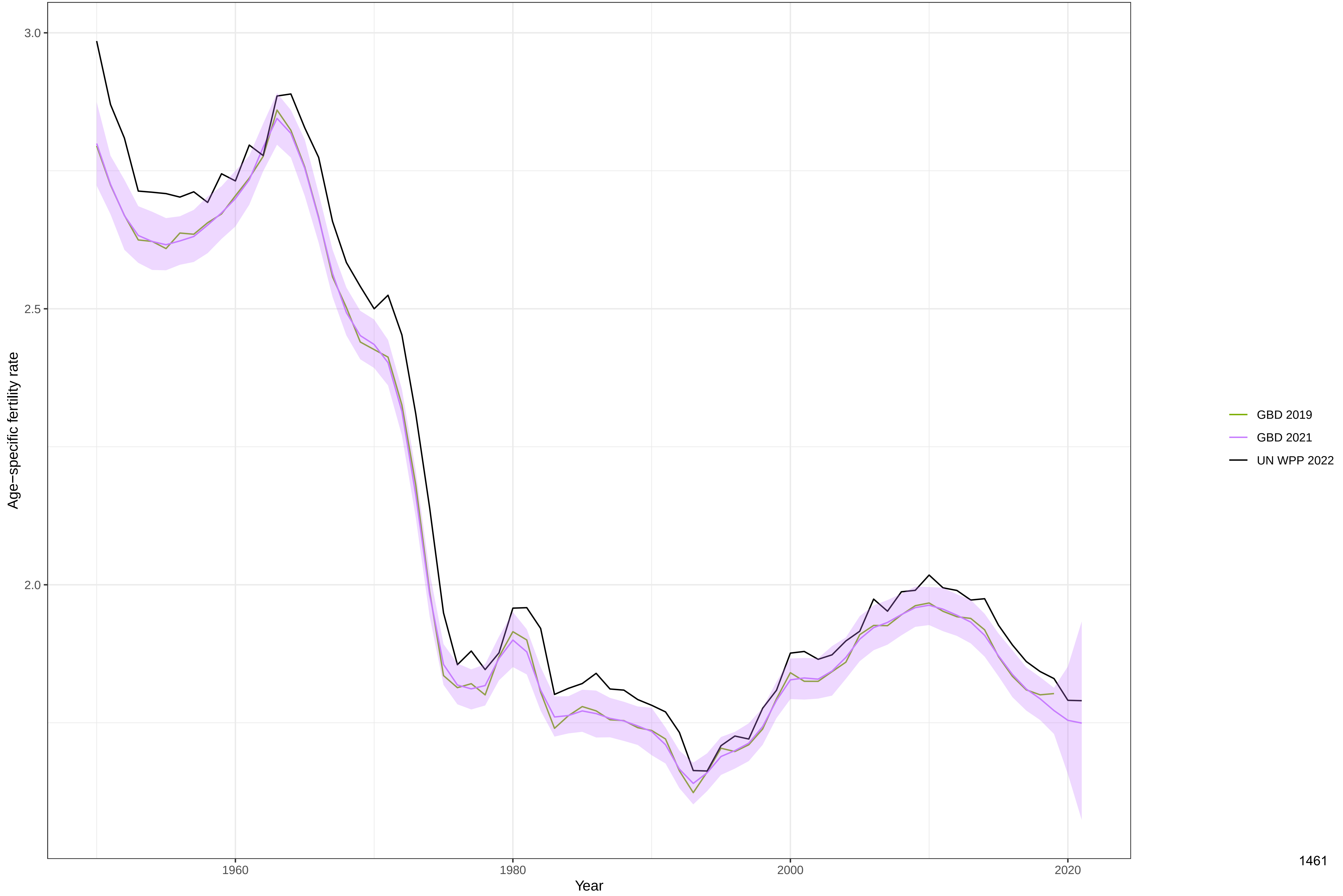

Age−specific fertility fitted trend lines, 1950−2021: Germany, Ages: 10−14
Data points enclosed with a diamond are reference sources

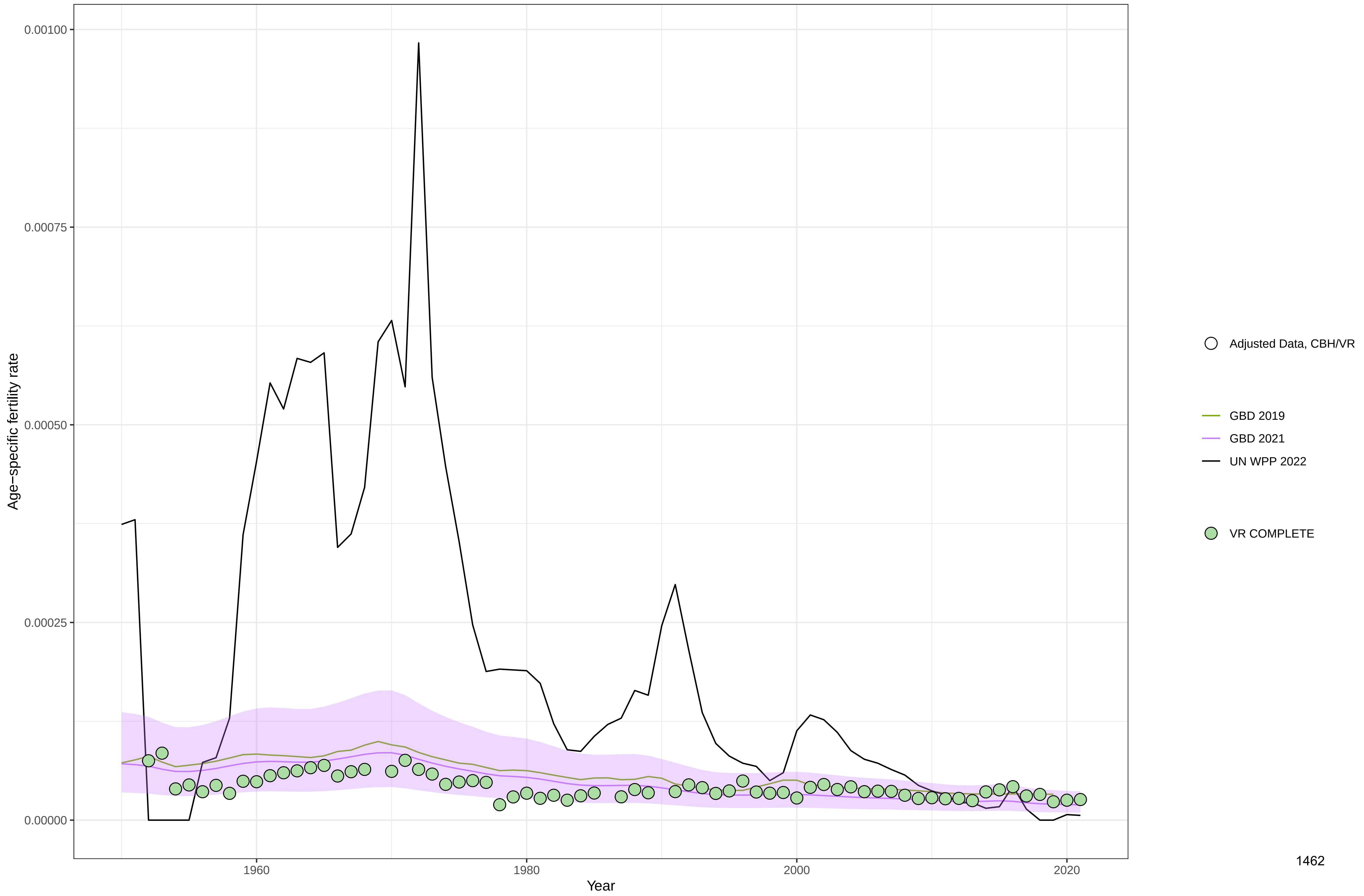

Age−specific fertility fitted trend lines, 1950−2021: Germany, Ages: 15−19
Data points enclosed with a diamond are reference sources

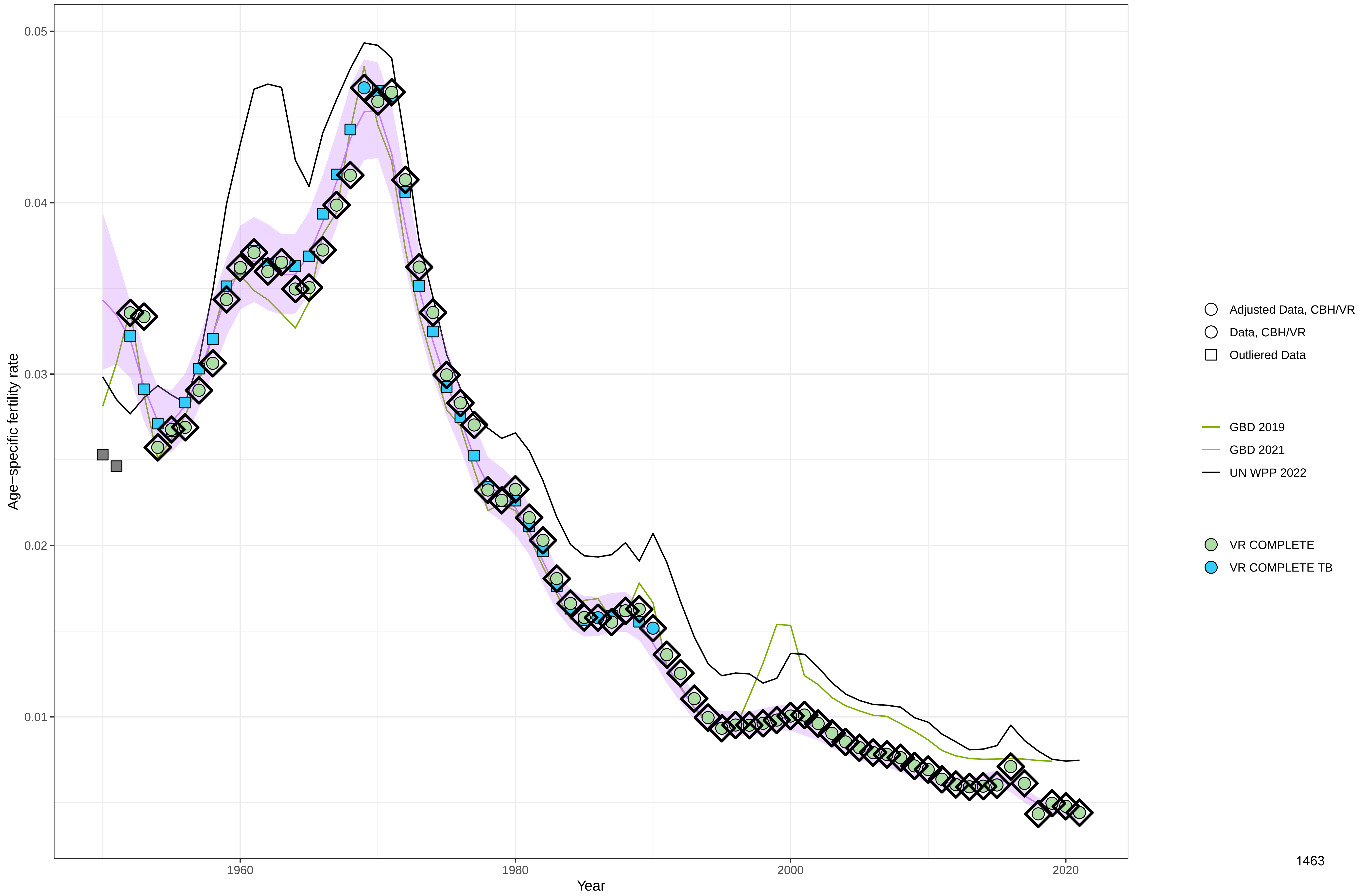

Age−specific fertility fitted trend lines, 1950−2021: Germany, Ages: 20−24
Data points enclosed with a diamond are reference sources

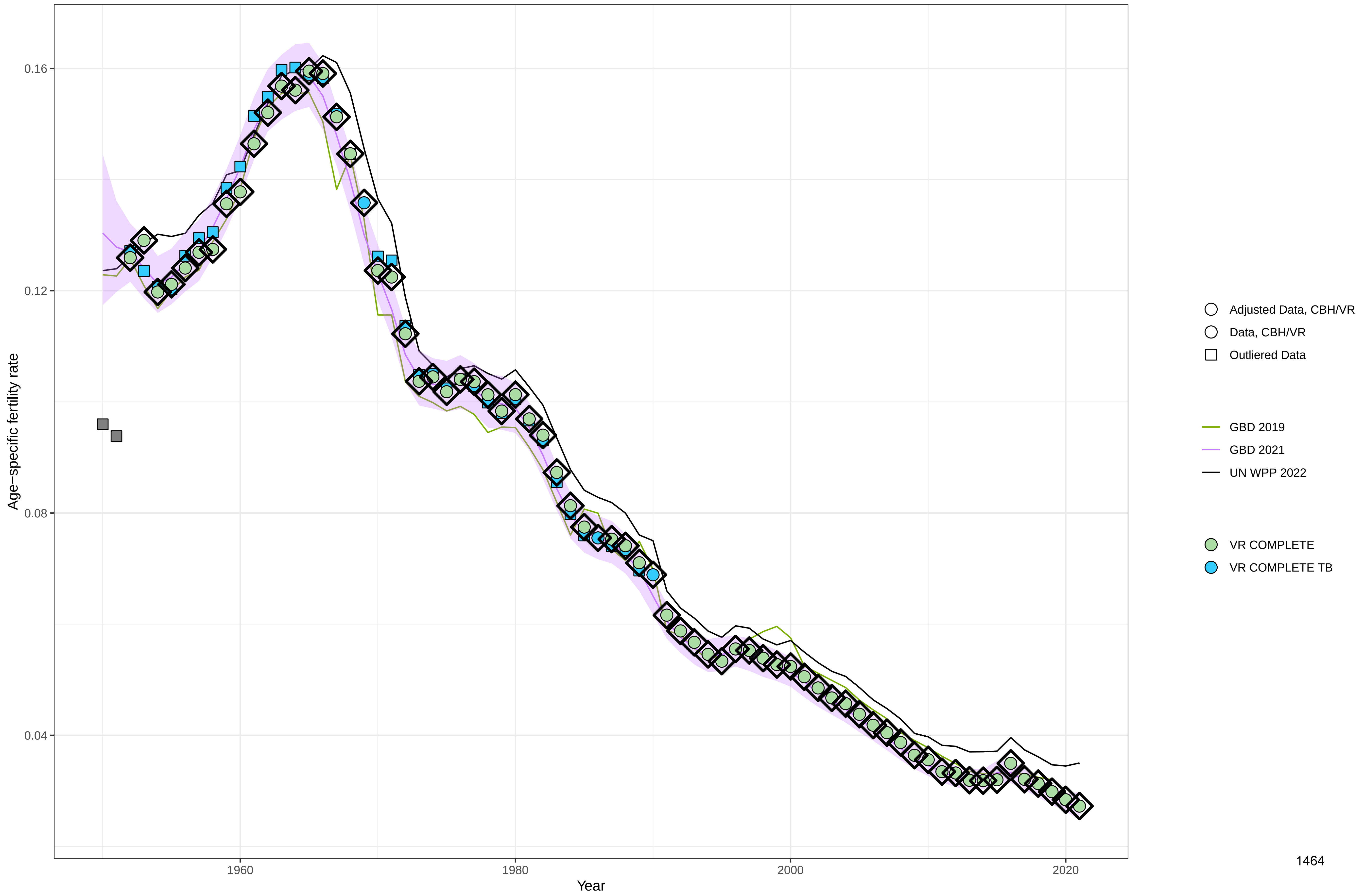

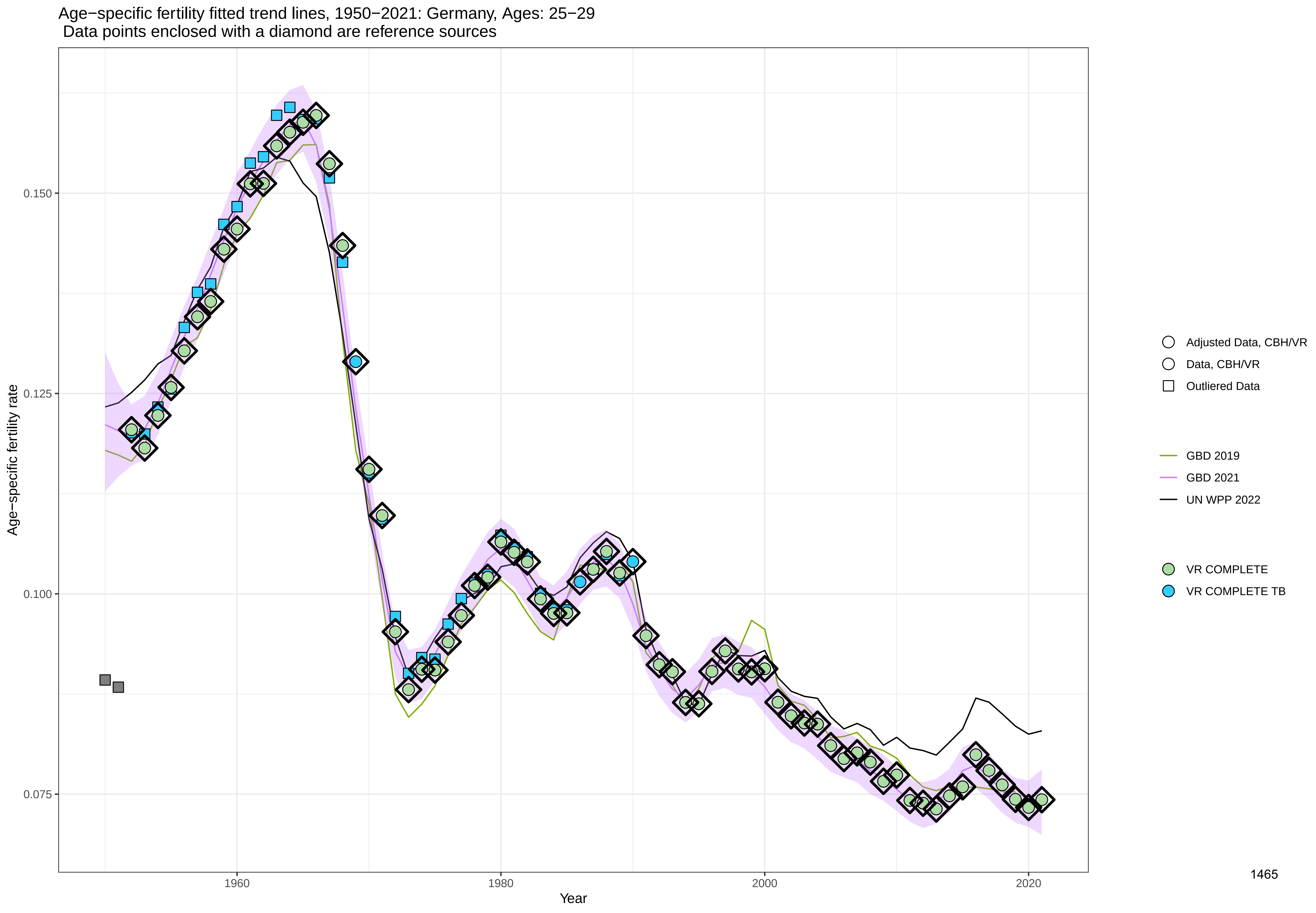

Age−specific fertility fitted trend lines, 1950−2021: Germany, Ages: 25−29
Data points enclosed with a diamond are reference sources
Age−specific fertility rate
0.150
0.125
0.100
0.075
1960
1980
2000
2020
Year
Adjusted Data, CBH/VR
Data, CBH/VR
Outliered Data
GBD 2019
GBD 2021
UN WPP 2022
VR COMPLETE
VR COMPLETE TB

Age−specific fertility fitted trend lines, 1950−2021: Germany, Ages: 30−34
Data points enclosed with a diamond are reference sources

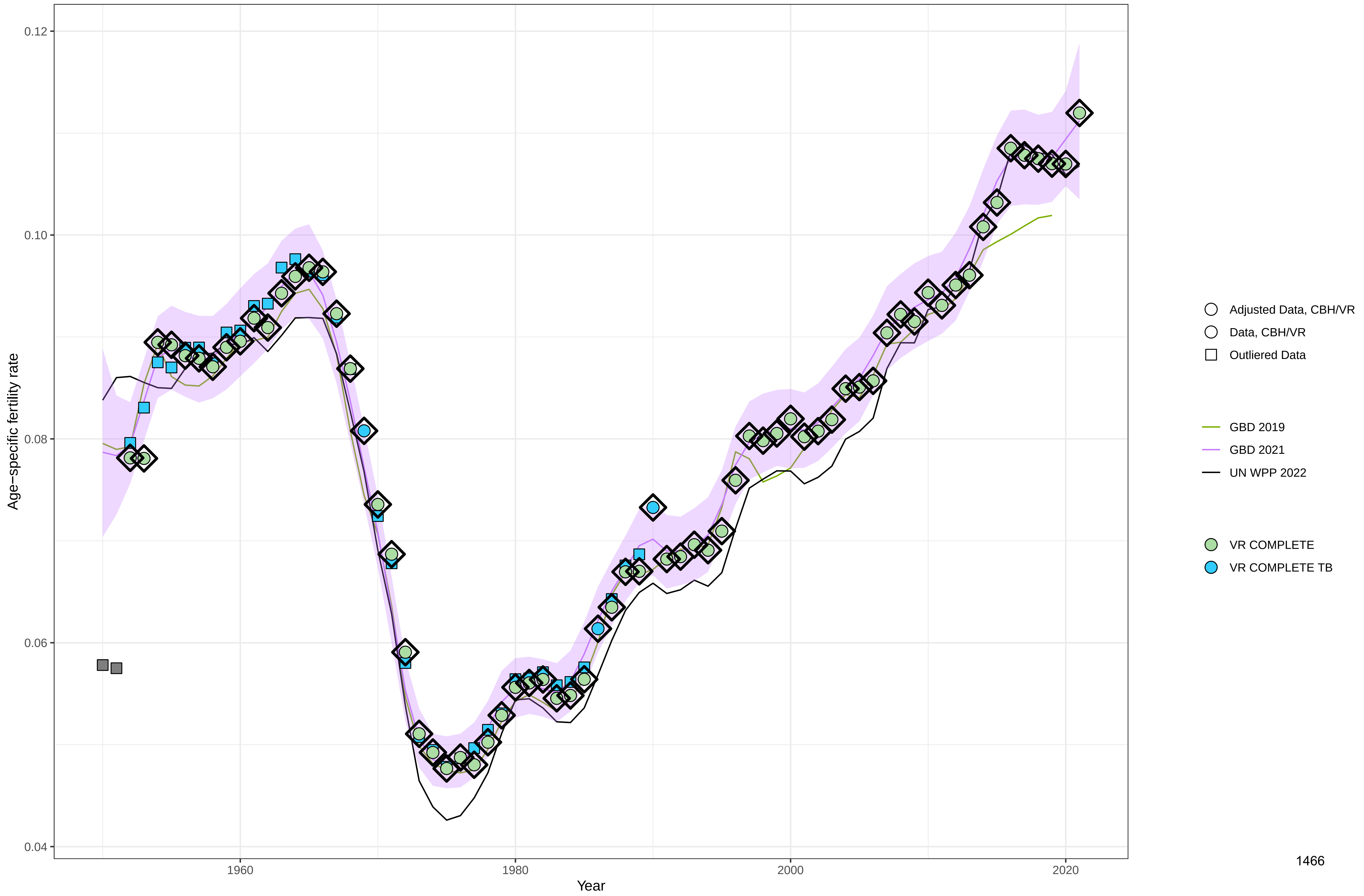

Age−specific fertility fitted trend lines, 1950−2021: Germany, Ages: 35−39
Data points enclosed with a diamond are reference sources

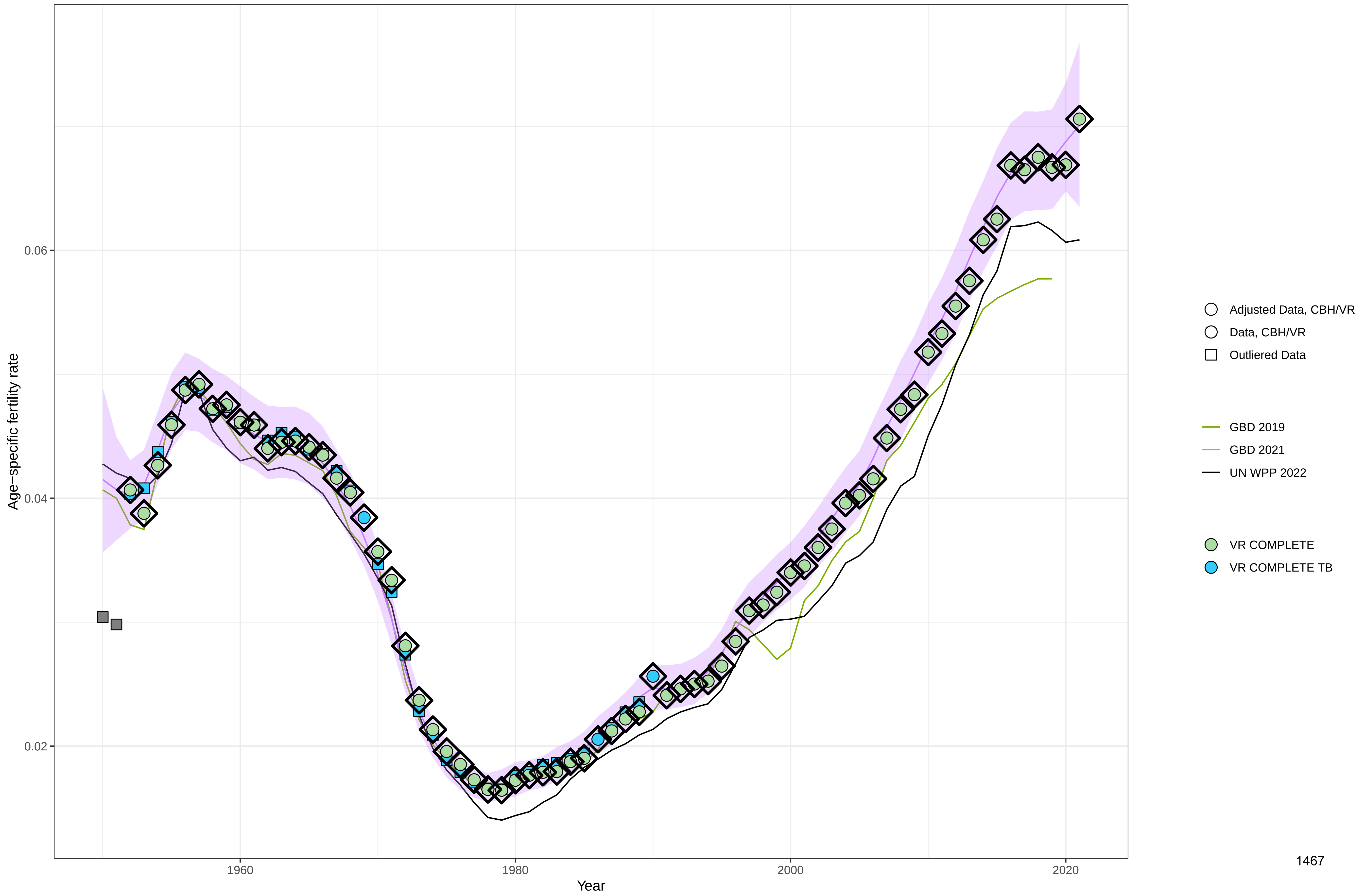

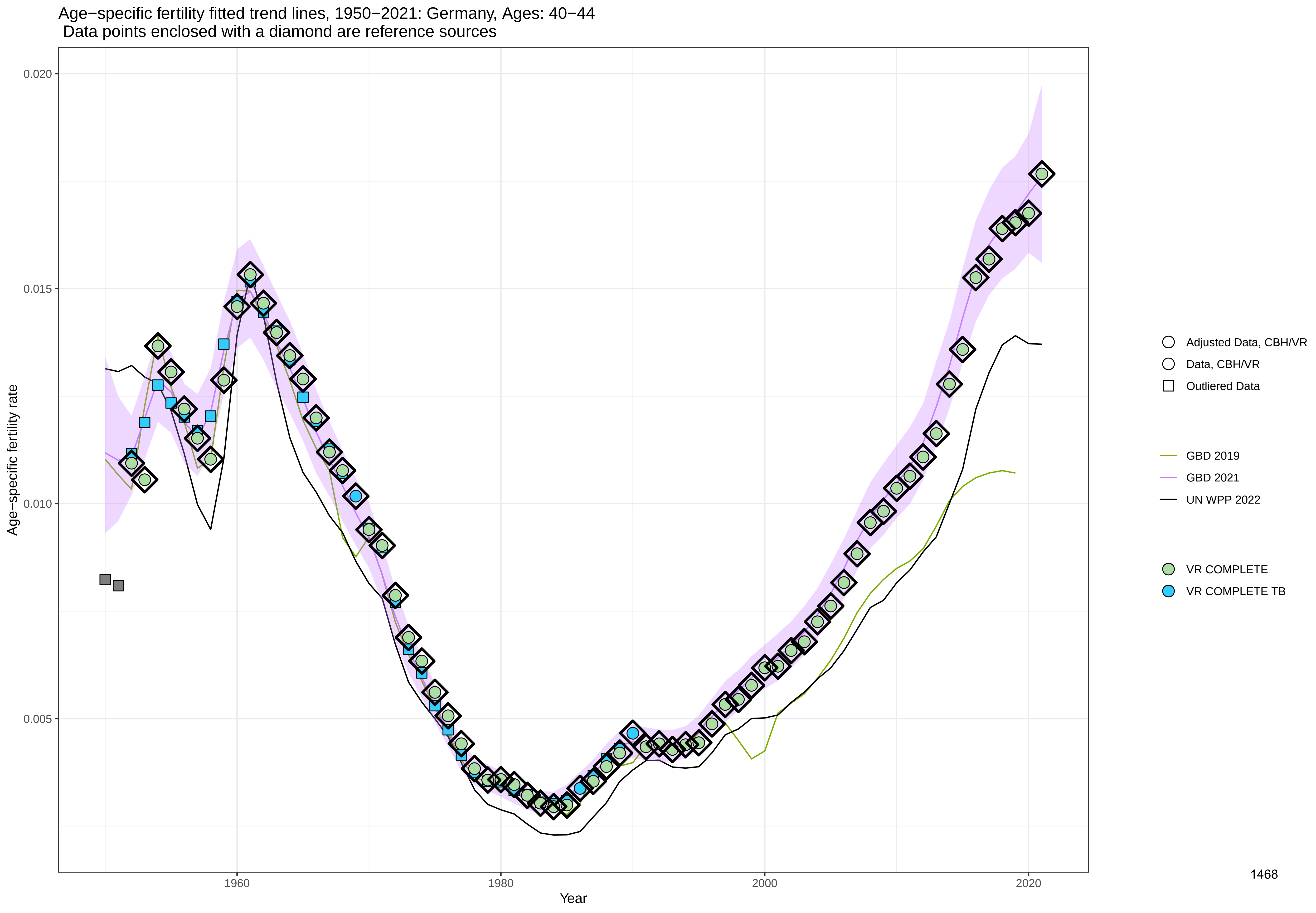

Age−specific fertility fitted trend lines, 1950−2021: Germany, Ages: 40−44
Data points enclosed with a diamond are reference sources
Age−specific fertility rate
0.020
0.015
0.010
0.005
1960
1980
2000
2020
Year
Adjusted Data, CBH/VR
Data, CBH/VR
Outliered Data
GBD 2019
GBD 2021
UN WPP 2022
VR COMPLETE
VR COMPLETE TB

Age−specific fertility fitted trend lines, 1950−2021: Germany, Ages: 45−49
Data points enclosed with a diamond are reference sources

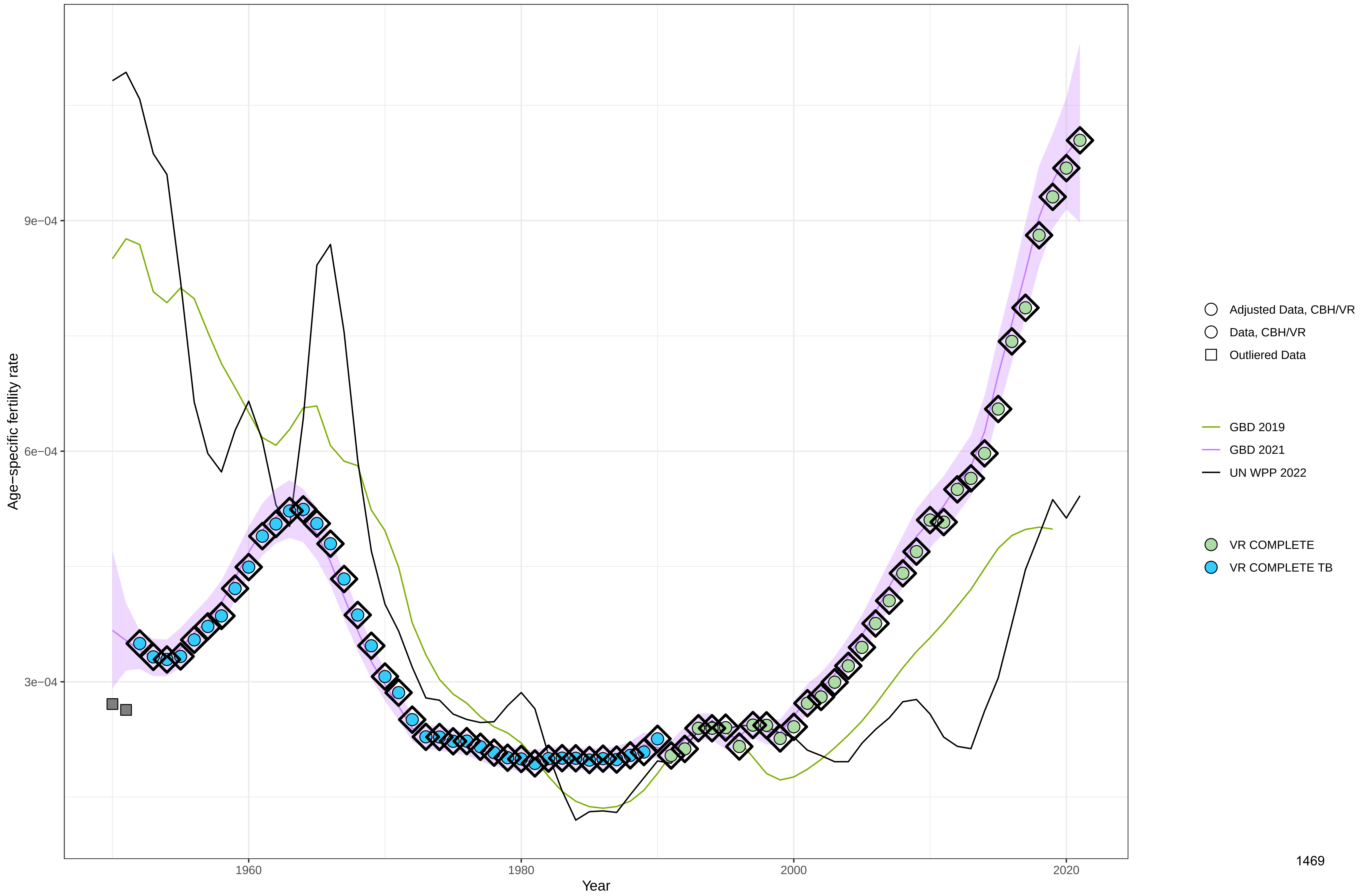

Age−specific fertility fitted trend lines, 1950−2021: Germany, Ages: 50−54
Data points enclosed with a diamond are reference sources

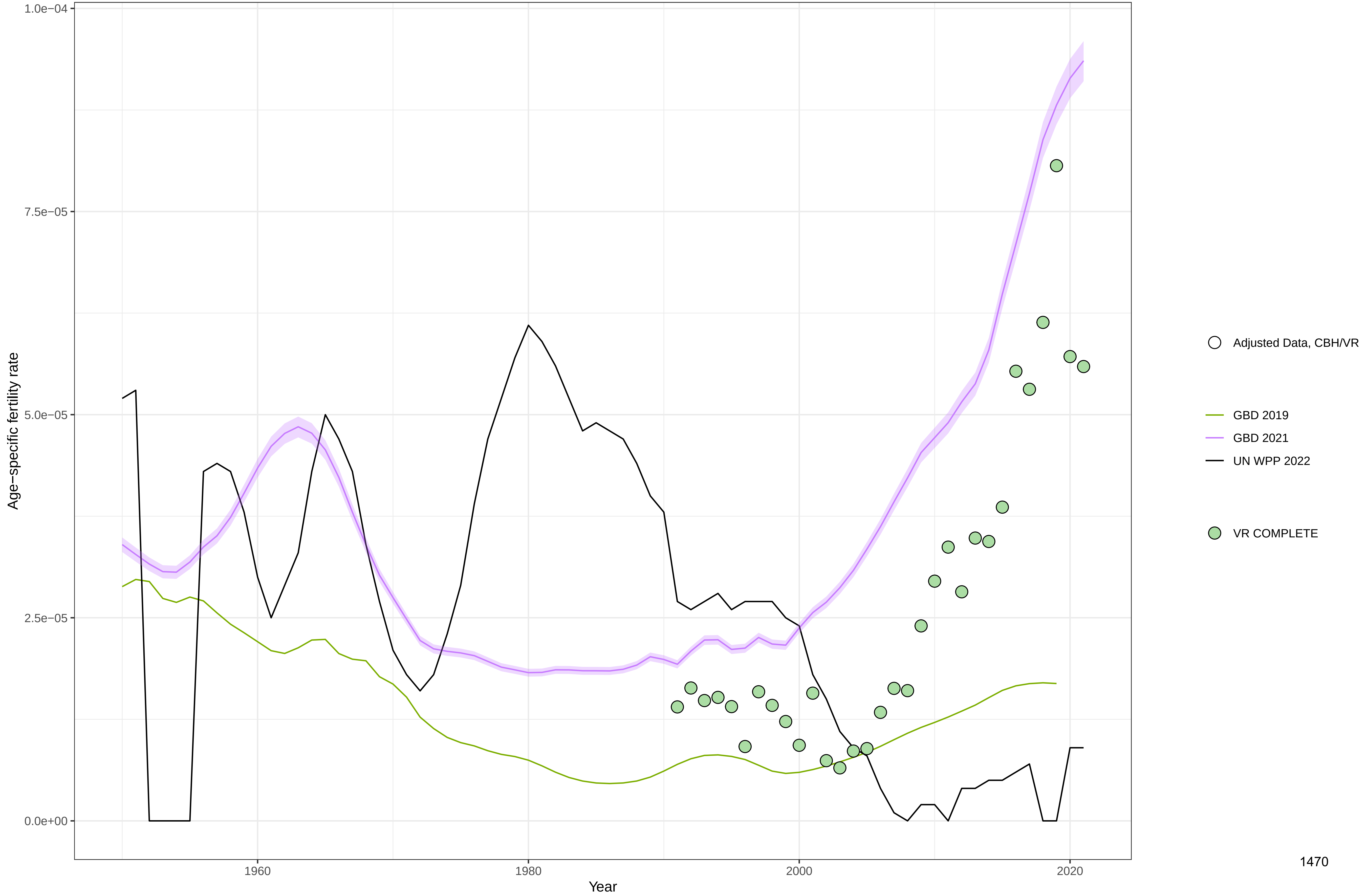

Total fertility rate fitted trend lines, 1950−2021: Germany TFR
Data points enclosed with a diamond are reference sources

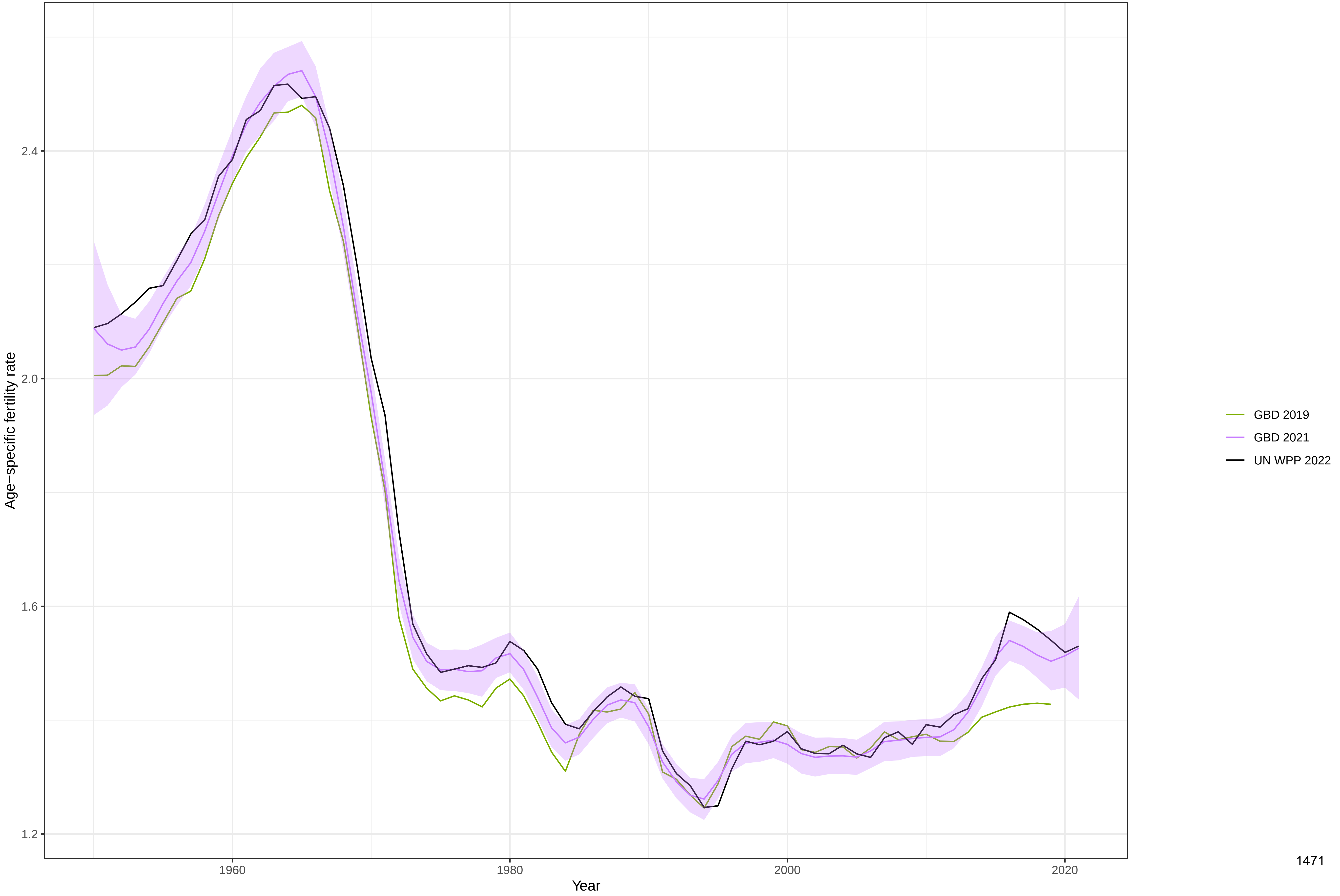

Age−specific fertility fitted trend lines, 1950−2021: Greece, Ages: 10−14
Data points enclosed with a diamond are reference sources

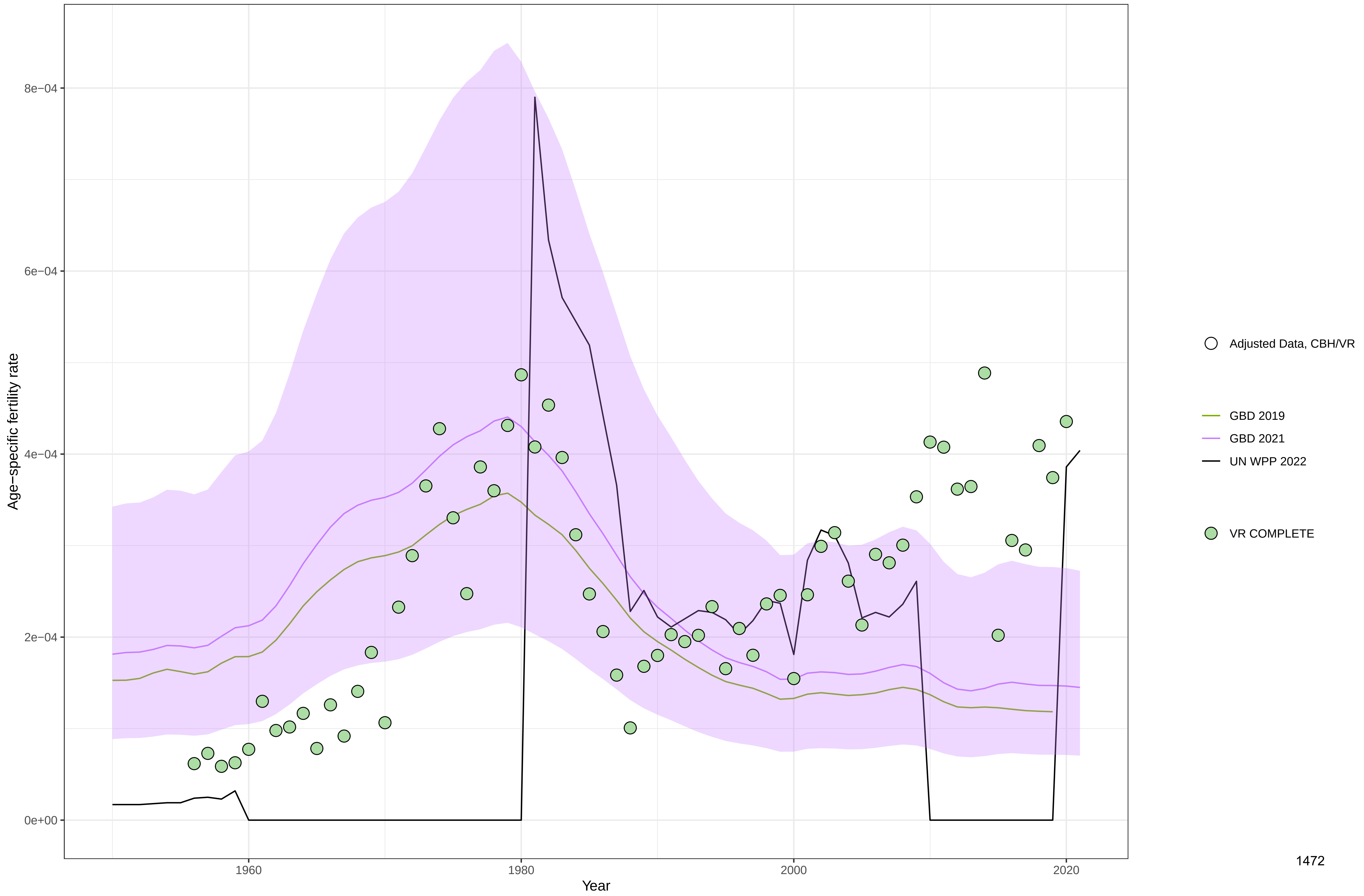

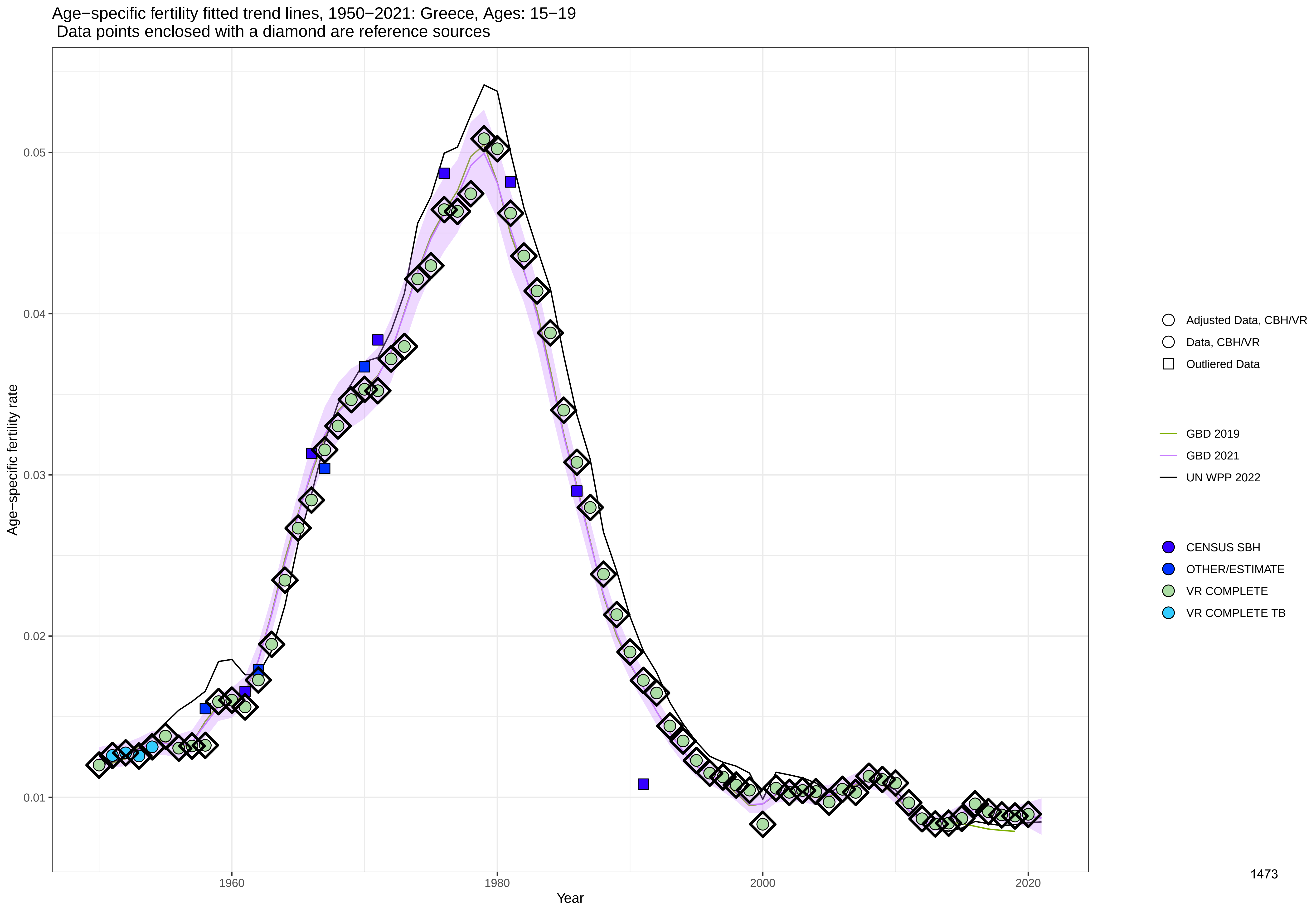
Age−specific fertility fitted trend lines, 1950−2021: Greece, Ages: 15−19
Data points enclosed with a diamond are reference sources
Age−specific fertility rate
0.01
0.02
0.03
0.04
0.05
Year
1960
1980
2000
2020
Adjusted Data, CBH/VR
Data, CBH/VR
Outliered Data
GBD 2019
GBD 2021
UN WPP 2022
CENSUS SBH
OTHER/ESTIMATE
VR COMPLETE
VR COMPLETE TB

Age−specific fertility fitted trend lines, 1950−2021: Greece, Ages: 20−24
Data points enclosed with a diamond are reference sources

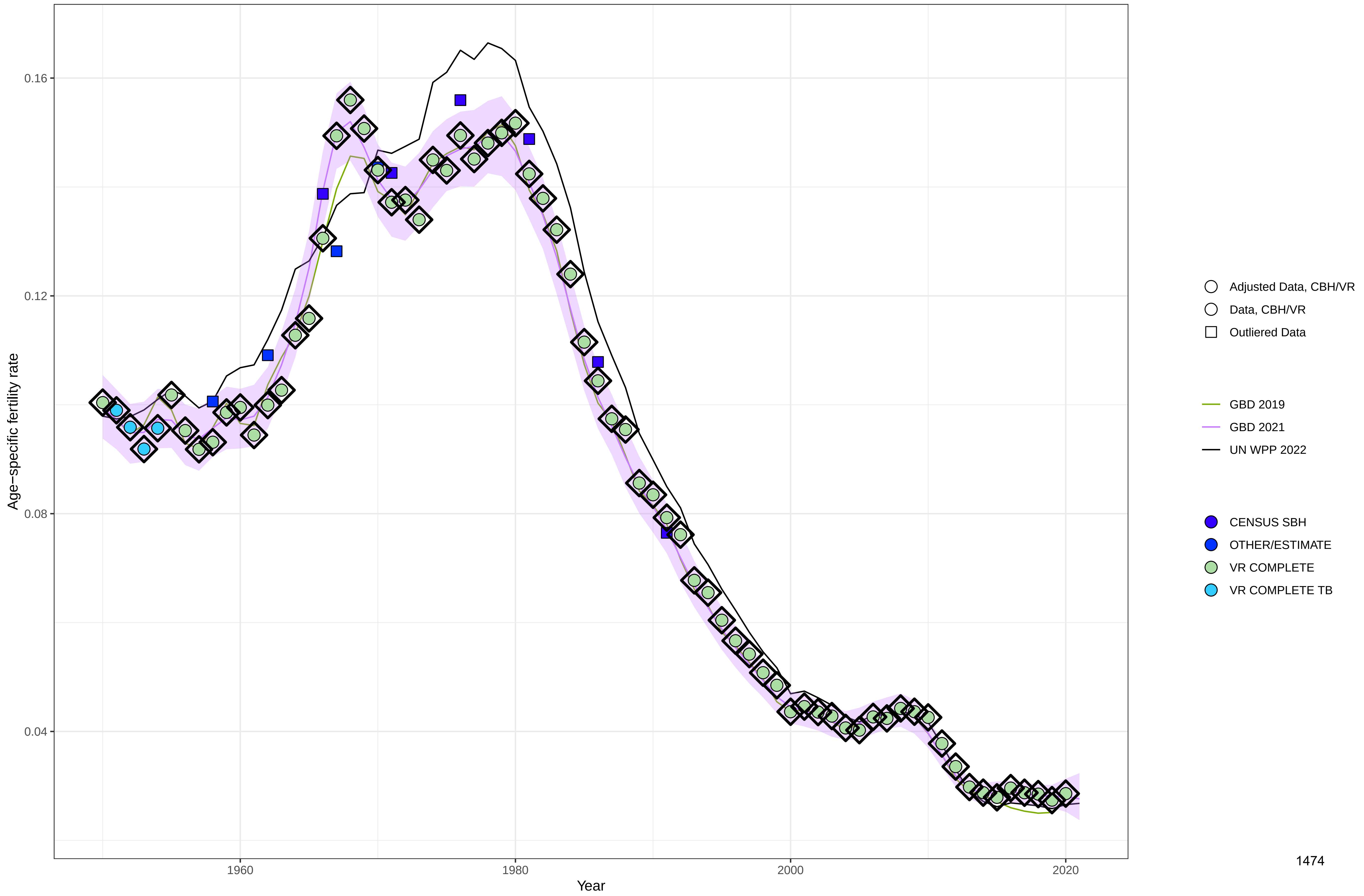

Age−specific fertility fitted trend lines, 1950−2021: Greece, Ages: 25−29
Data points enclosed with a diamond are reference sources

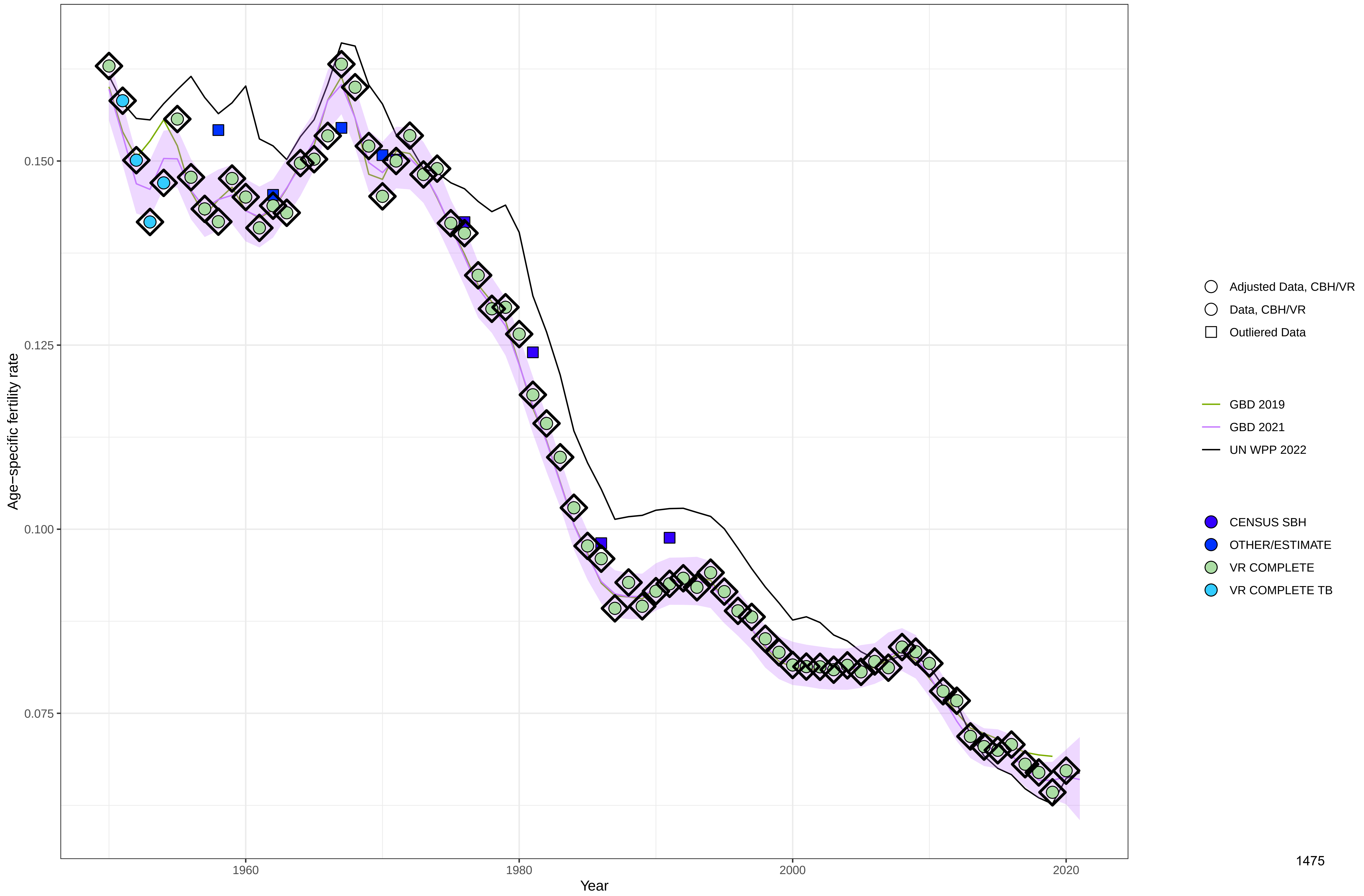

Age−specific fertility fitted trend lines, 1950−2021: Greece, Ages: 30−34
Data points enclosed with a diamond are reference sources

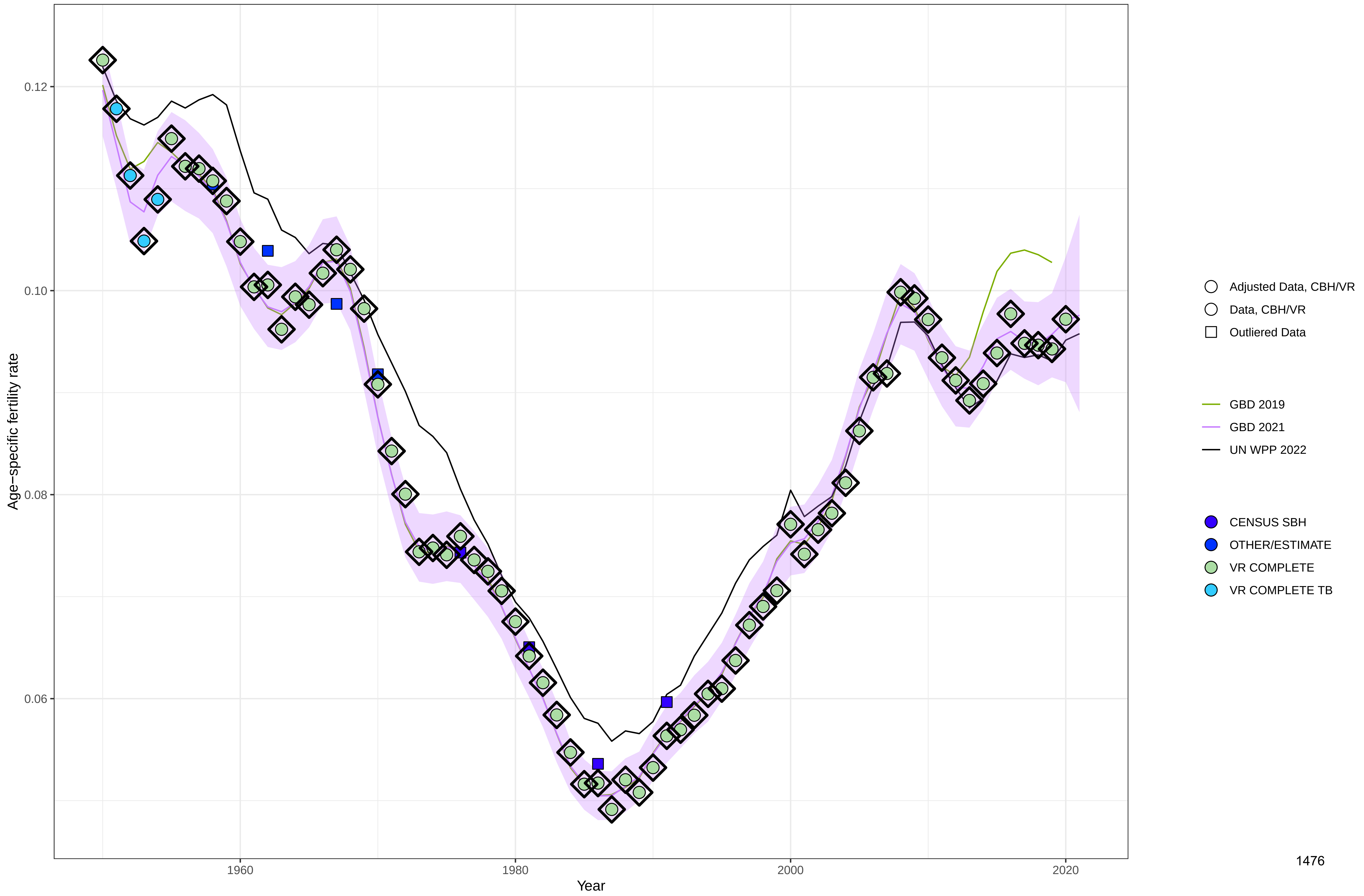

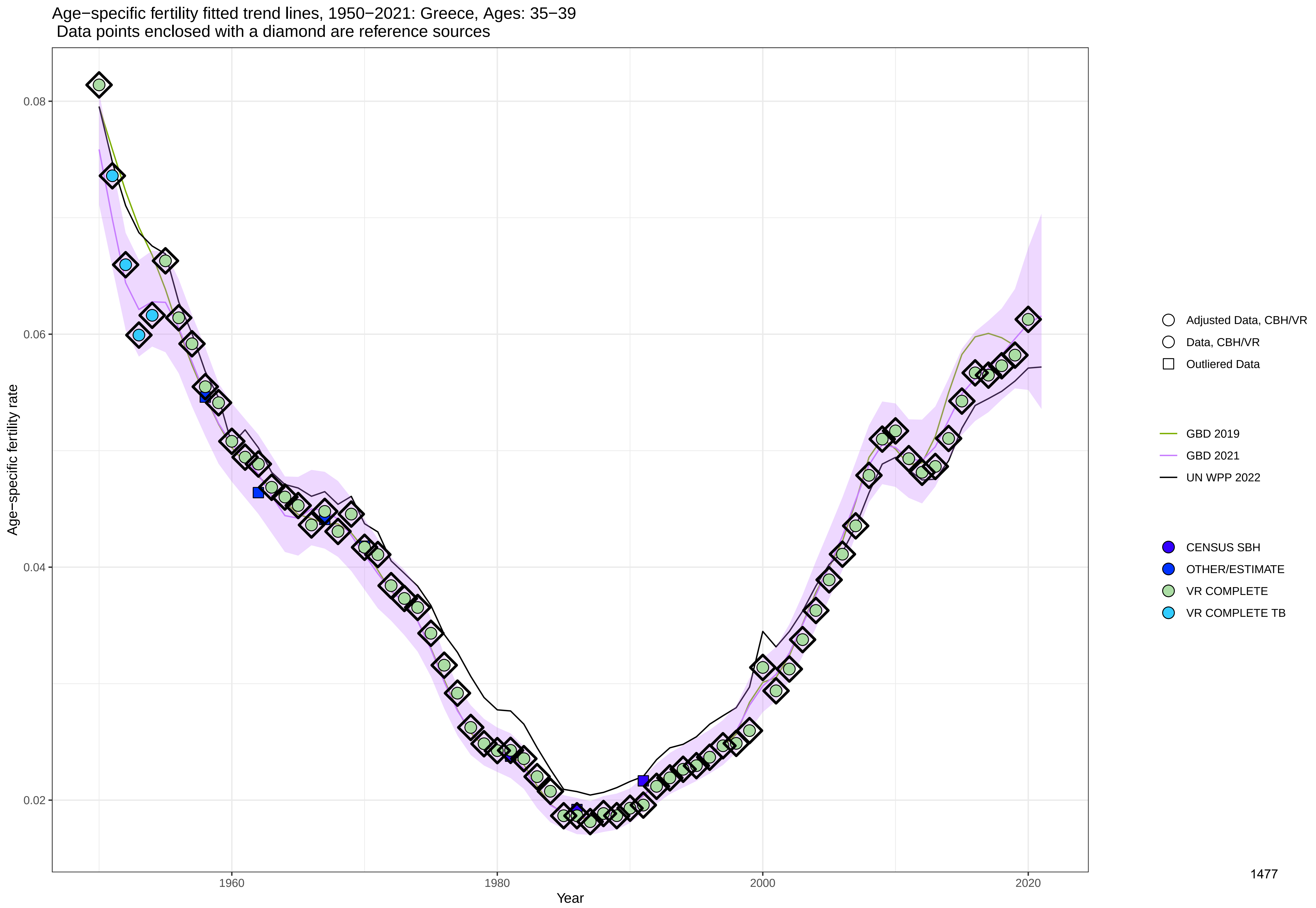

Age−specific fertility fitted trend lines, 1950−2021: Greece, Ages: 35−39
Data points enclosed with a diamond are reference sources
Age−specific fertility rate
0.08
0.06
0.04
0.02
1960
1980
2000
2020
Year
Adjusted Data, CBH/VR
Data, CBH/VR
Outliered Data
GBD 2019
GBD 2021
UN WPP 2022
CENSUS SBH
OTHER/ESTIMATE
VR COMPLETE
VR COMPLETE TB

Age−specific fertility fitted trend lines, 1950−2021: Greece, Ages: 40−44
Data points enclosed with a diamond are reference sources

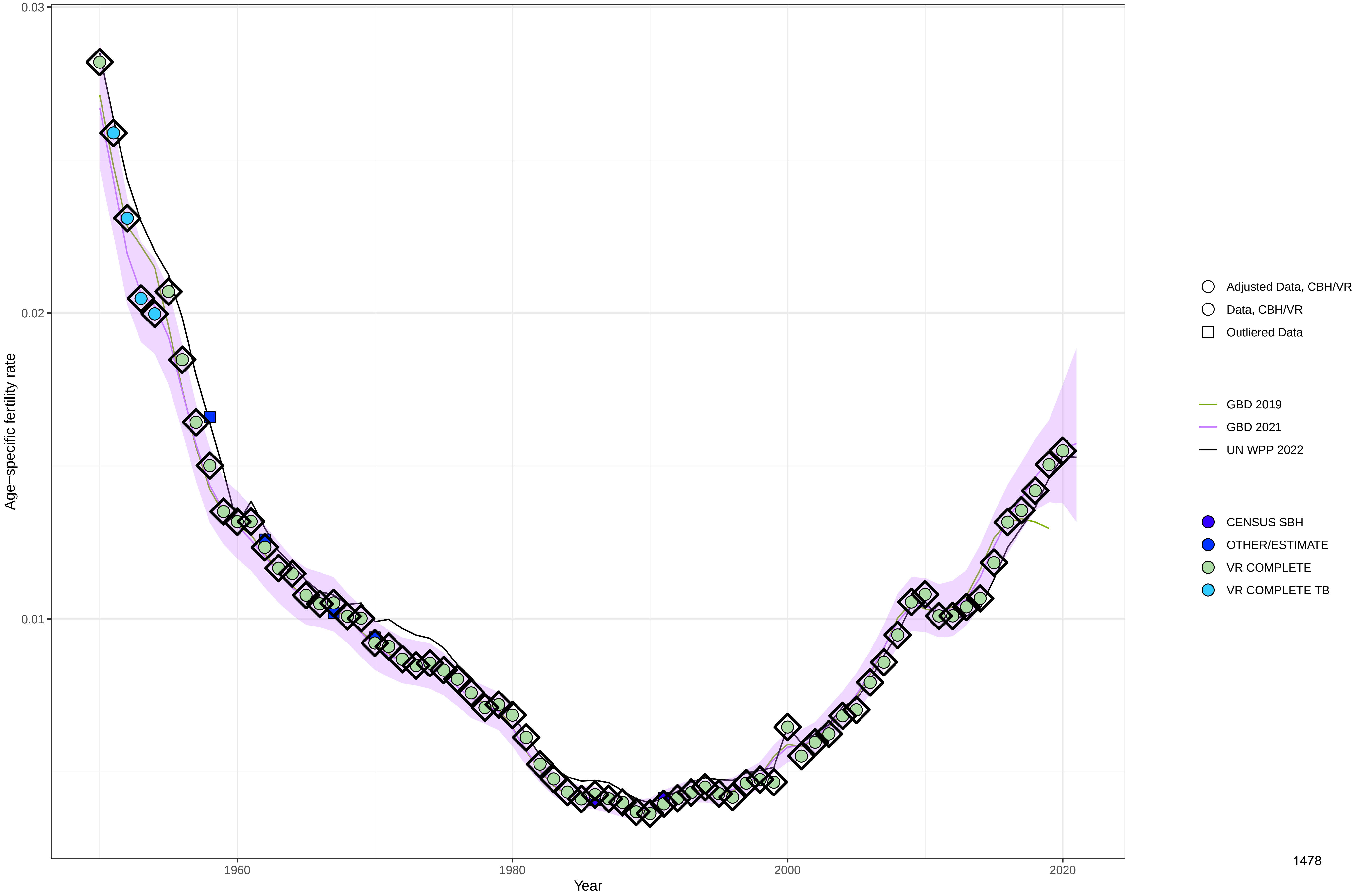

Age−specific fertility fitted trend lines, 1950−2021: Greece, Ages: 45−49
Data points enclosed with a diamond are reference sources

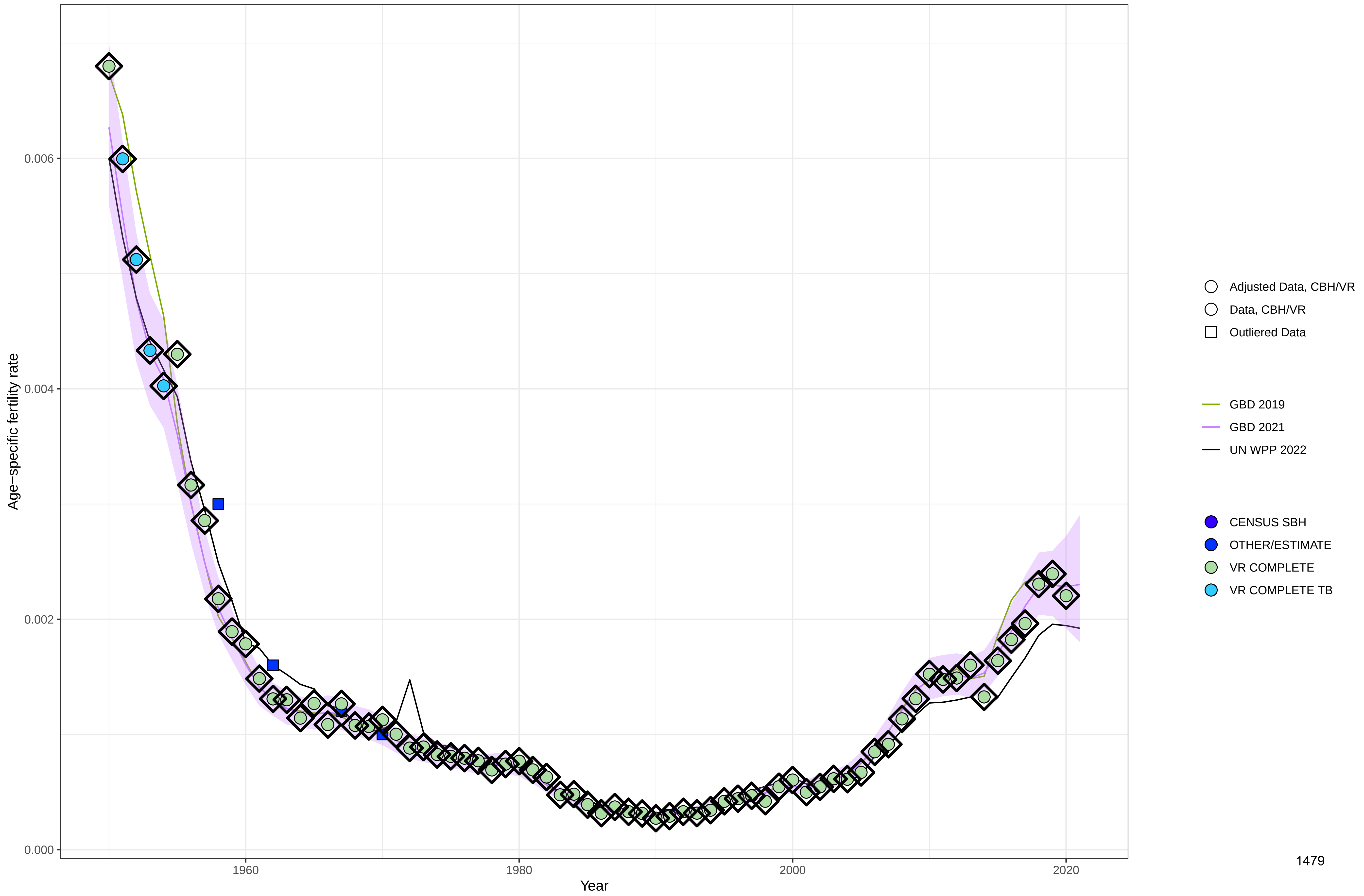

Age−specific fertility fitted trend lines, 1950−2021: Greece, Ages: 50−54
Data points enclosed with a diamond are reference sources

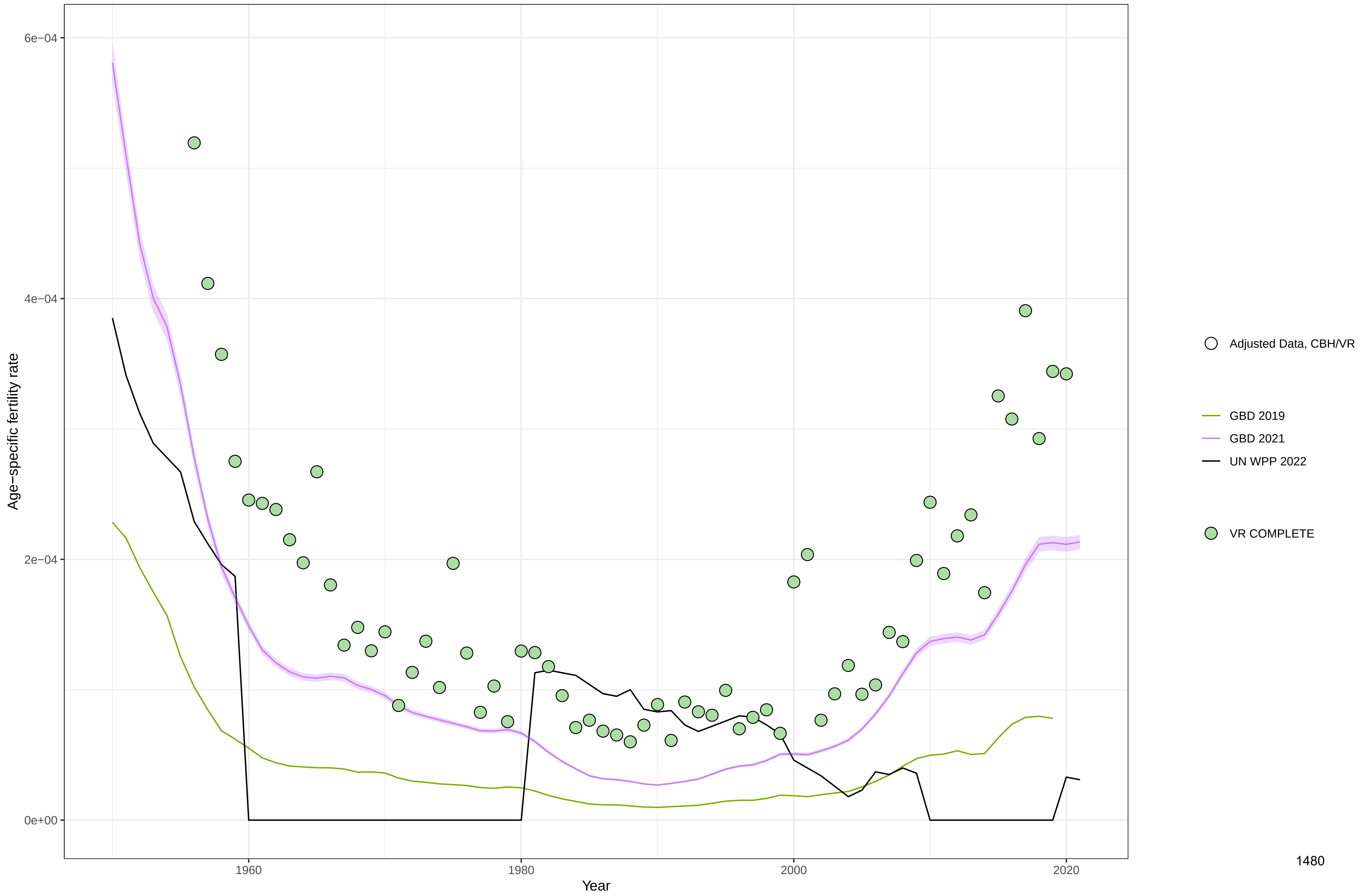

Total fertility rate fitted trend lines, 1950−2021: Greece TFR
Data points enclosed with a diamond are reference sources

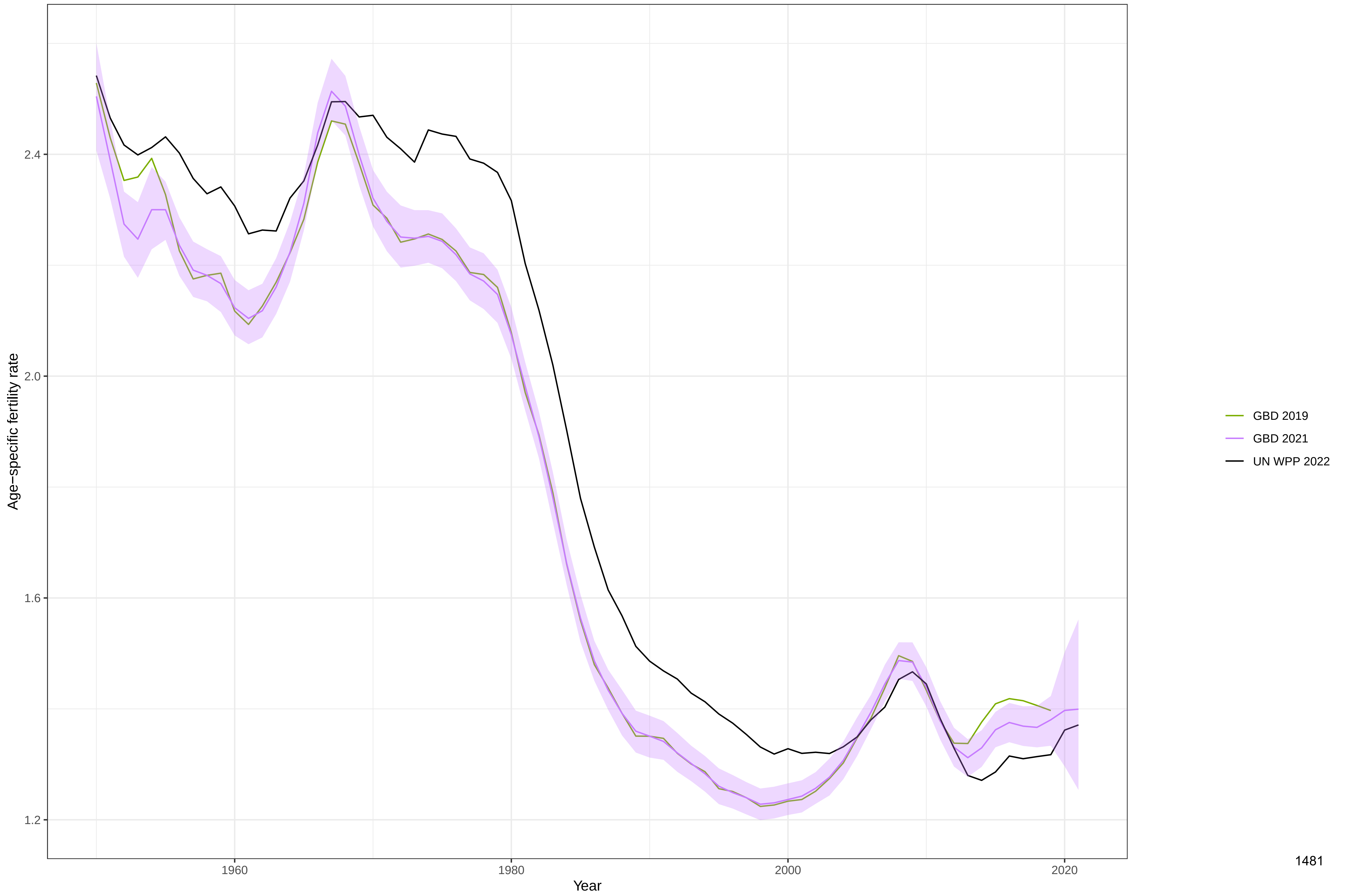

Age−specific fertility fitted trend lines, 1950−2021: Iceland, Ages: 10−14
Data points enclosed with a diamond are reference sources

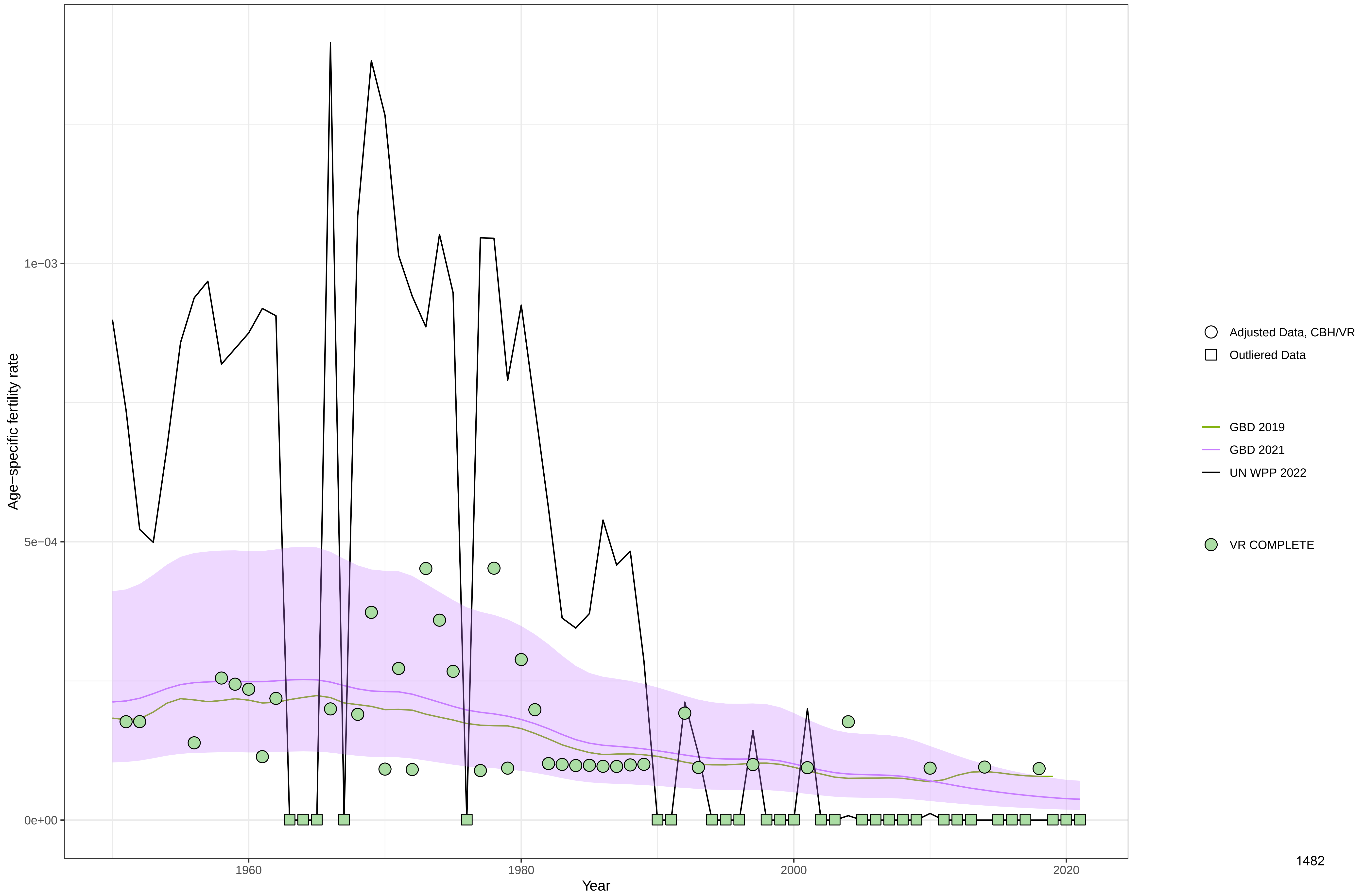

Age−specific fertility fitted trend lines, 1950−2021: Iceland, Ages: 15−19
Data points enclosed with a diamond are reference sources

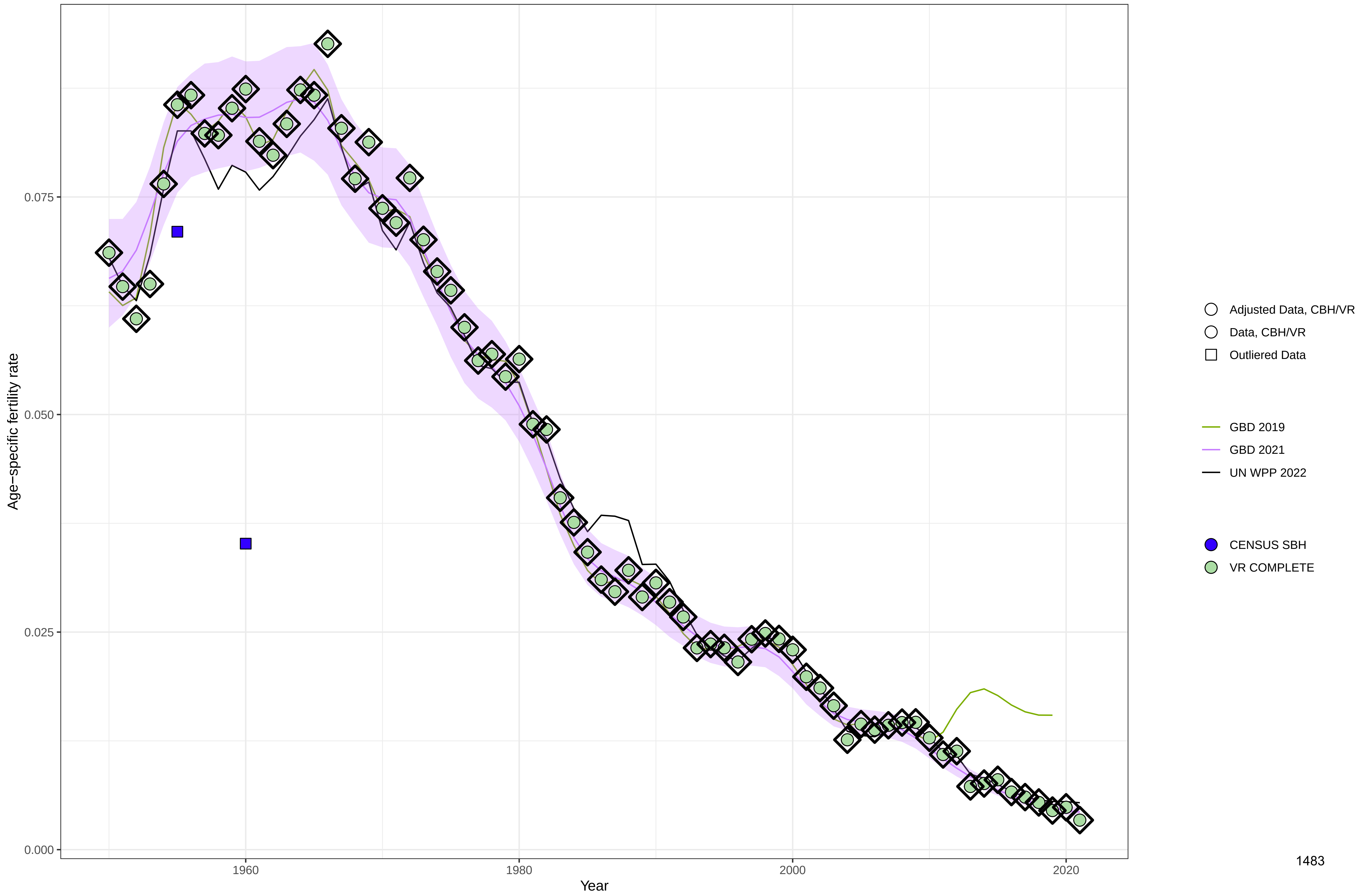

Age−specific fertility fitted trend lines, 1950−2021: Iceland, Ages: 20−24
Data points enclosed with a diamond are reference sources

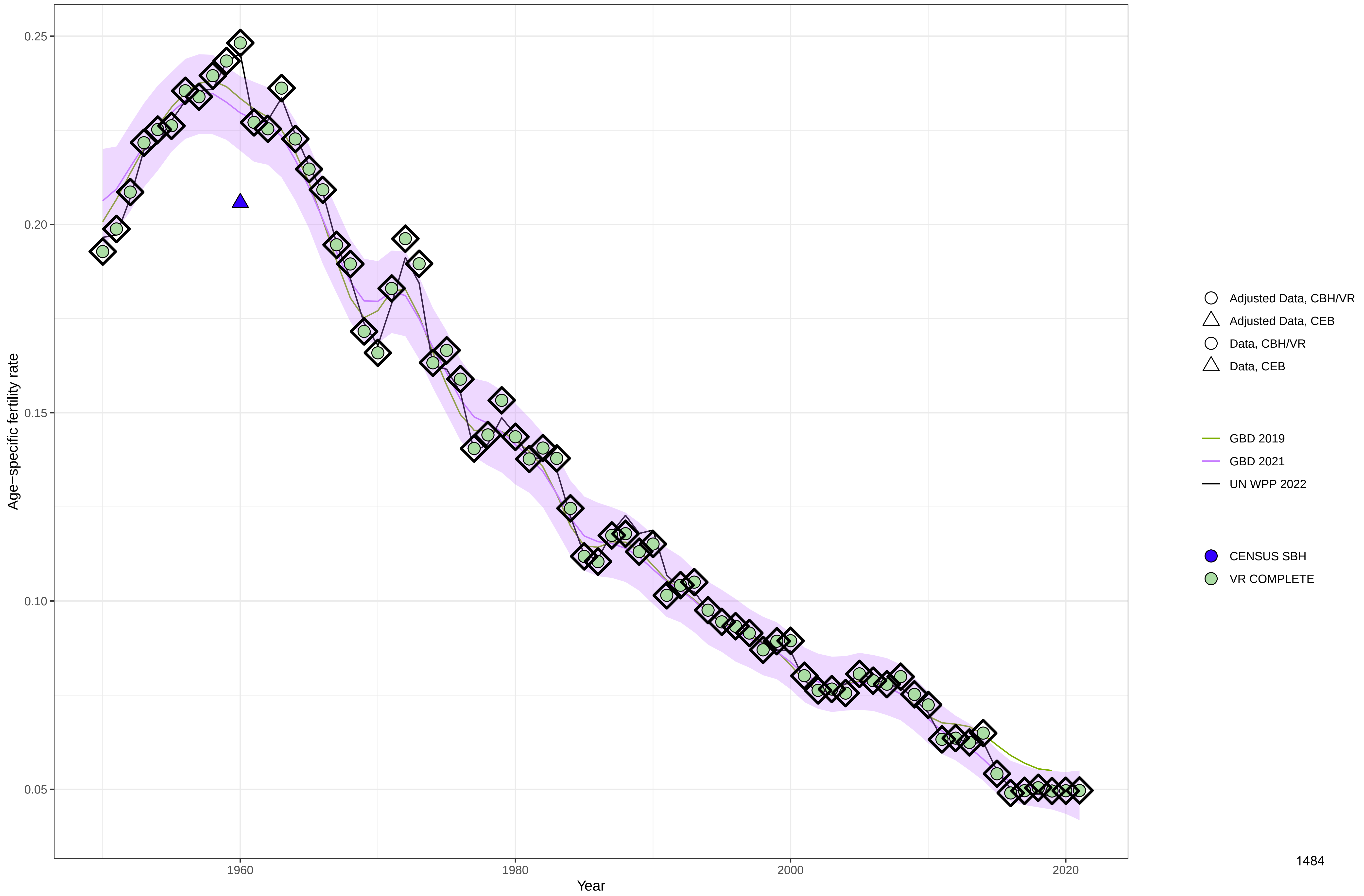

Age−specific fertility fitted trend lines, 1950−2021: Iceland, Ages: 25−29
Data points enclosed with a diamond are reference sources

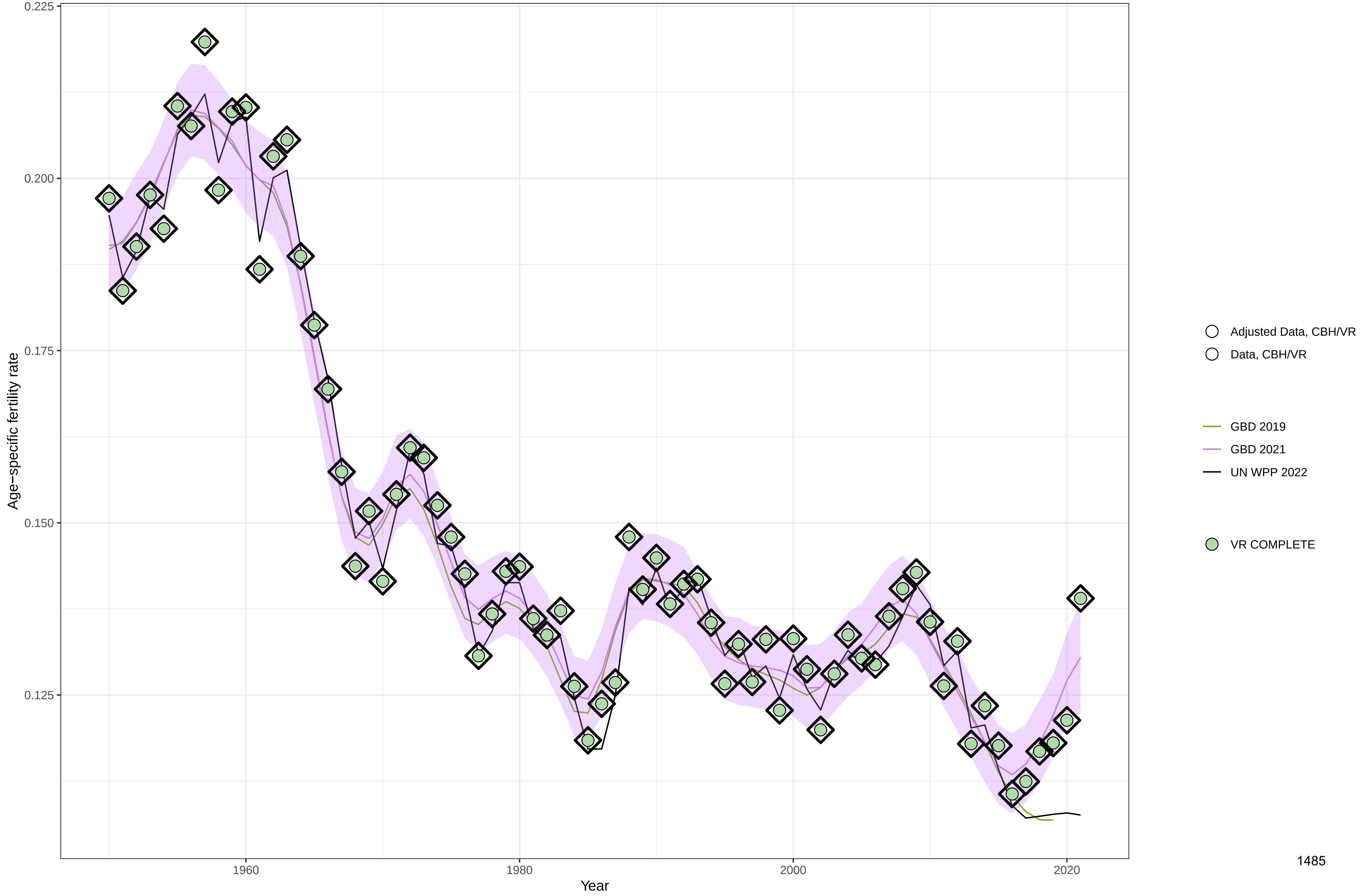

Age−specific fertility fitted trend lines, 1950−2021: Iceland, Ages: 30−34
Data points enclosed with a diamond are reference sources

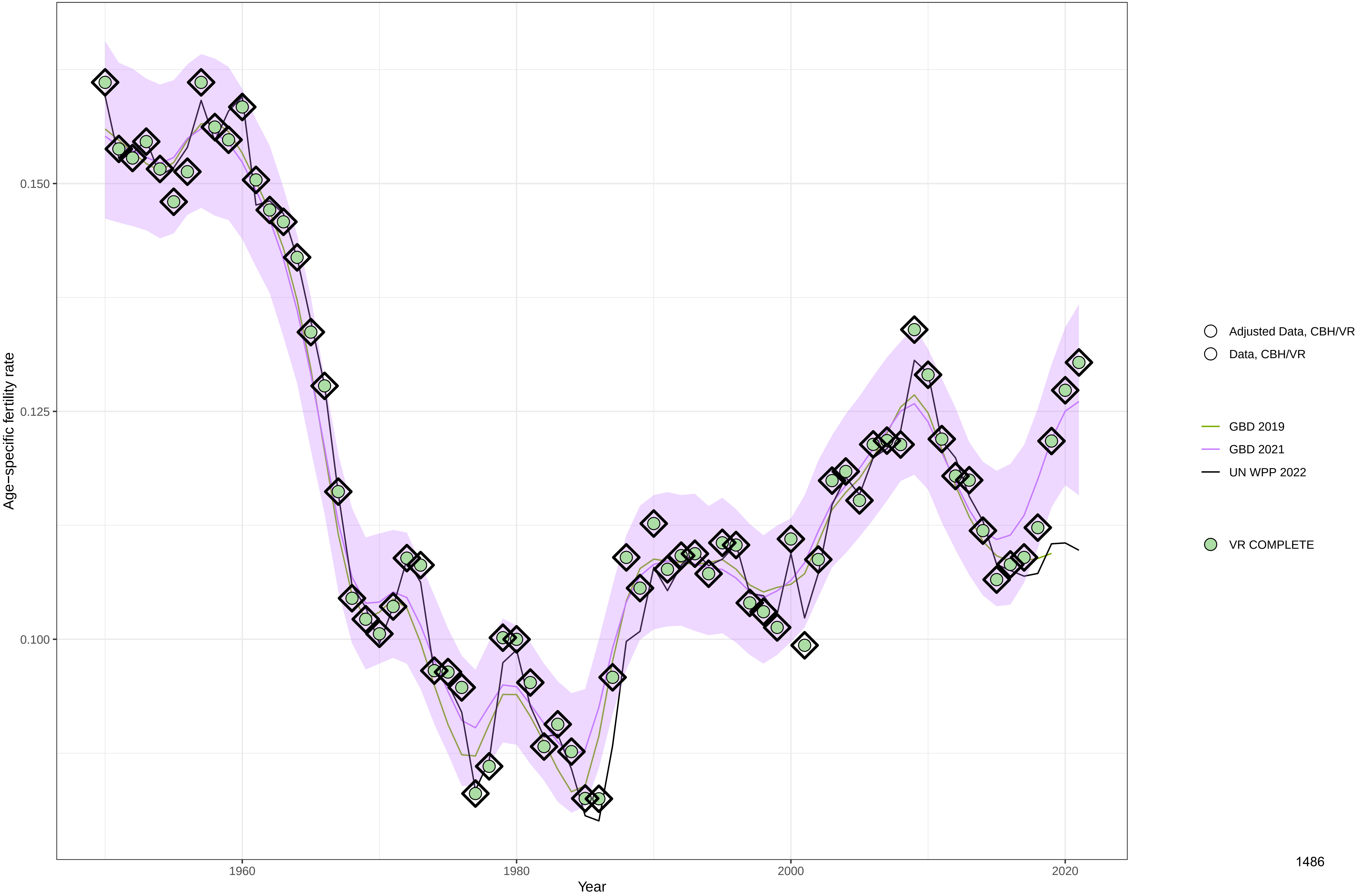

Age−specific fertility fitted trend lines, 1950−2021: Iceland, Ages: 35−39
Data points enclosed with a diamond are reference sources

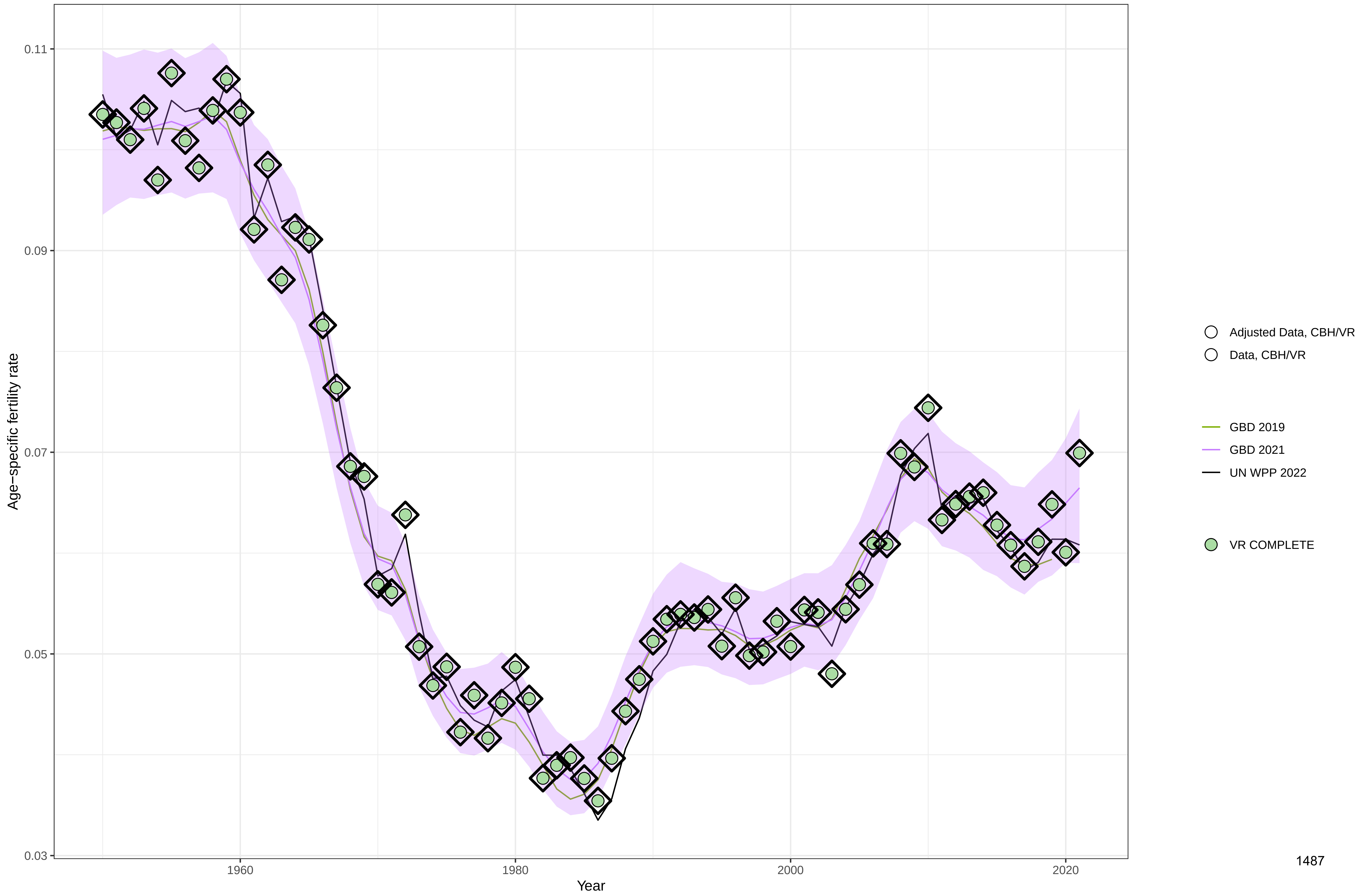

Age−specific fertility fitted trend lines, 1950−2021: Iceland, Ages: 40−44
Data points enclosed with a diamond are reference sources

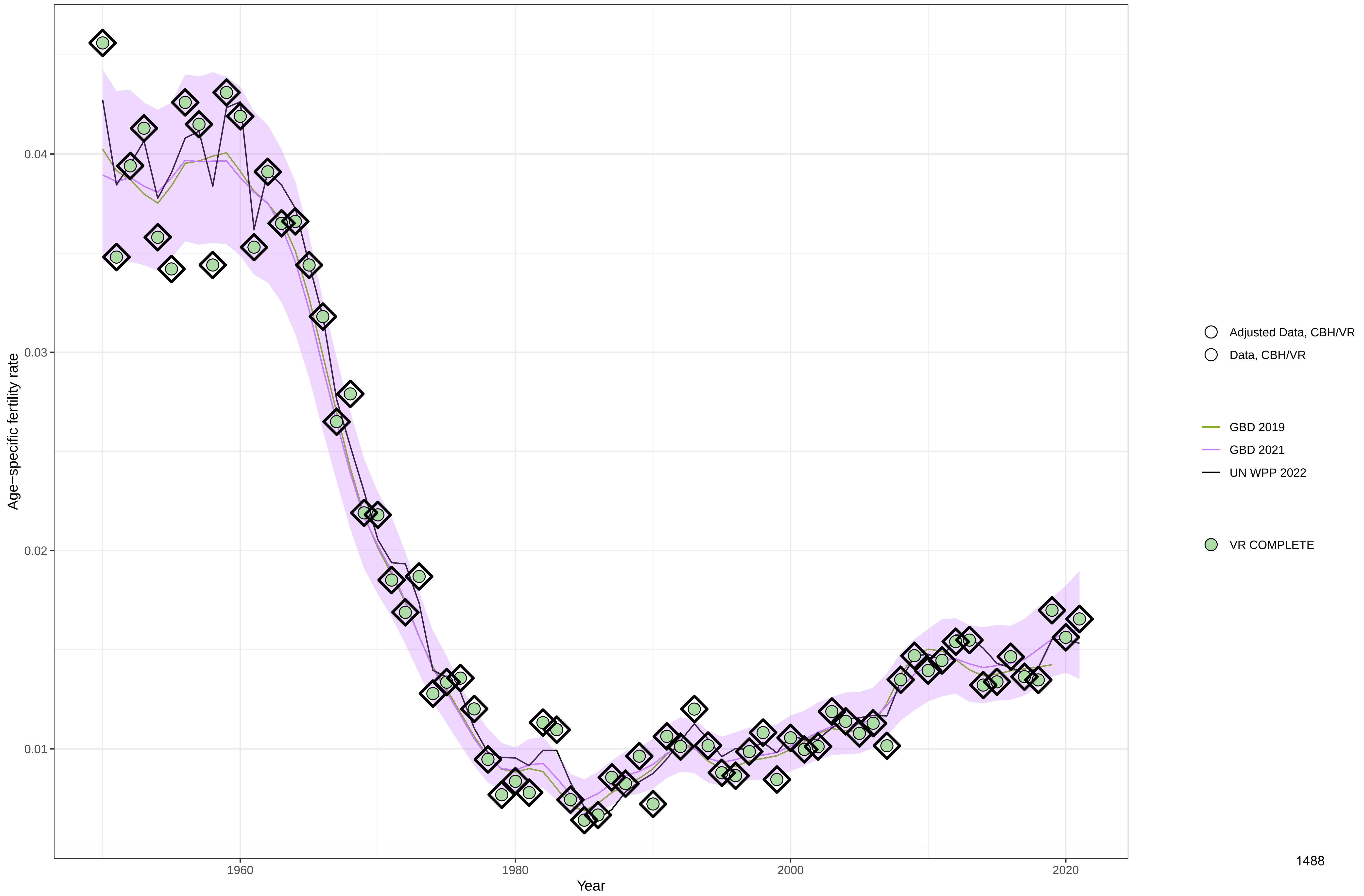

Age−specific fertility fitted trend lines, 1950−2021: Iceland, Ages: 45−49
Data points enclosed with a diamond are reference sources

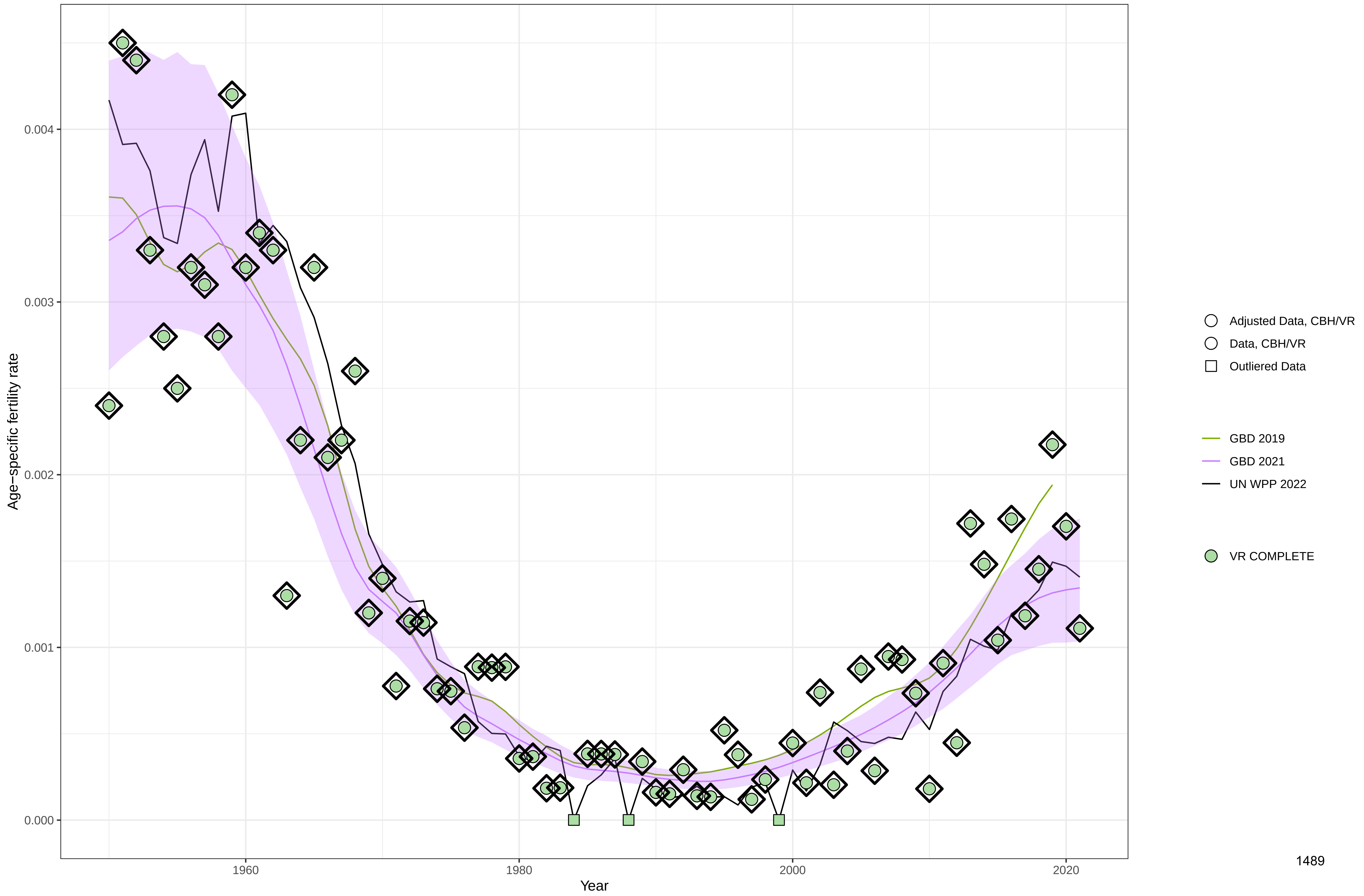

Age−specific fertility fitted trend lines, 1950−2021: Iceland, Ages: 50−54
Data points enclosed with a diamond are reference sources

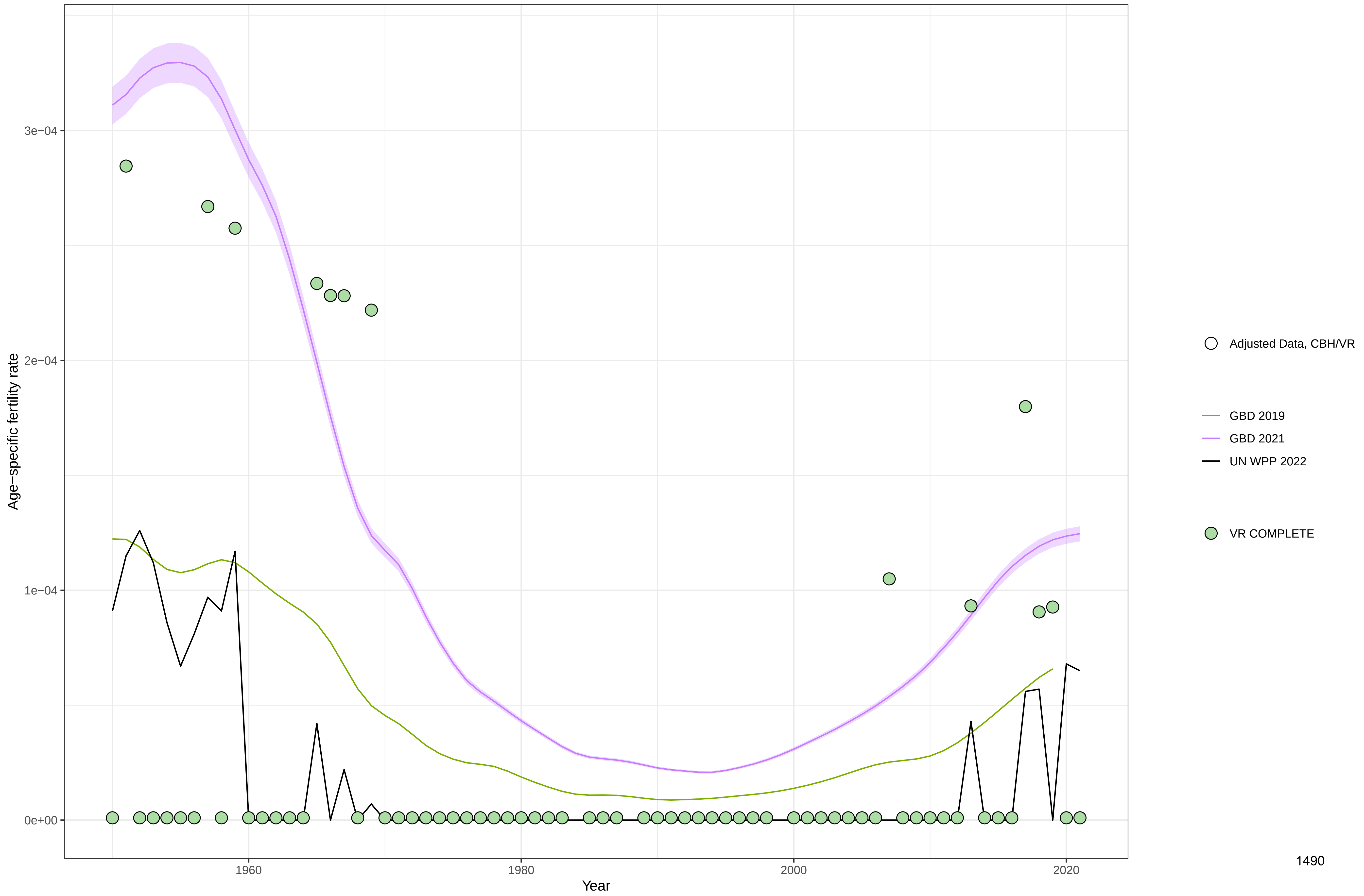

Total fertility rate fitted trend lines, 1950−2021: Iceland TFR
Data points enclosed with a diamond are reference sources

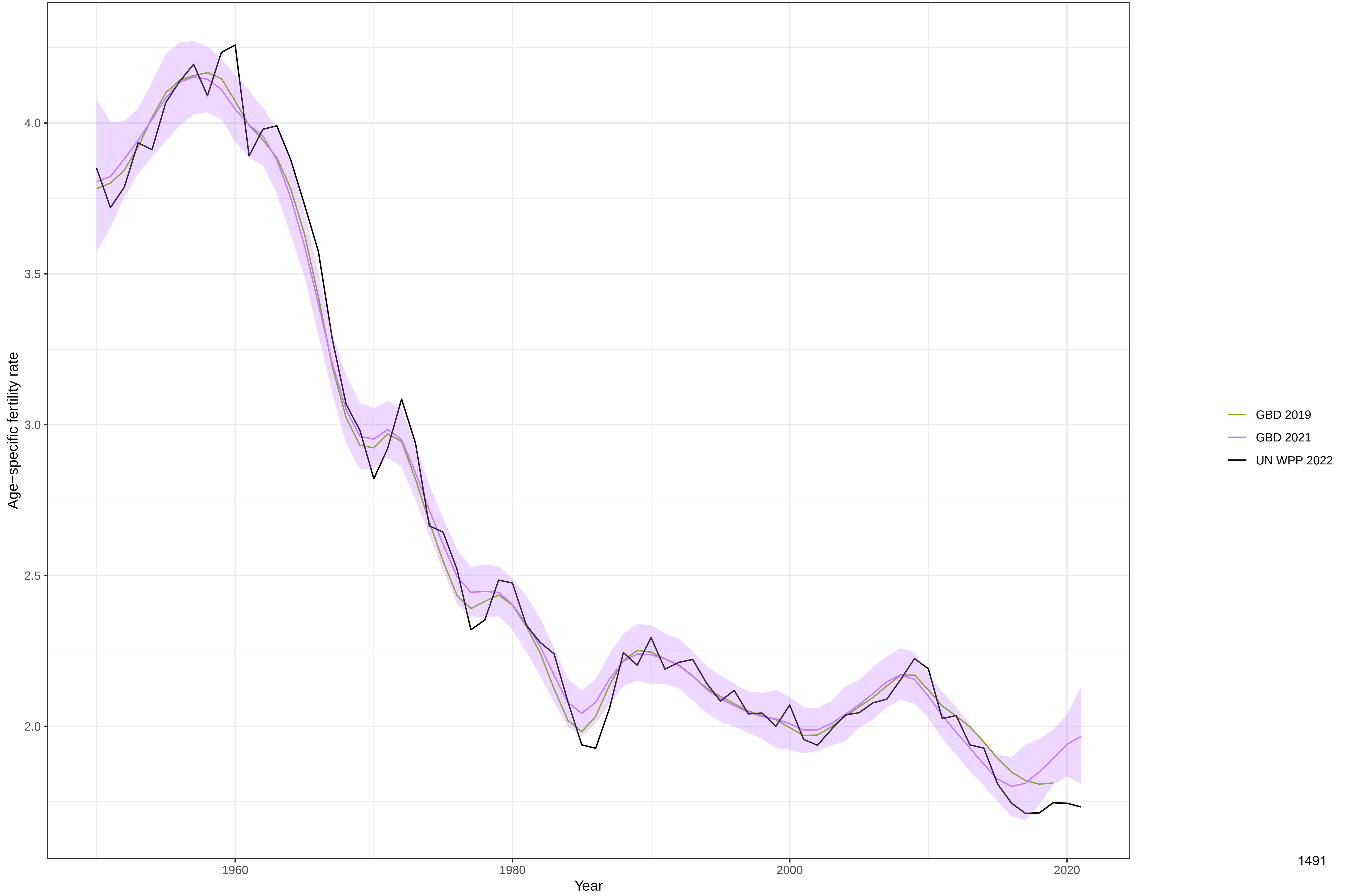

Age−specific fertility fitted trend lines, 1950−2021: Ireland, Ages: 10−14
Data points enclosed with a diamond are reference sources

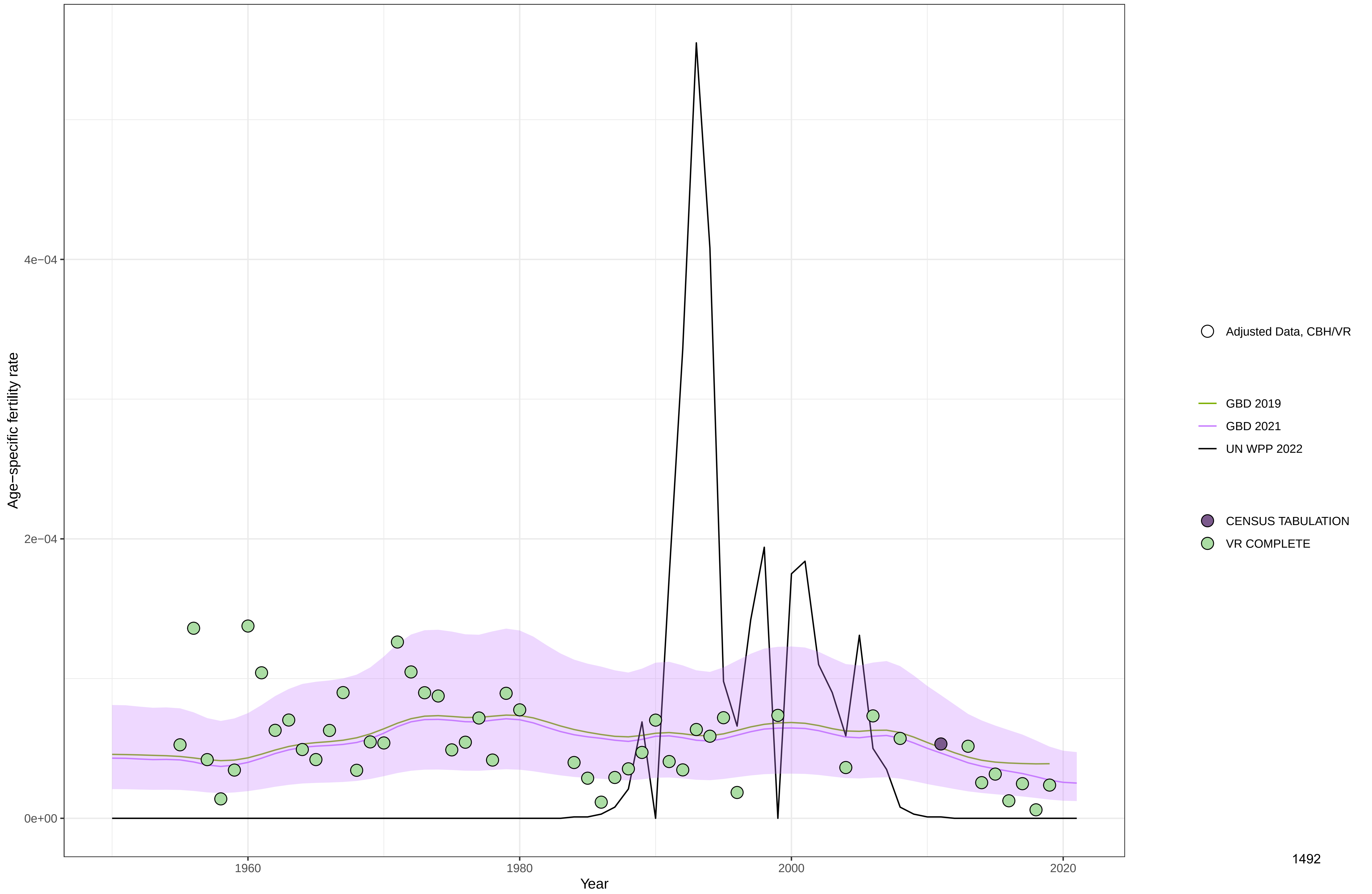

Age−specific fertility fitted trend lines, 1950−2021: Ireland, Ages: 15−19
Data points enclosed with a diamond are reference sources

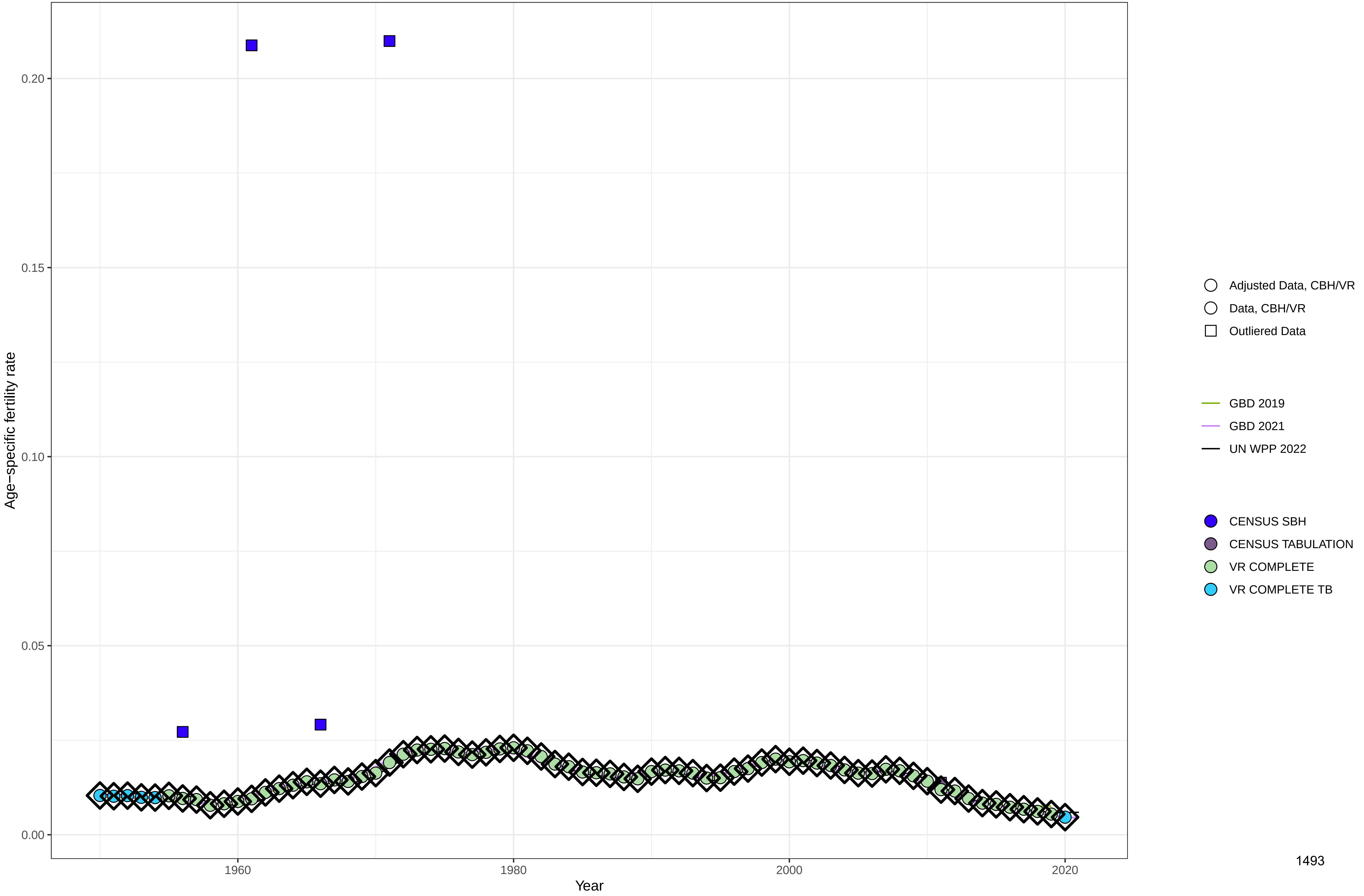

Age−specific fertility fitted trend lines, 1950−2021: Ireland, Ages: 20−24
Data points enclosed with a diamond are reference sources

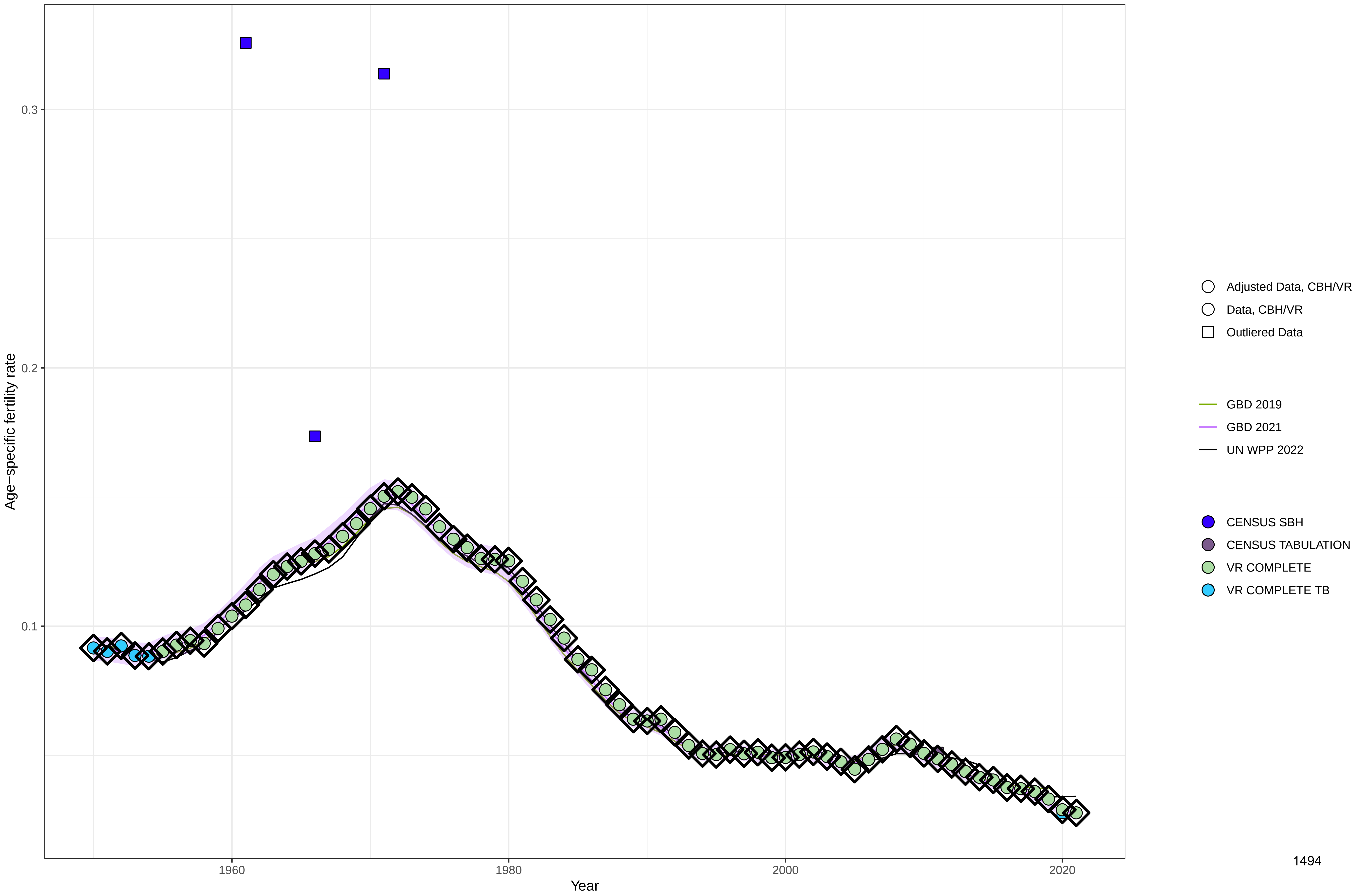

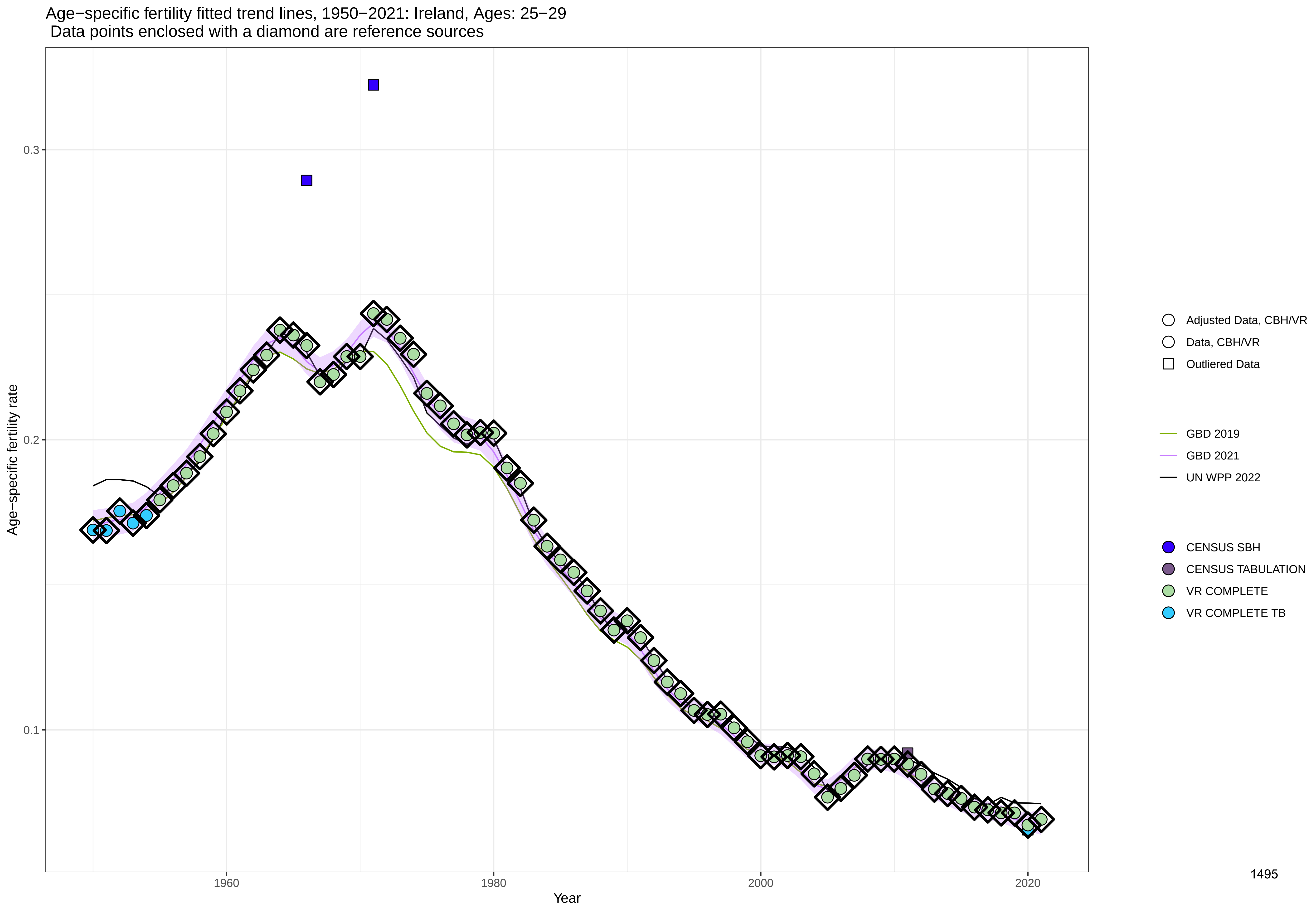

Age−specific fertility fitted trend lines, 1950−2021: Ireland, Ages: 25−29
Data points enclosed with a diamond are reference sources
Age−specific fertility rate
Year
0.1
0.2
0.3
1960
1980
2000
2020
Adjusted Data, CBH/VR
Data, CBH/VR
Outliered Data
GBD 2019
GBD 2021
UN WPP 2022
CENSUS SBH
CENSUS TABULATION
VR COMPLETE
VR COMPLETE TB

Age−specific fertility fitted trend lines, 1950−2021: Ireland, Ages: 30−34
Data points enclosed with a diamond are reference sources

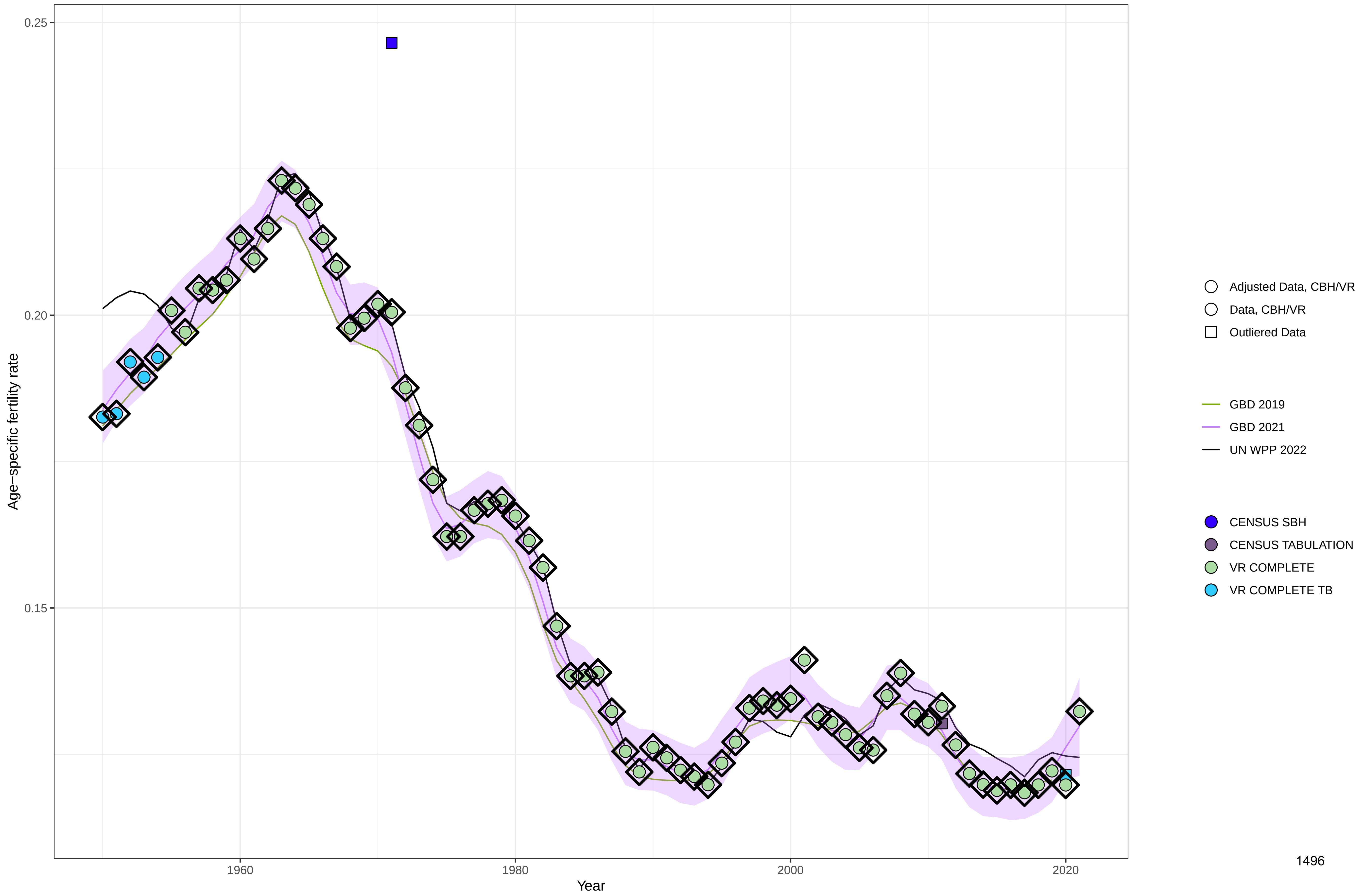

Age−specific fertility fitted trend lines, 1950−2021: Ireland, Ages: 35−39
Data points enclosed with a diamond are reference sources

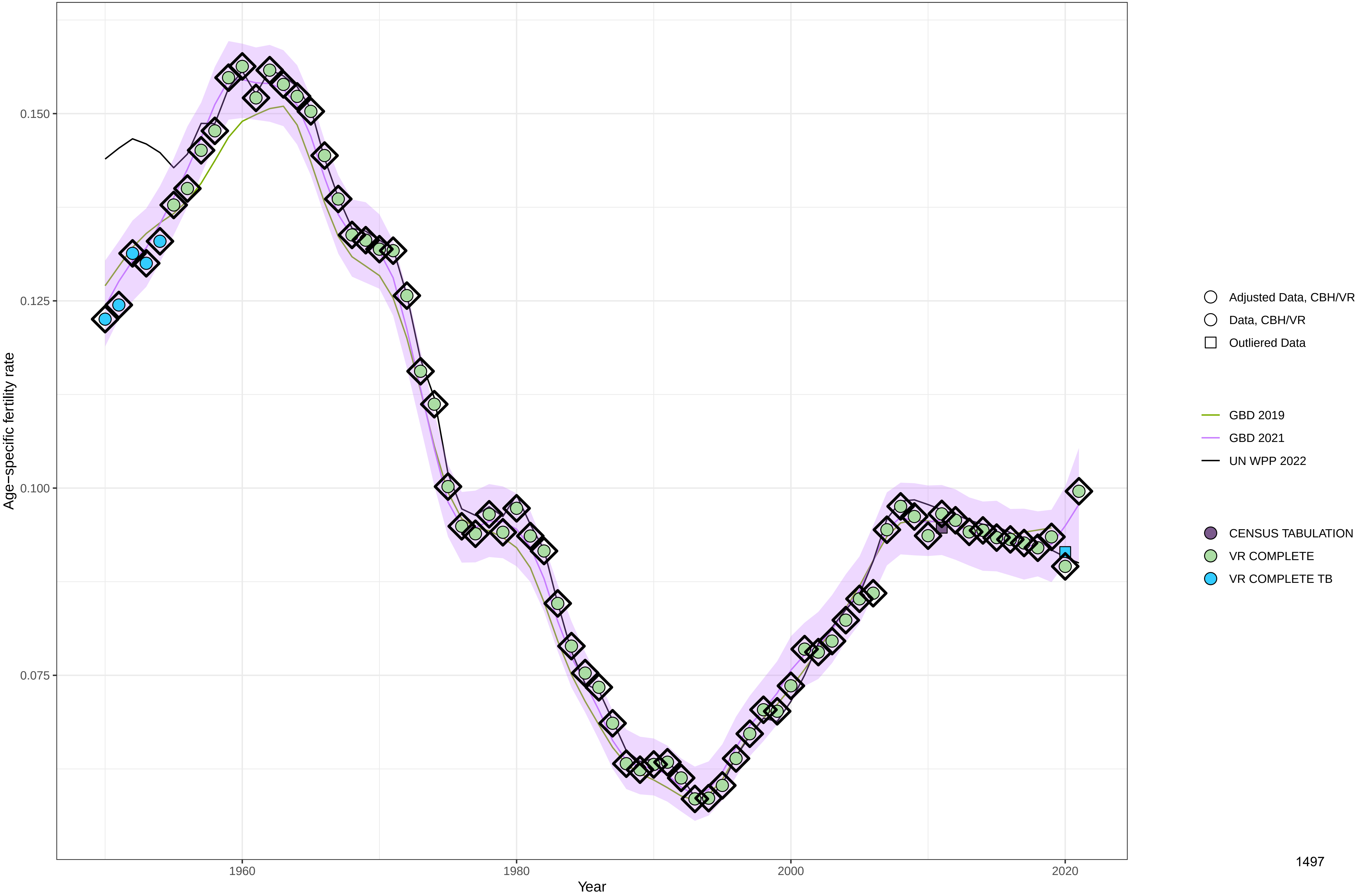

Age−specific fertility fitted trend lines, 1950−2021: Ireland, Ages: 40−44
Data points enclosed with a diamond are reference sources

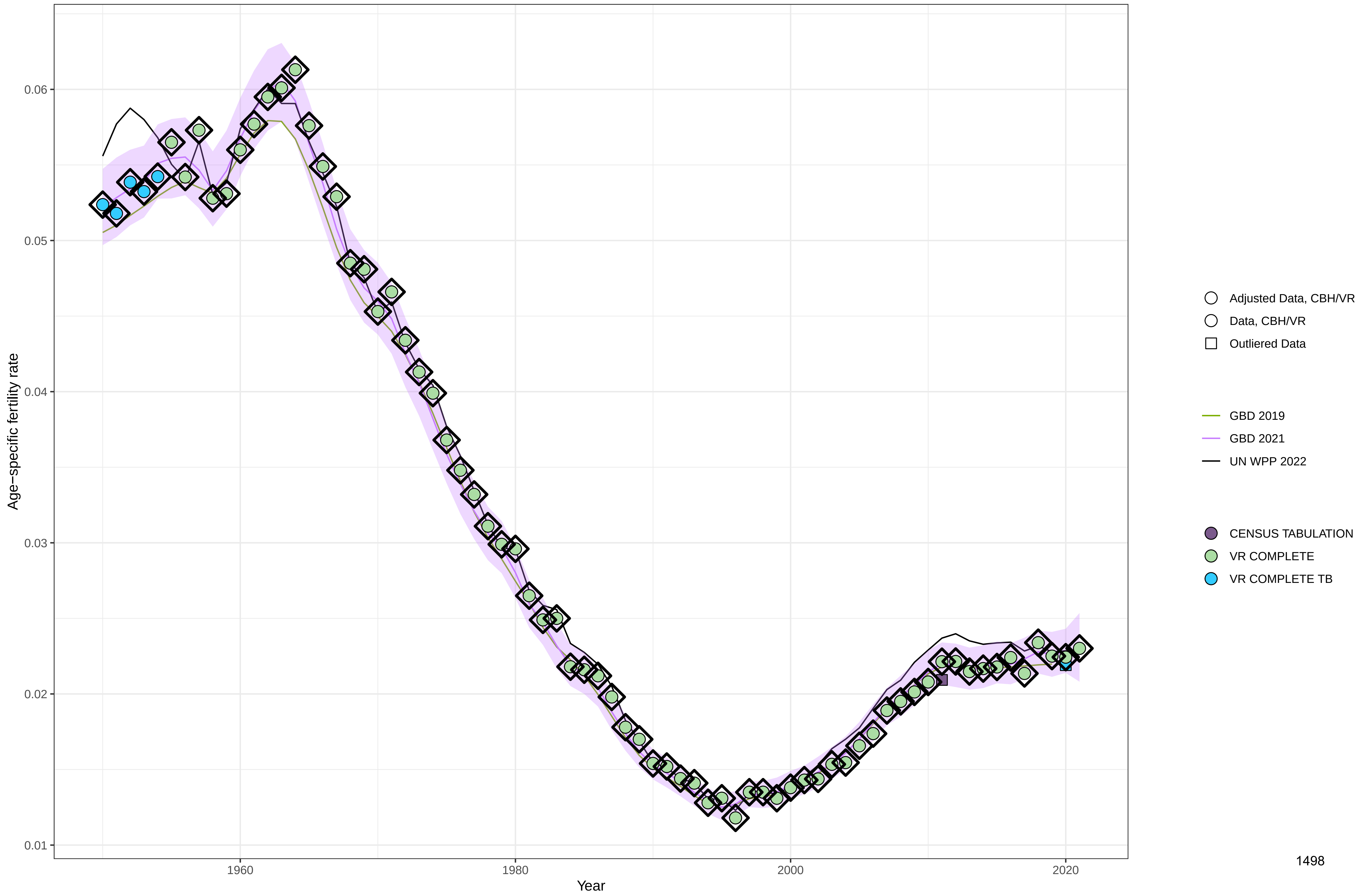

Age−specific fertility fitted trend lines, 1950−2021: Ireland, Ages: 45−49
Data points enclosed with a diamond are reference sources

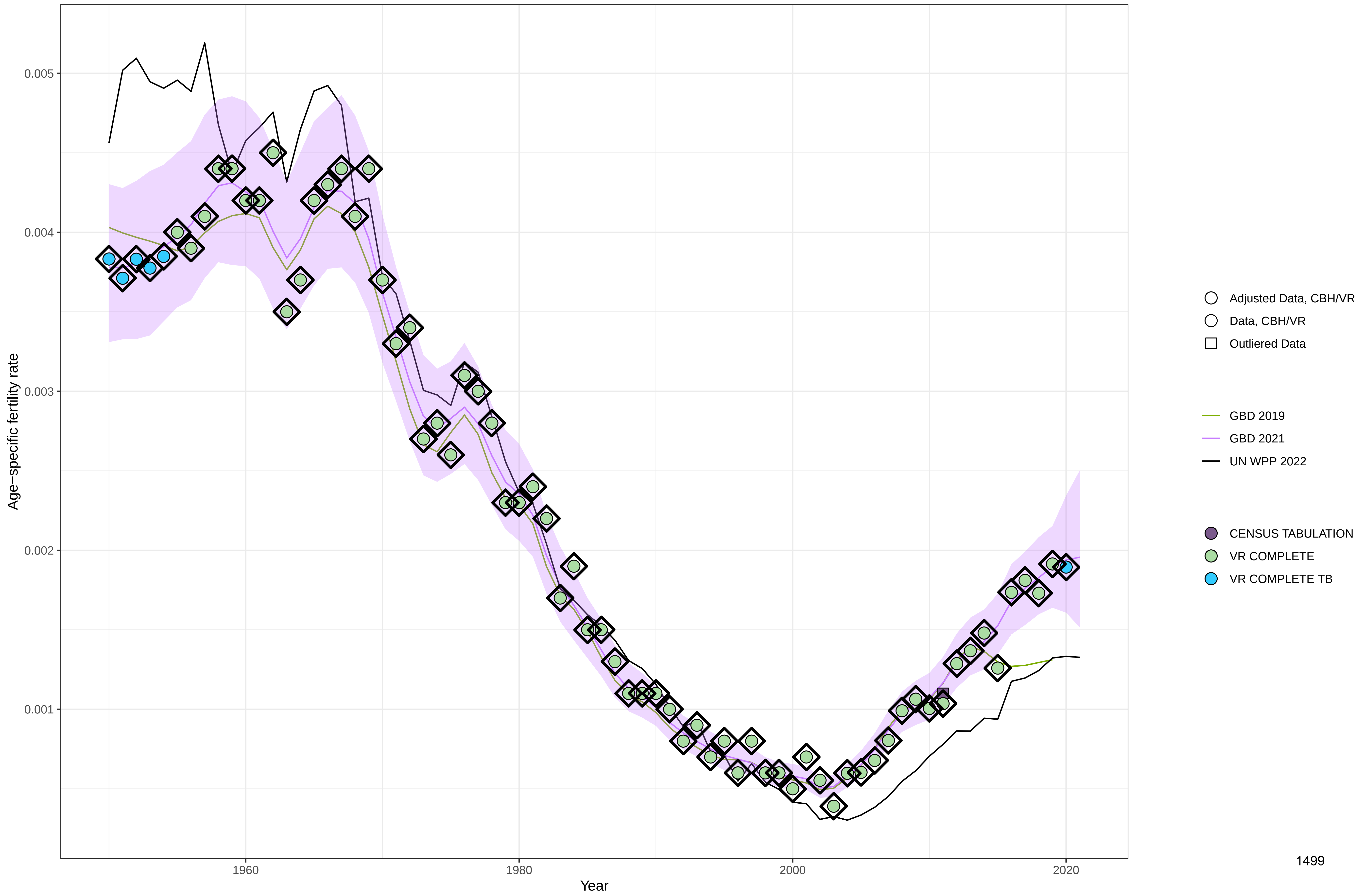

Age−specific fertility fitted trend lines, 1950−2021: Ireland, Ages: 50−54
Data points enclosed with a diamond are reference sources

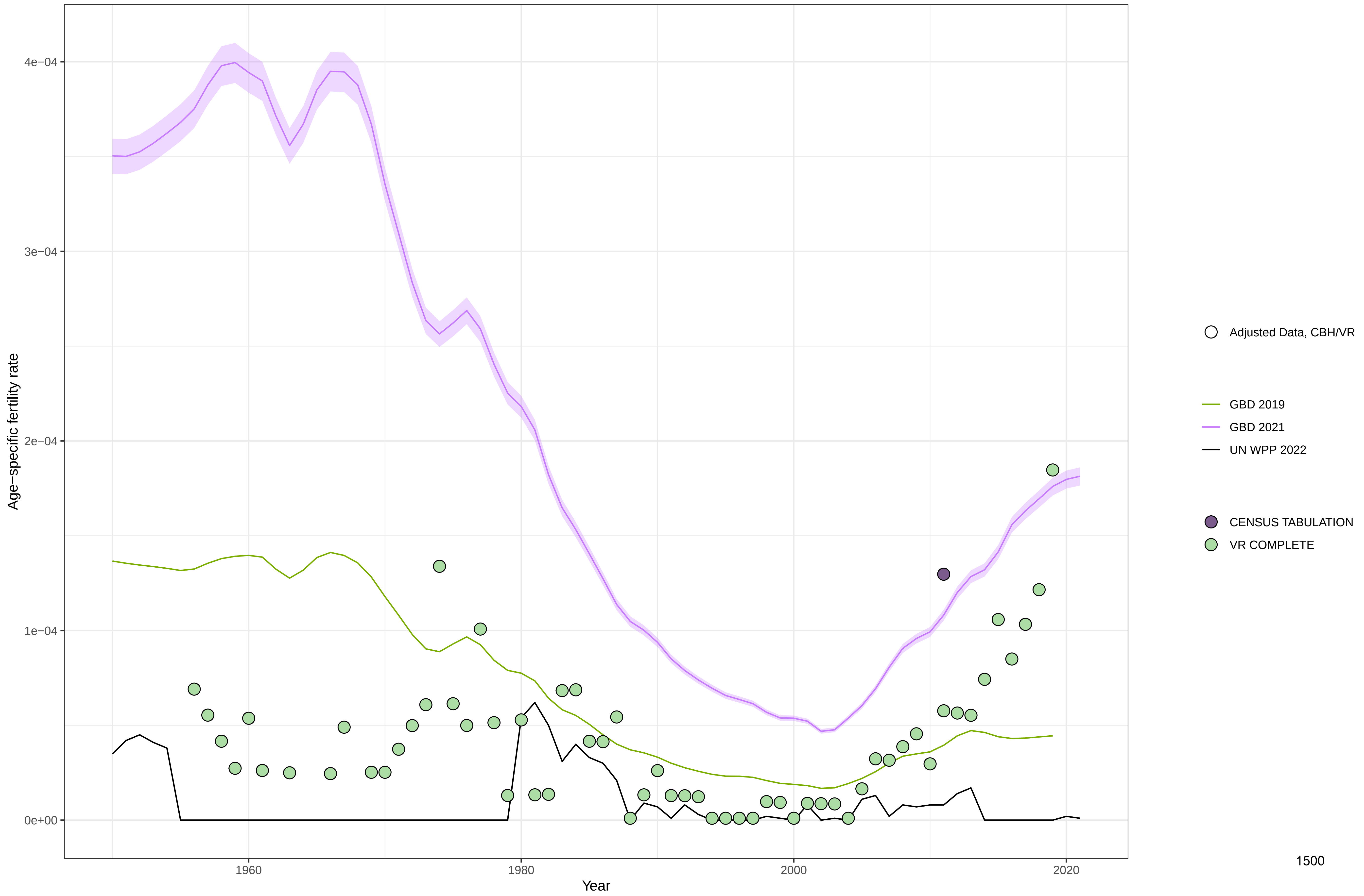

Total fertility rate fitted trend lines, 1950−2021: Ireland TFR
Data points enclosed with a diamond are reference sources

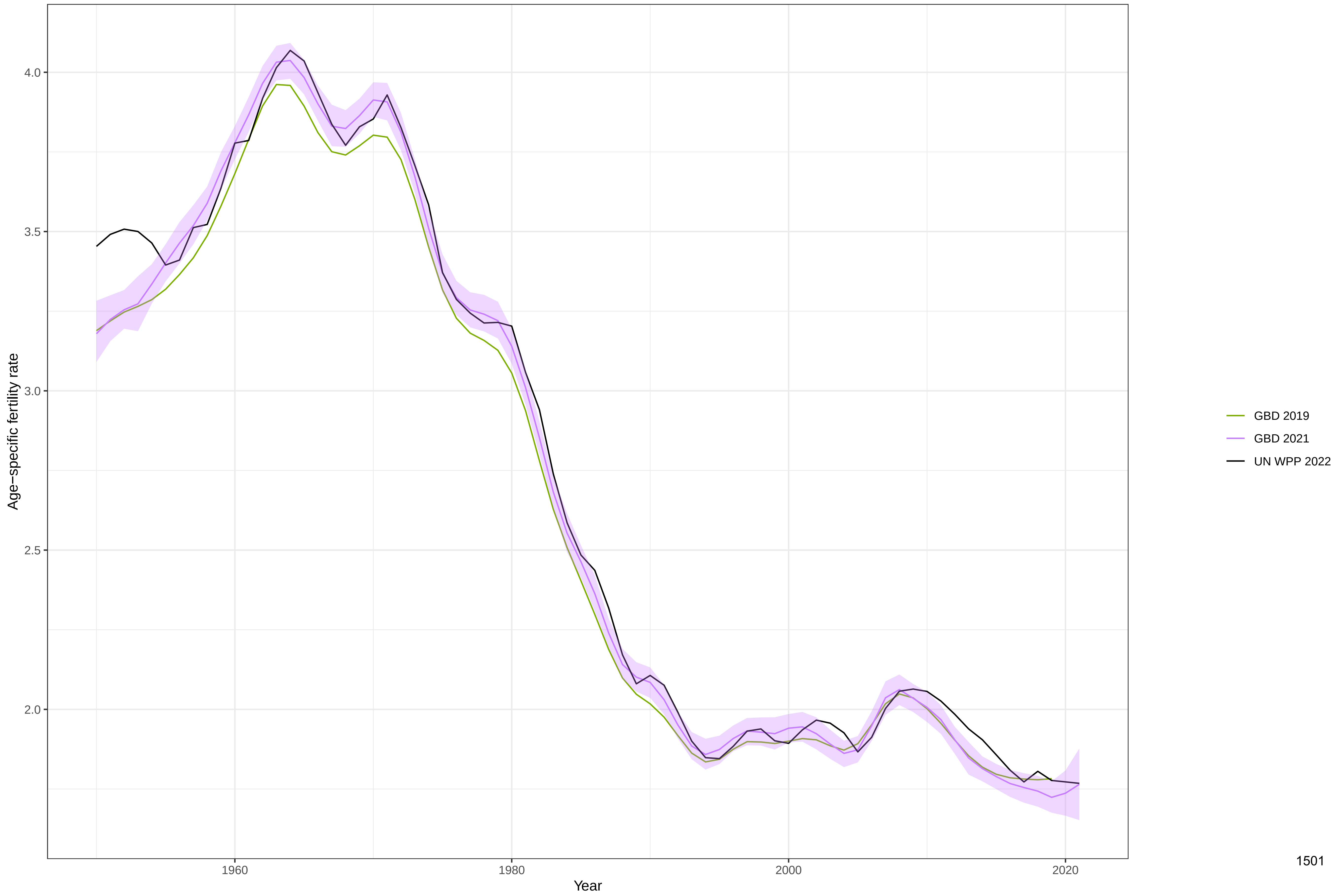

Age−specific fertility fitted trend lines, 1950−2021: Israel, Ages: 10−14
Data points enclosed with a diamond are reference sources

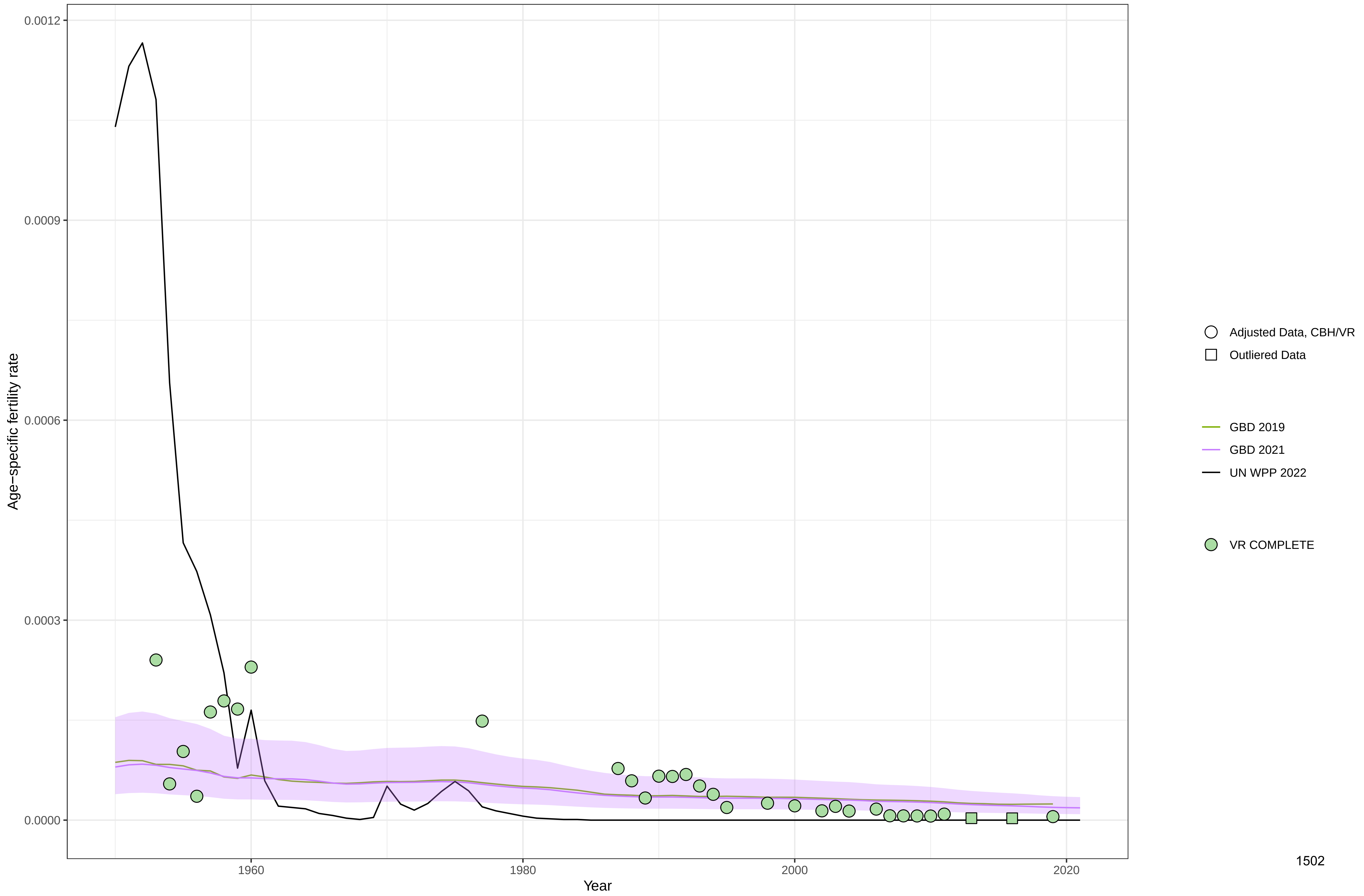

Age−specific fertility fitted trend lines, 1950−2021: Israel, Ages: 15−19
Data points enclosed with a diamond are reference sources

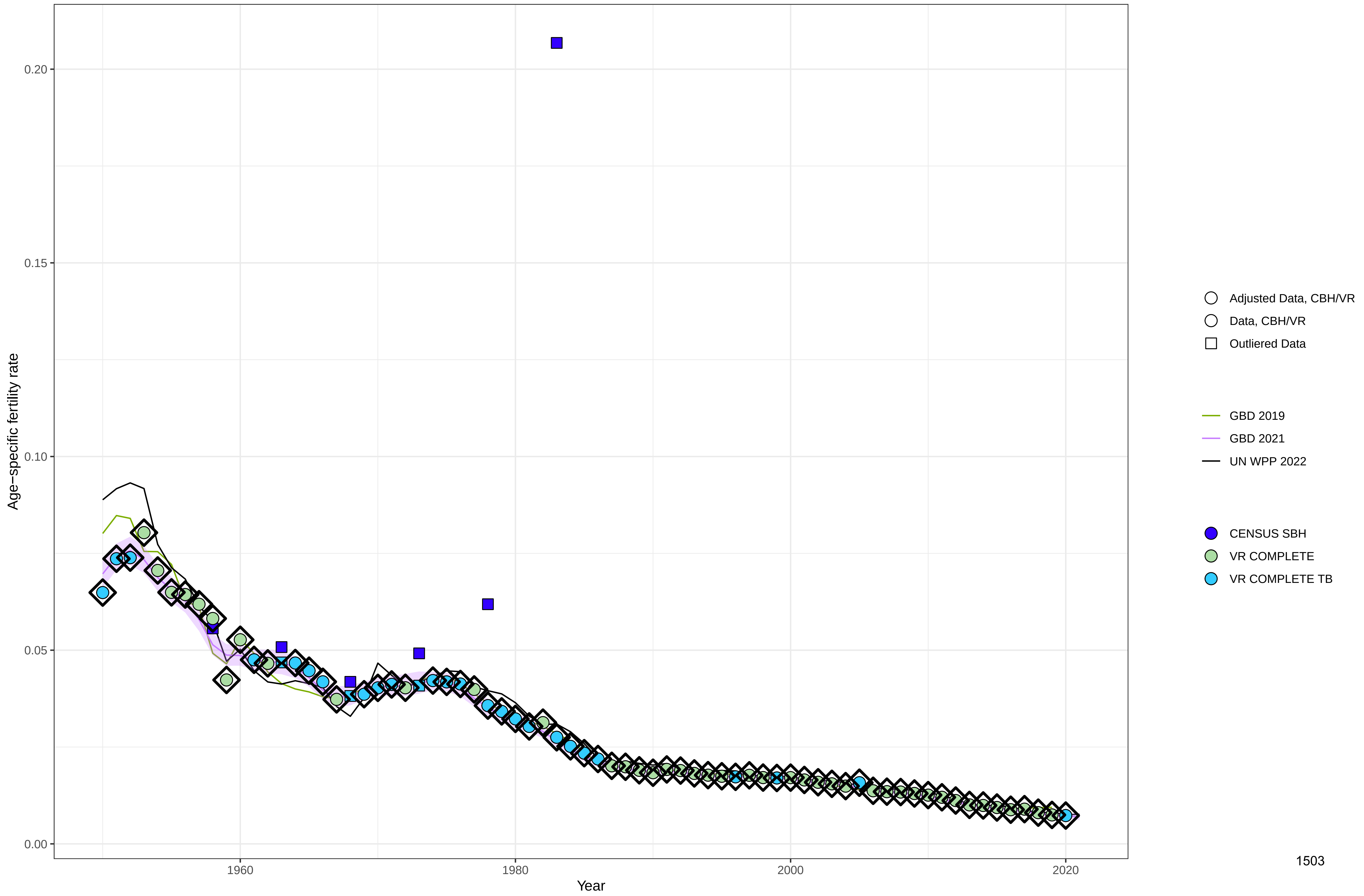

Age−specific fertility fitted trend lines, 1950−2021: Israel, Ages: 20−24
Data points enclosed with a diamond are reference sources

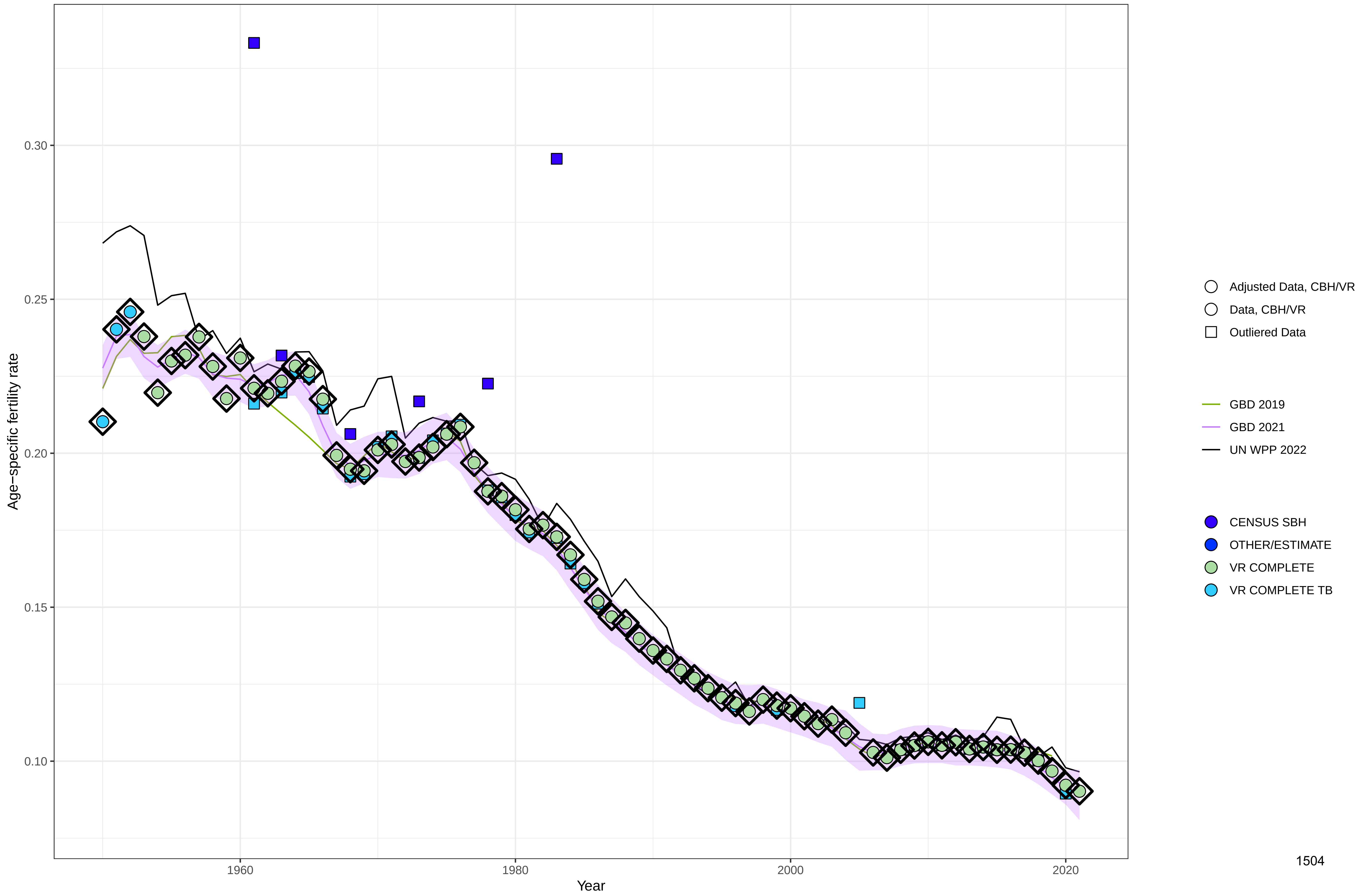

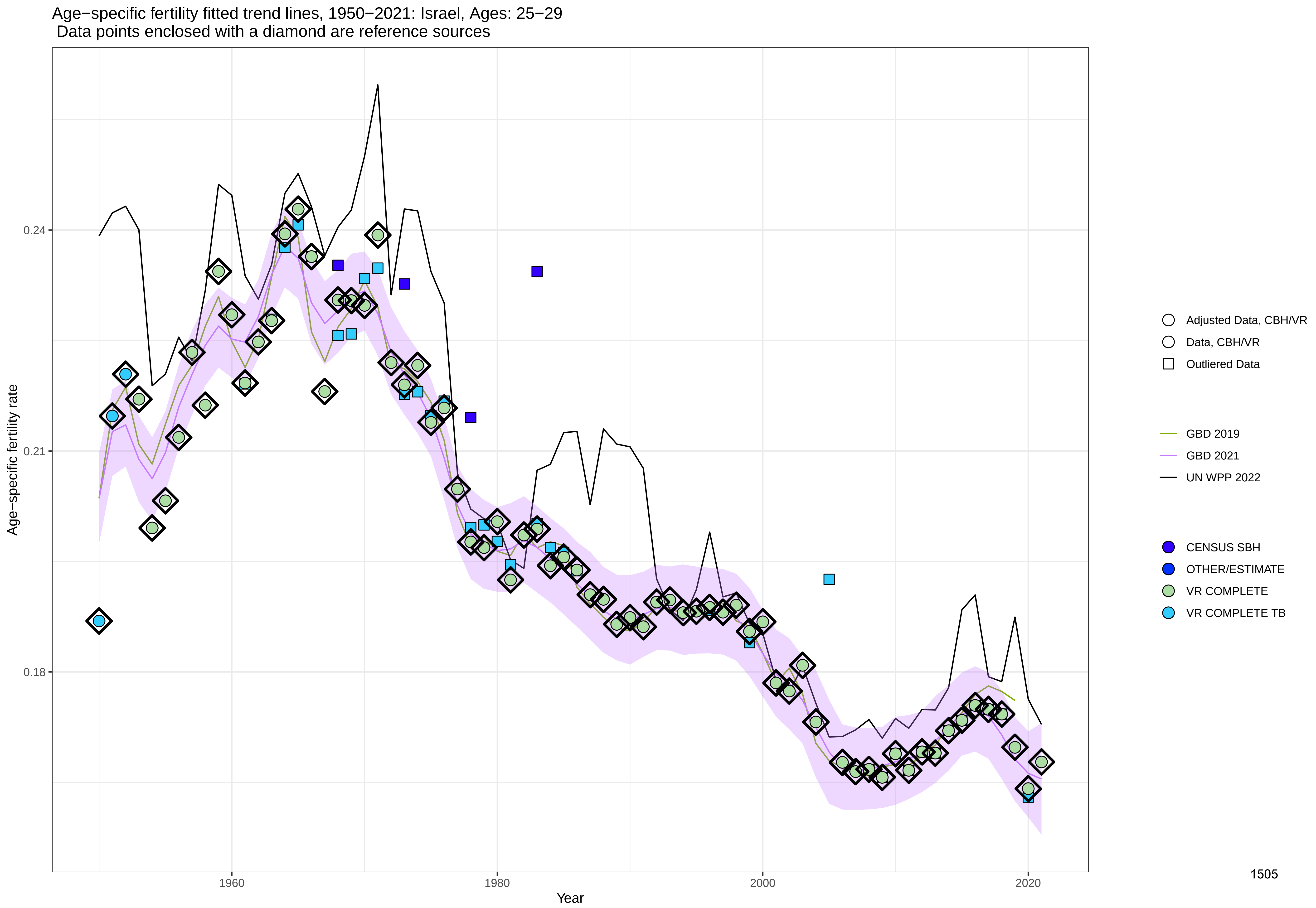
Age−specific fertility fitted trend lines, 1950−2021: Israel, Ages: 25−29
Data points enclosed with a diamond are reference sources
Age−specific fertility rate
0.24
0.21
0.18
1960
1980
2000
2020
Year
Adjusted Data, CBH/VR
Data, CBH/VR
Outliered Data
GBD 2019
GBD 2021
UN WPP 2022
CENSUS SBH
OTHER/ESTIMATE
VR COMPLETE
VR COMPLETE TB

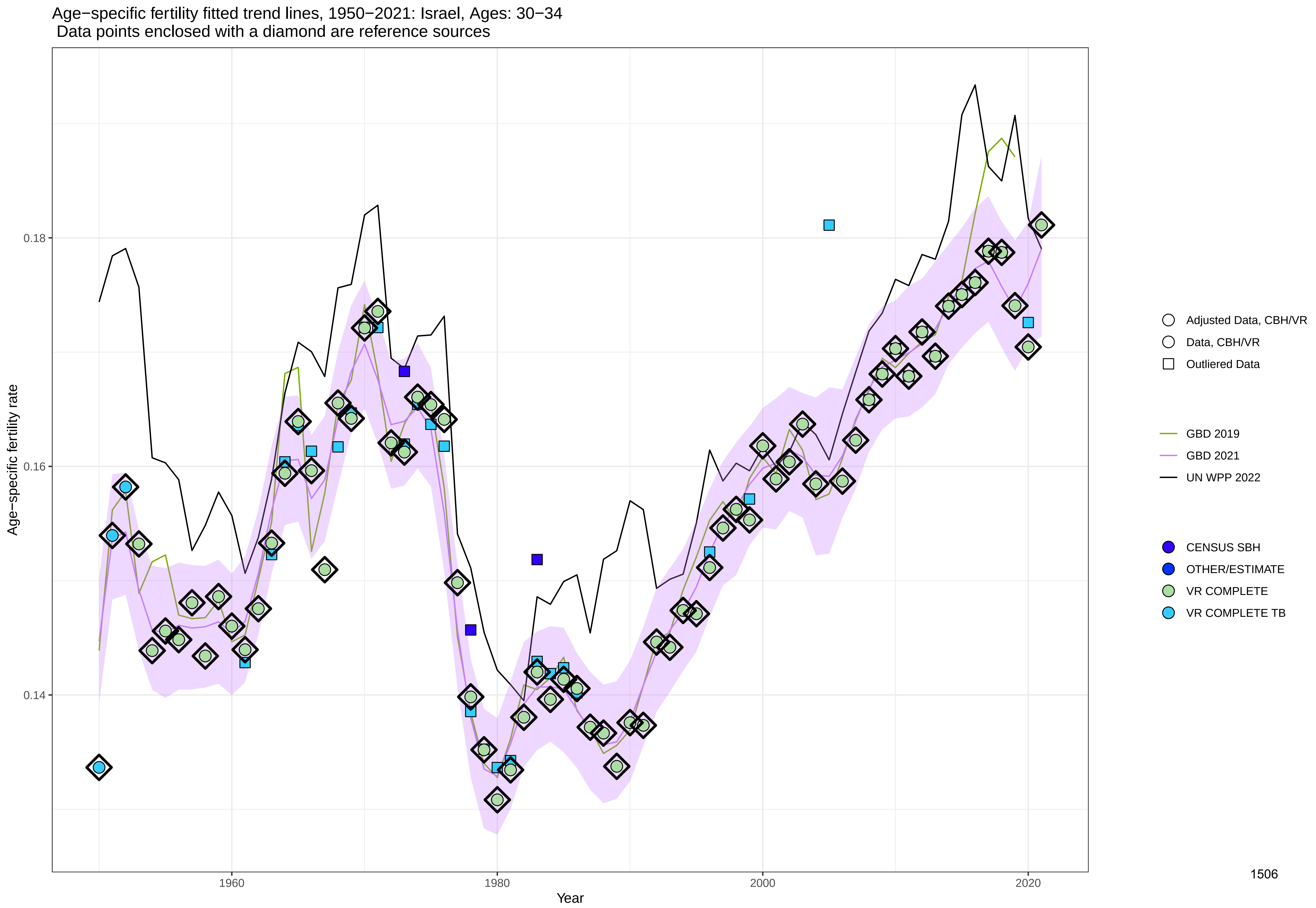

Age−specific fertility fitted trend lines, 1950−2021: Israel, Ages: 30−34
Data points enclosed with a diamond are reference sources
Age−specific fertility rate
0.18
0.16
0.14
1960
1980
2000
2020
Year
Adjusted Data, CBH/VR
Data, CBH/VR
Outliered Data
GBD 2019
GBD 2021
UN WPP 2022
CENSUS SBH
OTHER/ESTIMATE
VR COMPLETE
VR COMPLETE TB

Age−specific fertility fitted trend lines, 1950−2021: Israel, Ages: 35−39
Data points enclosed with a diamond are reference sources

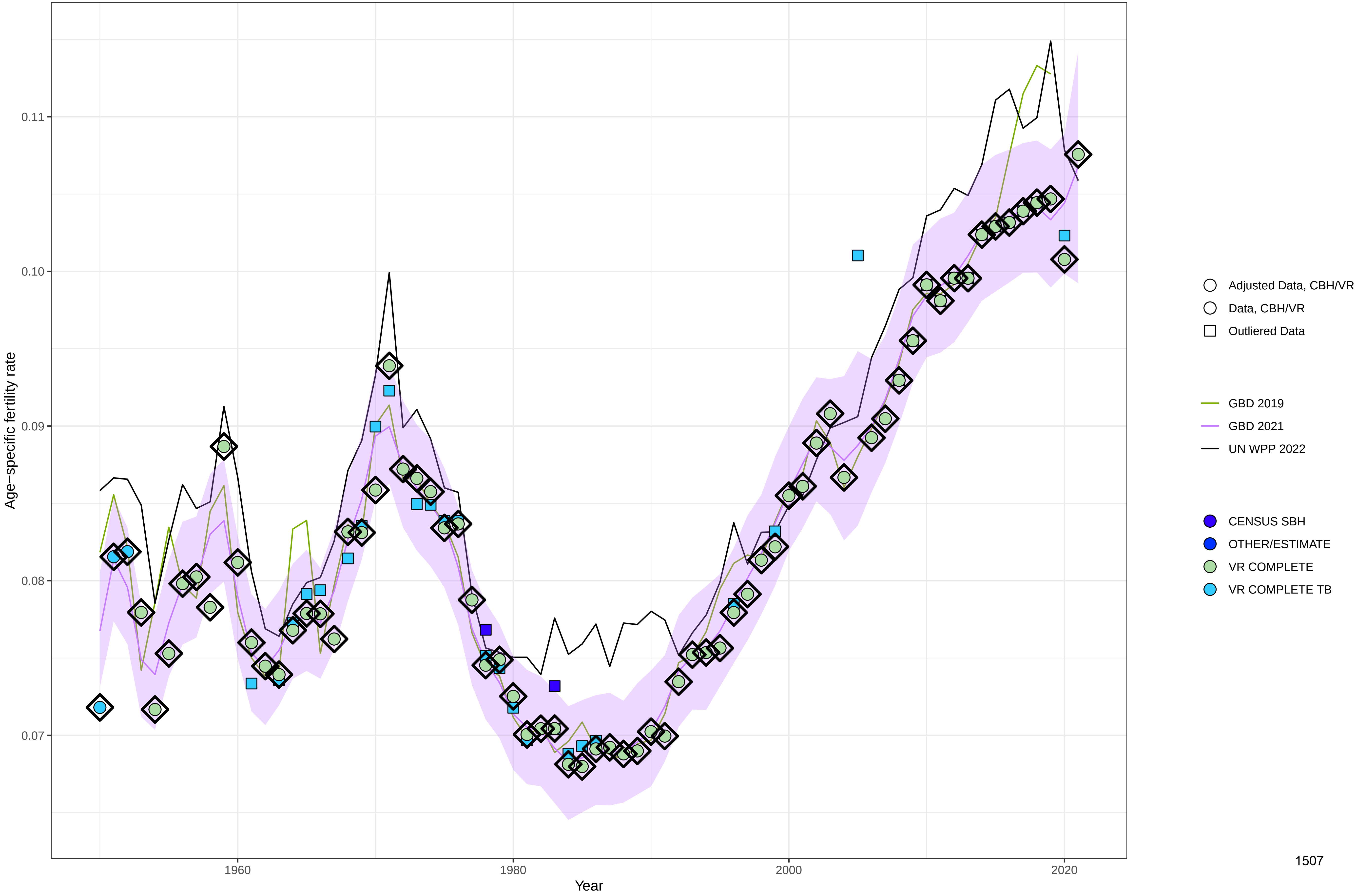

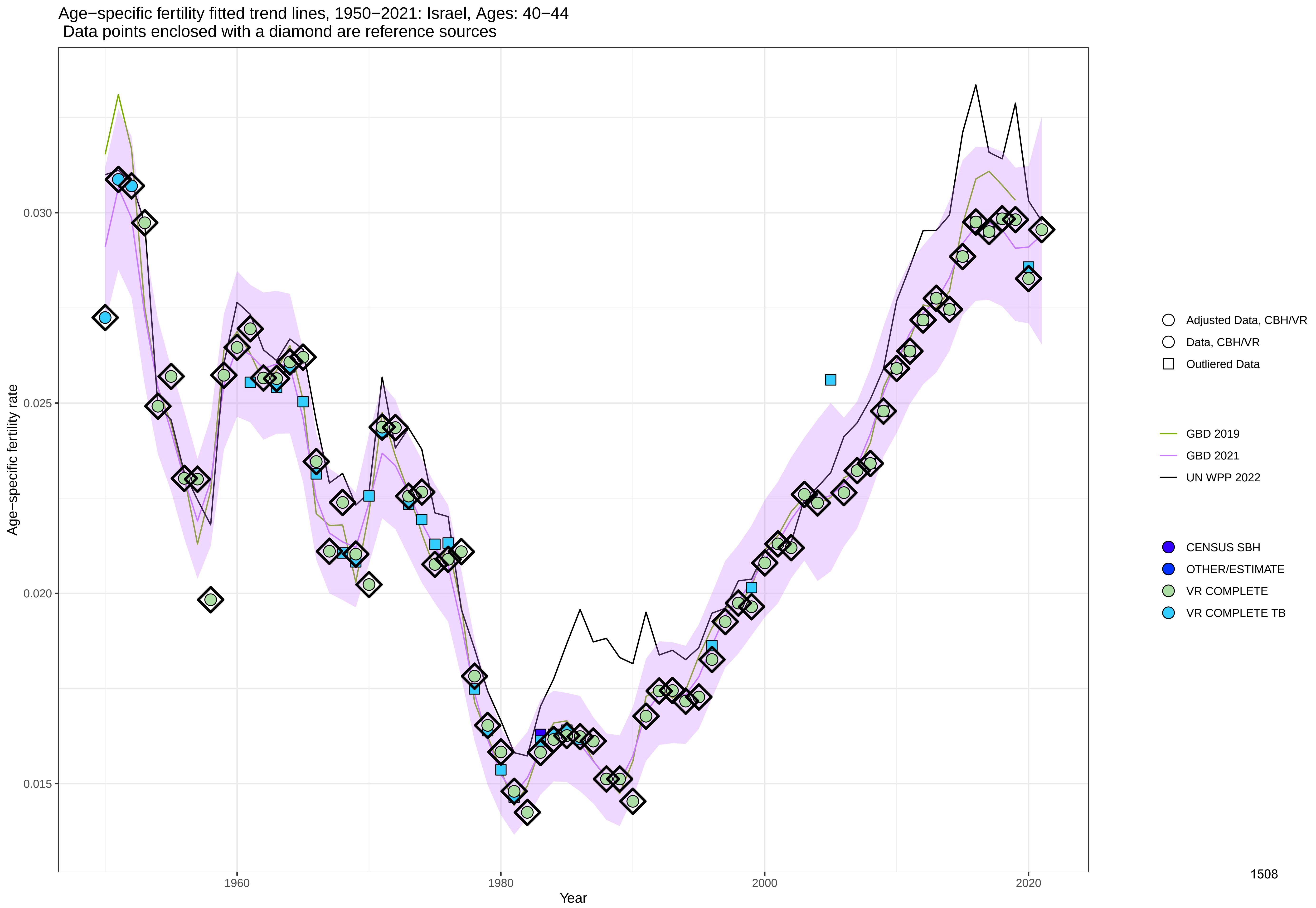

Age−specific fertility fitted trend lines, 1950−2021: Israel, Ages: 40−44
Data points enclosed with a diamond are reference sources
Age−specific fertility rate
0.030
0.025
0.020
0.015
1960
1980
2000
2020
Year
Adjusted Data, CBH/VR
Data, CBH/VR
Outliered Data
GBD 2019
GBD 2021
UN WPP 2022
CENSUS SBH
OTHER/ESTIMATE
VR COMPLETE
VR COMPLETE TB

Age−specific fertility fitted trend lines, 1950−2021: Israel, Ages: 45−49
Data points enclosed with a diamond are reference sources

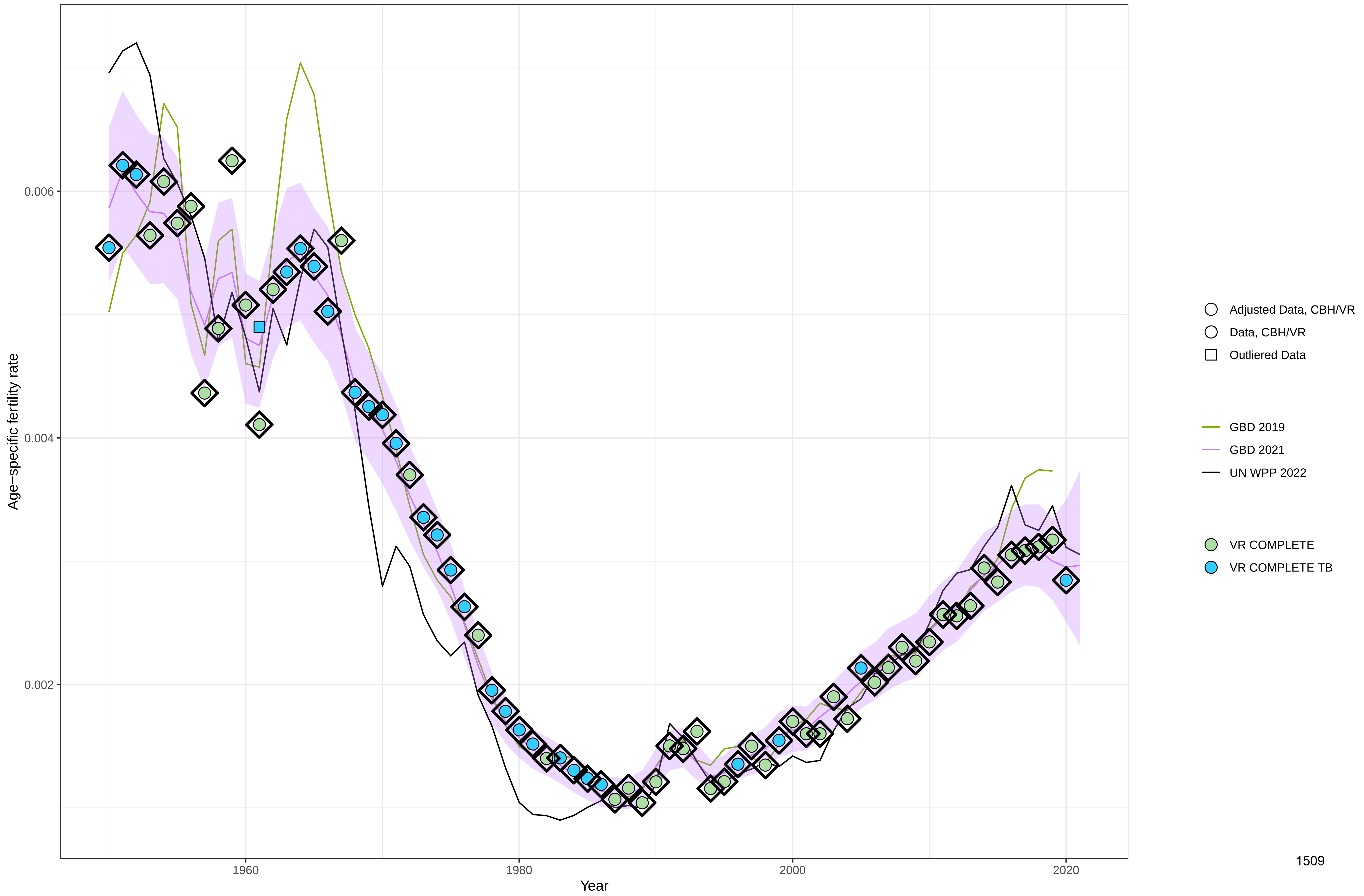

Age−specific fertility fitted trend lines, 1950−2021: Israel, Ages: 50−54
Data points enclosed with a diamond are reference sources

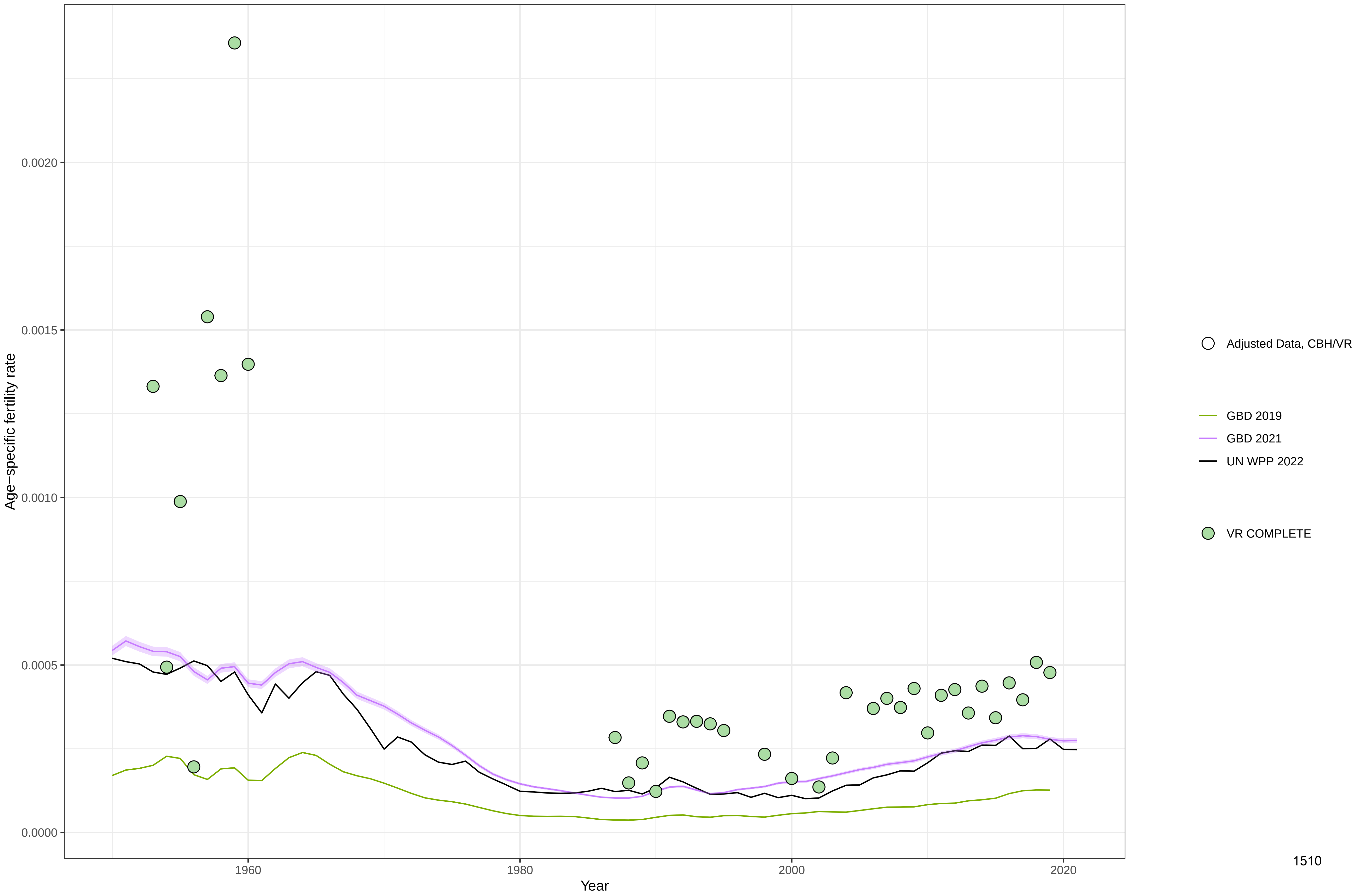

Total fertility rate fitted trend lines, 1950−2021: Israel TFR
Data points enclosed with a diamond are reference sources

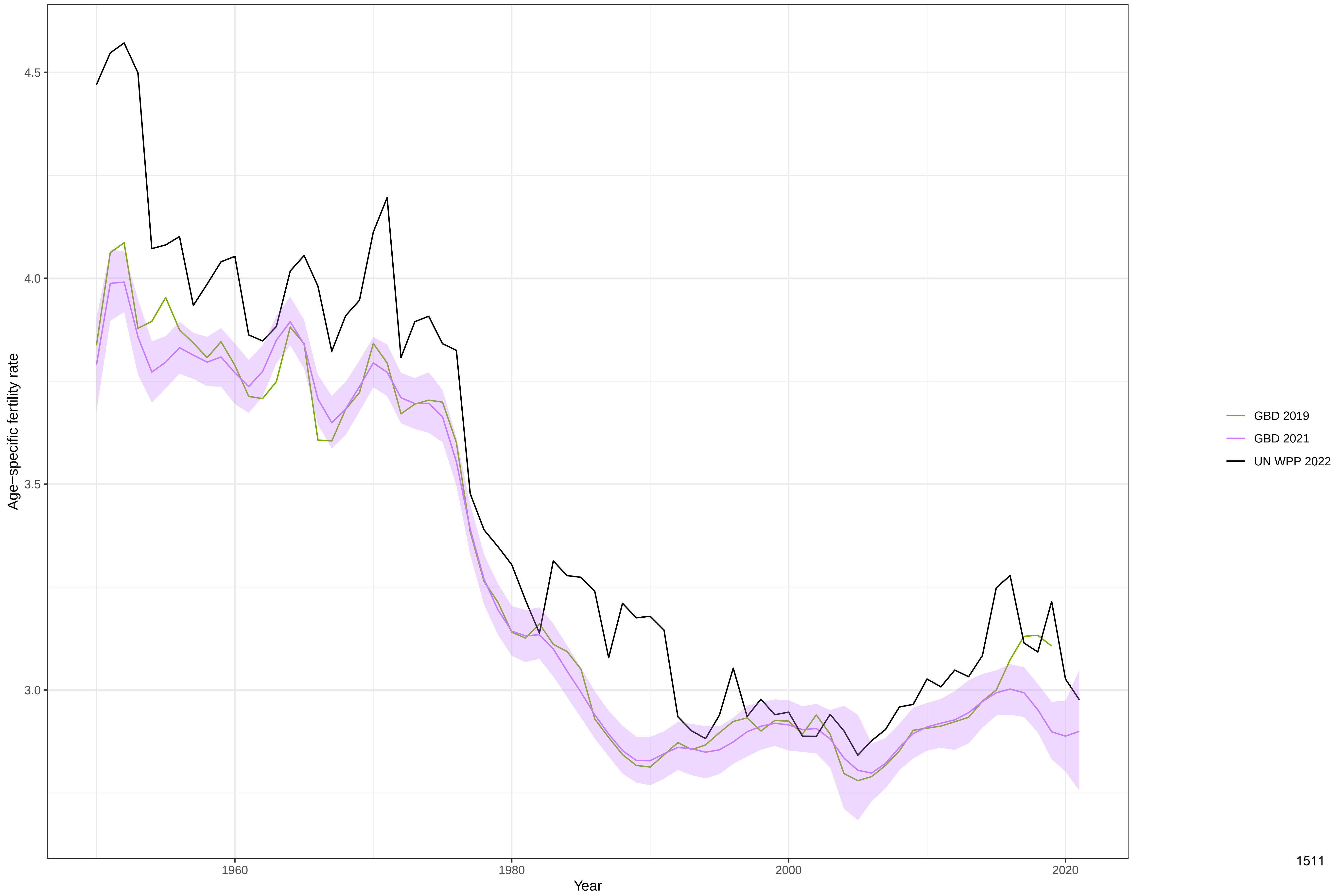

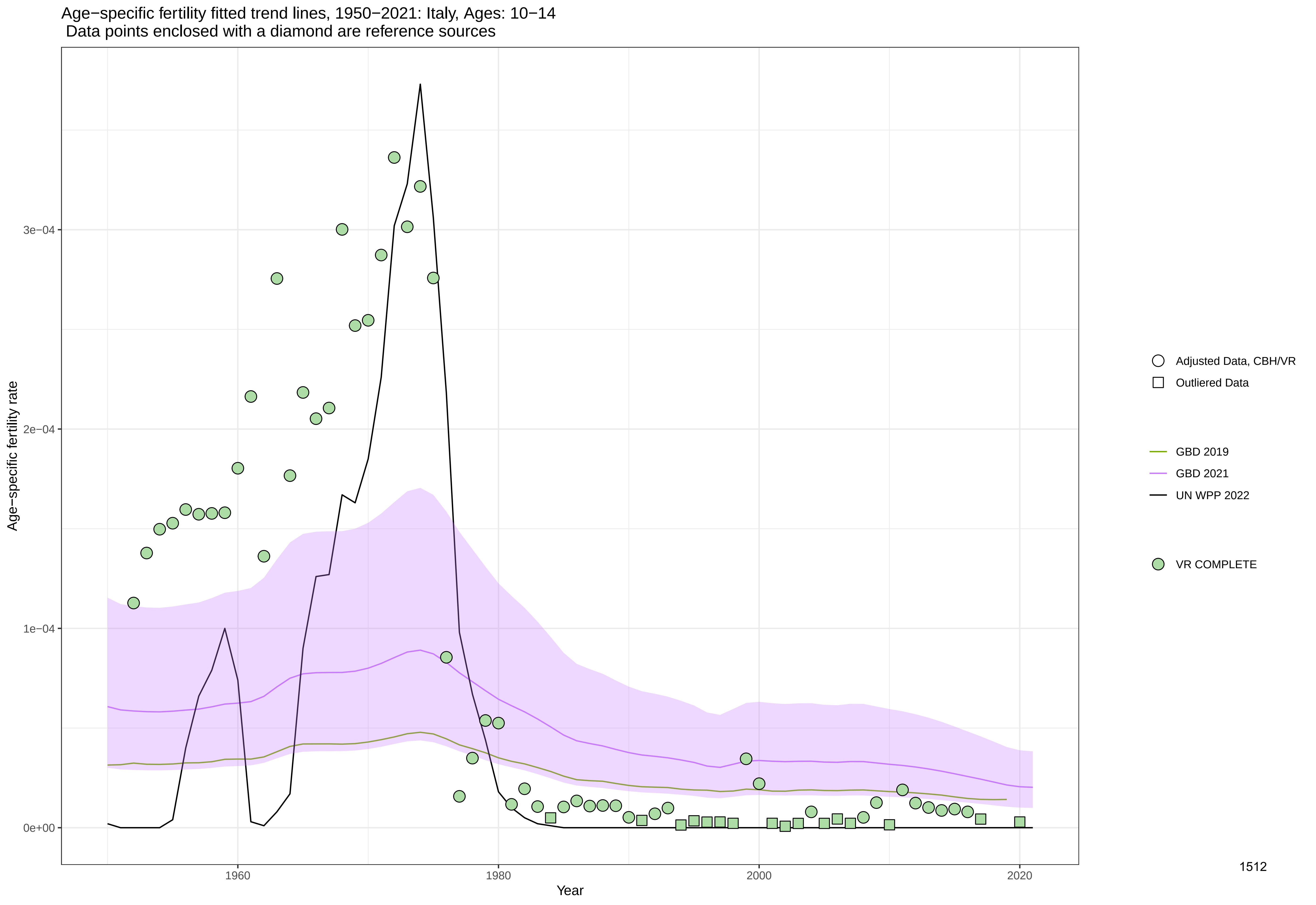

Age−specific fertility fitted trend lines, 1950−2021: Italy, Ages: 10−14
Data points enclosed with a diamond are reference sources
Age−specific fertility rate
3e−04
2e−04
1e−04
0e+00
1960
1980
2000
2020
Year
Adjusted Data, CBH/VR
Outliered Data
GBD 2019
GBD 2021
UN WPP 2022
VR COMPLETE

Age−specific fertility fitted trend lines, 1950−2021: Italy, Ages: 15−19
Data points enclosed with a diamond are reference sources

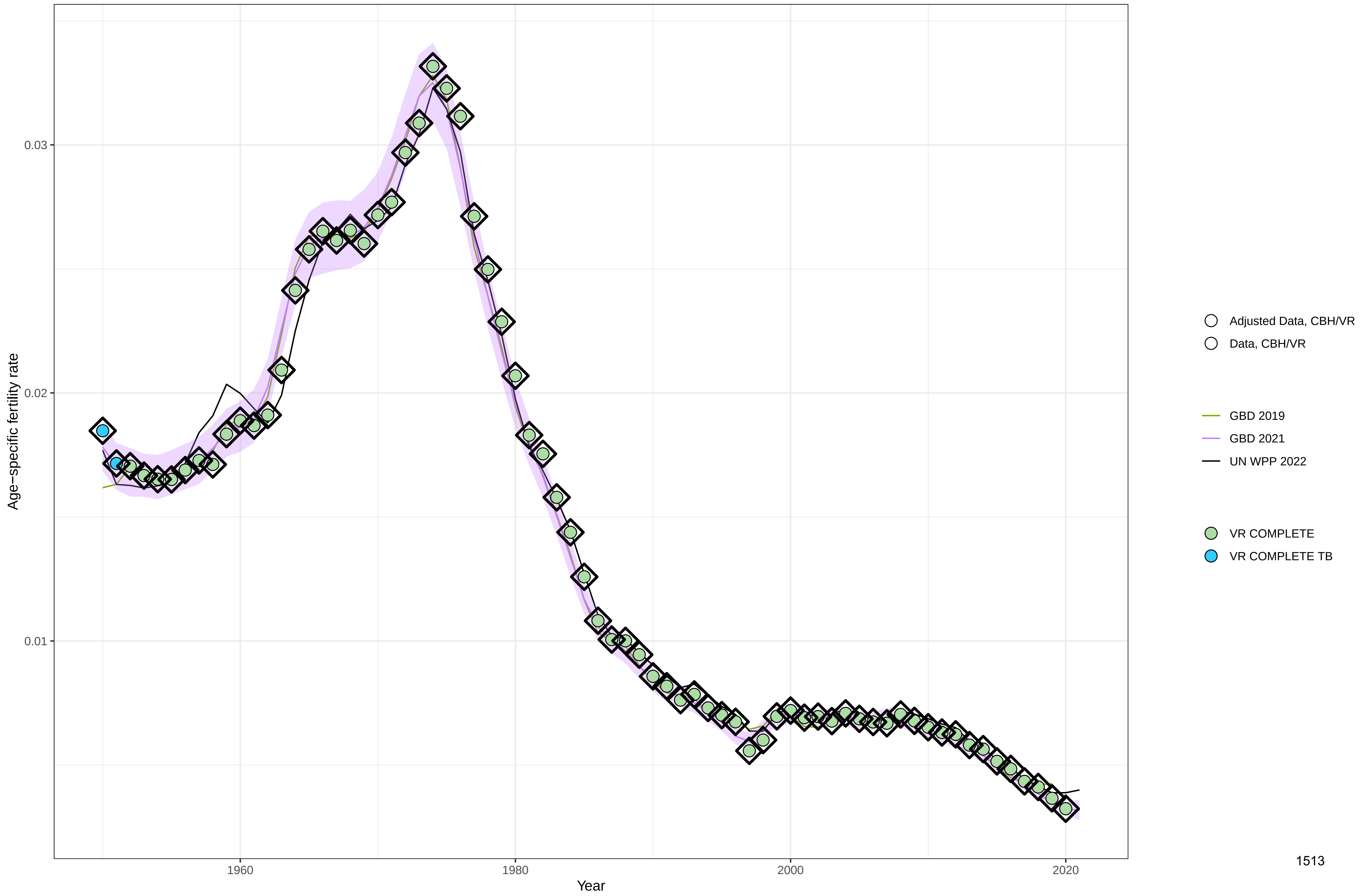

Age−specific fertility fitted trend lines, 1950−2021: Italy, Ages: 20−24
Data points enclosed with a diamond are reference sources

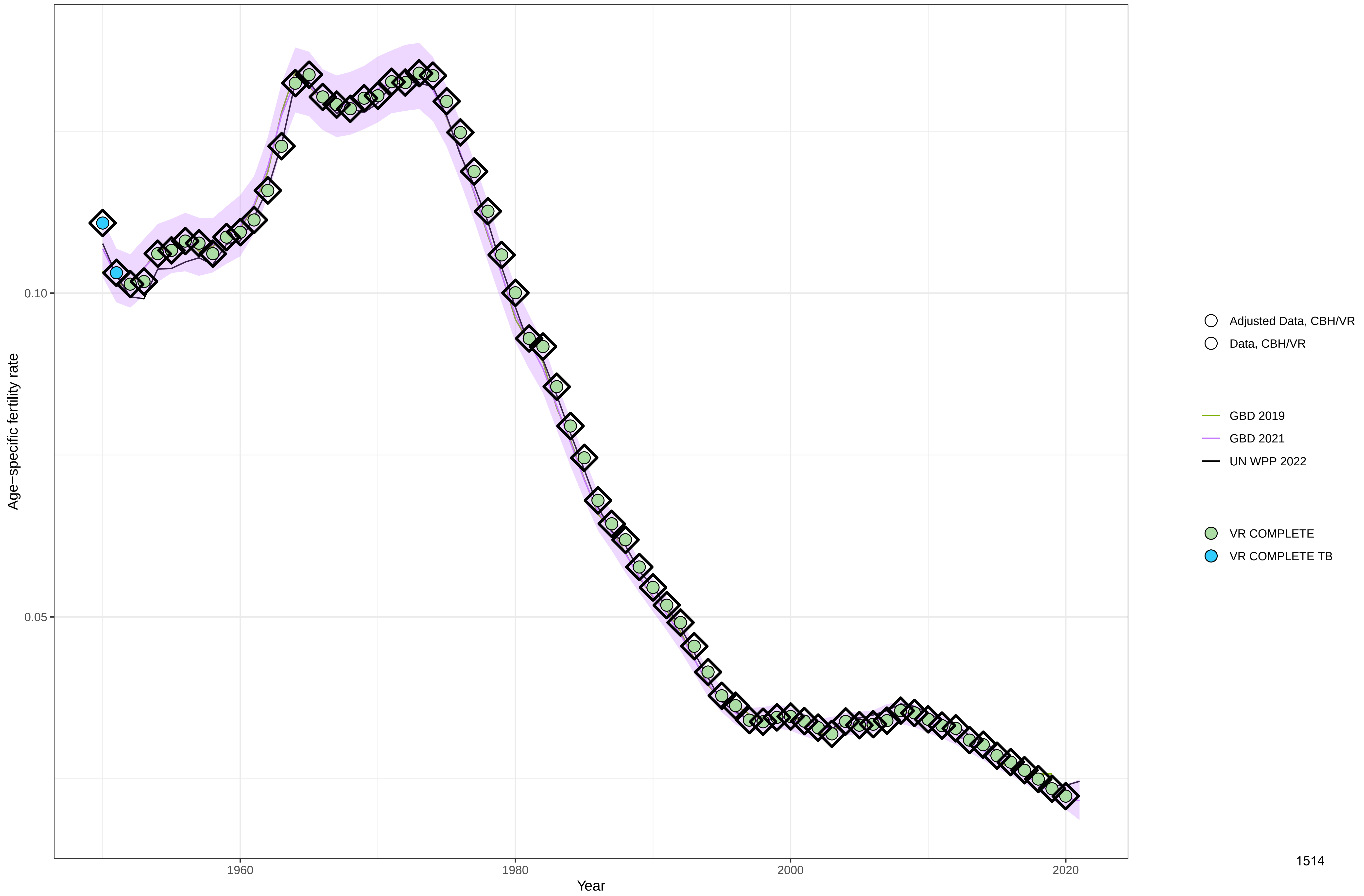

Age−specific fertility fitted trend lines, 1950−2021: Italy, Ages: 25−29
Data points enclosed with a diamond are reference sources

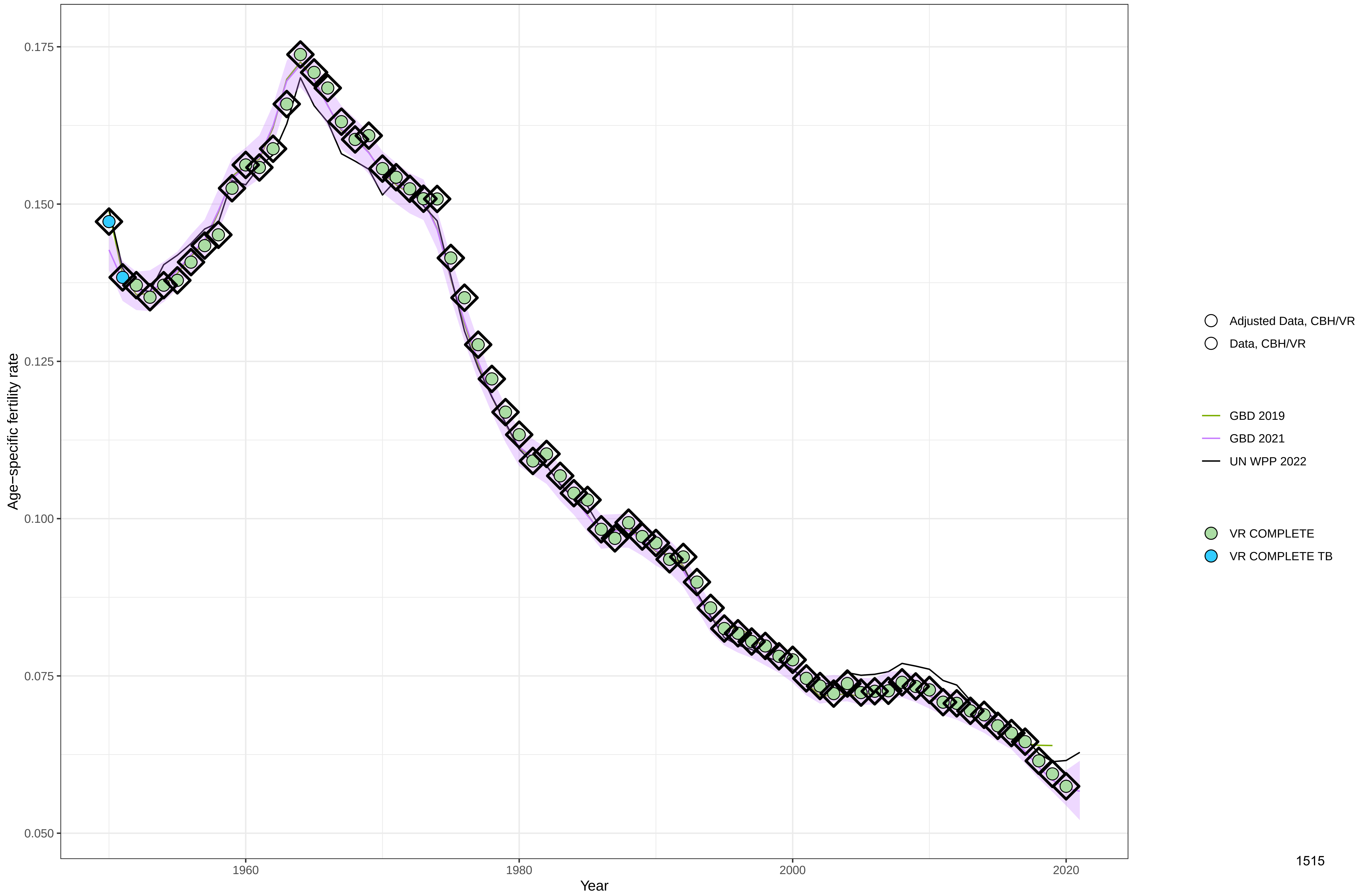

Age−specific fertility fitted trend lines, 1950−2021: Italy, Ages: 30−34
Data points enclosed with a diamond are reference sources

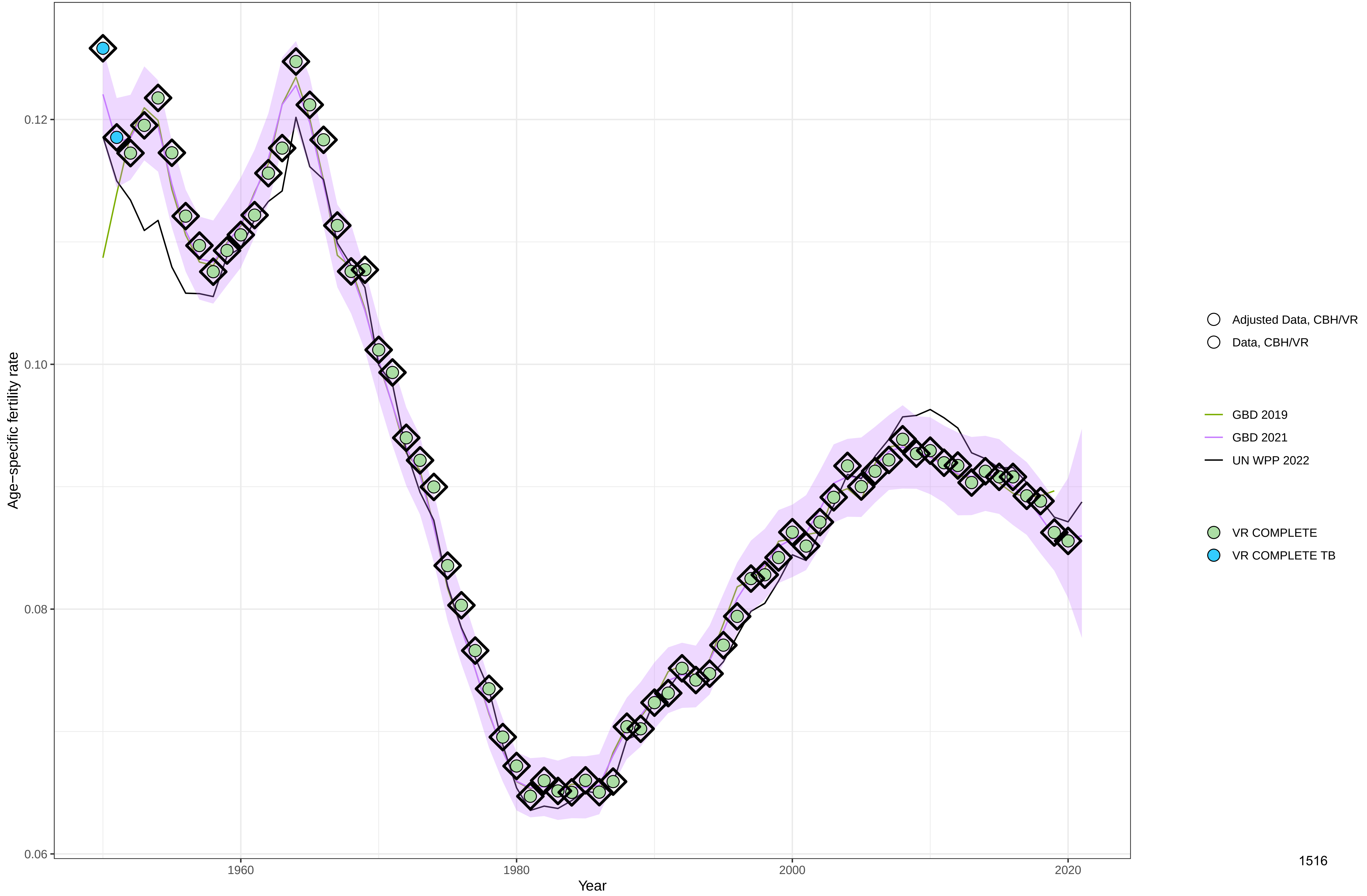

Age−specific fertility fitted trend lines, 1950−2021: Italy, Ages: 35−39
Data points enclosed with a diamond are reference sources

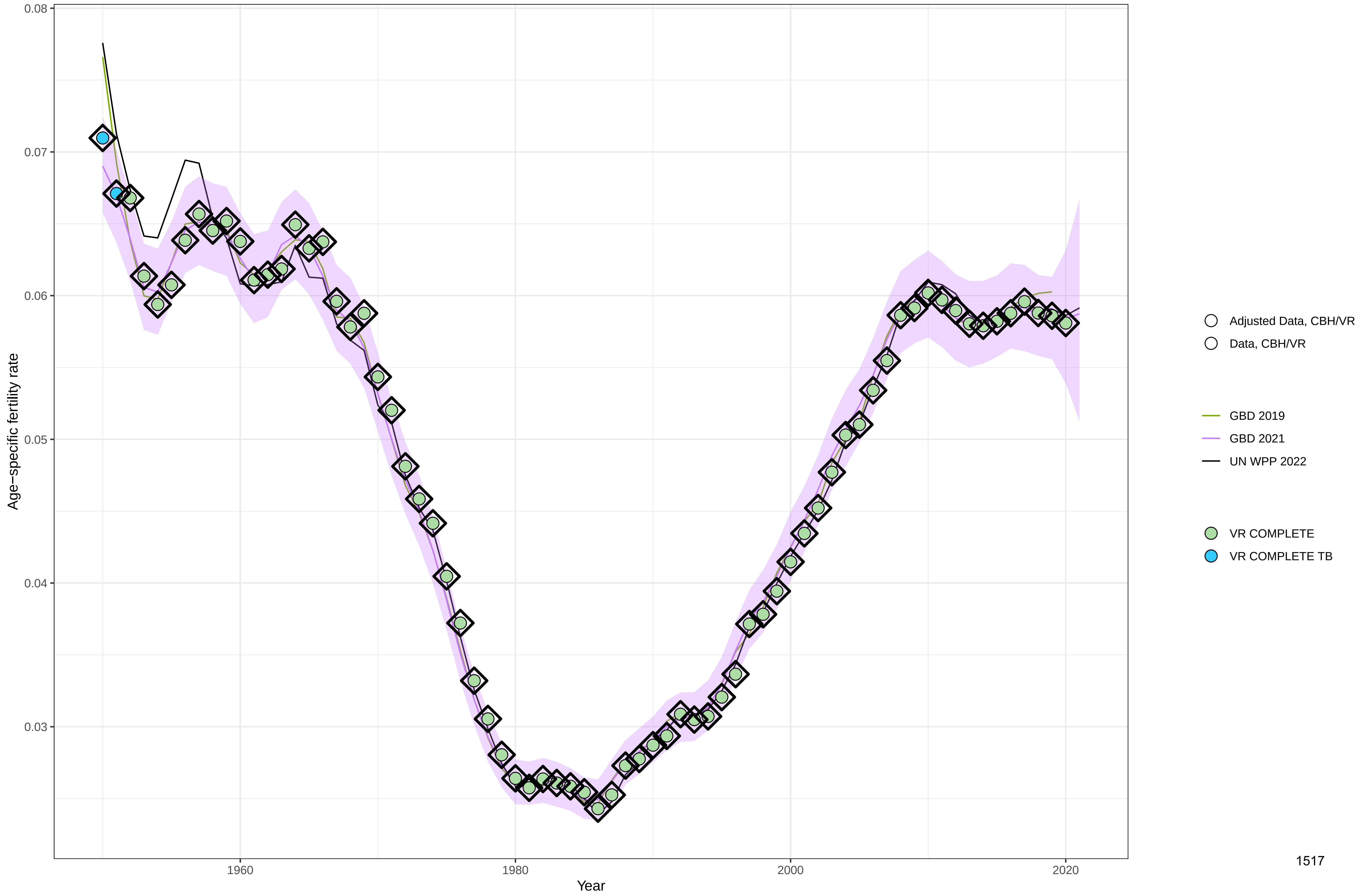

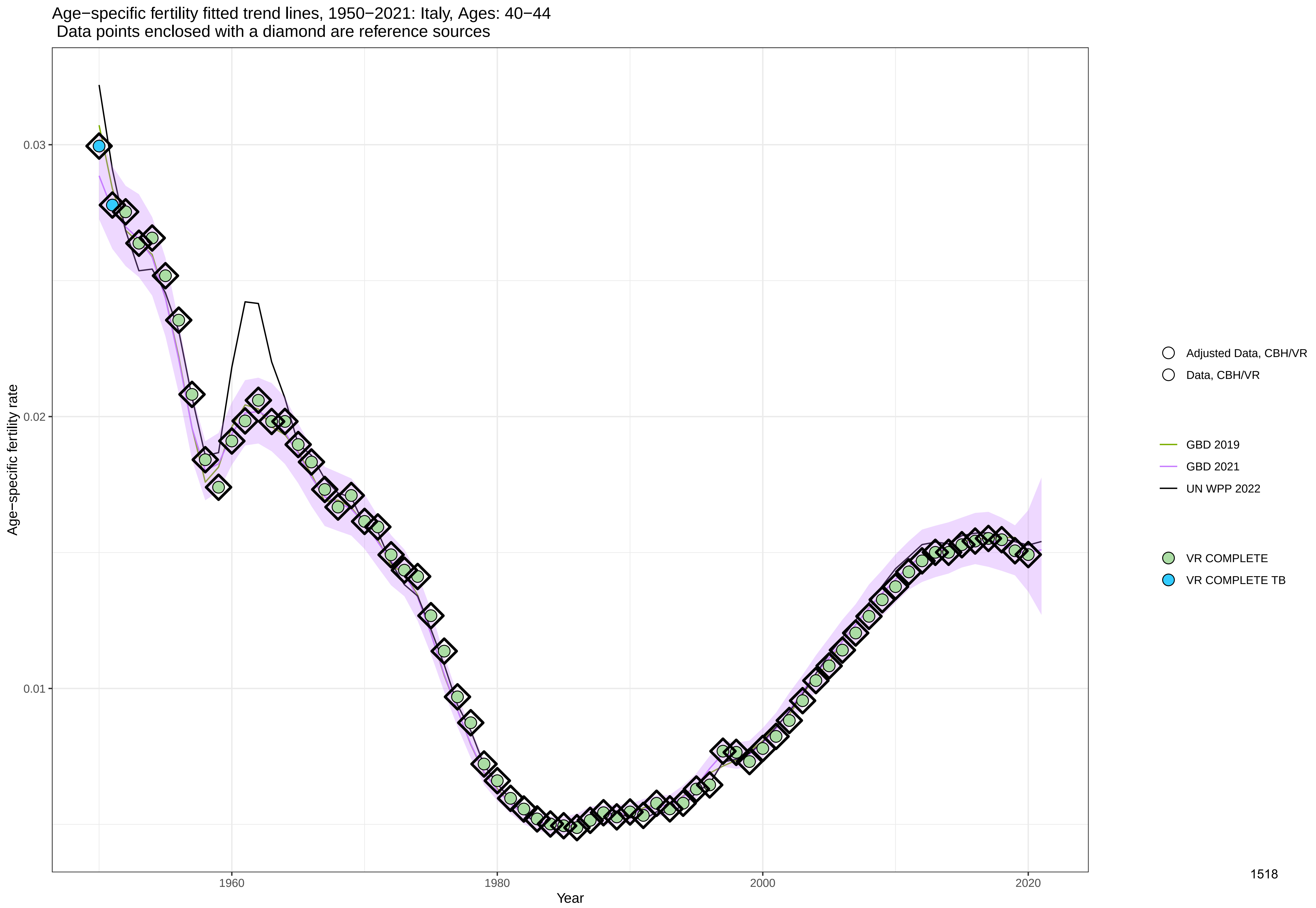
Age−specific fertility fitted trend lines, 1950−2021: Italy, Ages: 40−44
Data points enclosed with a diamond are reference sources
0.03
0.02
0.01
1960
1980
2000
2020
Year
Age−specific fertility rate
Adjusted Data, CBH/VR
Data, CBH/VR
GBD 2019
GBD 2021
UN WPP 2022
VR COMPLETE
VR COMPLETE TB

Age−specific fertility fitted trend lines, 1950−2021: Italy, Ages: 45−49
Data points enclosed with a diamond are reference sources

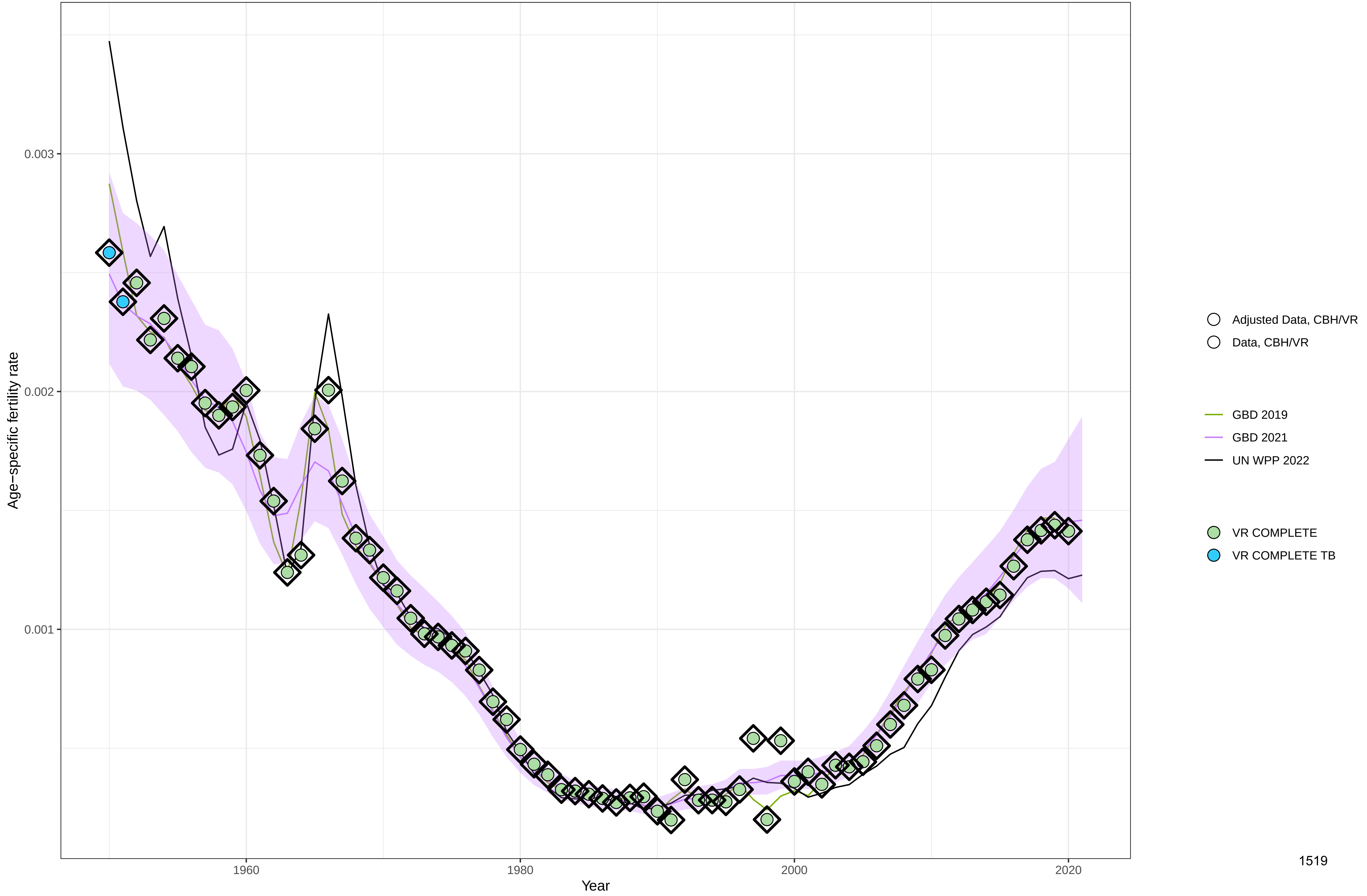

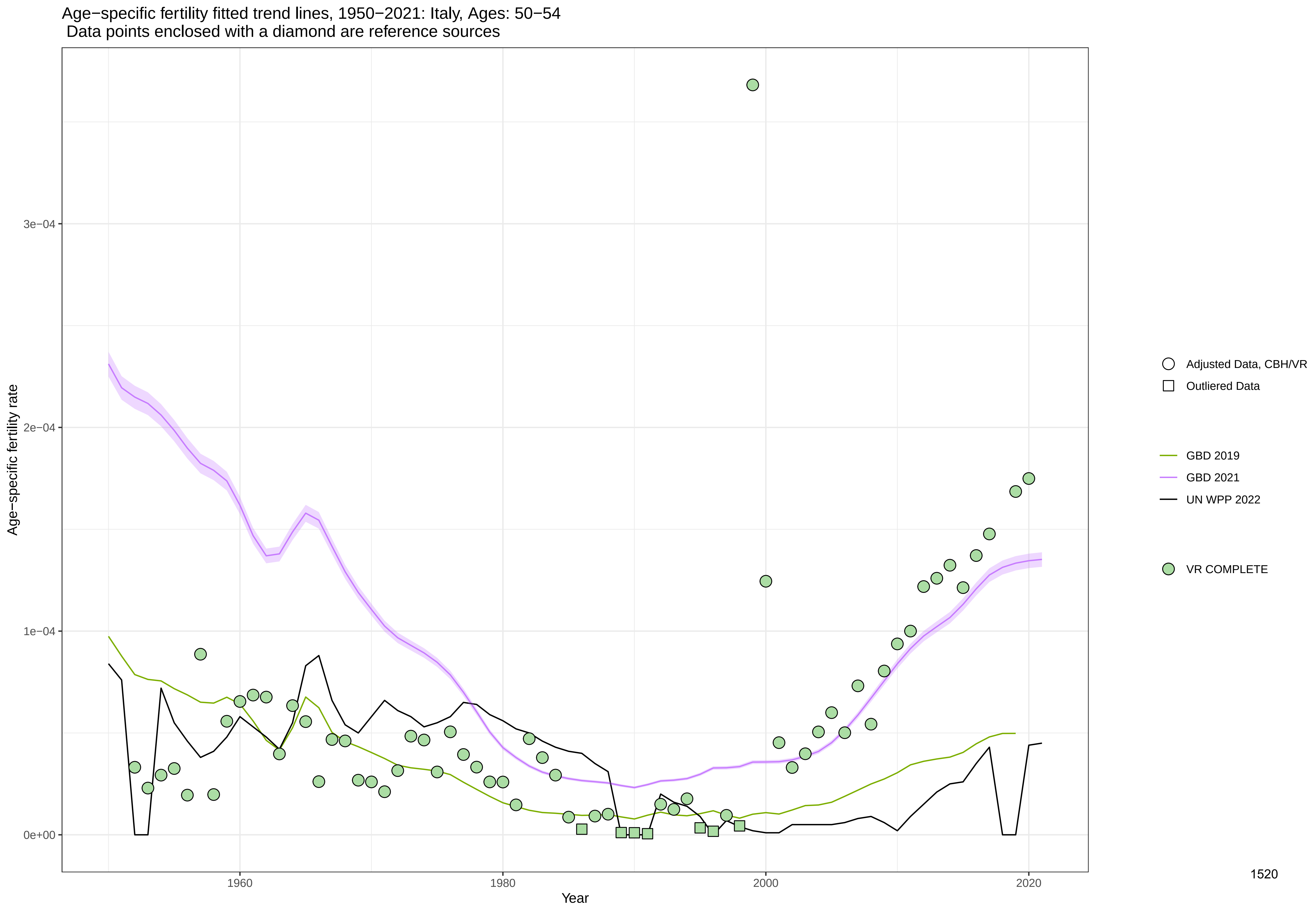

Age−specific fertility fitted trend lines, 1950−2021: Italy, Ages: 50−54
Data points enclosed with a diamond are reference sources
Age−specific fertility rate
3e−04
2e−04
1e−04
0e+00
1960
1980
2000
2020
Year
Adjusted Data, CBH/VR
Outliered Data
GBD 2019
GBD 2021
UN WPP 2022
VR COMPLETE

Total fertility rate fitted trend lines, 1950−2021: Italy TFR
Data points enclosed with a diamond are reference sources

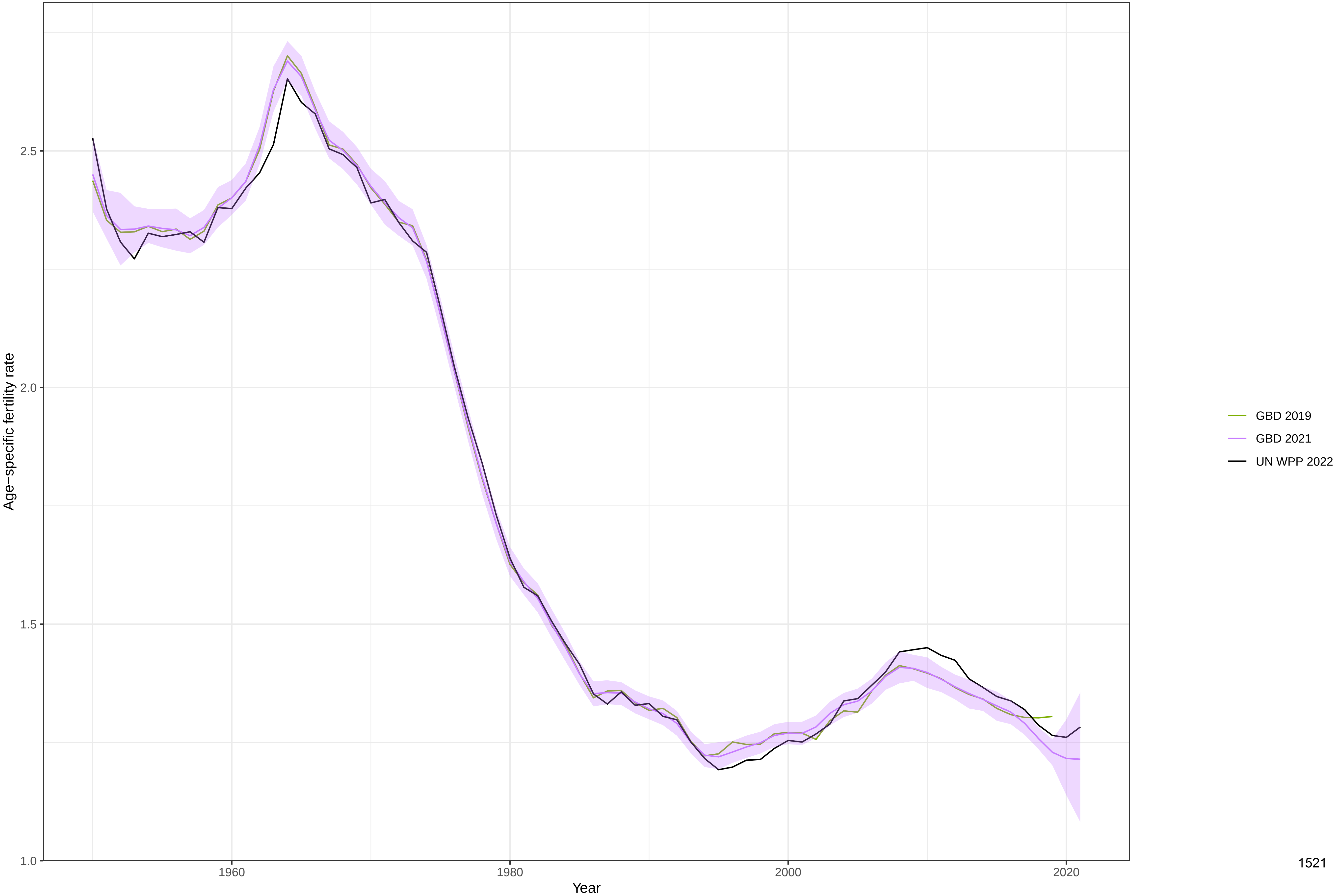

Age−specific fertility fitted trend lines, 1950−2021: Abruzzo, Ages: 10−14
Data points enclosed with a diamond are reference sources

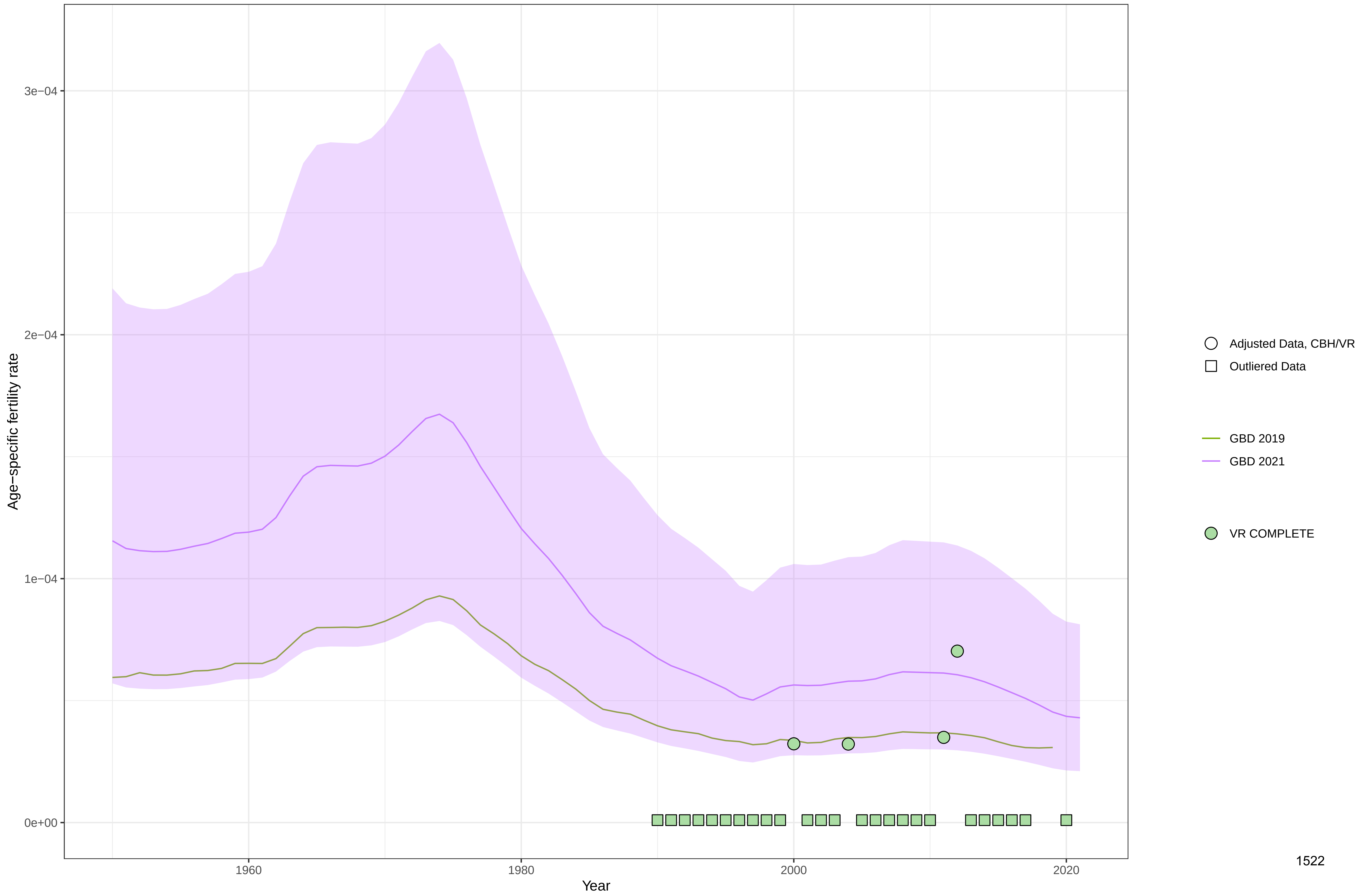

Age−specific fertility fitted trend lines, 1950−2021: Abruzzo, Ages: 15−19
Data points enclosed with a diamond are reference sources

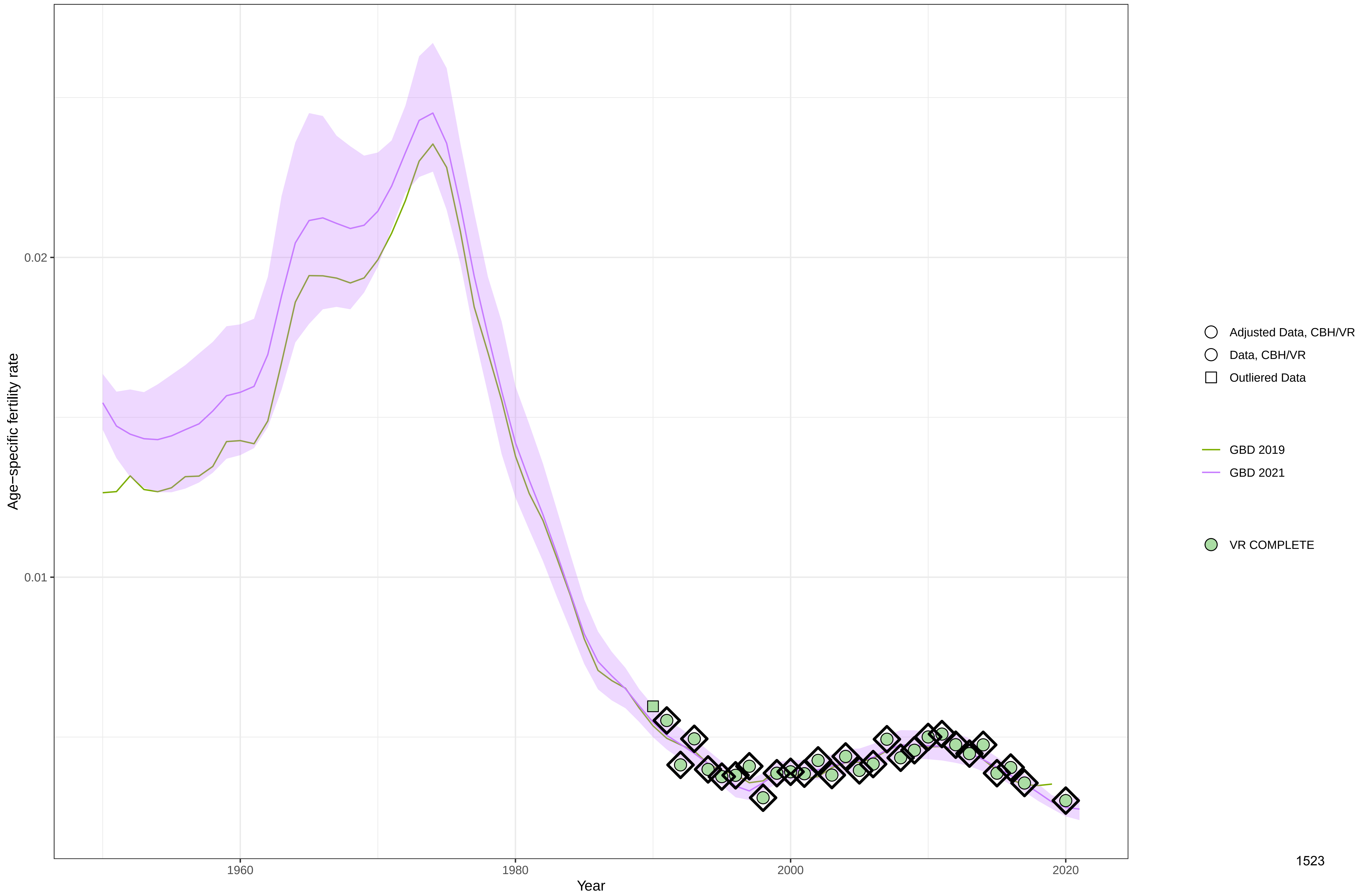

Age−specific fertility fitted trend lines, 1950−2021: Abruzzo, Ages: 20−24
Data points enclosed with a diamond are reference sources

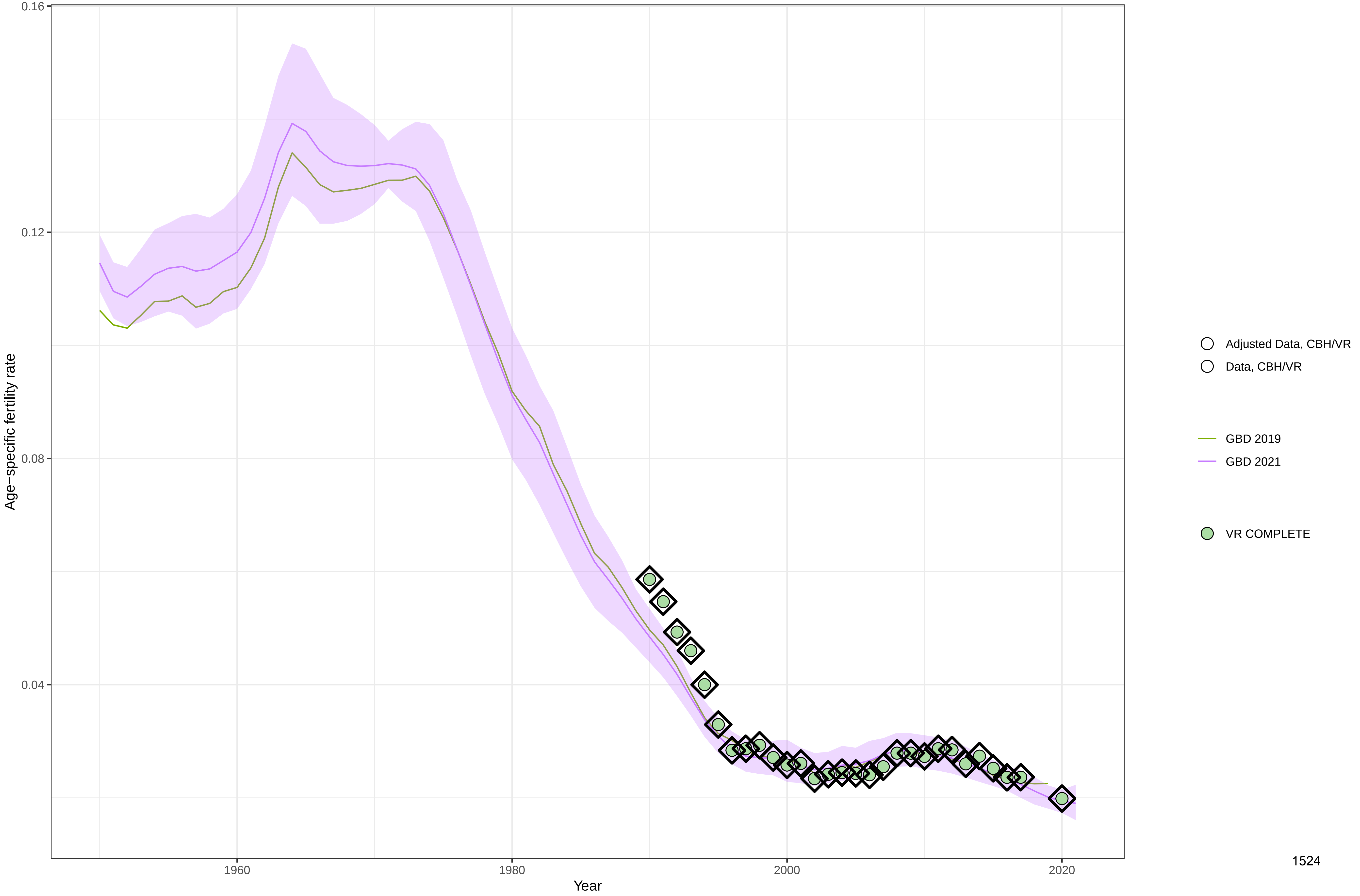

Age−specific fertility fitted trend lines, 1950−2021: Abruzzo, Ages: 25−29
Data points enclosed with a diamond are reference sources

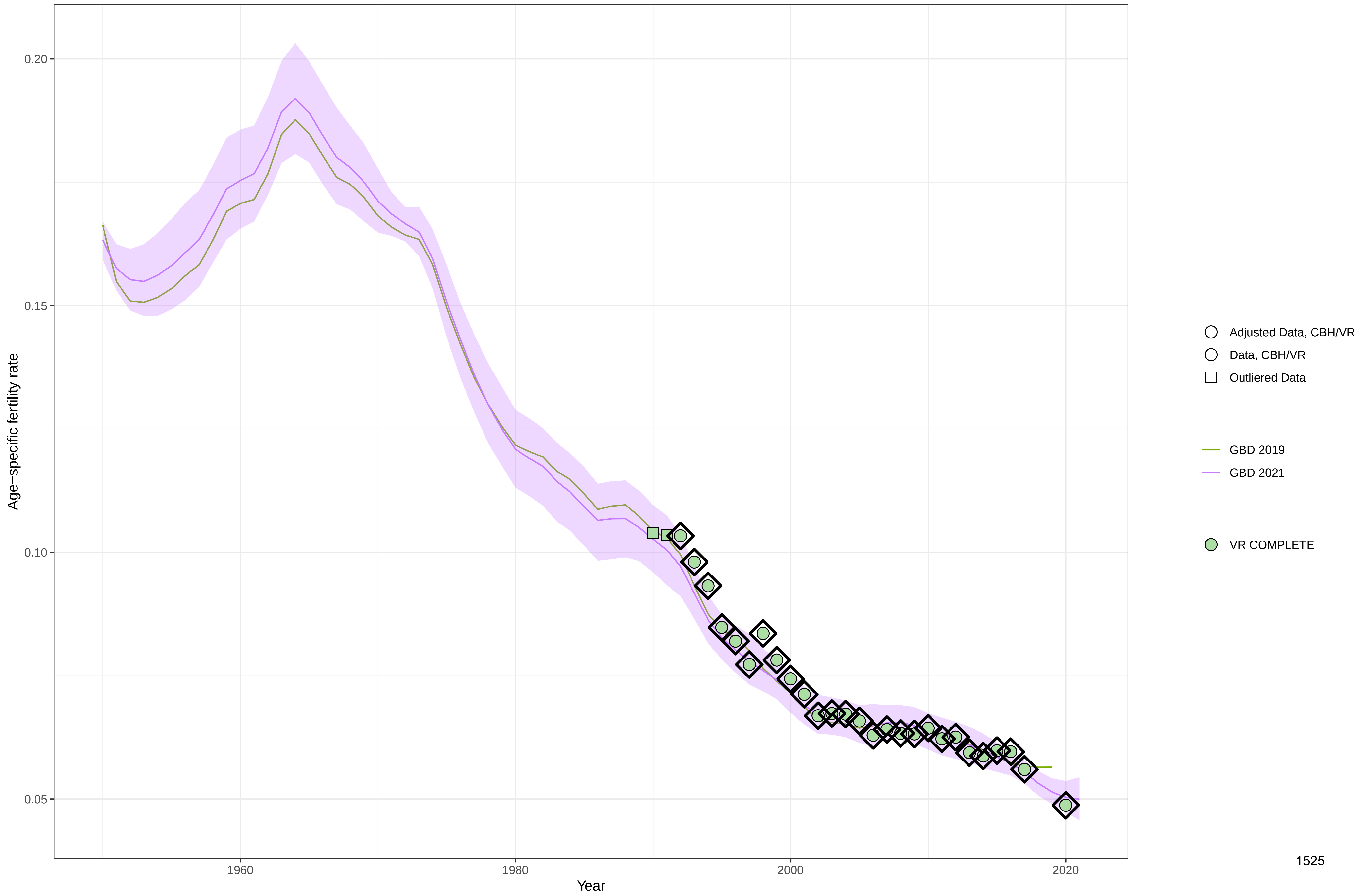

Age−specific fertility fitted trend lines, 1950−2021: Abruzzo, Ages: 30−34
Data points enclosed with a diamond are reference sources

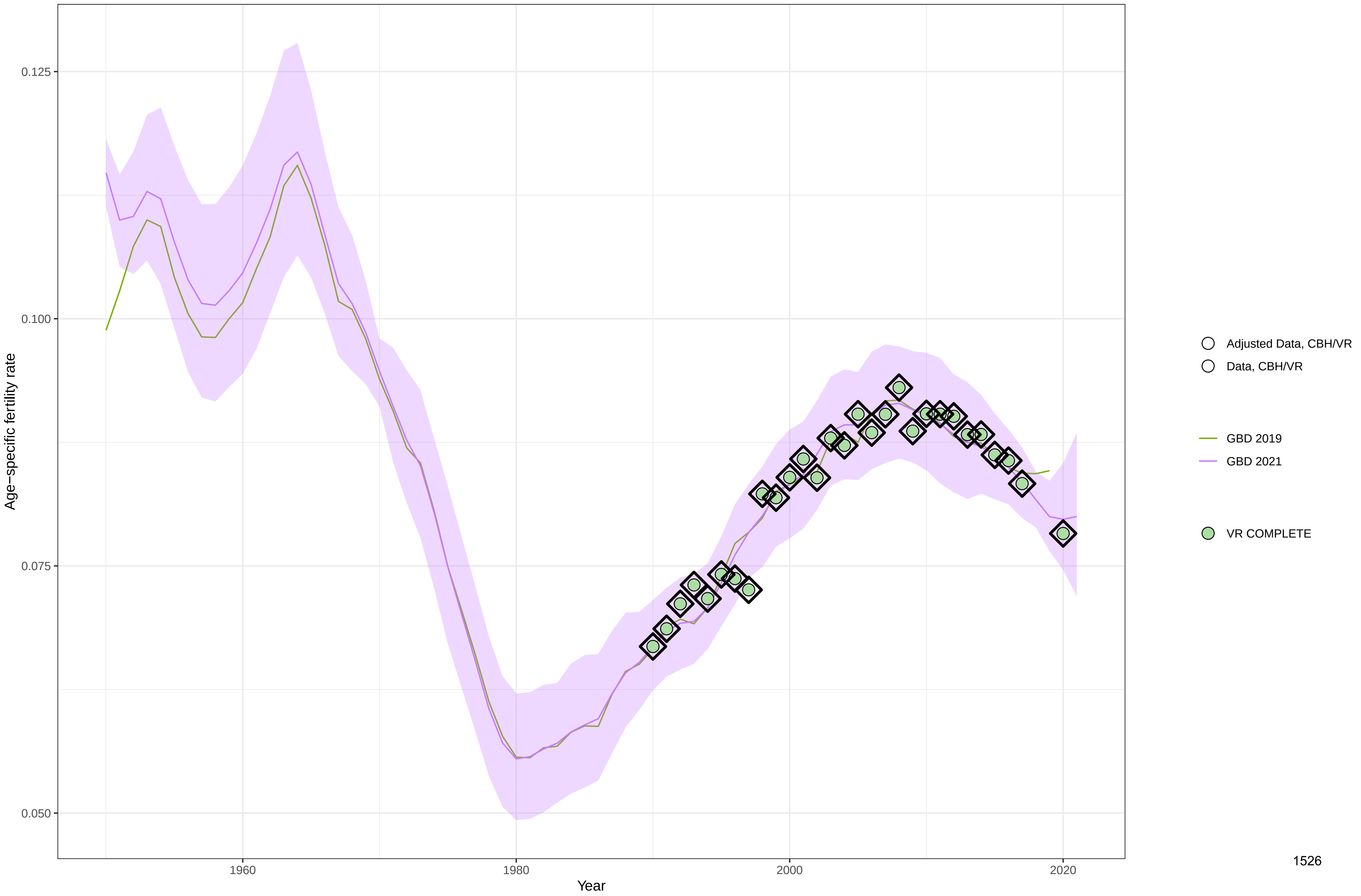

Age−specific fertility fitted trend lines, 1950−2021: Abruzzo, Ages: 35−39
Data points enclosed with a diamond are reference sources

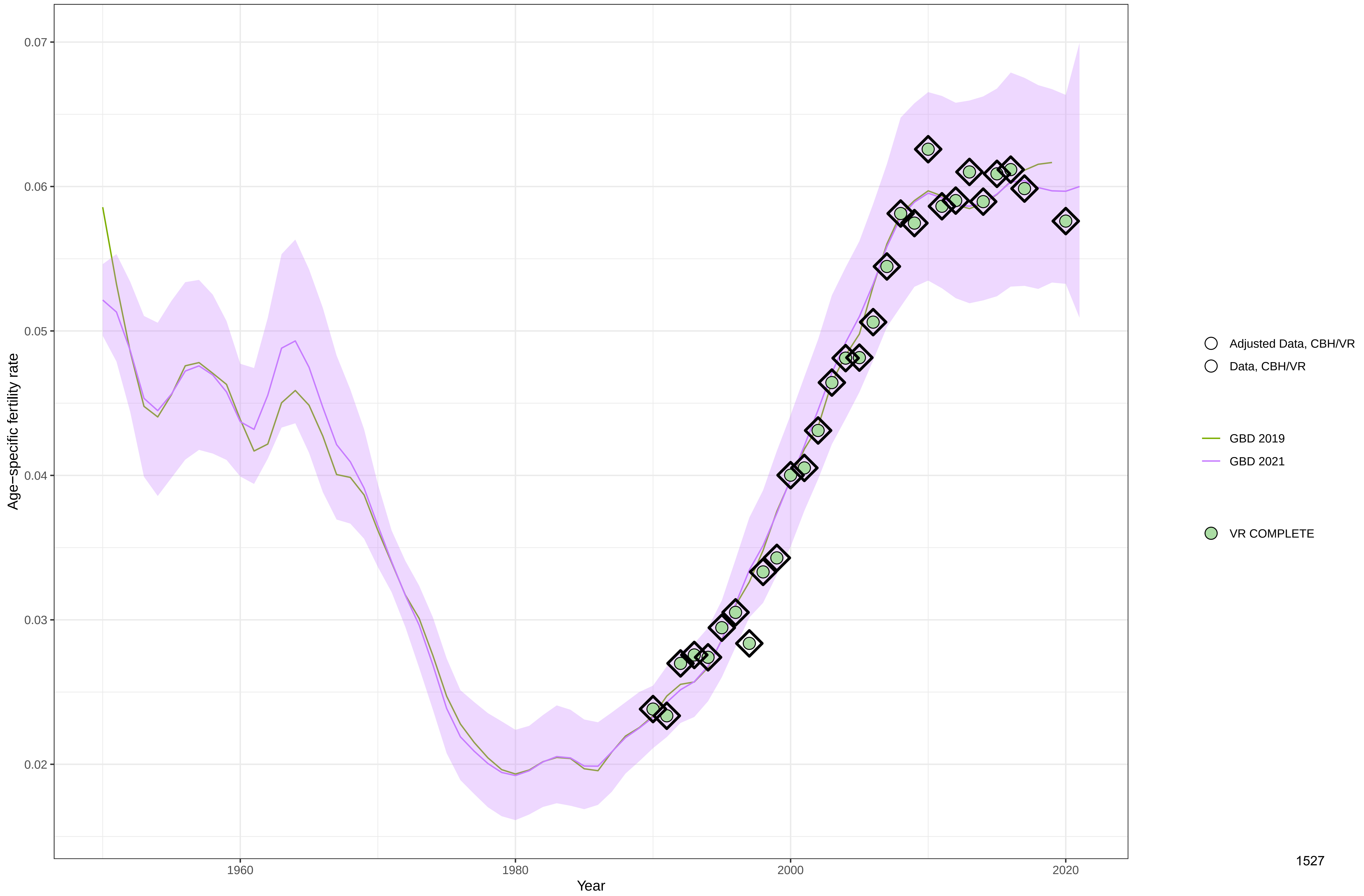

Age−specific fertility fitted trend lines, 1950−2021: Abruzzo, Ages: 40−44
Data points enclosed with a diamond are reference sources

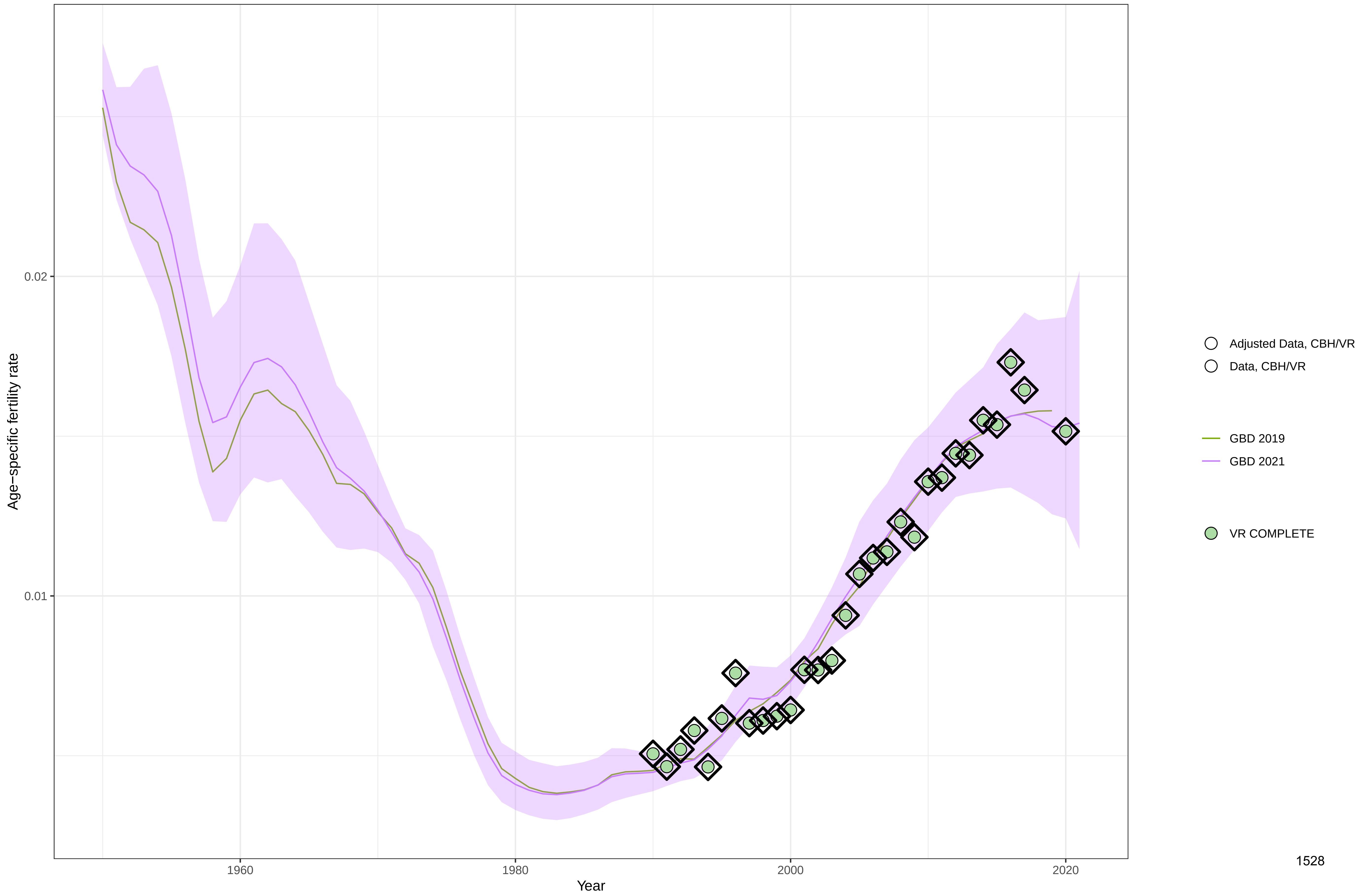

Age−specific fertility fitted trend lines, 1950−2021: Abruzzo, Ages: 45−49
Data points enclosed with a diamond are reference sources

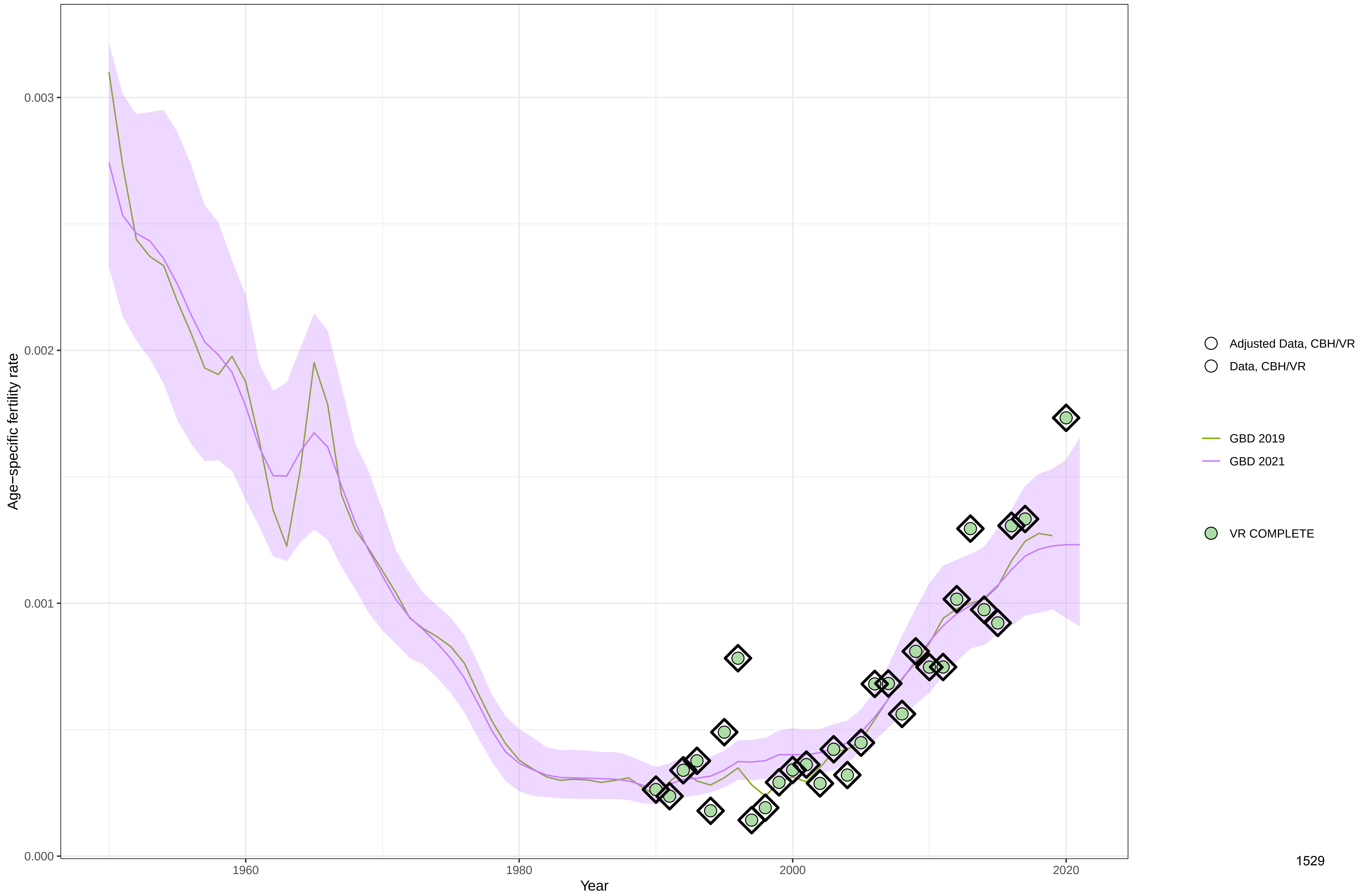

Age−specific fertility fitted trend lines, 1950−2021: Abruzzo, Ages: 50−54
 Data points enclosed with a diamond are reference sources

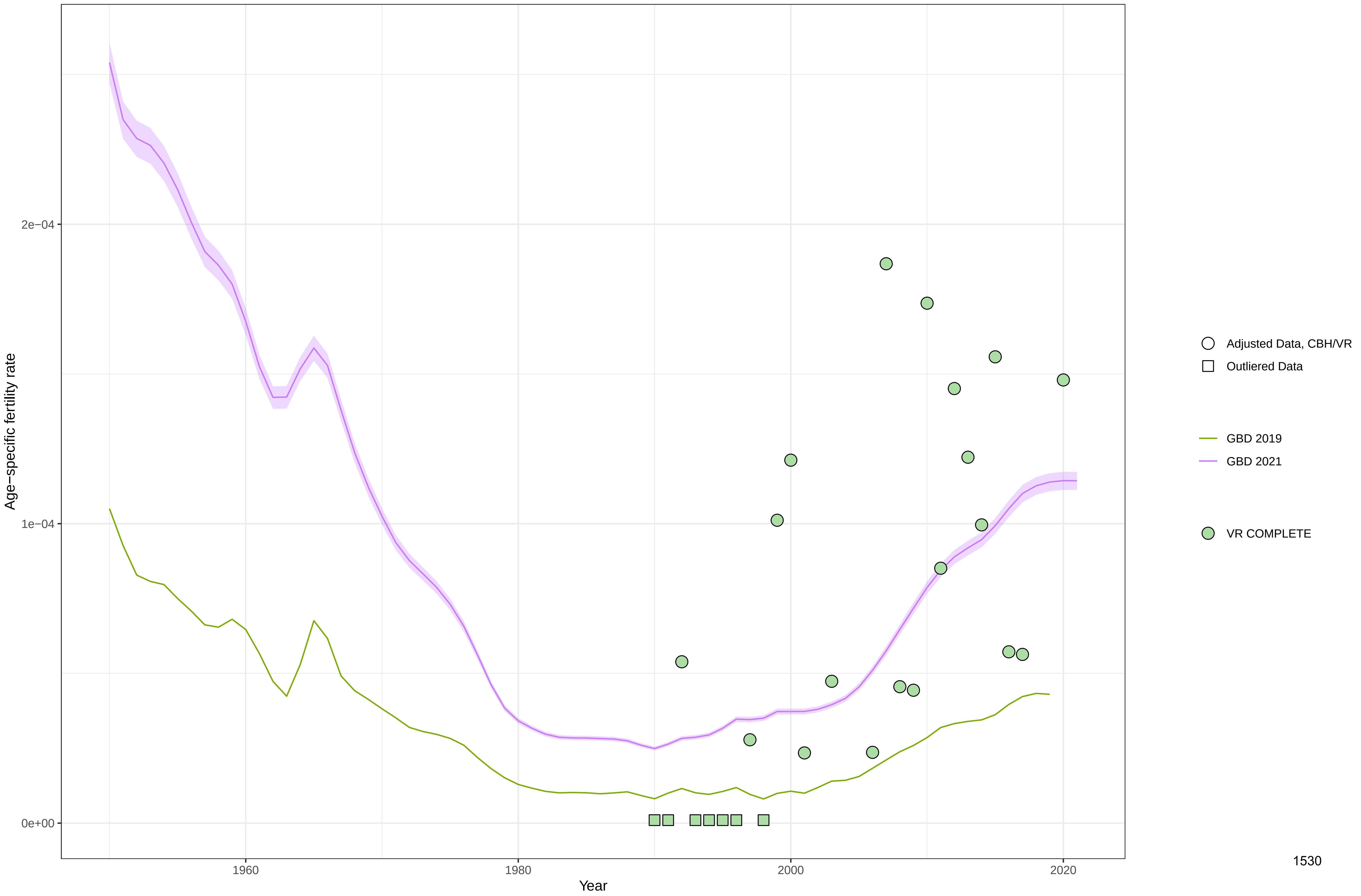

Total fertility rate fitted trend lines, 1950−2021: Abruzzo TFR
Data points enclosed with a diamond are reference sources

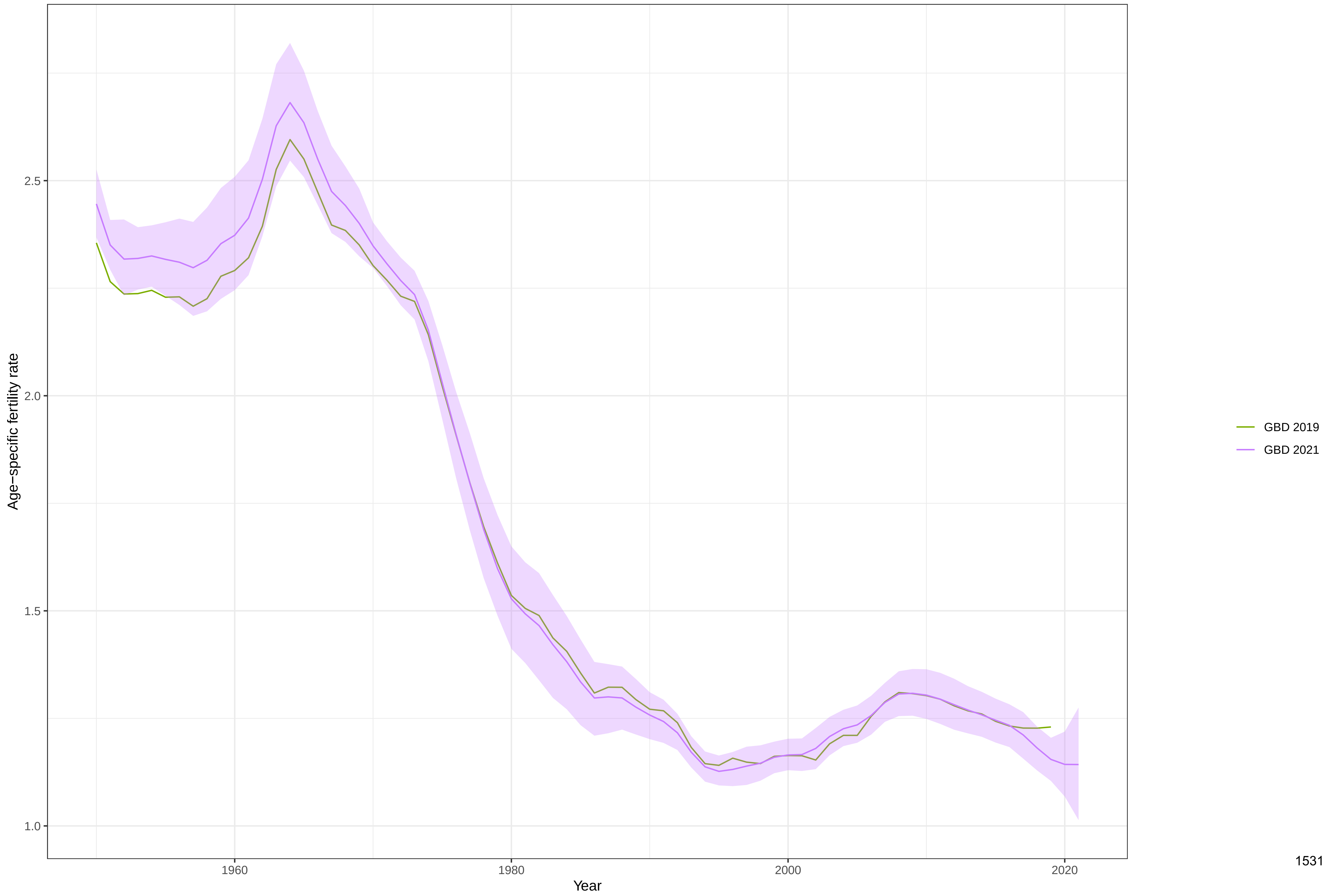

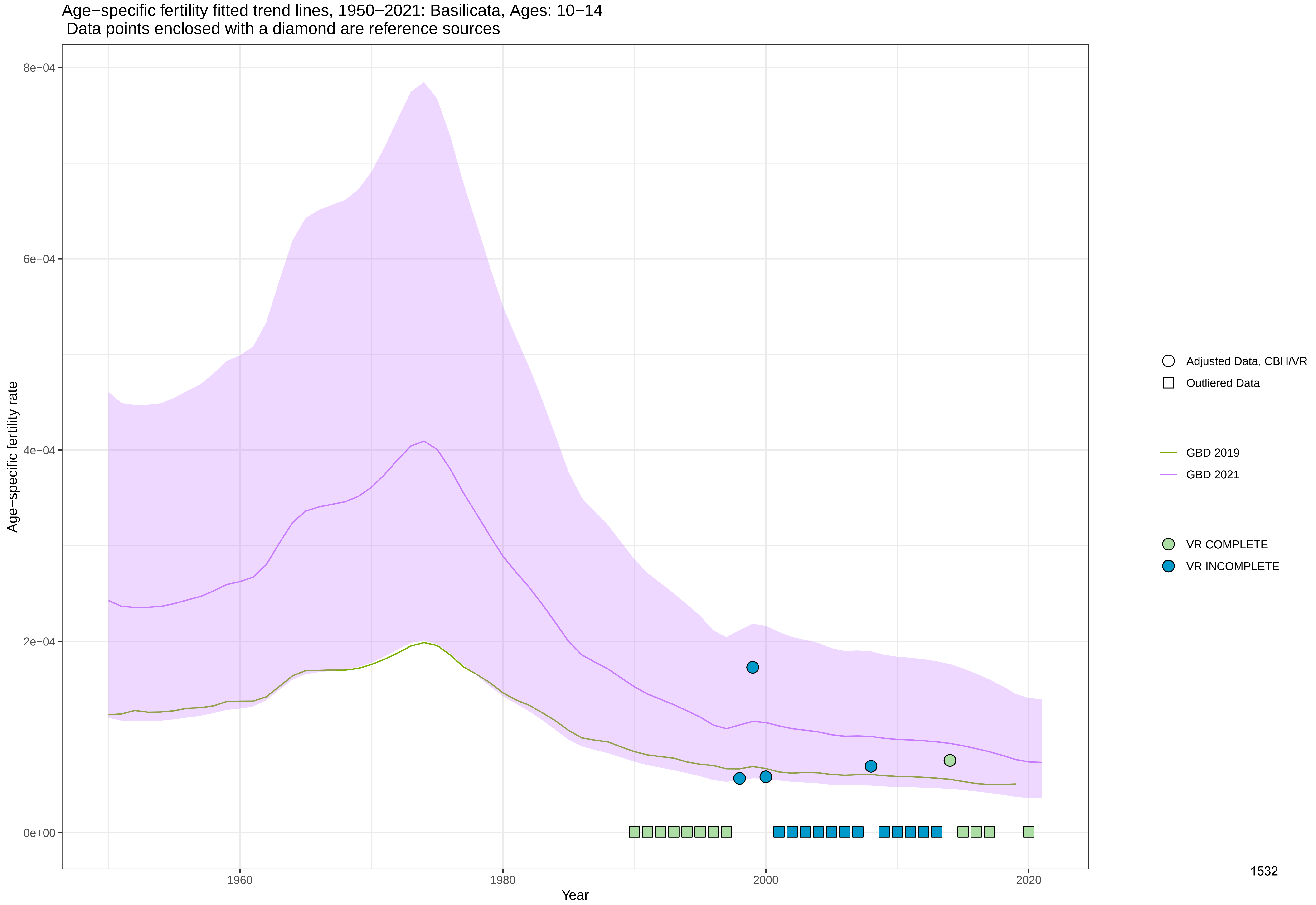

Age−specific fertility fitted trend lines, 1950−2021: Basilicata, Ages: 10−14
Data points enclosed with a diamond are reference sources
Age−specific fertility rate
Year
8e−04
6e−04
4e−04
2e−04
0e+00
1960
1980
2000
2020
Adjusted Data, CBH/VR
Outliered Data
GBD 2019
GBD 2021
VR COMPLETE
VR INCOMPLETE

Age−specific fertility fitted trend lines, 1950−2021: Basilicata, Ages: 15−19
Data points enclosed with a diamond are reference sources

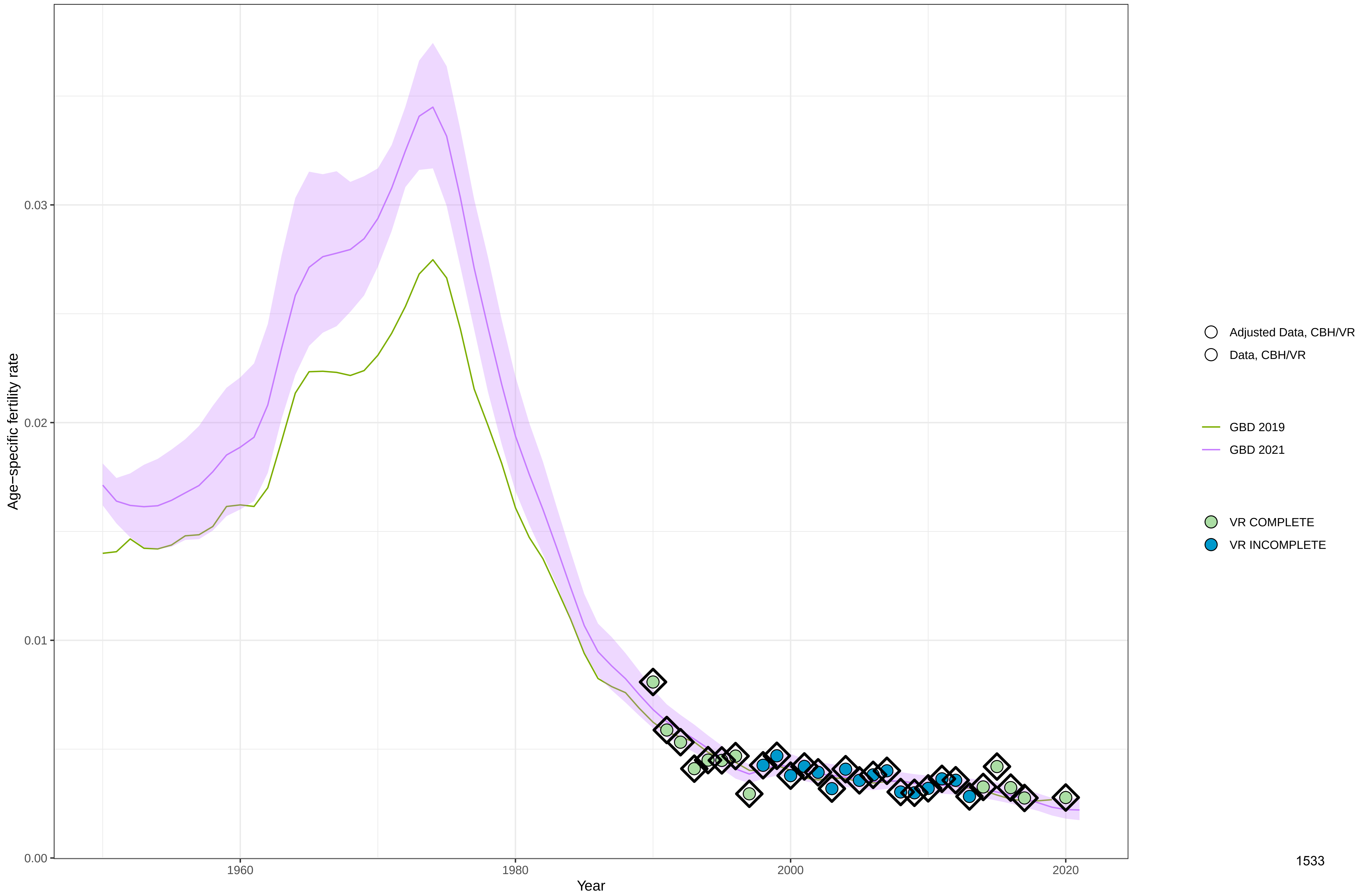

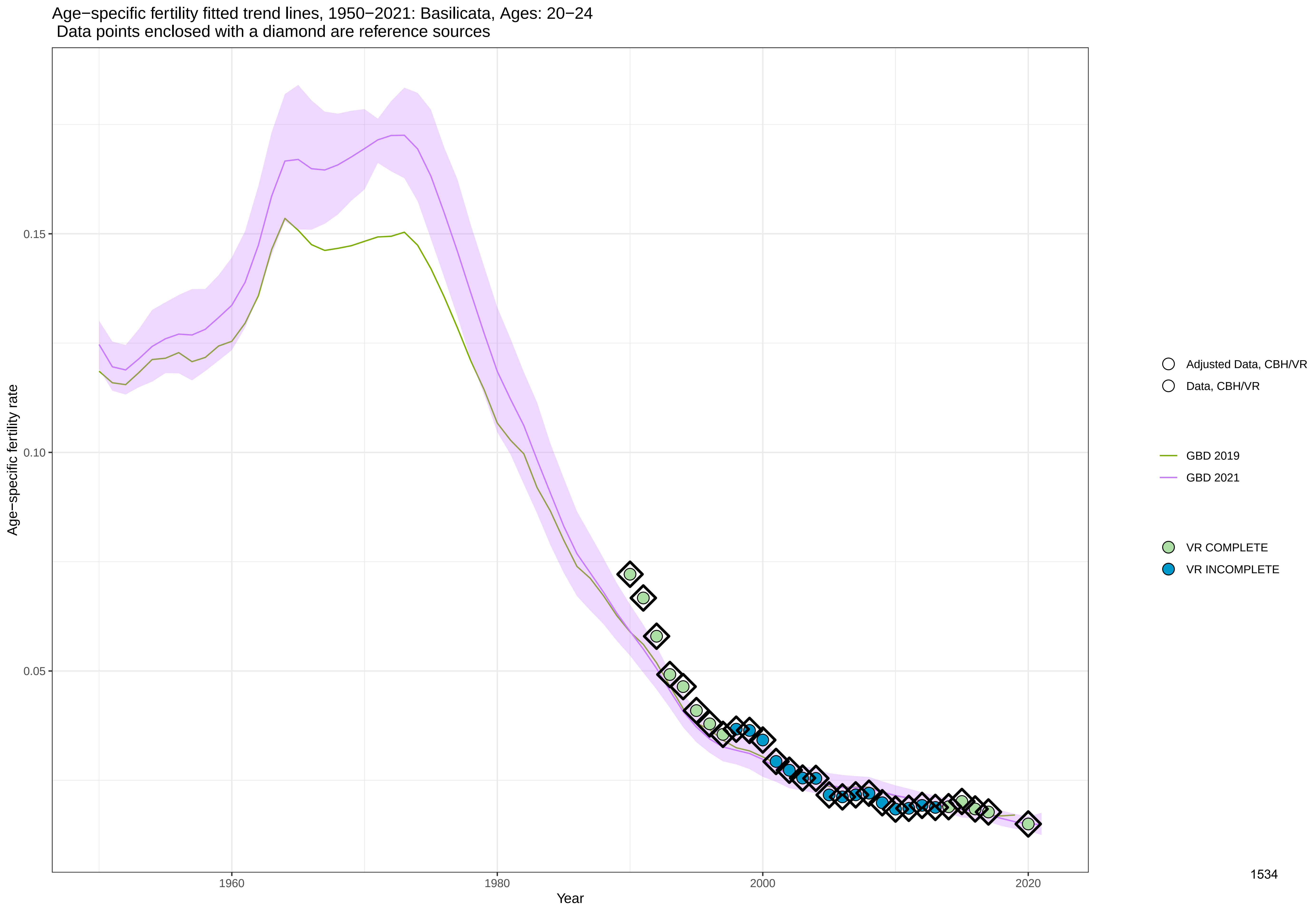

Age−specific fertility fitted trend lines, 1950−2021: Basilicata, Ages: 20−24
Data points enclosed with a diamond are reference sources
Age−specific fertility rate
0.15
0.10
0.05
1960
1980
2000
2020
Year
Adjusted Data, CBH/VR
Data, CBH/VR
GBD 2019
GBD 2021
VR COMPLETE
VR INCOMPLETE

Age−specific fertility fitted trend lines, 1950−2021: Basilicata, Ages: 25−29
Data points enclosed with a diamond are reference sources

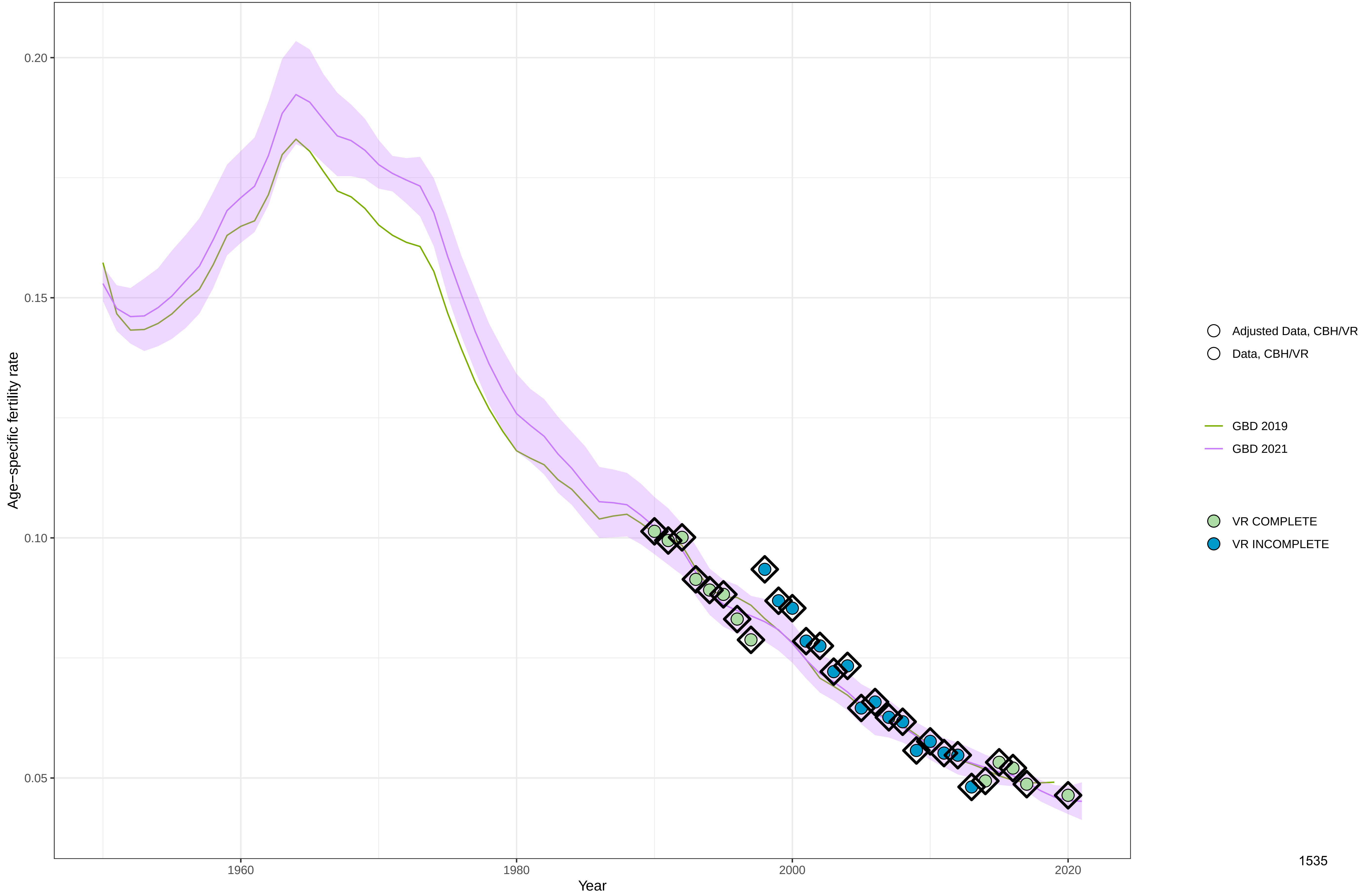

Age−specific fertility fitted trend lines, 1950−2021: Basilicata, Ages: 30−34
Data points enclosed with a diamond are reference sources

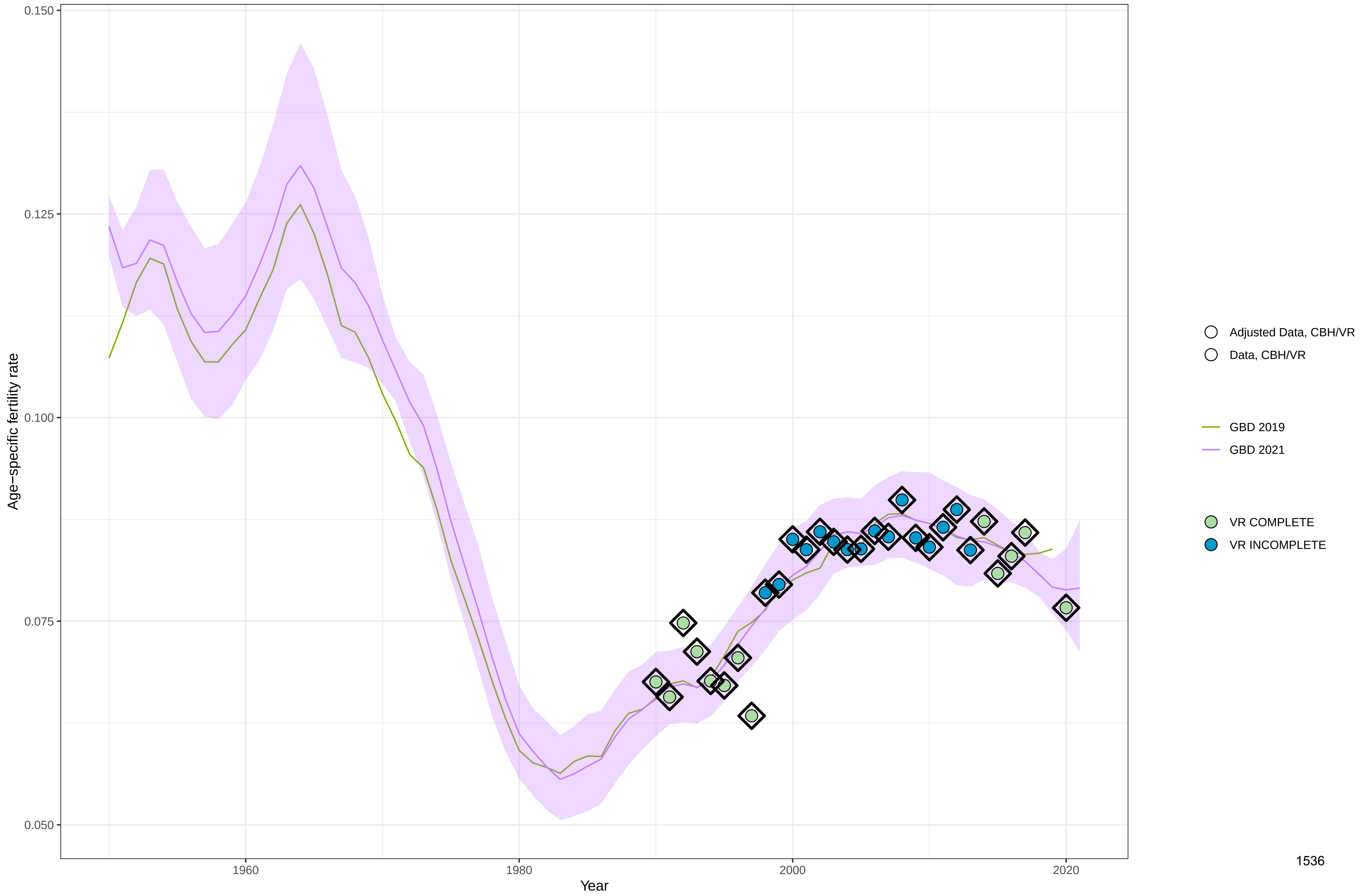

Age−specific fertility fitted trend lines, 1950−2021: Basilicata, Ages: 35−39
Data points enclosed with a diamond are reference sources

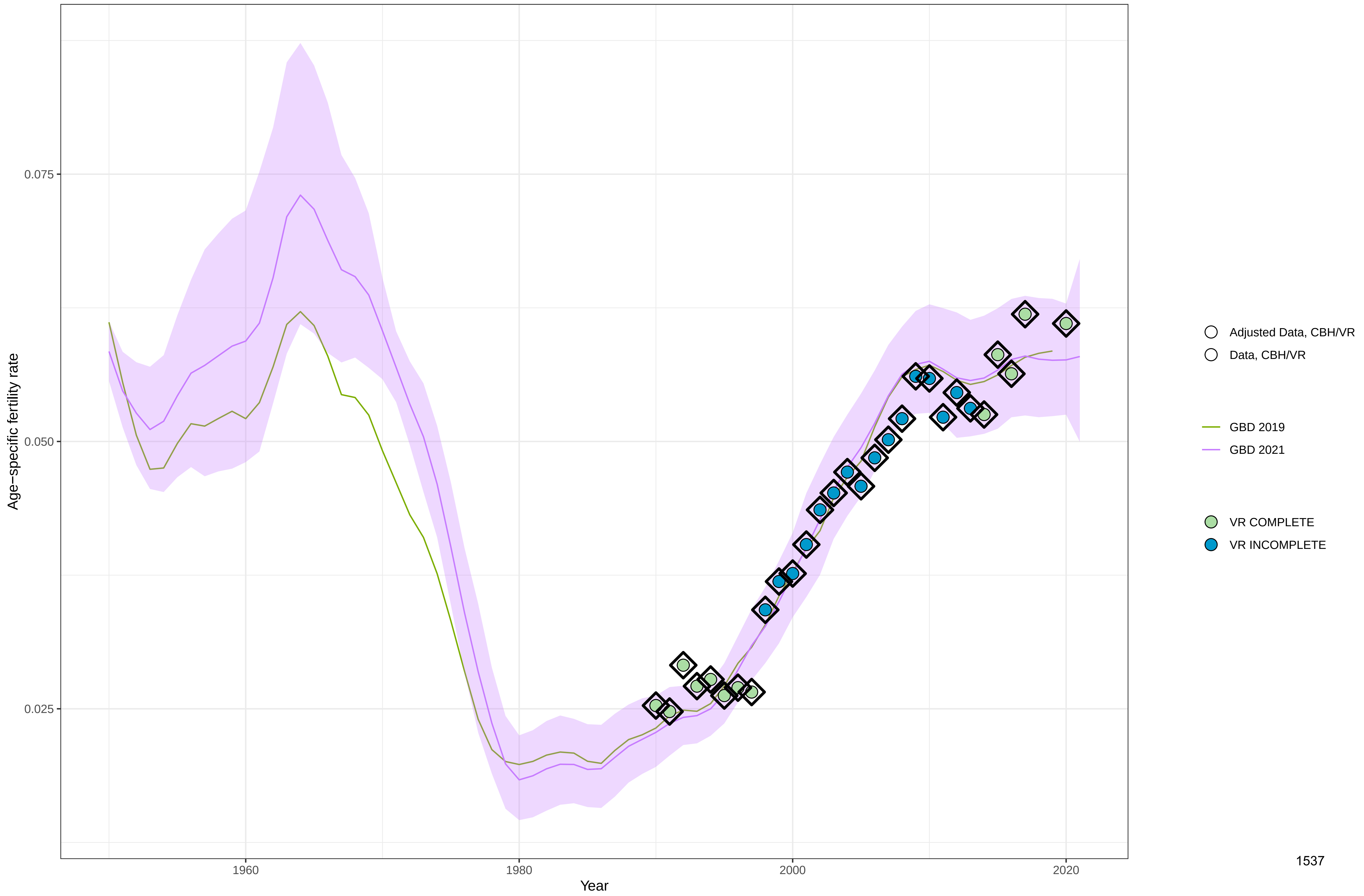

Age−specific fertility fitted trend lines, 1950−2021: Basilicata, Ages: 40−44
Data points enclosed with a diamond are reference sources

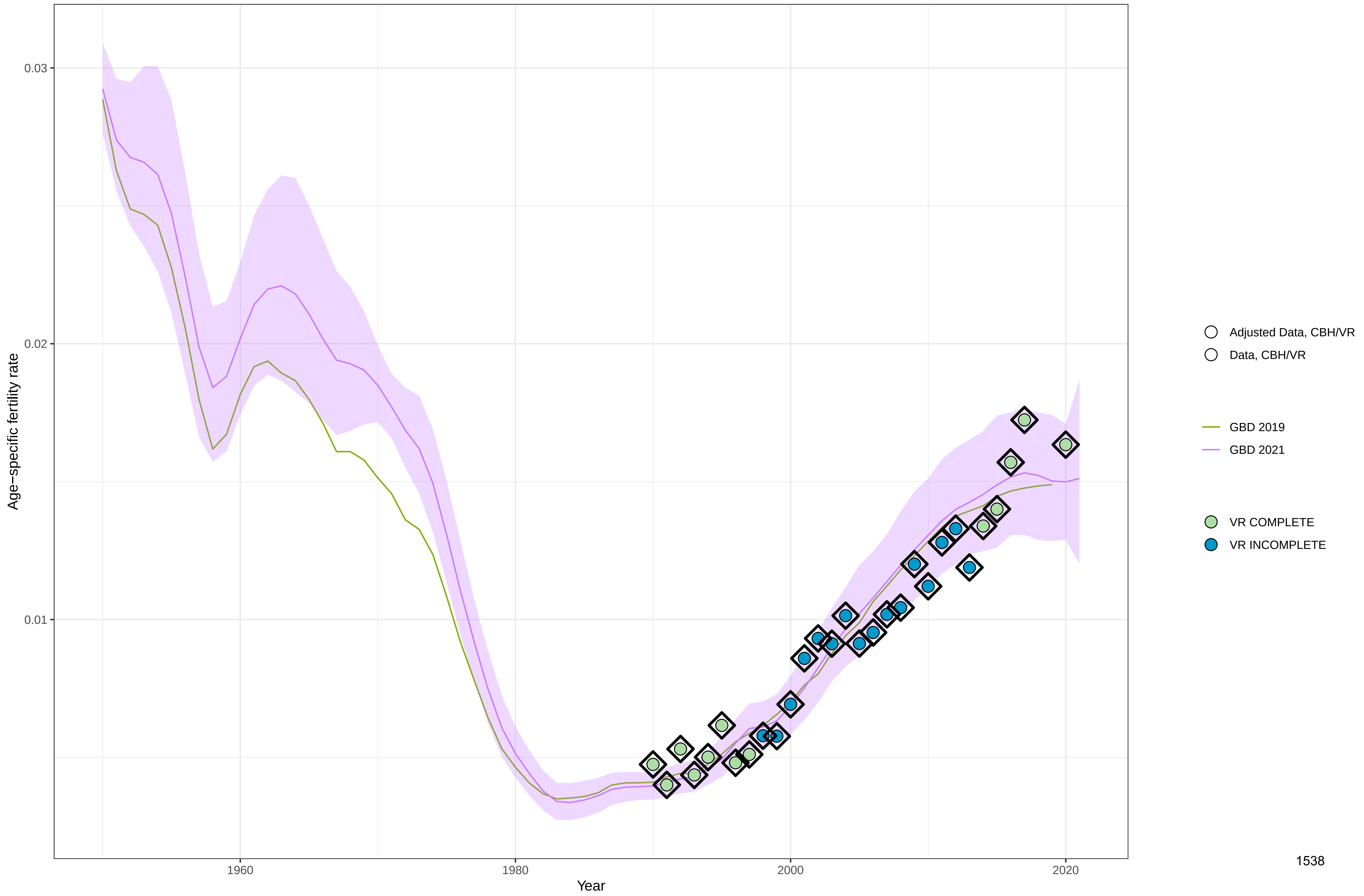

Age−specific fertility fitted trend lines, 1950−2021: Basilicata, Ages: 45−49
Data points enclosed with a diamond are reference sources

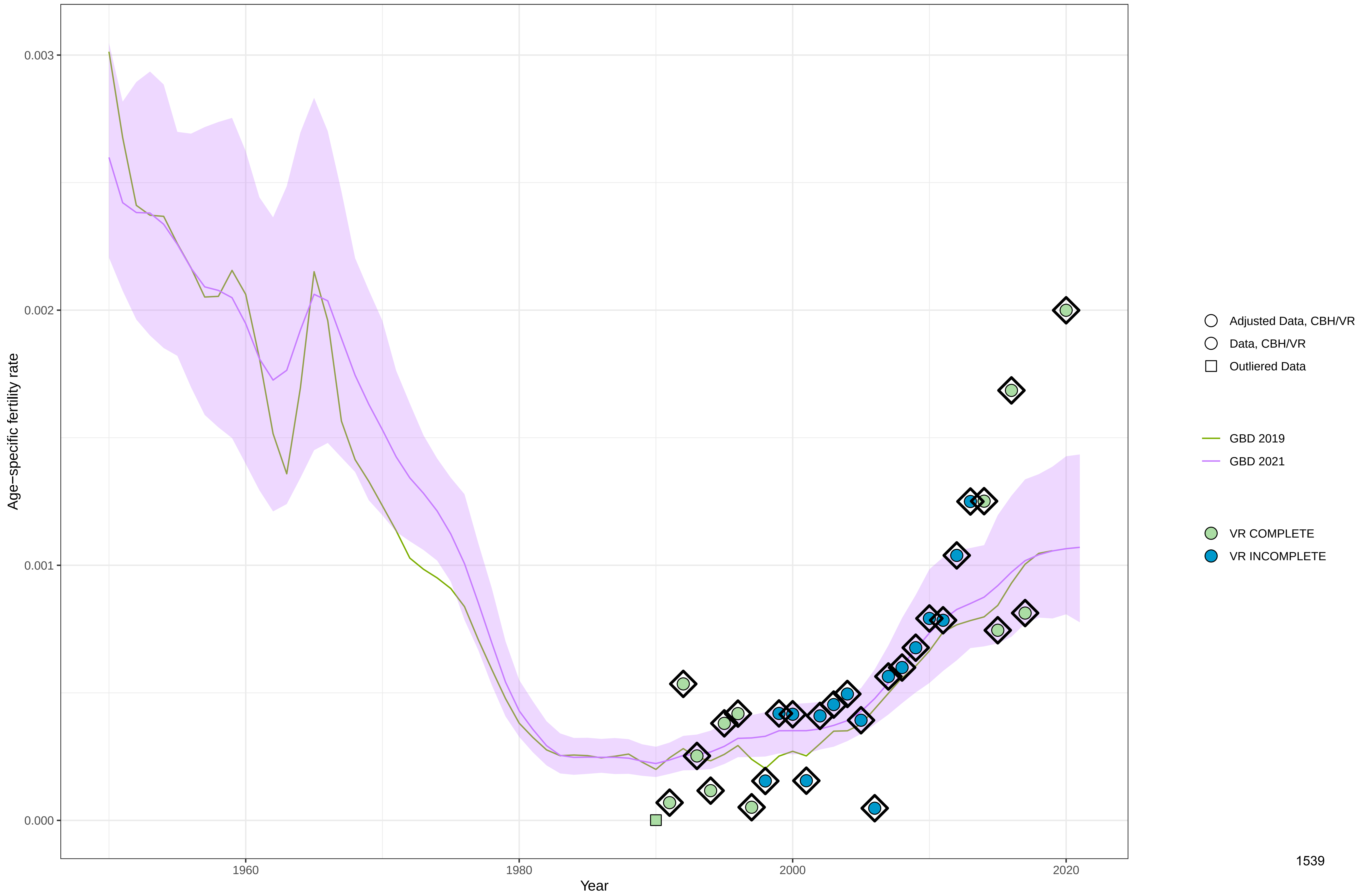

Age−specific fertility fitted trend lines, 1950−2021: Basilicata, Ages: 50−54
Data points enclosed with a diamond are reference sources

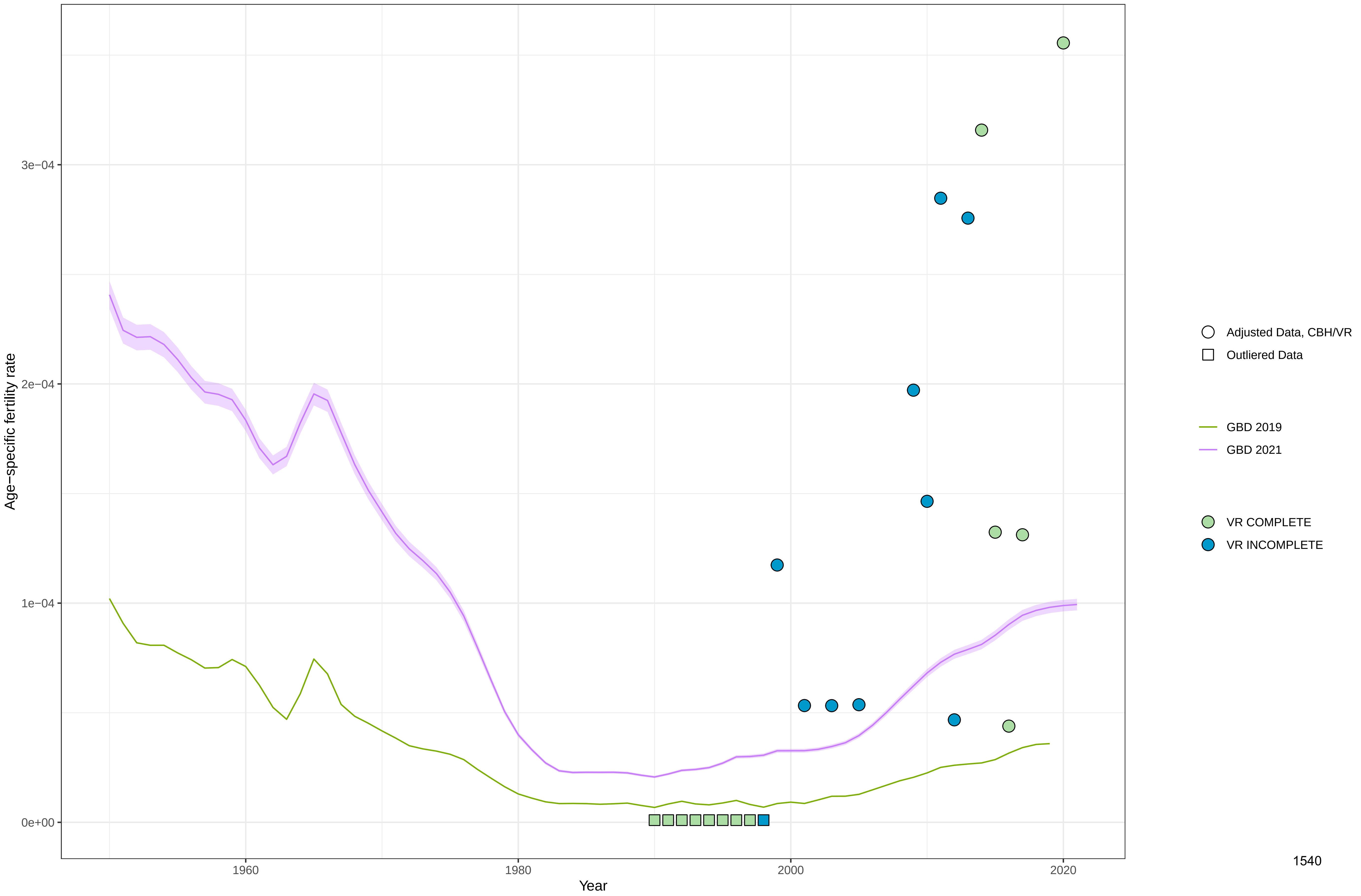

Total fertility rate fitted trend lines, 1950−2021: Basilicata TFR
Data points enclosed with a diamond are reference sources

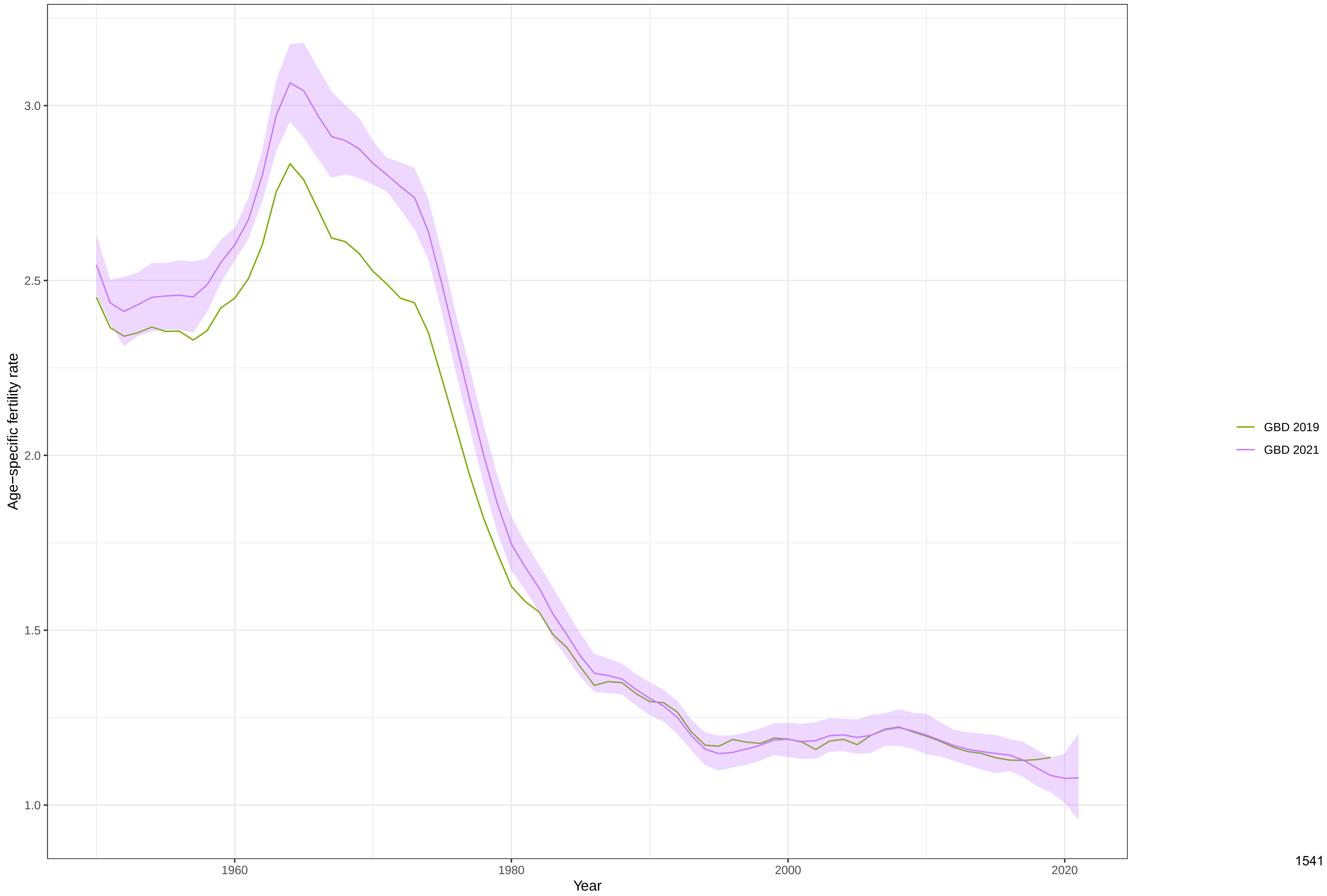

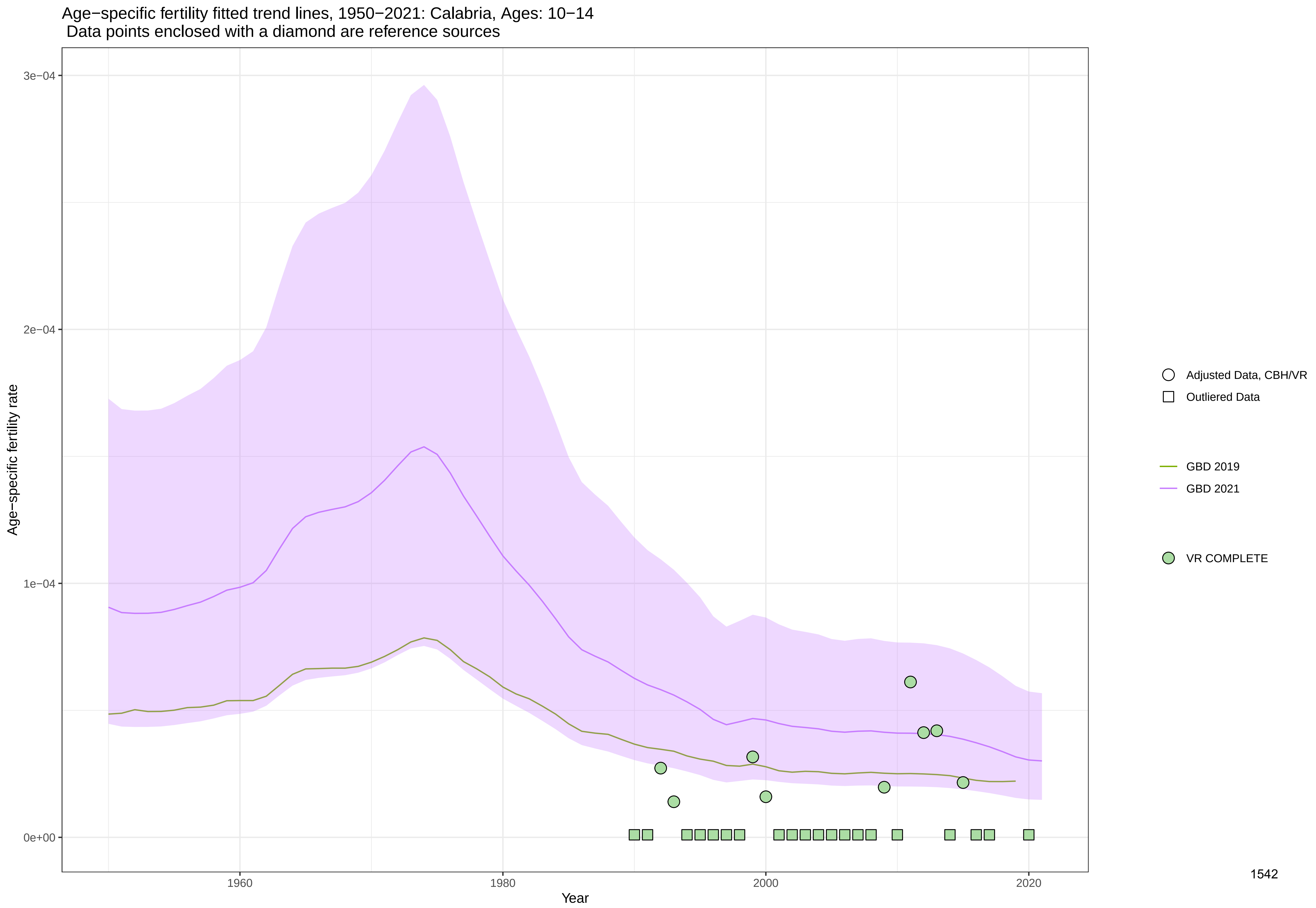

Age−specific fertility fitted trend lines, 1950−2021: Calabria, Ages: 10−14
Data points enclosed with a diamond are reference sources
3e−04
2e−04
1e−04
0e+00
Age−specific fertility rate
1960
1980
2000
2020
Year
Adjusted Data, CBH/VR
Outliered Data
GBD 2019
GBD 2021
VR COMPLETE

1542

Age−specific fertility fitted trend lines, 1950−2021: Calabria, Ages: 15−19
Data points enclosed with a diamond are reference sources

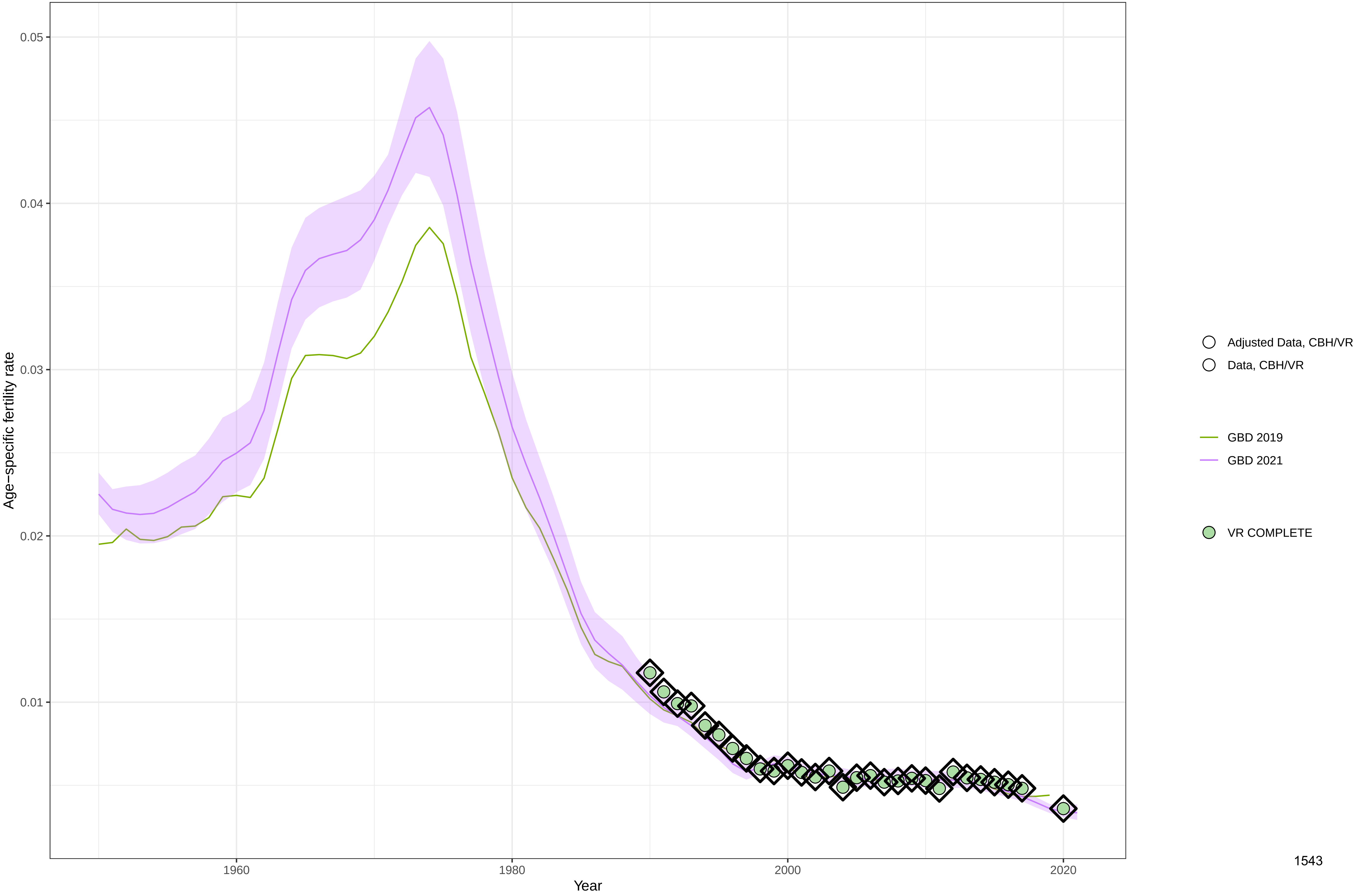

Age−specific fertility fitted trend lines, 1950−2021: Calabria, Ages: 20−24
Data points enclosed with a diamond are reference sources

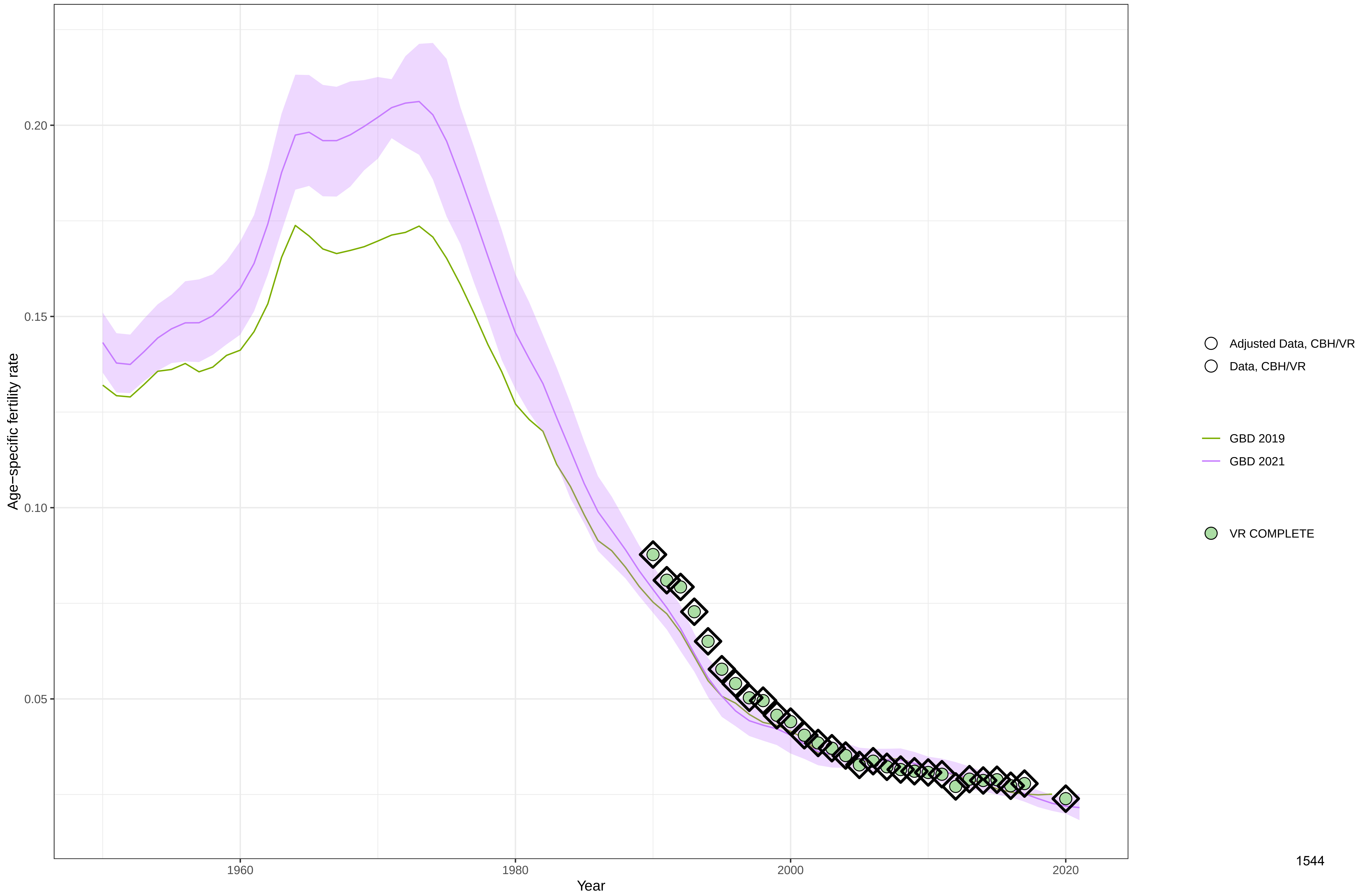

Age−specific fertility fitted trend lines, 1950−2021: Calabria, Ages: 25−29
Data points enclosed with a diamond are reference sources

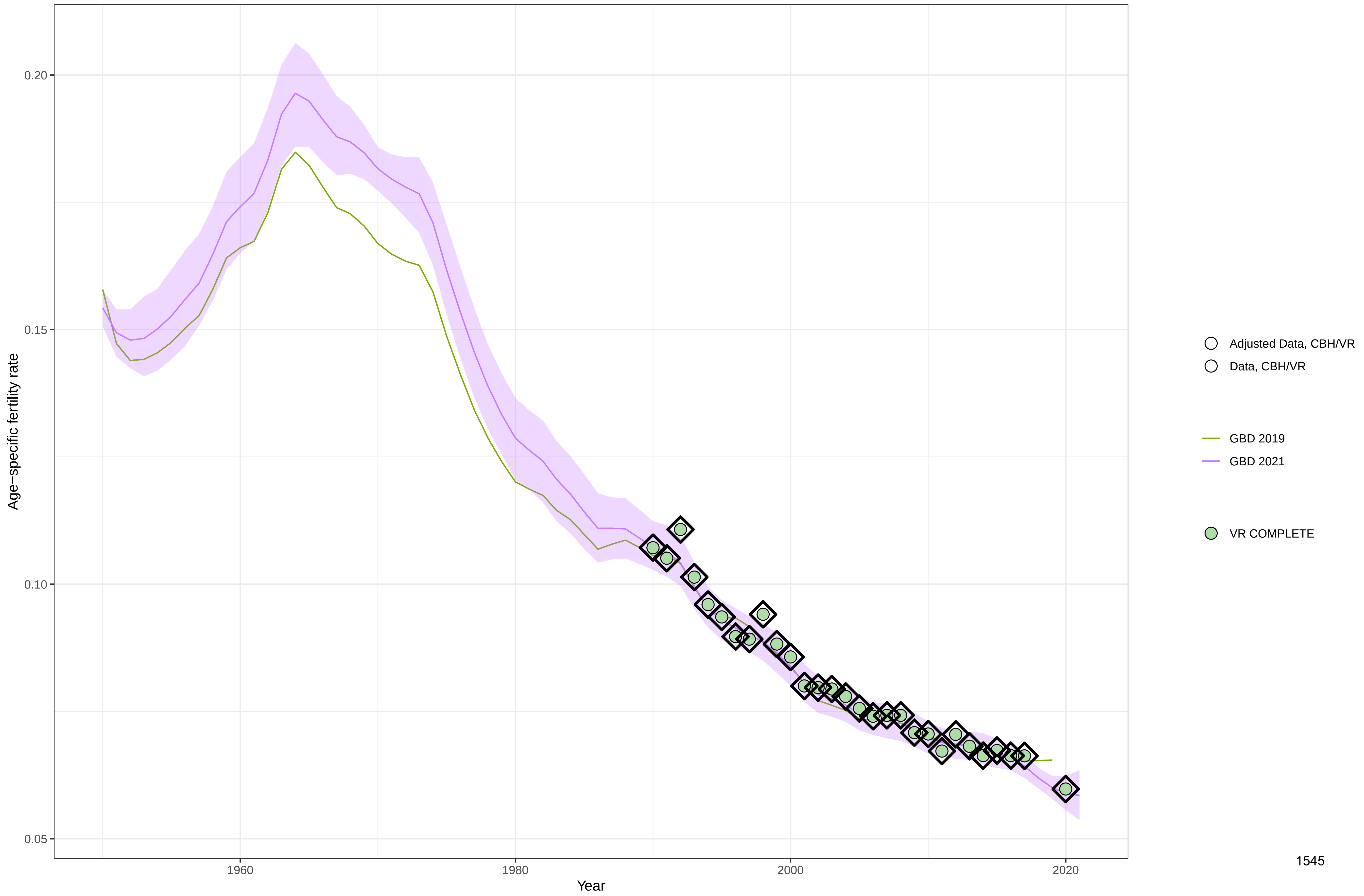

Age−specific fertility fitted trend lines, 1950−2021: Calabria, Ages: 30−34
Data points enclosed with a diamond are reference sources

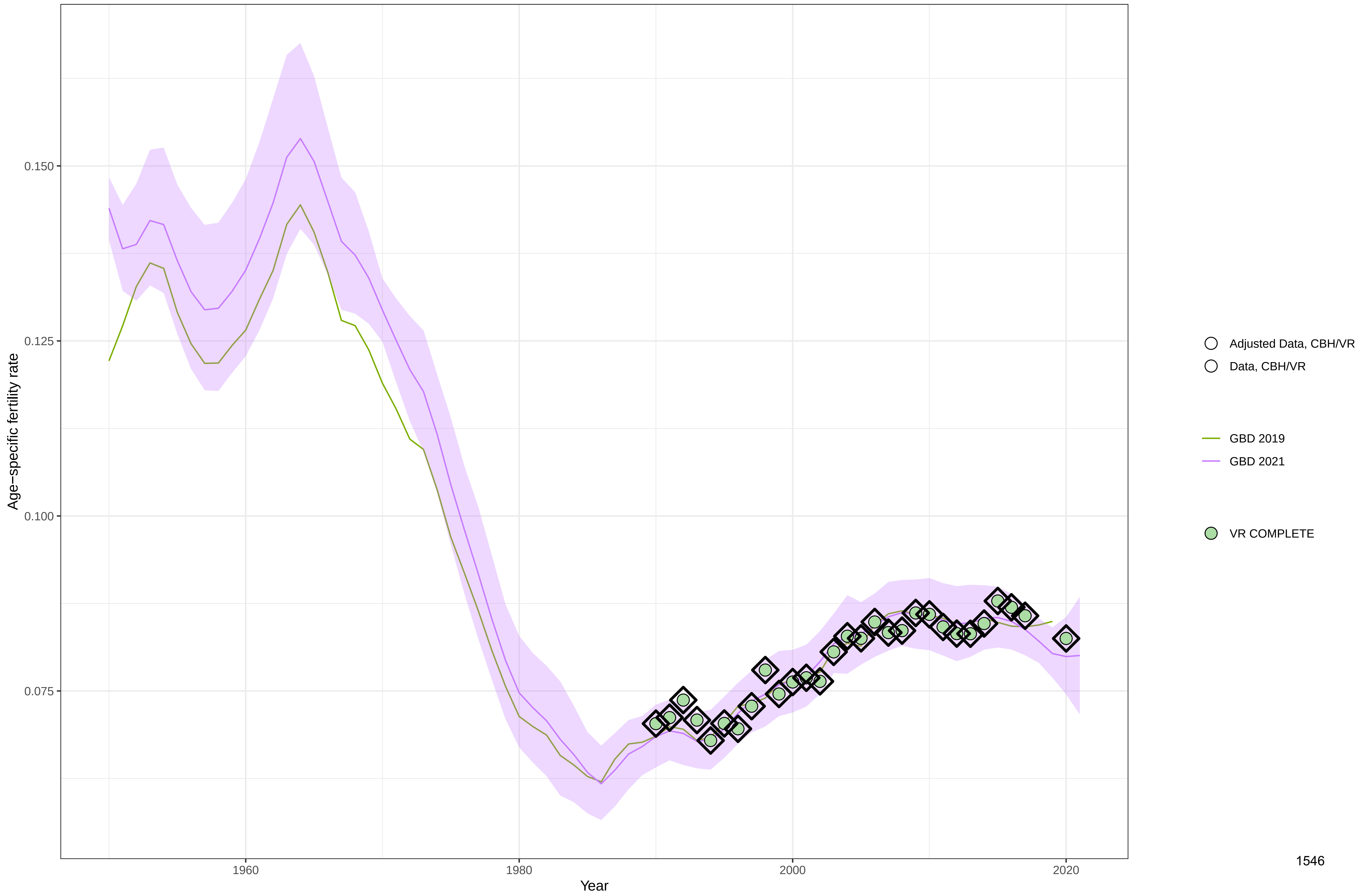

Age−specific fertility fitted trend lines, 1950−2021: Calabria, Ages: 35−39
Data points enclosed with a diamond are reference sources

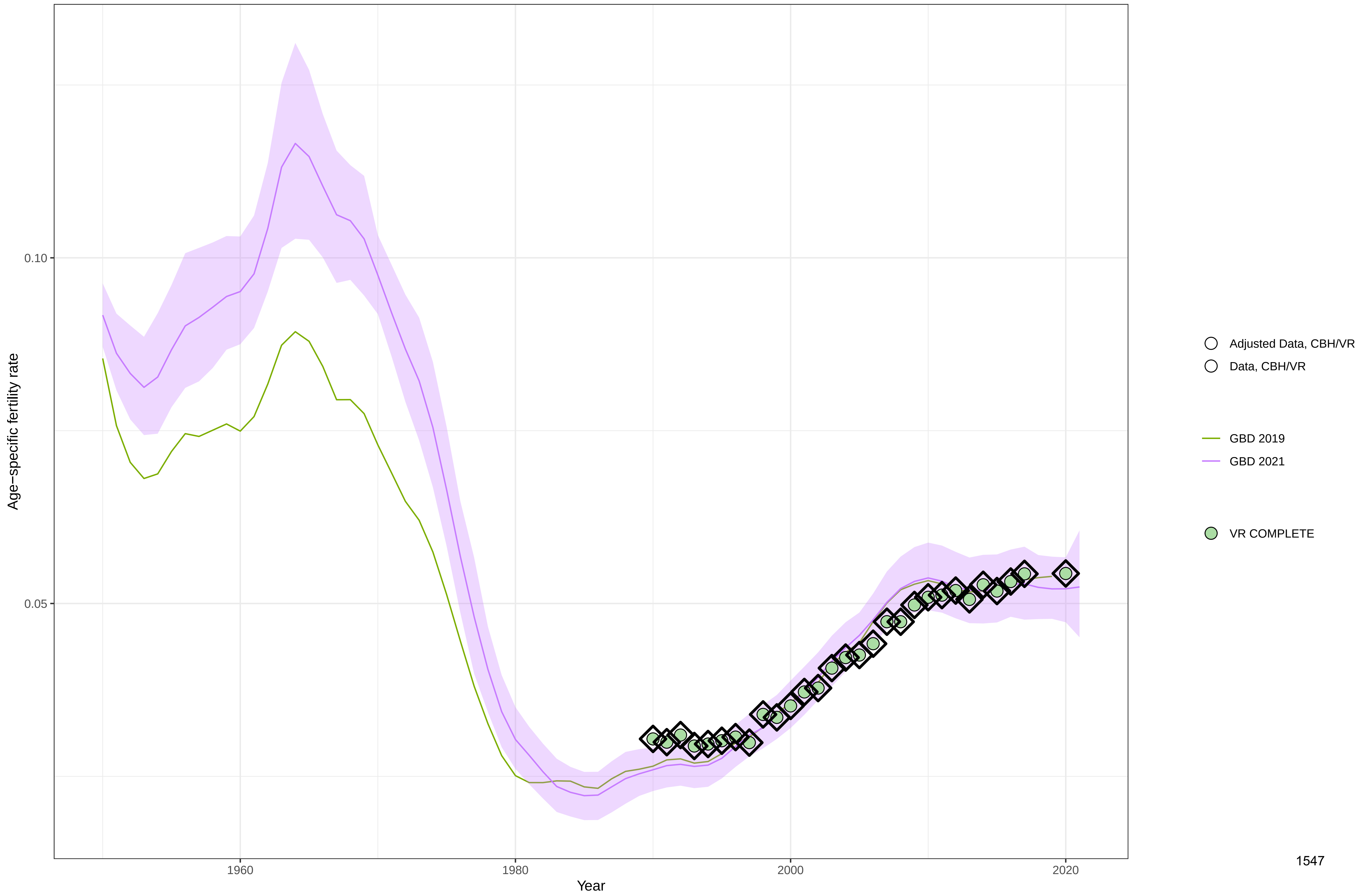

Age−specific fertility fitted trend lines, 1950−2021: Calabria, Ages: 40−44
Data points enclosed with a diamond are reference sources

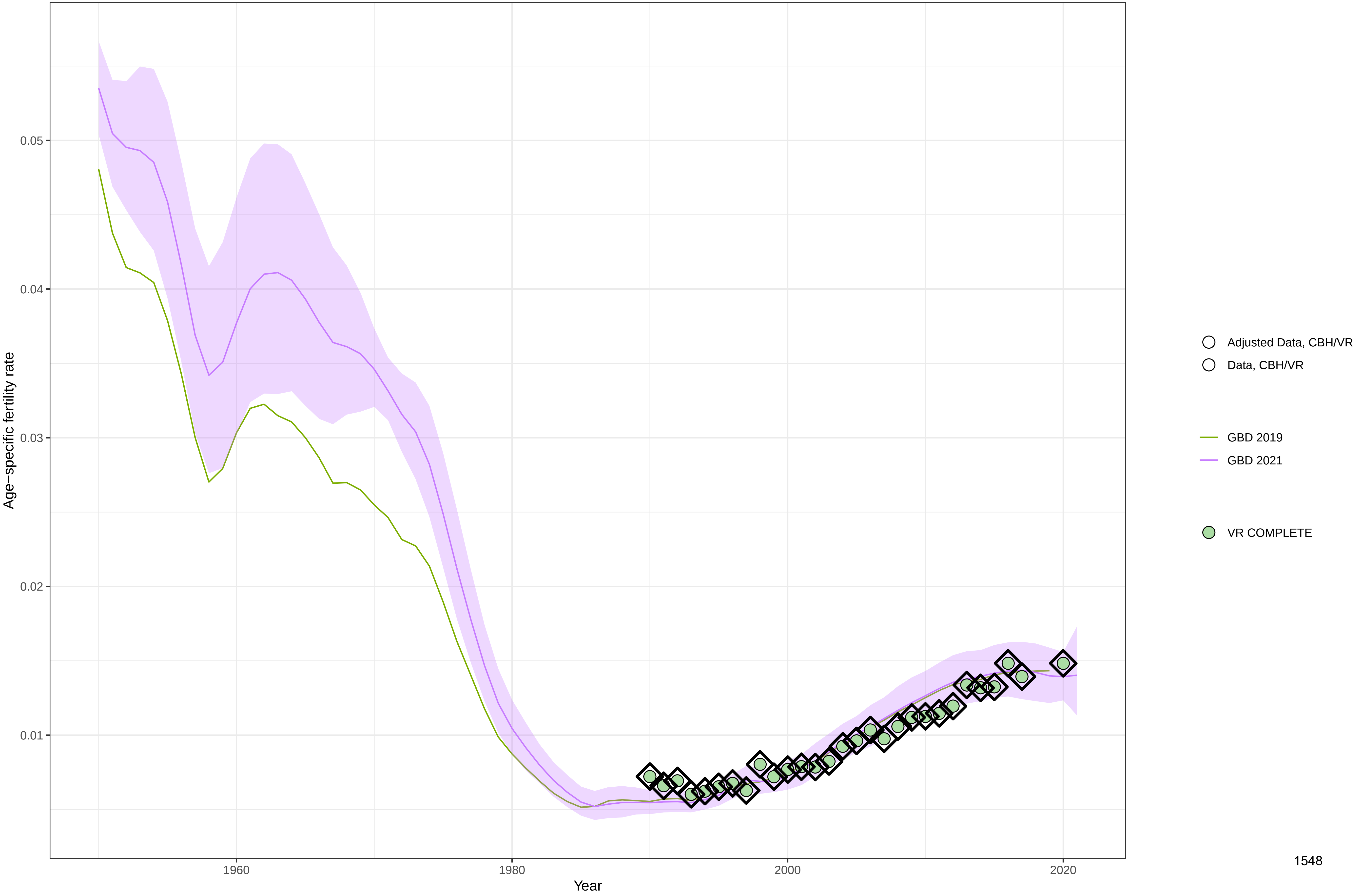

Age−specific fertility fitted trend lines, 1950−2021: Calabria, Ages: 45−49
Data points enclosed with a diamond are reference sources

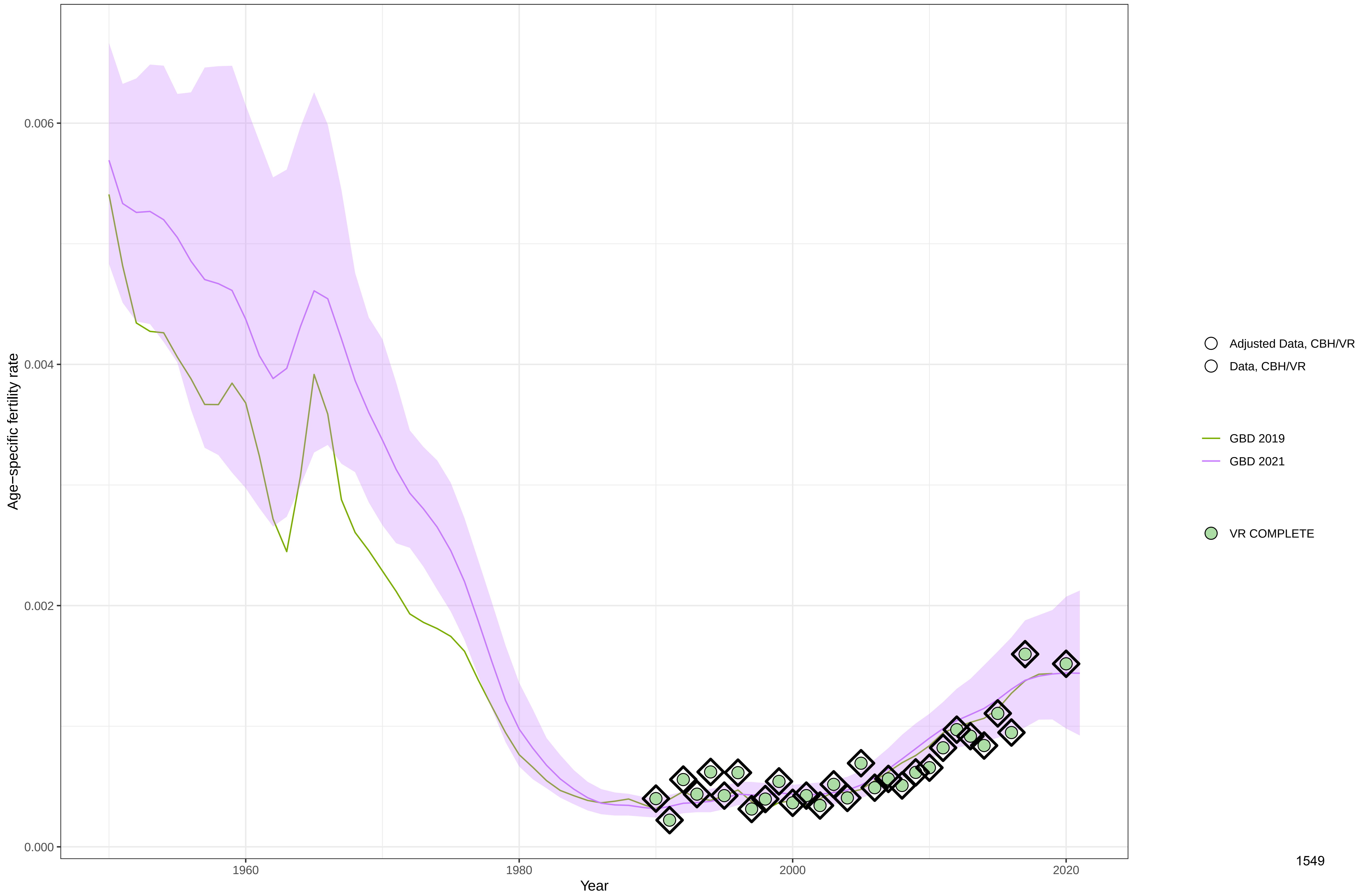

Age−specific fertility fitted trend lines, 1950−2021: Calabria, Ages: 50−54
Data points enclosed with a diamond are reference sources

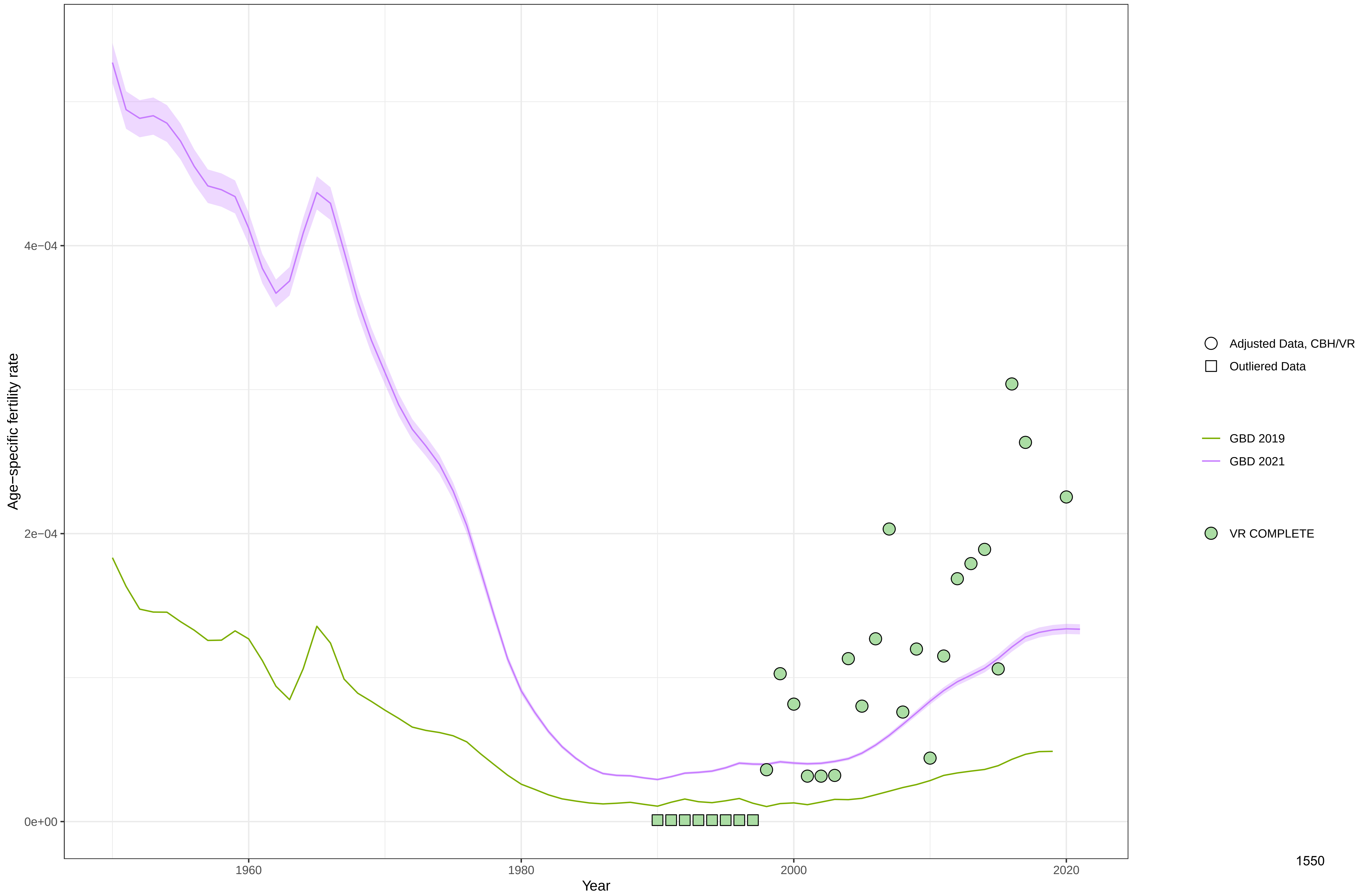

Total fertility rate fitted trend lines, 1950−2021: Calabria TFR
Data points enclosed with a diamond are reference sources

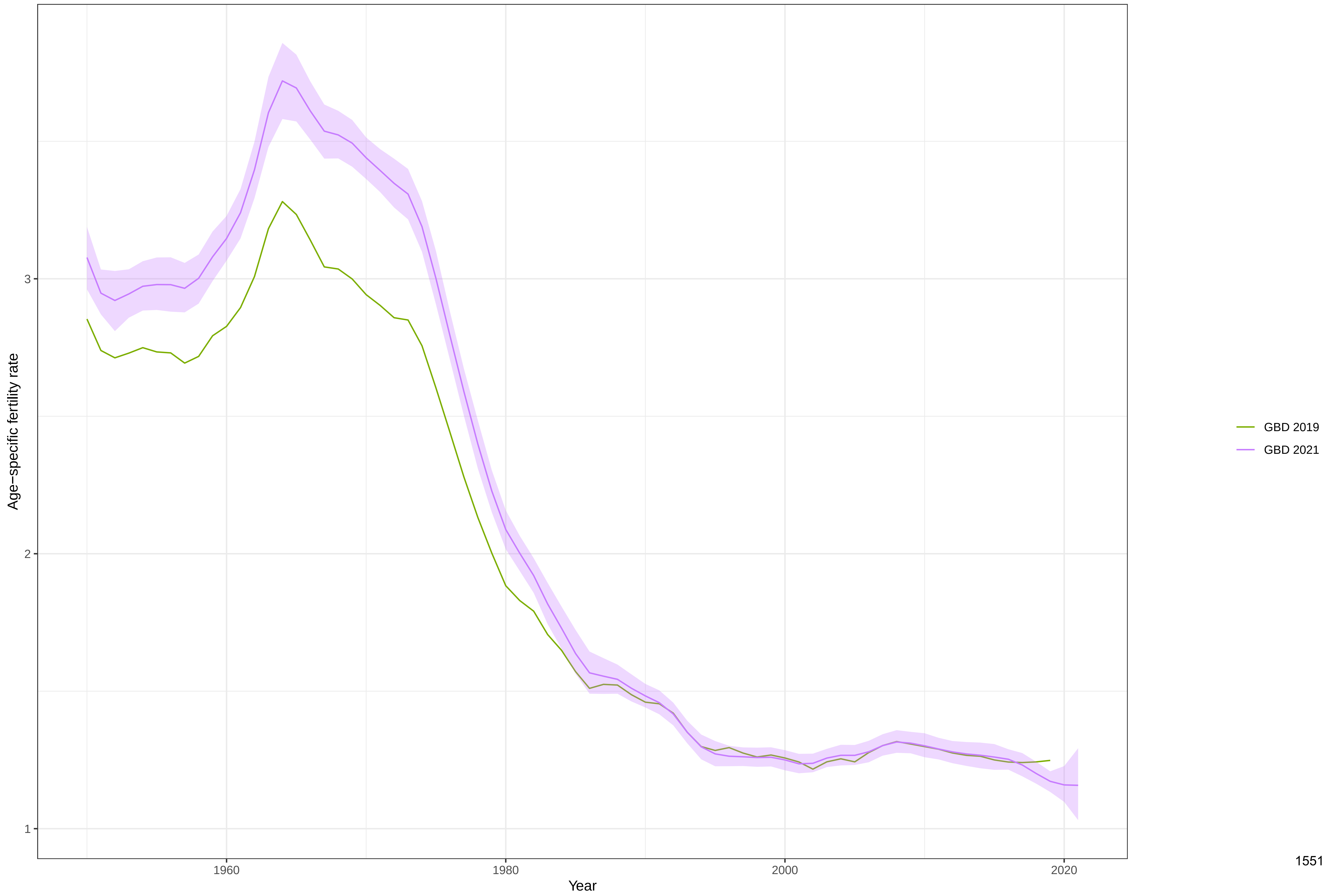

Age−specific fertility fitted trend lines, 1950−2021: Campania, Ages: 10−14
Data points enclosed with a diamond are reference sources

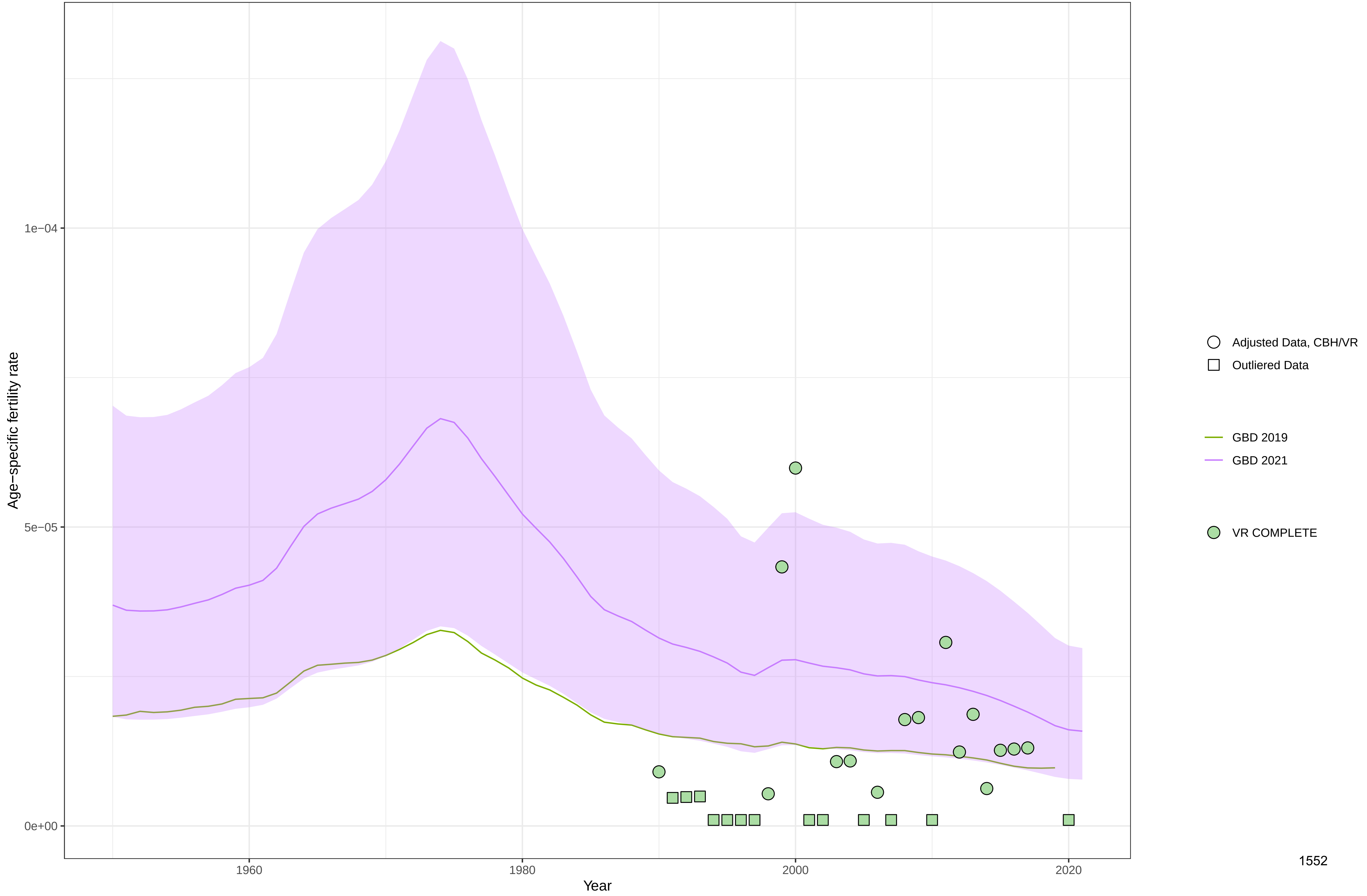

Age−specific fertility fitted trend lines, 1950−2021: Campania, Ages: 15−19
Data points enclosed with a diamond are reference sources

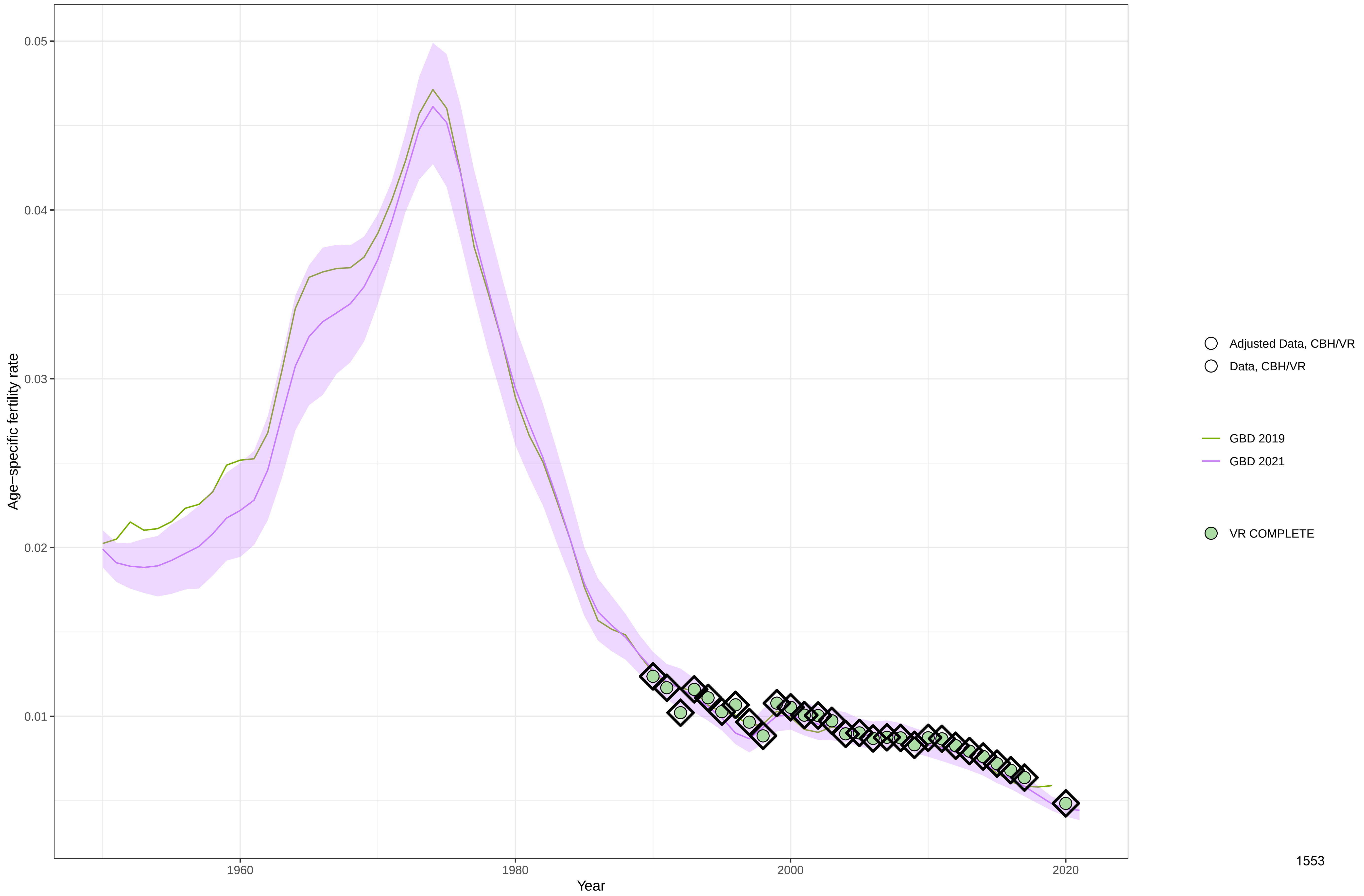

Age−specific fertility fitted trend lines, 1950−2021: Campania, Ages: 20−24
Data points enclosed with a diamond are reference sources

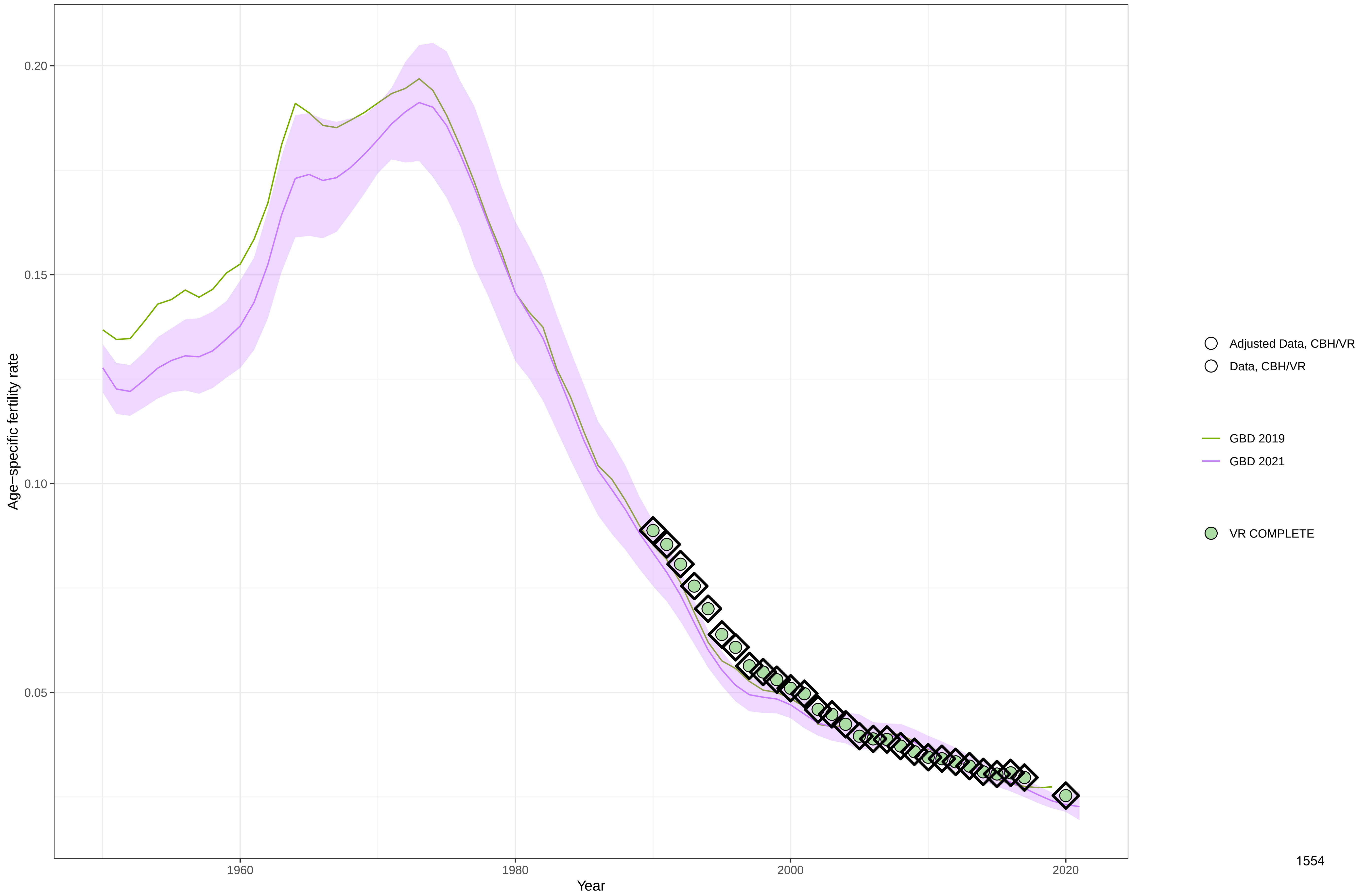

Age−specific fertility fitted trend lines, 1950−2021: Campania, Ages: 25−29
Data points enclosed with a diamond are reference sources

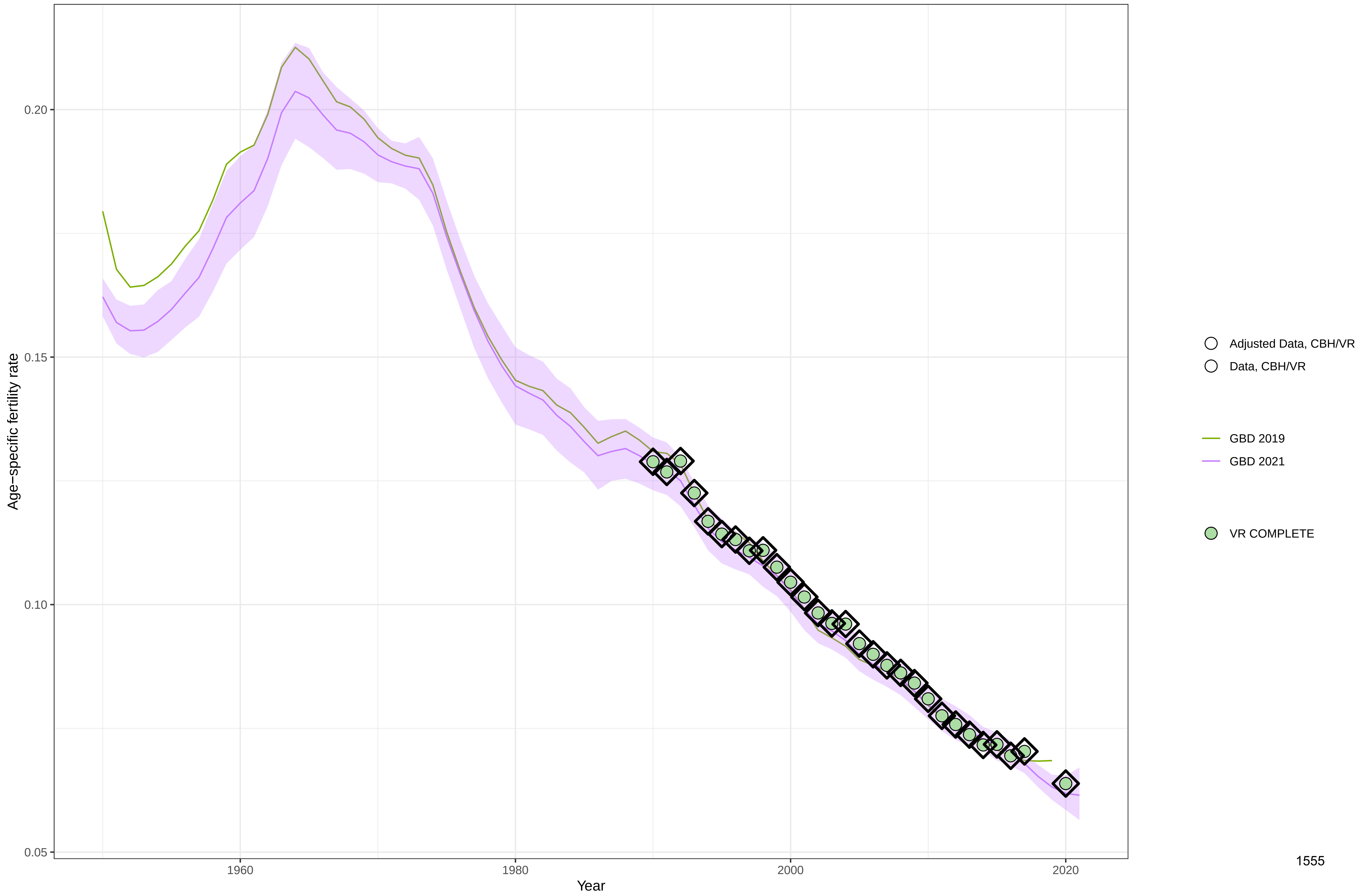

Age−specific fertility fitted trend lines, 1950−2021: Campania, Ages: 30−34
Data points enclosed with a diamond are reference sources

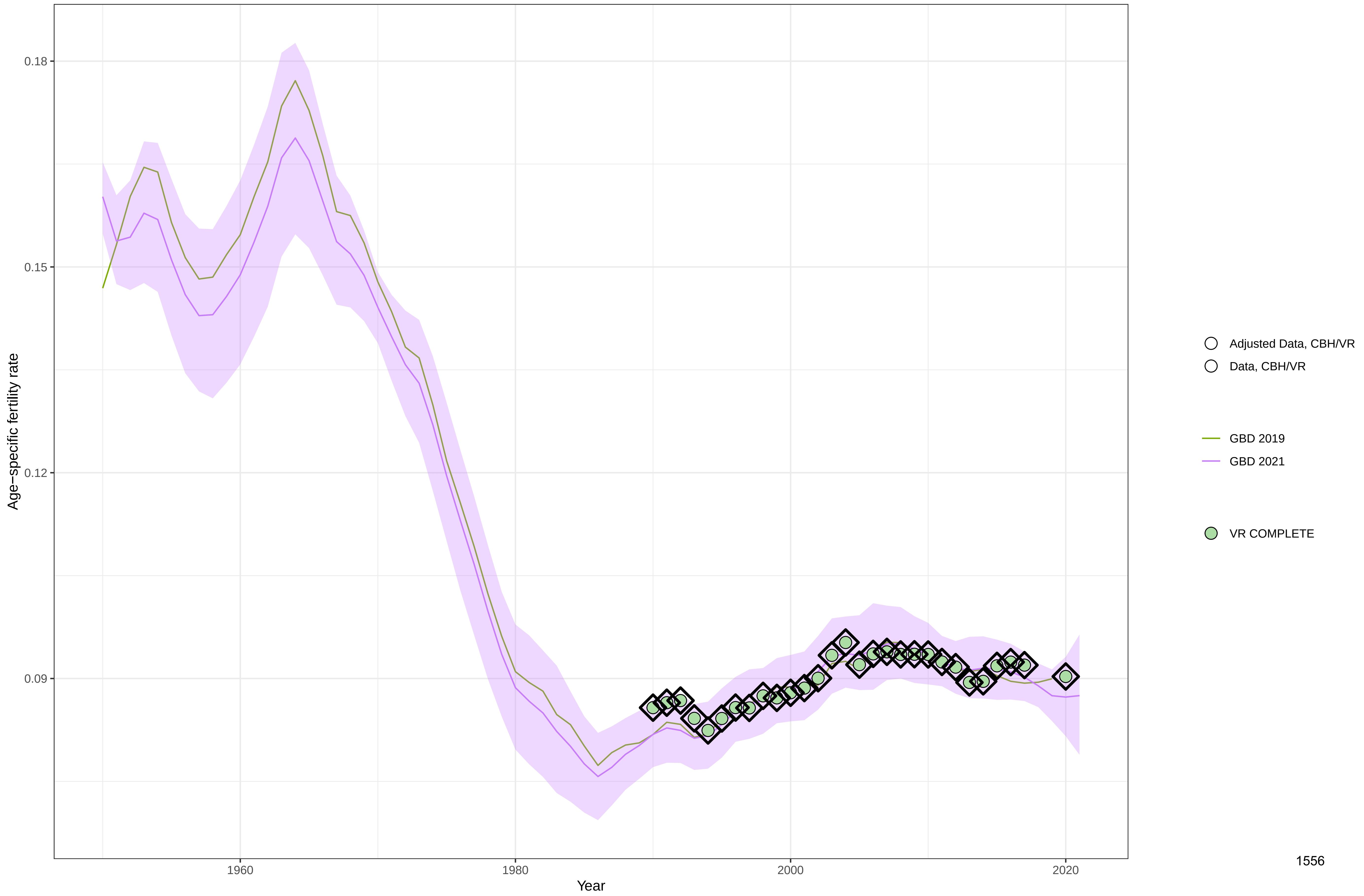

Age−specific fertility fitted trend lines, 1950−2021: Campania, Ages: 35−39
Data points enclosed with a diamond are reference sources

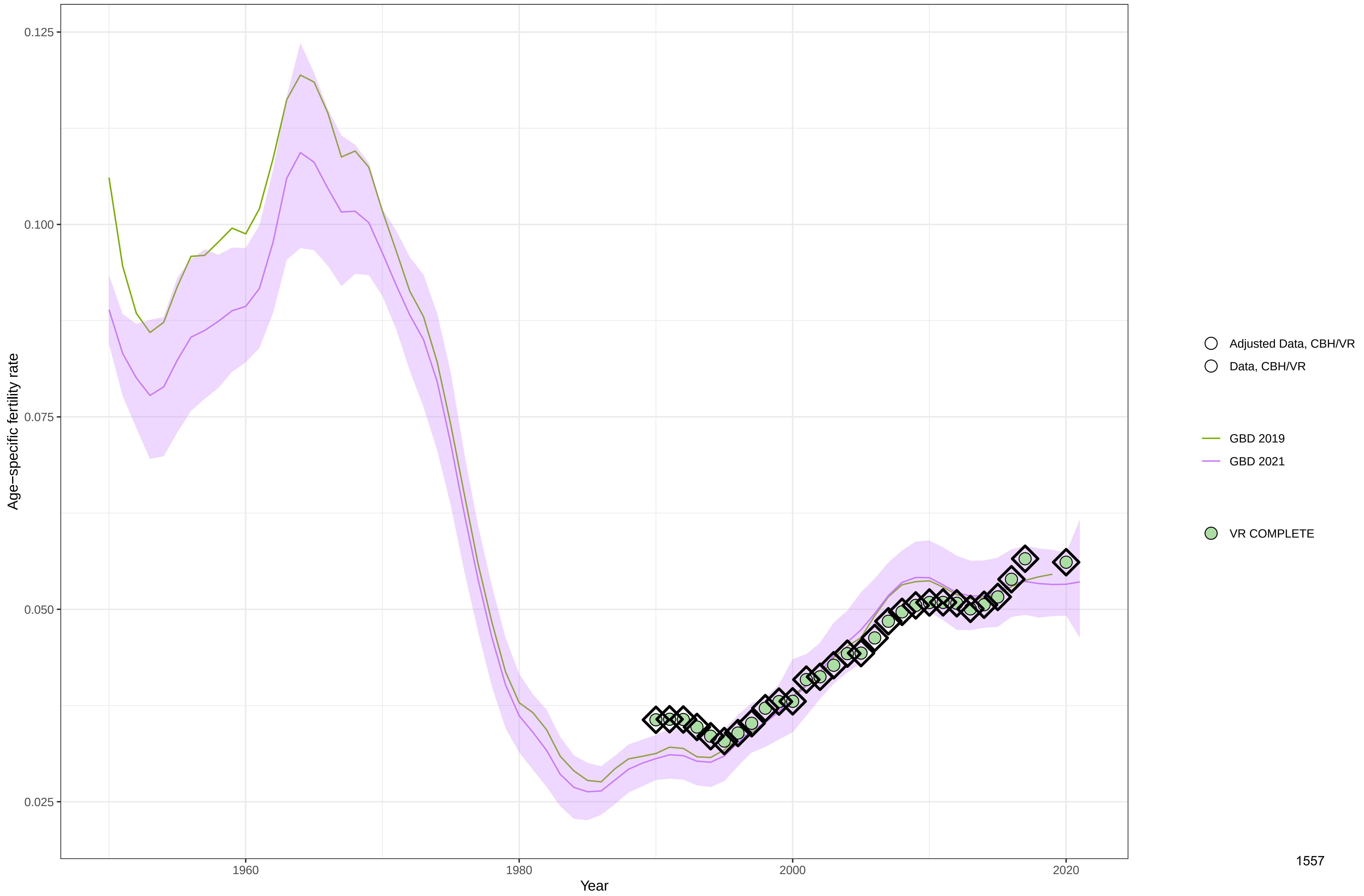

Age−specific fertility fitted trend lines, 1950−2021: Campania, Ages: 40−44
Data points enclosed with a diamond are reference sources

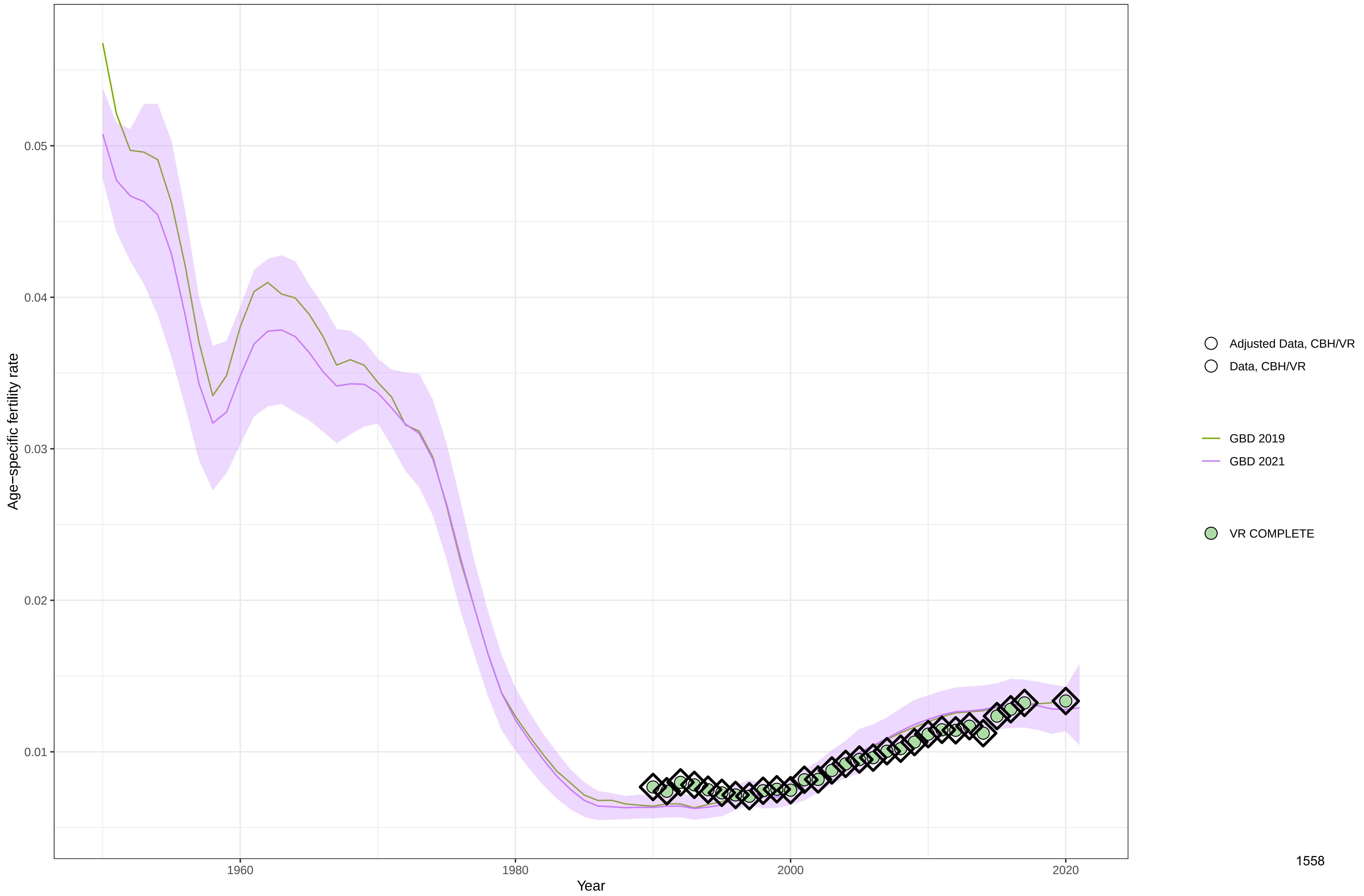

Age−specific fertility fitted trend lines, 1950−2021: Campania, Ages: 45−49
Data points enclosed with a diamond are reference sources

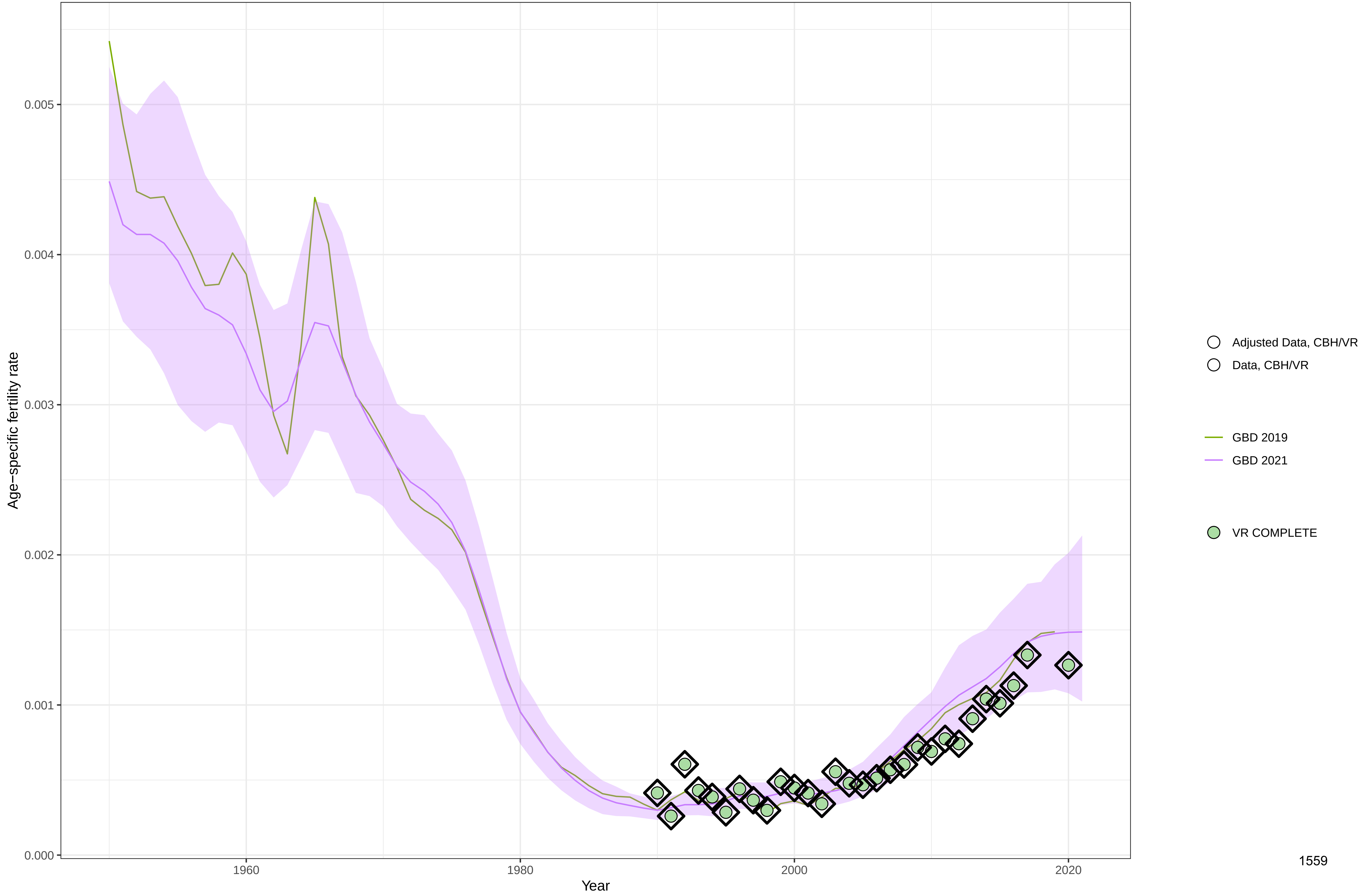

Age−specific fertility fitted trend lines, 1950−2021: Campania, Ages: 50−54
Data points enclosed with a diamond are reference sources

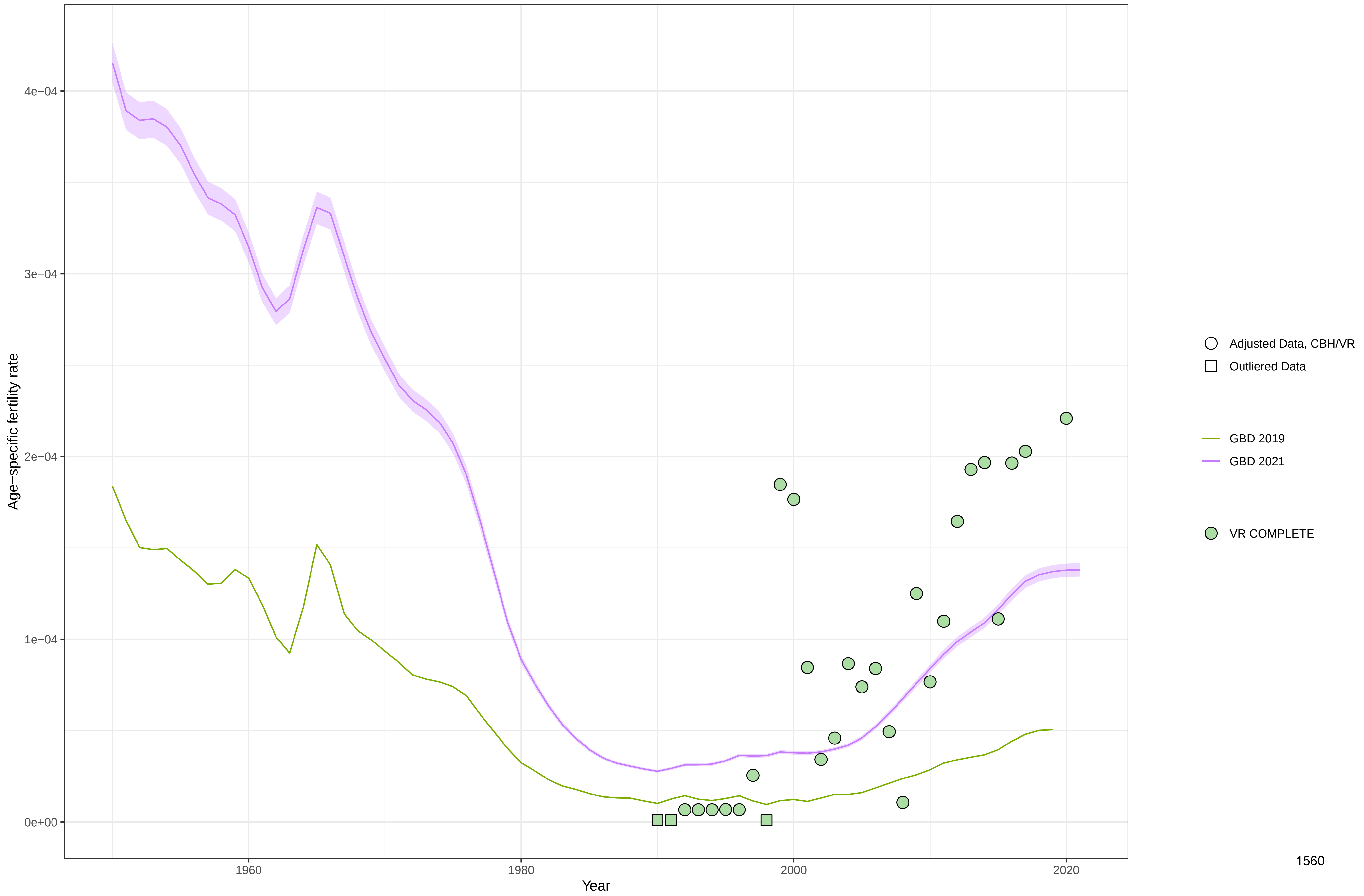

Total fertility rate fitted trend lines, 1950−2021: Campania TFR
Data points enclosed with a diamond are reference sources

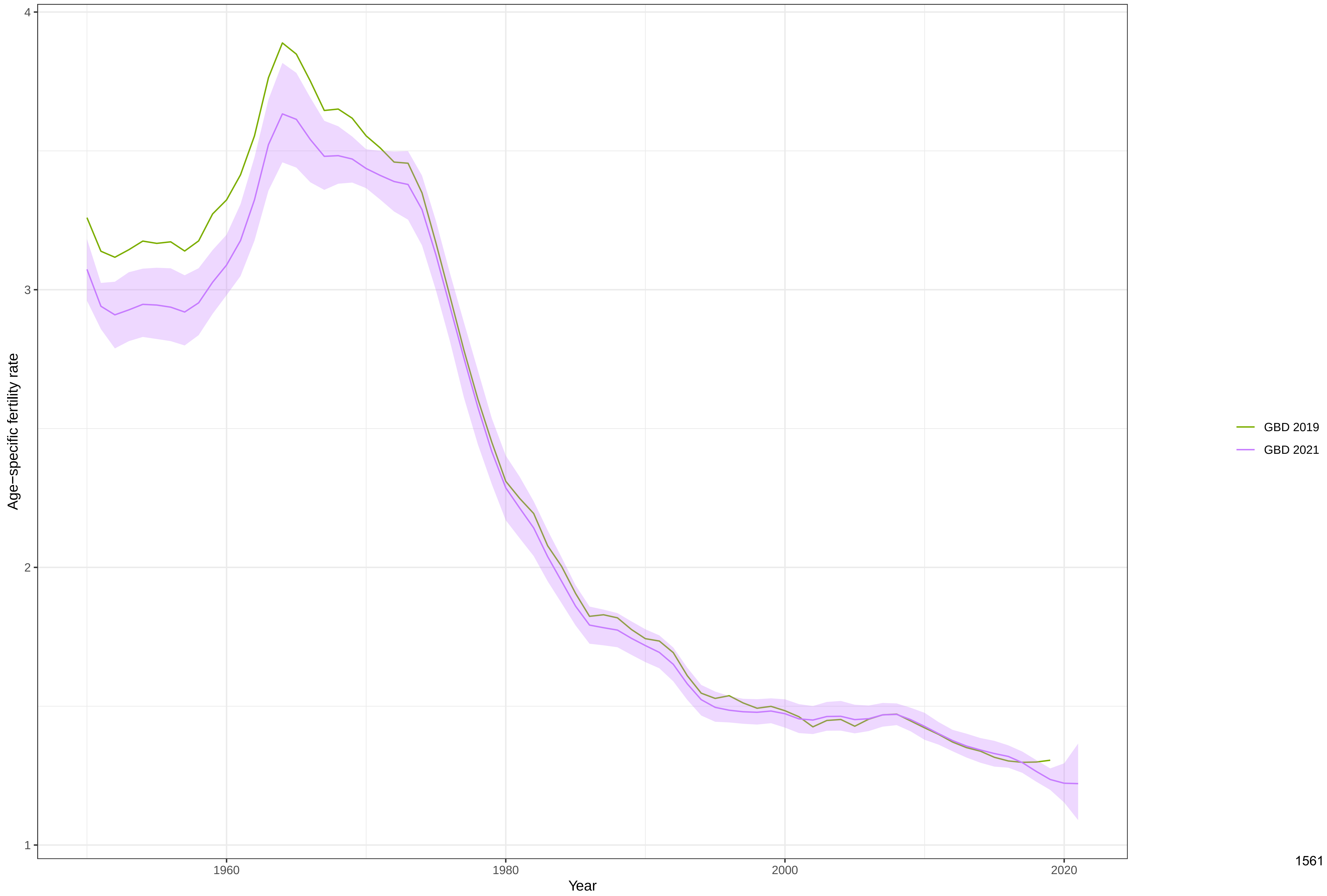

Age−specific fertility fitted trend lines, 1950−2021: Emilia−Romagna, Ages: 10−14
Data points enclosed with a diamond are reference sources

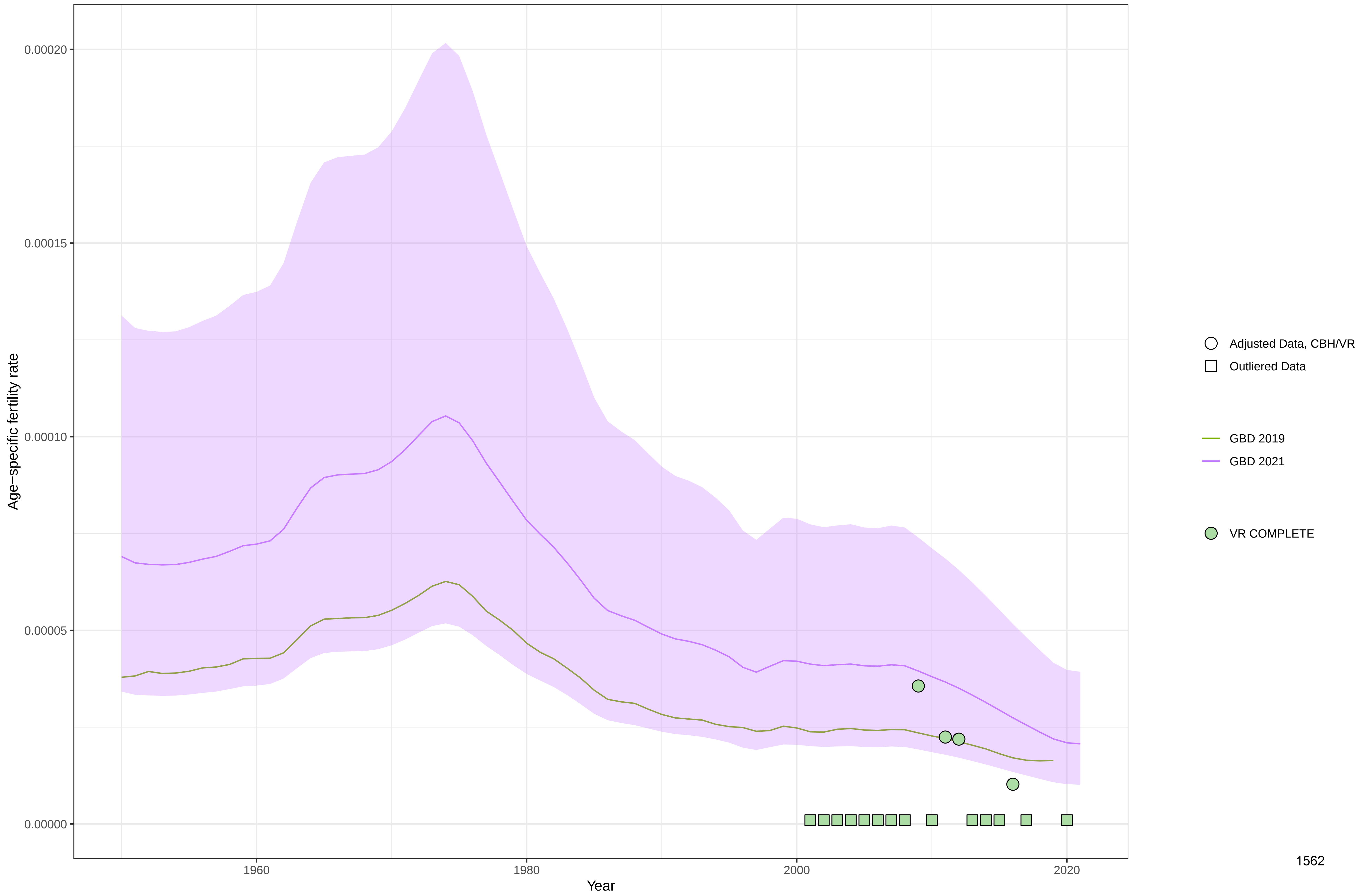

Age−specific fertility fitted trend lines, 1950−2021: Emilia−Romagna, Ages: 15−19
Data points enclosed with a diamond are reference sources

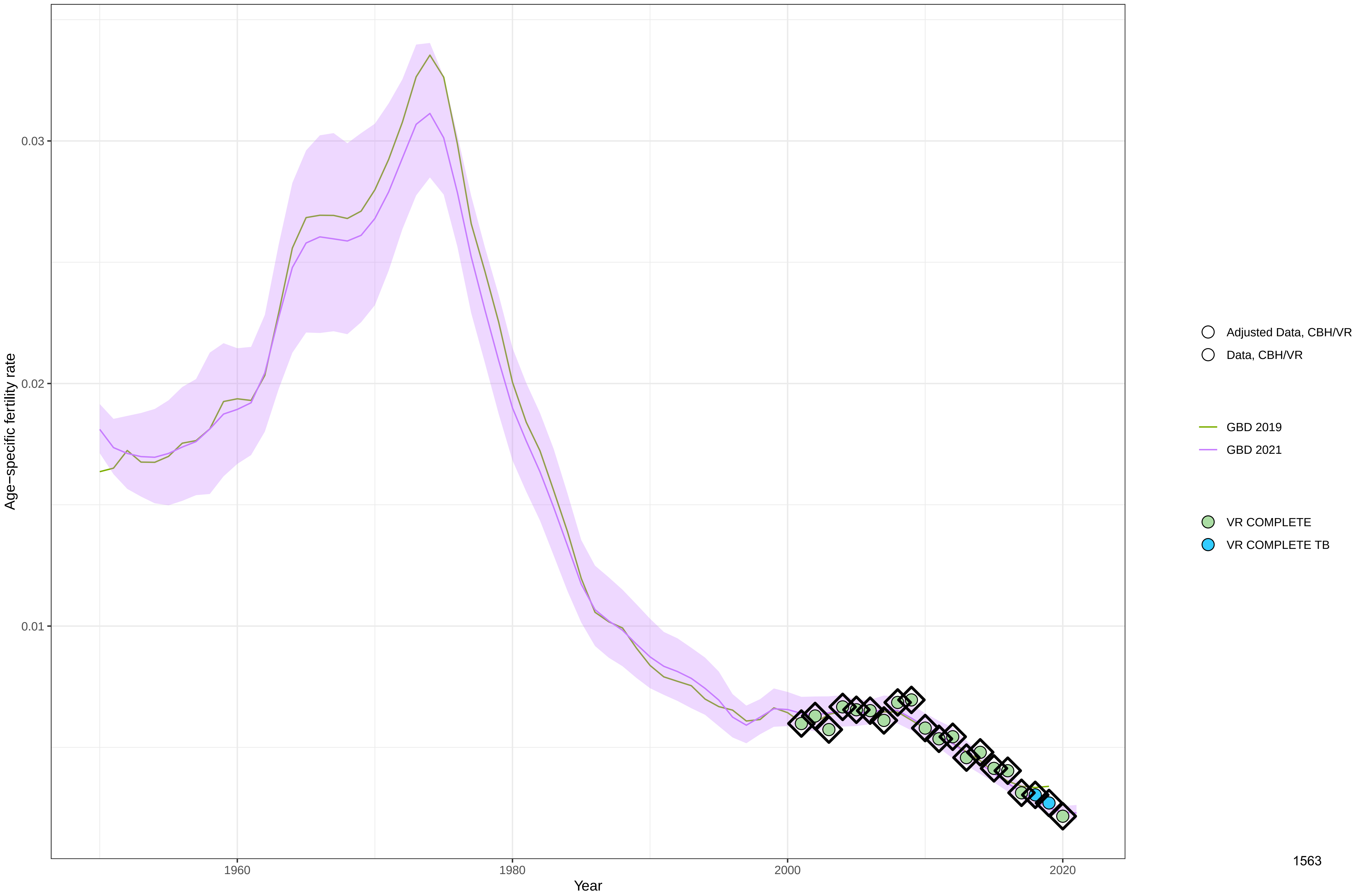

Age−specific fertility fitted trend lines, 1950−2021: Emilia−Romagna, Ages: 20−24
Data points enclosed with a diamond are reference sources

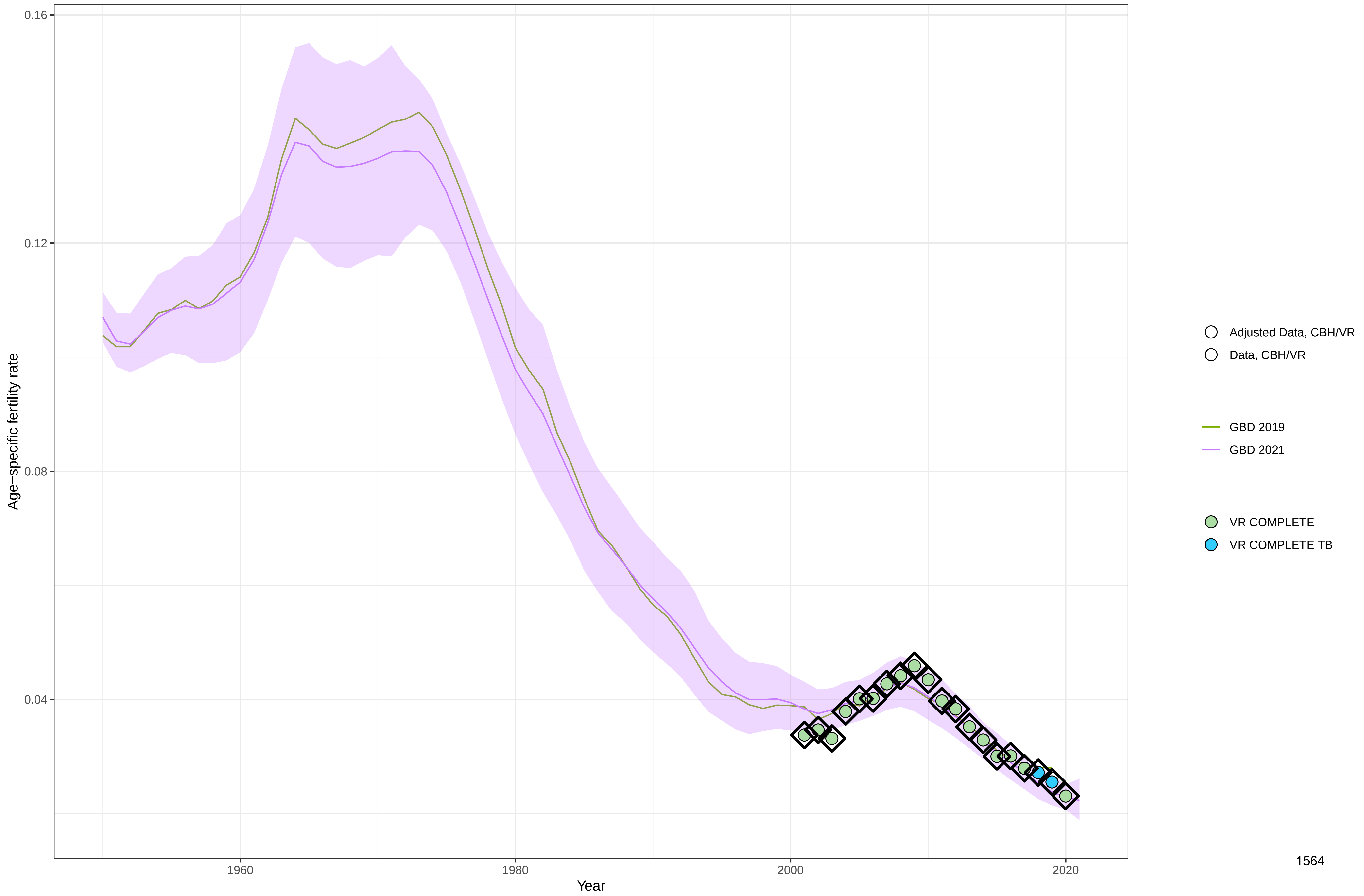

Age−specific fertility fitted trend lines, 1950−2021: Emilia−Romagna, Ages: 25−29
Data points enclosed with a diamond are reference sources

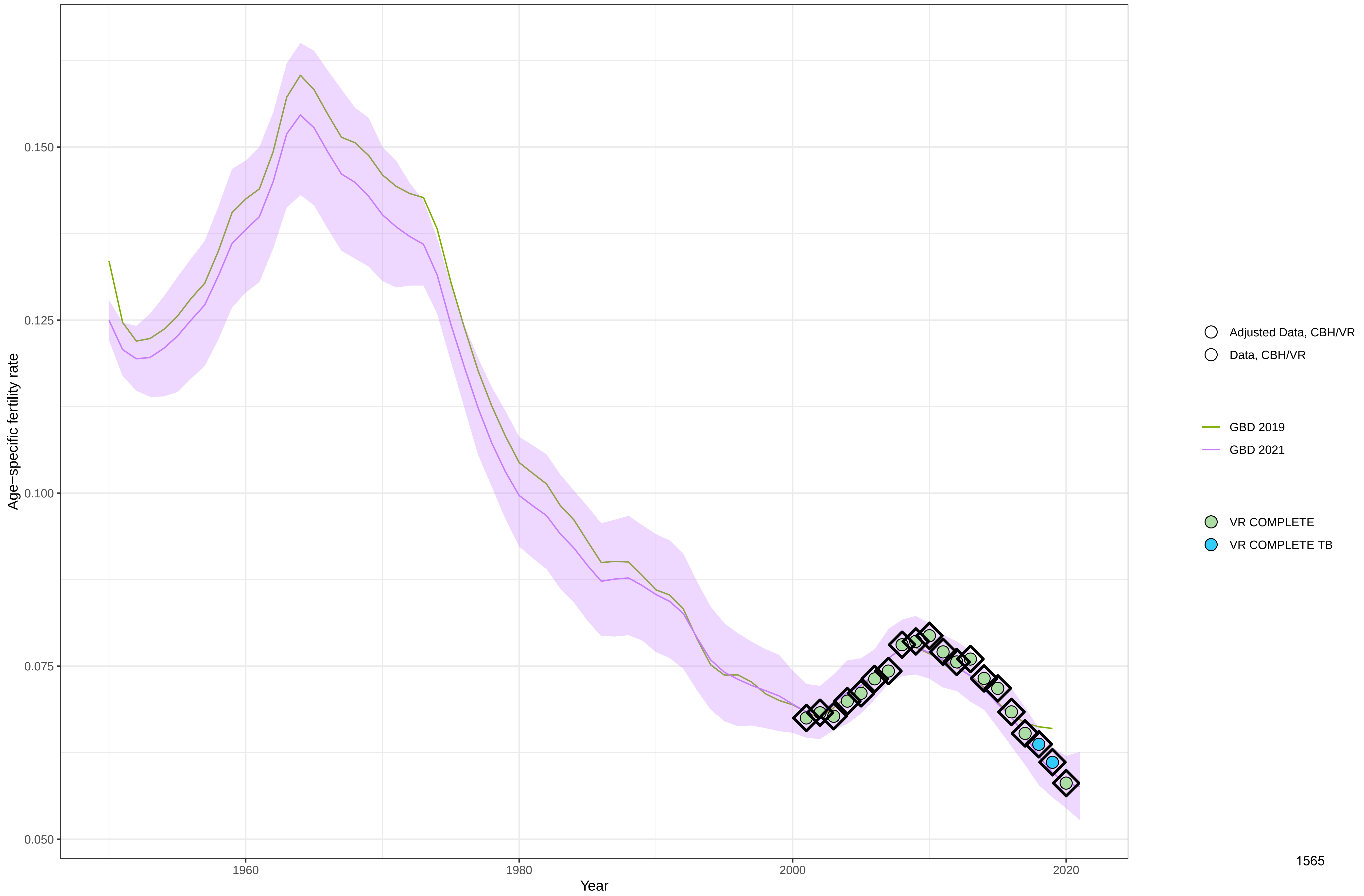

Age−specific fertility fitted trend lines, 1950−2021: Emilia−Romagna, Ages: 30−34
Data points enclosed with a diamond are reference sources

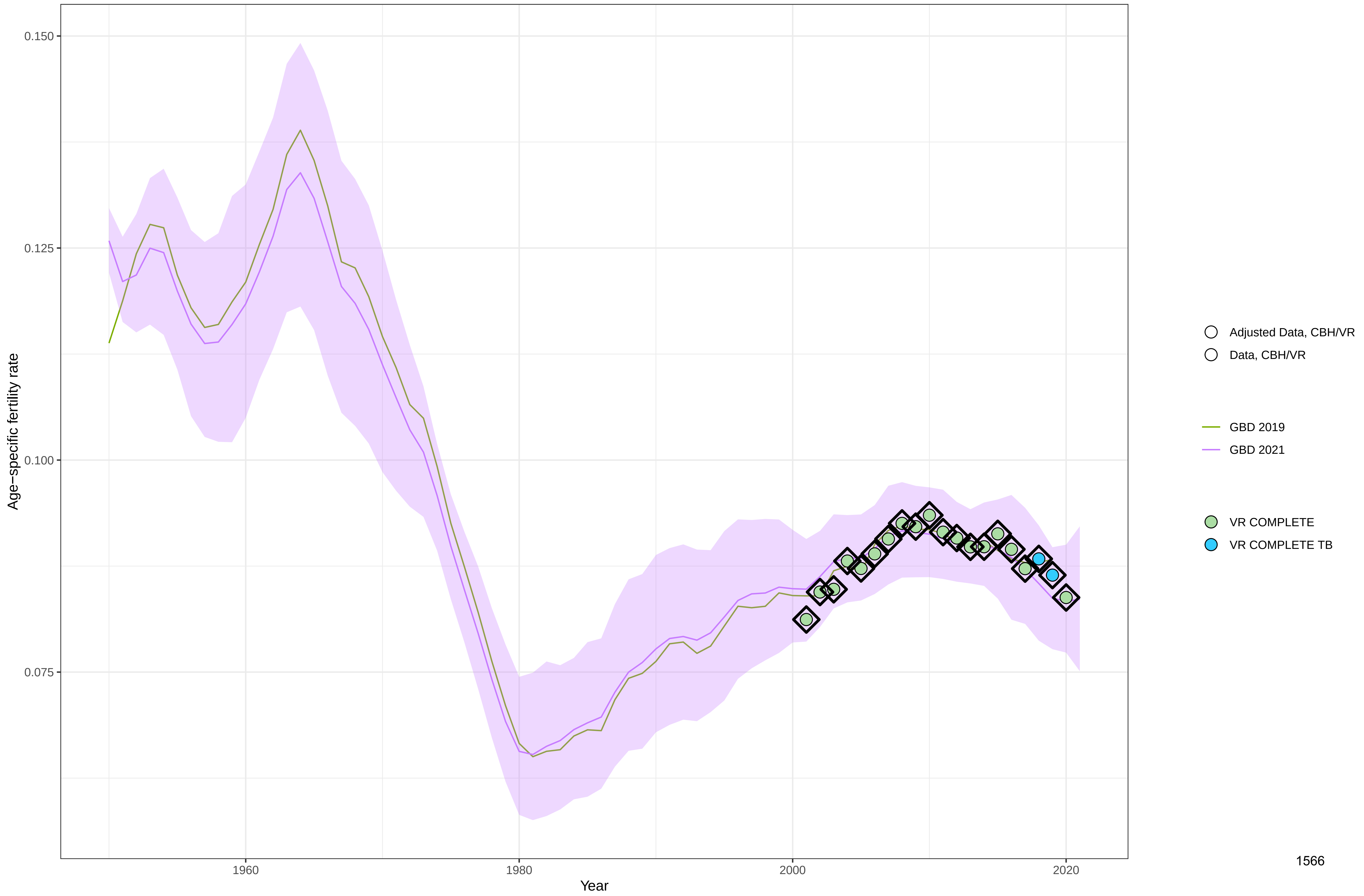

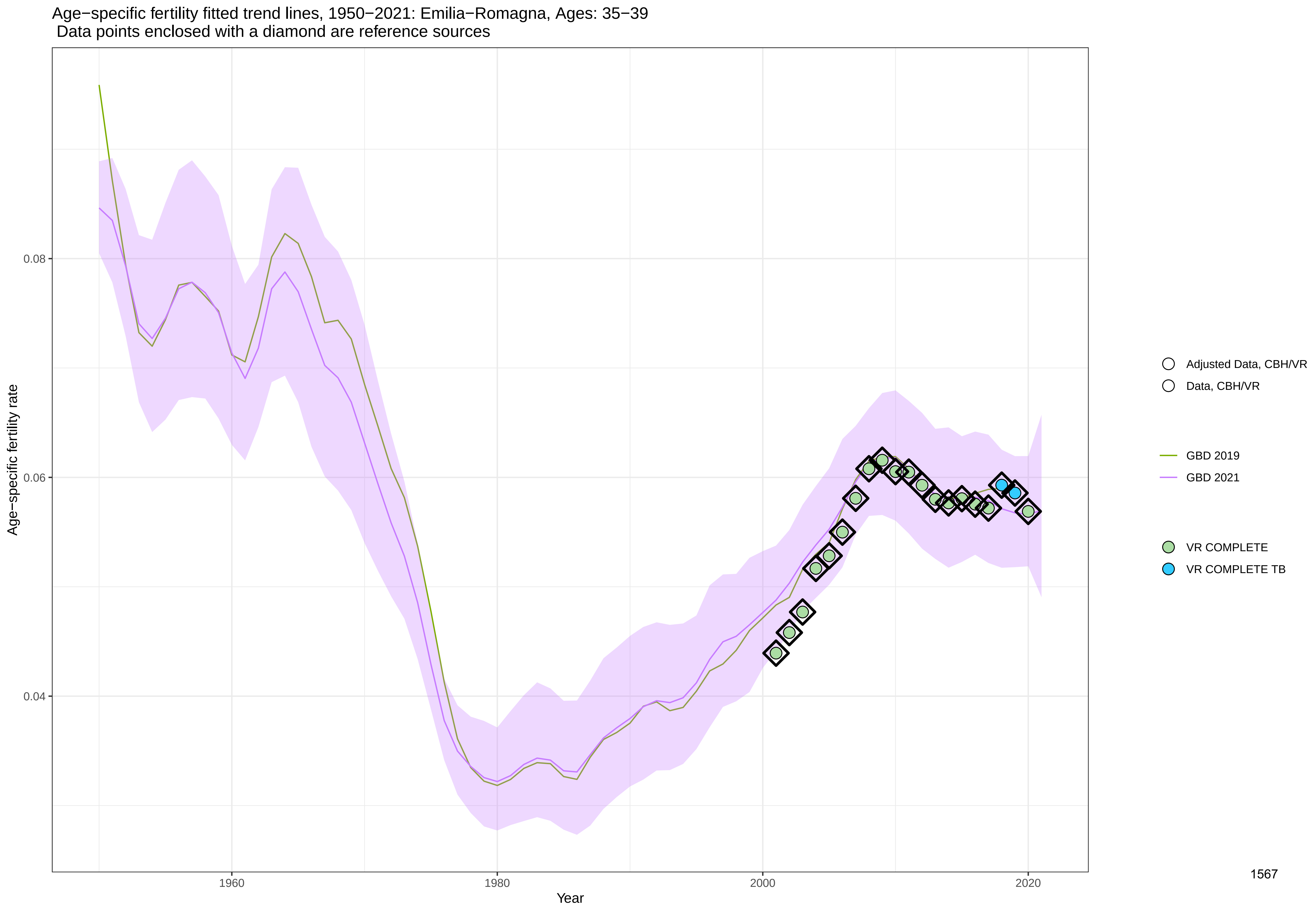

Age−specific fertility fitted trend lines, 1950−2021: Emilia−Romagna, Ages: 35−39
Data points enclosed with a diamond are reference sources
Age−specific fertility rate
0.08
0.06
0.04
Year
1960
1980
2000
2020
Adjusted Data, CBH/VR
Data, CBH/VR
GBD 2019
GBD 2021
VR COMPLETE
VR COMPLETE TB

Age−specific fertility fitted trend lines, 1950−2021: Emilia−Romagna, Ages: 40−44
Data points enclosed with a diamond are reference sources

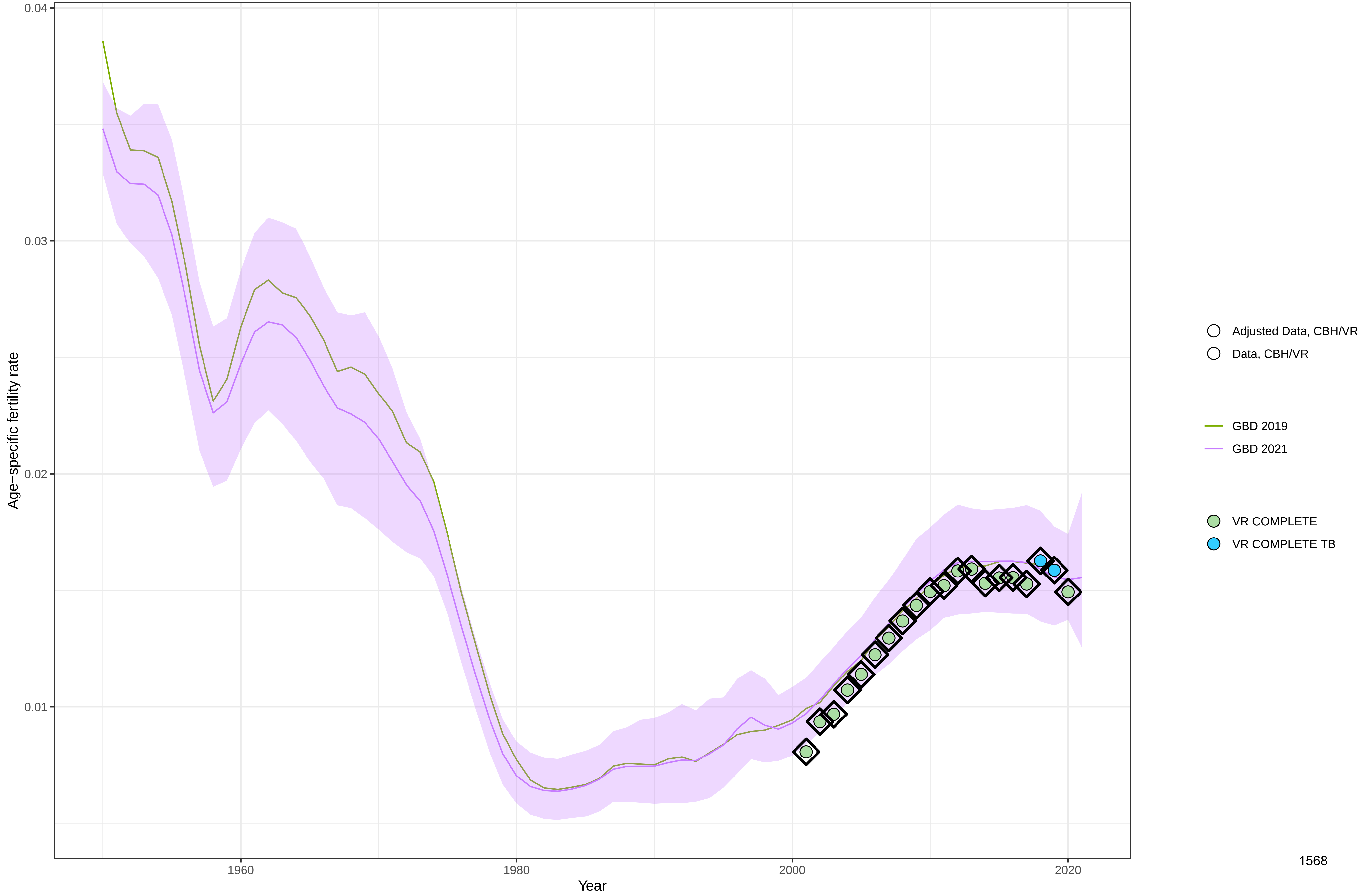

Age−specific fertility fitted trend lines, 1950−2021: Emilia−Romagna, Ages: 45−49
Data points enclosed with a diamond are reference sources

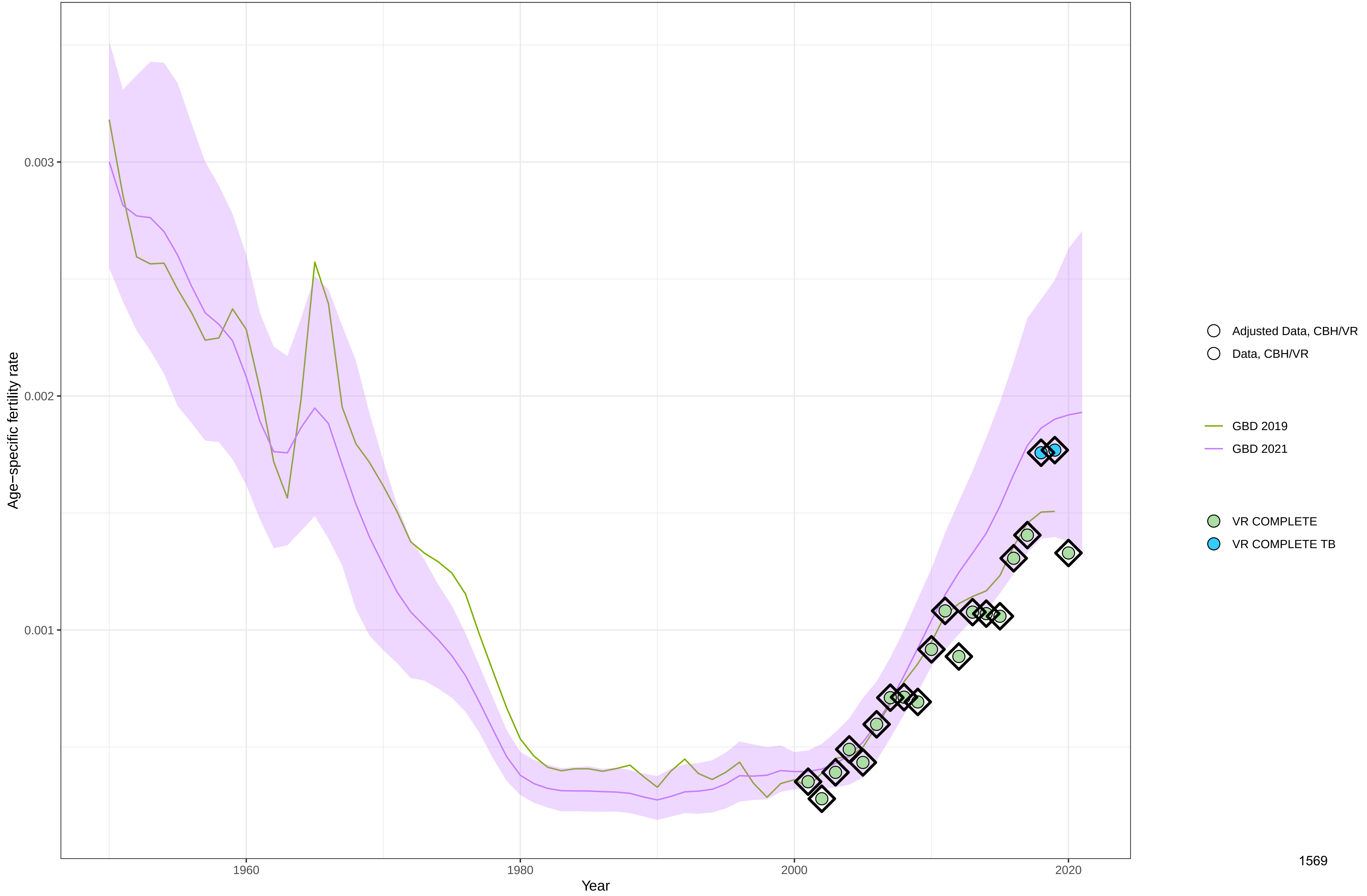

Age−specific fertility fitted trend lines, 1950−2021: Emilia−Romagna, Ages: 50−54
Data points enclosed with a diamond are reference sources

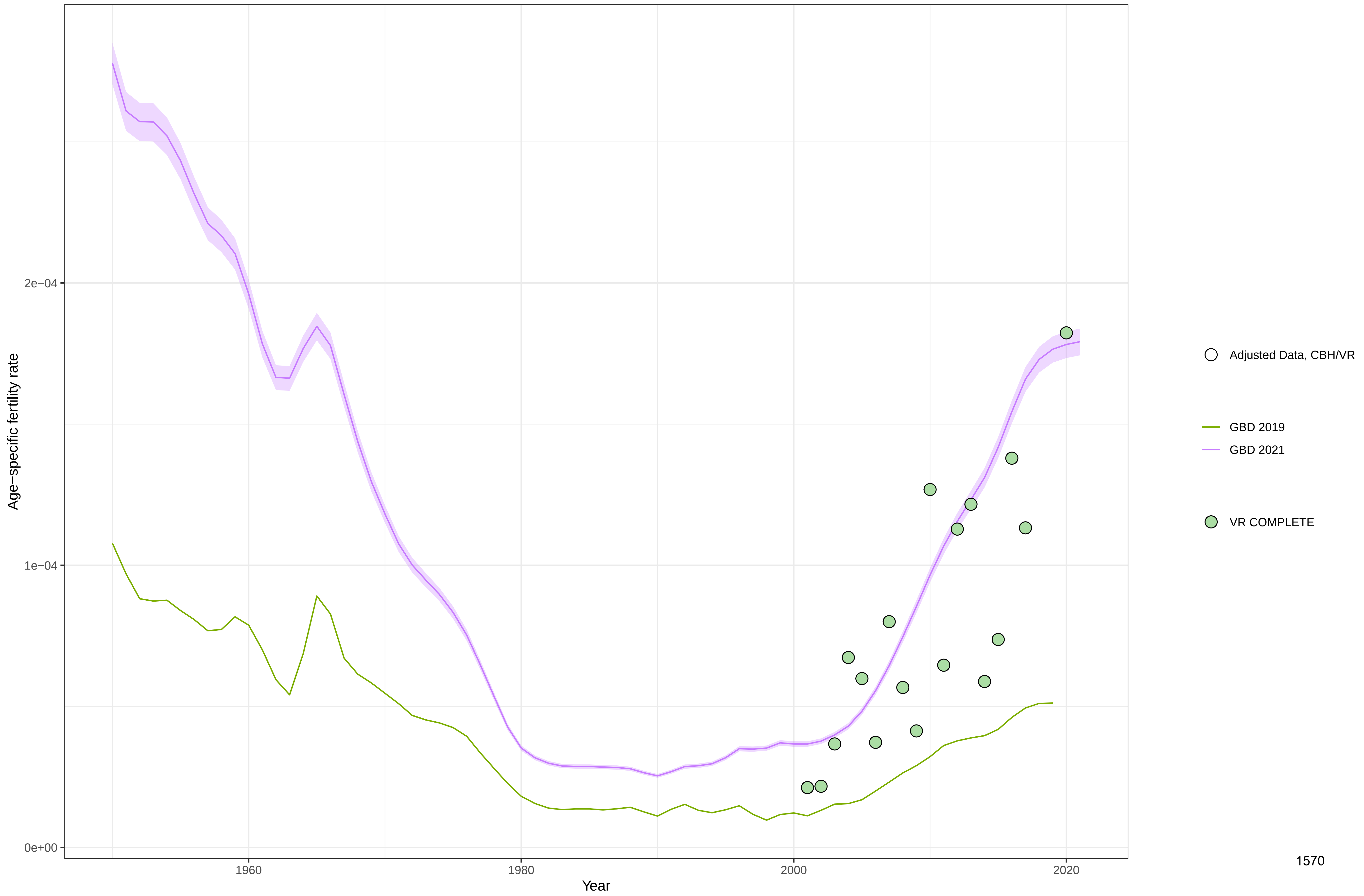

Total fertility rate fitted trend lines, 1950−2021: Emilia−Romagna TFR
Data points enclosed with a diamond are reference sources

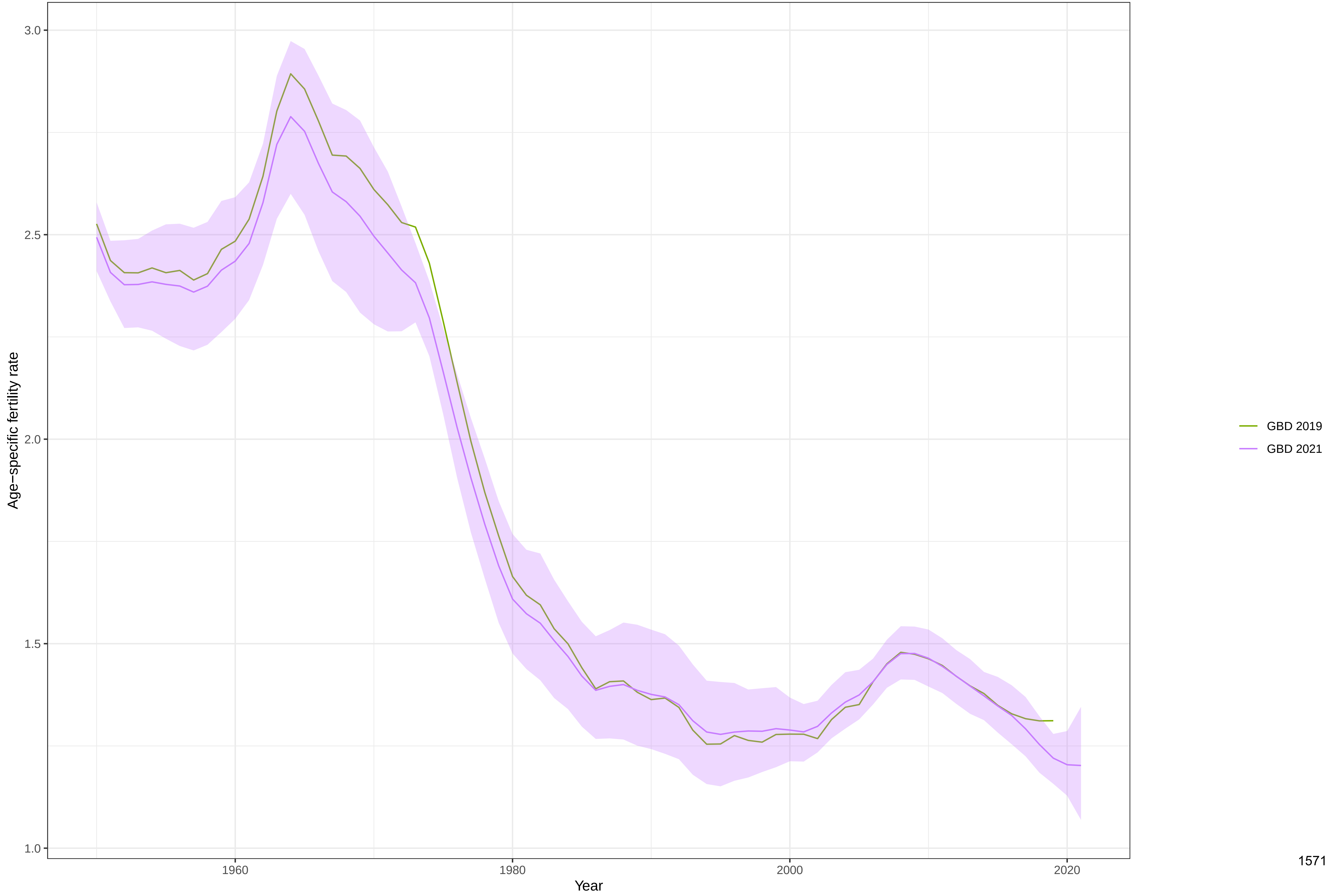

Age−specific fertility fitted trend lines, 1950−2021: Friuli−Venezia Giulia, Ages: 10−14
Data points enclosed with a diamond are reference sources

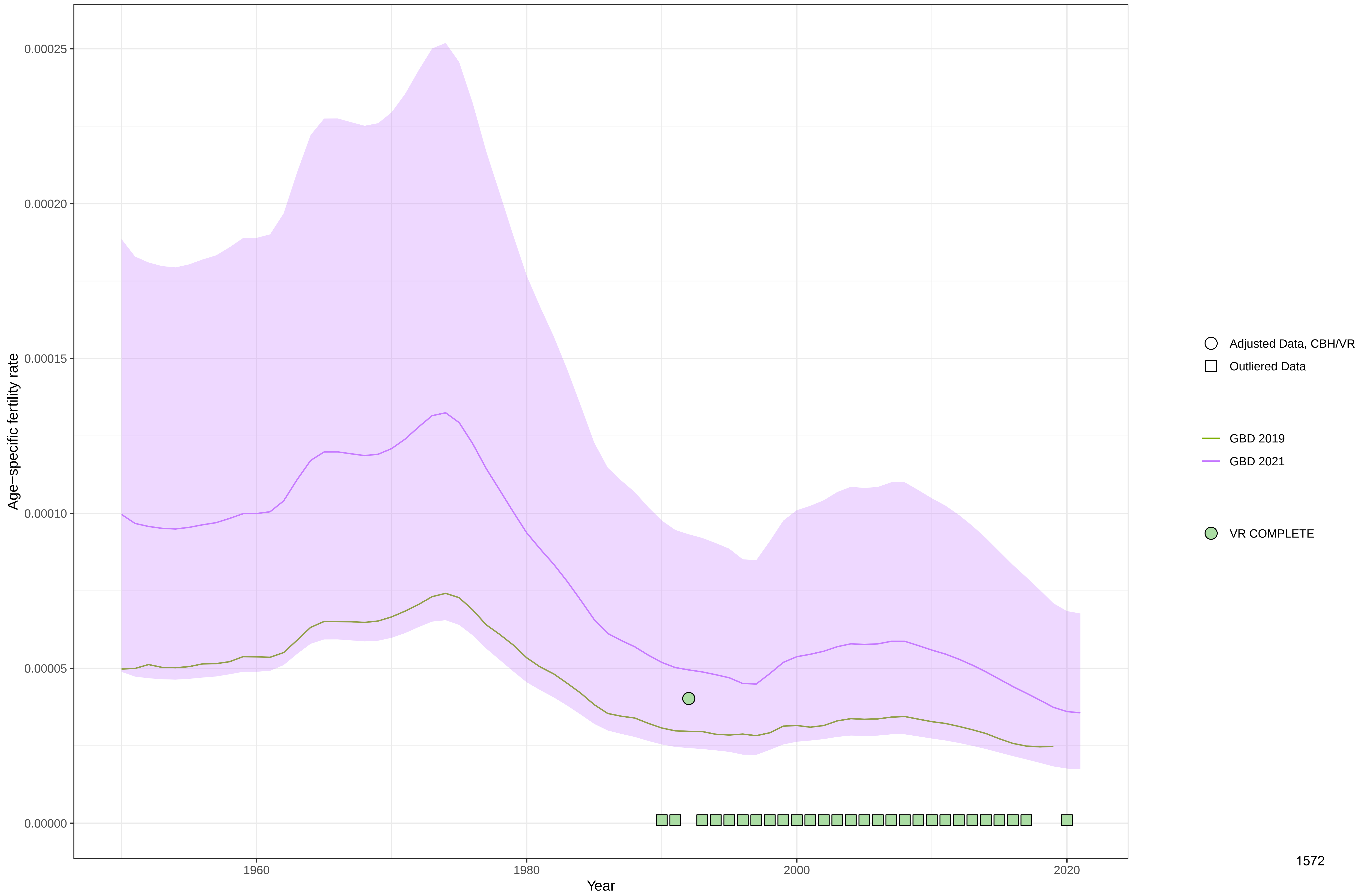

Age−specific fertility fitted trend lines, 1950−2021: Friuli−Venezia Giulia, Ages: 15−19
Data points enclosed with a diamond are reference sources

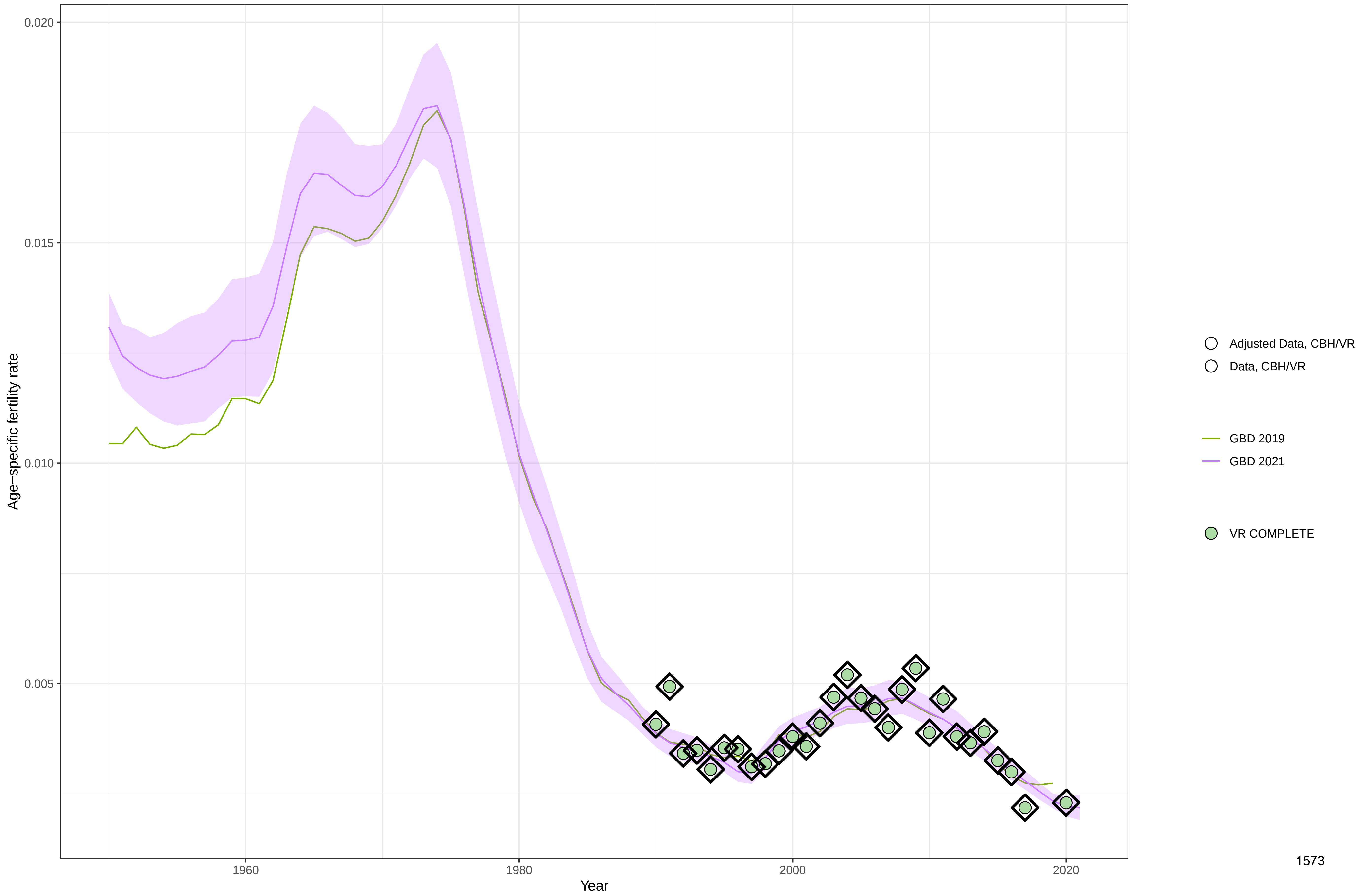

Age−specific fertility fitted trend lines, 1950−2021: Friuli−Venezia Giulia, Ages: 20−24
Data points enclosed with a diamond are reference sources

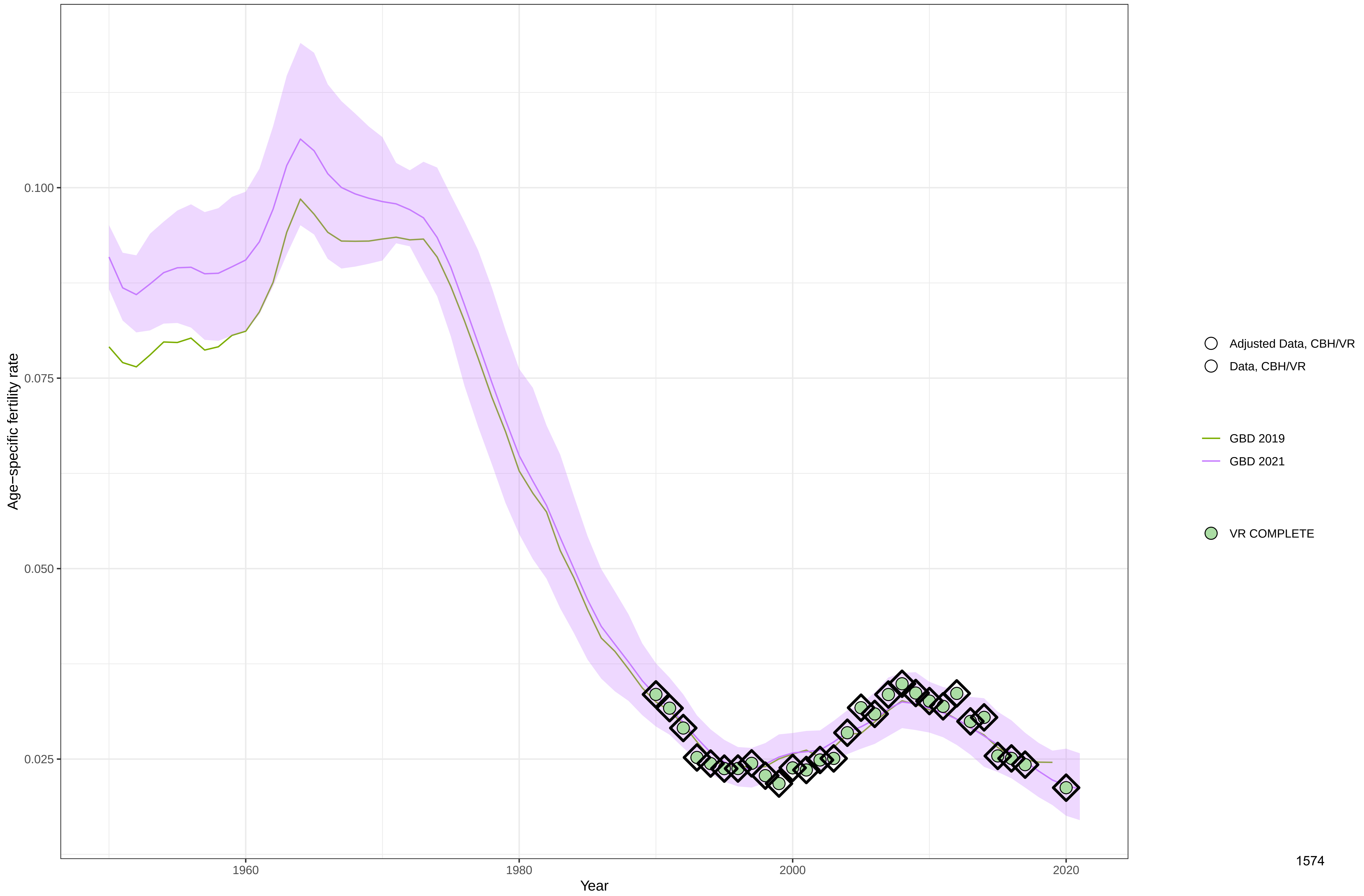

Age−specific fertility fitted trend lines, 1950−2021: Friuli−Venezia Giulia, Ages: 25−29
Data points enclosed with a diamond are reference sources

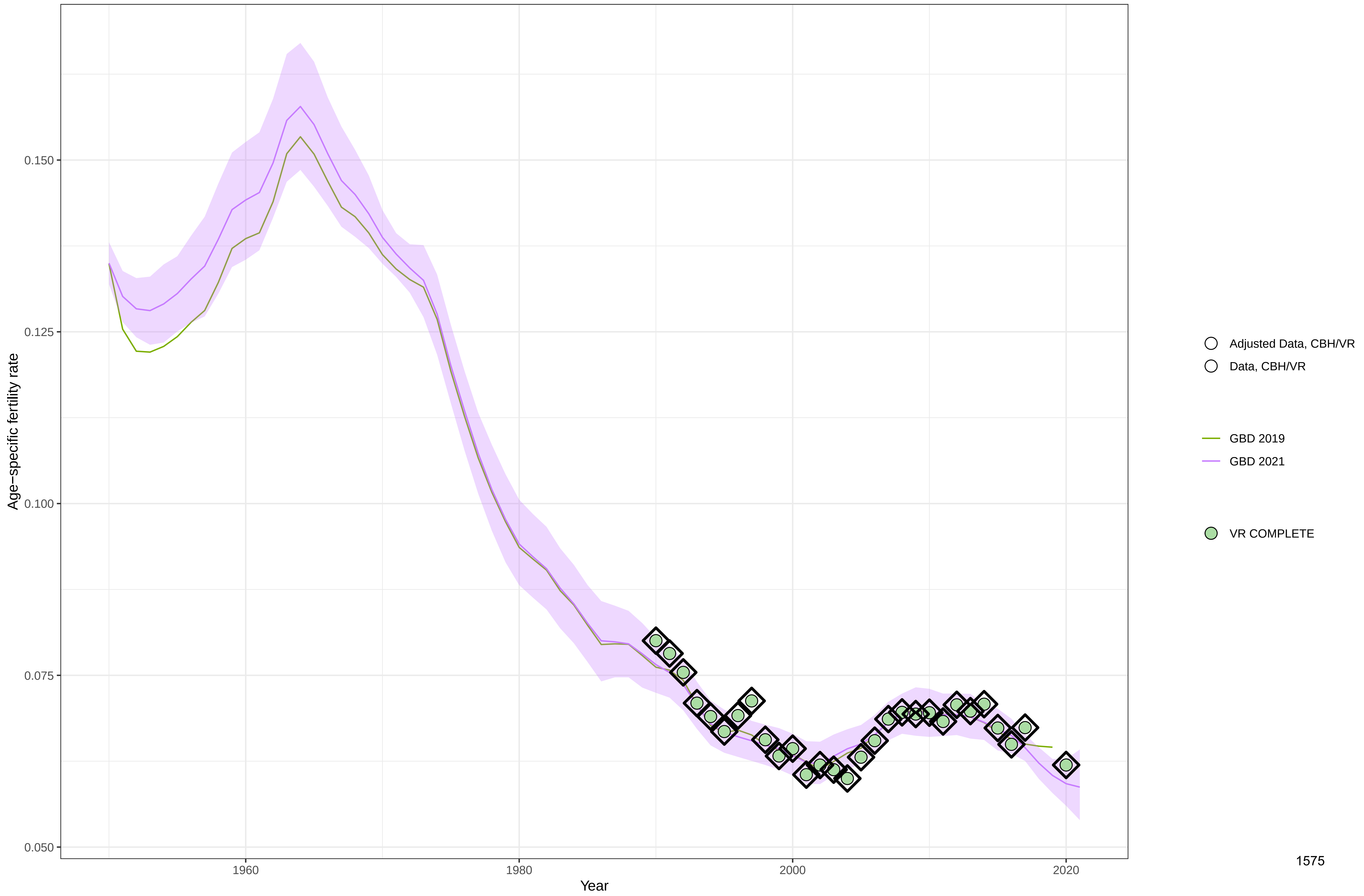

Age−specific fertility fitted trend lines, 1950−2021: Friuli−Venezia Giulia, Ages: 30−34
Data points enclosed with a diamond are reference sources

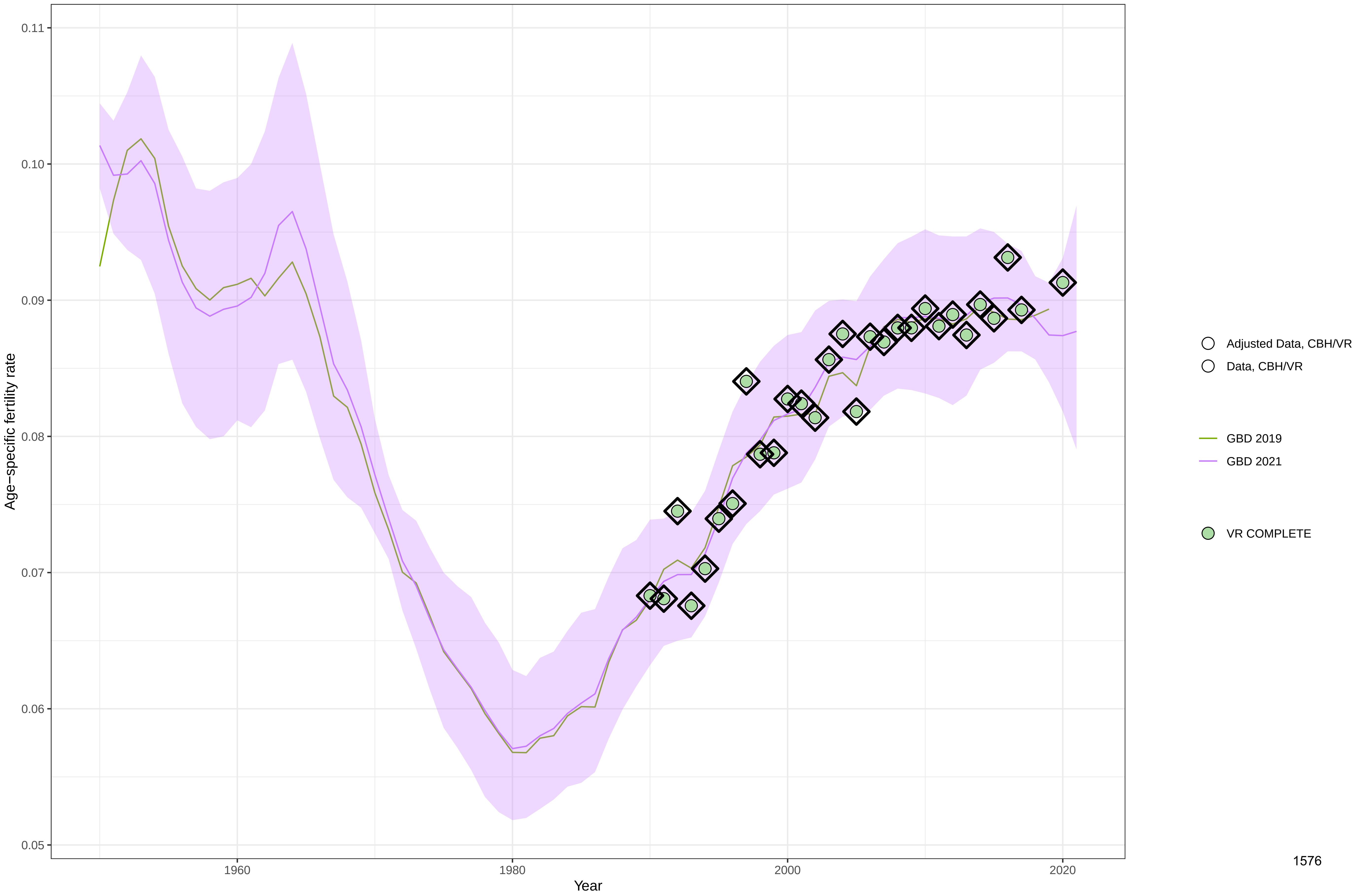

Age−specific fertility fitted trend lines, 1950−2021: Friuli−Venezia Giulia, Ages: 35−39
Data points enclosed with a diamond are reference sources

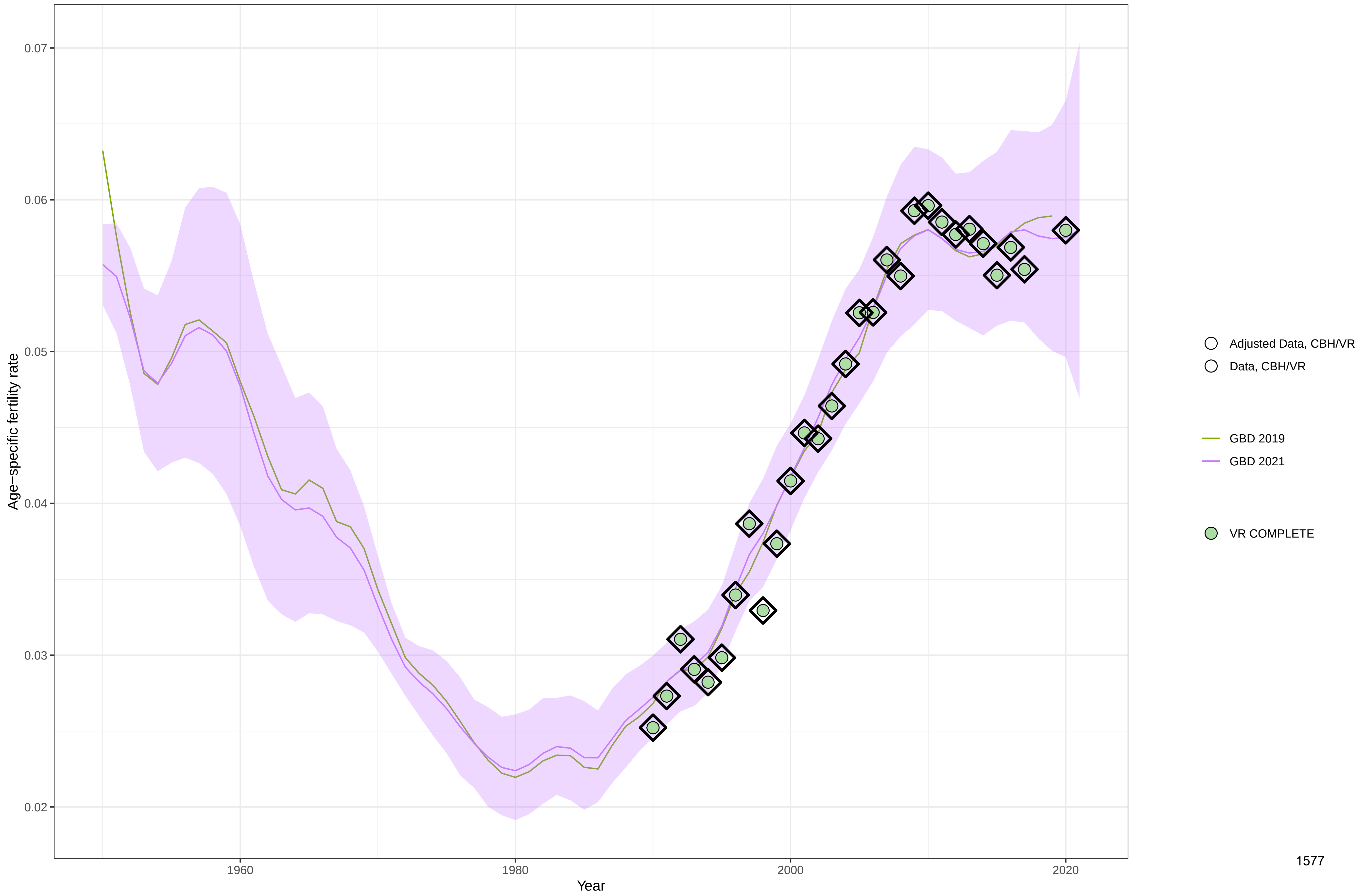

Age−specific fertility fitted trend lines, 1950−2021: Friuli−Venezia Giulia, Ages: 40−44
Data points enclosed with a diamond are reference sources

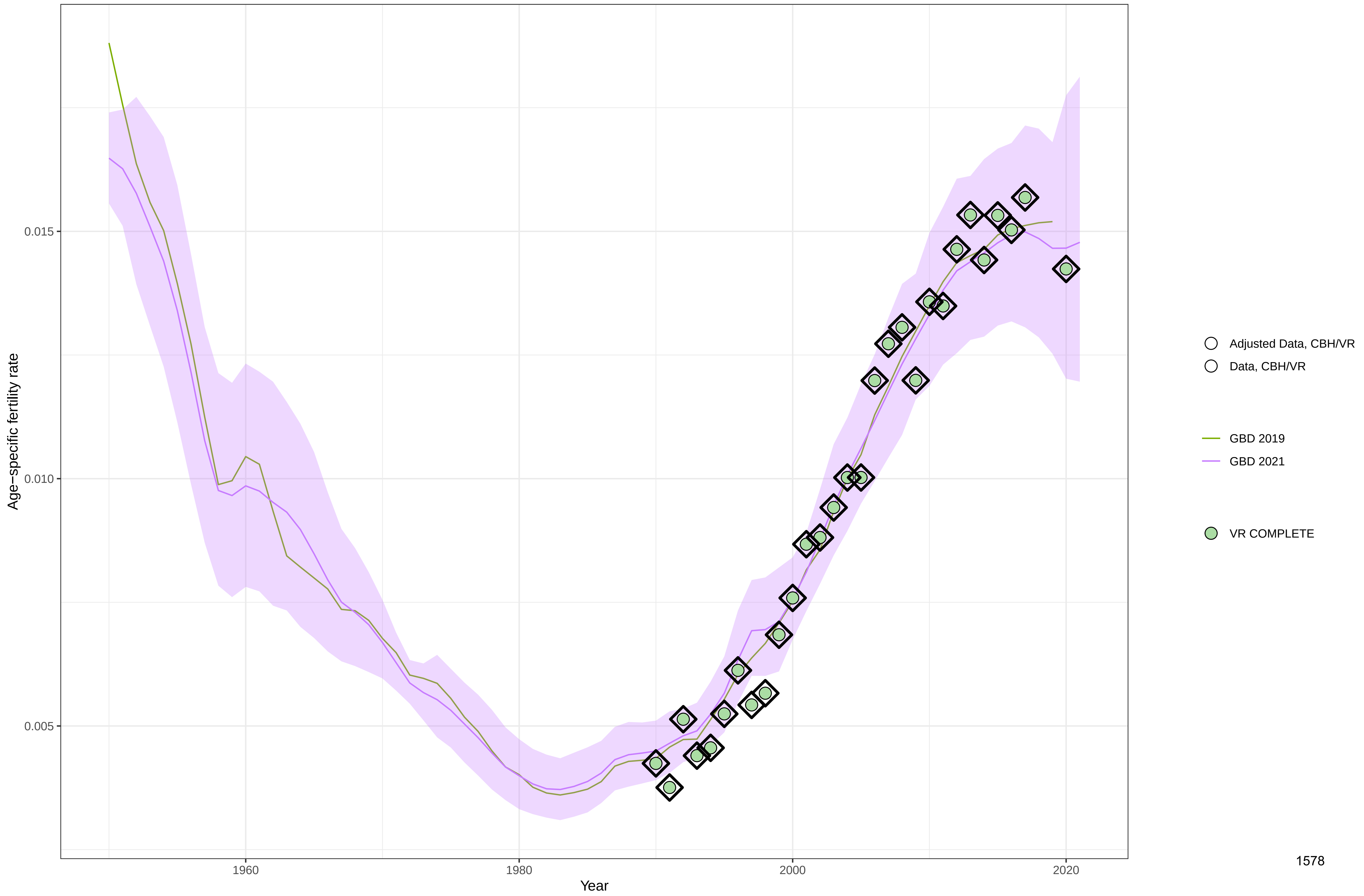

Age−specific fertility fitted trend lines, 1950−2021: Friuli−Venezia Giulia, Ages: 45−49
Data points enclosed with a diamond are reference sources

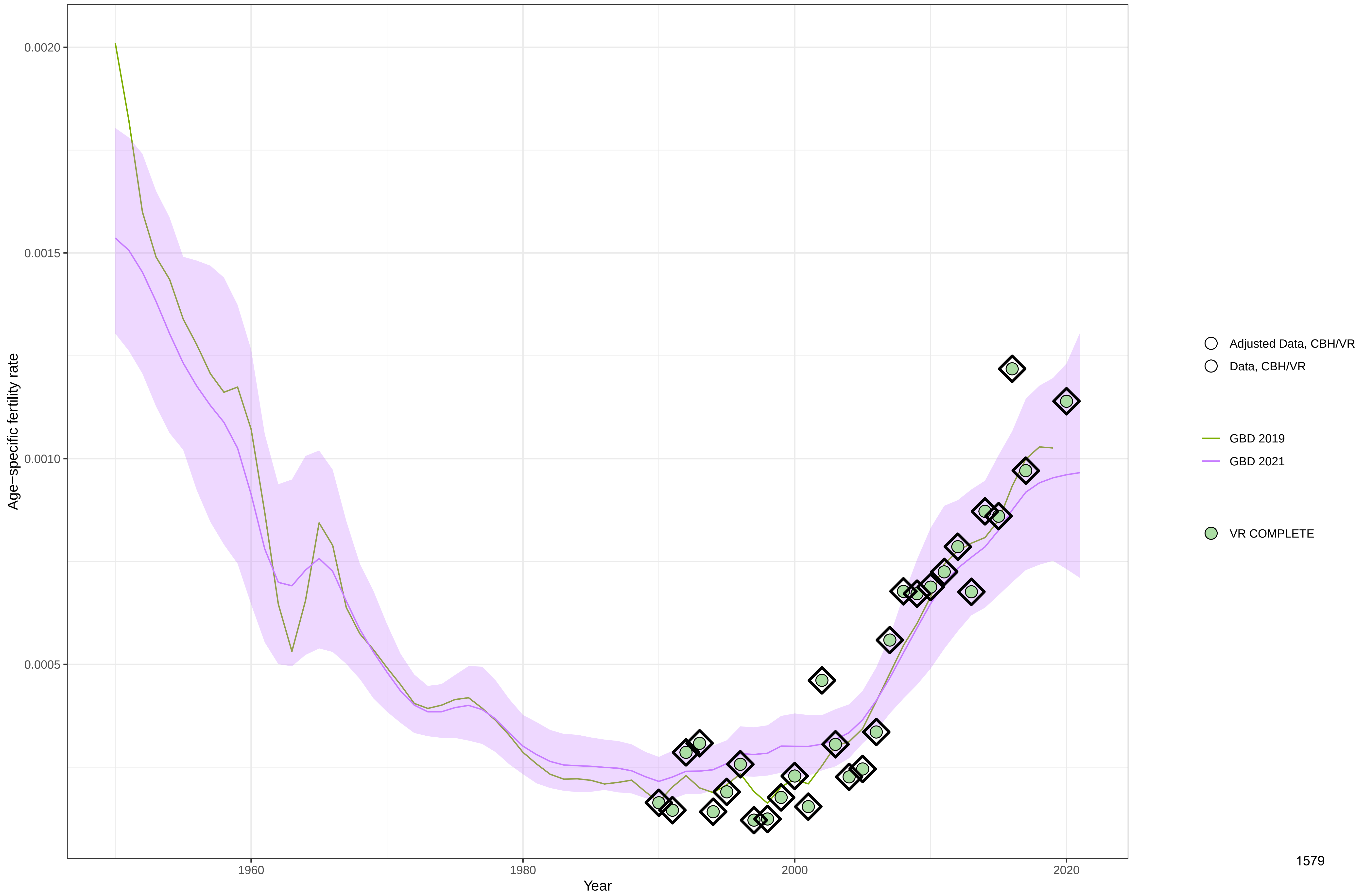

Age−specific fertility fitted trend lines, 1950−2021: Friuli−Venezia Giulia, Ages: 50−54
Data points enclosed with a diamond are reference sources

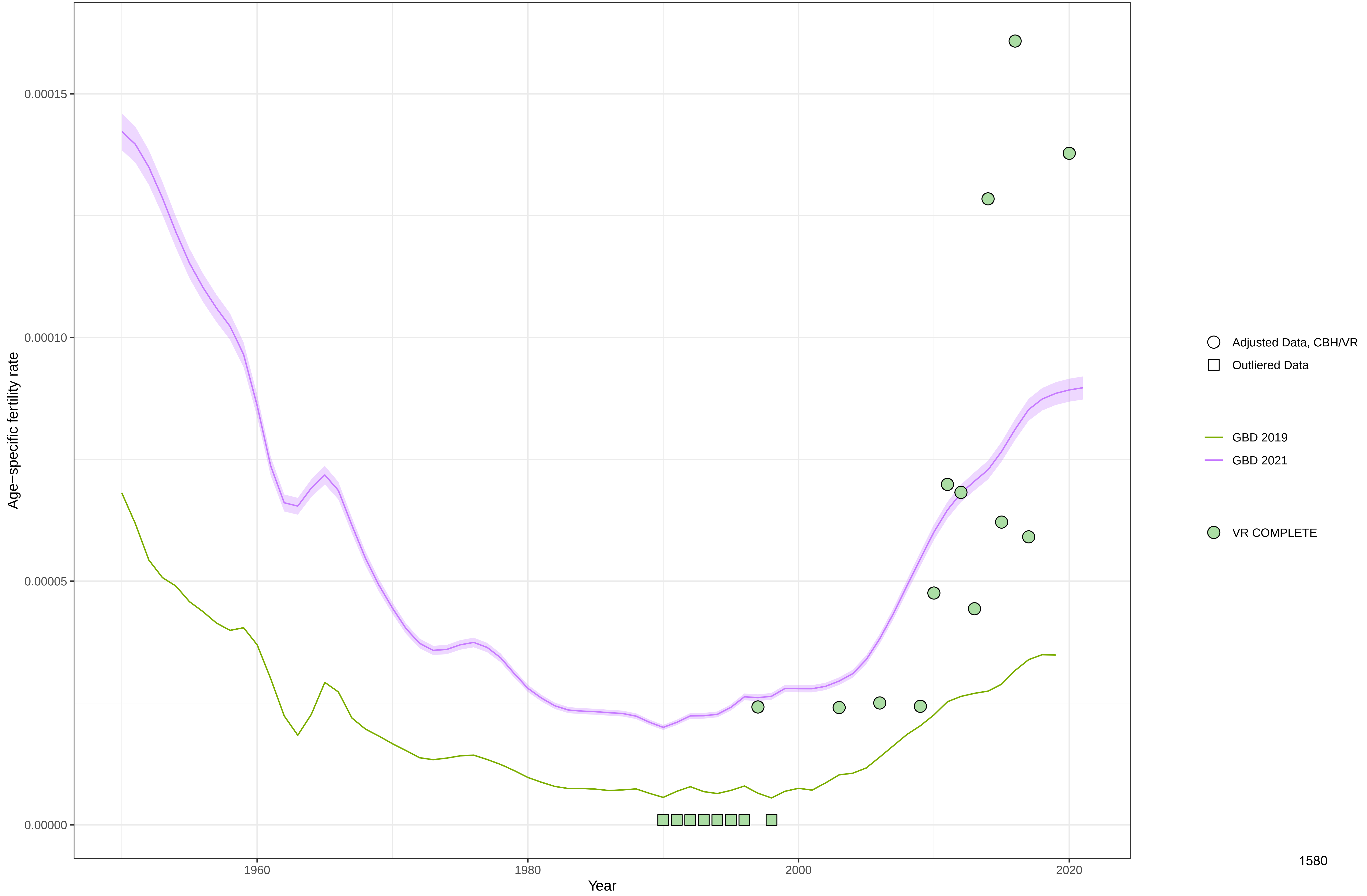

Total fertility rate fitted trend lines, 1950−2021: Friuli−Venezia Giulia TFR
Data points enclosed with a diamond are reference sources

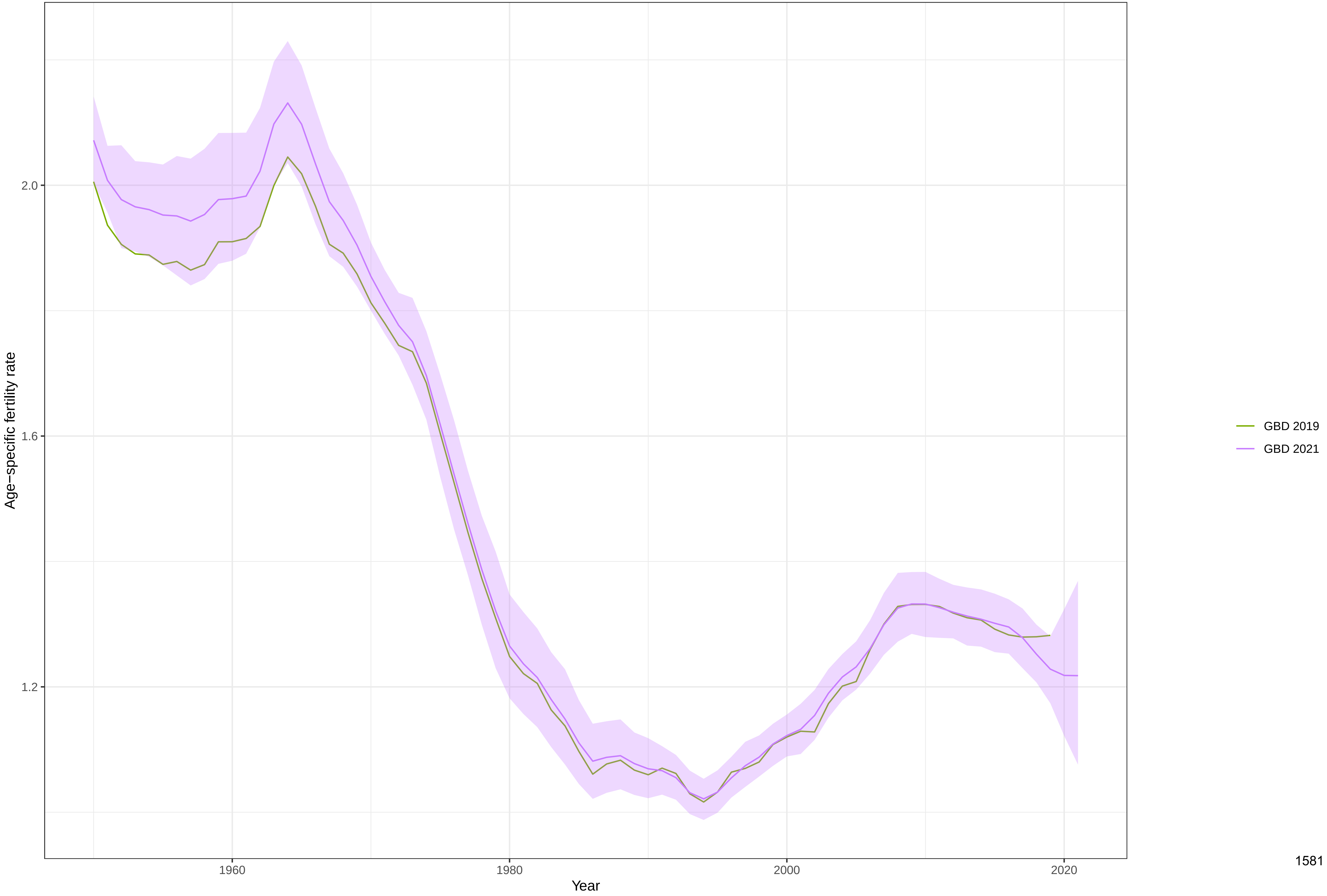

Age−specific fertility fitted trend lines, 1950−2021: Lazio, Ages: 10−14
Data points enclosed with a diamond are reference sources

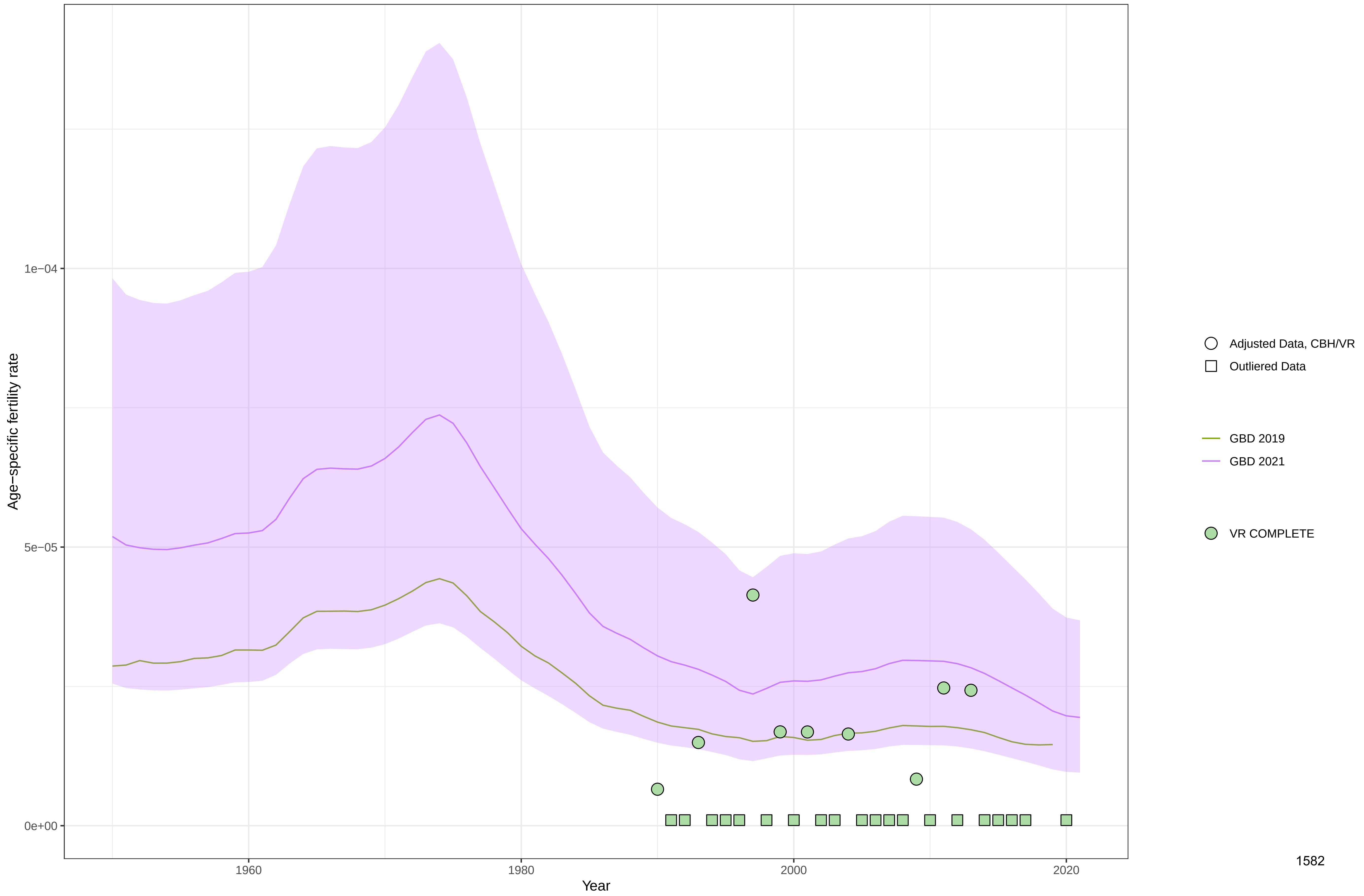

Age−specific fertility fitted trend lines, 1950−2021: Lazio, Ages: 15−19
Data points enclosed with a diamond are reference sources

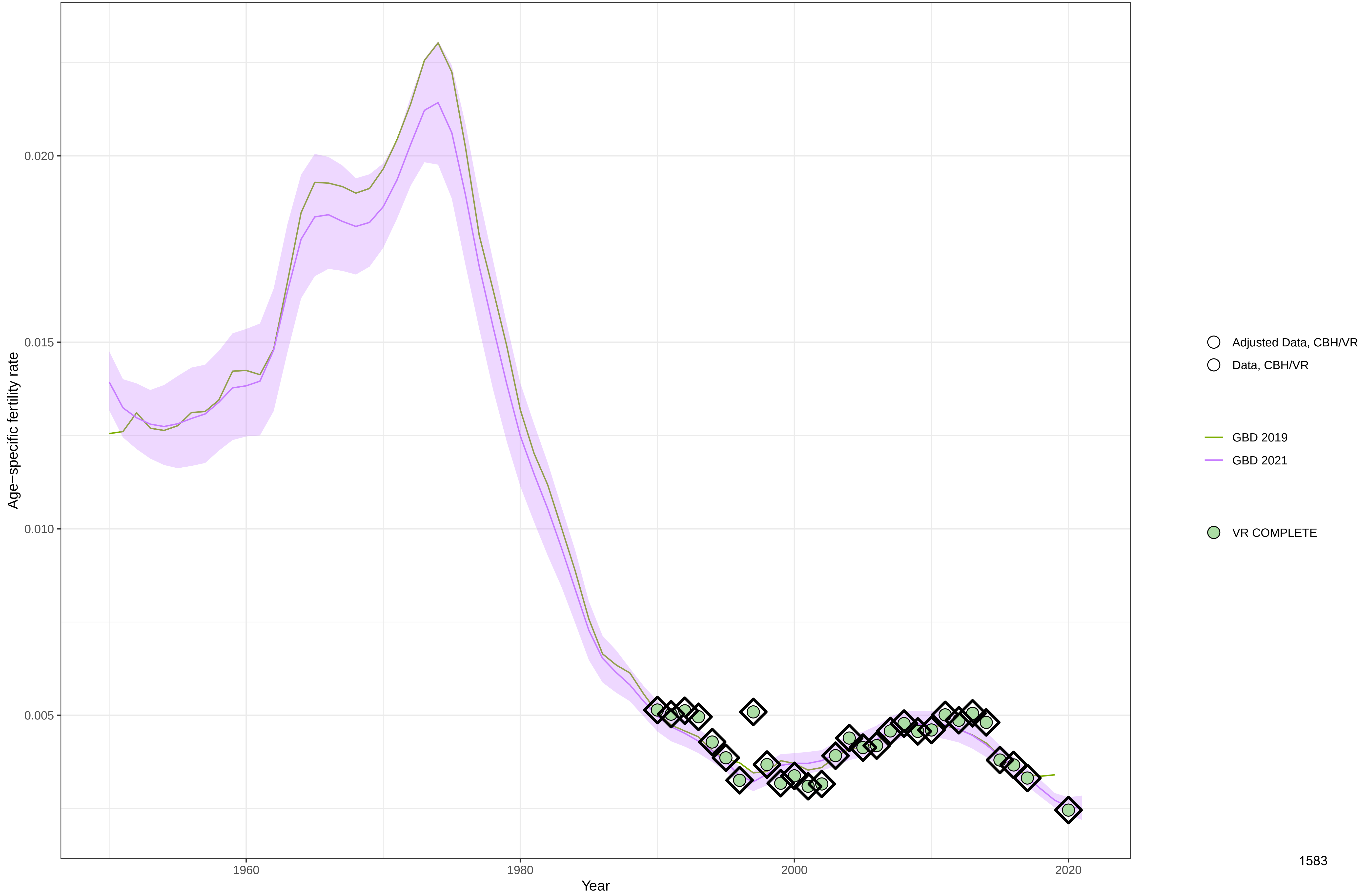

Age−specific fertility fitted trend lines, 1950−2021: Lazio, Ages: 20−24
Data points enclosed with a diamond are reference sources

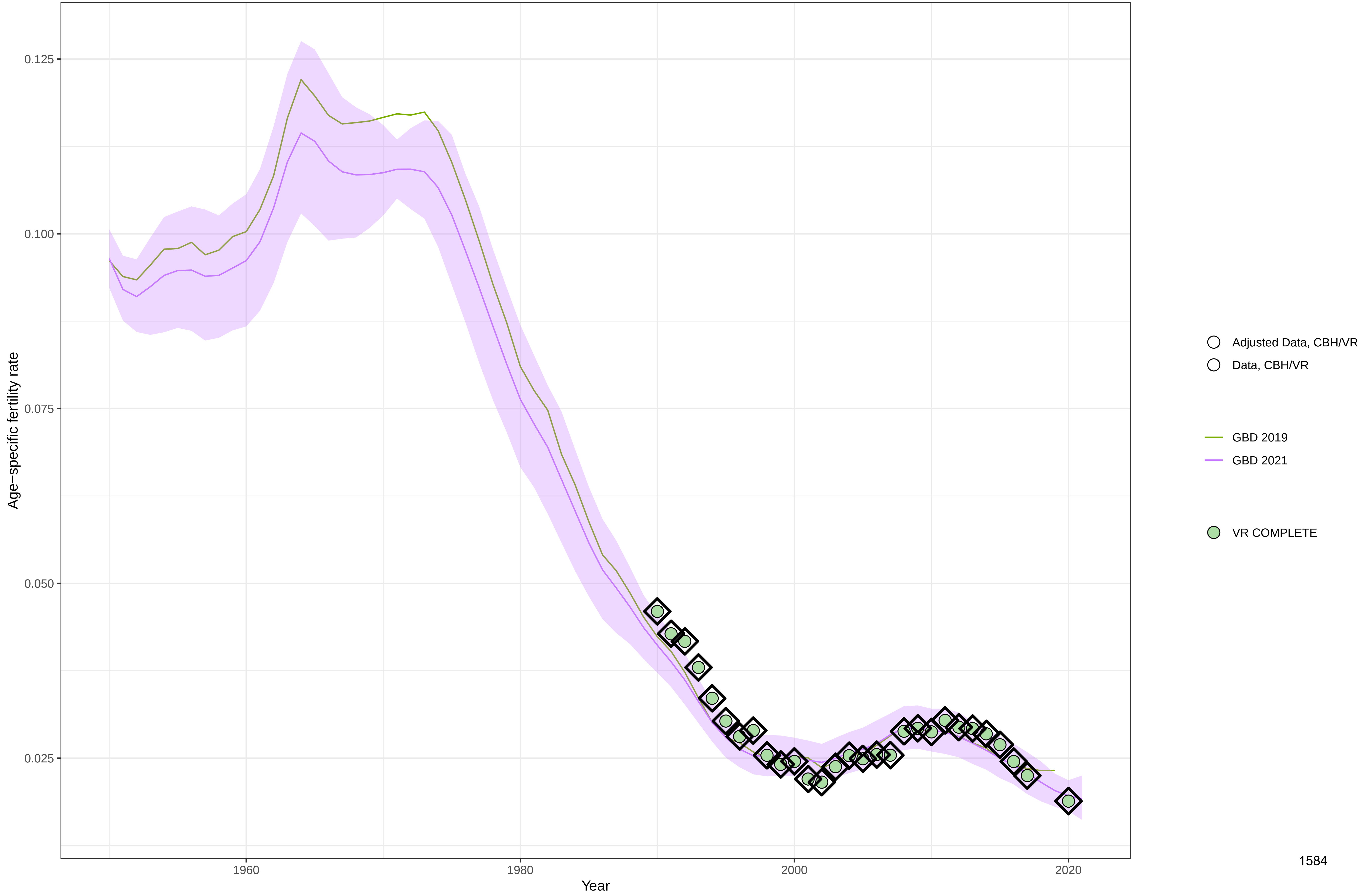

Age−specific fertility fitted trend lines, 1950−2021: Lazio, Ages: 25−29
Data points enclosed with a diamond are reference sources

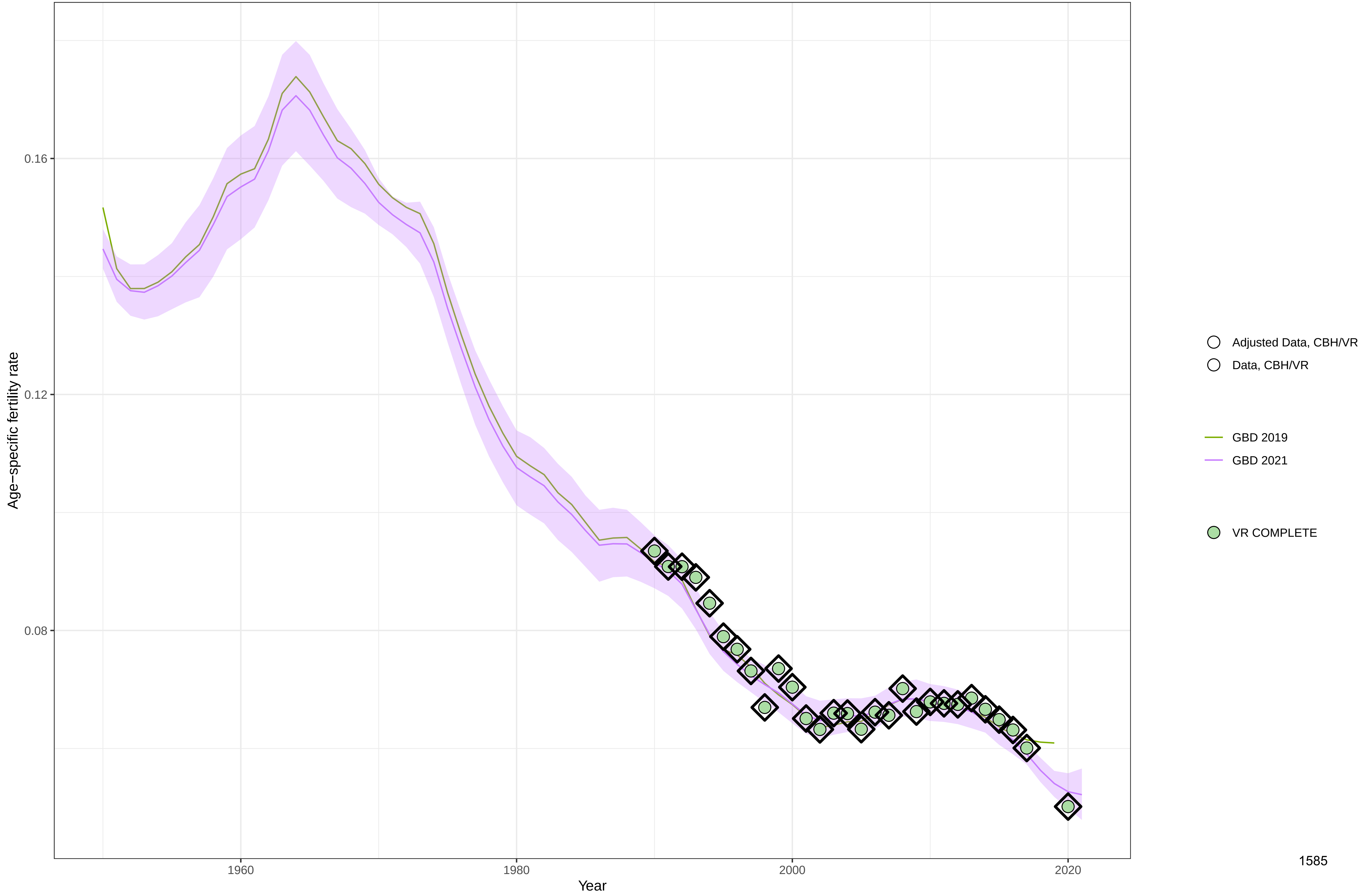

Age−specific fertility fitted trend lines, 1950−2021: Lazio, Ages: 30−34
Data points enclosed with a diamond are reference sources

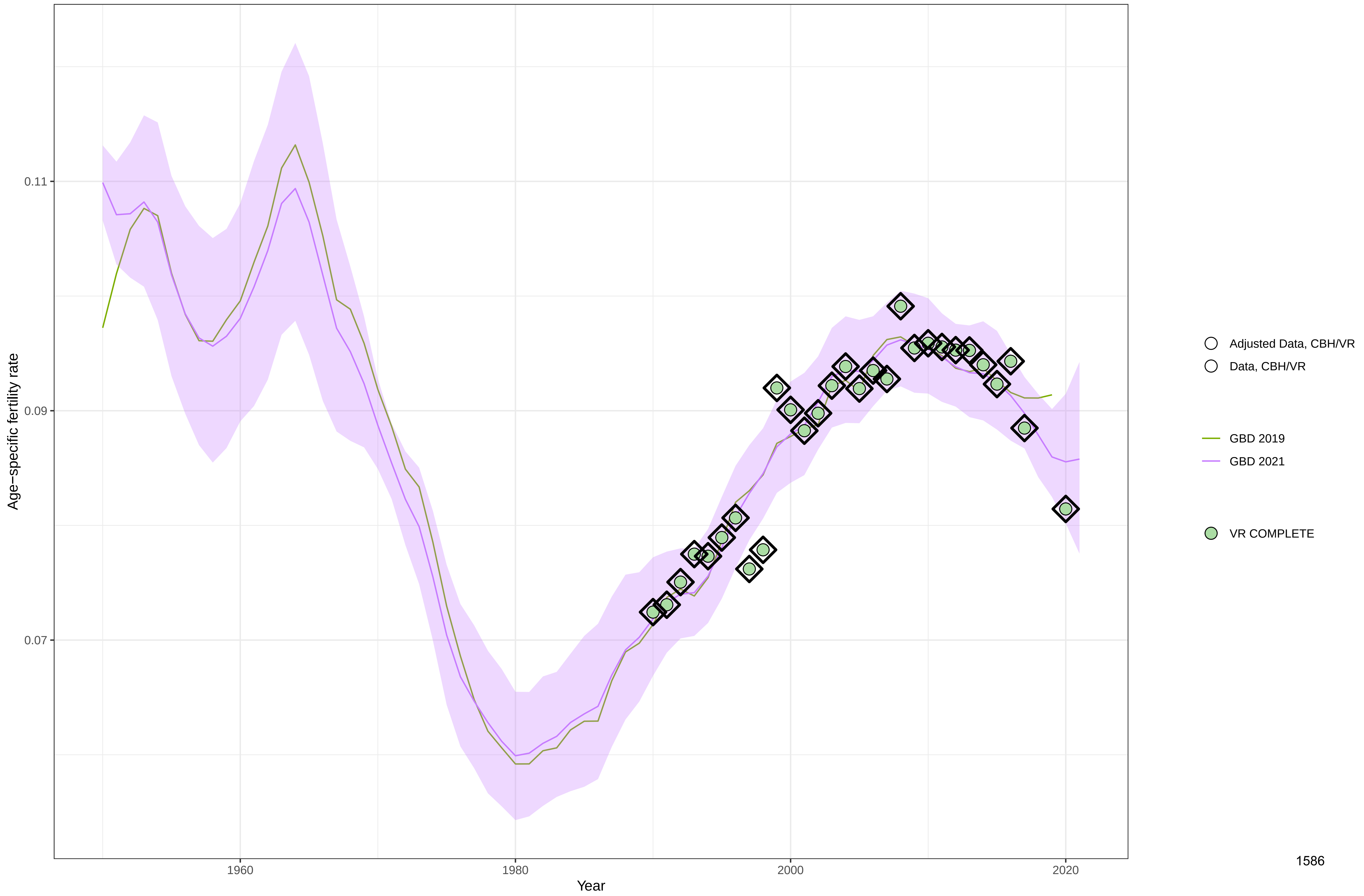

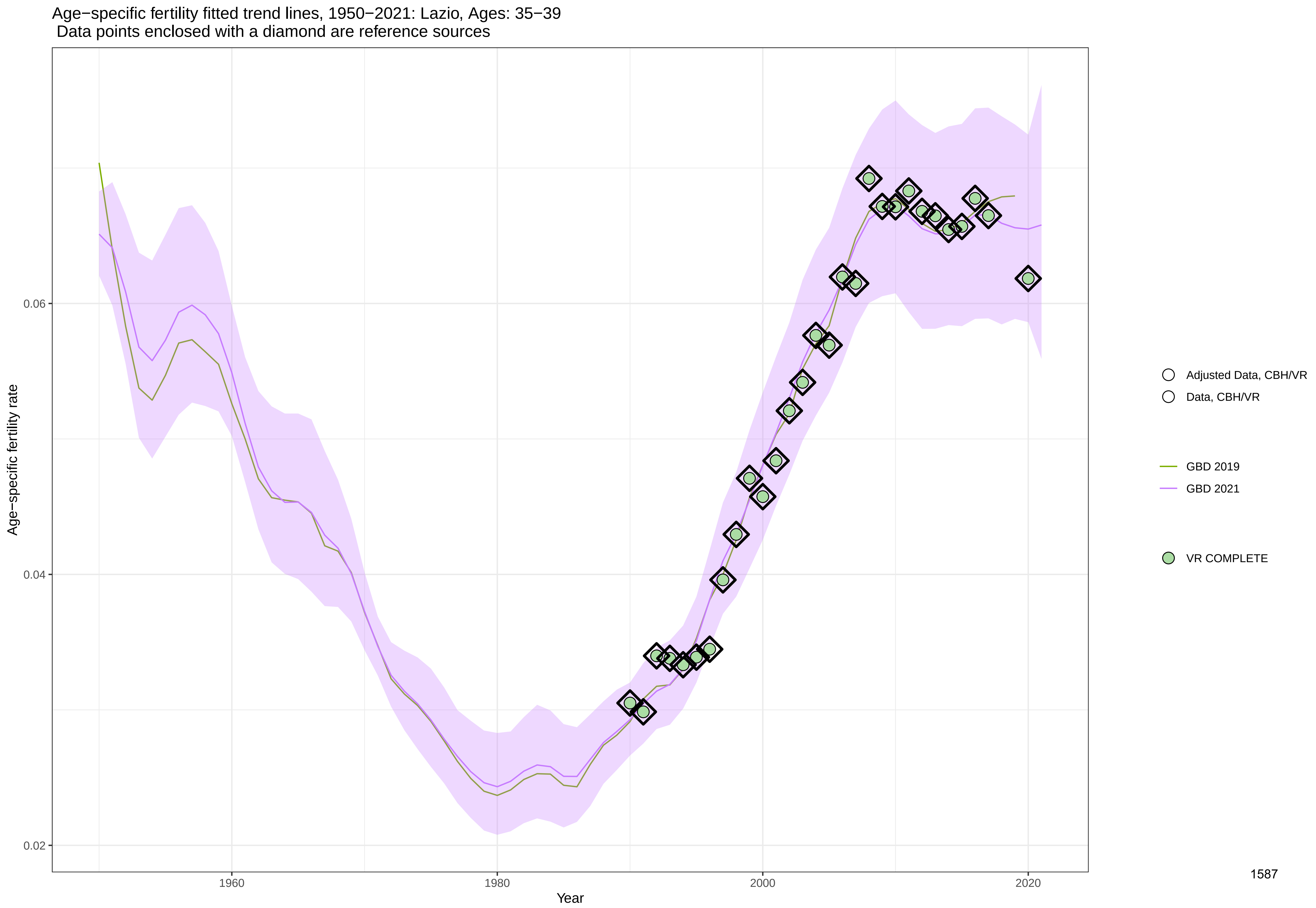

Age−specific fertility fitted trend lines, 1950−2021: Lazio, Ages: 35−39
Data points enclosed with a diamond are reference sources
Age−specific fertility rate
0.06
0.04
0.02
1960
1980
2000
2020
Year
Adjusted Data, CBH/VR
Data, CBH/VR
GBD 2019
GBD 2021
VR COMPLETE

Age−specific fertility fitted trend lines, 1950−2021: Lazio, Ages: 40−44
Data points enclosed with a diamond are reference sources

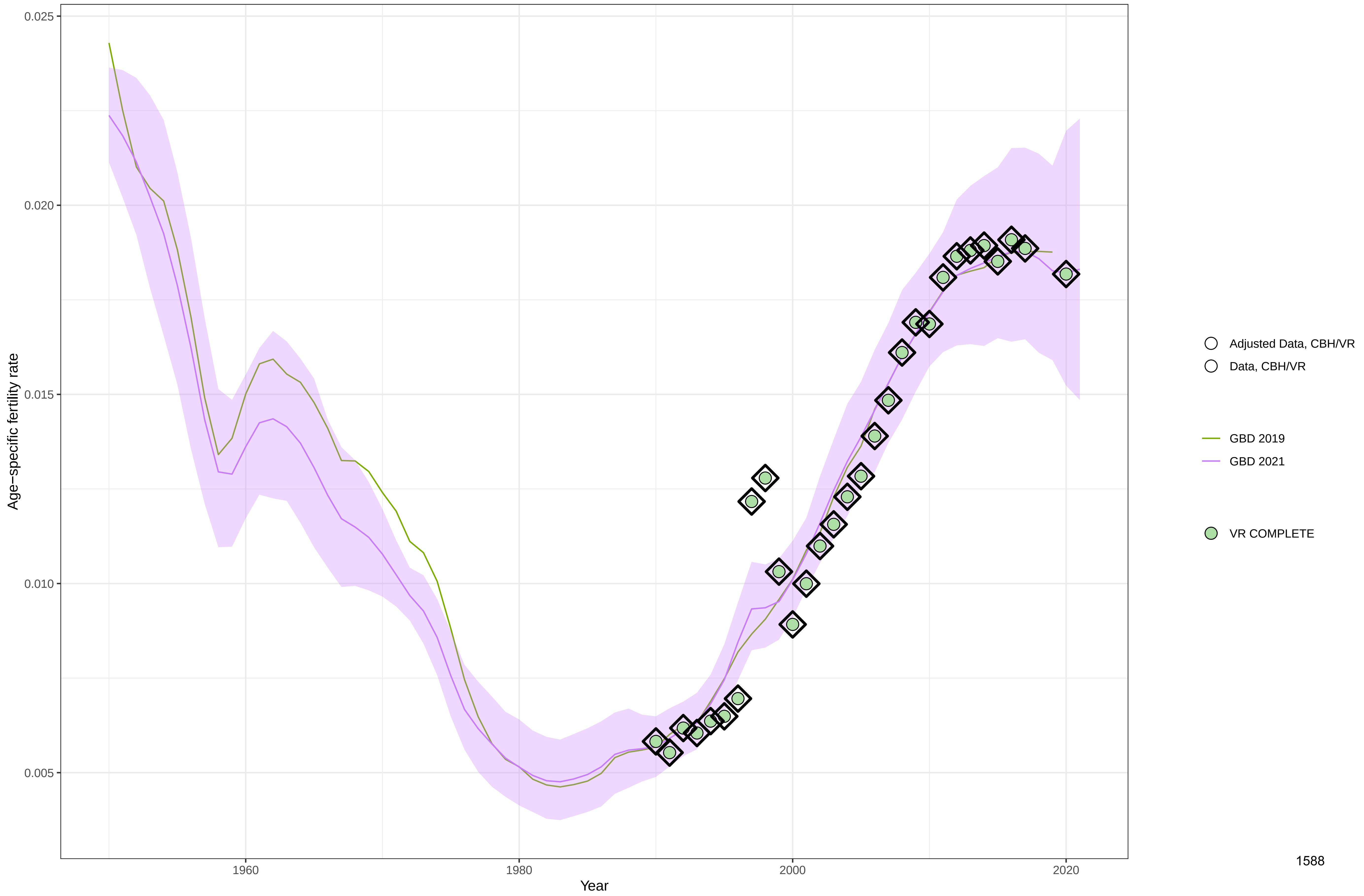

Age−specific fertility fitted trend lines, 1950−2021: Lazio, Ages: 45−49
Data points enclosed with a diamond are reference sources

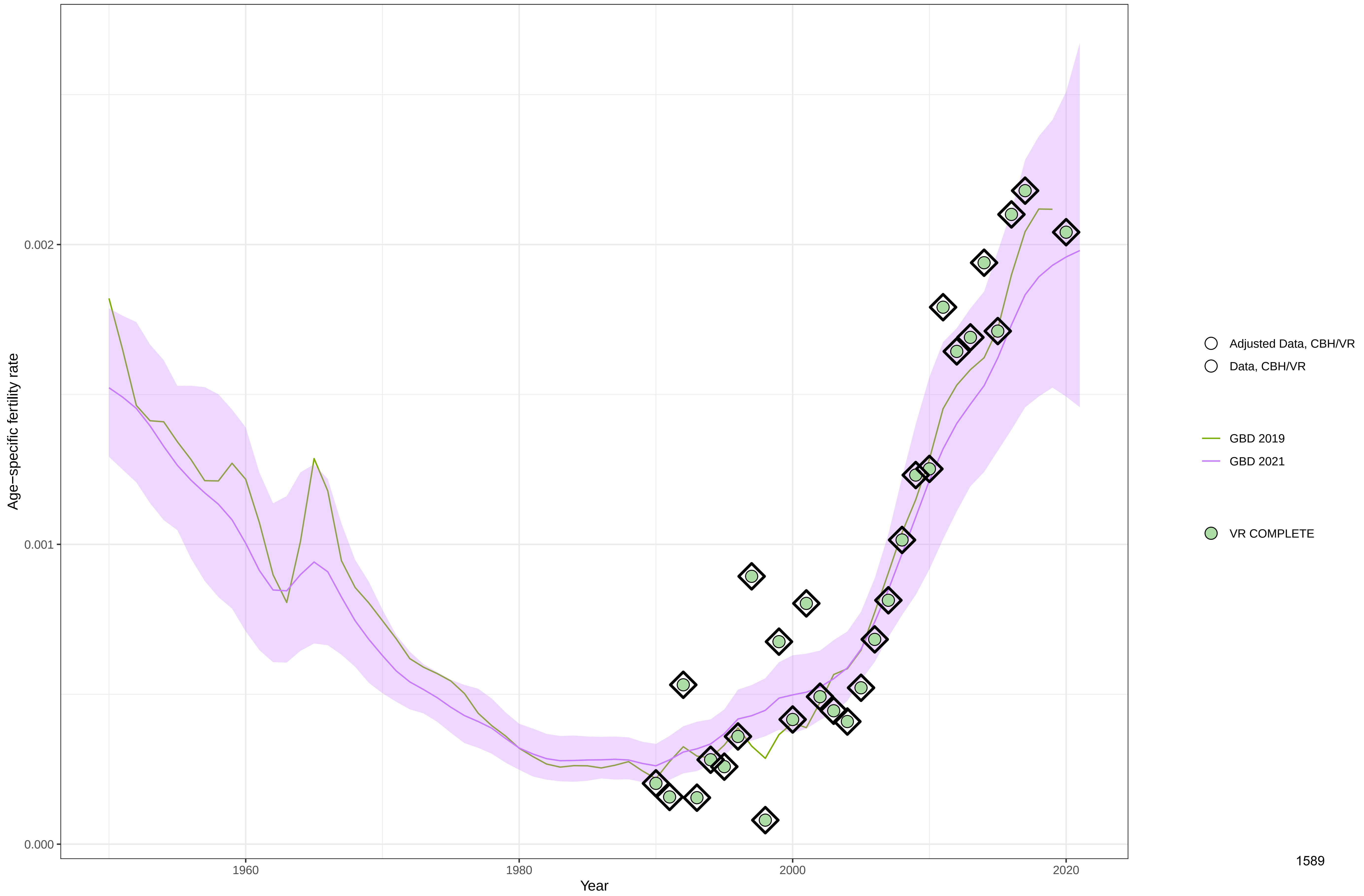

Age−specific fertility fitted trend lines, 1950−2021: Lazio, Ages: 50−54
Data points enclosed with a diamond are reference sources

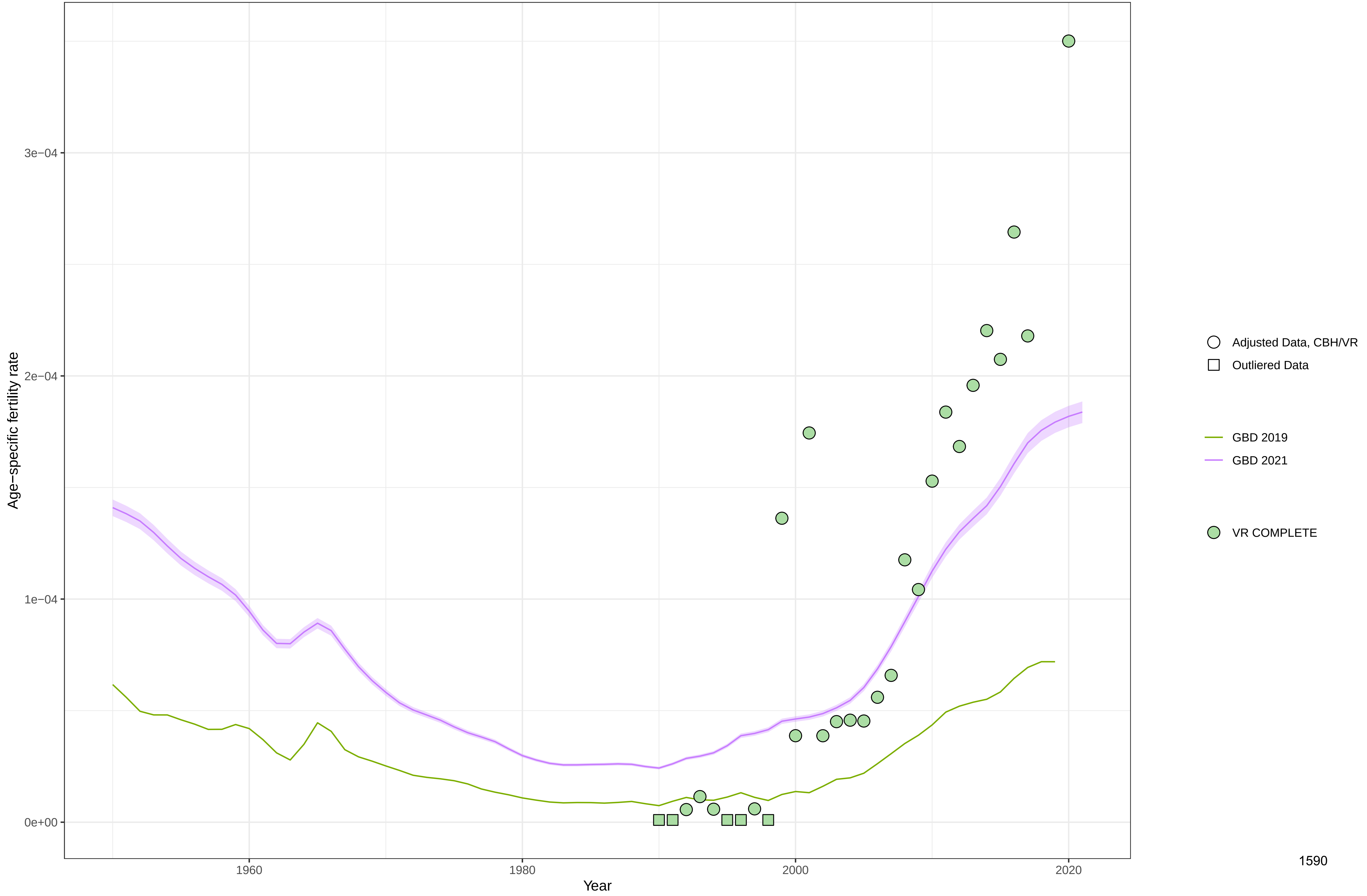

Total fertility rate fitted trend lines, 1950−2021: Lazio TFR
Data points enclosed with a diamond are reference sources

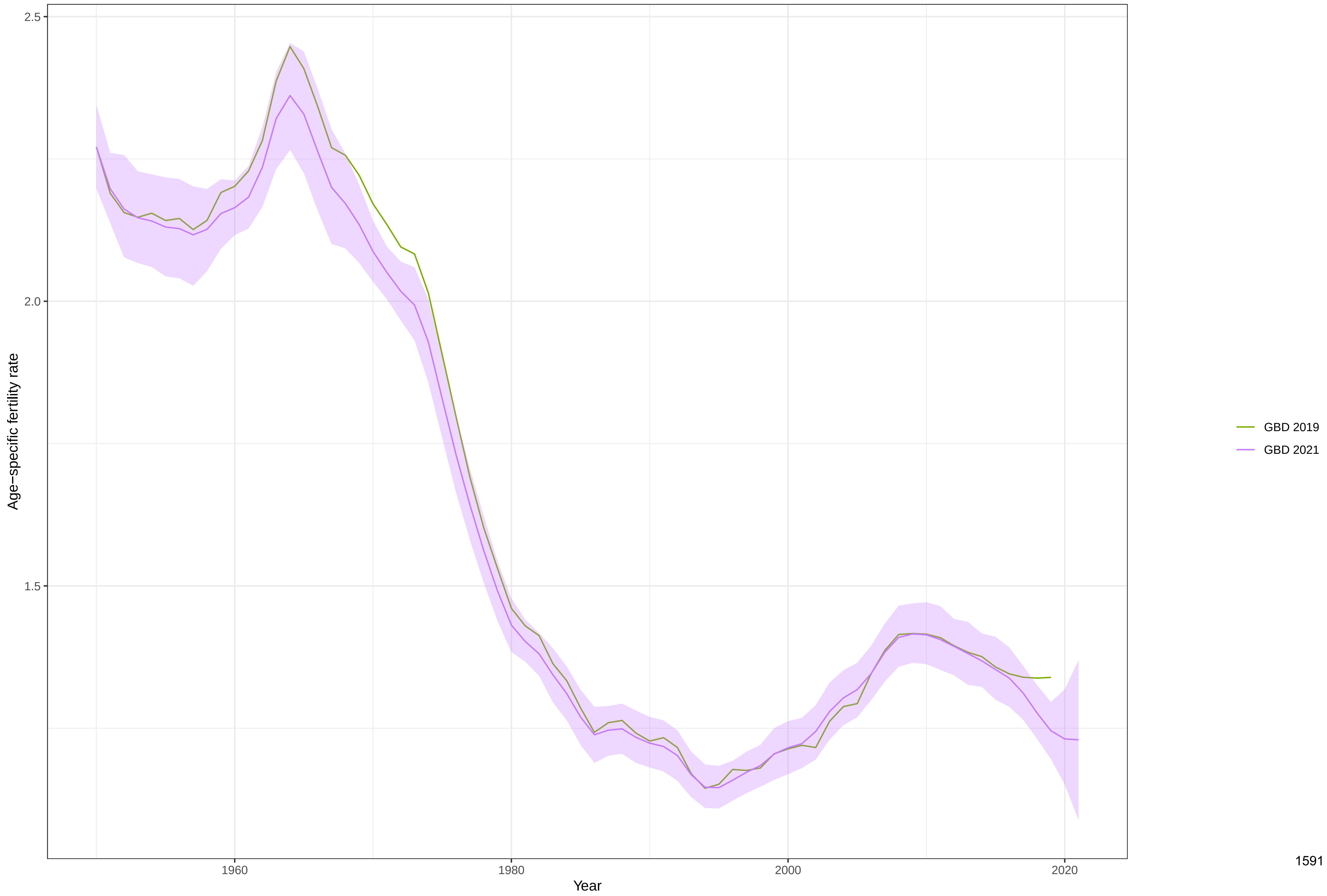

Age−specific fertility fitted trend lines, 1950−2021: Liguria, Ages: 10−14
Data points enclosed with a diamond are reference sources

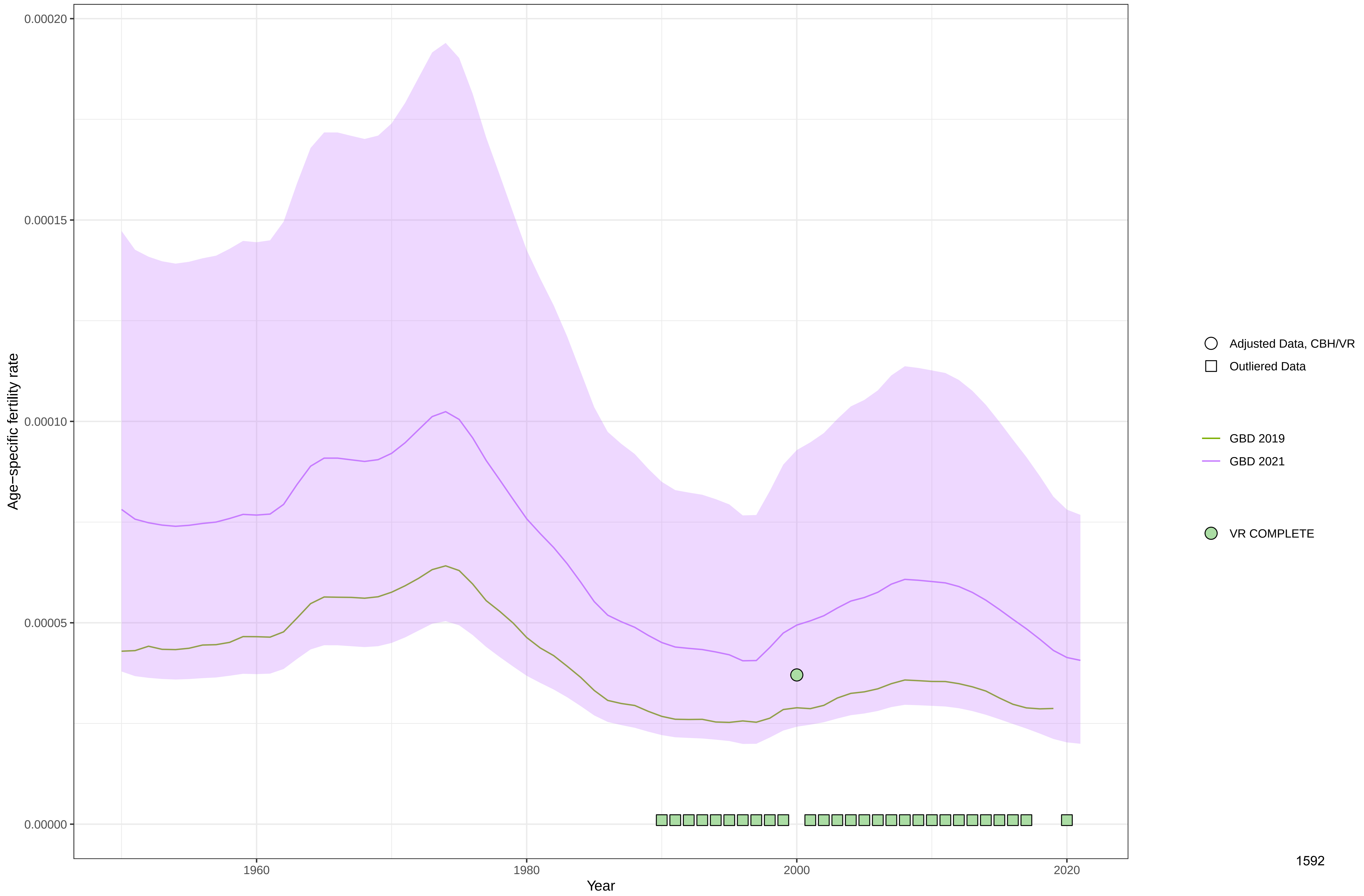

Age−specific fertility fitted trend lines, 1950−2021: Liguria, Ages: 15−19
Data points enclosed with a diamond are reference sources

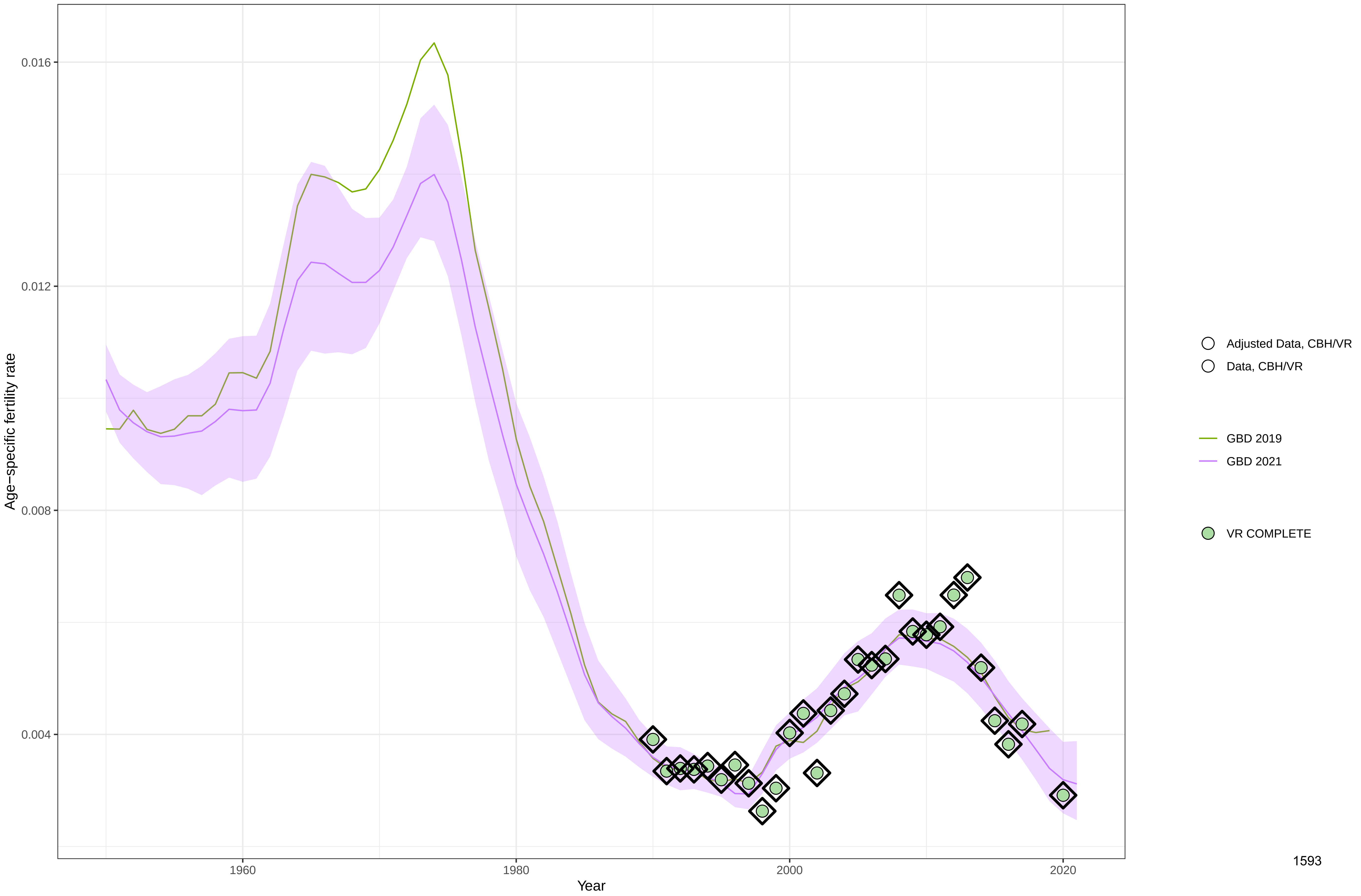

Age−specific fertility fitted trend lines, 1950−2021: Liguria, Ages: 20−24
Data points enclosed with a diamond are reference sources

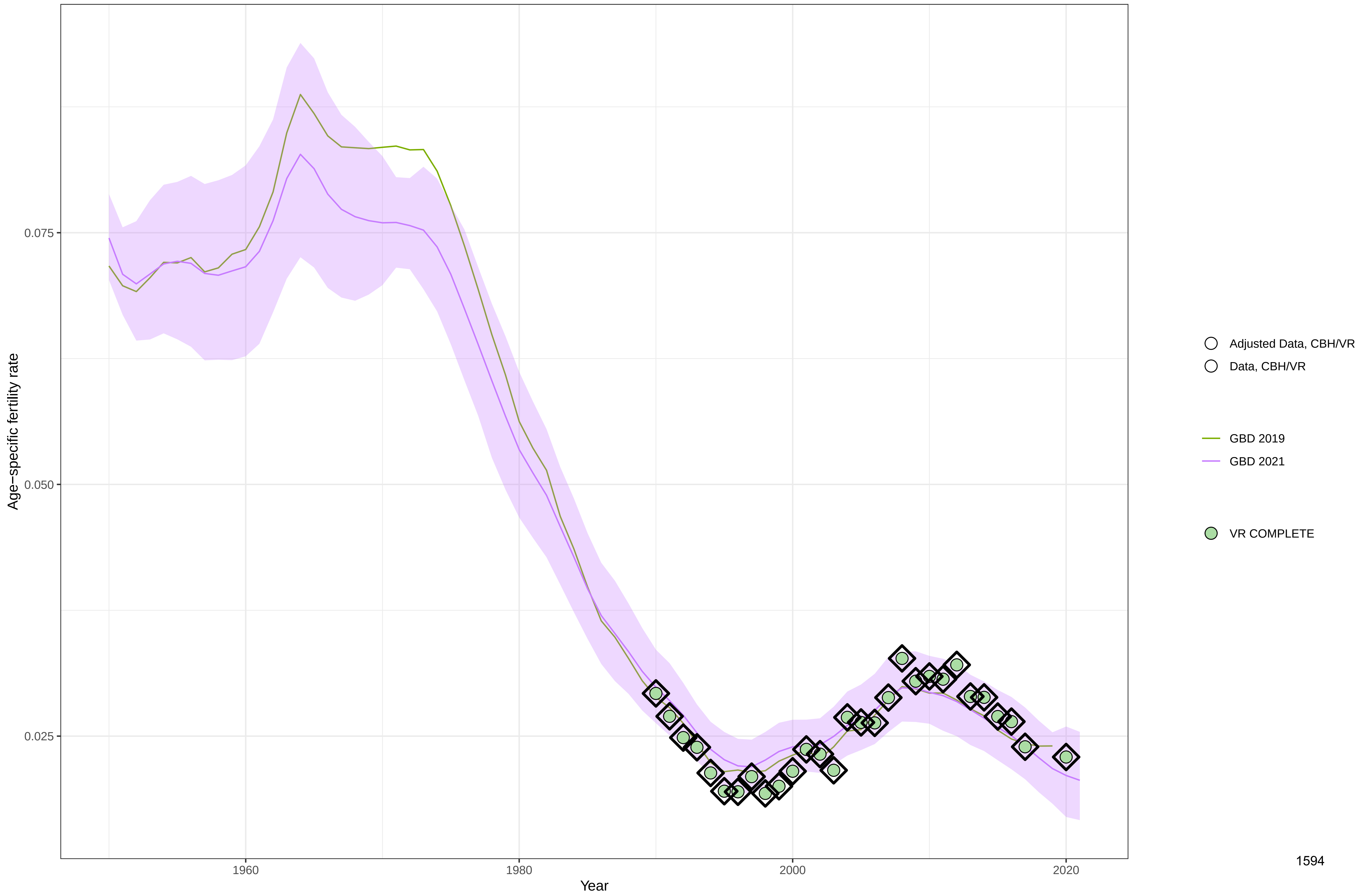

Age−specific fertility fitted trend lines, 1950−2021: Liguria, Ages: 25−29
Data points enclosed with a diamond are reference sources

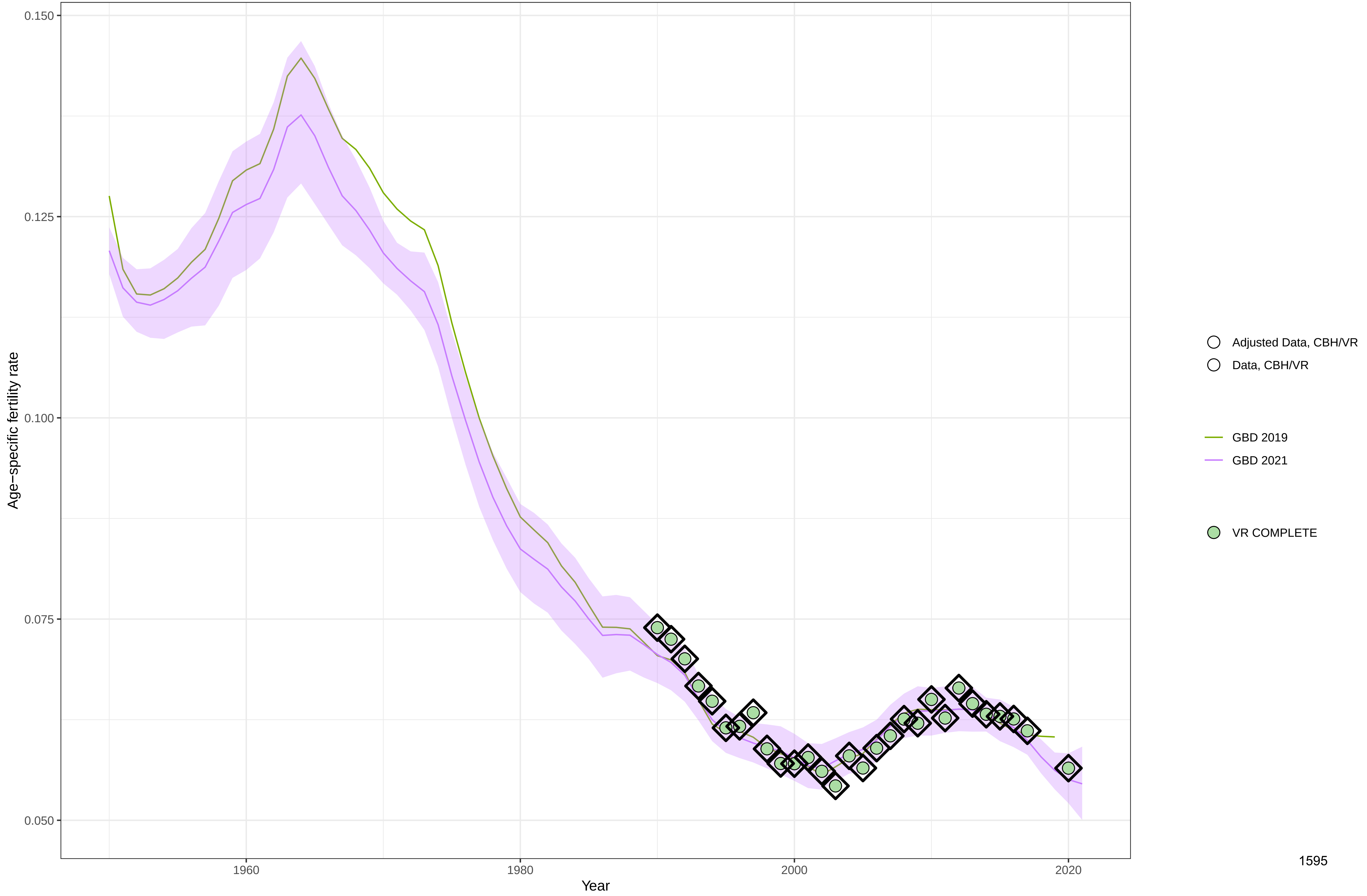

Age−specific fertility fitted trend lines, 1950−2021: Liguria, Ages: 30−34
Data points enclosed with a diamond are reference sources

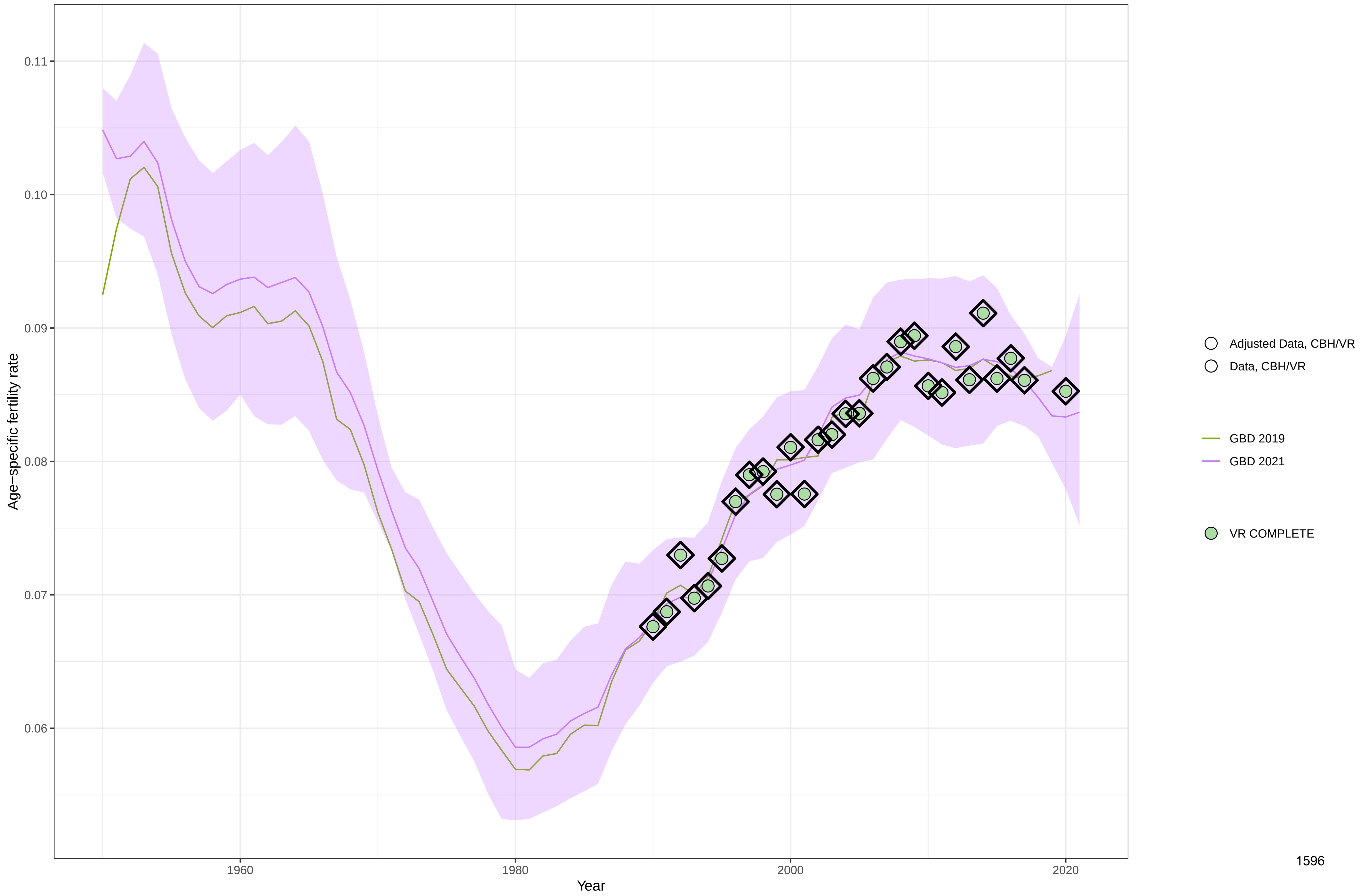

Age−specific fertility fitted trend lines, 1950−2021: Liguria, Ages: 35−39
Data points enclosed with a diamond are reference sources

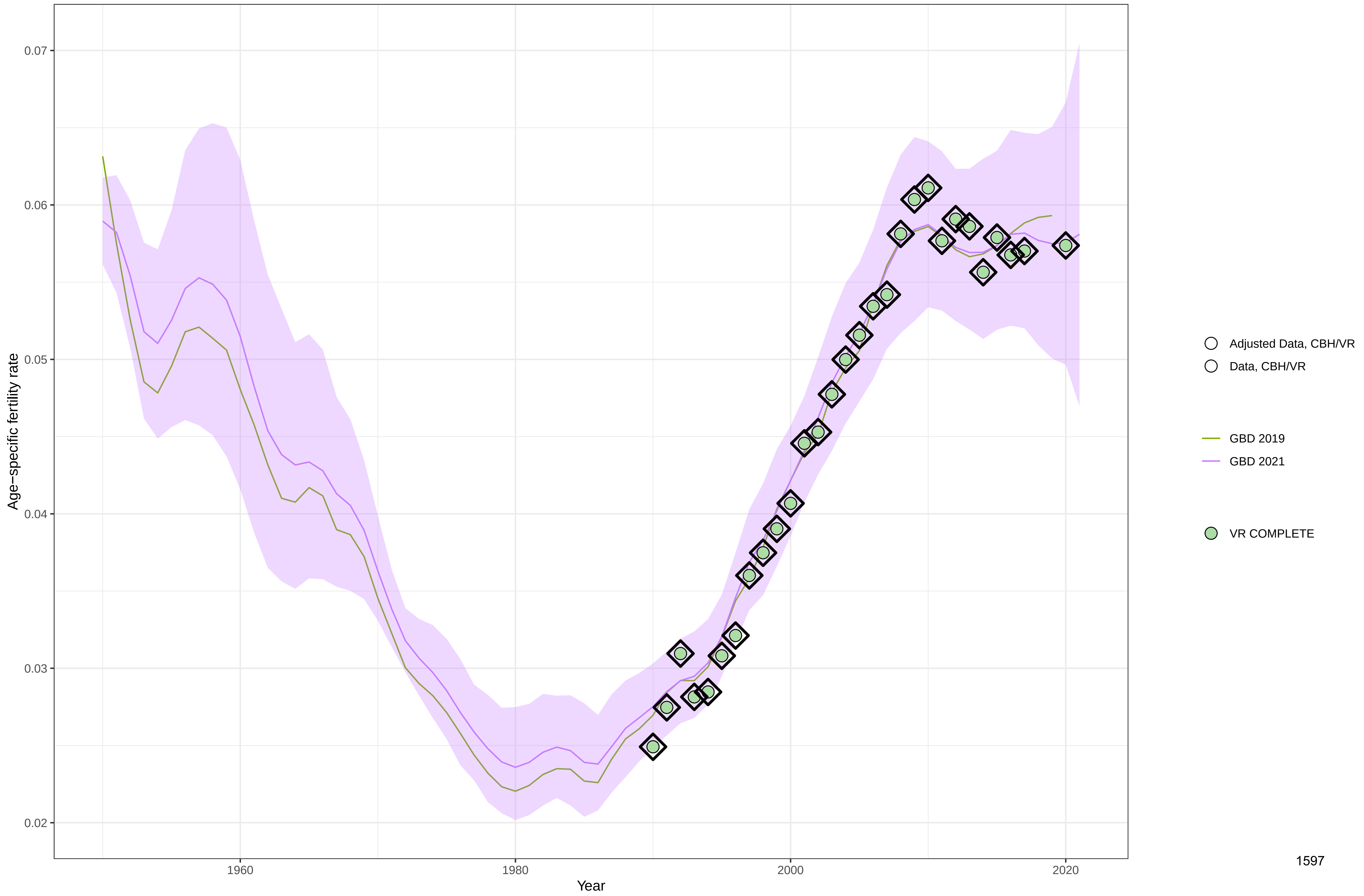

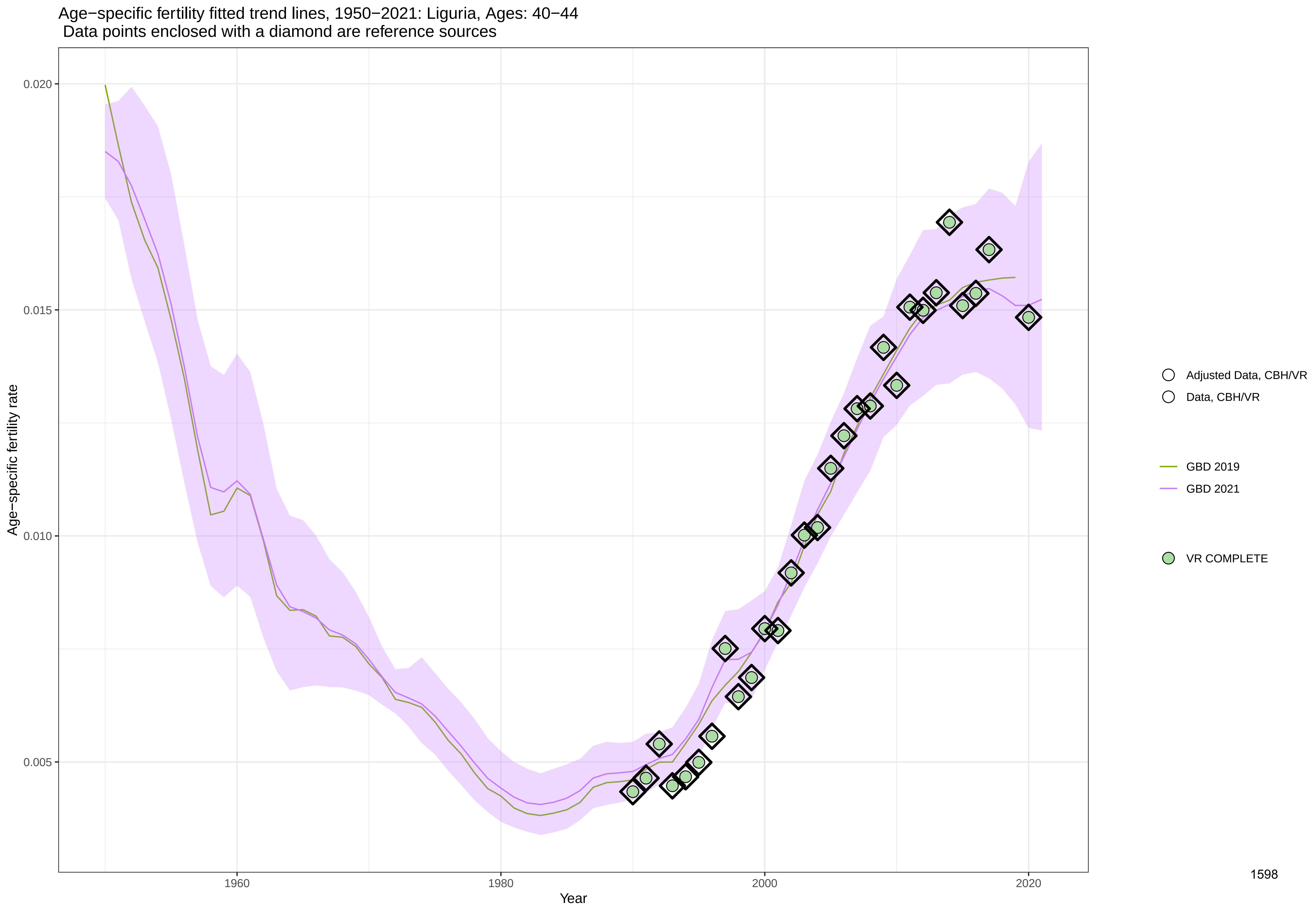
Age−specific fertility fitted trend lines, 1950−2021: Liguria, Ages: 40−44
Data points enclosed with a diamond are reference sources
Age−specific fertility rate
0.020
0.015
0.010
0.005
1960
1980
2000
2020
Year
Adjusted Data, CBH/VR
Data, CBH/VR
GBD 2019
GBD 2021
VR COMPLETE

Age−specific fertility fitted trend lines, 1950−2021: Liguria, Ages: 45−49
Data points enclosed with a diamond are reference sources

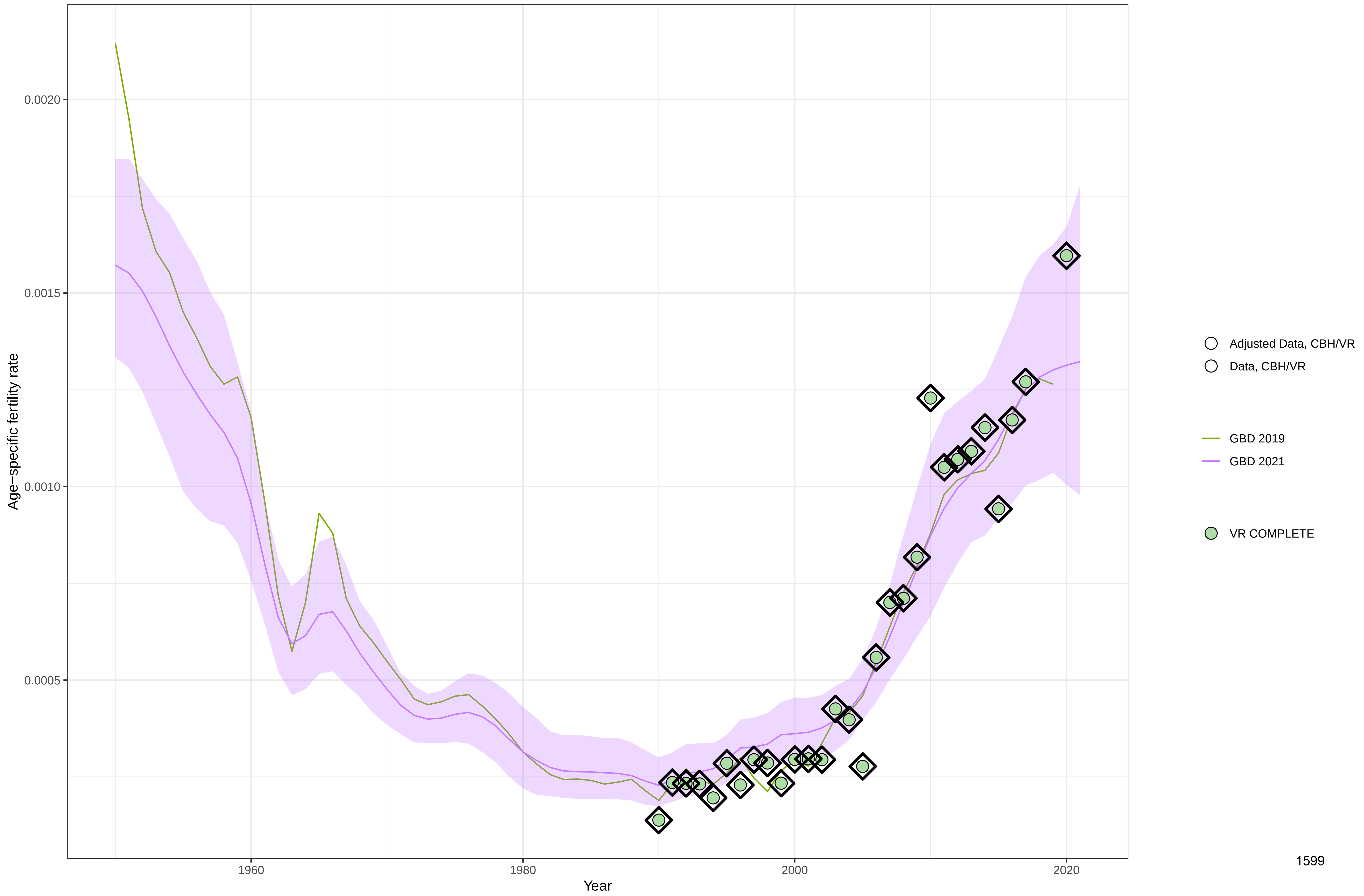

Age−specific fertility fitted trend lines, 1950−2021: Liguria, Ages: 50−54
Data points enclosed with a diamond are reference sources

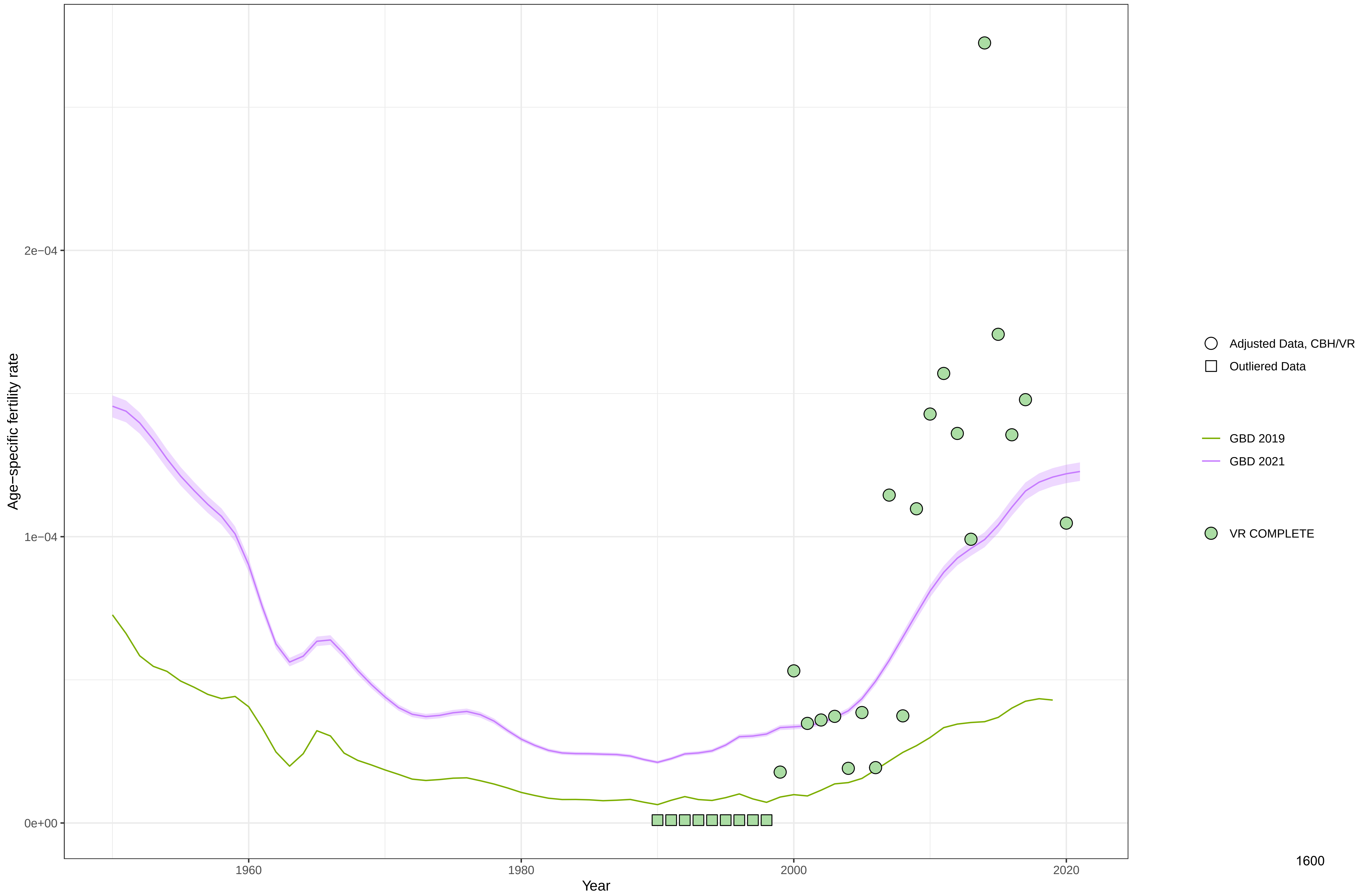

Total fertility rate fitted trend lines, 1950−2021: Liguria TFR
Data points enclosed with a diamond are reference sources

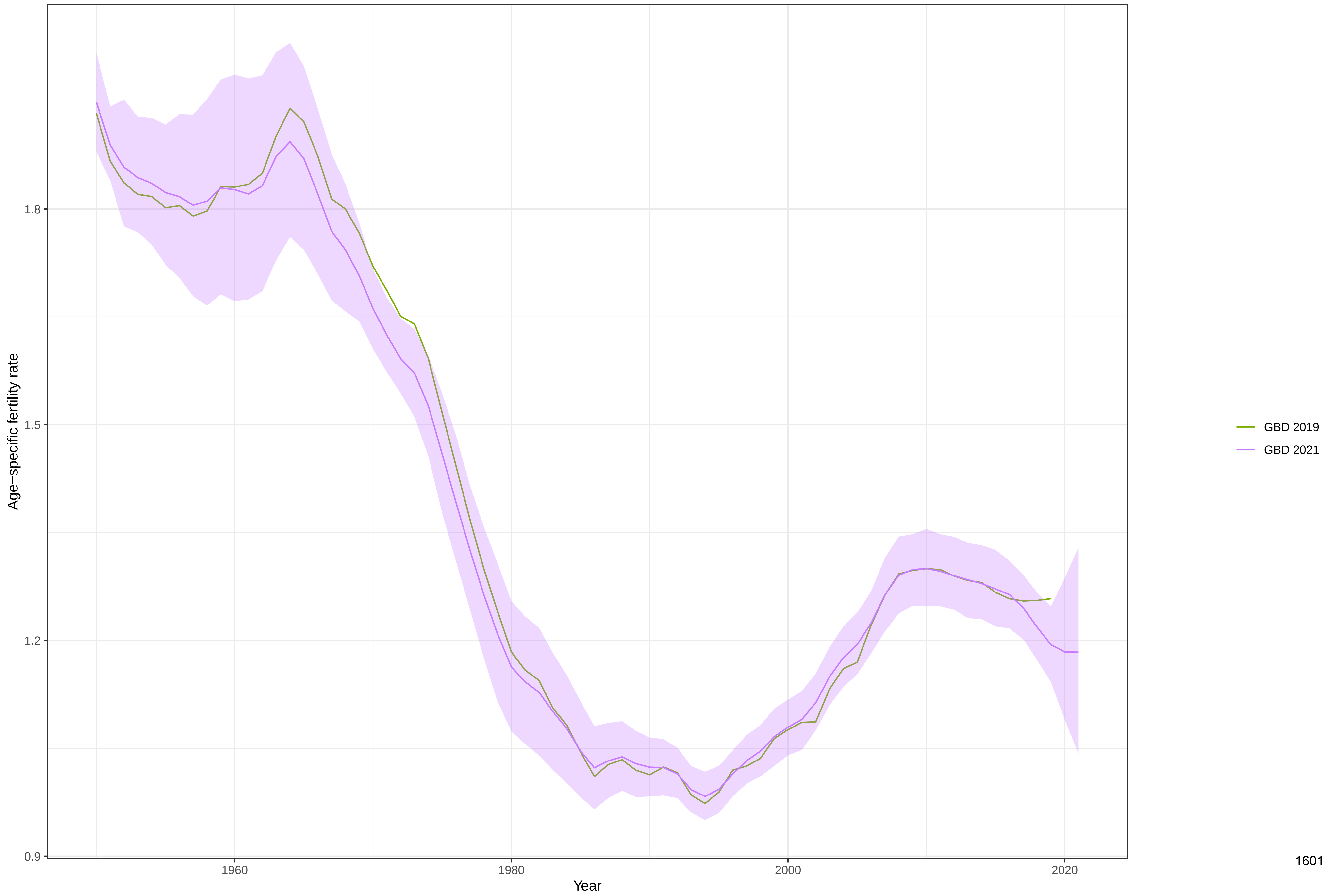

Age−specific fertility fitted trend lines, 1950−2021: Lombardia, Ages: 10−14
Data points enclosed with a diamond are reference sources

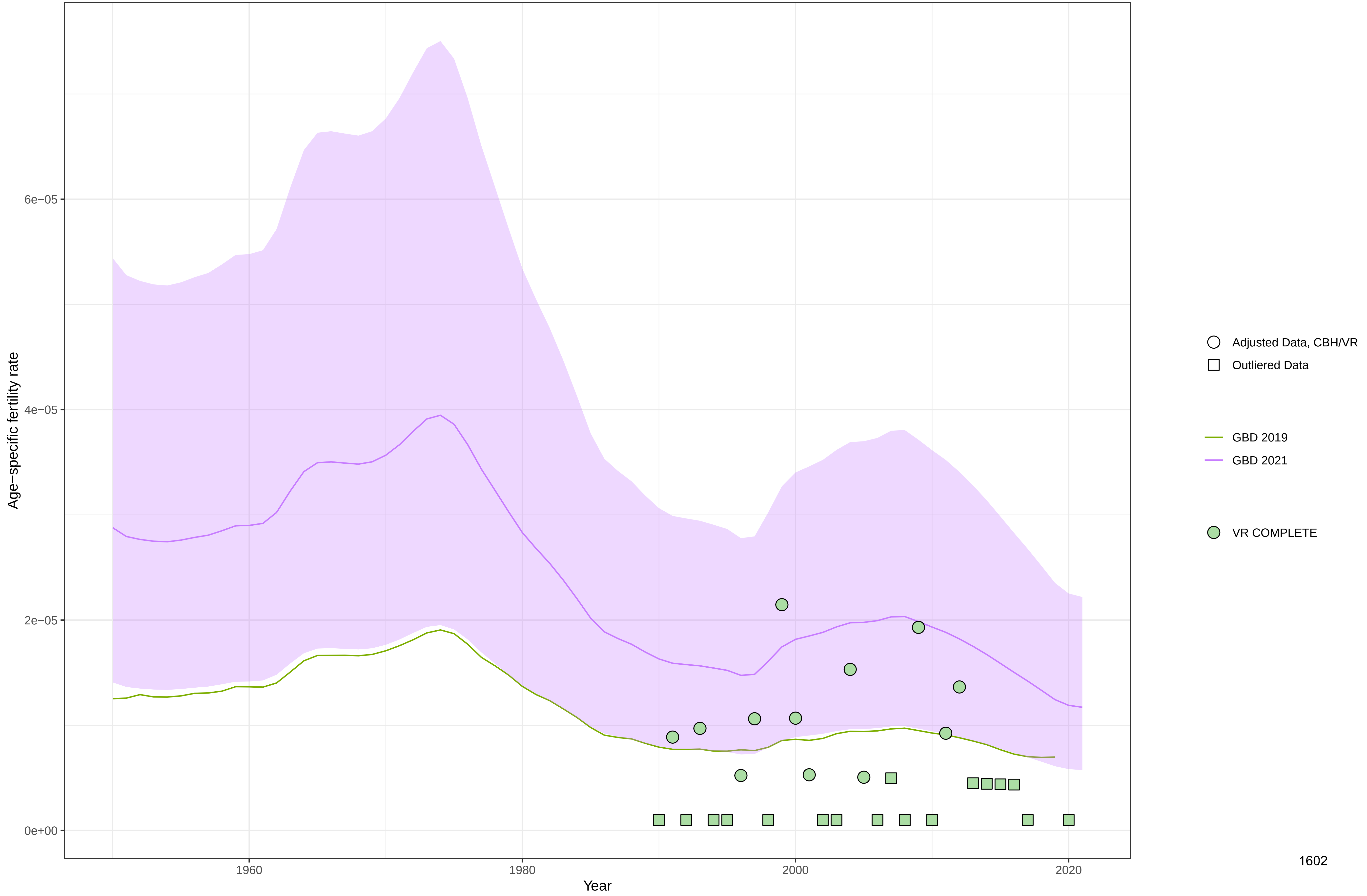

Age−specific fertility fitted trend lines, 1950−2021: Lombardia, Ages: 15−19
Data points enclosed with a diamond are reference sources

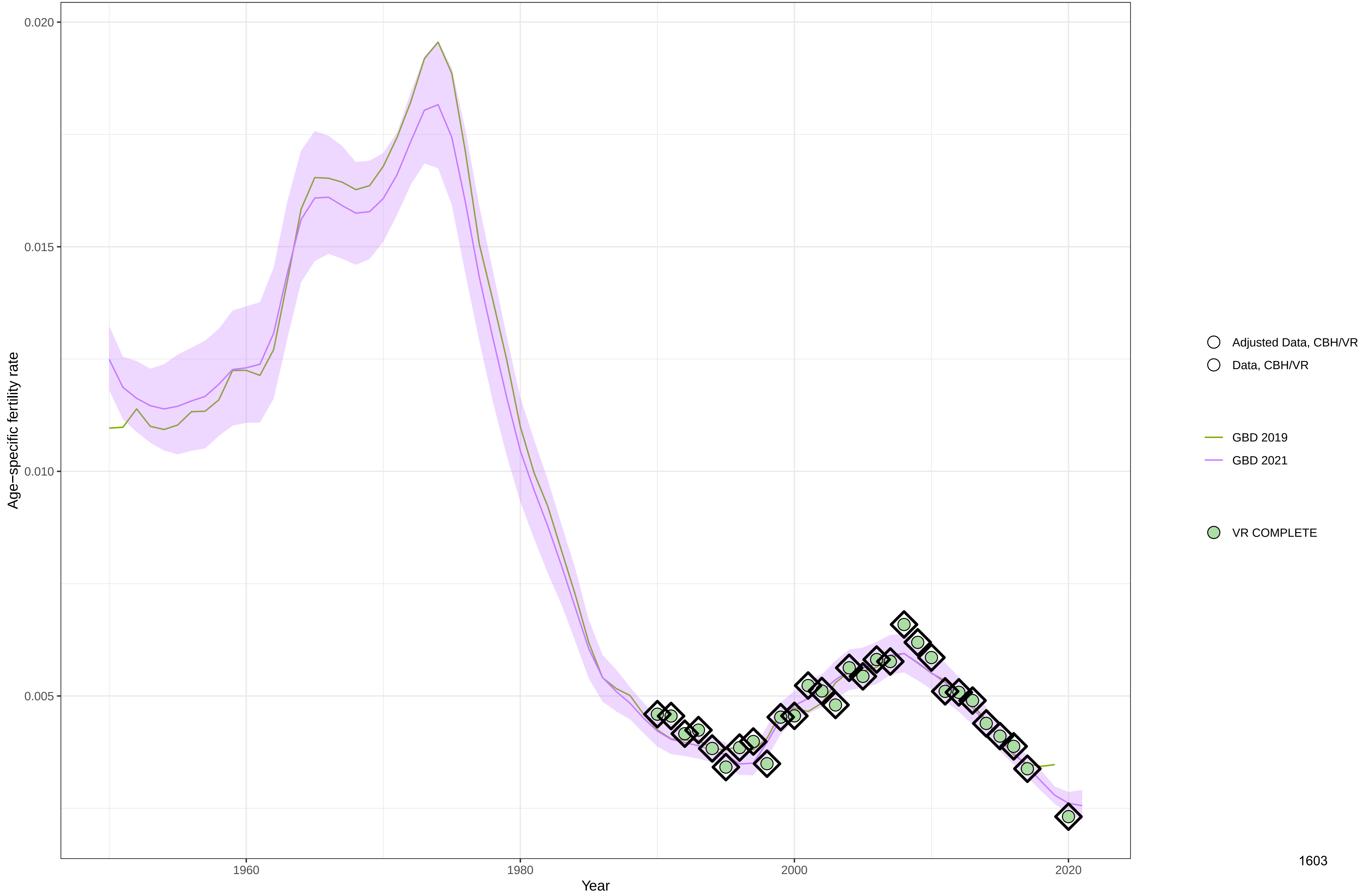

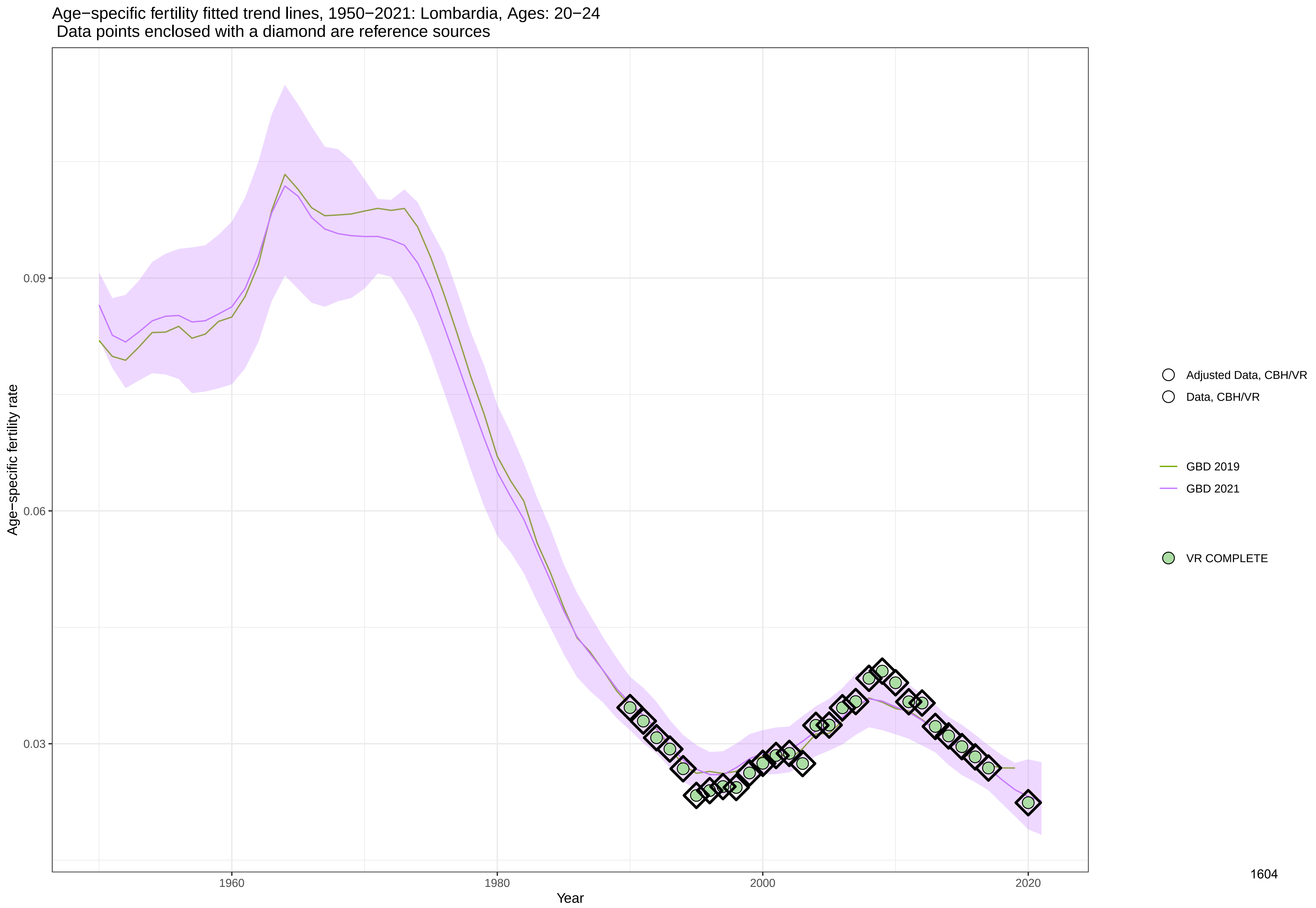

Age−specific fertility fitted trend lines, 1950−2021: Lombardia, Ages: 20−24
Data points enclosed with a diamond are reference sources
Age−specific fertility rate
0.09
0.06
0.03
1960
1980
2000
2020
Year
Adjusted Data, CBH/VR
Data, CBH/VR
GBD 2019
GBD 2021
VR COMPLETE

Age−specific fertility fitted trend lines, 1950−2021: Lombardia, Ages: 25−29
Data points enclosed with a diamond are reference sources

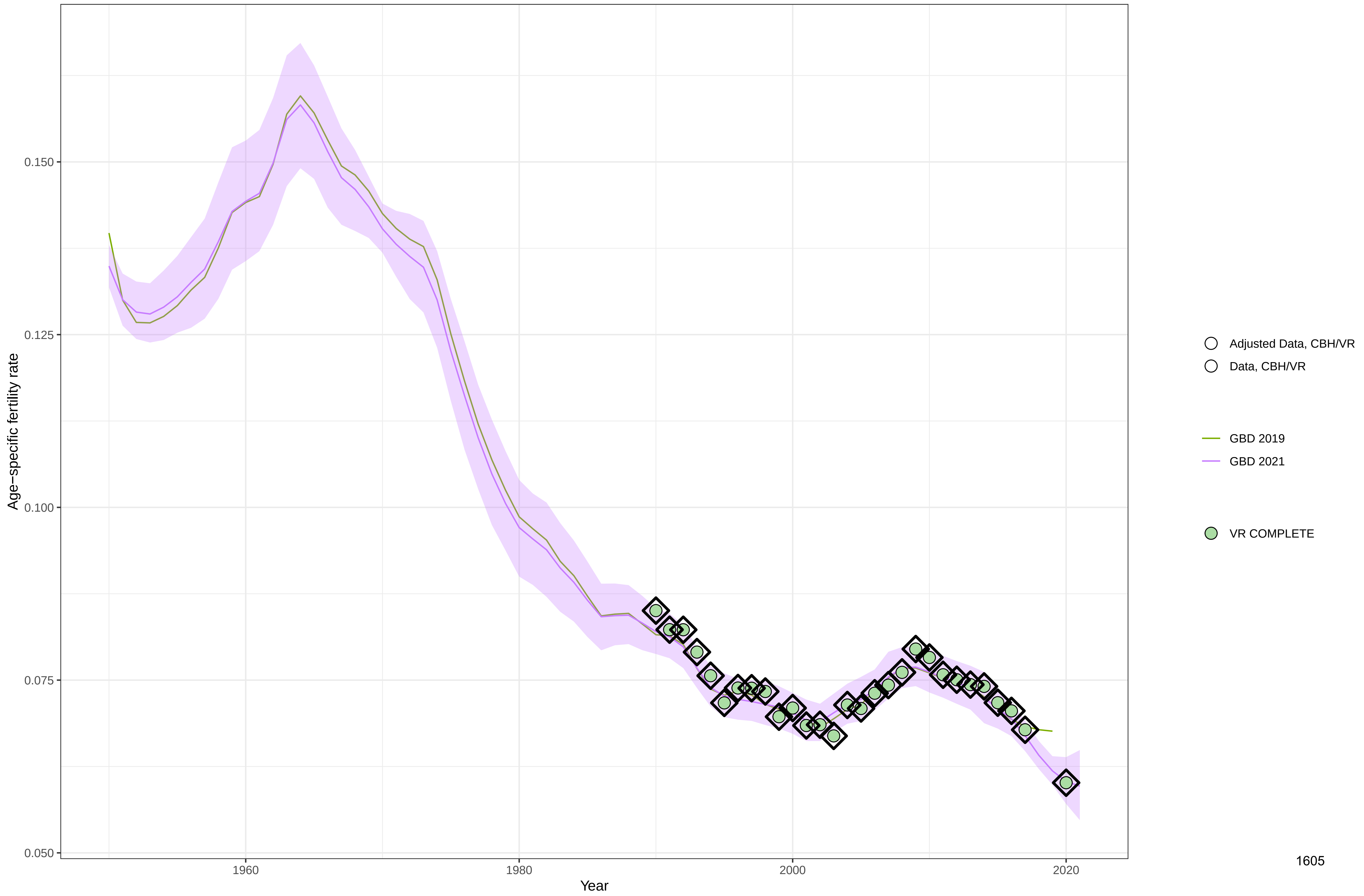

Age−specific fertility fitted trend lines, 1950−2021: Lombardia, Ages: 30−34
Data points enclosed with a diamond are reference sources

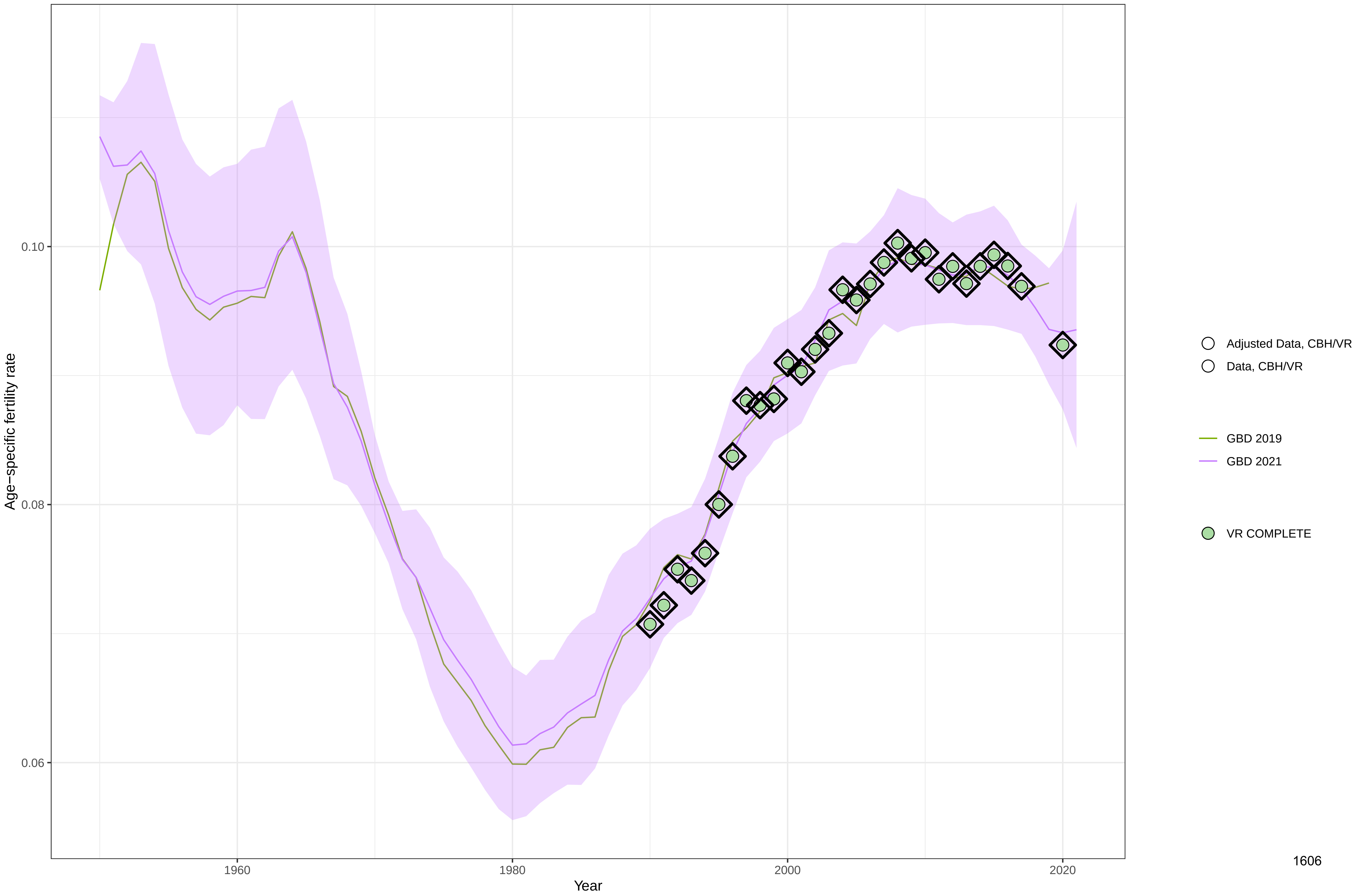

Age−specific fertility fitted trend lines, 1950−2021: Lombardia, Ages: 35−39
Data points enclosed with a diamond are reference sources

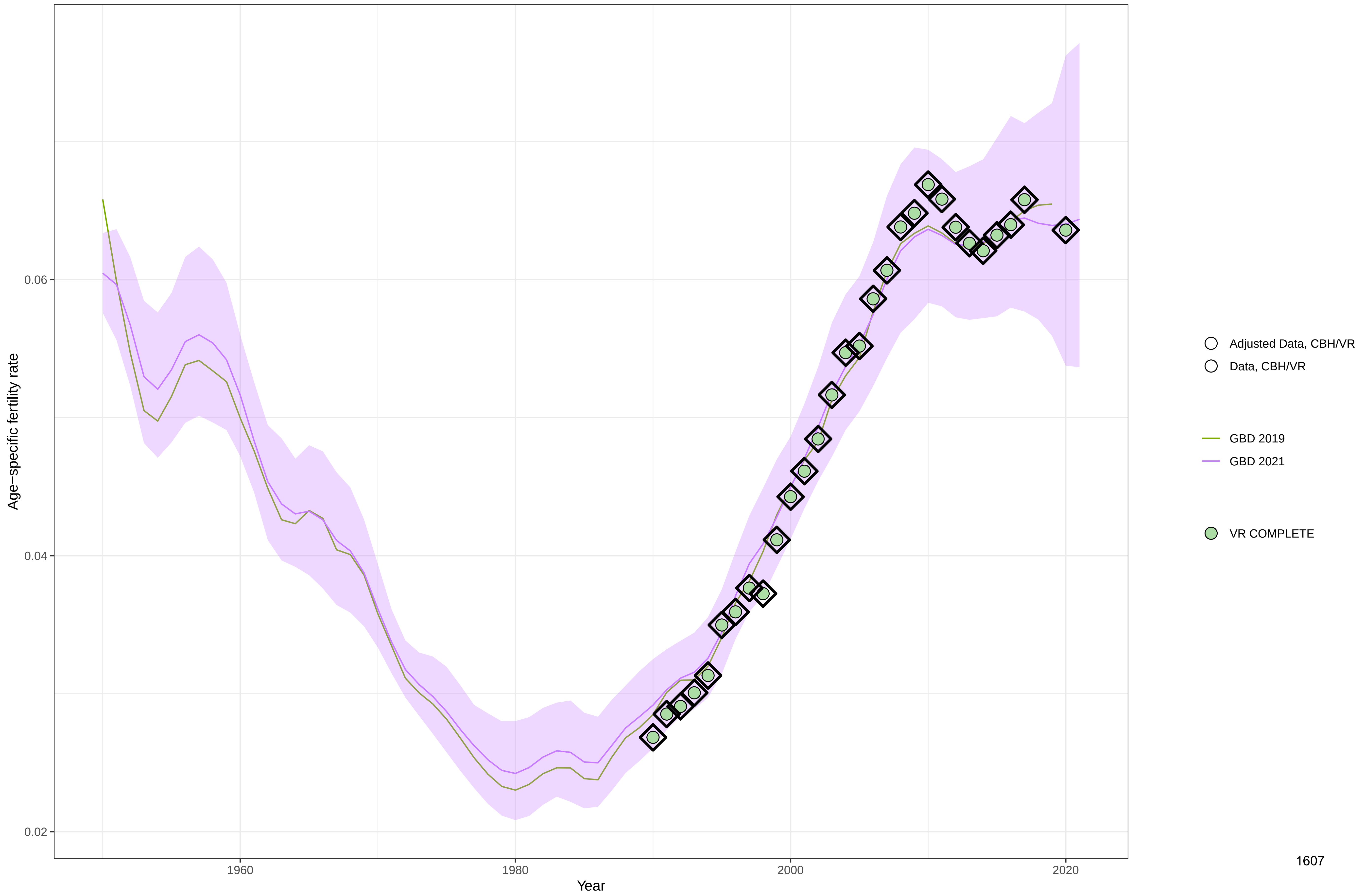

Age−specific fertility fitted trend lines, 1950−2021: Lombardia, Ages: 40−44
Data points enclosed with a diamond are reference sources

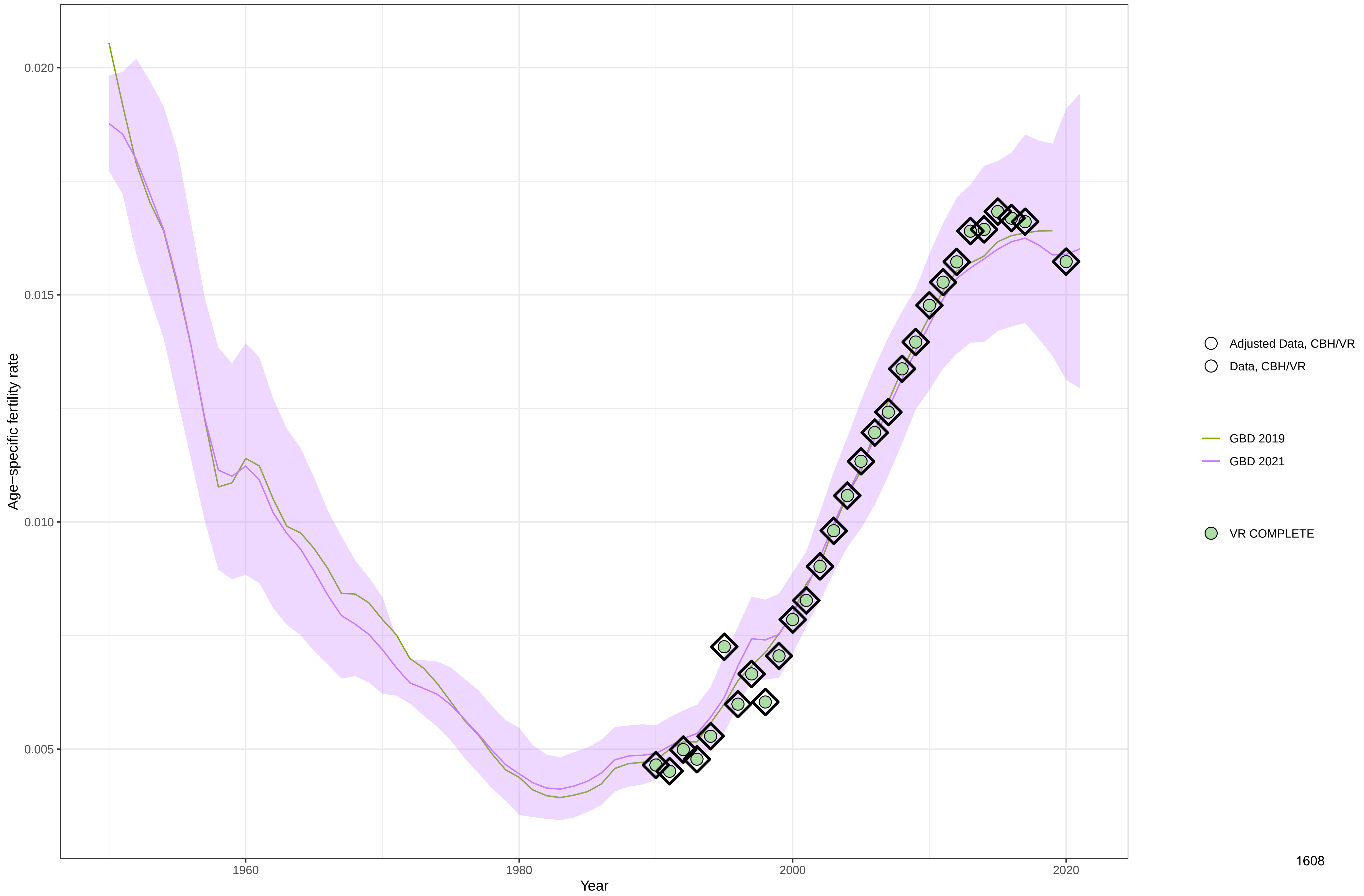

Age−specific fertility fitted trend lines, 1950−2021: Lombardia, Ages: 45−49
Data points enclosed with a diamond are reference sources

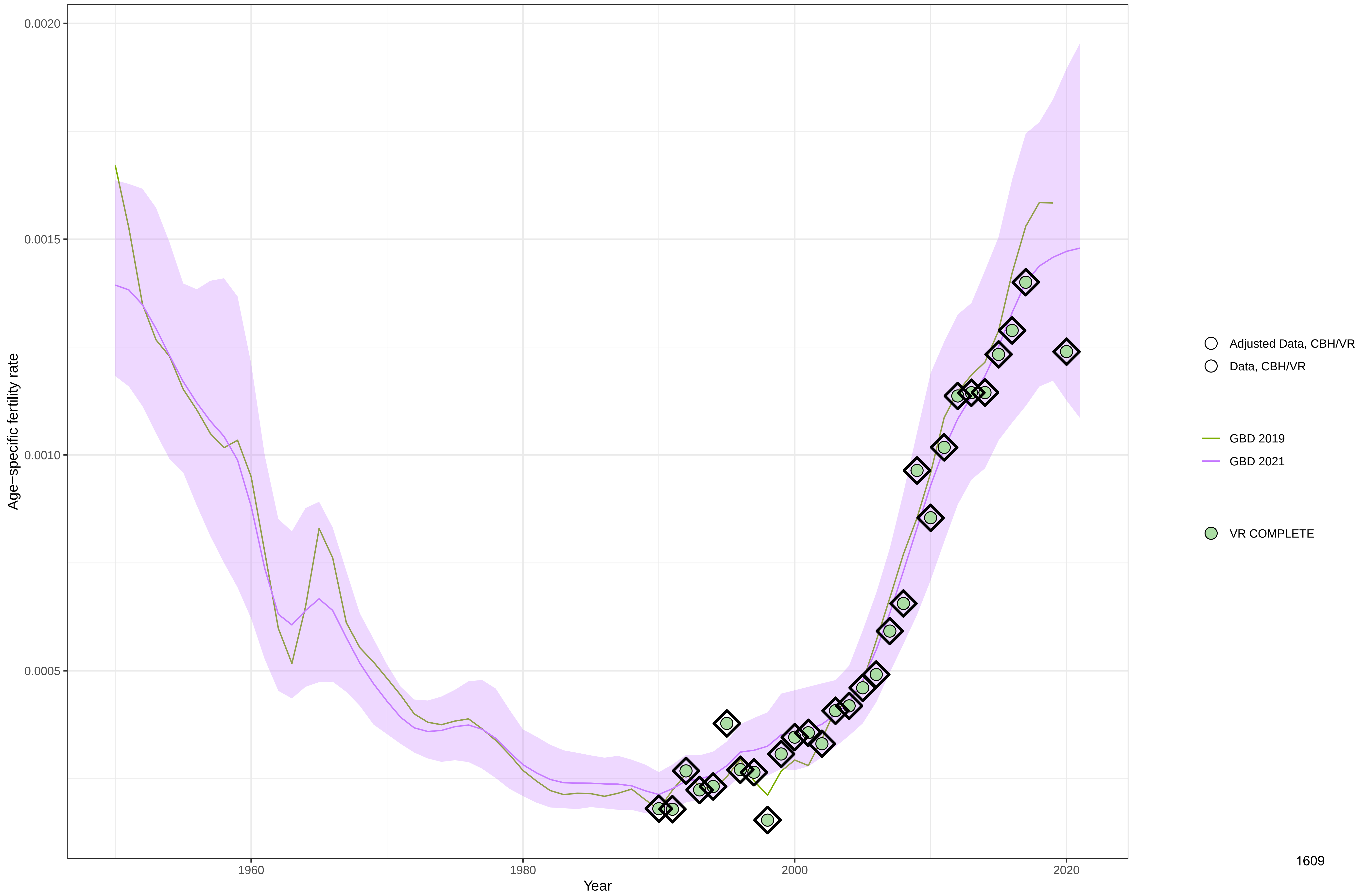

Age−specific fertility fitted trend lines, 1950−2021: Lombardia, Ages: 50−54
Data points enclosed with a diamond are reference sources

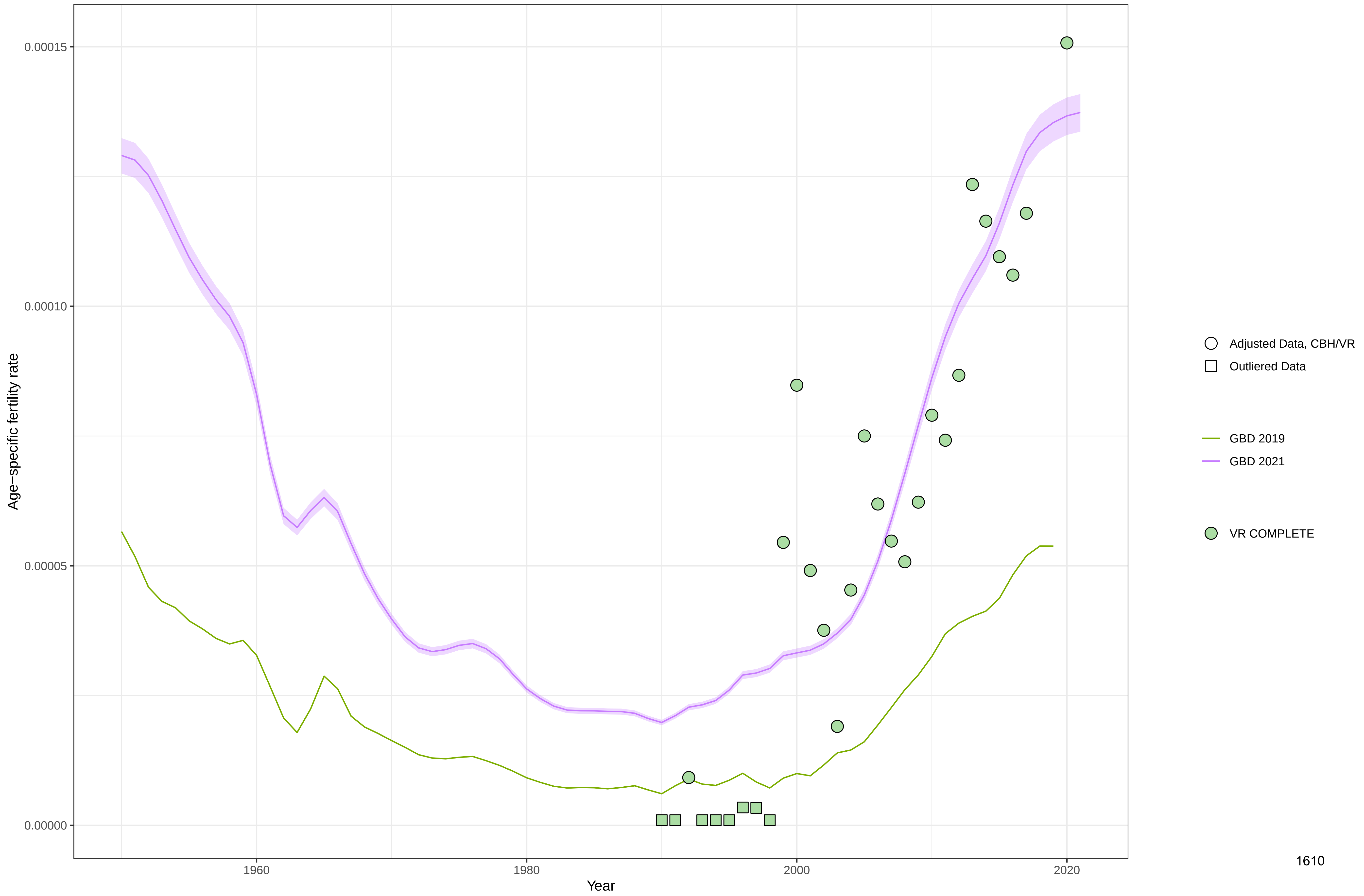

Total fertility rate fitted trend lines, 1950−2021: Lombardia TFR
Data points enclosed with a diamond are reference sources

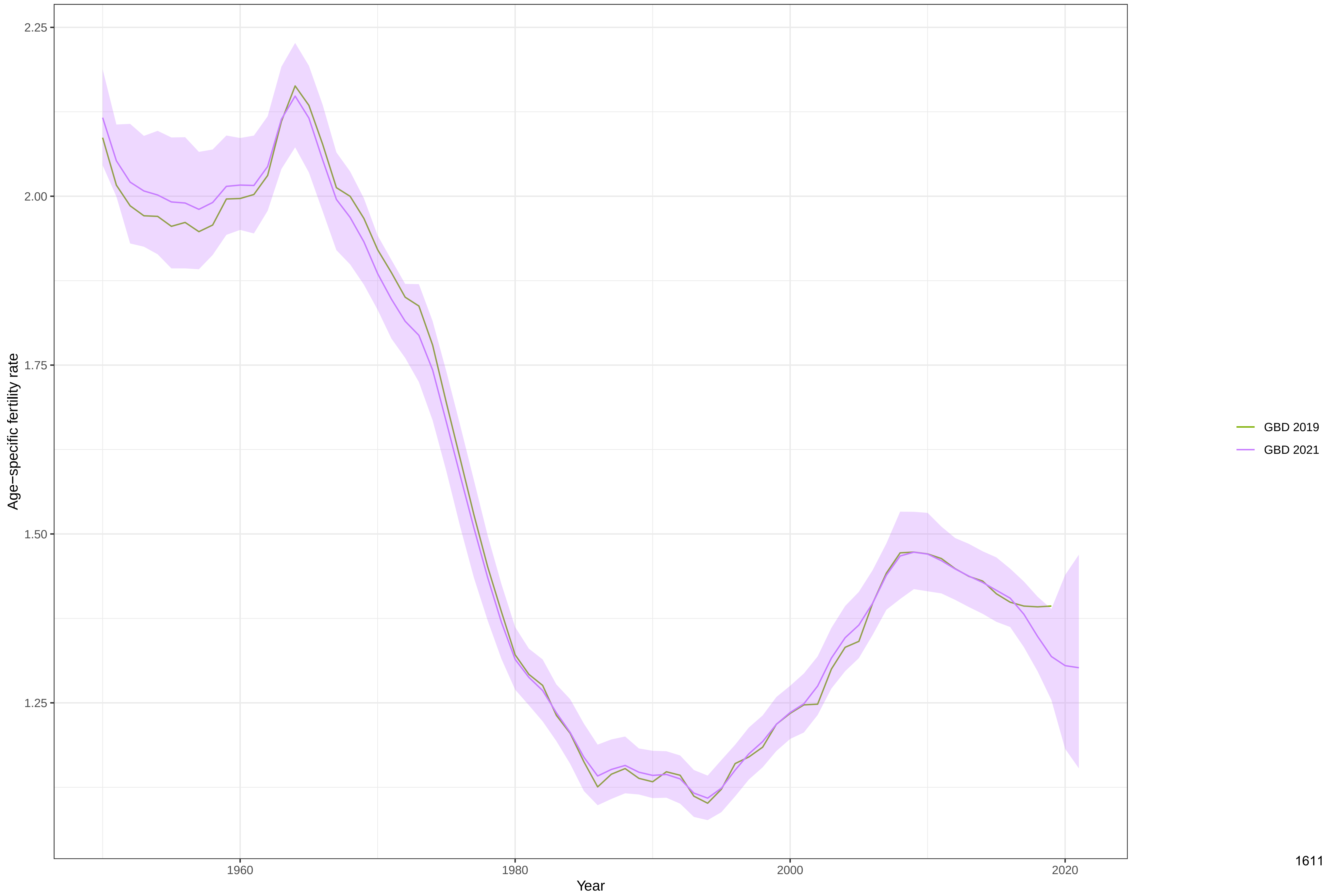

Age−specific fertility fitted trend lines, 1950−2021: Marche, Ages: 10−14
Data points enclosed with a diamond are reference sources

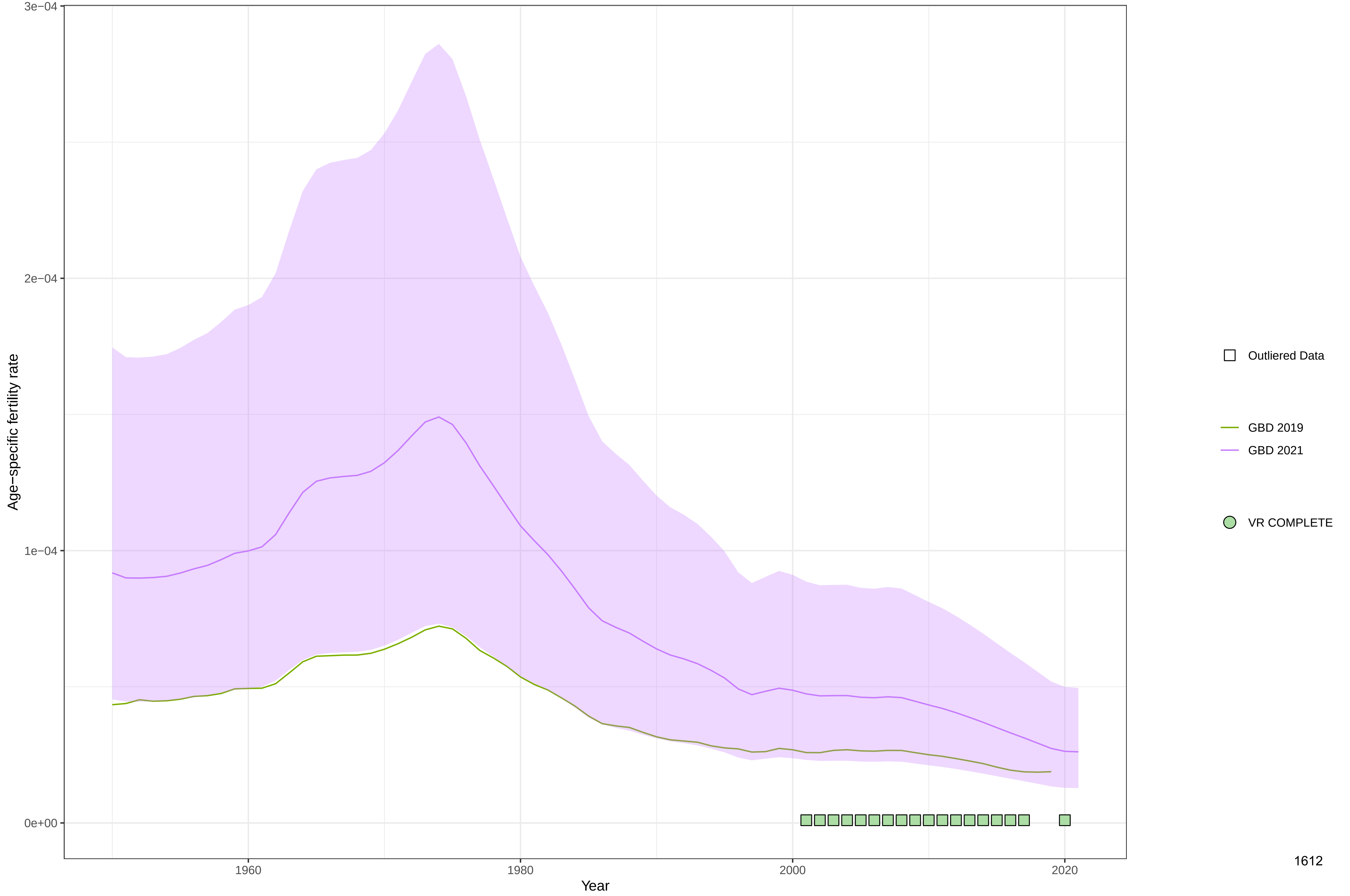

Age−specific fertility fitted trend lines, 1950−2021: Marche, Ages: 15−19
Data points enclosed with a diamond are reference sources

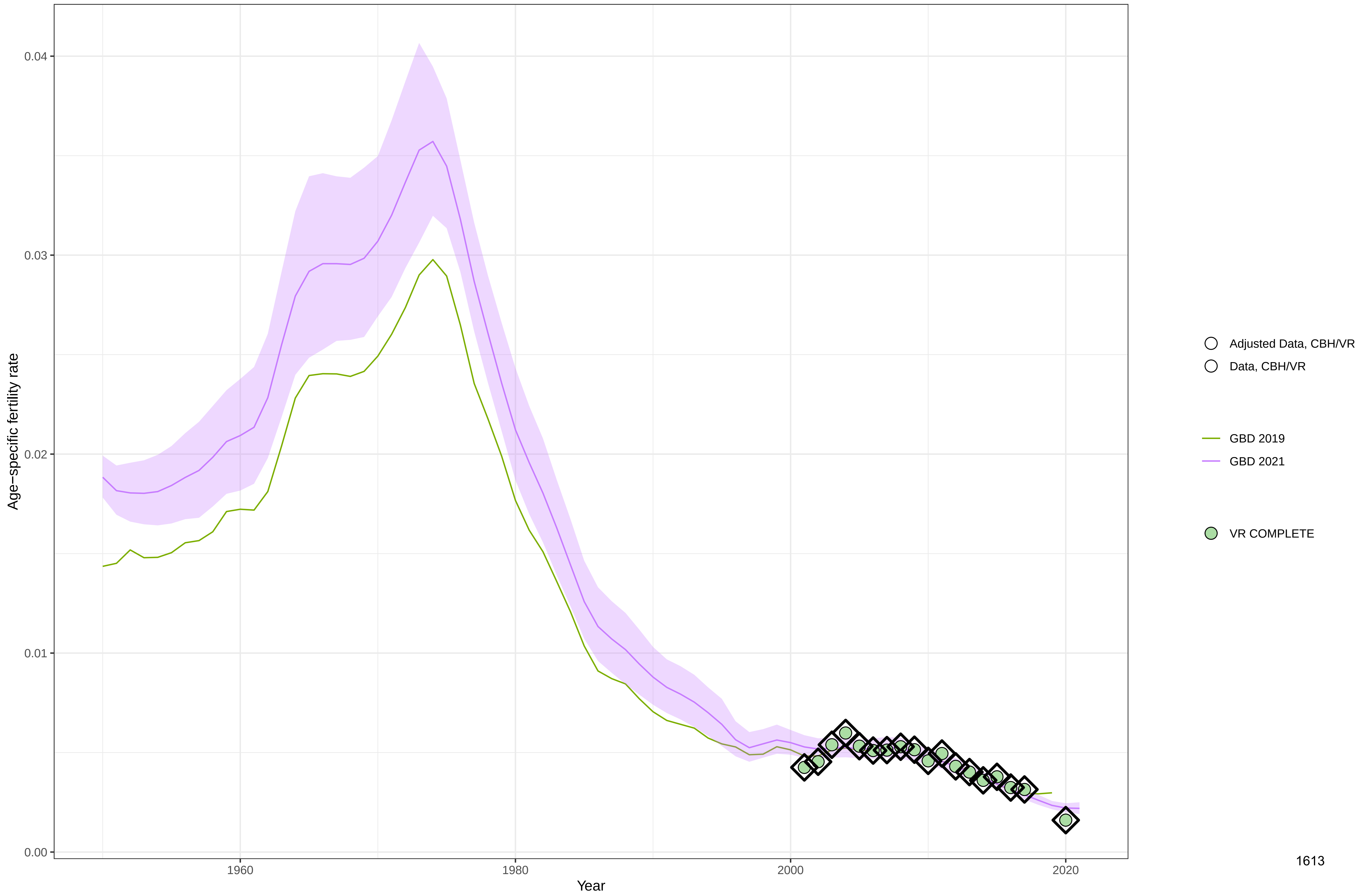

Age−specific fertility fitted trend lines, 1950−2021: Marche, Ages: 20−24
Data points enclosed with a diamond are reference sources

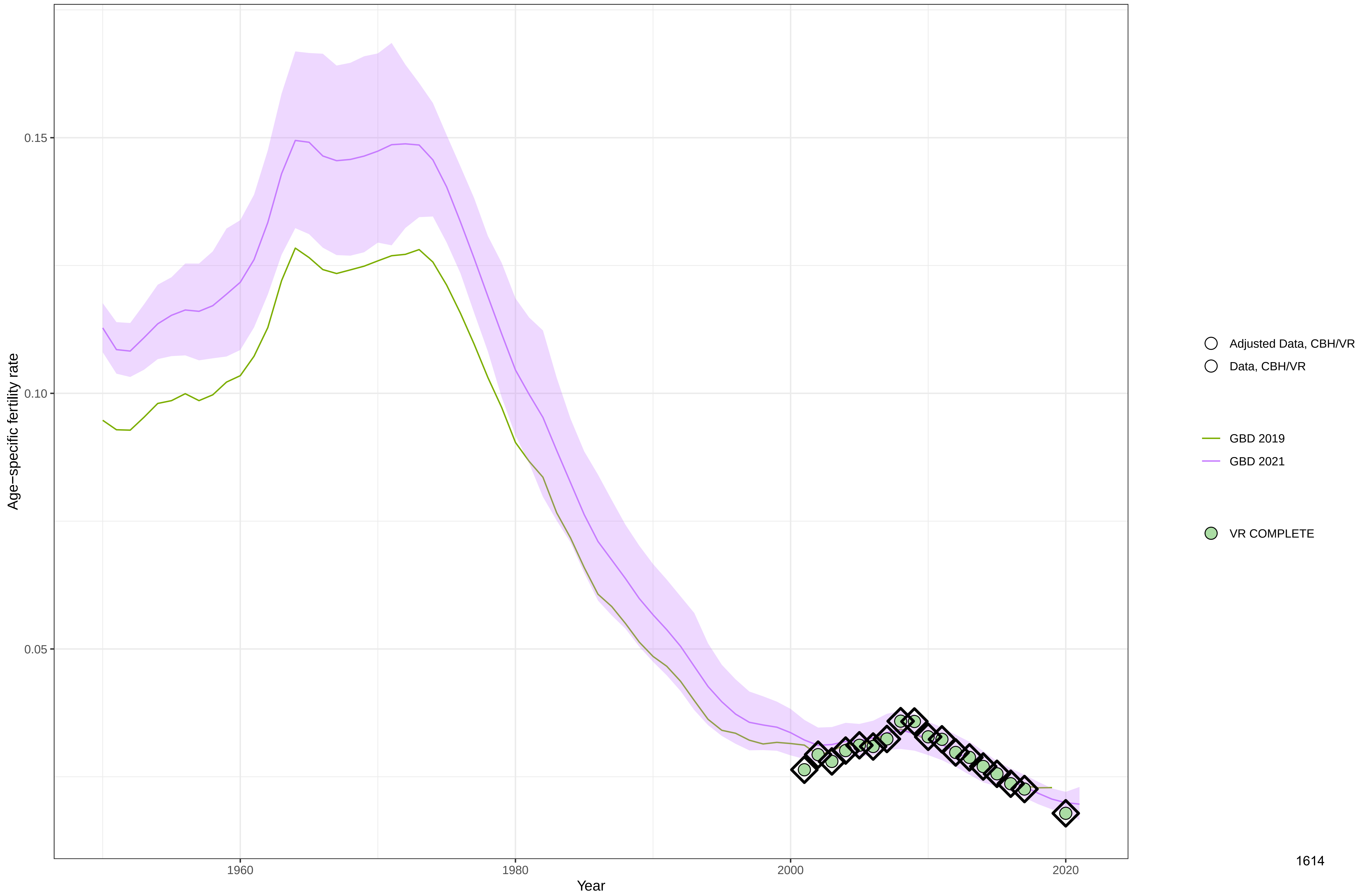

Age−specific fertility fitted trend lines, 1950−2021: Marche, Ages: 25−29
Data points enclosed with a diamond are reference sources

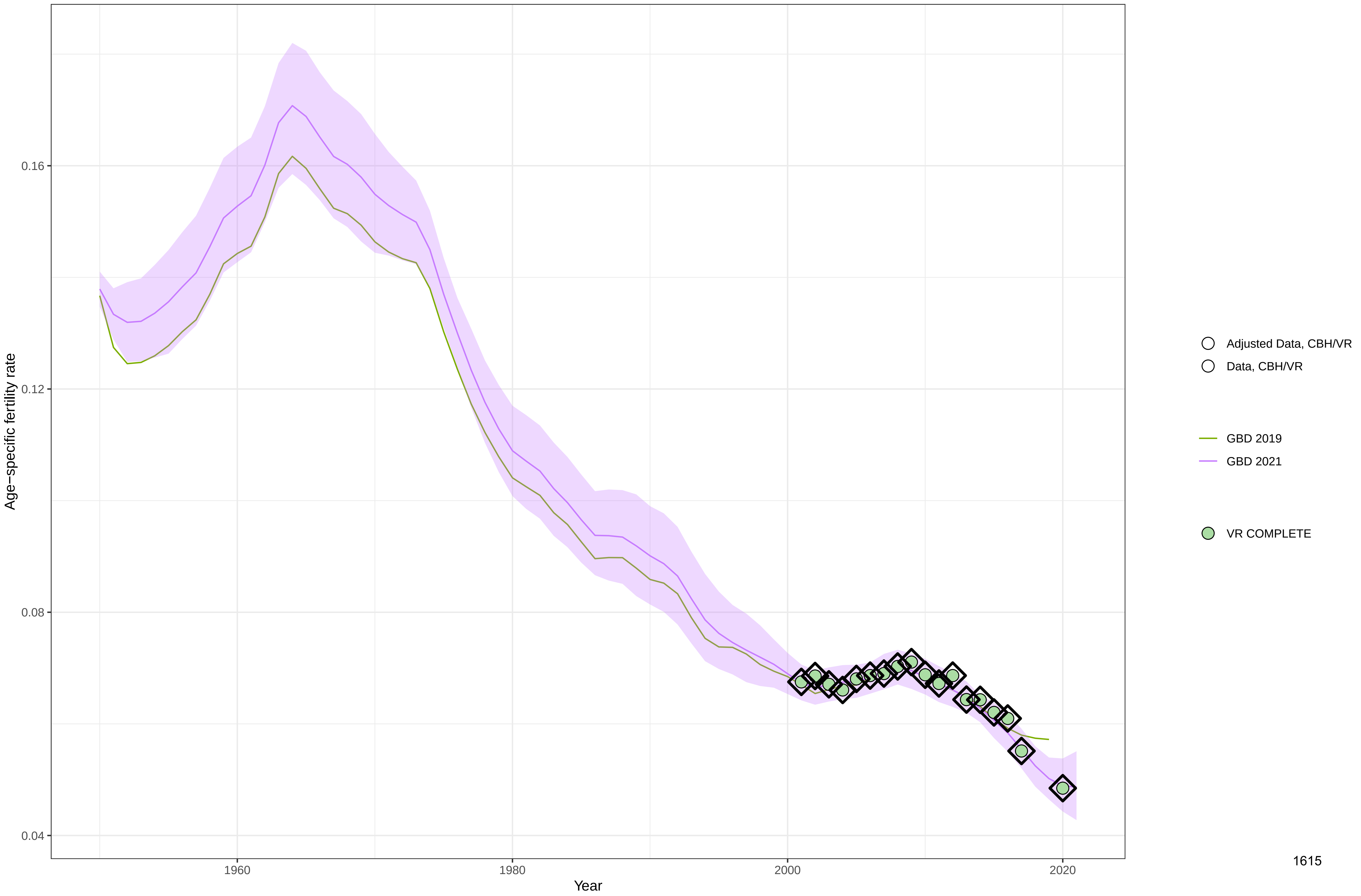

Age−specific fertility fitted trend lines, 1950−2021: Marche, Ages: 30−34
Data points enclosed with a diamond are reference sources

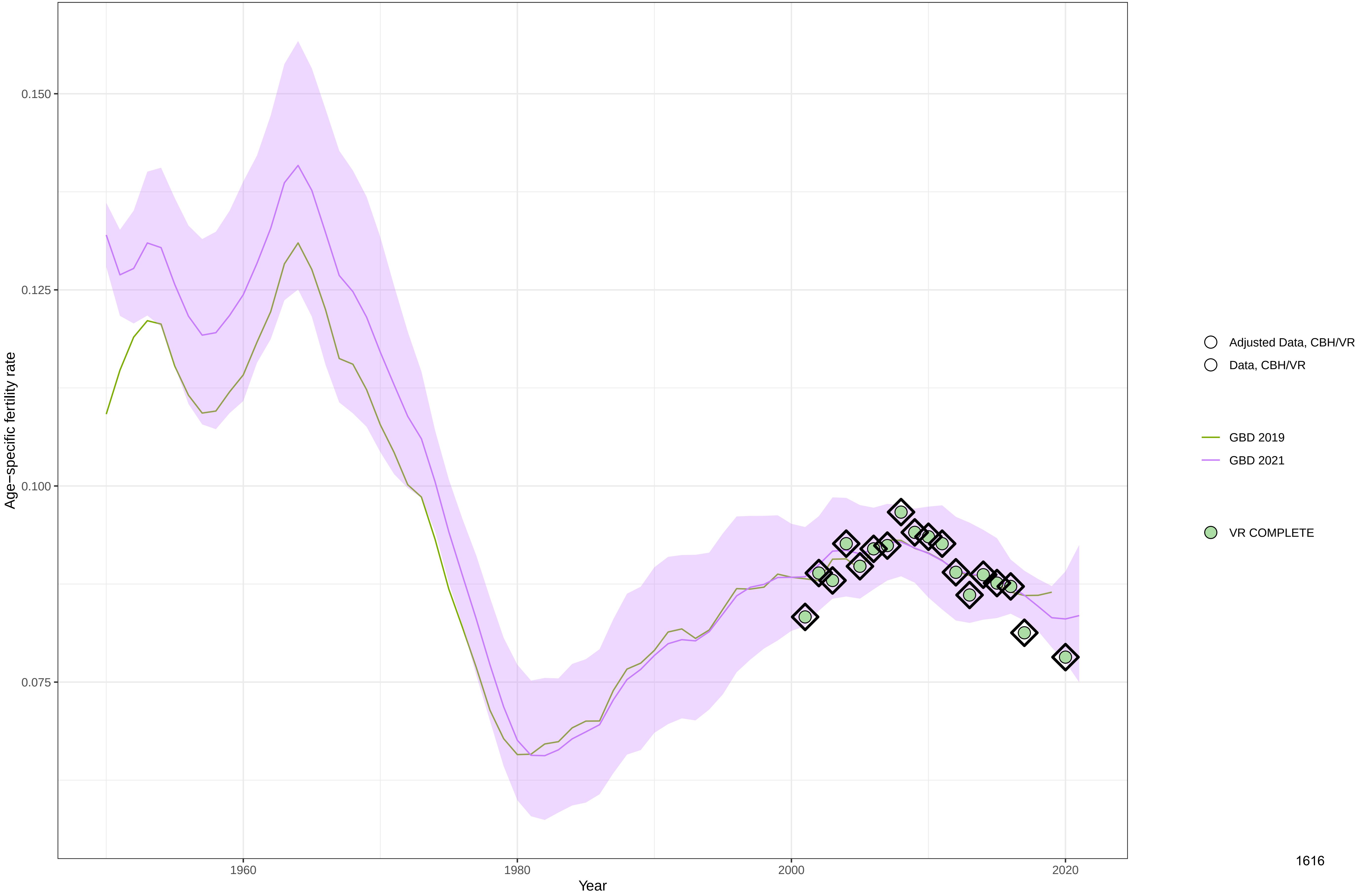

Age−specific fertility fitted trend lines, 1950−2021: Marche, Ages: 35−39
Data points enclosed with a diamond are reference sources

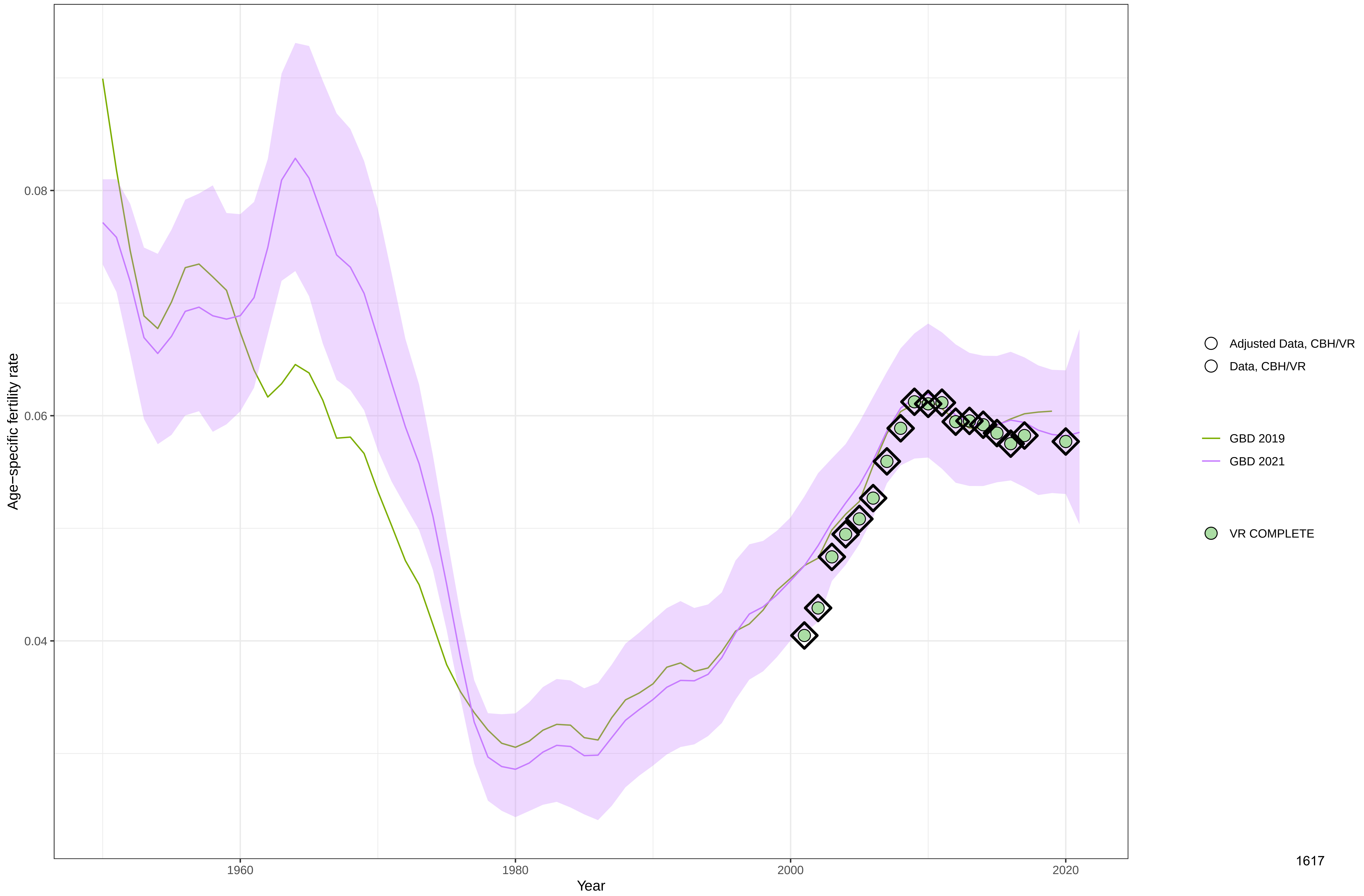

Age−specific fertility fitted trend lines, 1950−2021: Marche, Ages: 40−44
Data points enclosed with a diamond are reference sources

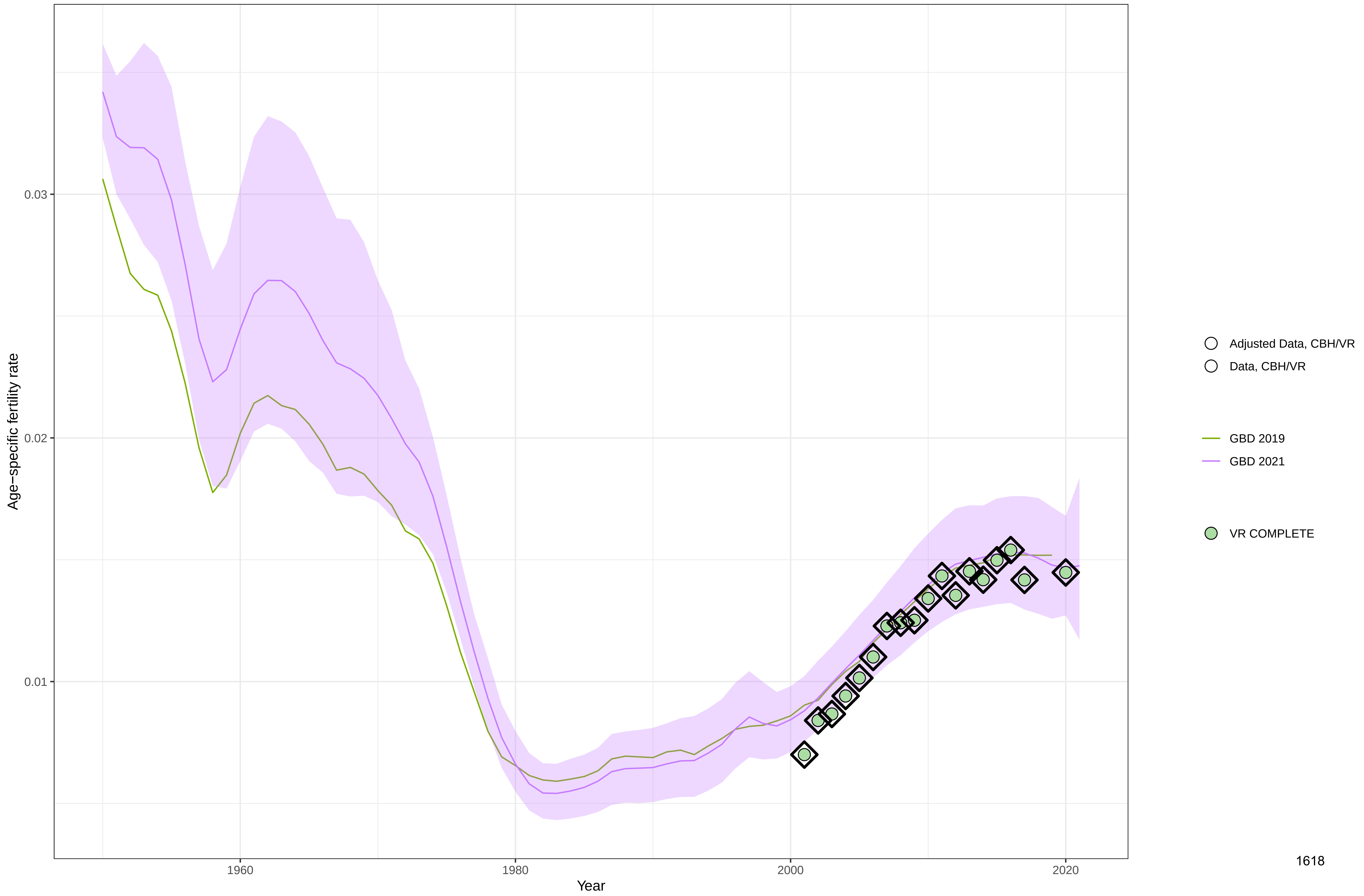

Age−specific fertility fitted trend lines, 1950−2021: Marche, Ages: 45−49
Data points enclosed with a diamond are reference sources

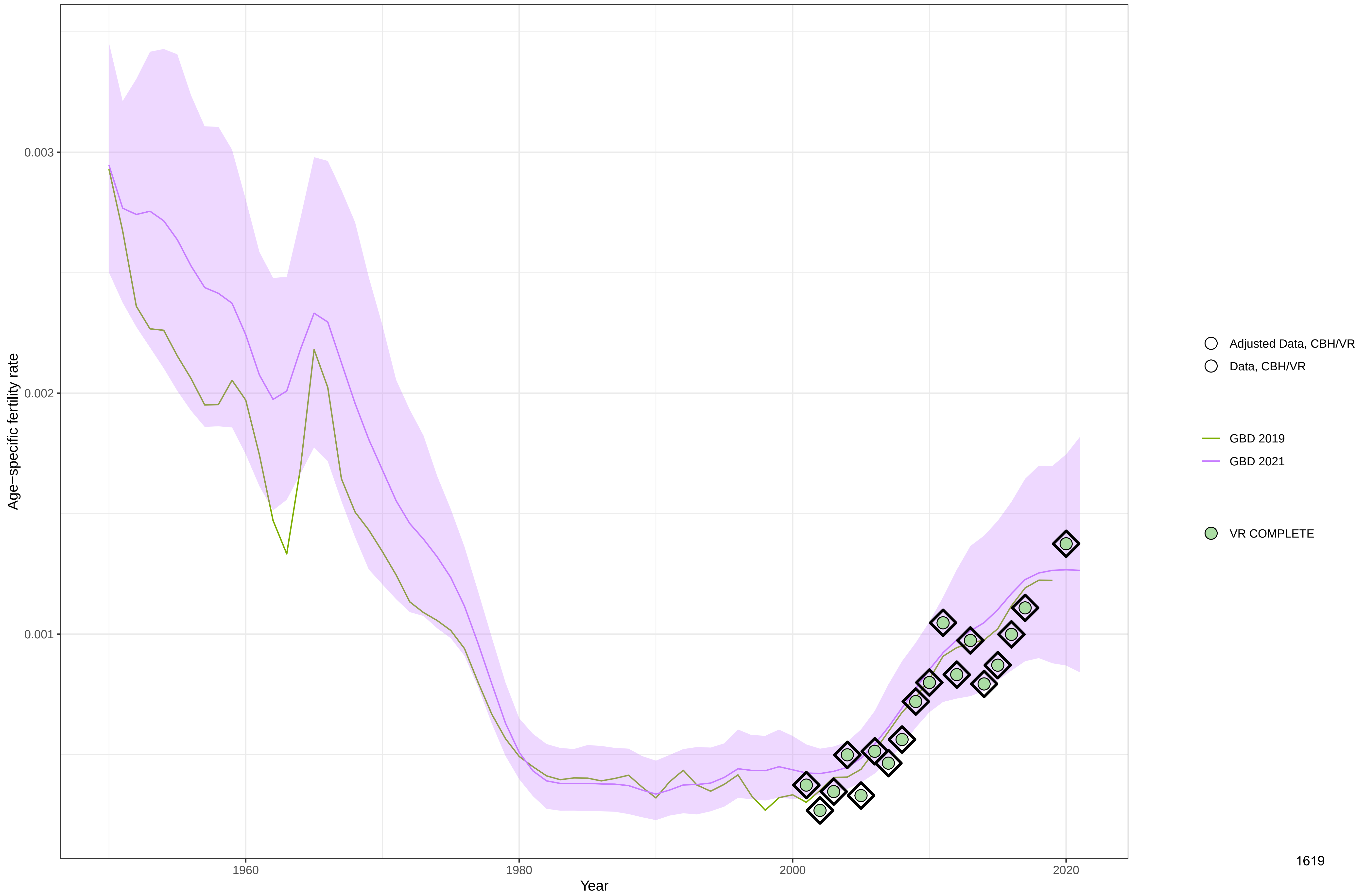

Age−specific fertility fitted trend lines, 1950−2021: Marche, Ages: 50−54
Data points enclosed with a diamond are reference sources

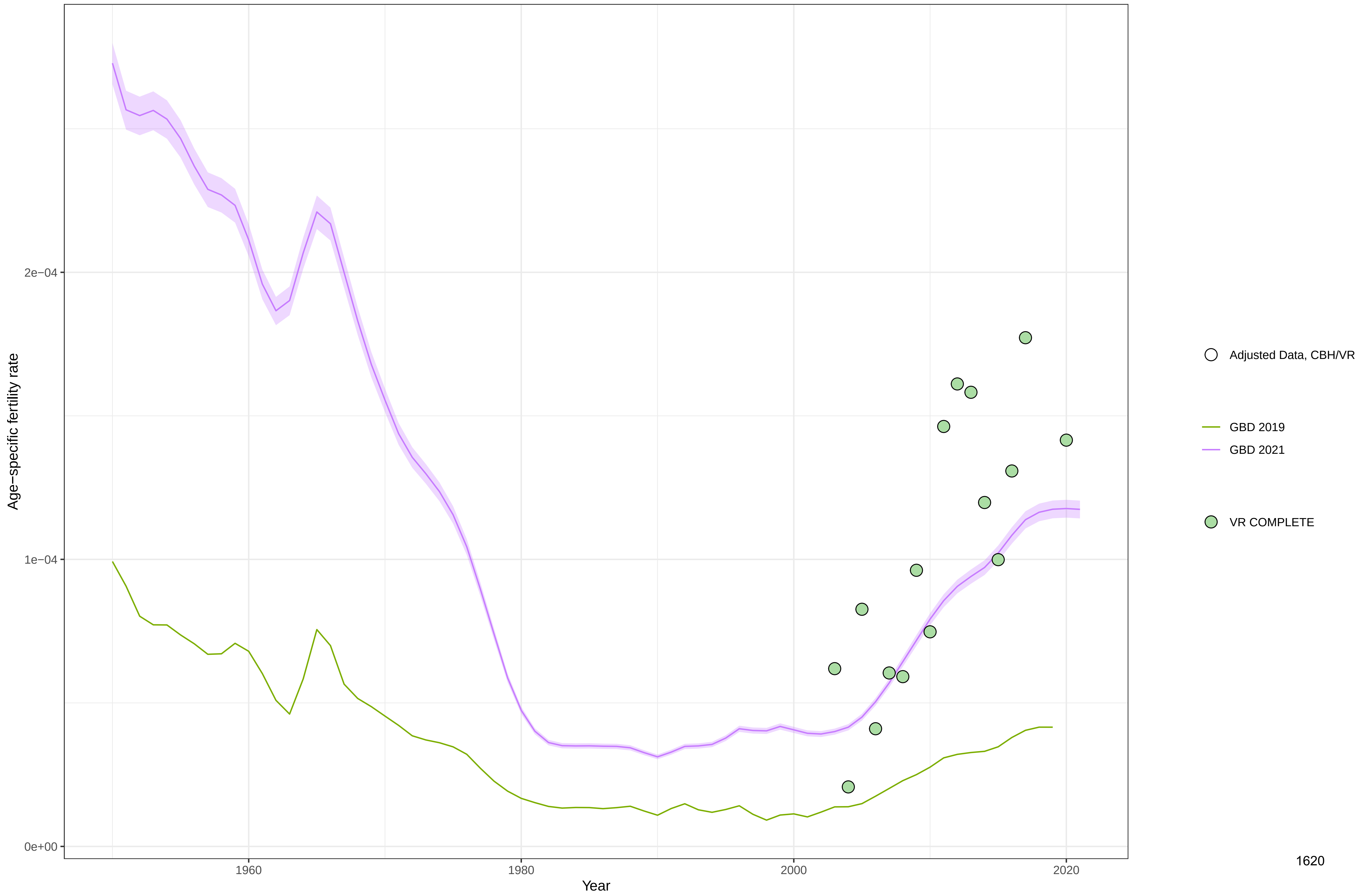

Total fertility rate fitted trend lines, 1950−2021: Marche TFR
Data points enclosed with a diamond are reference sources

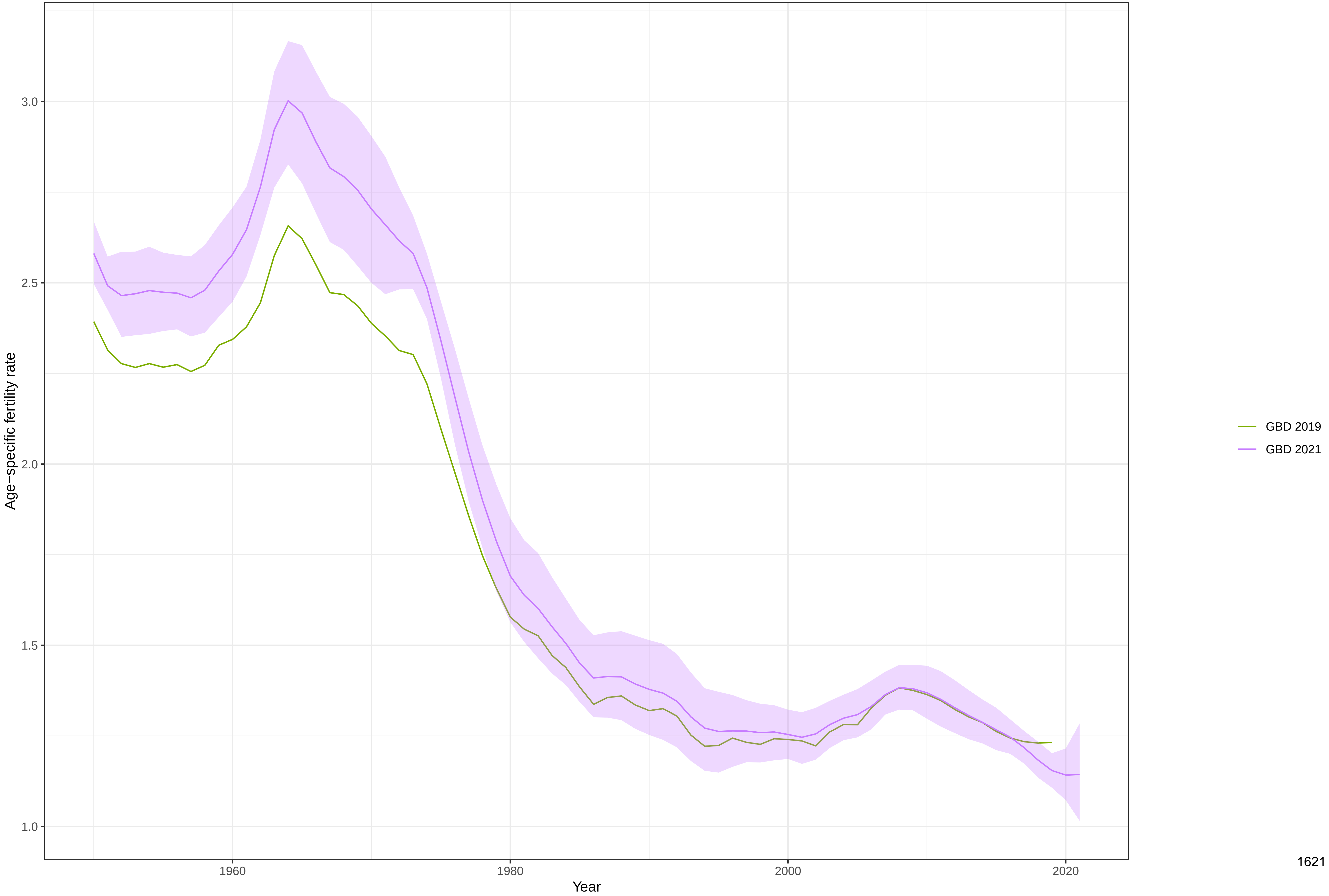

Age−specific fertility fitted trend lines, 1950−2021: Molise, Ages: 10−14
Data points enclosed with a diamond are reference sources

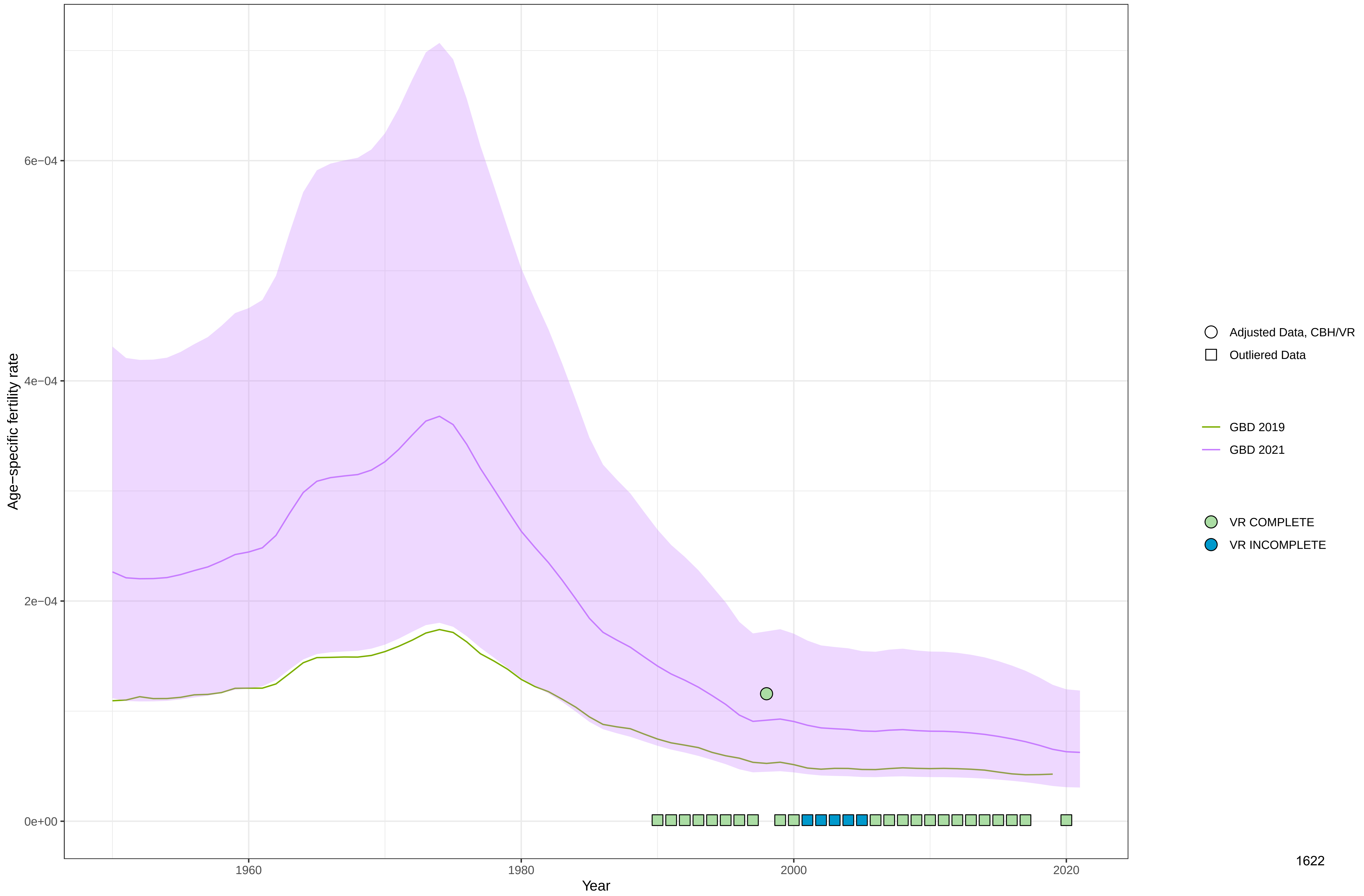

Age−specific fertility fitted trend lines, 1950−2021: Molise, Ages: 15−19
Data points enclosed with a diamond are reference sources

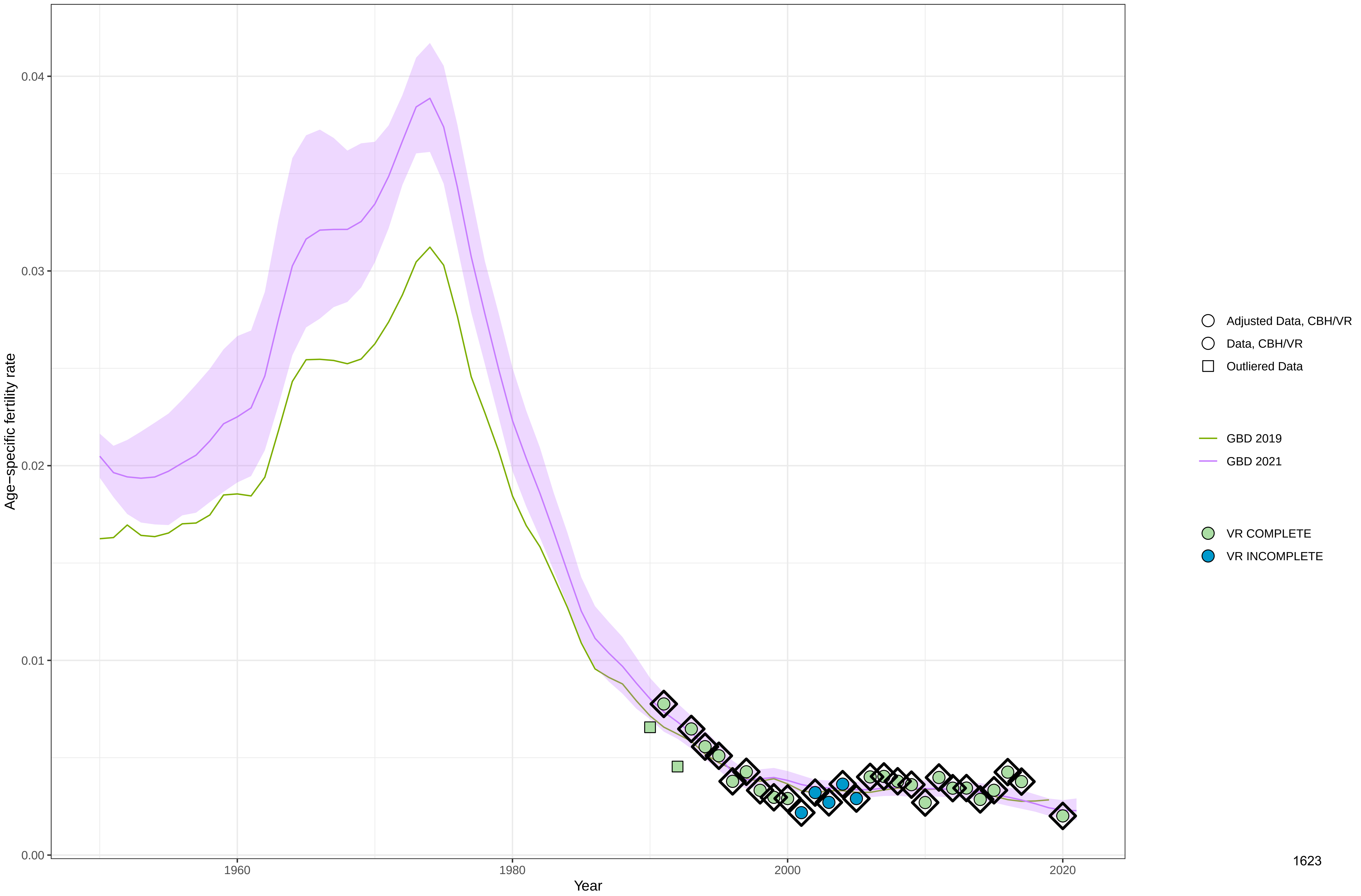

Age−specific fertility fitted trend lines, 1950−2021: Molise, Ages: 20−24
Data points enclosed with a diamond are reference sources

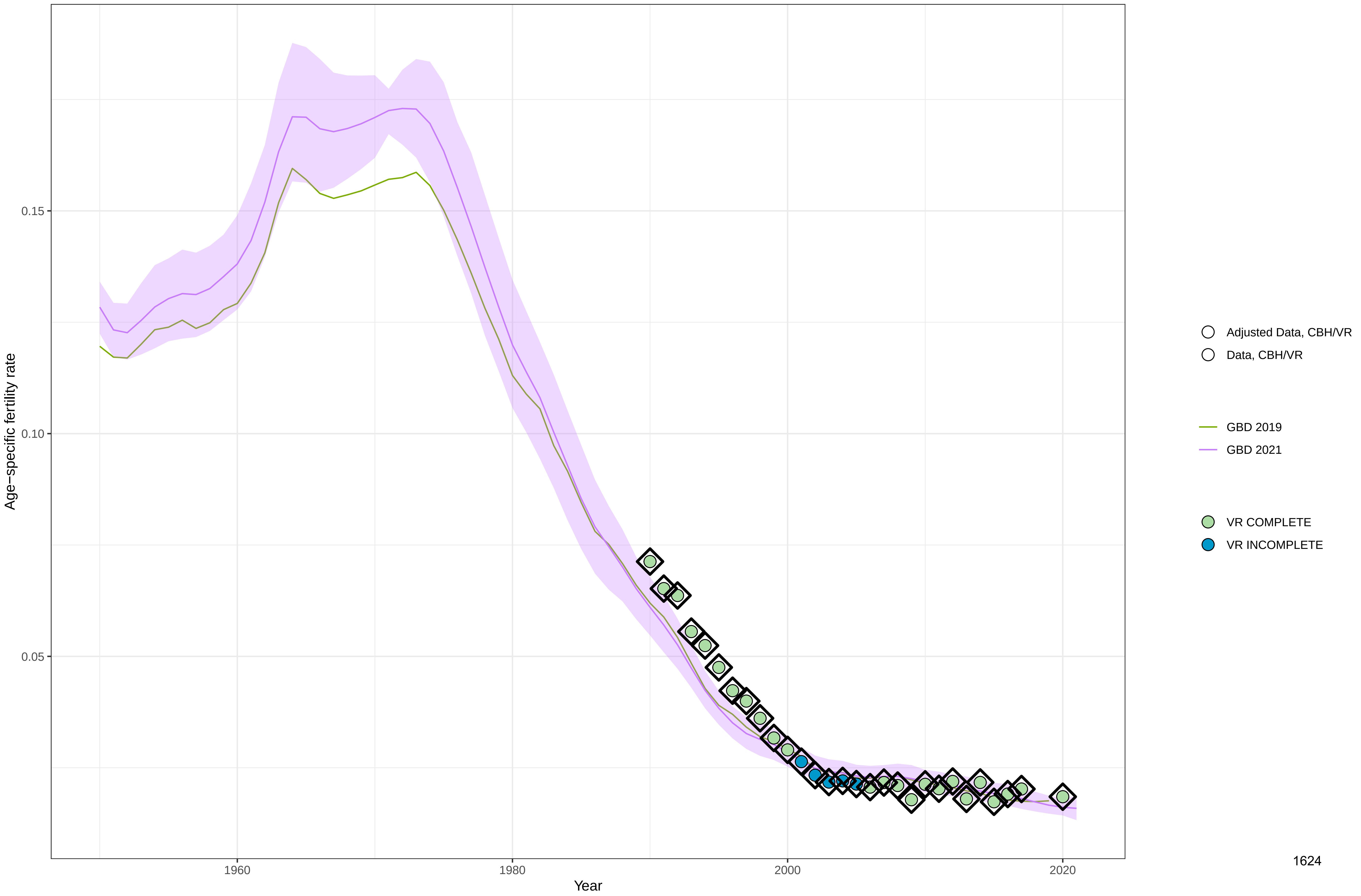

Age−specific fertility fitted trend lines, 1950−2021: Molise, Ages: 25−29
Data points enclosed with a diamond are reference sources

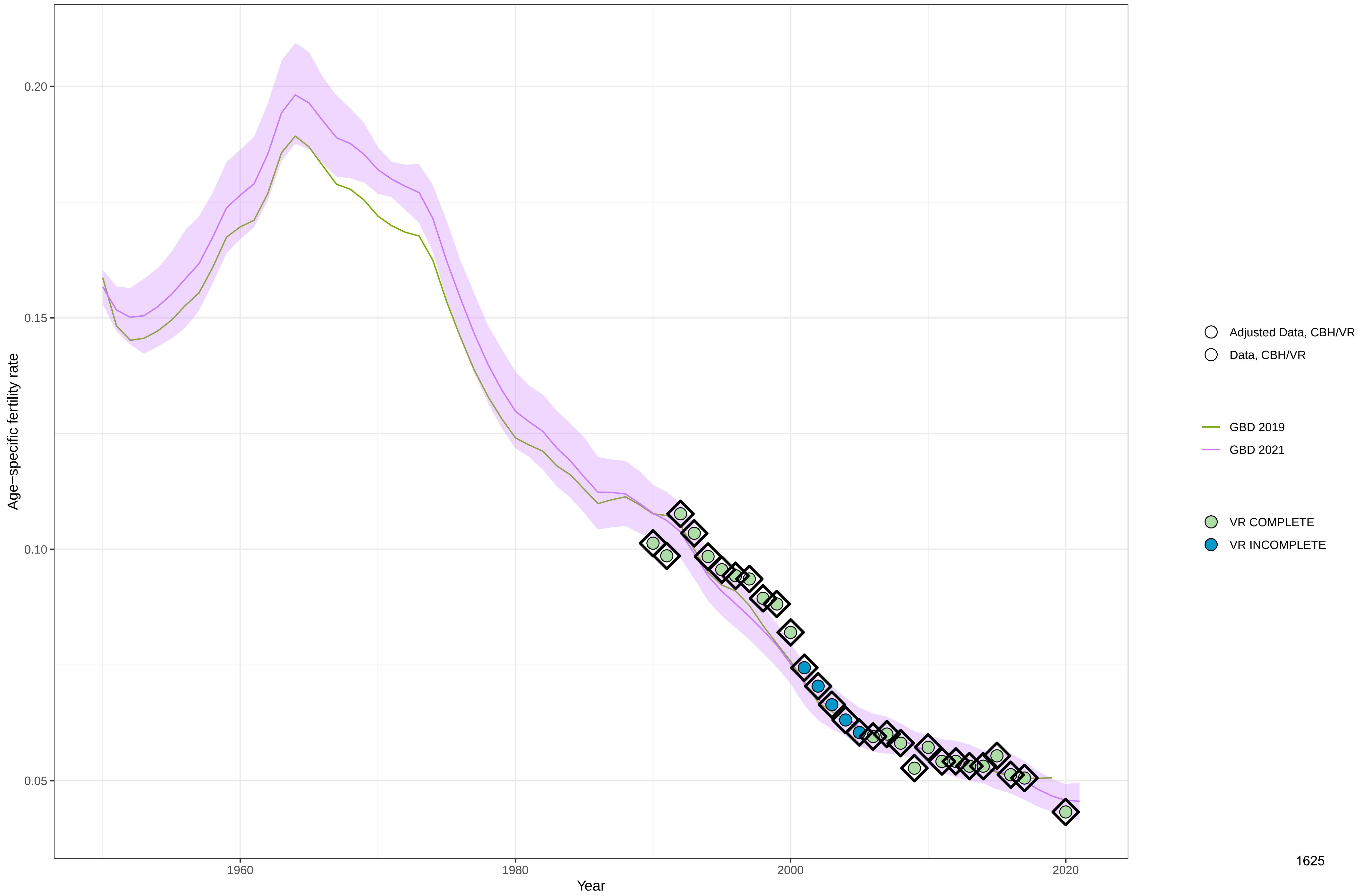

Age−specific fertility fitted trend lines, 1950−2021: Molise, Ages: 30−34
Data points enclosed with a diamond are reference sources

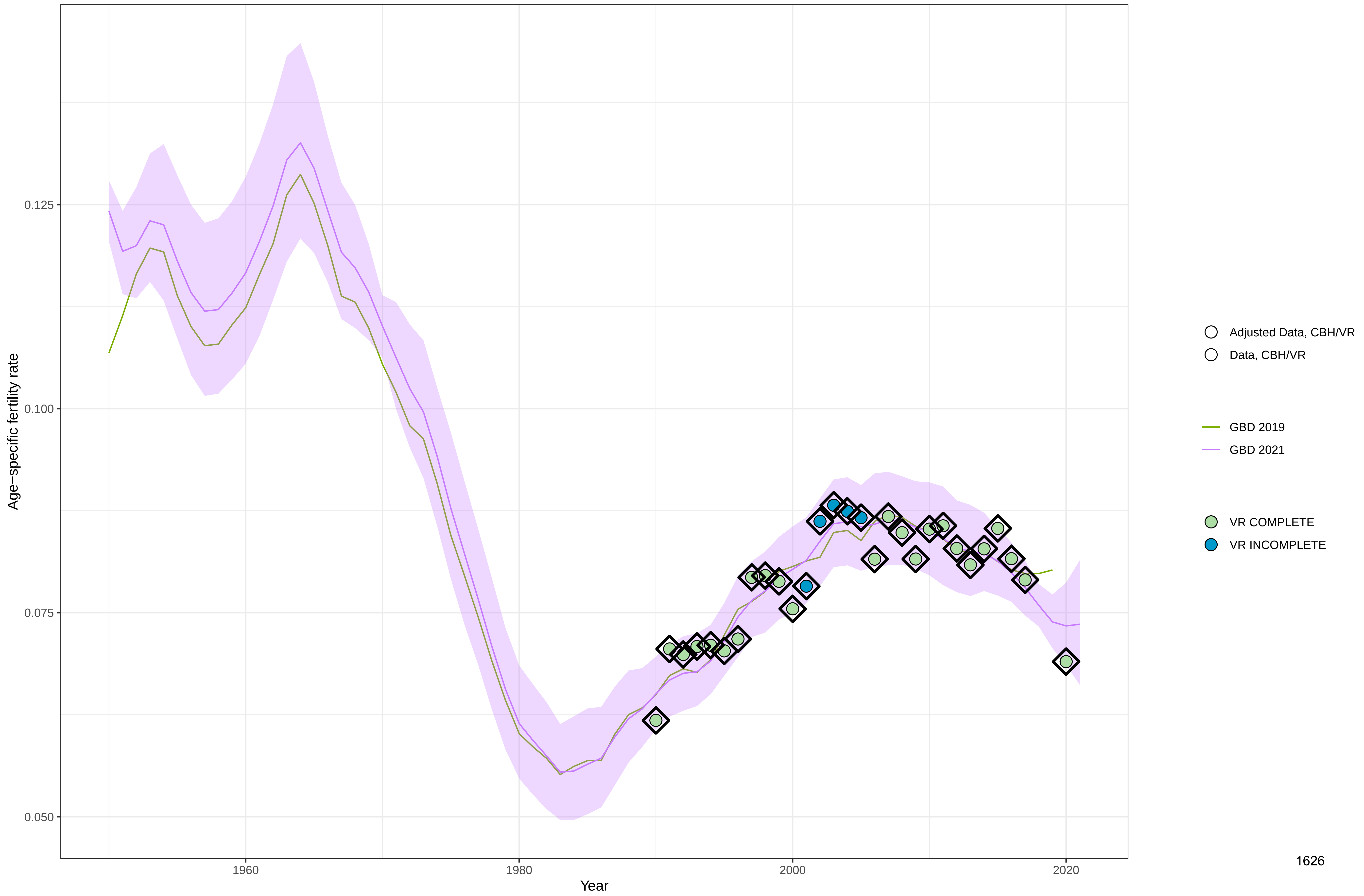

Age−specific fertility fitted trend lines, 1950−2021: Molise, Ages: 35−39
Data points enclosed with a diamond are reference sources

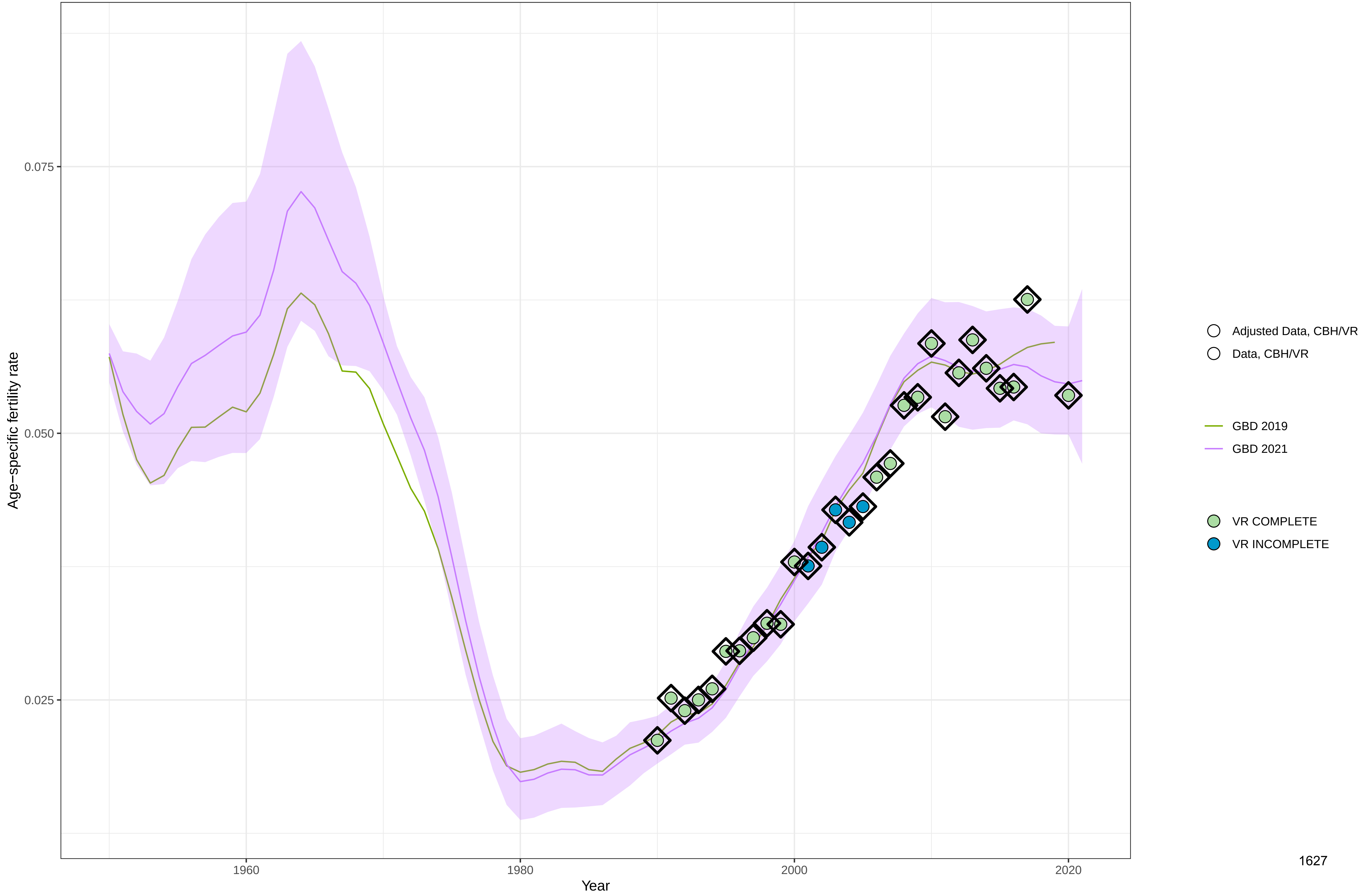

Age−specific fertility fitted trend lines, 1950−2021: Molise, Ages: 40−44
Data points enclosed with a diamond are reference sources

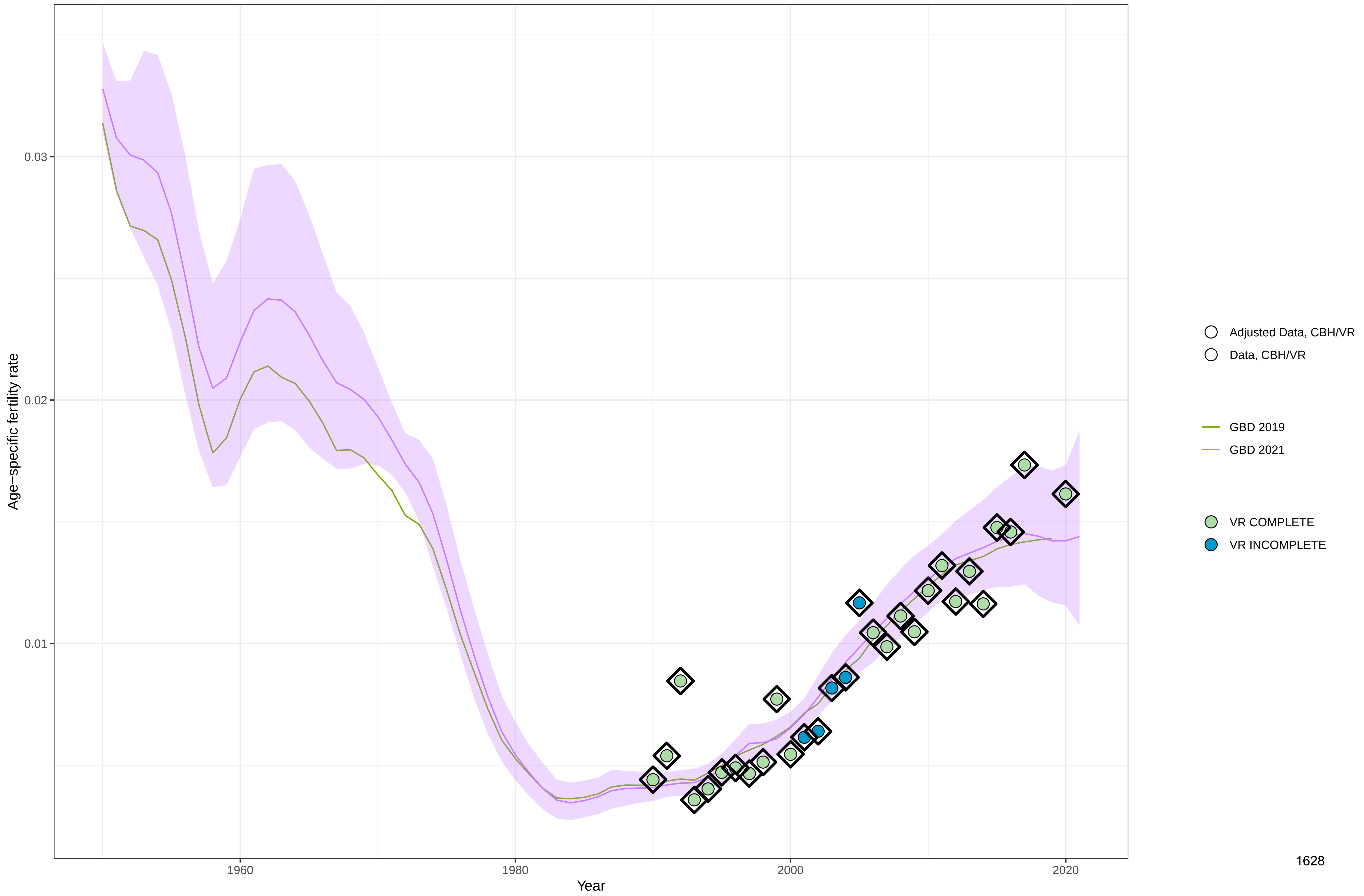

Age−specific fertility fitted trend lines, 1950−2021: Molise, Ages: 45−49
Data points enclosed with a diamond are reference sources

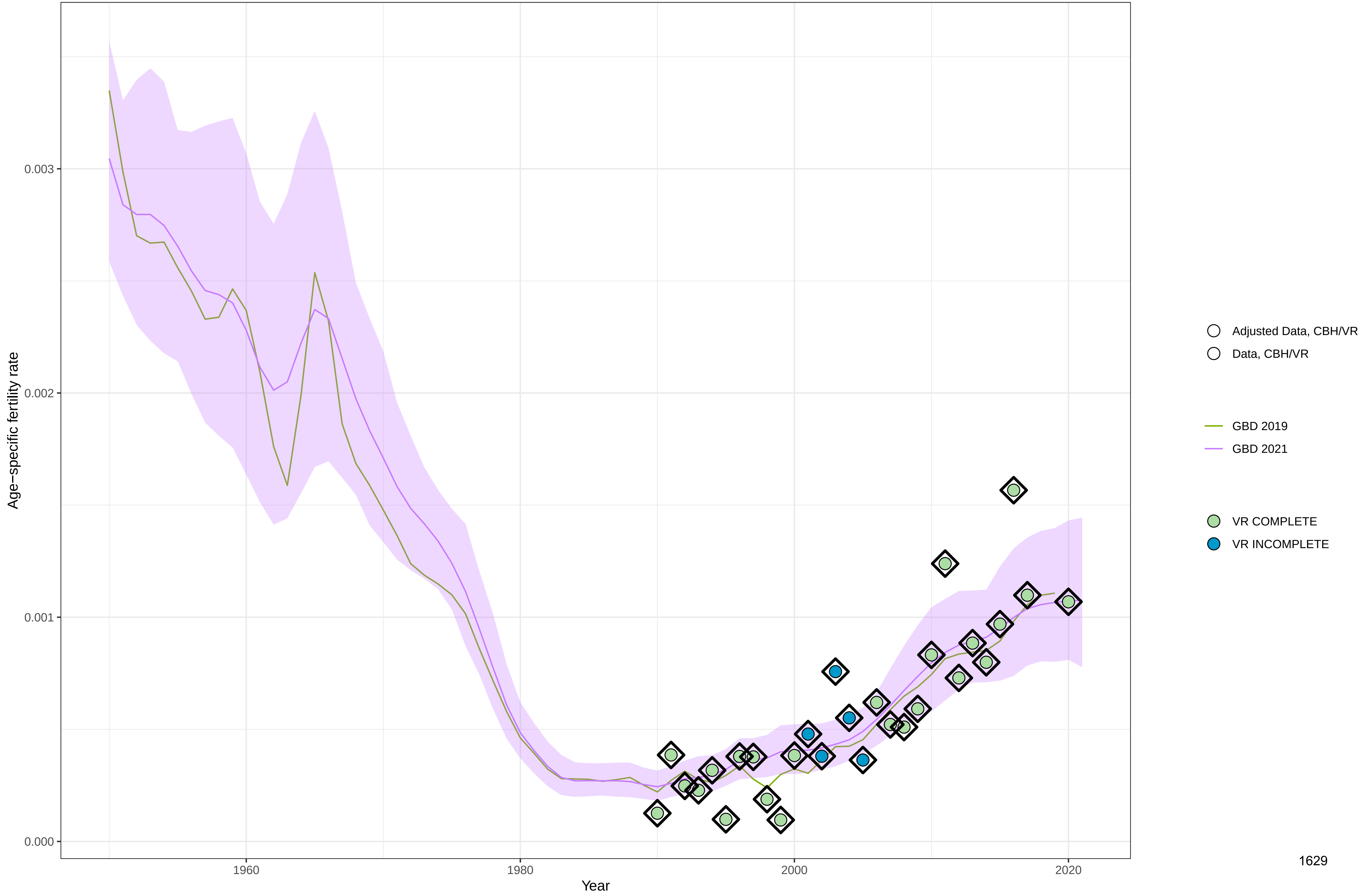

Age−specific fertility fitted trend lines, 1950−2021: Molise, Ages: 50−54
Data points enclosed with a diamond are reference sources

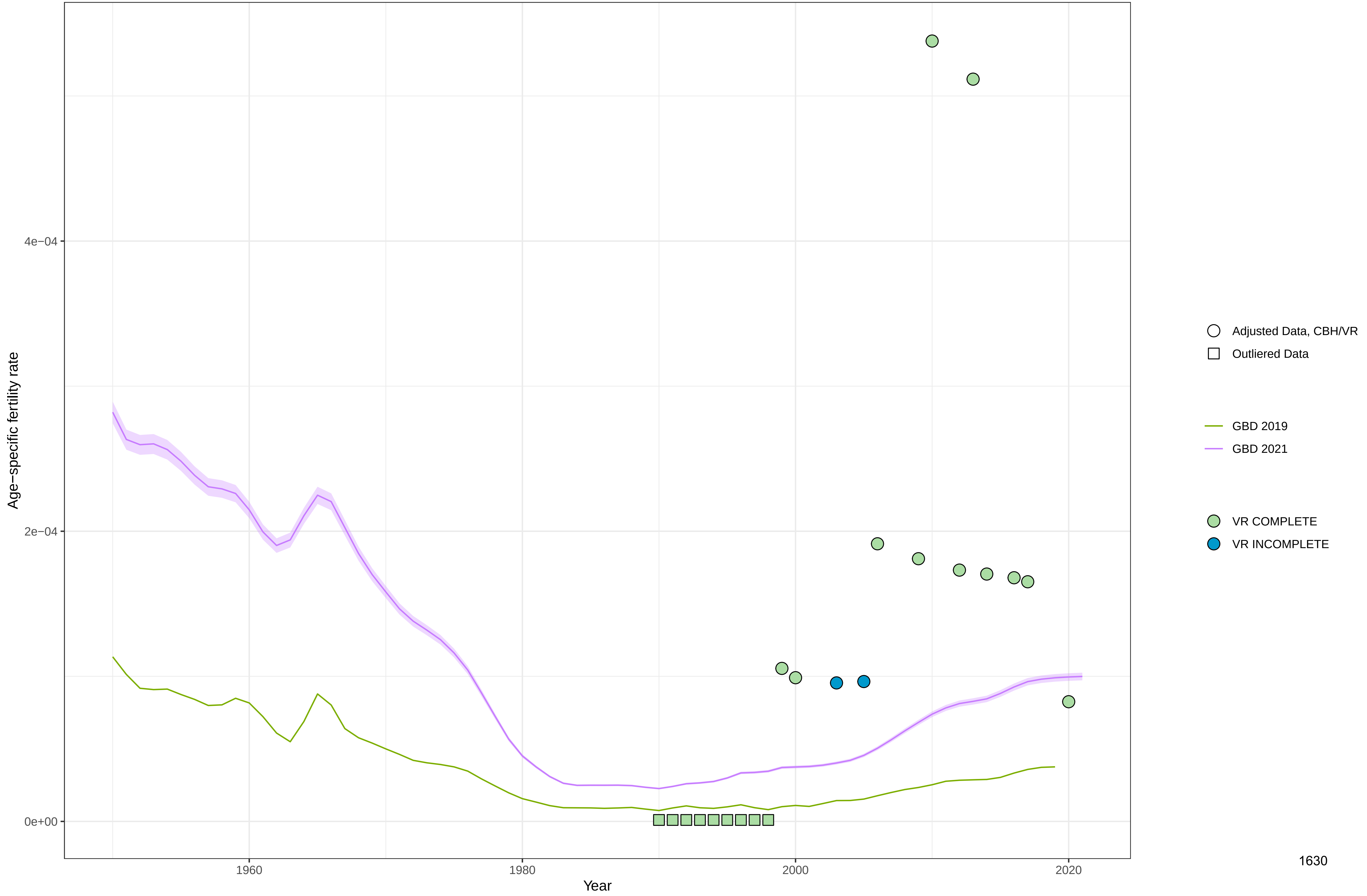

Total fertility rate fitted trend lines, 1950−2021: Molise TFR
Data points enclosed with a diamond are reference sources

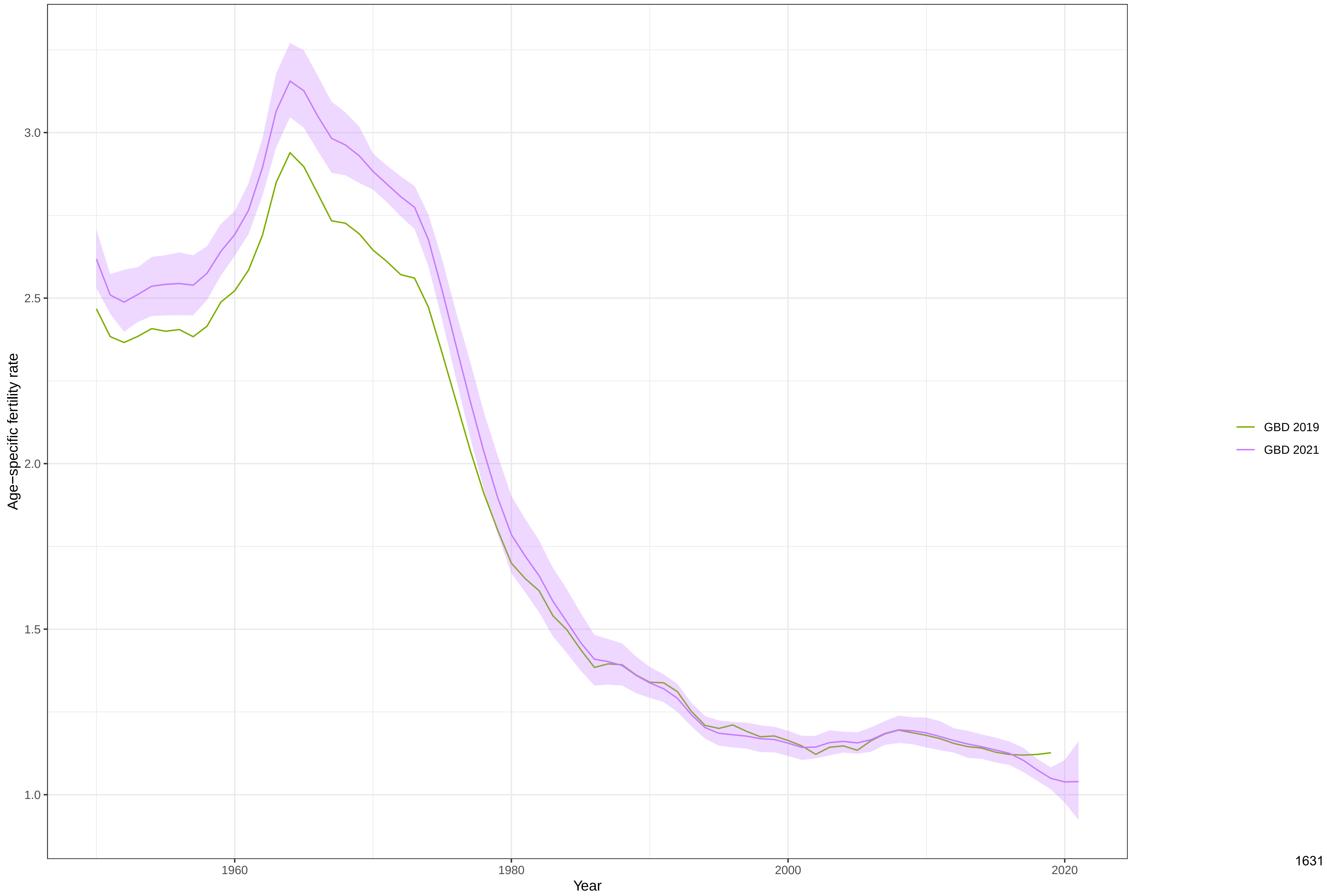

Age−specific fertility fitted trend lines, 1950−2021: Piemonte, Ages: 10−14
Data points enclosed with a diamond are reference sources

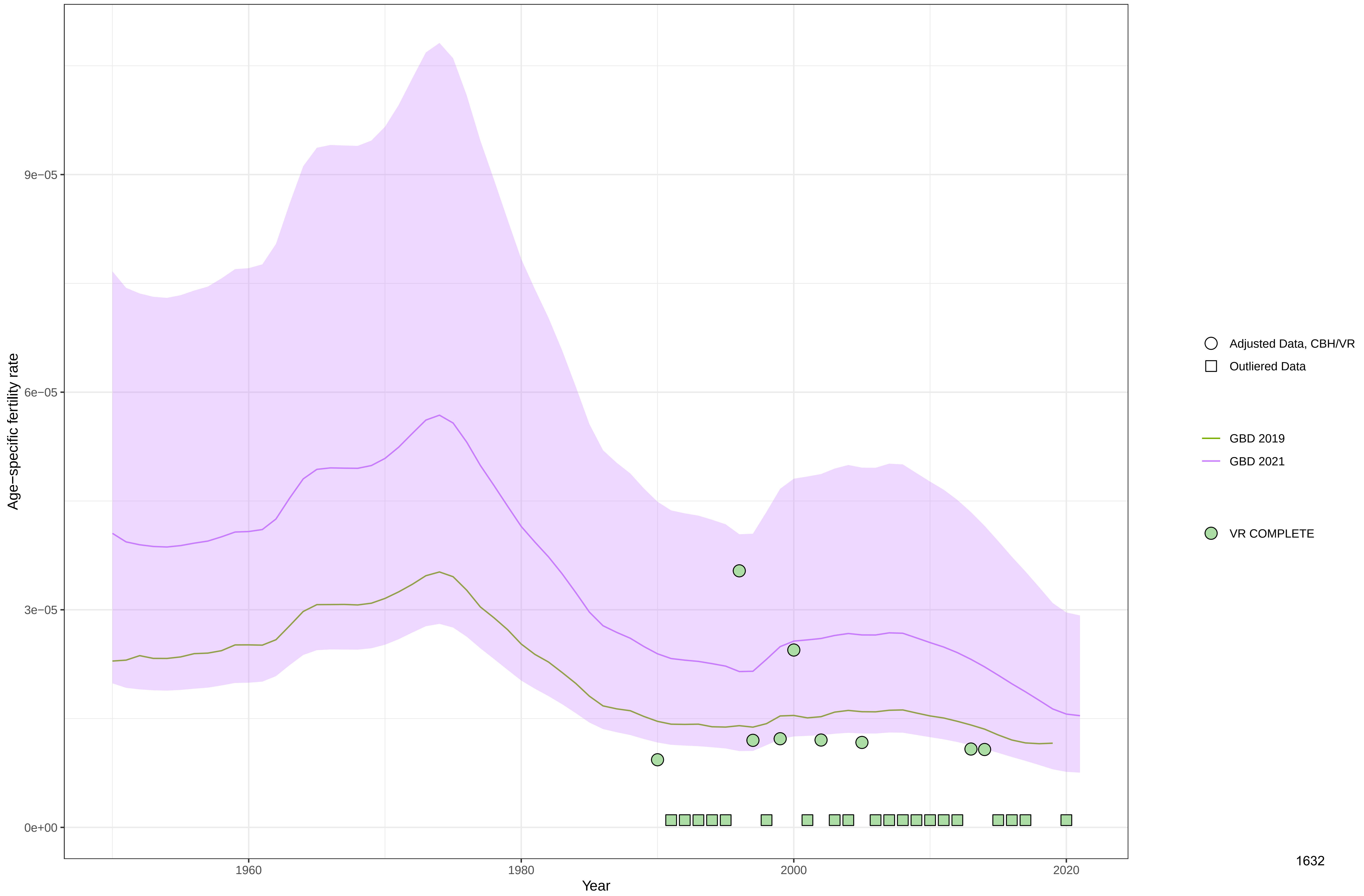

Age−specific fertility fitted trend lines, 1950−2021: Piemonte, Ages: 15−19
Data points enclosed with a diamond are reference sources

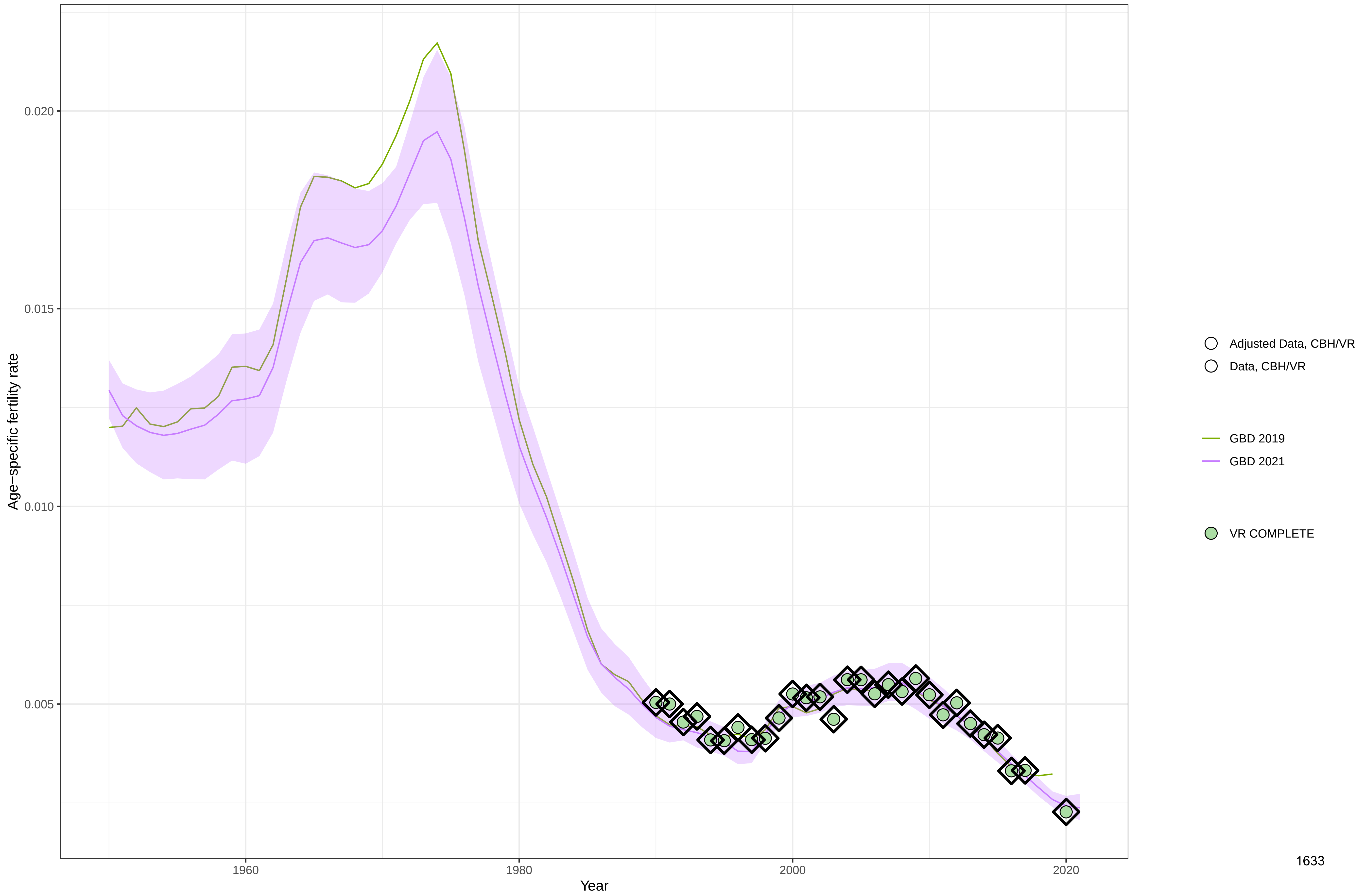

Age−specific fertility fitted trend lines, 1950−2021: Piemonte, Ages: 20−24
Data points enclosed with a diamond are reference sources

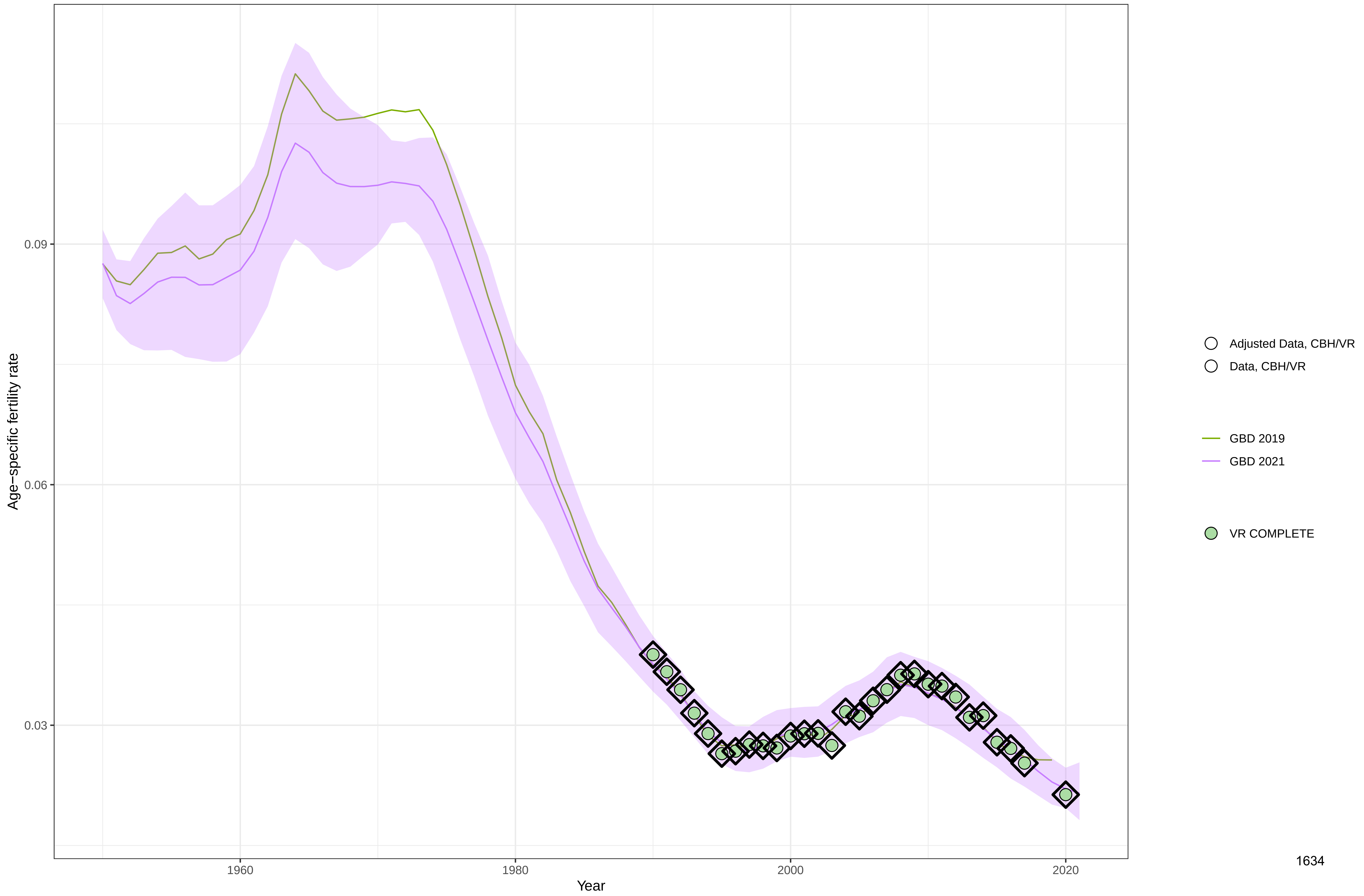

Age−specific fertility fitted trend lines, 1950−2021: Piemonte, Ages: 25−29
Data points enclosed with a diamond are reference sources

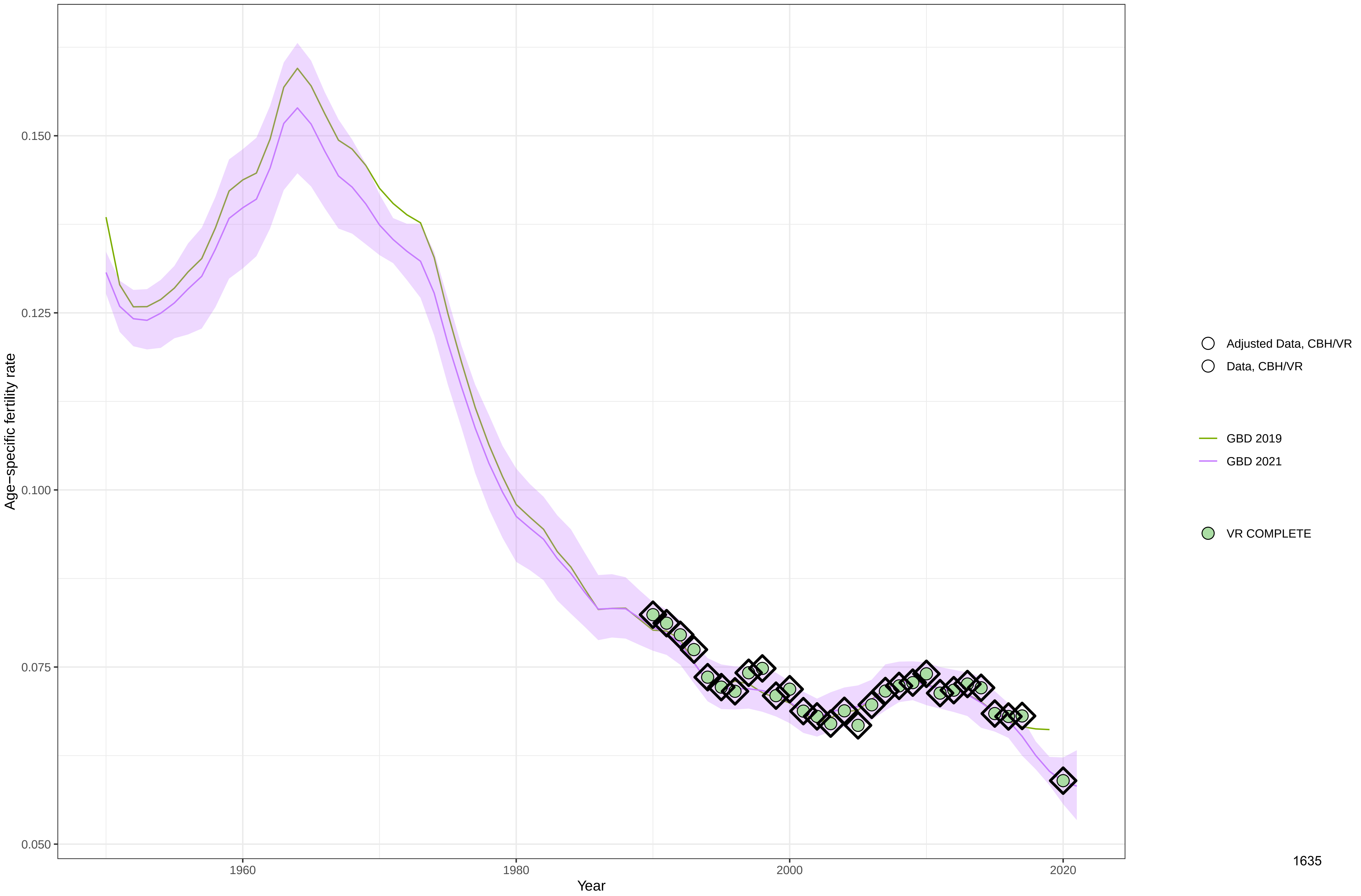

Age−specific fertility fitted trend lines, 1950−2021: Piemonte, Ages: 30−34
Data points enclosed with a diamond are reference sources

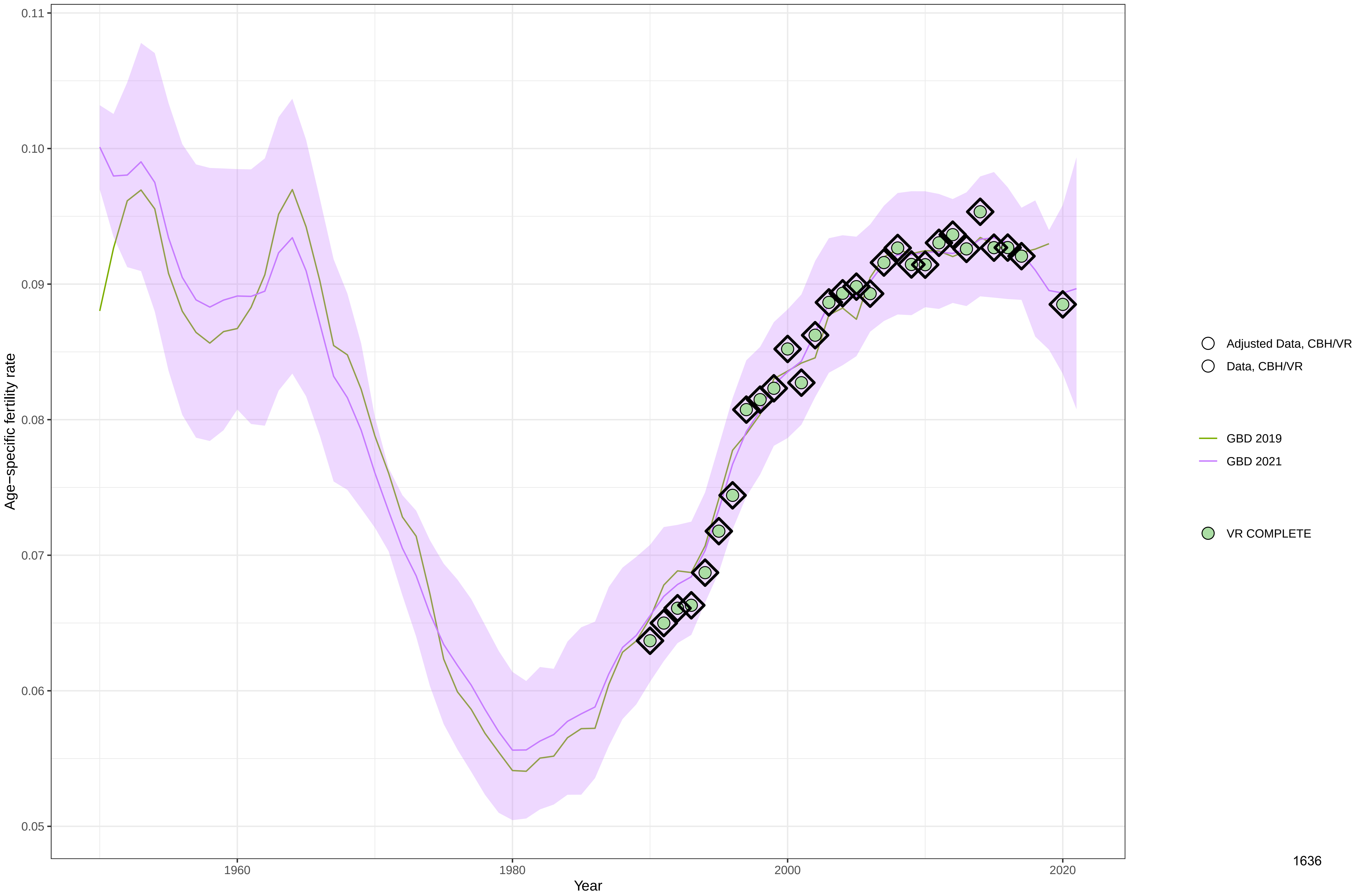

Age−specific fertility fitted trend lines, 1950−2021: Piemonte, Ages: 35−39
Data points enclosed with a diamond are reference sources

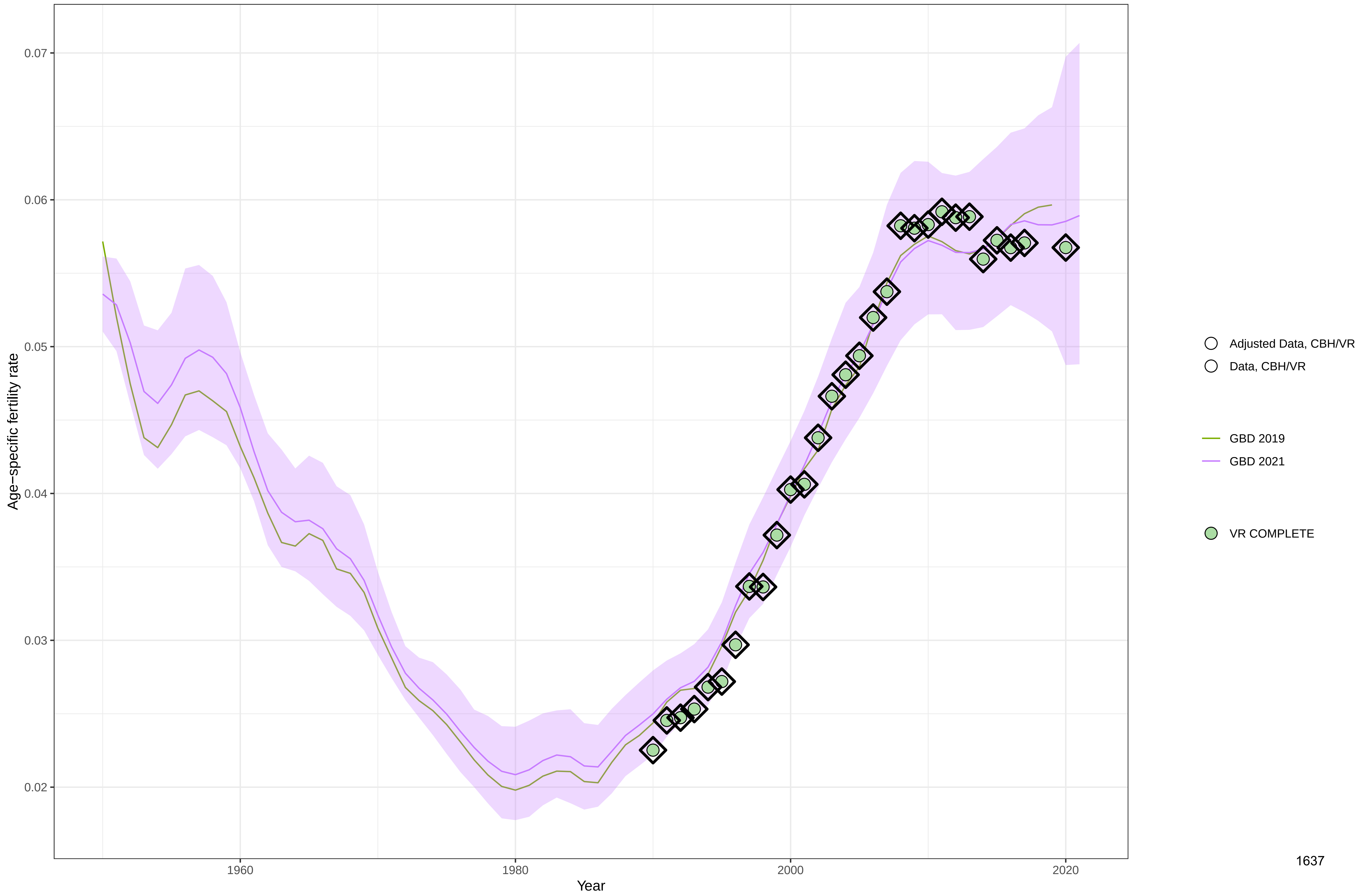

Age−specific fertility fitted trend lines, 1950−2021: Piemonte, Ages: 40−44
Data points enclosed with a diamond are reference sources

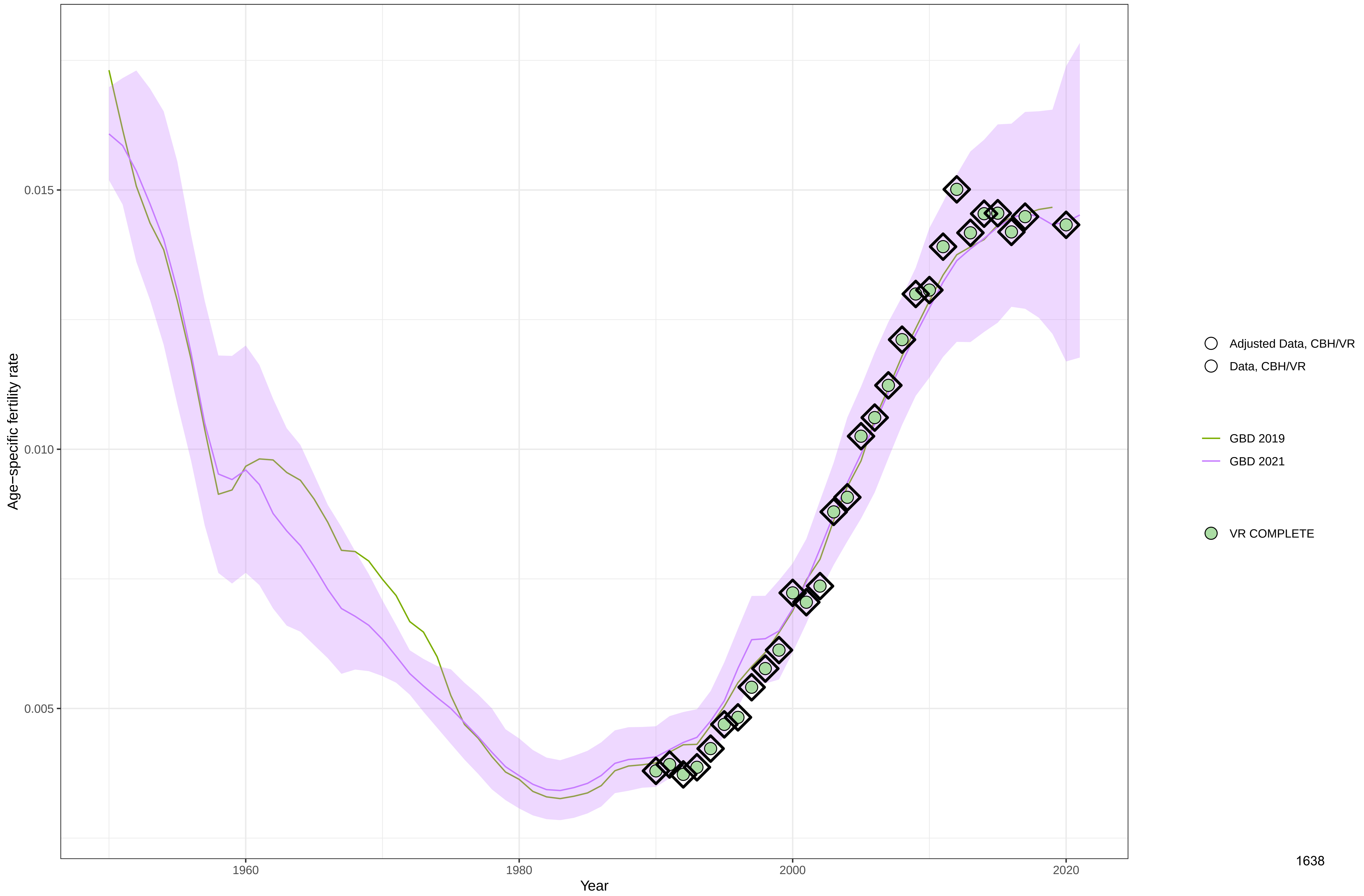

Age−specific fertility fitted trend lines, 1950−2021: Piemonte, Ages: 45−49
Data points enclosed with a diamond are reference sources

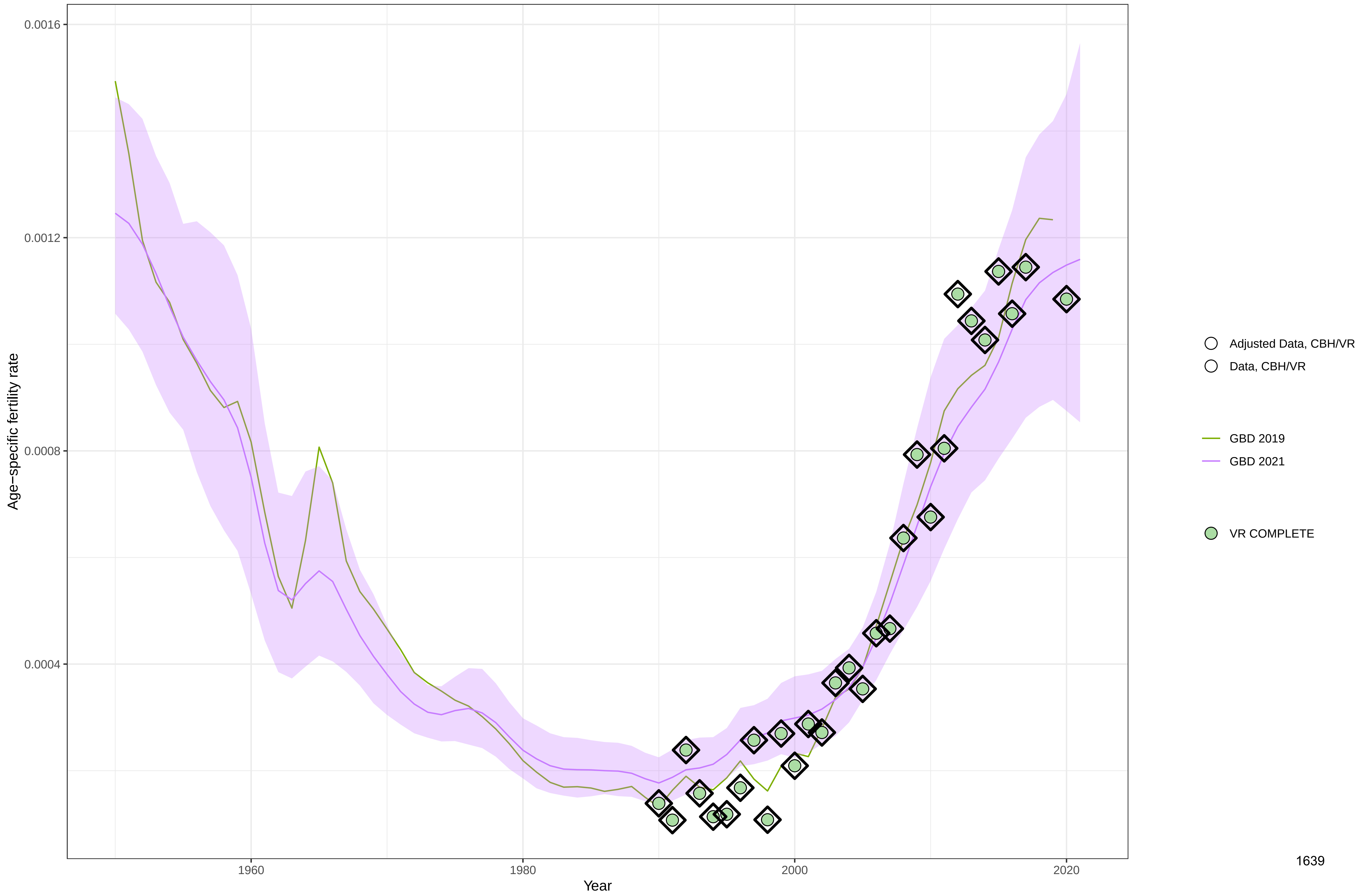

Age−specific fertility fitted trend lines, 1950−2021: Piemonte, Ages: 50−54
Data points enclosed with a diamond are reference sources

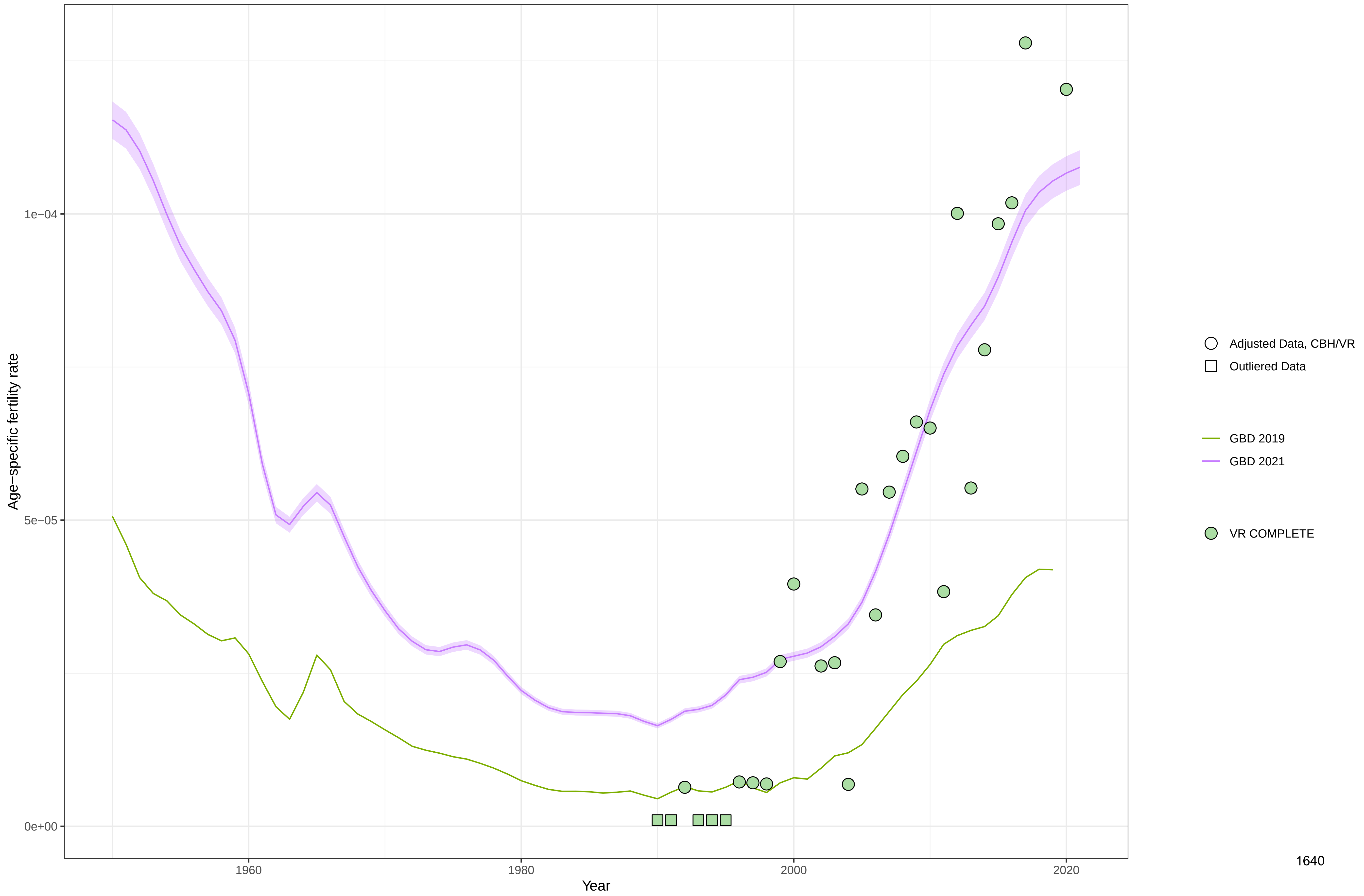

Total fertility rate fitted trend lines, 1950−2021: Piemonte TFR
Data points enclosed with a diamond are reference sources

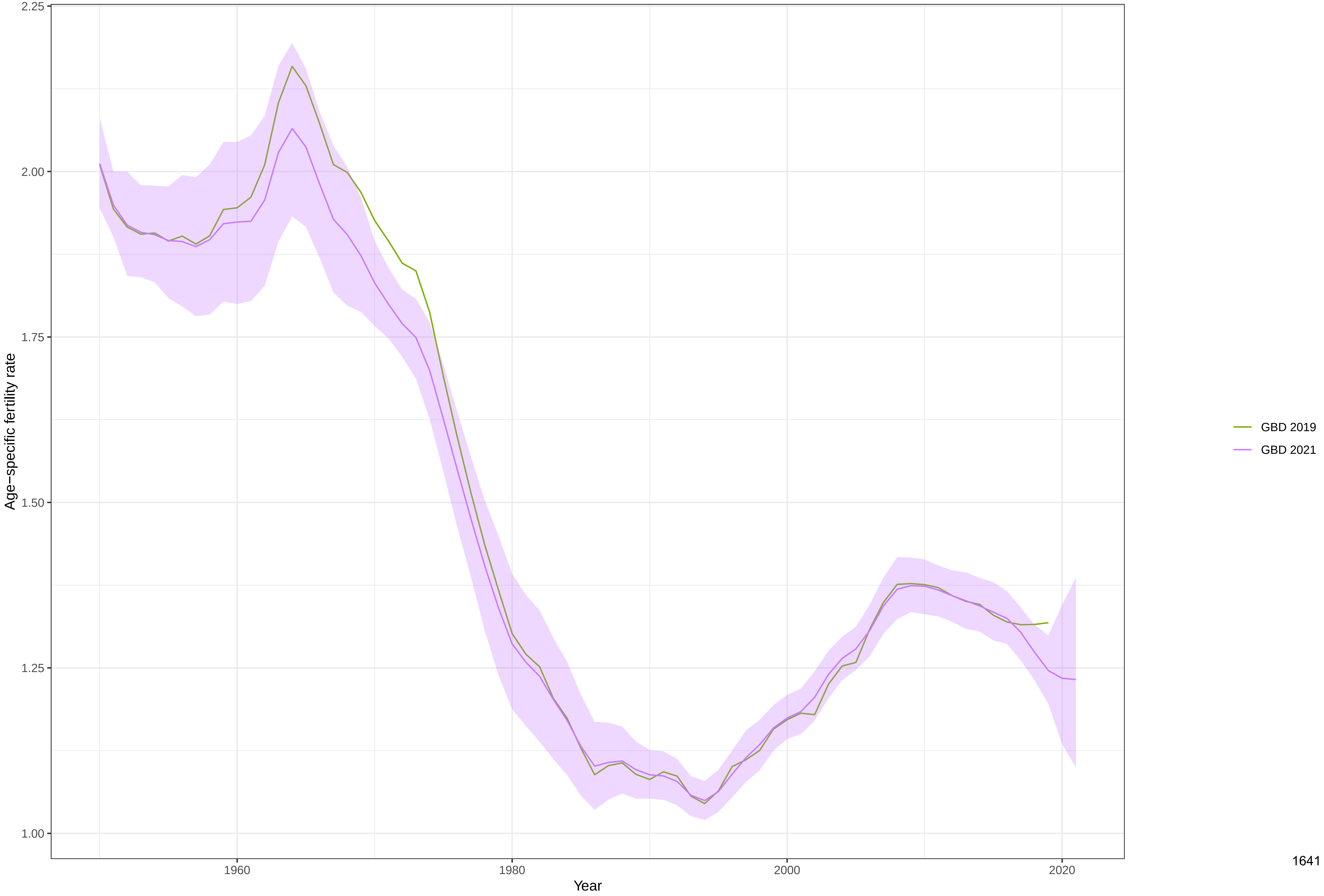

Age−specific fertility fitted trend lines, 1950−2021: Provincia autonoma di Bolzano, Ages: 10−14
Data points enclosed with a diamond are reference sources

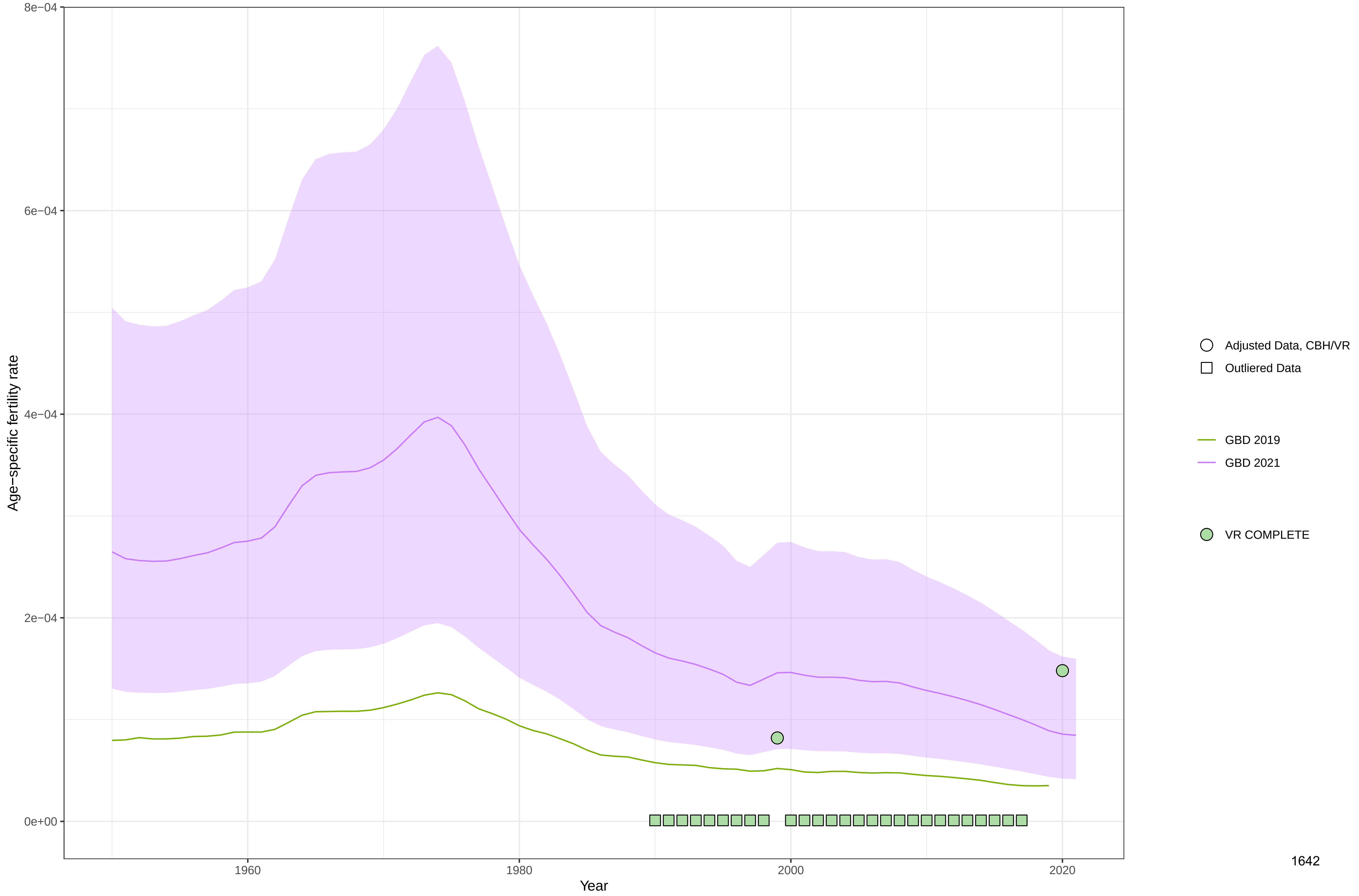

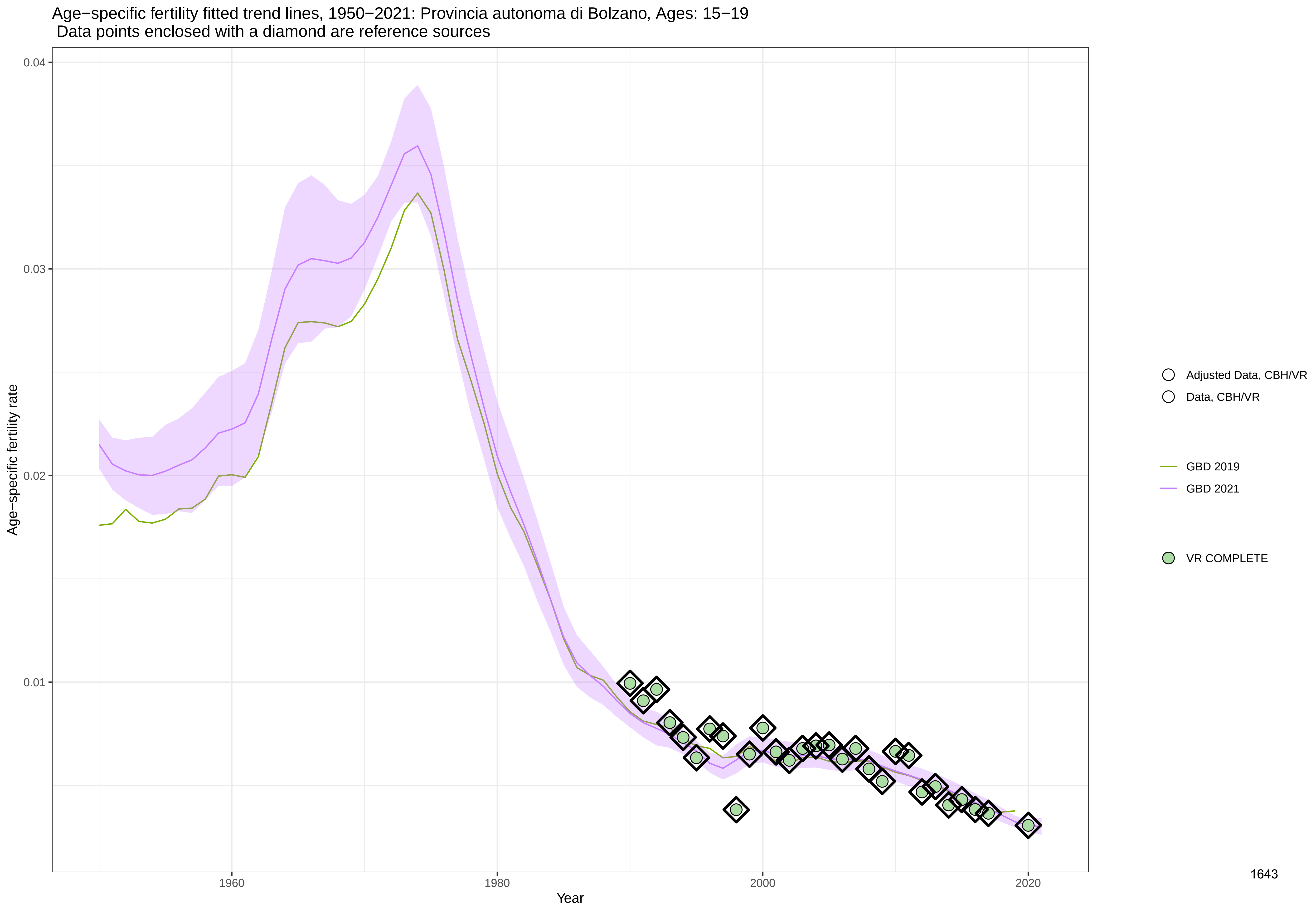
Age−specific fertility fitted trend lines, 1950−2021: Provincia autonoma di Bolzano, Ages: 15−19
Data points enclosed with a diamond are reference sources
Age−specific fertility rate
0.04
0.03
0.02
0.01
1960
1980
2000
2020
Year
Adjusted Data, CBH/VR
Data, CBH/VR
GBD 2019
GBD 2021
VR COMPLETE

Age−specific fertility fitted trend lines, 1950−2021: Provincia autonoma di Bolzano, Ages: 20−24
Data points enclosed with a diamond are reference sources

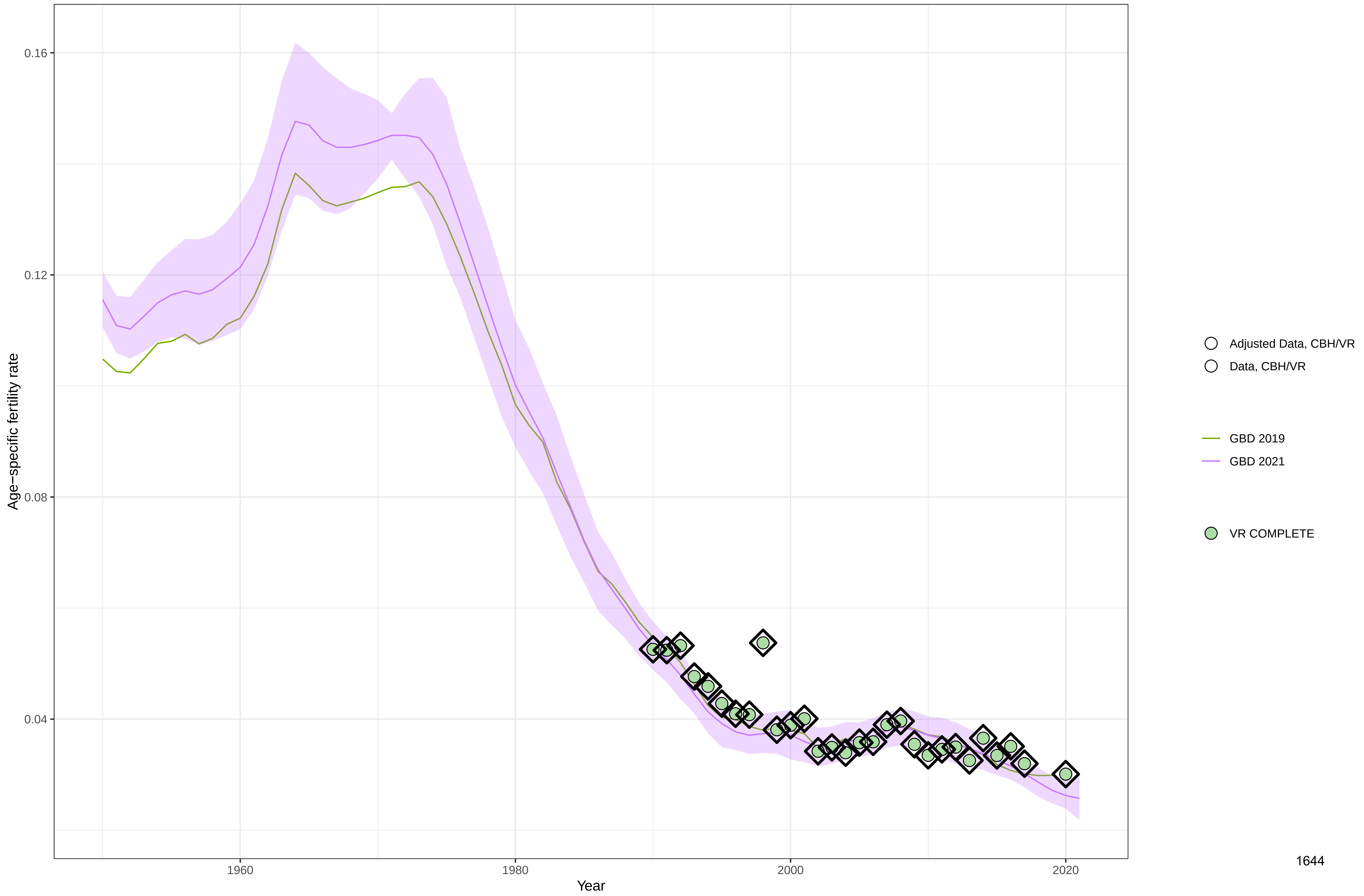

Age−specific fertility fitted trend lines, 1950−2021: Provincia autonoma di Bolzano, Ages: 25−29
Data points enclosed with a diamond are reference sources

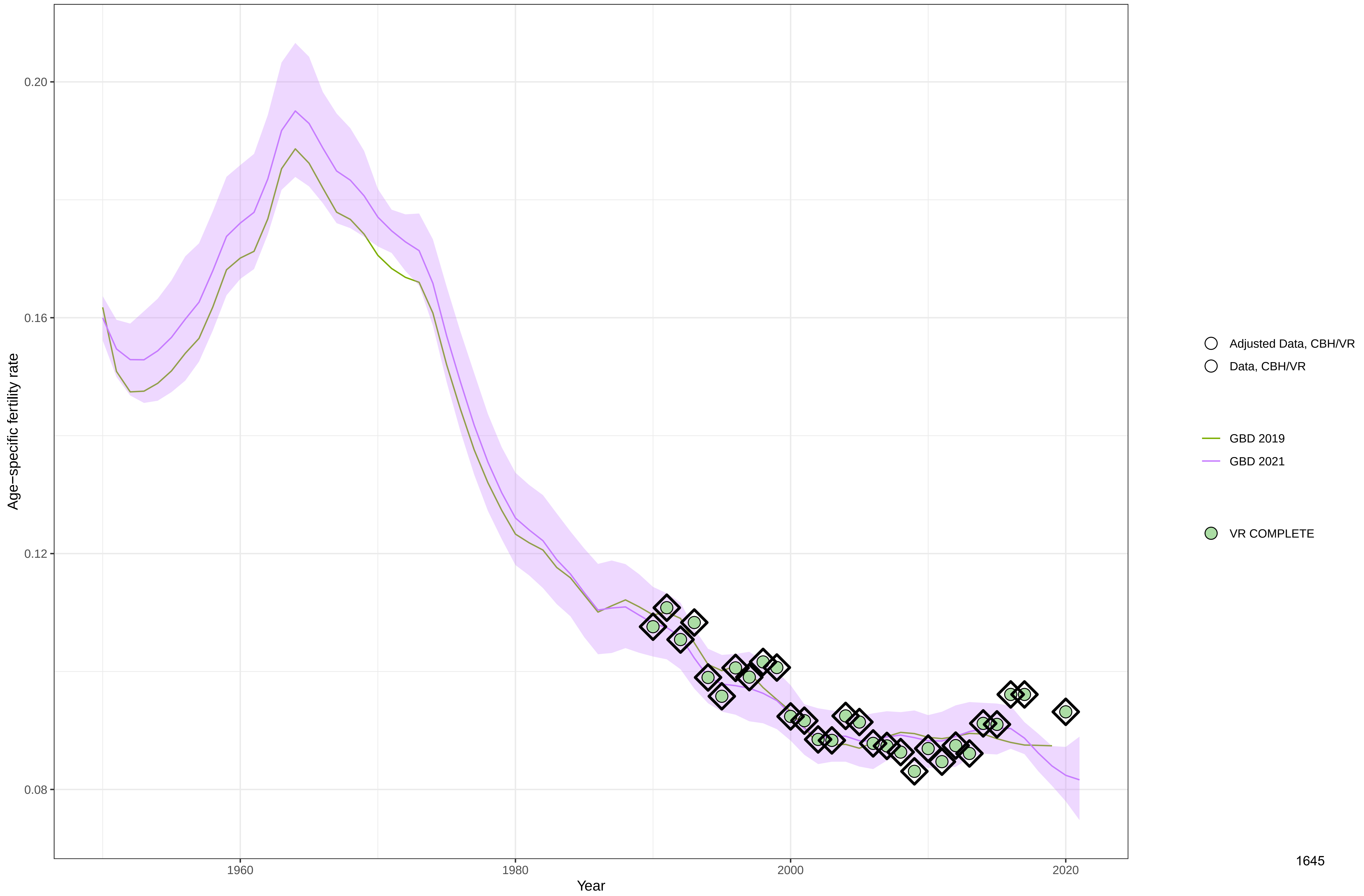

Age−specific fertility fitted trend lines, 1950−2021: Provincia autonoma di Bolzano, Ages: 30−34
Data points enclosed with a diamond are reference sources

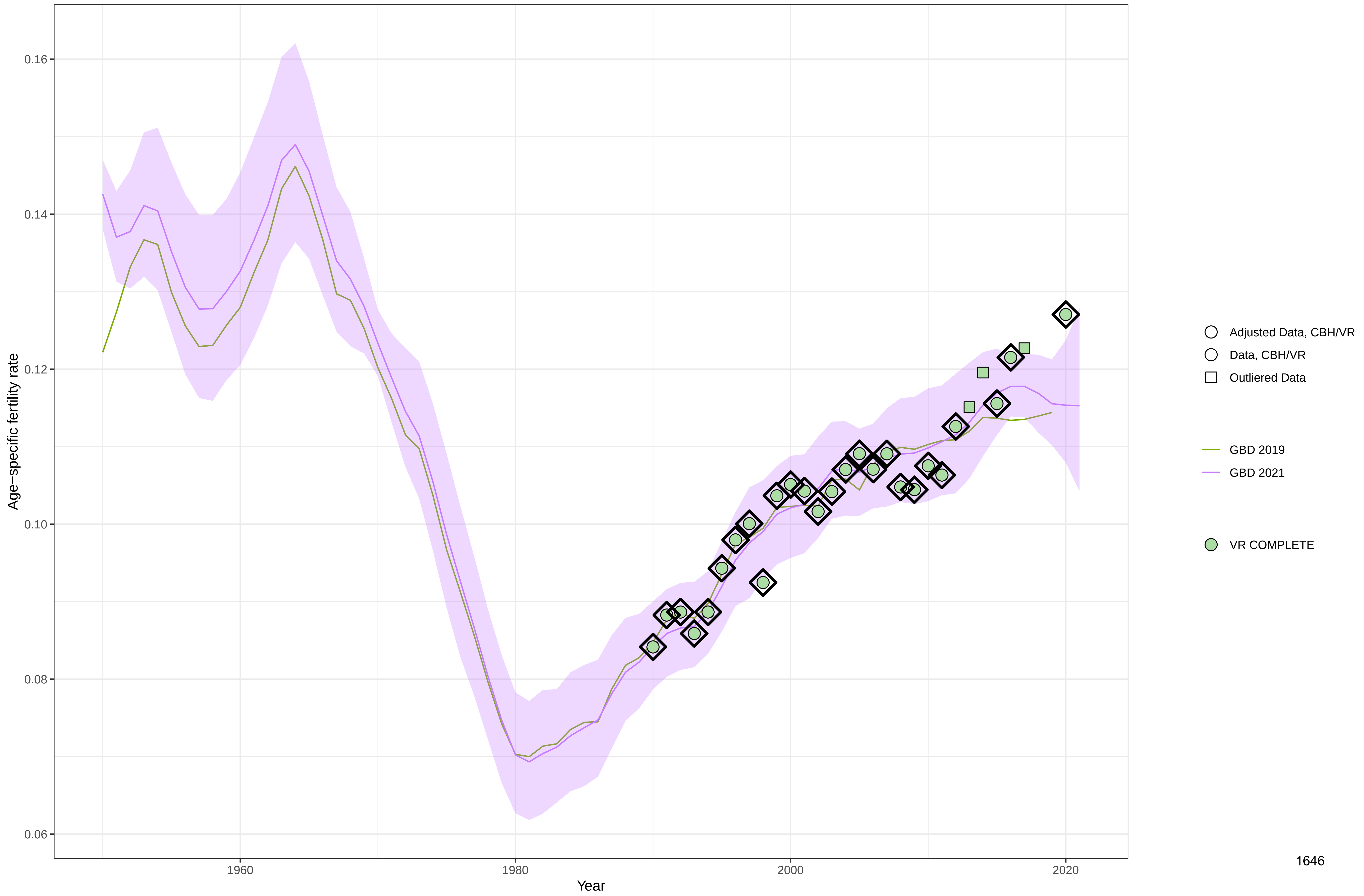

Age−specific fertility fitted trend lines, 1950−2021: Provincia autonoma di Bolzano, Ages: 35−39
Data points enclosed with a diamond are reference sources

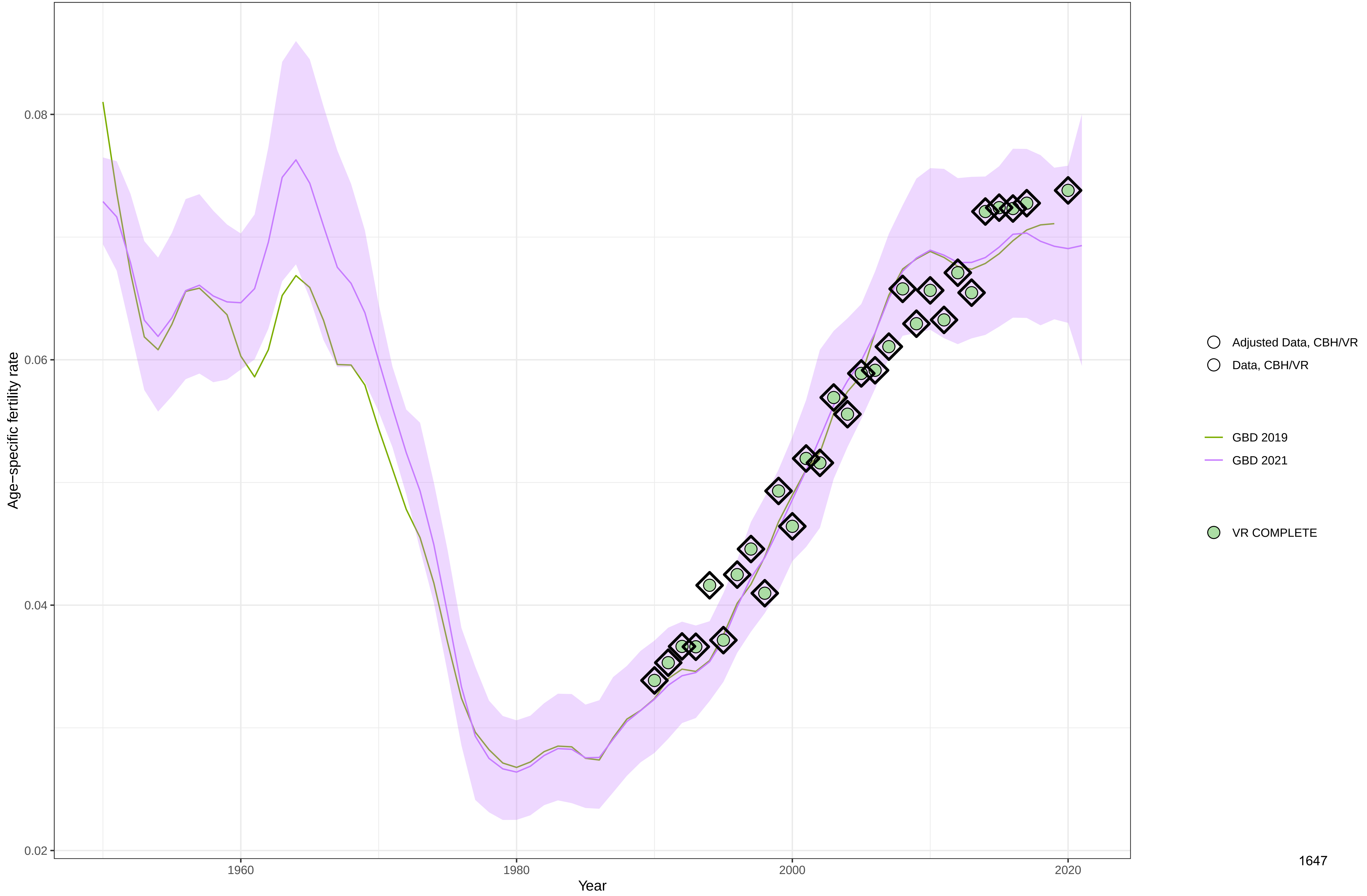

Age−specific fertility fitted trend lines, 1950−2021: Provincia autonoma di Bolzano, Ages: 40−44
Data points enclosed with a diamond are reference sources

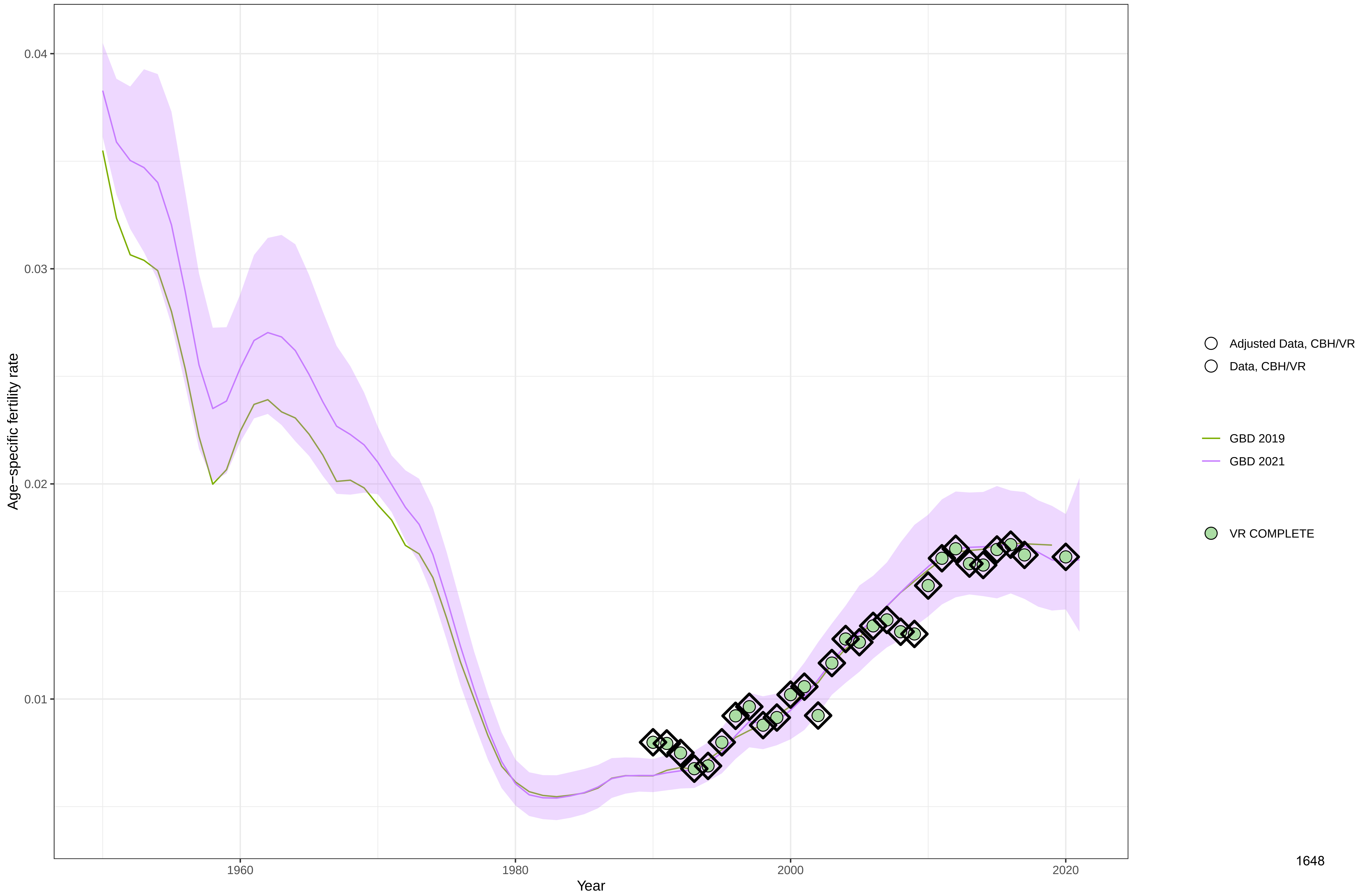

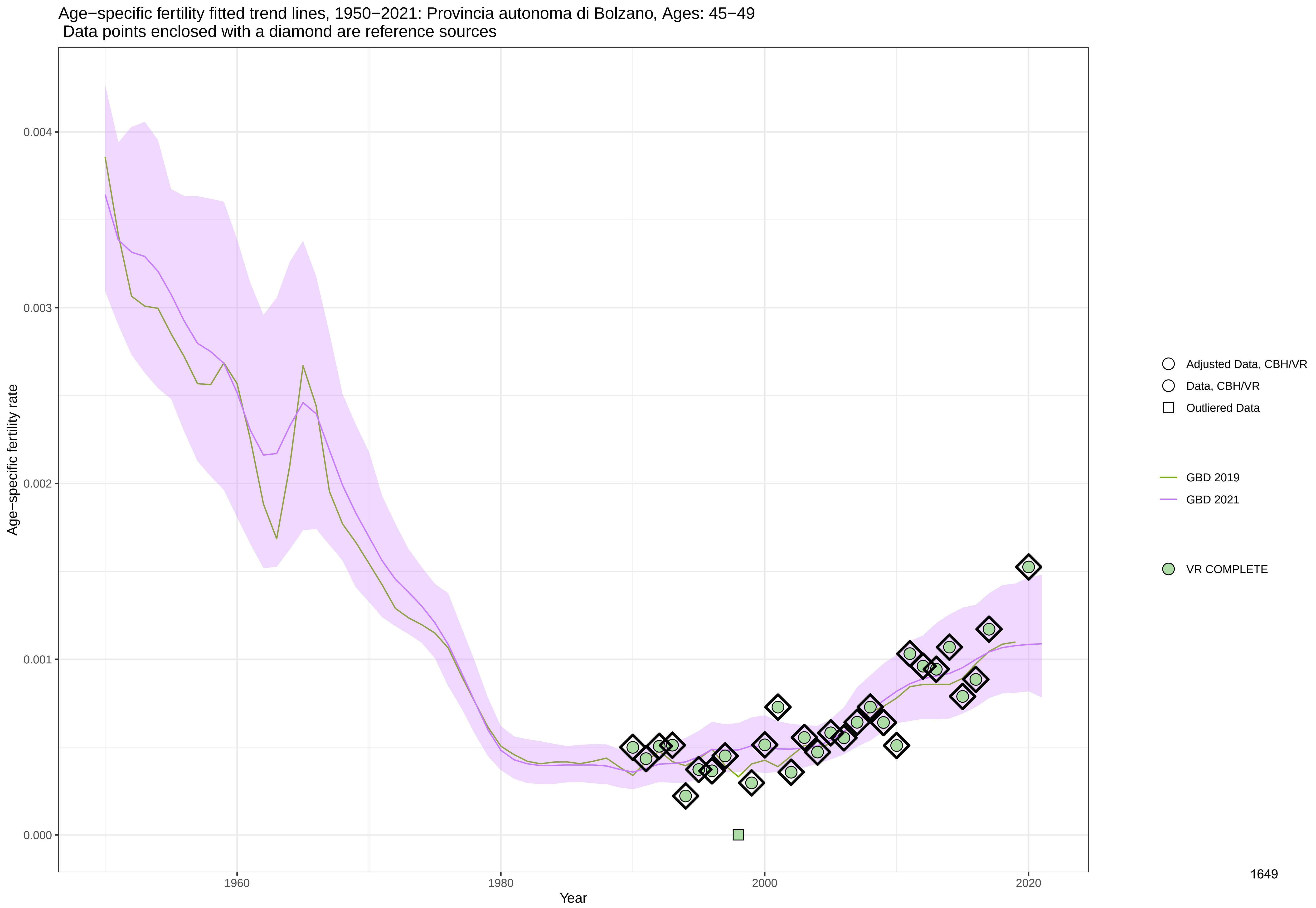

Age−specific fertility fitted trend lines, 1950−2021: Provincia autonoma di Bolzano, Ages: 45−49
Data points enclosed with a diamond are reference sources
Age−specific fertility rate
0.000
0.001
0.002
0.003
0.004
1960
1980
2000
2020
Year
Adjusted Data, CBH/VR
Data, CBH/VR
Outliered Data
GBD 2019
GBD 2021
VR COMPLETE

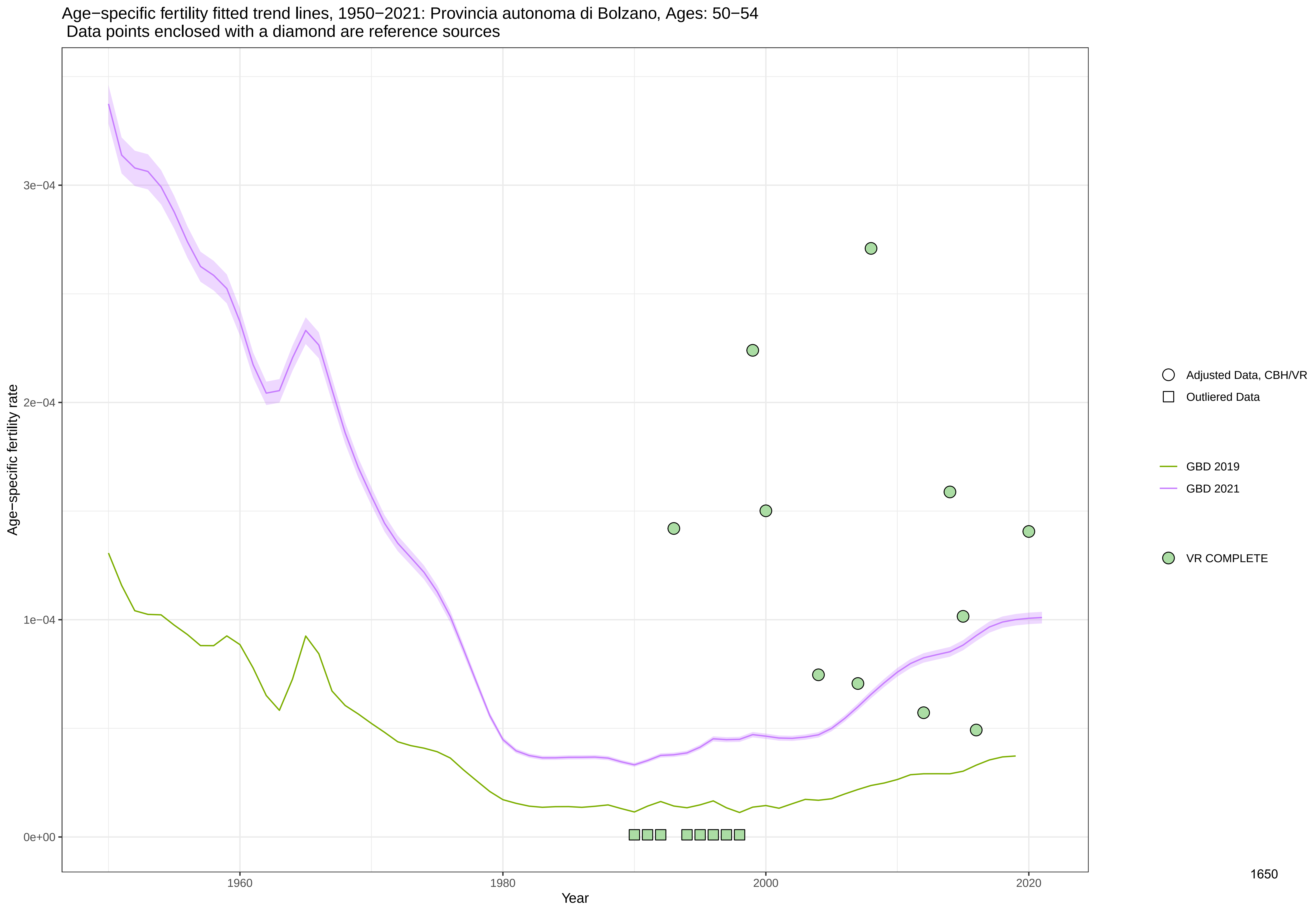
Age−specific fertility fitted trend lines, 1950−2021: Provincia autonoma di Bolzano, Ages: 50−54
Data points enclosed with a diamond are reference sources
Age−specific fertility rate
3e−04
2e−04
1e−04
0e+00
1960
1980
2000
2020
Year
Adjusted Data, CBH/VR
Outliered Data
GBD 2019
GBD 2021
VR COMPLETE

Total fertility rate fitted trend lines, 1950−2021: Provincia autonoma di Bolzano TFR
Data points enclosed with a diamond are reference sources

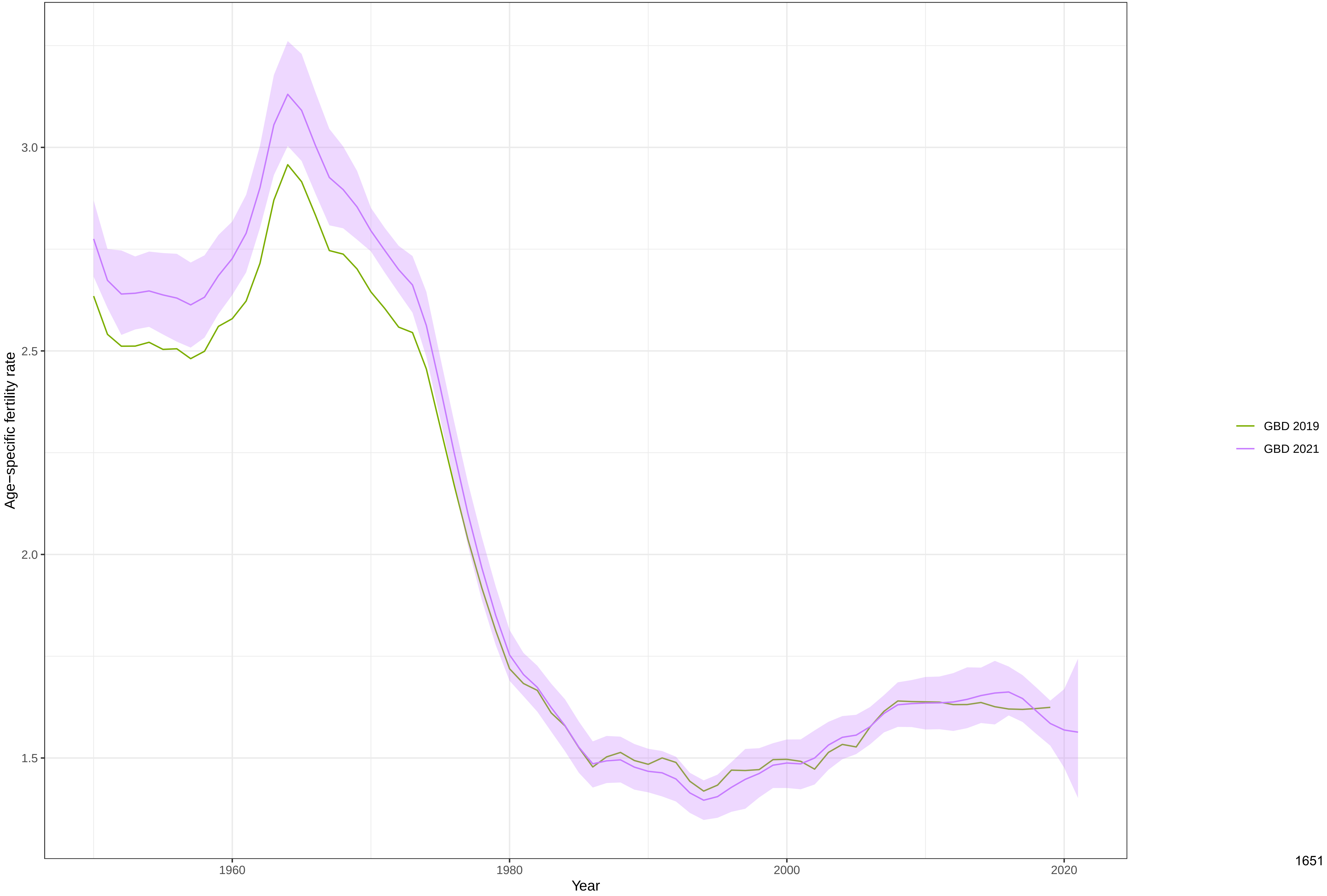

Age−specific fertility fitted trend lines, 1950−2021: Provincia autonoma di Trento, Ages: 10−14
Data points enclosed with a diamond are reference sources

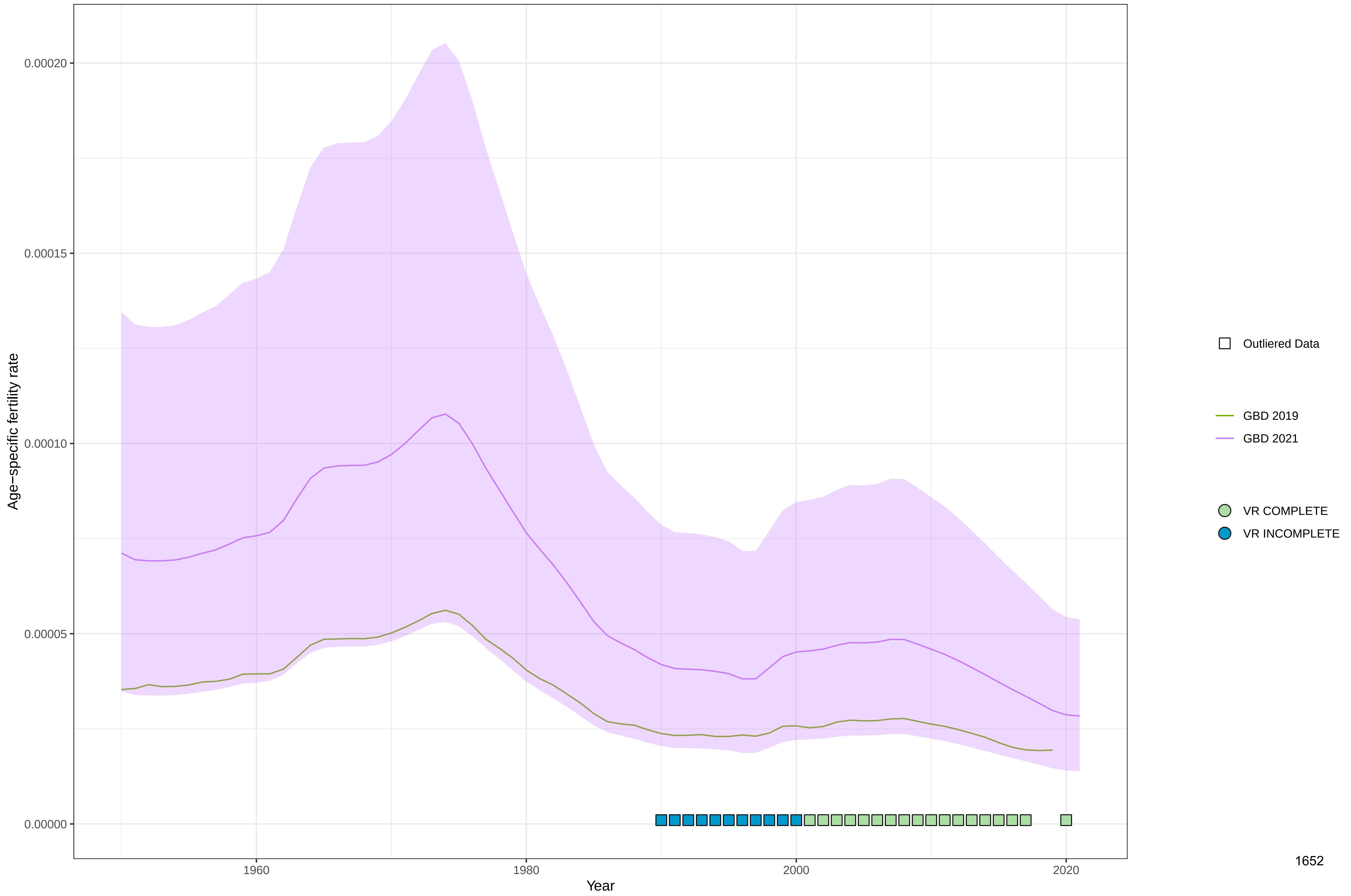

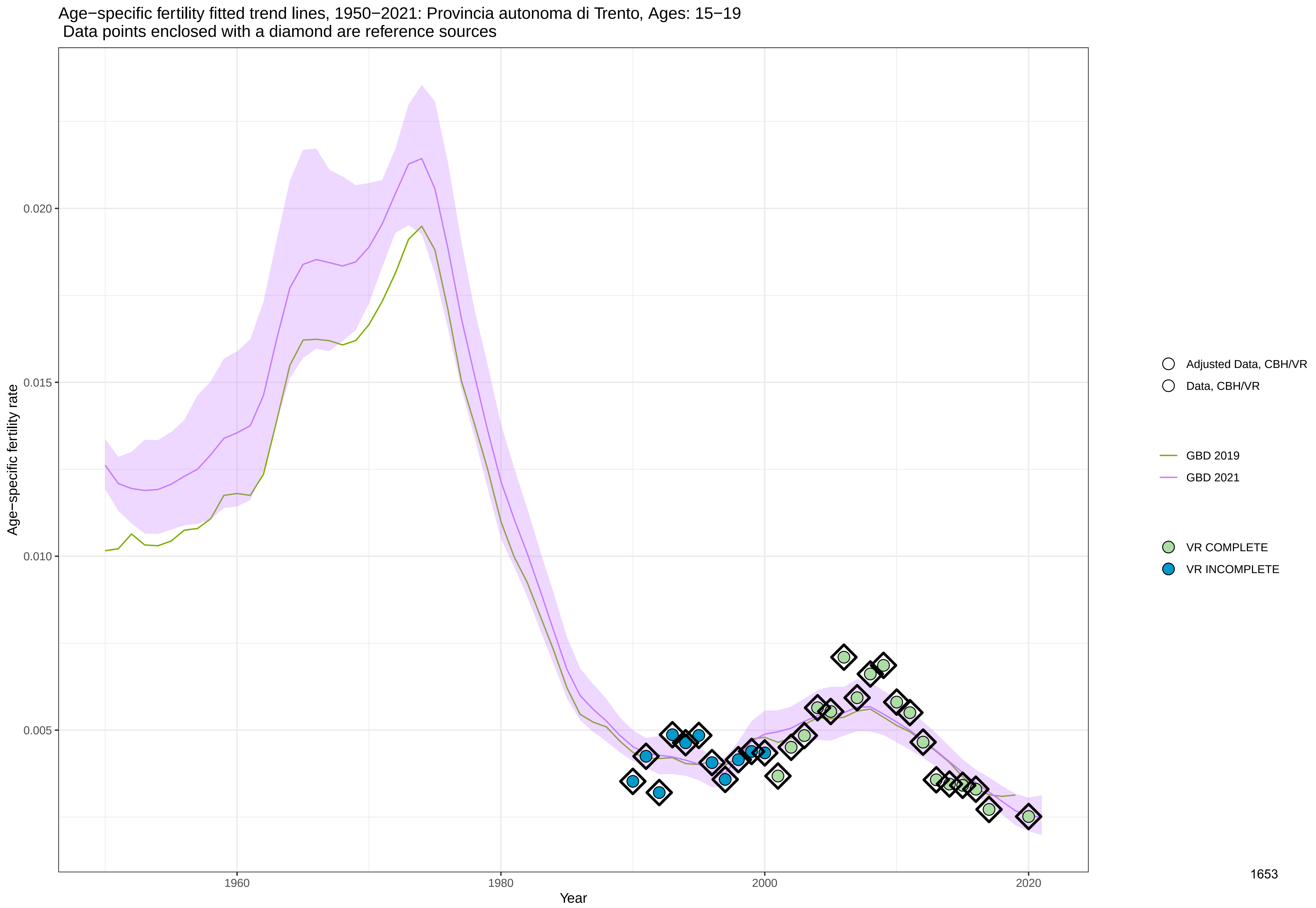

Age−specific fertility fitted trend lines, 1950−2021: Provincia autonoma di Trento, Ages: 15−19
Data points enclosed with a diamond are reference sources
Age−specific fertility rate
0.020
0.015
0.010
0.005
1960
1980
2000
2020
Year
Adjusted Data, CBH/VR
Data, CBH/VR
GBD 2019
GBD 2021
VR COMPLETE
VR INCOMPLETE

Age−specific fertility fitted trend lines, 1950−2021: Provincia autonoma di Trento, Ages: 20−24
Data points enclosed with a diamond are reference sources

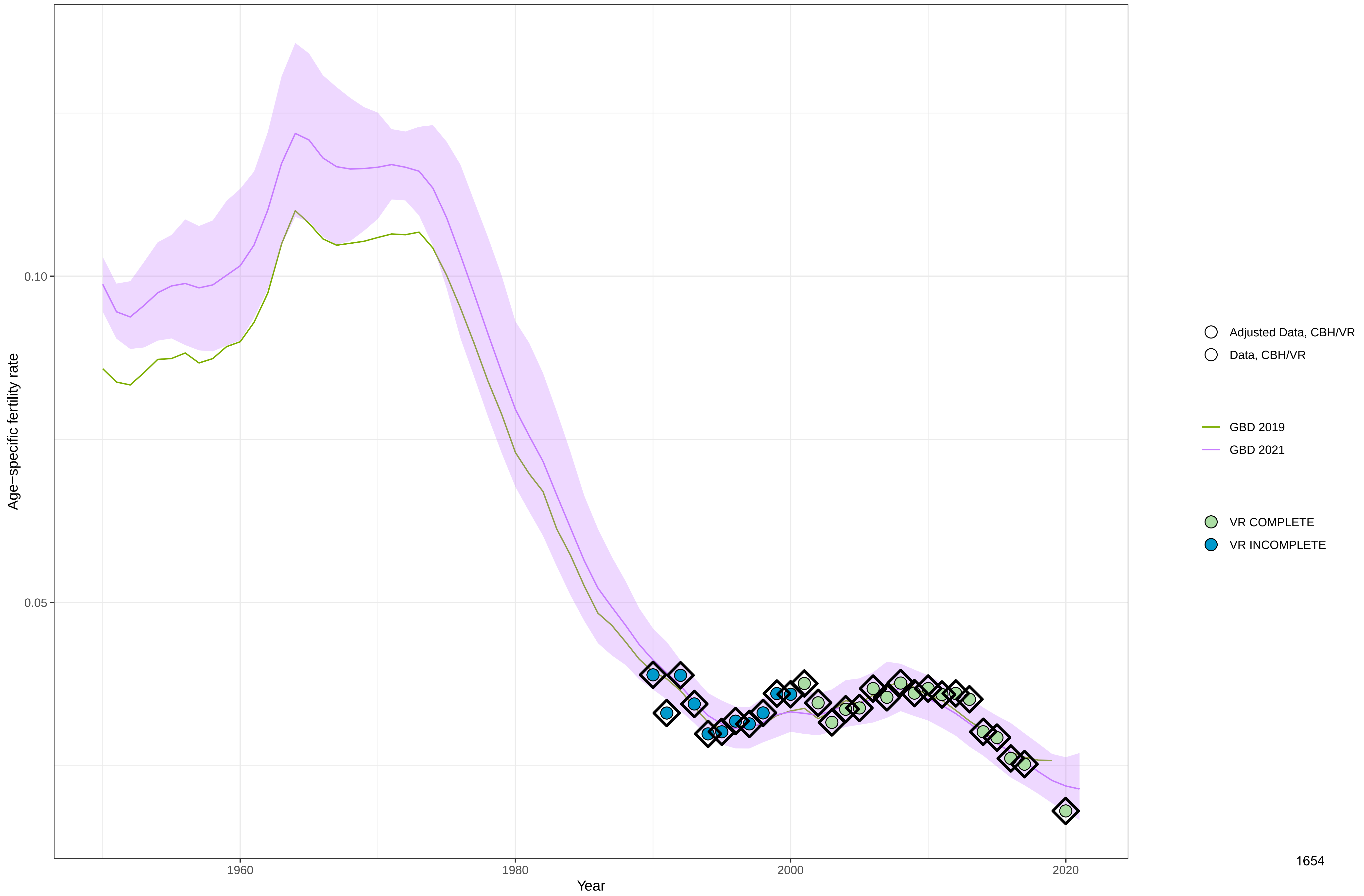

Age−specific fertility fitted trend lines, 1950−2021: Provincia autonoma di Trento, Ages: 25−29
Data points enclosed with a diamond are reference sources

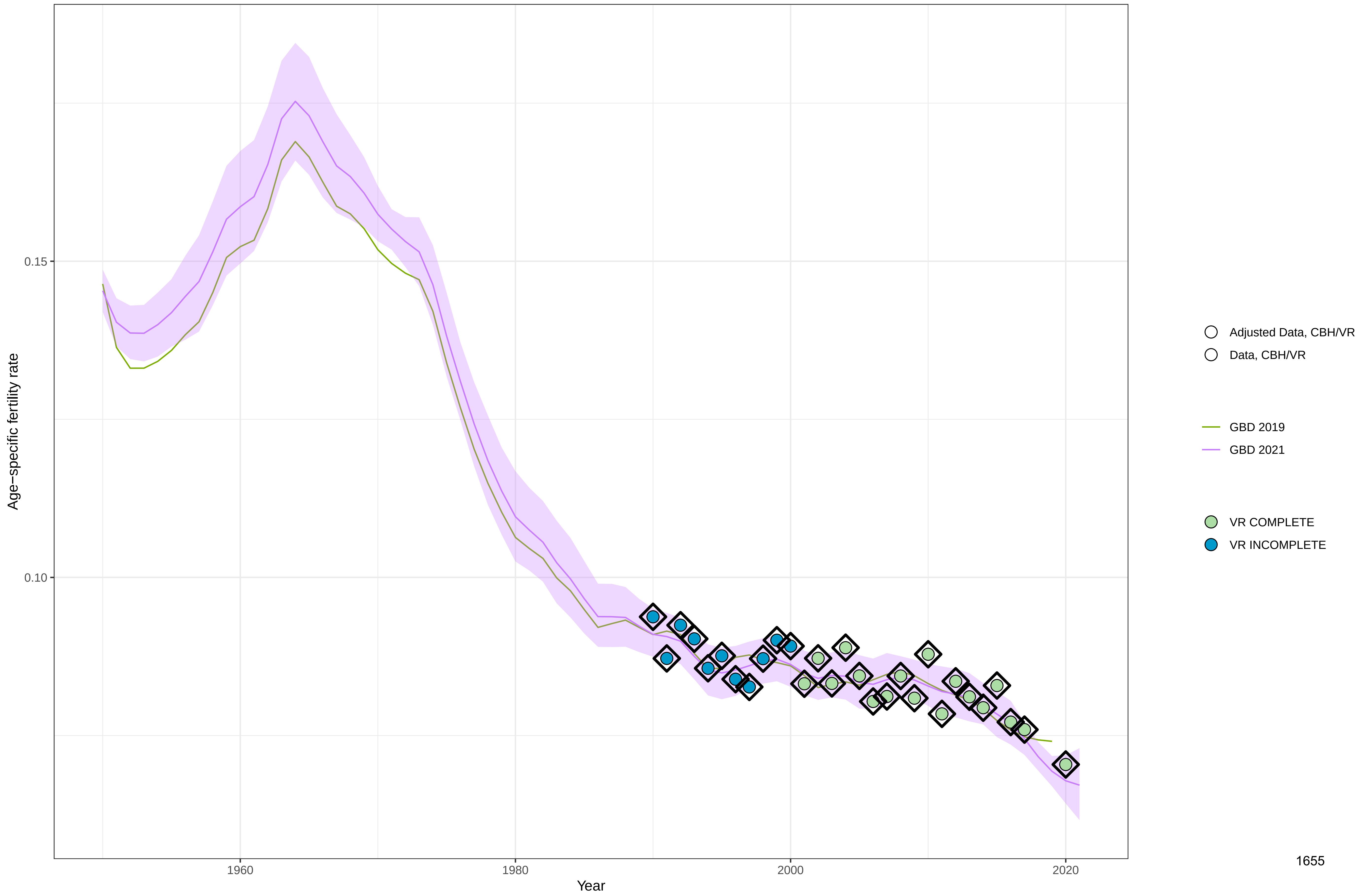

Age−specific fertility fitted trend lines, 1950−2021: Provincia autonoma di Trento, Ages: 30−34
Data points enclosed with a diamond are reference sources

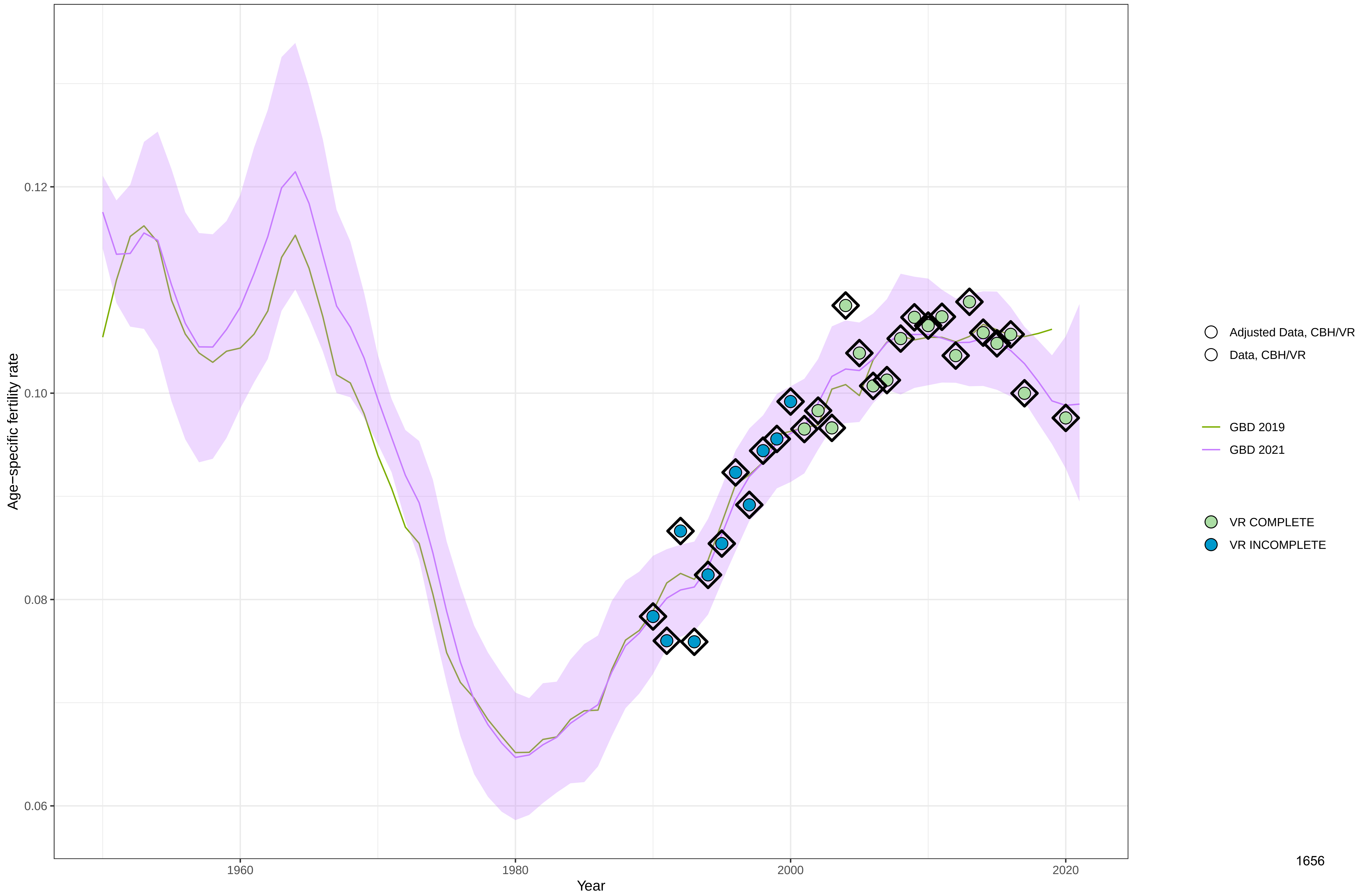

Age−specific fertility fitted trend lines, 1950−2021: Provincia autonoma di Trento, Ages: 35−39
Data points enclosed with a diamond are reference sources

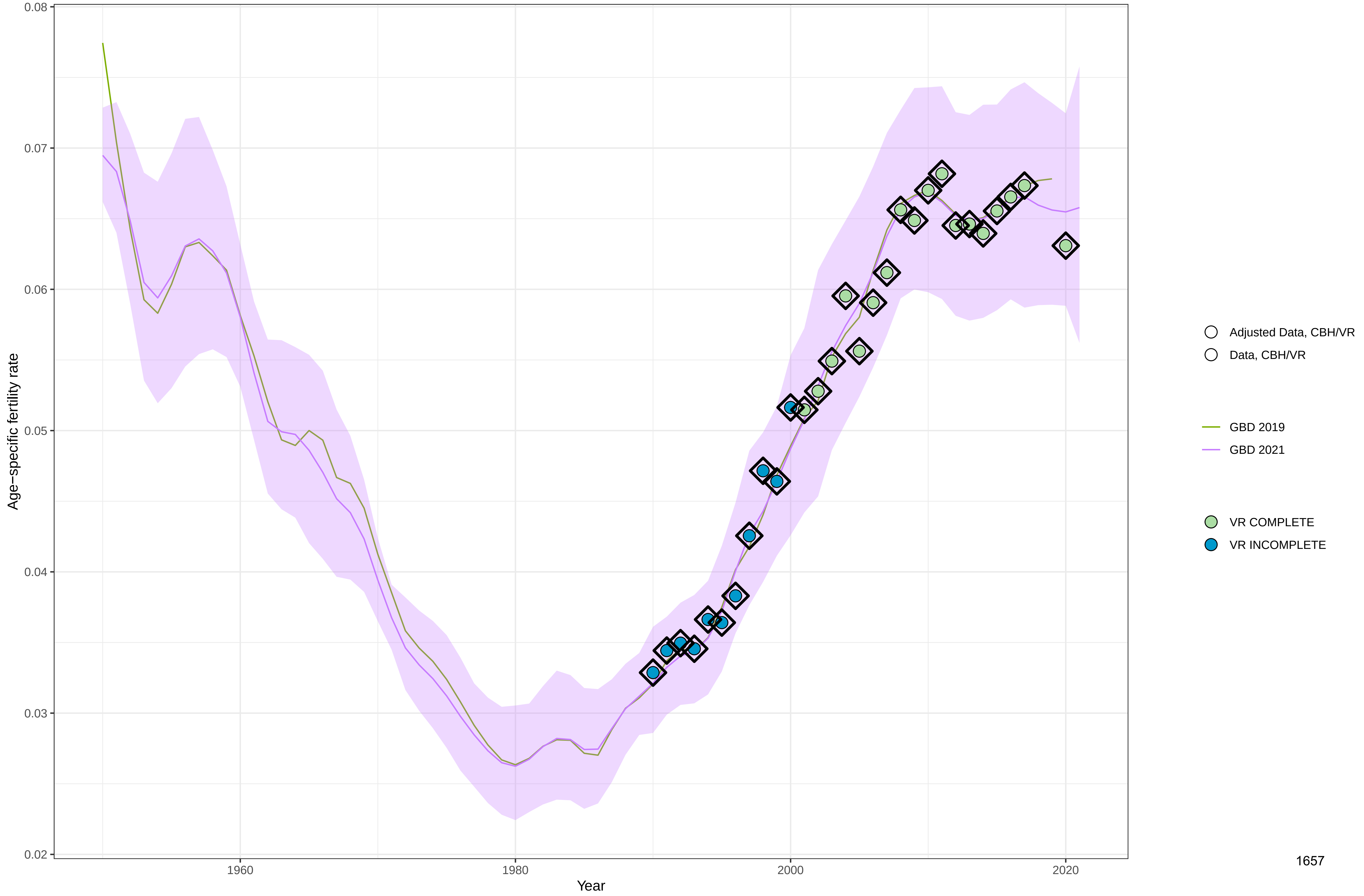

Age−specific fertility fitted trend lines, 1950−2021: Provincia autonoma di Trento, Ages: 40−44
Data points enclosed with a diamond are reference sources

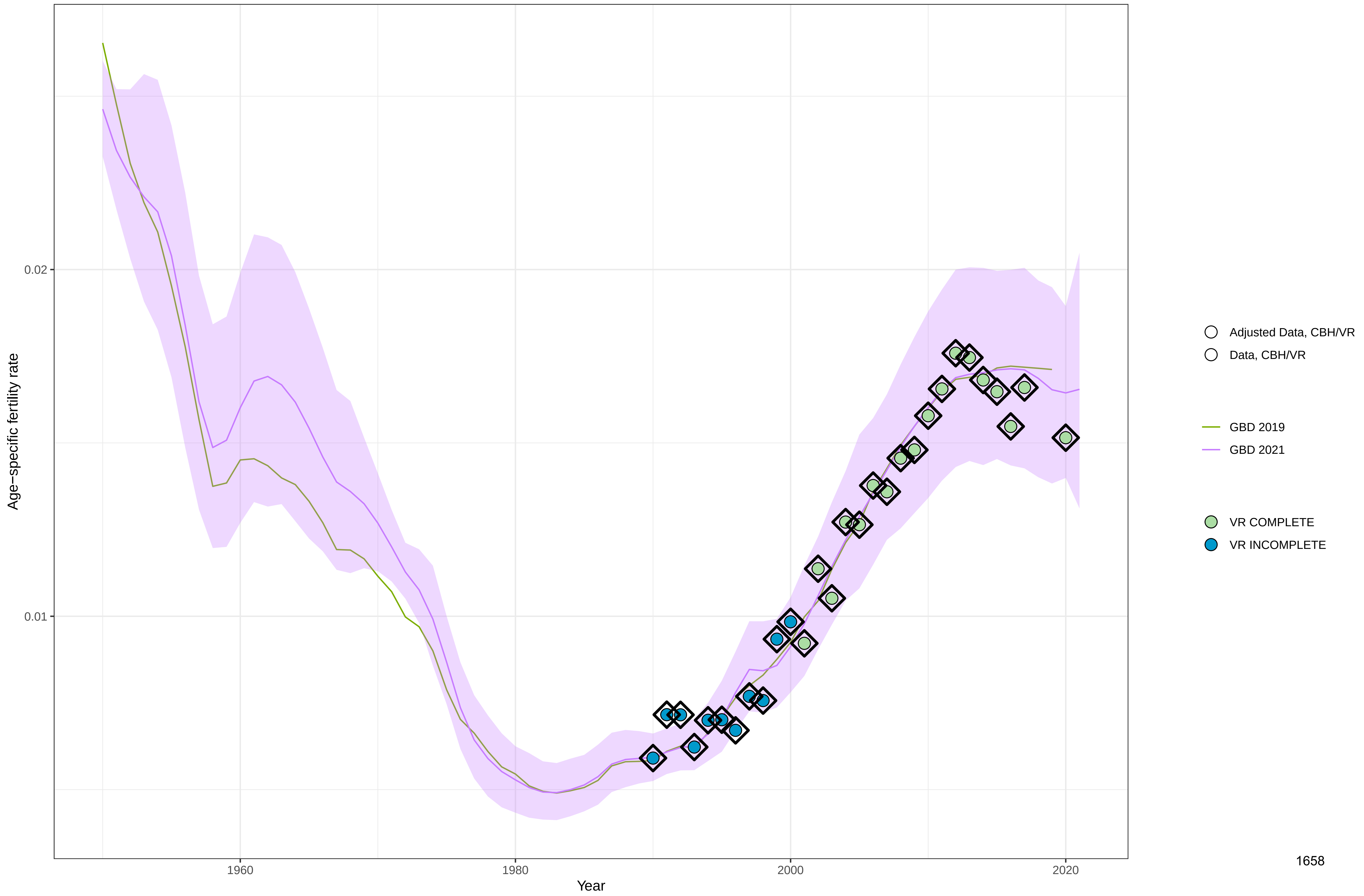

Age−specific fertility fitted trend lines, 1950−2021: Provincia autonoma di Trento, Ages: 45−49
Data points enclosed with a diamond are reference sources

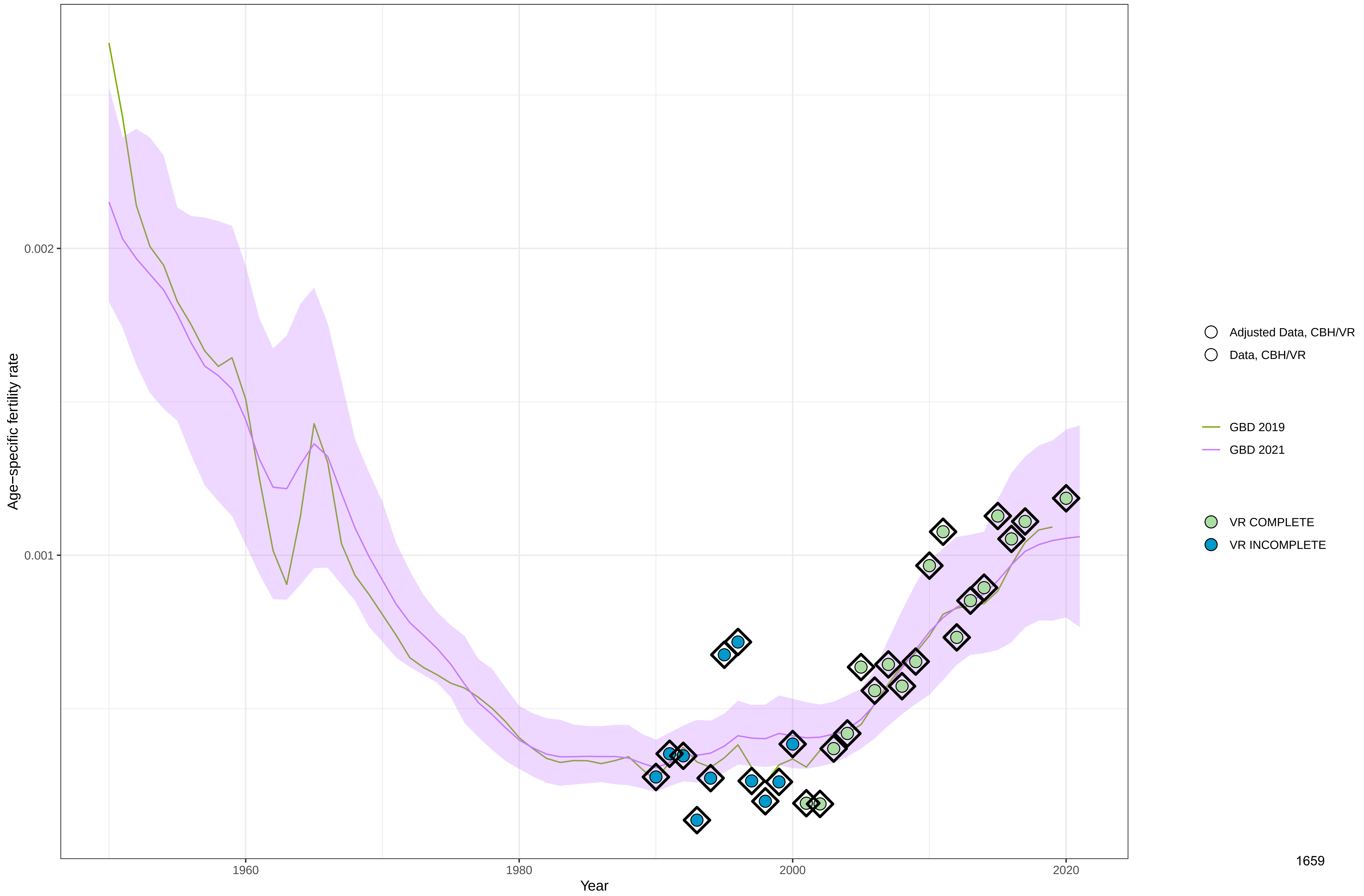

Age−specific fertility fitted trend lines, 1950−2021: Provincia autonoma di Trento, Ages: 50−54
Data points enclosed with a diamond are reference sources

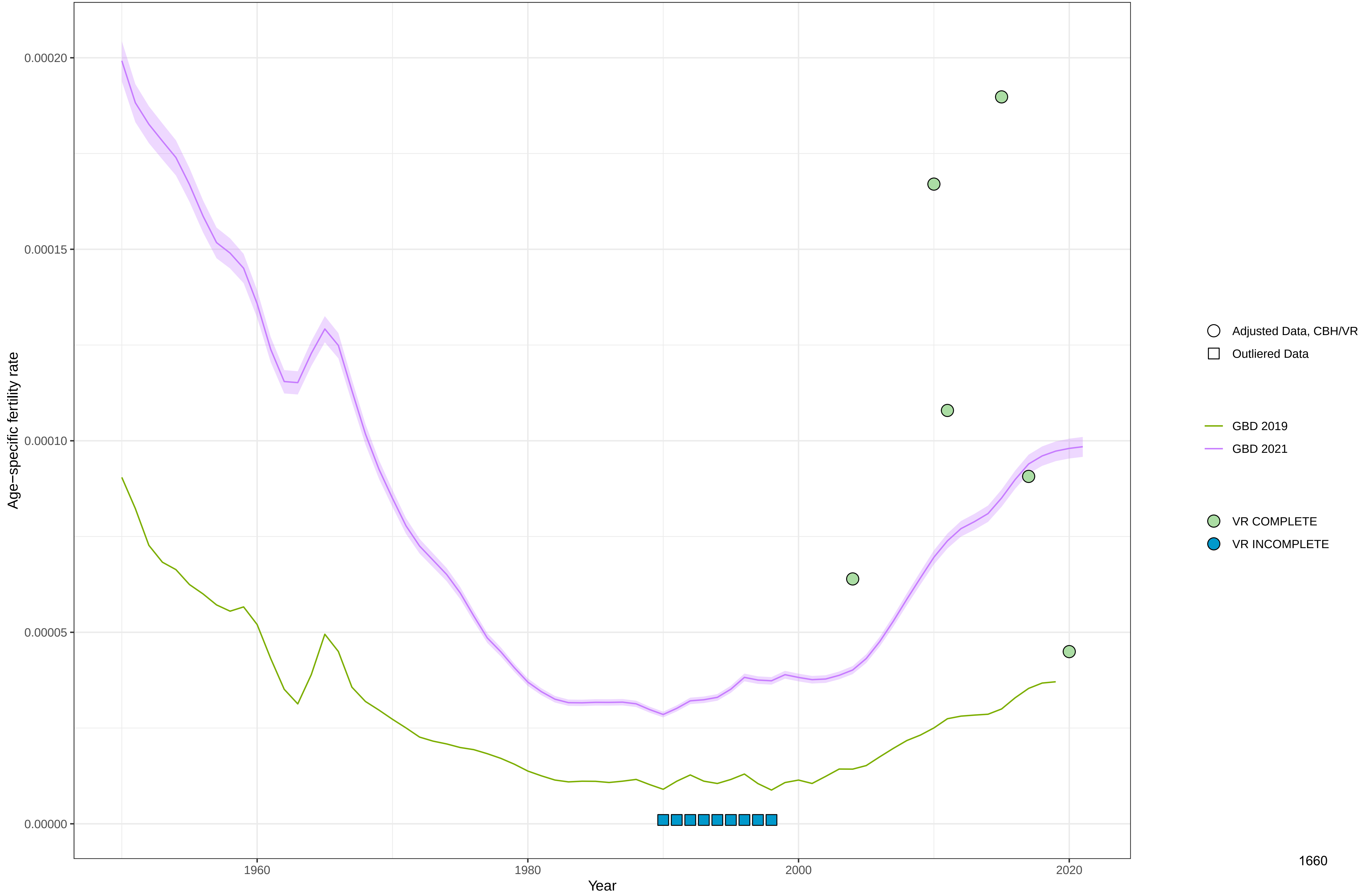

Total fertility rate fitted trend lines, 1950−2021: Provincia autonoma di Trento TFR
Data points enclosed with a diamond are reference sources

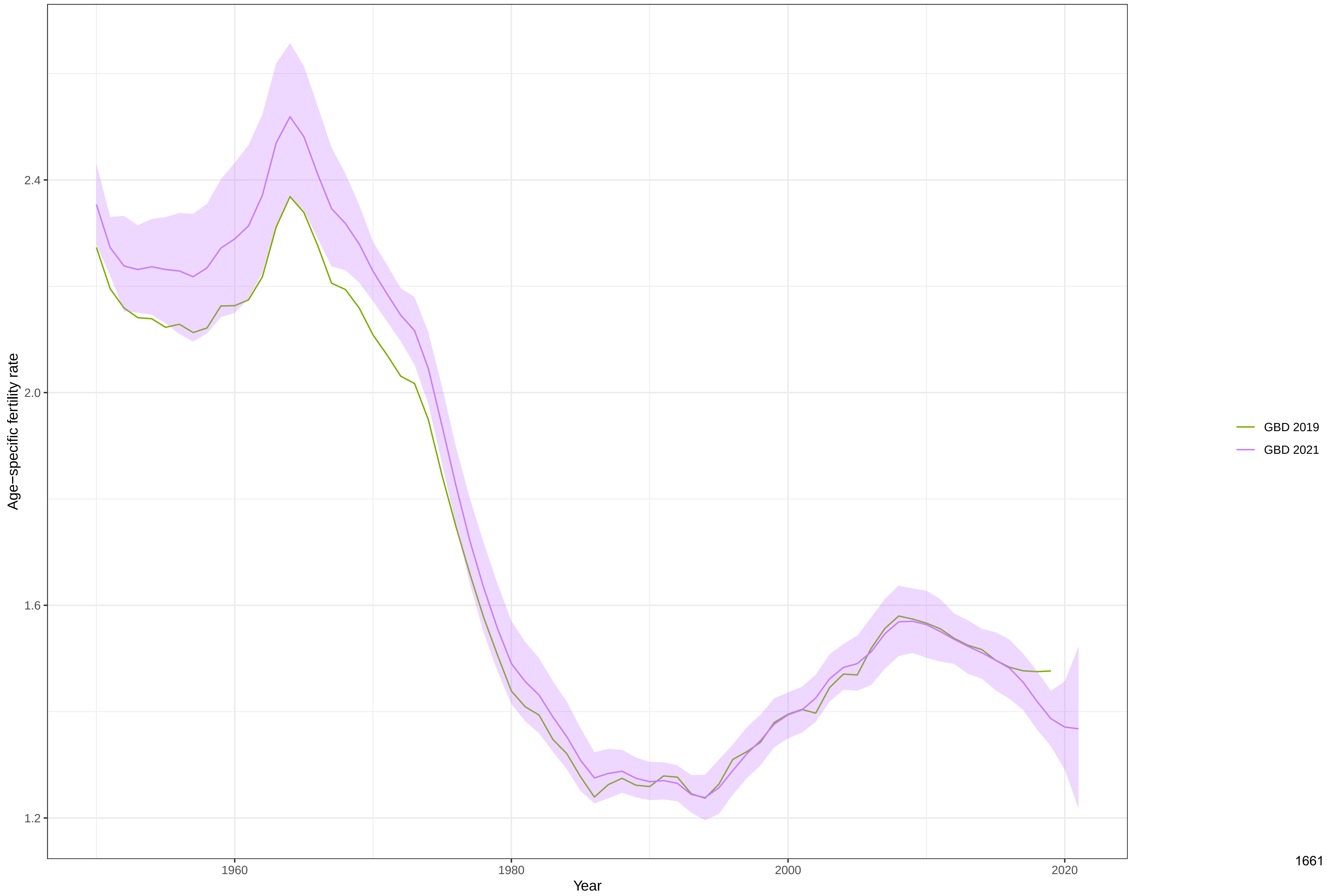

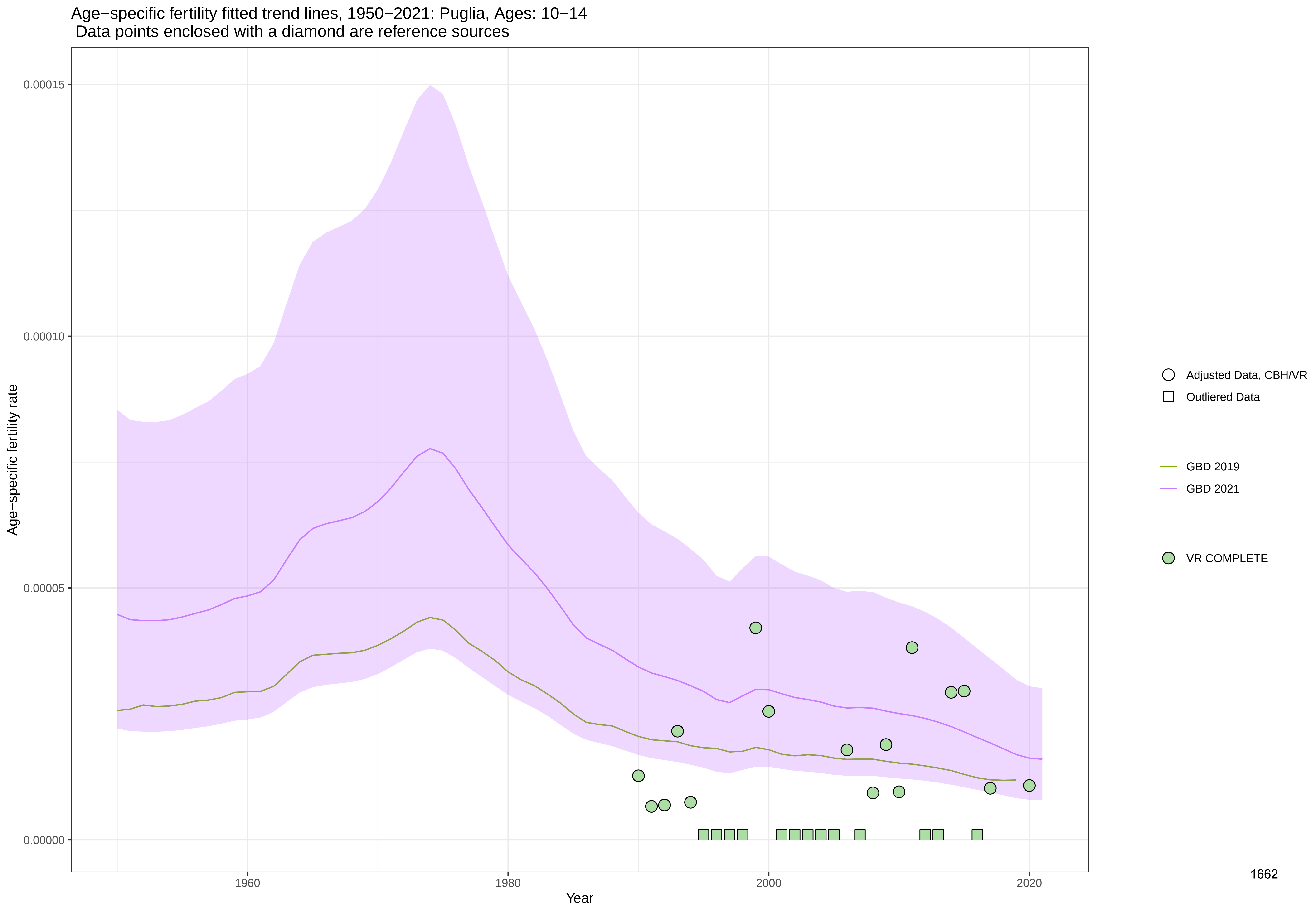

Age−specific fertility fitted trend lines, 1950−2021: Puglia, Ages: 10−14
Data points enclosed with a diamond are reference sources
Age−specific fertility rate
0.00015
0.00010
0.00005
0.00000
1960
1980
2000
2020
Year
Adjusted Data, CBH/VR
Outliered Data
GBD 2019
GBD 2021
VR COMPLETE

Age−specific fertility fitted trend lines, 1950−2021: Puglia, Ages: 15−19
Data points enclosed with a diamond are reference sources

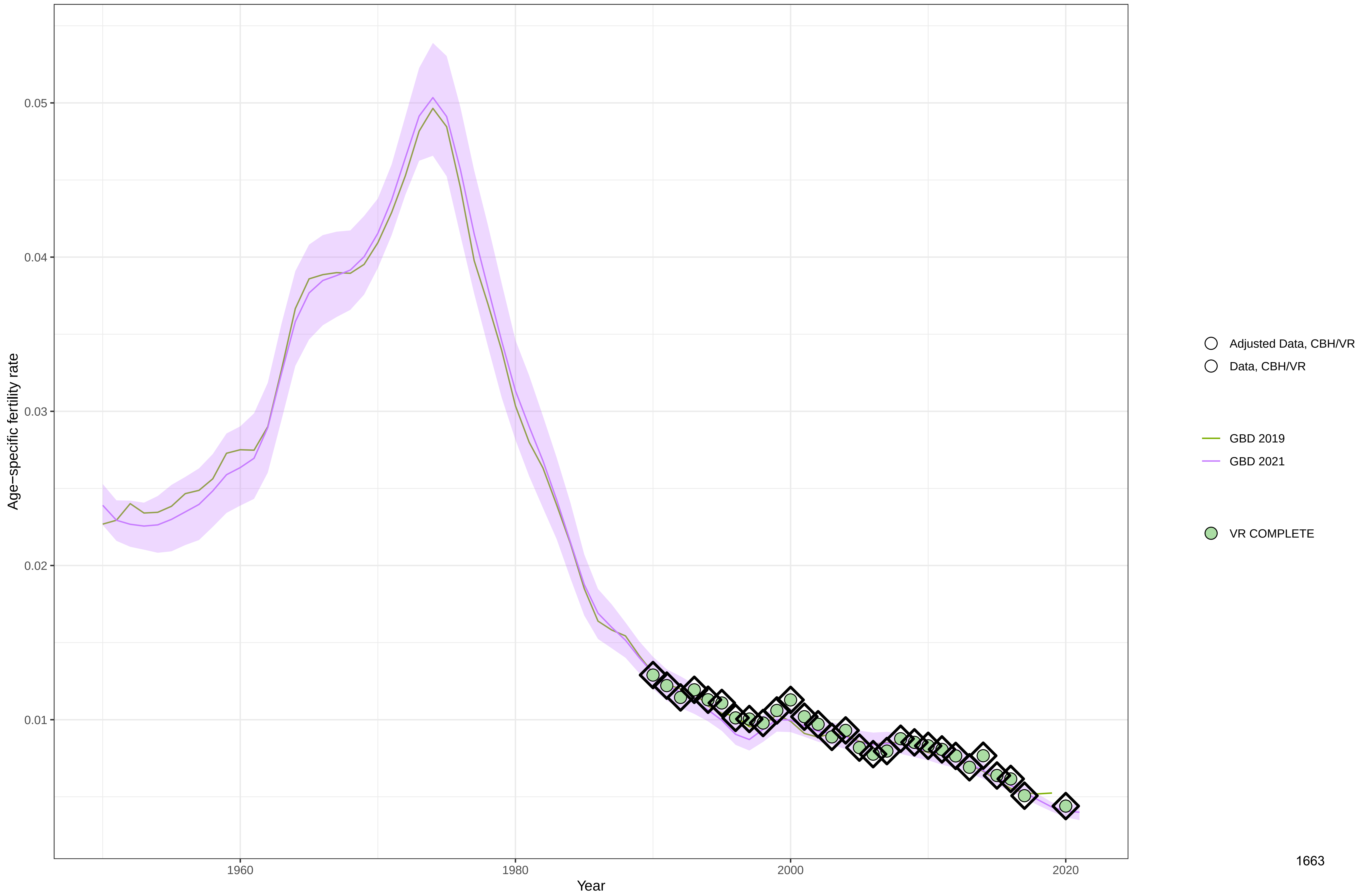

Age−specific fertility fitted trend lines, 1950−2021: Puglia, Ages: 20−24
Data points enclosed with a diamond are reference sources

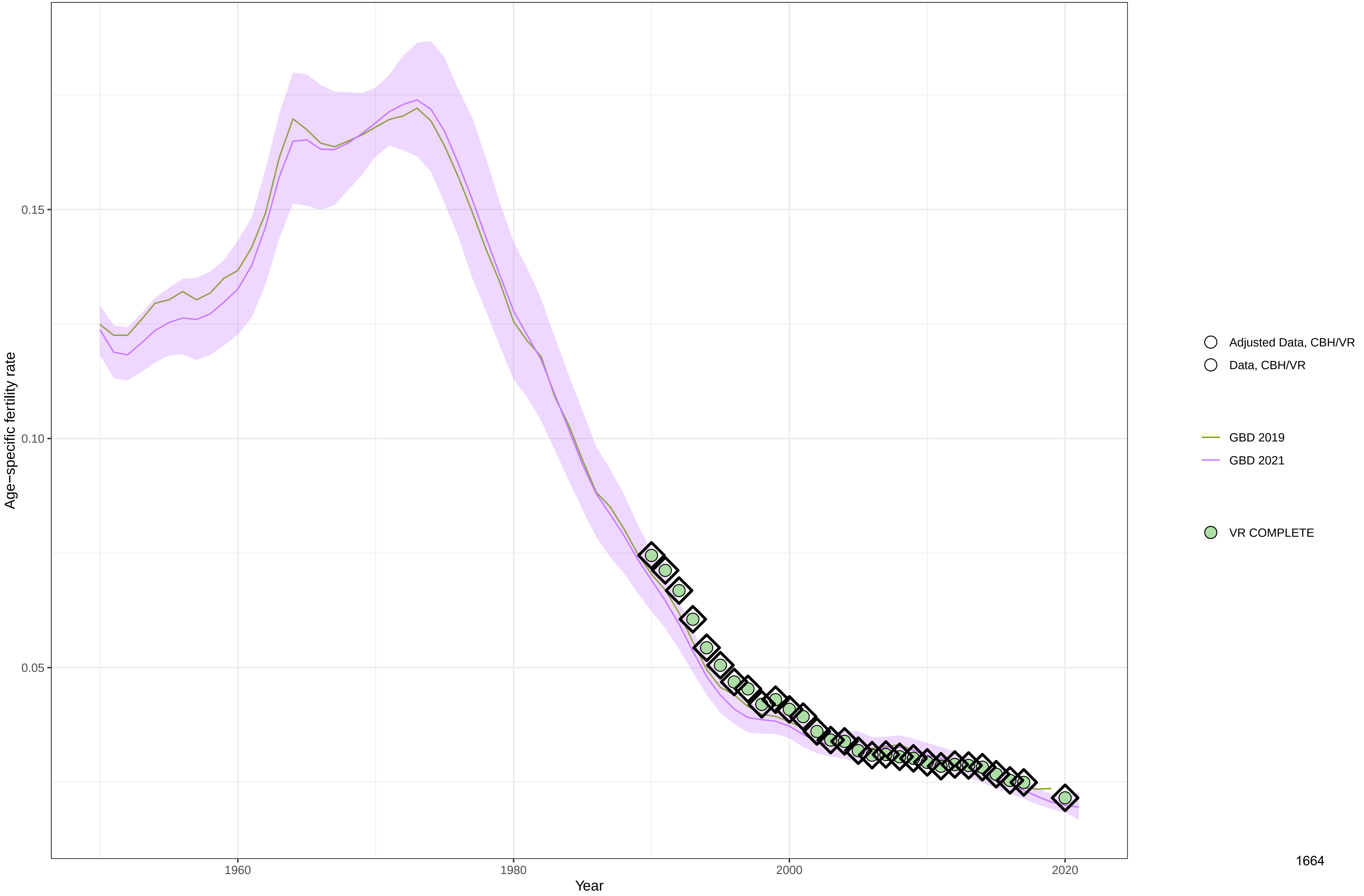

Age−specific fertility fitted trend lines, 1950−2021: Puglia, Ages: 25−29
Data points enclosed with a diamond are reference sources

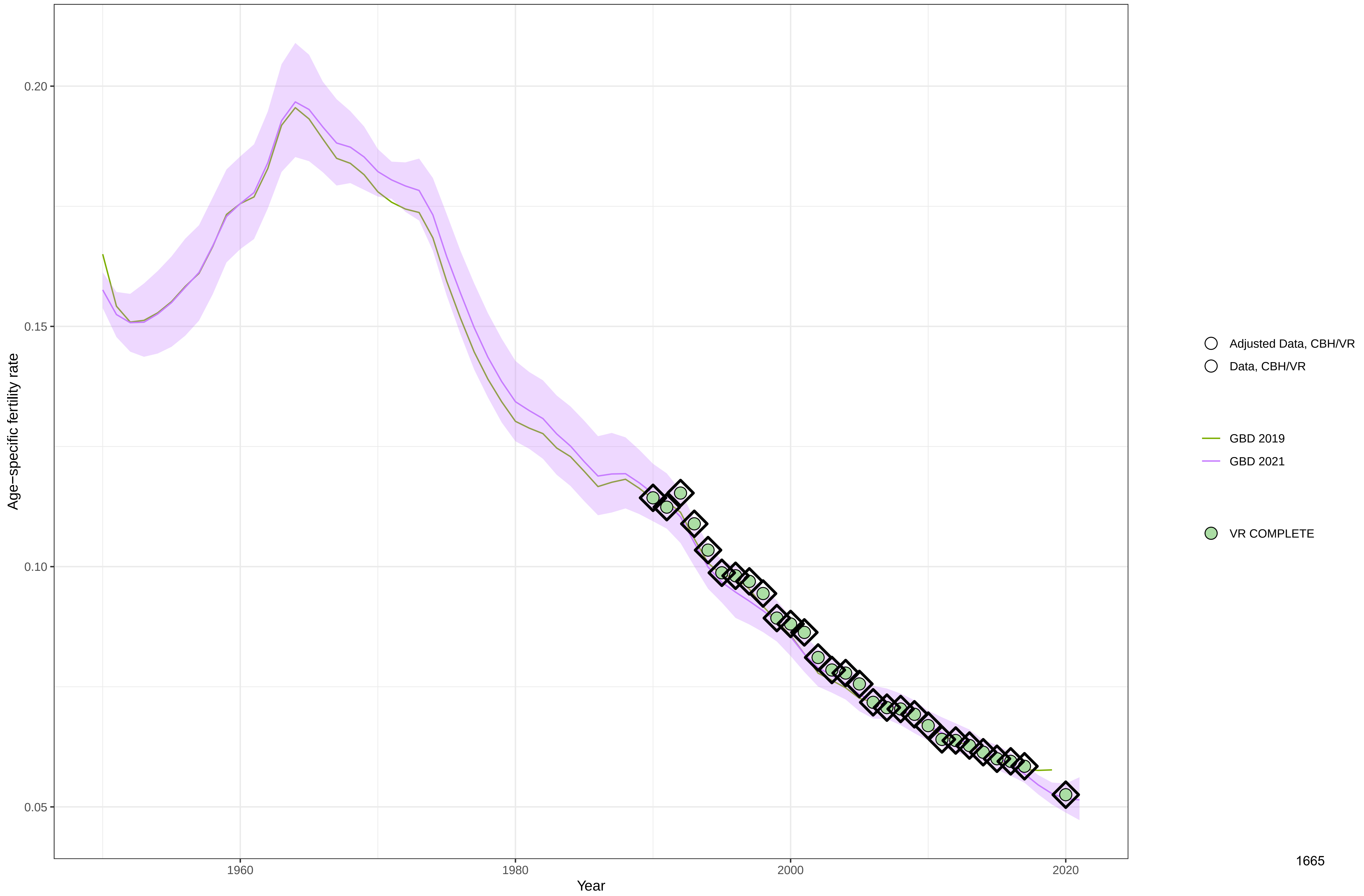

Age−specific fertility fitted trend lines, 1950−2021: Puglia, Ages: 30−34
Data points enclosed with a diamond are reference sources

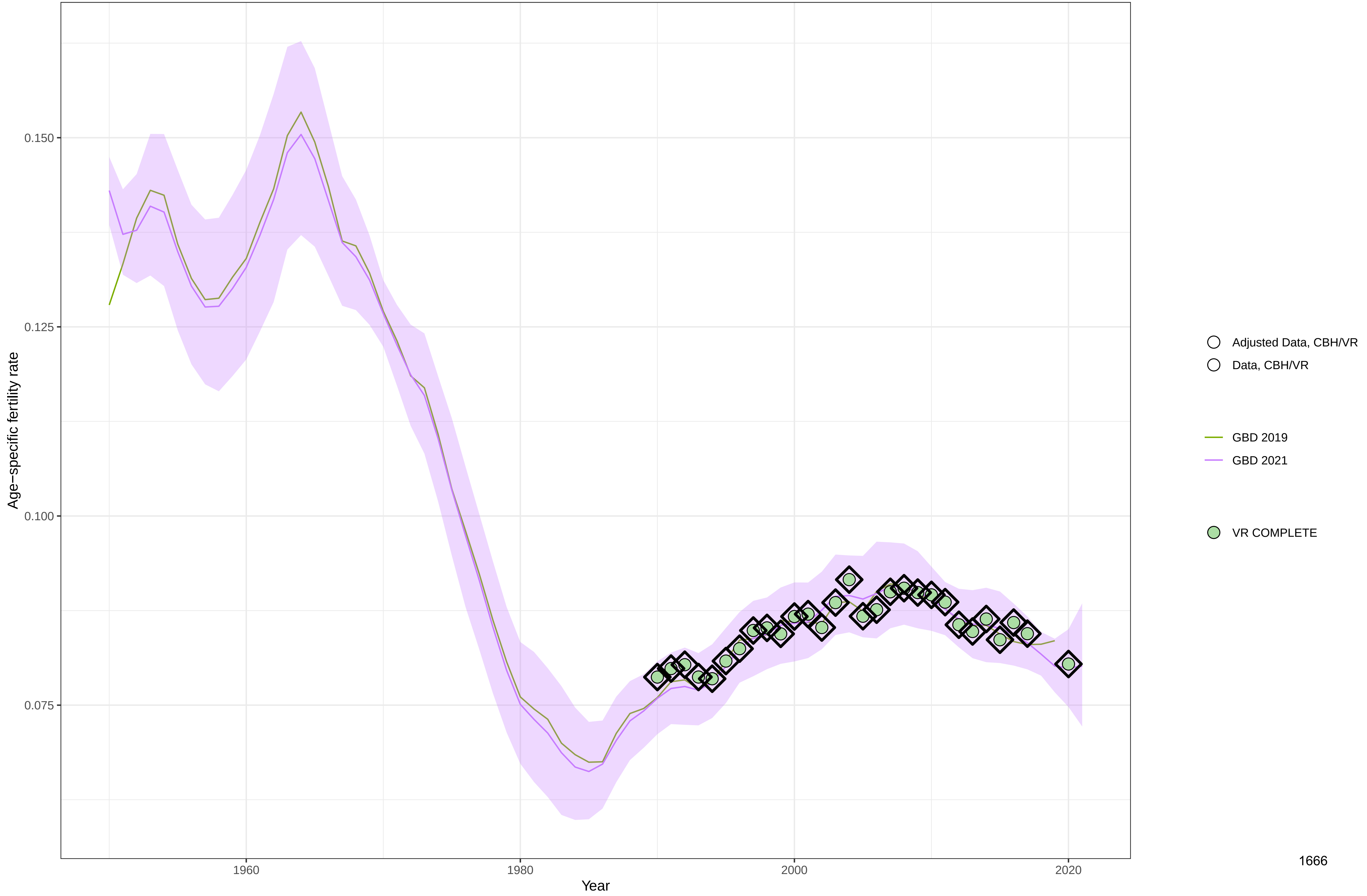

Age−specific fertility fitted trend lines, 1950−2021: Puglia, Ages: 35−39
Data points enclosed with a diamond are reference sources

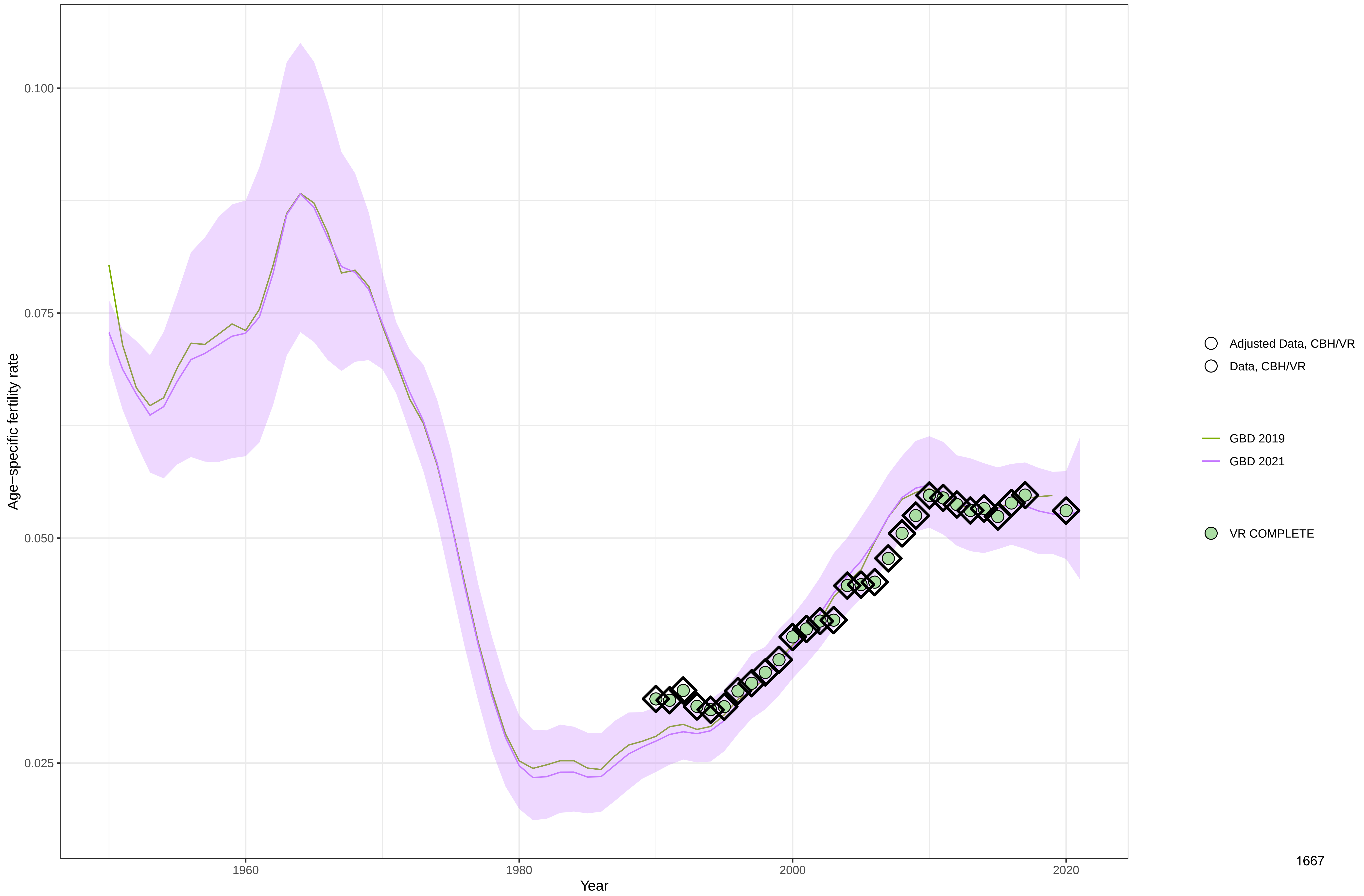

Age−specific fertility fitted trend lines, 1950−2021: Puglia, Ages: 40−44
Data points enclosed with a diamond are reference sources

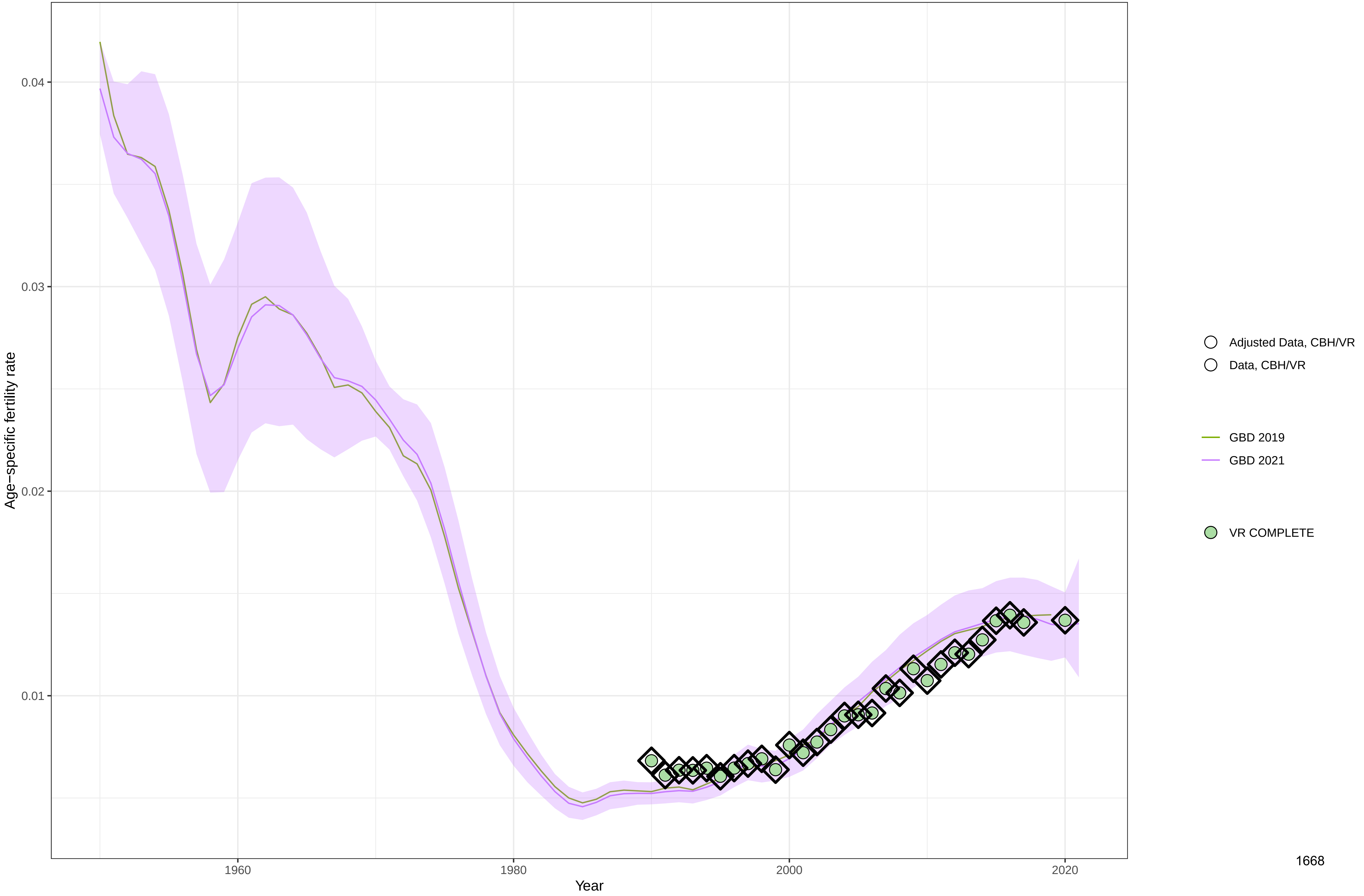

Age−specific fertility fitted trend lines, 1950−2021: Puglia, Ages: 45−49
Data points enclosed with a diamond are reference sources

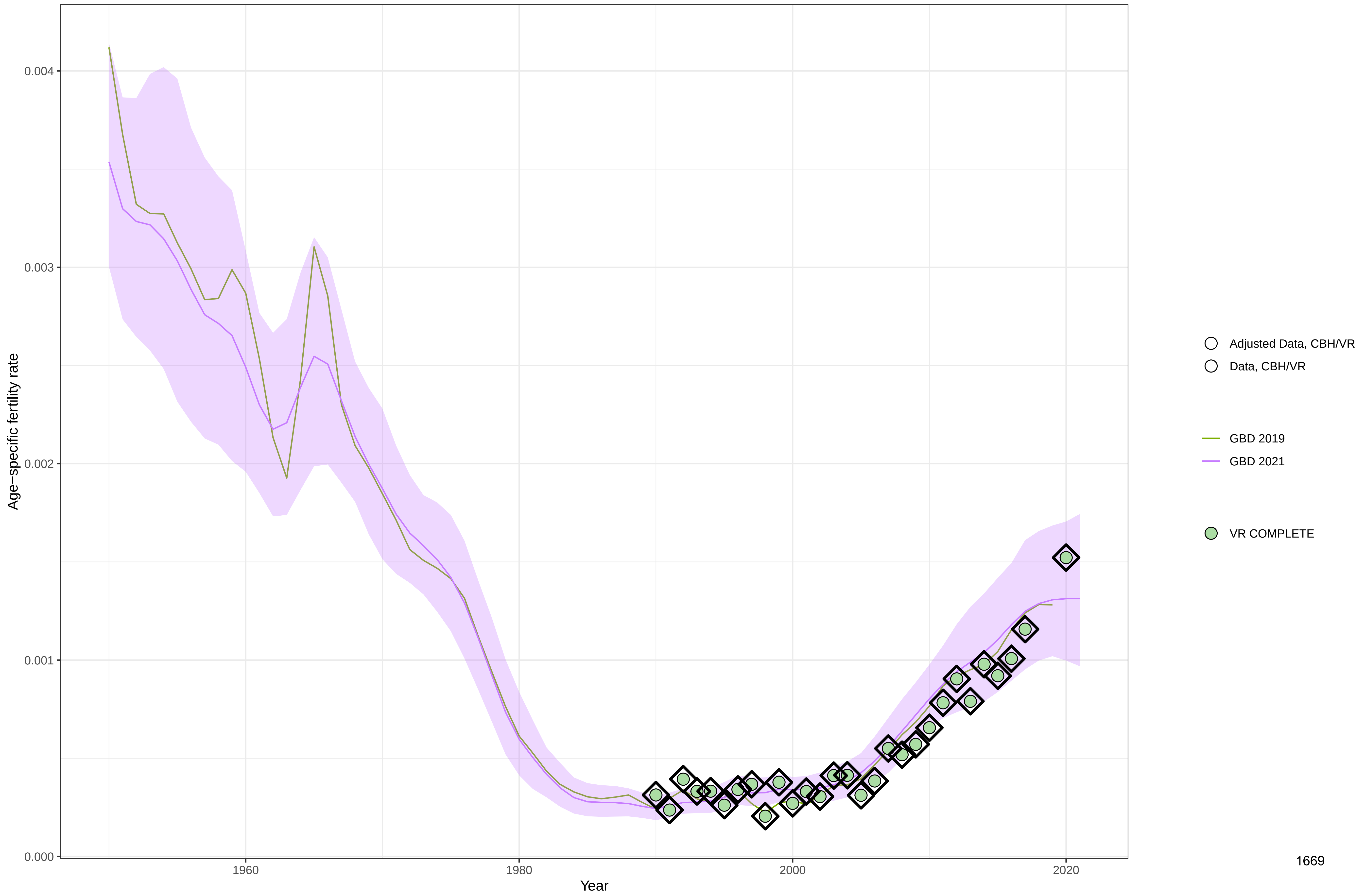

Age−specific fertility fitted trend lines, 1950−2021: Puglia, Ages: 50−54
Data points enclosed with a diamond are reference sources

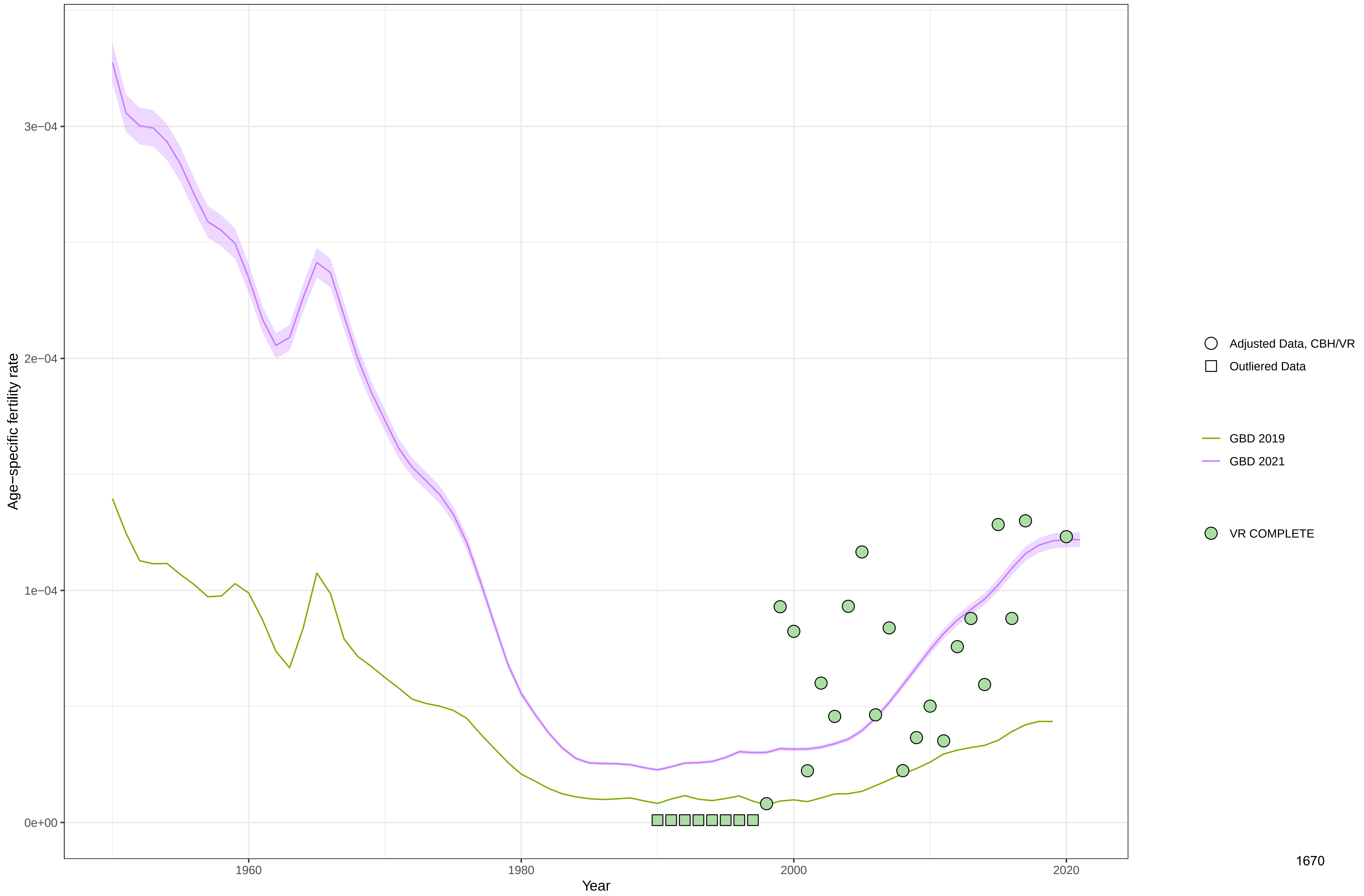

Total fertility rate fitted trend lines, 1950−2021: Puglia TFR
Data points enclosed with a diamond are reference sources

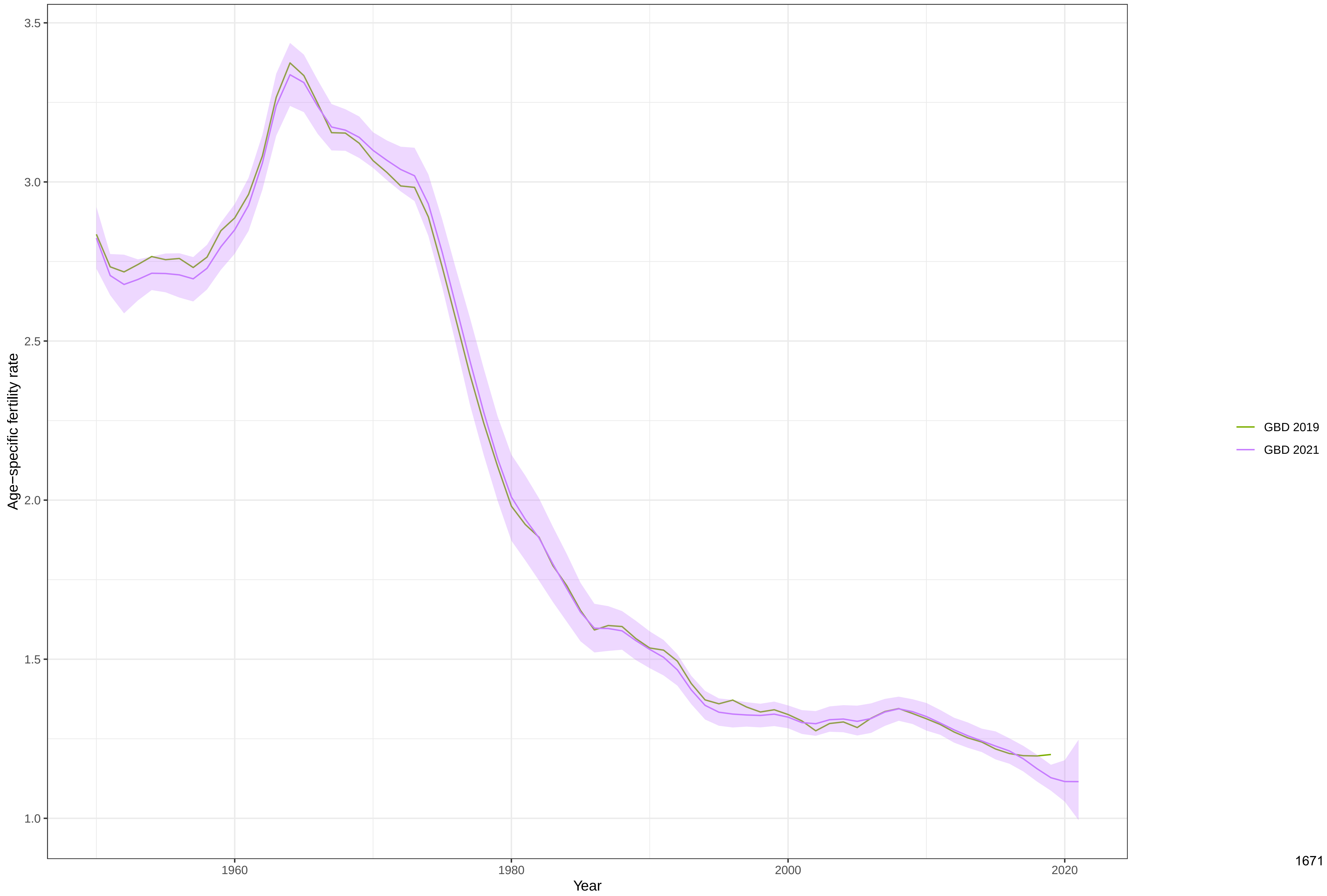

Age−specific fertility fitted trend lines, 1950−2021: Sardegna, Ages: 10−14
Data points enclosed with a diamond are reference sources

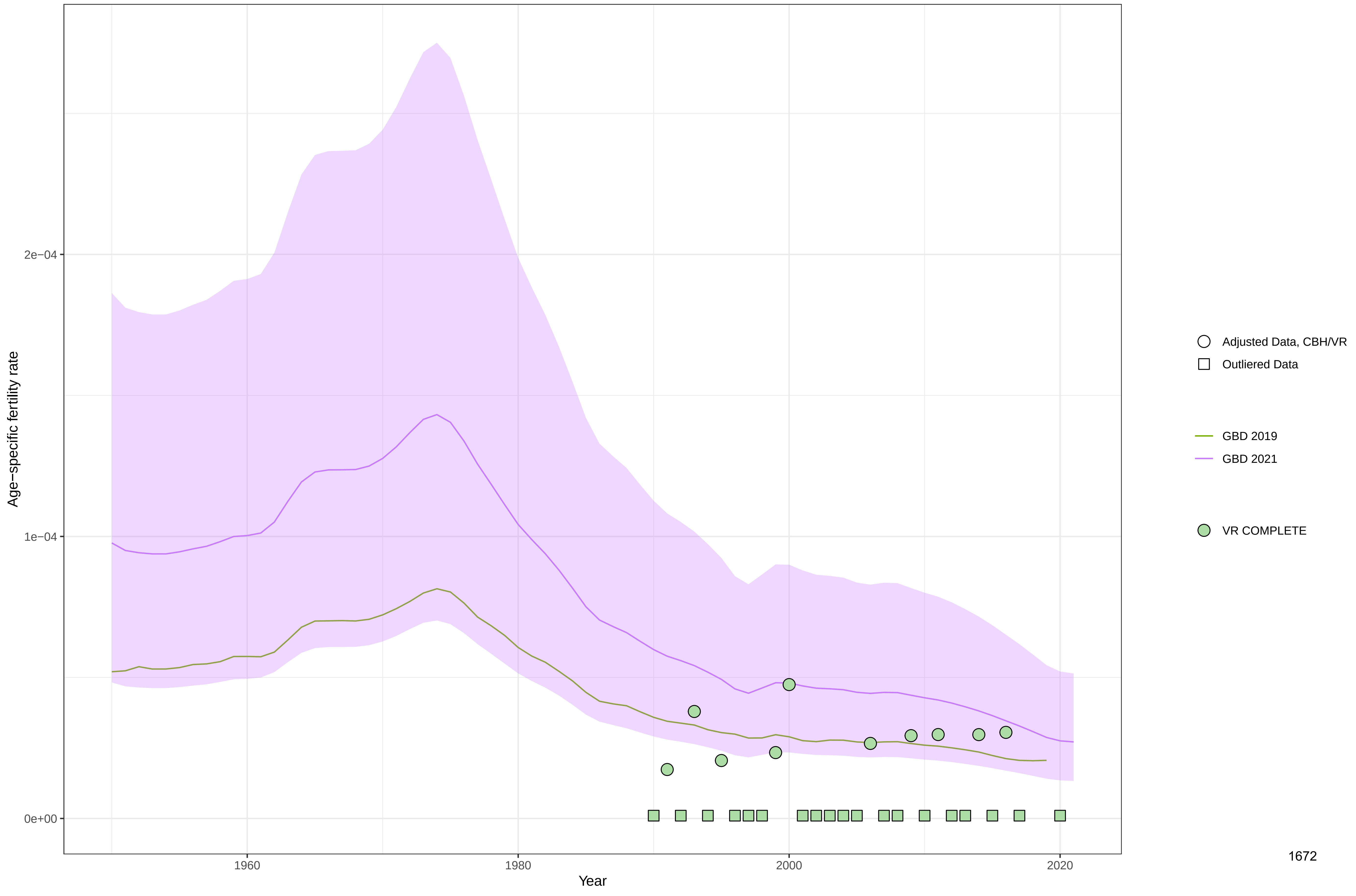

Age−specific fertility fitted trend lines, 1950−2021: Sardegna, Ages: 15−19
Data points enclosed with a diamond are reference sources

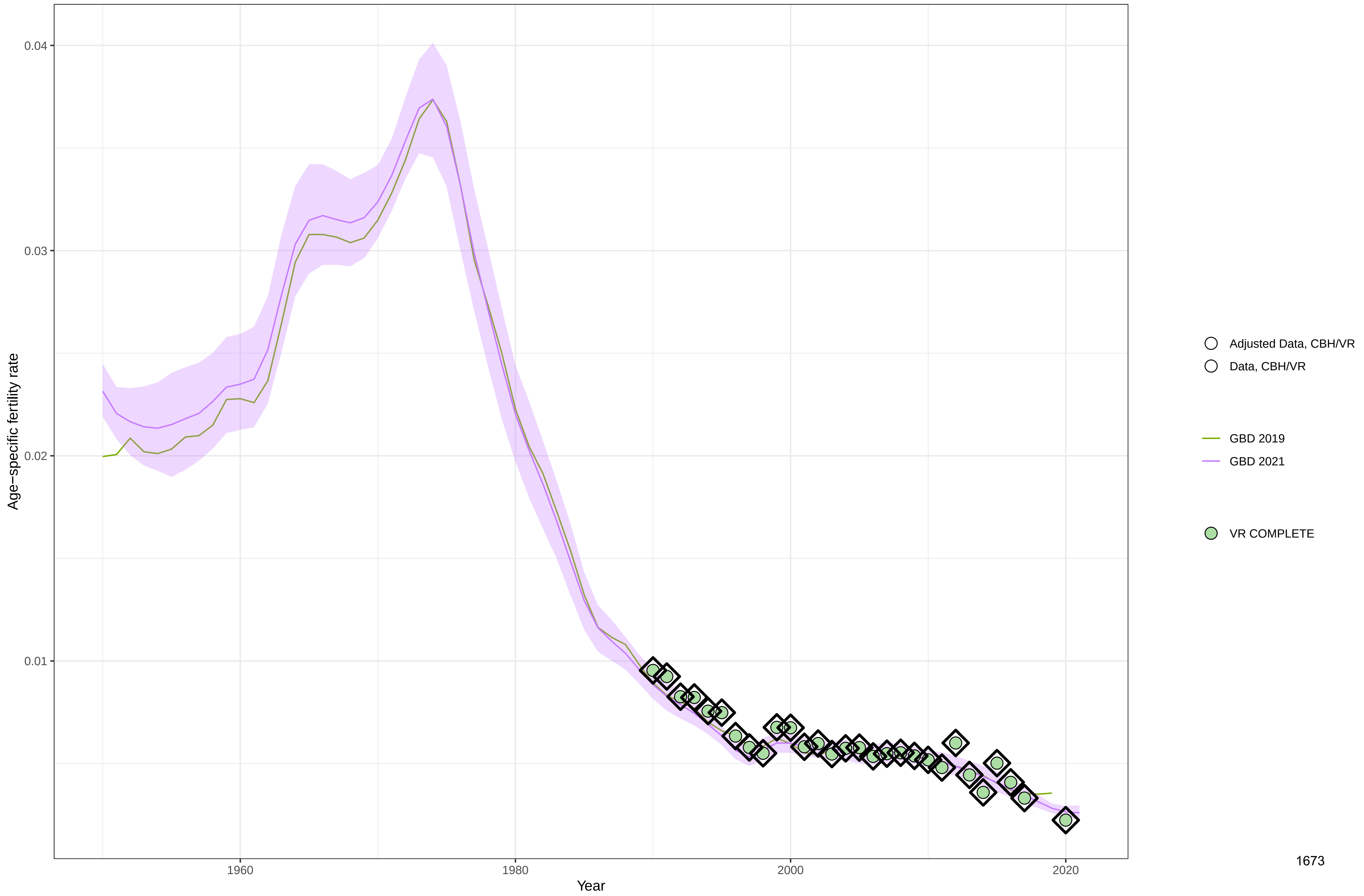

Age−specific fertility fitted trend lines, 1950−2021: Sardegna, Ages: 20−24
Data points enclosed with a diamond are reference sources

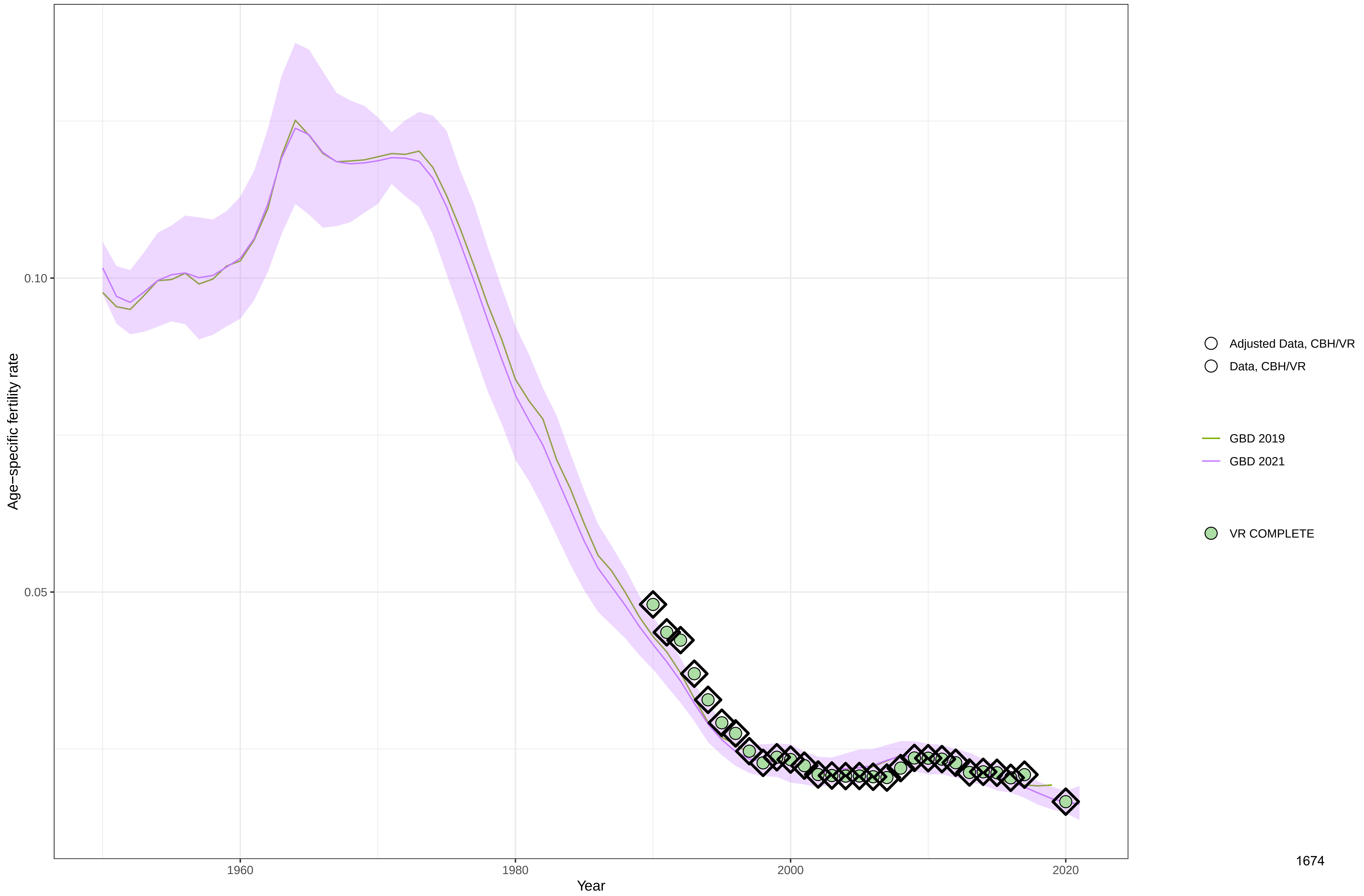

Age−specific fertility fitted trend lines, 1950−2021: Sardegna, Ages: 25−29
Data points enclosed with a diamond are reference sources

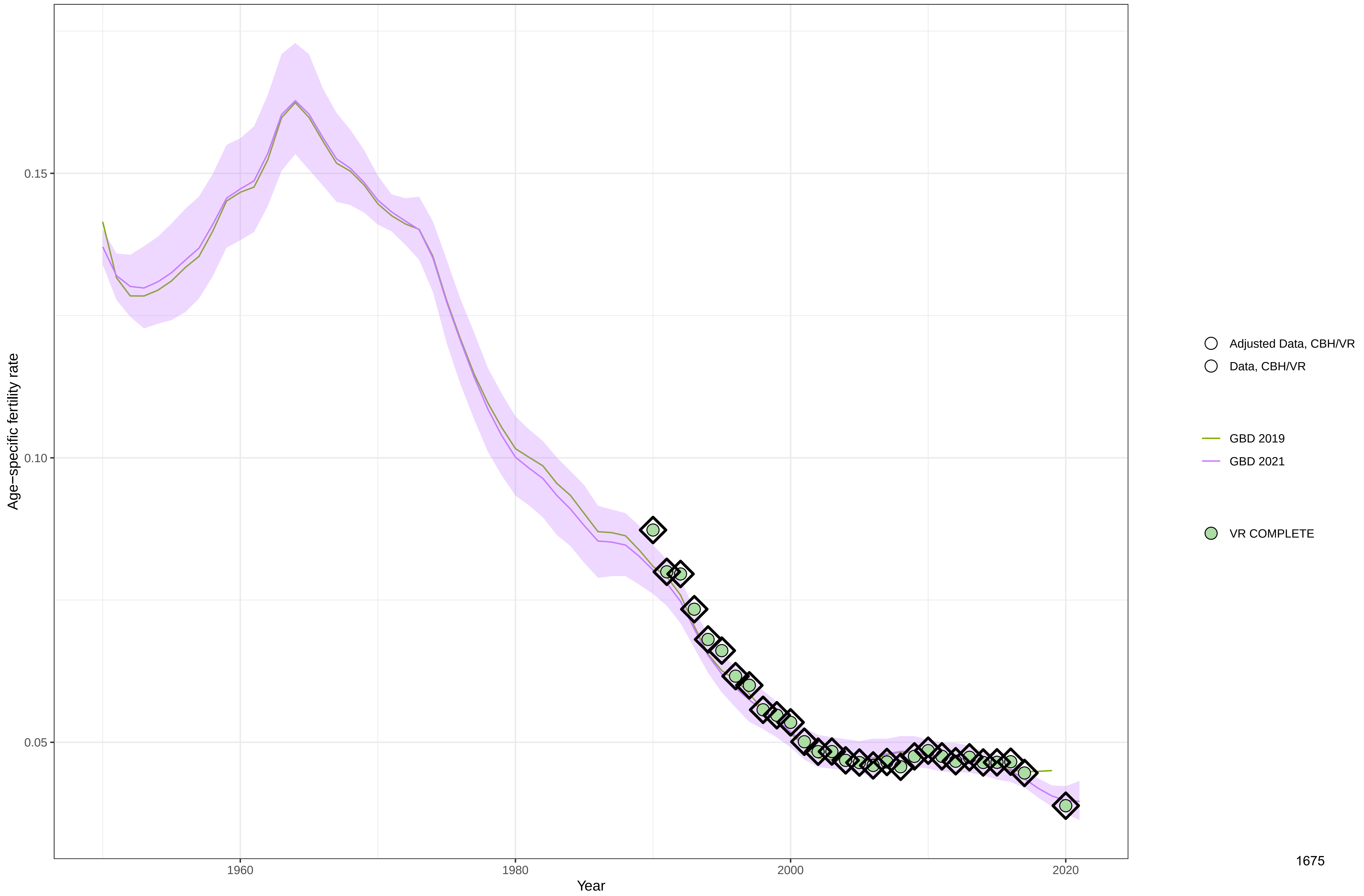

Age−specific fertility fitted trend lines, 1950−2021: Sardegna, Ages: 30−34
Data points enclosed with a diamond are reference sources

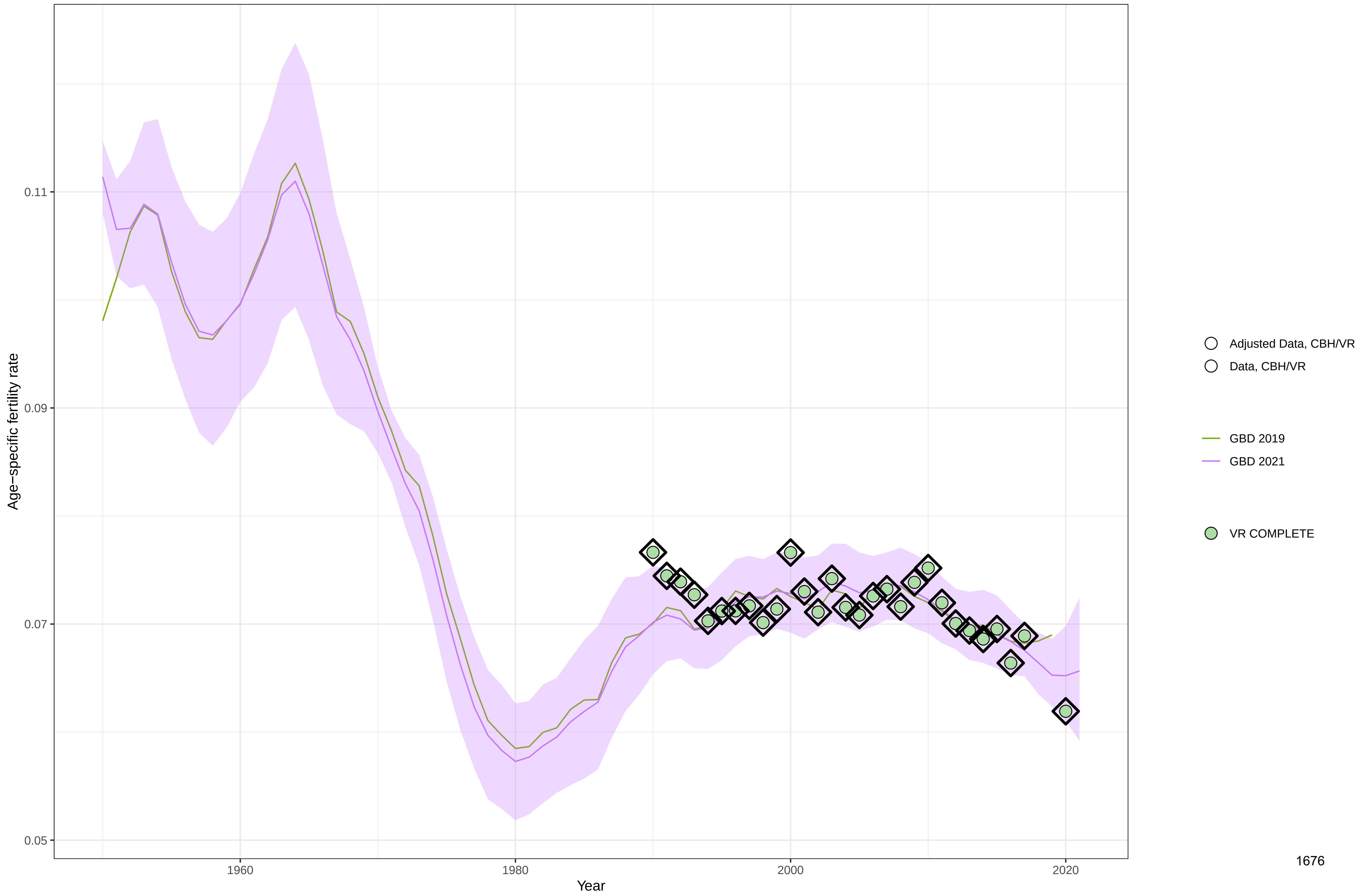

Age−specific fertility fitted trend lines, 1950−2021: Sardegna, Ages: 35−39
Data points enclosed with a diamond are reference sources

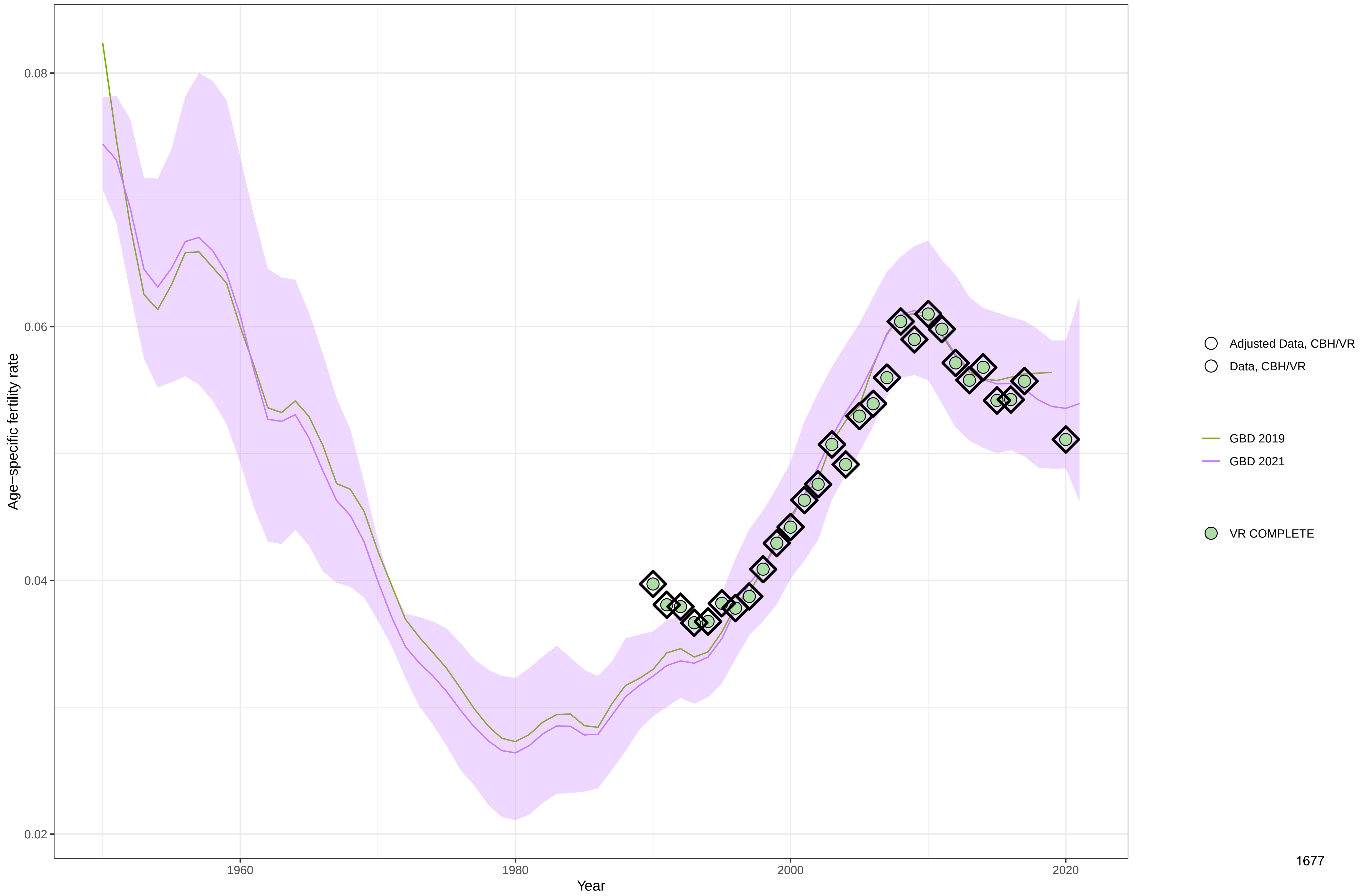

Age−specific fertility fitted trend lines, 1950−2021: Sardegna, Ages: 40−44
Data points enclosed with a diamond are reference sources

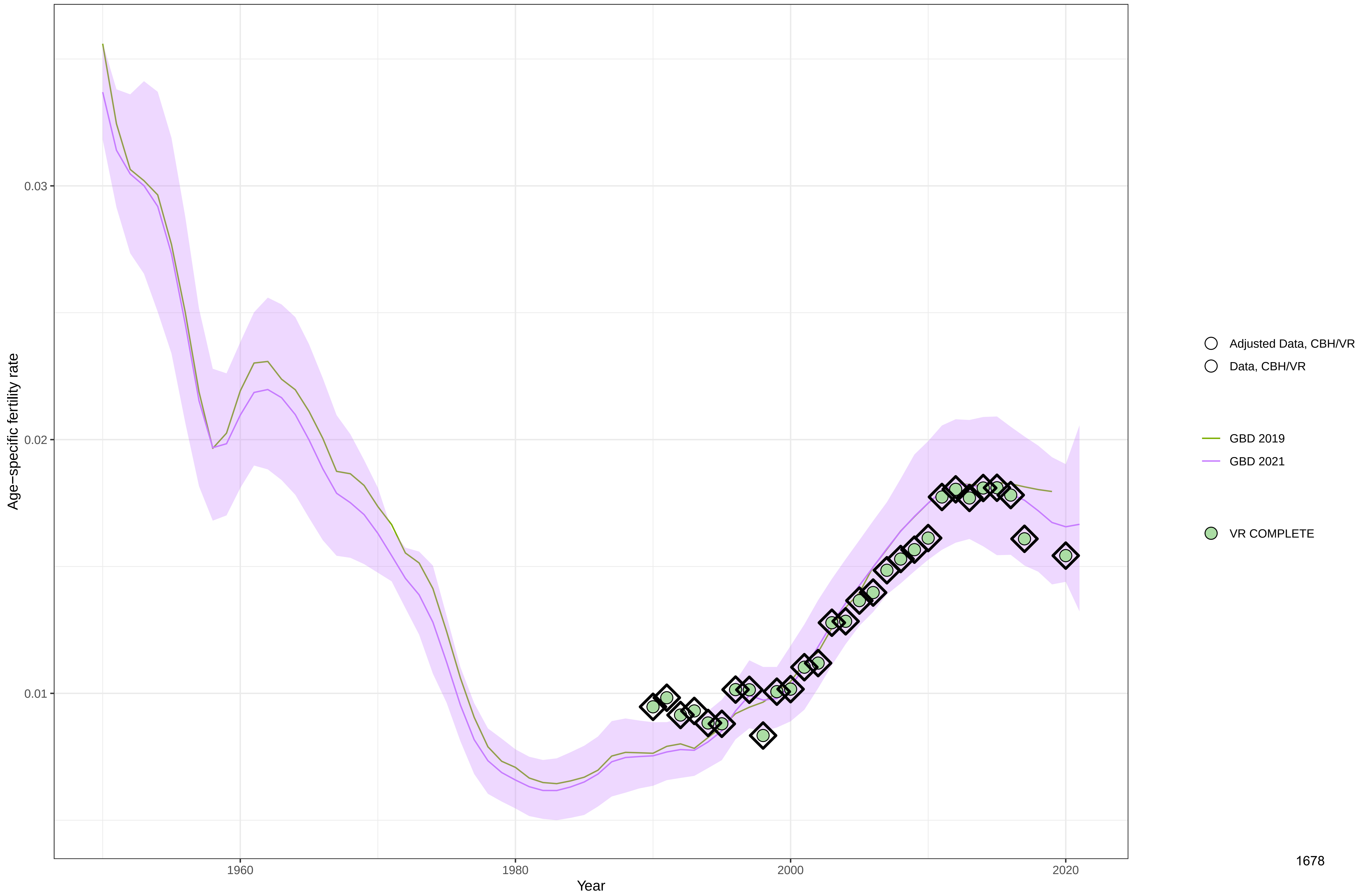

Age−specific fertility fitted trend lines, 1950−2021: Sardegna, Ages: 45−49
Data points enclosed with a diamond are reference sources

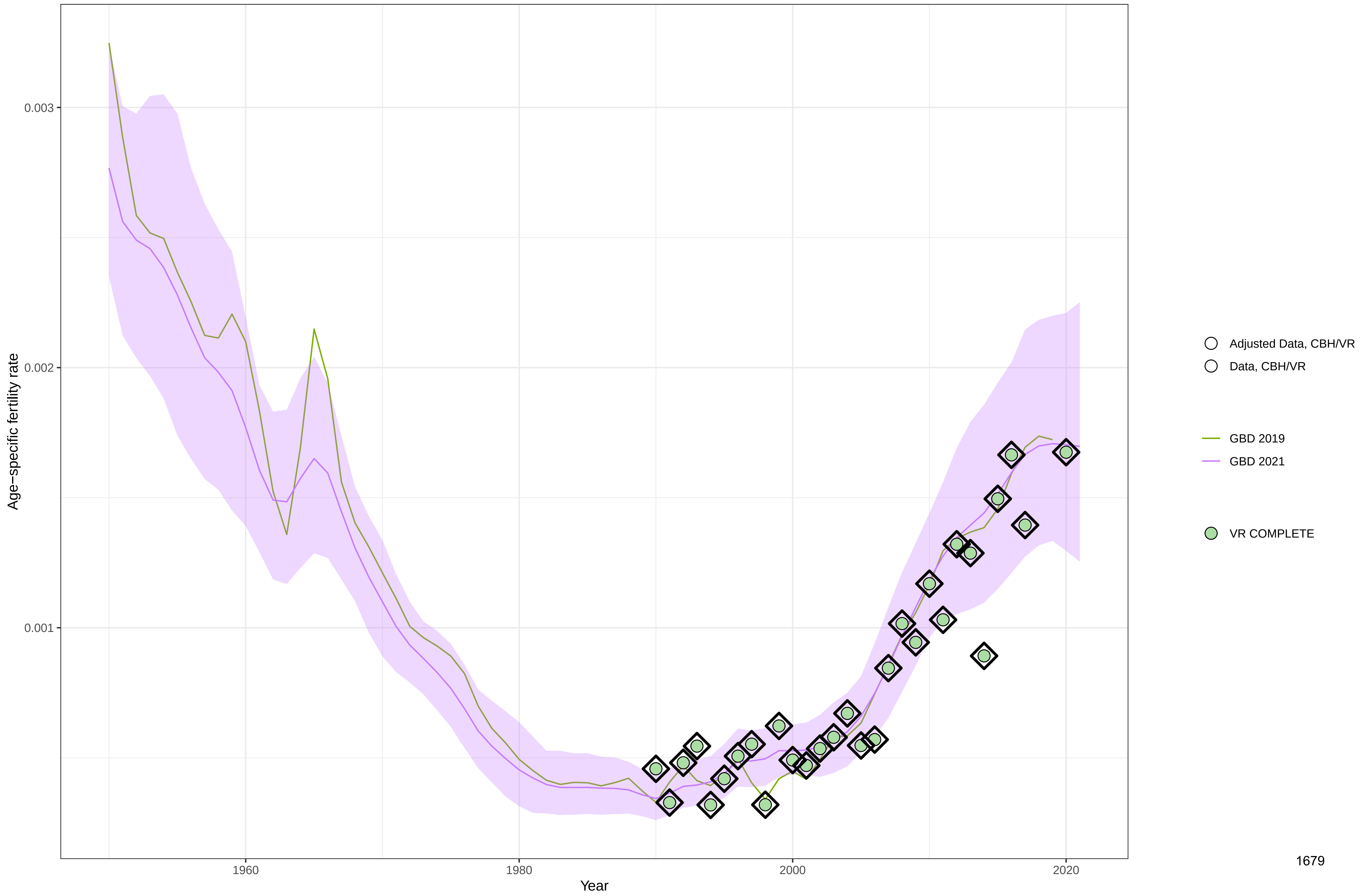

Age−specific fertility fitted trend lines, 1950−2021: Sardegna, Ages: 50−54
Data points enclosed with a diamond are reference sources

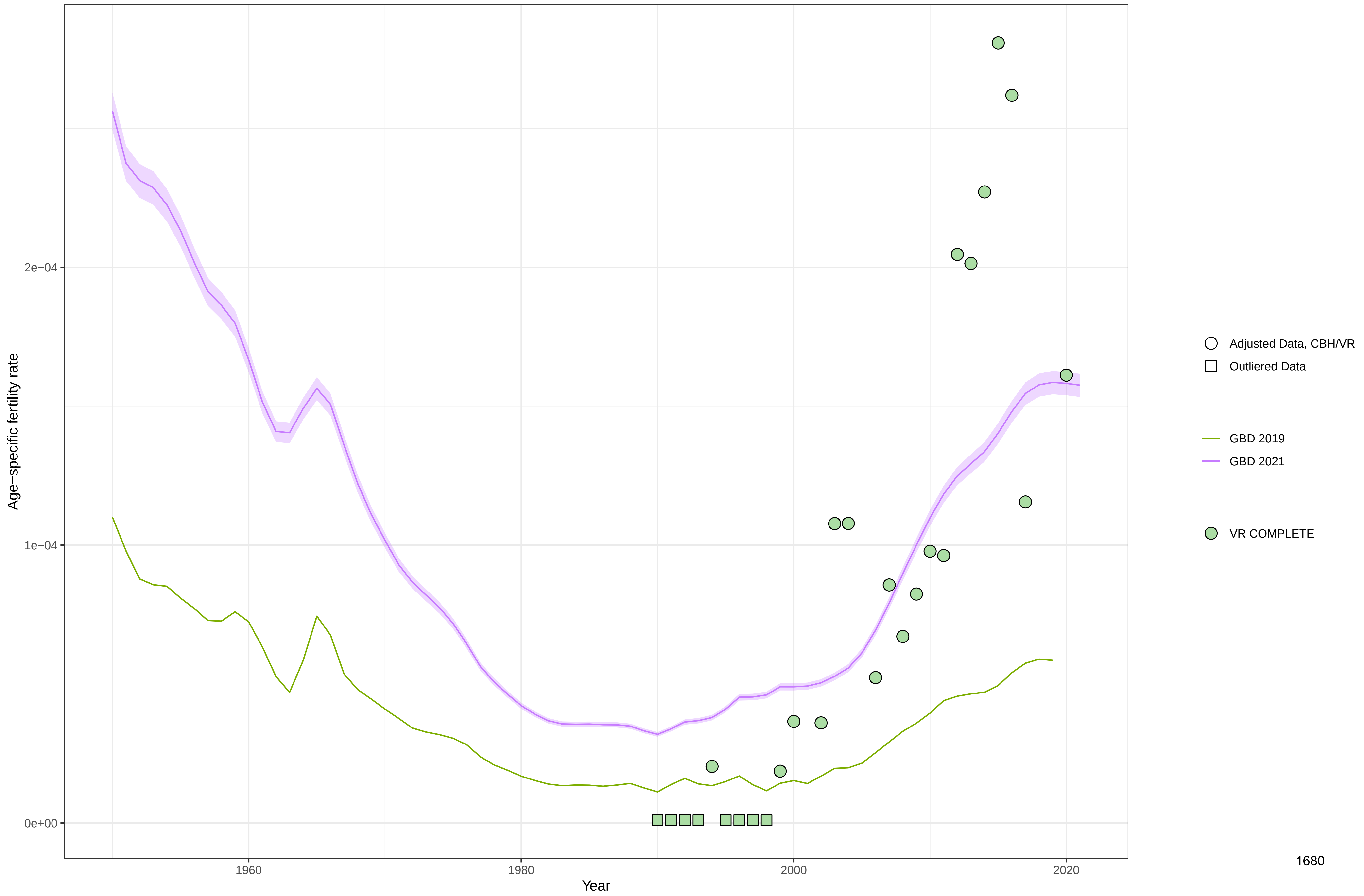

Total fertility rate fitted trend lines, 1950−2021: Sardegna TFR
Data points enclosed with a diamond are reference sources

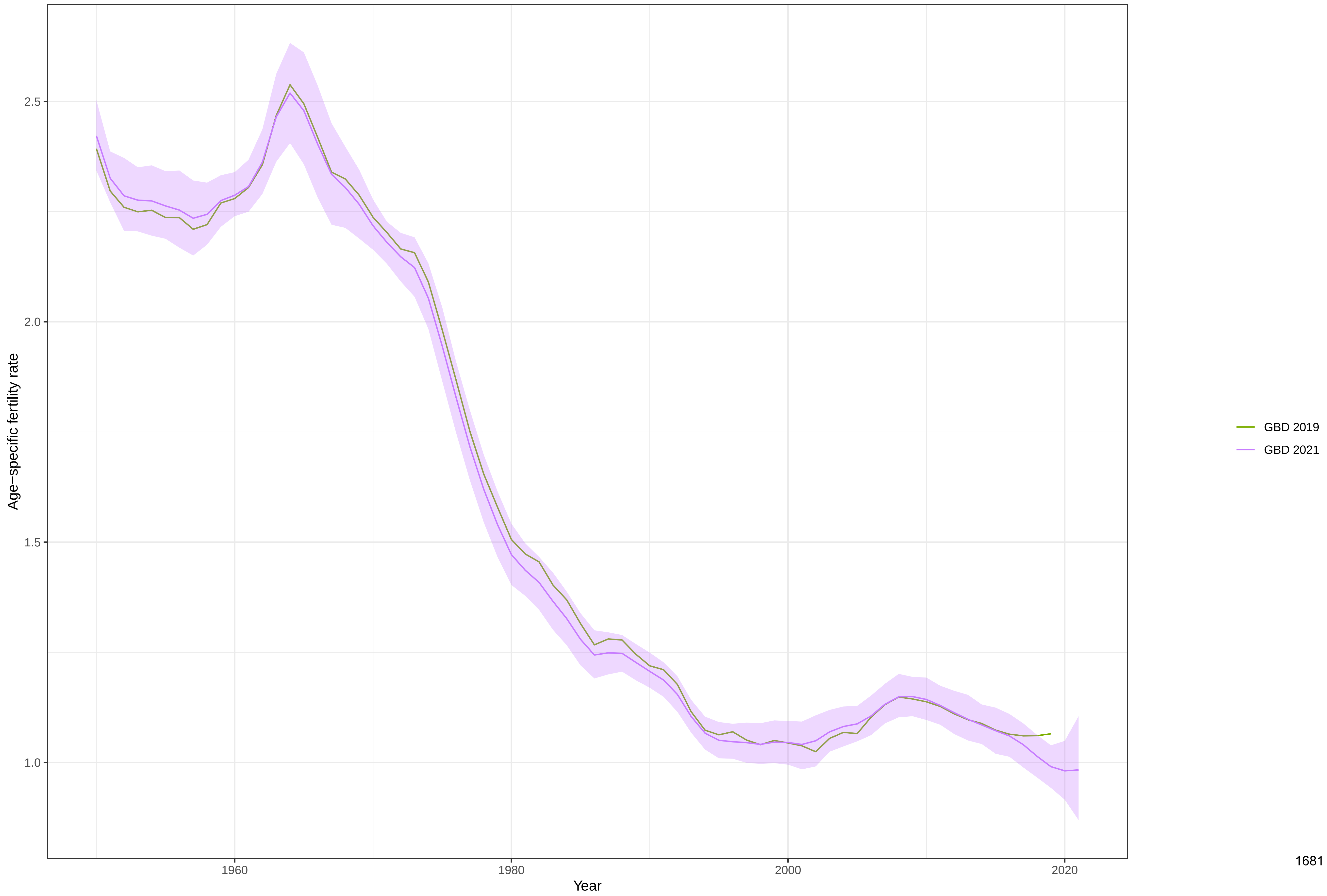

Age−specific fertility fitted trend lines, 1950−2021: Sicilia, Ages: 10−14
Data points enclosed with a diamond are reference sources

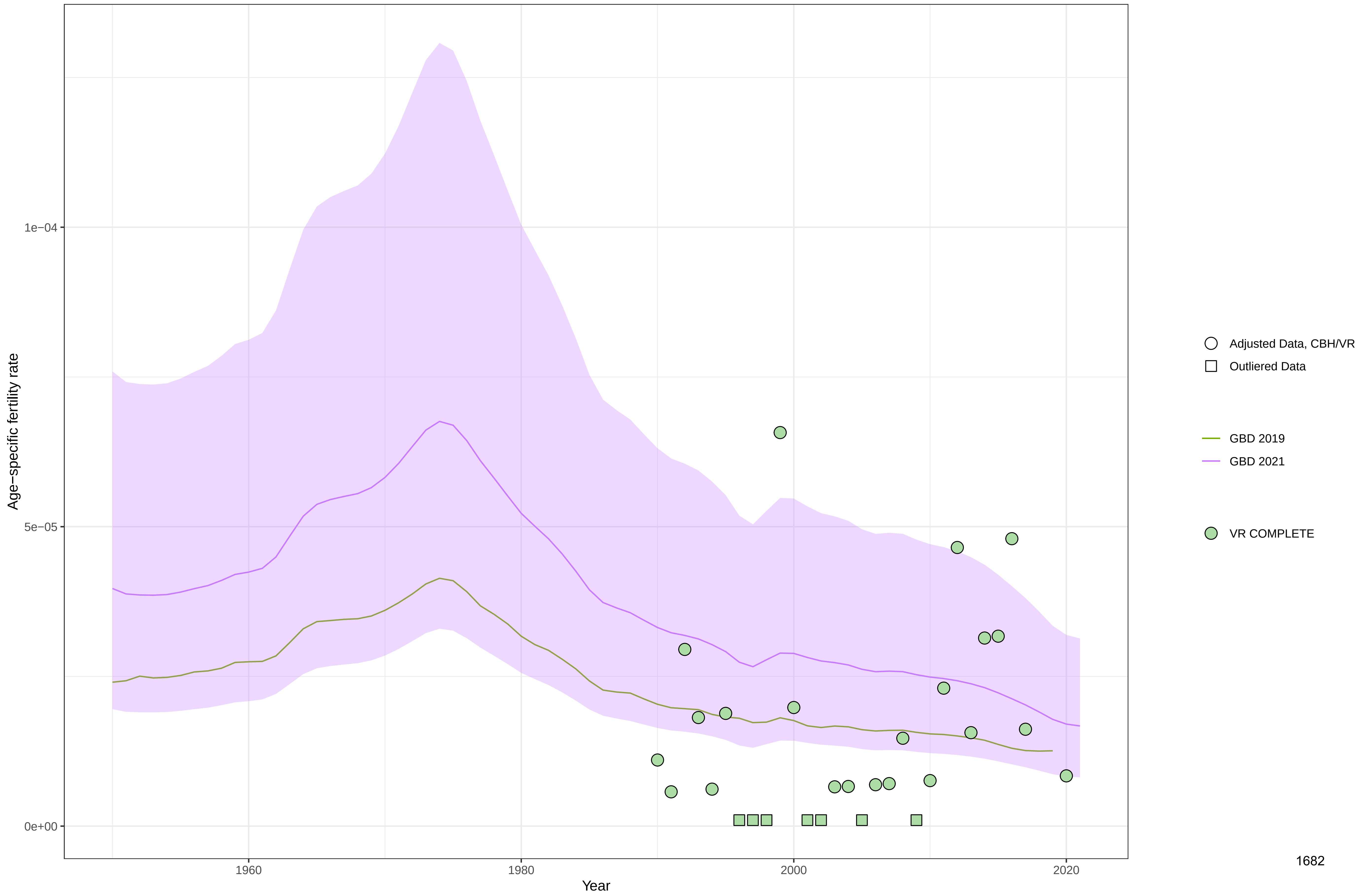

Age−specific fertility fitted trend lines, 1950−2021: Sicilia, Ages: 15−19
Data points enclosed with a diamond are reference sources

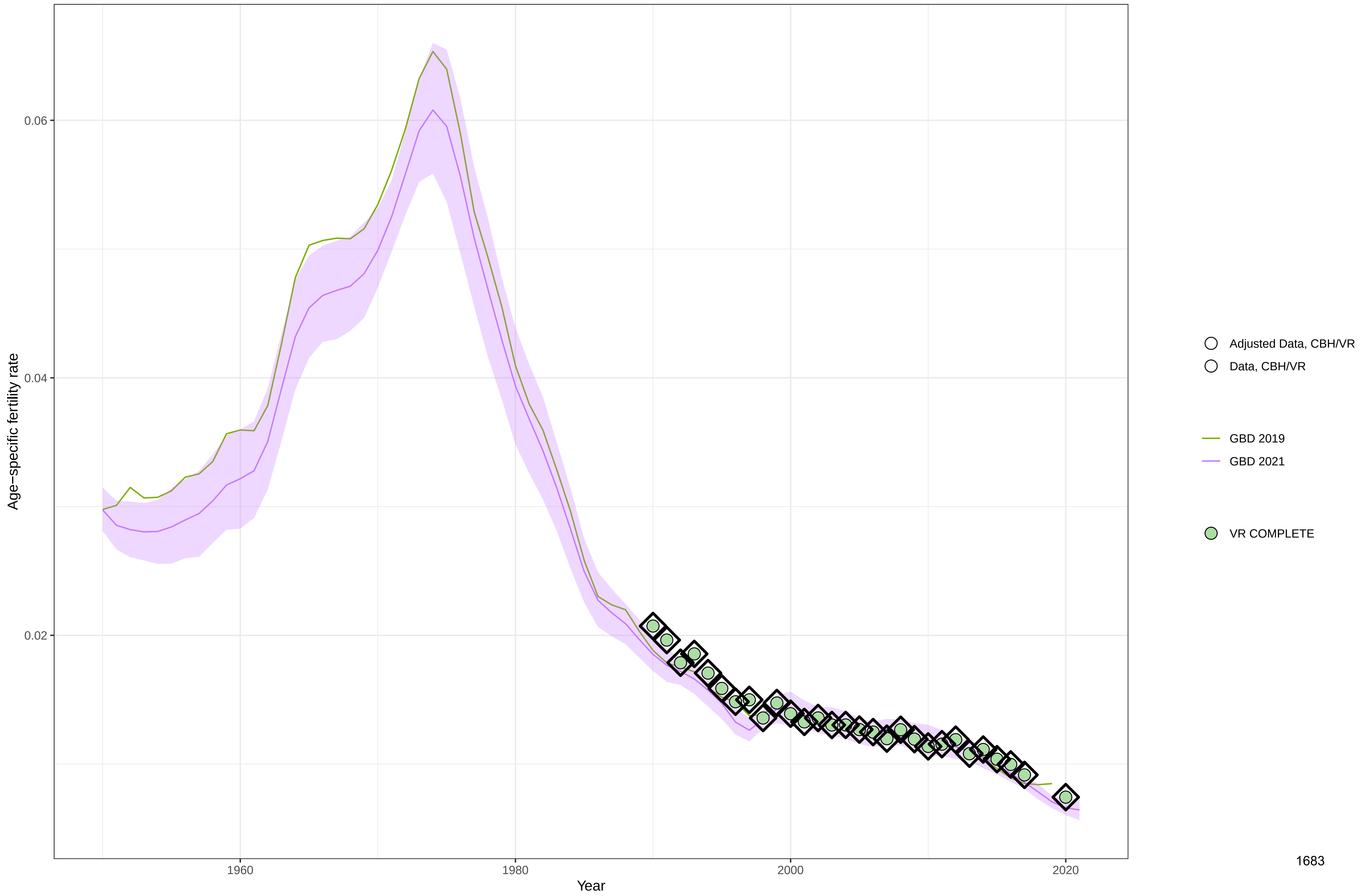

1683

Age−specific fertility fitted trend lines, 1950−2021: Sicilia, Ages: 20−24
Data points enclosed with a diamond are reference sources

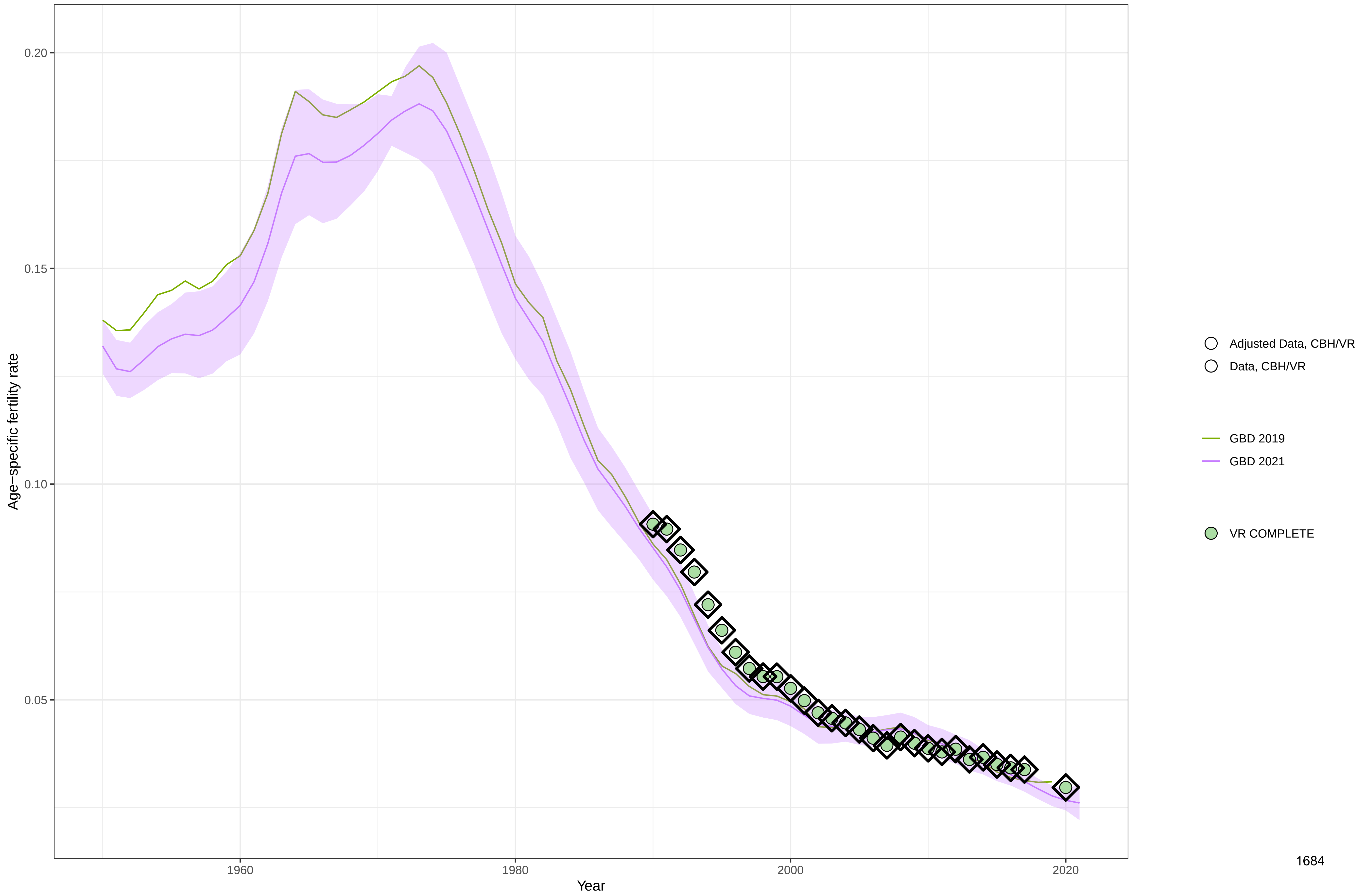

Age−specific fertility fitted trend lines, 1950−2021: Sicilia, Ages: 25−29
Data points enclosed with a diamond are reference sources

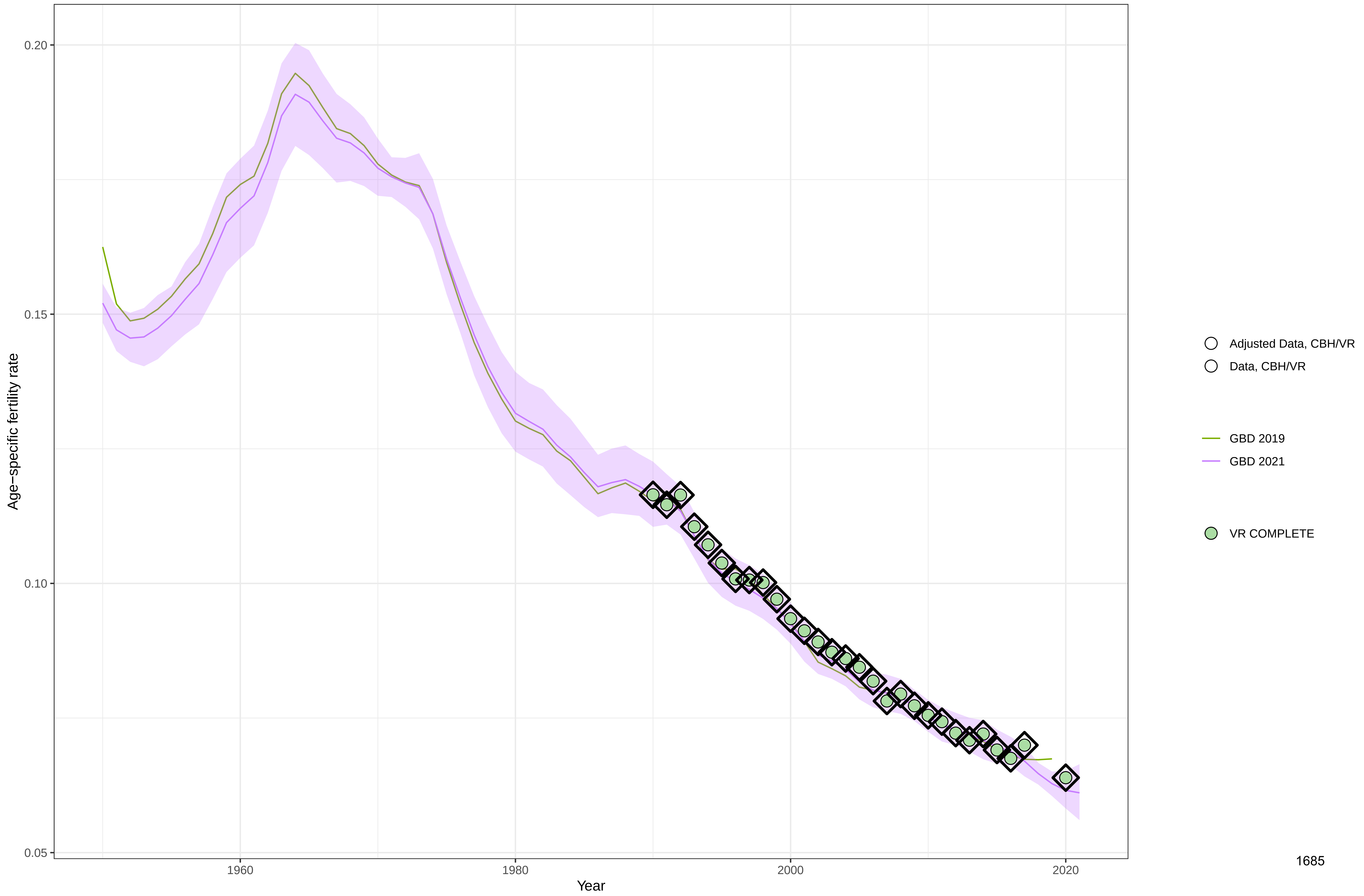

Age−specific fertility fitted trend lines, 1950−2021: Sicilia, Ages: 30−34
Data points enclosed with a diamond are reference sources

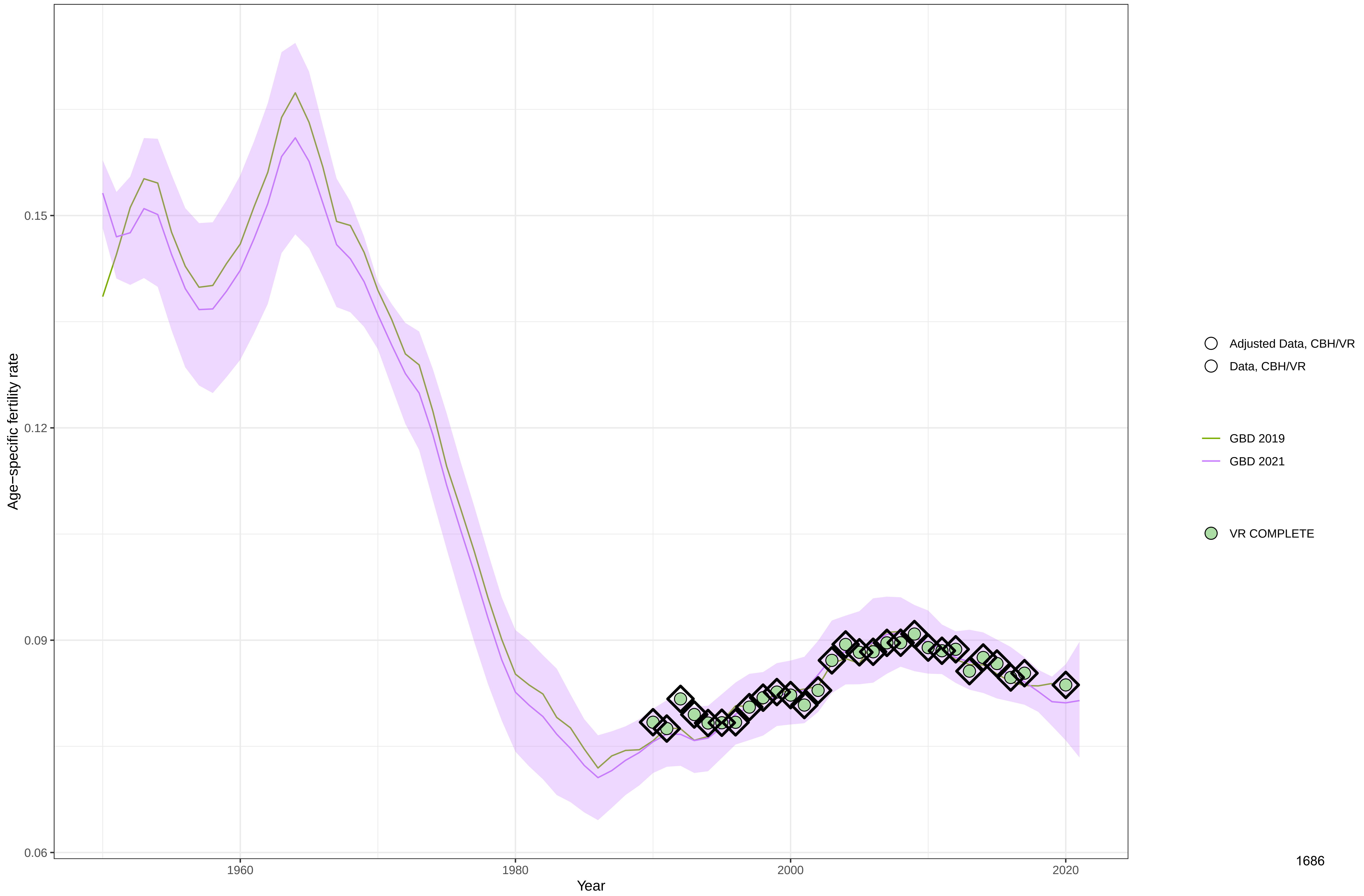

Age−specific fertility fitted trend lines, 1950−2021: Sicilia, Ages: 35−39
Data points enclosed with a diamond are reference sources

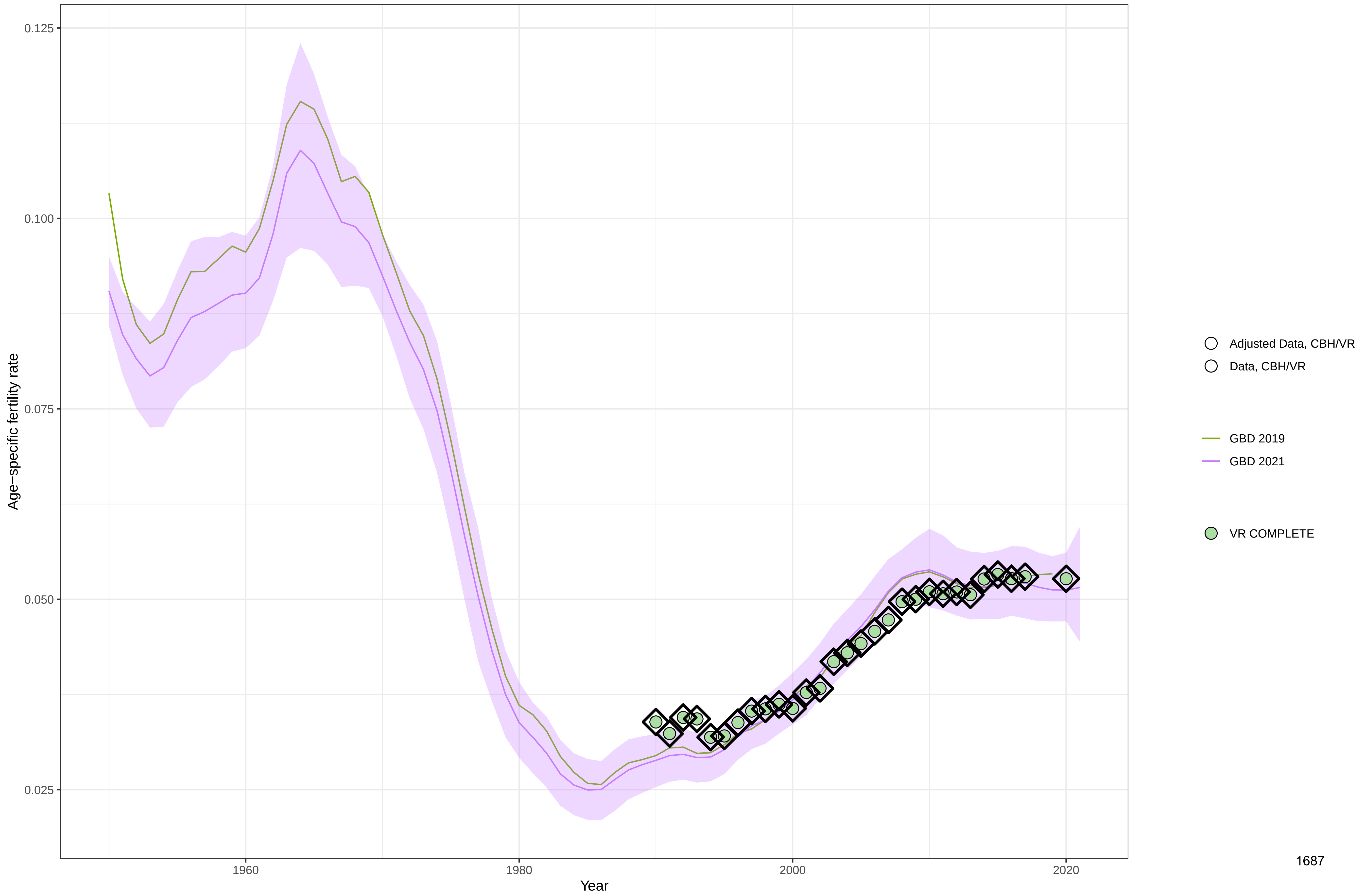

Age−specific fertility fitted trend lines, 1950−2021: Sicilia, Ages: 40−44
Data points enclosed with a diamond are reference sources

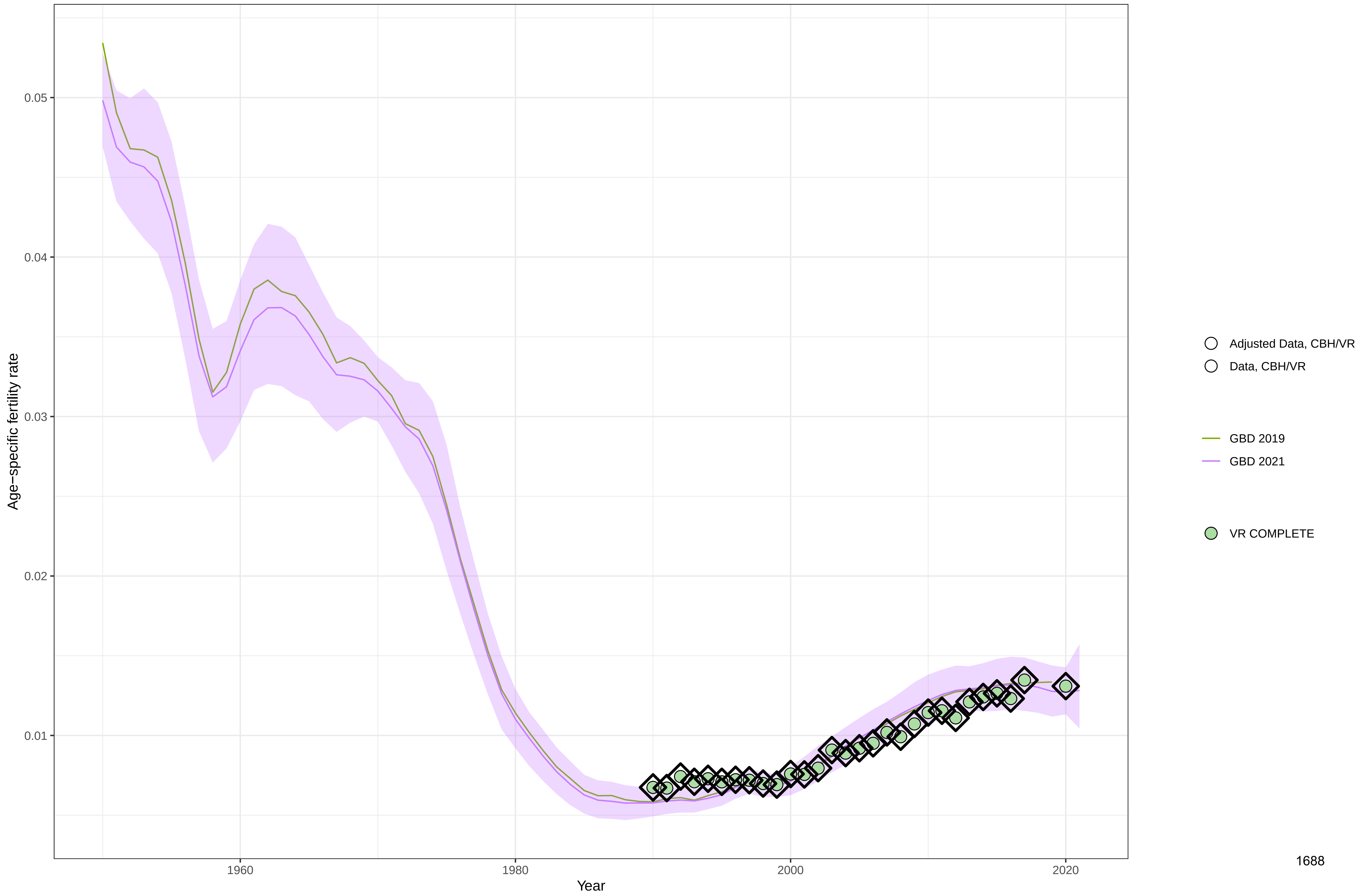

Age−specific fertility fitted trend lines, 1950−2021: Sicilia, Ages: 45−49
Data points enclosed with a diamond are reference sources

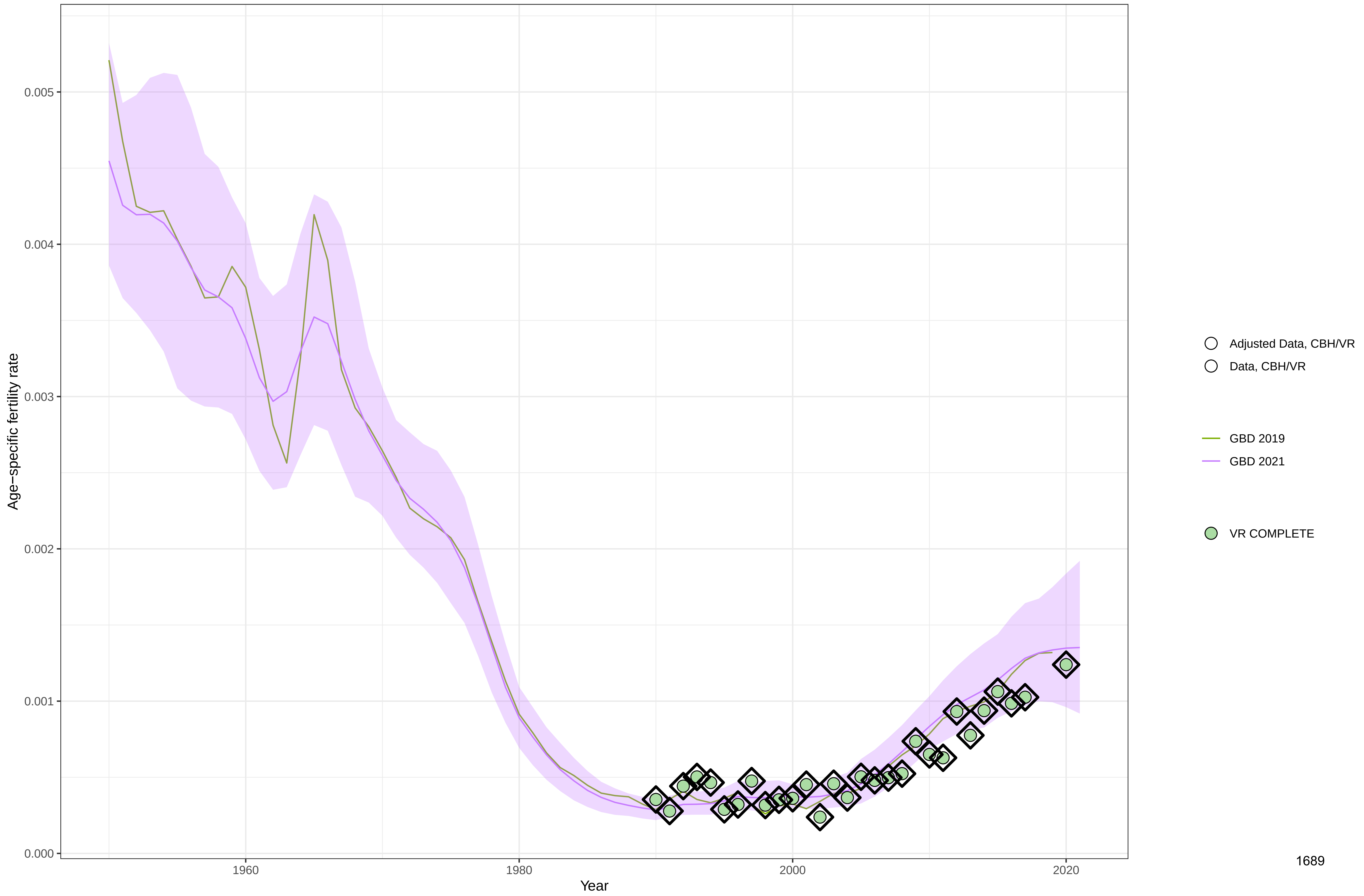

Age−specific fertility fitted trend lines, 1950−2021: Sicilia, Ages: 50−54
Data points enclosed with a diamond are reference sources

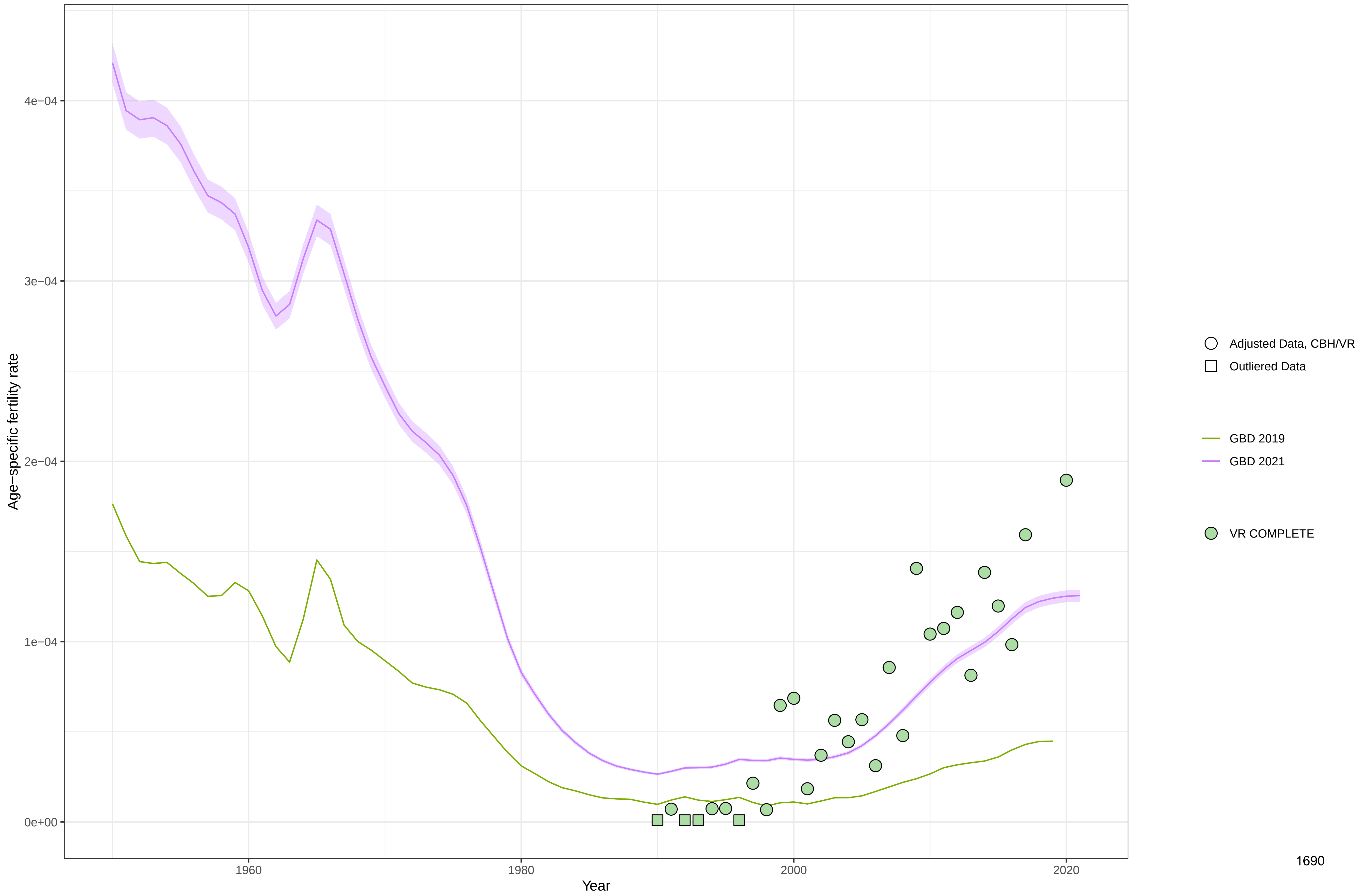

Total fertility rate fitted trend lines, 1950−2021: Sicilia TFR
Data points enclosed with a diamond are reference sources

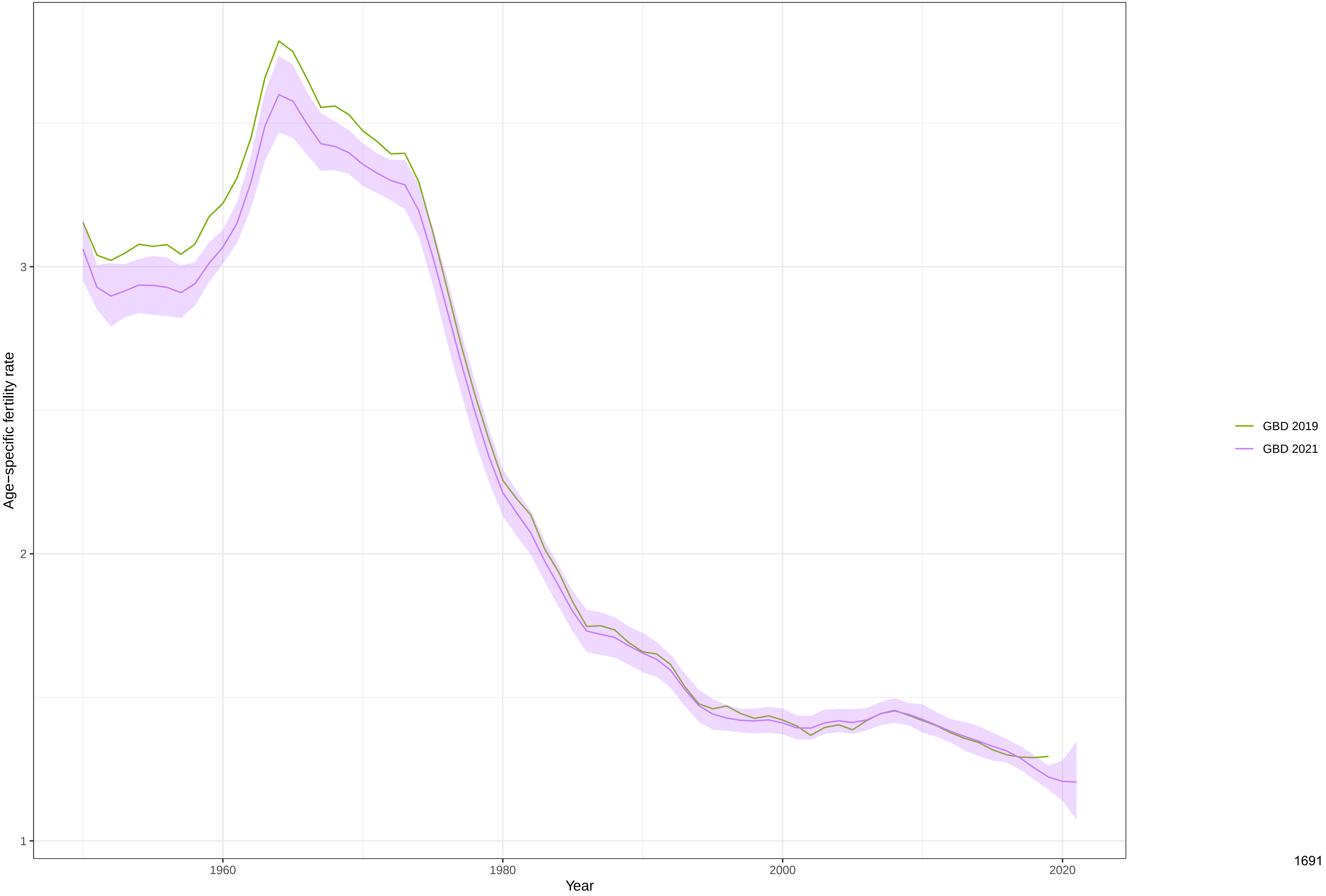

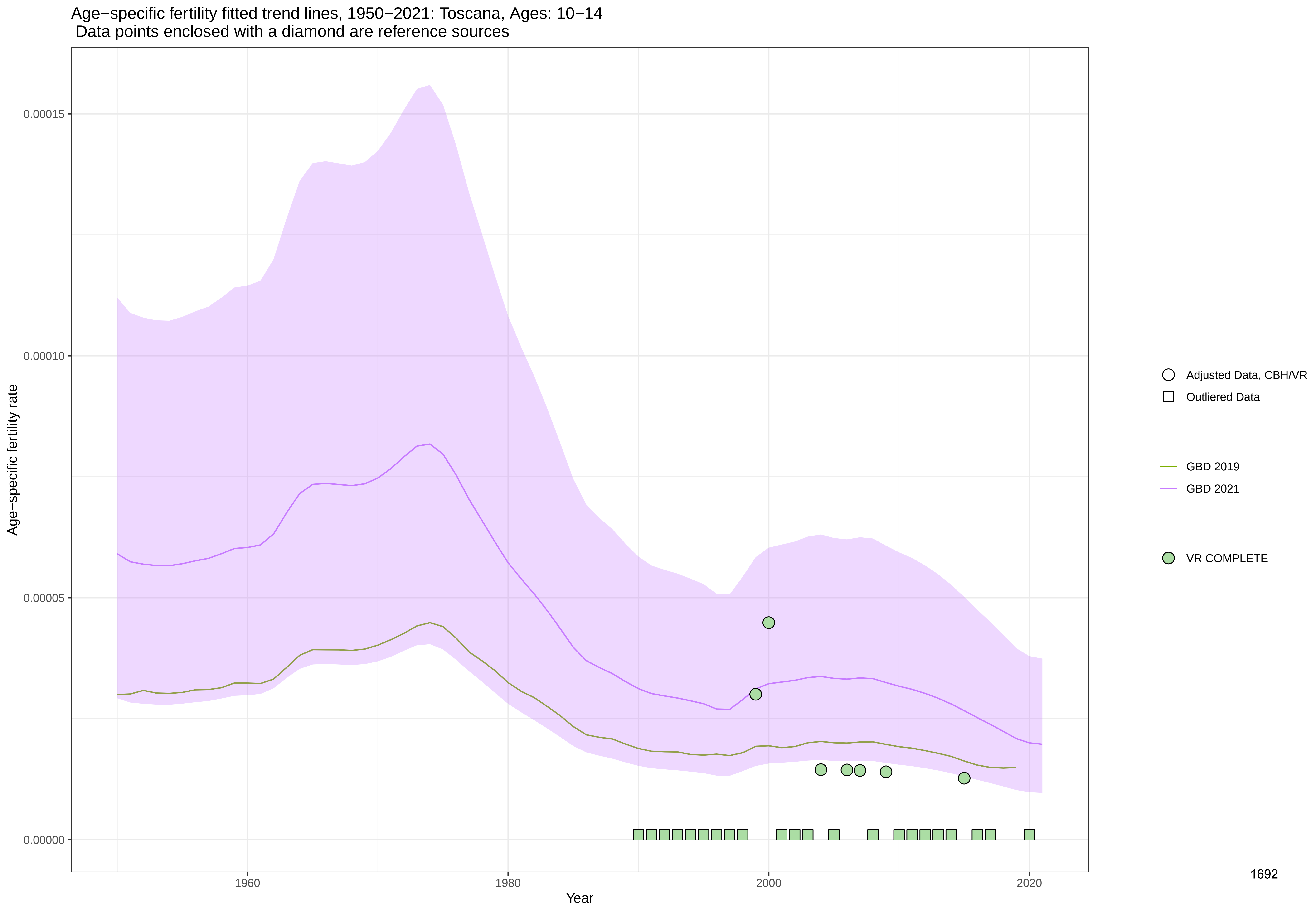

Age−specific fertility fitted trend lines, 1950−2021: Toscana, Ages: 10−14
Data points enclosed with a diamond are reference sources
0.00015
0.00010
0.00005
0.00000
Age−specific fertility rate
1960
1980
2000
2020
Year
Adjusted Data, CBH/VR
Outliered Data
GBD 2019
GBD 2021
VR COMPLETE

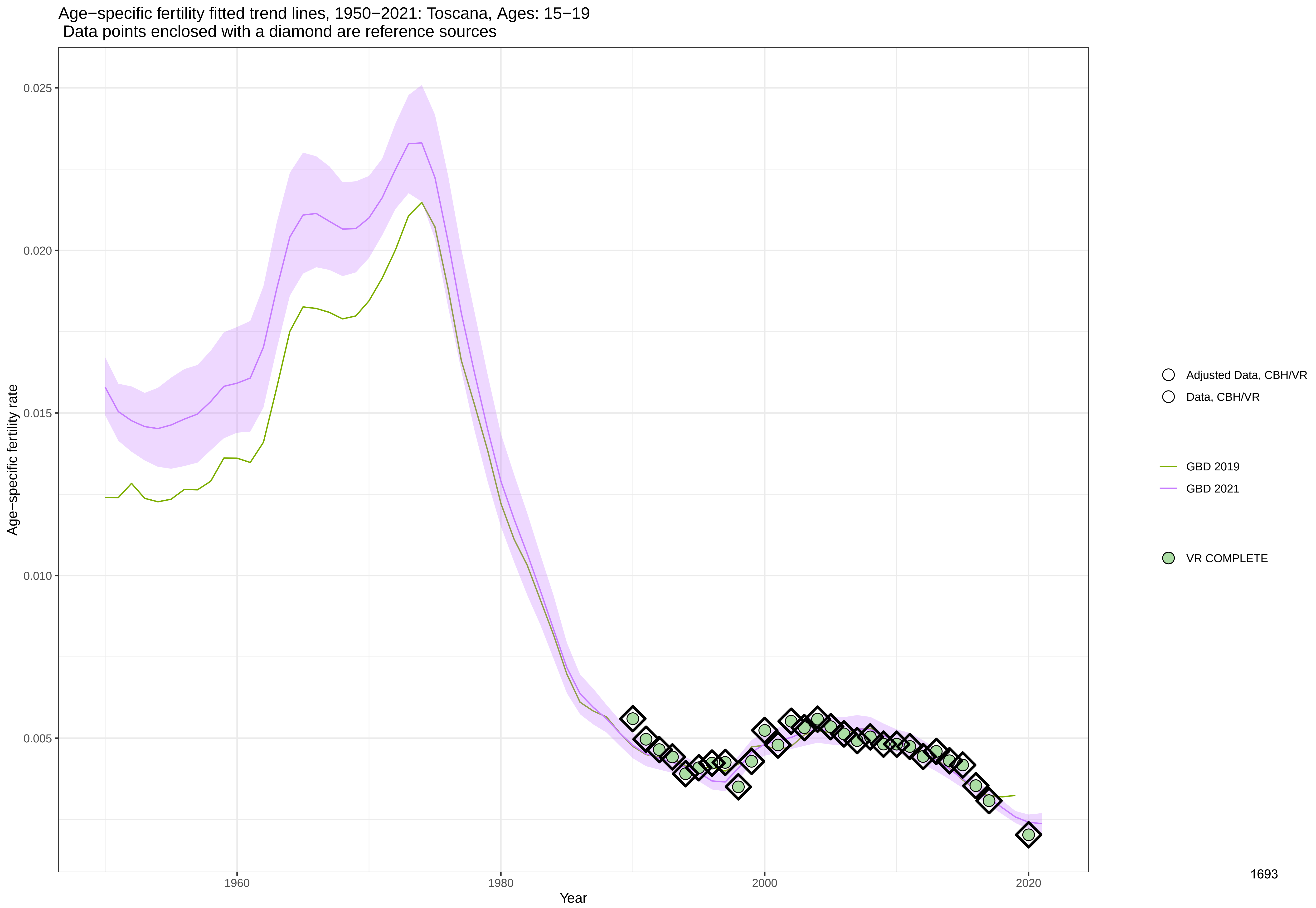

Age−specific fertility fitted trend lines, 1950−2021: Toscana, Ages: 15−19
Data points enclosed with a diamond are reference sources
Age−specific fertility rate
0.025
0.020
0.015
0.010
0.005
1960
1980
2000
2020
Year
Adjusted Data, CBH/VR
Data, CBH/VR
GBD 2019
GBD 2021
VR COMPLETE

Age−specific fertility fitted trend lines, 1950−2021: Toscana, Ages: 20−24
Data points enclosed with a diamond are reference sources

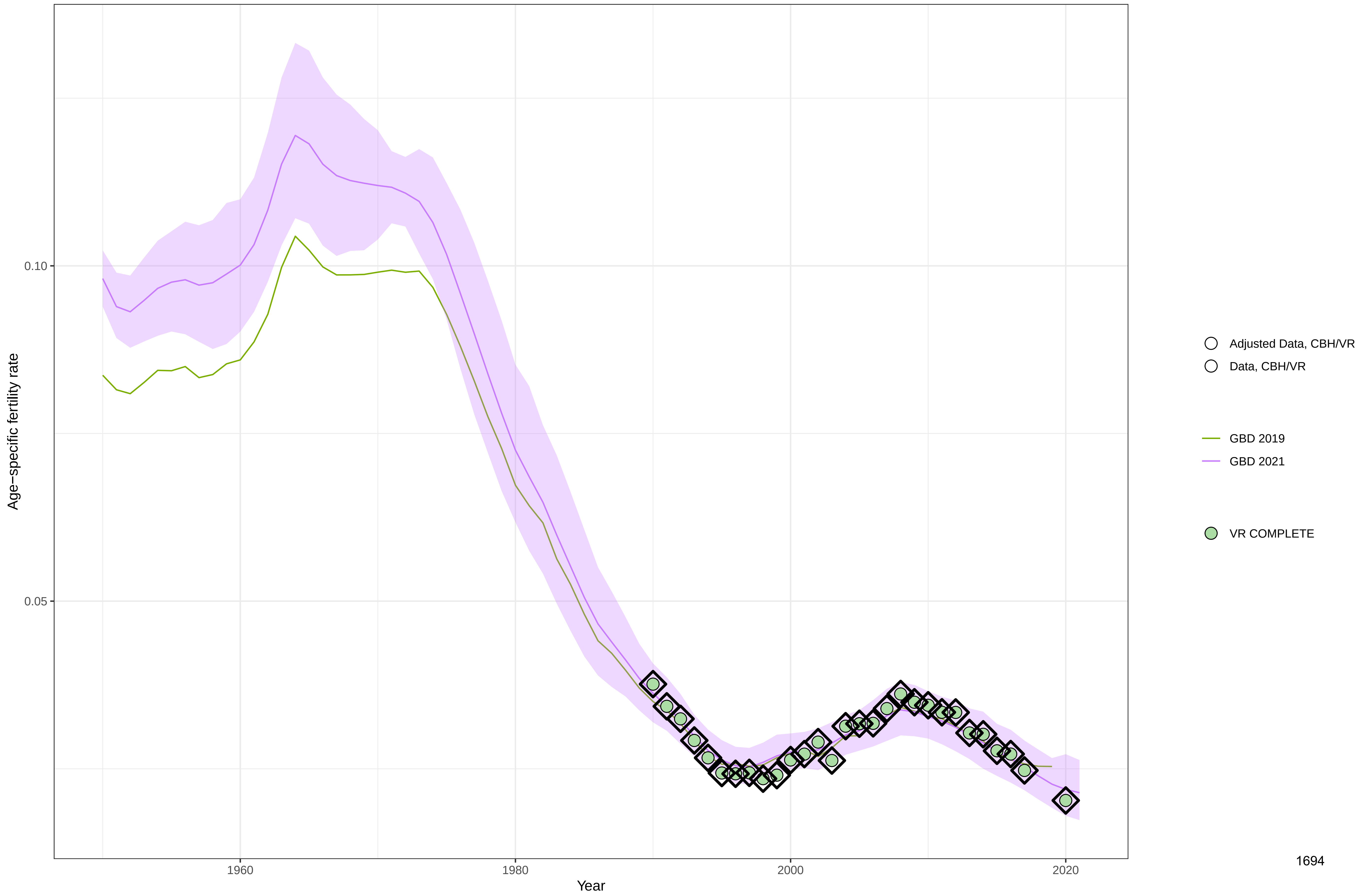

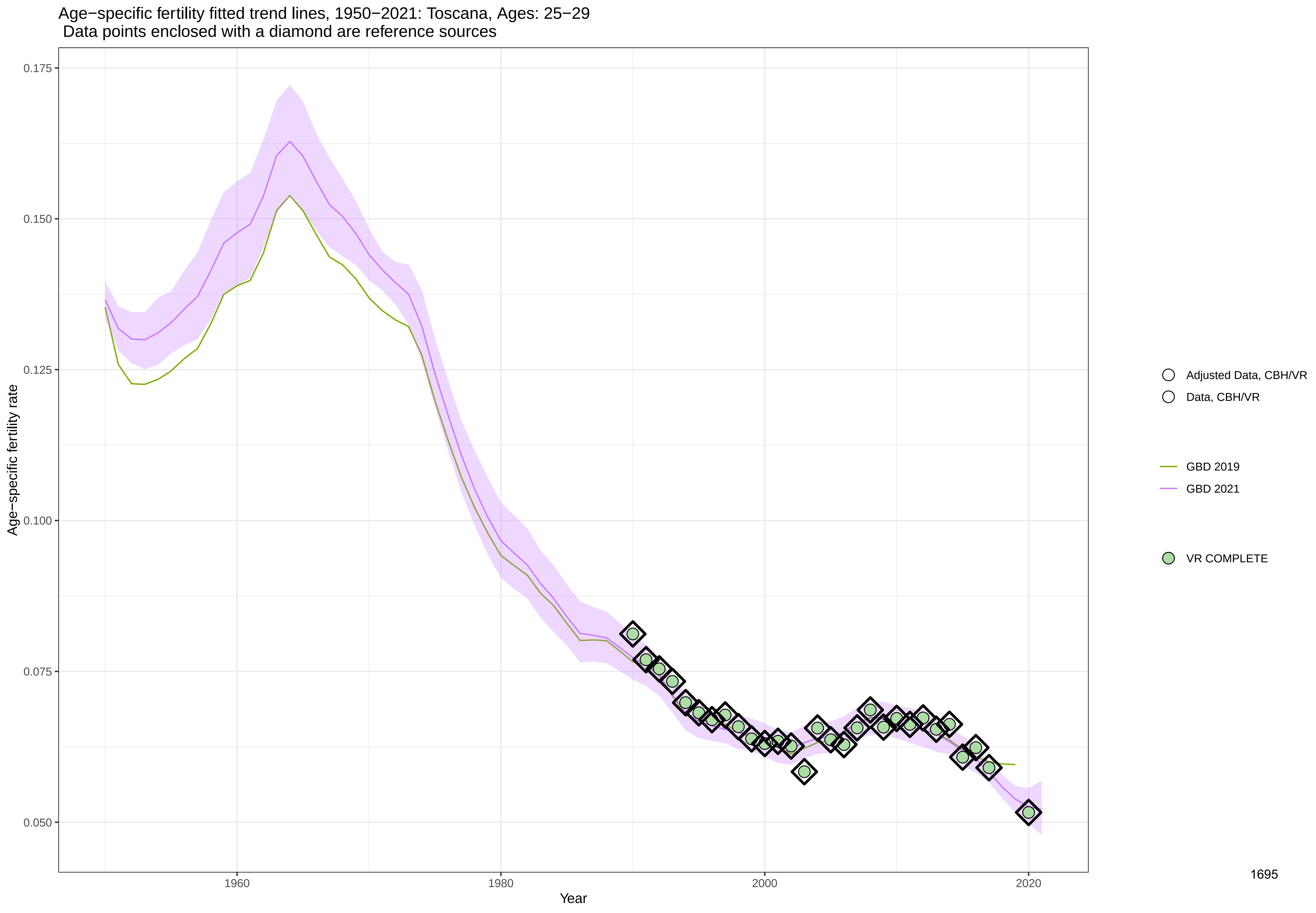

Age−specific fertility fitted trend lines, 1950−2021: Toscana, Ages: 25−29
Data points enclosed with a diamond are reference sources
Age−specific fertility rate
0.175
0.150
0.125
0.100
0.075
0.050
1960
1980
2000
2020
Year
Adjusted Data, CBH/VR
Data, CBH/VR
GBD 2019
GBD 2021
VR COMPLETE

Age−specific fertility fitted trend lines, 1950−2021: Toscana, Ages: 30−34
Data points enclosed with a diamond are reference sources

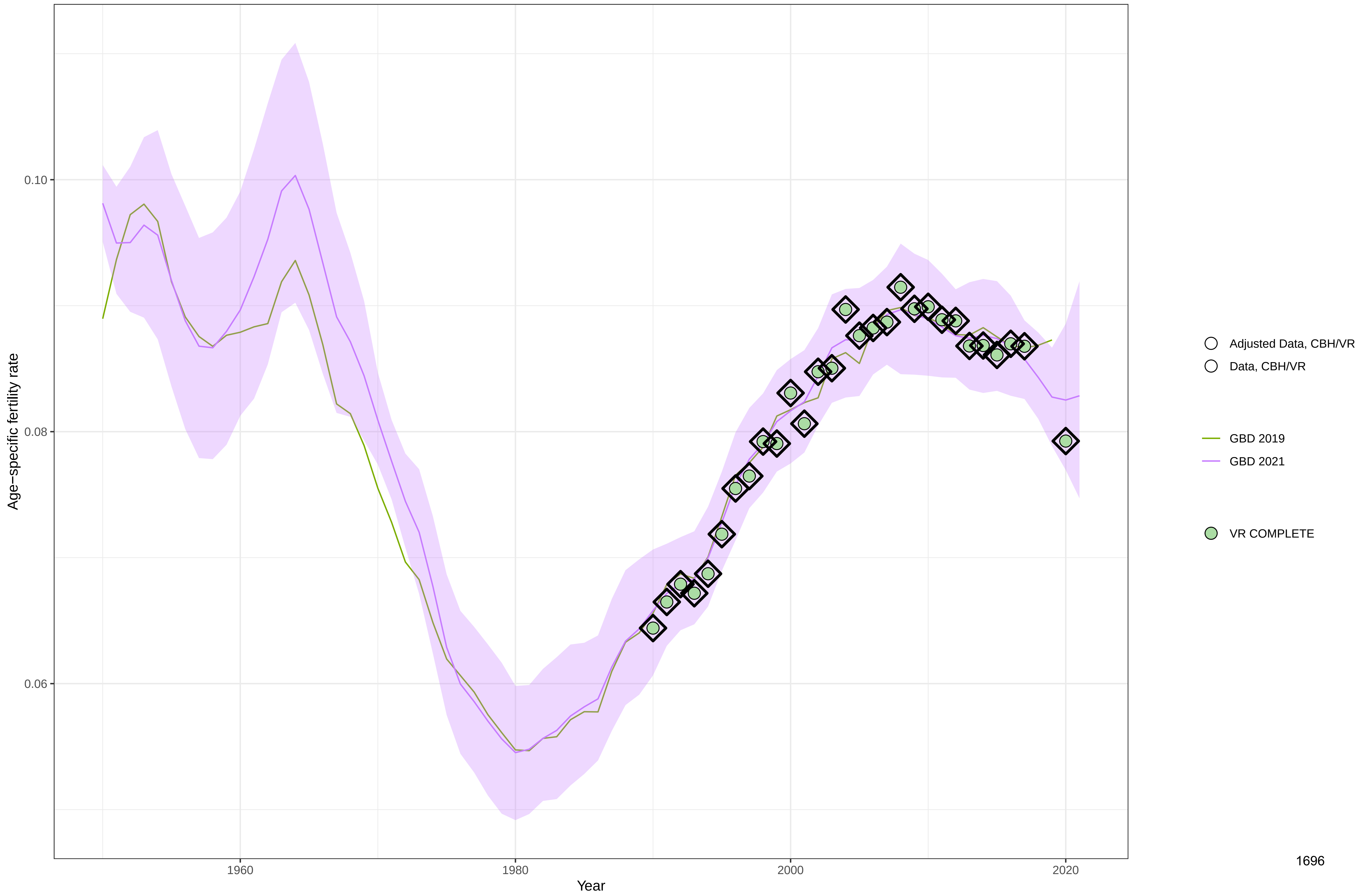

Age−specific fertility fitted trend lines, 1950−2021: Toscana, Ages: 35−39
Data points enclosed with a diamond are reference sources

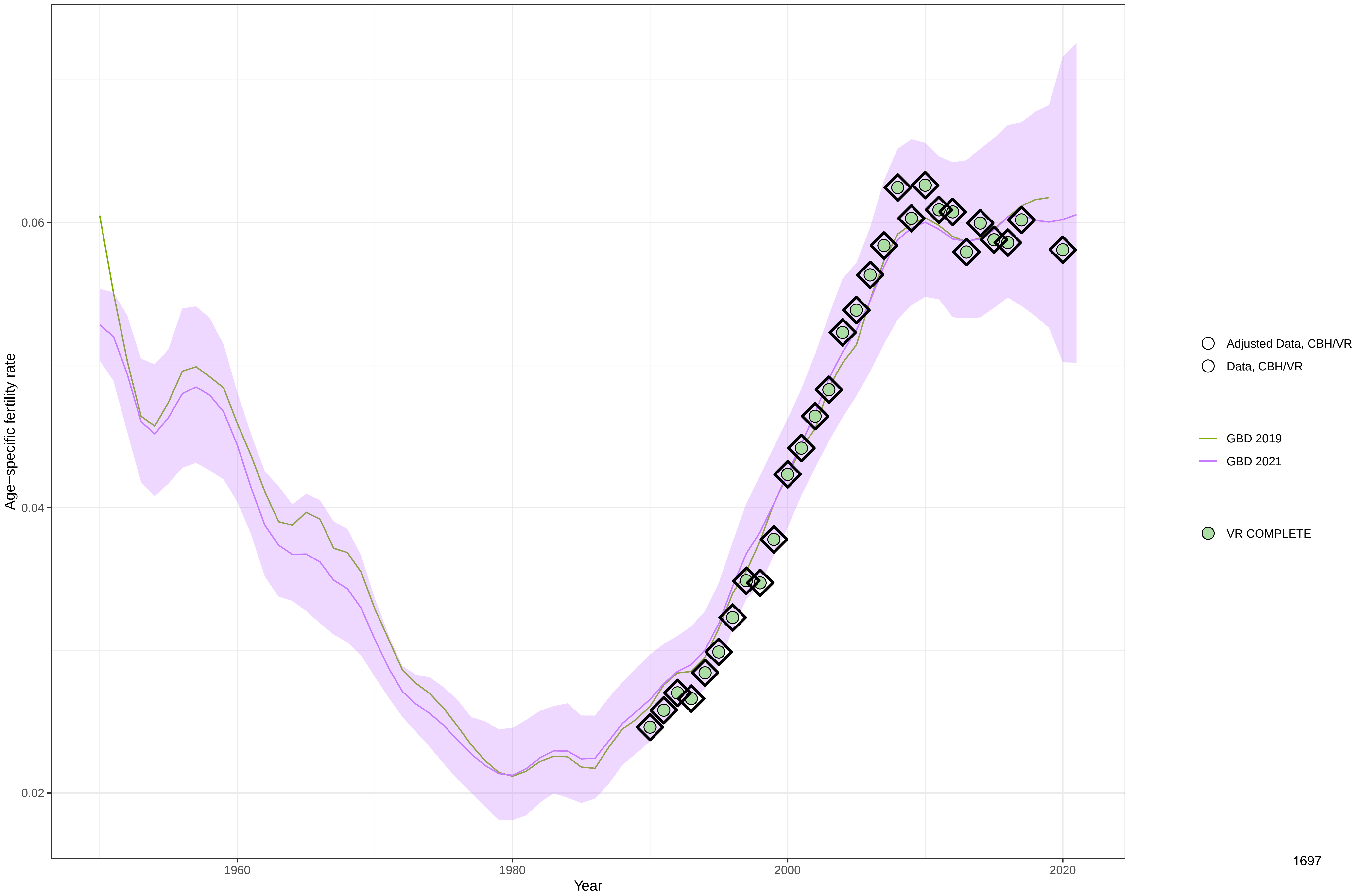

Age−specific fertility fitted trend lines, 1950−2021: Toscana, Ages: 40−44
Data points enclosed with a diamond are reference sources

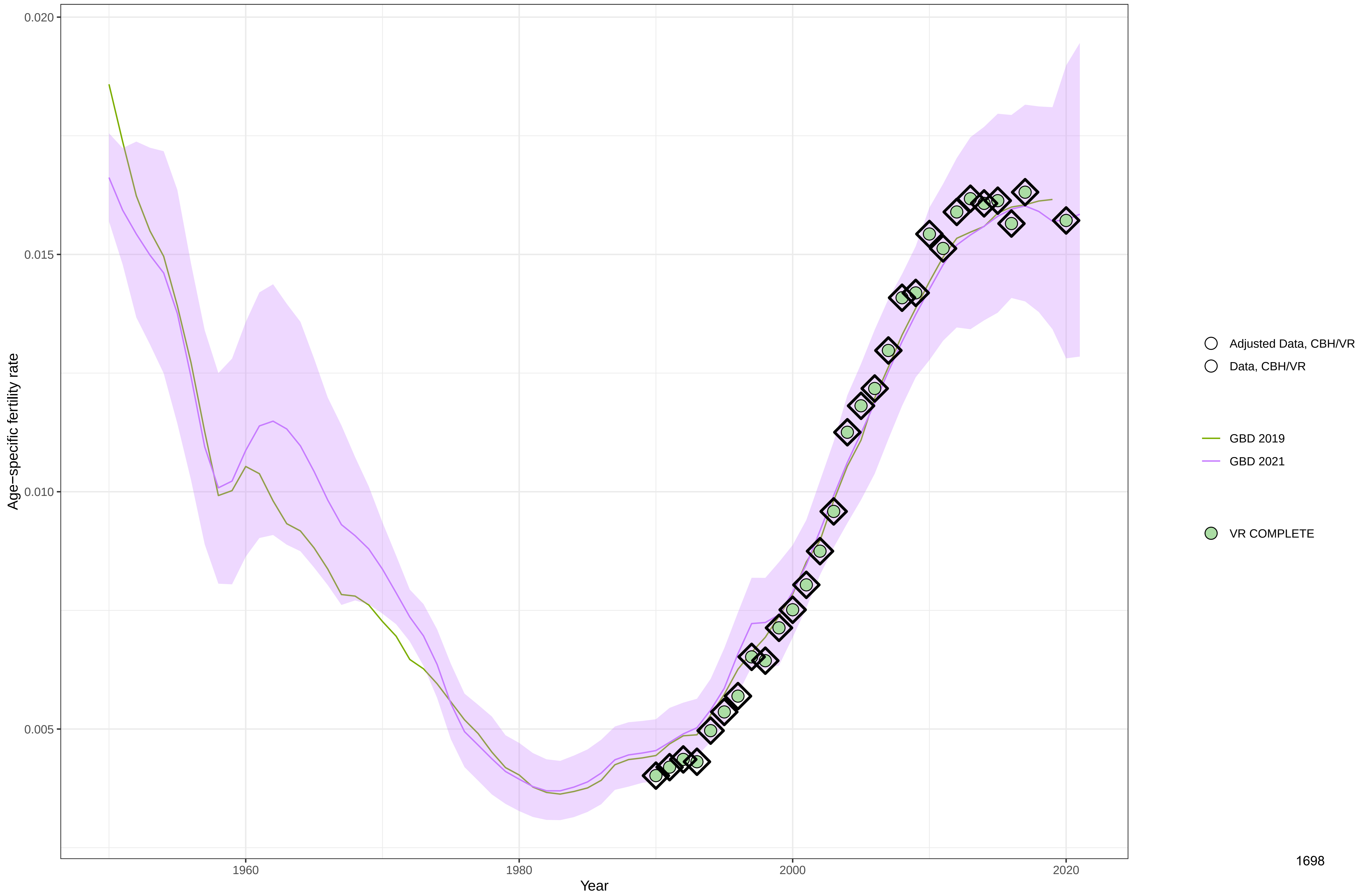

Age−specific fertility fitted trend lines, 1950−2021: Toscana, Ages: 45−49
Data points enclosed with a diamond are reference sources

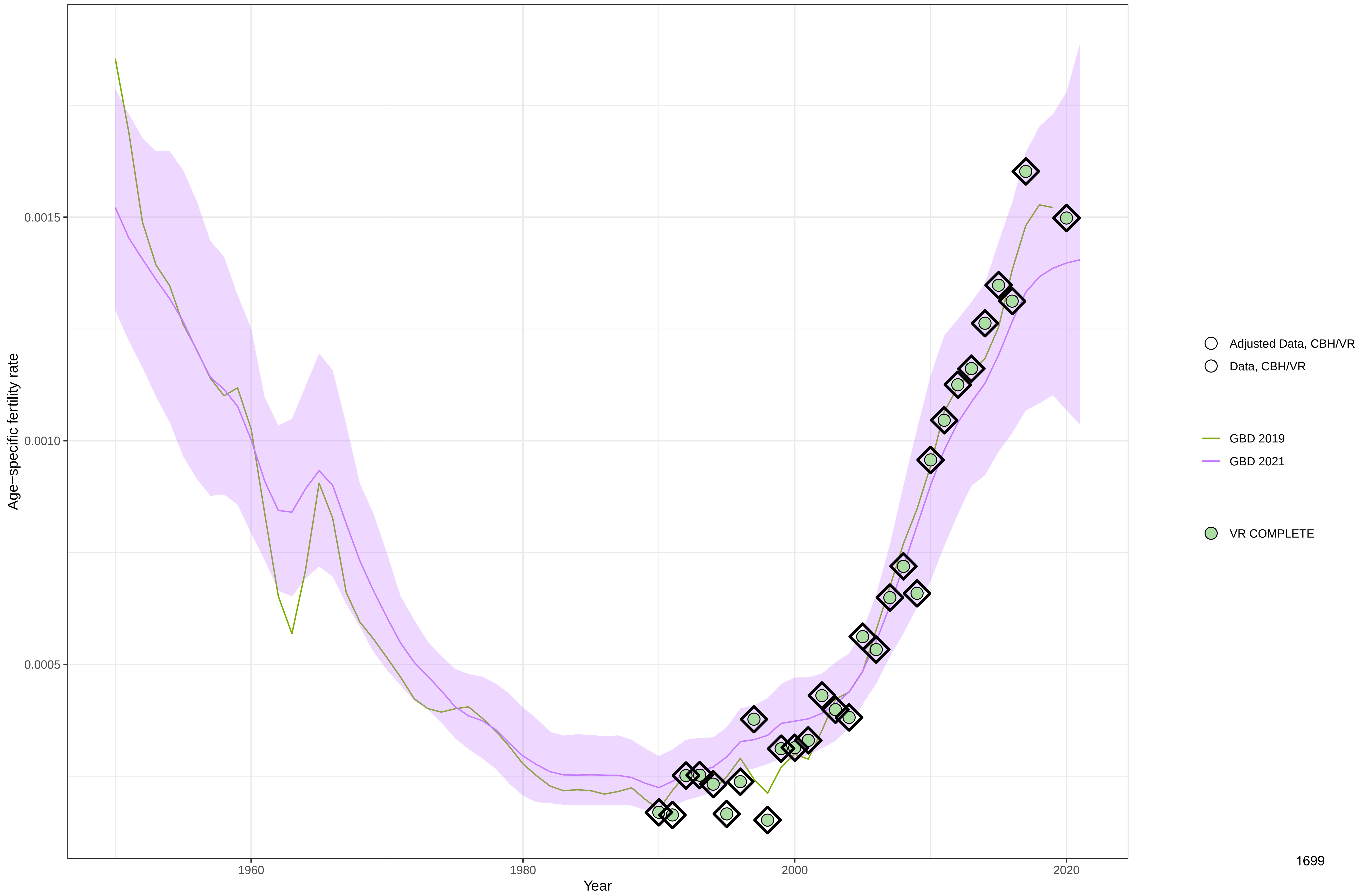

Age−specific fertility fitted trend lines, 1950−2021: Toscana, Ages: 50−54
Data points enclosed with a diamond are reference sources

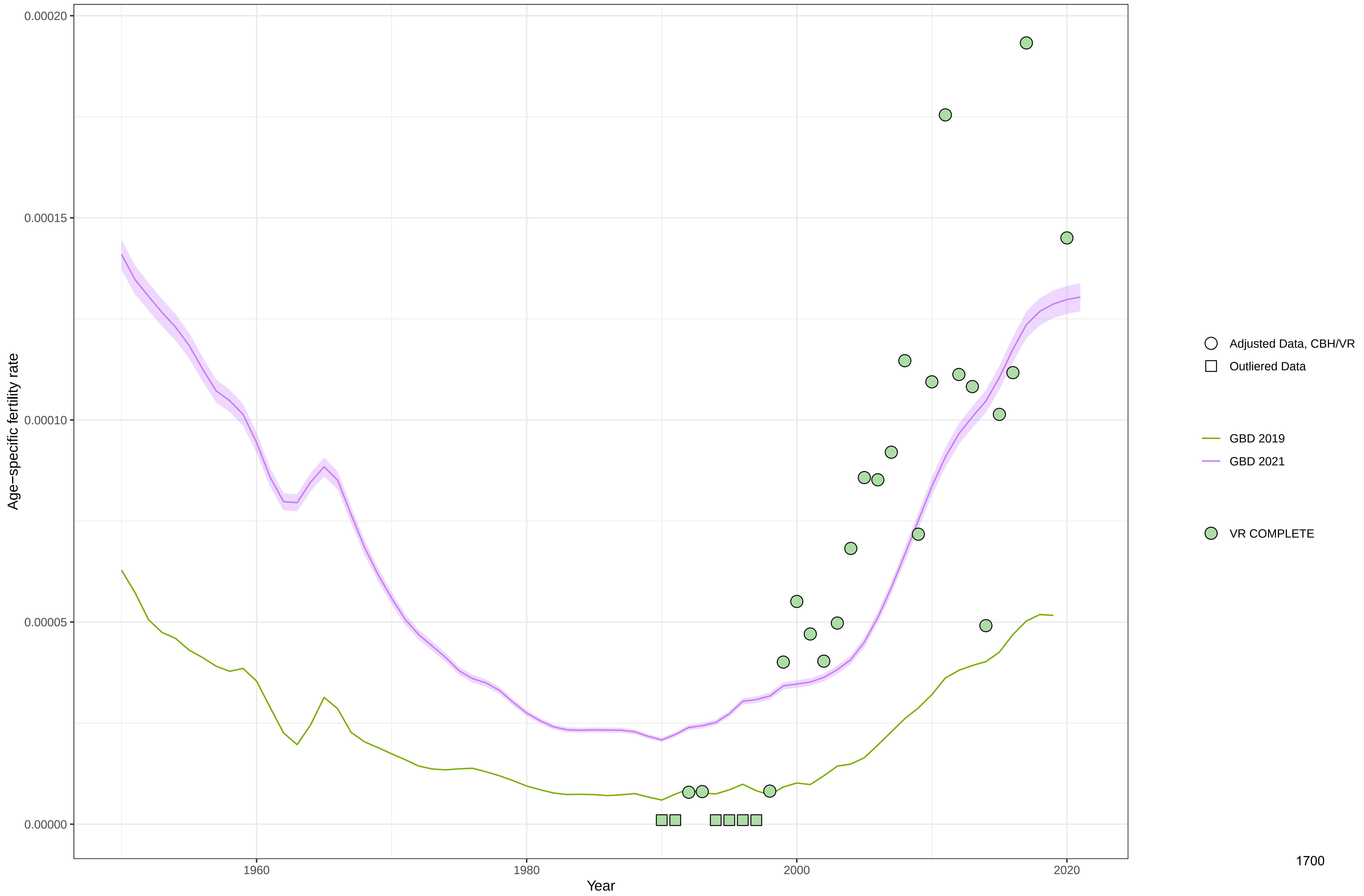

Total fertility rate fitted trend lines, 1950−2021: Toscana TFR
Data points enclosed with a diamond are reference sources

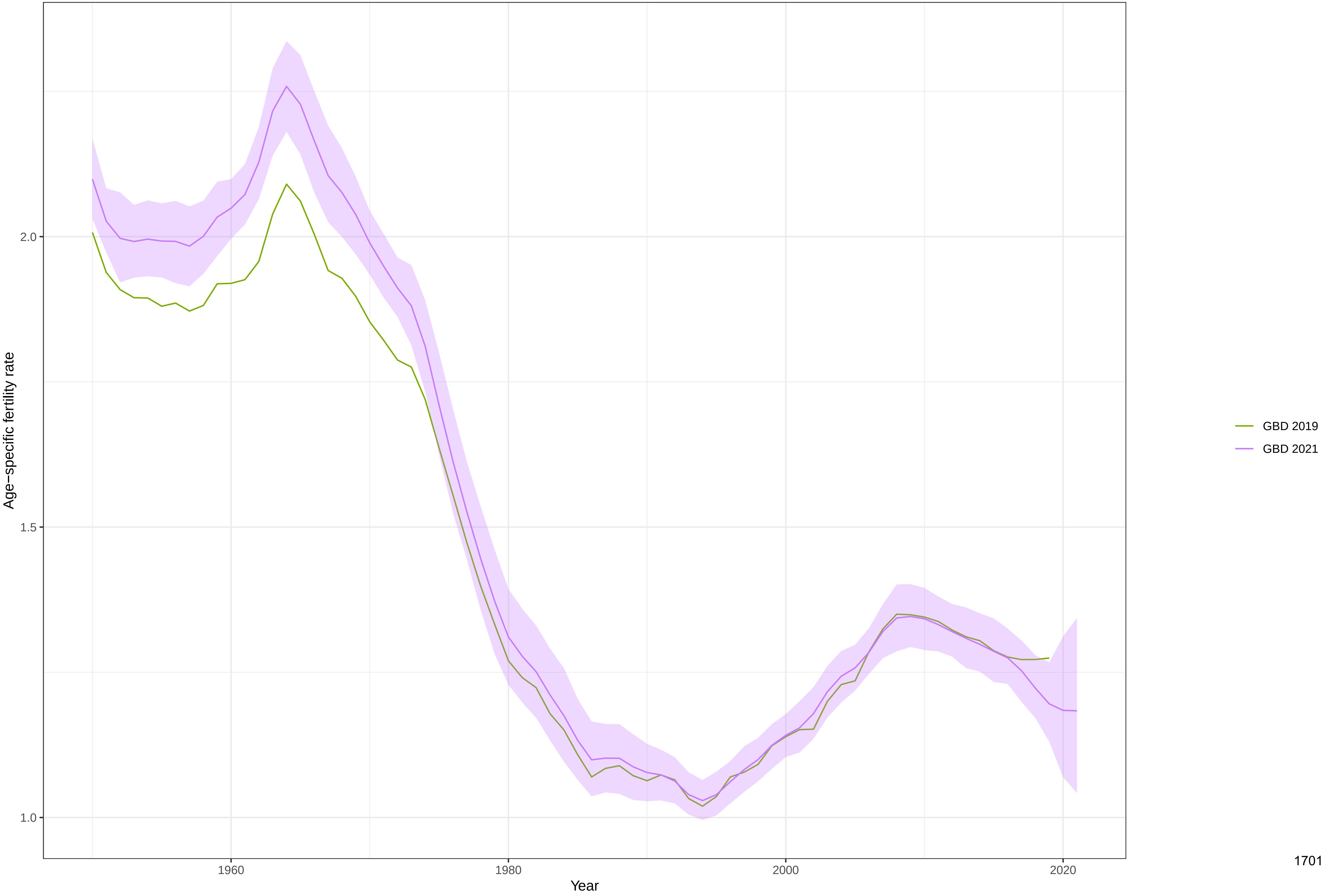

Age−specific fertility fitted trend lines, 1950−2021: Umbria, Ages: 10−14
Data points enclosed with a diamond are reference sources

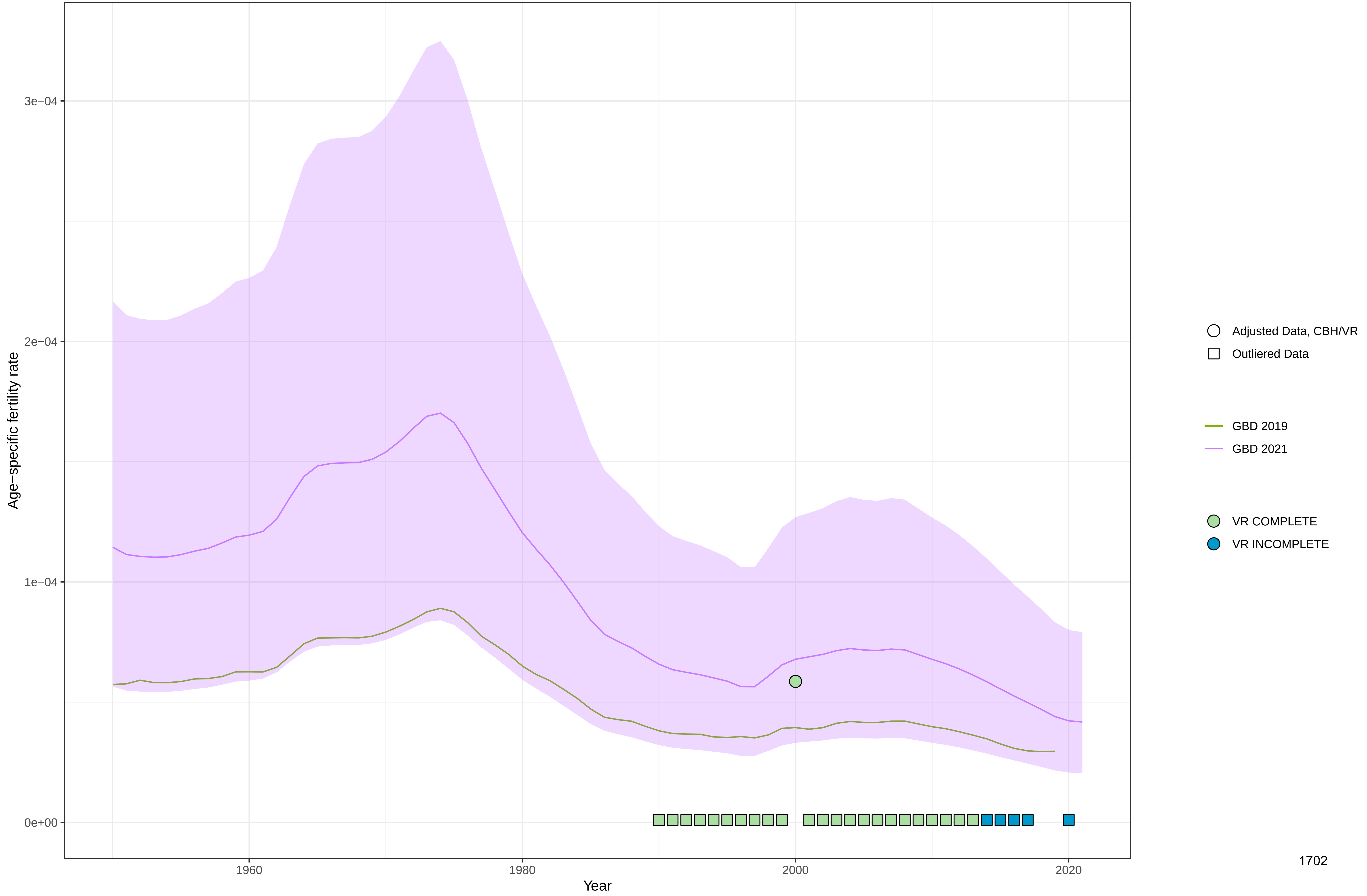

Age−specific fertility fitted trend lines, 1950−2021: Umbria, Ages: 15−19
Data points enclosed with a diamond are reference sources

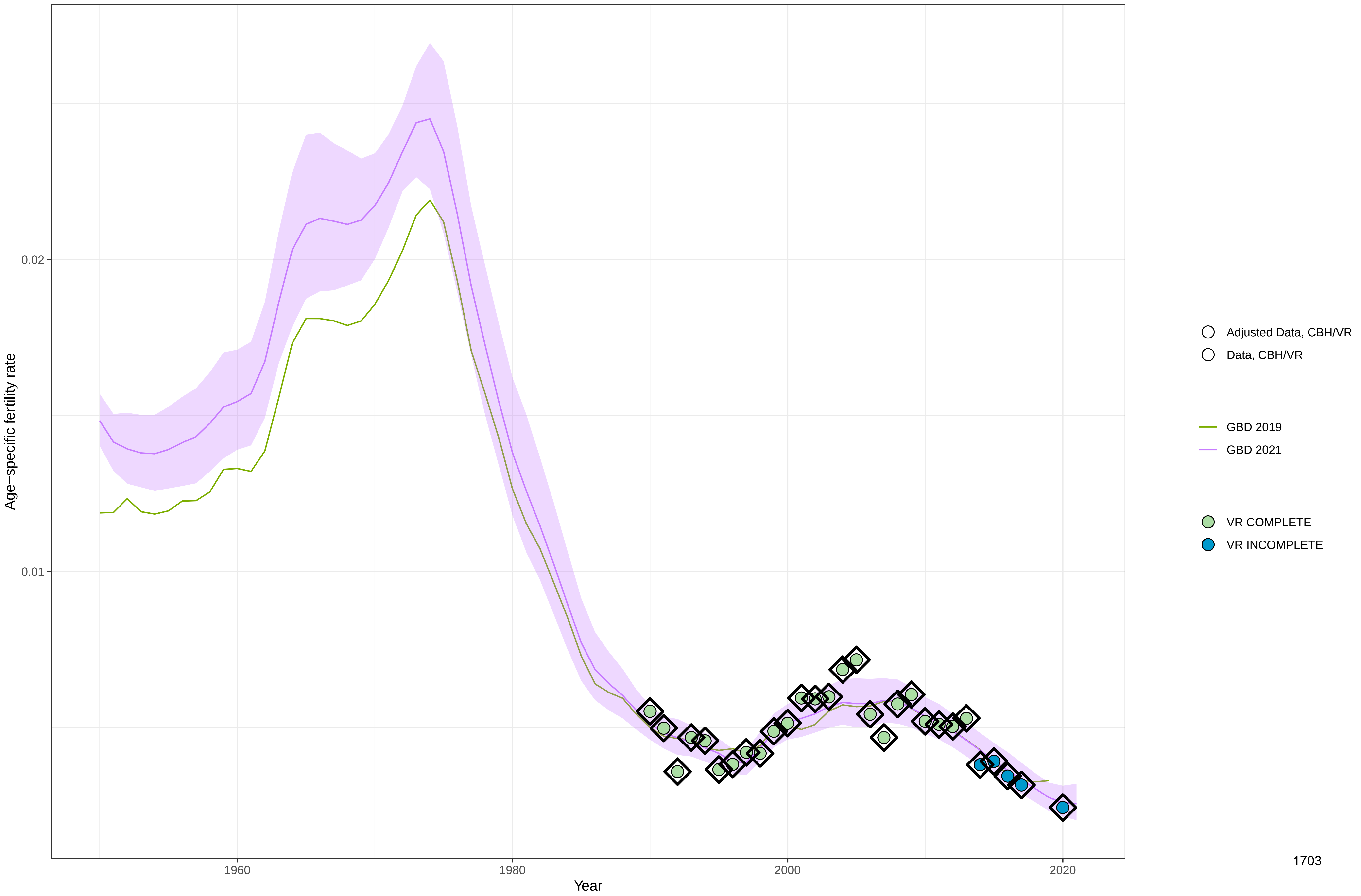

Age−specific fertility fitted trend lines, 1950−2021: Umbria, Ages: 20−24
Data points enclosed with a diamond are reference sources

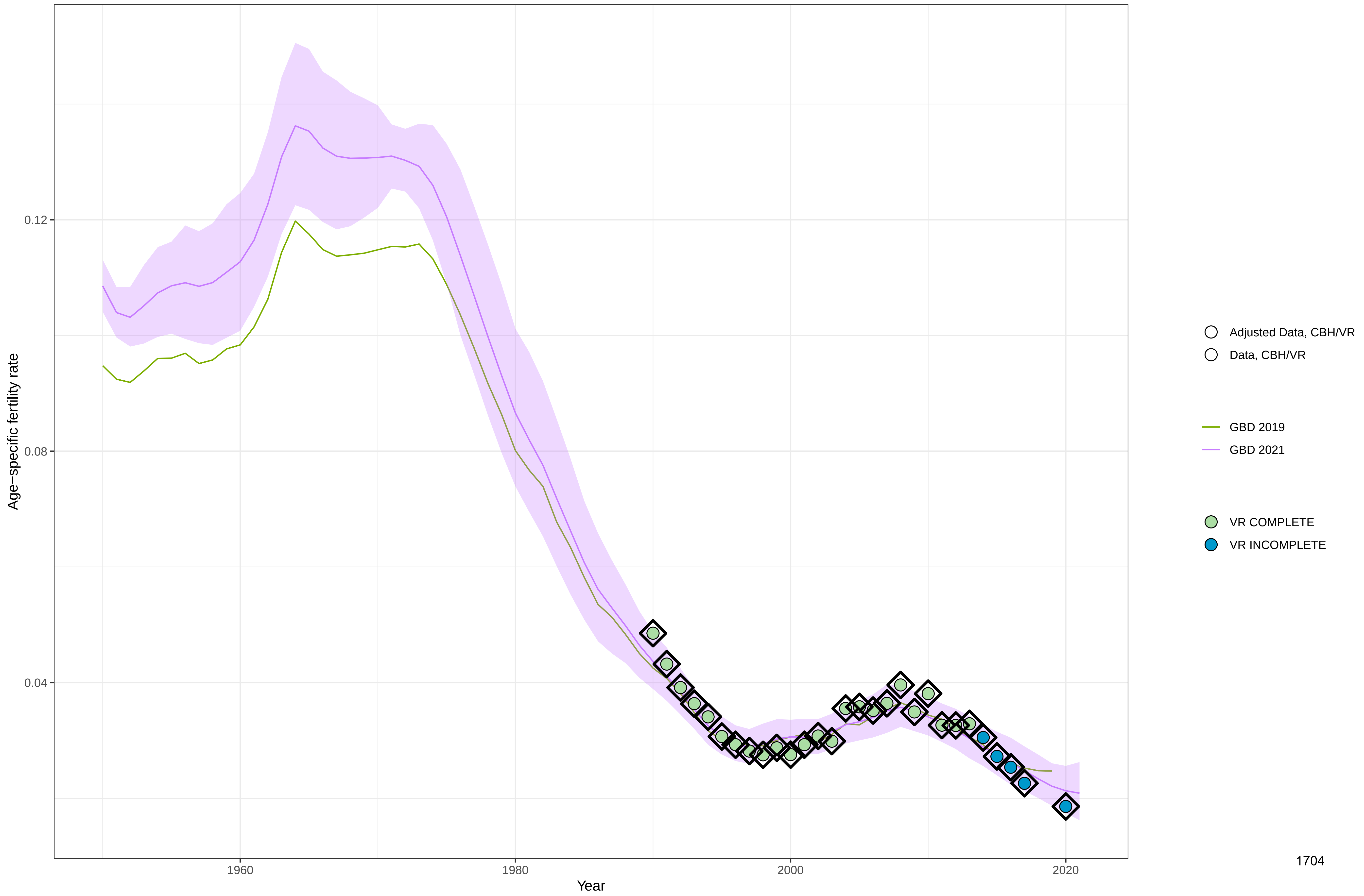

Age−specific fertility fitted trend lines, 1950−2021: Umbria, Ages: 25−29
Data points enclosed with a diamond are reference sources

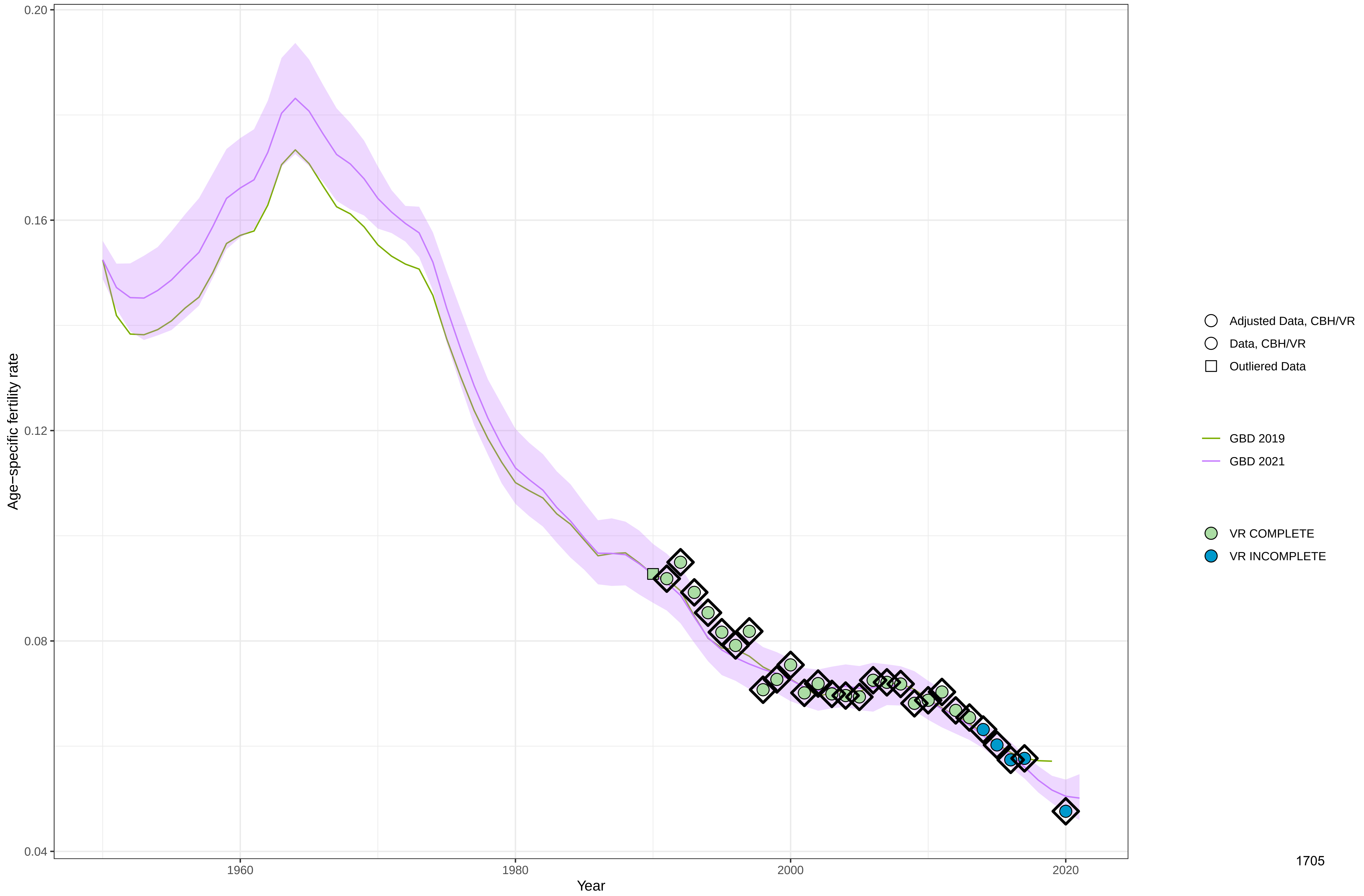

Age−specific fertility fitted trend lines, 1950−2021: Umbria, Ages: 30−34
Data points enclosed with a diamond are reference sources

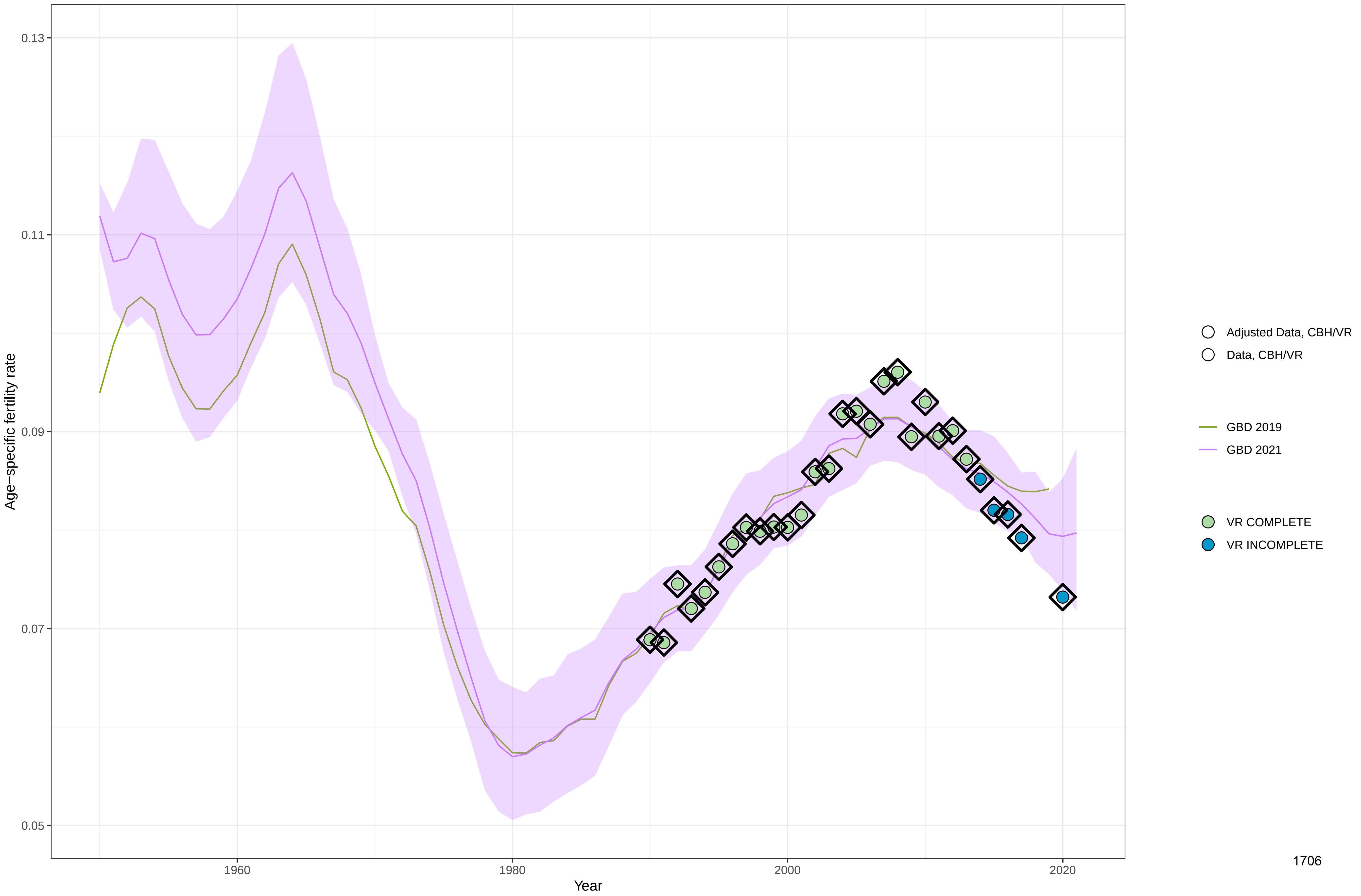

Age−specific fertility fitted trend lines, 1950−2021: Umbria, Ages: 35−39
Data points enclosed with a diamond are reference sources

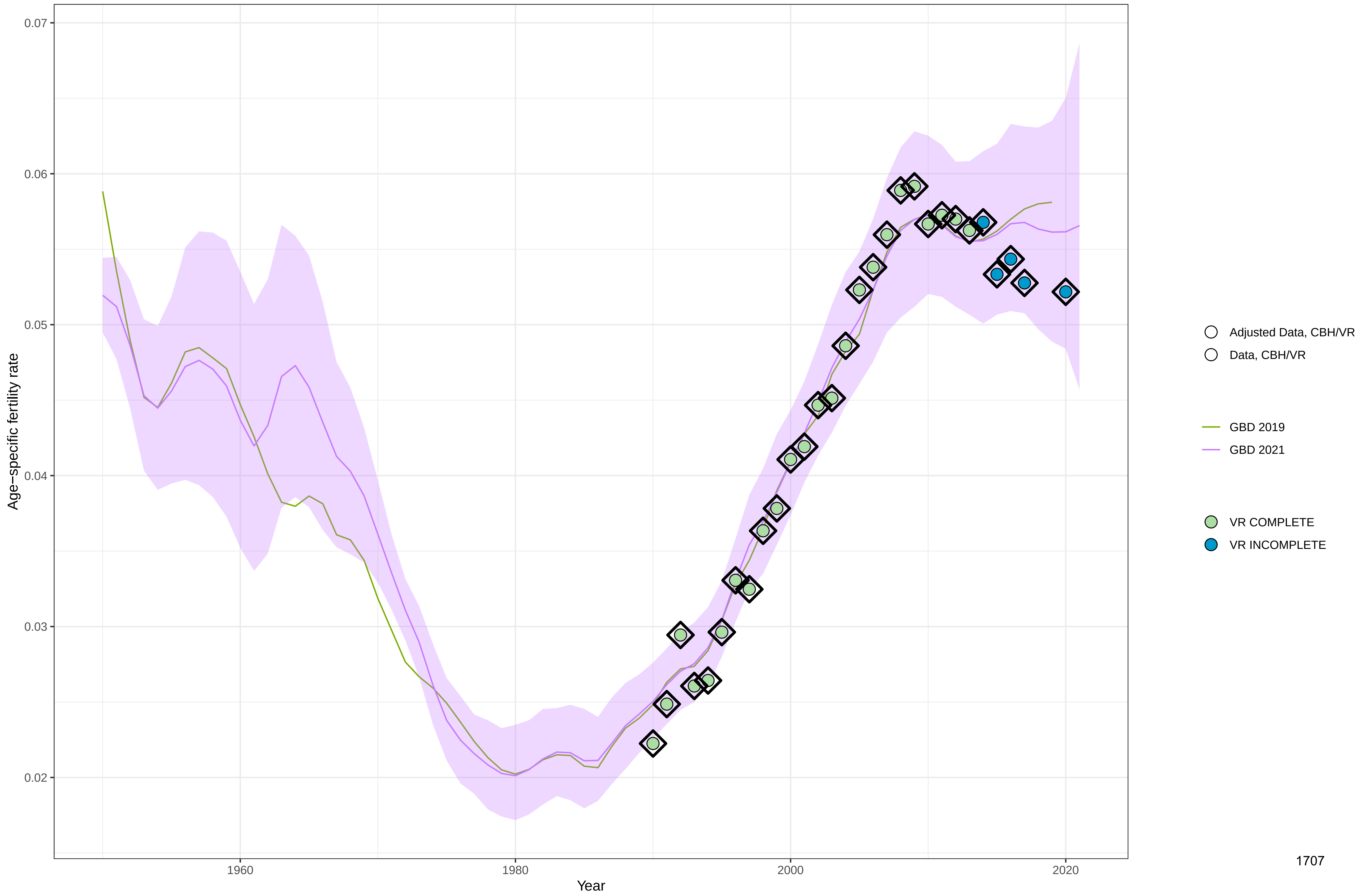

Age−specific fertility fitted trend lines, 1950−2021: Umbria, Ages: 40−44
Data points enclosed with a diamond are reference sources

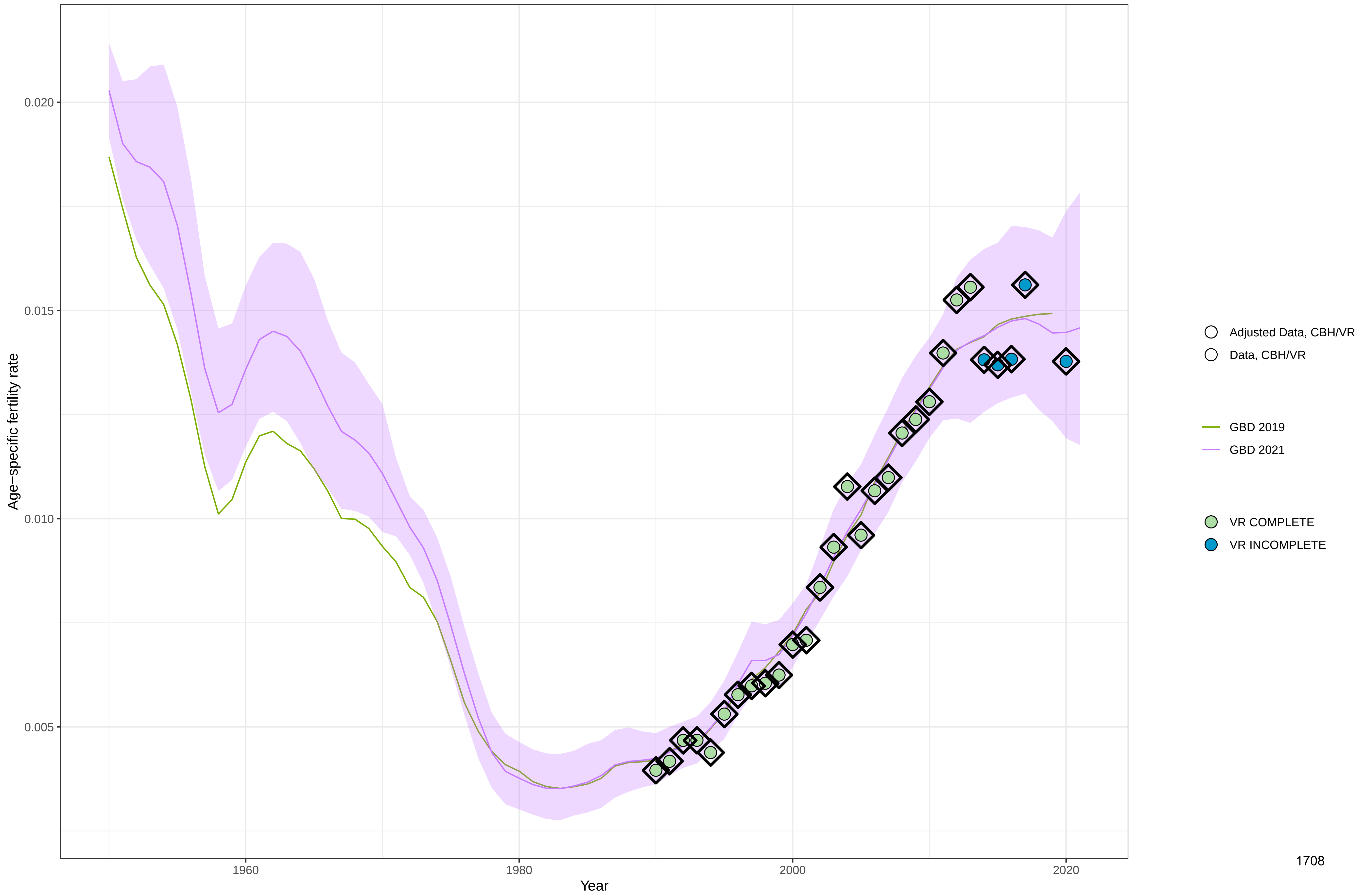

Age−specific fertility fitted trend lines, 1950−2021: Umbria, Ages: 45−49
Data points enclosed with a diamond are reference sources

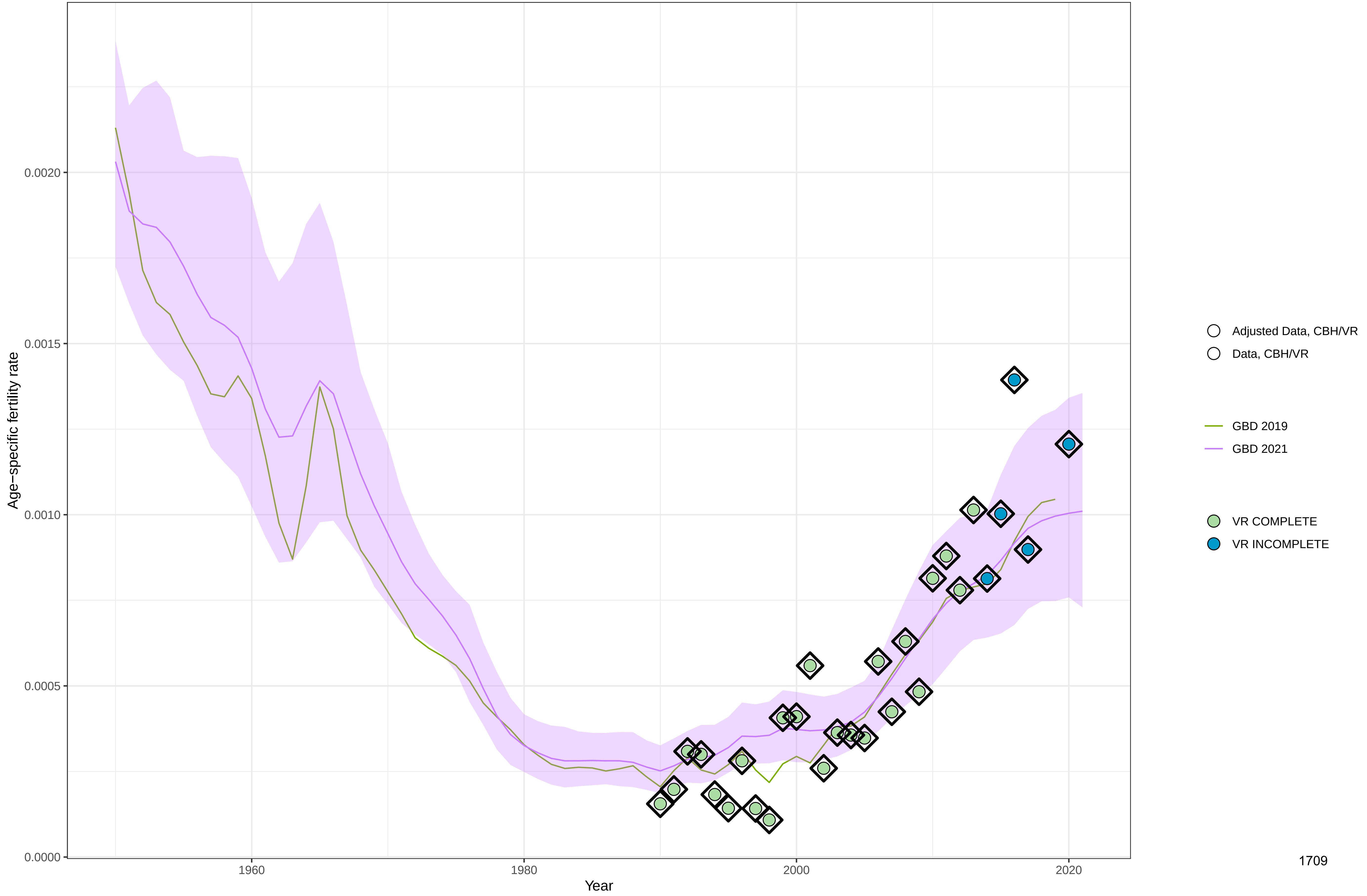

Age−specific fertility fitted trend lines, 1950−2021: Umbria, Ages: 50−54
Data points enclosed with a diamond are reference sources

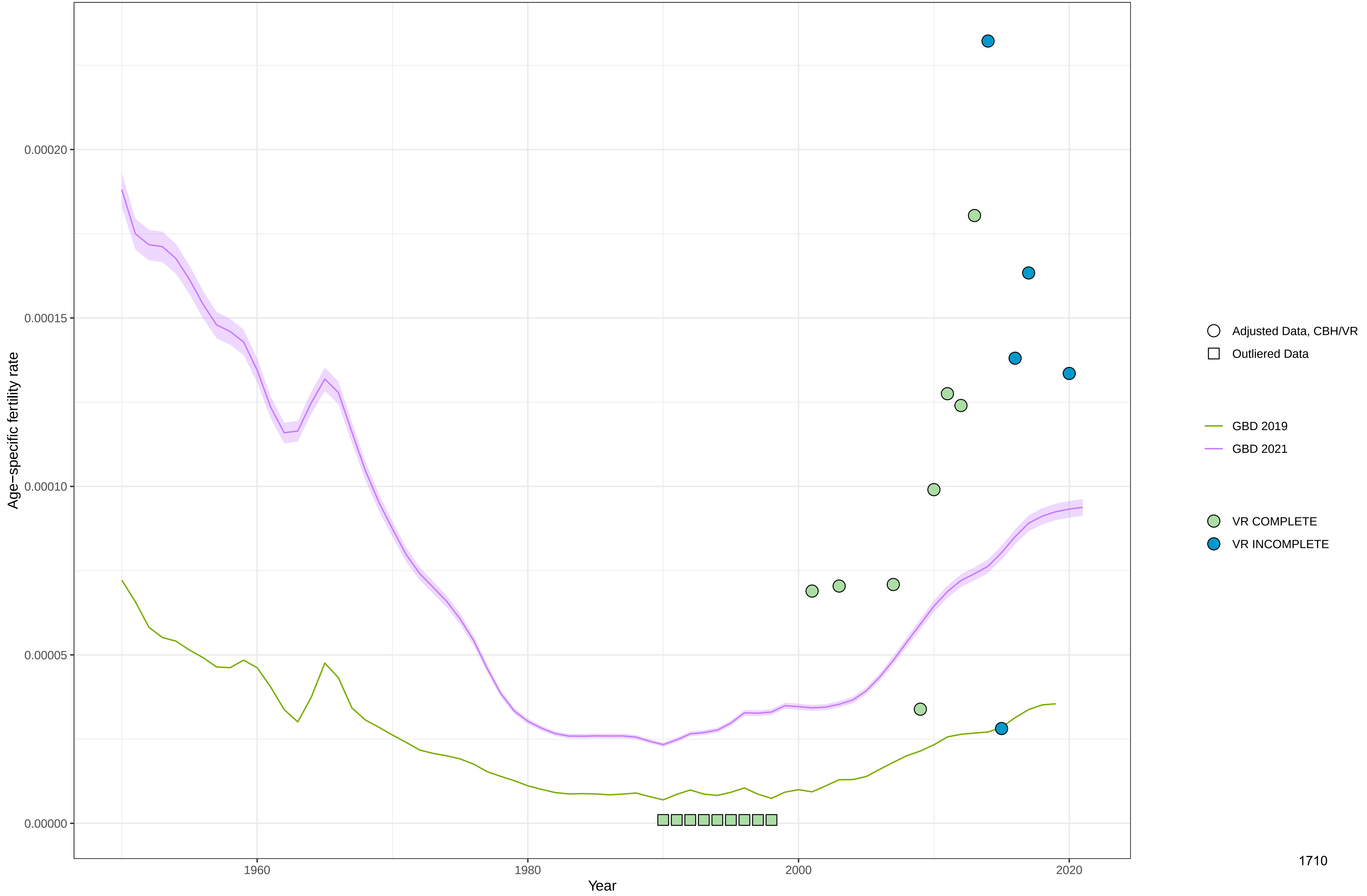

Total fertility rate fitted trend lines, 1950−2021: Umbria TFR
Data points enclosed with a diamond are reference sources

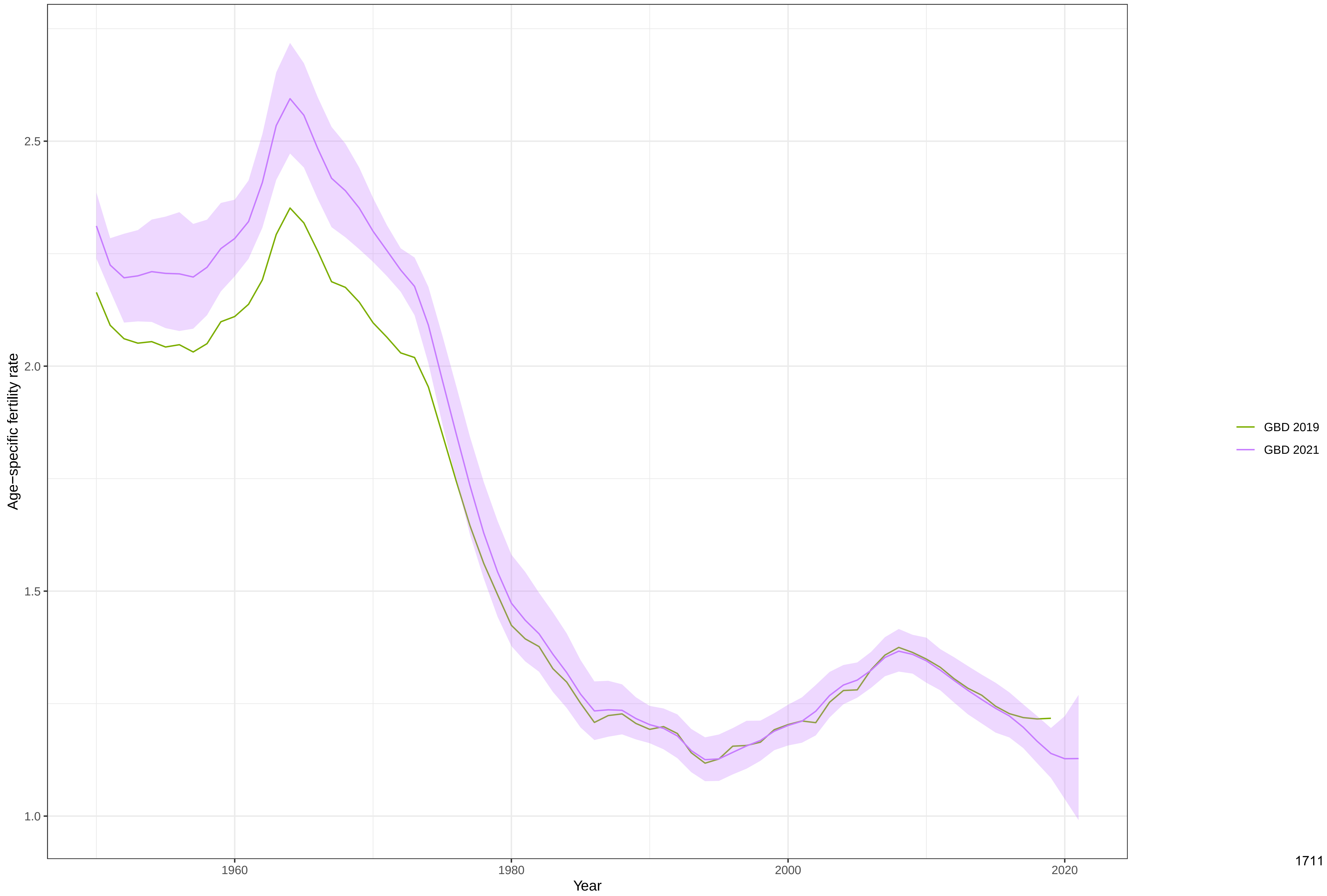

Age−specific fertility fitted trend lines, 1950−2021: Valle d'Aosta, Ages: 10−14
Data points enclosed with a diamond are reference sources

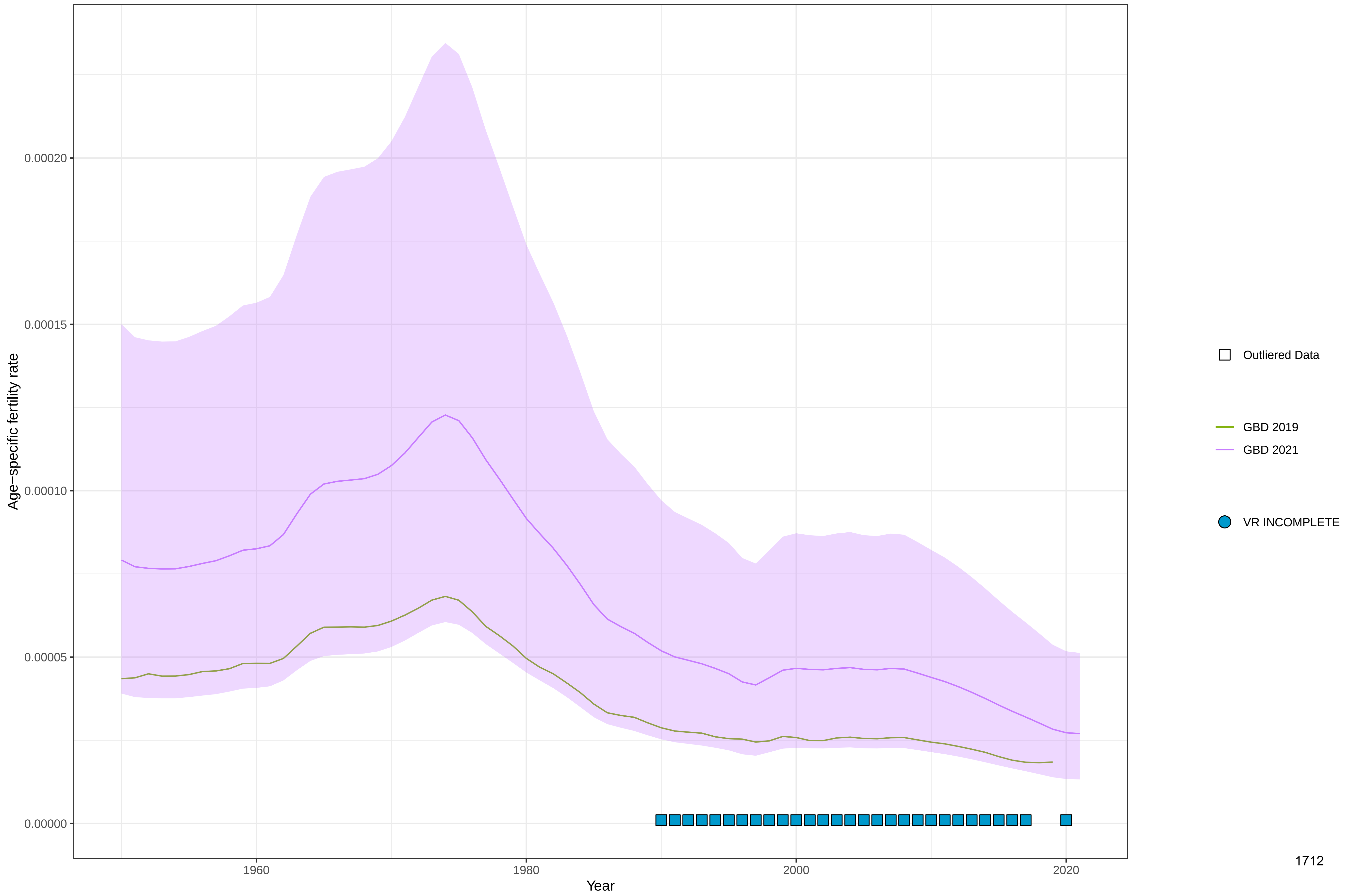

Age−specific fertility fitted trend lines, 1950−2021: Valle d'Aosta, Ages: 15−19
Data points enclosed with a diamond are reference sources

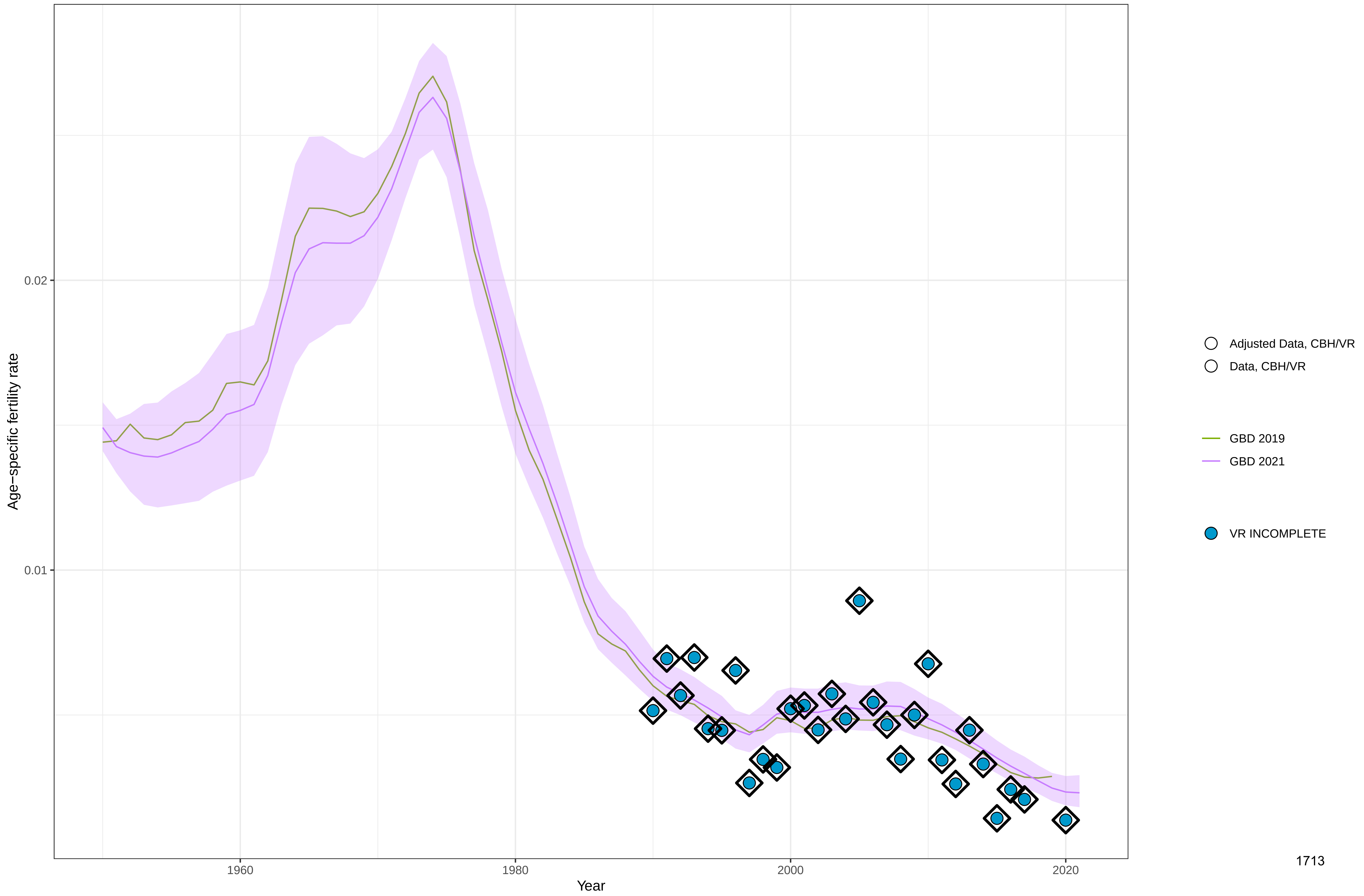

Age−specific fertility fitted trend lines, 1950−2021: Valle d'Aosta, Ages: 20−24
Data points enclosed with a diamond are reference sources

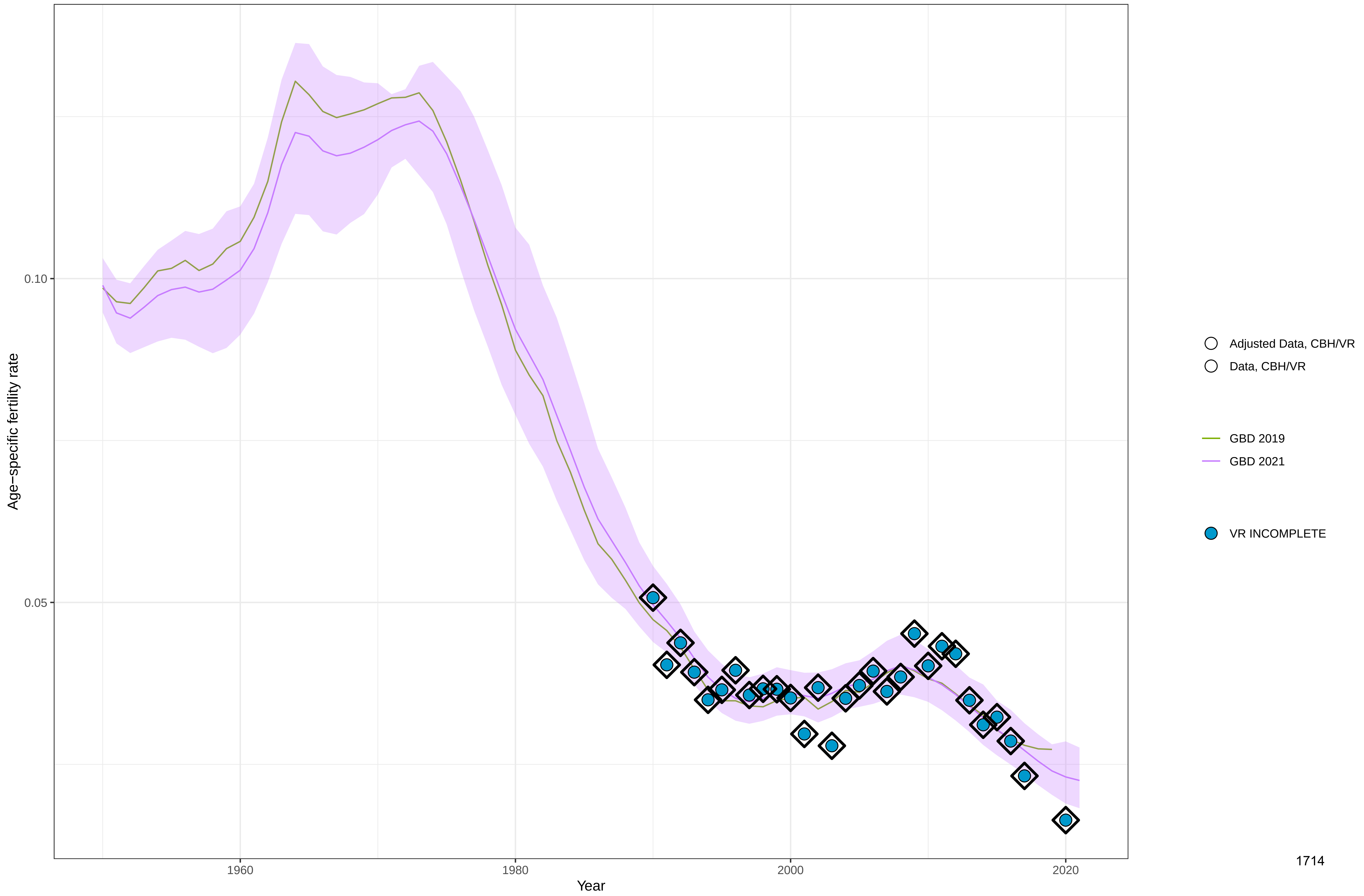

1714

Age−specific fertility fitted trend lines, 1950−2021: Valle d'Aosta, Ages: 25−29
Data points enclosed with a diamond are reference sources

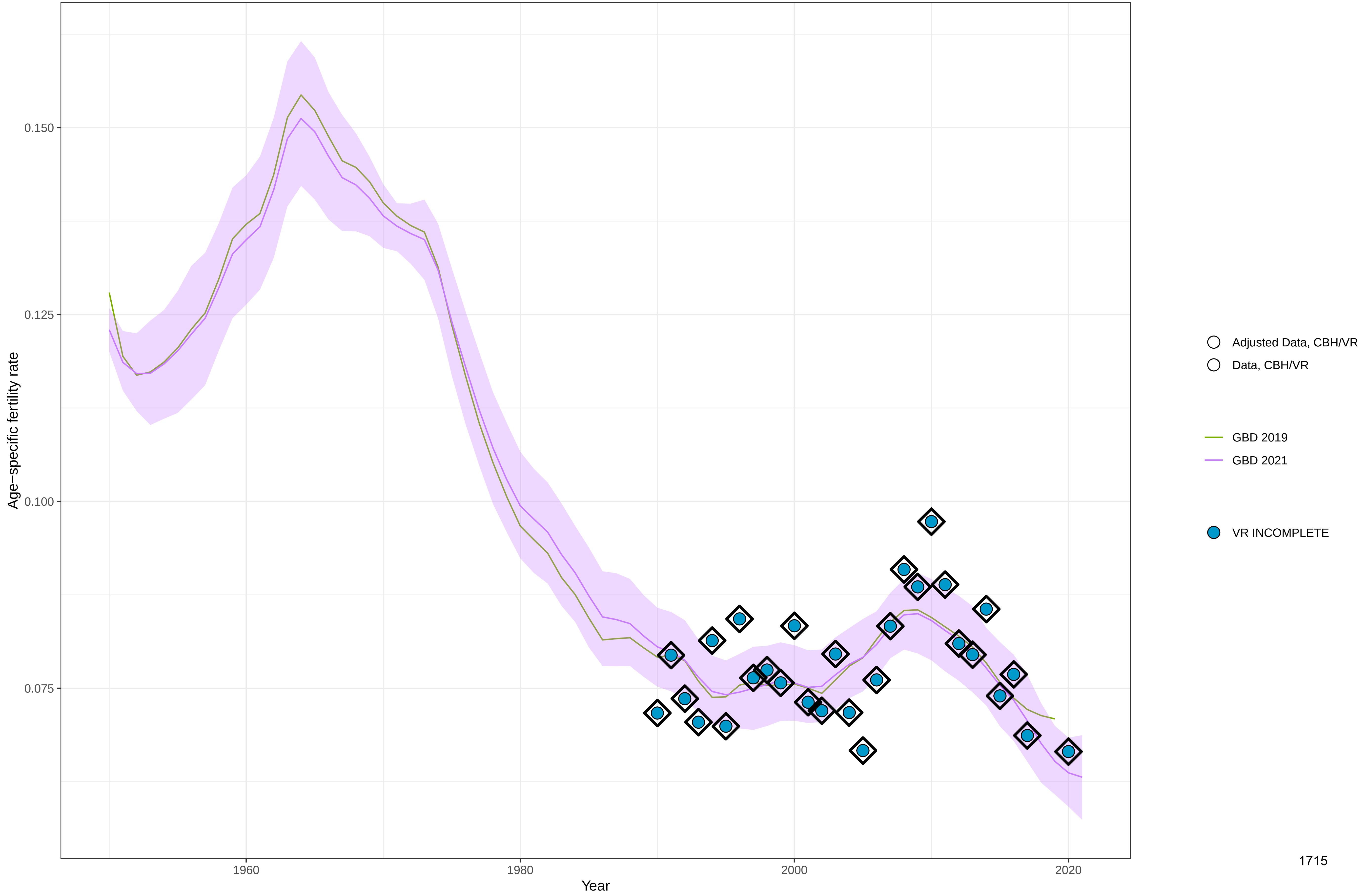

Age−specific fertility fitted trend lines, 1950−2021: Valle d'Aosta, Ages: 30−34
Data points enclosed with a diamond are reference sources

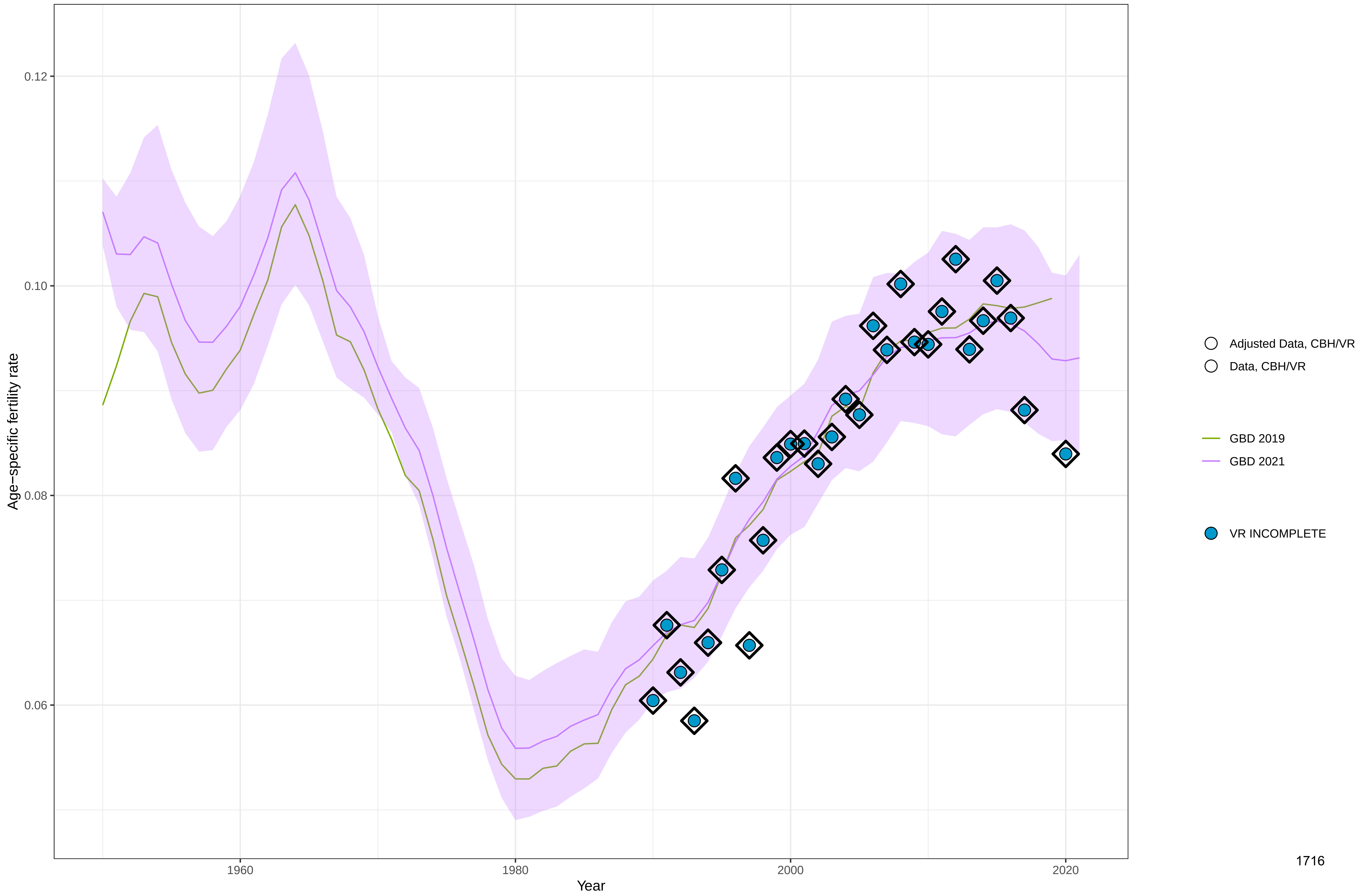

Age−specific fertility fitted trend lines, 1950−2021: Valle d'Aosta, Ages: 35−39
Data points enclosed with a diamond are reference sources

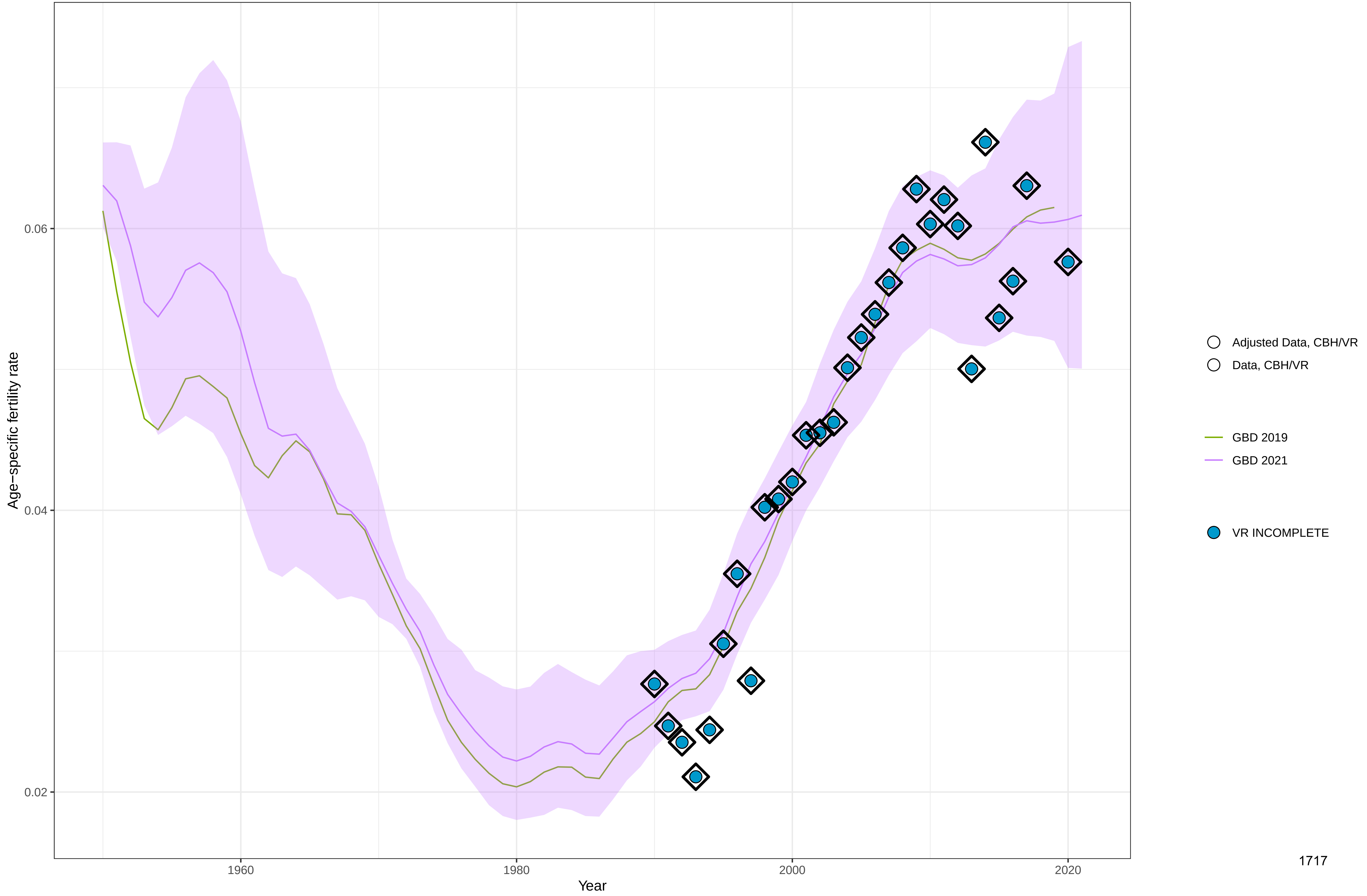

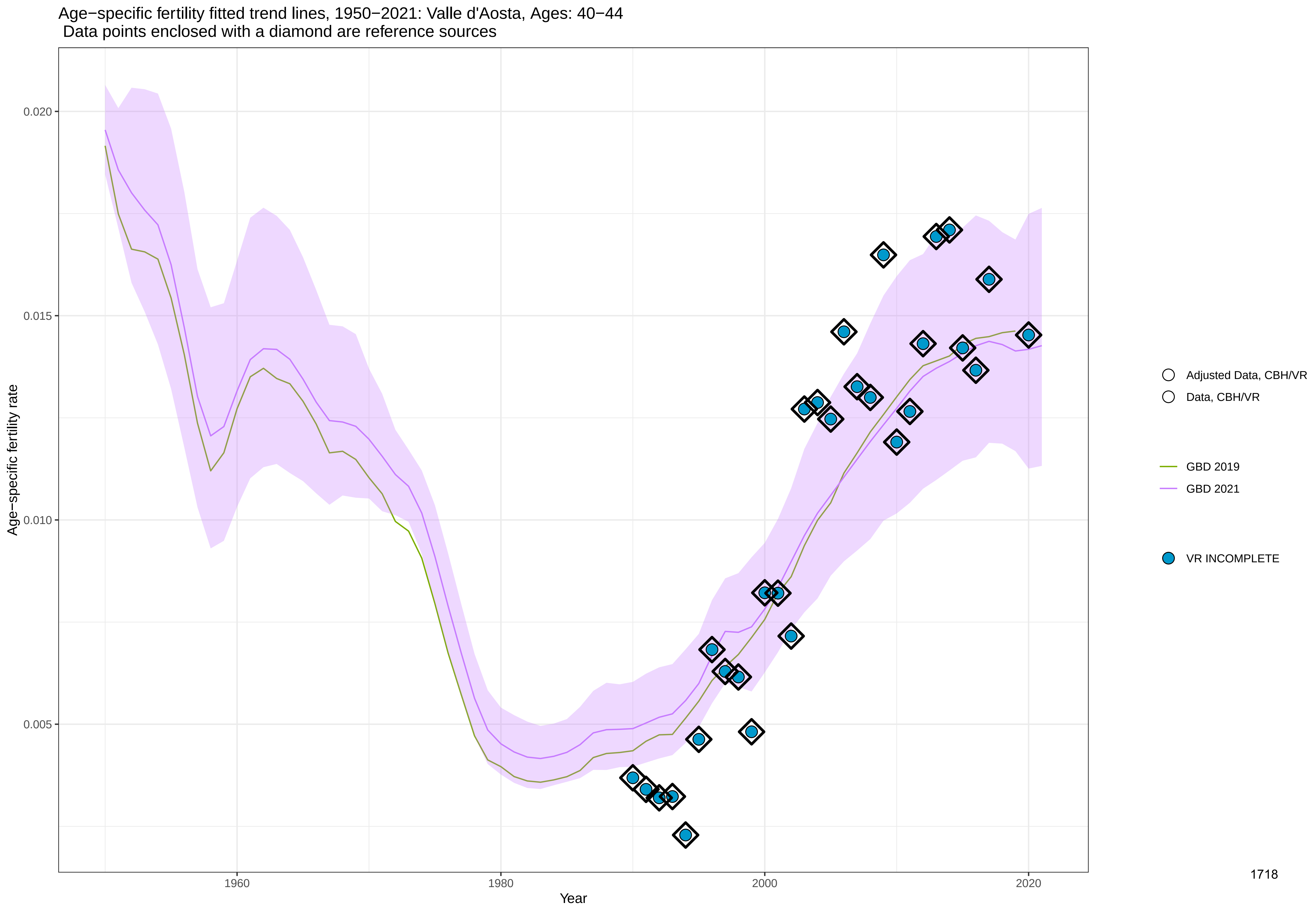
Age−specific fertility fitted trend lines, 1950−2021: Valle d'Aosta, Ages: 40−44
Data points enclosed with a diamond are reference sources
0.020
0.015
0.010
0.005
Age−specific fertility rate
1960
1980
2000
2020
Year
Adjusted Data, CBH/VR
Data, CBH/VR
GBD 2019
GBD 2021
VR INCOMPLETE

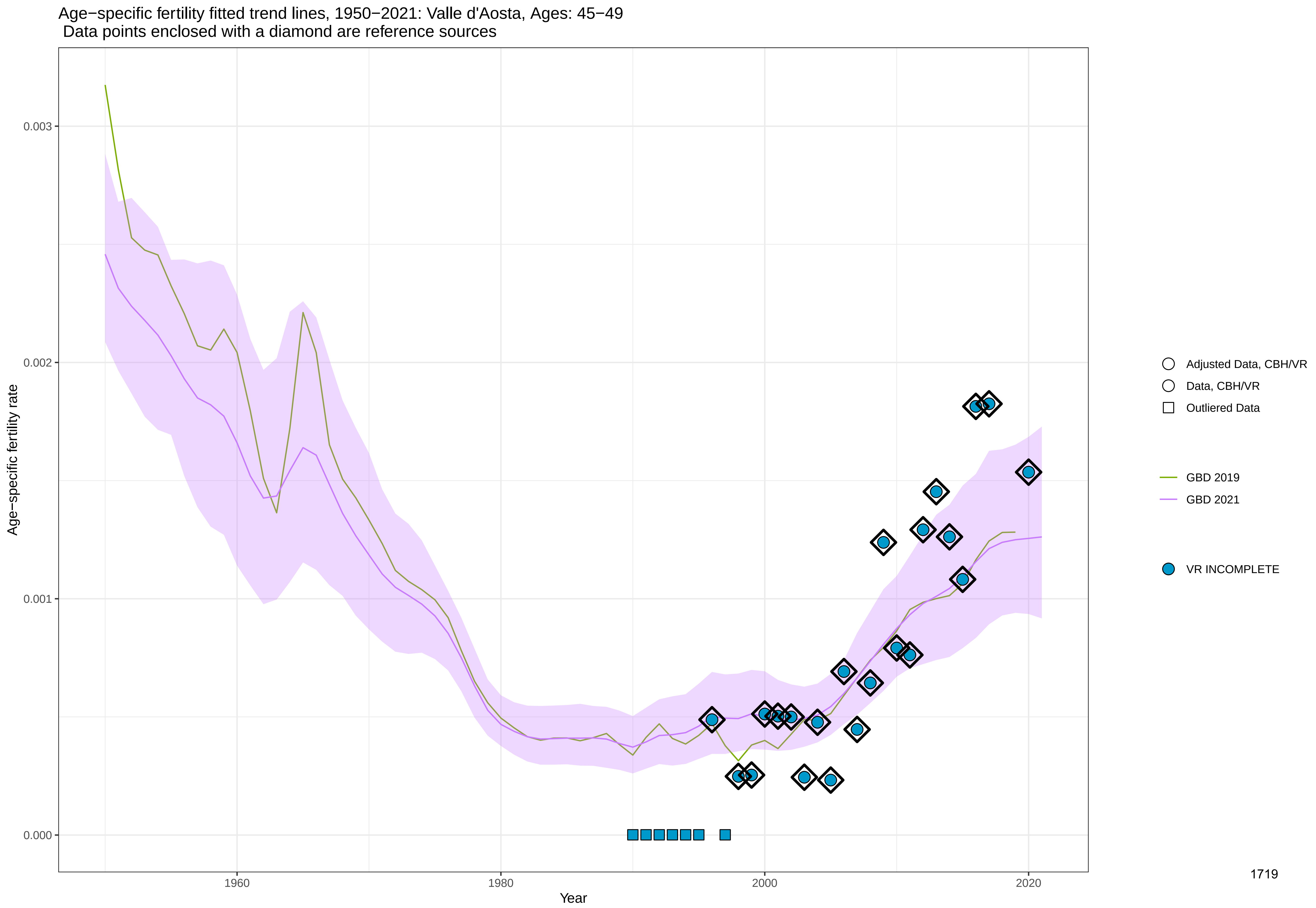

Age−specific fertility fitted trend lines, 1950−2021: Valle d'Aosta, Ages: 45−49
Data points enclosed with a diamond are reference sources
Age−specific fertility rate
0.000
0.001
0.002
0.003
1960
1980
2000
2020
Year
Adjusted Data, CBH/VR
Data, CBH/VR
Outliered Data
GBD 2019
GBD 2021
VR INCOMPLETE

1719

Age−specific fertility fitted trend lines, 1950−2021: Valle d'Aosta, Ages: 50−54
Data points enclosed with a diamond are reference sources

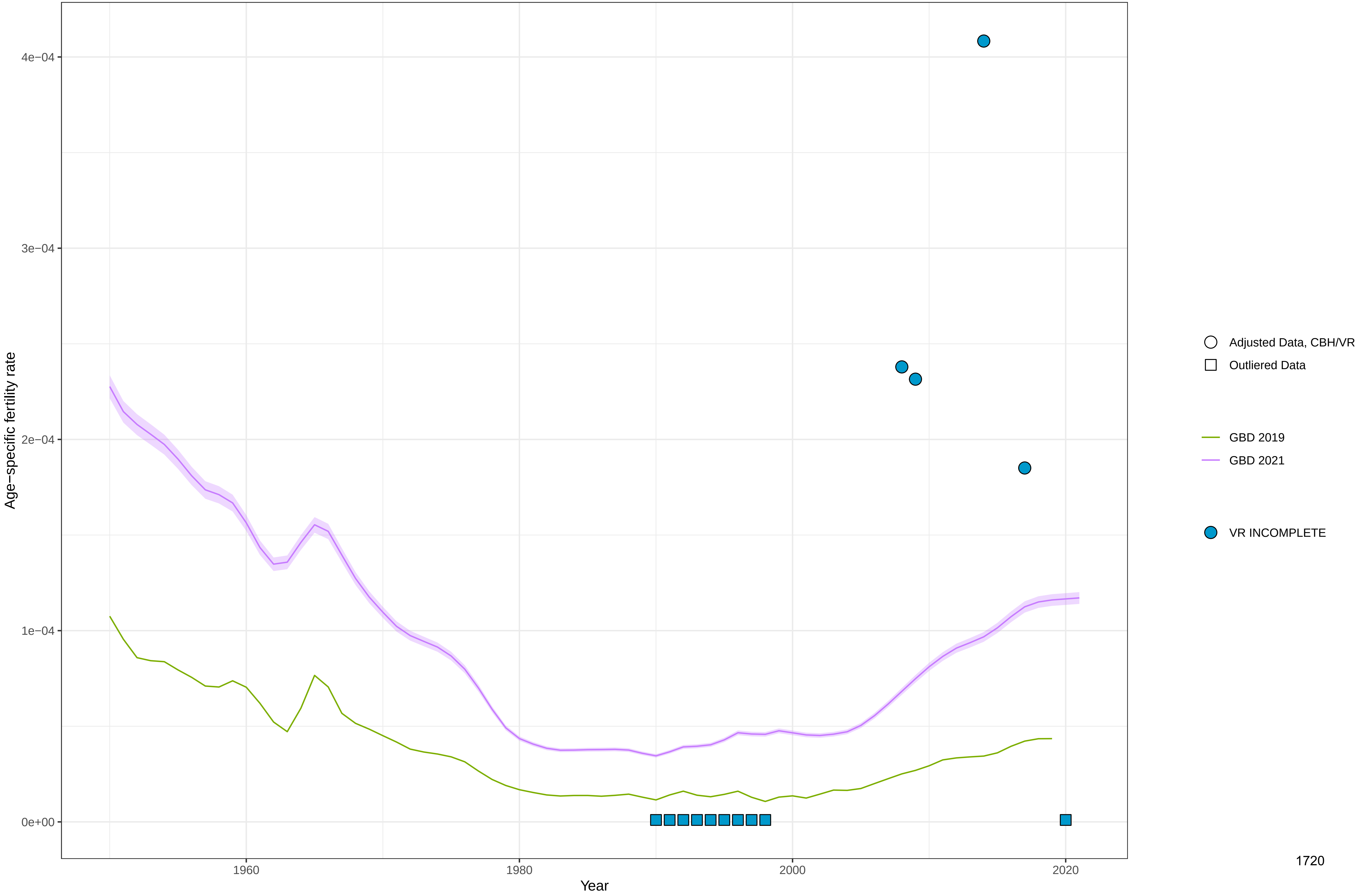

Total fertility rate fitted trend lines, 1950−2021: Valle d'Aosta TFR
Data points enclosed with a diamond are reference sources

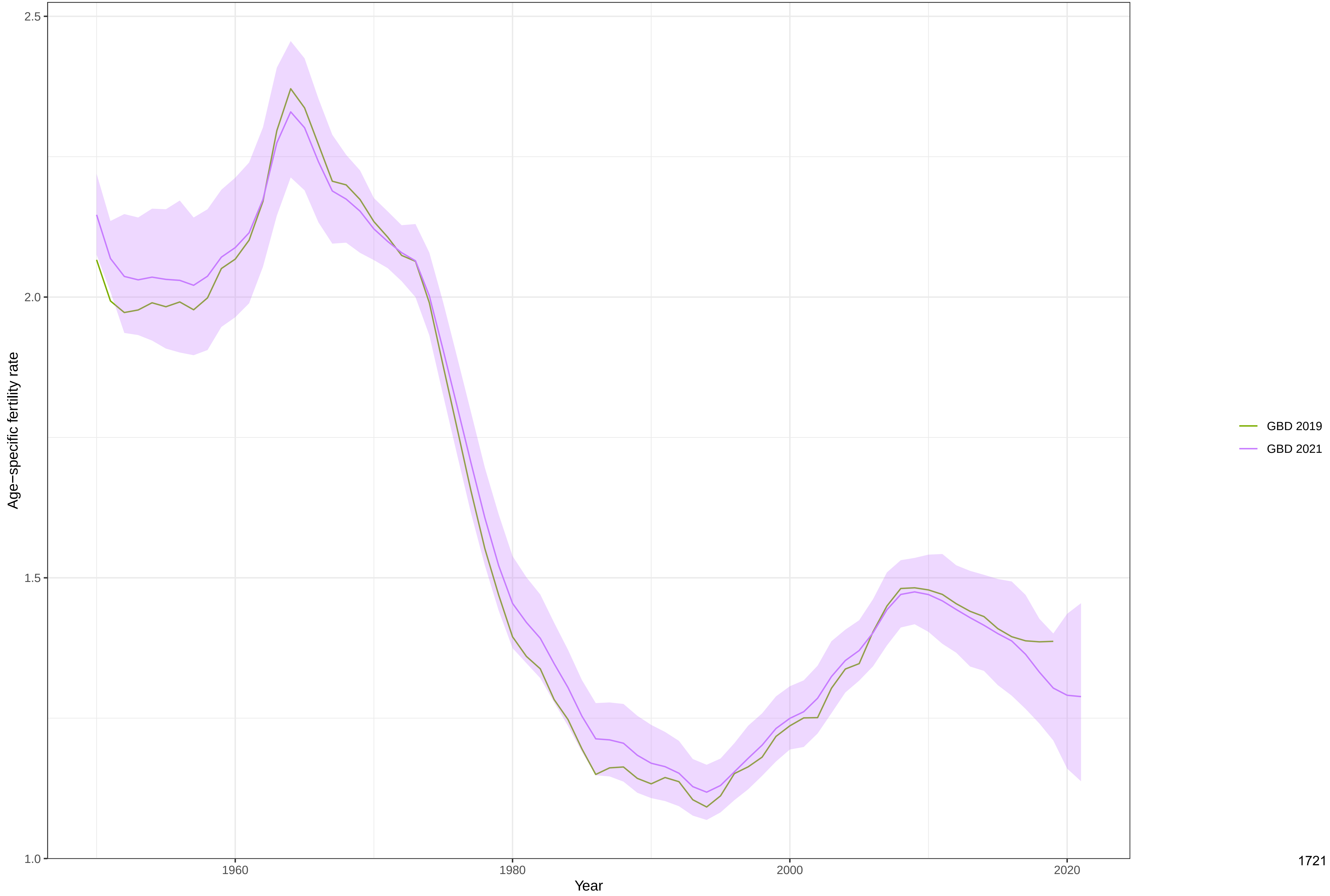

Age−specific fertility fitted trend lines, 1950−2021: Veneto, Ages: 10−14
Data points enclosed with a diamond are reference sources

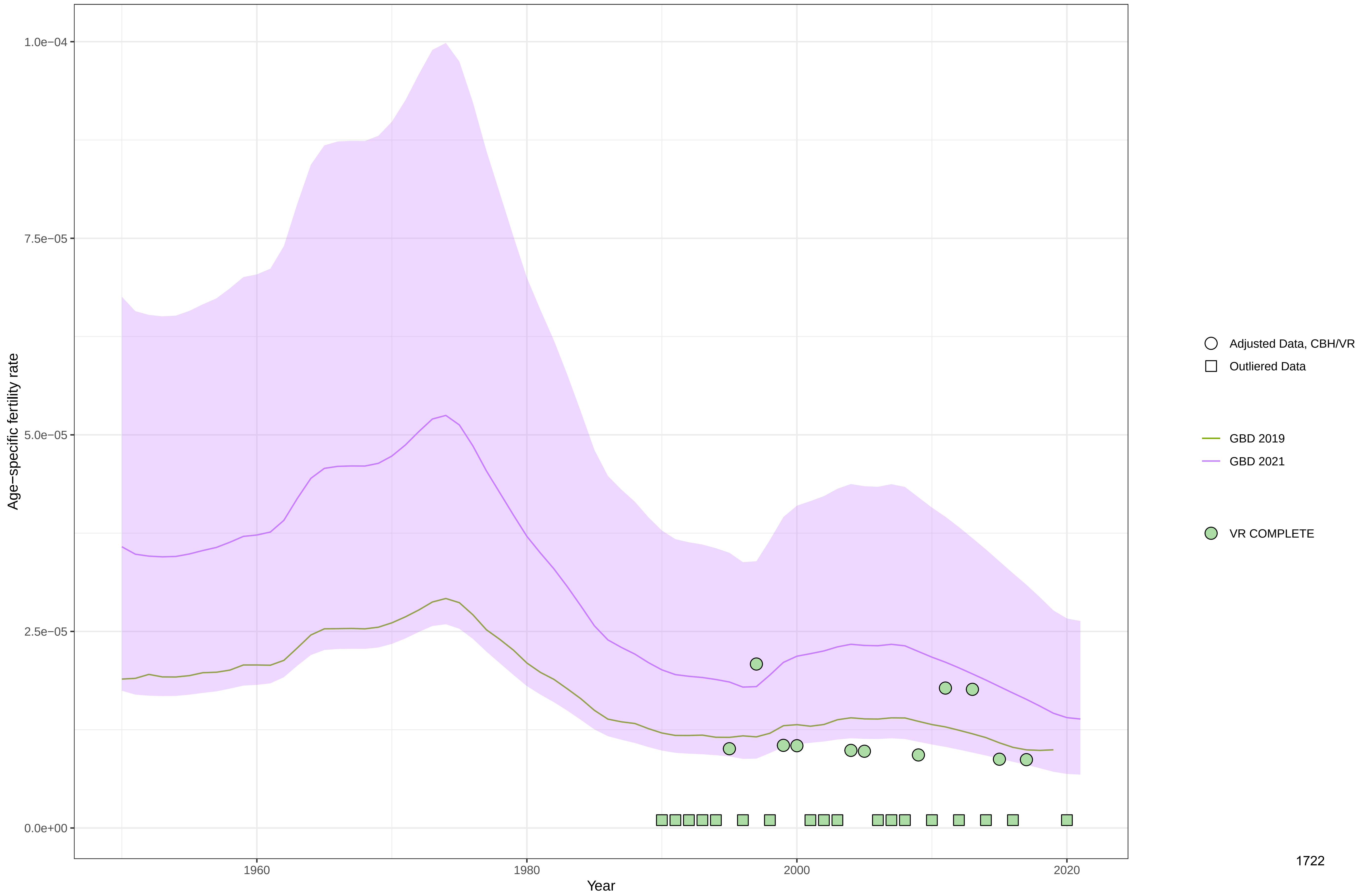

1722

Age−specific fertility fitted trend lines, 1950−2021: Veneto, Ages: 15−19
Data points enclosed with a diamond are reference sources

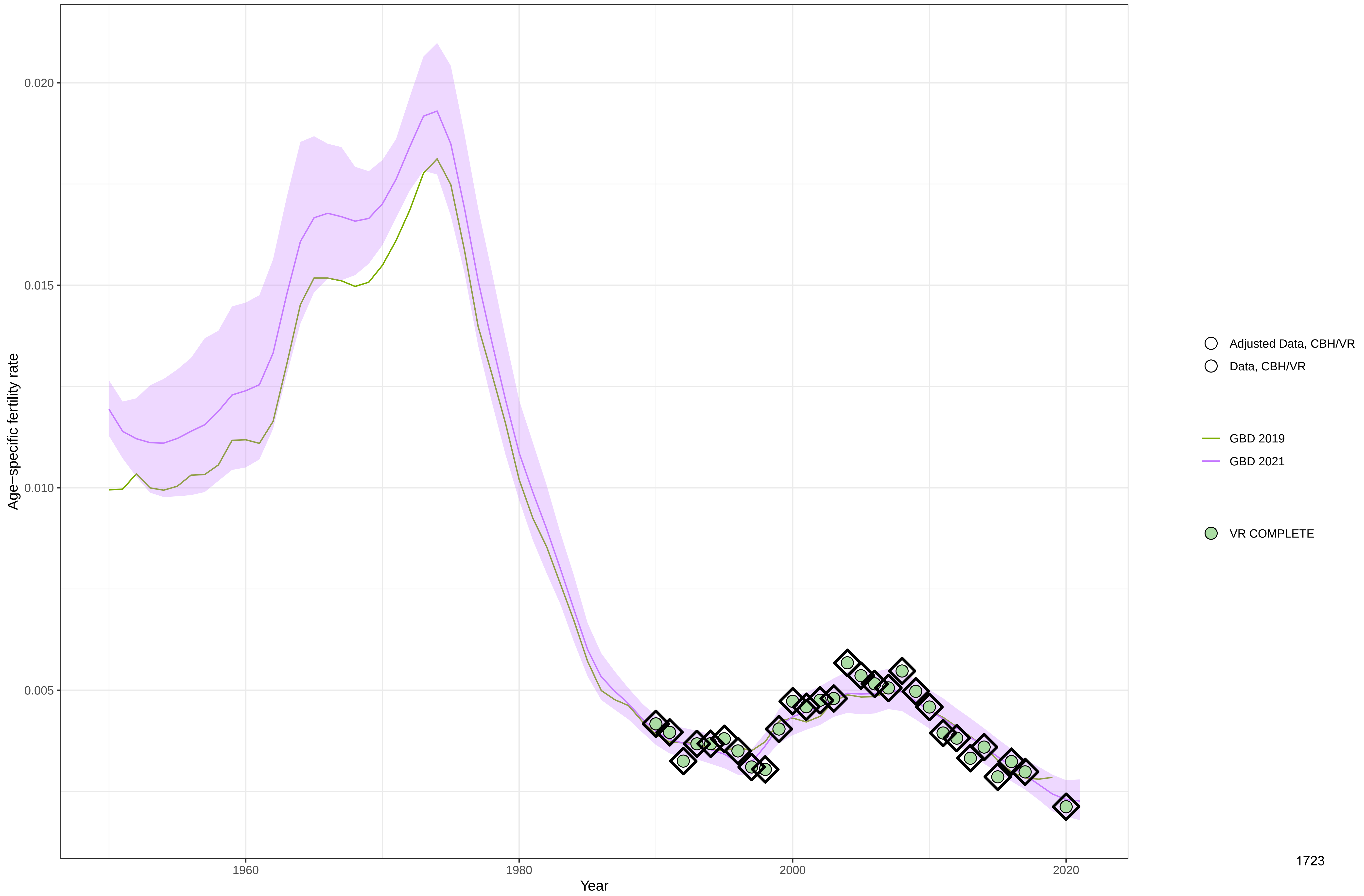

Age−specific fertility fitted trend lines, 1950−2021: Veneto, Ages: 20−24
Data points enclosed with a diamond are reference sources

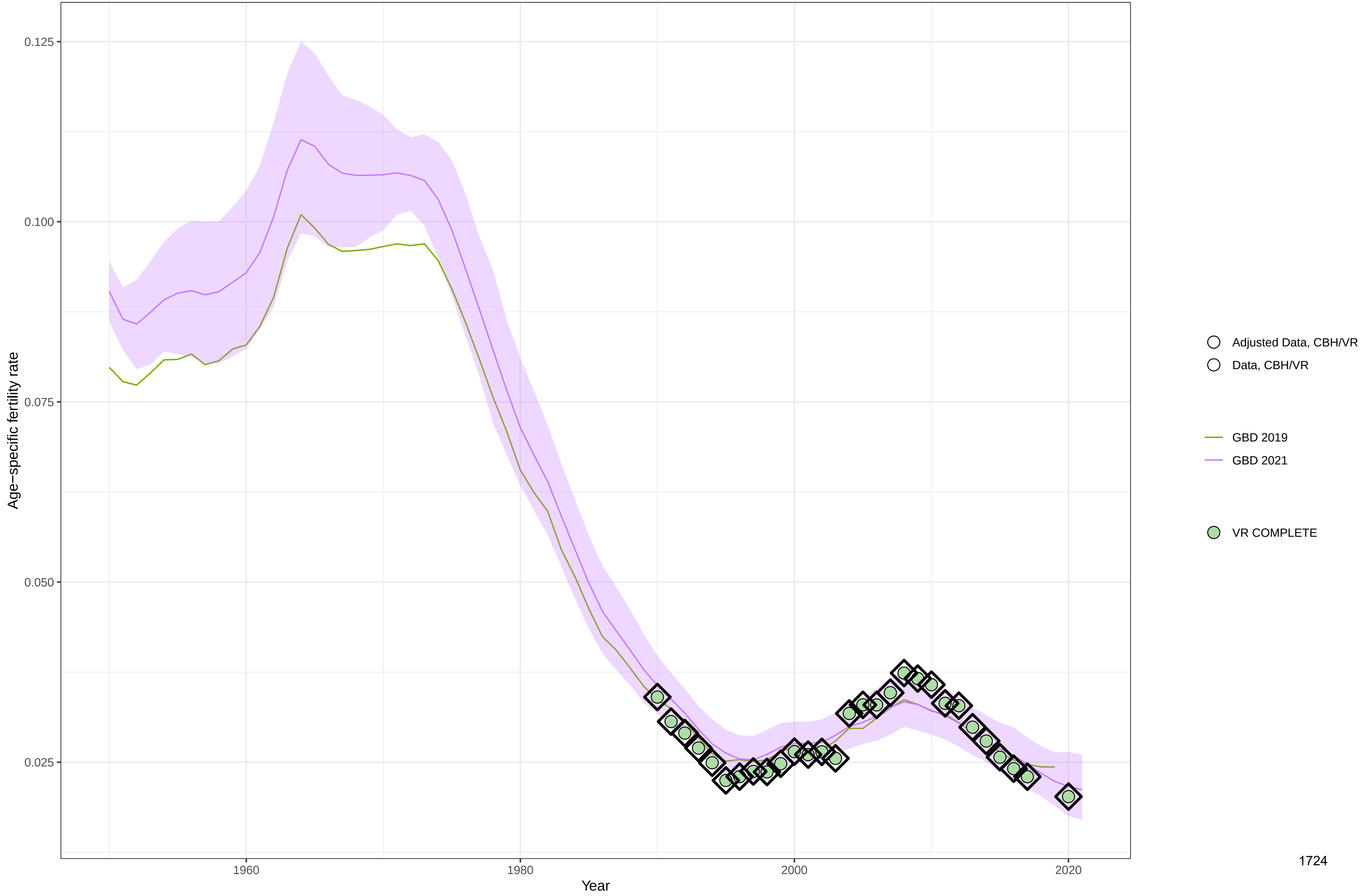

Age−specific fertility fitted trend lines, 1950−2021: Veneto, Ages: 25−29
Data points enclosed with a diamond are reference sources

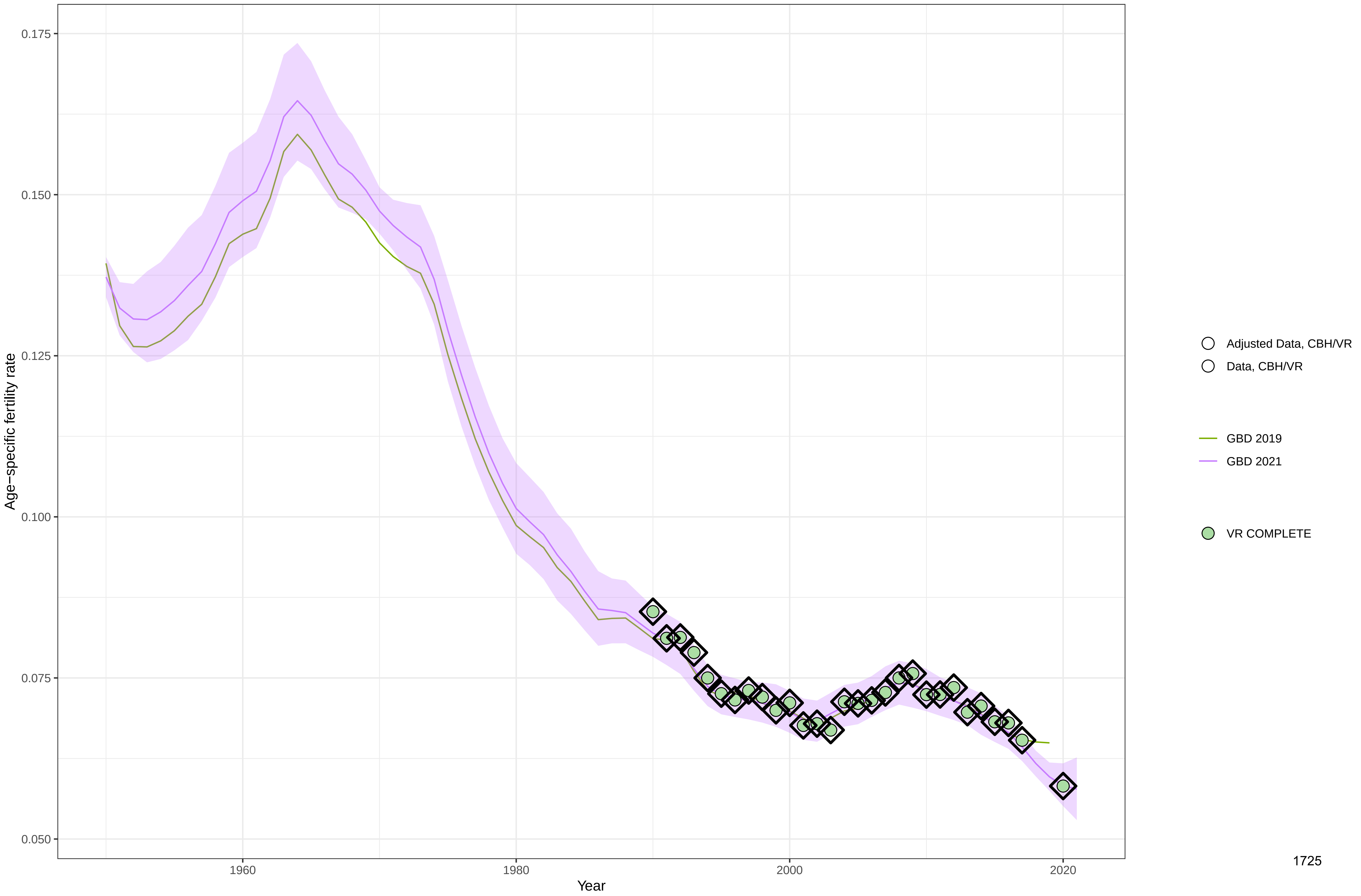

Age−specific fertility fitted trend lines, 1950−2021: Veneto, Ages: 30−34
Data points enclosed with a diamond are reference sources

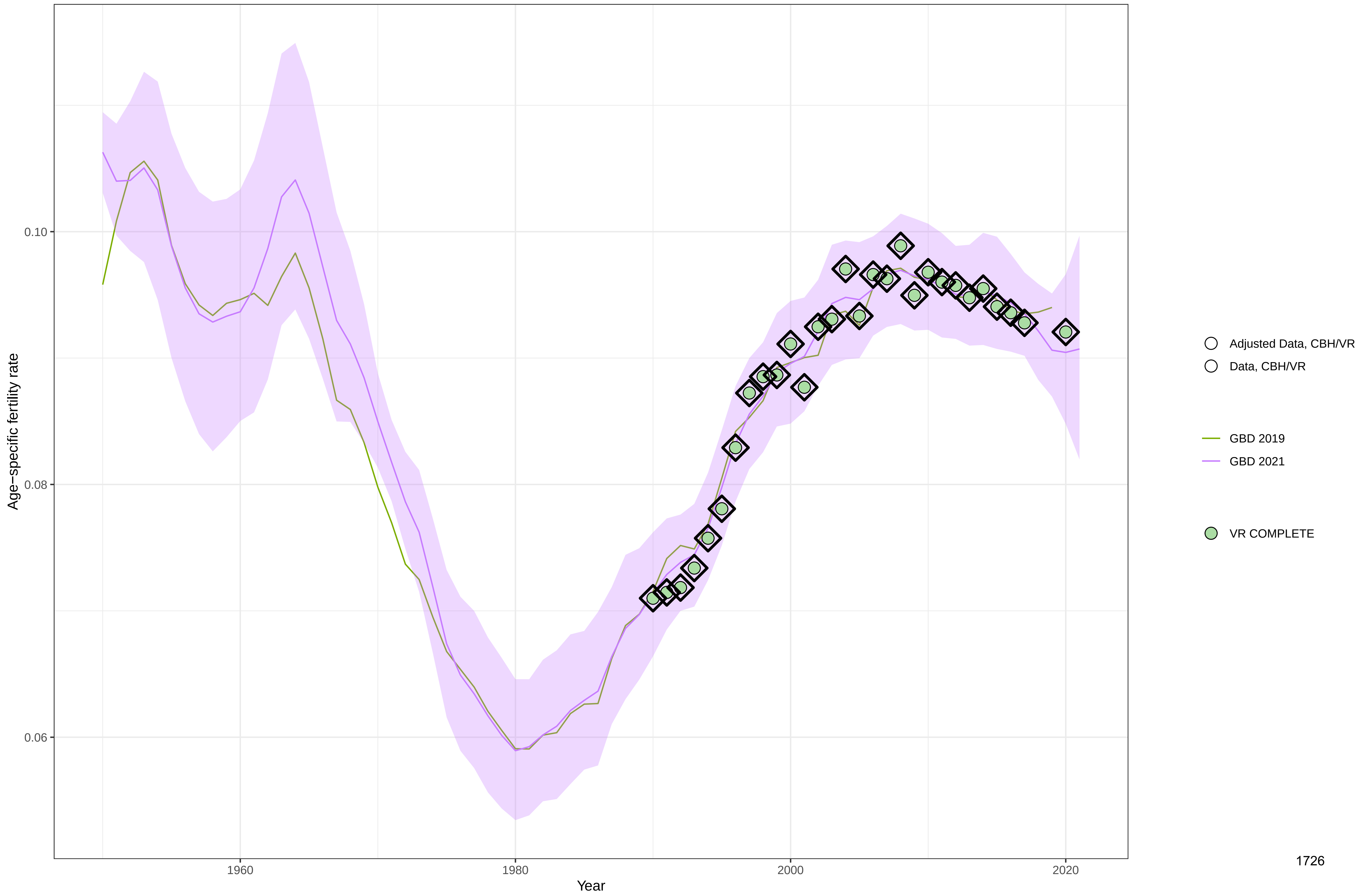

Age−specific fertility fitted trend lines, 1950−2021: Veneto, Ages: 35−39
Data points enclosed with a diamond are reference sources

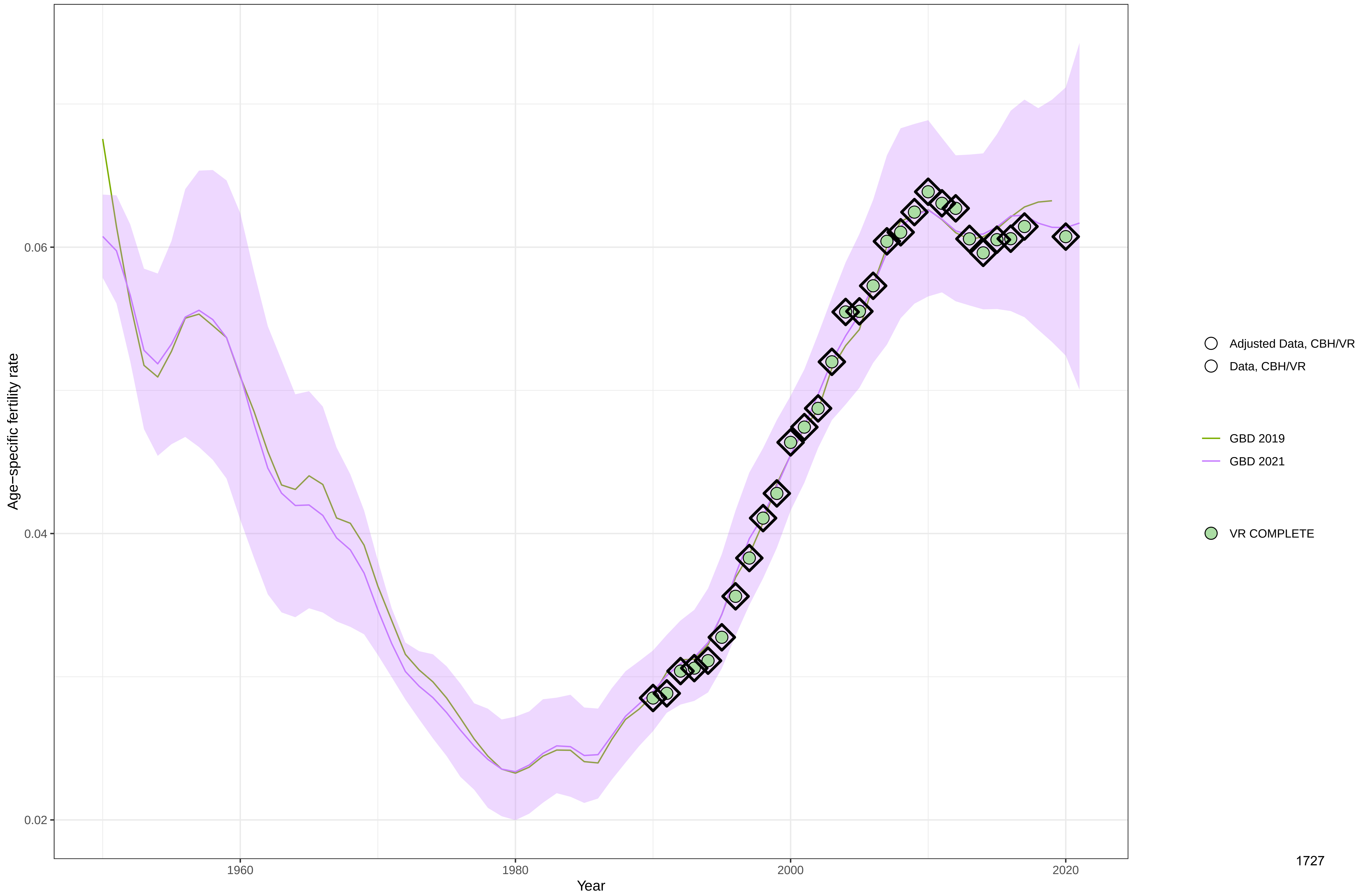

Age−specific fertility fitted trend lines, 1950−2021: Veneto, Ages: 40−44
Data points enclosed with a diamond are reference sources

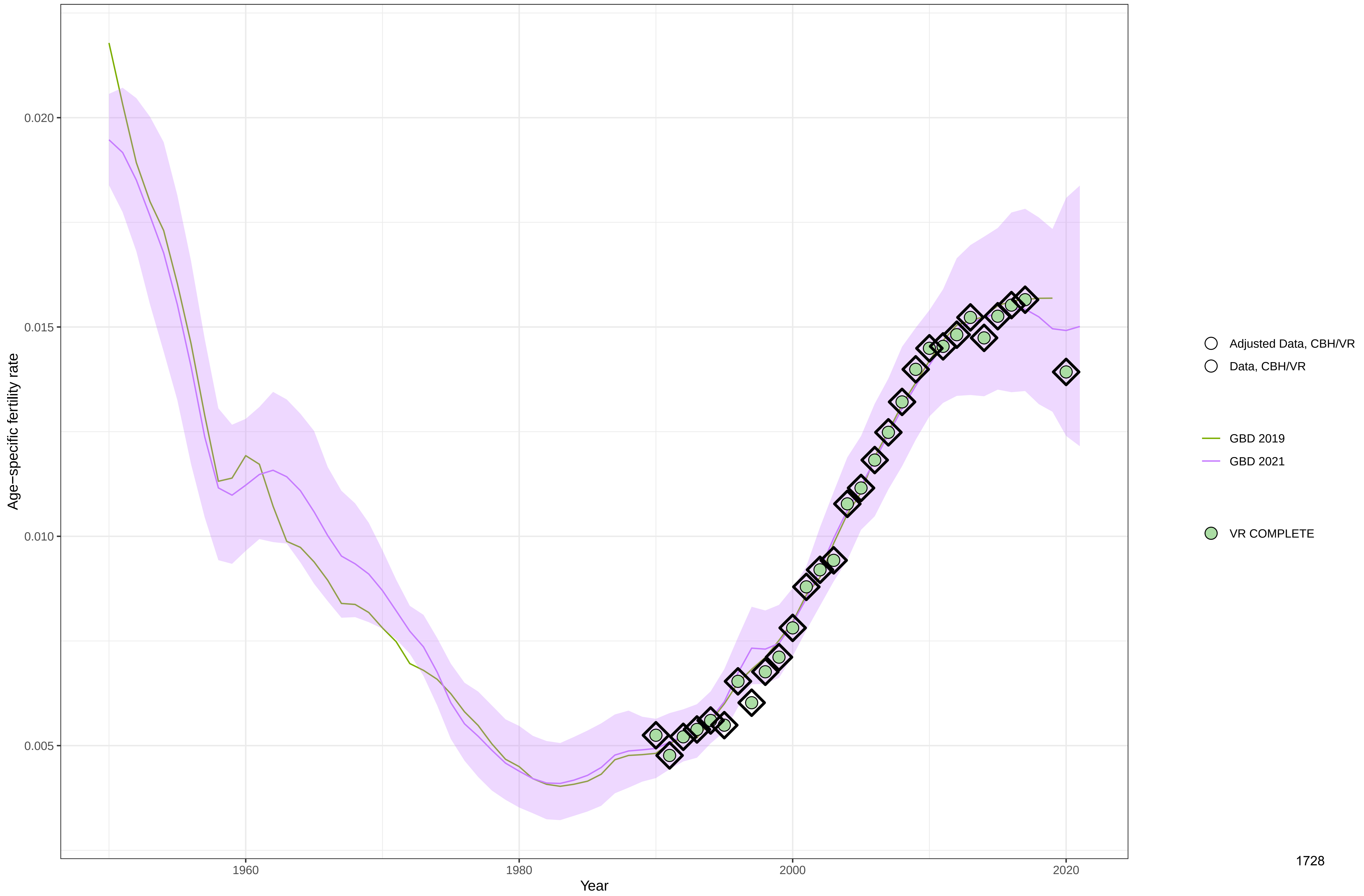

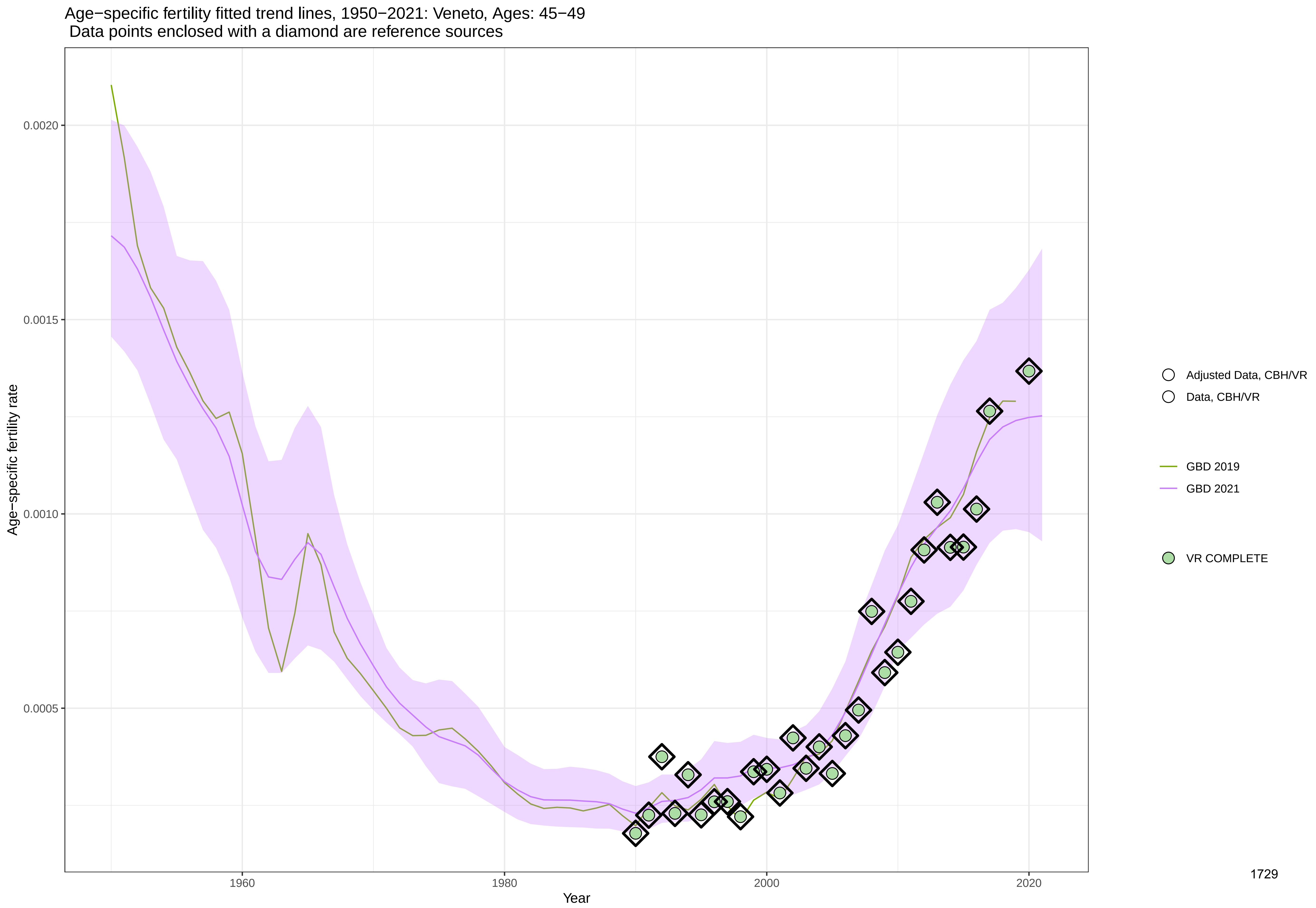
Age−specific fertility fitted trend lines, 1950−2021: Veneto, Ages: 45−49
Data points enclosed with a diamond are reference sources
0.0020
0.0015
0.0010
0.0005
Age−specific fertility rate
1960
1980
2000
2020
Year
Adjusted Data, CBH/VR
Data, CBH/VR
GBD 2019
GBD 2021
VR COMPLETE

Age−specific fertility fitted trend lines, 1950−2021: Veneto, Ages: 50−54
Data points enclosed with a diamond are reference sources

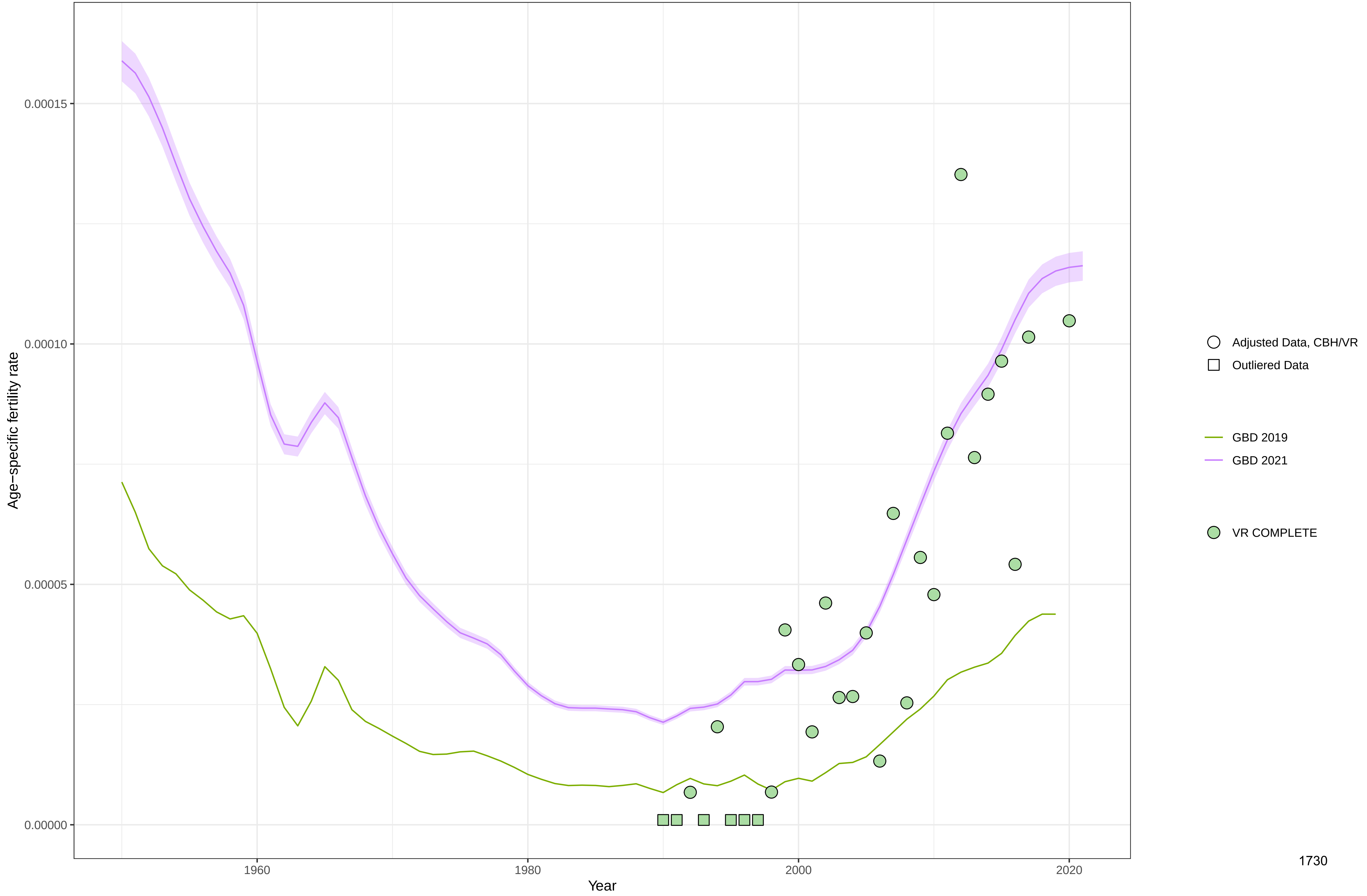

Total fertility rate fitted trend lines, 1950−2021: Veneto TFR
Data points enclosed with a diamond are reference sources

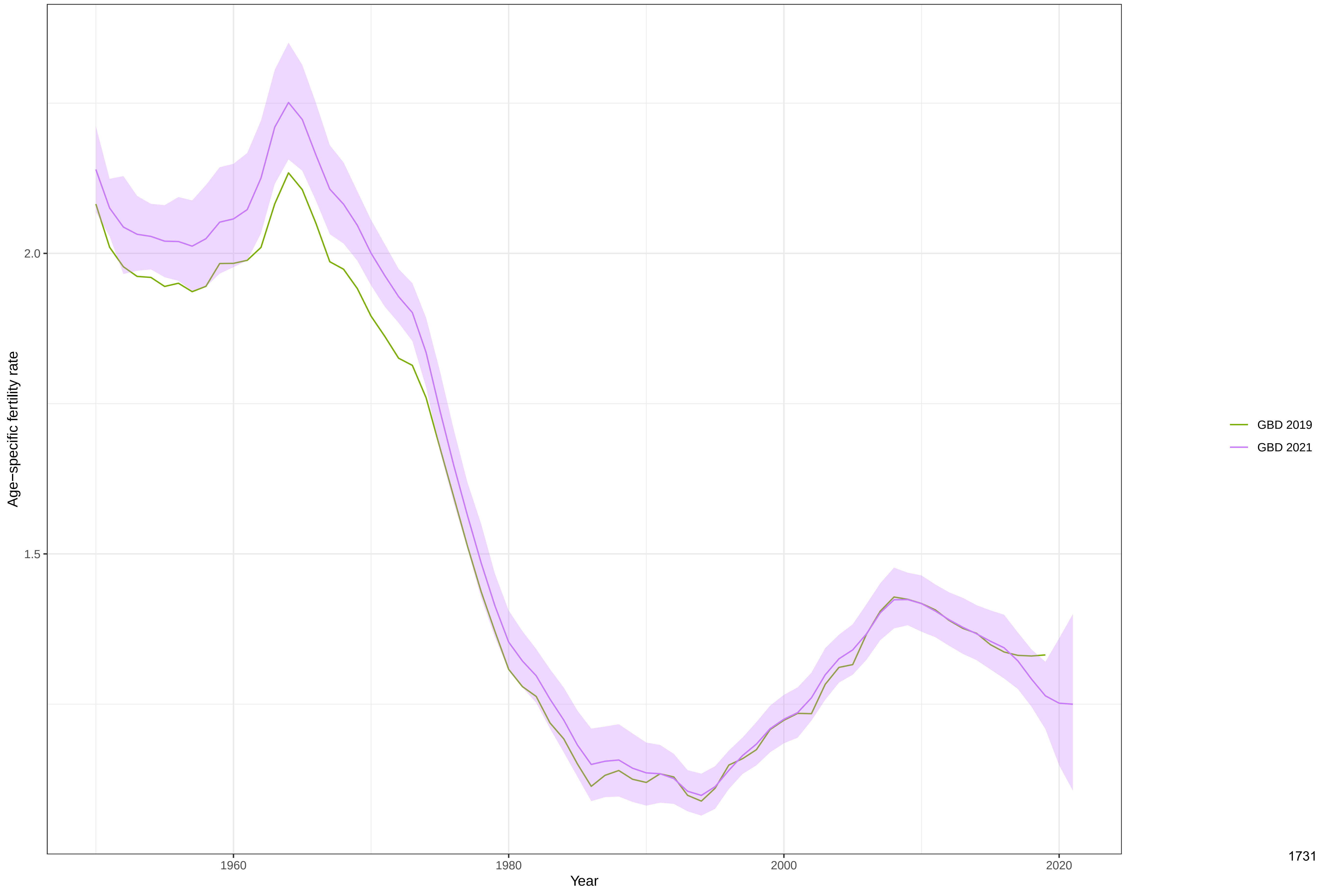

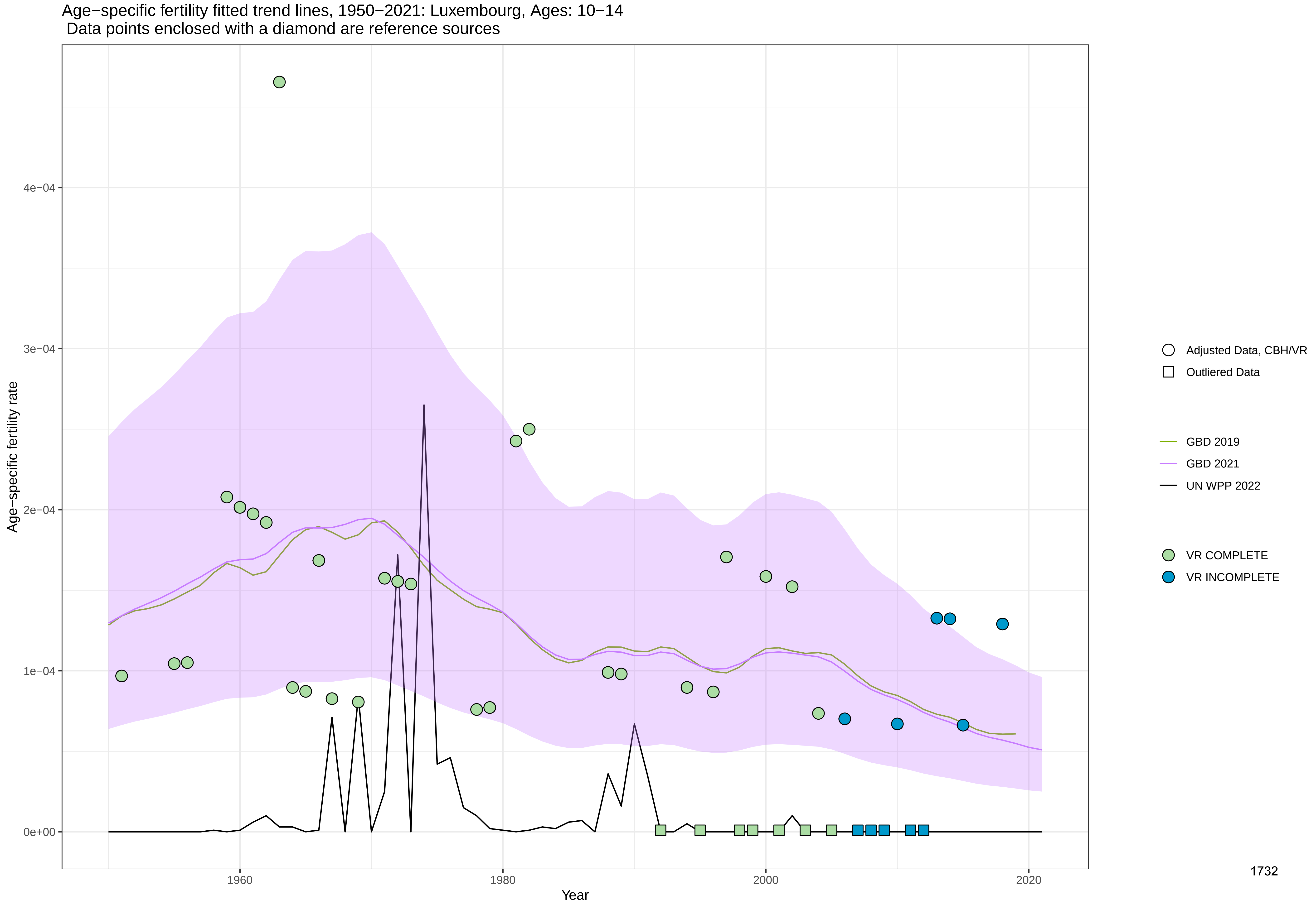

Age−specific fertility fitted trend lines, 1950−2021: Luxembourg, Ages: 10−14
Data points enclosed with a diamond are reference sources
Age−specific fertility rate
4e−04
3e−04
2e−04
1e−04
0e+00
1960
1980
2000
2020
Year
Adjusted Data, CBH/VR
Outliered Data
GBD 2019
GBD 2021
UN WPP 2022
VR COMPLETE
VR INCOMPLETE

Age−specific fertility fitted trend lines, 1950−2021: Luxembourg, Ages: 15−19
Data points enclosed with a diamond are reference sources

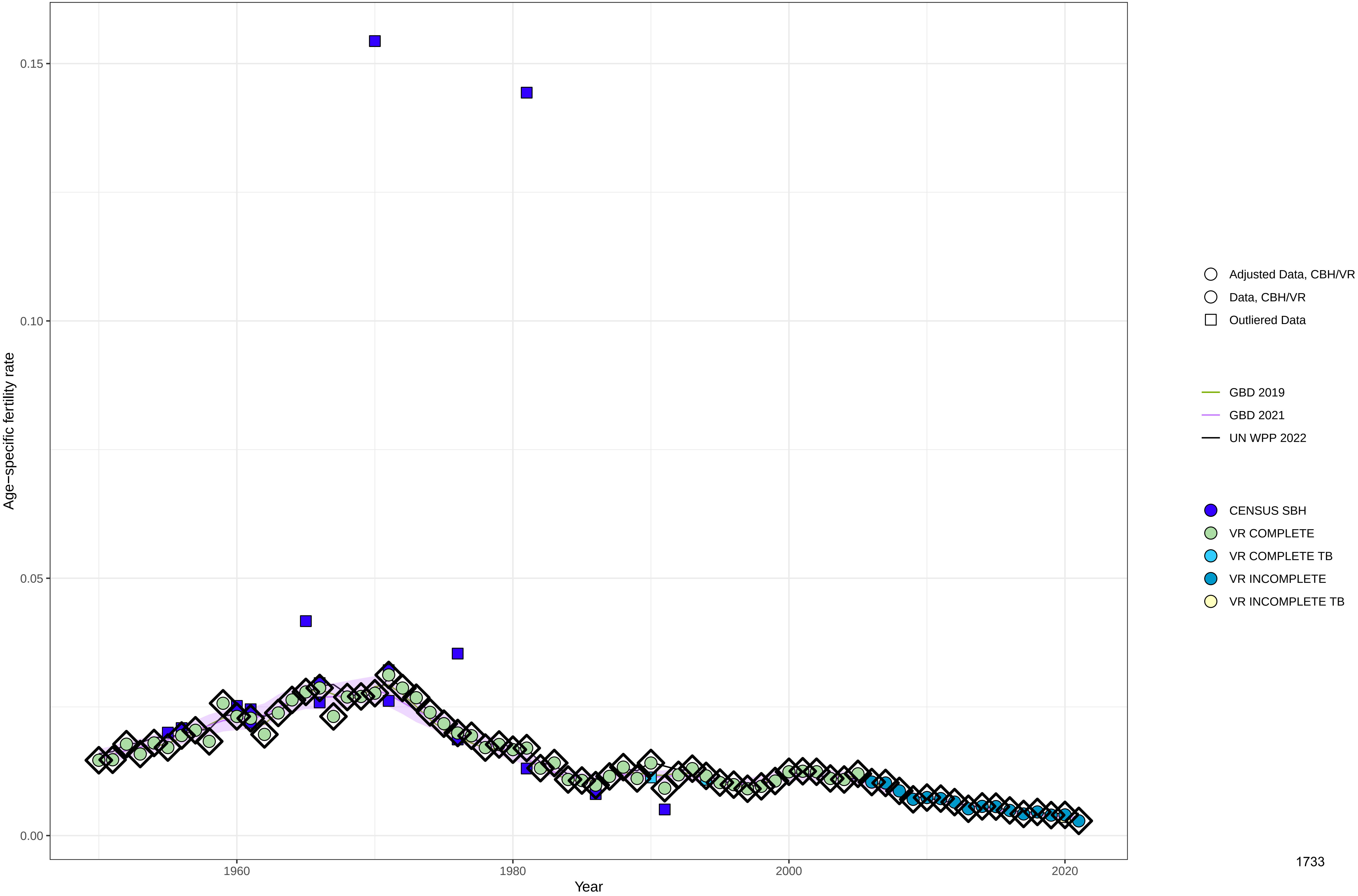

Age−specific fertility fitted trend lines, 1950−2021: Luxembourg, Ages: 20−24
Data points enclosed with a diamond are reference sources

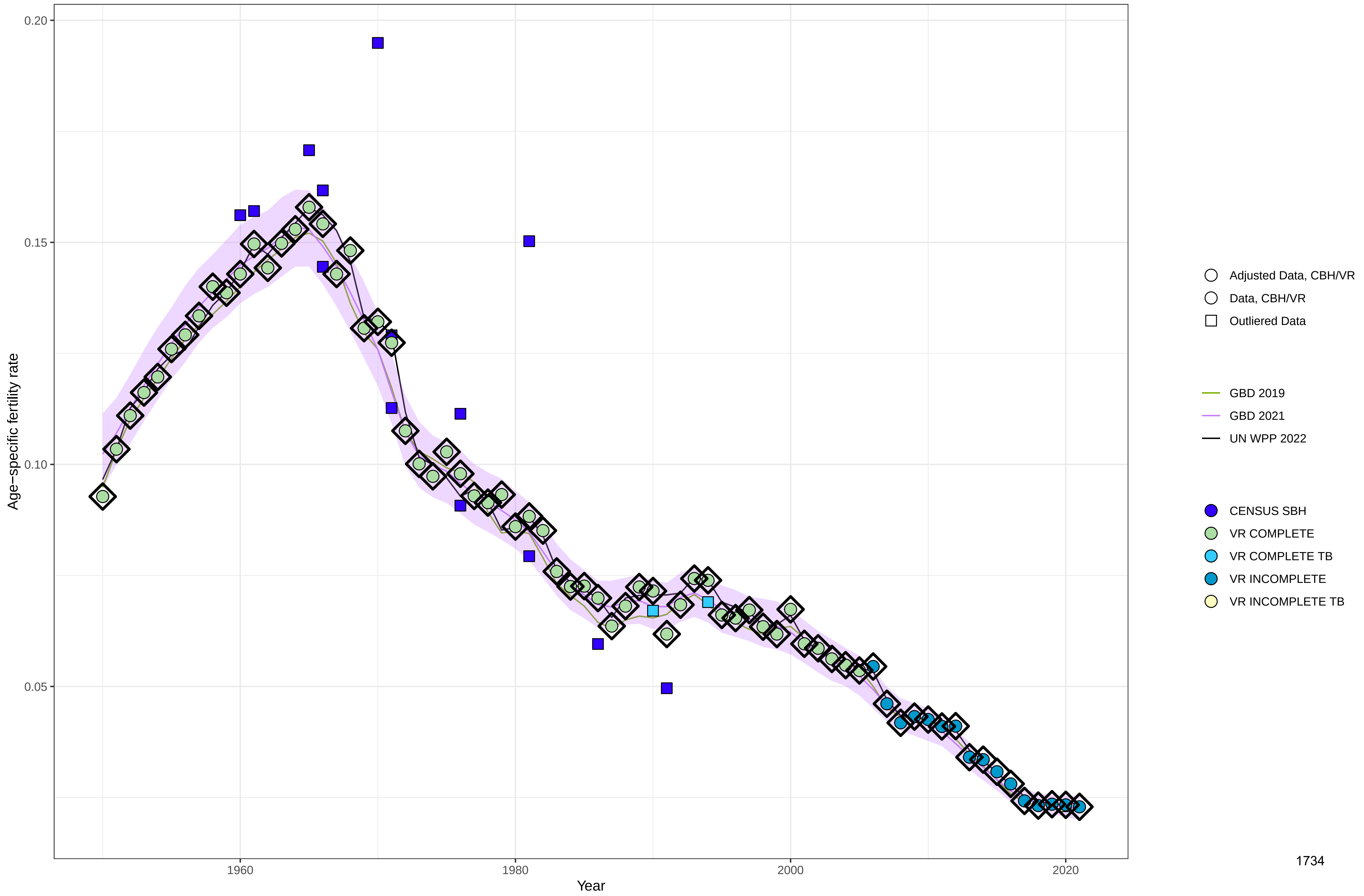

Age−specific fertility fitted trend lines, 1950−2021: Luxembourg, Ages: 25−29
Data points enclosed with a diamond are reference sources

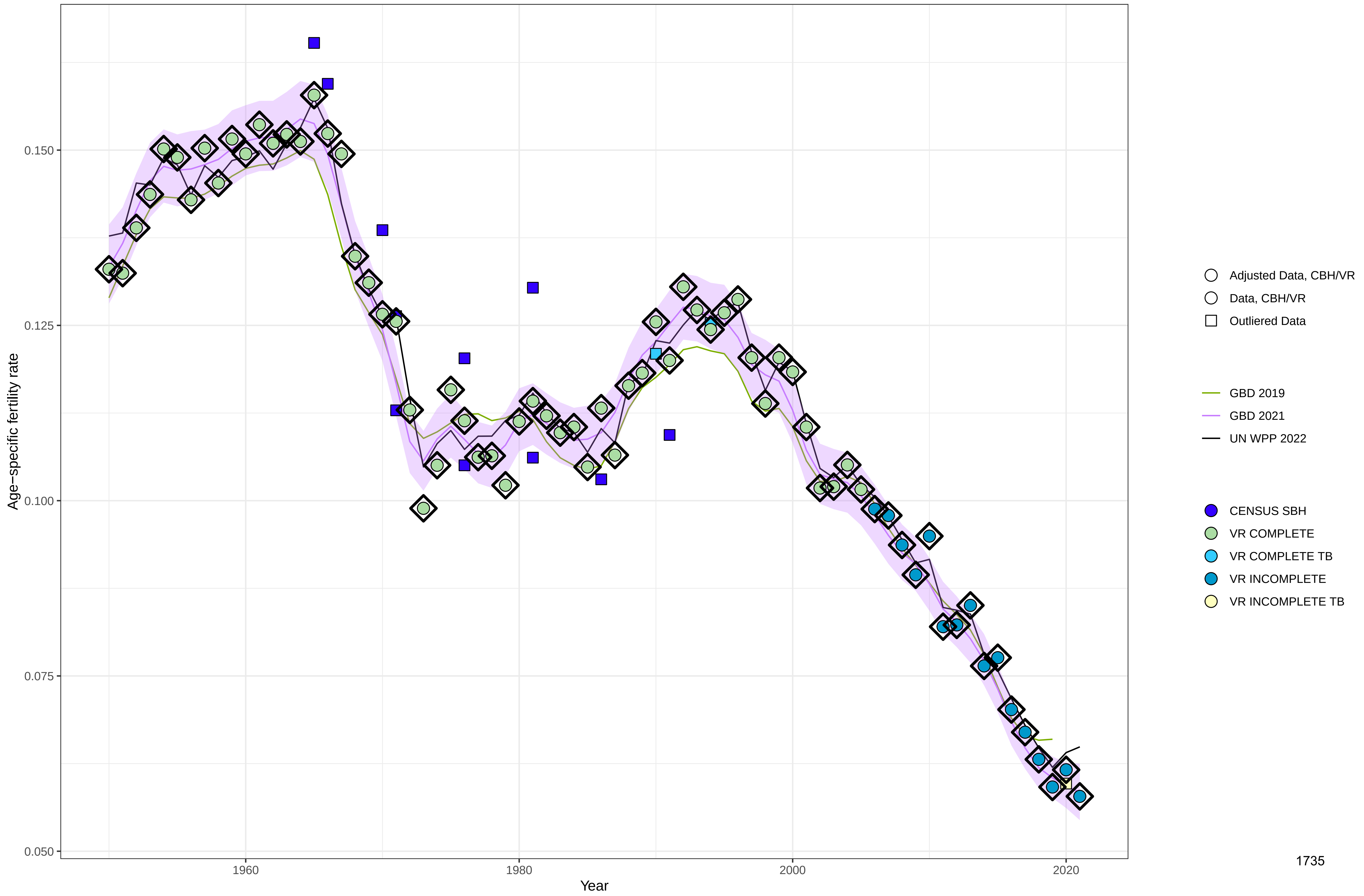

Age−specific fertility fitted trend lines, 1950−2021: Luxembourg, Ages: 30−34
Data points enclosed with a diamond are reference sources

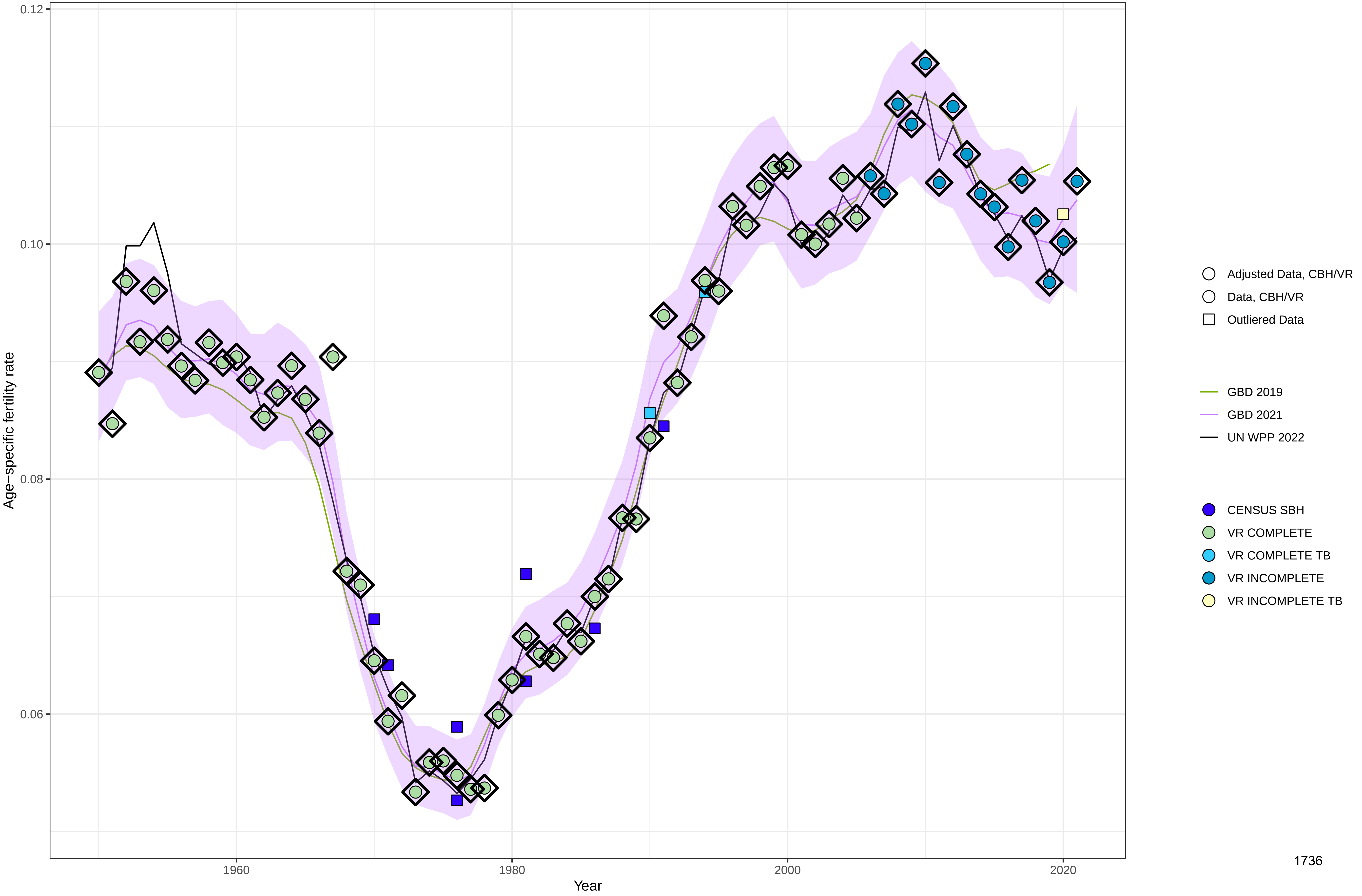

Age−specific fertility fitted trend lines, 1950−2021: Luxembourg, Ages: 35−39
Data points enclosed with a diamond are reference sources

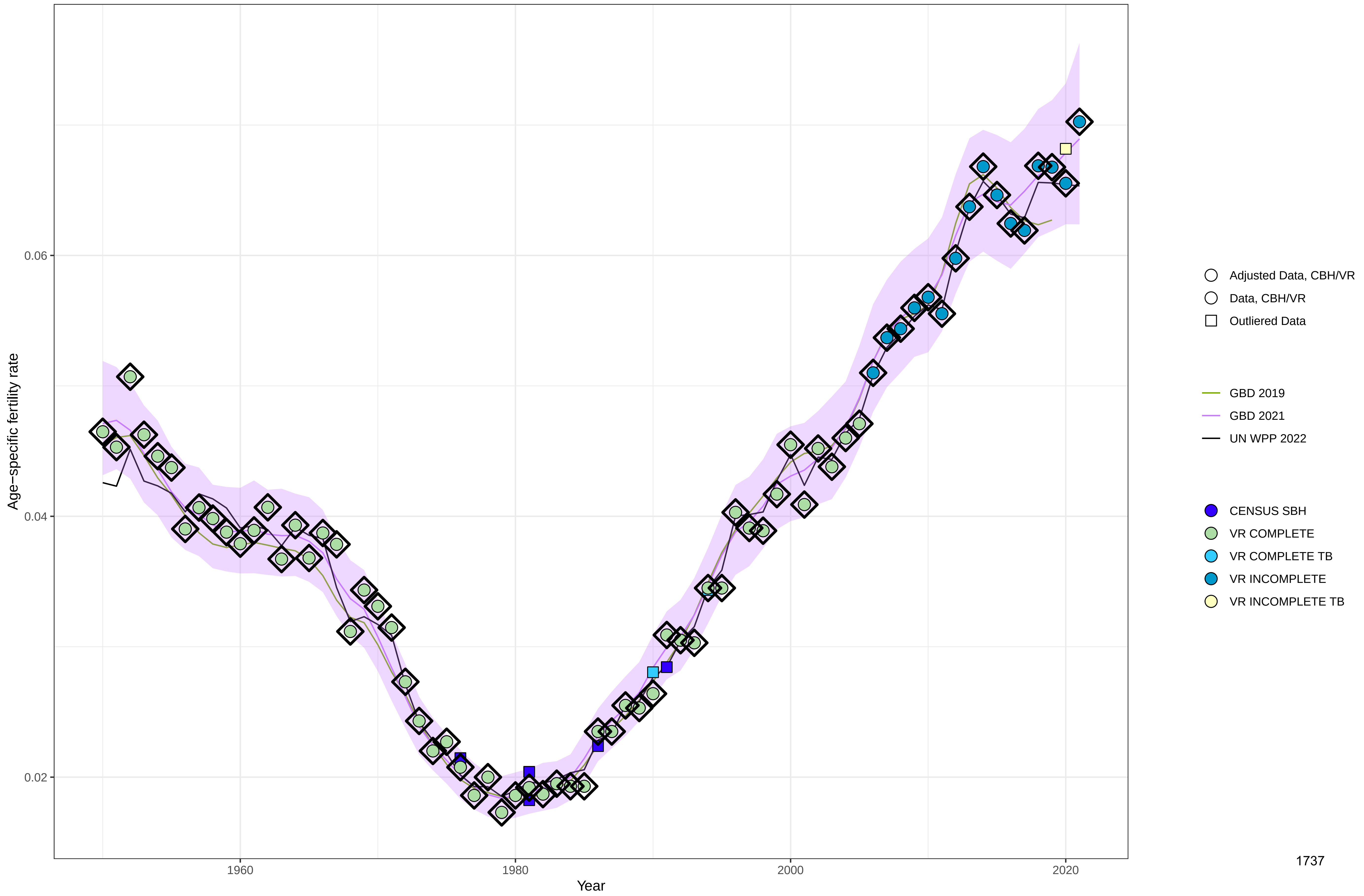

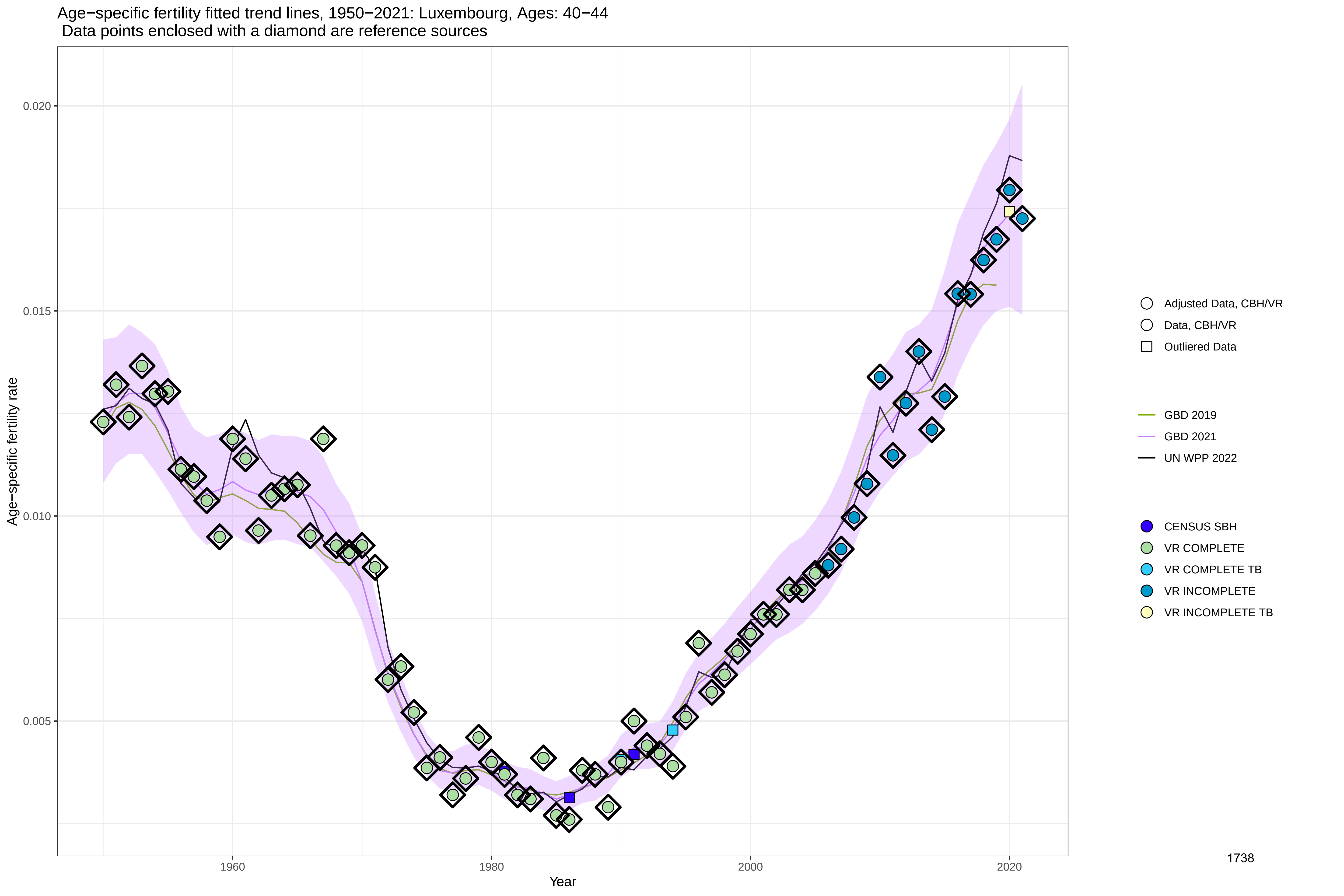
Age−specific fertility fitted trend lines, 1950−2021: Luxembourg, Ages: 40−44
Data points enclosed with a diamond are reference sources
Age−specific fertility rate
0.020
0.015
0.010
0.005
1960
1980
2000
2020
Year
Adjusted Data, CBH/VR
Data, CBH/VR
Outliered Data
GBD 2019
GBD 2021
UN WPP 2022
CENSUS SBH
VR COMPLETE
VR COMPLETE TB
VR INCOMPLETE
VR INCOMPLETE TB
1738

Age−specific fertility fitted trend lines, 1950−2021: Luxembourg, Ages: 45−49
Data points enclosed with a diamond are reference sources

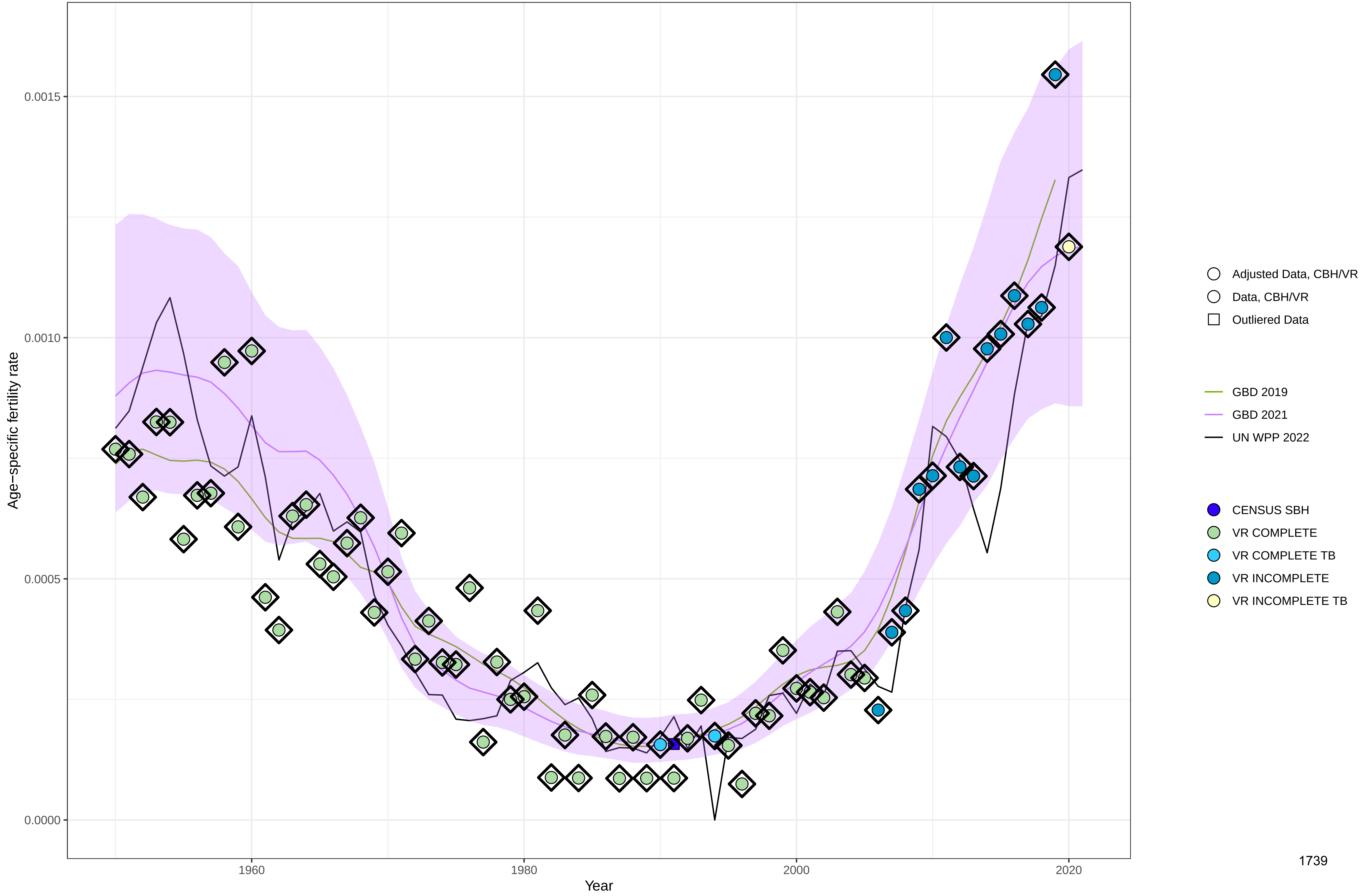

Age−specific fertility fitted trend lines, 1950−2021: Luxembourg, Ages: 50−54
Data points enclosed with a diamond are reference sources

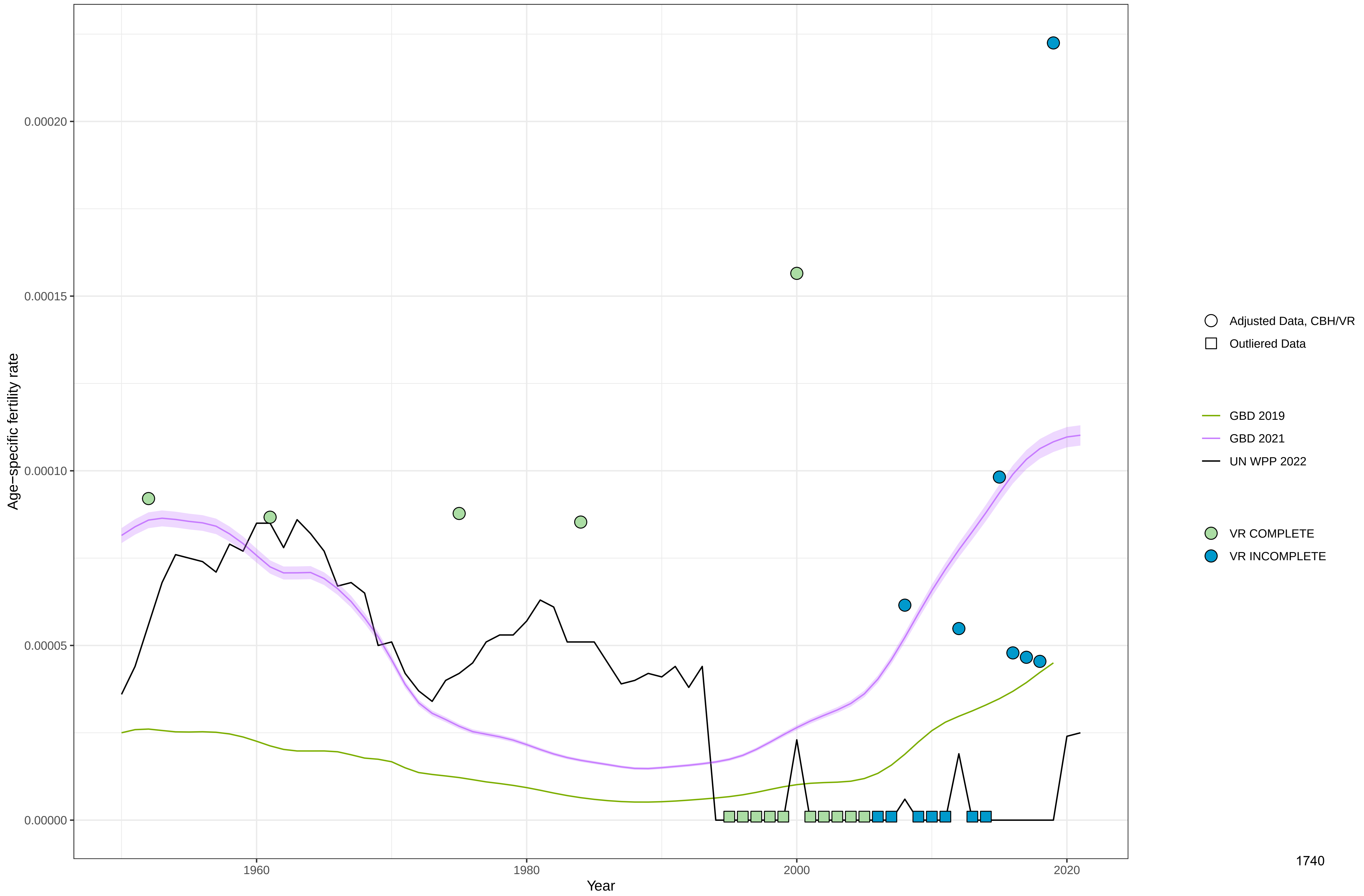

Total fertility rate fitted trend lines, 1950−2021: Luxembourg TFR
Data points enclosed with a diamond are reference sources

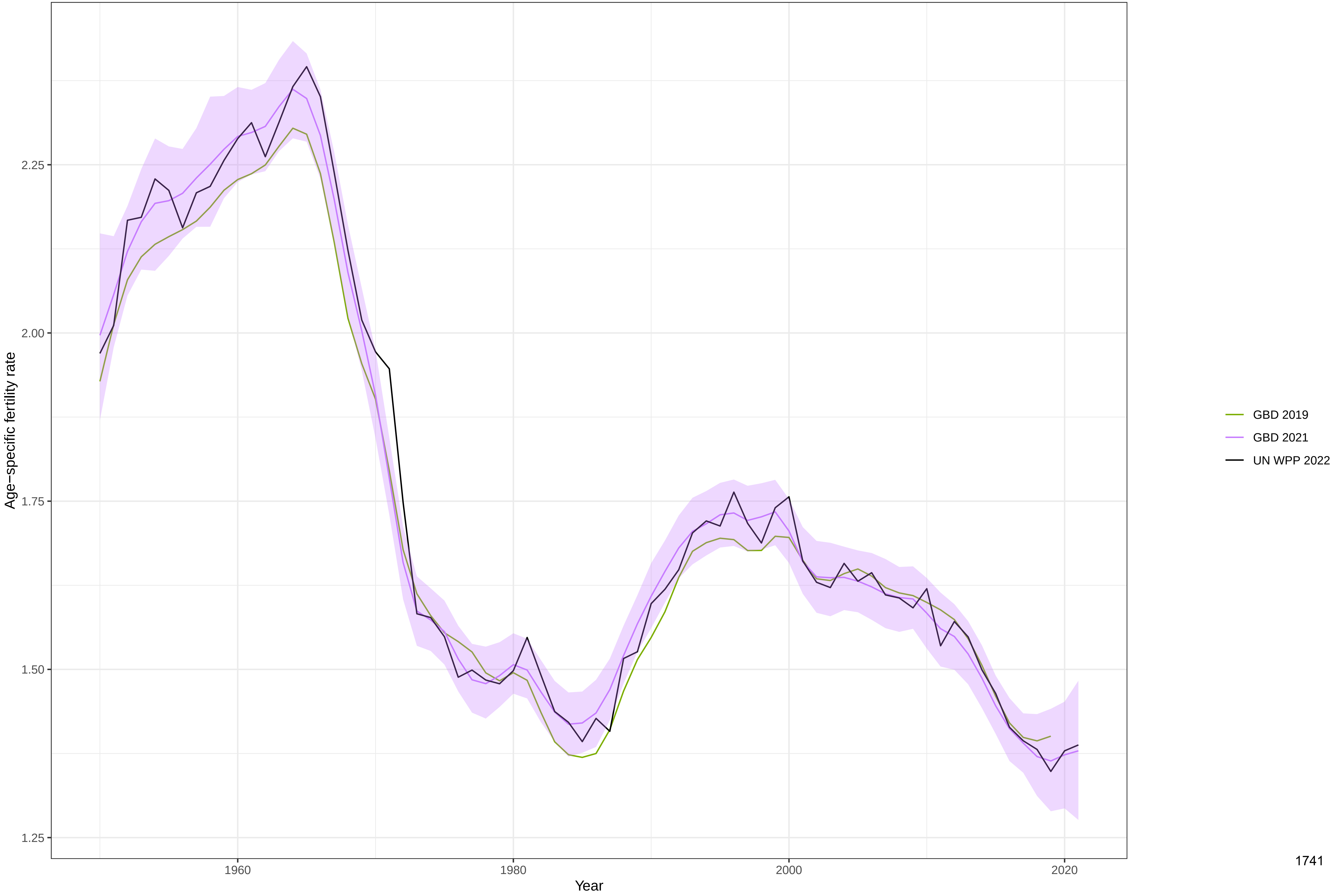

Age−specific fertility fitted trend lines, 1950−2021: Malta, Ages: 10−14
Data points enclosed with a diamond are reference sources

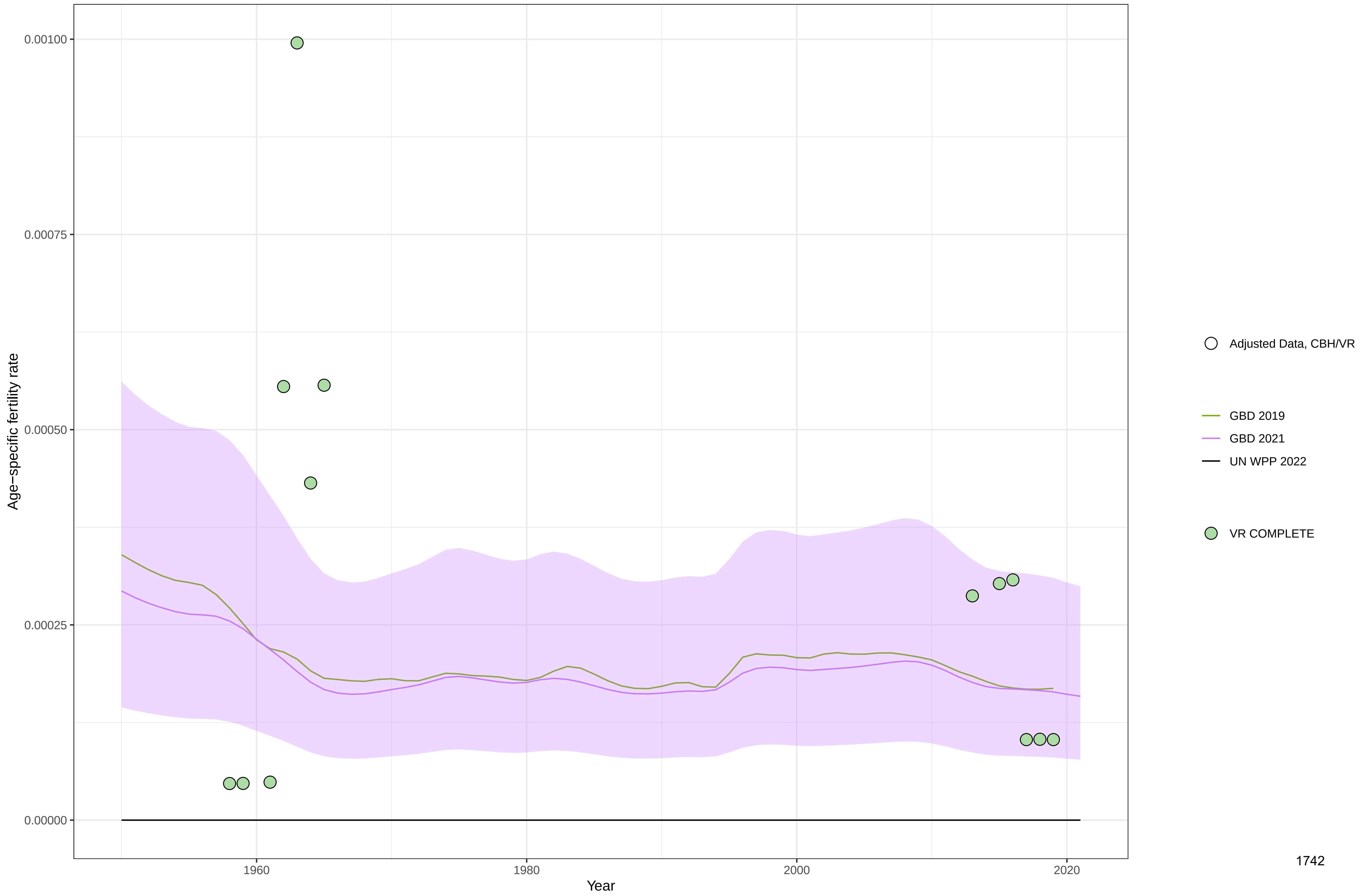

Age−specific fertility fitted trend lines, 1950−2021: Malta, Ages: 15−19
Data points enclosed with a diamond are reference sources

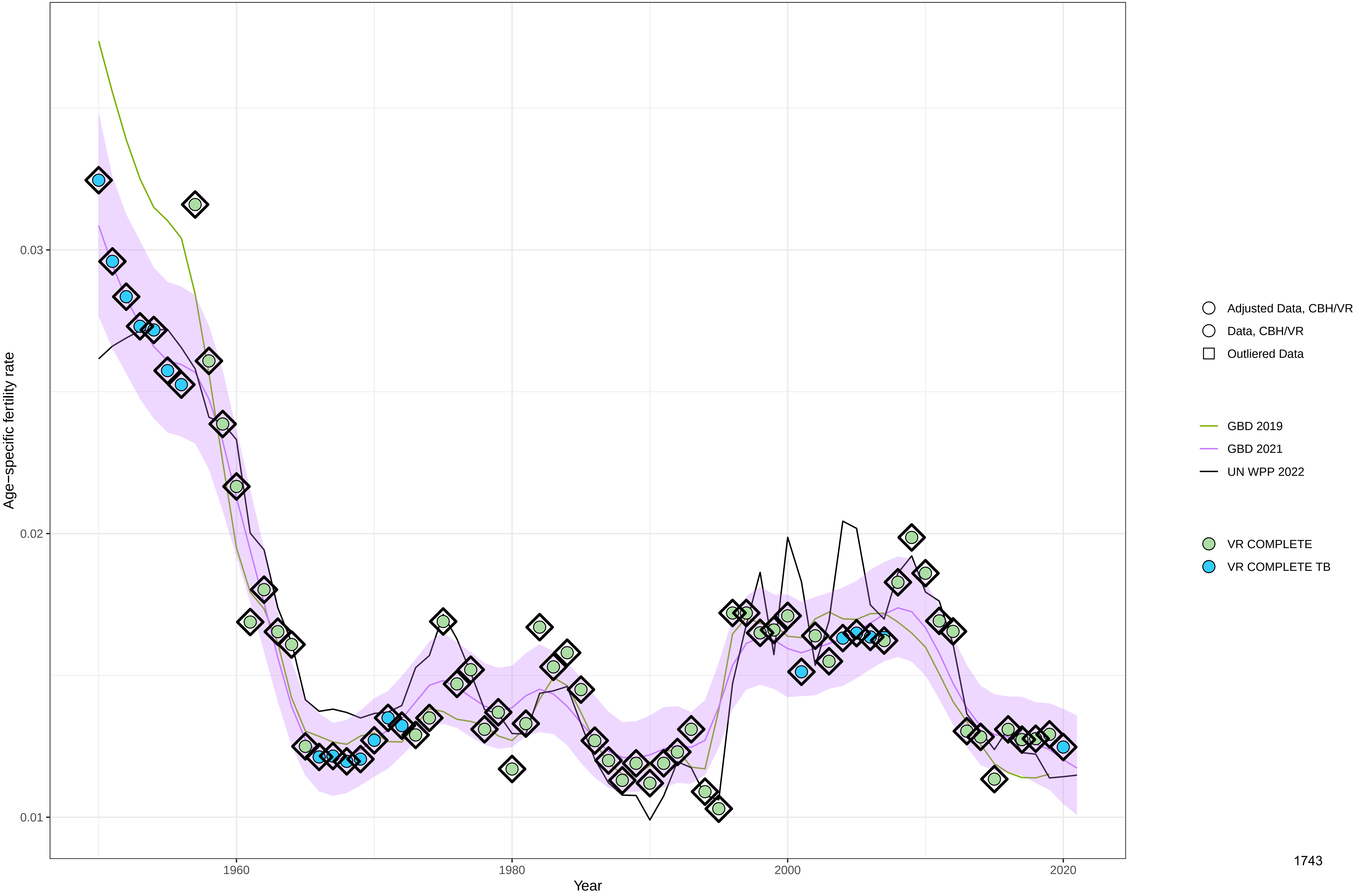

Age−specific fertility fitted trend lines, 1950−2021: Malta, Ages: 20−24
Data points enclosed with a diamond are reference sources

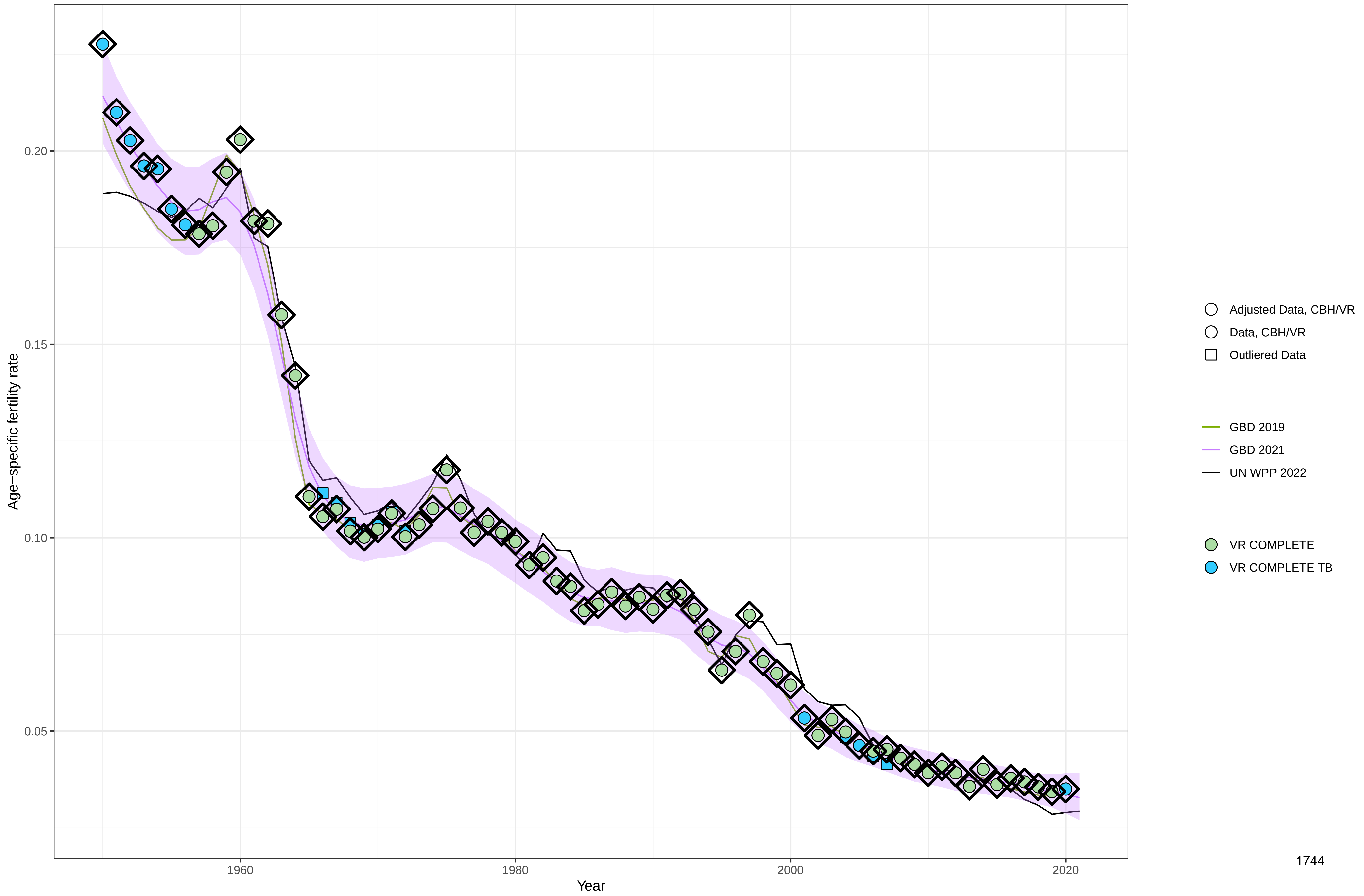

Age−specific fertility fitted trend lines, 1950−2021: Malta, Ages: 25−29
Data points enclosed with a diamond are reference sources

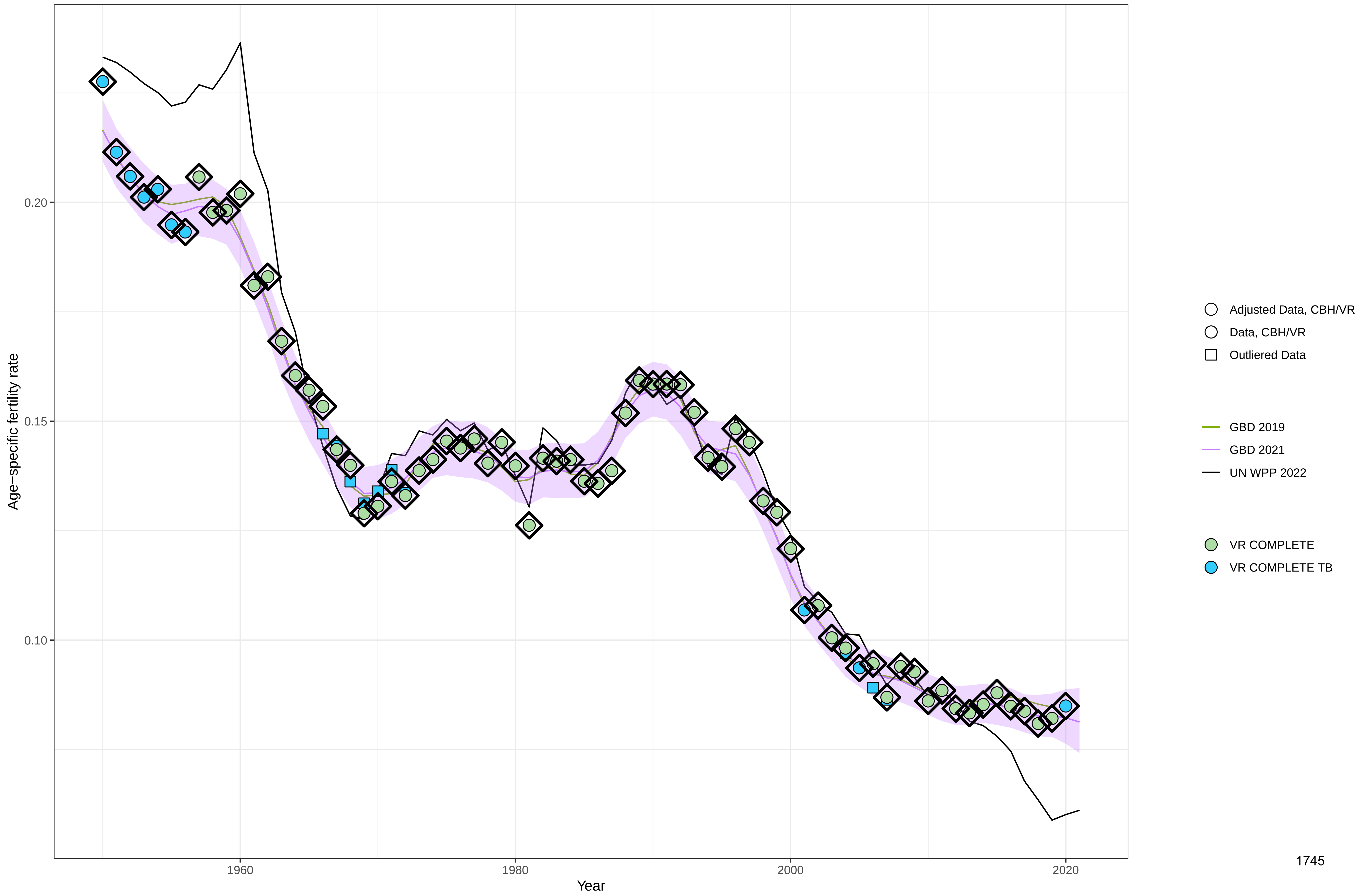

Age−specific fertility fitted trend lines, 1950−2021: Malta, Ages: 30−34
Data points enclosed with a diamond are reference sources

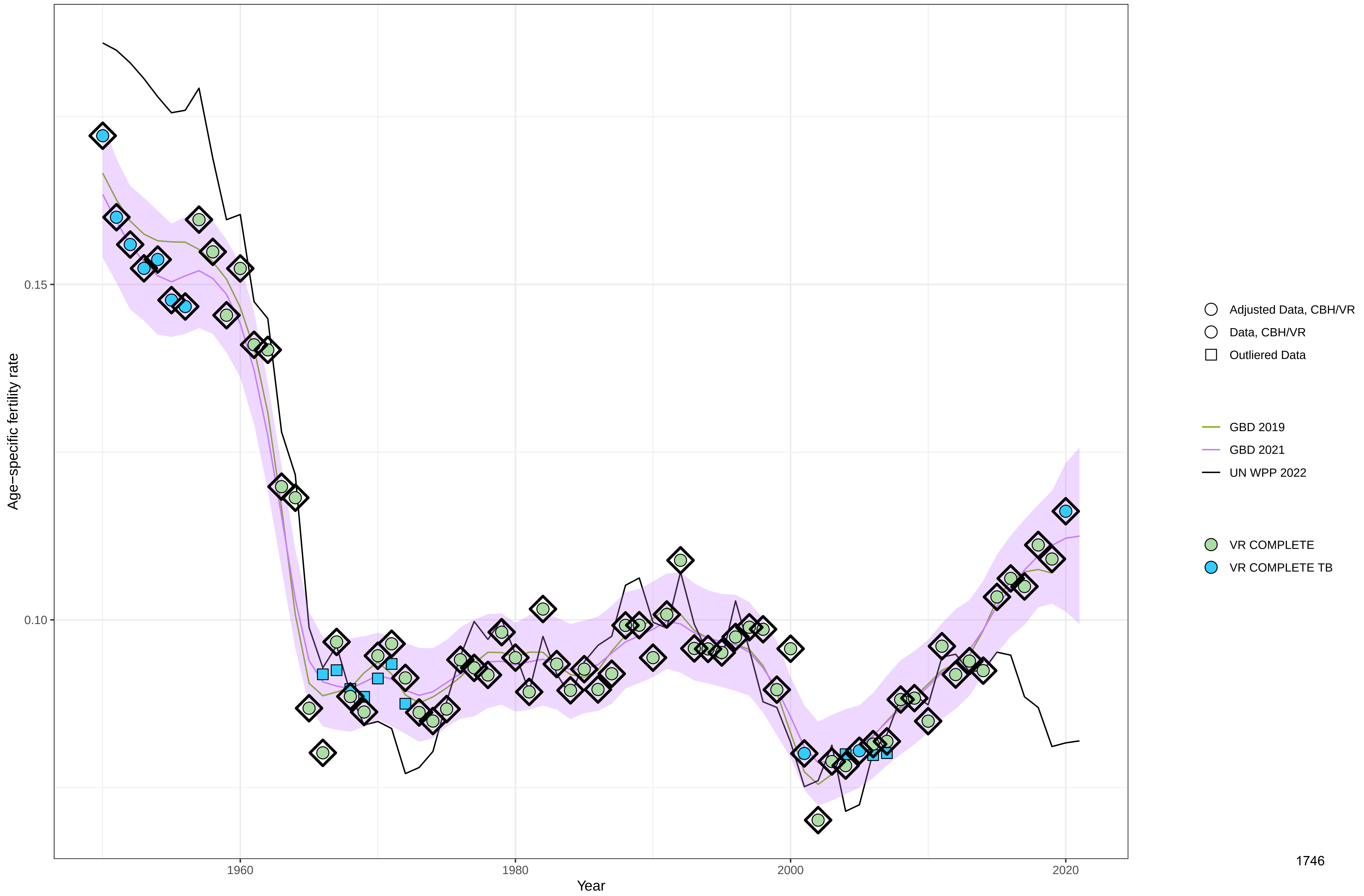

Age−specific fertility fitted trend lines, 1950−2021: Malta, Ages: 35−39
Data points enclosed with a diamond are reference sources

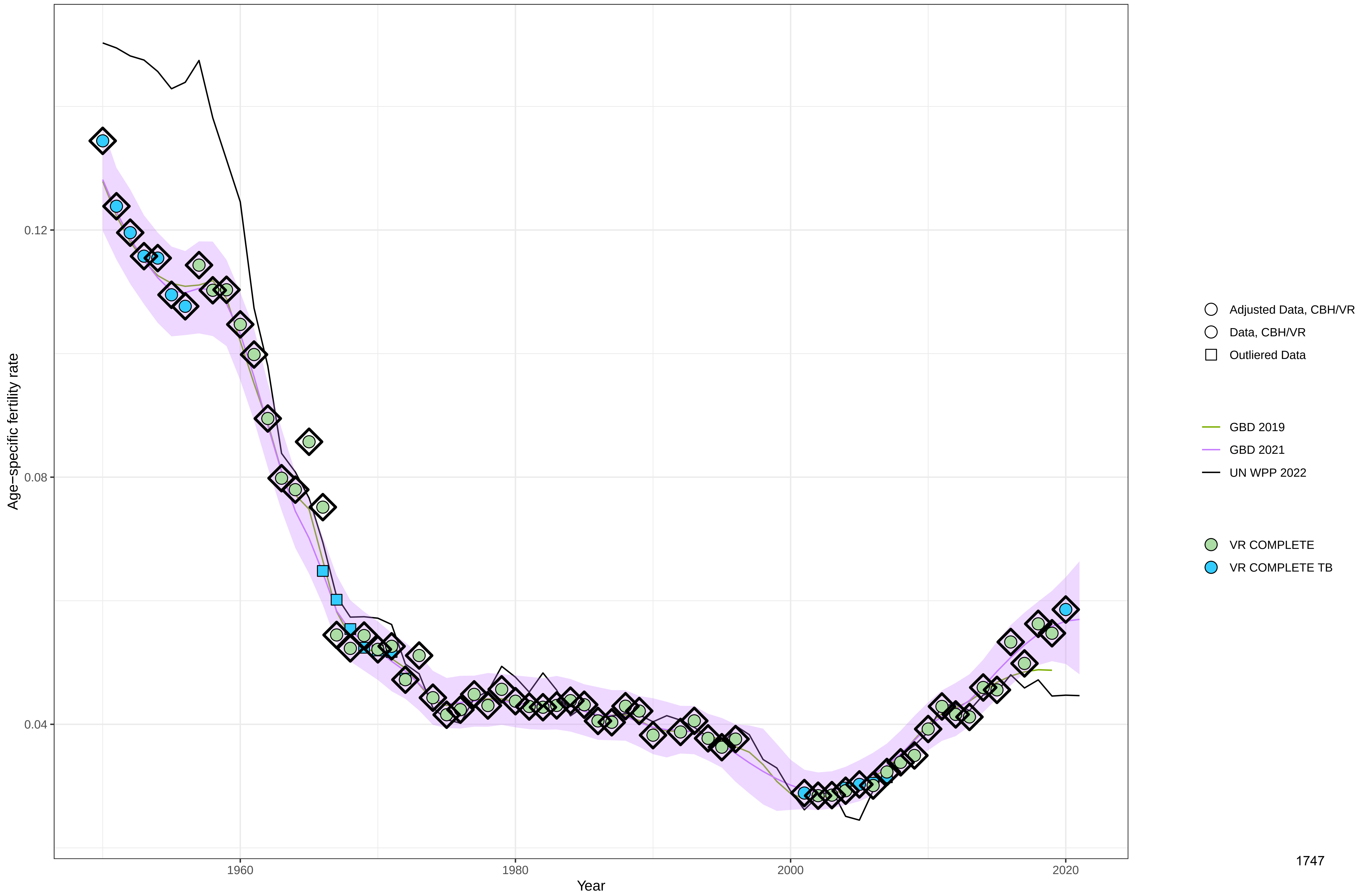

Age−specific fertility fitted trend lines, 1950−2021: Malta, Ages: 40−44
Data points enclosed with a diamond are reference sources

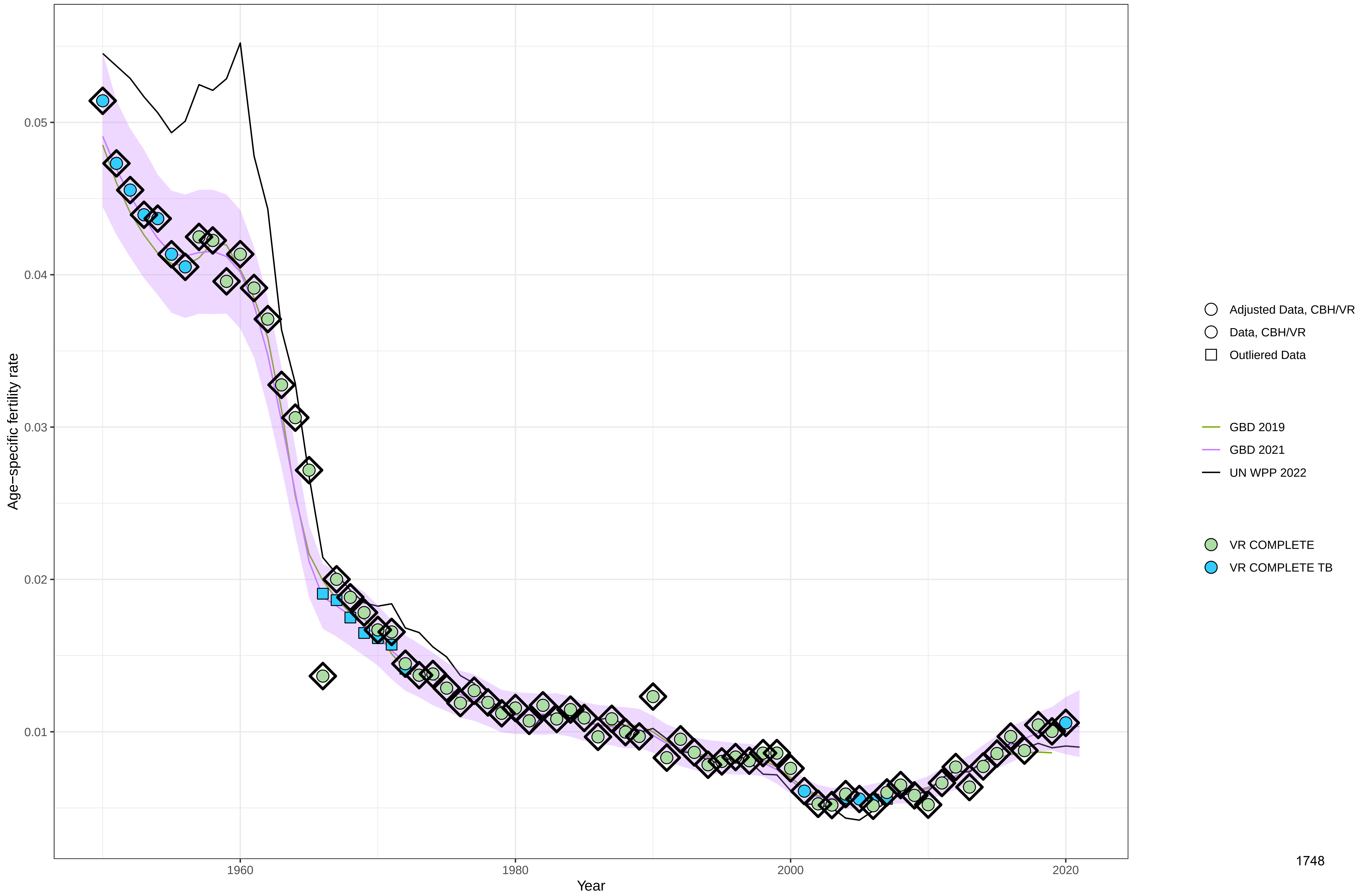

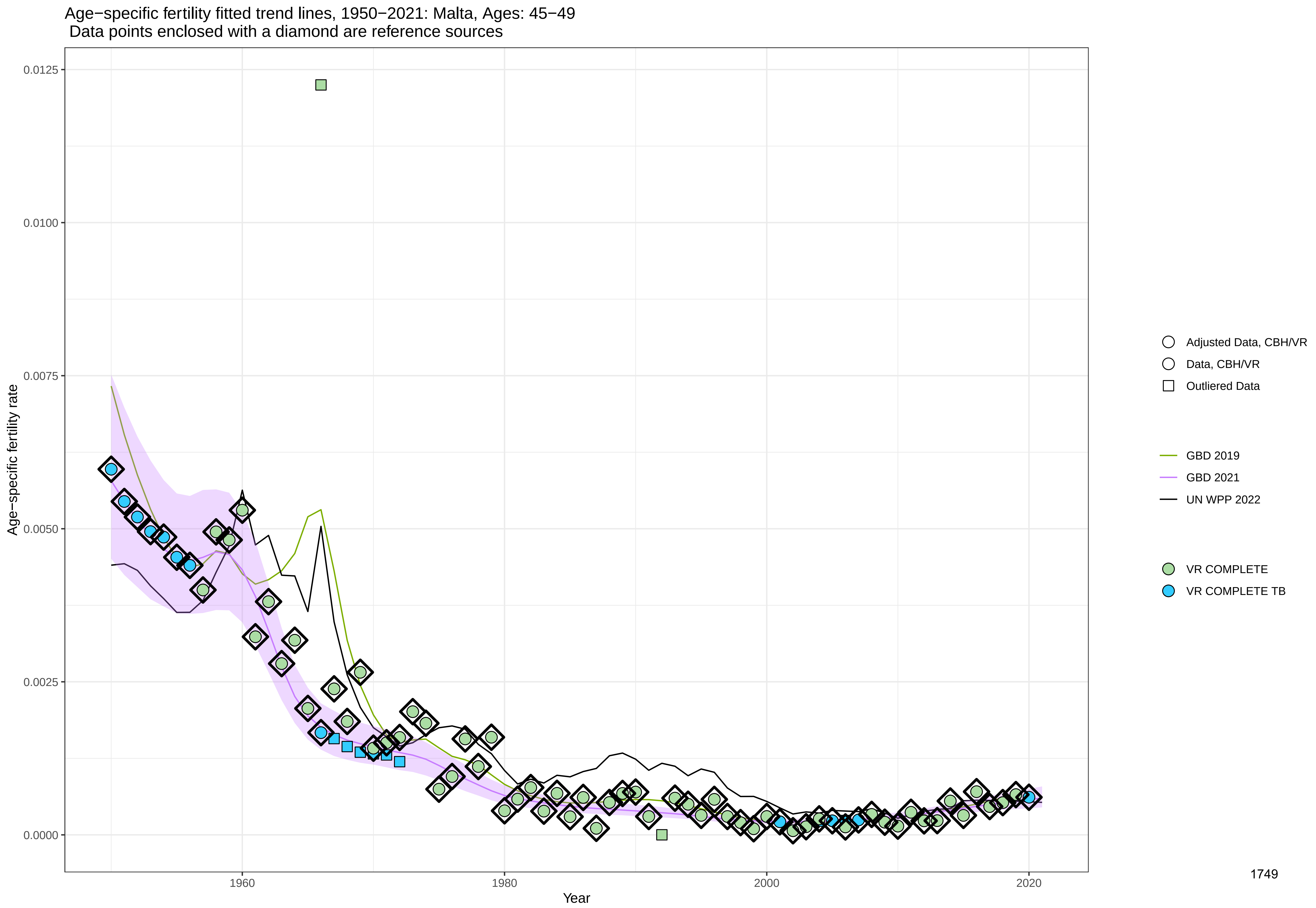

Age−specific fertility fitted trend lines, 1950−2021: Malta, Ages: 45−49
Data points enclosed with a diamond are reference sources
Age−specific fertility rate
Year
0.0000
0.0025
0.0050
0.0075
0.0100
0.0125
1960
1980
2000
2020
Adjusted Data, CBH/VR
Data, CBH/VR
Outliered Data
GBD 2019
GBD 2021
UN WPP 2022
VR COMPLETE
VR COMPLETE TB

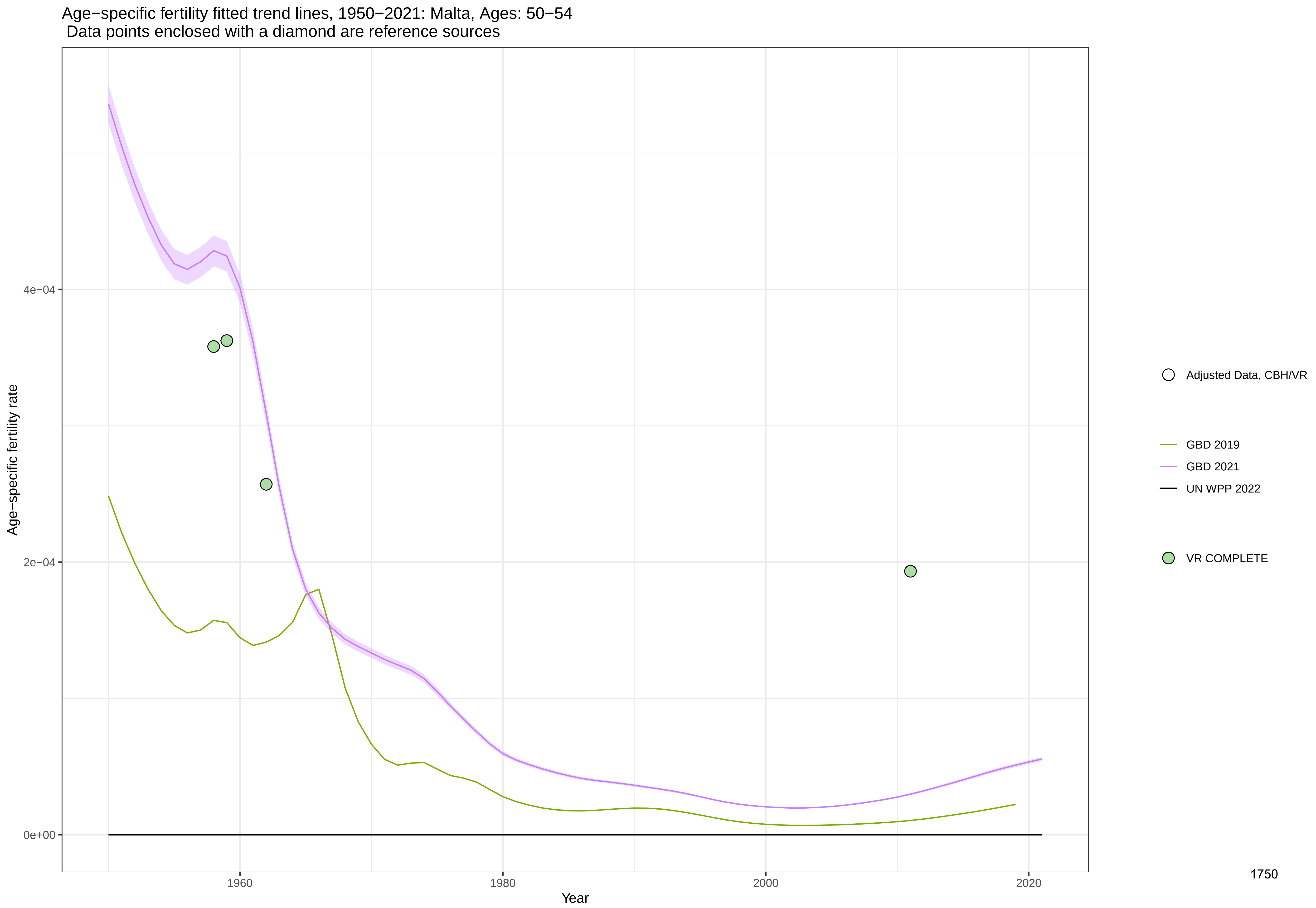

Age−specific fertility fitted trend lines, 1950−2021: Malta, Ages: 50−54
Data points enclosed with a diamond are reference sources
Age−specific fertility rate
4e−04
2e−04
0e+00
1960
1980
2000
2020
Year
Adjusted Data, CBH/VR
GBD 2019
GBD 2021
UN WPP 2022
VR COMPLETE

Total fertility rate fitted trend lines, 1950−2021: Malta TFR
Data points enclosed with a diamond are reference sources

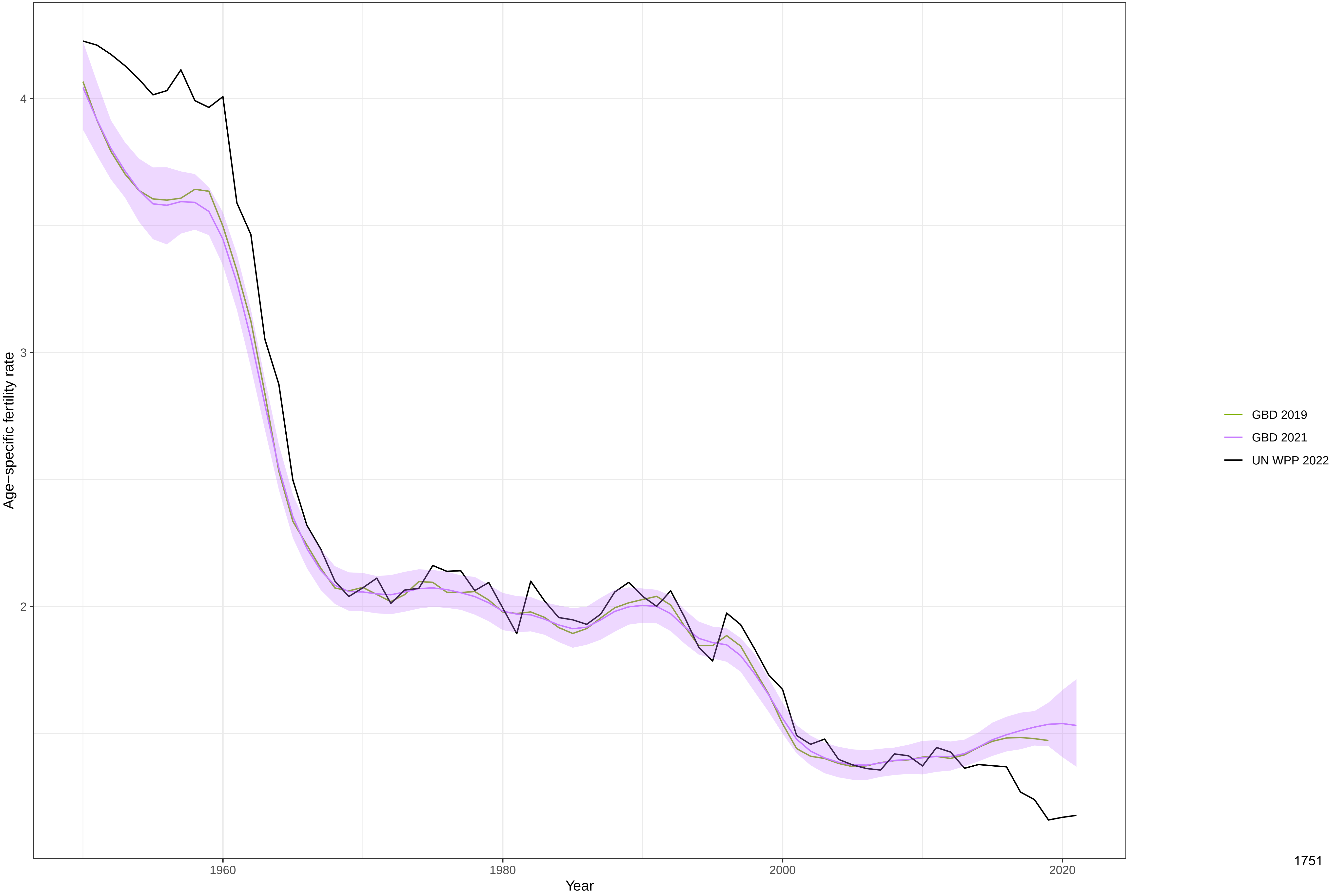

Age−specific fertility fitted trend lines, 1950−2021: Monaco, Ages: 10−14
Data points enclosed with a diamond are reference sources

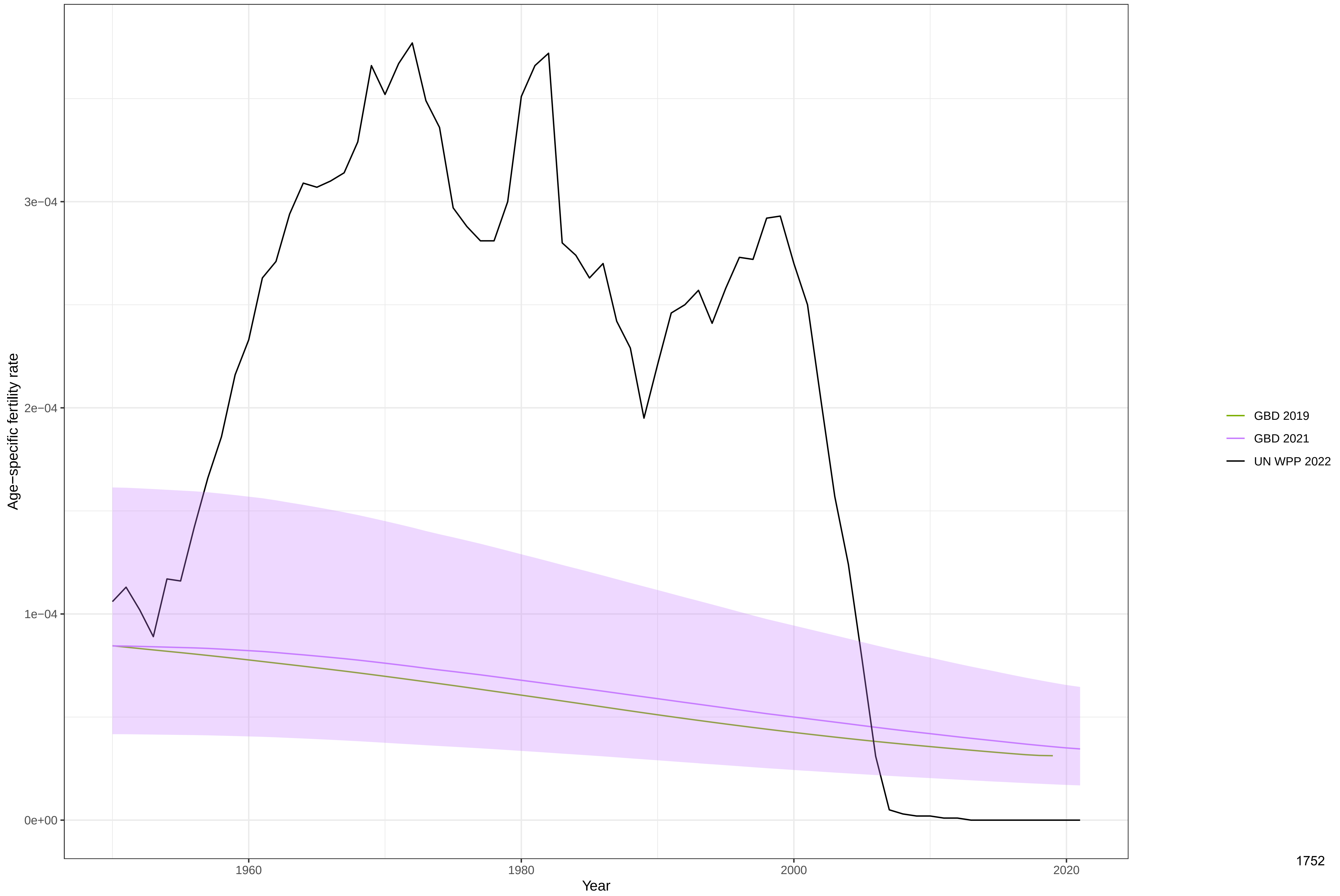

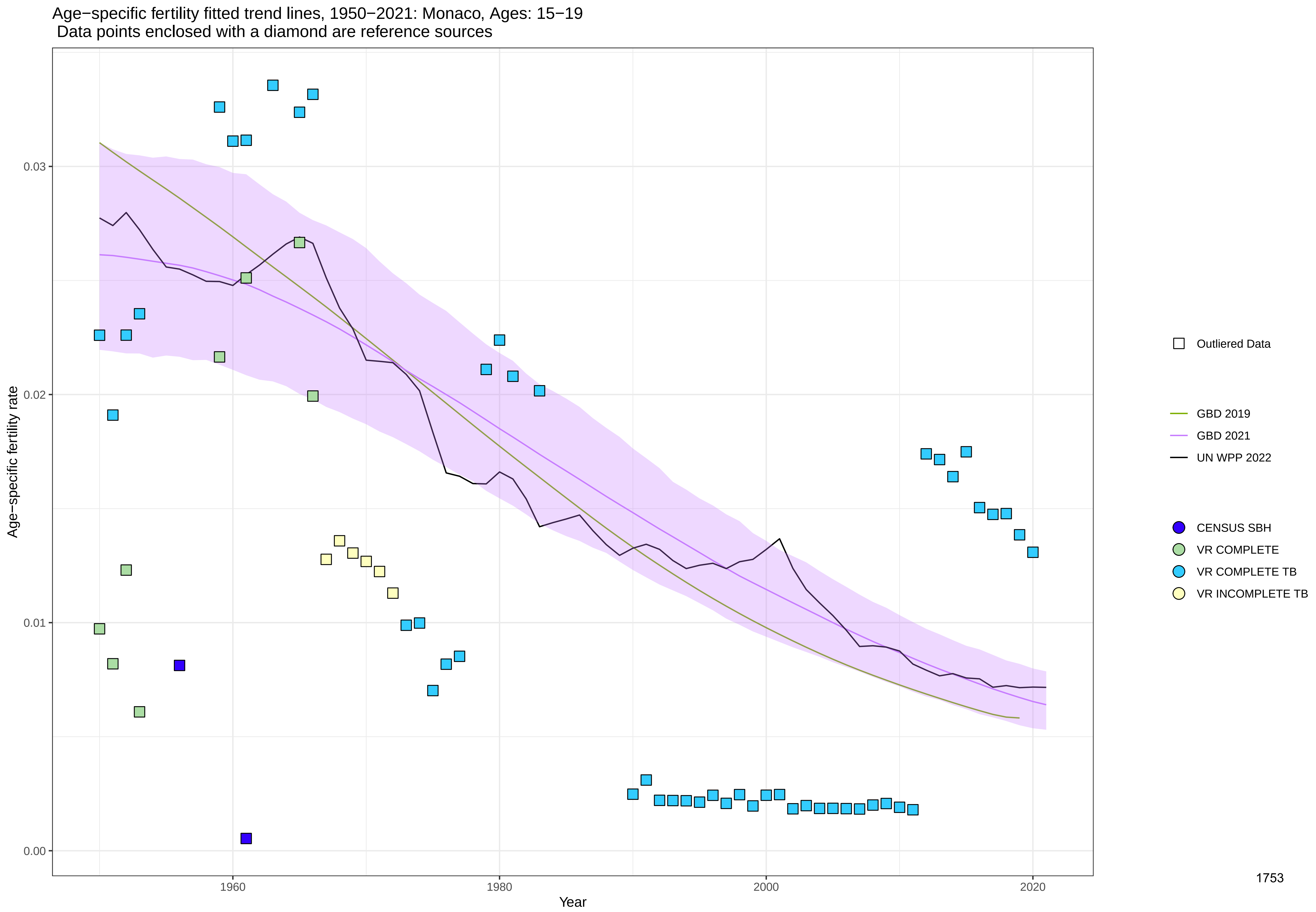

Age−specific fertility fitted trend lines, 1950−2021: Monaco, Ages: 15−19
Data points enclosed with a diamond are reference sources
Age−specific fertility rate
0.00
0.01
0.02
0.03
Year
1960
1980
2000
2020
Outliered Data
GBD 2019
GBD 2021
UN WPP 2022
CENSUS SBH
VR COMPLETE
VR COMPLETE TB
VR INCOMPLETE TB

Age−specific fertility fitted trend lines, 1950−2021: Monaco, Ages: 20−24
Data points enclosed with a diamond are reference sources

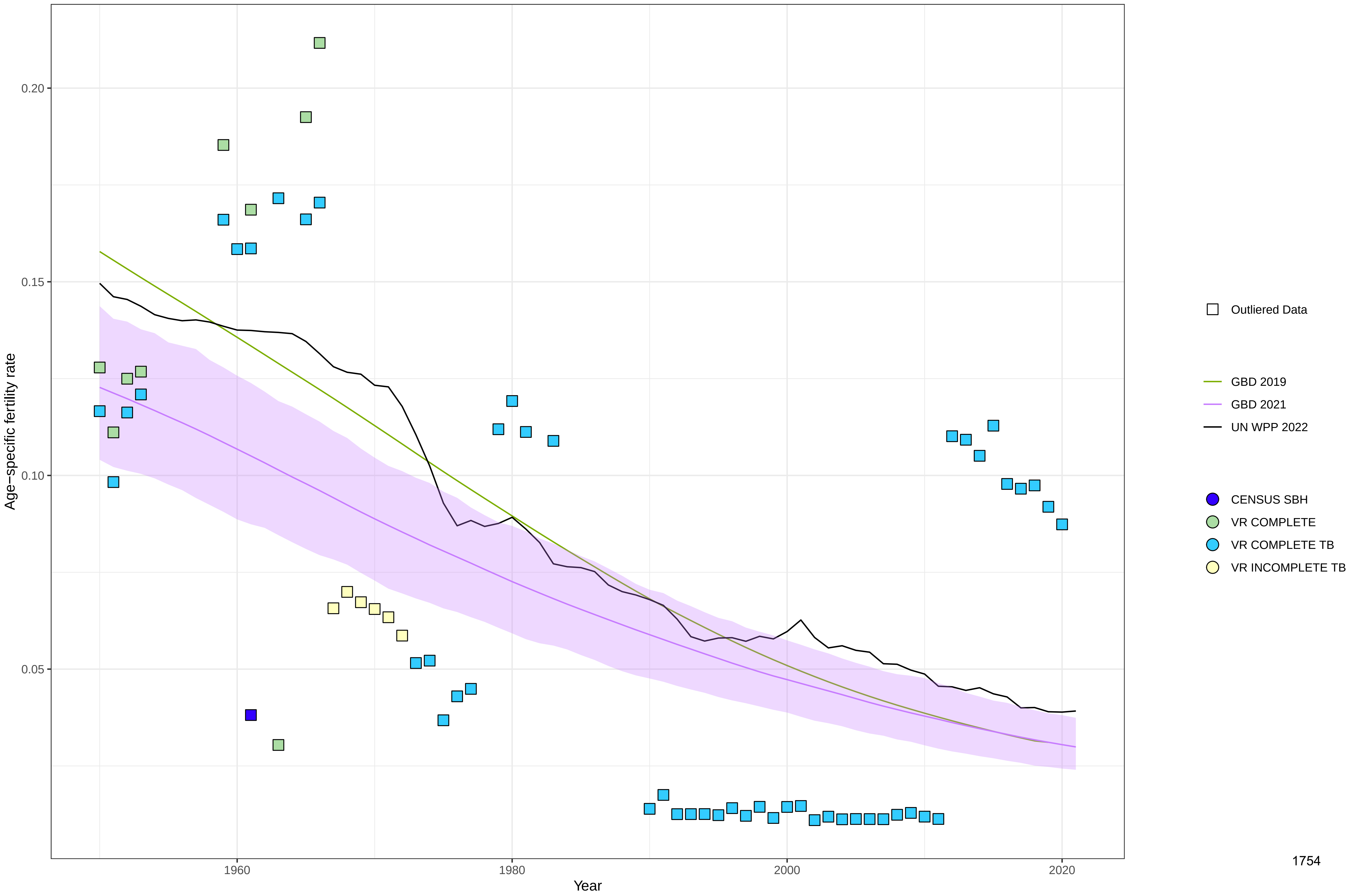

Age−specific fertility fitted trend lines, 1950−2021: Monaco, Ages: 25−29
Data points enclosed with a diamond are reference sources

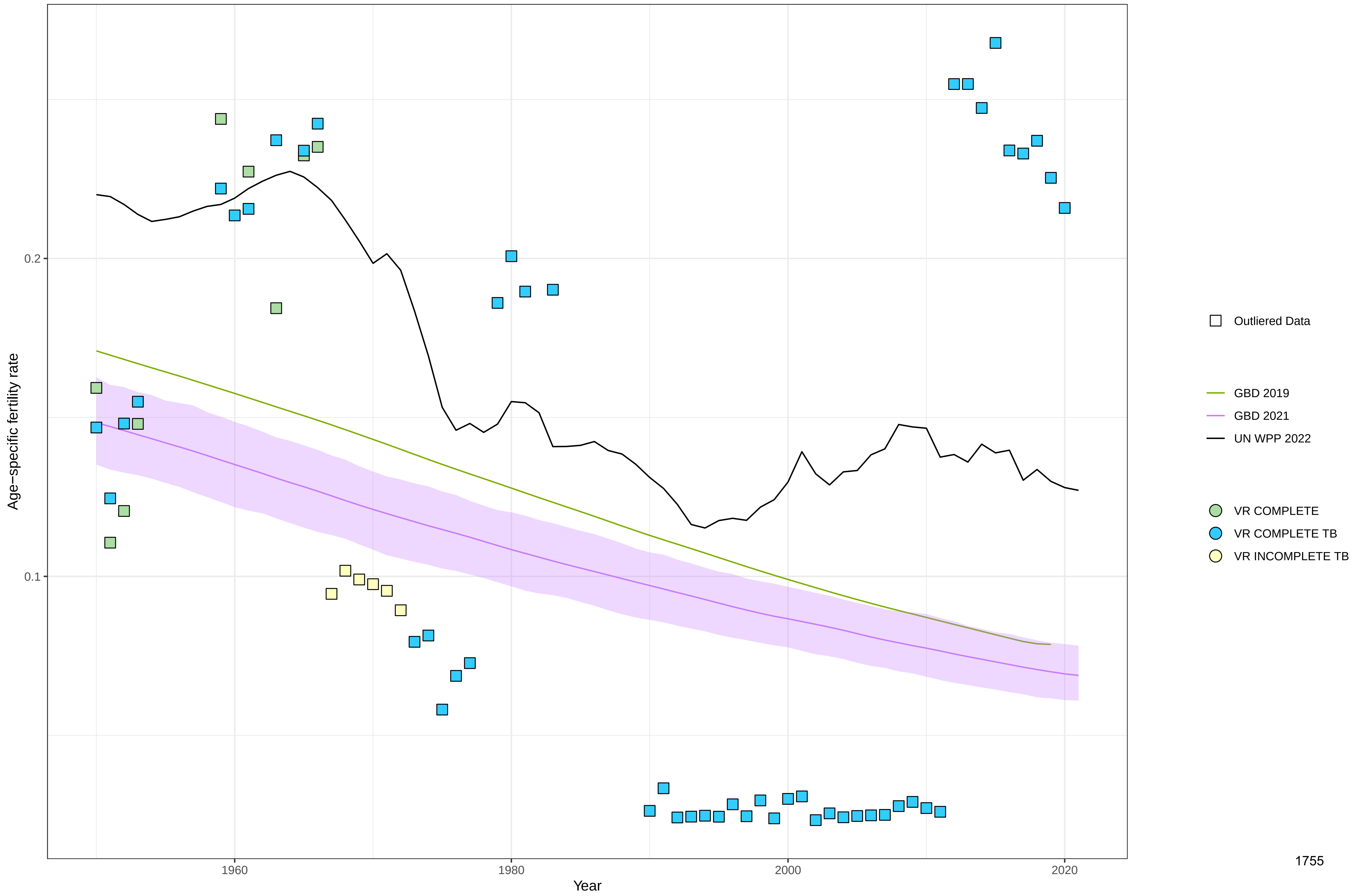

Age−specific fertility fitted trend lines, 1950−2021: Monaco, Ages: 30−34
Data points enclosed with a diamond are reference sources

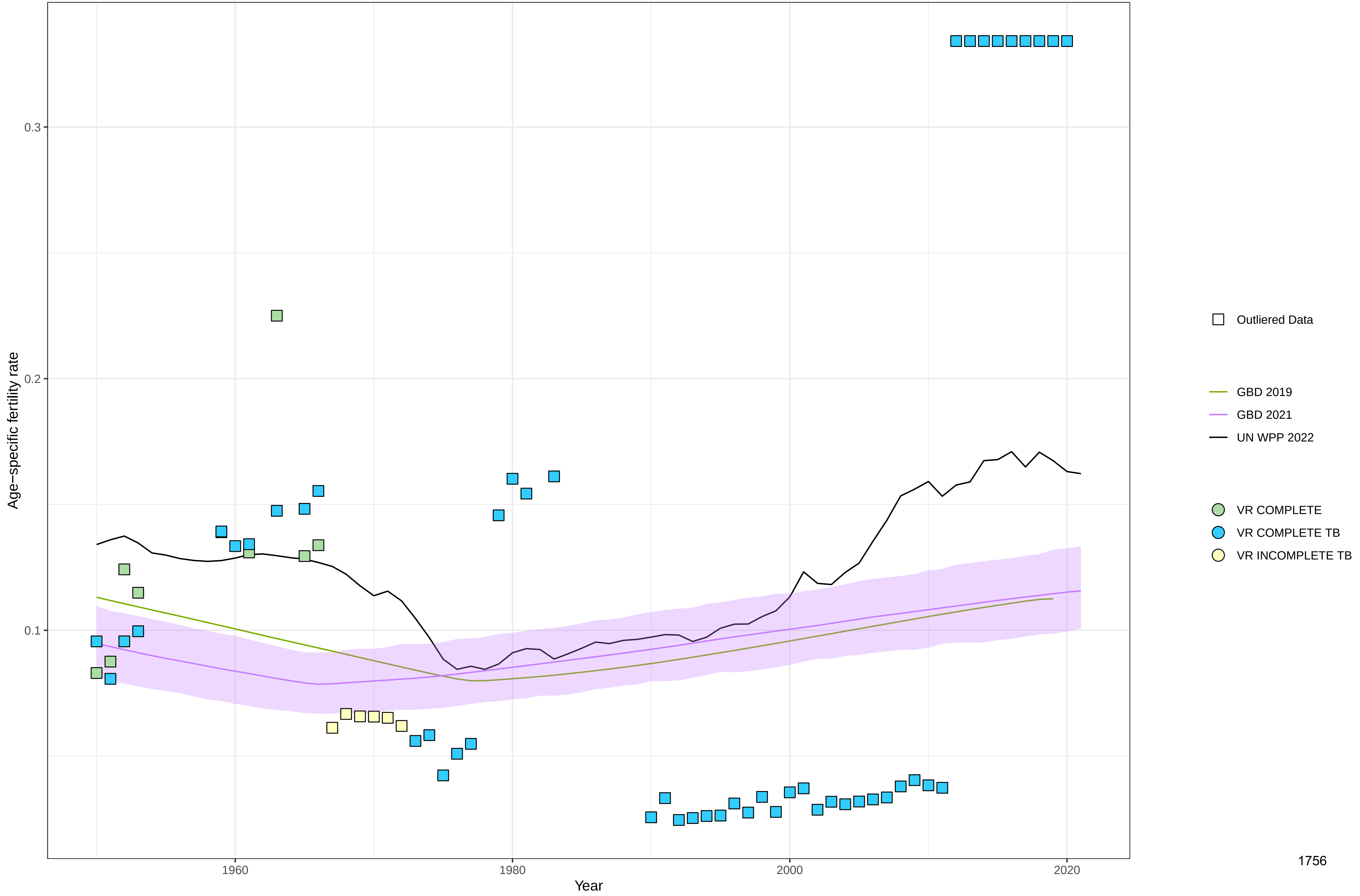

Age−specific fertility fitted trend lines, 1950−2021: Monaco, Ages: 35−39
Data points enclosed with a diamond are reference sources

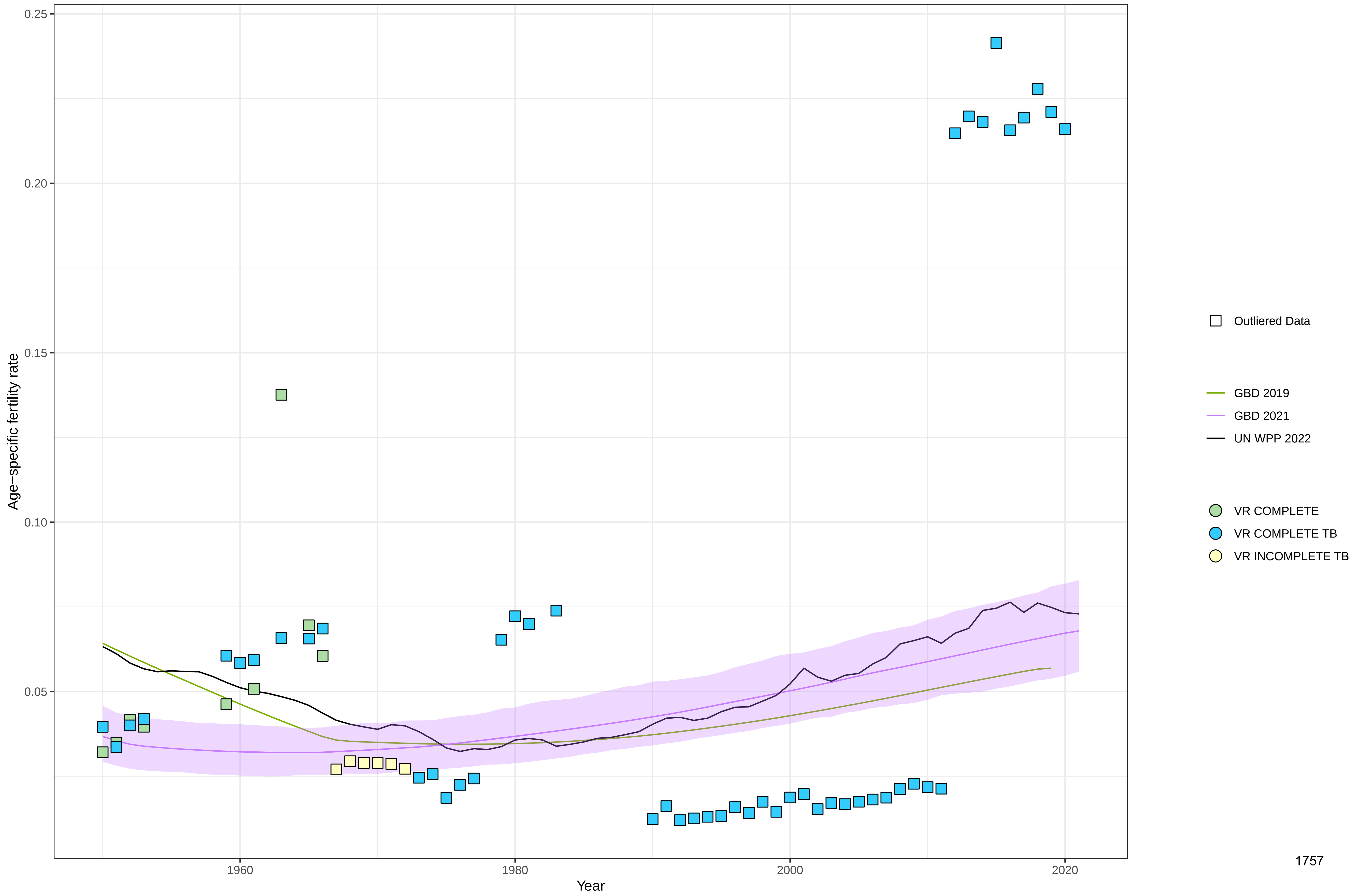

Age−specific fertility fitted trend lines, 1950−2021: Monaco, Ages: 40−44
Data points enclosed with a diamond are reference sources

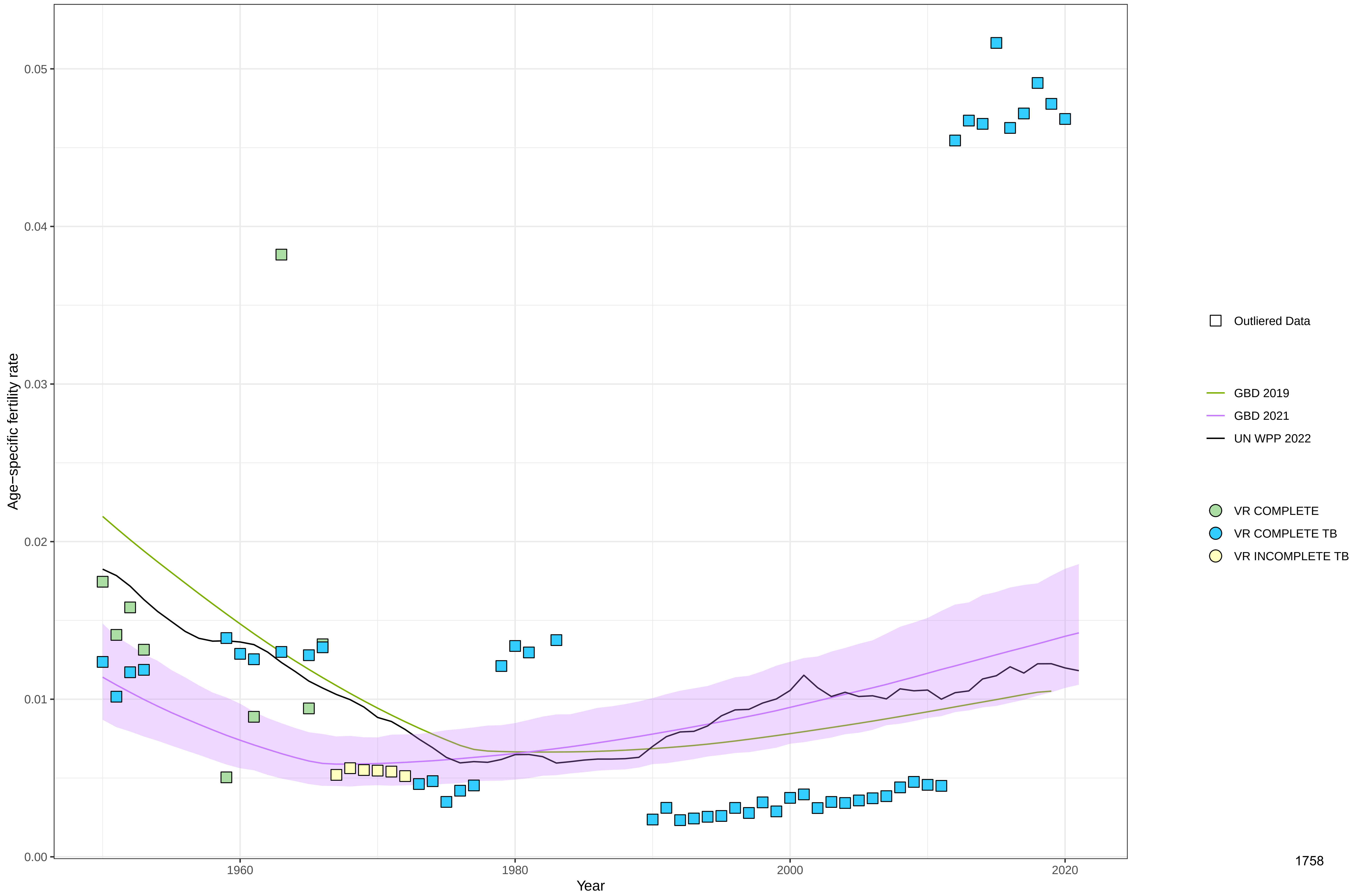

Age−specific fertility fitted trend lines, 1950−2021: Monaco, Ages: 45−49
Data points enclosed with a diamond are reference sources

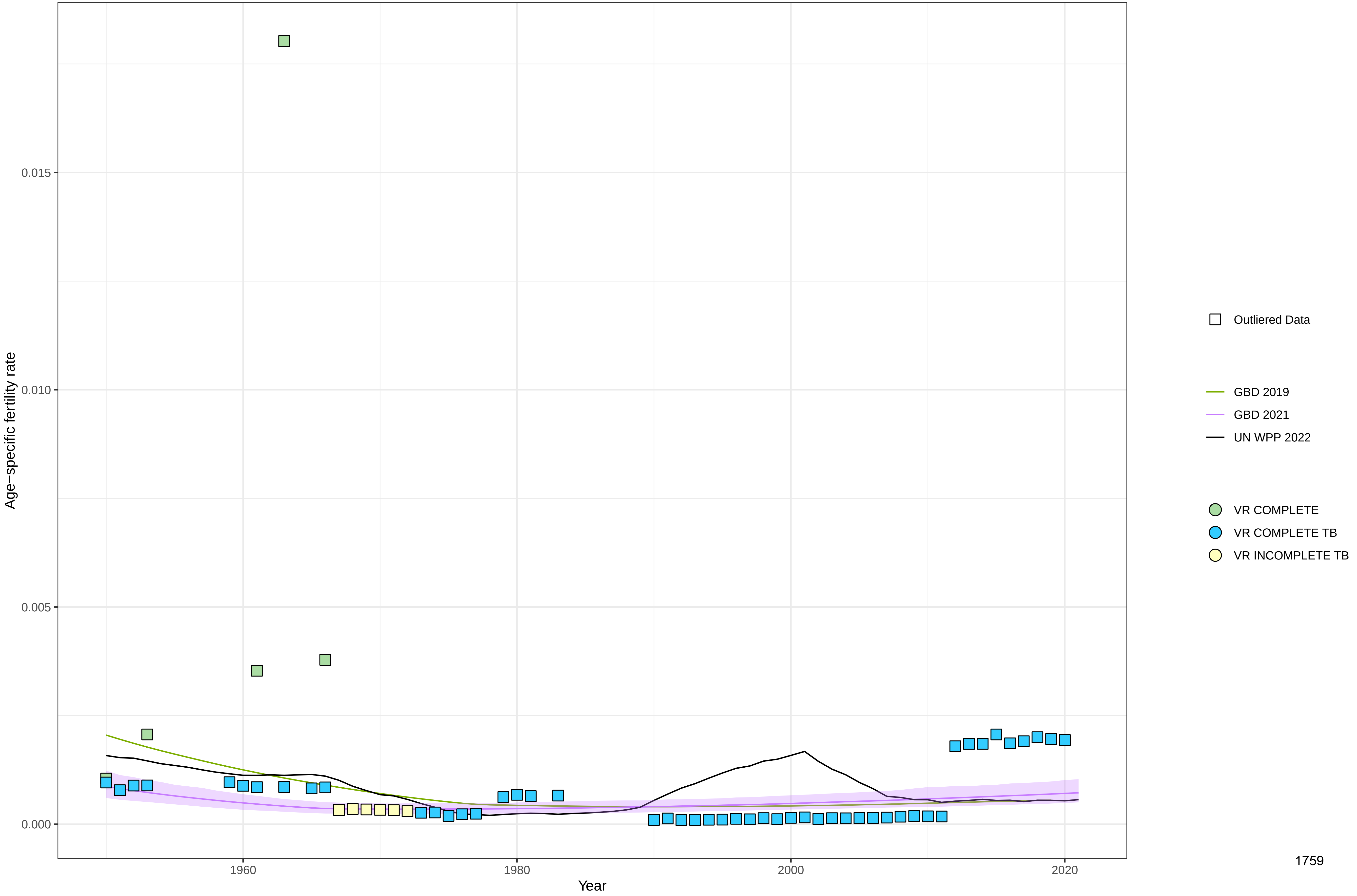

Age−specific fertility fitted trend lines, 1950−2021: Monaco, Ages: 50−54
Data points enclosed with a diamond are reference sources

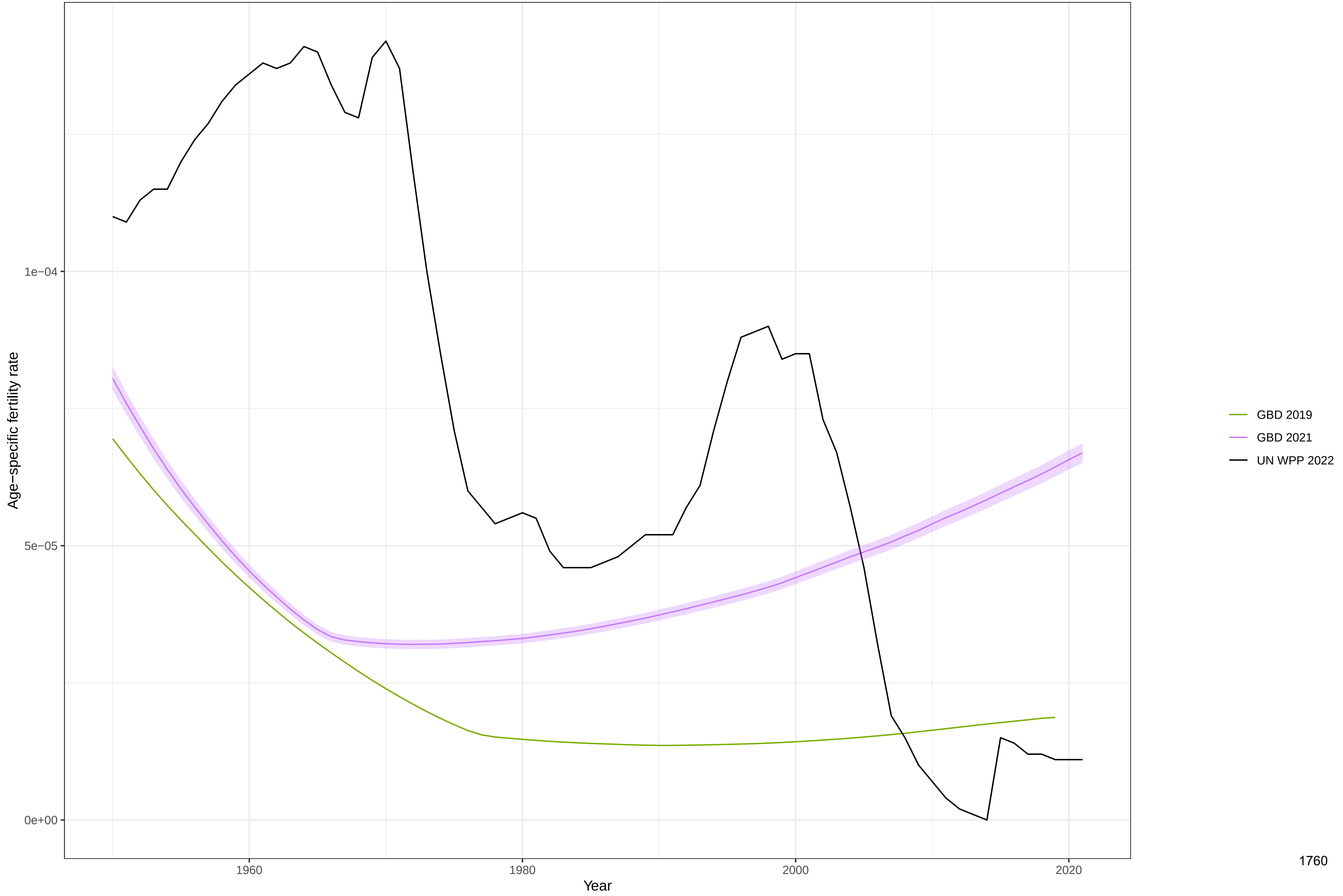

Total fertility rate fitted trend lines, 1950−2021: Monaco TFR
Data points enclosed with a diamond are reference sources

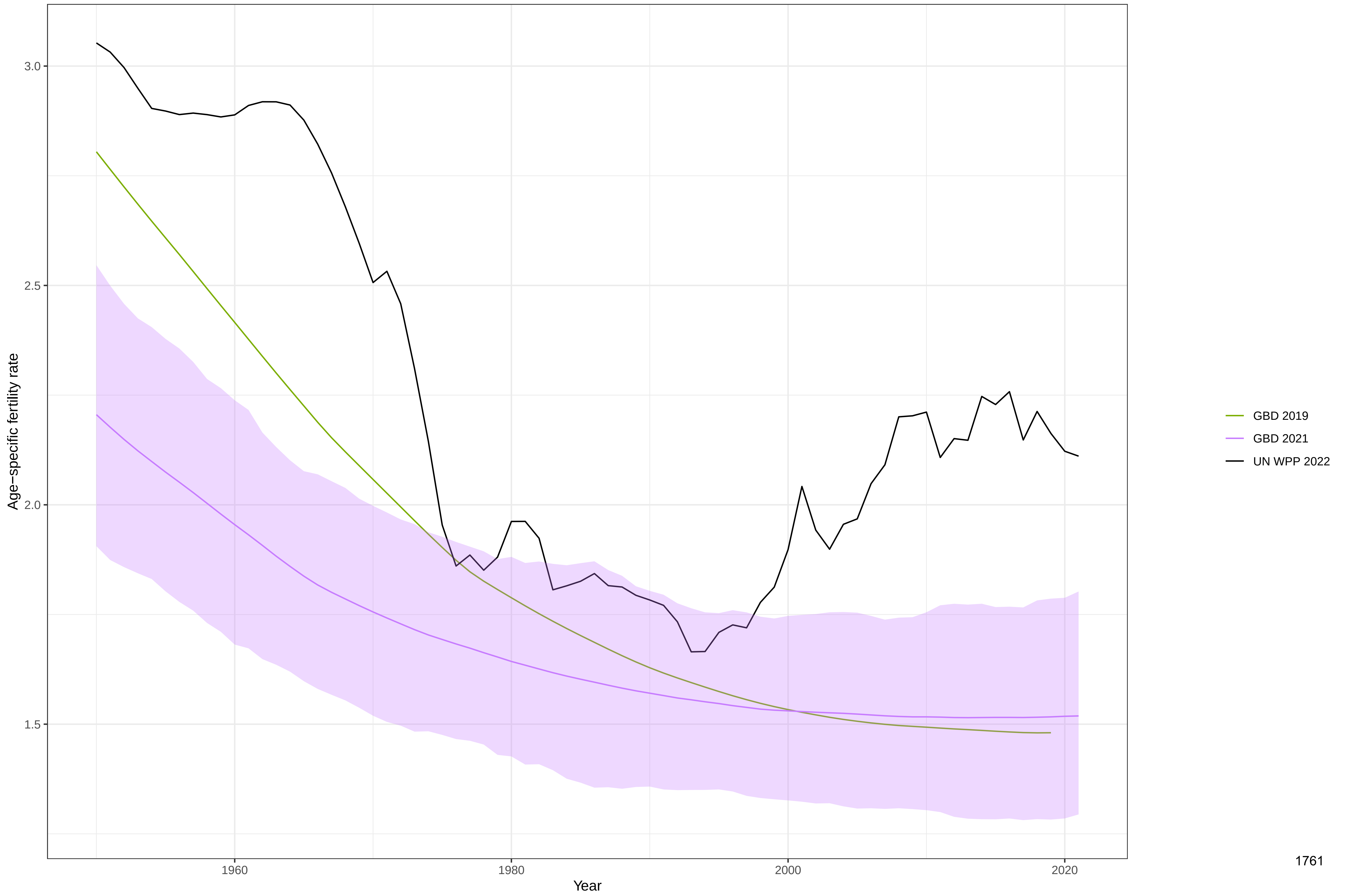

1761

Age−specific fertility fitted trend lines, 1950−2021: Netherlands, Ages: 10−14
Data points enclosed with a diamond are reference sources

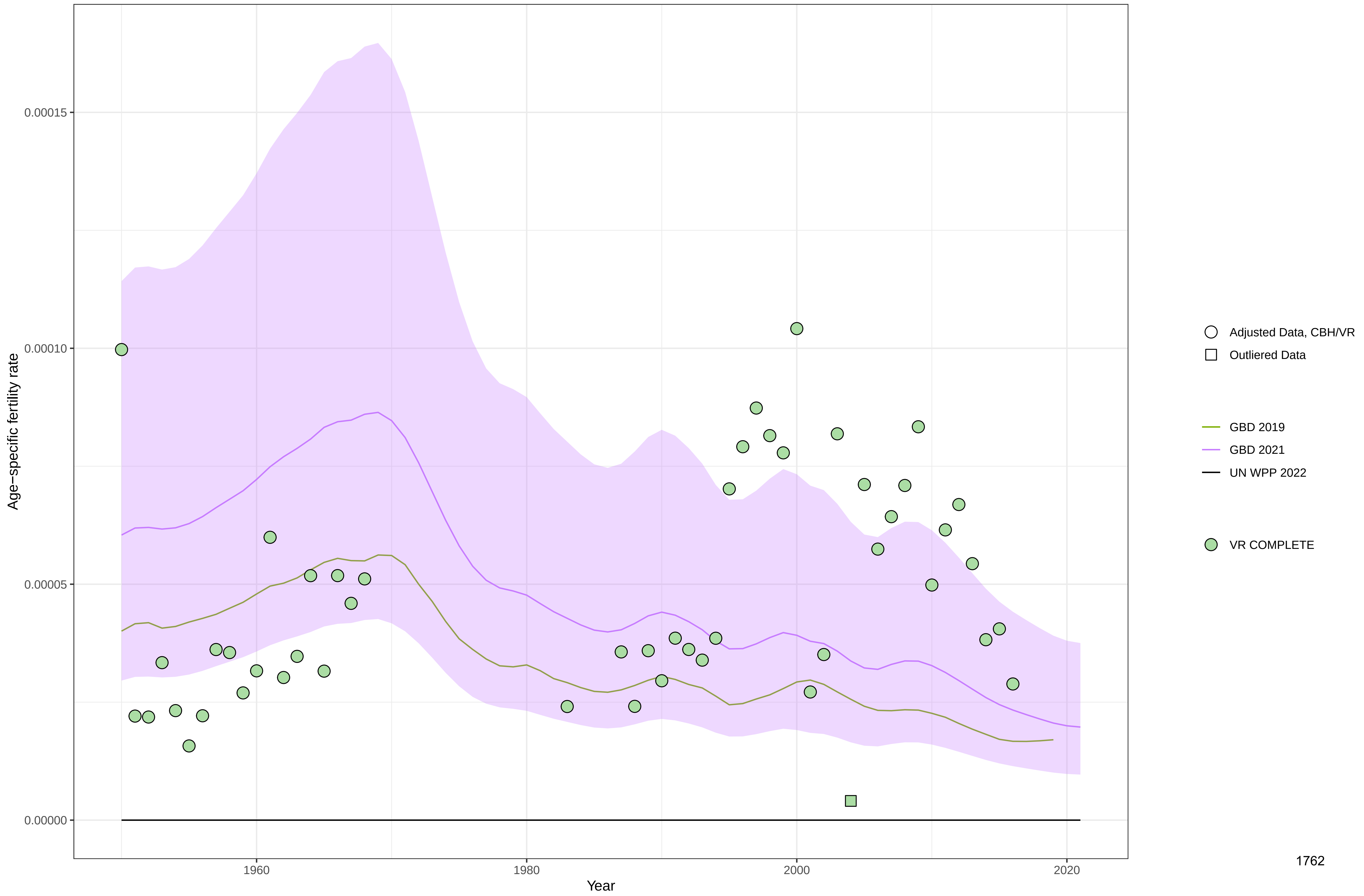

Age−specific fertility fitted trend lines, 1950−2021: Netherlands, Ages: 15−19
Data points enclosed with a diamond are reference sources

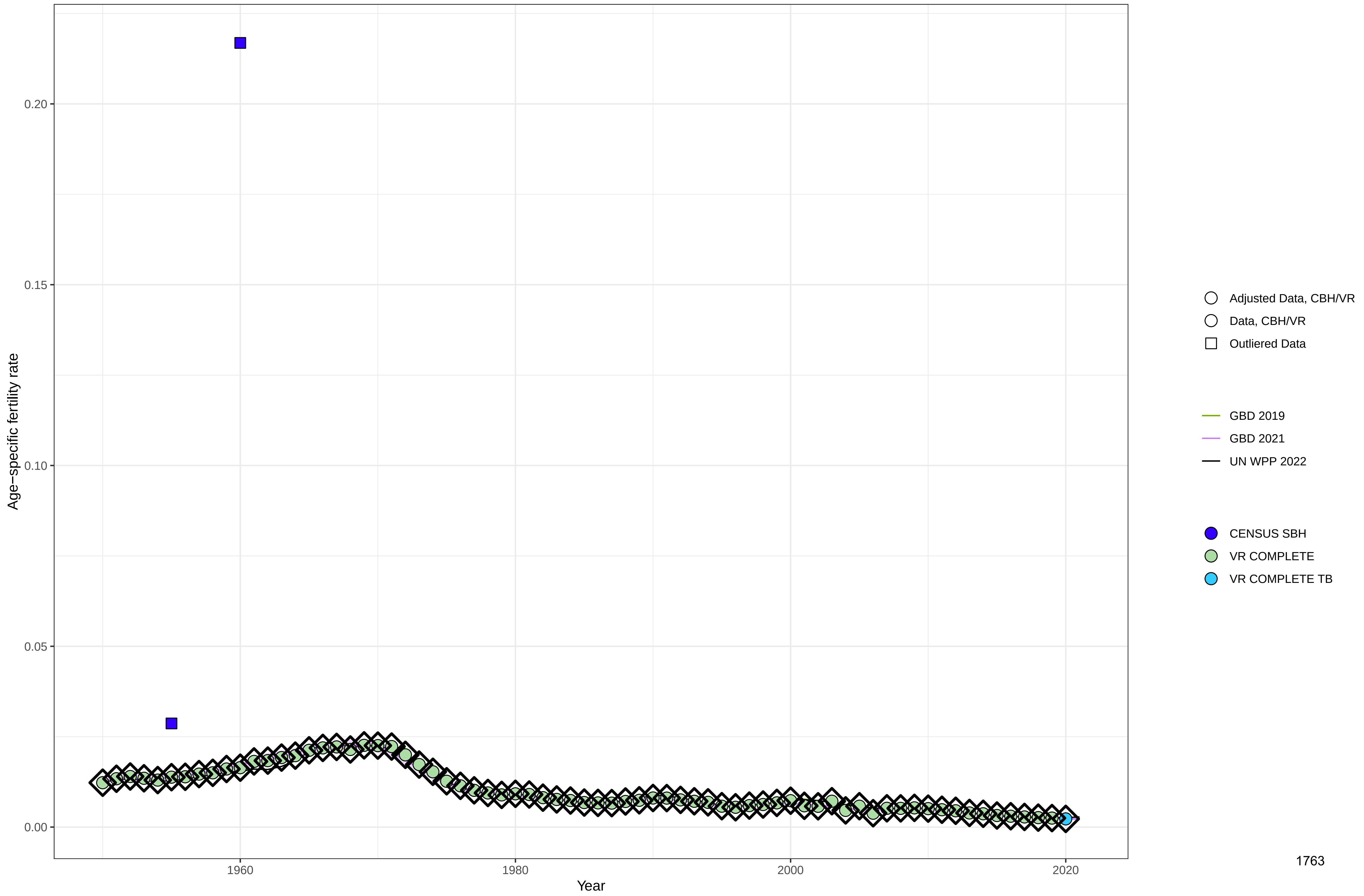

Age−specific fertility fitted trend lines, 1950−2021: Netherlands, Ages: 20−24
Data points enclosed with a diamond are reference sources

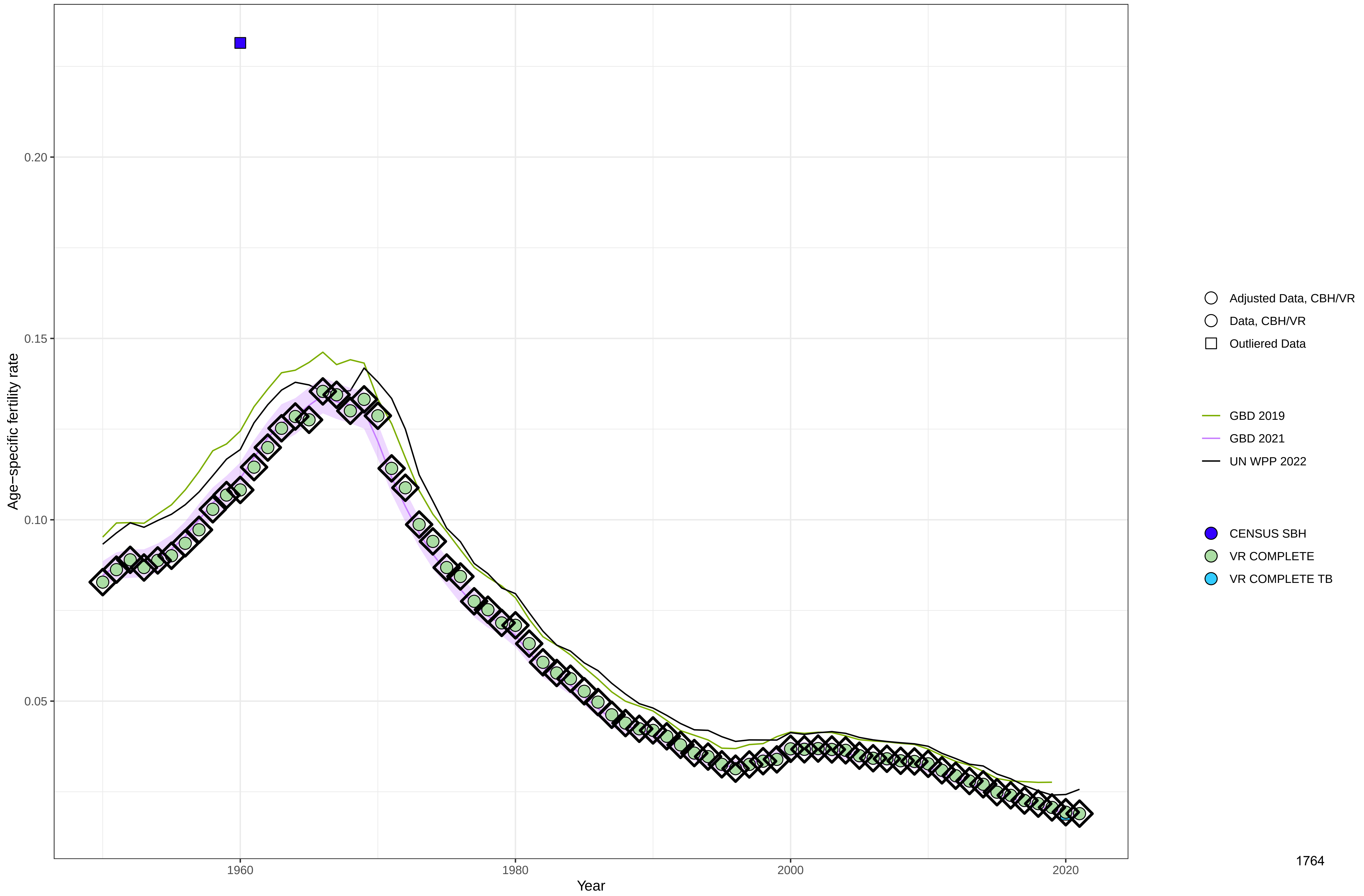

Age−specific fertility fitted trend lines, 1950−2021: Netherlands, Ages: 25−29
Data points enclosed with a diamond are reference sources

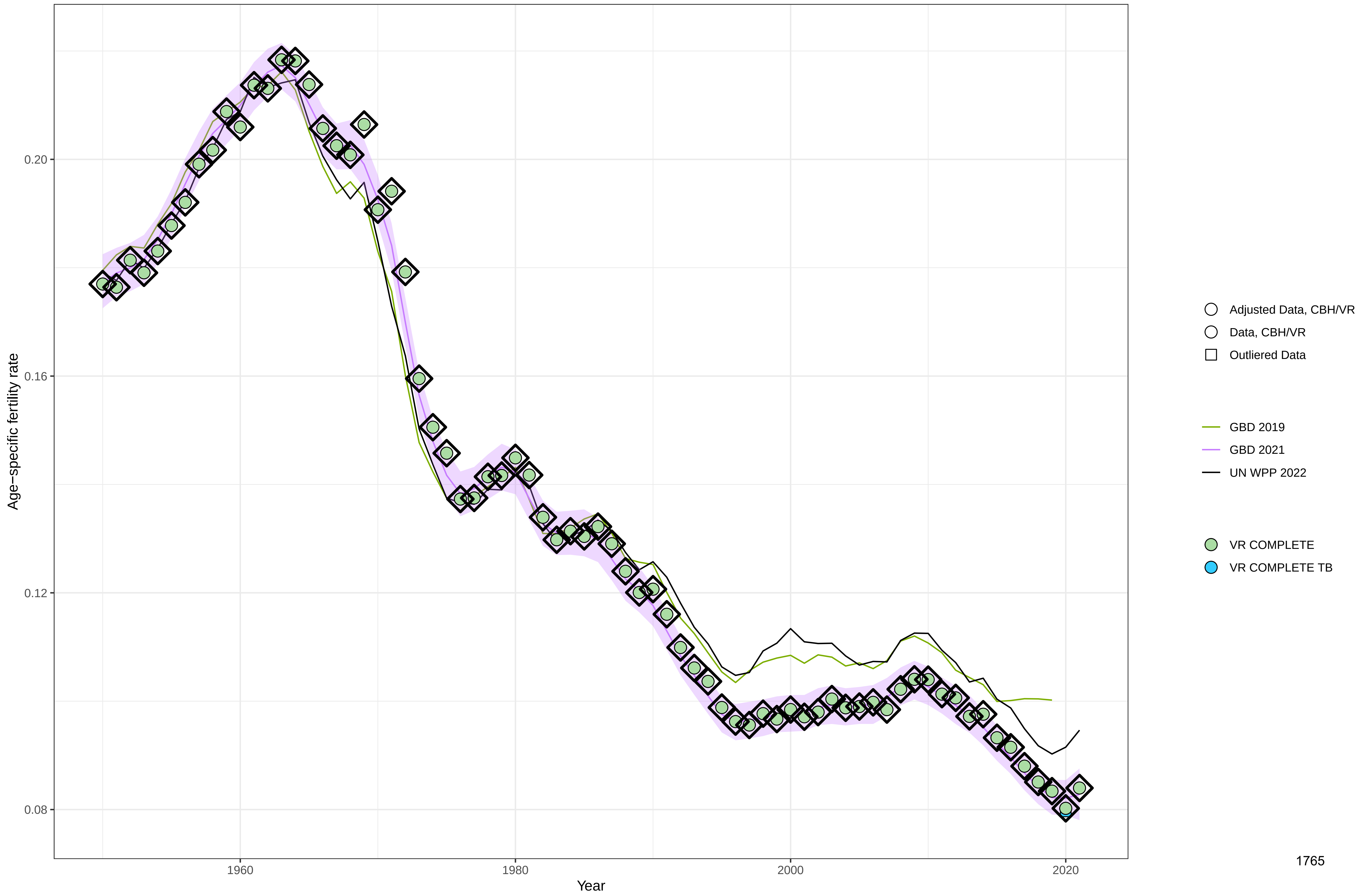

Age−specific fertility fitted trend lines, 1950−2021: Netherlands, Ages: 30−34
Data points enclosed with a diamond are reference sources

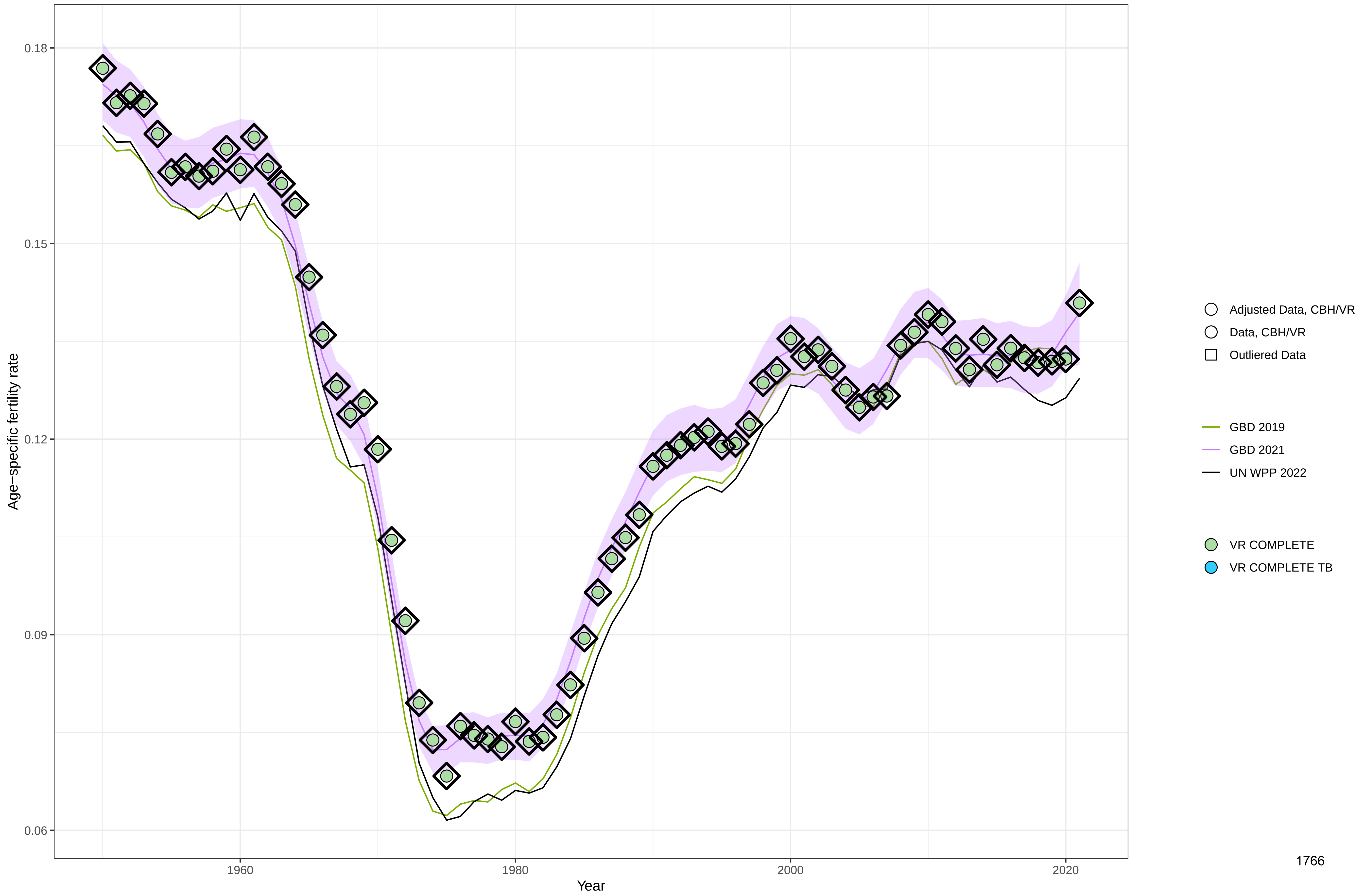

Age−specific fertility fitted trend lines, 1950−2021: Netherlands, Ages: 35−39
Data points enclosed with a diamond are reference sources

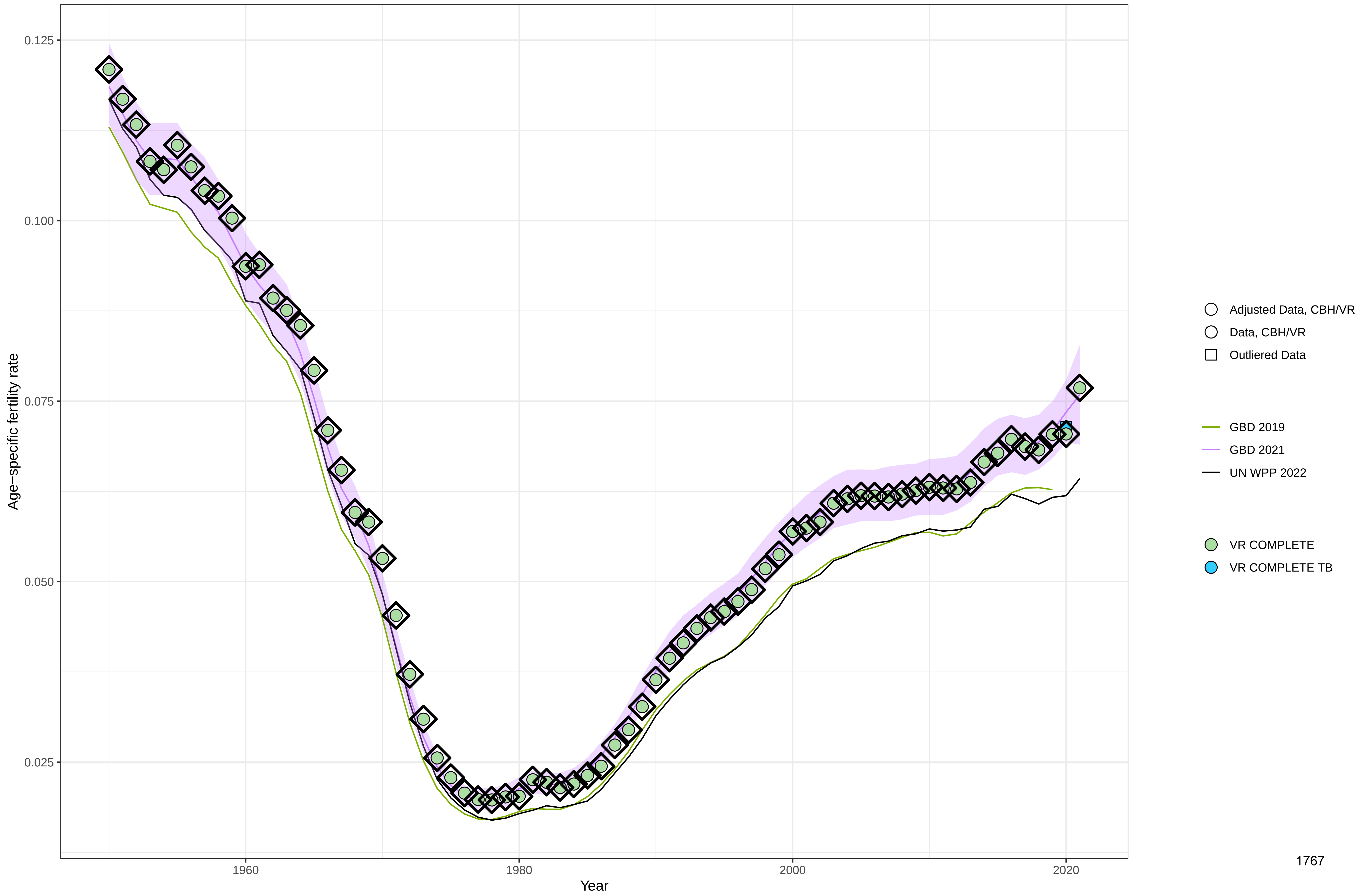

Age−specific fertility fitted trend lines, 1950−2021: Netherlands, Ages: 40−44
Data points enclosed with a diamond are reference sources

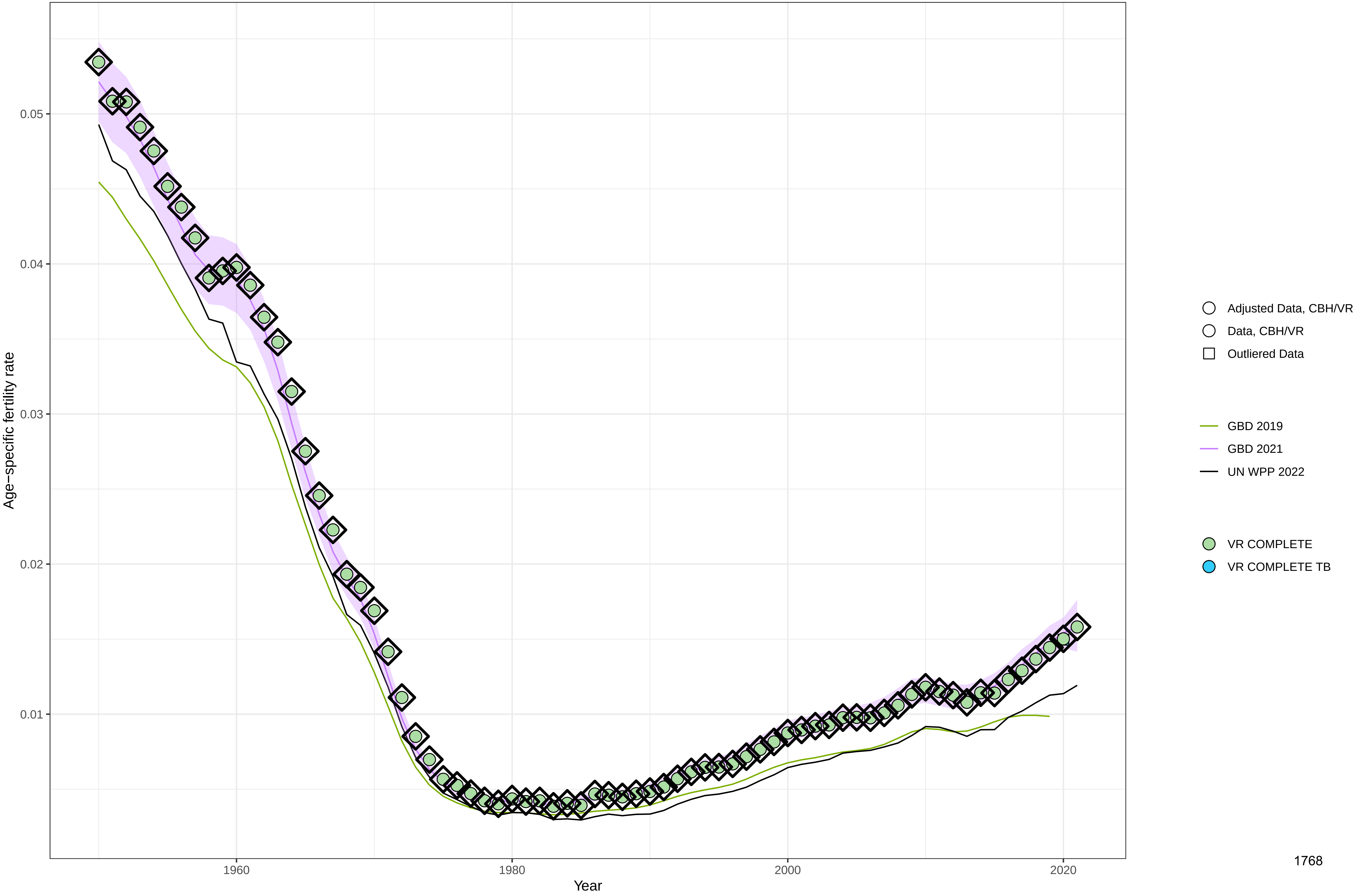

Age−specific fertility fitted trend lines, 1950−2021: Netherlands, Ages: 45−49
Data points enclosed with a diamond are reference sources

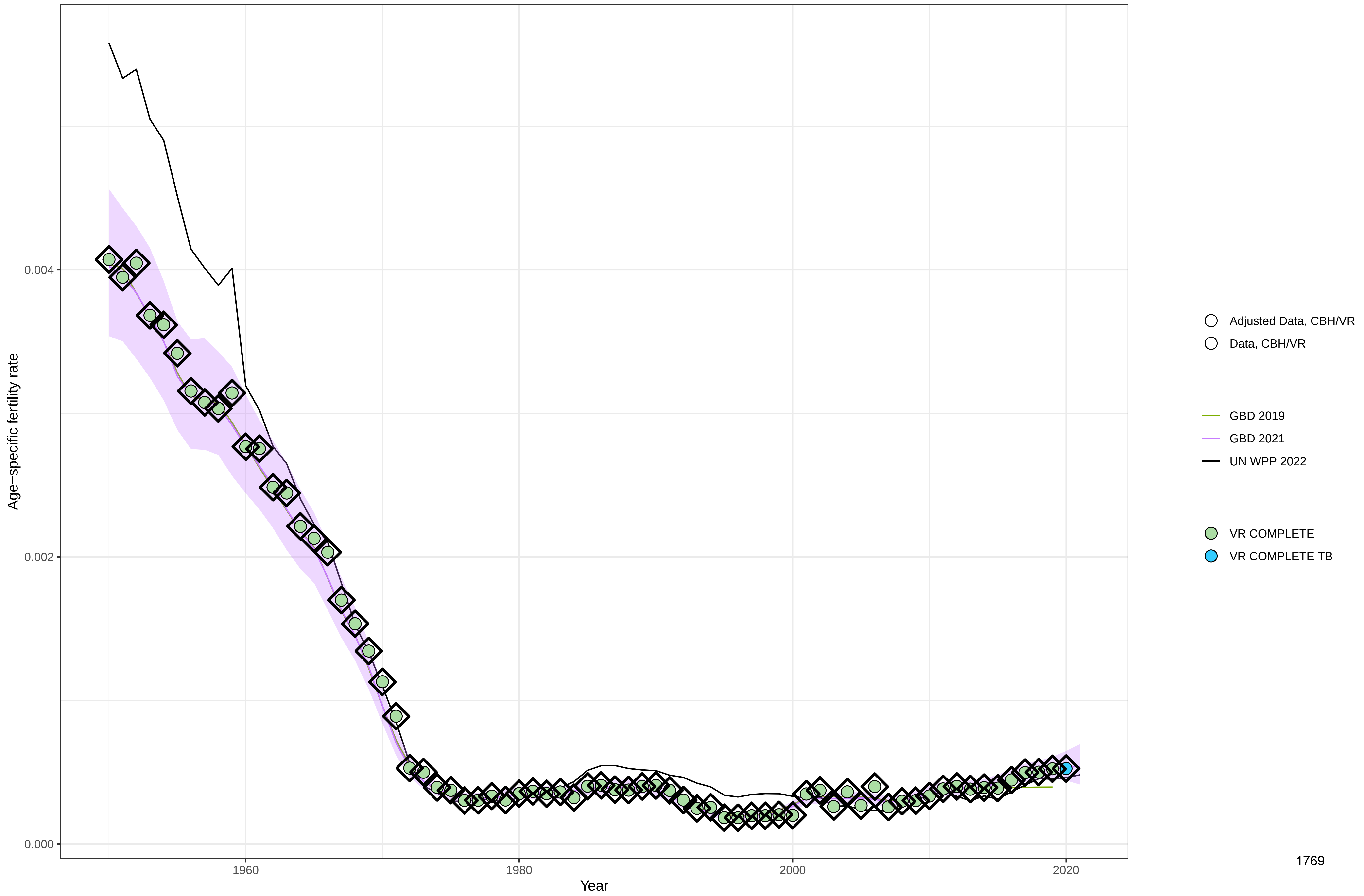

Age−specific fertility fitted trend lines, 1950−2021: Netherlands, Ages: 50−54
Data points enclosed with a diamond are reference sources

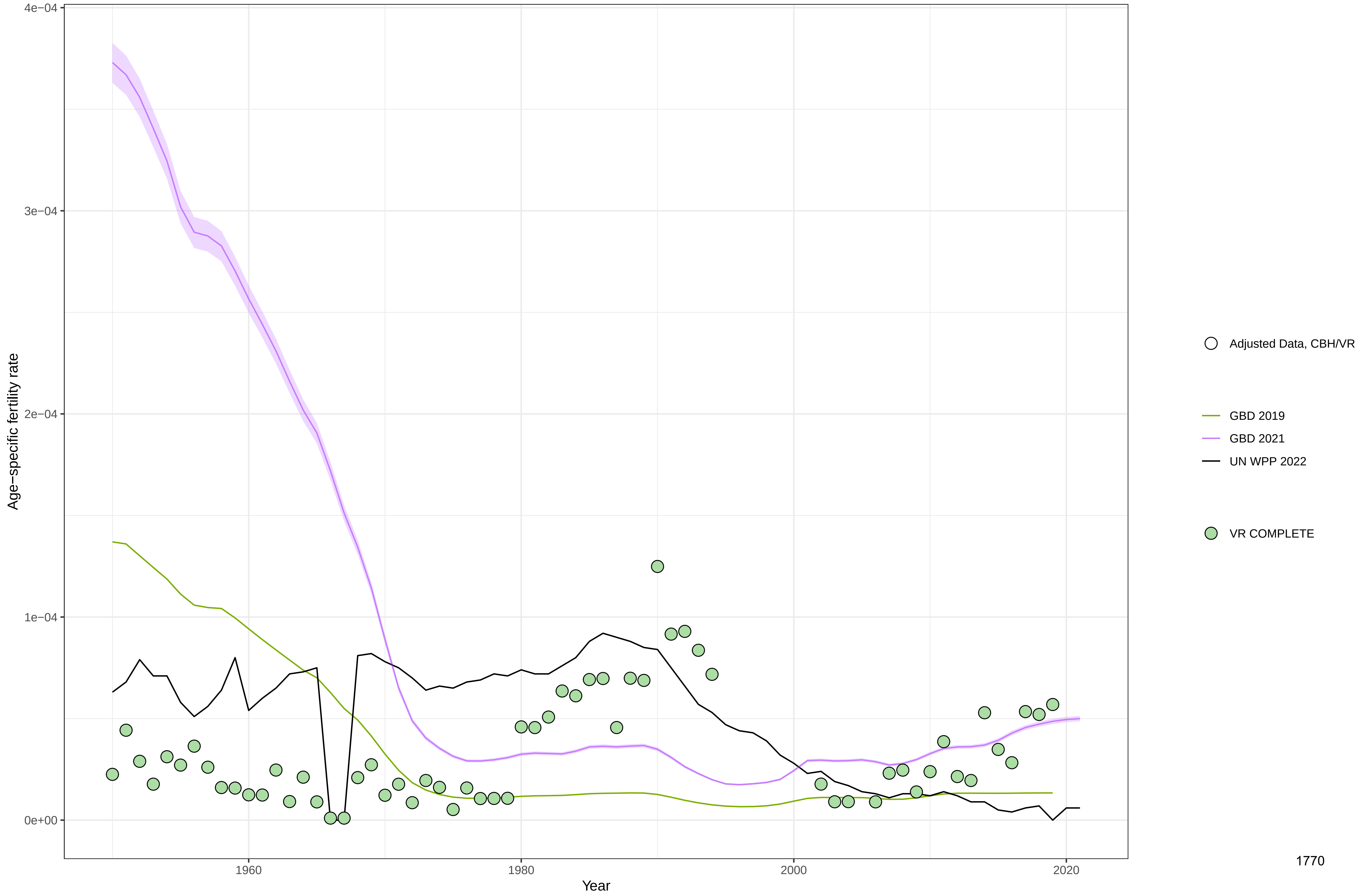

Total fertility rate fitted trend lines, 1950−2021: Netherlands TFR
Data points enclosed with a diamond are reference sources

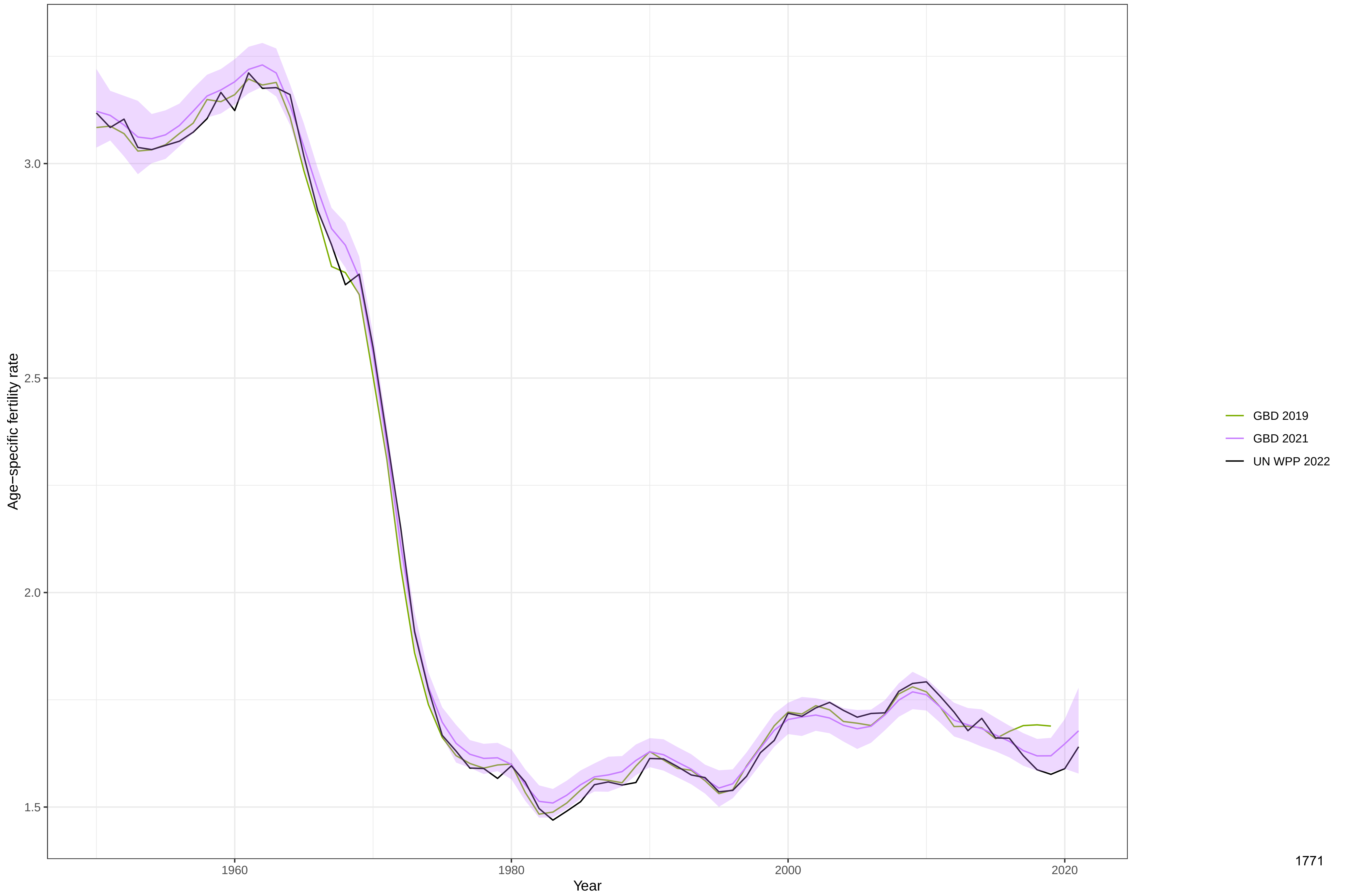

Age−specific fertility fitted trend lines, 1950−2021: Norway, Ages: 10−14
Data points enclosed with a diamond are reference sources

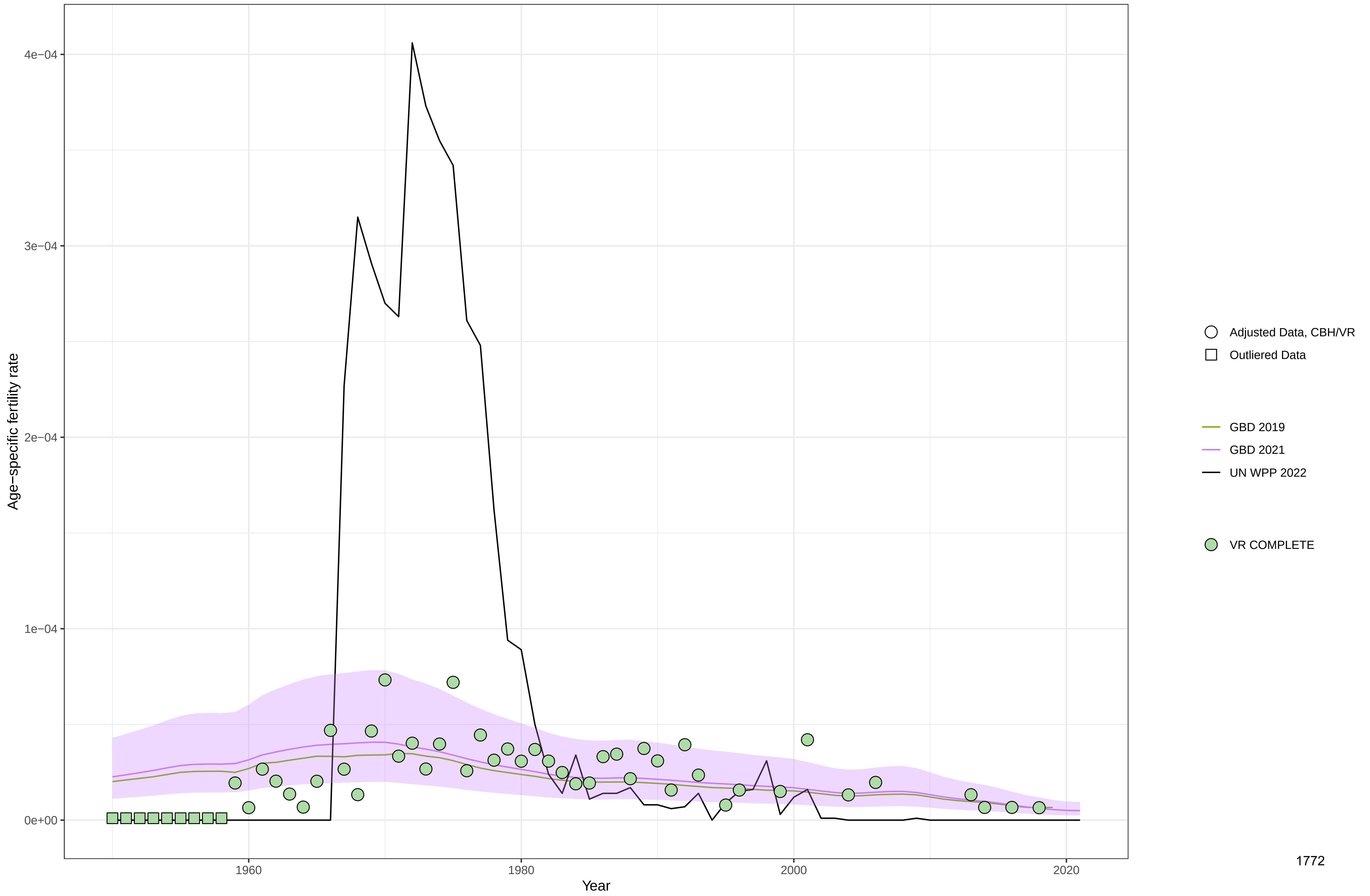

Age−specific fertility fitted trend lines, 1950−2021: Norway, Ages: 15−19
Data points enclosed with a diamond are reference sources

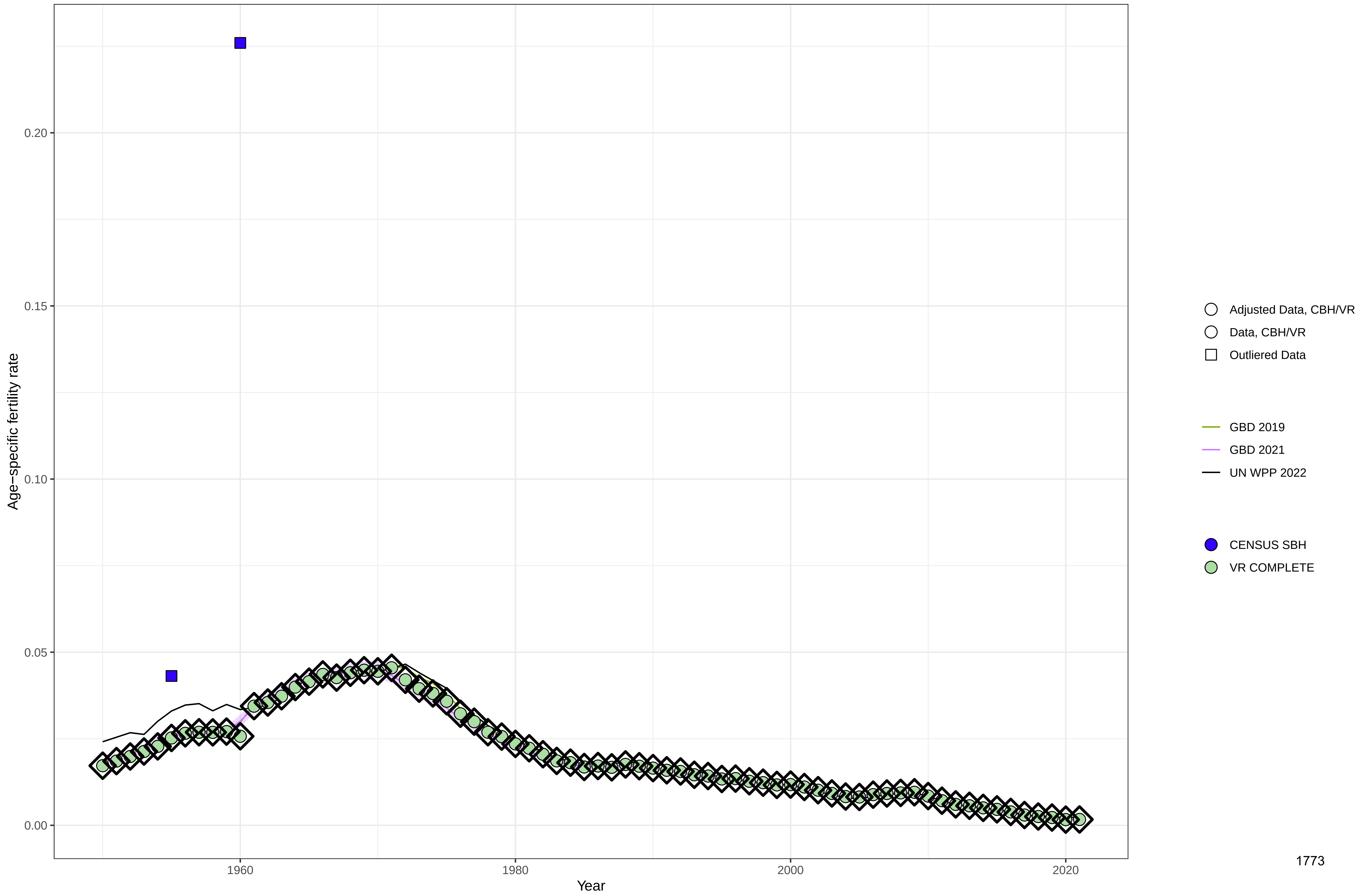

1773

Age−specific fertility fitted trend lines, 1950−2021: Norway, Ages: 20−24
Data points enclosed with a diamond are reference sources

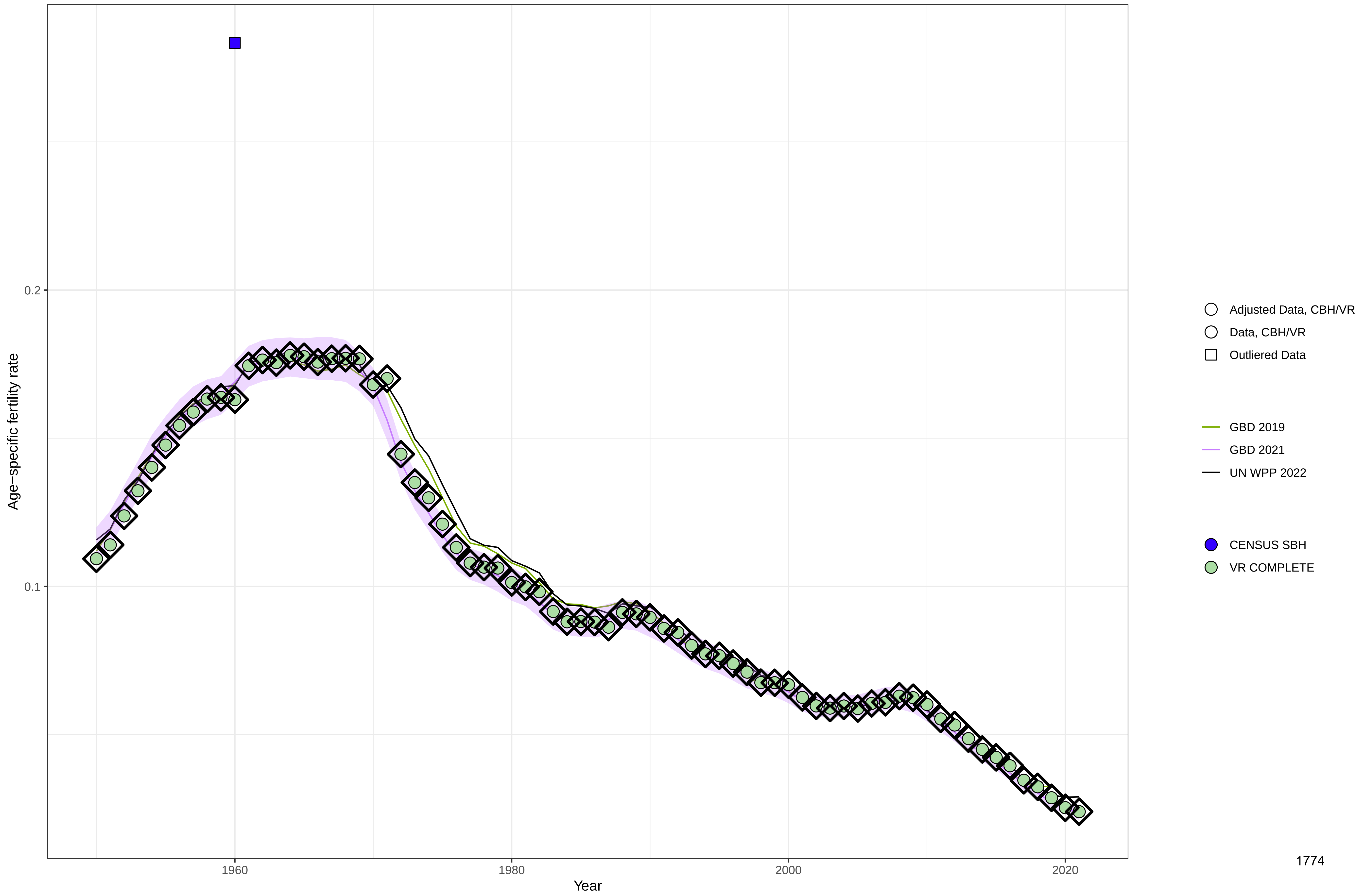

1774

Age−specific fertility fitted trend lines, 1950−2021: Norway, Ages: 25−29
Data points enclosed with a diamond are reference sources

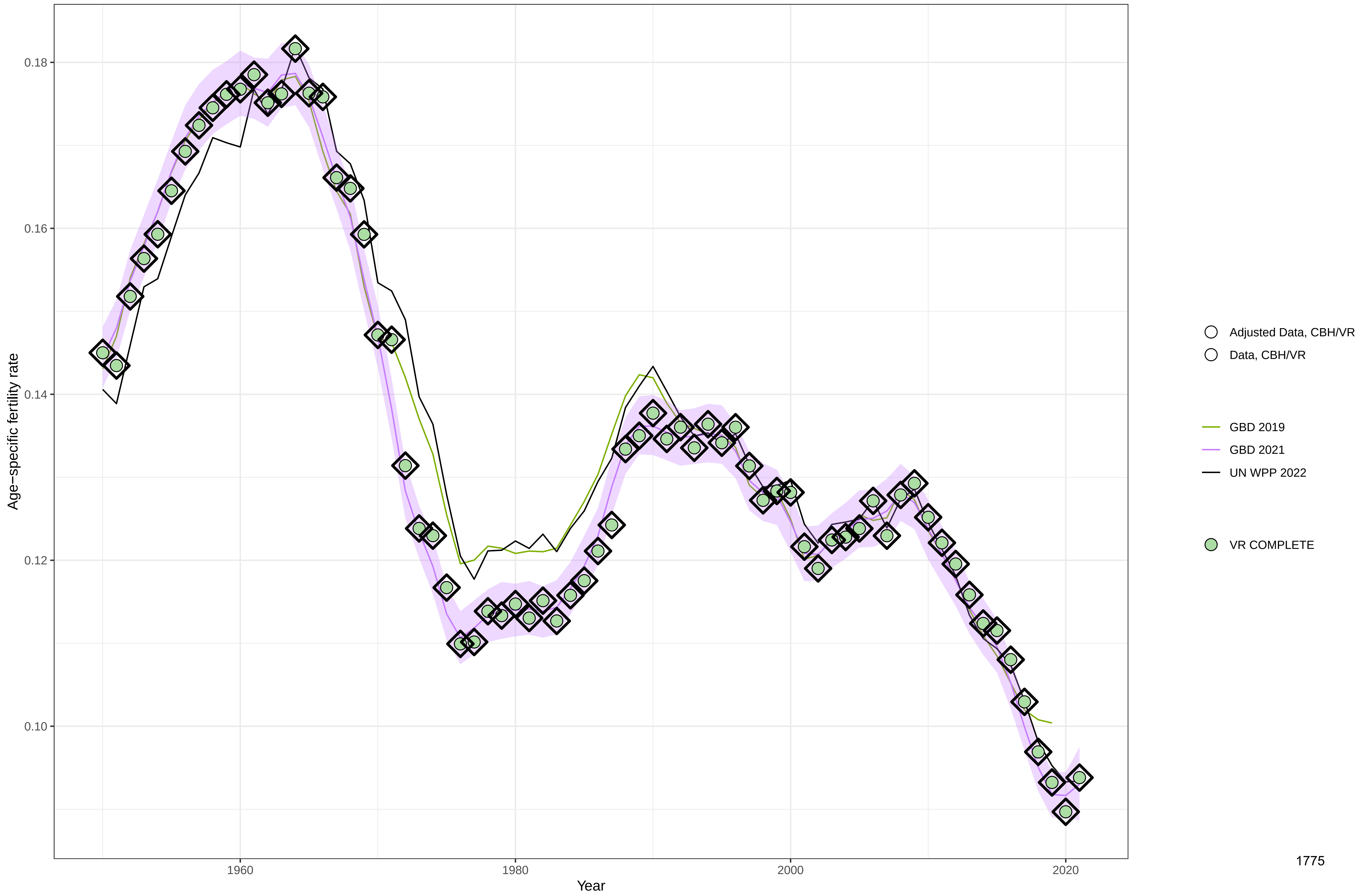

Age−specific fertility fitted trend lines, 1950−2021: Norway, Ages: 30−34
Data points enclosed with a diamond are reference sources

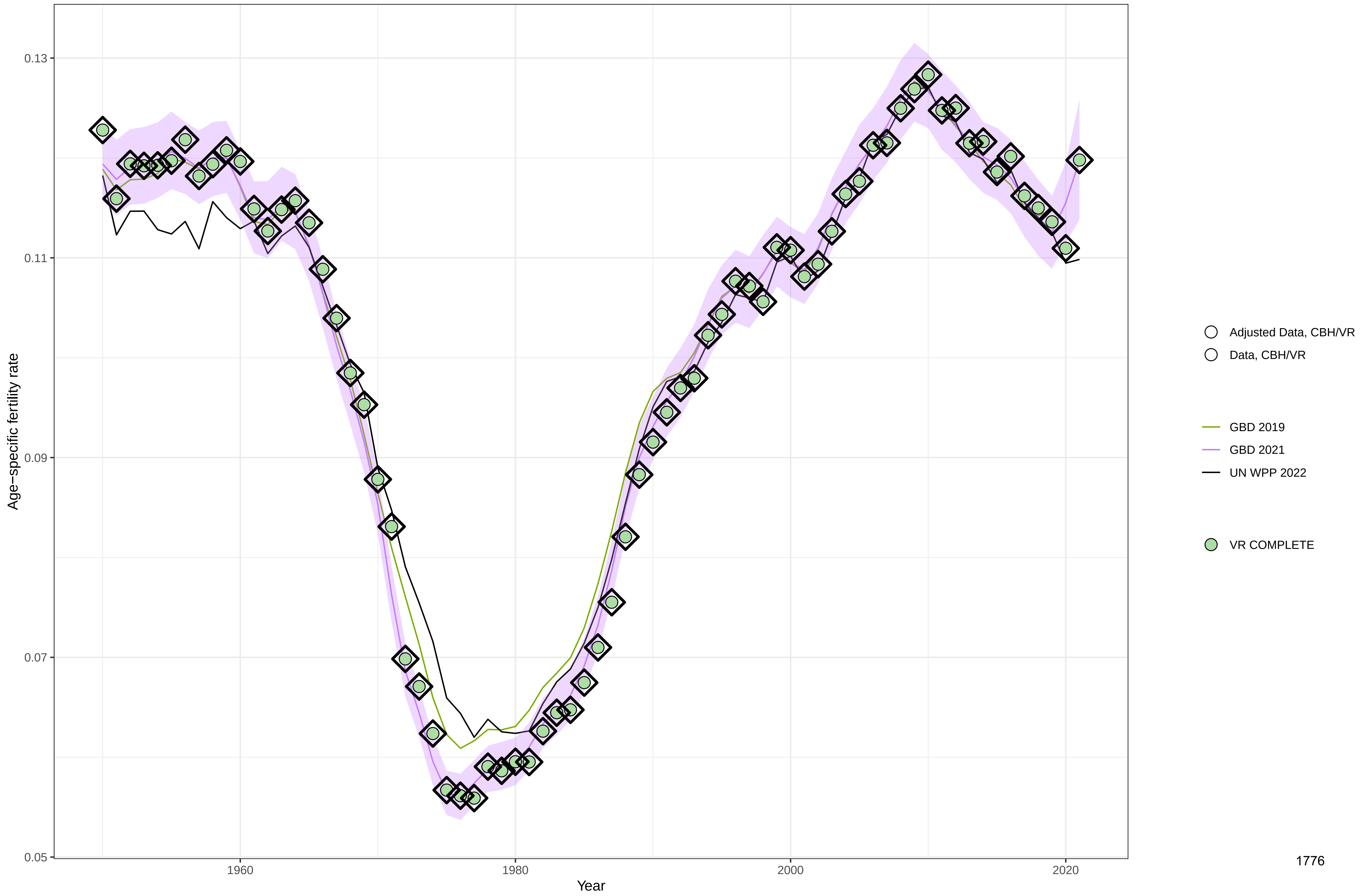

Age−specific fertility fitted trend lines, 1950−2021: Norway, Ages: 35−39
Data points enclosed with a diamond are reference sources

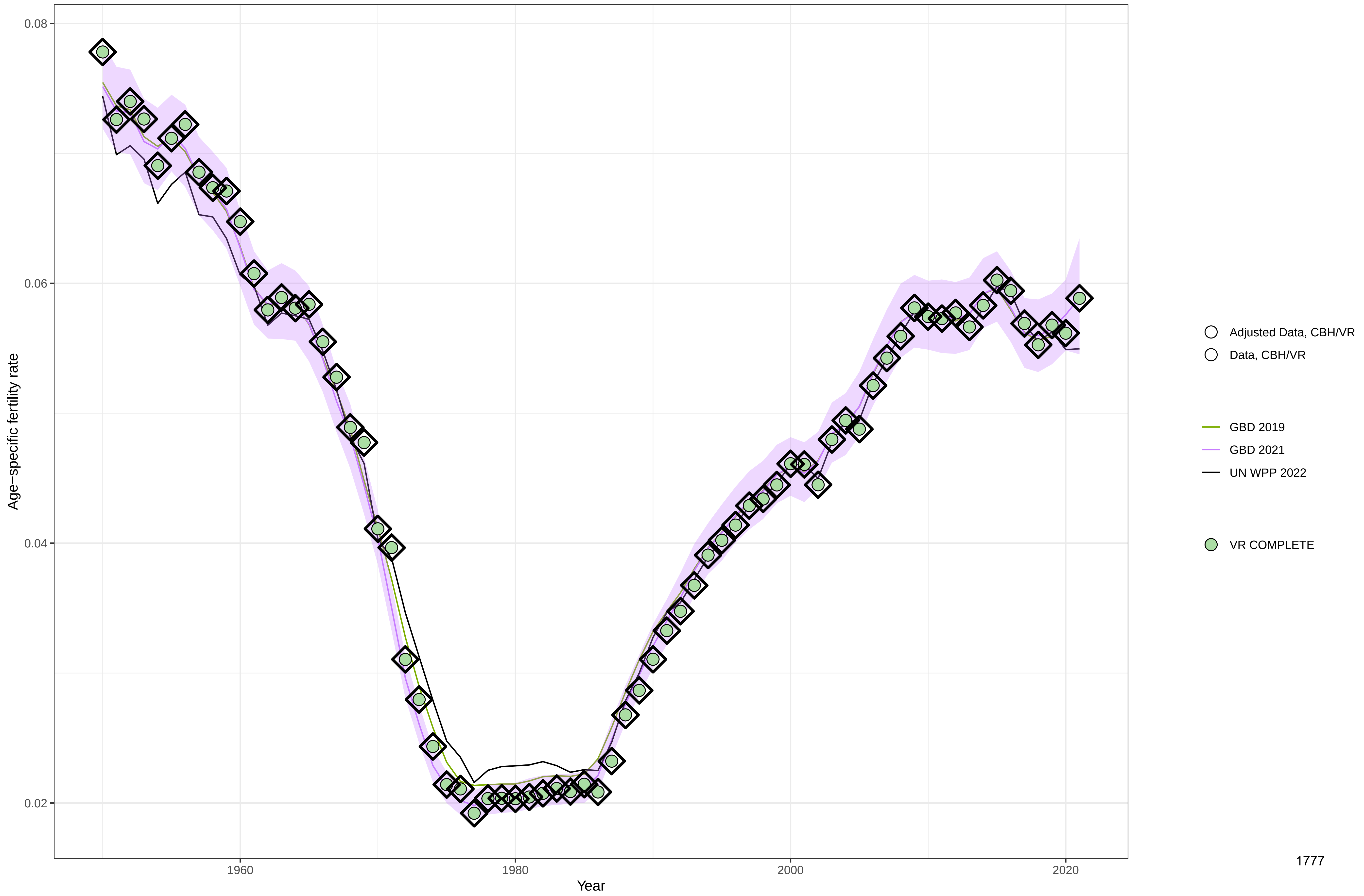

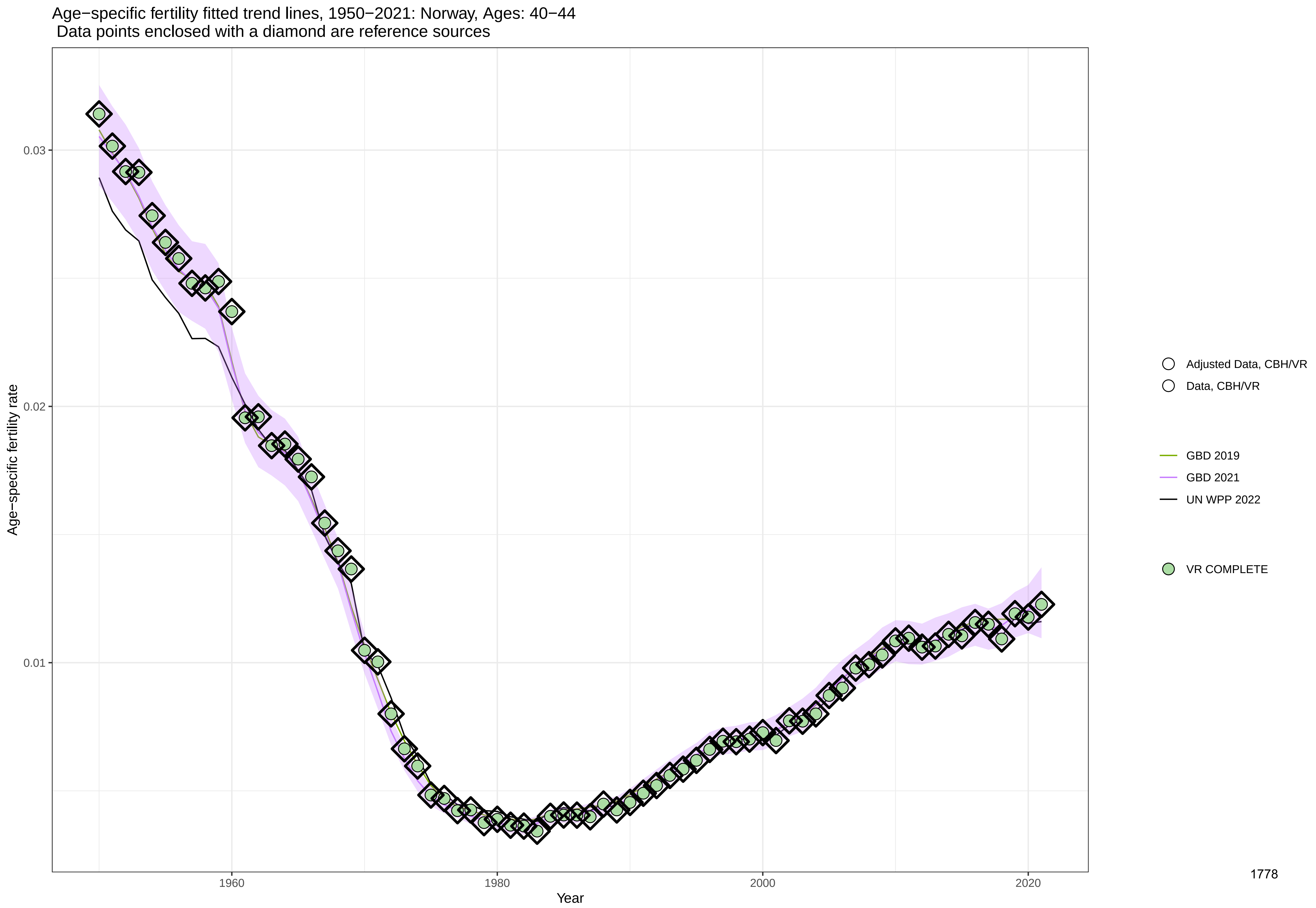

Age−specific fertility fitted trend lines, 1950−2021: Norway, Ages: 40−44
Data points enclosed with a diamond are reference sources
0.03
0.02
0.01
Age−specific fertility rate
1960
1980
2000
2020
Year
Adjusted Data, CBH/VR
Data, CBH/VR
GBD 2019
GBD 2021
UN WPP 2022
VR COMPLETE

Age−specific fertility fitted trend lines, 1950−2021: Norway, Ages: 45−49
Data points enclosed with a diamond are reference sources

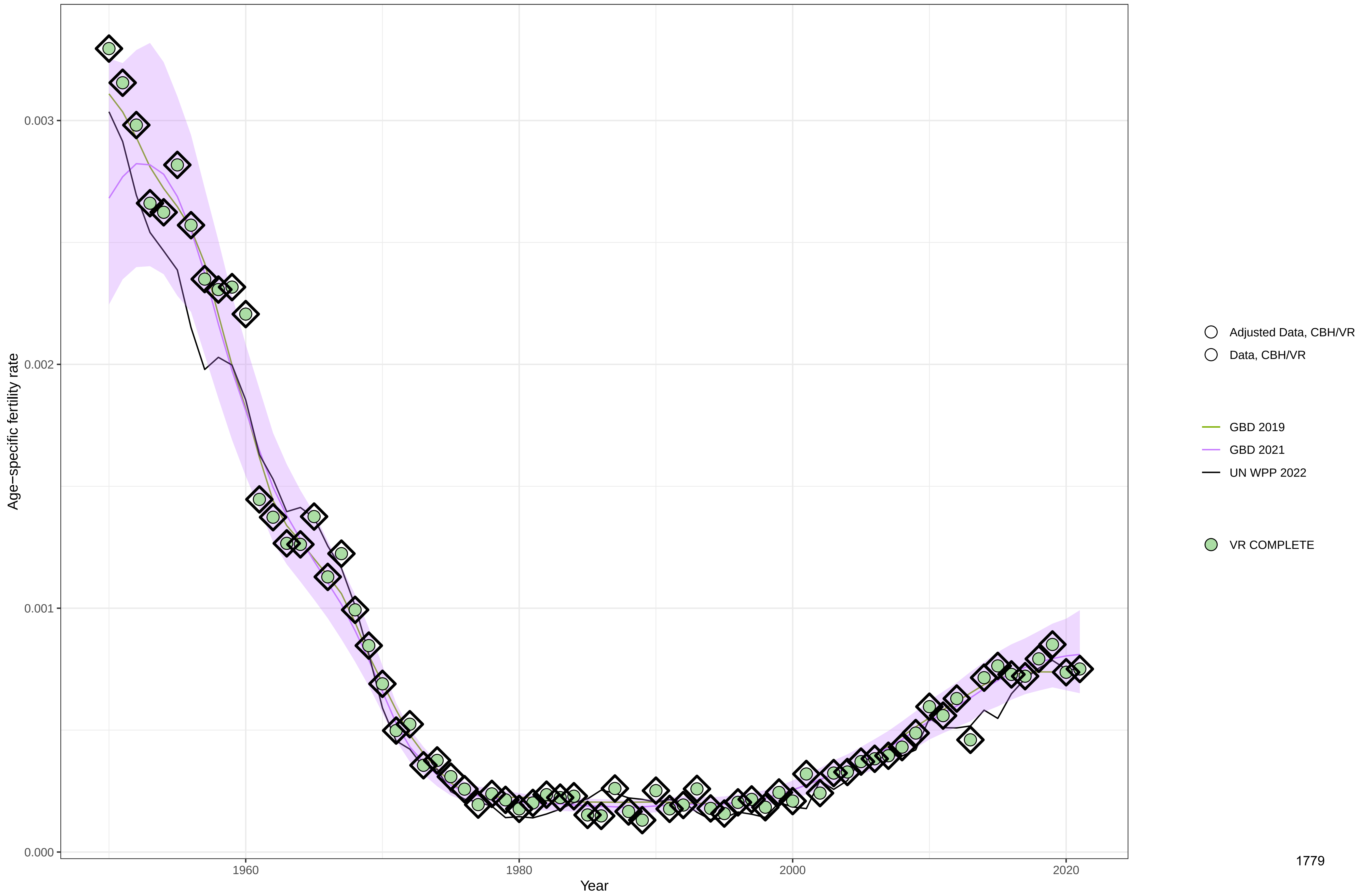

Age−specific fertility fitted trend lines, 1950−2021: Norway, Ages: 50−54
Data points enclosed with a diamond are reference sources

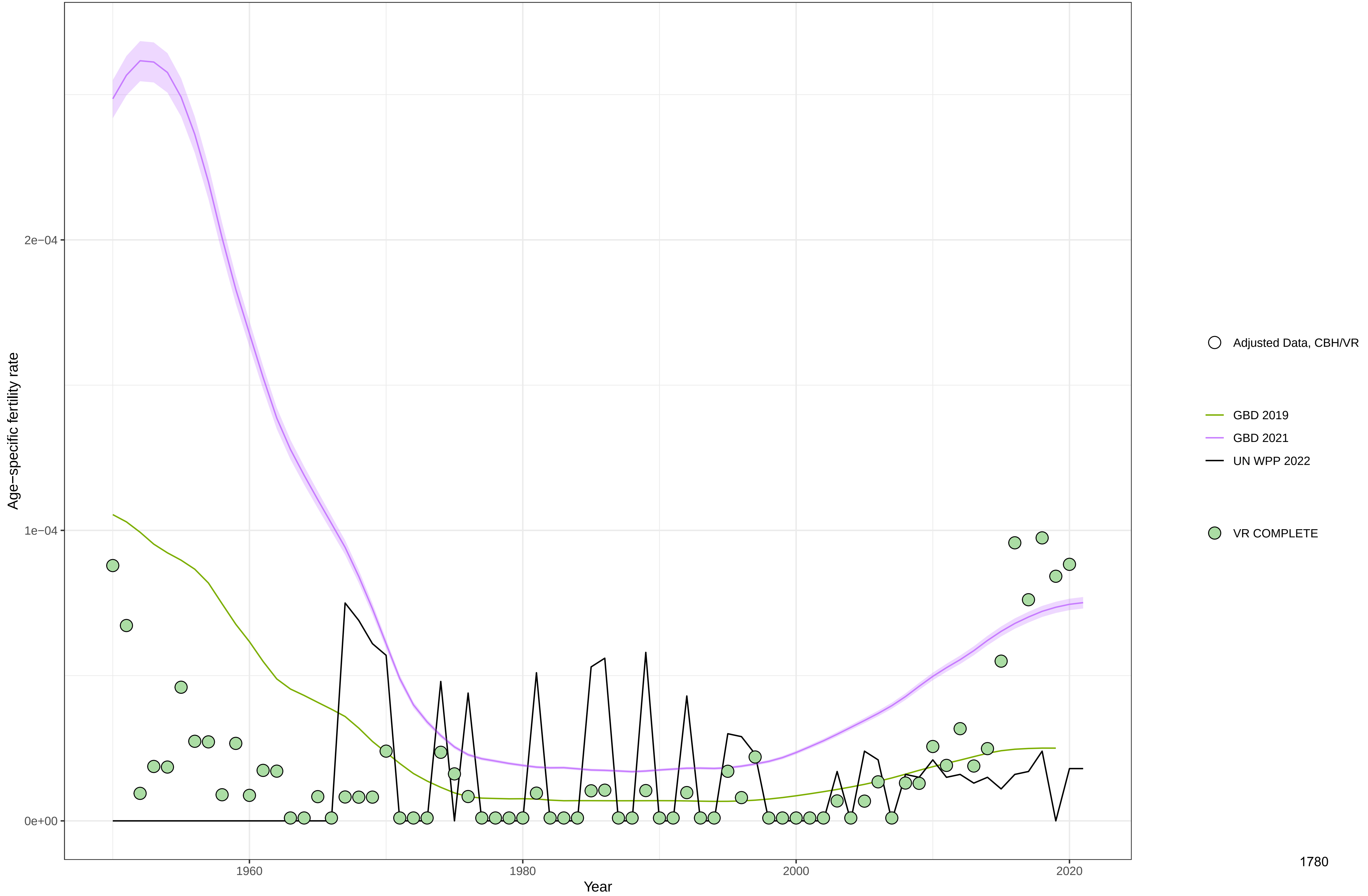

Total fertility rate fitted trend lines, 1950−2021: Norway TFR
Data points enclosed with a diamond are reference sources

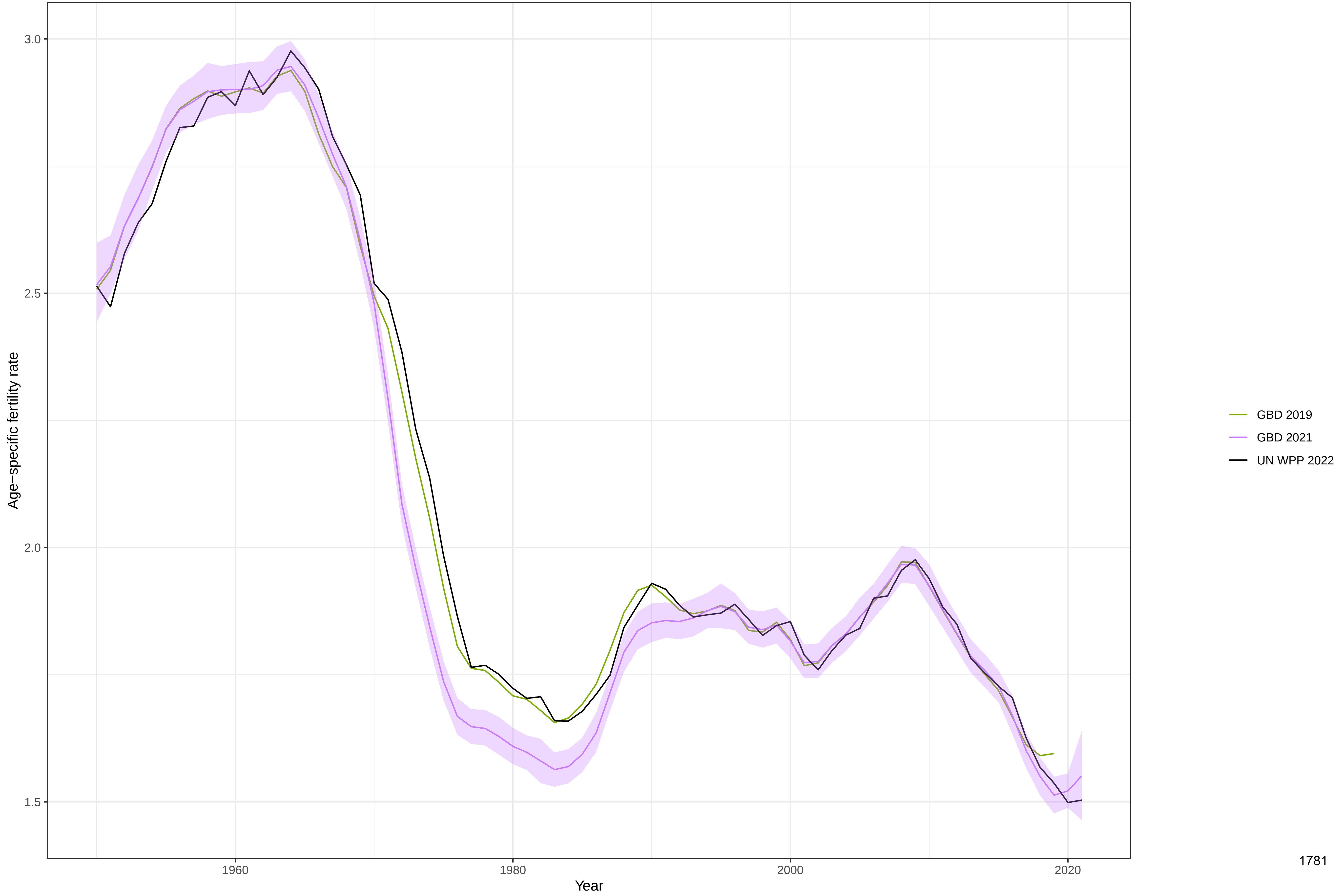

Age−specific fertility fitted trend lines, 1950−2021: Agder, Ages: 10−14
Data points enclosed with a diamond are reference sources

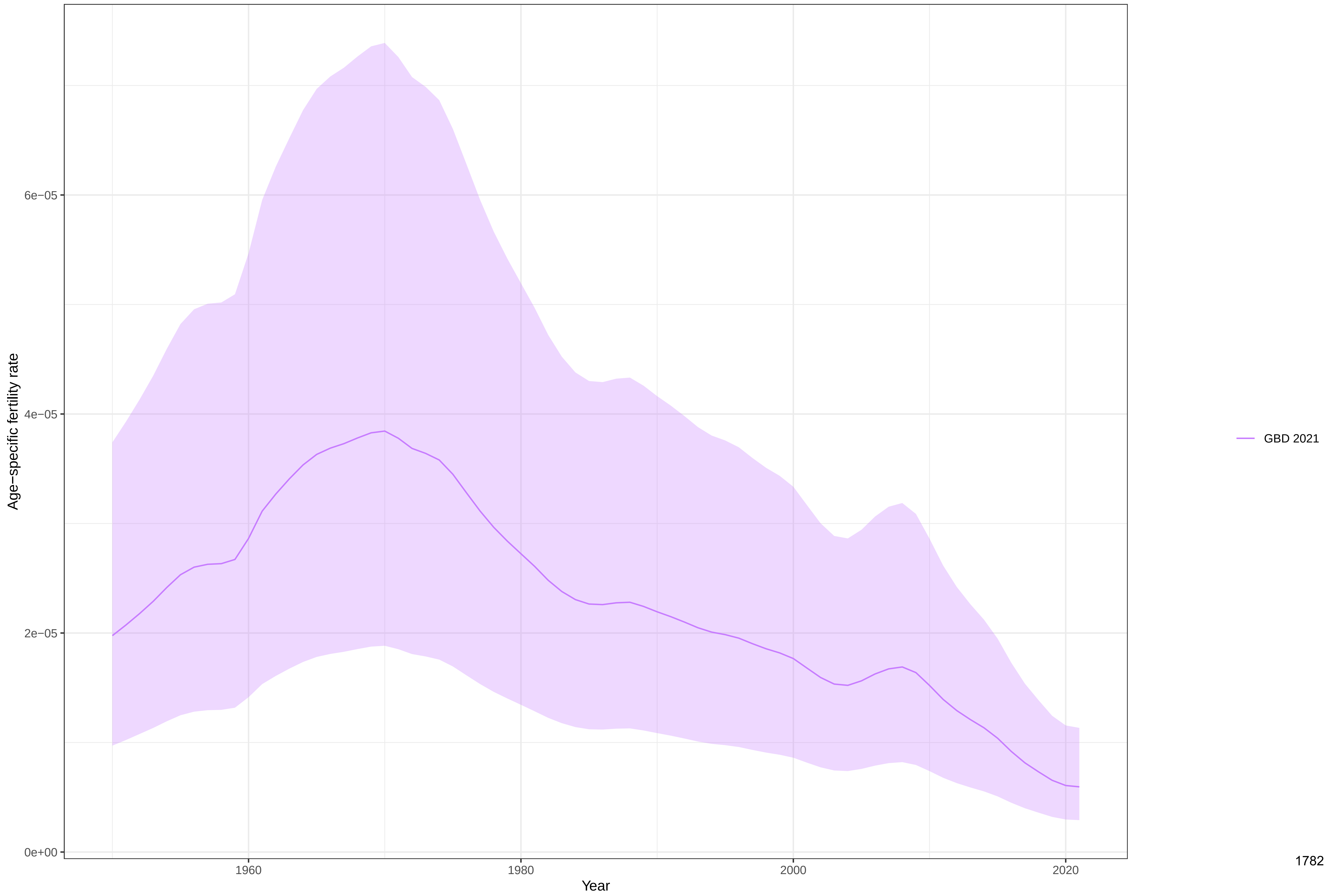

Age−specific fertility fitted trend lines, 1950−2021: Agder, Ages: 15−19
Data points enclosed with a diamond are reference sources

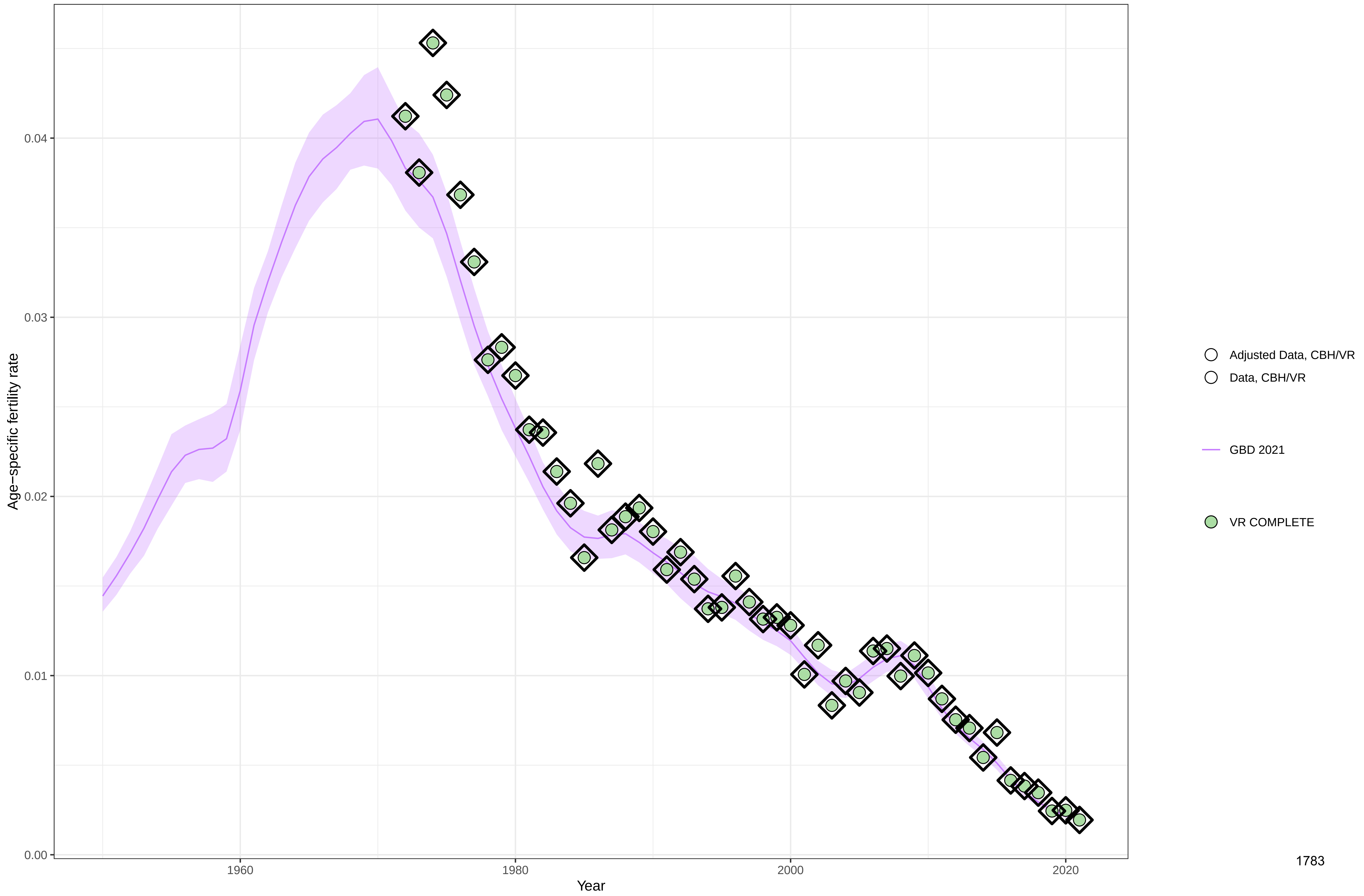

Age−specific fertility fitted trend lines, 1950−2021: Agder, Ages: 20−24
Data points enclosed with a diamond are reference sources

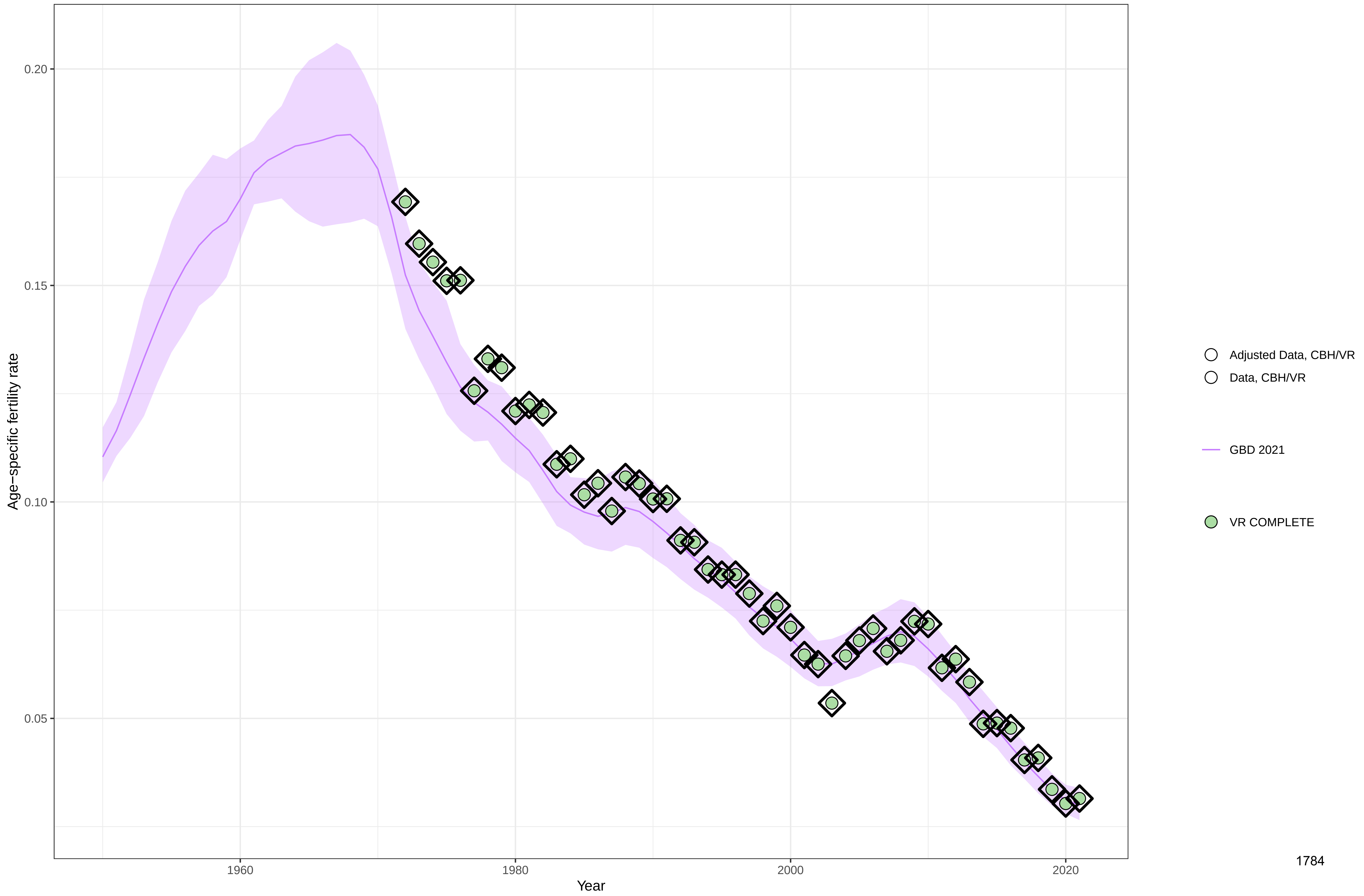

Age−specific fertility fitted trend lines, 1950−2021: Agder, Ages: 25−29
Data points enclosed with a diamond are reference sources

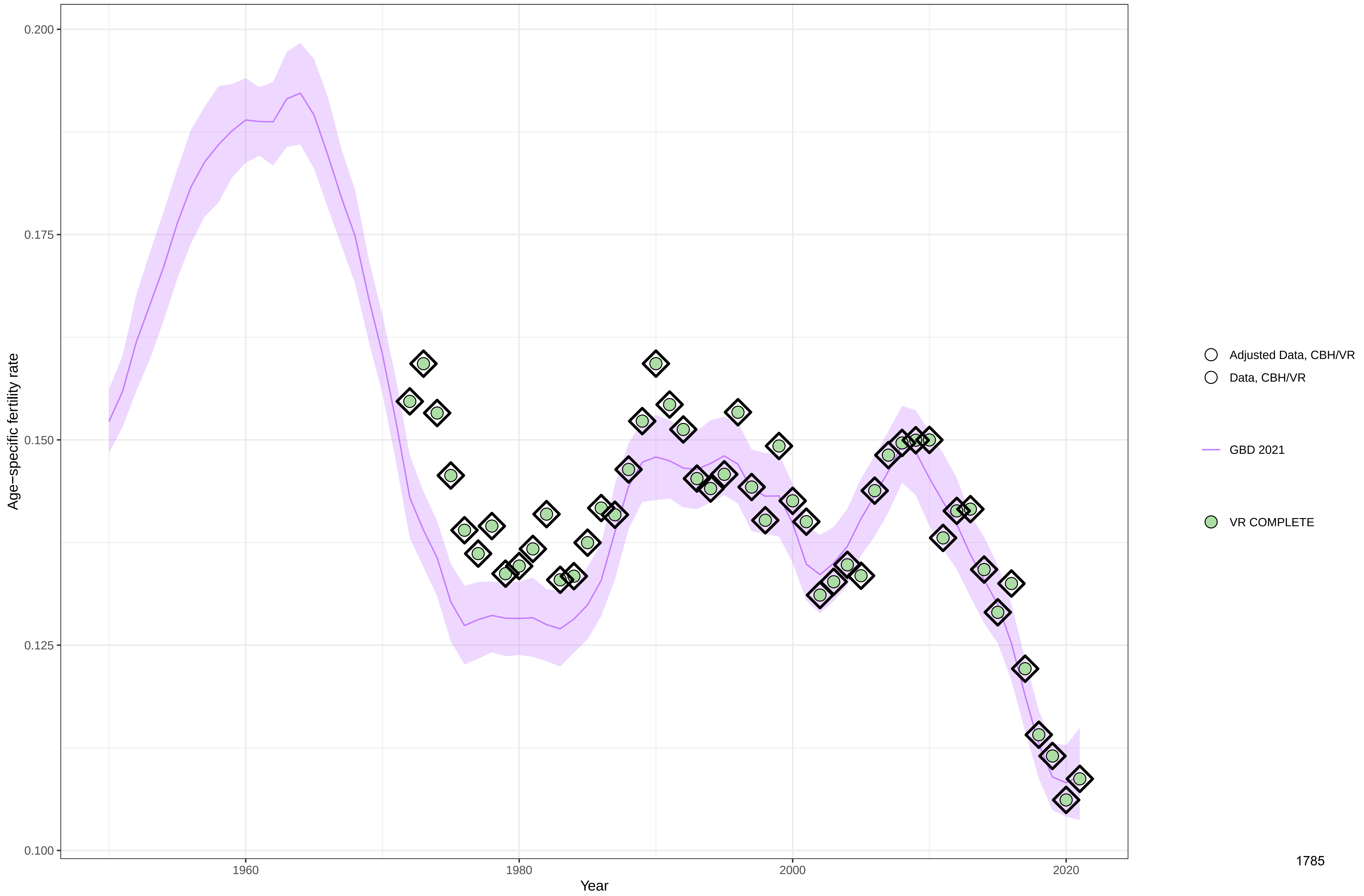

Age−specific fertility fitted trend lines, 1950−2021: Agder, Ages: 30−34
Data points enclosed with a diamond are reference sources

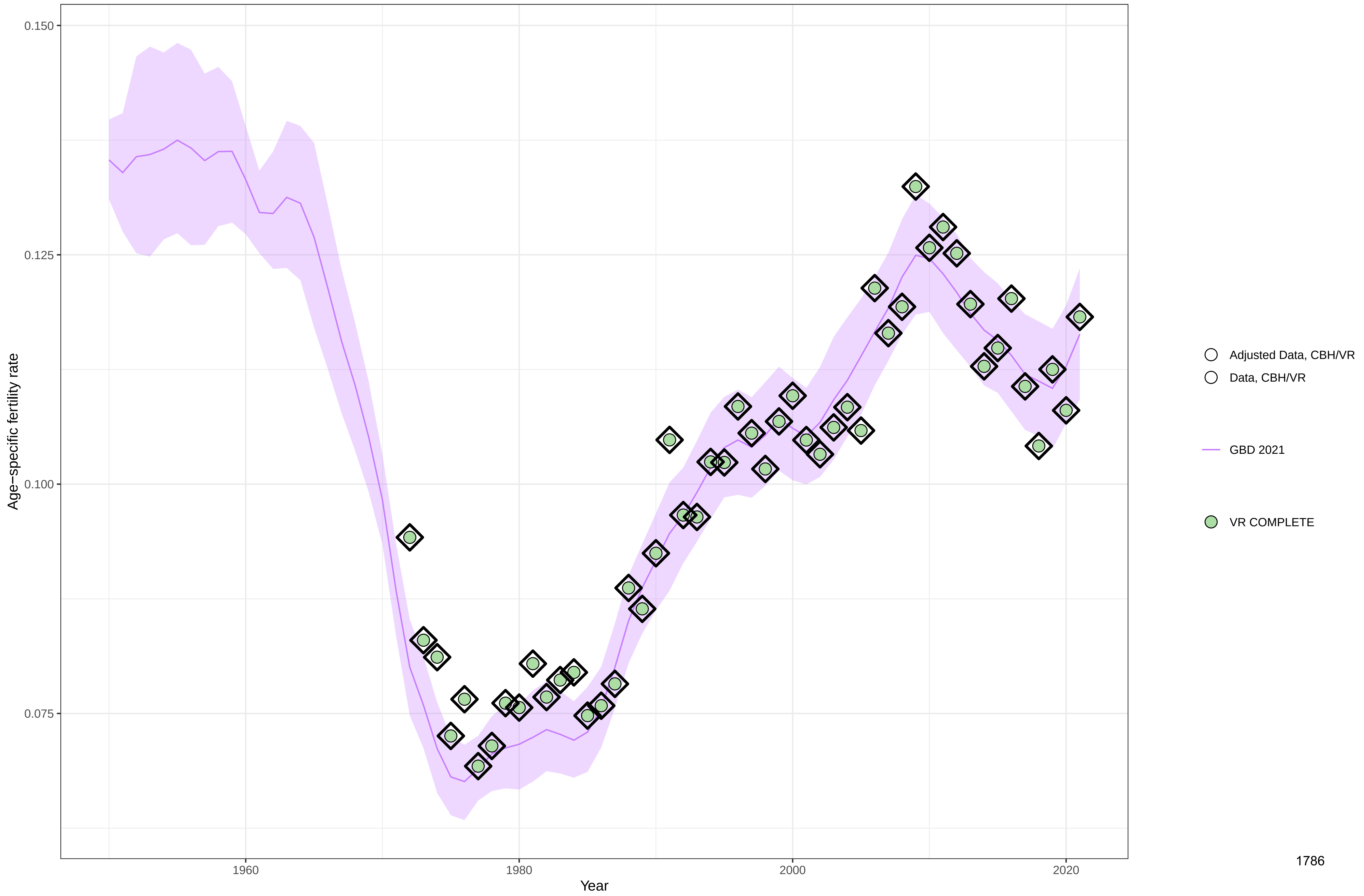

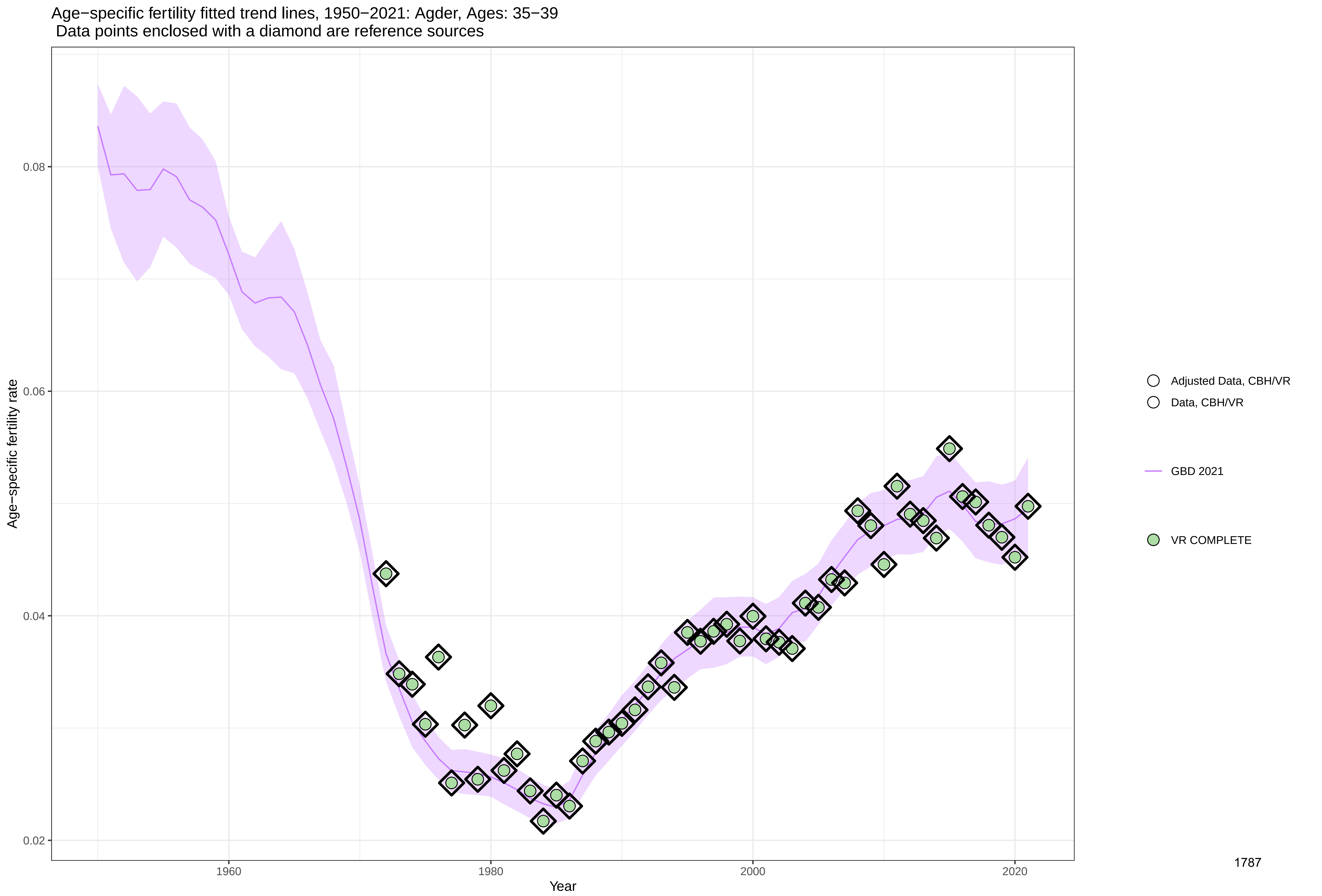
Age−specific fertility fitted trend lines, 1950−2021: Agder, Ages: 35−39
Data points enclosed with a diamond are reference sources
Age−specific fertility rate
0.08
0.06
0.04
0.02
1960
1980
2000
2020
Year
Adjusted Data, CBH/VR
Data, CBH/VR
GBD 2021
VR COMPLETE

Age−specific fertility fitted trend lines, 1950−2021: Agder, Ages: 40−44
Data points enclosed with a diamond are reference sources

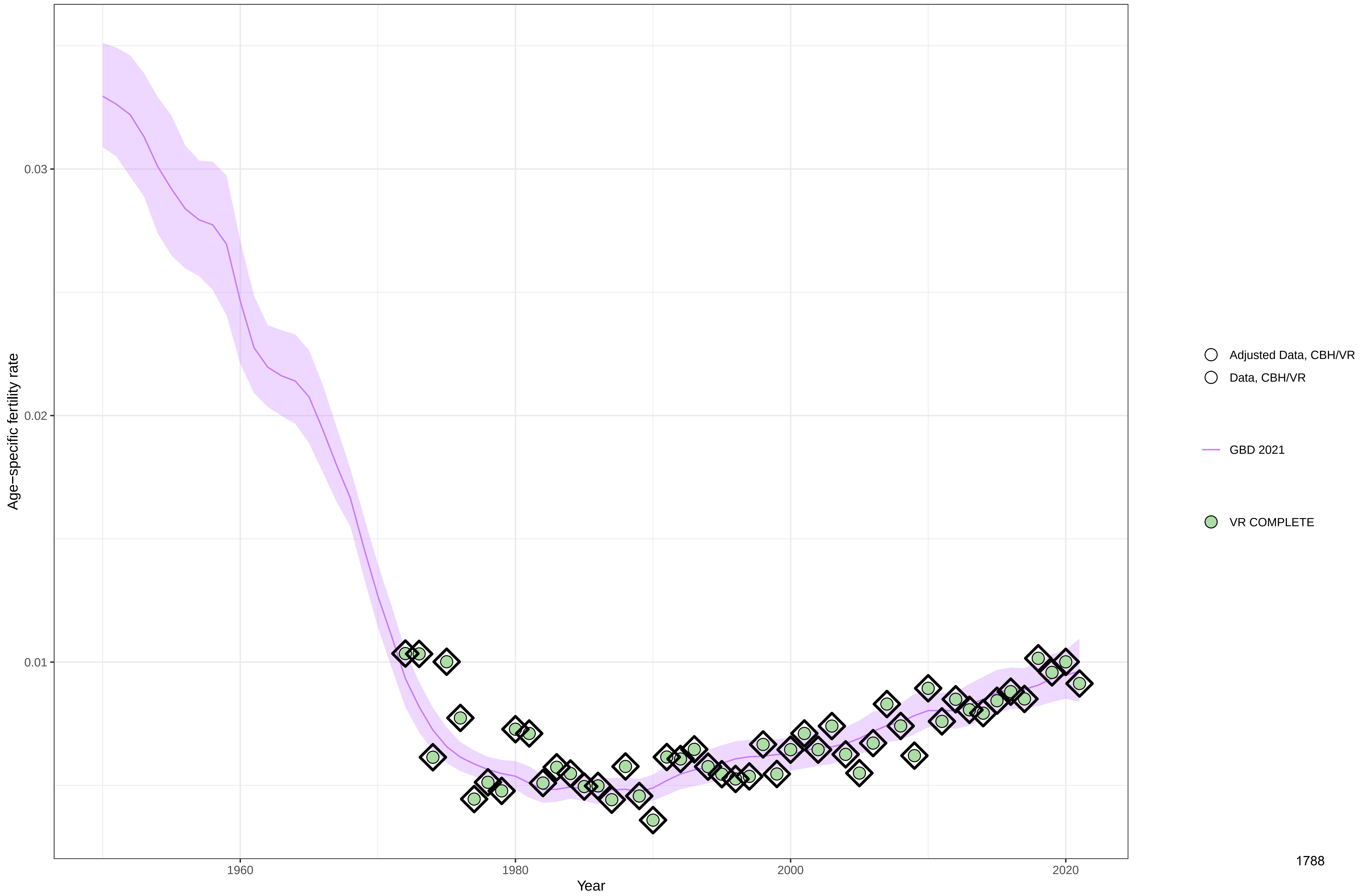

Age−specific fertility fitted trend lines, 1950−2021: Agder, Ages: 45−49
Data points enclosed with a diamond are reference sources

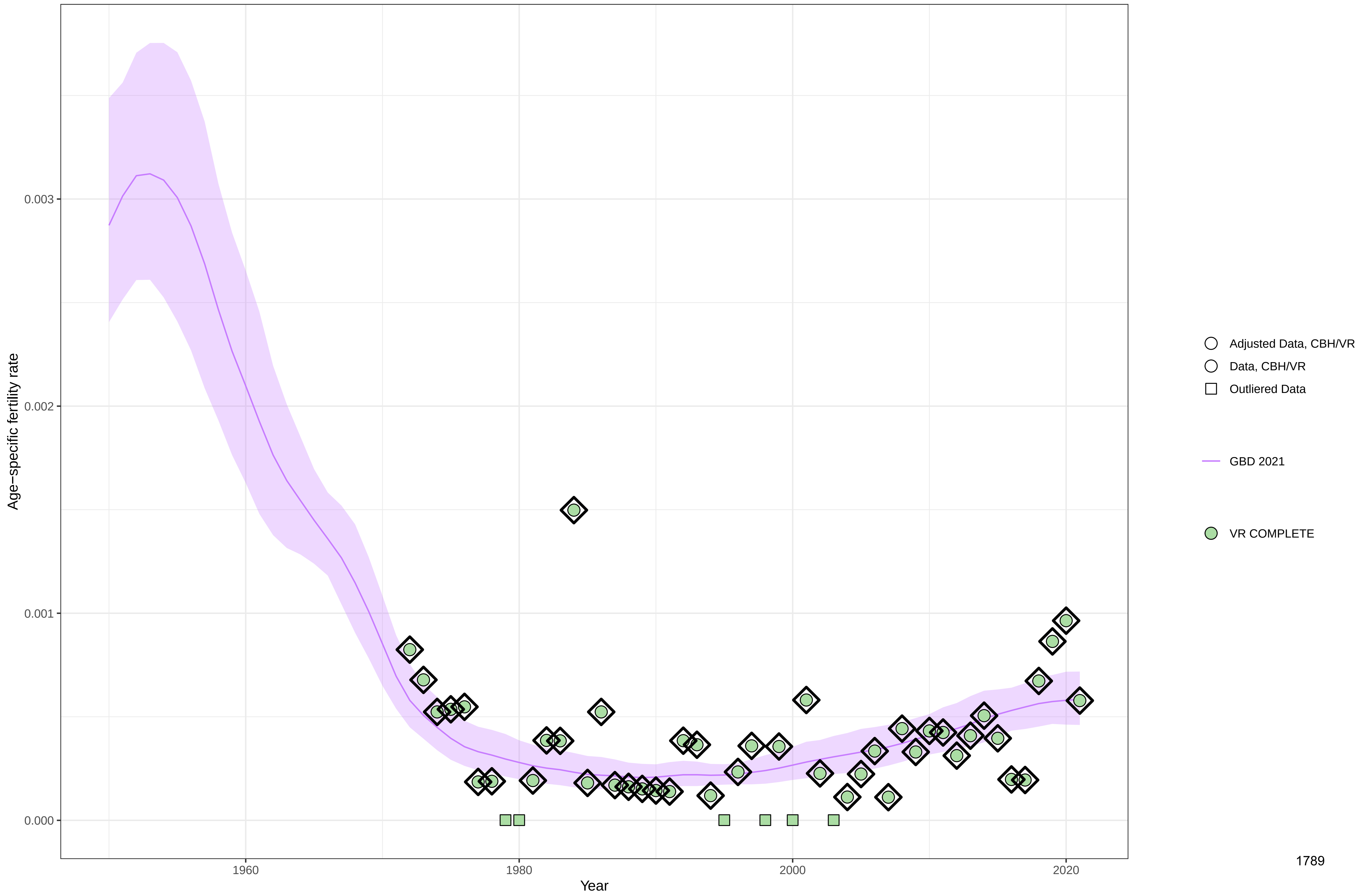

Age−specific fertility fitted trend lines, 1950−2021: Agder, Ages: 50−54
Data points enclosed with a diamond are reference sources

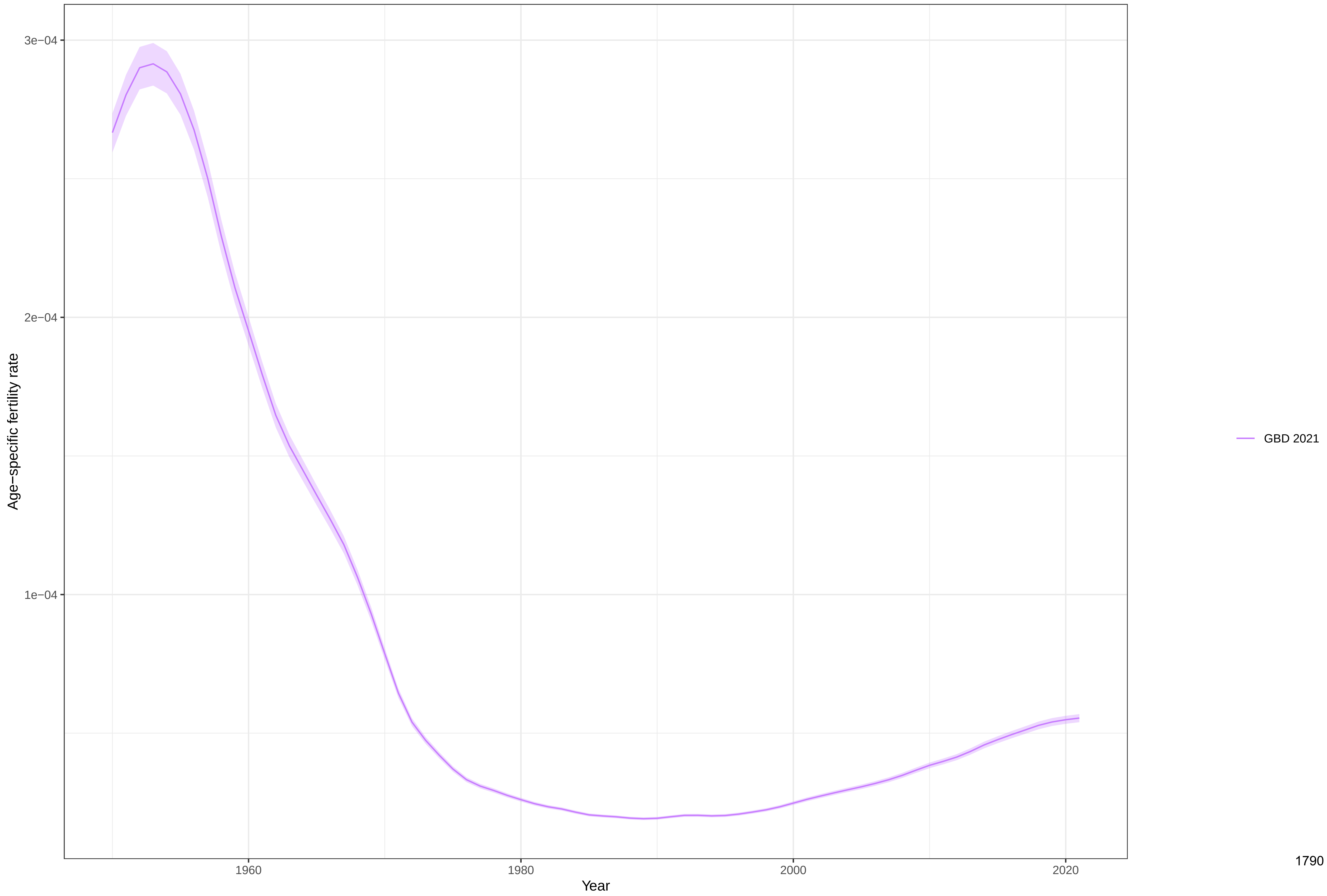

Total fertility rate fitted trend lines, 1950−2021: Agder TFR
Data points enclosed with a diamond are reference sources

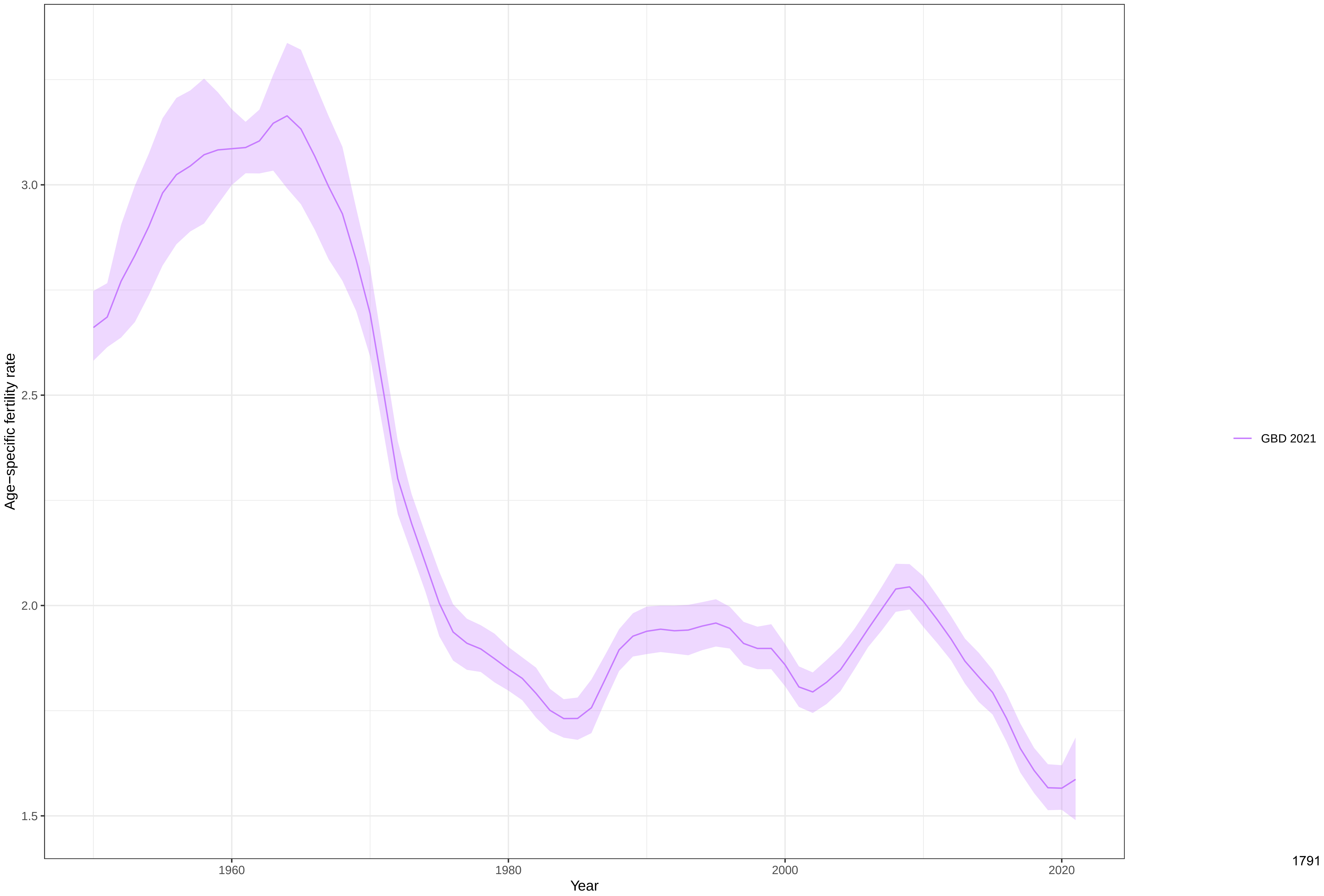

1791

Age−specific fertility fitted trend lines, 1950−2021: Innlandet, Ages: 10−14
 Data points enclosed with a diamond are reference sources

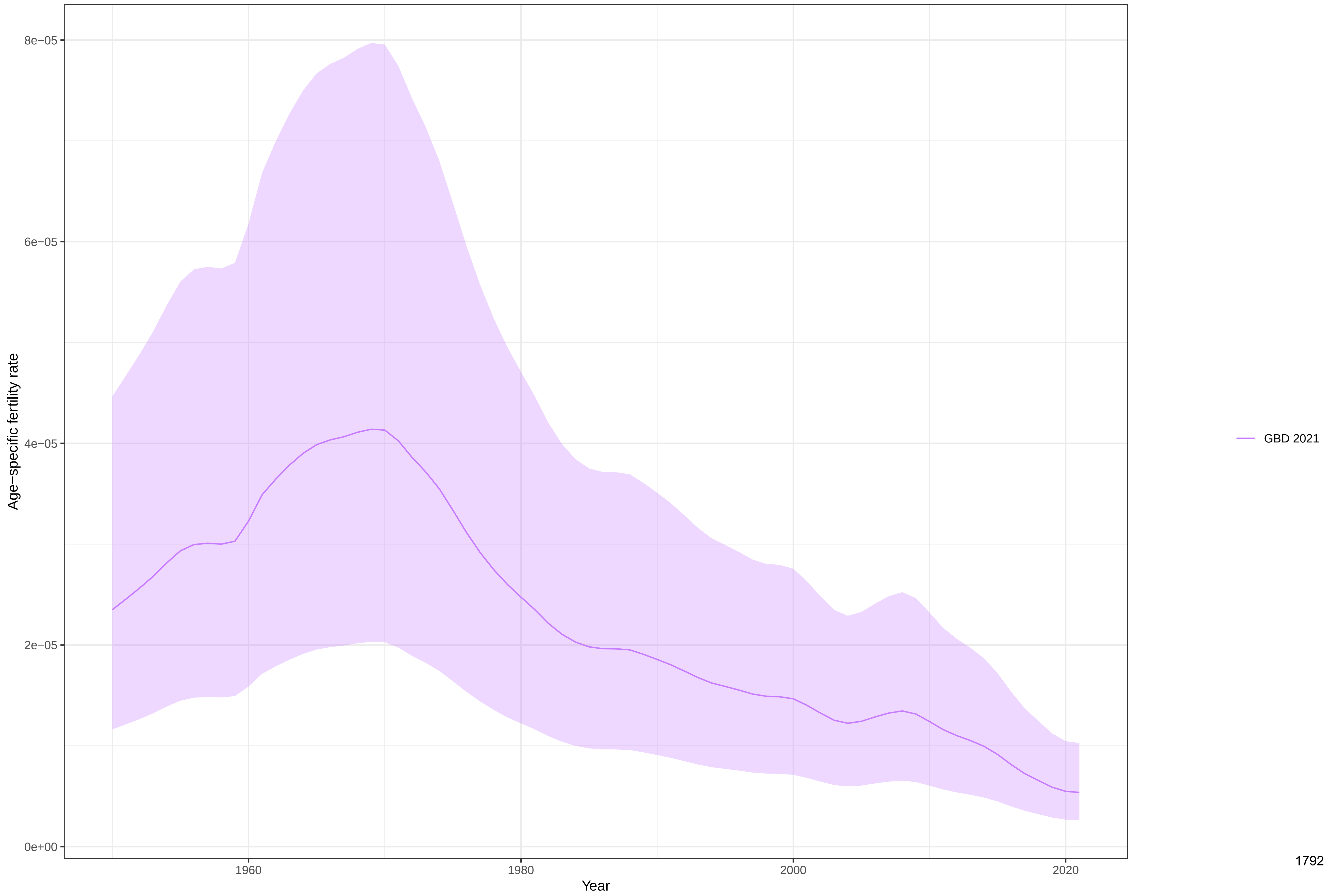

Age−specific fertility fitted trend lines, 1950−2021: Innlandet, Ages: 15−19
Data points enclosed with a diamond are reference sources

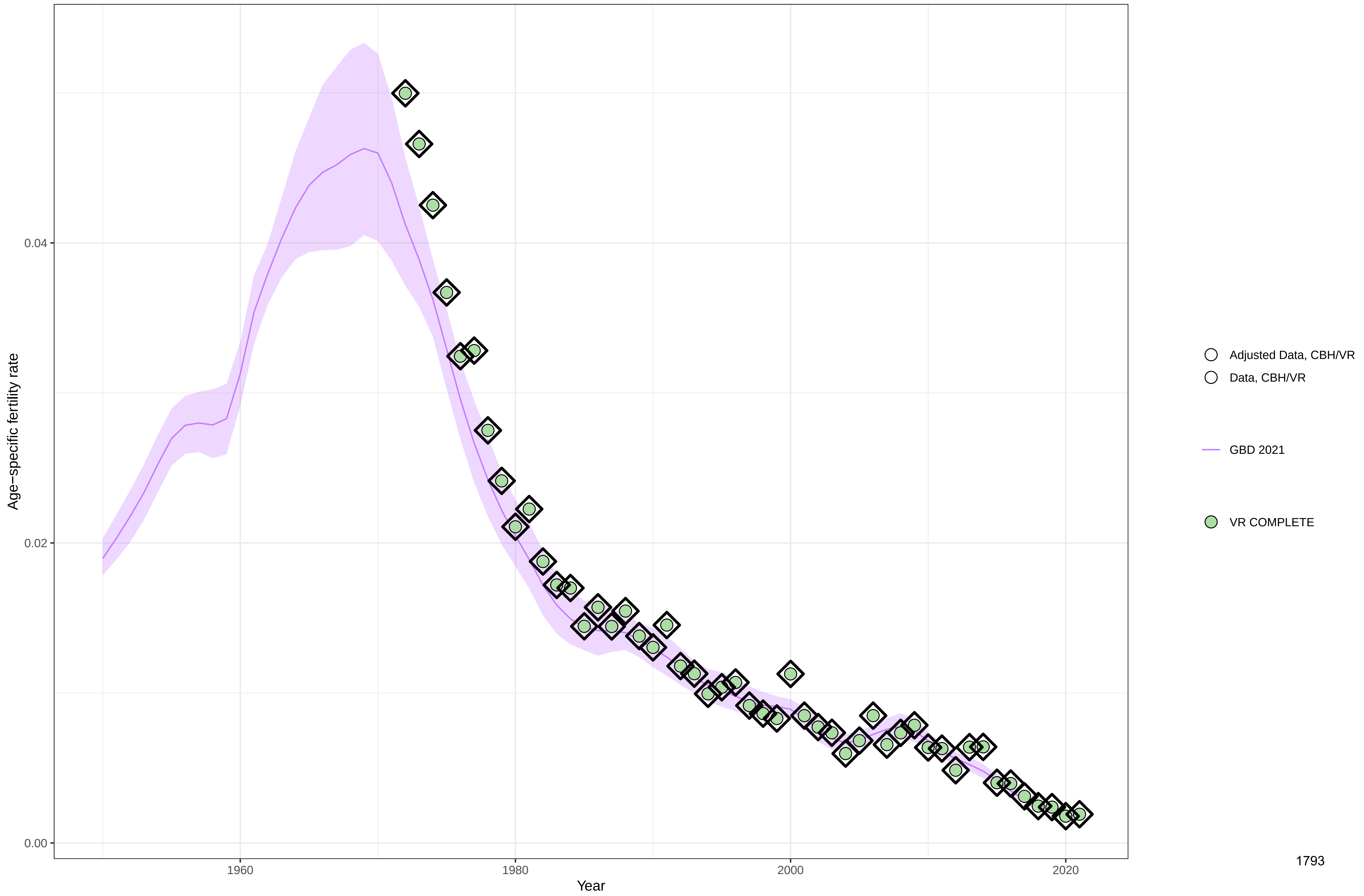

Age−specific fertility fitted trend lines, 1950−2021: Innlandet, Ages: 20−24
Data points enclosed with a diamond are reference sources

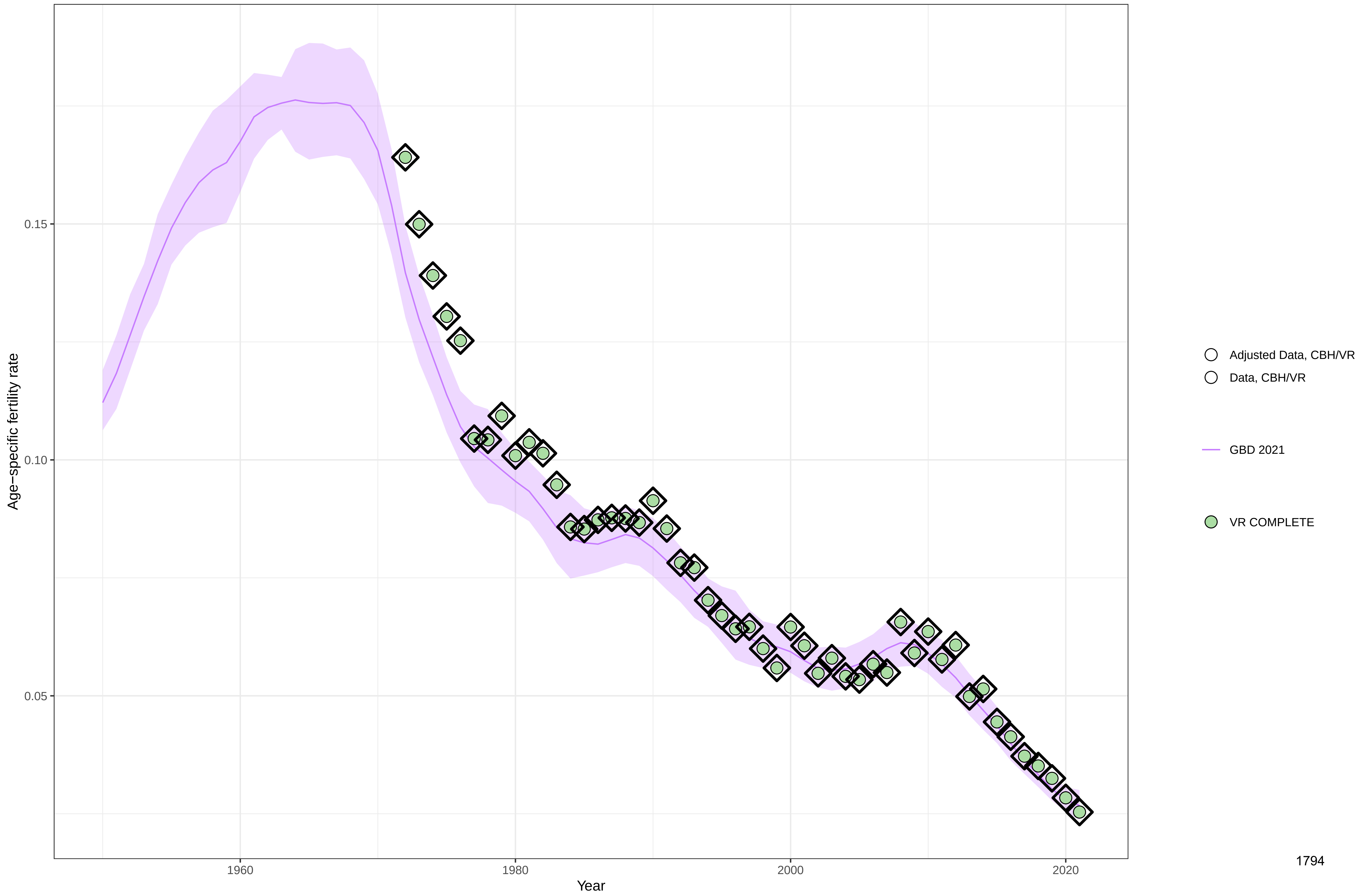

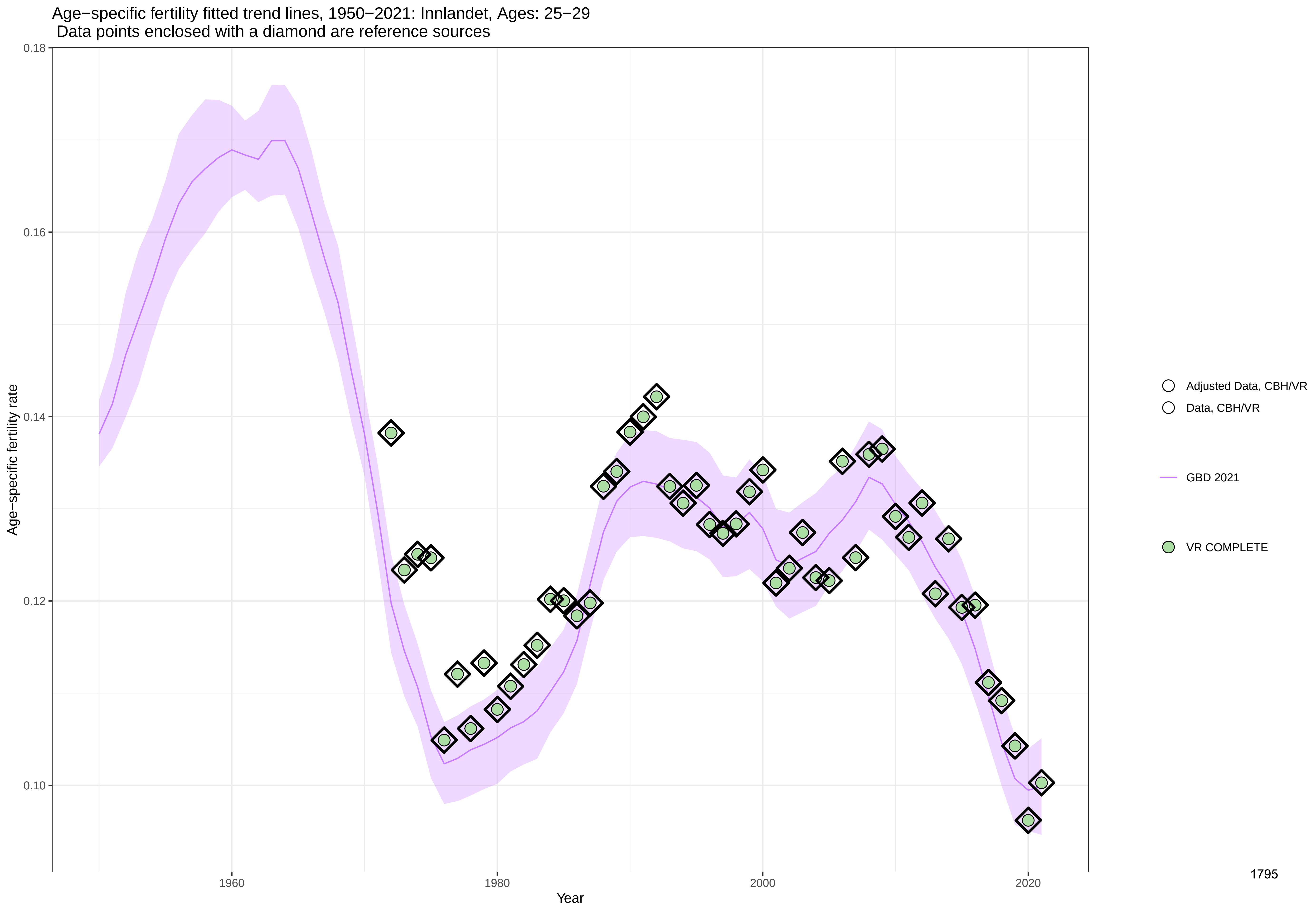

Age−specific fertility fitted trend lines, 1950−2021: Innlandet, Ages: 25−29
Data points enclosed with a diamond are reference sources
Age−specific fertility rate
Year
0.18
0.16
0.14
0.12
0.10
1960
1980
2000
2020
Adjusted Data, CBH/VR
Data, CBH/VR
GBD 2021
VR COMPLETE

Age−specific fertility fitted trend lines, 1950−2021: Innlandet, Ages: 30−34
Data points enclosed with a diamond are reference sources

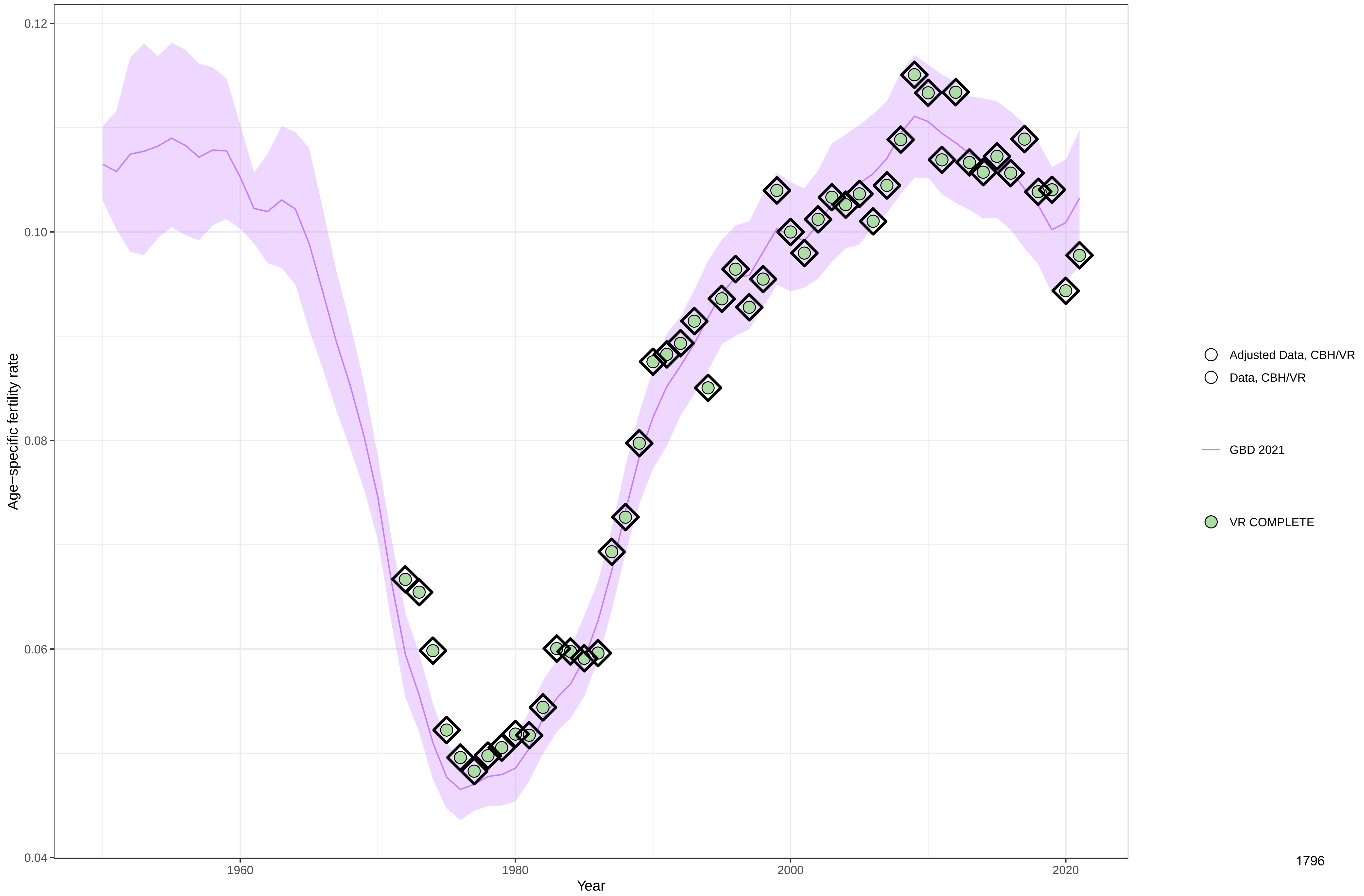

Age−specific fertility fitted trend lines, 1950−2021: Innlandet, Ages: 35−39
Data points enclosed with a diamond are reference sources

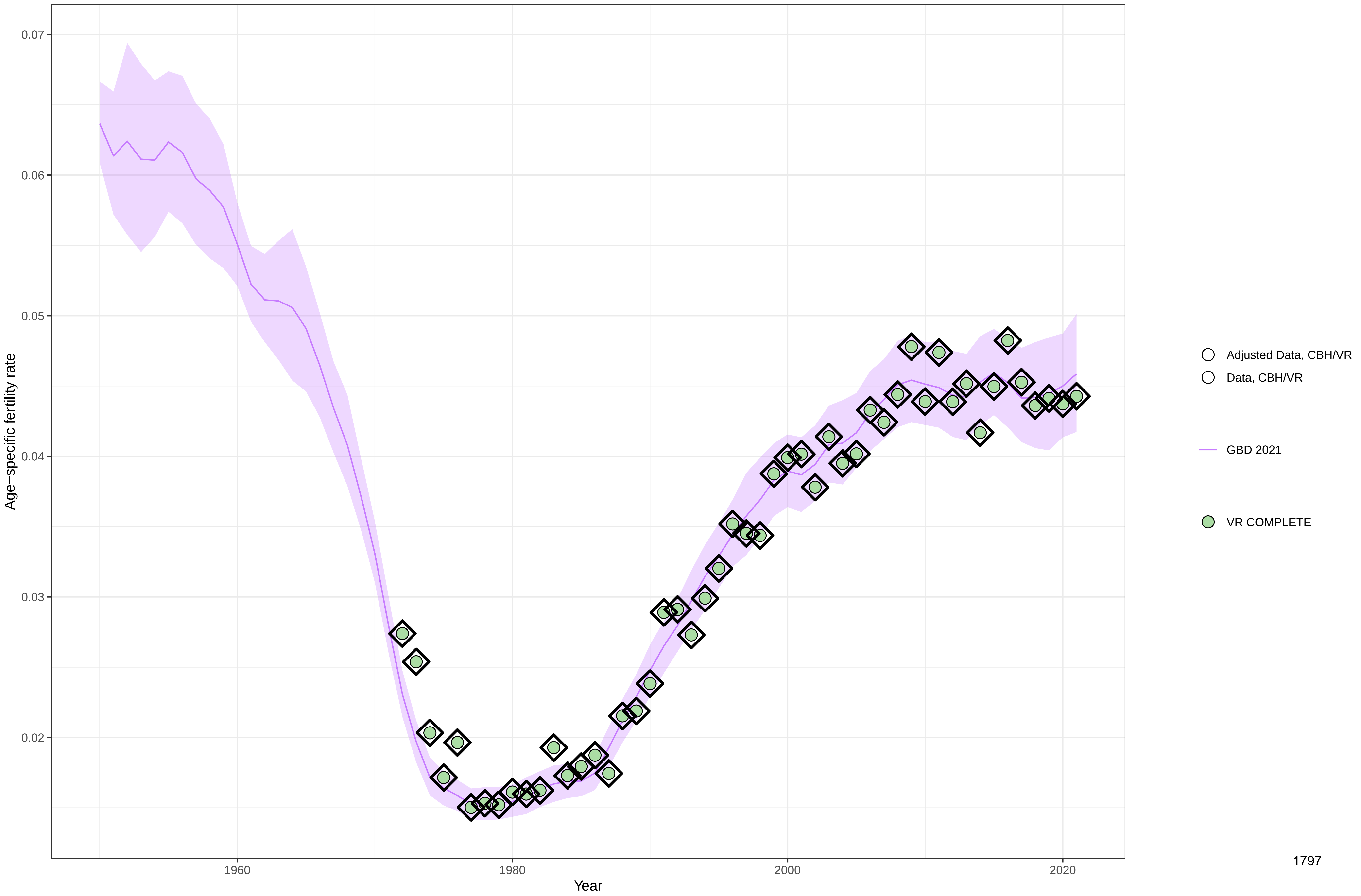

Age−specific fertility fitted trend lines, 1950−2021: Innlandet, Ages: 40−44
Data points enclosed with a diamond are reference sources

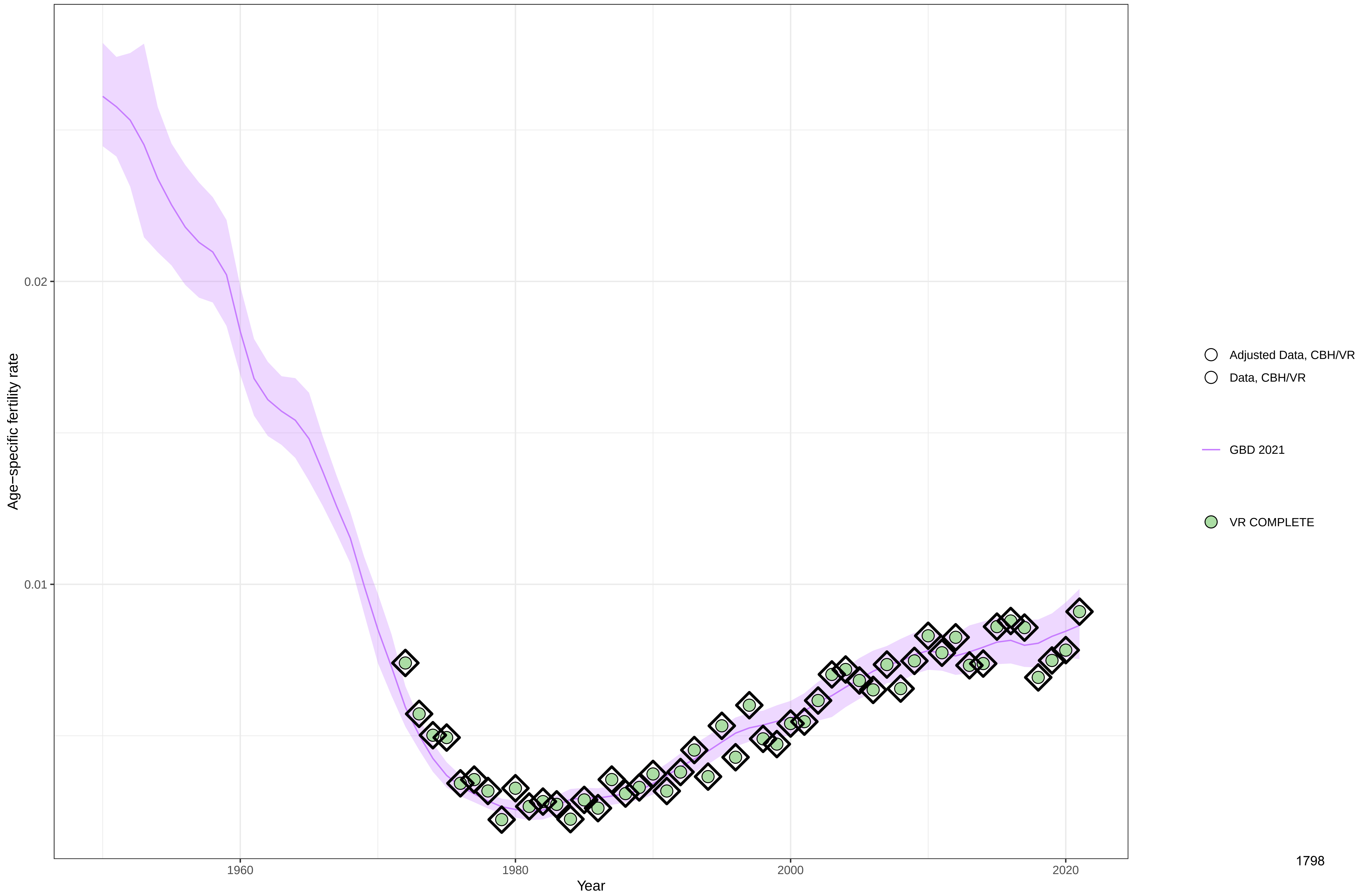

Age−specific fertility fitted trend lines, 1950−2021: Innlandet, Ages: 45−49
Data points enclosed with a diamond are reference sources

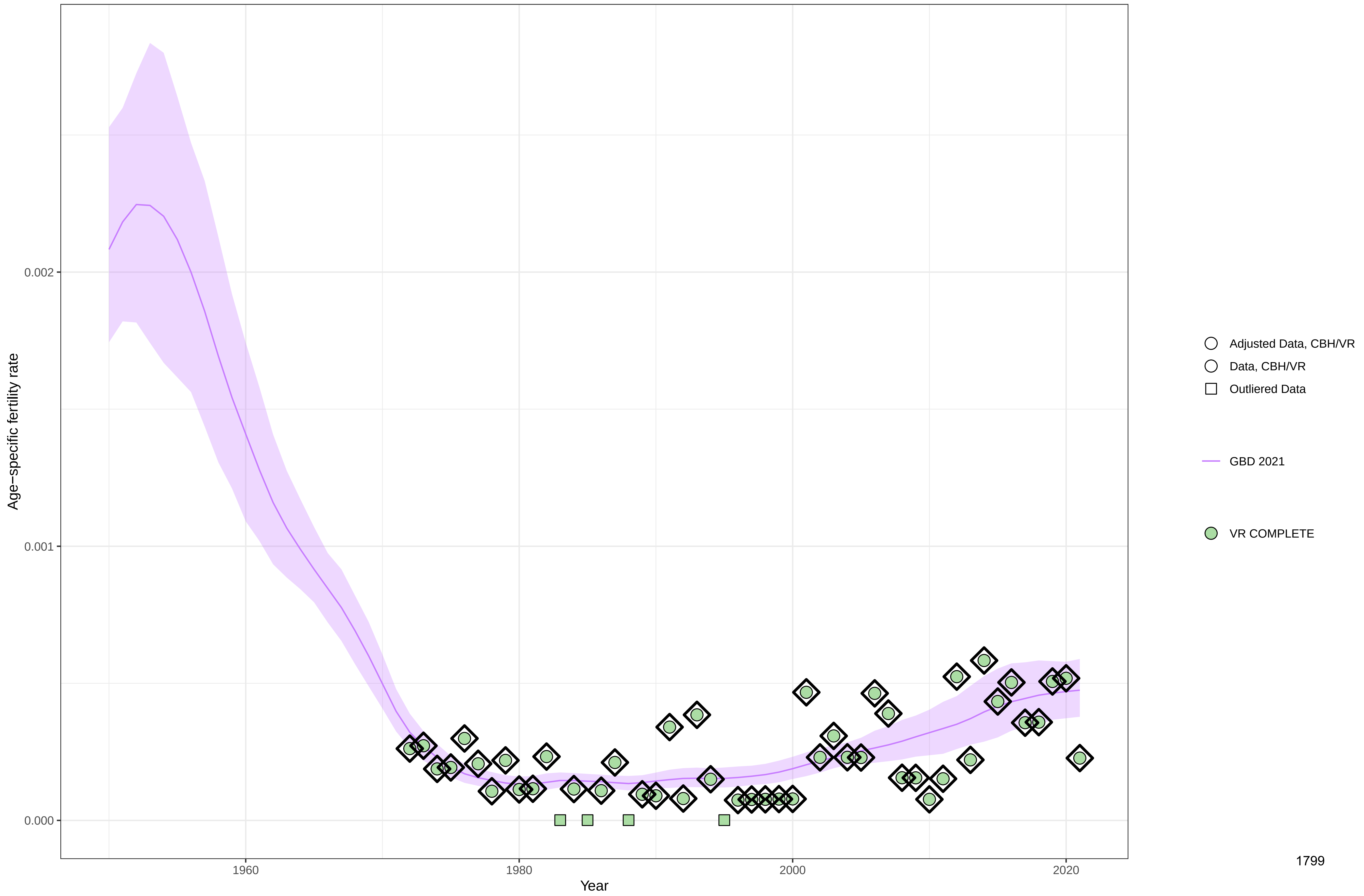

Age−specific fertility fitted trend lines, 1950−2021: Innlandet, Ages: 50−54
Data points enclosed with a diamond are reference sources

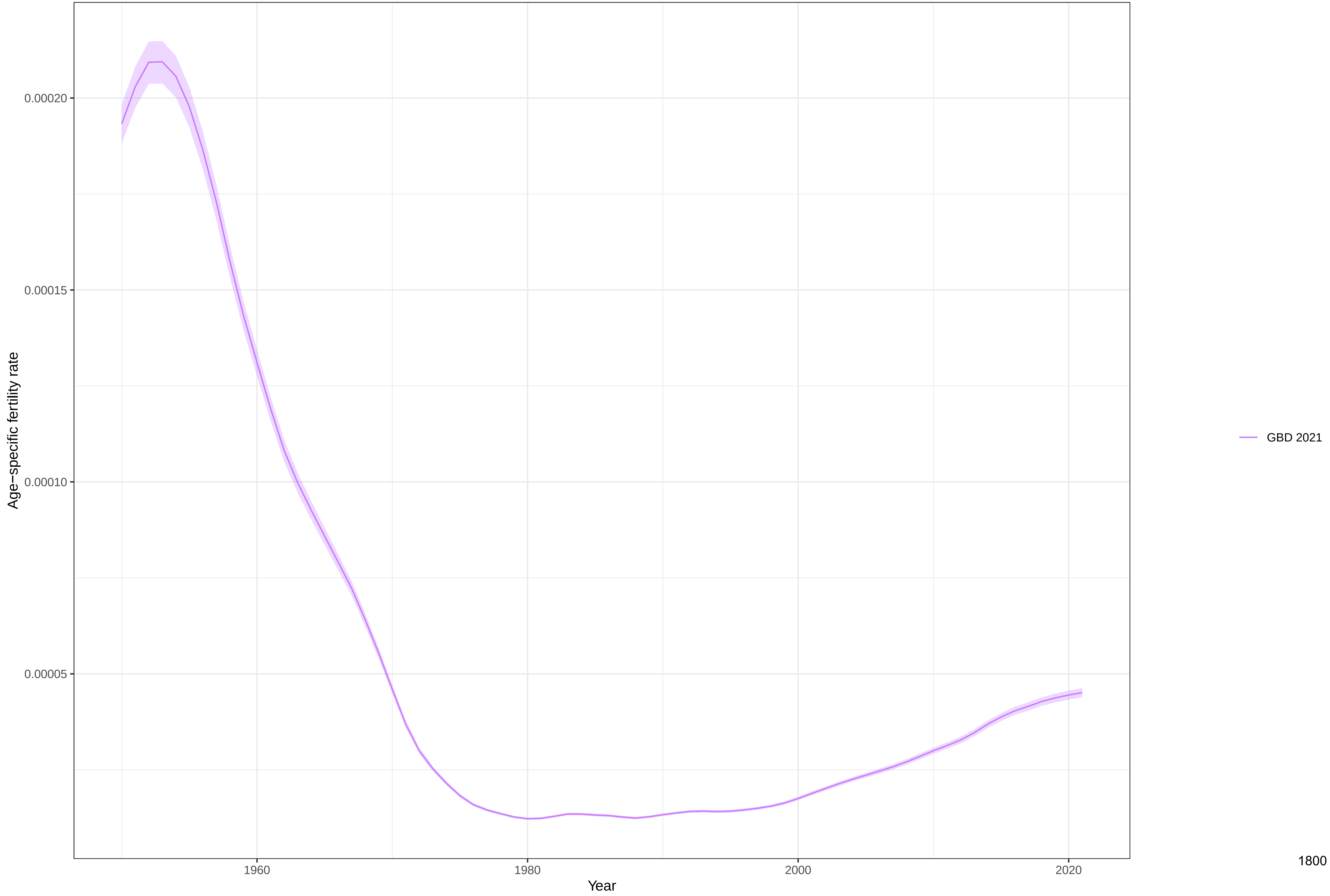

Total fertility rate fitted trend lines, 1950−2021: Innlandet TFR
Data points enclosed with a diamond are reference sources

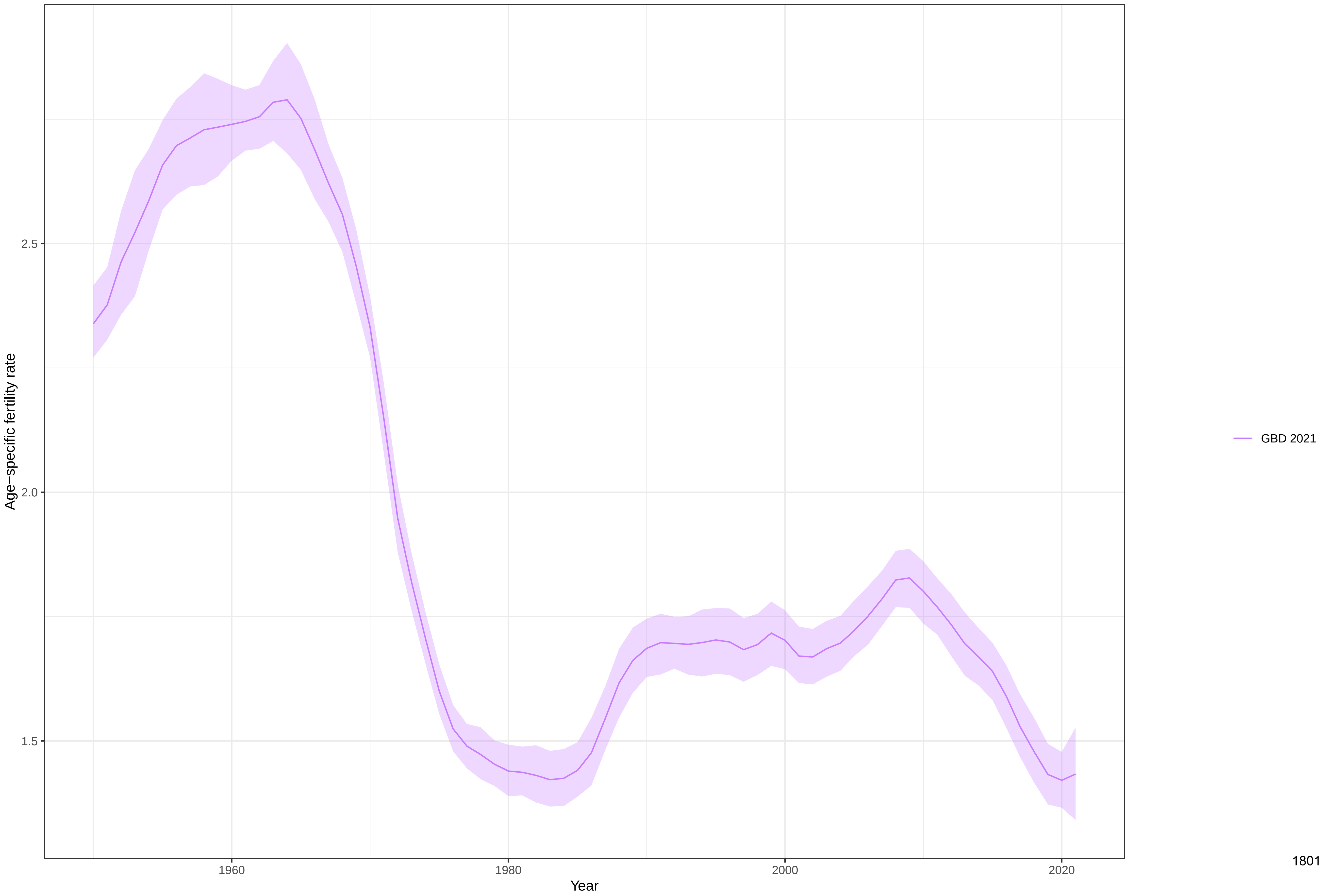

Age−specific fertility fitted trend lines, 1950−2021: Møre og Romsdal, Ages: 10−14
Data points enclosed with a diamond are reference sources

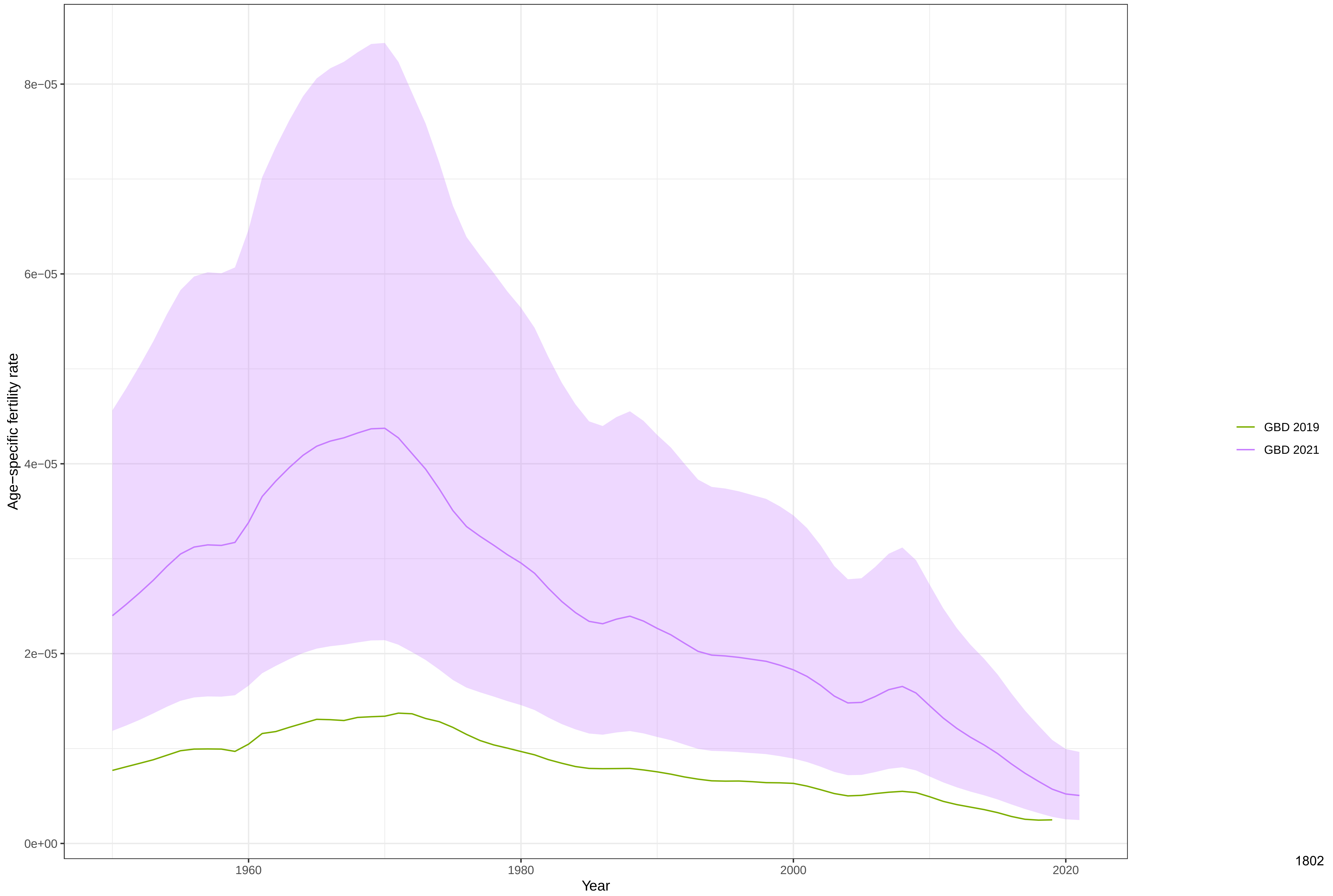

Age−specific fertility fitted trend lines, 1950−2021: Møre og Romsdal, Ages: 15−19
Data points enclosed with a diamond are reference sources

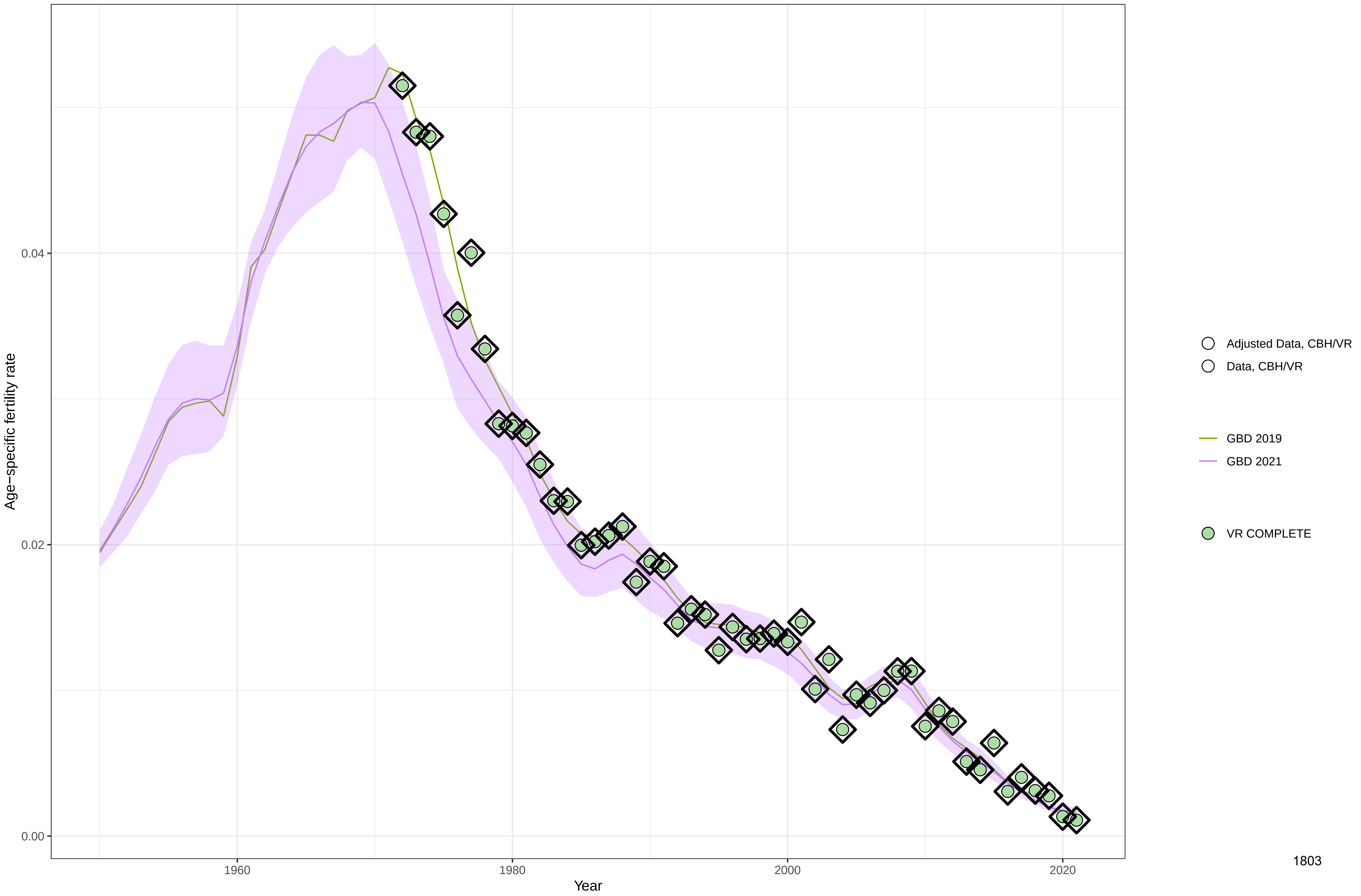

Age−specific fertility fitted trend lines, 1950−2021: Møre og Romsdal, Ages: 20−24
Data points enclosed with a diamond are reference sources

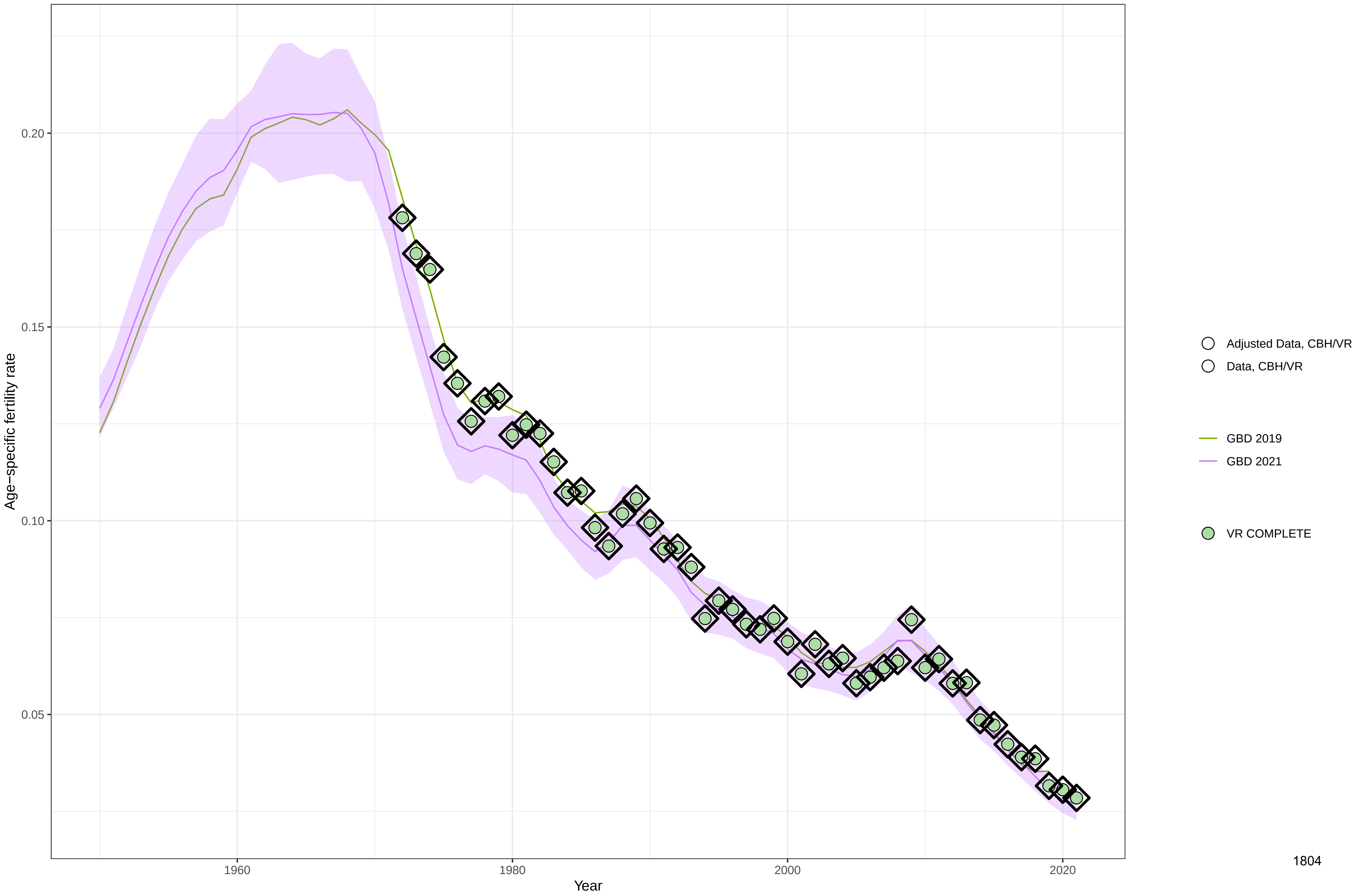

Age−specific fertility fitted trend lines, 1950−2021: Møre og Romsdal, Ages: 25−29
Data points enclosed with a diamond are reference sources

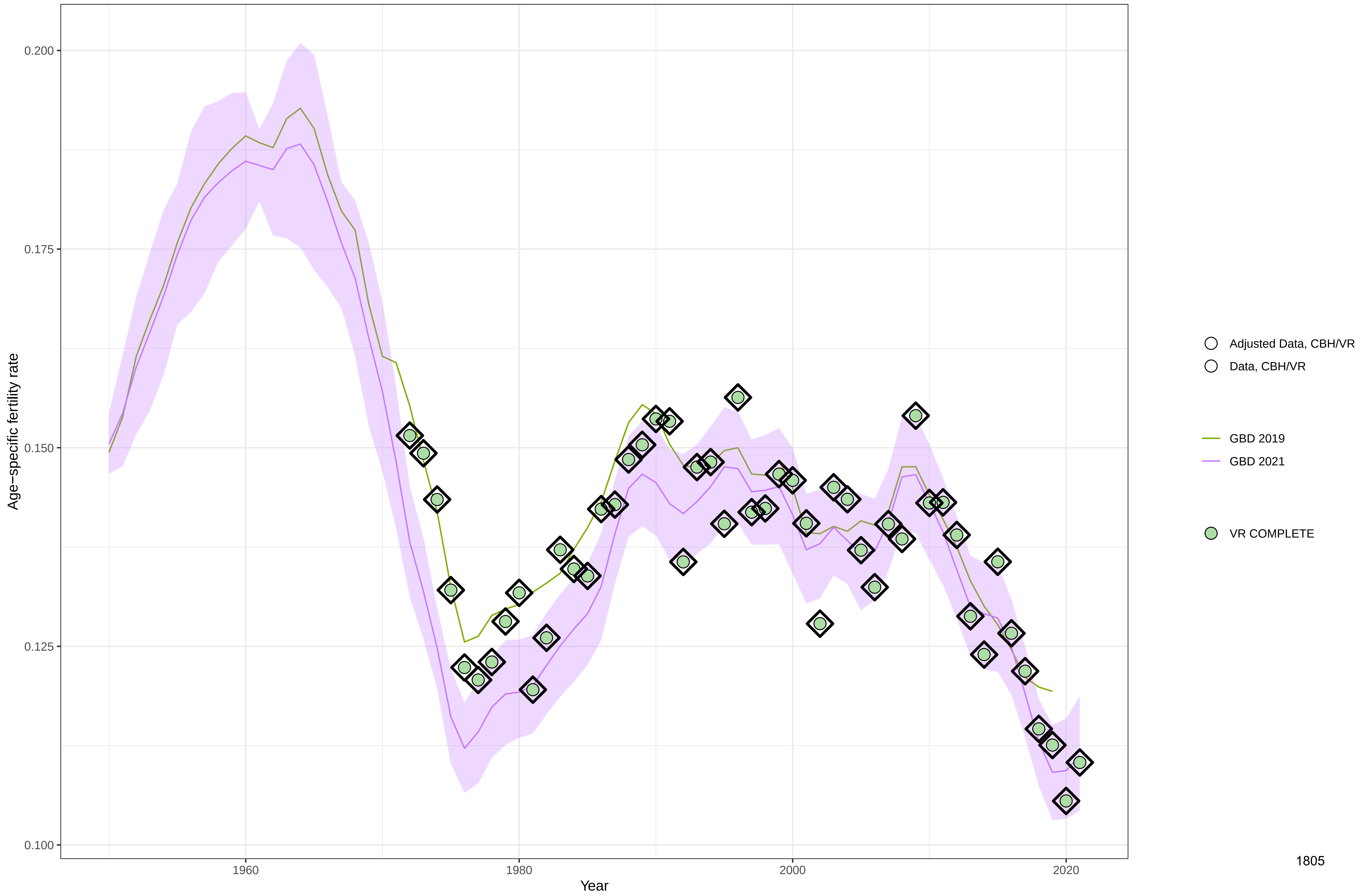

Age−specific fertility fitted trend lines, 1950−2021: Møre og Romsdal, Ages: 30−34
Data points enclosed with a diamond are reference sources

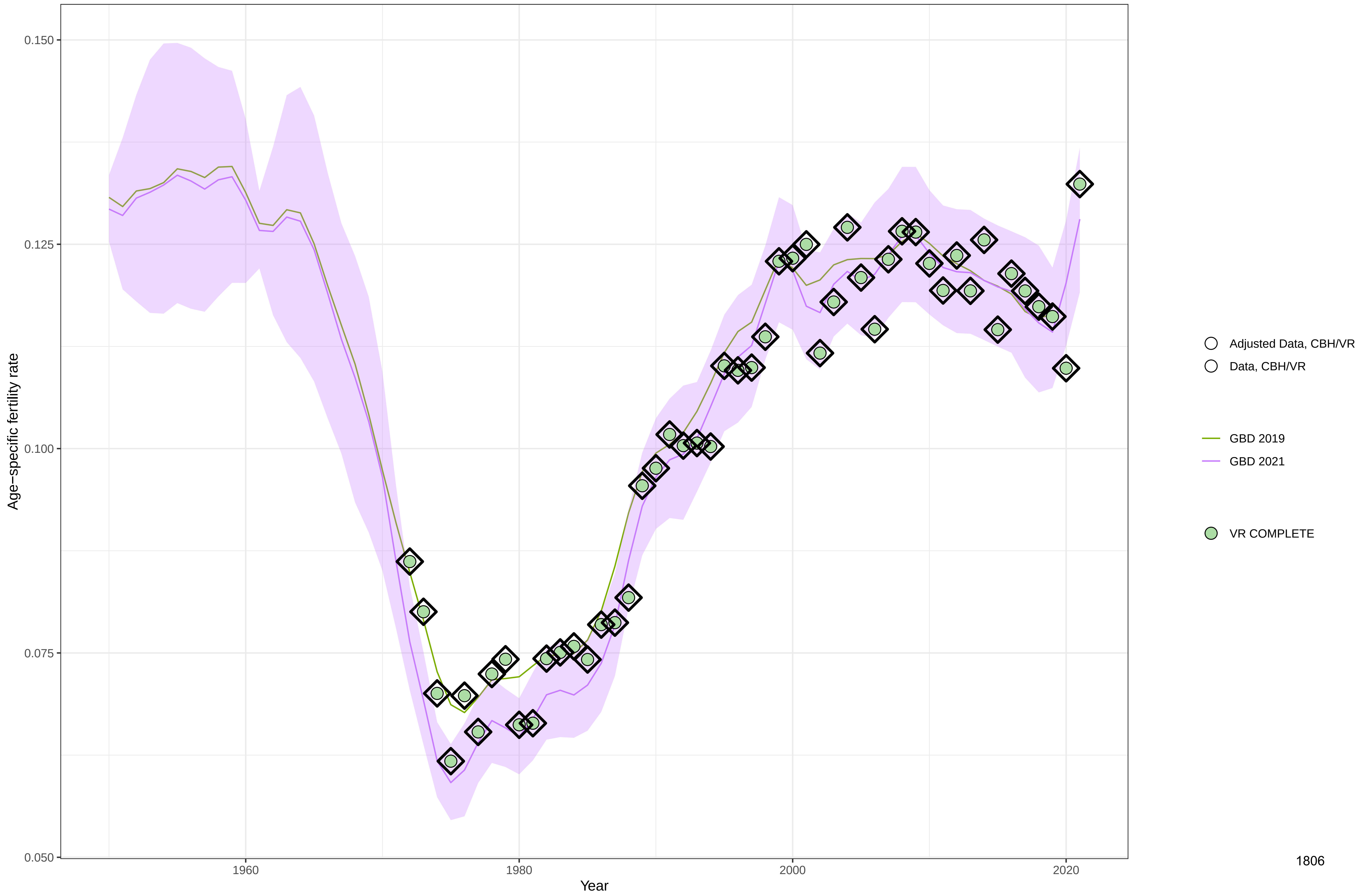

Age−specific fertility fitted trend lines, 1950−2021: Møre og Romsdal, Ages: 35−39
Data points enclosed with a diamond are reference sources

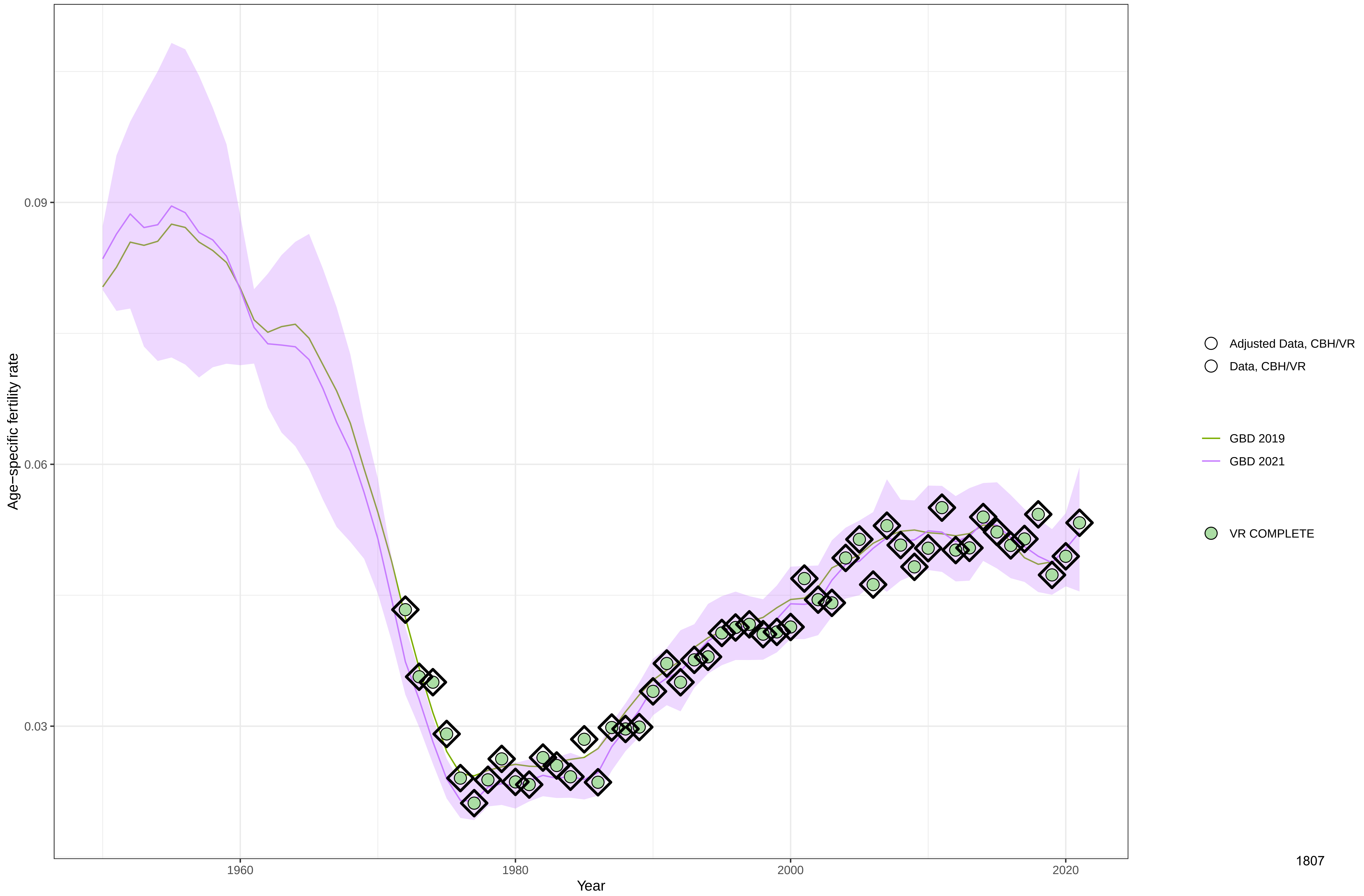

Age−specific fertility fitted trend lines, 1950−2021: Møre og Romsdal, Ages: 40−44
Data points enclosed with a diamond are reference sources

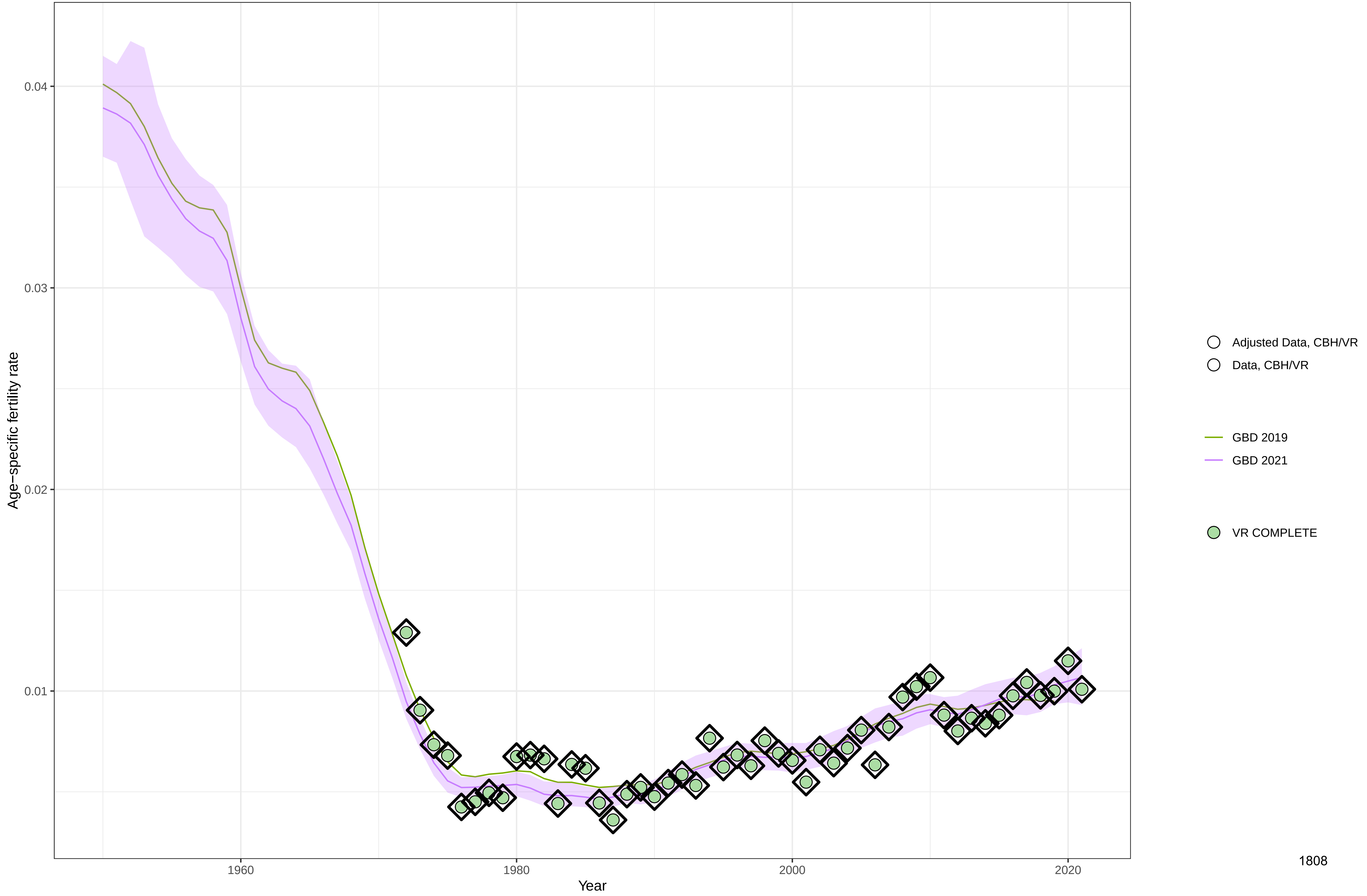

Age−specific fertility fitted trend lines, 1950−2021: Møre og Romsdal, Ages: 45−49
Data points enclosed with a diamond are reference sources

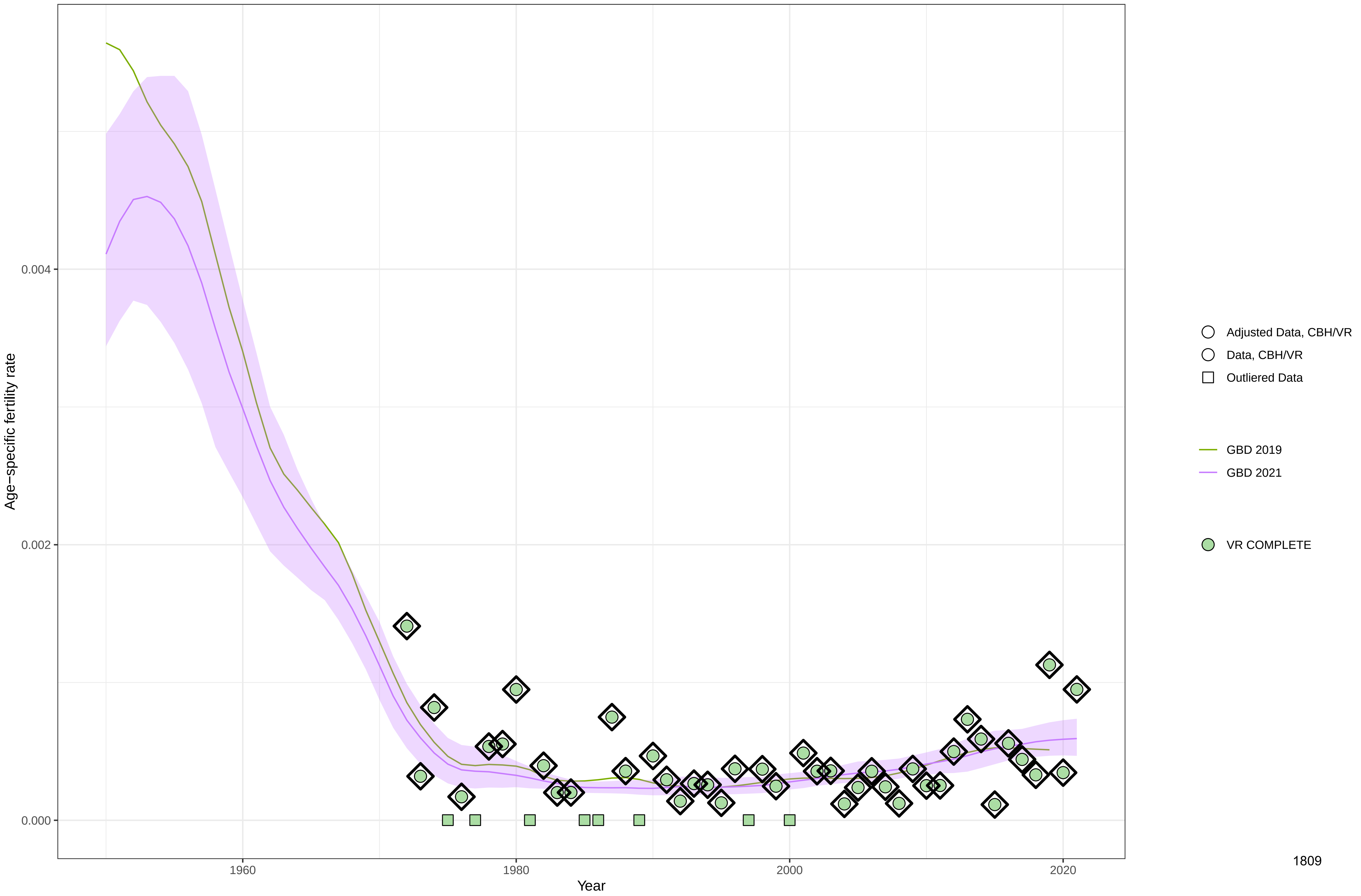

Age−specific fertility fitted trend lines, 1950−2021: Møre og Romsdal, Ages: 50−54
Data points enclosed with a diamond are reference sources

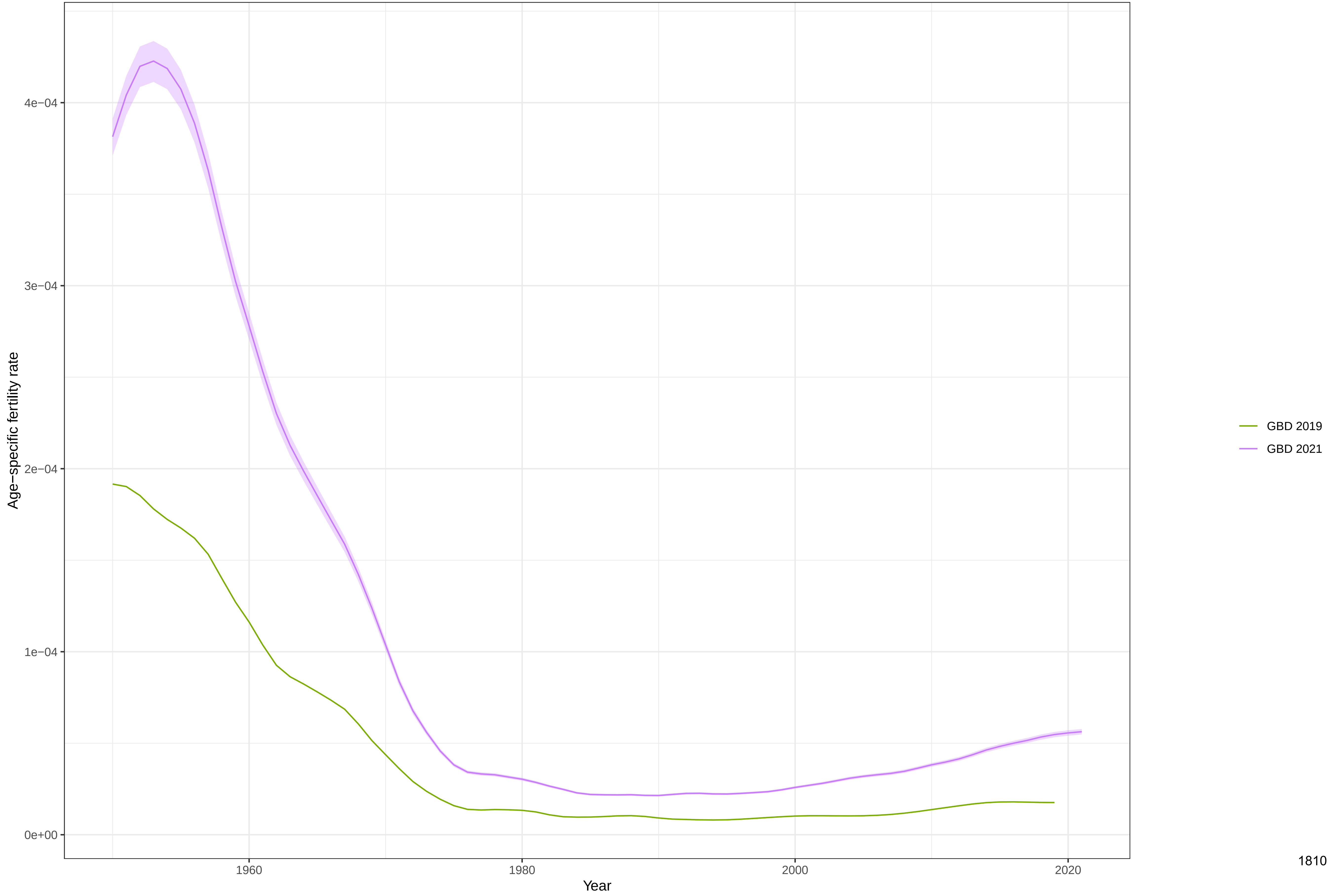

Total fertility rate fitted trend lines, 1950−2021: Møre og Romsdal TFR
Data points enclosed with a diamond are reference sources

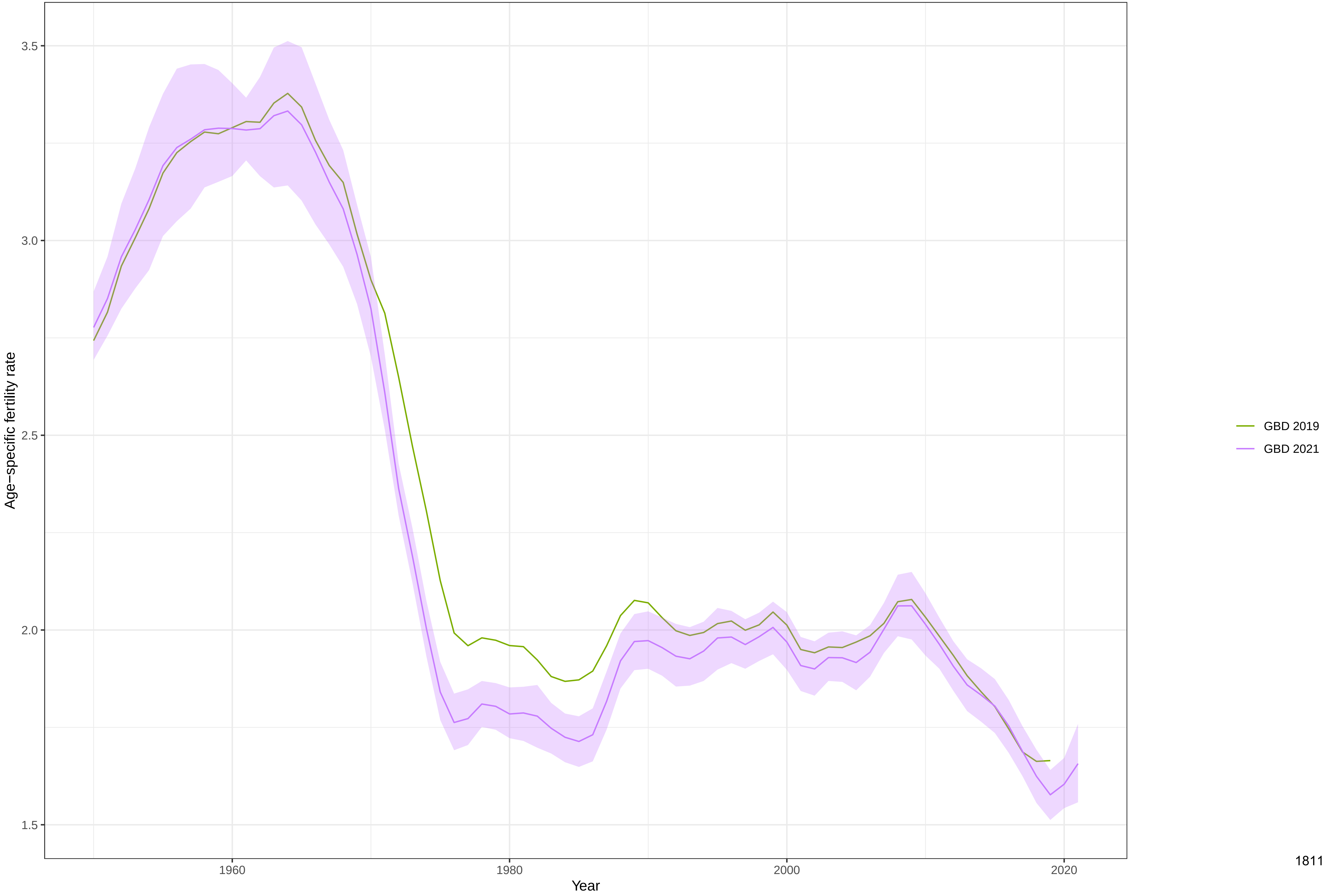

Age−specific fertility fitted trend lines, 1950−2021: Nordland, Ages: 10−14
Data points enclosed with a diamond are reference sources

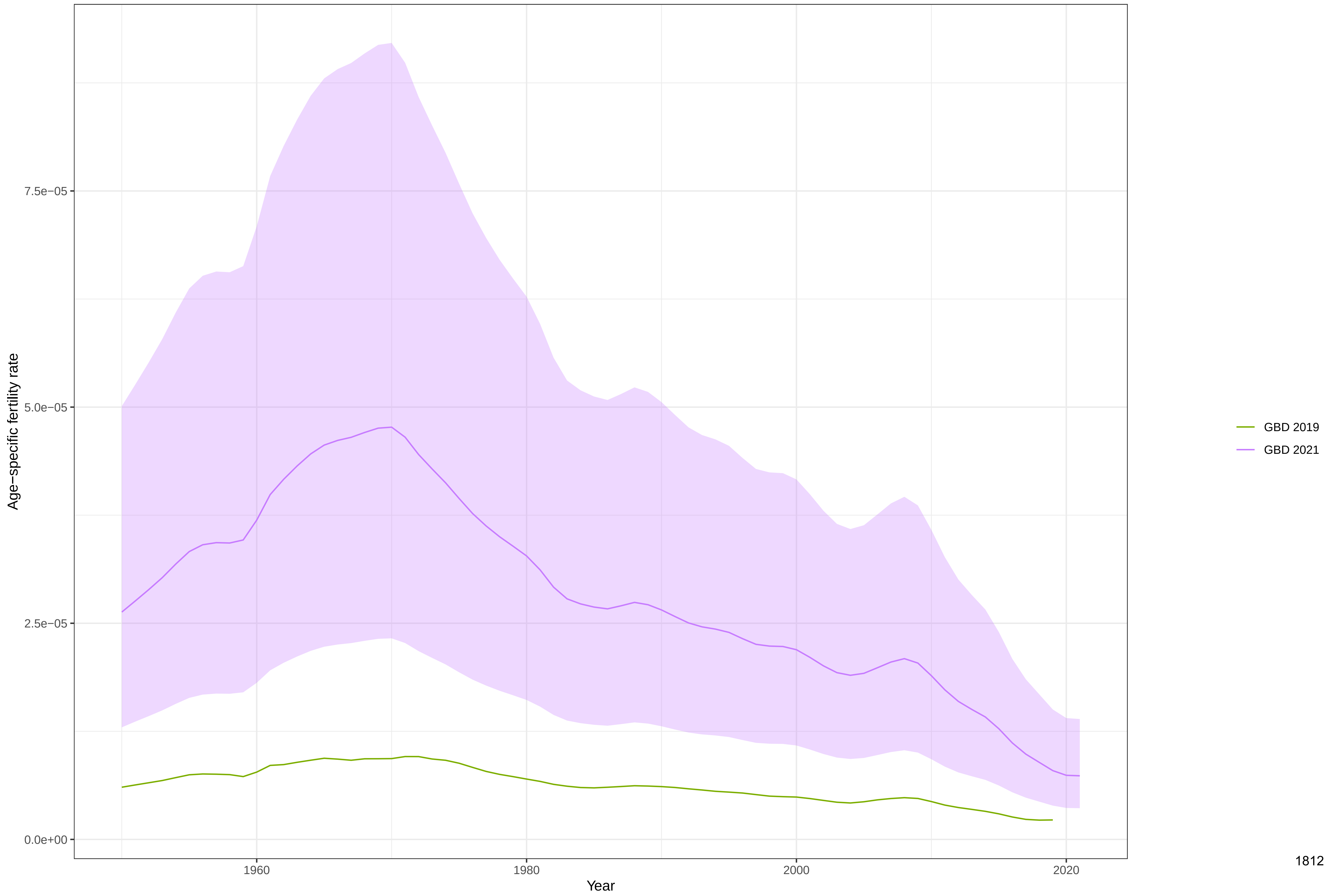

Age−specific fertility fitted trend lines, 1950−2021: Nordland, Ages: 15−19
Data points enclosed with a diamond are reference sources

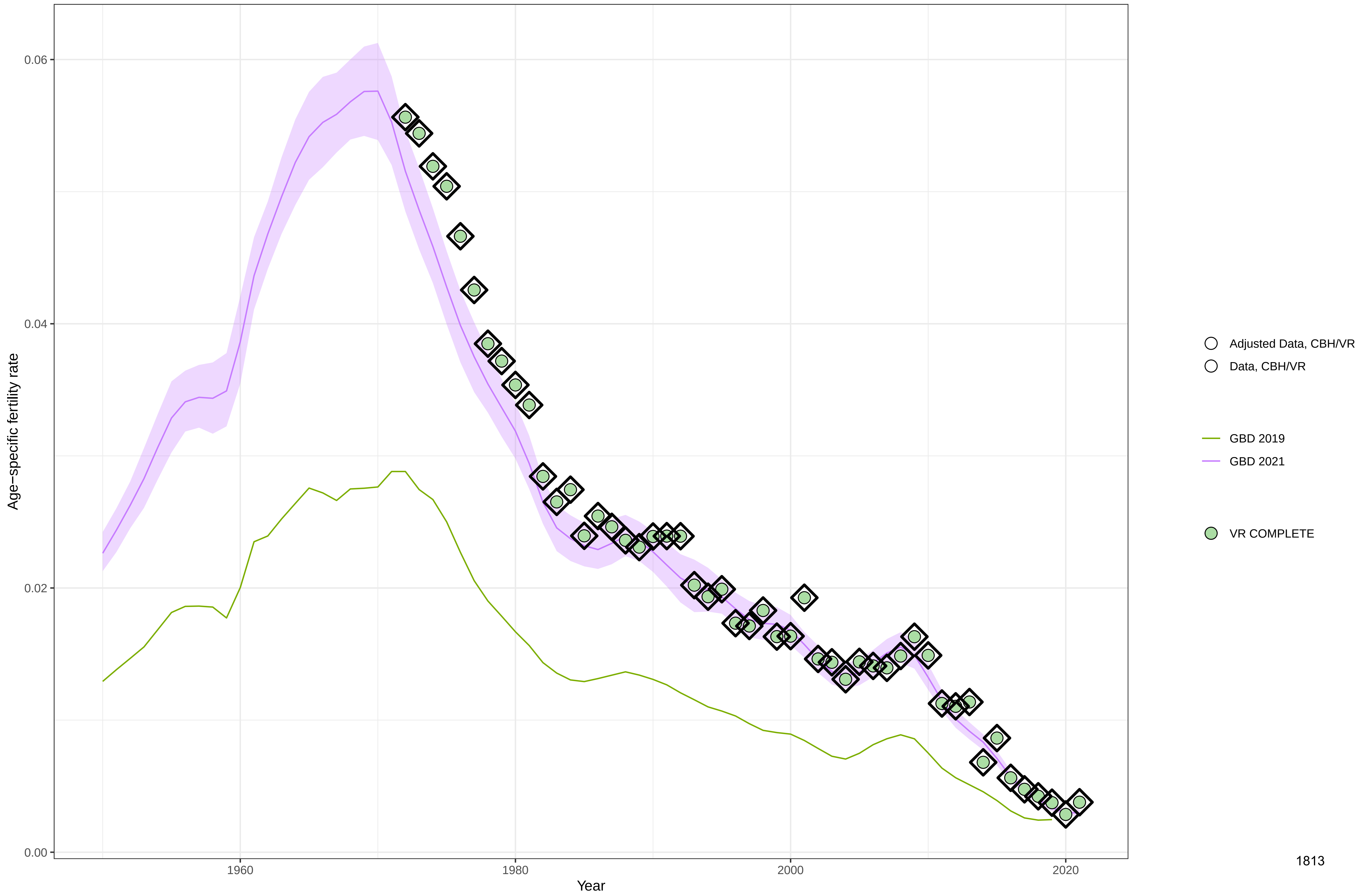

Age−specific fertility fitted trend lines, 1950−2021: Nordland, Ages: 20−24
Data points enclosed with a diamond are reference sources

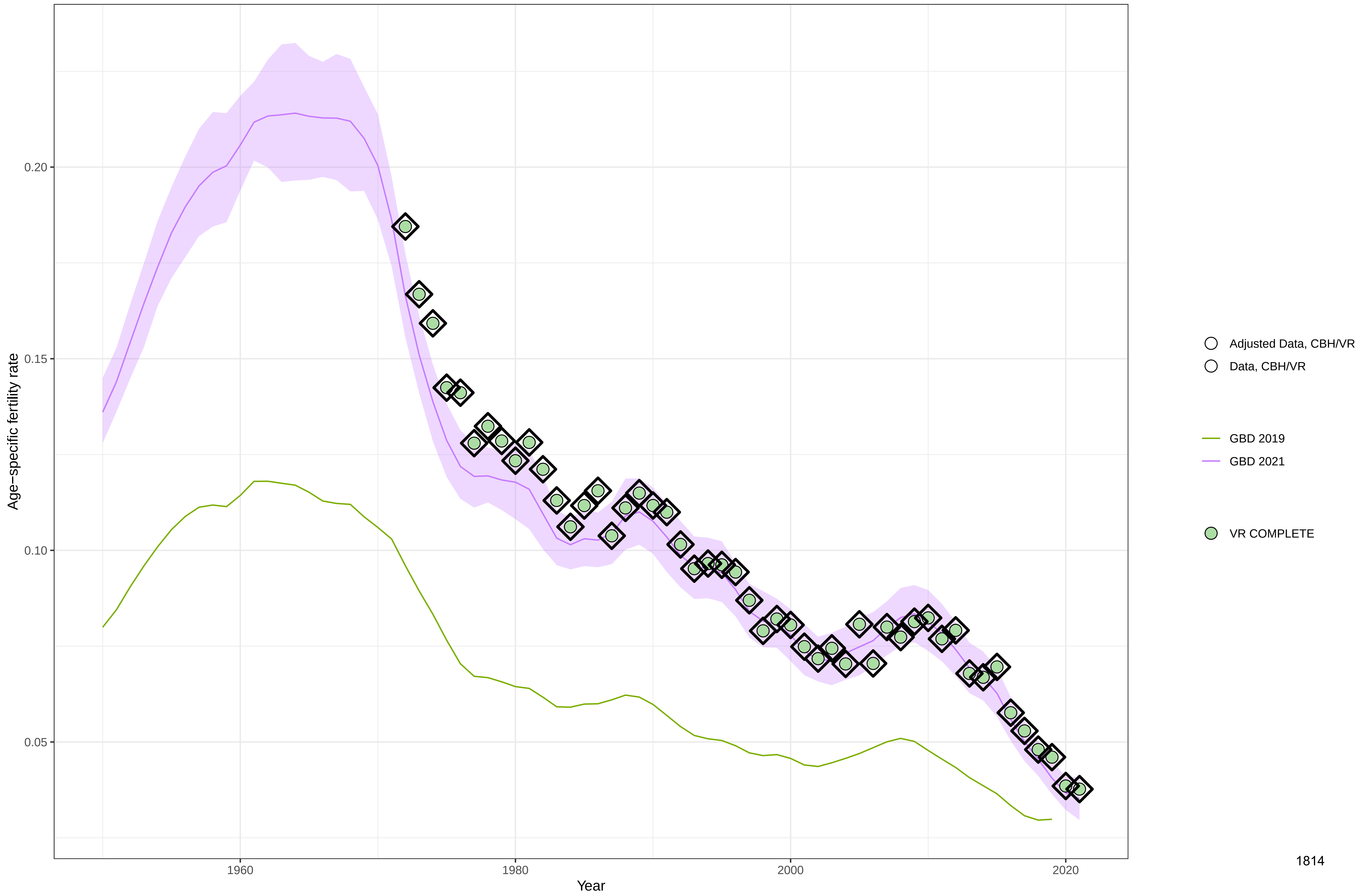

Age−specific fertility fitted trend lines, 1950−2021: Nordland, Ages: 25−29
Data points enclosed with a diamond are reference sources

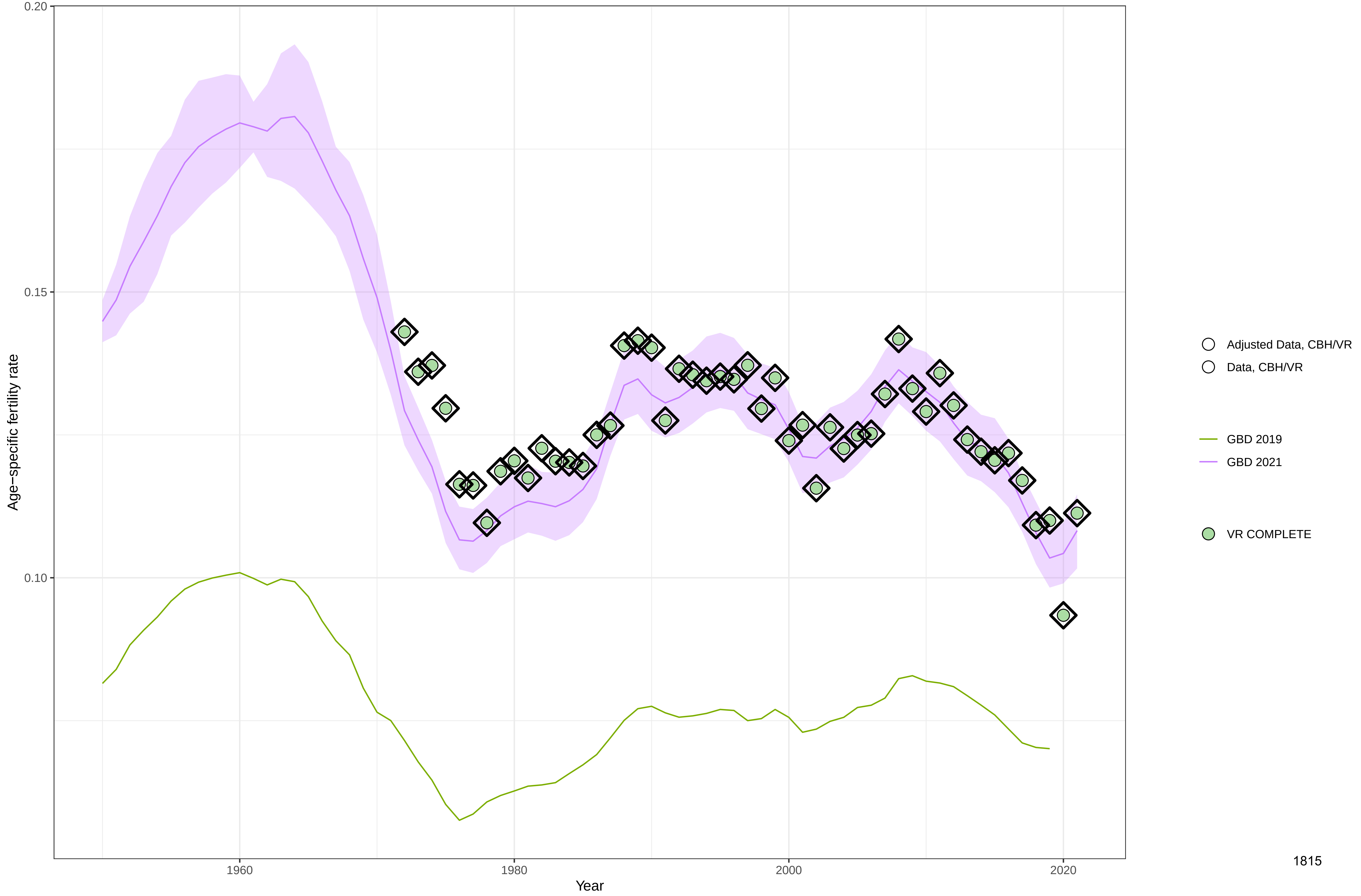

Age−specific fertility fitted trend lines, 1950−2021: Nordland, Ages: 30−34
Data points enclosed with a diamond are reference sources

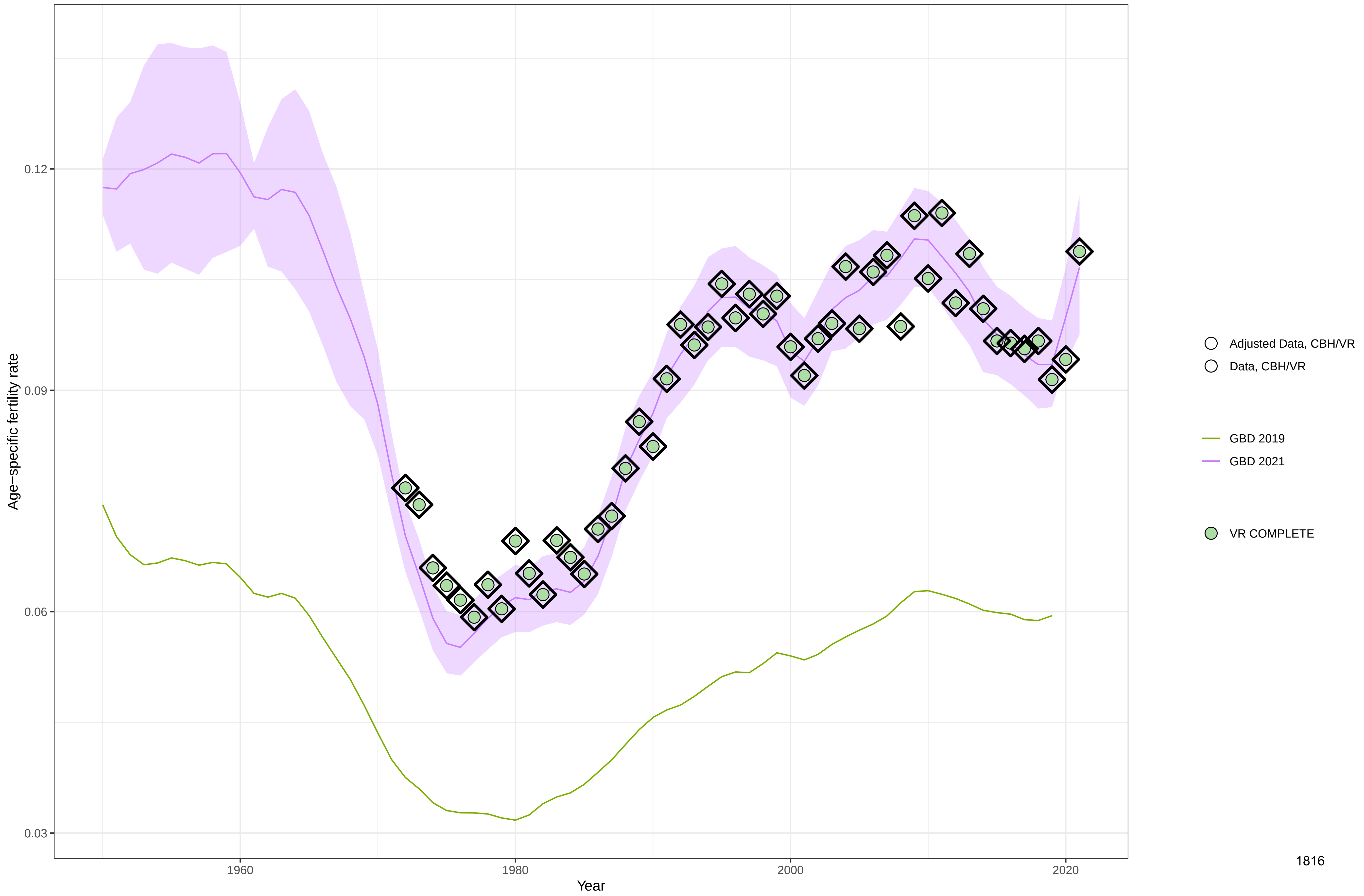

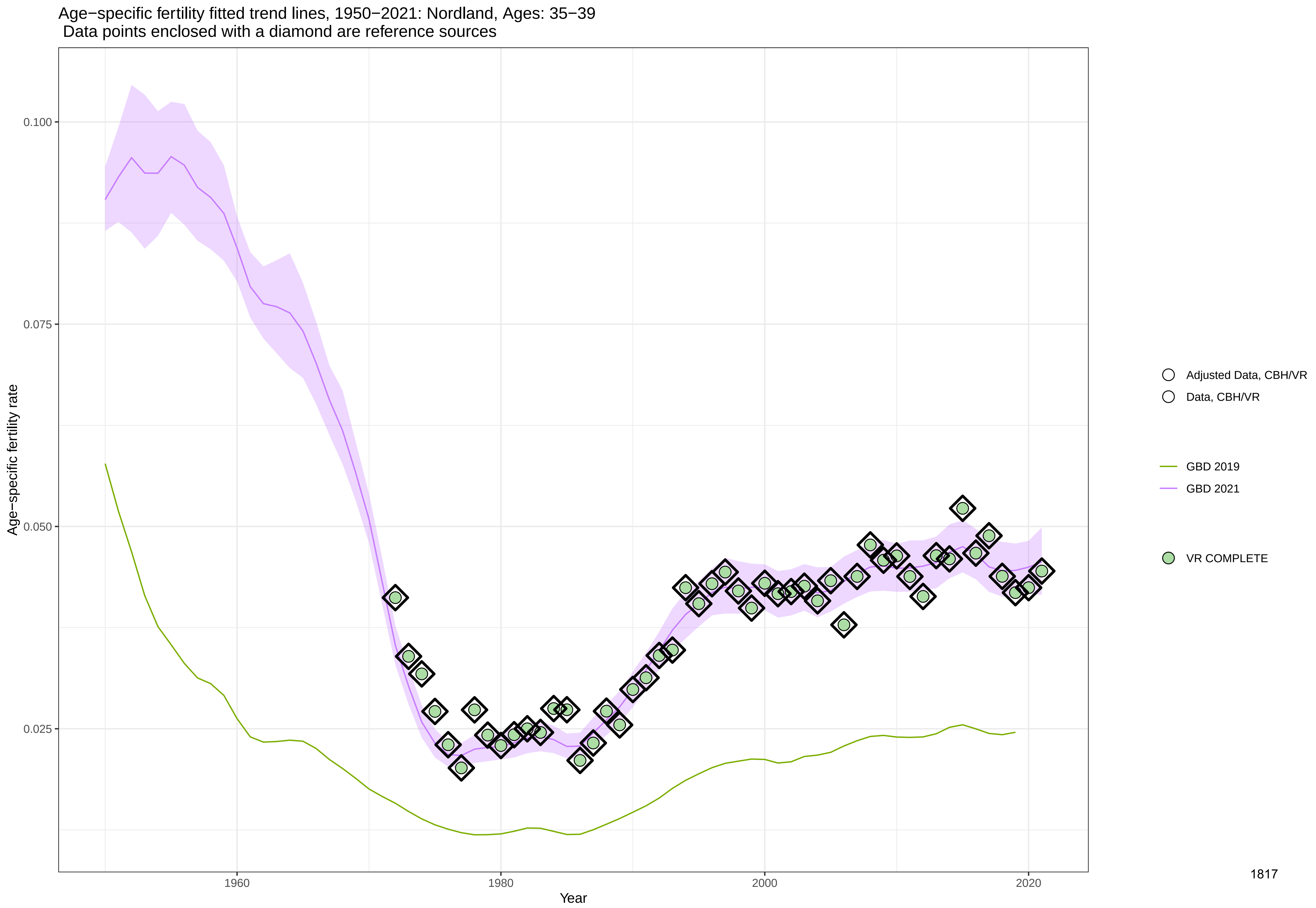
Age−specific fertility fitted trend lines, 1950−2021: Nordland, Ages: 35−39
Data points enclosed with a diamond are reference sources
Age−specific fertility rate
0.100
0.075
0.050
0.025
1960
1980
2000
2020
Year
Adjusted Data, CBH/VR
Data, CBH/VR
GBD 2019
GBD 2021
VR COMPLETE

Age−specific fertility fitted trend lines, 1950−2021: Nordland, Ages: 40−44
Data points enclosed with a diamond are reference sources

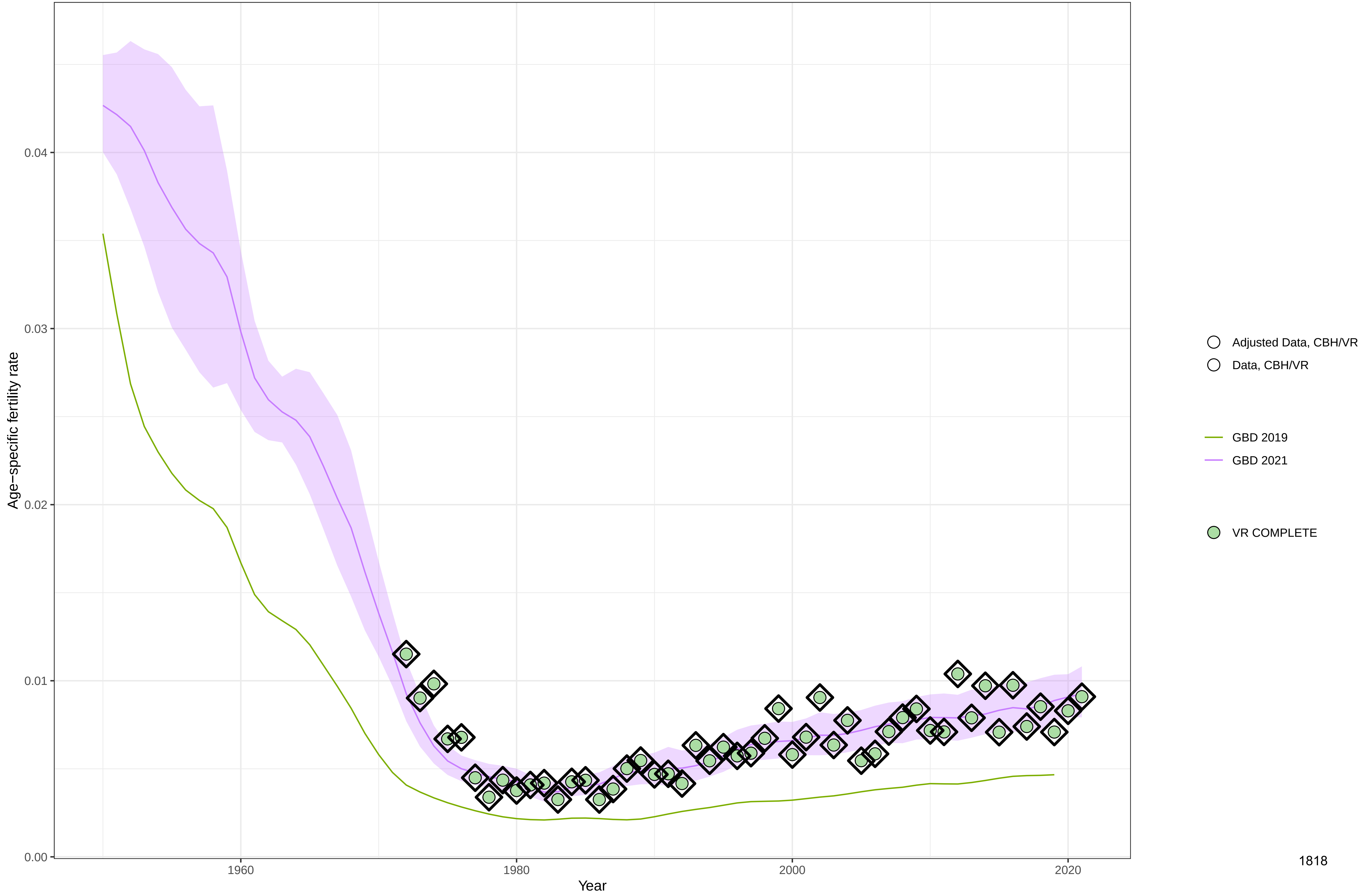

1818

Age−specific fertility fitted trend lines, 1950−2021: Nordland, Ages: 45−49
Data points enclosed with a diamond are reference sources

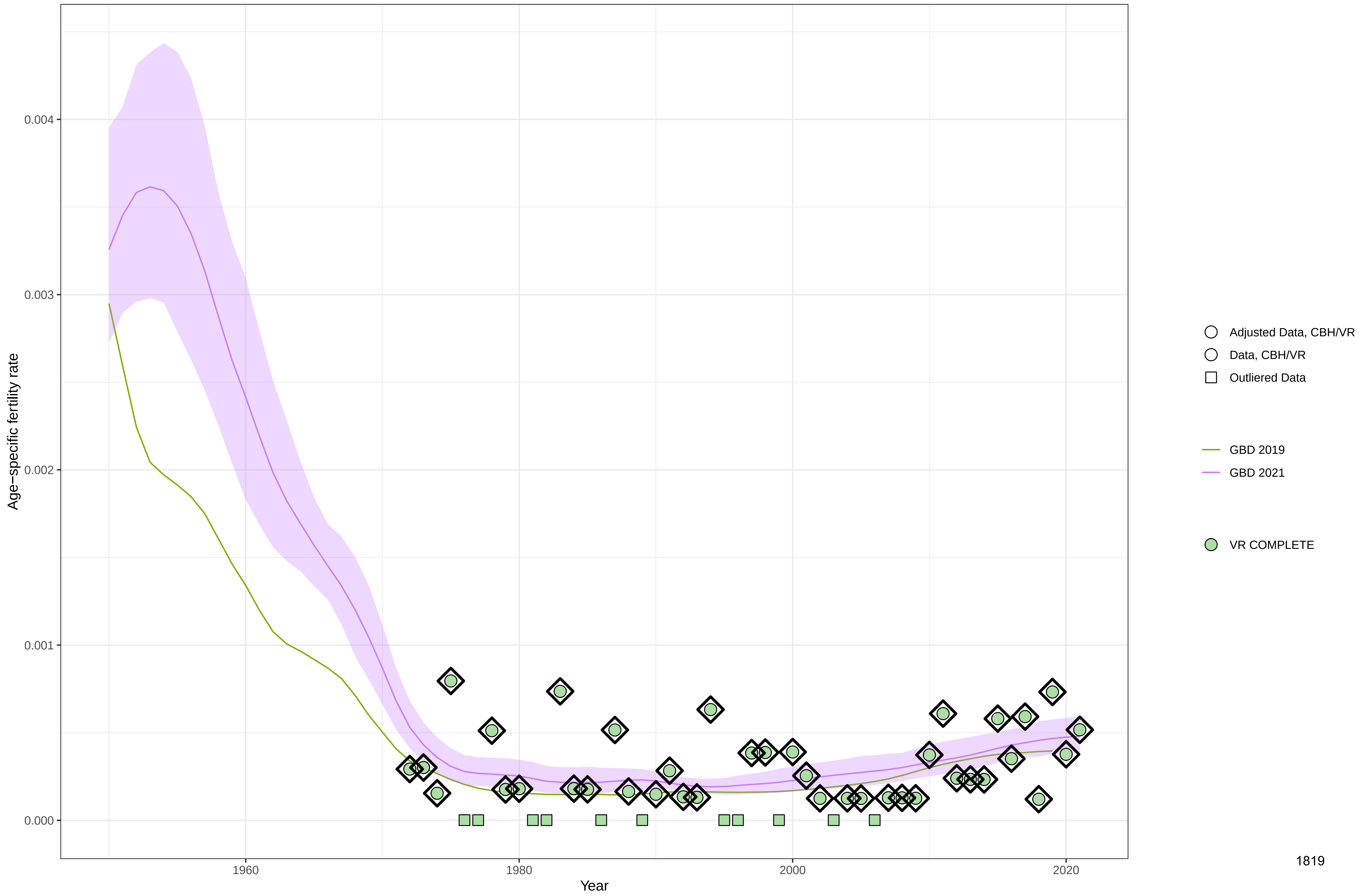

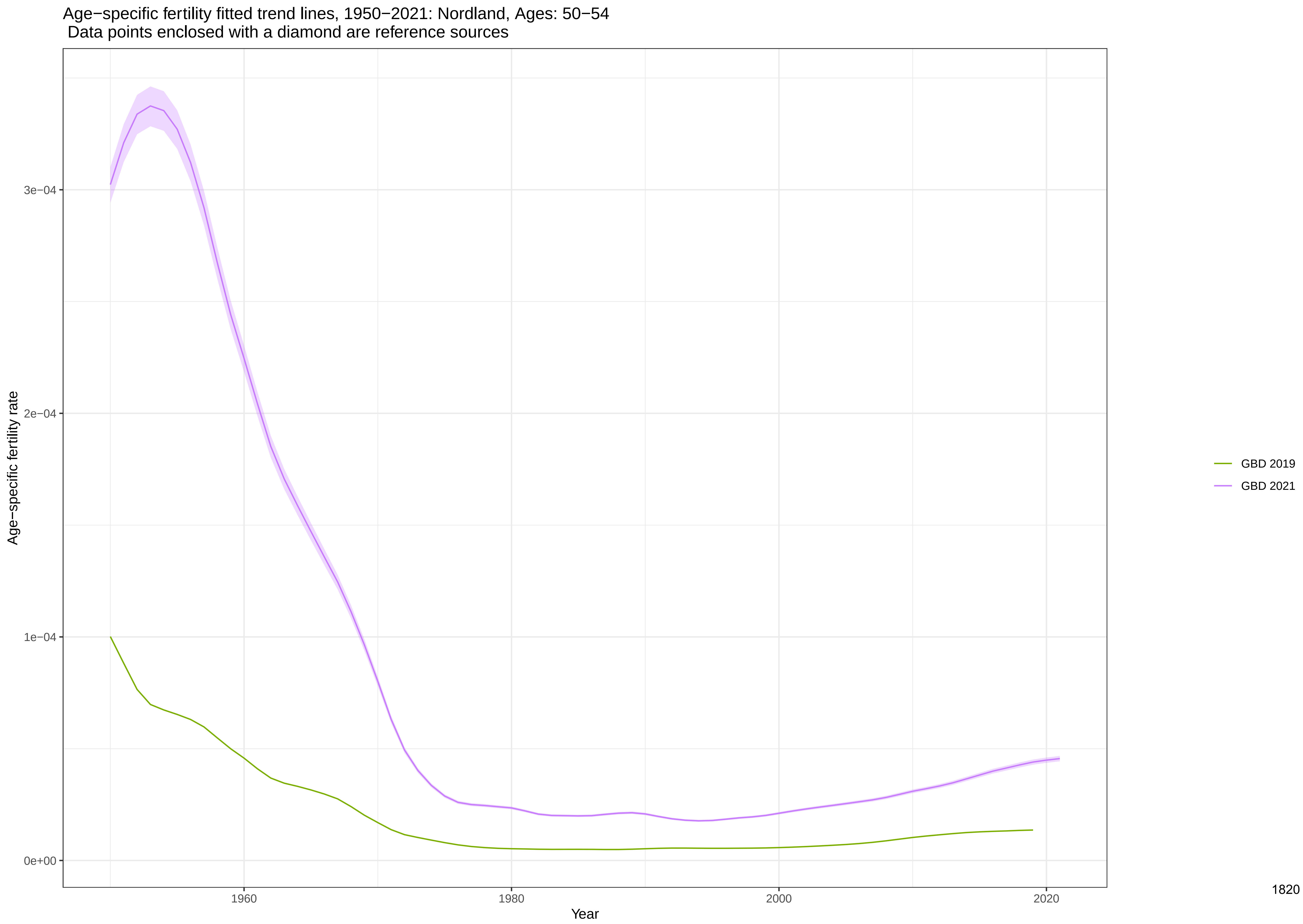
Age−specific fertility fitted trend lines, 1950−2021: Nordland, Ages: 50−54
Data points enclosed with a diamond are reference sources
Age−specific fertility rate
3e−04
2e−04
1e−04
0e+00
1960
1980
2000
2020
Year
GBD 2019
GBD 2021

Total fertility rate fitted trend lines, 1950−2021: Nordland TFR
Data points enclosed with a diamond are reference sources

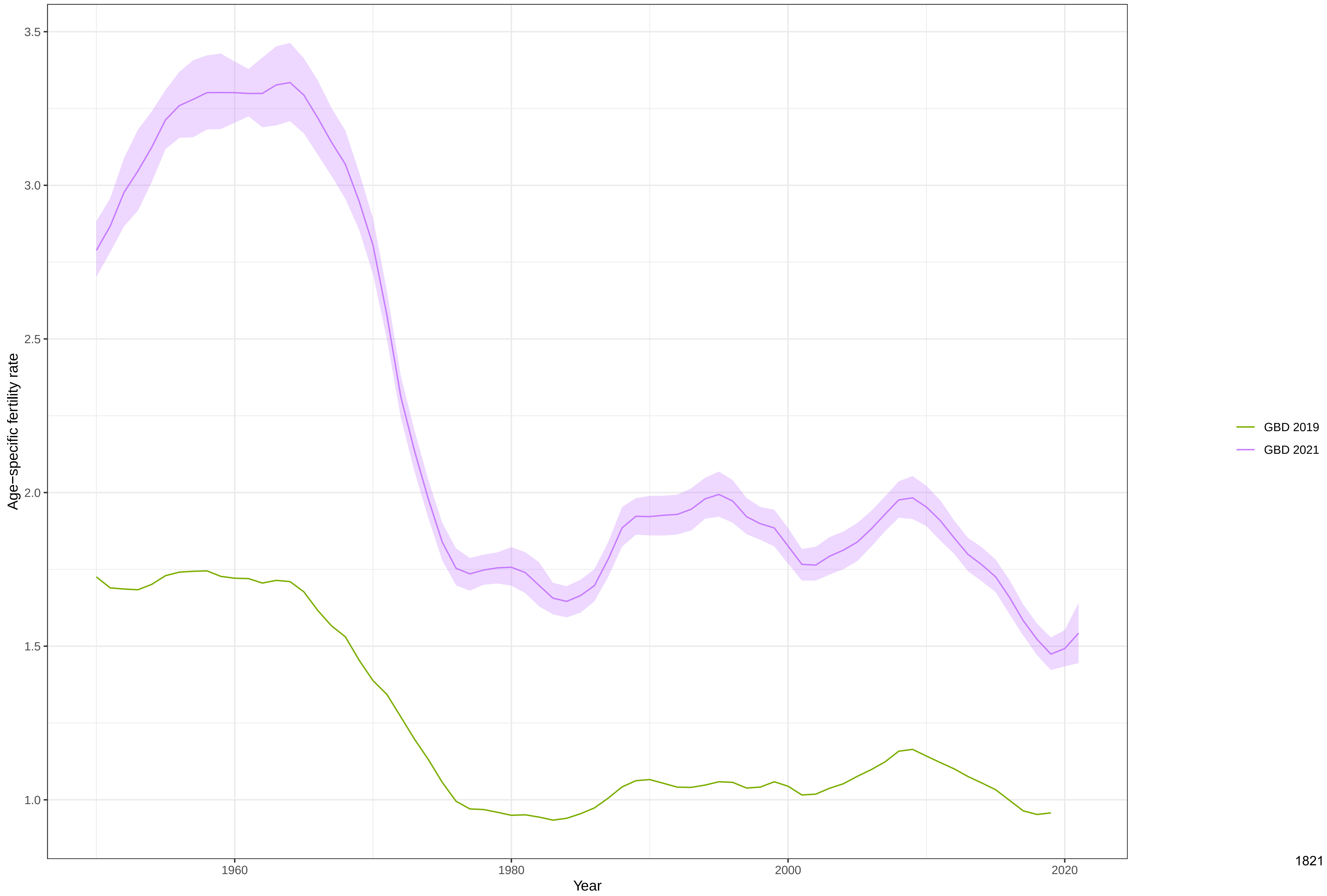

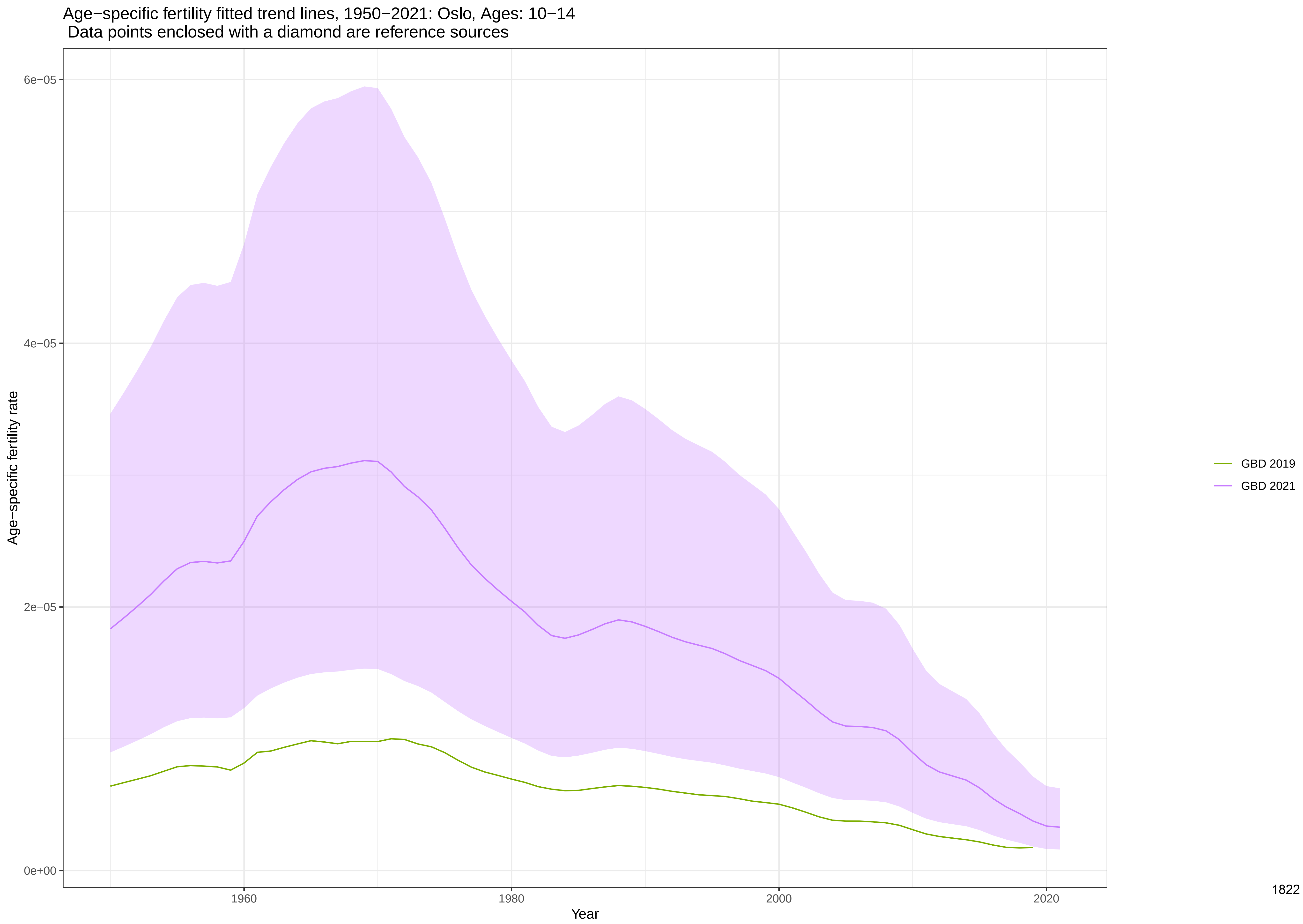

Age−specific fertility fitted trend lines, 1950−2021: Oslo, Ages: 10−14
Data points enclosed with a diamond are reference sources
6e−05
4e−05
2e−05
0e+00
Age−specific fertility rate
1960
1980
2000
2020
Year
GBD 2019
GBD 2021

Age−specific fertility fitted trend lines, 1950−2021: Oslo, Ages: 15−19
Data points enclosed with a diamond are reference sources

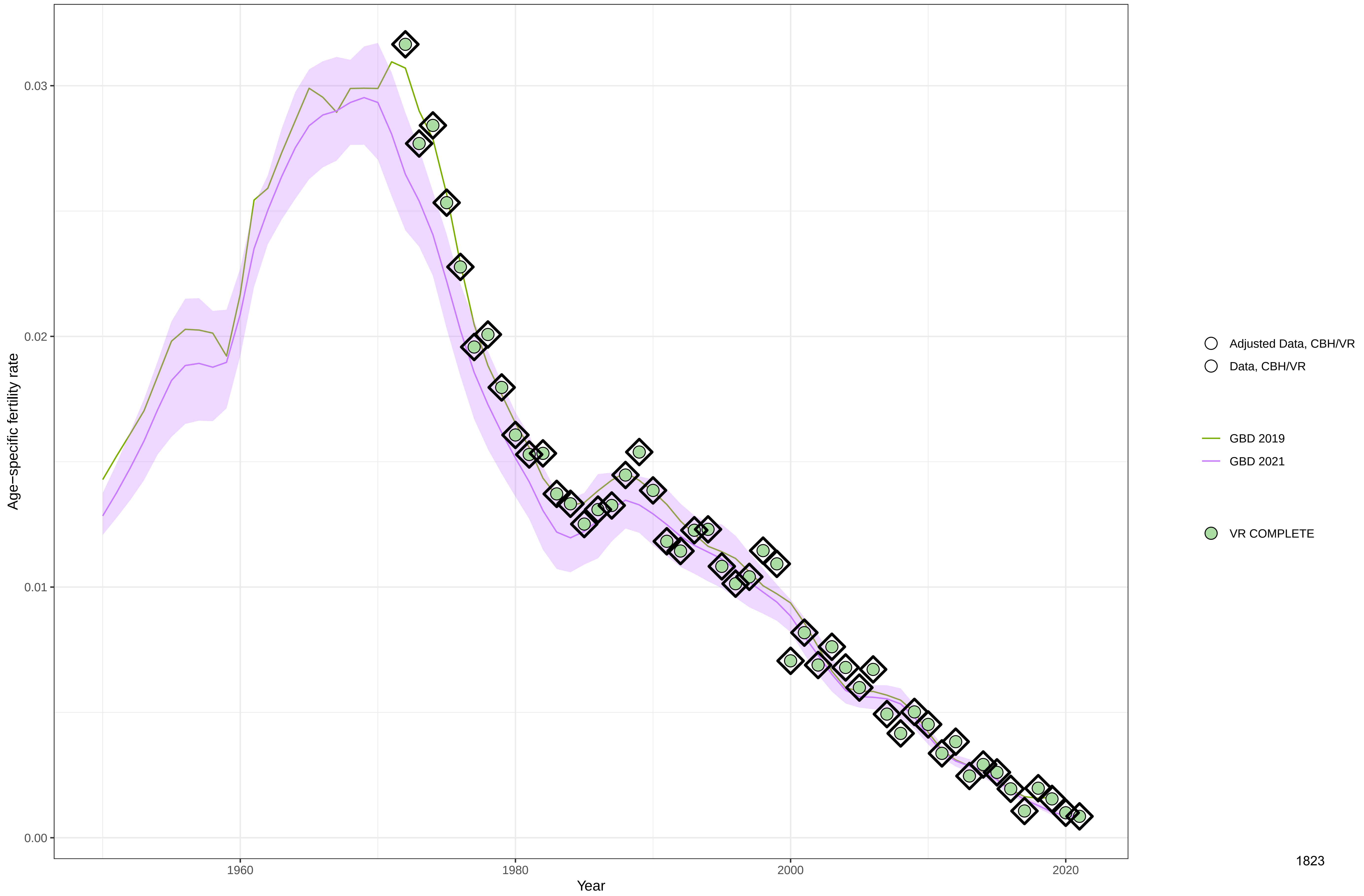

Age−specific fertility fitted trend lines, 1950−2021: Oslo, Ages: 20−24
Data points enclosed with a diamond are reference sources

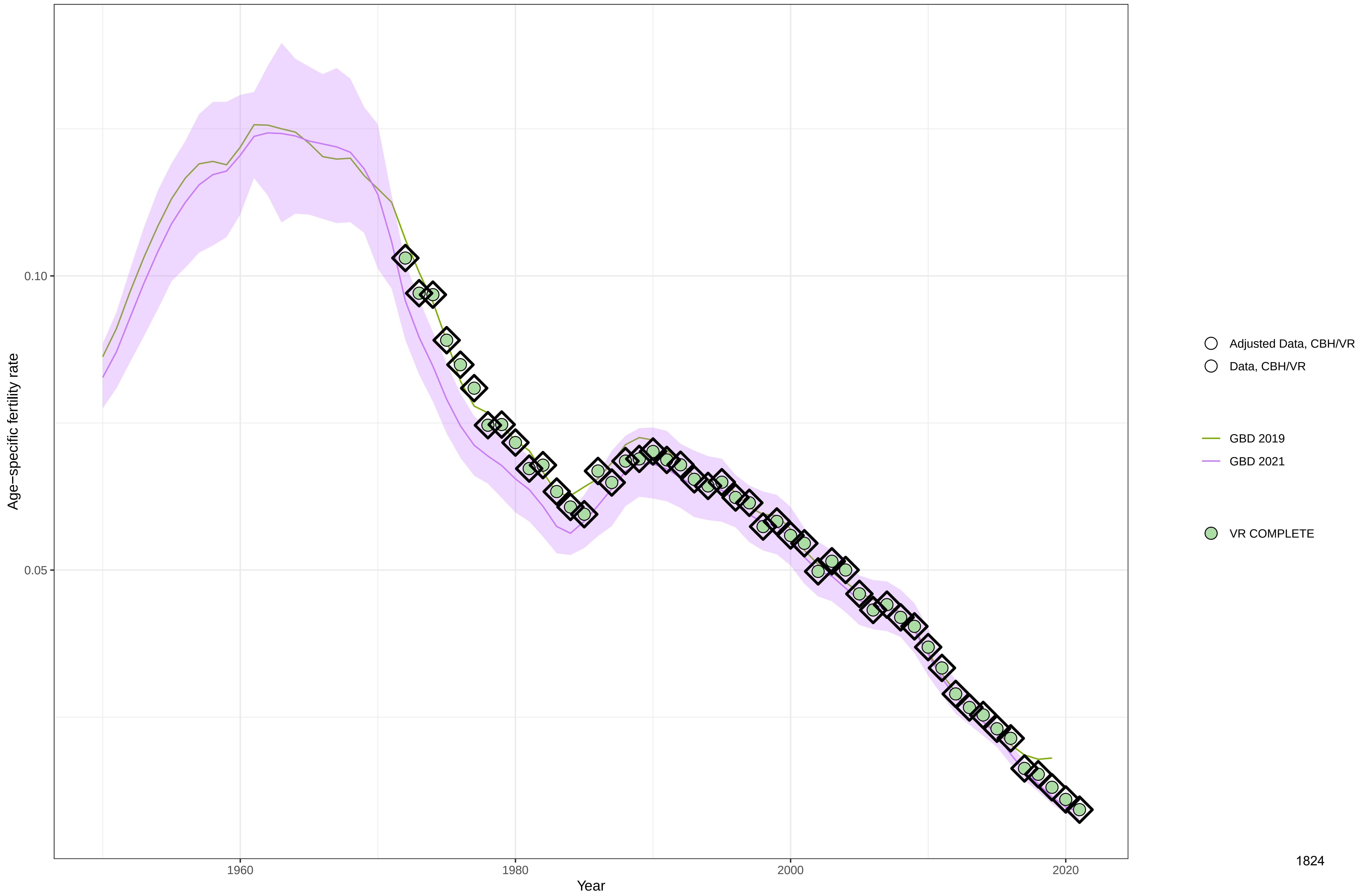

1824

Age−specific fertility fitted trend lines, 1950−2021: Oslo, Ages: 25−29
Data points enclosed with a diamond are reference sources

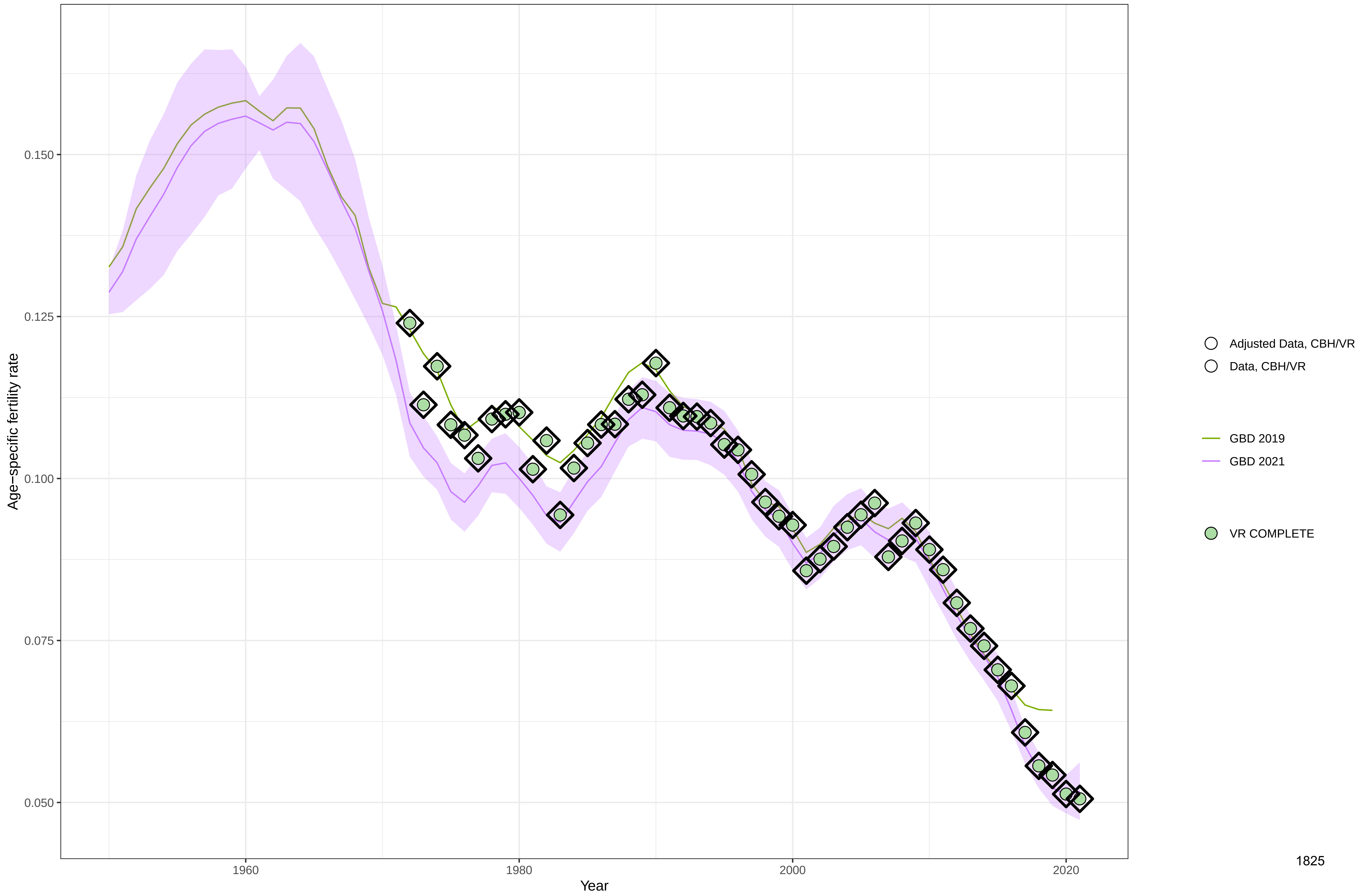

Age−specific fertility fitted trend lines, 1950−2021: Oslo, Ages: 30−34
Data points enclosed with a diamond are reference sources

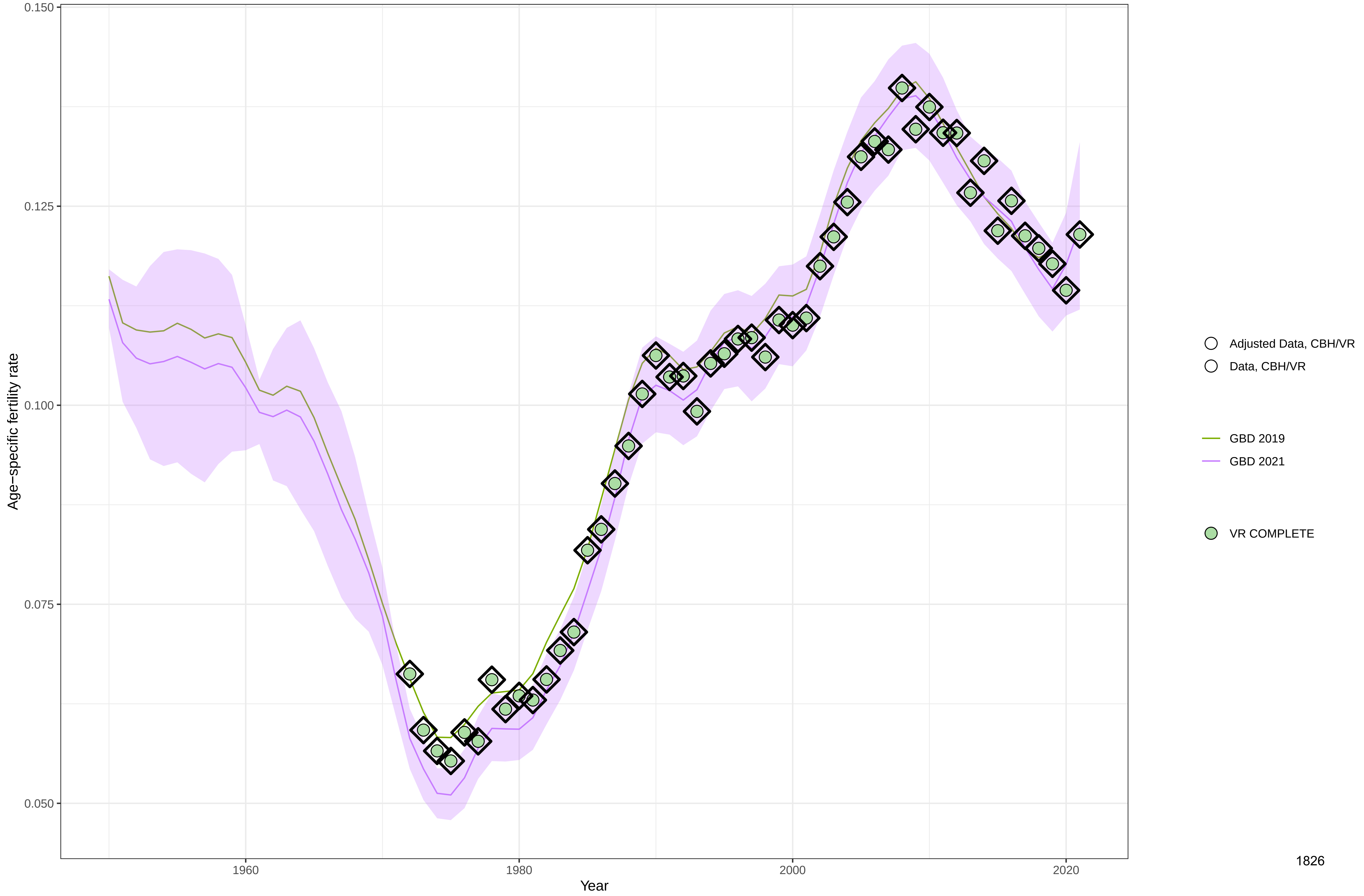

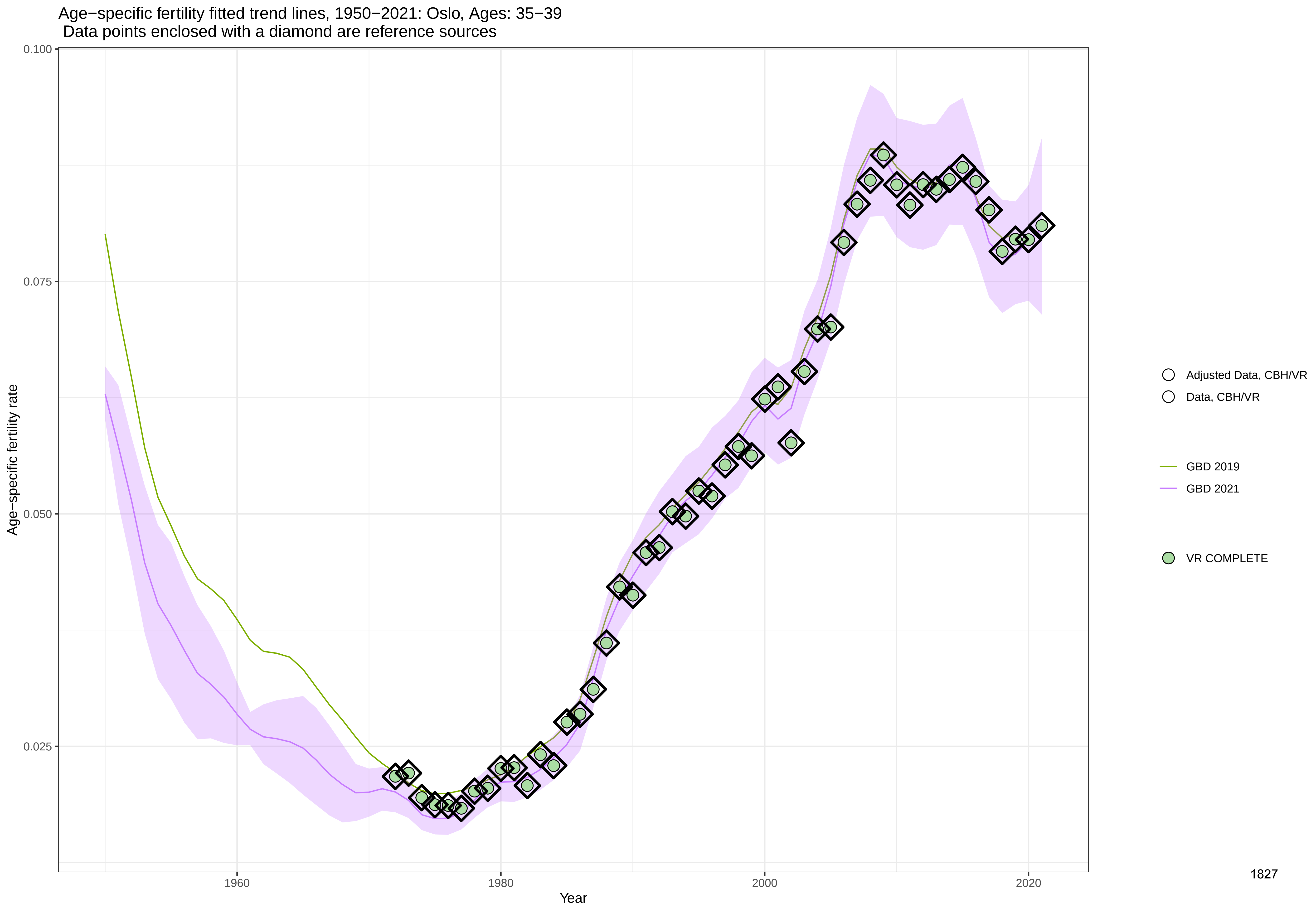
Age−specific fertility fitted trend lines, 1950−2021: Oslo, Ages: 35−39
Data points enclosed with a diamond are reference sources
0.100
0.075
0.050
0.025
Age−specific fertility rate
1960
1980
2000
2020
Year
Adjusted Data, CBH/VR
Data, CBH/VR
GBD 2019
GBD 2021
VR COMPLETE

Age−specific fertility fitted trend lines, 1950−2021: Oslo, Ages: 40−44
Data points enclosed with a diamond are reference sources

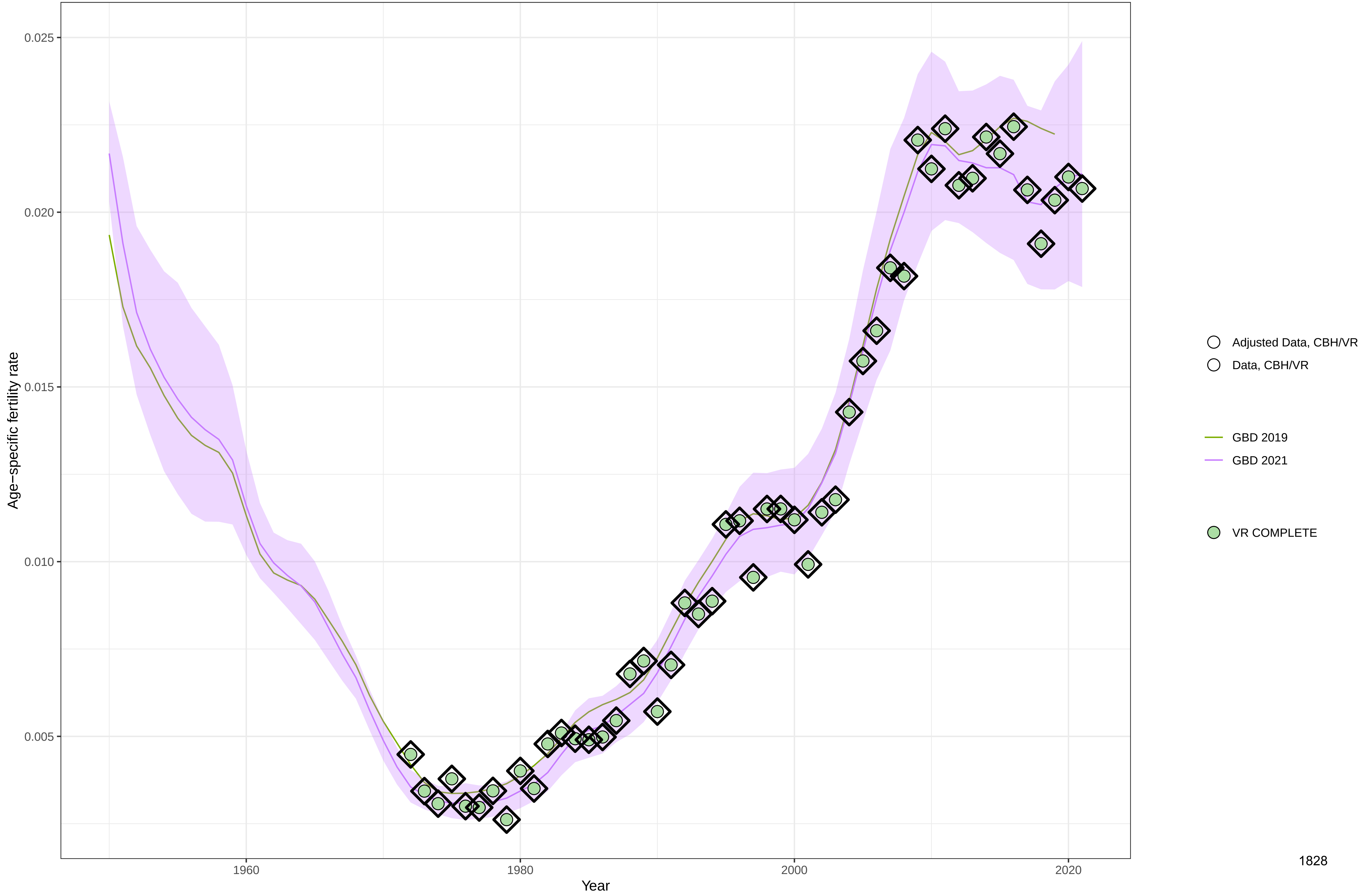

Age−specific fertility fitted trend lines, 1950−2021: Oslo, Ages: 45−49
Data points enclosed with a diamond are reference sources

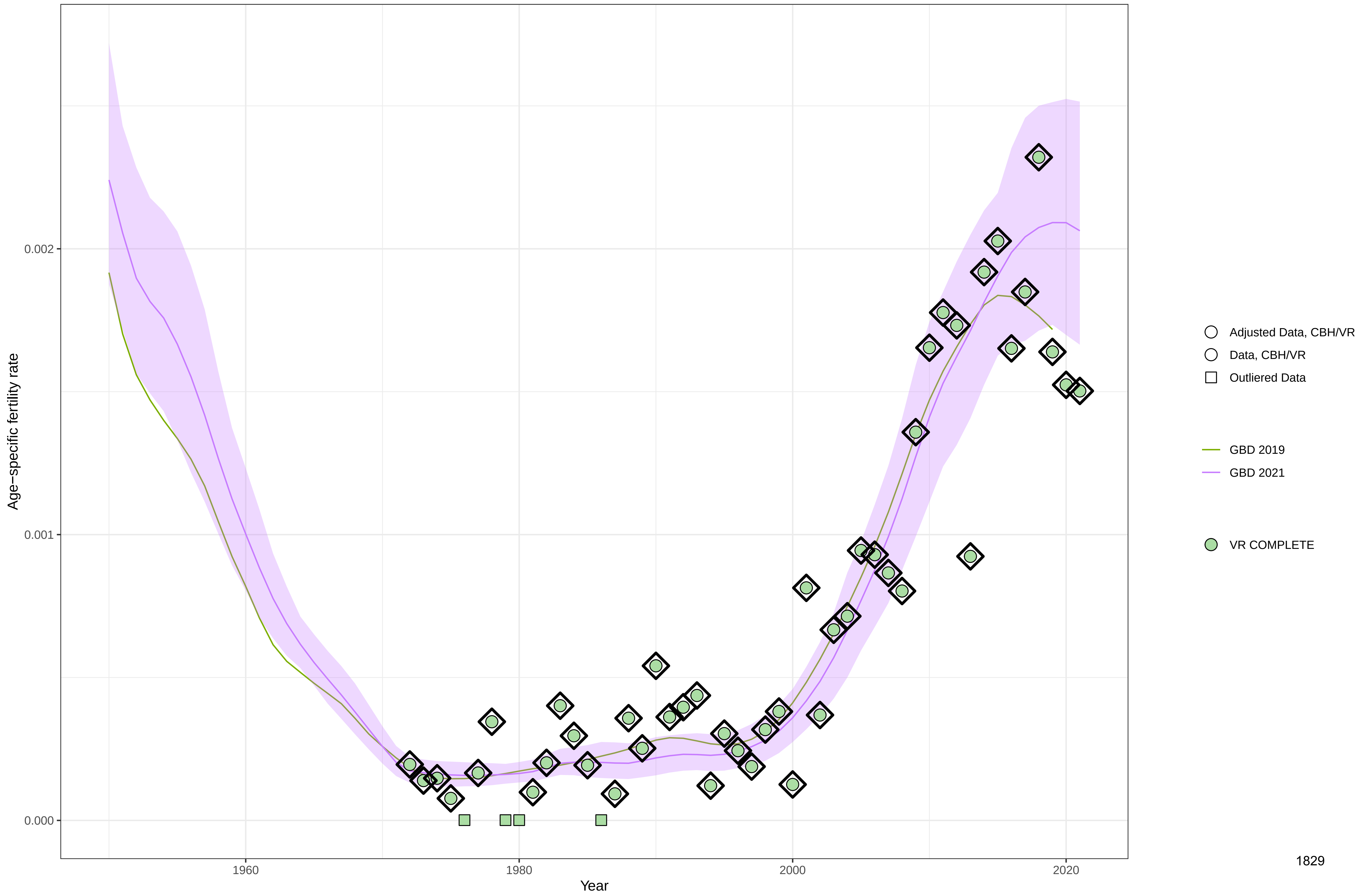

Age−specific fertility fitted trend lines, 1950−2021: Oslo, Ages: 50−54
Data points enclosed with a diamond are reference sources

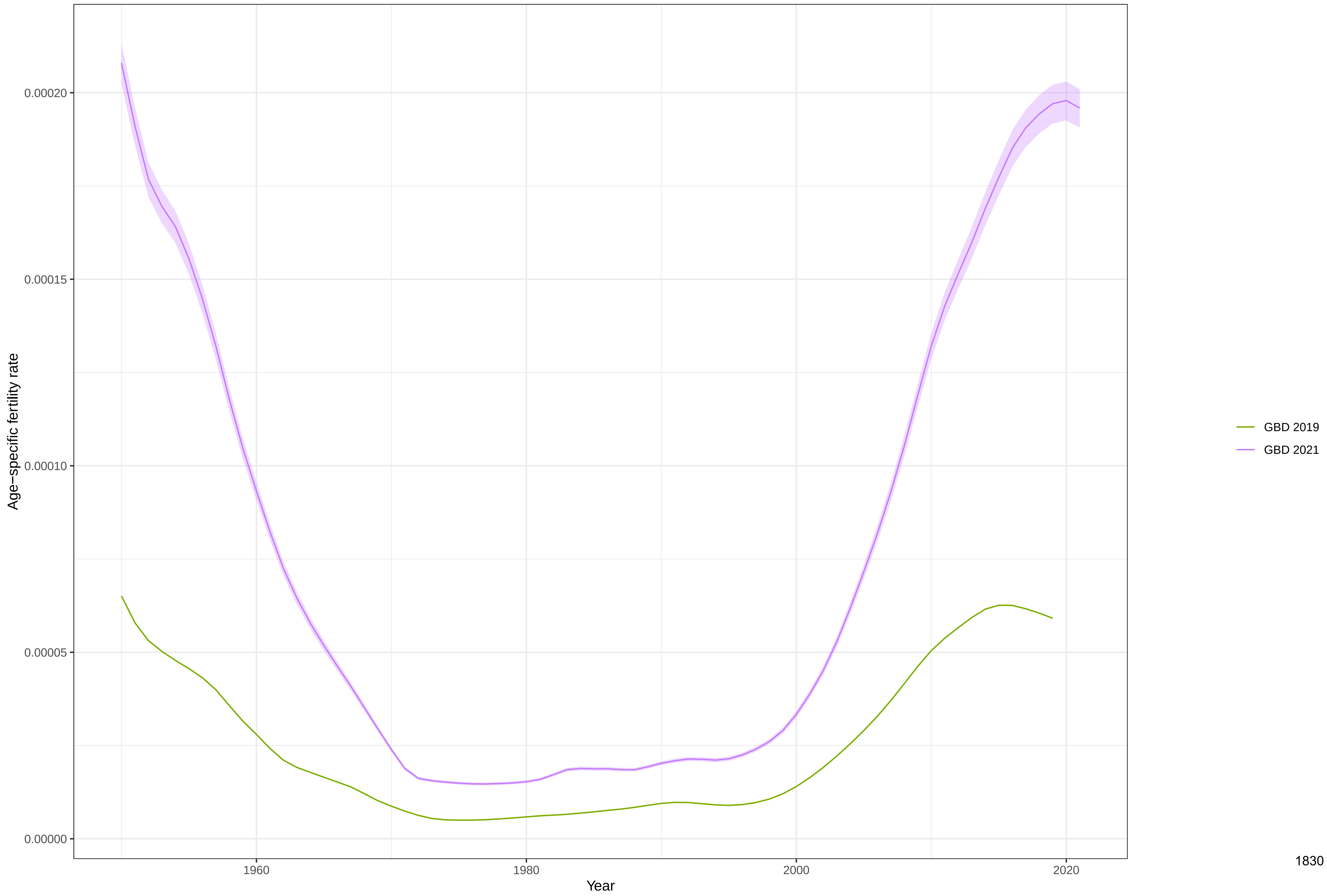

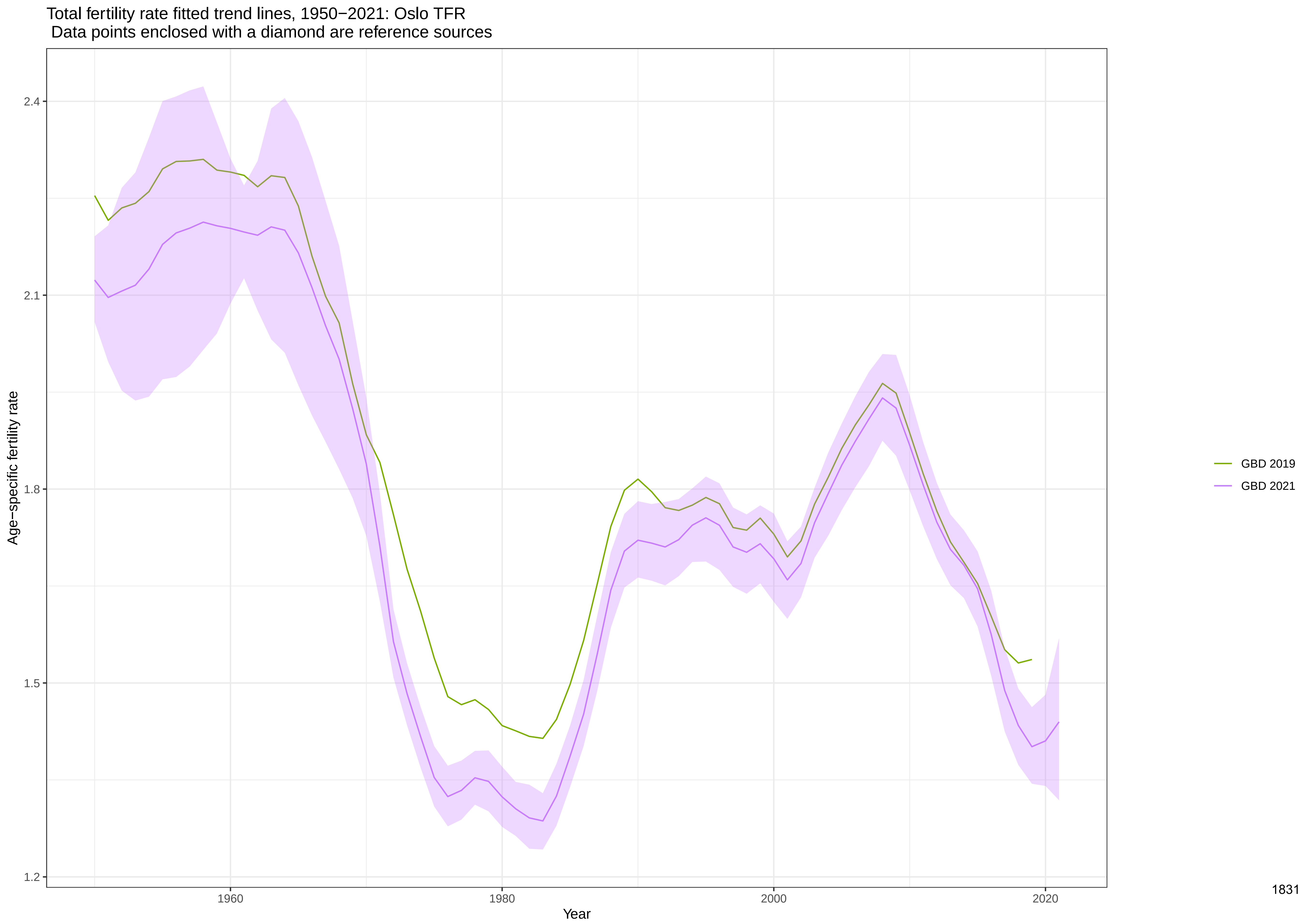
Total fertility rate fitted trend lines, 1950−2021: Oslo TFR
Data points enclosed with a diamond are reference sources
Age−specific fertility rate
2.4
2.1
1.8
1.5
1.2
1960
1980
2000
2020
Year
GBD 2019
GBD 2021

Age−specific fertility fitted trend lines, 1950−2021: Rogaland, Ages: 10−14
Data points enclosed with a diamond are reference sources

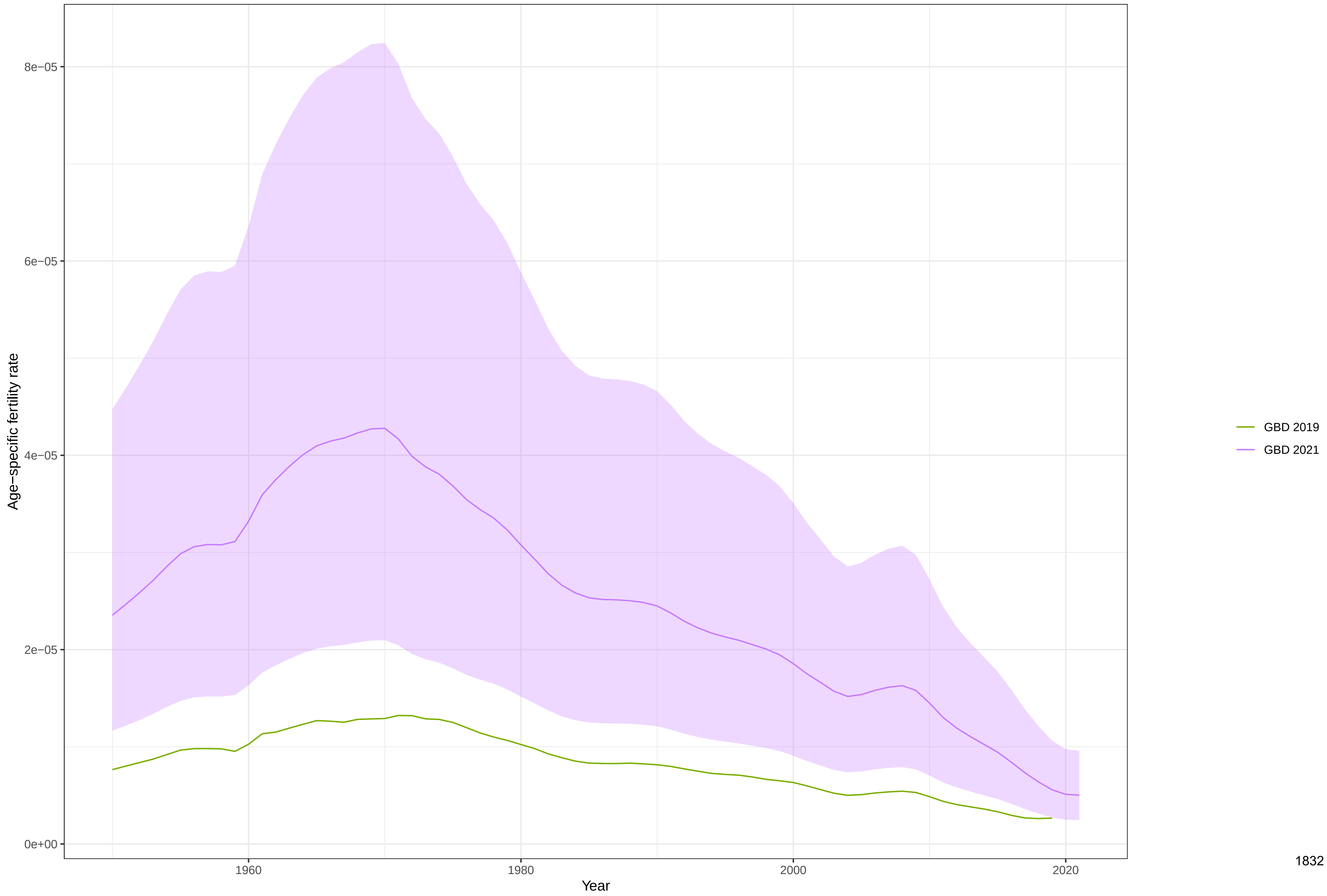

Age−specific fertility fitted trend lines, 1950−2021: Rogaland, Ages: 15−19
Data points enclosed with a diamond are reference sources

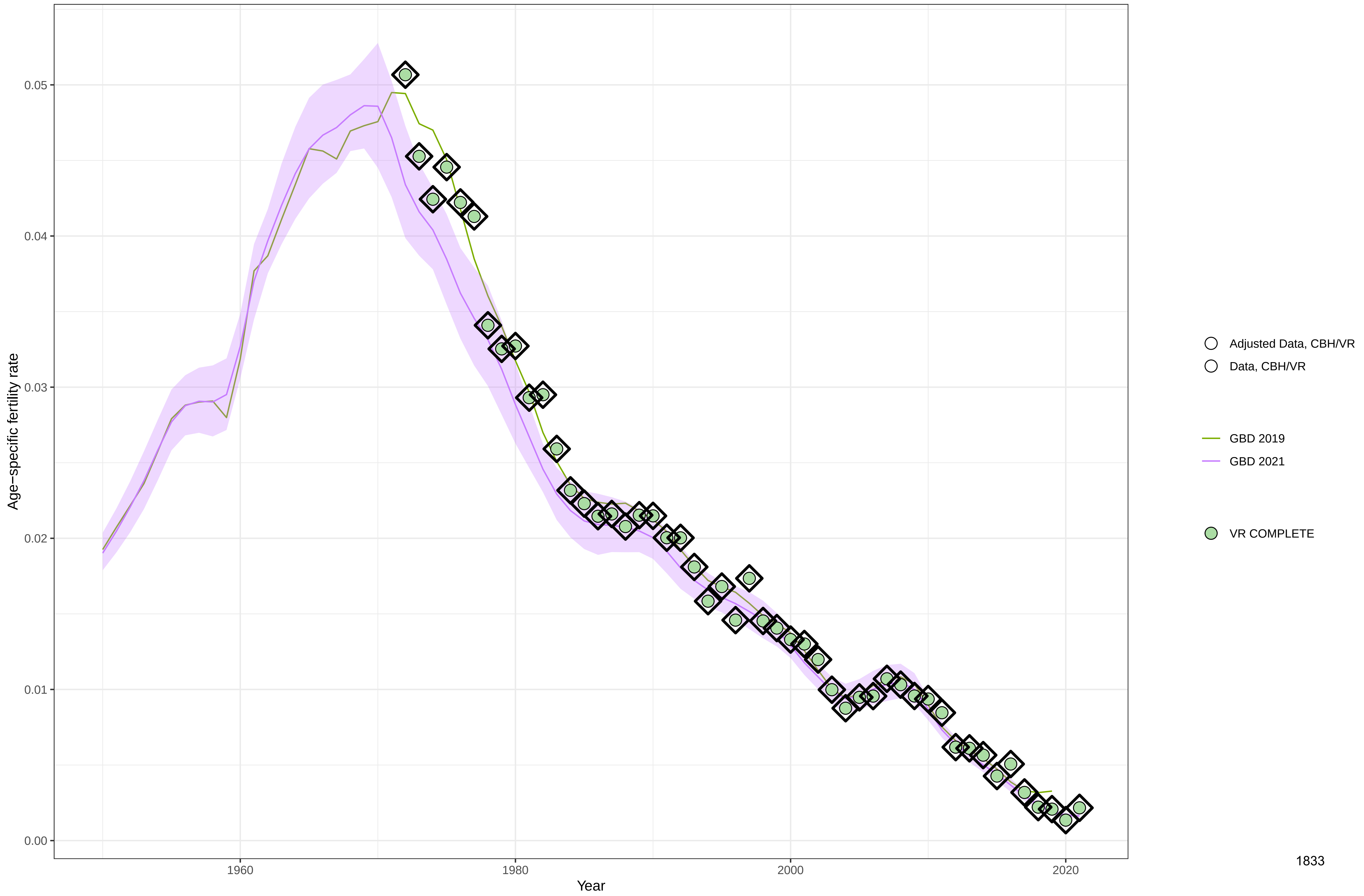

Age−specific fertility fitted trend lines, 1950−2021: Rogaland, Ages: 20−24
Data points enclosed with a diamond are reference sources

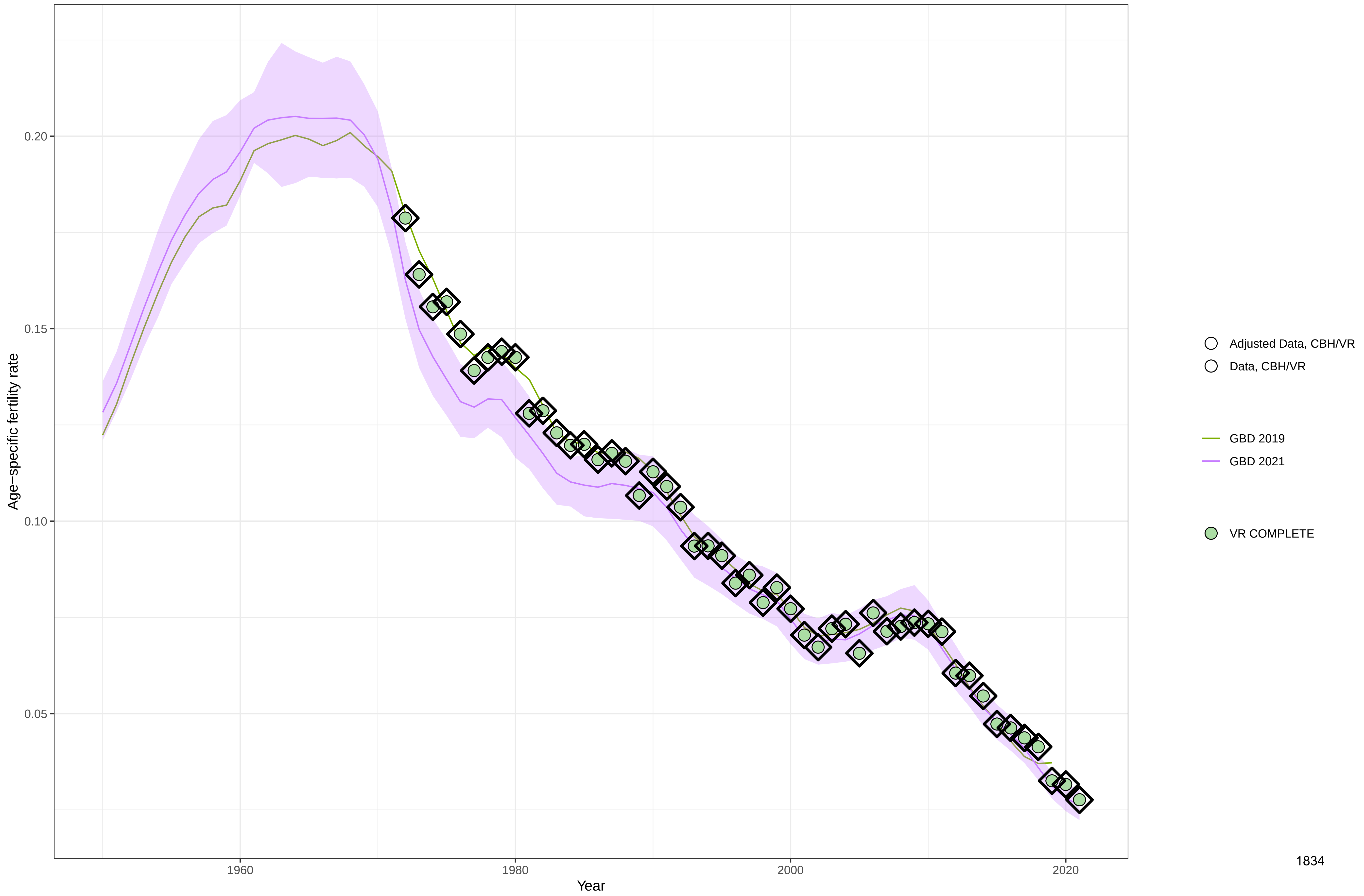

Age−specific fertility fitted trend lines, 1950−2021: Rogaland, Ages: 25−29
Data points enclosed with a diamond are reference sources

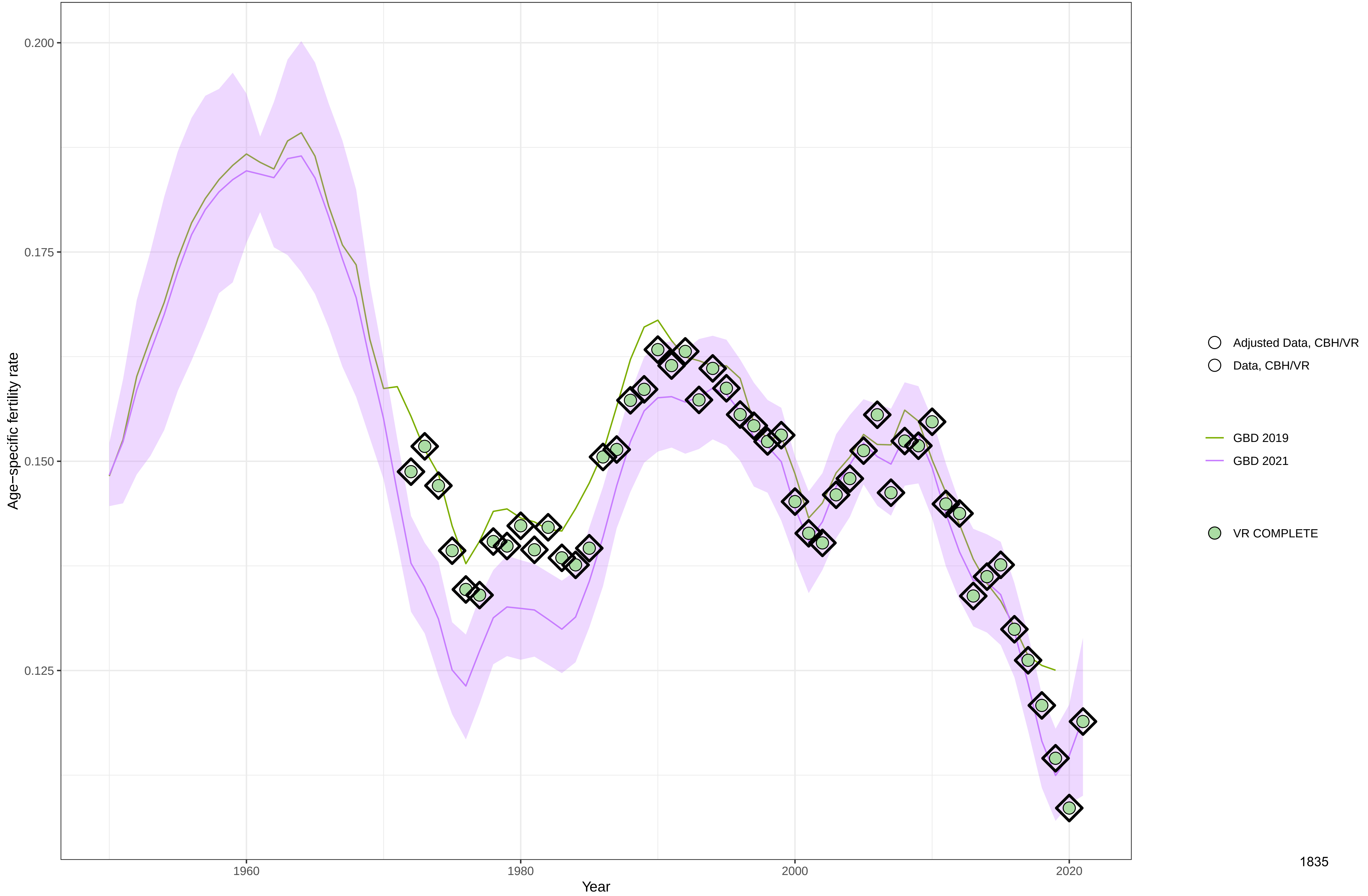

Age−specific fertility fitted trend lines, 1950−2021: Rogaland, Ages: 30−34
Data points enclosed with a diamond are reference sources

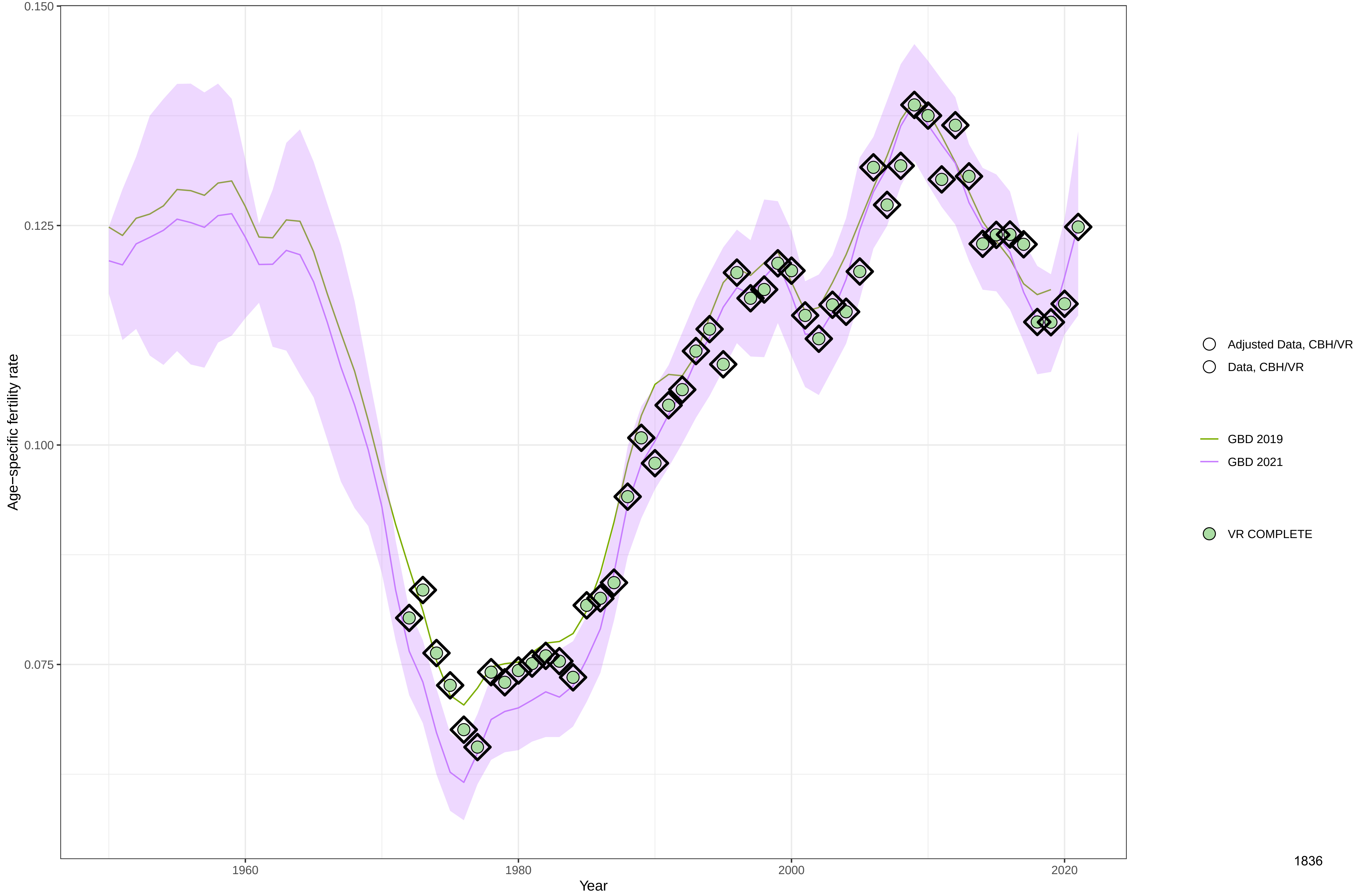

Age−specific fertility fitted trend lines, 1950−2021: Rogaland, Ages: 35−39
Data points enclosed with a diamond are reference sources

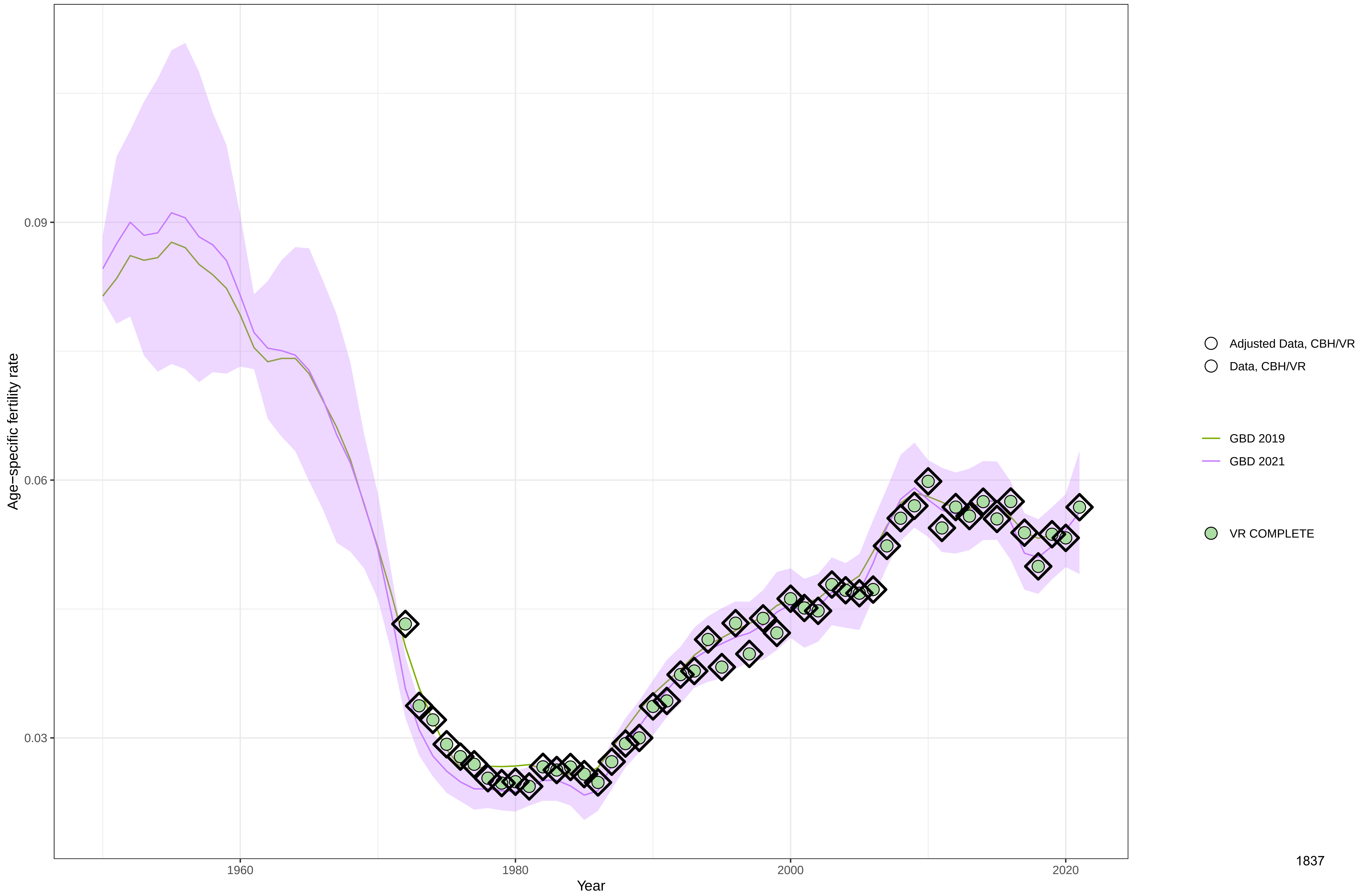

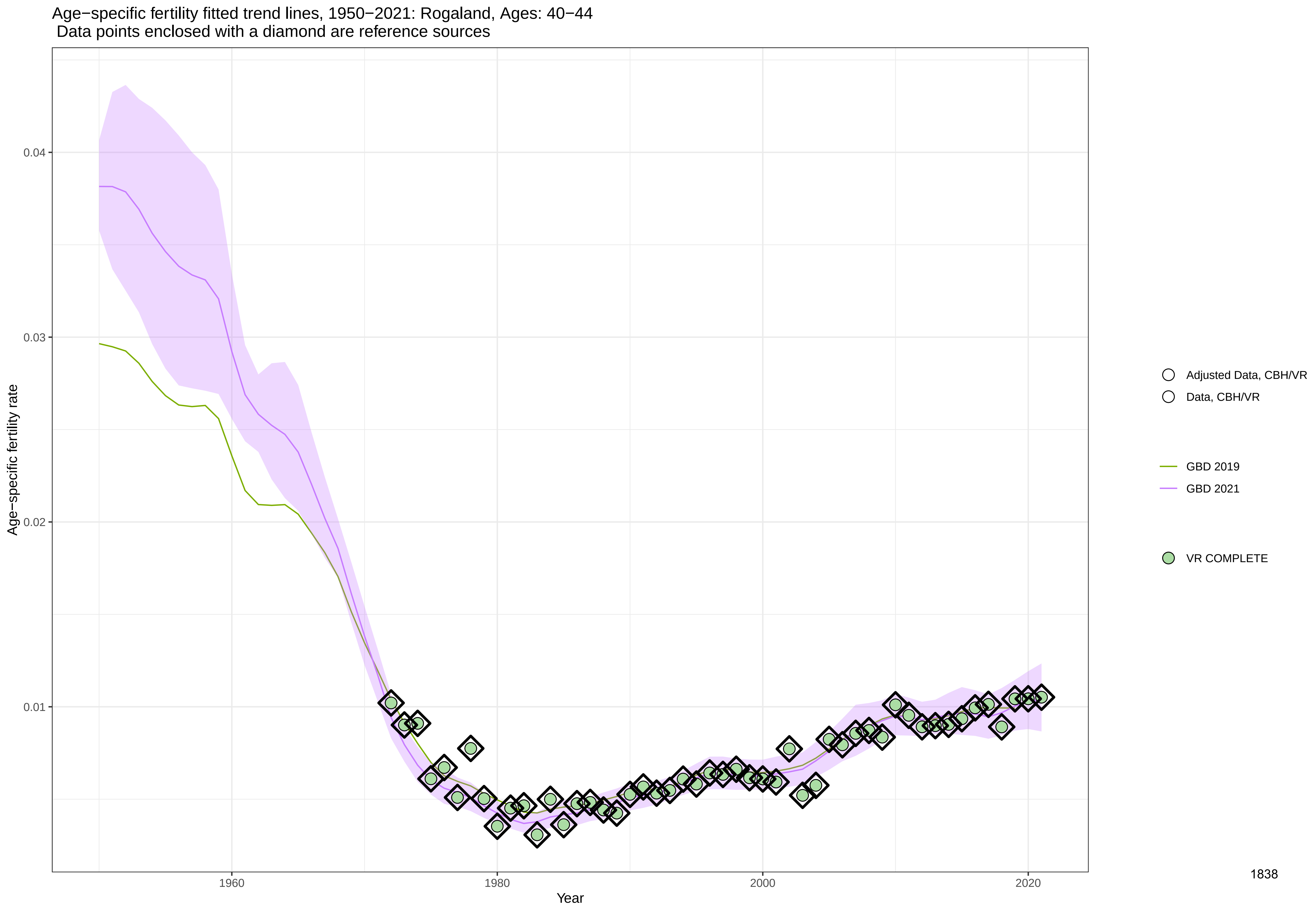
Age−specific fertility fitted trend lines, 1950−2021: Rogaland, Ages: 40−44
Data points enclosed with a diamond are reference sources
Age−specific fertility rate
0.04
0.03
0.02
0.01
1960
1980
2000
2020
Year
Adjusted Data, CBH/VR
Data, CBH/VR
GBD 2019
GBD 2021
VR COMPLETE

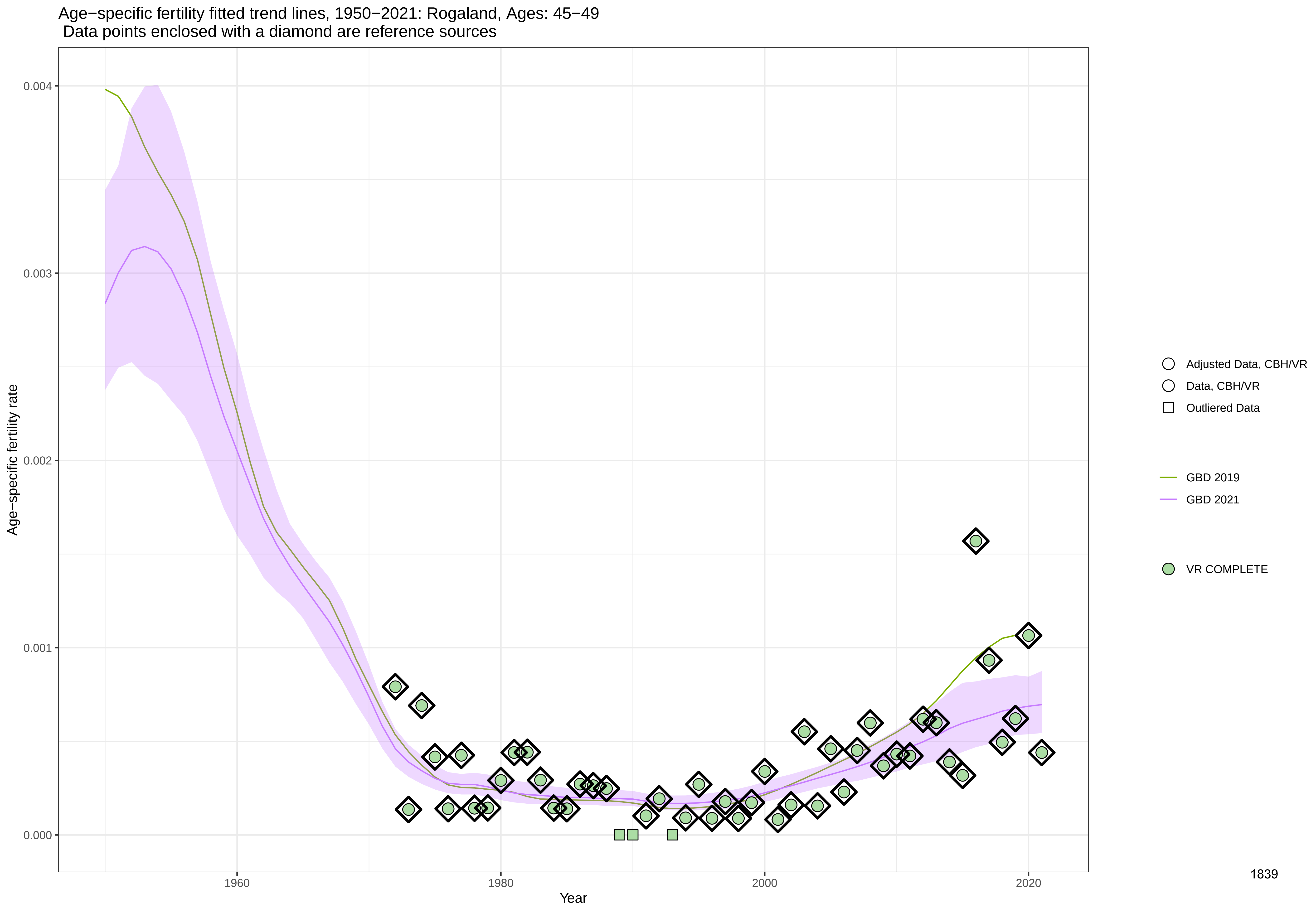

Age−specific fertility fitted trend lines, 1950−2021: Rogaland, Ages: 45−49
Data points enclosed with a diamond are reference sources
Age−specific fertility rate
0.004
0.003
0.002
0.001
0.000
1960
1980
2000
2020
Year
Adjusted Data, CBH/VR
Data, CBH/VR
Outliered Data
GBD 2019
GBD 2021
VR COMPLETE

Age−specific fertility fitted trend lines, 1950−2021: Rogaland, Ages: 50−54
Data points enclosed with a diamond are reference sources

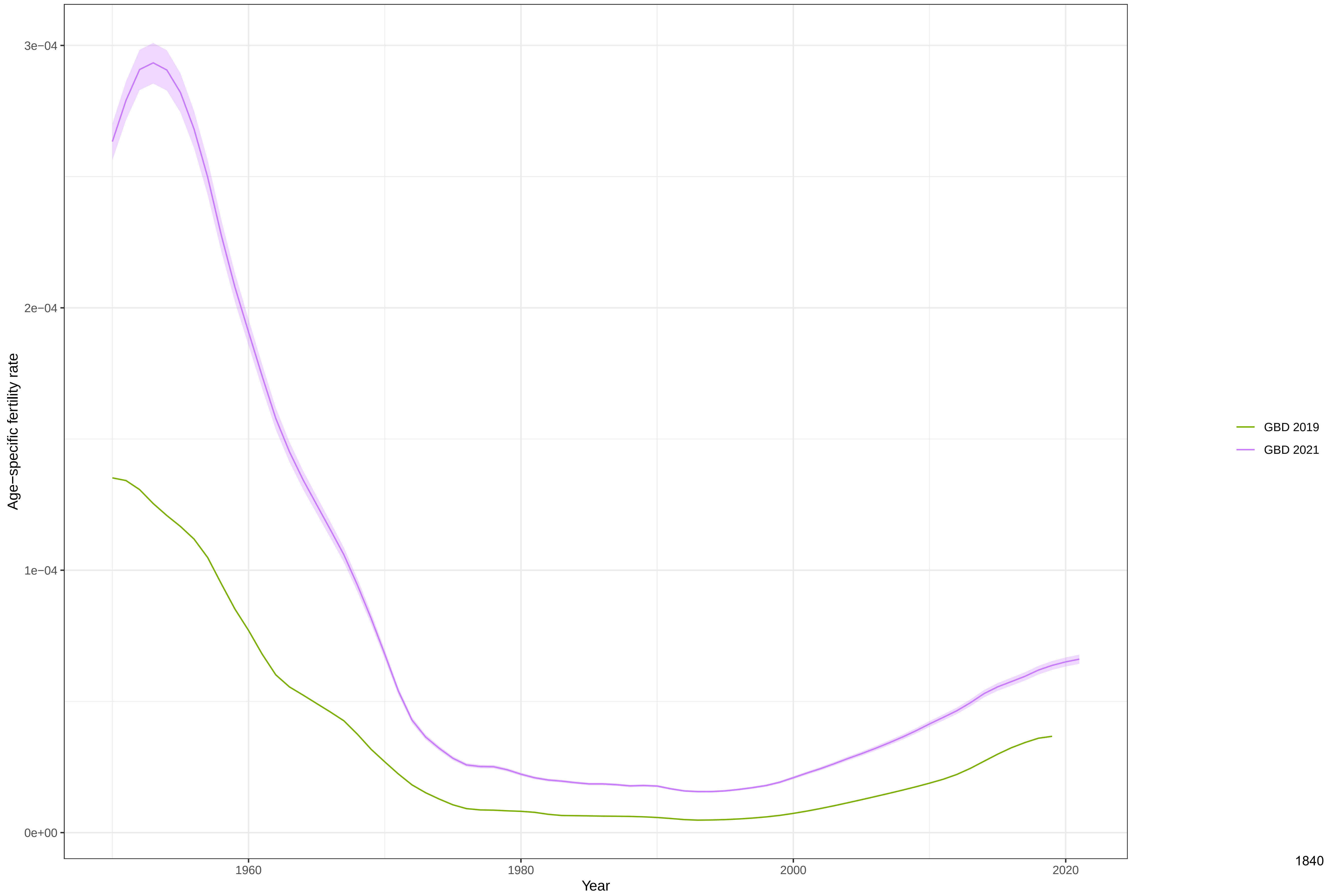

Total fertility rate fitted trend lines, 1950−2021: Rogaland TFR
Data points enclosed with a diamond are reference sources

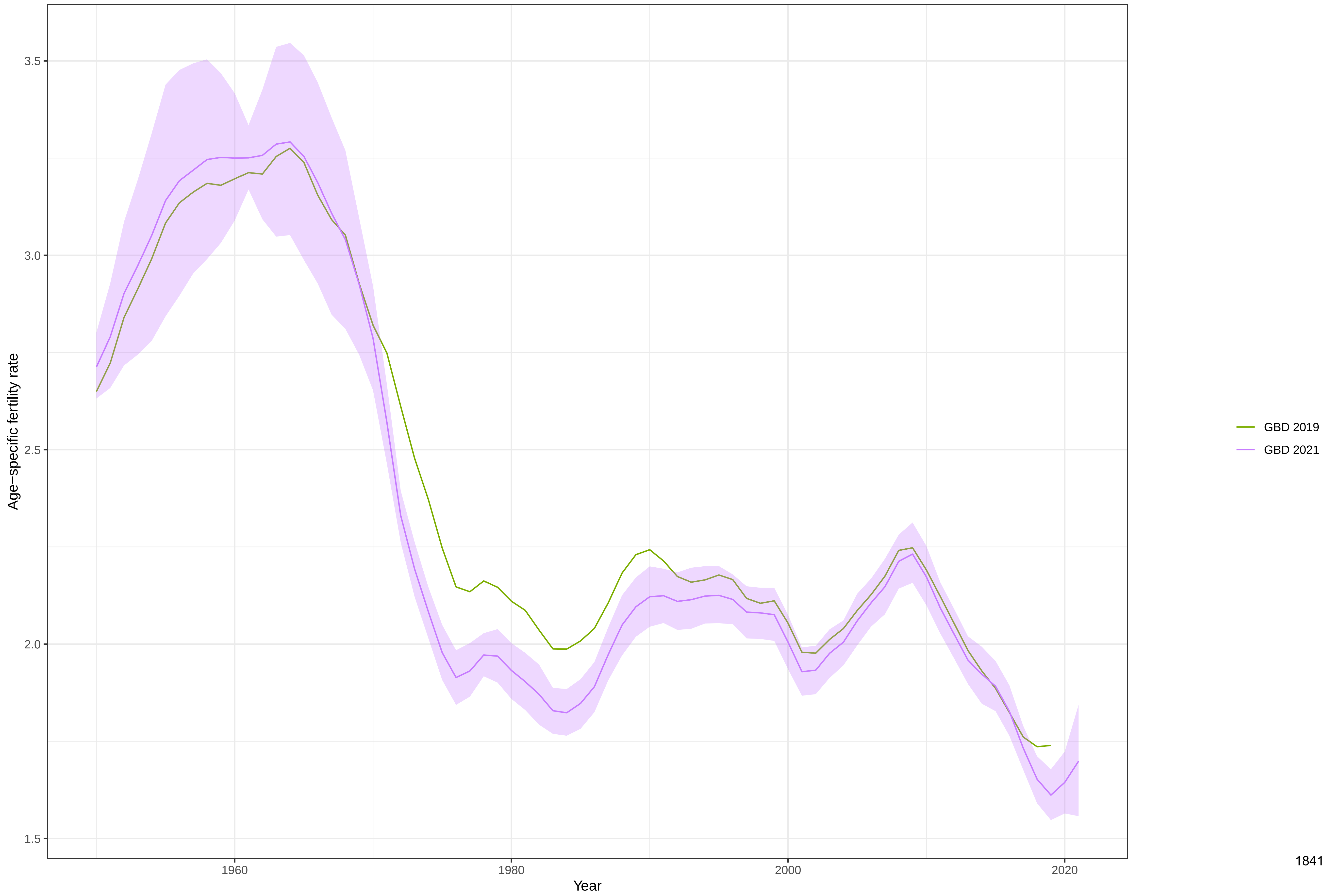

Age−specific fertility fitted trend lines, 1950−2021: Troms og Finnmark, Ages: 10−14
Data points enclosed with a diamond are reference sources

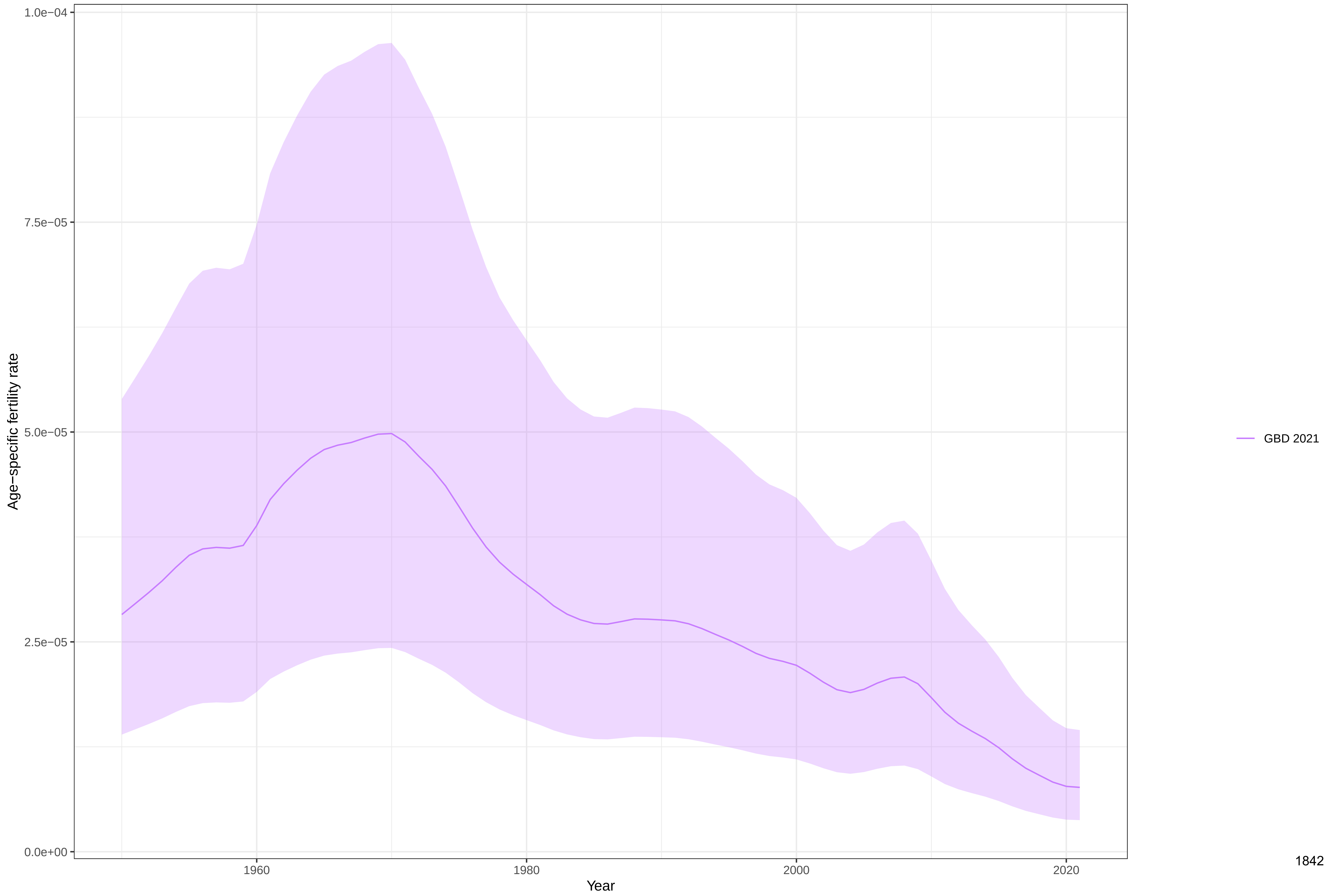

Age−specific fertility fitted trend lines, 1950−2021: Troms og Finnmark, Ages: 15−19
Data points enclosed with a diamond are reference sources

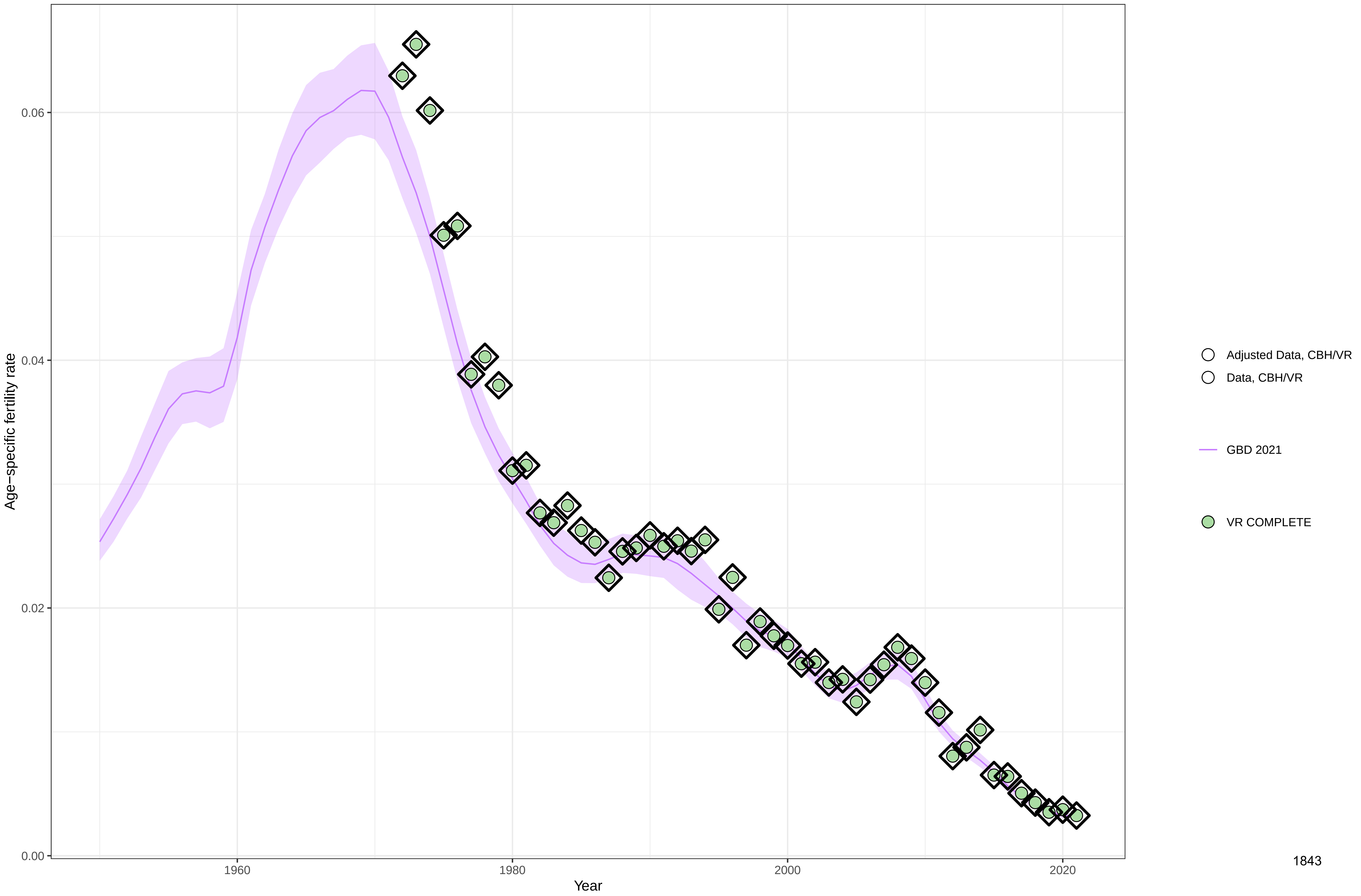

Age−specific fertility fitted trend lines, 1950−2021: Troms og Finnmark, Ages: 20−24
Data points enclosed with a diamond are reference sources

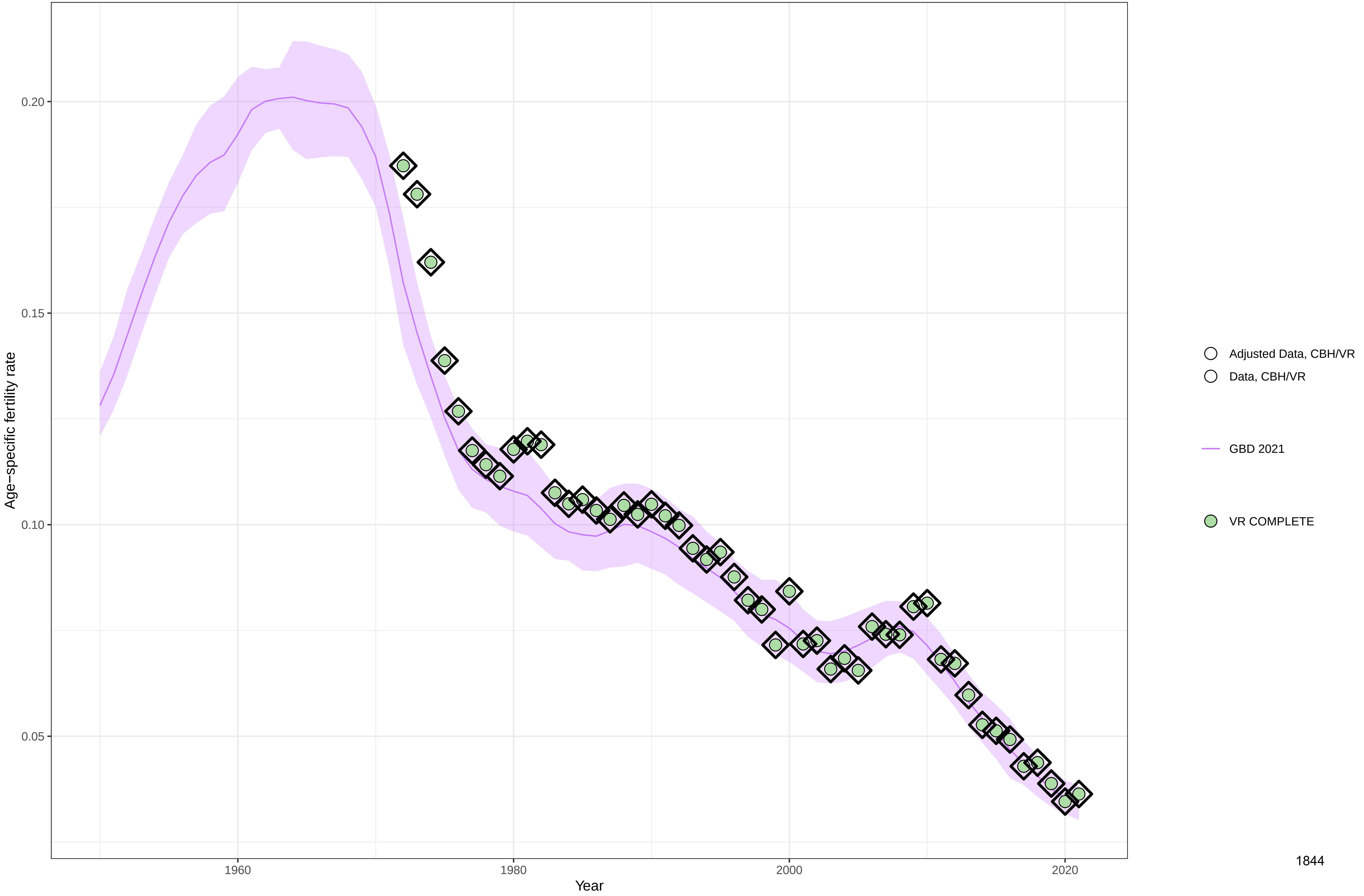

Age−specific fertility fitted trend lines, 1950−2021: Troms og Finnmark, Ages: 25−29
Data points enclosed with a diamond are reference sources

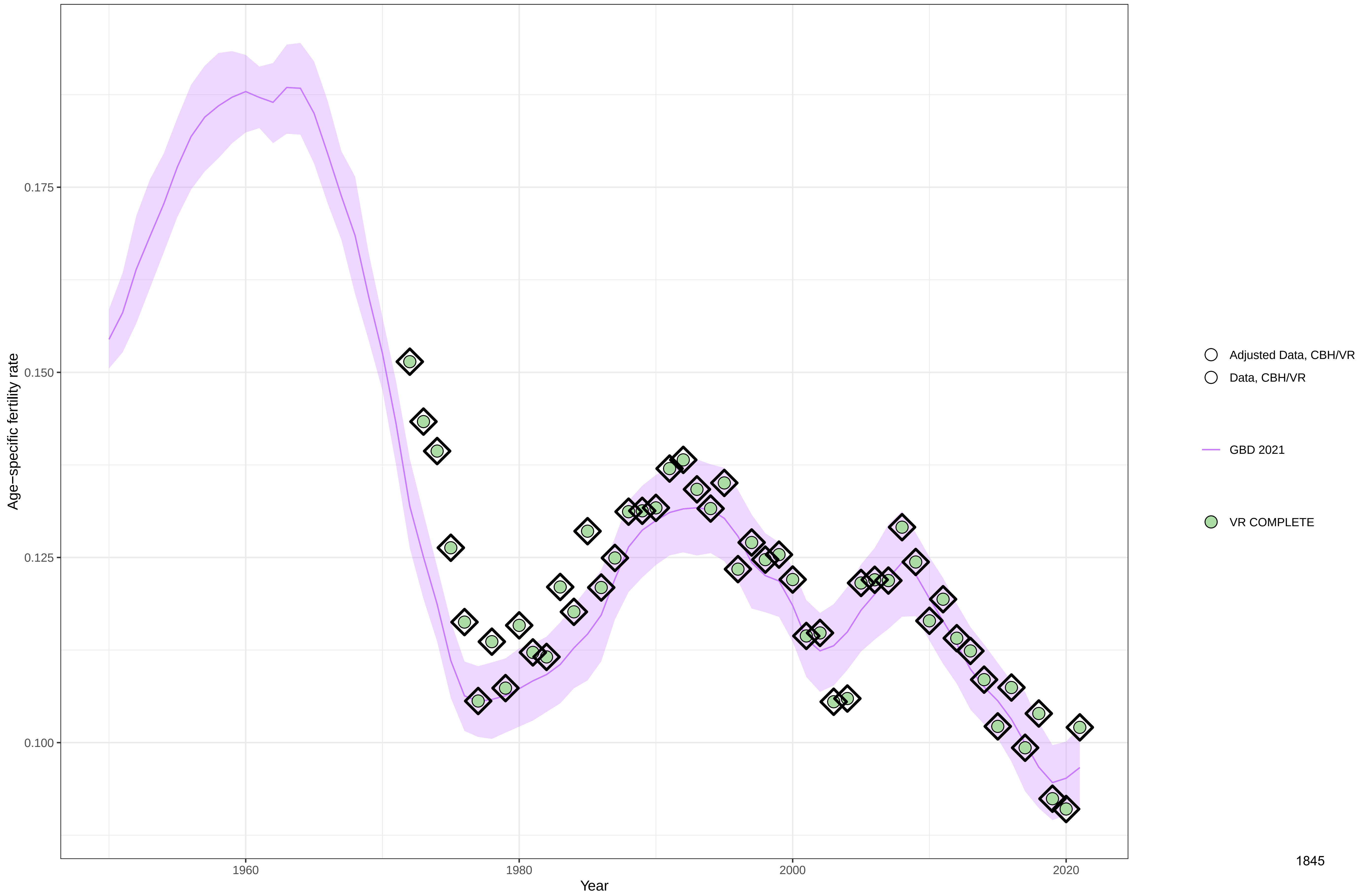

Age−specific fertility fitted trend lines, 1950−2021: Troms og Finnmark, Ages: 30−34
Data points enclosed with a diamond are reference sources

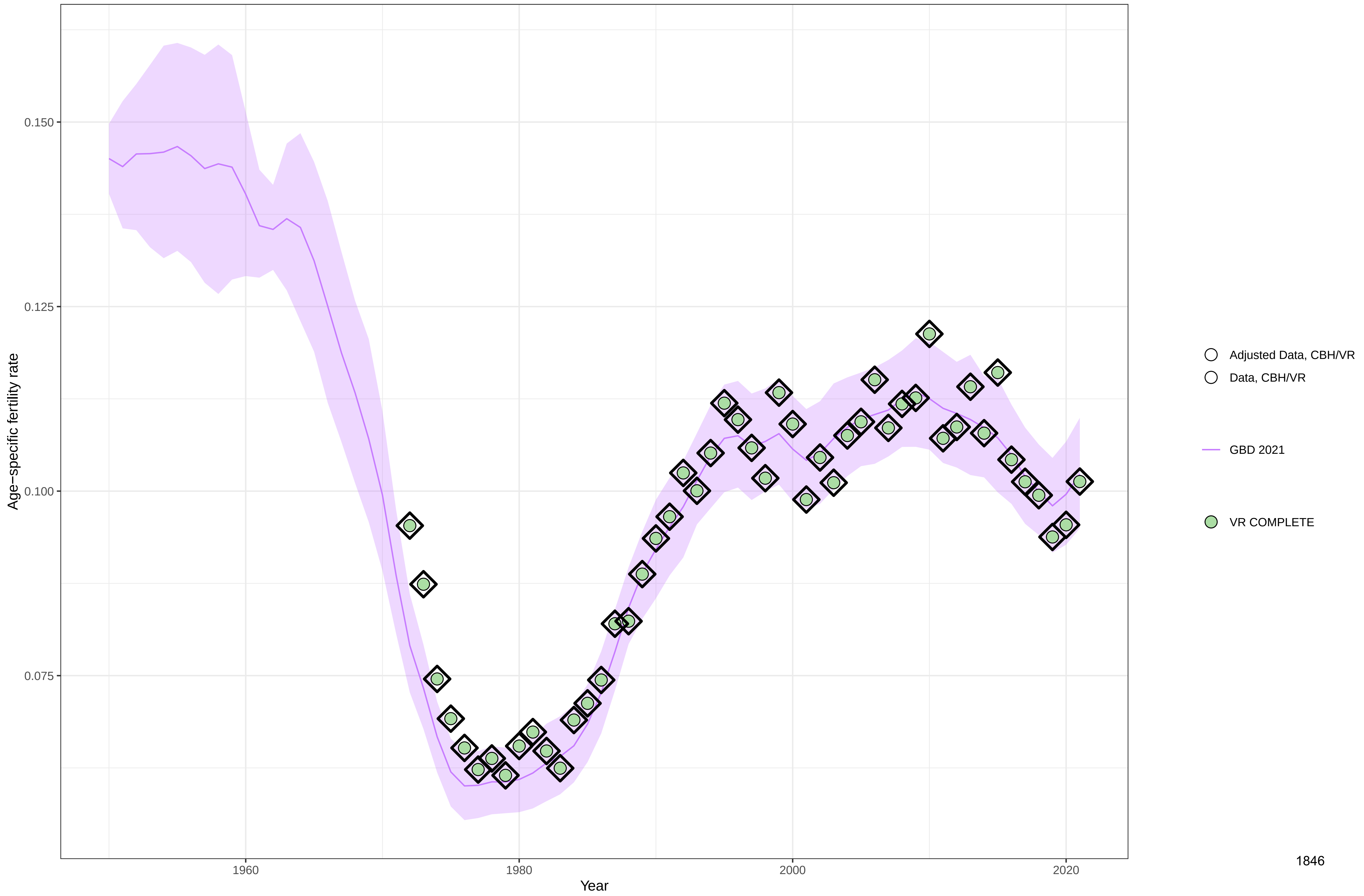

Age−specific fertility fitted trend lines, 1950−2021: Troms og Finnmark, Ages: 35−39
Data points enclosed with a diamond are reference sources

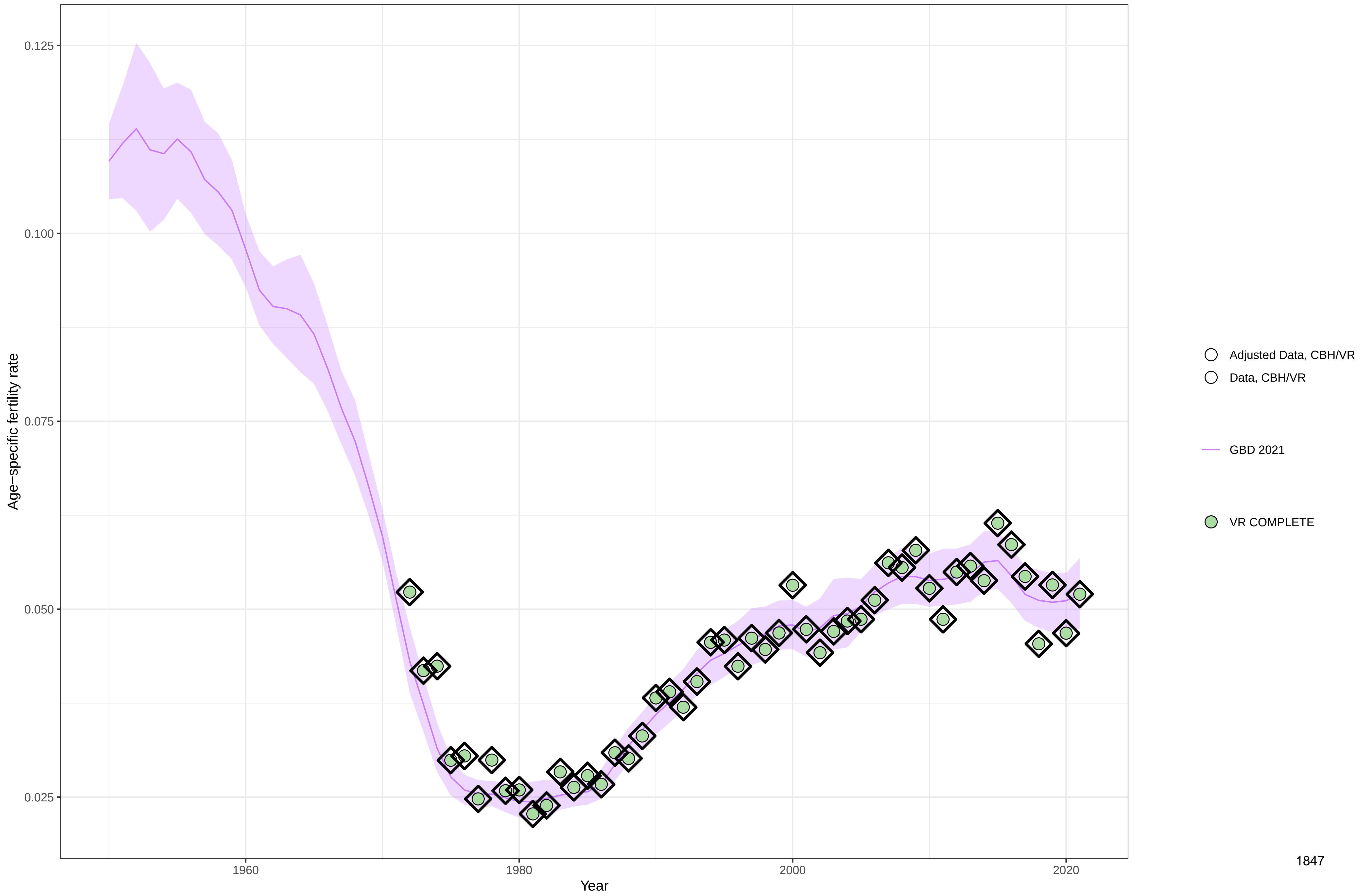

Age−specific fertility fitted trend lines, 1950−2021: Troms og Finnmark, Ages: 40−44
Data points enclosed with a diamond are reference sources

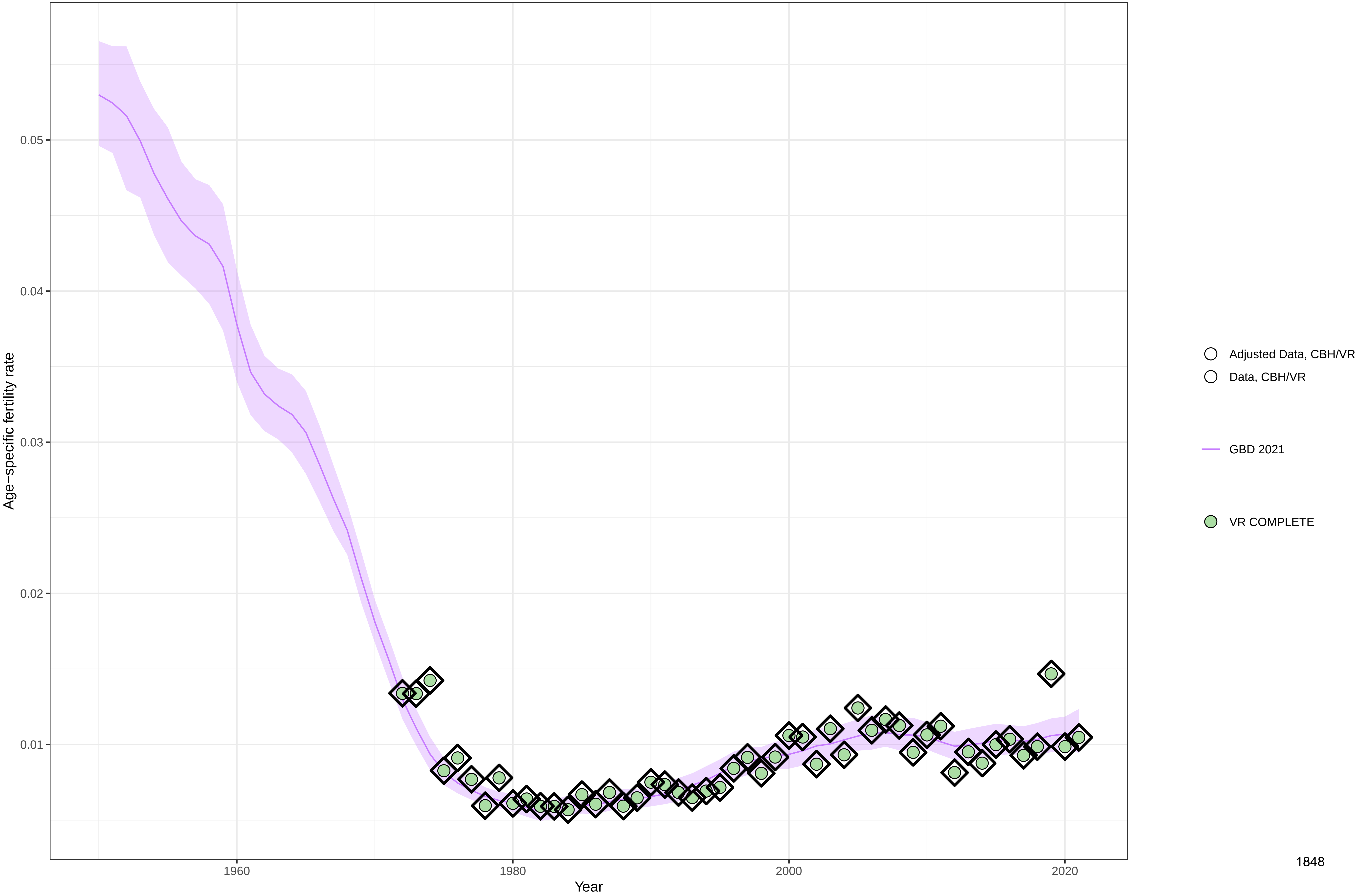

Age−specific fertility fitted trend lines, 1950−2021: Troms og Finnmark, Ages: 45−49
Data points enclosed with a diamond are reference sources

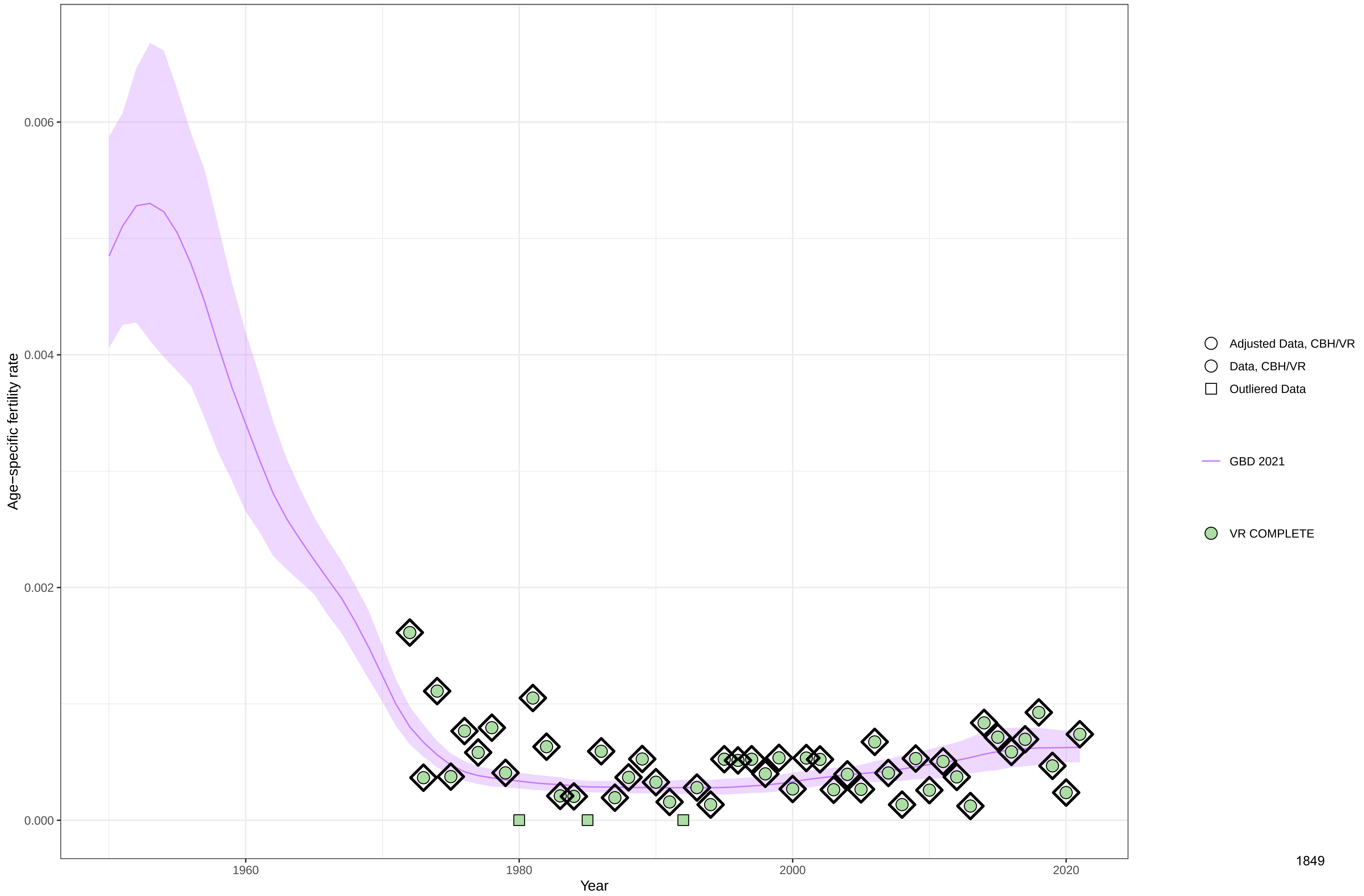

Age−specific fertility fitted trend lines, 1950−2021: Troms og Finnmark, Ages: 50−54
Data points enclosed with a diamond are reference sources

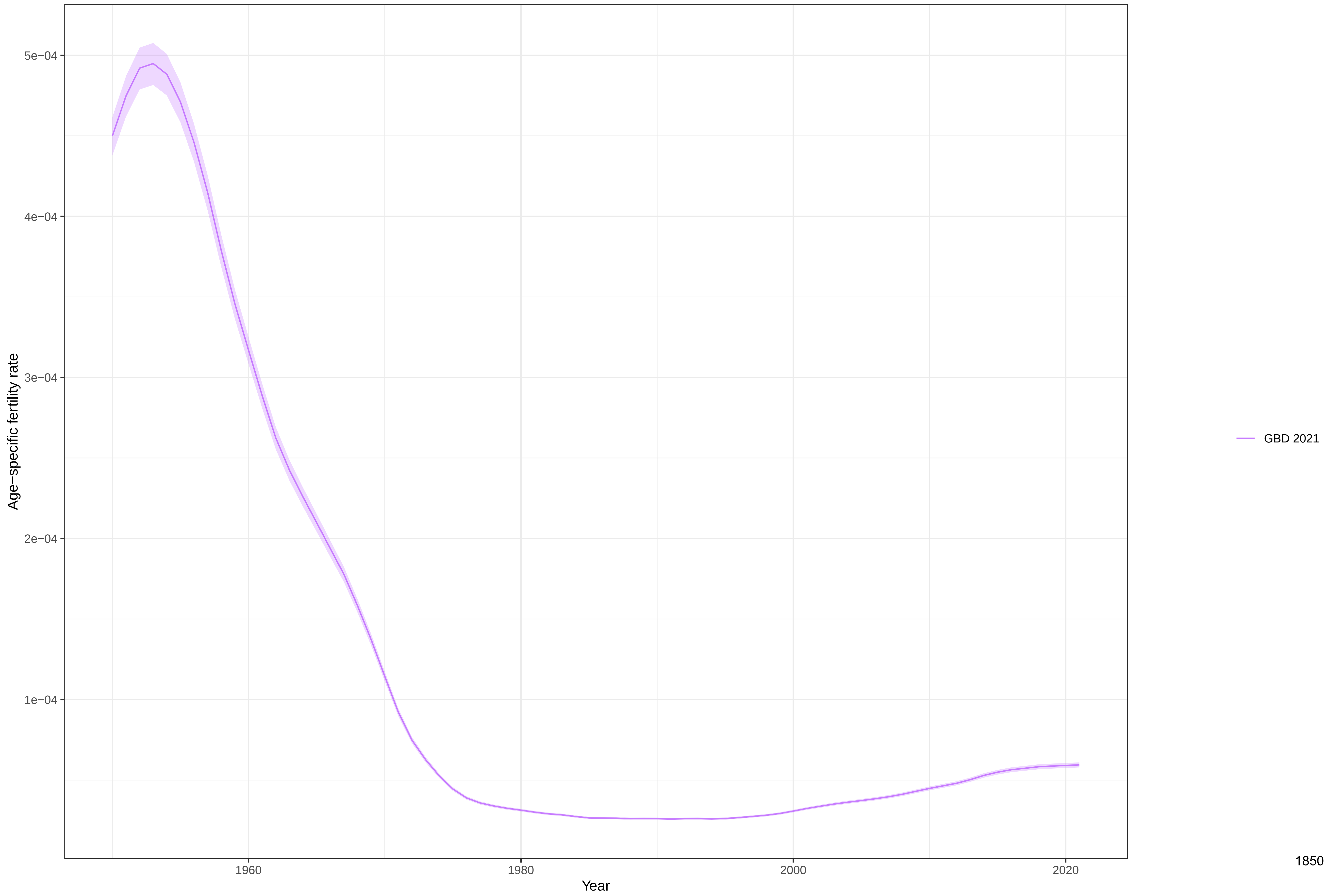

Total fertility rate fitted trend lines, 1950−2021: Troms og Finnmark TFR
Data points enclosed with a diamond are reference sources

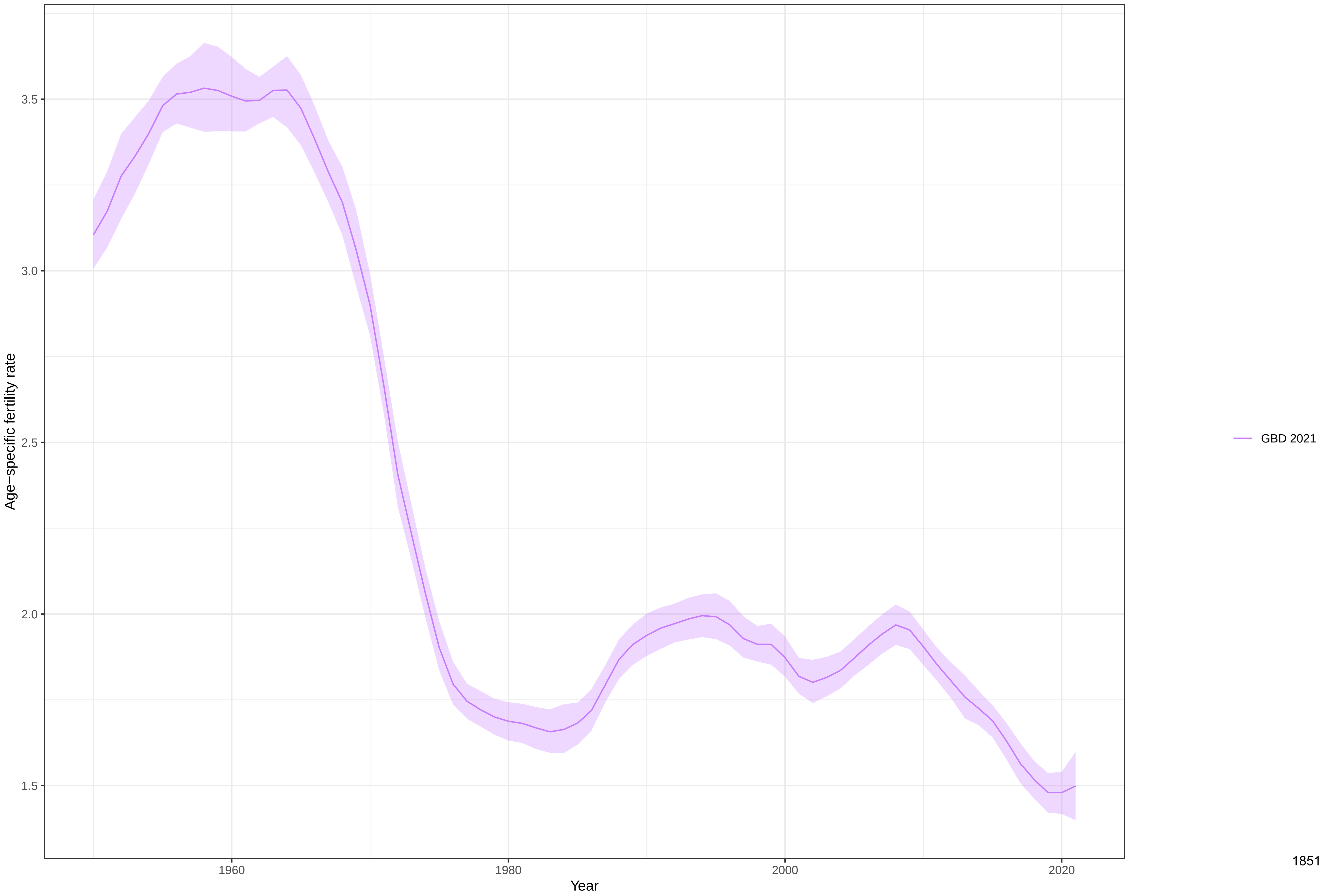

Age−specific fertility fitted trend lines, 1950−2021: Trøndelag, Ages: 10−14
Data points enclosed with a diamond are reference sources

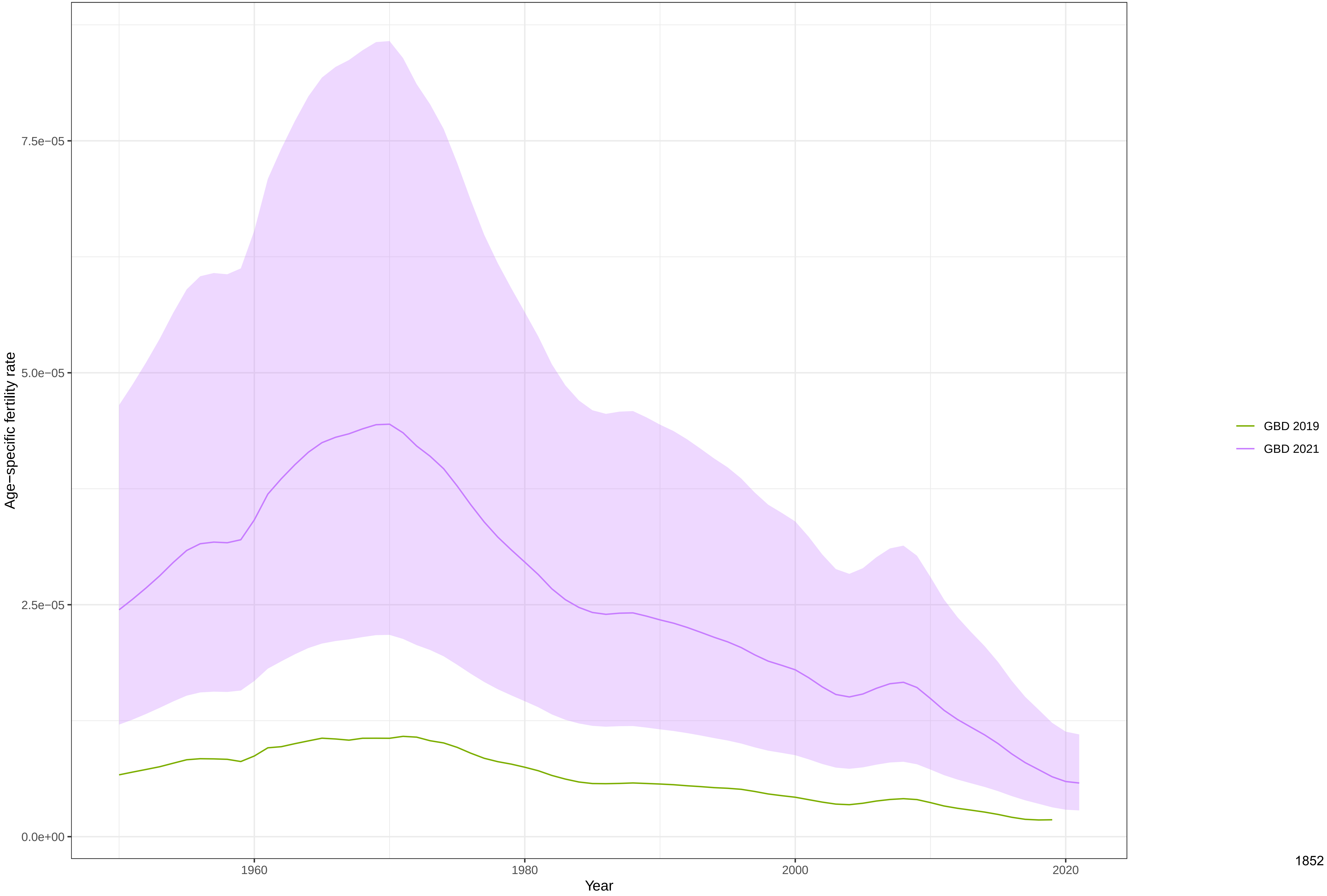

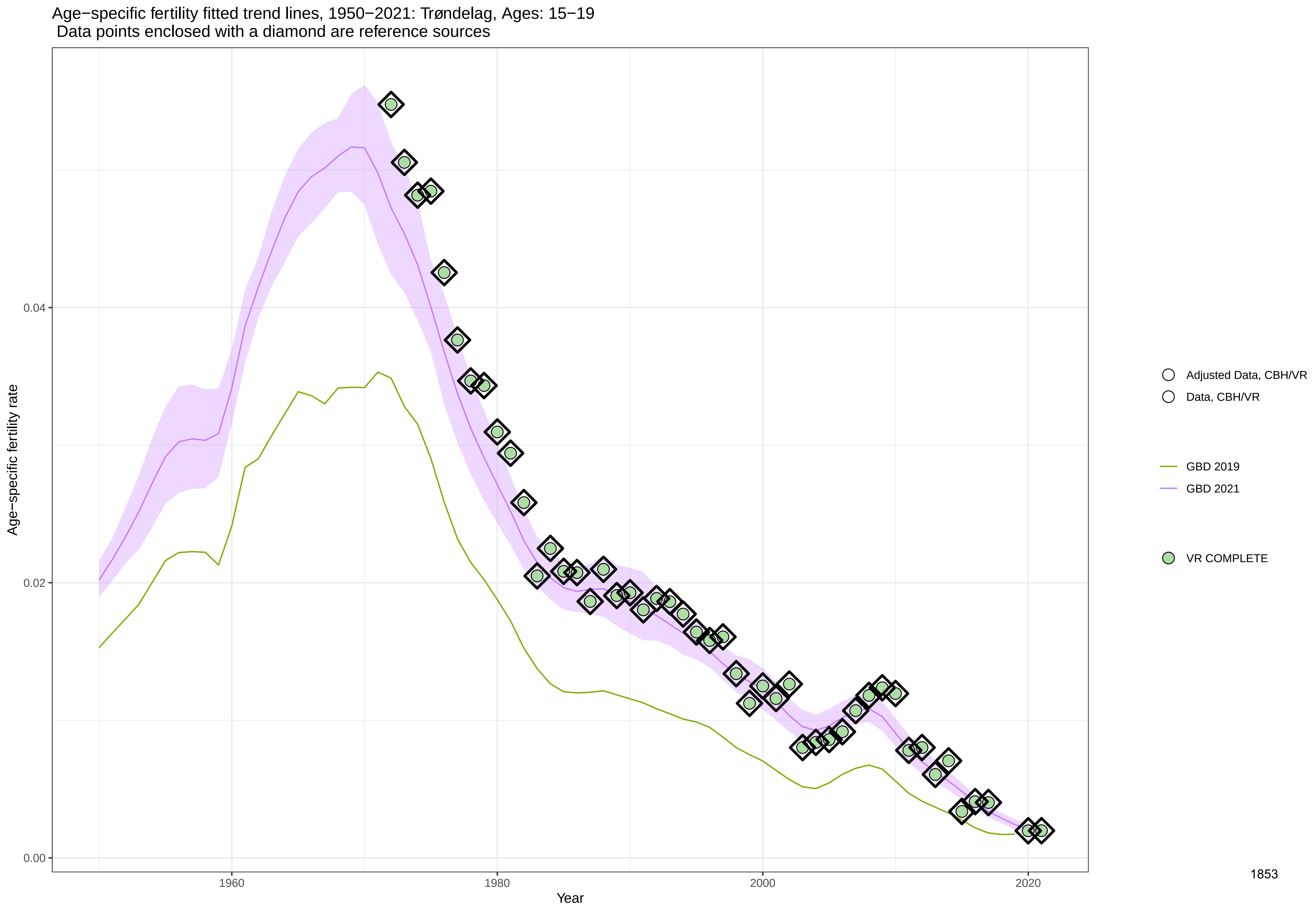
Age−specific fertility fitted trend lines, 1950−2021: Trøndelag, Ages: 15−19
Data points enclosed with a diamond are reference sources
Age−specific fertility rate
0.00
0.02
0.04
1960
1980
2000
2020
Year
Adjusted Data, CBH/VR
Data, CBH/VR
GBD 2019
GBD 2021
VR COMPLETE

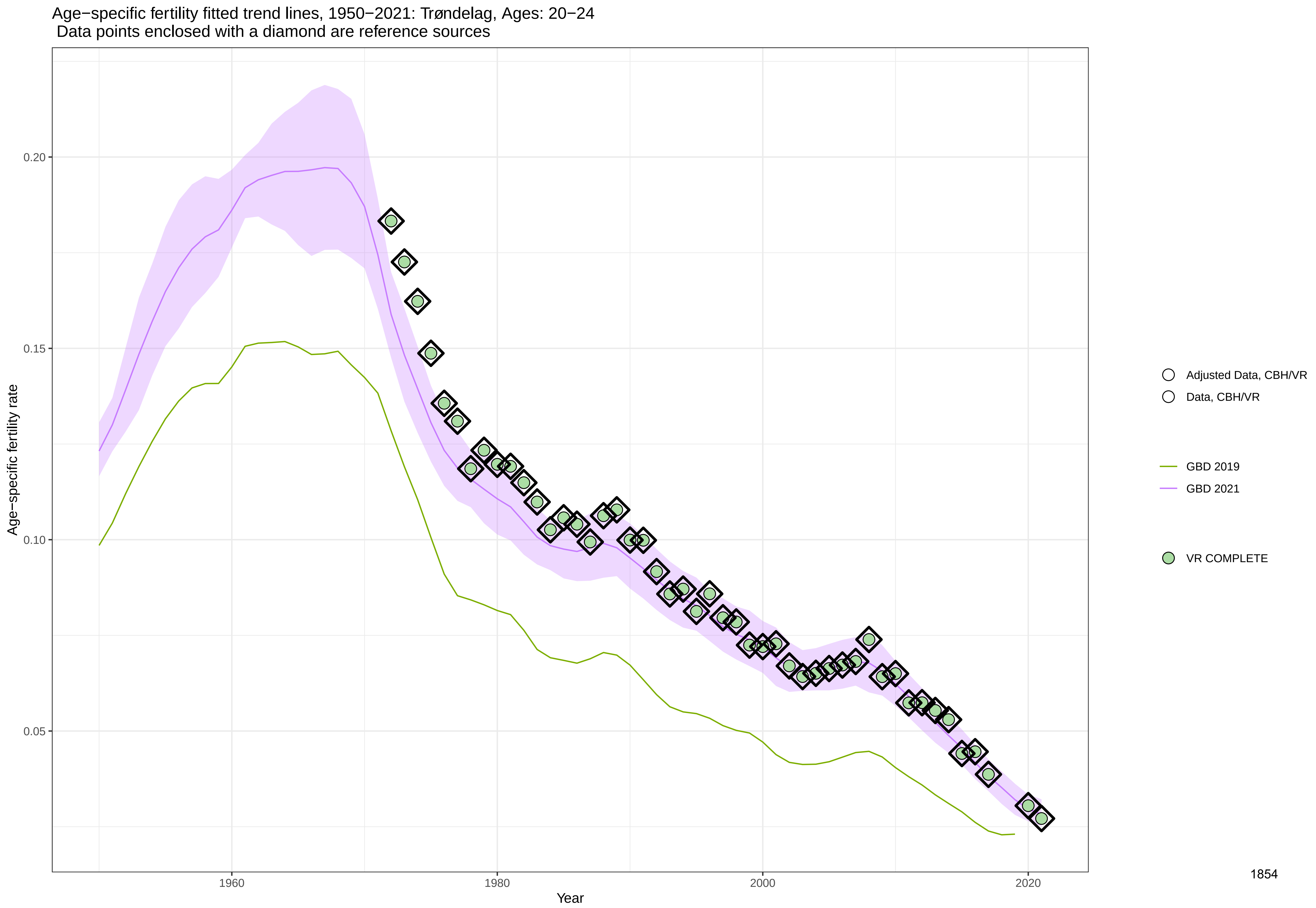

Age−specific fertility fitted trend lines, 1950−2021: Trøndelag, Ages: 20−24
Data points enclosed with a diamond are reference sources
Age−specific fertility rate
0.20
0.15
0.10
0.05
1960
1980
2000
2020
Year
Adjusted Data, CBH/VR
Data, CBH/VR
GBD 2019
GBD 2021
VR COMPLETE

Age−specific fertility fitted trend lines, 1950−2021: Trøndelag, Ages: 25−29
Data points enclosed with a diamond are reference sources

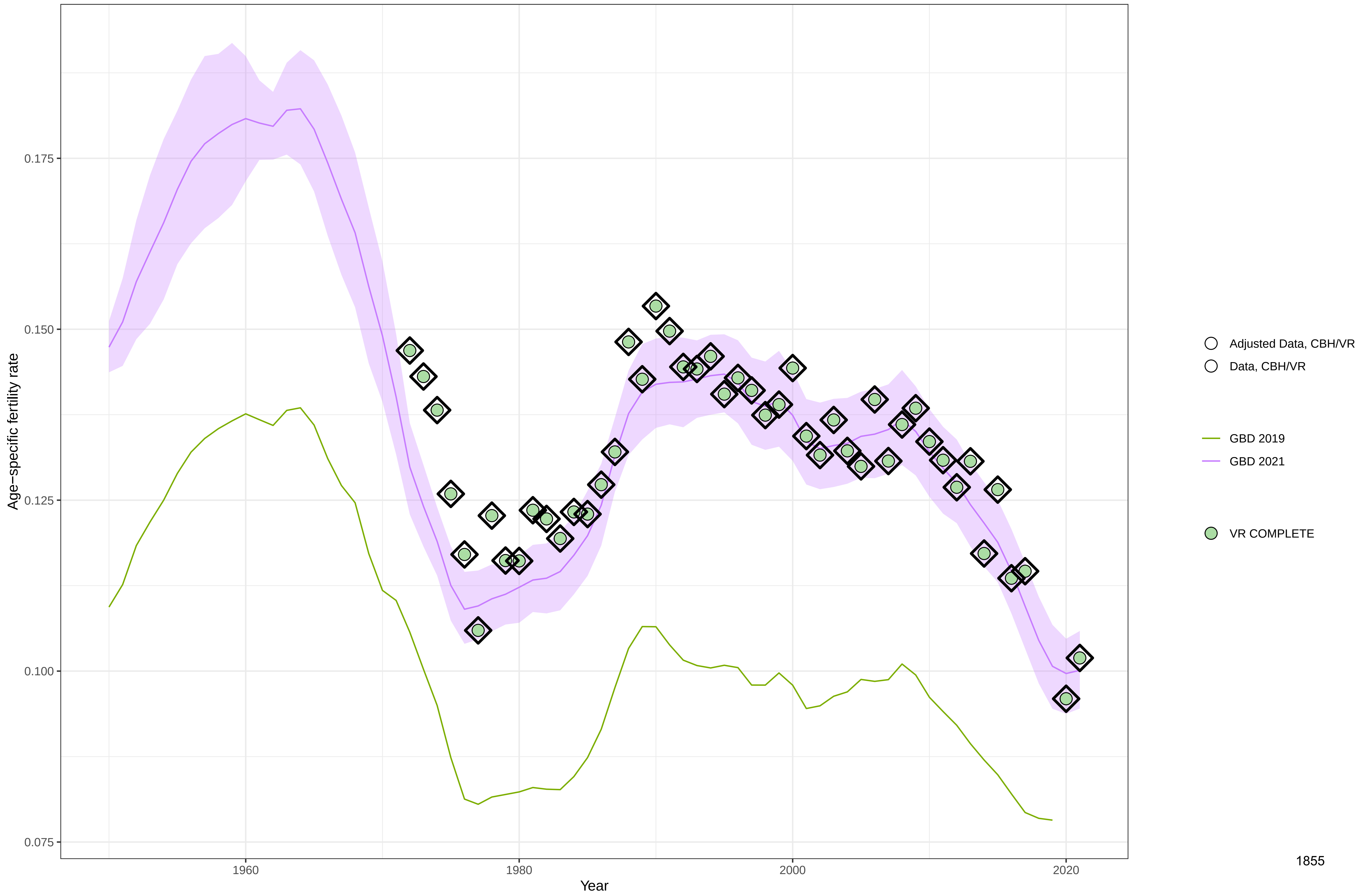

Age−specific fertility fitted trend lines, 1950−2021: Trøndelag, Ages: 30−34
Data points enclosed with a diamond are reference sources

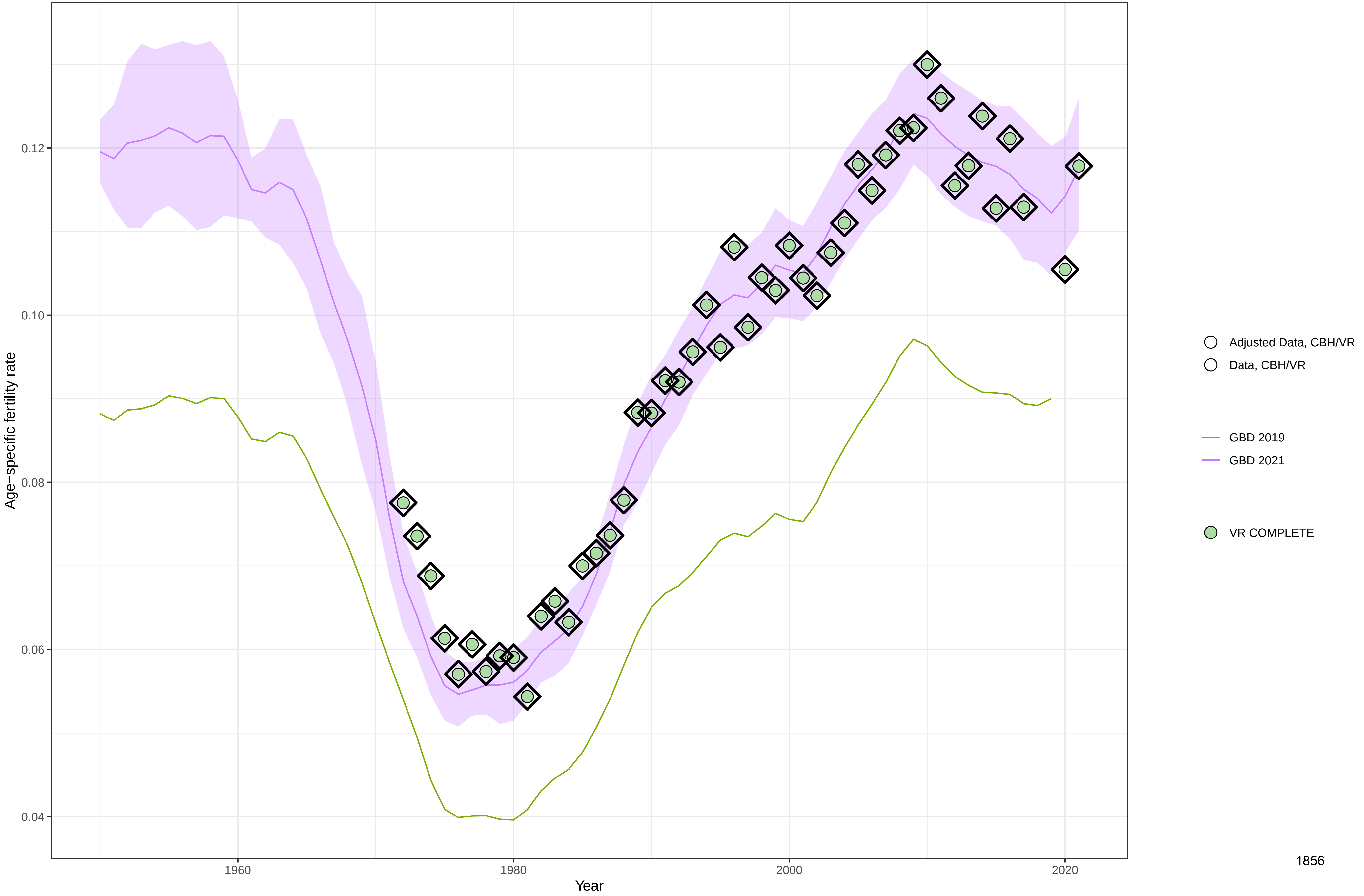

Age−specific fertility fitted trend lines, 1950−2021: Trøndelag, Ages: 35−39
Data points enclosed with a diamond are reference sources

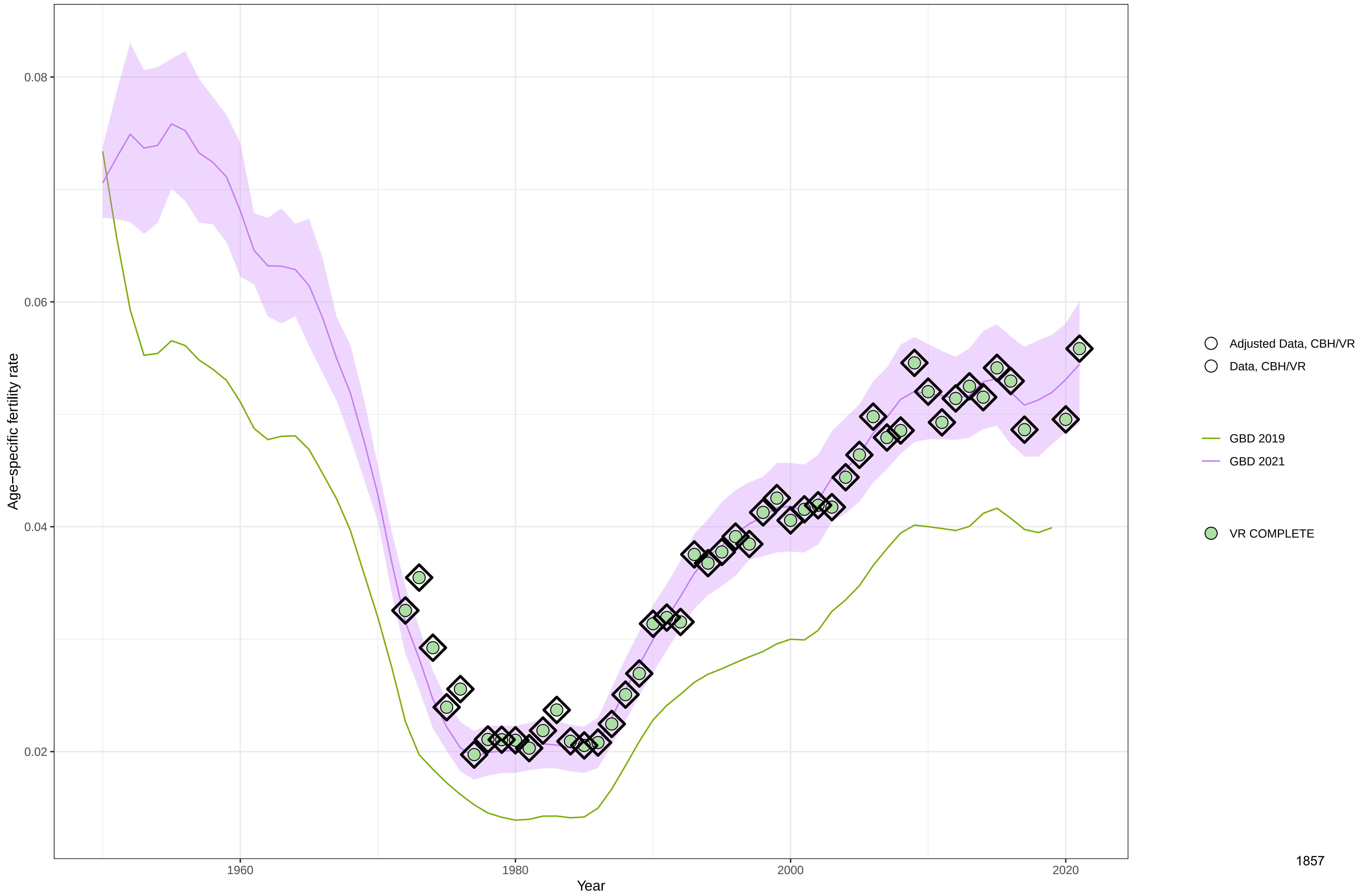

Age−specific fertility fitted trend lines, 1950−2021: Trøndelag, Ages: 40−44
Data points enclosed with a diamond are reference sources

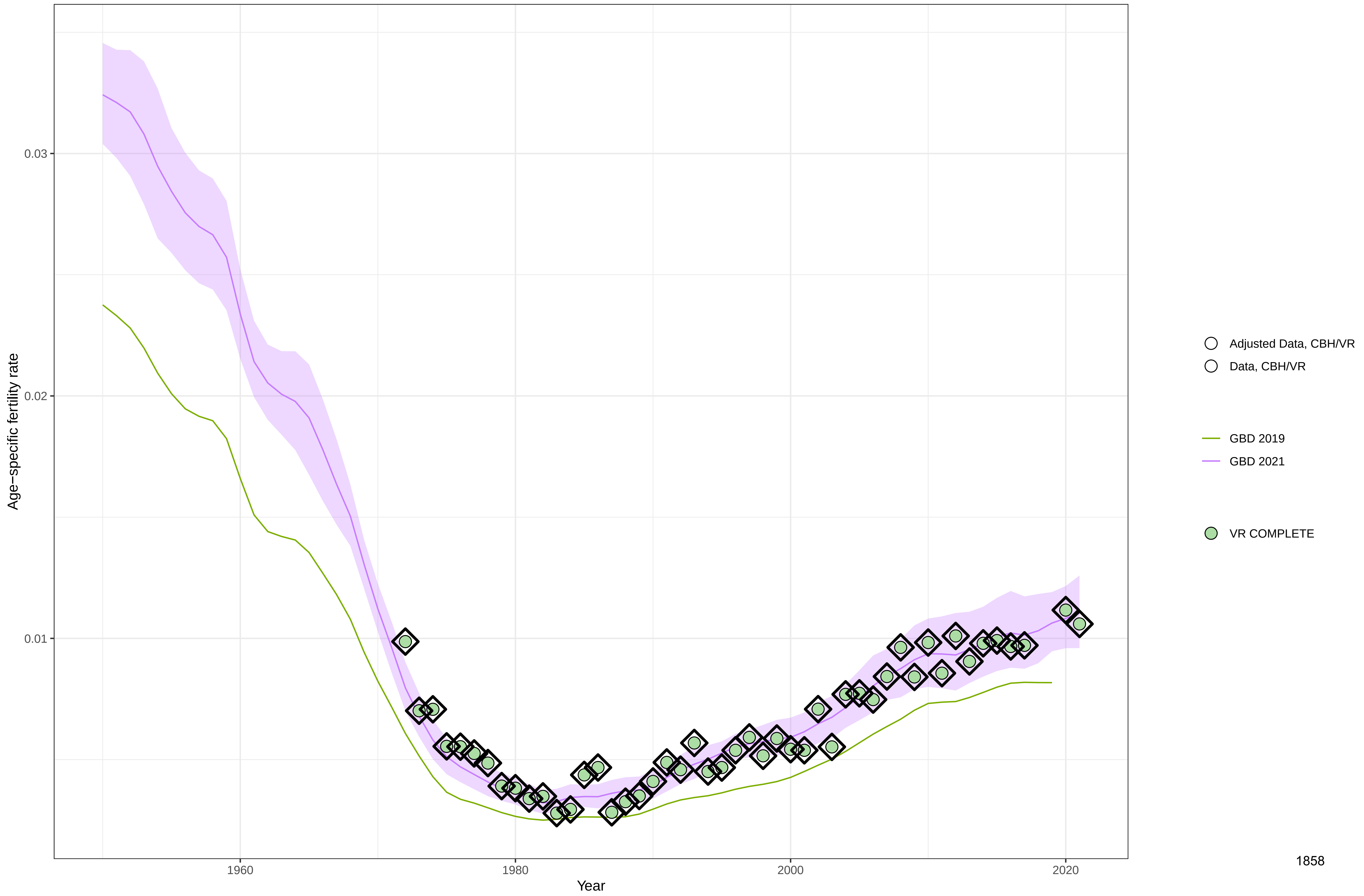

Age−specific fertility fitted trend lines, 1950−2021: Trøndelag, Ages: 45−49
Data points enclosed with a diamond are reference sources

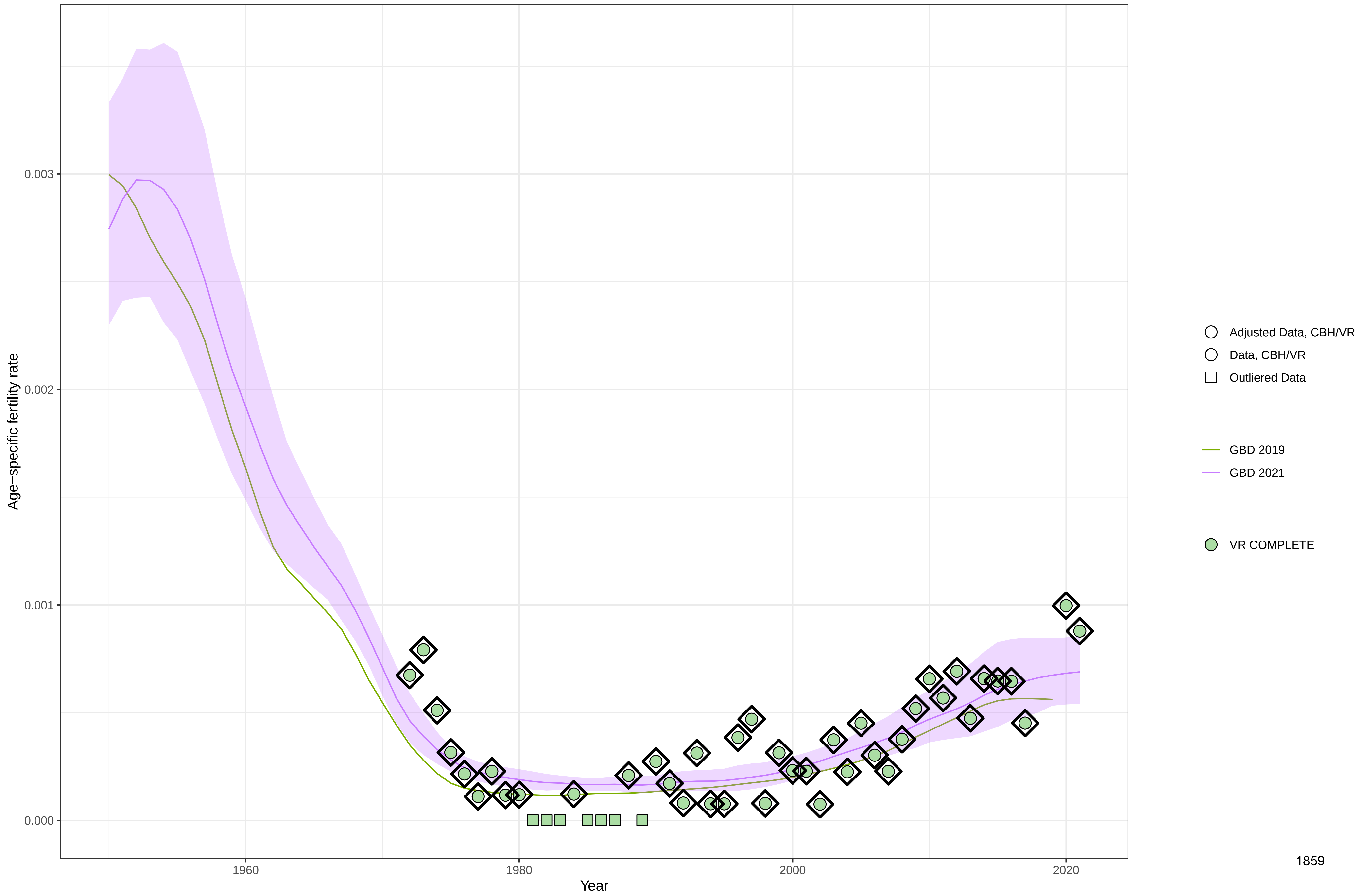

Age−specific fertility fitted trend lines, 1950−2021: Trøndelag, Ages: 50−54
Data points enclosed with a diamond are reference sources

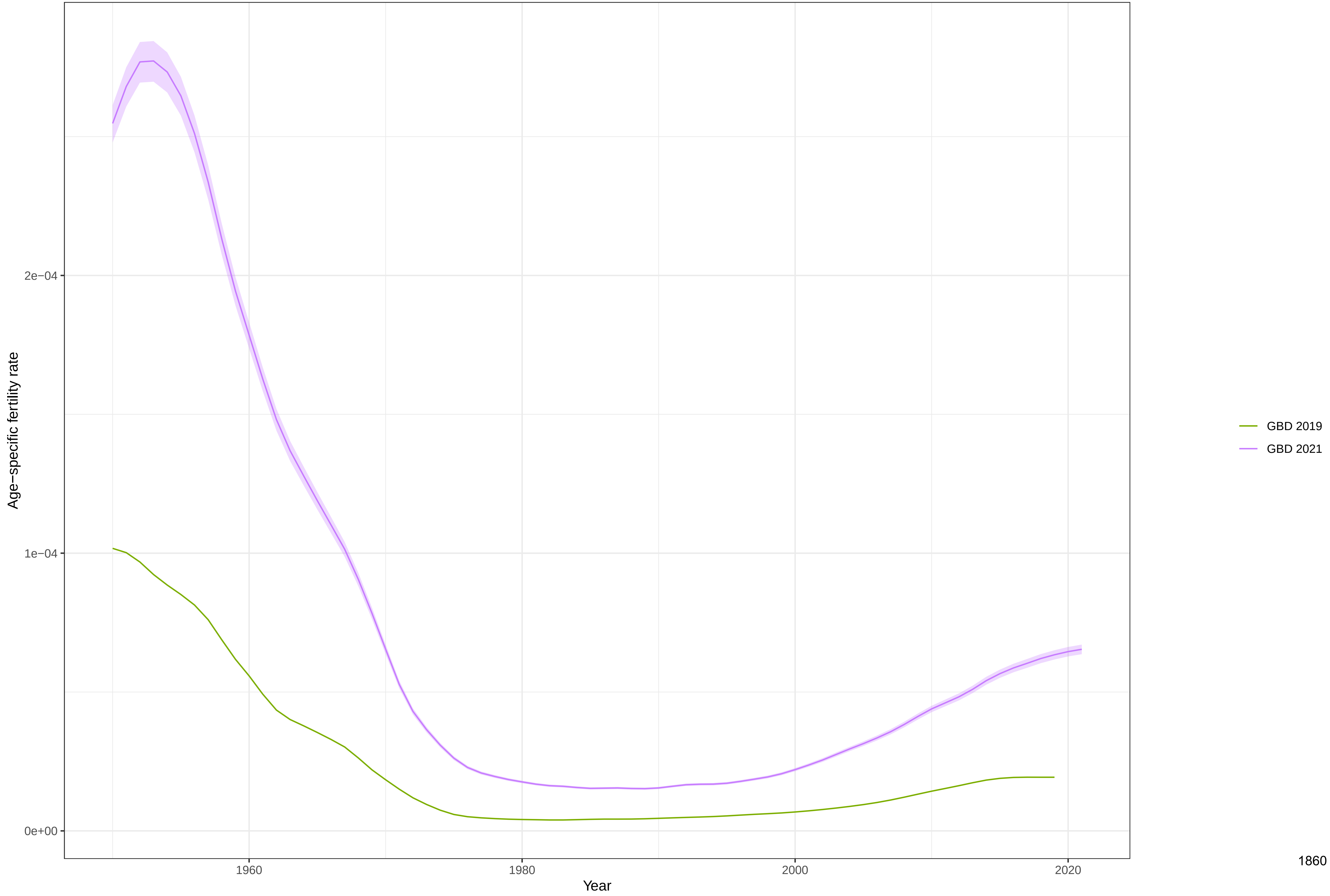

Total fertility rate fitted trend lines, 1950−2021: Trøndelag TFR
Data points enclosed with a diamond are reference sources

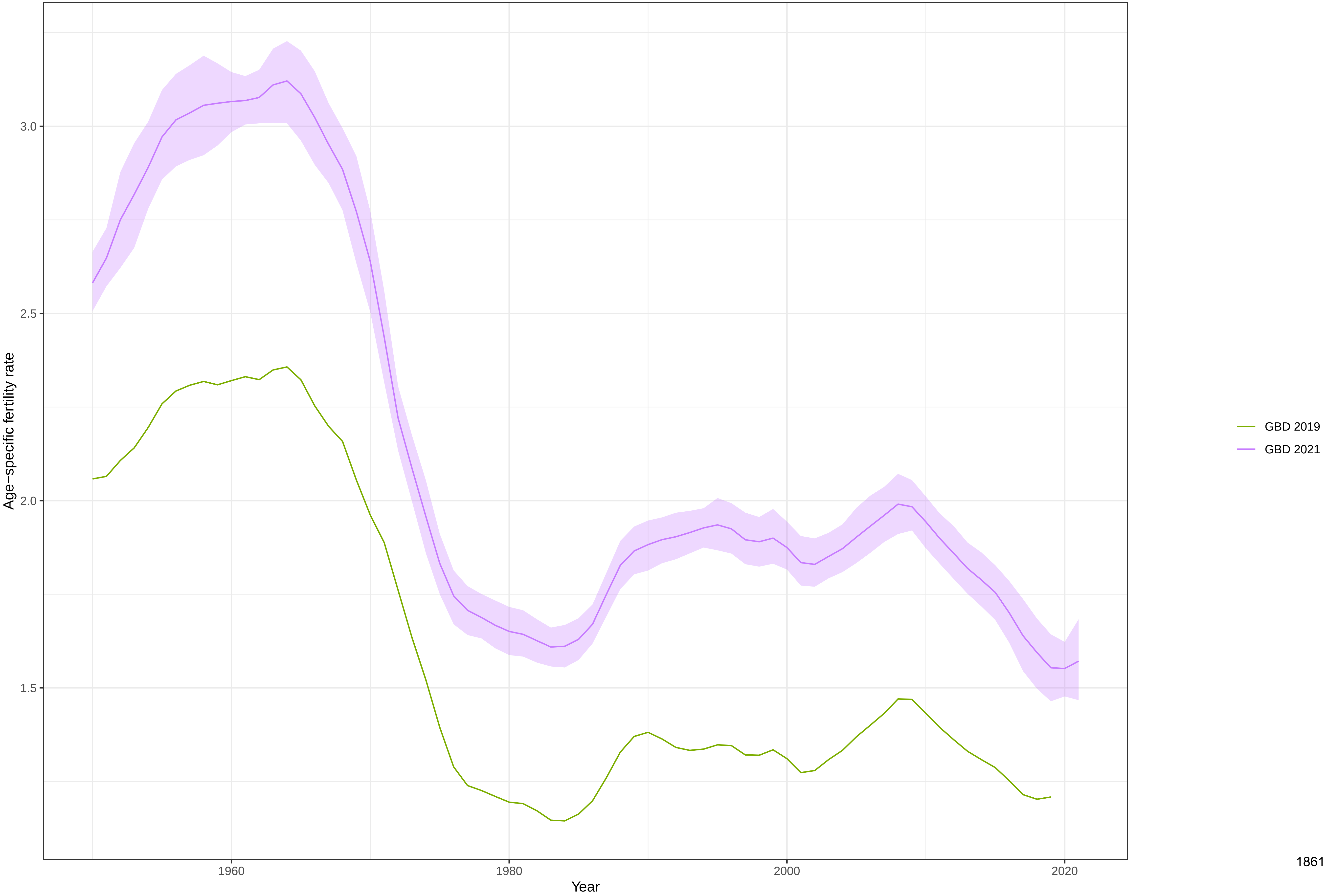

Age−specific fertility fitted trend lines, 1950−2021: Vestfold og Telemark, Ages: 10−14
Data points enclosed with a diamond are reference sources

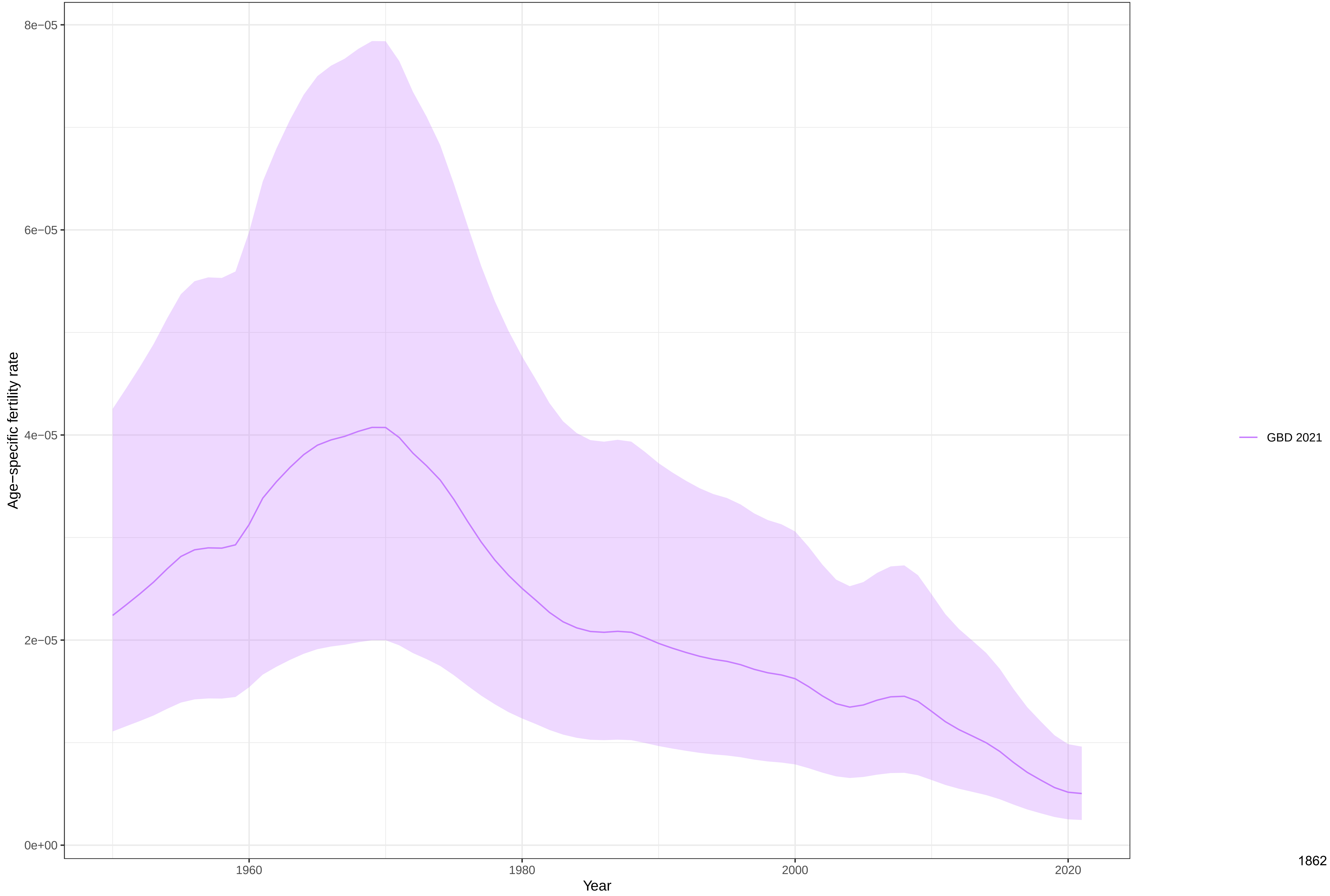

Age−specific fertility fitted trend lines, 1950−2021: Vestfold og Telemark, Ages: 15−19
Data points enclosed with a diamond are reference sources

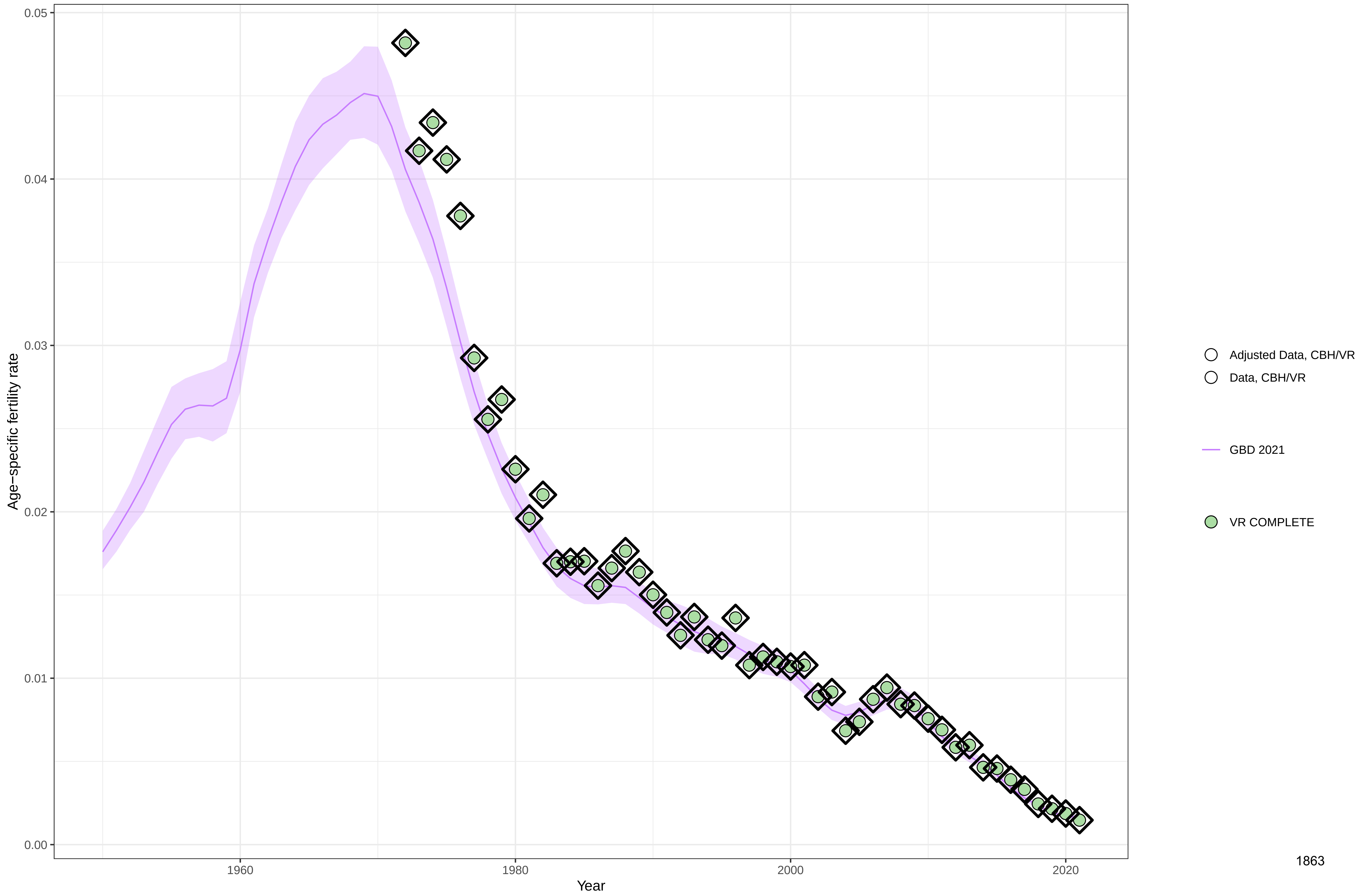

Age−specific fertility fitted trend lines, 1950−2021: Vestfold og Telemark, Ages: 20−24
Data points enclosed with a diamond are reference sources

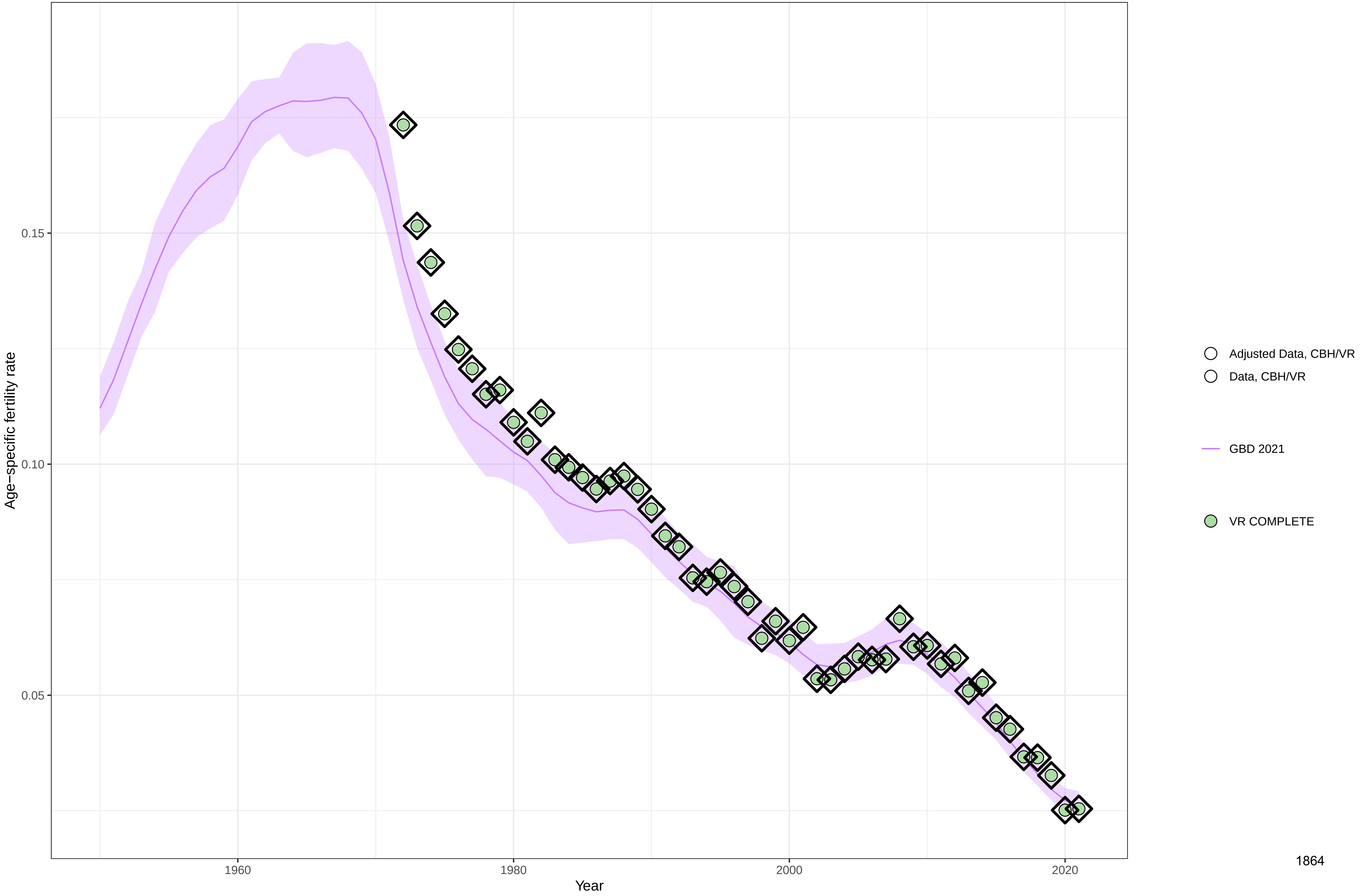

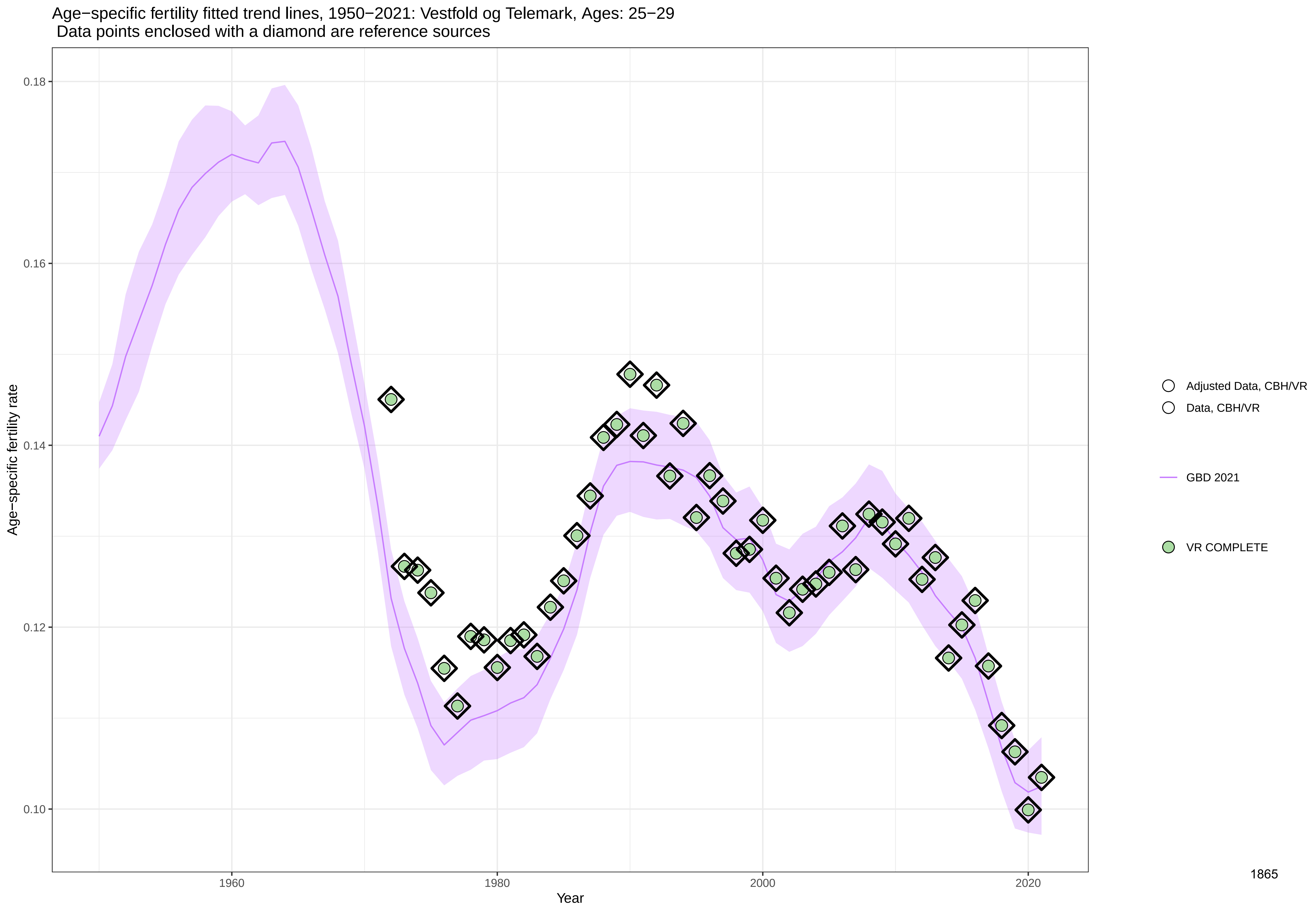
Age−specific fertility fitted trend lines, 1950−2021: Vestfold og Telemark, Ages: 25−29
Data points enclosed with a diamond are reference sources
Age−specific fertility rate
Year
0.18
0.16
0.14
0.12
0.10
1960
1980
2000
2020
Adjusted Data, CBH/VR
Data, CBH/VR
GBD 2021
VR COMPLETE

Age−specific fertility fitted trend lines, 1950−2021: Vestfold og Telemark, Ages: 30−34
Data points enclosed with a diamond are reference sources

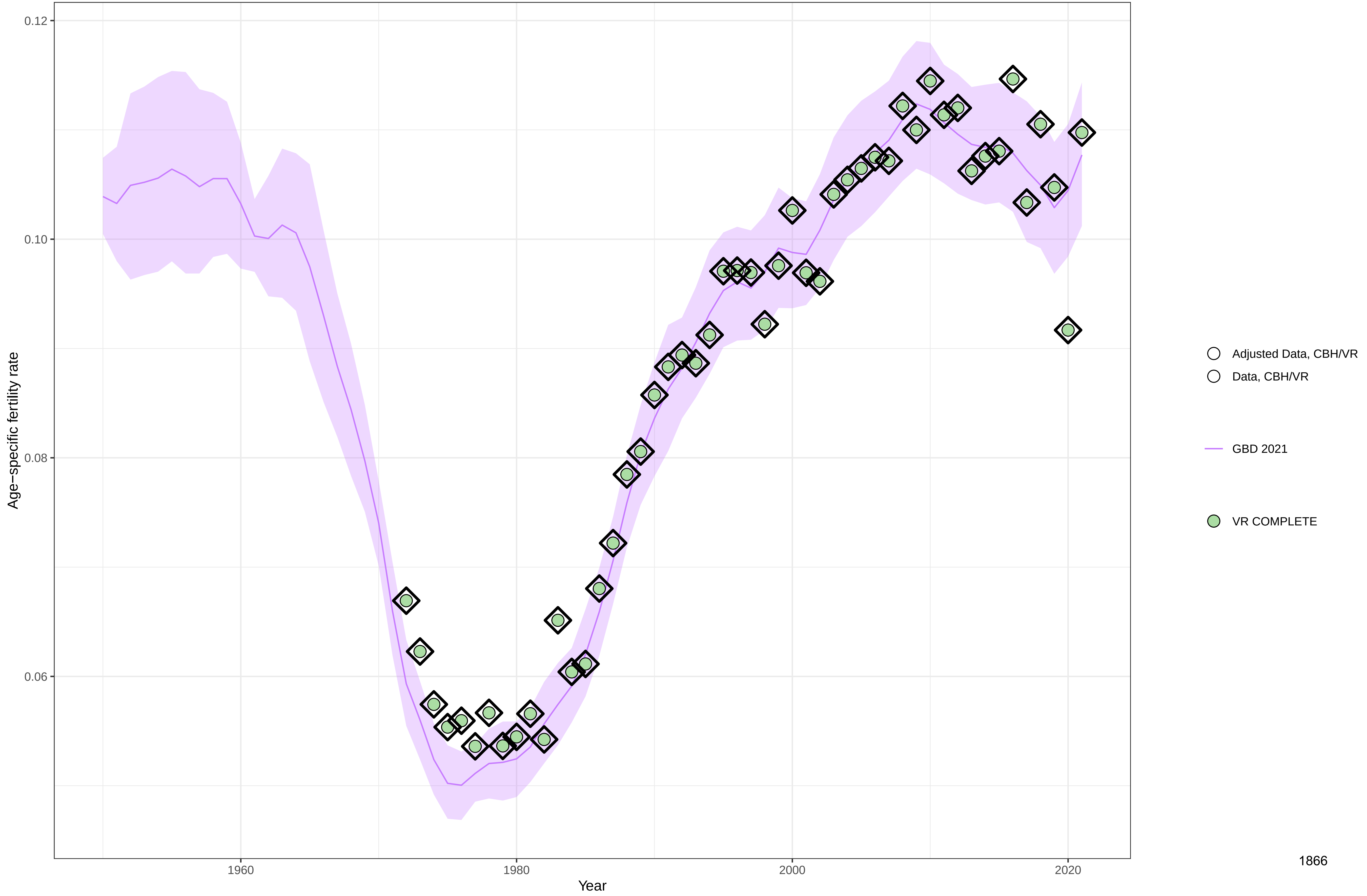

Age−specific fertility fitted trend lines, 1950−2021: Vestfold og Telemark, Ages: 35−39
Data points enclosed with a diamond are reference sources

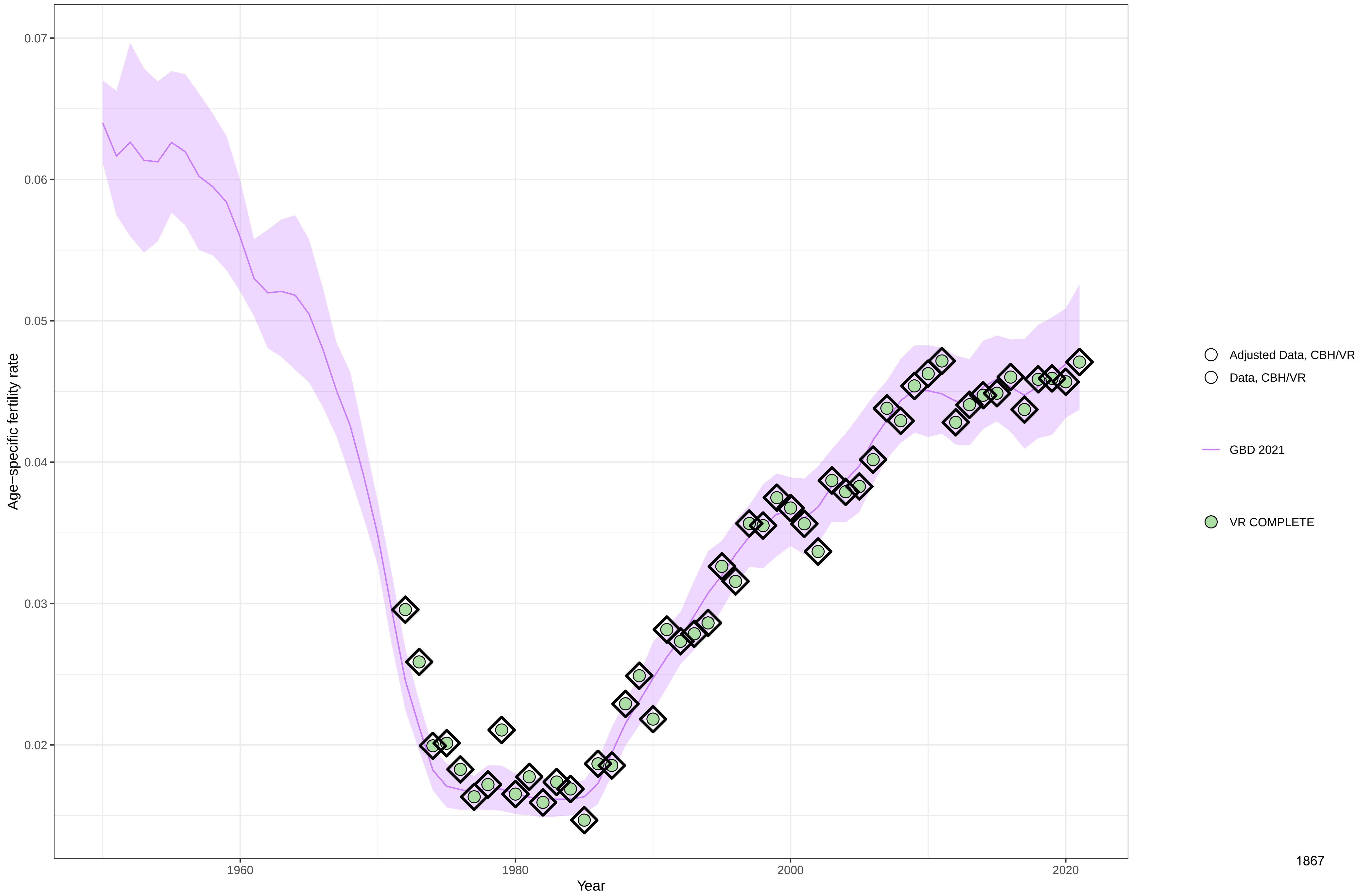

Age−specific fertility fitted trend lines, 1950−2021: Vestfold og Telemark, Ages: 40−44
Data points enclosed with a diamond are reference sources

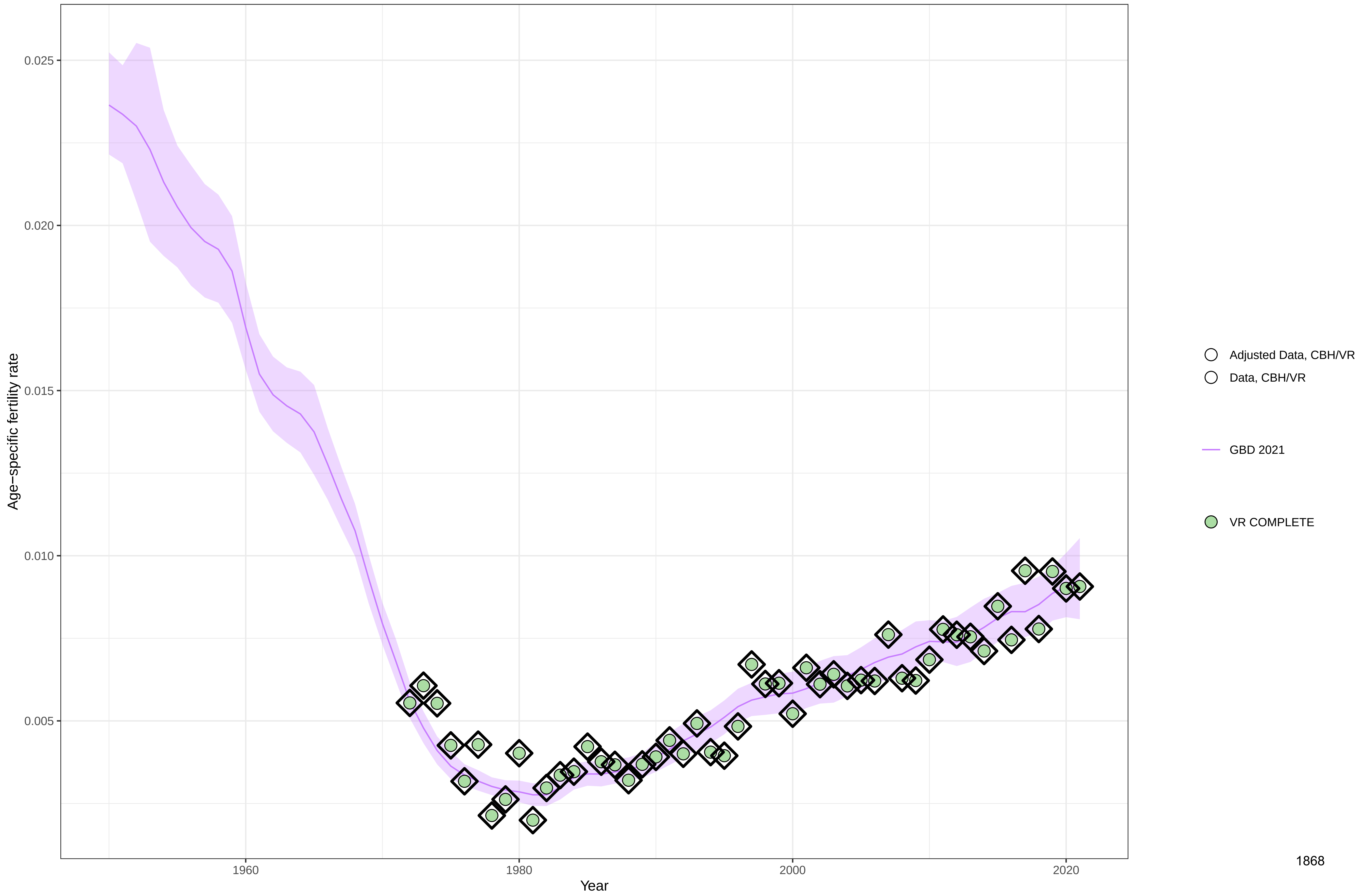

Age−specific fertility fitted trend lines, 1950−2021: Vestfold og Telemark, Ages: 45−49
Data points enclosed with a diamond are reference sources

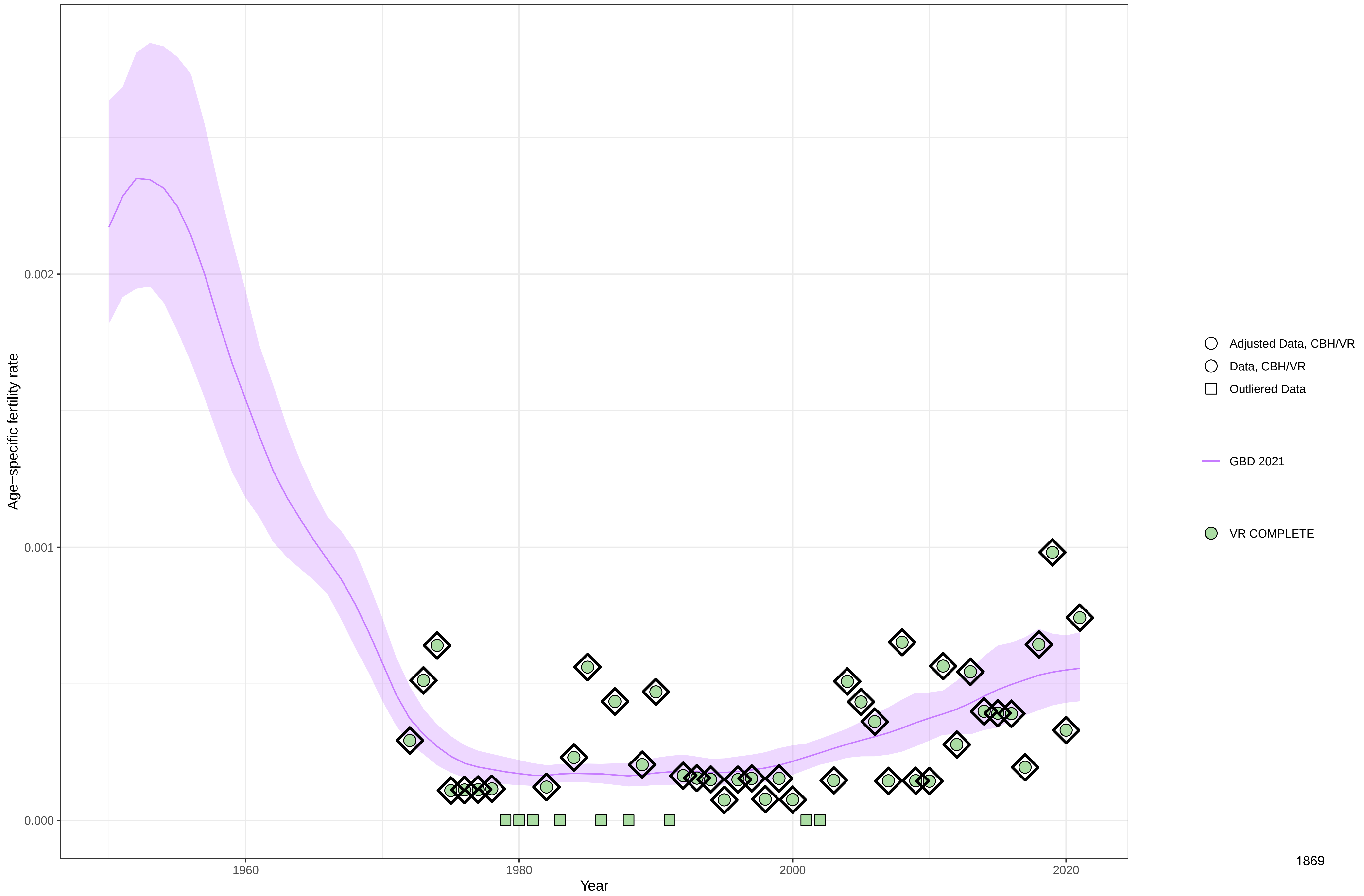

Age−specific fertility fitted trend lines, 1950−2021: Vestfold og Telemark, Ages: 50−54
Data points enclosed with a diamond are reference sources

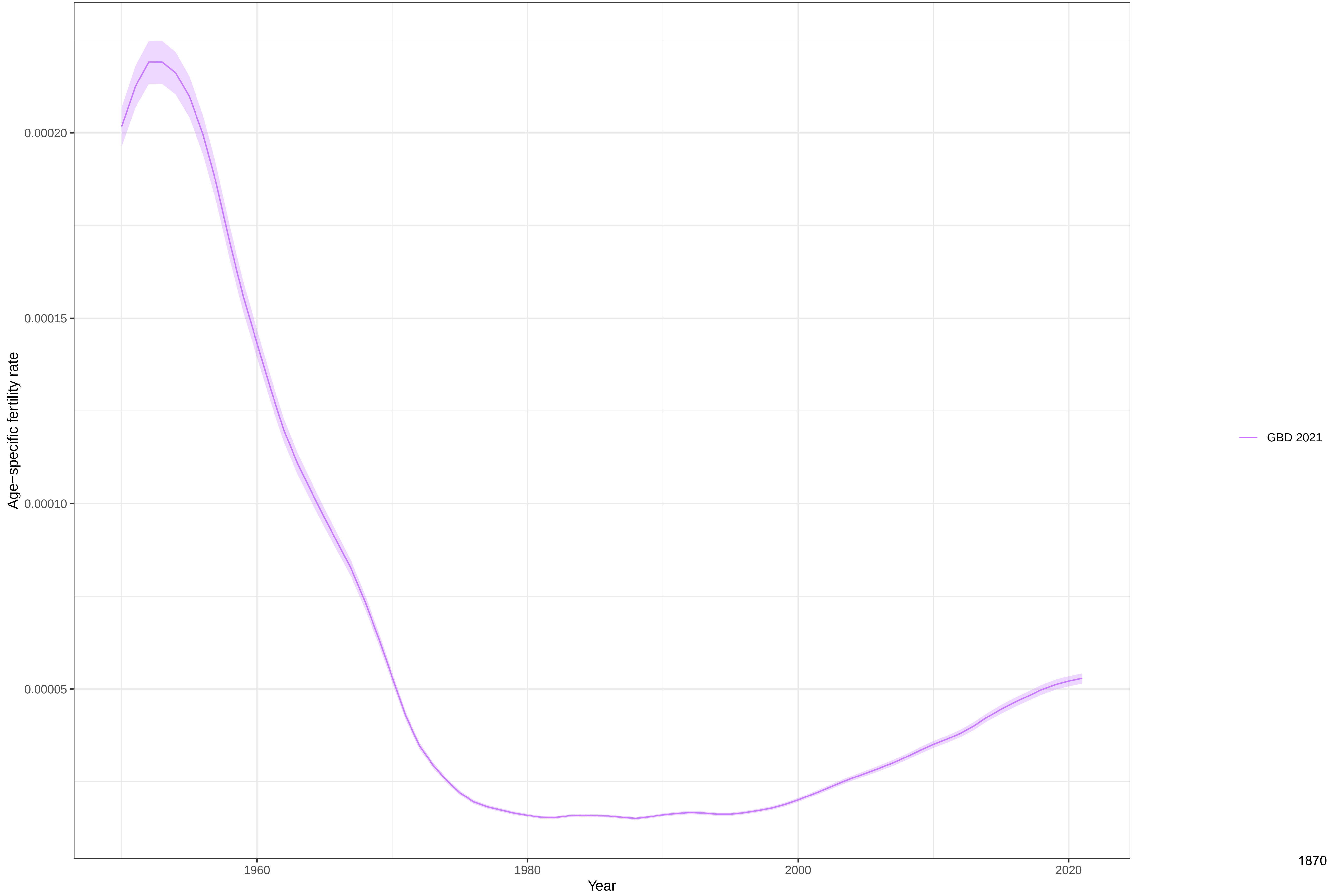

Total fertility rate fitted trend lines, 1950−2021: Vestfold og Telemark TFR
Data points enclosed with a diamond are reference sources

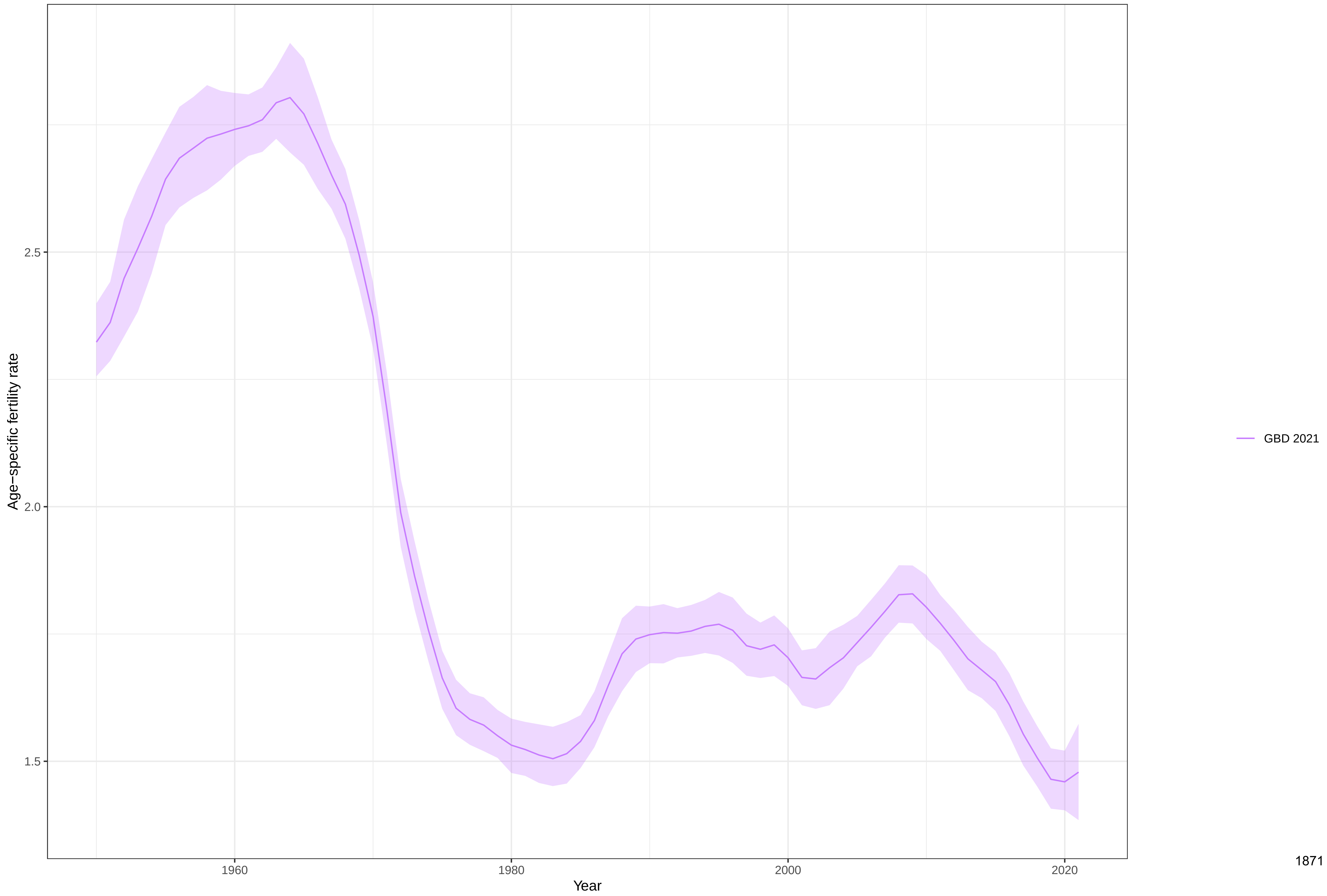

Age−specific fertility fitted trend lines, 1950−2021: Vestland, Ages: 10−14
Data points enclosed with a diamond are reference sources

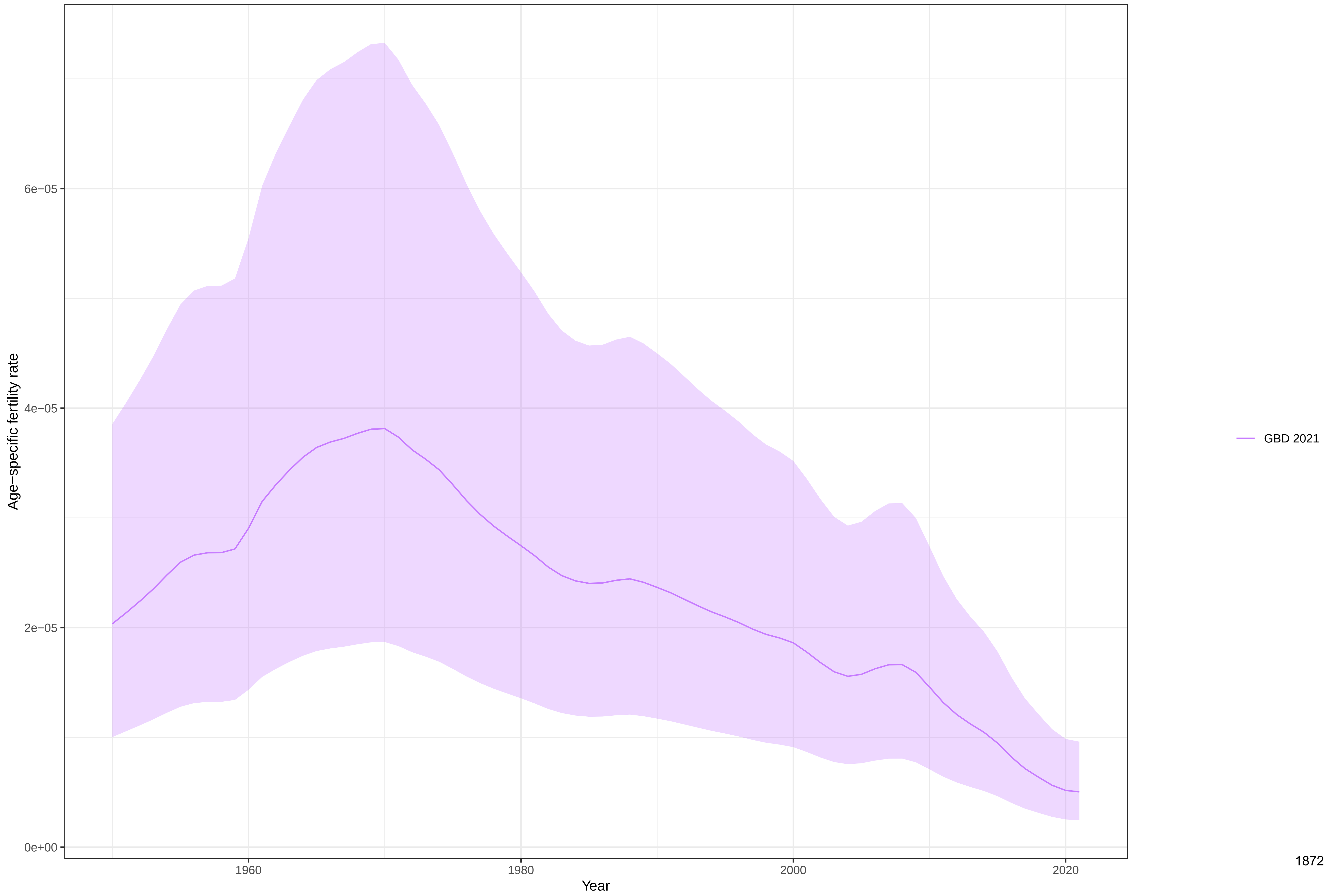

Age−specific fertility fitted trend lines, 1950−2021: Vestland, Ages: 15−19
Data points enclosed with a diamond are reference sources

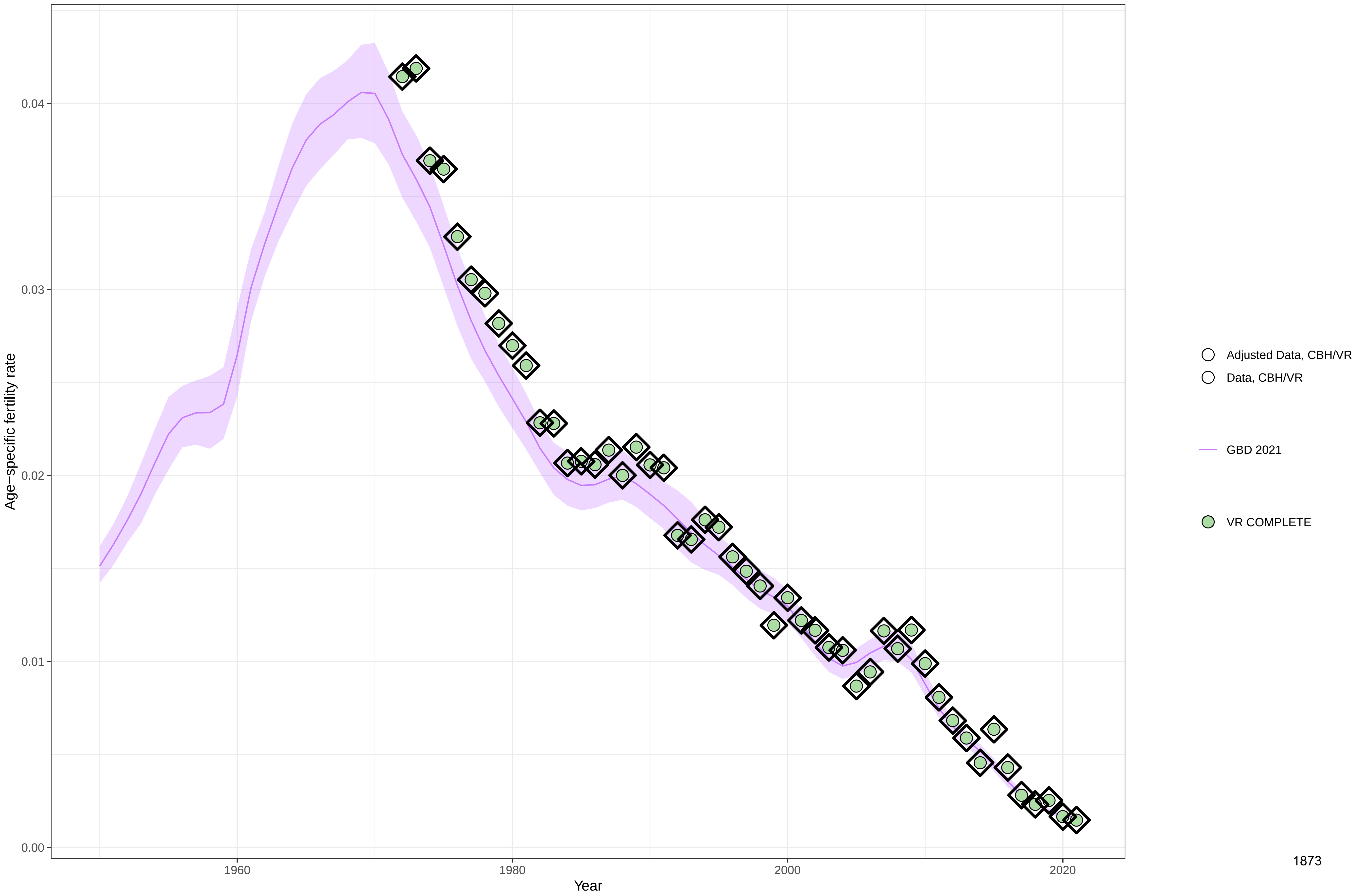

1873

Age−specific fertility fitted trend lines, 1950−2021: Vestland, Ages: 20−24
Data points enclosed with a diamond are reference sources

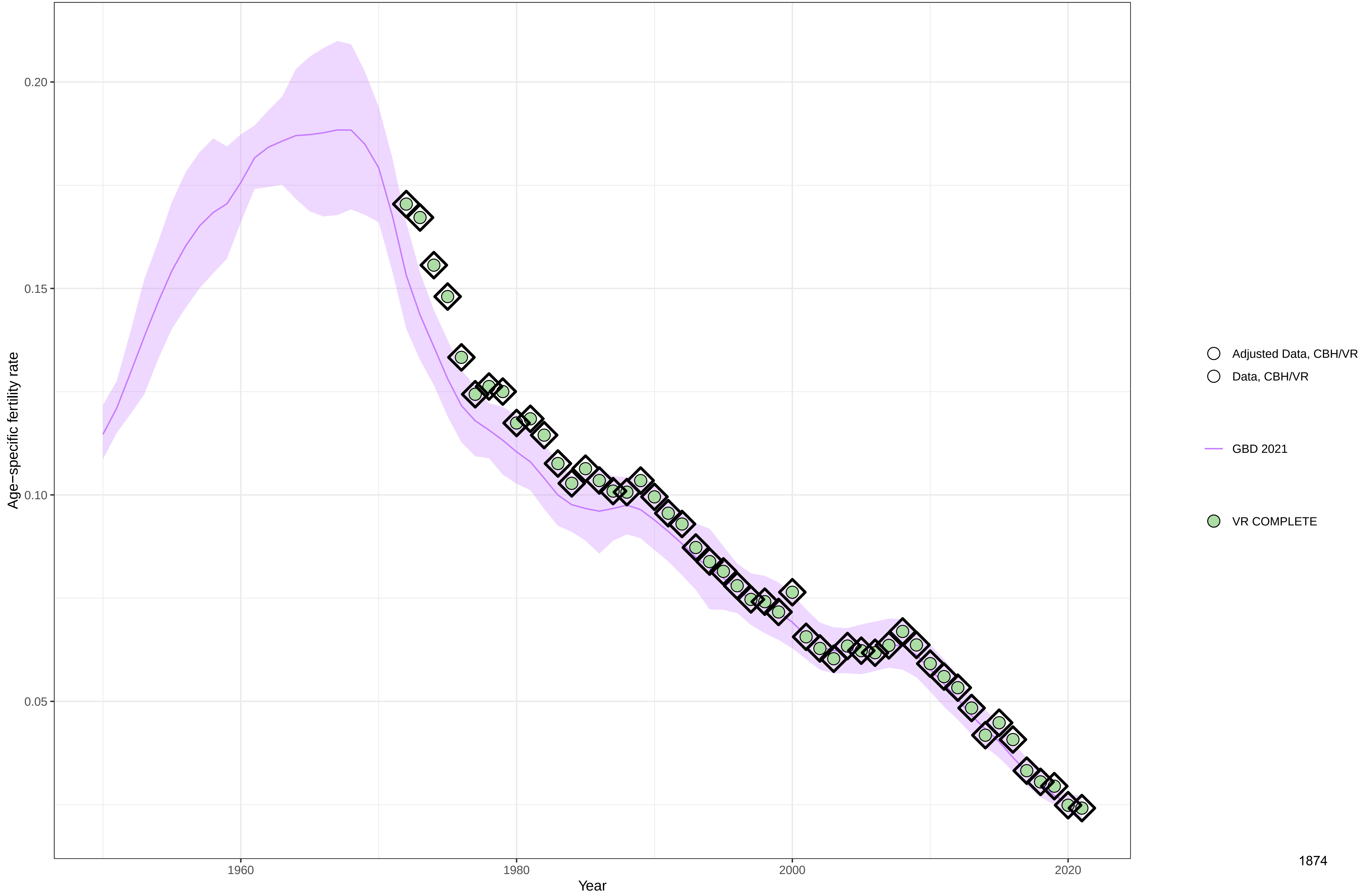

Age−specific fertility fitted trend lines, 1950−2021: Vestland, Ages: 25−29
Data points enclosed with a diamond are reference sources

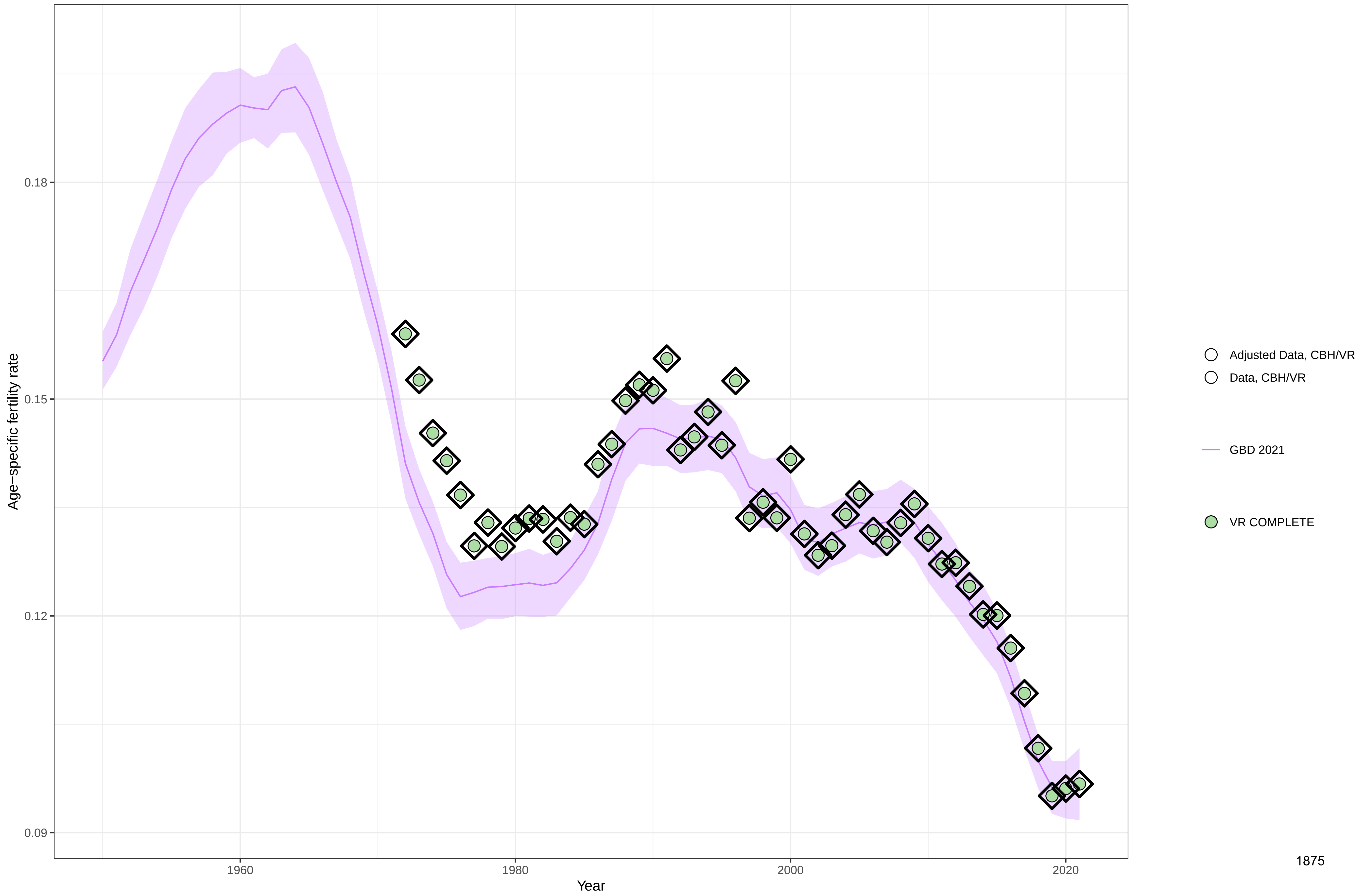

1875

Age−specific fertility fitted trend lines, 1950−2021: Vestland, Ages: 30−34
Data points enclosed with a diamond are reference sources

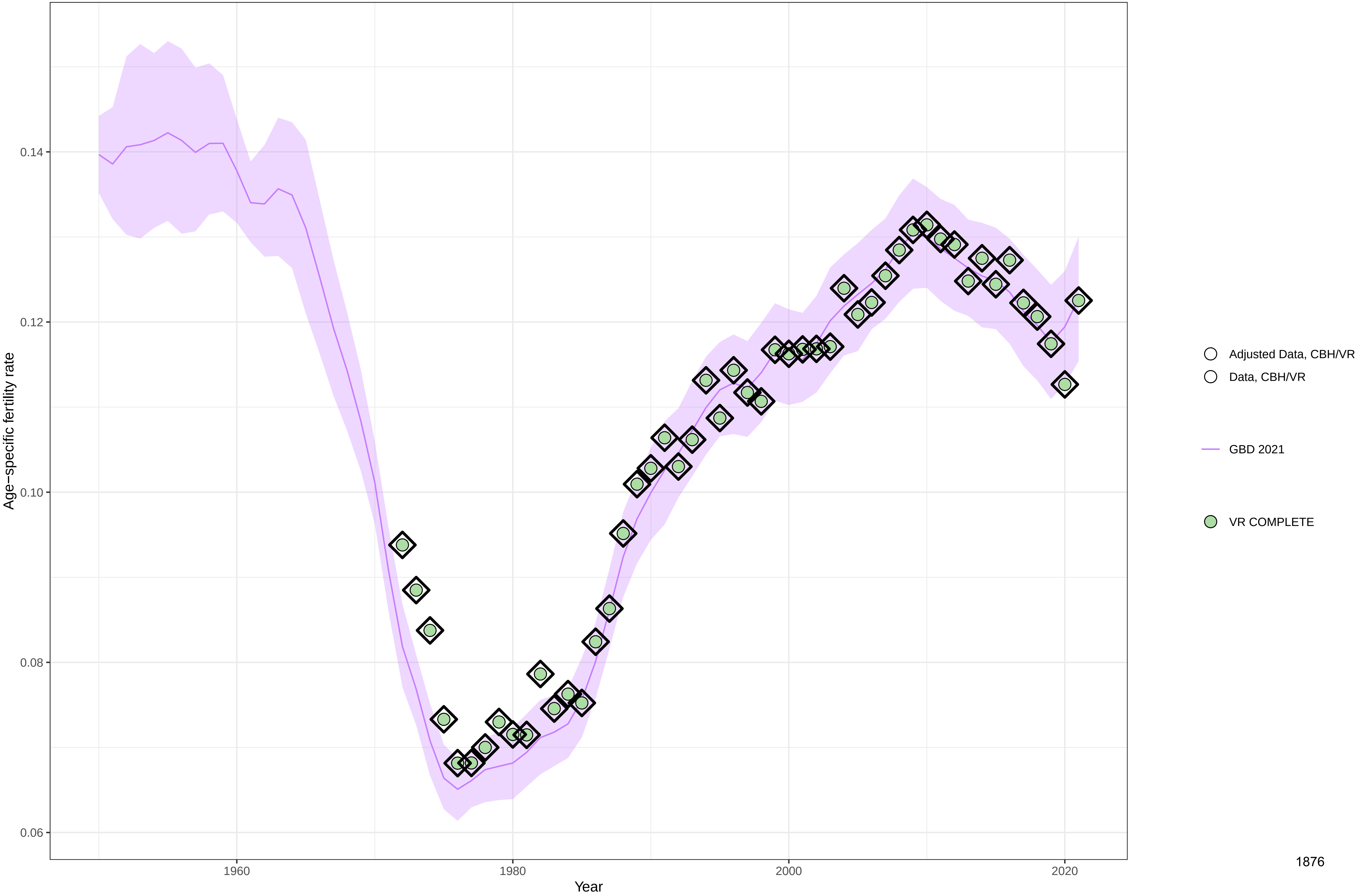

Age−specific fertility fitted trend lines, 1950−2021: Vestland, Ages: 35−39
Data points enclosed with a diamond are reference sources

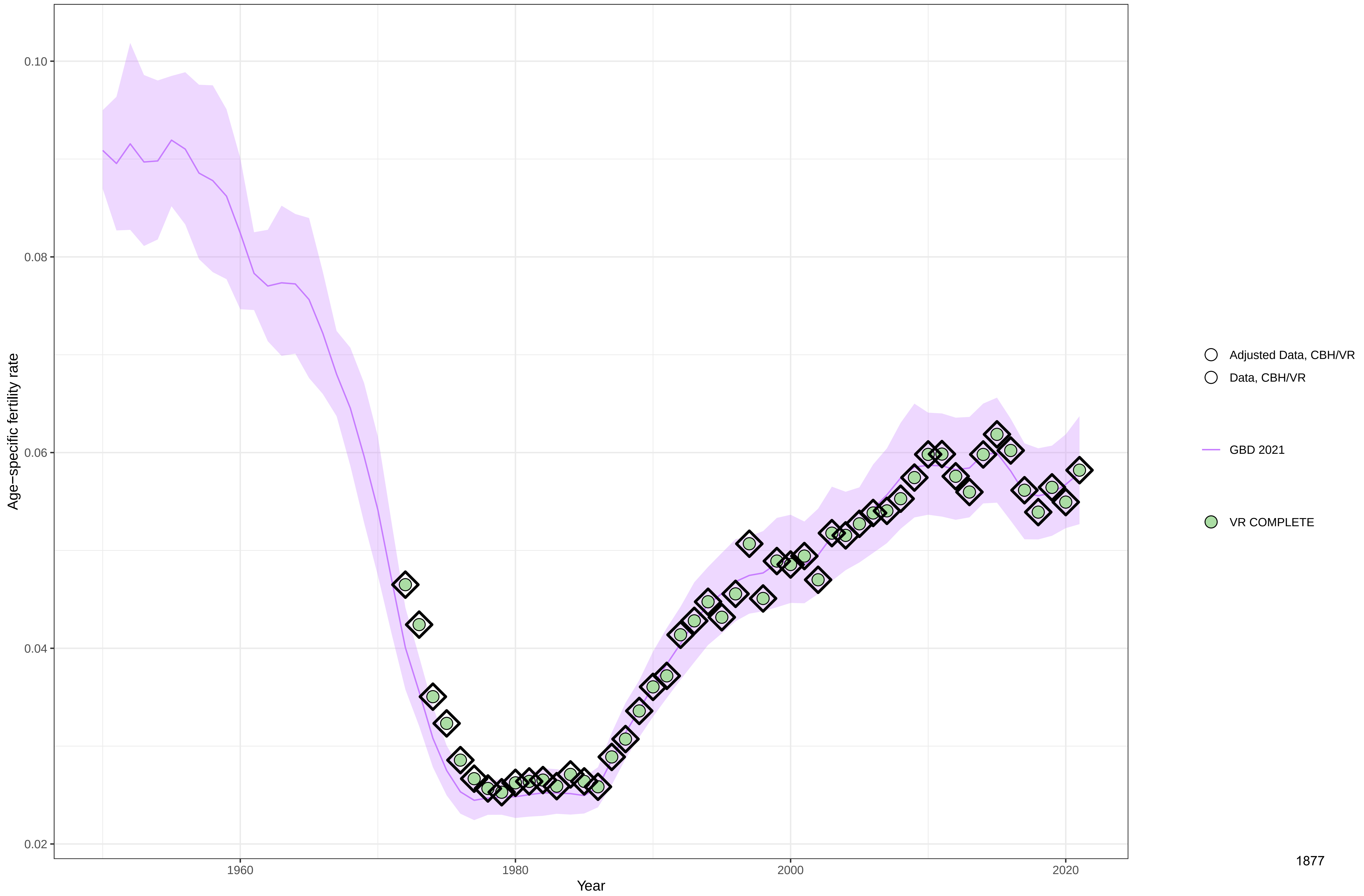

Age−specific fertility fitted trend lines, 1950−2021: Vestland, Ages: 40−44
Data points enclosed with a diamond are reference sources

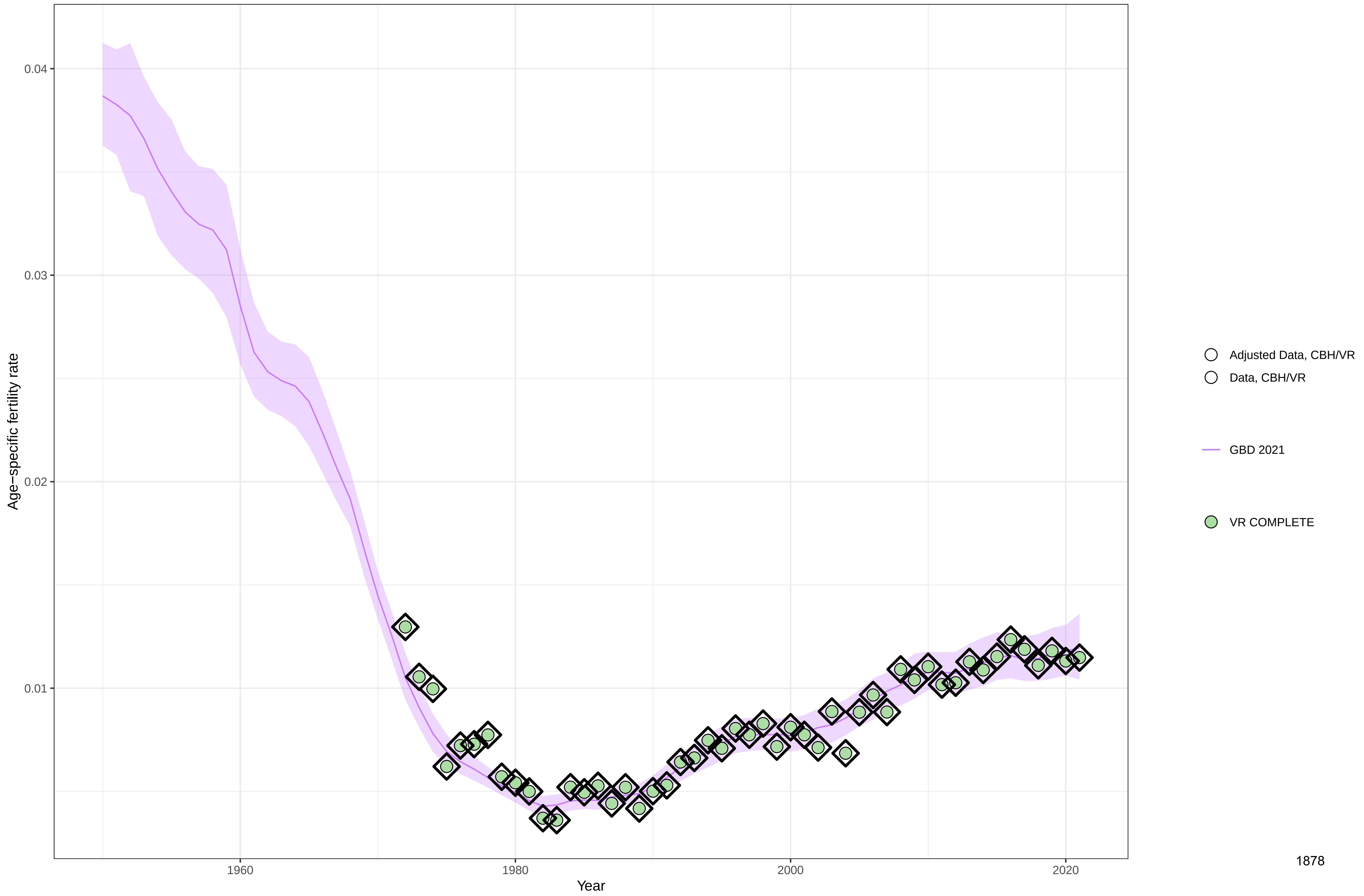

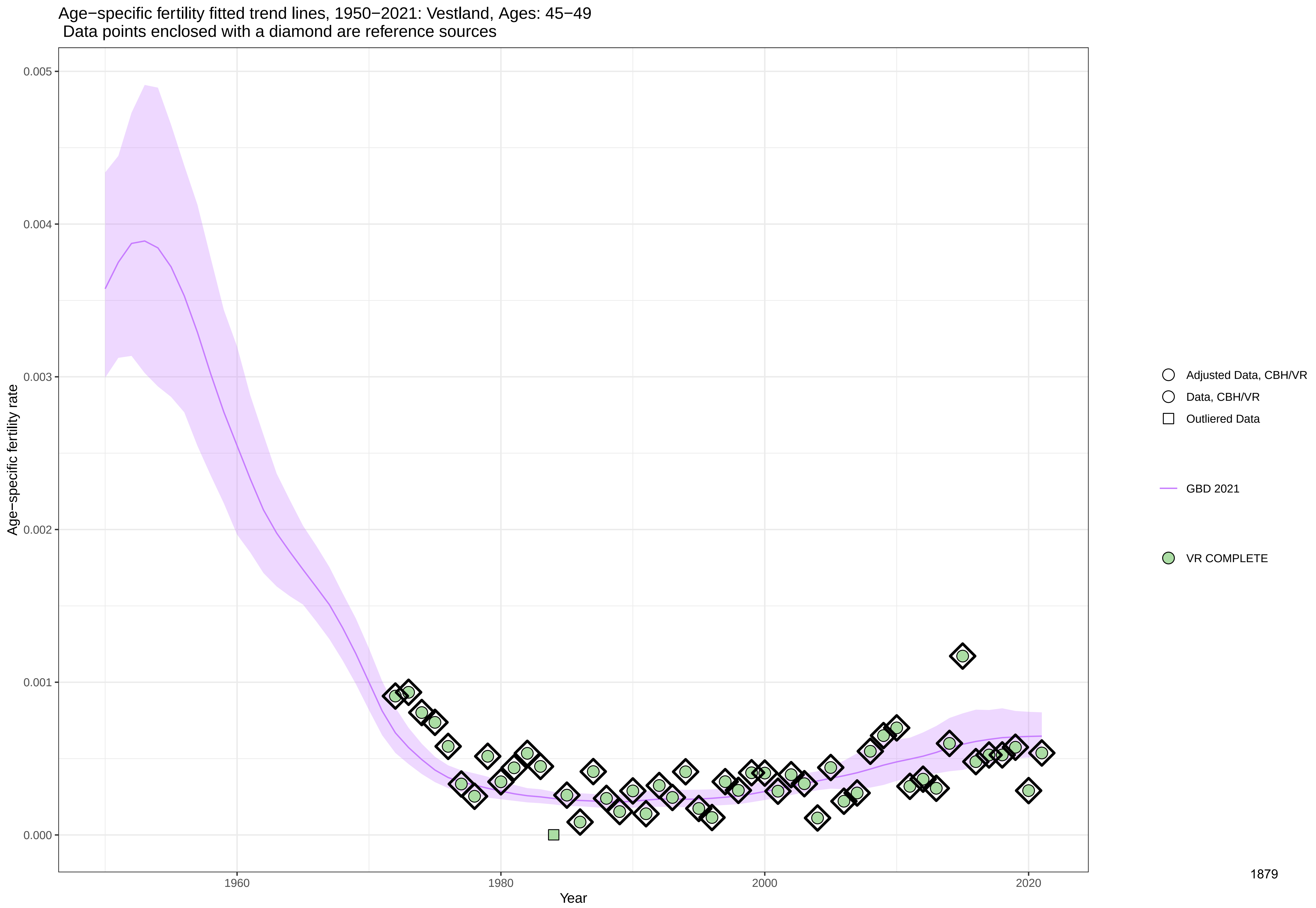

Age−specific fertility fitted trend lines, 1950−2021: Vestland, Ages: 45−49
Data points enclosed with a diamond are reference sources
0.005
0.004
0.003
0.002
0.001
0.000
Age−specific fertility rate
1960
1980
2000
2020
Year
Adjusted Data, CBH/VR
Data, CBH/VR
Outliered Data
GBD 2021
VR COMPLETE

Age−specific fertility fitted trend lines, 1950−2021: Vestland, Ages: 50−54
Data points enclosed with a diamond are reference sources

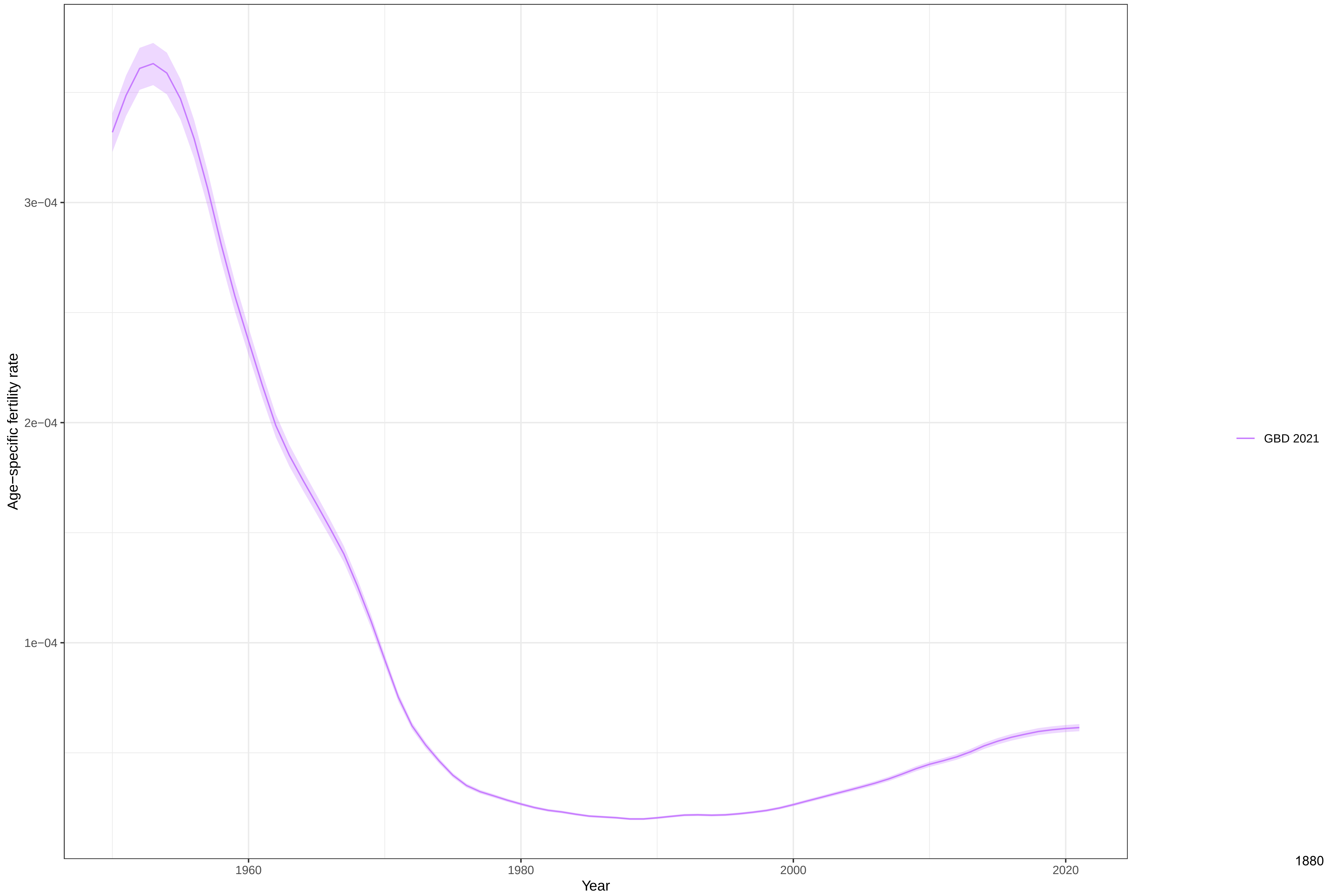

Total fertility rate fitted trend lines, 1950−2021: Vestland TFR
Data points enclosed with a diamond are reference sources

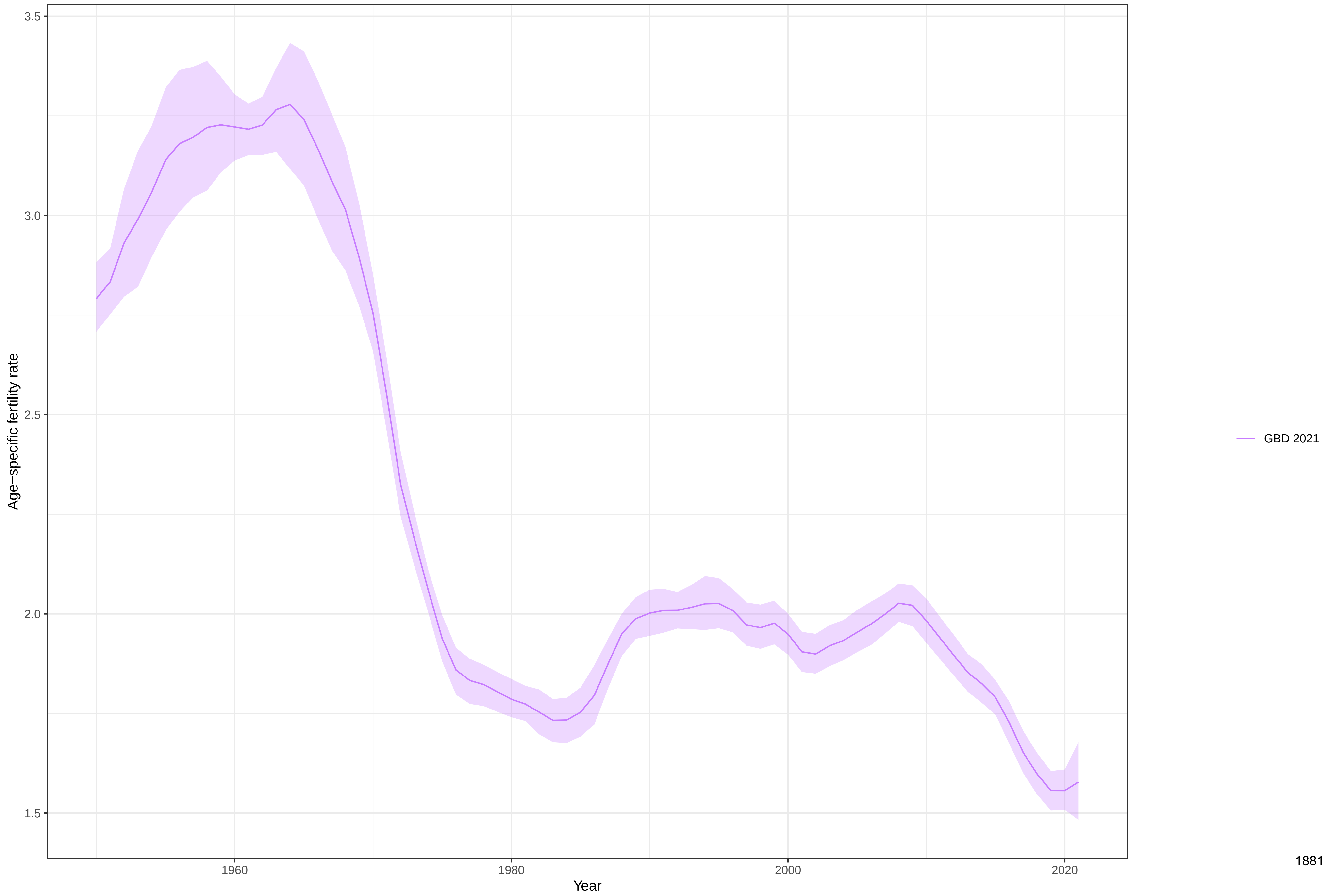

Age−specific fertility fitted trend lines, 1950−2021: Viken, Ages: 10−14
Data points enclosed with a diamond are reference sources

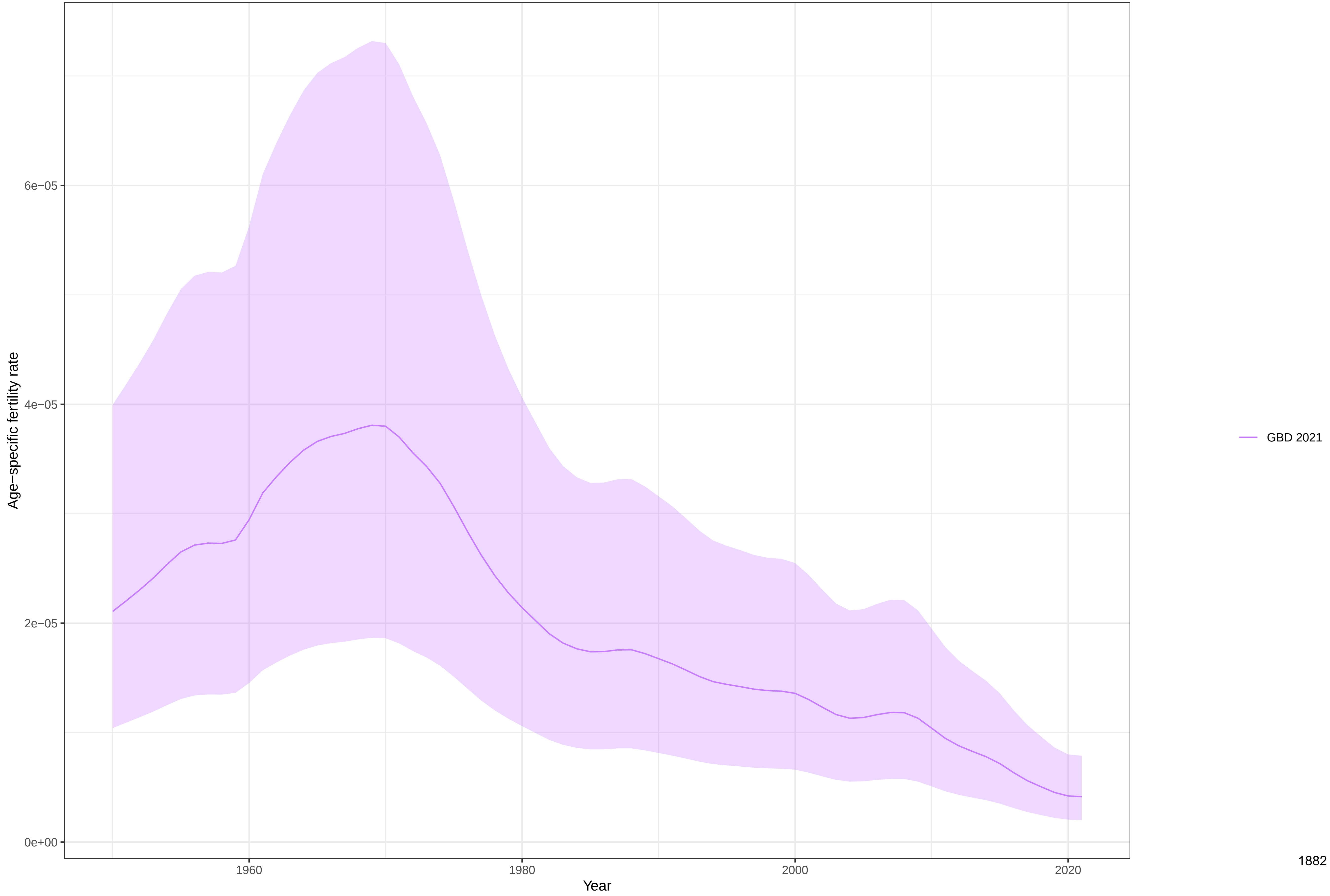

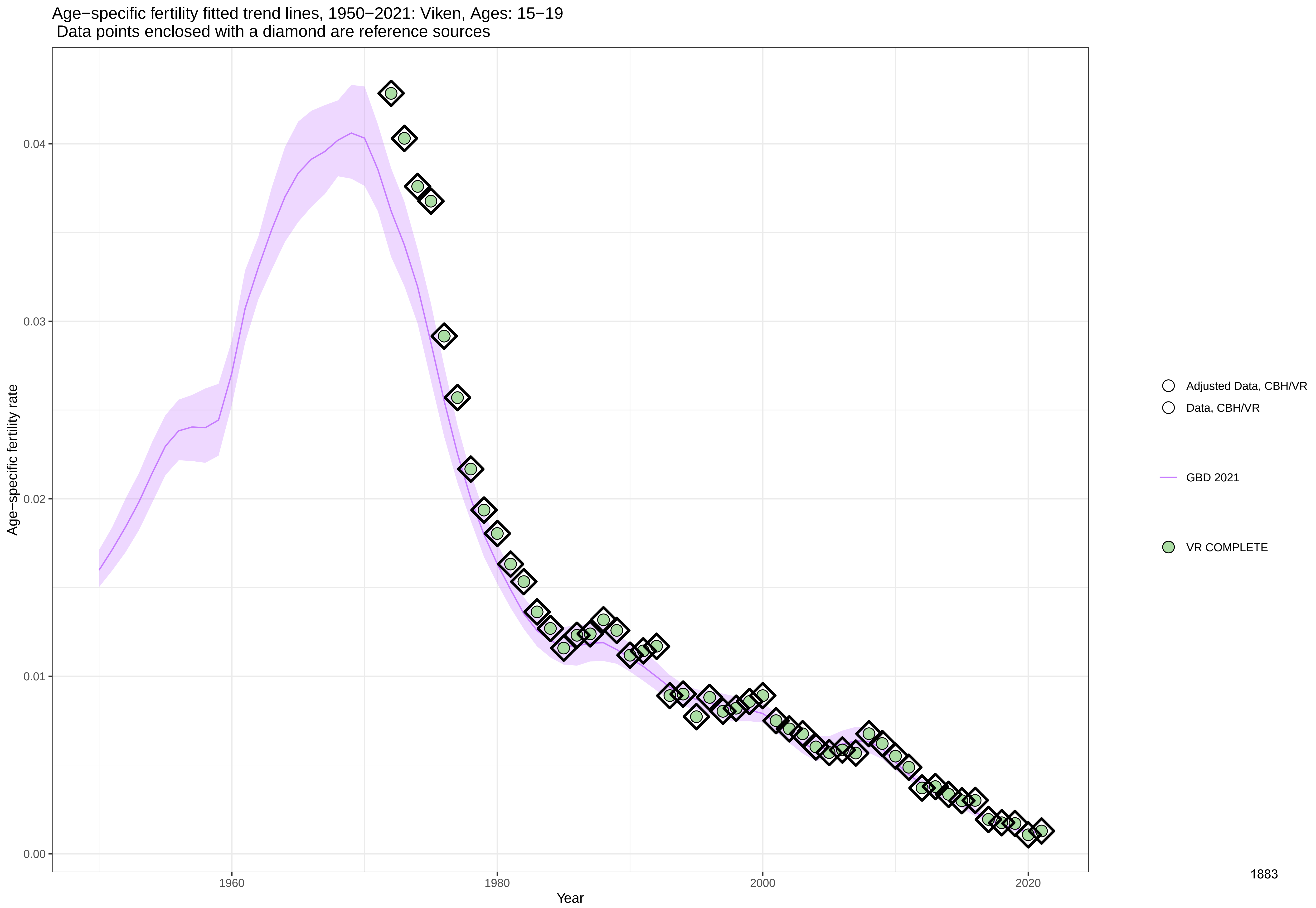

Age−specific fertility fitted trend lines, 1950−2021: Viken, Ages: 15−19
Data points enclosed with a diamond are reference sources
Age−specific fertility rate
0.00
0.01
0.02
0.03
0.04
1960
1980
2000
2020
Year
Adjusted Data, CBH/VR
Data, CBH/VR
GBD 2021
VR COMPLETE

1883

Age−specific fertility fitted trend lines, 1950−2021: Viken, Ages: 20−24
Data points enclosed with a diamond are reference sources

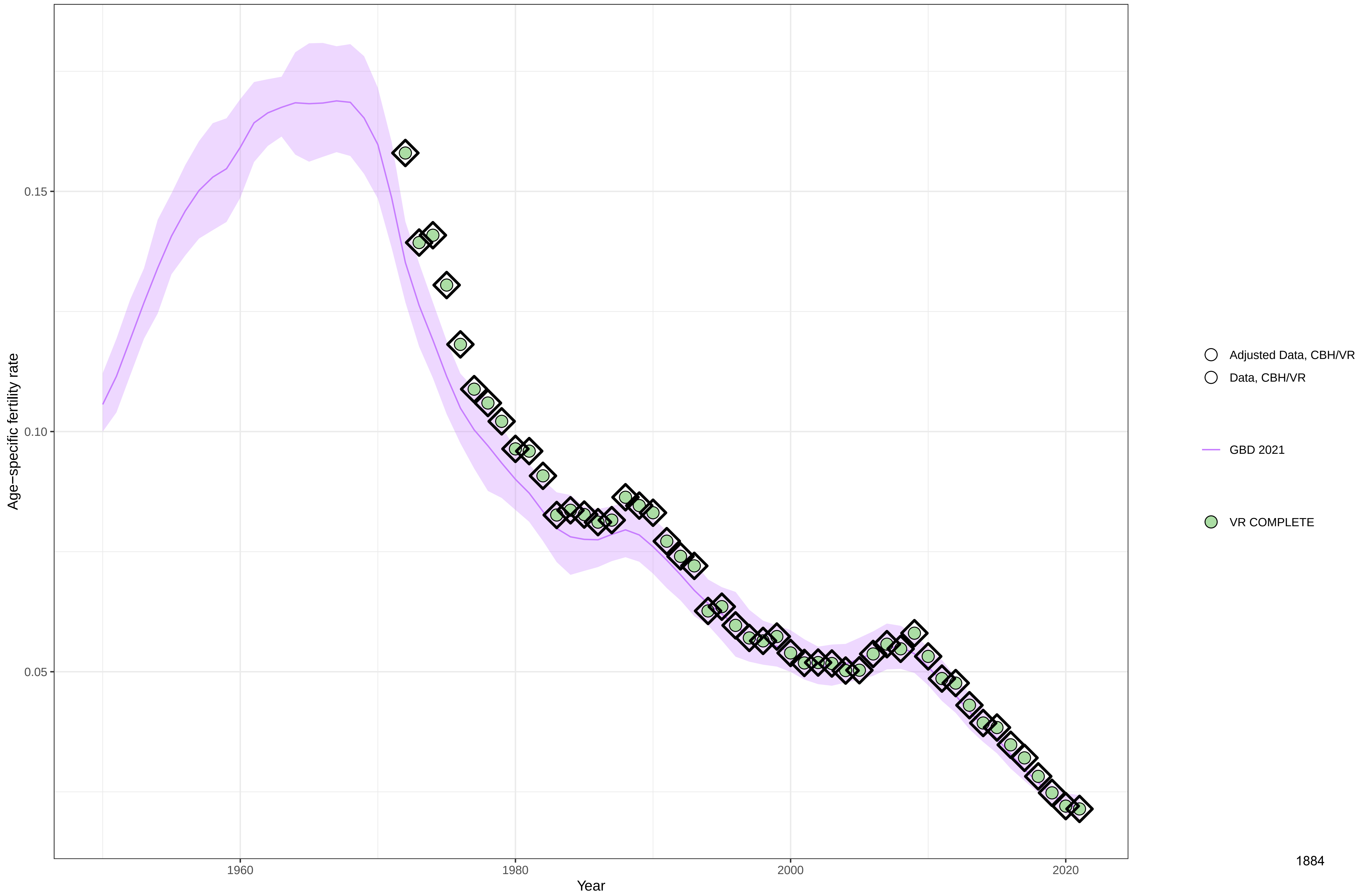

Age−specific fertility fitted trend lines, 1950−2021: Viken, Ages: 25−29
Data points enclosed with a diamond are reference sources

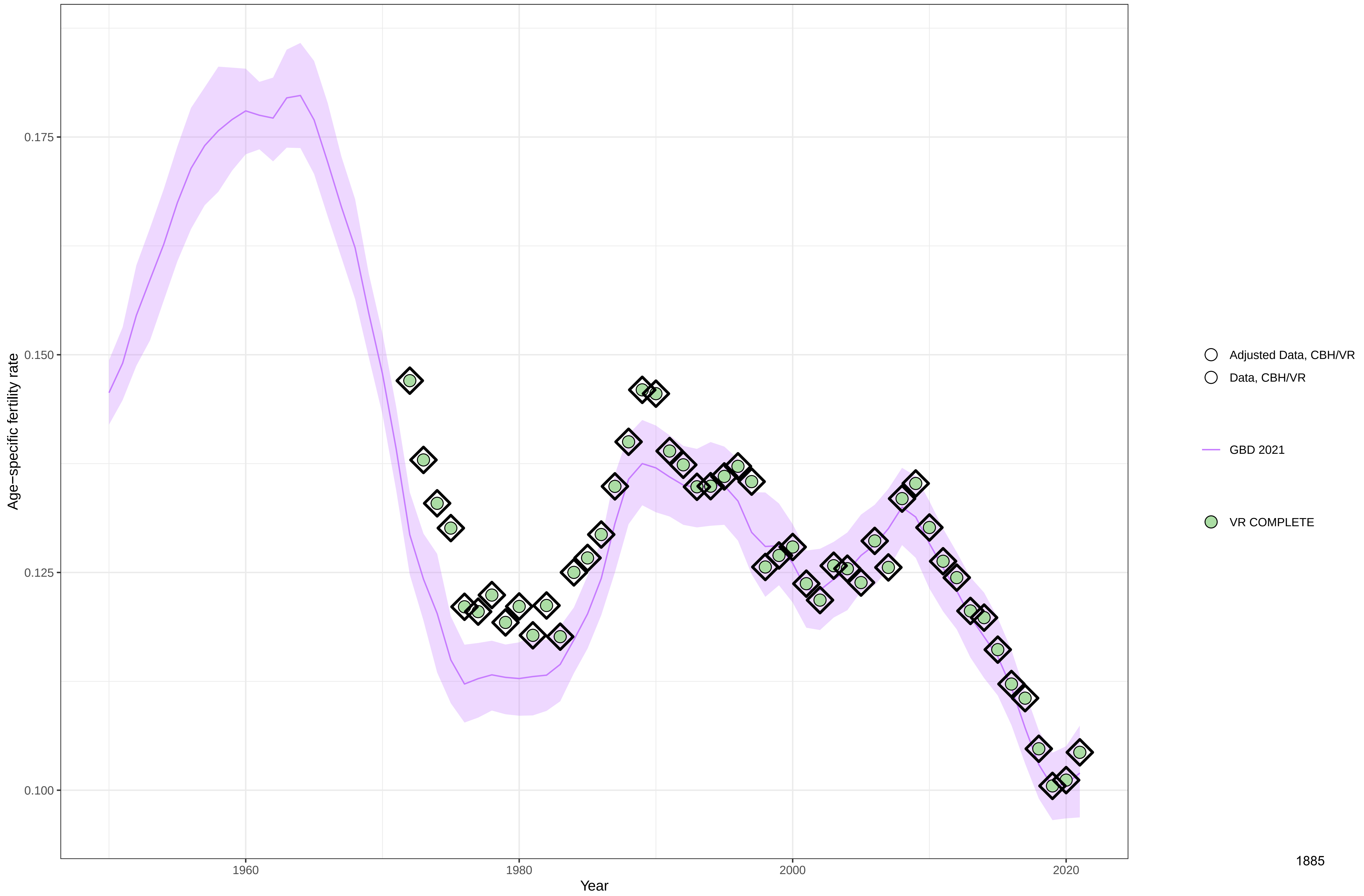

Age−specific fertility fitted trend lines, 1950−2021: Viken, Ages: 30−34
Data points enclosed with a diamond are reference sources

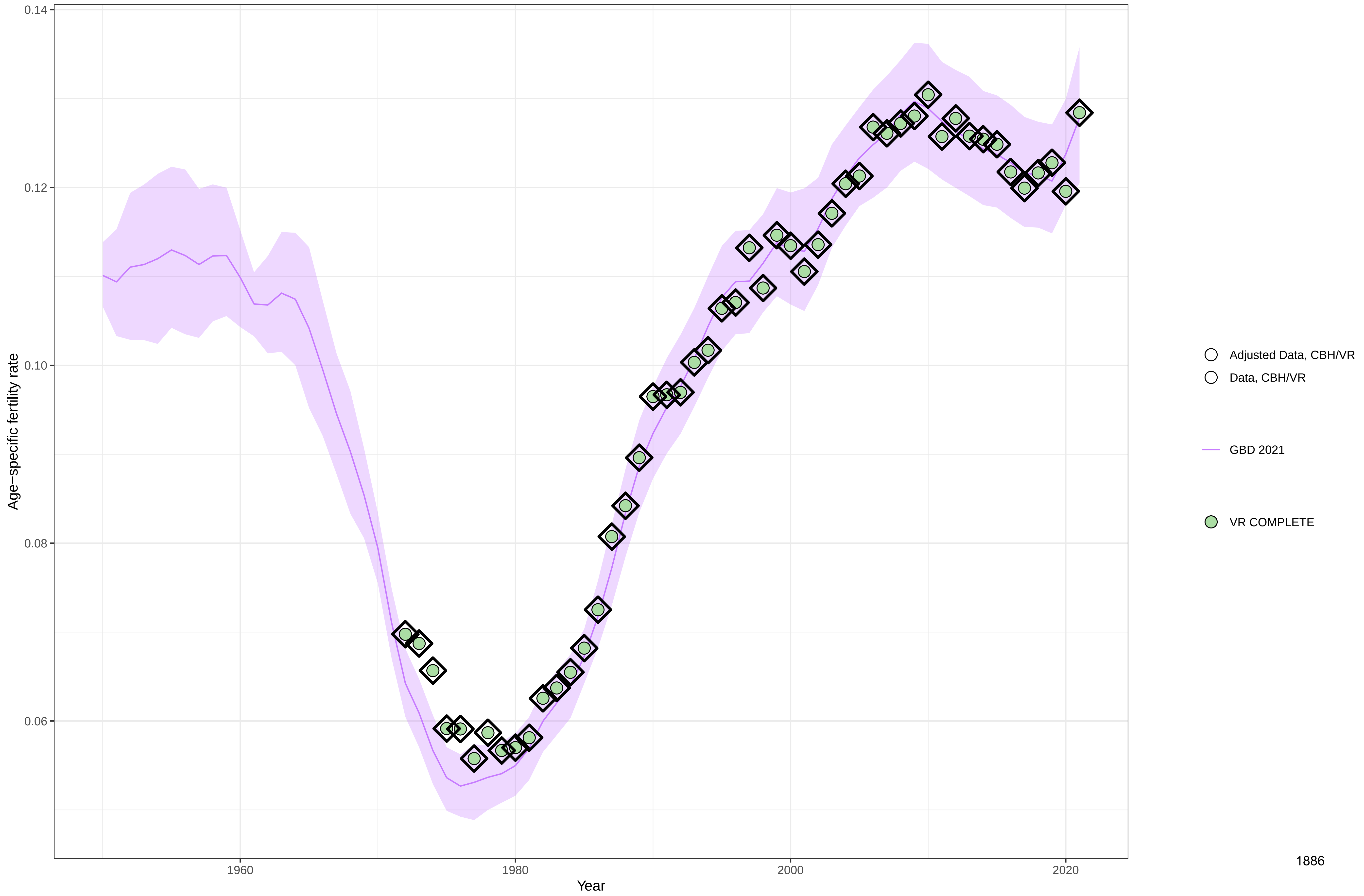

Age−specific fertility fitted trend lines, 1950−2021: Viken, Ages: 35−39
Data points enclosed with a diamond are reference sources

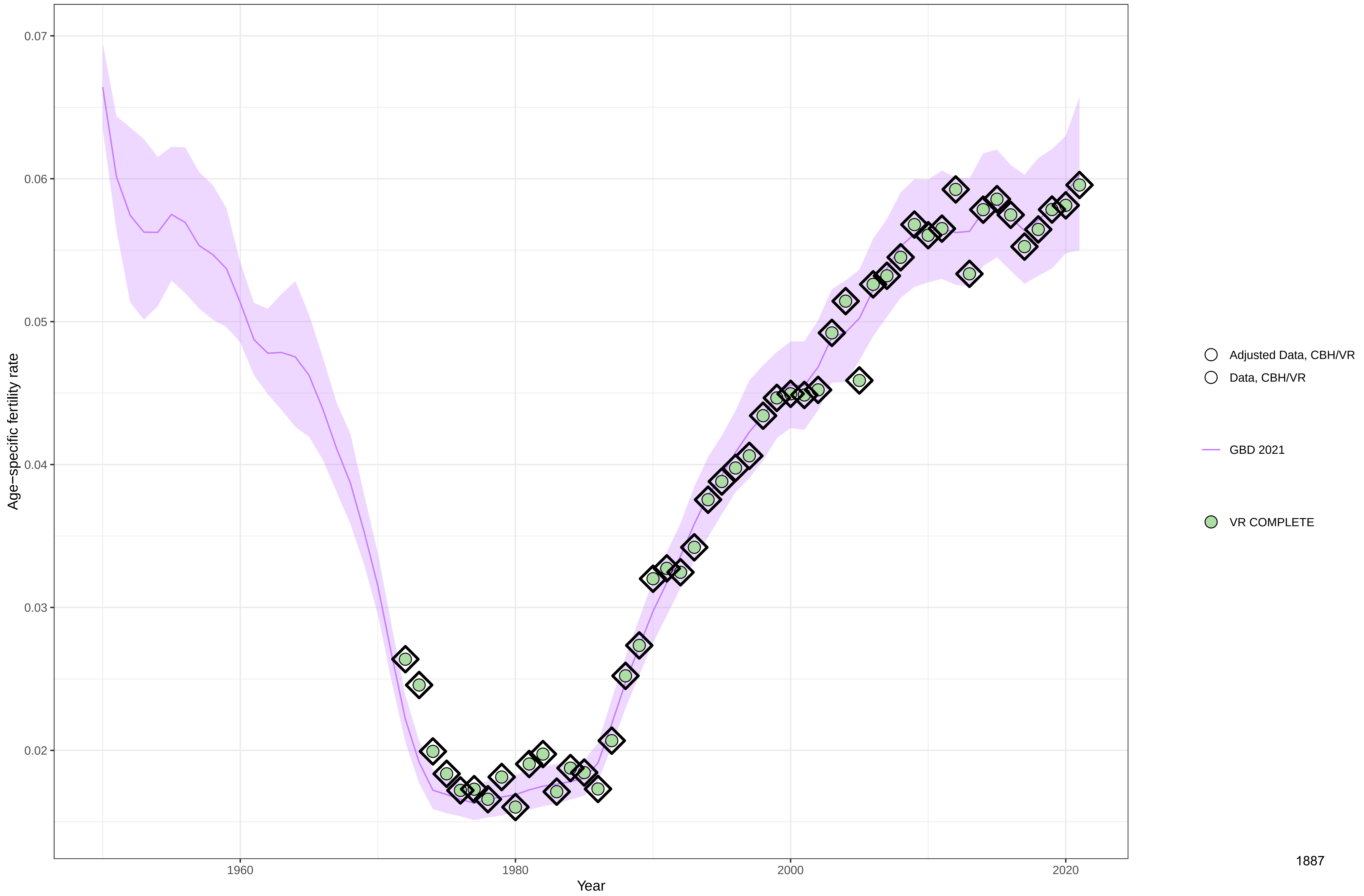

Age−specific fertility fitted trend lines, 1950−2021: Viken, Ages: 40−44
Data points enclosed with a diamond are reference sources

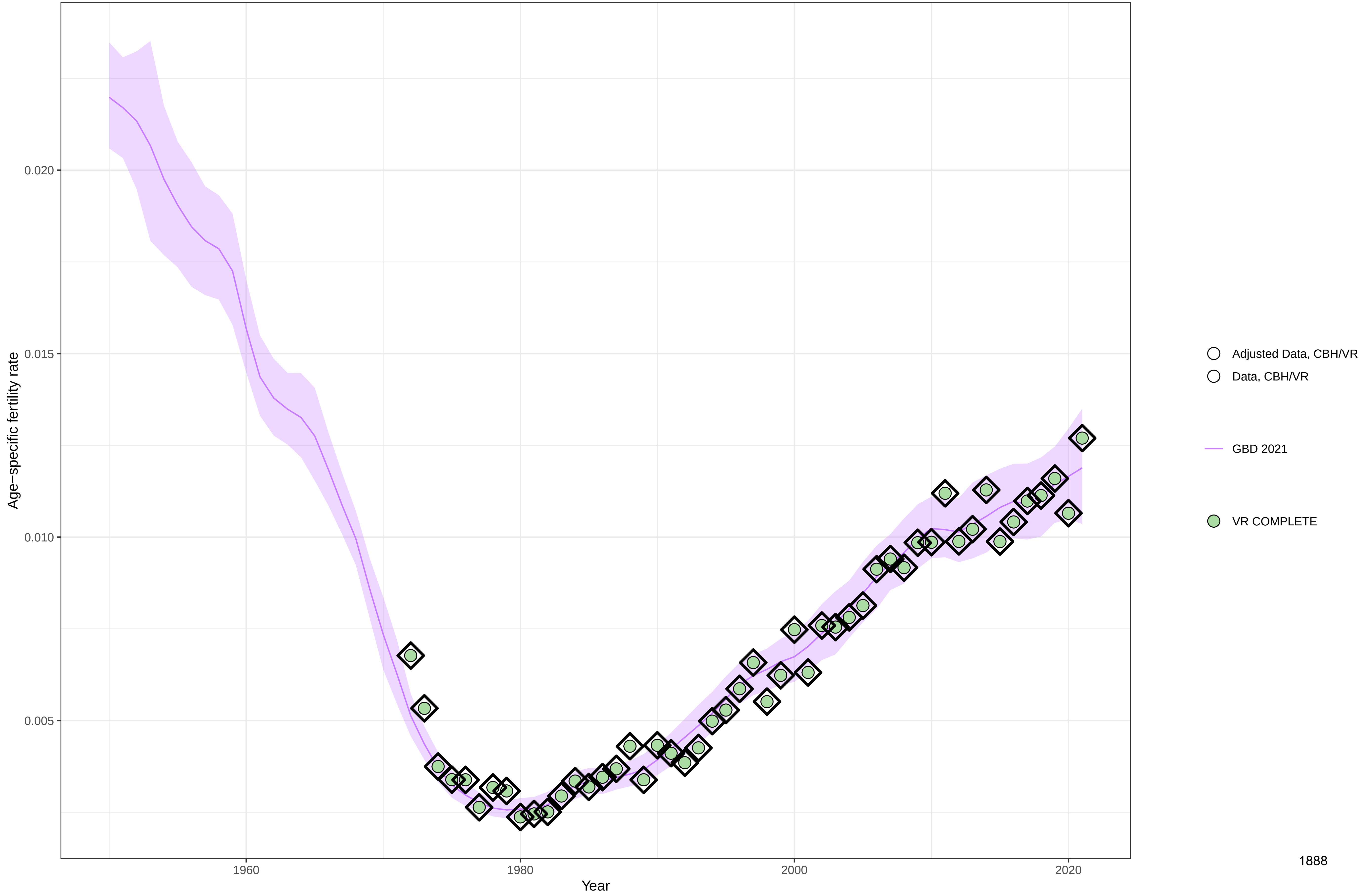

1888

Age−specific fertility fitted trend lines, 1950−2021: Viken, Ages: 45−49
Data points enclosed with a diamond are reference sources

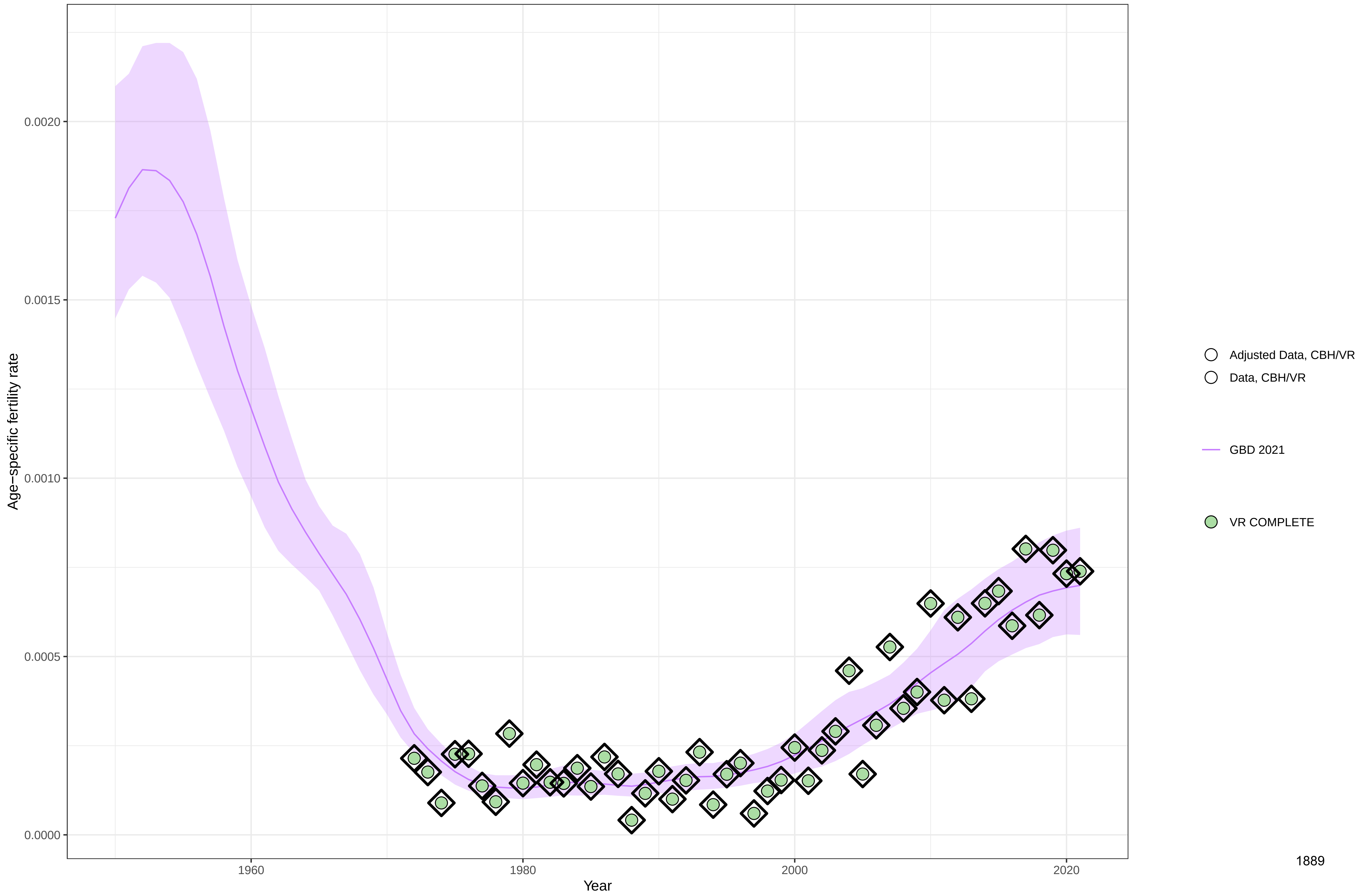

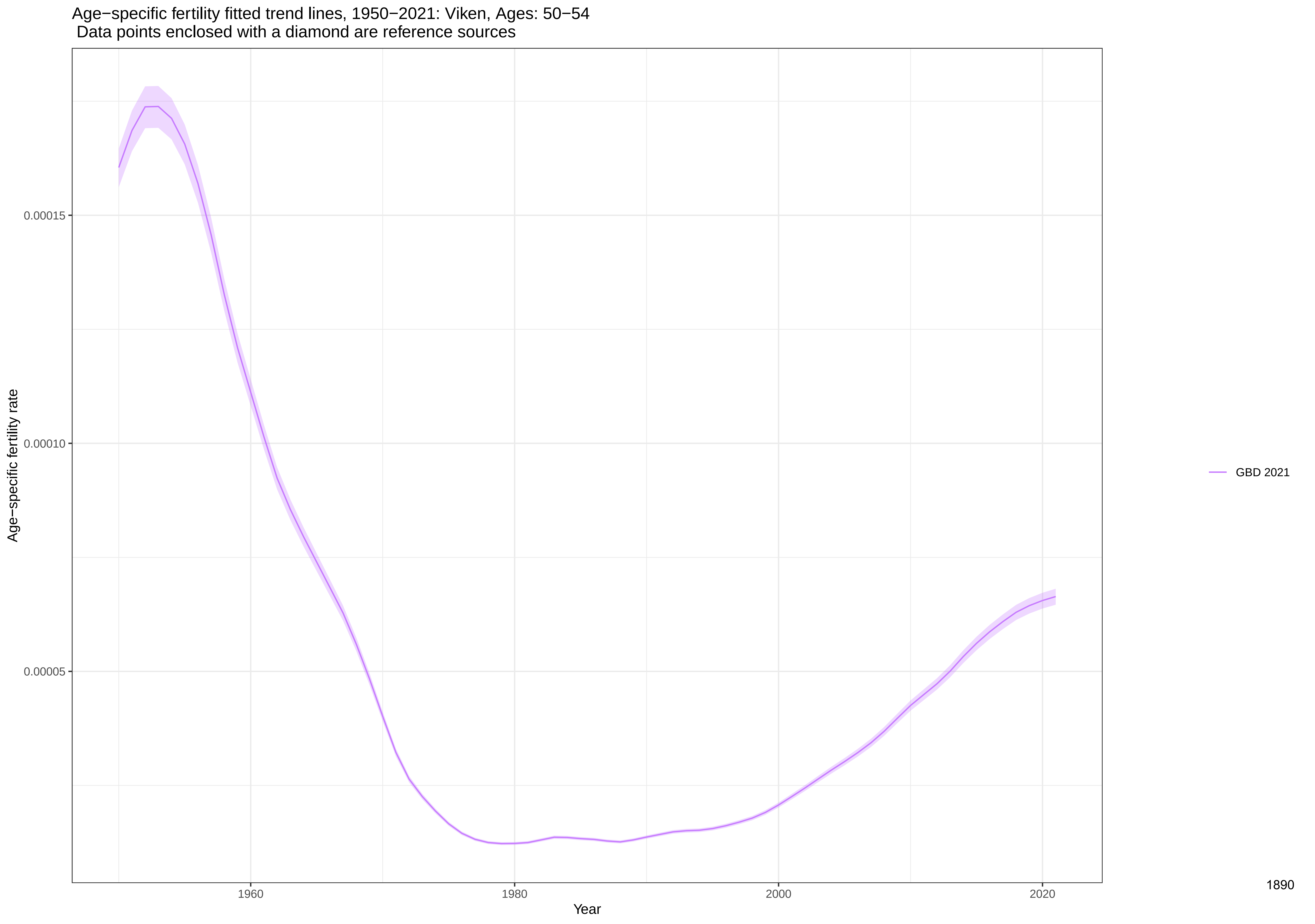

Age−specific fertility fitted trend lines, 1950−2021: Viken, Ages: 50−54
Data points enclosed with a diamond are reference sources
0.00015
0.00010
0.00005
1960
1980
2000
2020
Year
Age−specific fertility rate
GBD 2021

Total fertility rate fitted trend lines, 1950−2021: Viken TFR
Data points enclosed with a diamond are reference sources

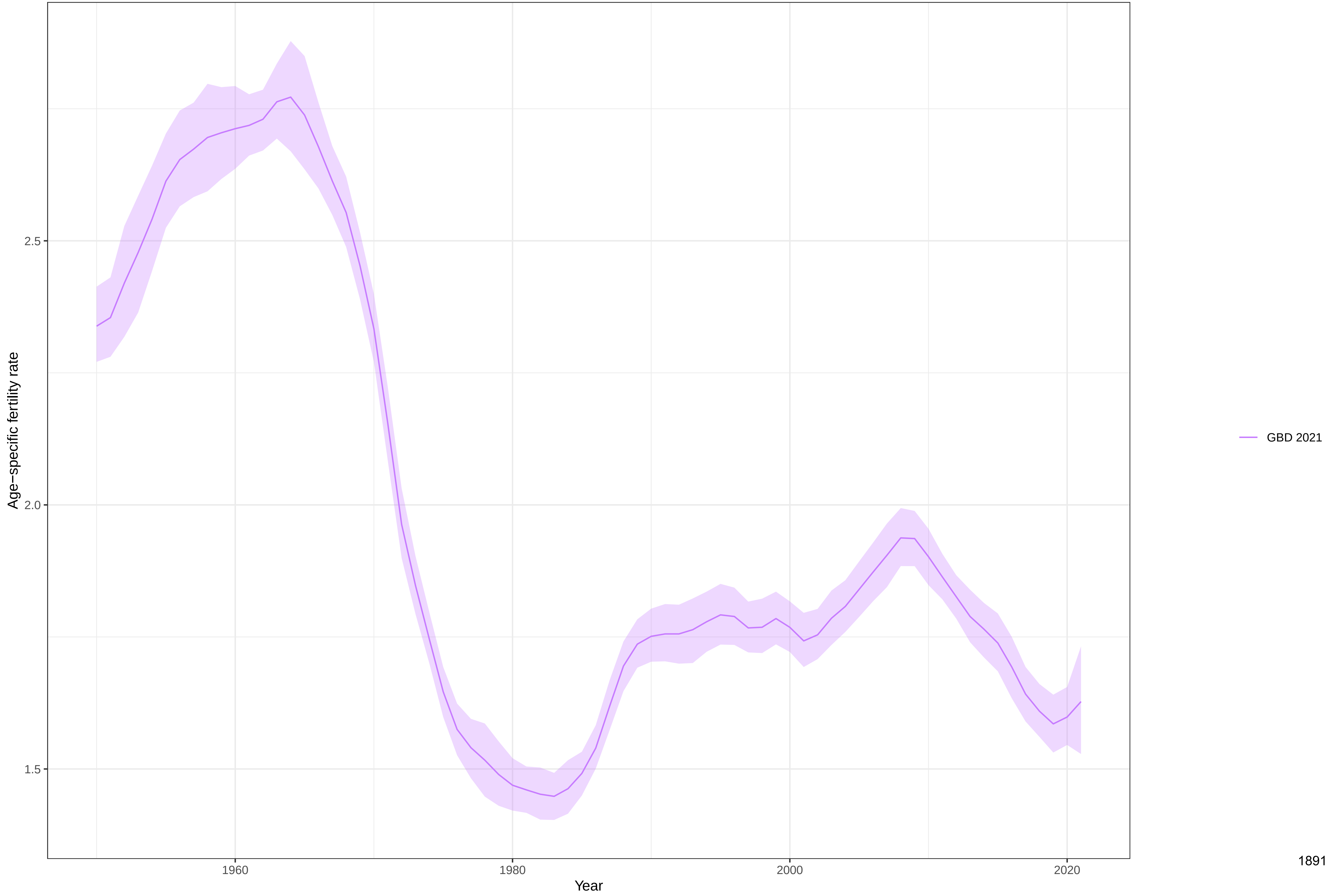

Age−specific fertility fitted trend lines, 1950−2021: Portugal, Ages: 10−14
Data points enclosed with a diamond are reference sources

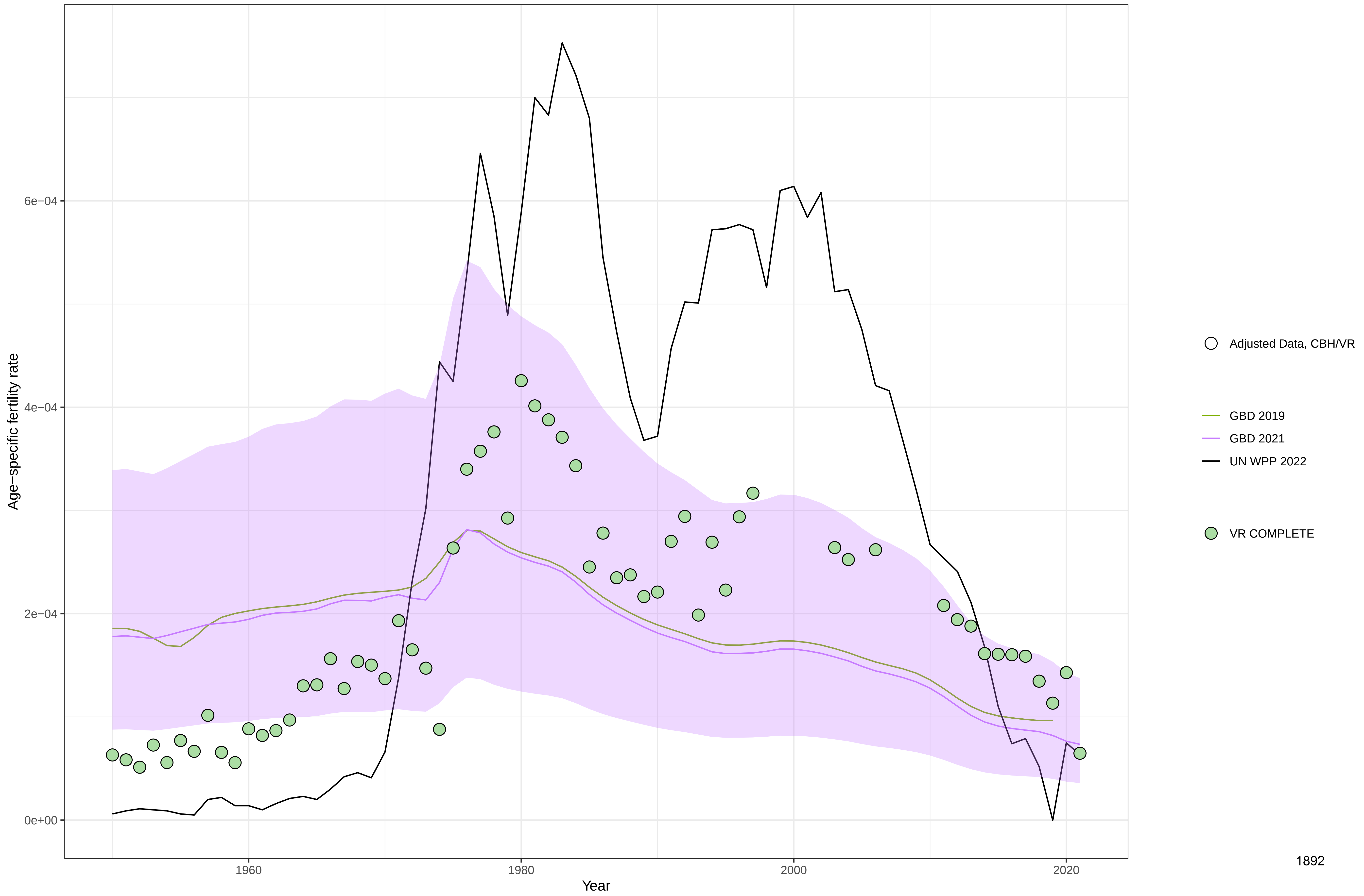

Age−specific fertility fitted trend lines, 1950−2021: Portugal, Ages: 15−19
Data points enclosed with a diamond are reference sources

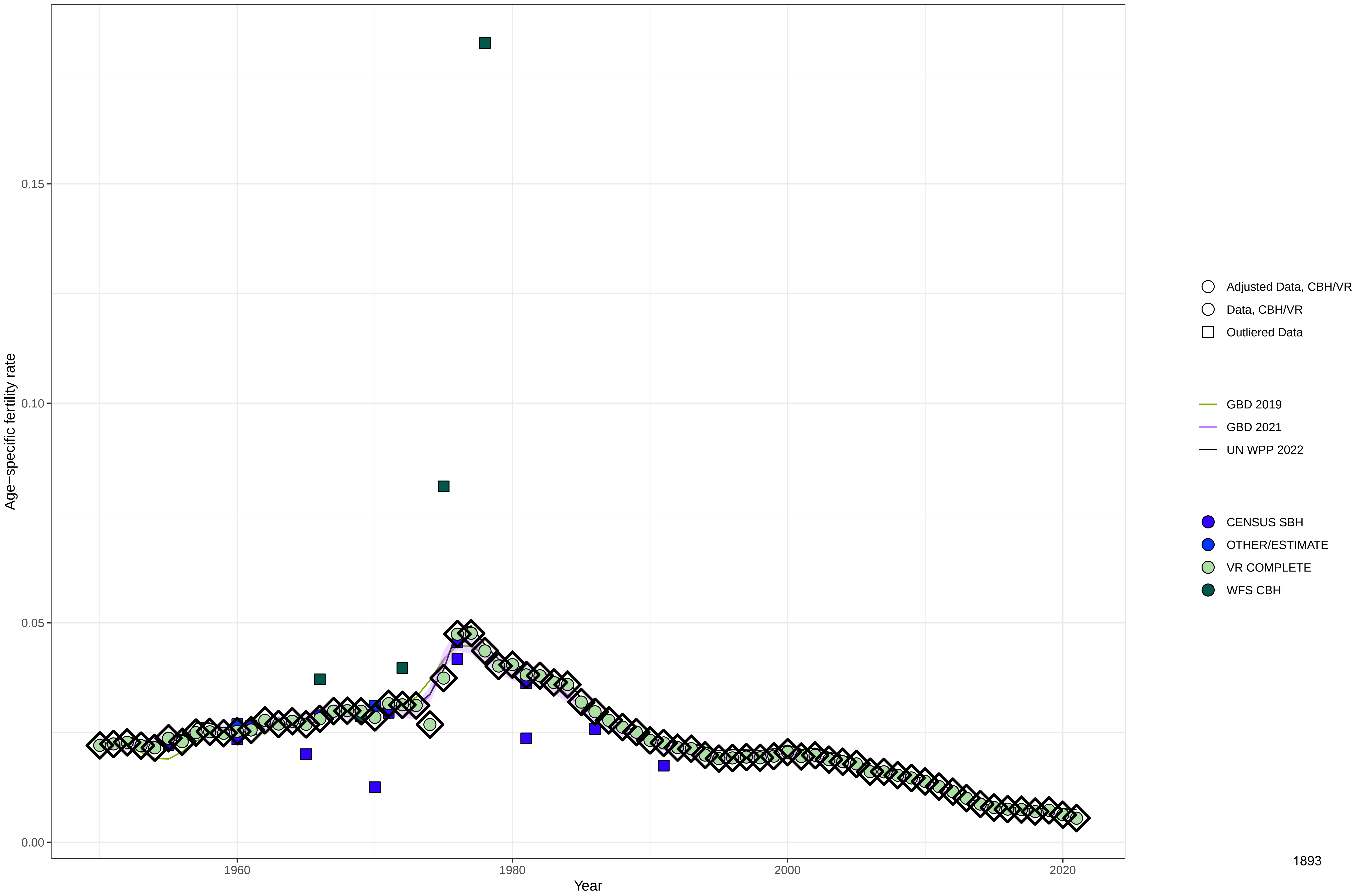

Age−specific fertility fitted trend lines, 1950−2021: Portugal, Ages: 20−24
Data points enclosed with a diamond are reference sources

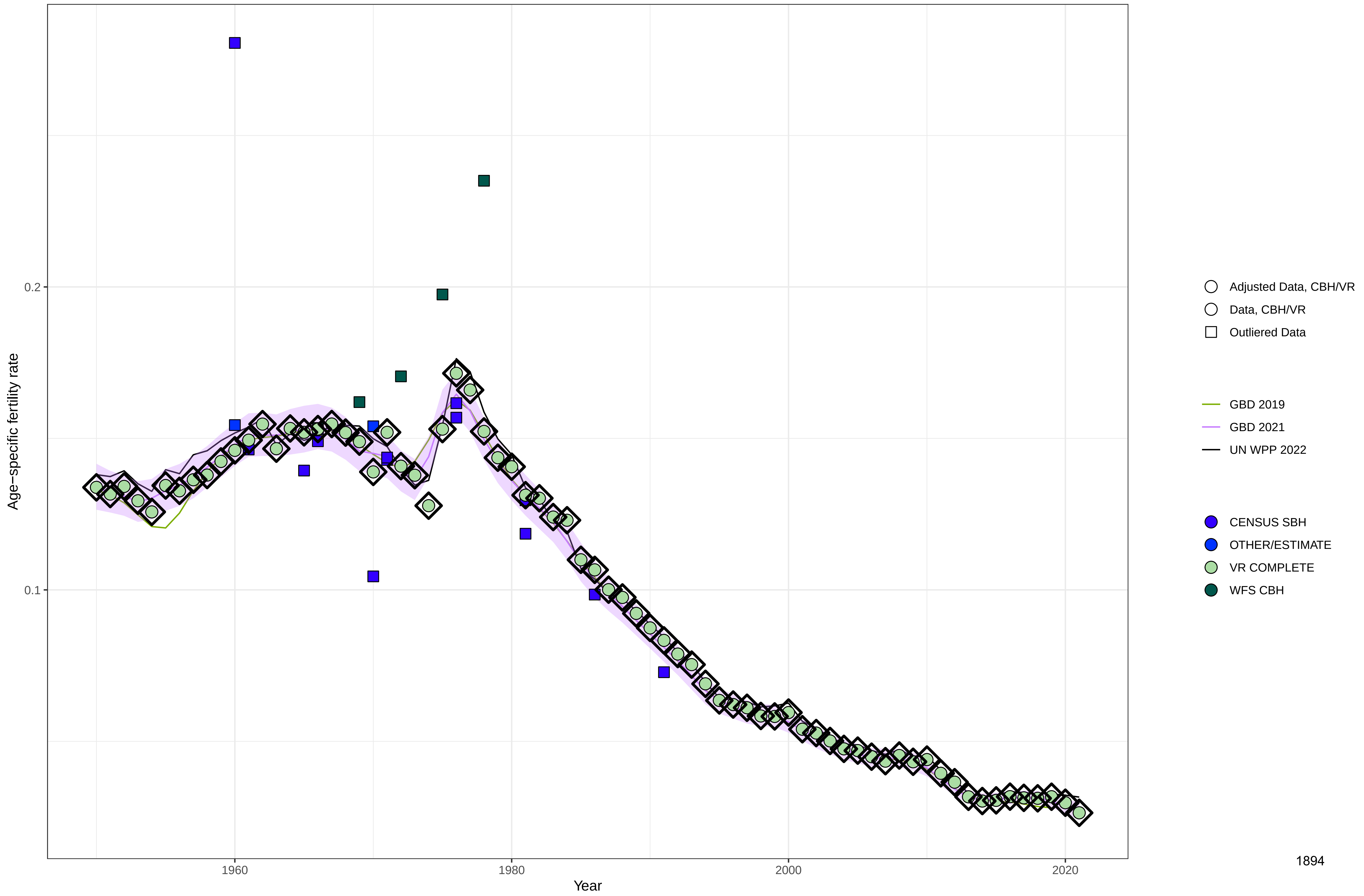

Age−specific fertility fitted trend lines, 1950−2021: Portugal, Ages: 25−29
Data points enclosed with a diamond are reference sources

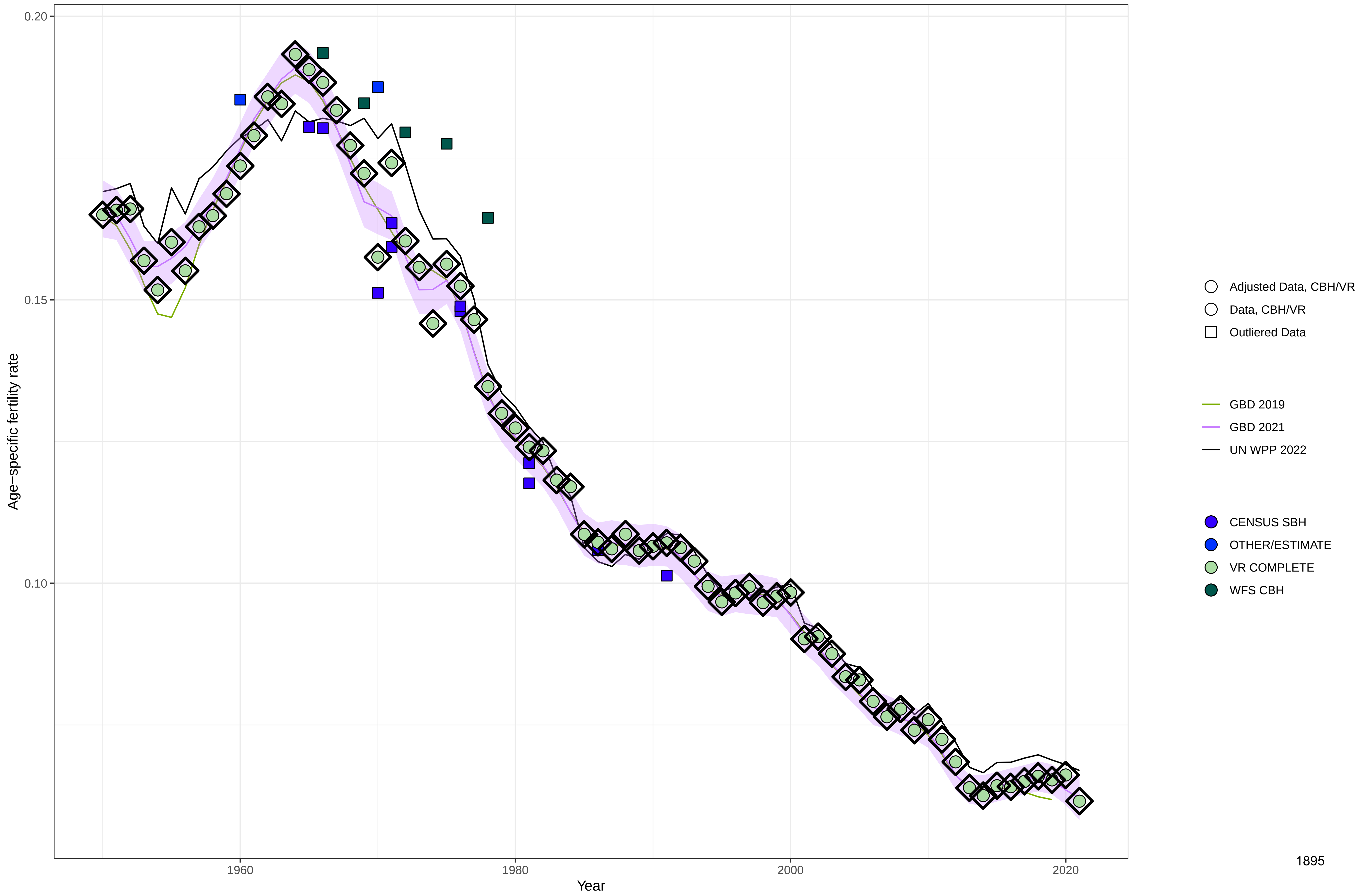

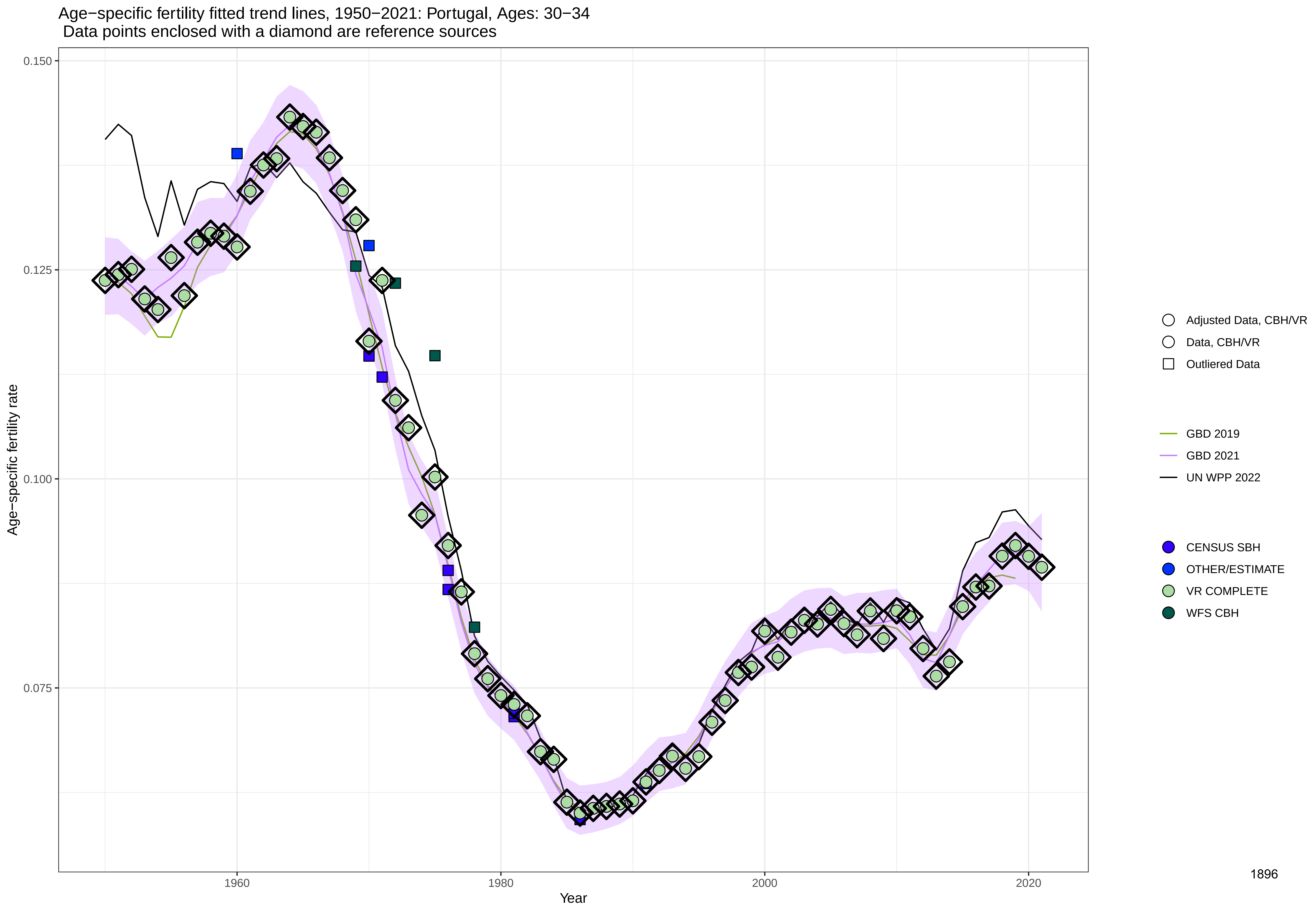

Age−specific fertility fitted trend lines, 1950−2021: Portugal, Ages: 30−34
Data points enclosed with a diamond are reference sources
Age−specific fertility rate
0.150
0.125
0.100
0.075
1960
1980
2000
2020
Year
Adjusted Data, CBH/VR
Data, CBH/VR
Outliered Data
GBD 2019
GBD 2021
UN WPP 2022
CENSUS SBH
OTHER/ESTIMATE
VR COMPLETE
WFS CBH

Age−specific fertility fitted trend lines, 1950−2021: Portugal, Ages: 35−39
Data points enclosed with a diamond are reference sources

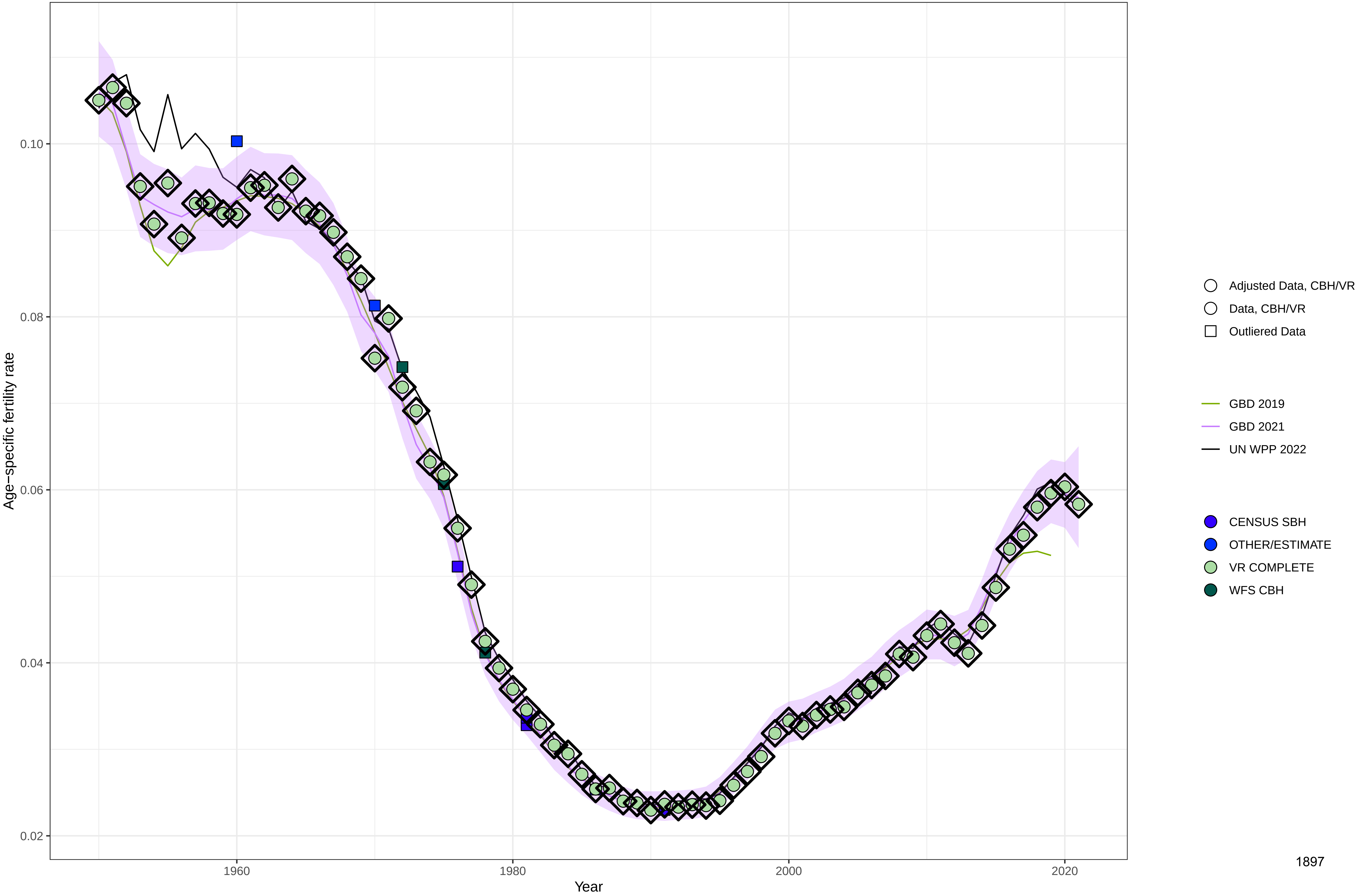

Age−specific fertility fitted trend lines, 1950−2021: Portugal, Ages: 40−44
Data points enclosed with a diamond are reference sources

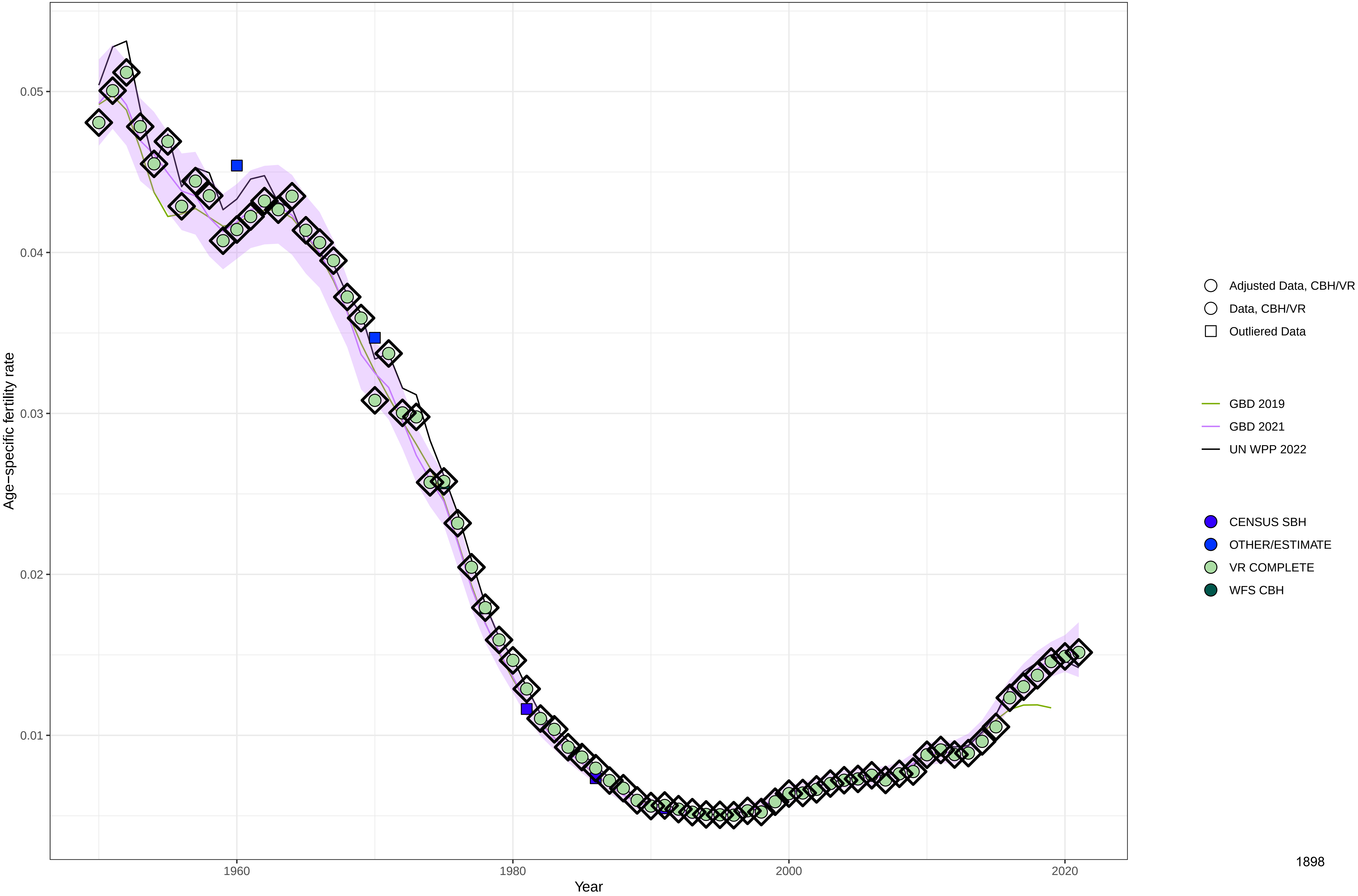

Age−specific fertility fitted trend lines, 1950−2021: Portugal, Ages: 45−49
Data points enclosed with a diamond are reference sources

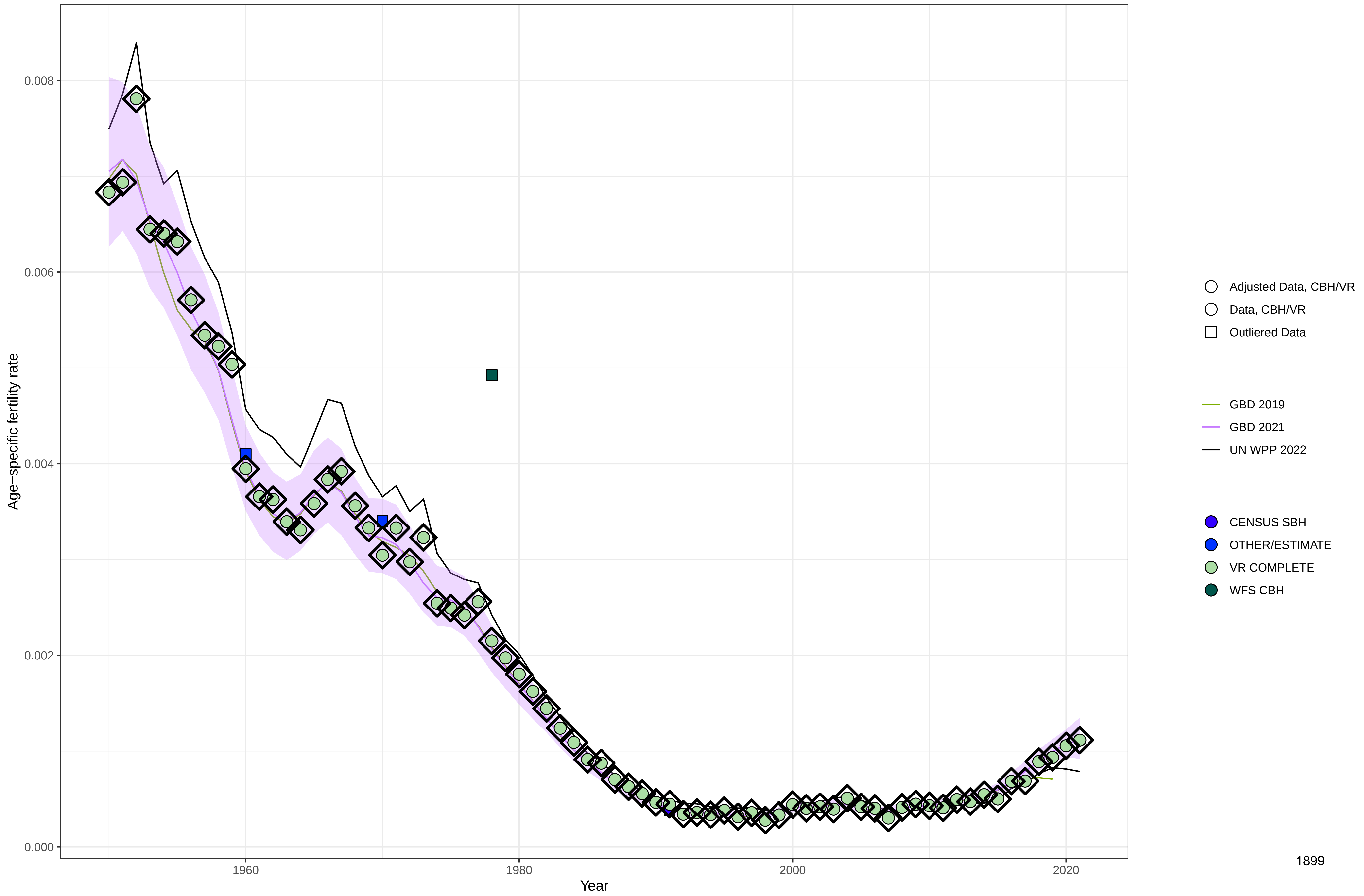

Age−specific fertility fitted trend lines, 1950−2021: Portugal, Ages: 50−54
Data points enclosed with a diamond are reference sources

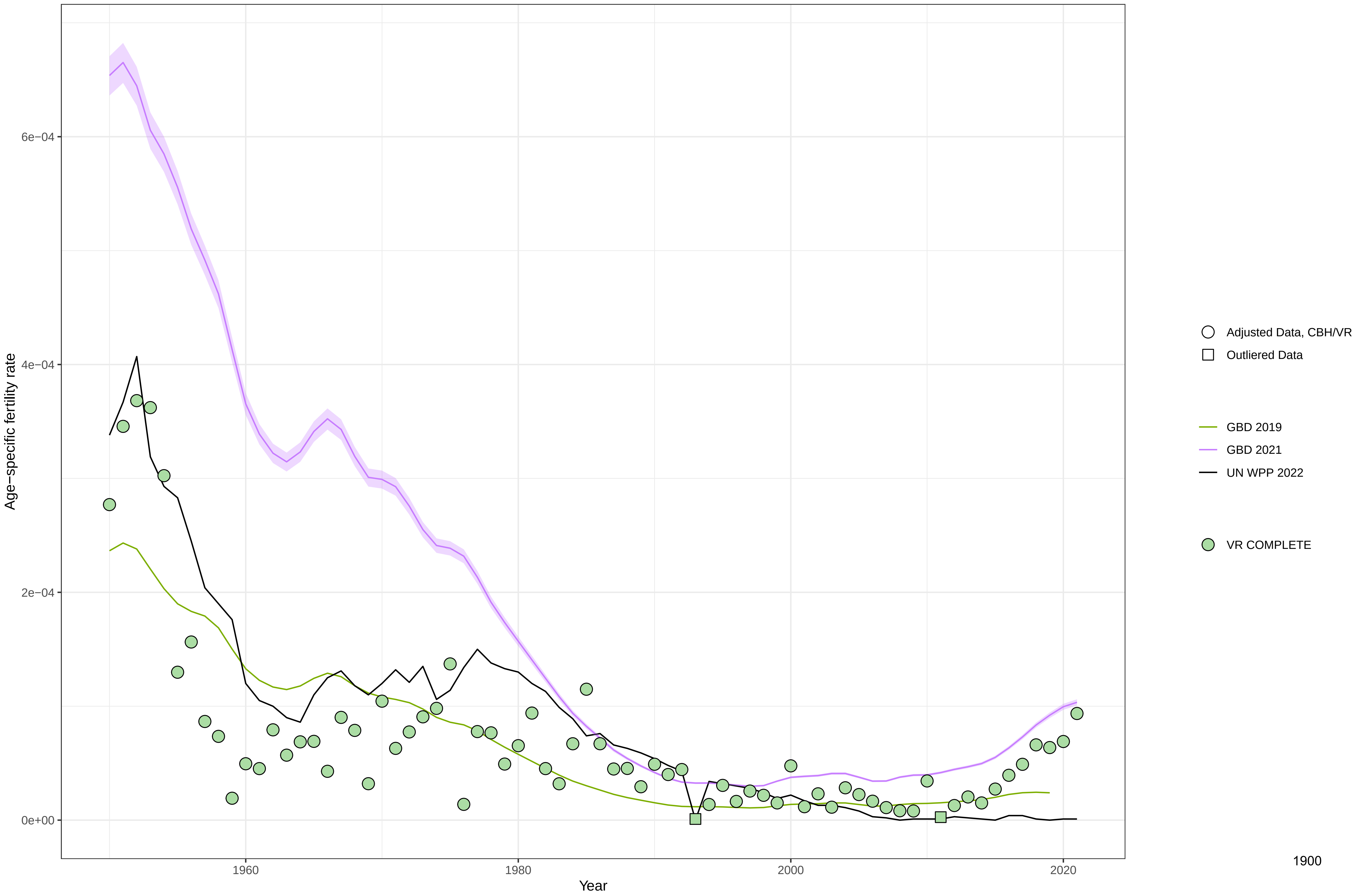

1900

Total fertility rate fitted trend lines, 1950−2021: Portugal TFR
Data points enclosed with a diamond are reference sources

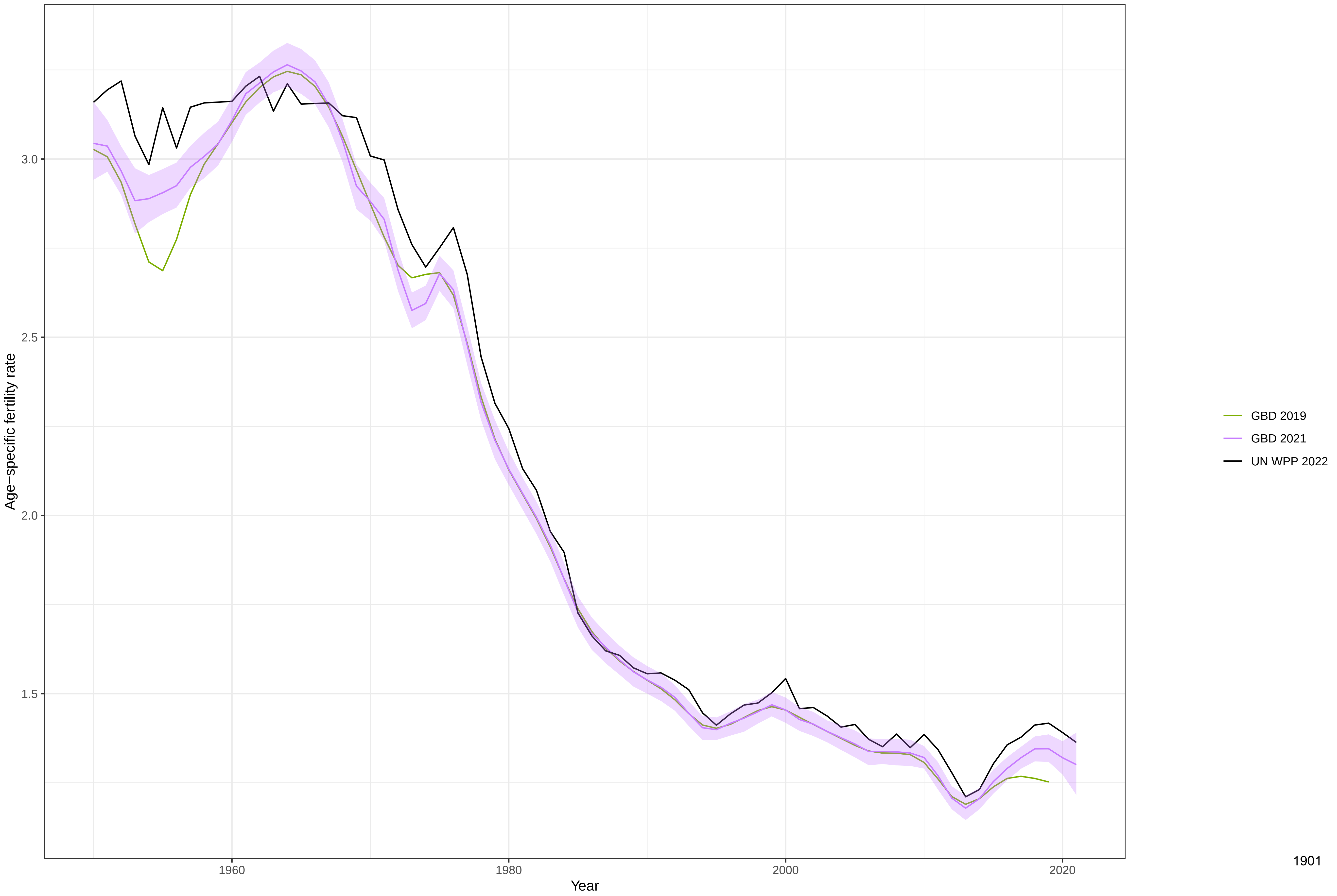

Age−specific fertility fitted trend lines, 1950−2021: San Marino, Ages: 10−14
Data points enclosed with a diamond are reference sources

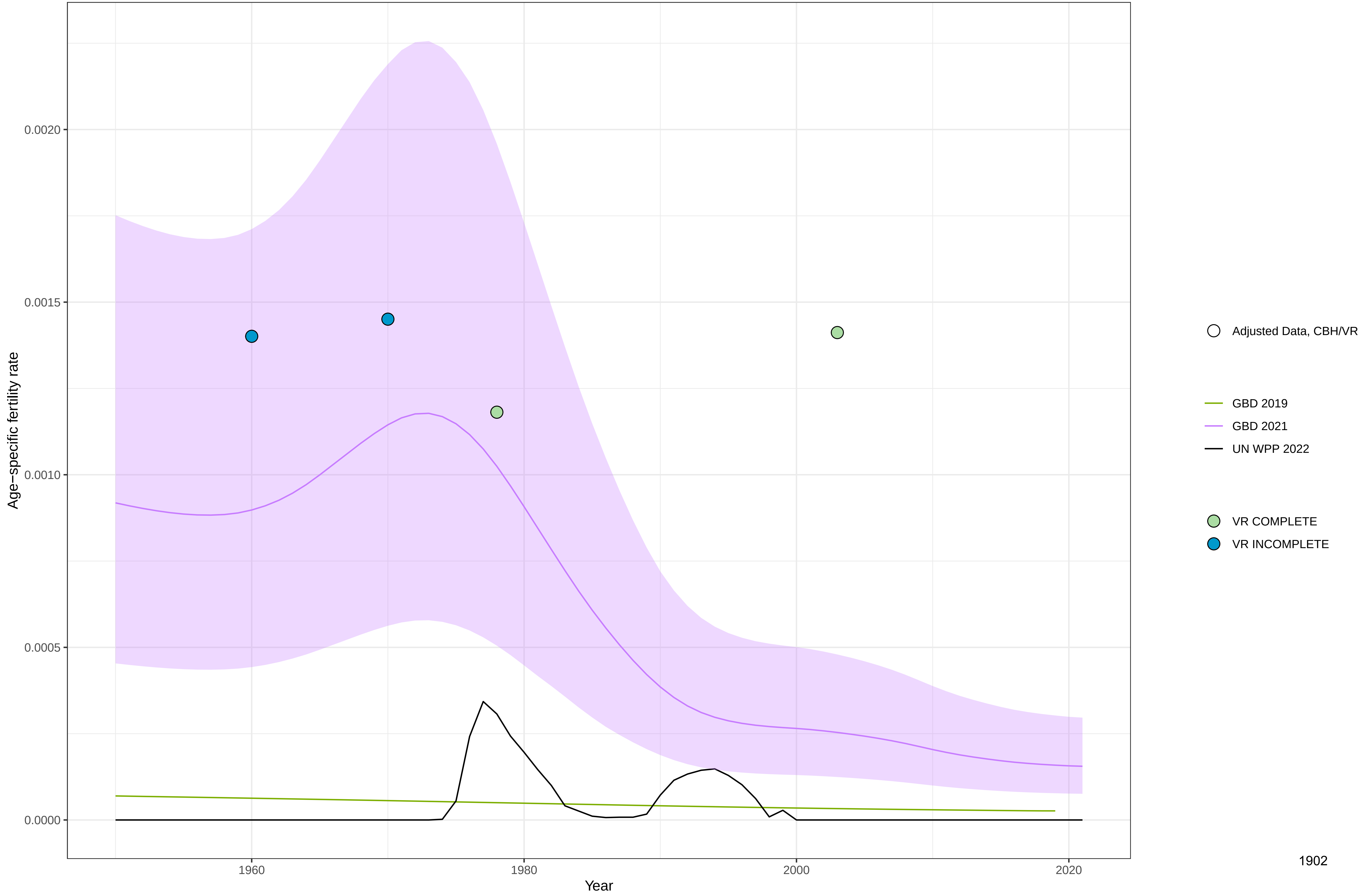

Age−specific fertility fitted trend lines, 1950−2021: San Marino, Ages: 15−19
Data points enclosed with a diamond are reference sources

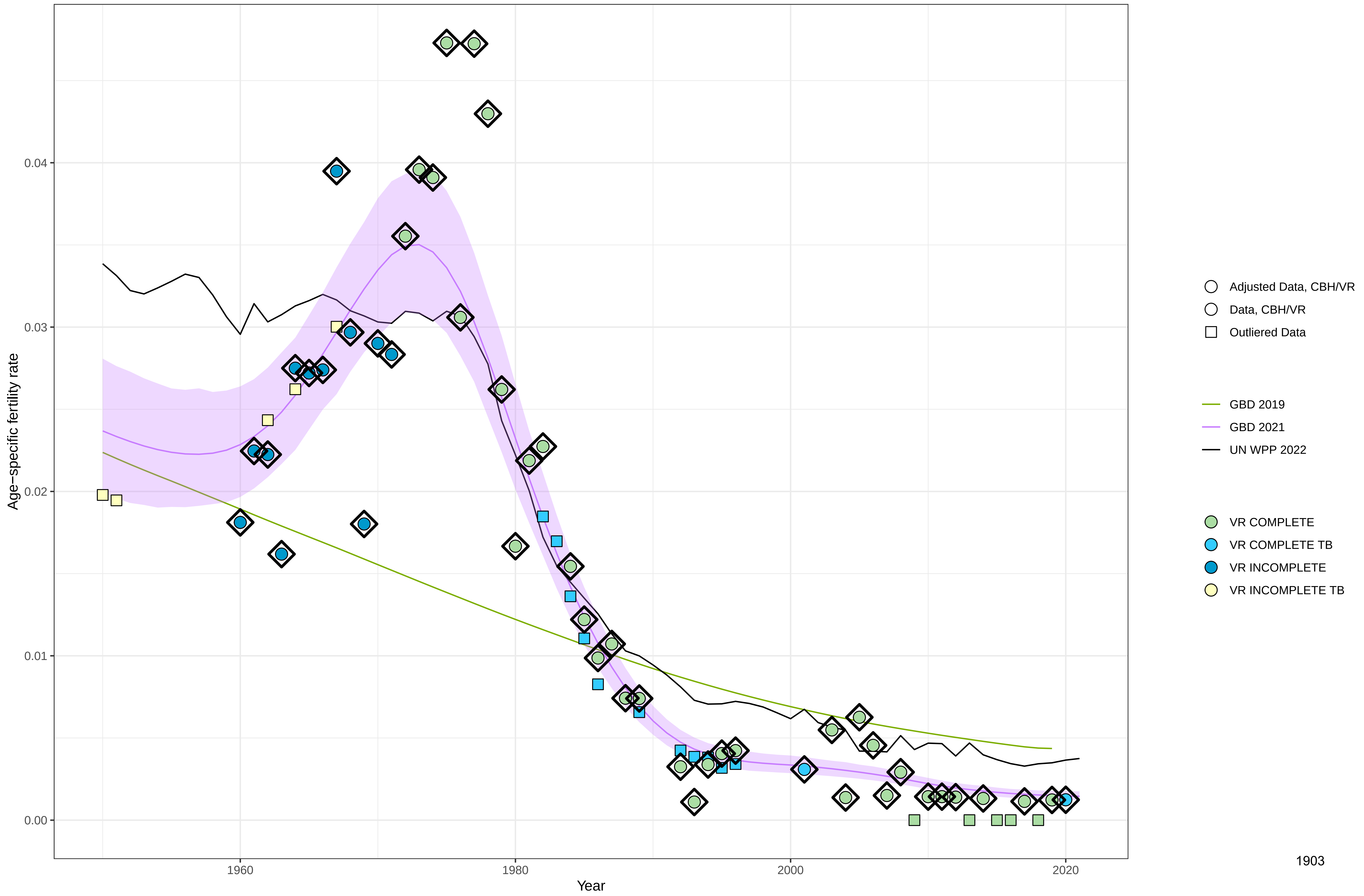

Age−specific fertility fitted trend lines, 1950−2021: San Marino, Ages: 20−24
Data points enclosed with a diamond are reference sources

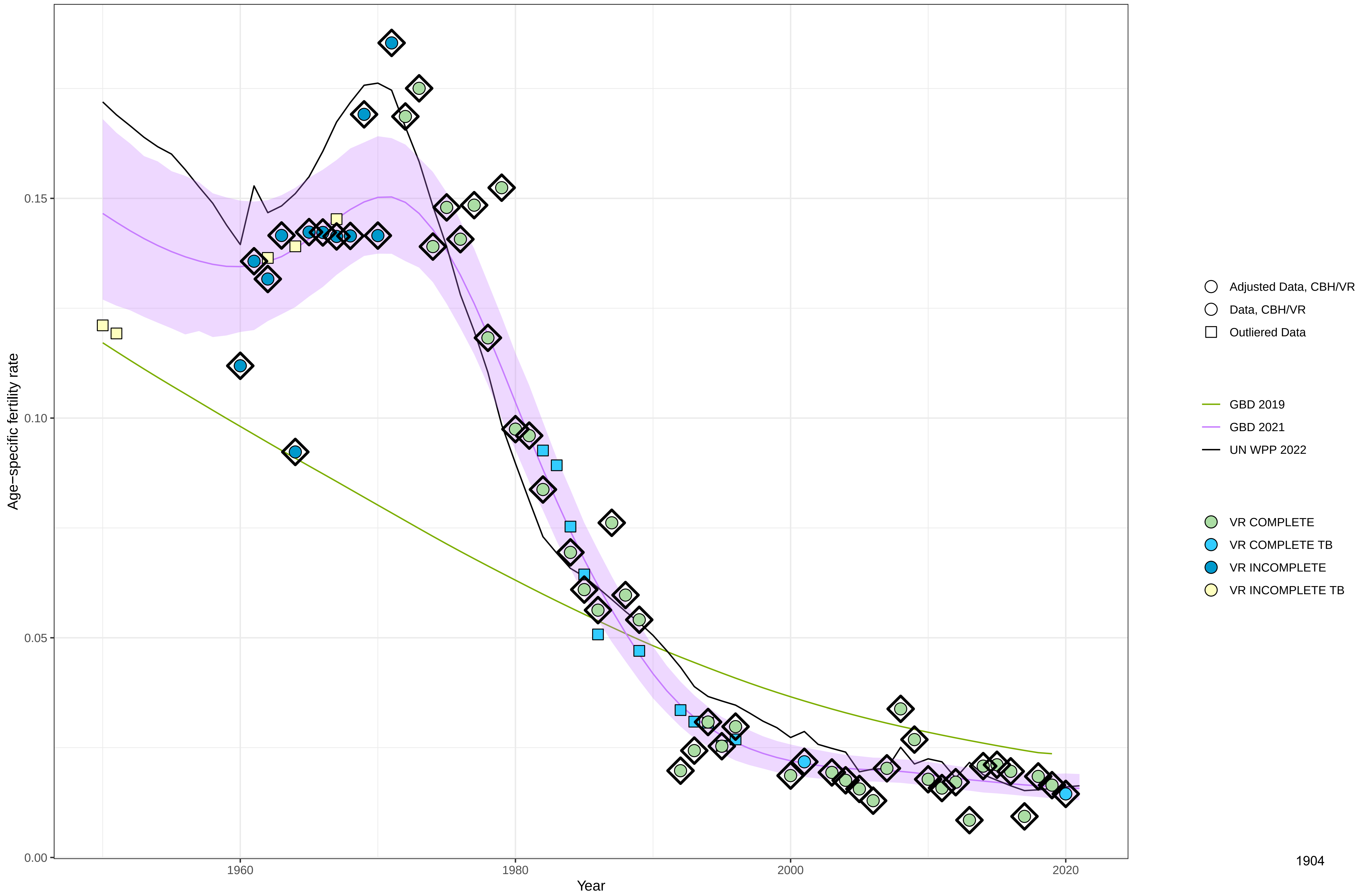

Age−specific fertility fitted trend lines, 1950−2021: San Marino, Ages: 25−29
Data points enclosed with a diamond are reference sources

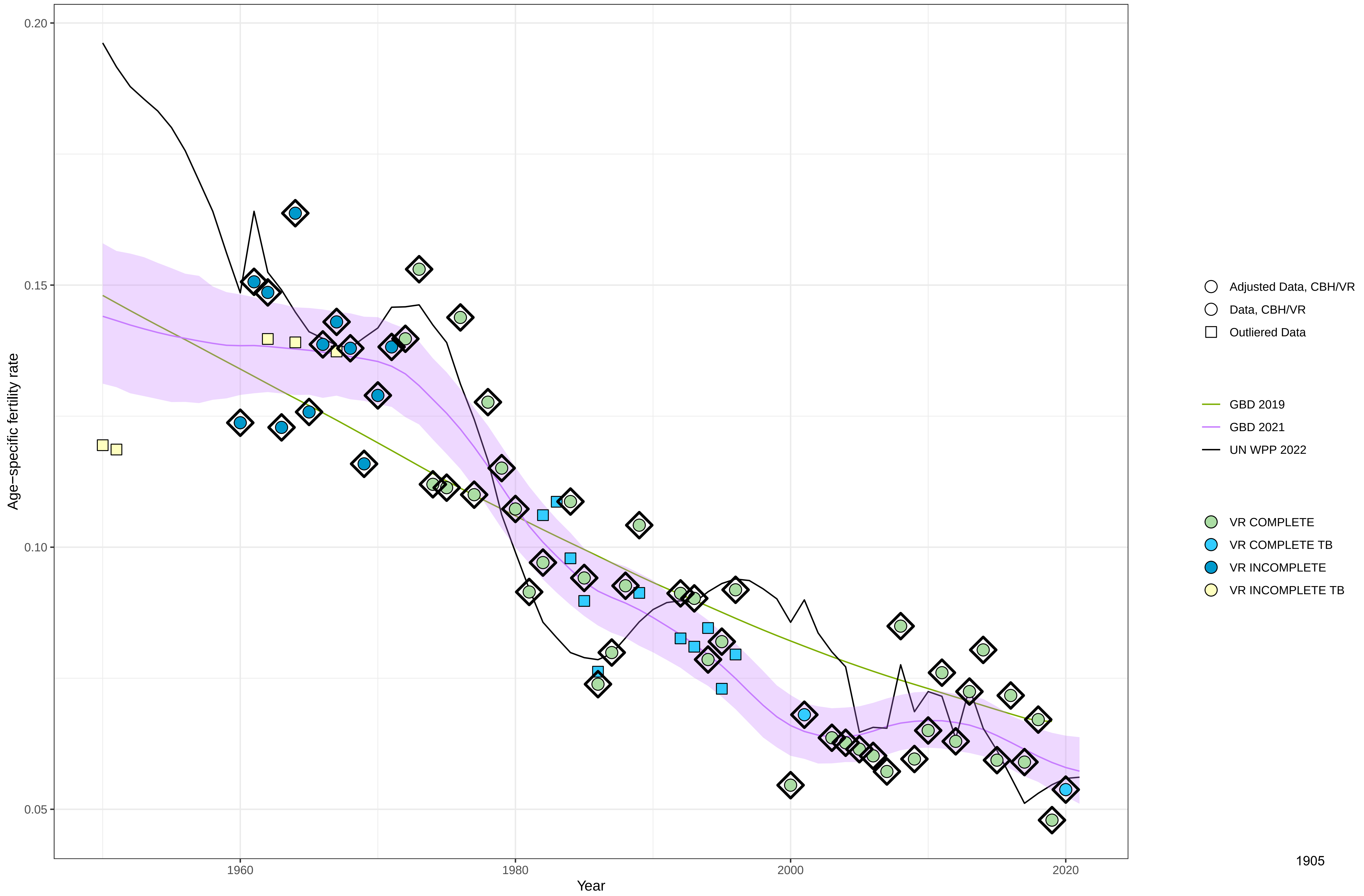

Age−specific fertility fitted trend lines, 1950−2021: San Marino, Ages: 30−34
Data points enclosed with a diamond are reference sources

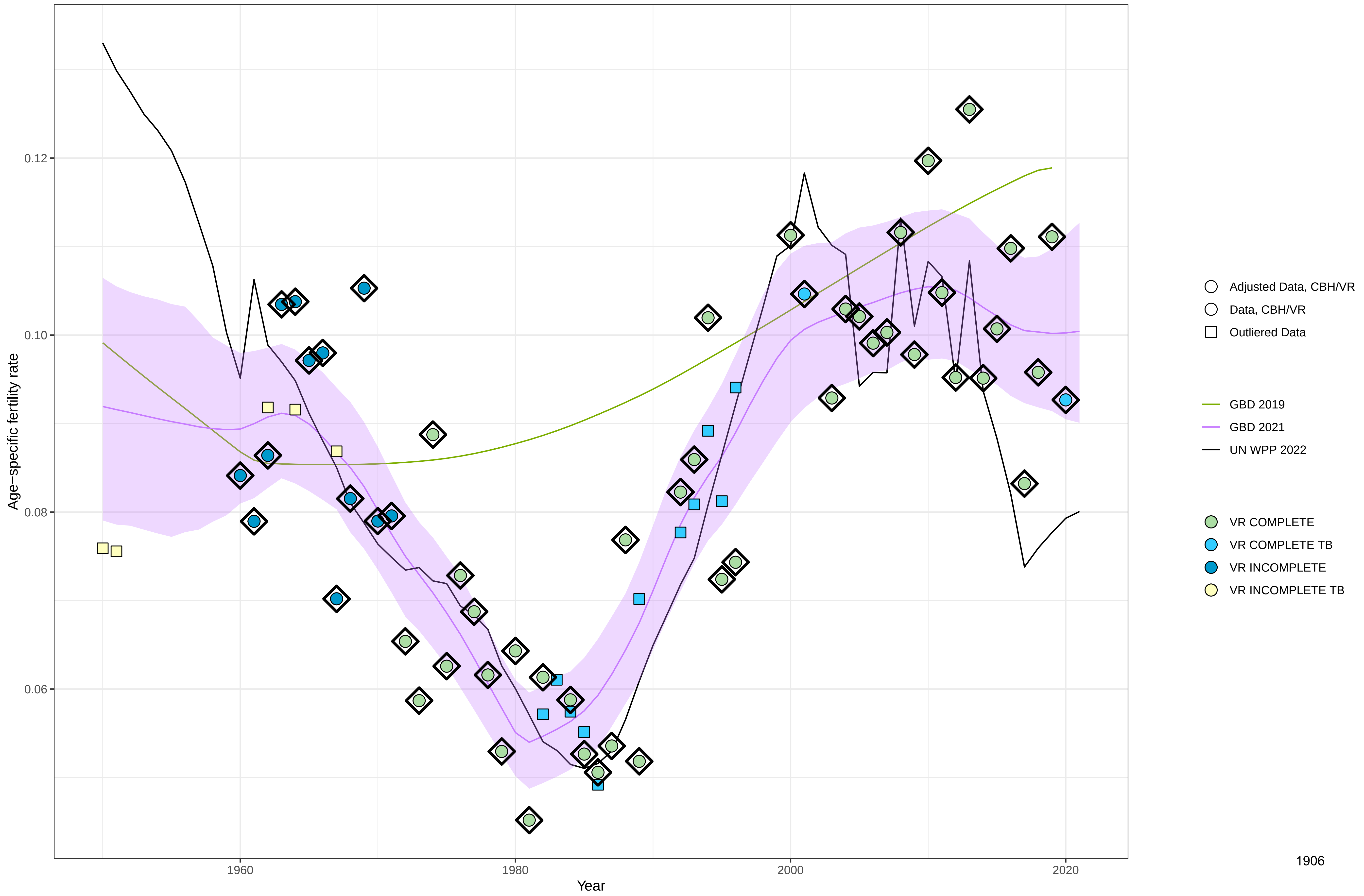

Age−specific fertility fitted trend lines, 1950−2021: San Marino, Ages: 35−39
Data points enclosed with a diamond are reference sources

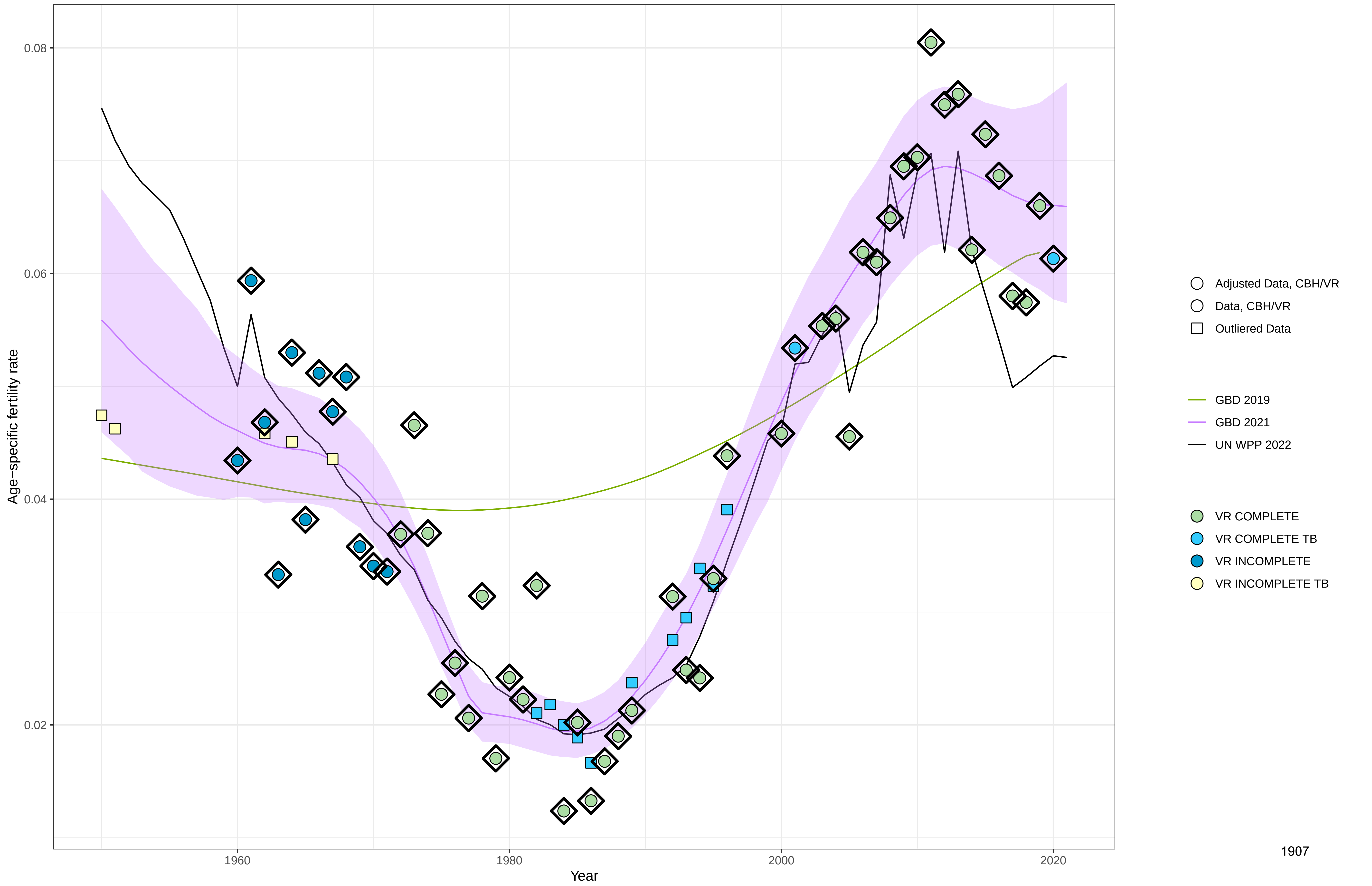

Age−specific fertility fitted trend lines, 1950−2021: San Marino, Ages: 40−44
Data points enclosed with a diamond are reference sources

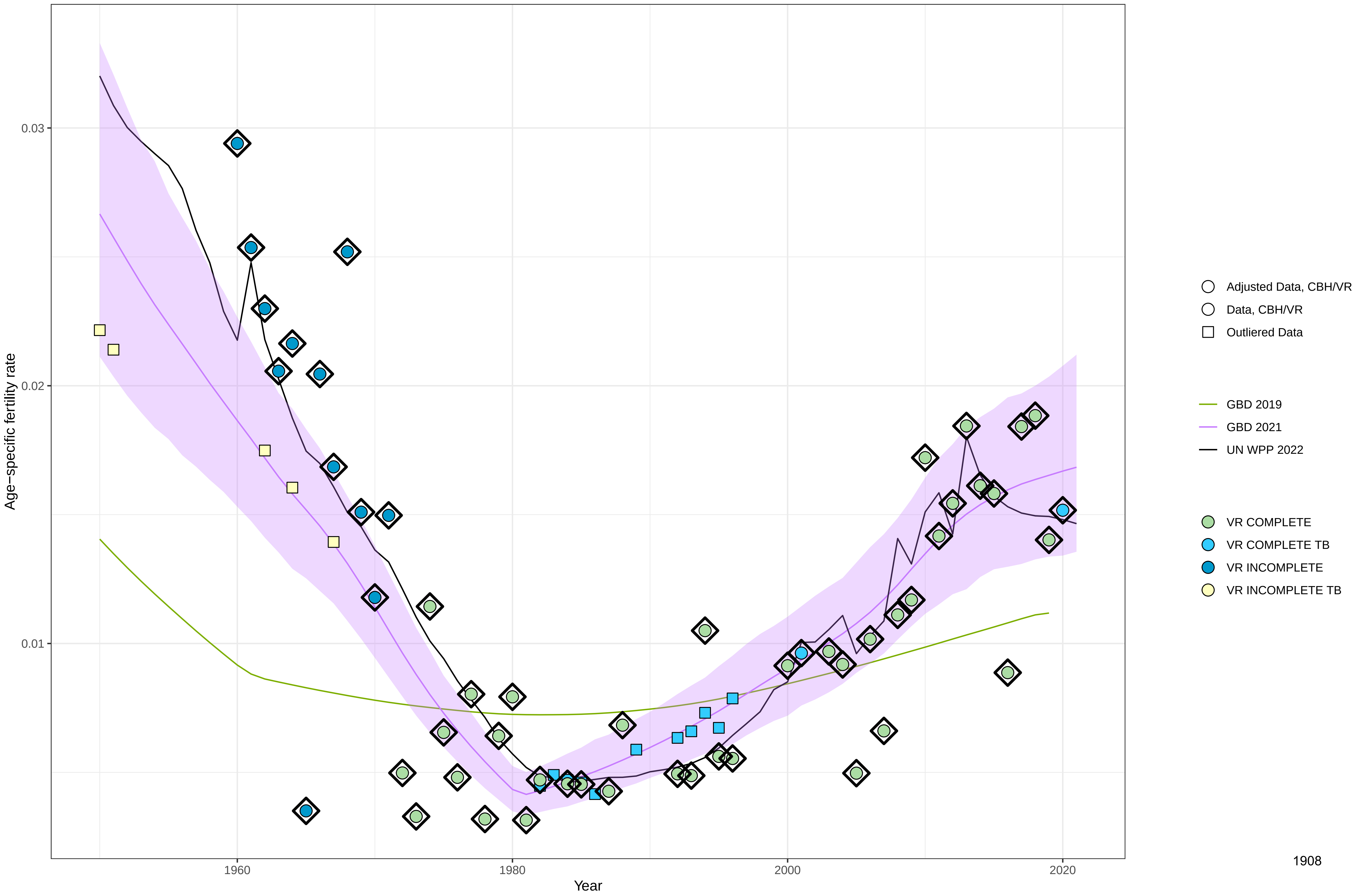

Age−specific fertility fitted trend lines, 1950−2021: San Marino, Ages: 45−49
Data points enclosed with a diamond are reference sources

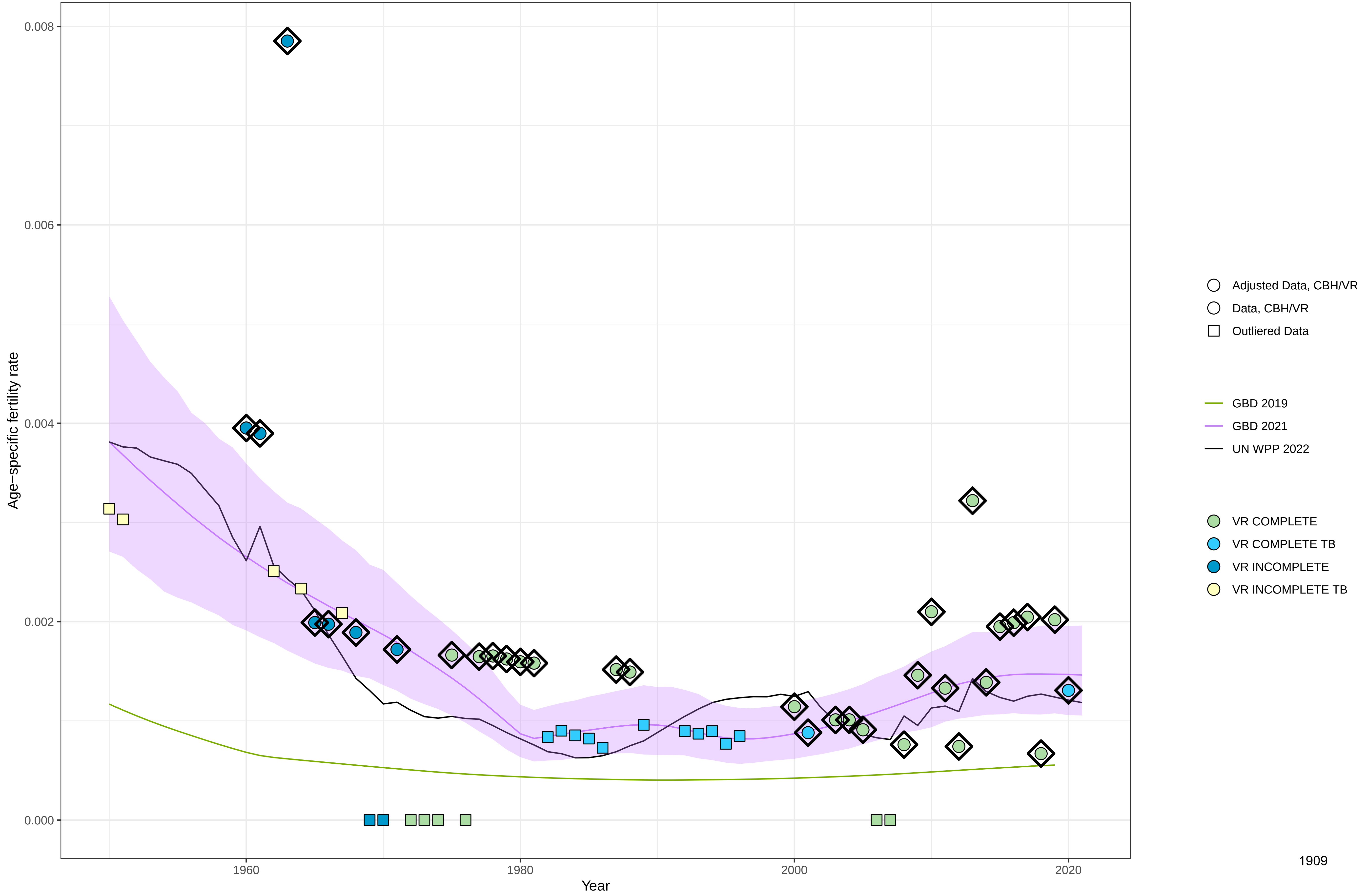

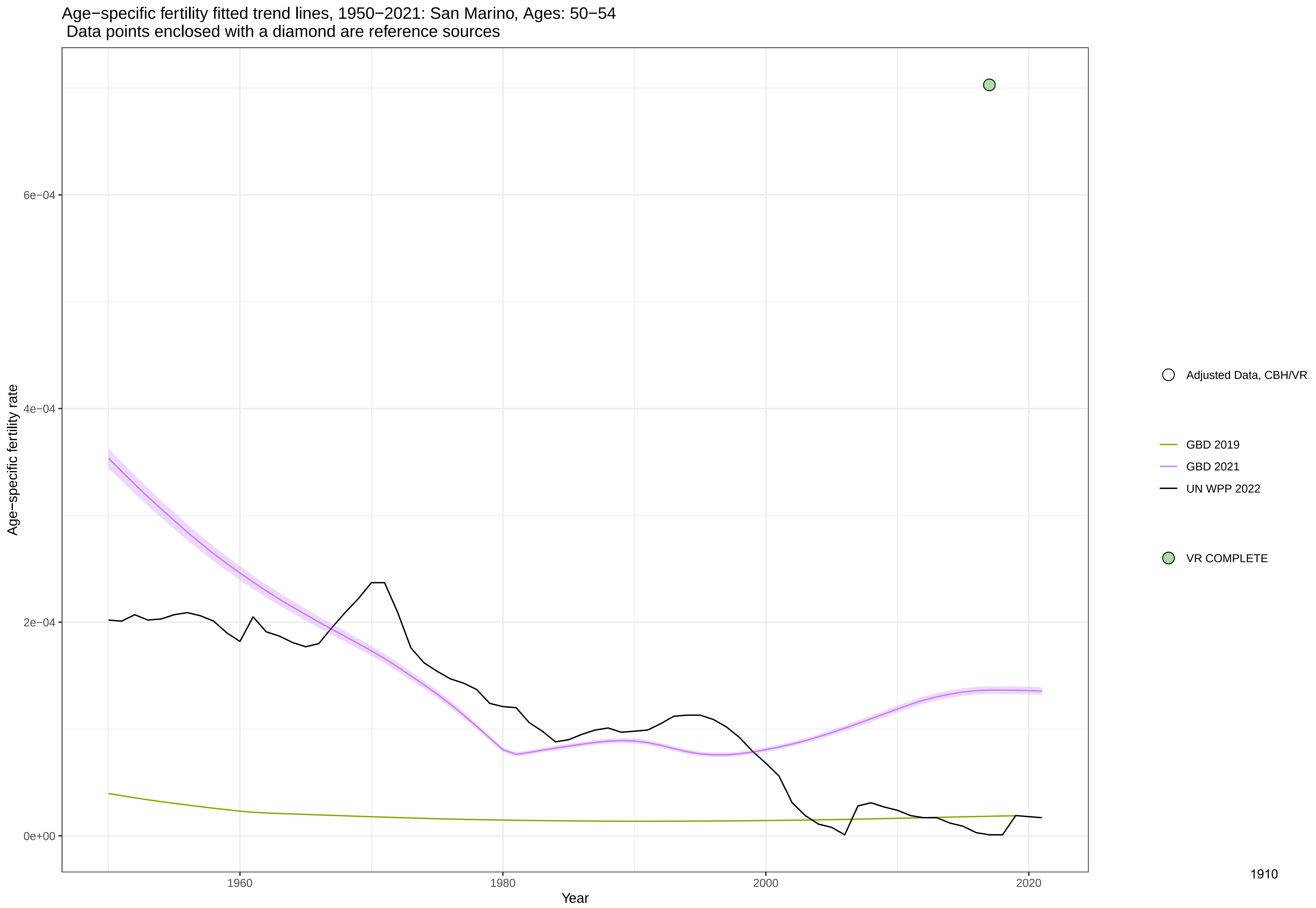
Age−specific fertility fitted trend lines, 1950−2021: San Marino, Ages: 50−54
Data points enclosed with a diamond are reference sources
Age−specific fertility rate
6e−04
4e−04
2e−04
0e+00
1960
1980
2000
2020
Year
Adjusted Data, CBH/VR
GBD 2019
GBD 2021
UN WPP 2022
VR COMPLETE

Total fertility rate fitted trend lines, 1950−2021: San Marino TFR
Data points enclosed with a diamond are reference sources

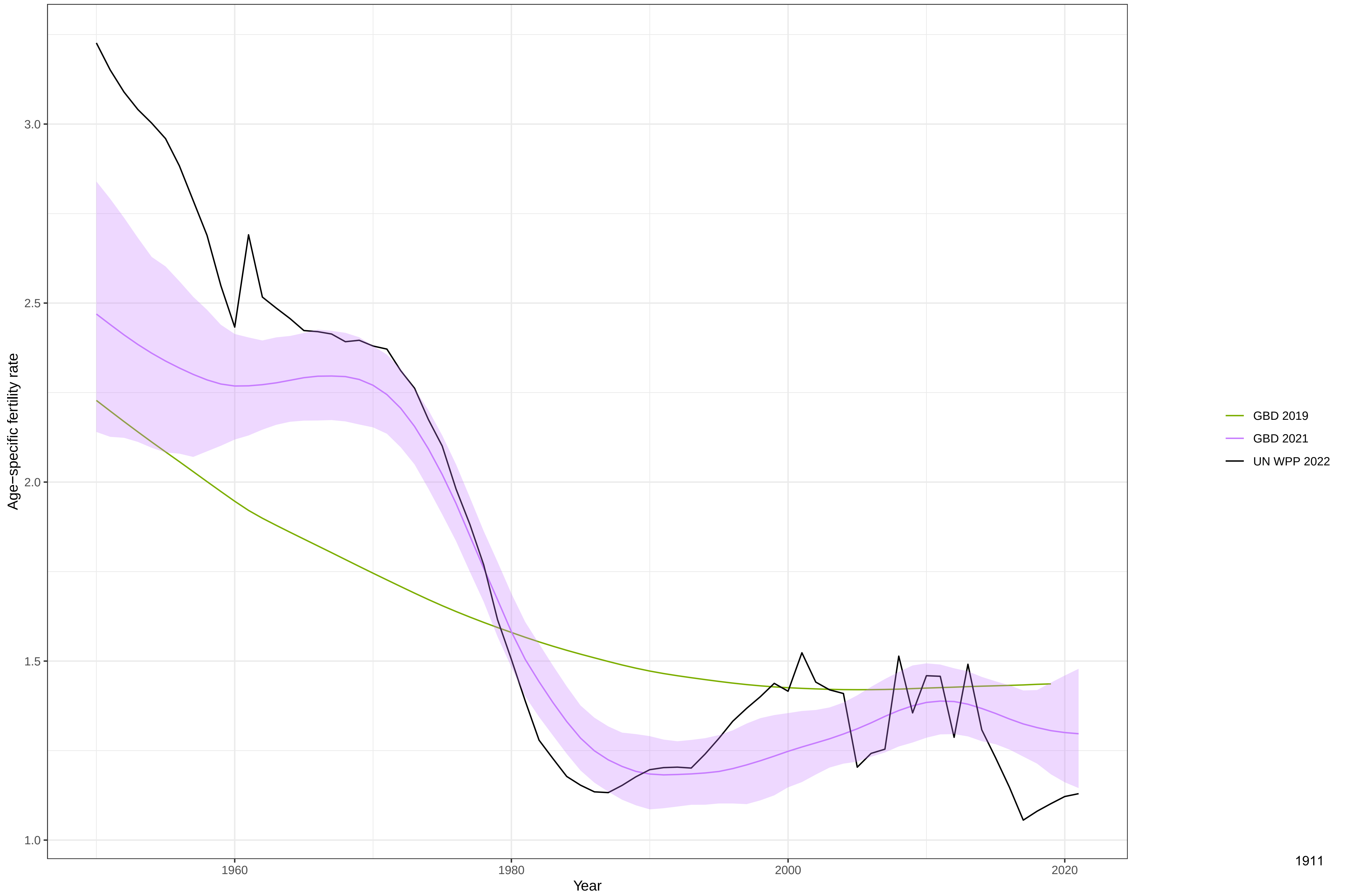

Age−specific fertility fitted trend lines, 1950−2021: Spain, Ages: 10−14
Data points enclosed with a diamond are reference sources

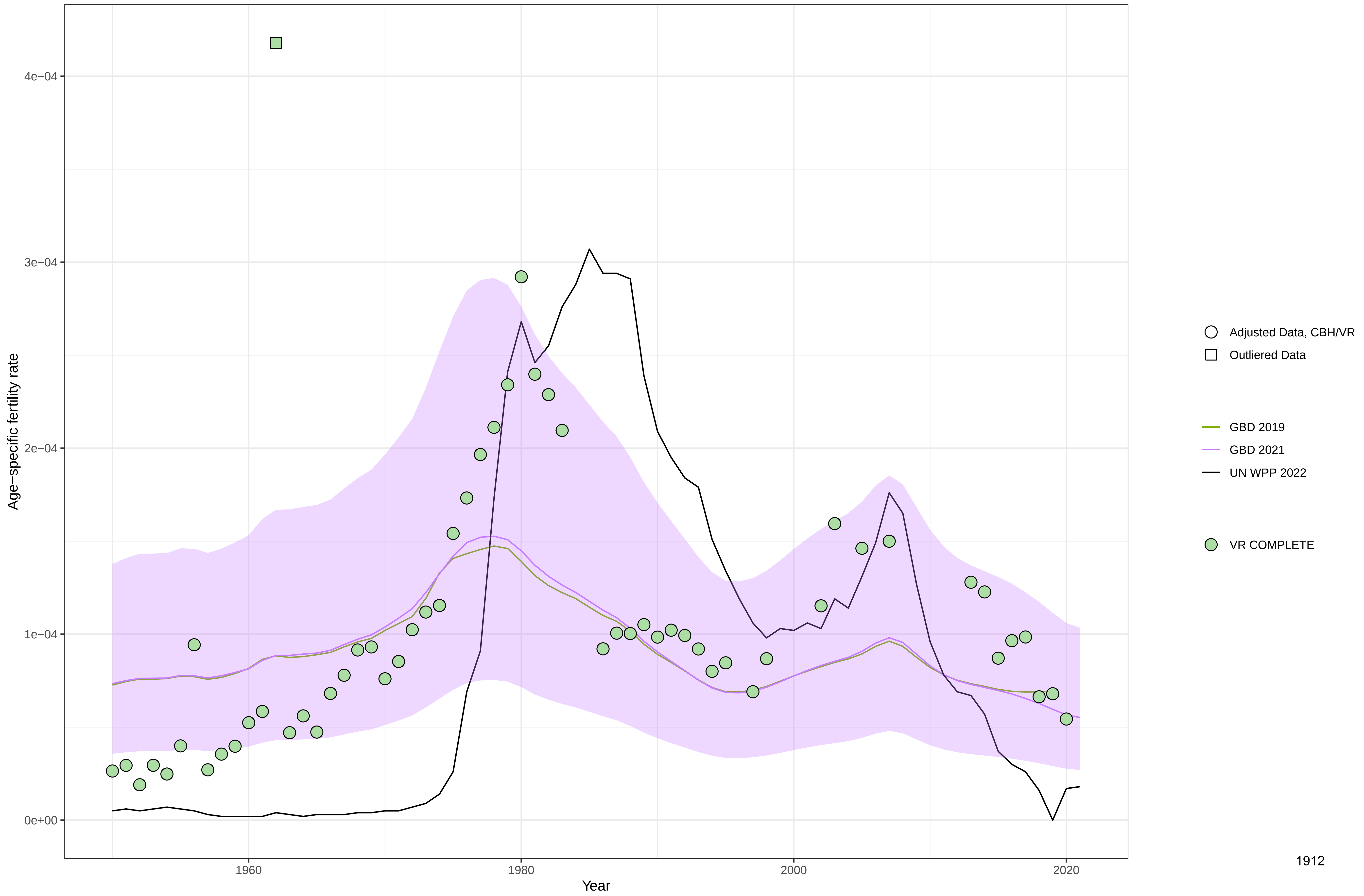

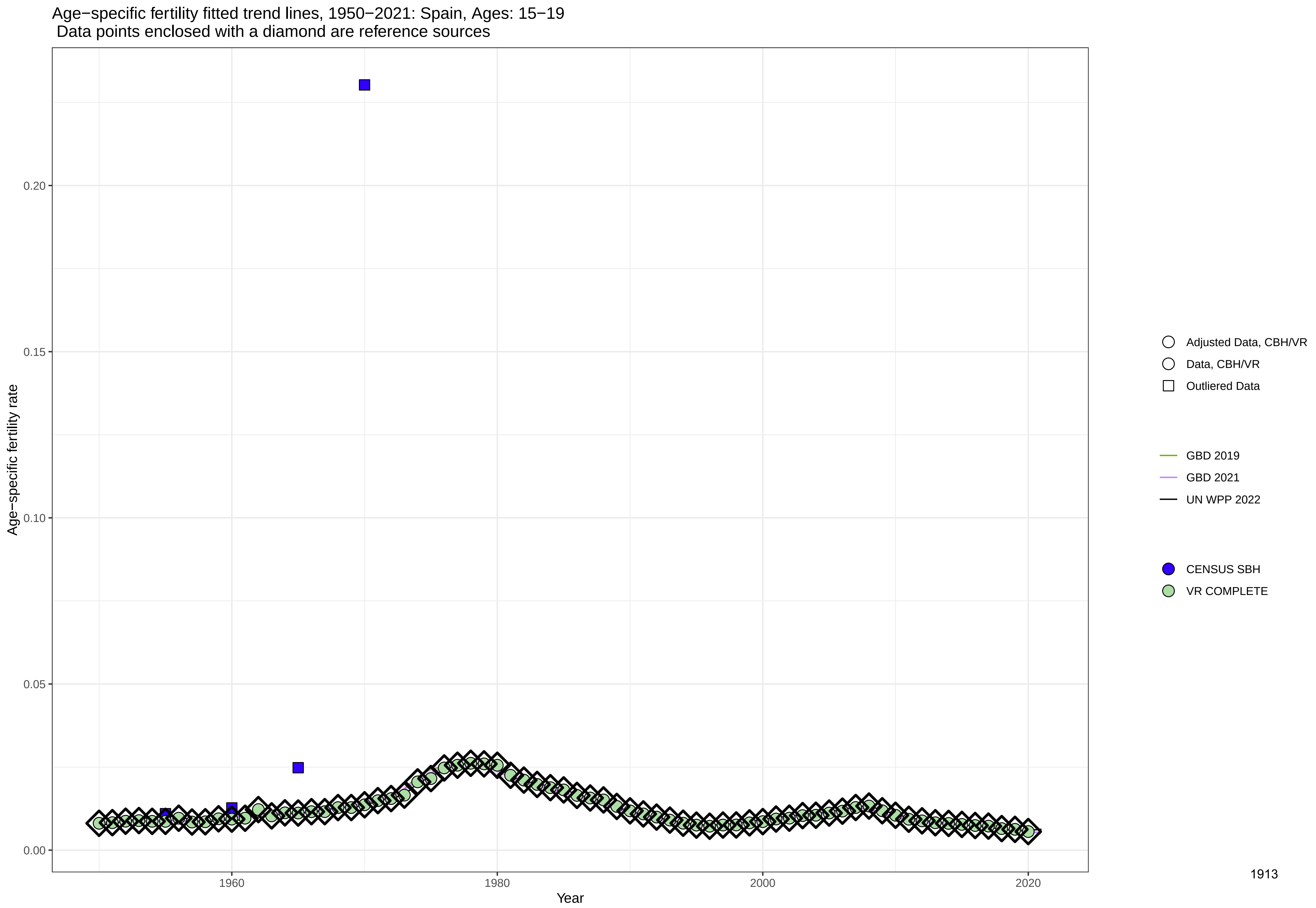

Age−specific fertility fitted trend lines, 1950−2021: Spain, Ages: 15−19
Data points enclosed with a diamond are reference sources
Age−specific fertility rate
0.00
0.05
0.10
0.15
0.20
1960
1980
2000
2020
Year
Adjusted Data, CBH/VR
Data, CBH/VR
Outliered Data
GBD 2019
GBD 2021
UN WPP 2022
CENSUS SBH
VR COMPLETE

Age−specific fertility fitted trend lines, 1950−2021: Spain, Ages: 20−24
Data points enclosed with a diamond are reference sources

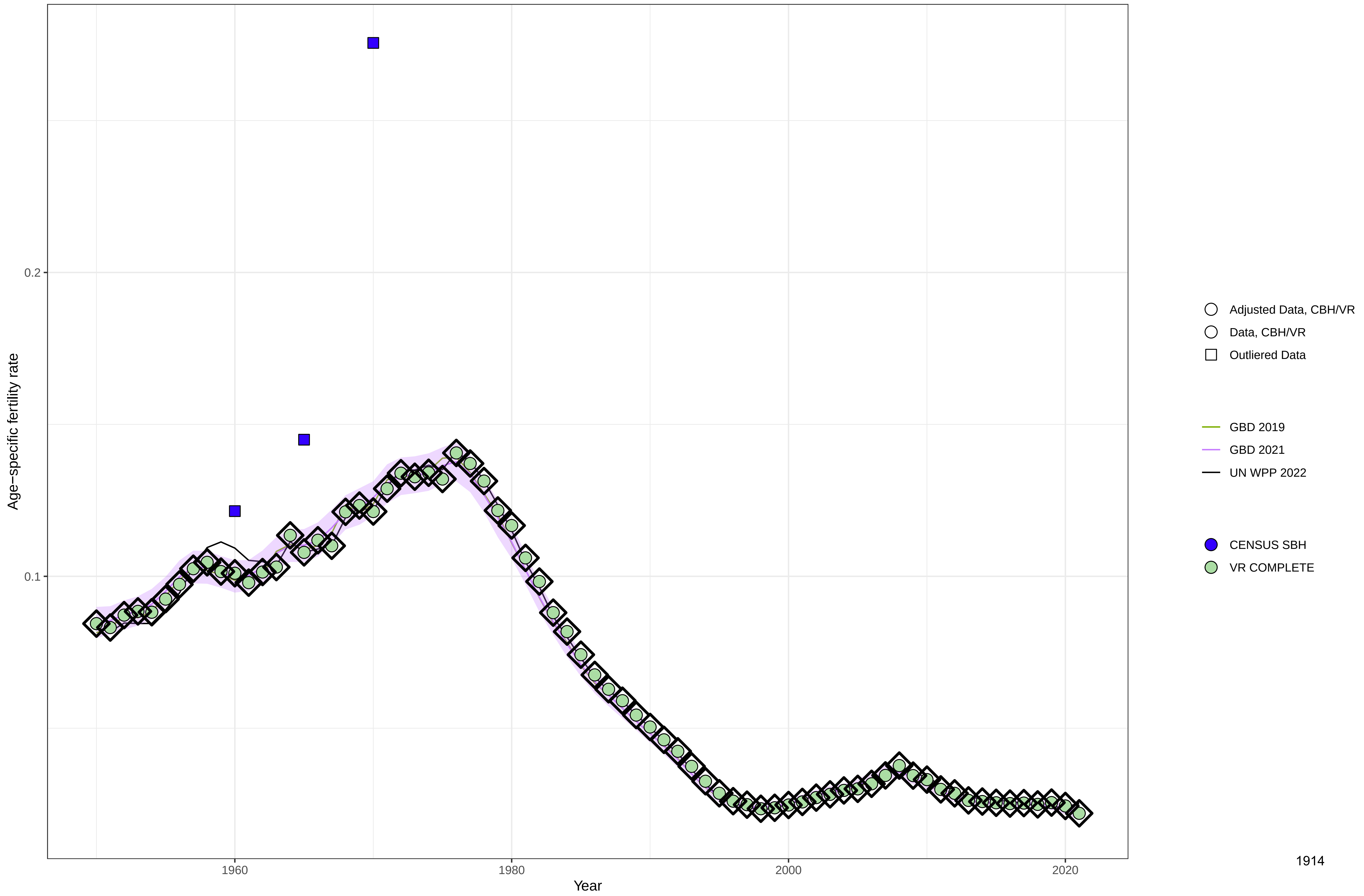

Age−specific fertility fitted trend lines, 1950−2021: Spain, Ages: 25−29
Data points enclosed with a diamond are reference sources

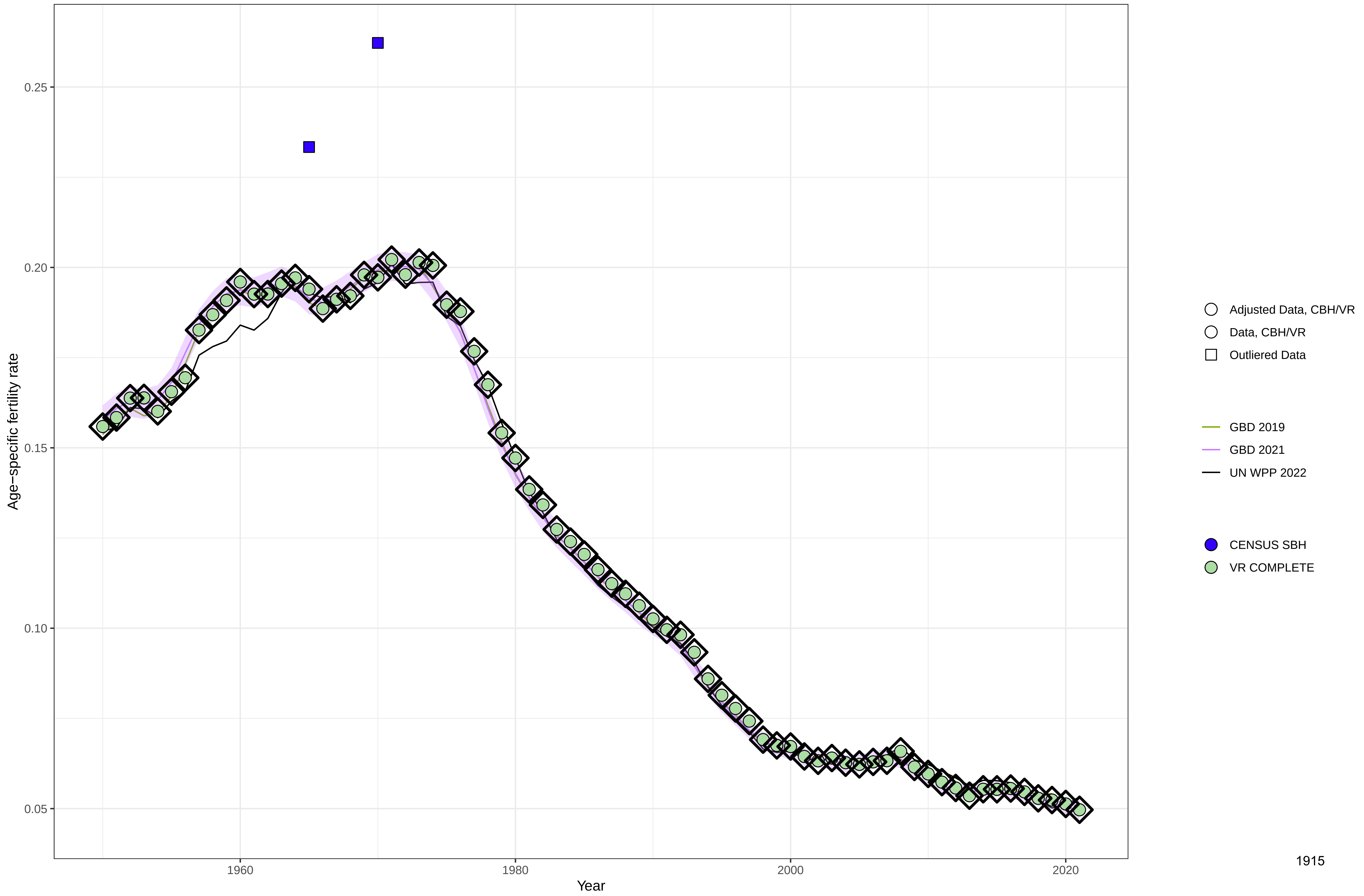

Age−specific fertility fitted trend lines, 1950−2021: Spain, Ages: 30−34
Data points enclosed with a diamond are reference sources

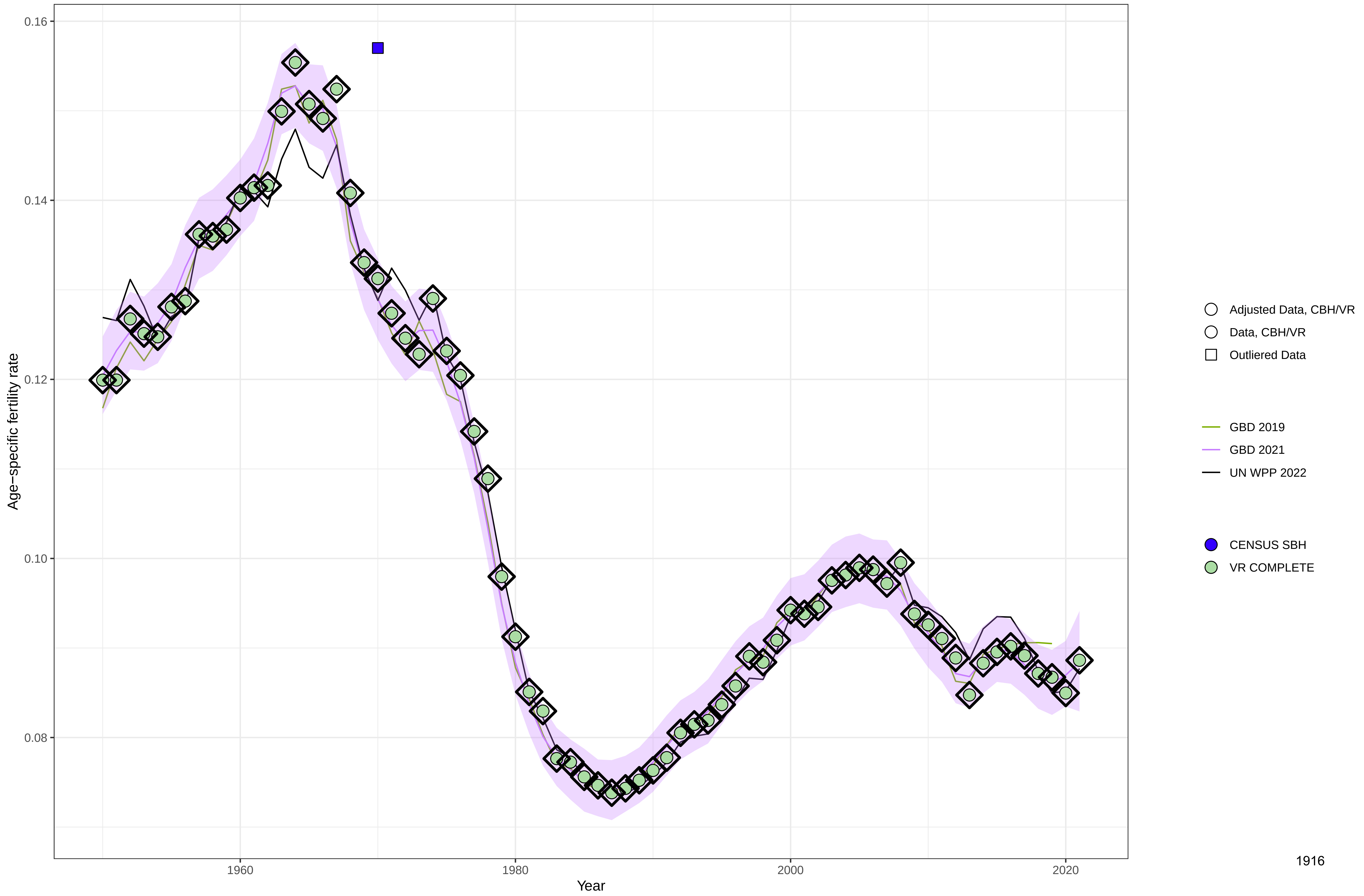

Age−specific fertility fitted trend lines, 1950−2021: Spain, Ages: 35−39
Data points enclosed with a diamond are reference sources

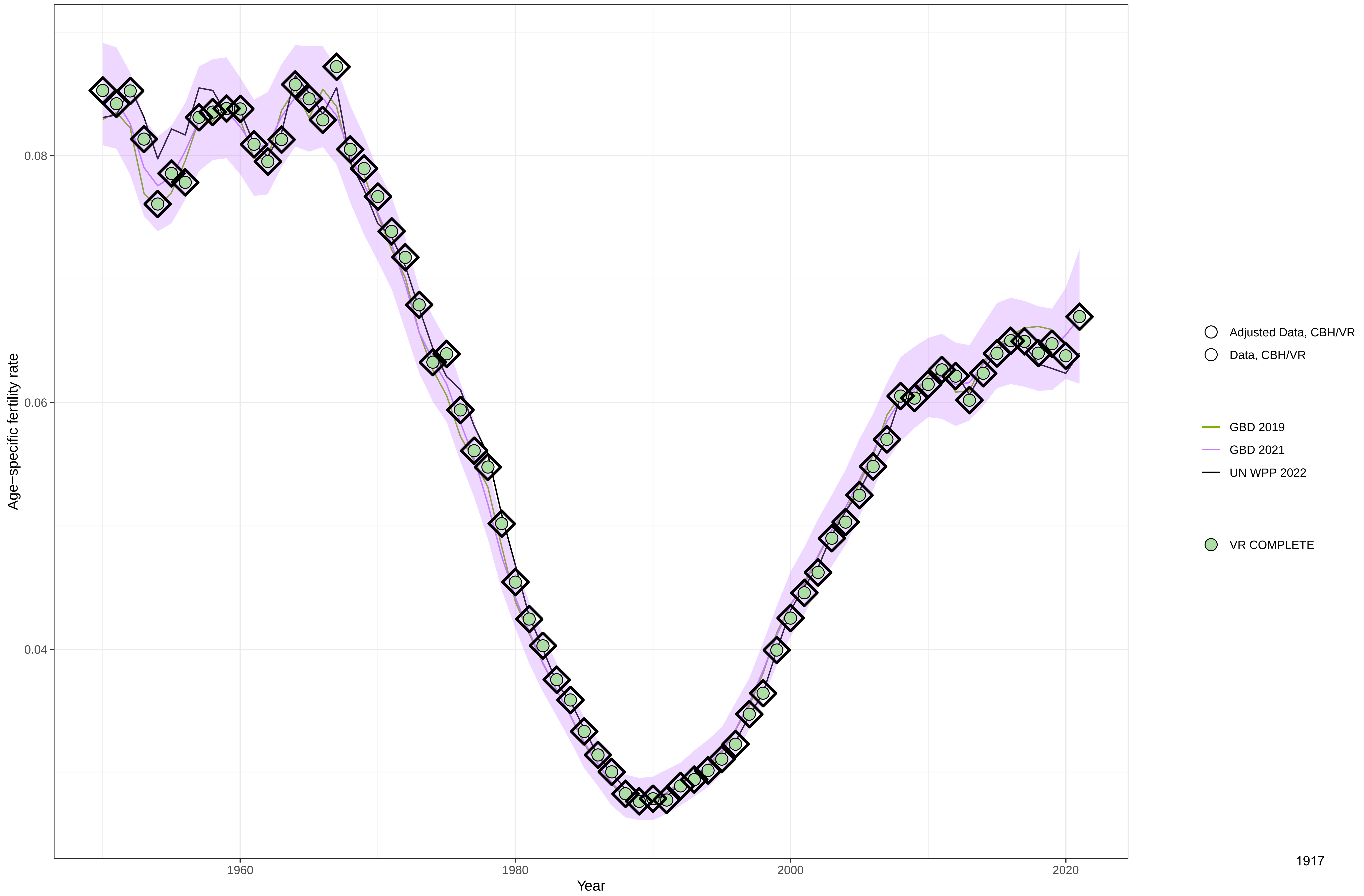

Age−specific fertility fitted trend lines, 1950−2021: Spain, Ages: 40−44
Data points enclosed with a diamond are reference sources

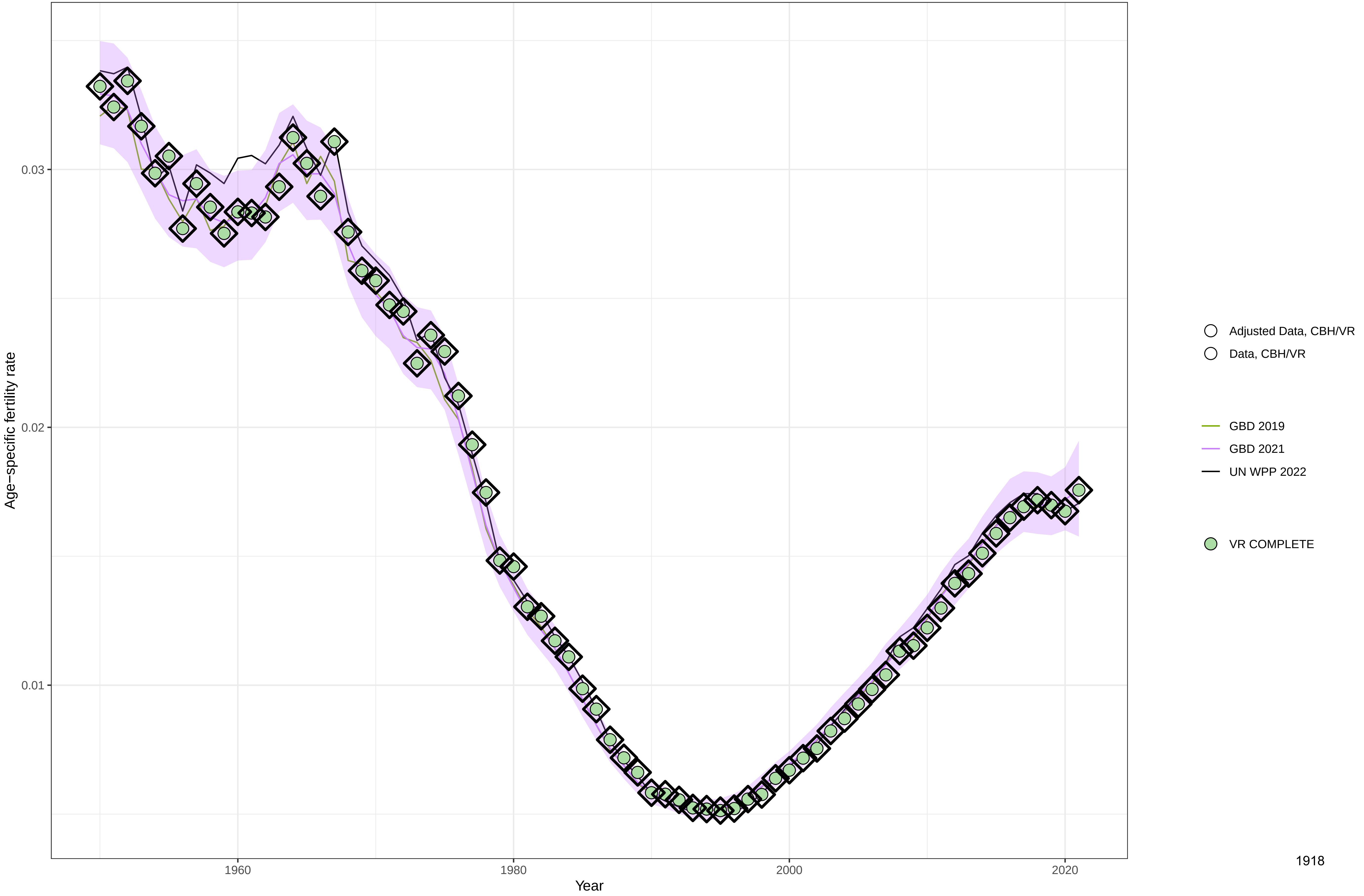

Age−specific fertility fitted trend lines, 1950−2021: Spain, Ages: 45−49
Data points enclosed with a diamond are reference sources

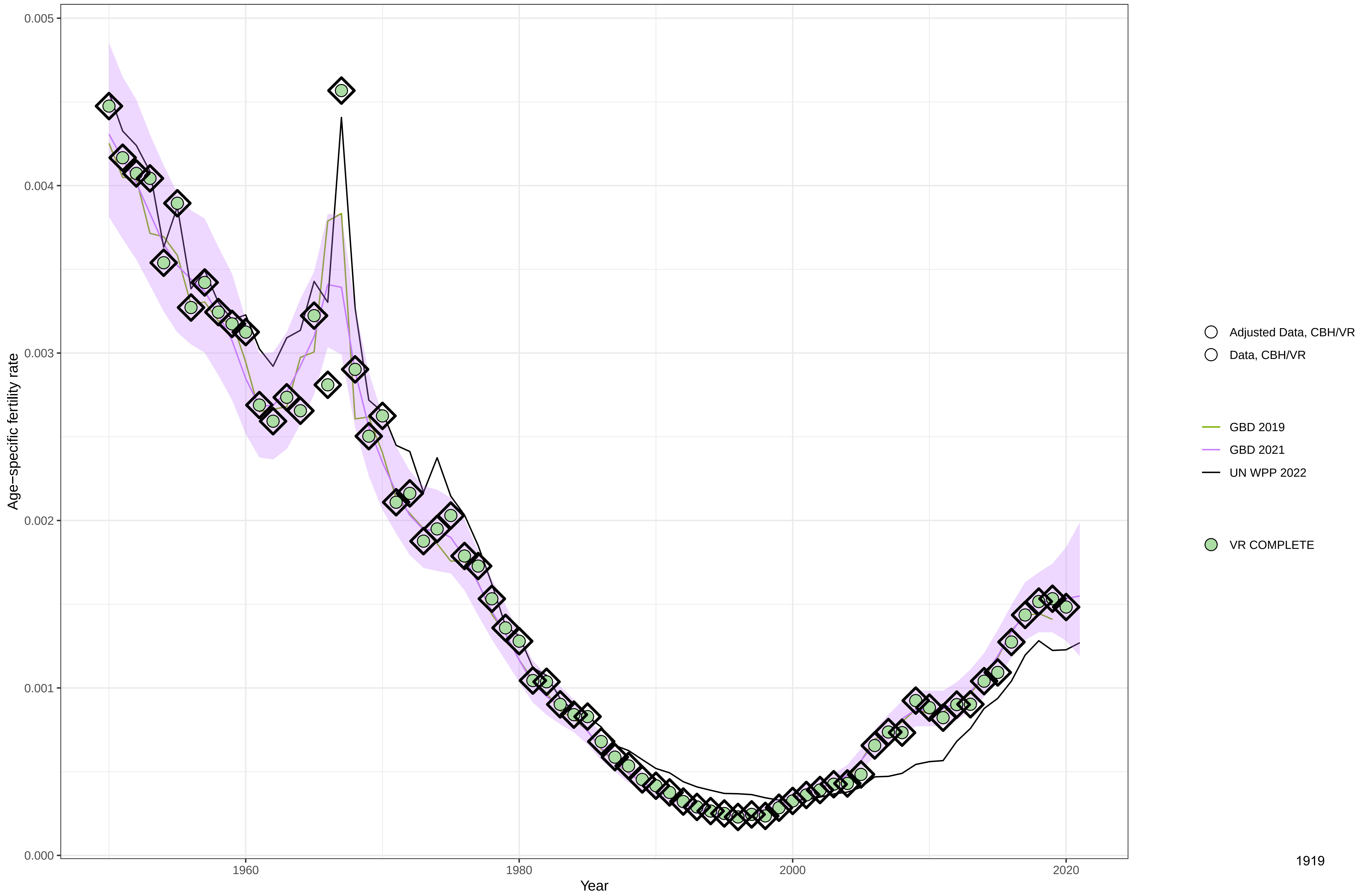

Age−specific fertility fitted trend lines, 1950−2021: Spain, Ages: 50−54
Data points enclosed with a diamond are reference sources

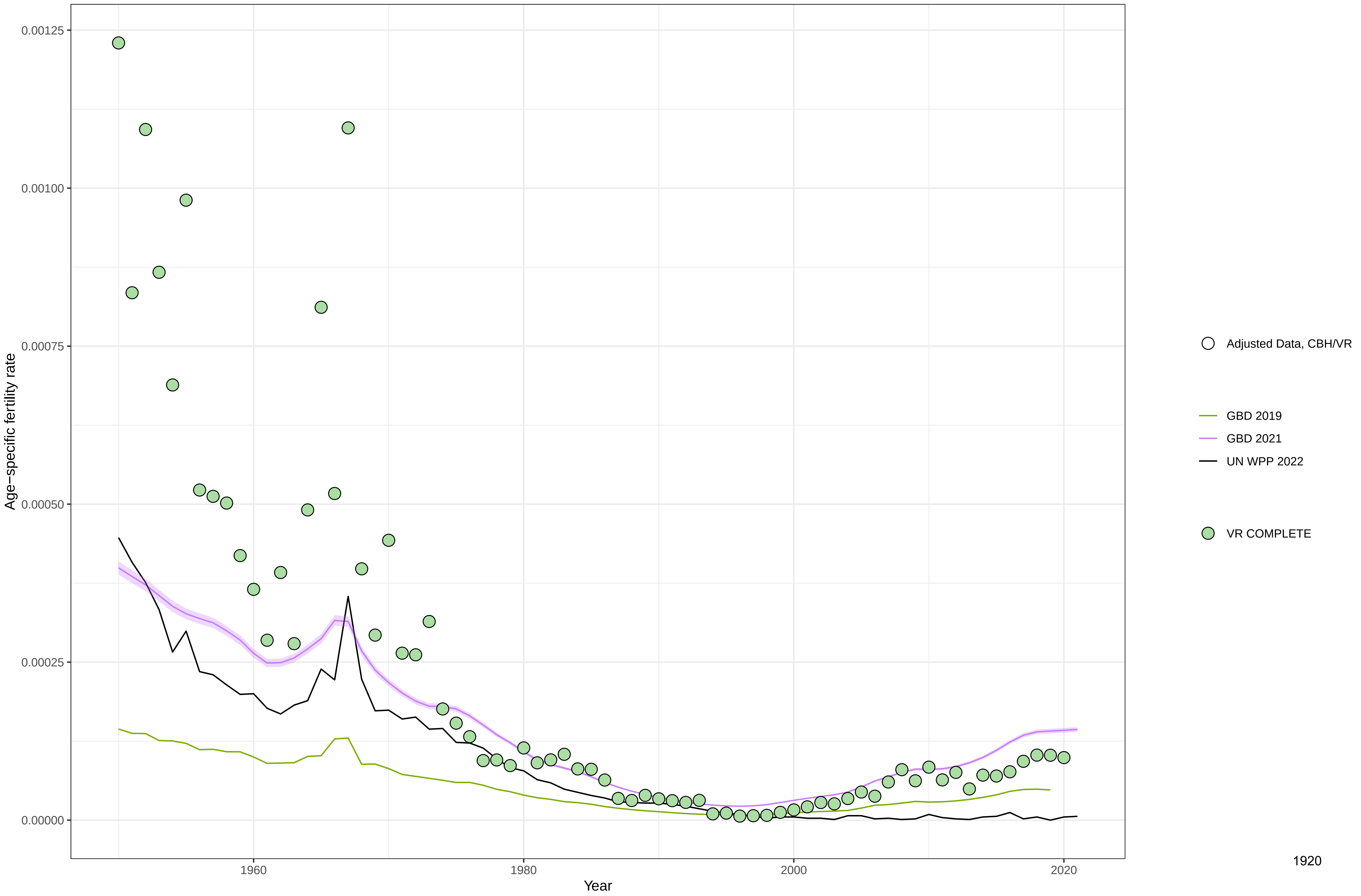

Total fertility rate fitted trend lines, 1950−2021: Spain TFR
Data points enclosed with a diamond are reference sources

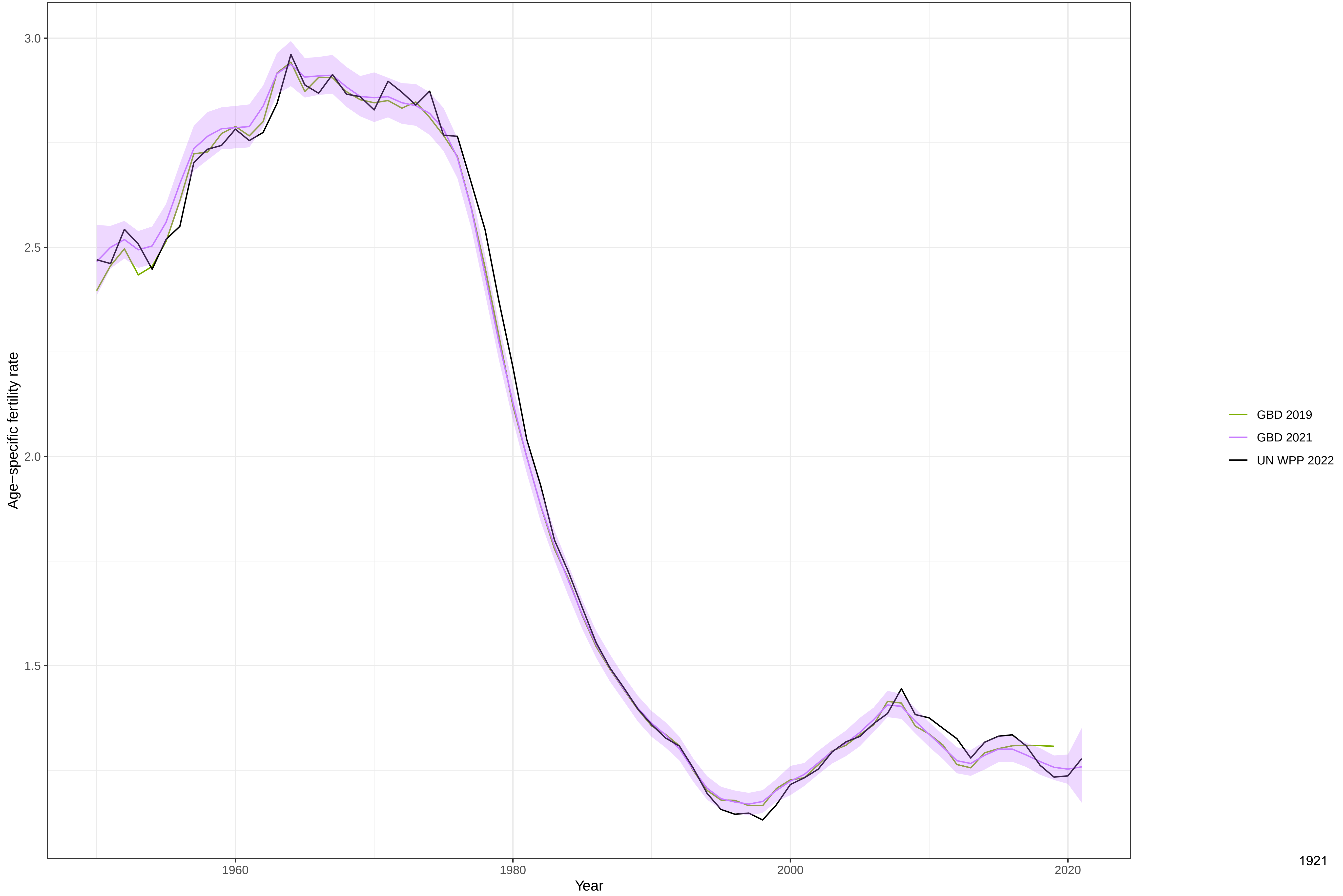

Age−specific fertility fitted trend lines, 1950−2021: Sweden, Ages: 10−14
Data points enclosed with a diamond are reference sources

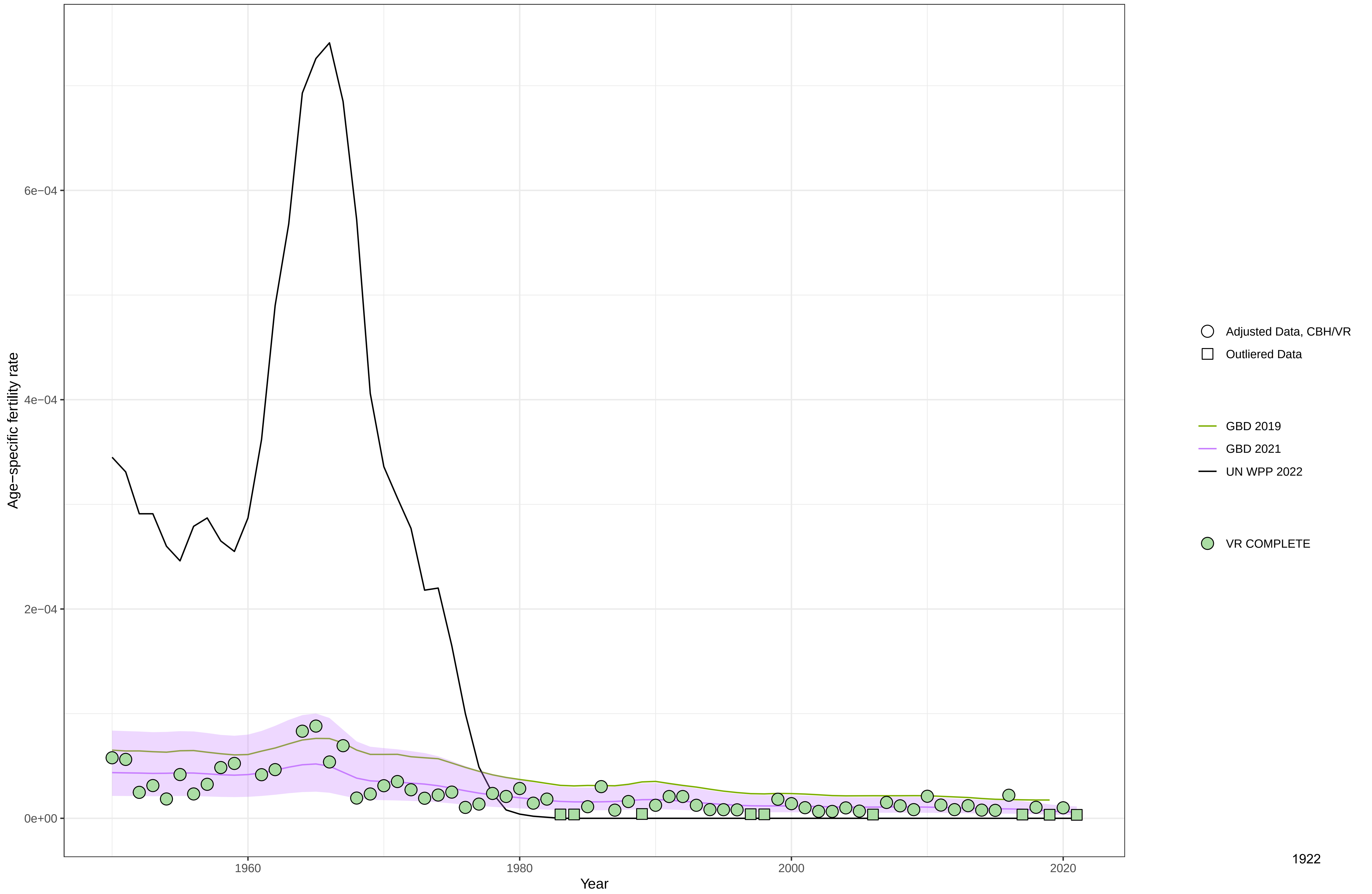

Age−specific fertility fitted trend lines, 1950−2021: Sweden, Ages: 15−19
Data points enclosed with a diamond are reference sources

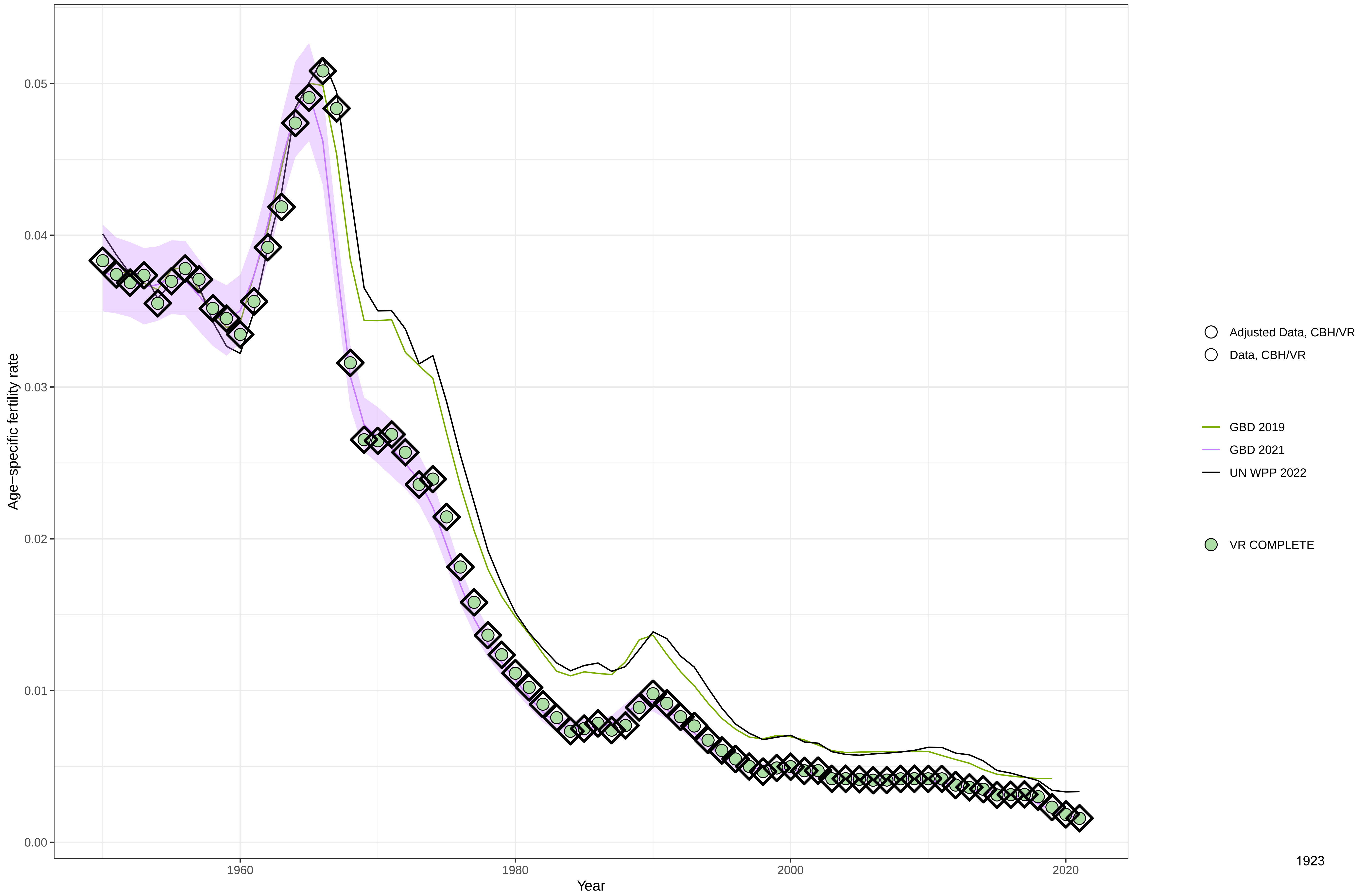

Age−specific fertility fitted trend lines, 1950−2021: Sweden, Ages: 20−24
Data points enclosed with a diamond are reference sources

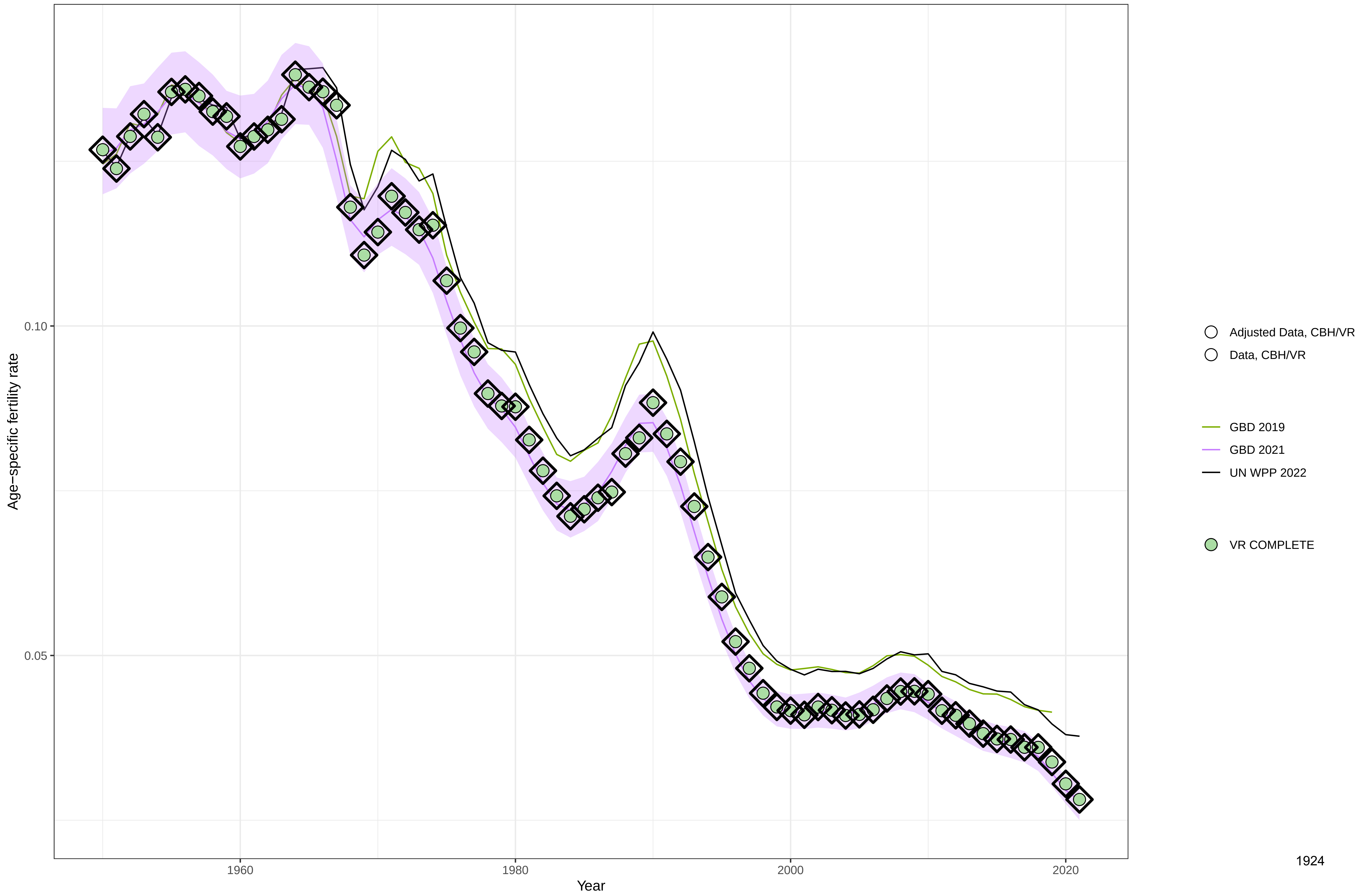

Age−specific fertility fitted trend lines, 1950−2021: Sweden, Ages: 25−29
Data points enclosed with a diamond are reference sources

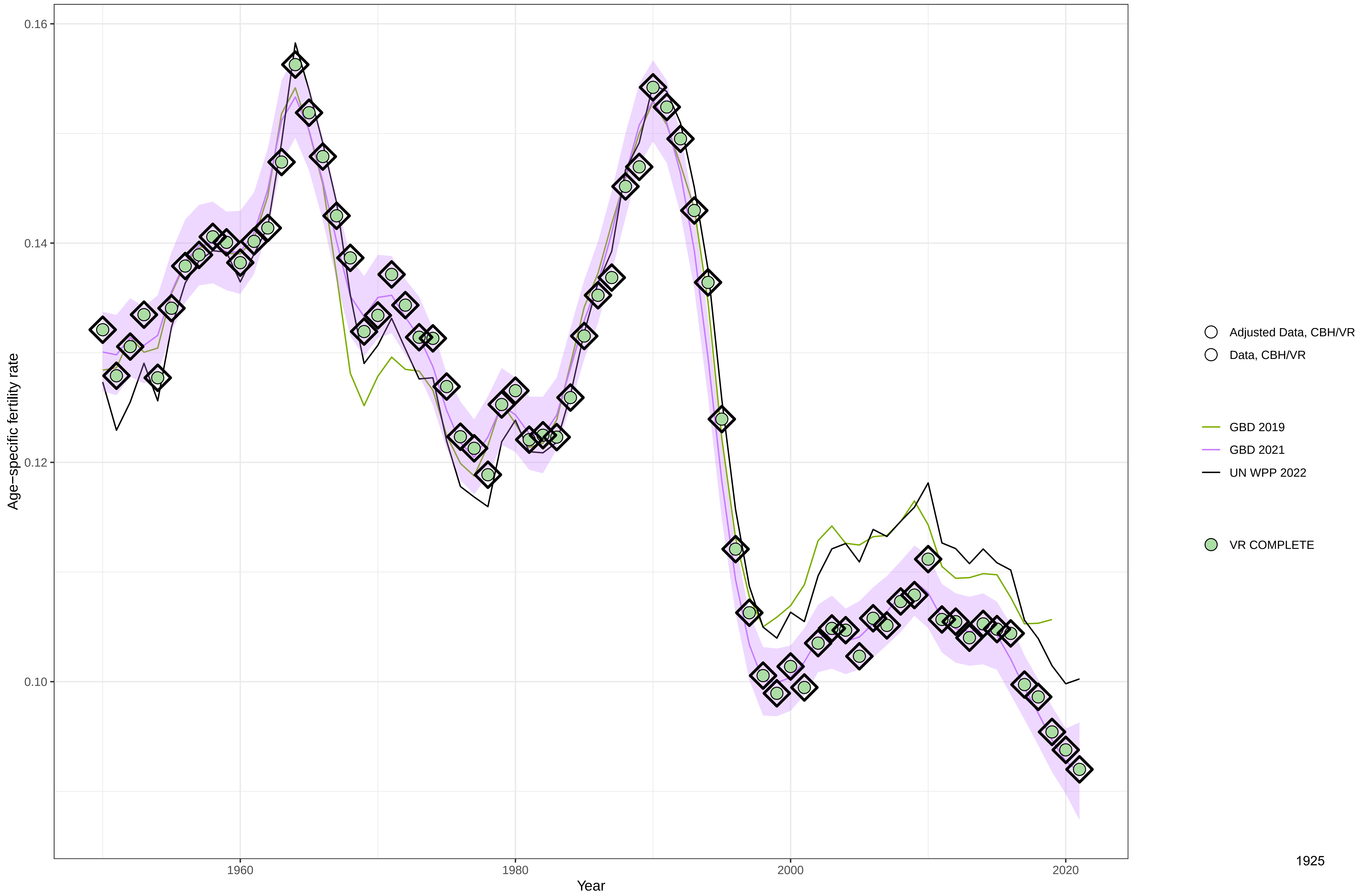

Age−specific fertility fitted trend lines, 1950−2021: Sweden, Ages: 30−34
Data points enclosed with a diamond are reference sources

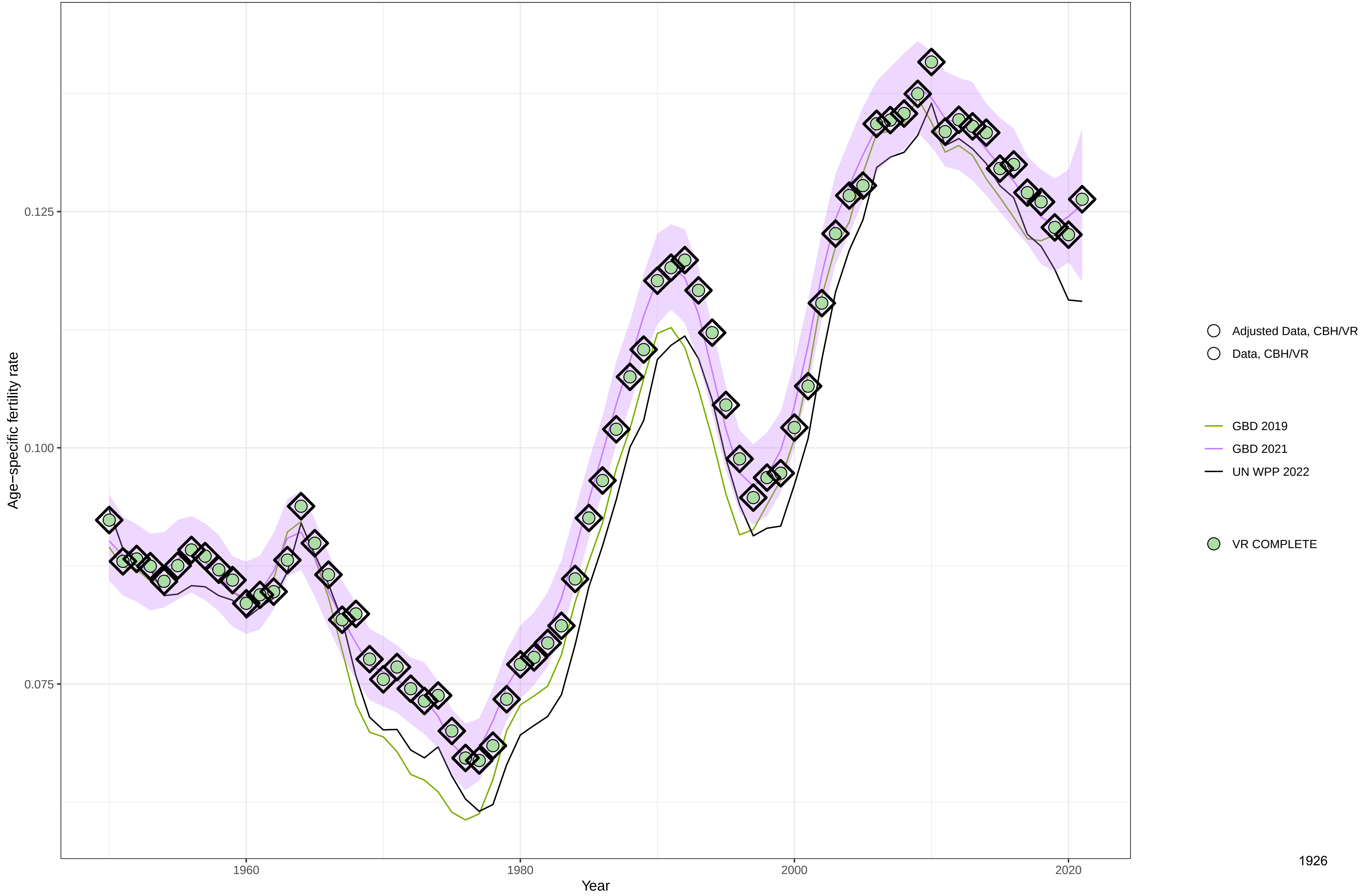

Age−specific fertility fitted trend lines, 1950−2021: Sweden, Ages: 35−39
Data points enclosed with a diamond are reference sources

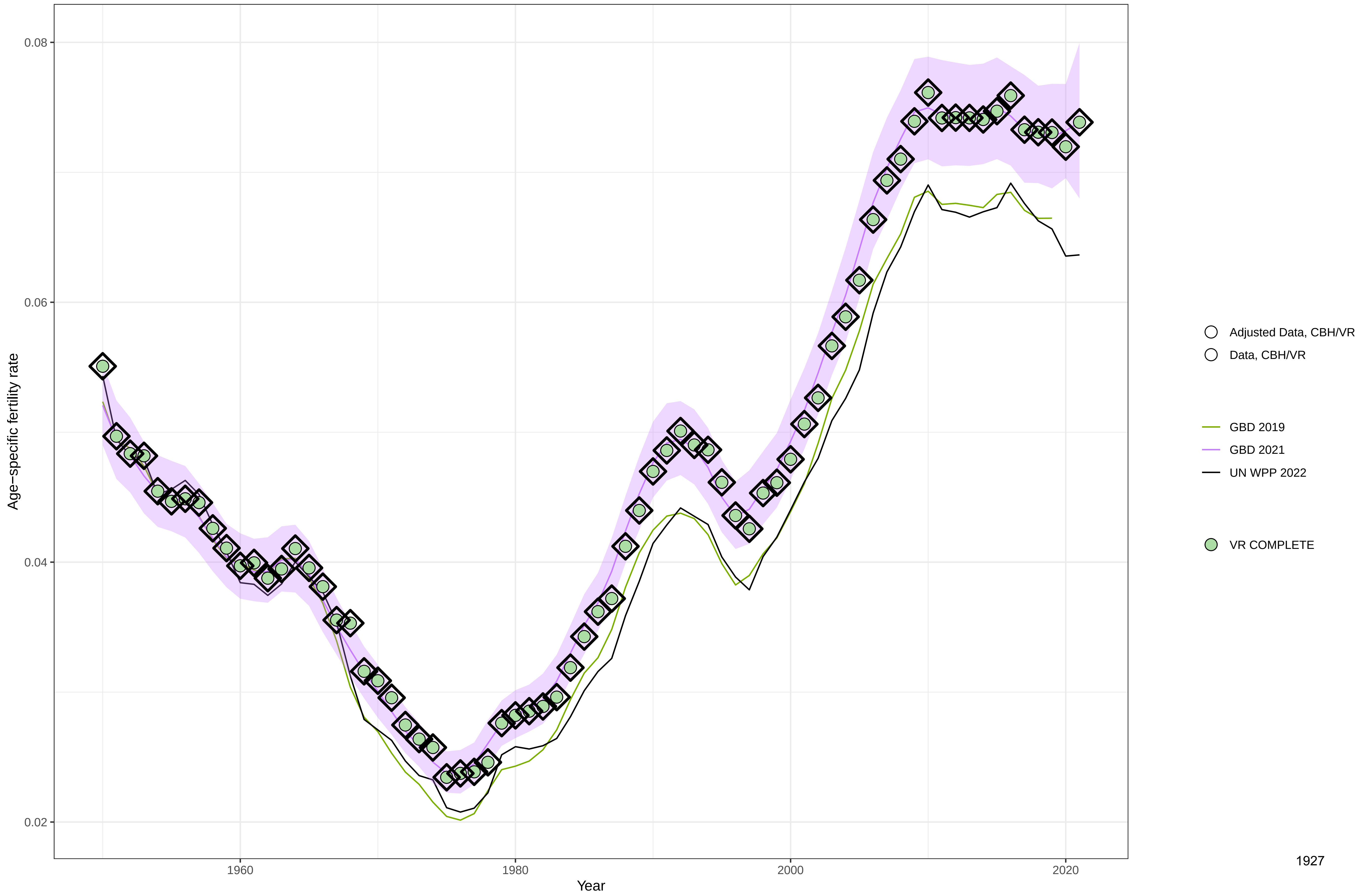

Age−specific fertility fitted trend lines, 1950−2021: Sweden, Ages: 40−44
Data points enclosed with a diamond are reference sources

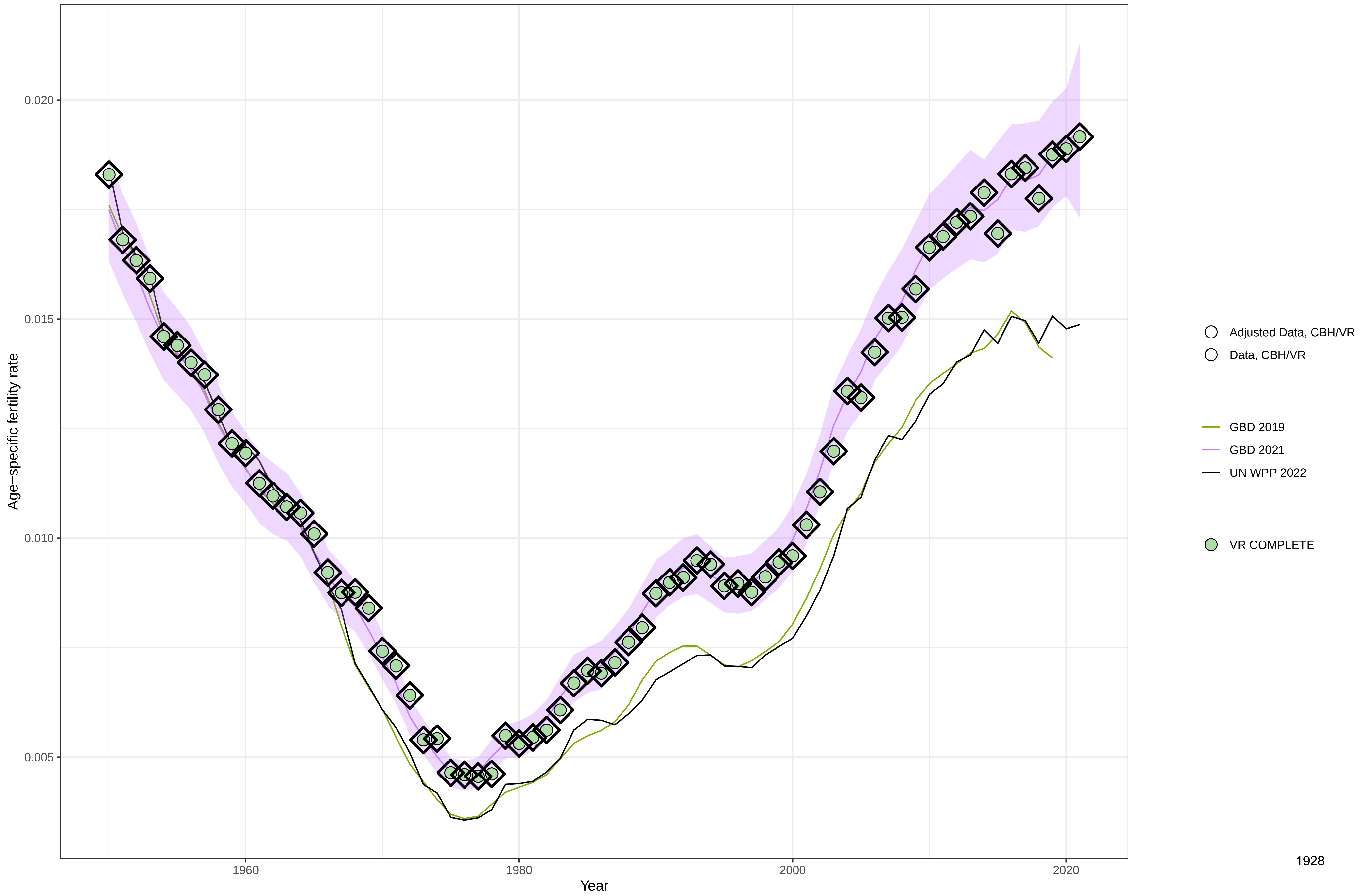

Age−specific fertility fitted trend lines, 1950−2021: Sweden, Ages: 45−49
Data points enclosed with a diamond are reference sources

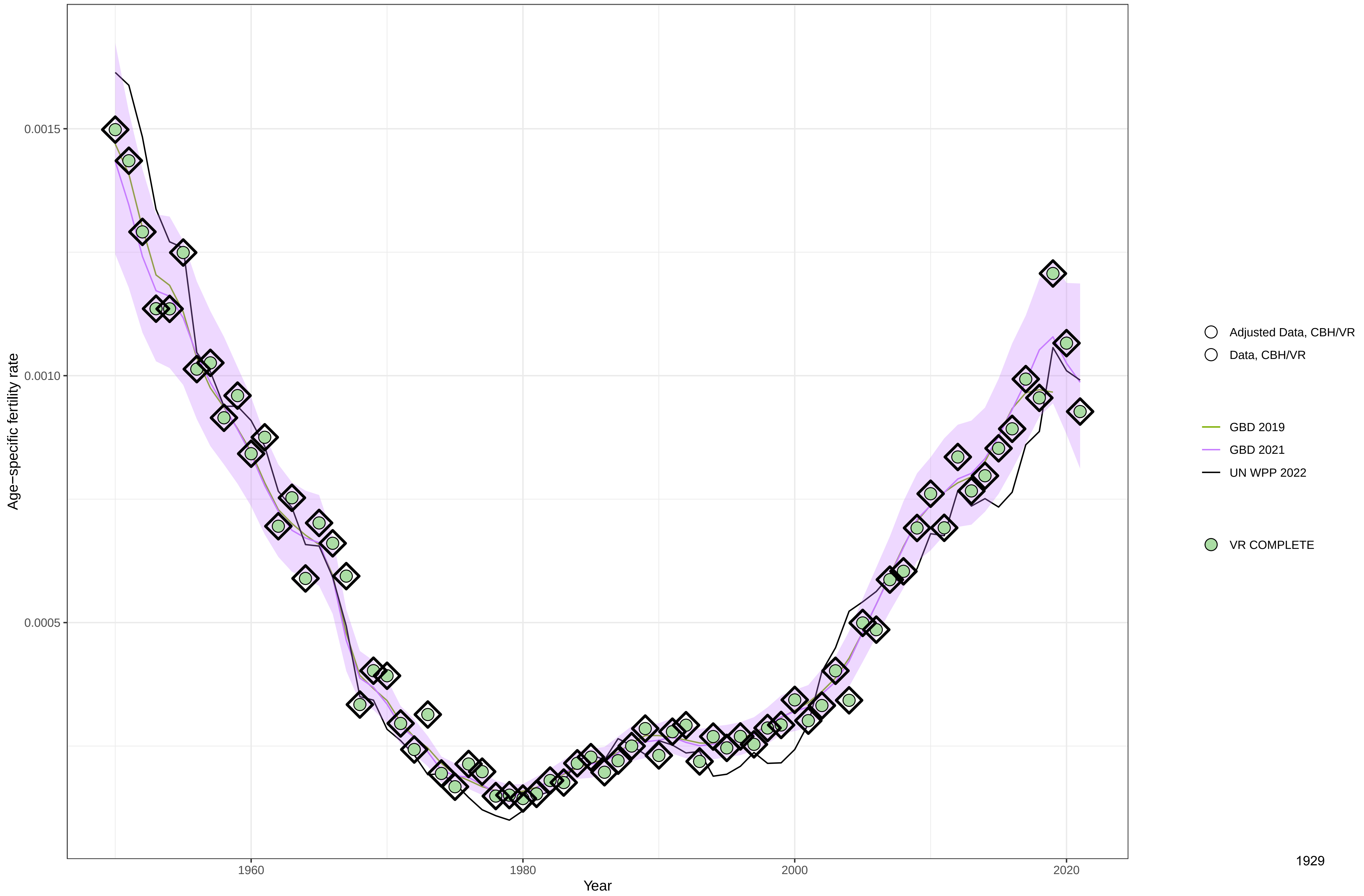

Age−specific fertility fitted trend lines, 1950−2021: Sweden, Ages: 50−54
Data points enclosed with a diamond are reference sources

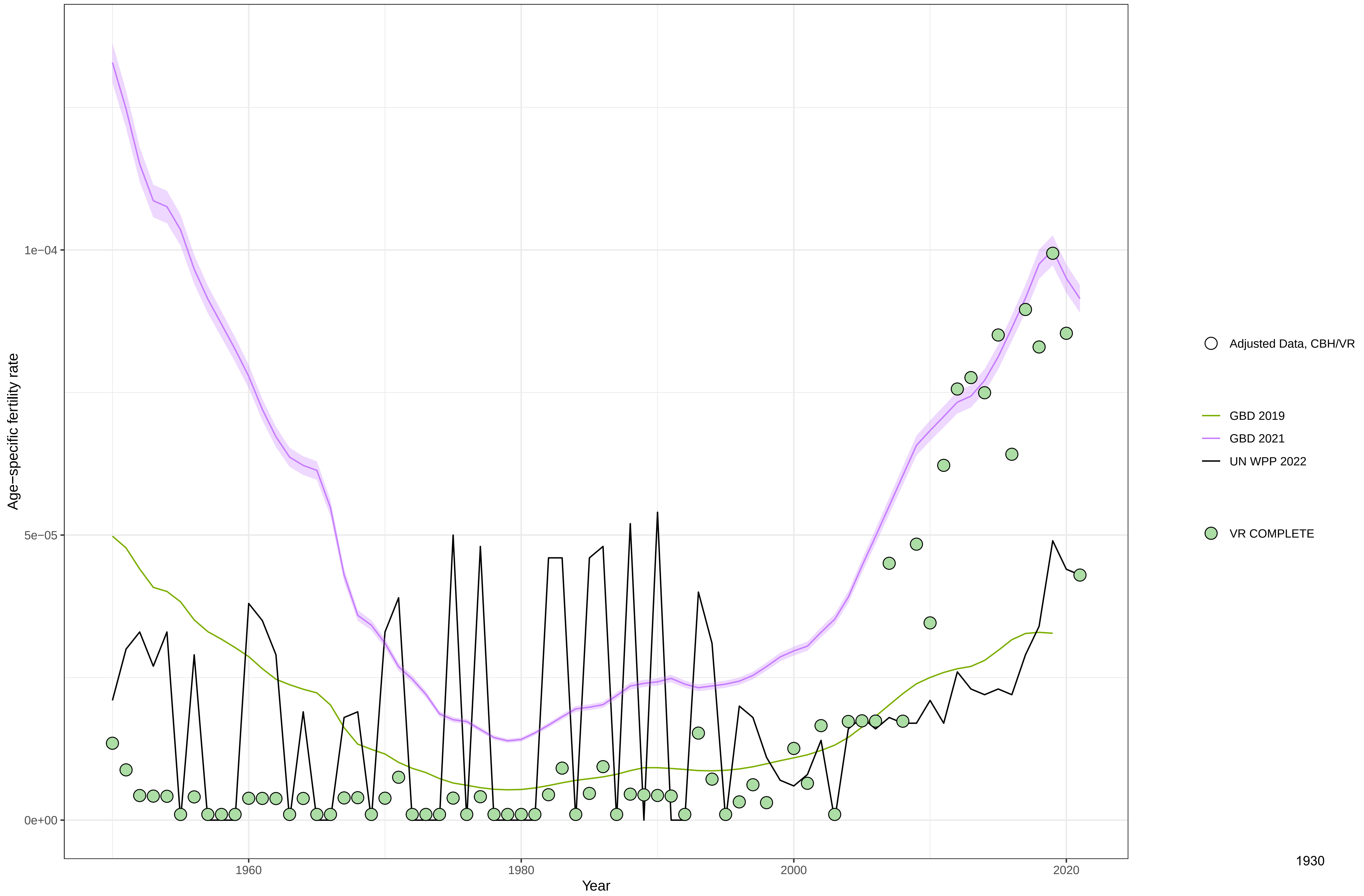

Total fertility rate fitted trend lines, 1950−2021: Sweden TFR
Data points enclosed with a diamond are reference sources

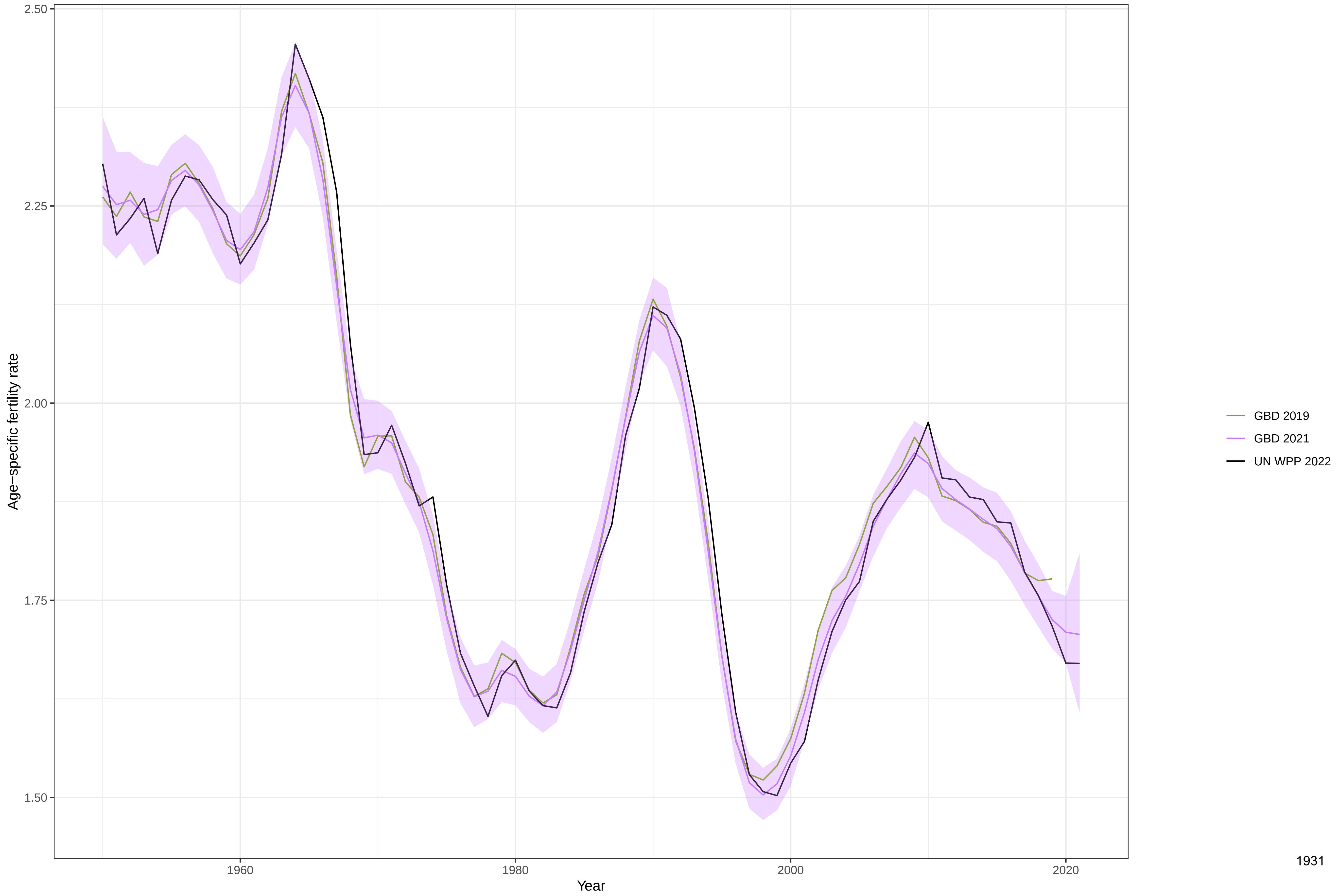

Age−specific fertility fitted trend lines, 1950−2021: Stockholm, Ages: 10−14
Data points enclosed with a diamond are reference sources

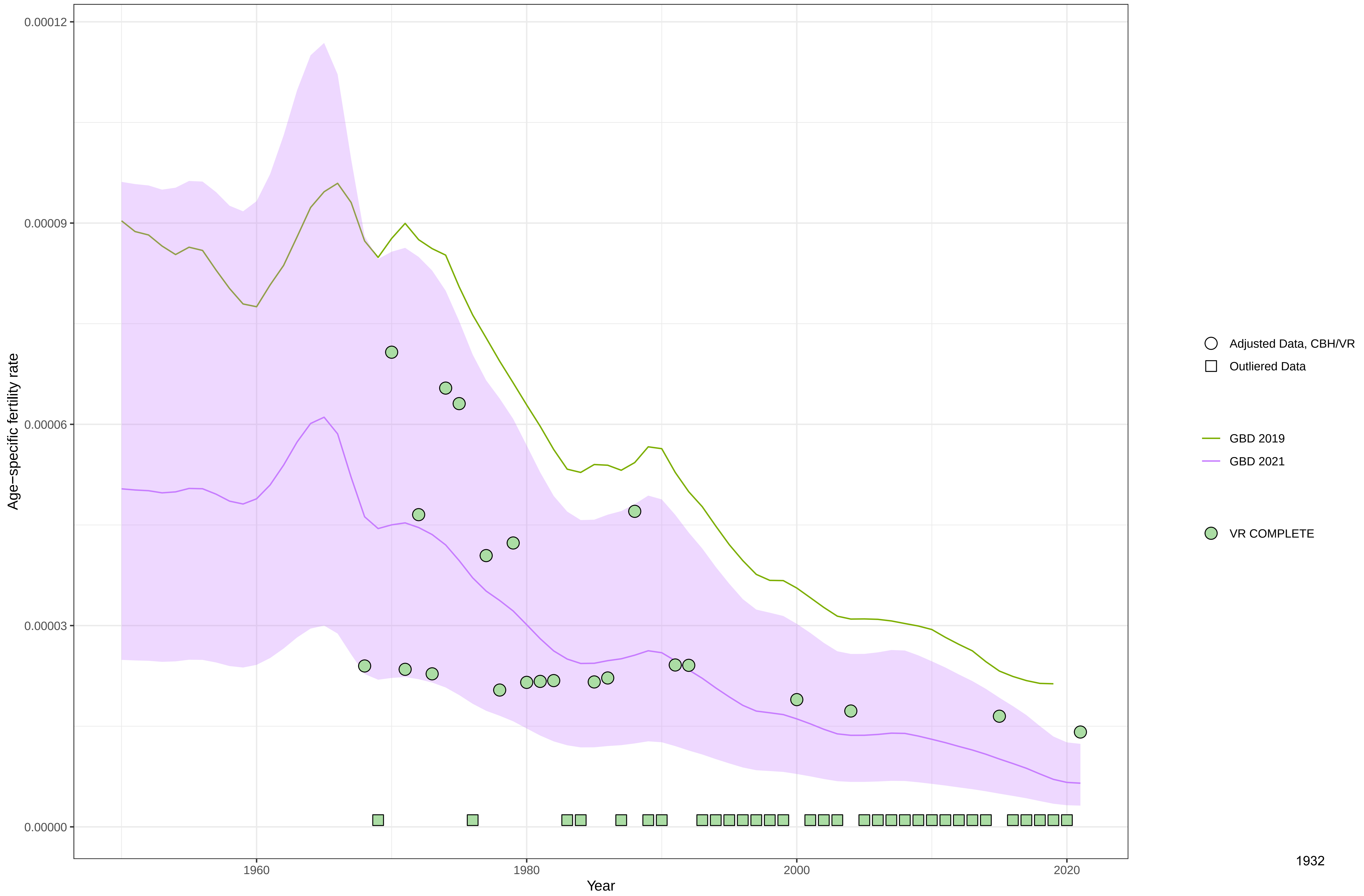

Age−specific fertility fitted trend lines, 1950−2021: Stockholm, Ages: 15−19
Data points enclosed with a diamond are reference sources

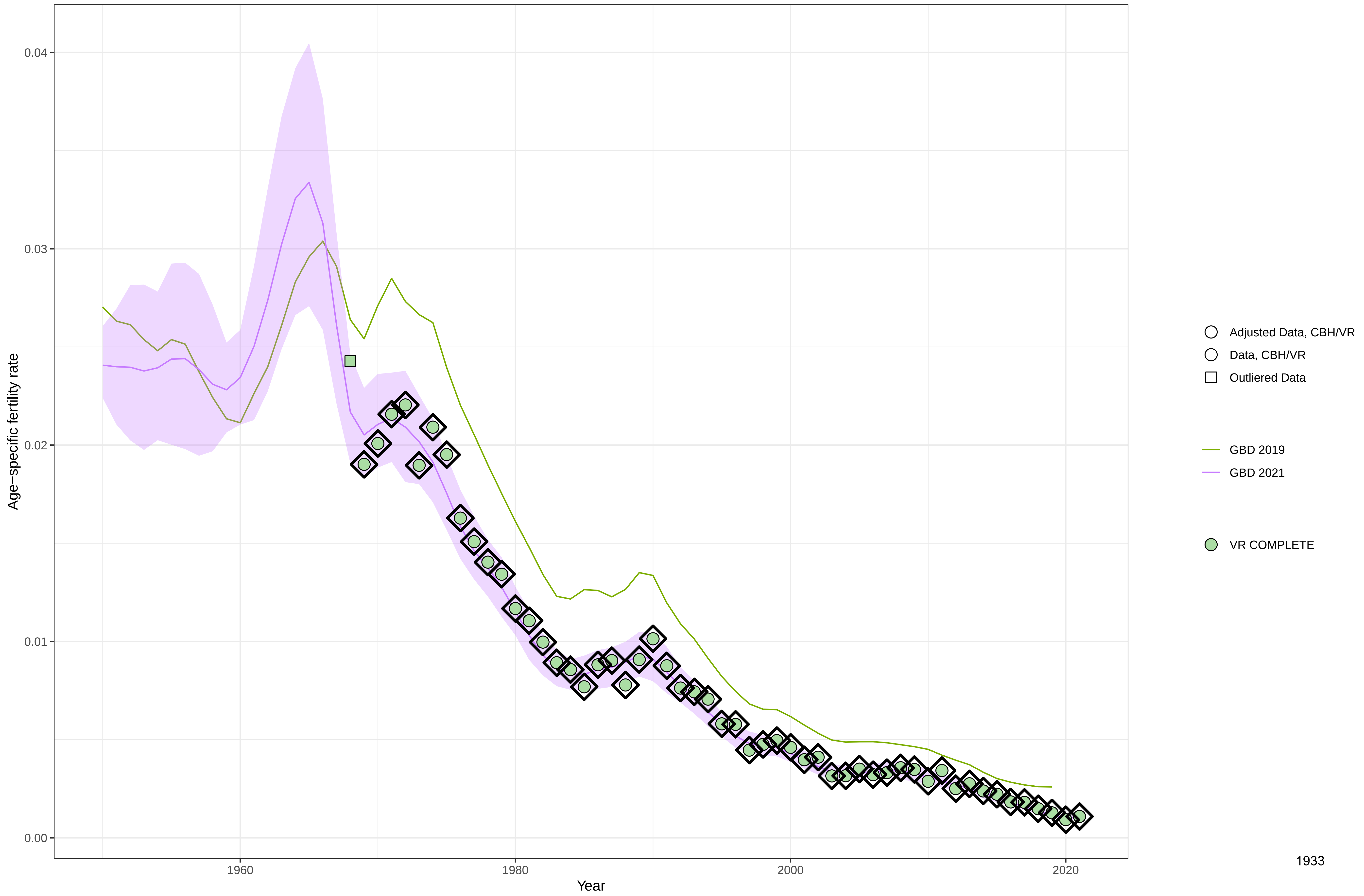

Age−specific fertility fitted trend lines, 1950−2021: Stockholm, Ages: 20−24
Data points enclosed with a diamond are reference sources

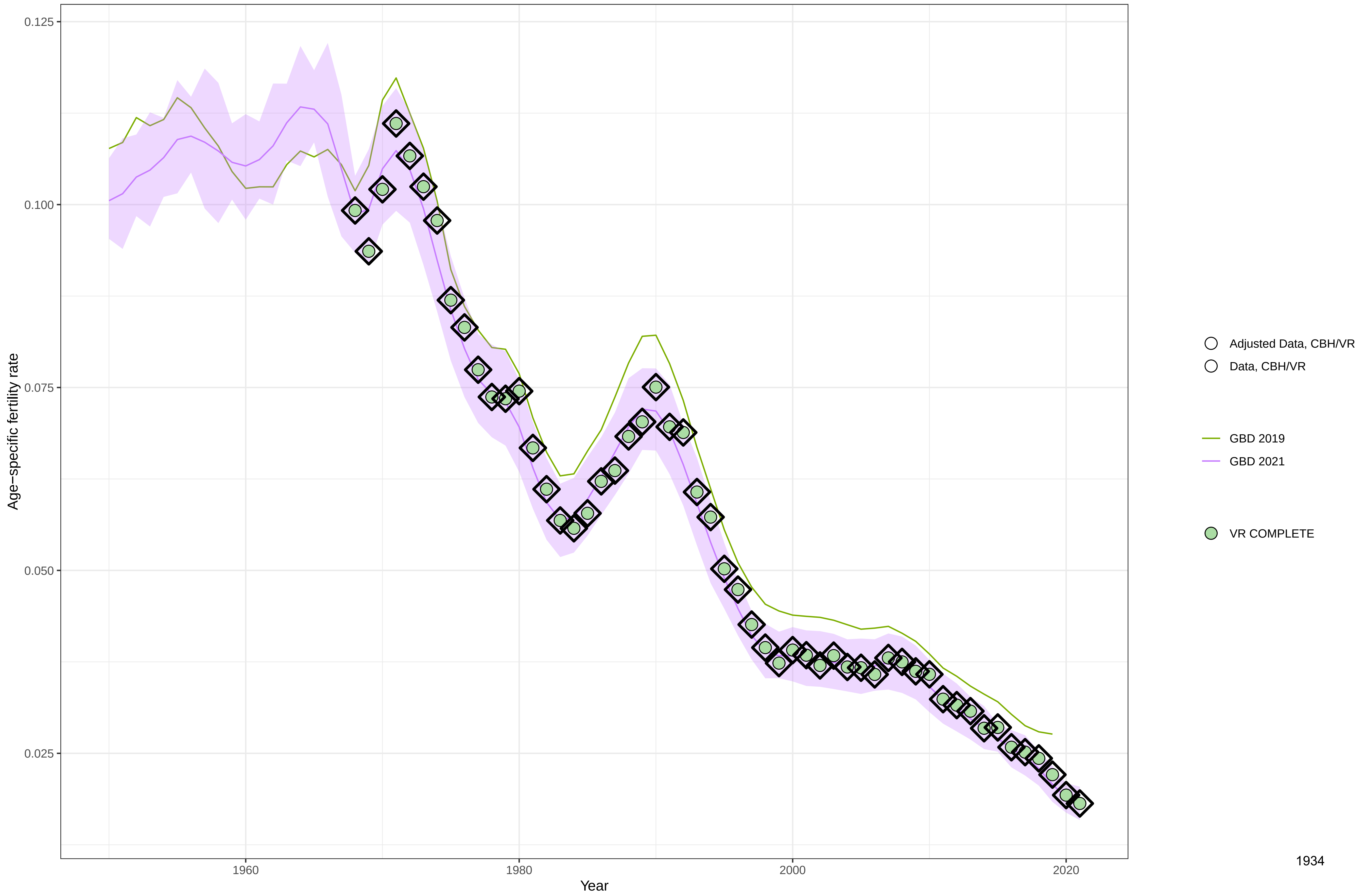

Age−specific fertility fitted trend lines, 1950−2021: Stockholm, Ages: 25−29
Data points enclosed with a diamond are reference sources

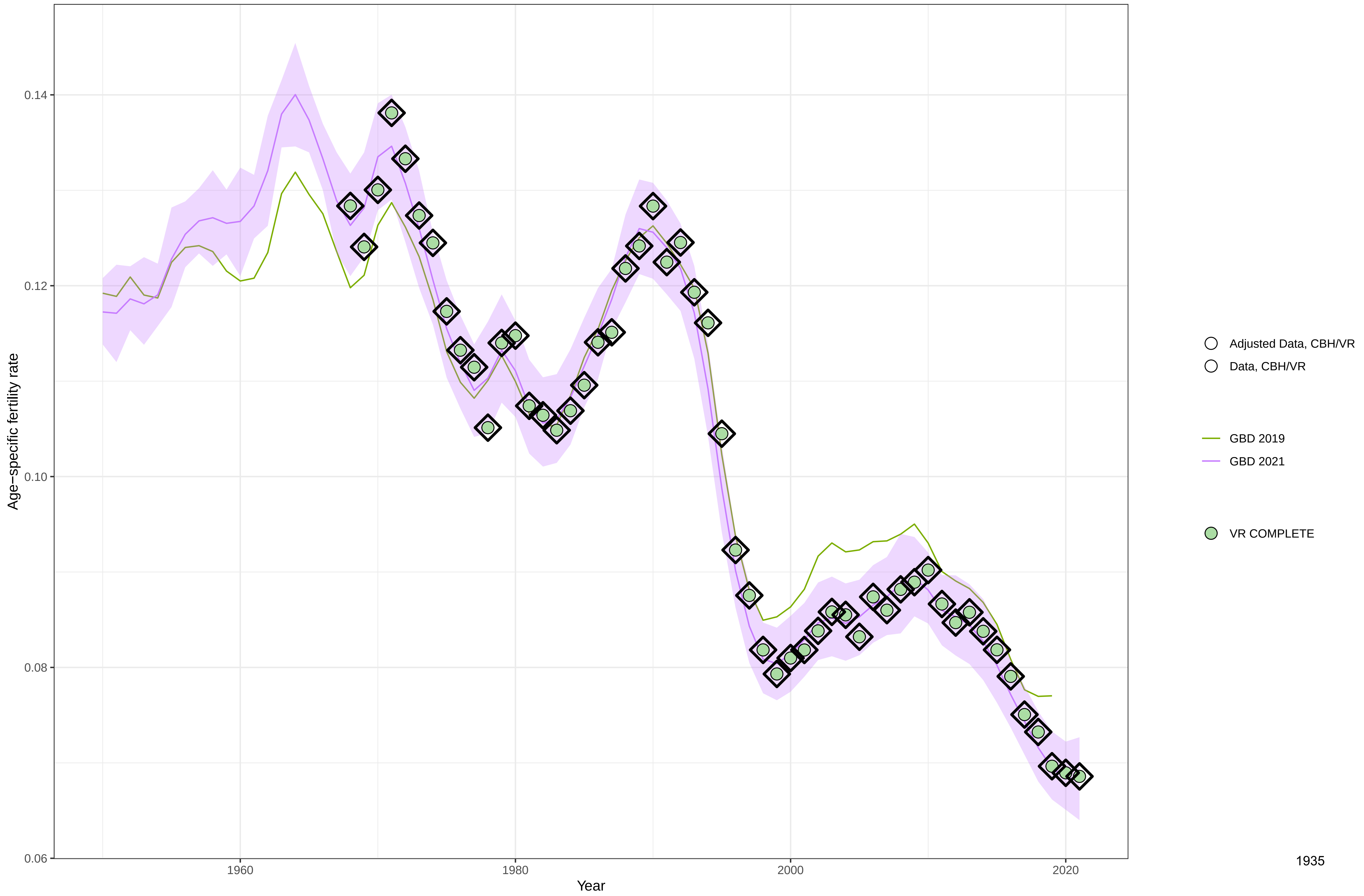

Age−specific fertility fitted trend lines, 1950−2021: Stockholm, Ages: 30−34
Data points enclosed with a diamond are reference sources

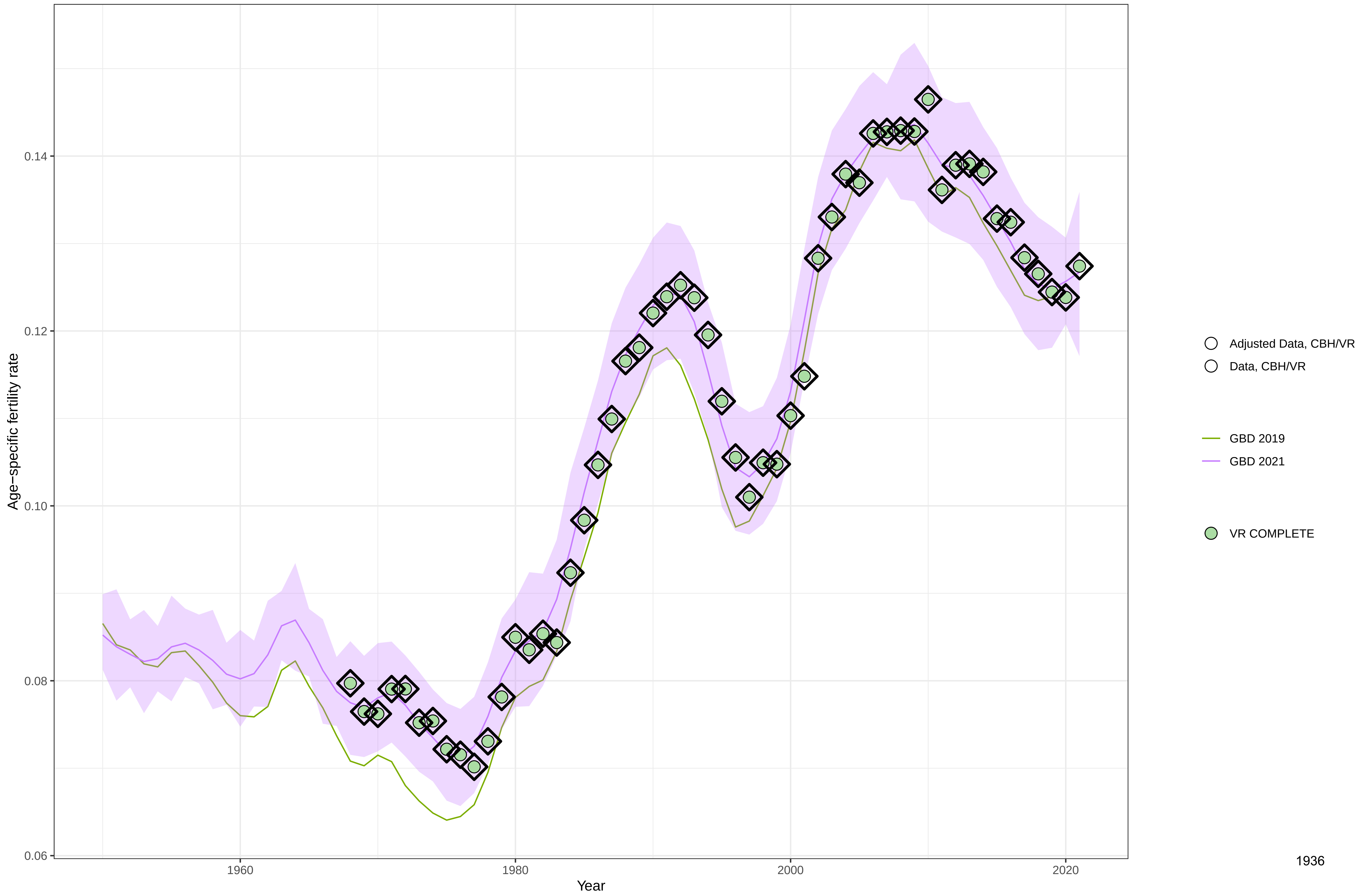

Age−specific fertility fitted trend lines, 1950−2021: Stockholm, Ages: 35−39
Data points enclosed with a diamond are reference sources

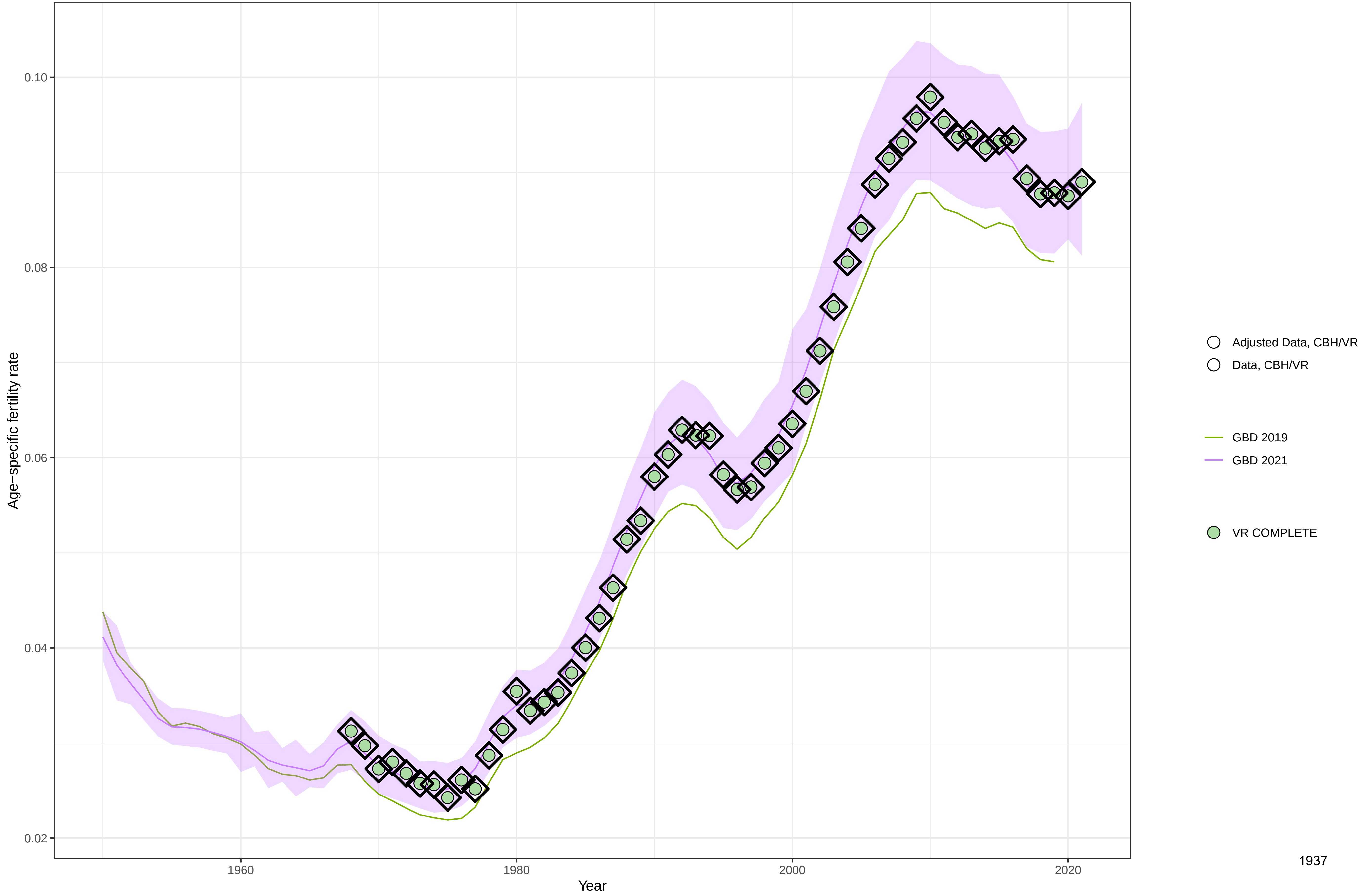

Age−specific fertility fitted trend lines, 1950−2021: Stockholm, Ages: 40−44
Data points enclosed with a diamond are reference sources

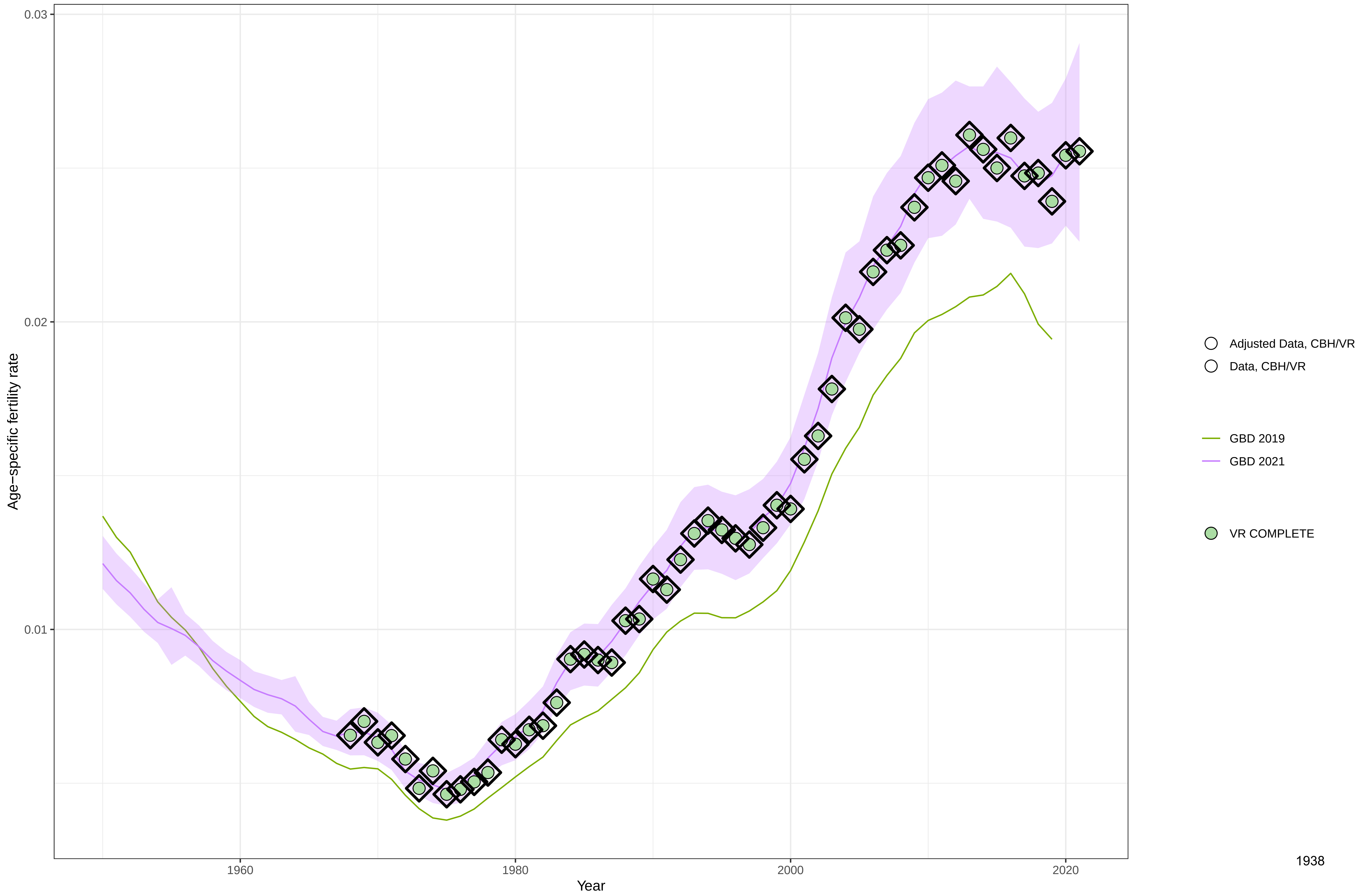

Age−specific fertility fitted trend lines, 1950−2021: Stockholm, Ages: 45−49
Data points enclosed with a diamond are reference sources

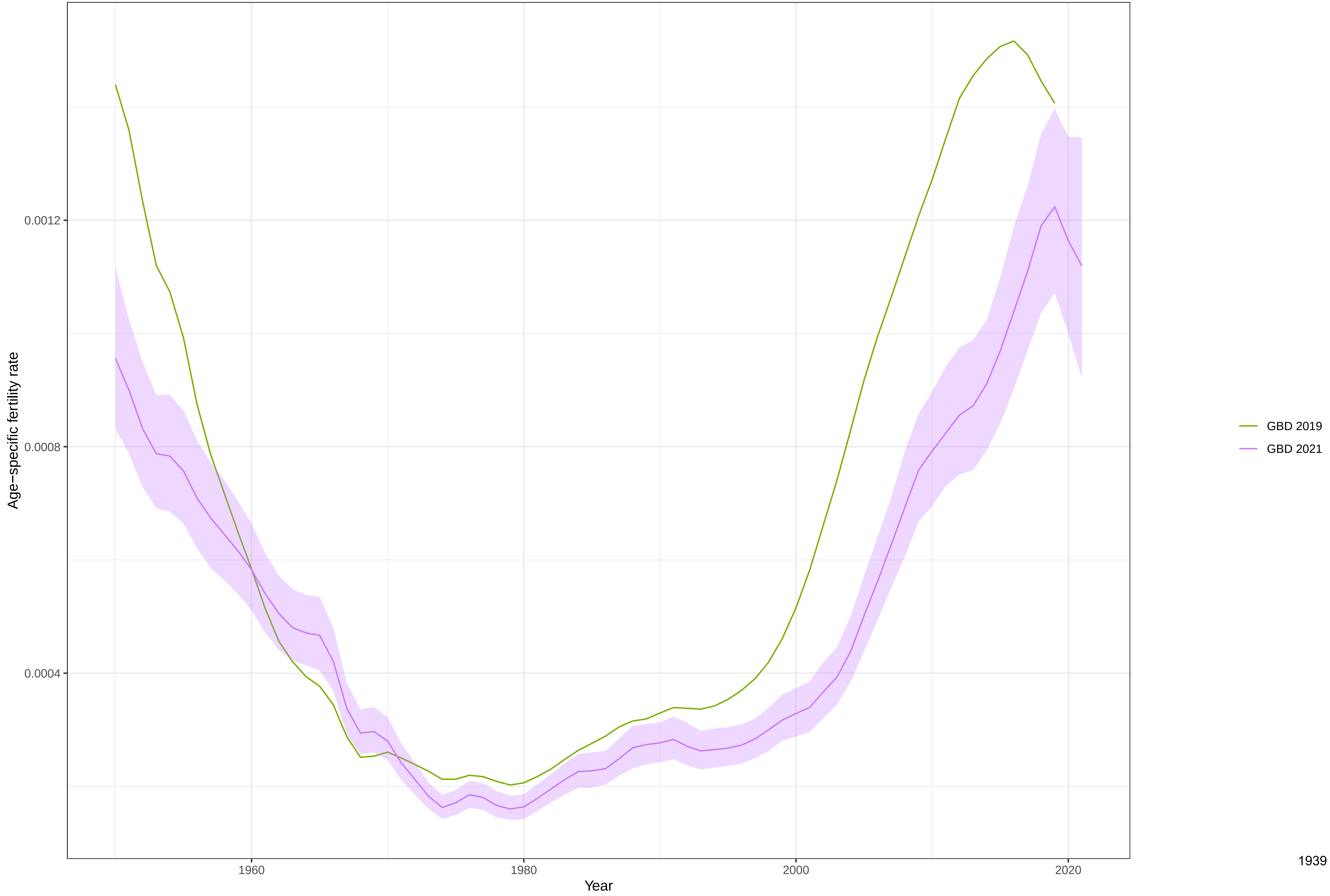

Age−specific fertility fitted trend lines, 1950−2021: Stockholm, Ages: 50−54
Data points enclosed with a diamond are reference sources

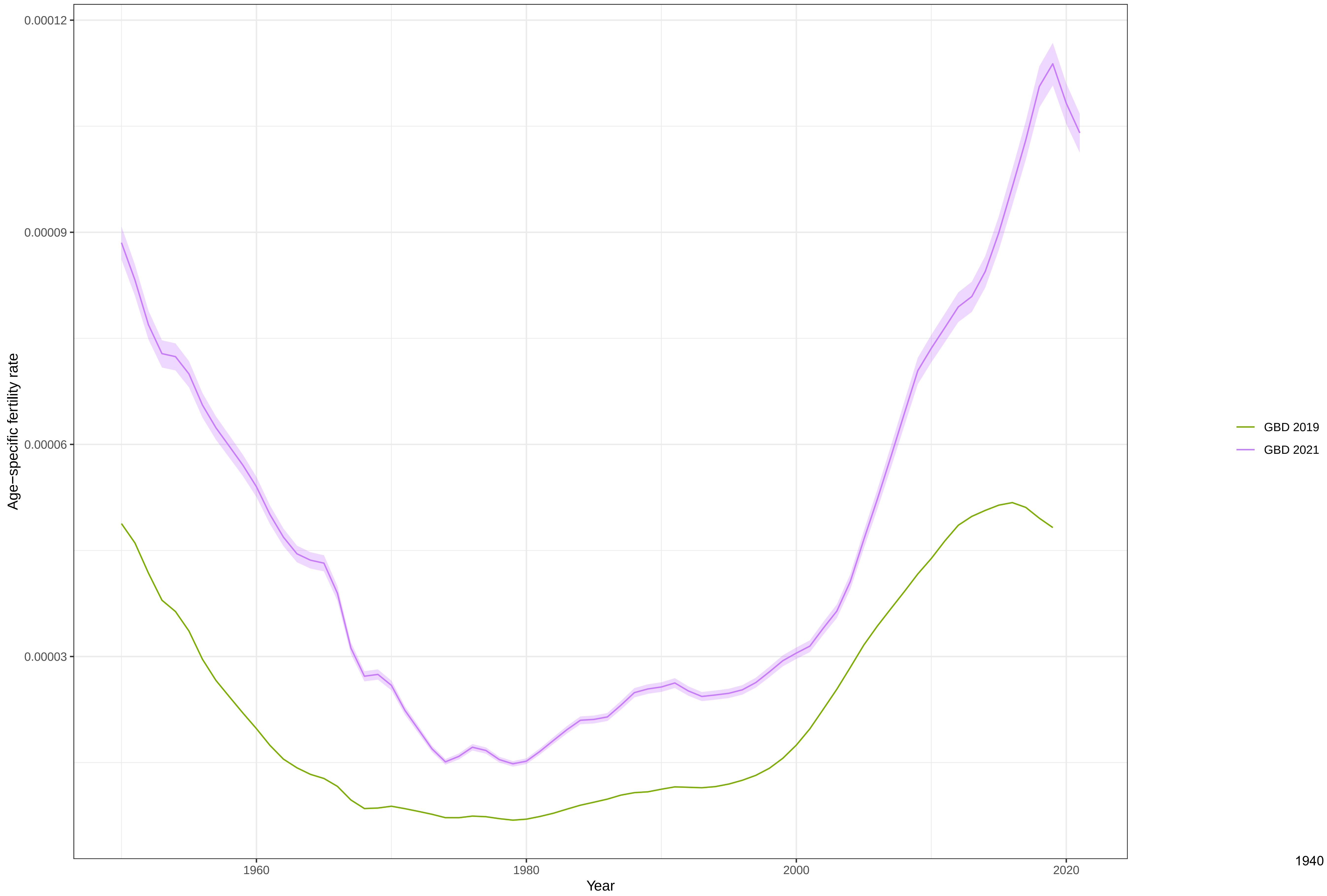

1940

Total fertility rate fitted trend lines, 1950−2021: Stockholm TFR
Data points enclosed with a diamond are reference sources

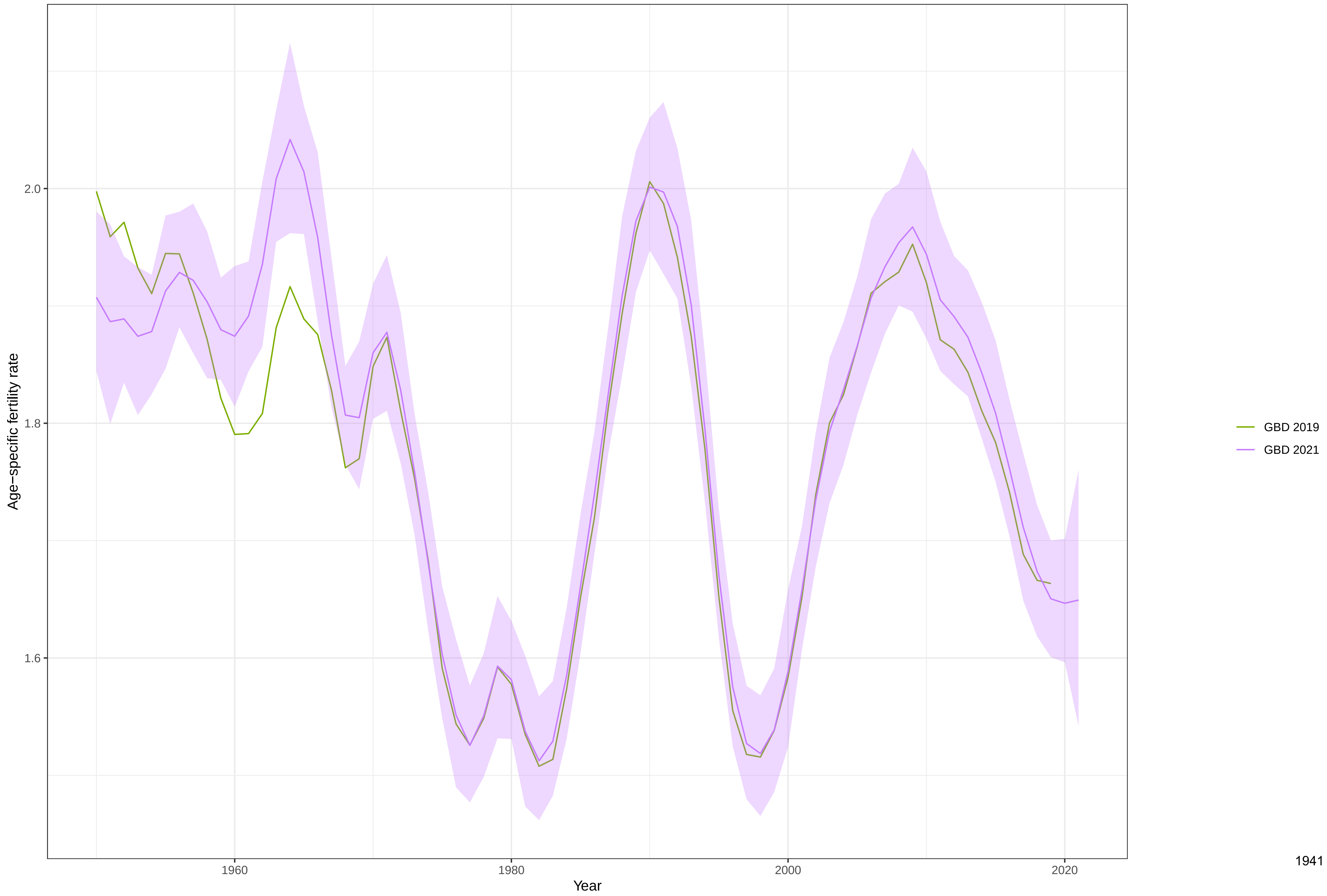

Age−specific fertility fitted trend lines, 1950−2021: Sweden except Stockholm, Ages: 10−14
Data points enclosed with a diamond are reference sources

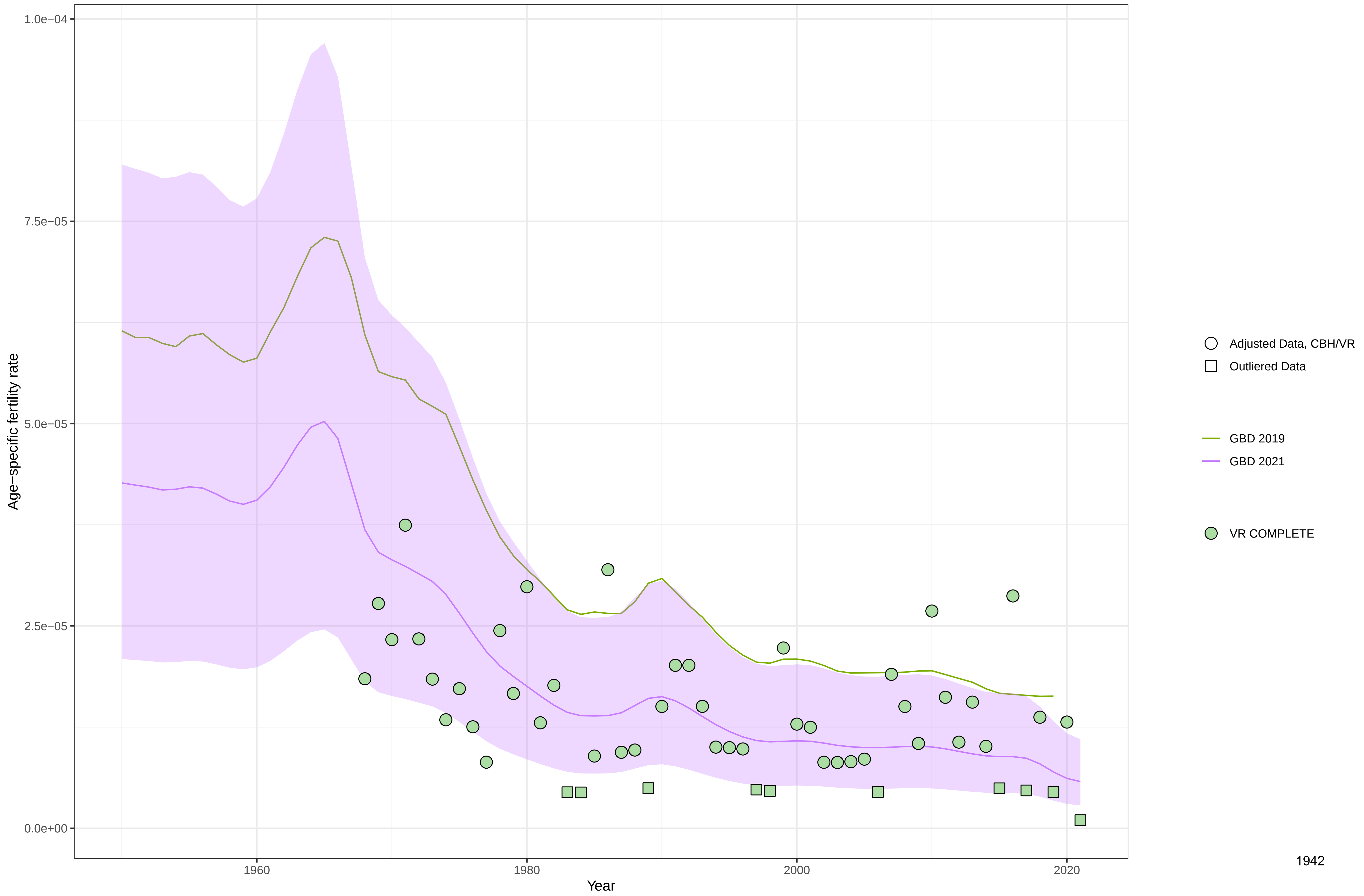

Age−specific fertility fitted trend lines, 1950−2021: Sweden except Stockholm, Ages: 15−19
Data points enclosed with a diamond are reference sources

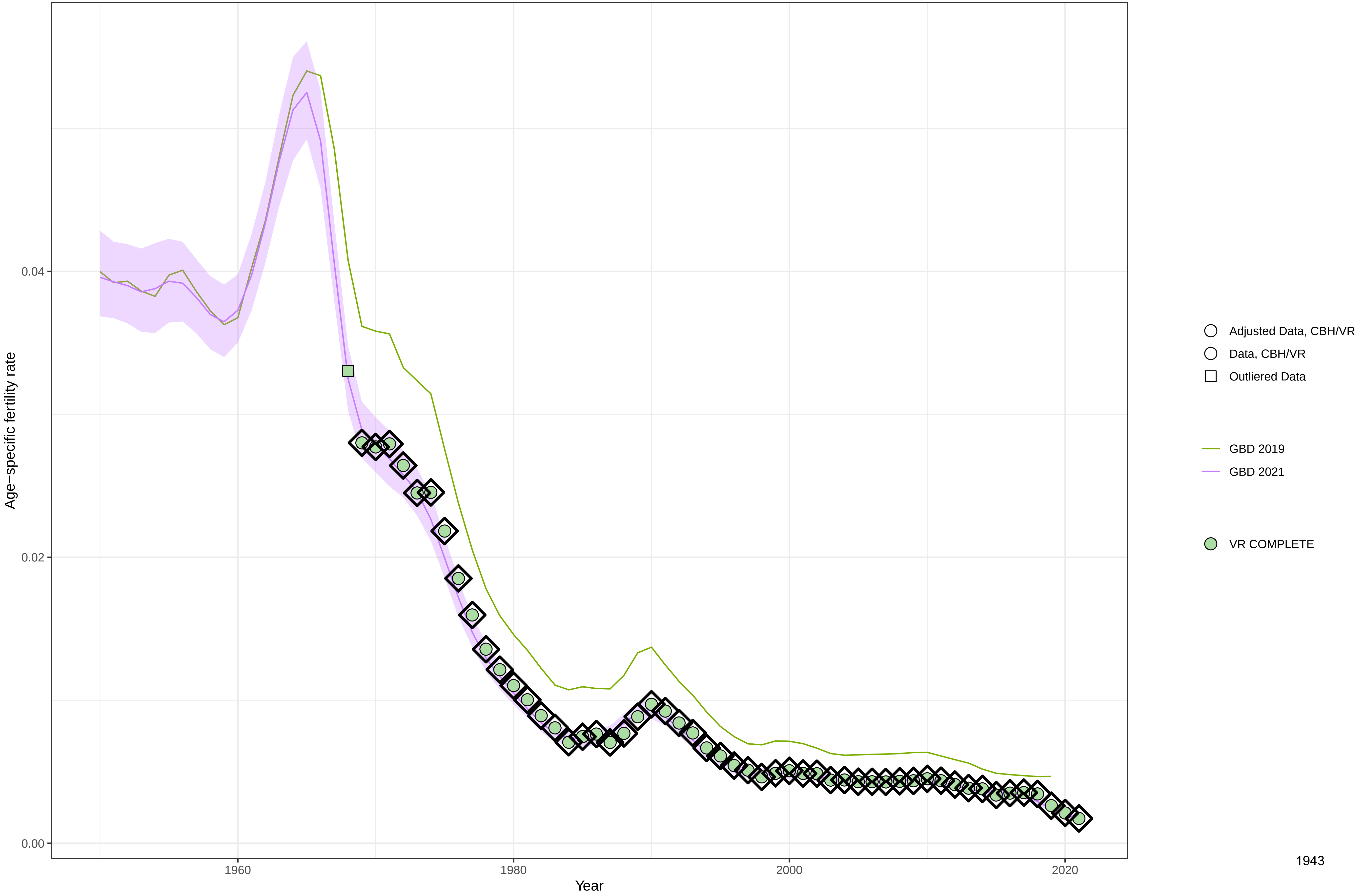

Age−specific fertility fitted trend lines, 1950−2021: Sweden except Stockholm, Ages: 20−24
Data points enclosed with a diamond are reference sources

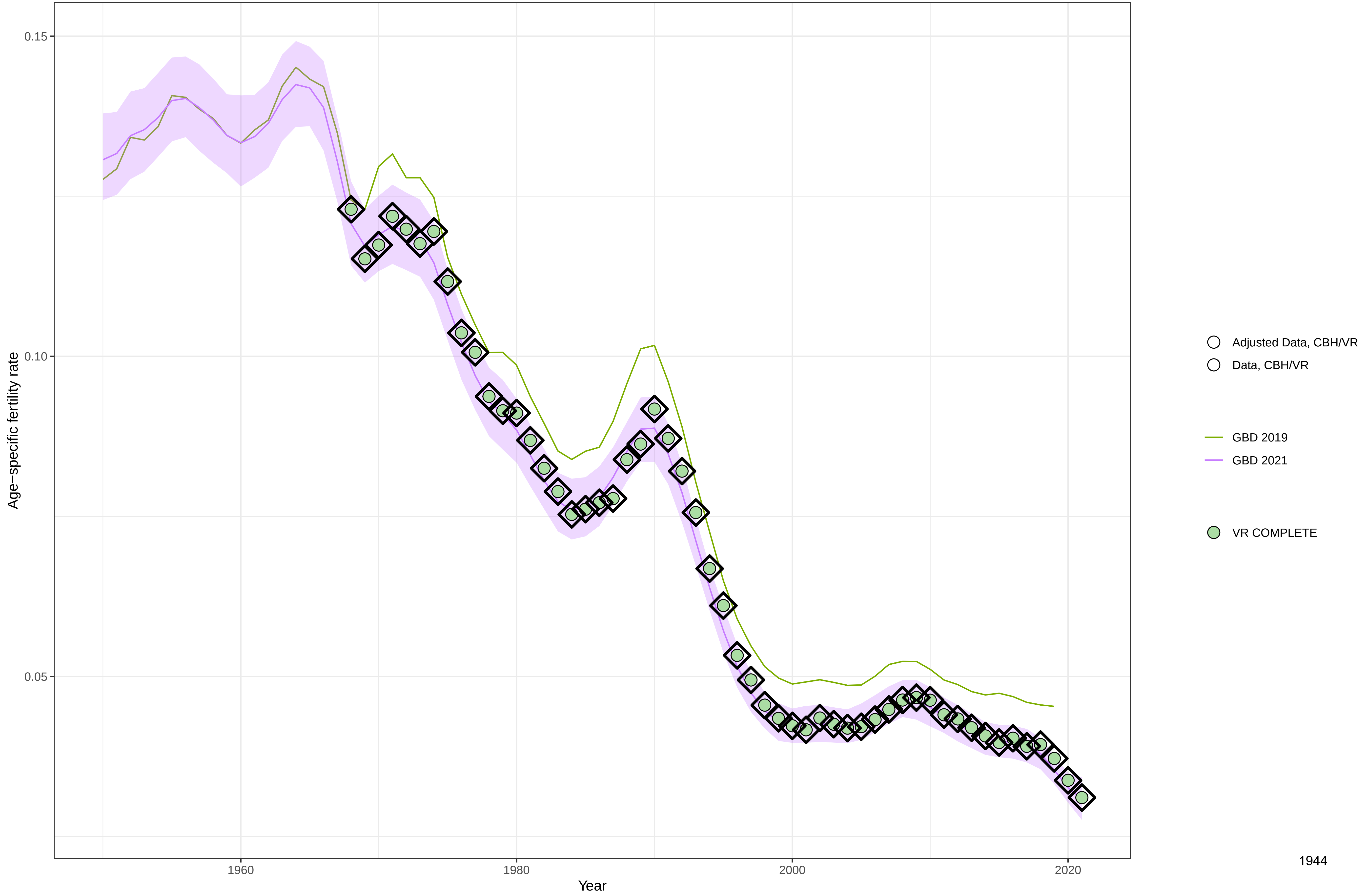

Age−specific fertility fitted trend lines, 1950−2021: Sweden except Stockholm, Ages: 25−29
Data points enclosed with a diamond are reference sources

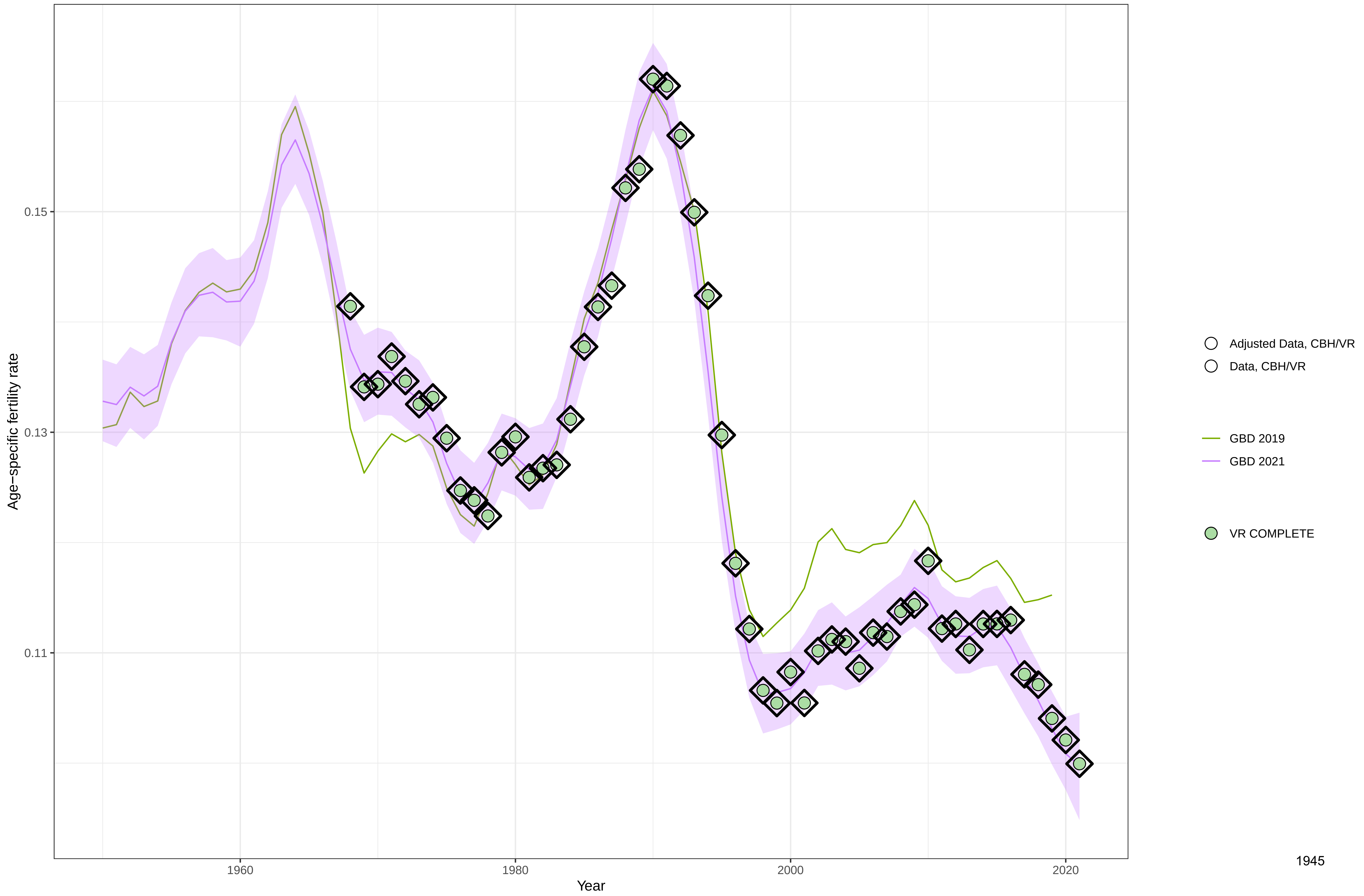

Age−specific fertility fitted trend lines, 1950−2021: Sweden except Stockholm, Ages: 30−34
Data points enclosed with a diamond are reference sources

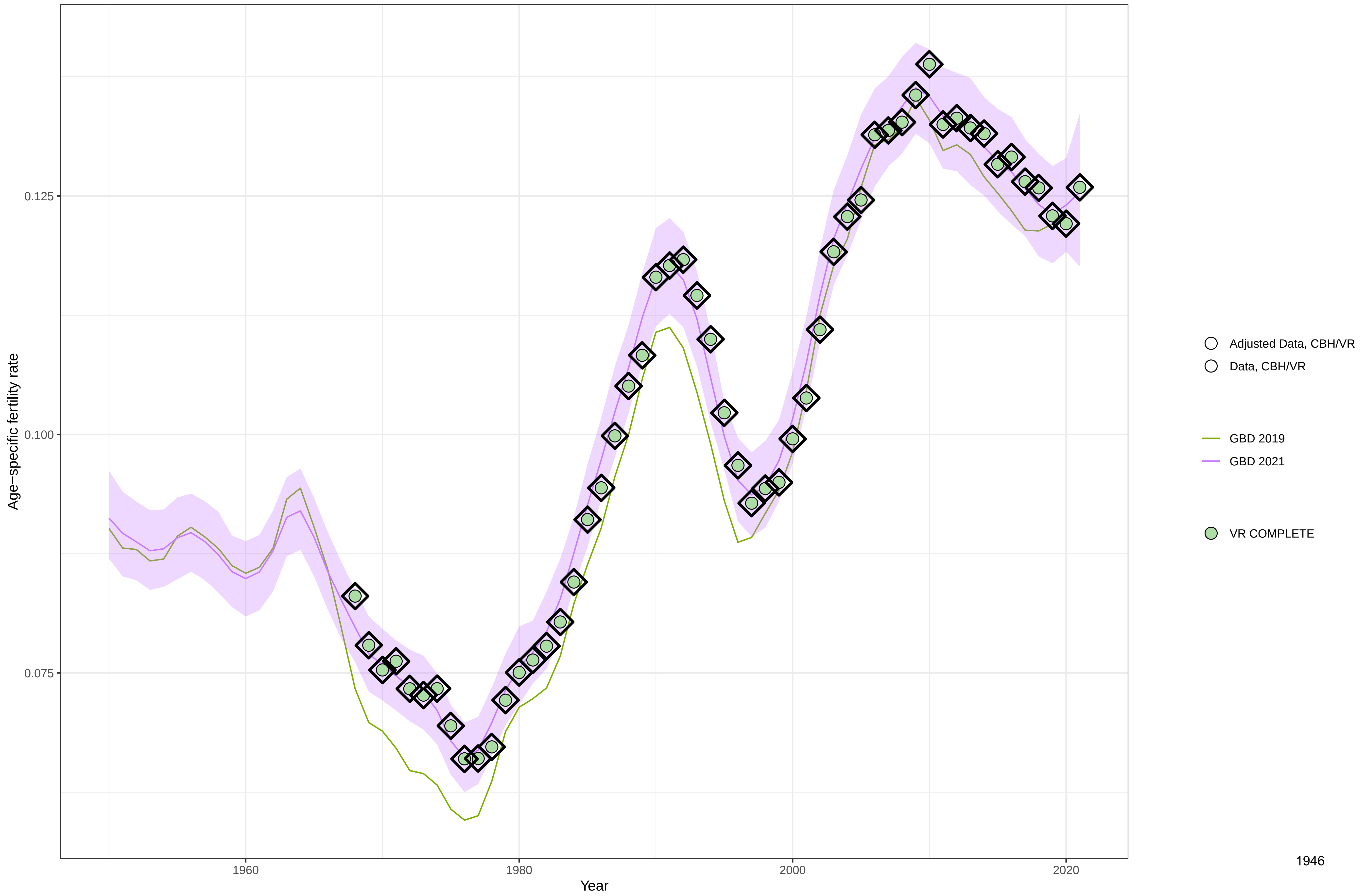

Age−specific fertility fitted trend lines, 1950−2021: Sweden except Stockholm, Ages: 35−39
Data points enclosed with a diamond are reference sources

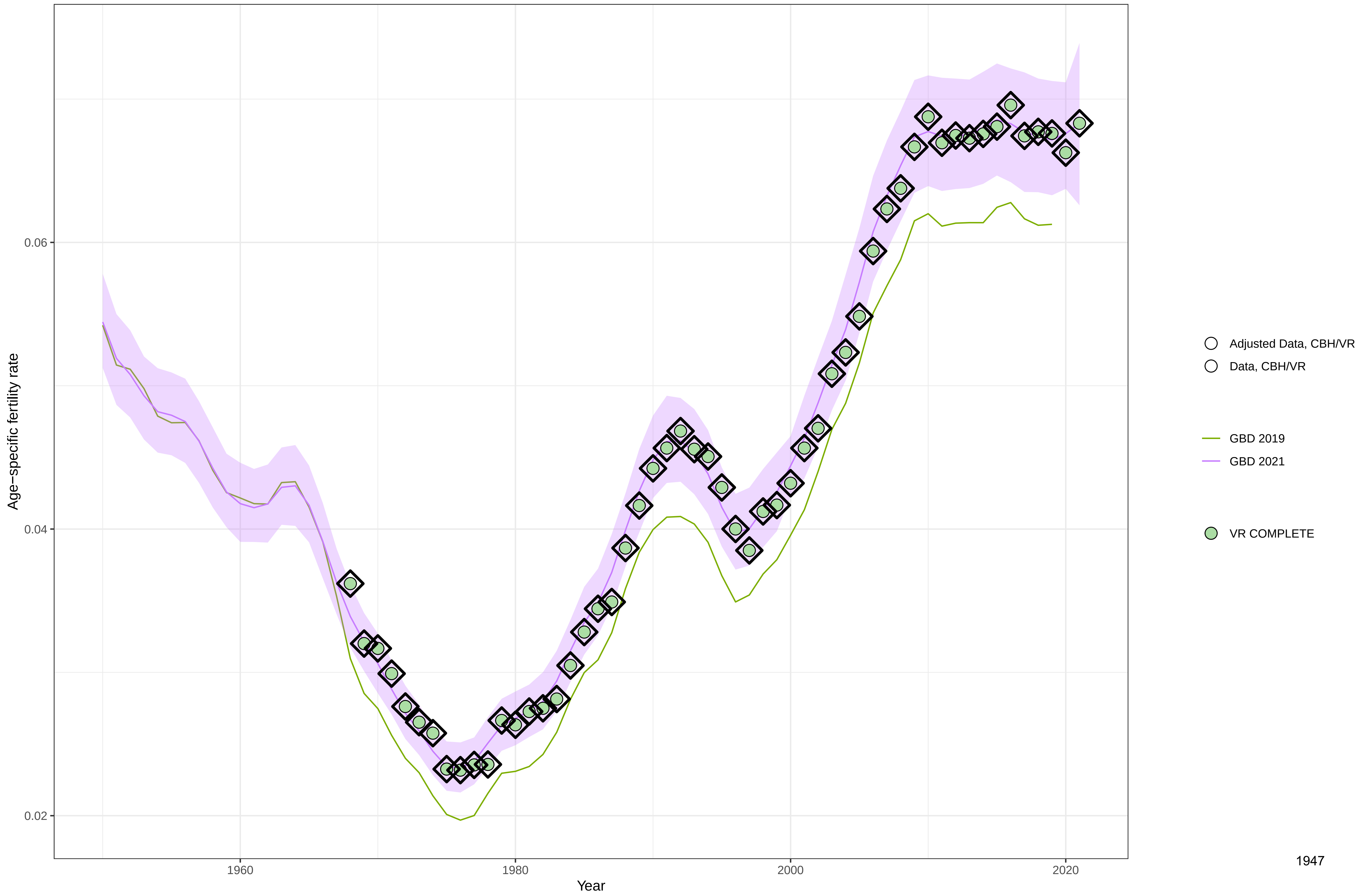

Age−specific fertility fitted trend lines, 1950−2021: Sweden except Stockholm, Ages: 40−44
Data points enclosed with a diamond are reference sources

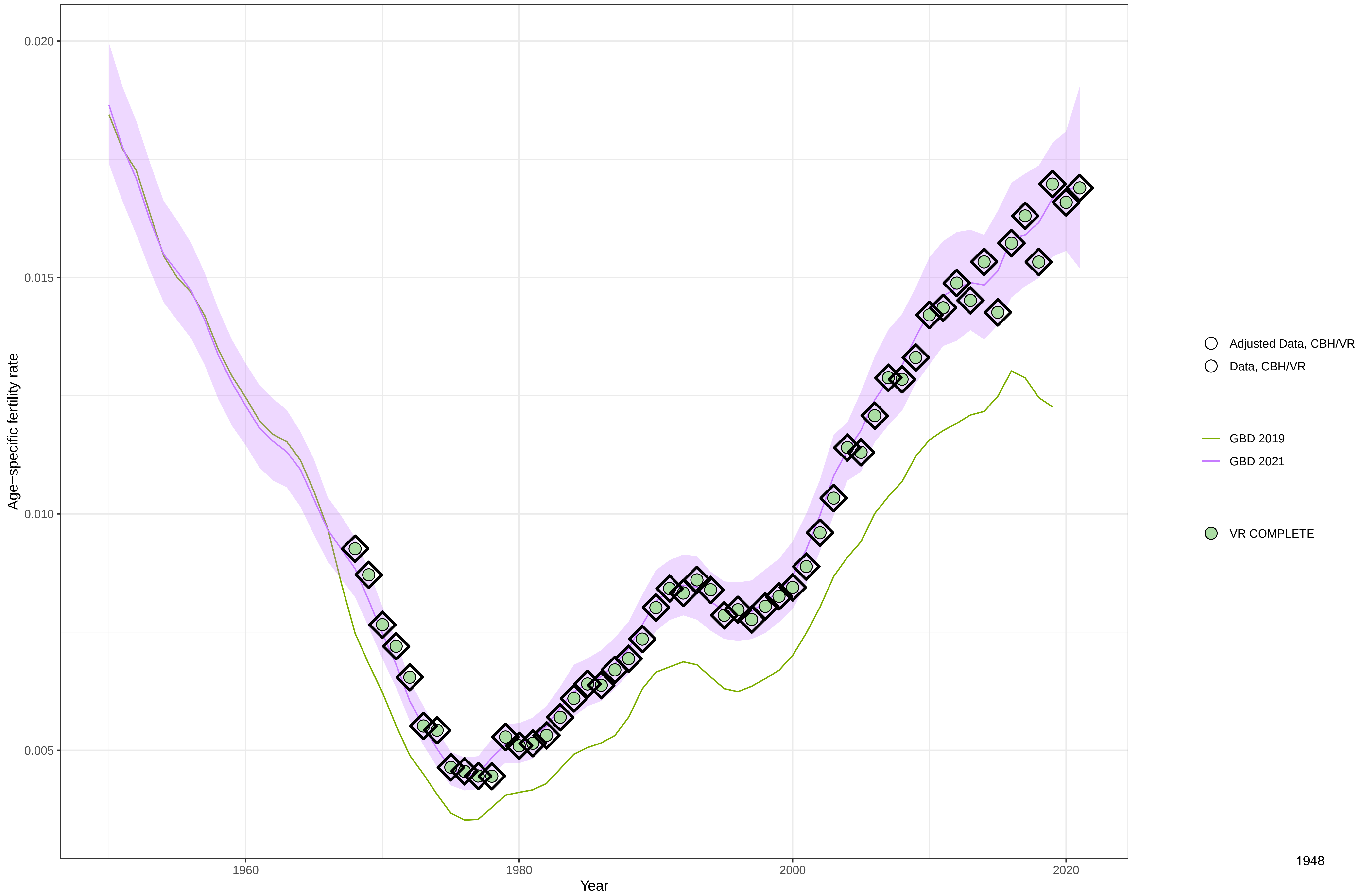

Age−specific fertility fitted trend lines, 1950−2021: Sweden except Stockholm, Ages: 45−49
Data points enclosed with a diamond are reference sources

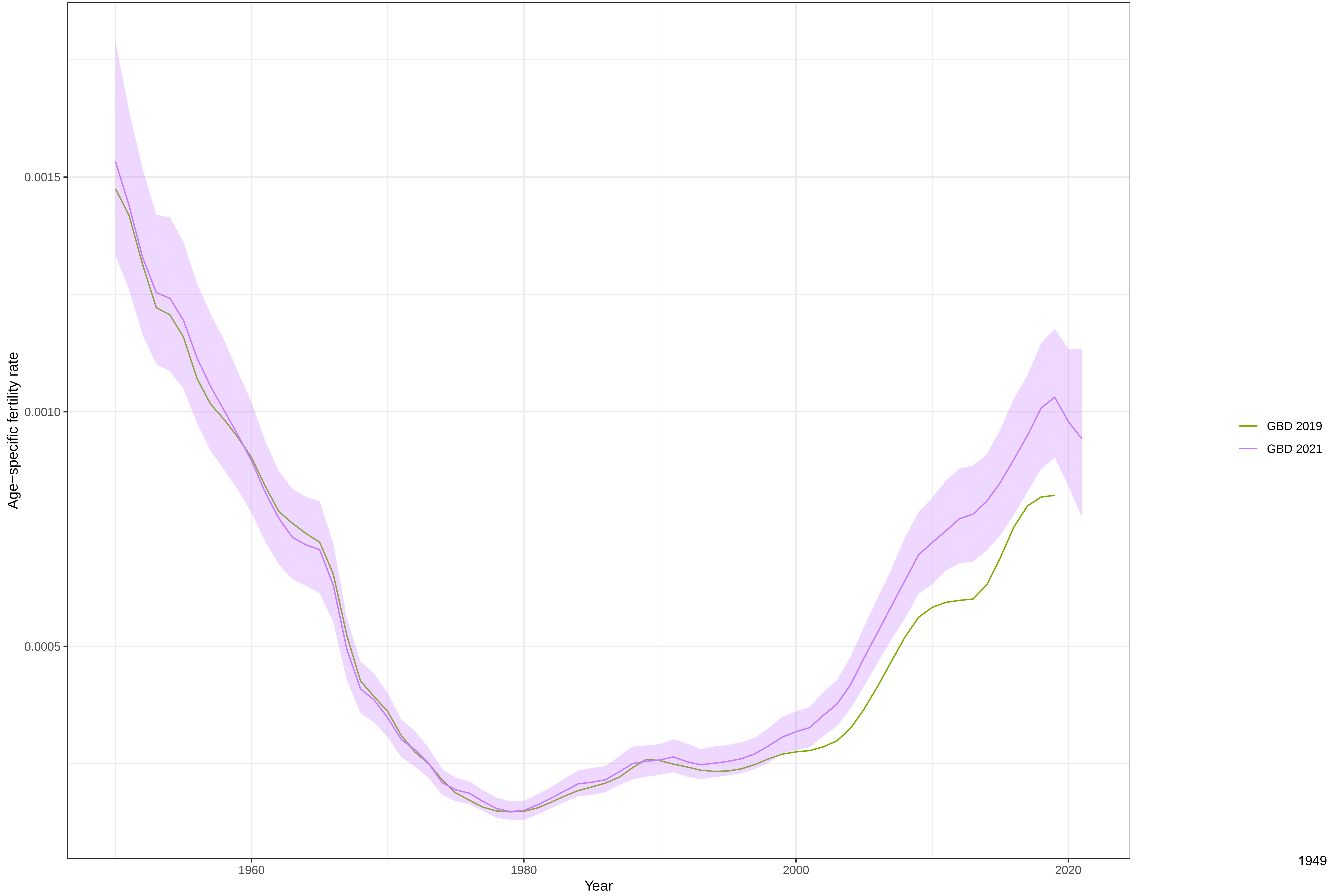

1949

Age−specific fertility fitted trend lines, 1950−2021: Sweden except Stockholm, Ages: 50−54
Data points enclosed with a diamond are reference sources

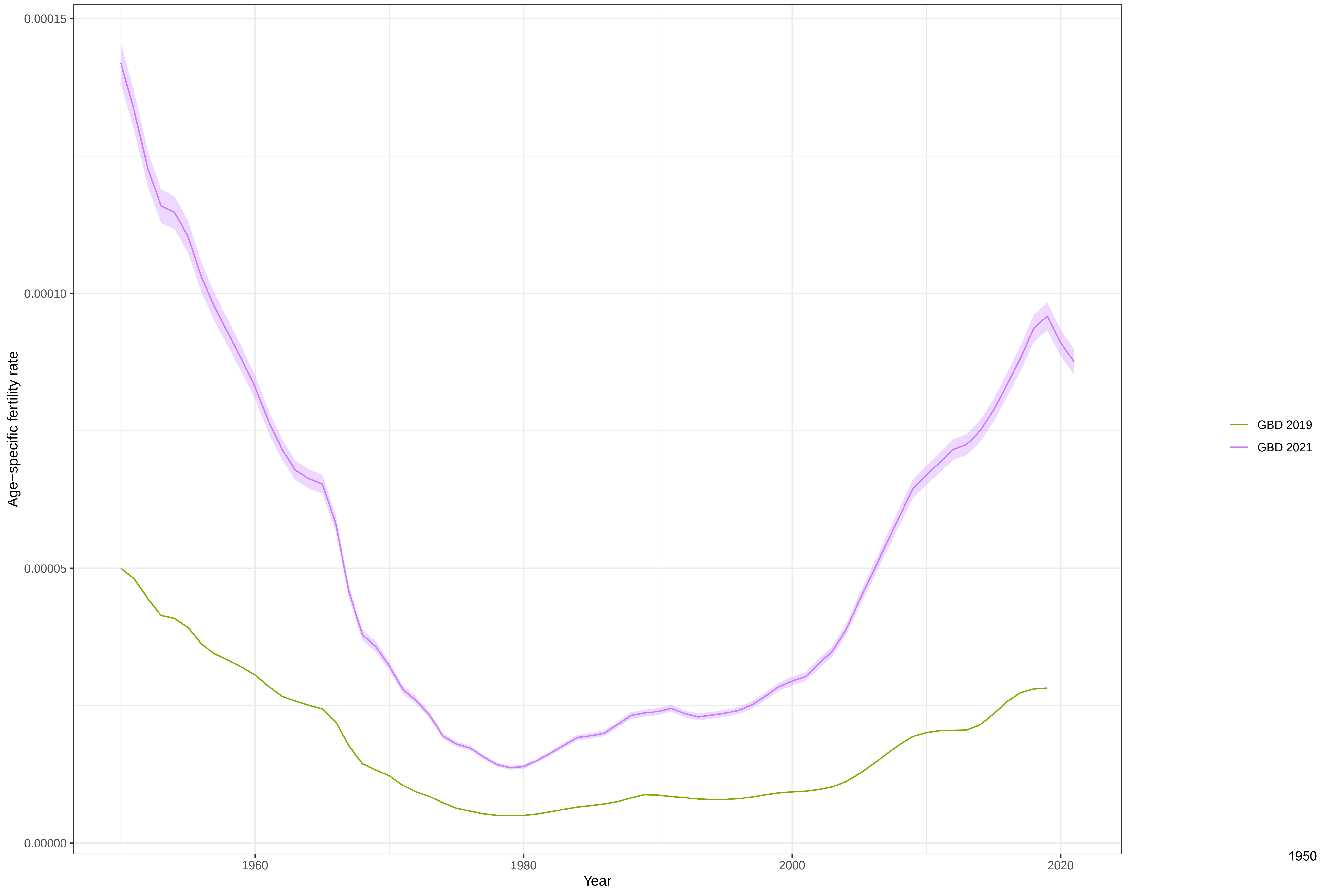

Total fertility rate fitted trend lines, 1950−2021: Sweden except Stockholm TFR
Data points enclosed with a diamond are reference sources

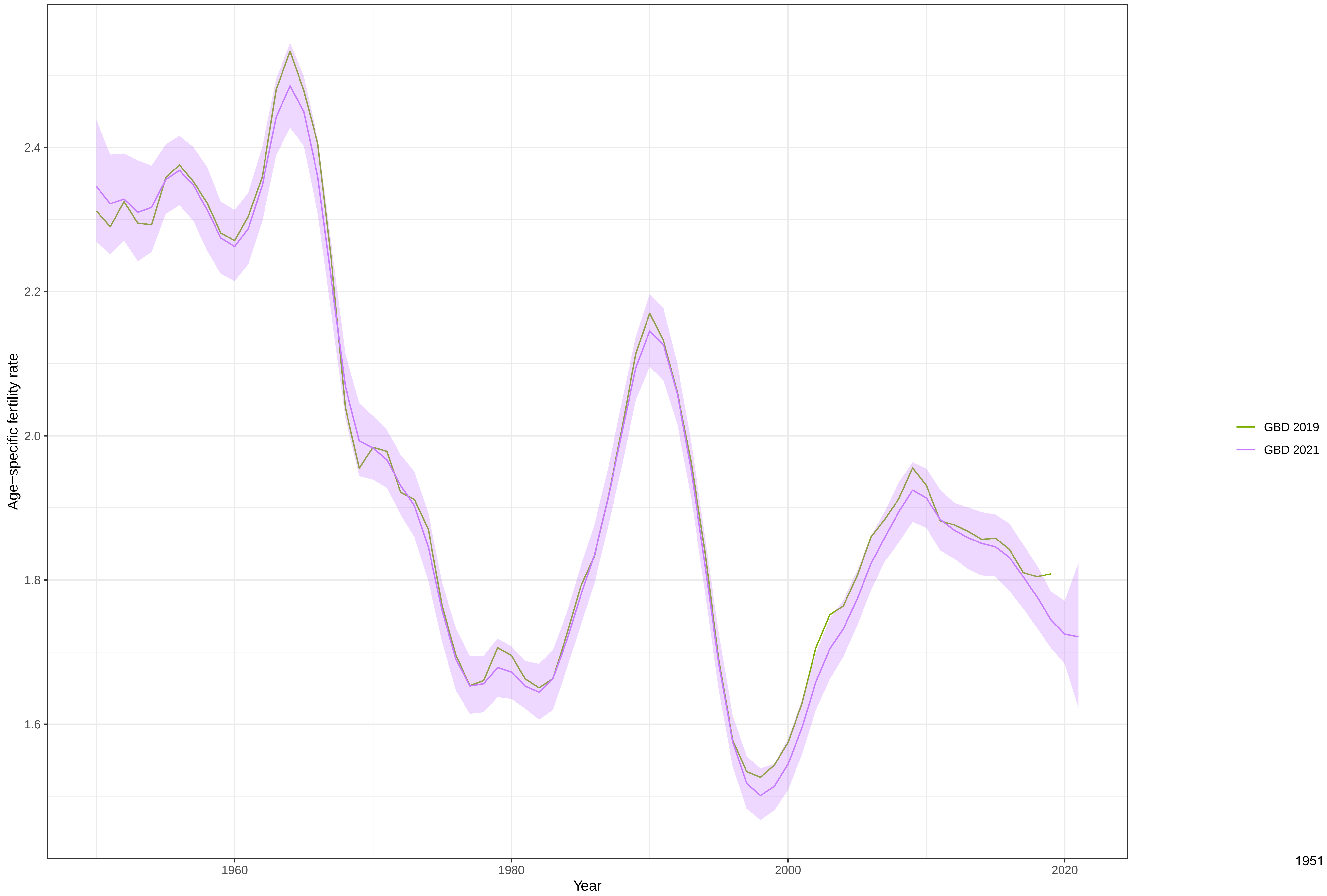

1951

Age−specific fertility fitted trend lines, 1950−2021: Switzerland, Ages: 10−14
Data points enclosed with a diamond are reference sources

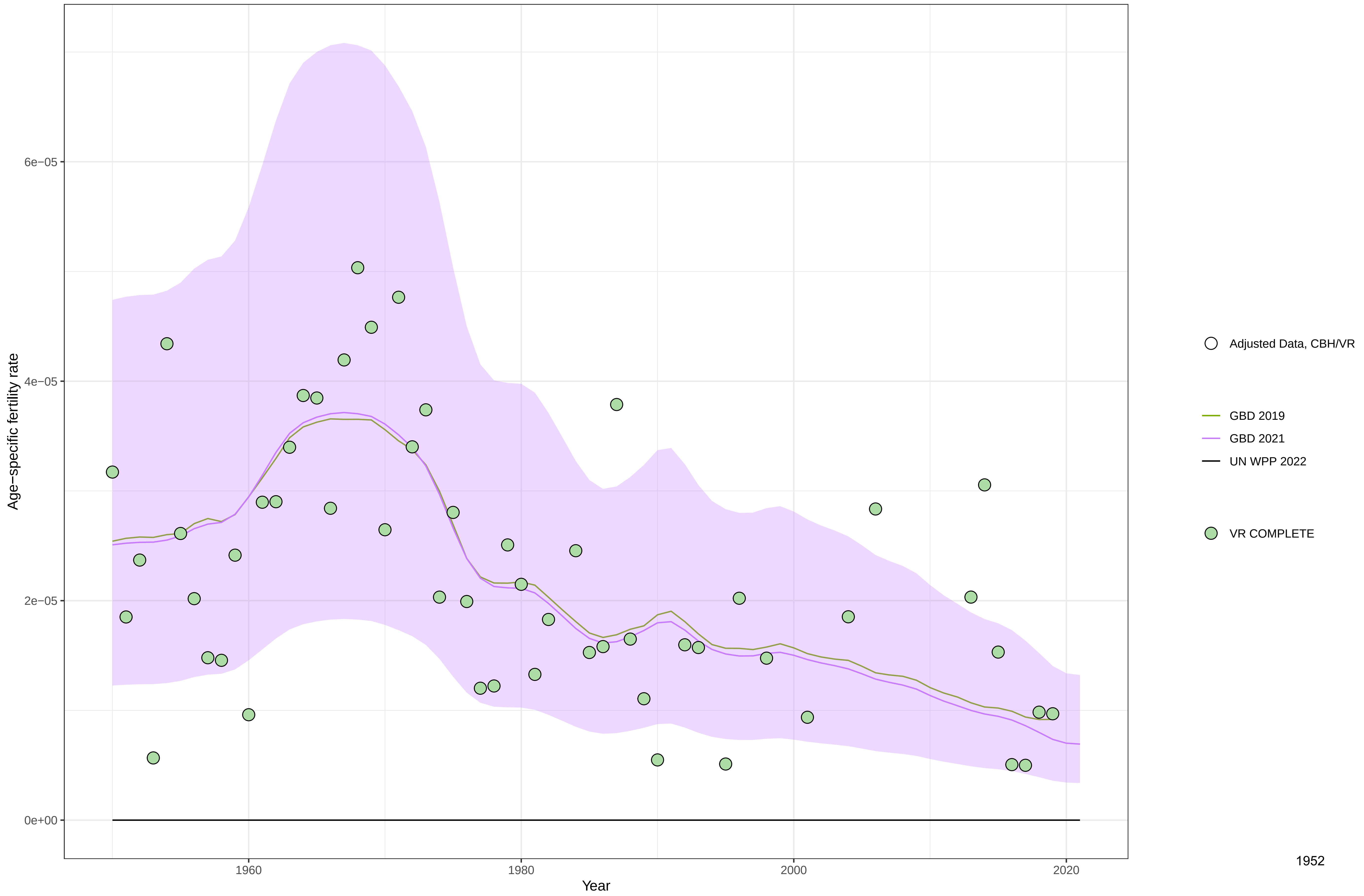

Age−specific fertility fitted trend lines, 1950−2021: Switzerland, Ages: 15−19
Data points enclosed with a diamond are reference sources

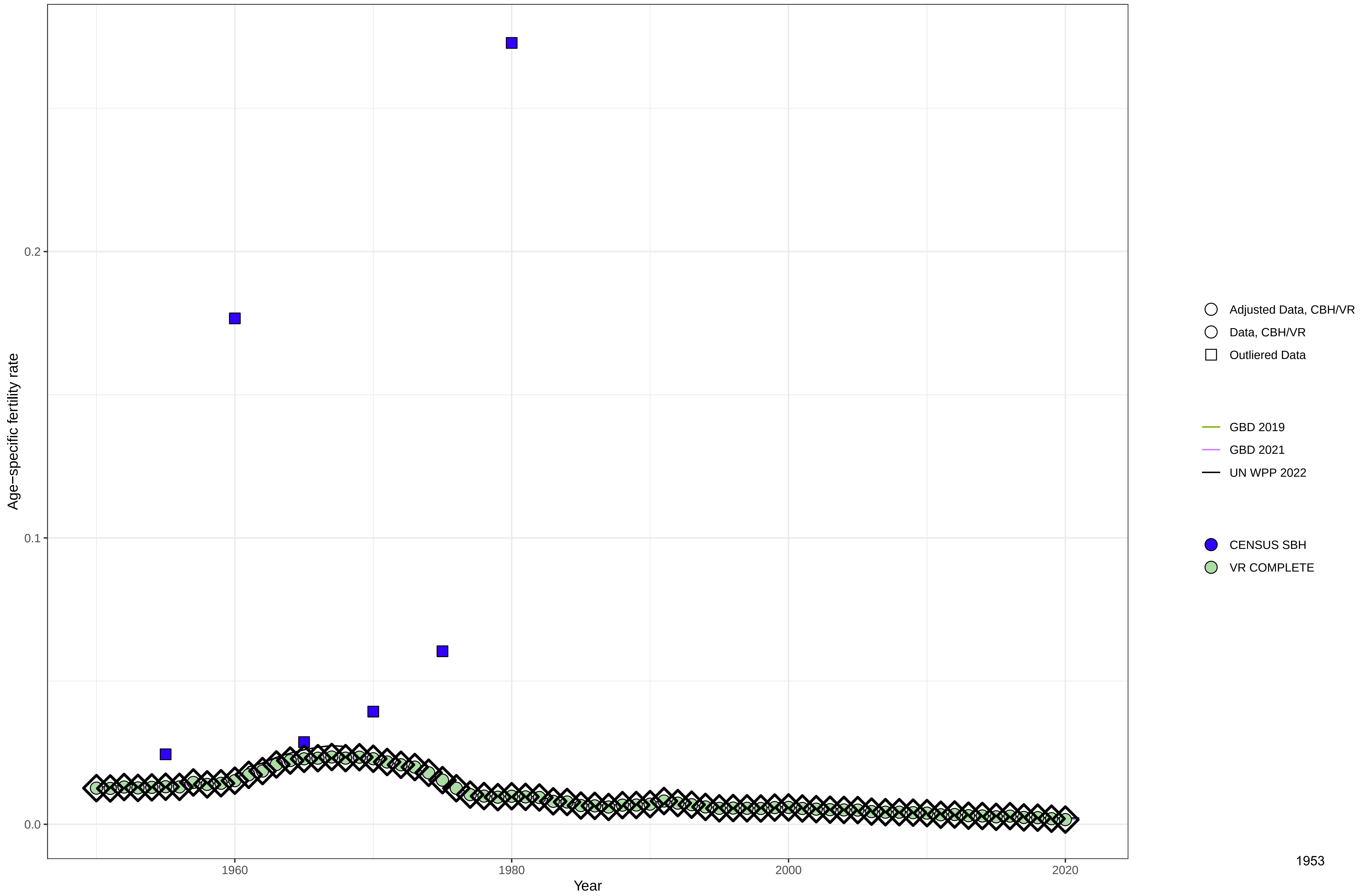

Age−specific fertility fitted trend lines, 1950−2021: Switzerland, Ages: 20−24
Data points enclosed with a diamond are reference sources

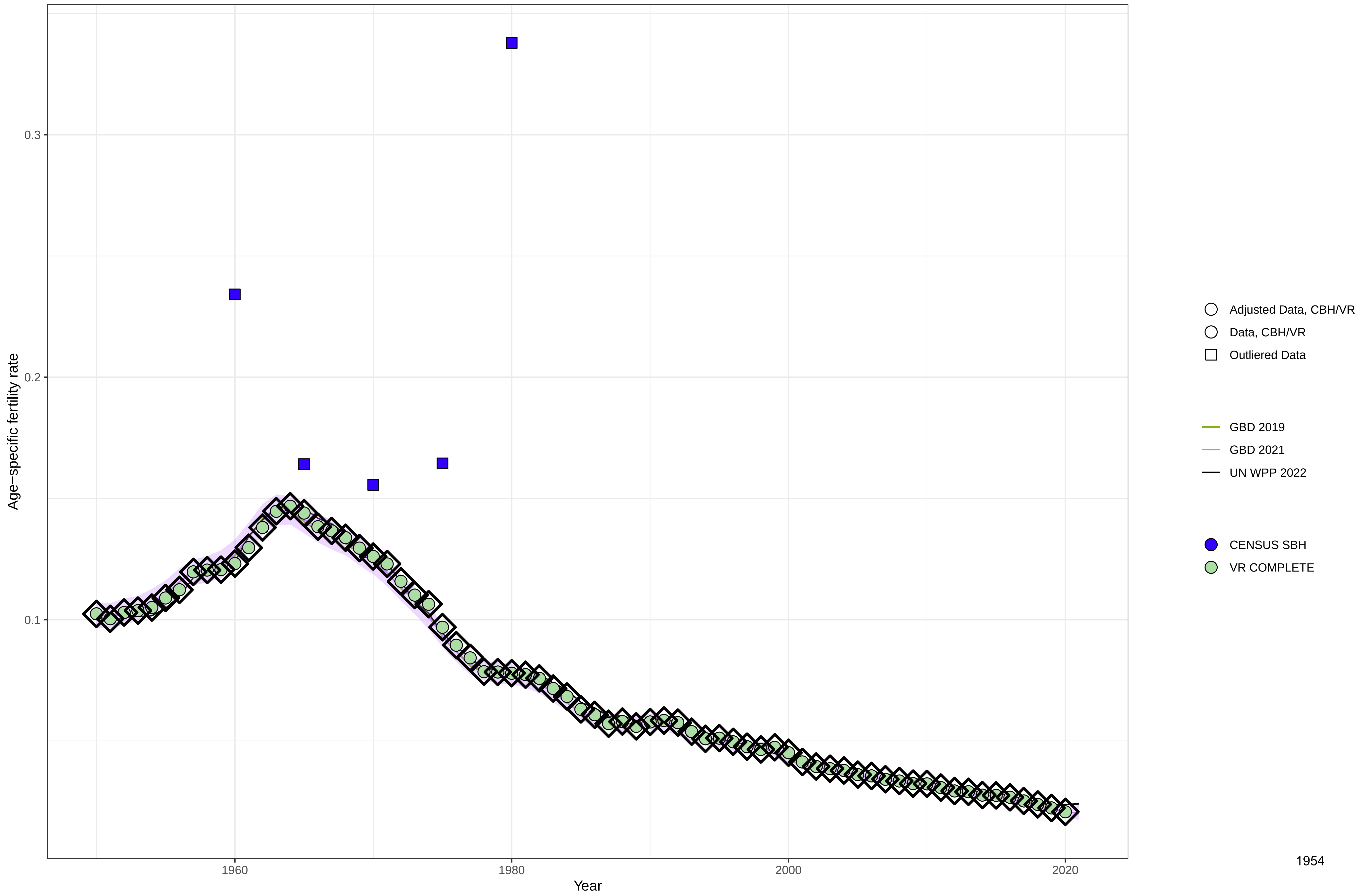

Age−specific fertility fitted trend lines, 1950−2021: Switzerland, Ages: 25−29
Data points enclosed with a diamond are reference sources

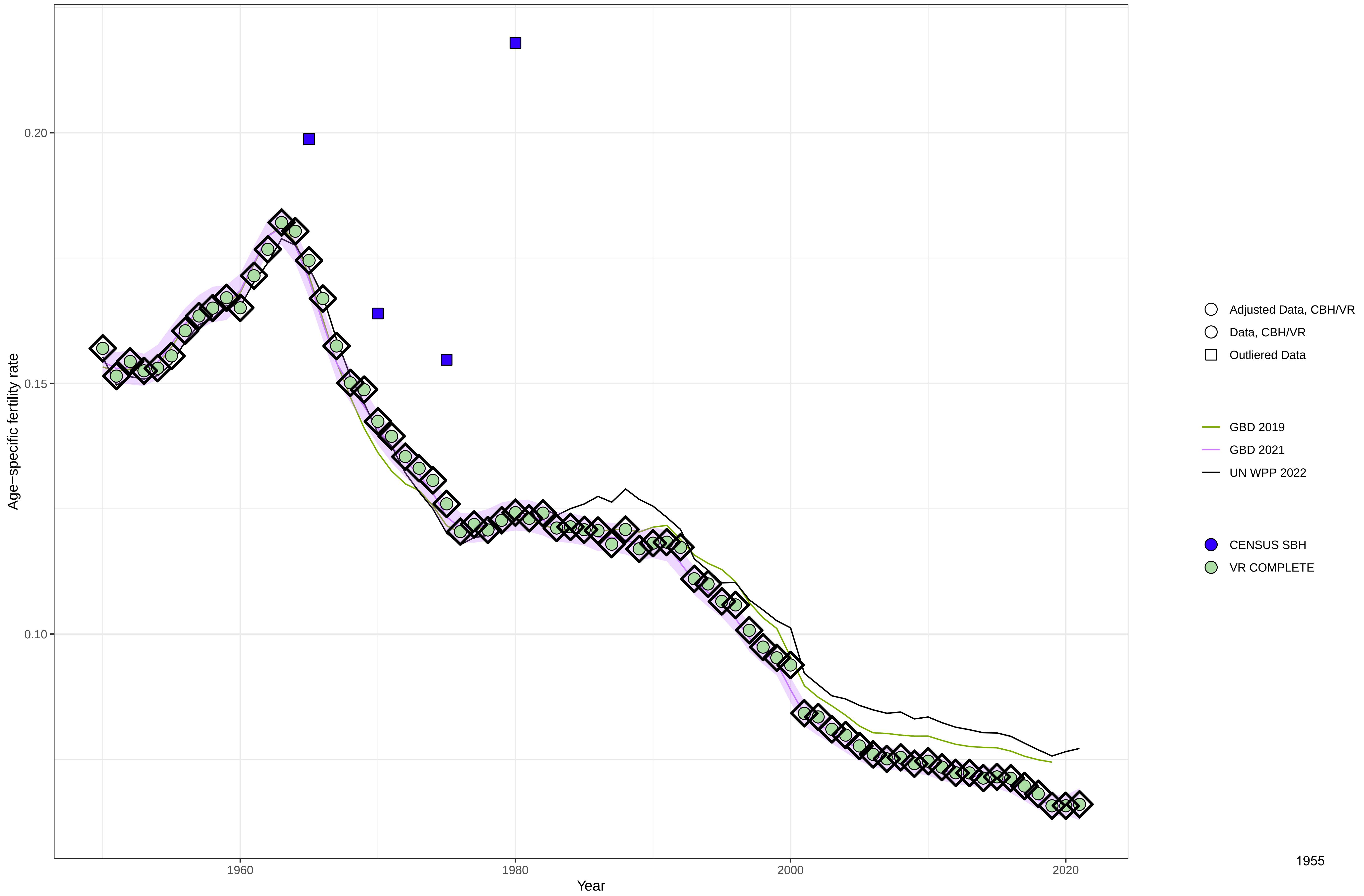

Age−specific fertility fitted trend lines, 1950−2021: Switzerland, Ages: 30−34
Data points enclosed with a diamond are reference sources

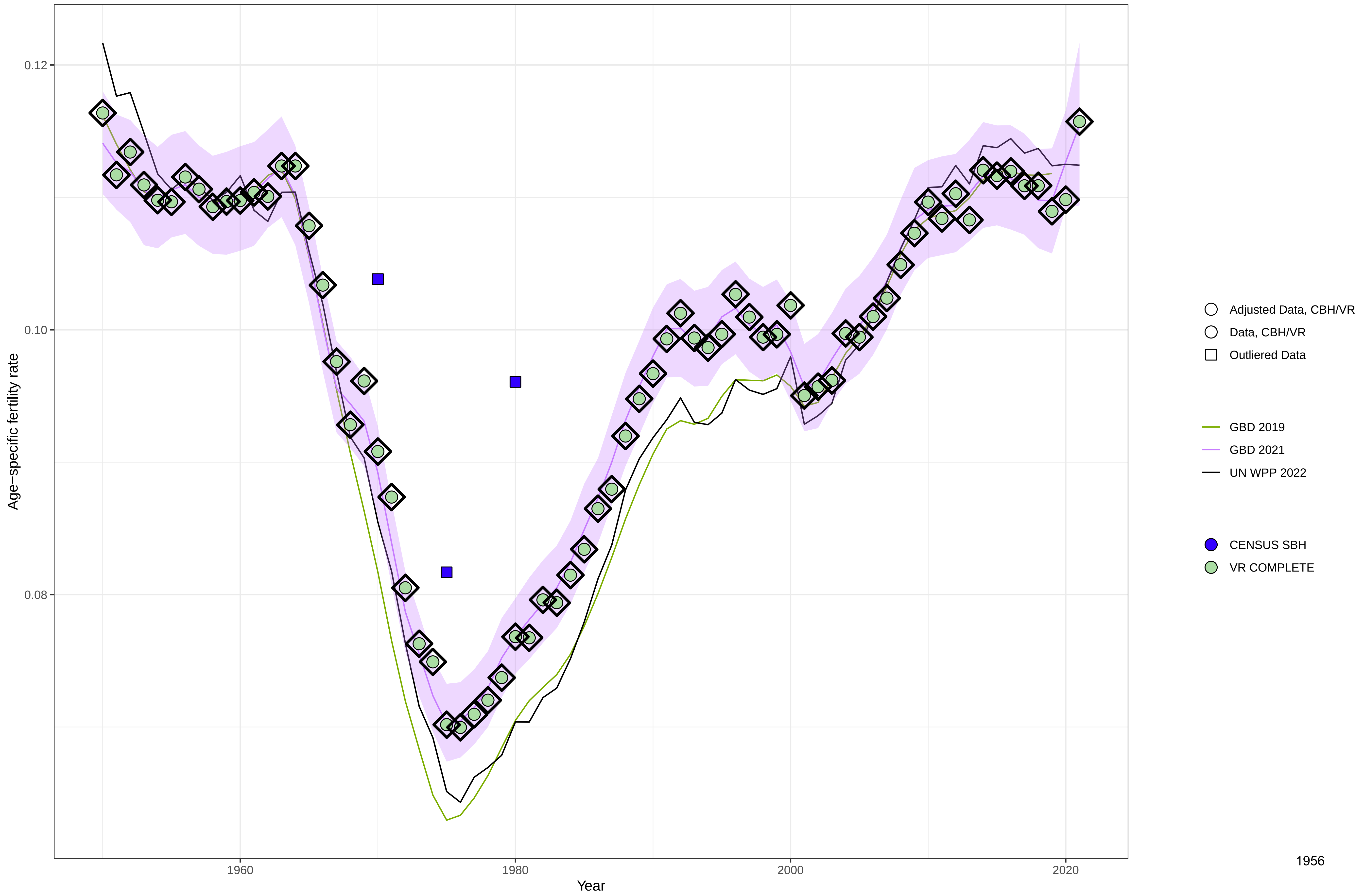

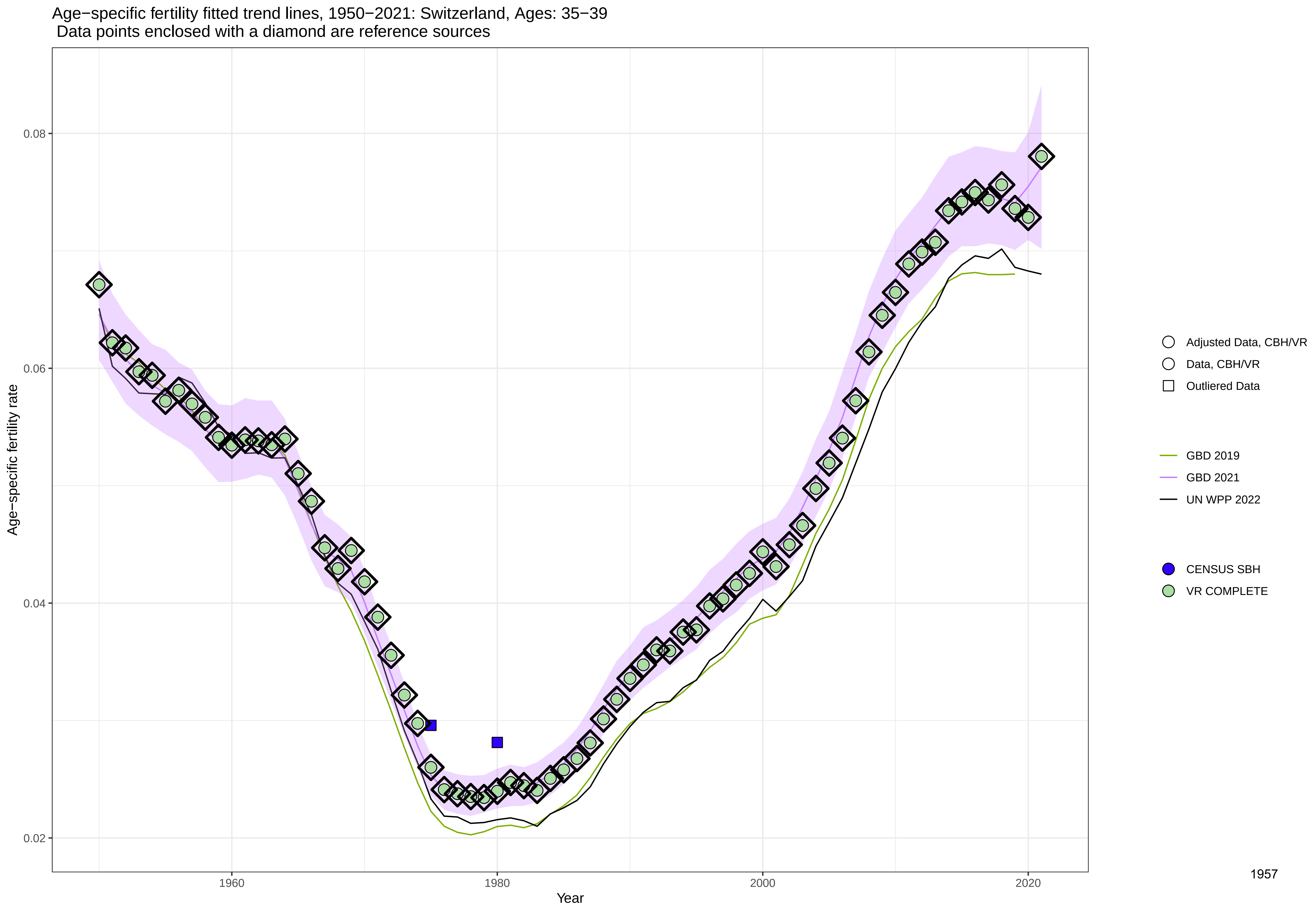

Age−specific fertility fitted trend lines, 1950−2021: Switzerland, Ages: 35−39
Data points enclosed with a diamond are reference sources
0.08
0.06
0.04
0.02
Age−specific fertility rate
1960
1980
2000
2020
Year
Adjusted Data, CBH/VR
Data, CBH/VR
Outliered Data
GBD 2019
GBD 2021
UN WPP 2022
CENSUS SBH
VR COMPLETE

Age−specific fertility fitted trend lines, 1950−2021: Switzerland, Ages: 40−44
Data points enclosed with a diamond are reference sources

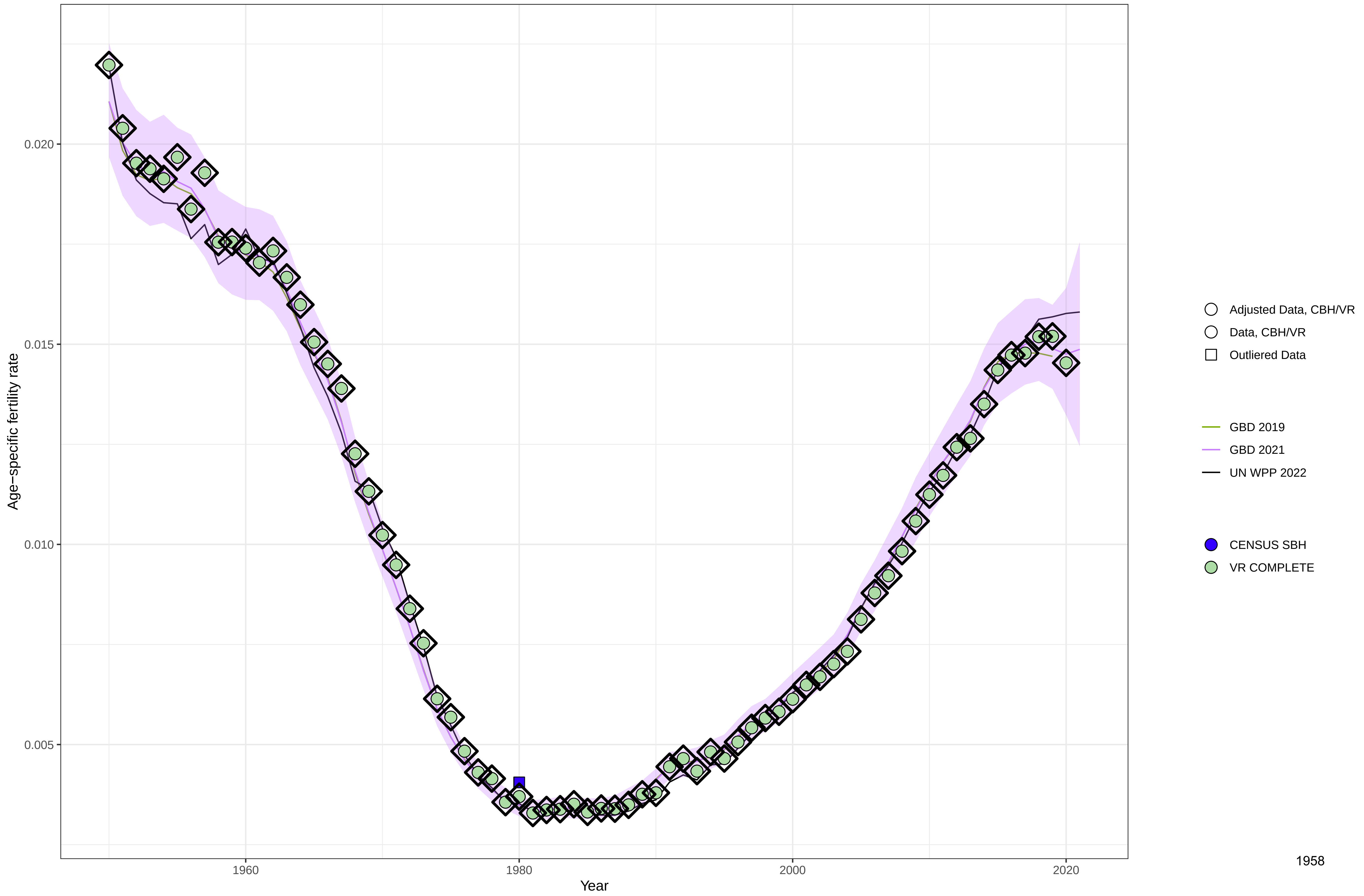

Age−specific fertility fitted trend lines, 1950−2021: Switzerland, Ages: 45−49
Data points enclosed with a diamond are reference sources

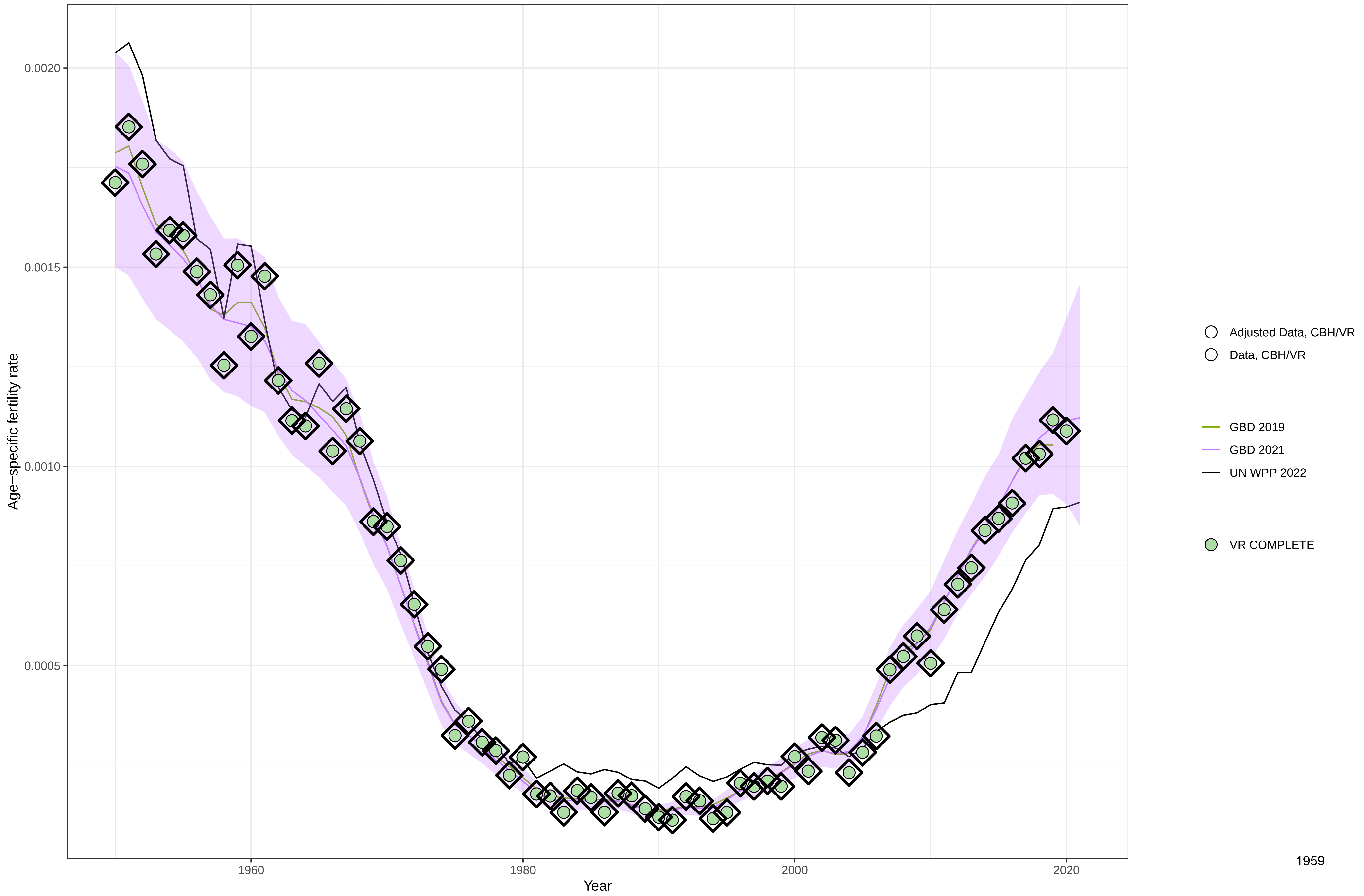

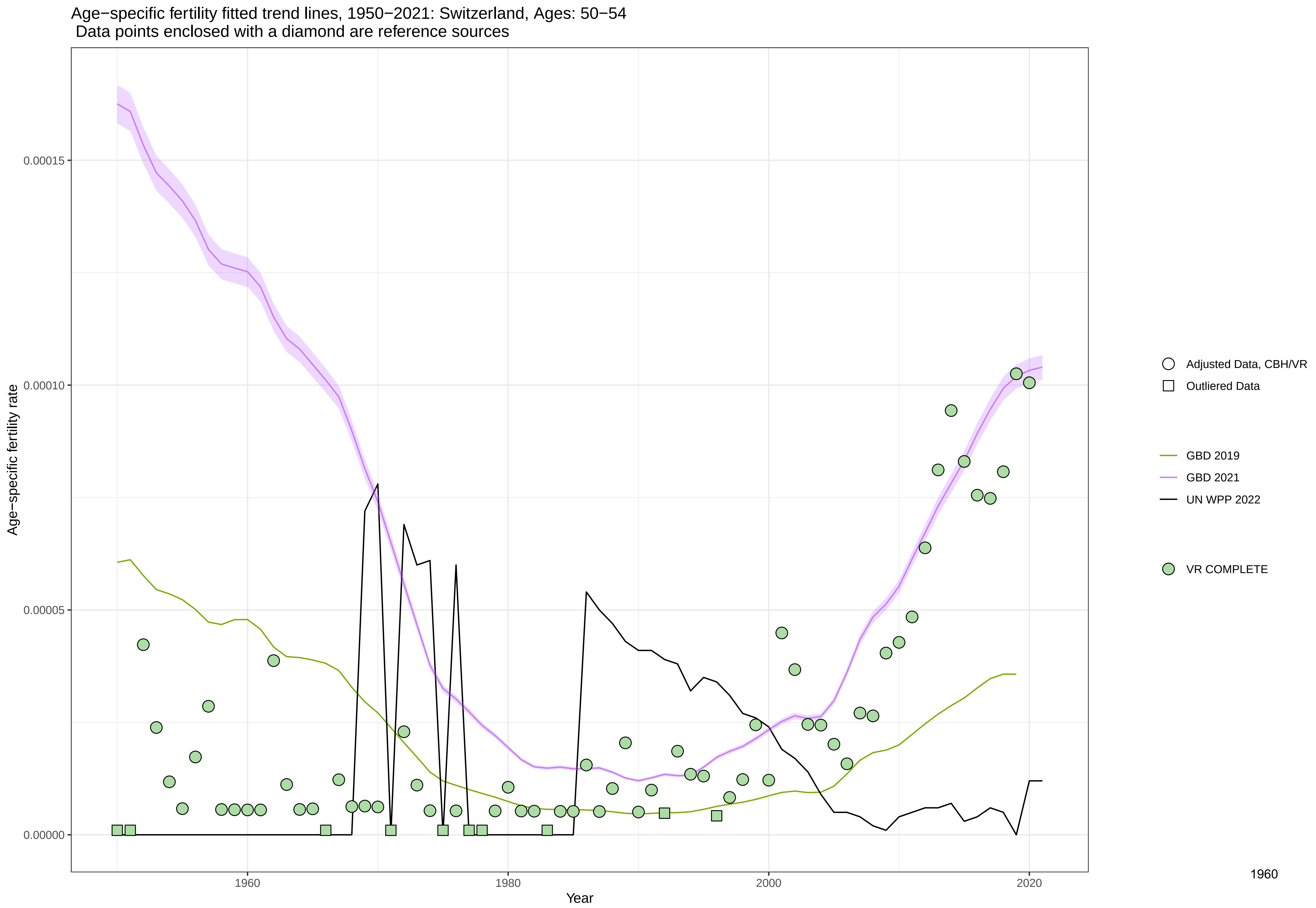

Age−specific fertility fitted trend lines, 1950−2021: Switzerland, Ages: 50−54
Data points enclosed with a diamond are reference sources
Age−specific fertility rate
0.00015
0.00010
0.00005
0.00000
1960
1980
2000
2020
Year
Adjusted Data, CBH/VR
Outliered Data
GBD 2019
GBD 2021
UN WPP 2022
VR COMPLETE

1960

Total fertility rate fitted trend lines, 1950−2021: Switzerland TFR
Data points enclosed with a diamond are reference sources

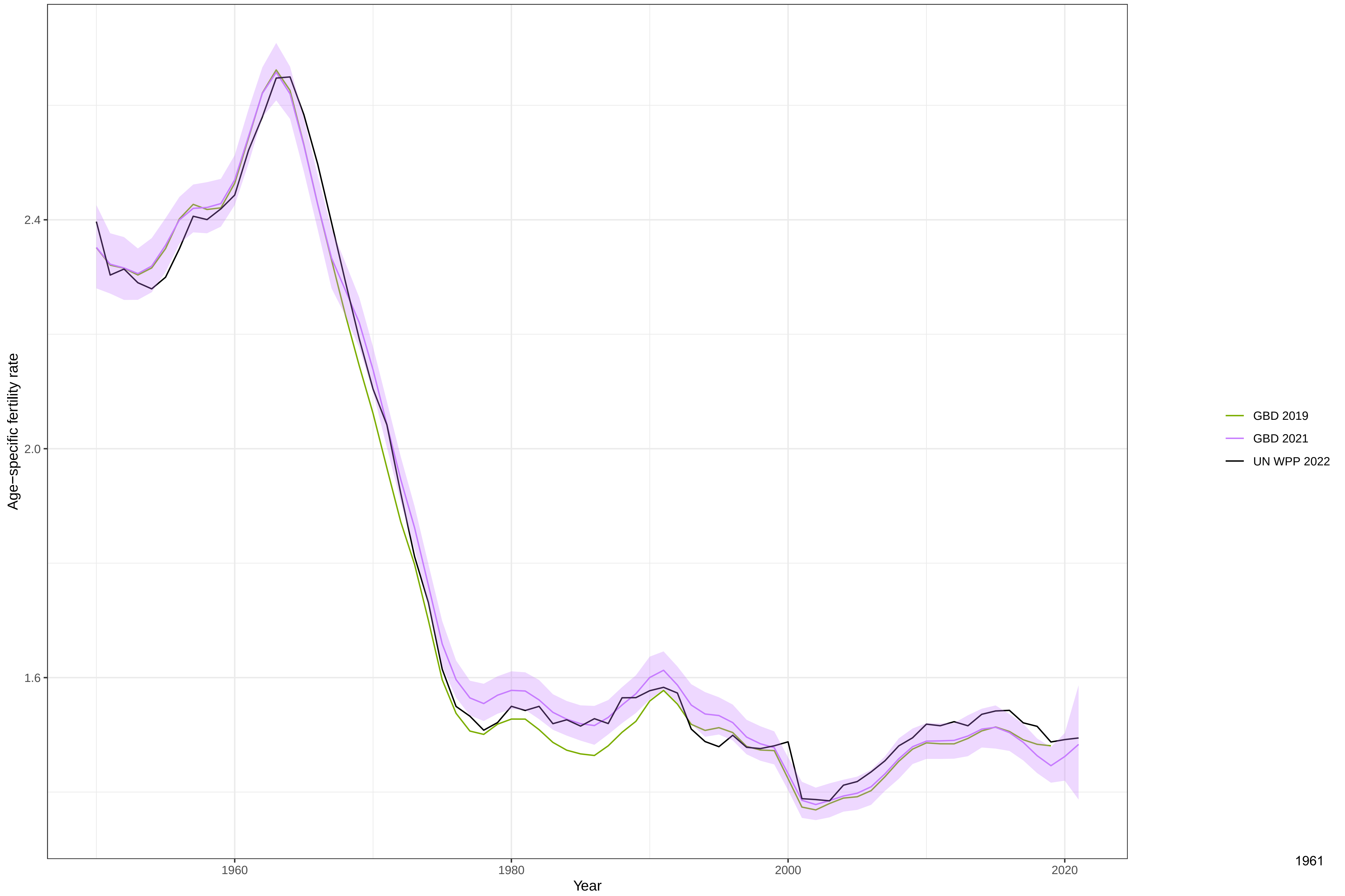

Age−specific fertility fitted trend lines, 1950−2021: UK, Ages: 10−14
Data points enclosed with a diamond are reference sources

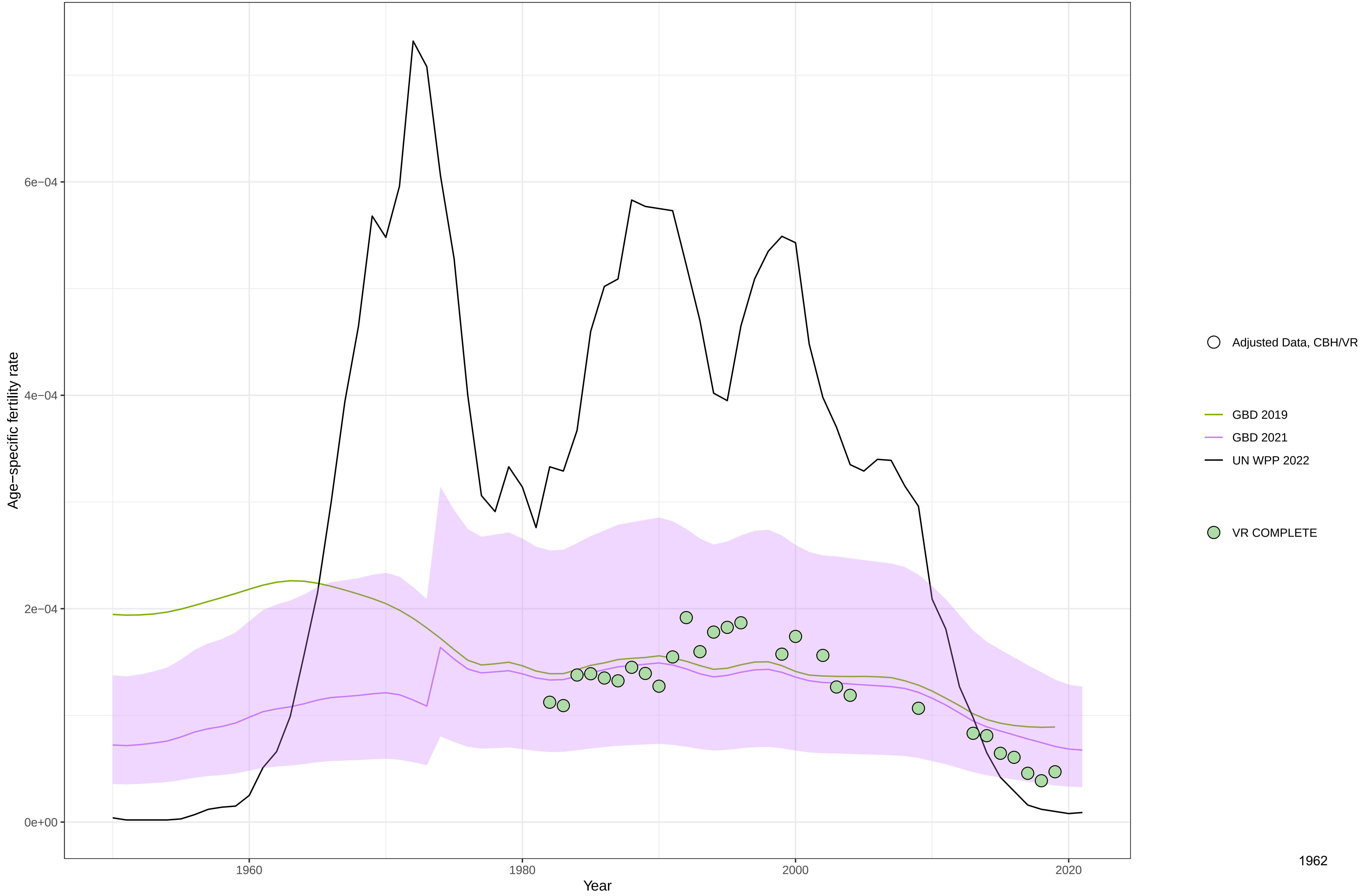

Age−specific fertility fitted trend lines, 1950−2021: UK, Ages: 15−19
Data points enclosed with a diamond are reference sources

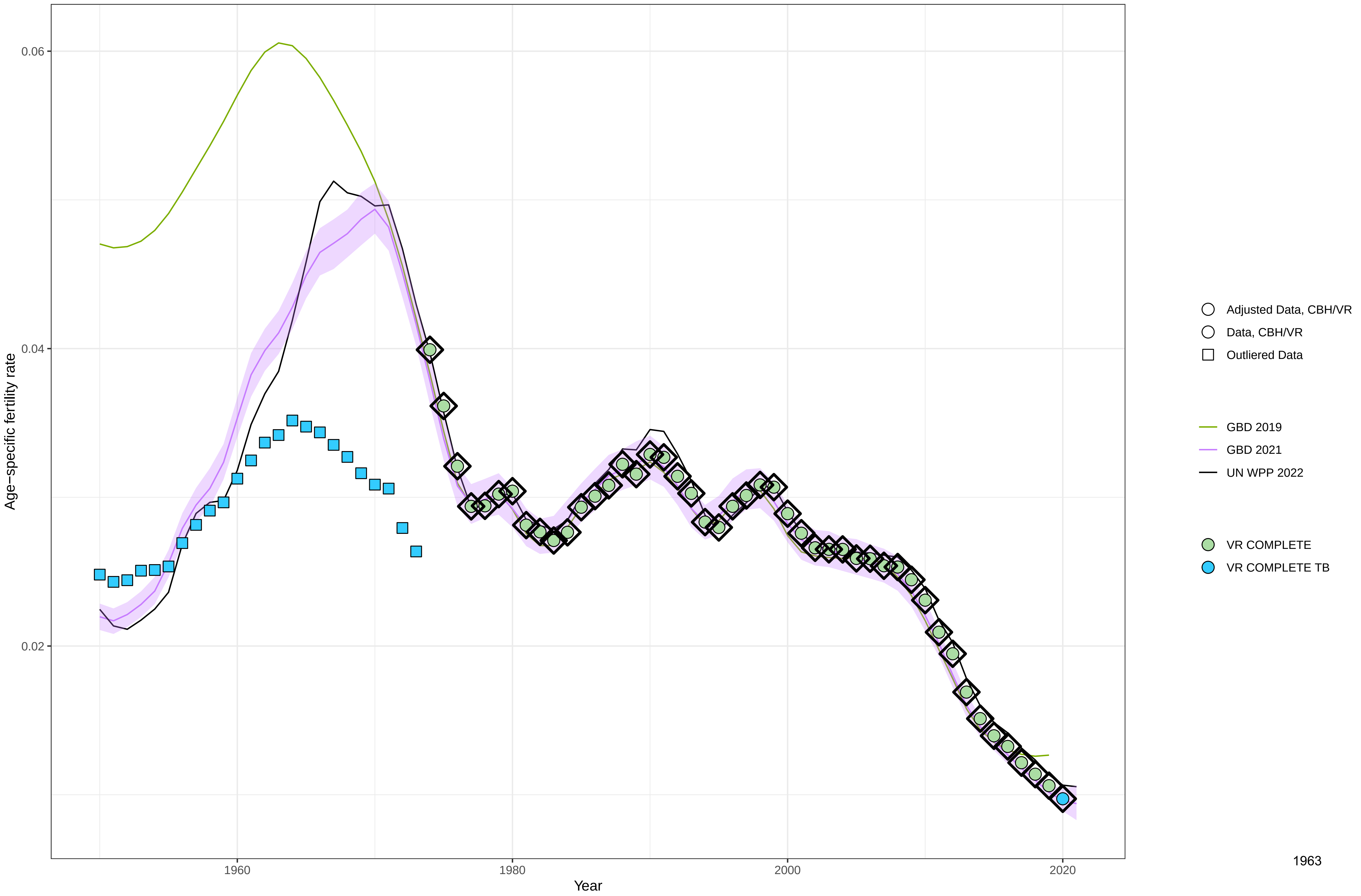

Age−specific fertility fitted trend lines, 1950−2021: UK, Ages: 20−24
Data points enclosed with a diamond are reference sources

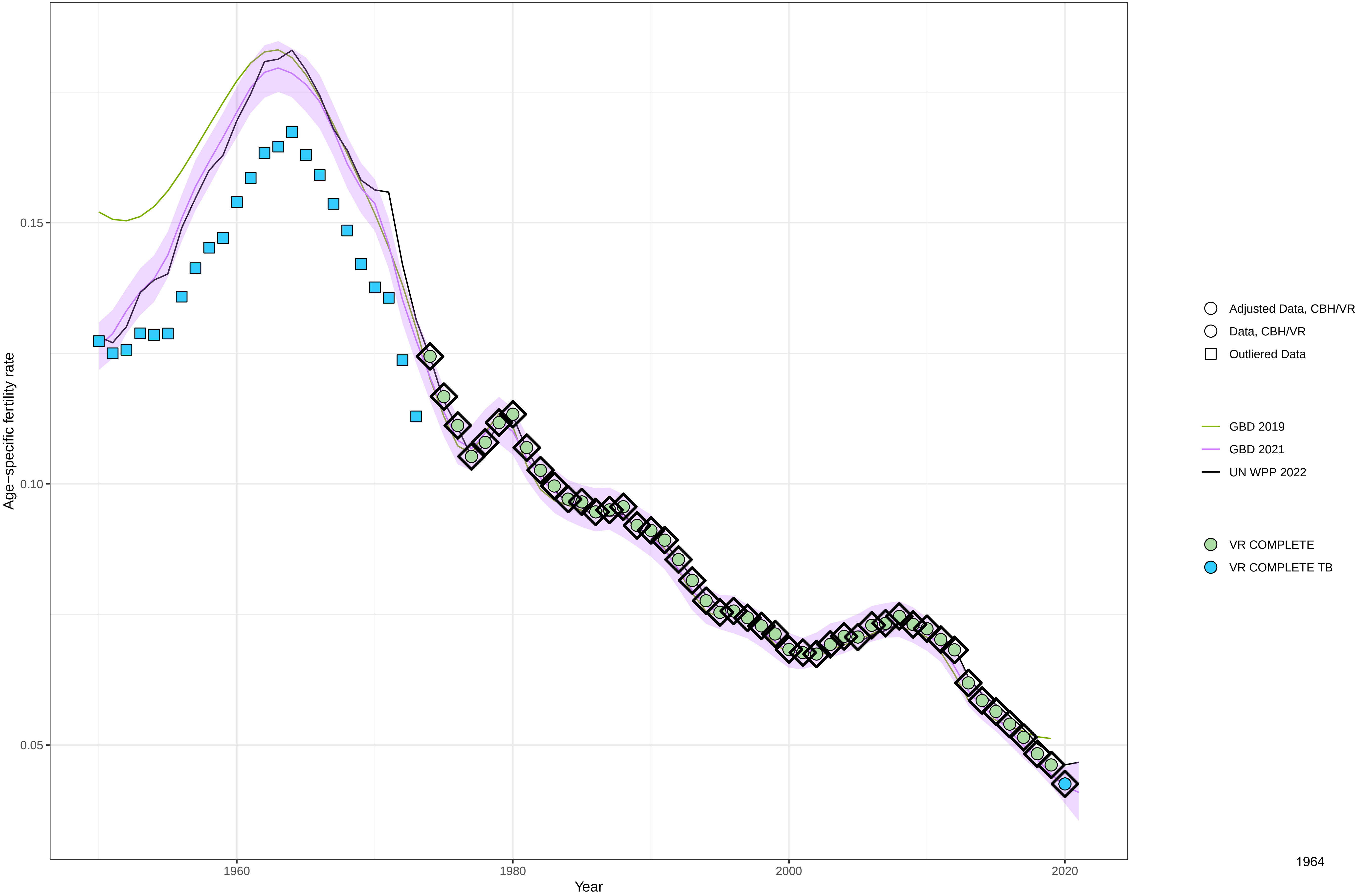

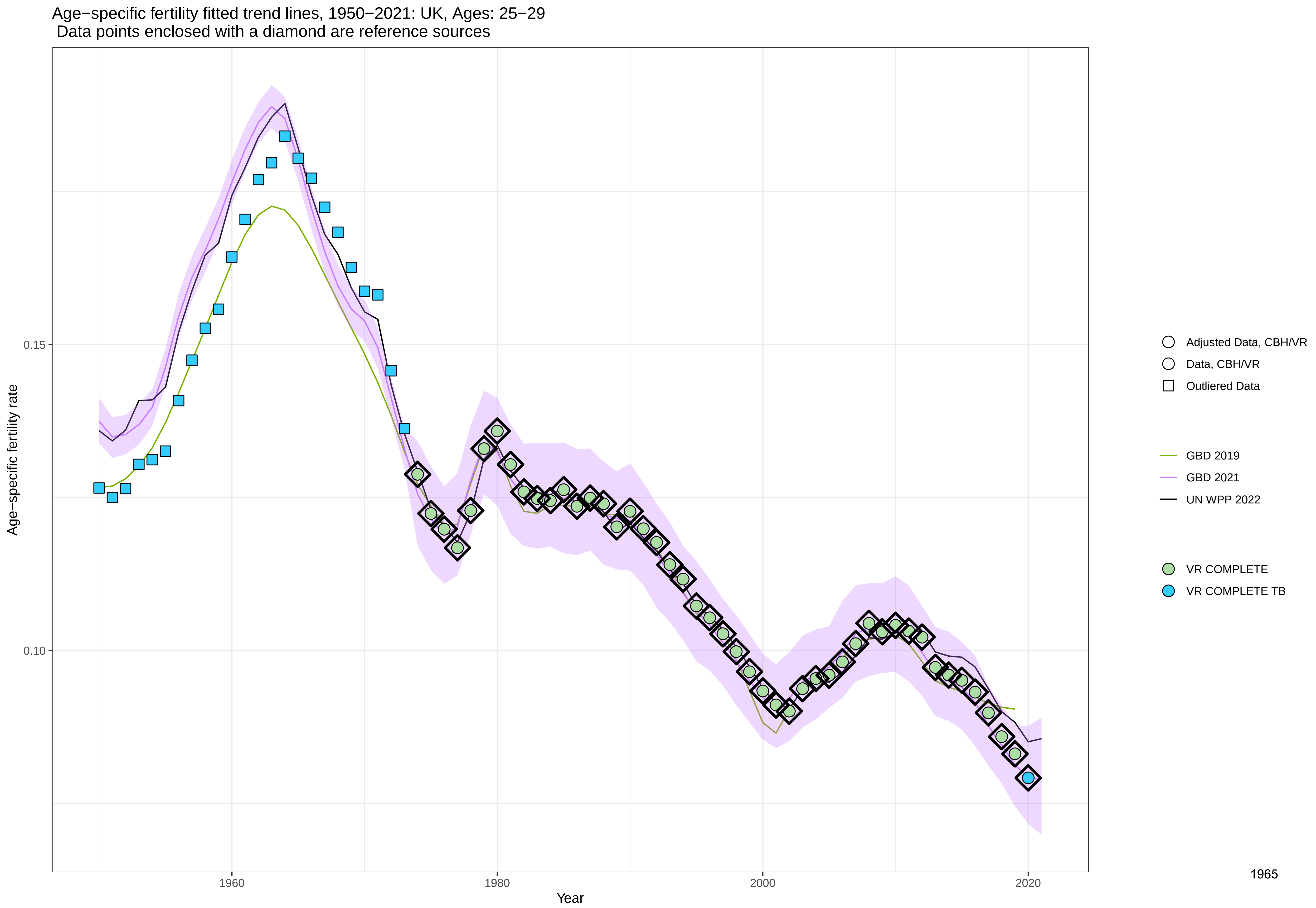

Age−specific fertility fitted trend lines, 1950−2021: UK, Ages: 25−29
Data points enclosed with a diamond are reference sources
Age−specific fertility rate
0.15
0.10
1960
1980
2000
2020
Year
Adjusted Data, CBH/VR
Data, CBH/VR
Outliered Data
GBD 2019
GBD 2021
UN WPP 2022
VR COMPLETE
VR COMPLETE TB

1965

Age−specific fertility fitted trend lines, 1950−2021: UK, Ages: 30−34
Data points enclosed with a diamond are reference sources

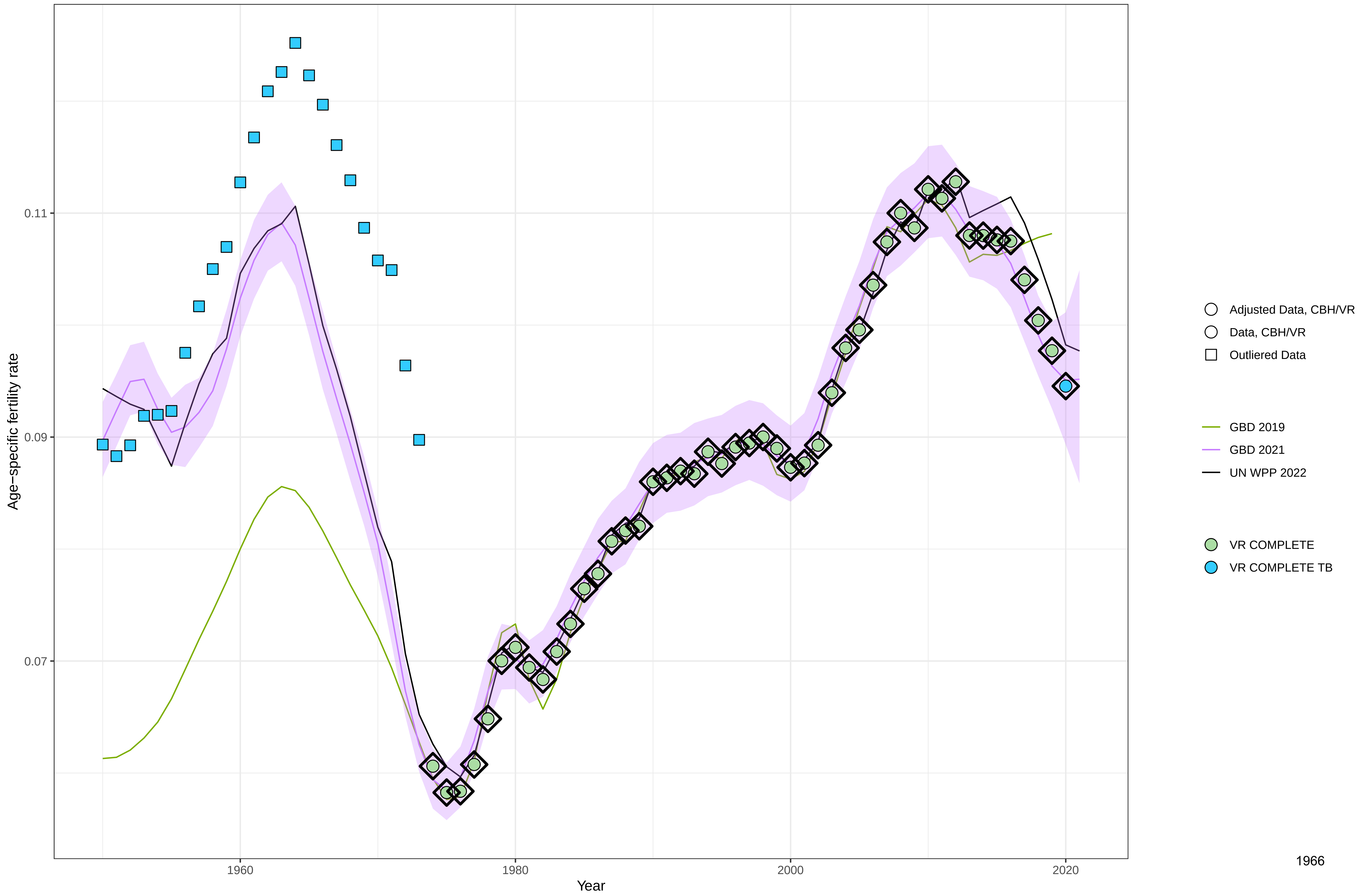

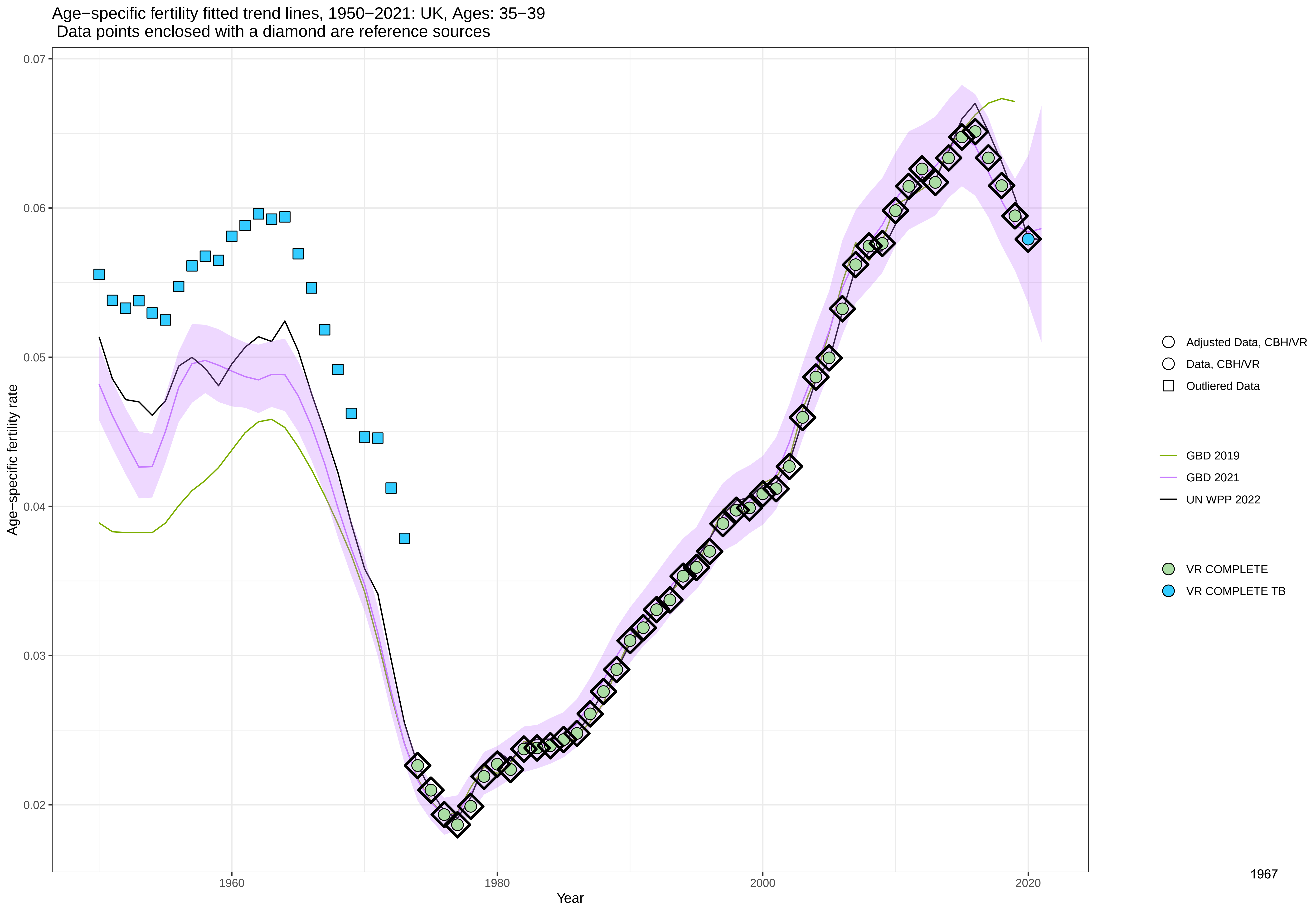

Age−specific fertility fitted trend lines, 1950−2021: UK, Ages: 35−39
Data points enclosed with a diamond are reference sources
0.07
0.06
0.05
0.04
0.03
0.02
Age−specific fertility rate
1960
1980
2000
2020
Year
Adjusted Data, CBH/VR
Data, CBH/VR
Outliered Data
GBD 2019
GBD 2021
UN WPP 2022
VR COMPLETE
VR COMPLETE TB

1967

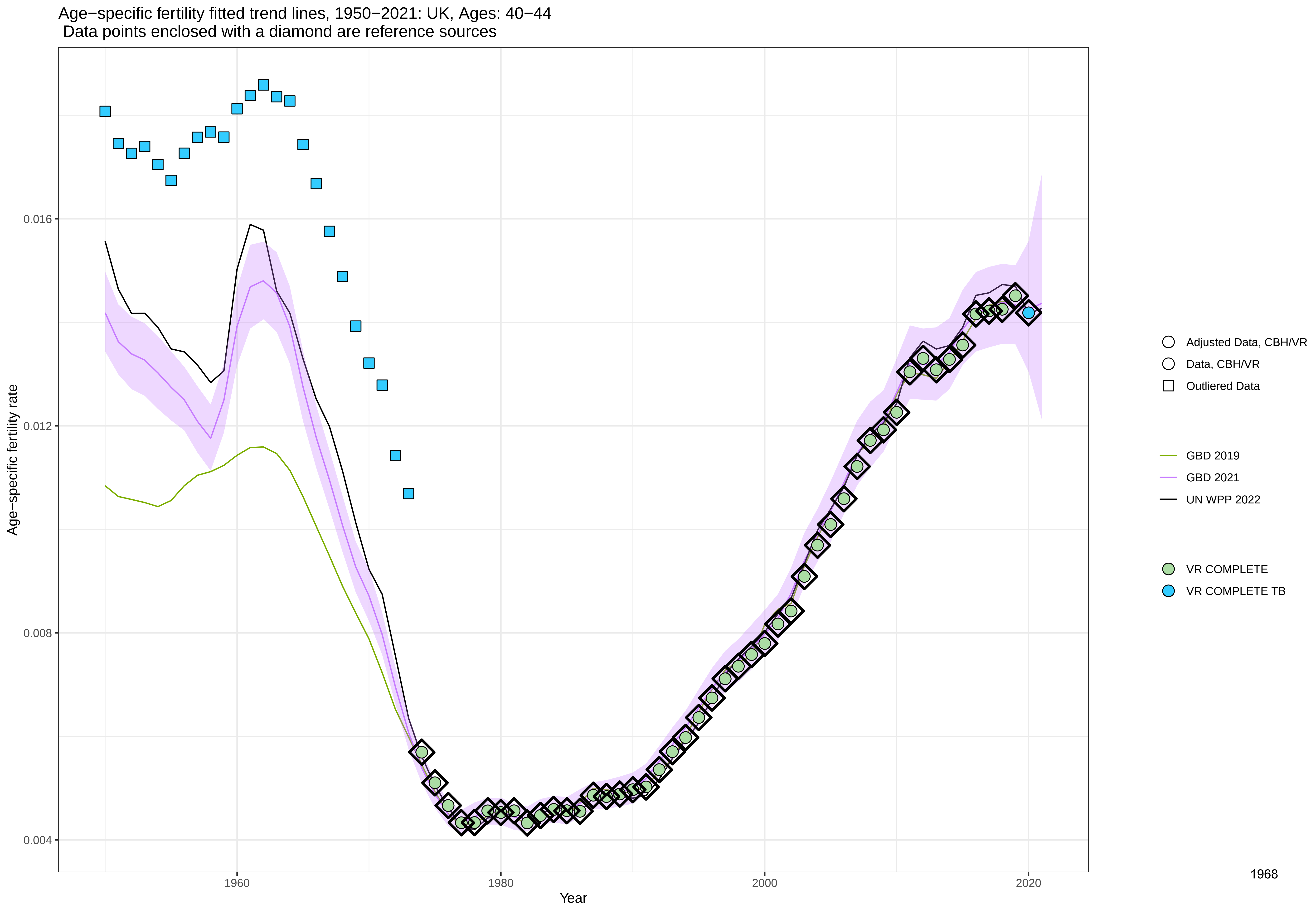

Age−specific fertility fitted trend lines, 1950−2021: UK, Ages: 40−44
Data points enclosed with a diamond are reference sources
Age−specific fertility rate
0.016
0.012
0.008
0.004
1960
1980
2000
2020
Year
Adjusted Data, CBH/VR
Data, CBH/VR
Outliered Data
GBD 2019
GBD 2021
UN WPP 2022
VR COMPLETE
VR COMPLETE TB

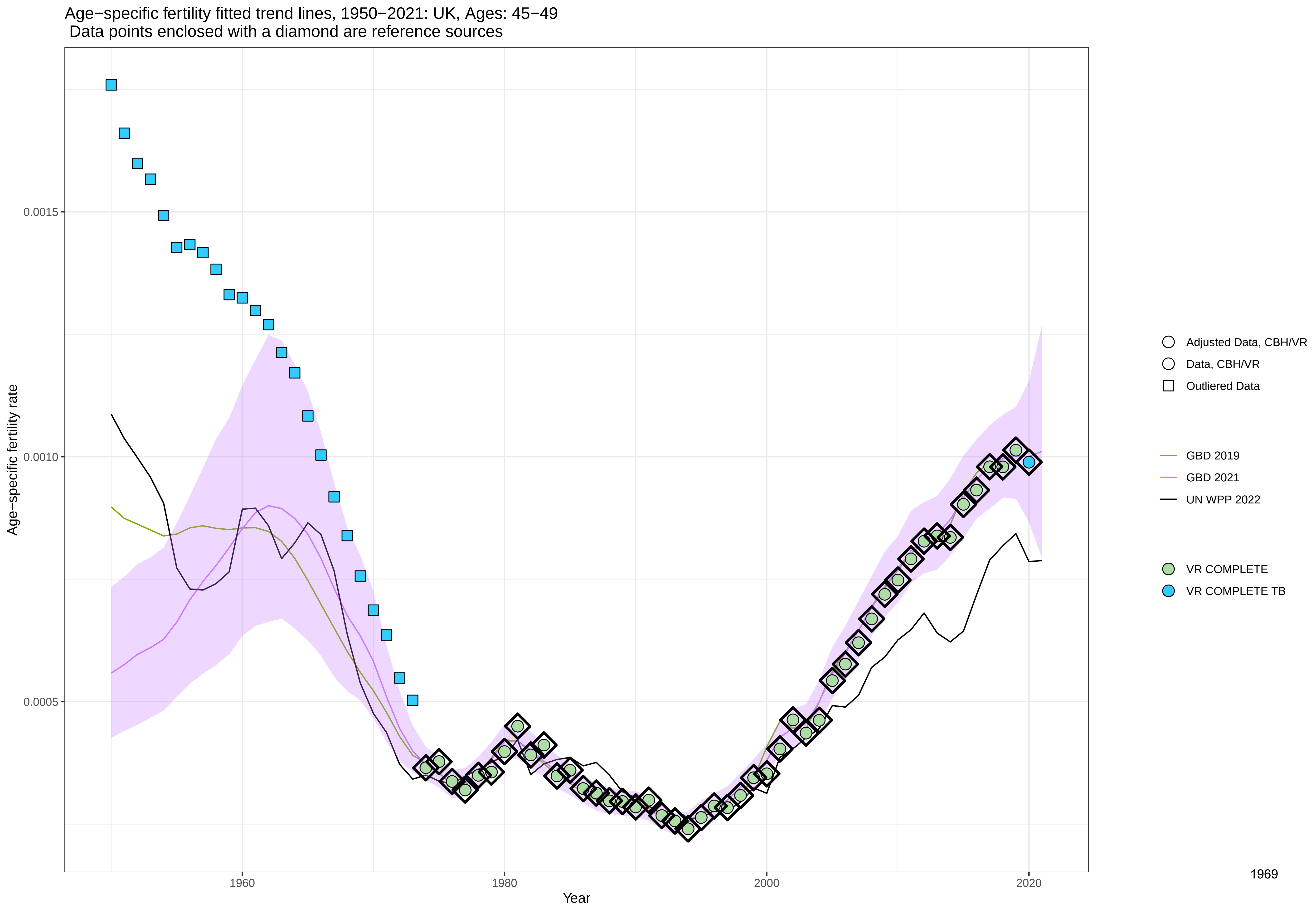

Age−specific fertility fitted trend lines, 1950−2021: UK, Ages: 45−49
Data points enclosed with a diamond are reference sources
Age−specific fertility rate
0.0015
0.0010
0.0005
1960
1980
2000
2020
Year
Adjusted Data, CBH/VR
Data, CBH/VR
Outliered Data
GBD 2019
GBD 2021
UN WPP 2022
VR COMPLETE
VR COMPLETE TB

Age−specific fertility fitted trend lines, 1950−2021: UK, Ages: 50−54
Data points enclosed with a diamond are reference sources

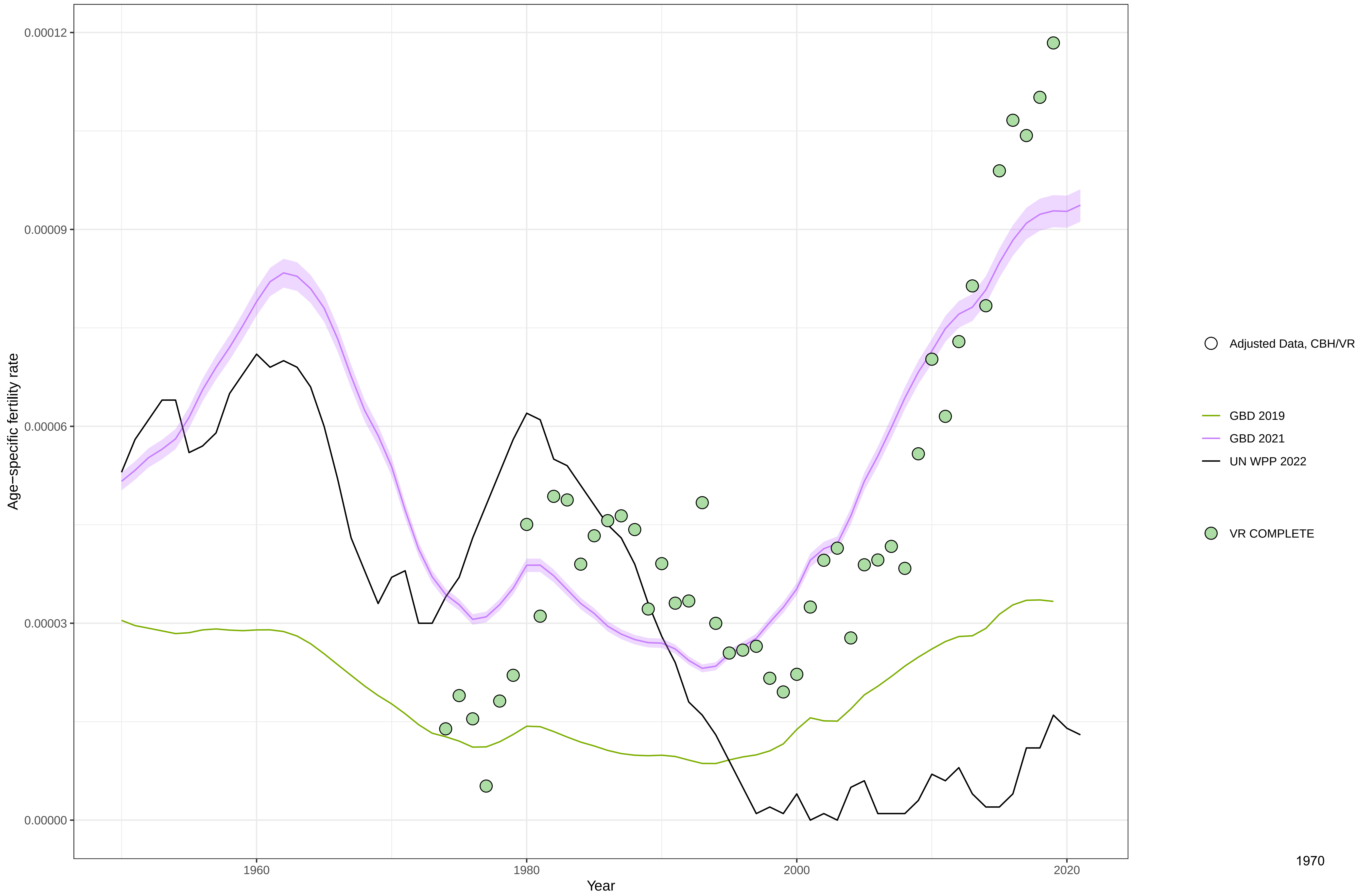

Total fertility rate fitted trend lines, 1950−2021: UK TFR
Data points enclosed with a diamond are reference sources

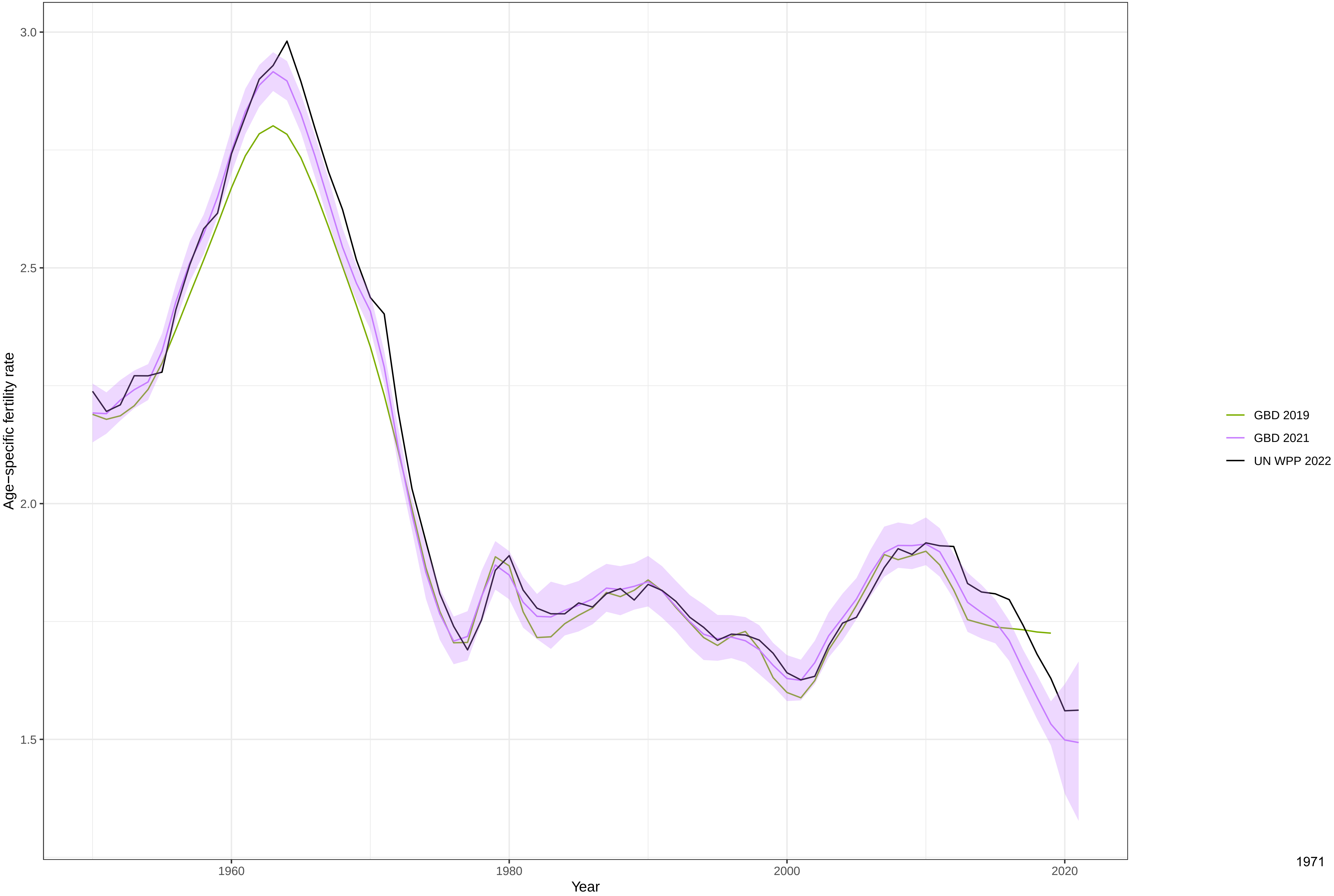

Age−specific fertility fitted trend lines, 1950−2021: England, Ages: 10−14
Data points enclosed with a diamond are reference sources

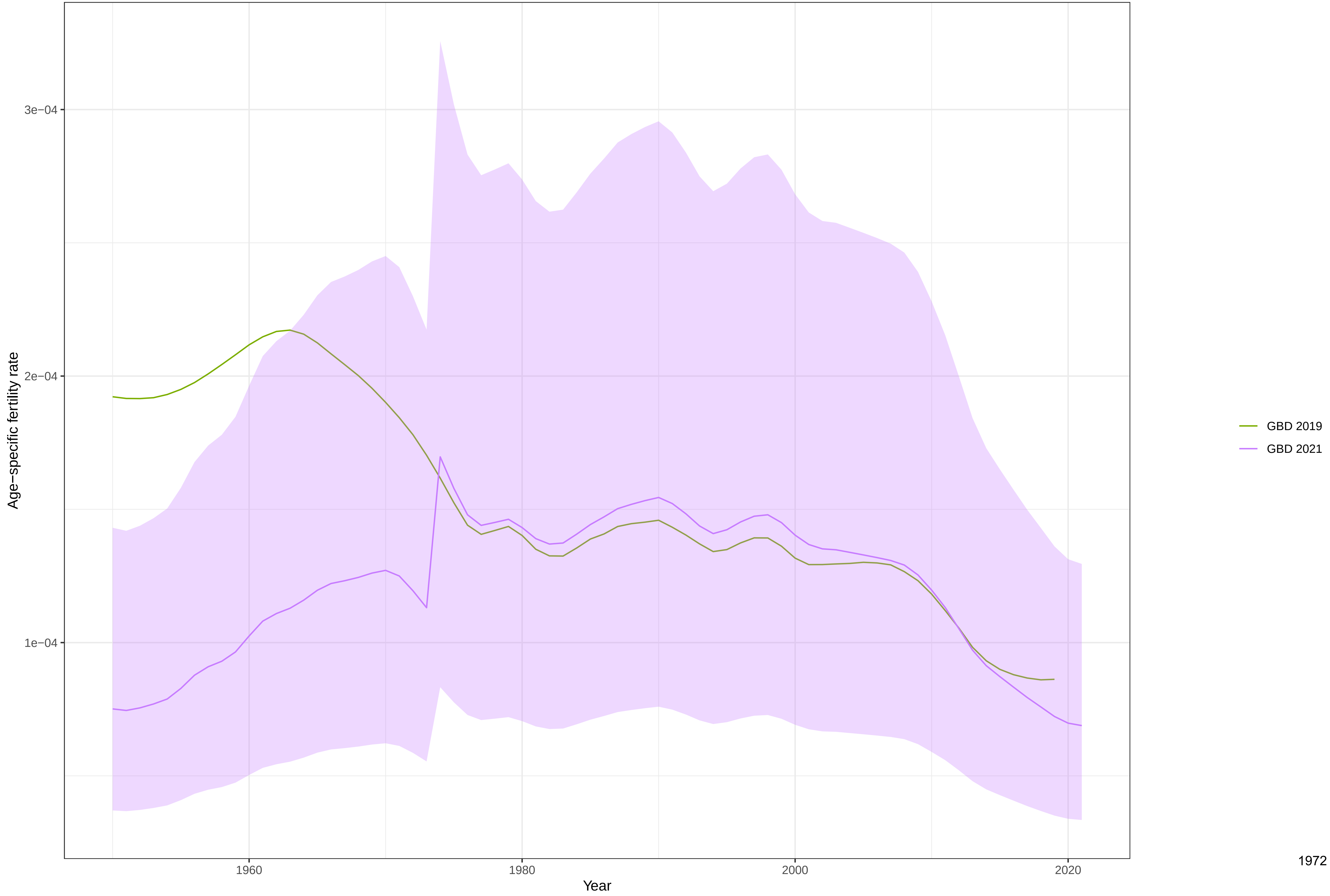

Age−specific fertility fitted trend lines, 1950−2021: England, Ages: 15−19
Data points enclosed with a diamond are reference sources

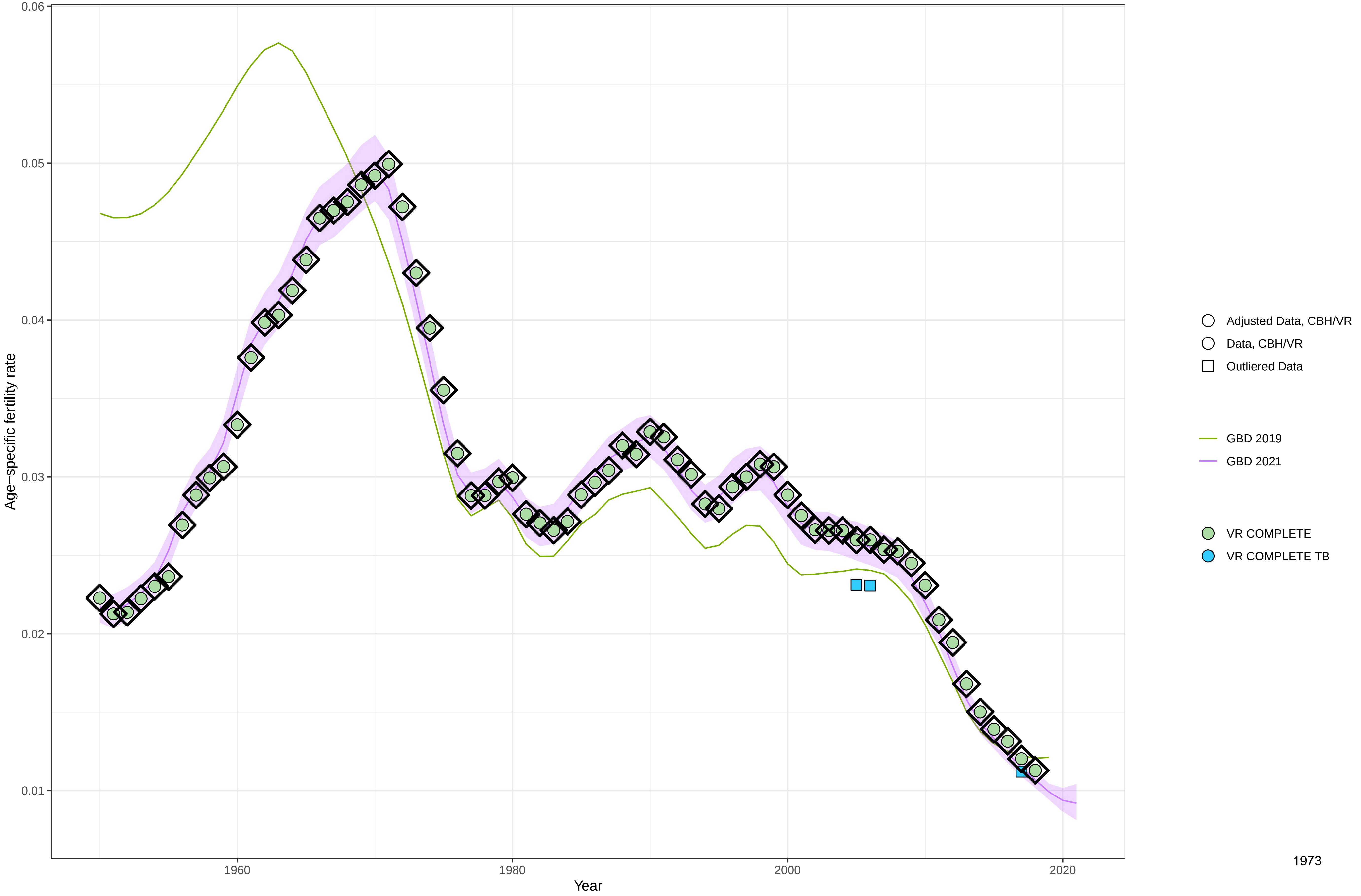

Age−specific fertility fitted trend lines, 1950−2021: England, Ages: 20−24
Data points enclosed with a diamond are reference sources

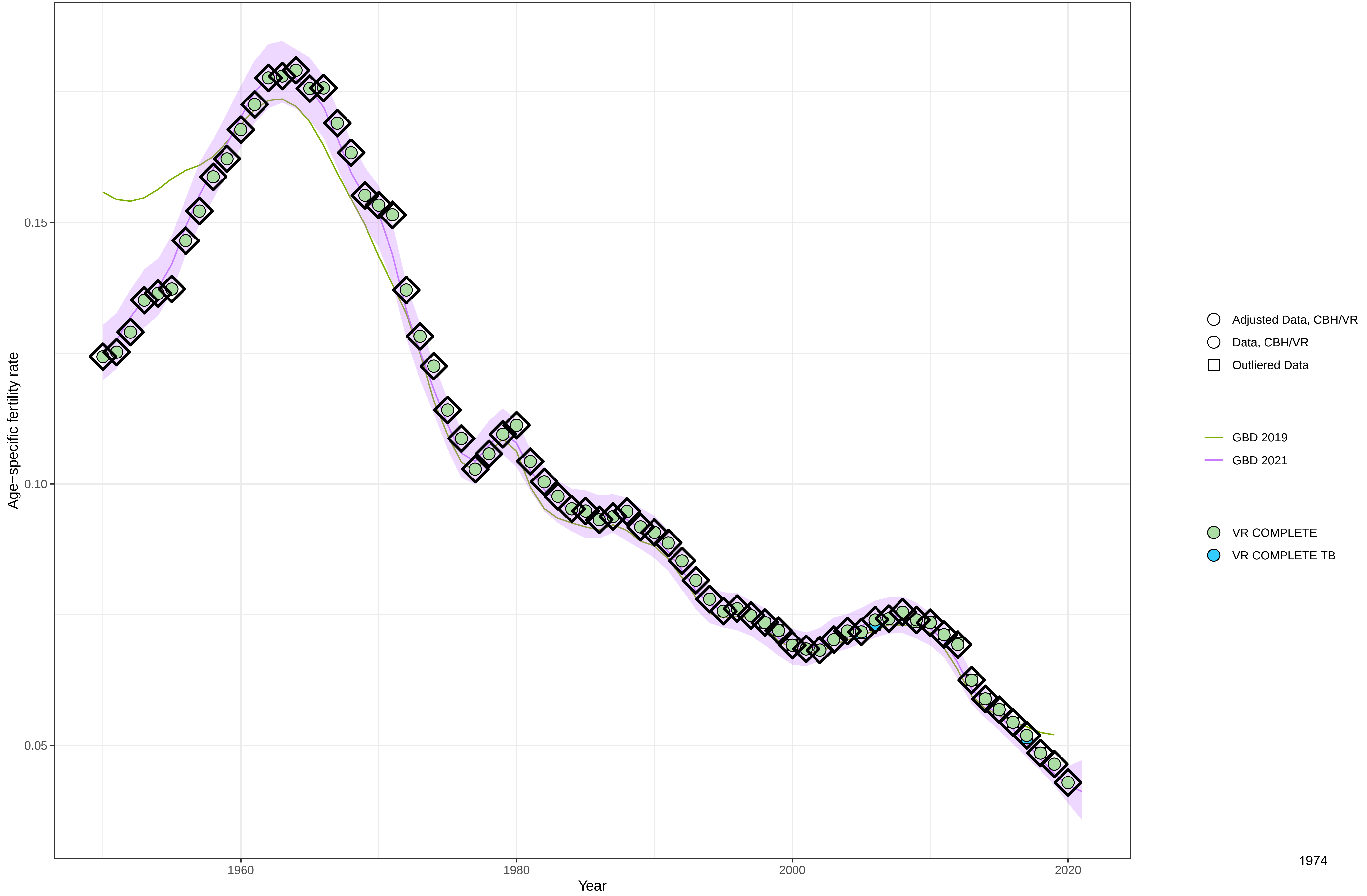

Age−specific fertility fitted trend lines, 1950−2021: England, Ages: 25−29
Data points enclosed with a diamond are reference sources

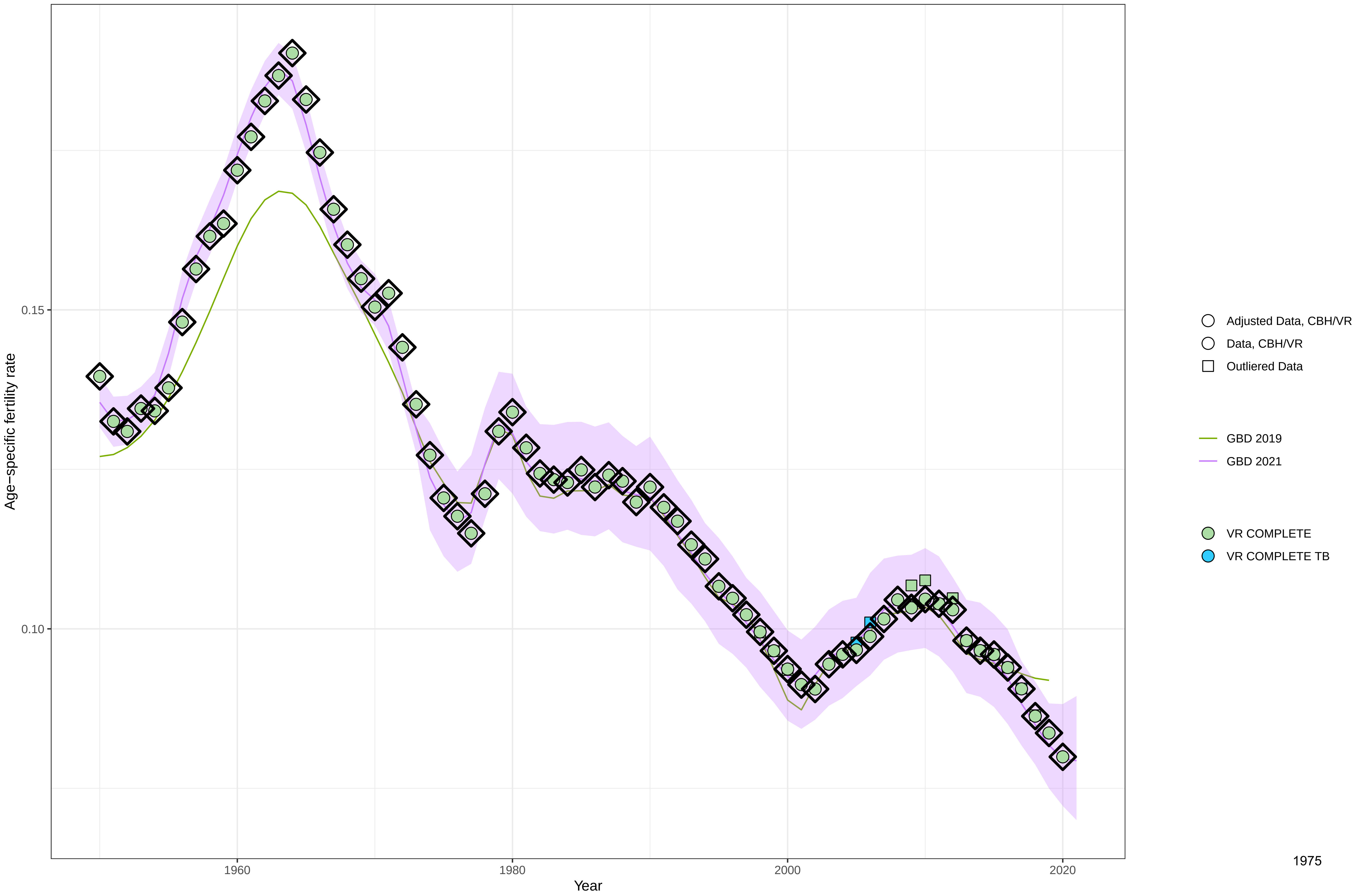

Age−specific fertility fitted trend lines, 1950−2021: England, Ages: 30−34
Data points enclosed with a diamond are reference sources

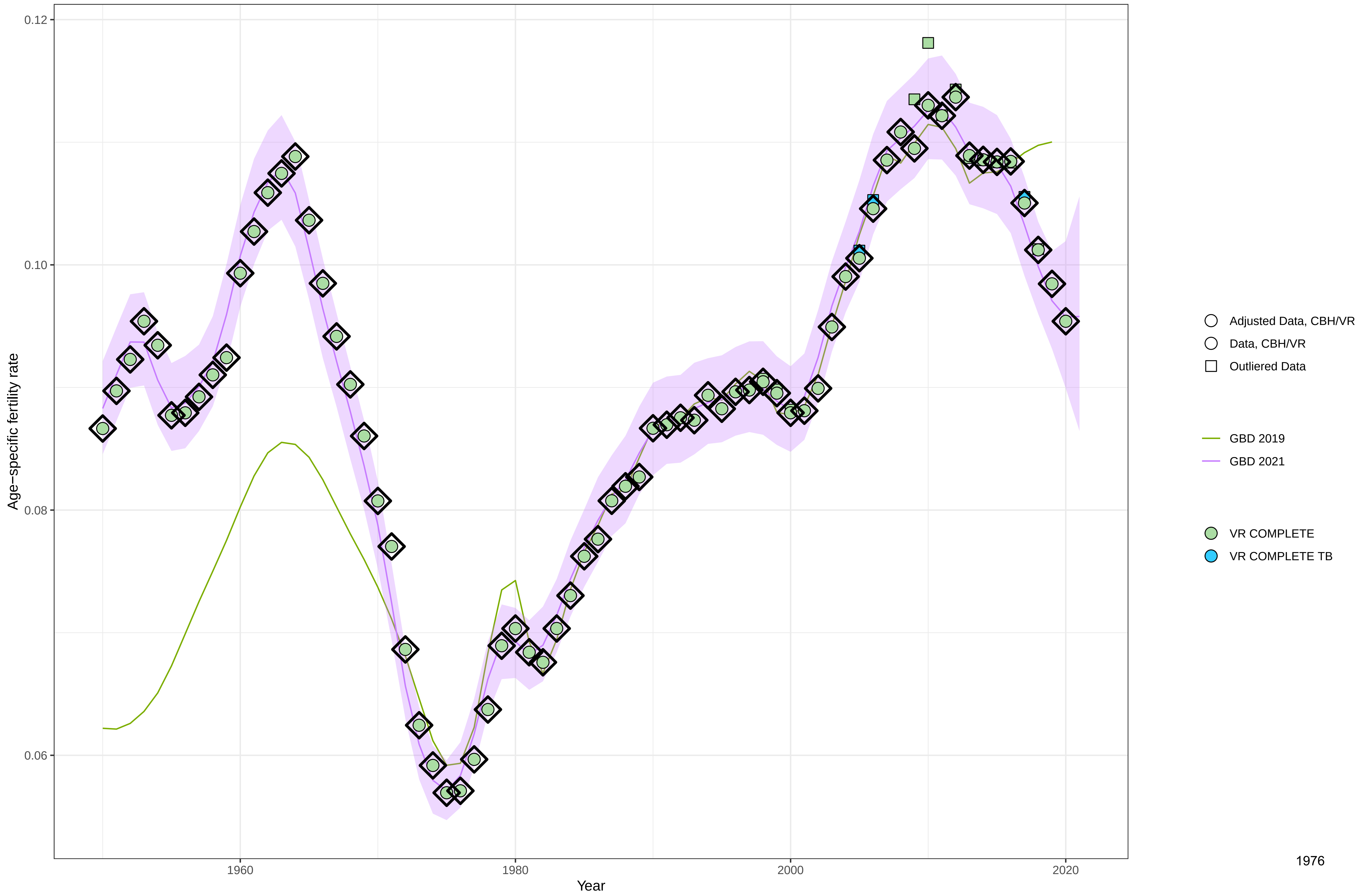

1976

Age−specific fertility fitted trend lines, 1950−2021: England, Ages: 35−39
Data points enclosed with a diamond are reference sources

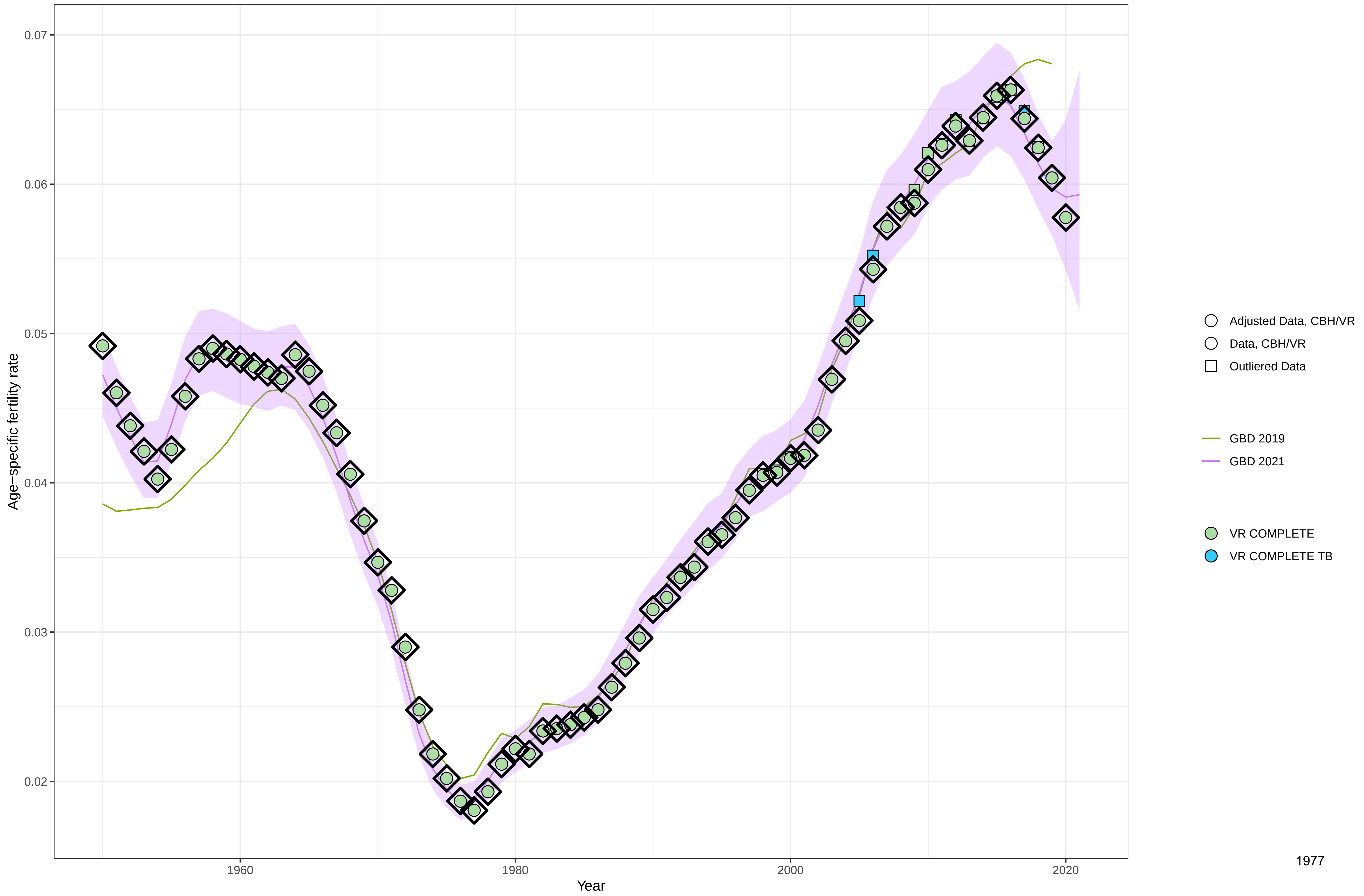

Age−specific fertility fitted trend lines, 1950−2021: England, Ages: 40−44
Data points enclosed with a diamond are reference sources

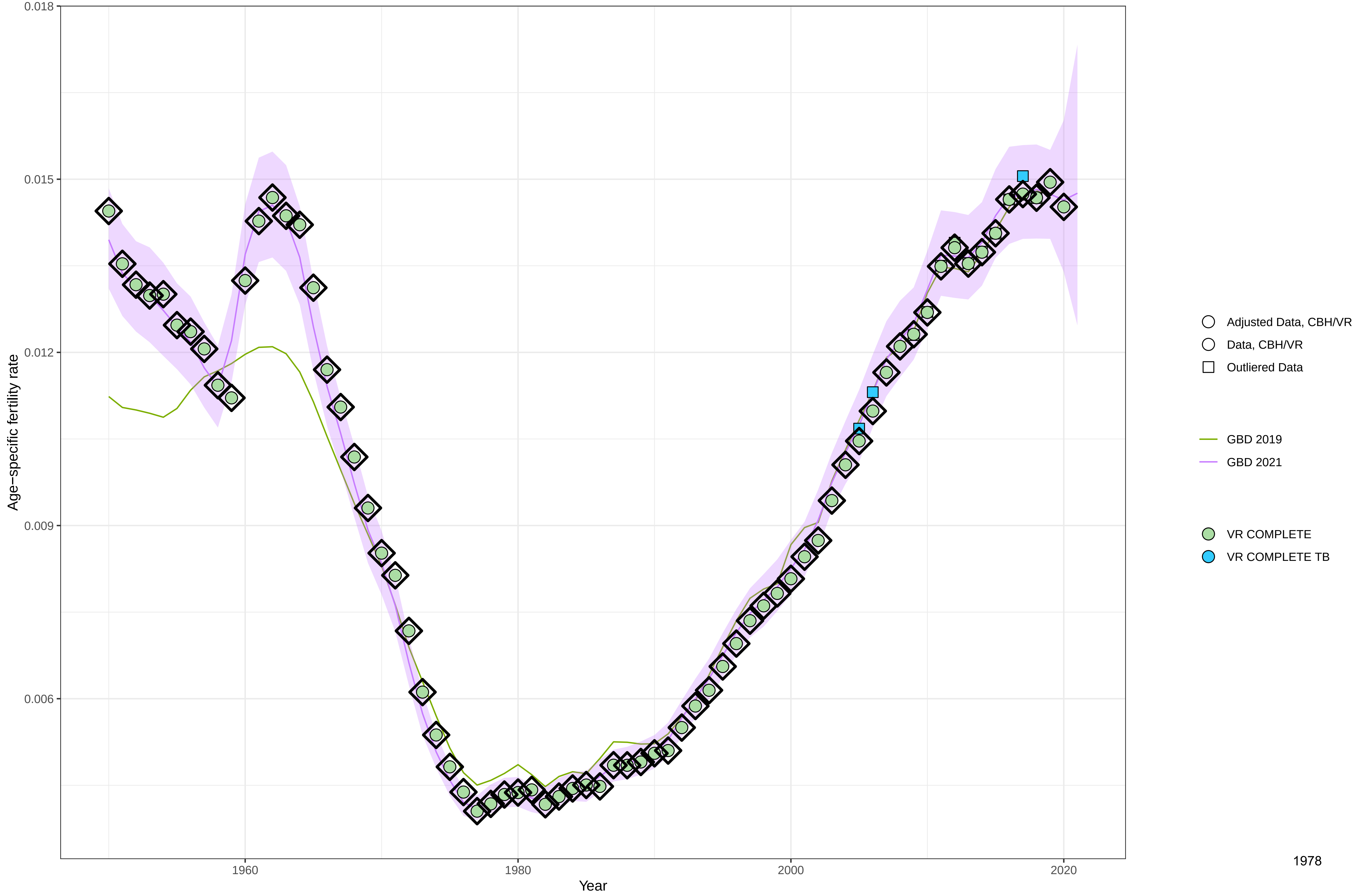

Age−specific fertility fitted trend lines, 1950−2021: England, Ages: 45−49
Data points enclosed with a diamond are reference sources

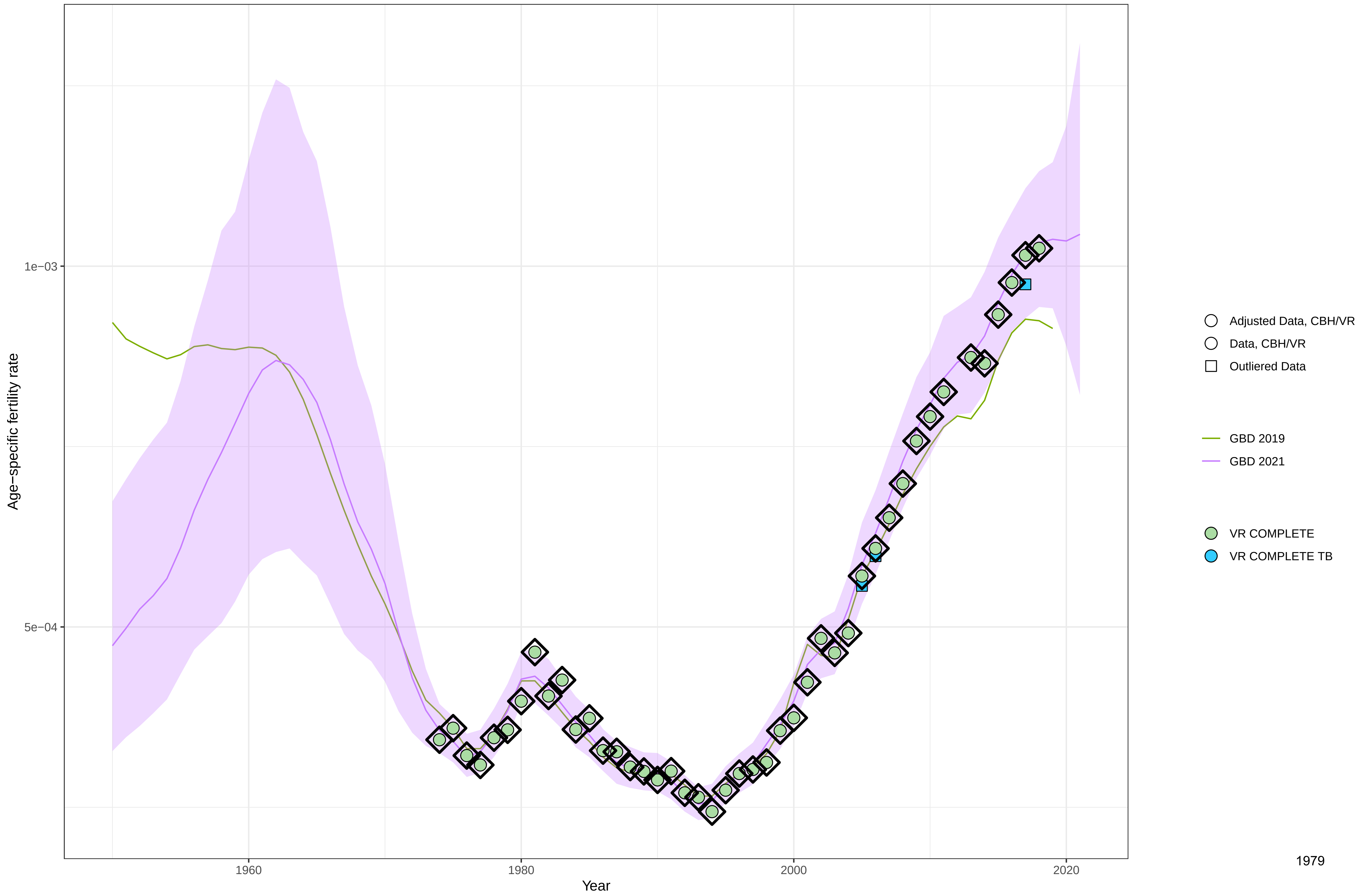

Age−specific fertility fitted trend lines, 1950−2021: England, Ages: 50−54
Data points enclosed with a diamond are reference sources

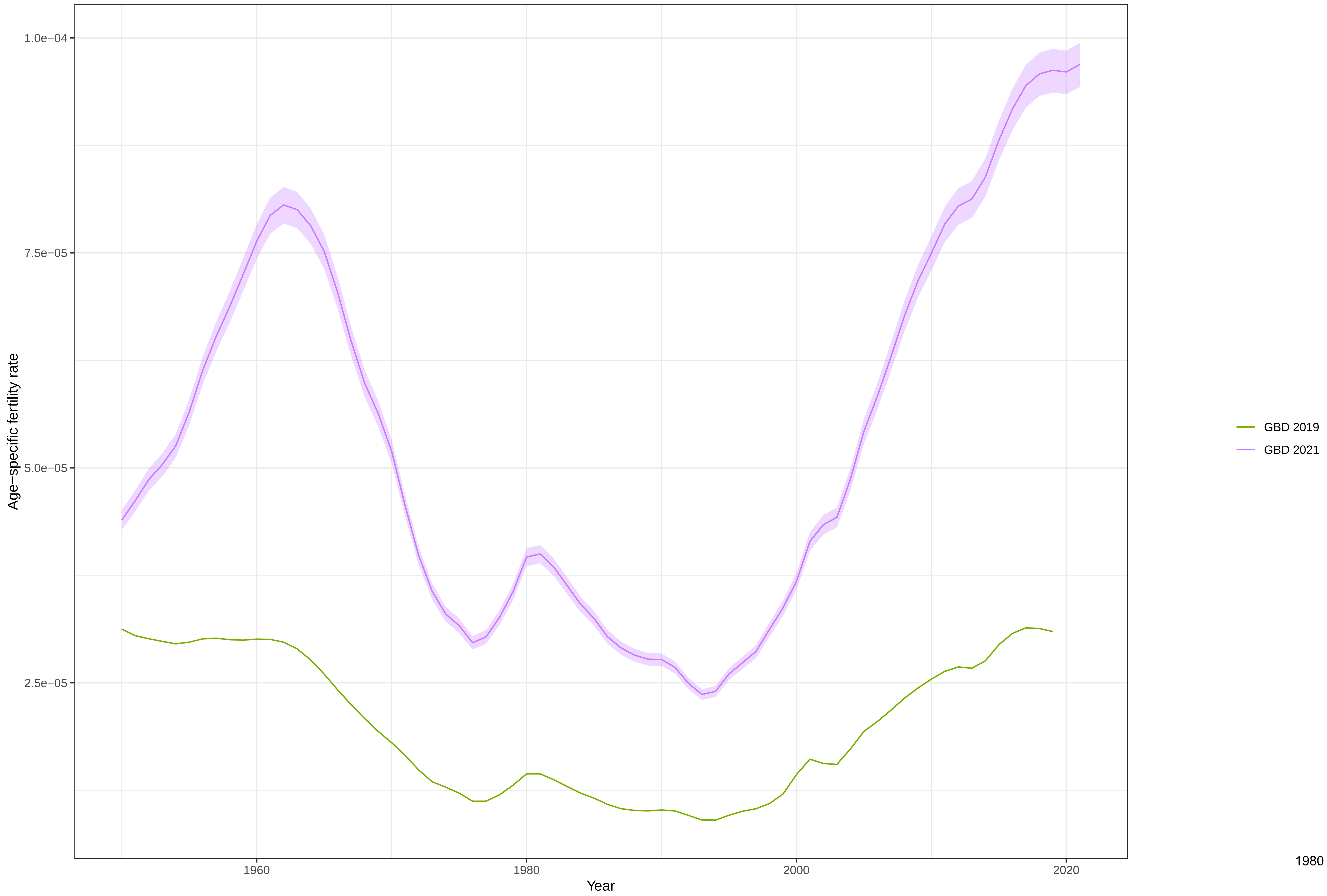

1980

Total fertility rate fitted trend lines, 1950−2021: England TFR
Data points enclosed with a diamond are reference sources

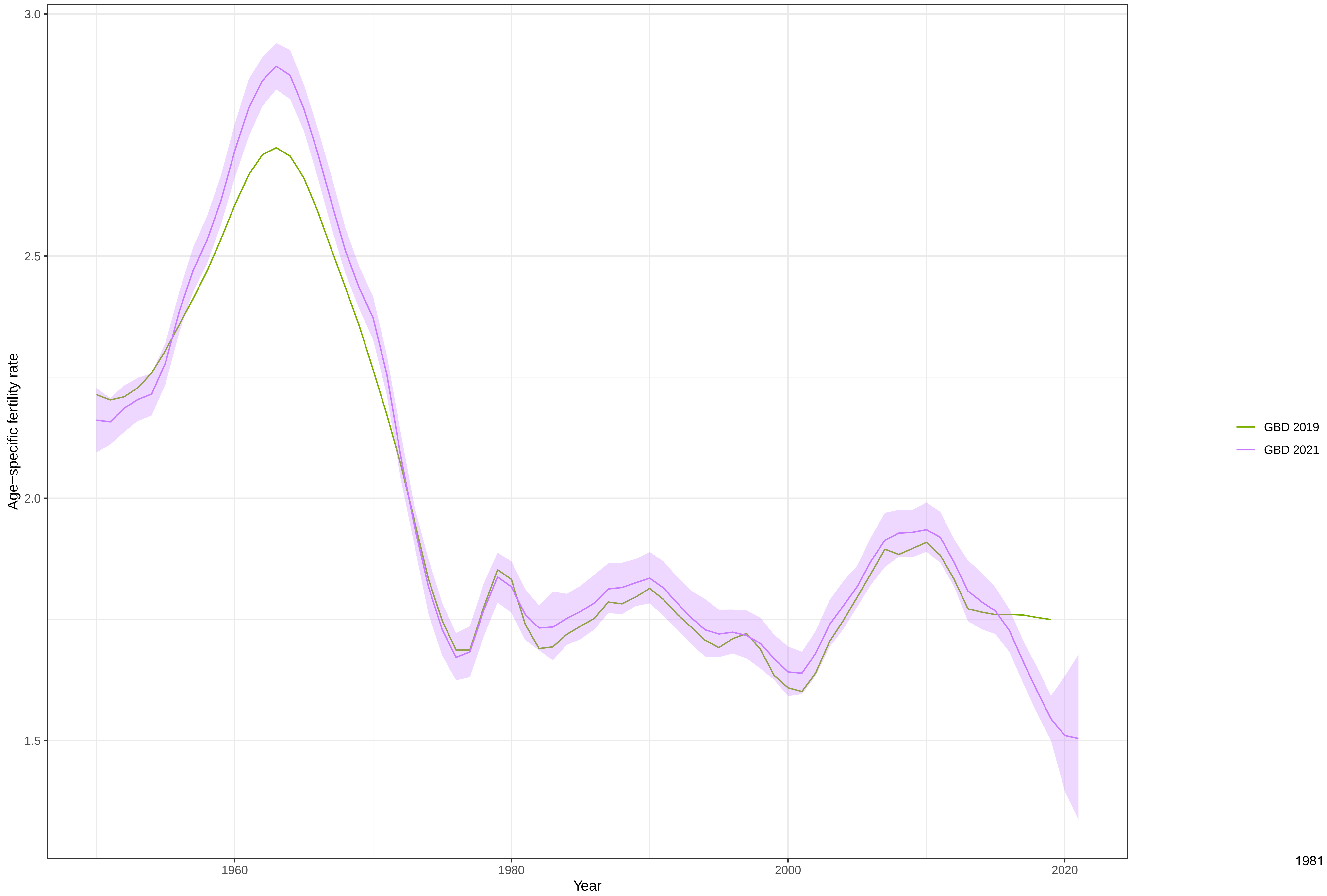

Age−specific fertility fitted trend lines, 1950−2021: East Midlands, Ages: 10−14
Data points enclosed with a diamond are reference sources

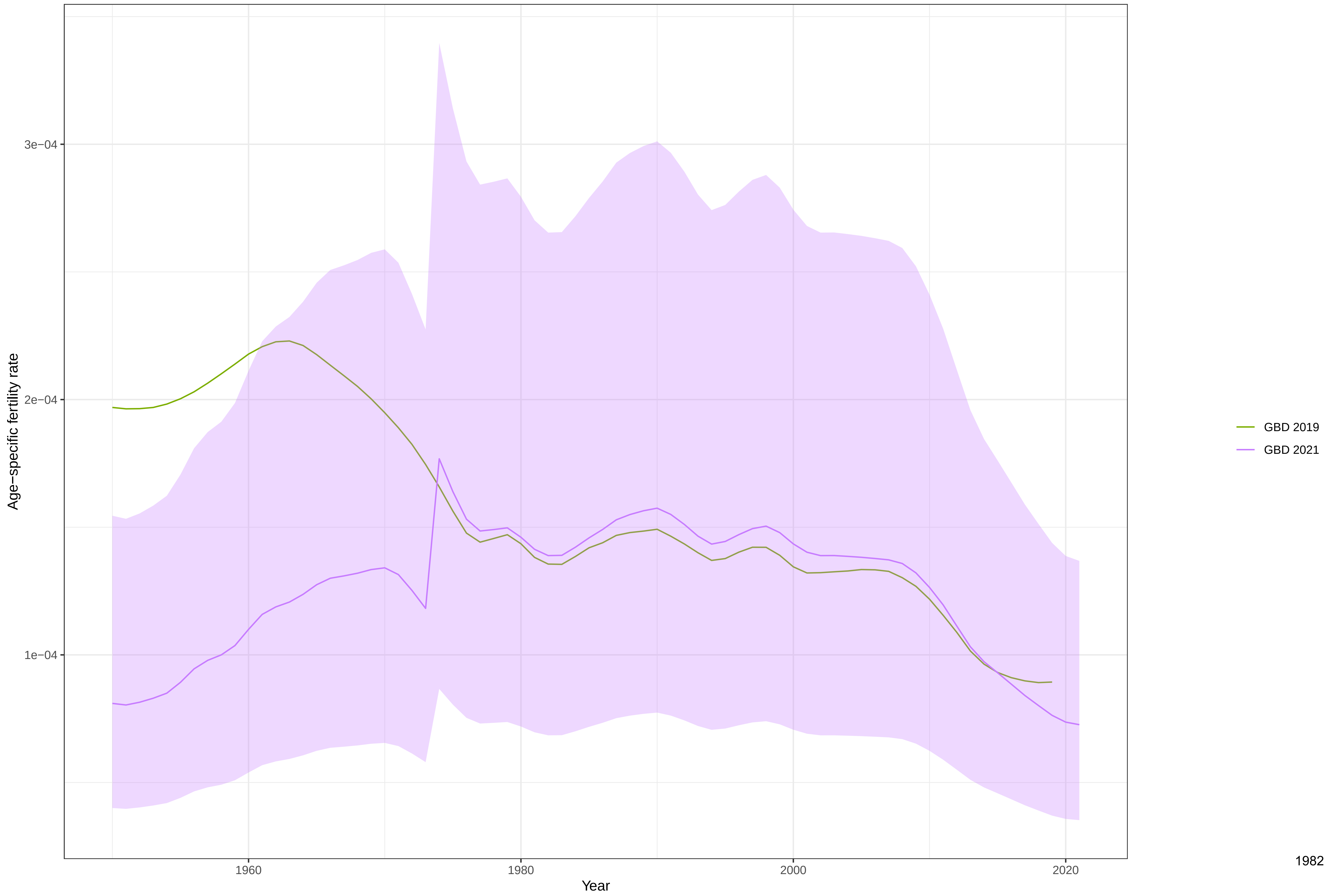

Age−specific fertility fitted trend lines, 1950−2021: East Midlands, Ages: 15−19
Data points enclosed with a diamond are reference sources

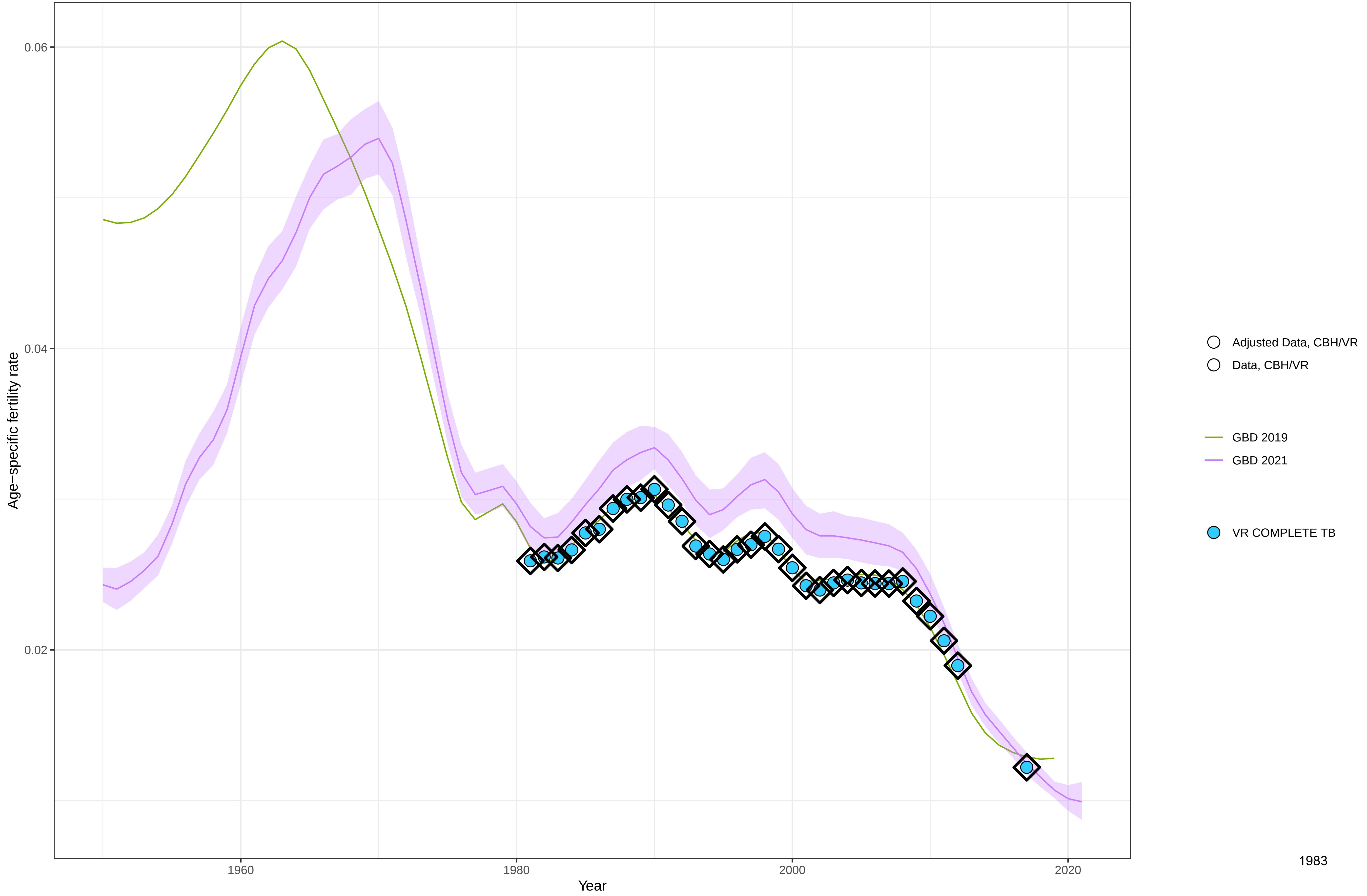

1983

Age−specific fertility fitted trend lines, 1950−2021: East Midlands, Ages: 20−24
Data points enclosed with a diamond are reference sources

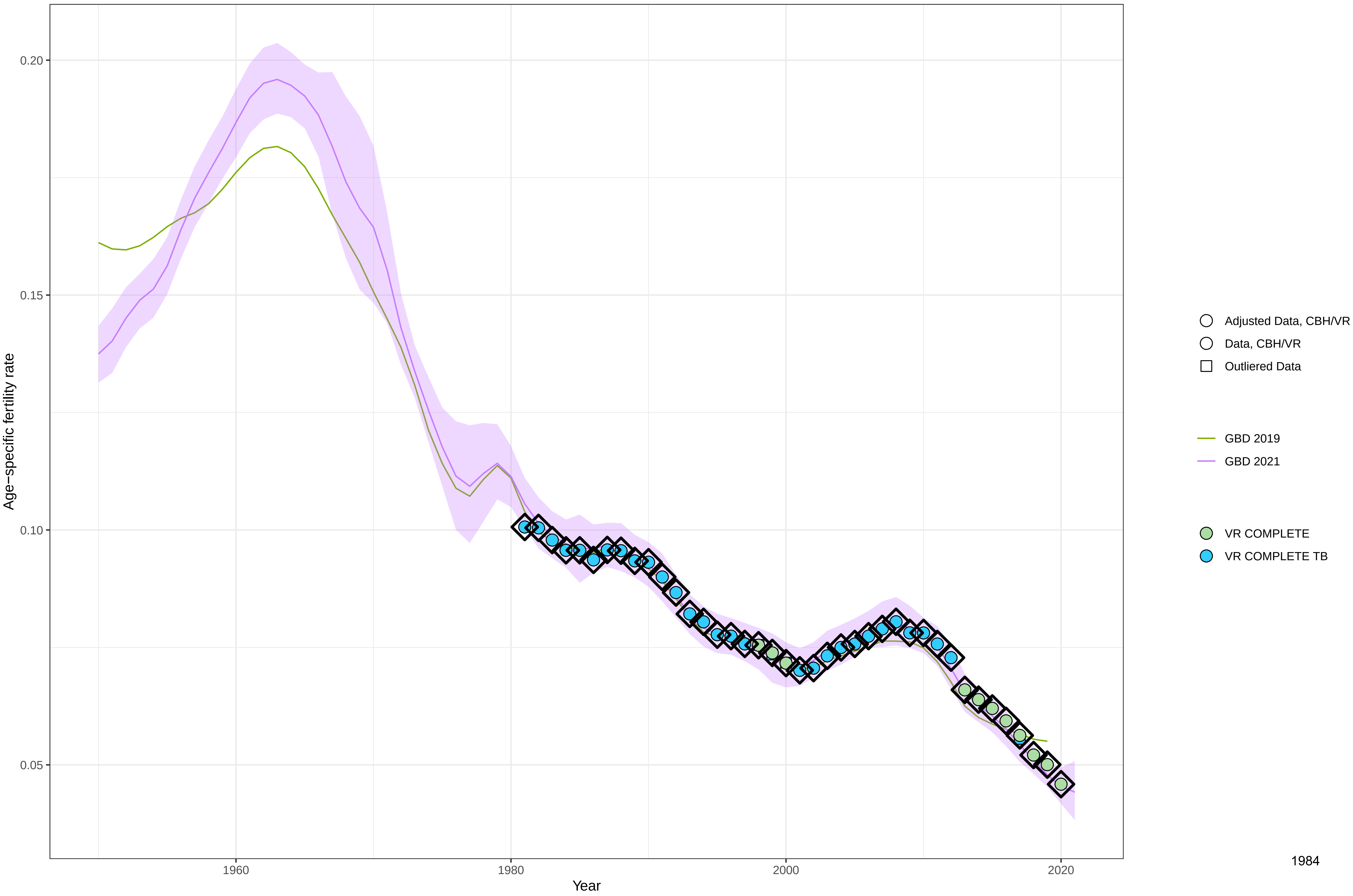

1984

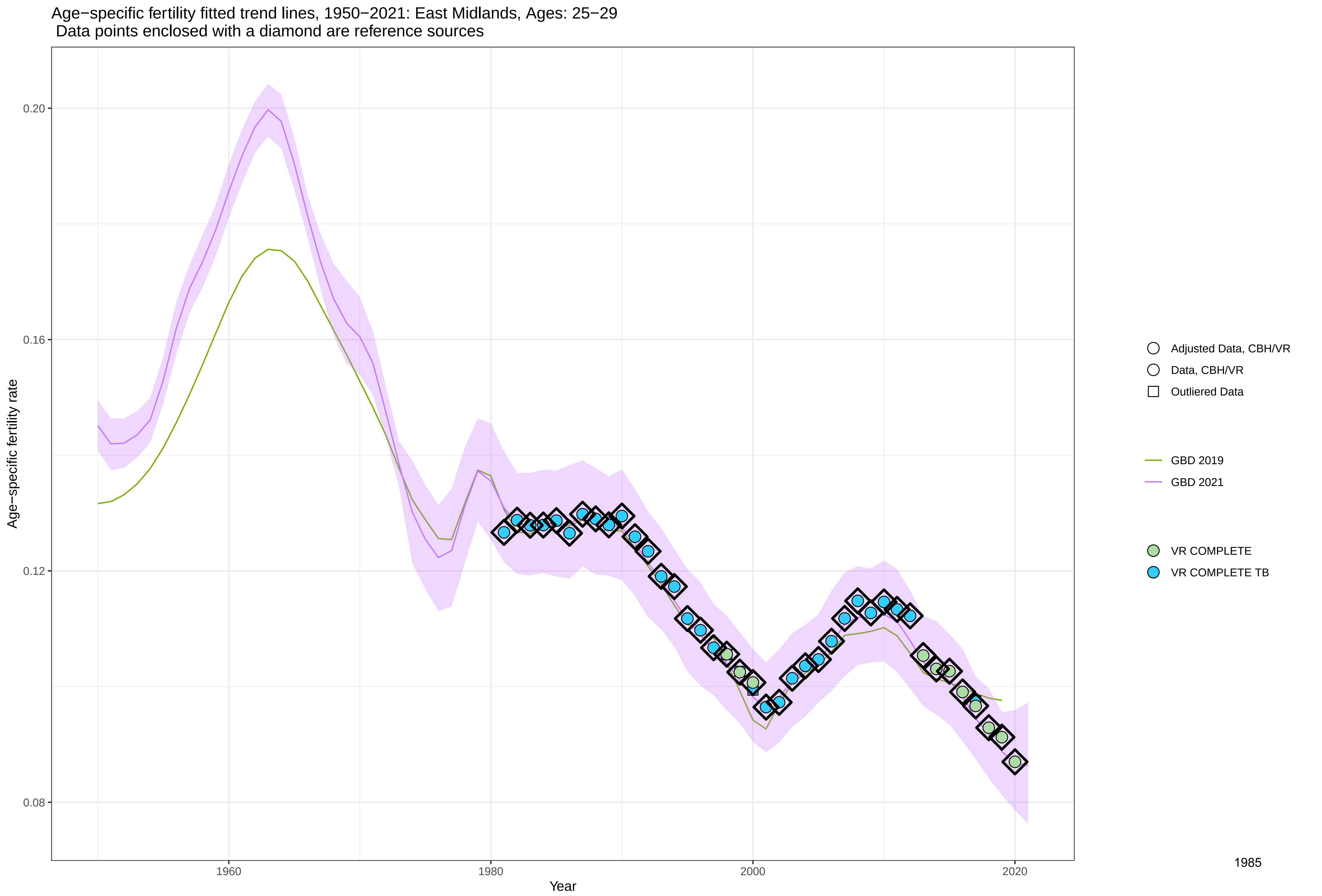

Age−specific fertility fitted trend lines, 1950−2021: East Midlands, Ages: 25−29
Data points enclosed with a diamond are reference sources
Age−specific fertility rate
0.20
0.16
0.12
0.08
1960
1980
2000
2020
Year
Adjusted Data, CBH/VR
Data, CBH/VR
Outliered Data
GBD 2019
GBD 2021
VR COMPLETE
VR COMPLETE TB

1985

Age−specific fertility fitted trend lines, 1950−2021: East Midlands, Ages: 30−34
Data points enclosed with a diamond are reference sources

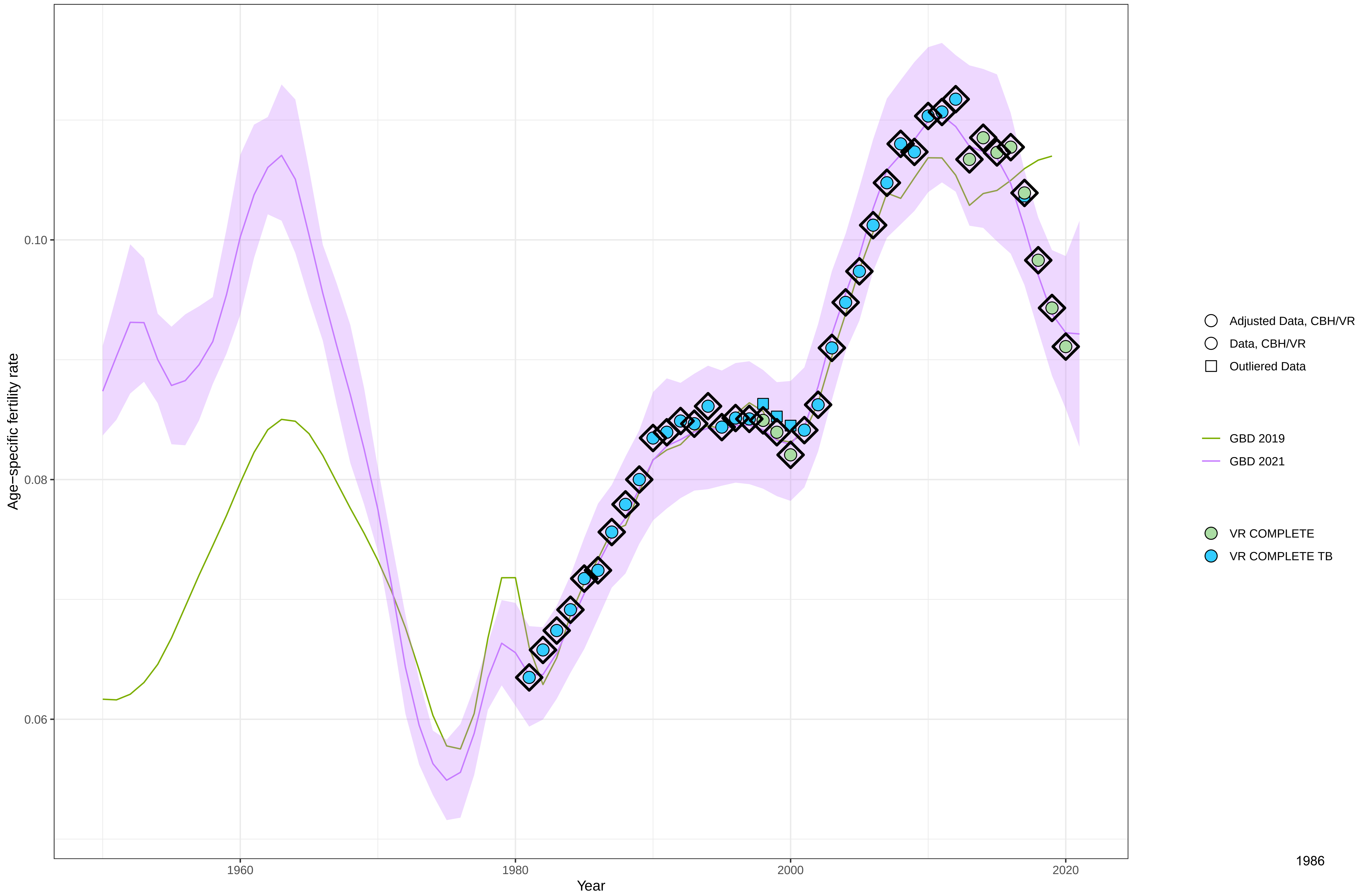

Age−specific fertility fitted trend lines, 1950−2021: East Midlands, Ages: 35−39
Data points enclosed with a diamond are reference sources

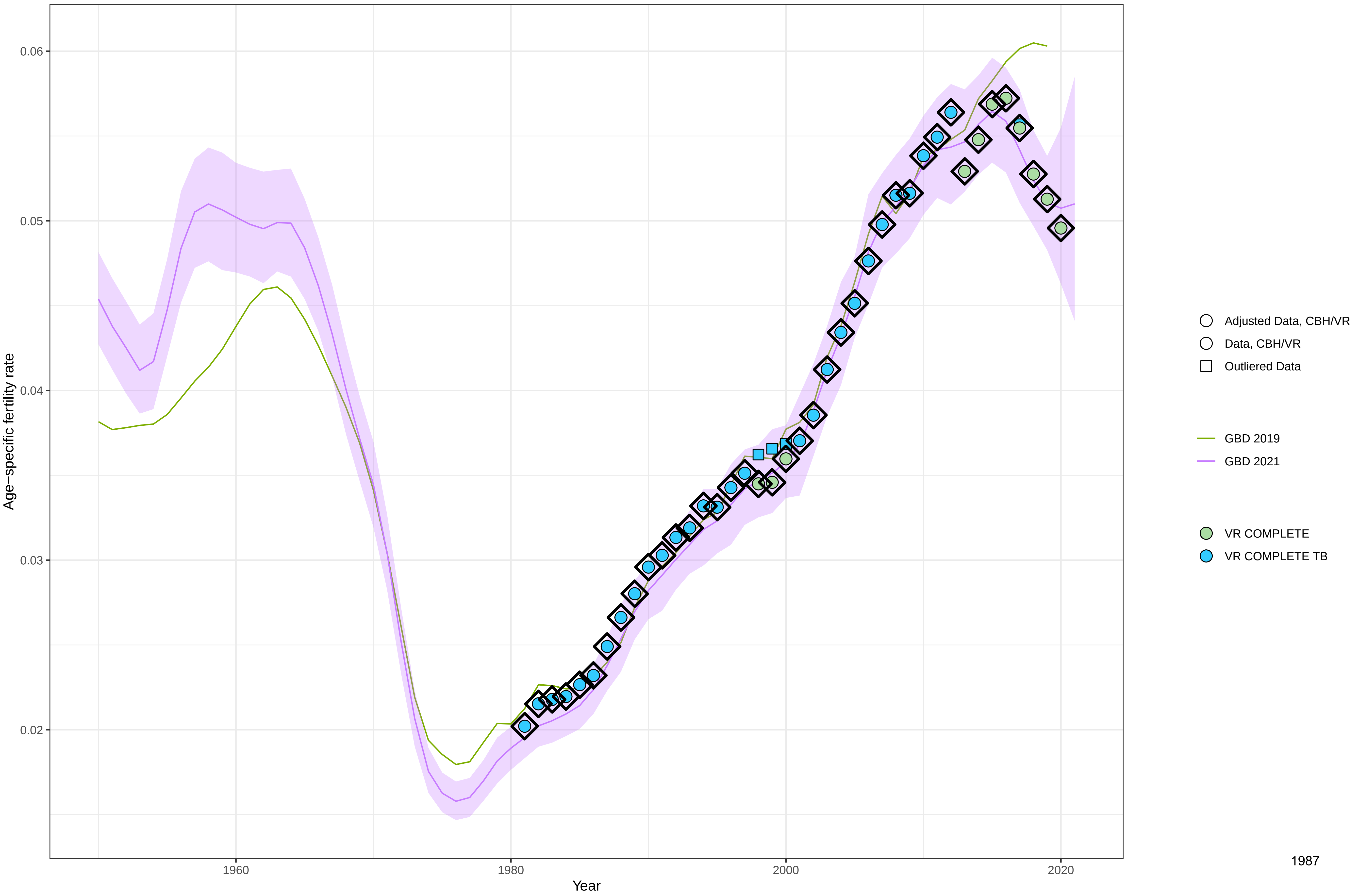

Age−specific fertility fitted trend lines, 1950−2021: East Midlands, Ages: 40−44
Data points enclosed with a diamond are reference sources

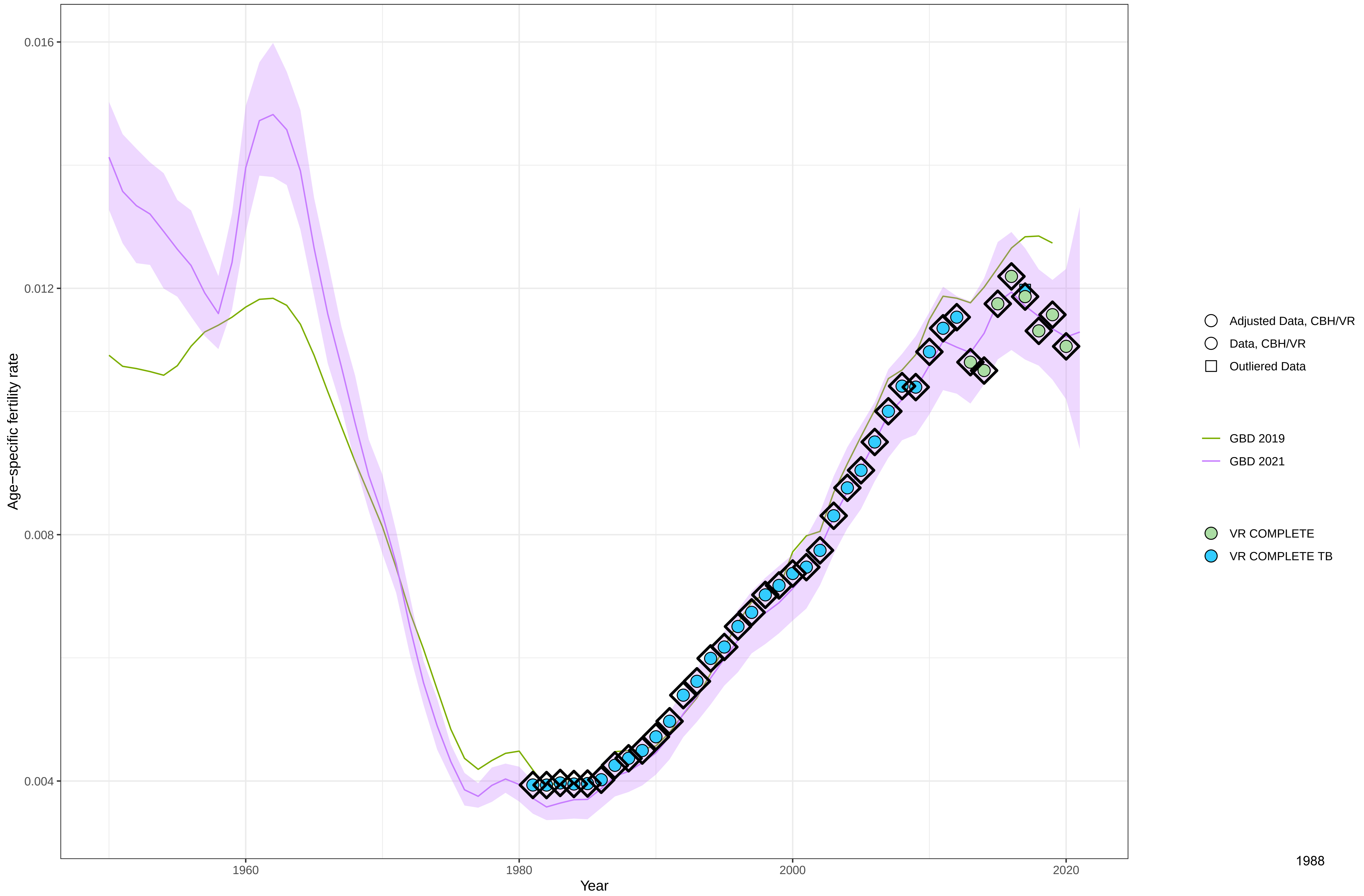

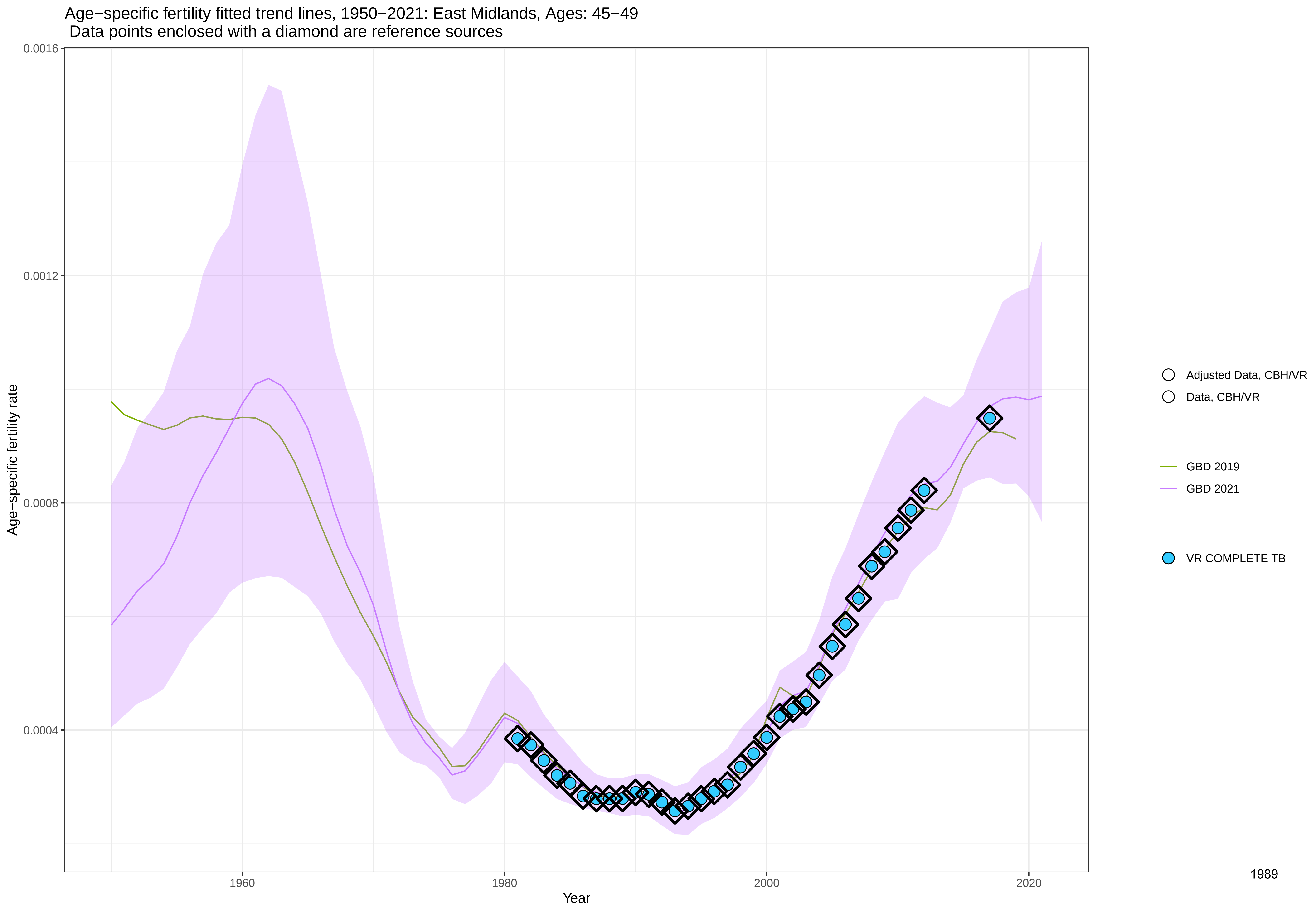

Age−specific fertility fitted trend lines, 1950−2021: East Midlands, Ages: 45−49
Data points enclosed with a diamond are reference sources
0.0016
0.0012
0.0008
0.0004
Age−specific fertility rate
1960
1980
2000
2020
Year
Adjusted Data, CBH/VR
Data, CBH/VR
GBD 2019
GBD 2021
VR COMPLETE TB

1989

Age−specific fertility fitted trend lines, 1950−2021: East Midlands, Ages: 50−54
Data points enclosed with a diamond are reference sources

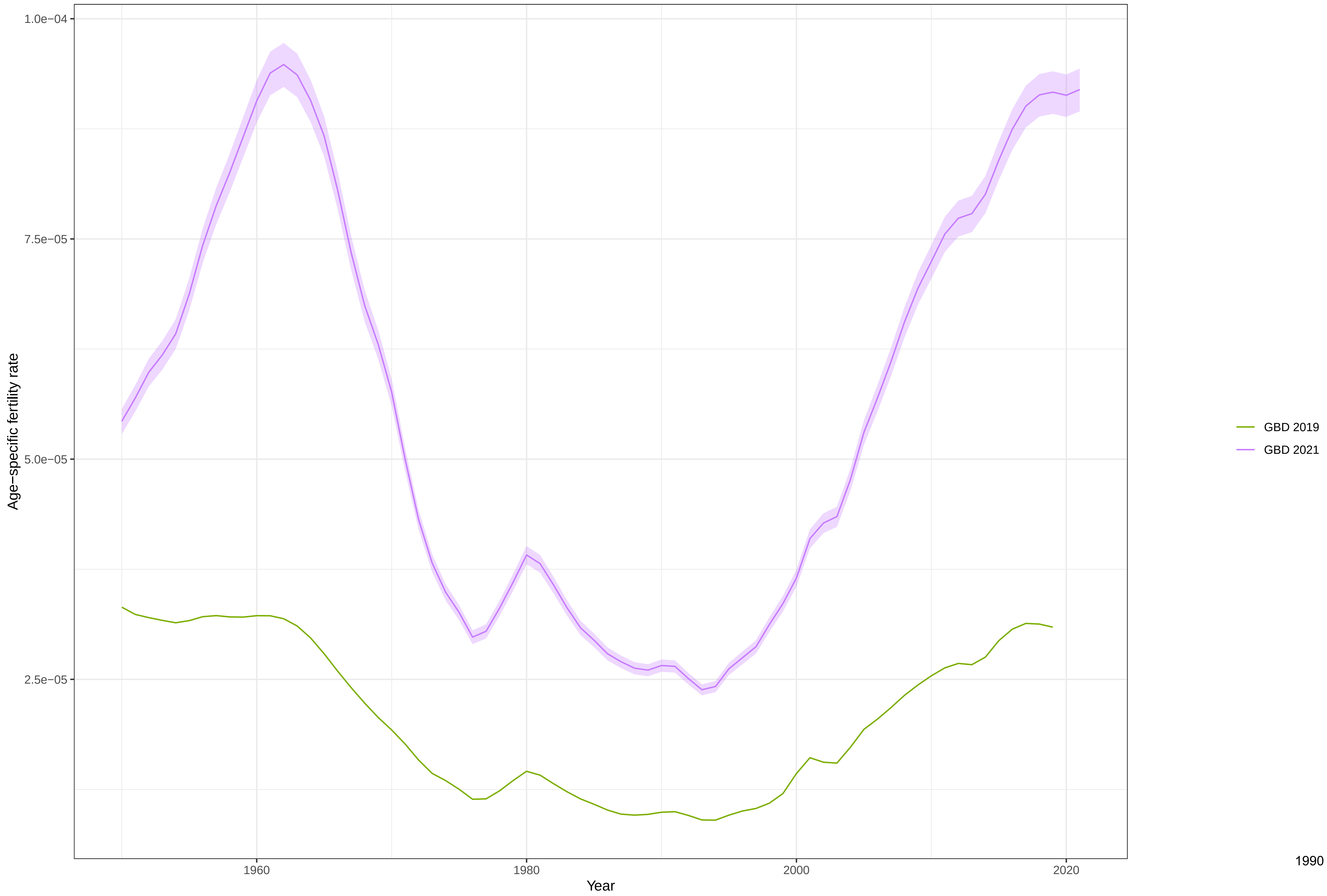

1990

Total fertility rate fitted trend lines, 1950−2021: East Midlands TFR
Data points enclosed with a diamond are reference sources

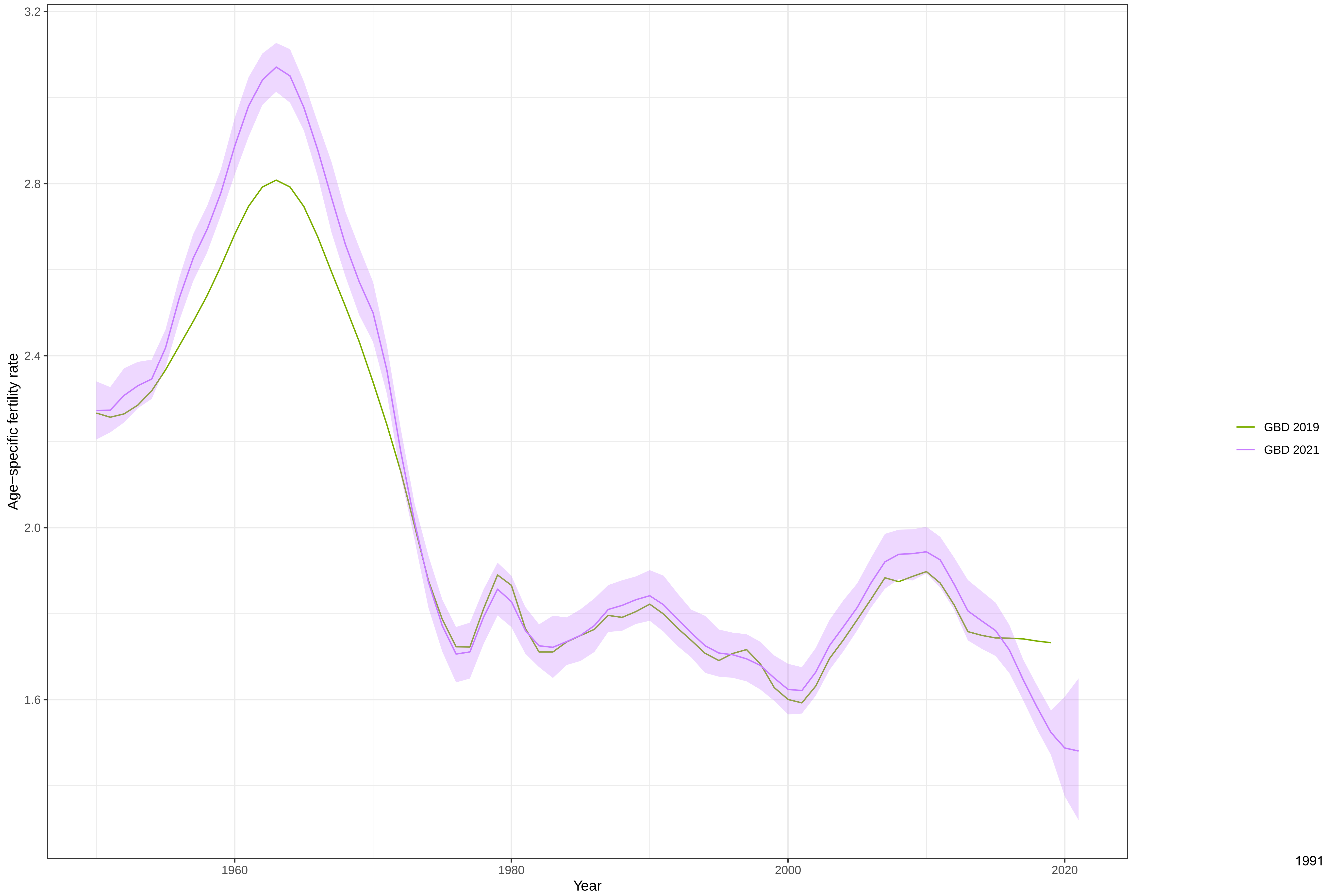

Age−specific fertility fitted trend lines, 1950−2021: Derby, Ages: 10−14
Data points enclosed with a diamond are reference sources

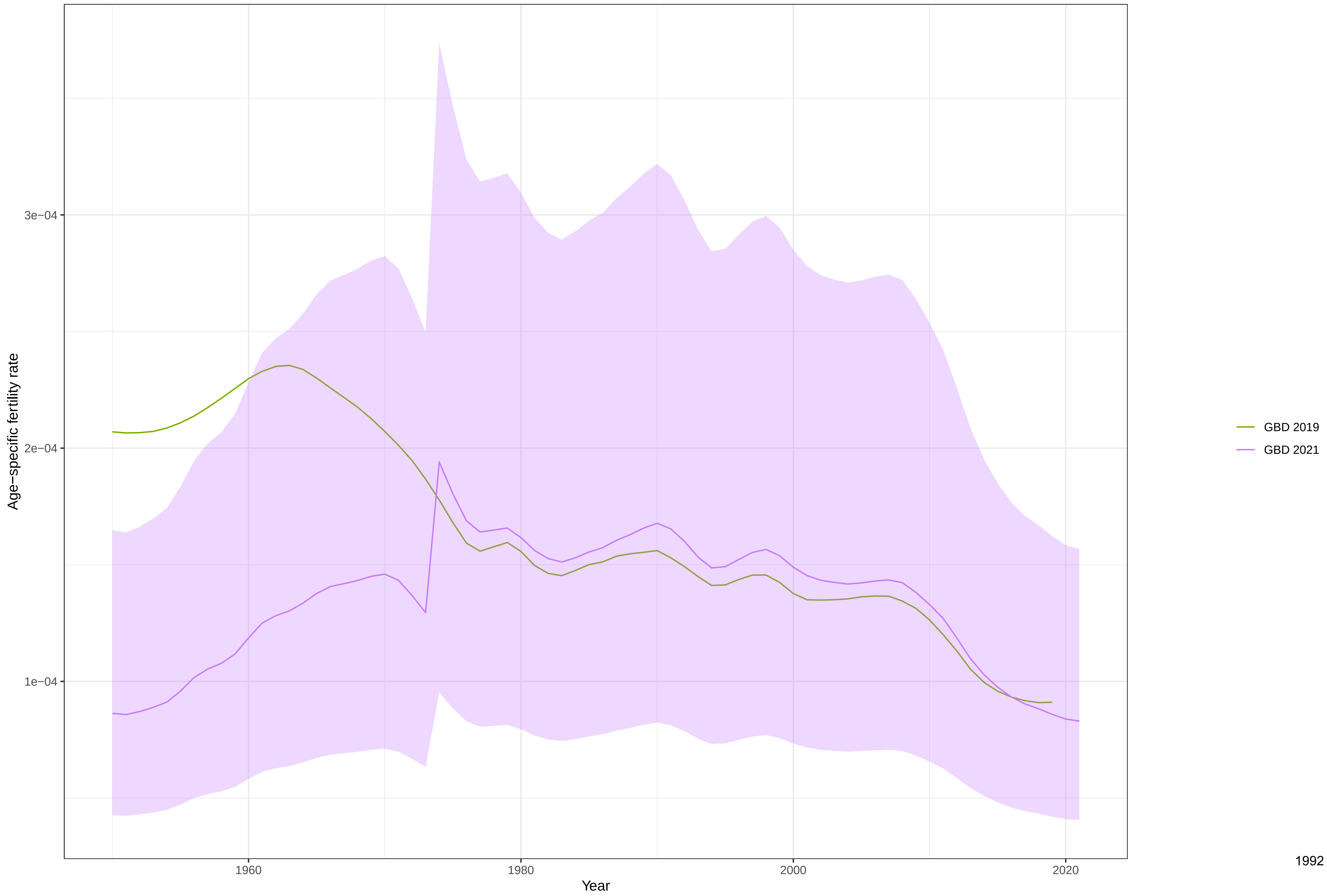

Age−specific fertility fitted trend lines, 1950−2021: Derby, Ages: 15−19
Data points enclosed with a diamond are reference sources

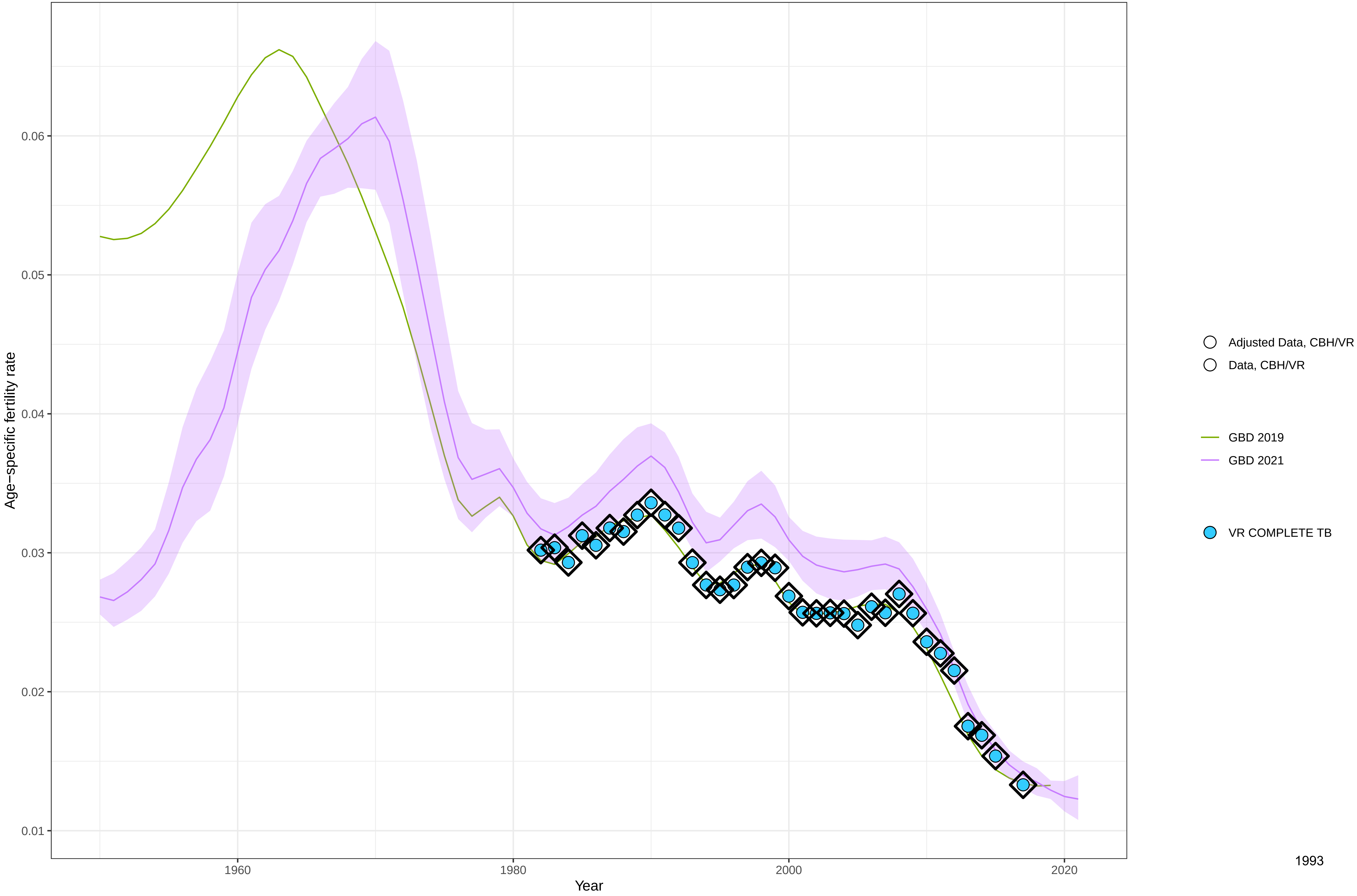

1993

Age−specific fertility fitted trend lines, 1950−2021: Derby, Ages: 20−24
Data points enclosed with a diamond are reference sources

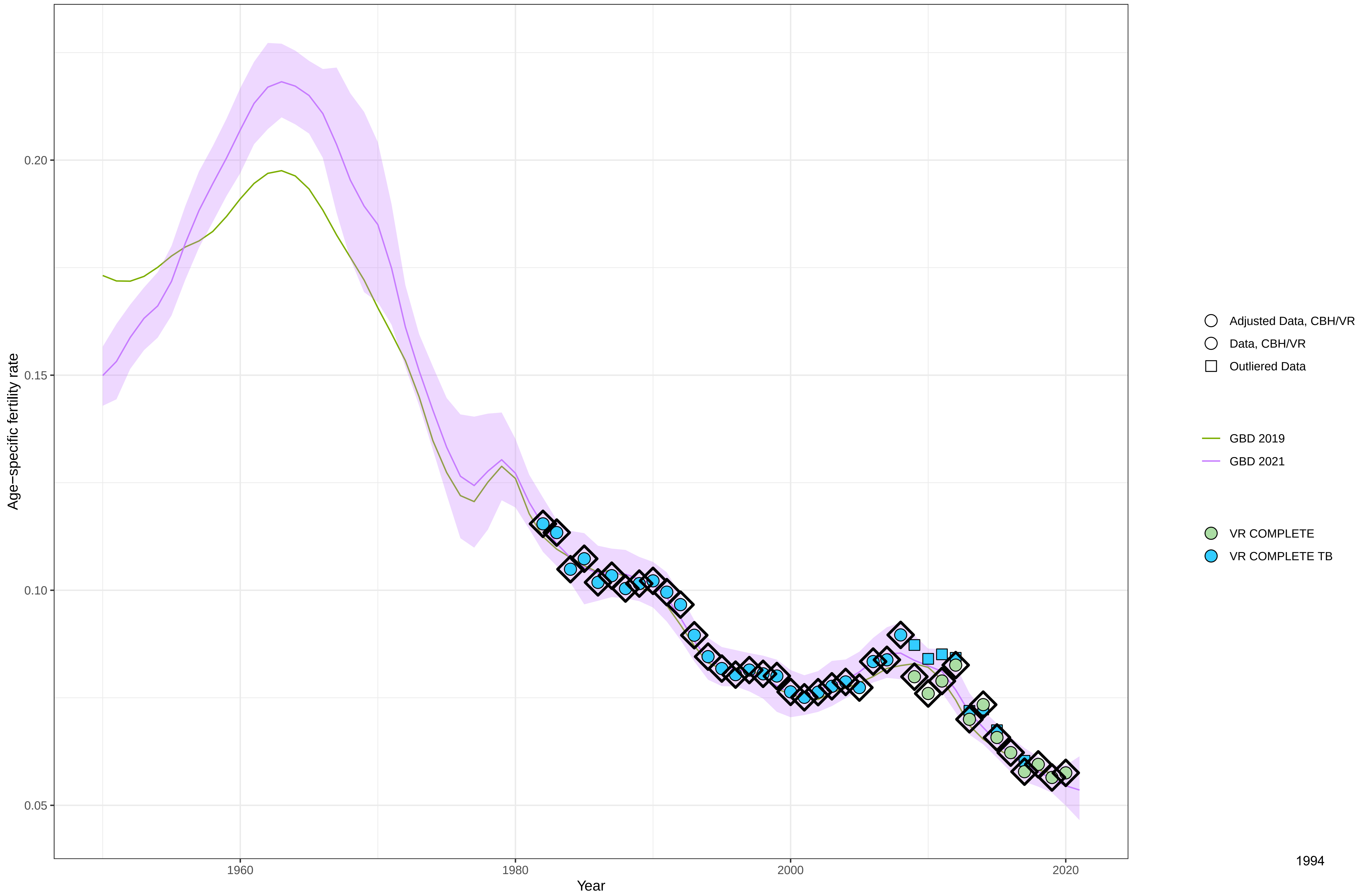

Age−specific fertility fitted trend lines, 1950−2021: Derby, Ages: 25−29
Data points enclosed with a diamond are reference sources

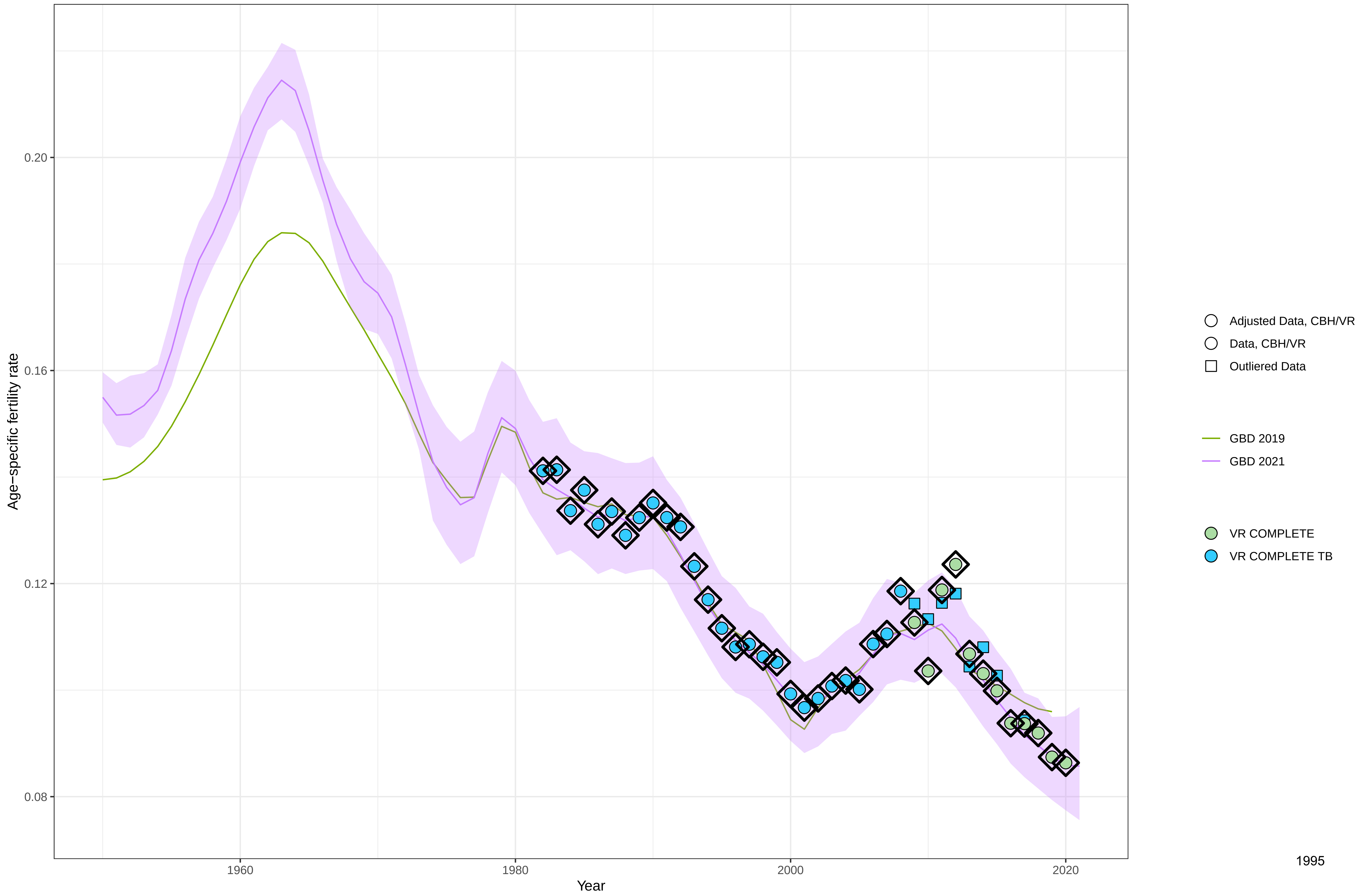

Age−specific fertility fitted trend lines, 1950−2021: Derby, Ages: 30−34
Data points enclosed with a diamond are reference sources

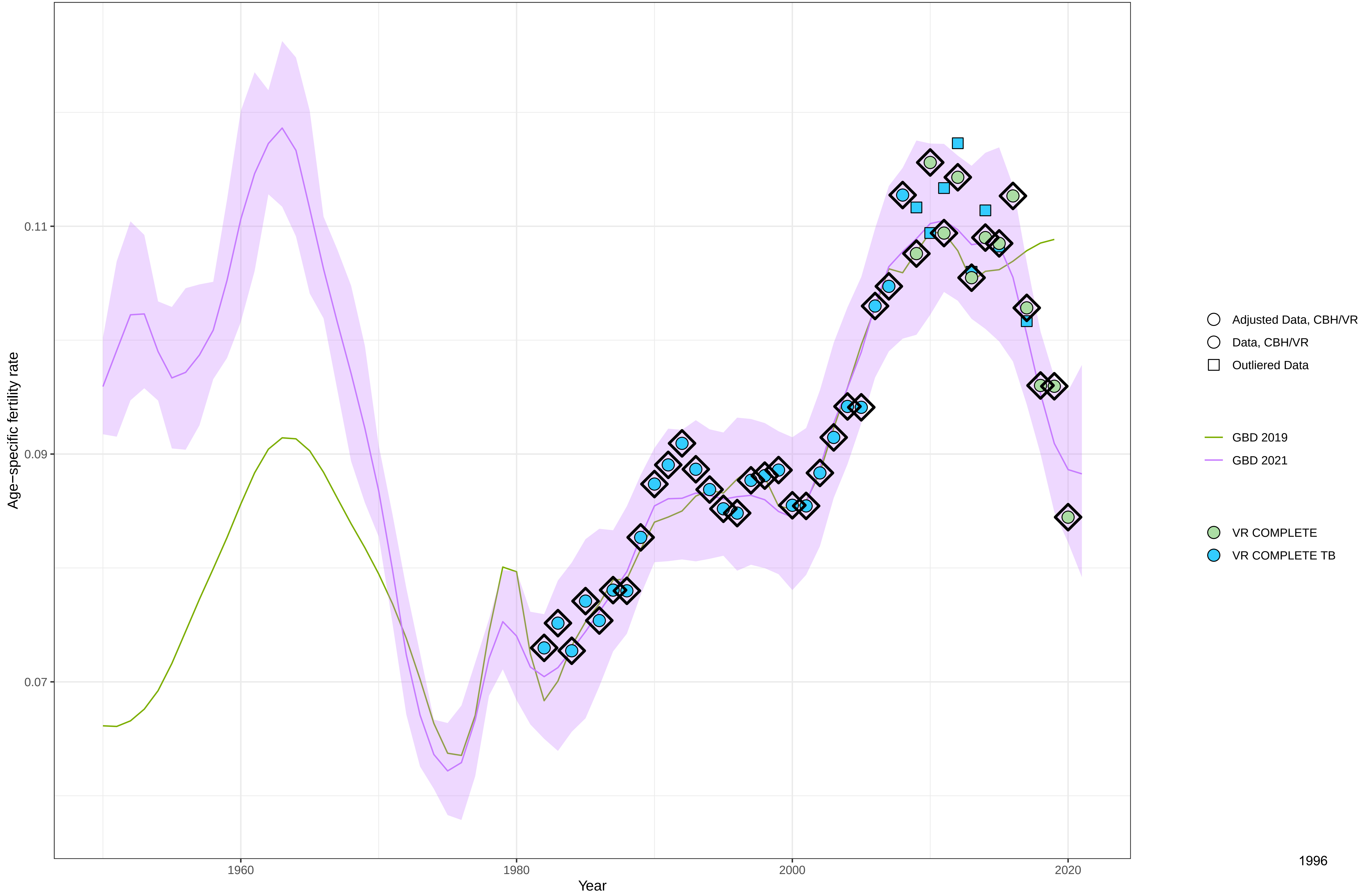

Age−specific fertility fitted trend lines, 1950−2021: Derby, Ages: 35−39
Data points enclosed with a diamond are reference sources

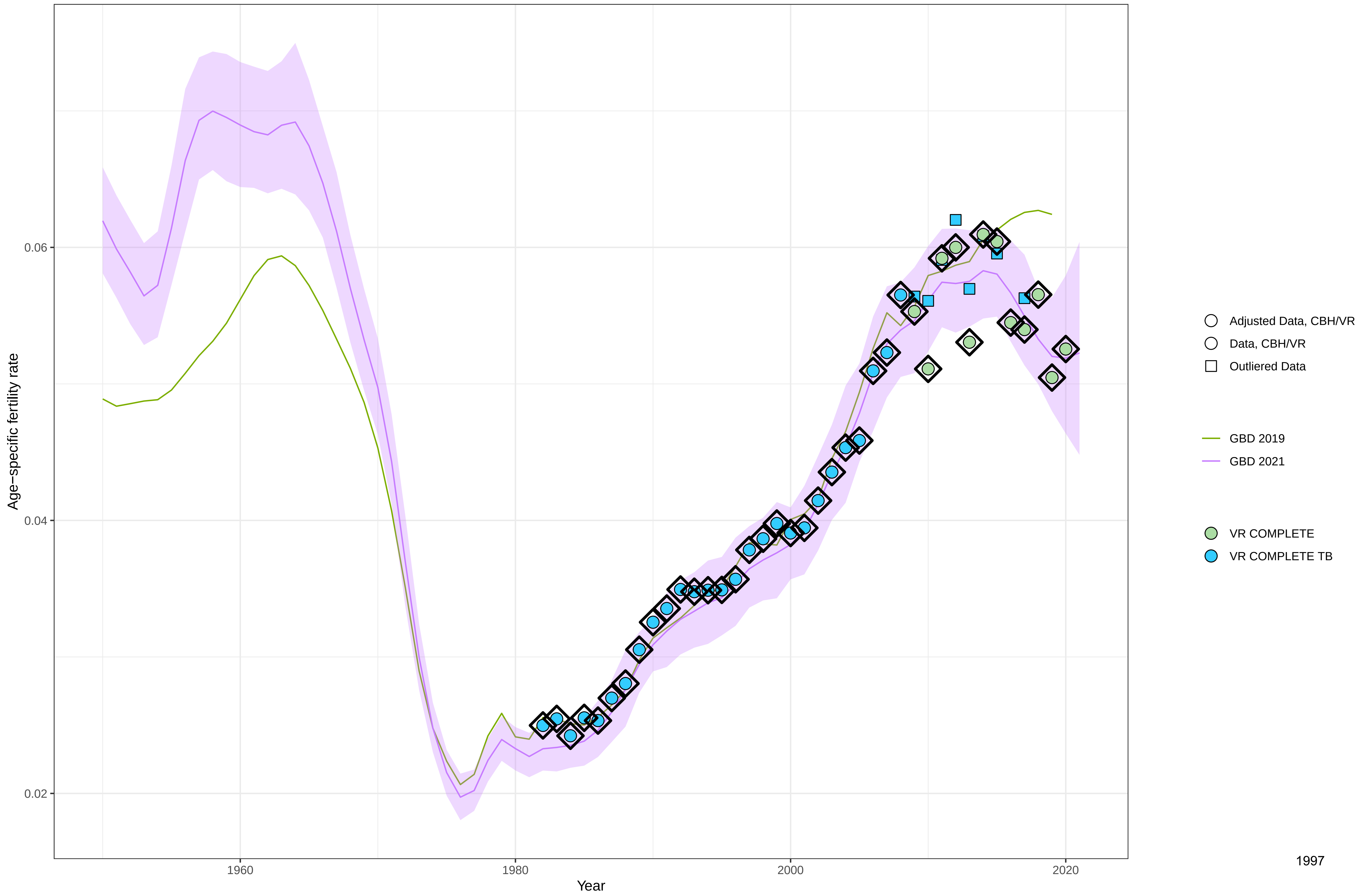

Age−specific fertility fitted trend lines, 1950−2021: Derby, Ages: 40−44
Data points enclosed with a diamond are reference sources

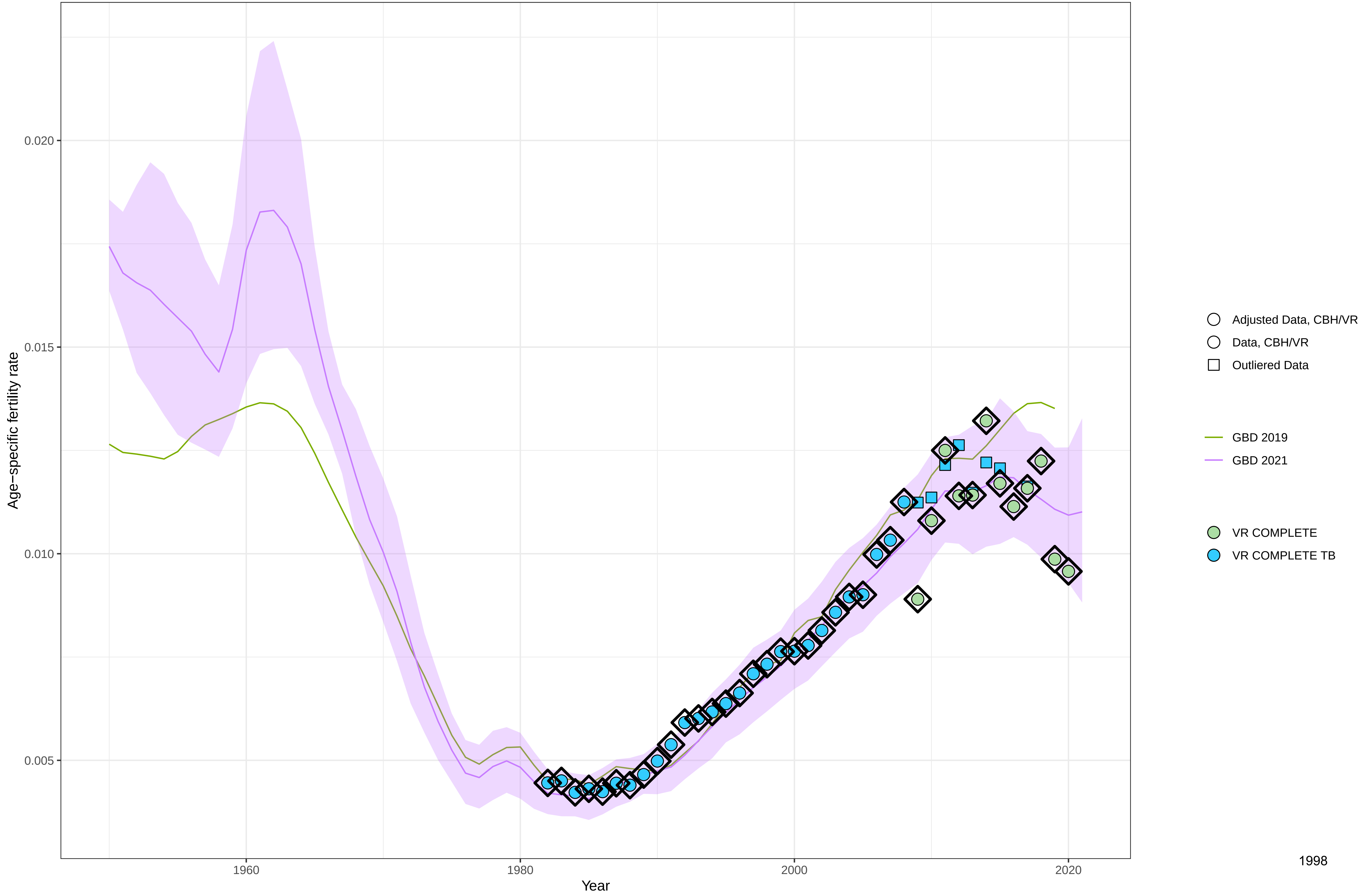

Age−specific fertility fitted trend lines, 1950−2021: Derby, Ages: 45−49
Data points enclosed with a diamond are reference sources

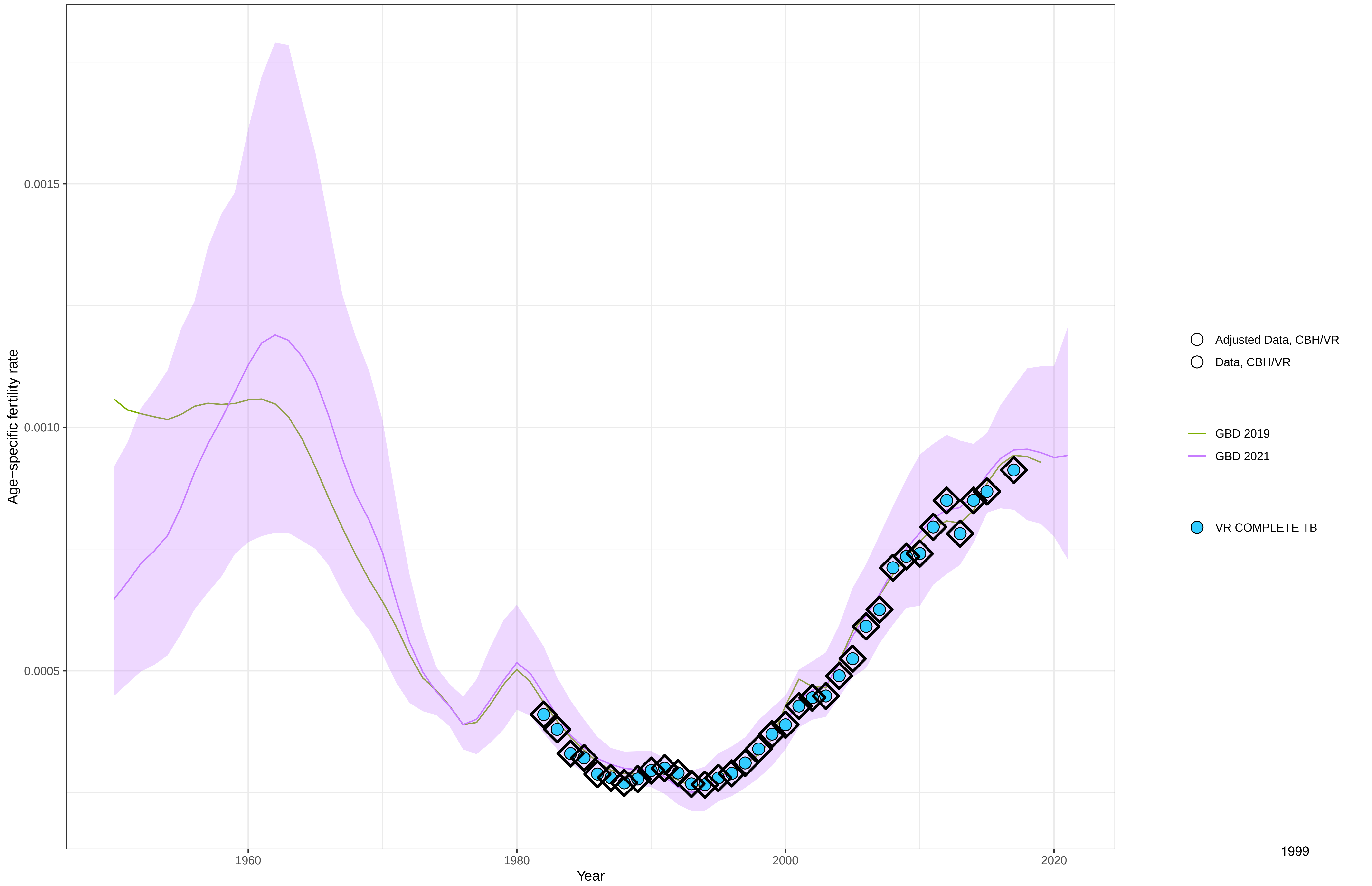

Age−specific fertility fitted trend lines, 1950−2021: Derby, Ages: 50−54
Data points enclosed with a diamond are reference sources

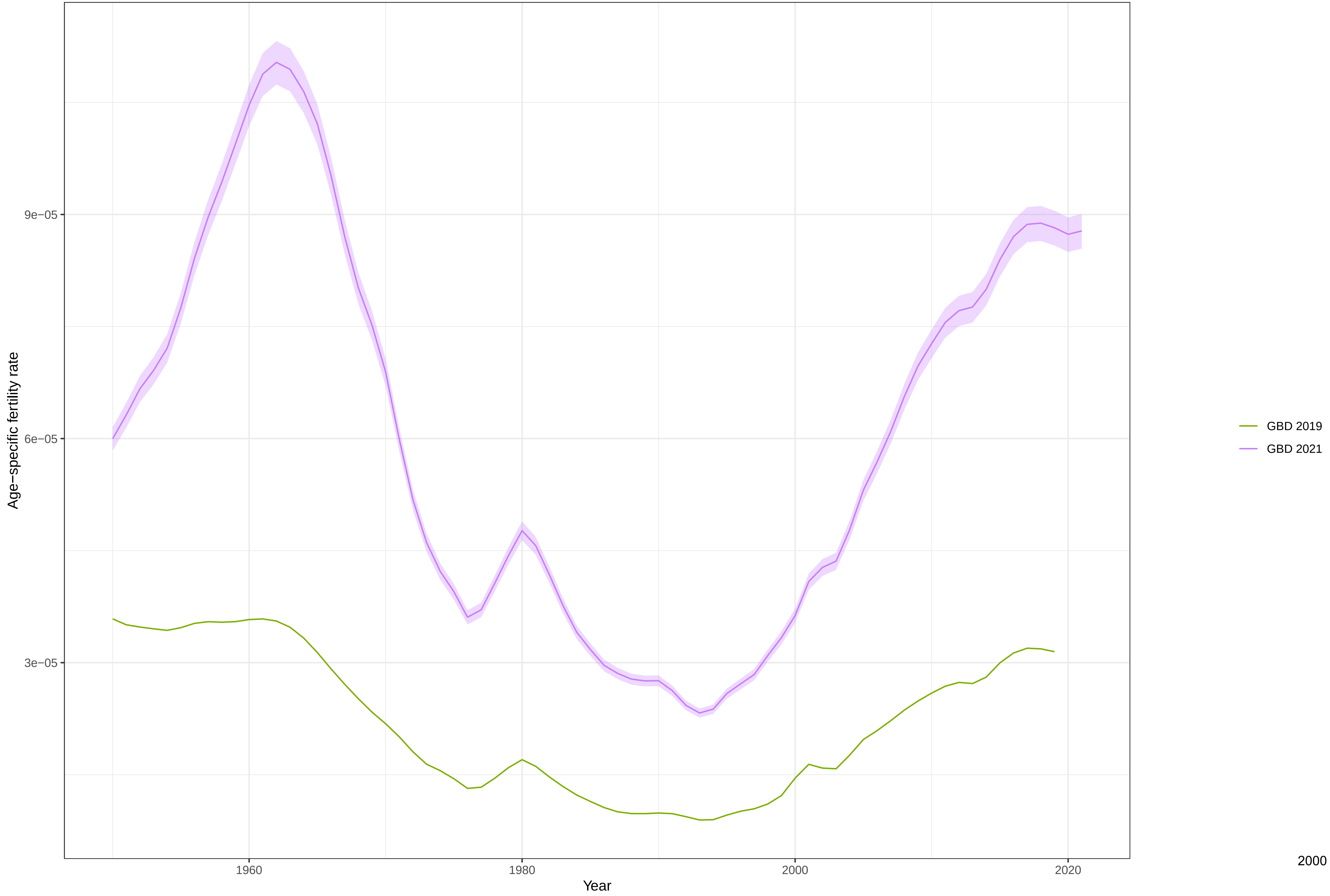

2000

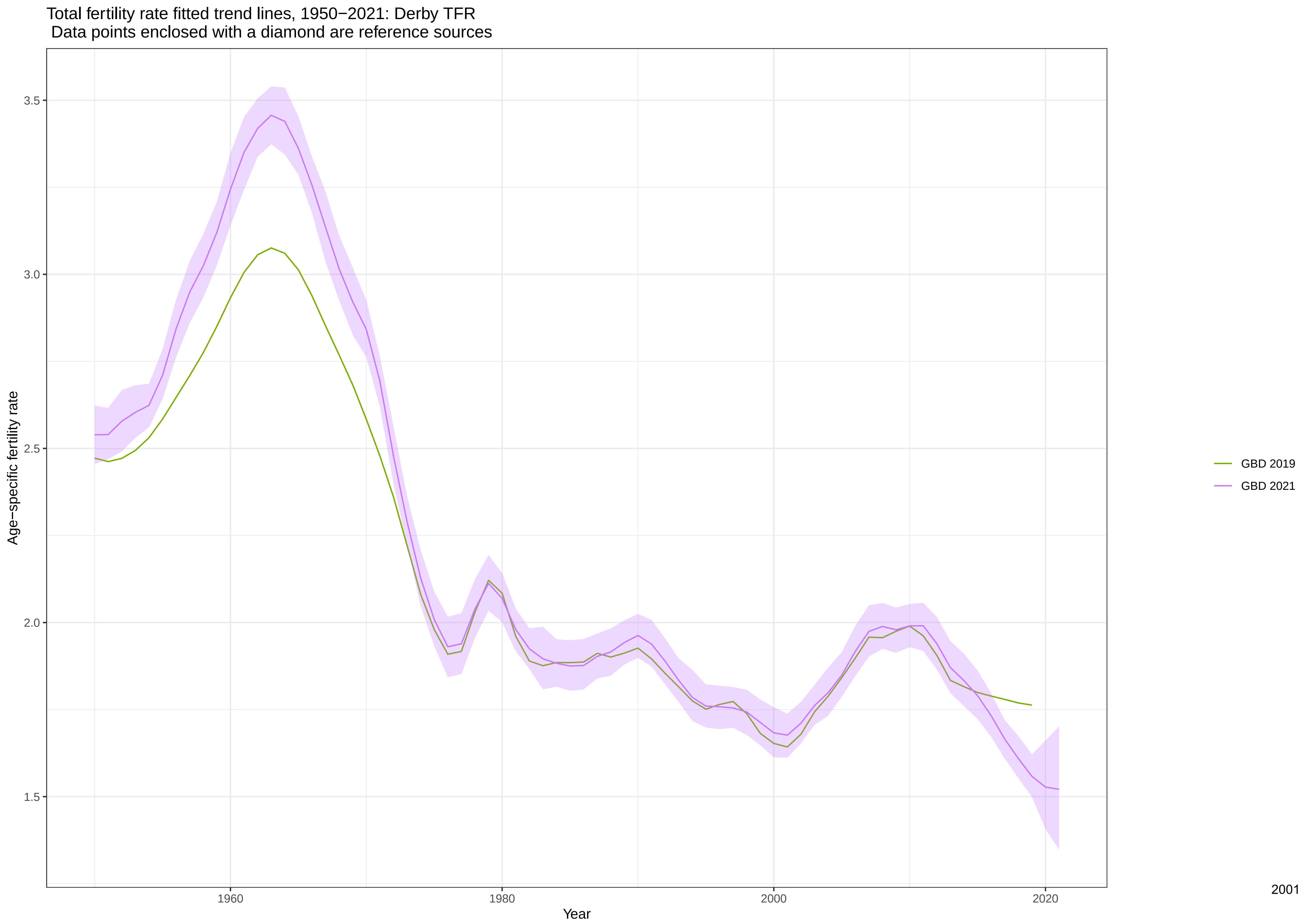

Total fertility rate fitted trend lines, 1950−2021: Derby TFR
Data points enclosed with a diamond are reference sources
Age−specific fertility rate
3.5
3.0
2.5
2.0
1.5
1960
1980
2000
2020
Year
GBD 2019
GBD 2021

Age−specific fertility fitted trend lines, 1950−2021: Derbyshire, Ages: 10−14
Data points enclosed with a diamond are reference sources

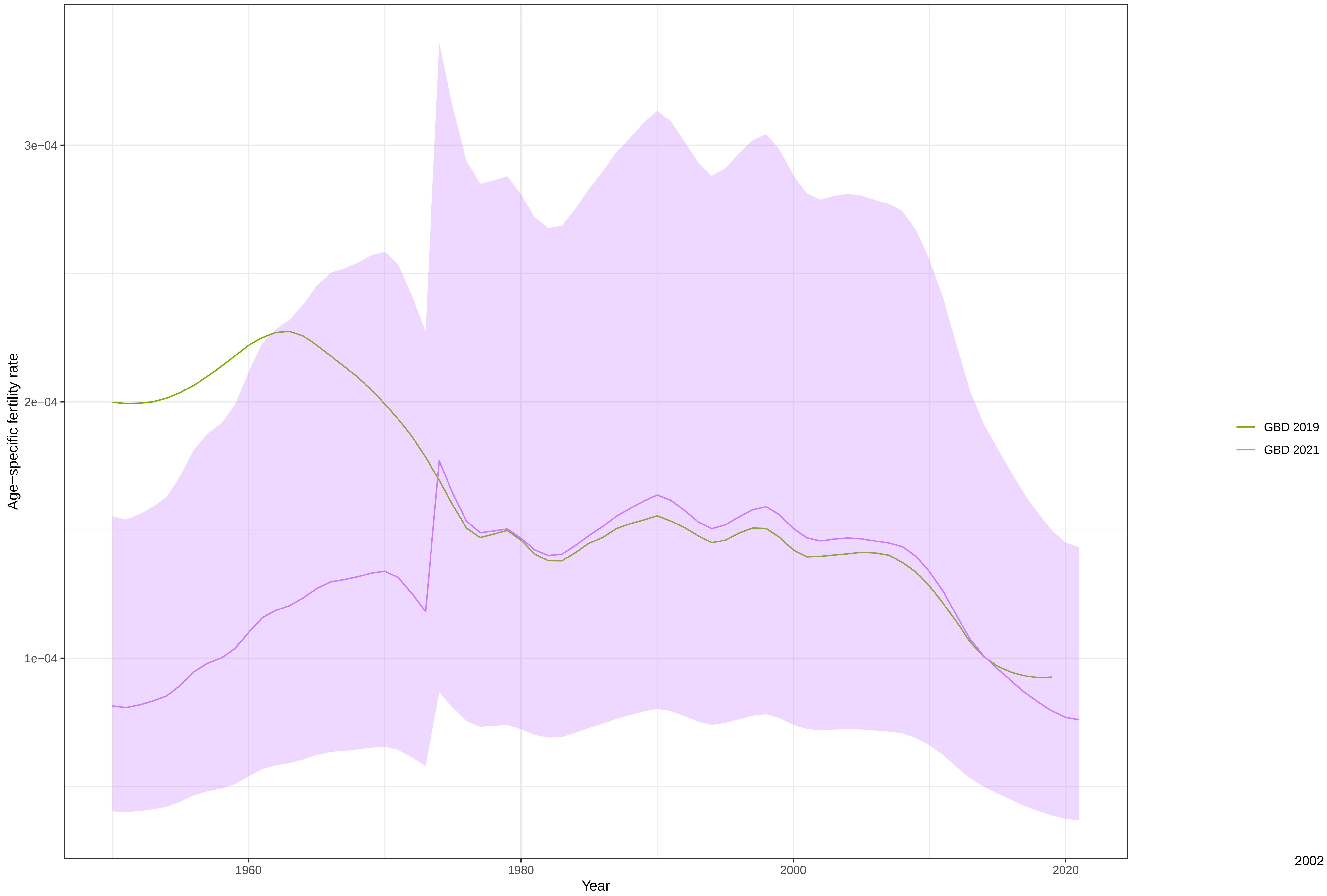

Age−specific fertility fitted trend lines, 1950−2021: Derbyshire, Ages: 15−19
Data points enclosed with a diamond are reference sources

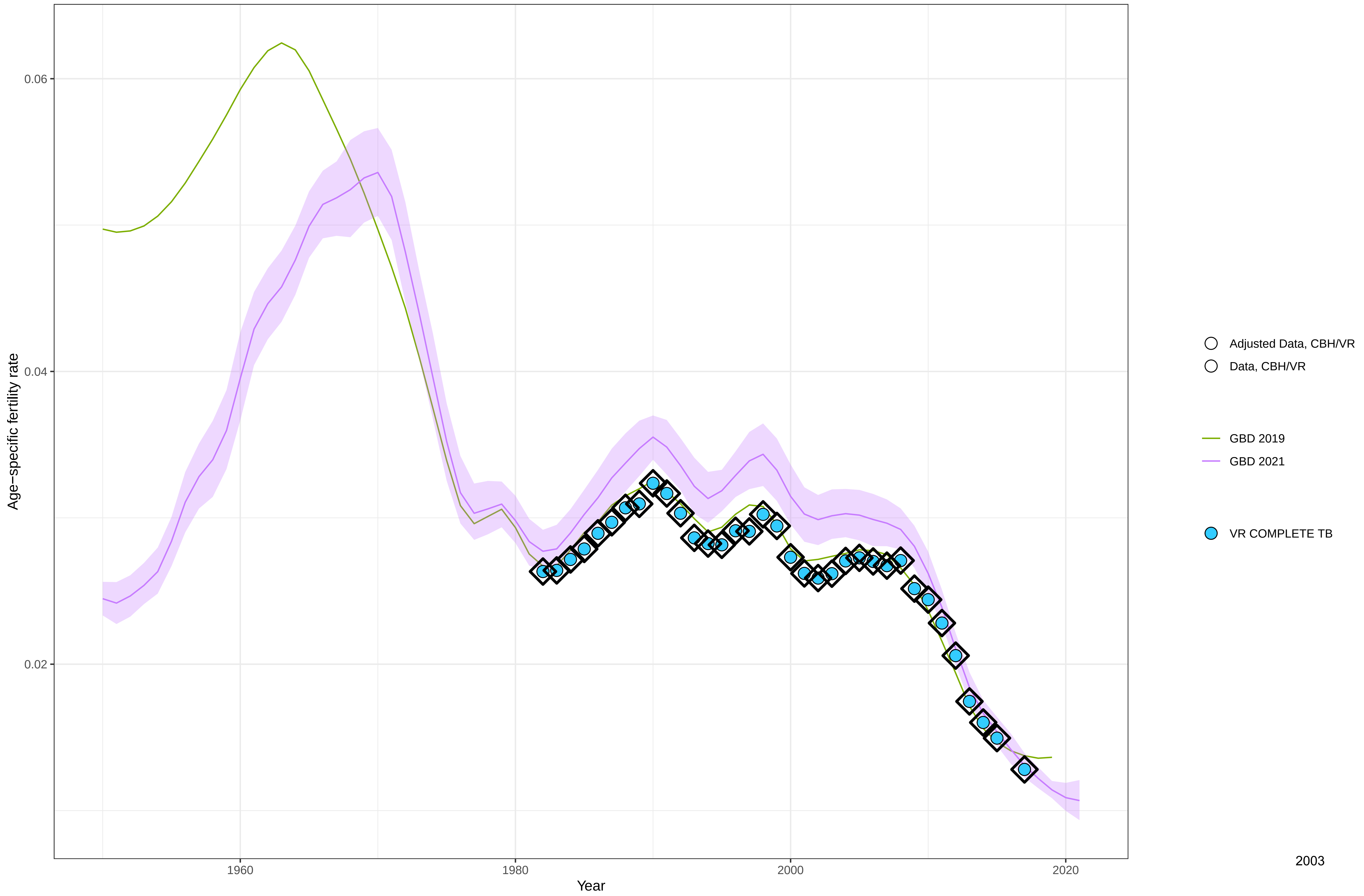

2003

Age−specific fertility fitted trend lines, 1950−2021: Derbyshire, Ages: 20−24
Data points enclosed with a diamond are reference sources

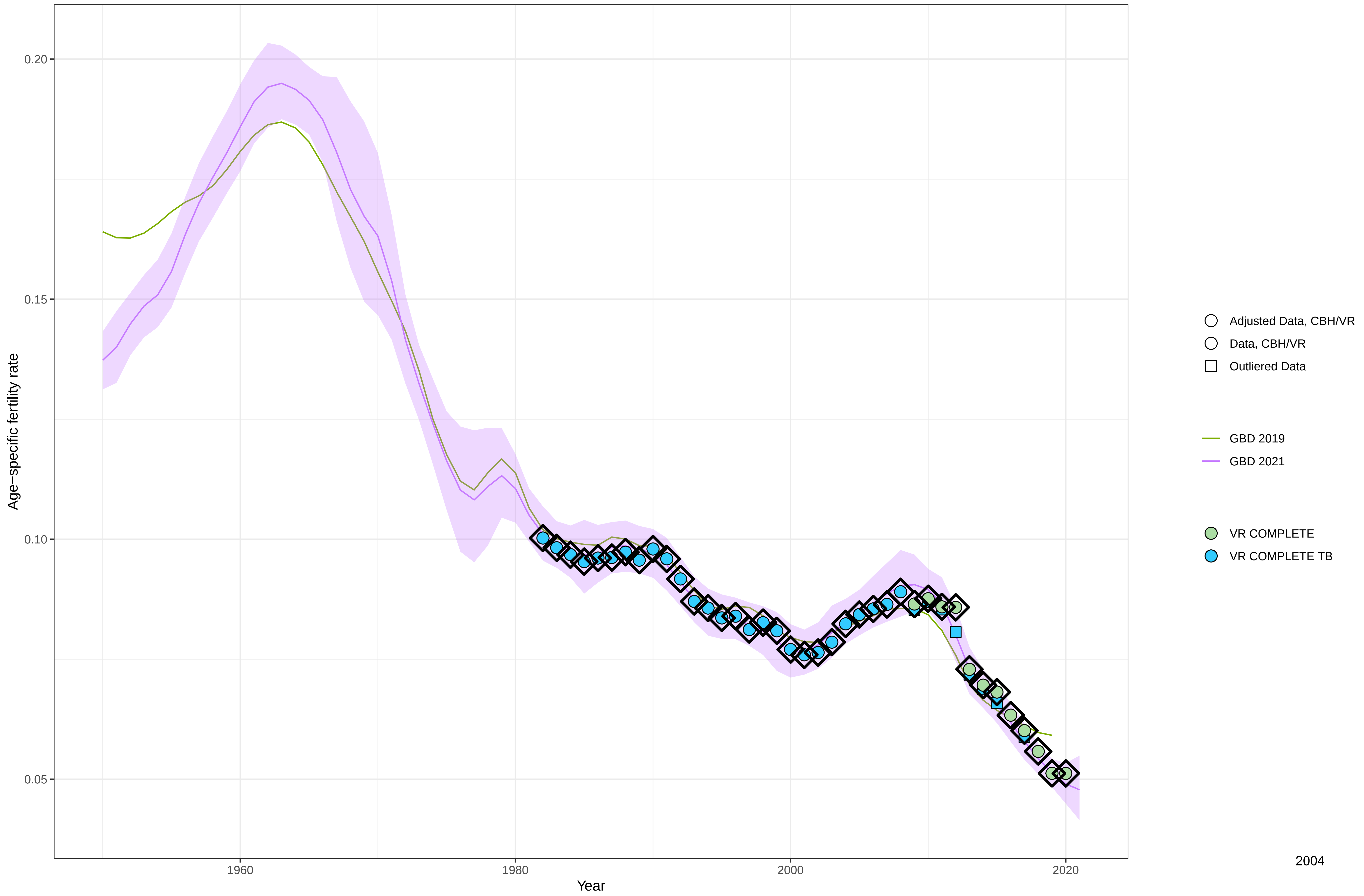

Age−specific fertility fitted trend lines, 1950−2021: Derbyshire, Ages: 25−29
Data points enclosed with a diamond are reference sources

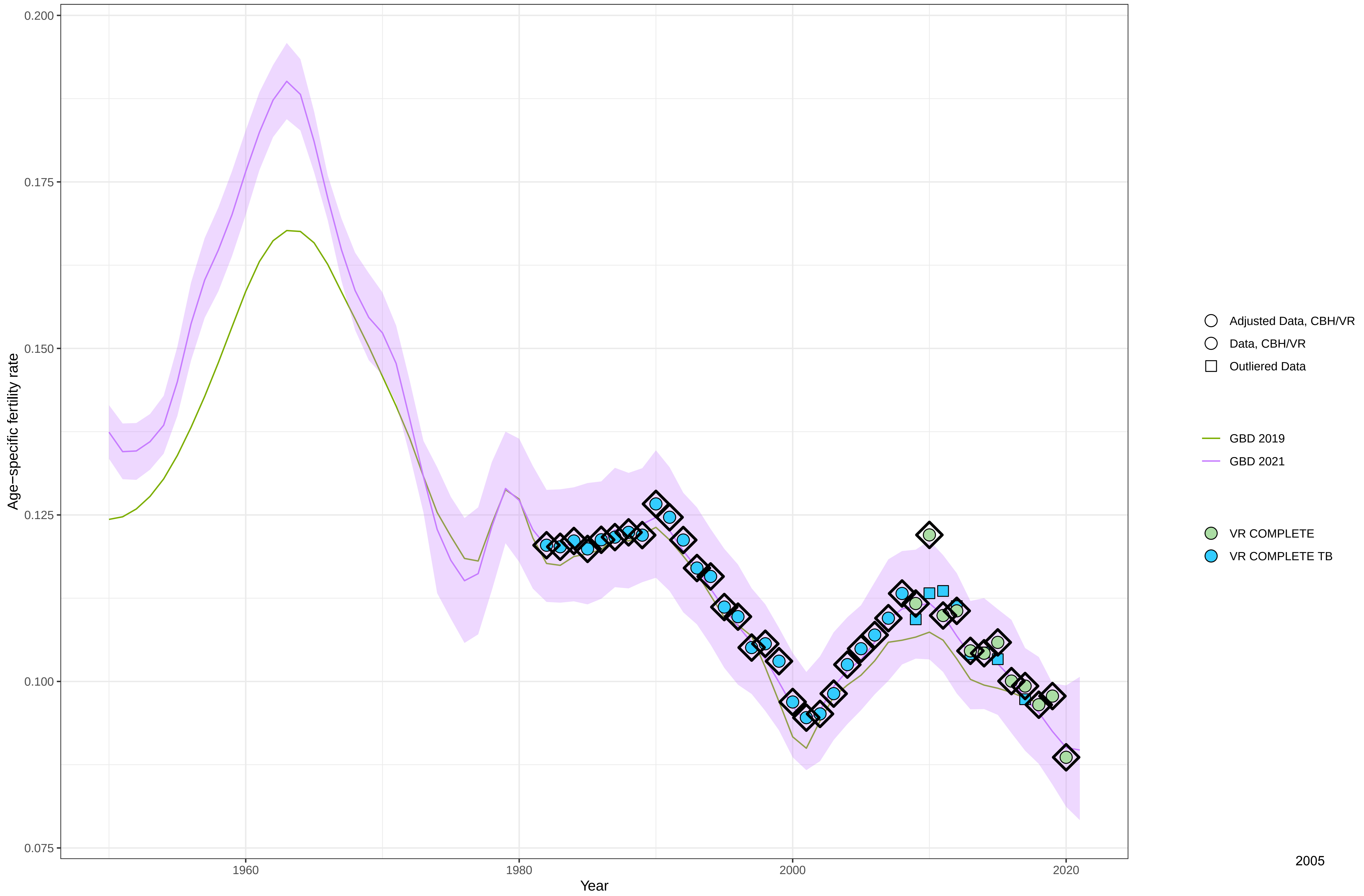

Age−specific fertility fitted trend lines, 1950−2021: Derbyshire, Ages: 30−34
Data points enclosed with a diamond are reference sources

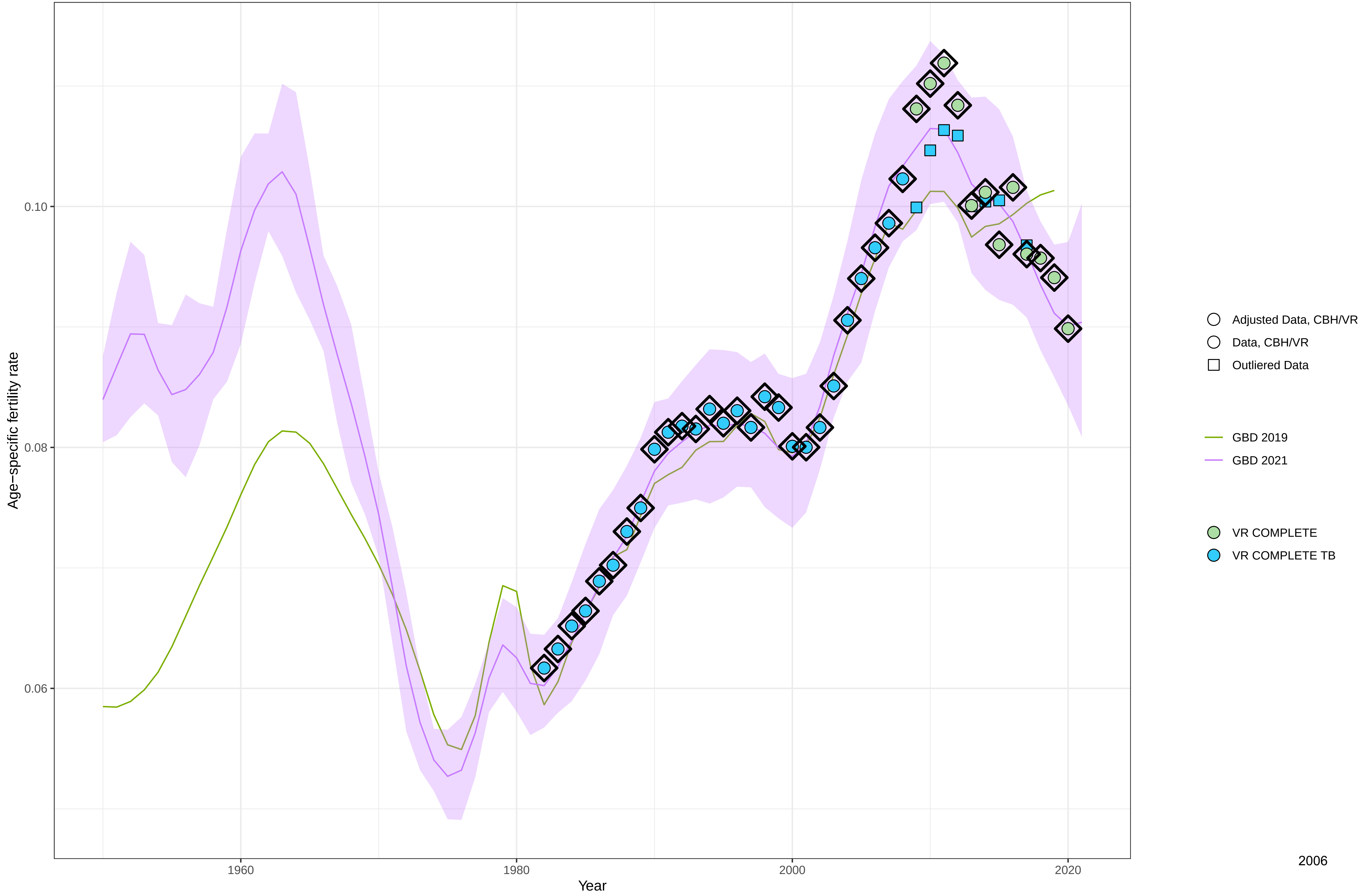

Age−specific fertility fitted trend lines, 1950−2021: Derbyshire, Ages: 35−39
Data points enclosed with a diamond are reference sources

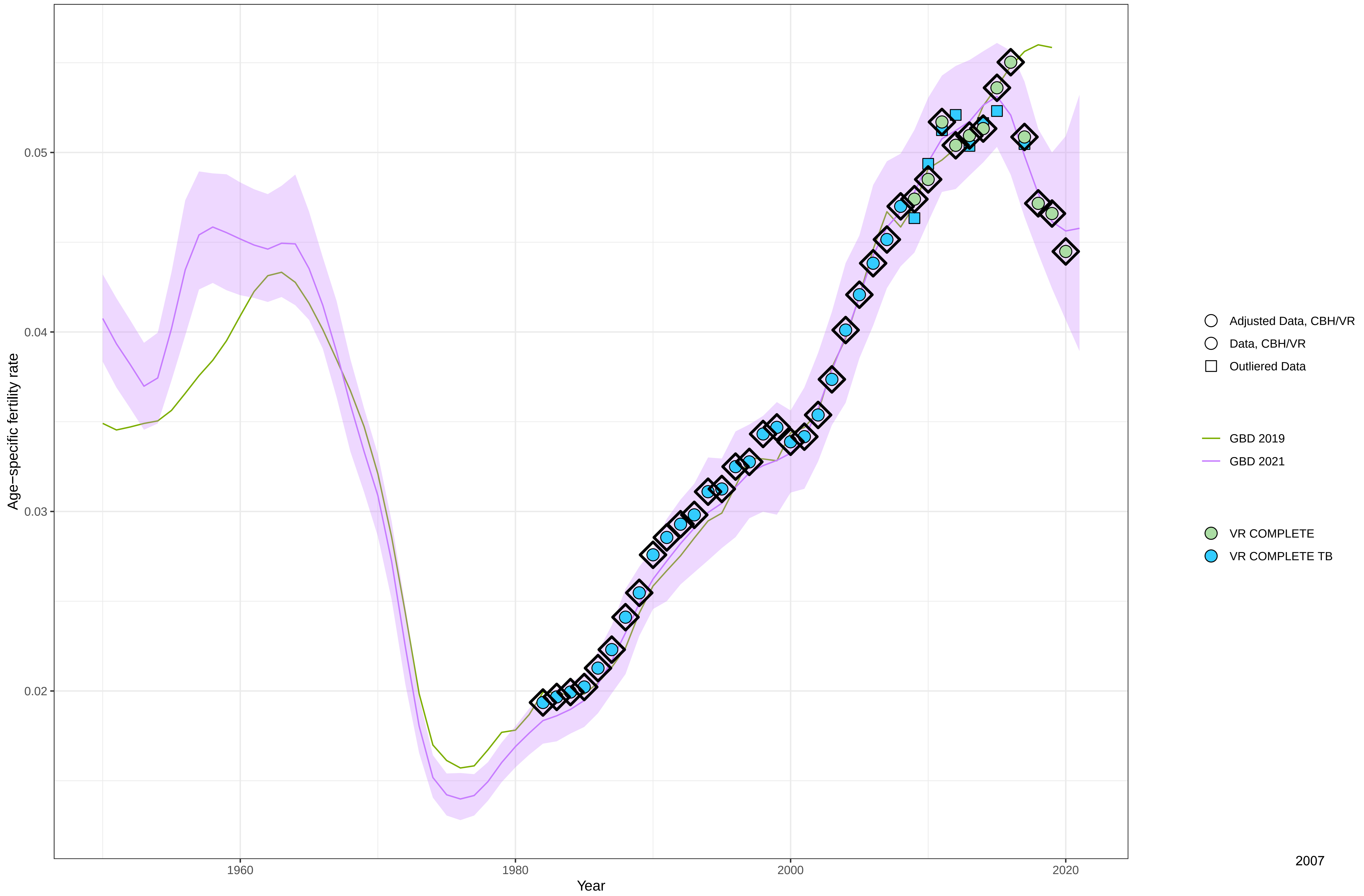

Age−specific fertility fitted trend lines, 1950−2021: Derbyshire, Ages: 40−44
Data points enclosed with a diamond are reference sources

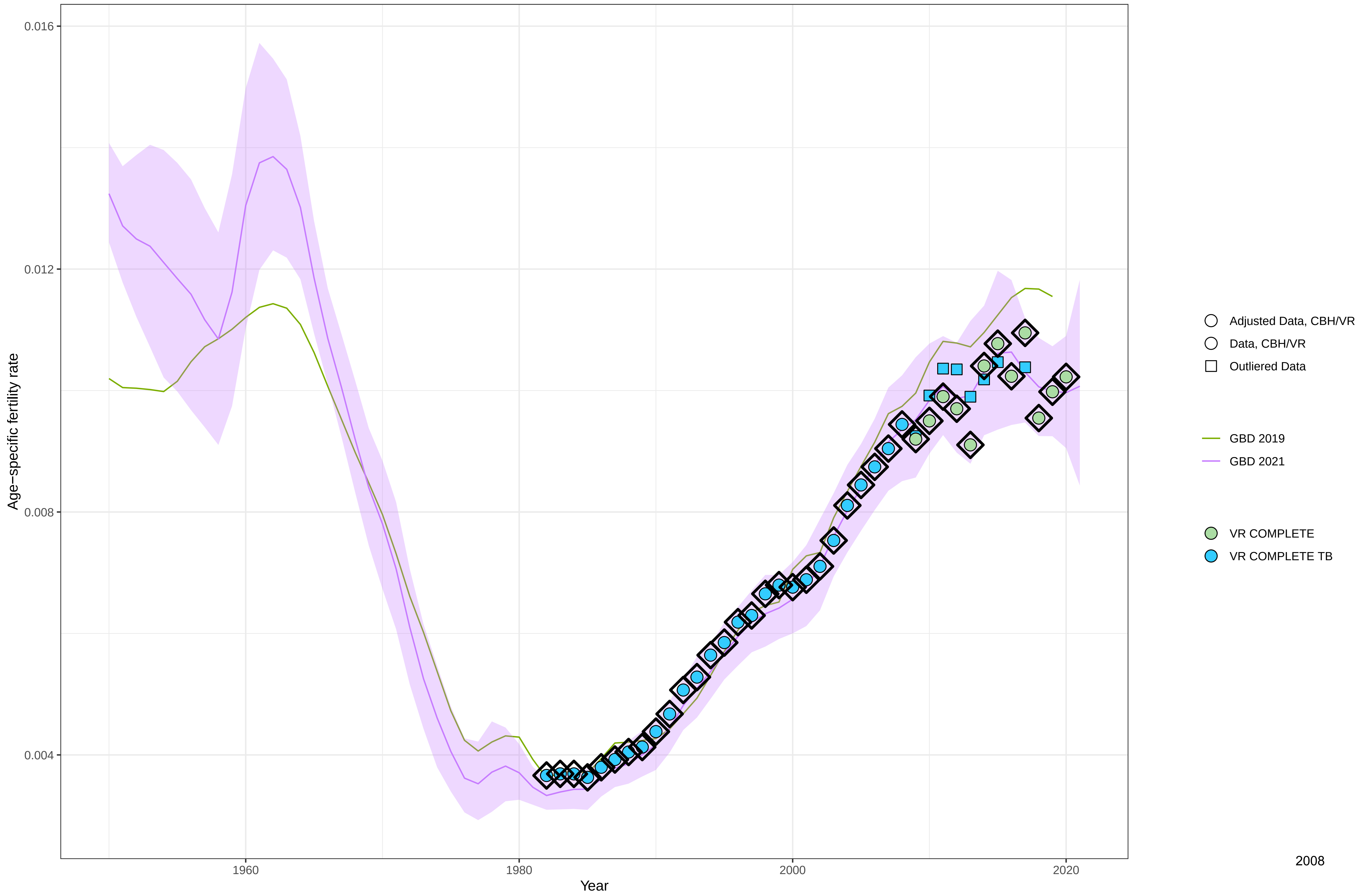

2008

Age−specific fertility fitted trend lines, 1950−2021: Derbyshire, Ages: 45−49
Data points enclosed with a diamond are reference sources

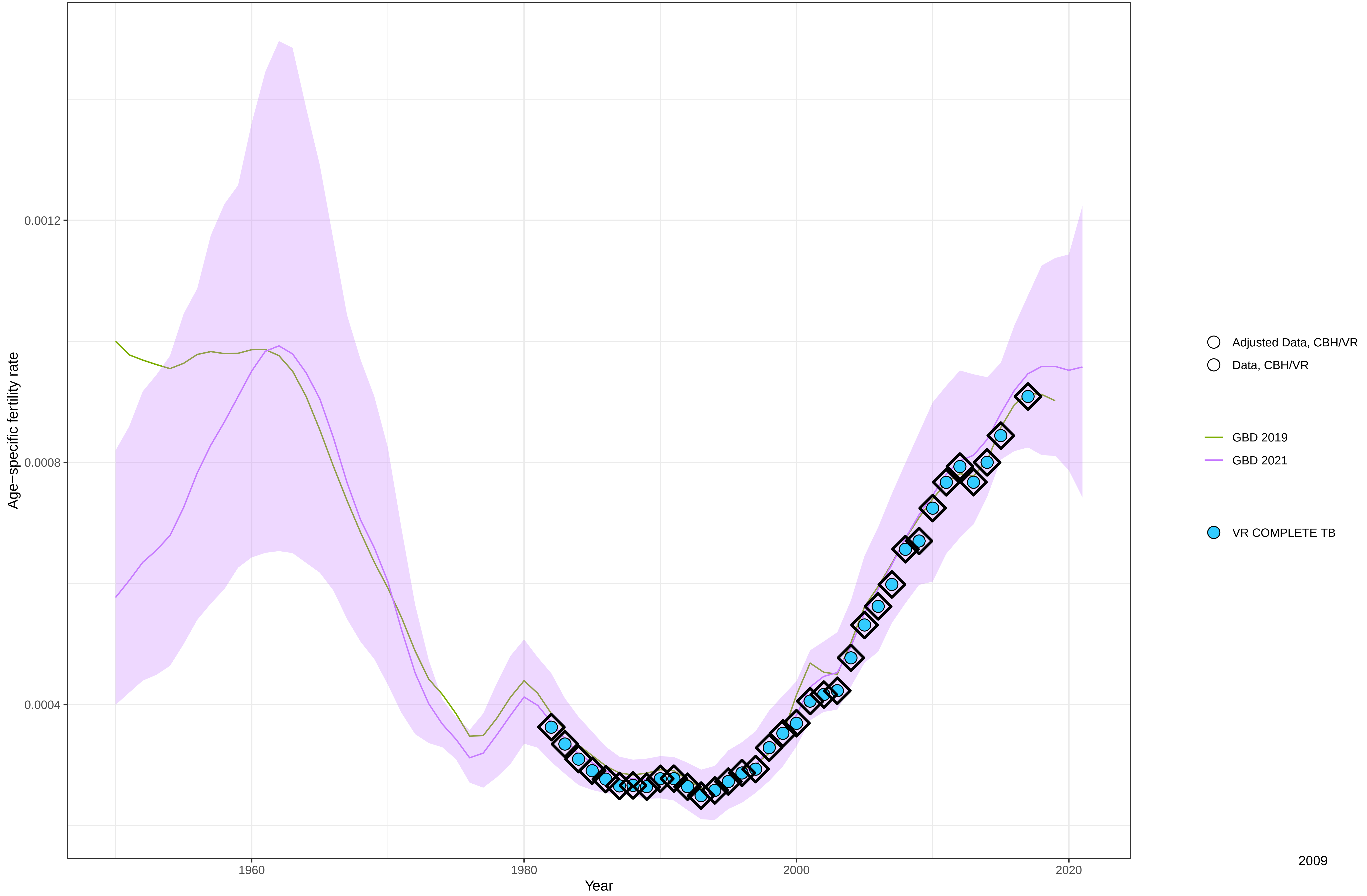

2009

Age−specific fertility fitted trend lines, 1950−2021: Derbyshire, Ages: 50−54
Data points enclosed with a diamond are reference sources

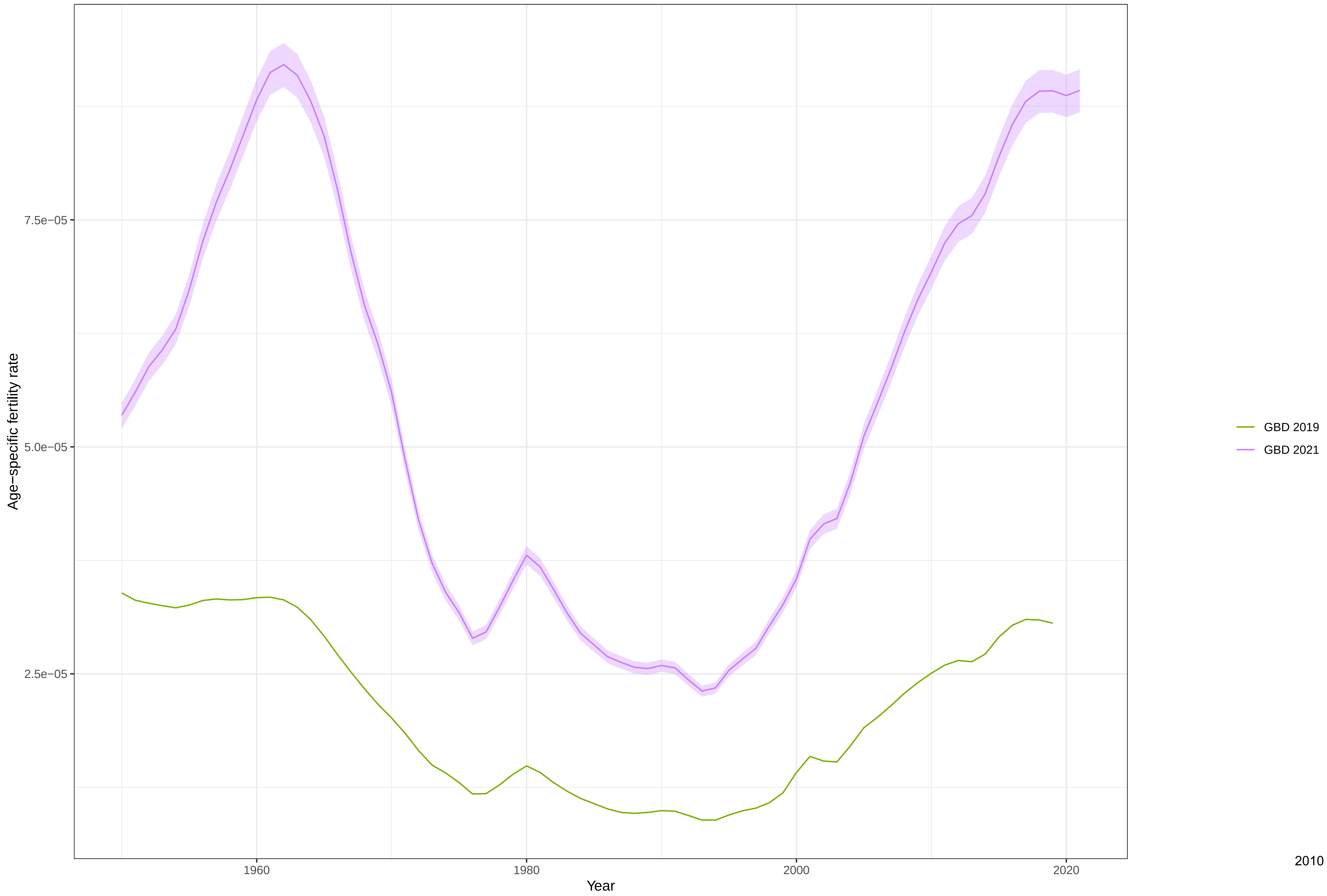

Total fertility rate fitted trend lines, 1950−2021: Derbyshire TFR
Data points enclosed with a diamond are reference sources

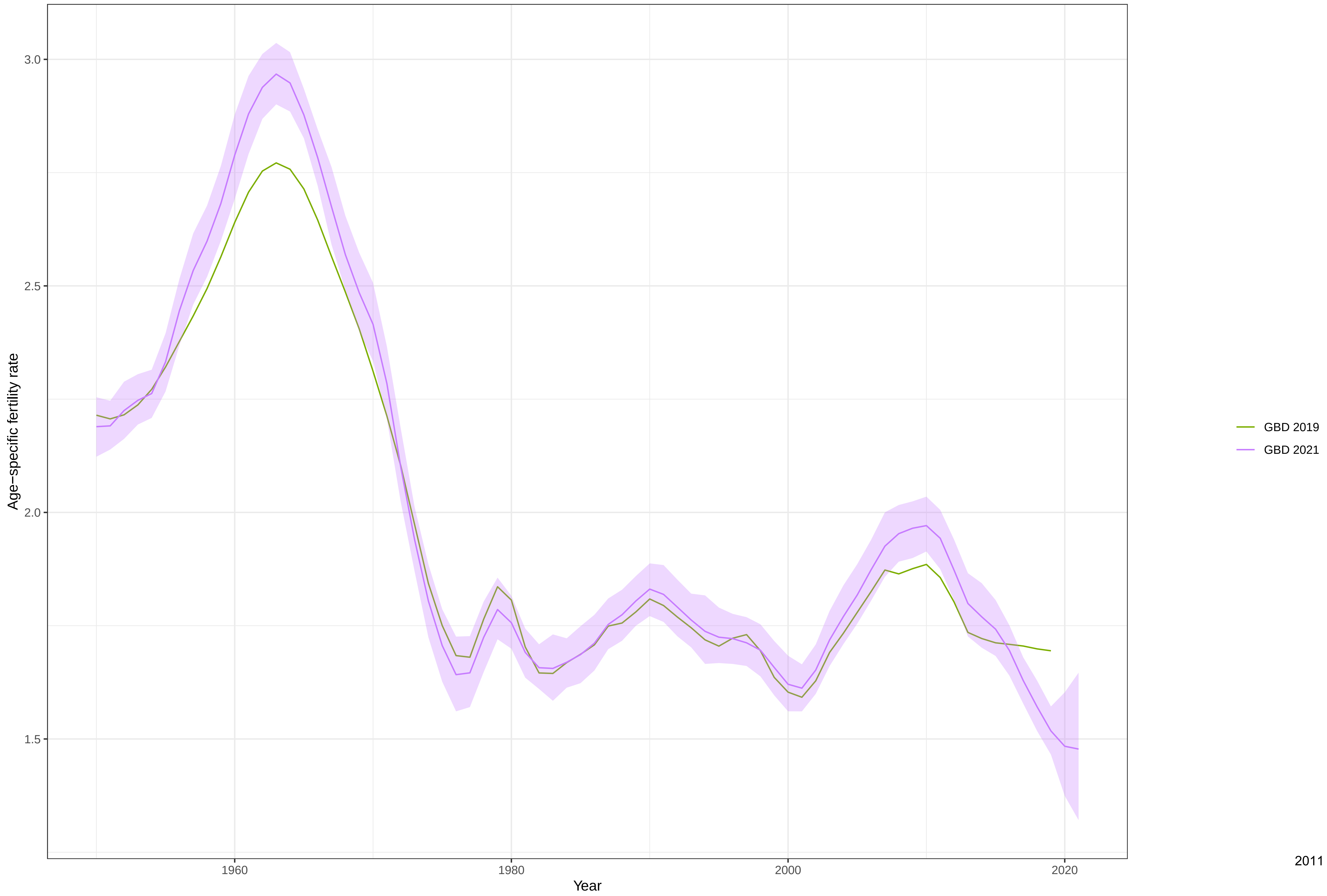

2011

Age−specific fertility fitted trend lines, 1950−2021: Leicester, Ages: 10−14
Data points enclosed with a diamond are reference sources

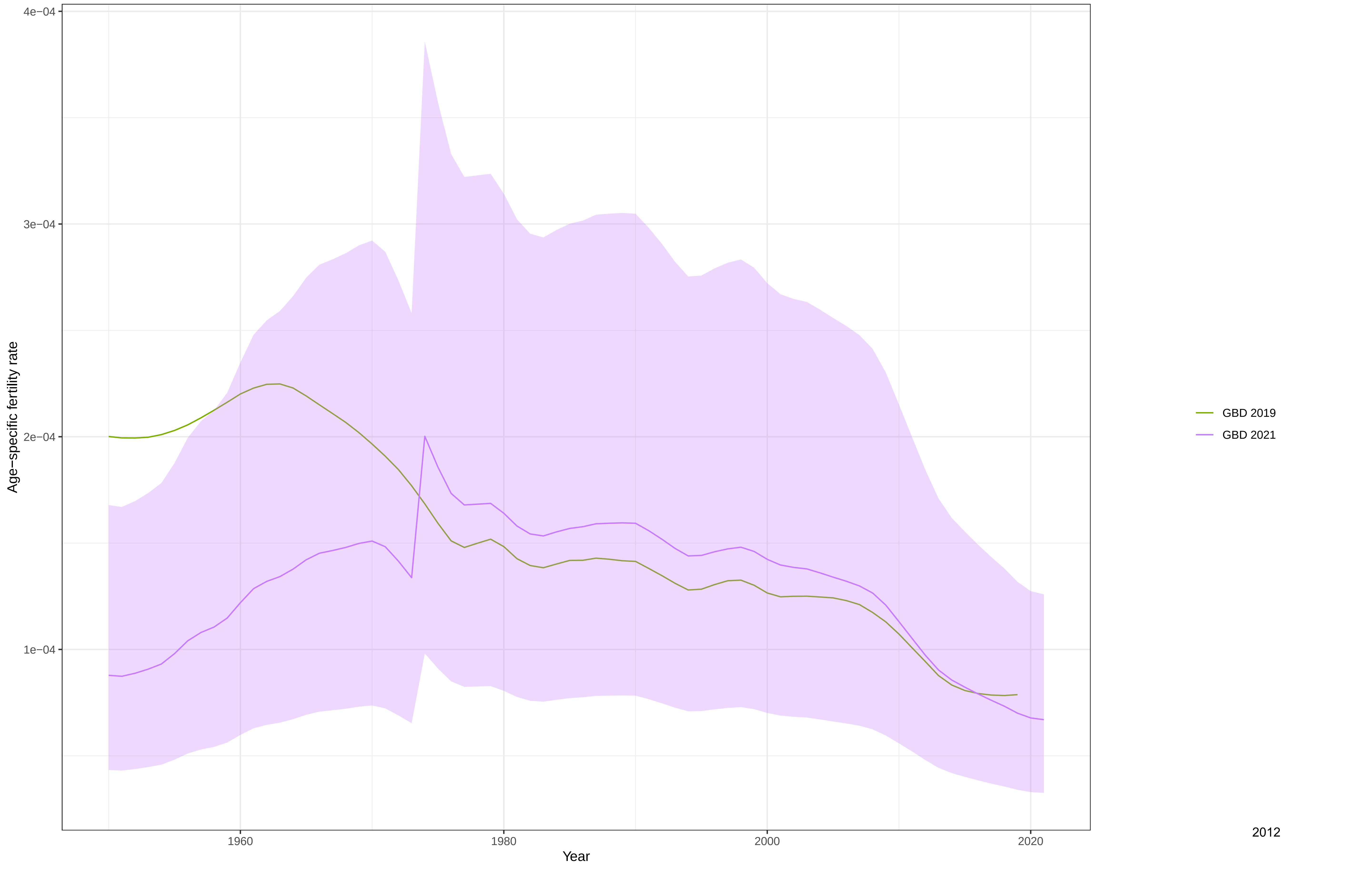

Age−specific fertility fitted trend lines, 1950−2021: Leicester, Ages: 15−19
Data points enclosed with a diamond are reference sources

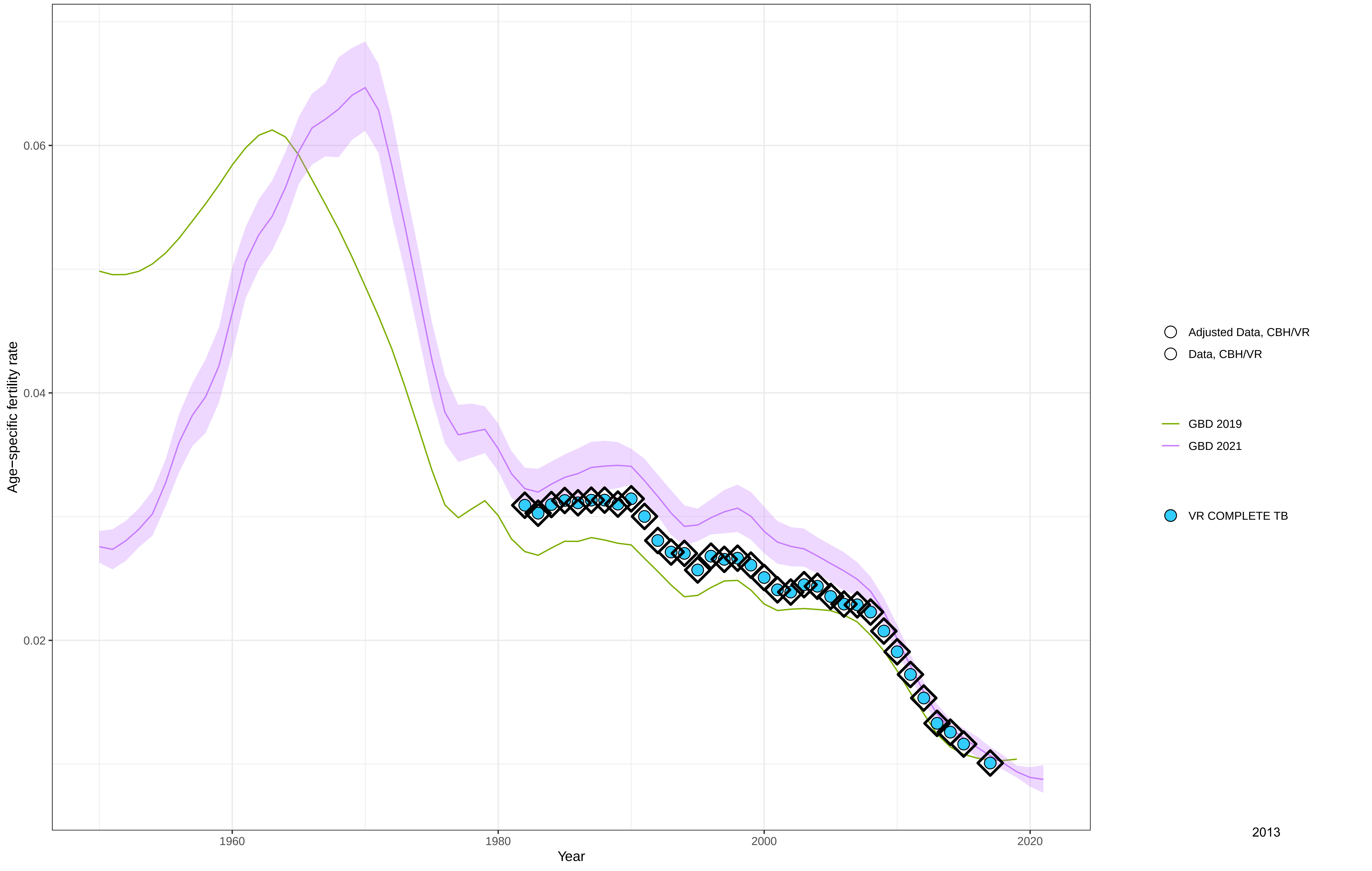

Age−specific fertility fitted trend lines, 1950−2021: Leicester, Ages: 20−24
Data points enclosed with a diamond are reference sources

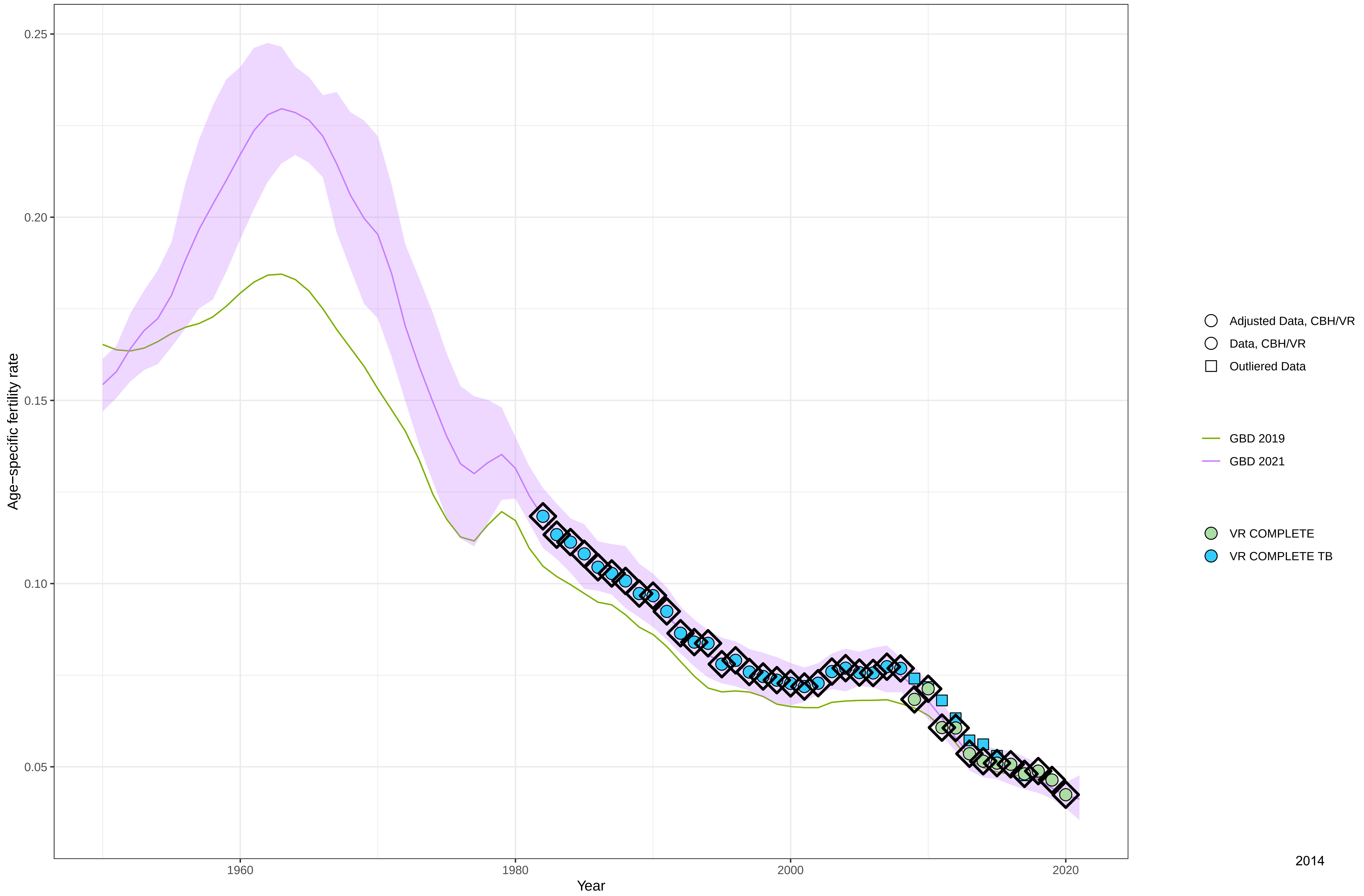

2014

Age−specific fertility fitted trend lines, 1950−2021: Leicester, Ages: 25−29
Data points enclosed with a diamond are reference sources

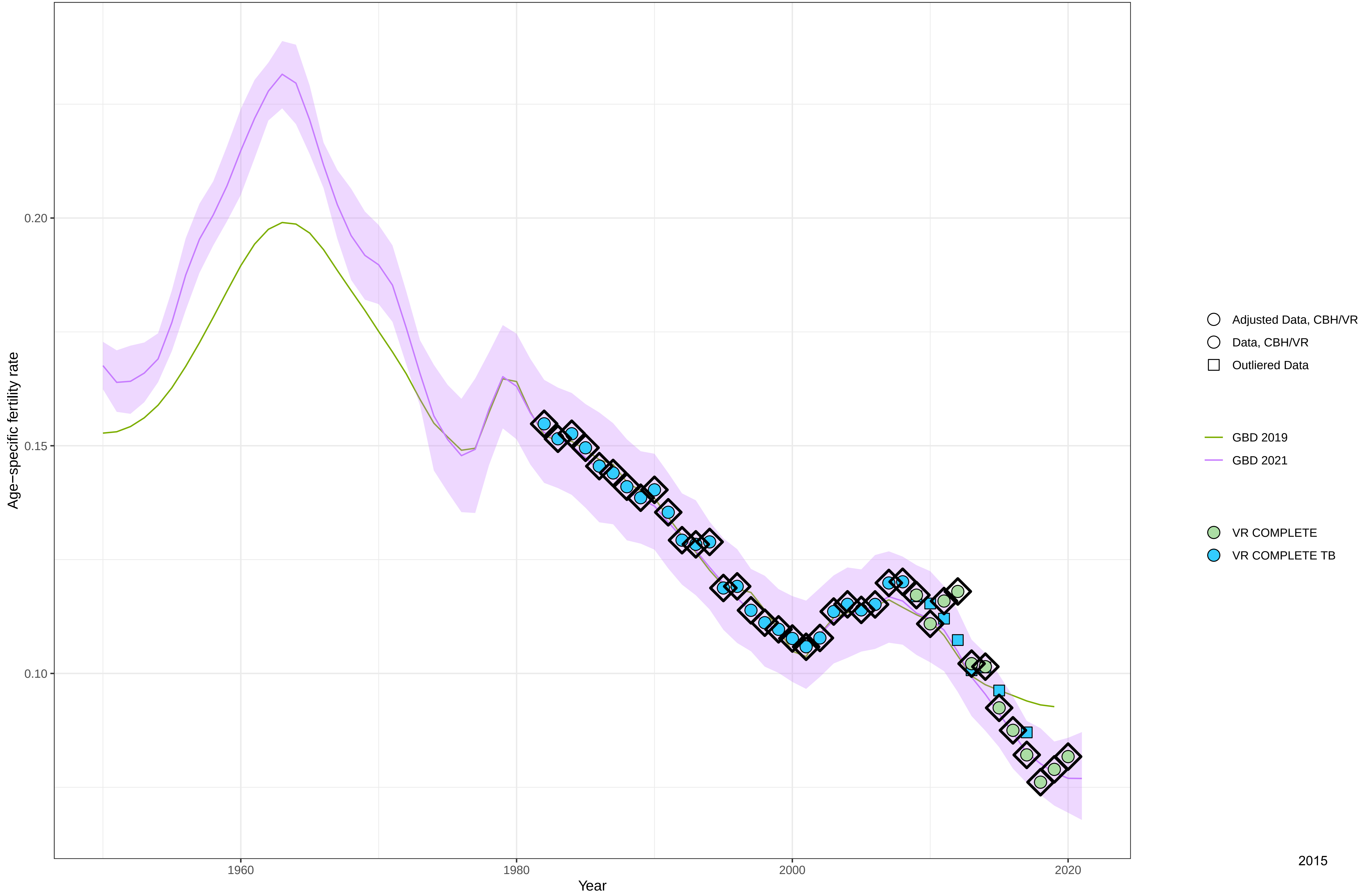

2015

Age−specific fertility fitted trend lines, 1950−2021: Leicester, Ages: 30−34
Data points enclosed with a diamond are reference sources

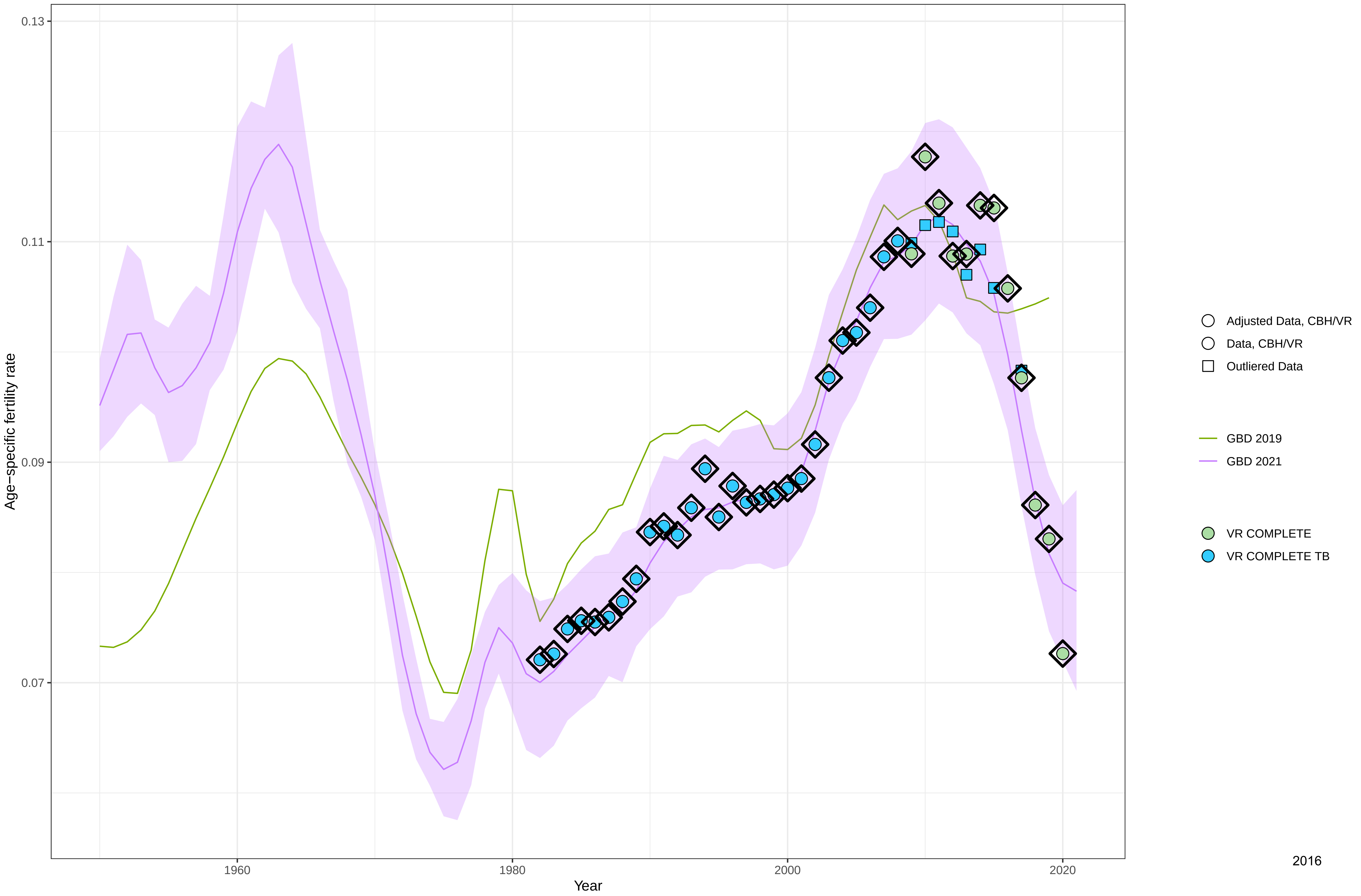

2016

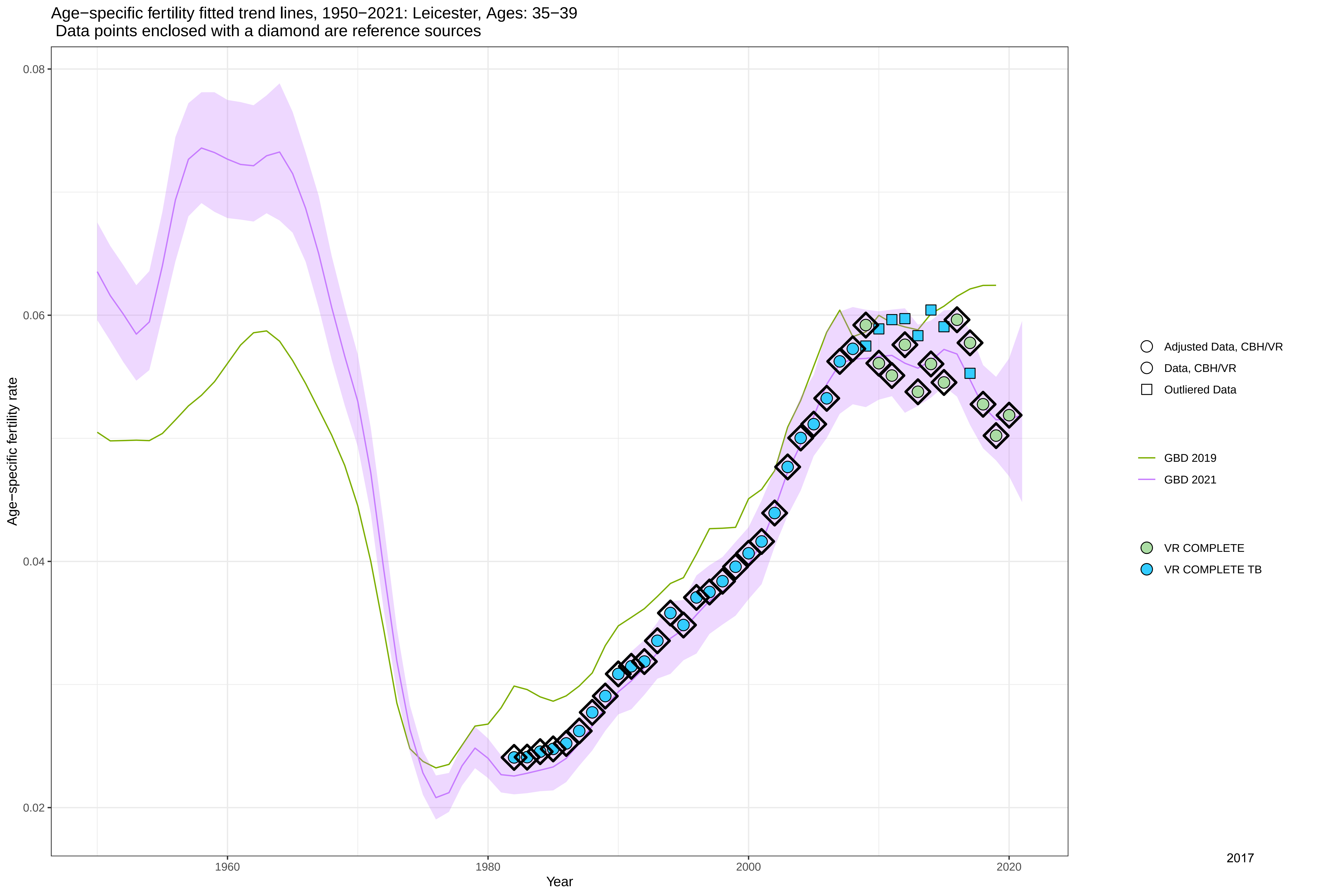
Age−specific fertility fitted trend lines, 1950−2021: Leicester, Ages: 35−39
Data points enclosed with a diamond are reference sources
0.08
0.06
0.04
0.02
Age−specific fertility rate
1960
1980
2000
2020
Year
Adjusted Data, CBH/VR
Data, CBH/VR
Outliered Data
GBD 2019
GBD 2021
VR COMPLETE
VR COMPLETE TB
2017

Age−specific fertility fitted trend lines, 1950−2021: Leicester, Ages: 40−44
Data points enclosed with a diamond are reference sources

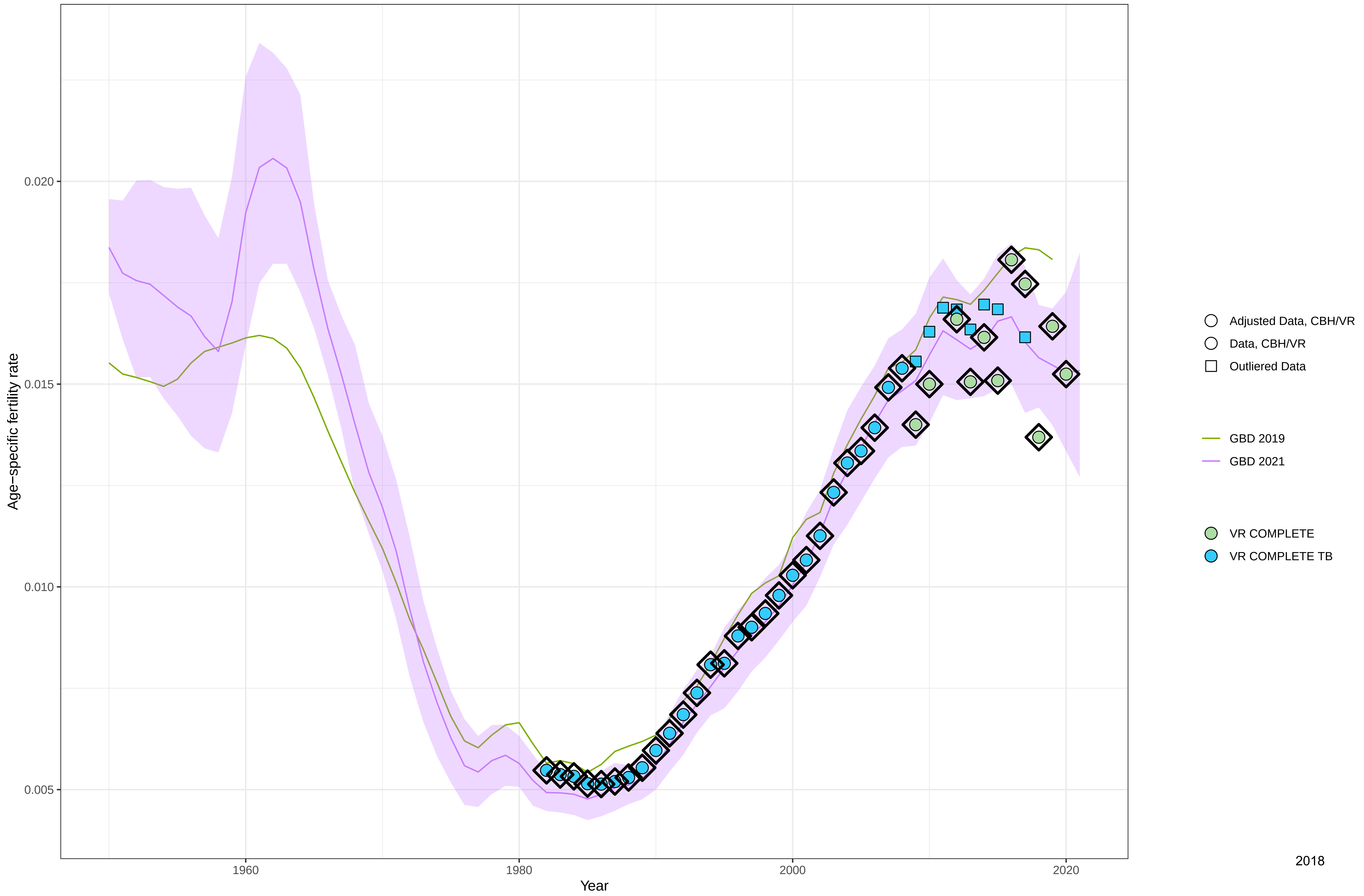

2018

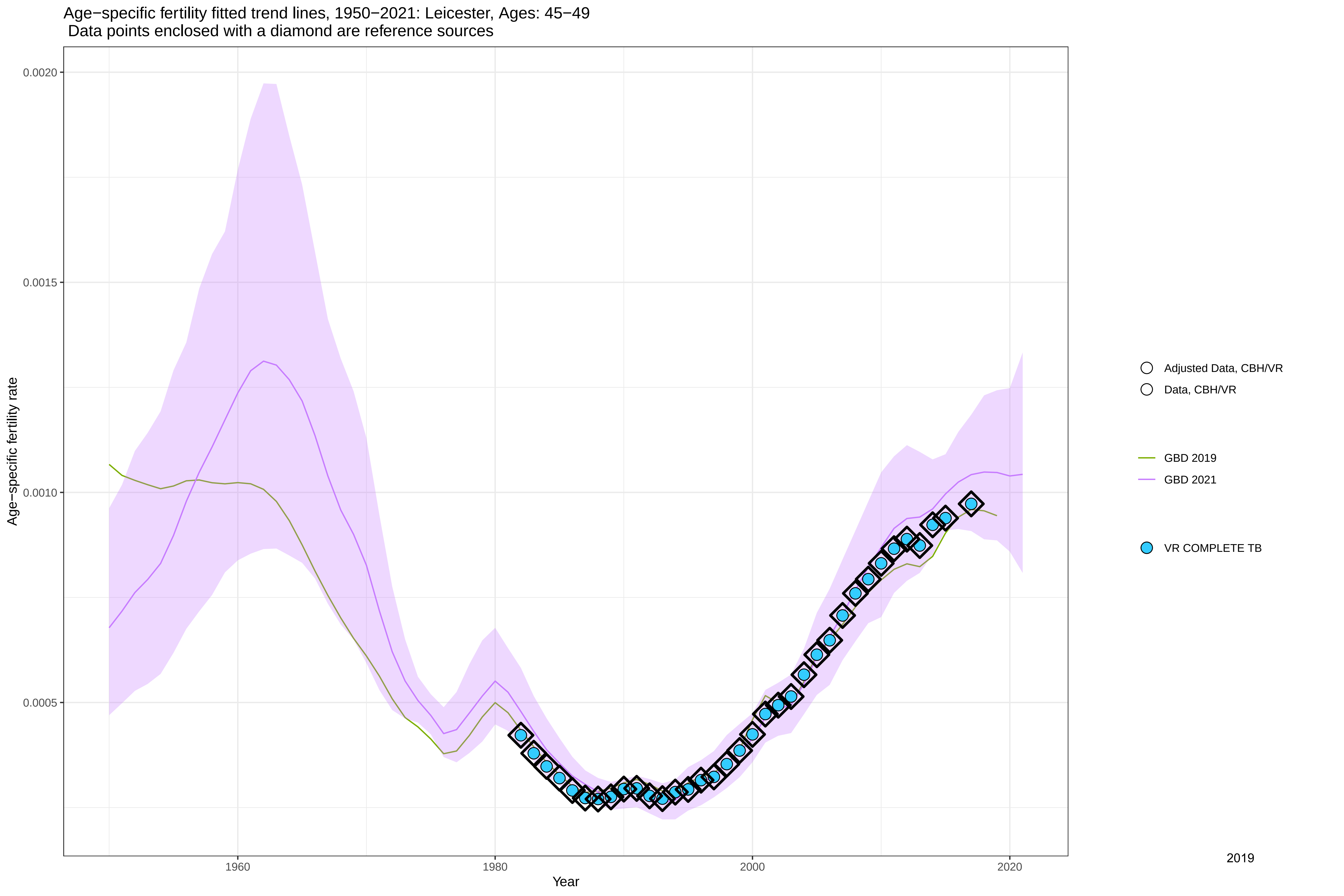

Age−specific fertility fitted trend lines, 1950−2021: Leicester, Ages: 45−49
Data points enclosed with a diamond are reference sources
0.0020
0.0015
0.0010
0.0005
Age−specific fertility rate
1960
1980
2000
2020
Year
Adjusted Data, CBH/VR
Data, CBH/VR
GBD 2019
GBD 2021
VR COMPLETE TB
2019

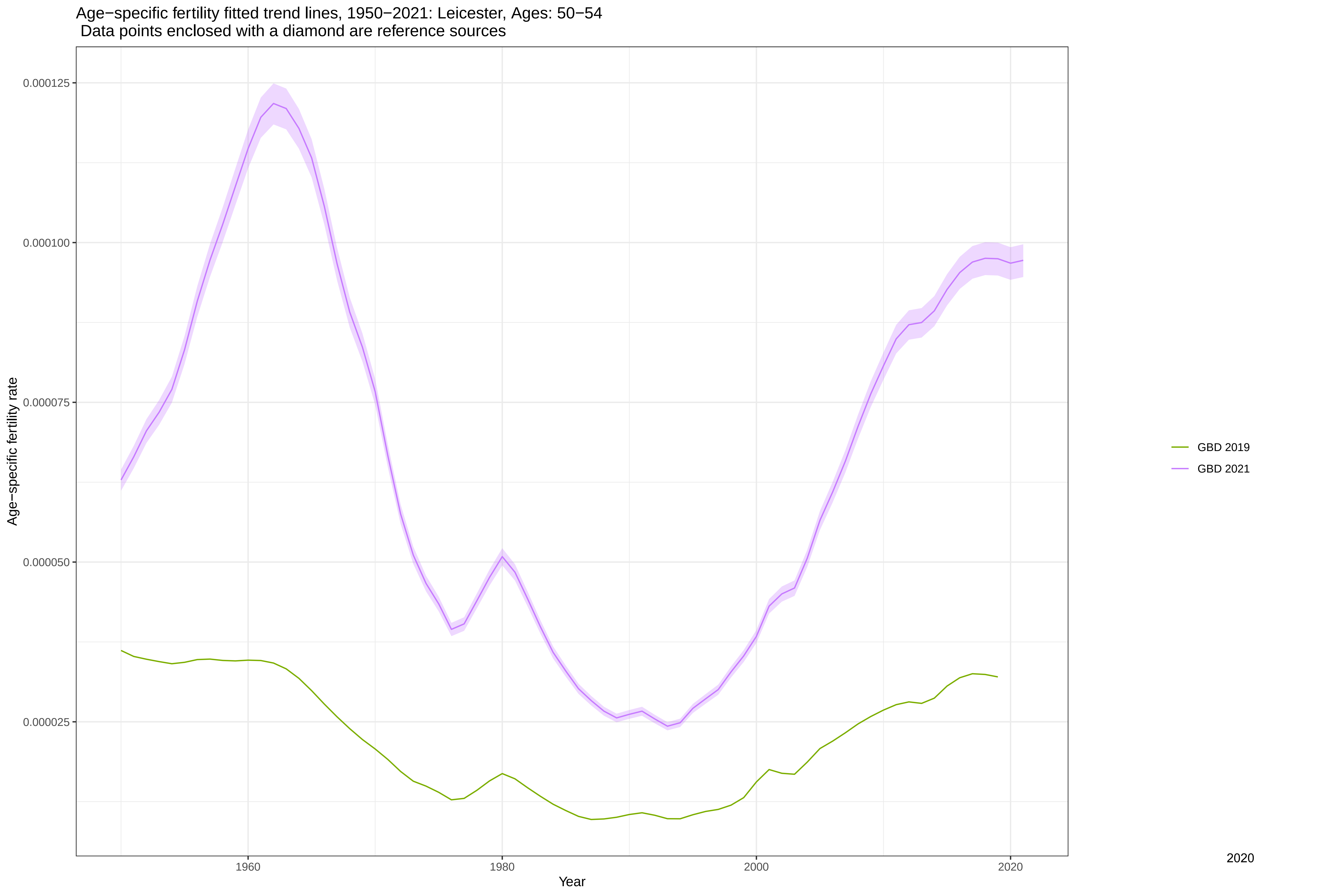

Age−specific fertility fitted trend lines, 1950−2021: Leicester, Ages: 50−54
Data points enclosed with a diamond are reference sources
0.000125
0.000100
0.000075
0.000050
0.000025
Age−specific fertility rate
1960
1980
2000
2020
Year
GBD 2019
GBD 2021
2020

Total fertility rate fitted trend lines, 1950−2021: Leicester TFR
Data points enclosed with a diamond are reference sources

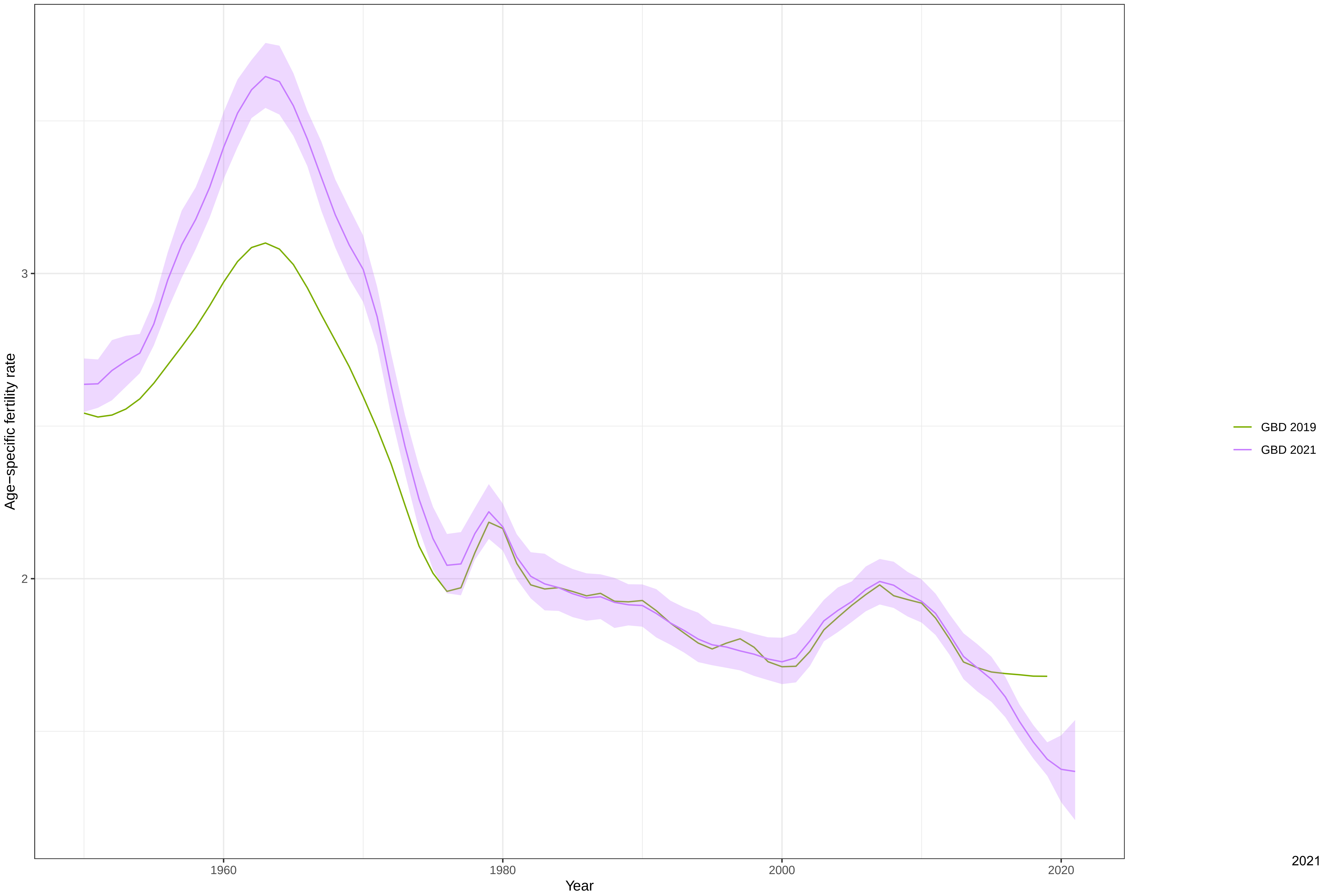

Age−specific fertility fitted trend lines, 1950−2021: Leicestershire, Ages: 10−14
Data points enclosed with a diamond are reference sources

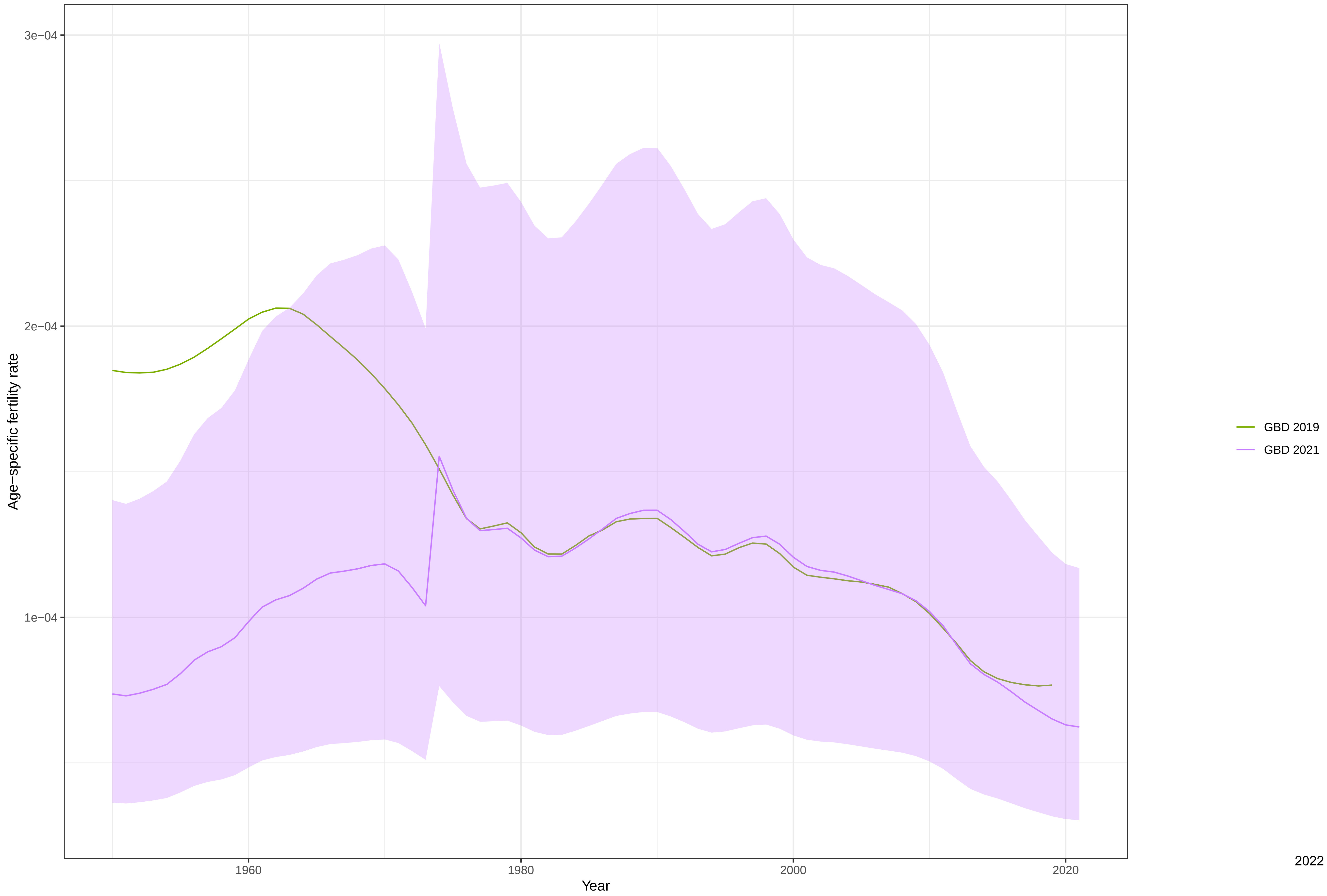

2022

Age−specific fertility fitted trend lines, 1950−2021: Leicestershire, Ages: 15−19
Data points enclosed with a diamond are reference sources

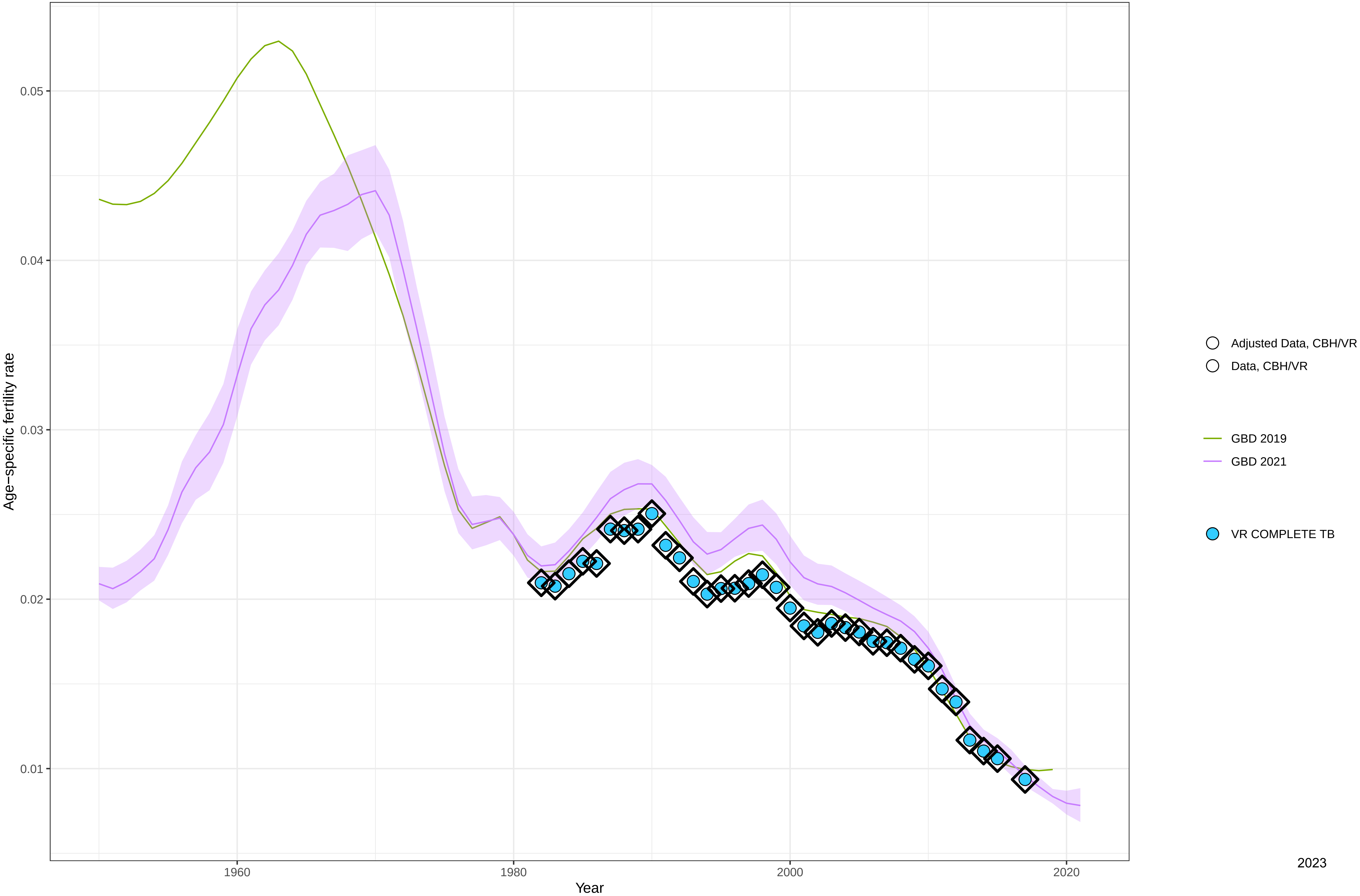

2023

Age−specific fertility fitted trend lines, 1950−2021: Leicestershire, Ages: 20−24
Data points enclosed with a diamond are reference sources

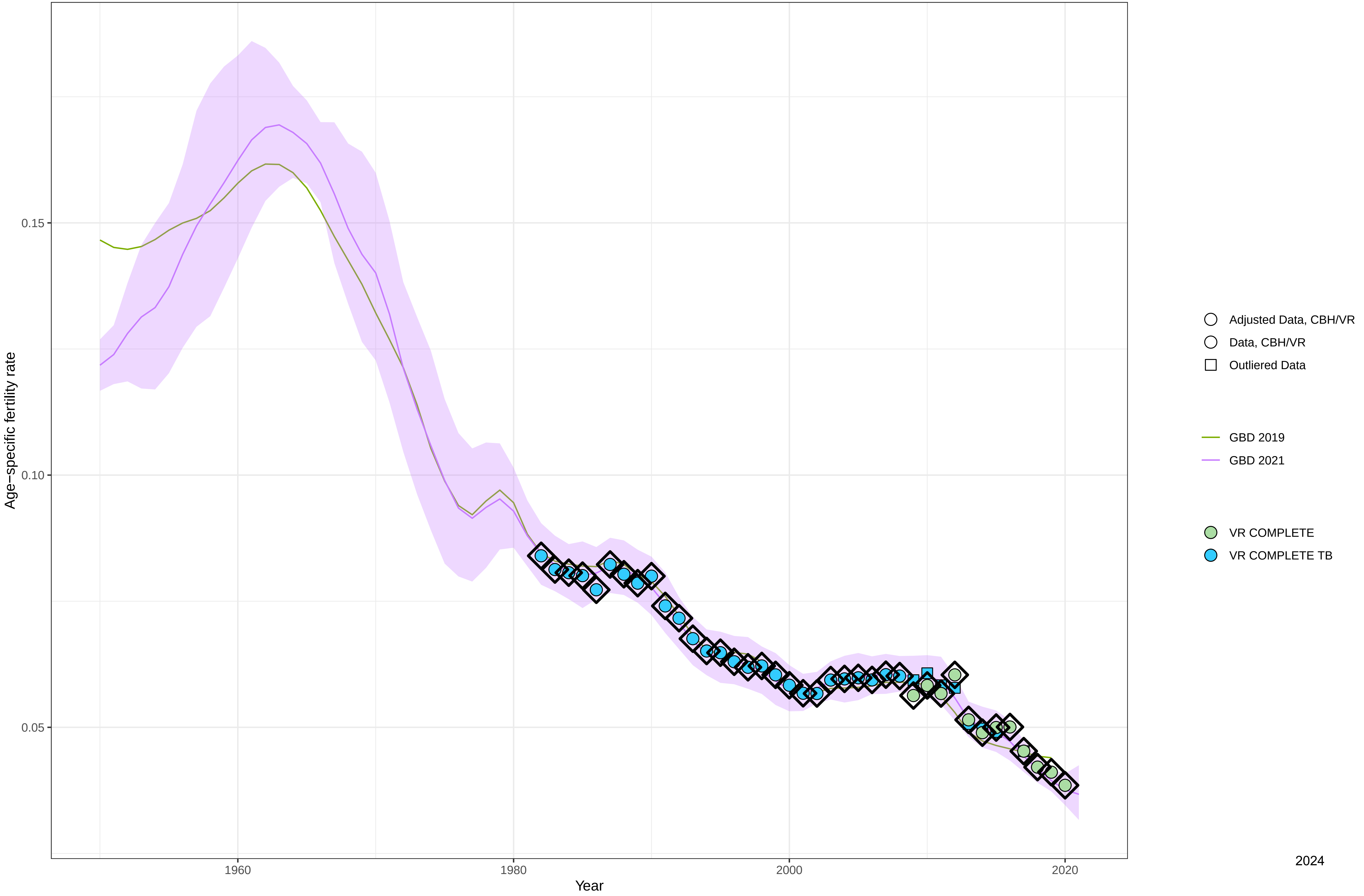

2024

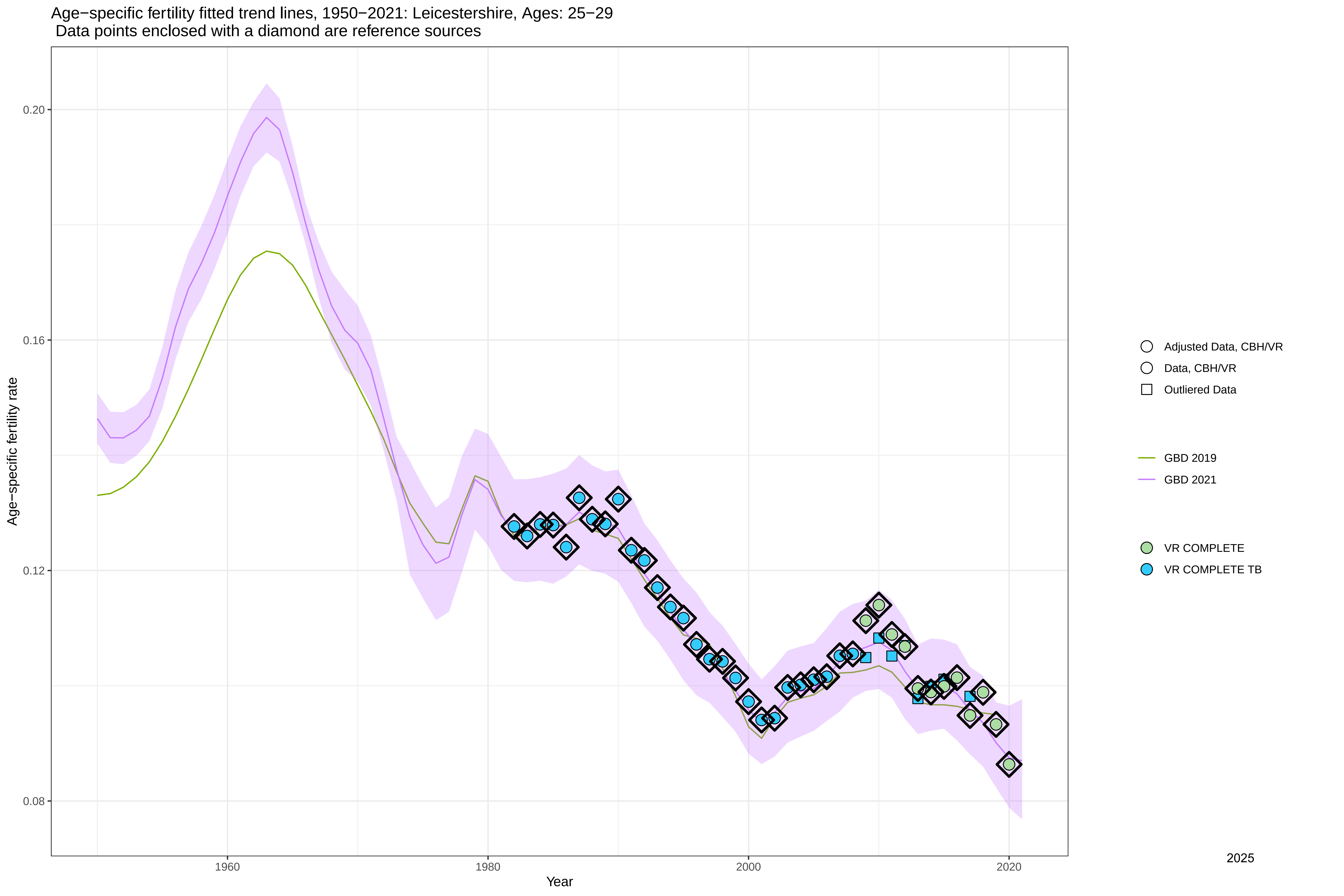

Age−specific fertility fitted trend lines, 1950−2021: Leicestershire, Ages: 25−29
Data points enclosed with a diamond are reference sources
Age−specific fertility rate
0.20
0.16
0.12
0.08
1960
1980
2000
2020
Year
Adjusted Data, CBH/VR
Data, CBH/VR
Outliered Data
GBD 2019
GBD 2021
VR COMPLETE
VR COMPLETE TB
2025

Age−specific fertility fitted trend lines, 1950−2021: Leicestershire, Ages: 30−34
Data points enclosed with a diamond are reference sources

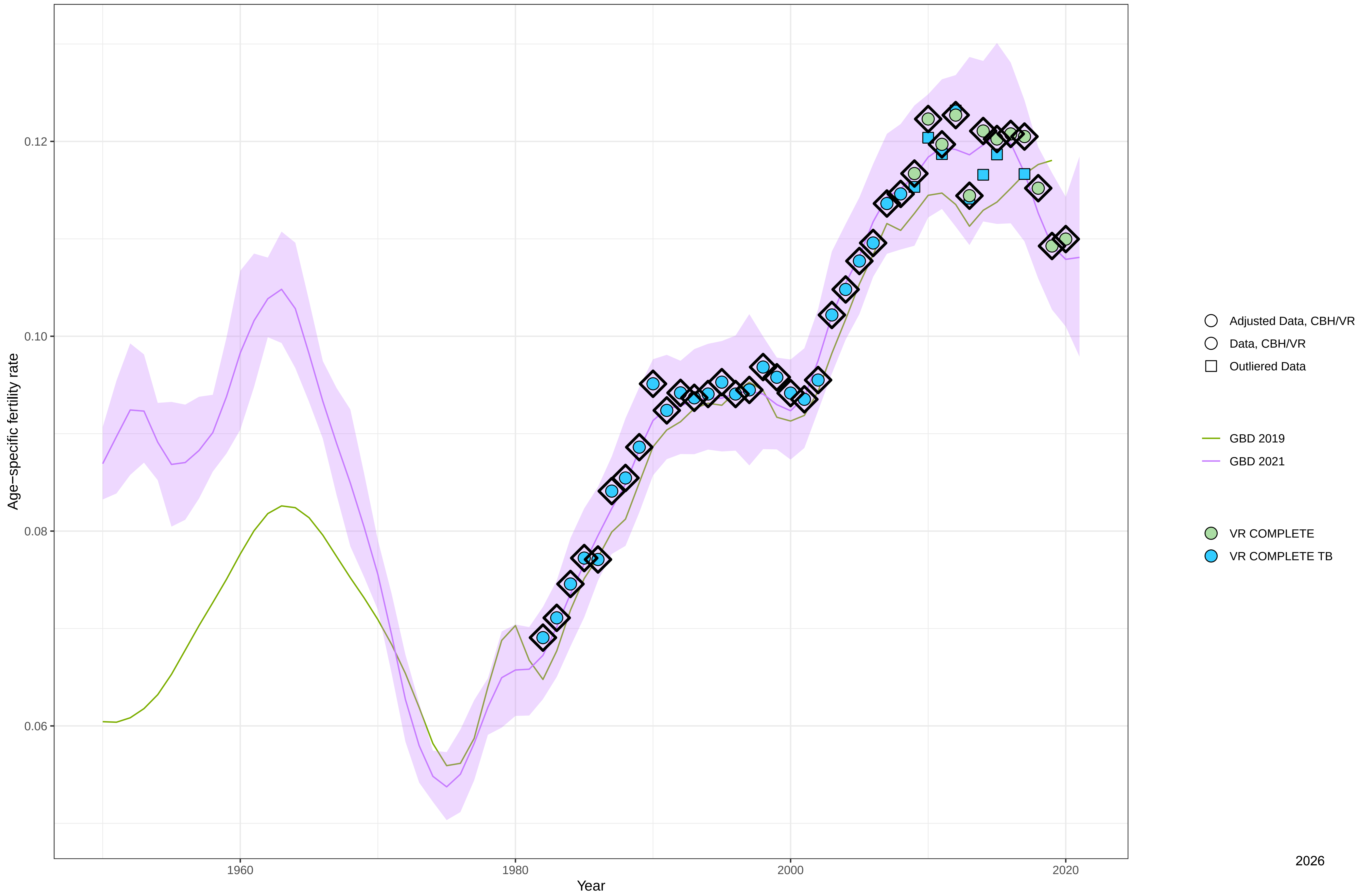

2026

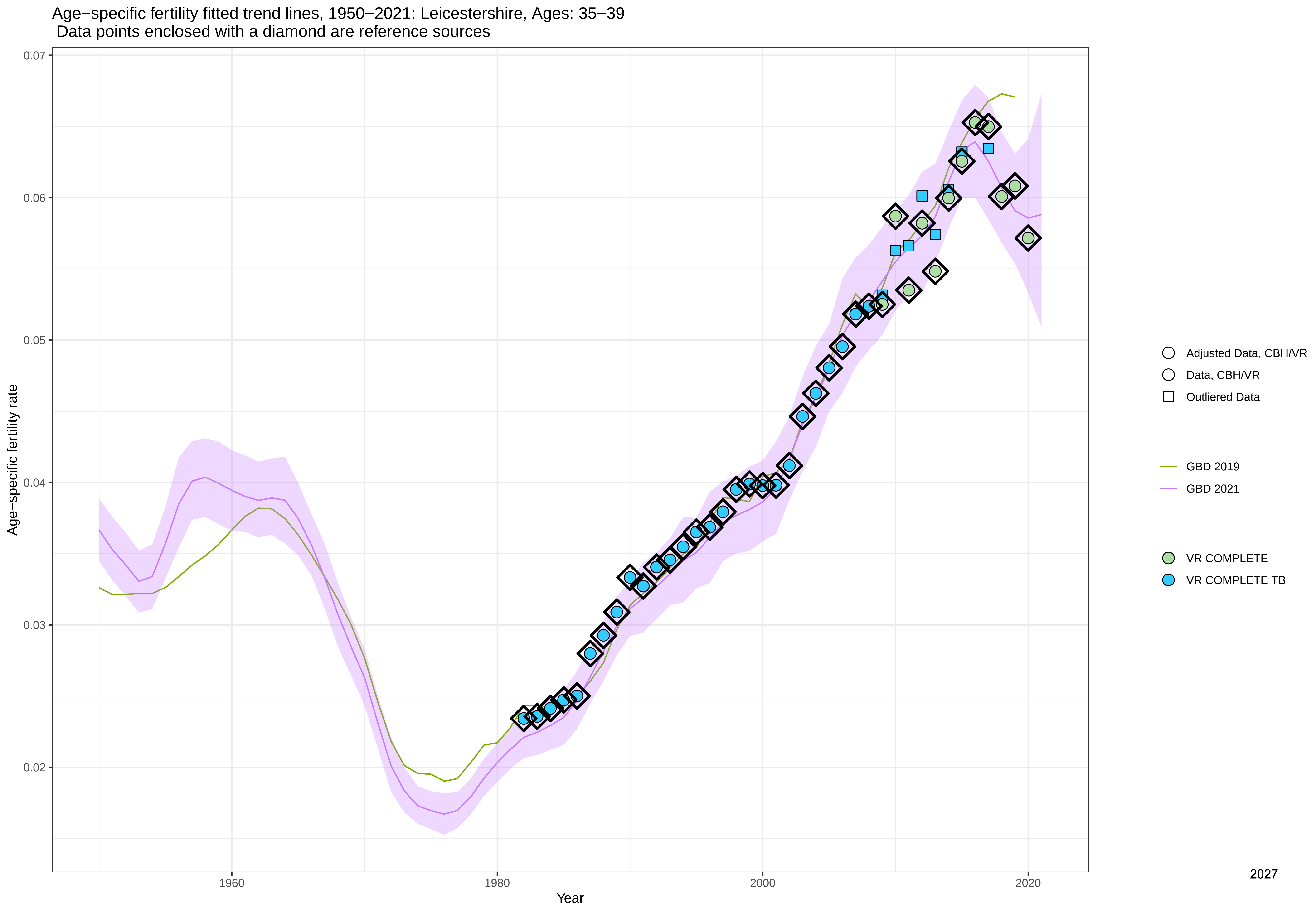

Age−specific fertility fitted trend lines, 1950−2021: Leicestershire, Ages: 35−39
Data points enclosed with a diamond are reference sources
Age−specific fertility rate
Year
0.07
0.06
0.05
0.04
0.03
0.02
1960
1980
2000
2020
Adjusted Data, CBH/VR
Data, CBH/VR
Outliered Data
GBD 2019
GBD 2021
VR COMPLETE
VR COMPLETE TB

Age−specific fertility fitted trend lines, 1950−2021: Leicestershire, Ages: 40−44
Data points enclosed with a diamond are reference sources

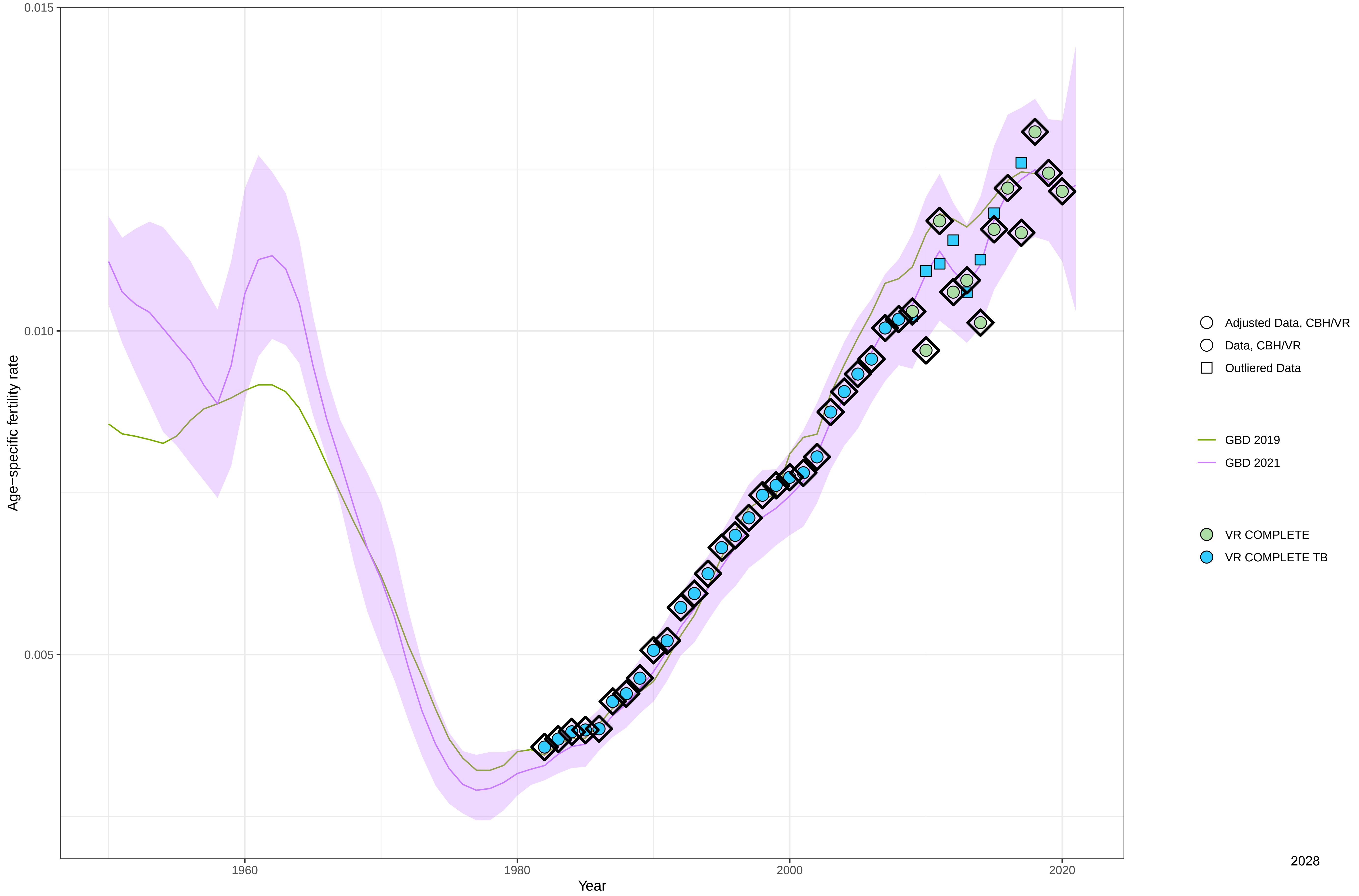

2028

Age−specific fertility fitted trend lines, 1950−2021: Leicestershire, Ages: 45−49
Data points enclosed with a diamond are reference sources

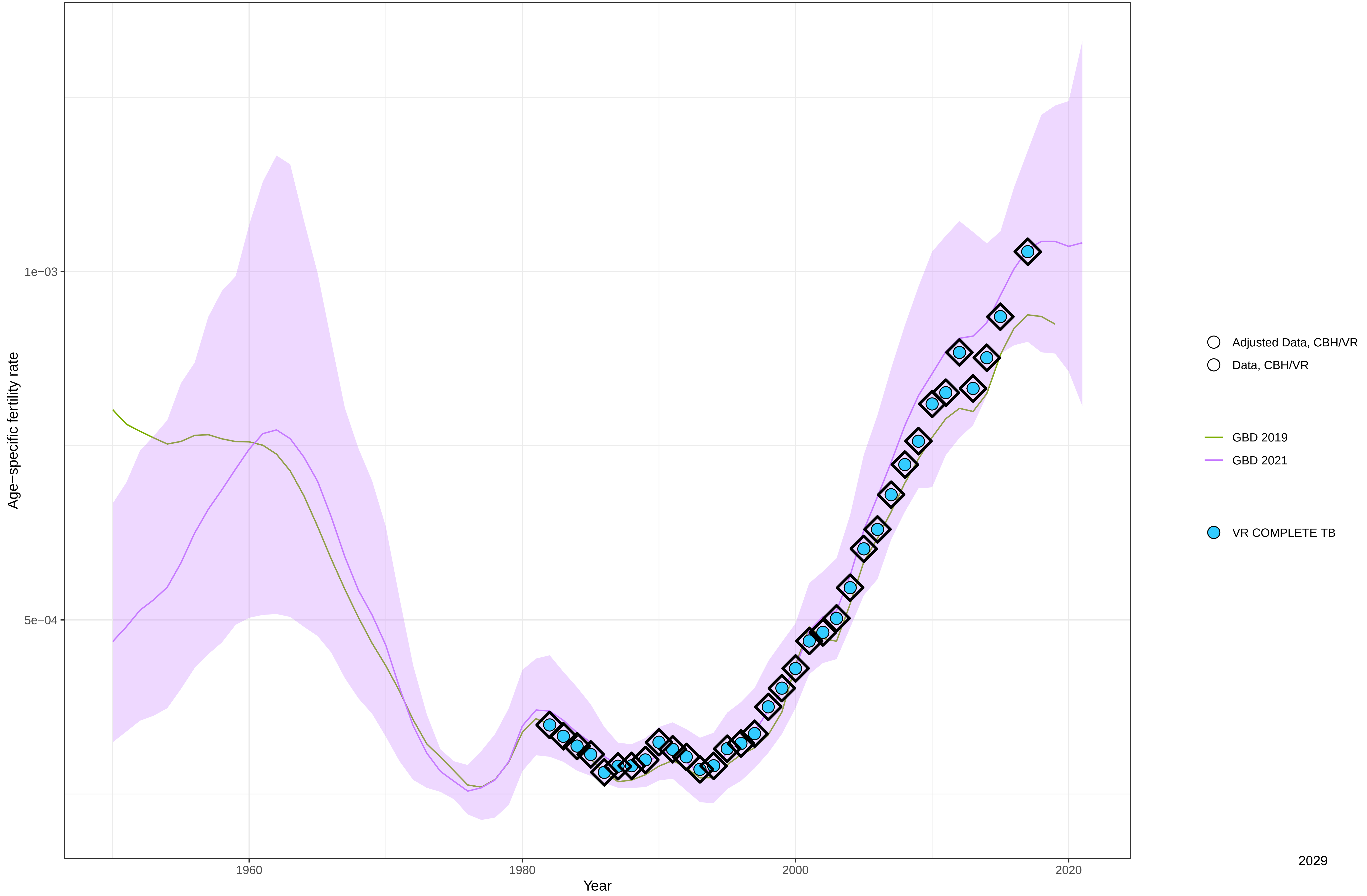

Age−specific fertility fitted trend lines, 1950−2021: Leicestershire, Ages: 50−54
Data points enclosed with a diamond are reference sources

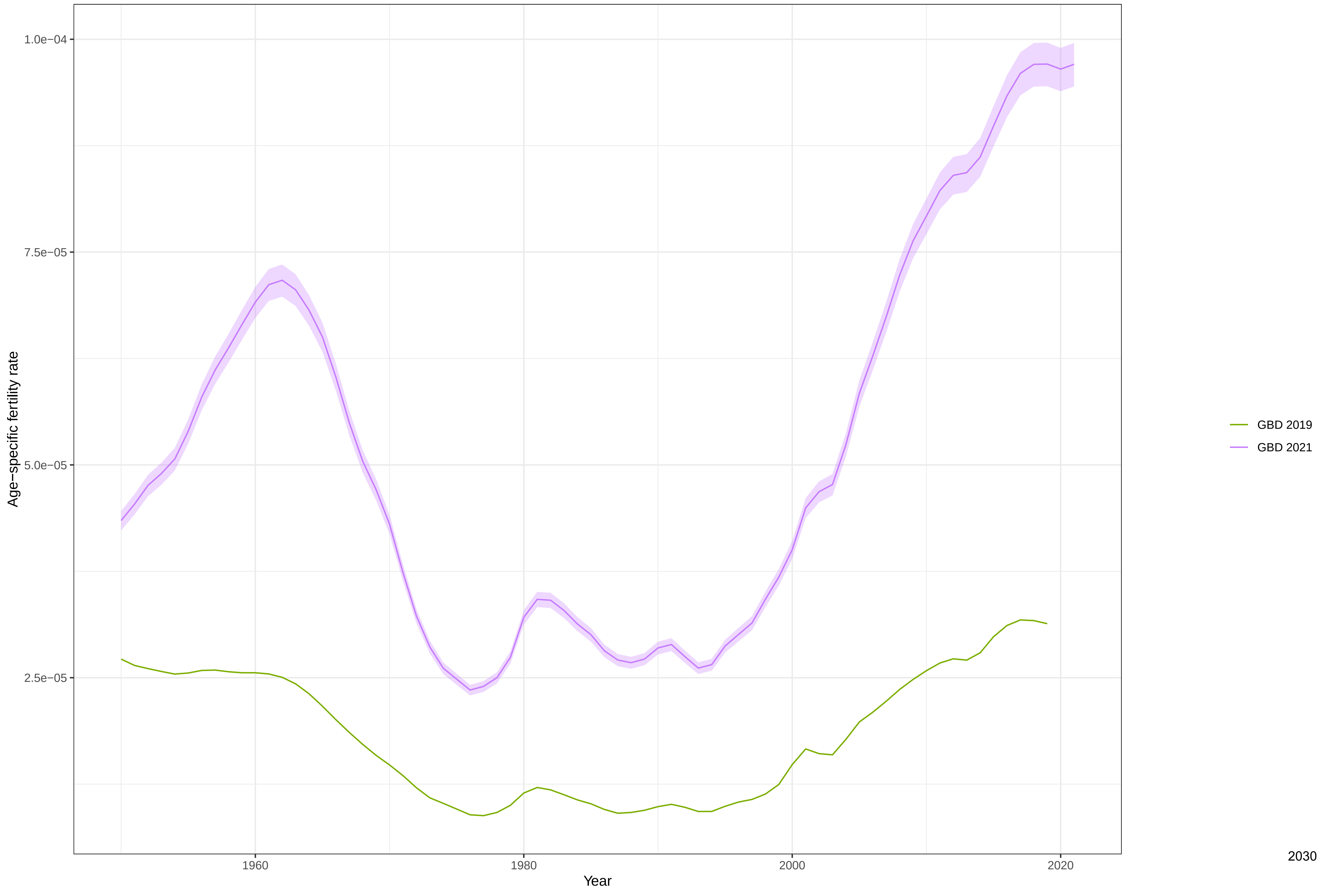

Total fertility rate fitted trend lines, 1950−2021: Leicestershire TFR
Data points enclosed with a diamond are reference sources

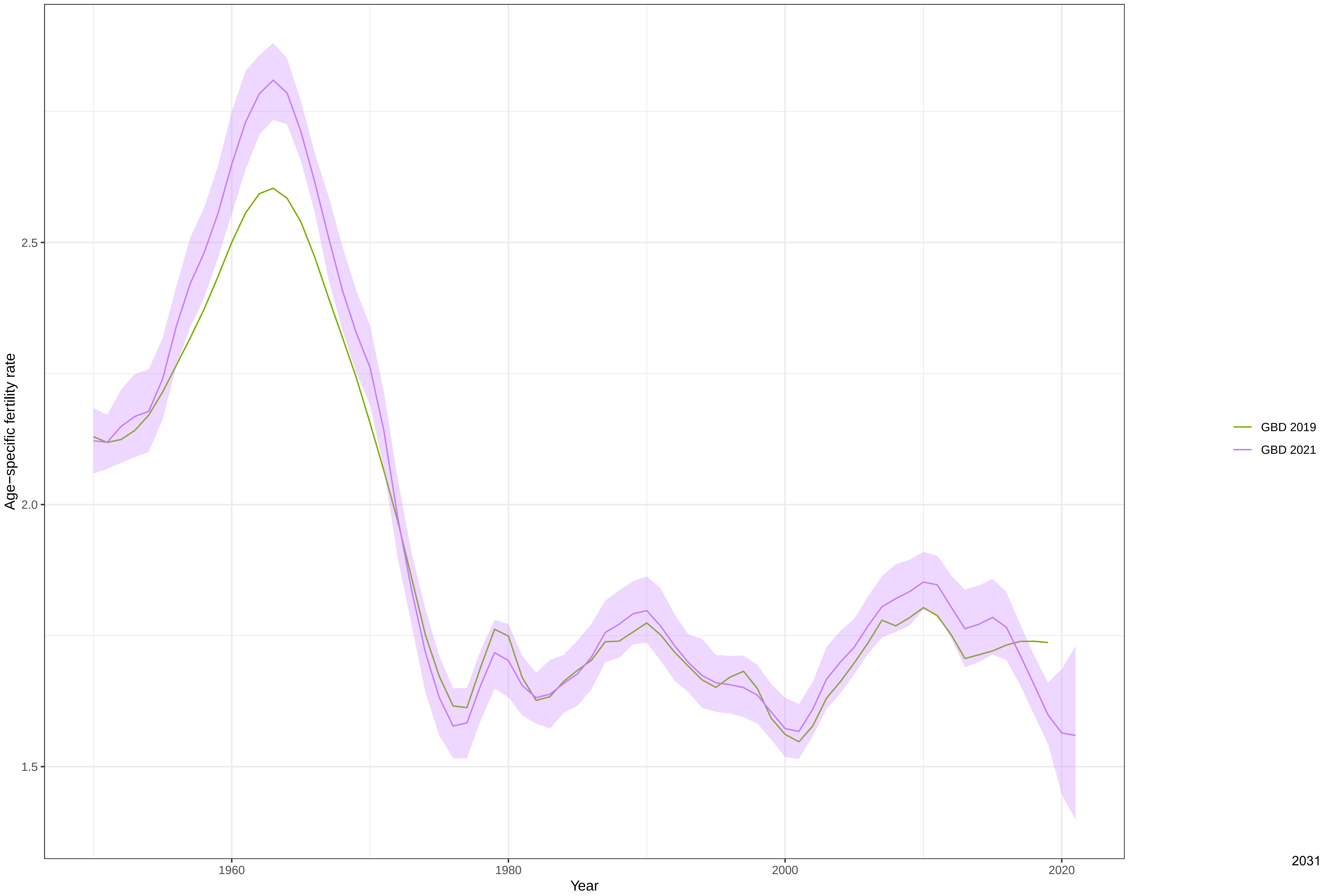

2031

Age−specific fertility fitted trend lines, 1950−2021: Lincolnshire, Ages: 10−14
Data points enclosed with a diamond are reference sources

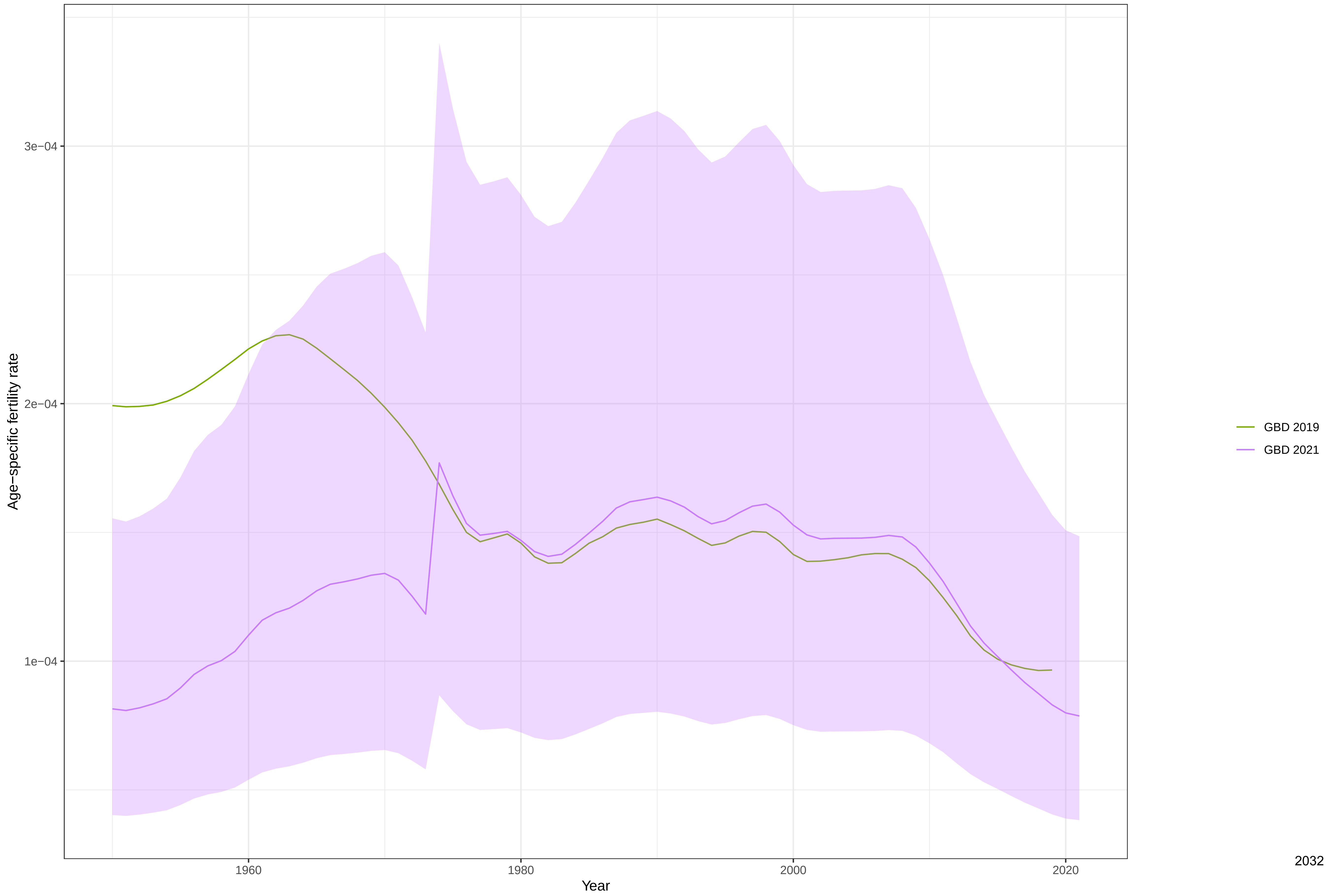

2032

Age−specific fertility fitted trend lines, 1950−2021: Lincolnshire, Ages: 15−19
Data points enclosed with a diamond are reference sources

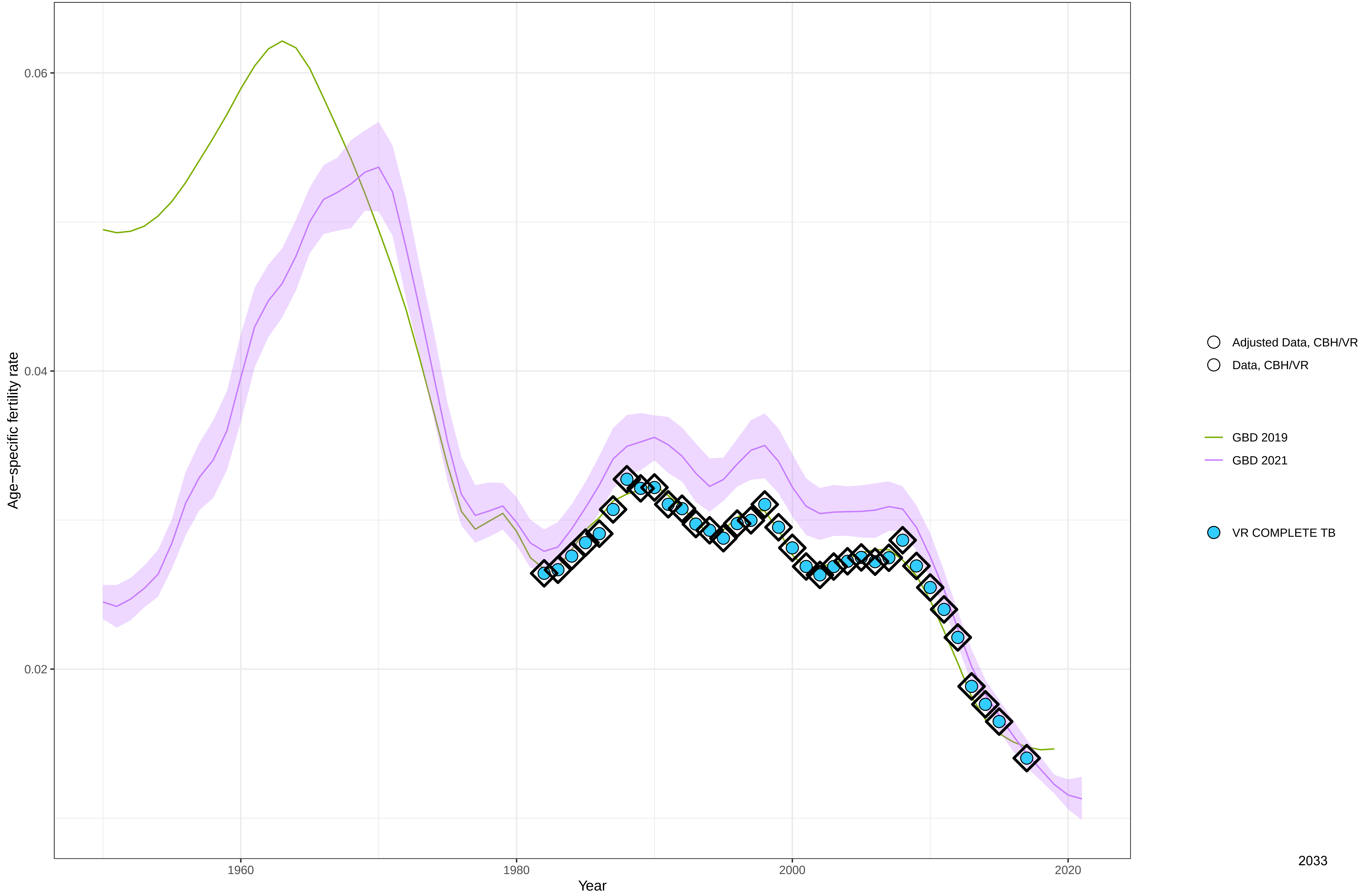

2033

Age−specific fertility fitted trend lines, 1950−2021: Lincolnshire, Ages: 20−24
Data points enclosed with a diamond are reference sources

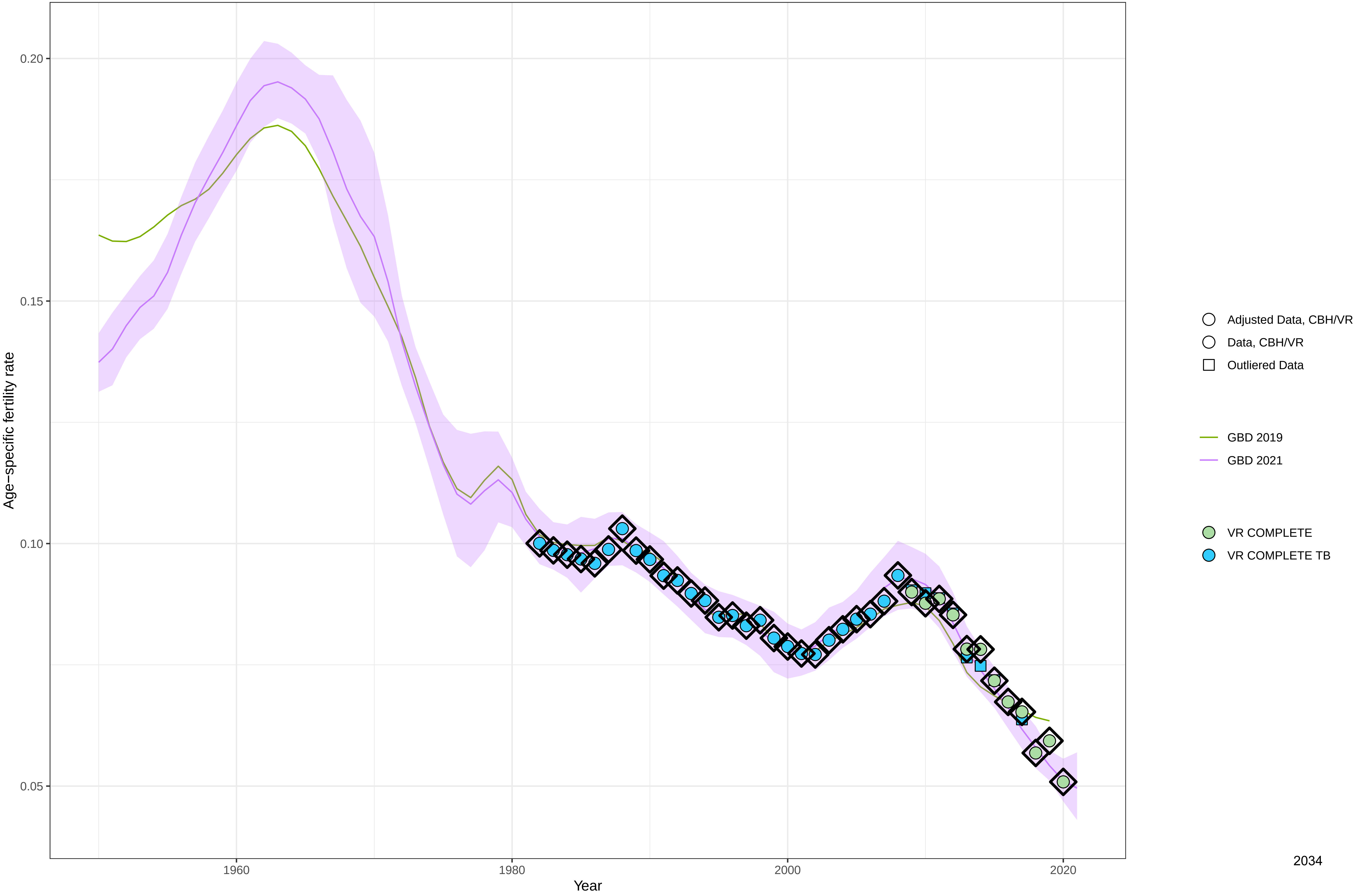

2034

Age−specific fertility fitted trend lines, 1950−2021: Lincolnshire, Ages: 25−29
Data points enclosed with a diamond are reference sources

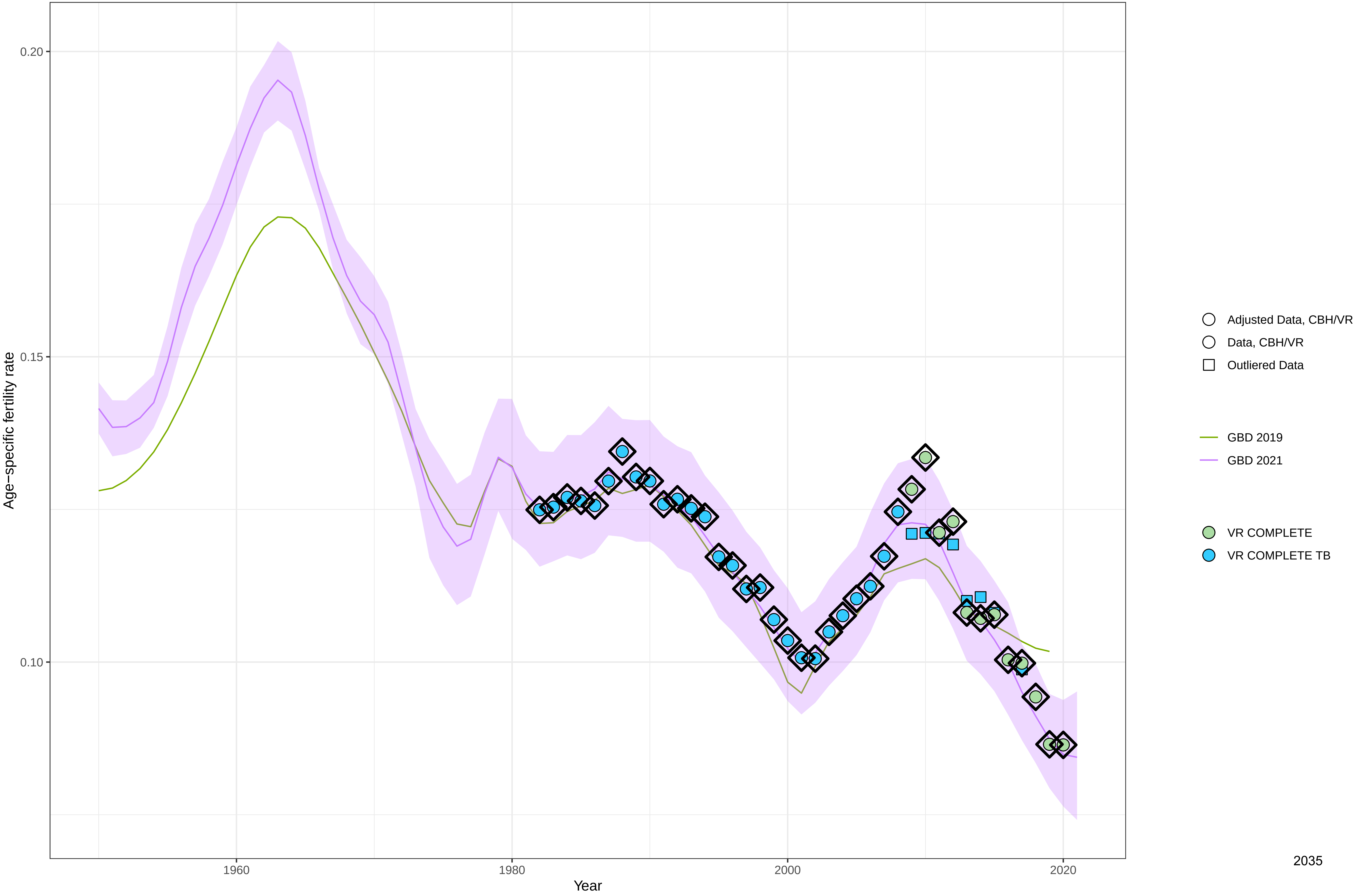

2035

Age−specific fertility fitted trend lines, 1950−2021: Lincolnshire, Ages: 30−34
Data points enclosed with a diamond are reference sources

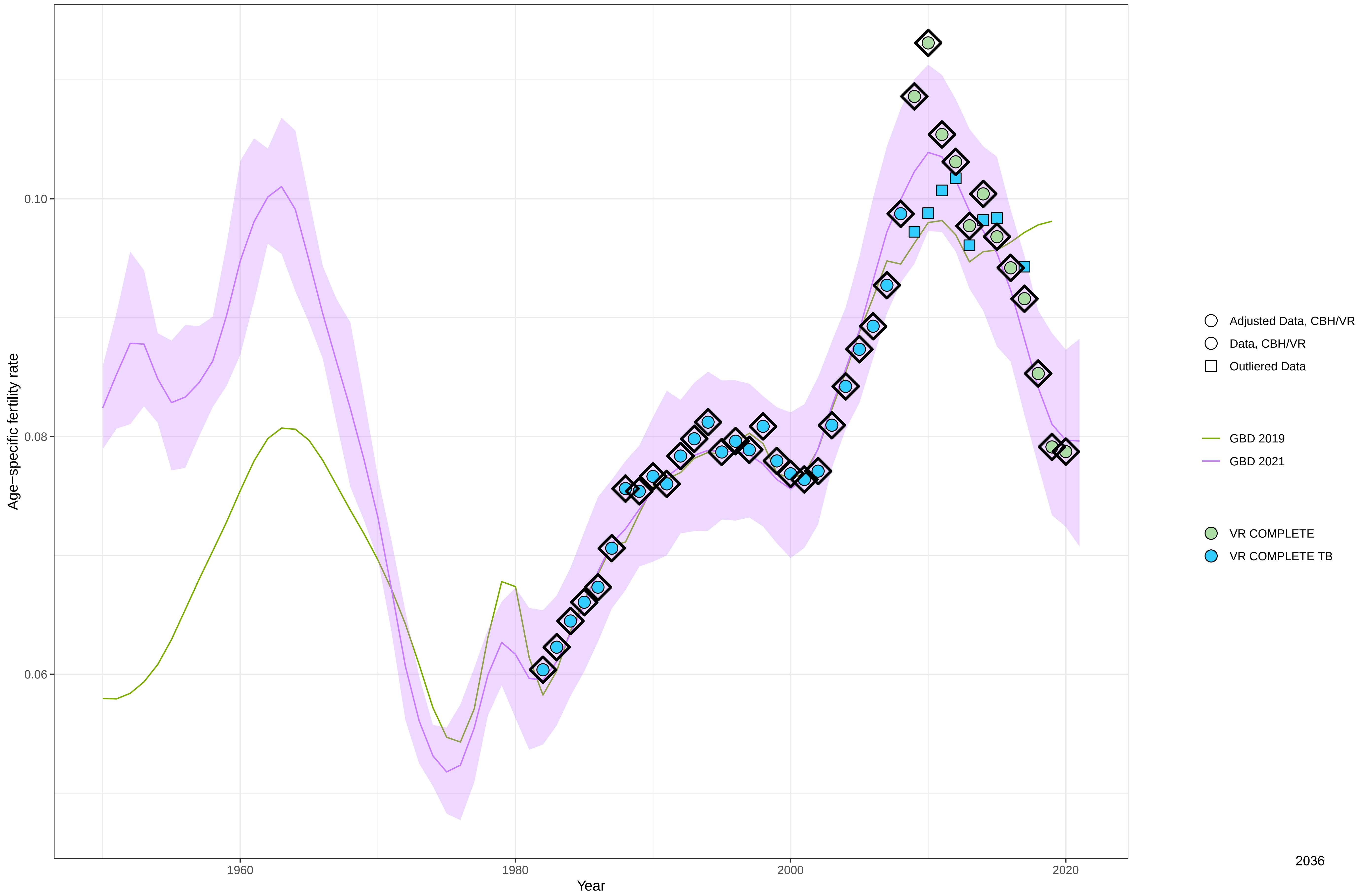

2036

Age−specific fertility fitted trend lines, 1950−2021: Lincolnshire, Ages: 35−39
Data points enclosed with a diamond are reference sources

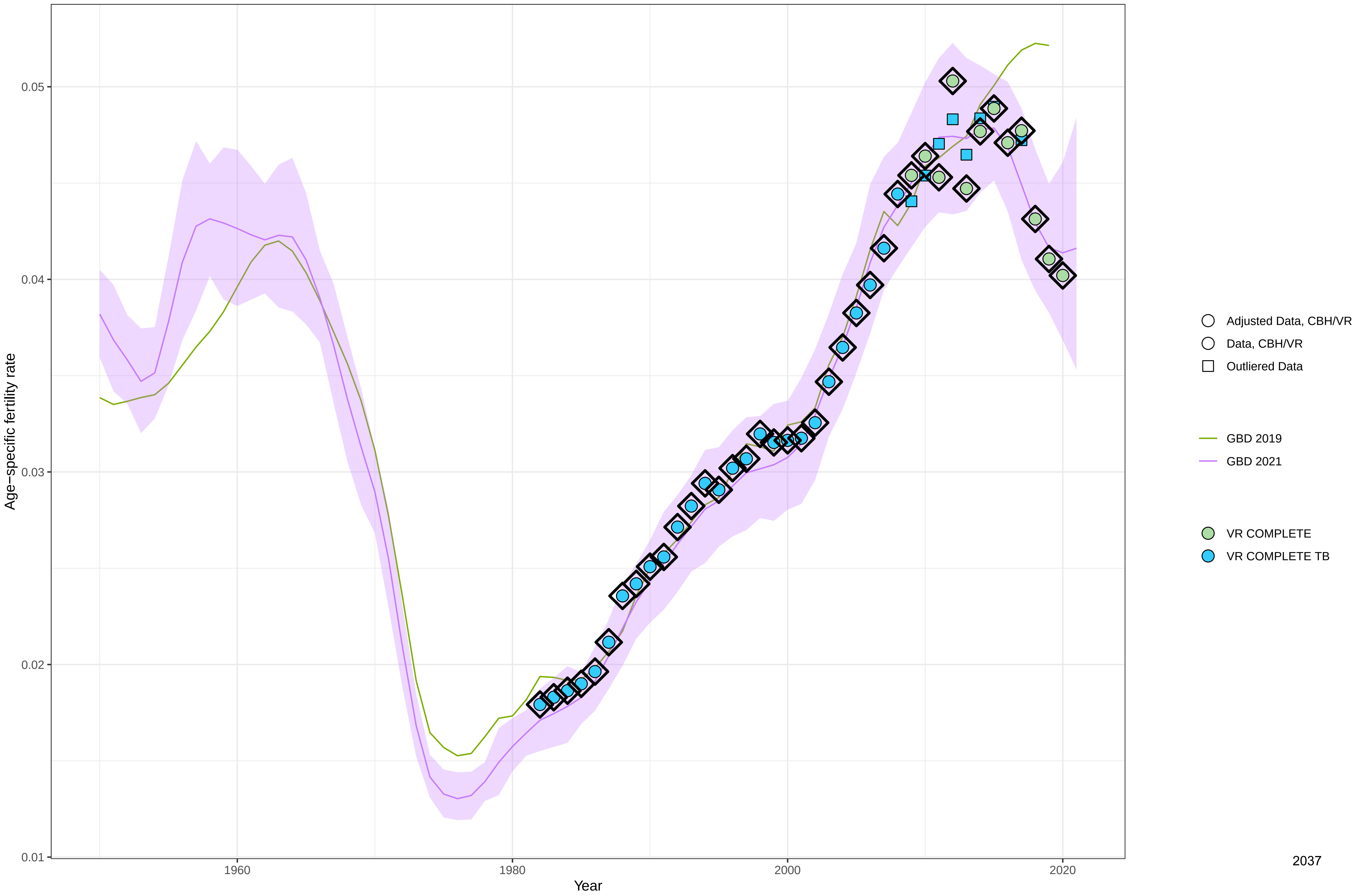

Age−specific fertility fitted trend lines, 1950−2021: Lincolnshire, Ages: 40−44
Data points enclosed with a diamond are reference sources

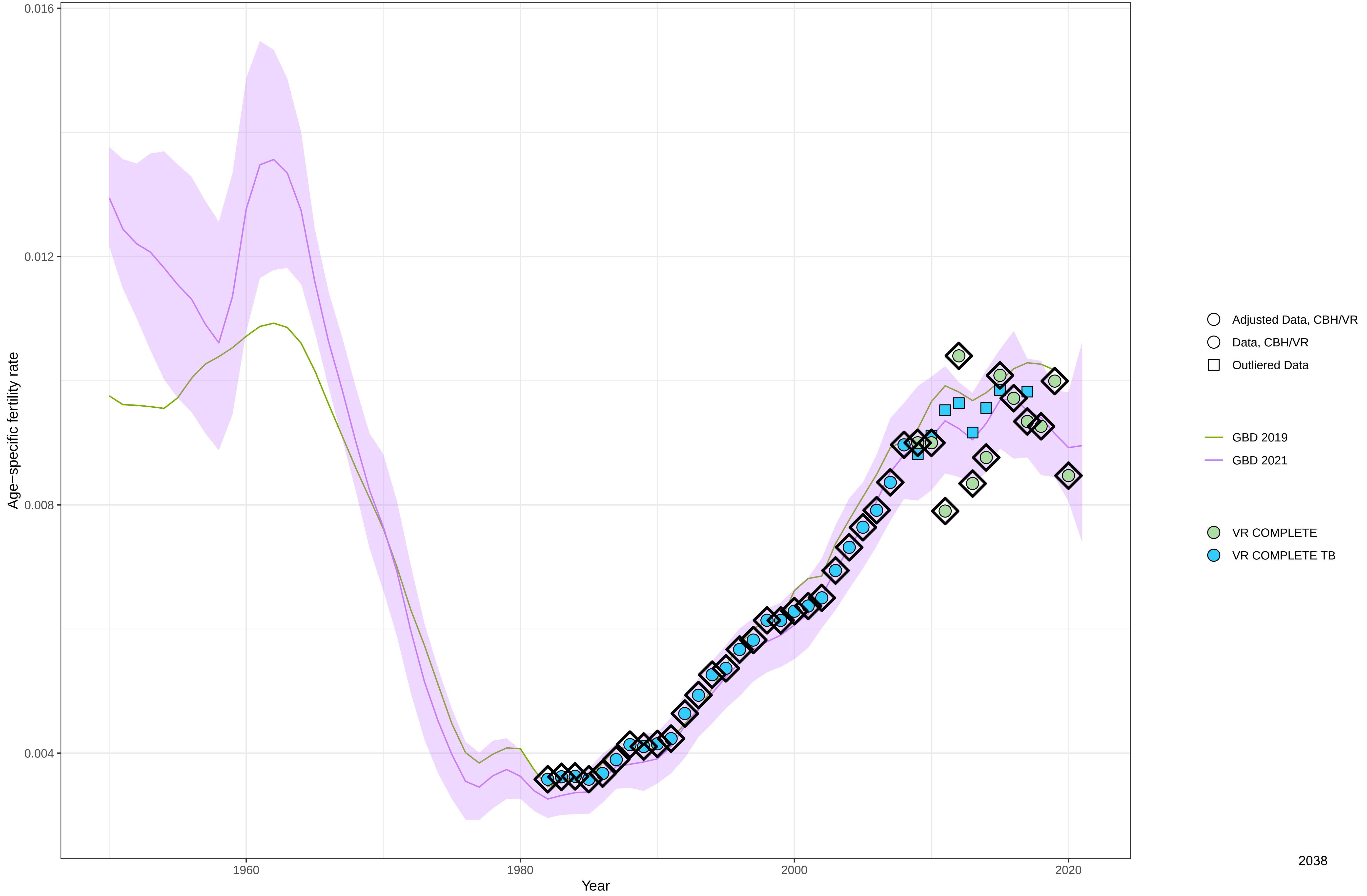

Age−specific fertility fitted trend lines, 1950−2021: Lincolnshire, Ages: 45−49
Data points enclosed with a diamond are reference sources

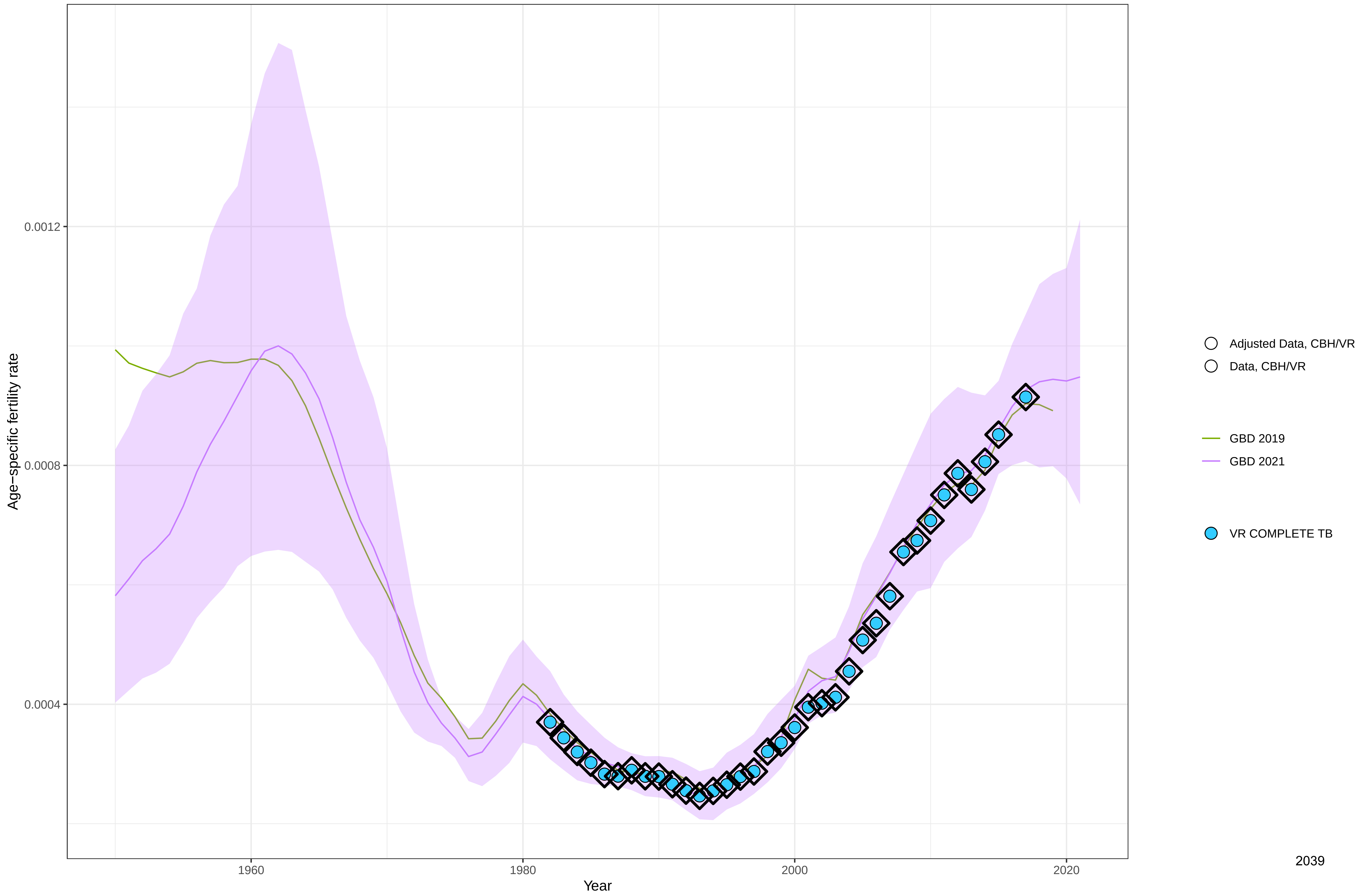

Age−specific fertility fitted trend lines, 1950−2021: Lincolnshire, Ages: 50−54
Data points enclosed with a diamond are reference sources

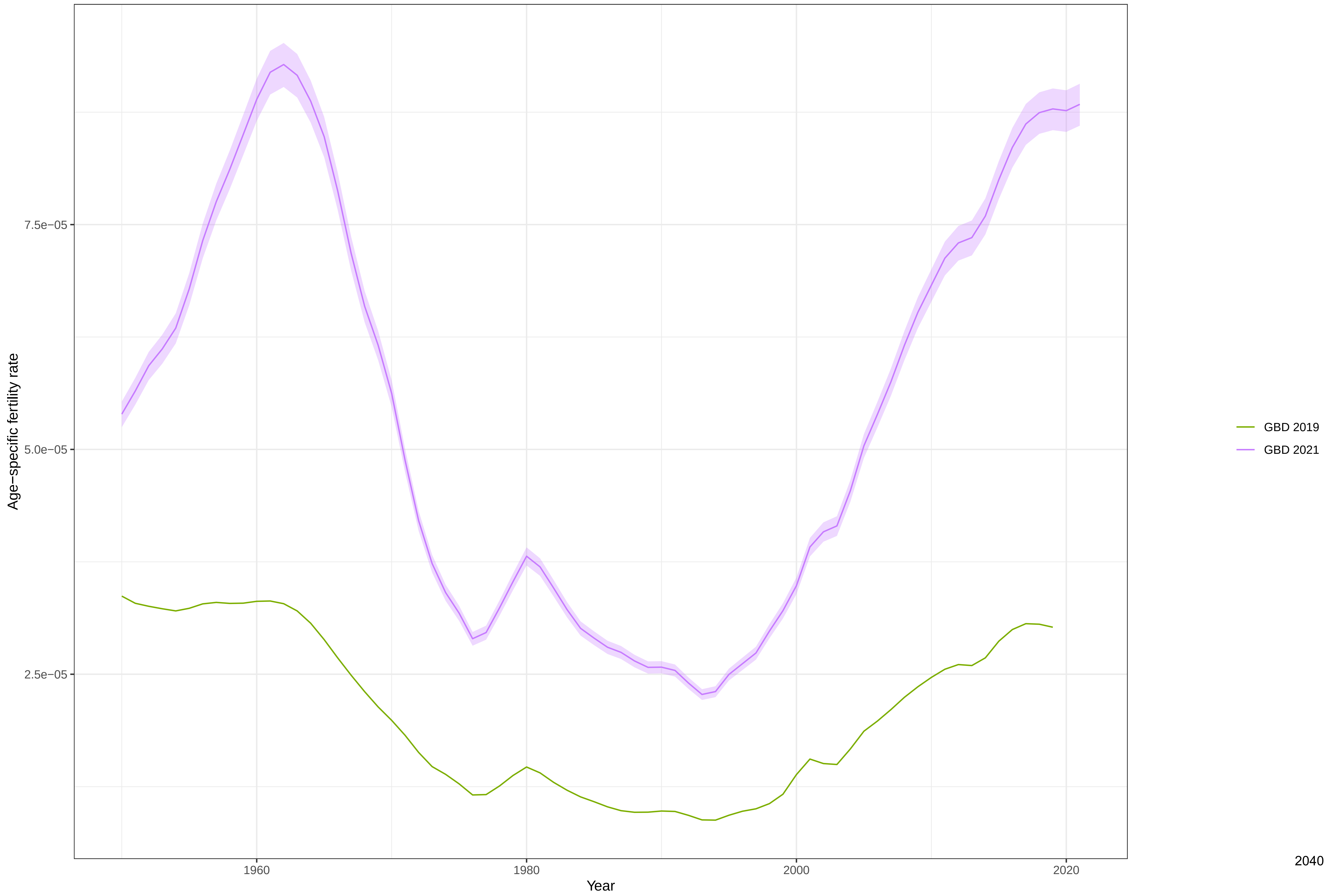

2040

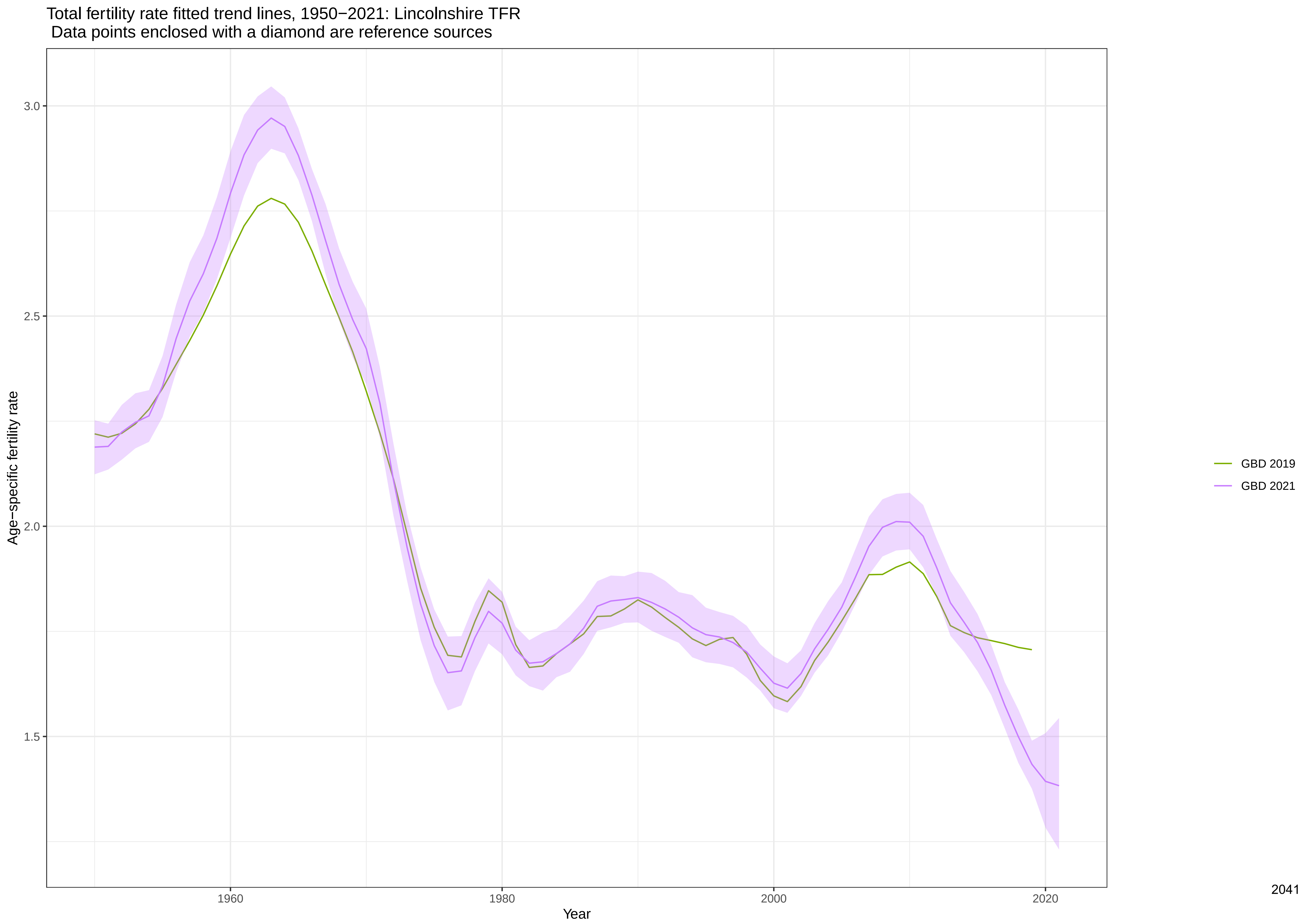

Total fertility rate fitted trend lines, 1950−2021: Lincolnshire TFR
Data points enclosed with a diamond are reference sources
Age−specific fertility rate
3.0
2.5
2.0
1.5
1960
1980
2000
2020
Year
GBD 2019
GBD 2021

Age−specific fertility fitted trend lines, 1950−2021: Northamptonshire, Ages: 10−14
Data points enclosed with a diamond are reference sources

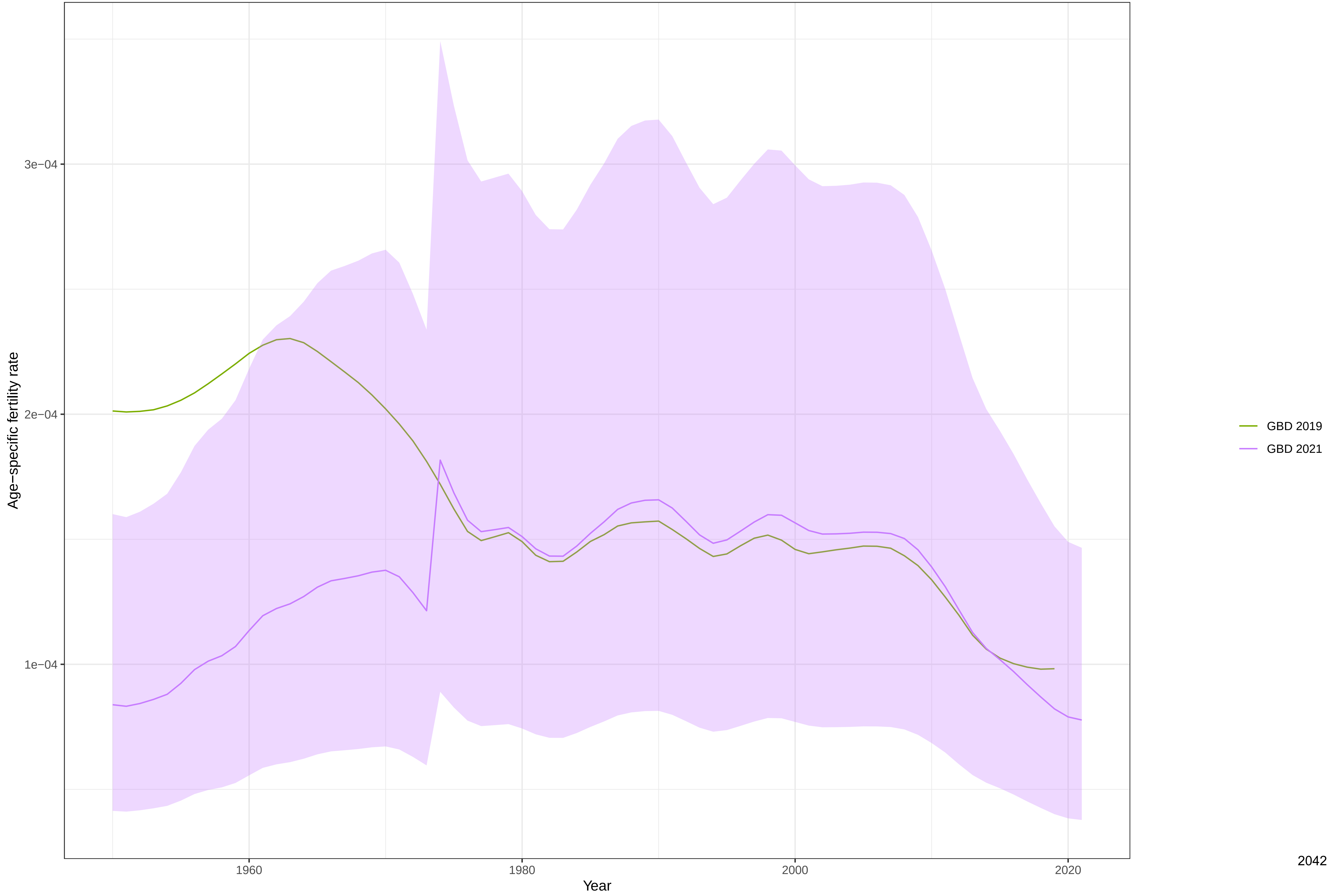

Age−specific fertility fitted trend lines, 1950−2021: Northamptonshire, Ages: 15−19
Data points enclosed with a diamond are reference sources

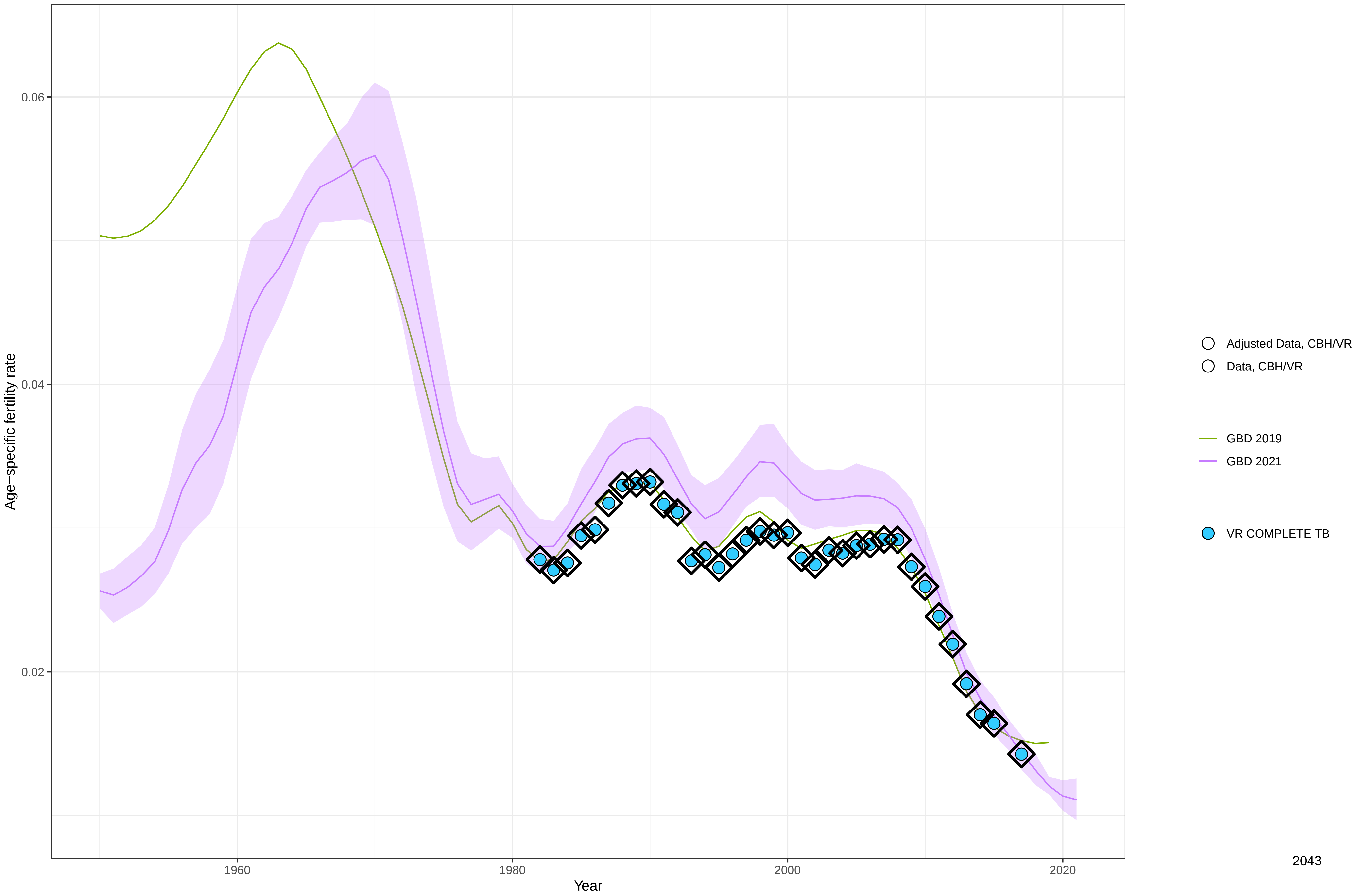

Age−specific fertility fitted trend lines, 1950−2021: Northamptonshire, Ages: 20−24
Data points enclosed with a diamond are reference sources

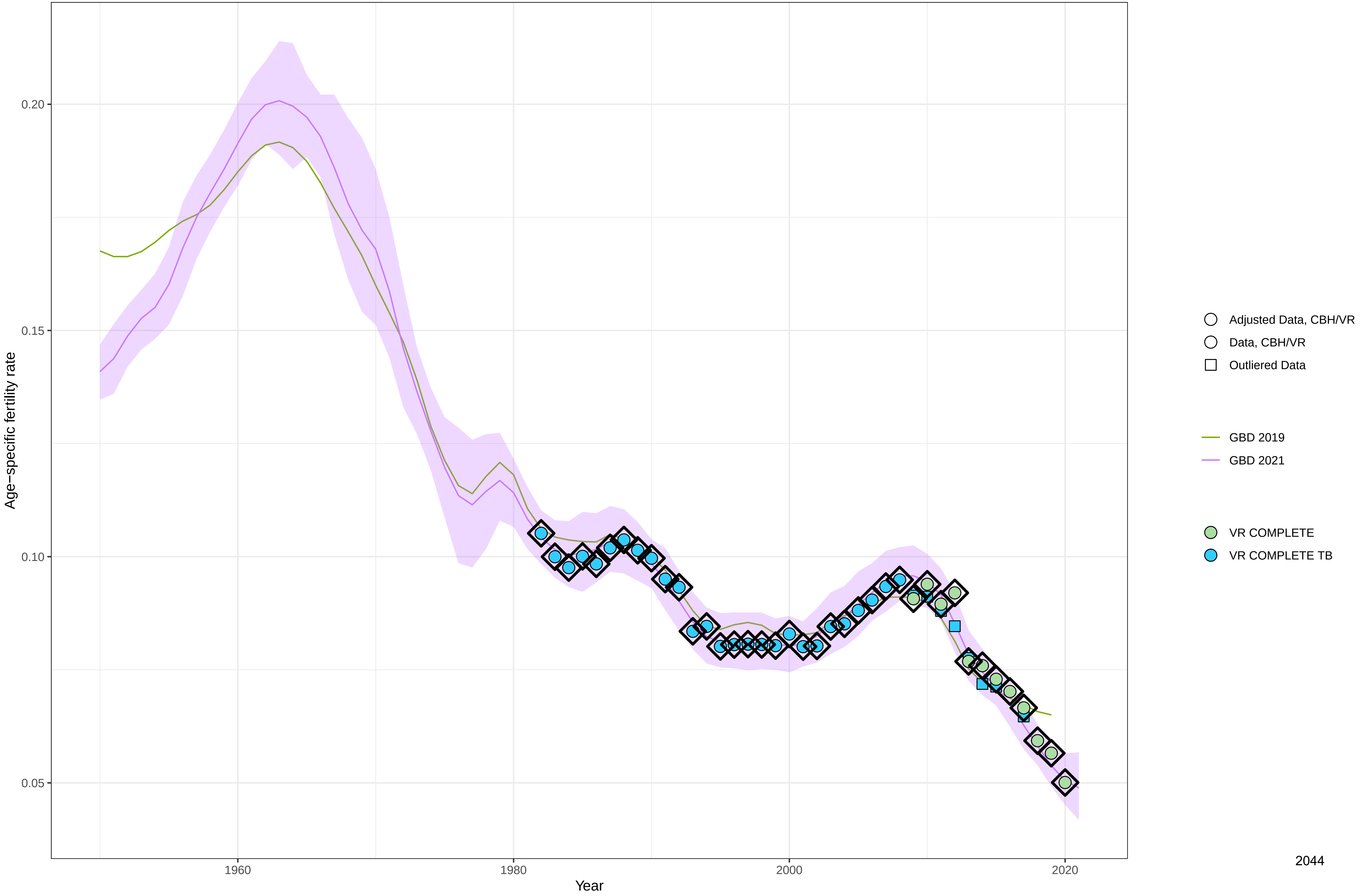

2044

Age−specific fertility fitted trend lines, 1950−2021: Northamptonshire, Ages: 25−29
Data points enclosed with a diamond are reference sources

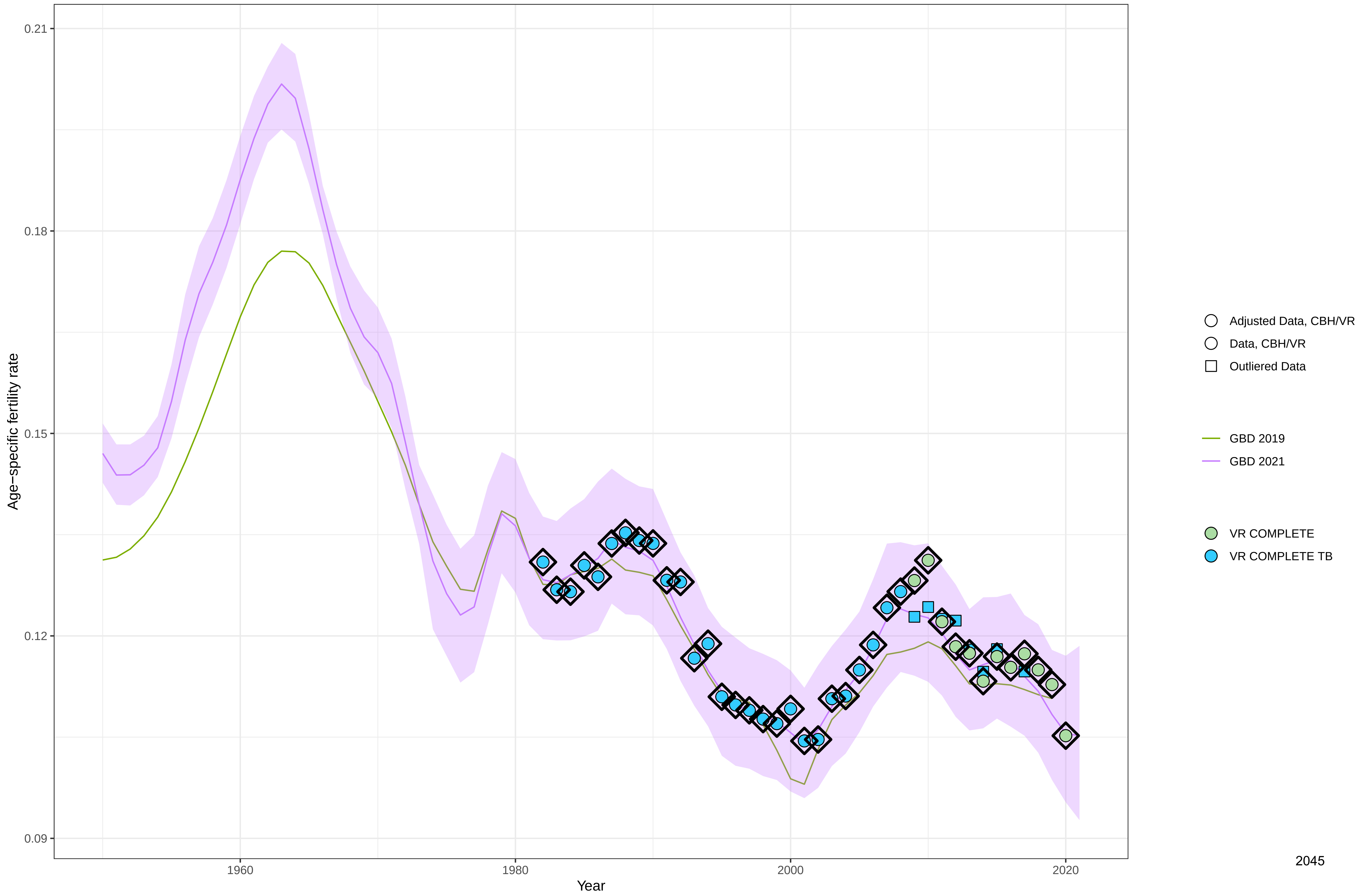

Age−specific fertility fitted trend lines, 1950−2021: Northamptonshire, Ages: 30−34
Data points enclosed with a diamond are reference sources

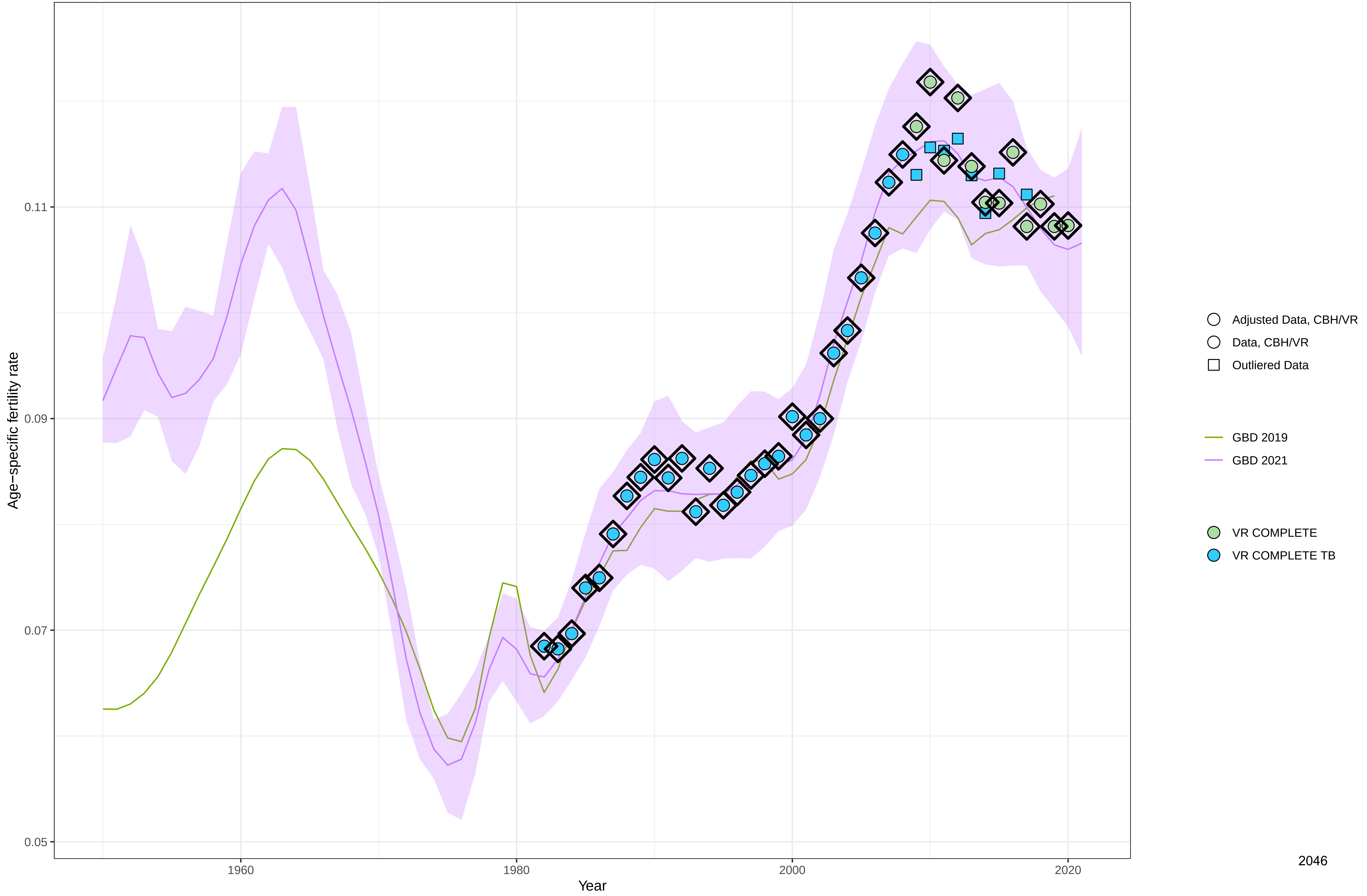

Age−specific fertility fitted trend lines, 1950−2021: Northamptonshire, Ages: 35−39
Data points enclosed with a diamond are reference sources

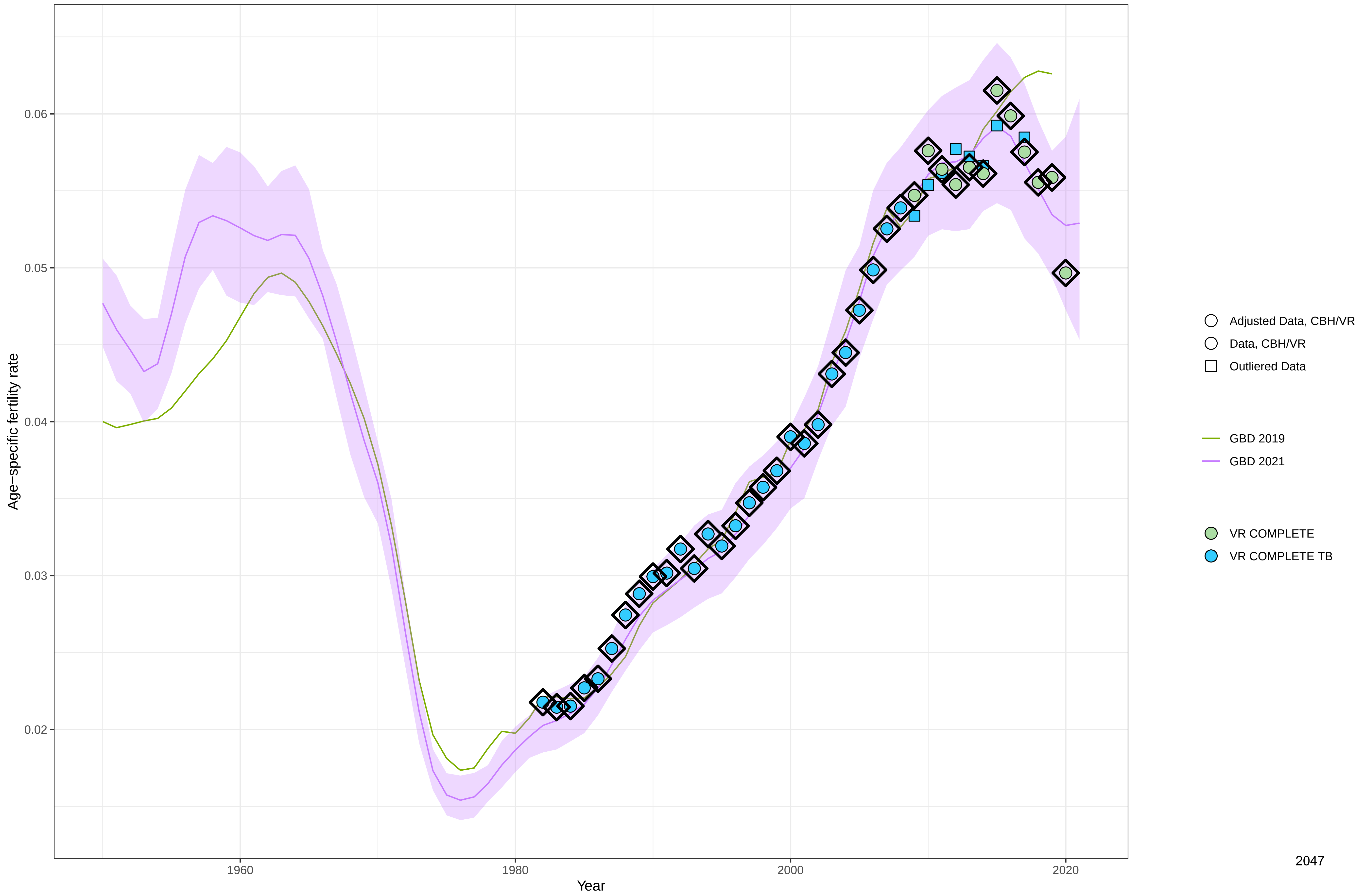

Age−specific fertility fitted trend lines, 1950−2021: Northamptonshire, Ages: 40−44
Data points enclosed with a diamond are reference sources

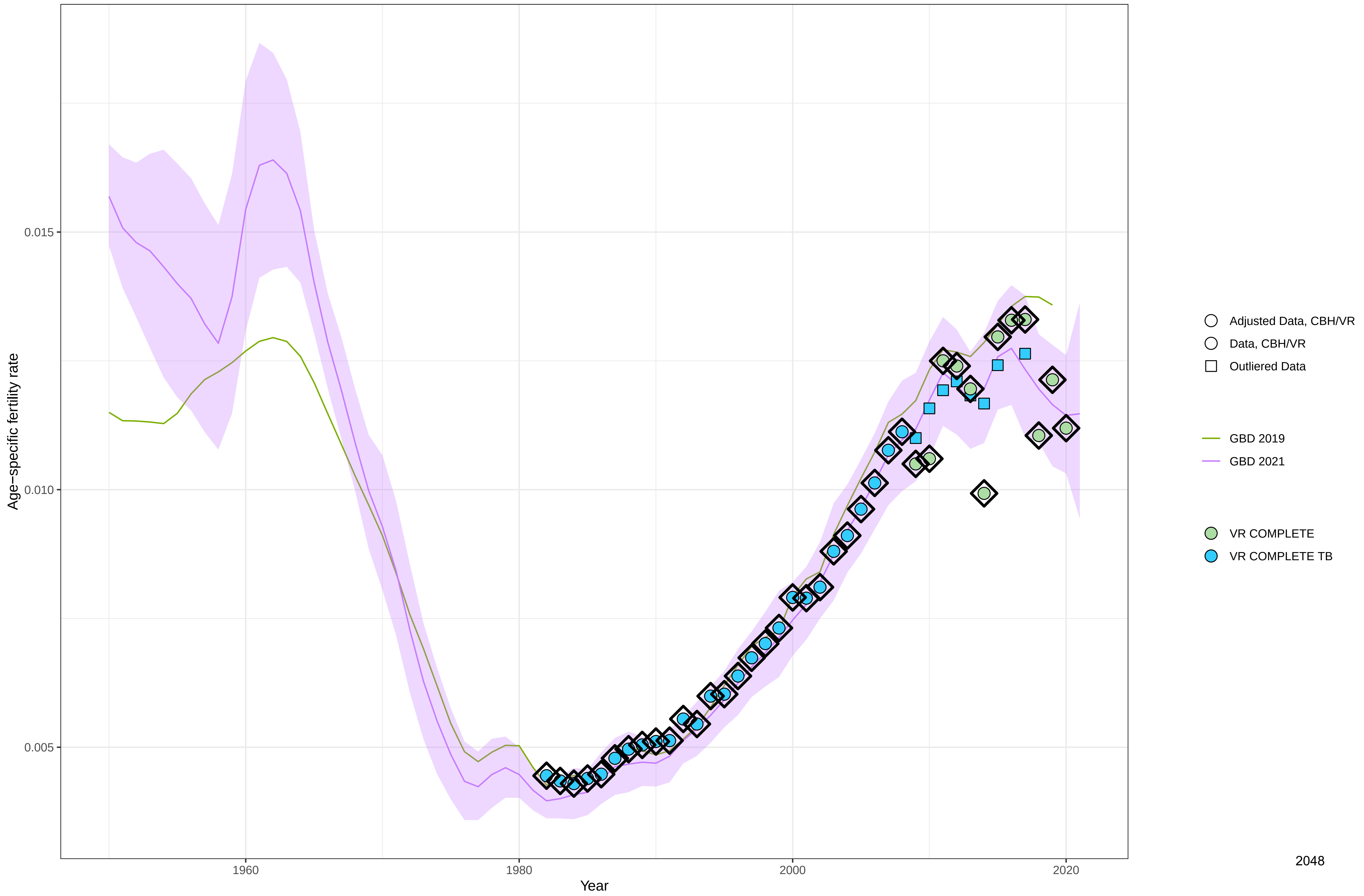

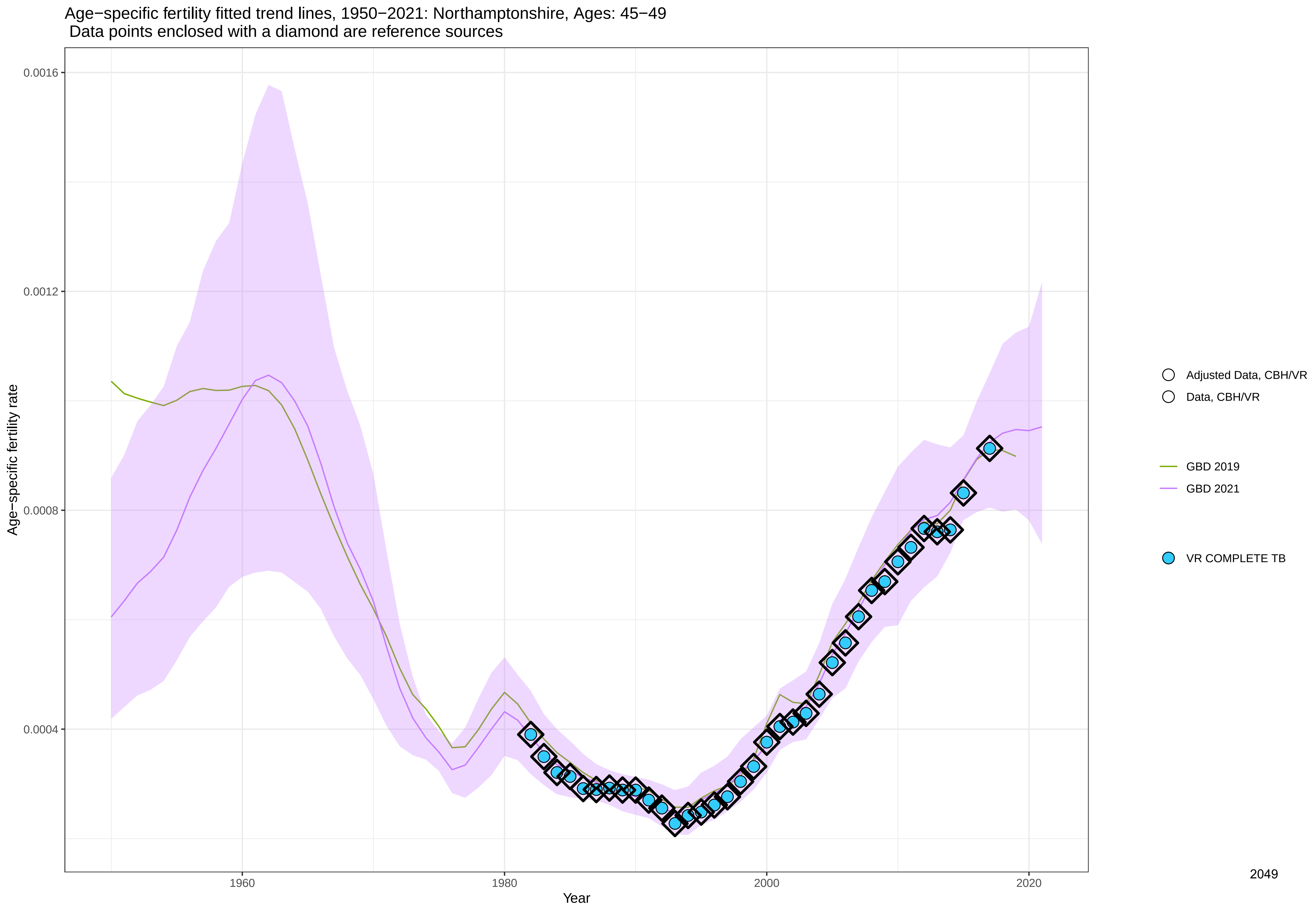

Age−specific fertility fitted trend lines, 1950−2021: Northamptonshire, Ages: 45−49
Data points enclosed with a diamond are reference sources
Age−specific fertility rate
0.0016
0.0012
0.0008
0.0004
1960
1980
2000
2020
Year
Adjusted Data, CBH/VR
Data, CBH/VR
GBD 2019
GBD 2021
VR COMPLETE TB

Age−specific fertility fitted trend lines, 1950−2021: Northamptonshire, Ages: 50−54
Data points enclosed with a diamond are reference sources

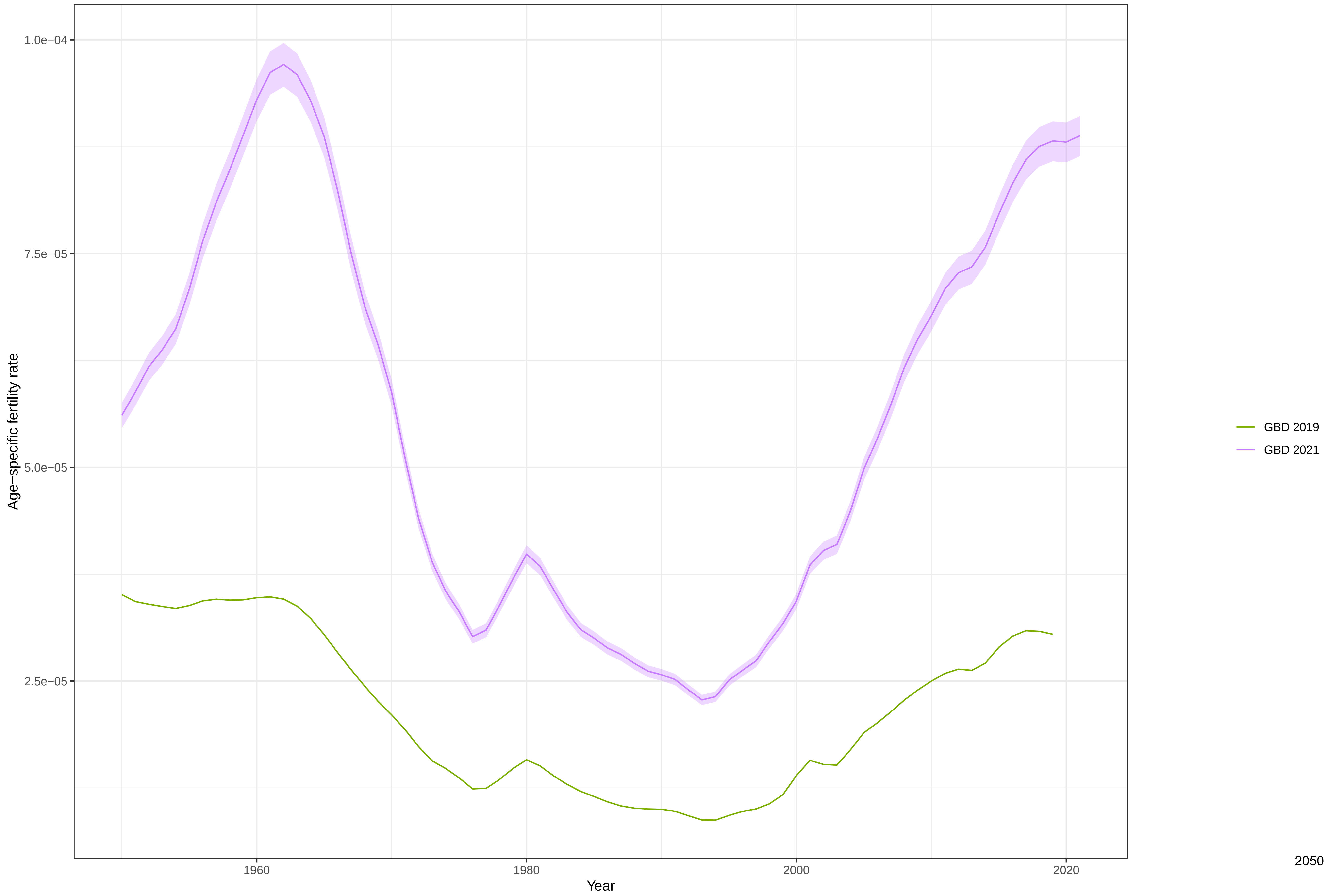

2050

Total fertility rate fitted trend lines, 1950−2021: Northamptonshire TFR
Data points enclosed with a diamond are reference sources

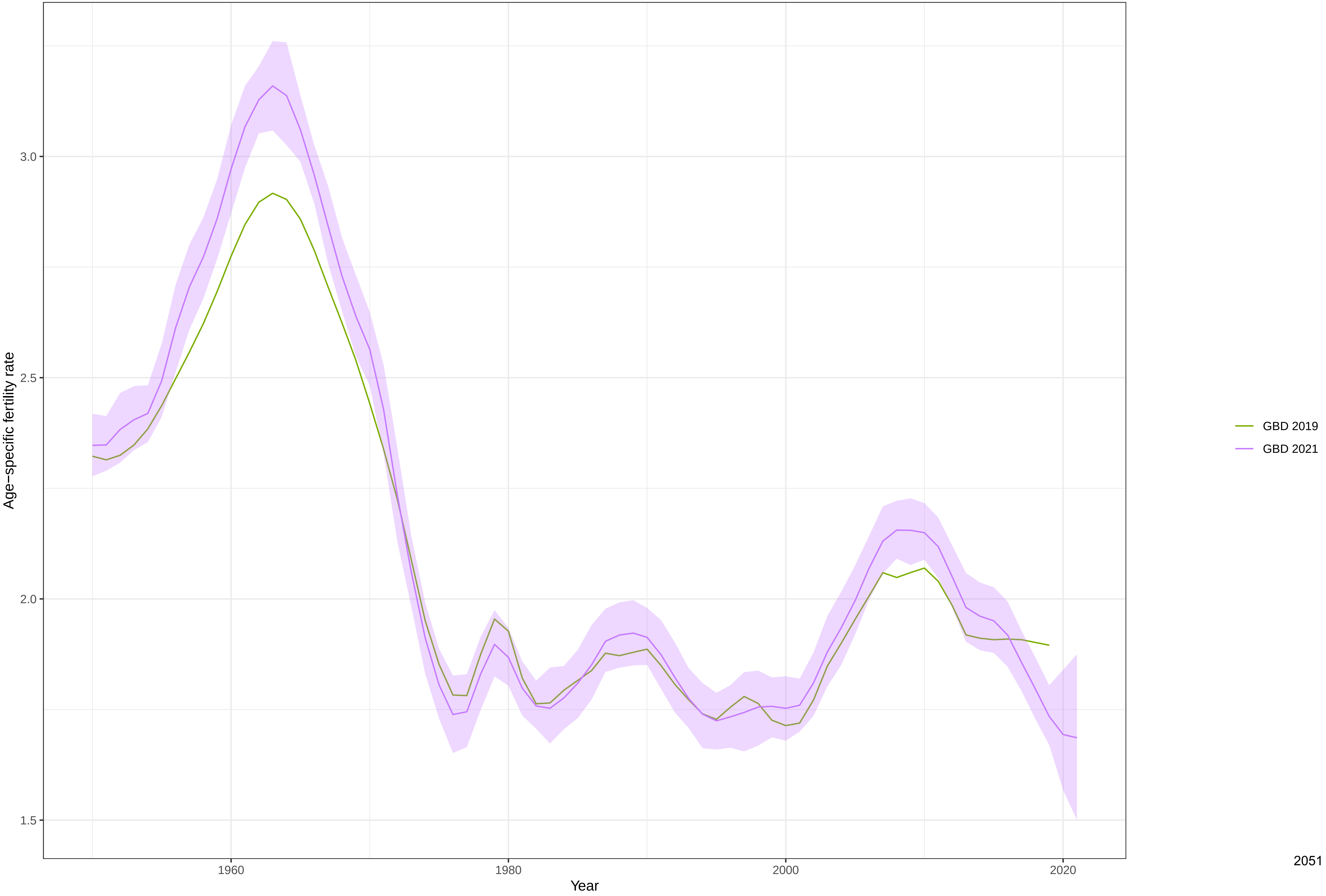

Age−specific fertility fitted trend lines, 1950−2021: Nottingham, Ages: 10−14
Data points enclosed with a diamond are reference sources

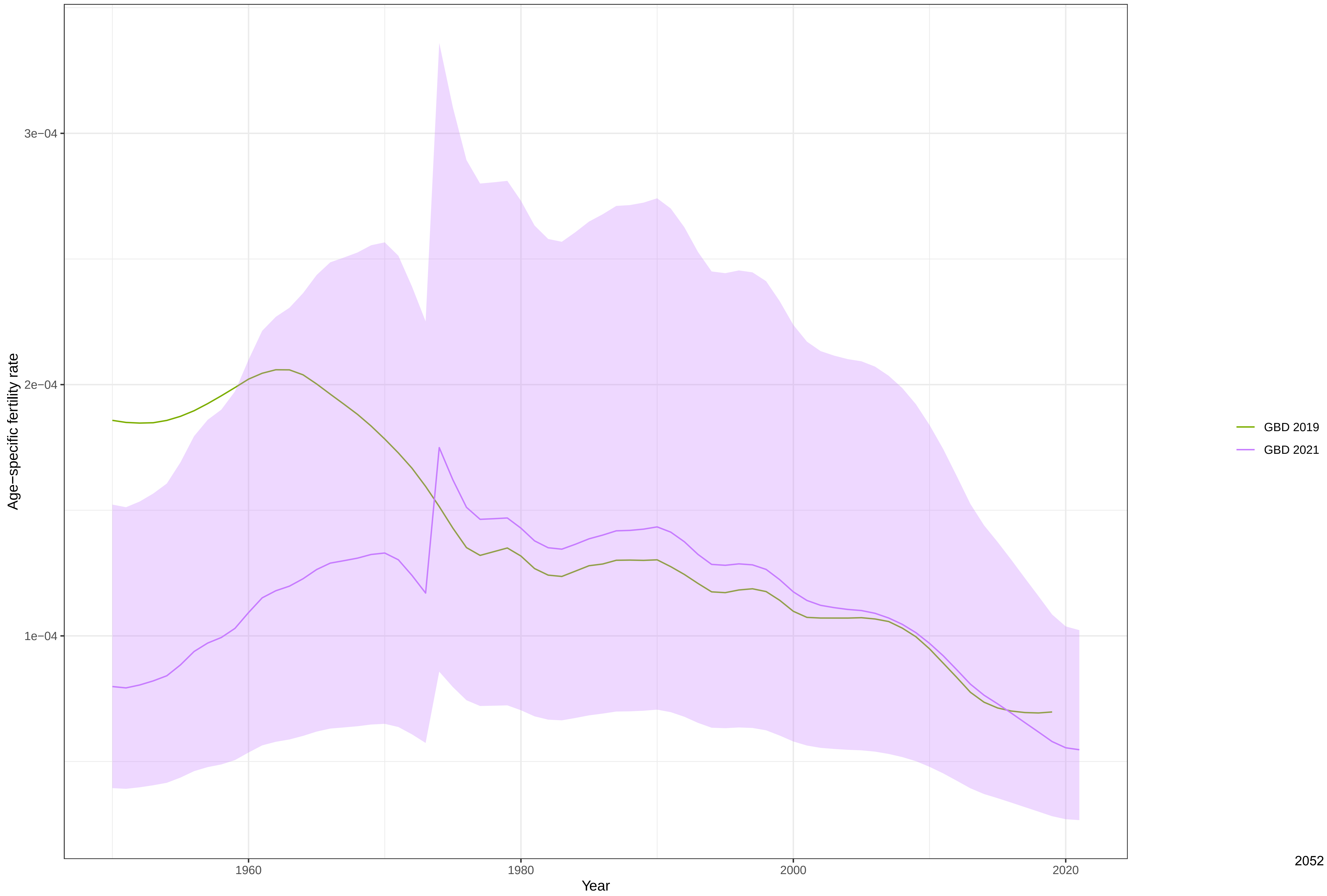

Age−specific fertility fitted trend lines, 1950−2021: Nottingham, Ages: 15−19
Data points enclosed with a diamond are reference sources

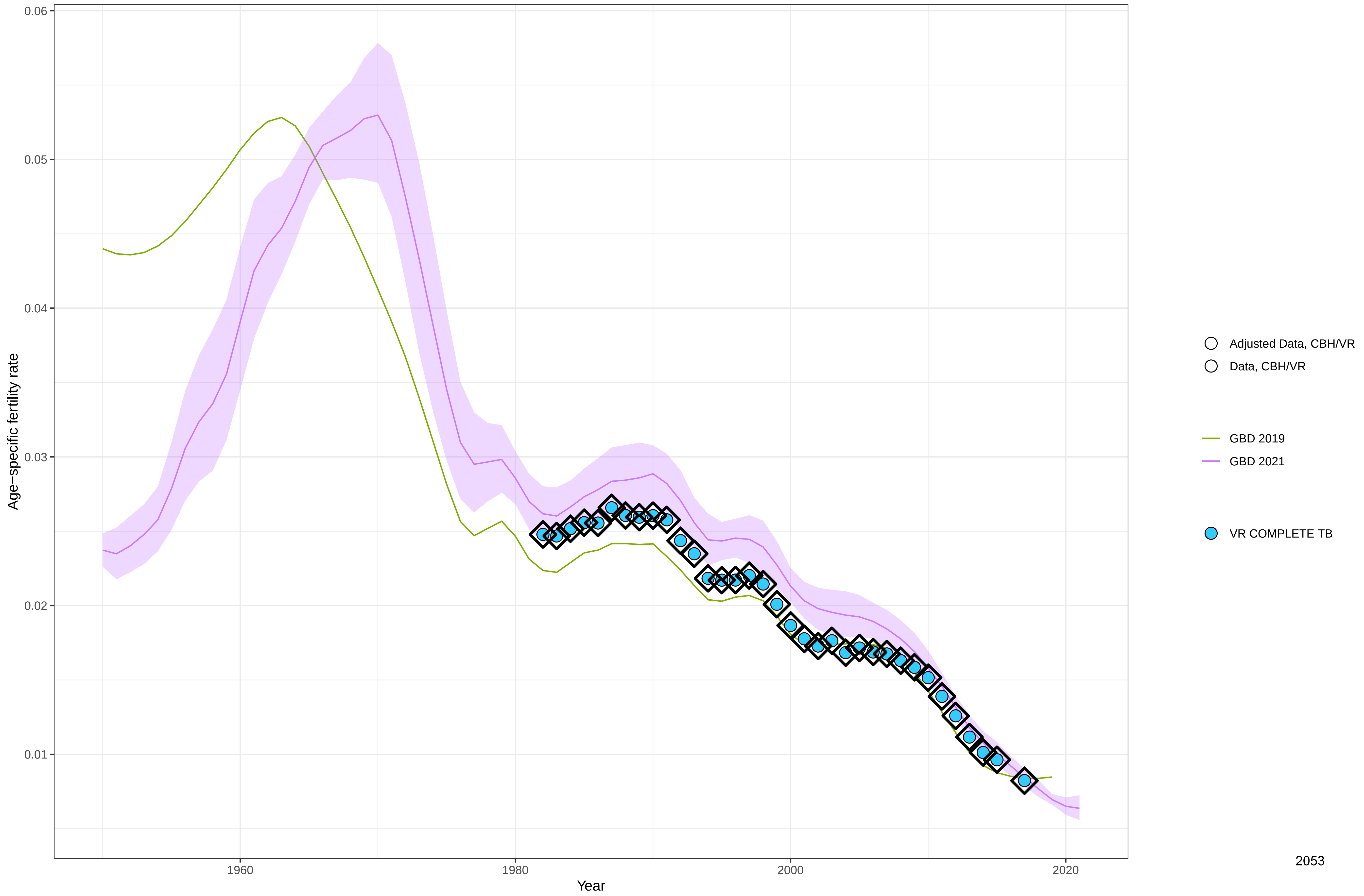

Age−specific fertility fitted trend lines, 1950−2021: Nottingham, Ages: 20−24
Data points enclosed with a diamond are reference sources

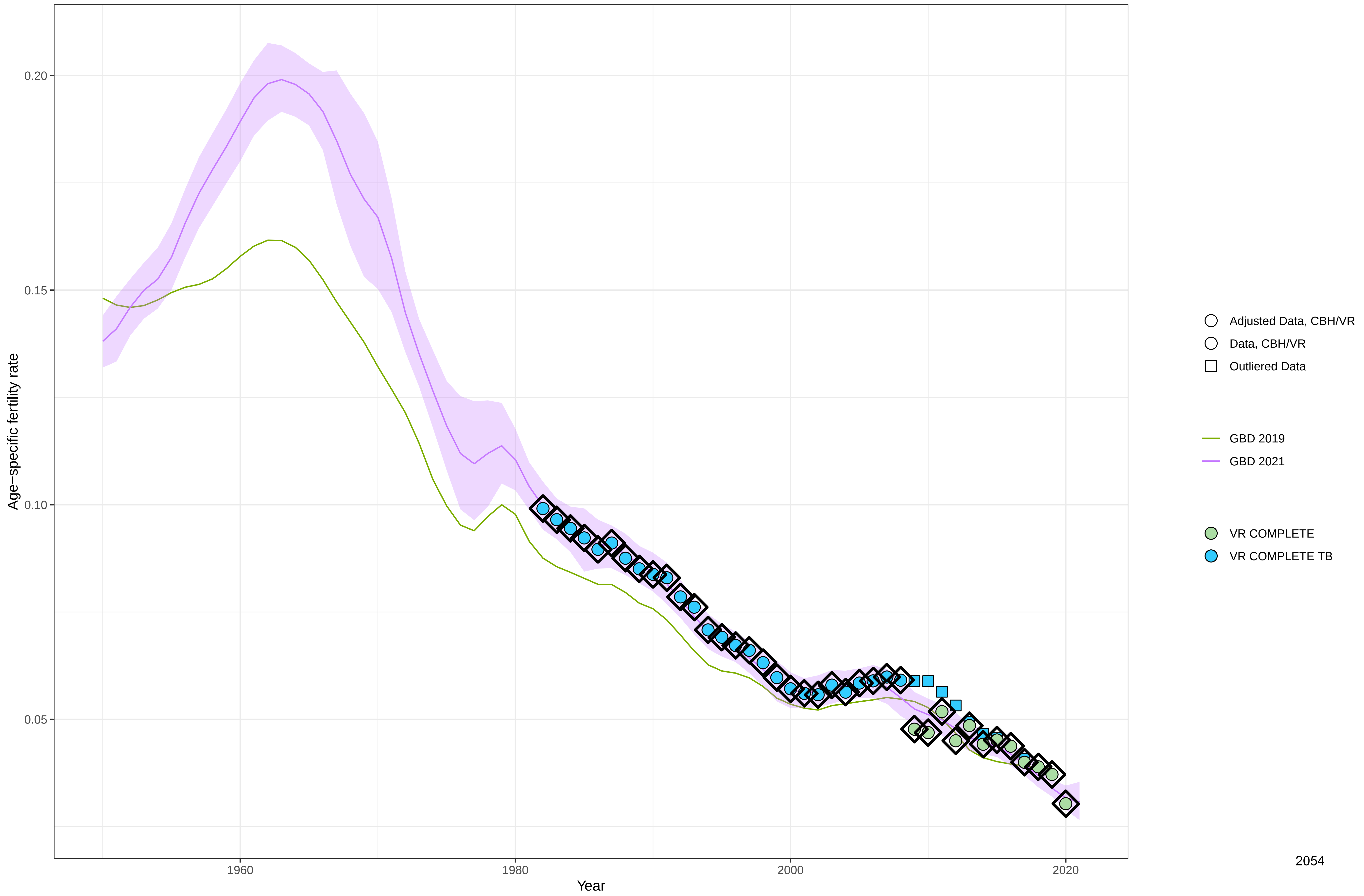

Age−specific fertility fitted trend lines, 1950−2021: Nottingham, Ages: 25−29
Data points enclosed with a diamond are reference sources

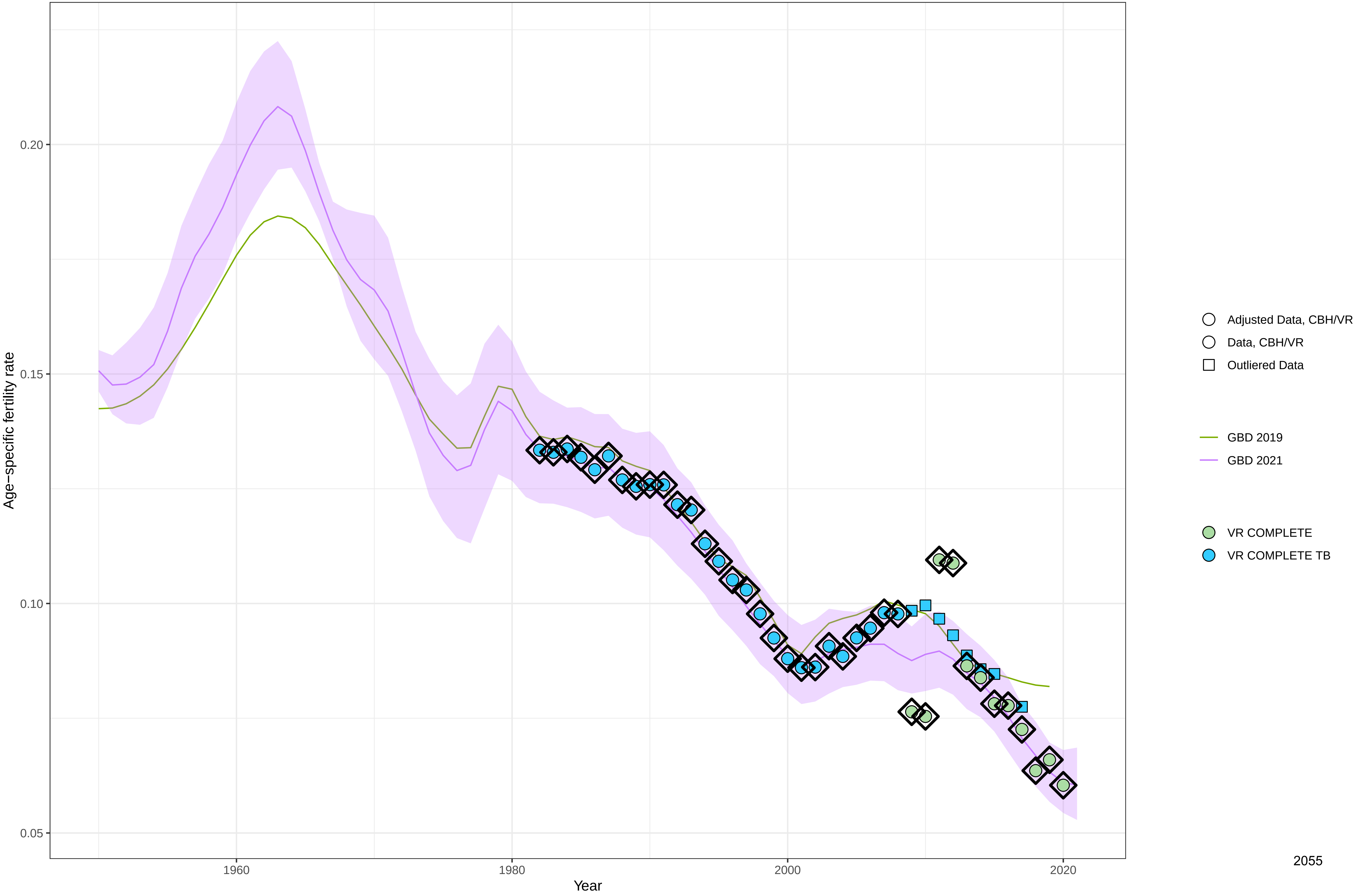

Age−specific fertility fitted trend lines, 1950−2021: Nottingham, Ages: 30−34
Data points enclosed with a diamond are reference sources

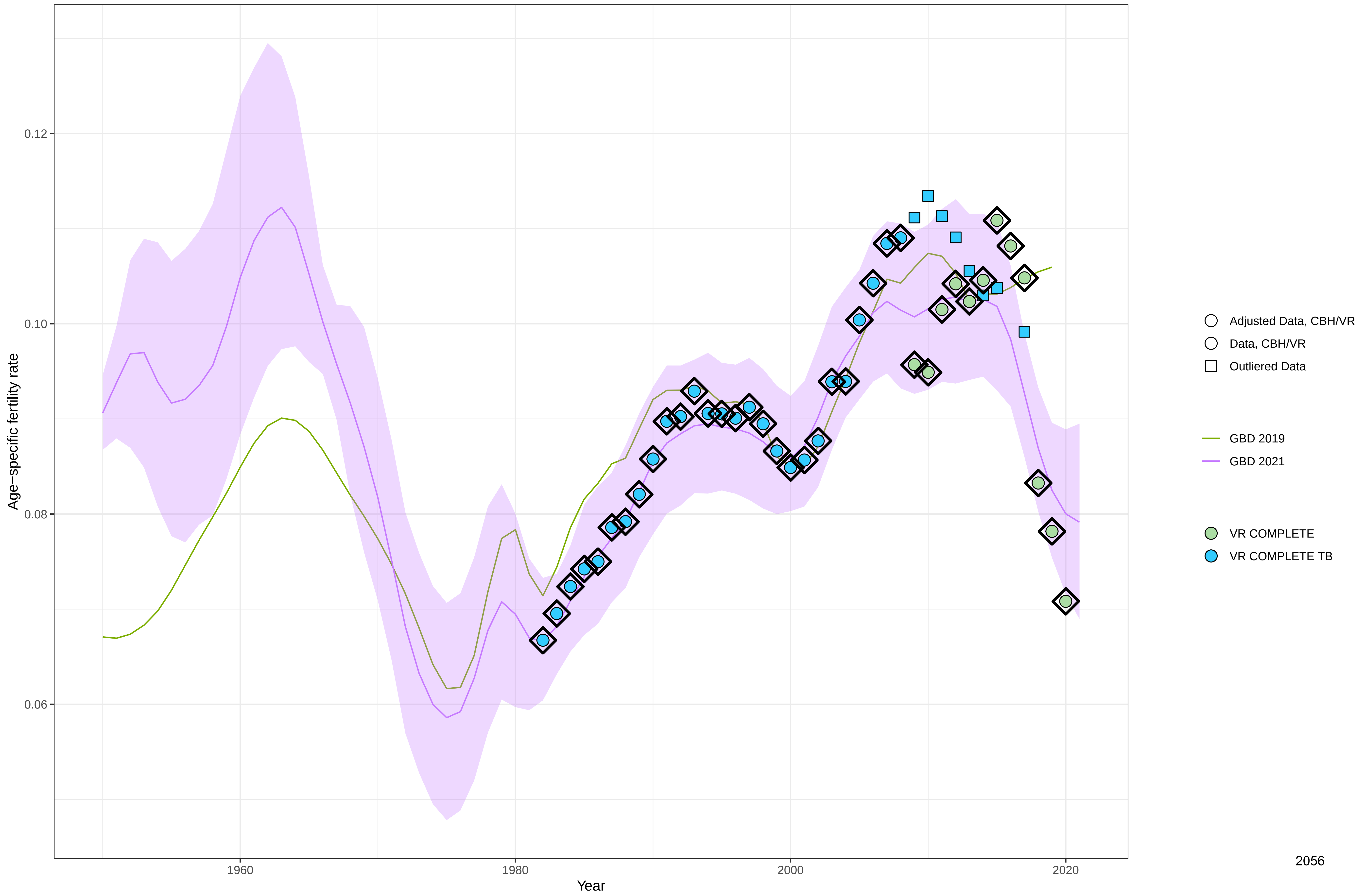

Age−specific fertility fitted trend lines, 1950−2021: Nottingham, Ages: 35−39
Data points enclosed with a diamond are reference sources

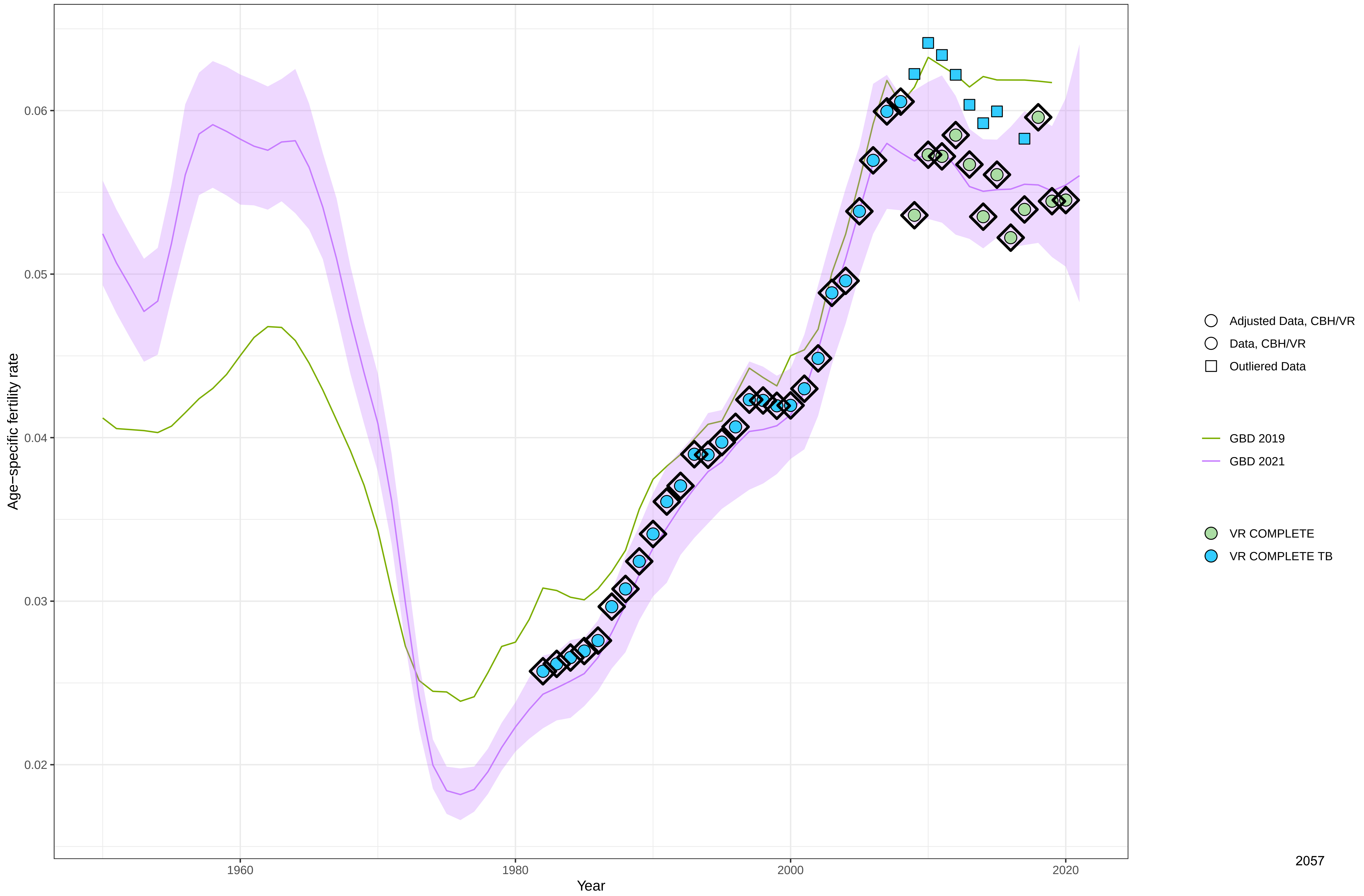

Age−specific fertility fitted trend lines, 1950−2021: Nottingham, Ages: 40−44
Data points enclosed with a diamond are reference sources

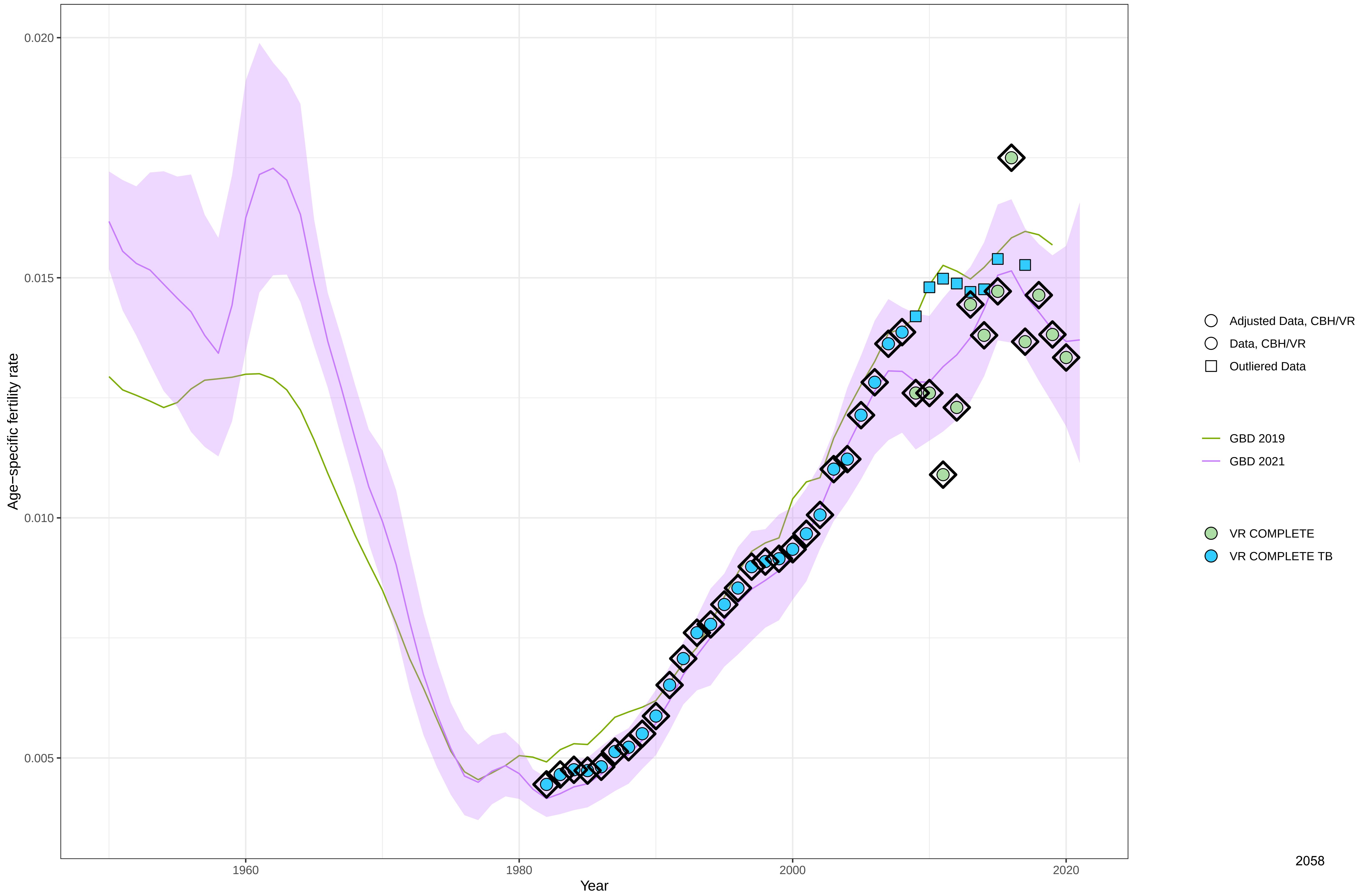

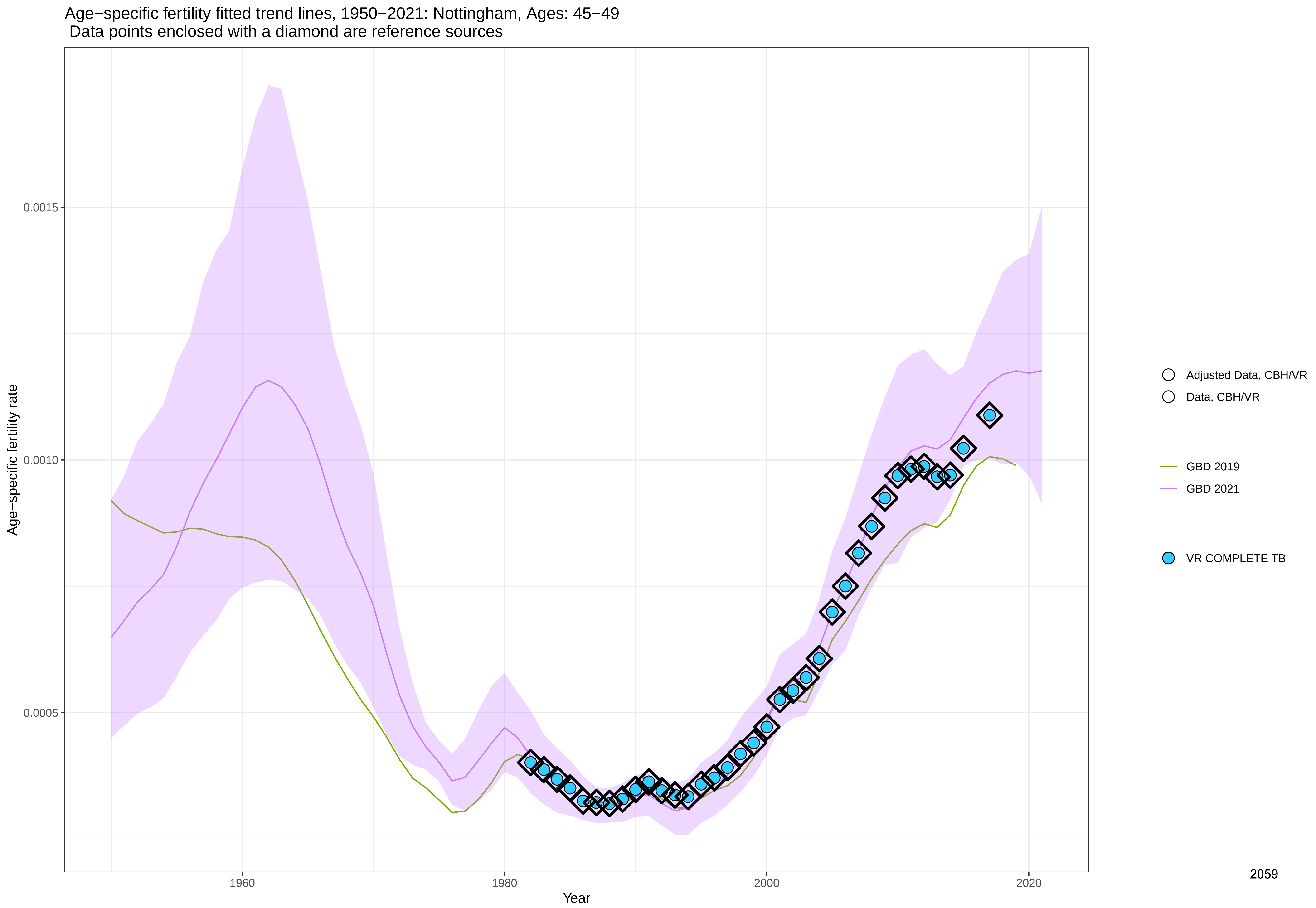
Age−specific fertility fitted trend lines, 1950−2021: Nottingham, Ages: 45−49
Data points enclosed with a diamond are reference sources
Age−specific fertility rate
0.0015
0.0010
0.0005
1960
1980
2000
2020
Year
Adjusted Data, CBH/VR
Data, CBH/VR
GBD 2019
GBD 2021
VR COMPLETE TB

Age−specific fertility fitted trend lines, 1950−2021: Nottingham, Ages: 50−54
Data points enclosed with a diamond are reference sources

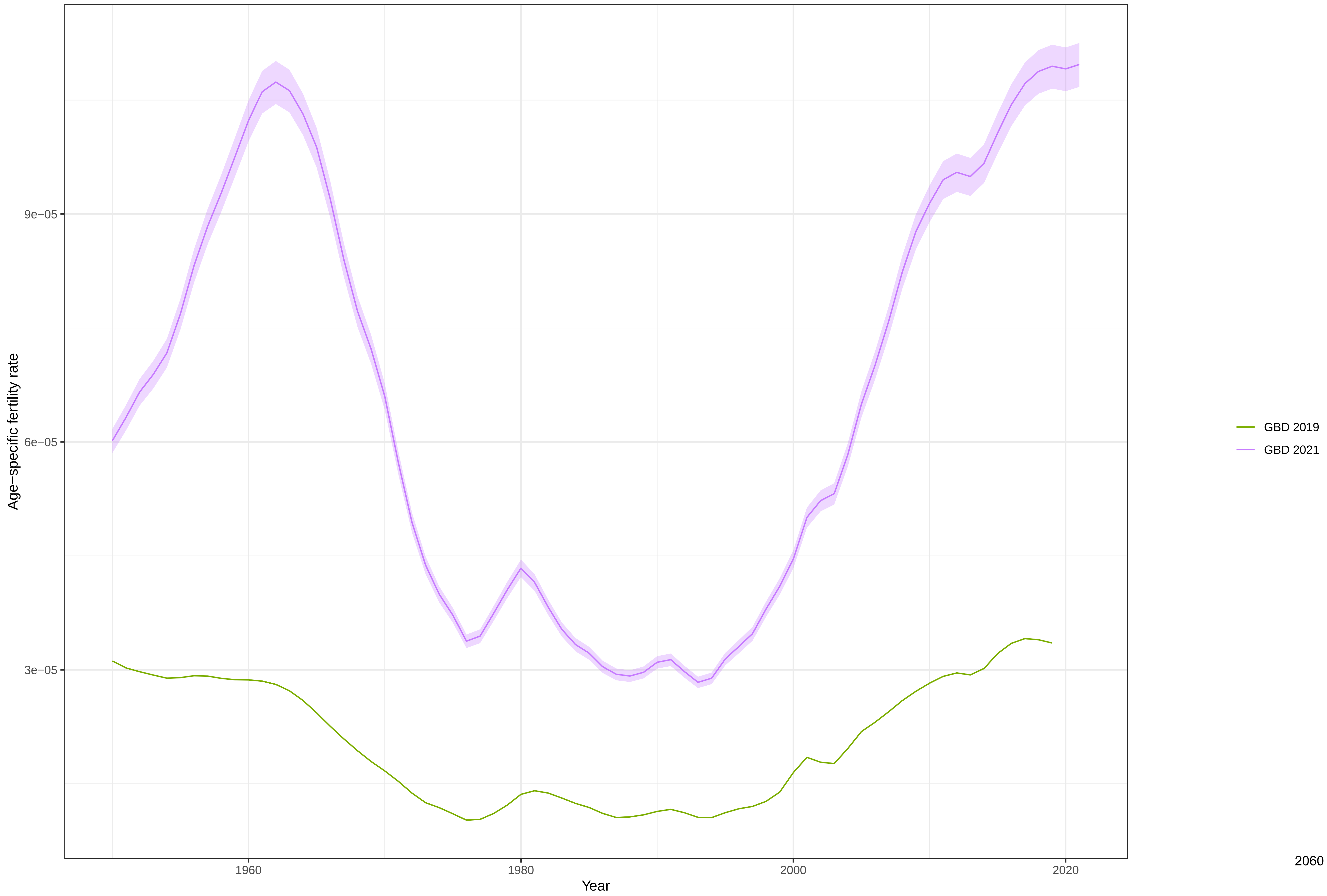

2060

Total fertility rate fitted trend lines, 1950−2021: Nottingham TFR
Data points enclosed with a diamond are reference sources

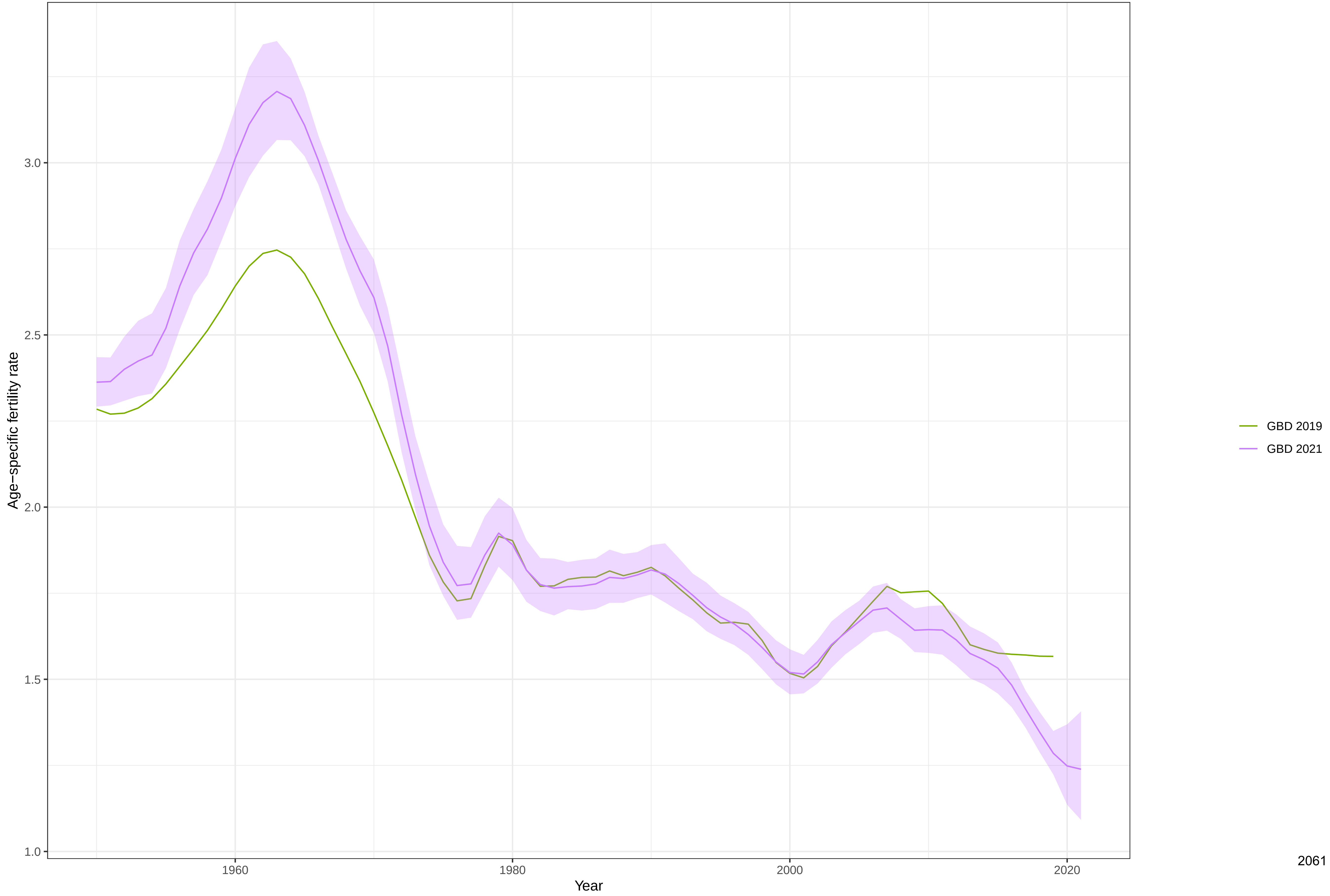

Age−specific fertility fitted trend lines, 1950−2021: Nottinghamshire, Ages: 10−14
Data points enclosed with a diamond are reference sources

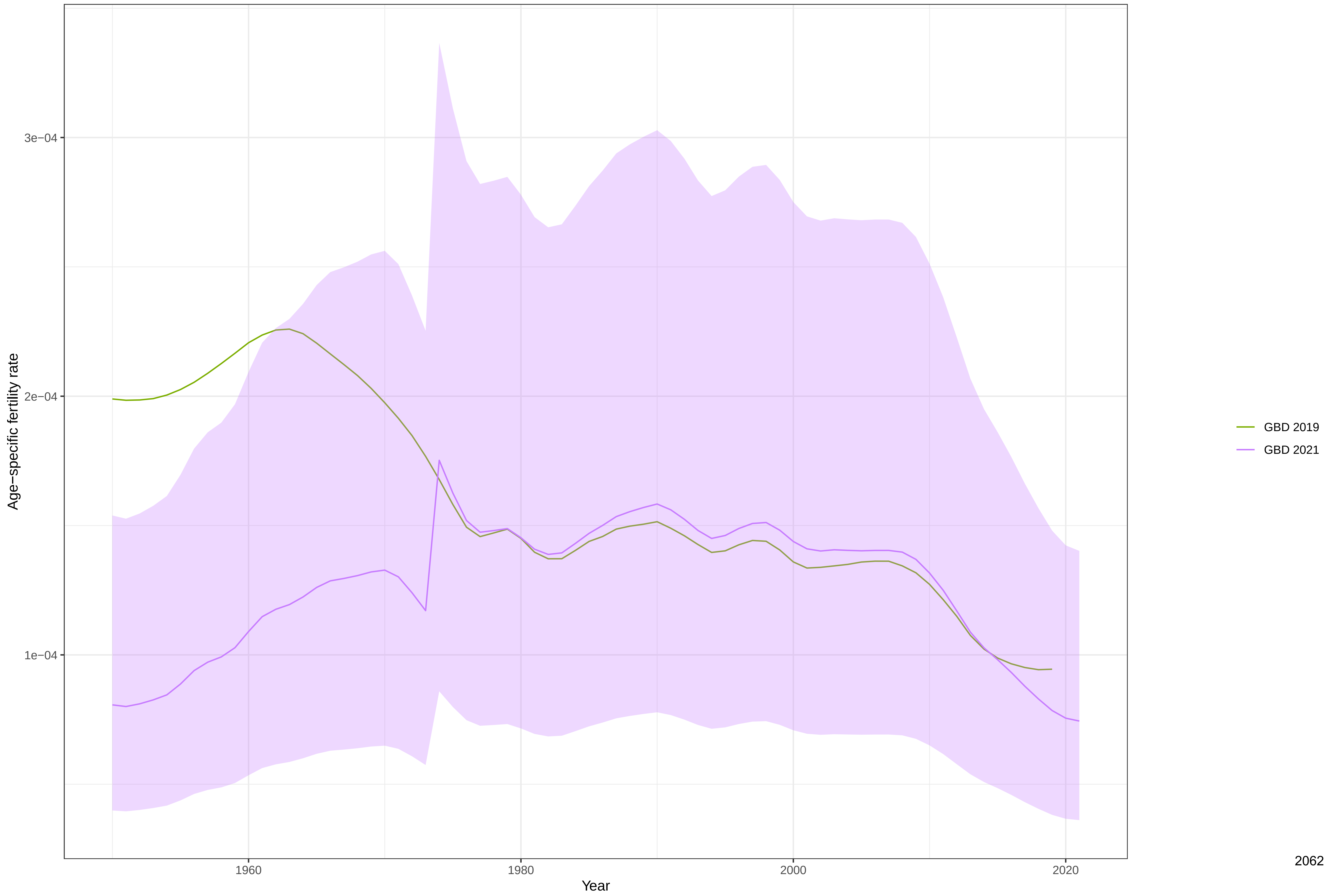

Age−specific fertility fitted trend lines, 1950−2021: Nottinghamshire, Ages: 15−19
Data points enclosed with a diamond are reference sources

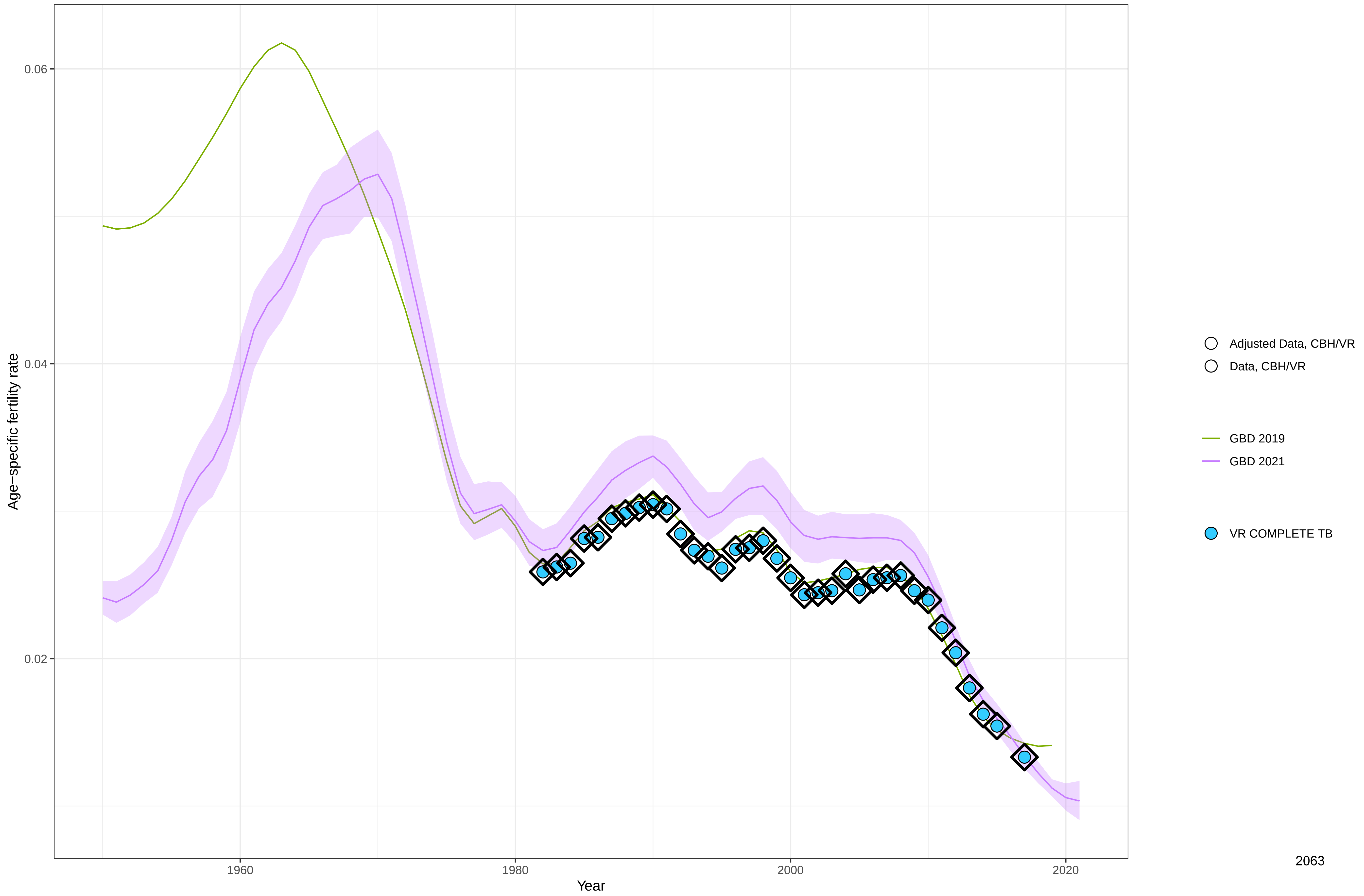

Age−specific fertility fitted trend lines, 1950−2021: Nottinghamshire, Ages: 20−24
Data points enclosed with a diamond are reference sources

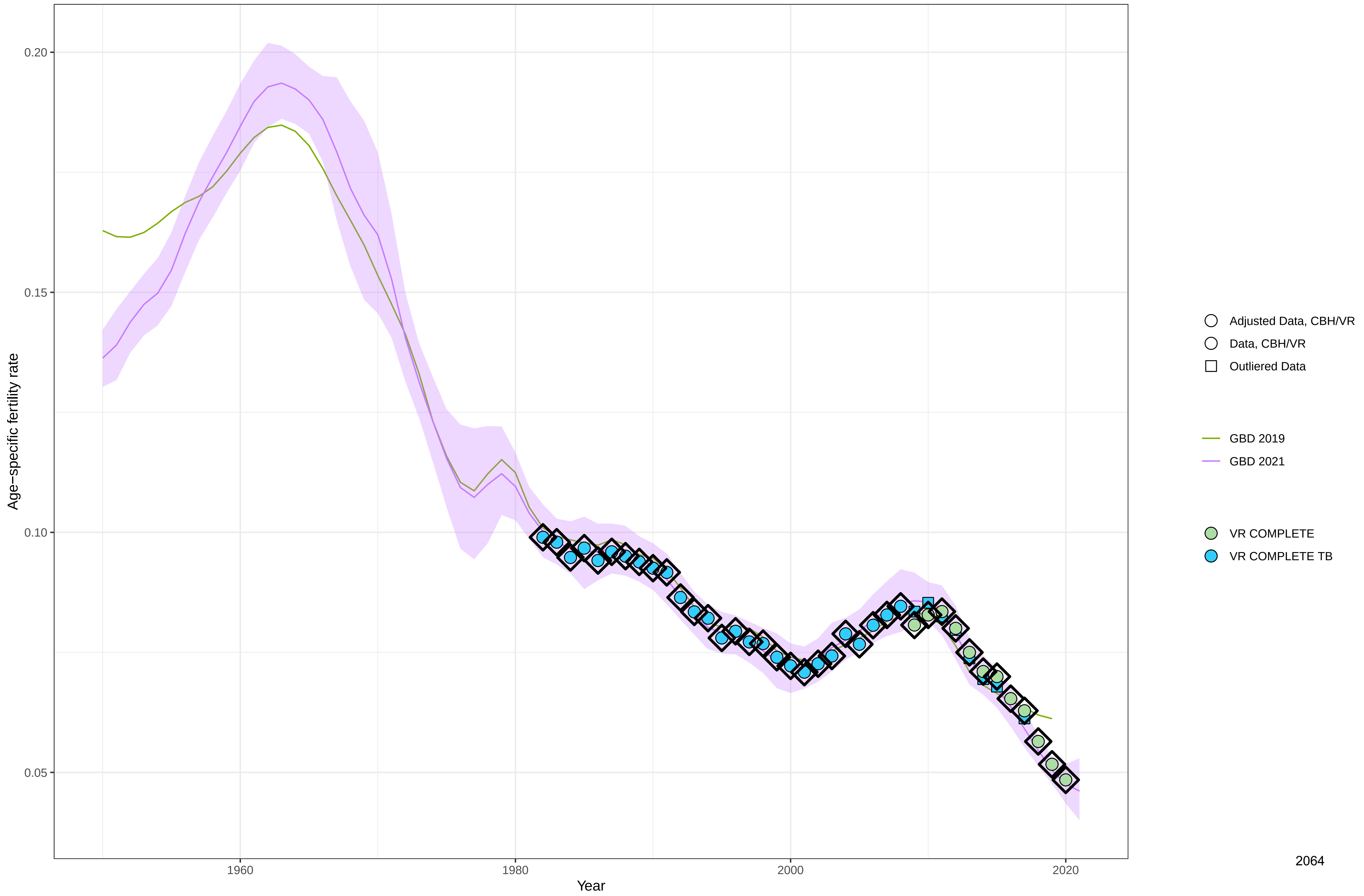

2064

Age−specific fertility fitted trend lines, 1950−2021: Nottinghamshire, Ages: 25−29
Data points enclosed with a diamond are reference sources

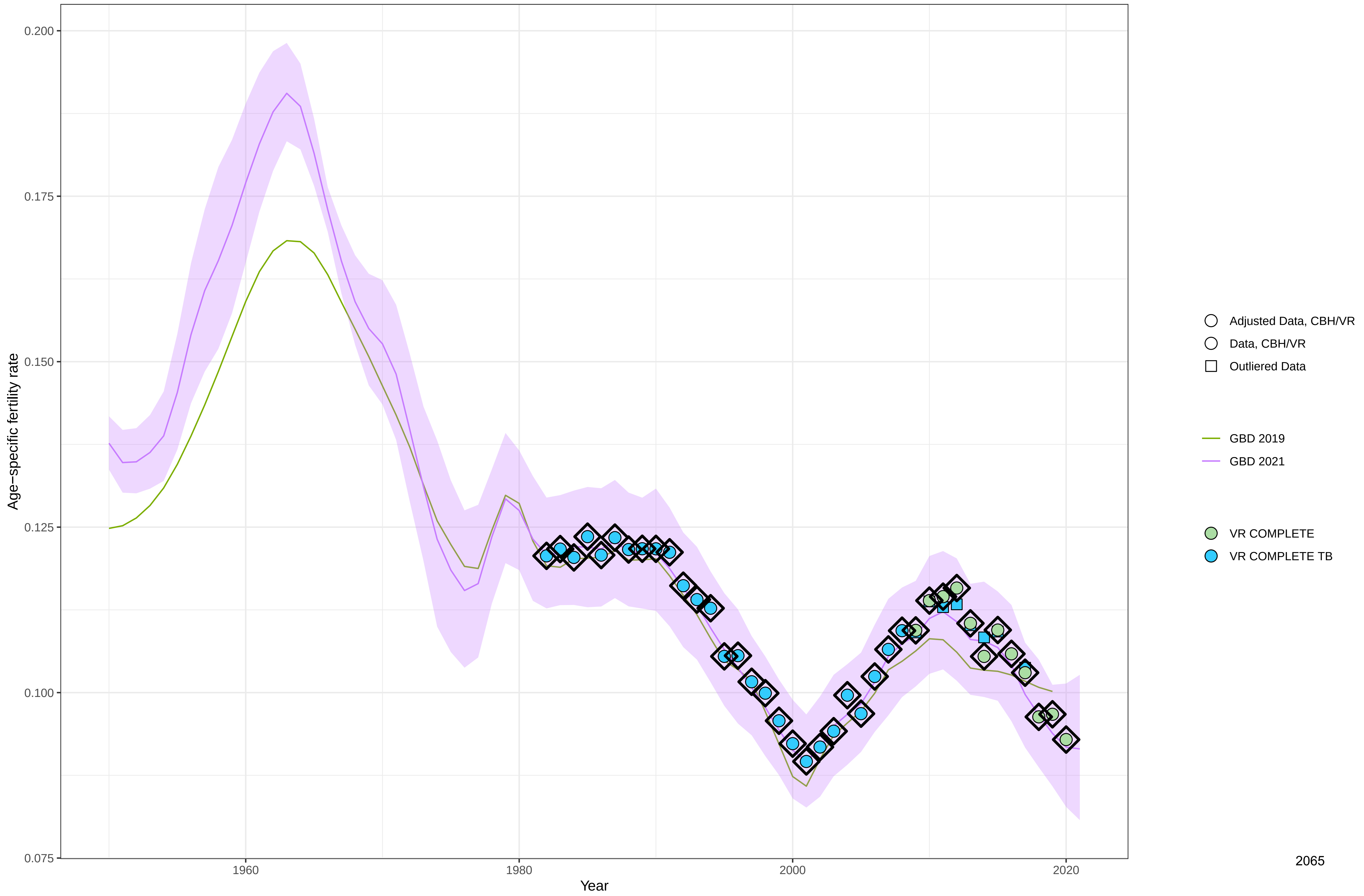

Age−specific fertility fitted trend lines, 1950−2021: Nottinghamshire, Ages: 30−34
Data points enclosed with a diamond are reference sources

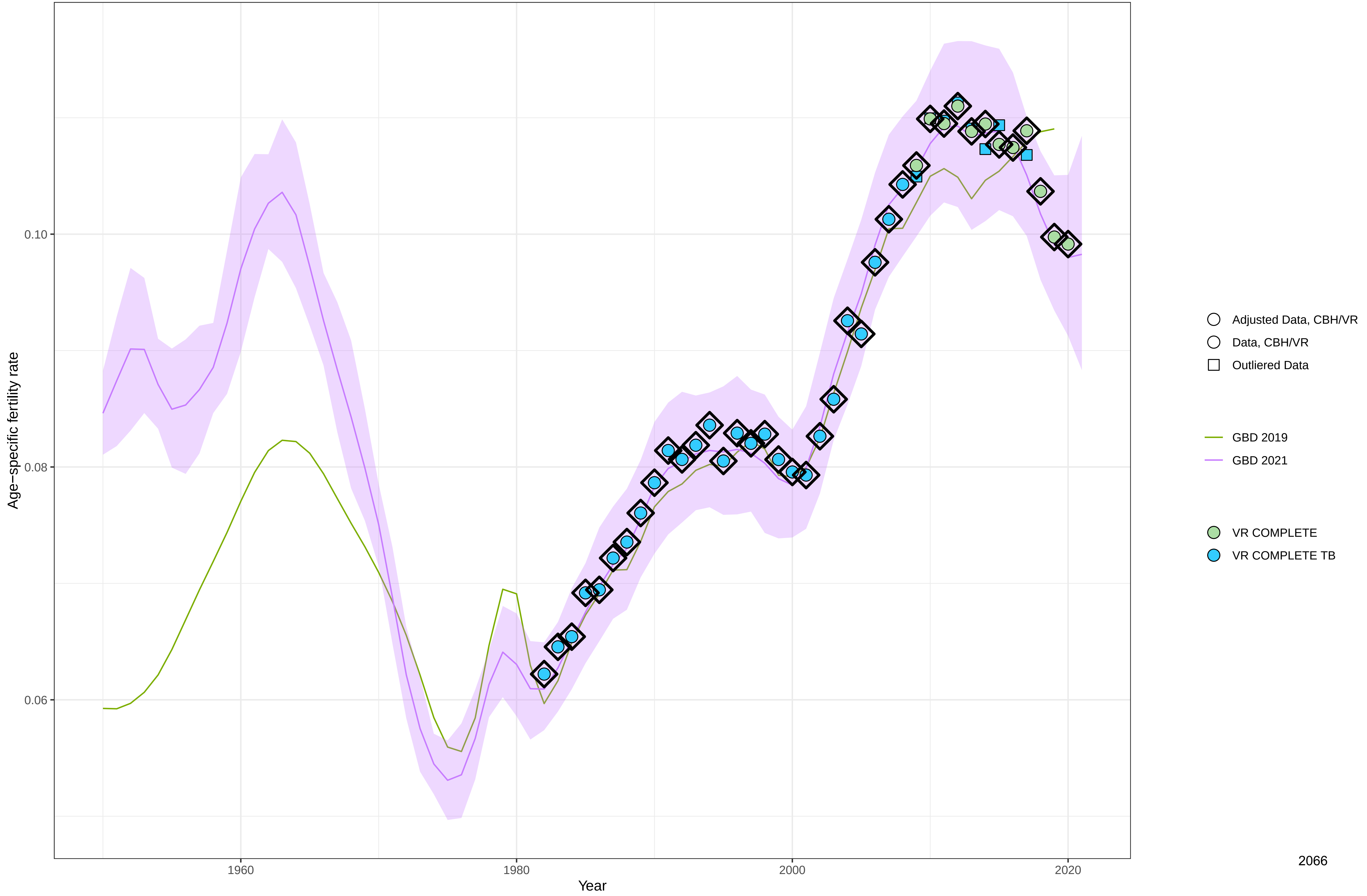

Age−specific fertility fitted trend lines, 1950−2021: Nottinghamshire, Ages: 35−39
Data points enclosed with a diamond are reference sources

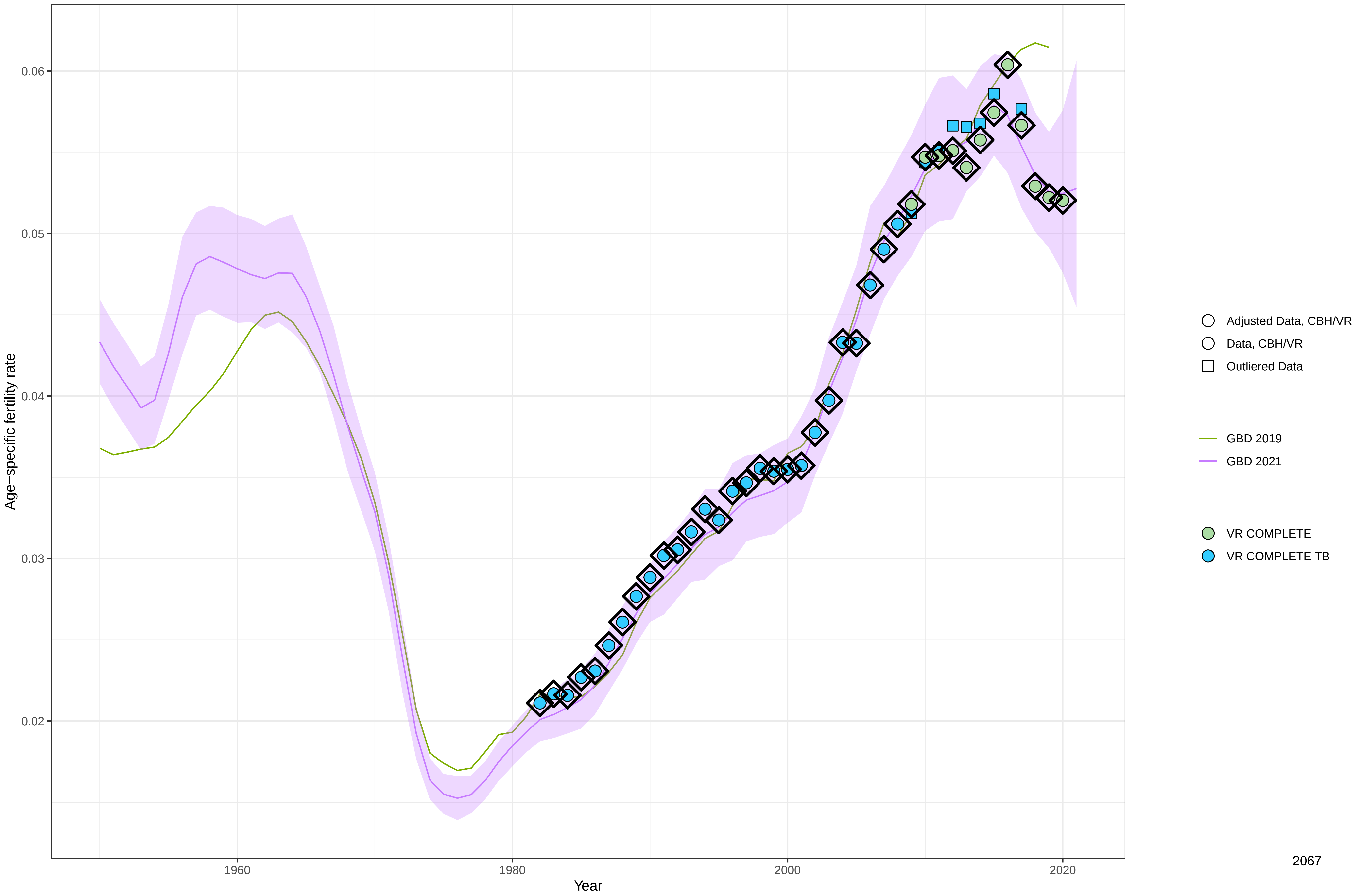

Age−specific fertility fitted trend lines, 1950−2021: Nottinghamshire, Ages: 40−44
Data points enclosed with a diamond are reference sources

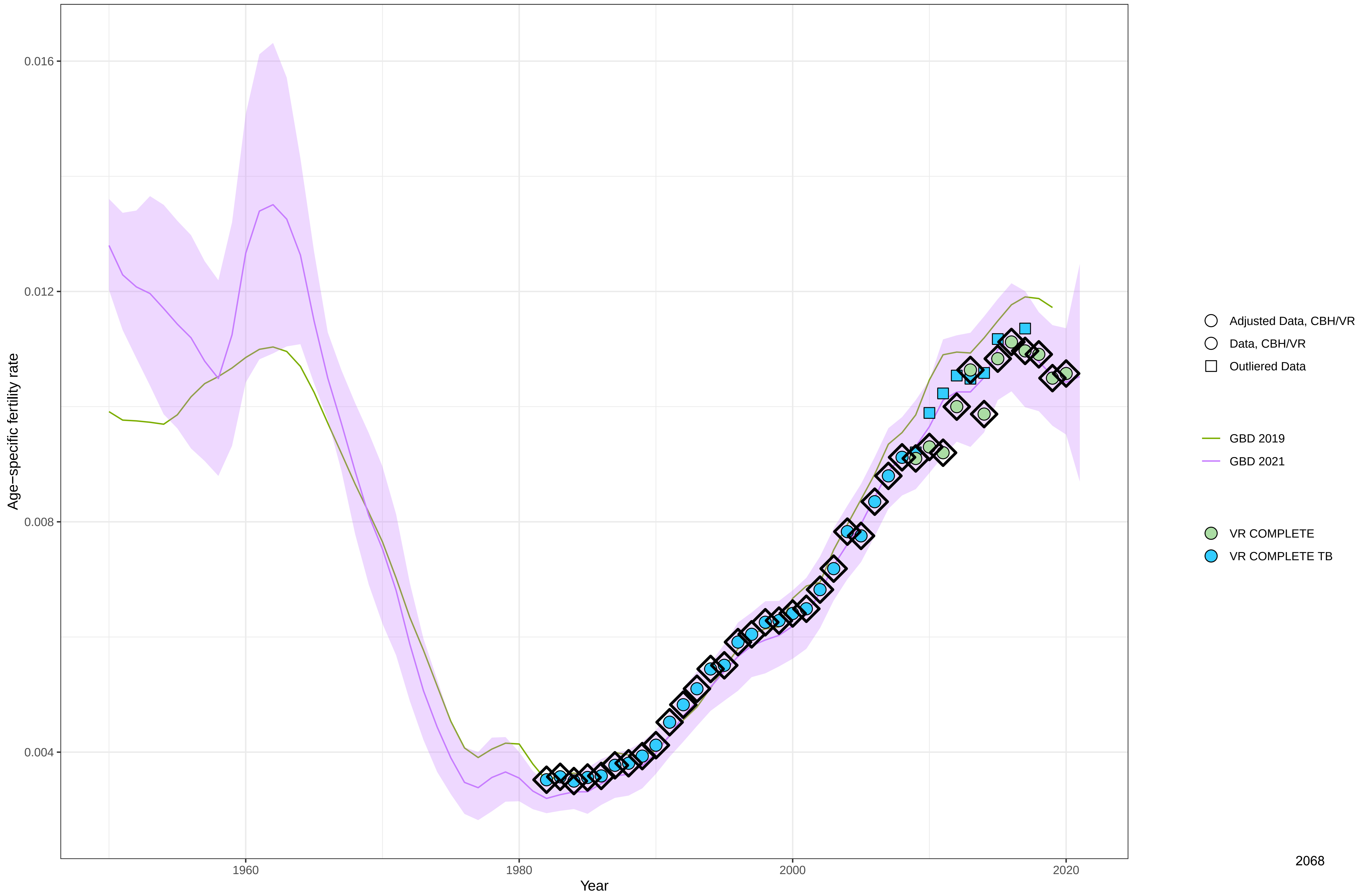

Age−specific fertility fitted trend lines, 1950−2021: Nottinghamshire, Ages: 45−49
Data points enclosed with a diamond are reference sources

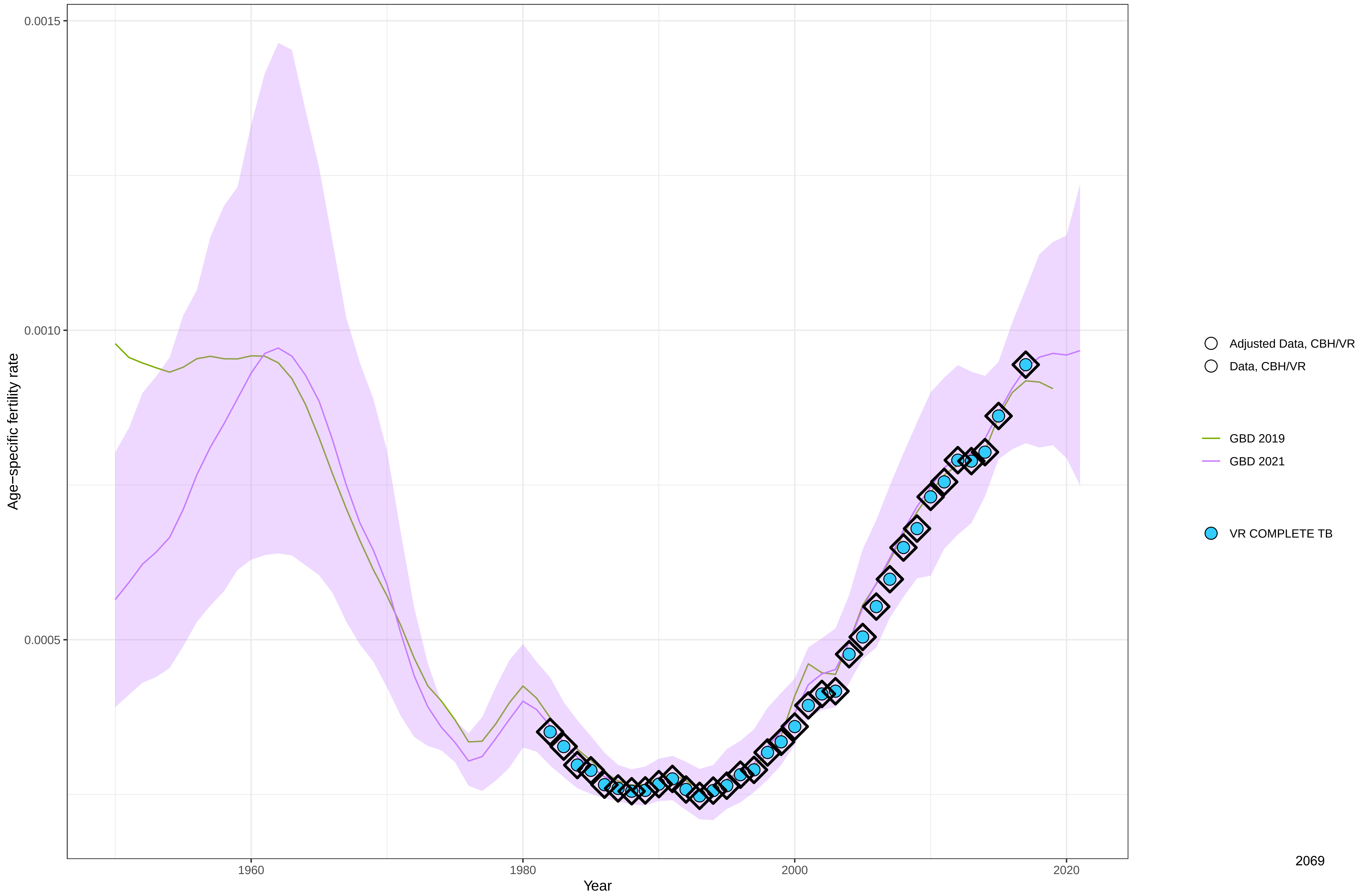

Age−specific fertility fitted trend lines, 1950−2021: Nottinghamshire, Ages: 50−54
Data points enclosed with a diamond are reference sources

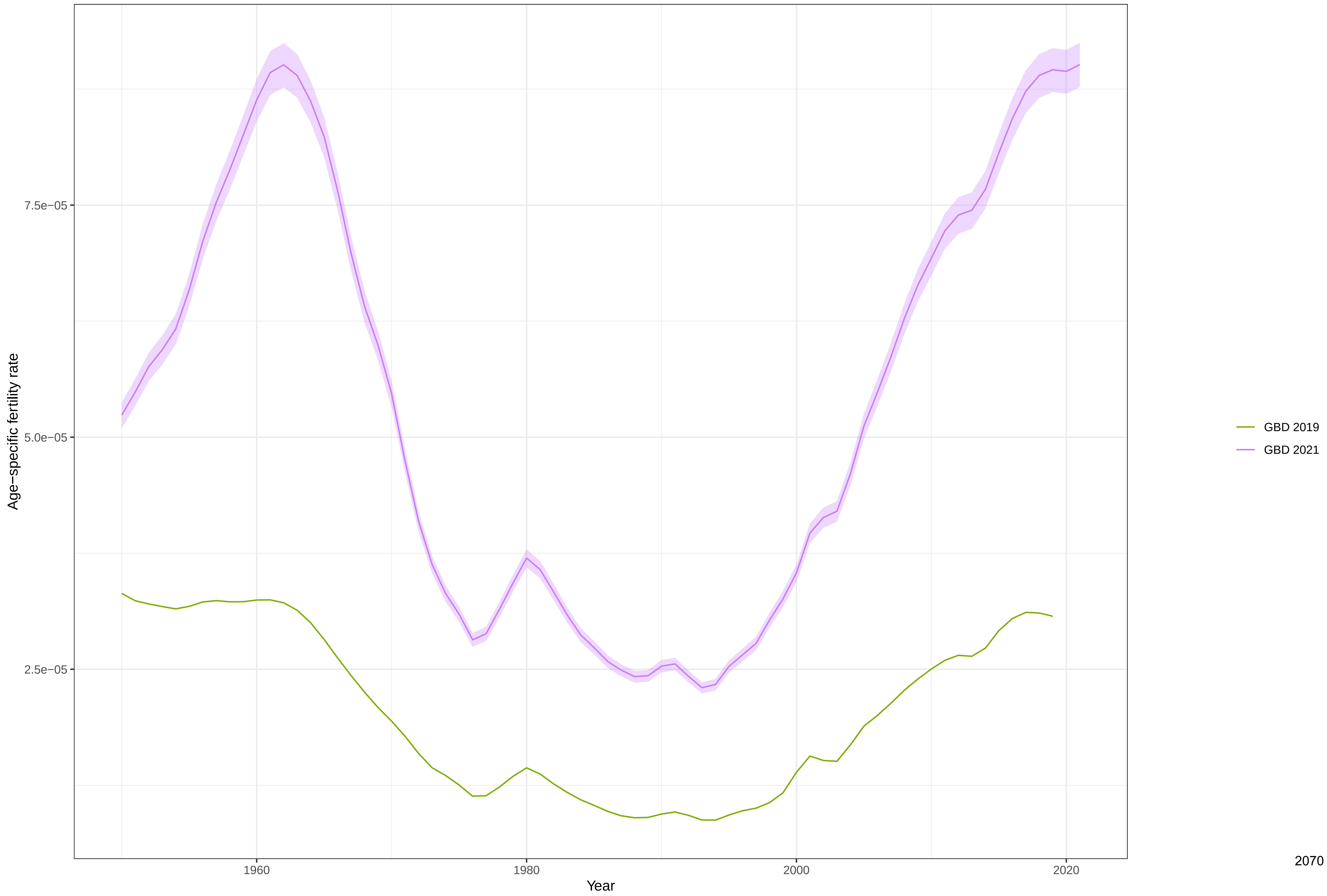

2070

Total fertility rate fitted trend lines, 1950−2021: Nottinghamshire TFR
Data points enclosed with a diamond are reference sources

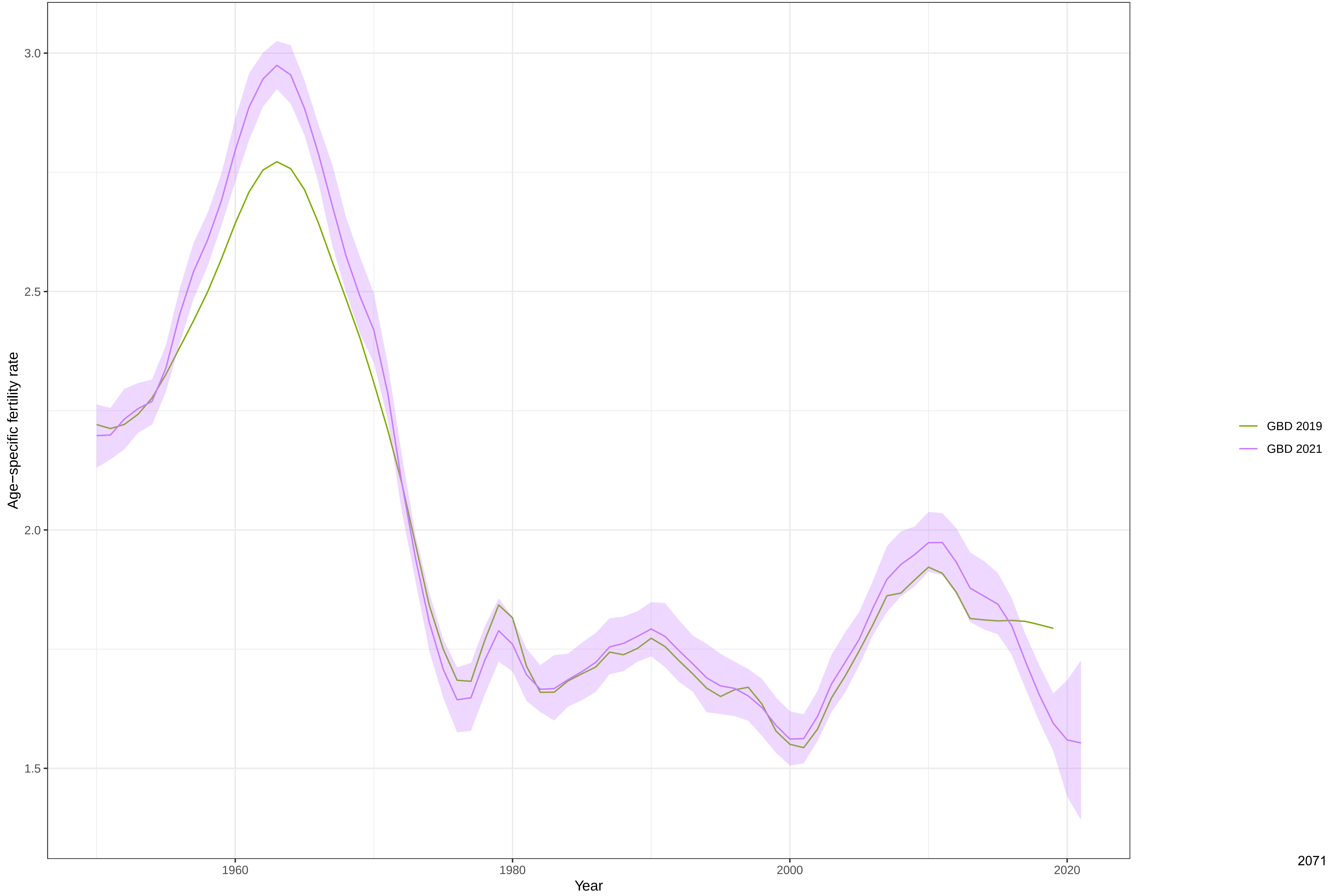

Age−specific fertility fitted trend lines, 1950−2021: Rutland, Ages: 10−14
Data points enclosed with a diamond are reference sources

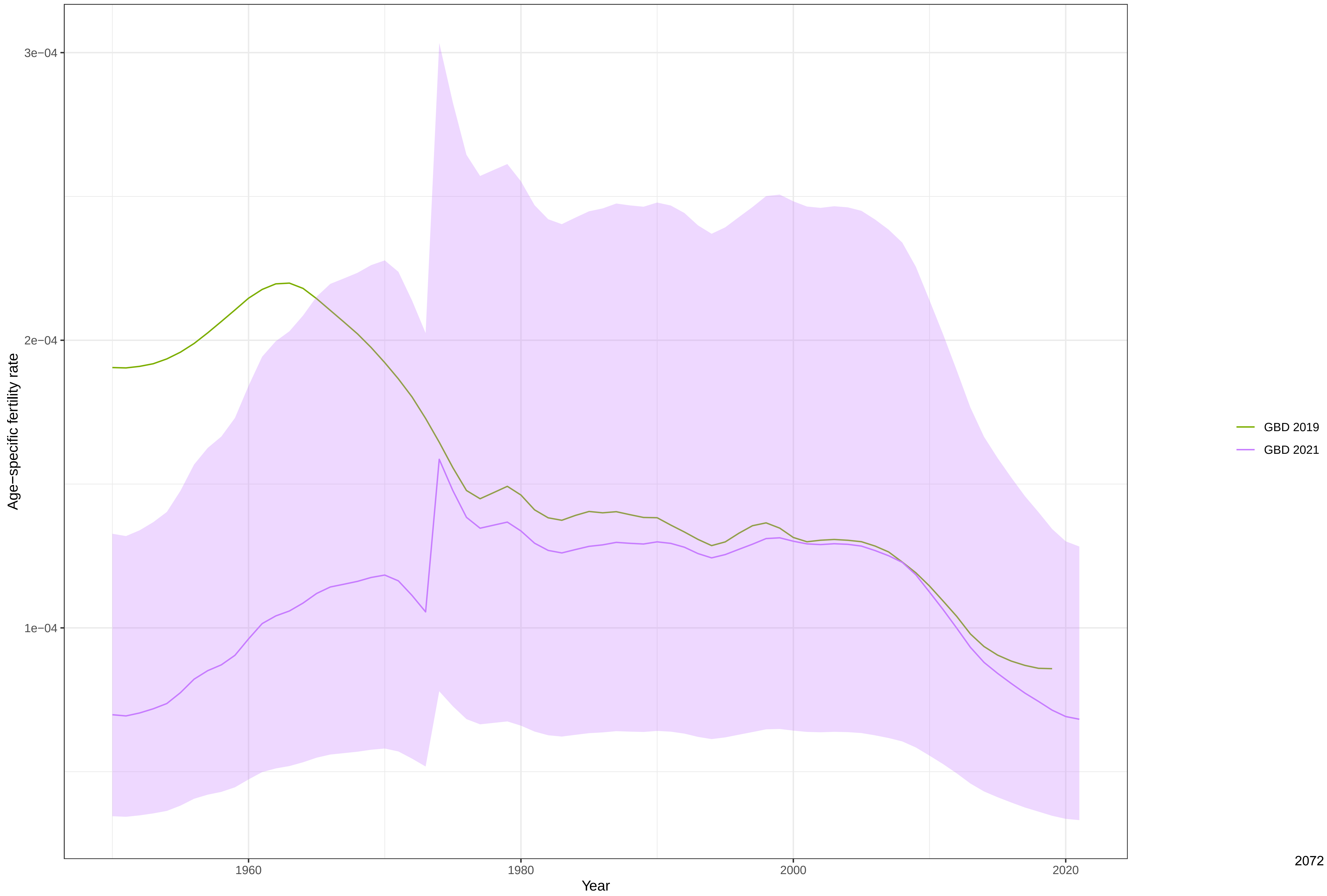

Age−specific fertility fitted trend lines, 1950−2021: Rutland, Ages: 15−19
Data points enclosed with a diamond are reference sources

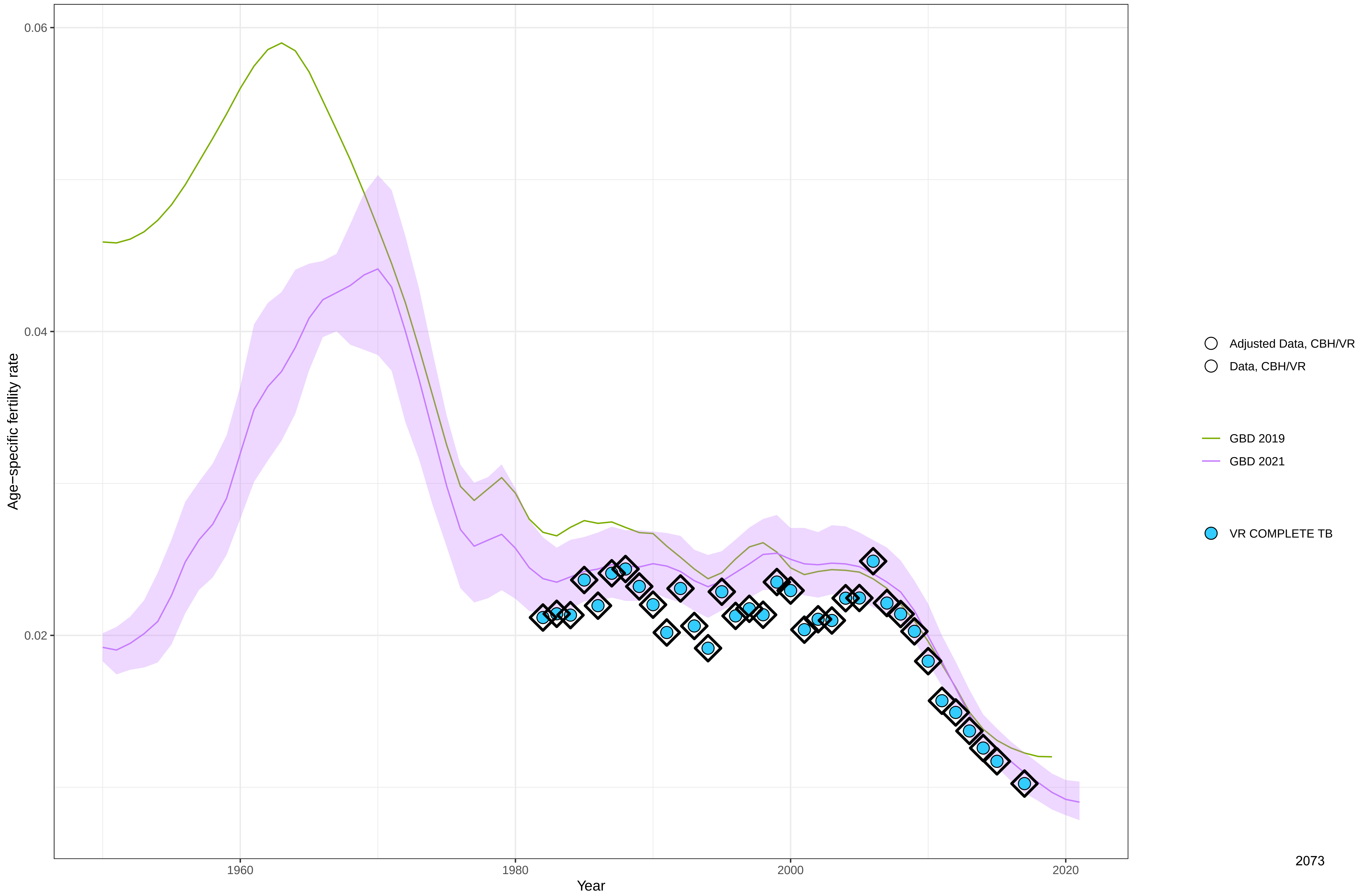

Age−specific fertility fitted trend lines, 1950−2021: Rutland, Ages: 20−24
Data points enclosed with a diamond are reference sources

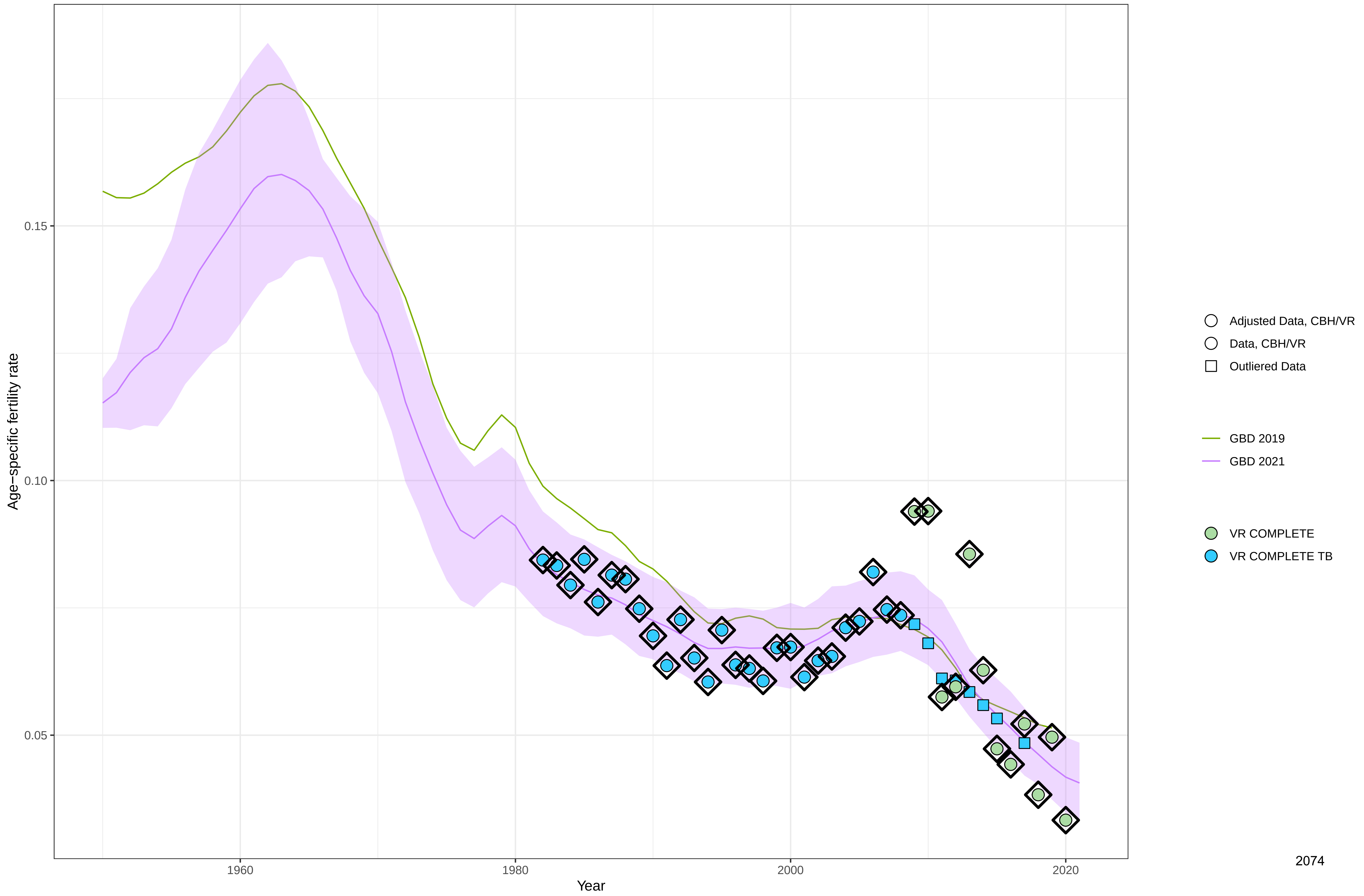

Age−specific fertility fitted trend lines, 1950−2021: Rutland, Ages: 25−29
Data points enclosed with a diamond are reference sources

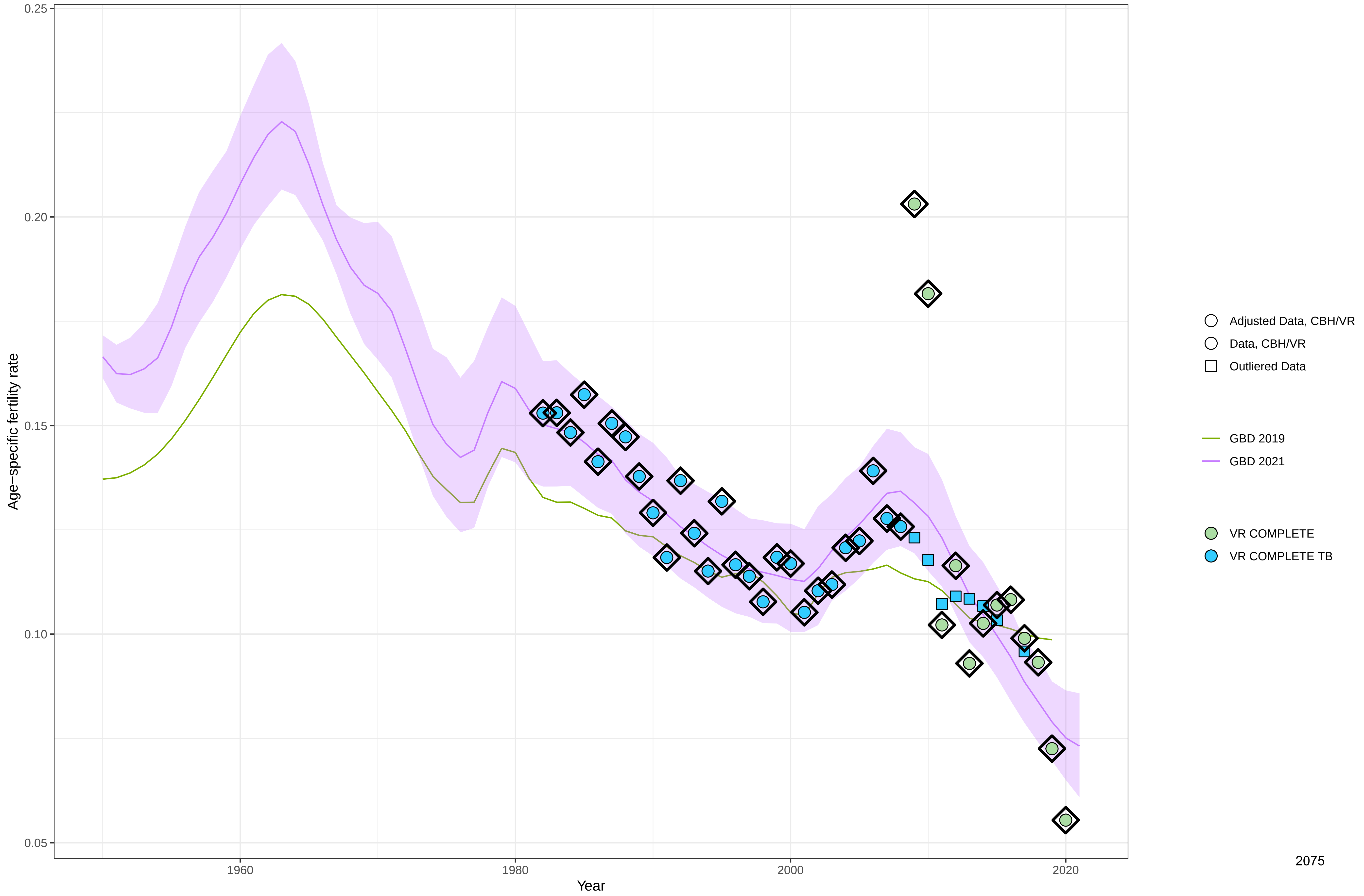

Age−specific fertility fitted trend lines, 1950−2021: Rutland, Ages: 30−34
Data points enclosed with a diamond are reference sources

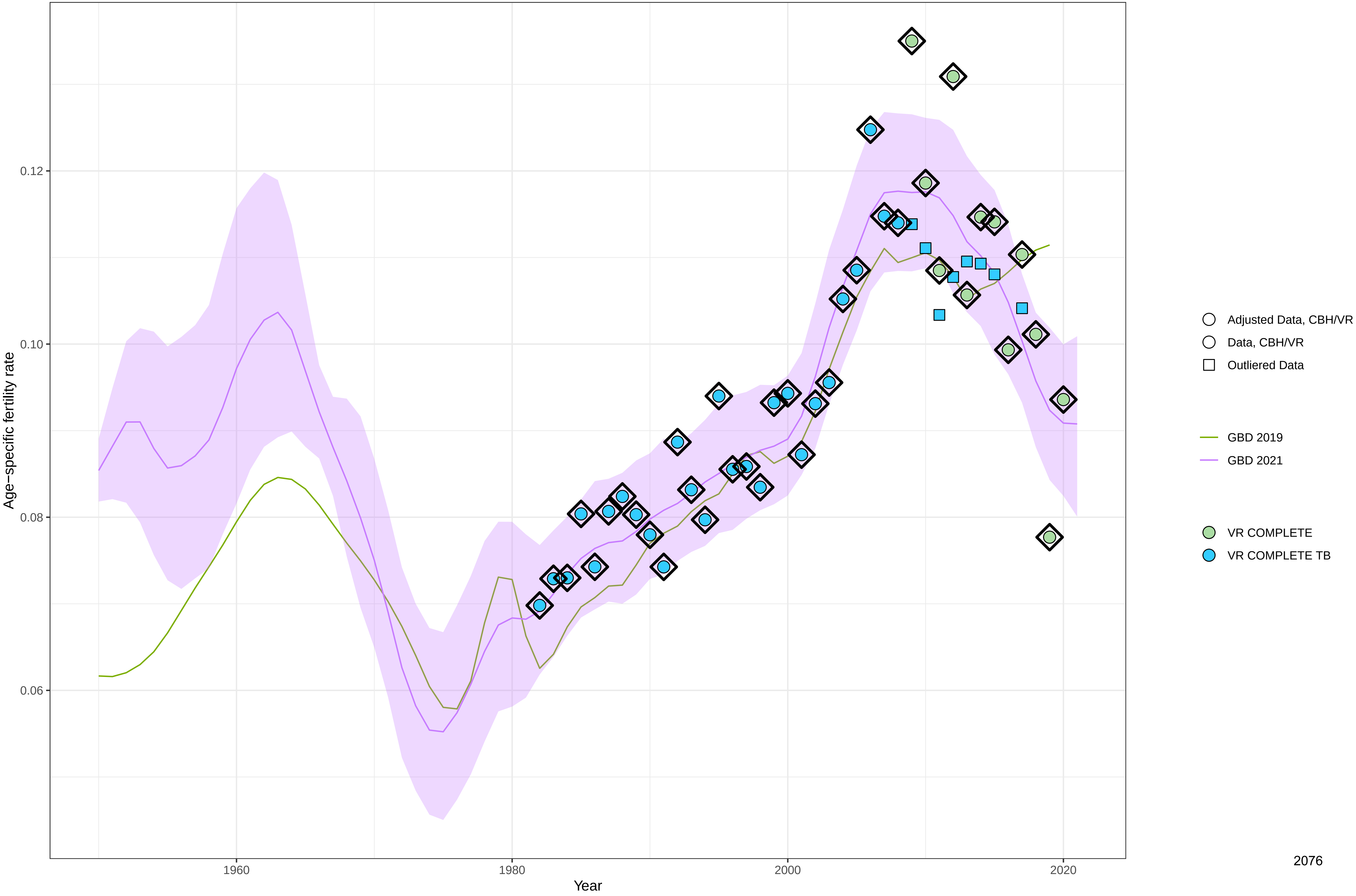

Age−specific fertility fitted trend lines, 1950−2021: Rutland, Ages: 35−39
Data points enclosed with a diamond are reference sources

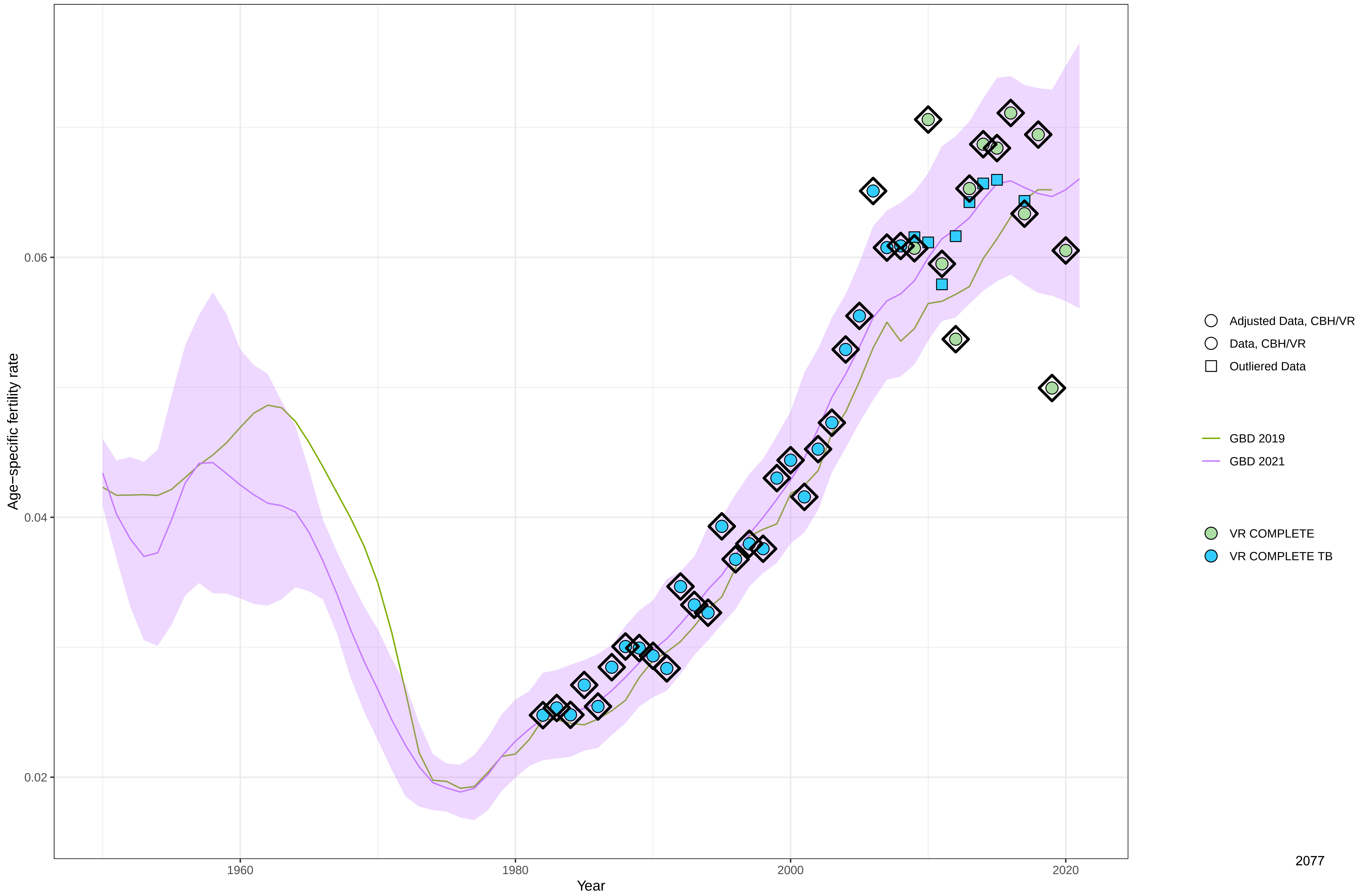

Age−specific fertility fitted trend lines, 1950−2021: Rutland, Ages: 40−44
Data points enclosed with a diamond are reference sources

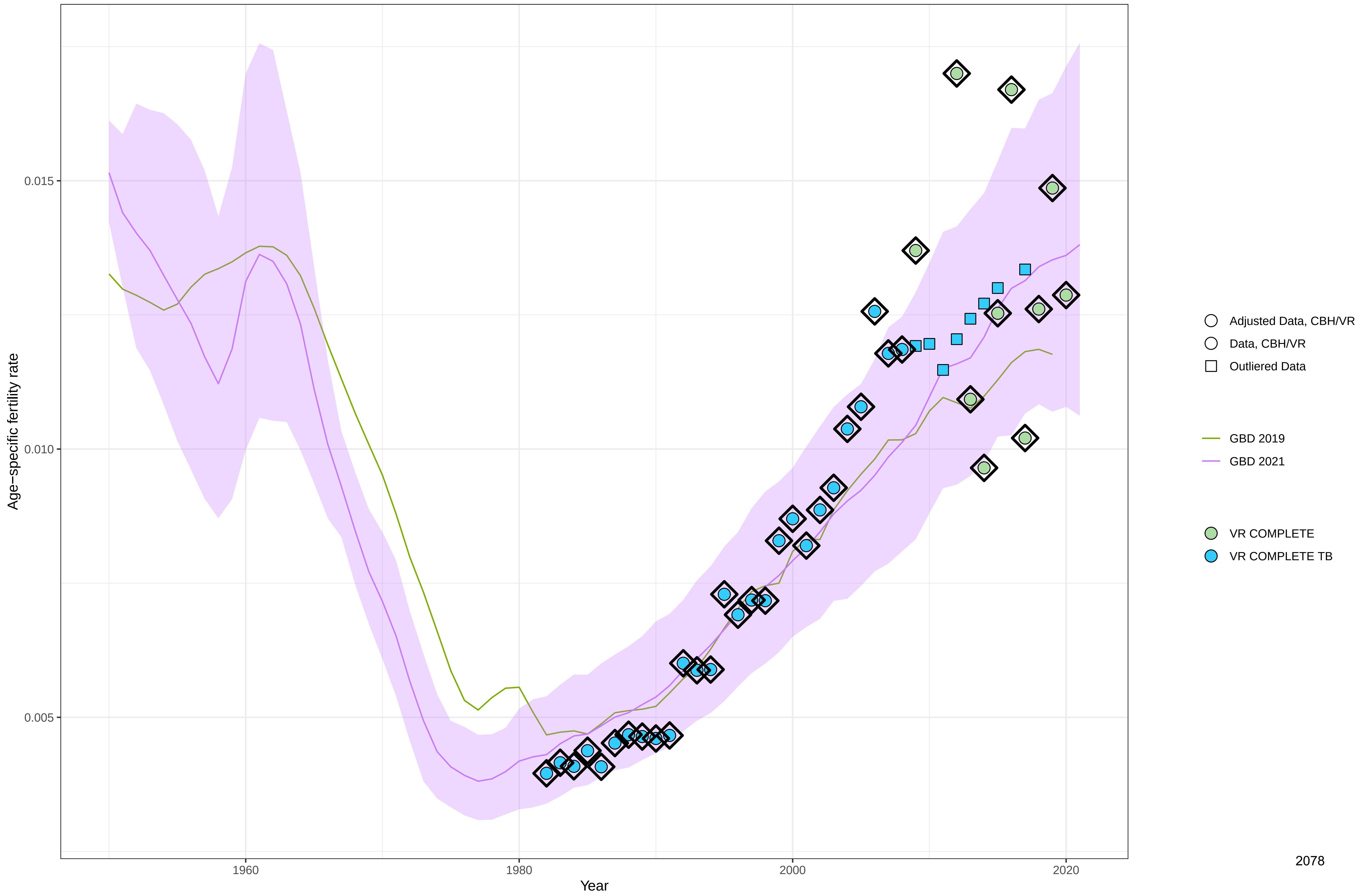

Age−specific fertility fitted trend lines, 1950−2021: Rutland, Ages: 45−49
Data points enclosed with a diamond are reference sources

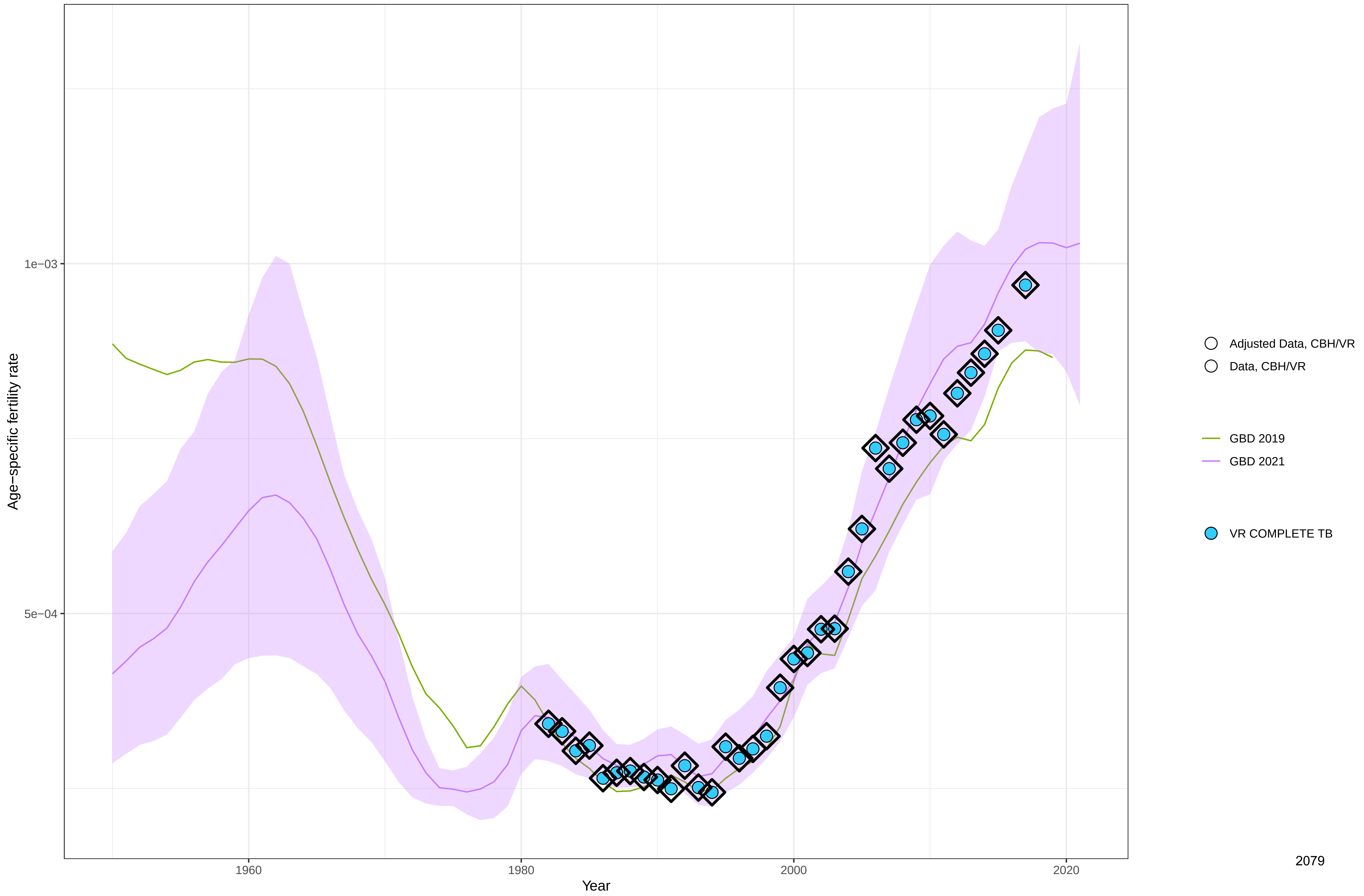

Age−specific fertility fitted trend lines, 1950−2021: Rutland, Ages: 50−54
Data points enclosed with a diamond are reference sources

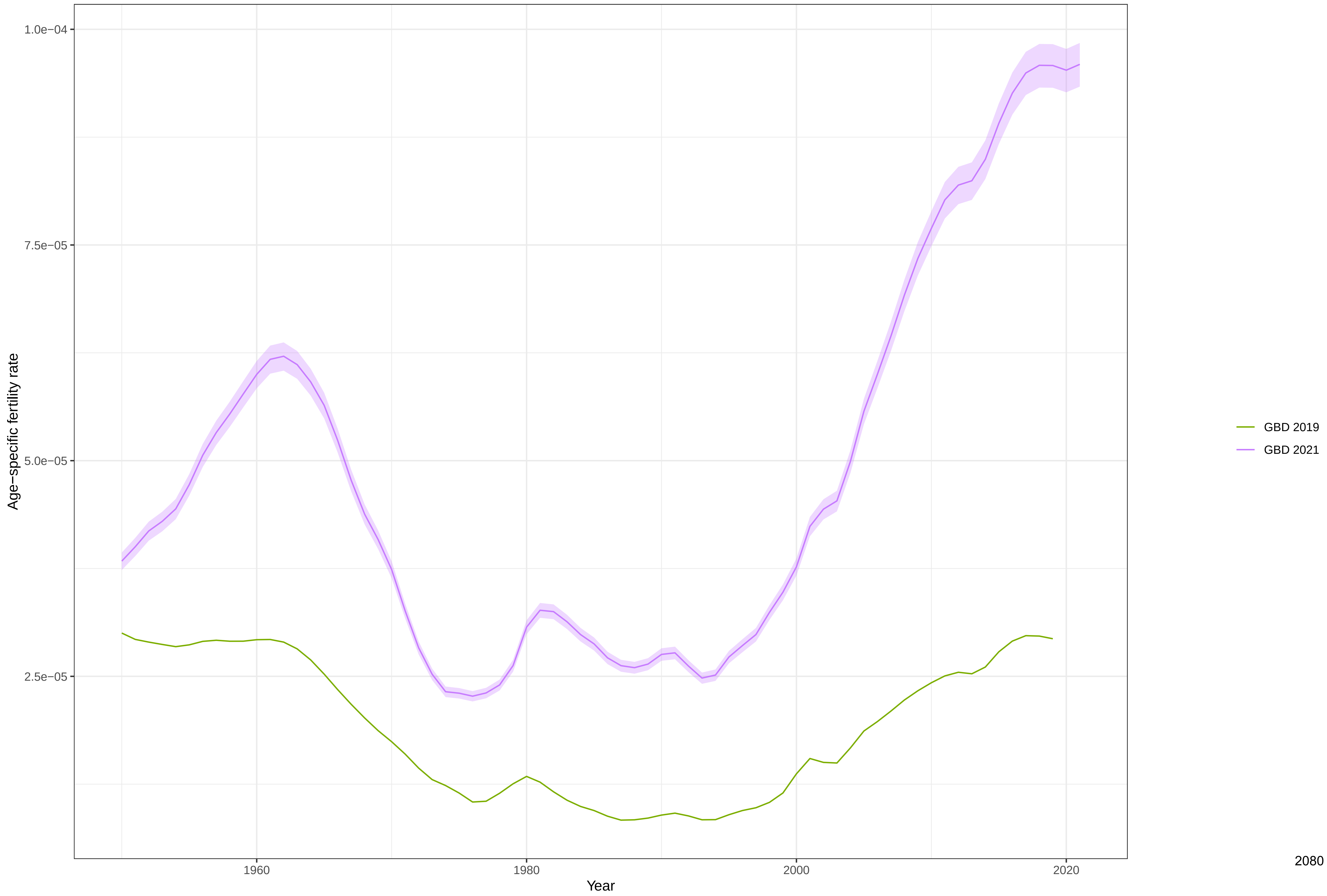

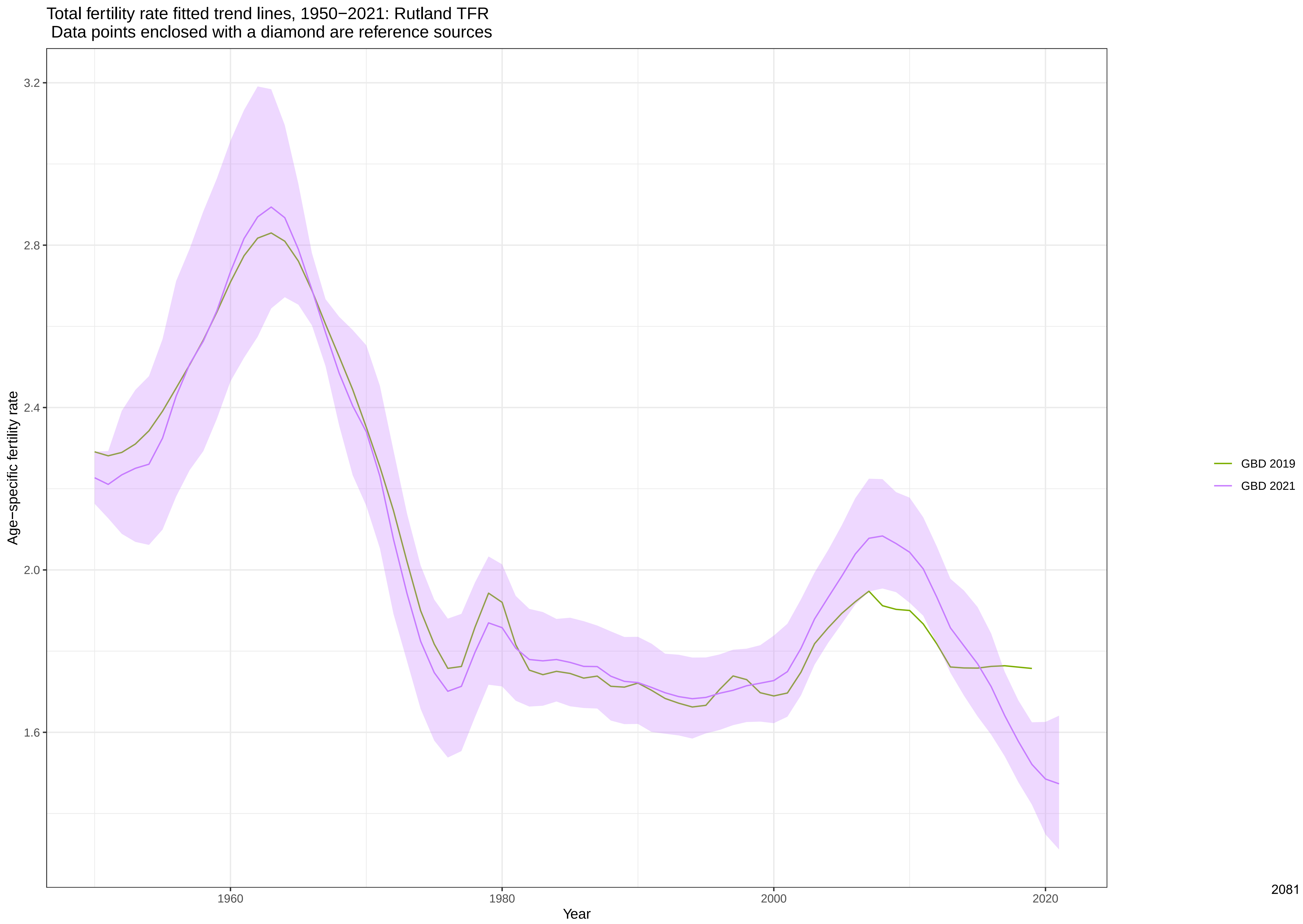

Total fertility rate fitted trend lines, 1950−2021: Rutland TFR
Data points enclosed with a diamond are reference sources
Age−specific fertility rate
Year
3.2
2.8
2.4
2.0
1.6
1960
1980
2000
2020
GBD 2019
GBD 2021

Age−specific fertility fitted trend lines, 1950−2021: East of England, Ages: 10−14
Data points enclosed with a diamond are reference sources

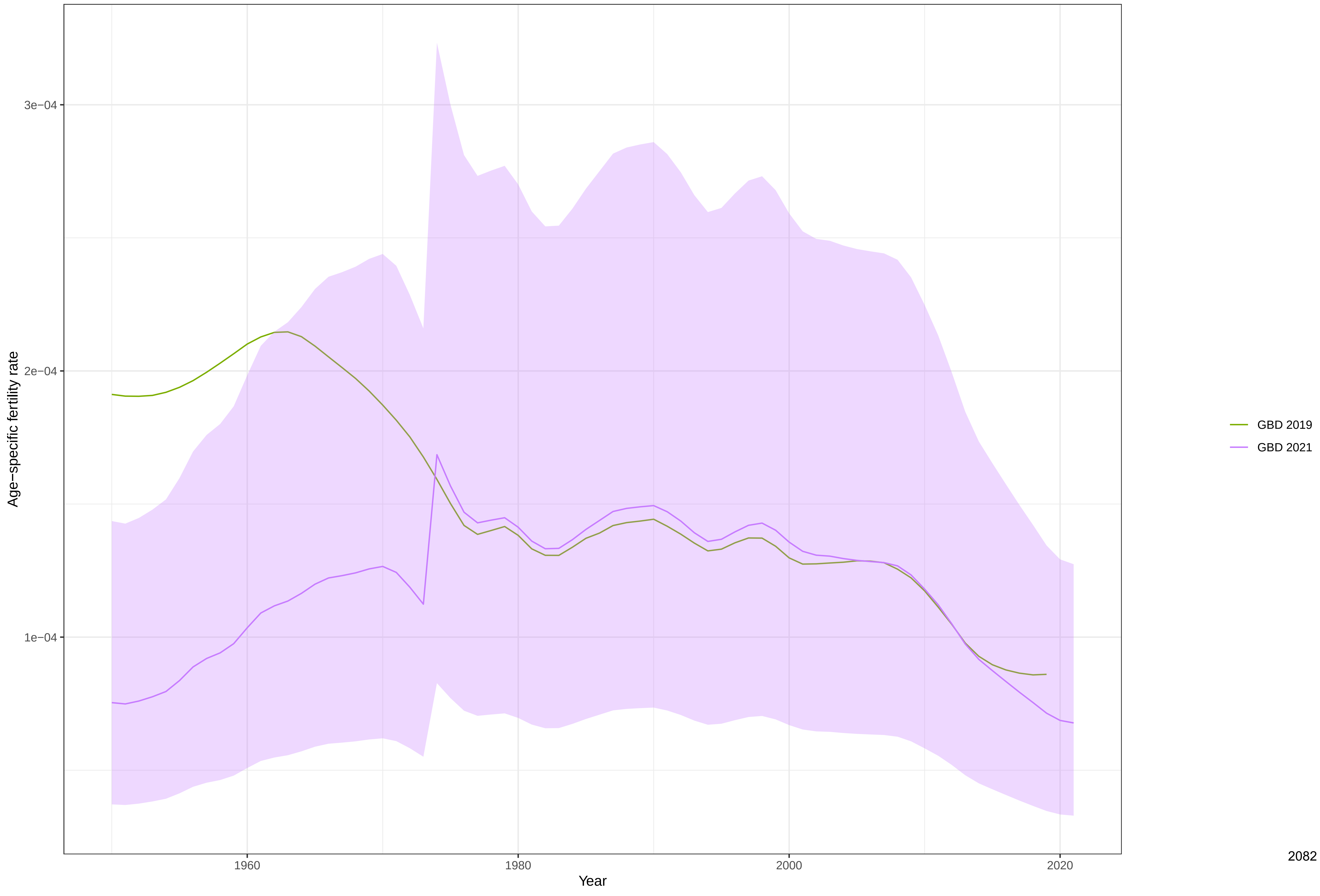

Age−specific fertility fitted trend lines, 1950−2021: East of England, Ages: 15−19
Data points enclosed with a diamond are reference sources

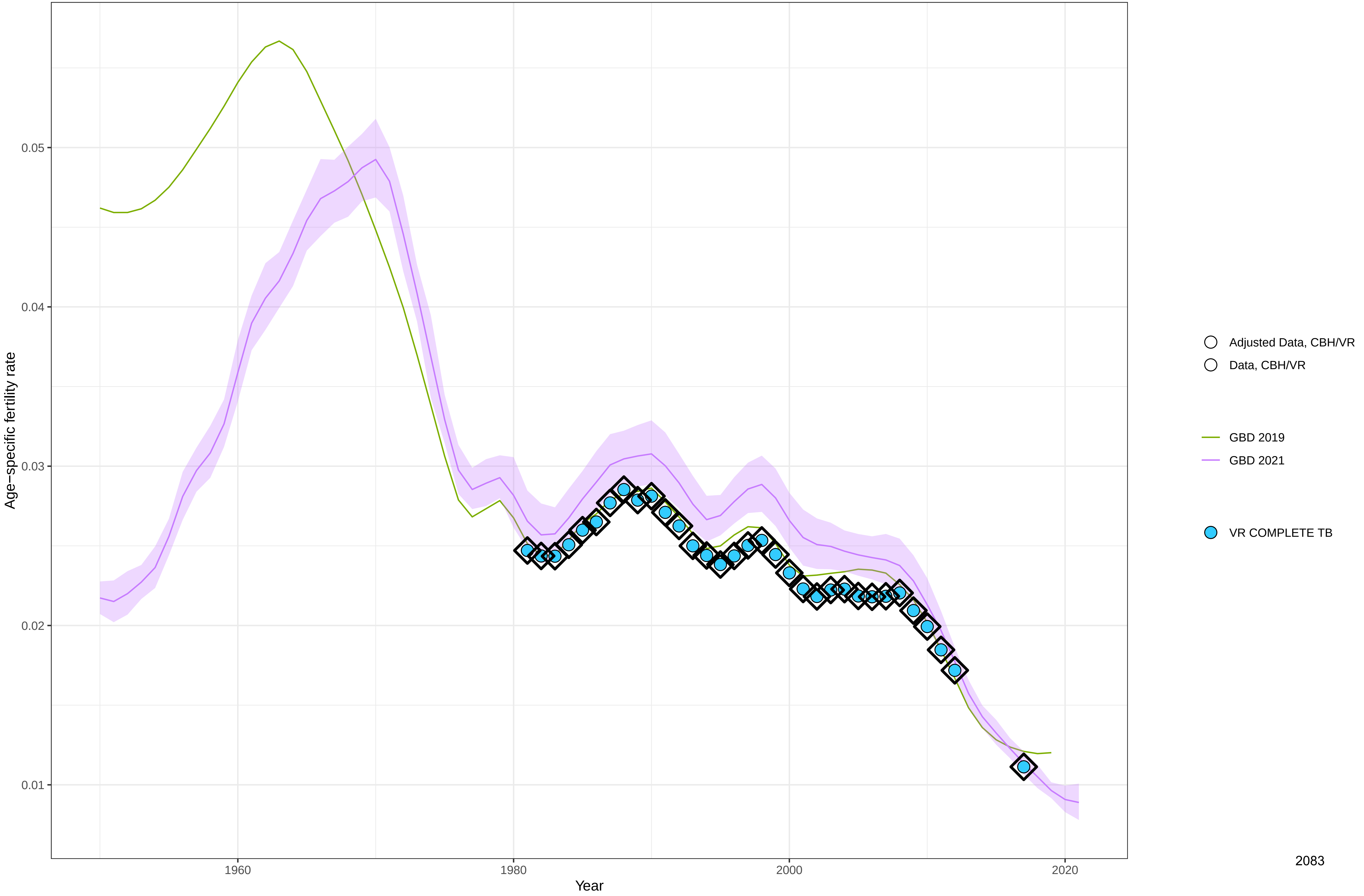

Age−specific fertility fitted trend lines, 1950−2021: East of England, Ages: 20−24
Data points enclosed with a diamond are reference sources

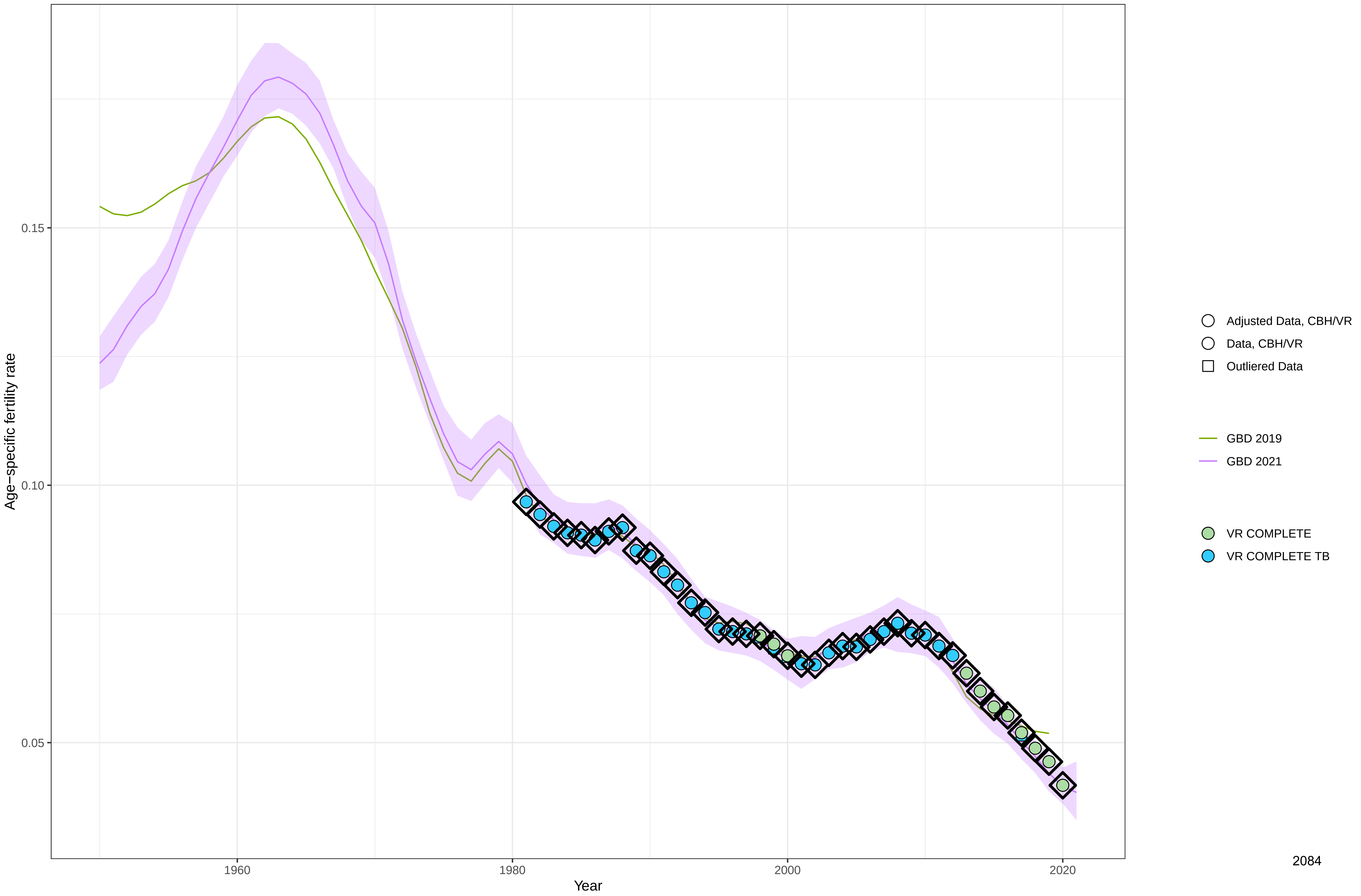

Age−specific fertility fitted trend lines, 1950−2021: East of England, Ages: 25−29
Data points enclosed with a diamond are reference sources

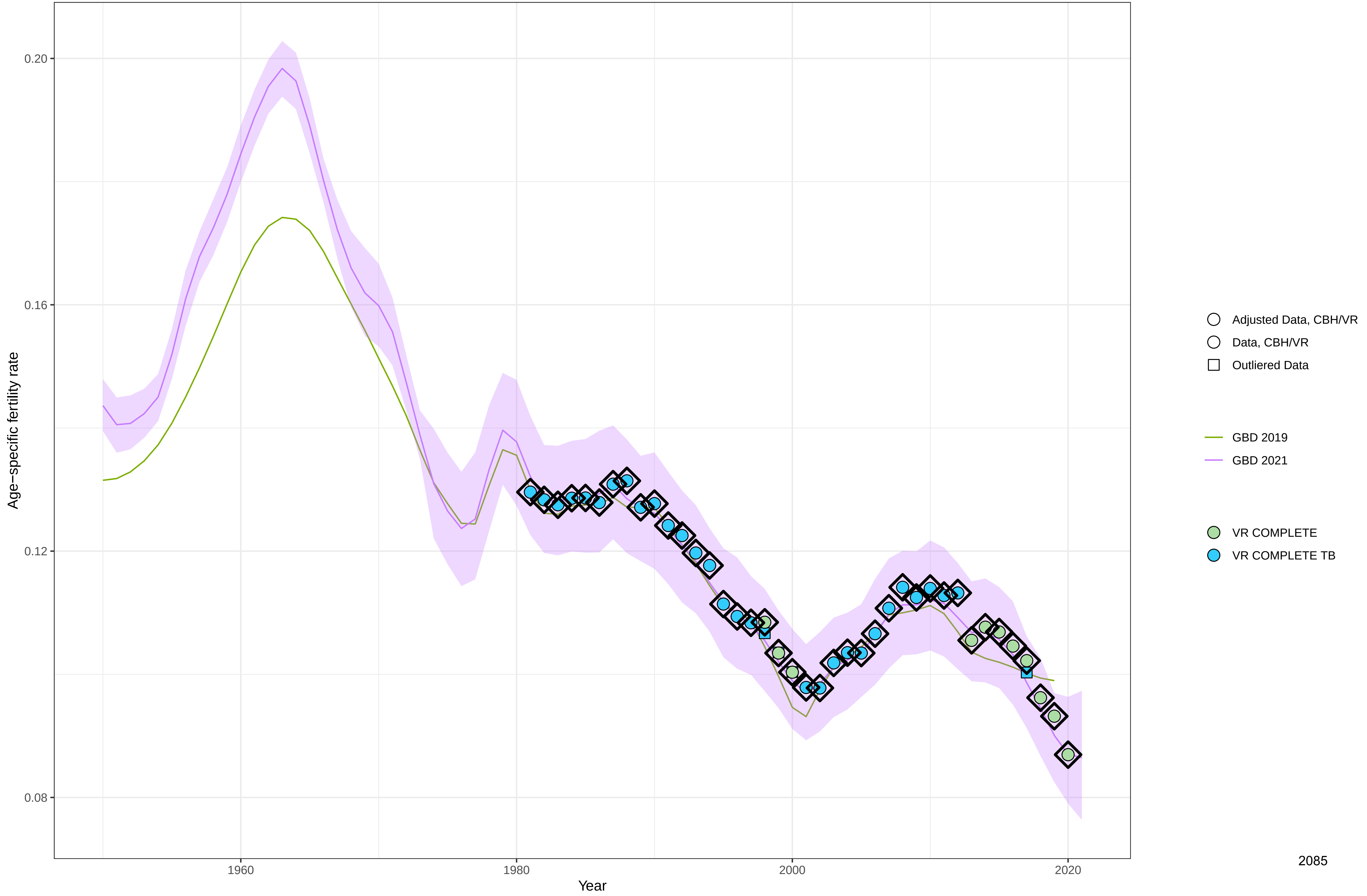

Age−specific fertility fitted trend lines, 1950−2021: East of England, Ages: 30−34
Data points enclosed with a diamond are reference sources

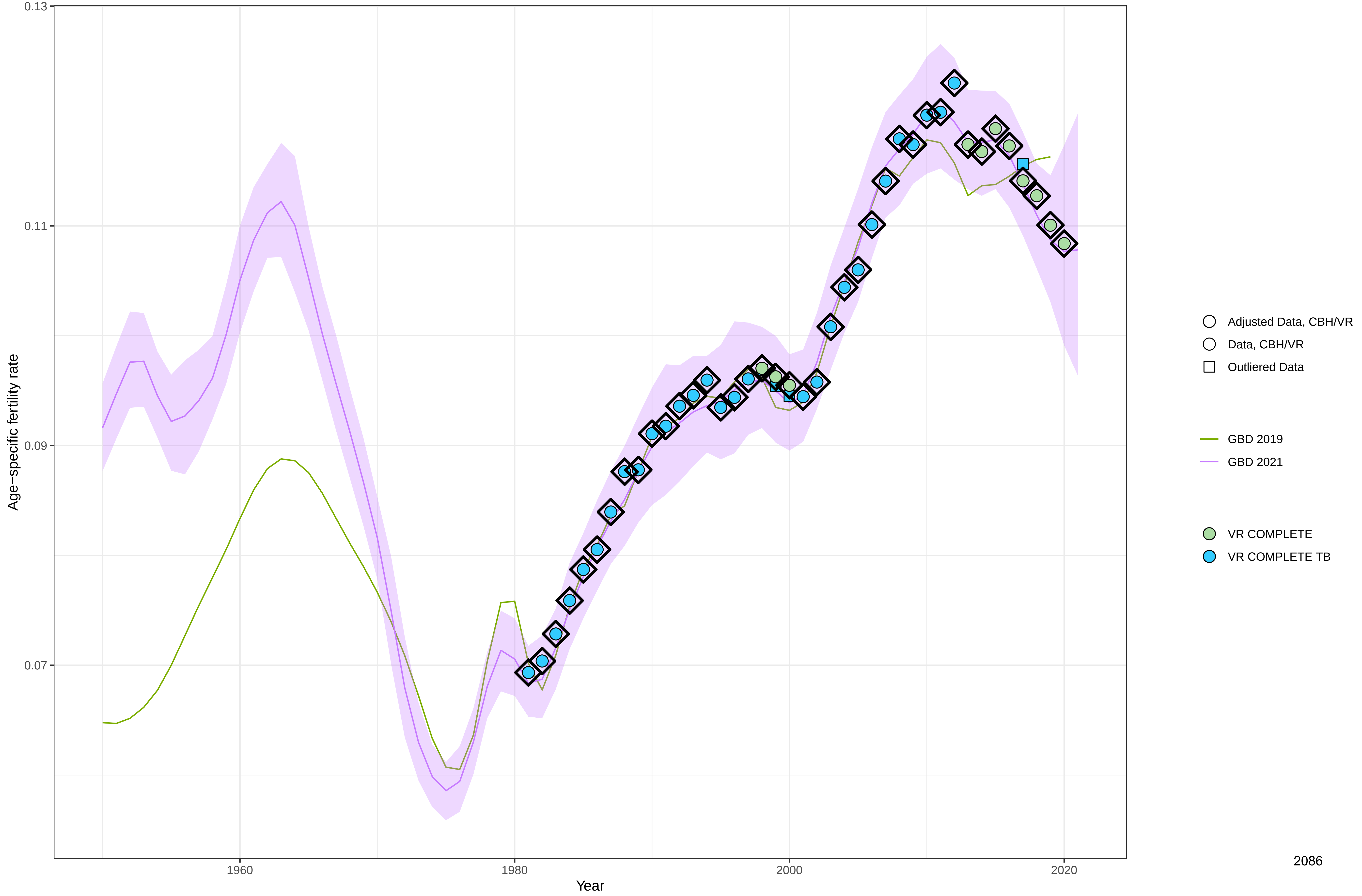

Age−specific fertility fitted trend lines, 1950−2021: East of England, Ages: 35−39
Data points enclosed with a diamond are reference sources

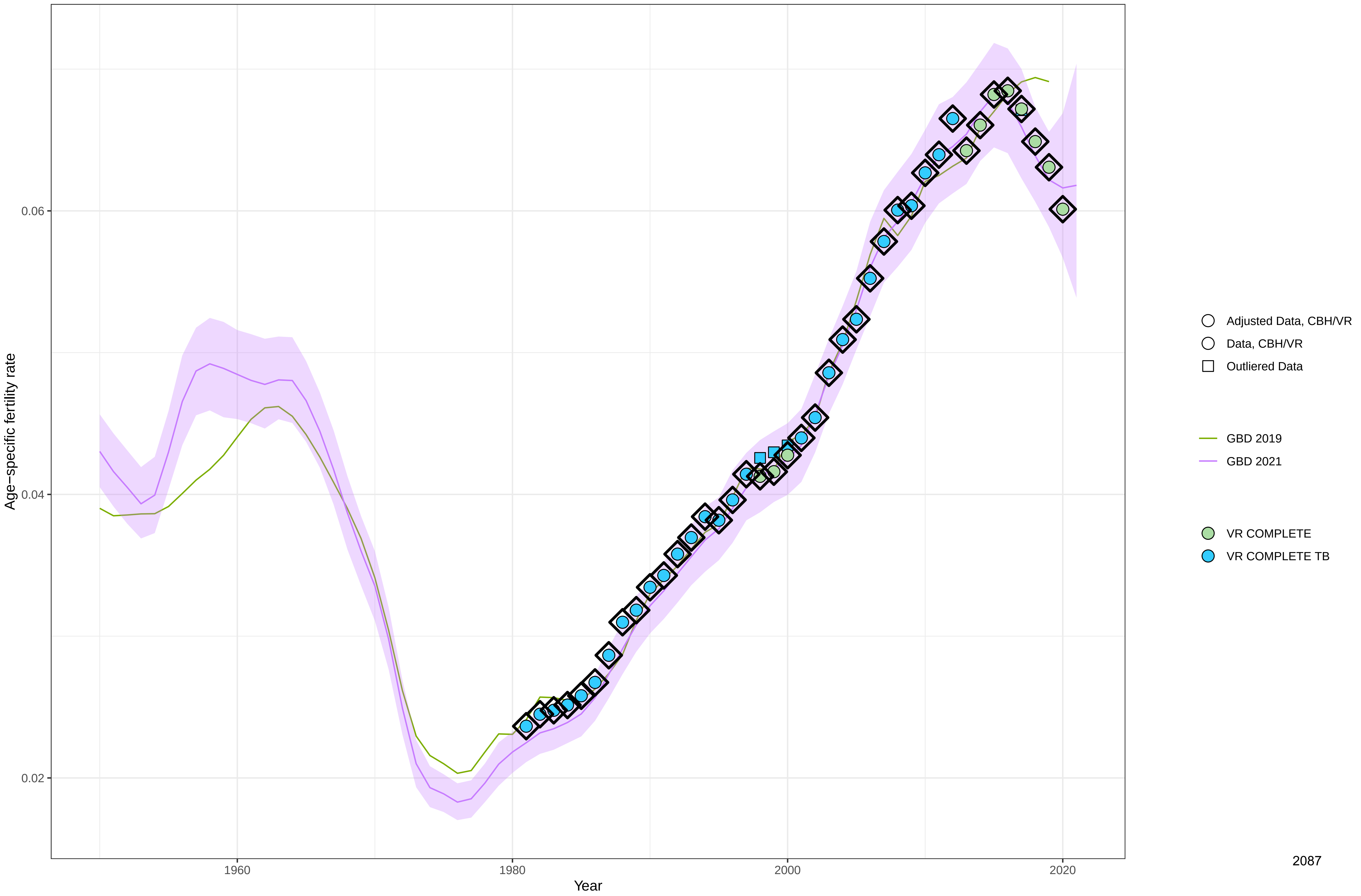

Age−specific fertility fitted trend lines, 1950−2021: East of England, Ages: 40−44
Data points enclosed with a diamond are reference sources

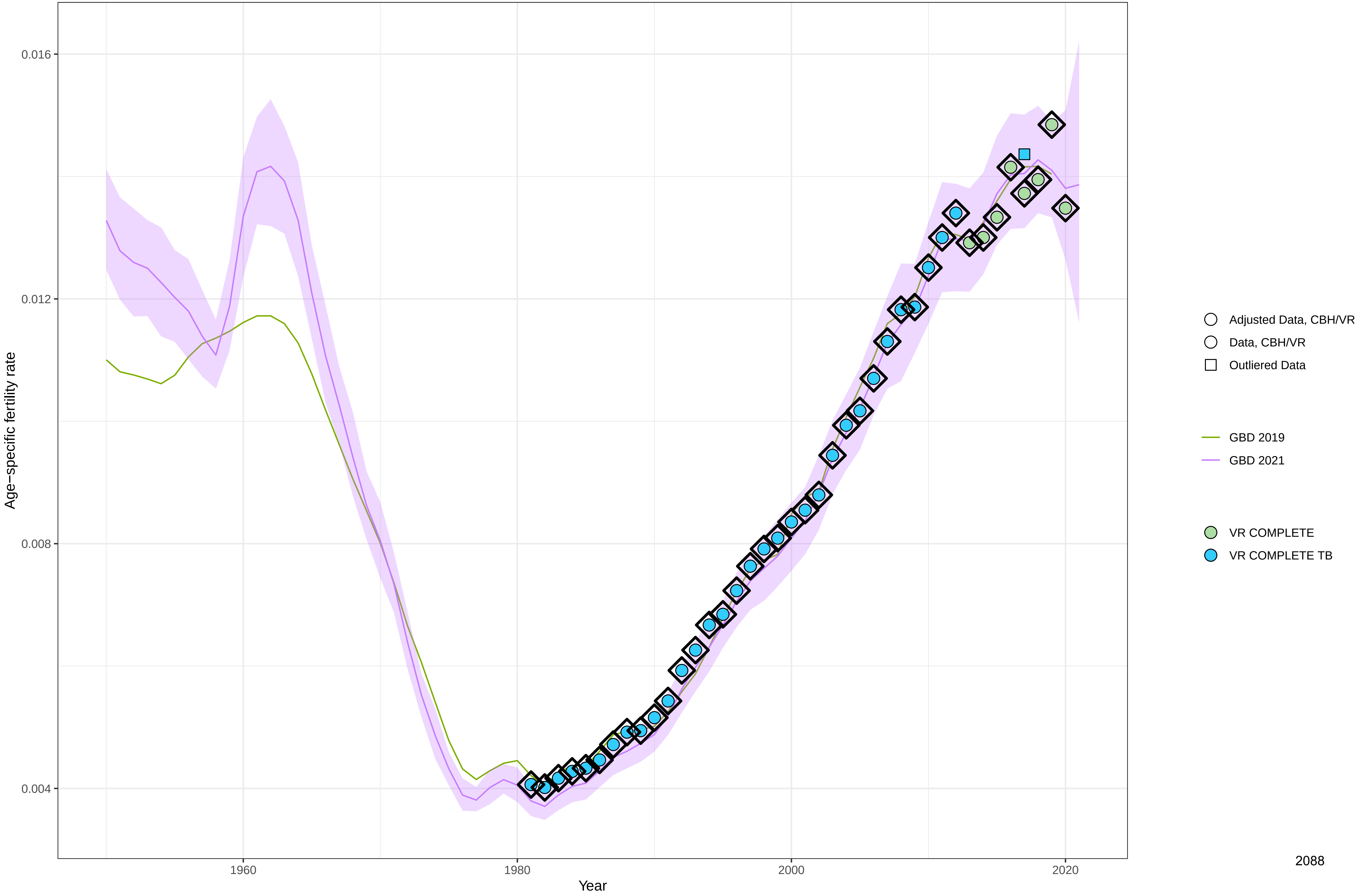

Age−specific fertility fitted trend lines, 1950−2021: East of England, Ages: 45−49
Data points enclosed with a diamond are reference sources

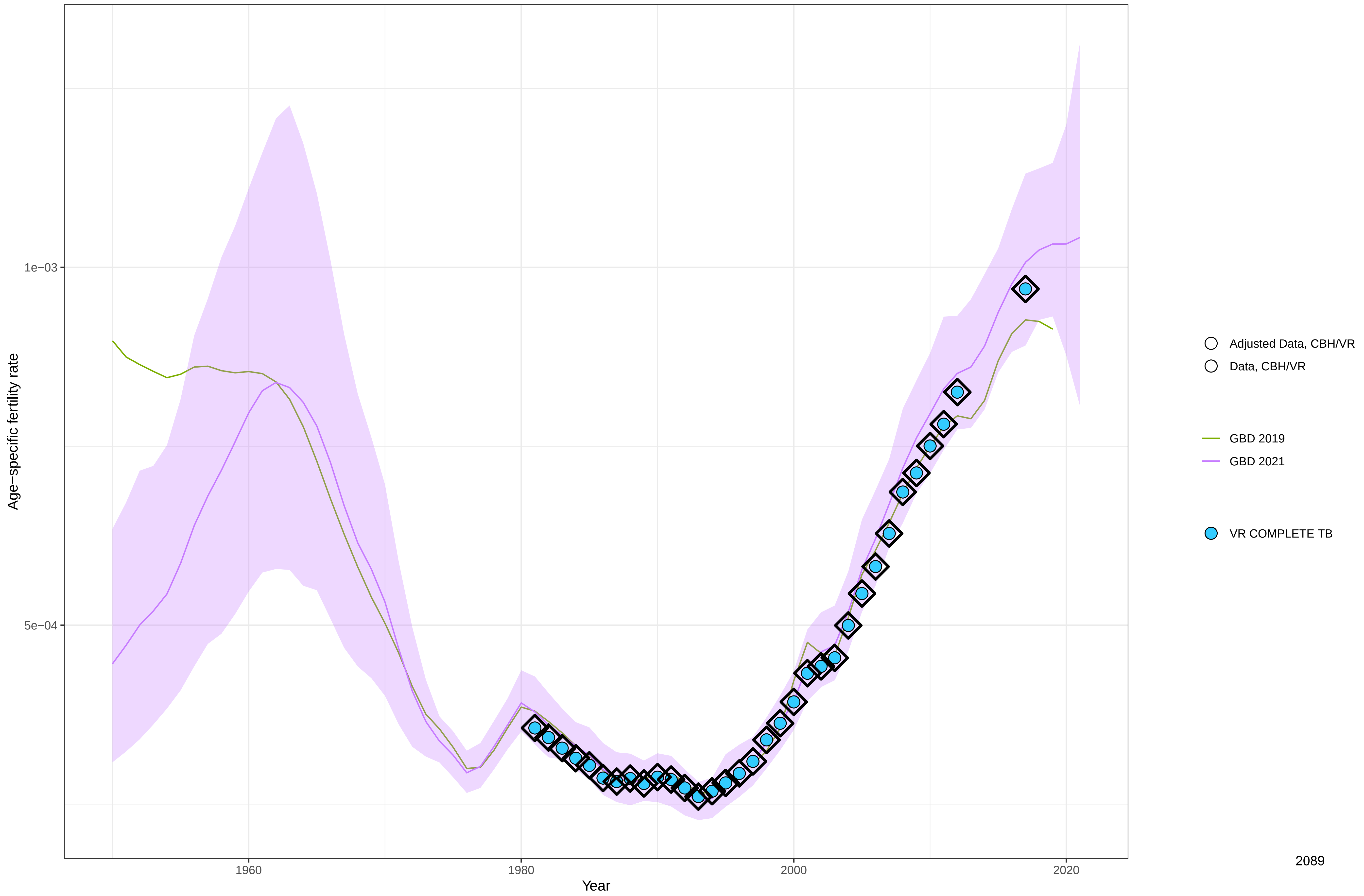

Age−specific fertility fitted trend lines, 1950−2021: East of England, Ages: 50−54
Data points enclosed with a diamond are reference sources

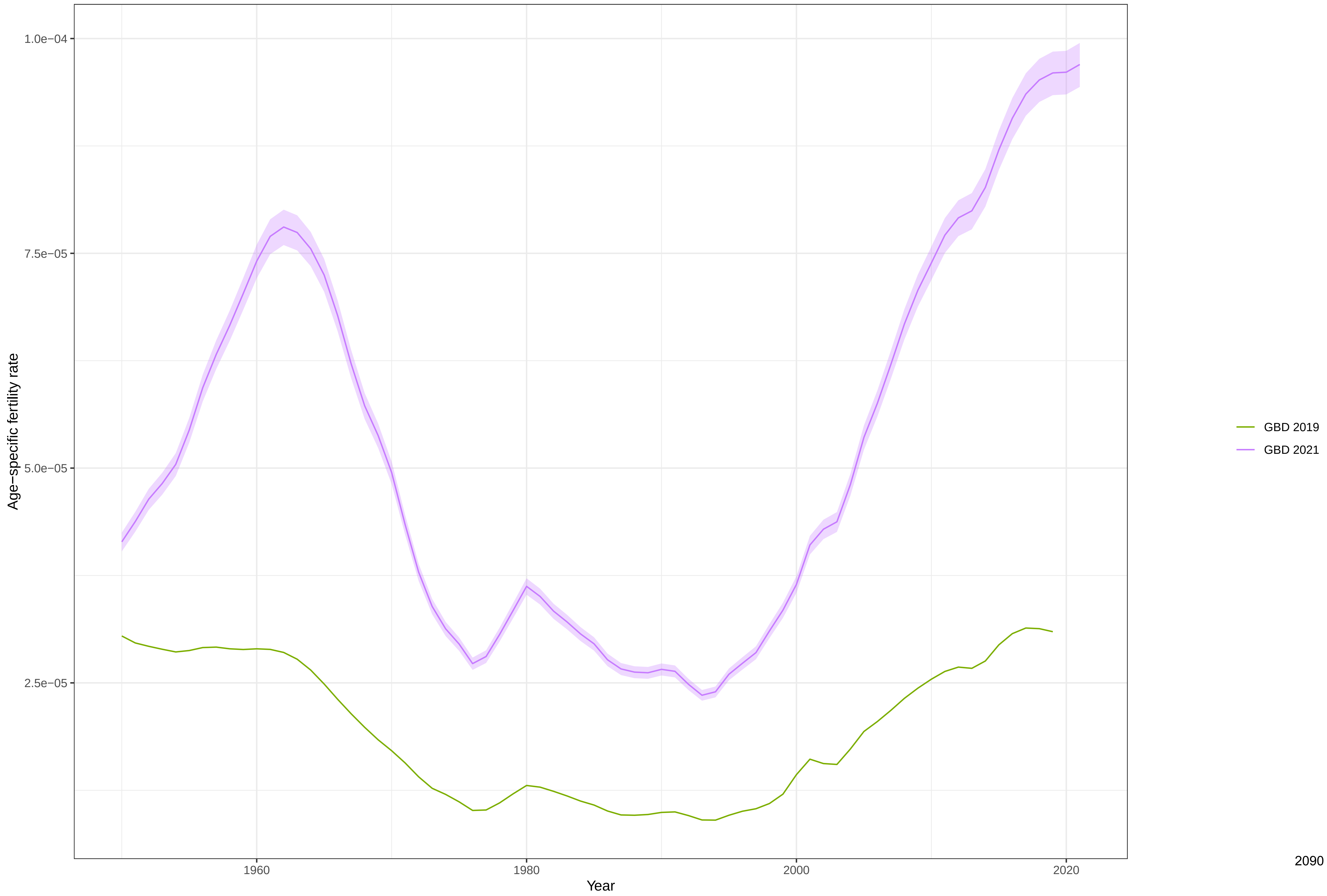

Total fertility rate fitted trend lines, 1950−2021: East of England TFR
Data points enclosed with a diamond are reference sources

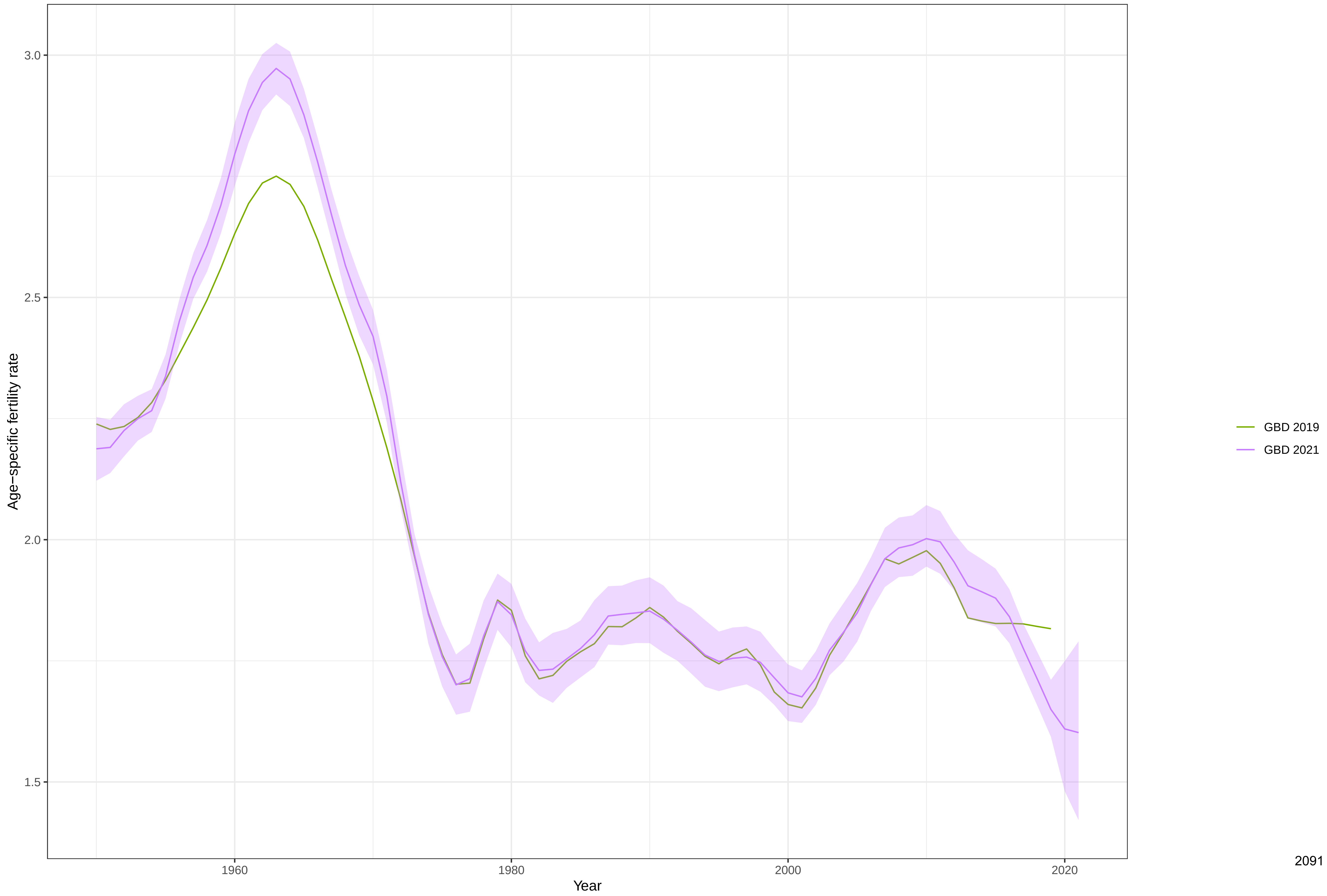

Age−specific fertility fitted trend lines, 1950−2021: Bedford, Ages: 10−14
Data points enclosed with a diamond are reference sources

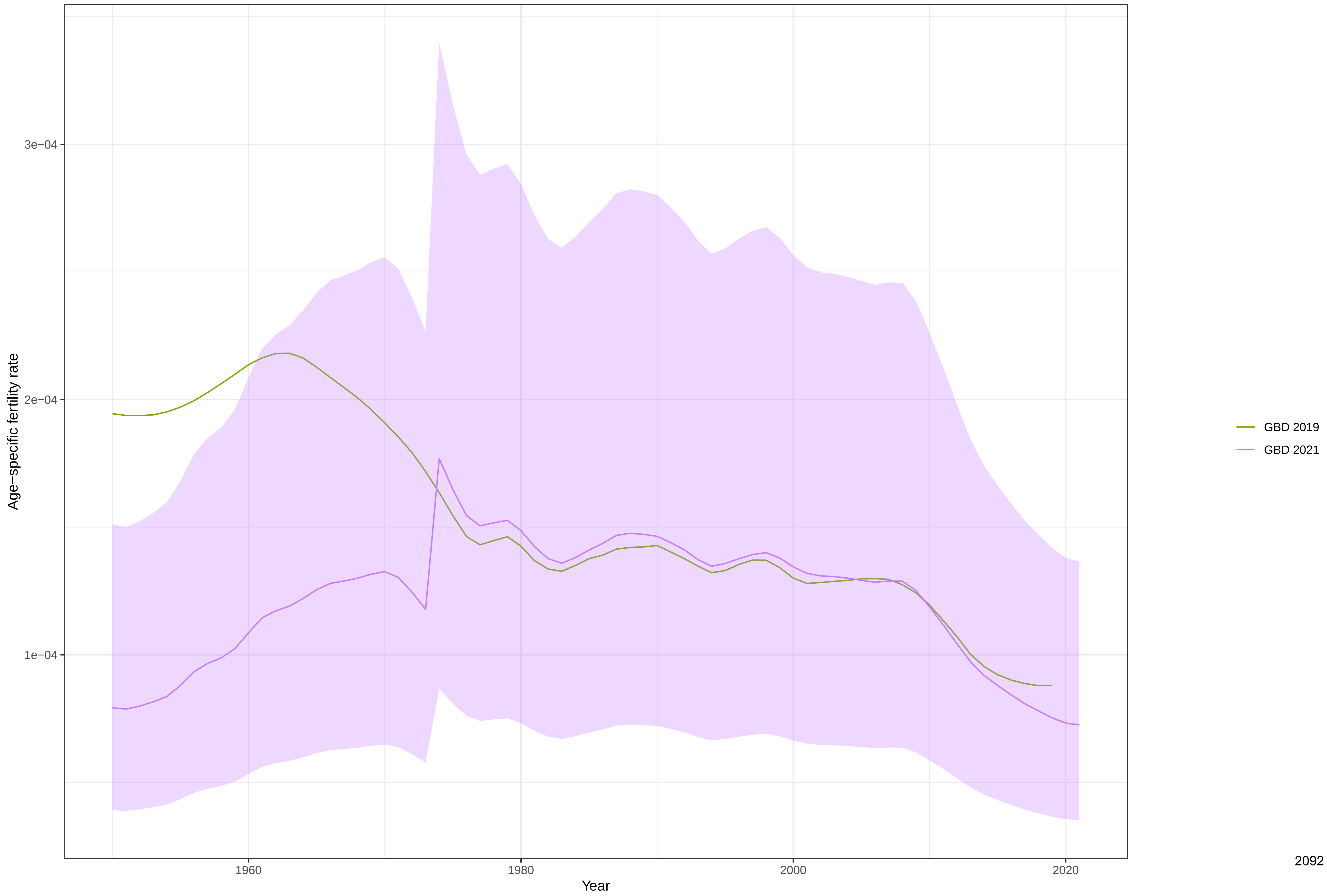

Age−specific fertility fitted trend lines, 1950−2021: Bedford, Ages: 15−19
Data points enclosed with a diamond are reference sources

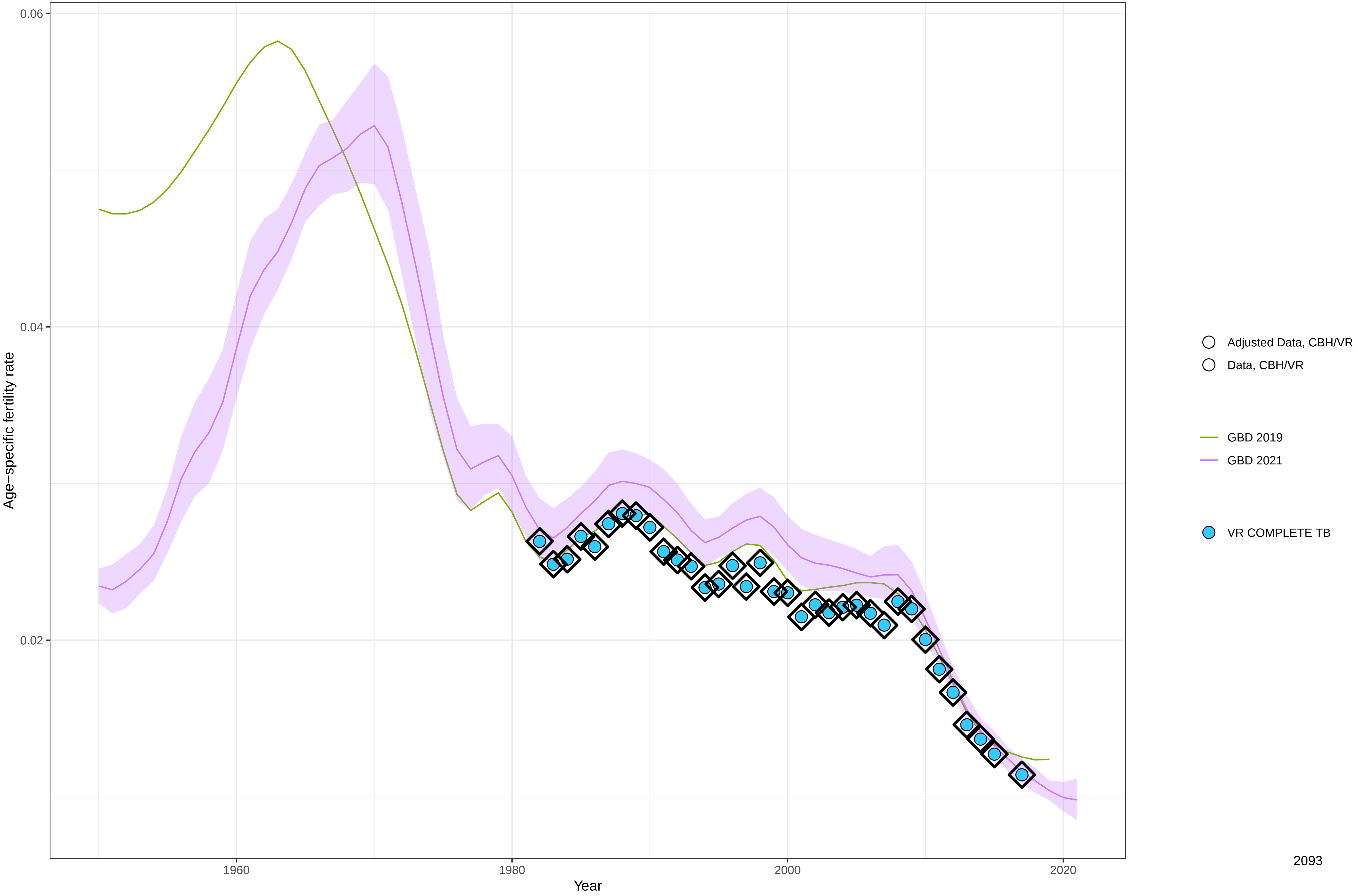

Age−specific fertility fitted trend lines, 1950−2021: Bedford, Ages: 20−24
Data points enclosed with a diamond are reference sources

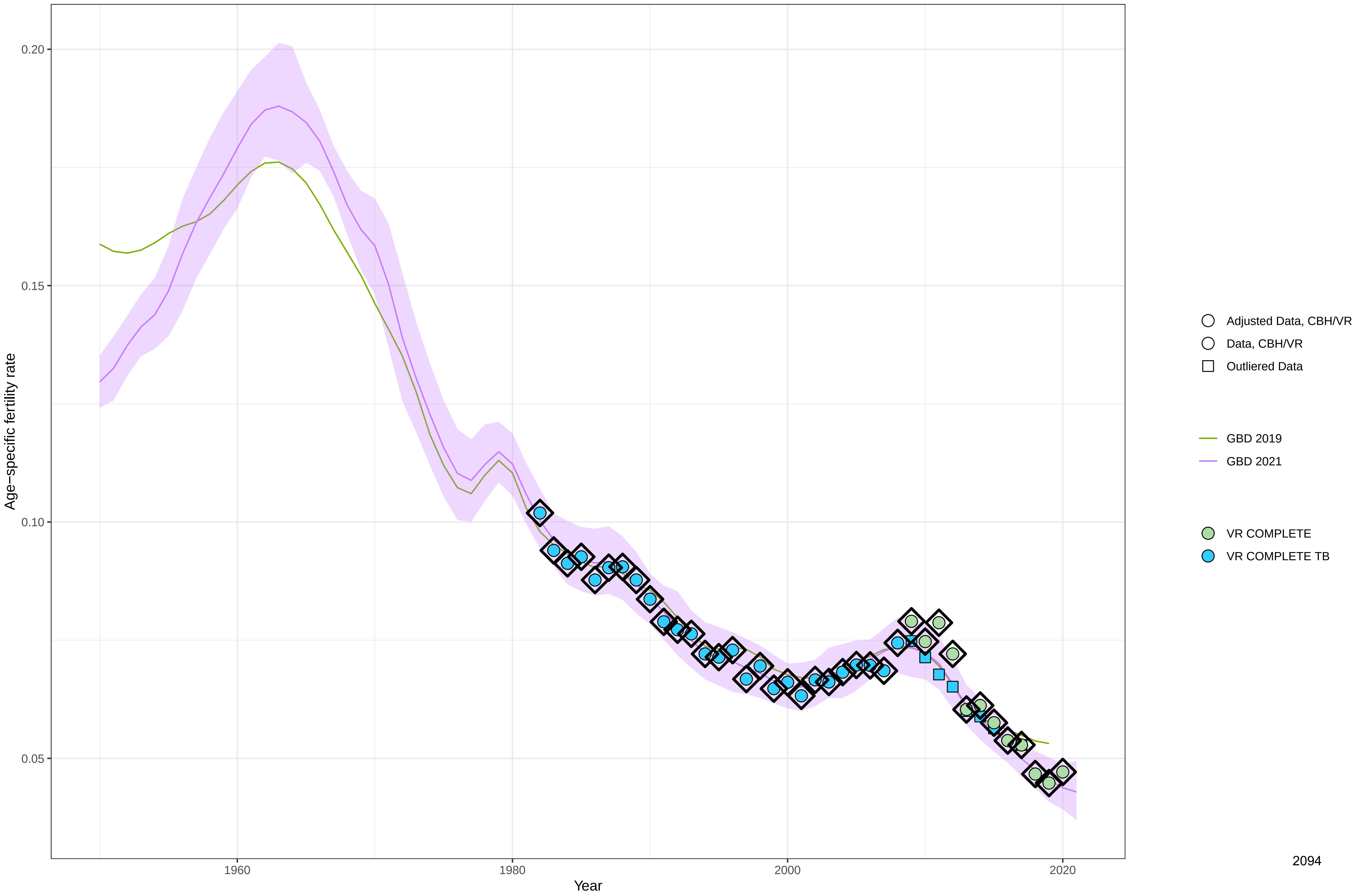

Age−specific fertility fitted trend lines, 1950−2021: Bedford, Ages: 25−29
Data points enclosed with a diamond are reference sources

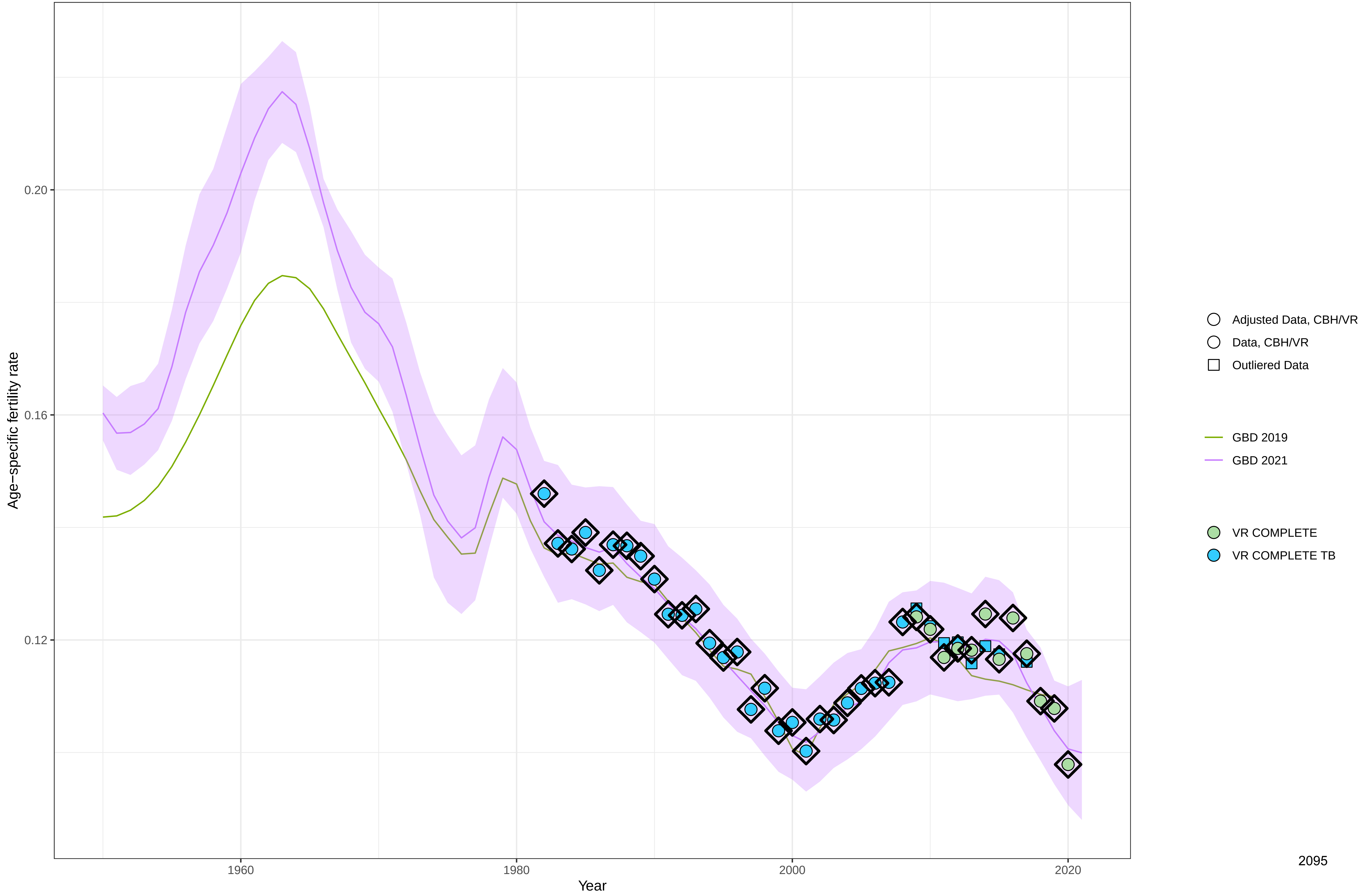

Age−specific fertility fitted trend lines, 1950−2021: Bedford, Ages: 30−34
Data points enclosed with a diamond are reference sources

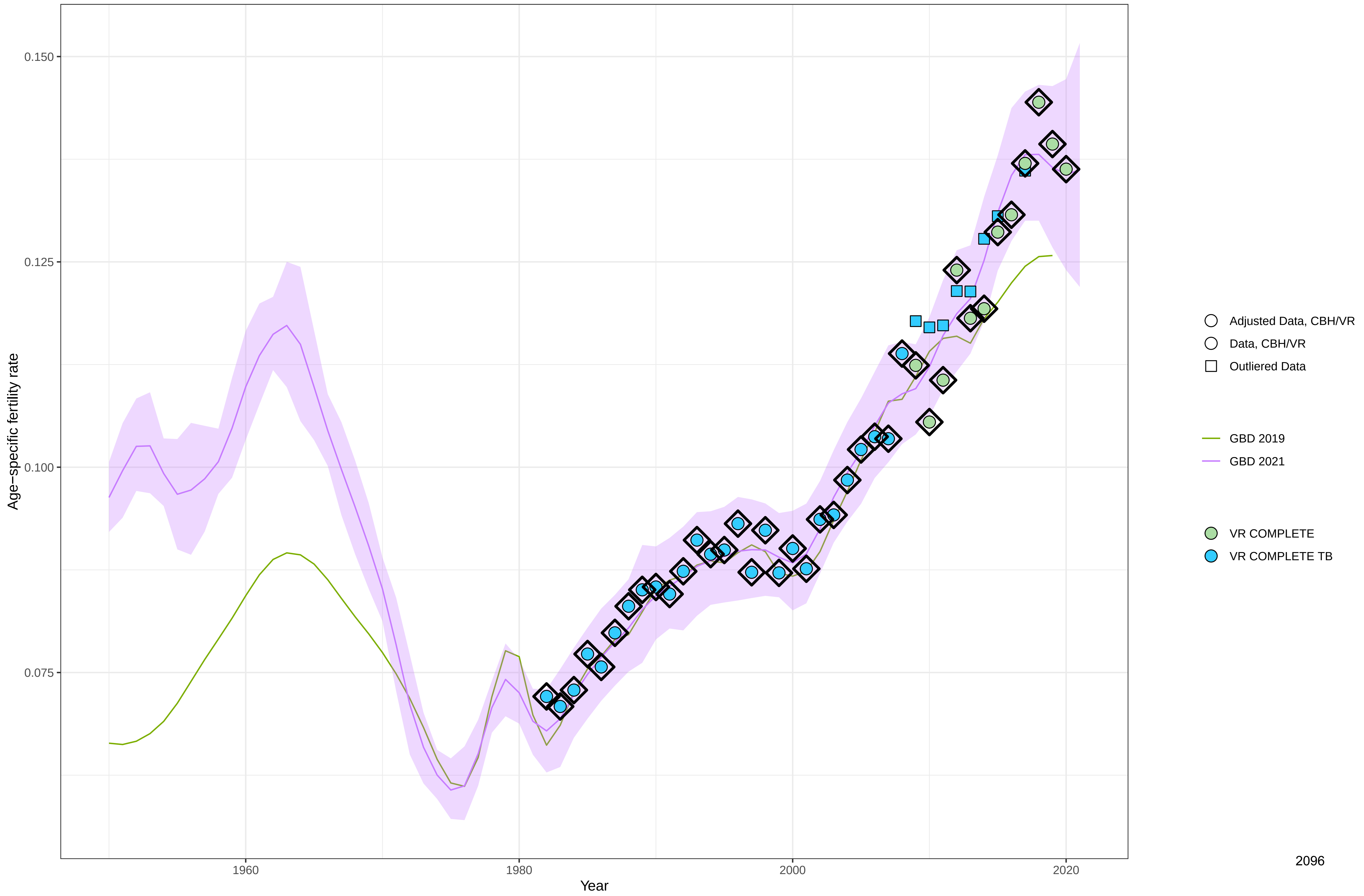

Age−specific fertility fitted trend lines, 1950−2021: Bedford, Ages: 35−39
Data points enclosed with a diamond are reference sources

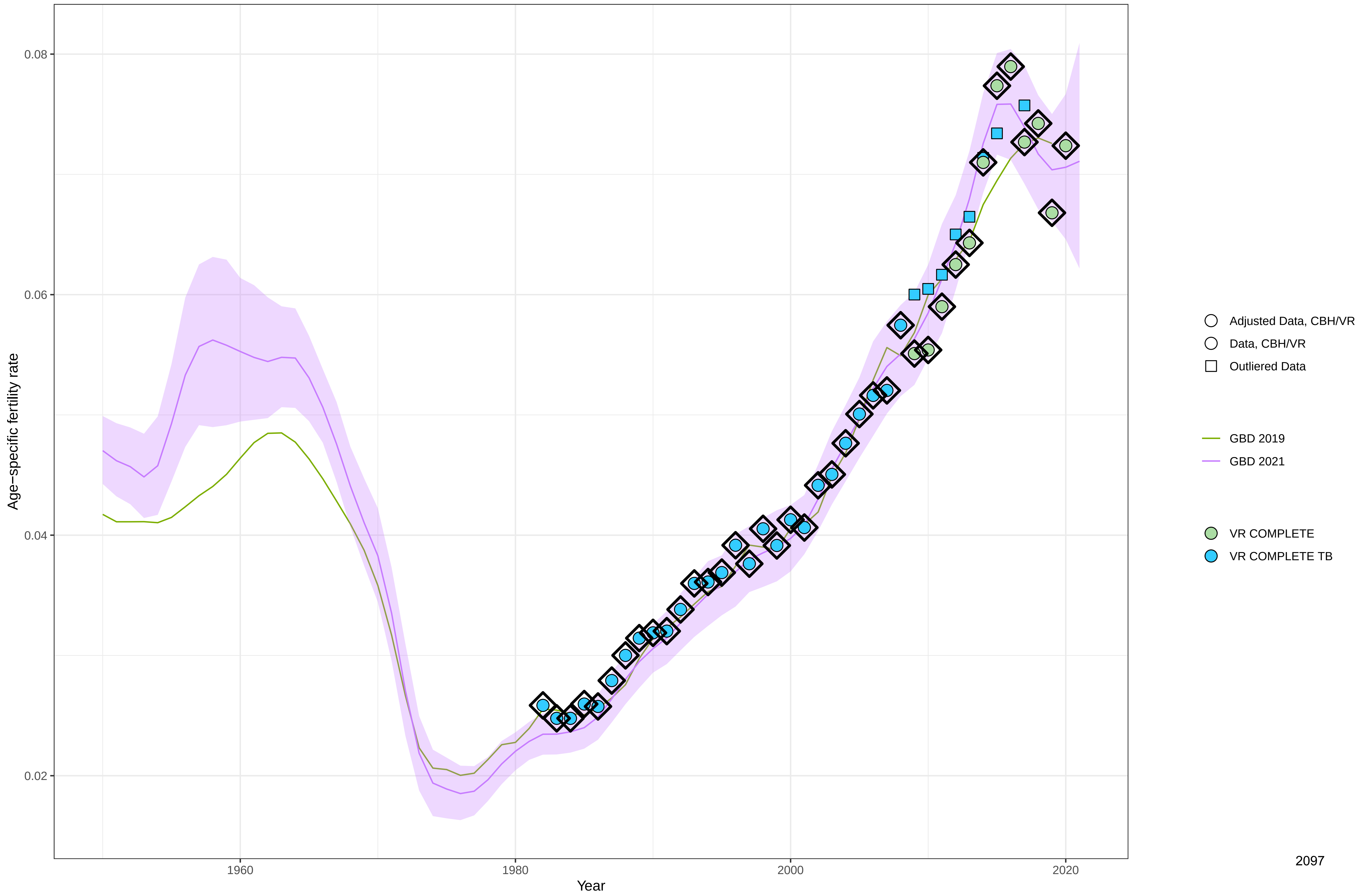

Age−specific fertility fitted trend lines, 1950−2021: Bedford, Ages: 40−44
Data points enclosed with a diamond are reference sources

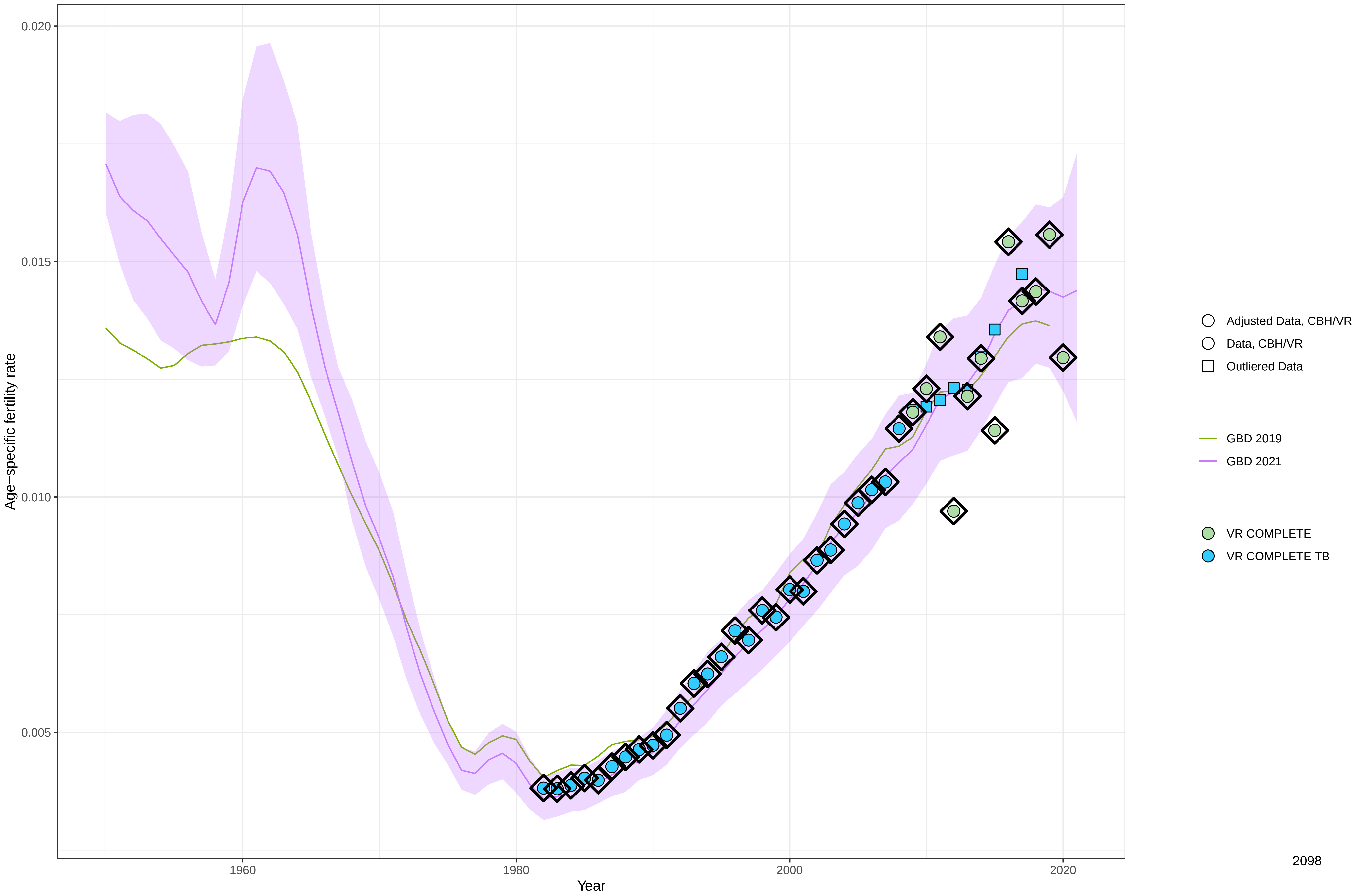

Age−specific fertility fitted trend lines, 1950−2021: Bedford, Ages: 45−49
Data points enclosed with a diamond are reference sources

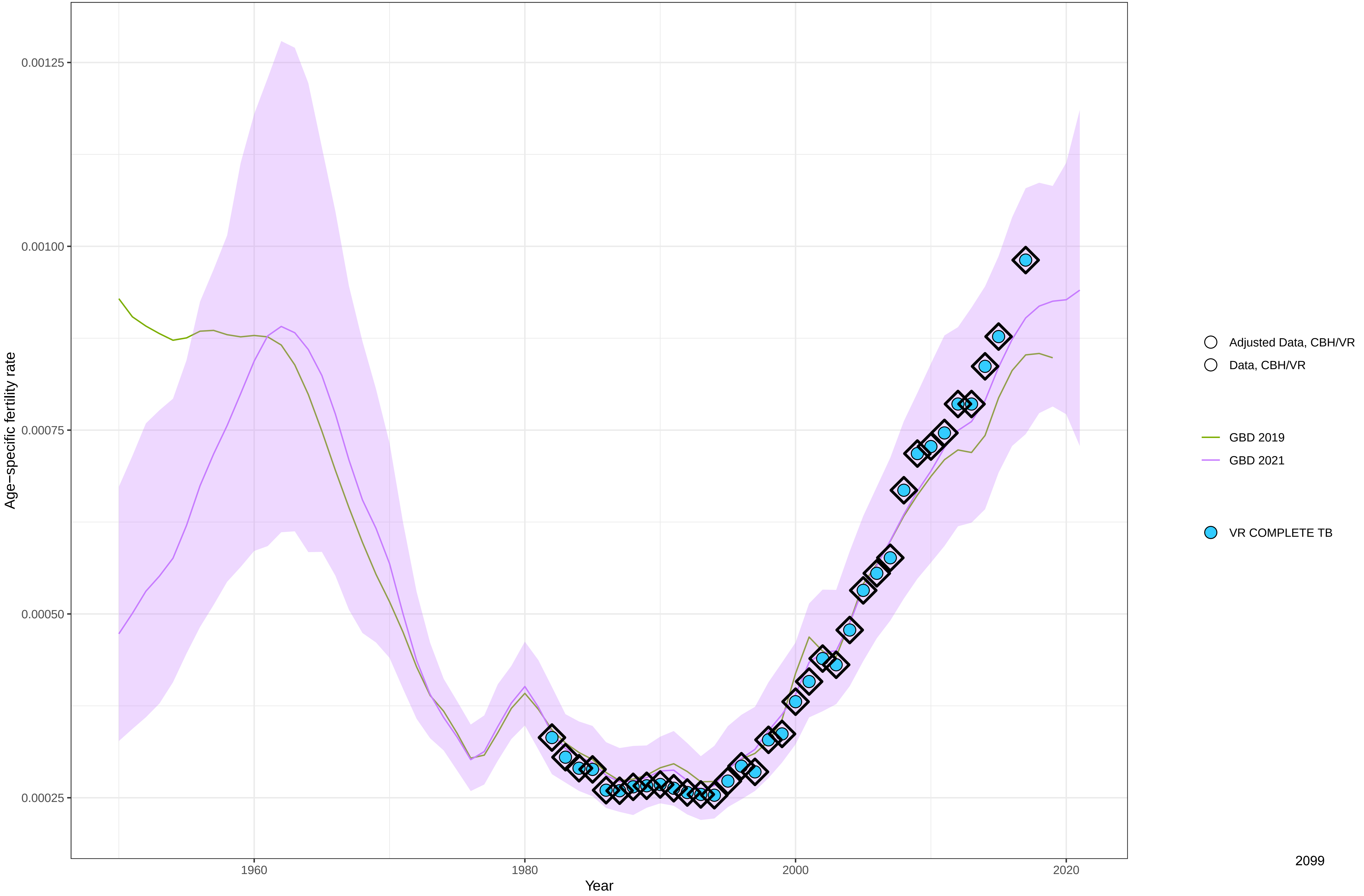

Age−specific fertility fitted trend lines, 1950−2021: Bedford, Ages: 50−54
Data points enclosed with a diamond are reference sources

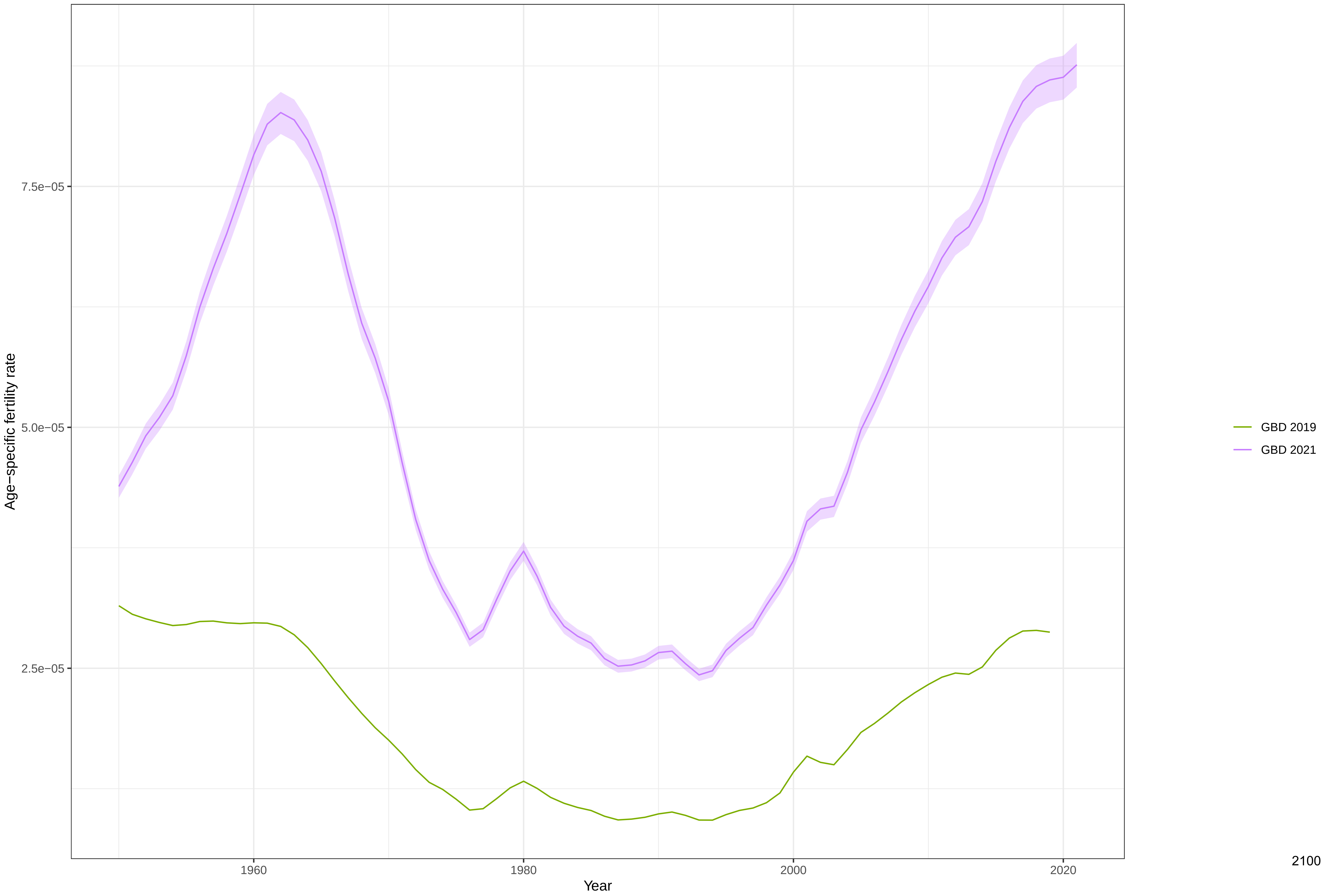

2100

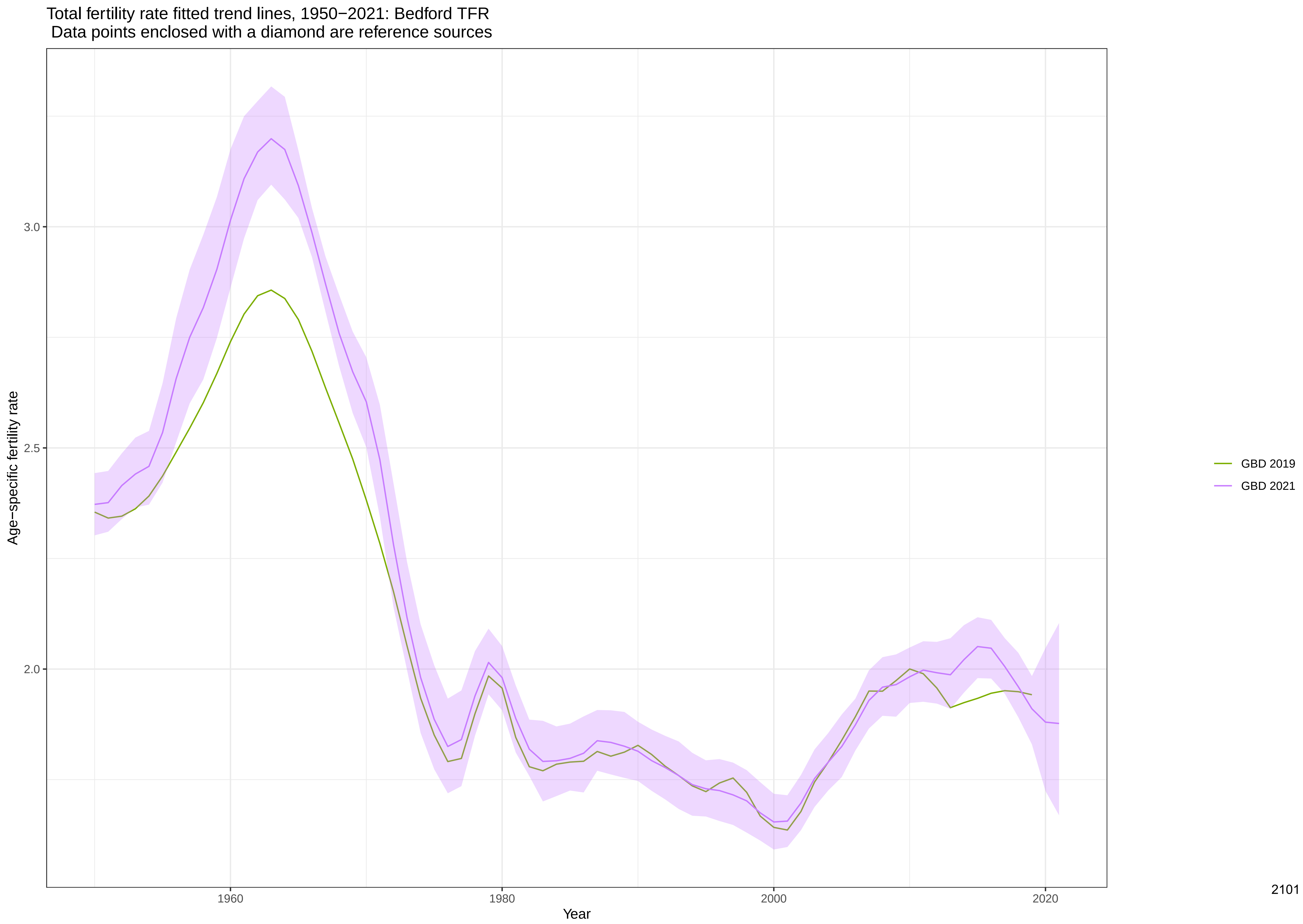

Total fertility rate fitted trend lines, 1950−2021: Bedford TFR
Data points enclosed with a diamond are reference sources
Age−specific fertility rate
3.0
2.5
2.0
1960
1980
2000
2020
Year
GBD 2019
GBD 2021

Age−specific fertility fitted trend lines, 1950−2021: Cambridgeshire, Ages: 10−14
Data points enclosed with a diamond are reference sources

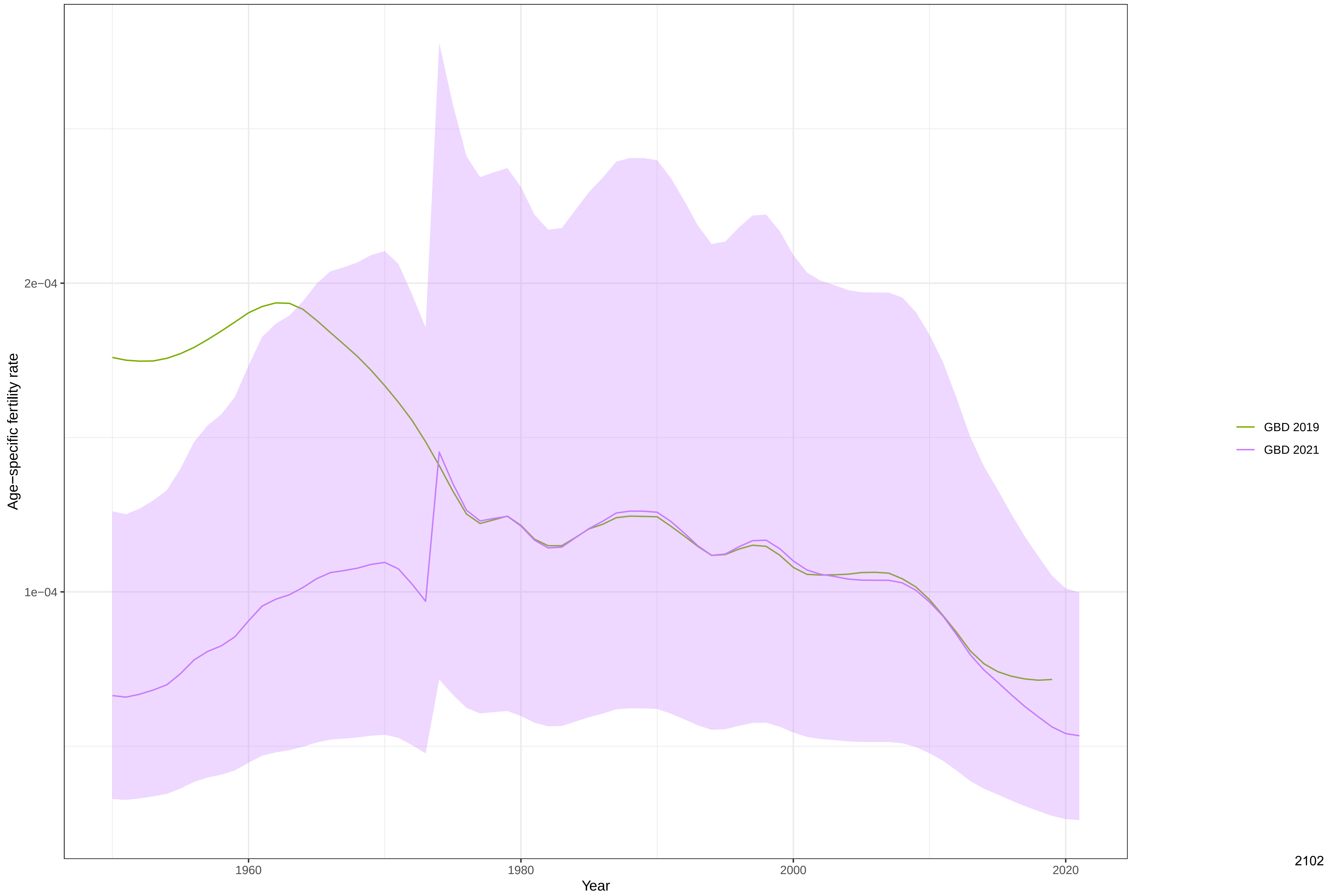

Age−specific fertility fitted trend lines, 1950−2021: Cambridgeshire, Ages: 15−19
Data points enclosed with a diamond are reference sources

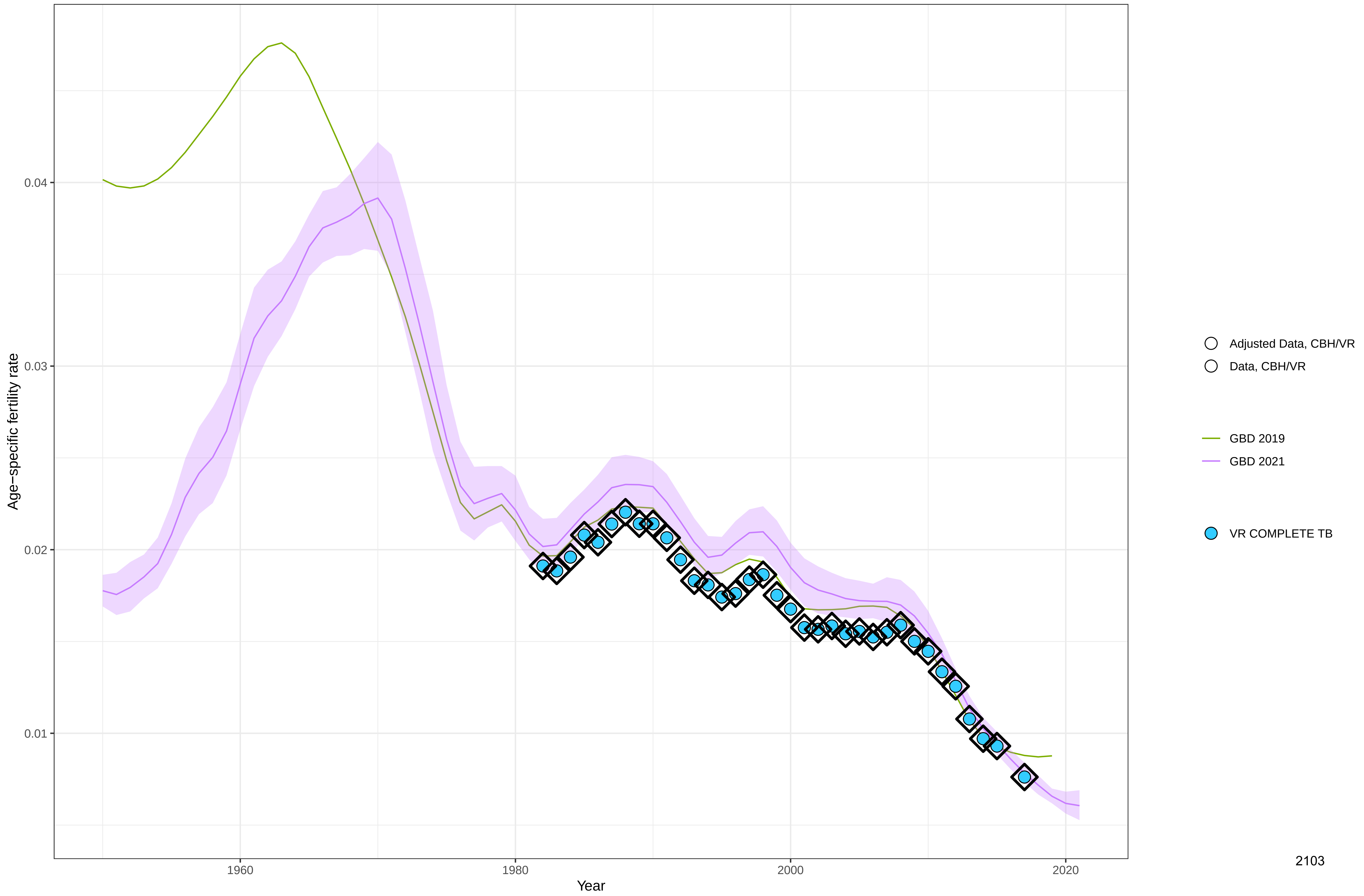

Age−specific fertility fitted trend lines, 1950−2021: Cambridgeshire, Ages: 20−24
Data points enclosed with a diamond are reference sources

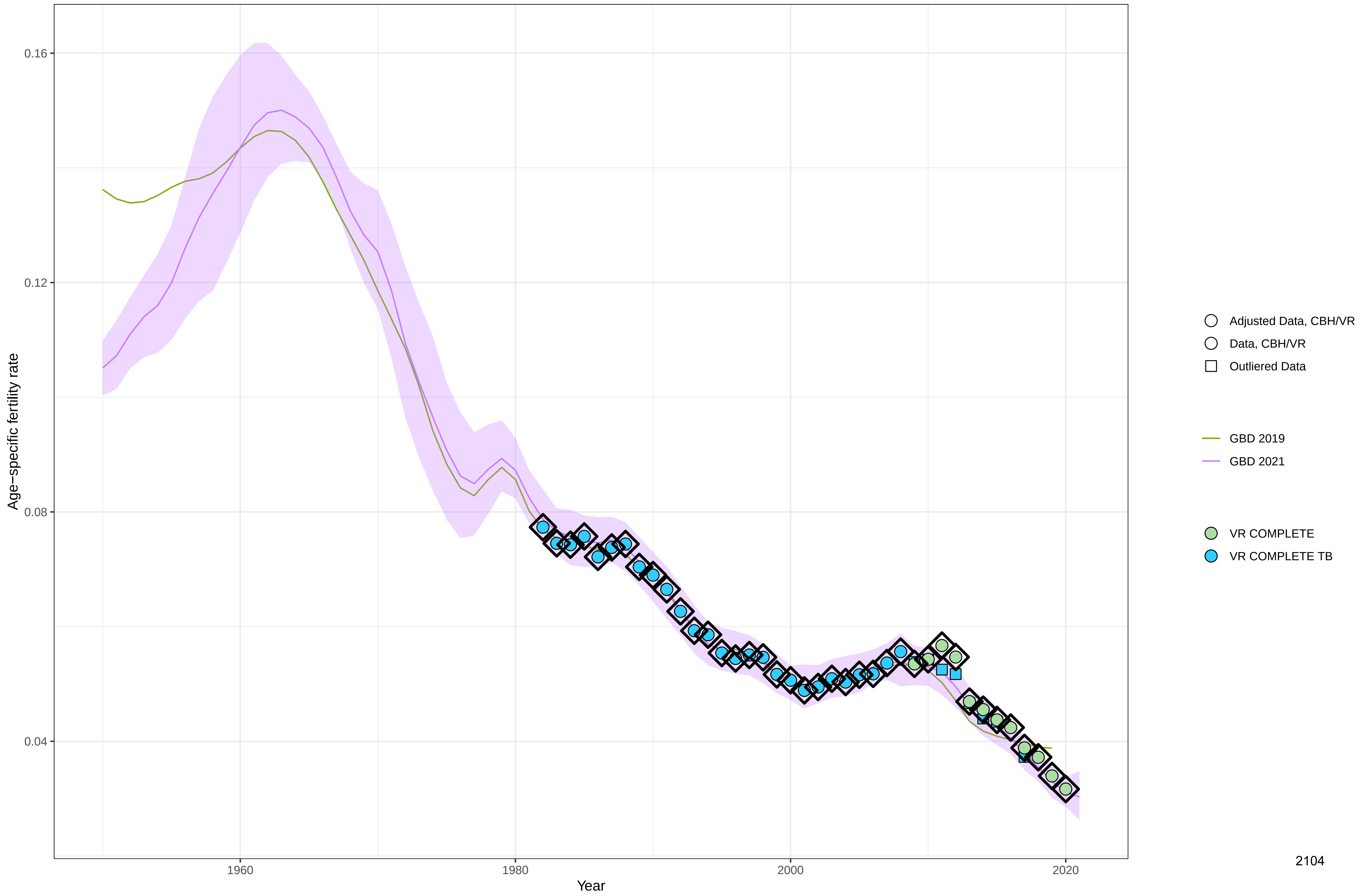

Age−specific fertility fitted trend lines, 1950−2021: Cambridgeshire, Ages: 25−29
Data points enclosed with a diamond are reference sources

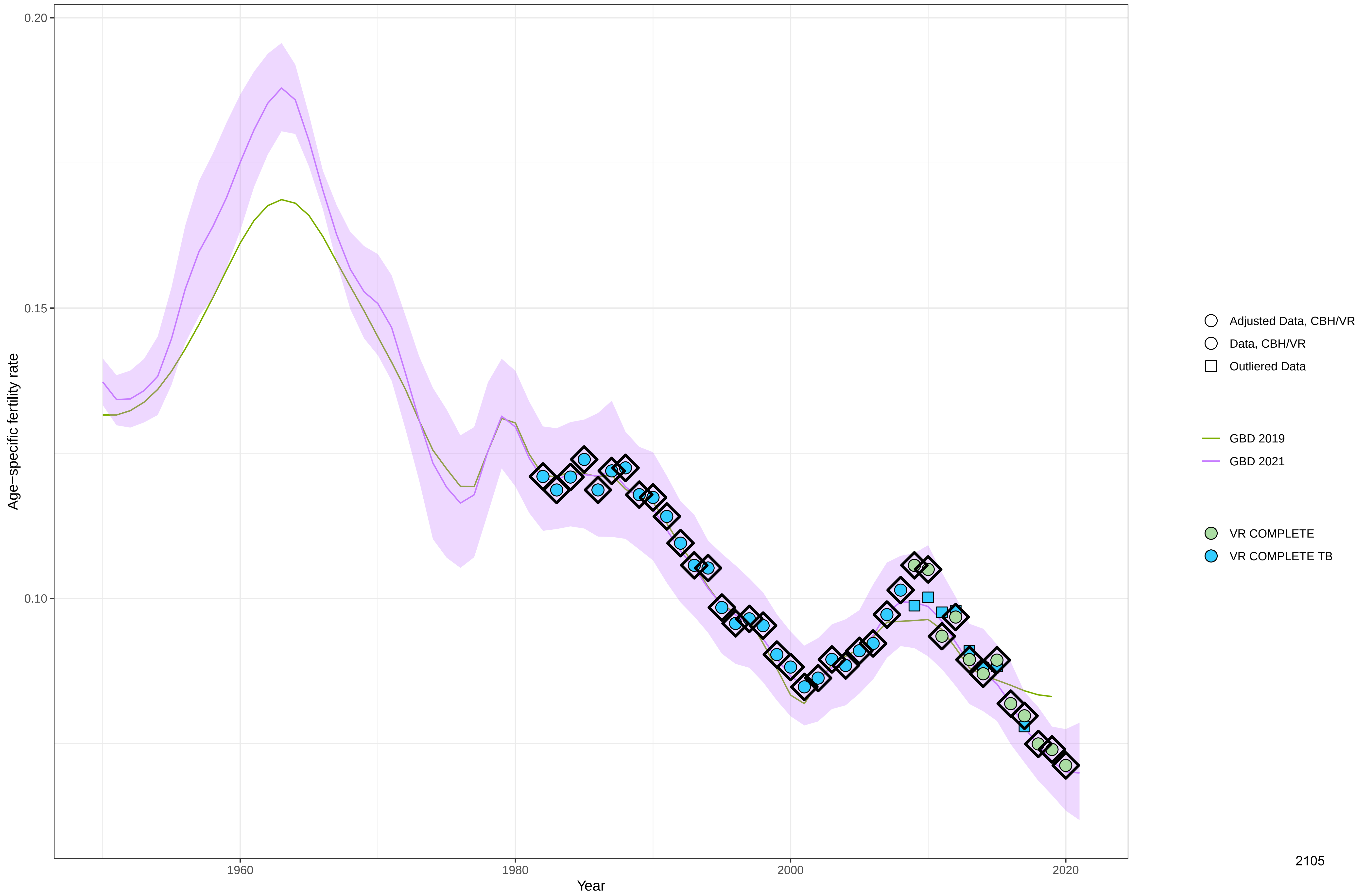

Age−specific fertility fitted trend lines, 1950−2021: Cambridgeshire, Ages: 30−34
Data points enclosed with a diamond are reference sources

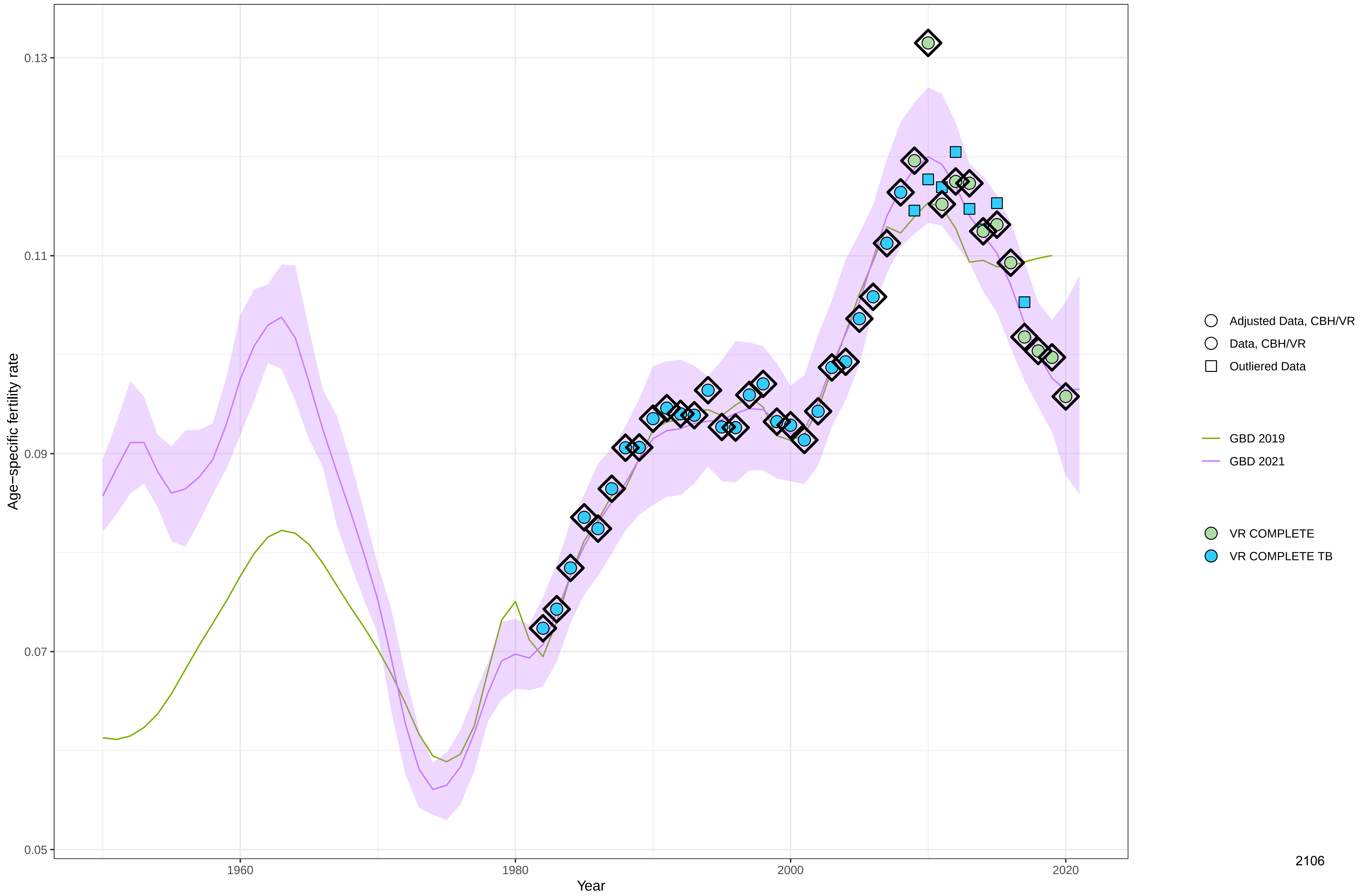

Age−specific fertility fitted trend lines, 1950−2021: Cambridgeshire, Ages: 35−39
Data points enclosed with a diamond are reference sources

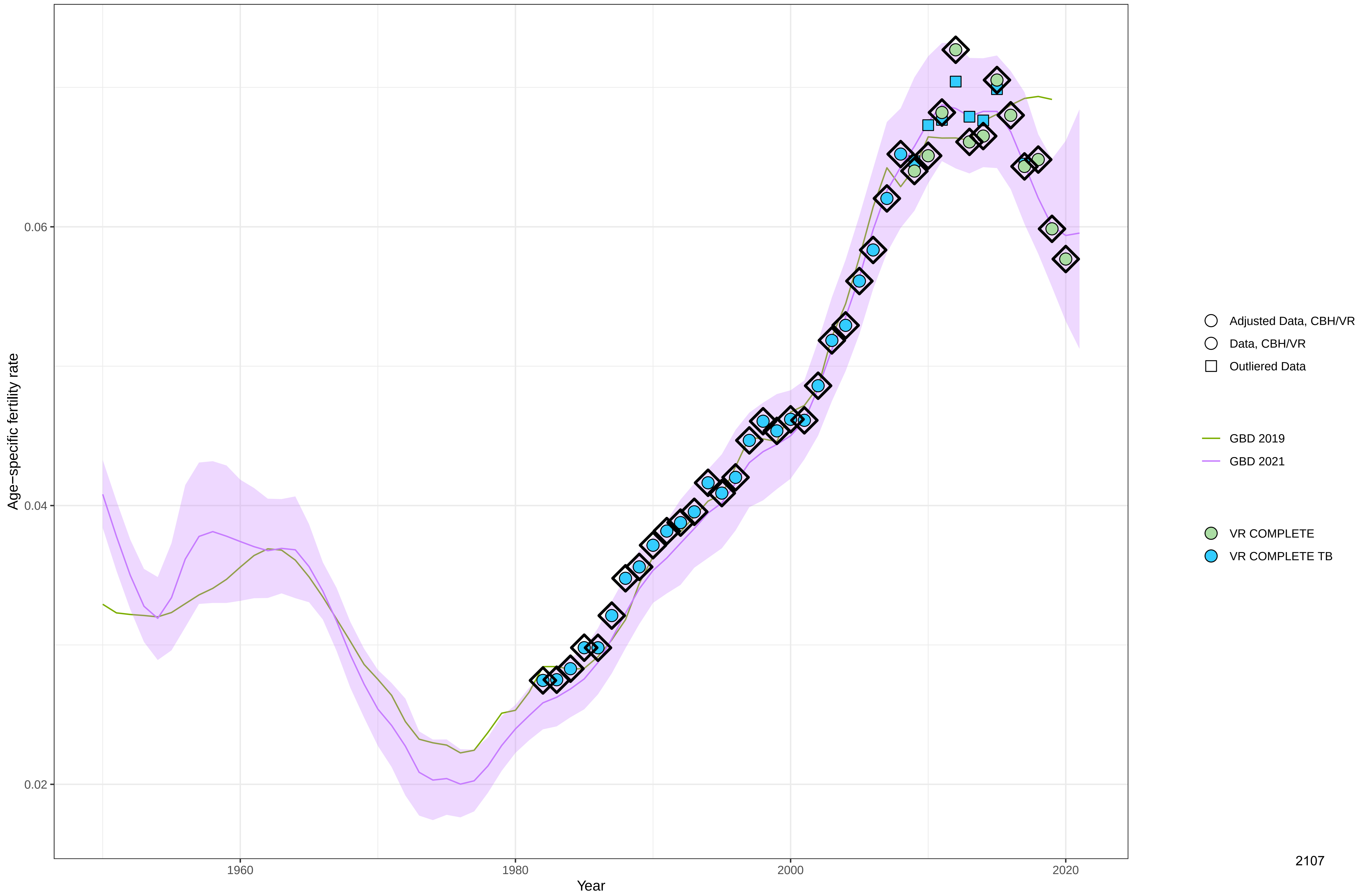

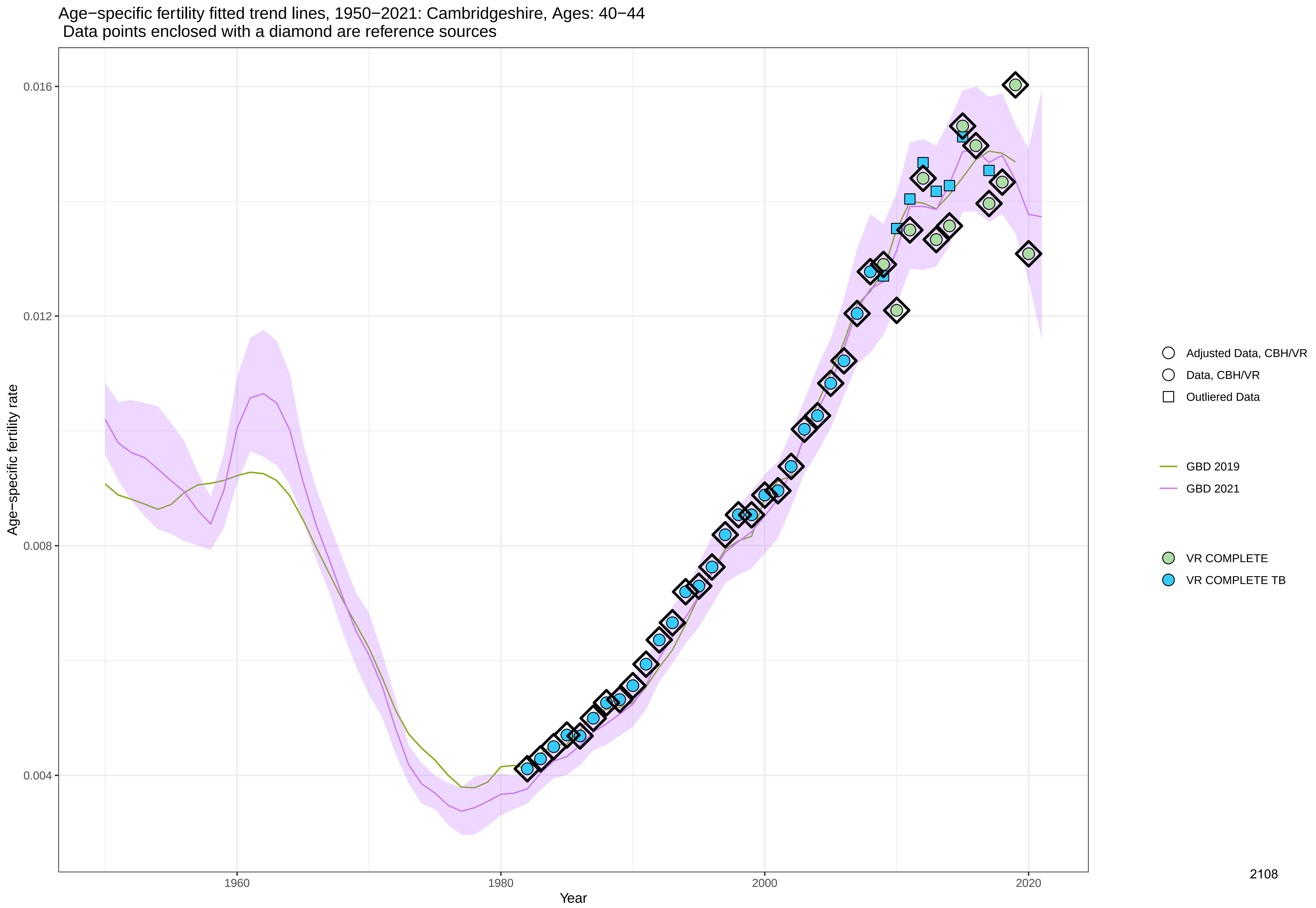

Age−specific fertility fitted trend lines, 1950−2021: Cambridgeshire, Ages: 40−44
Data points enclosed with a diamond are reference sources
0.016
0.012
0.008
0.004
Age−specific fertility rate
1960
1980
2000
2020
Year
Adjusted Data, CBH/VR
Data, CBH/VR
Outliered Data
GBD 2019
GBD 2021
VR COMPLETE
VR COMPLETE TB

Age−specific fertility fitted trend lines, 1950−2021: Cambridgeshire, Ages: 45−49
Data points enclosed with a diamond are reference sources

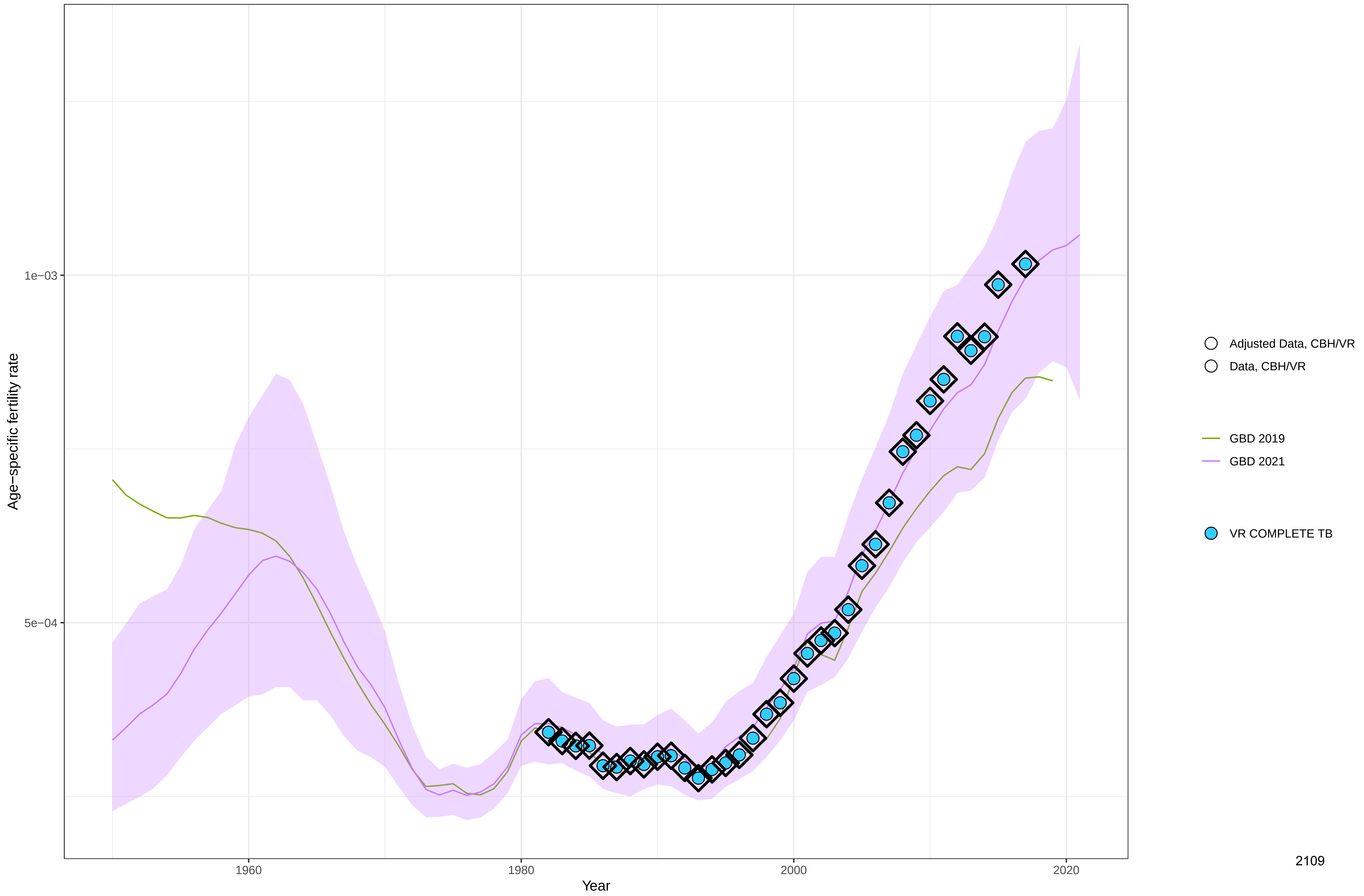

Age−specific fertility fitted trend lines, 1950−2021: Cambridgeshire, Ages: 50−54
Data points enclosed with a diamond are reference sources

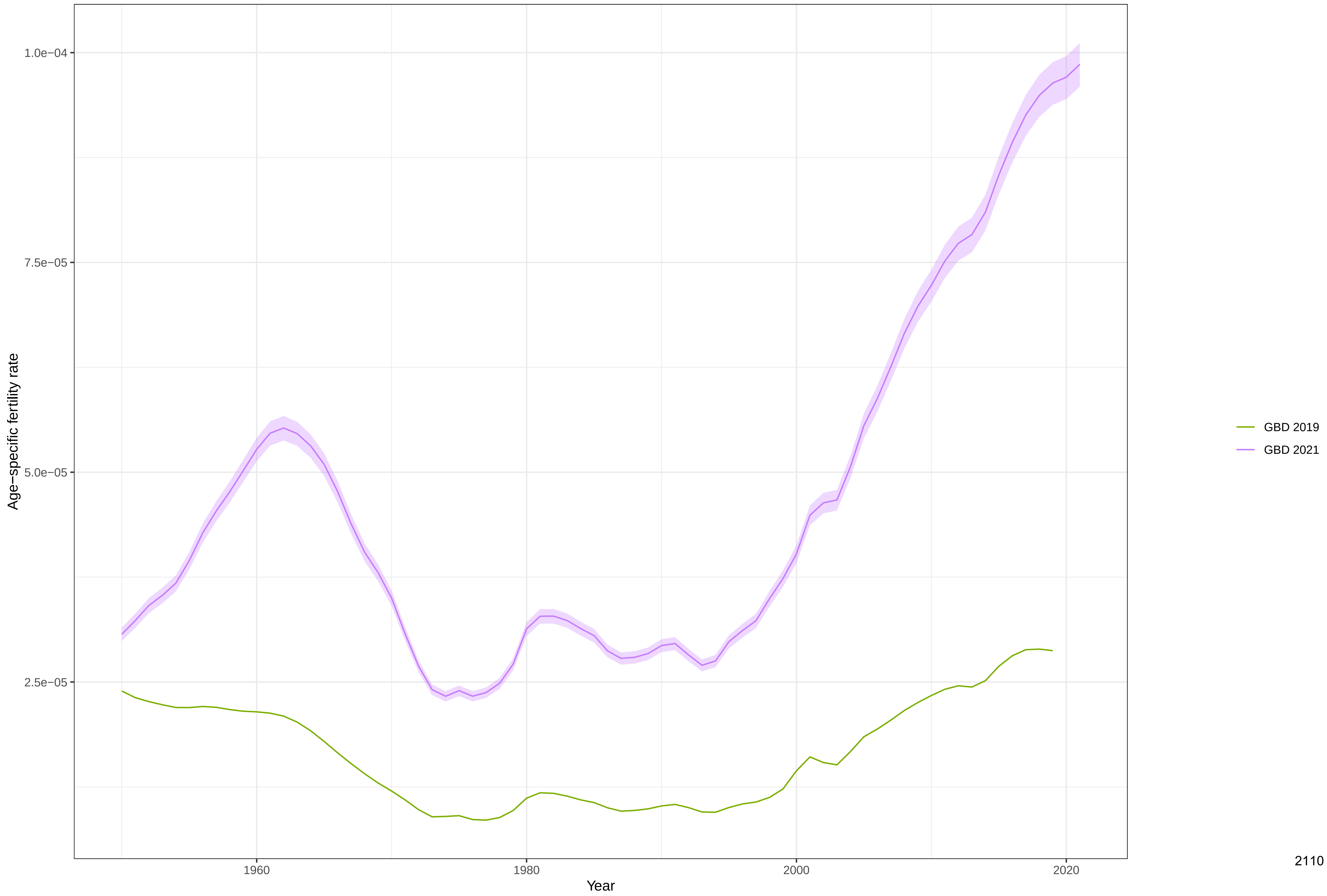

Total fertility rate fitted trend lines, 1950−2021: Cambridgeshire TFR
Data points enclosed with a diamond are reference sources

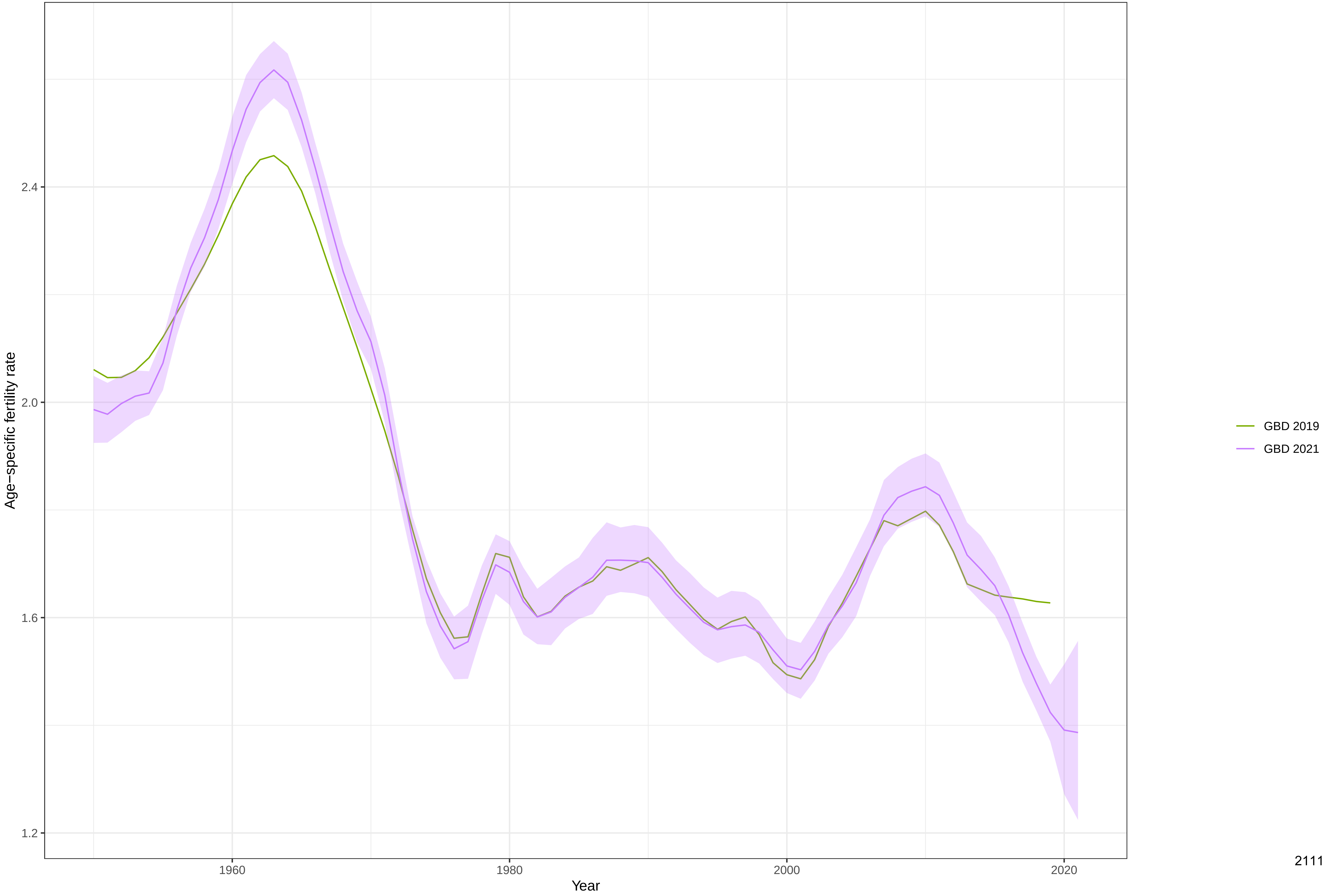

Age−specific fertility fitted trend lines, 1950−2021: Central Bedfordshire, Ages: 10−14
Data points enclosed with a diamond are reference sources

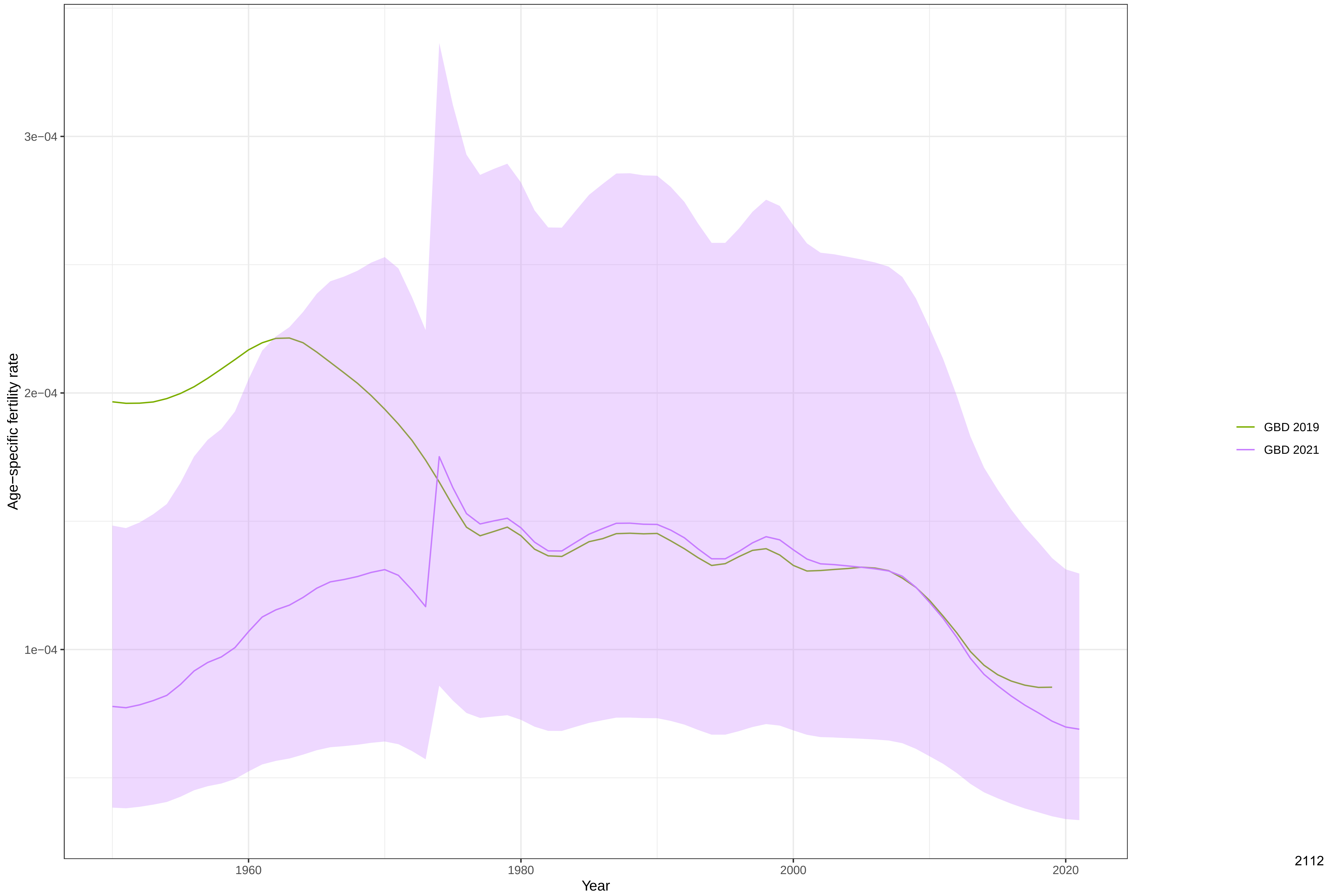

Age−specific fertility fitted trend lines, 1950−2021: Central Bedfordshire, Ages: 15−19
Data points enclosed with a diamond are reference sources

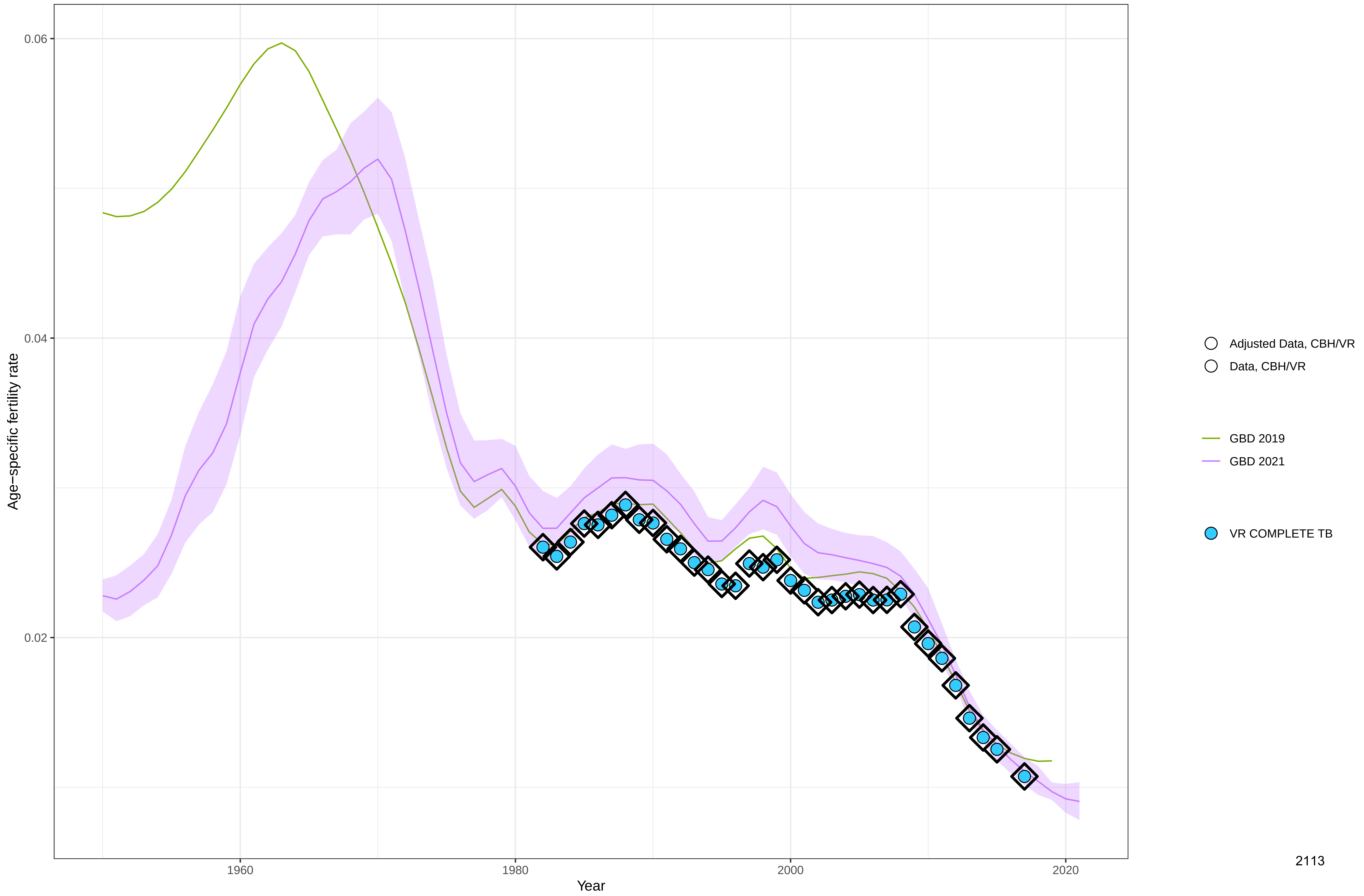

Age−specific fertility fitted trend lines, 1950−2021: Central Bedfordshire, Ages: 20−24
Data points enclosed with a diamond are reference sources

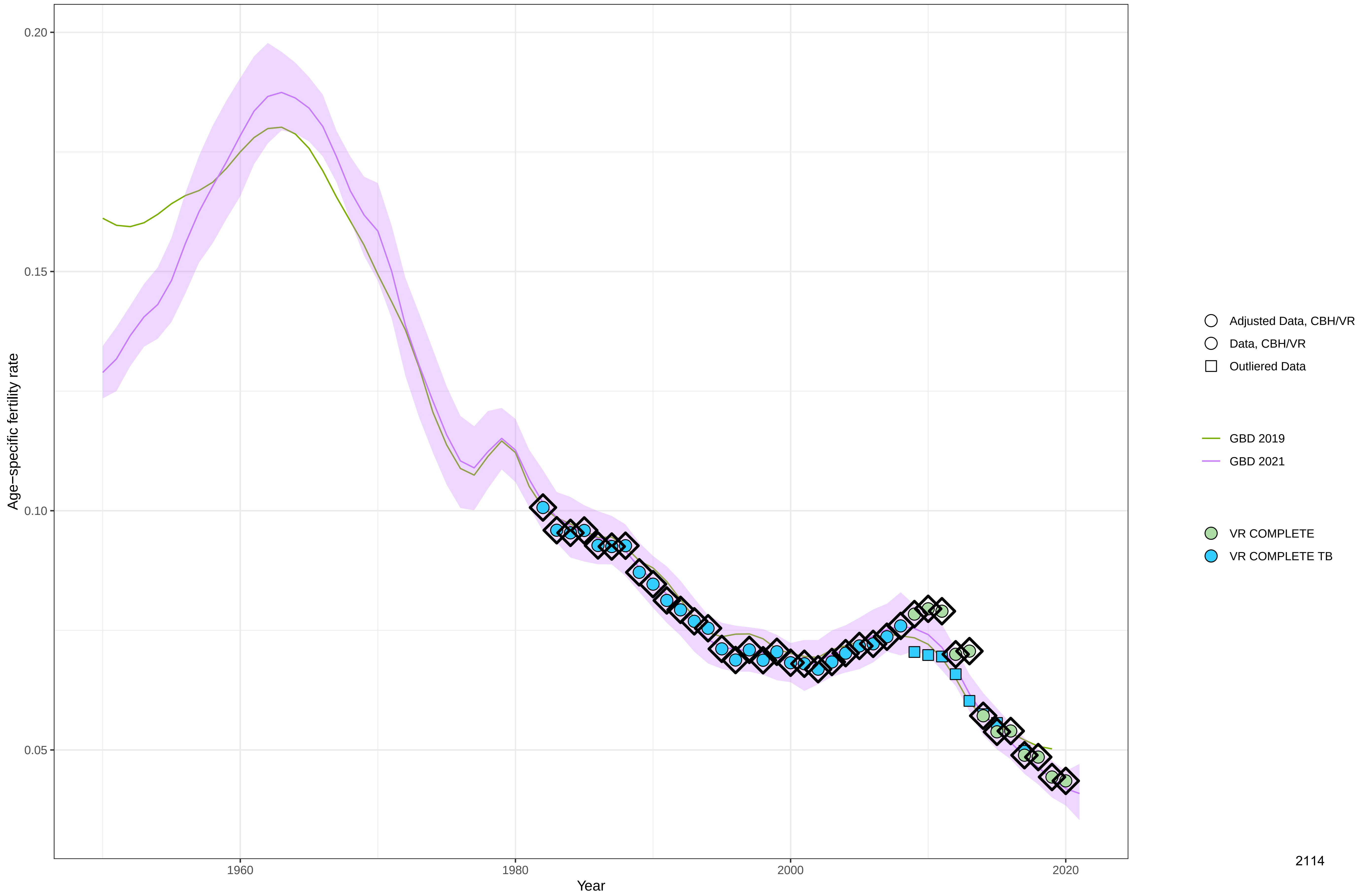

Age−specific fertility fitted trend lines, 1950−2021: Central Bedfordshire, Ages: 25−29
Data points enclosed with a diamond are reference sources

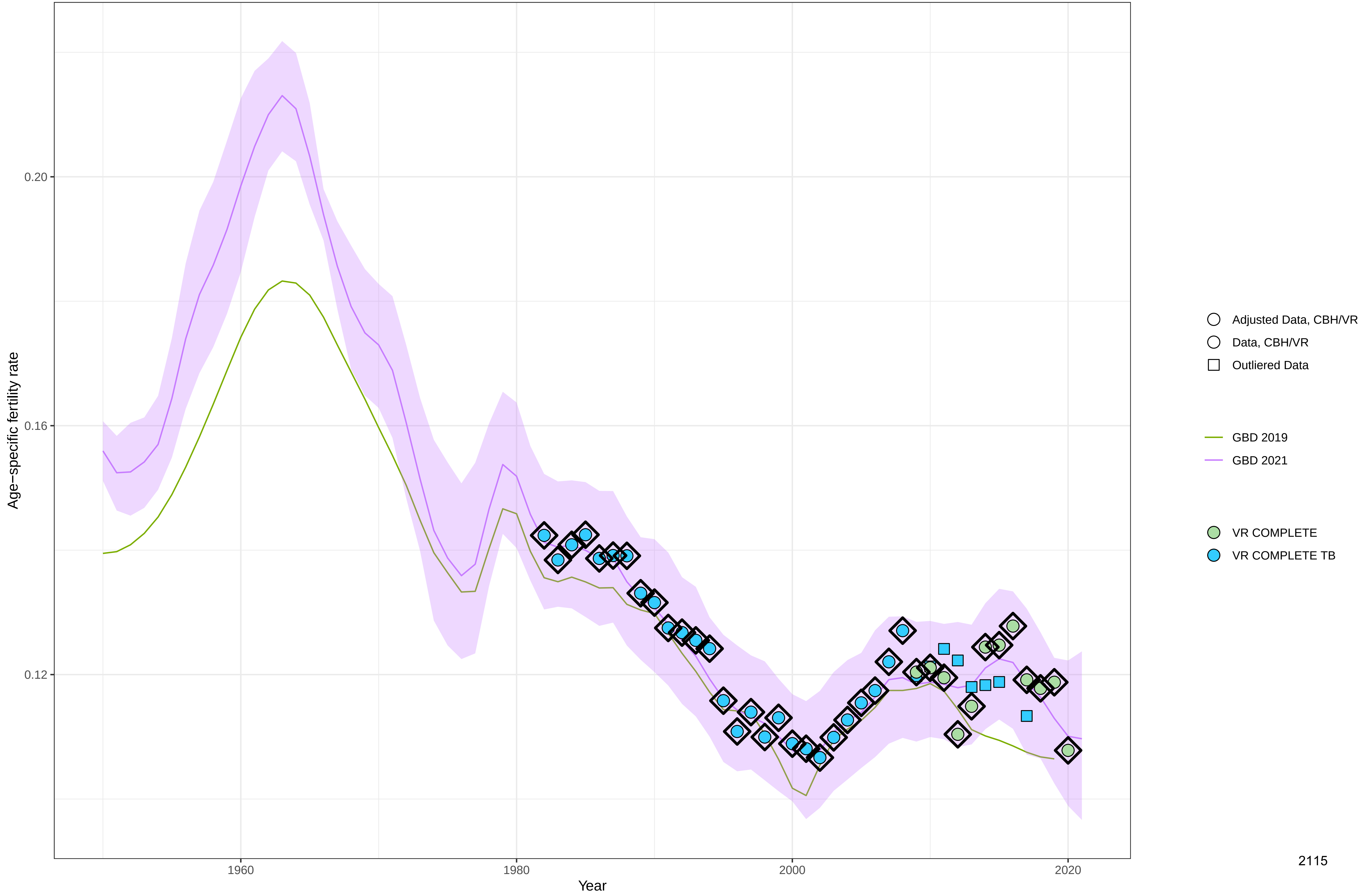

Age−specific fertility fitted trend lines, 1950−2021: Central Bedfordshire, Ages: 30−34
Data points enclosed with a diamond are reference sources

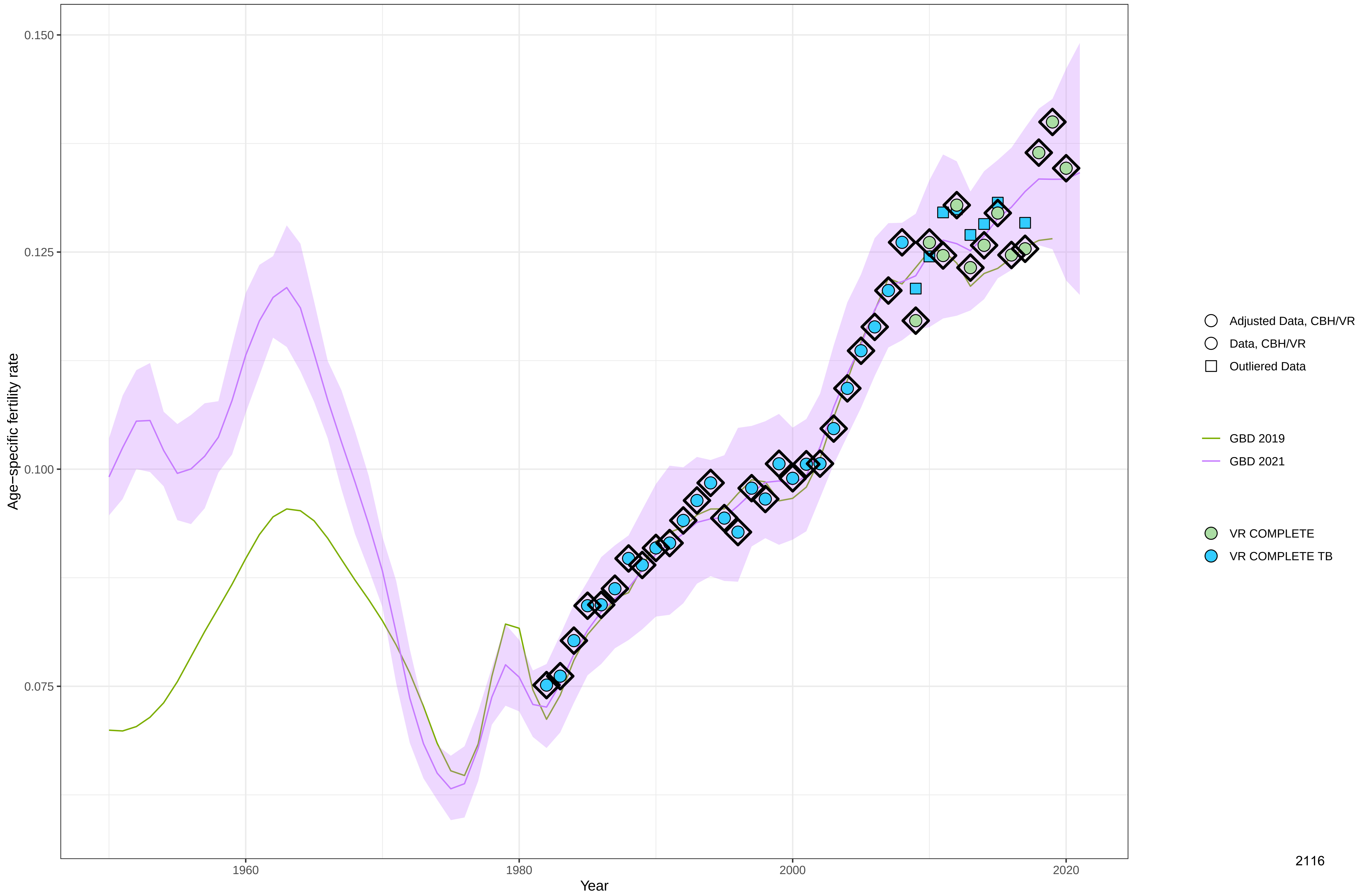

Age−specific fertility fitted trend lines, 1950−2021: Central Bedfordshire, Ages: 35−39
Data points enclosed with a diamond are reference sources

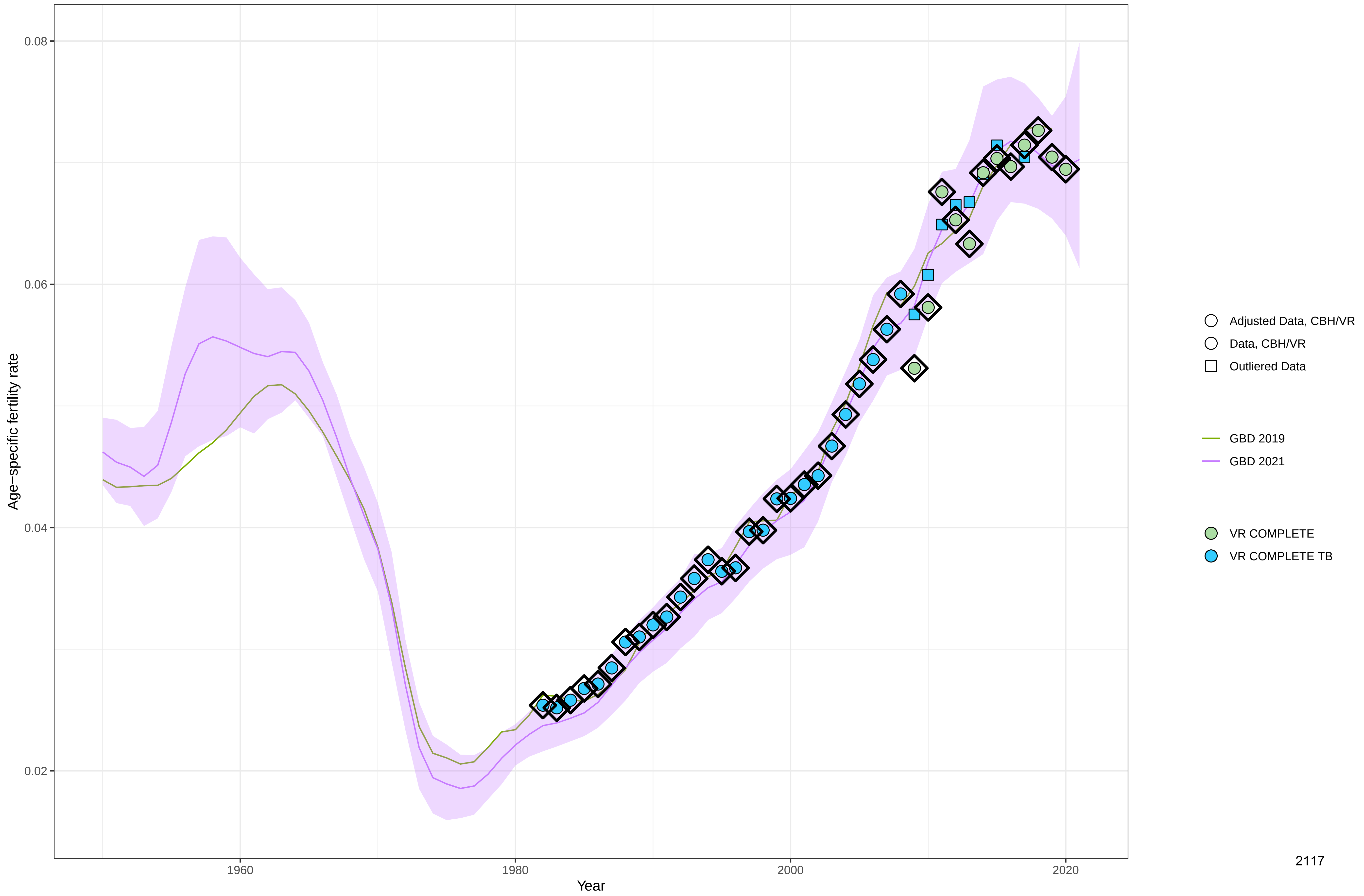

Age−specific fertility fitted trend lines, 1950−2021: Central Bedfordshire, Ages: 40−44
Data points enclosed with a diamond are reference sources

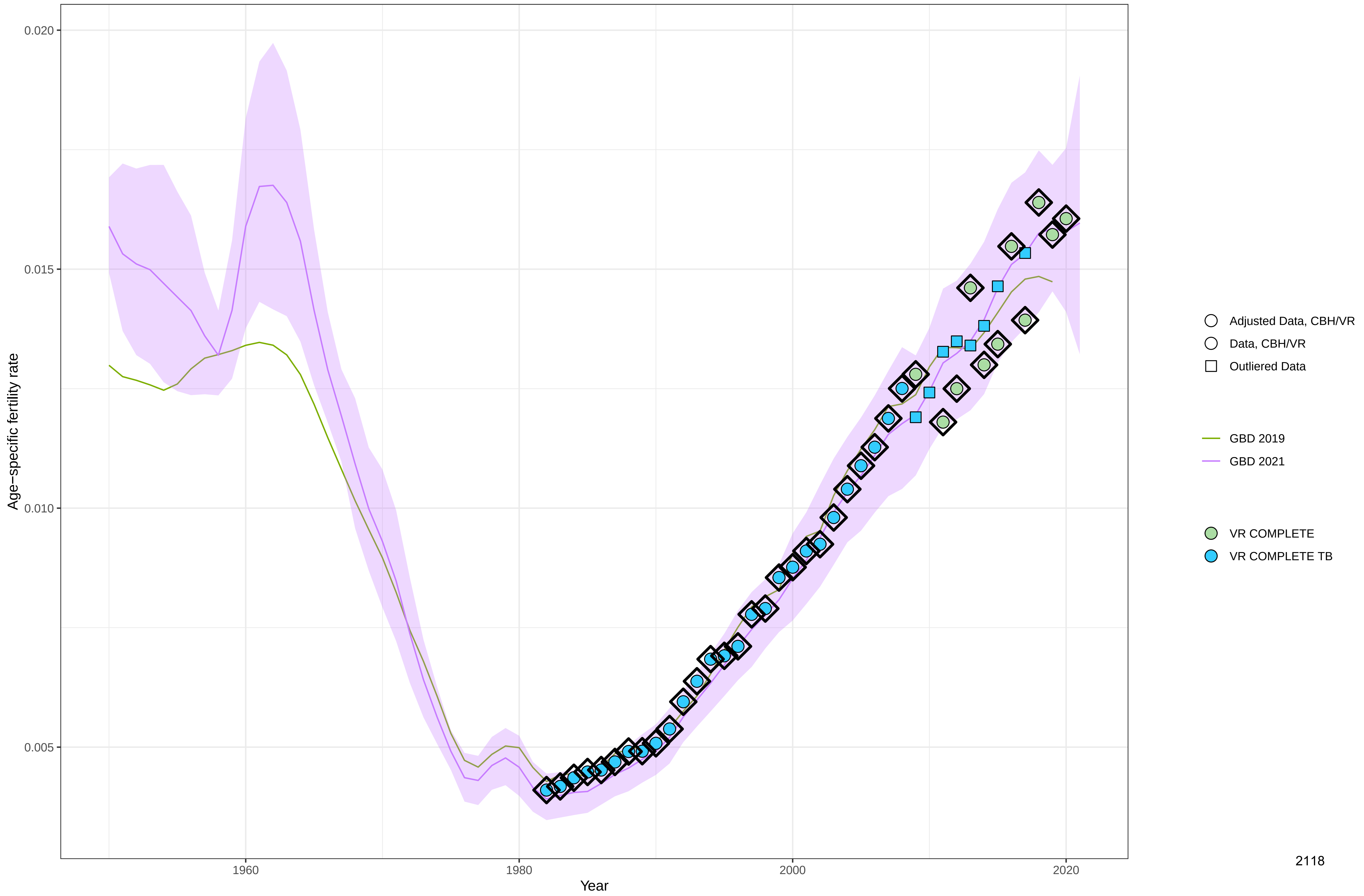

Age−specific fertility fitted trend lines, 1950−2021: Central Bedfordshire, Ages: 45−49
Data points enclosed with a diamond are reference sources

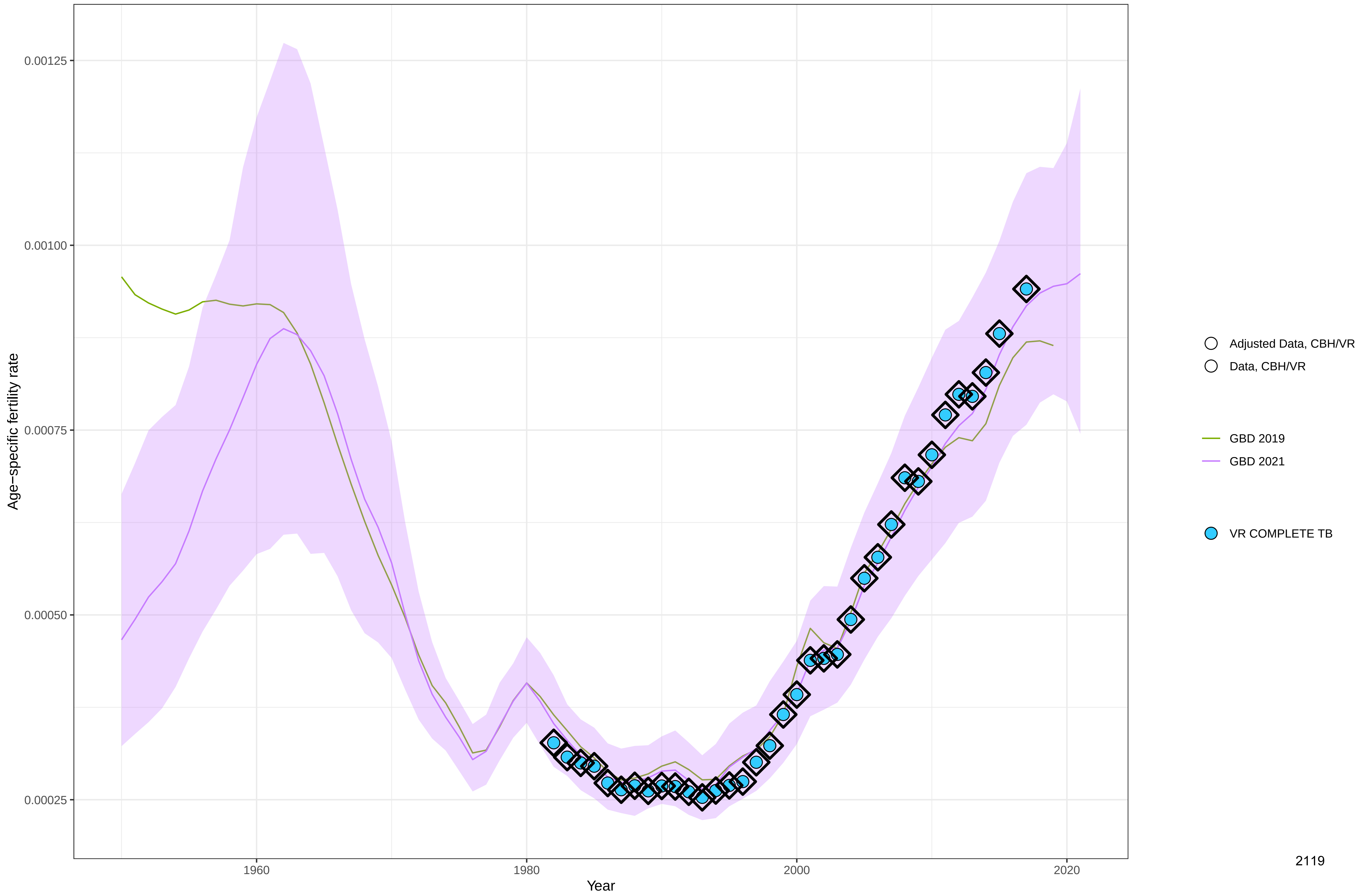

Age−specific fertility fitted trend lines, 1950−2021: Central Bedfordshire, Ages: 50−54
Data points enclosed with a diamond are reference sources

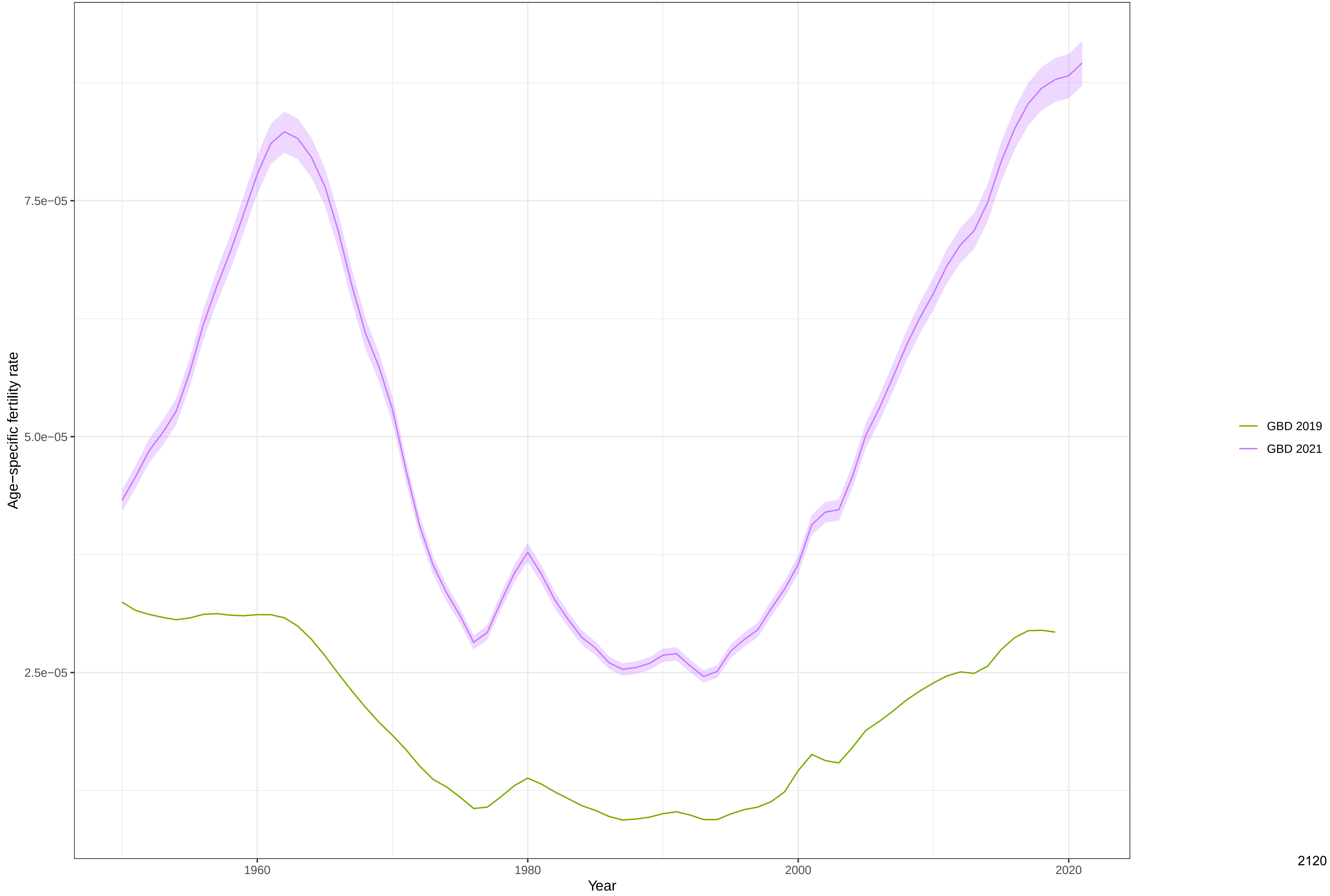

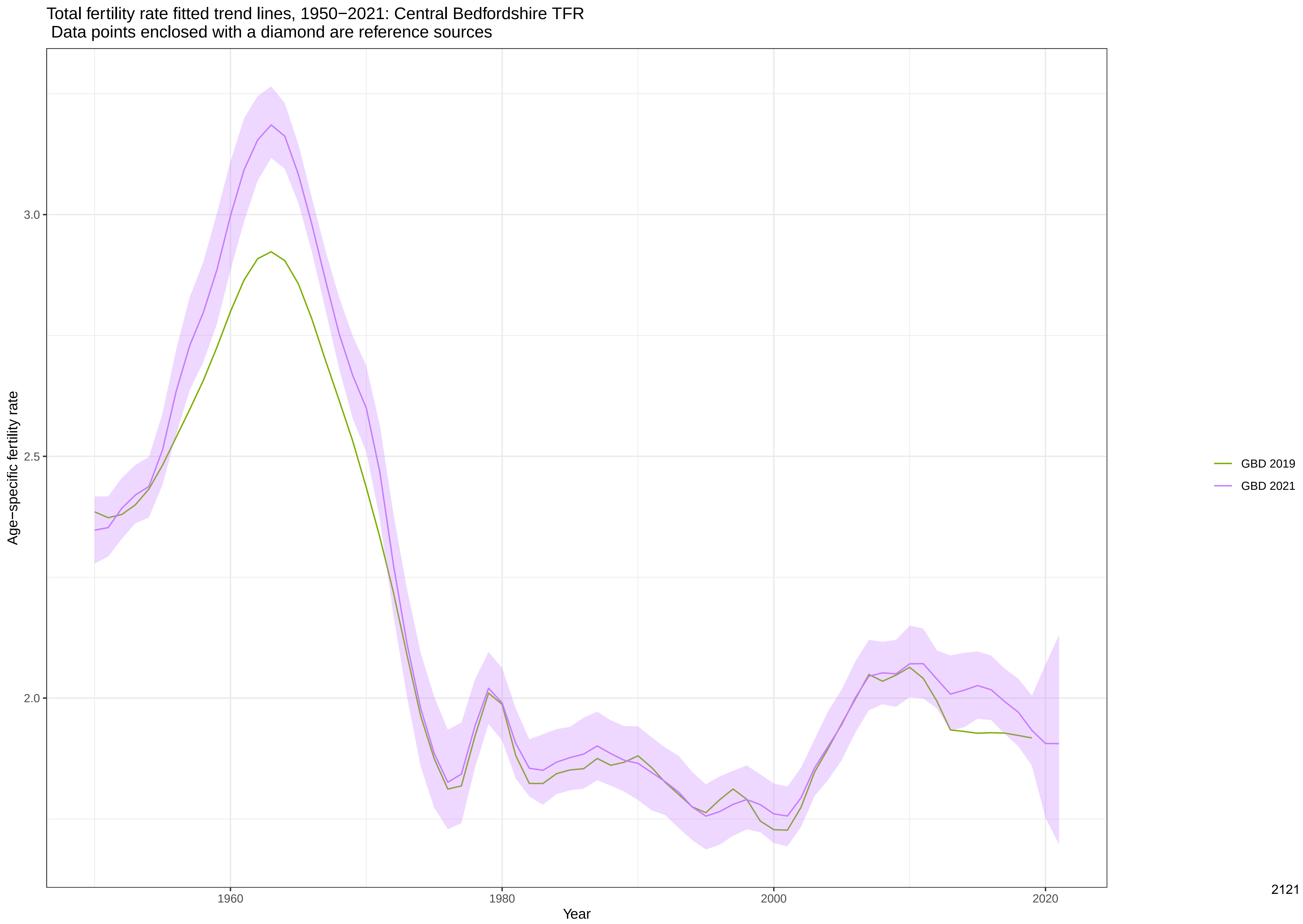

Total fertility rate fitted trend lines, 1950−2021: Central Bedfordshire TFR
Data points enclosed with a diamond are reference sources
Age−specific fertility rate
3.0
2.5
2.0
1960
1980
2000
2020
Year
GBD 2019
GBD 2021

Age−specific fertility fitted trend lines, 1950−2021: Essex, Ages: 10−14
Data points enclosed with a diamond are reference sources

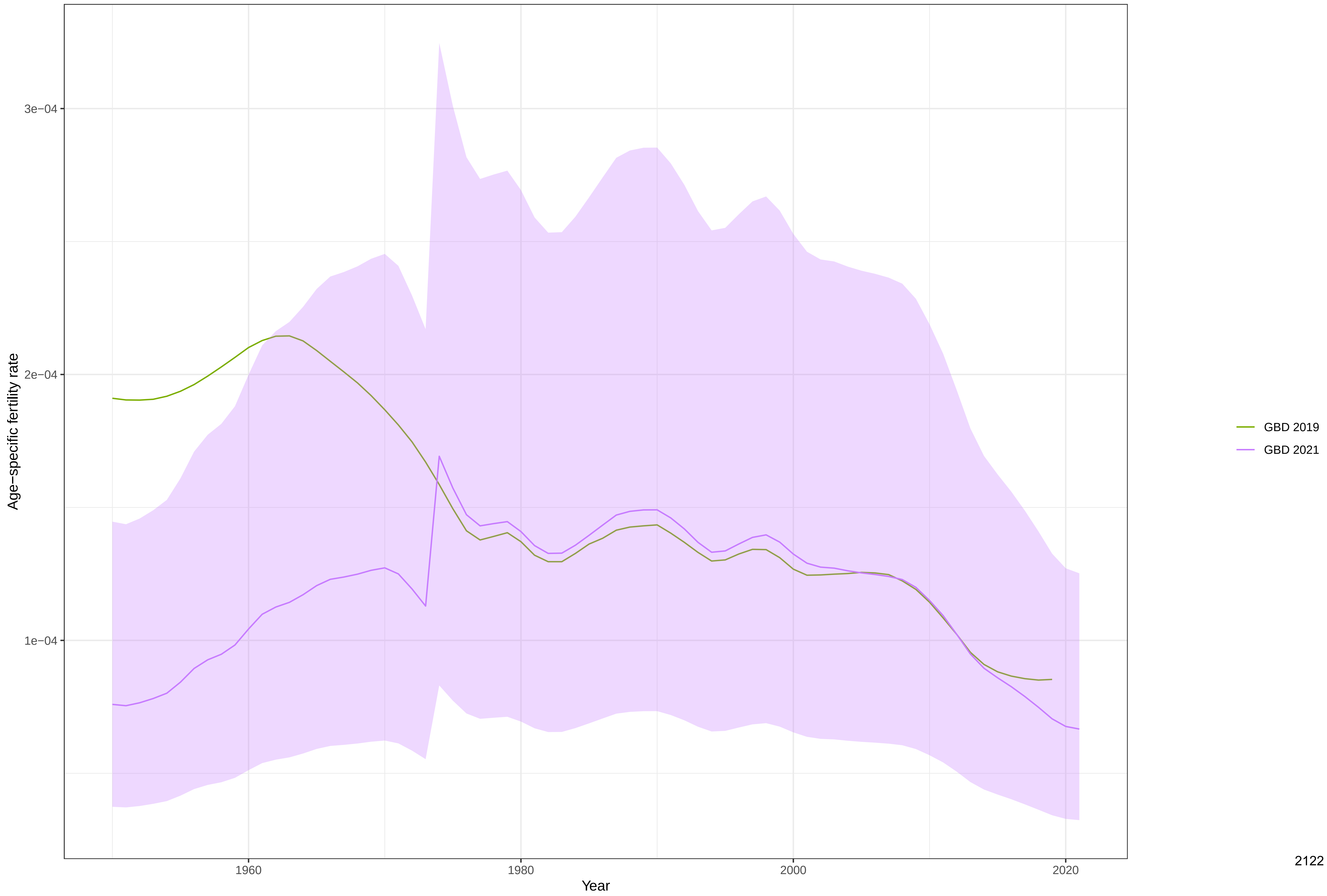

Age−specific fertility fitted trend lines, 1950−2021: Essex, Ages: 15−19
Data points enclosed with a diamond are reference sources

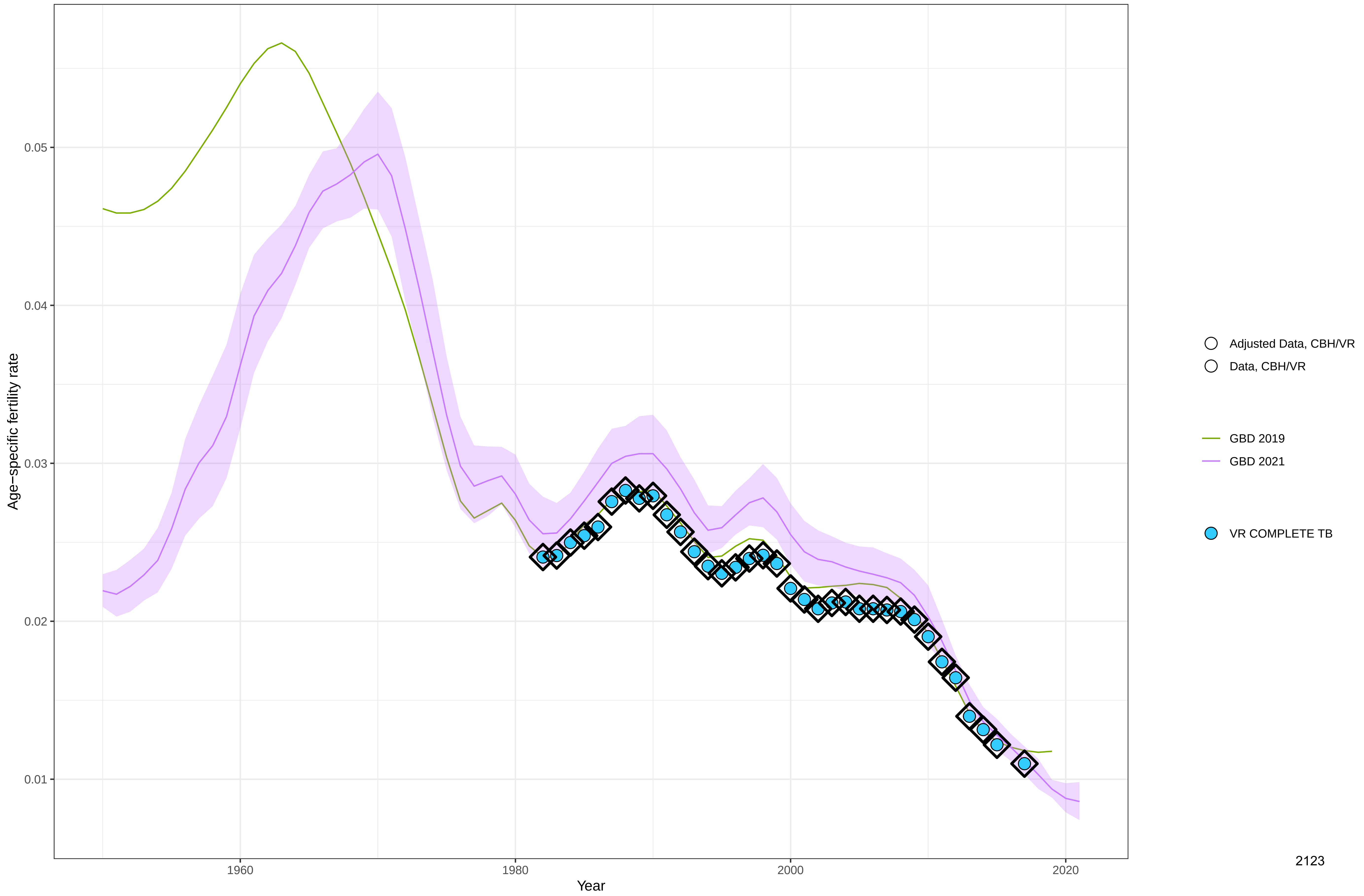

Age−specific fertility fitted trend lines, 1950−2021: Essex, Ages: 20−24
Data points enclosed with a diamond are reference sources

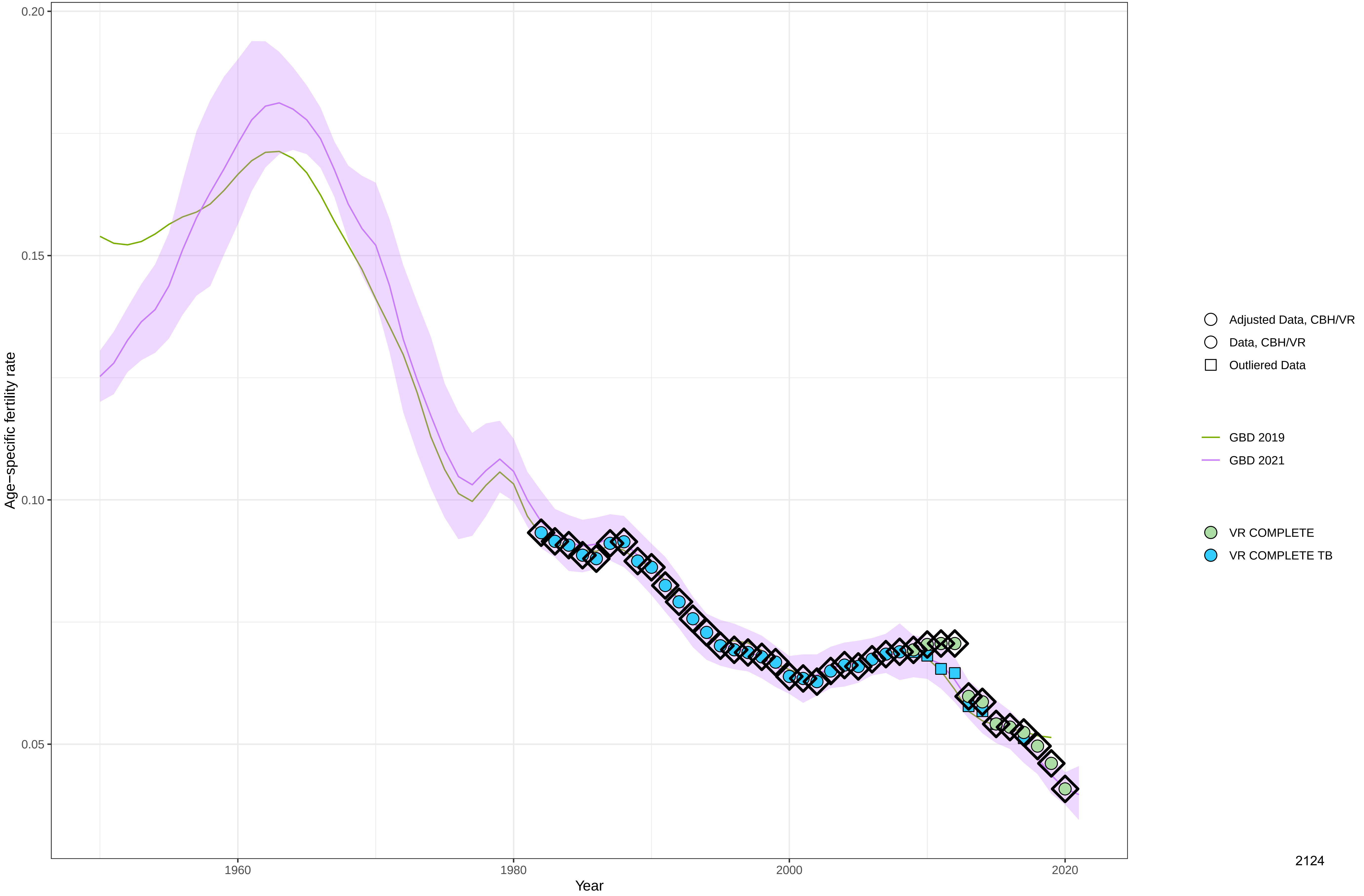

Age−specific fertility fitted trend lines, 1950−2021: Essex, Ages: 25−29
Data points enclosed with a diamond are reference sources

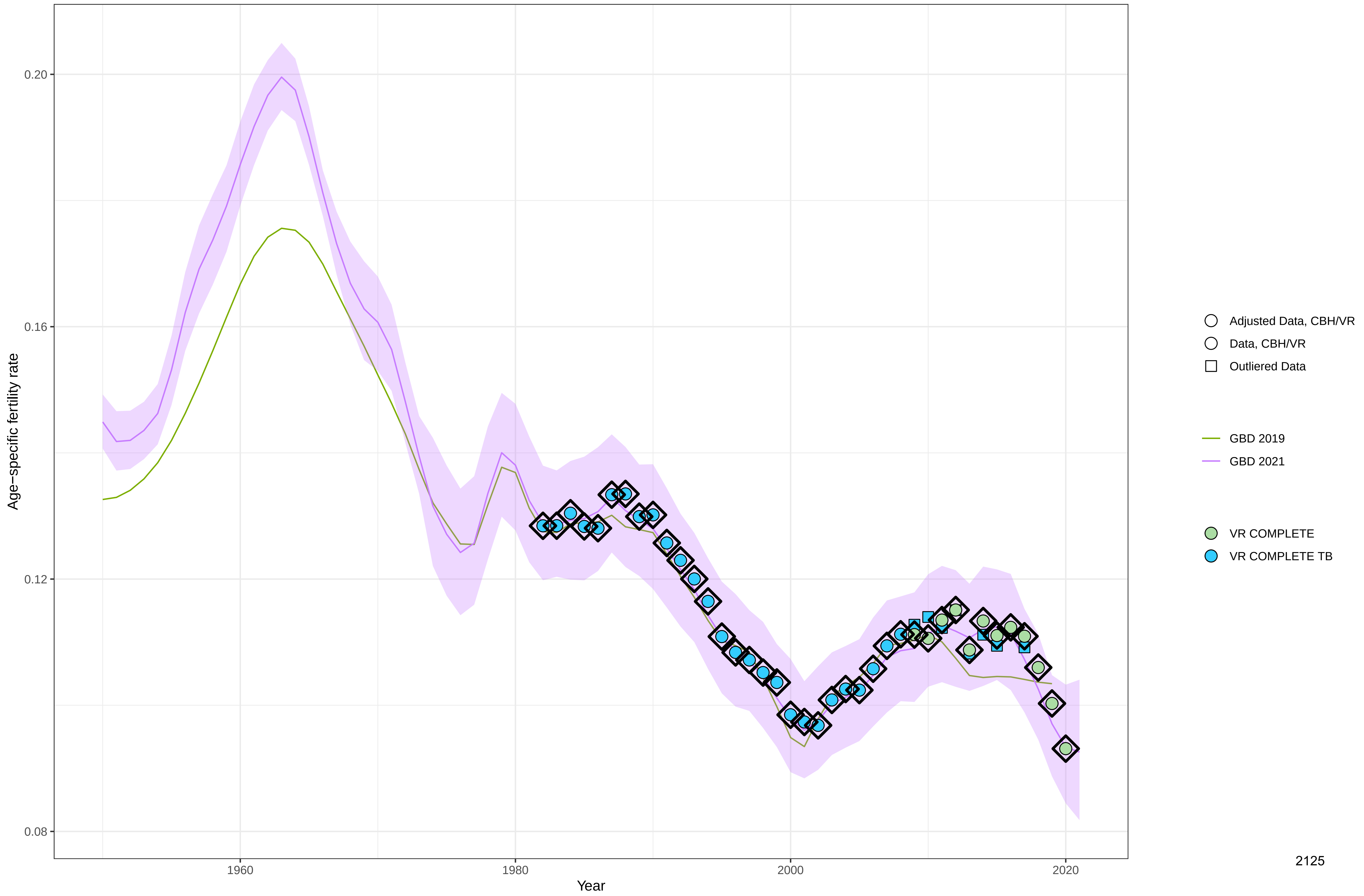

Age−specific fertility fitted trend lines, 1950−2021: Essex, Ages: 30−34
Data points enclosed with a diamond are reference sources

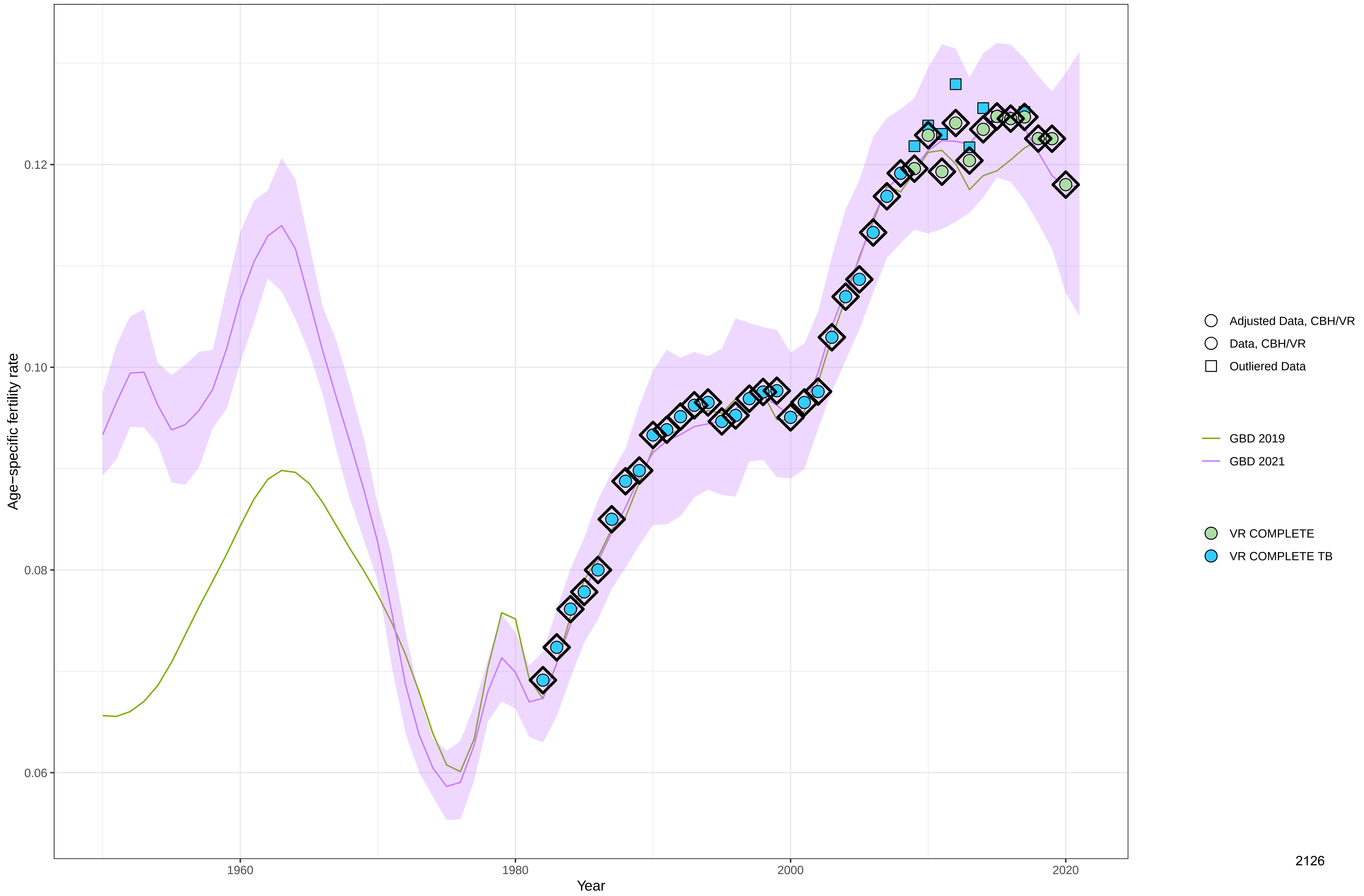

Age−specific fertility fitted trend lines, 1950−2021: Essex, Ages: 35−39
Data points enclosed with a diamond are reference sources

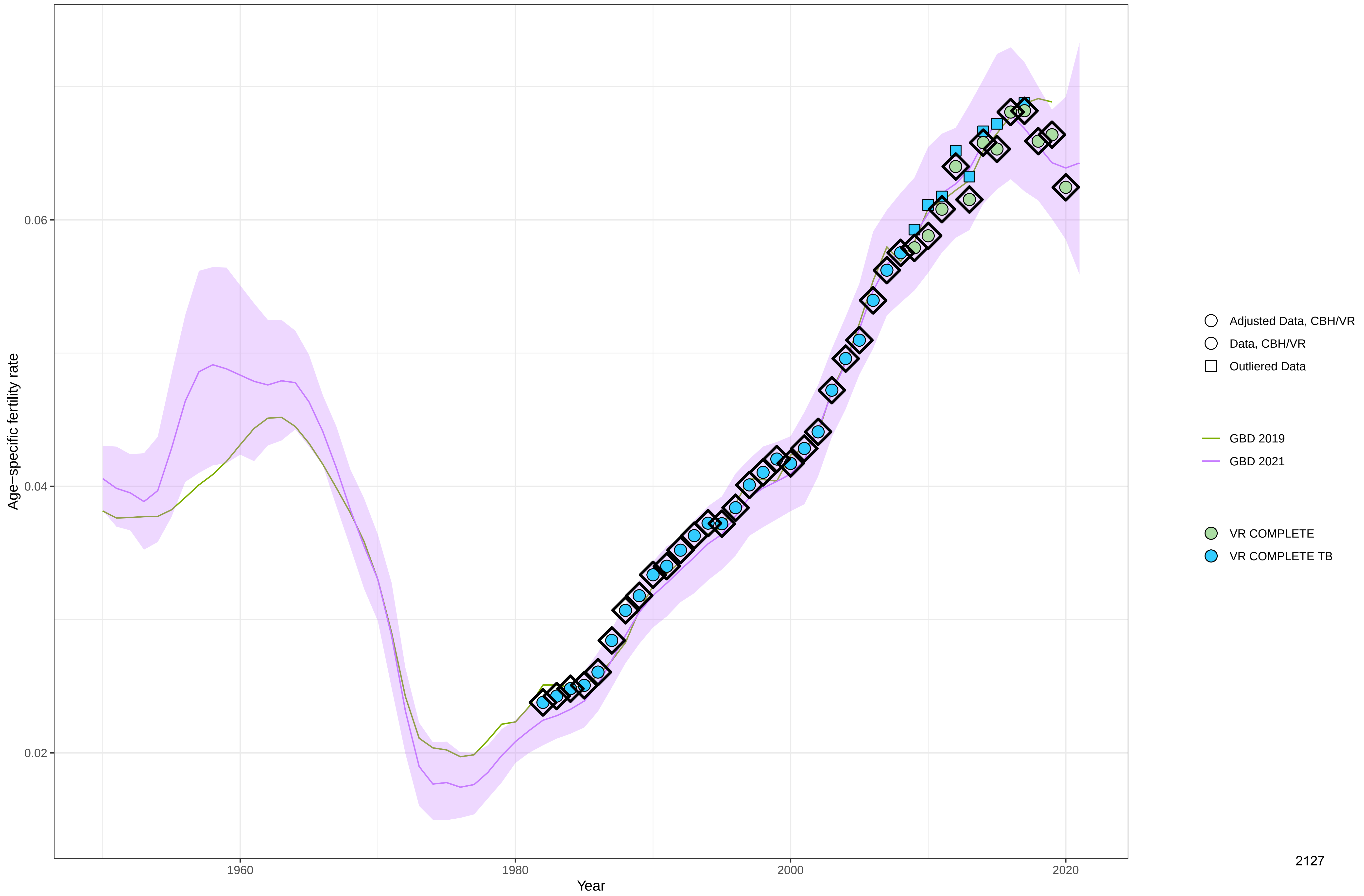

Age−specific fertility fitted trend lines, 1950−2021: Essex, Ages: 40−44
Data points enclosed with a diamond are reference sources

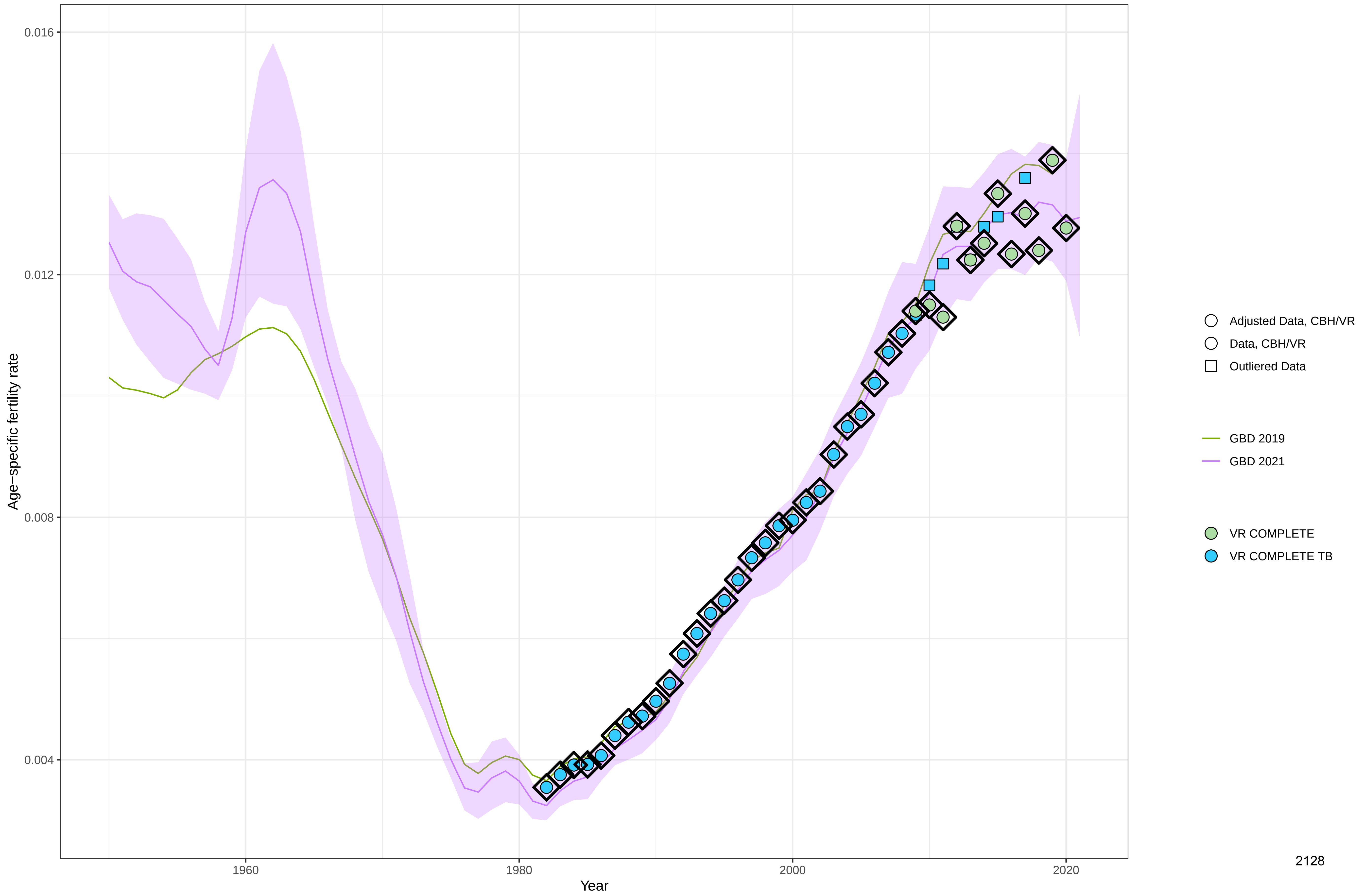

Age−specific fertility fitted trend lines, 1950−2021: Essex, Ages: 45−49
Data points enclosed with a diamond are reference sources

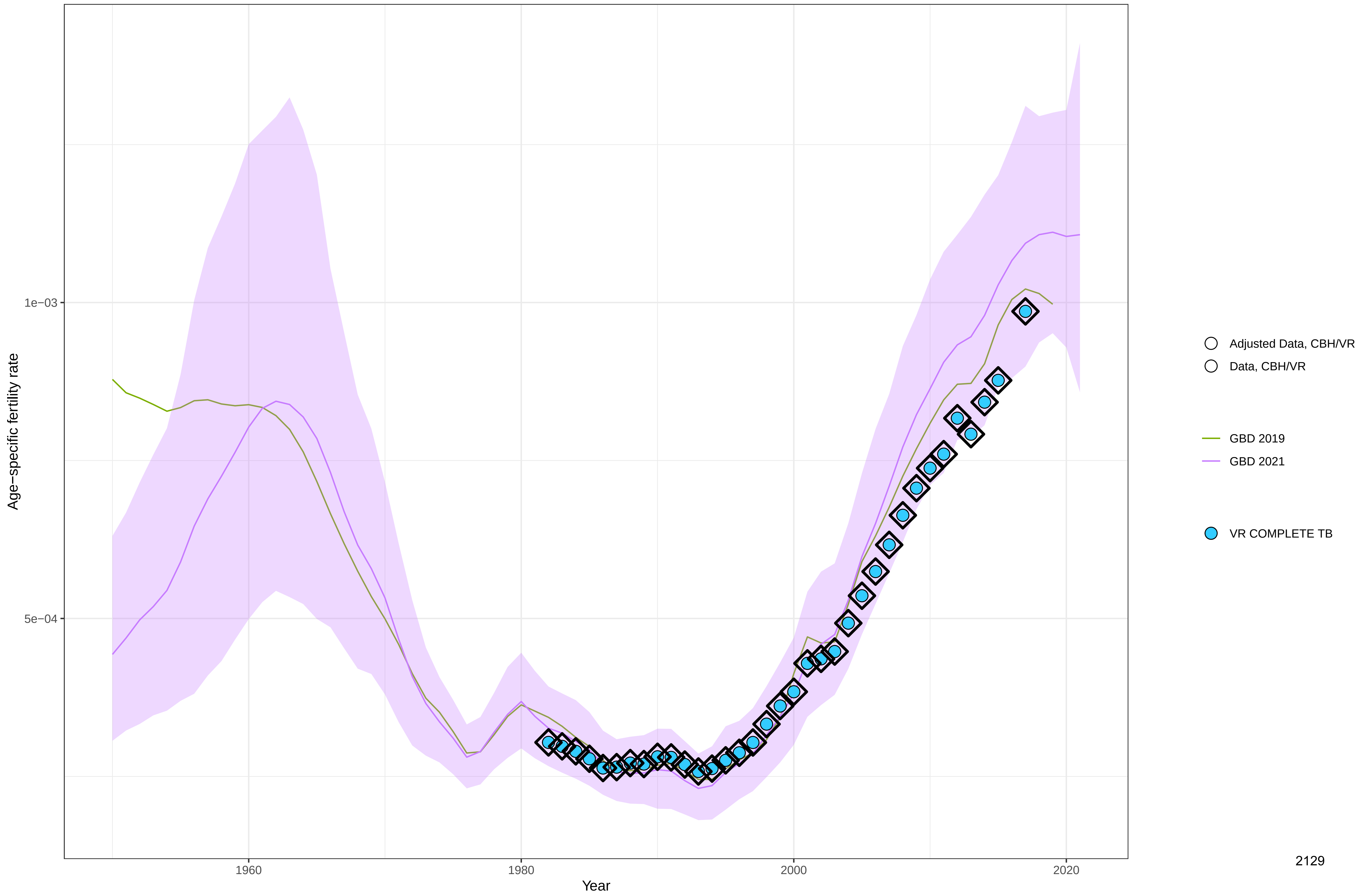

Age−specific fertility fitted trend lines, 1950−2021: Essex, Ages: 50−54
Data points enclosed with a diamond are reference sources

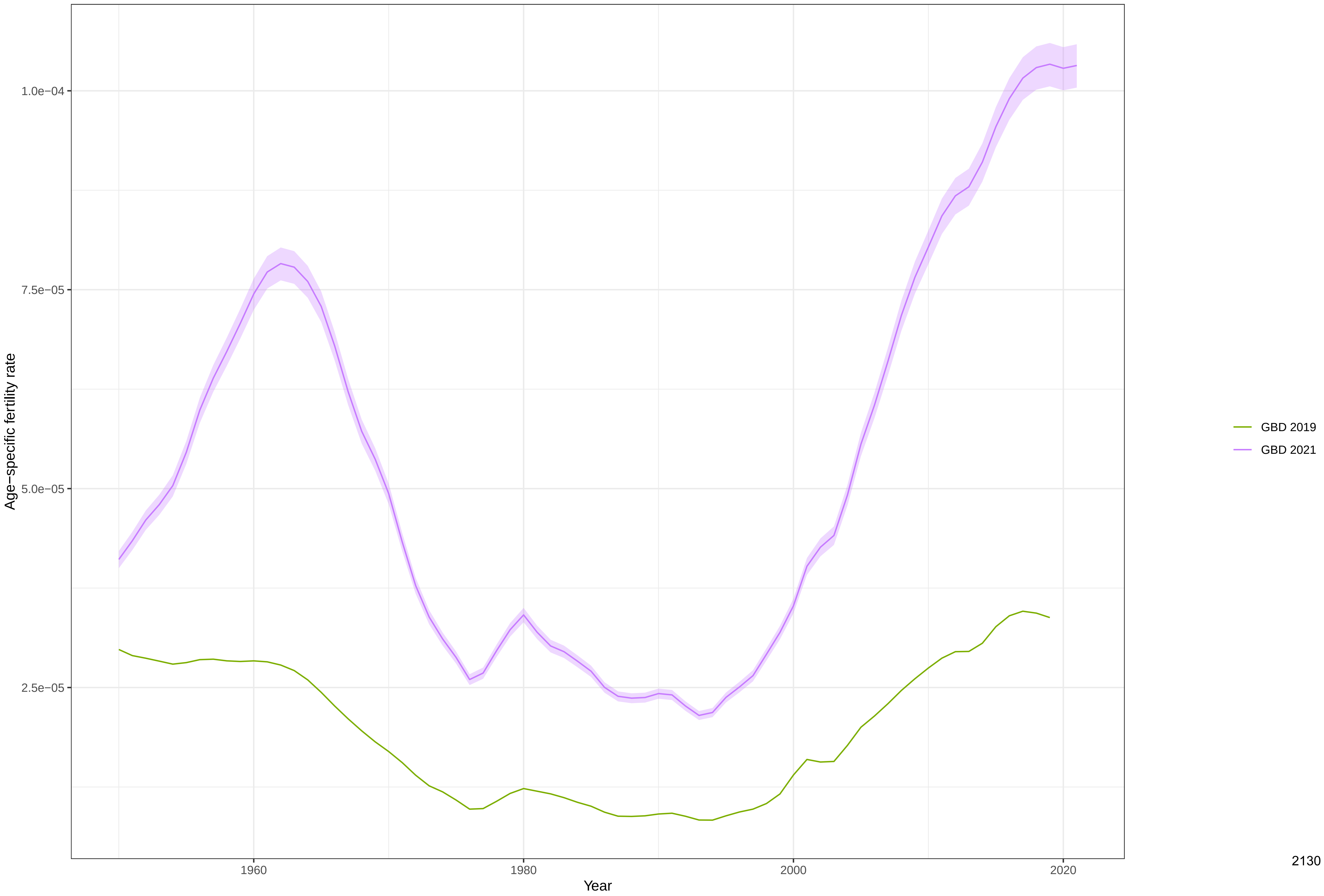

Total fertility rate fitted trend lines, 1950−2021: Essex TFR
Data points enclosed with a diamond are reference sources

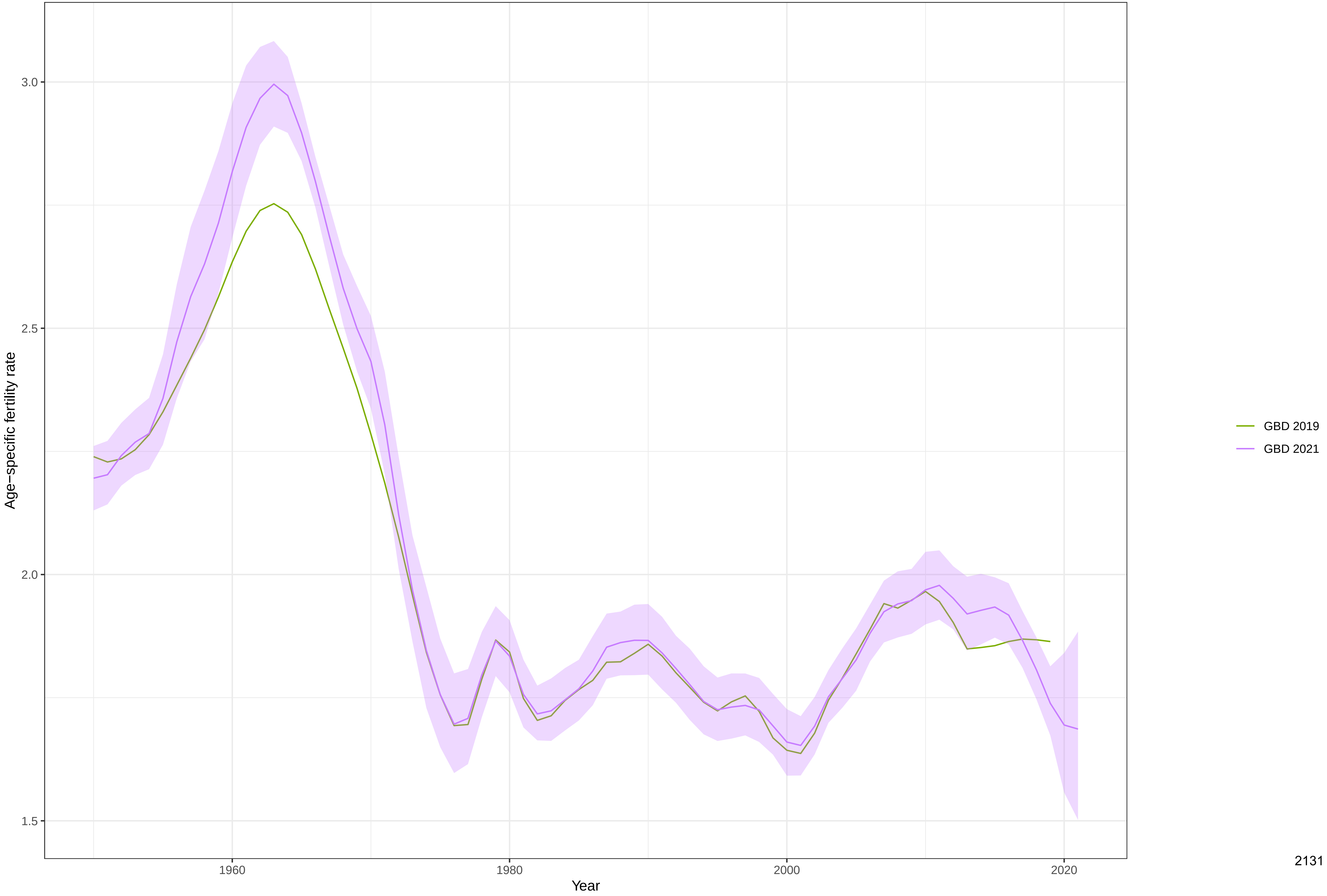

Age−specific fertility fitted trend lines, 1950−2021: Hertfordshire, Ages: 10−14
Data points enclosed with a diamond are reference sources

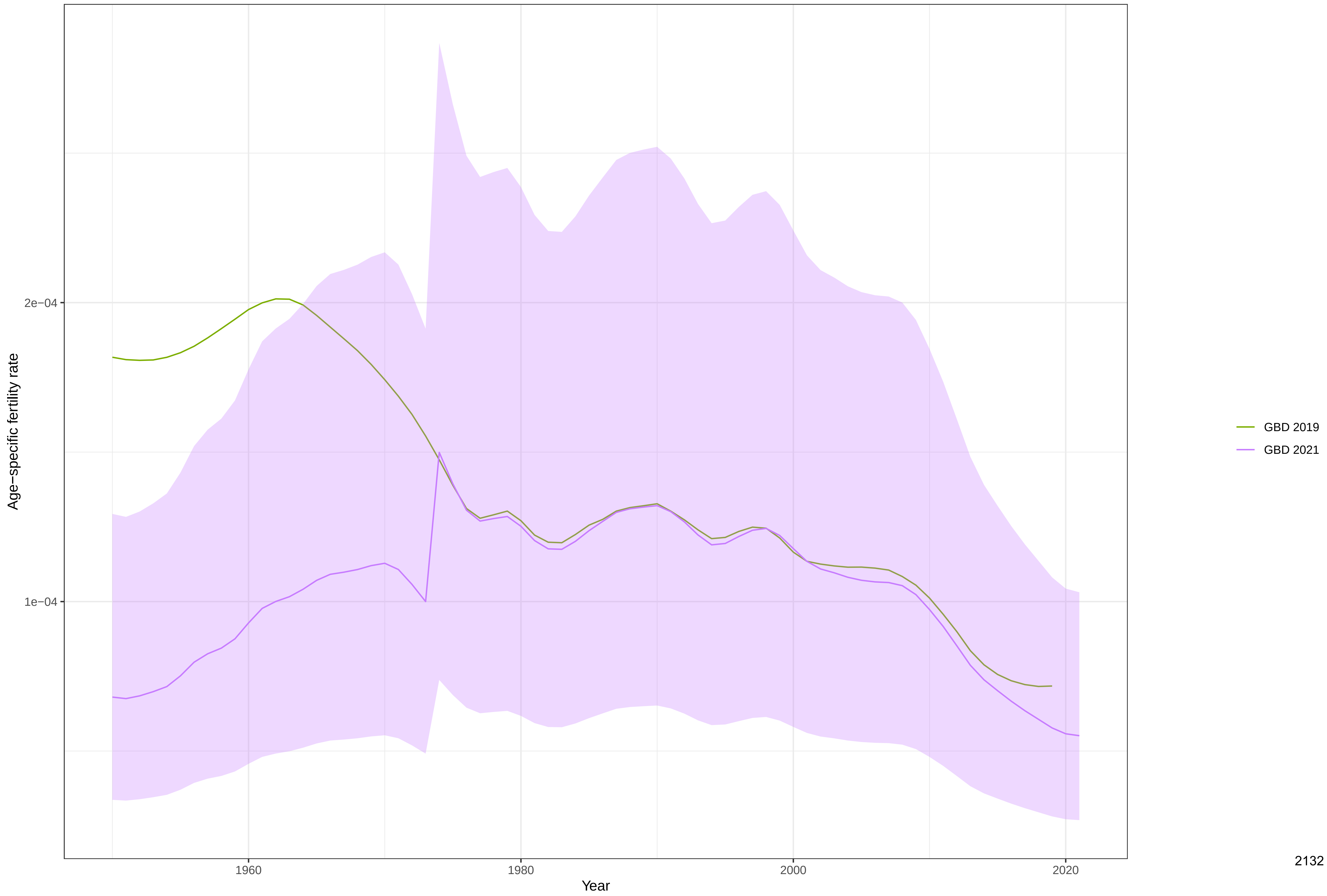

Age−specific fertility fitted trend lines, 1950−2021: Hertfordshire, Ages: 15−19
Data points enclosed with a diamond are reference sources

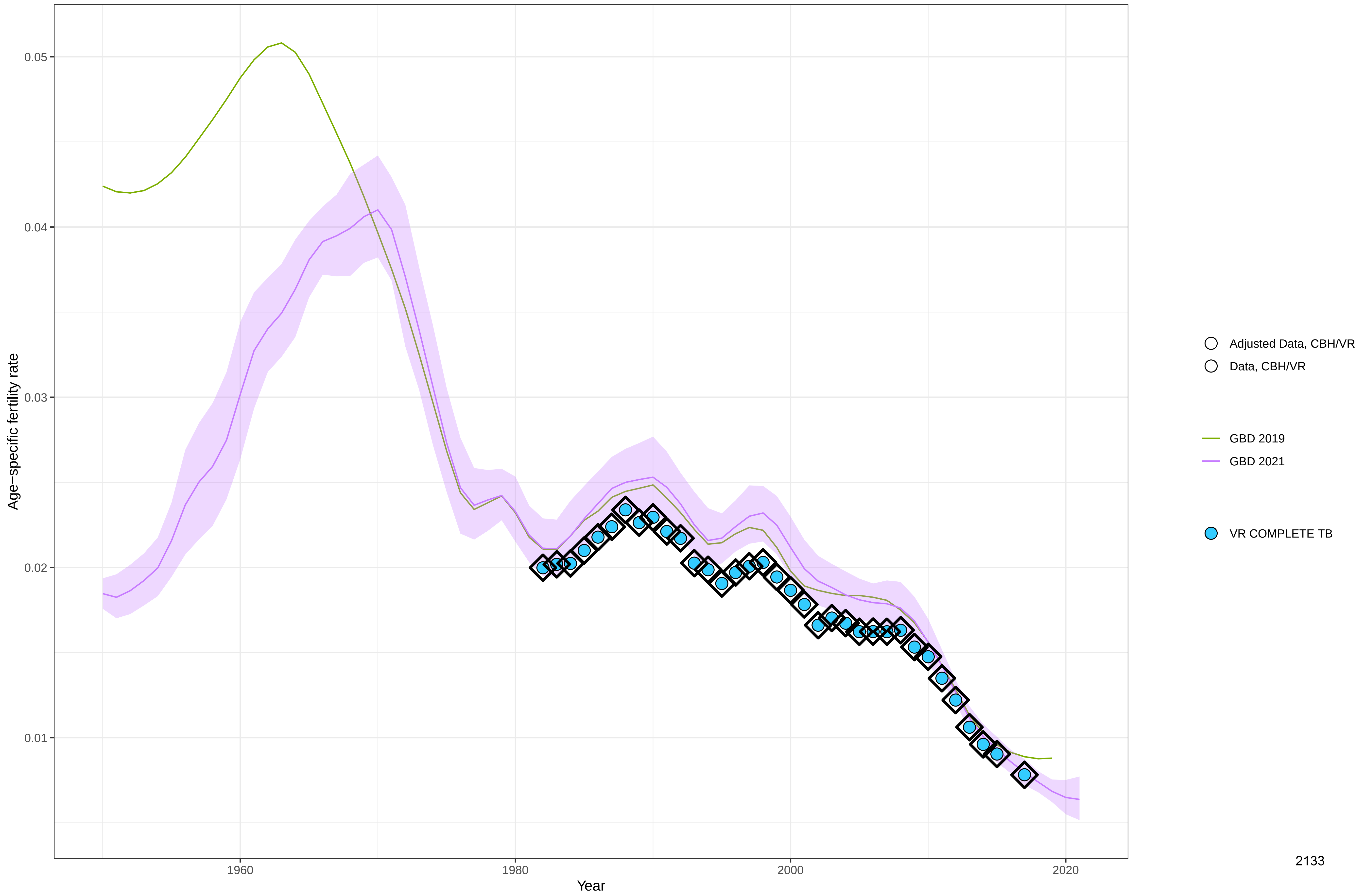

Age−specific fertility fitted trend lines, 1950−2021: Hertfordshire, Ages: 20−24
Data points enclosed with a diamond are reference sources

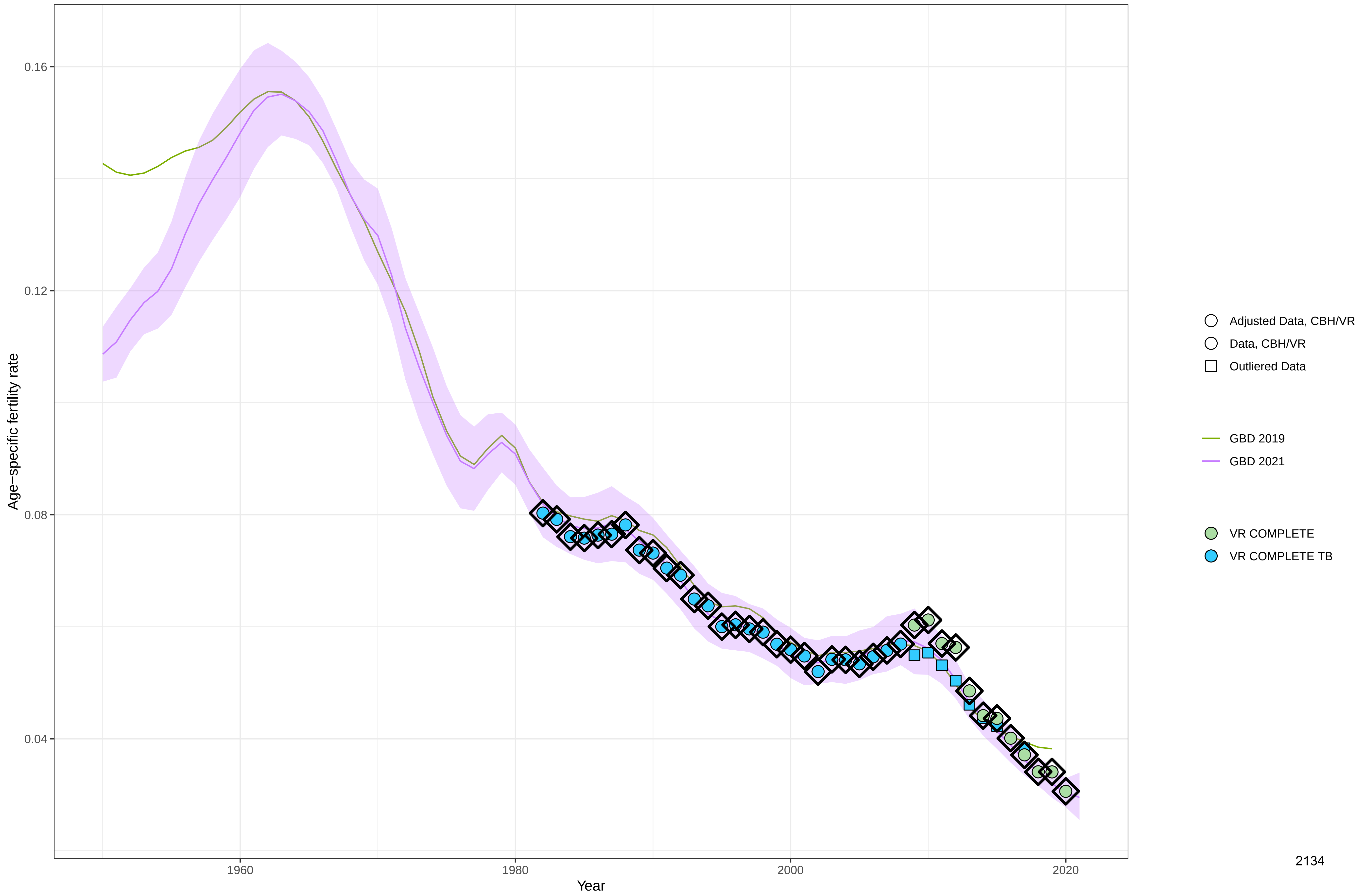

Age−specific fertility fitted trend lines, 1950−2021: Hertfordshire, Ages: 25−29
Data points enclosed with a diamond are reference sources

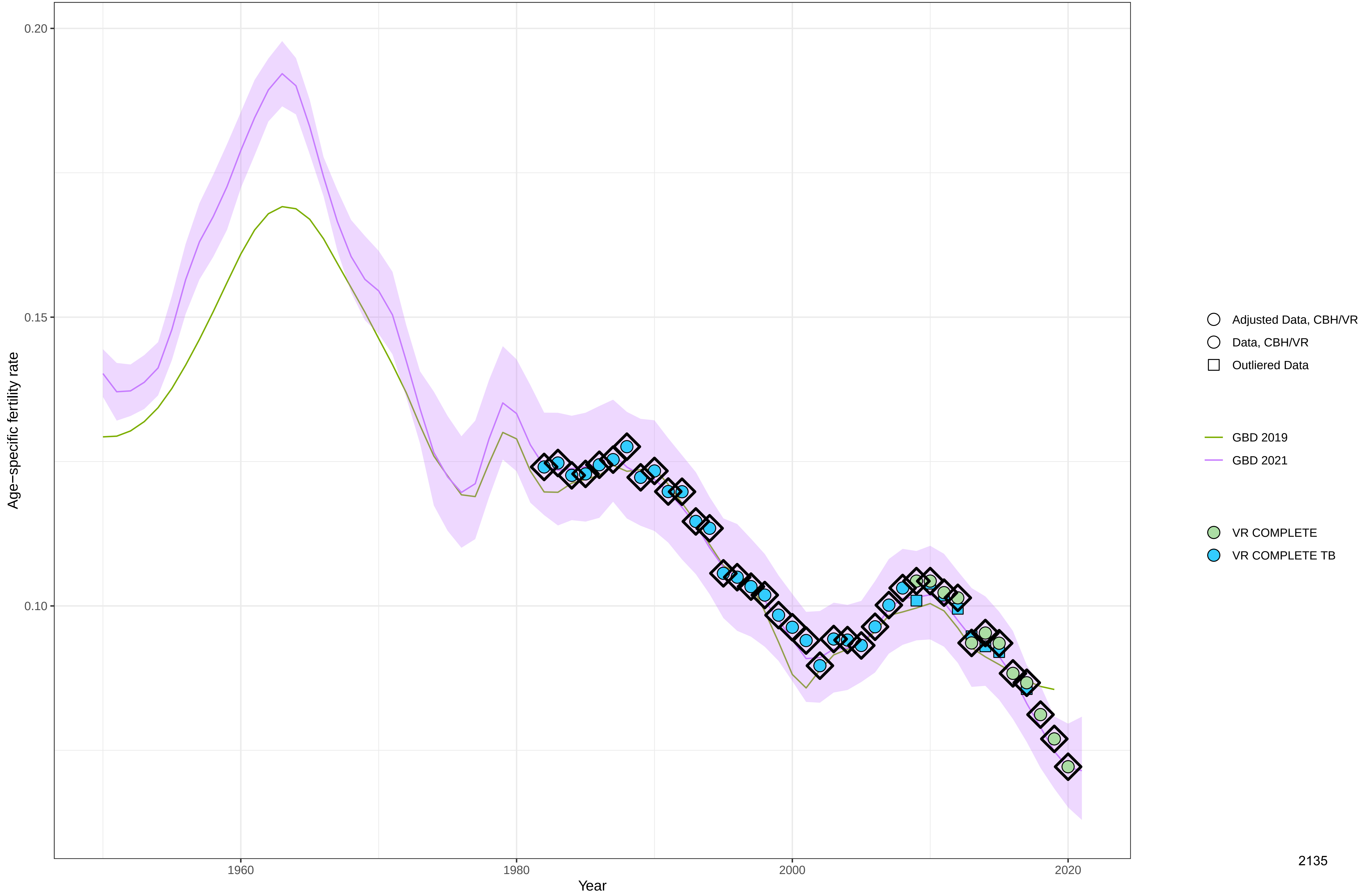

Age−specific fertility fitted trend lines, 1950−2021: Hertfordshire, Ages: 30−34
Data points enclosed with a diamond are reference sources

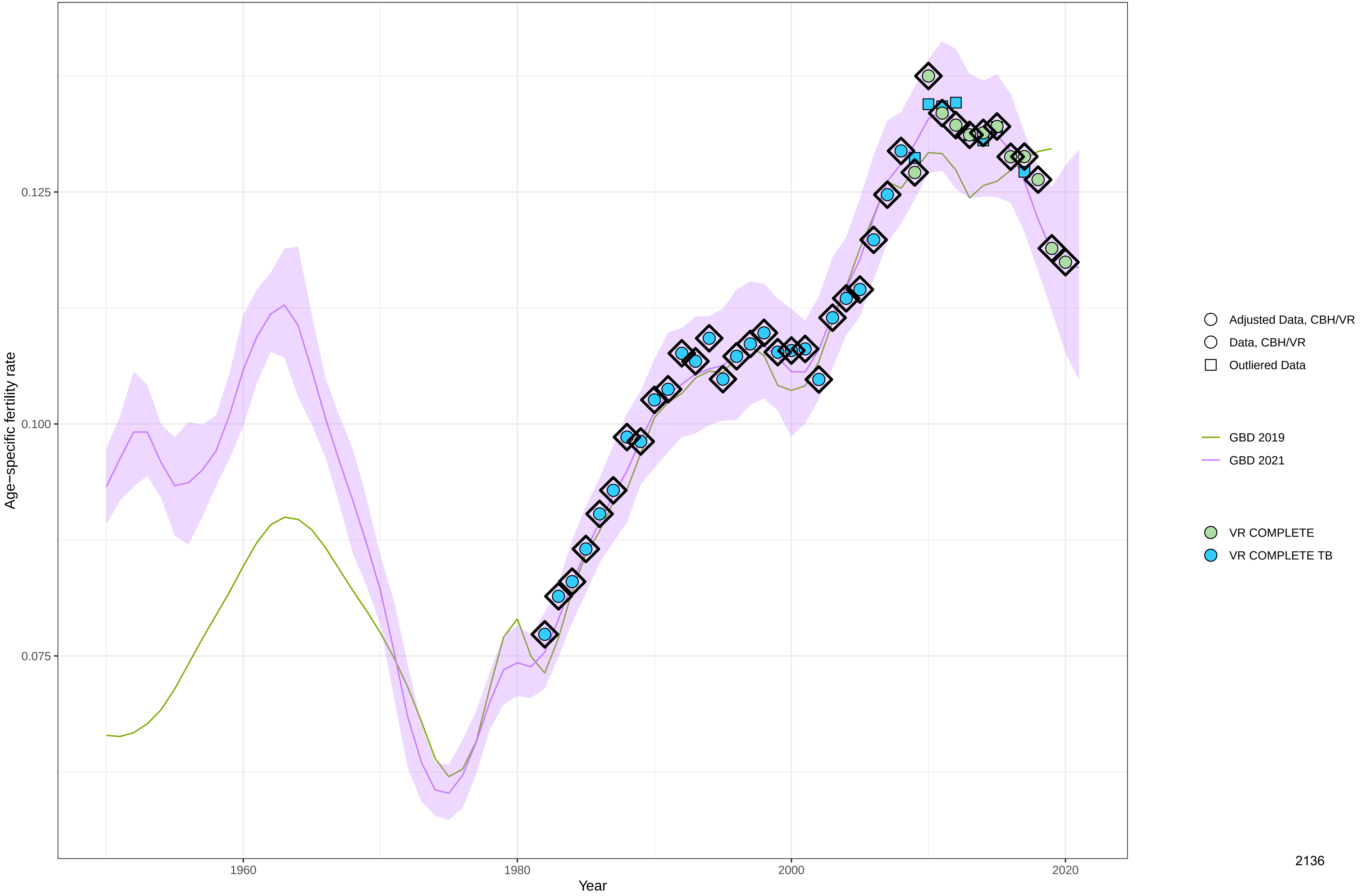

Age−specific fertility fitted trend lines, 1950−2021: Hertfordshire, Ages: 35−39
Data points enclosed with a diamond are reference sources

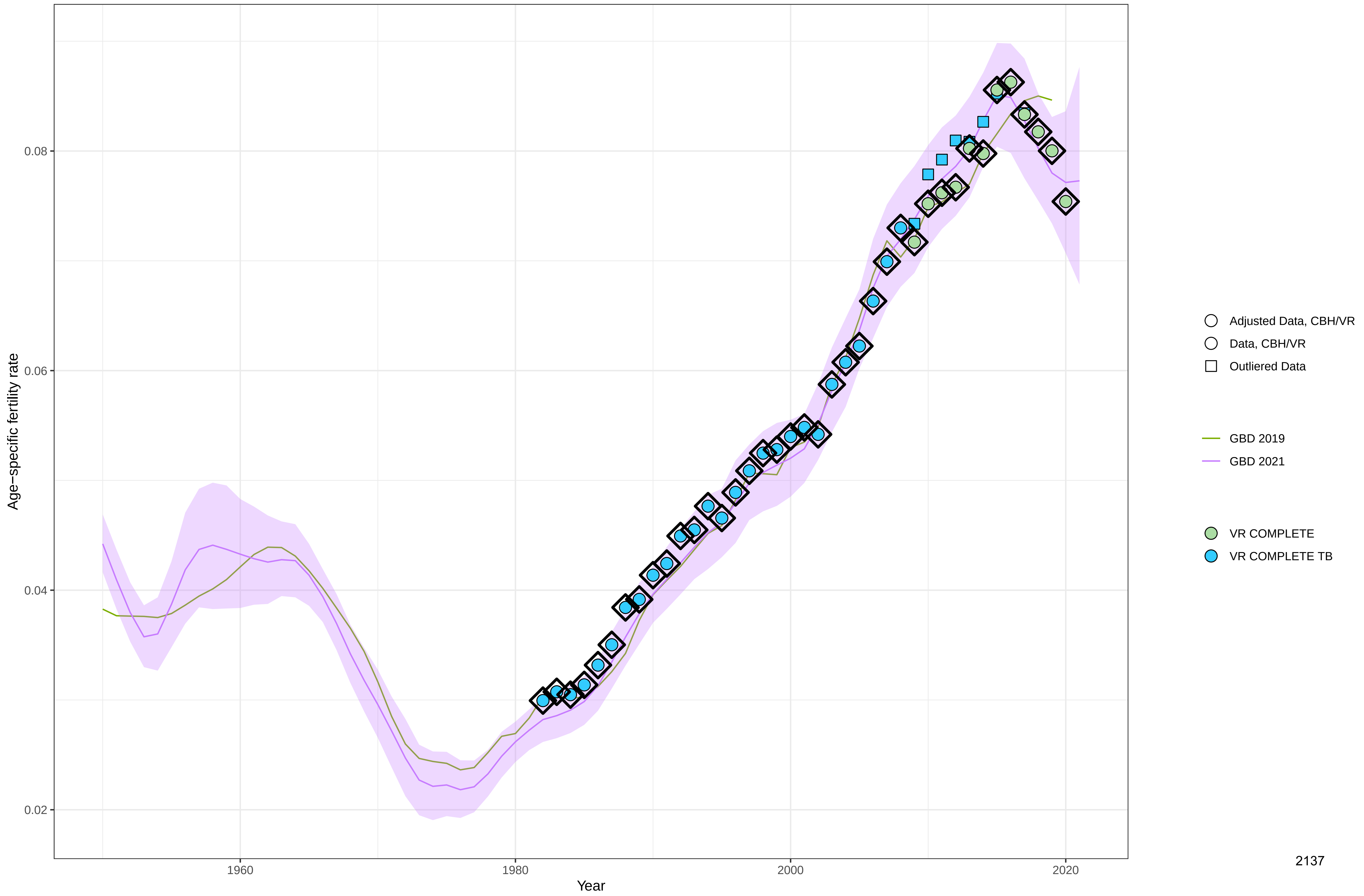

Age−specific fertility fitted trend lines, 1950−2021: Hertfordshire, Ages: 40−44
Data points enclosed with a diamond are reference sources

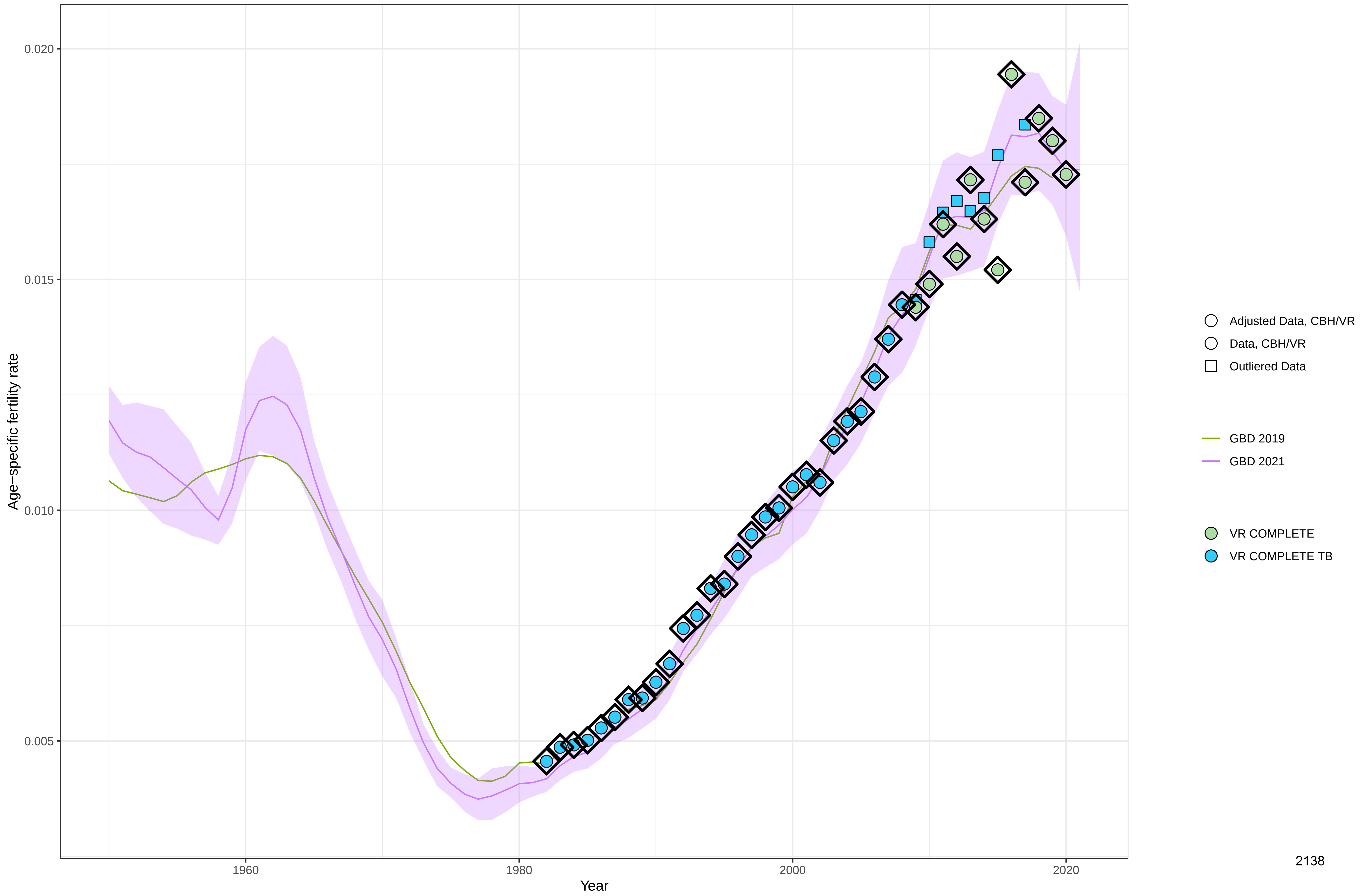

Age−specific fertility fitted trend lines, 1950−2021: Hertfordshire, Ages: 45−49
Data points enclosed with a diamond are reference sources

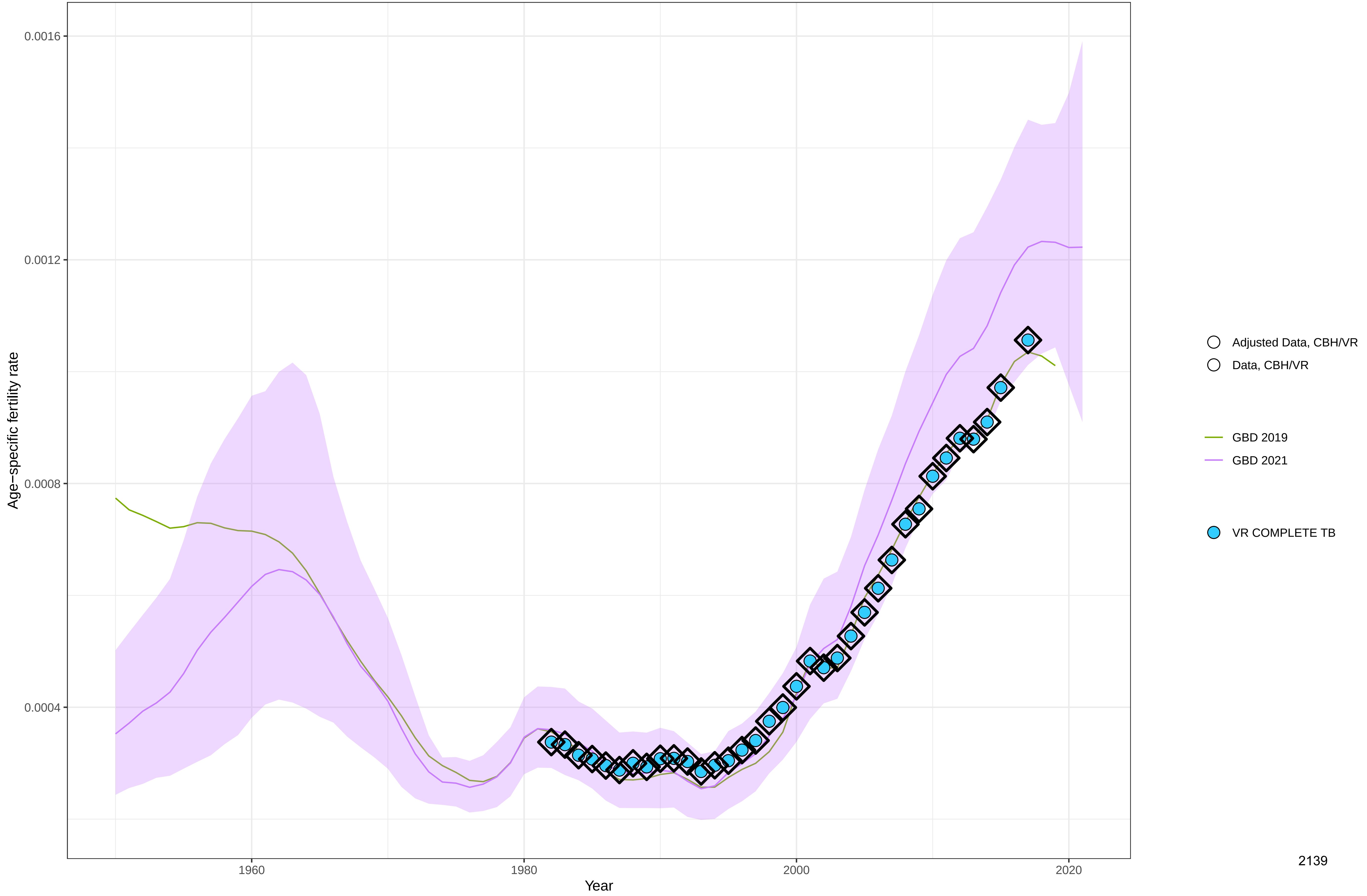

Age−specific fertility fitted trend lines, 1950−2021: Hertfordshire, Ages: 50−54
Data points enclosed with a diamond are reference sources

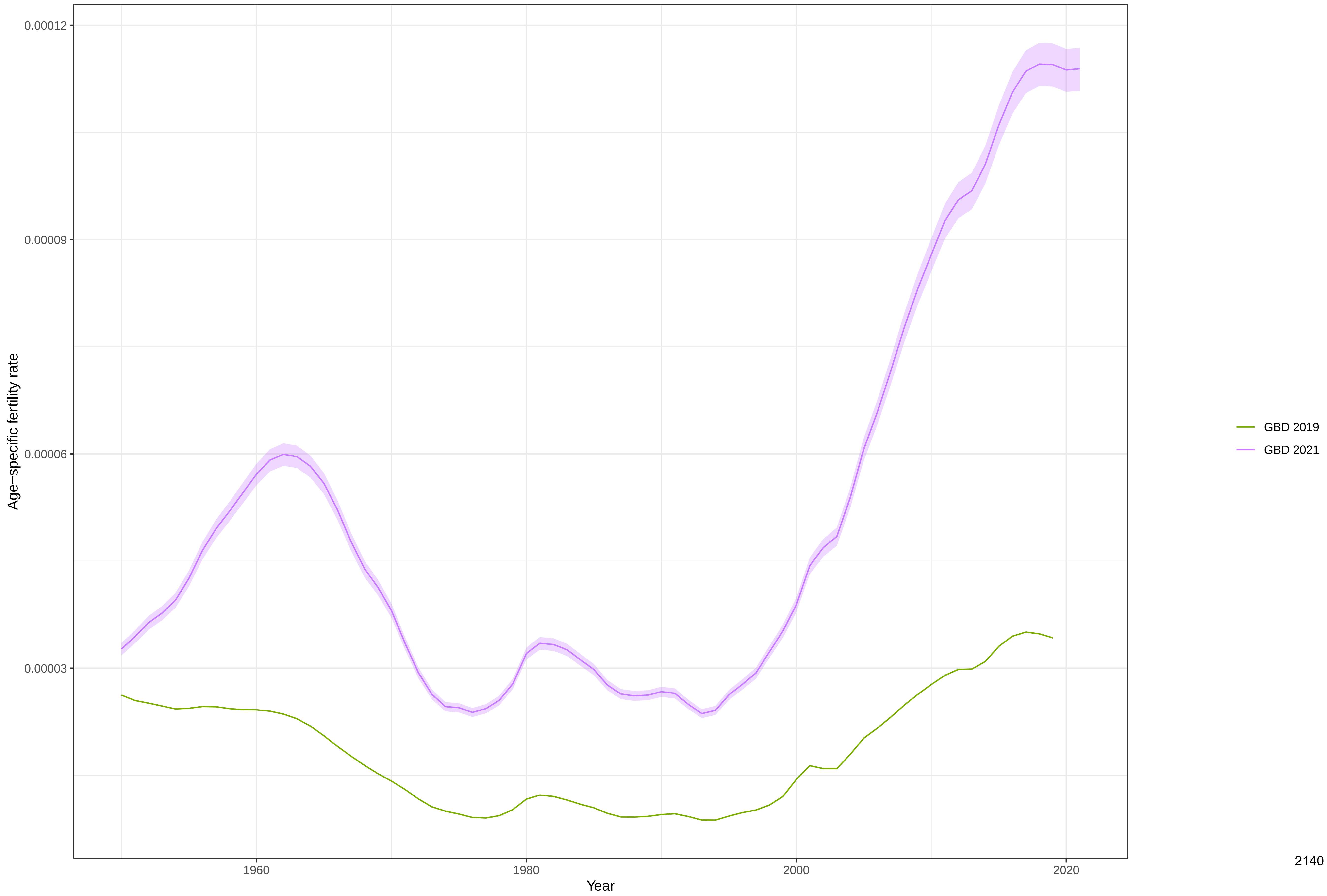

Total fertility rate fitted trend lines, 1950−2021: Hertfordshire TFR
Data points enclosed with a diamond are reference sources

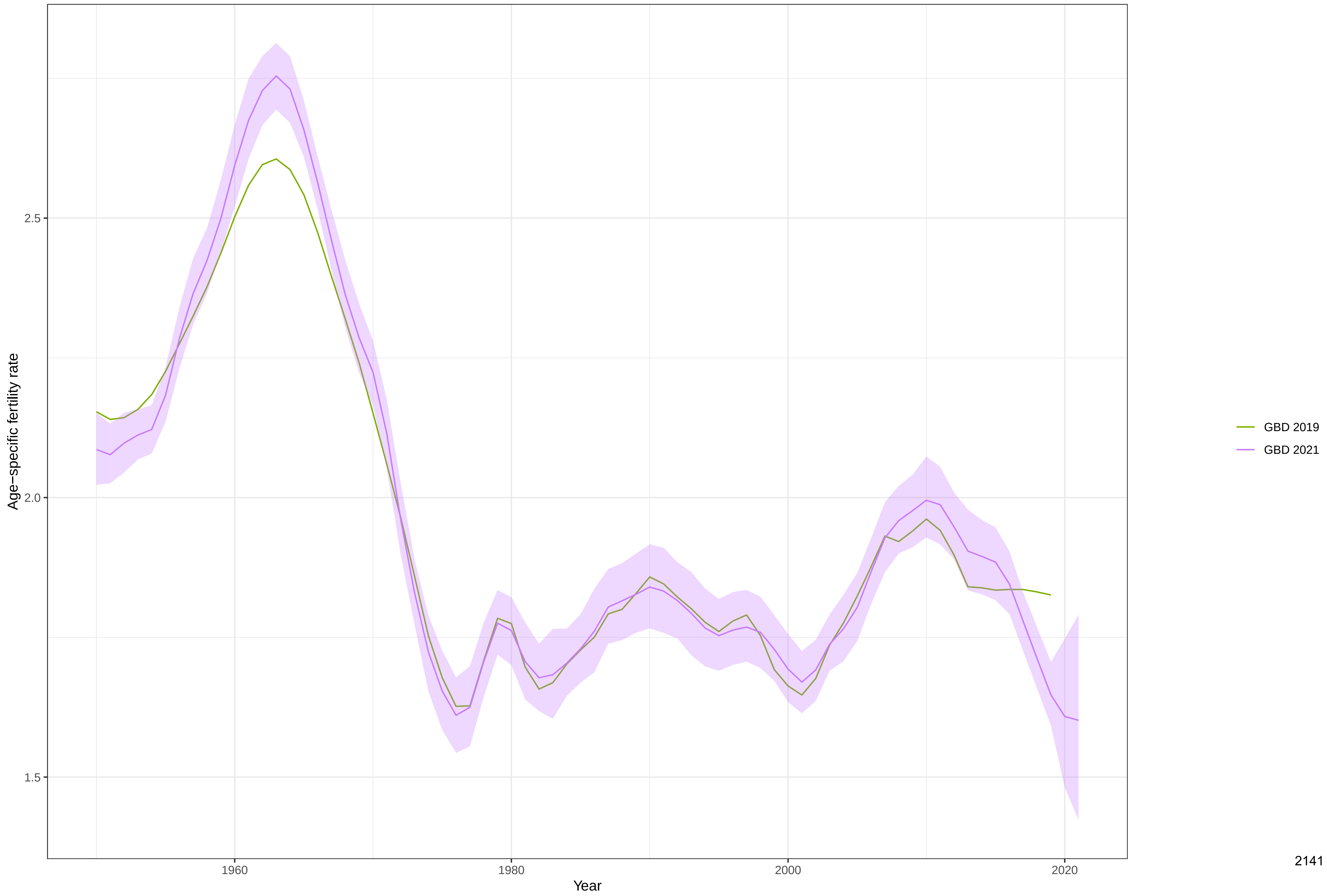

Age−specific fertility fitted trend lines, 1950−2021: Luton, Ages: 10−14
Data points enclosed with a diamond are reference sources

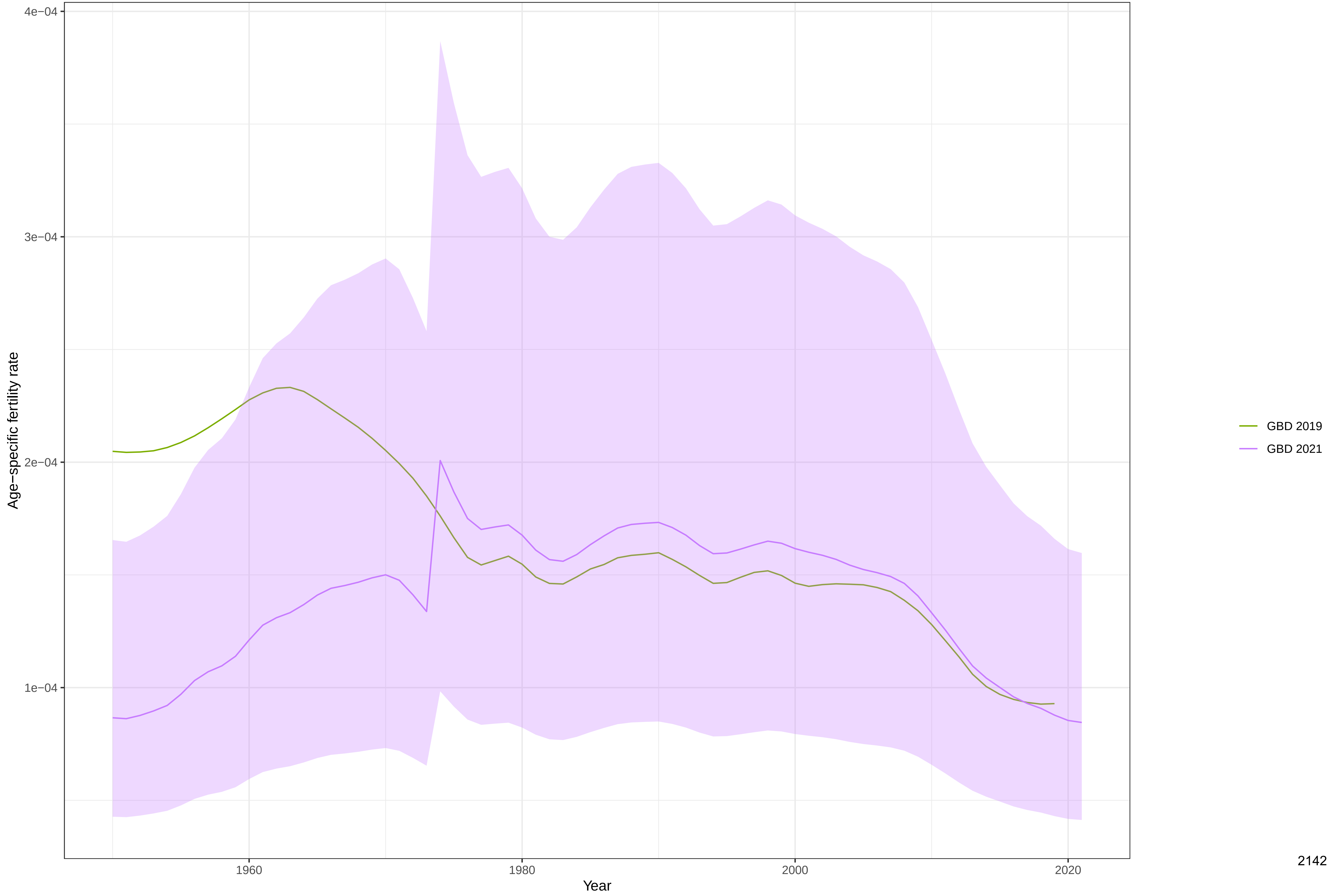

Age−specific fertility fitted trend lines, 1950−2021: Luton, Ages: 15−19
Data points enclosed with a diamond are reference sources

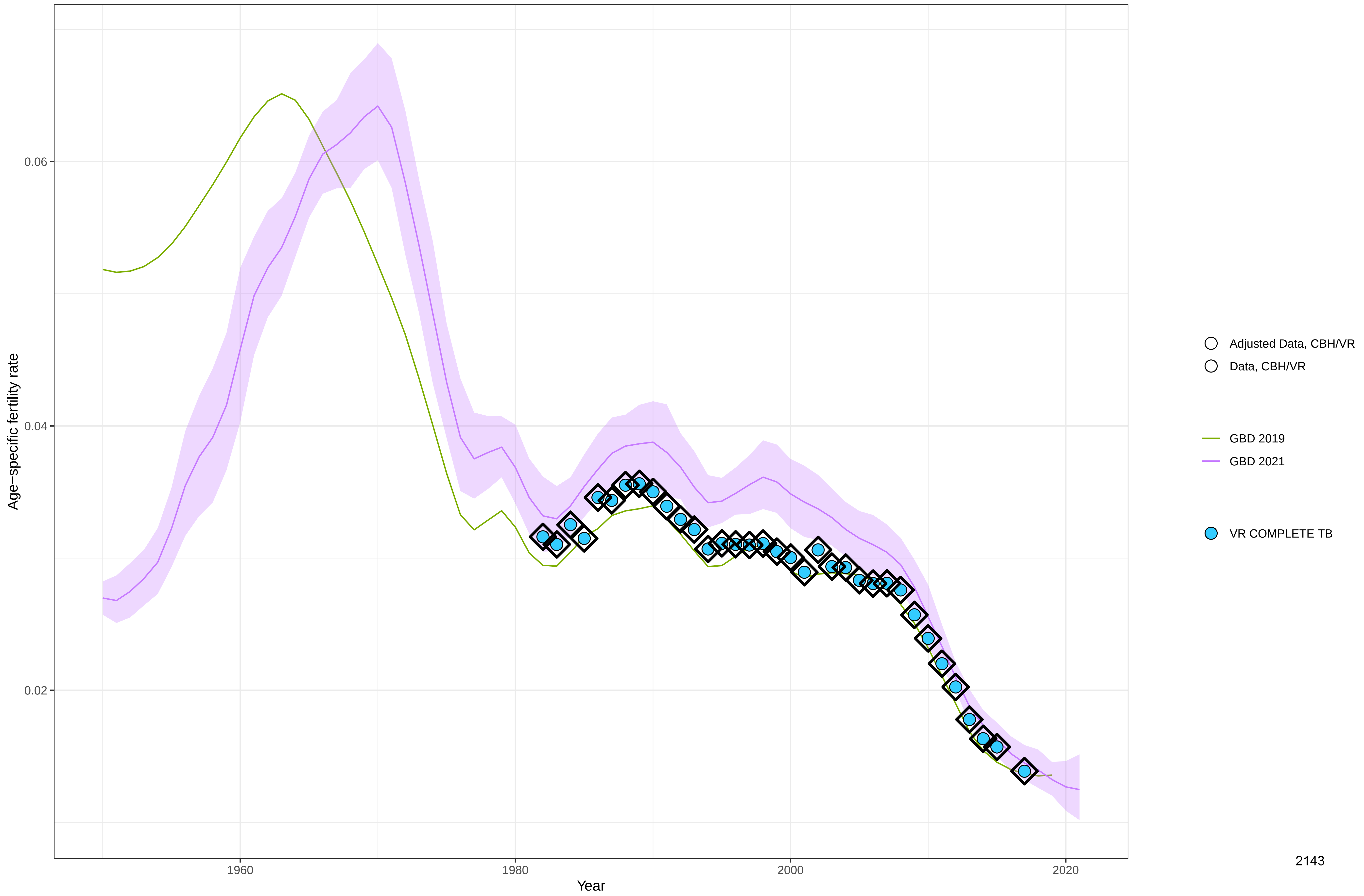

Age−specific fertility fitted trend lines, 1950−2021: Luton, Ages: 20−24
Data points enclosed with a diamond are reference sources

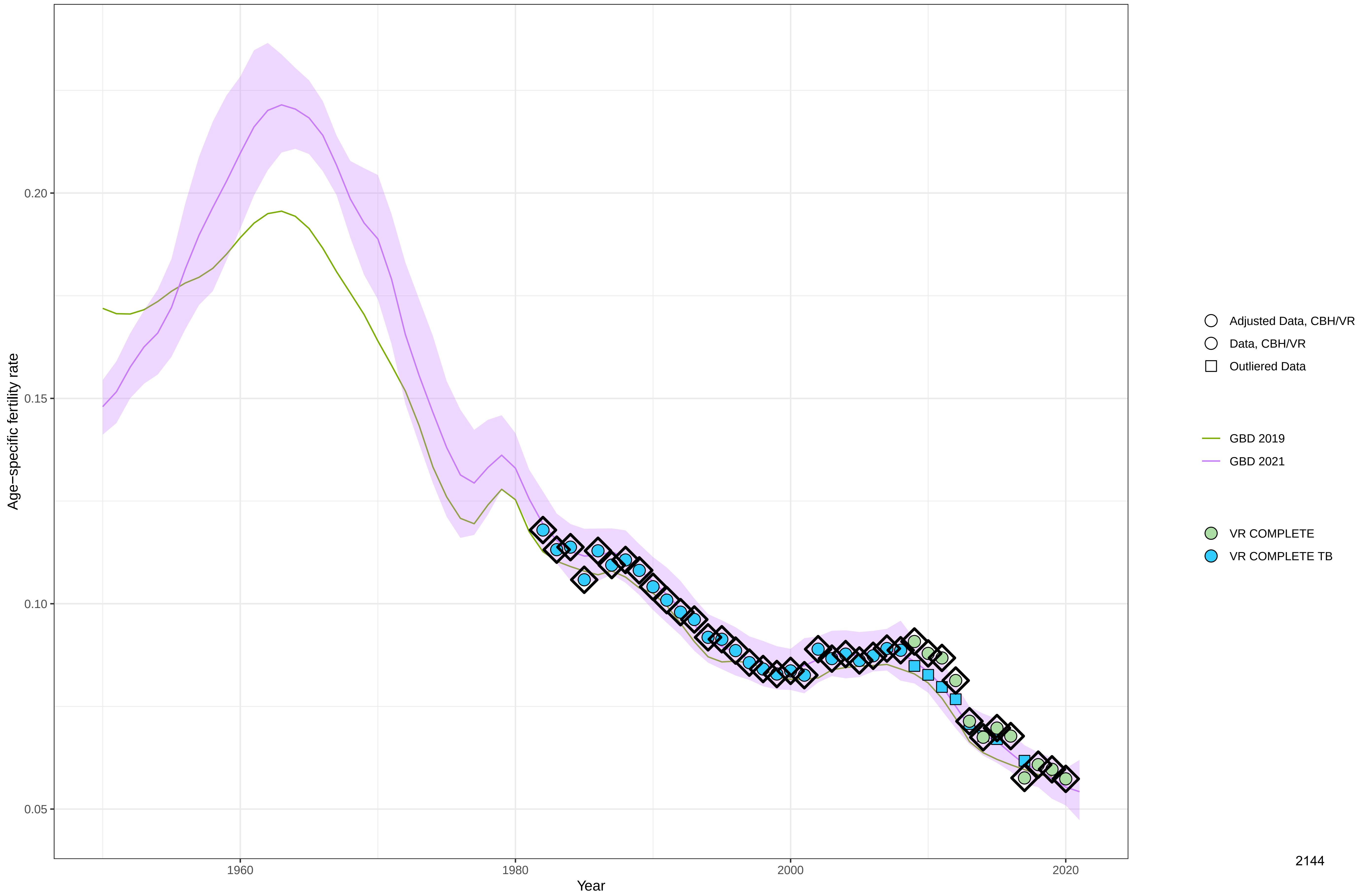

Age−specific fertility fitted trend lines, 1950−2021: Luton, Ages: 25−29
Data points enclosed with a diamond are reference sources

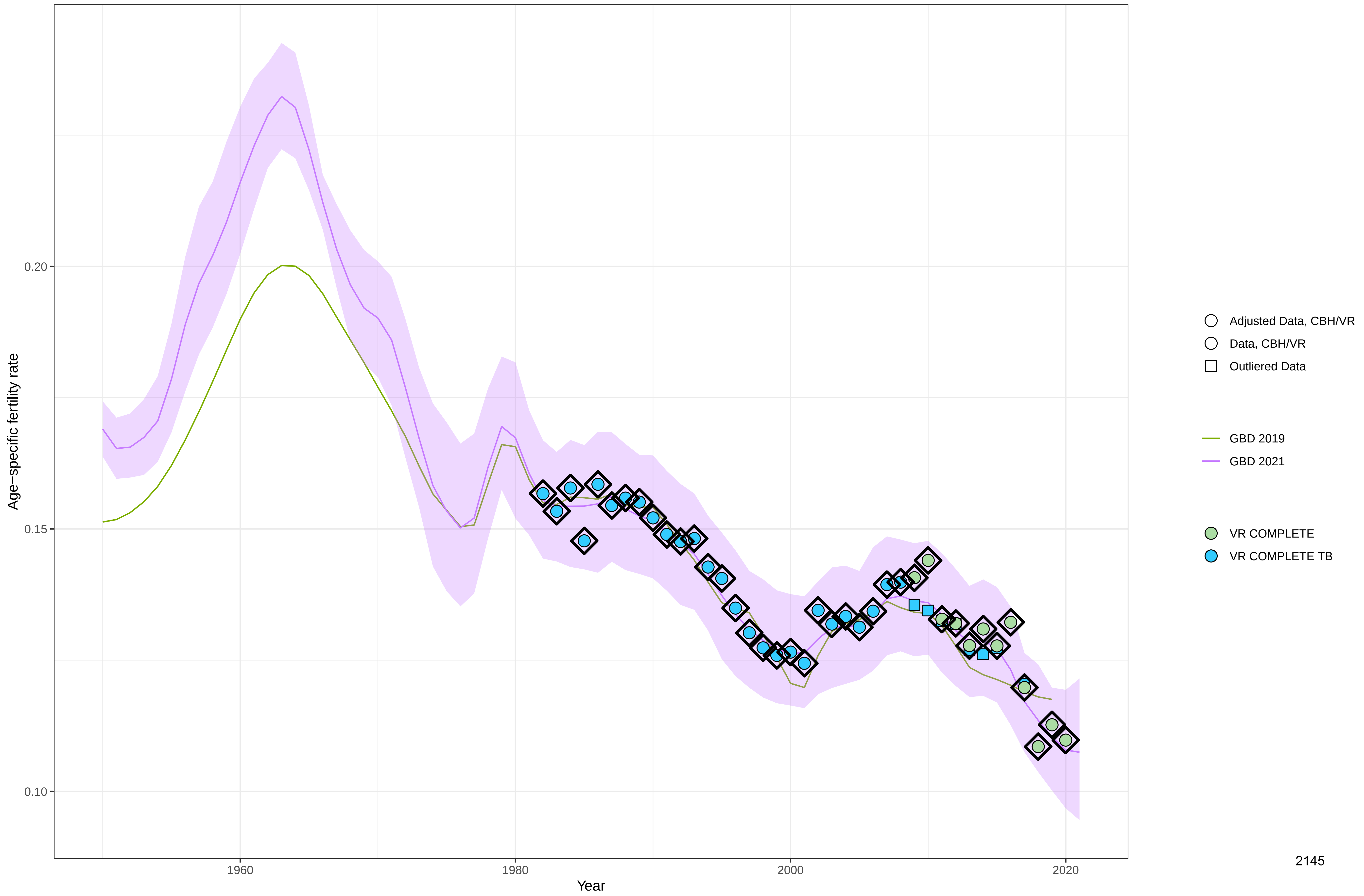

Age−specific fertility fitted trend lines, 1950−2021: Luton, Ages: 30−34
Data points enclosed with a diamond are reference sources

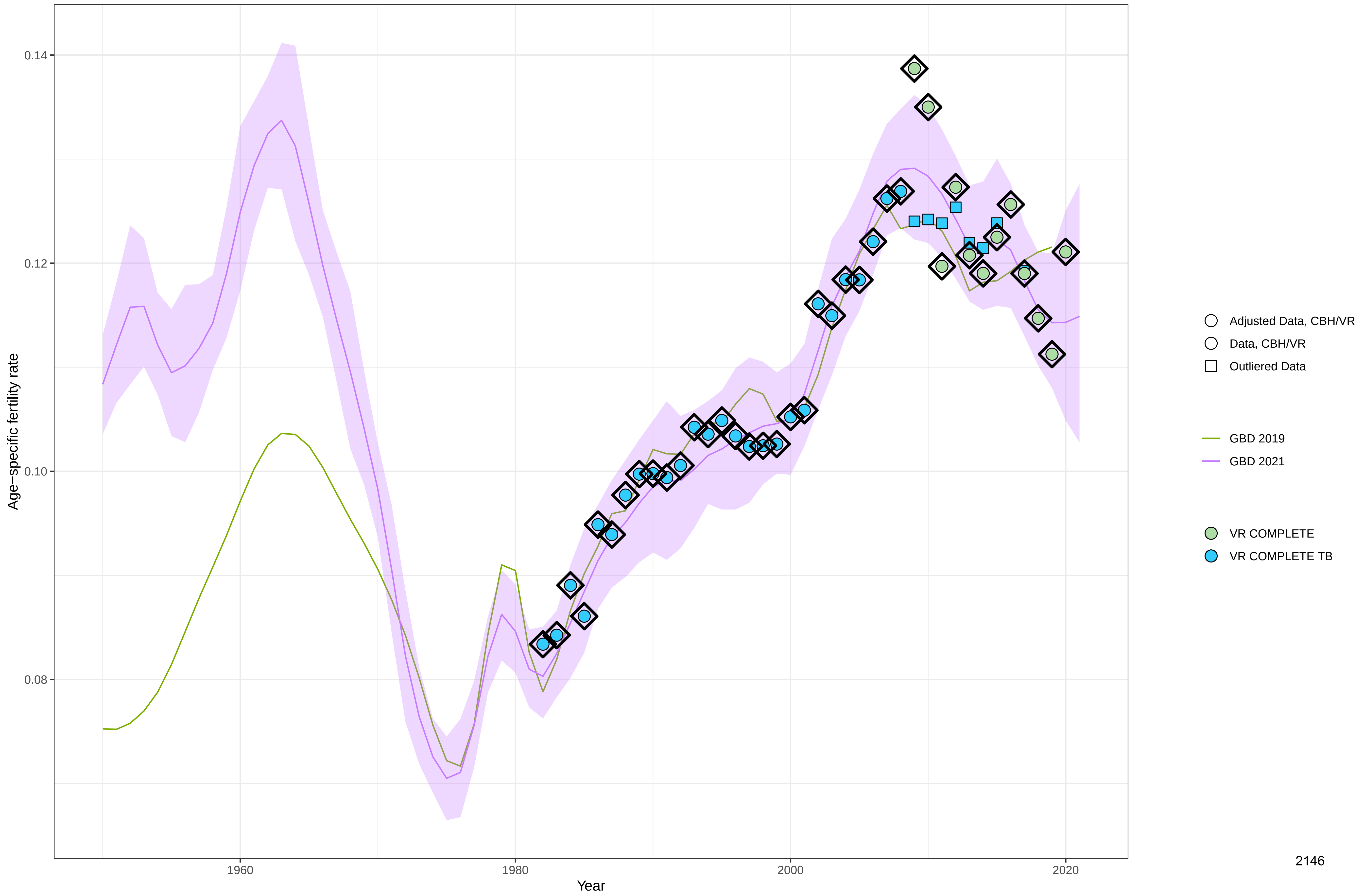

Age−specific fertility fitted trend lines, 1950−2021: Luton, Ages: 35−39
Data points enclosed with a diamond are reference sources

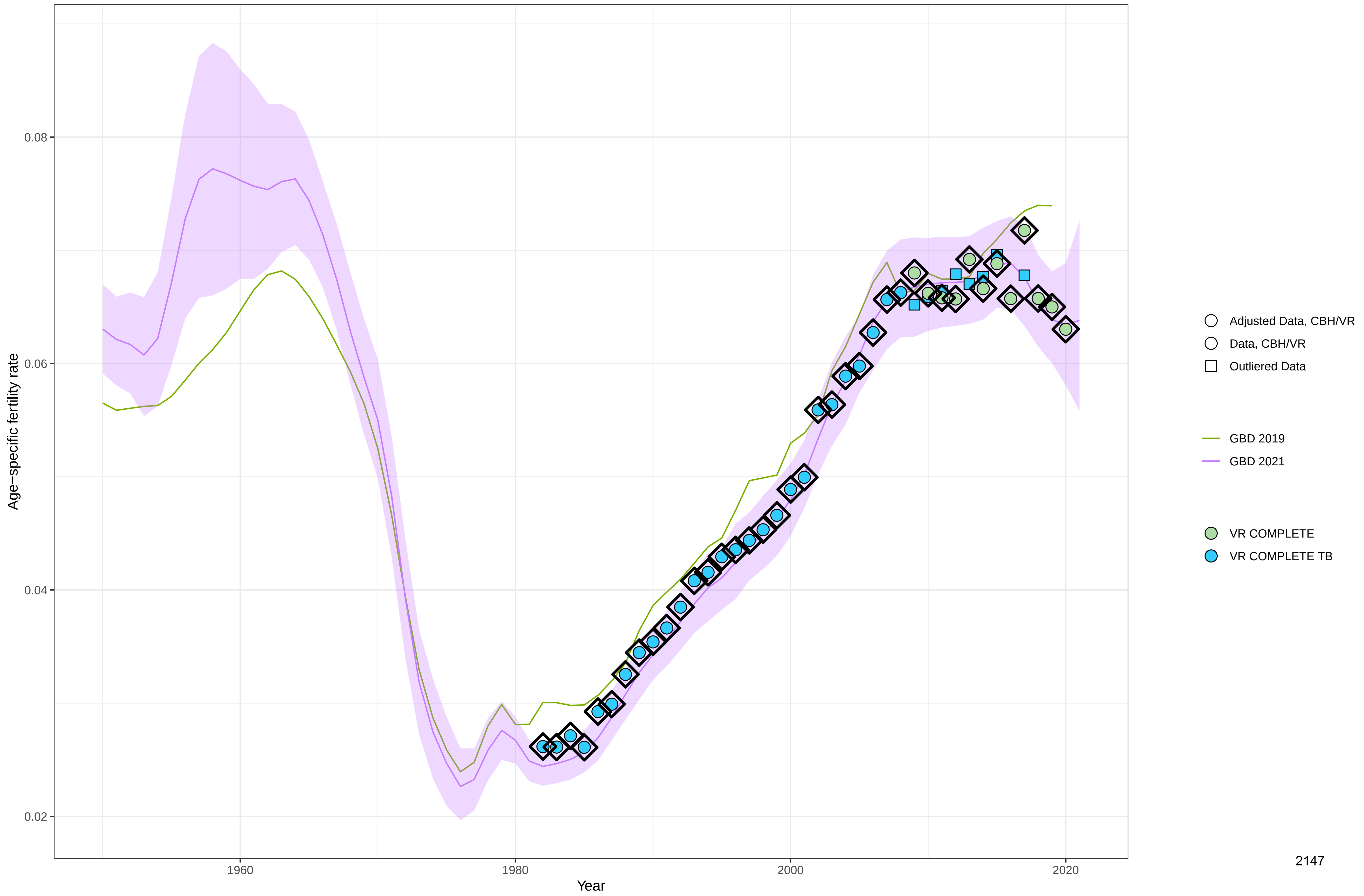

Age−specific fertility fitted trend lines, 1950−2021: Luton, Ages: 40−44
Data points enclosed with a diamond are reference sources

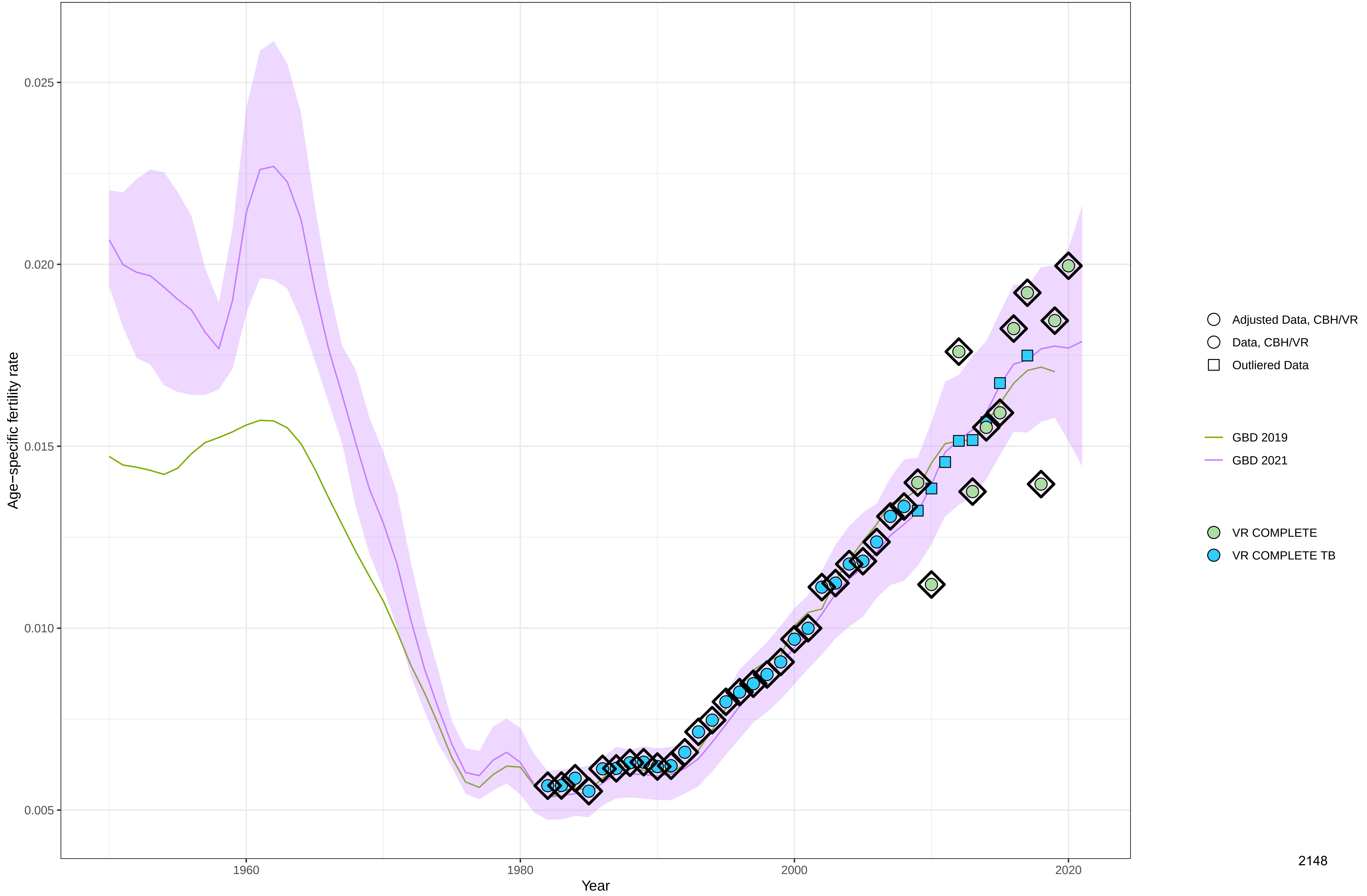

Age−specific fertility fitted trend lines, 1950−2021: Luton, Ages: 45−49
Data points enclosed with a diamond are reference sources

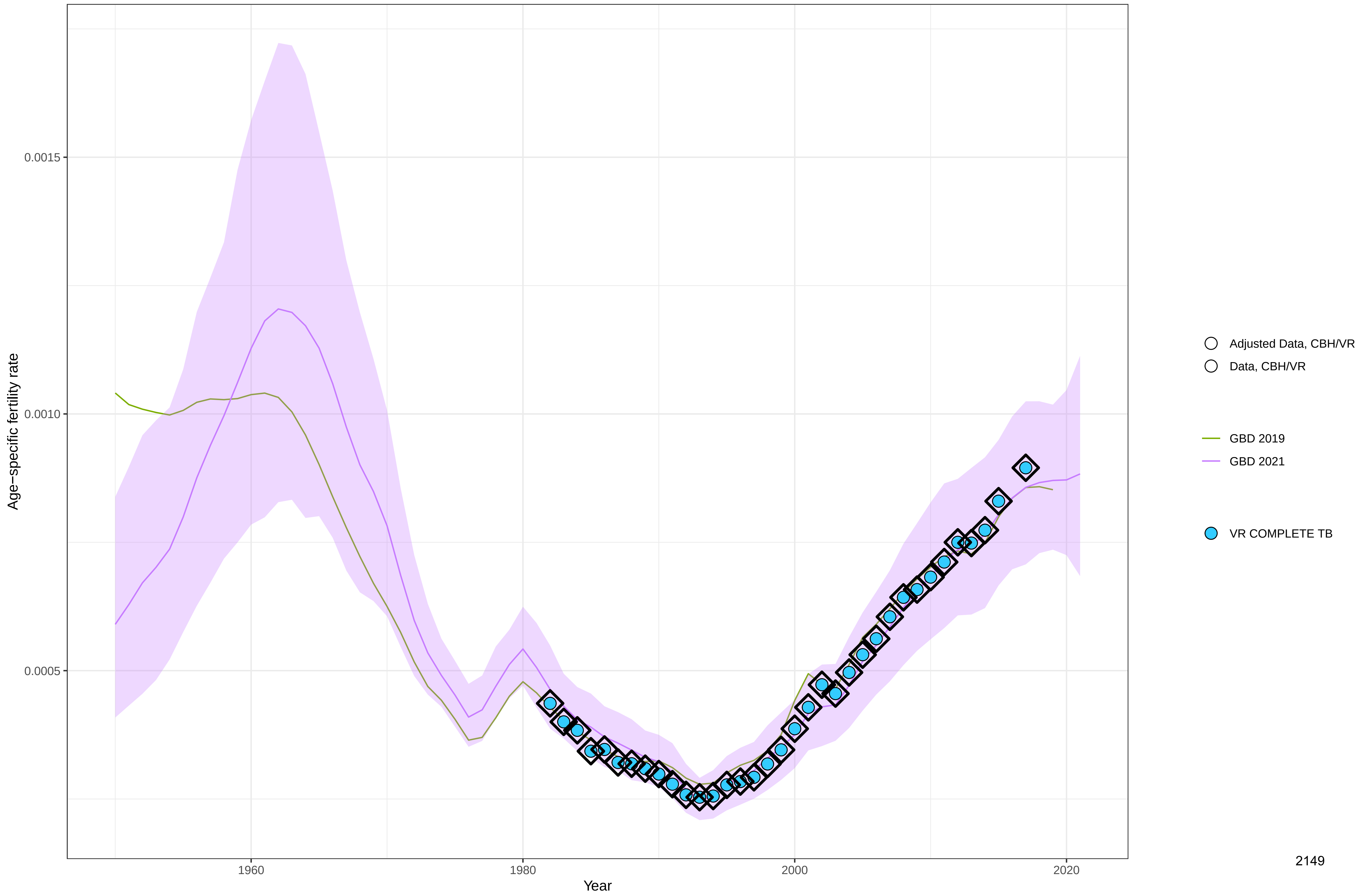

Age−specific fertility fitted trend lines, 1950−2021: Luton, Ages: 50−54
Data points enclosed with a diamond are reference sources

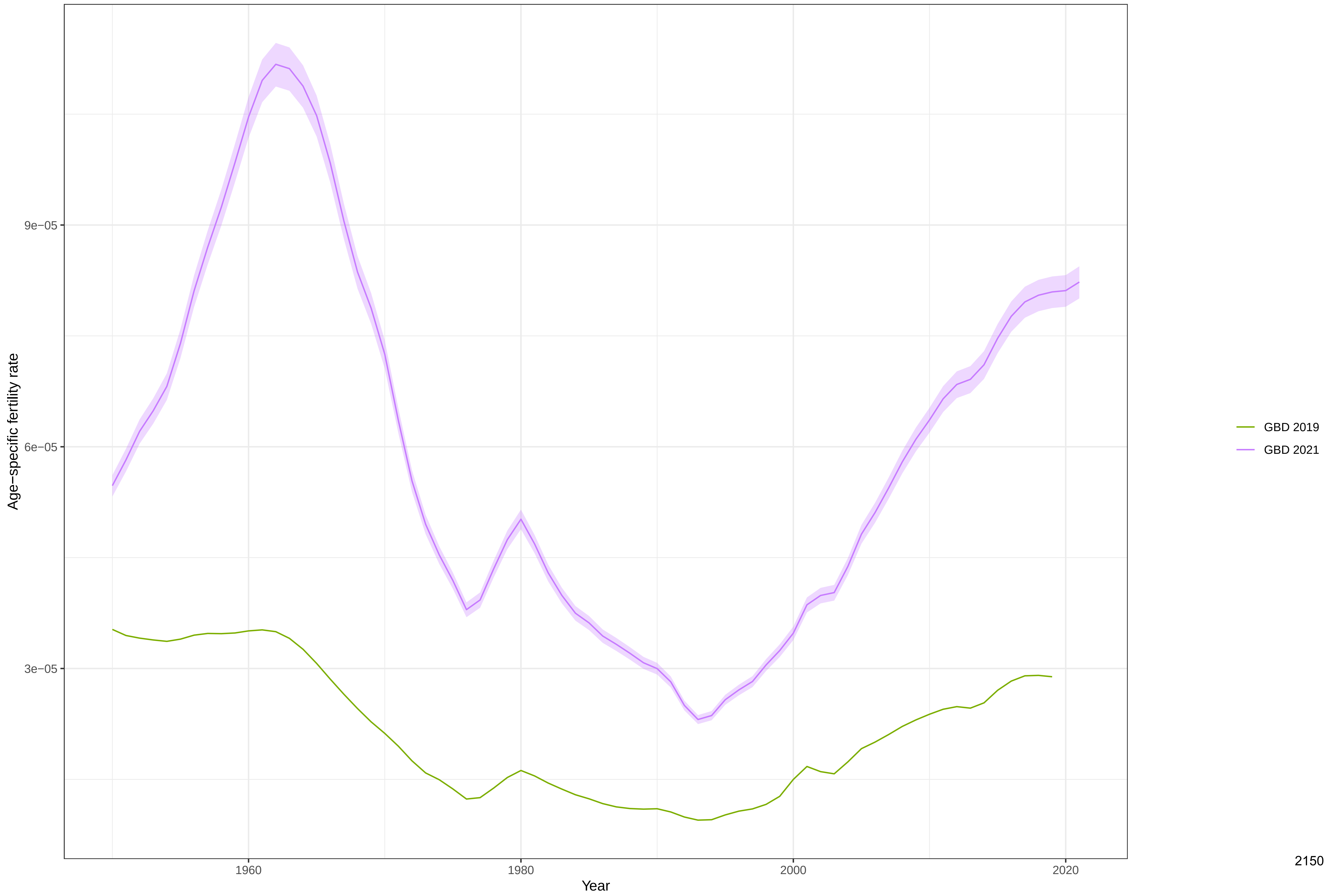

2150

Total fertility rate fitted trend lines, 1950−2021: Luton TFR
Data points enclosed with a diamond are reference sources

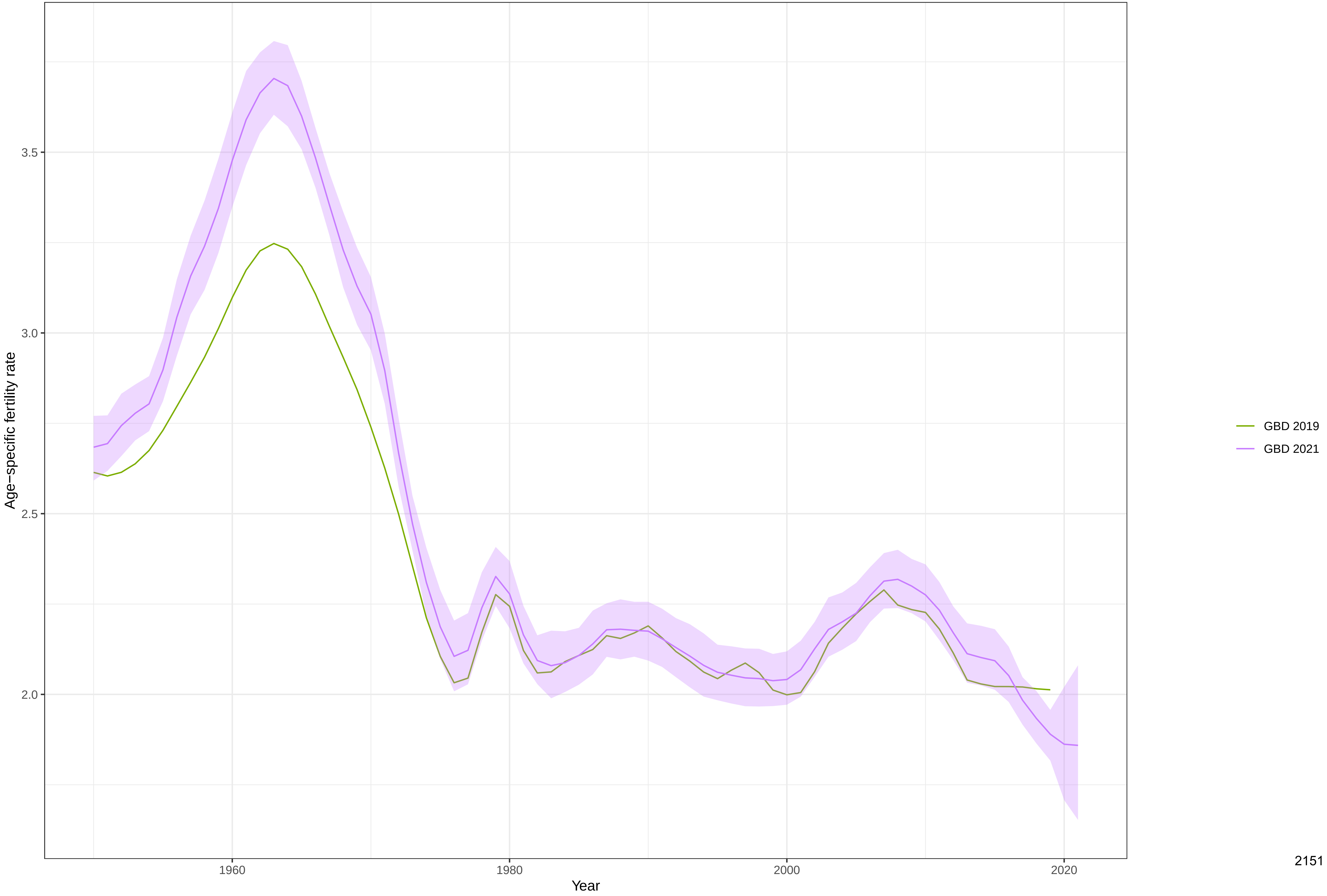

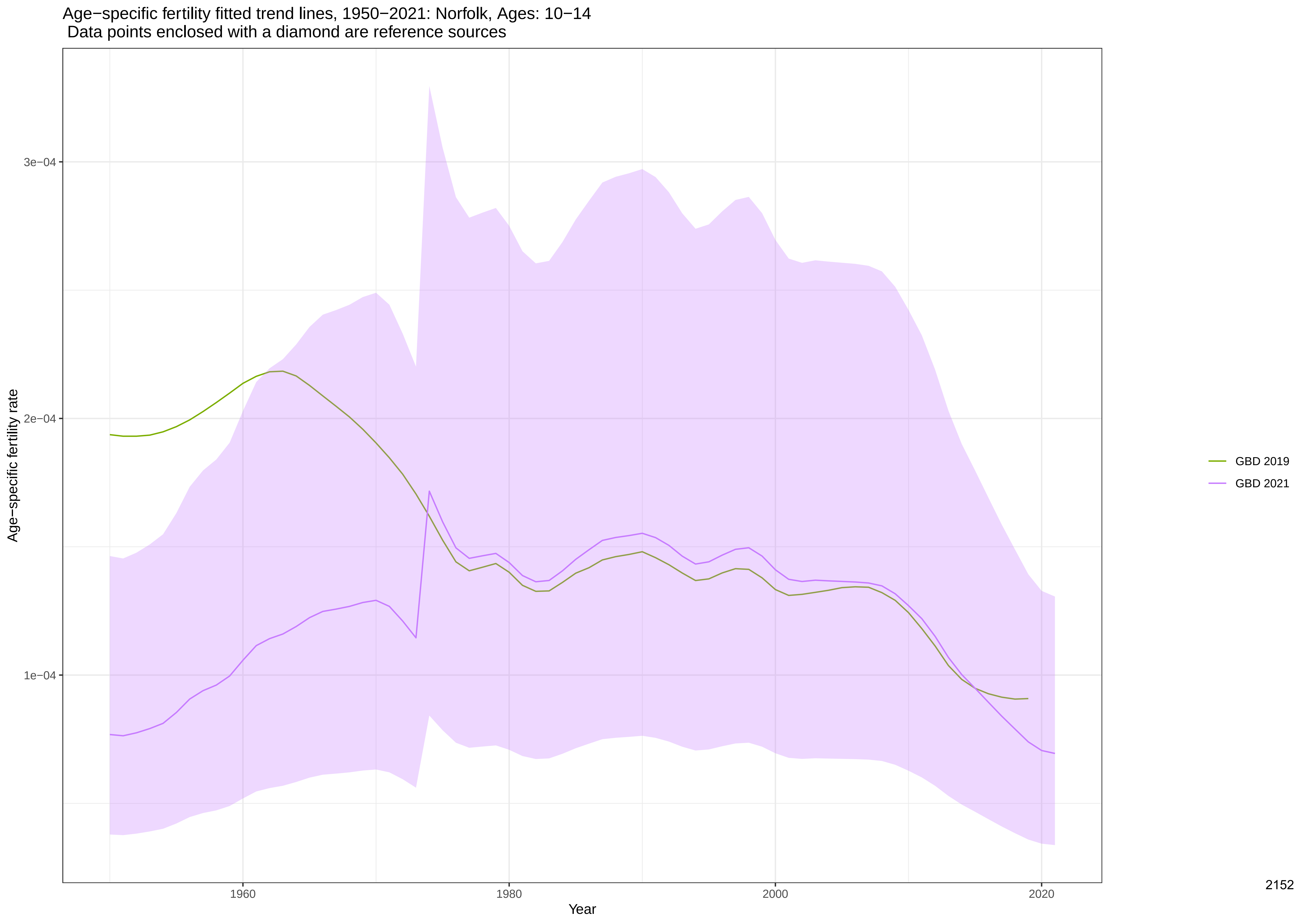
Age−specific fertility fitted trend lines, 1950−2021: Norfolk, Ages: 10−14
Data points enclosed with a diamond are reference sources
3e−04
2e−04
1e−04
Age−specific fertility rate
1960
1980
2000
2020
Year
GBD 2019
GBD 2021

Age−specific fertility fitted trend lines, 1950−2021: Norfolk, Ages: 15−19
Data points enclosed with a diamond are reference sources

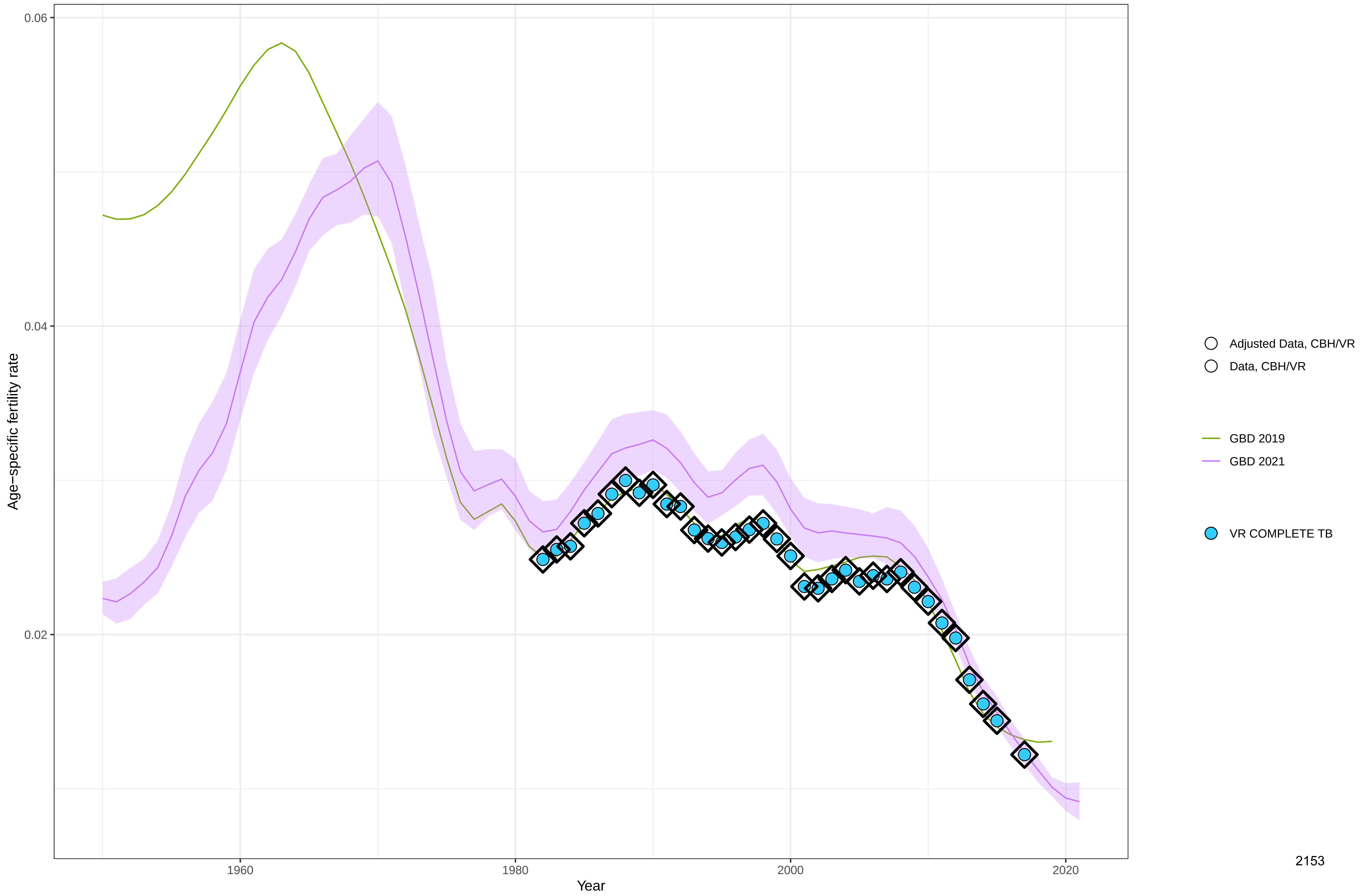

Age−specific fertility fitted trend lines, 1950−2021: Norfolk, Ages: 20−24
Data points enclosed with a diamond are reference sources

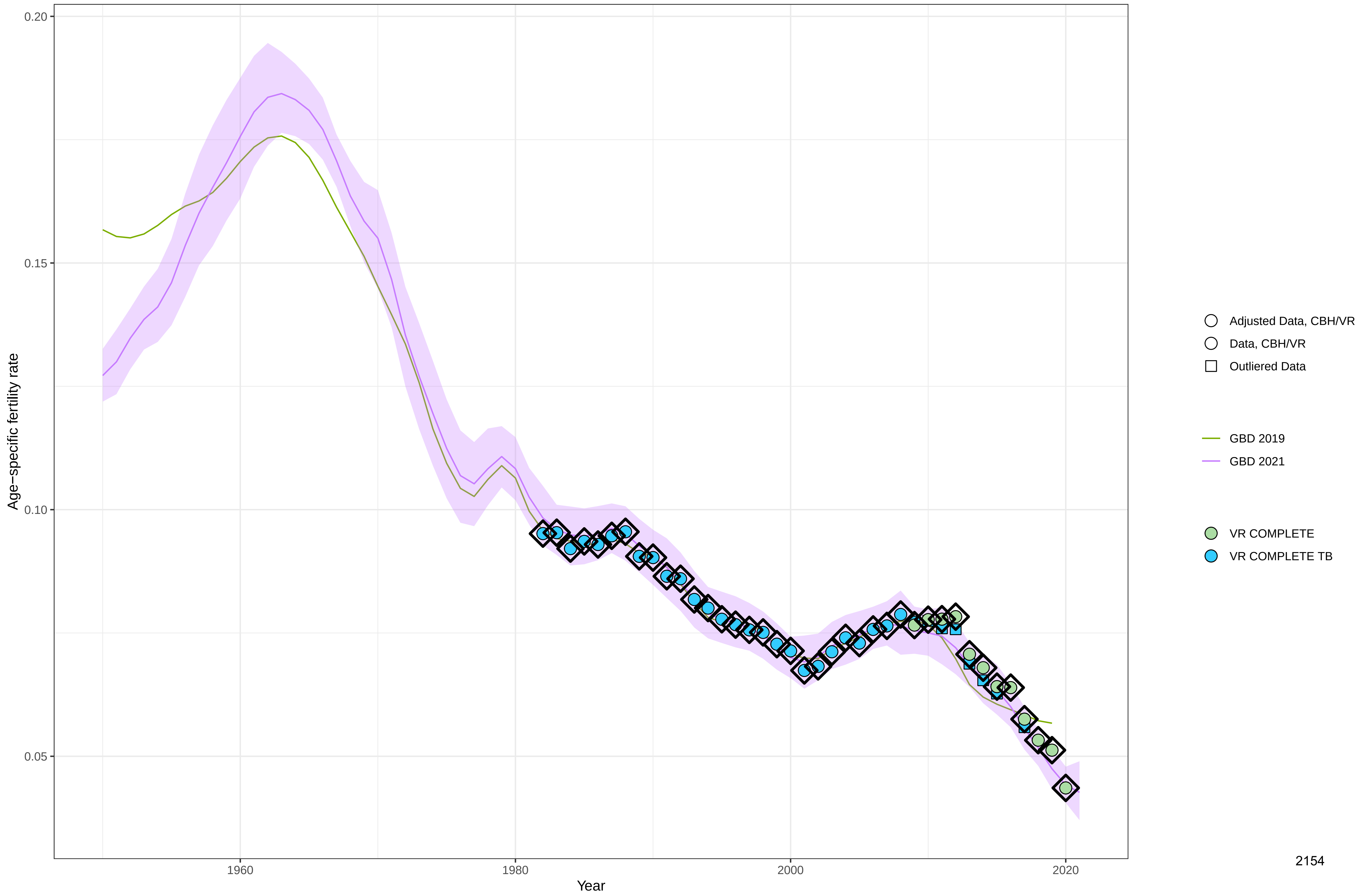

Age−specific fertility fitted trend lines, 1950−2021: Norfolk, Ages: 25−29
Data points enclosed with a diamond are reference sources

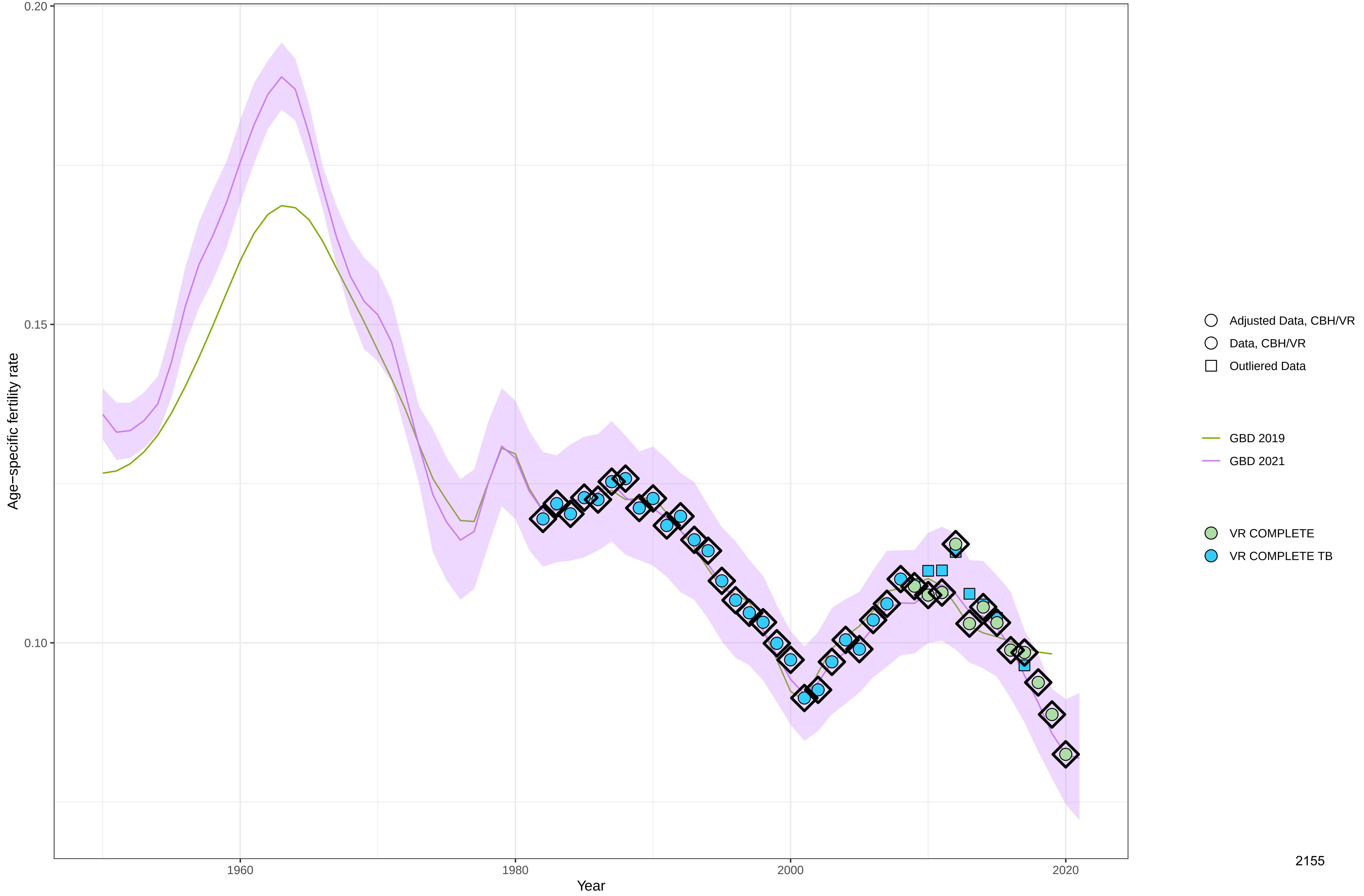

Age−specific fertility fitted trend lines, 1950−2021: Norfolk, Ages: 30−34
Data points enclosed with a diamond are reference sources

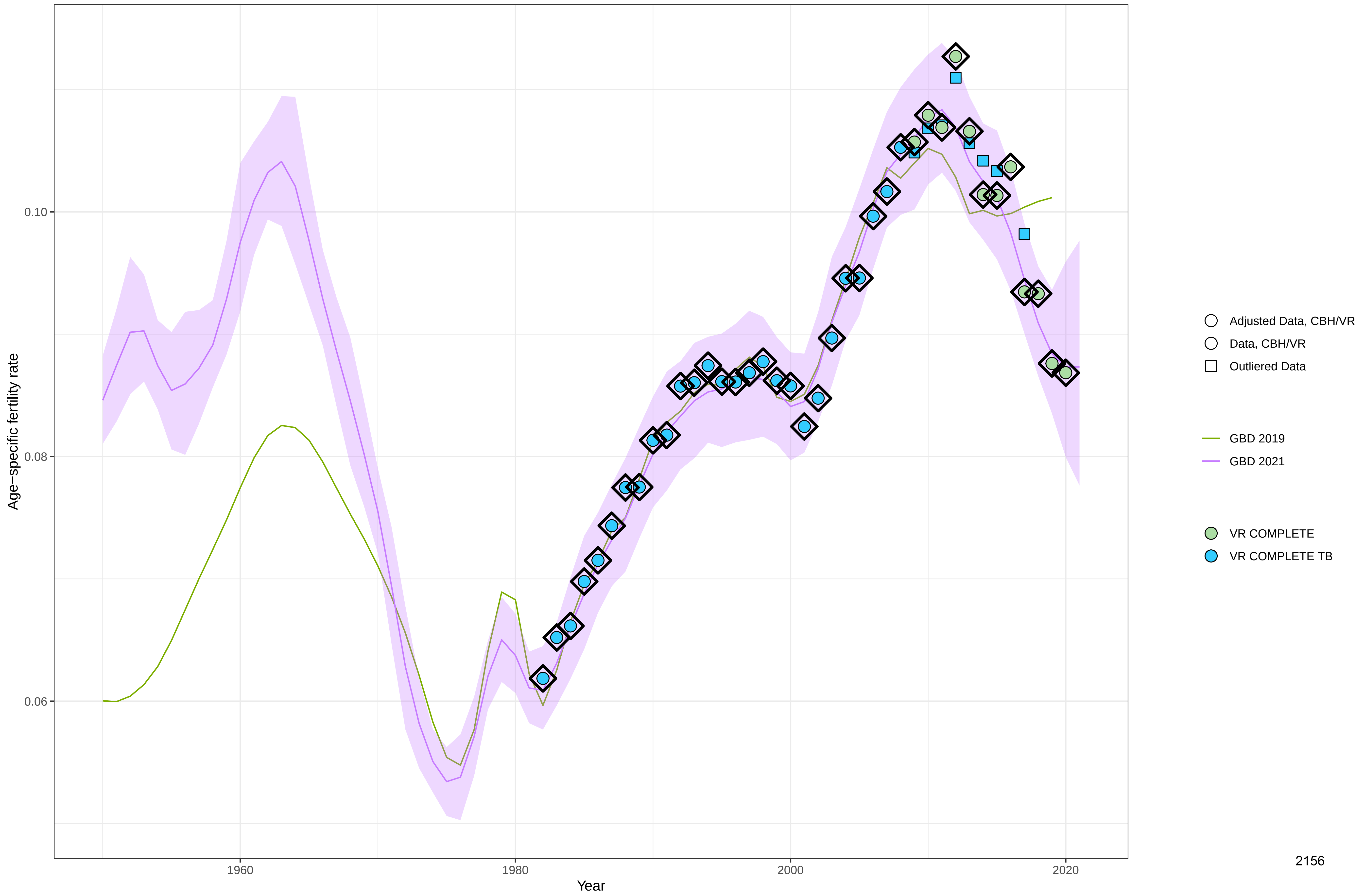

Age−specific fertility fitted trend lines, 1950−2021: Norfolk, Ages: 35−39
Data points enclosed with a diamond are reference sources

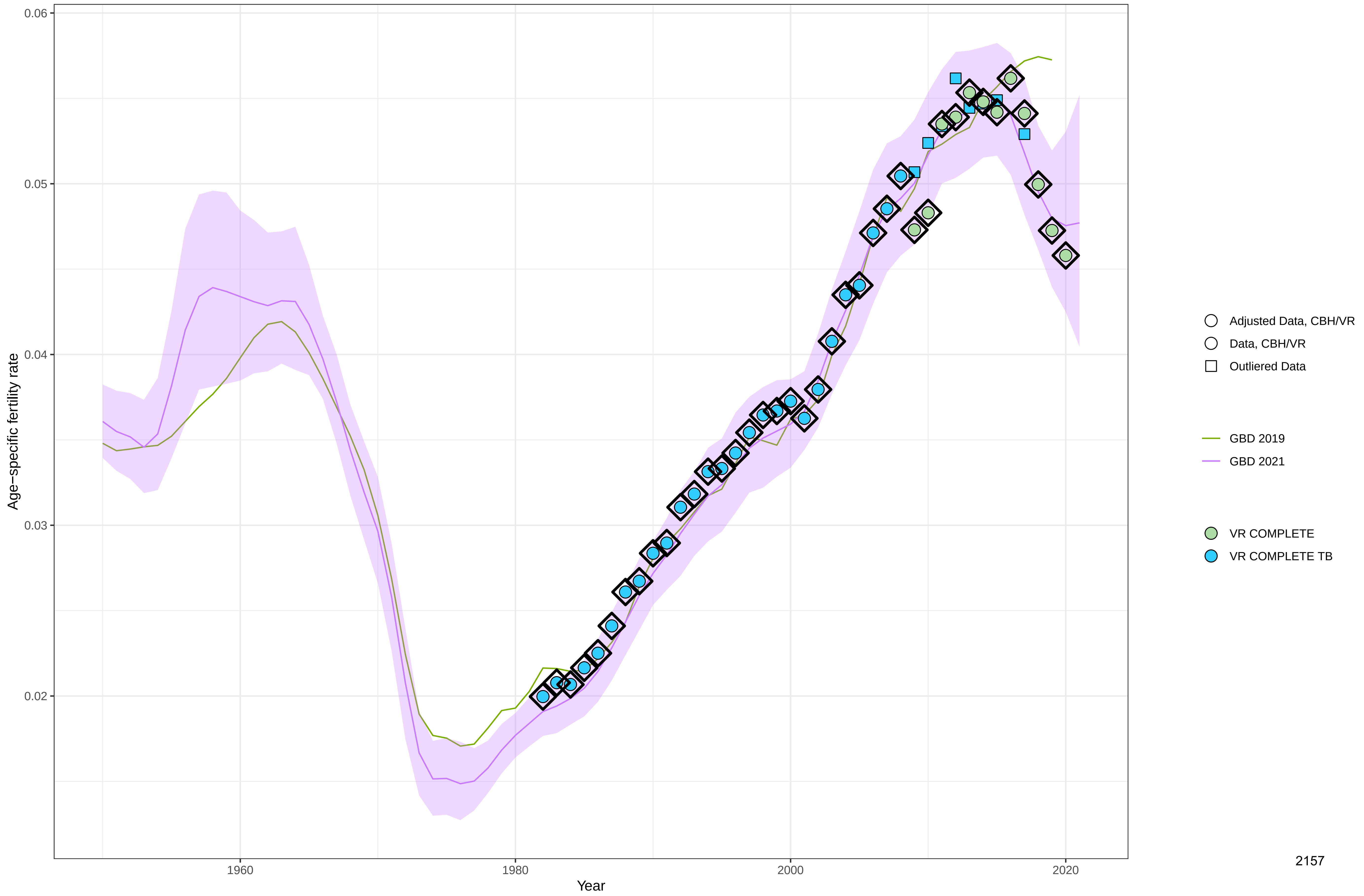

Age−specific fertility fitted trend lines, 1950−2021: Norfolk, Ages: 40−44
Data points enclosed with a diamond are reference sources

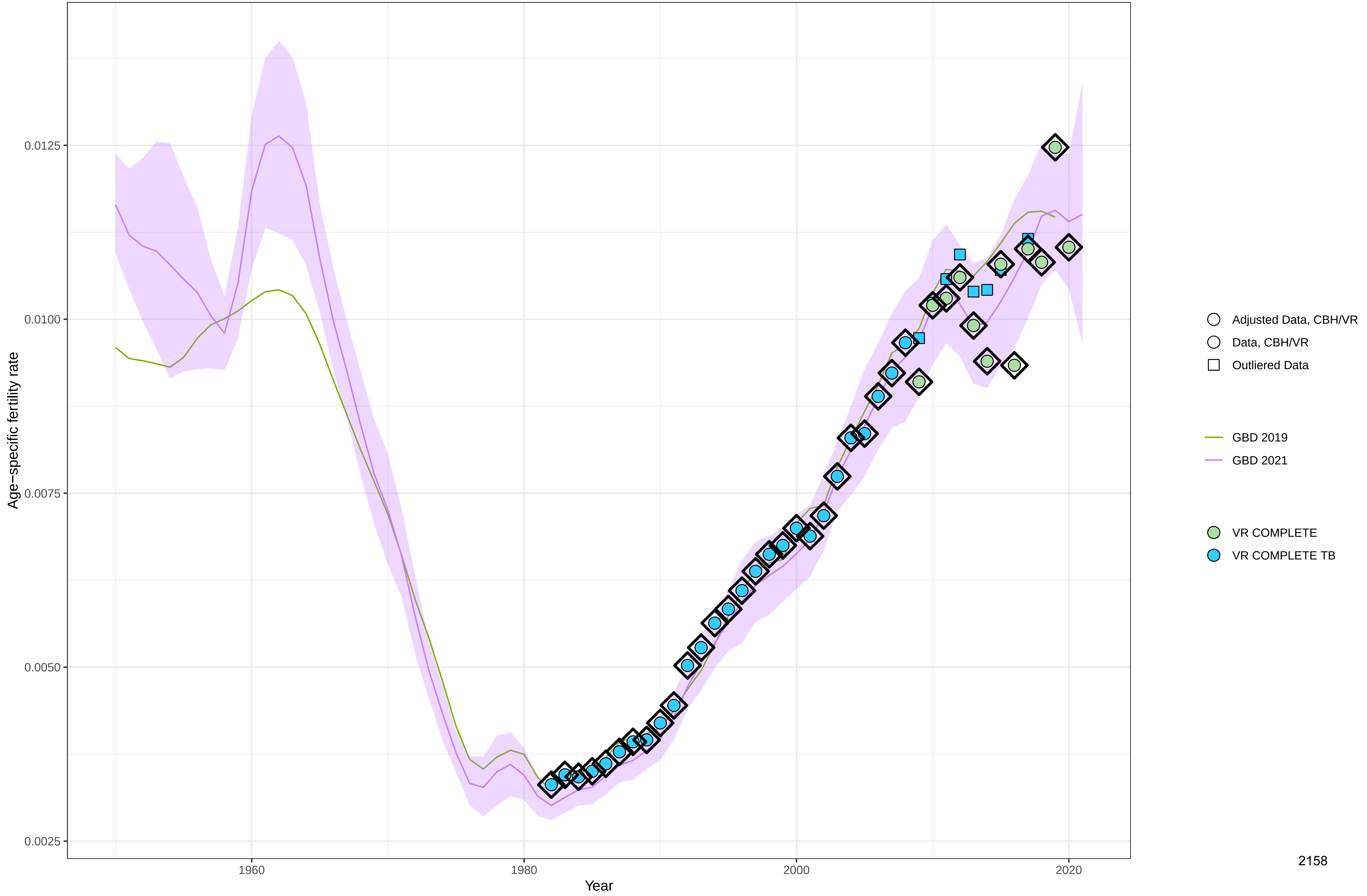

Age−specific fertility fitted trend lines, 1950−2021: Norfolk, Ages: 45−49
Data points enclosed with a diamond are reference sources

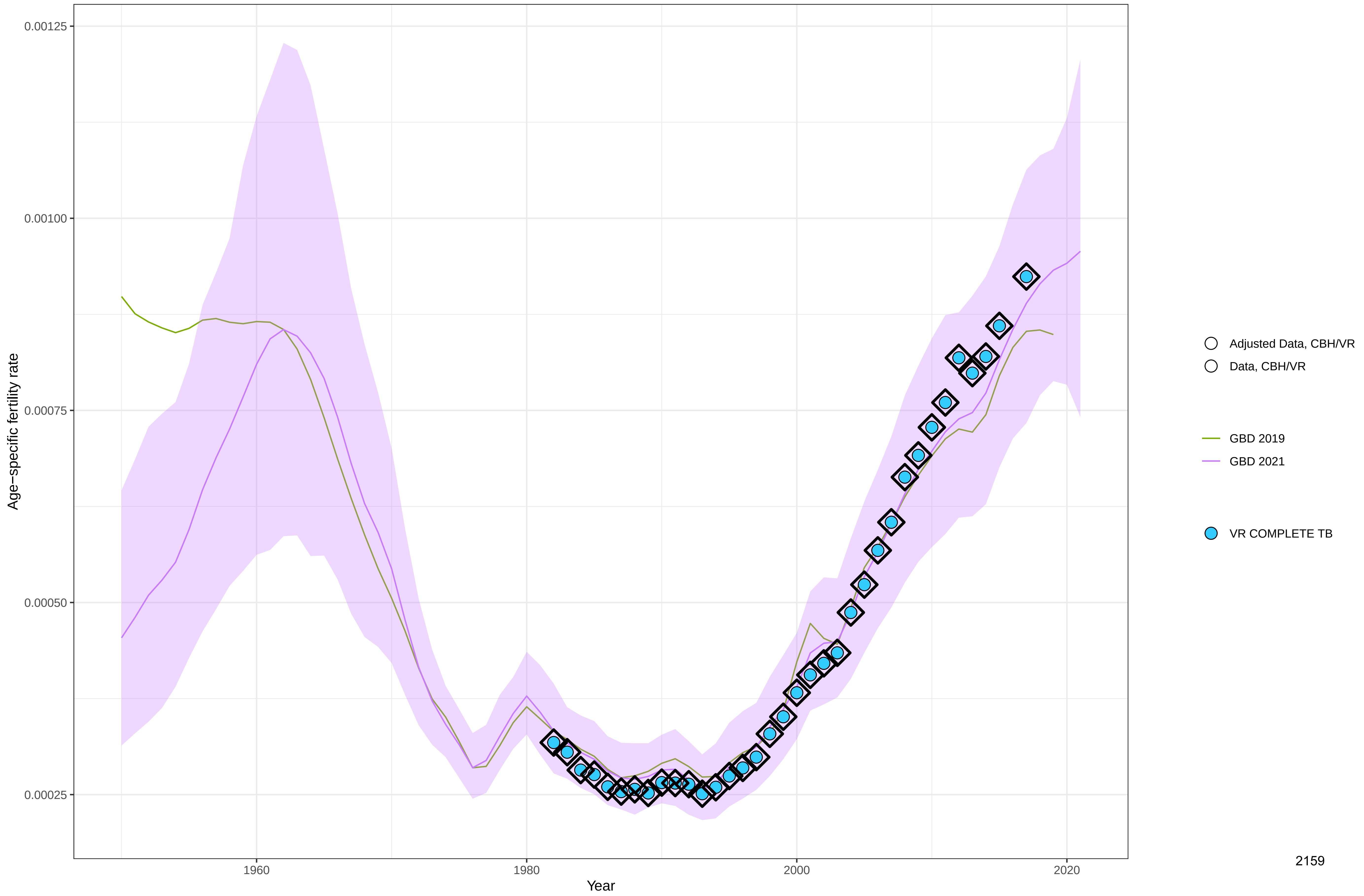

Age−specific fertility fitted trend lines, 1950−2021: Norfolk, Ages: 50−54
Data points enclosed with a diamond are reference sources

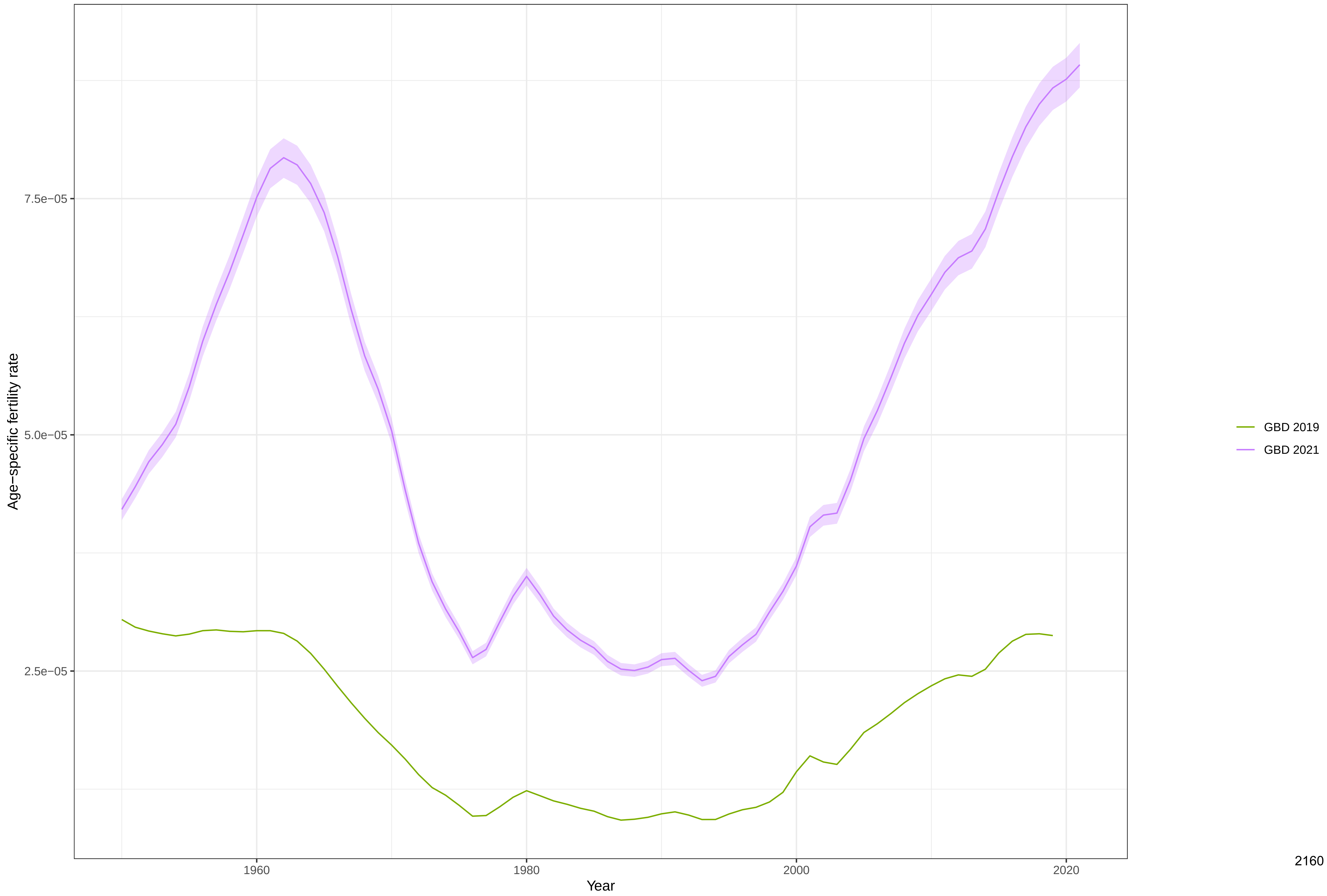

Total fertility rate fitted trend lines, 1950−2021: Norfolk TFR
Data points enclosed with a diamond are reference sources

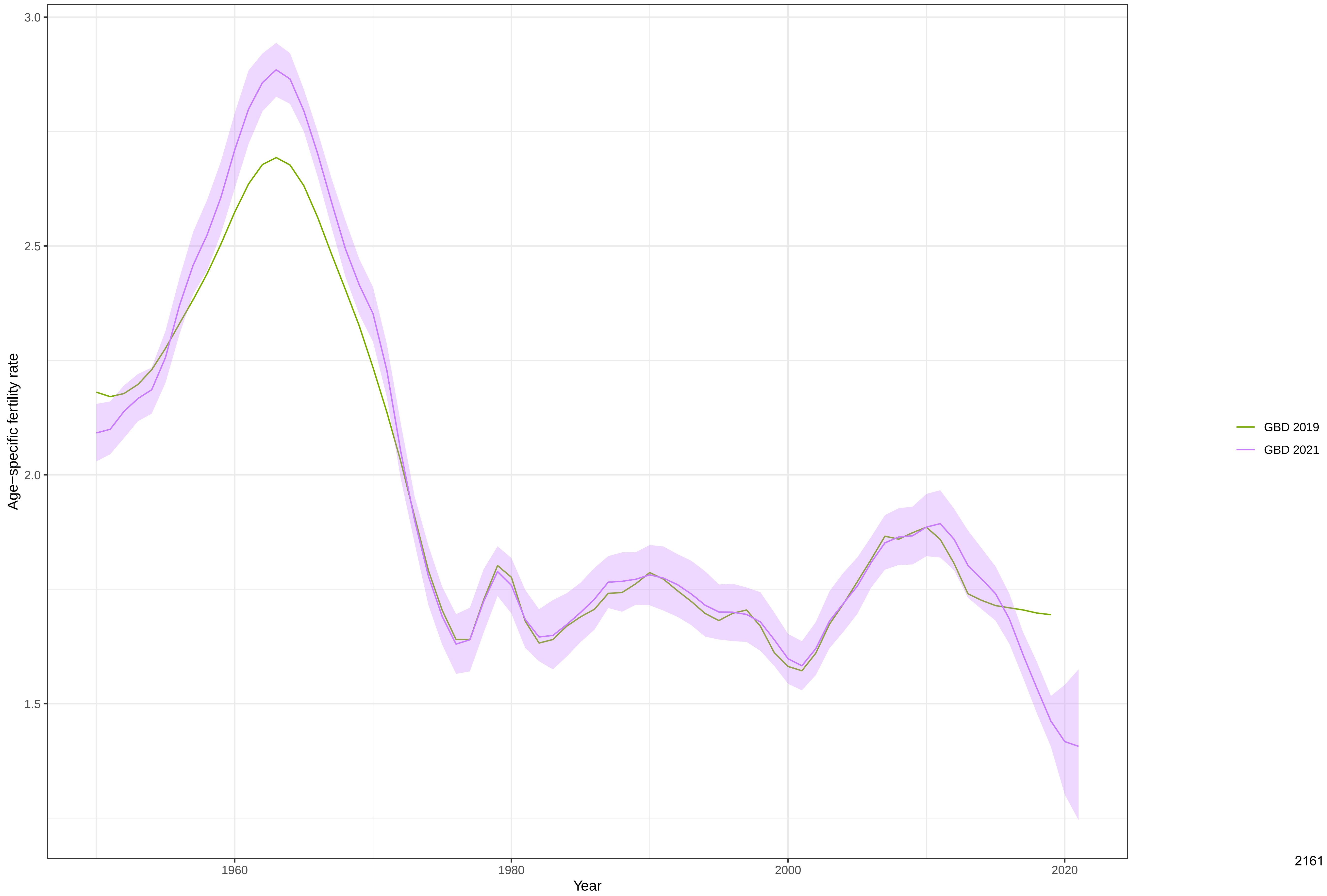

Age−specific fertility fitted trend lines, 1950−2021: Peterborough, Ages: 10−14
Data points enclosed with a diamond are reference sources

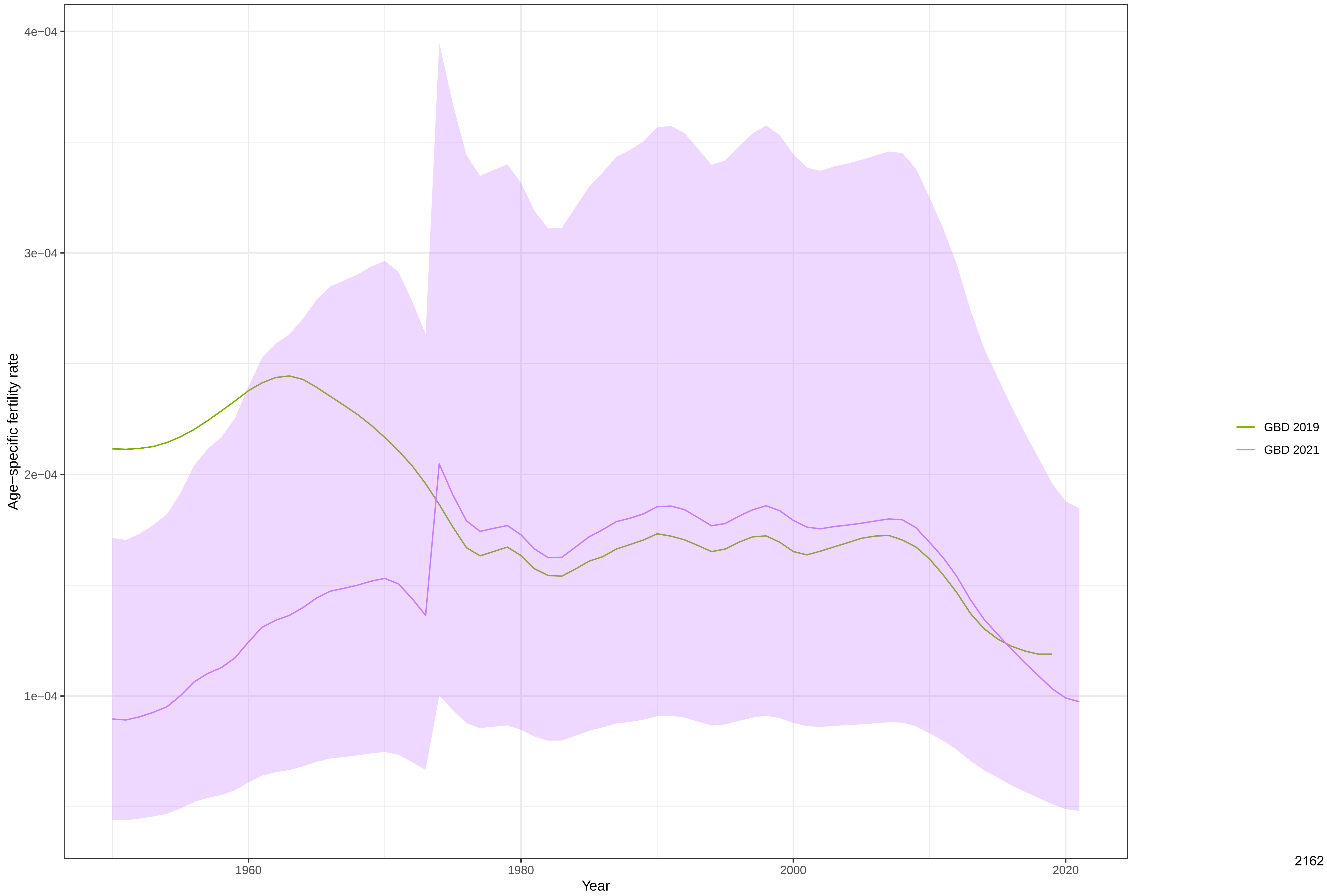

Age−specific fertility fitted trend lines, 1950−2021: Peterborough, Ages: 15−19
Data points enclosed with a diamond are reference sources

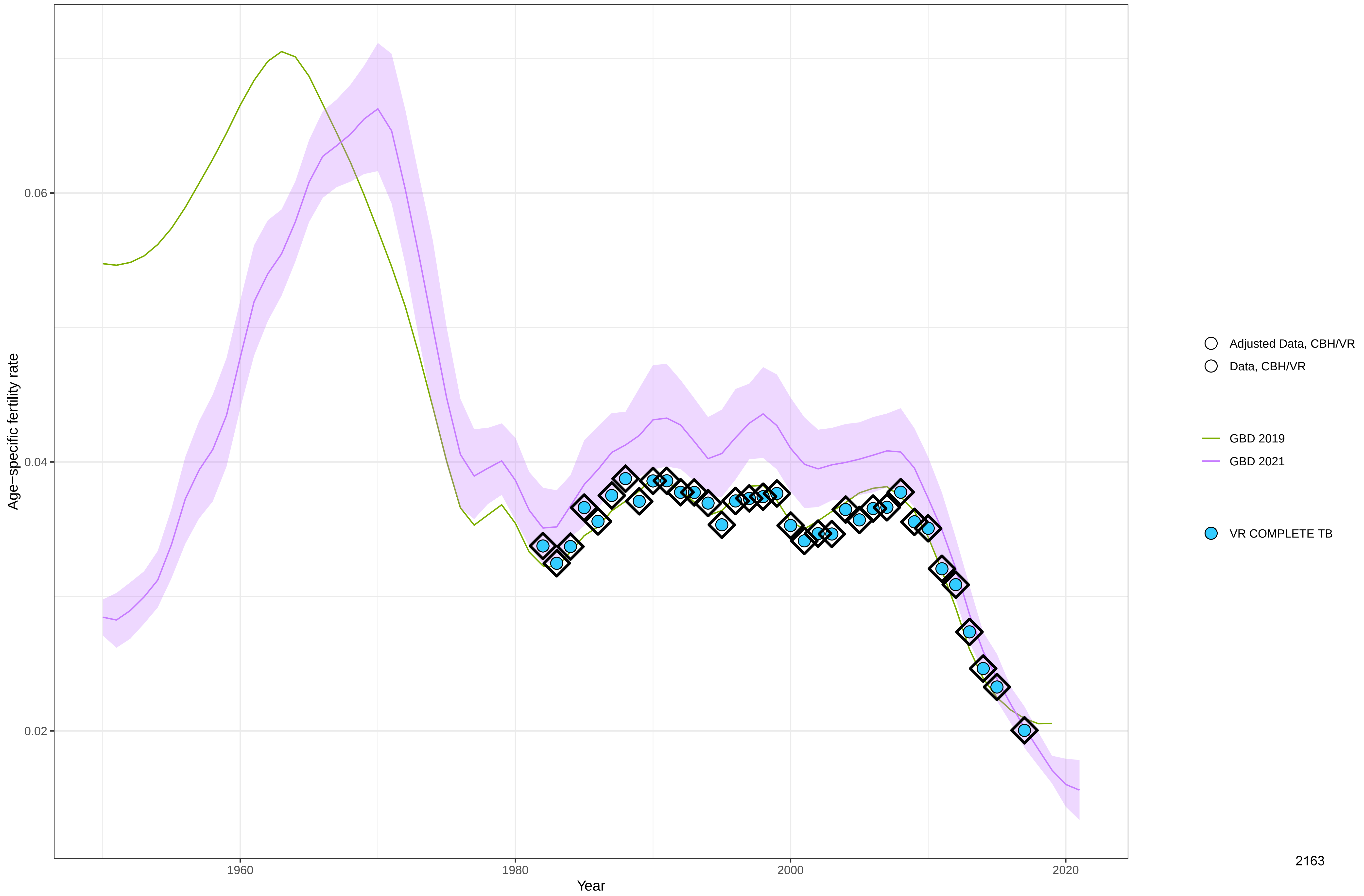

Age−specific fertility fitted trend lines, 1950−2021: Peterborough, Ages: 20−24
Data points enclosed with a diamond are reference sources

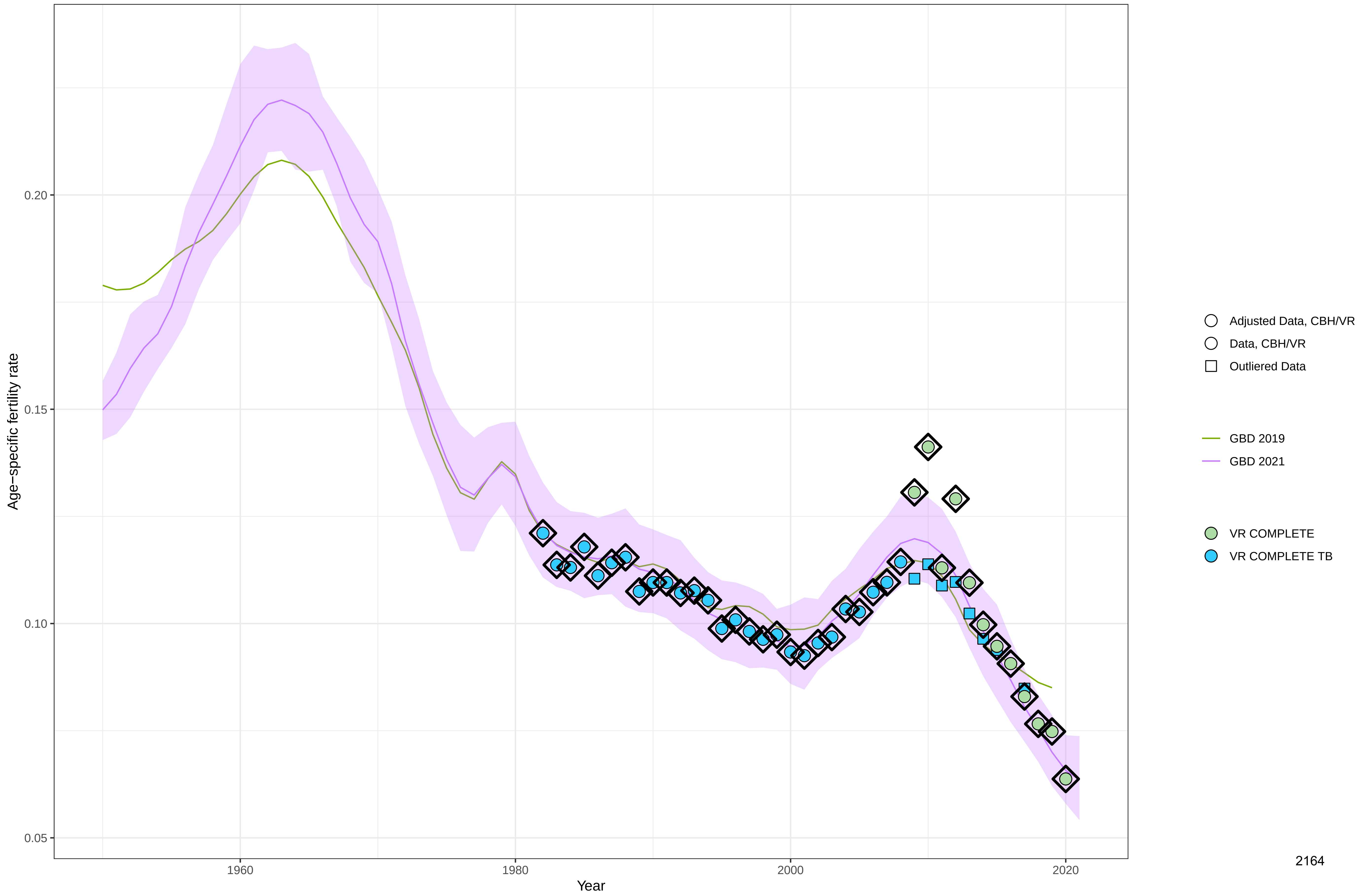

Age−specific fertility fitted trend lines, 1950−2021: Peterborough, Ages: 25−29
Data points enclosed with a diamond are reference sources

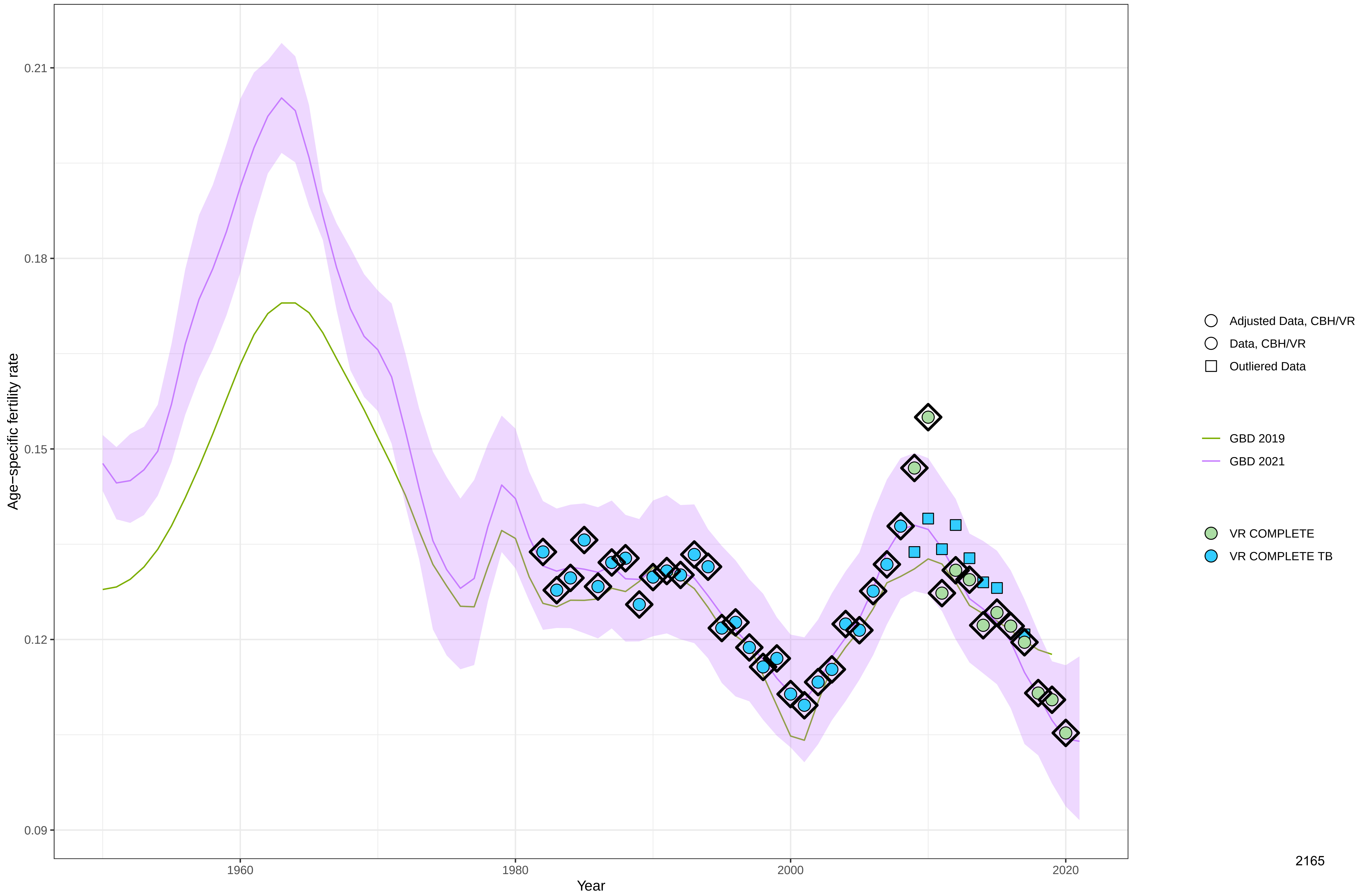

Age−specific fertility fitted trend lines, 1950−2021: Peterborough, Ages: 30−34
Data points enclosed with a diamond are reference sources

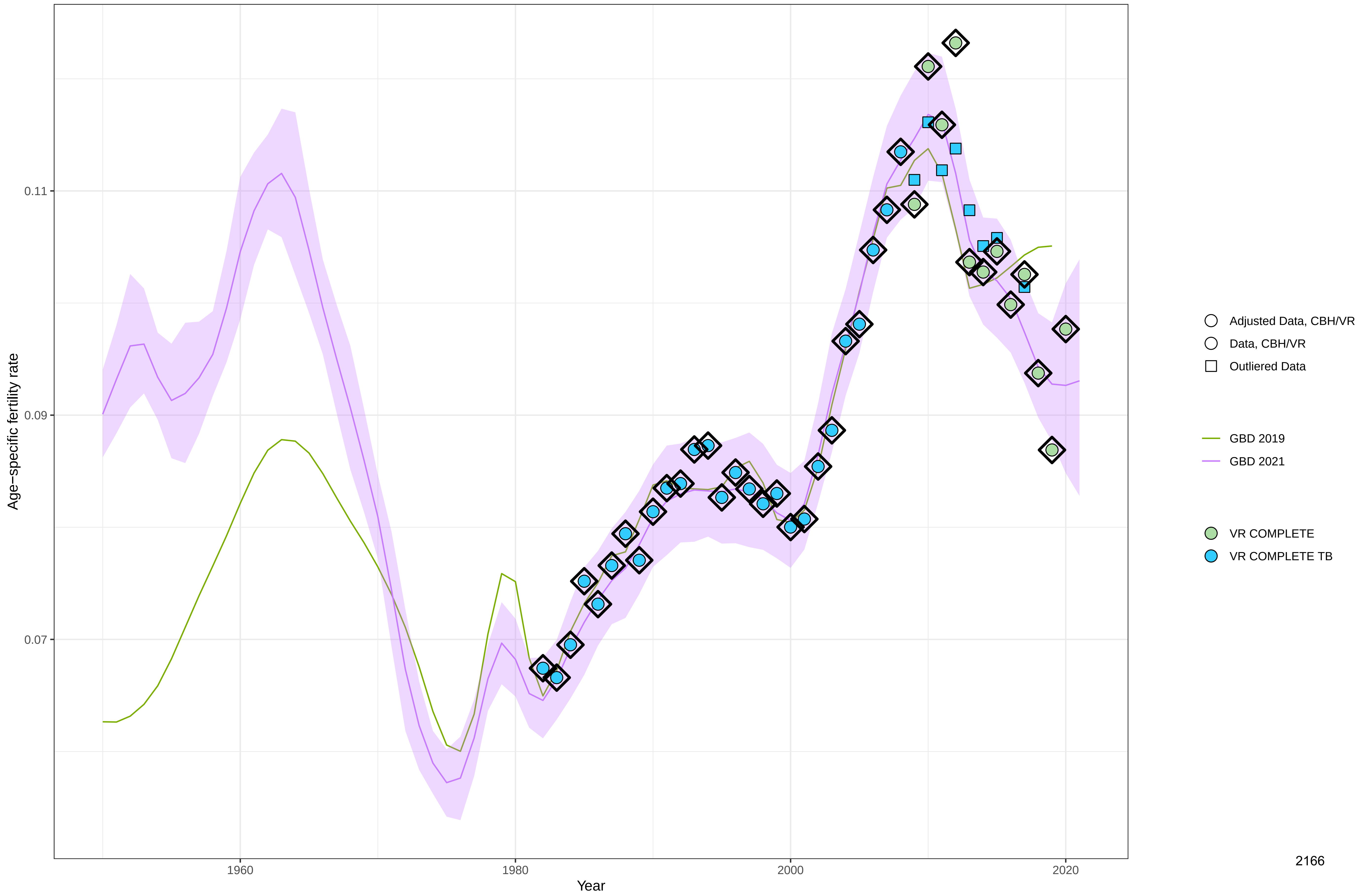

Age−specific fertility fitted trend lines, 1950−2021: Peterborough, Ages: 35−39
Data points enclosed with a diamond are reference sources

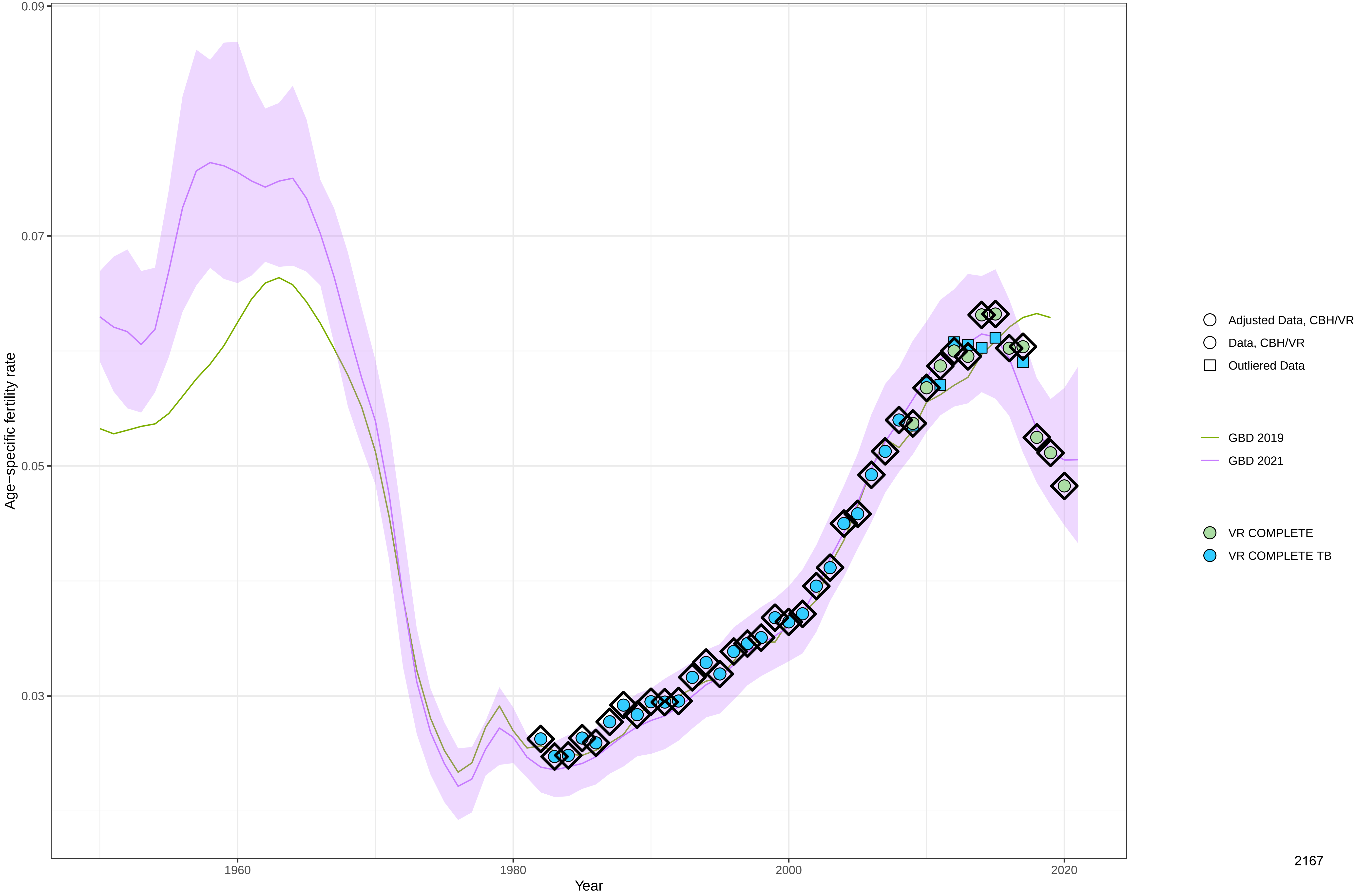

Age−specific fertility fitted trend lines, 1950−2021: Peterborough, Ages: 40−44
Data points enclosed with a diamond are reference sources

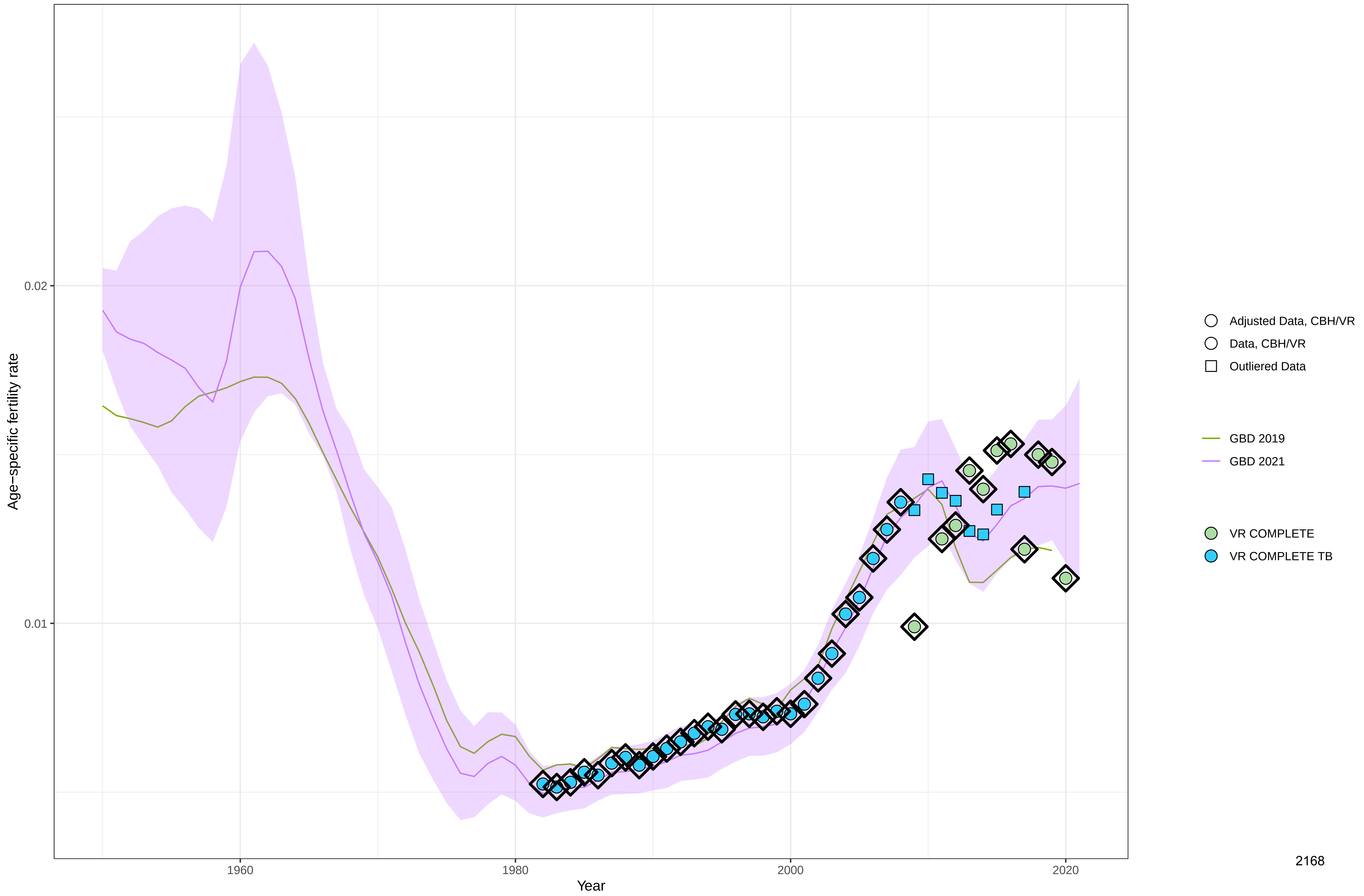

Age−specific fertility fitted trend lines, 1950−2021: Peterborough, Ages: 45−49
Data points enclosed with a diamond are reference sources

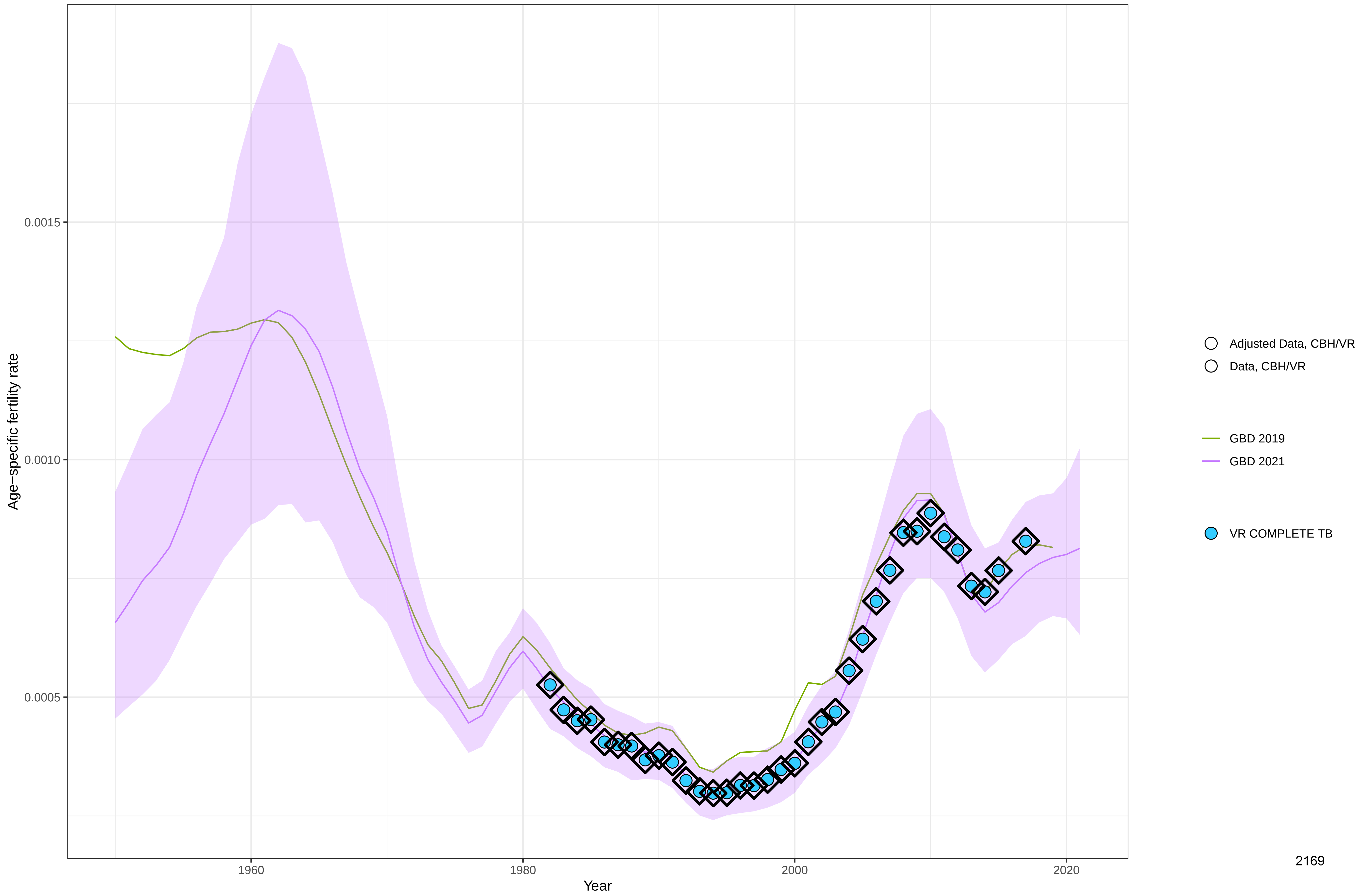

Age−specific fertility fitted trend lines, 1950−2021: Peterborough, Ages: 50−54
Data points enclosed with a diamond are reference sources

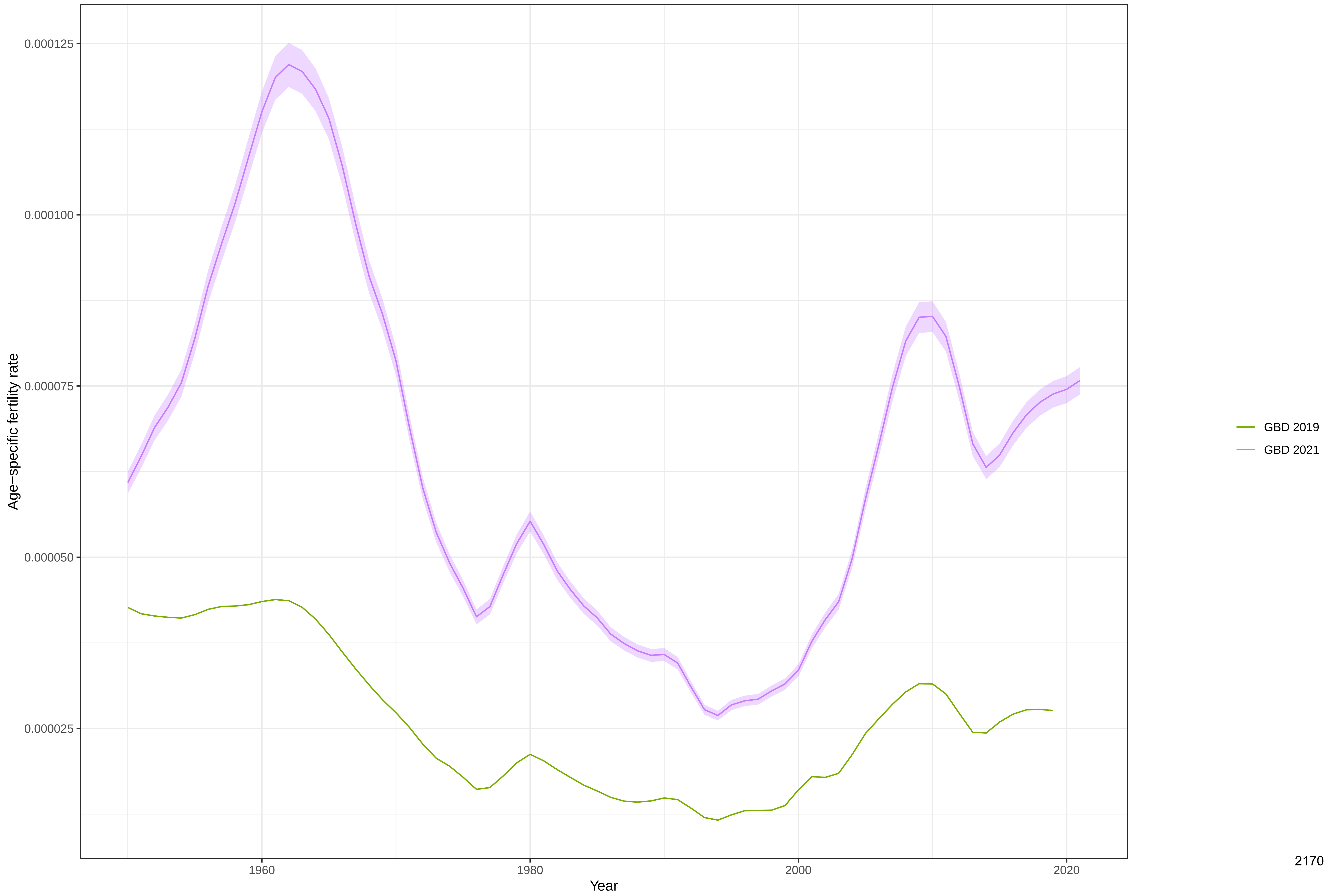

Total fertility rate fitted trend lines, 1950−2021: Peterborough TFR
Data points enclosed with a diamond are reference sources

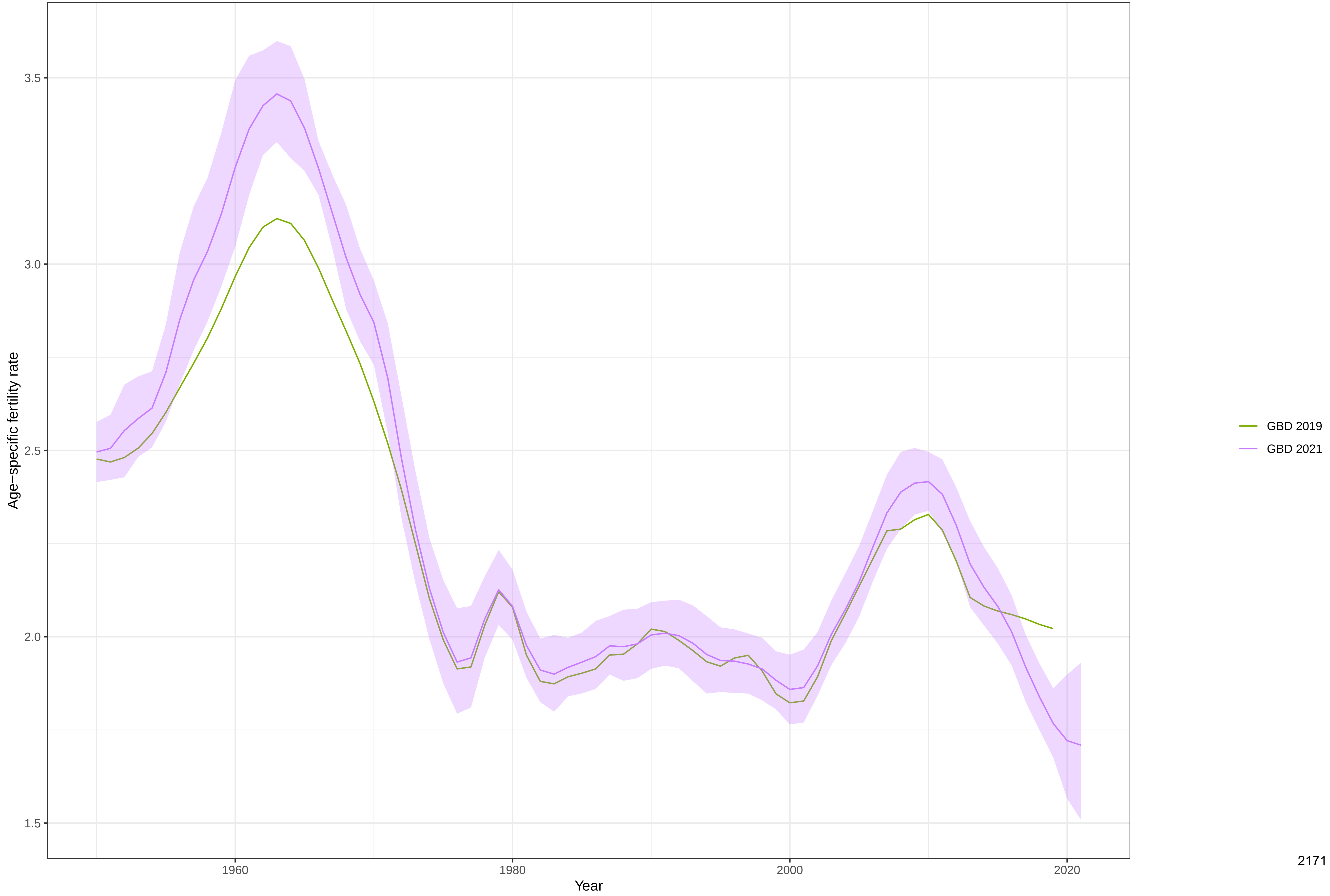

Age−specific fertility fitted trend lines, 1950−2021: Southend−on−Sea, Ages: 10−14
Data points enclosed with a diamond are reference sources

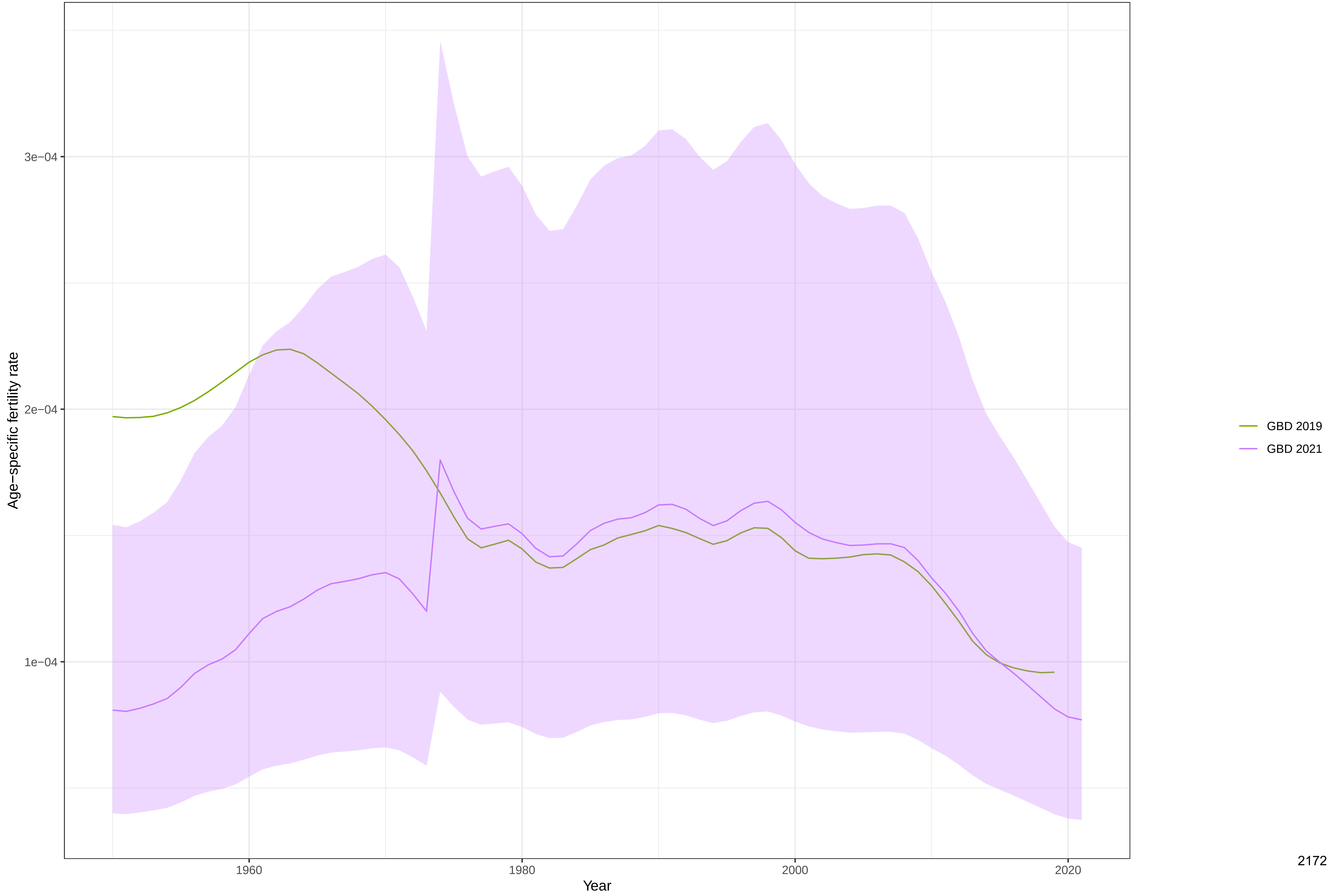

Age−specific fertility fitted trend lines, 1950−2021: Southend−on−Sea, Ages: 15−19
Data points enclosed with a diamond are reference sources

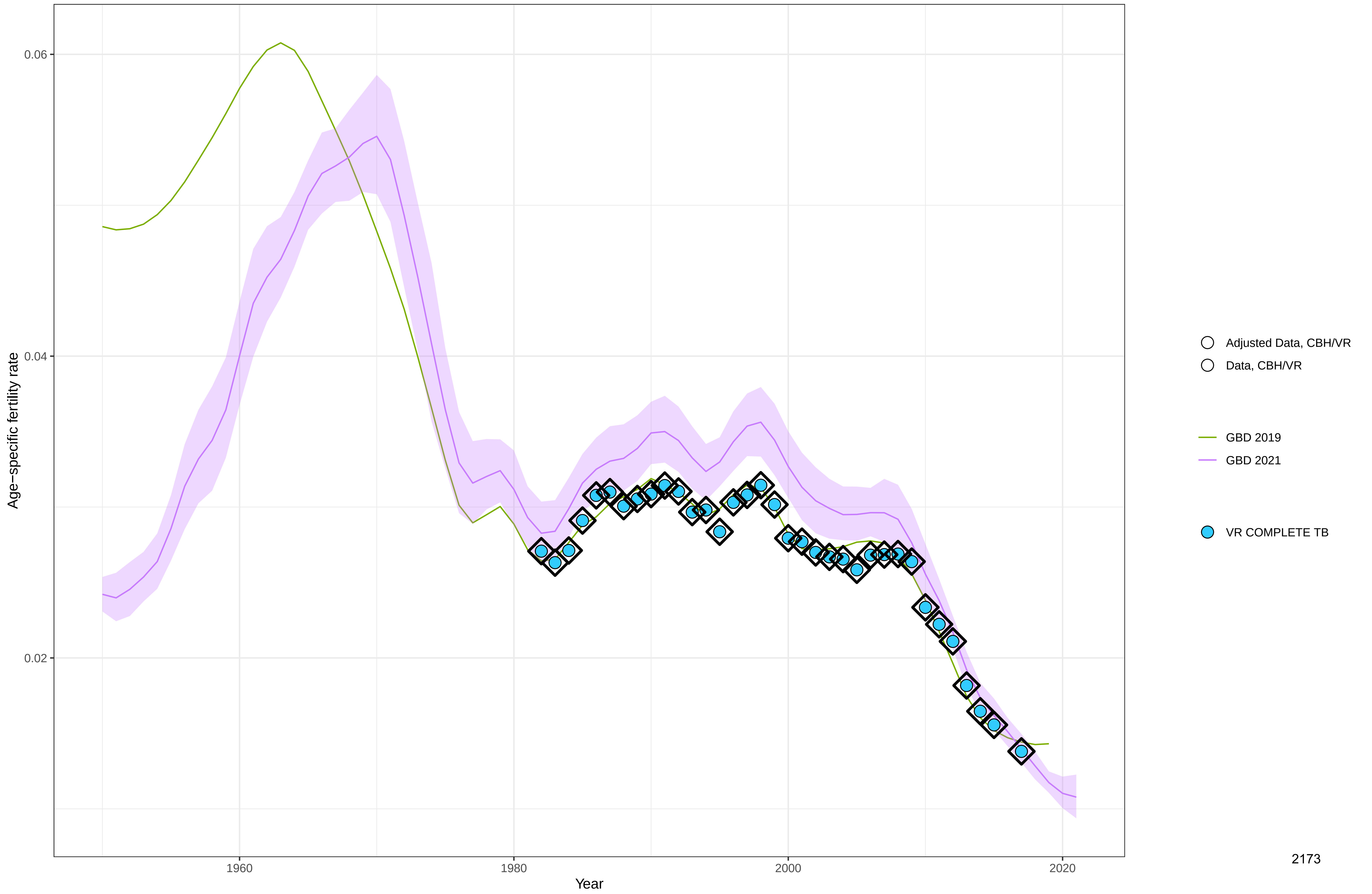

Age−specific fertility fitted trend lines, 1950−2021: Southend−on−Sea, Ages: 20−24
Data points enclosed with a diamond are reference sources

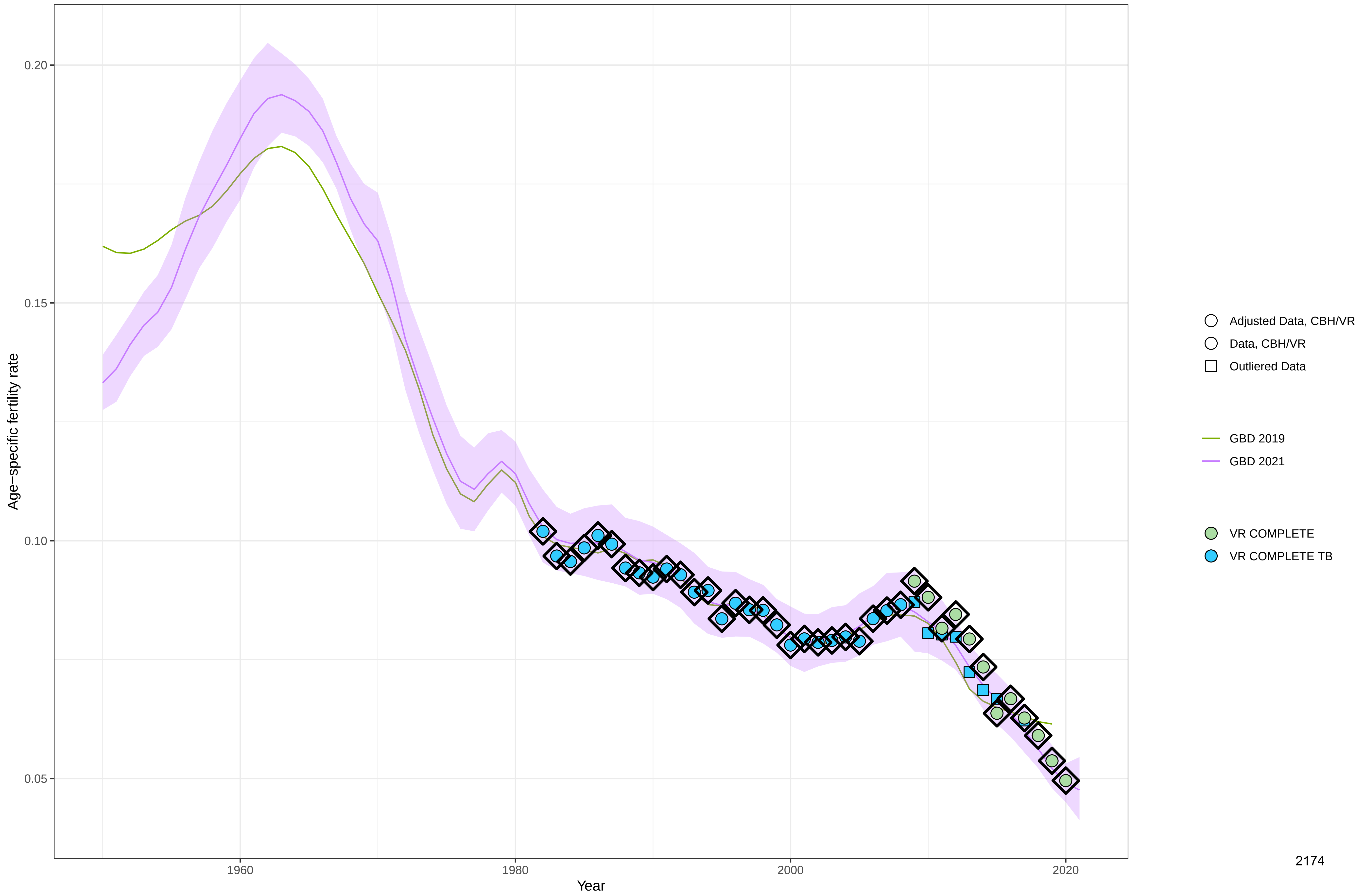

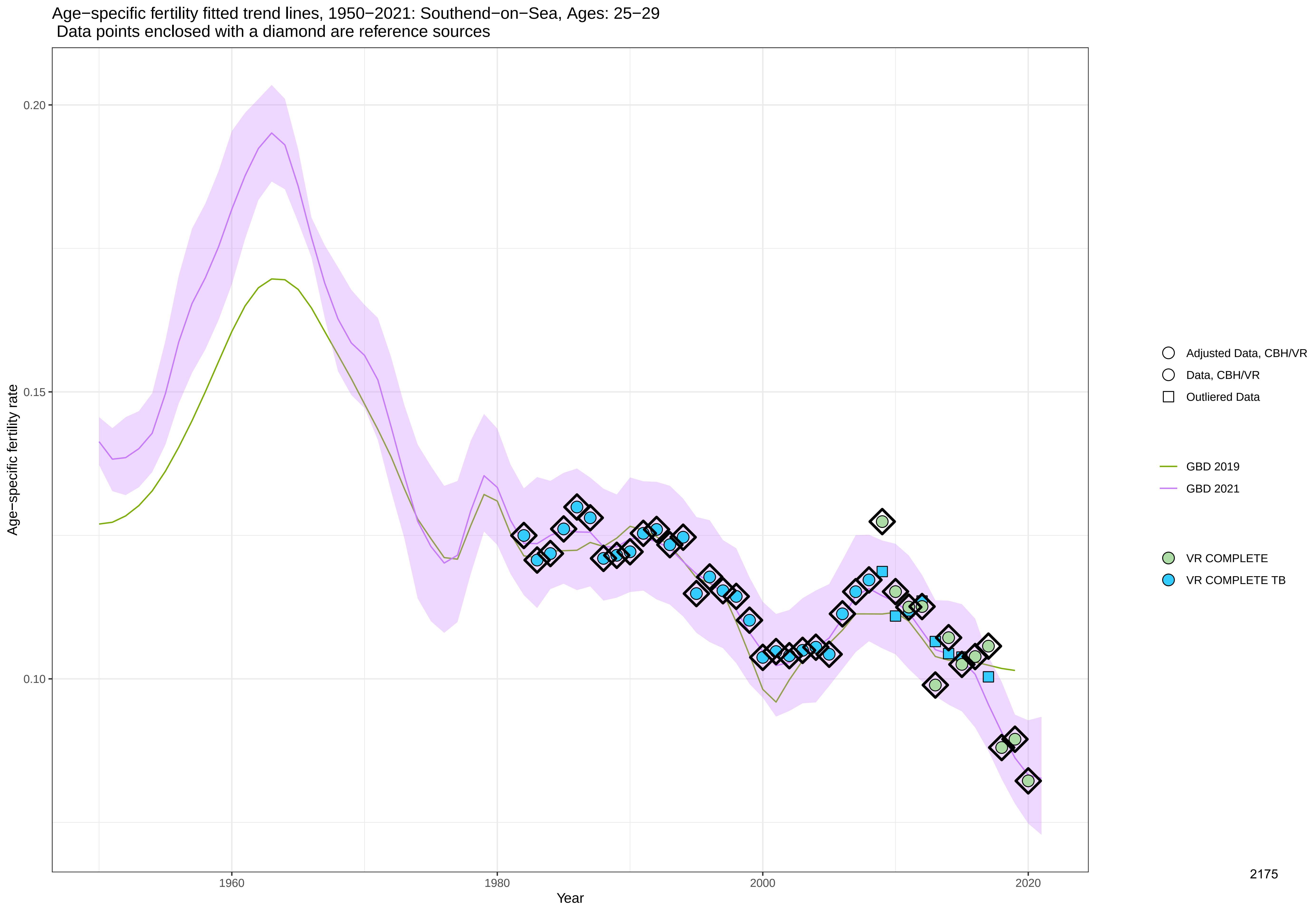

Age−specific fertility fitted trend lines, 1950−2021: Southend−on−Sea, Ages: 25−29
Data points enclosed with a diamond are reference sources
Age−specific fertility rate
0.20
0.15
0.10
1960
1980
2000
2020
Year
Adjusted Data, CBH/VR
Data, CBH/VR
Outliered Data
GBD 2019
GBD 2021
VR COMPLETE
VR COMPLETE TB

Age−specific fertility fitted trend lines, 1950−2021: Southend−on−Sea, Ages: 30−34
Data points enclosed with a diamond are reference sources

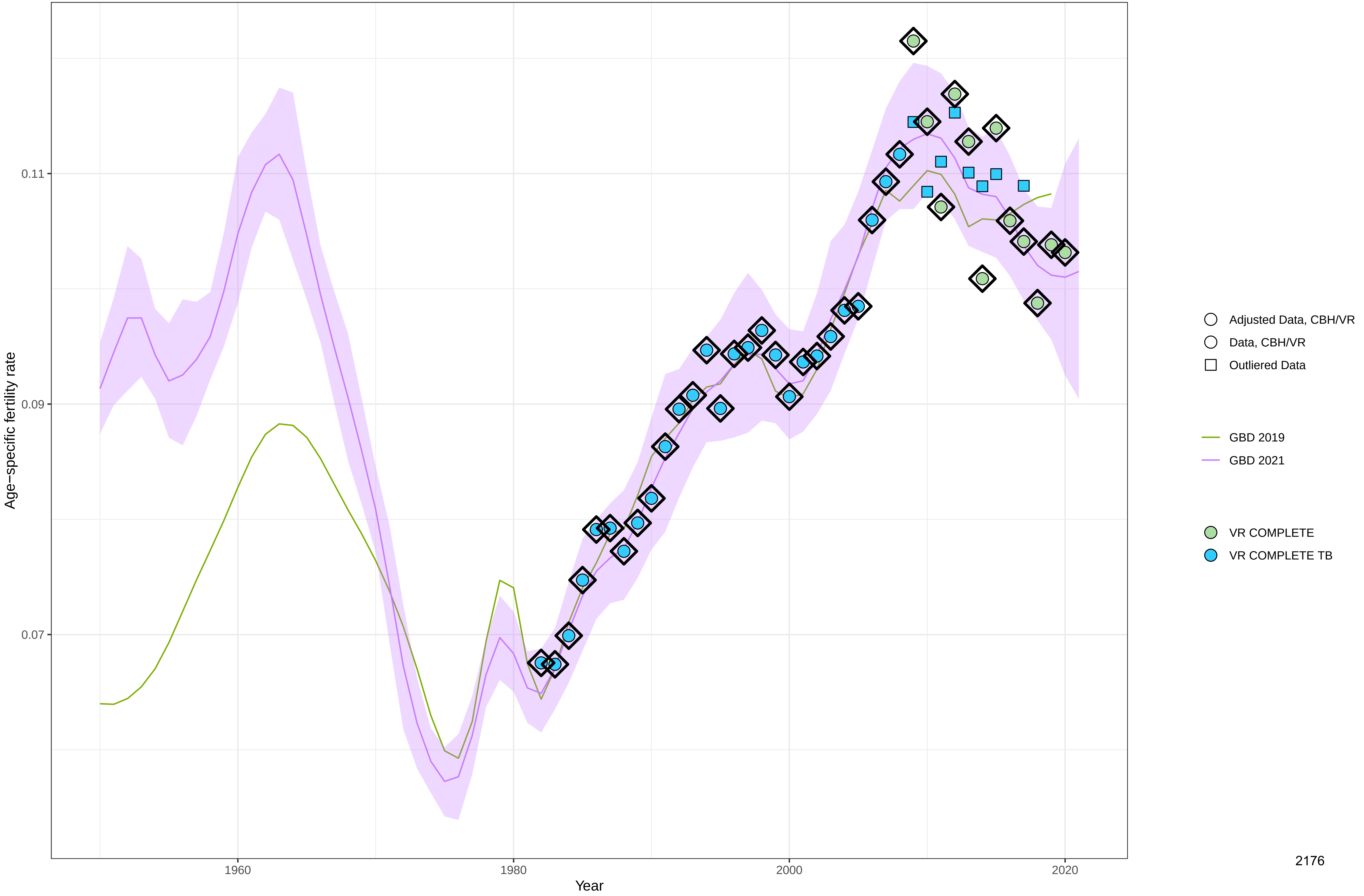

Age−specific fertility fitted trend lines, 1950−2021: Southend−on−Sea, Ages: 35−39
Data points enclosed with a diamond are reference sources

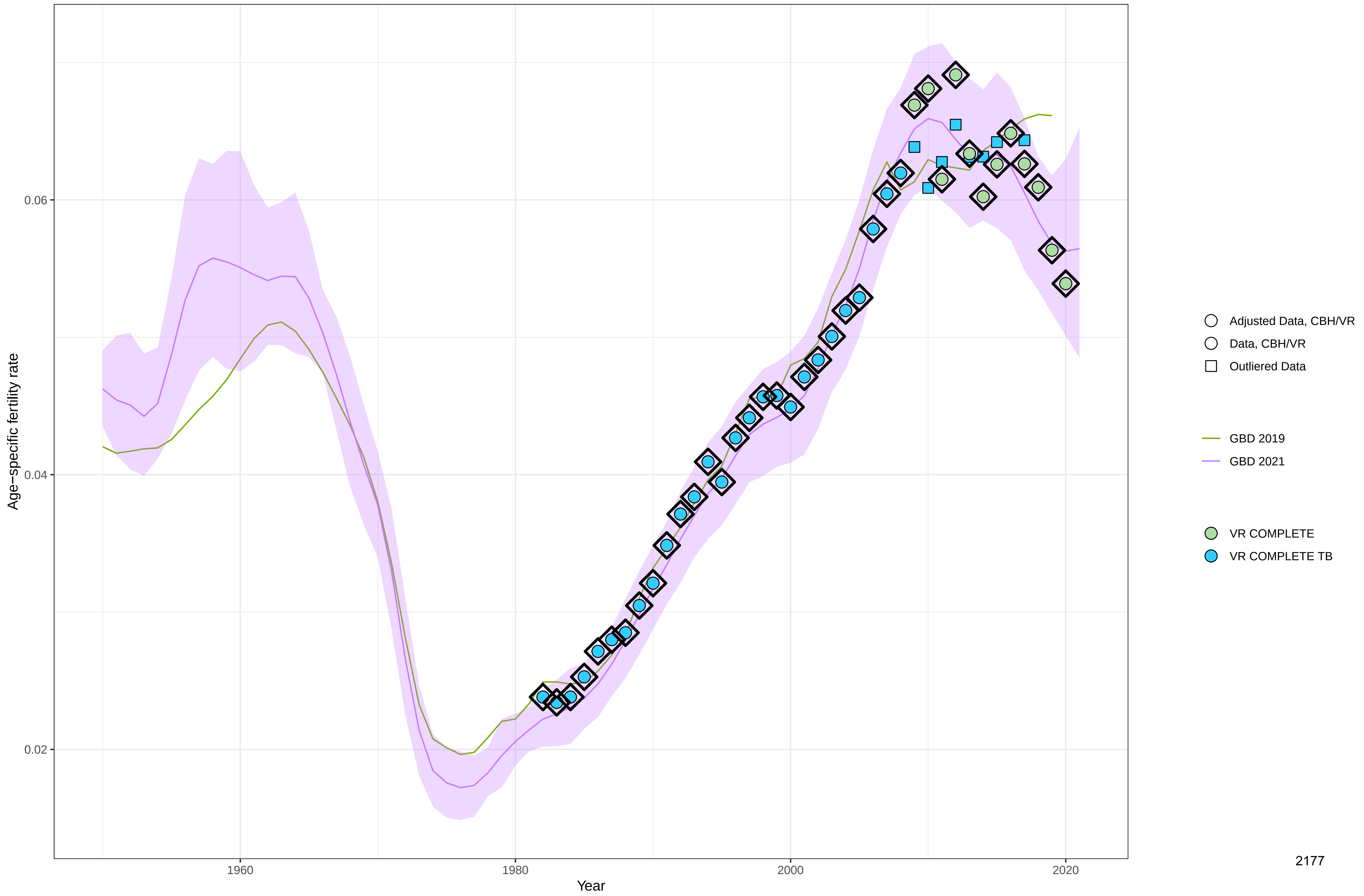

Age−specific fertility fitted trend lines, 1950−2021: Southend−on−Sea, Ages: 40−44
Data points enclosed with a diamond are reference sources

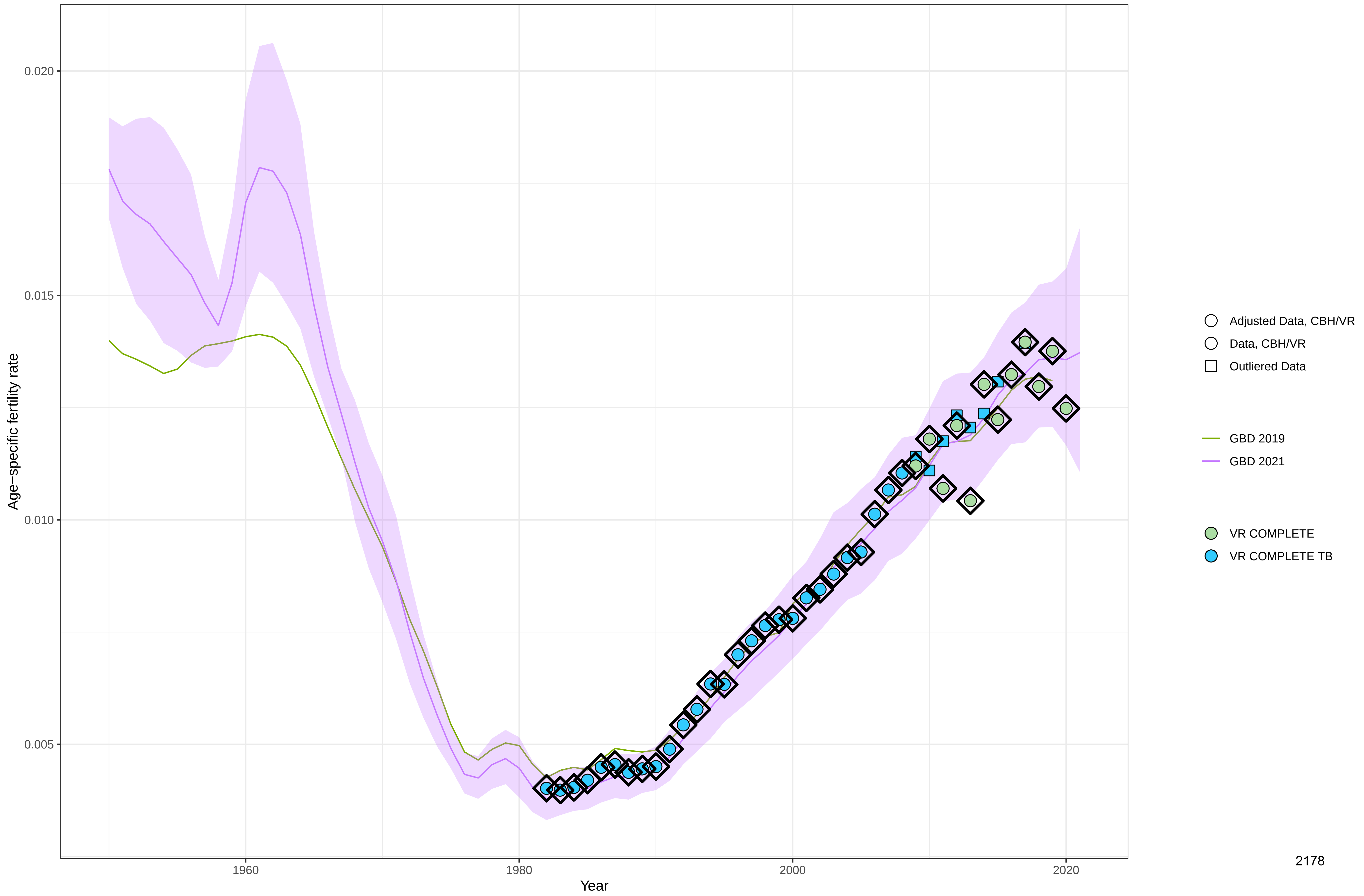

Age−specific fertility fitted trend lines, 1950−2021: Southend−on−Sea, Ages: 45−49
Data points enclosed with a diamond are reference sources

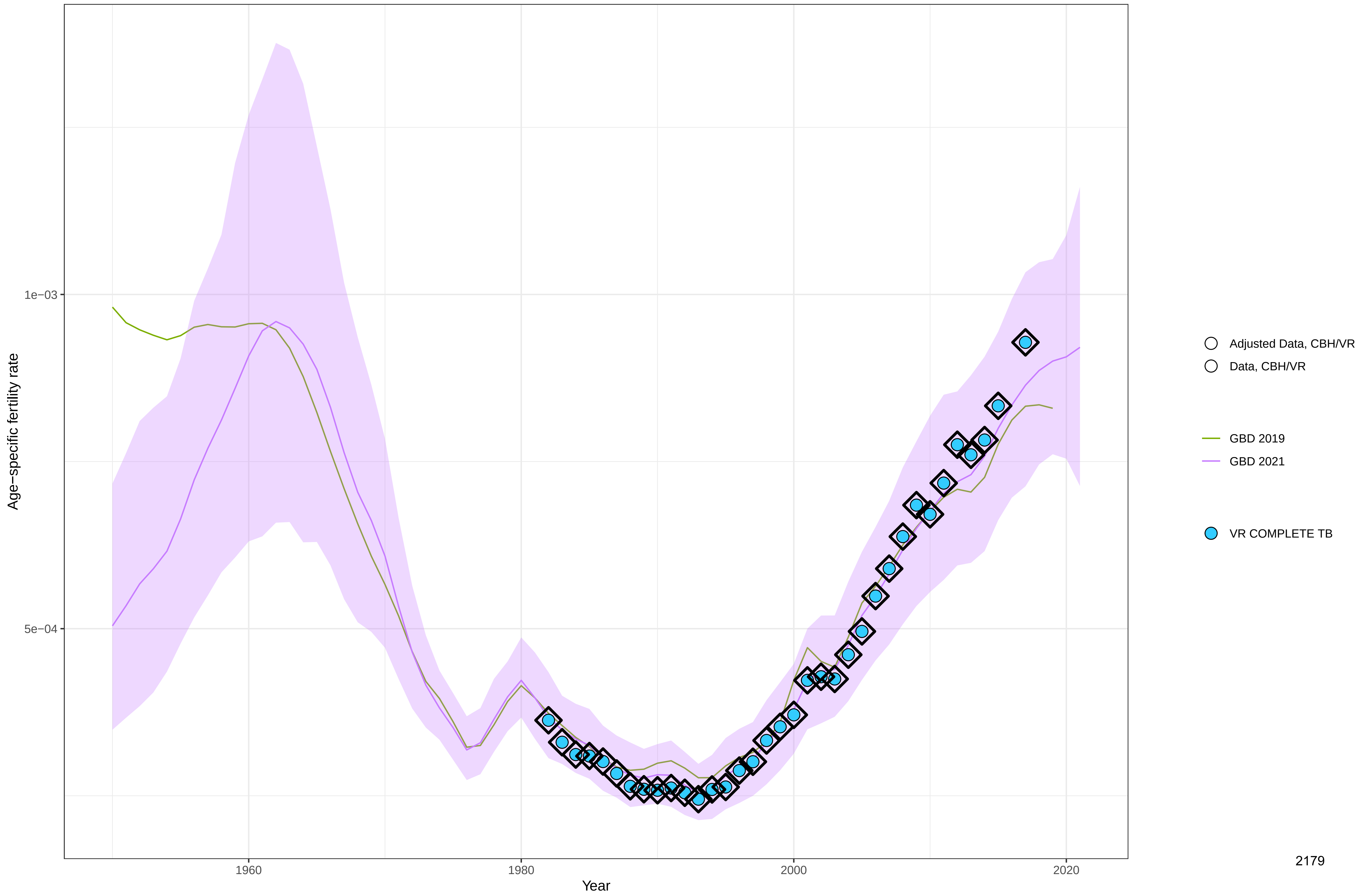

Age−specific fertility fitted trend lines, 1950−2021: Southend−on−Sea, Ages: 50−54
Data points enclosed with a diamond are reference sources

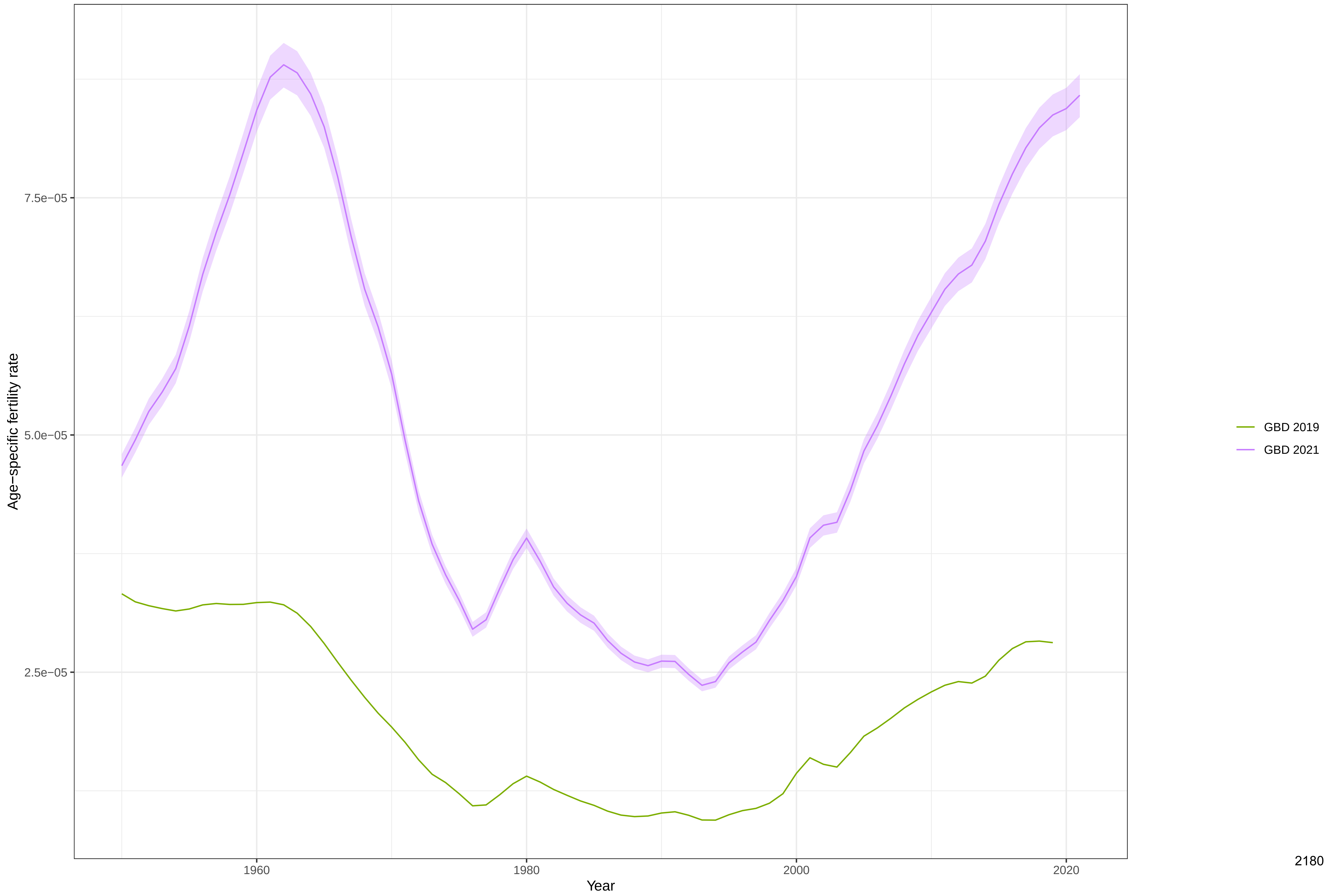

Total fertility rate fitted trend lines, 1950−2021: Southend−on−Sea TFR
Data points enclosed with a diamond are reference sources

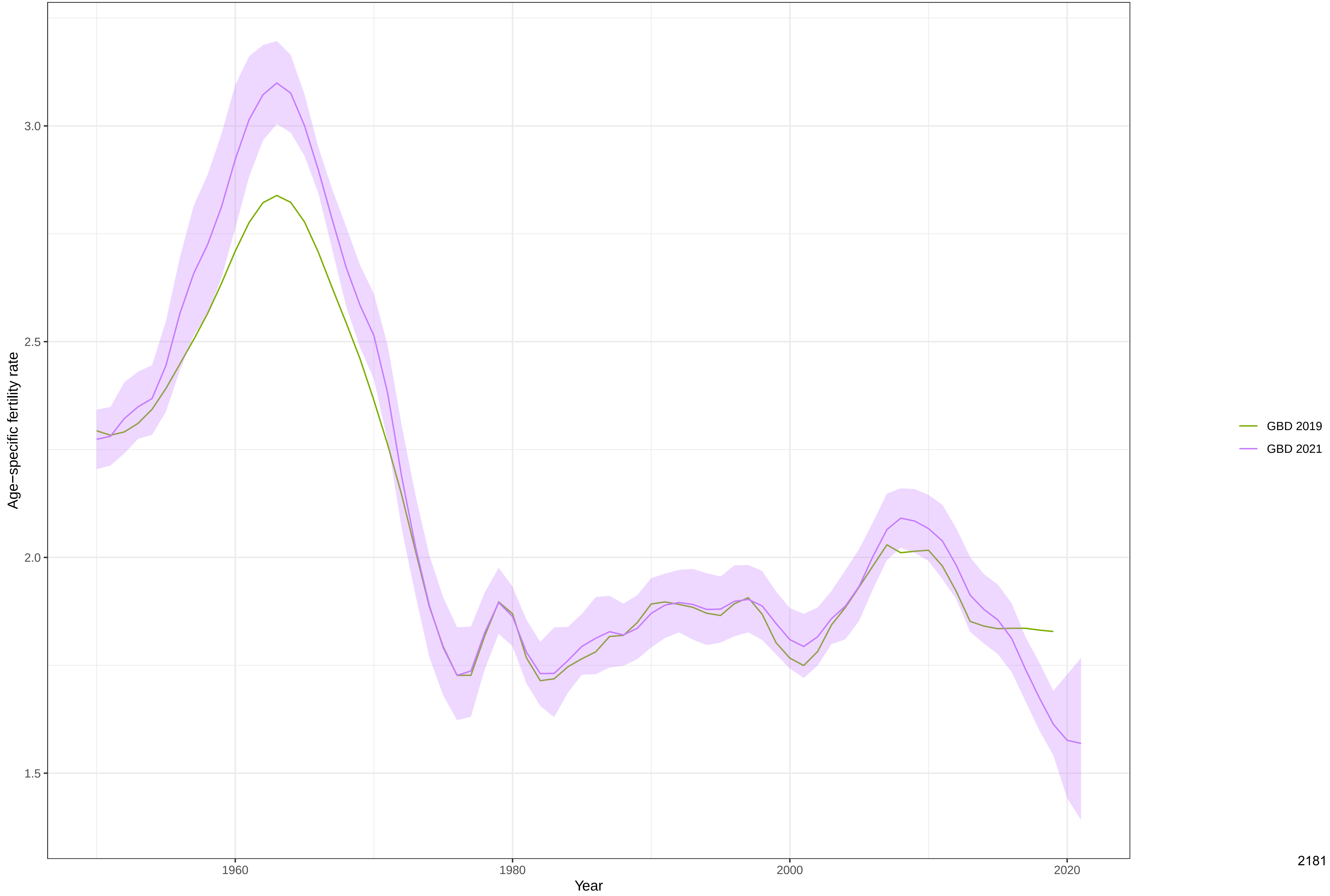

Age−specific fertility fitted trend lines, 1950−2021: Suffolk, Ages: 10−14
Data points enclosed with a diamond are reference sources

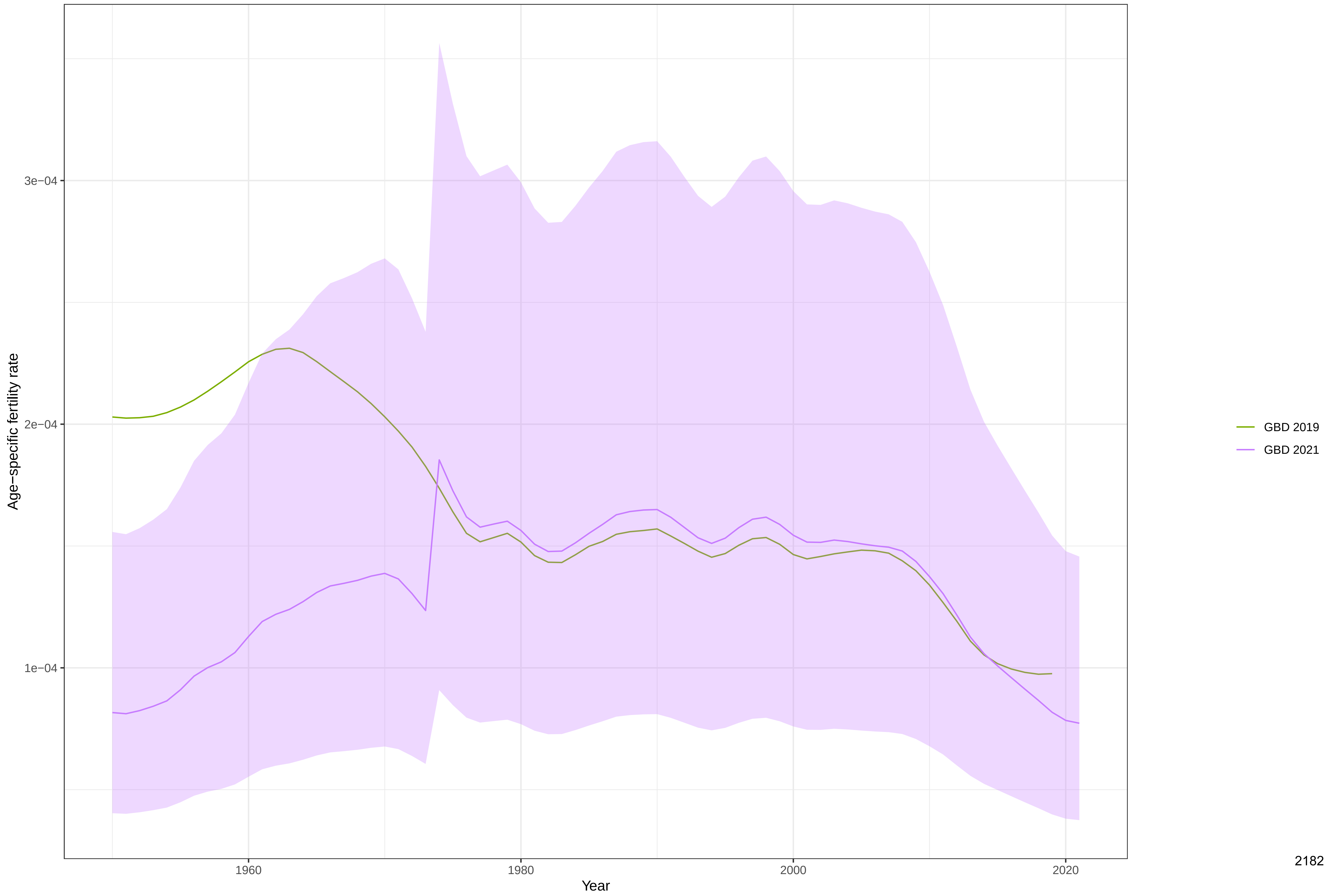

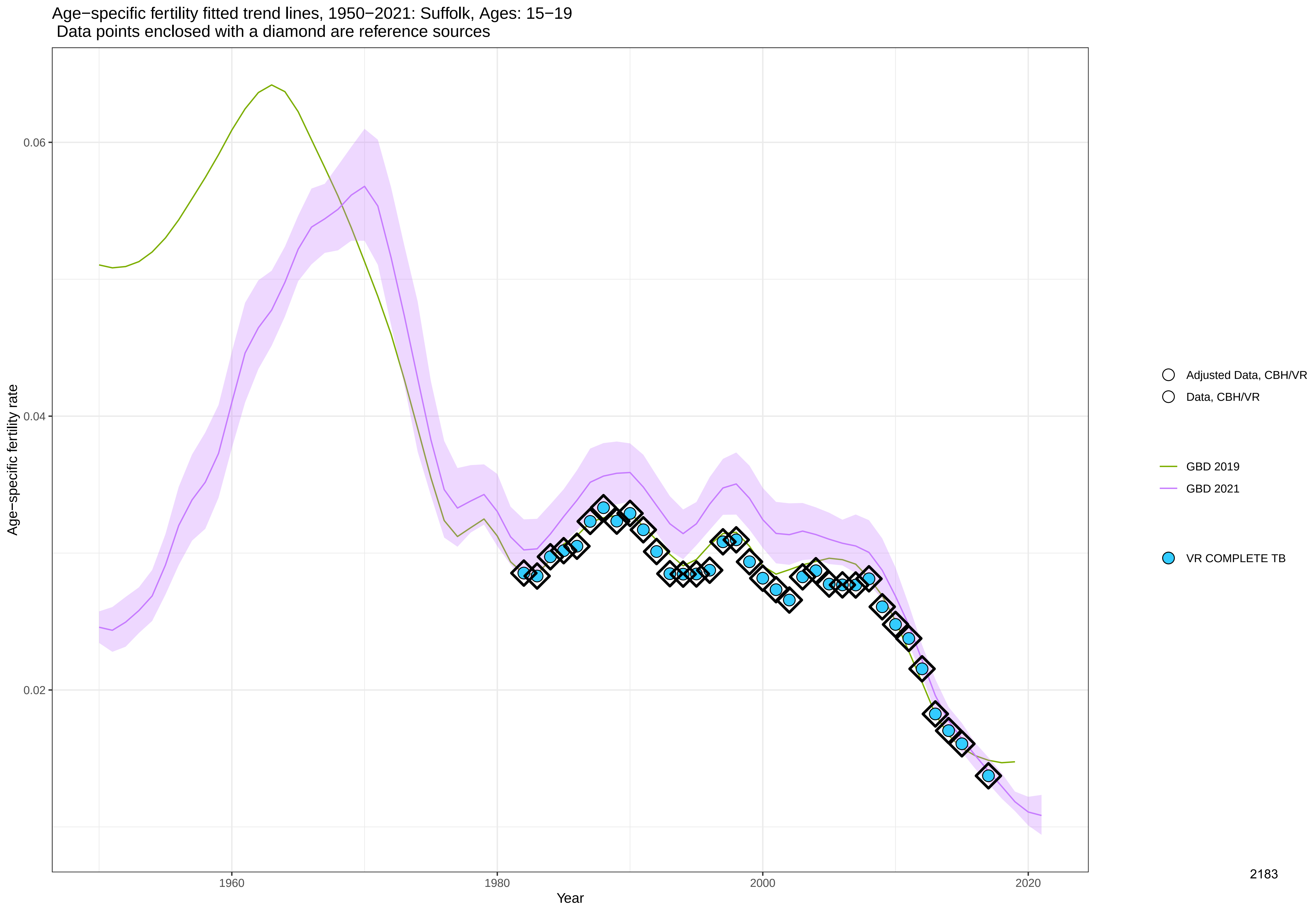
Age−specific fertility fitted trend lines, 1950−2021: Suffolk, Ages: 15−19
Data points enclosed with a diamond are reference sources
Age−specific fertility rate
0.06
0.04
0.02
1960
1980
2000
2020
Year
Adjusted Data, CBH/VR
Data, CBH/VR
GBD 2019
GBD 2021
VR COMPLETE TB

Age−specific fertility fitted trend lines, 1950−2021: Suffolk, Ages: 20−24
Data points enclosed with a diamond are reference sources

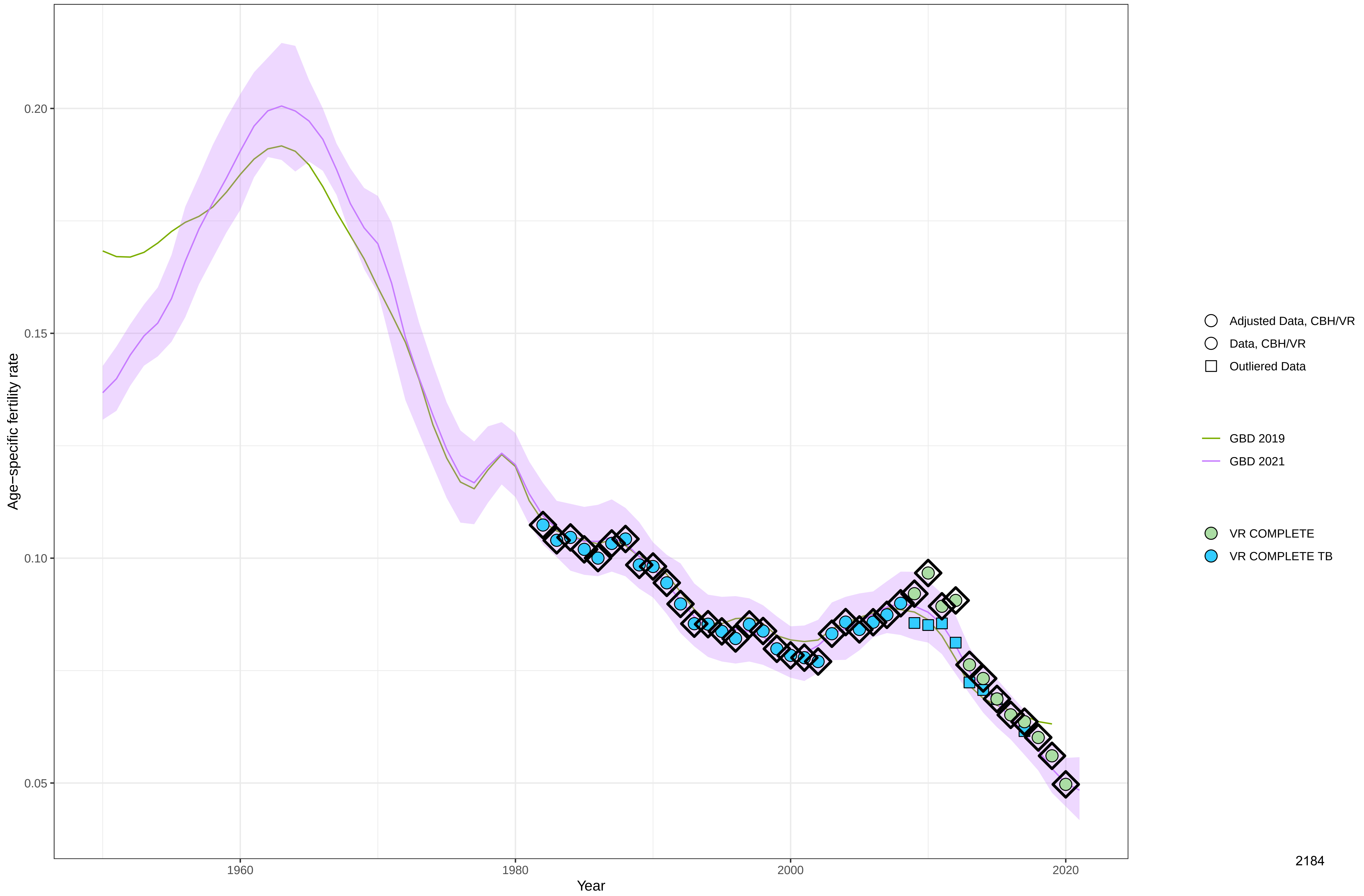

Age−specific fertility fitted trend lines, 1950−2021: Suffolk, Ages: 25−29
Data points enclosed with a diamond are reference sources

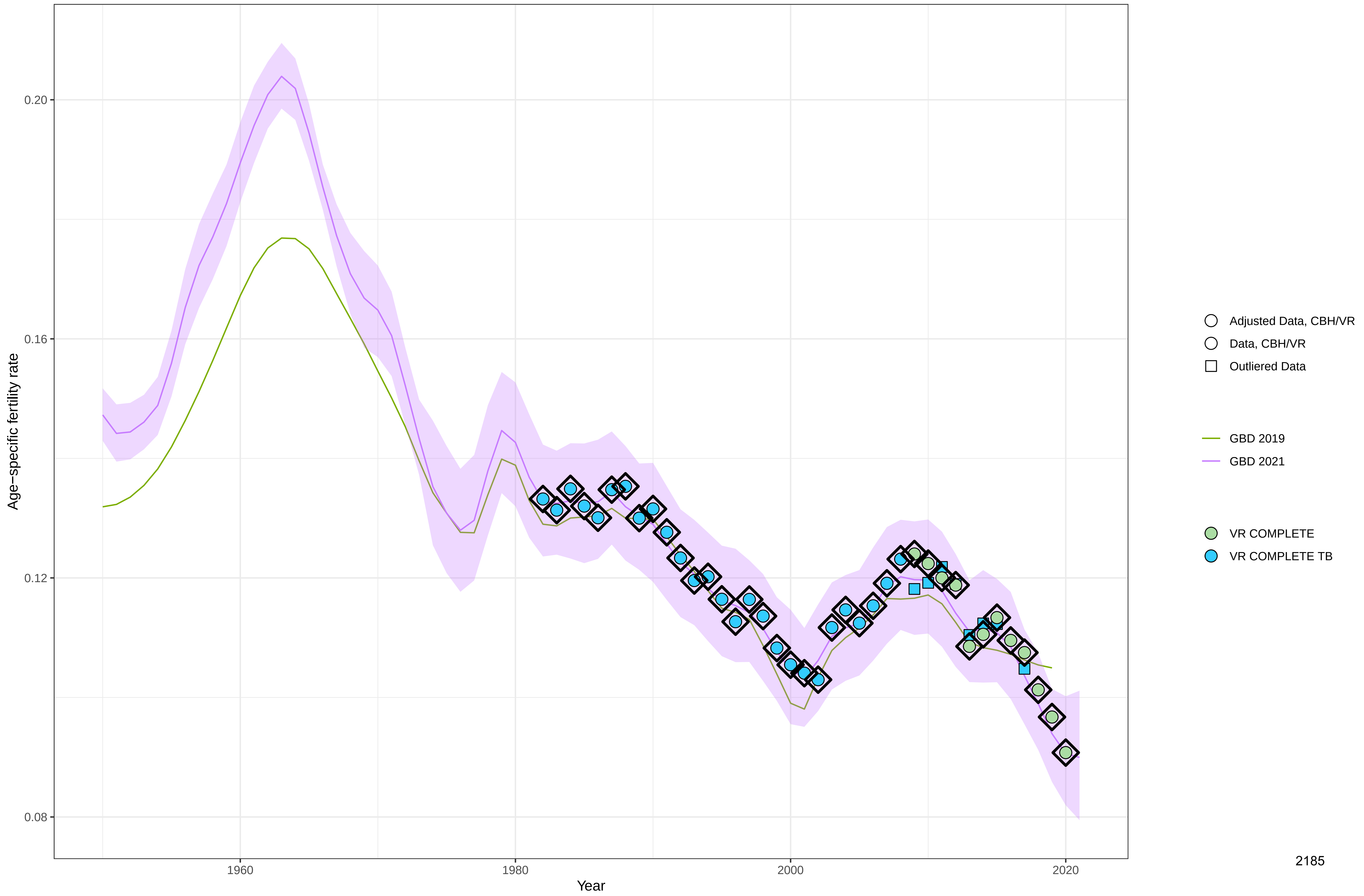

Age−specific fertility fitted trend lines, 1950−2021: Suffolk, Ages: 30−34
Data points enclosed with a diamond are reference sources

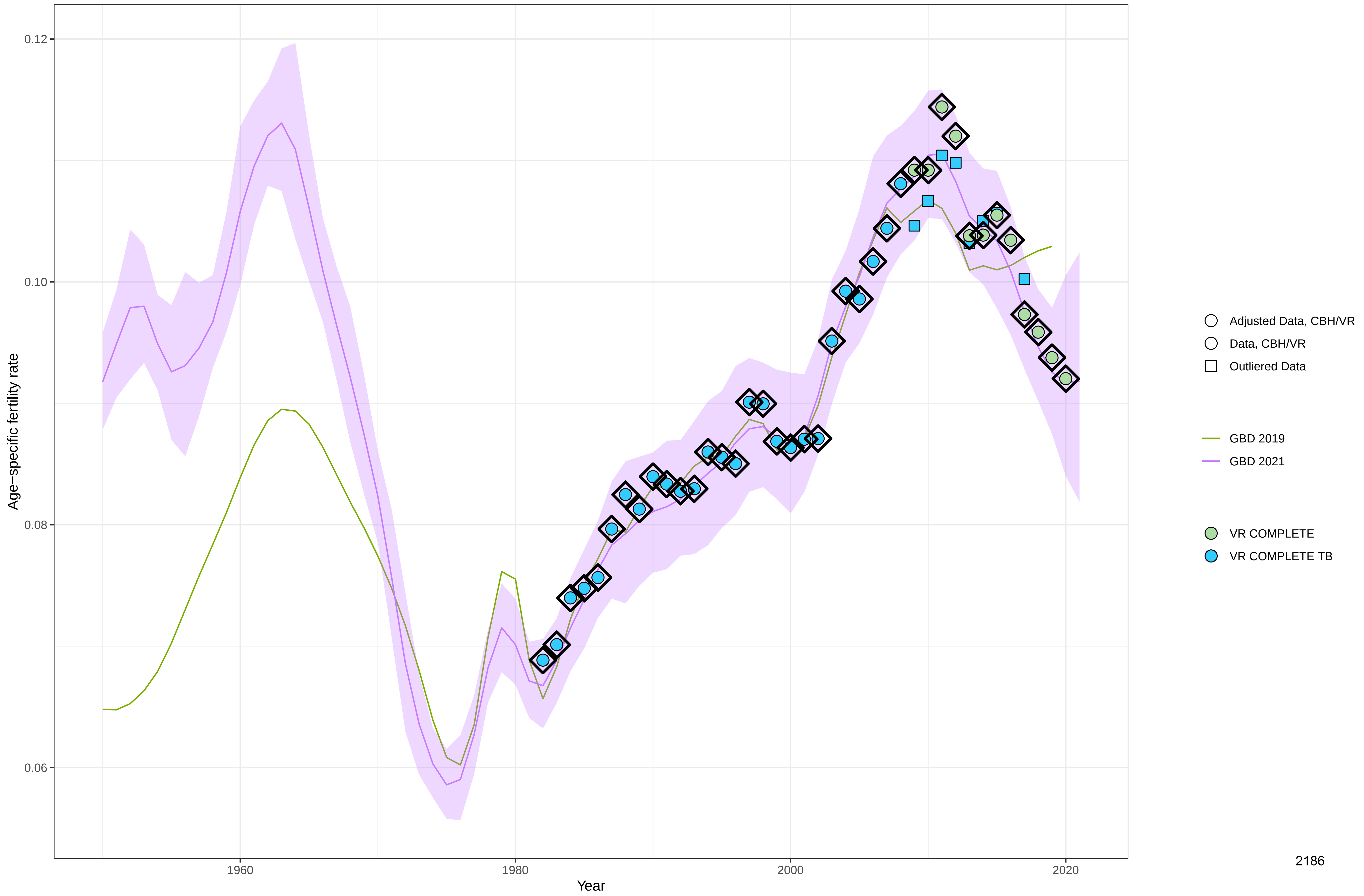

Age−specific fertility fitted trend lines, 1950−2021: Suffolk, Ages: 35−39
Data points enclosed with a diamond are reference sources

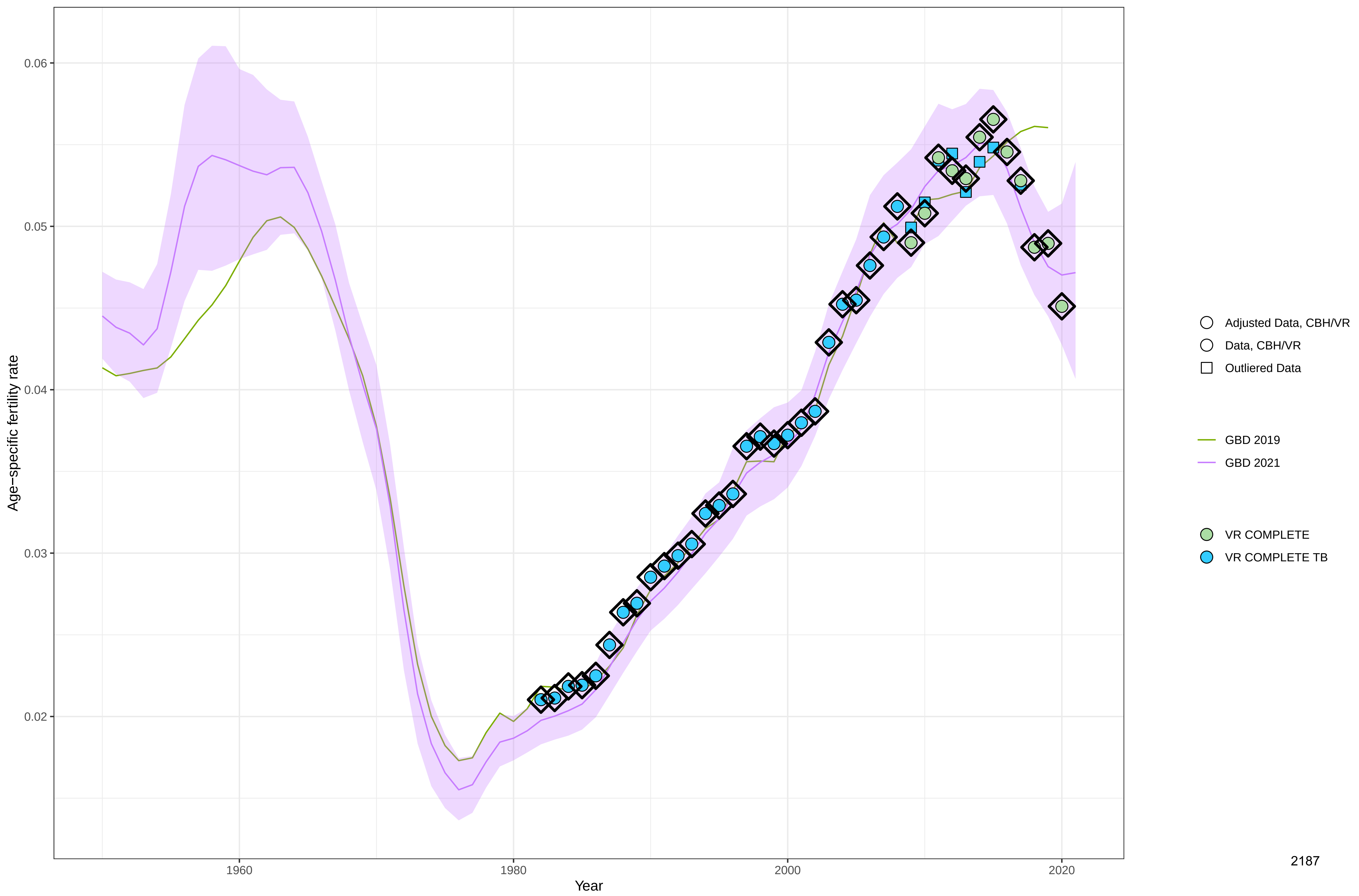

Age−specific fertility fitted trend lines, 1950−2021: Suffolk, Ages: 40−44
Data points enclosed with a diamond are reference sources

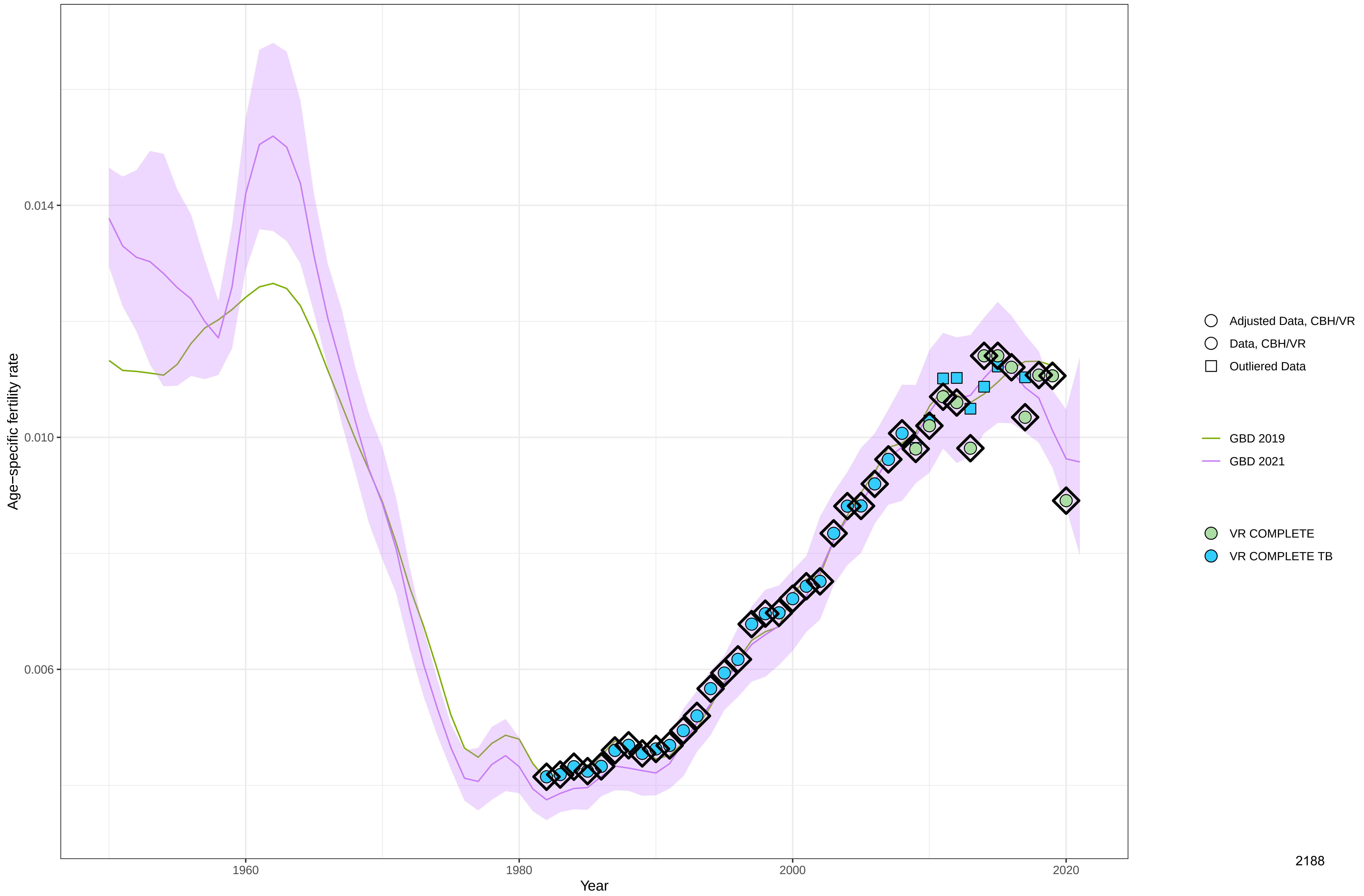

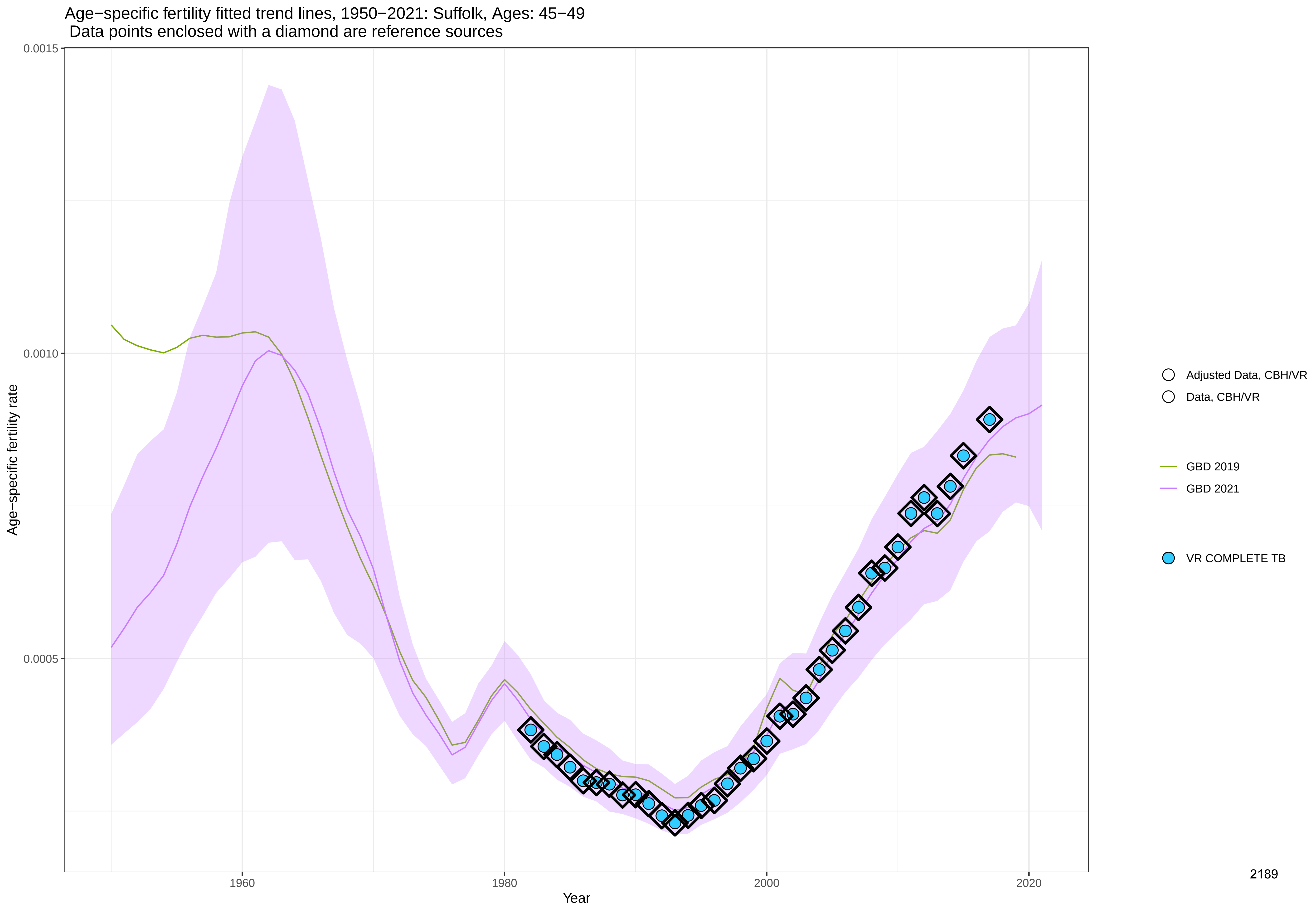

Age−specific fertility fitted trend lines, 1950−2021: Suffolk, Ages: 45−49
Data points enclosed with a diamond are reference sources
0.0015
0.0010
0.0005
Age−specific fertility rate
1960
1980
2000
2020
Year
Adjusted Data, CBH/VR
Data, CBH/VR
GBD 2019
GBD 2021
VR COMPLETE TB

Age−specific fertility fitted trend lines, 1950−2021: Suffolk, Ages: 50−54
Data points enclosed with a diamond are reference sources

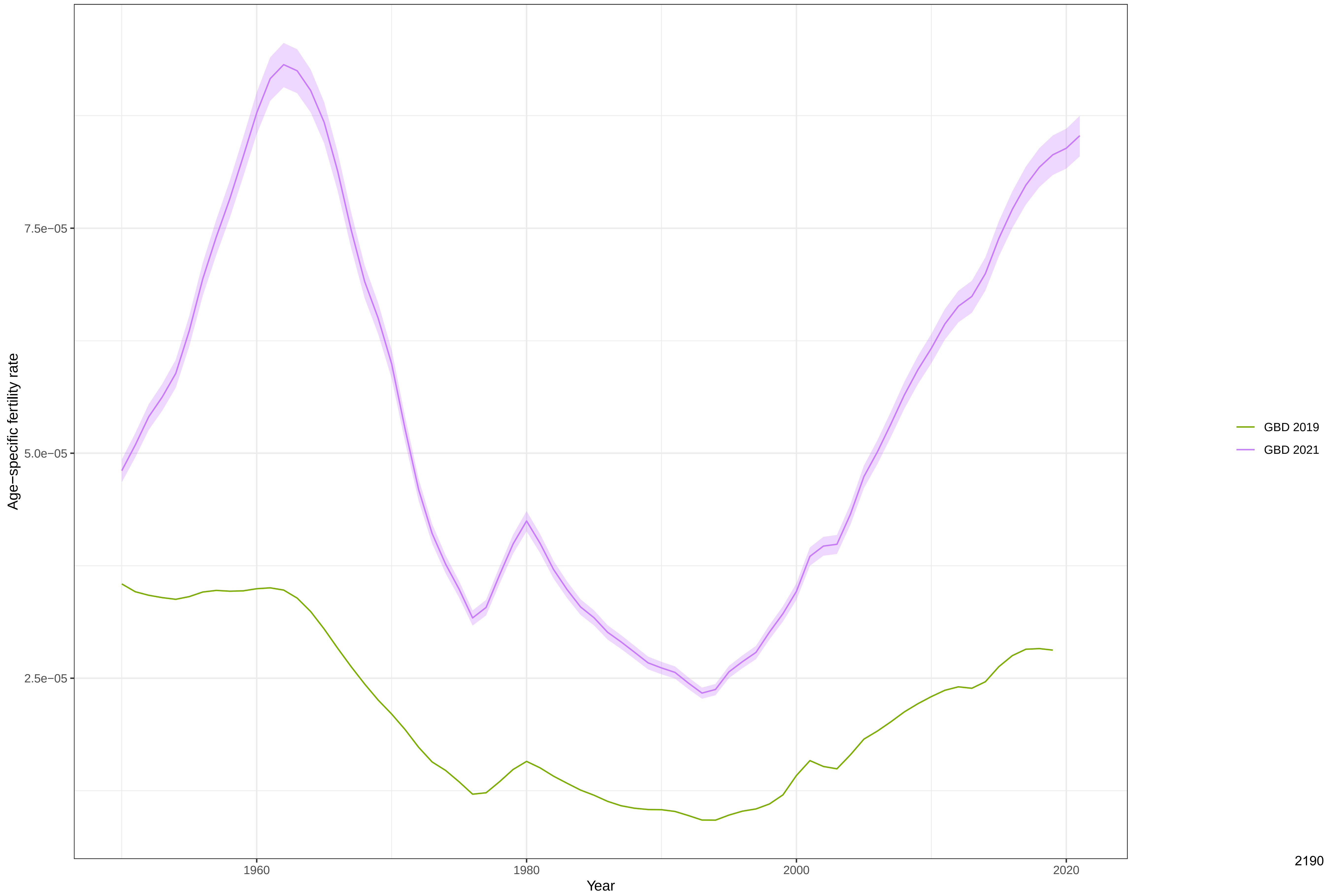

Total fertility rate fitted trend lines, 1950−2021: Suffolk TFR
Data points enclosed with a diamond are reference sources

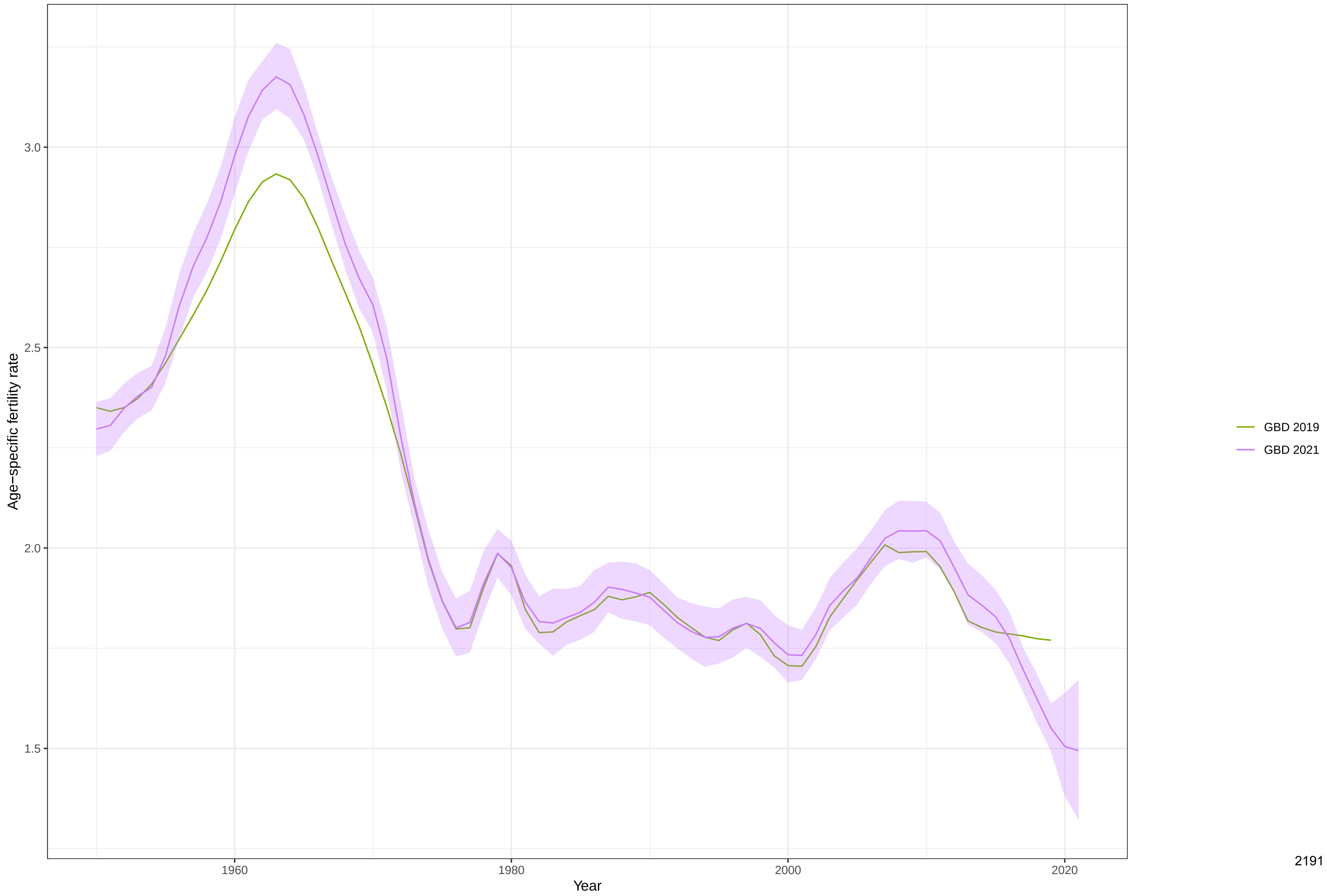

Age−specific fertility fitted trend lines, 1950−2021: Thurrock, Ages: 10−14
Data points enclosed with a diamond are reference sources

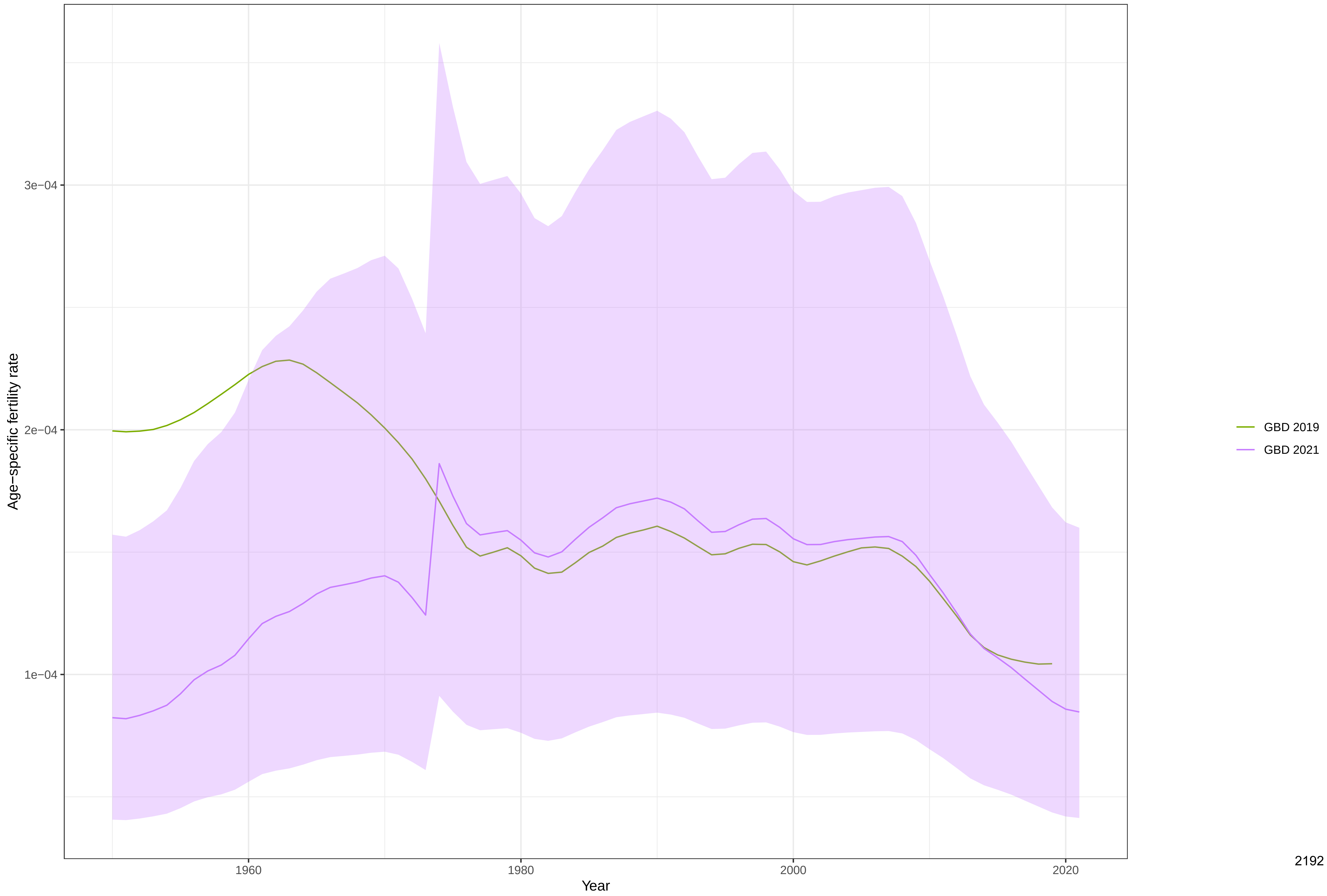

Age−specific fertility fitted trend lines, 1950−2021: Thurrock, Ages: 15−19
Data points enclosed with a diamond are reference sources

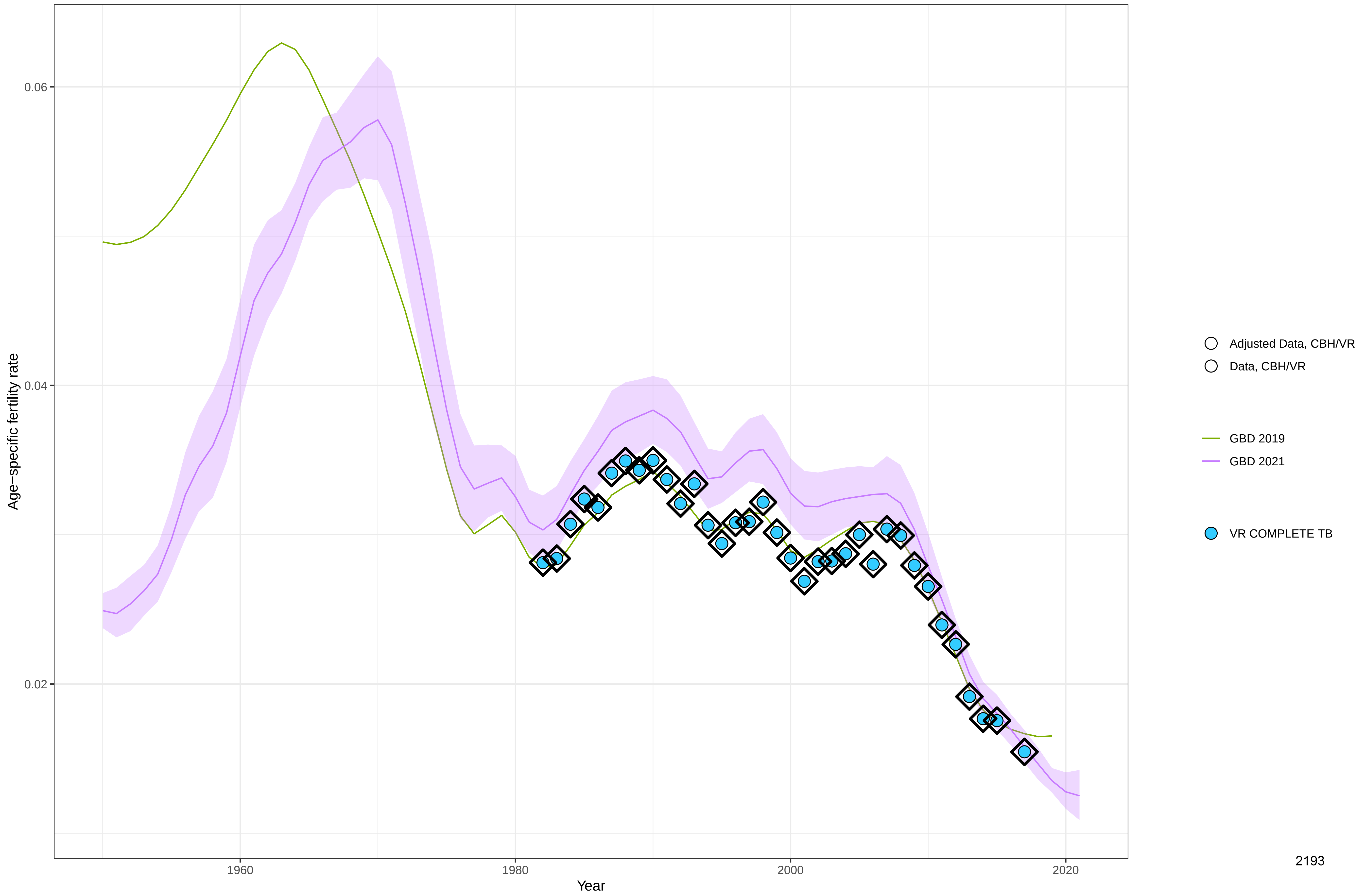

Age−specific fertility fitted trend lines, 1950−2021: Thurrock, Ages: 20−24
Data points enclosed with a diamond are reference sources

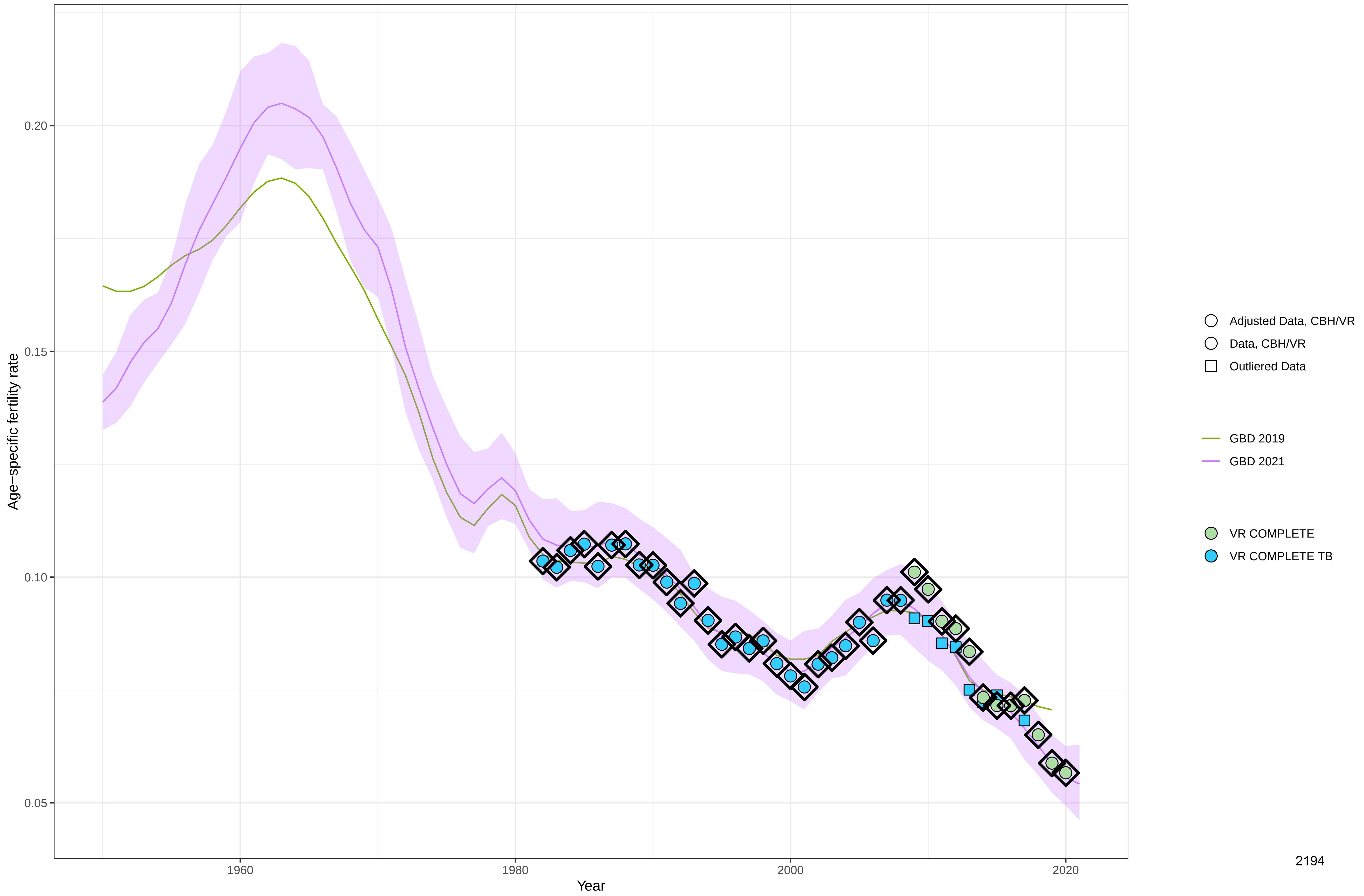

Age−specific fertility fitted trend lines, 1950−2021: Thurrock, Ages: 25−29
Data points enclosed with a diamond are reference sources

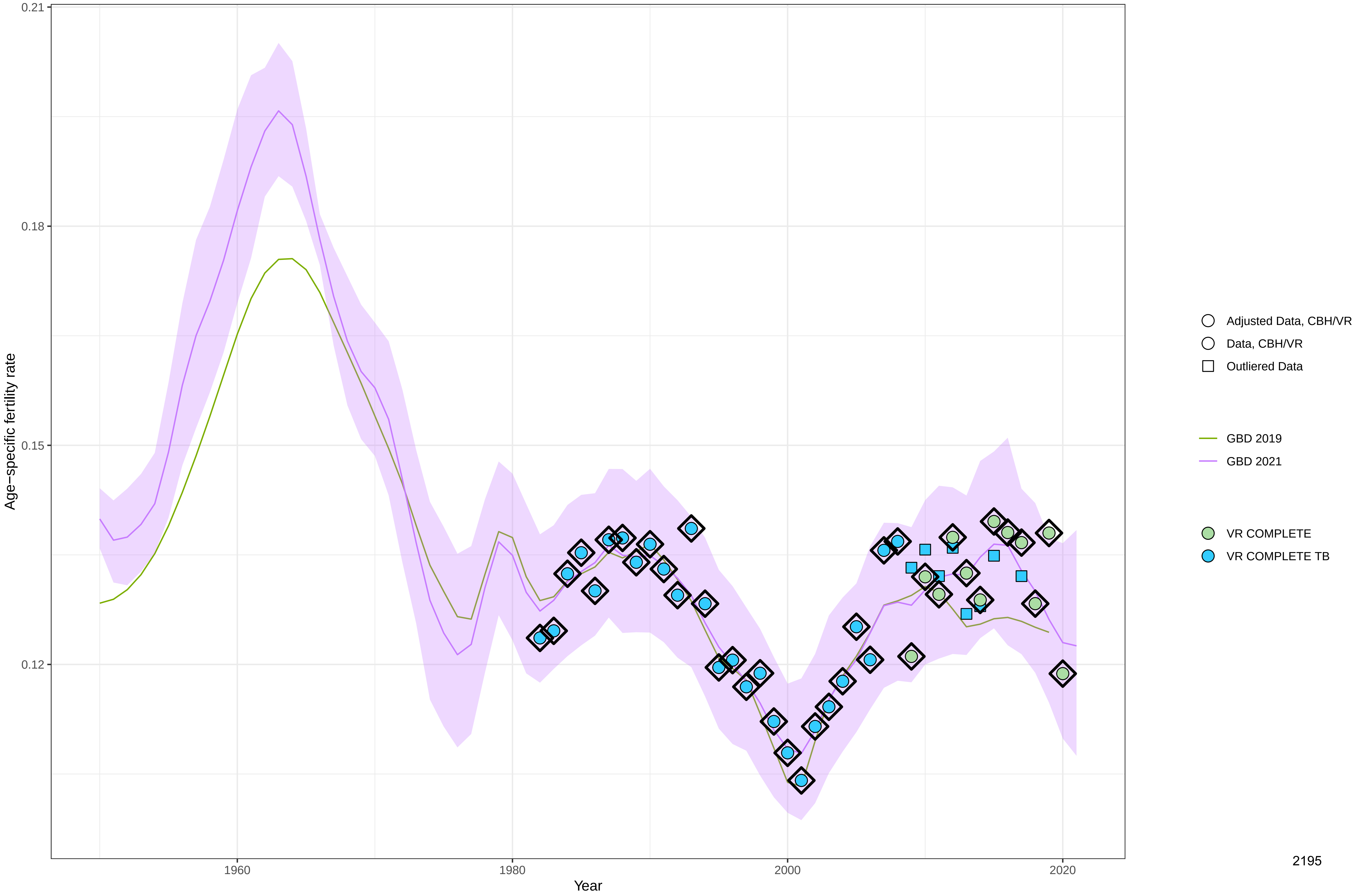

Age−specific fertility fitted trend lines, 1950−2021: Thurrock, Ages: 30−34
Data points enclosed with a diamond are reference sources

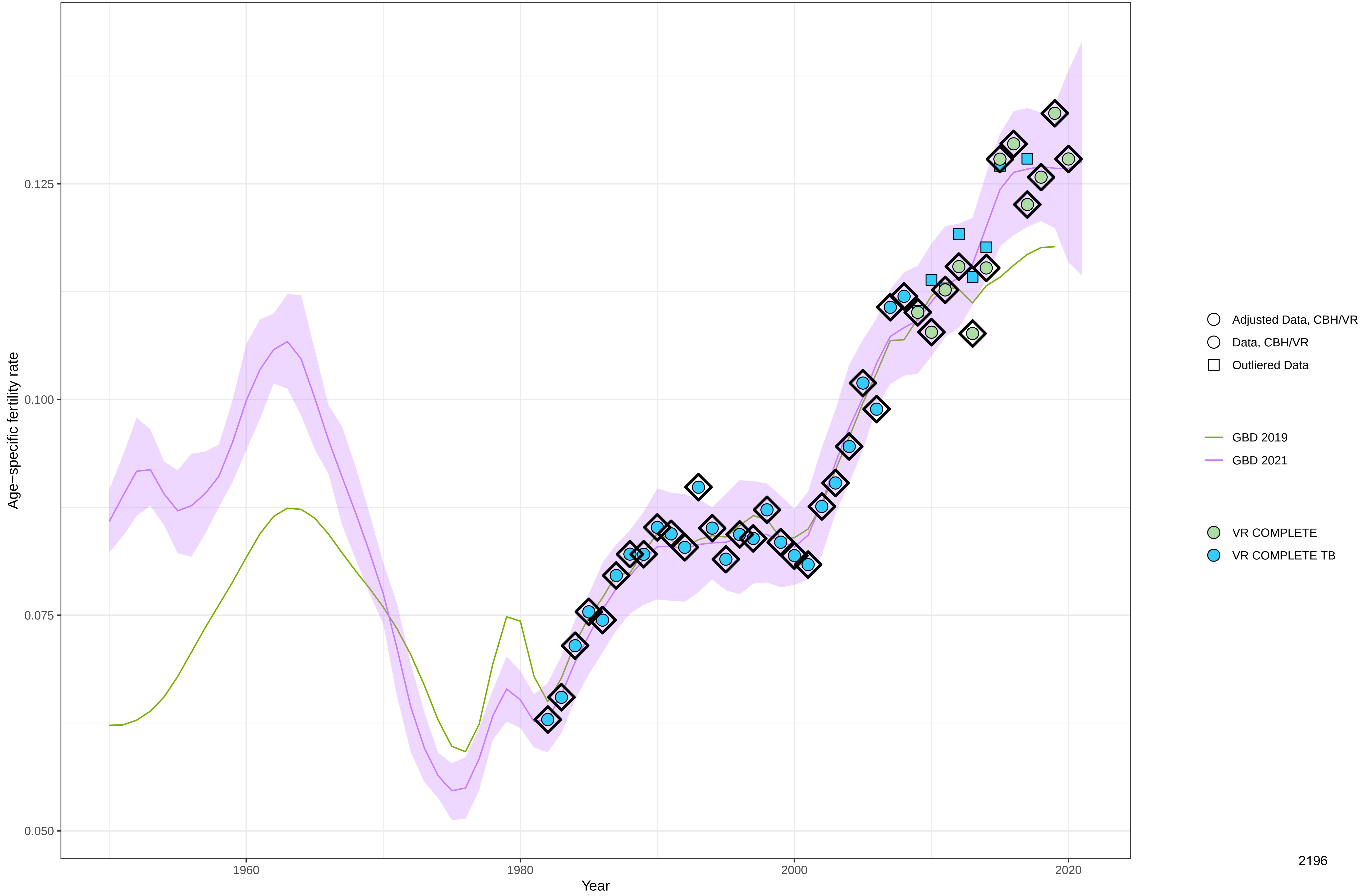

Age−specific fertility fitted trend lines, 1950−2021: Thurrock, Ages: 35−39
Data points enclosed with a diamond are reference sources

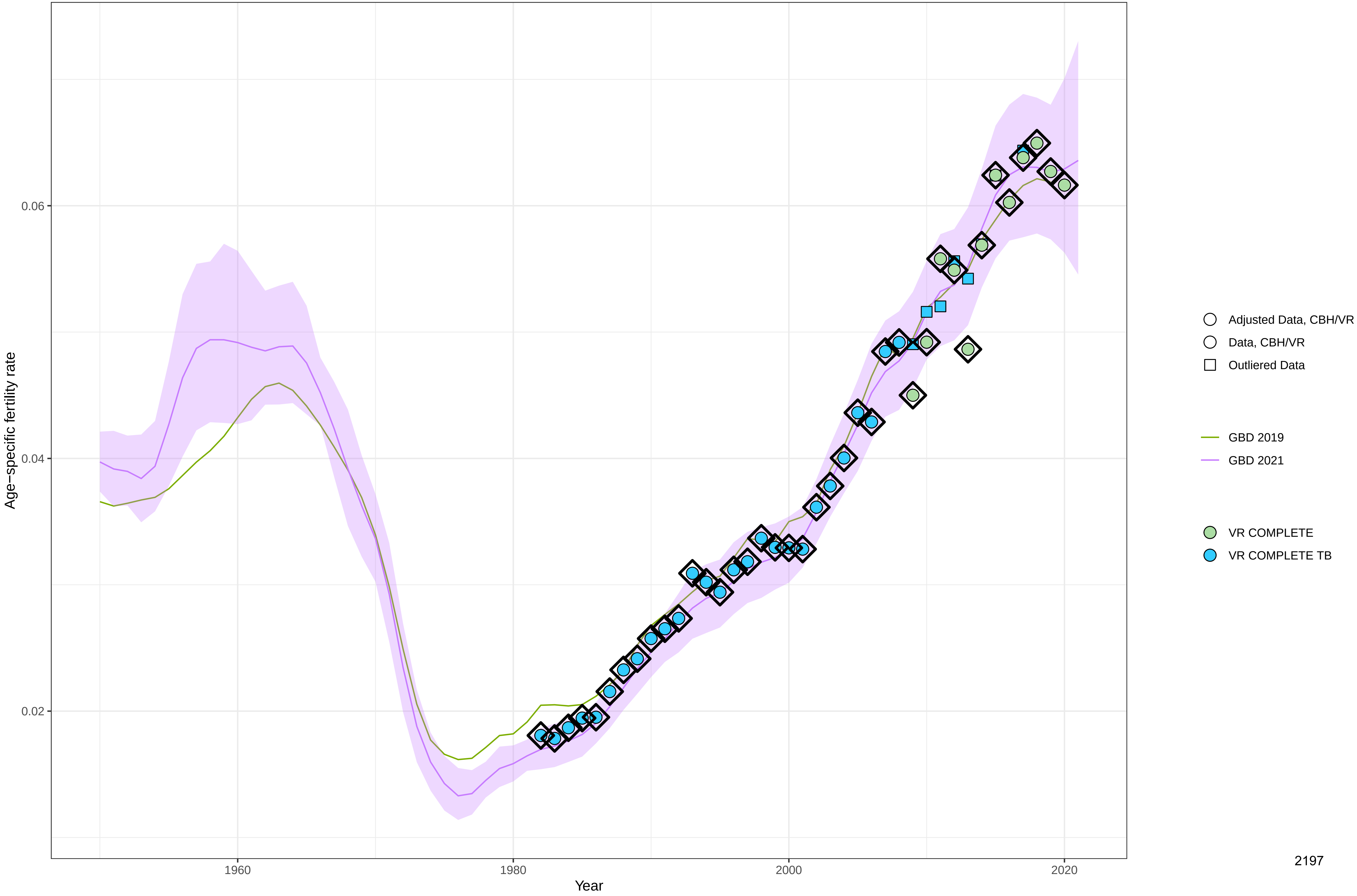

Age−specific fertility fitted trend lines, 1950−2021: Thurrock, Ages: 40−44
Data points enclosed with a diamond are reference sources

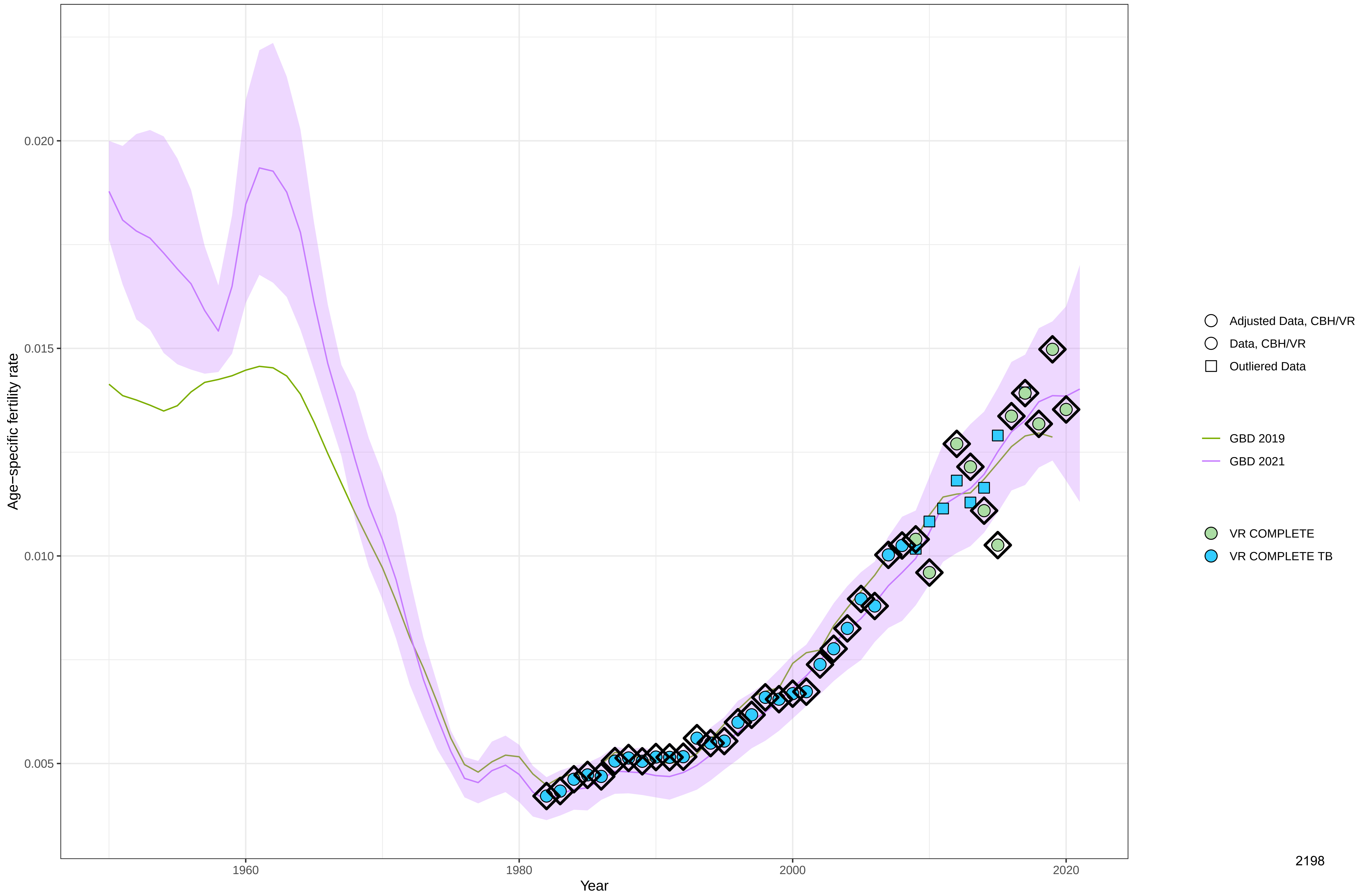

Age−specific fertility fitted trend lines, 1950−2021: Thurrock, Ages: 45−49
Data points enclosed with a diamond are reference sources

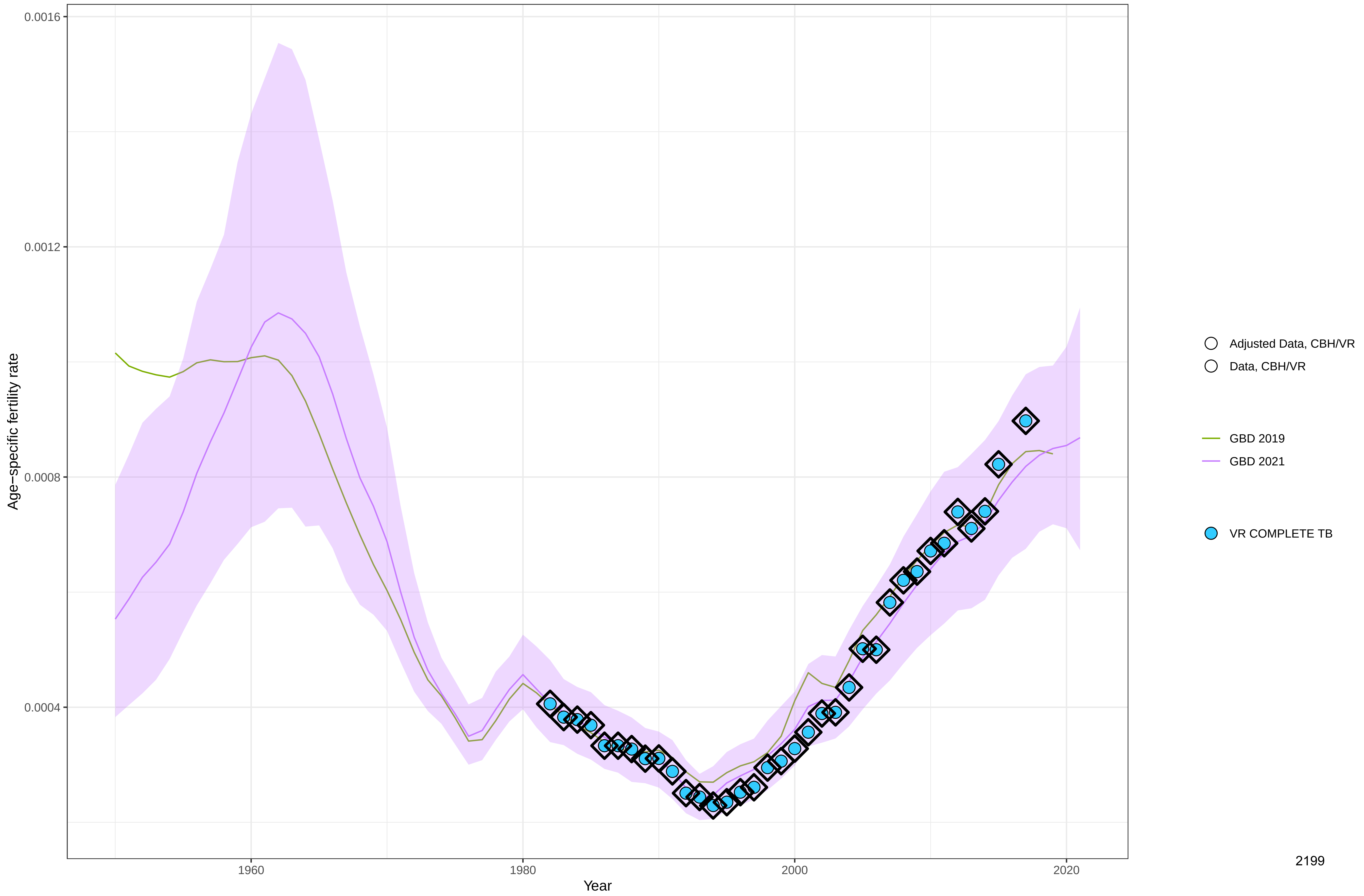

Age−specific fertility fitted trend lines, 1950−2021: Thurrock, Ages: 50−54
Data points enclosed with a diamond are reference sources

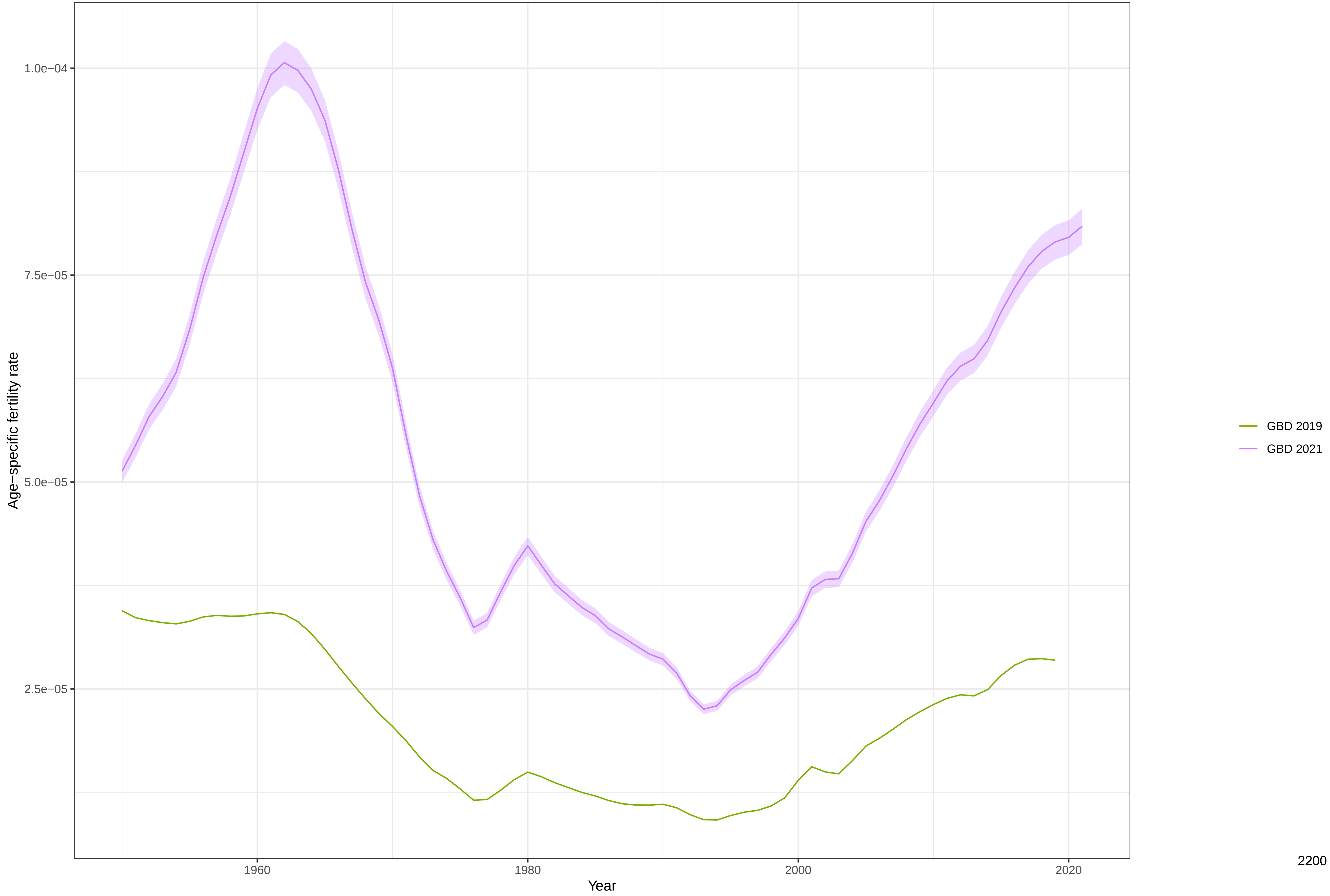

Total fertility rate fitted trend lines, 1950−2021: Thurrock TFR
Data points enclosed with a diamond are reference sources

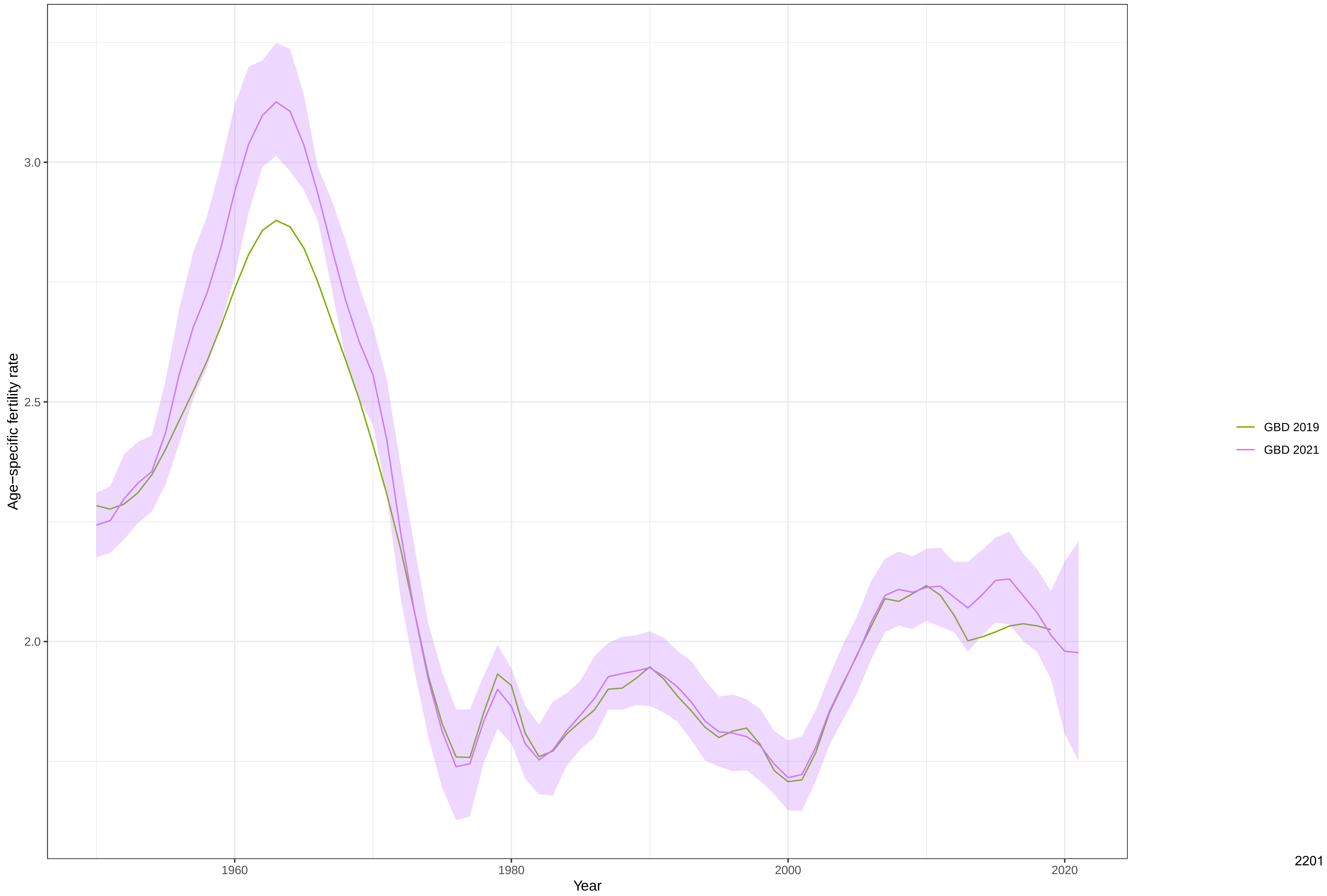

Age−specific fertility fitted trend lines, 1950−2021: Greater London, Ages: 10−14
Data points enclosed with a diamond are reference sources

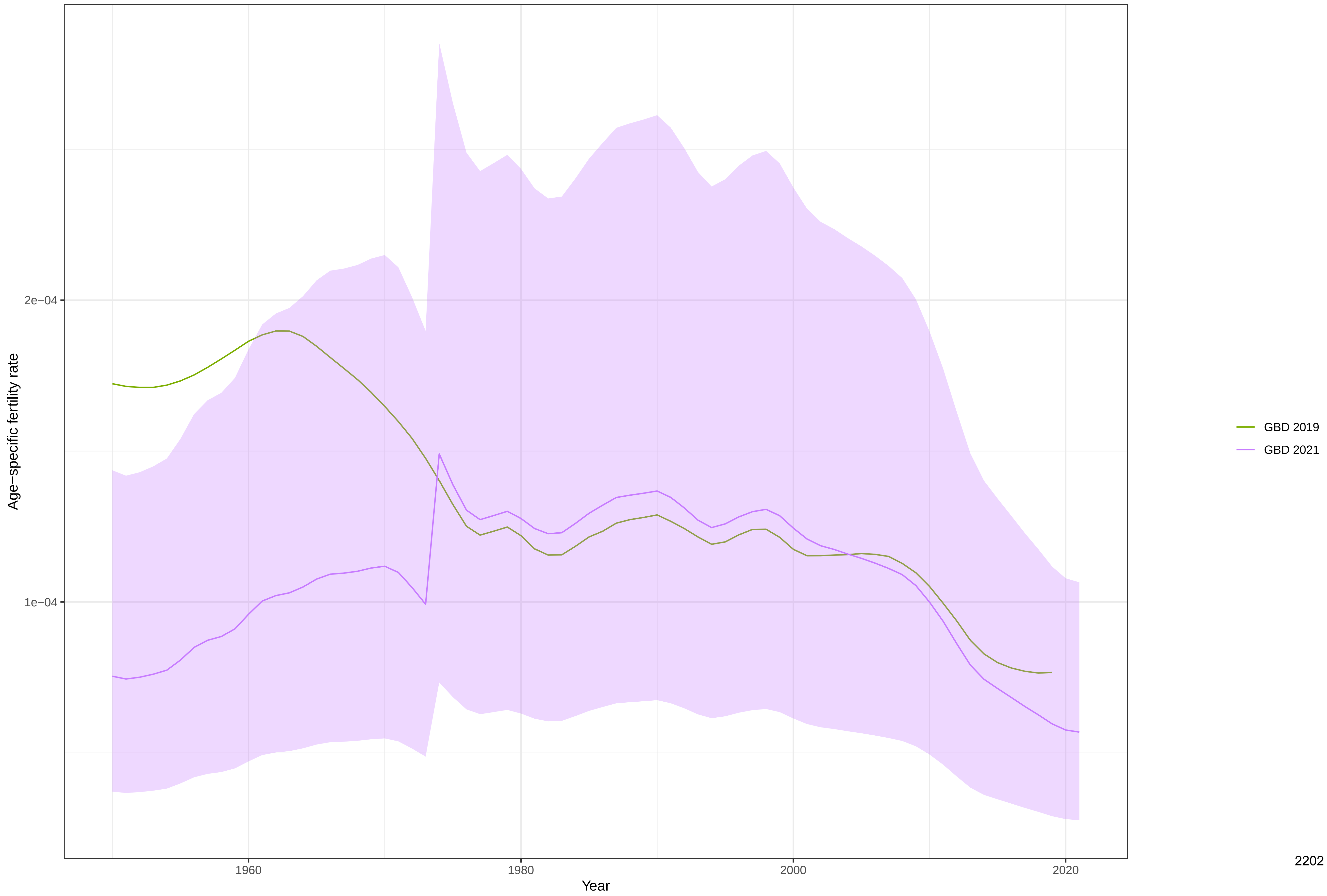

Age−specific fertility fitted trend lines, 1950−2021: Greater London, Ages: 15−19
Data points enclosed with a diamond are reference sources

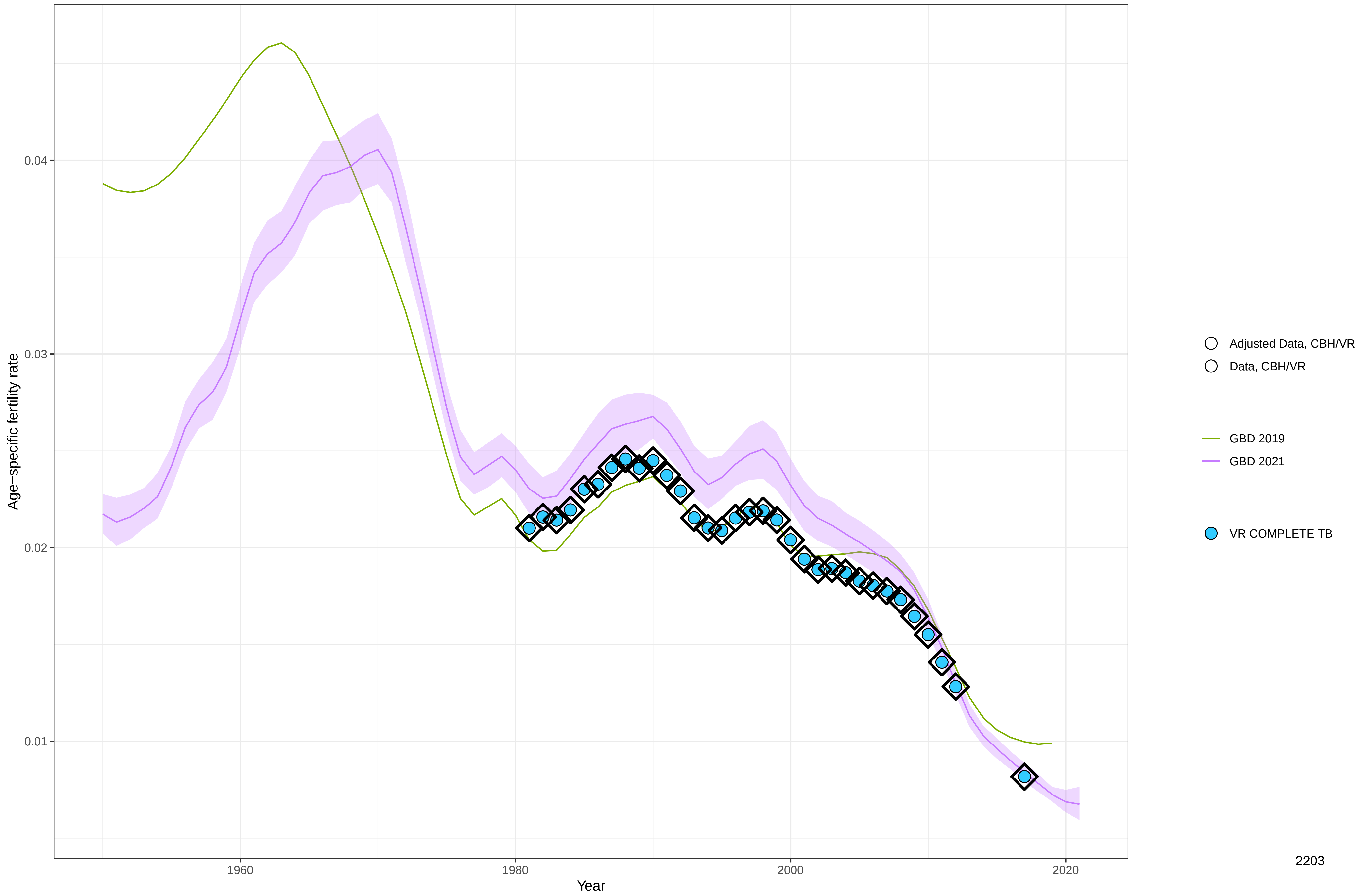

Age−specific fertility fitted trend lines, 1950−2021: Greater London, Ages: 20−24
Data points enclosed with a diamond are reference sources

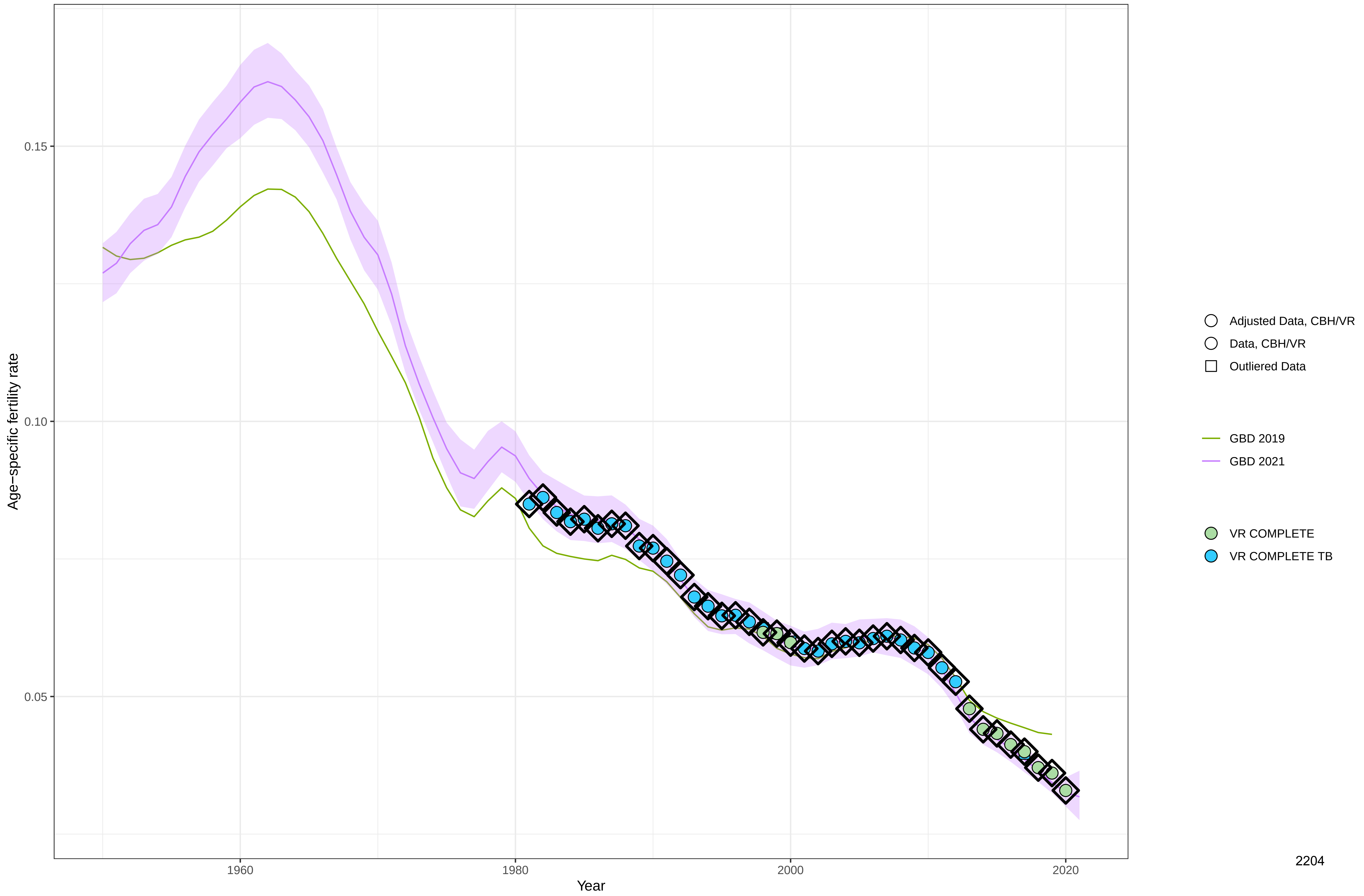

Age−specific fertility fitted trend lines, 1950−2021: Greater London, Ages: 25−29
Data points enclosed with a diamond are reference sources

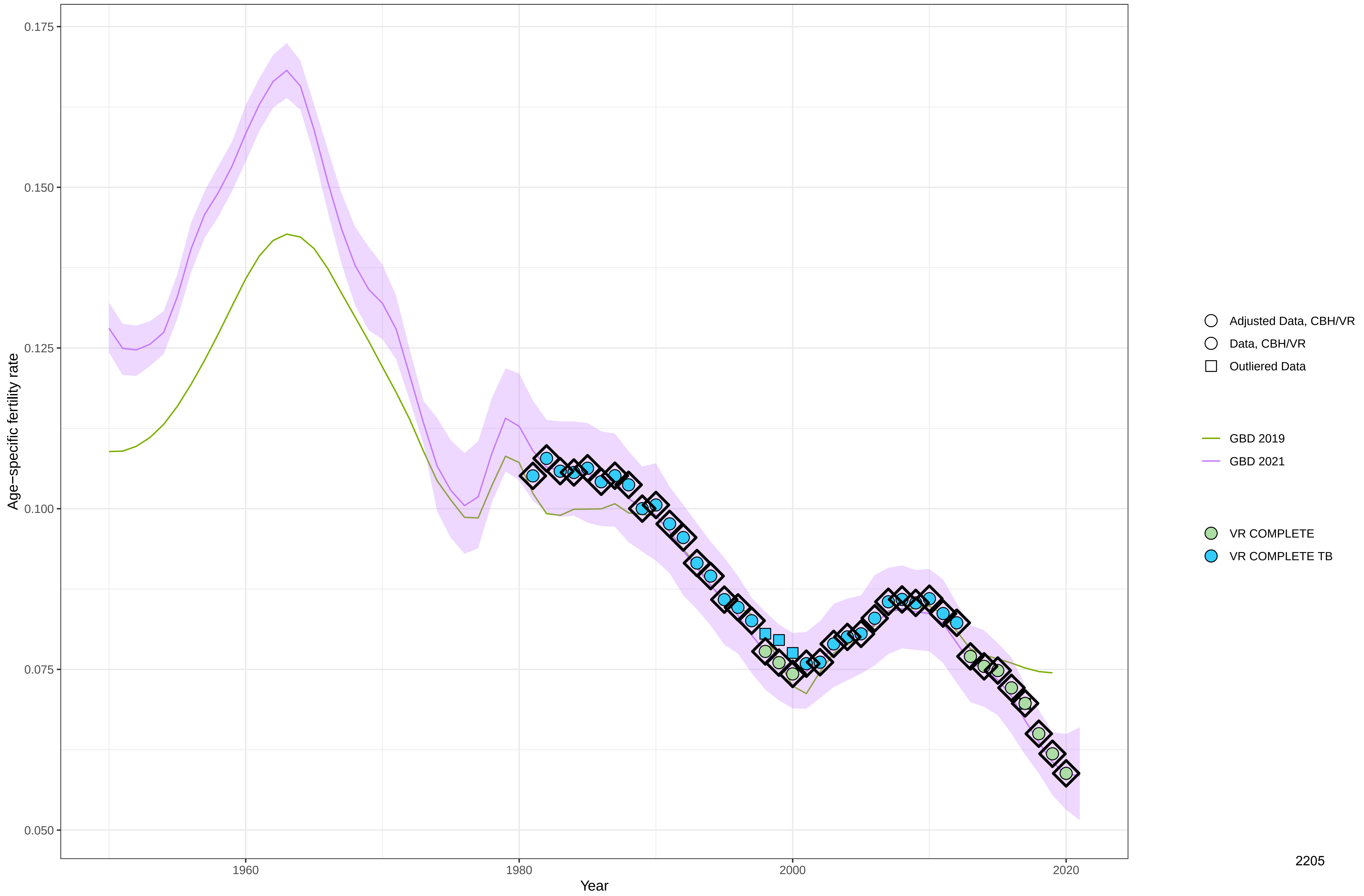

Age−specific fertility fitted trend lines, 1950−2021: Greater London, Ages: 30−34
Data points enclosed with a diamond are reference sources

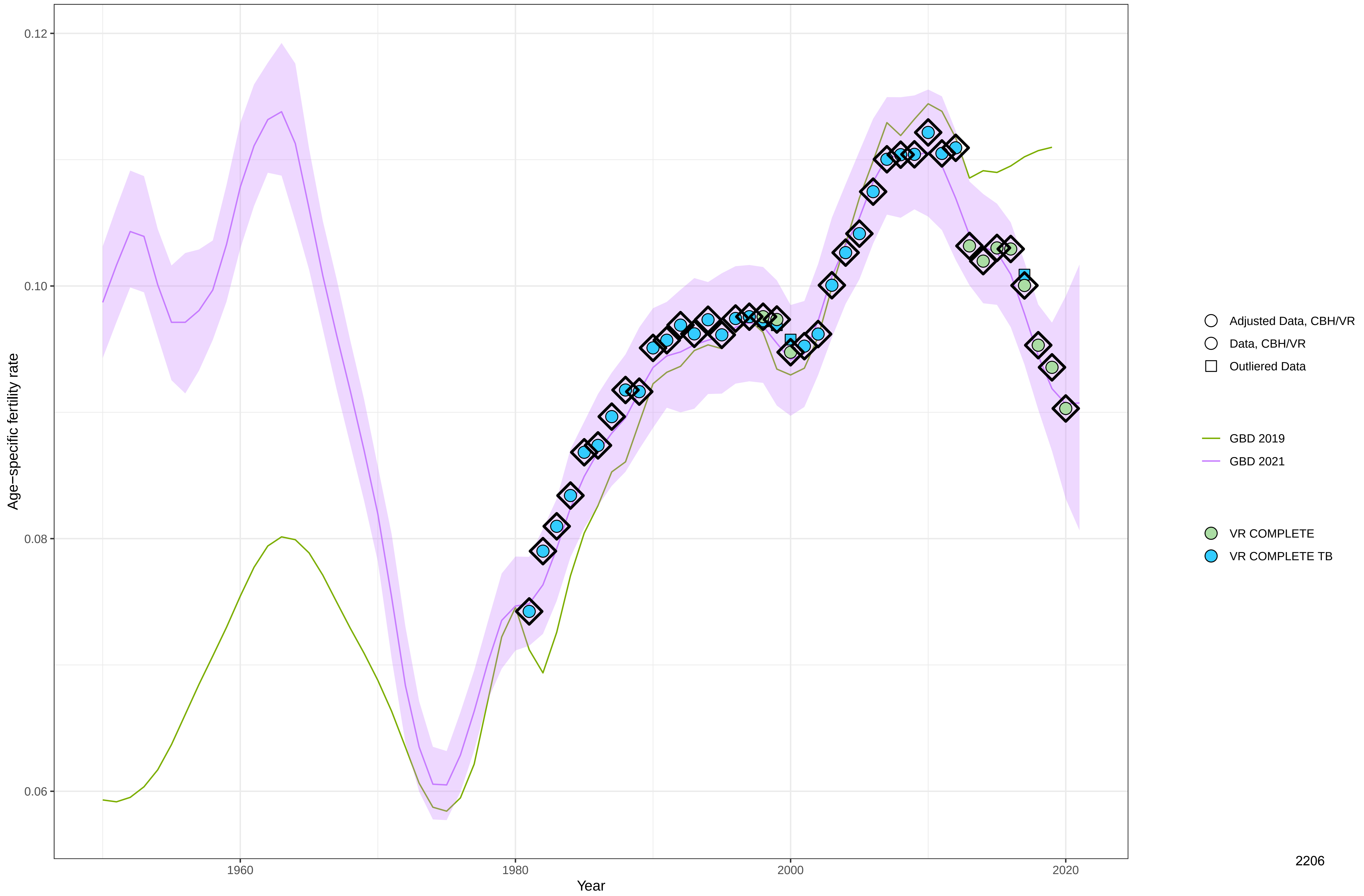

Age−specific fertility fitted trend lines, 1950−2021: Greater London, Ages: 35−39
Data points enclosed with a diamond are reference sources

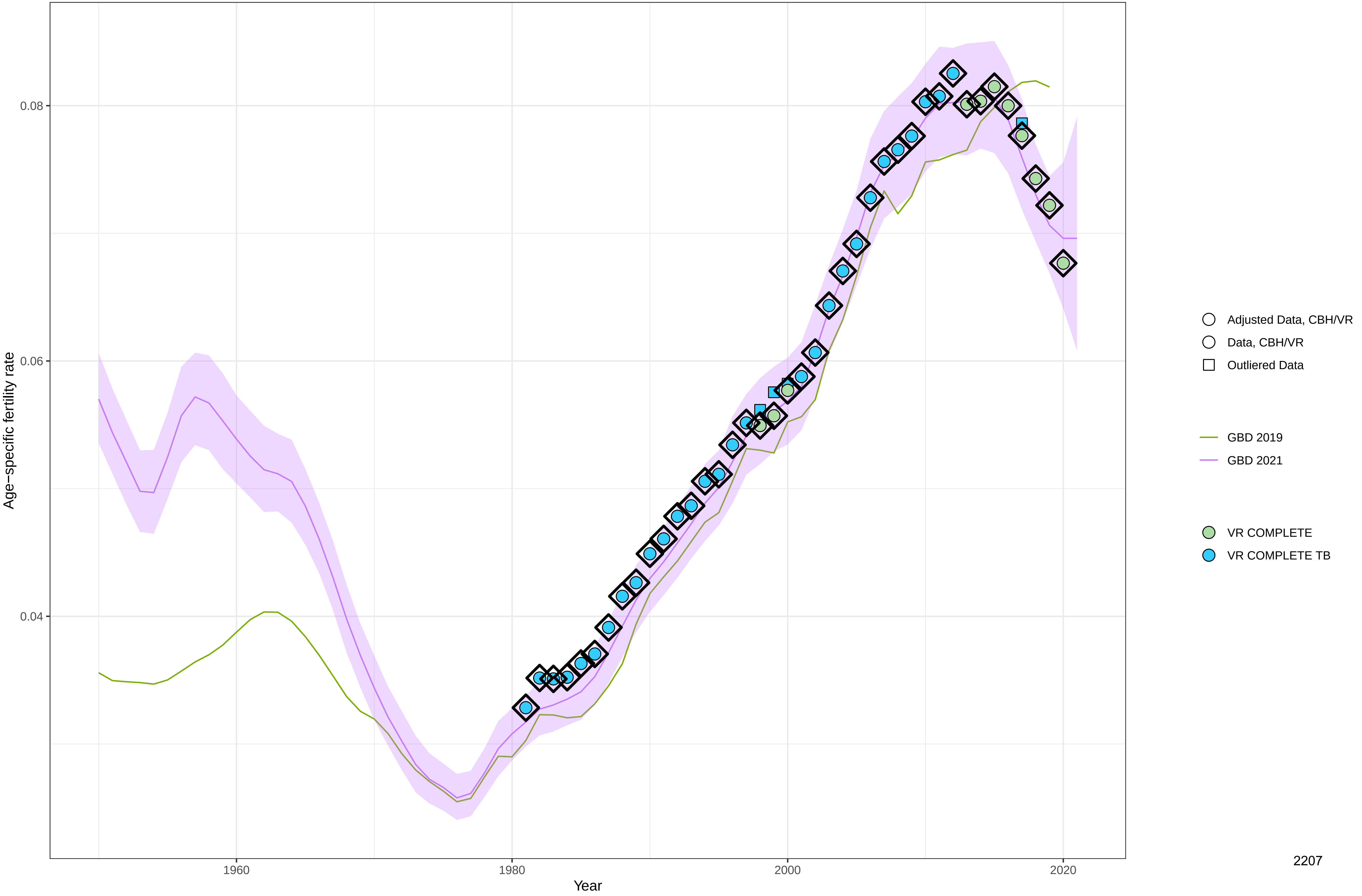

Age−specific fertility fitted trend lines, 1950−2021: Greater London, Ages: 40−44
Data points enclosed with a diamond are reference sources

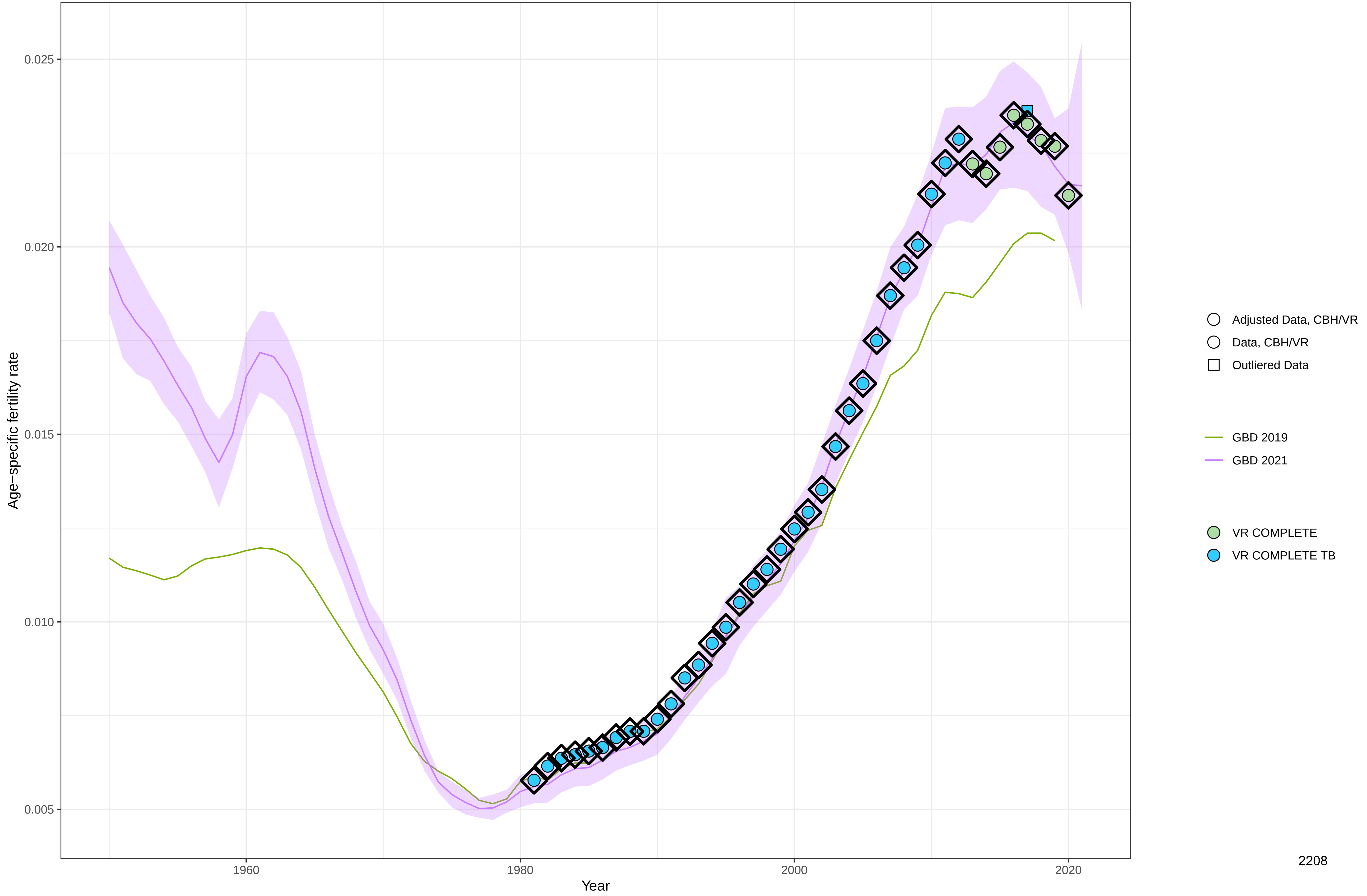

Age−specific fertility fitted trend lines, 1950−2021: Greater London, Ages: 45−49
Data points enclosed with a diamond are reference sources

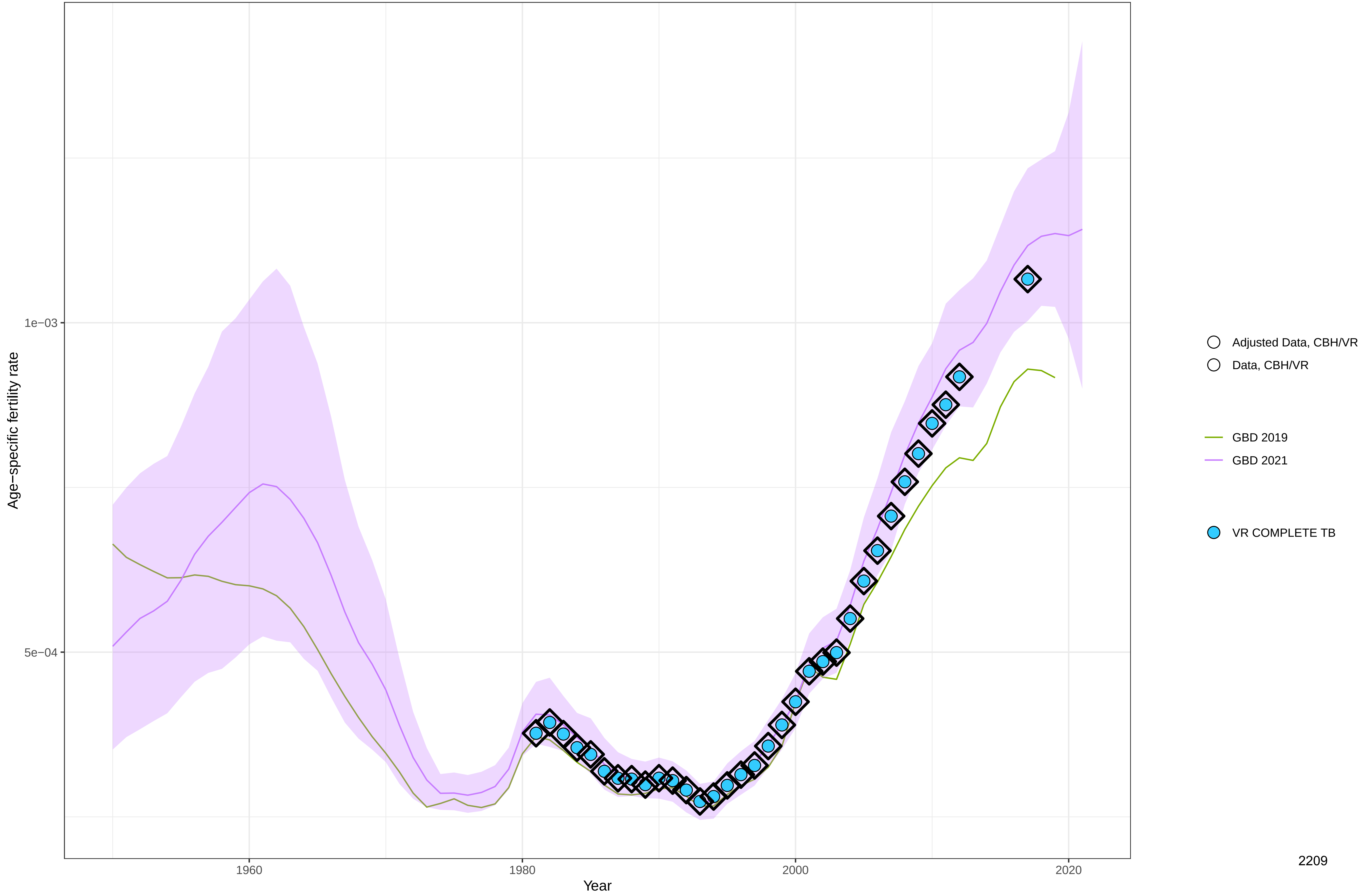

Age−specific fertility fitted trend lines, 1950−2021: Greater London, Ages: 50−54
Data points enclosed with a diamond are reference sources

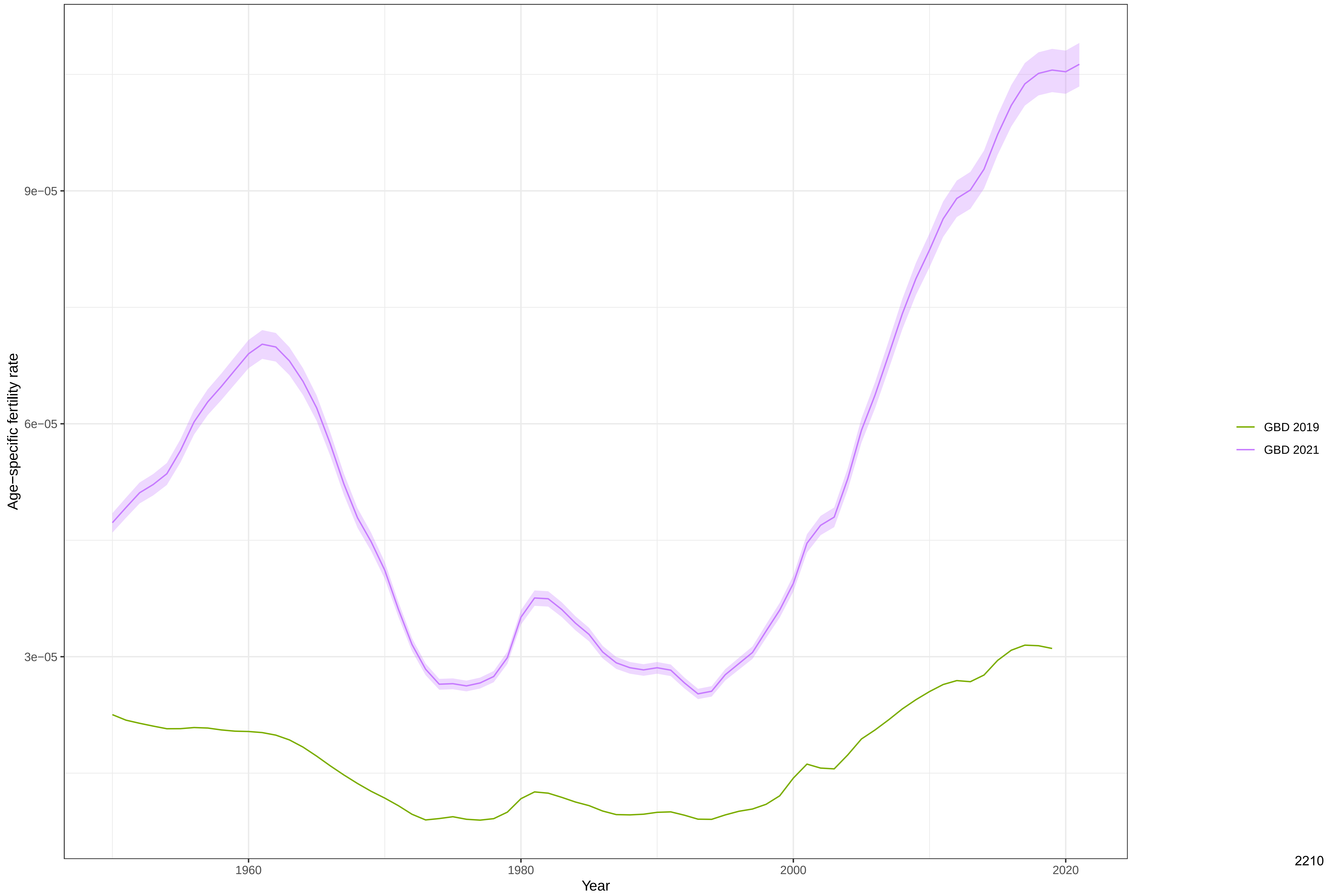

Total fertility rate fitted trend lines, 1950−2021: Greater London TFR
Data points enclosed with a diamond are reference sources

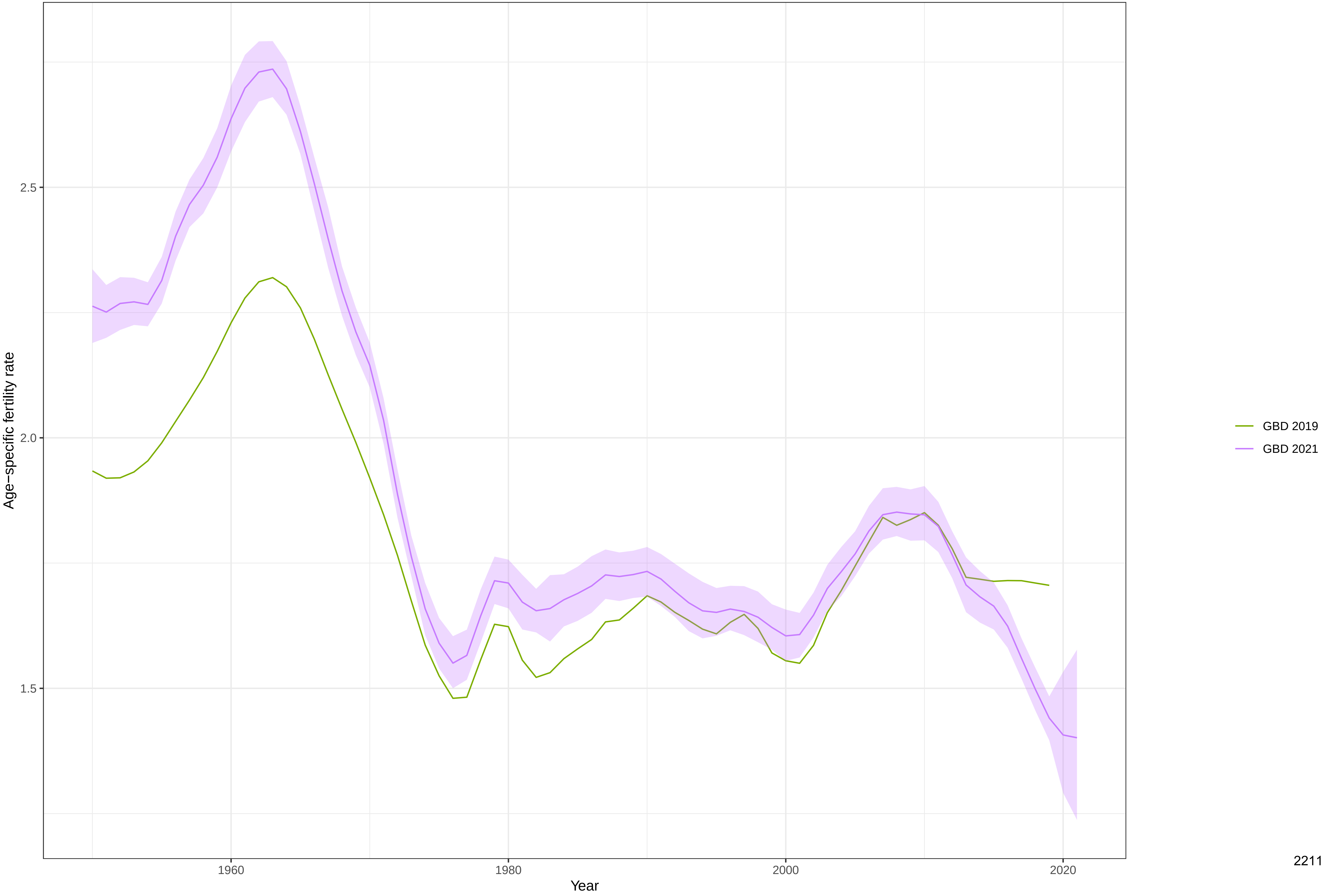

Age−specific fertility fitted trend lines, 1950−2021: Barking and Dagenham, Ages: 10−14
Data points enclosed with a diamond are reference sources

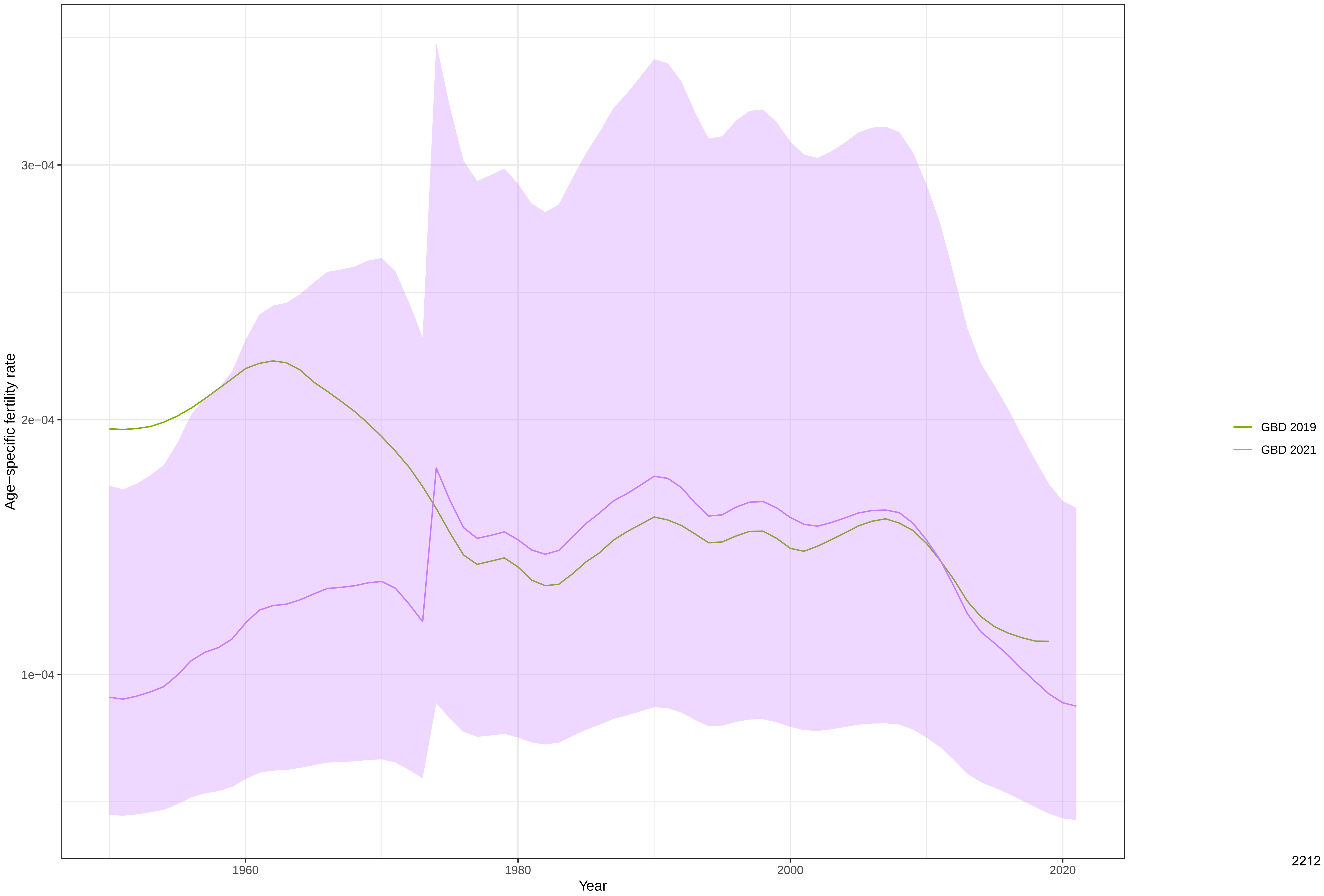

Age−specific fertility fitted trend lines, 1950−2021: Barking and Dagenham, Ages: 15−19
Data points enclosed with a diamond are reference sources

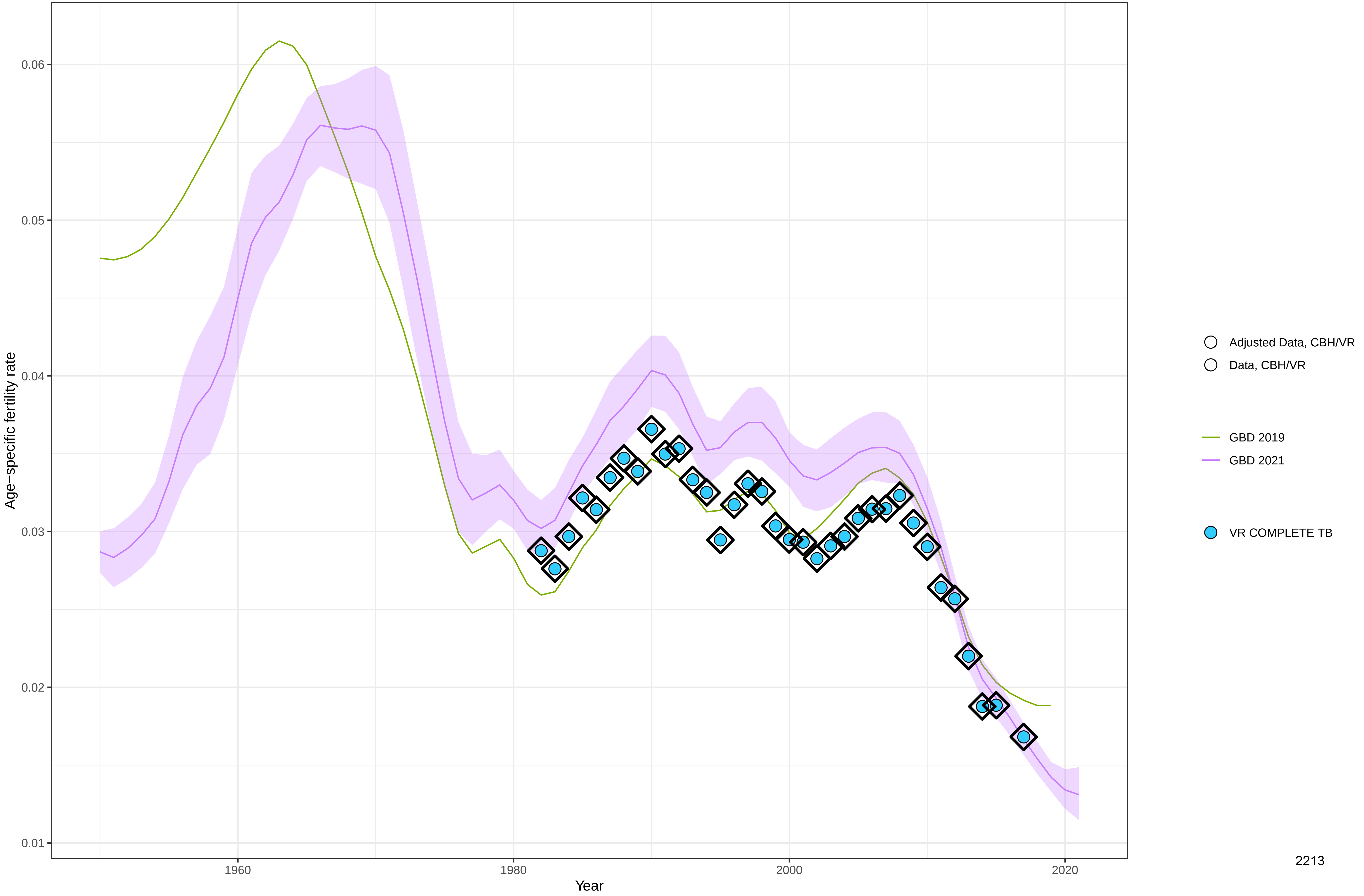

Age−specific fertility fitted trend lines, 1950−2021: Barking and Dagenham, Ages: 20−24
Data points enclosed with a diamond are reference sources

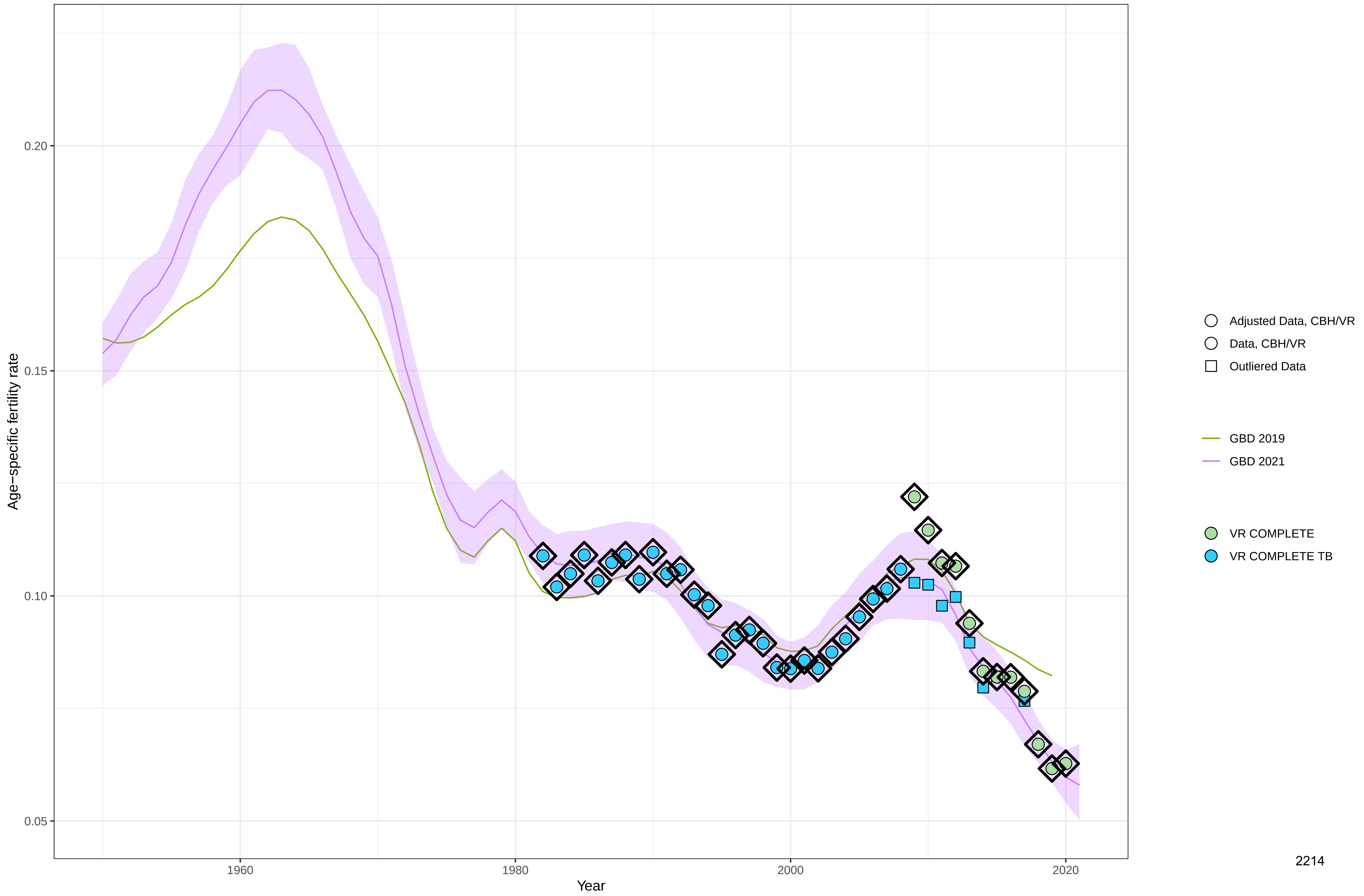

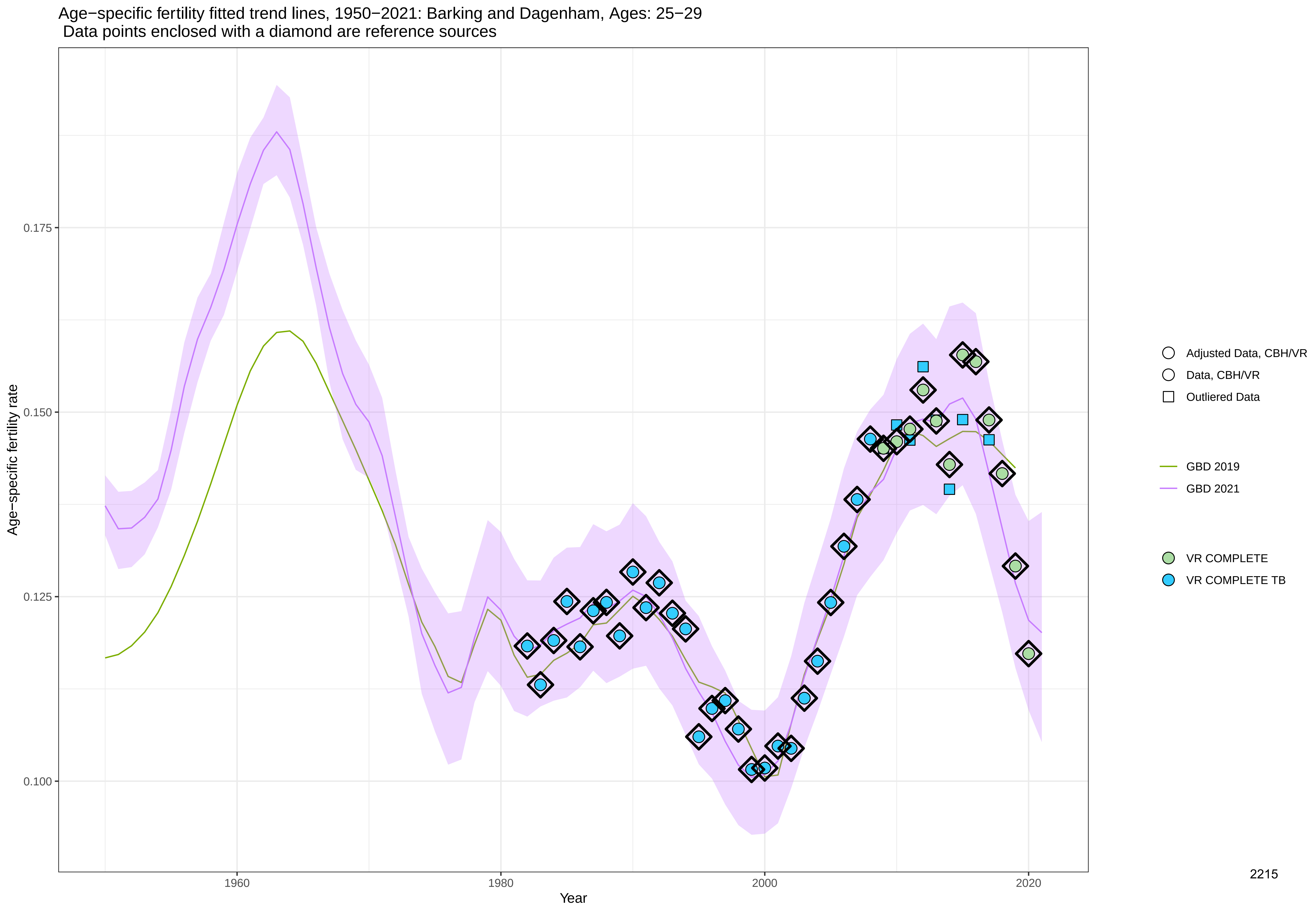

Age−specific fertility fitted trend lines, 1950−2021: Barking and Dagenham, Ages: 25−29
Data points enclosed with a diamond are reference sources
Age−specific fertility rate
0.175
0.150
0.125
0.100
1960
1980
2000
2020
Year
Adjusted Data, CBH/VR
Data, CBH/VR
Outliered Data
GBD 2019
GBD 2021
VR COMPLETE
VR COMPLETE TB

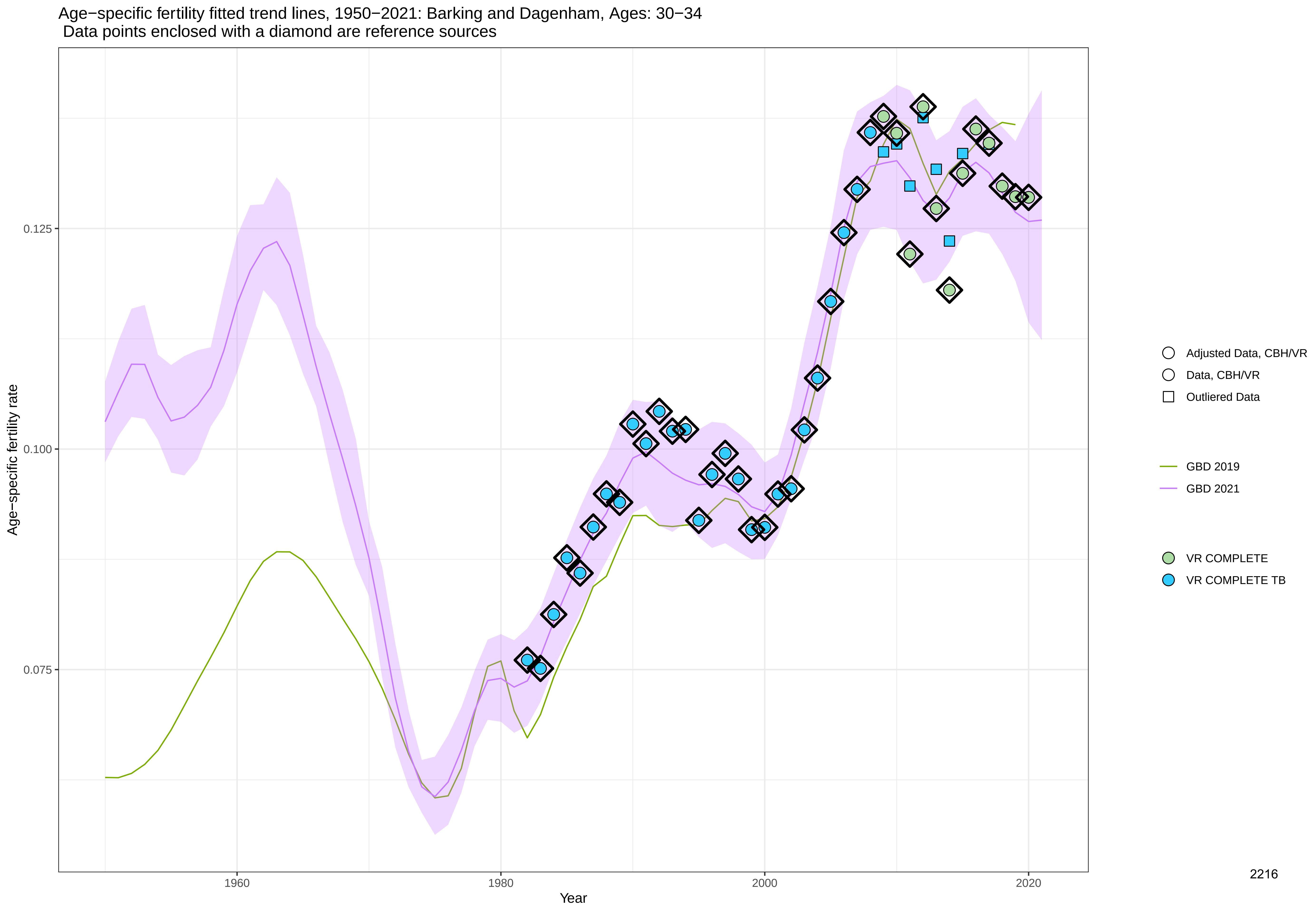

Age−specific fertility fitted trend lines, 1950−2021: Barking and Dagenham, Ages: 30−34
Data points enclosed with a diamond are reference sources
Age−specific fertility rate
0.125
0.100
0.075
1960
1980
2000
2020
Year
Adjusted Data, CBH/VR
Data, CBH/VR
Outliered Data
GBD 2019
GBD 2021
VR COMPLETE
VR COMPLETE TB

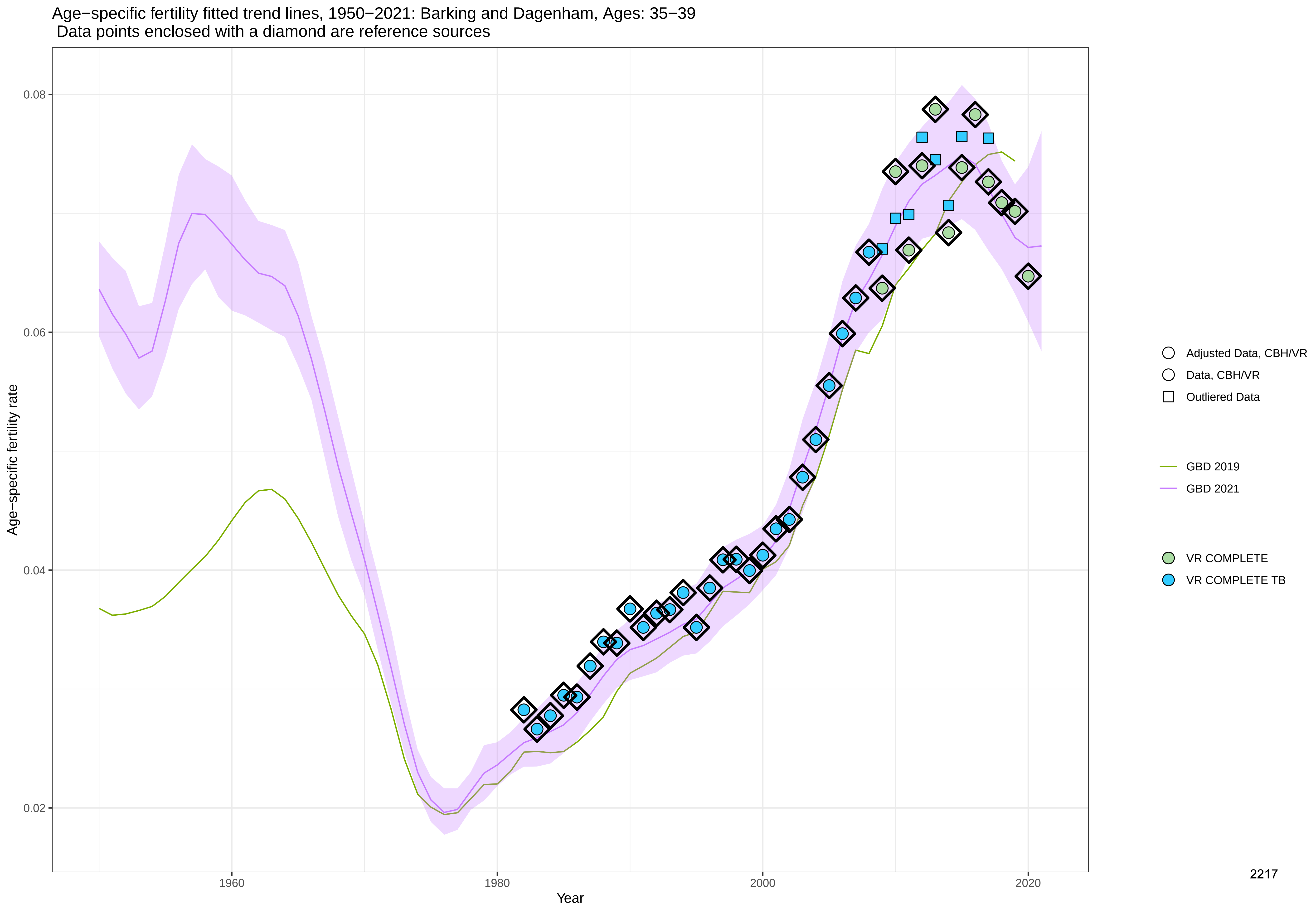

Age−specific fertility fitted trend lines, 1950−2021: Barking and Dagenham, Ages: 35−39
Data points enclosed with a diamond are reference sources
Age−specific fertility rate
0.02
0.04
0.06
0.08
1960
1980
2000
2020
Year
Adjusted Data, CBH/VR
Data, CBH/VR
Outliered Data
GBD 2019
GBD 2021
VR COMPLETE
VR COMPLETE TB

Age−specific fertility fitted trend lines, 1950−2021: Barking and Dagenham, Ages: 40−44
Data points enclosed with a diamond are reference sources

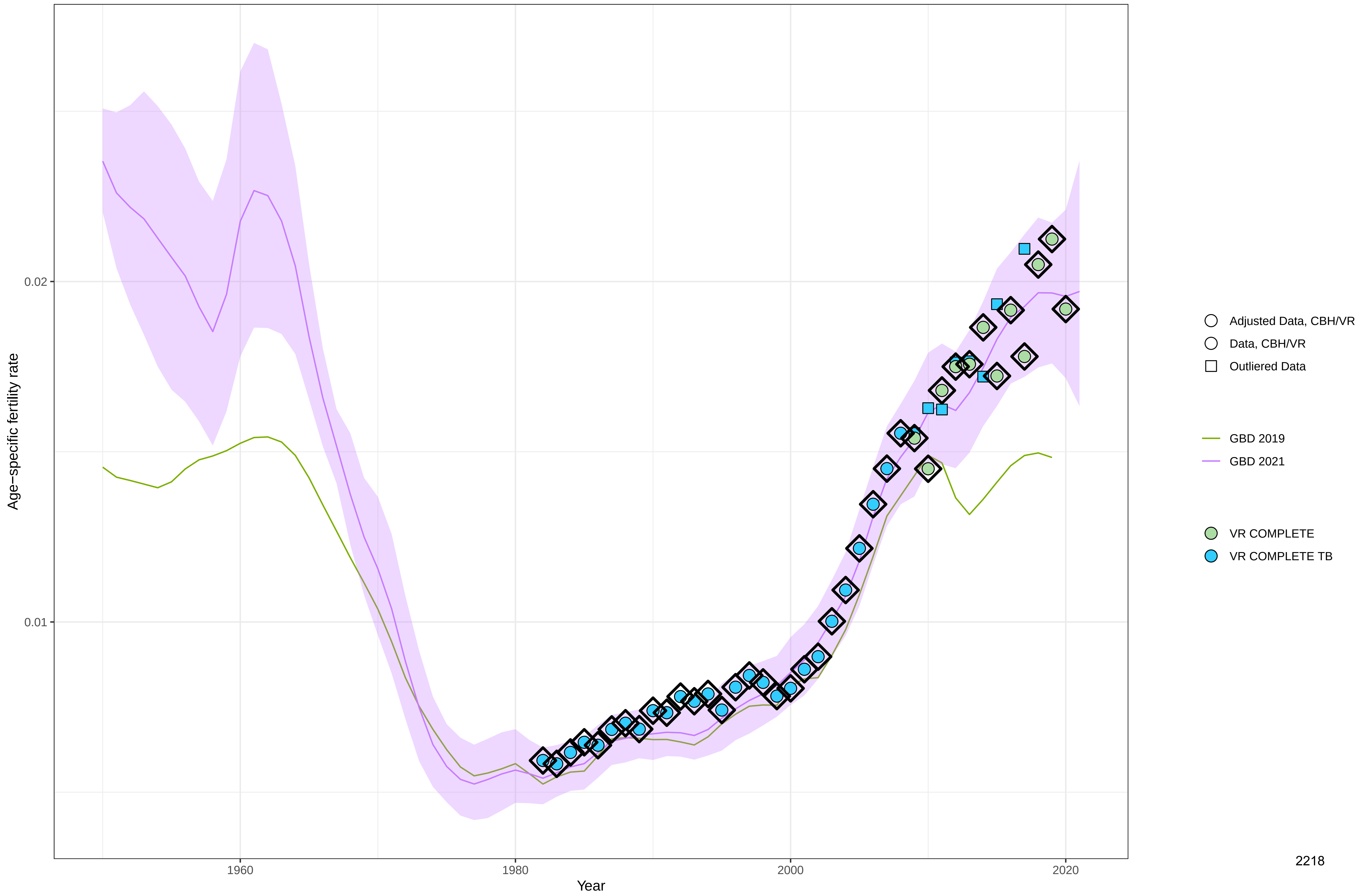

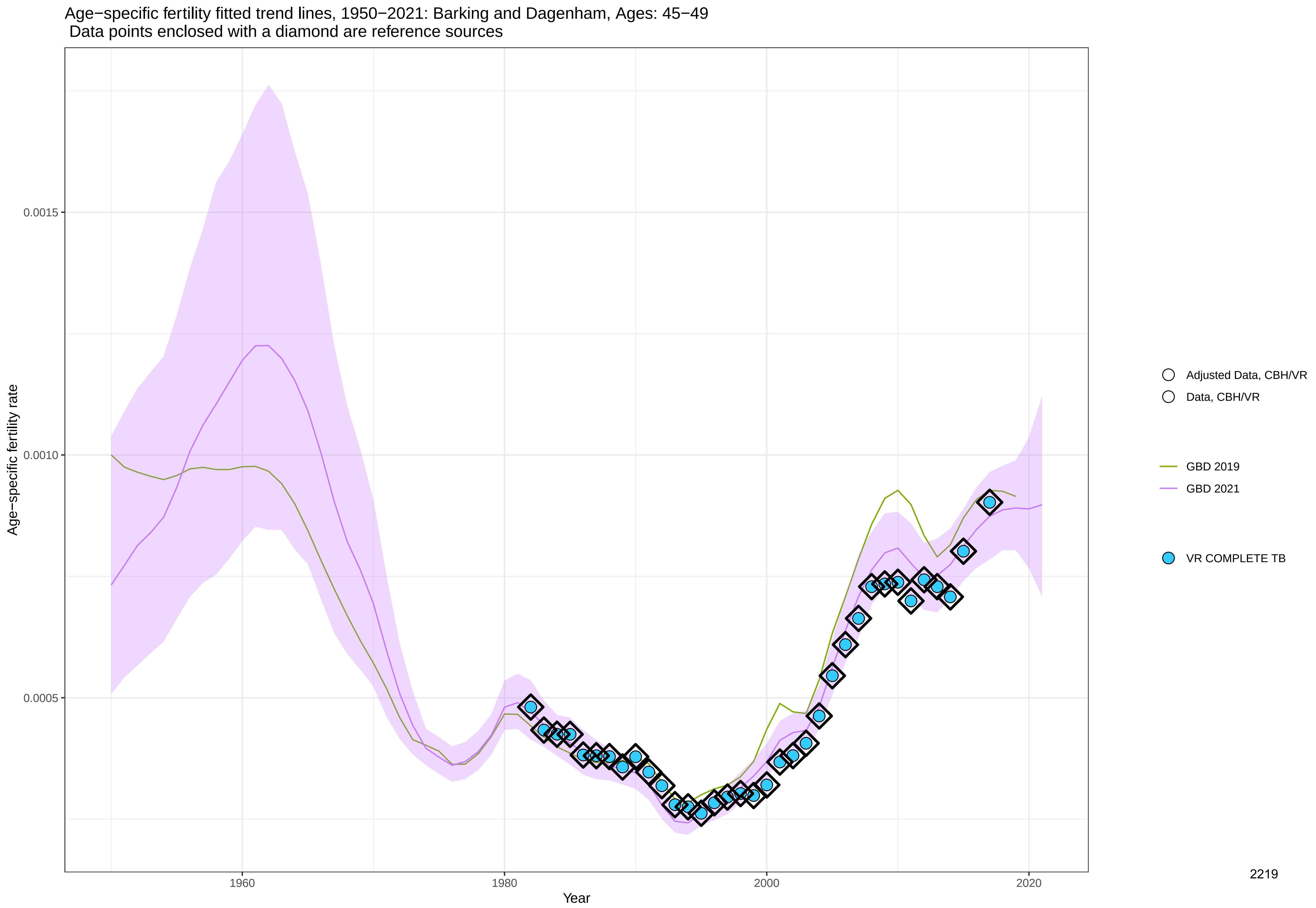

Age−specific fertility fitted trend lines, 1950−2021: Barking and Dagenham, Ages: 45−49
Data points enclosed with a diamond are reference sources
0.0015
0.0010
0.0005
Age−specific fertility rate
1960
1980
2000
2020
Year
Adjusted Data, CBH/VR
Data, CBH/VR
GBD 2019
GBD 2021
VR COMPLETE TB

Age−specific fertility fitted trend lines, 1950−2021: Barking and Dagenham, Ages: 50−54
Data points enclosed with a diamond are reference sources

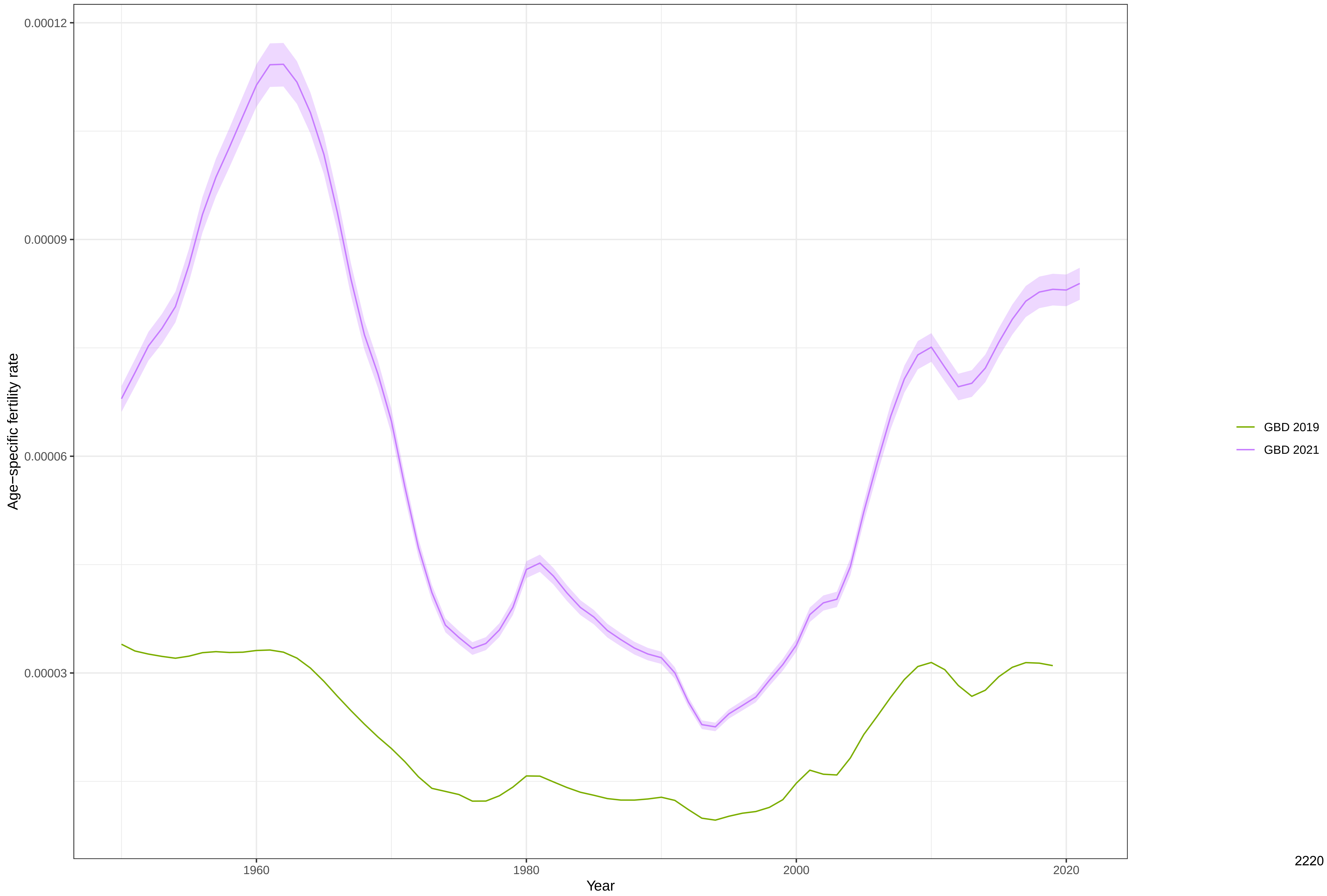

Total fertility rate fitted trend lines, 1950−2021: Barking and Dagenham TFR
Data points enclosed with a diamond are reference sources

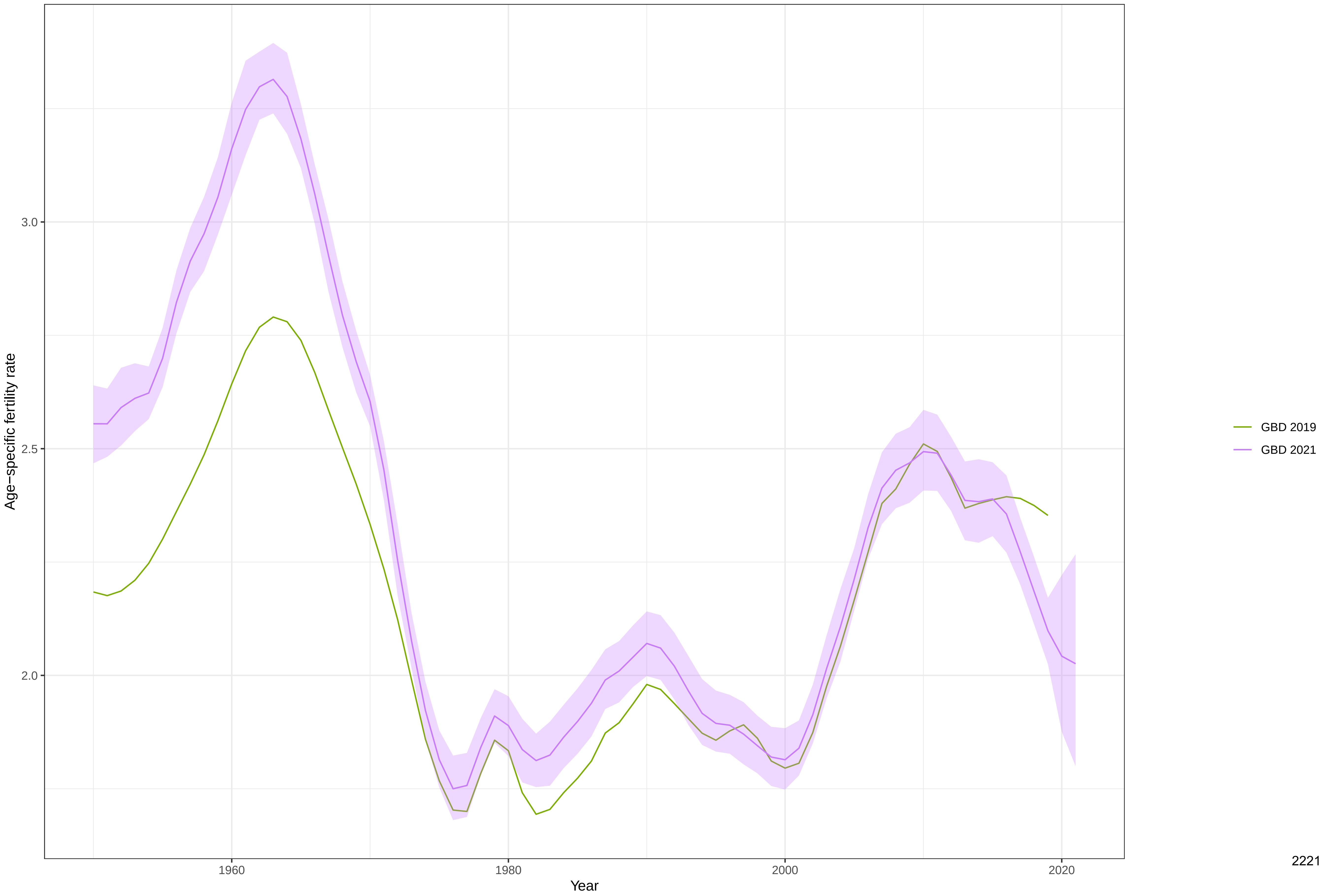

Age−specific fertility fitted trend lines, 1950−2021: Barnet, Ages: 10−14
Data points enclosed with a diamond are reference sources

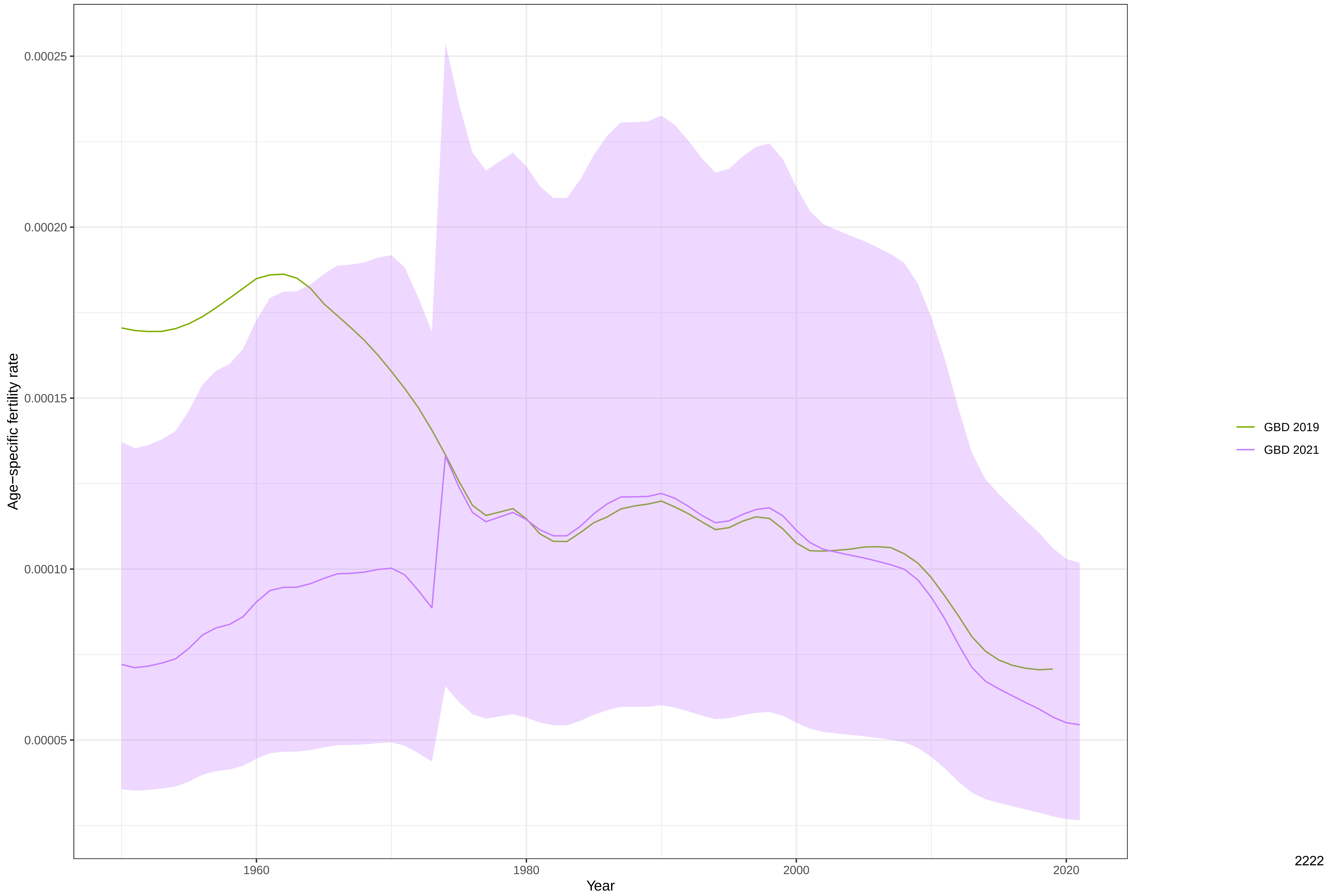

Age−specific fertility fitted trend lines, 1950−2021: Barnet, Ages: 15−19
Data points enclosed with a diamond are reference sources

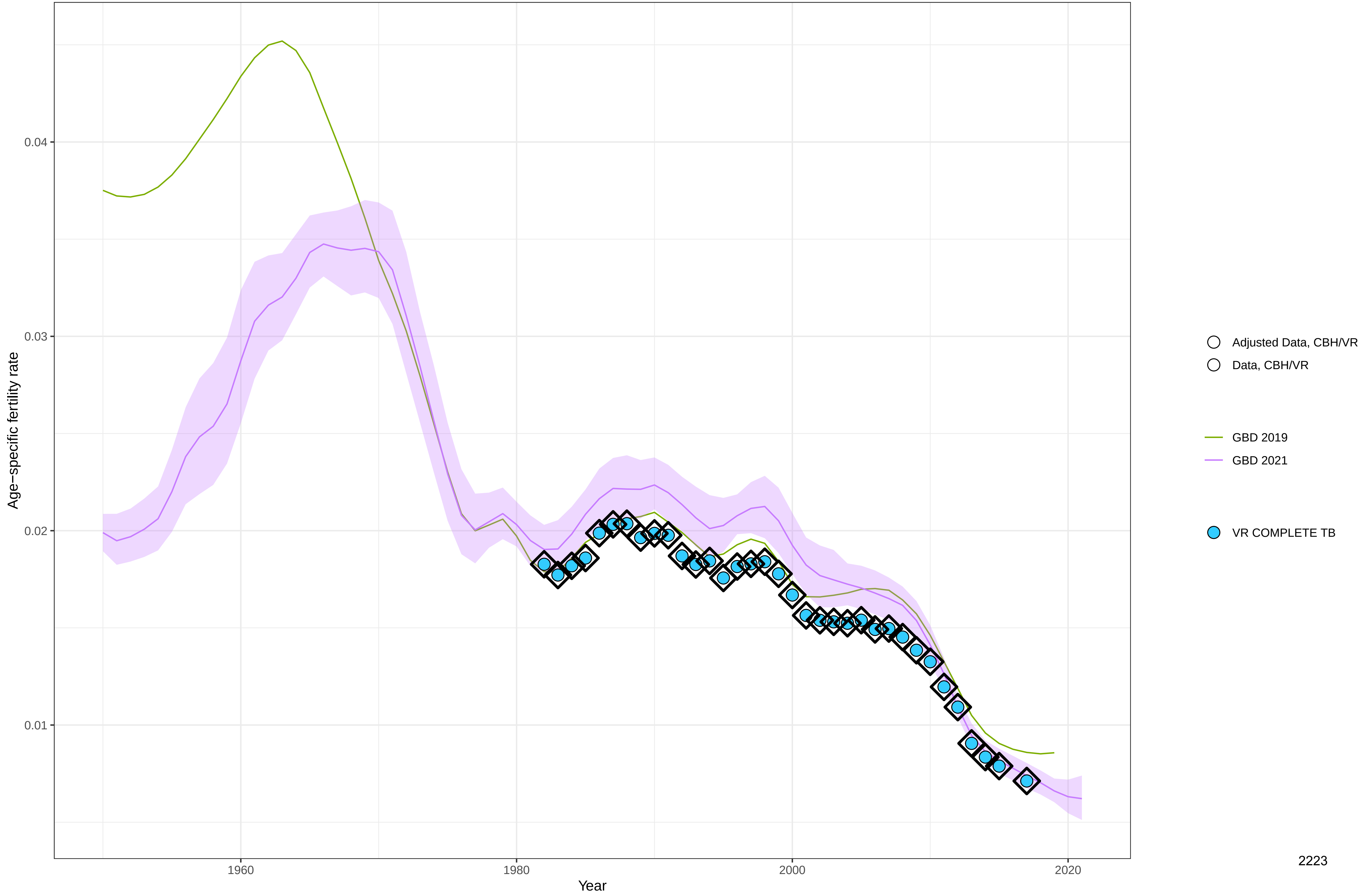

Age−specific fertility fitted trend lines, 1950−2021: Barnet, Ages: 20−24
Data points enclosed with a diamond are reference sources

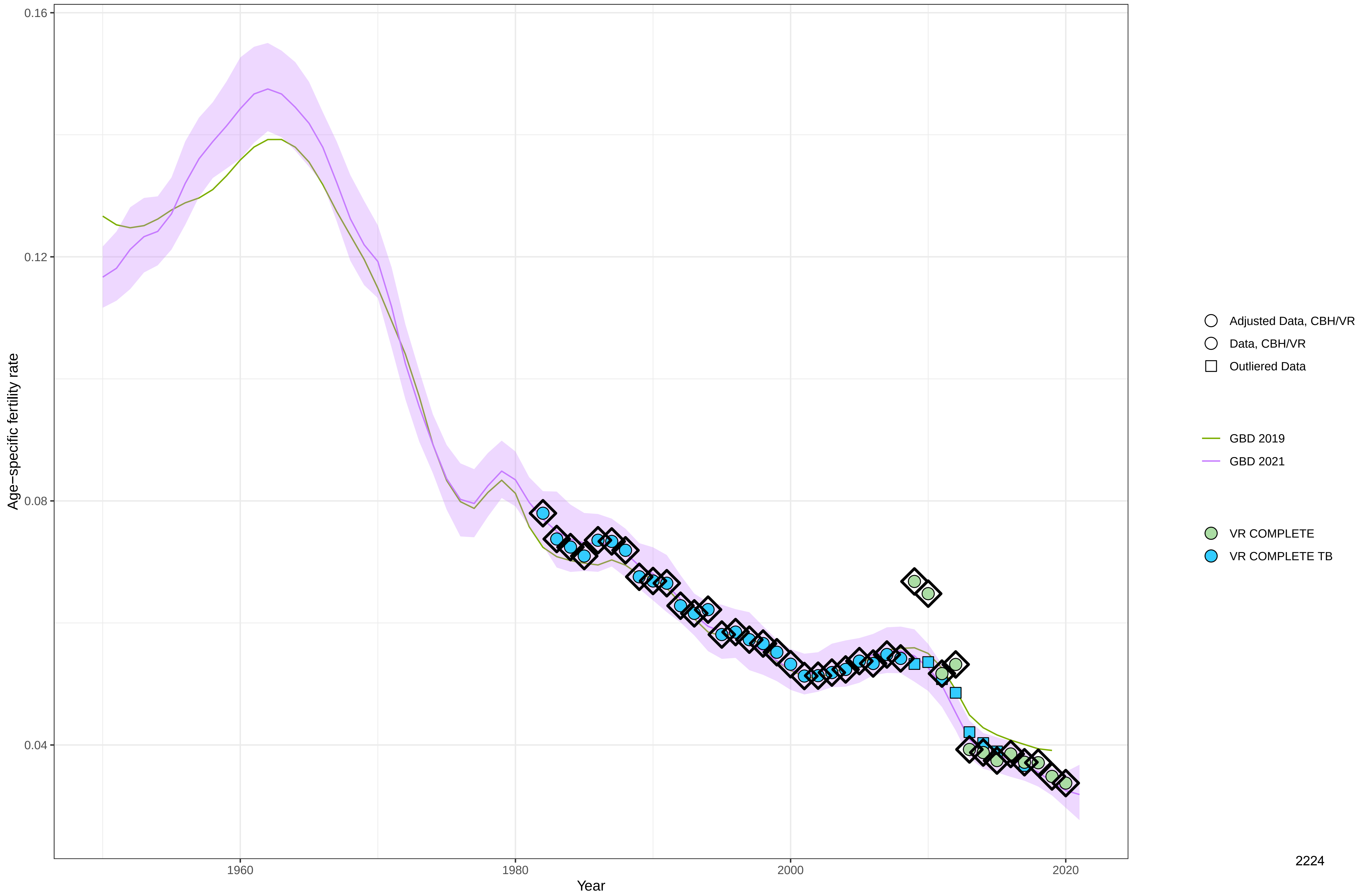

Age−specific fertility fitted trend lines, 1950−2021: Barnet, Ages: 25−29
Data points enclosed with a diamond are reference sources

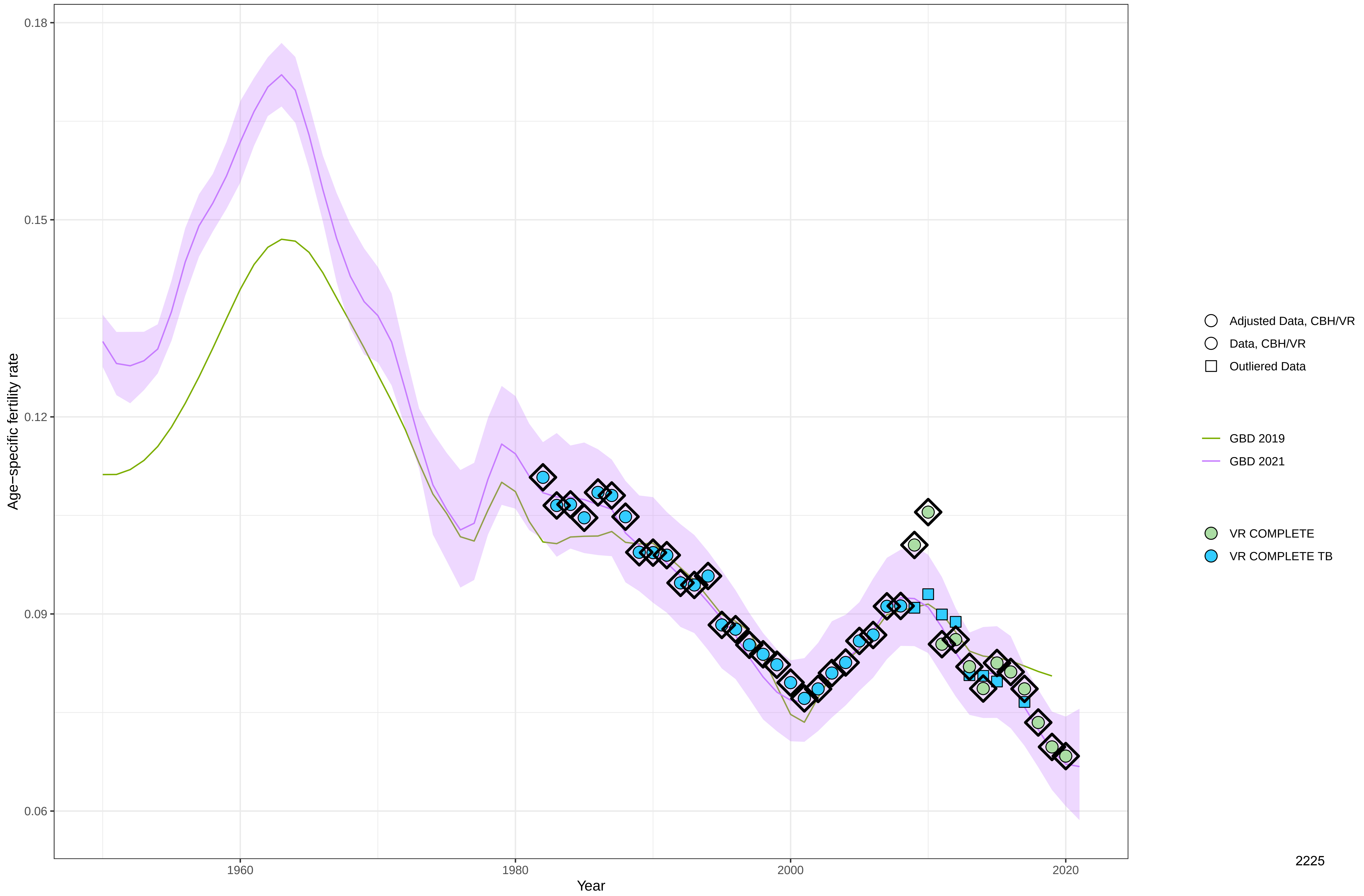

Age−specific fertility fitted trend lines, 1950−2021: Barnet, Ages: 30−34
Data points enclosed with a diamond are reference sources

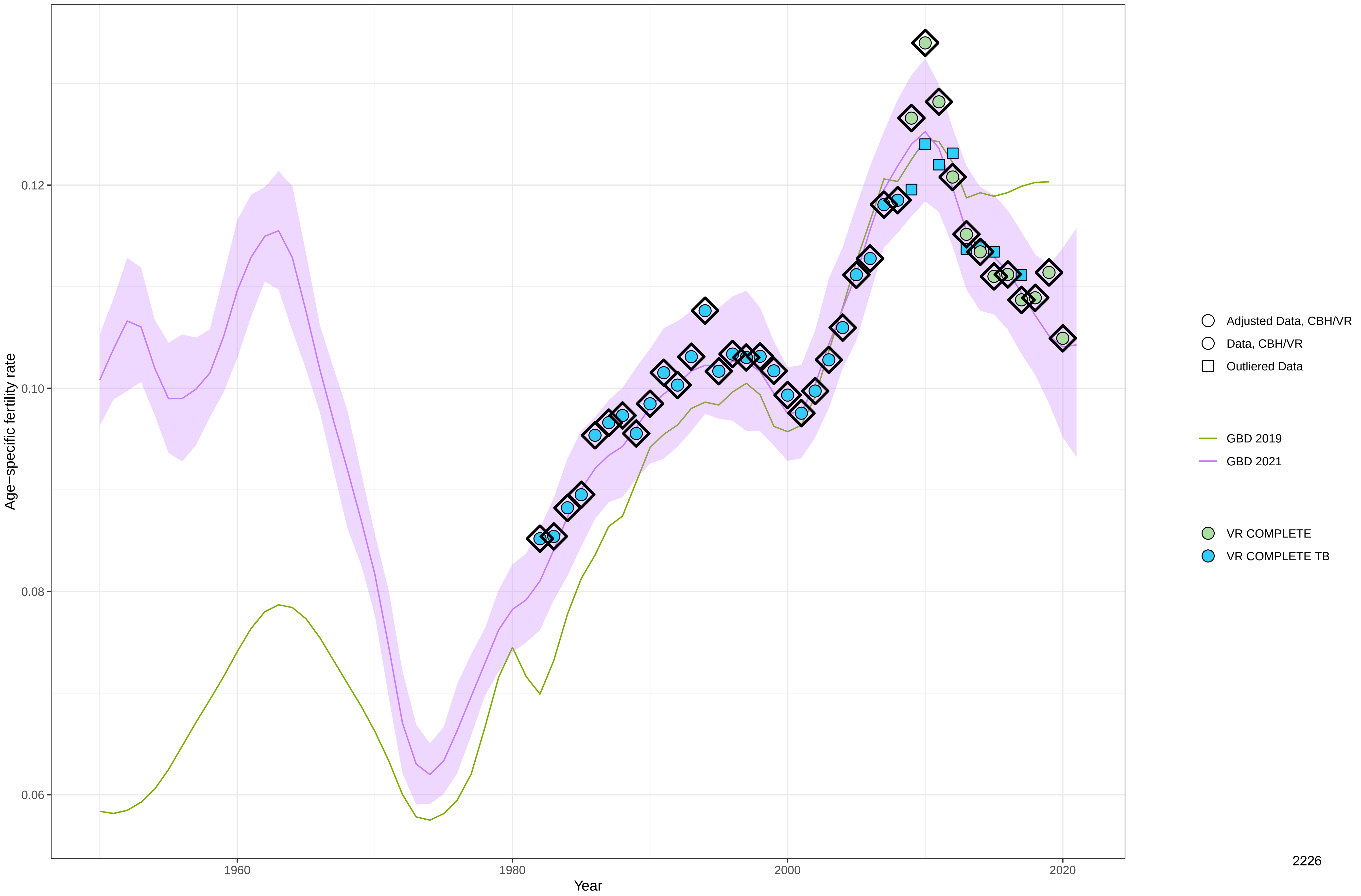

Age−specific fertility fitted trend lines, 1950−2021: Barnet, Ages: 35−39
Data points enclosed with a diamond are reference sources

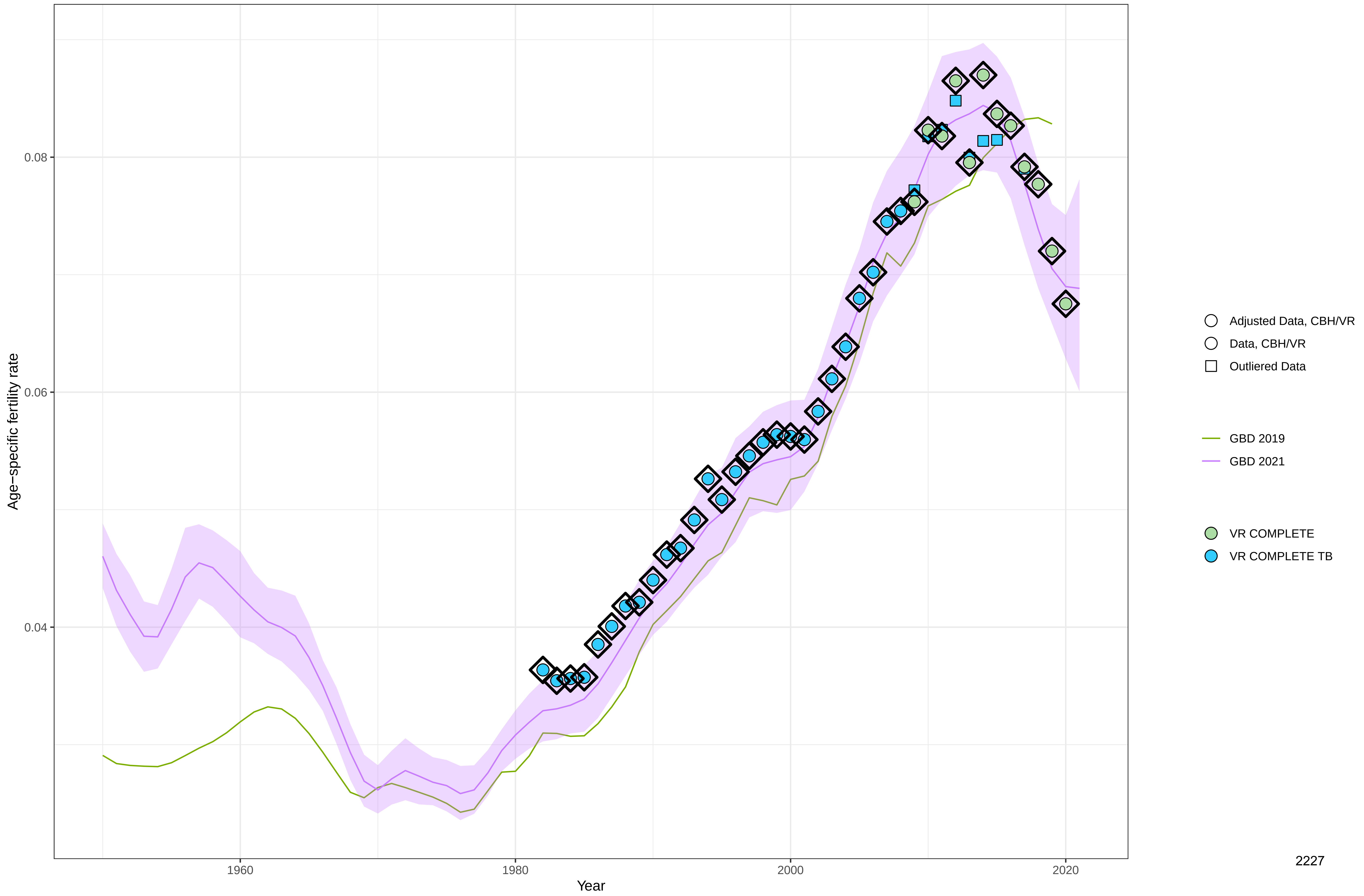

Age−specific fertility fitted trend lines, 1950−2021: Barnet, Ages: 40−44
Data points enclosed with a diamond are reference sources

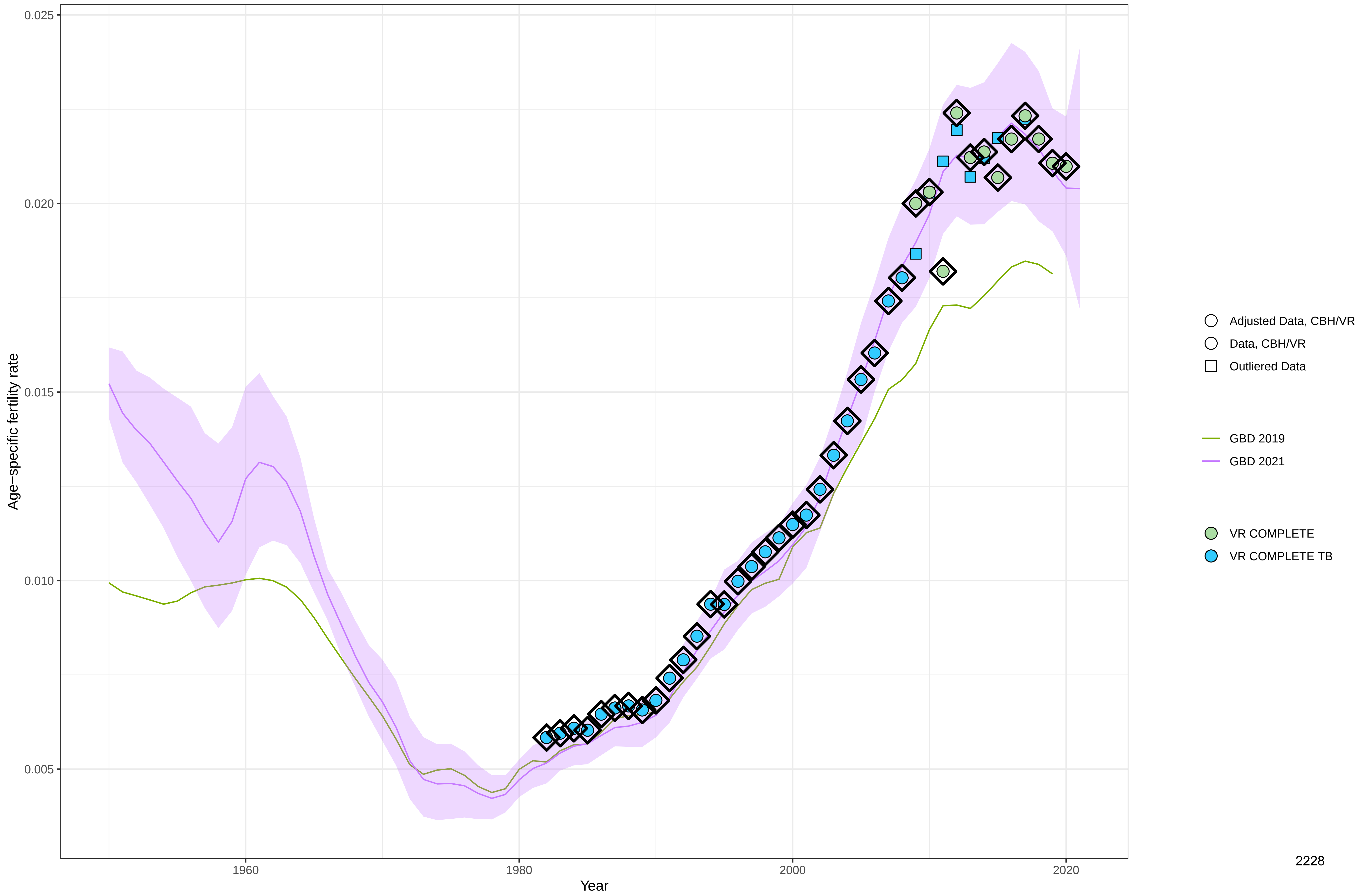

Age−specific fertility fitted trend lines, 1950−2021: Barnet, Ages: 45−49
Data points enclosed with a diamond are reference sources

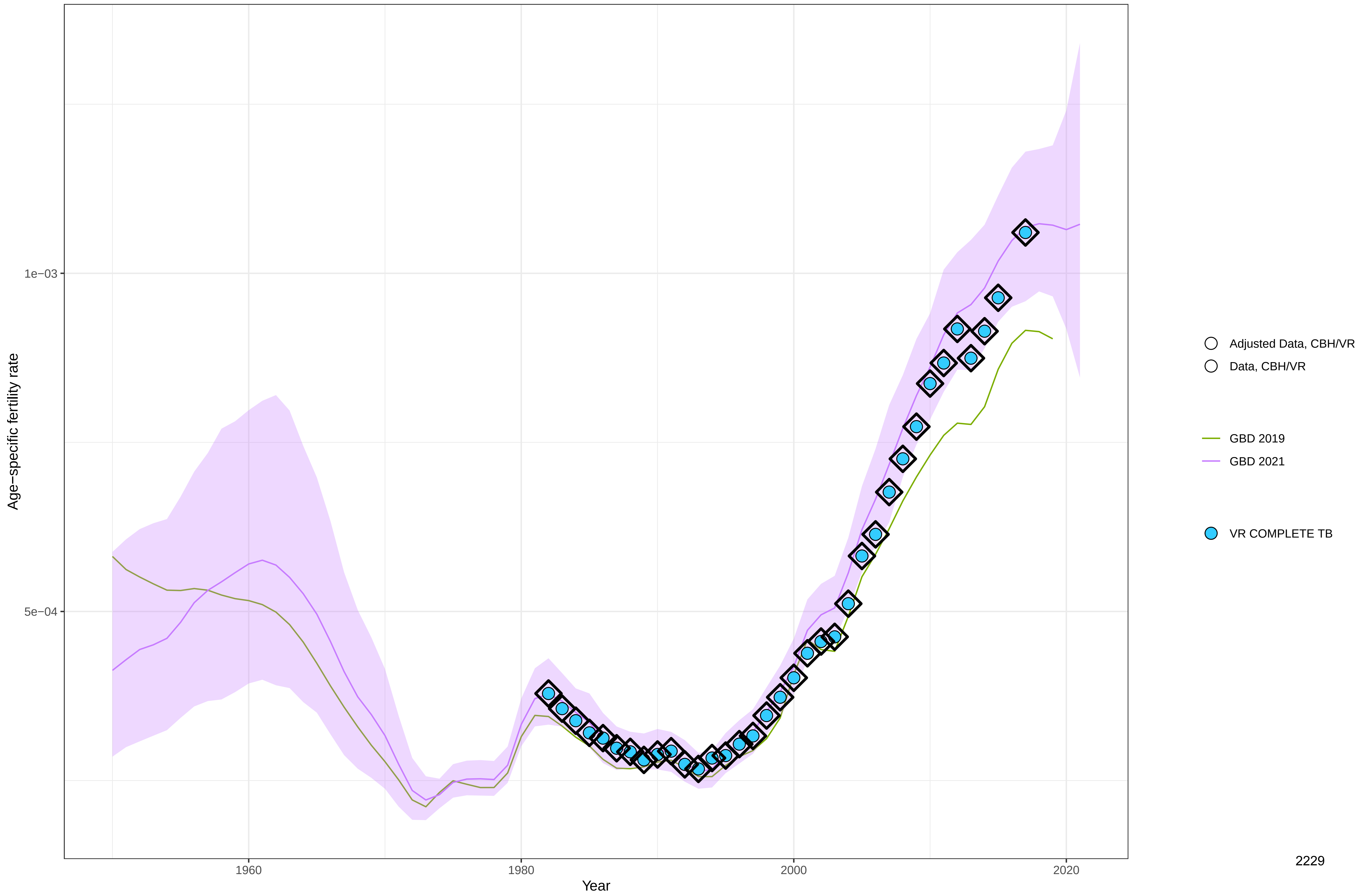

Age−specific fertility fitted trend lines, 1950−2021: Barnet, Ages: 50−54
Data points enclosed with a diamond are reference sources

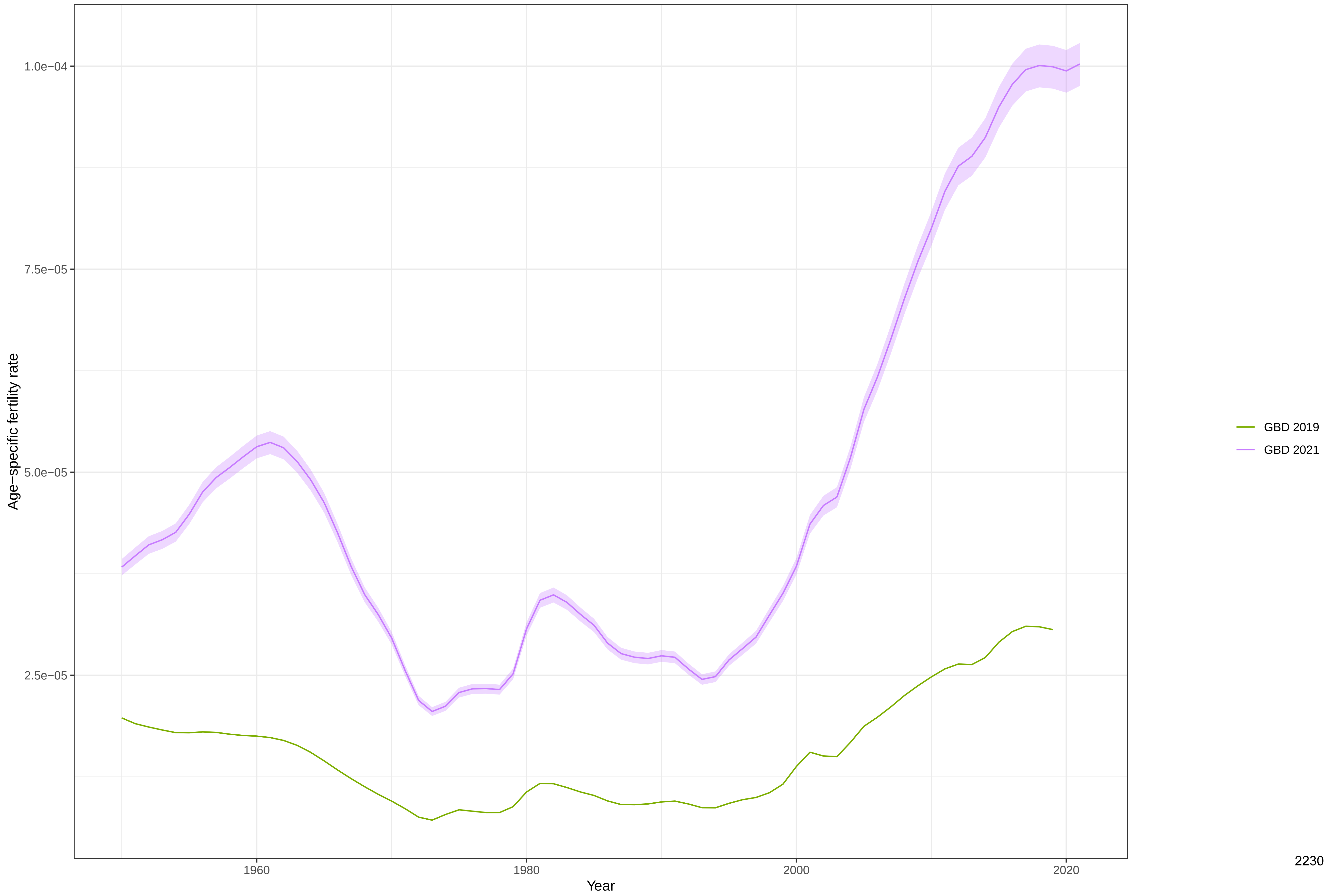

Total fertility rate fitted trend lines, 1950−2021: Barnet TFR
Data points enclosed with a diamond are reference sources

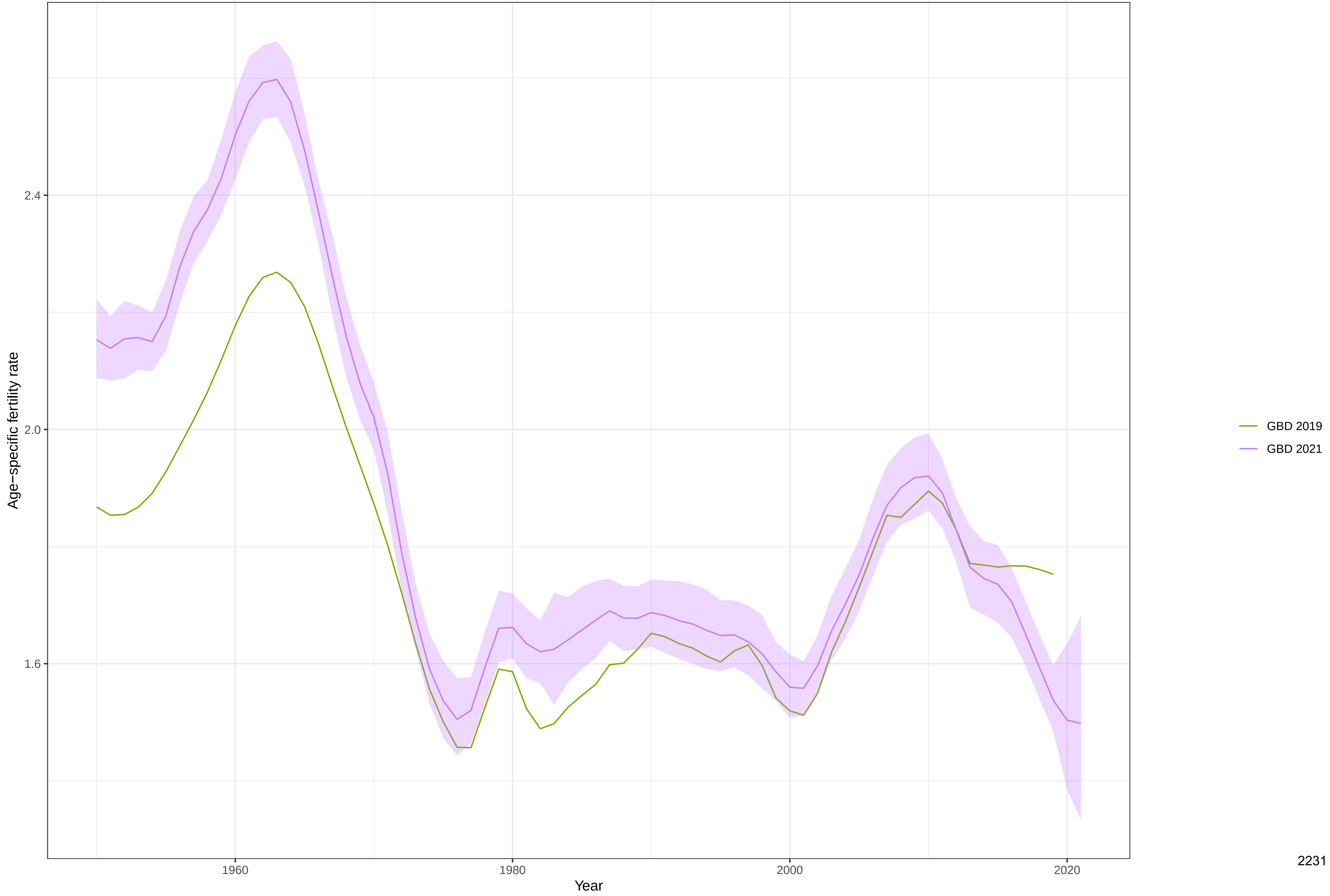

Age−specific fertility fitted trend lines, 1950−2021: Bexley, Ages: 10−14
Data points enclosed with a diamond are reference sources

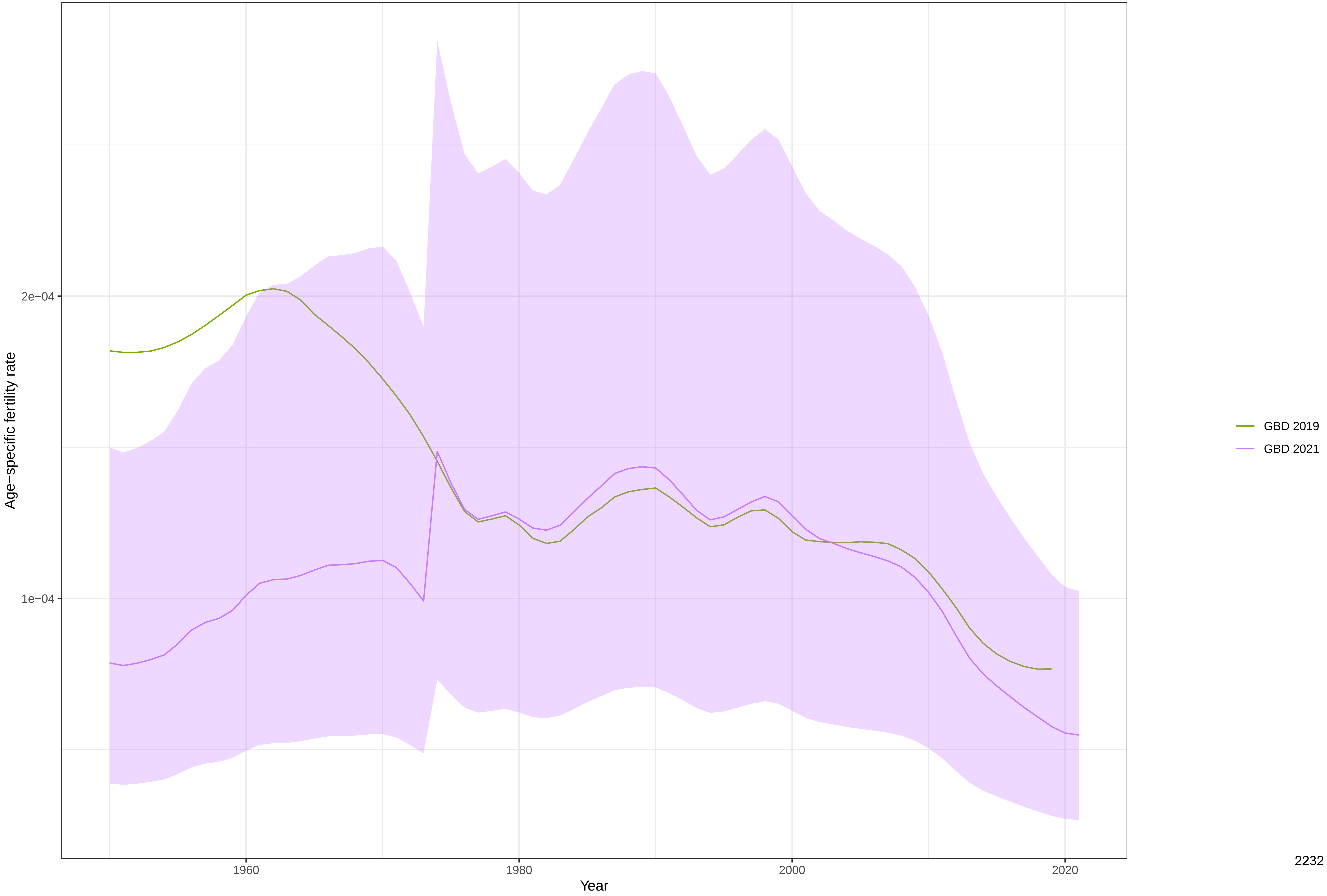

Age−specific fertility fitted trend lines, 1950−2021: Bexley, Ages: 15−19
Data points enclosed with a diamond are reference sources

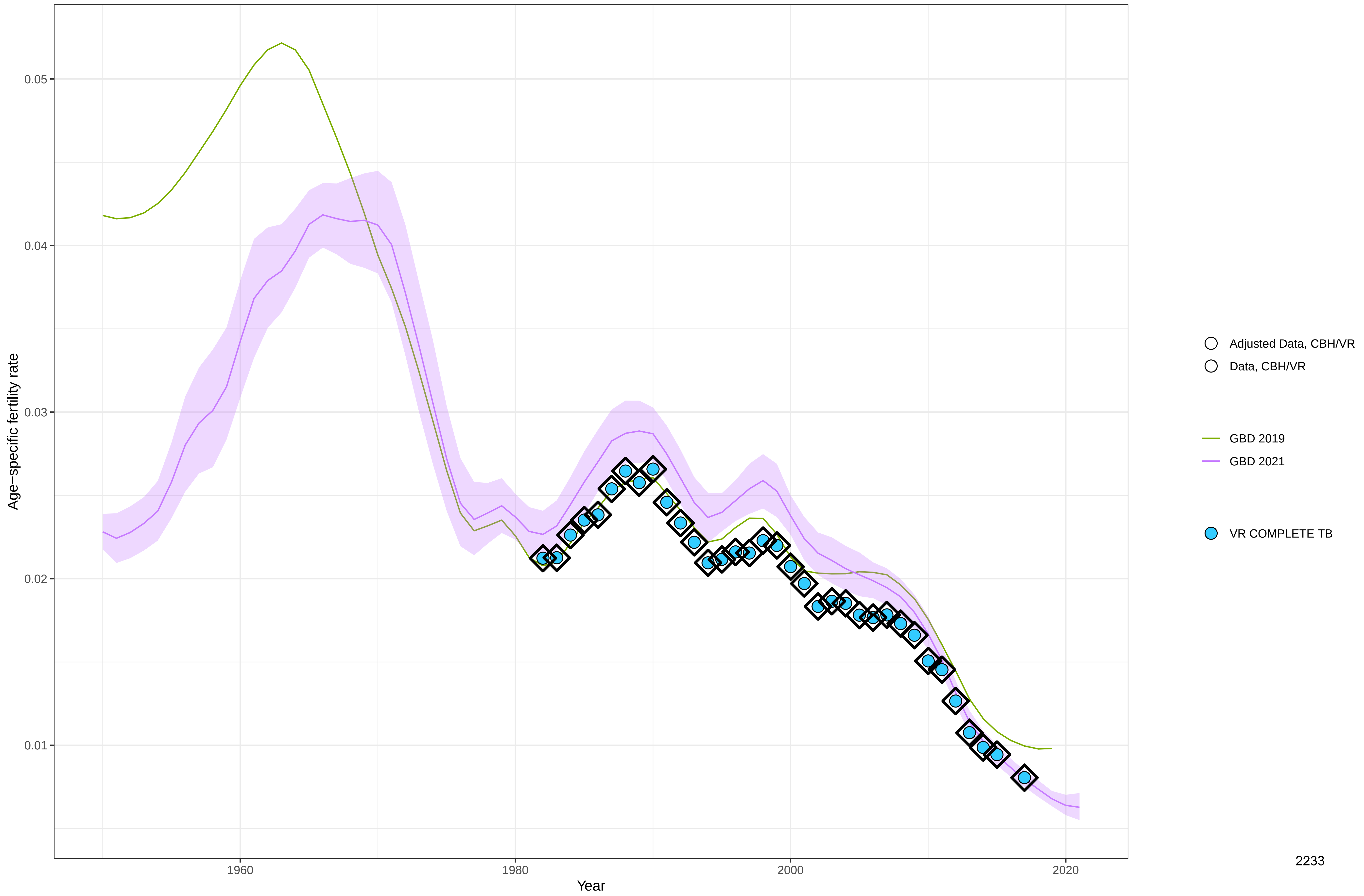

Age−specific fertility fitted trend lines, 1950−2021: Bexley, Ages: 20−24
Data points enclosed with a diamond are reference sources

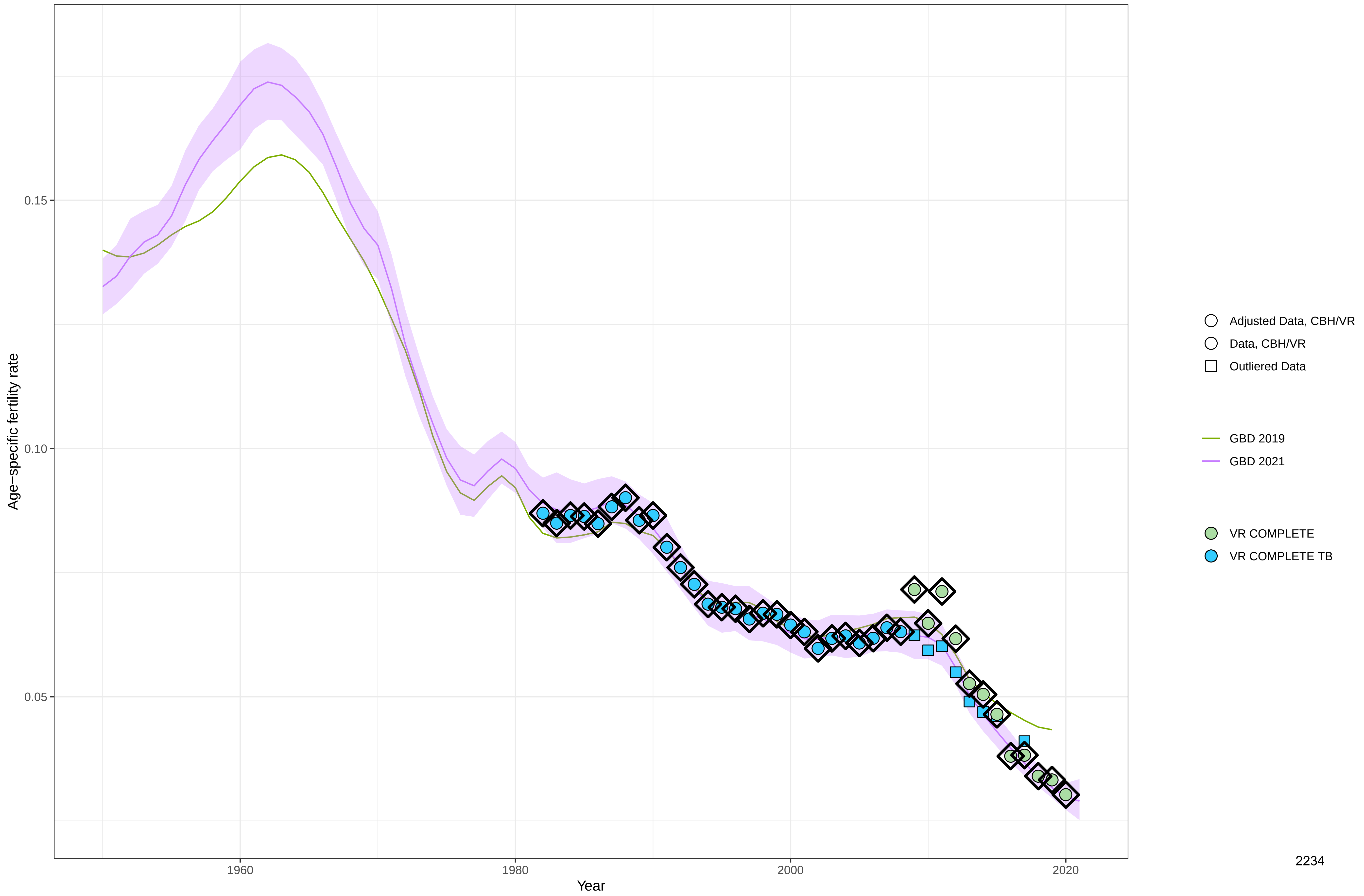

Age−specific fertility fitted trend lines, 1950−2021: Bexley, Ages: 25−29
Data points enclosed with a diamond are reference sources

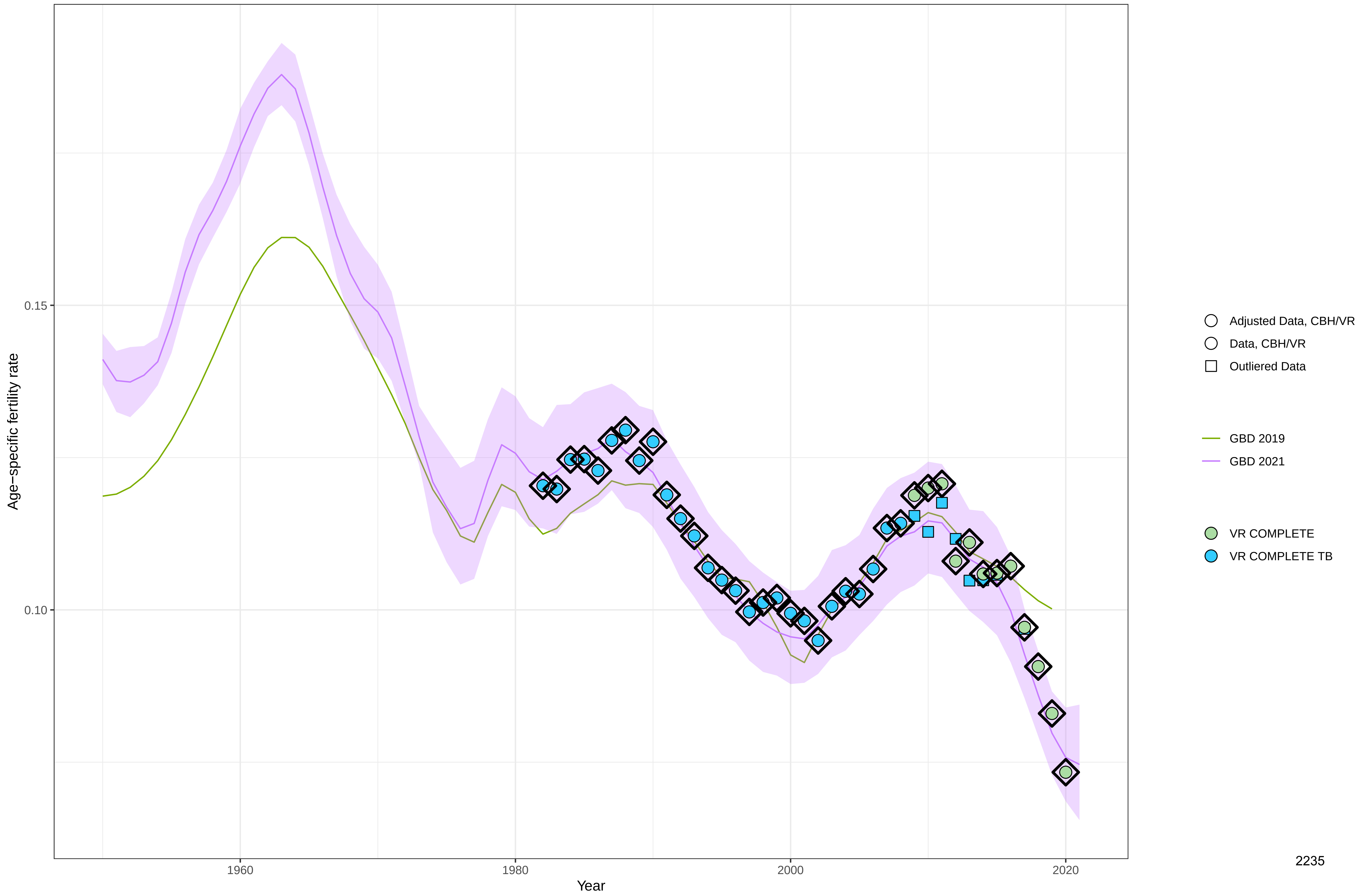

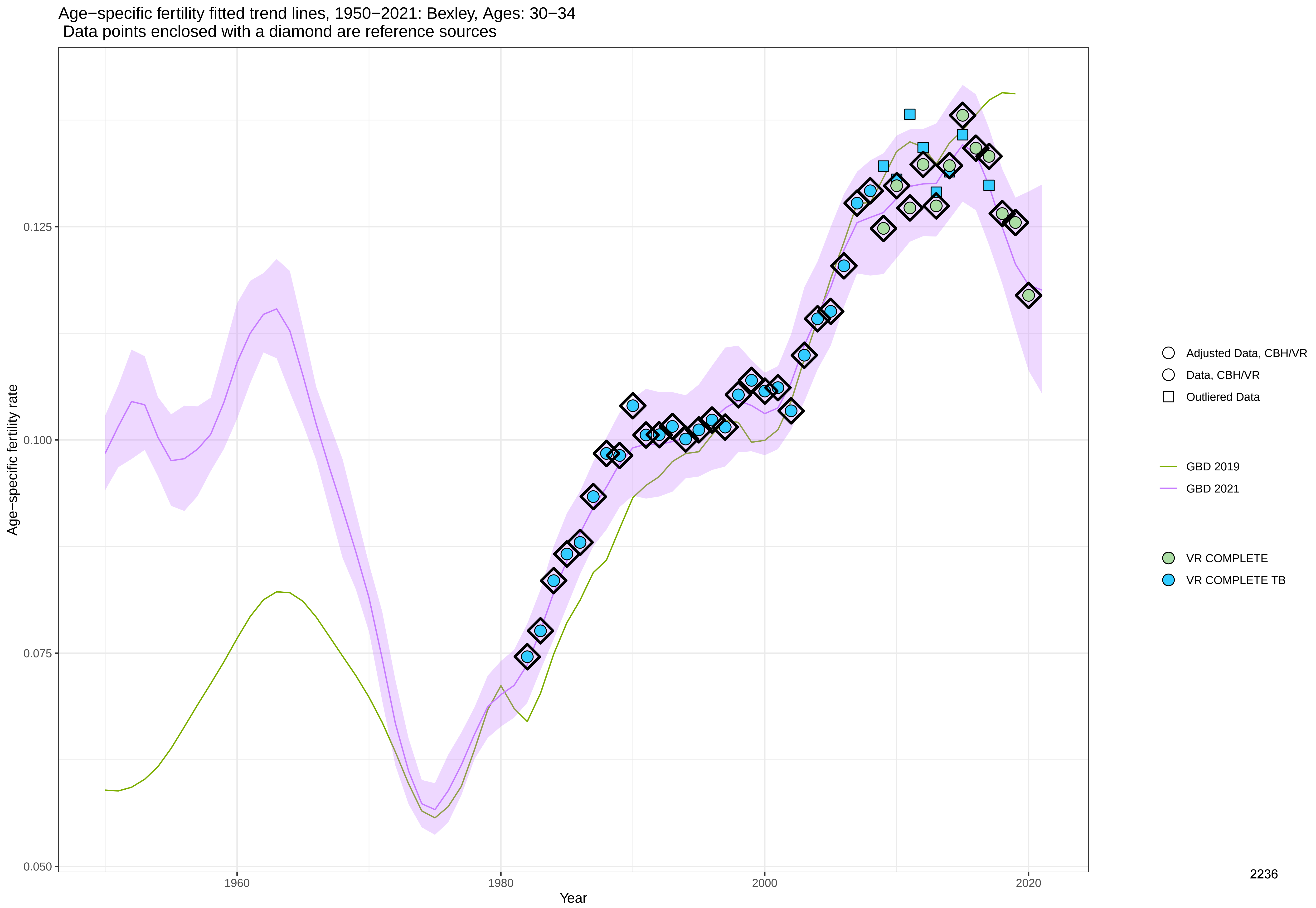

Age−specific fertility fitted trend lines, 1950−2021: Bexley, Ages: 30−34
Data points enclosed with a diamond are reference sources
Age−specific fertility rate
0.050
0.075
0.100
0.125
1960
1980
2000
2020
Year
Adjusted Data, CBH/VR
Data, CBH/VR
Outliered Data
GBD 2019
GBD 2021
VR COMPLETE
VR COMPLETE TB

Age−specific fertility fitted trend lines, 1950−2021: Bexley, Ages: 35−39
Data points enclosed with a diamond are reference sources

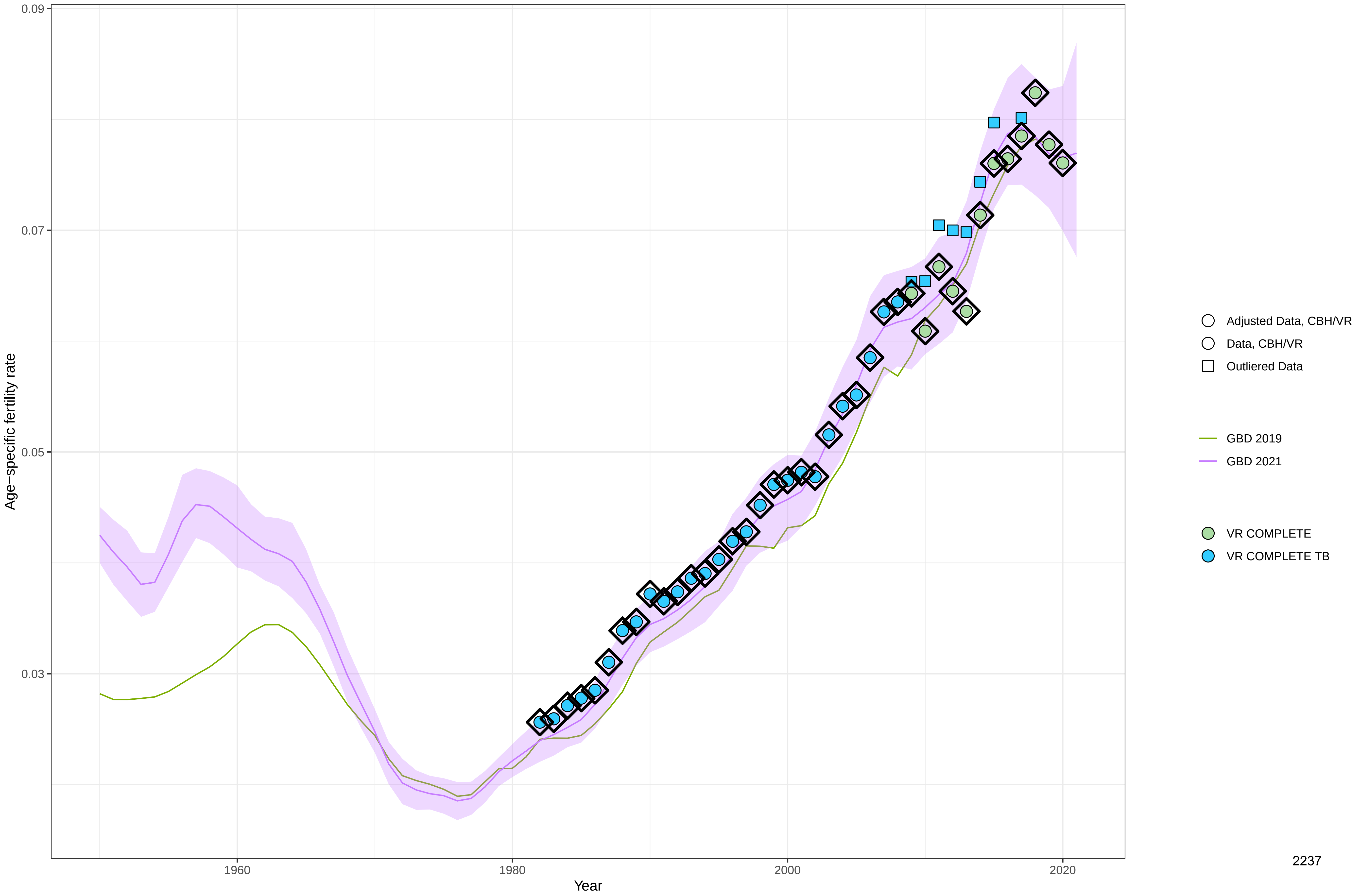

Age−specific fertility fitted trend lines, 1950−2021: Bexley, Ages: 40−44
Data points enclosed with a diamond are reference sources

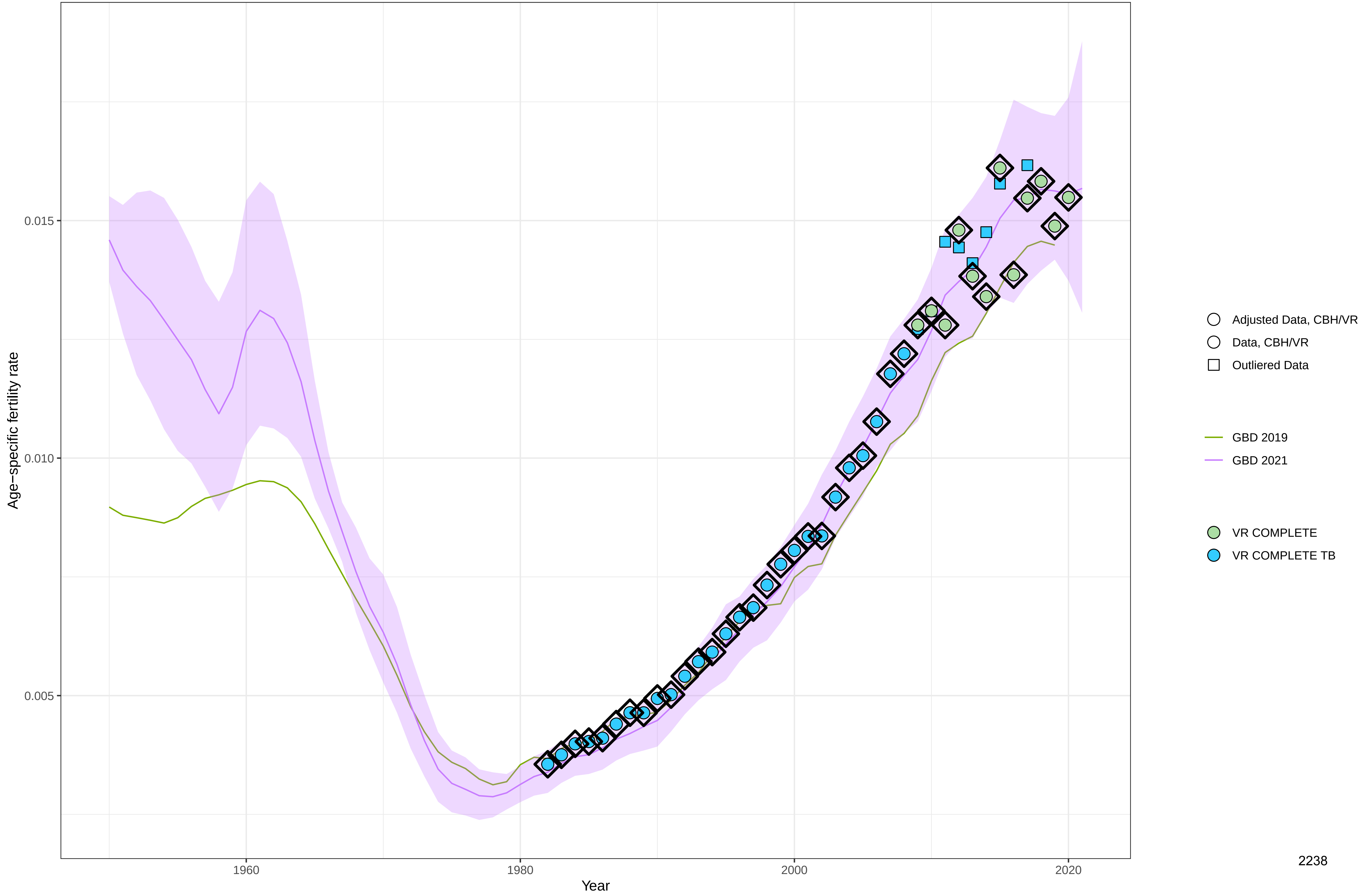

Age−specific fertility fitted trend lines, 1950−2021: Bexley, Ages: 45−49
Data points enclosed with a diamond are reference sources

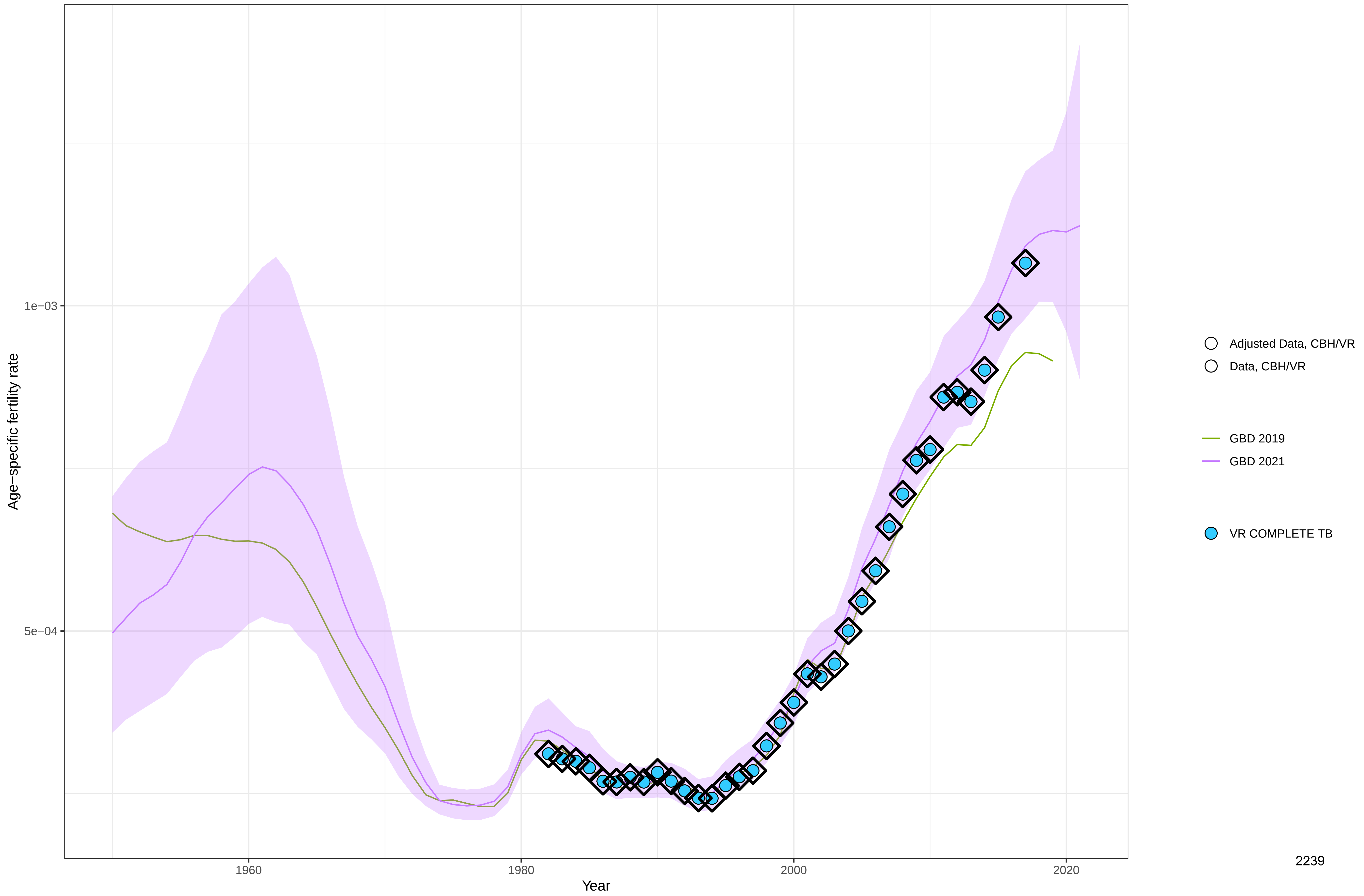

Age−specific fertility fitted trend lines, 1950−2021: Bexley, Ages: 50−54
Data points enclosed with a diamond are reference sources

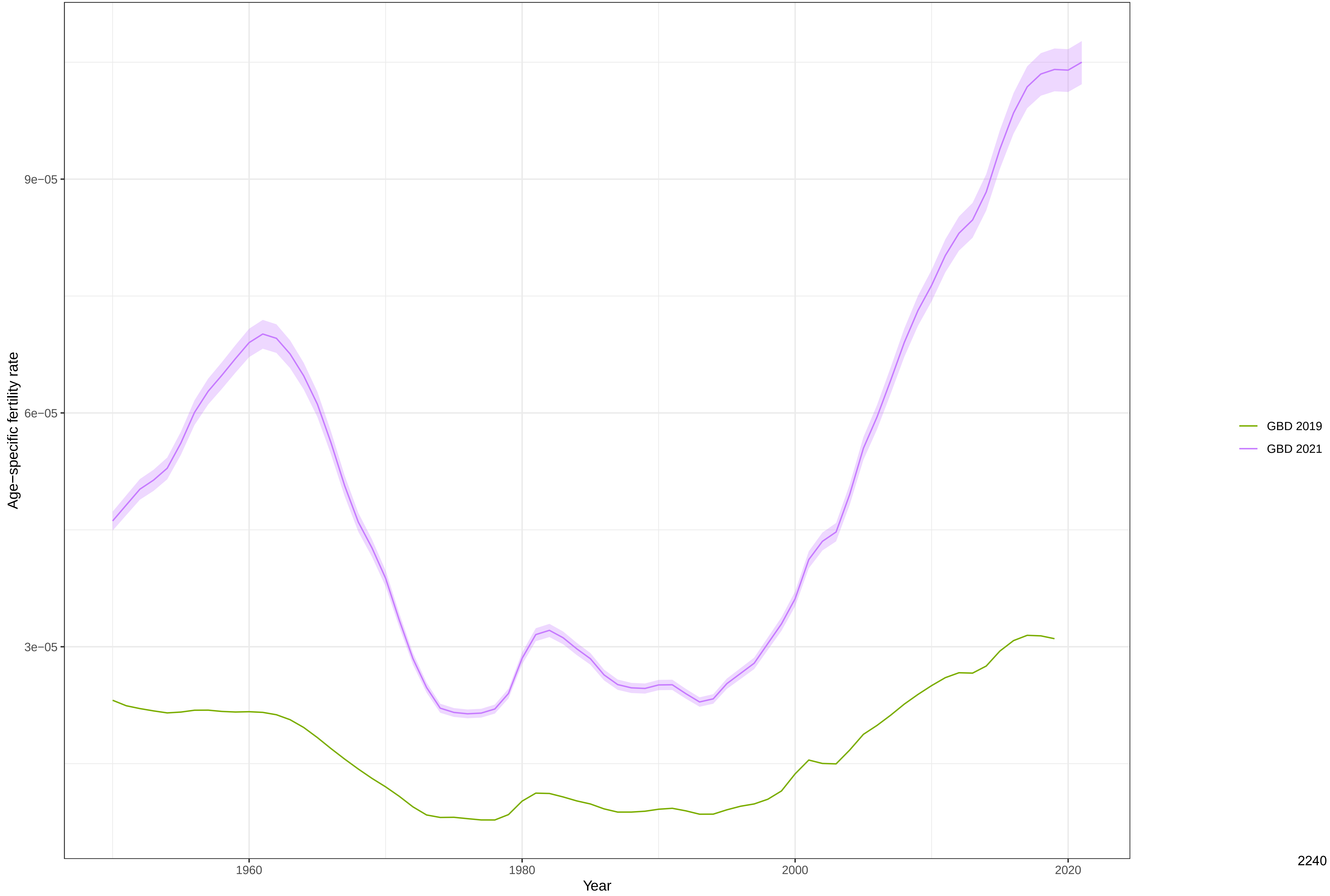

Total fertility rate fitted trend lines, 1950−2021: Bexley TFR
Data points enclosed with a diamond are reference sources

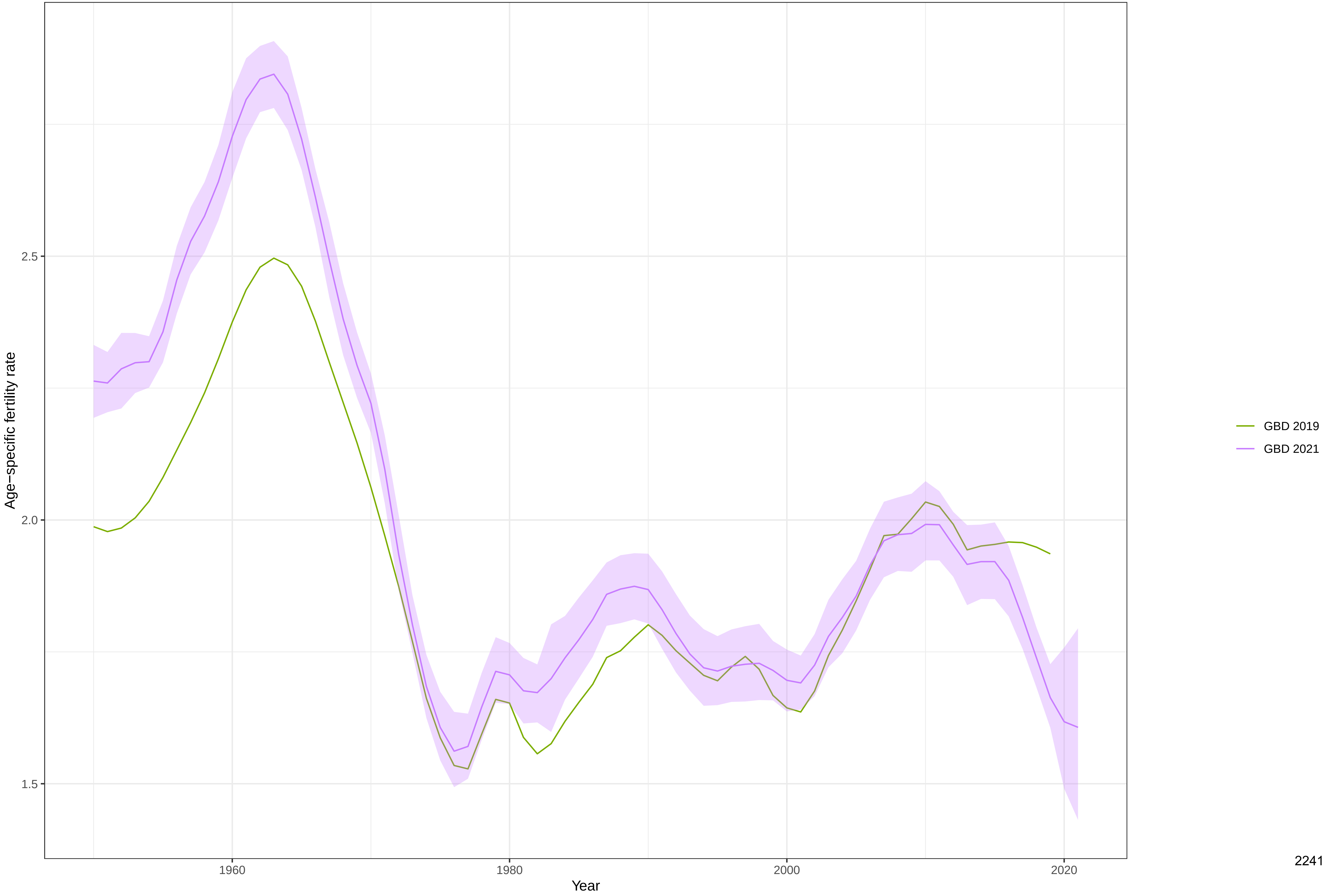

Age−specific fertility fitted trend lines, 1950−2021: Brent, Ages: 10−14
Data points enclosed with a diamond are reference sources

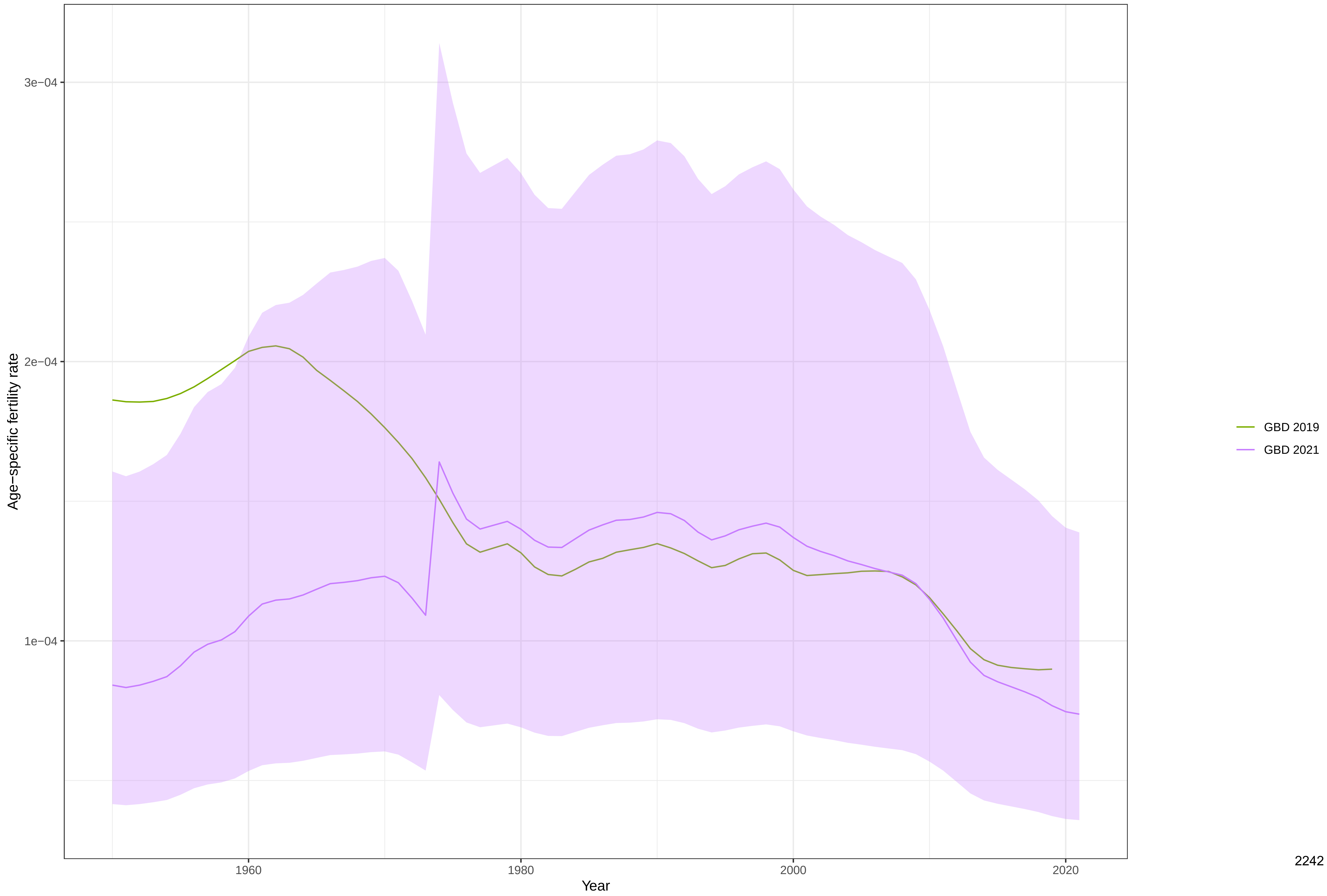

Age−specific fertility fitted trend lines, 1950−2021: Brent, Ages: 15−19
Data points enclosed with a diamond are reference sources

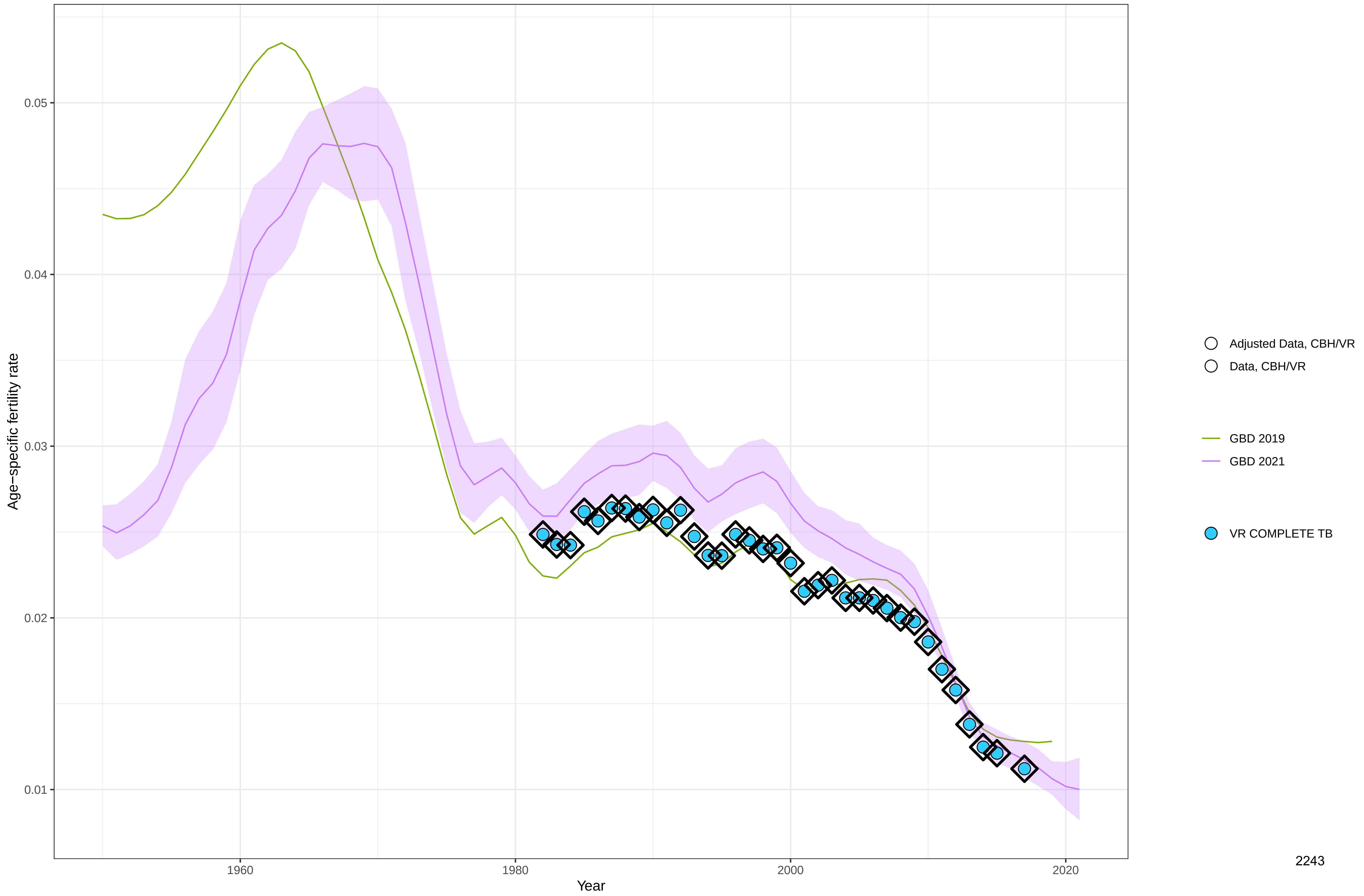

Age−specific fertility fitted trend lines, 1950−2021: Brent, Ages: 20−24
Data points enclosed with a diamond are reference sources

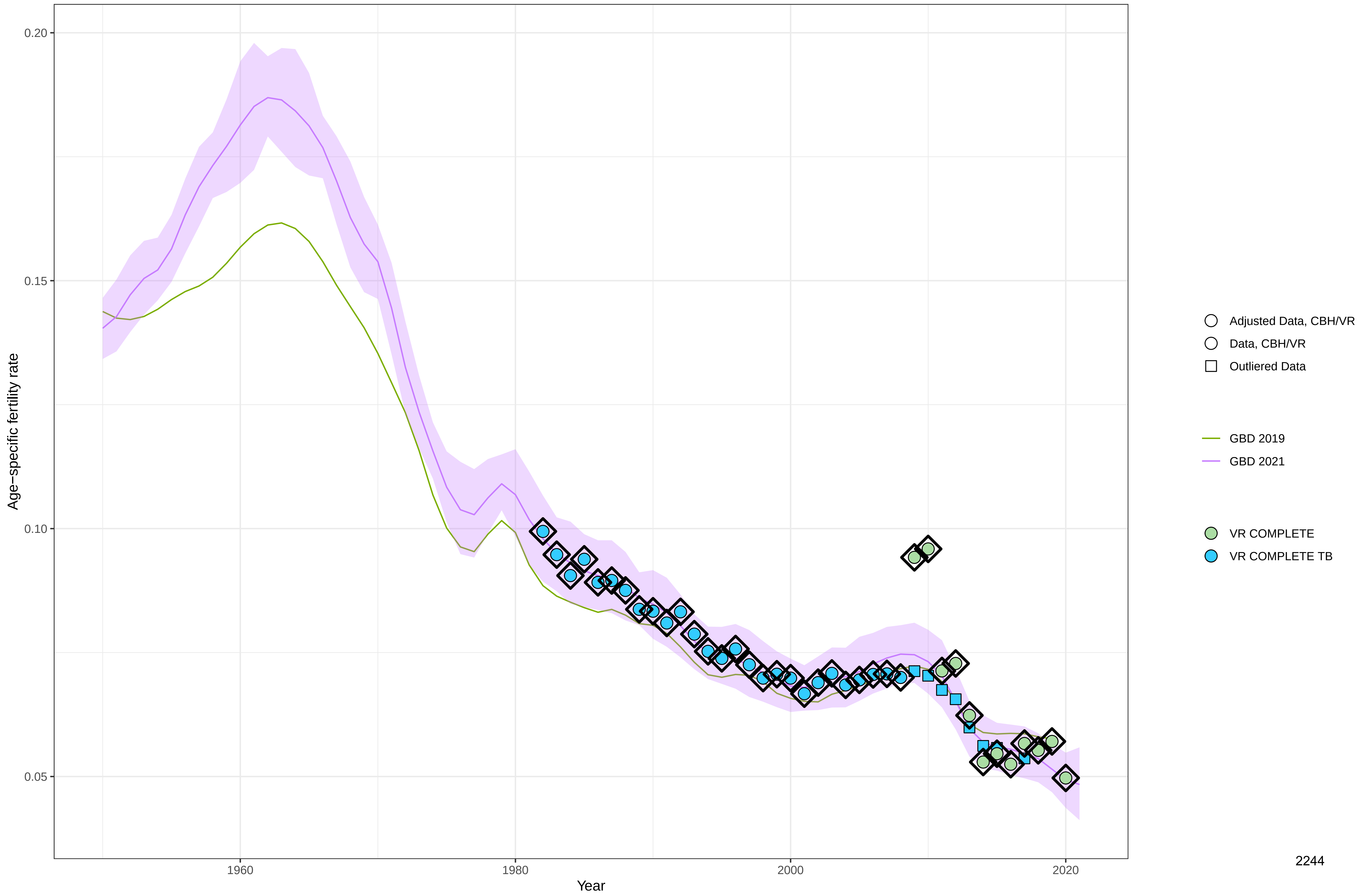

Age−specific fertility fitted trend lines, 1950−2021: Brent, Ages: 25−29
Data points enclosed with a diamond are reference sources

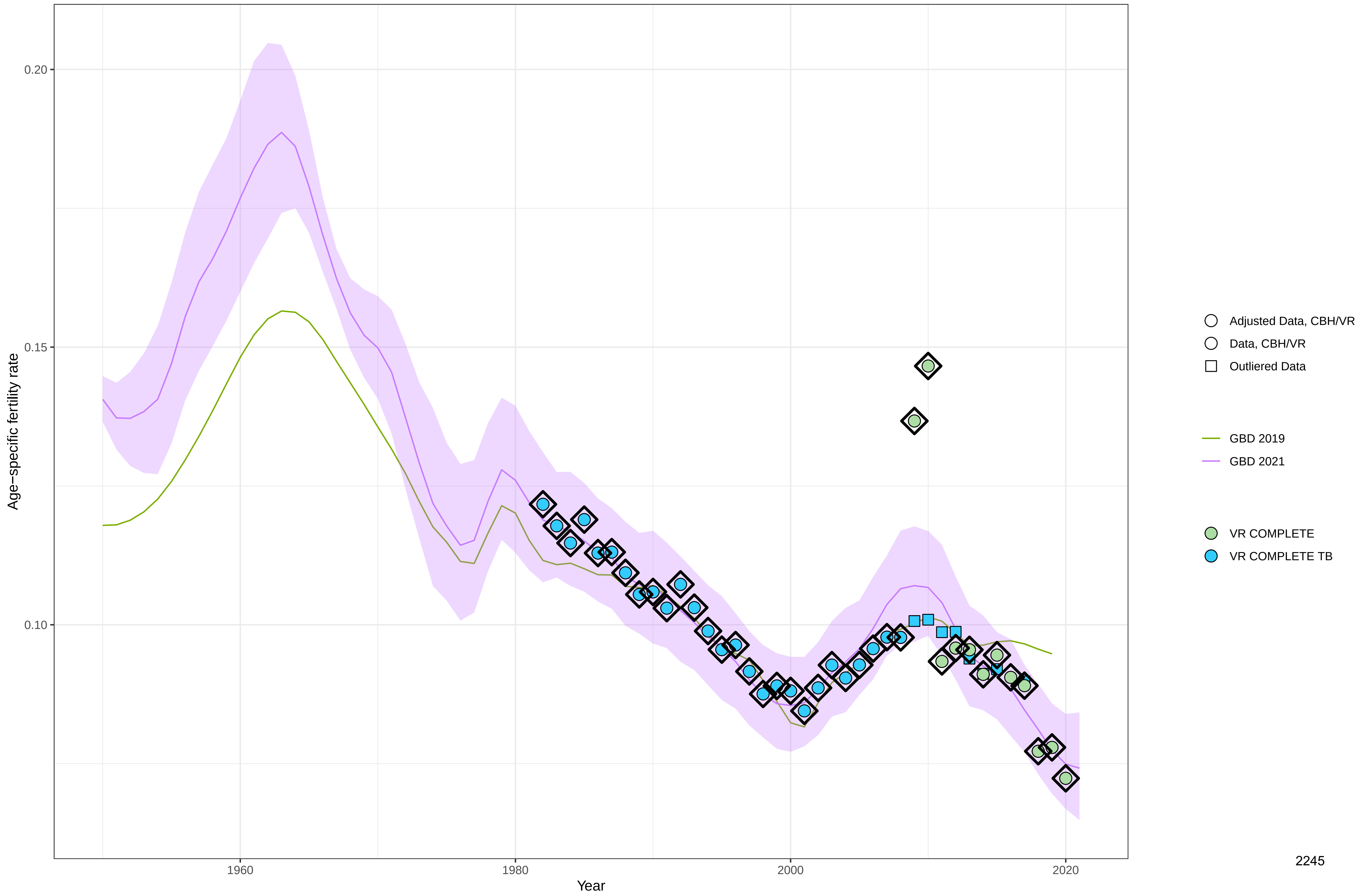

Age−specific fertility fitted trend lines, 1950−2021: Brent, Ages: 30−34
Data points enclosed with a diamond are reference sources

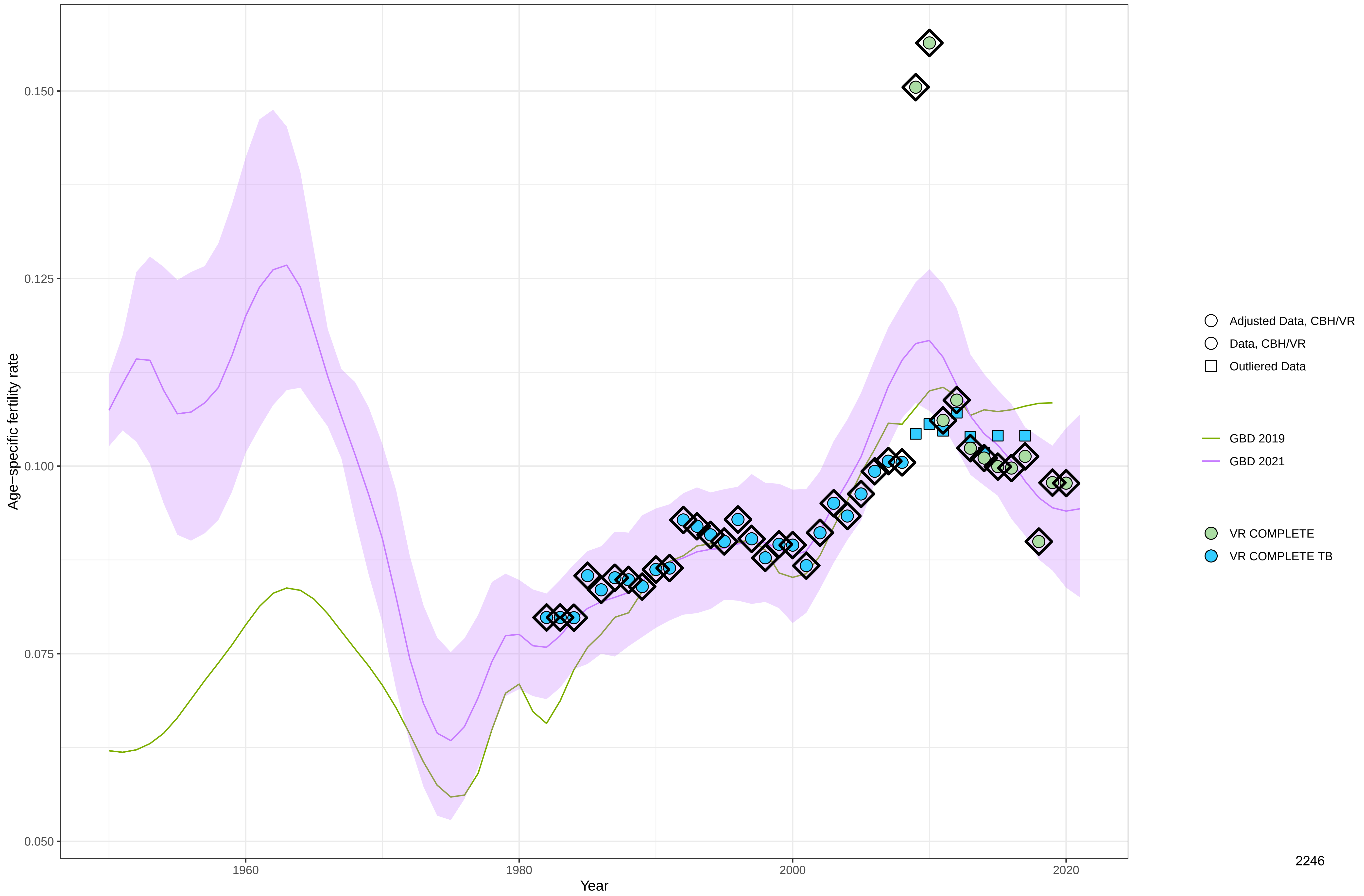

Age−specific fertility fitted trend lines, 1950−2021: Brent, Ages: 35−39
Data points enclosed with a diamond are reference sources

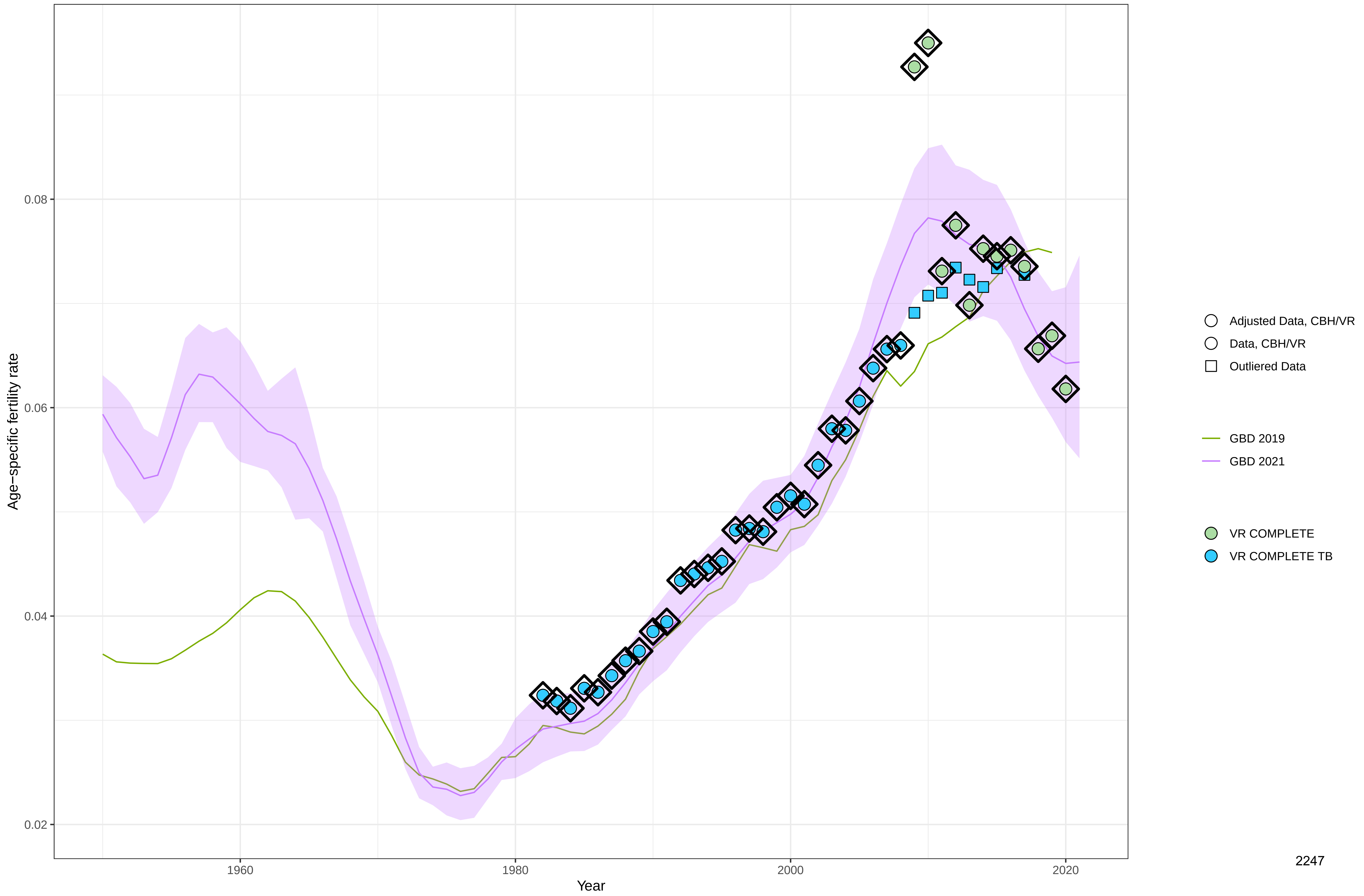

Age−specific fertility fitted trend lines, 1950−2021: Brent, Ages: 40−44
Data points enclosed with a diamond are reference sources

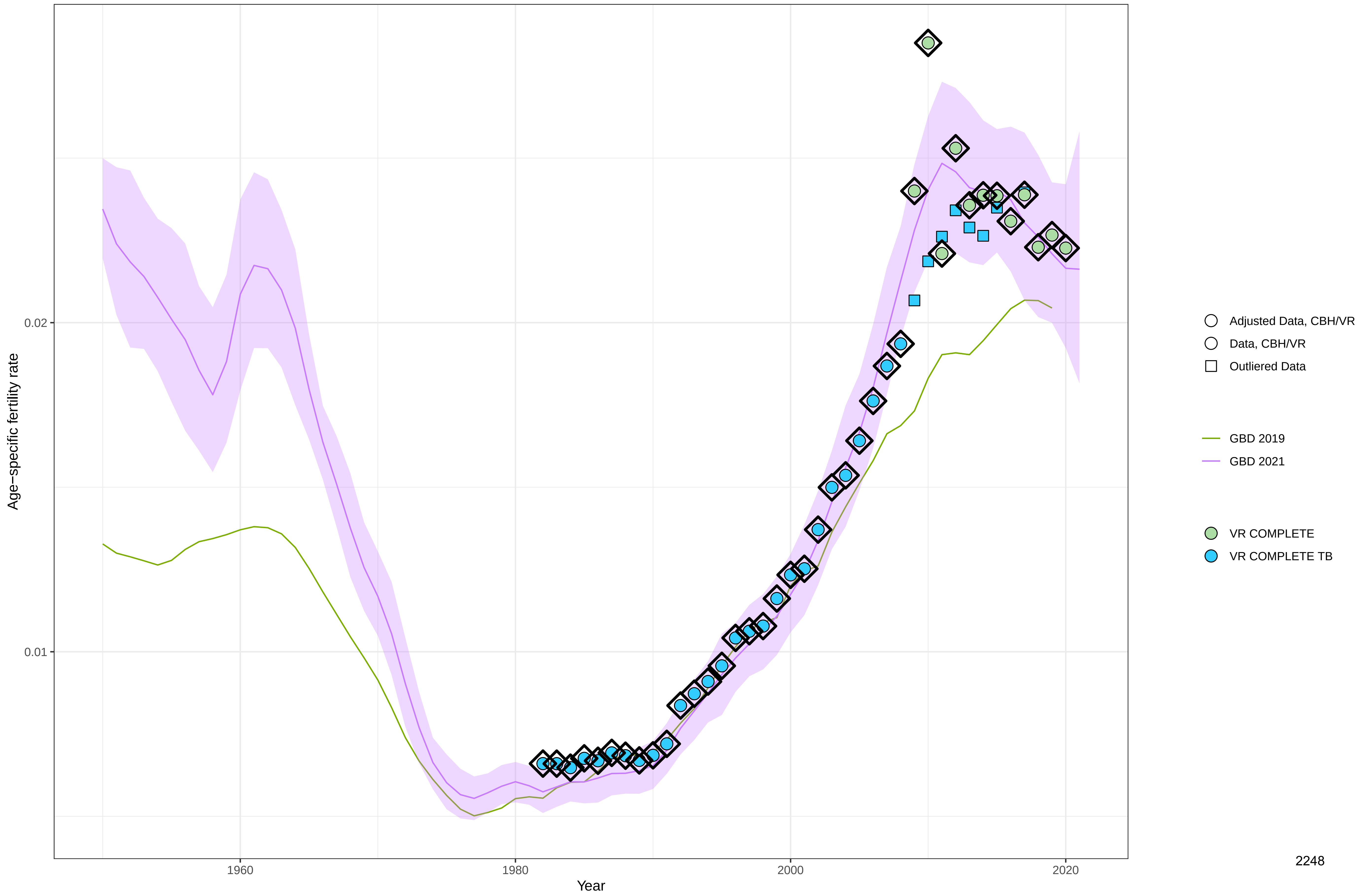

Age−specific fertility fitted trend lines, 1950−2021: Brent, Ages: 45−49
Data points enclosed with a diamond are reference sources

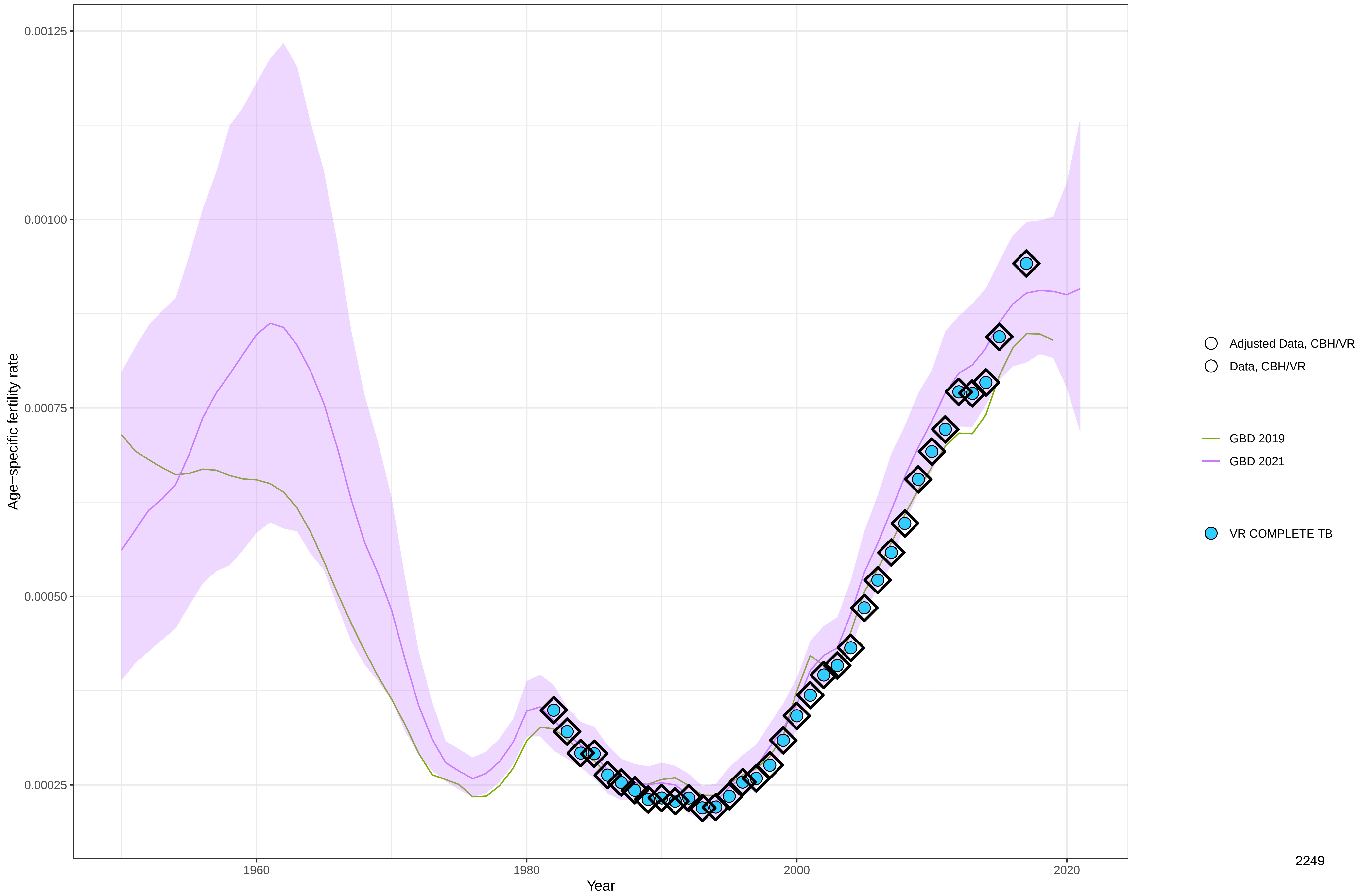

Age−specific fertility fitted trend lines, 1950−2021: Brent, Ages: 50−54
Data points enclosed with a diamond are reference sources

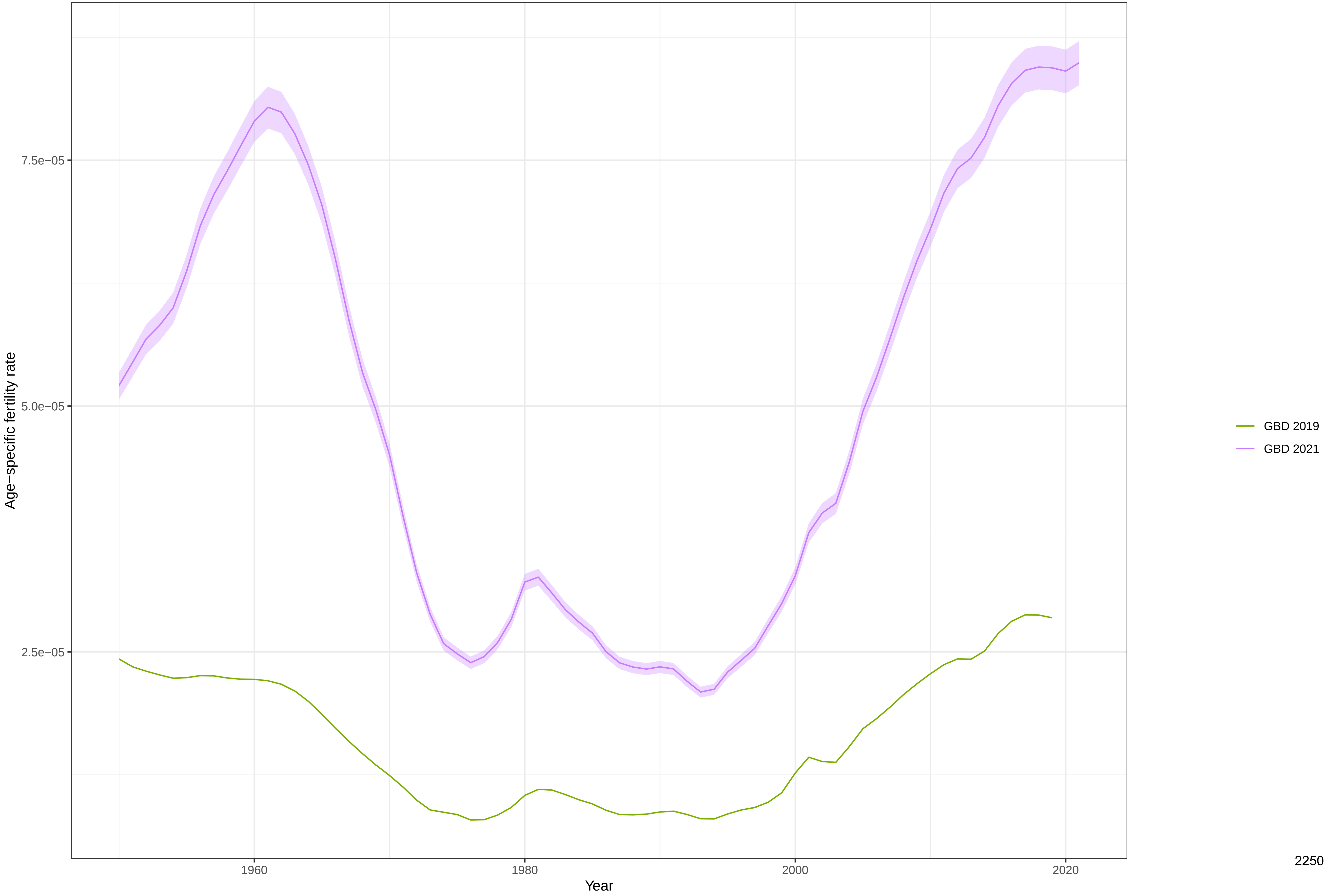

Total fertility rate fitted trend lines, 1950−2021: Brent TFR
Data points enclosed with a diamond are reference sources

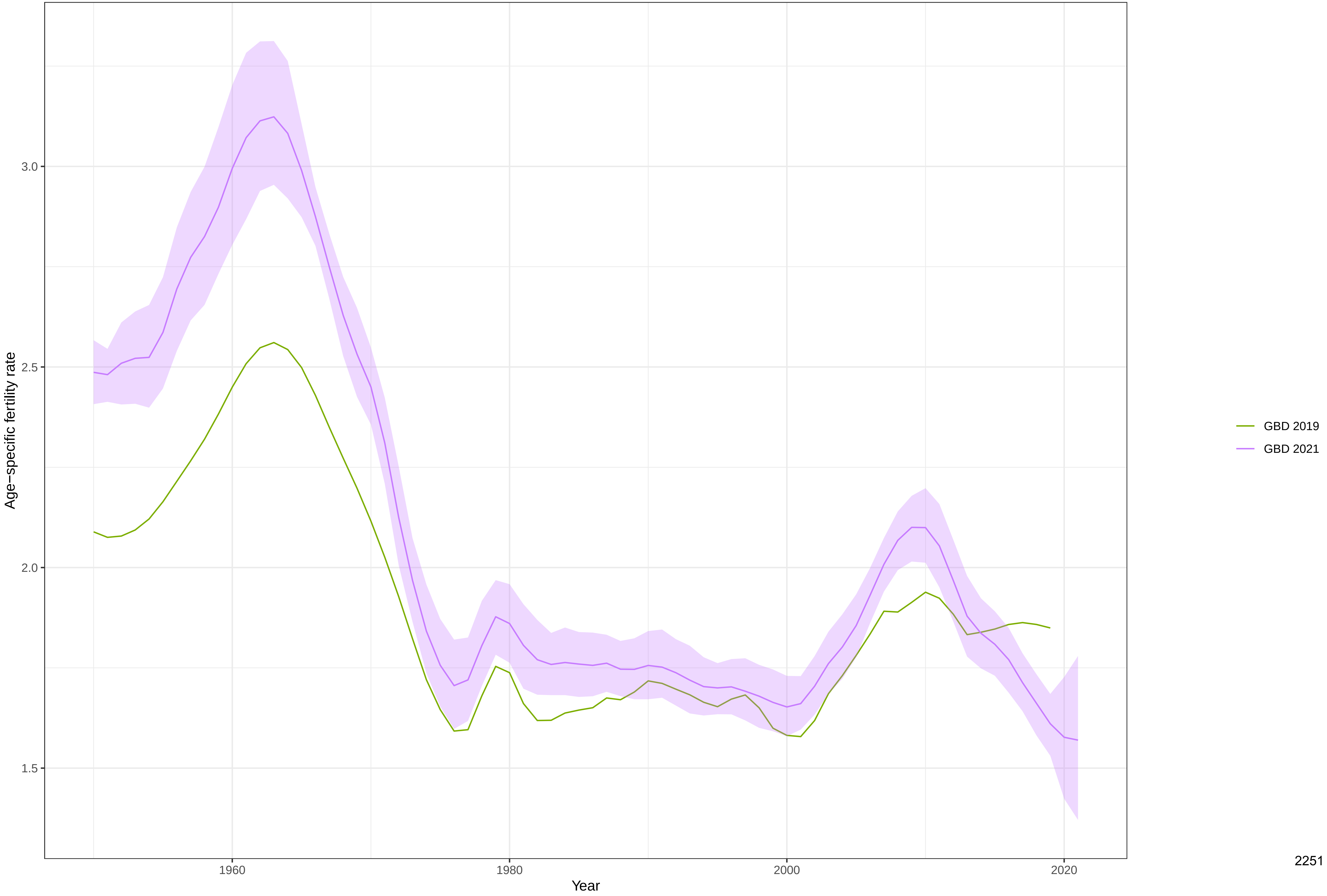

Age−specific fertility fitted trend lines, 1950−2021: Bromley, Ages: 10−14
Data points enclosed with a diamond are reference sources

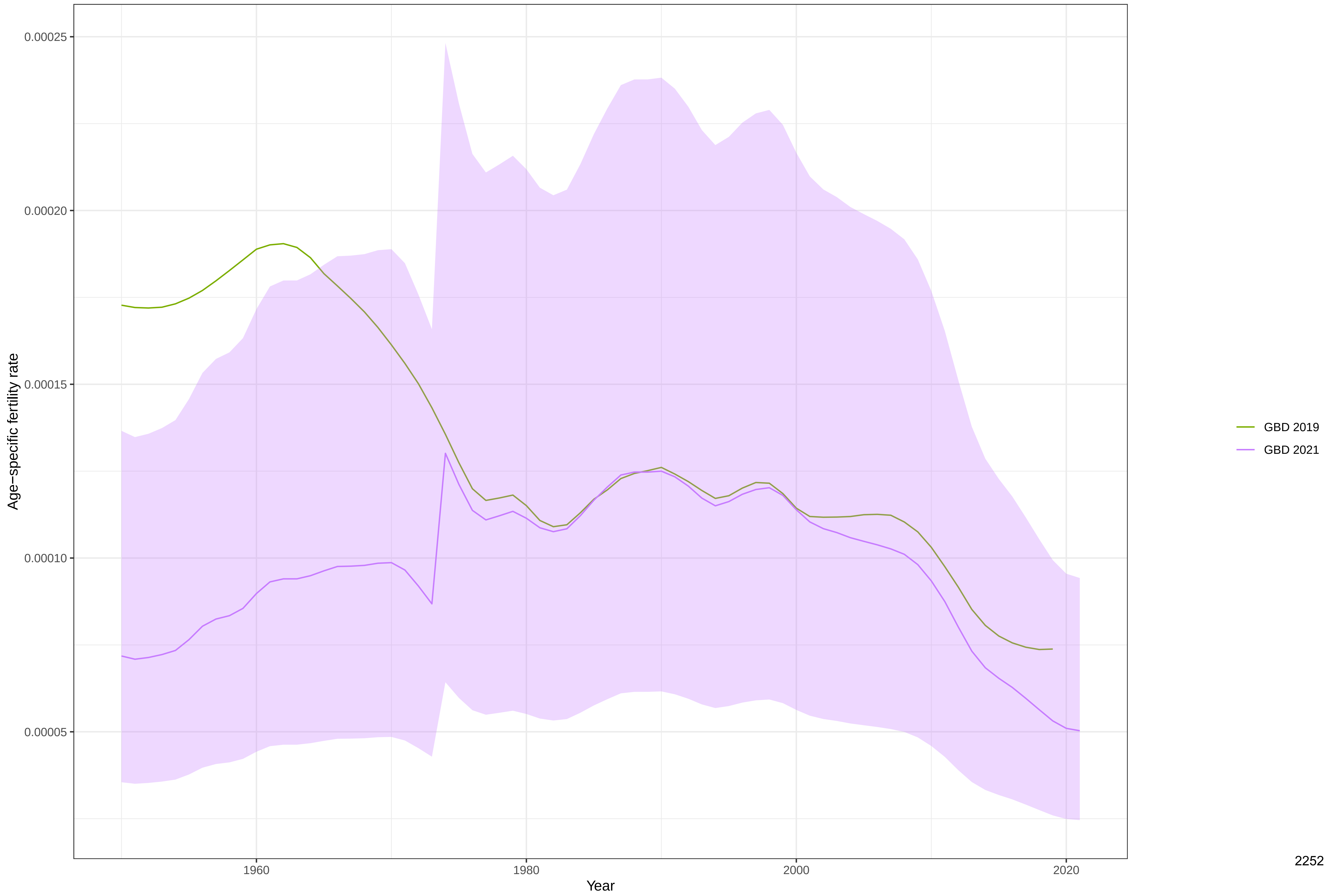

Age−specific fertility fitted trend lines, 1950−2021: Bromley, Ages: 15−19
Data points enclosed with a diamond are reference sources

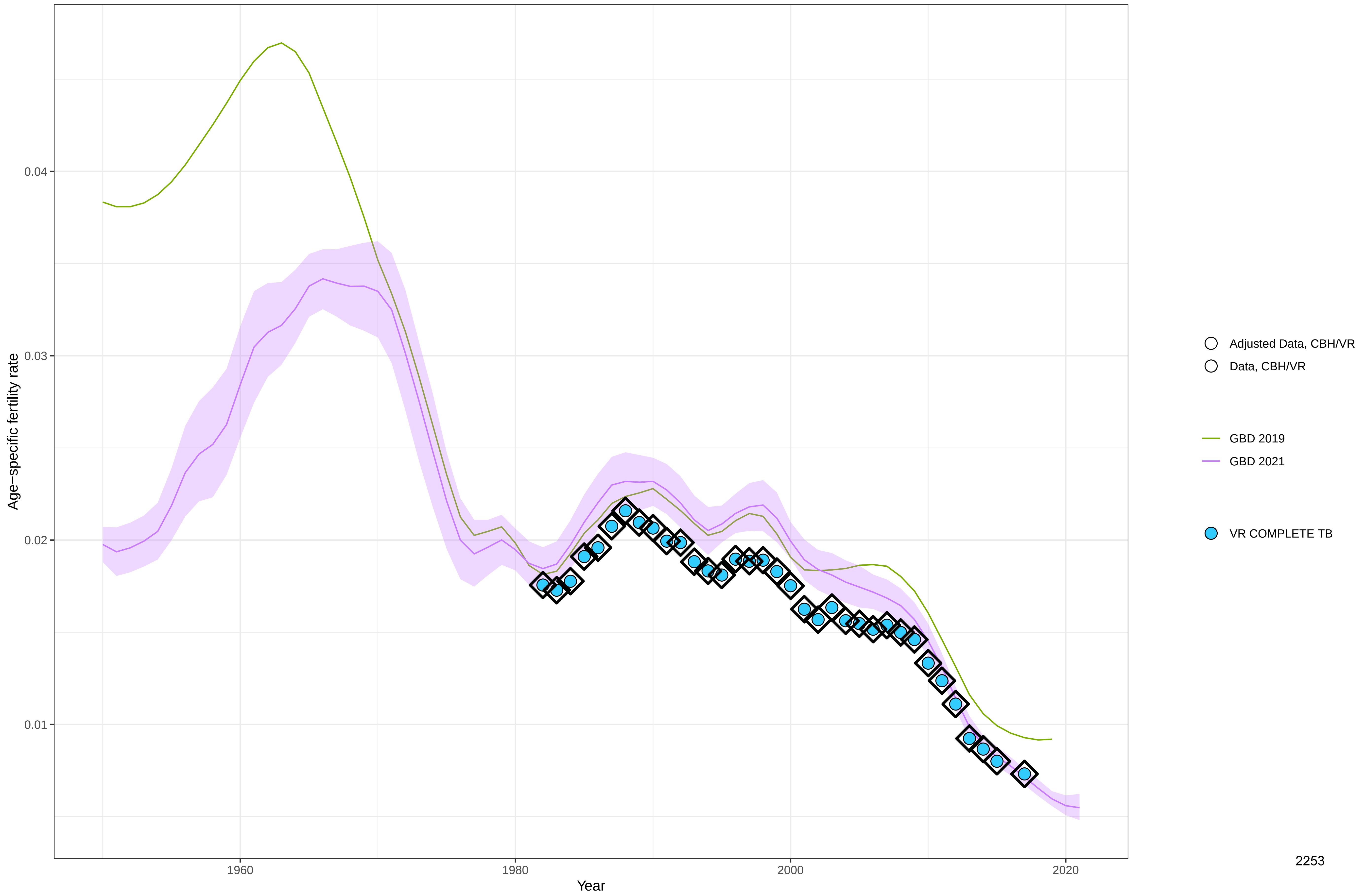

Age−specific fertility fitted trend lines, 1950−2021: Bromley, Ages: 20−24
Data points enclosed with a diamond are reference sources

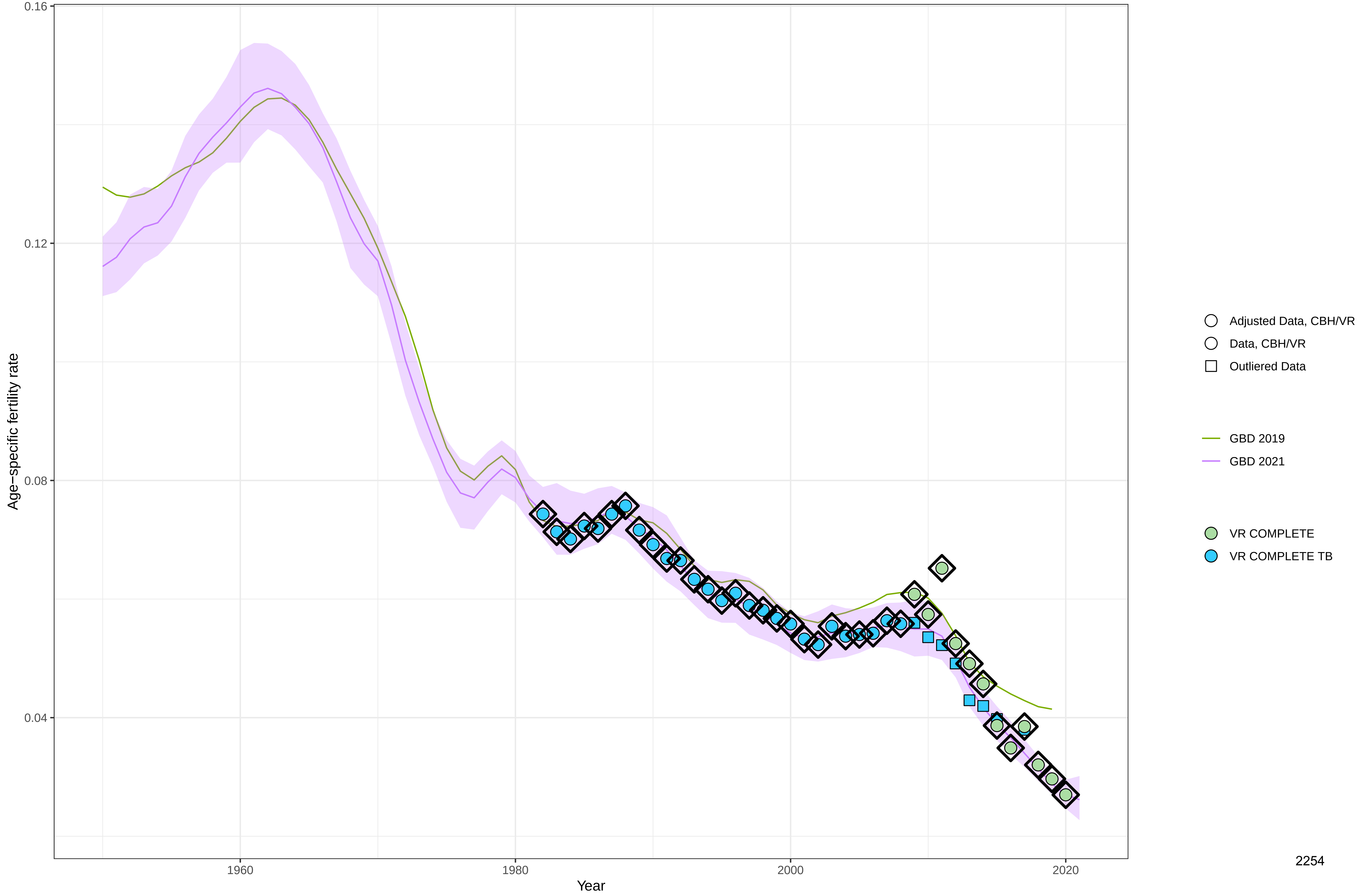

Age−specific fertility fitted trend lines, 1950−2021: Bromley, Ages: 25−29
Data points enclosed with a diamond are reference sources

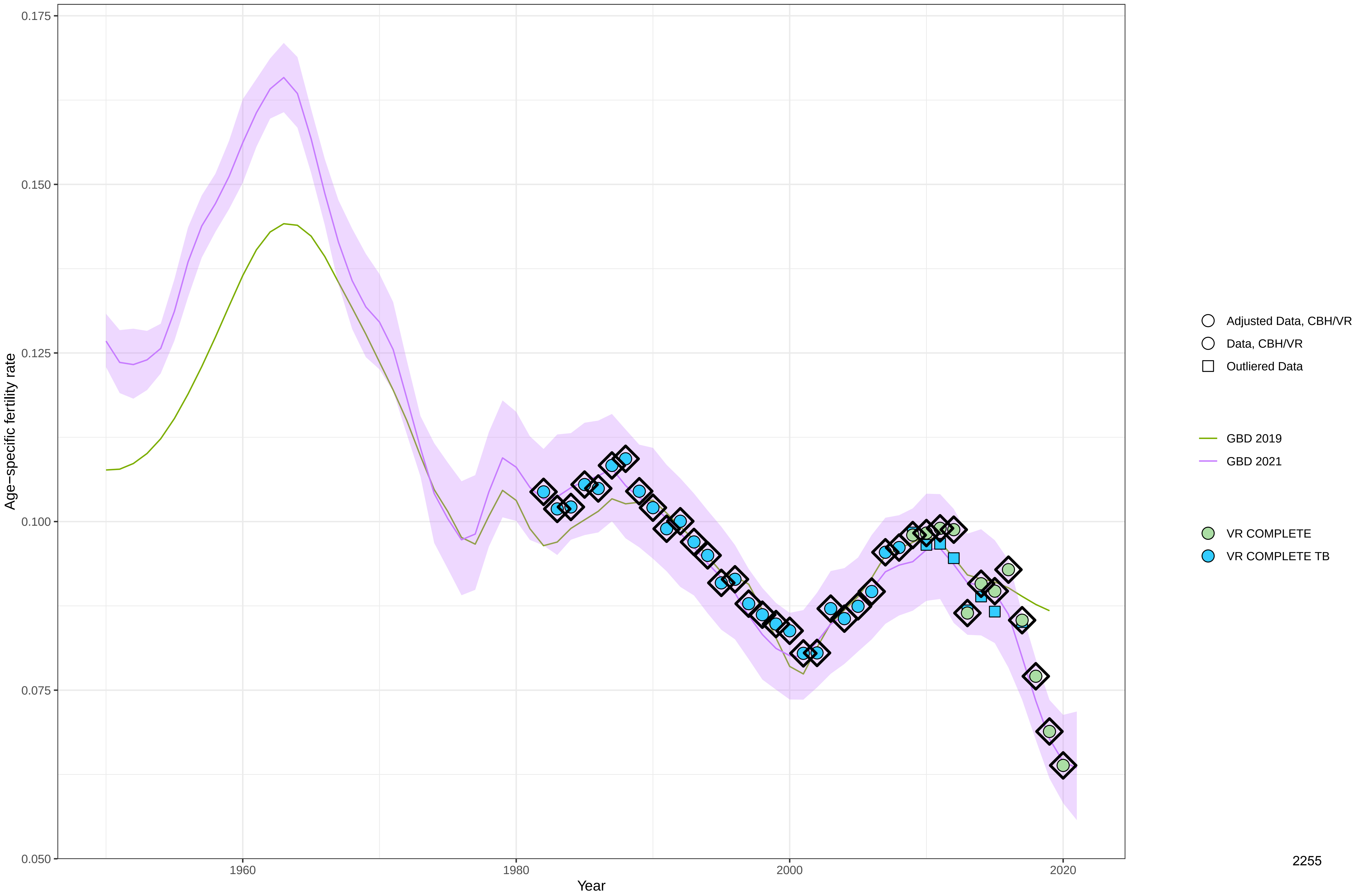

Age−specific fertility fitted trend lines, 1950−2021: Bromley, Ages: 30−34
Data points enclosed with a diamond are reference sources

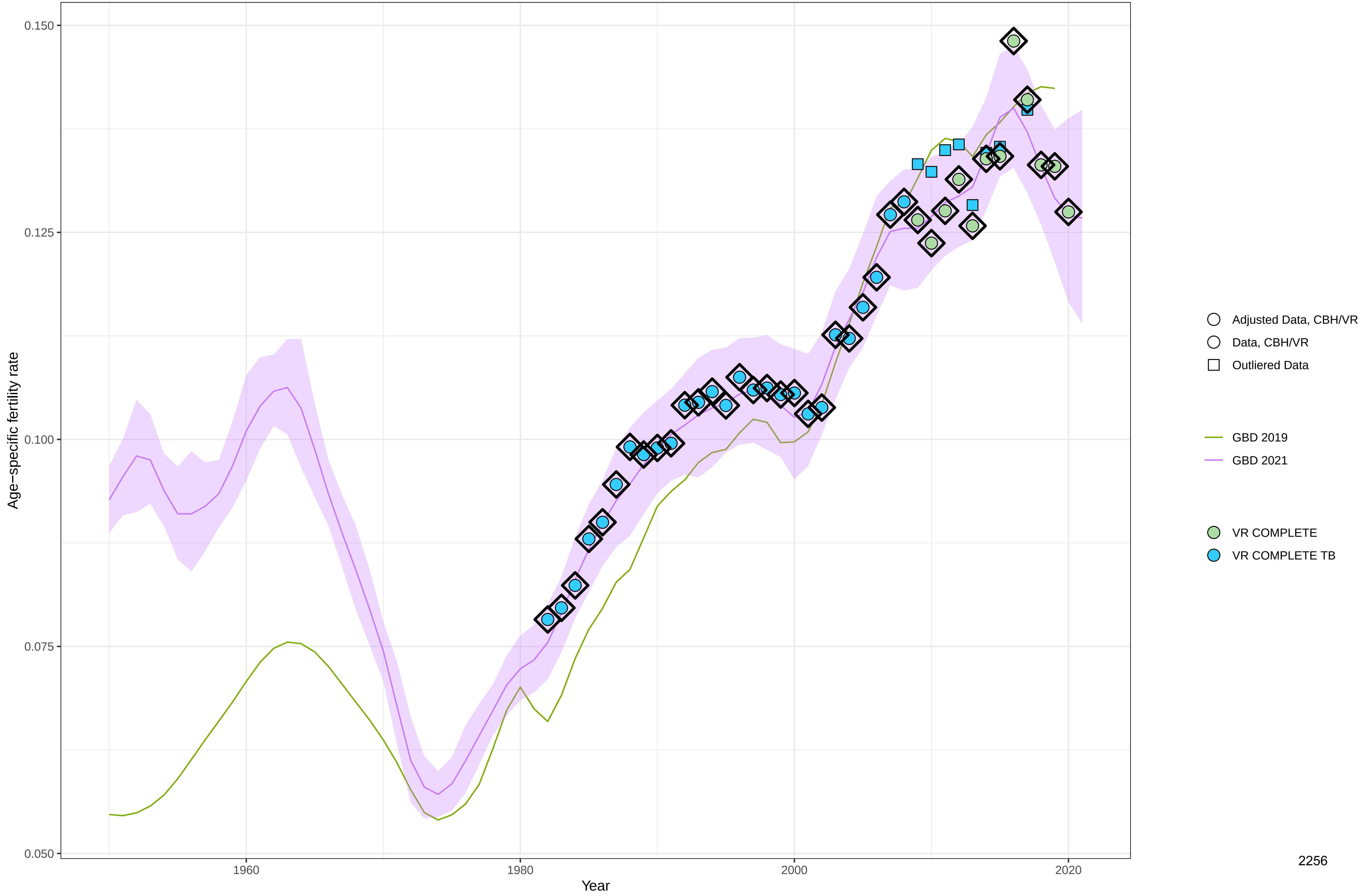

Age−specific fertility fitted trend lines, 1950−2021: Bromley, Ages: 35−39
Data points enclosed with a diamond are reference sources

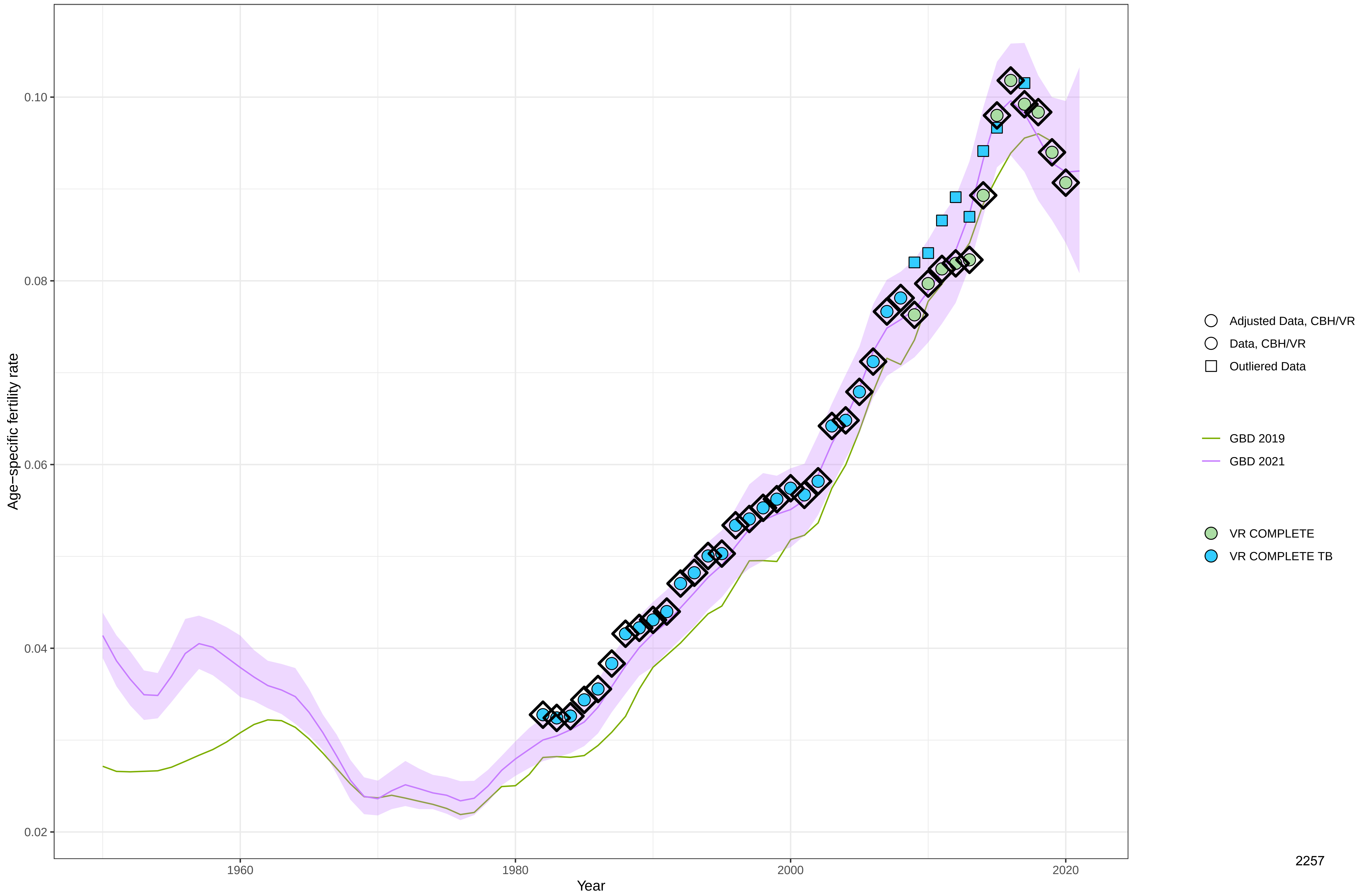

Age−specific fertility fitted trend lines, 1950−2021: Bromley, Ages: 40−44
Data points enclosed with a diamond are reference sources

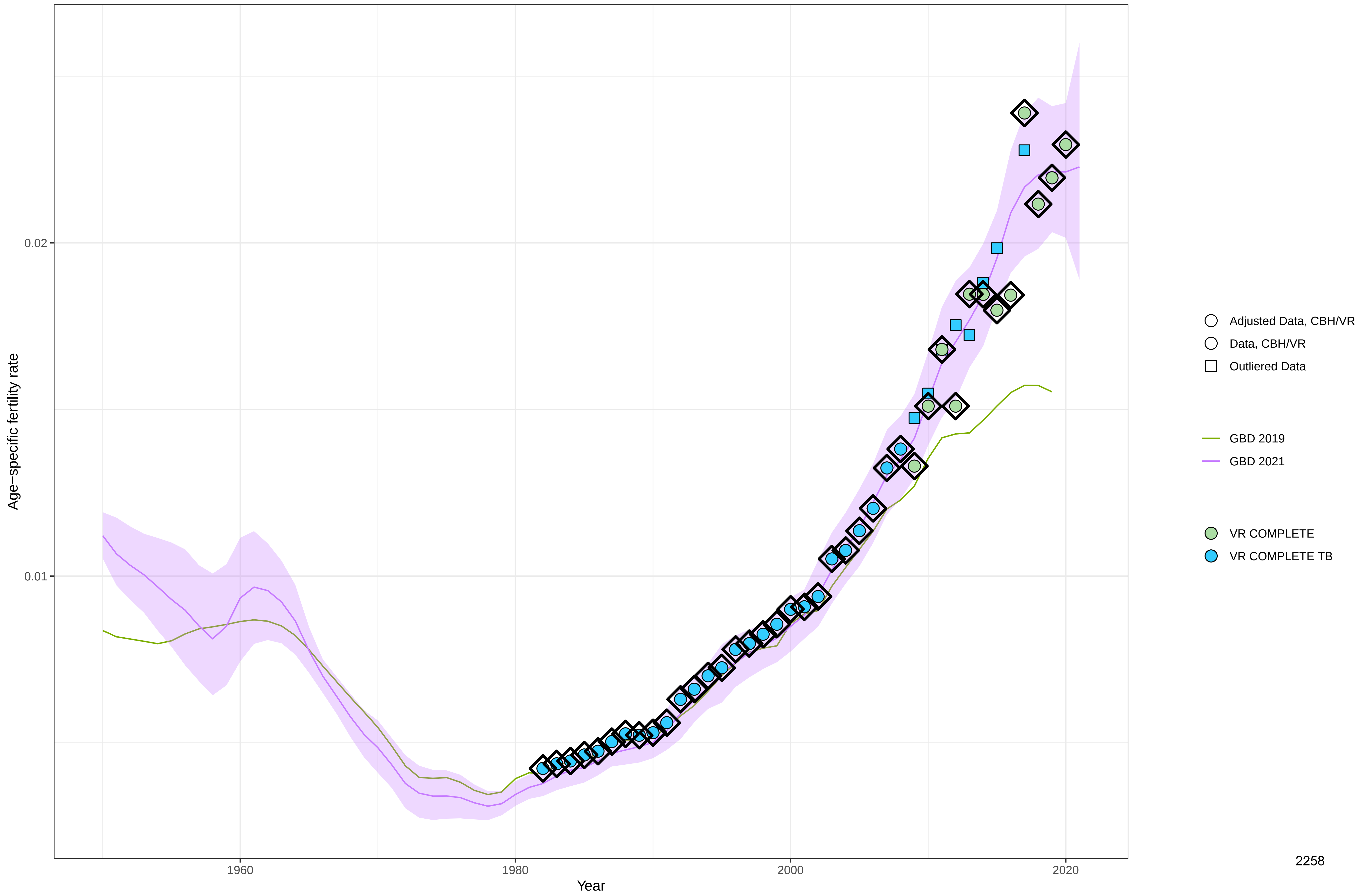

Age−specific fertility fitted trend lines, 1950−2021: Bromley, Ages: 45−49
Data points enclosed with a diamond are reference sources

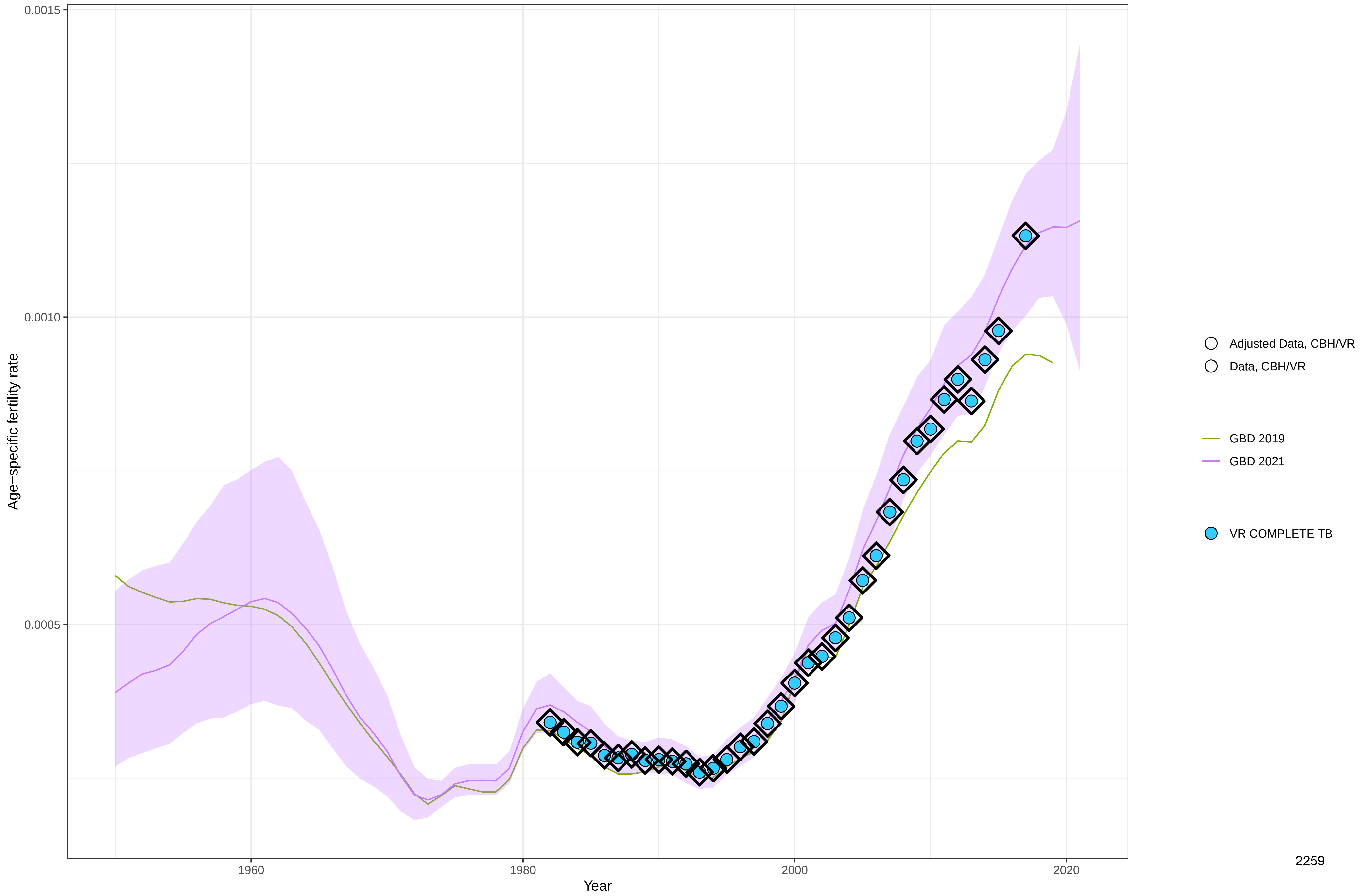

Age−specific fertility fitted trend lines, 1950−2021: Bromley, Ages: 50−54
Data points enclosed with a diamond are reference sources

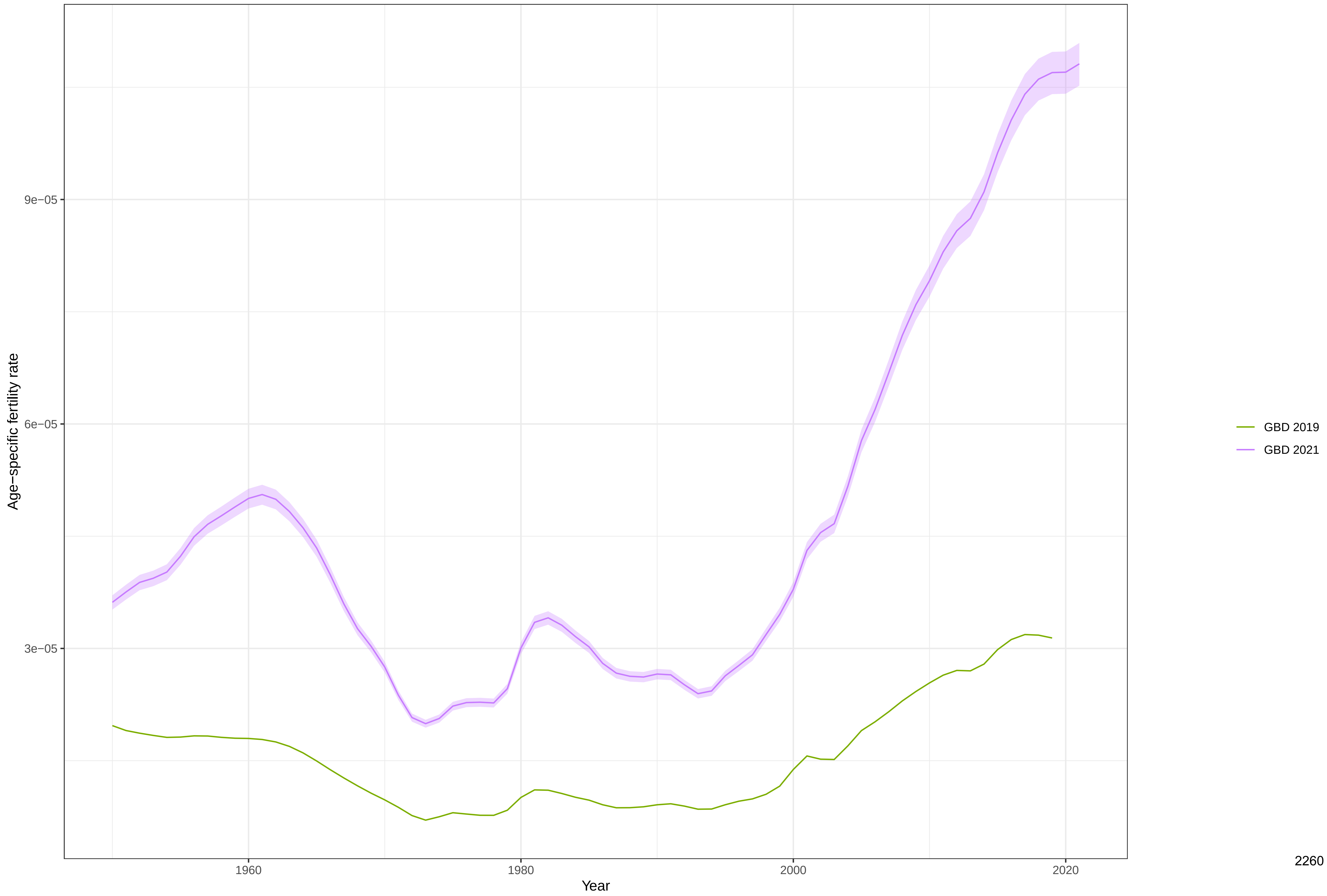

Total fertility rate fitted trend lines, 1950−2021: Bromley TFR
Data points enclosed with a diamond are reference sources

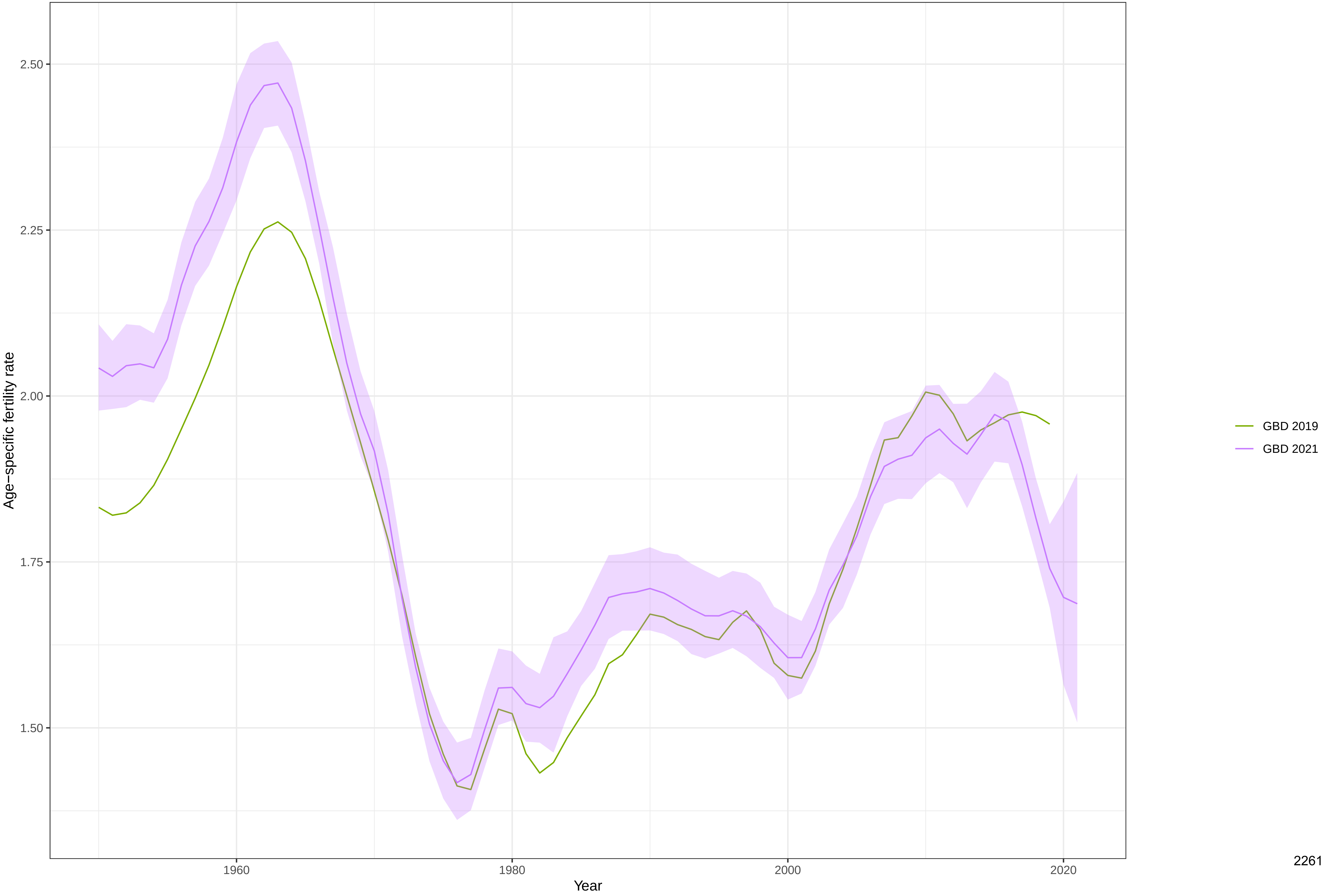

Age−specific fertility fitted trend lines, 1950−2021: Camden, Ages: 10−14
Data points enclosed with a diamond are reference sources

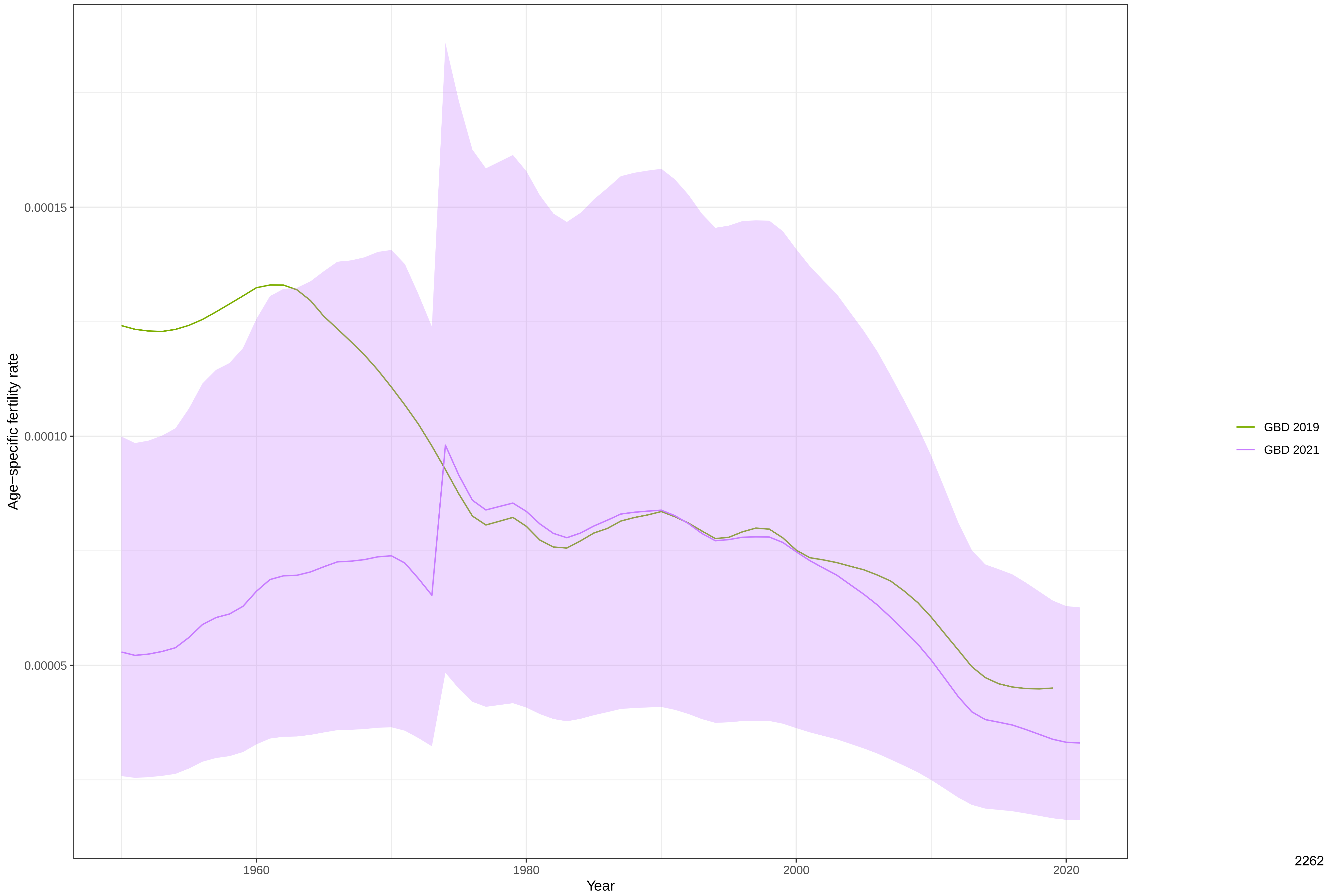

Age−specific fertility fitted trend lines, 1950−2021: Camden, Ages: 15−19
Data points enclosed with a diamond are reference sources

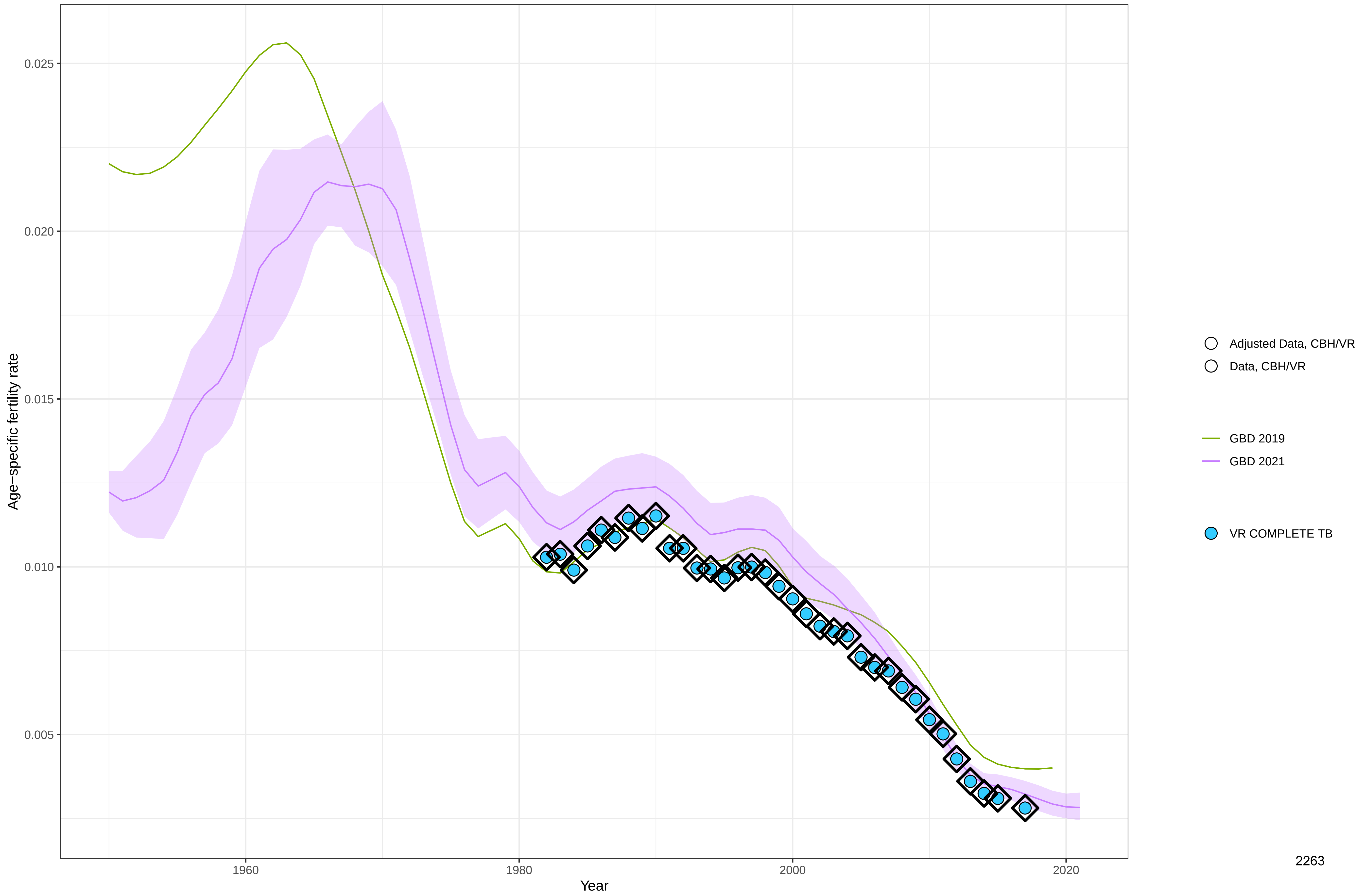

Age−specific fertility fitted trend lines, 1950−2021: Camden, Ages: 20−24
Data points enclosed with a diamond are reference sources

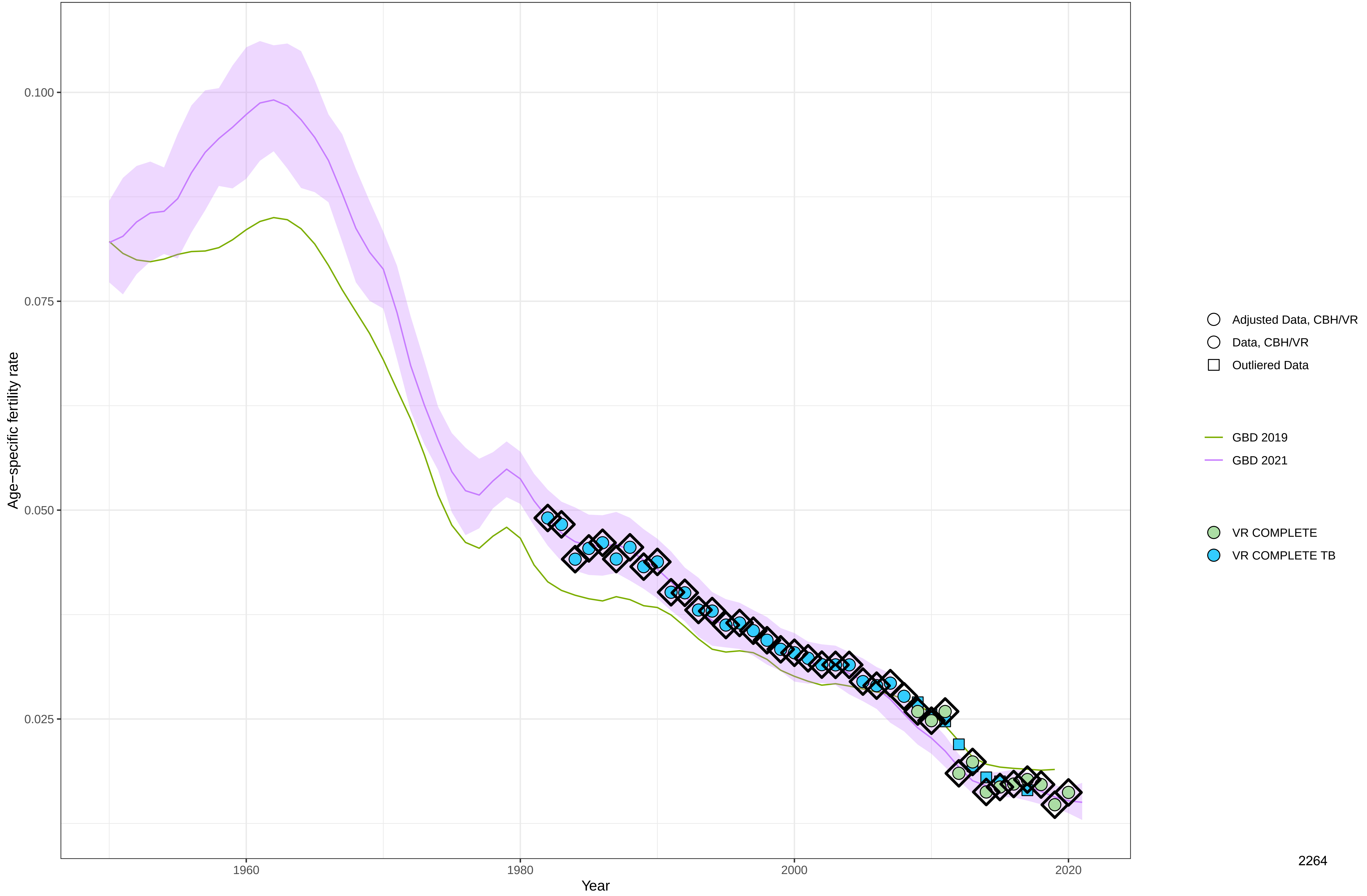

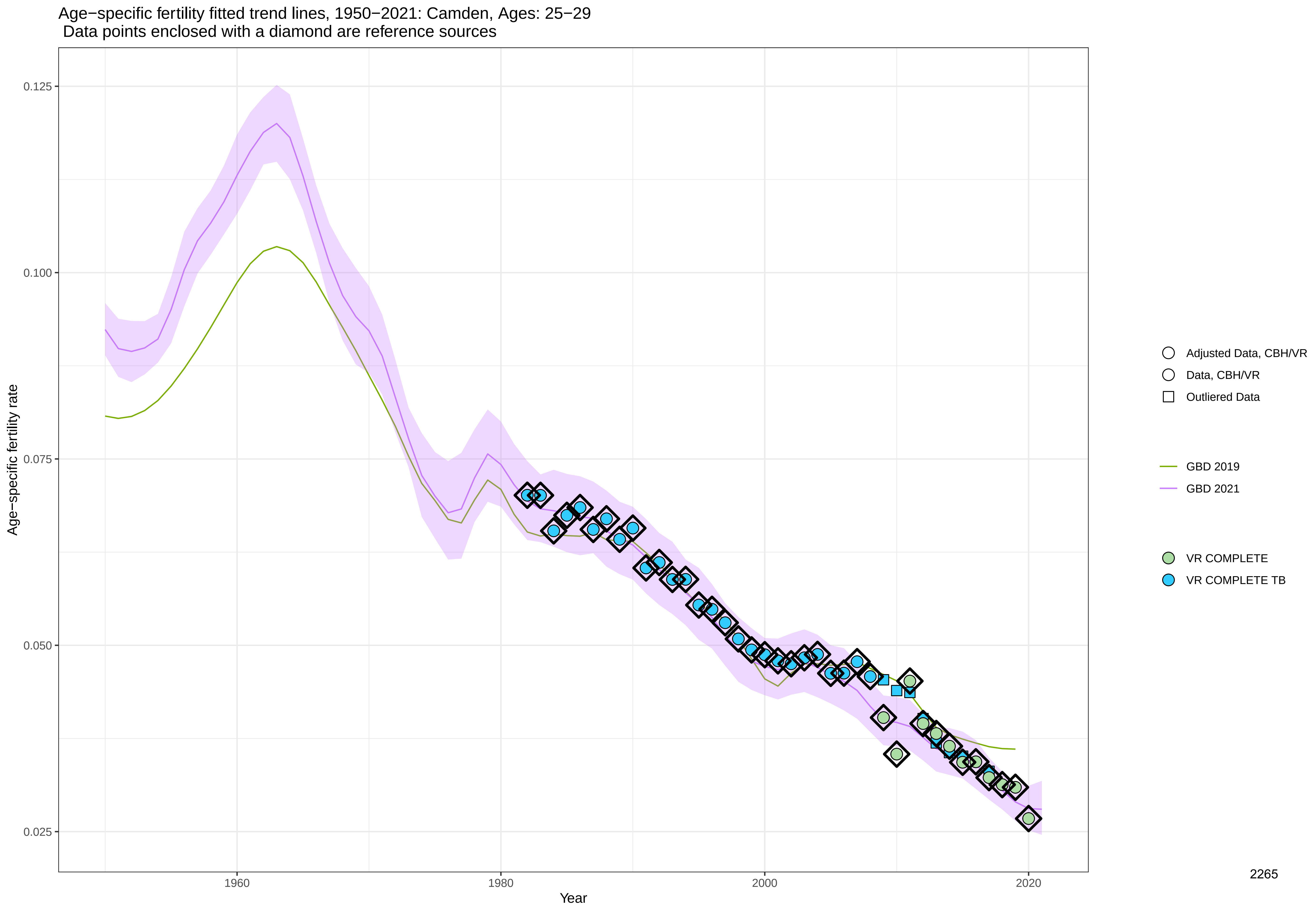

Age−specific fertility fitted trend lines, 1950−2021: Camden, Ages: 25−29
Data points enclosed with a diamond are reference sources
Age−specific fertility rate
0.125
0.100
0.075
0.050
0.025
1960
1980
2000
2020
Year
Adjusted Data, CBH/VR
Data, CBH/VR
Outliered Data
GBD 2019
GBD 2021
VR COMPLETE
VR COMPLETE TB

2265

Age−specific fertility fitted trend lines, 1950−2021: Camden, Ages: 30−34
Data points enclosed with a diamond are reference sources

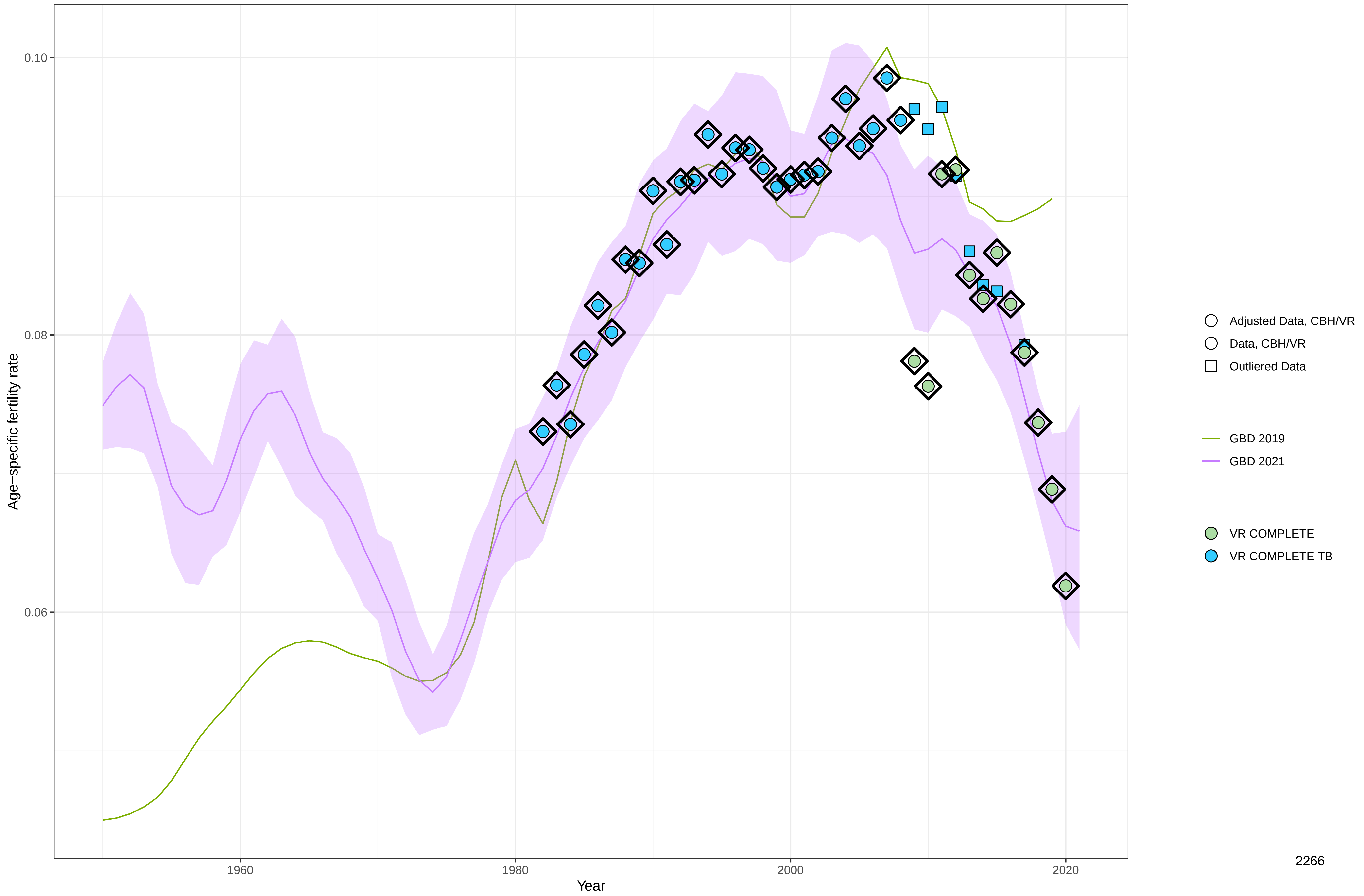

Age−specific fertility fitted trend lines, 1950−2021: Camden, Ages: 35−39
Data points enclosed with a diamond are reference sources

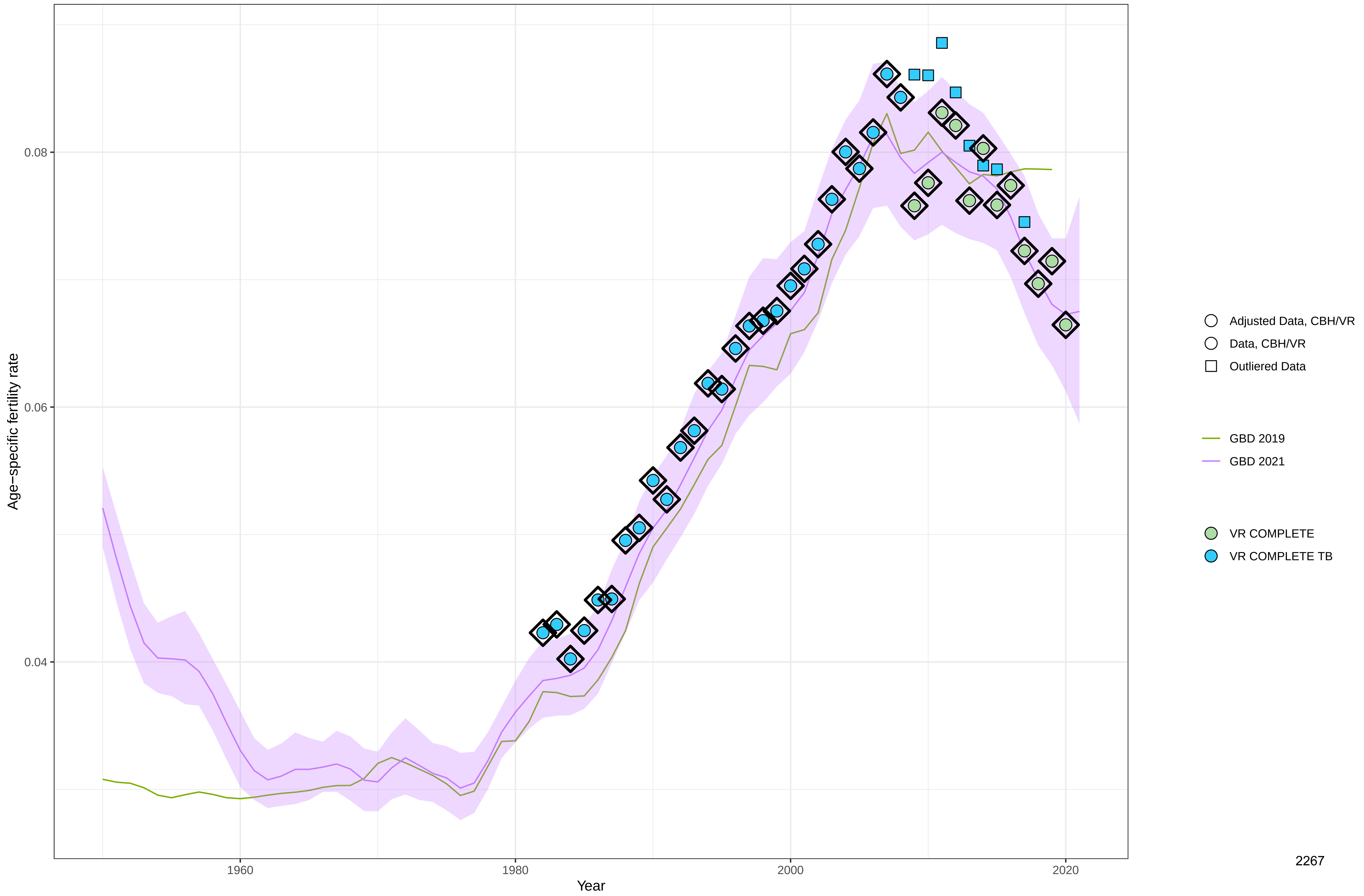

Age−specific fertility fitted trend lines, 1950−2021: Camden, Ages: 40−44
Data points enclosed with a diamond are reference sources

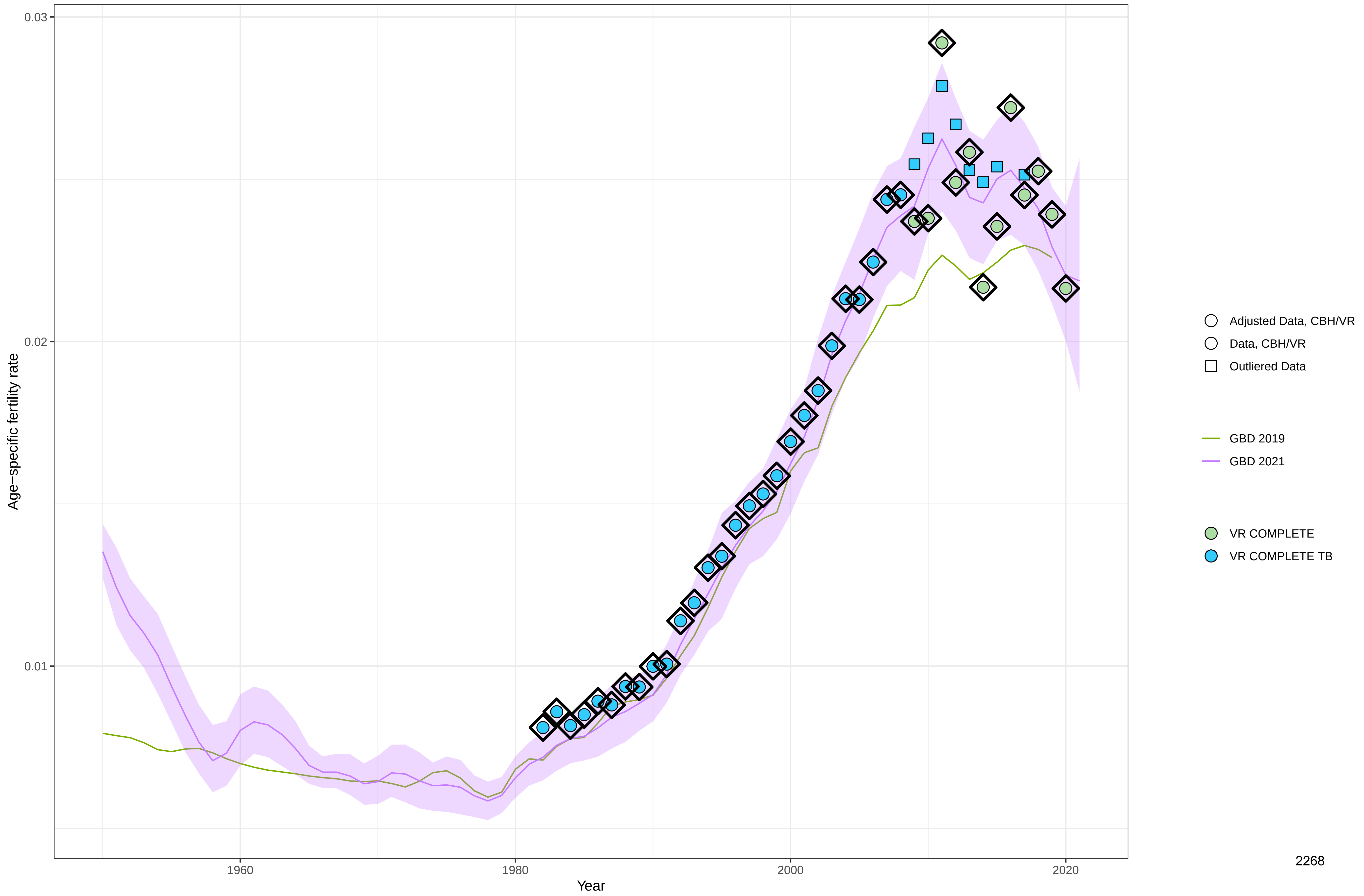

Age−specific fertility fitted trend lines, 1950−2021: Camden, Ages: 45−49
Data points enclosed with a diamond are reference sources

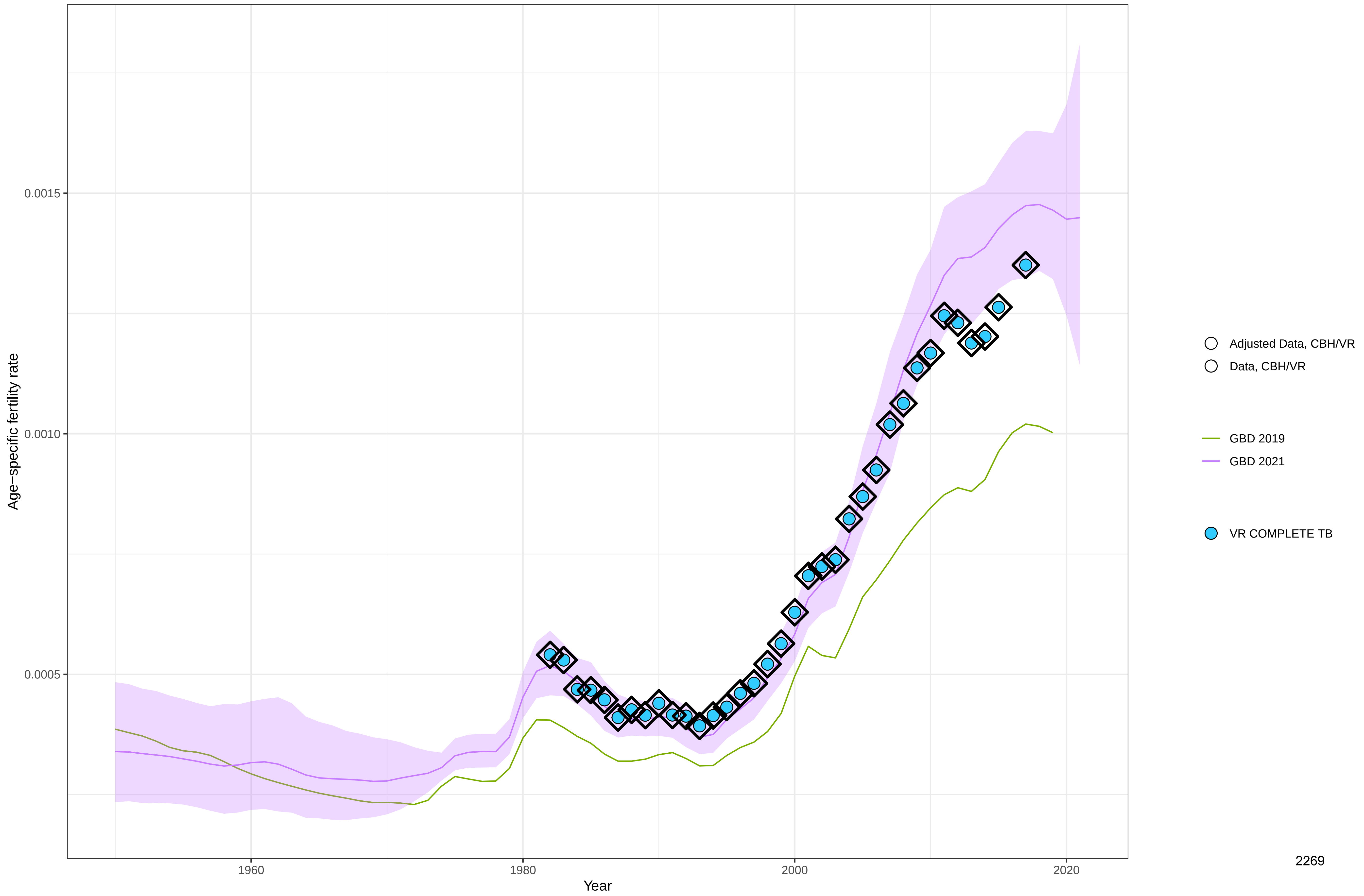

Age−specific fertility fitted trend lines, 1950−2021: Camden, Ages: 50−54
Data points enclosed with a diamond are reference sources

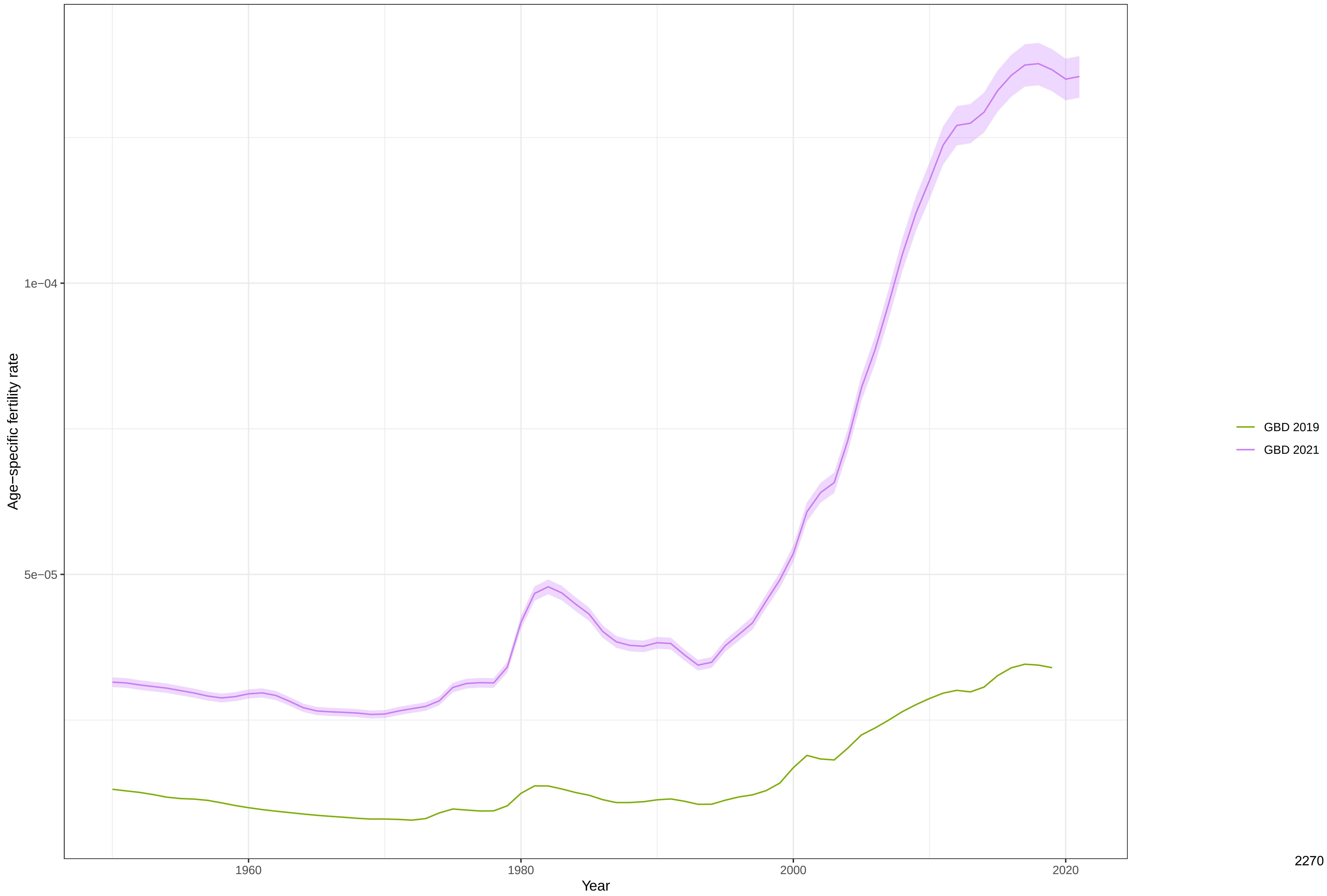

Total fertility rate fitted trend lines, 1950−2021: Camden TFR
Data points enclosed with a diamond are reference sources

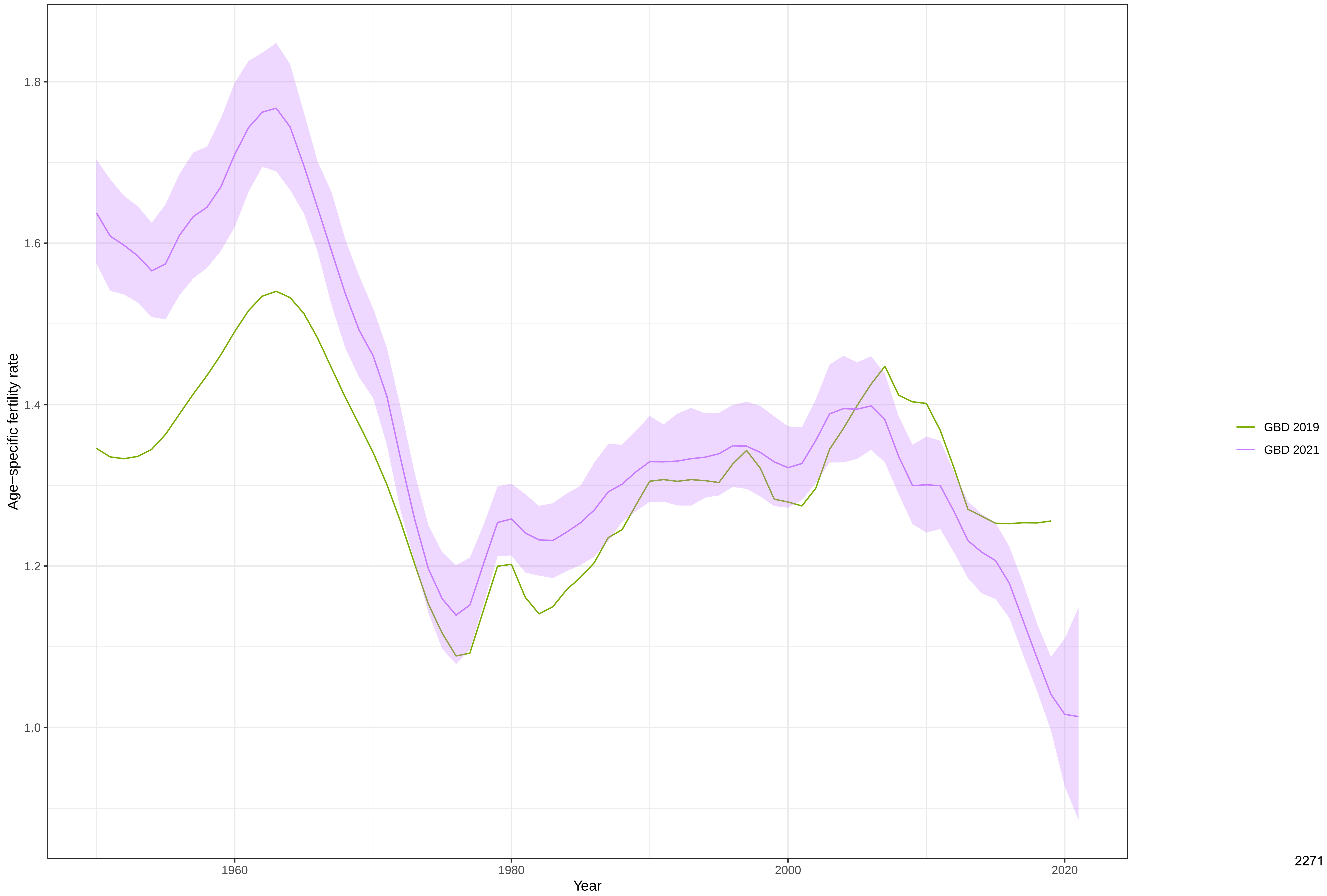

Age−specific fertility fitted trend lines, 1950−2021: Croydon, Ages: 10−14
Data points enclosed with a diamond are reference sources

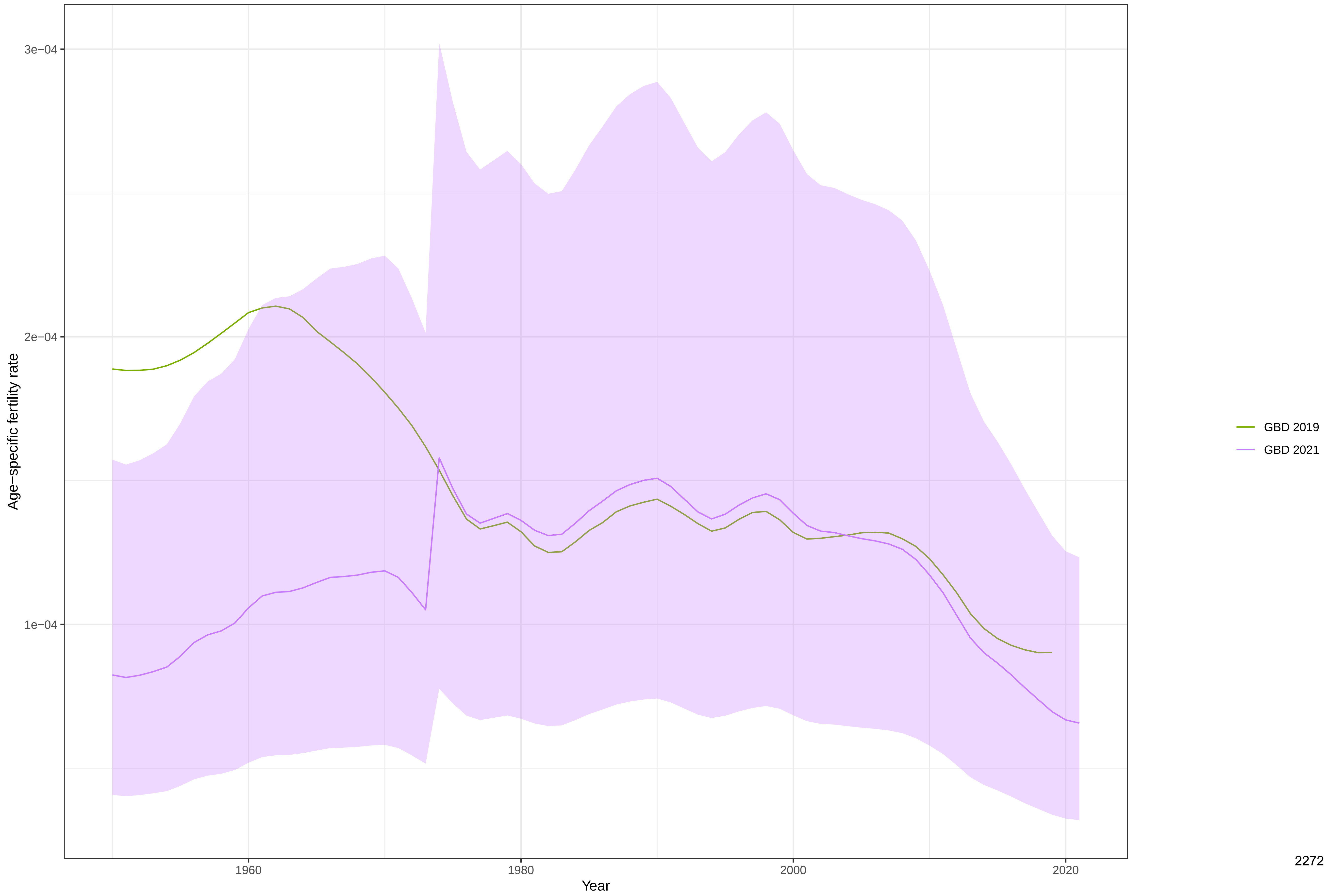

Age−specific fertility fitted trend lines, 1950−2021: Croydon, Ages: 15−19
Data points enclosed with a diamond are reference sources

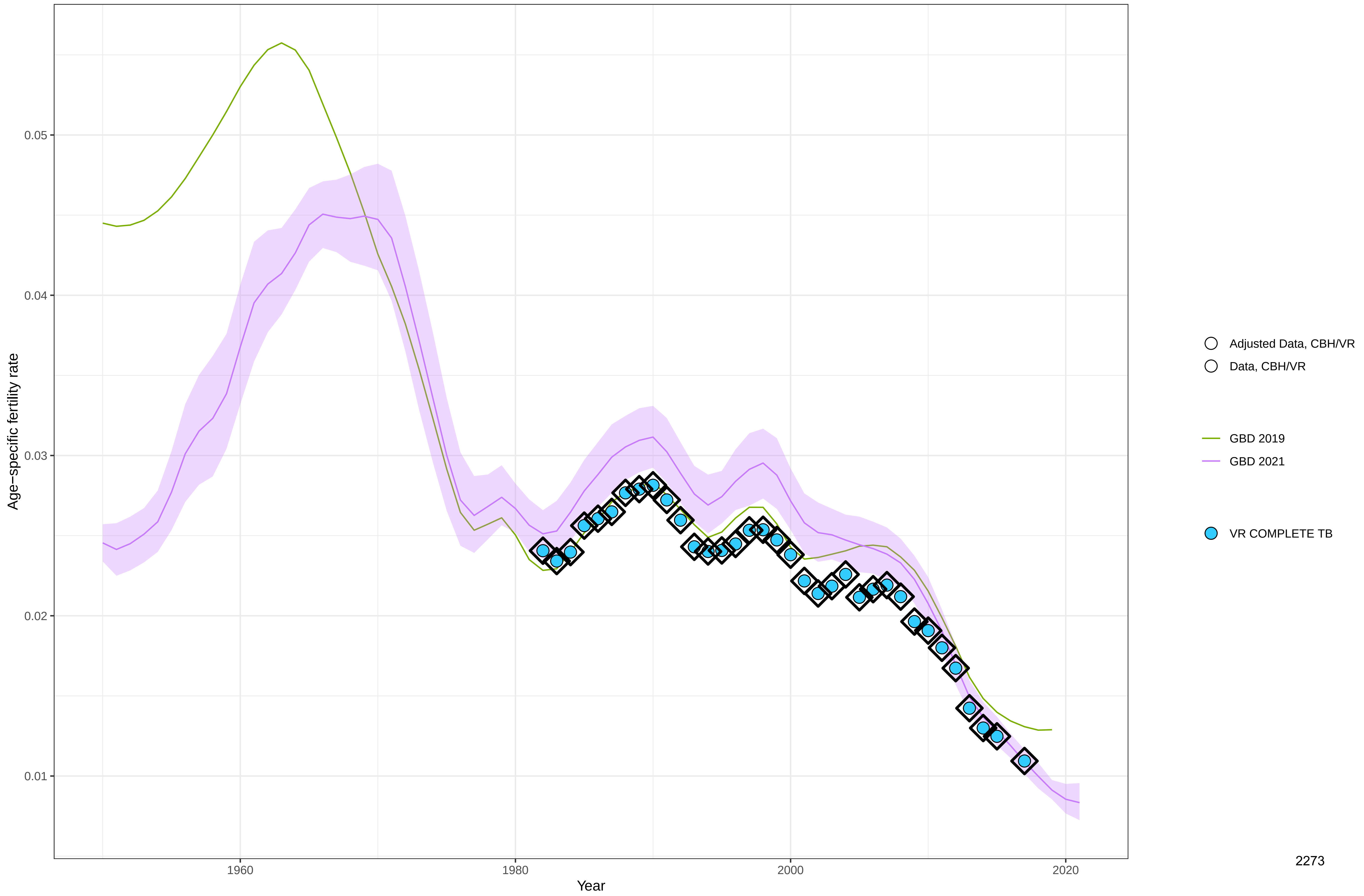

Age−specific fertility fitted trend lines, 1950−2021: Croydon, Ages: 20−24
Data points enclosed with a diamond are reference sources

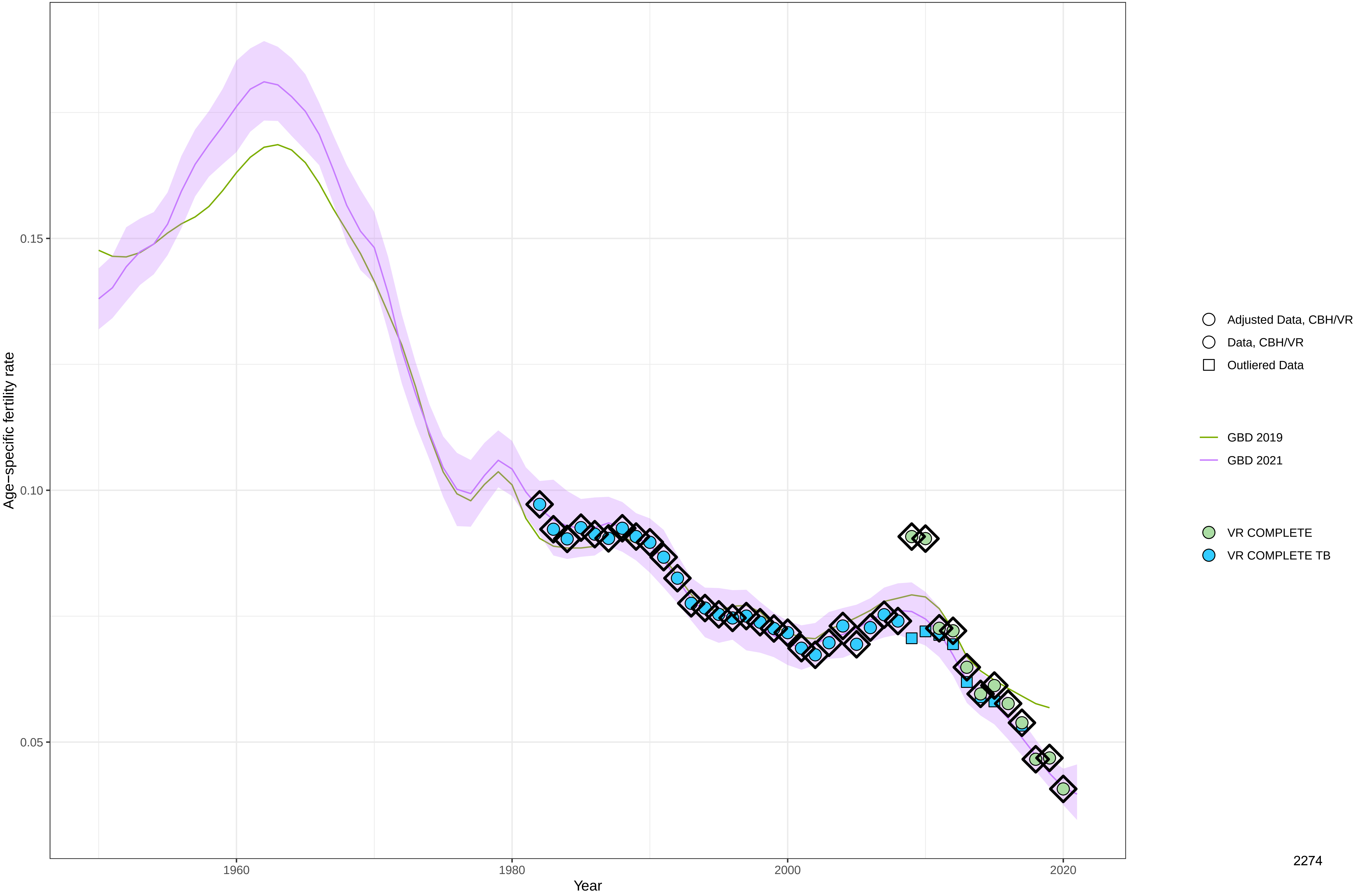

Age−specific fertility fitted trend lines, 1950−2021: Croydon, Ages: 25−29
Data points enclosed with a diamond are reference sources

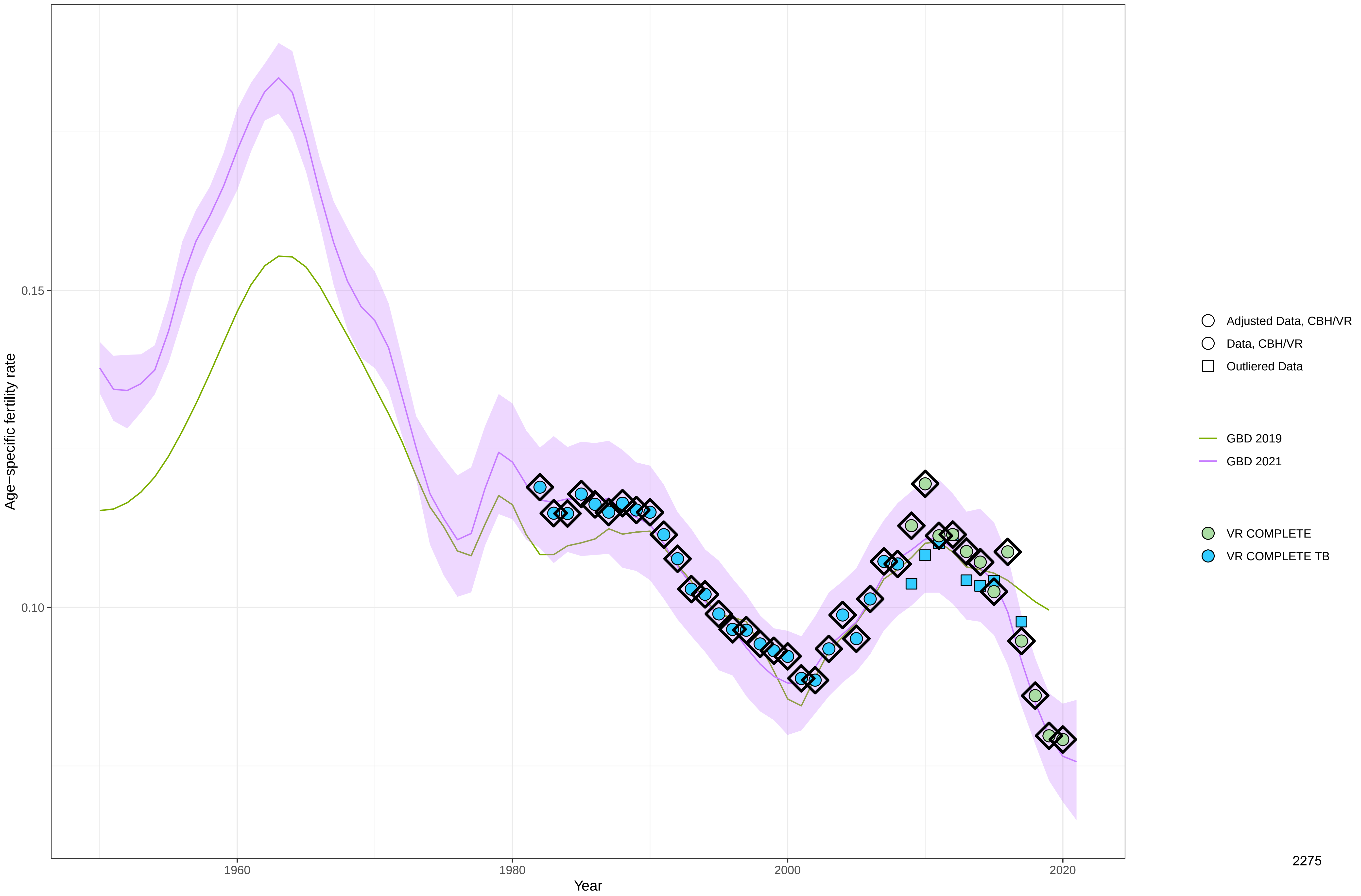

Age−specific fertility fitted trend lines, 1950−2021: Croydon, Ages: 30−34
Data points enclosed with a diamond are reference sources

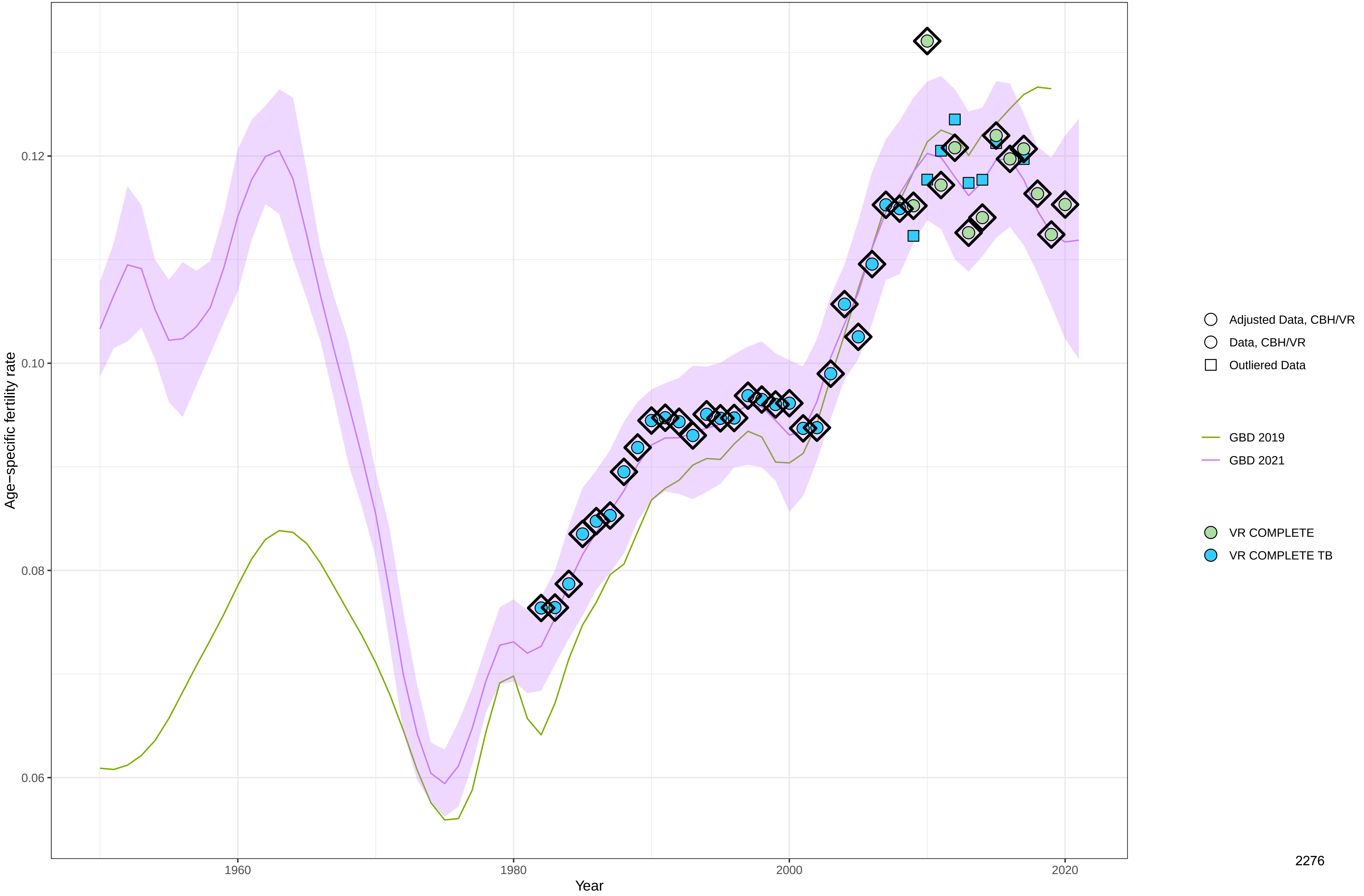

Age−specific fertility fitted trend lines, 1950−2021: Croydon, Ages: 35−39
Data points enclosed with a diamond are reference sources

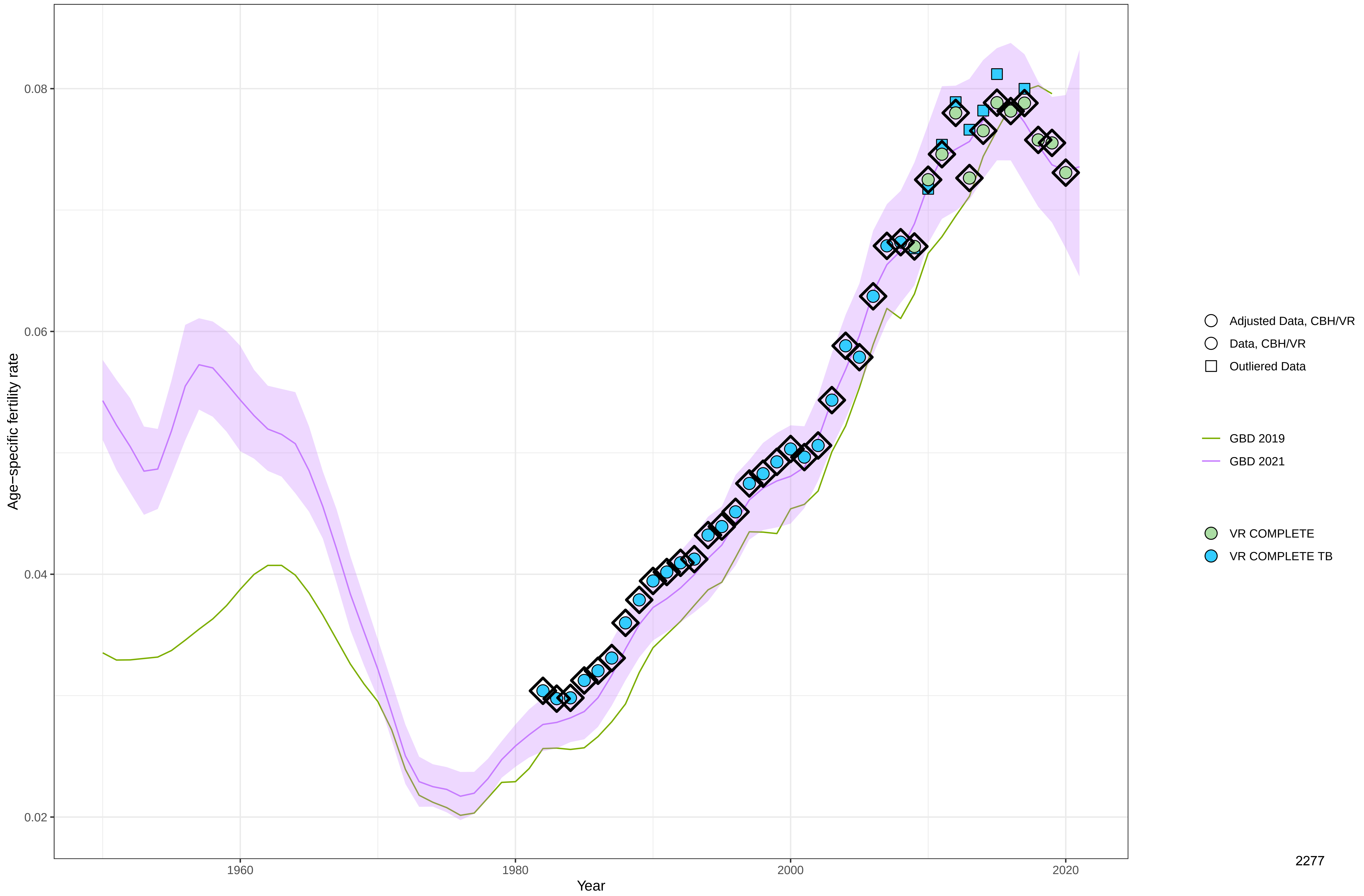

Age−specific fertility fitted trend lines, 1950−2021: Croydon, Ages: 40−44
Data points enclosed with a diamond are reference sources

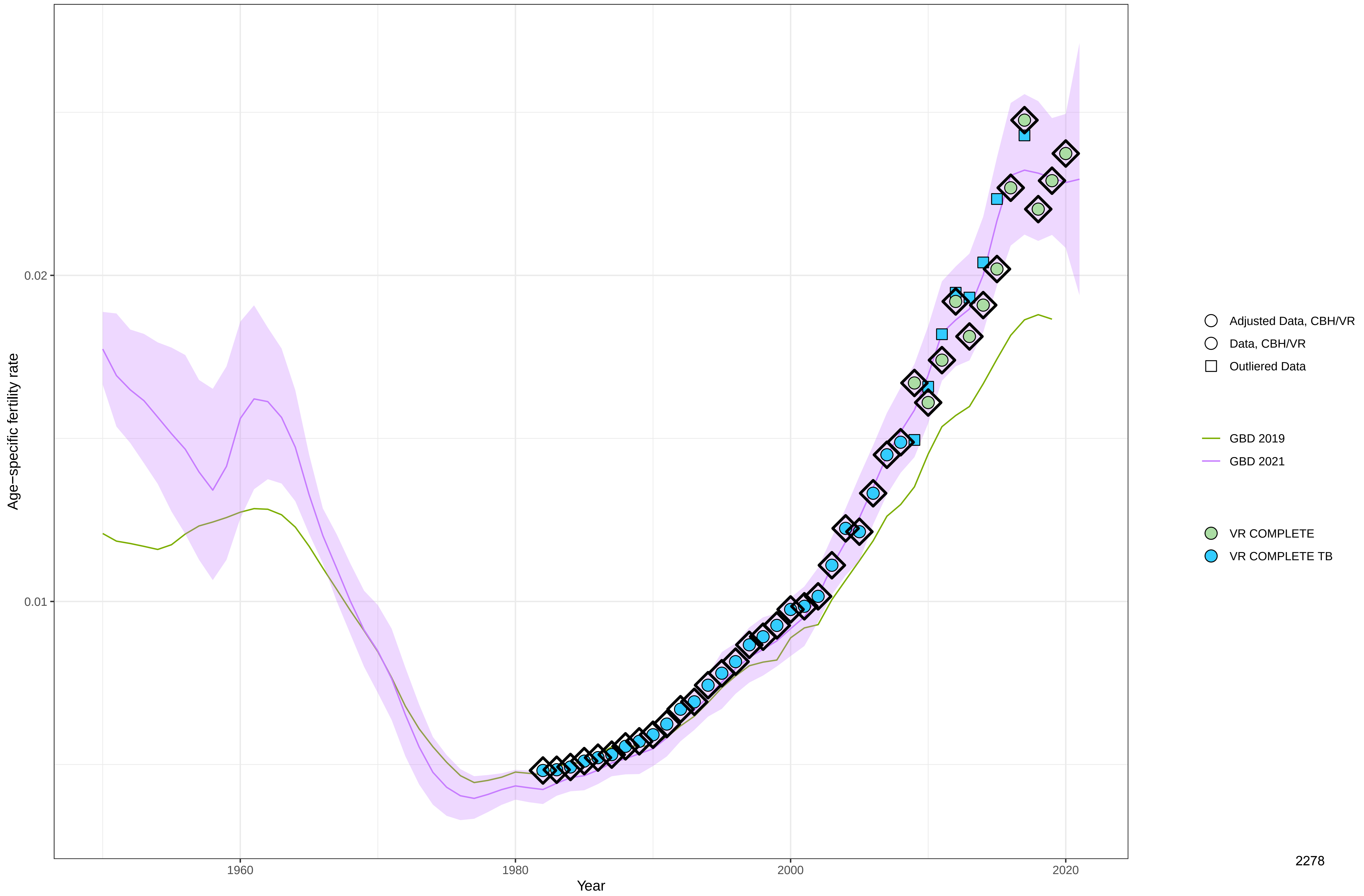

Age−specific fertility fitted trend lines, 1950−2021: Croydon, Ages: 45−49
Data points enclosed with a diamond are reference sources

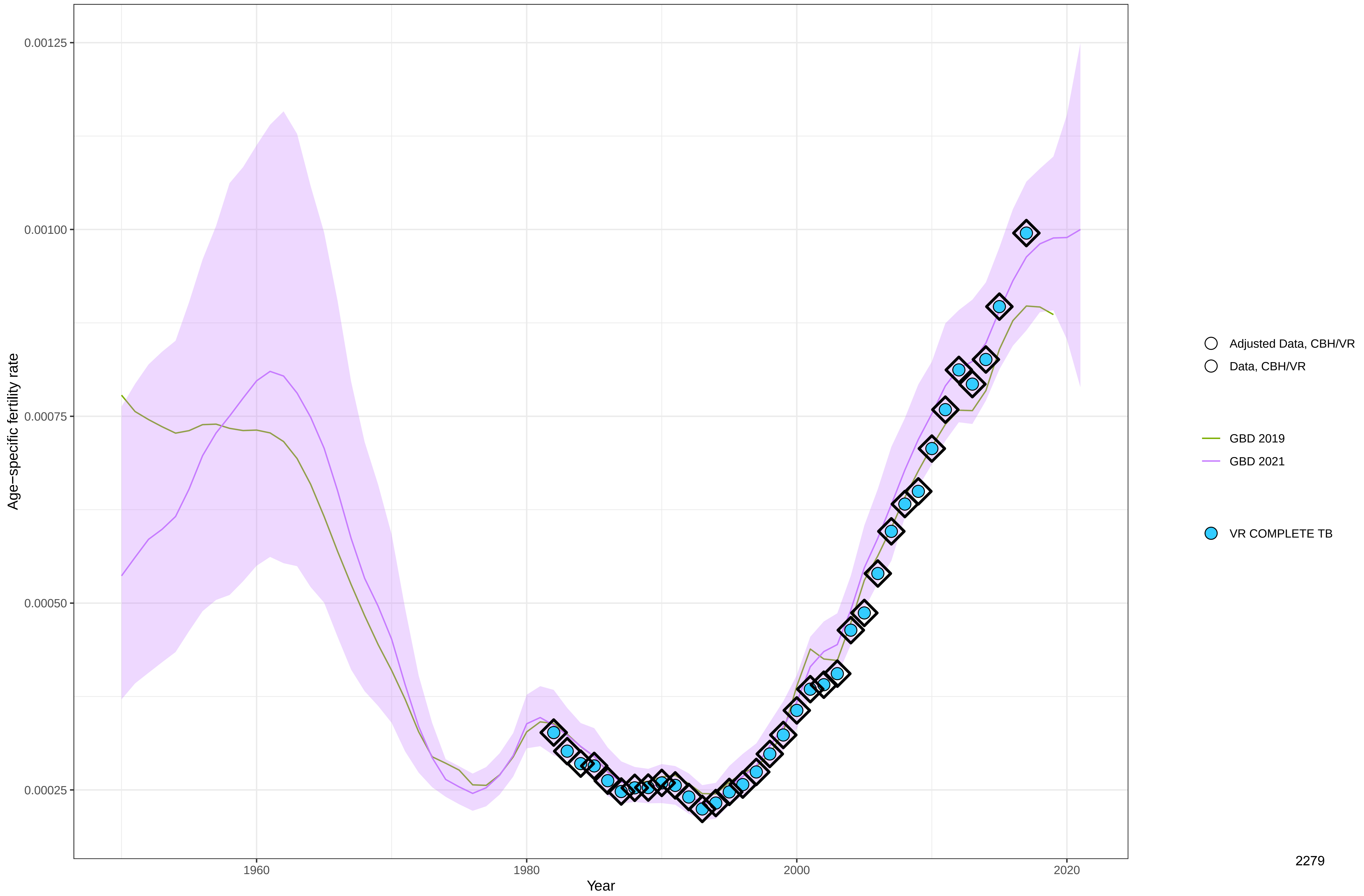

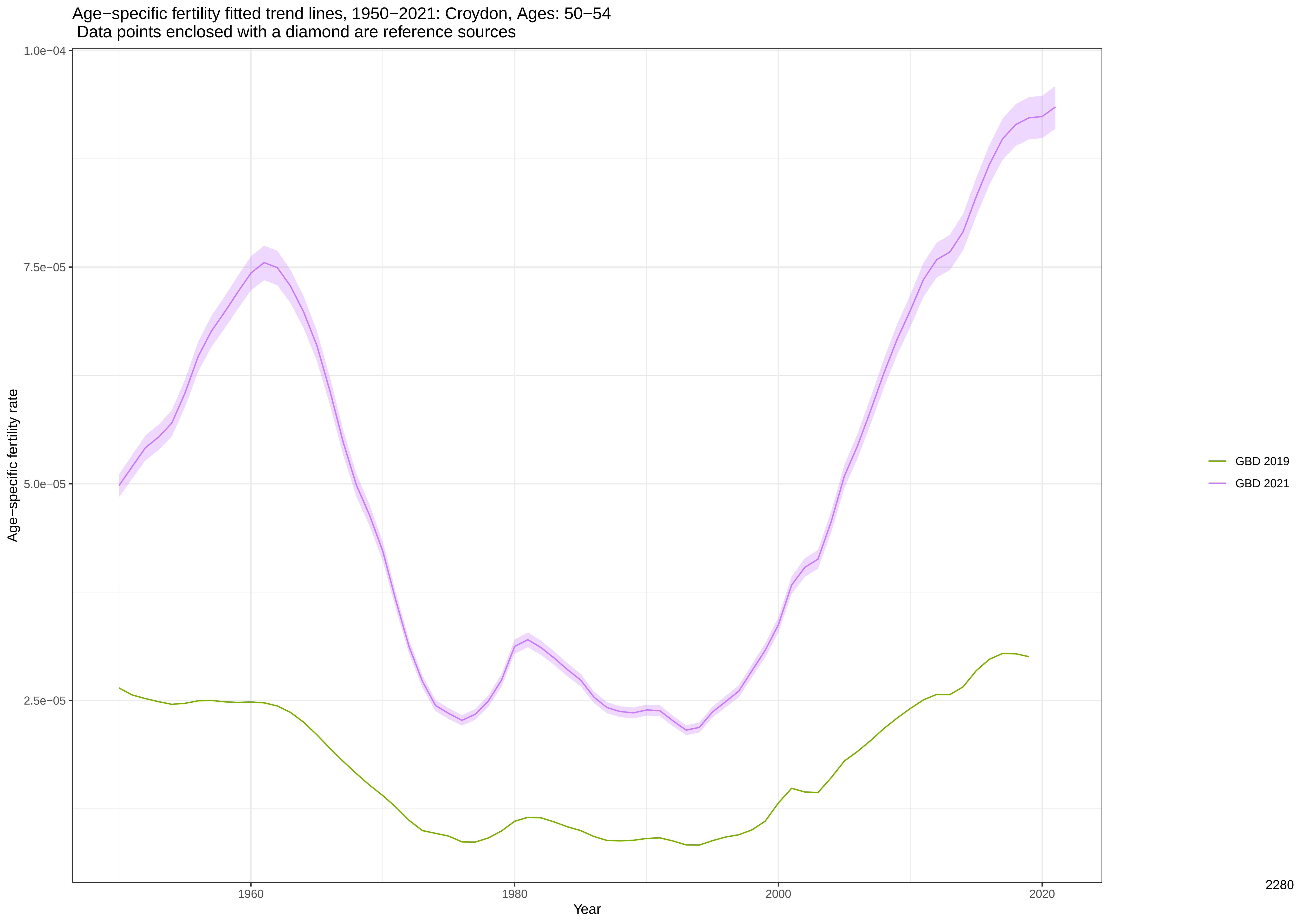

Age−specific fertility fitted trend lines, 1950−2021: Croydon, Ages: 50−54
Data points enclosed with a diamond are reference sources
1.0e−04
7.5e−05
5.0e−05
2.5e−05
Age−specific fertility rate
1960
1980
2000
2020
Year
GBD 2019
GBD 2021

Total fertility rate fitted trend lines, 1950−2021: Croydon TFR
Data points enclosed with a diamond are reference sources

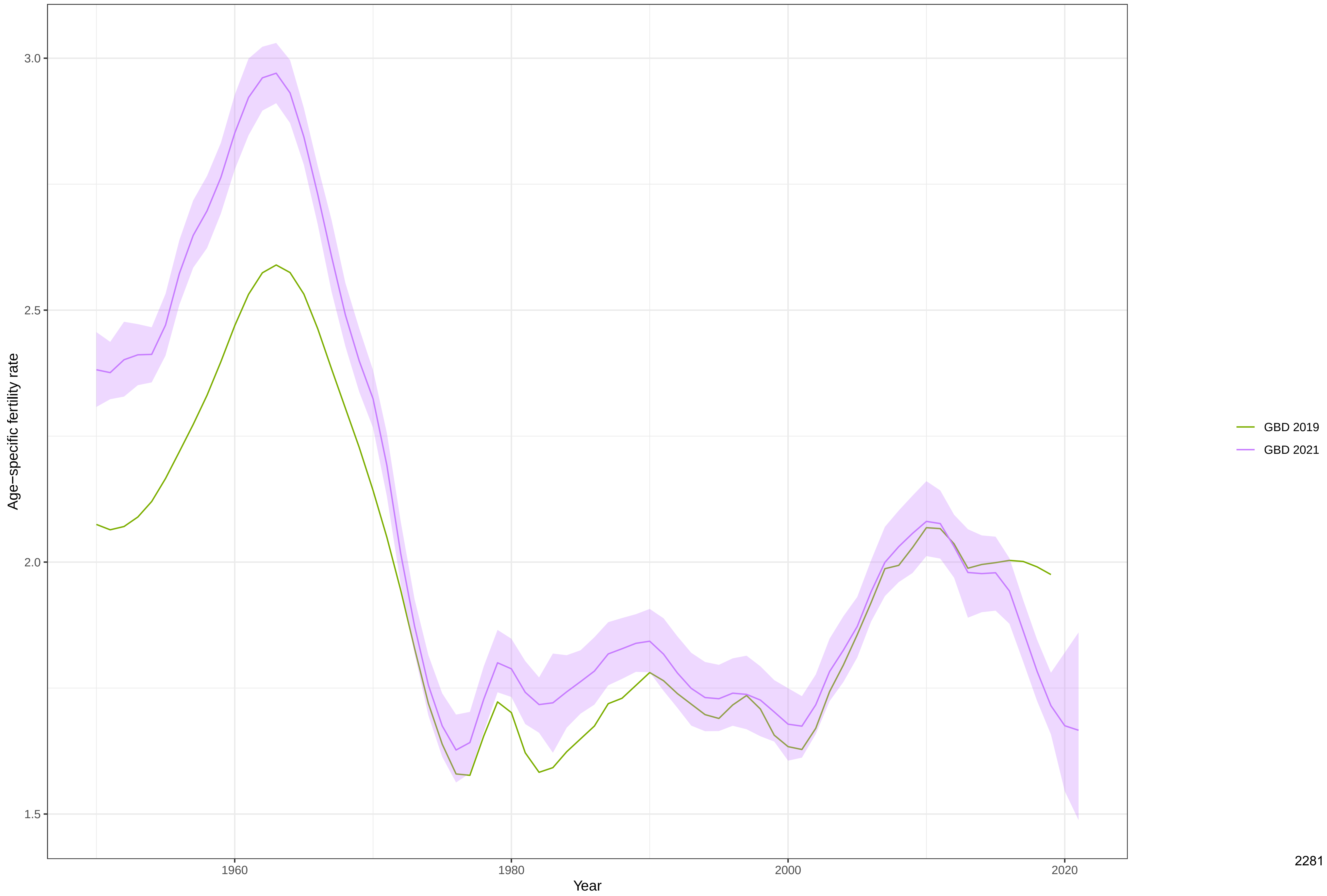

Age−specific fertility fitted trend lines, 1950−2021: Ealing, Ages: 10−14
Data points enclosed with a diamond are reference sources

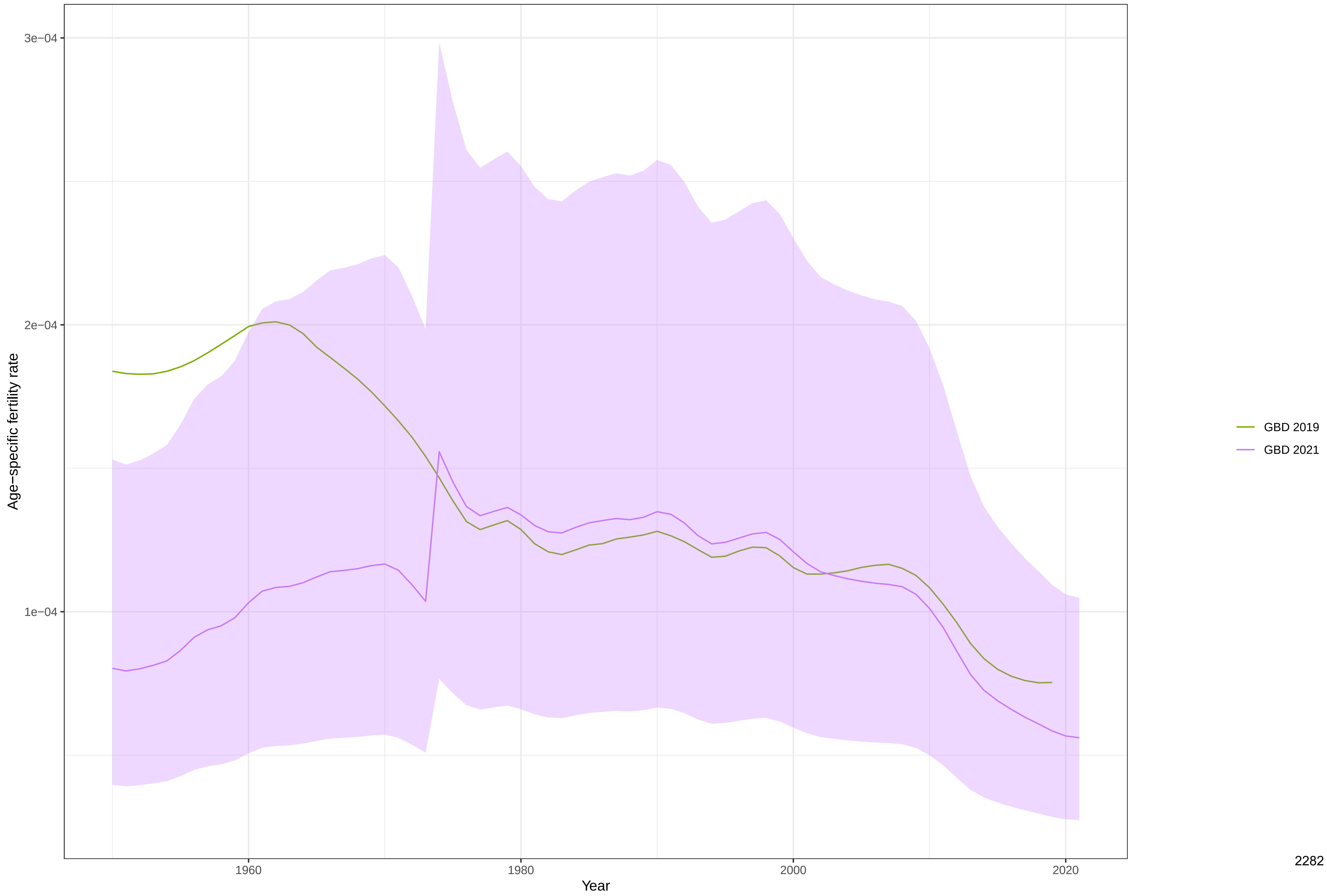

Age−specific fertility fitted trend lines, 1950−2021: Ealing, Ages: 15−19
Data points enclosed with a diamond are reference sources

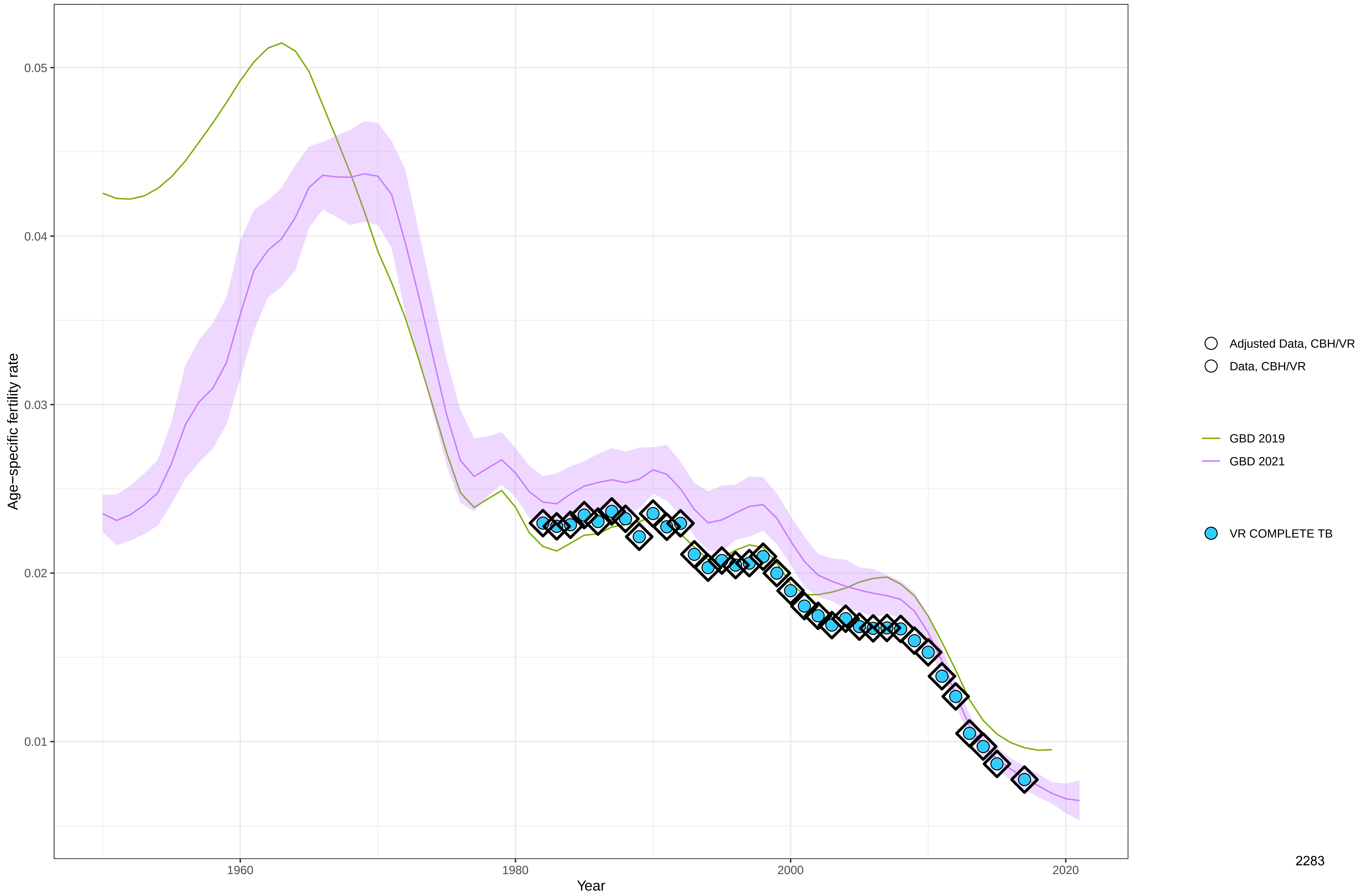

Age−specific fertility fitted trend lines, 1950−2021: Ealing, Ages: 20−24
Data points enclosed with a diamond are reference sources

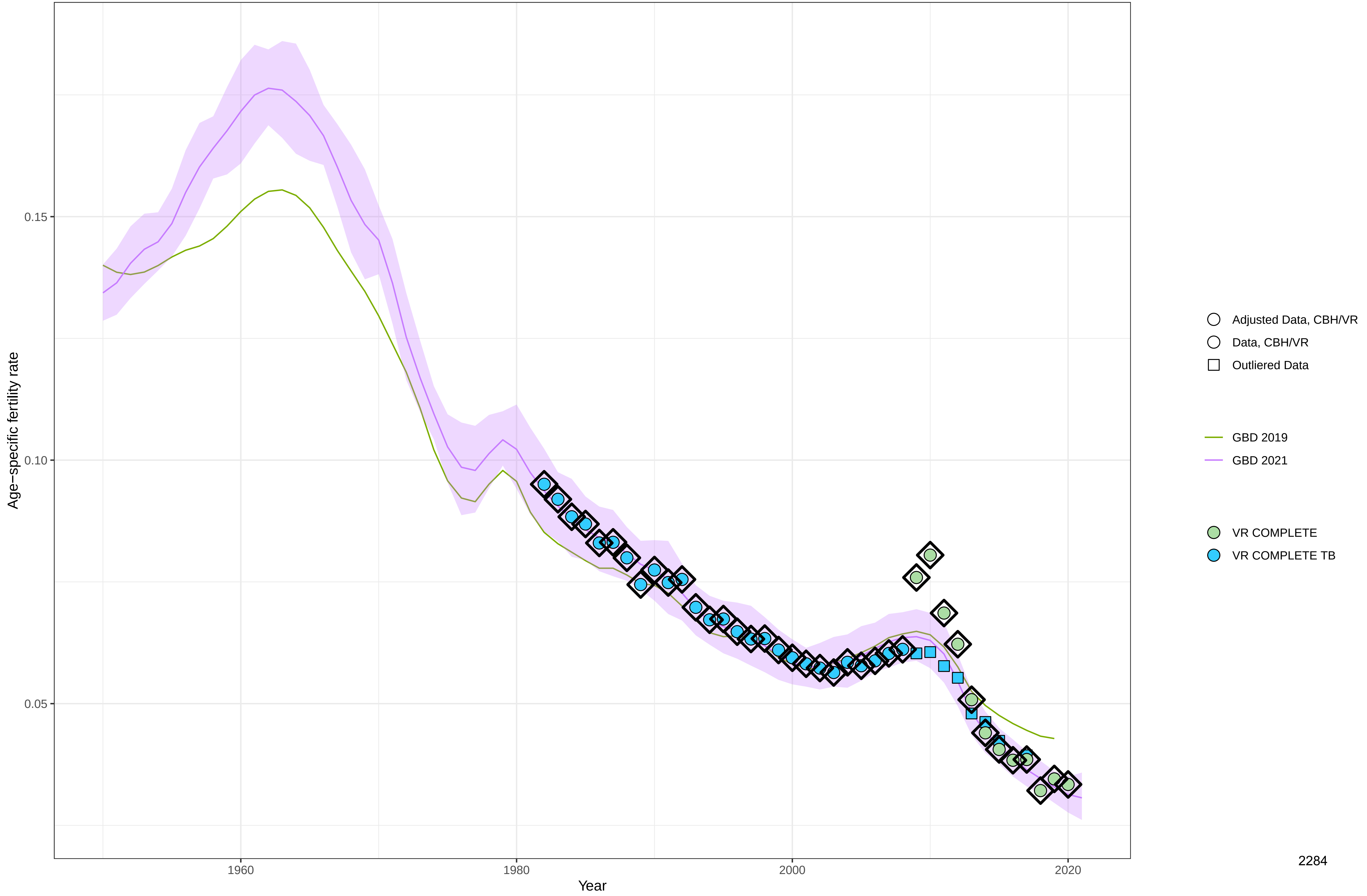

Age−specific fertility fitted trend lines, 1950−2021: Ealing, Ages: 25−29
Data points enclosed with a diamond are reference sources

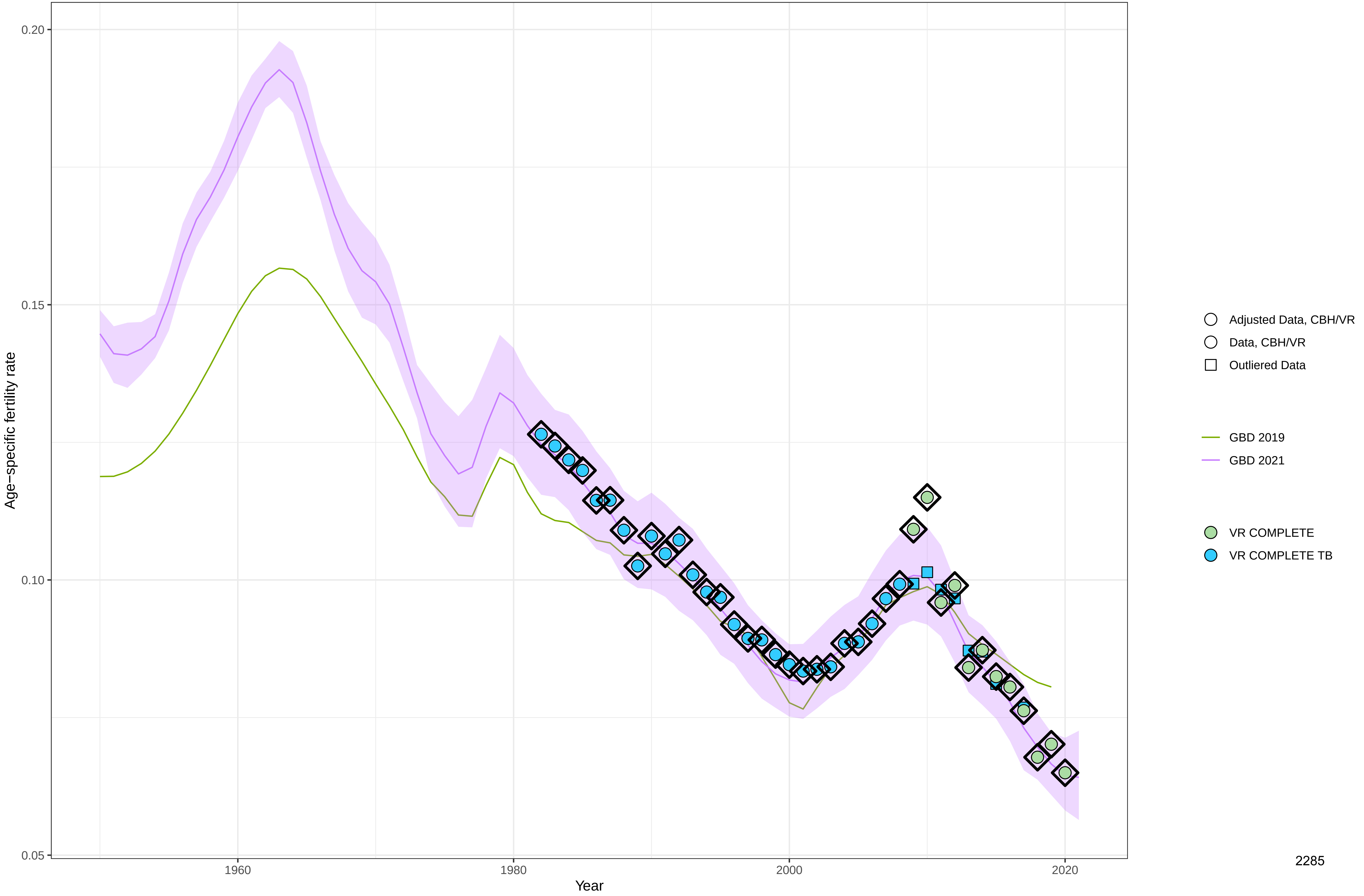

Age−specific fertility fitted trend lines, 1950−2021: Ealing, Ages: 30−34
Data points enclosed with a diamond are reference sources

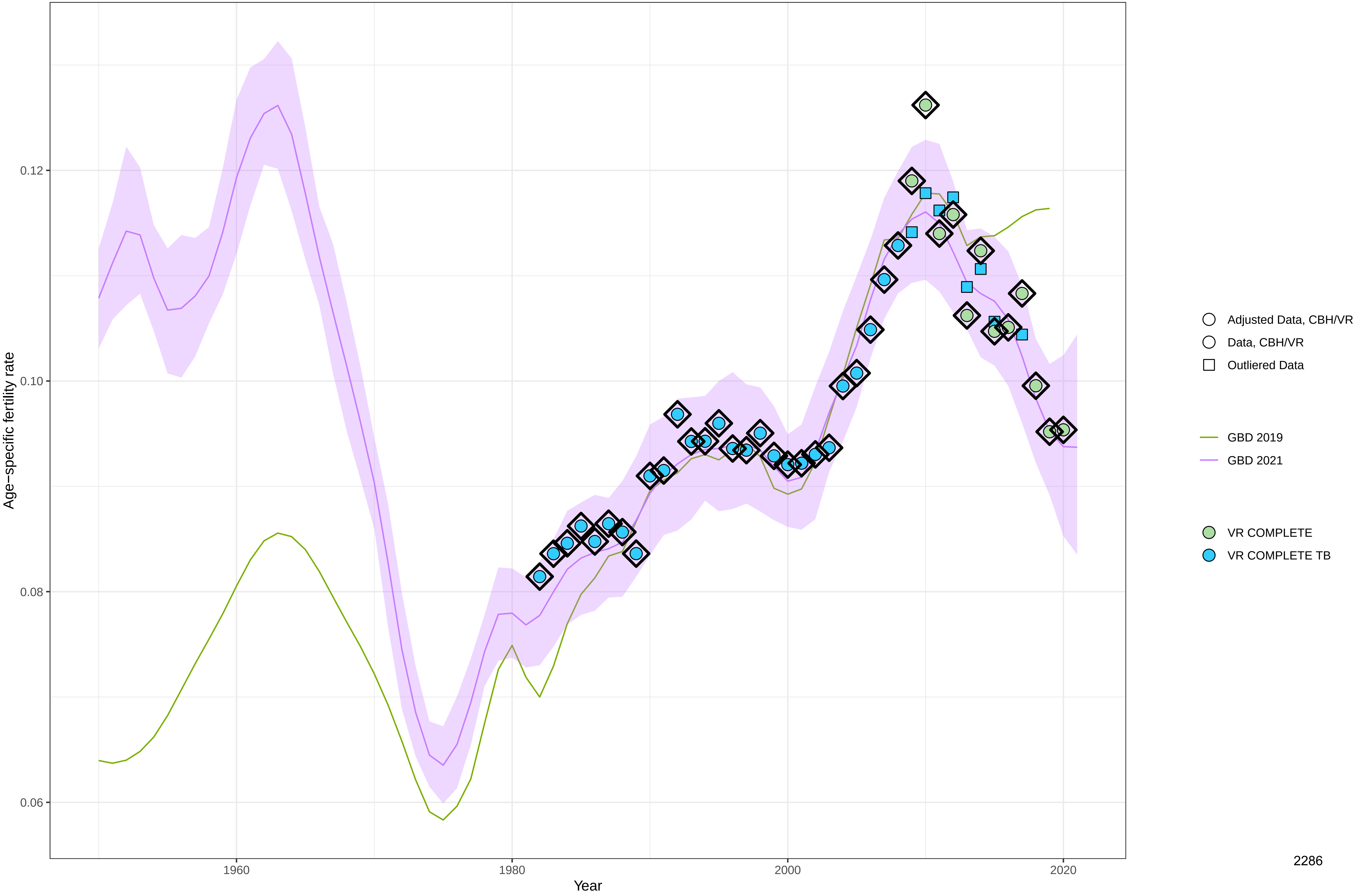

Age−specific fertility fitted trend lines, 1950−2021: Ealing, Ages: 35−39
Data points enclosed with a diamond are reference sources

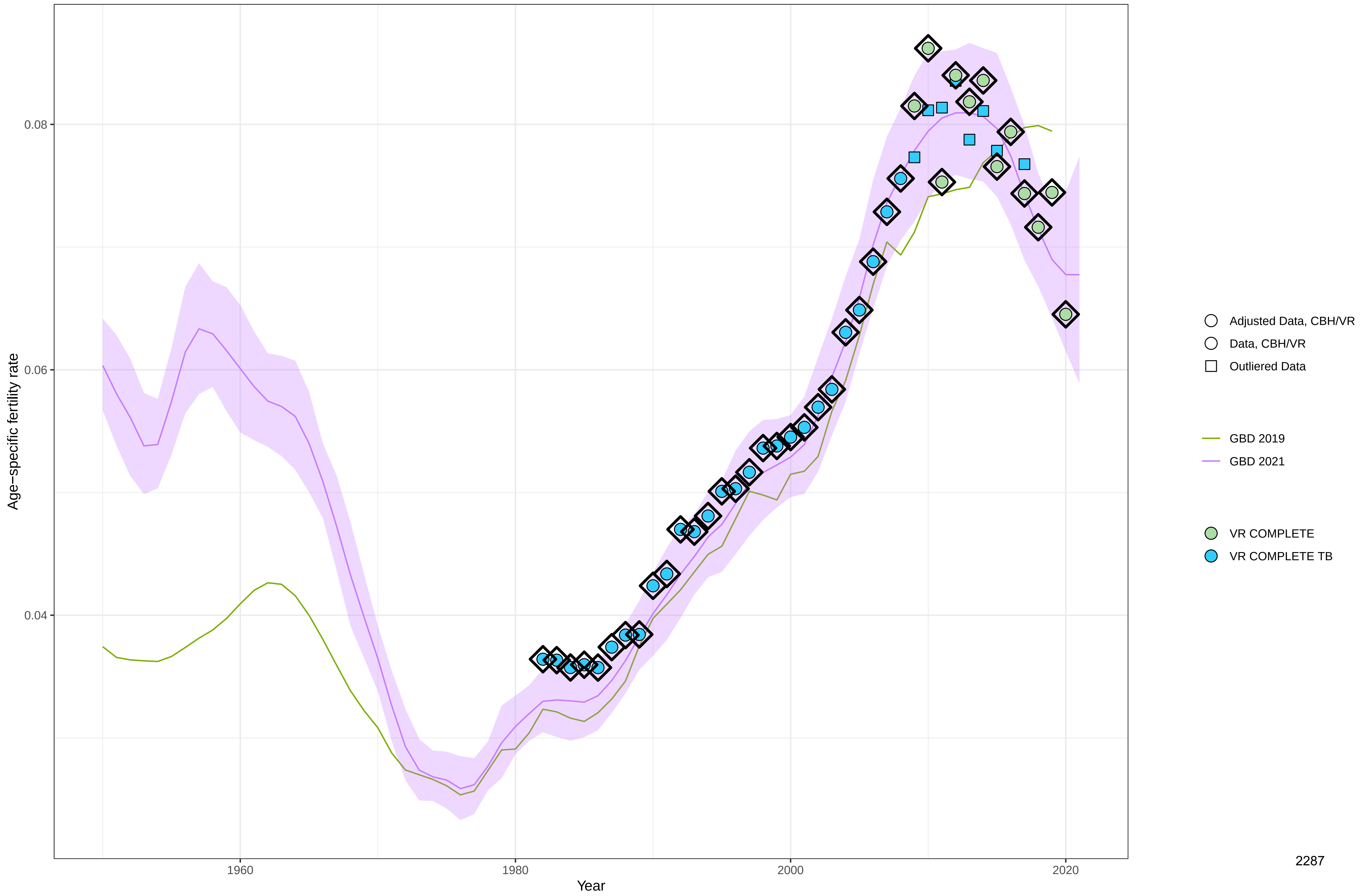

Age−specific fertility fitted trend lines, 1950−2021: Ealing, Ages: 40−44
Data points enclosed with a diamond are reference sources

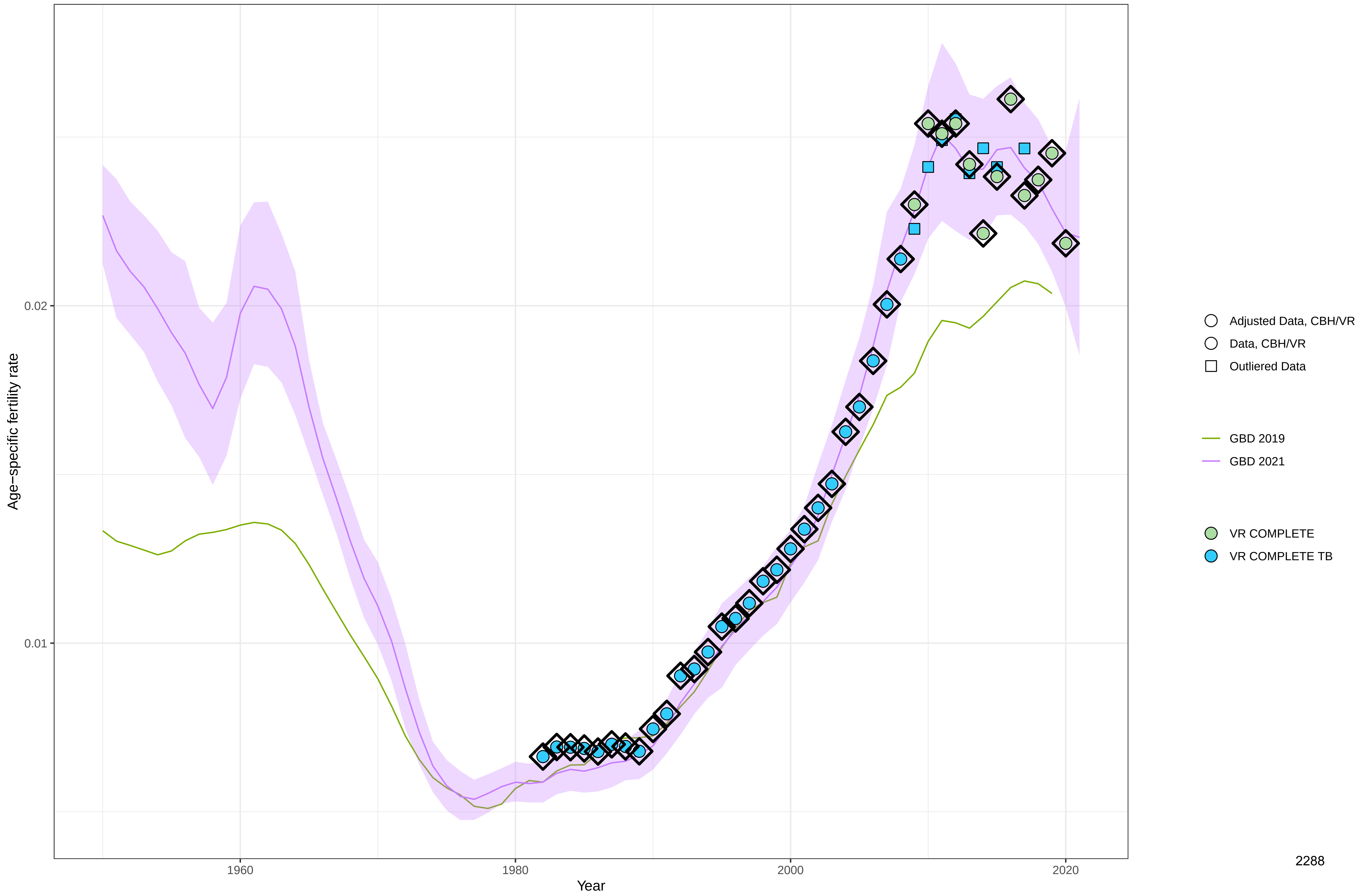

Age−specific fertility fitted trend lines, 1950−2021: Ealing, Ages: 45−49
Data points enclosed with a diamond are reference sources

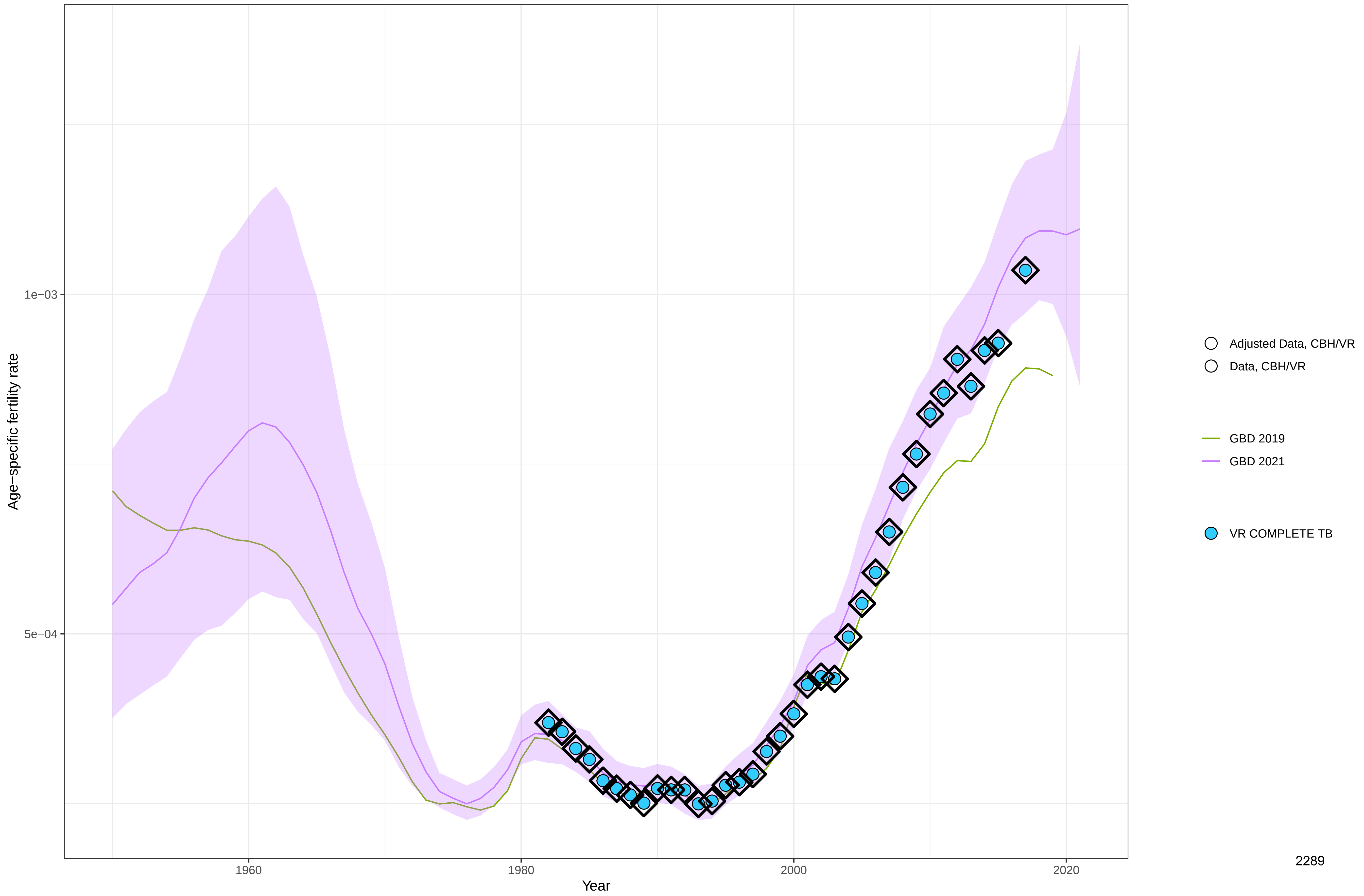

Age−specific fertility fitted trend lines, 1950−2021: Ealing, Ages: 50−54
Data points enclosed with a diamond are reference sources

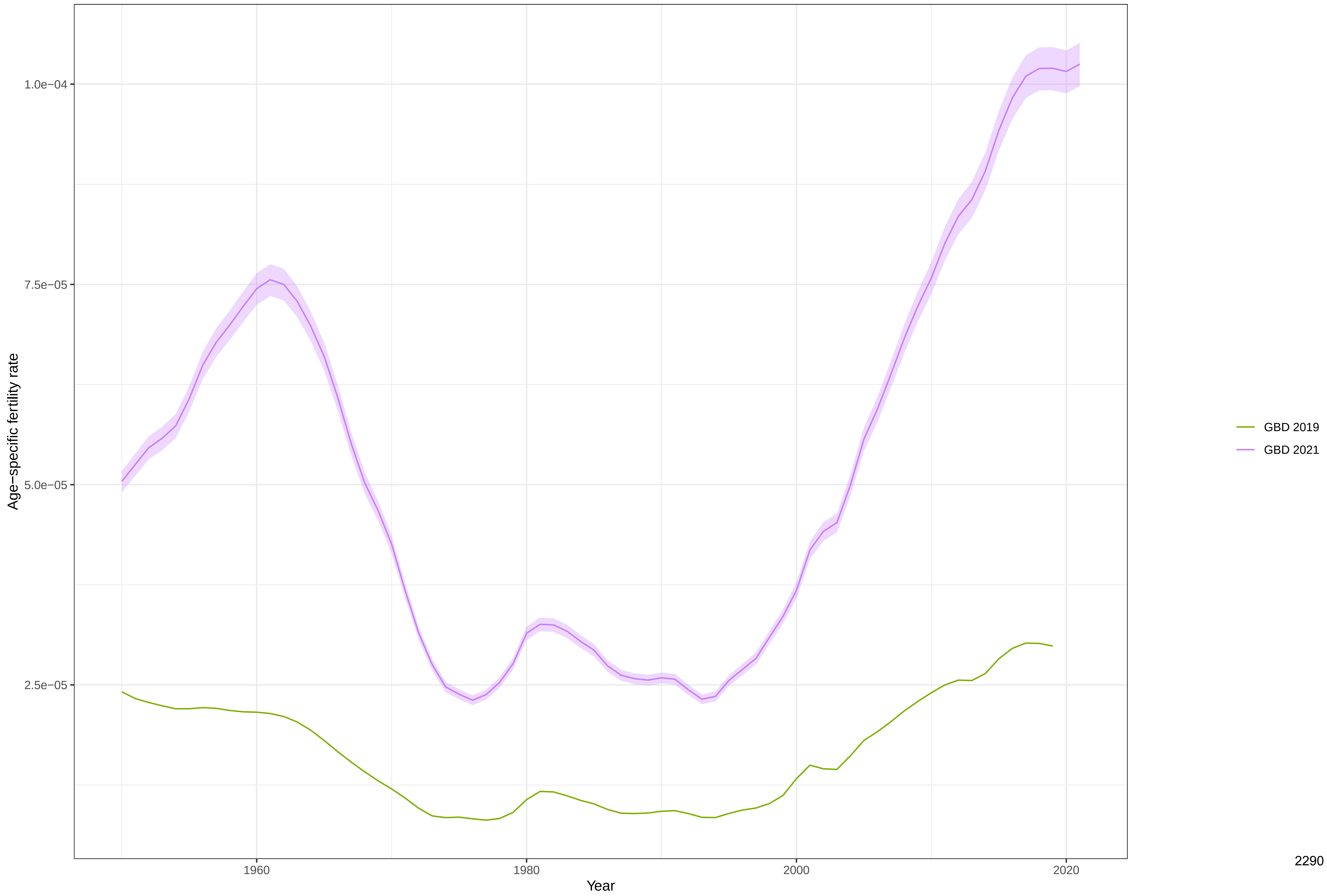

Total fertility rate fitted trend lines, 1950−2021: Ealing TFR
Data points enclosed with a diamond are reference sources

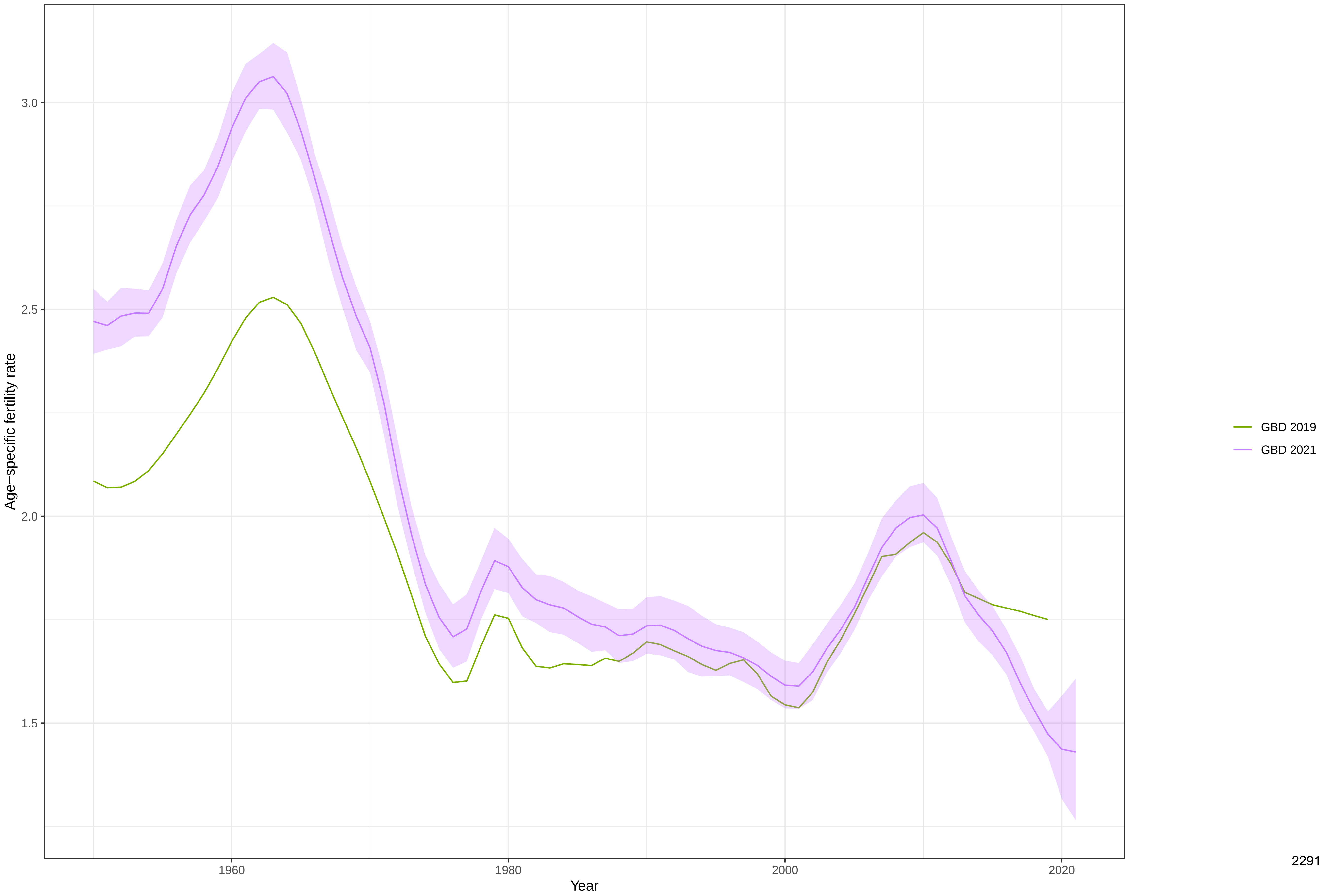

Age−specific fertility fitted trend lines, 1950−2021: Enfield, Ages: 10−14
Data points enclosed with a diamond are reference sources

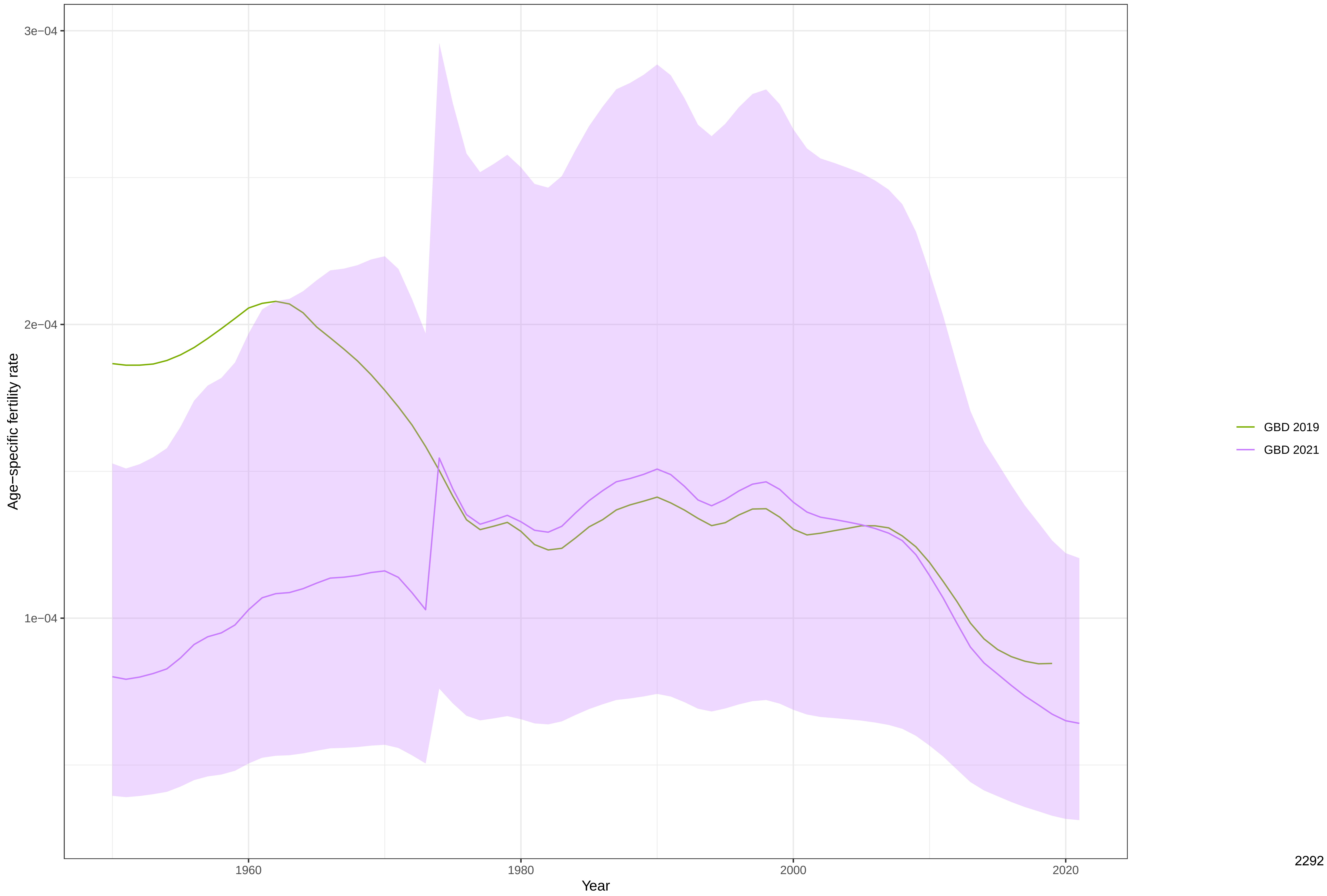

Age−specific fertility fitted trend lines, 1950−2021: Enfield, Ages: 15−19
Data points enclosed with a diamond are reference sources

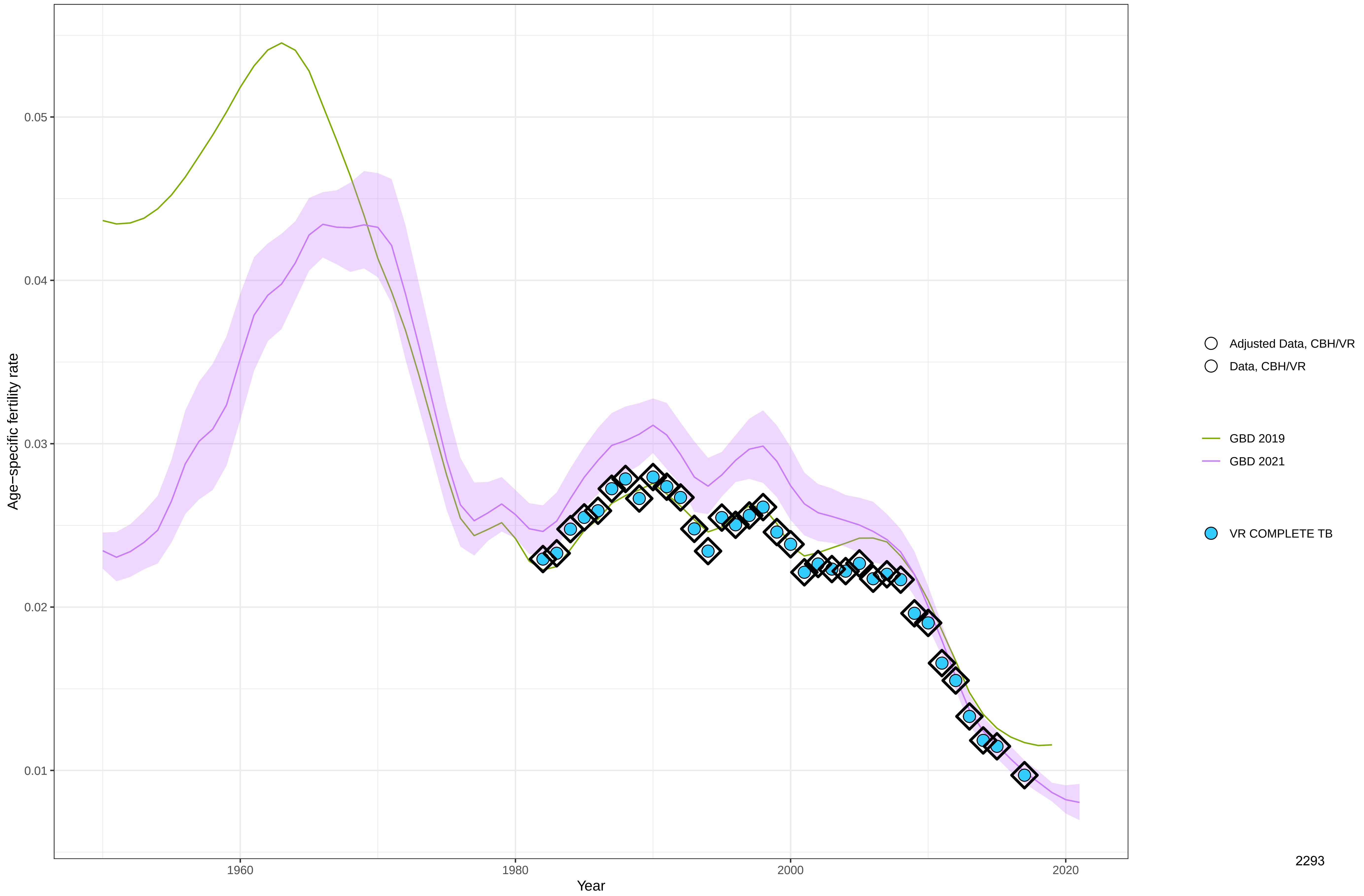

Age−specific fertility fitted trend lines, 1950−2021: Enfield, Ages: 20−24
Data points enclosed with a diamond are reference sources

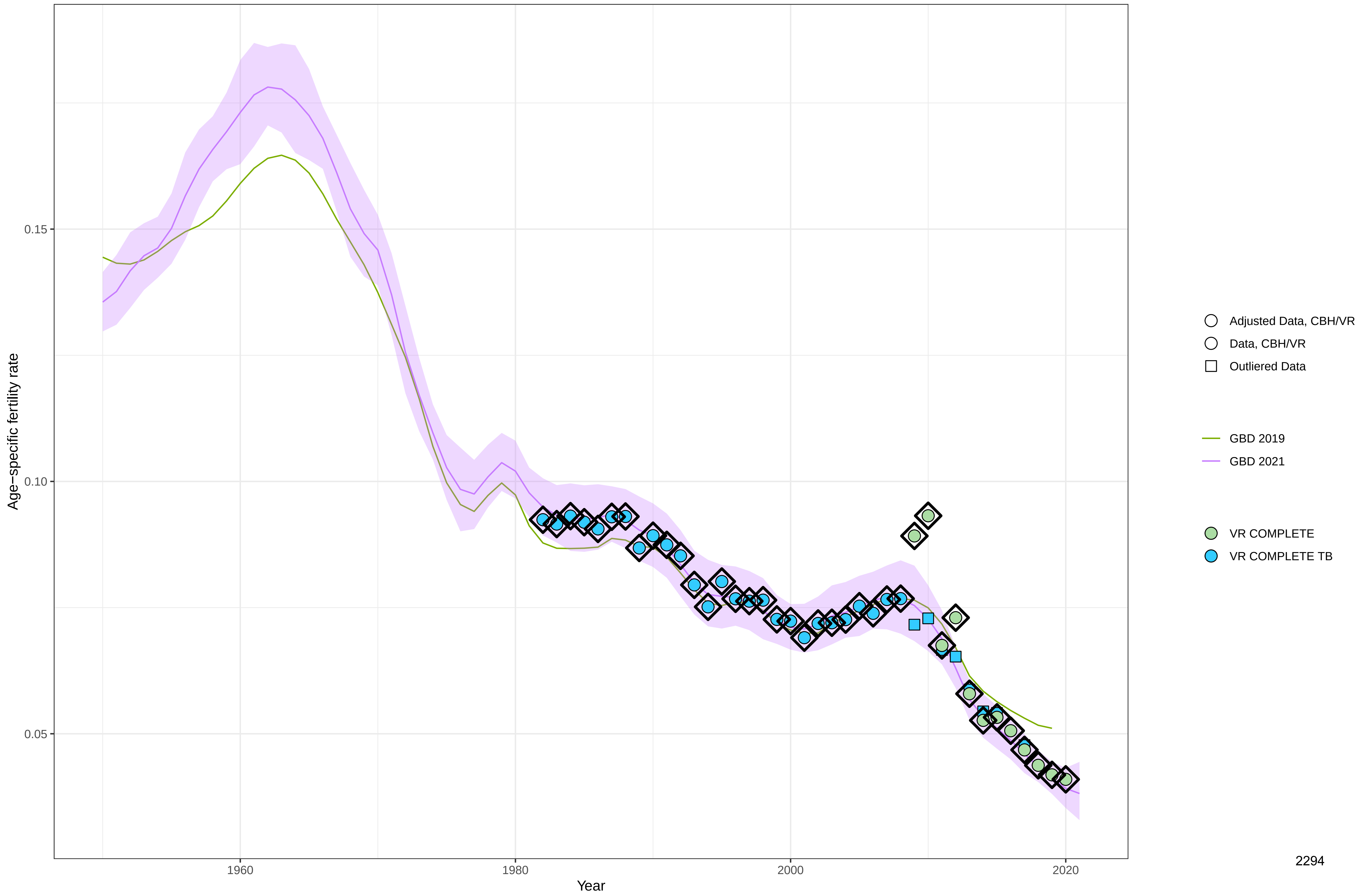

2294

Age−specific fertility fitted trend lines, 1950−2021: Enfield, Ages: 25−29
Data points enclosed with a diamond are reference sources

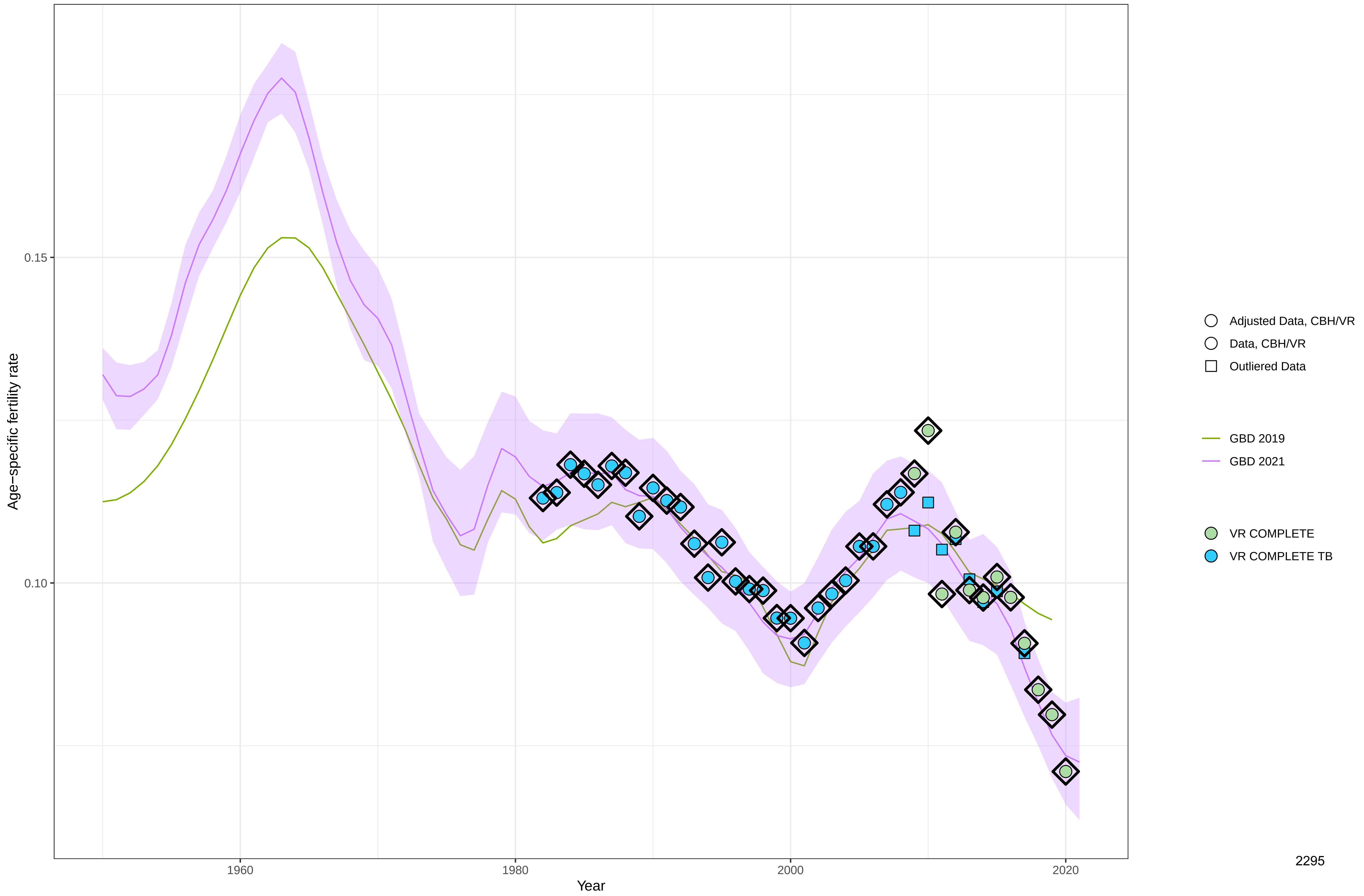

Age−specific fertility fitted trend lines, 1950−2021: Enfield, Ages: 30−34
Data points enclosed with a diamond are reference sources

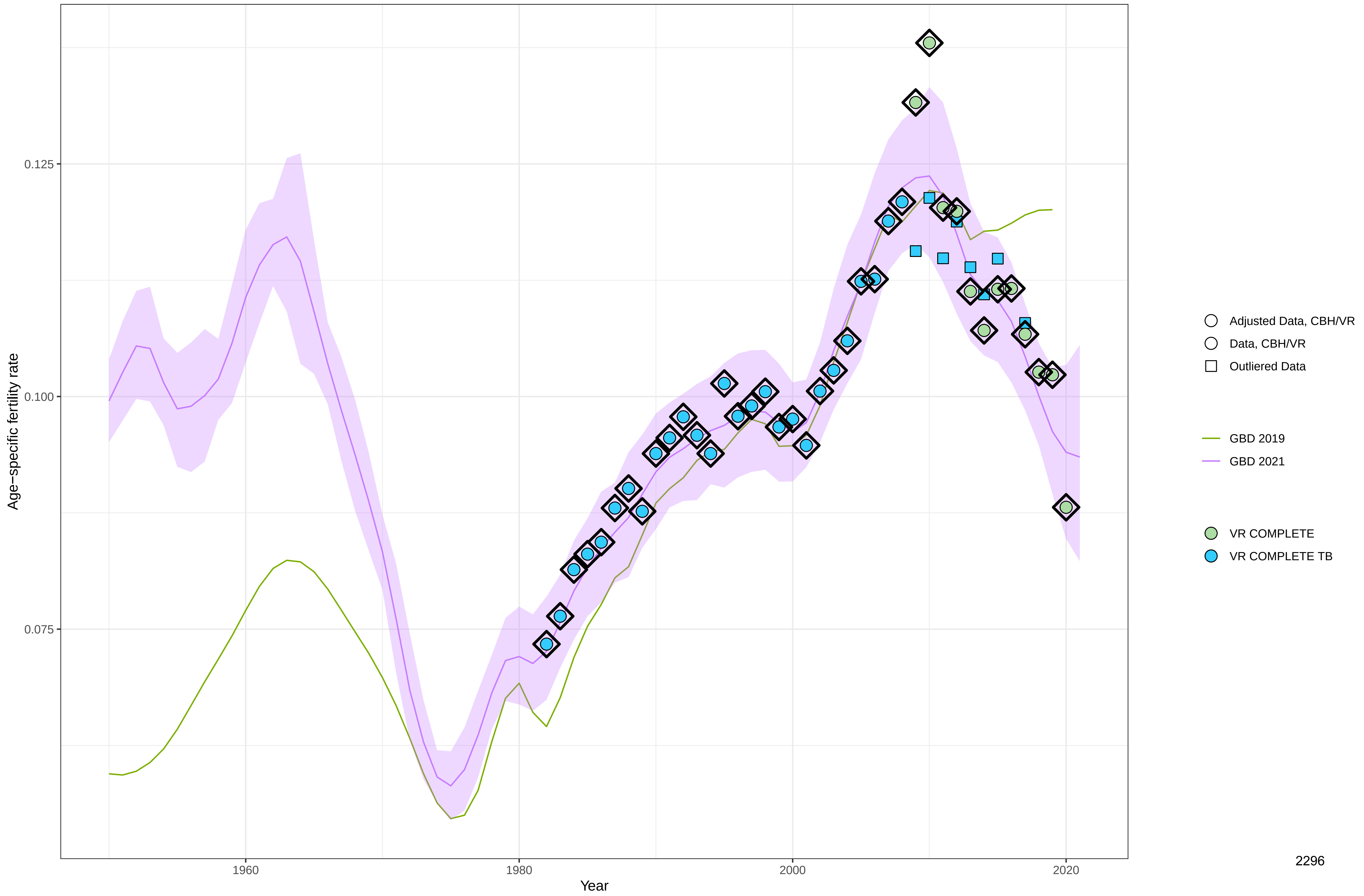

Age−specific fertility fitted trend lines, 1950−2021: Enfield, Ages: 35−39
Data points enclosed with a diamond are reference sources

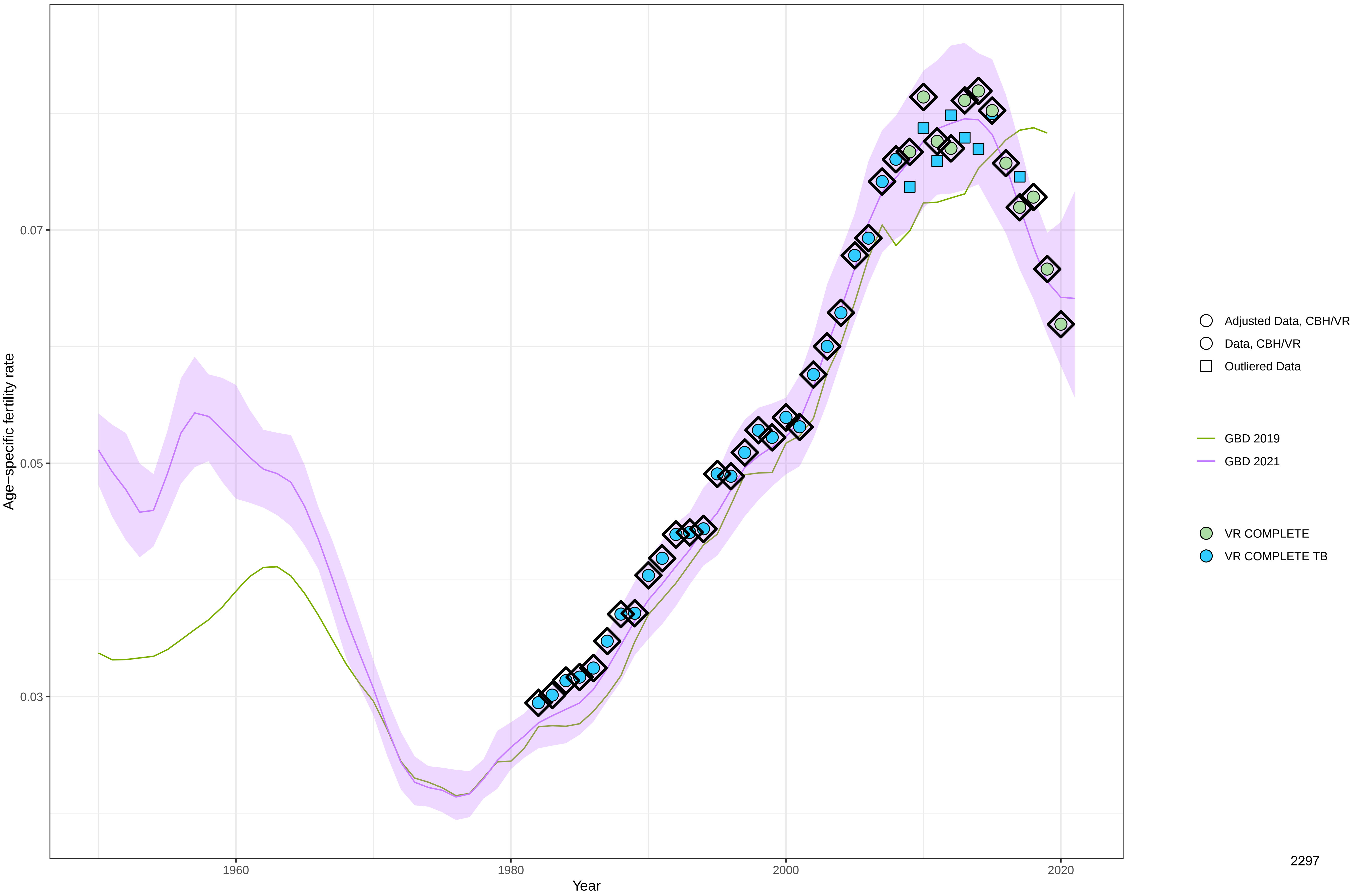

Age−specific fertility fitted trend lines, 1950−2021: Enfield, Ages: 40−44
Data points enclosed with a diamond are reference sources

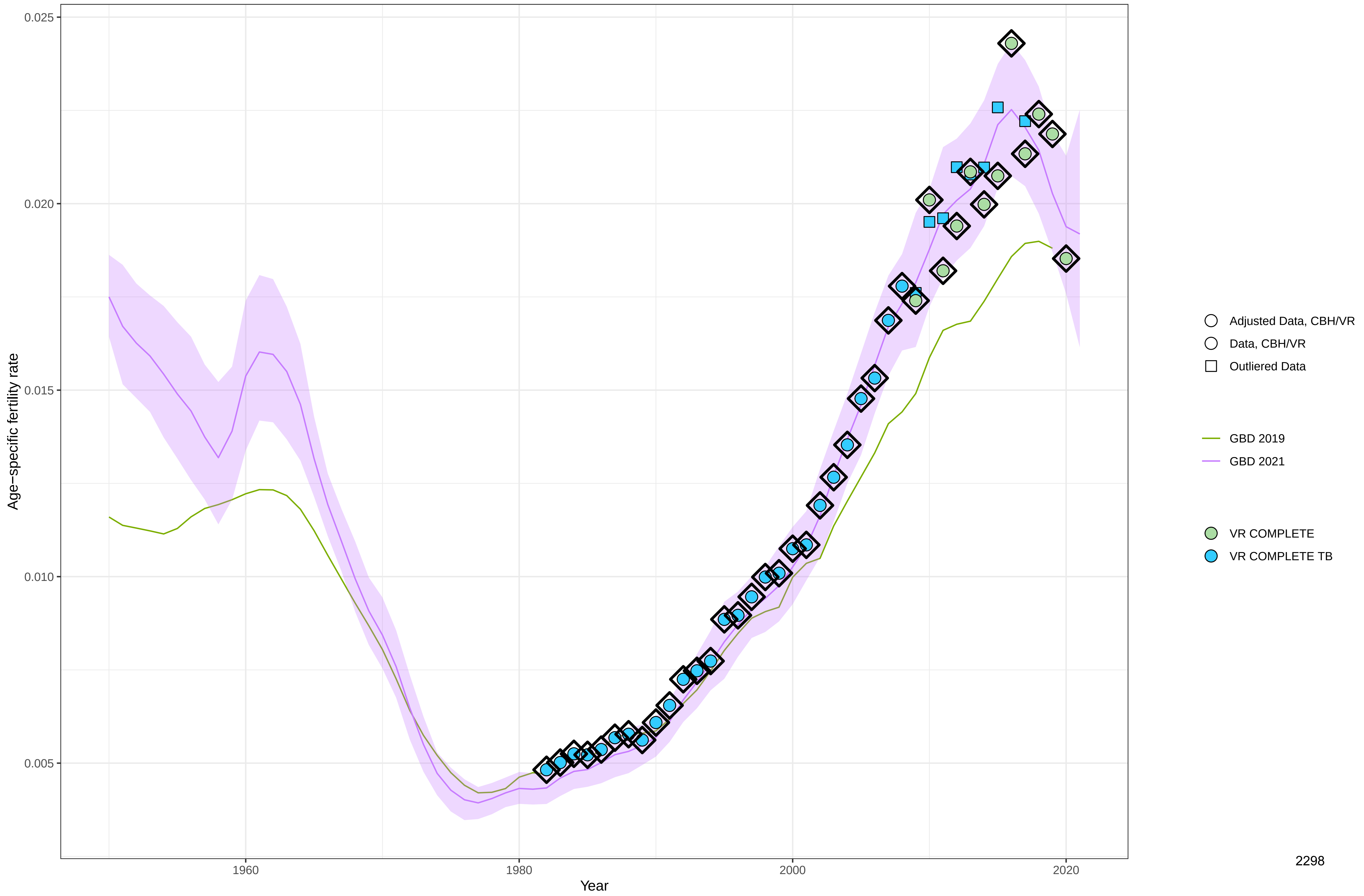

Age−specific fertility fitted trend lines, 1950−2021: Enfield, Ages: 45−49
Data points enclosed with a diamond are reference sources

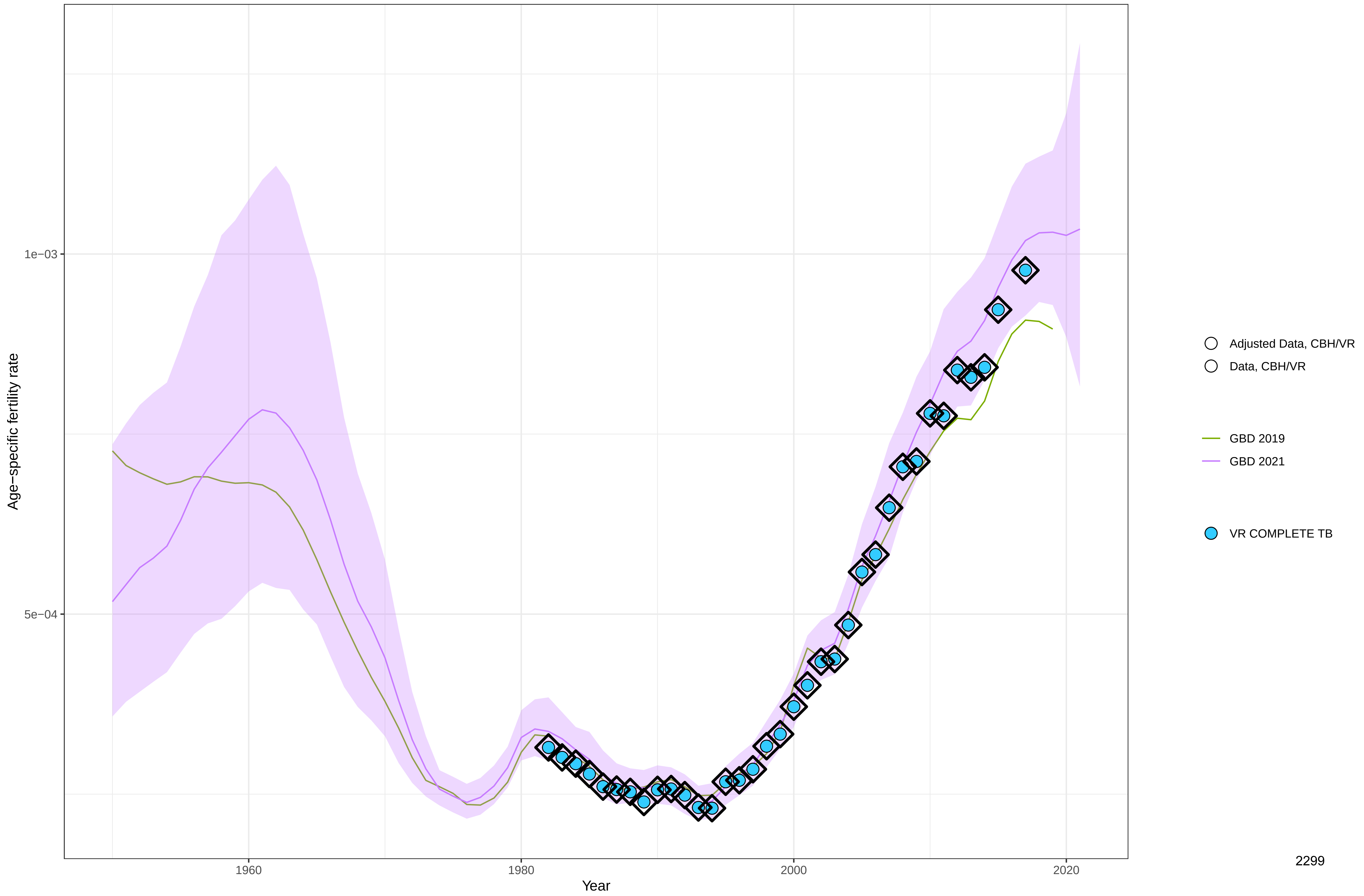

Age−specific fertility fitted trend lines, 1950−2021: Enfield, Ages: 50−54
Data points enclosed with a diamond are reference sources

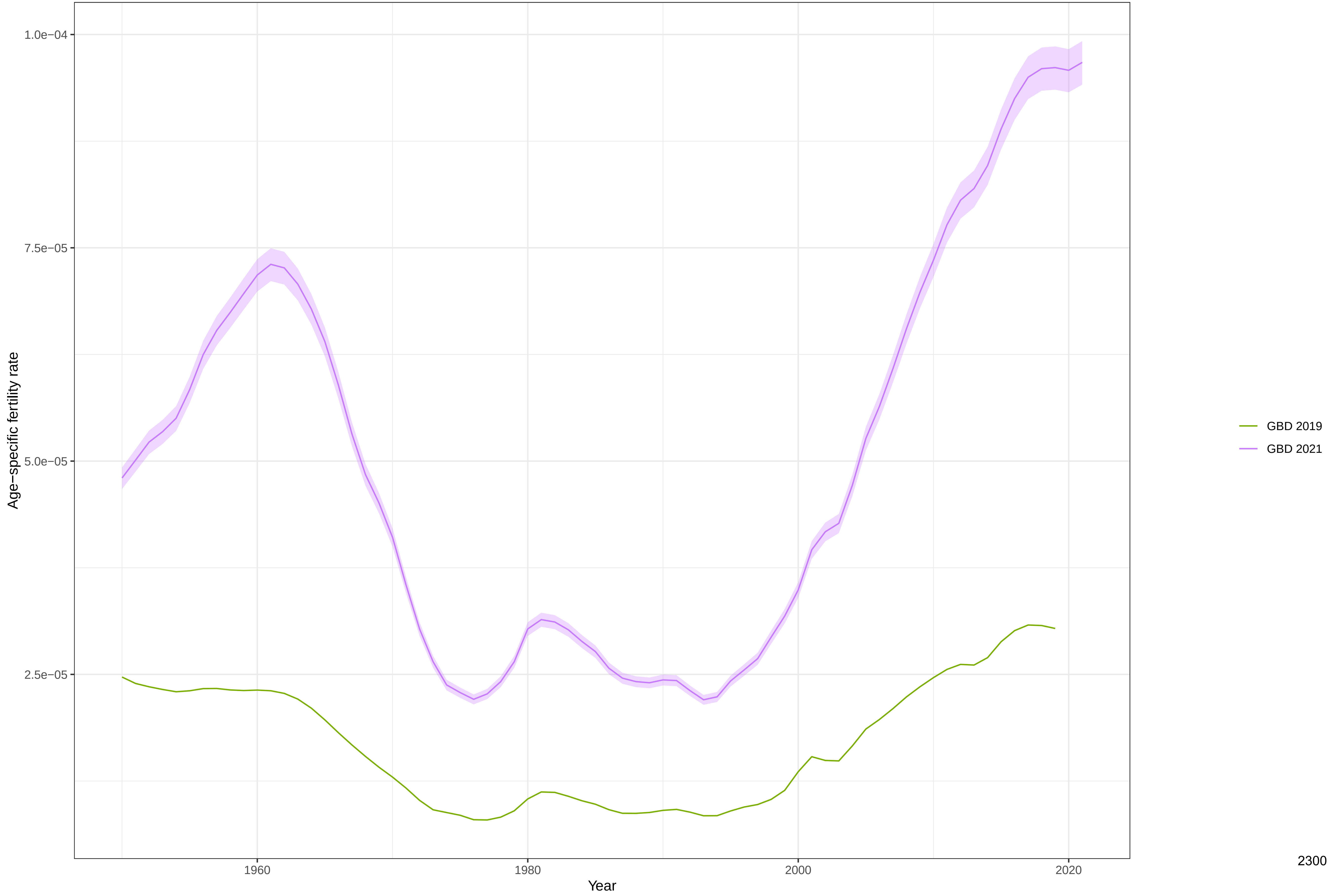

Total fertility rate fitted trend lines, 1950−2021: Enfield TFR
Data points enclosed with a diamond are reference sources

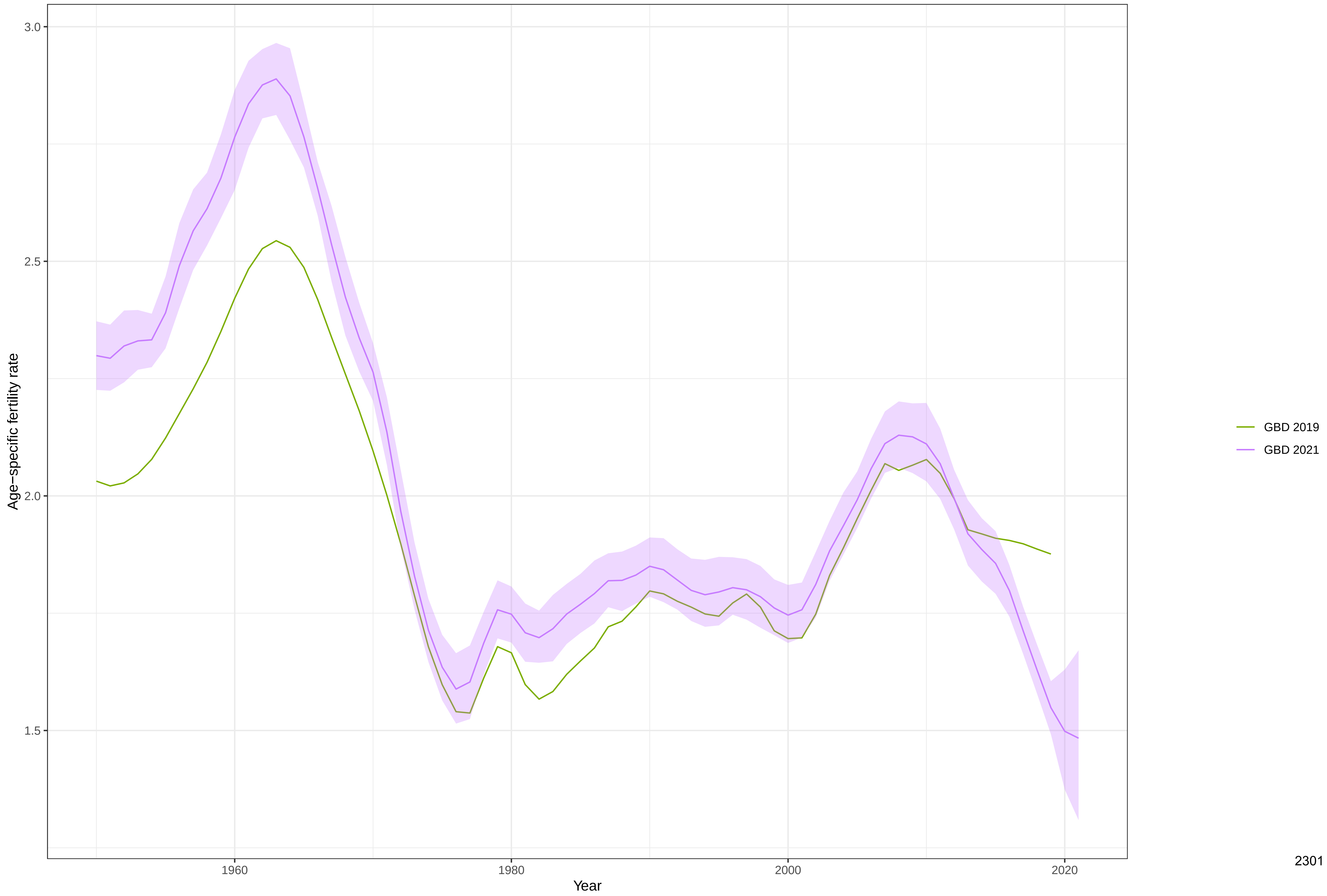

Age−specific fertility fitted trend lines, 1950−2021: Greenwich, Ages: 10−14
Data points enclosed with a diamond are reference sources

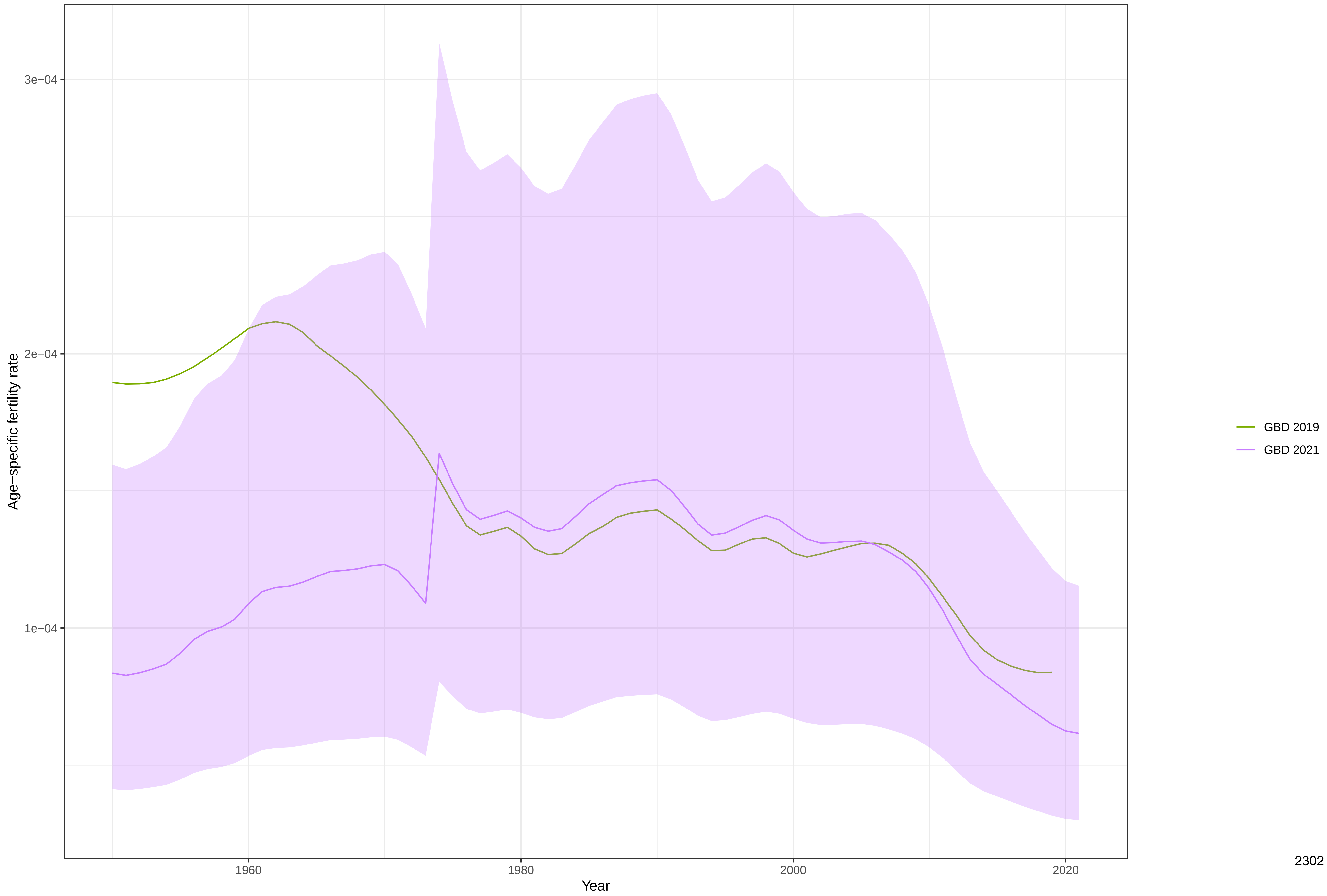

Age−specific fertility fitted trend lines, 1950−2021: Greenwich, Ages: 15−19
Data points enclosed with a diamond are reference sources

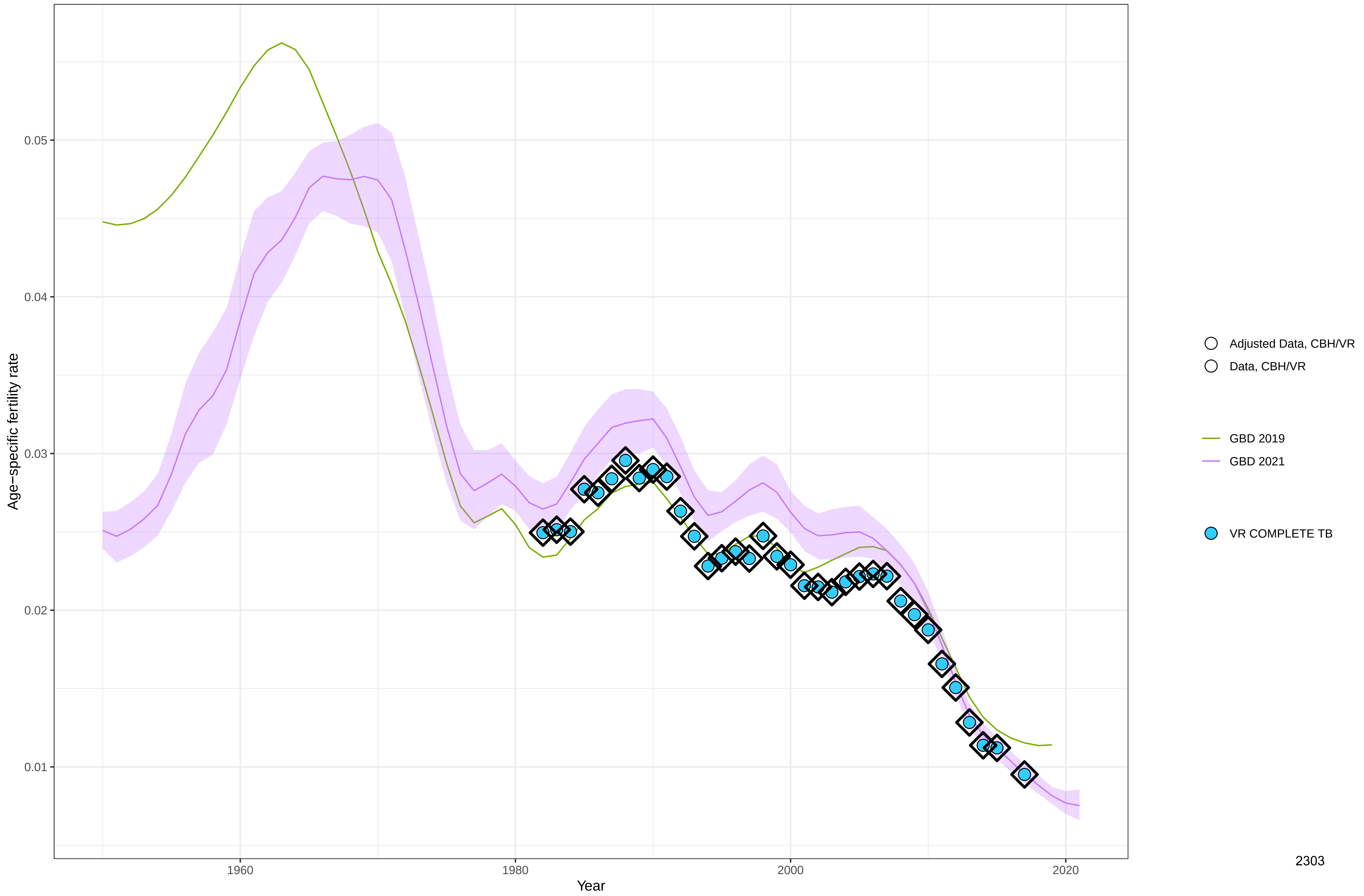

Age−specific fertility fitted trend lines, 1950−2021: Greenwich, Ages: 20−24
Data points enclosed with a diamond are reference sources

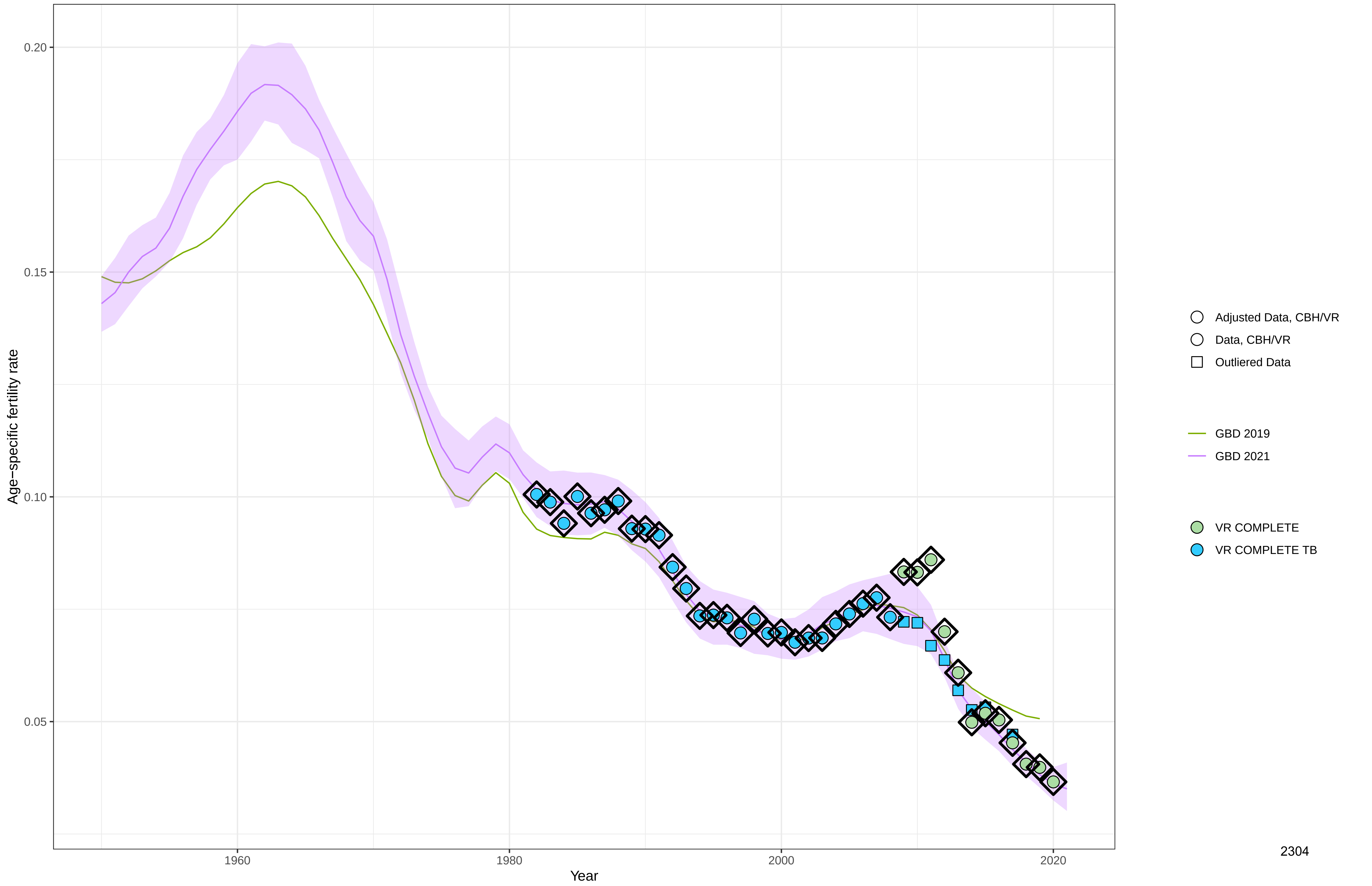

Age−specific fertility fitted trend lines, 1950−2021: Greenwich, Ages: 25−29
Data points enclosed with a diamond are reference sources

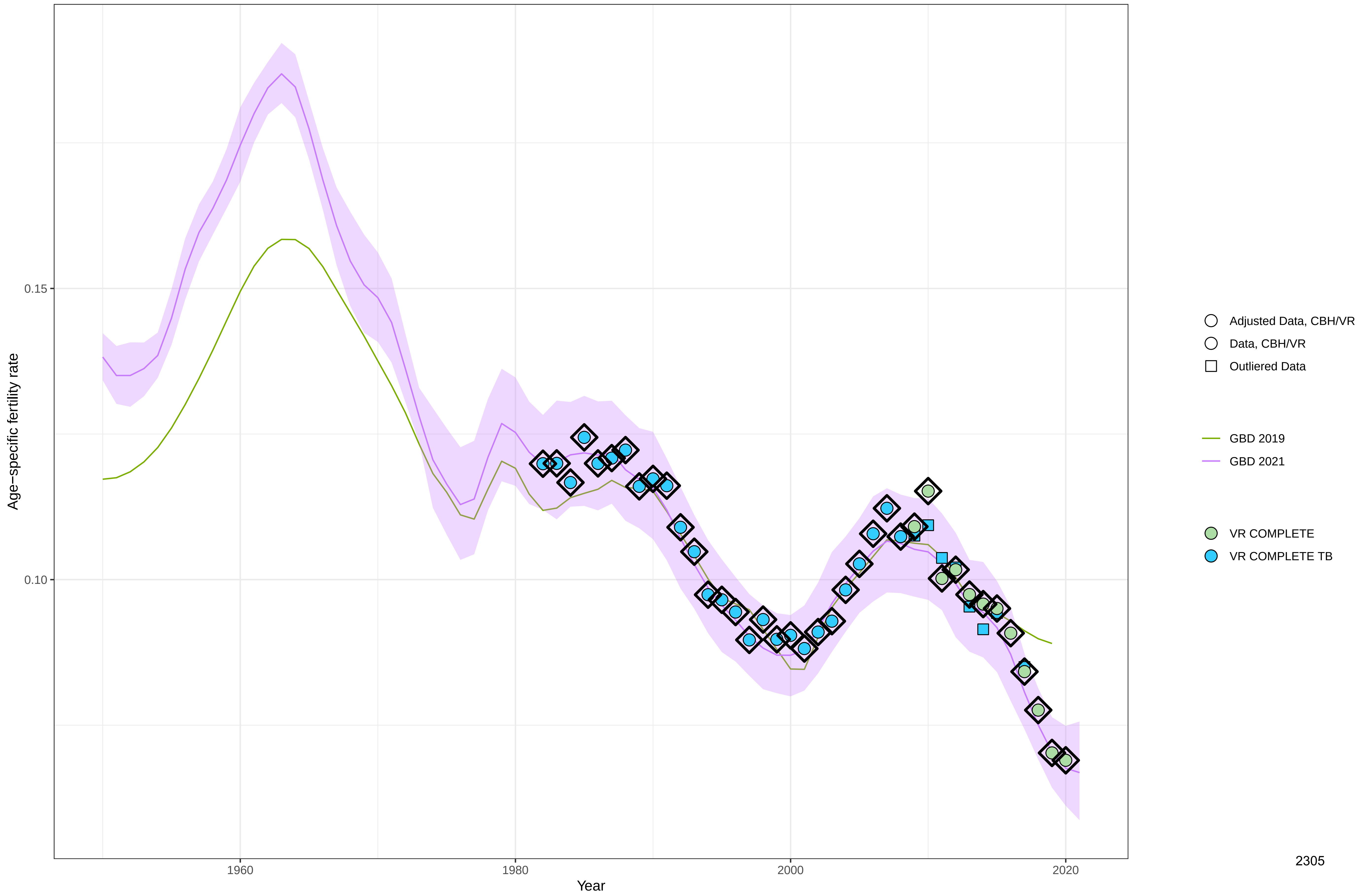

Age−specific fertility fitted trend lines, 1950−2021: Greenwich, Ages: 30−34
Data points enclosed with a diamond are reference sources

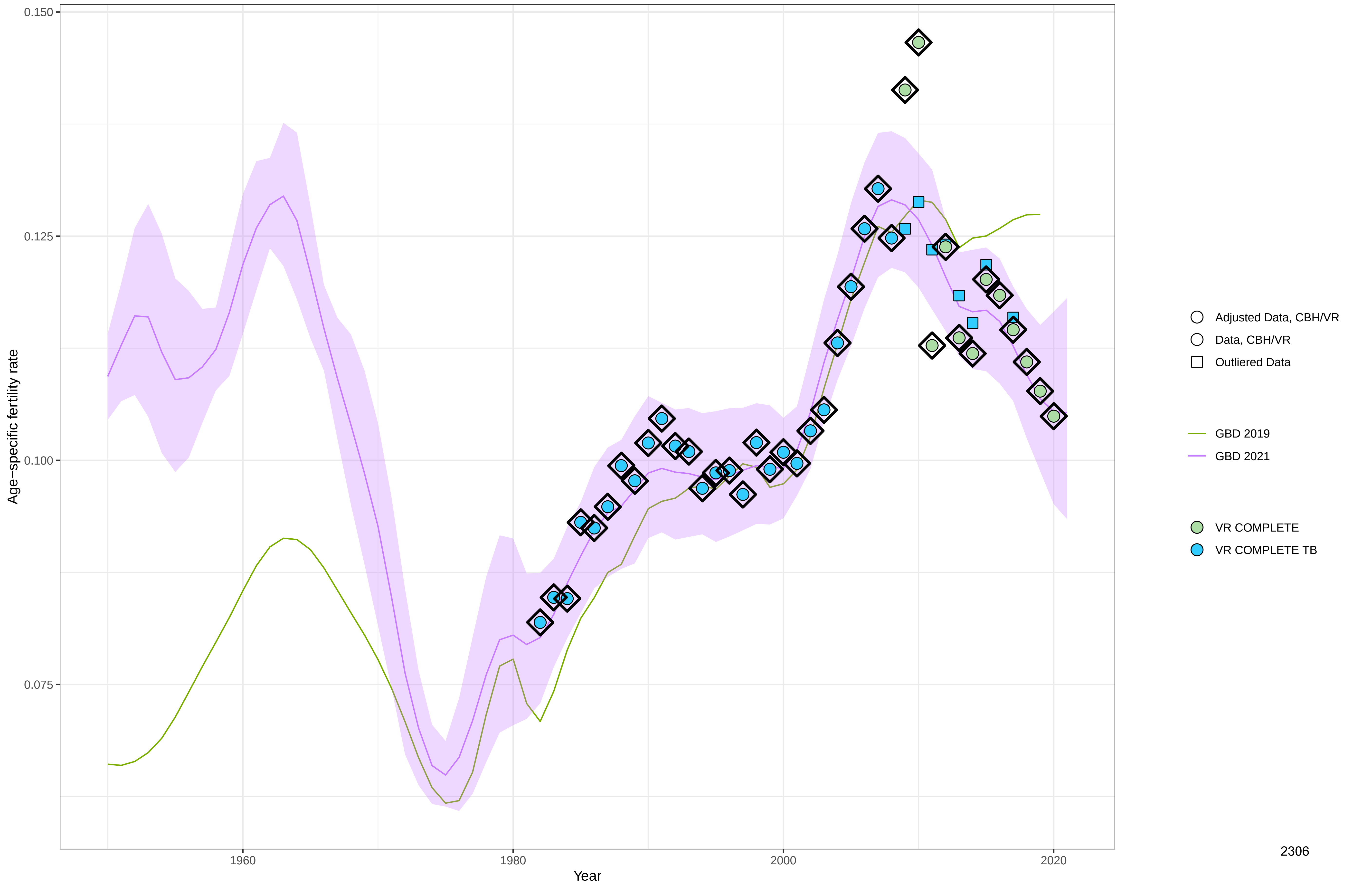

Age−specific fertility fitted trend lines, 1950−2021: Greenwich, Ages: 35−39
Data points enclosed with a diamond are reference sources

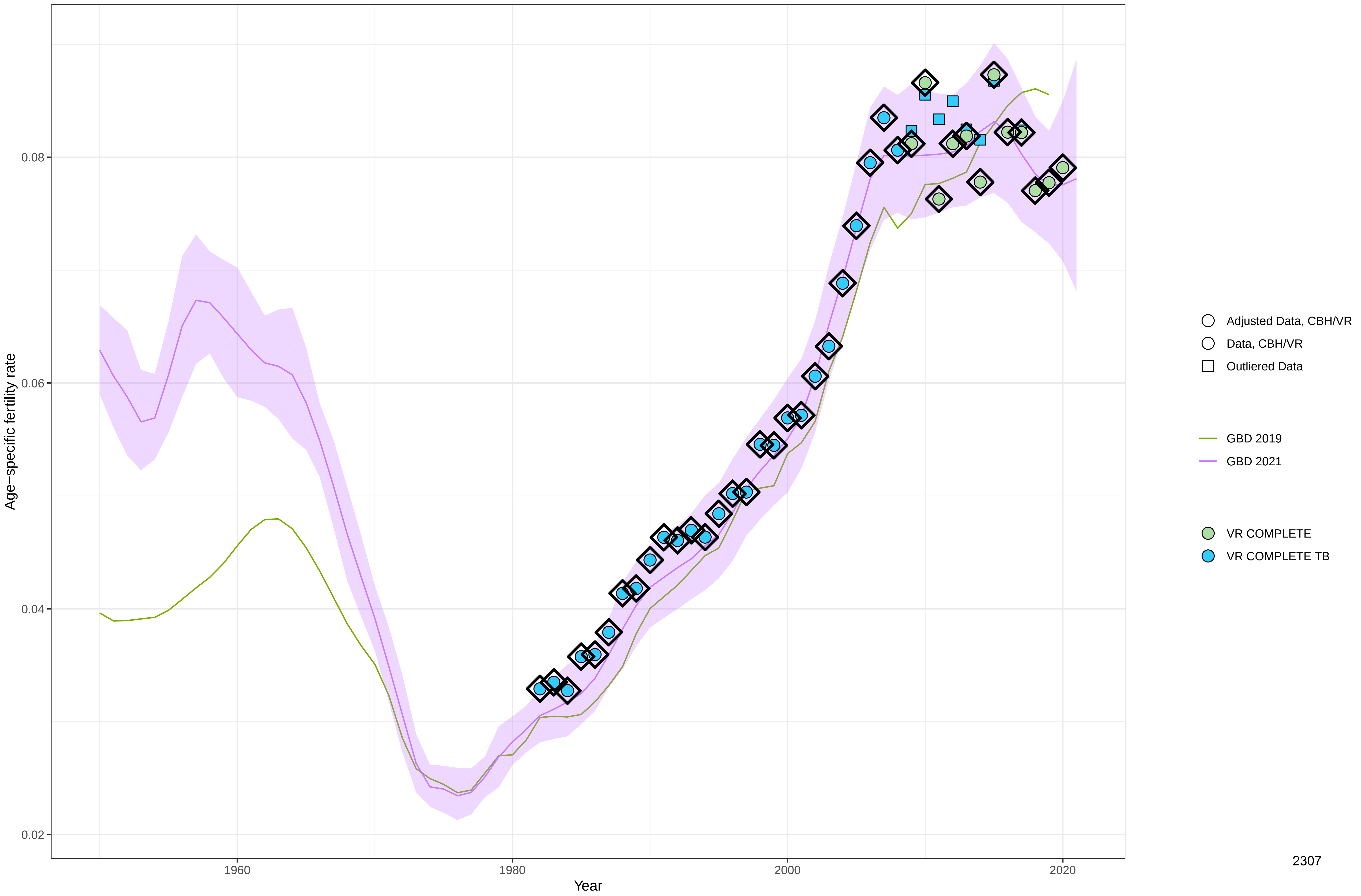

Age−specific fertility fitted trend lines, 1950−2021: Greenwich, Ages: 40−44
Data points enclosed with a diamond are reference sources

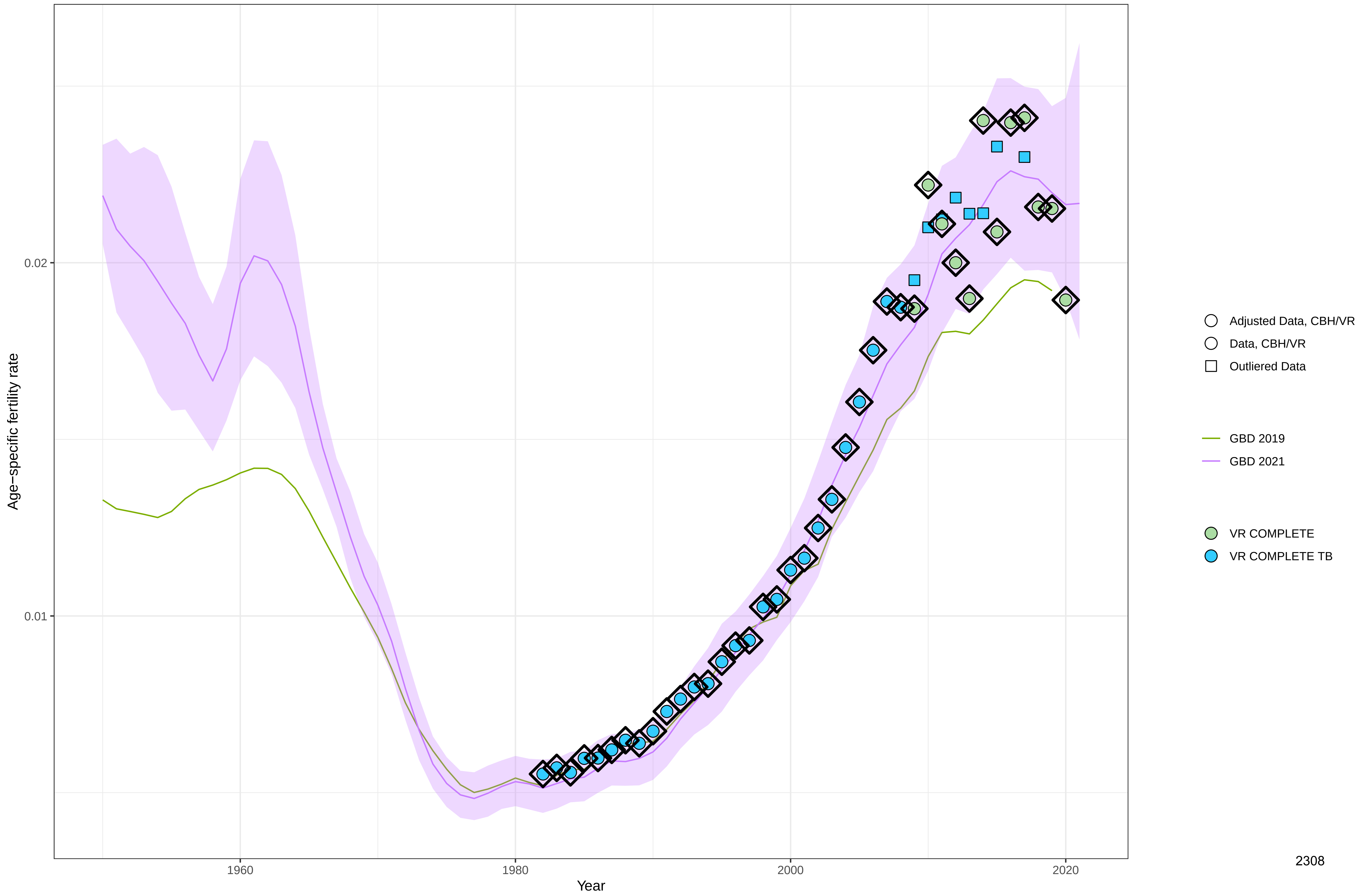

Age−specific fertility fitted trend lines, 1950−2021: Greenwich, Ages: 45−49
Data points enclosed with a diamond are reference sources

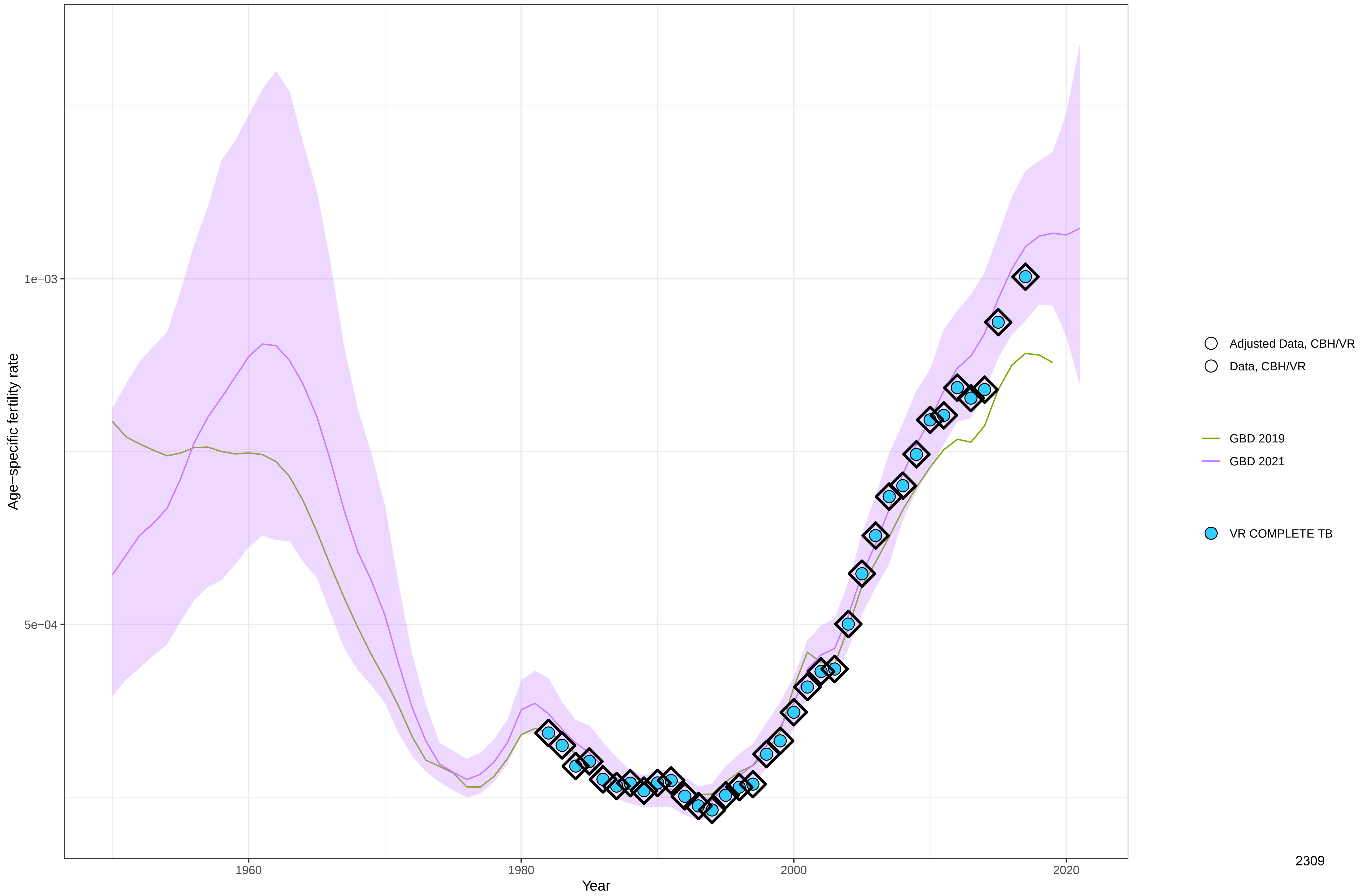

Age−specific fertility fitted trend lines, 1950−2021: Greenwich, Ages: 50−54
Data points enclosed with a diamond are reference sources

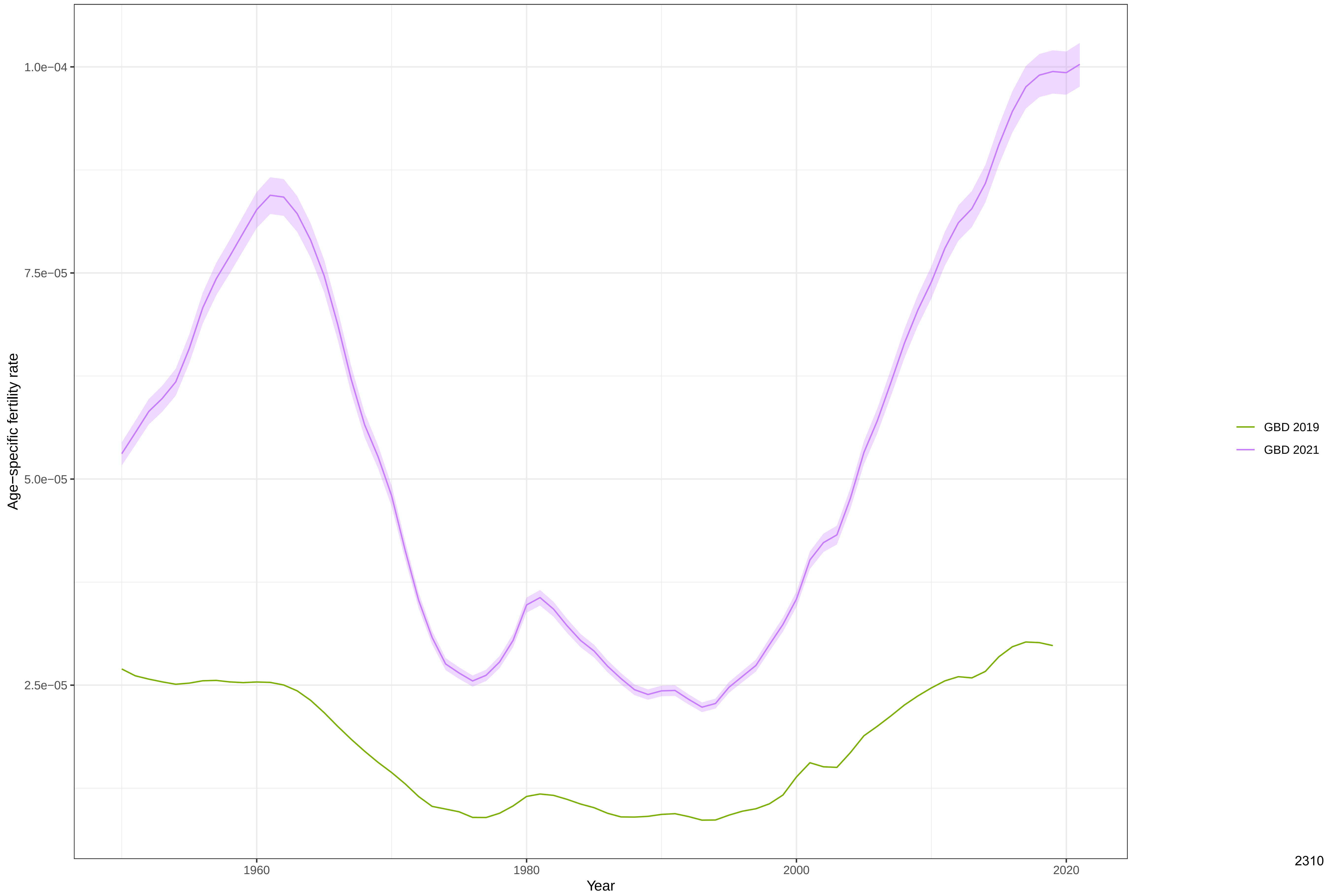

Total fertility rate fitted trend lines, 1950−2021: Greenwich TFR
Data points enclosed with a diamond are reference sources

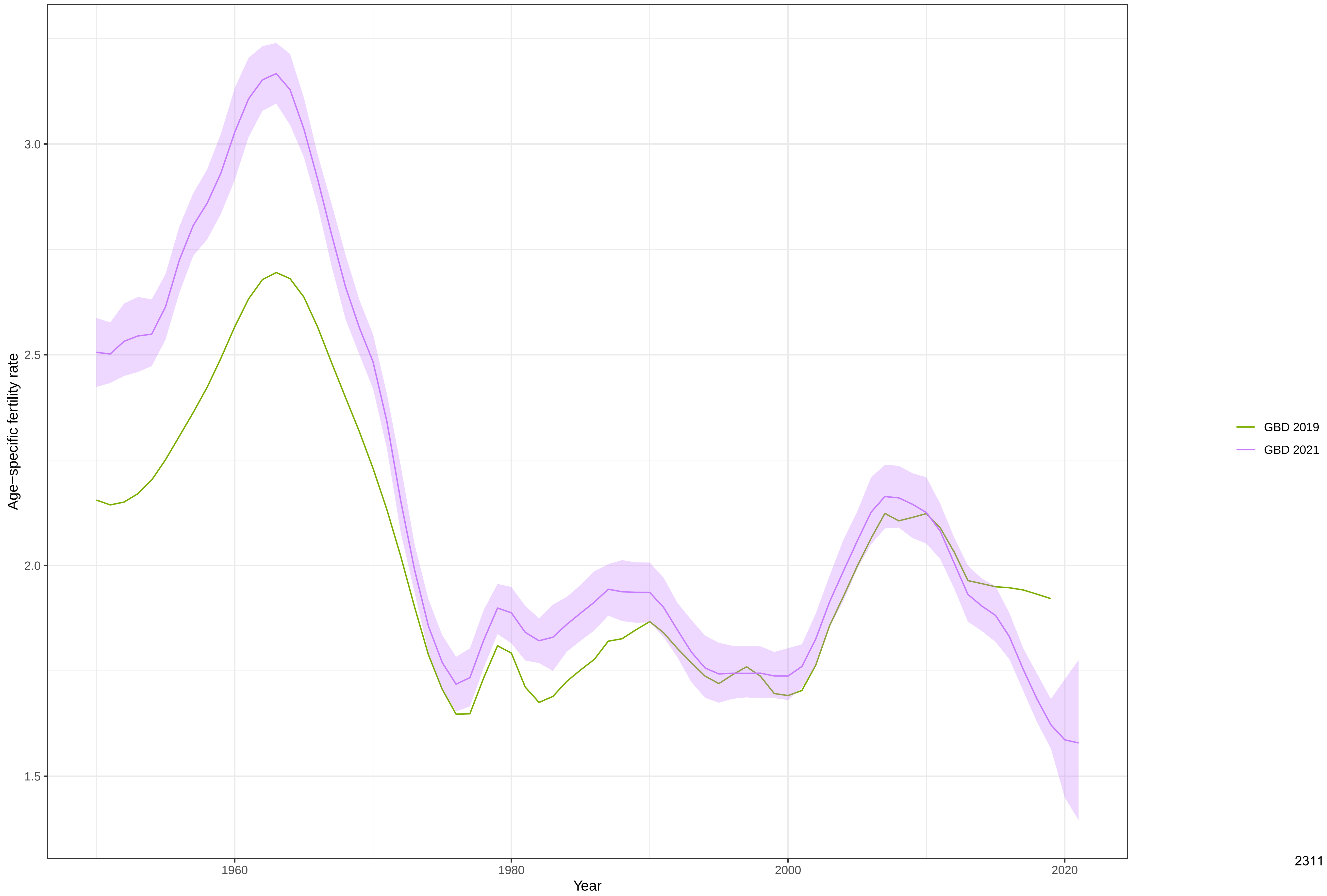

Age−specific fertility fitted trend lines, 1950−2021: Hackney, Ages: 10−14
Data points enclosed with a diamond are reference sources

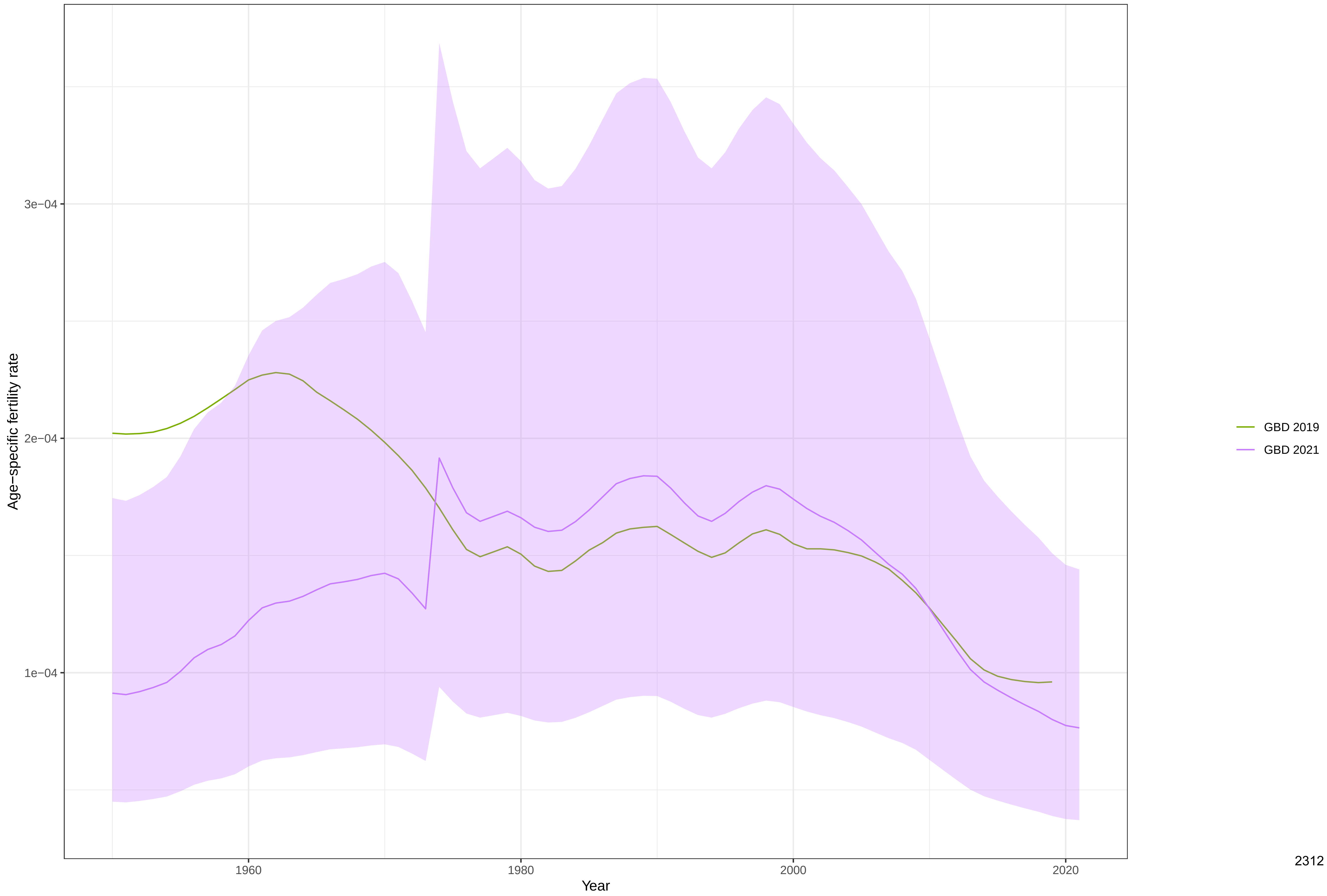

Age−specific fertility fitted trend lines, 1950−2021: Hackney, Ages: 15−19
Data points enclosed with a diamond are reference sources

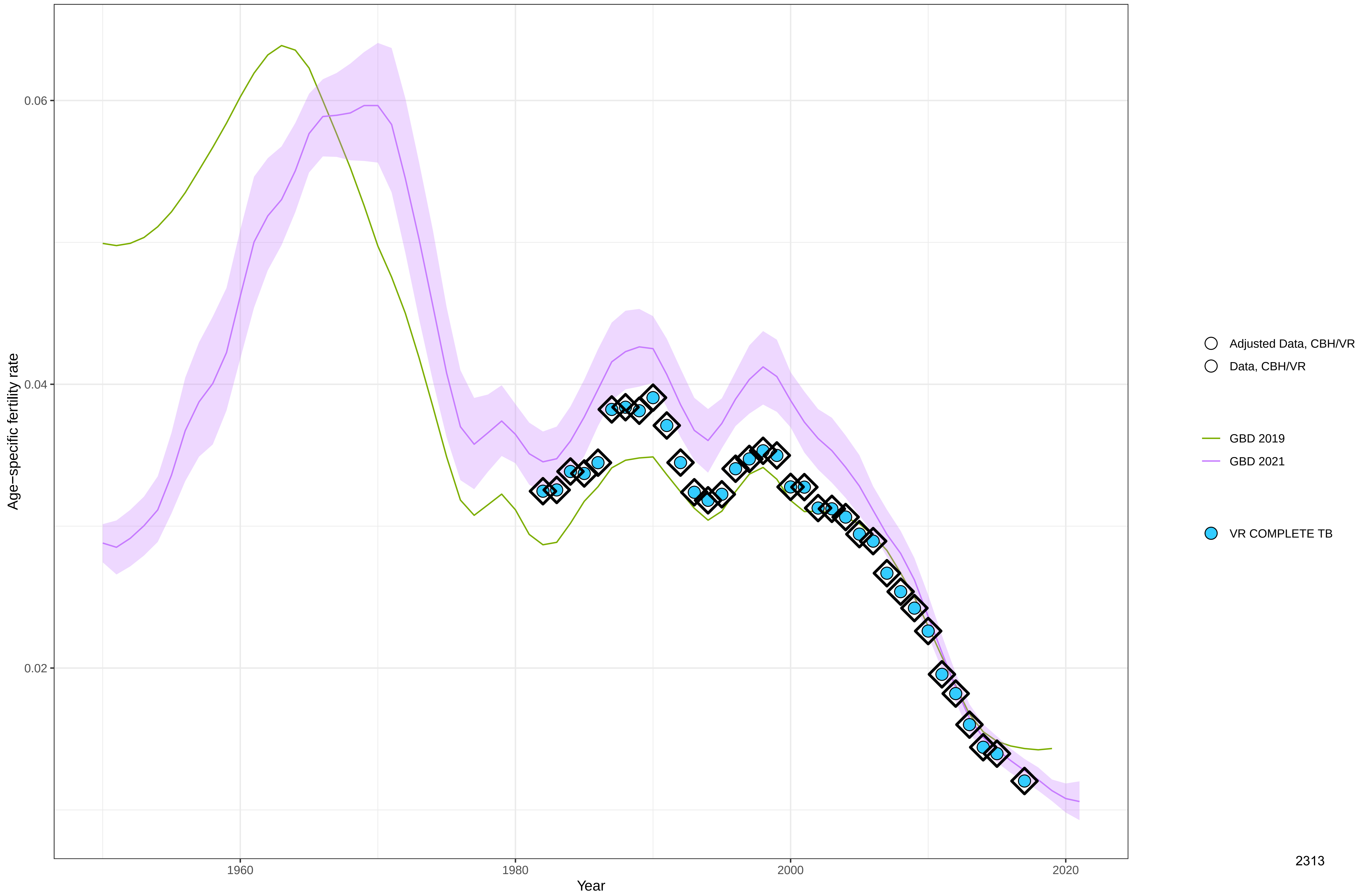

Age−specific fertility fitted trend lines, 1950−2021: Hackney, Ages: 20−24
Data points enclosed with a diamond are reference sources

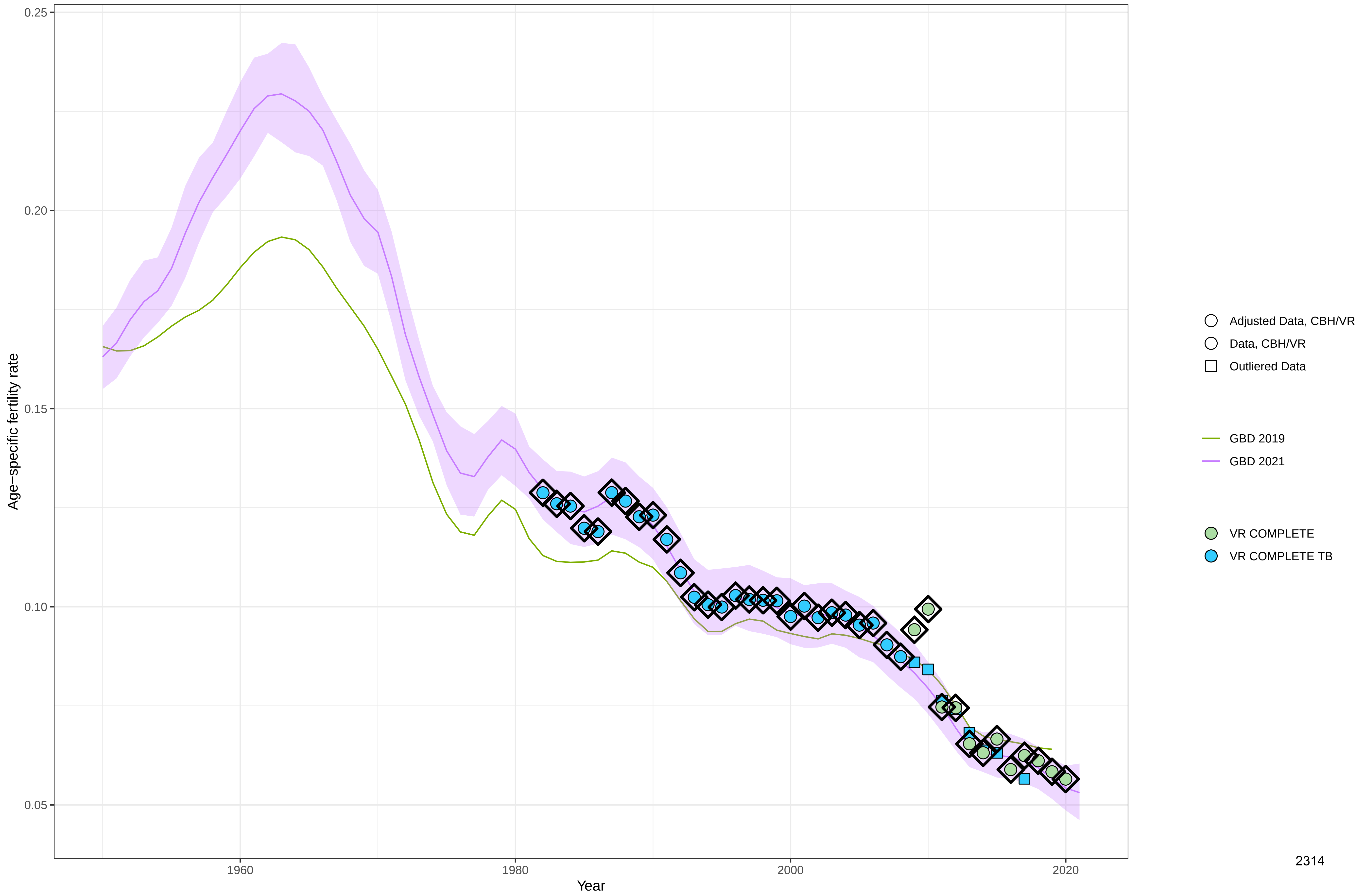

Age−specific fertility fitted trend lines, 1950−2021: Hackney, Ages: 25−29
Data points enclosed with a diamond are reference sources

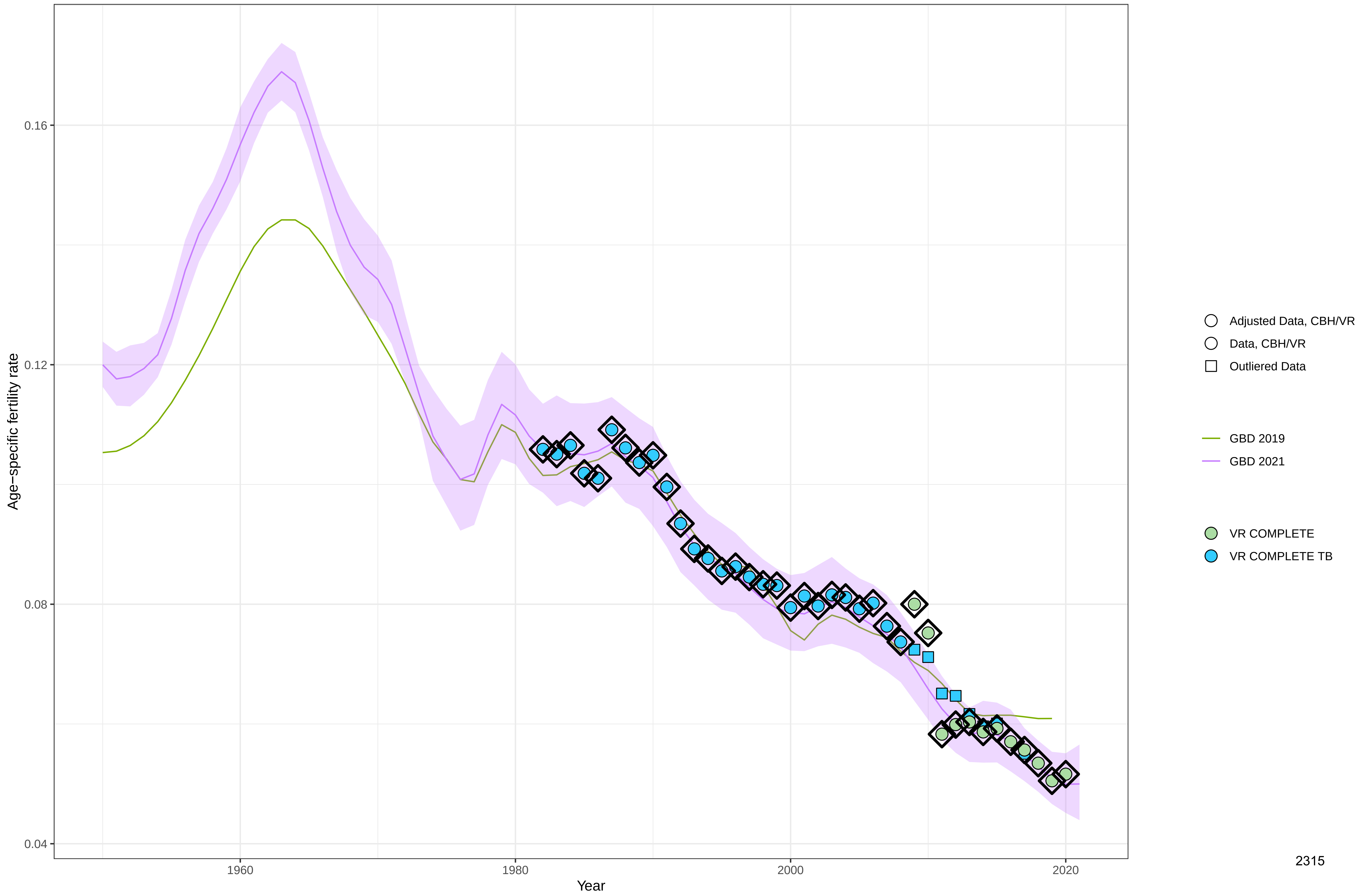

Age−specific fertility fitted trend lines, 1950−2021: Hackney, Ages: 30−34
Data points enclosed with a diamond are reference sources

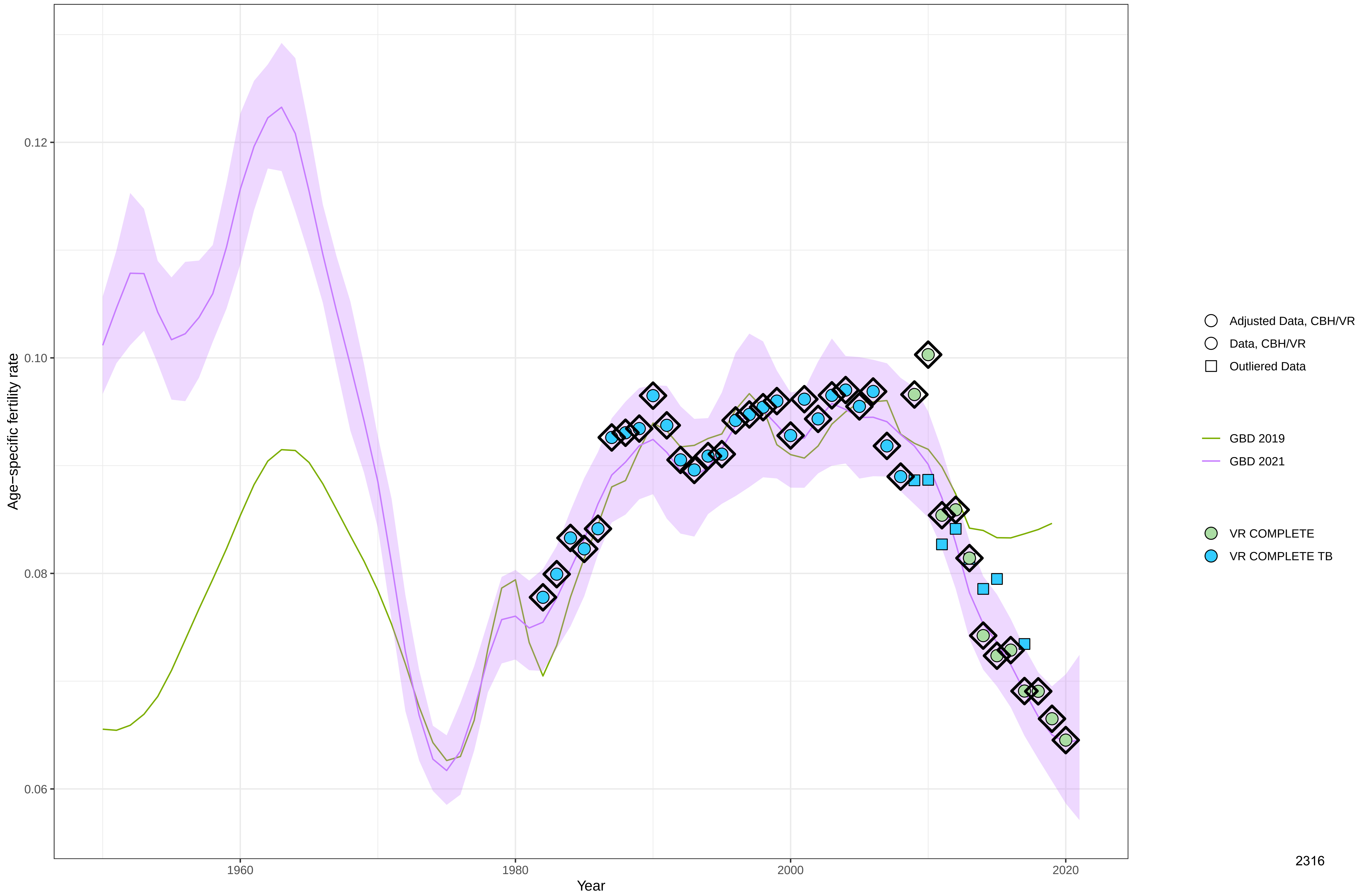

Age−specific fertility fitted trend lines, 1950−2021: Hackney, Ages: 35−39
Data points enclosed with a diamond are reference sources

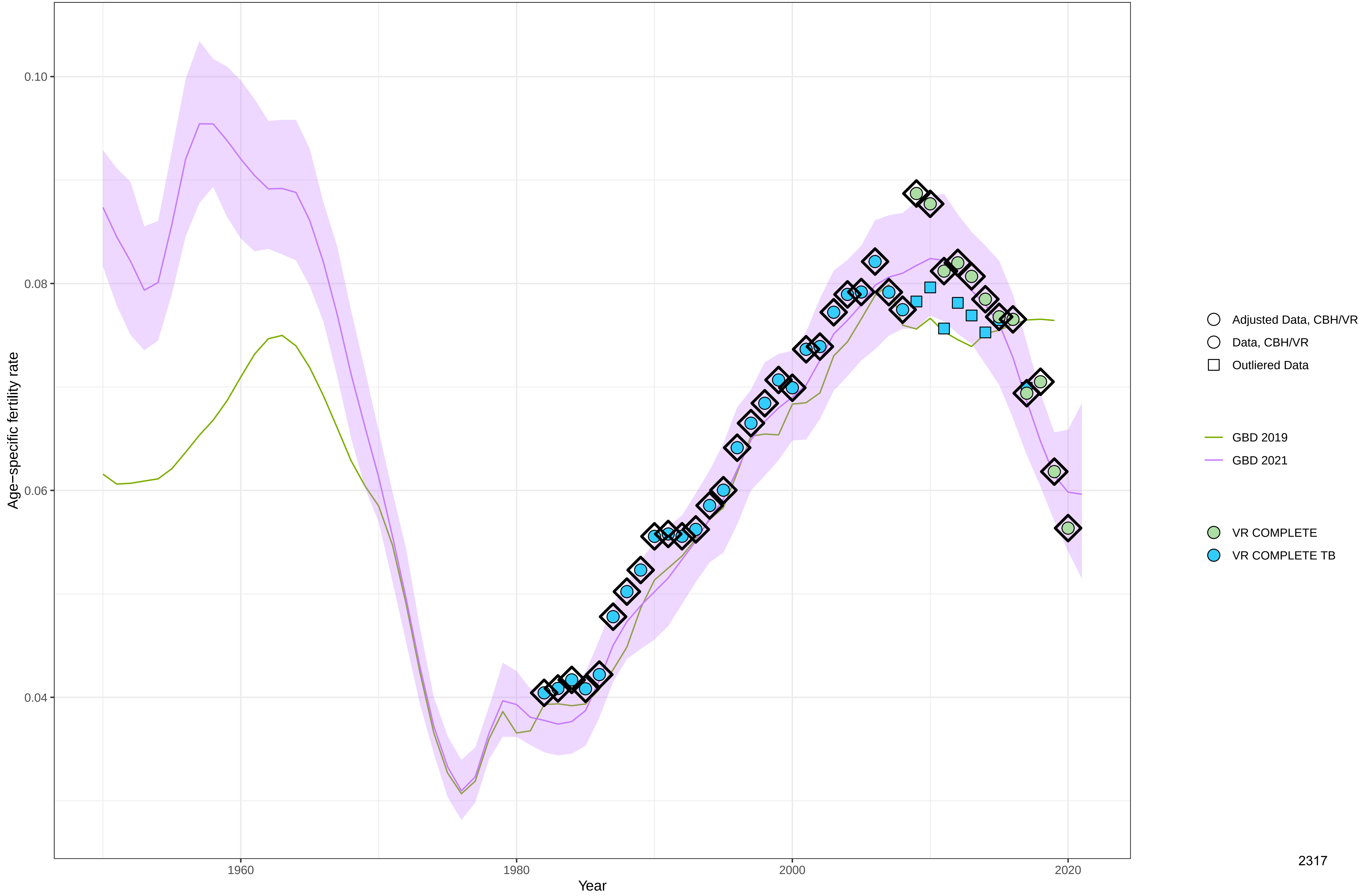

Age−specific fertility fitted trend lines, 1950−2021: Hackney, Ages: 40−44
Data points enclosed with a diamond are reference sources

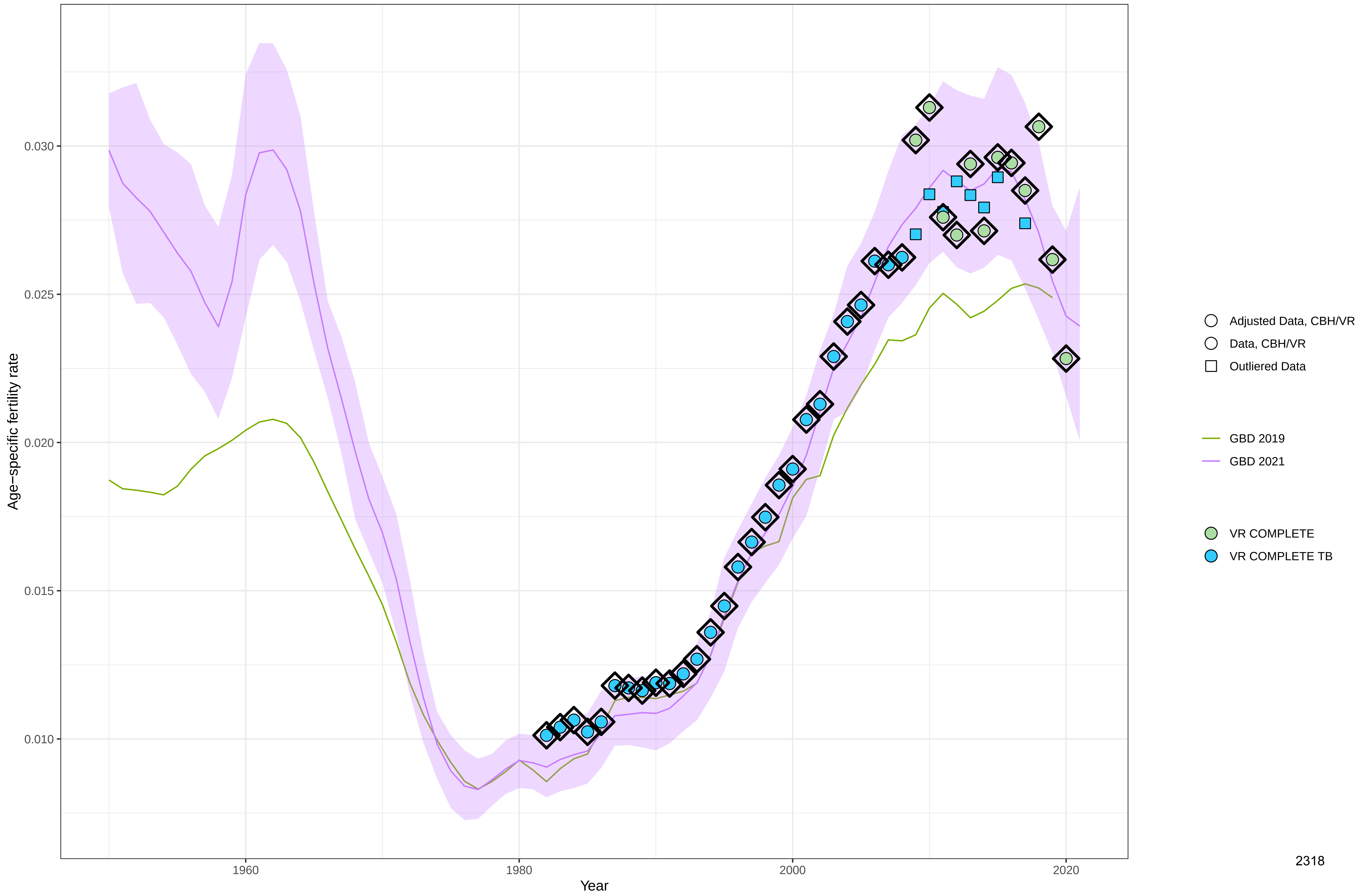

Age−specific fertility fitted trend lines, 1950−2021: Hackney, Ages: 45−49
Data points enclosed with a diamond are reference sources

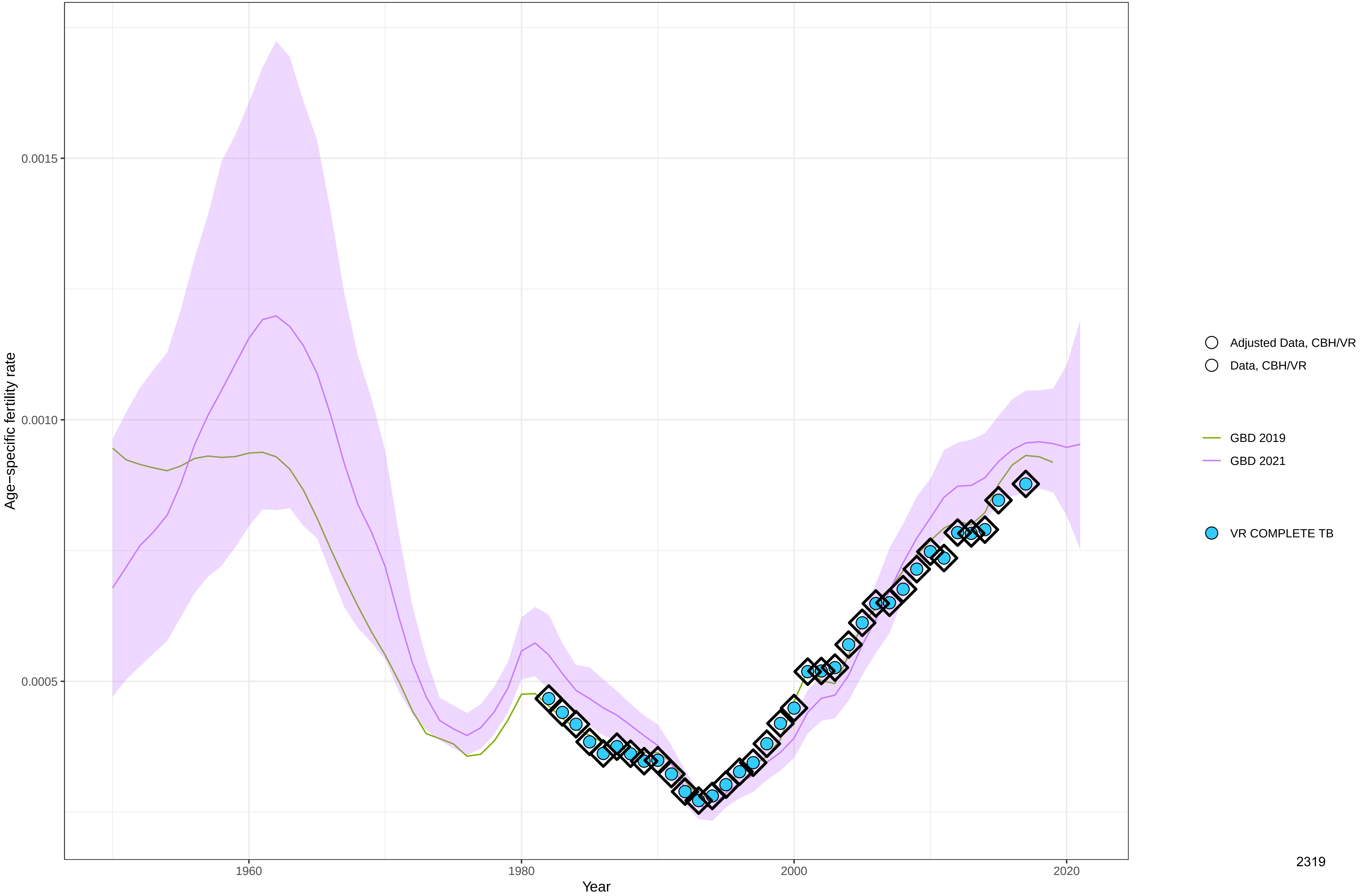

Age−specific fertility fitted trend lines, 1950−2021: Hackney, Ages: 50−54
Data points enclosed with a diamond are reference sources

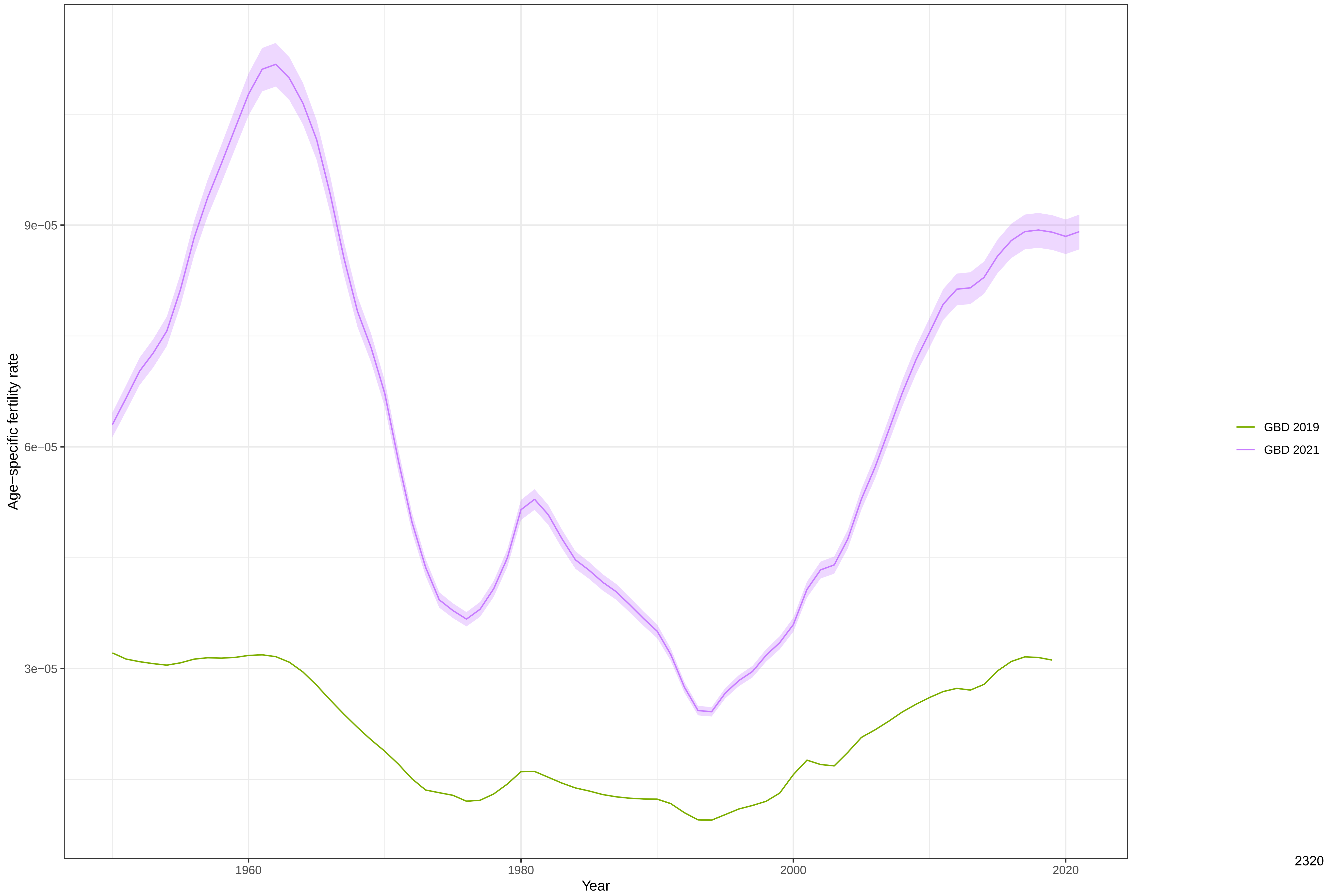

Total fertility rate fitted trend lines, 1950−2021: Hackney TFR
Data points enclosed with a diamond are reference sources

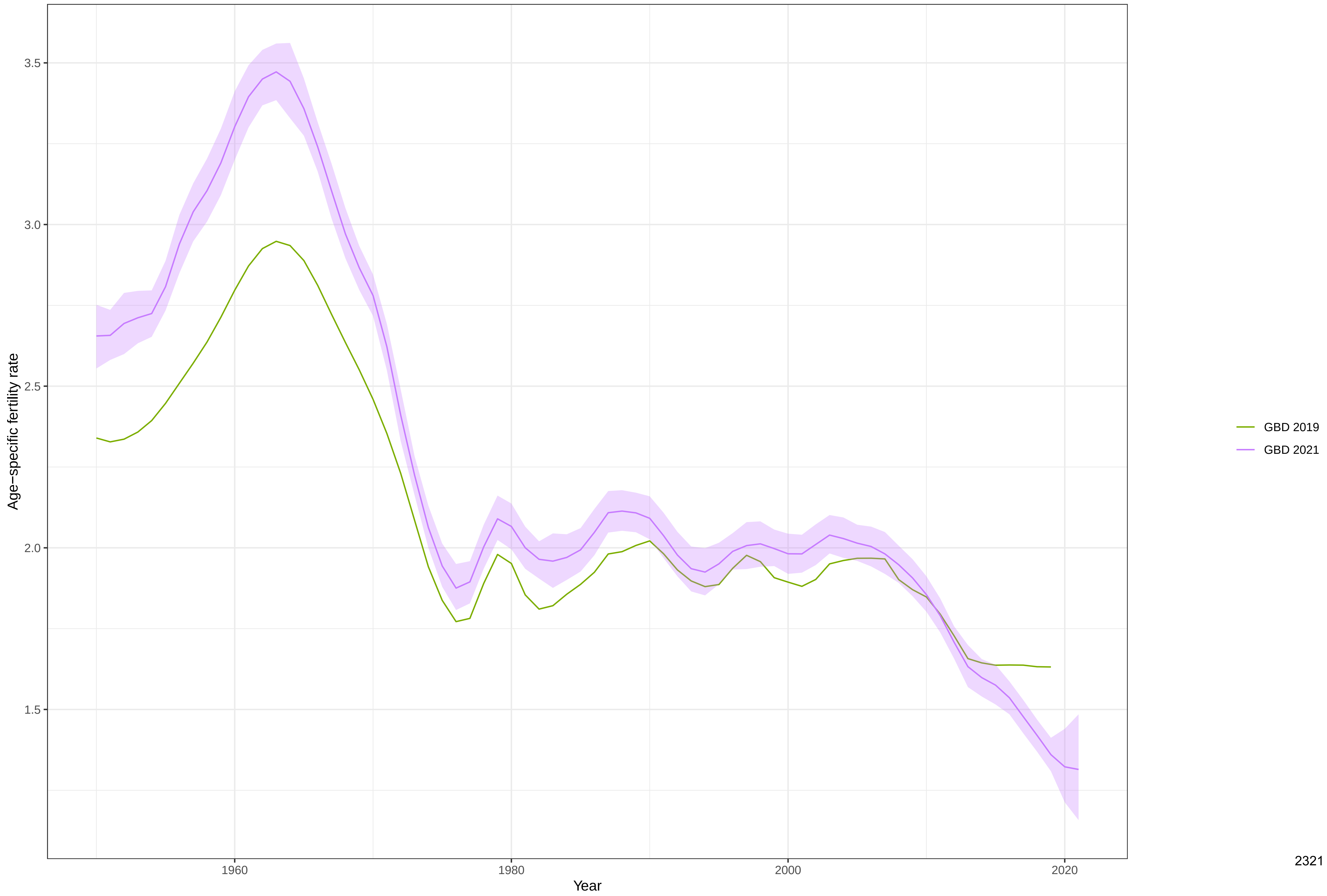

Age−specific fertility fitted trend lines, 1950−2021: Hammersmith and Fulham, Ages: 10−14
Data points enclosed with a diamond are reference sources

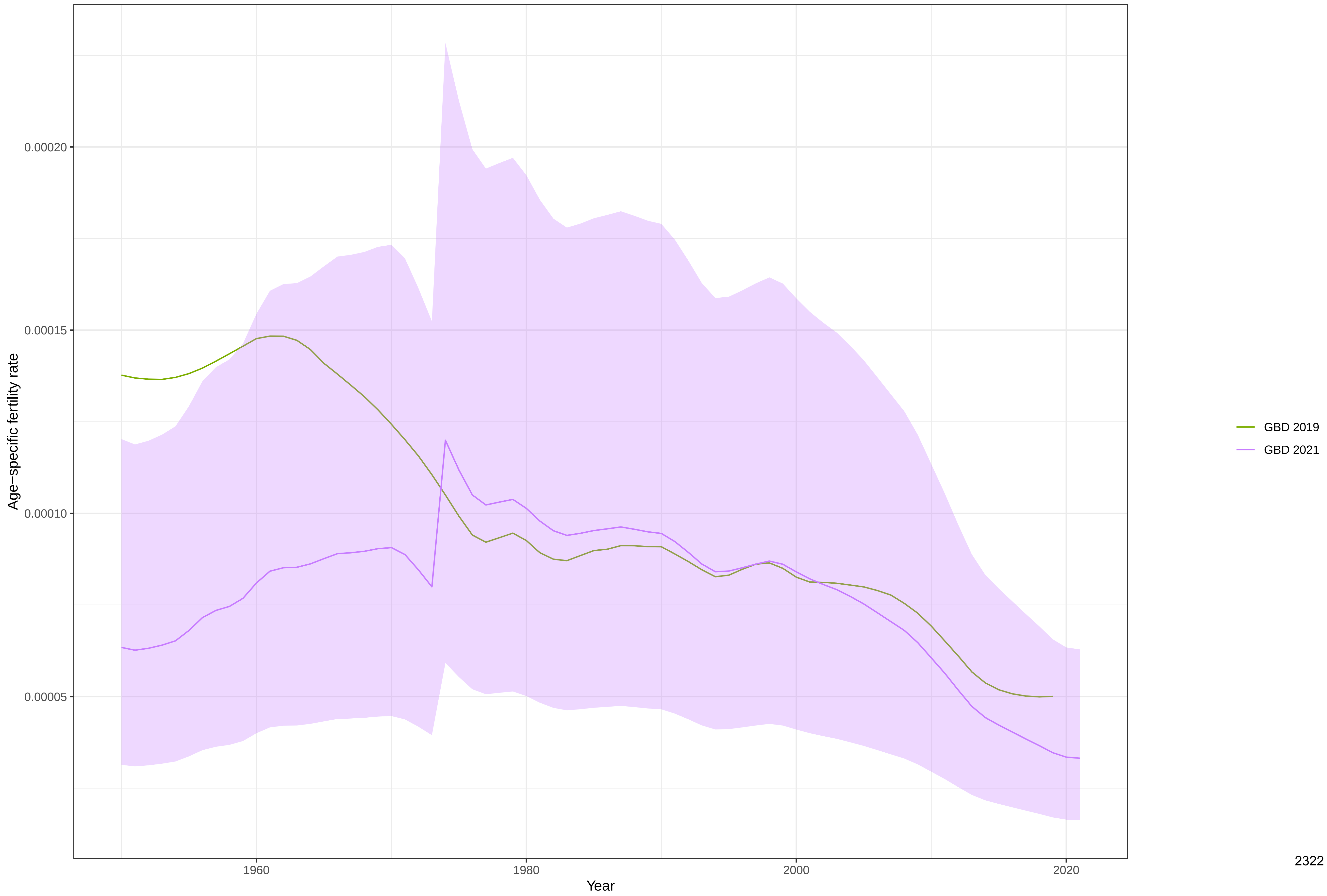

Age−specific fertility fitted trend lines, 1950−2021: Hammersmith and Fulham, Ages: 15−19
Data points enclosed with a diamond are reference sources

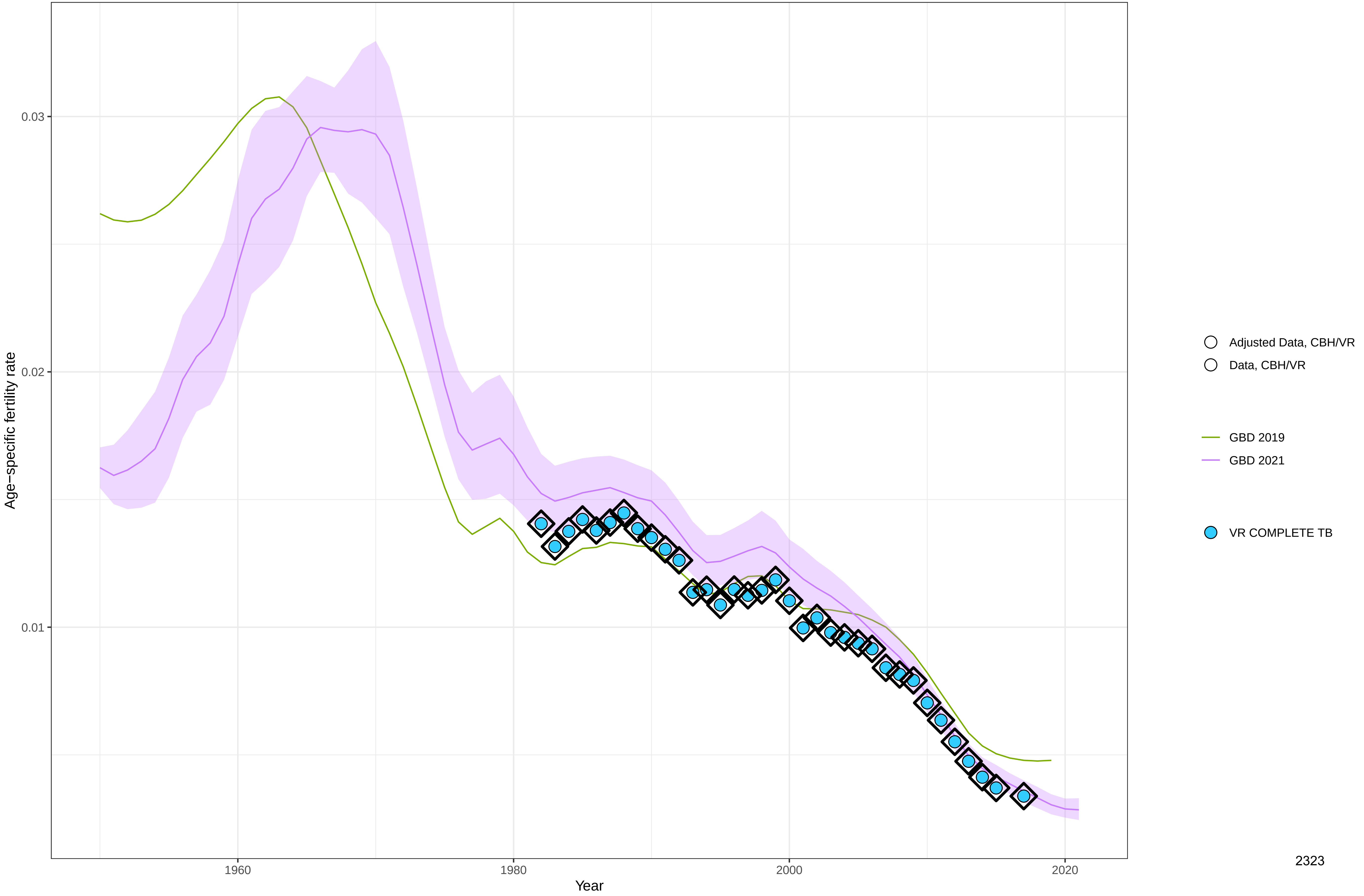

Age−specific fertility fitted trend lines, 1950−2021: Hammersmith and Fulham, Ages: 20−24
Data points enclosed with a diamond are reference sources

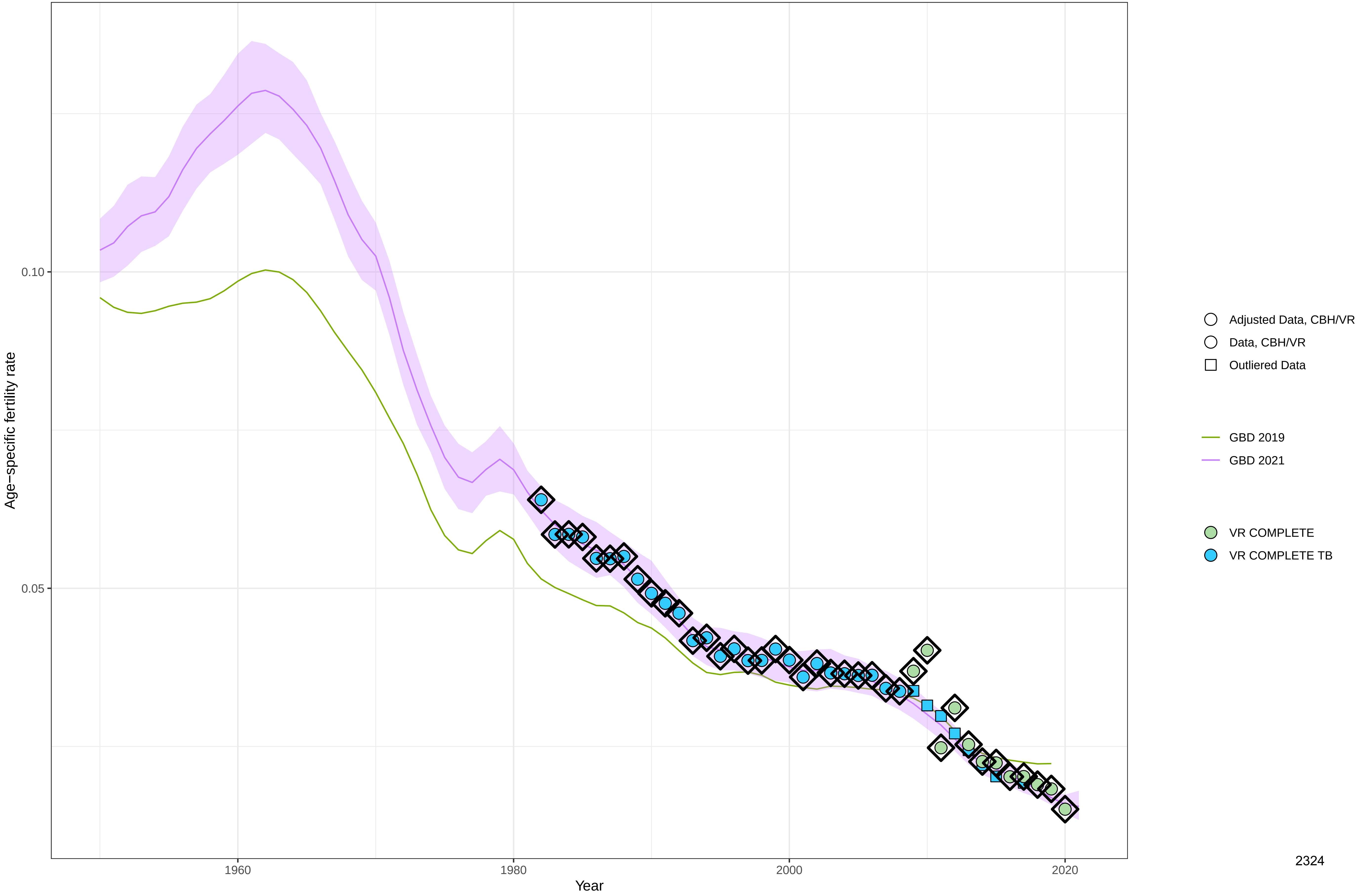

Age−specific fertility fitted trend lines, 1950−2021: Hammersmith and Fulham, Ages: 25−29
Data points enclosed with a diamond are reference sources

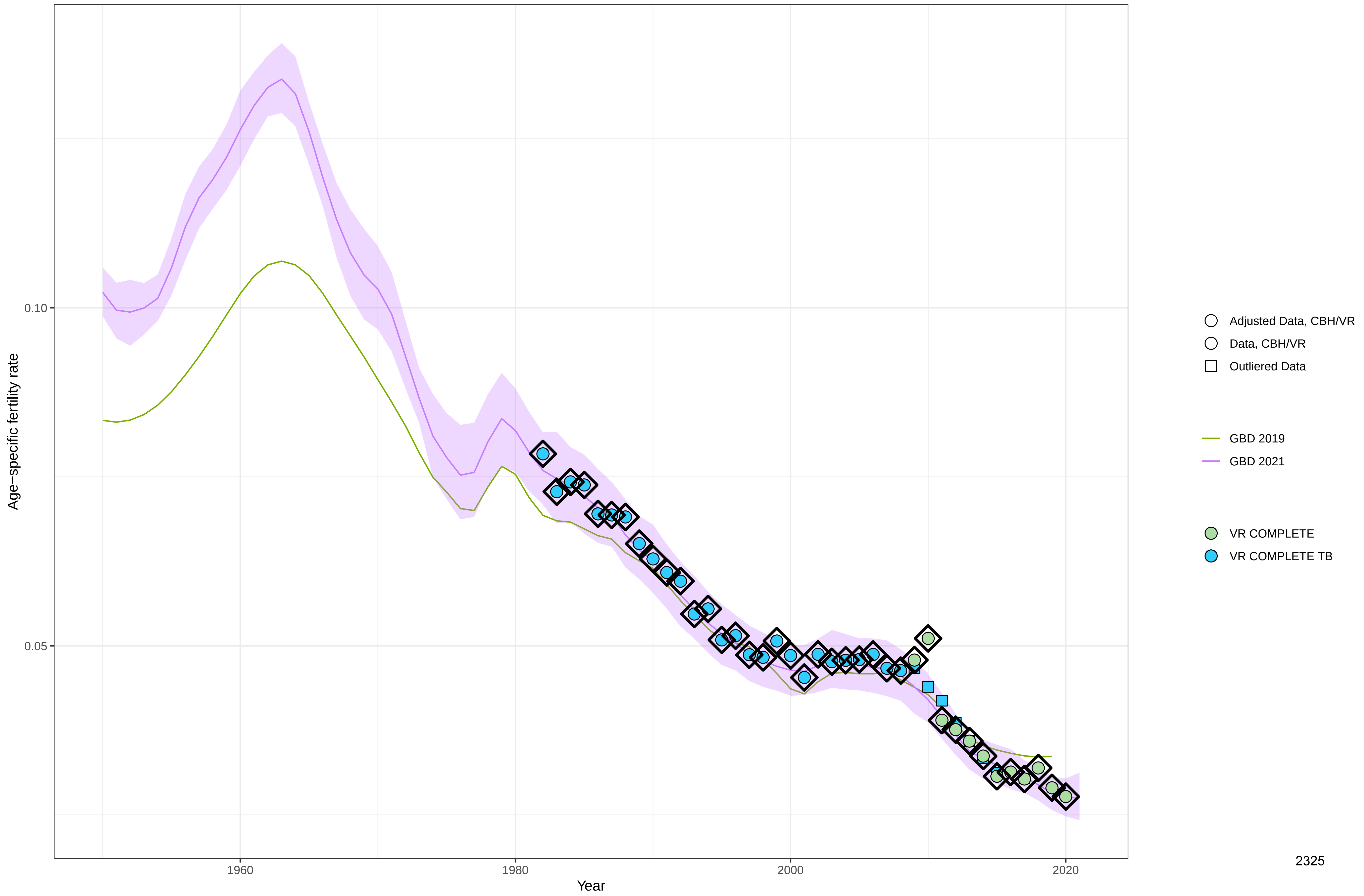

Age−specific fertility fitted trend lines, 1950−2021: Hammersmith and Fulham, Ages: 30−34
Data points enclosed with a diamond are reference sources

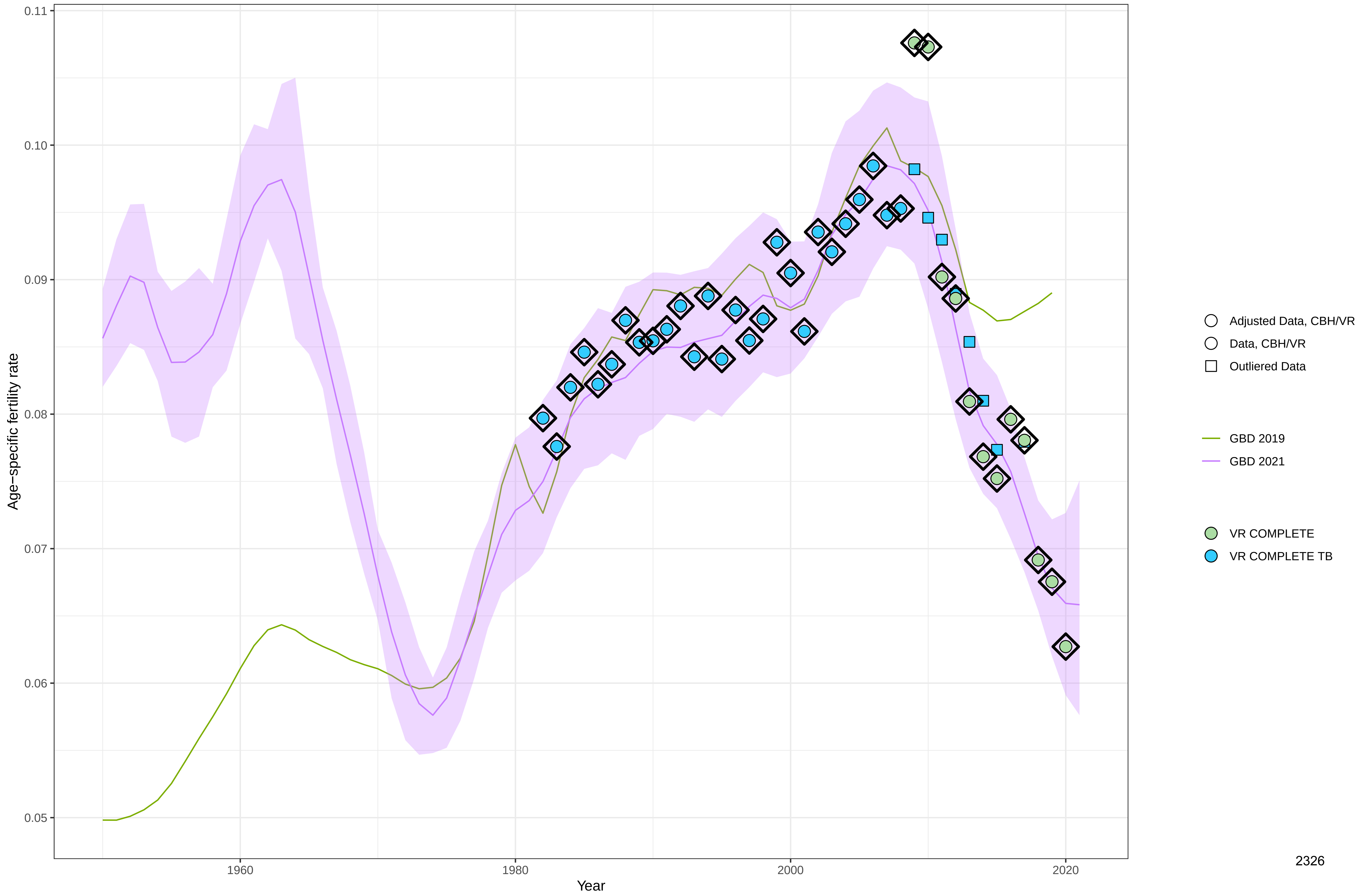

Age−specific fertility fitted trend lines, 1950−2021: Hammersmith and Fulham, Ages: 35−39
Data points enclosed with a diamond are reference sources

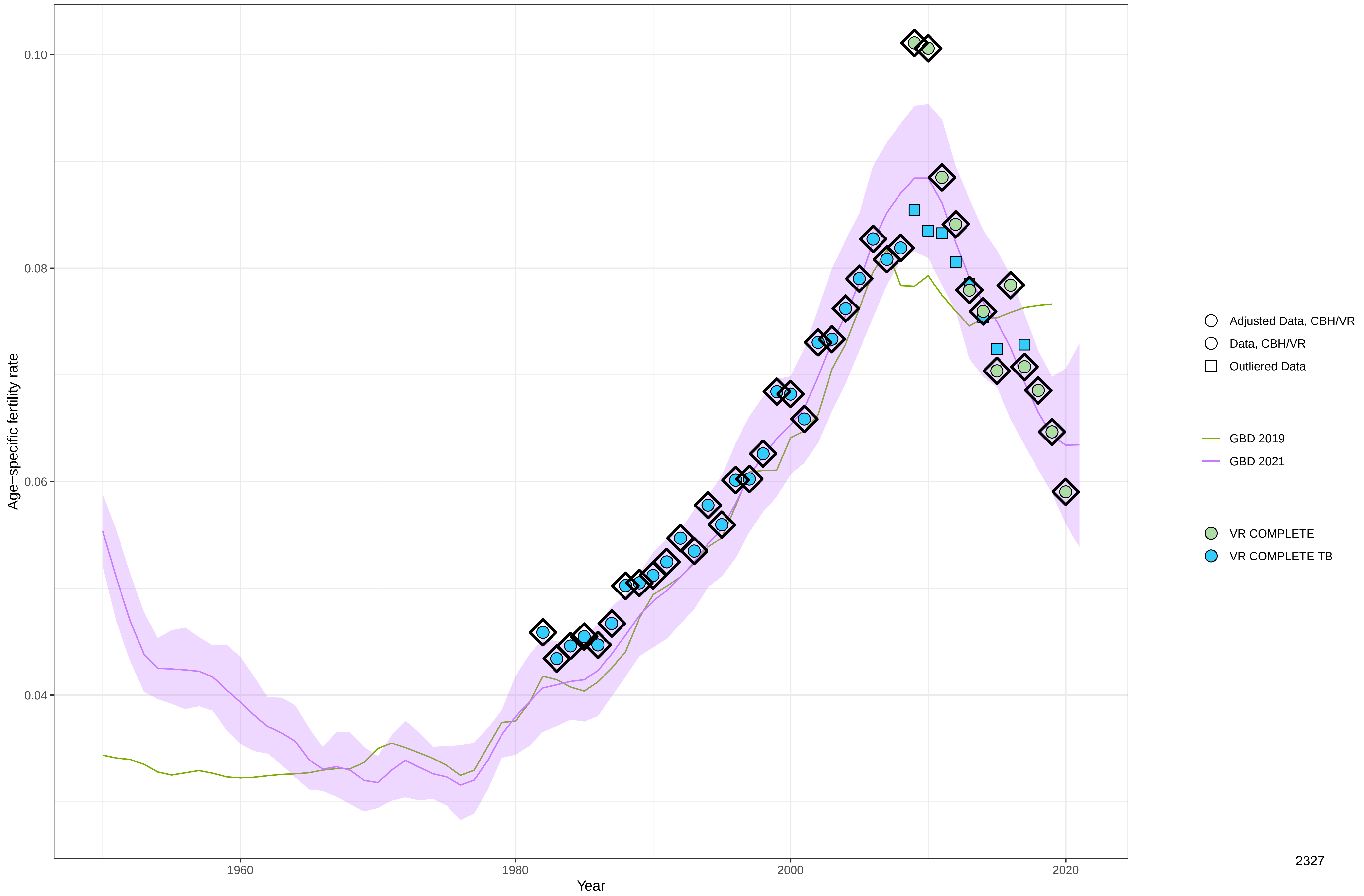

Age−specific fertility fitted trend lines, 1950−2021: Hammersmith and Fulham, Ages: 40−44
Data points enclosed with a diamond are reference sources

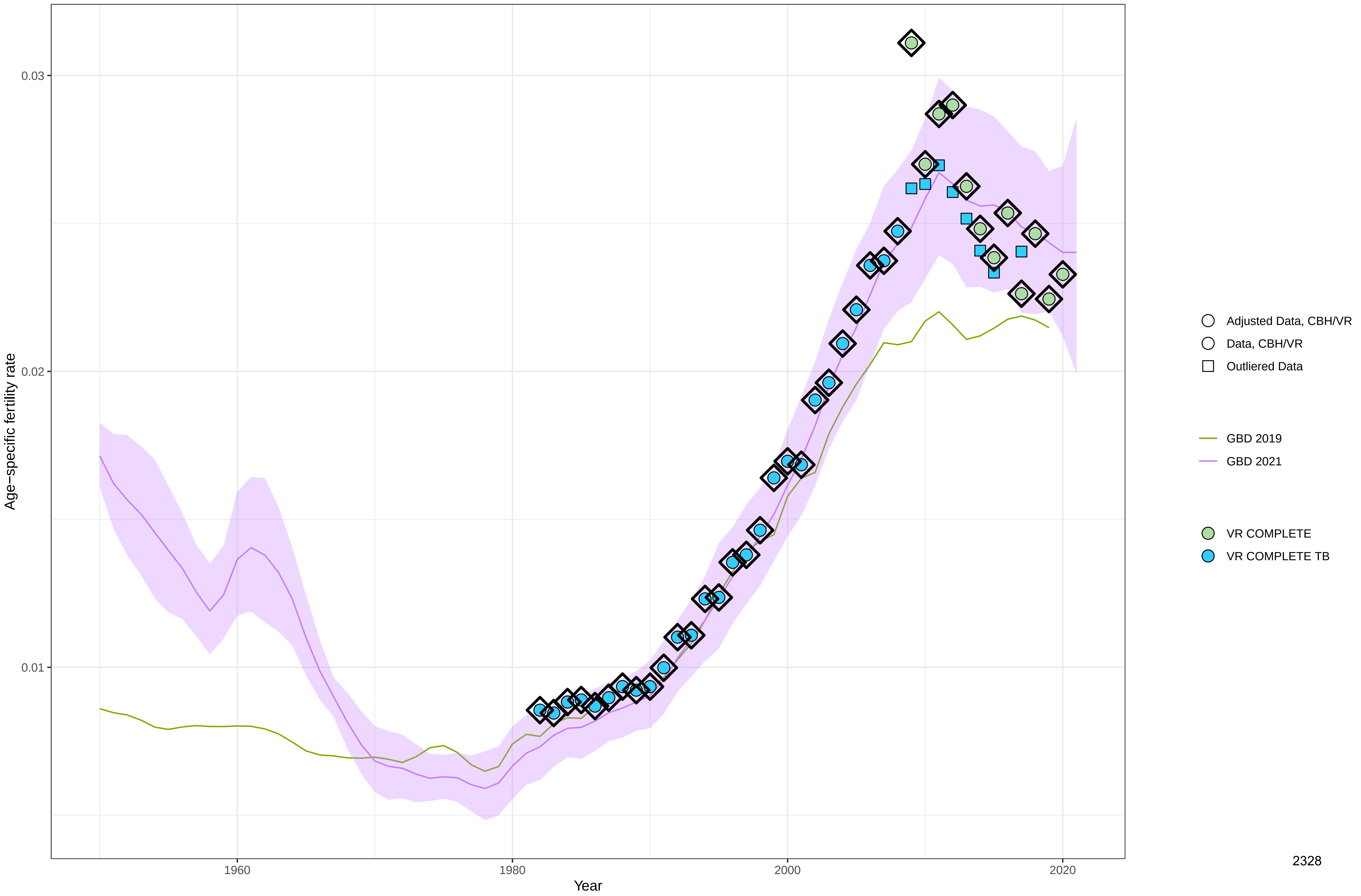

Age−specific fertility fitted trend lines, 1950−2021: Hammersmith and Fulham, Ages: 45−49
Data points enclosed with a diamond are reference sources

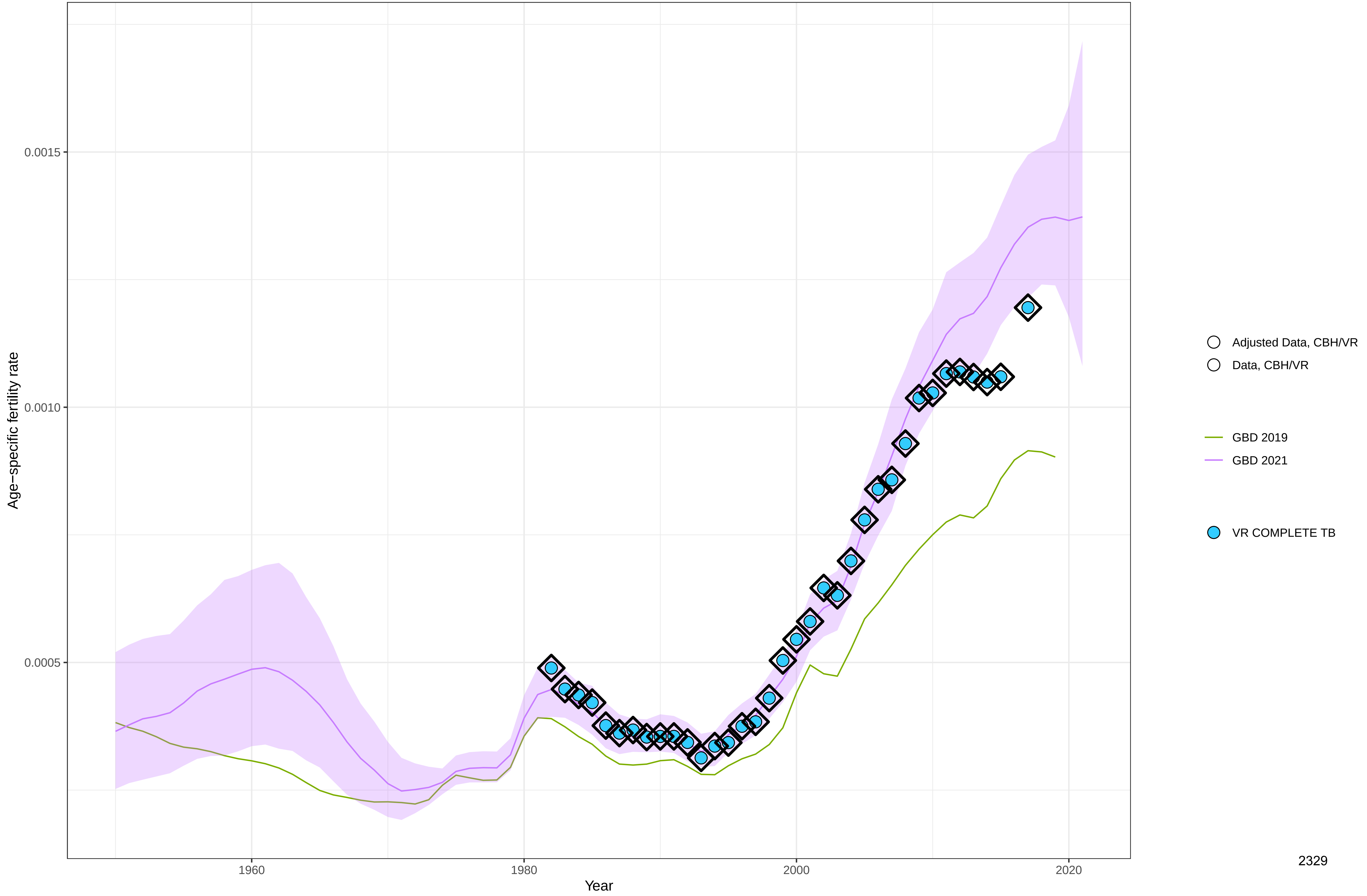

Age−specific fertility fitted trend lines, 1950−2021: Hammersmith and Fulham, Ages: 50−54
Data points enclosed with a diamond are reference sources

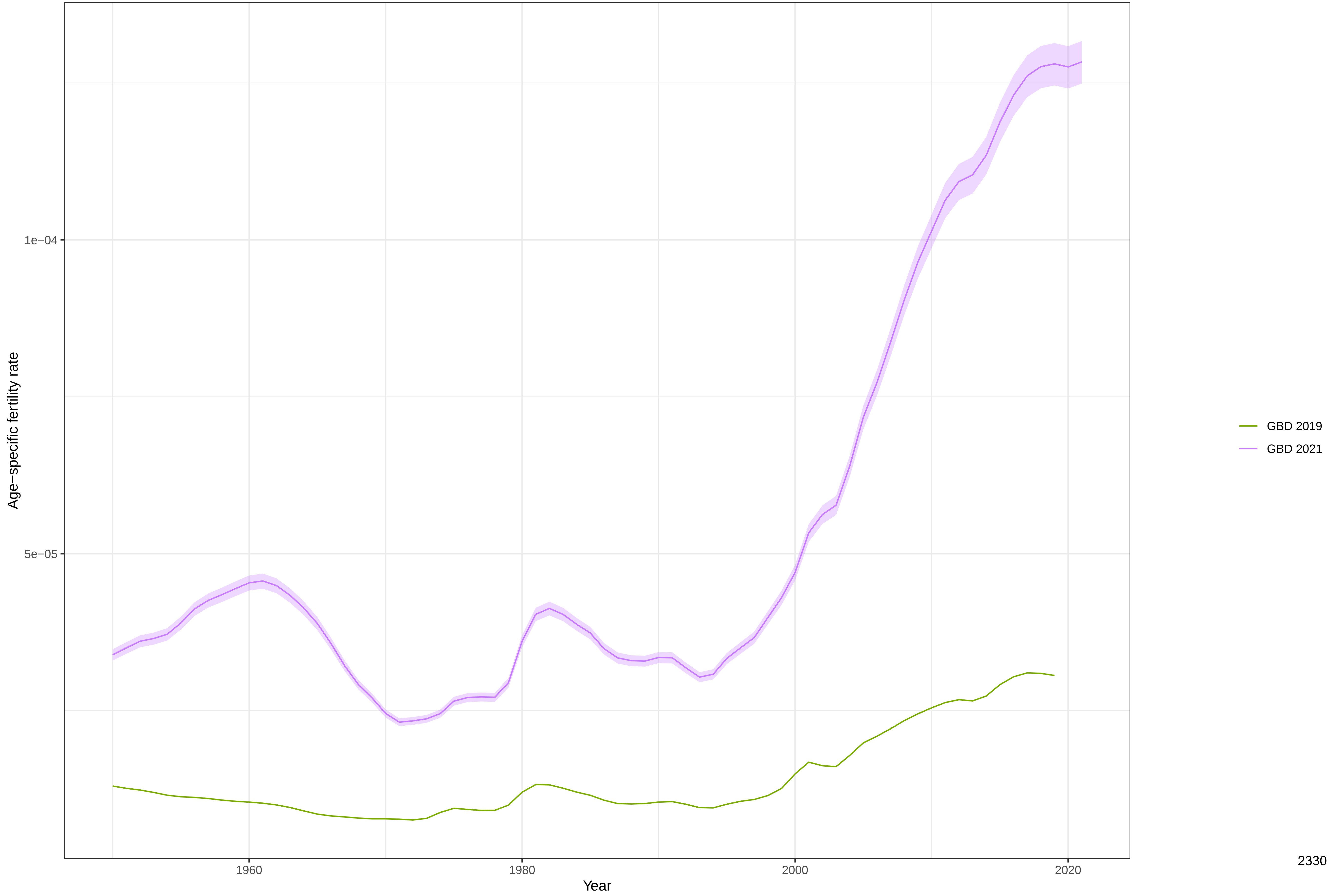

Total fertility rate fitted trend lines, 1950−2021: Hammersmith and Fulham TFR
Data points enclosed with a diamond are reference sources

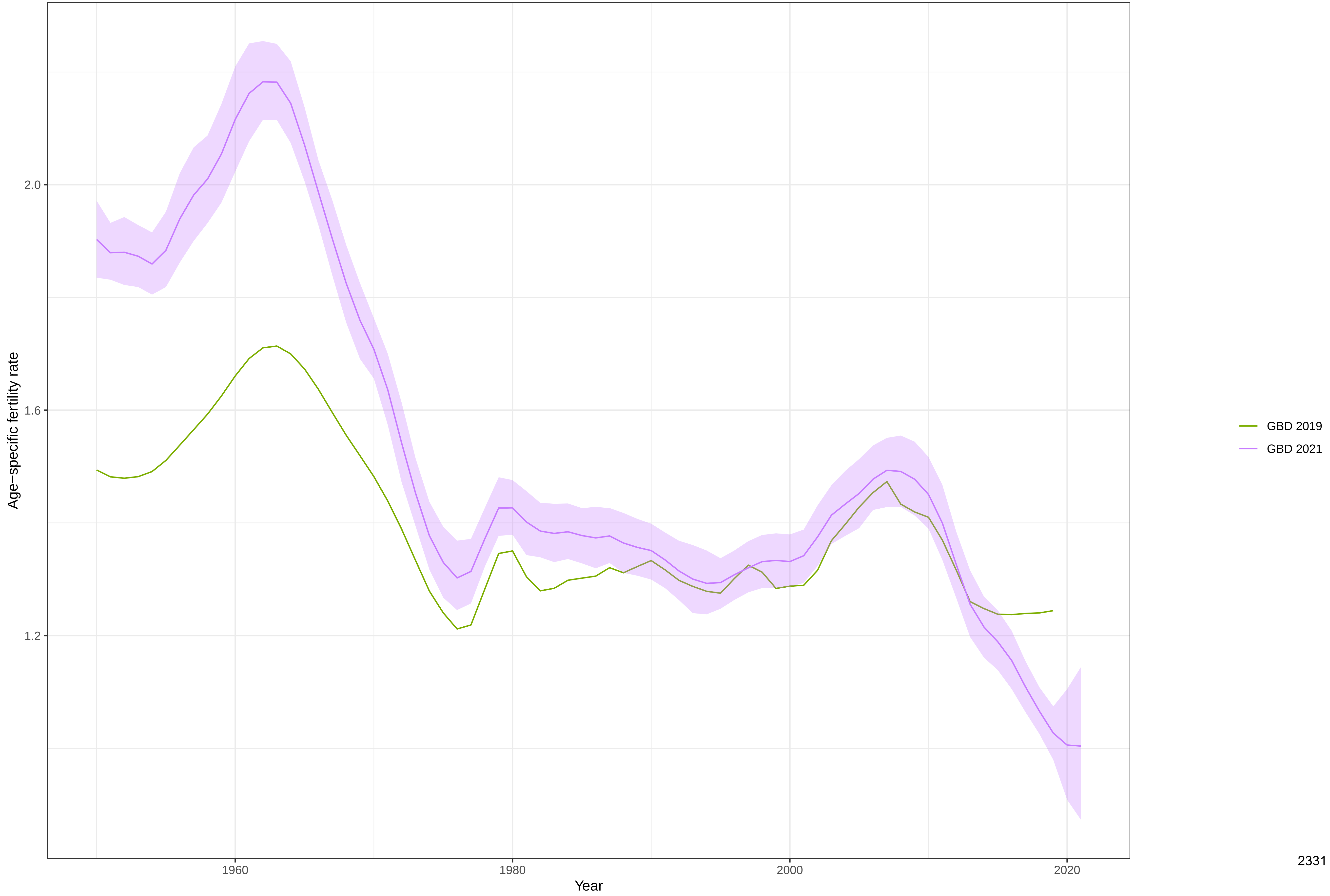

Age−specific fertility fitted trend lines, 1950−2021: Haringey, Ages: 10−14
Data points enclosed with a diamond are reference sources

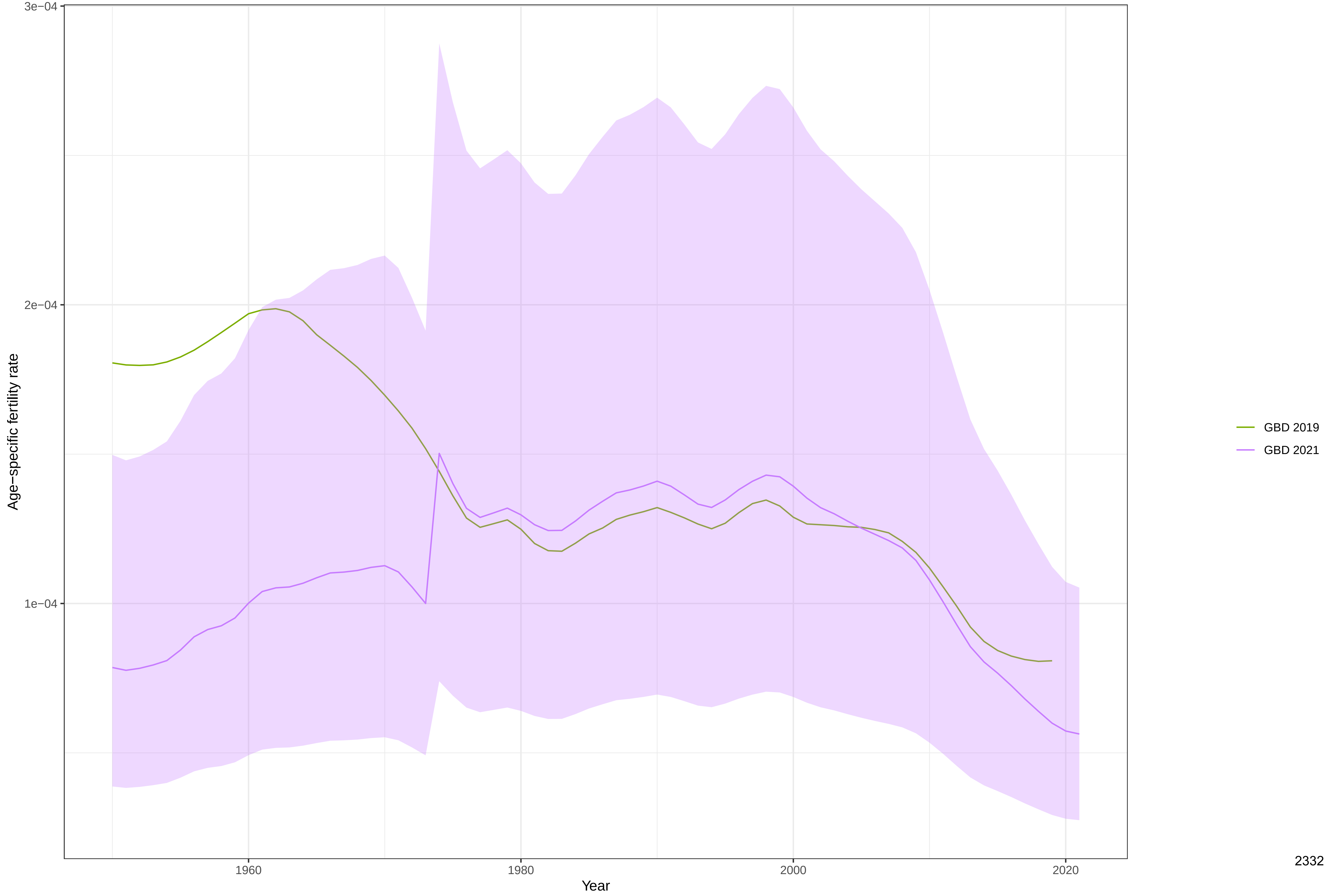

Age−specific fertility fitted trend lines, 1950−2021: Haringey, Ages: 15−19
Data points enclosed with a diamond are reference sources

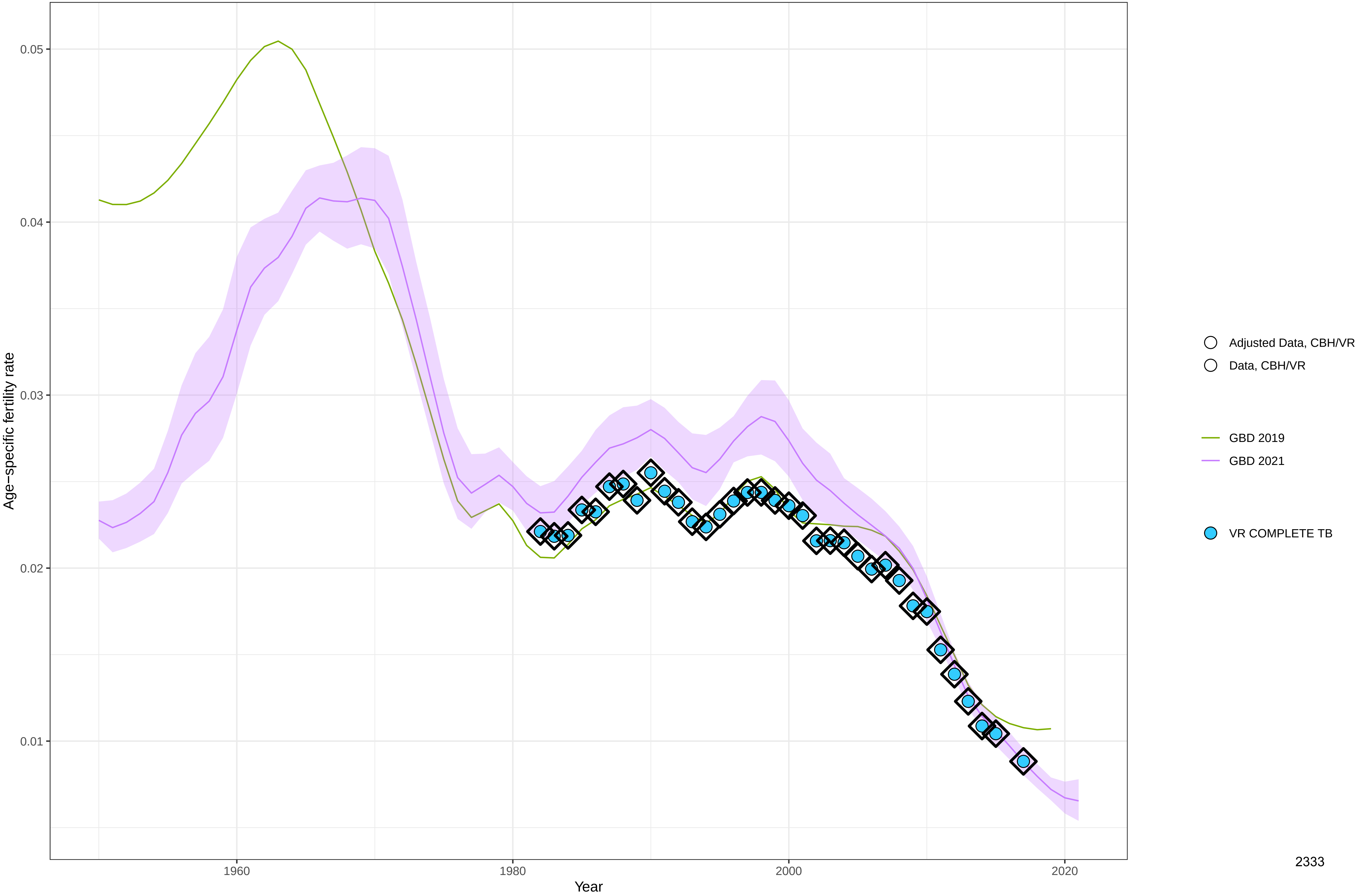

Age−specific fertility fitted trend lines, 1950−2021: Haringey, Ages: 20−24
Data points enclosed with a diamond are reference sources

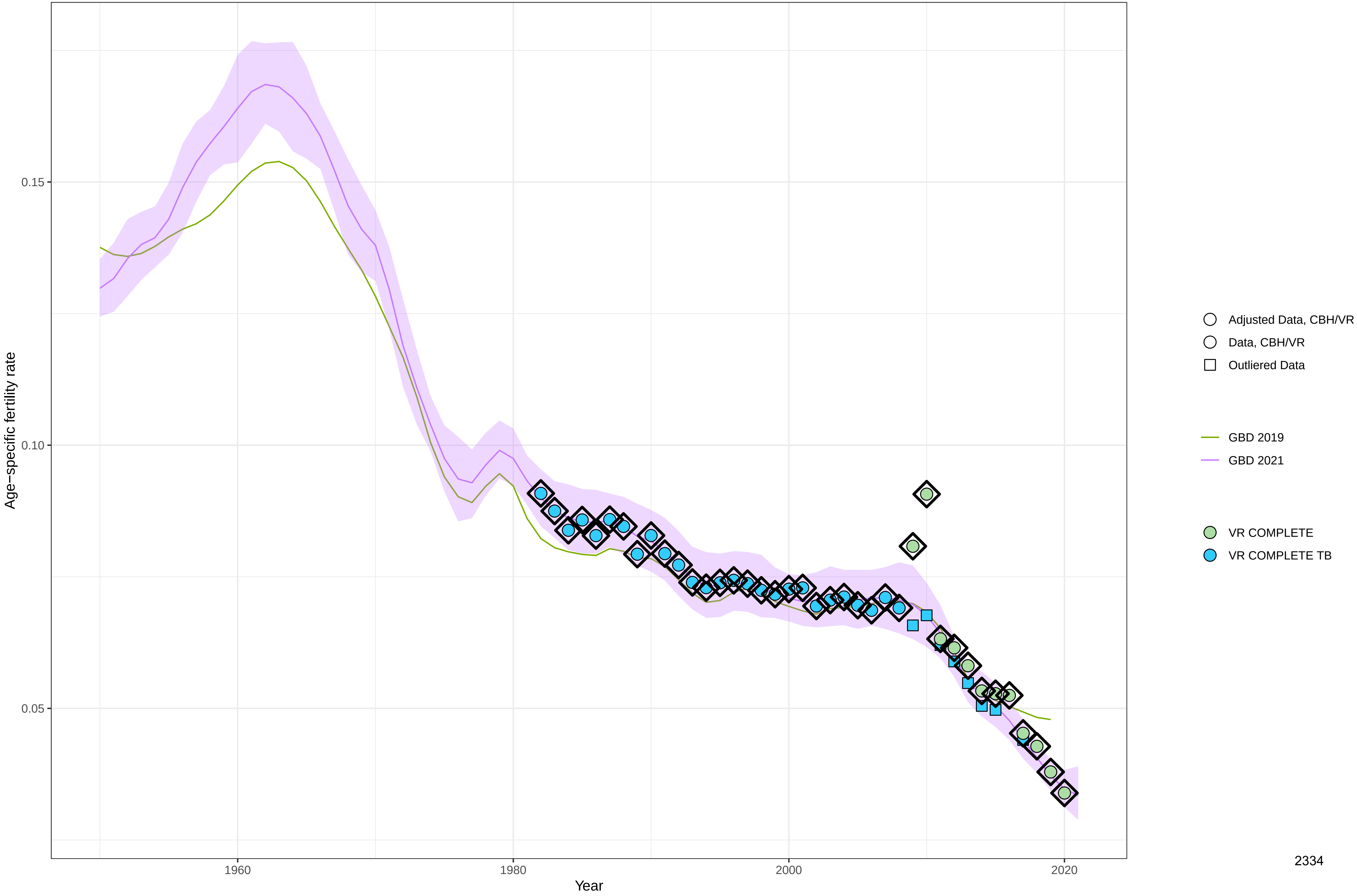

Age−specific fertility fitted trend lines, 1950−2021: Haringey, Ages: 25−29
Data points enclosed with a diamond are reference sources

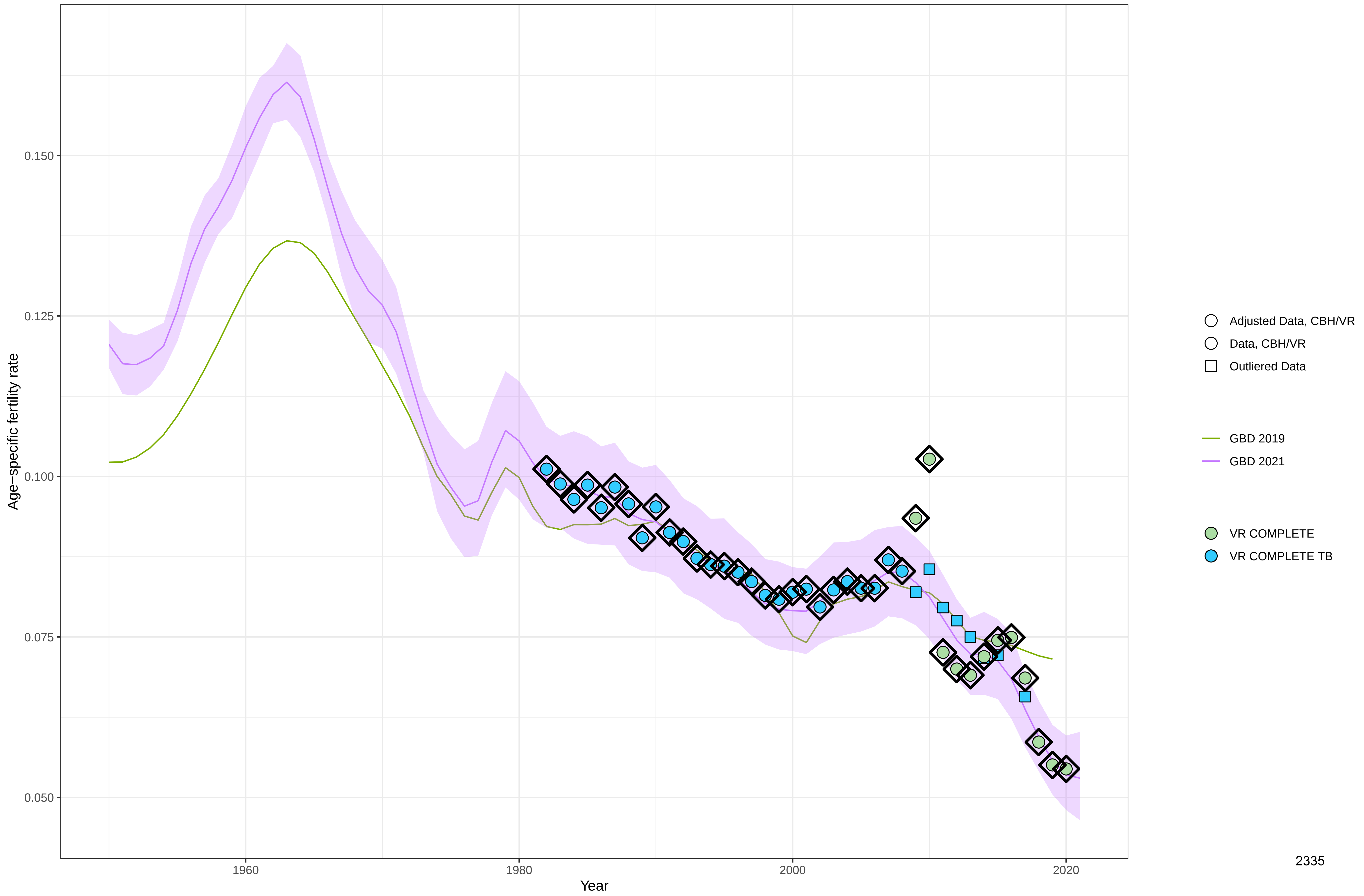

Age−specific fertility fitted trend lines, 1950−2021: Haringey, Ages: 30−34
Data points enclosed with a diamond are reference sources

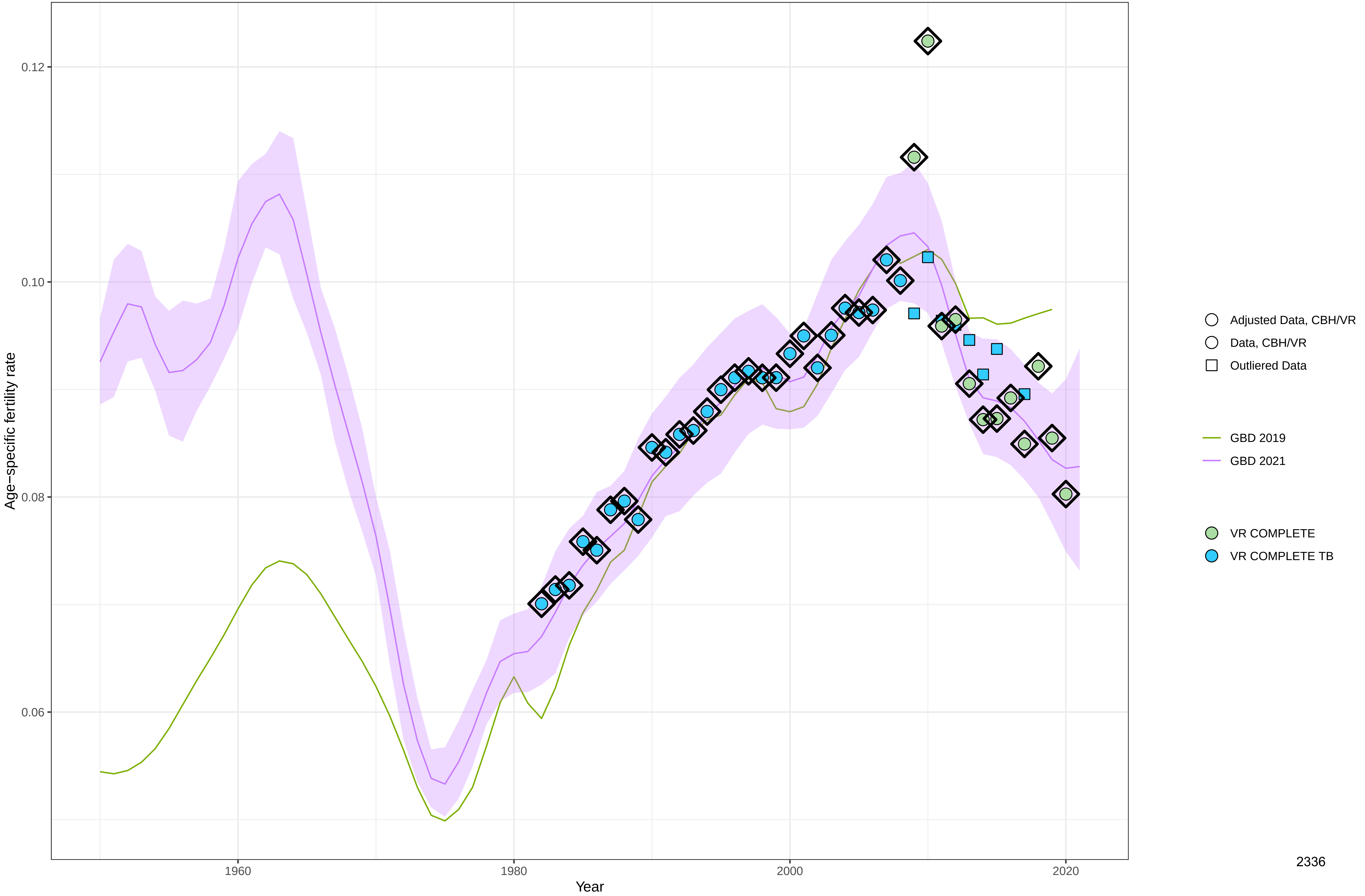

Age−specific fertility fitted trend lines, 1950−2021: Haringey, Ages: 35−39
Data points enclosed with a diamond are reference sources

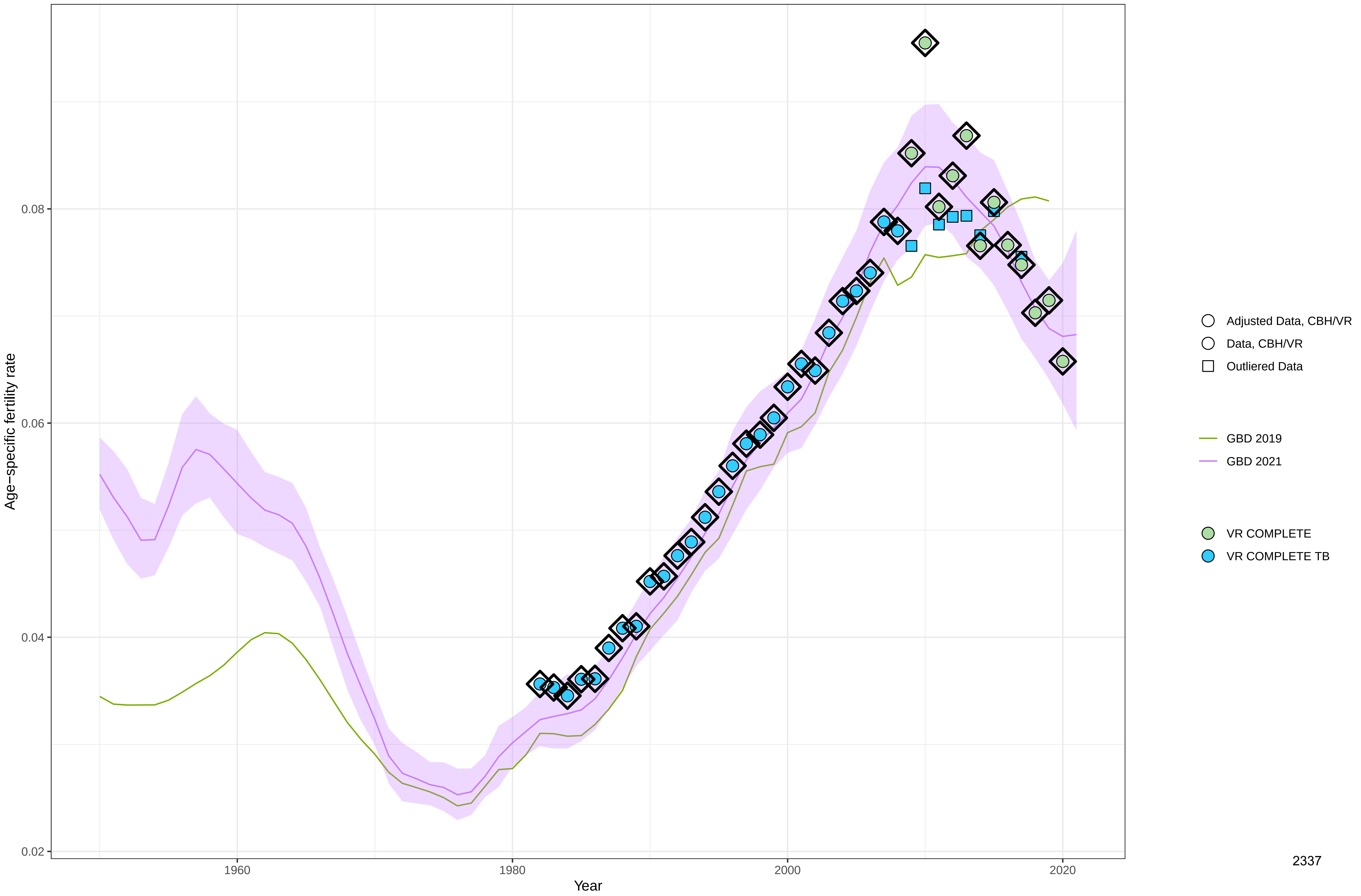

Age−specific fertility fitted trend lines, 1950−2021: Haringey, Ages: 40−44
Data points enclosed with a diamond are reference sources

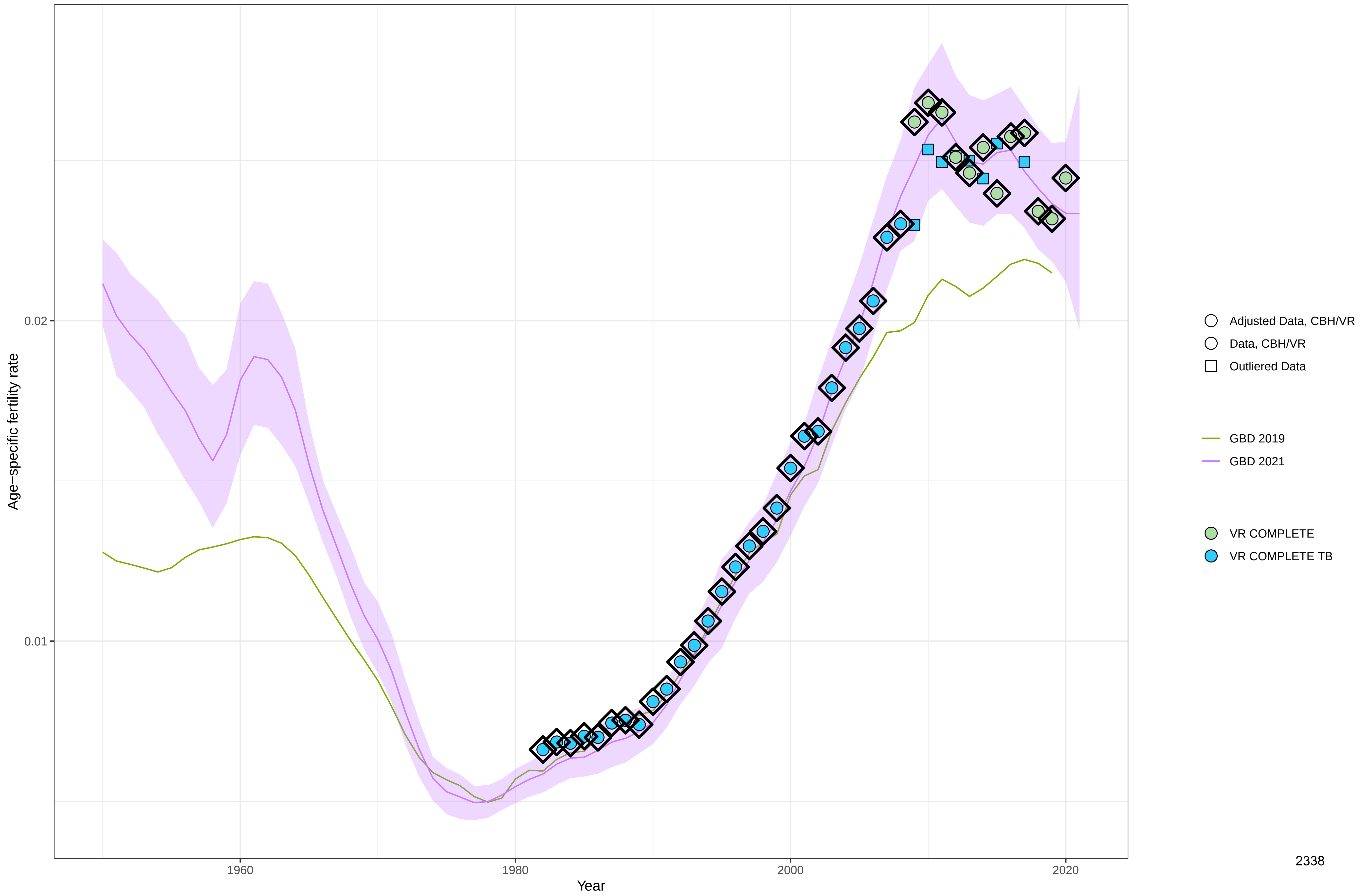

Age−specific fertility fitted trend lines, 1950−2021: Haringey, Ages: 45−49
Data points enclosed with a diamond are reference sources

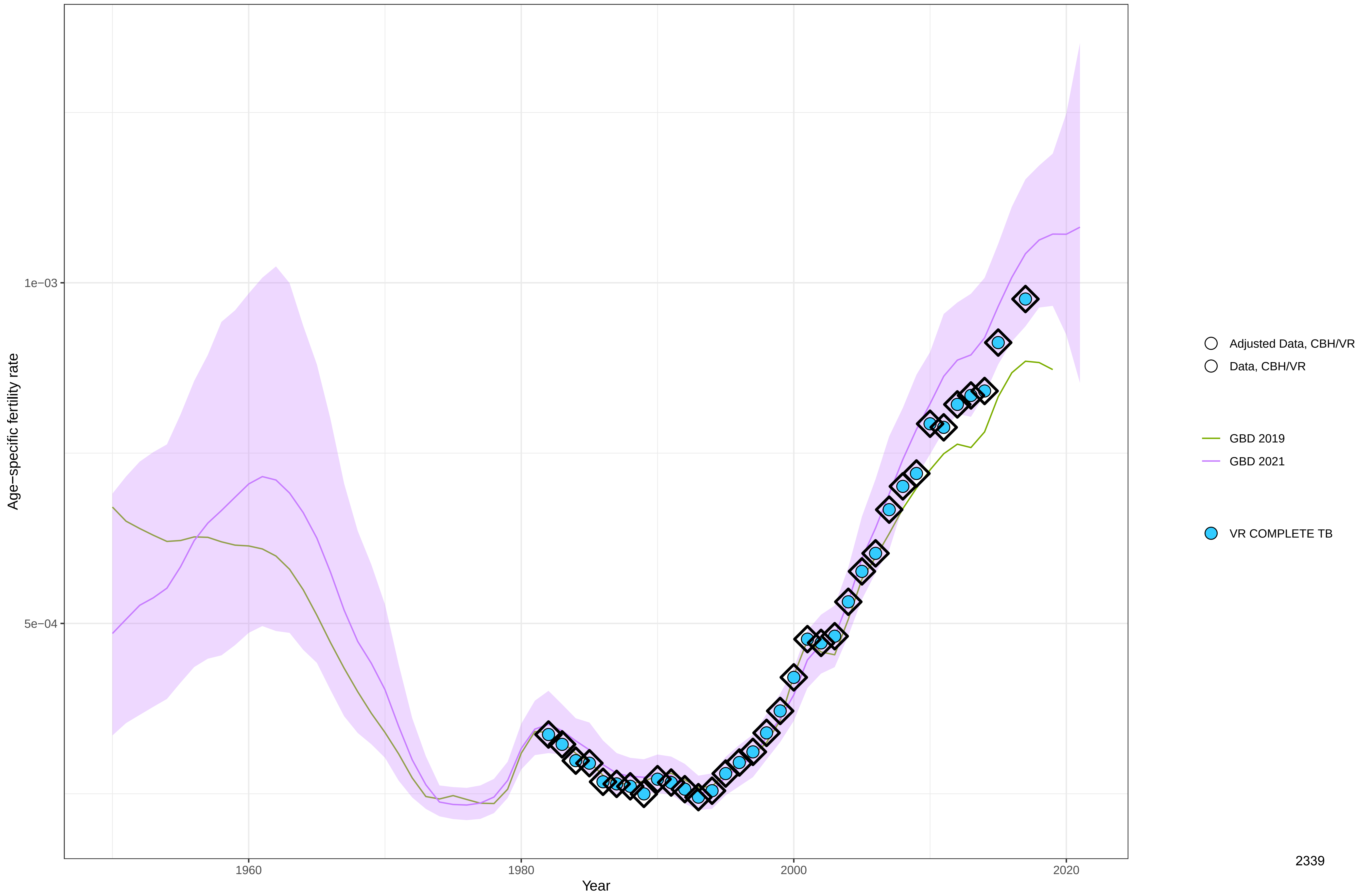

Age−specific fertility fitted trend lines, 1950−2021: Haringey, Ages: 50−54
Data points enclosed with a diamond are reference sources

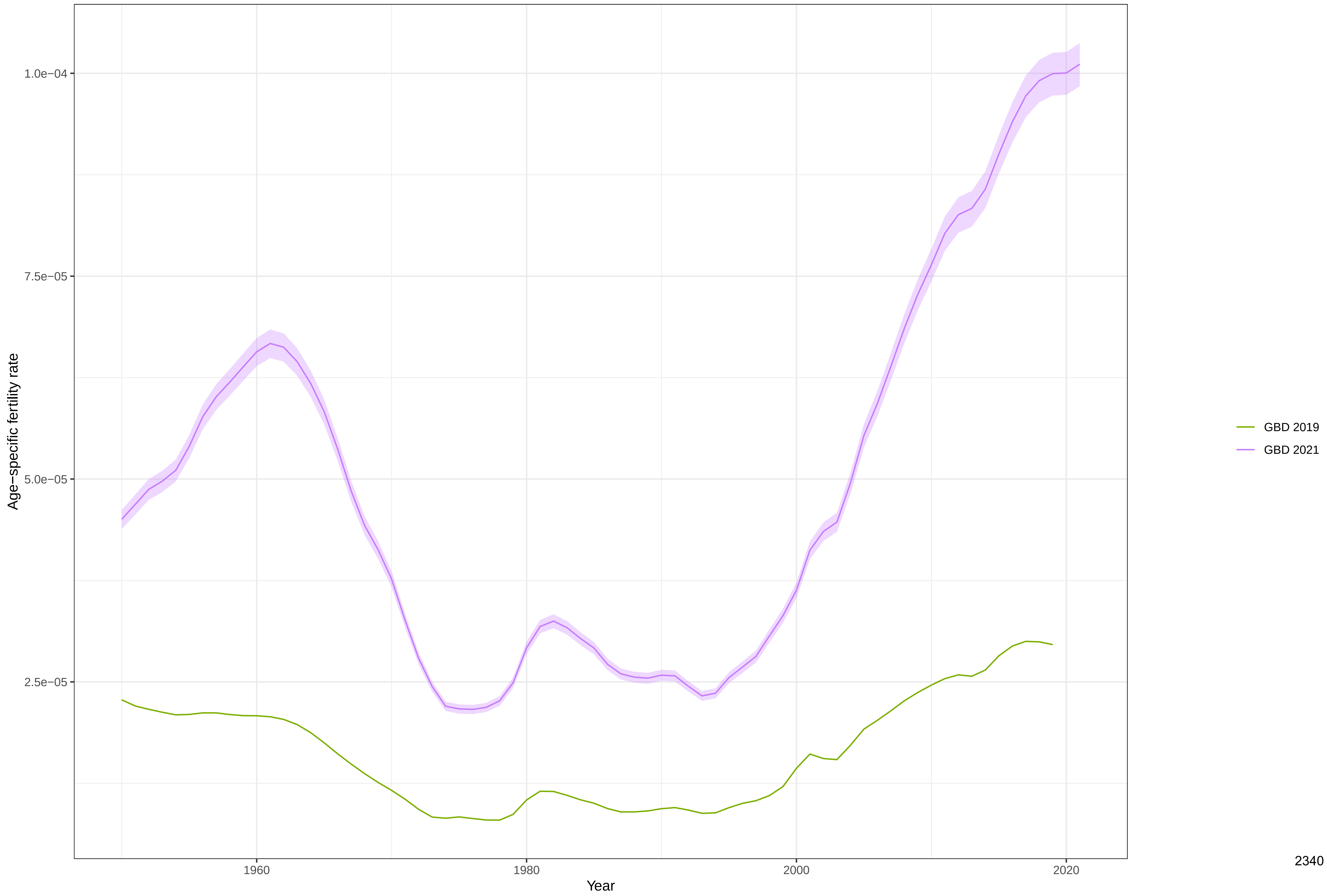

Total fertility rate fitted trend lines, 1950−2021: Haringey TFR
Data points enclosed with a diamond are reference sources

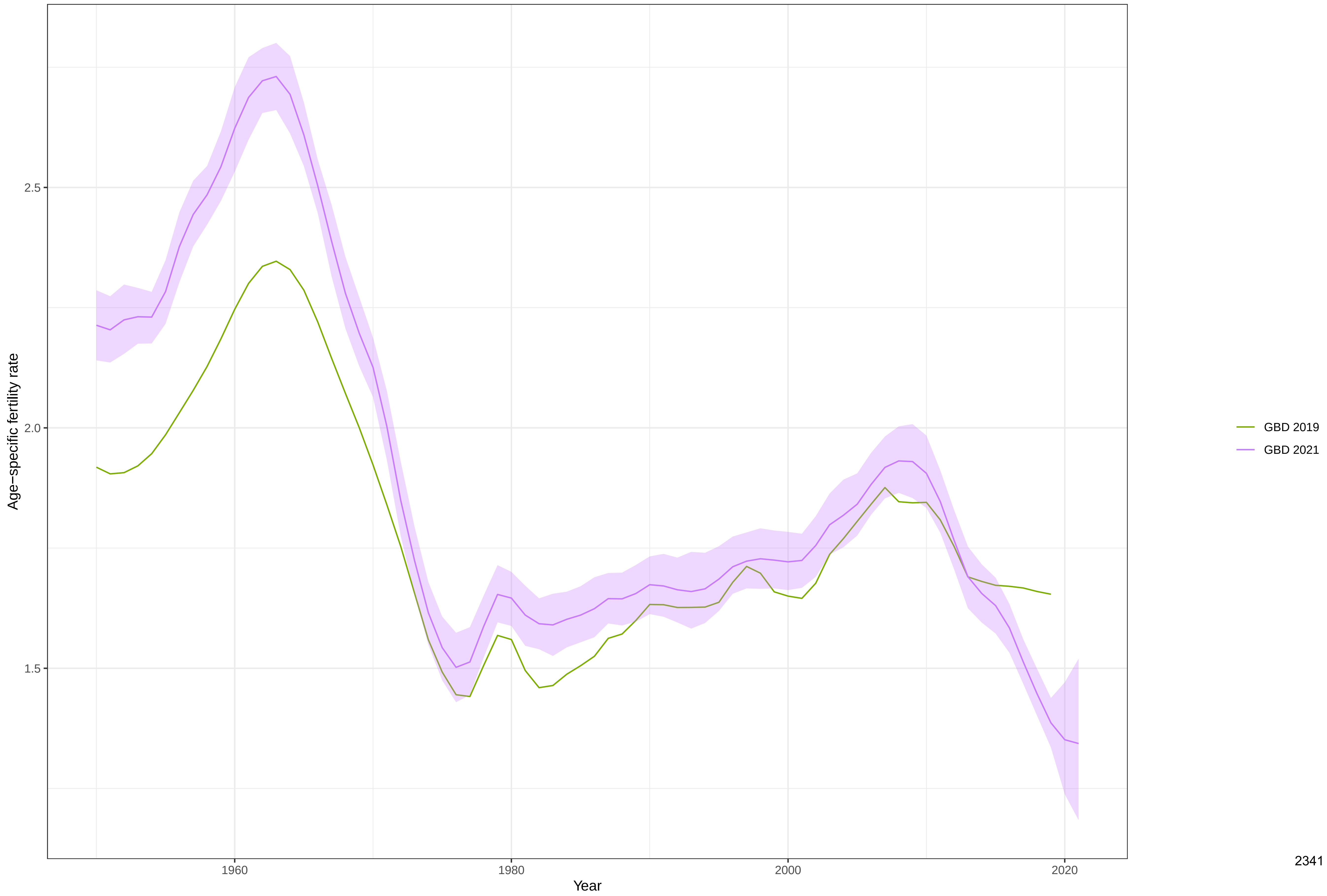

2341

Age−specific fertility fitted trend lines, 1950−2021: Harrow, Ages: 10−14
Data points enclosed with a diamond are reference sources

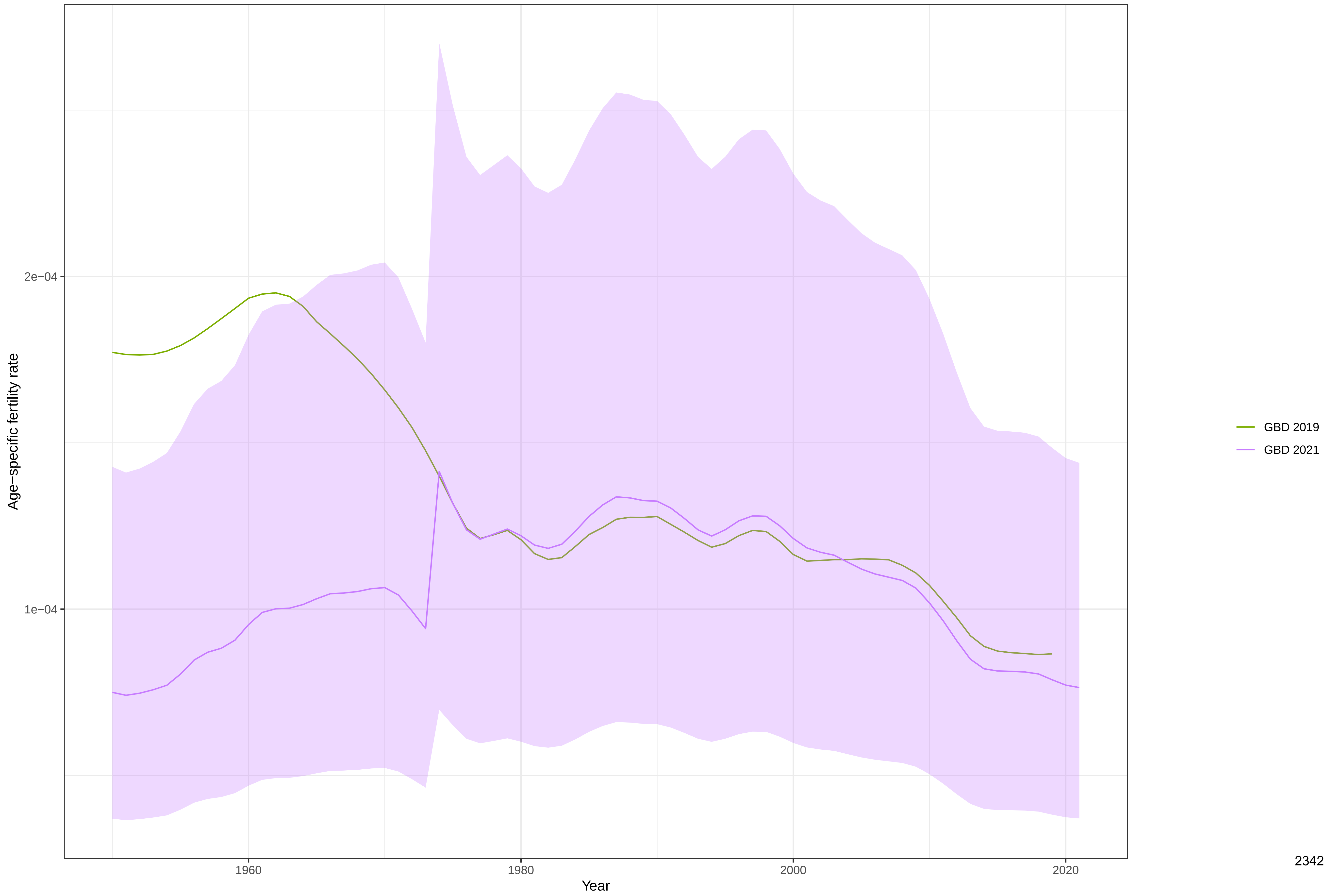

Age−specific fertility fitted trend lines, 1950−2021: Harrow, Ages: 15−19
Data points enclosed with a diamond are reference sources

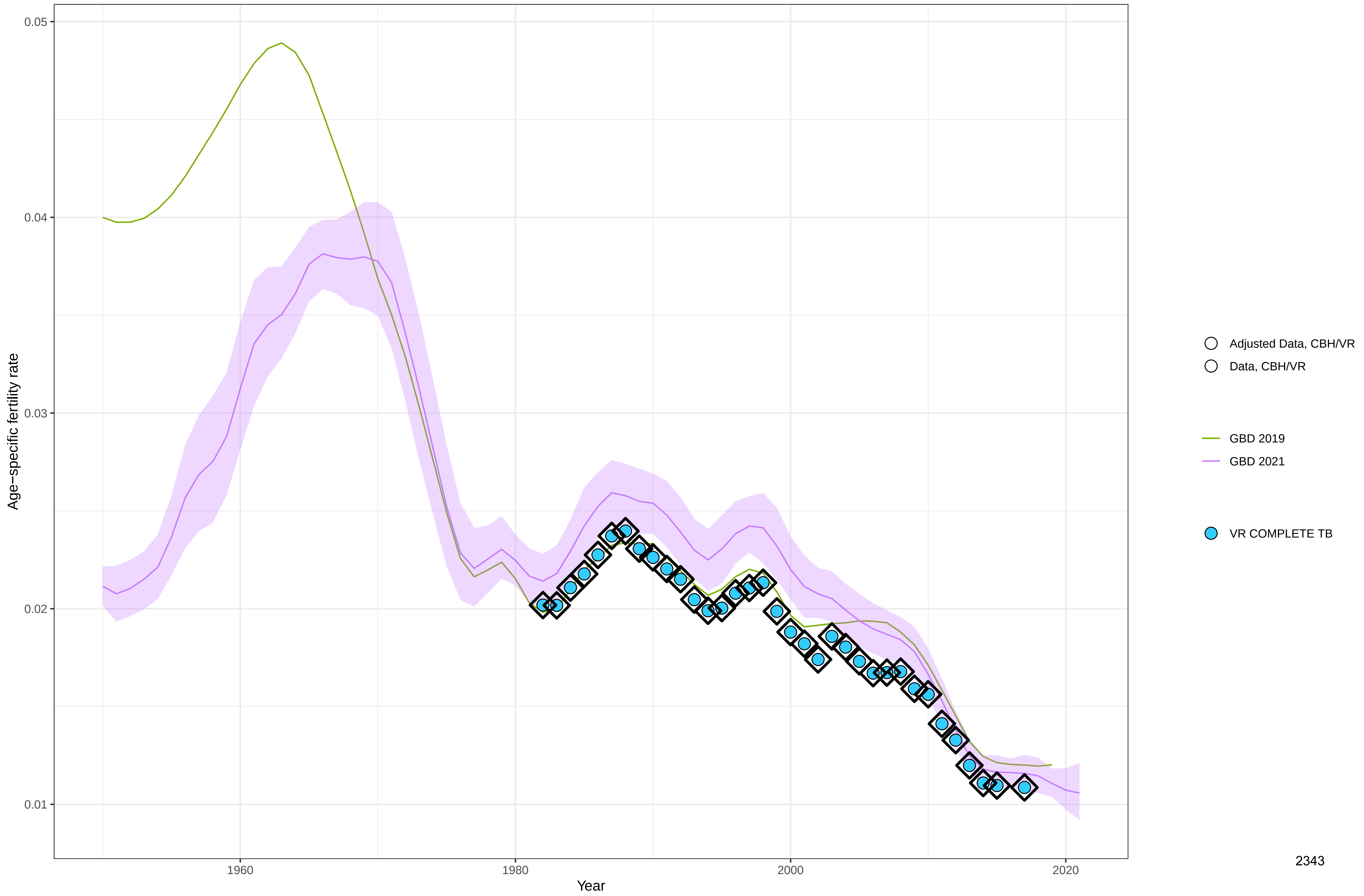

Age−specific fertility fitted trend lines, 1950−2021: Harrow, Ages: 20−24
Data points enclosed with a diamond are reference sources

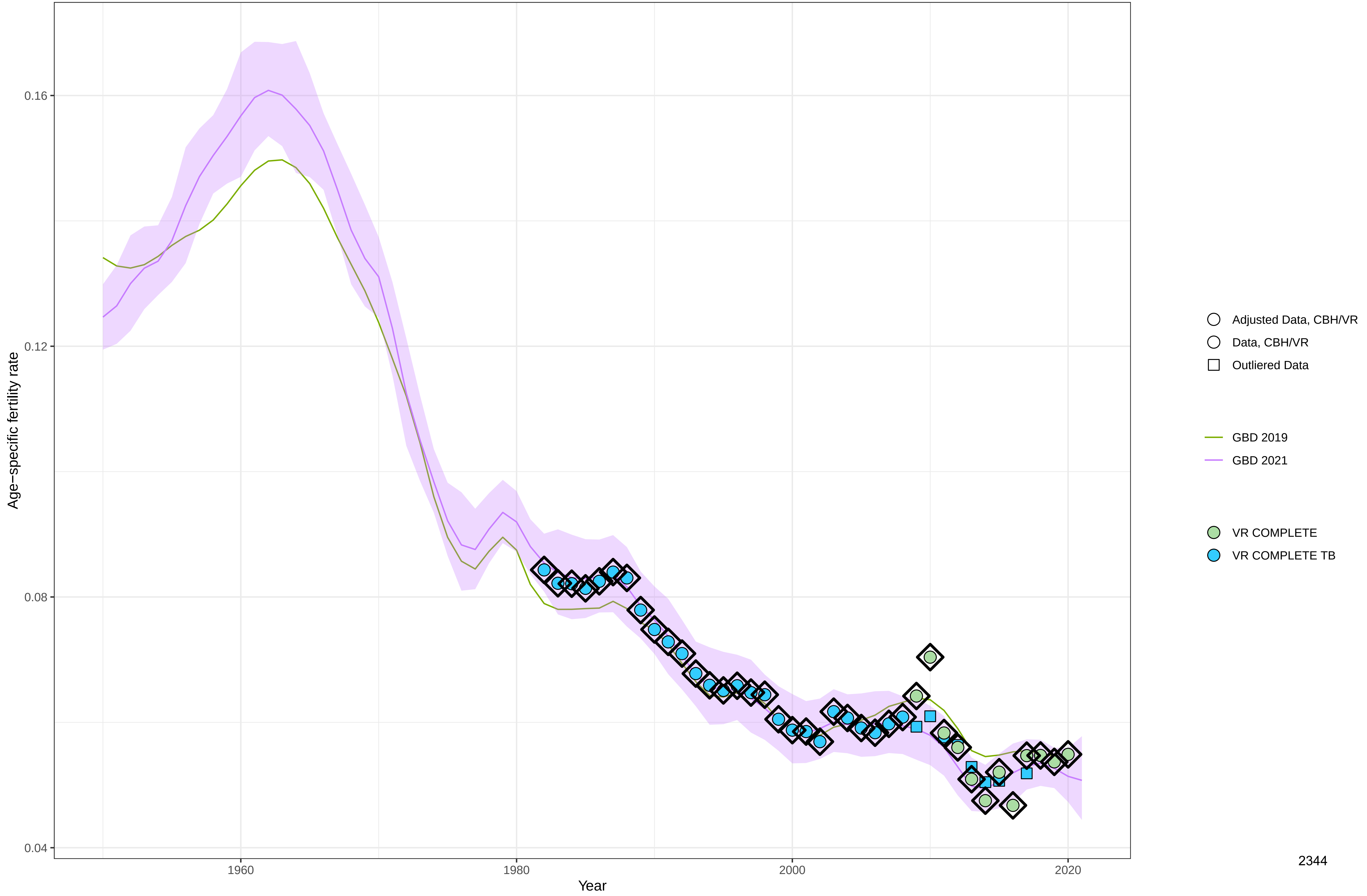

Age−specific fertility fitted trend lines, 1950−2021: Harrow, Ages: 25−29
Data points enclosed with a diamond are reference sources

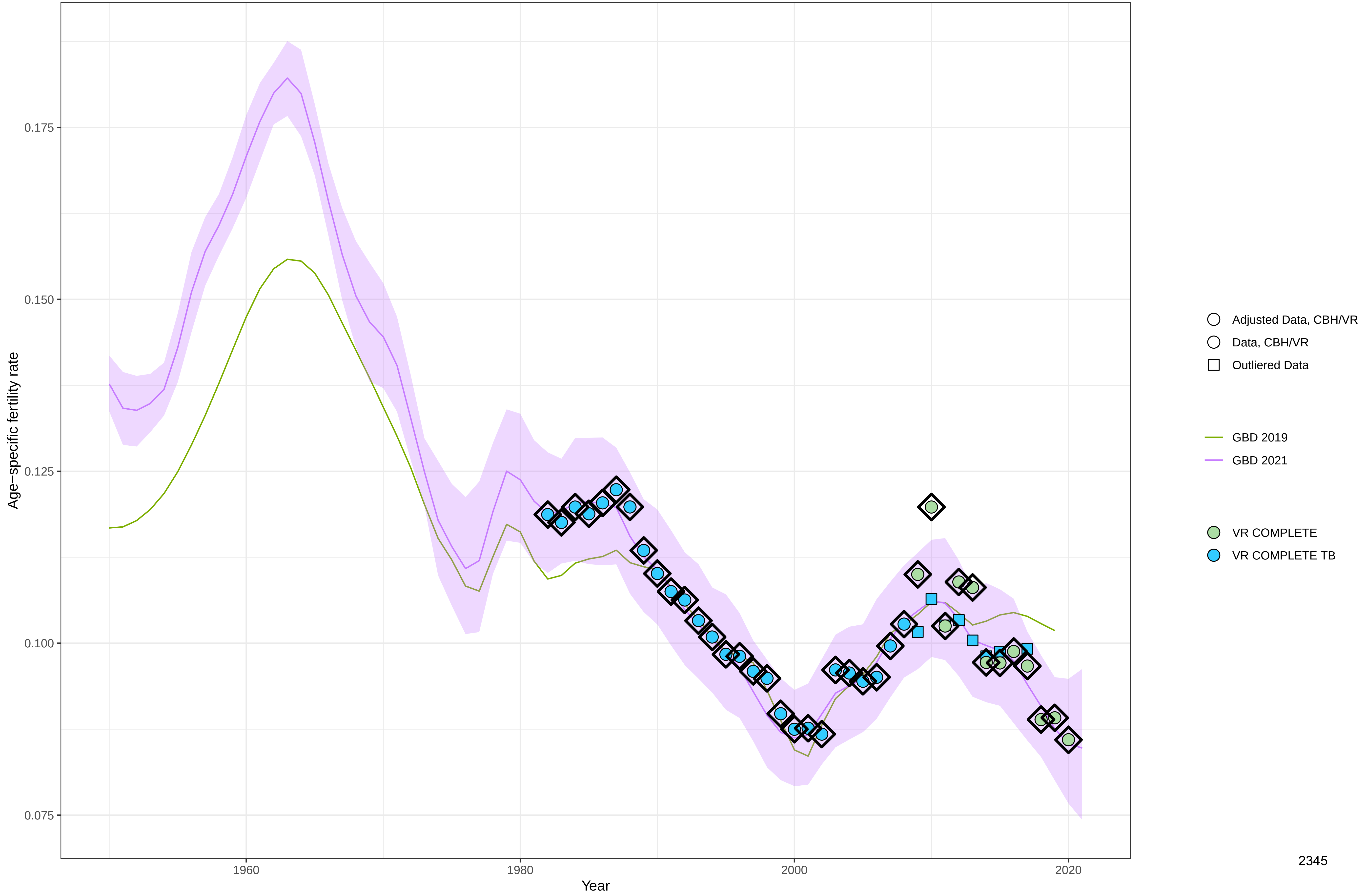

Age−specific fertility fitted trend lines, 1950−2021: Harrow, Ages: 30−34
Data points enclosed with a diamond are reference sources

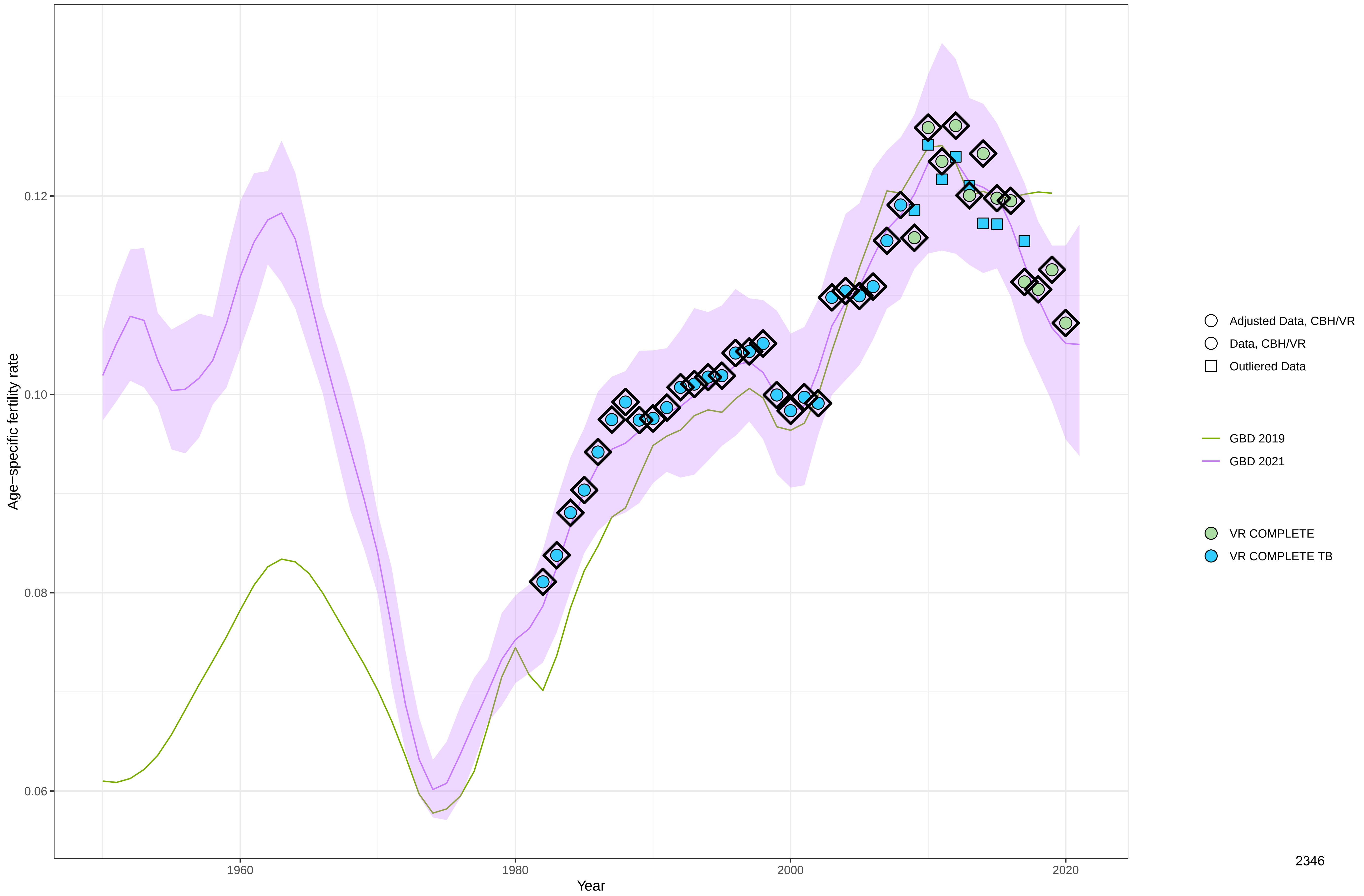

Age−specific fertility fitted trend lines, 1950−2021: Harrow, Ages: 35−39
Data points enclosed with a diamond are reference sources

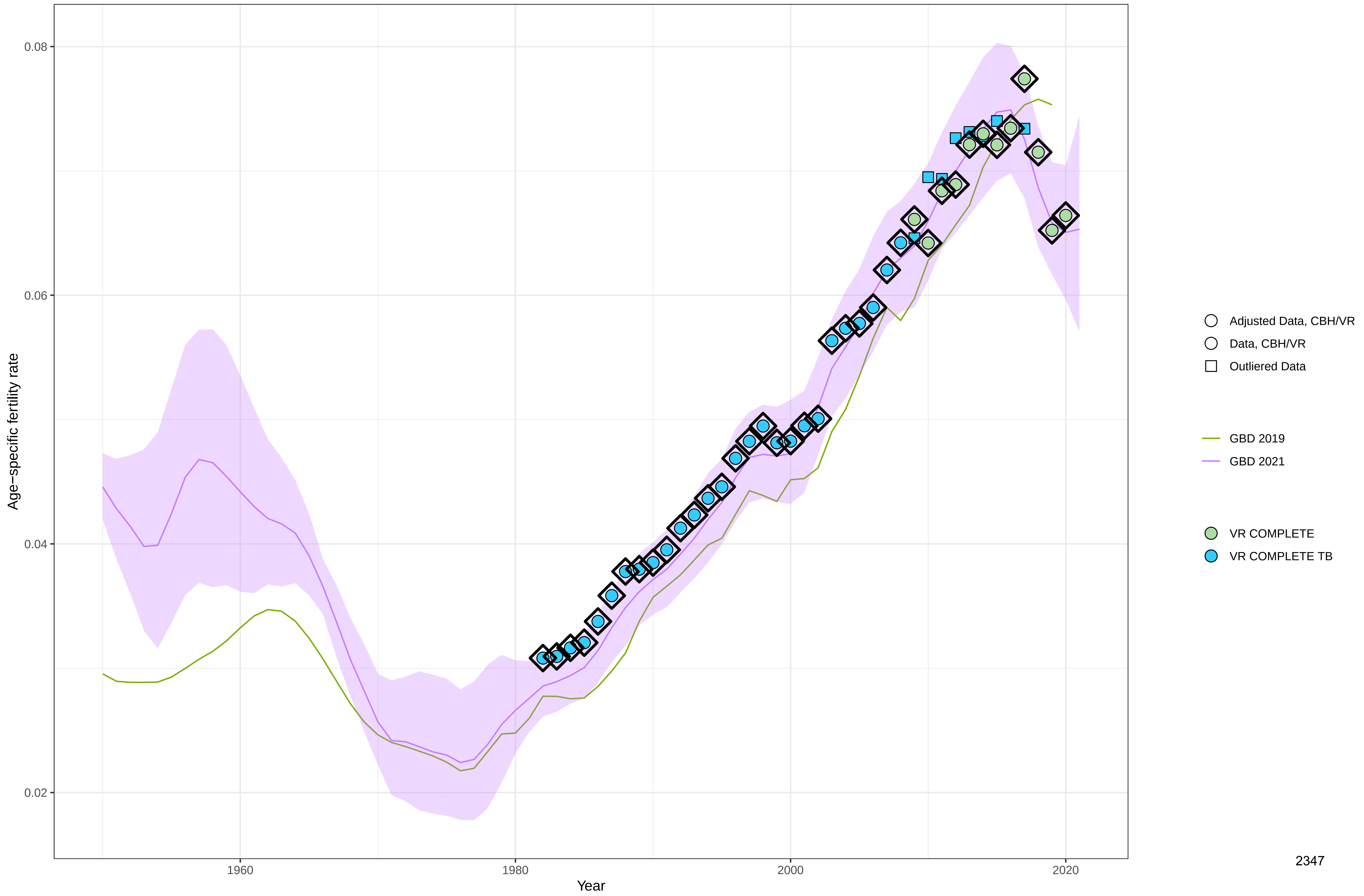

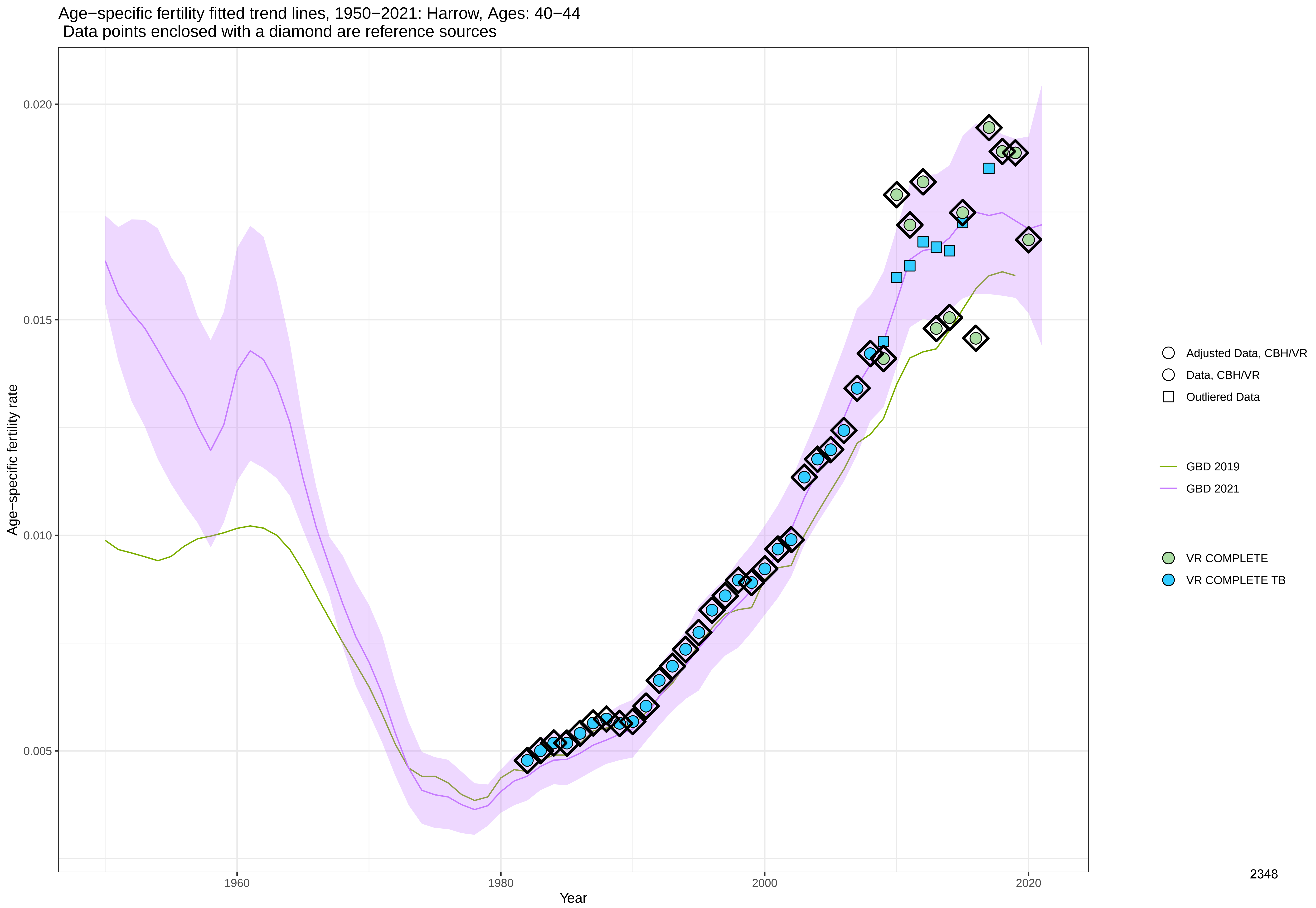

Age−specific fertility fitted trend lines, 1950−2021: Harrow, Ages: 40−44
Data points enclosed with a diamond are reference sources
Age−specific fertility rate
0.020
0.015
0.010
0.005
1960
1980
2000
2020
Year
Adjusted Data, CBH/VR
Data, CBH/VR
Outliered Data
GBD 2019
GBD 2021
VR COMPLETE
VR COMPLETE TB

Age−specific fertility fitted trend lines, 1950−2021: Harrow, Ages: 45−49
Data points enclosed with a diamond are reference sources

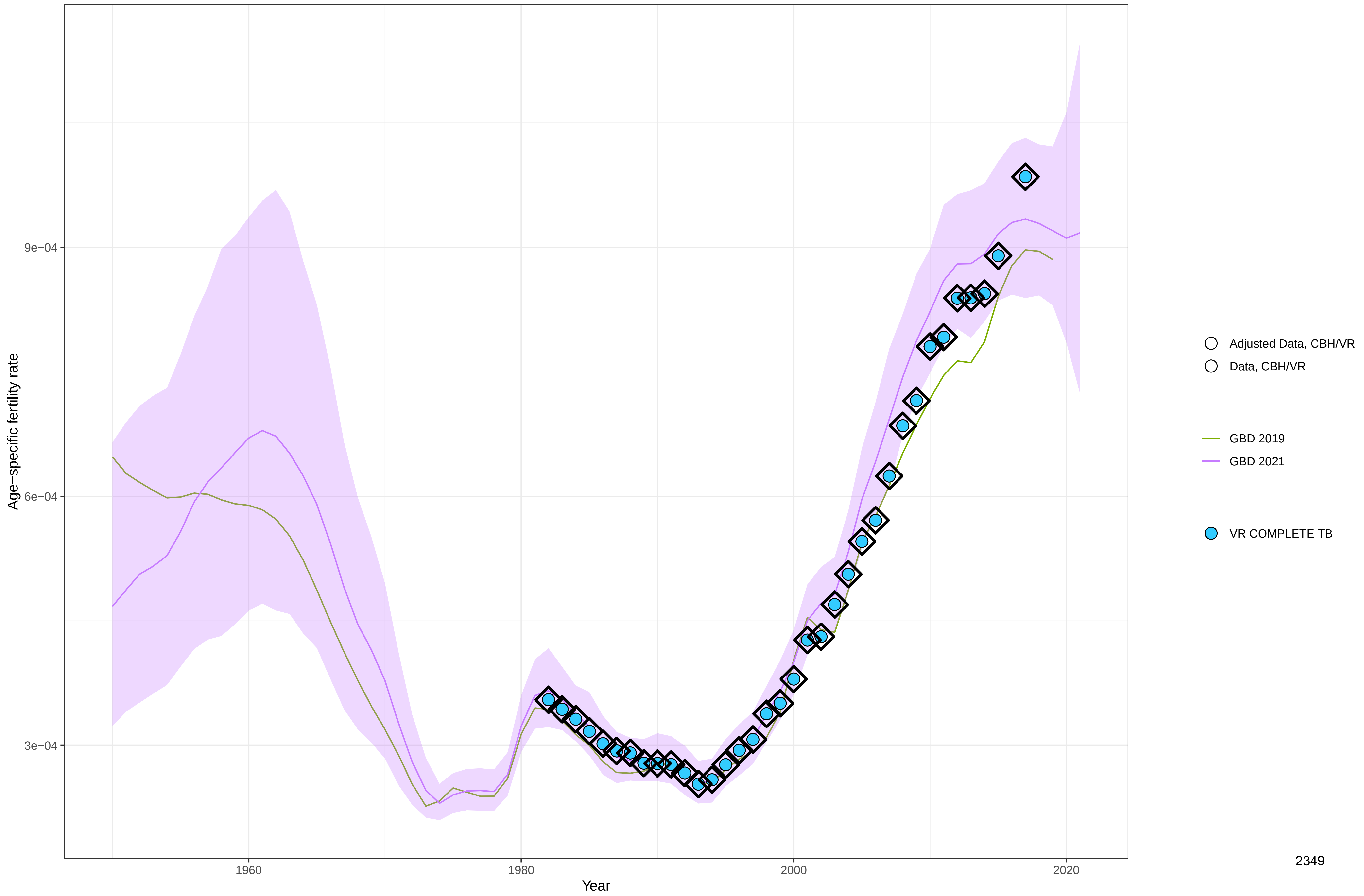

Age−specific fertility fitted trend lines, 1950−2021: Harrow, Ages: 50−54
Data points enclosed with a diamond are reference sources

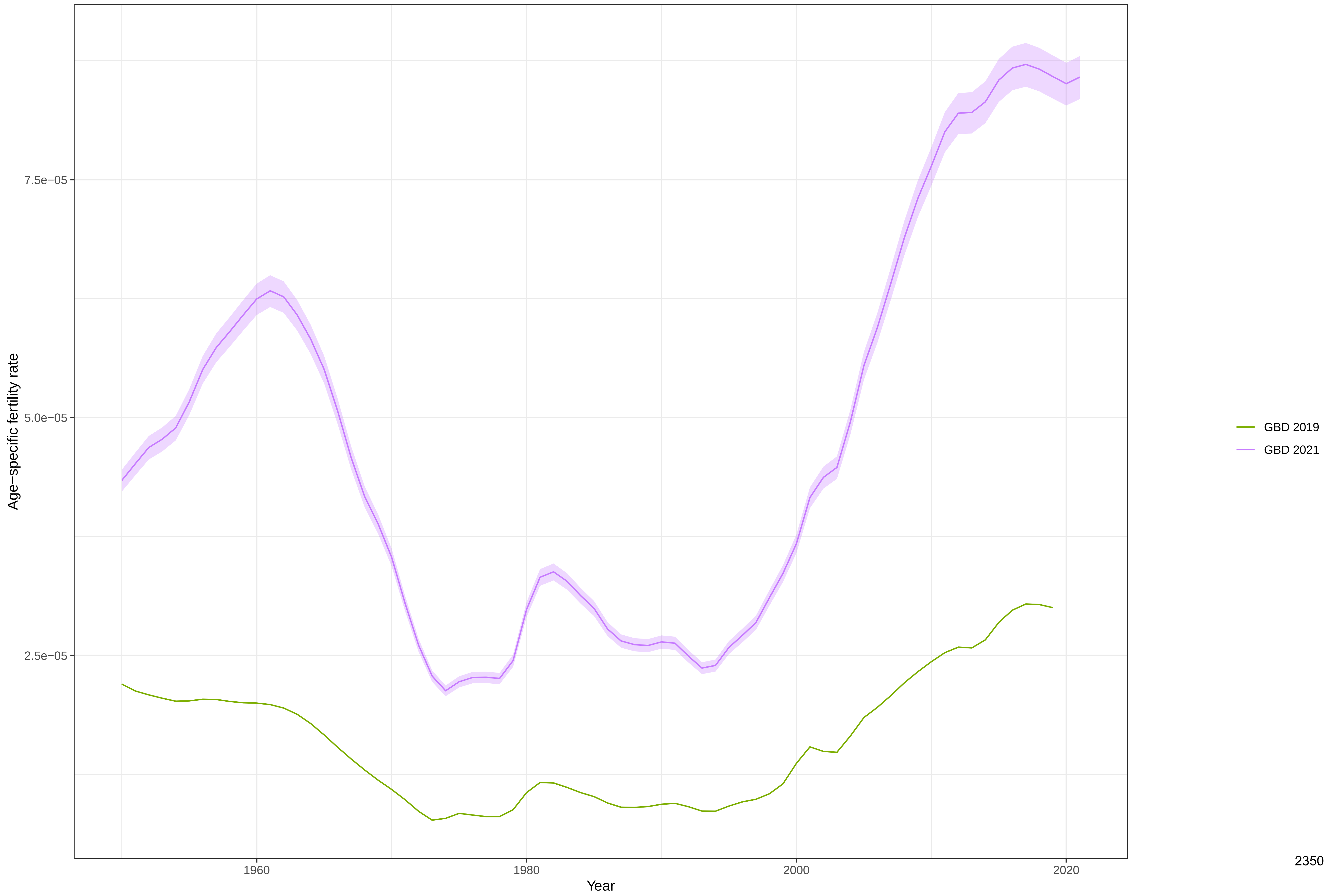

Total fertility rate fitted trend lines, 1950−2021: Harrow TFR
Data points enclosed with a diamond are reference sources

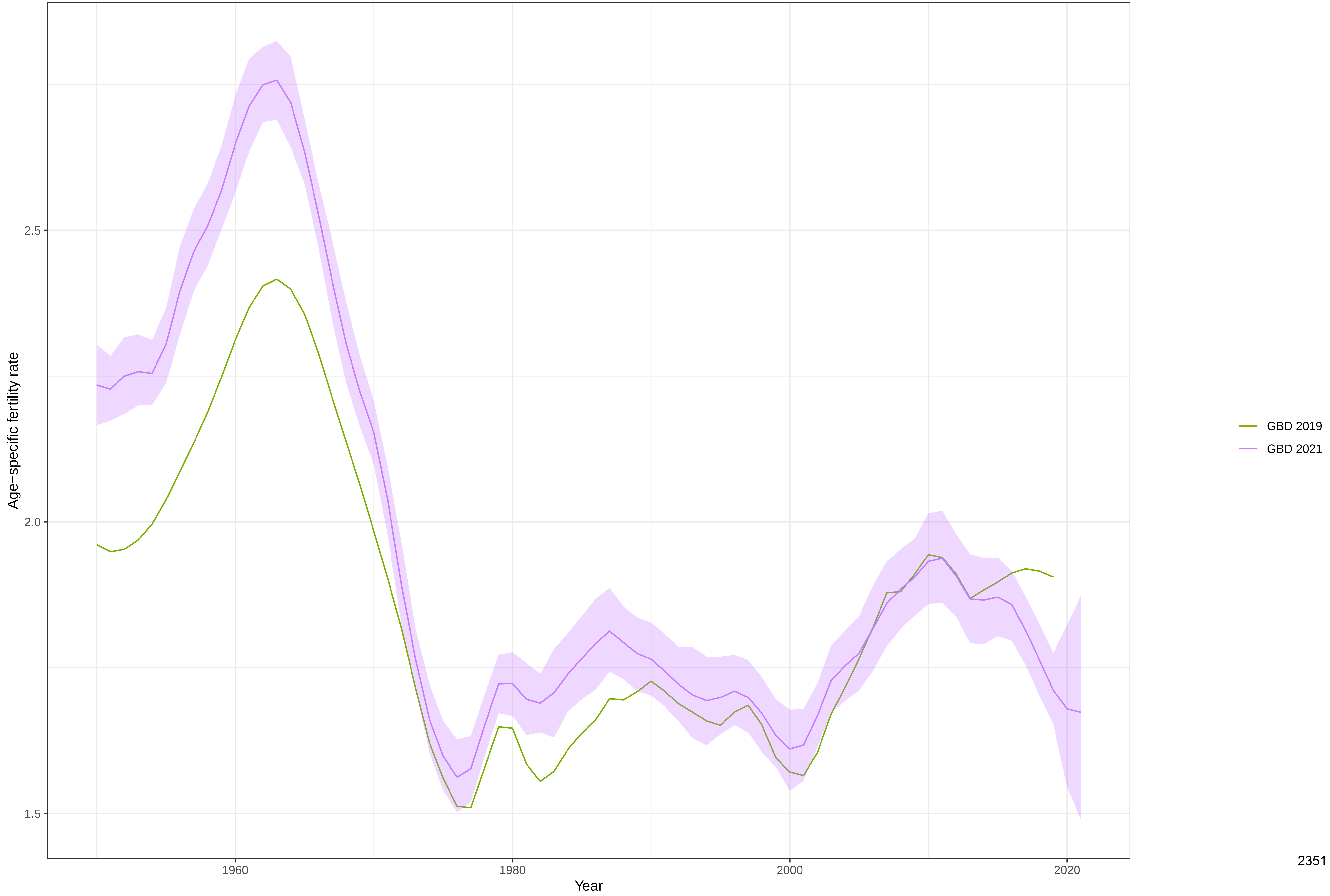

Age−specific fertility fitted trend lines, 1950−2021: Havering, Ages: 10−14
Data points enclosed with a diamond are reference sources

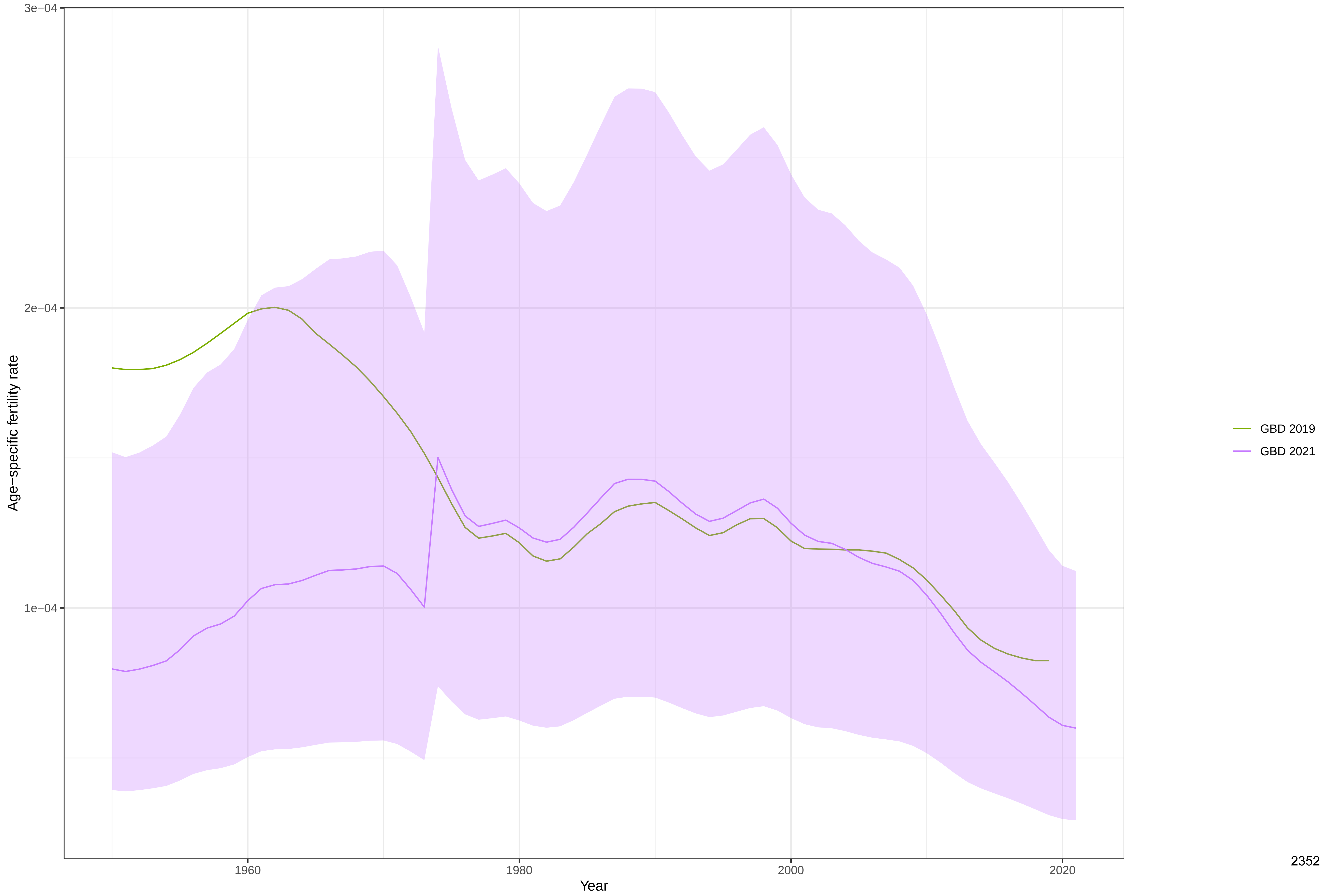

Age−specific fertility fitted trend lines, 1950−2021: Havering, Ages: 15−19
Data points enclosed with a diamond are reference sources

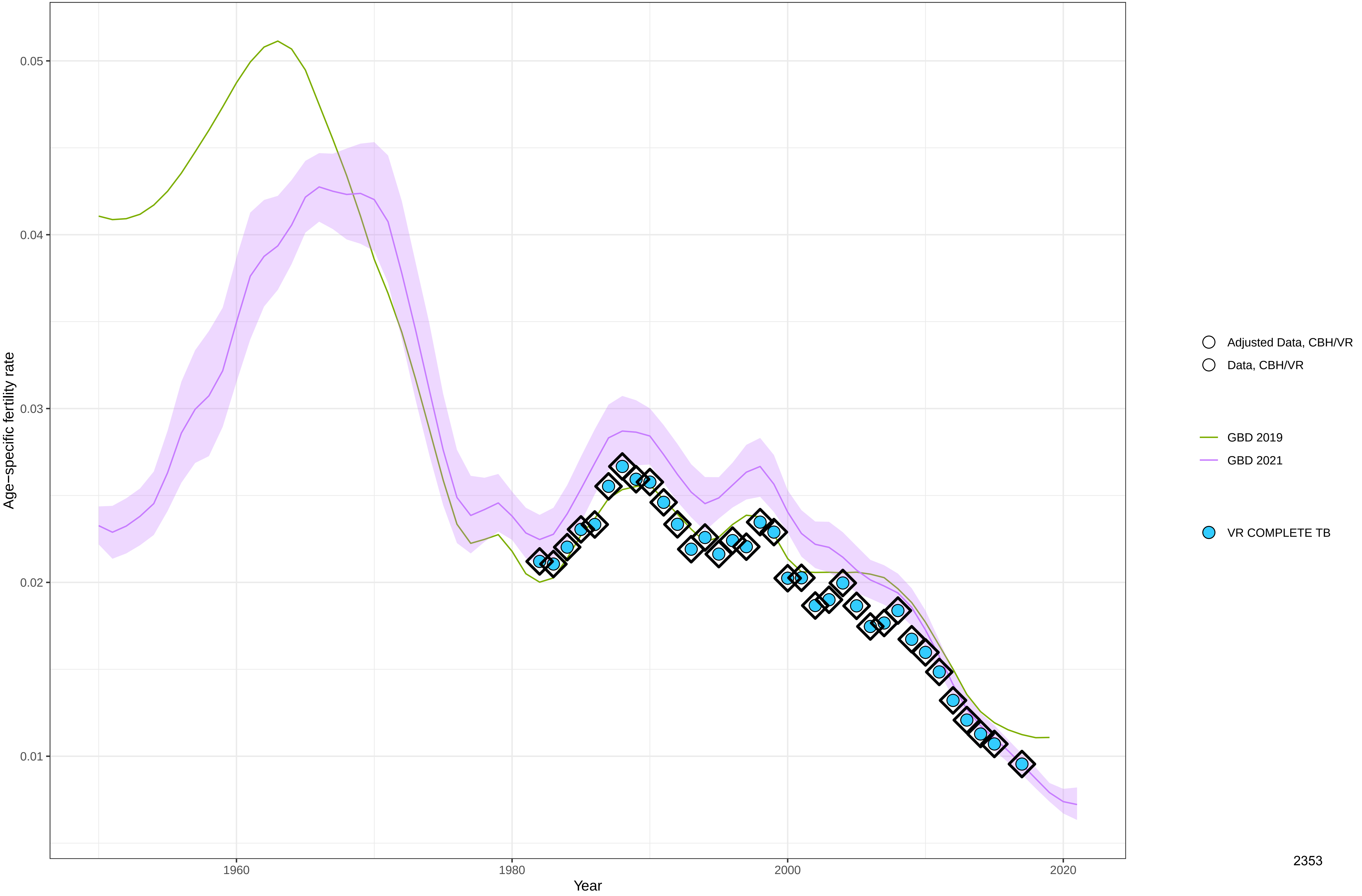

Age−specific fertility fitted trend lines, 1950−2021: Havering, Ages: 20−24
Data points enclosed with a diamond are reference sources

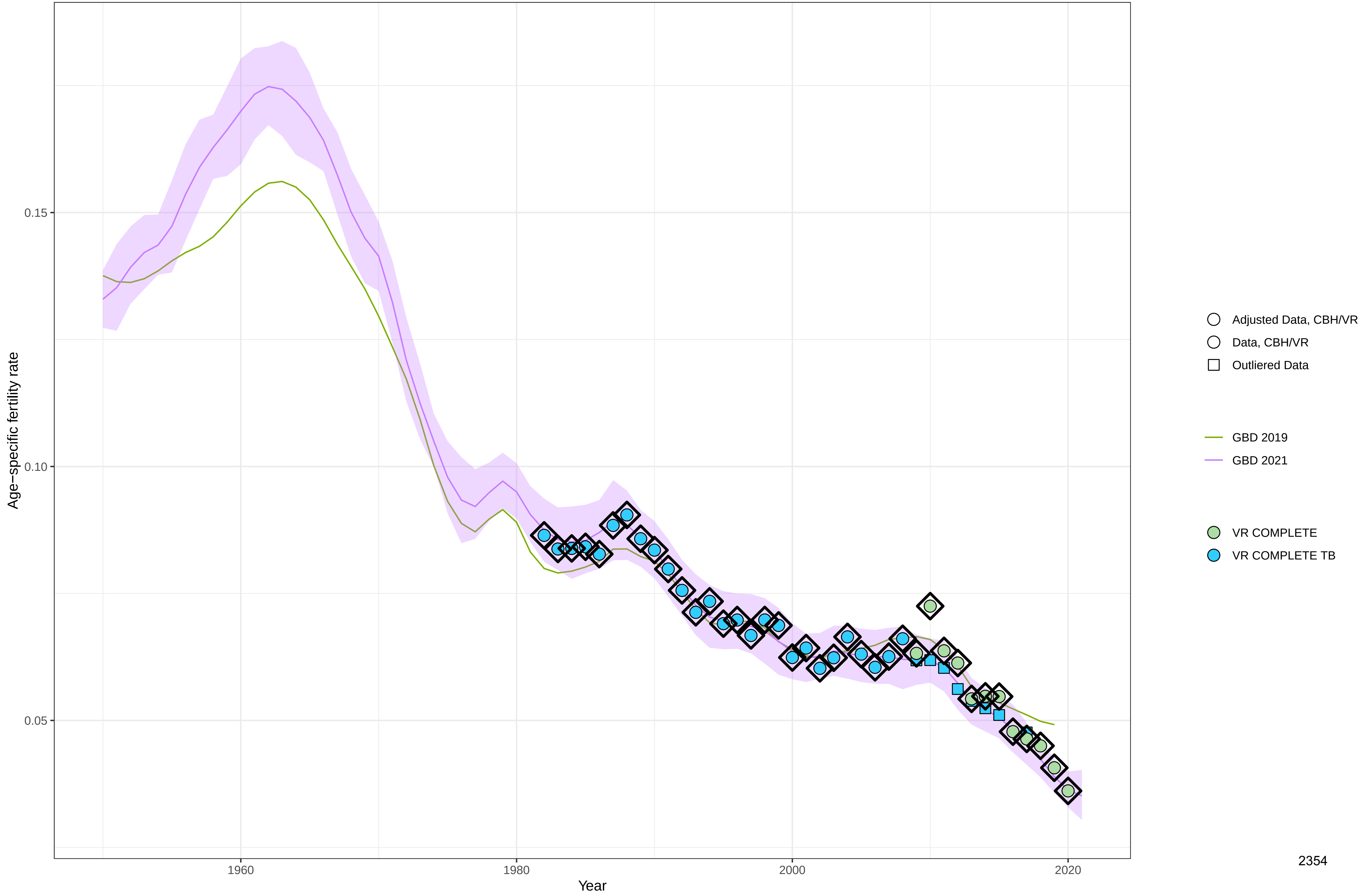

Age−specific fertility fitted trend lines, 1950−2021: Havering, Ages: 25−29
Data points enclosed with a diamond are reference sources

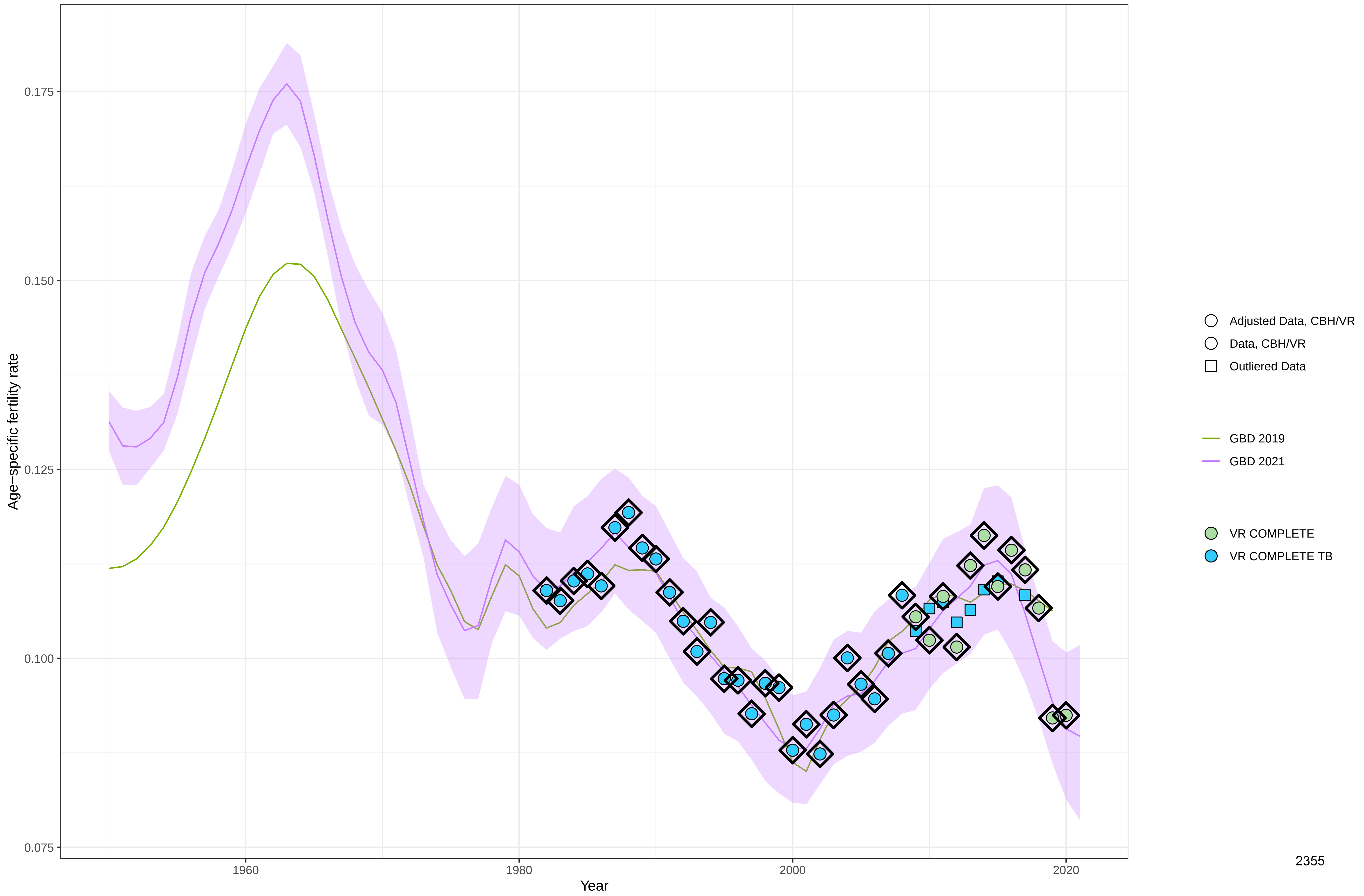

Age−specific fertility fitted trend lines, 1950−2021: Havering, Ages: 30−34
Data points enclosed with a diamond are reference sources

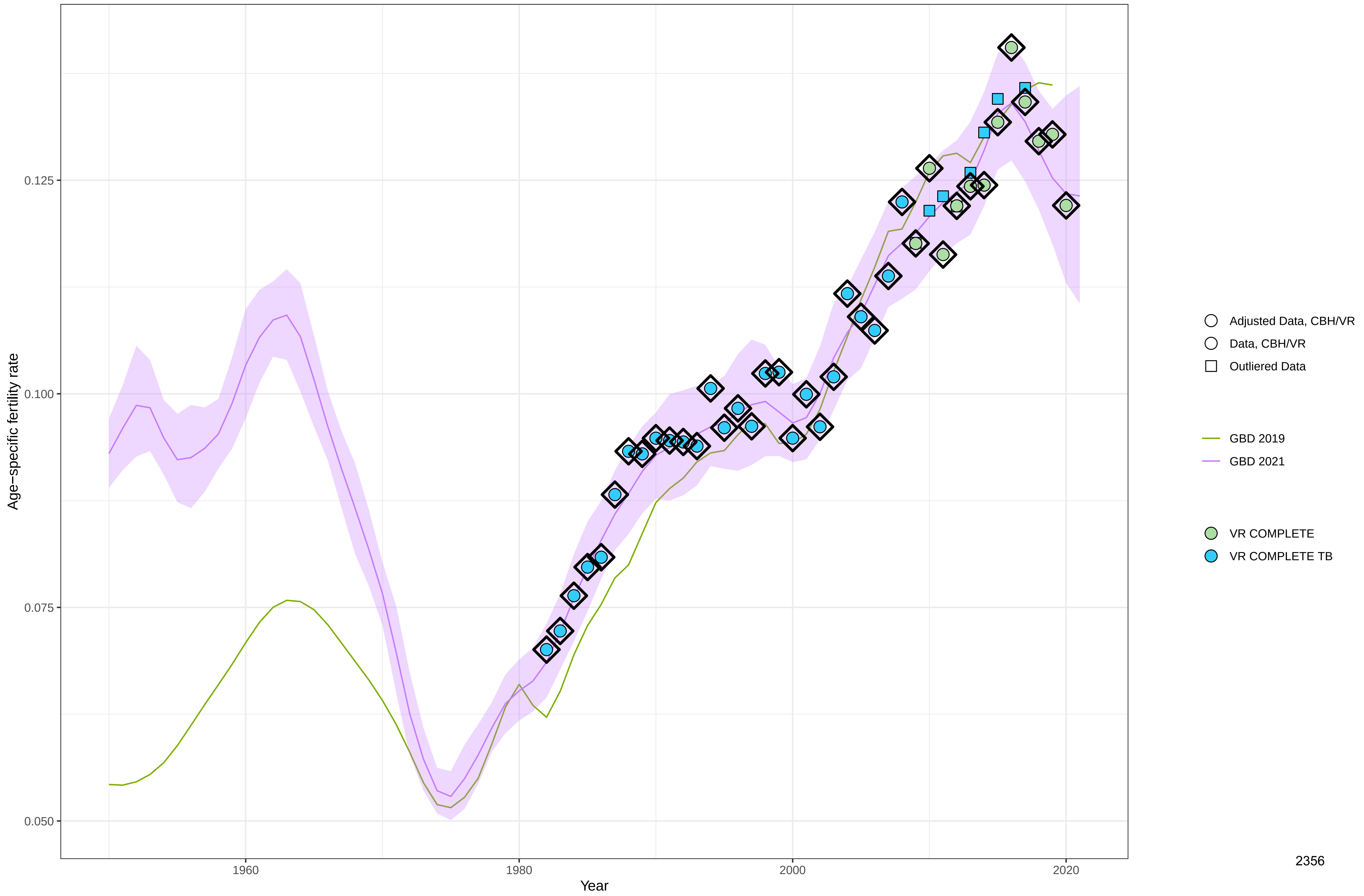

Age−specific fertility fitted trend lines, 1950−2021: Havering, Ages: 35−39
Data points enclosed with a diamond are reference sources

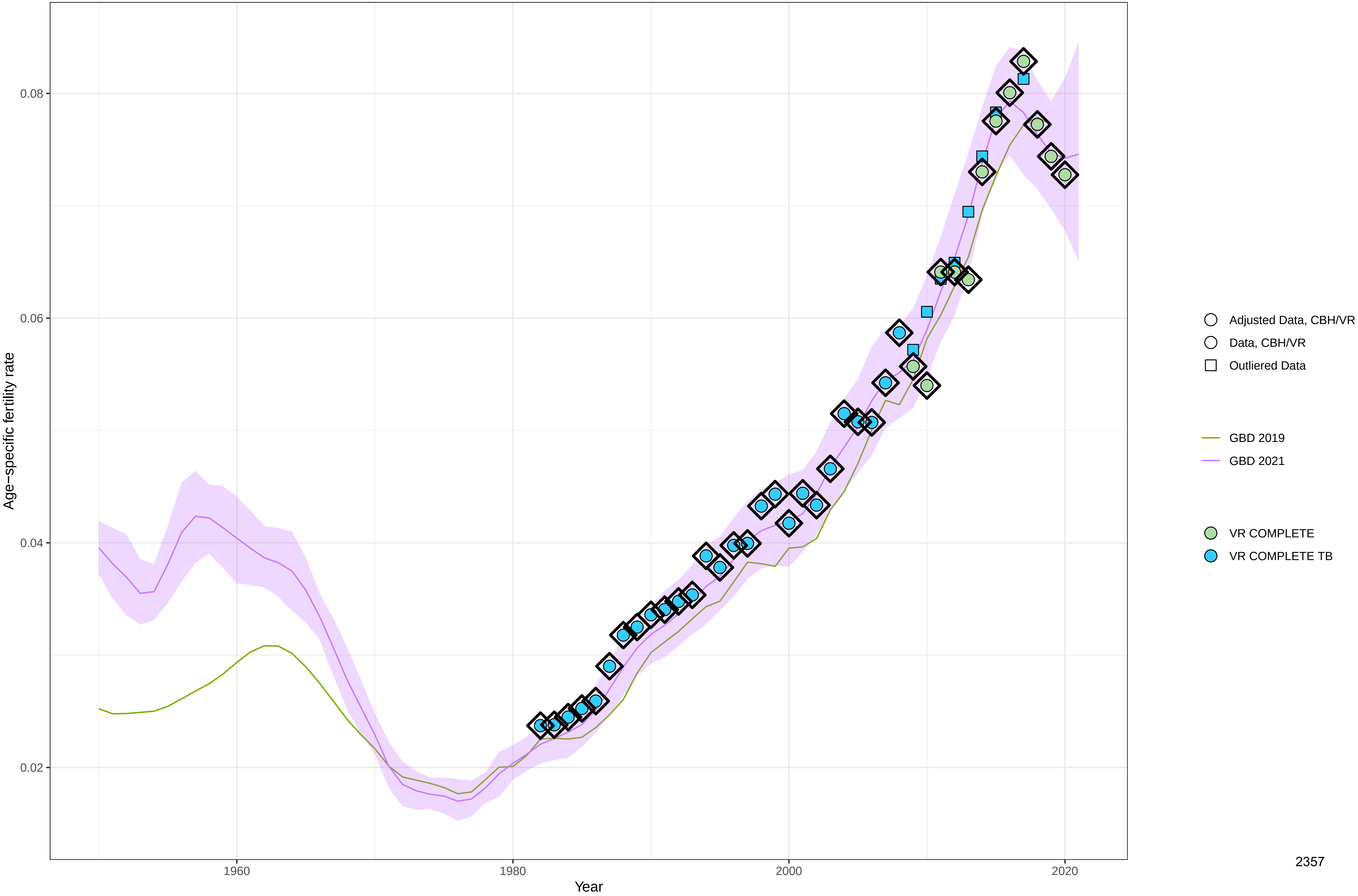

Age−specific fertility fitted trend lines, 1950−2021: Havering, Ages: 40−44
Data points enclosed with a diamond are reference sources

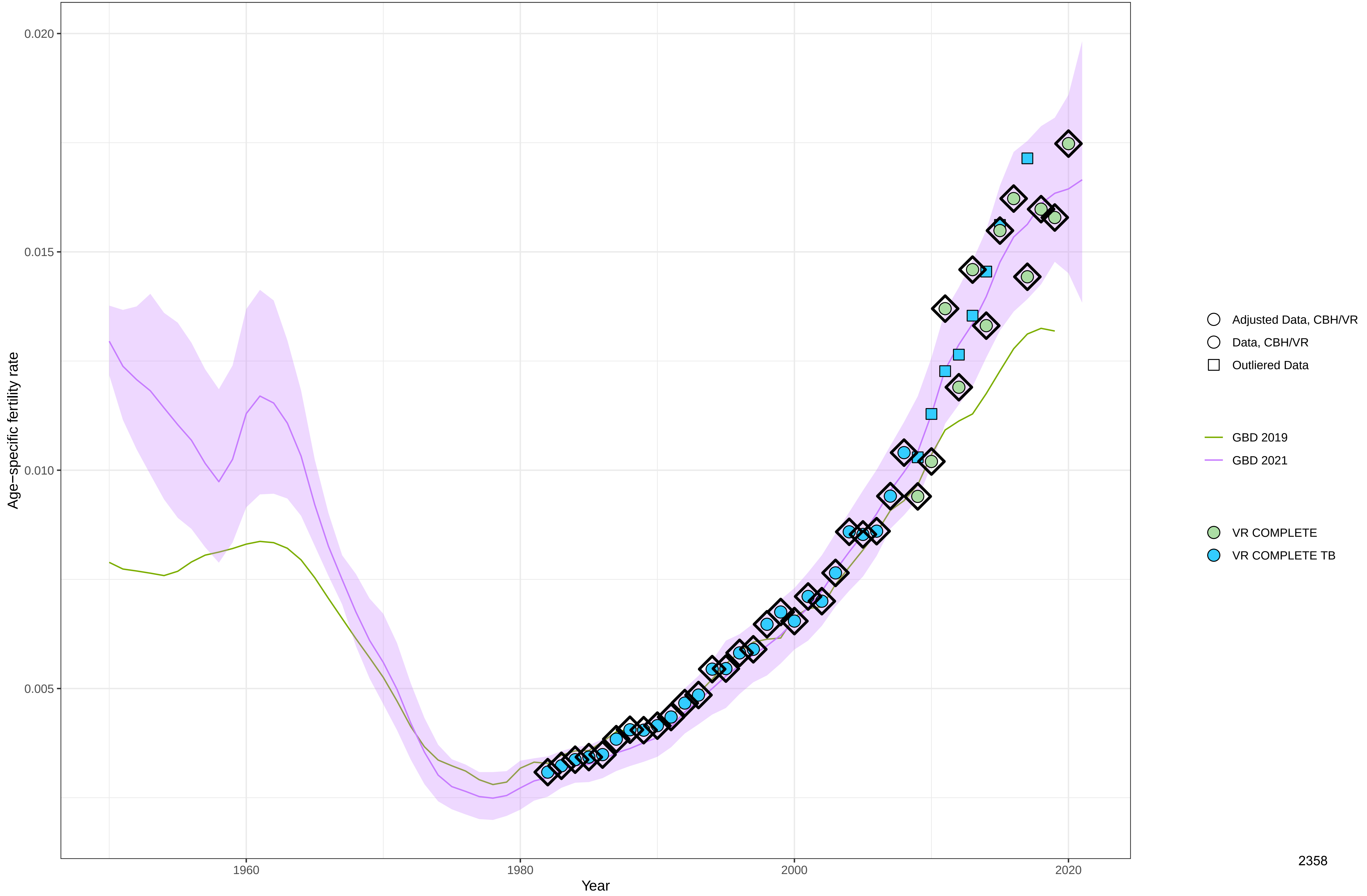

Age−specific fertility fitted trend lines, 1950−2021: Havering, Ages: 45−49
Data points enclosed with a diamond are reference sources

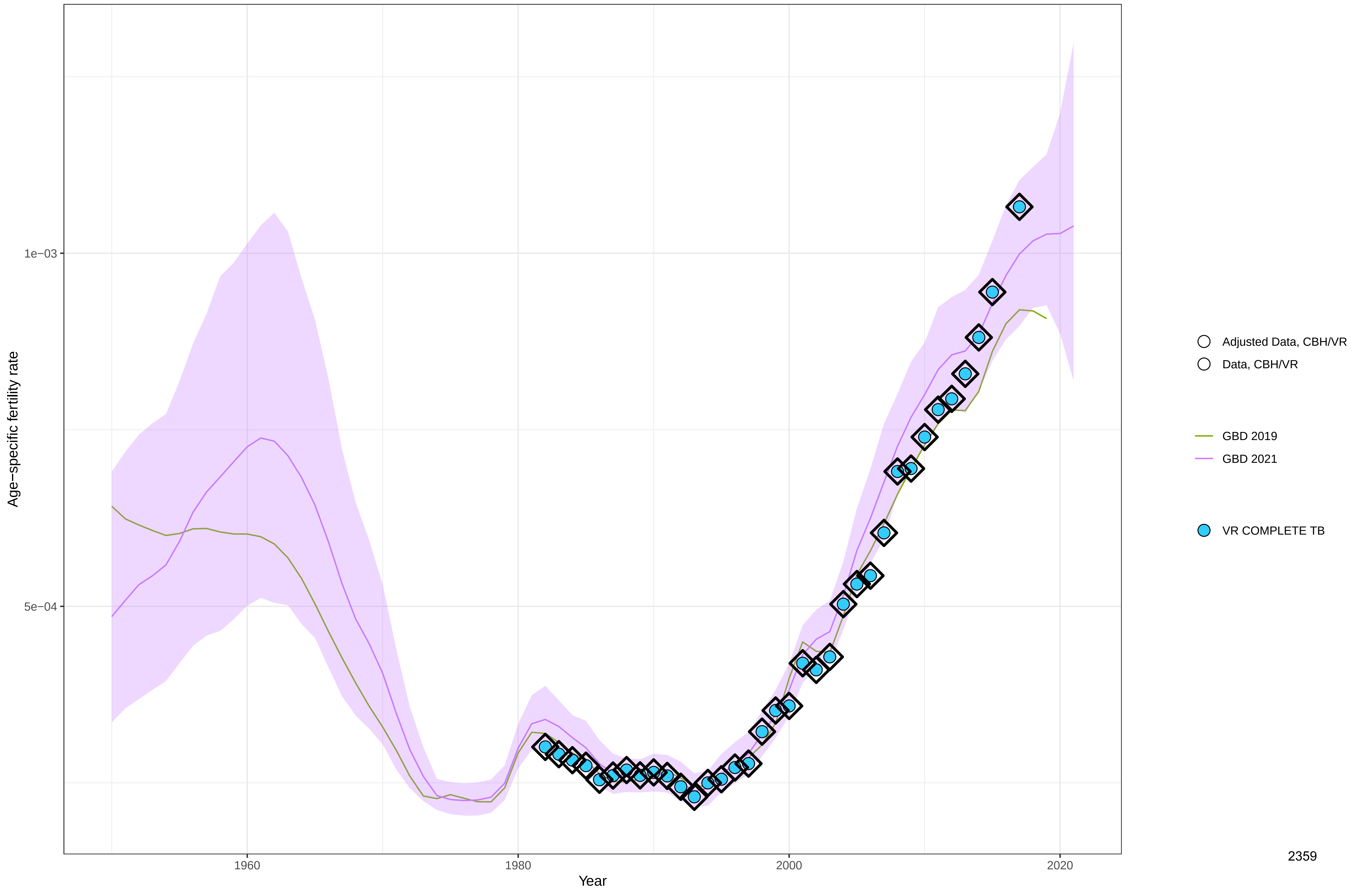

Age−specific fertility fitted trend lines, 1950−2021: Havering, Ages: 50−54
Data points enclosed with a diamond are reference sources

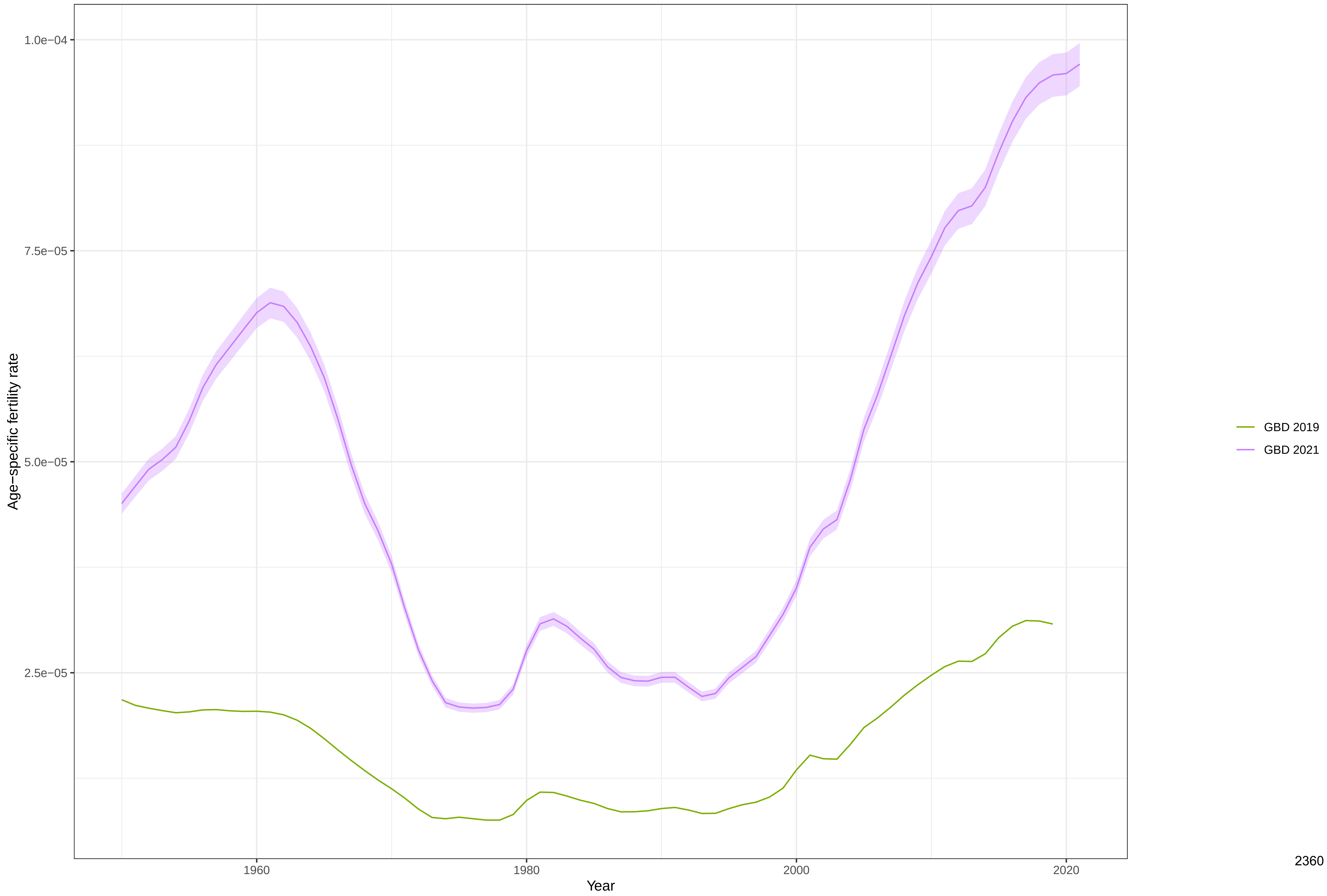

Total fertility rate fitted trend lines, 1950−2021: Havering TFR
Data points enclosed with a diamond are reference sources

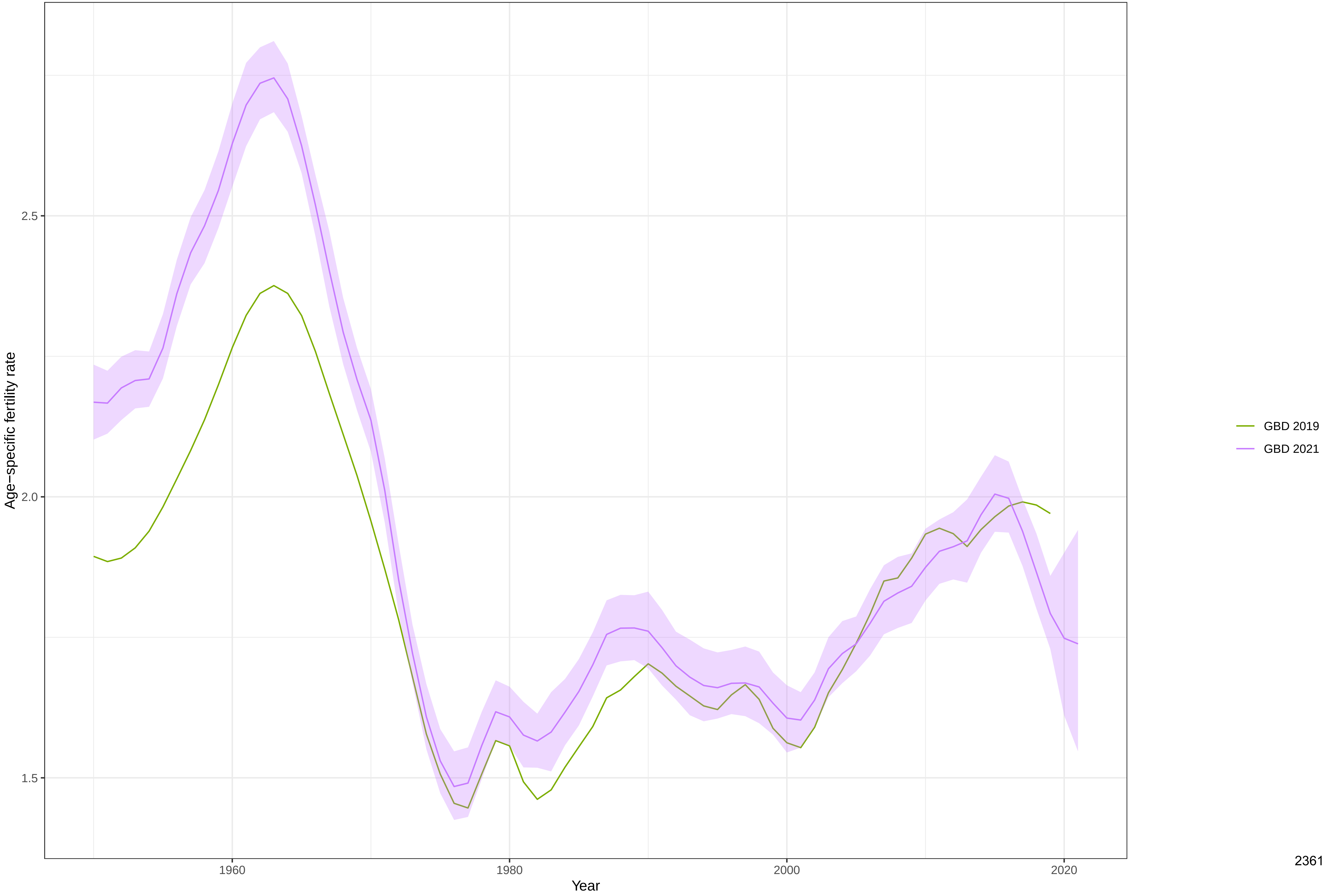

Age−specific fertility fitted trend lines, 1950−2021: Hillingdon, Ages: 10−14
Data points enclosed with a diamond are reference sources

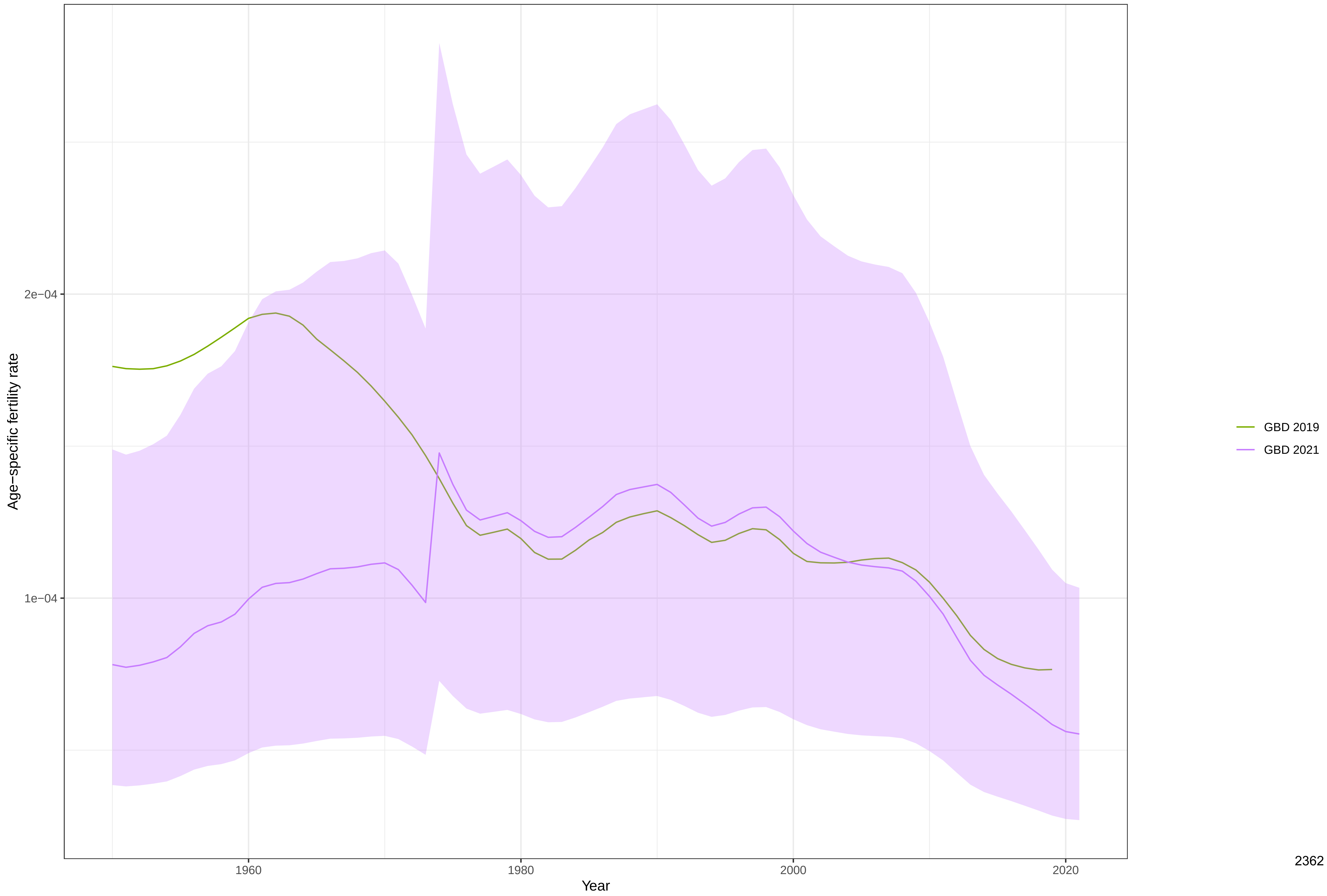

Age−specific fertility fitted trend lines, 1950−2021: Hillingdon, Ages: 15−19
Data points enclosed with a diamond are reference sources

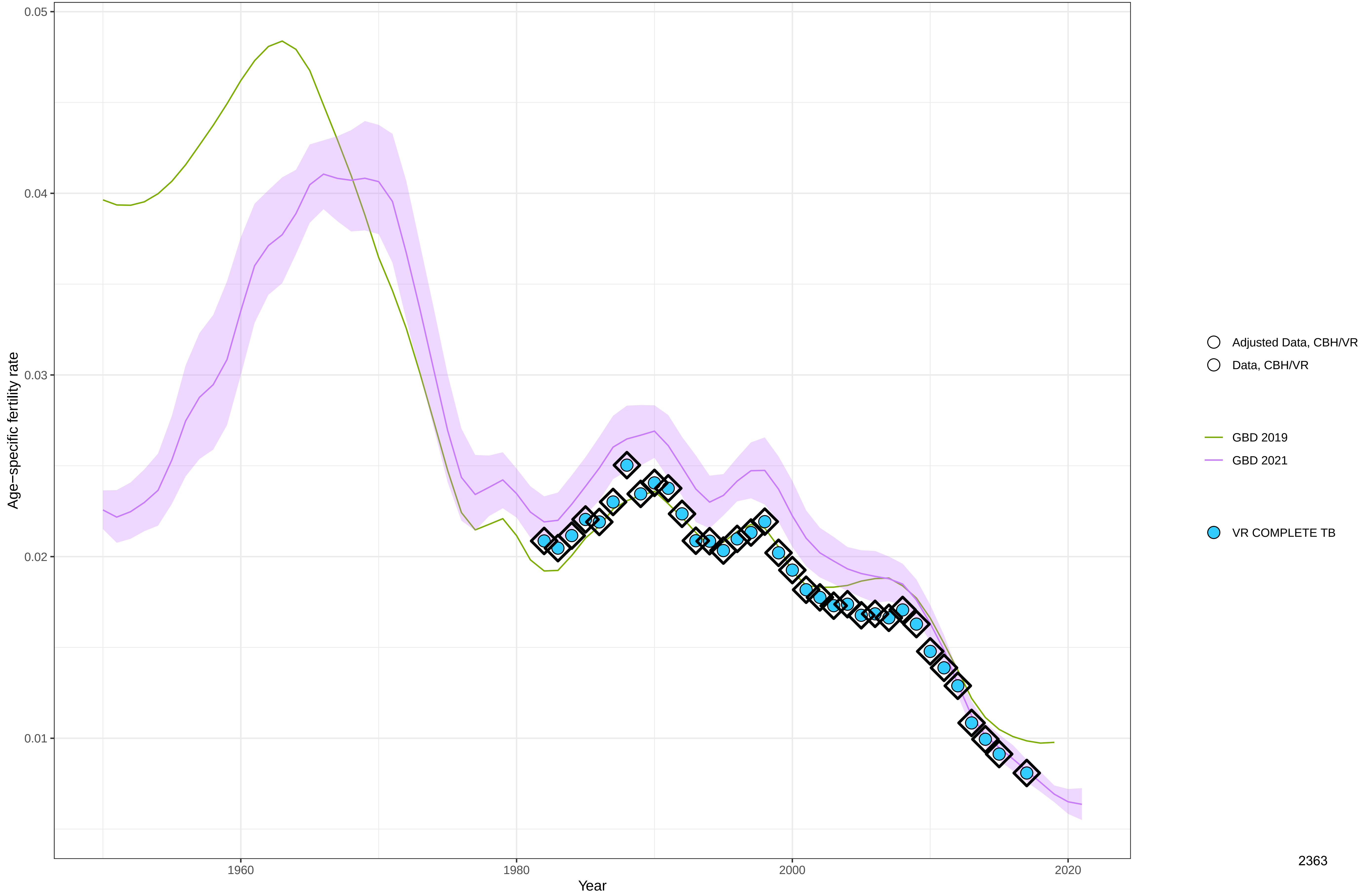

Age−specific fertility fitted trend lines, 1950−2021: Hillingdon, Ages: 20−24
Data points enclosed with a diamond are reference sources

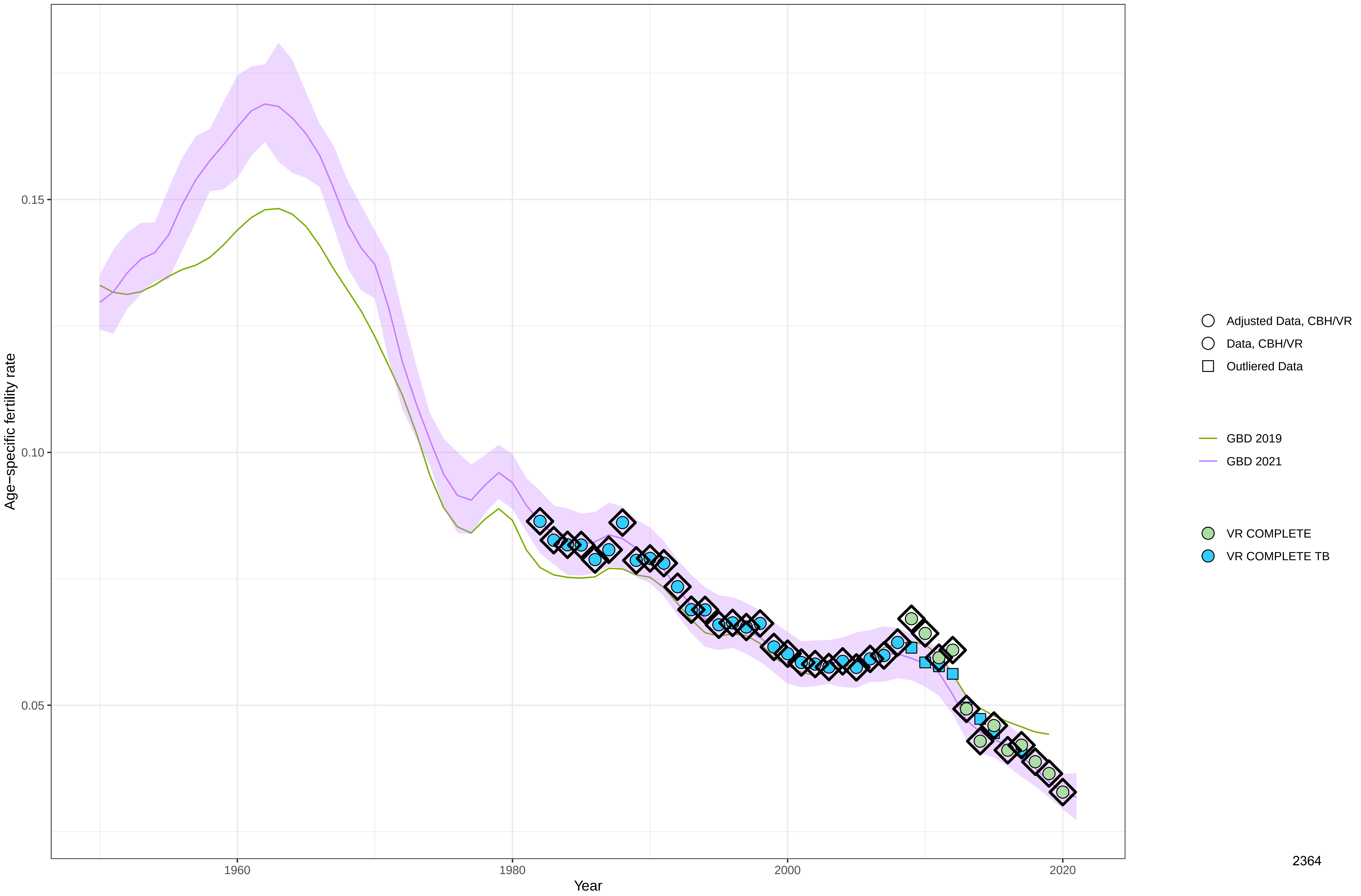

Age−specific fertility fitted trend lines, 1950−2021: Hillingdon, Ages: 25−29
Data points enclosed with a diamond are reference sources

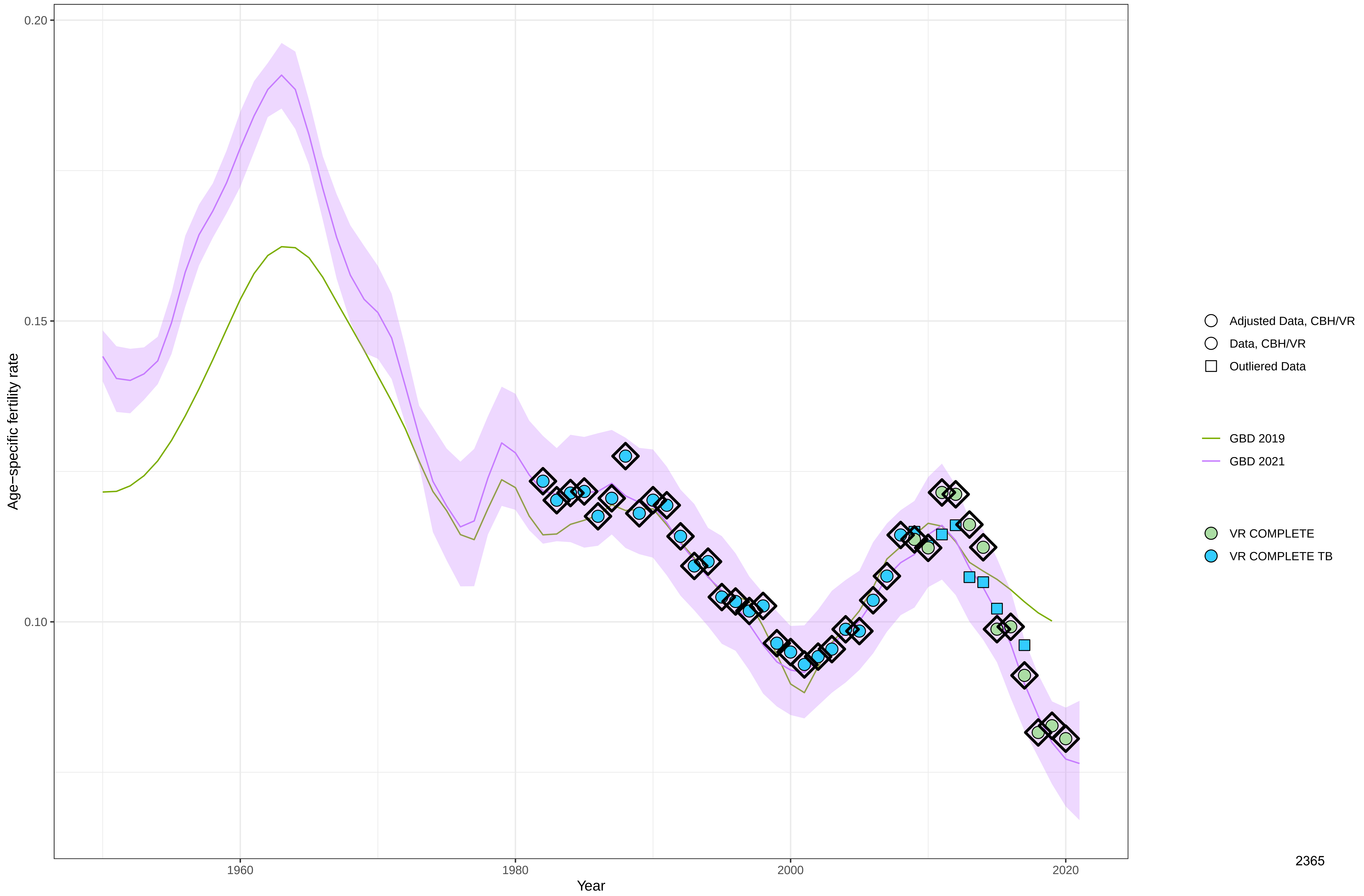

Age−specific fertility fitted trend lines, 1950−2021: Hillingdon, Ages: 30−34
Data points enclosed with a diamond are reference sources

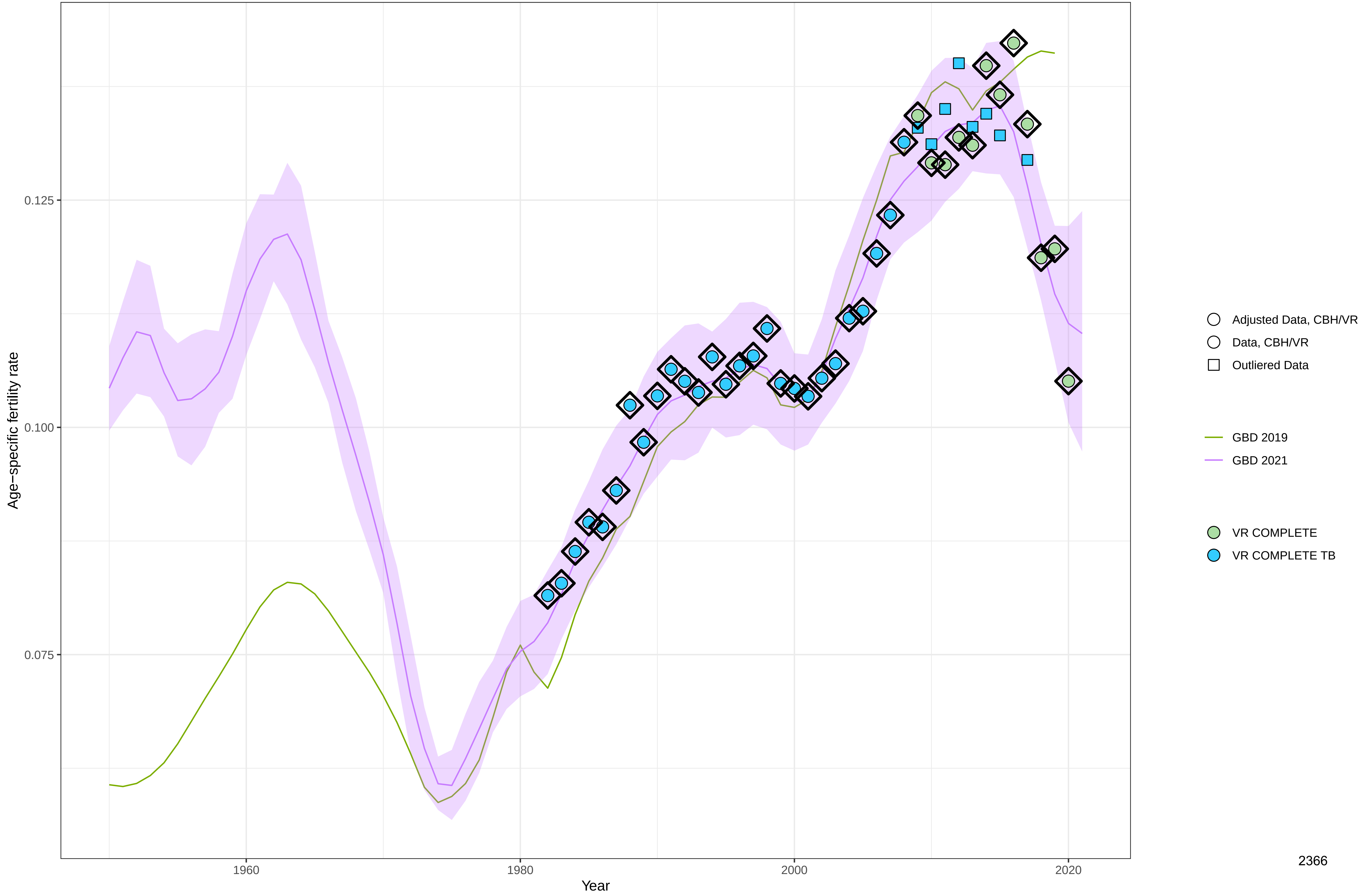

Age−specific fertility fitted trend lines, 1950−2021: Hillingdon, Ages: 35−39
Data points enclosed with a diamond are reference sources

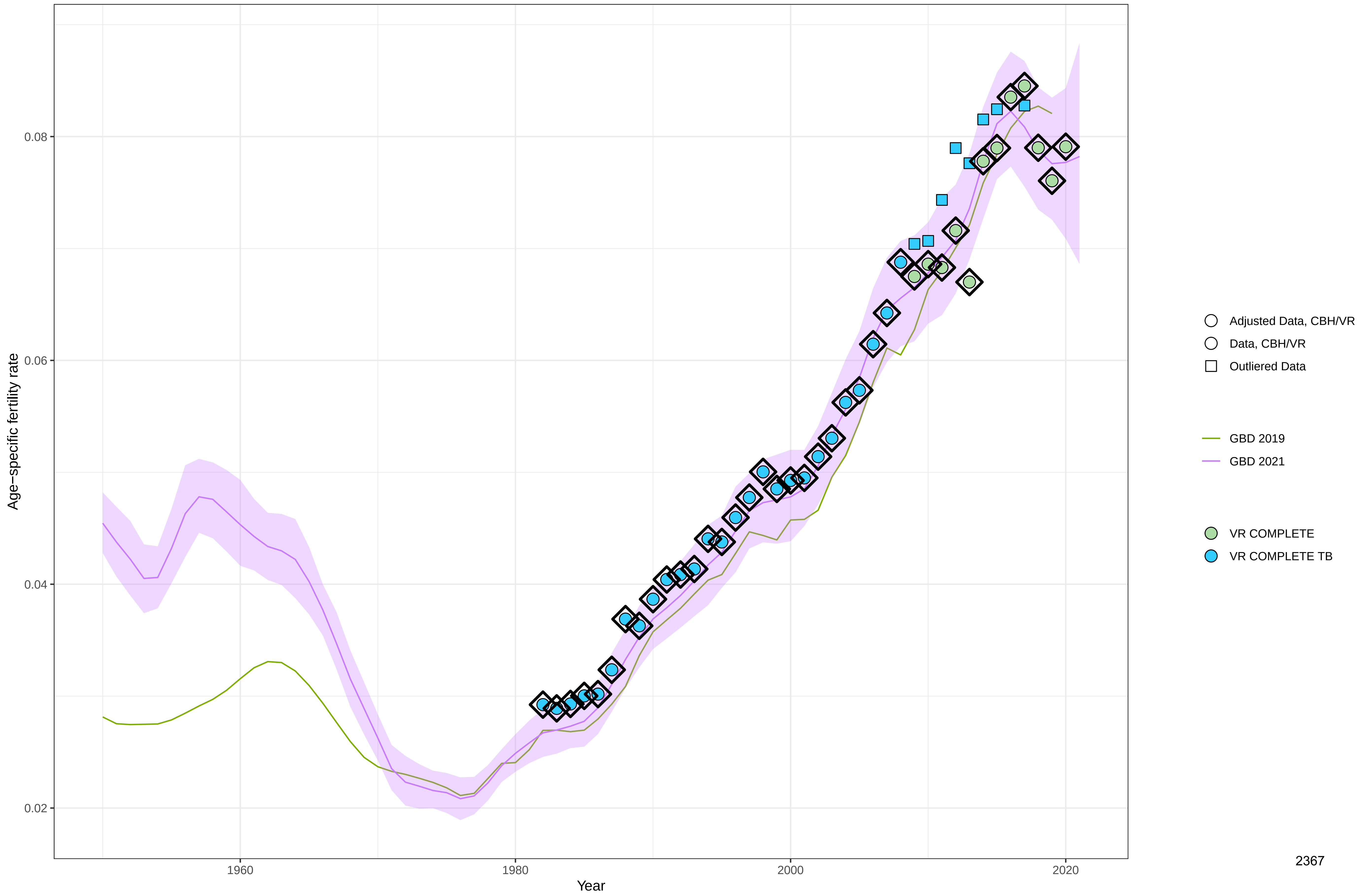

Age−specific fertility fitted trend lines, 1950−2021: Hillingdon, Ages: 40−44
Data points enclosed with a diamond are reference sources

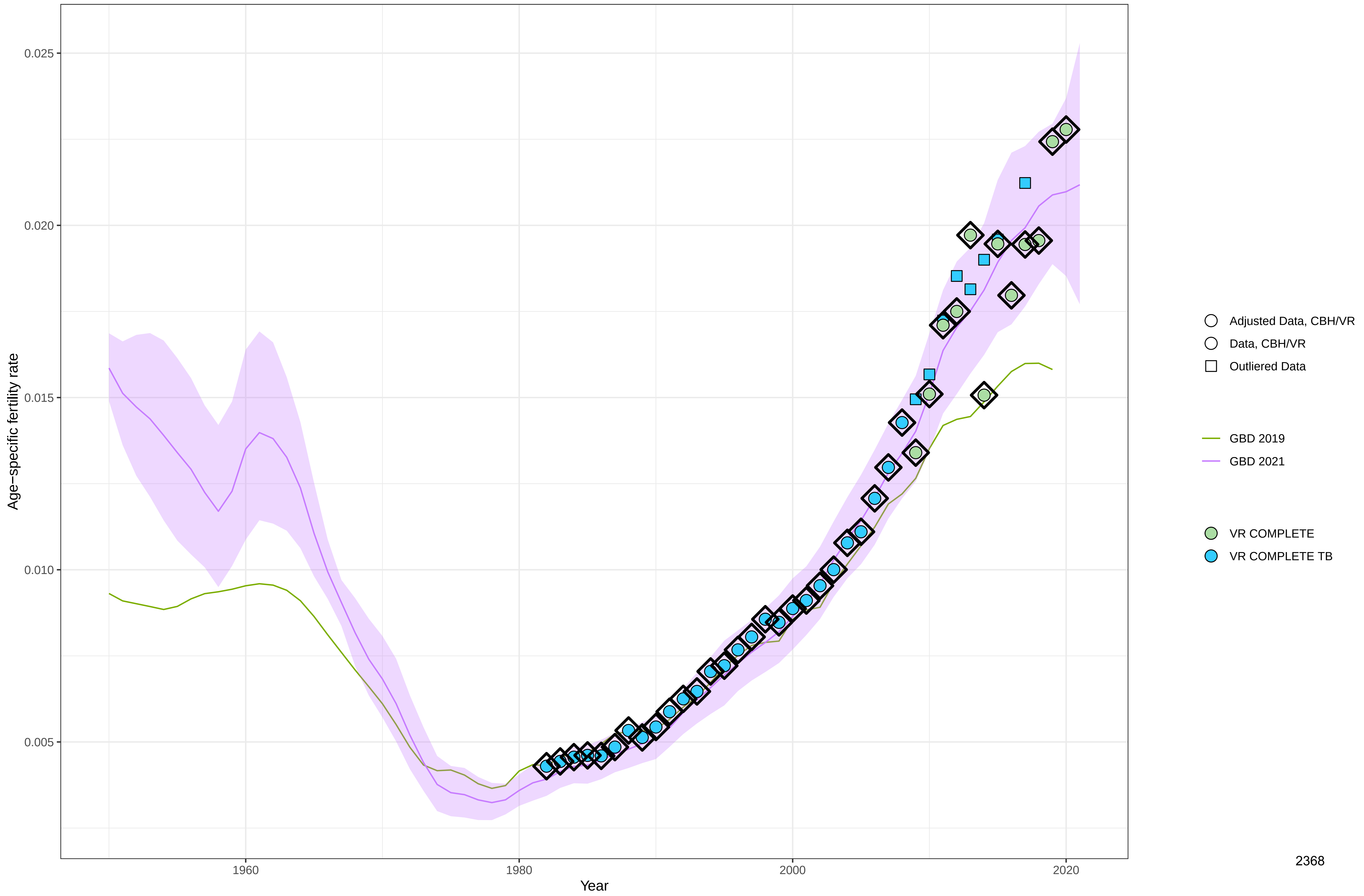

Age−specific fertility fitted trend lines, 1950−2021: Hillingdon, Ages: 45−49
Data points enclosed with a diamond are reference sources

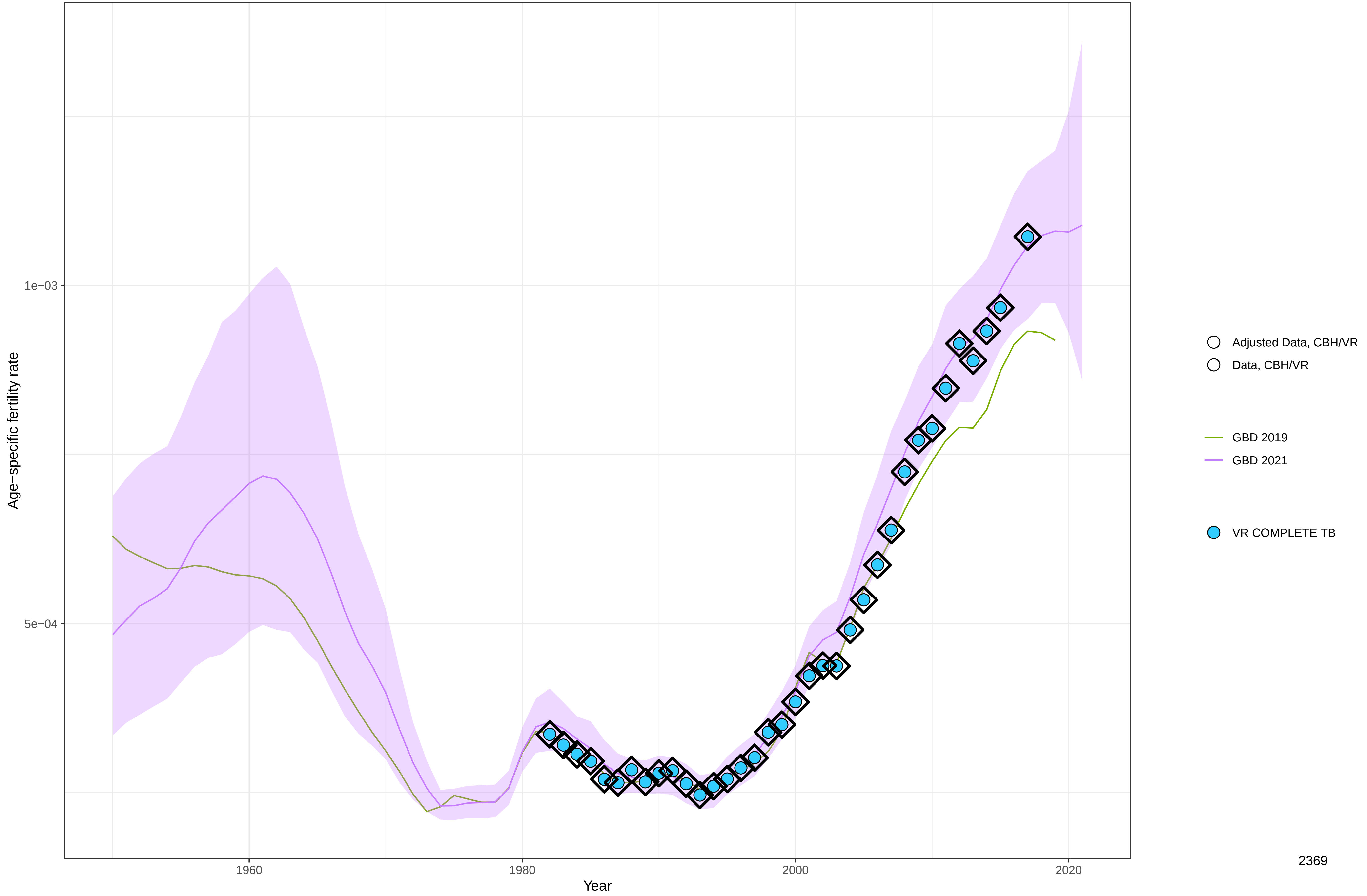

Age−specific fertility fitted trend lines, 1950−2021: Hillingdon, Ages: 50−54
Data points enclosed with a diamond are reference sources

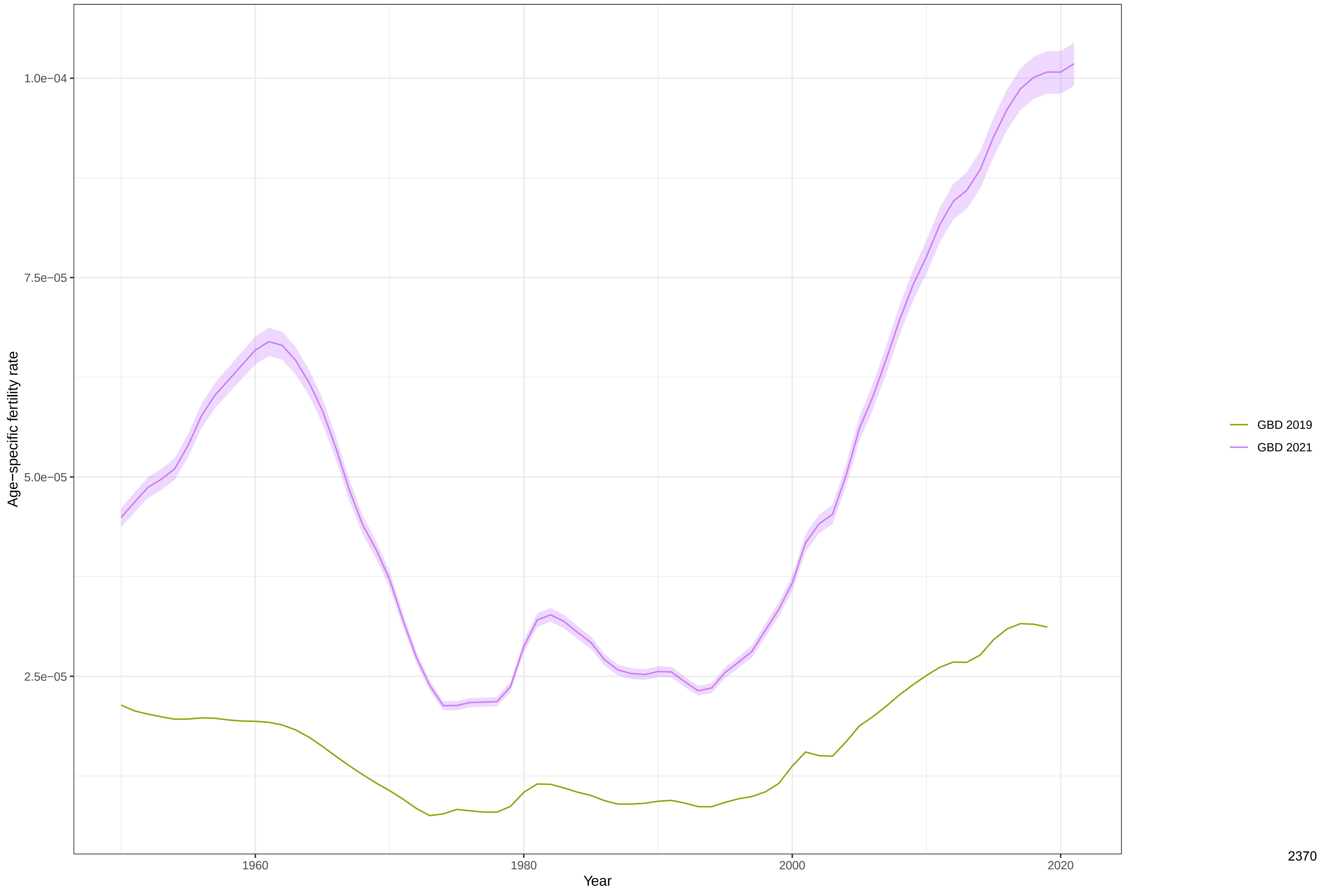

Total fertility rate fitted trend lines, 1950−2021: Hillingdon TFR
Data points enclosed with a diamond are reference sources

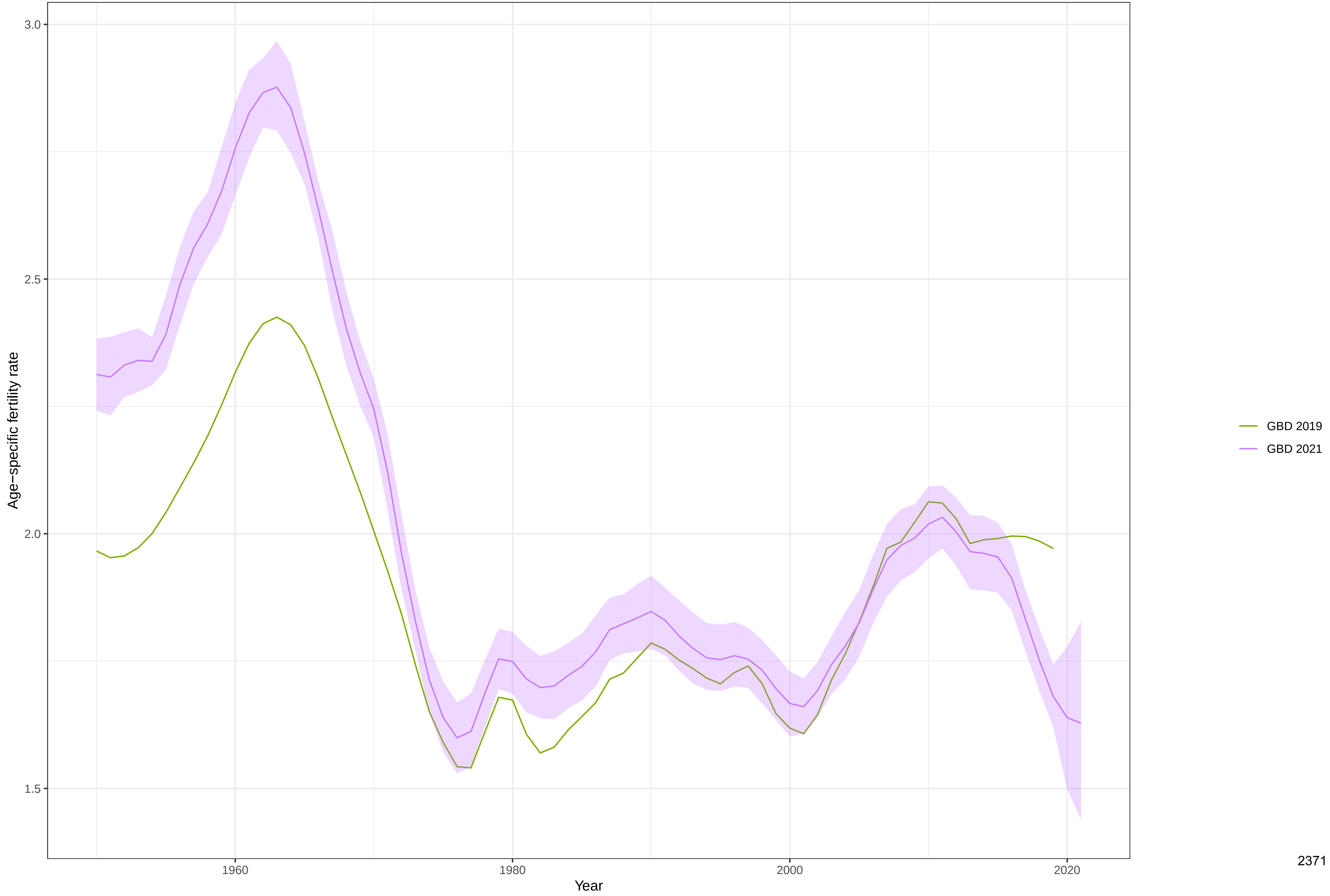

Age−specific fertility fitted trend lines, 1950−2021: Hounslow, Ages: 10−14
Data points enclosed with a diamond are reference sources

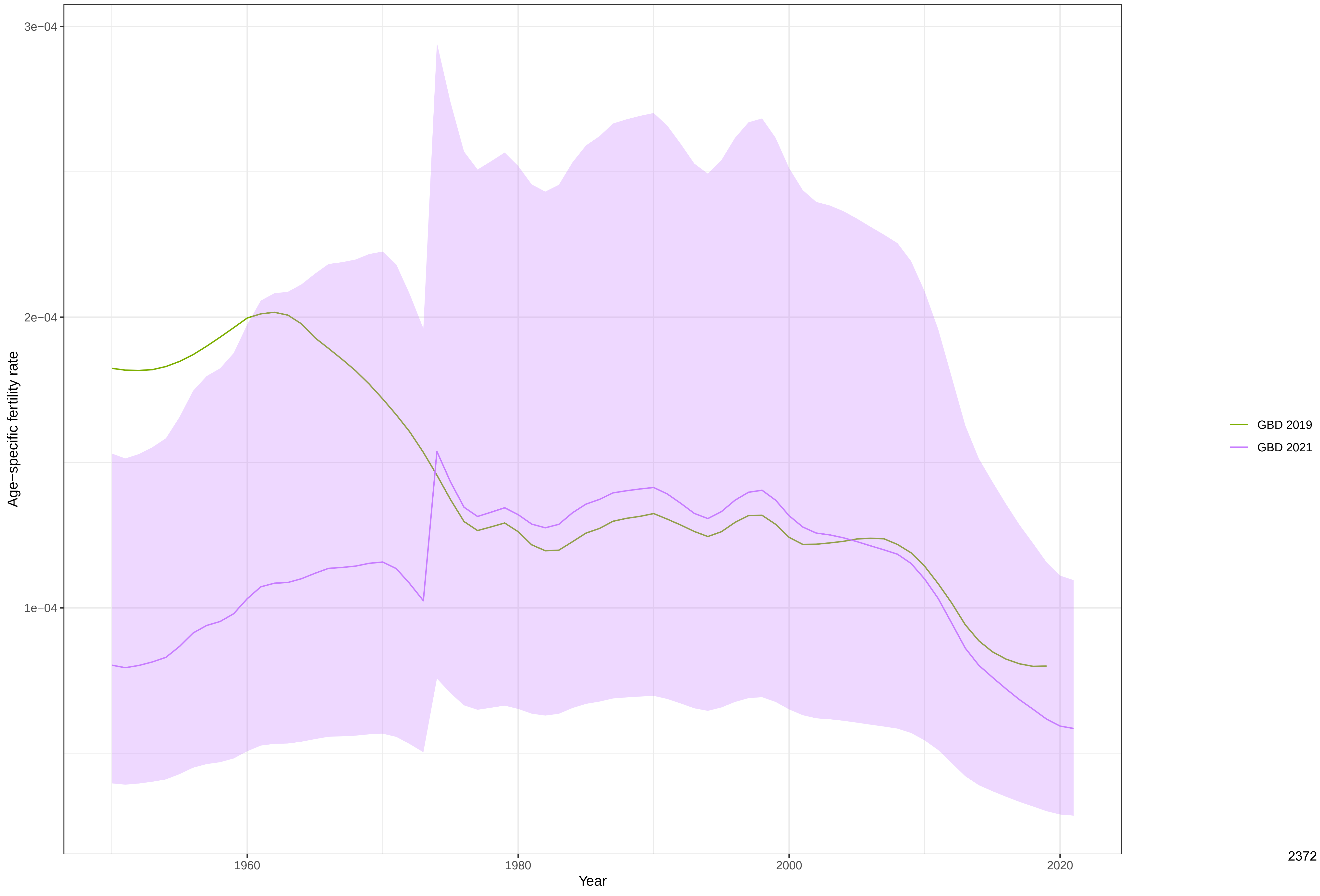

Age−specific fertility fitted trend lines, 1950−2021: Hounslow, Ages: 15−19
Data points enclosed with a diamond are reference sources

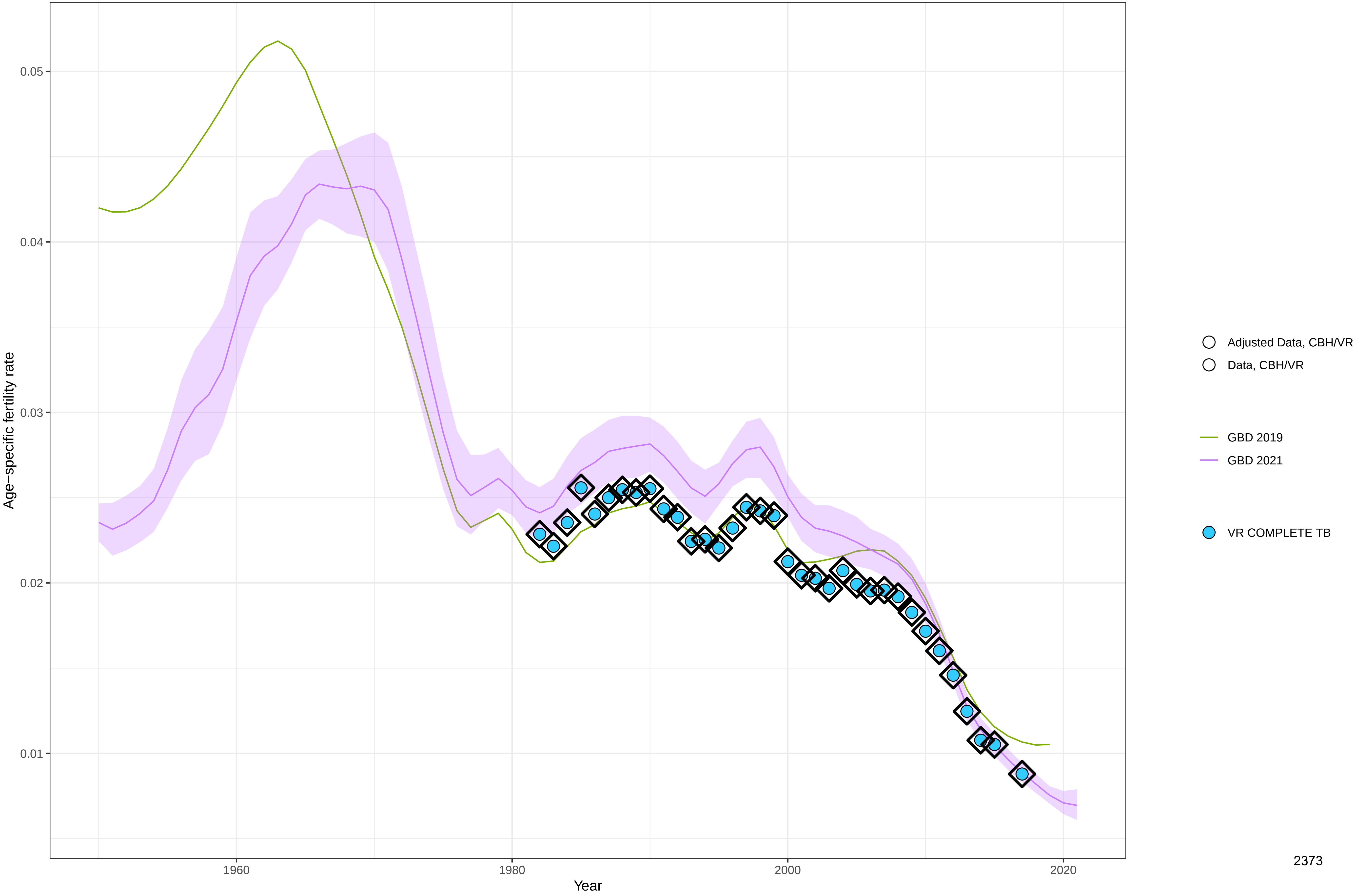

Age−specific fertility fitted trend lines, 1950−2021: Hounslow, Ages: 20−24
Data points enclosed with a diamond are reference sources

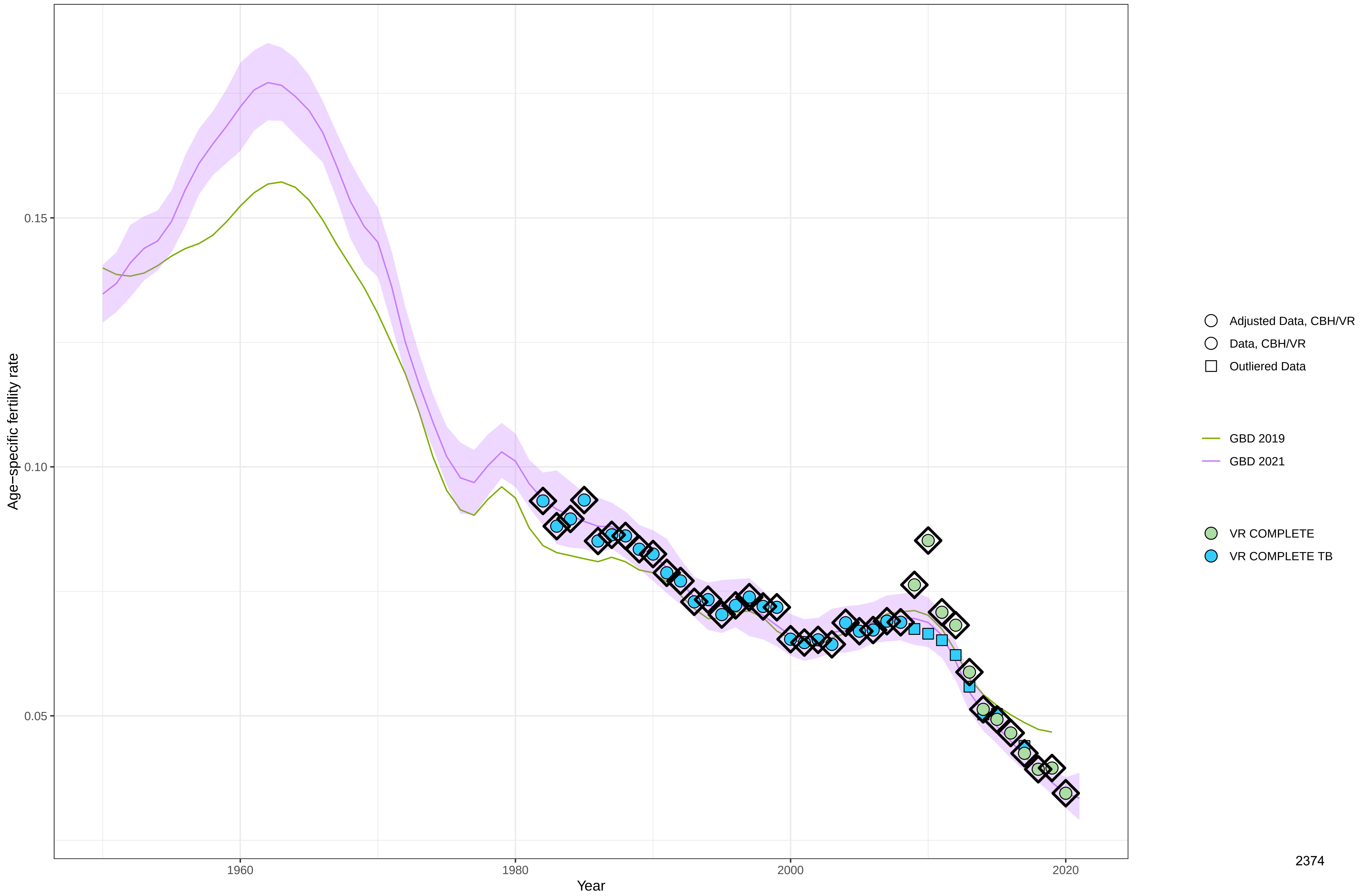

Age−specific fertility fitted trend lines, 1950−2021: Hounslow, Ages: 25−29
Data points enclosed with a diamond are reference sources

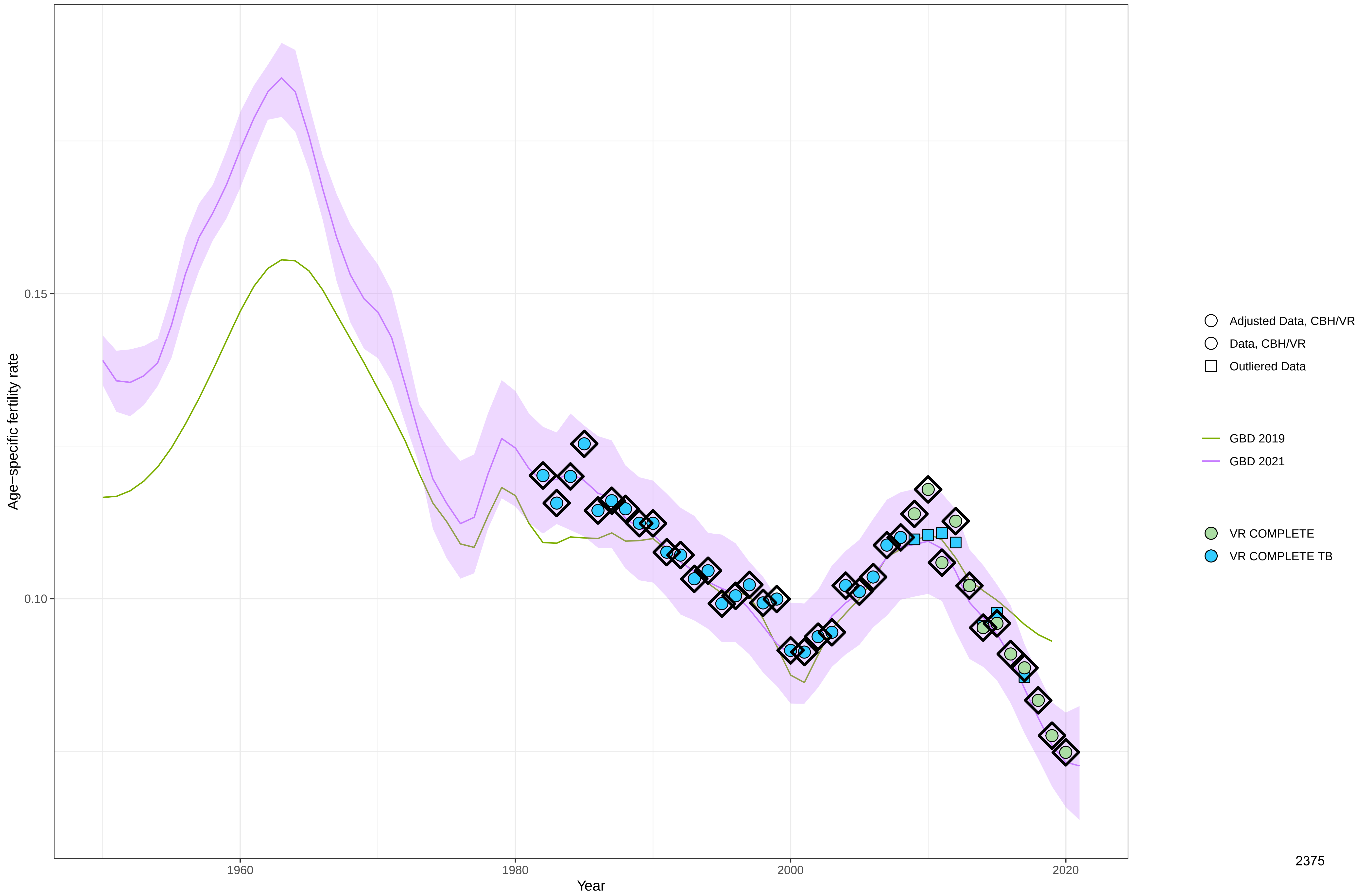

Age−specific fertility fitted trend lines, 1950−2021: Hounslow, Ages: 30−34
Data points enclosed with a diamond are reference sources

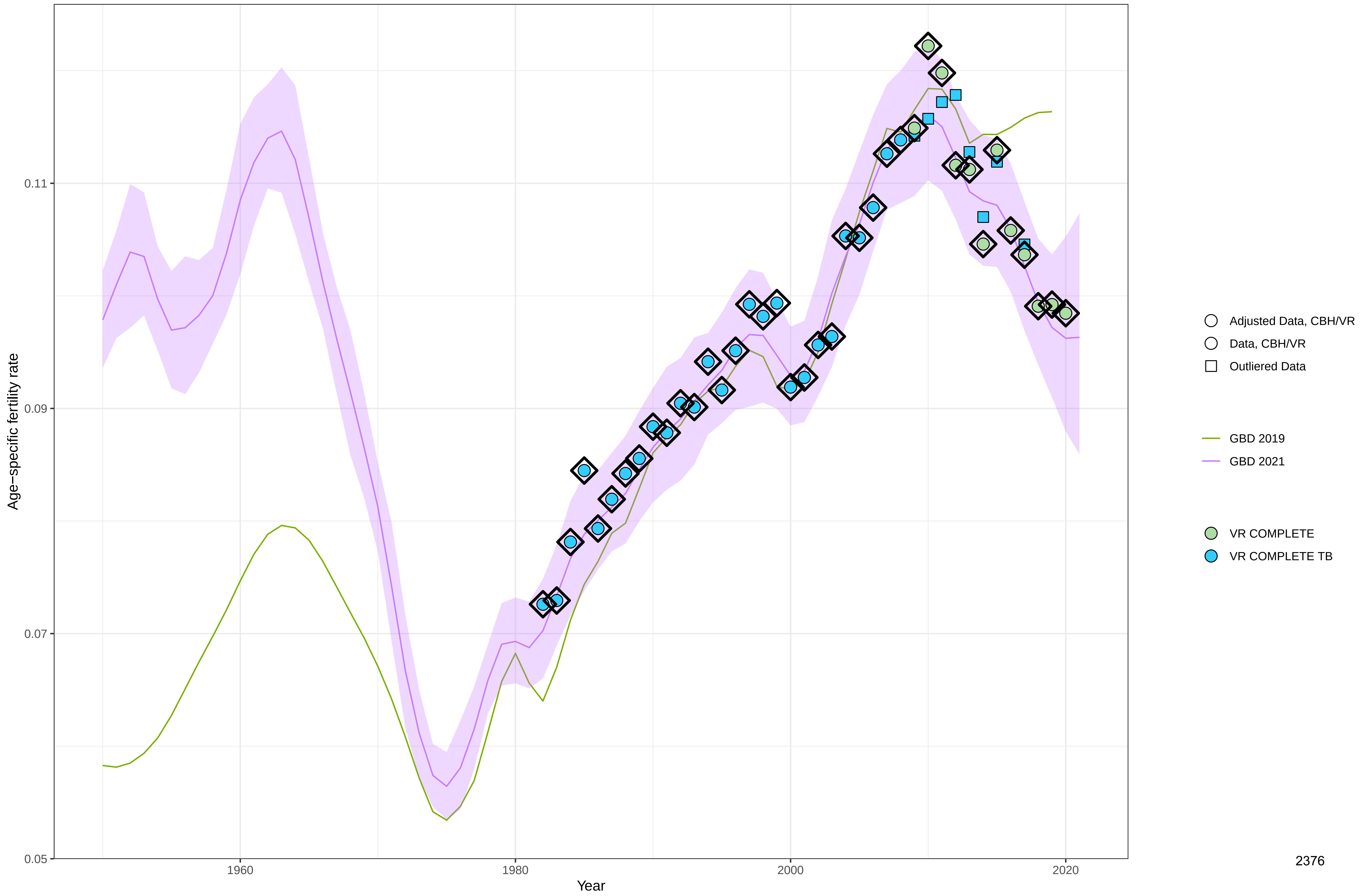

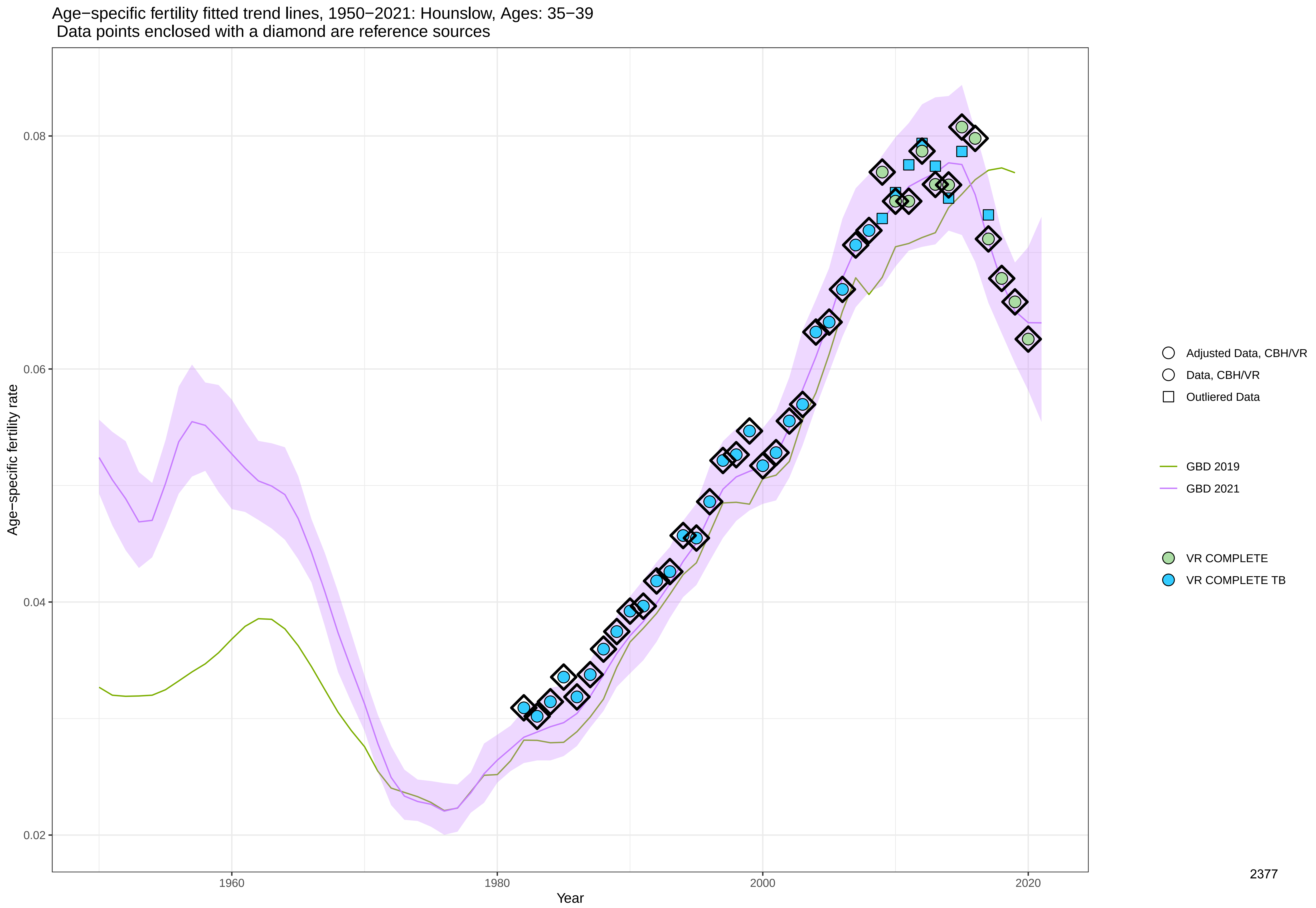

Age−specific fertility fitted trend lines, 1950−2021: Hounslow, Ages: 35−39
Data points enclosed with a diamond are reference sources
Age−specific fertility rate
0.08
0.06
0.04
0.02
Year
1960
1980
2000
2020
Adjusted Data, CBH/VR
Data, CBH/VR
Outliered Data
GBD 2019
GBD 2021
VR COMPLETE
VR COMPLETE TB

Age−specific fertility fitted trend lines, 1950−2021: Hounslow, Ages: 40−44
Data points enclosed with a diamond are reference sources

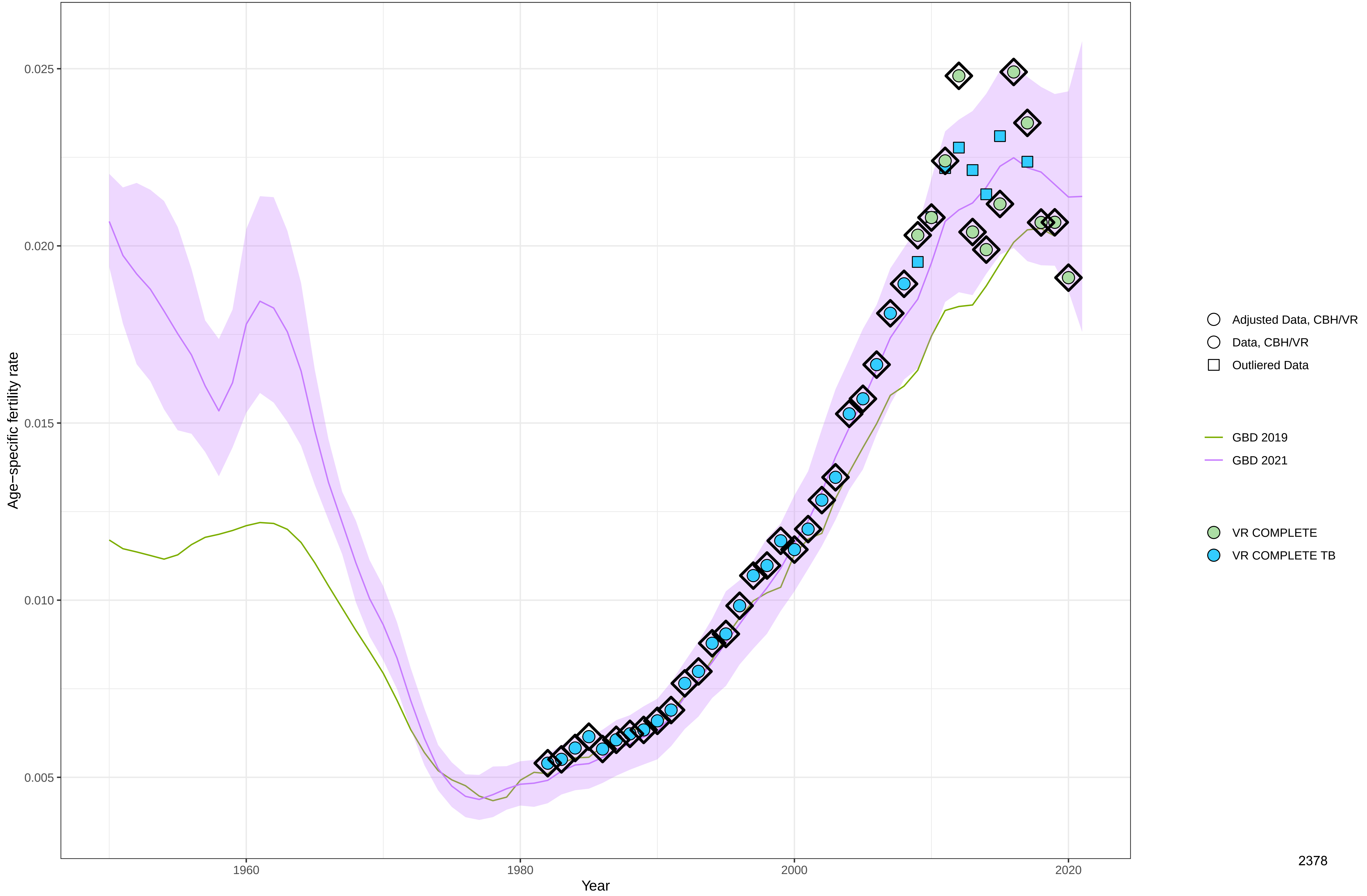

Age−specific fertility fitted trend lines, 1950−2021: Hounslow, Ages: 45−49
Data points enclosed with a diamond are reference sources

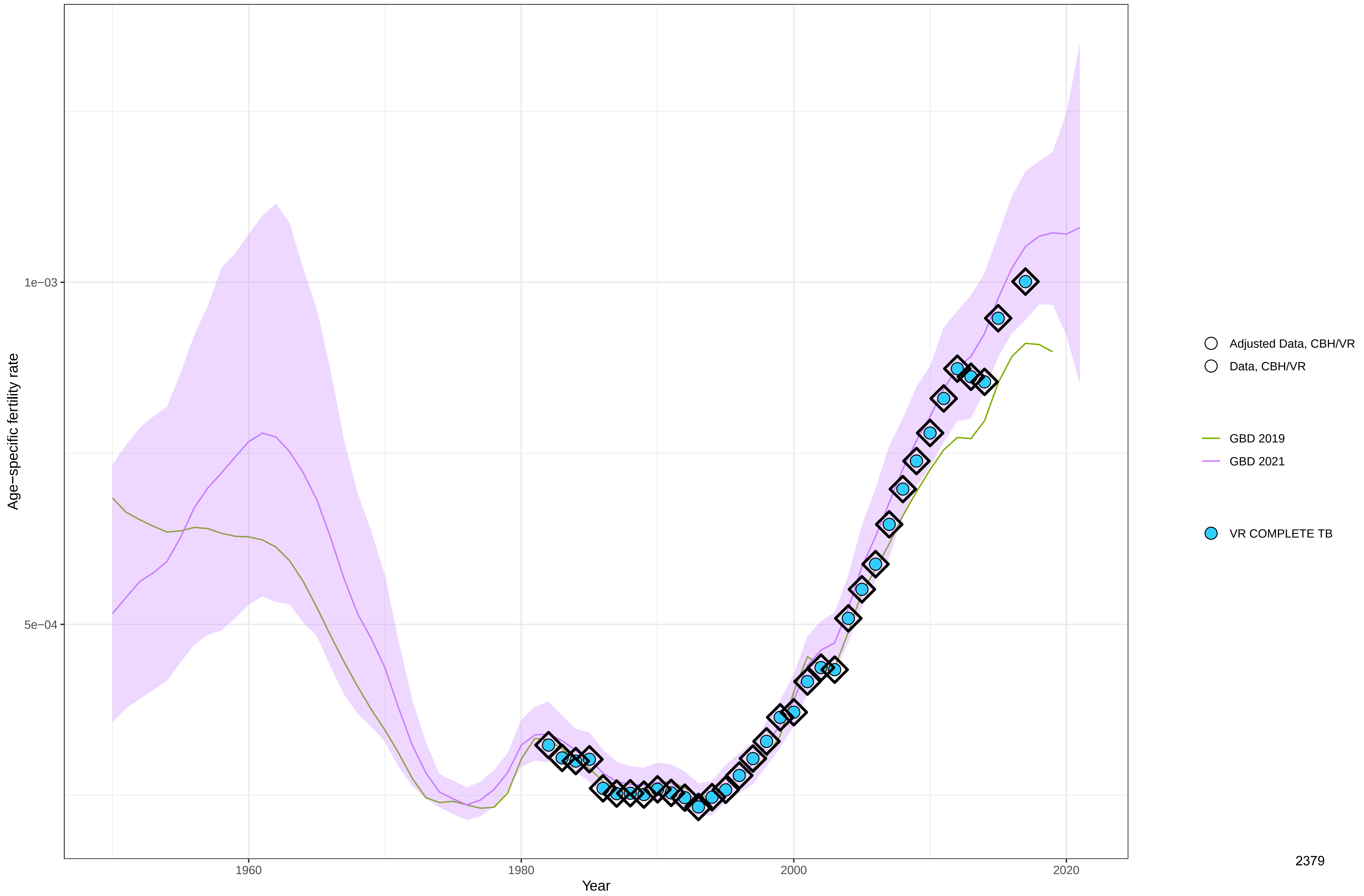

Age−specific fertility fitted trend lines, 1950−2021: Hounslow, Ages: 50−54
Data points enclosed with a diamond are reference sources

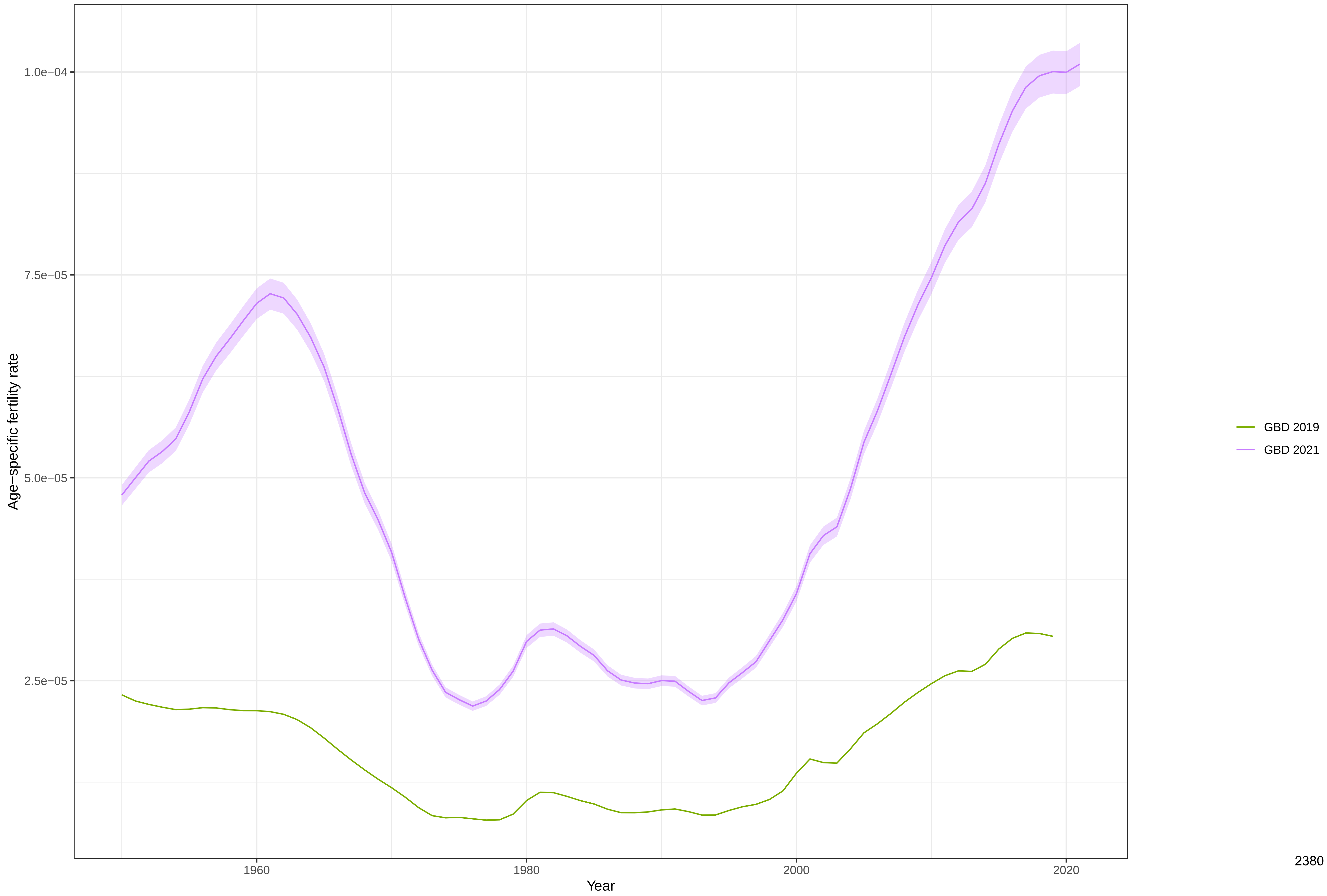

Total fertility rate fitted trend lines, 1950−2021: Hounslow TFR
Data points enclosed with a diamond are reference sources

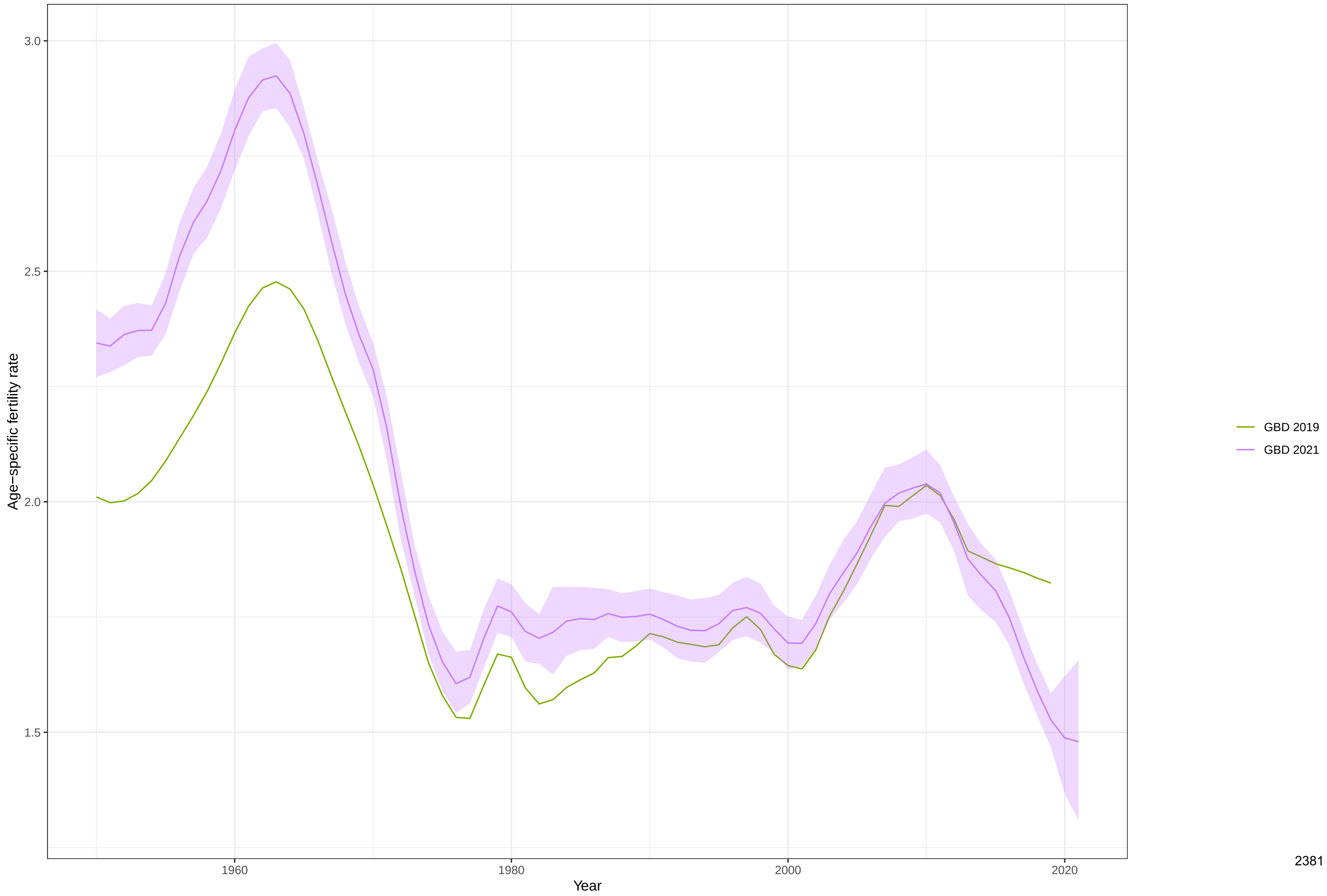

Age−specific fertility fitted trend lines, 1950−2021: Islington, Ages: 10−14
Data points enclosed with a diamond are reference sources

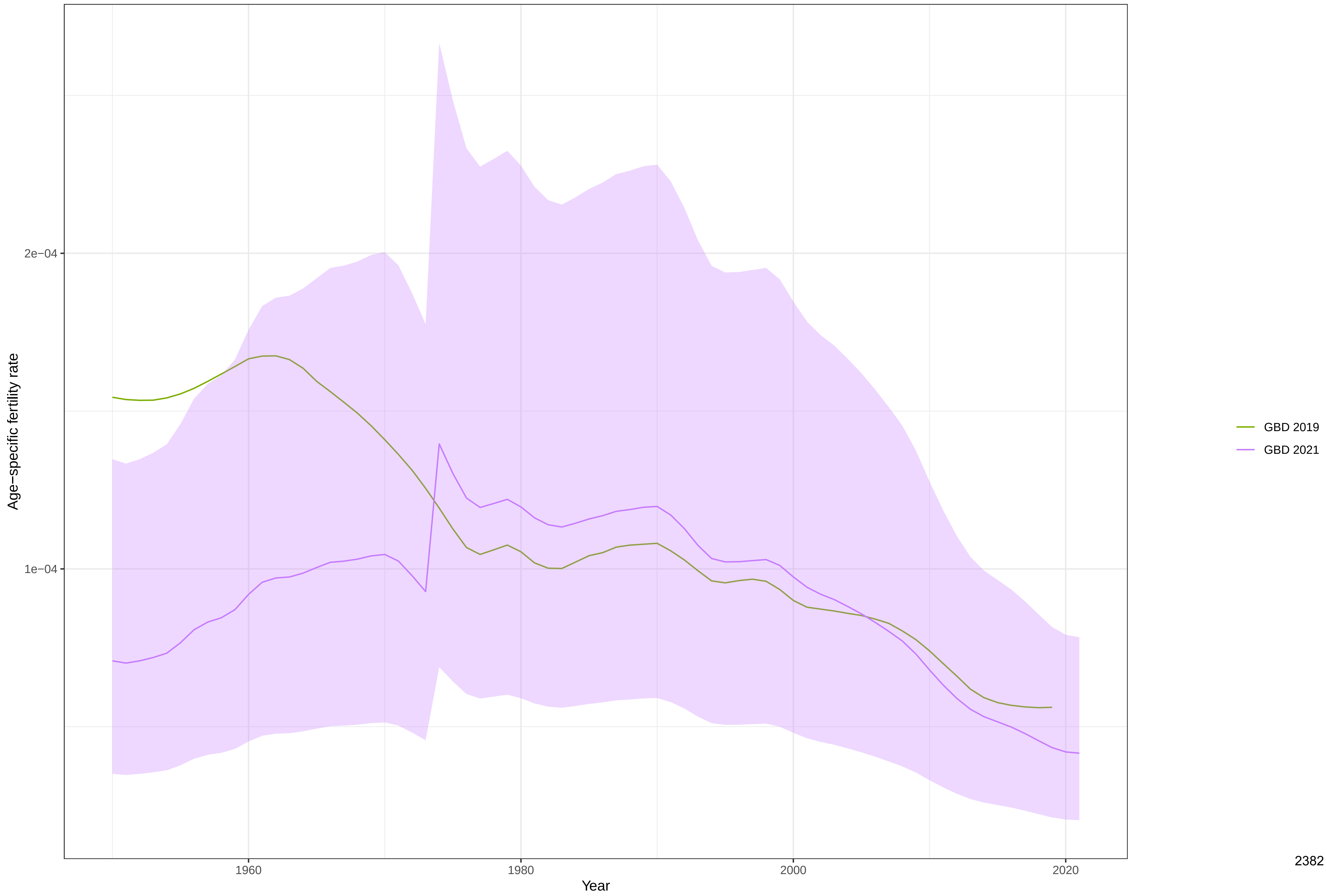

Age−specific fertility fitted trend lines, 1950−2021: Islington, Ages: 15−19
Data points enclosed with a diamond are reference sources

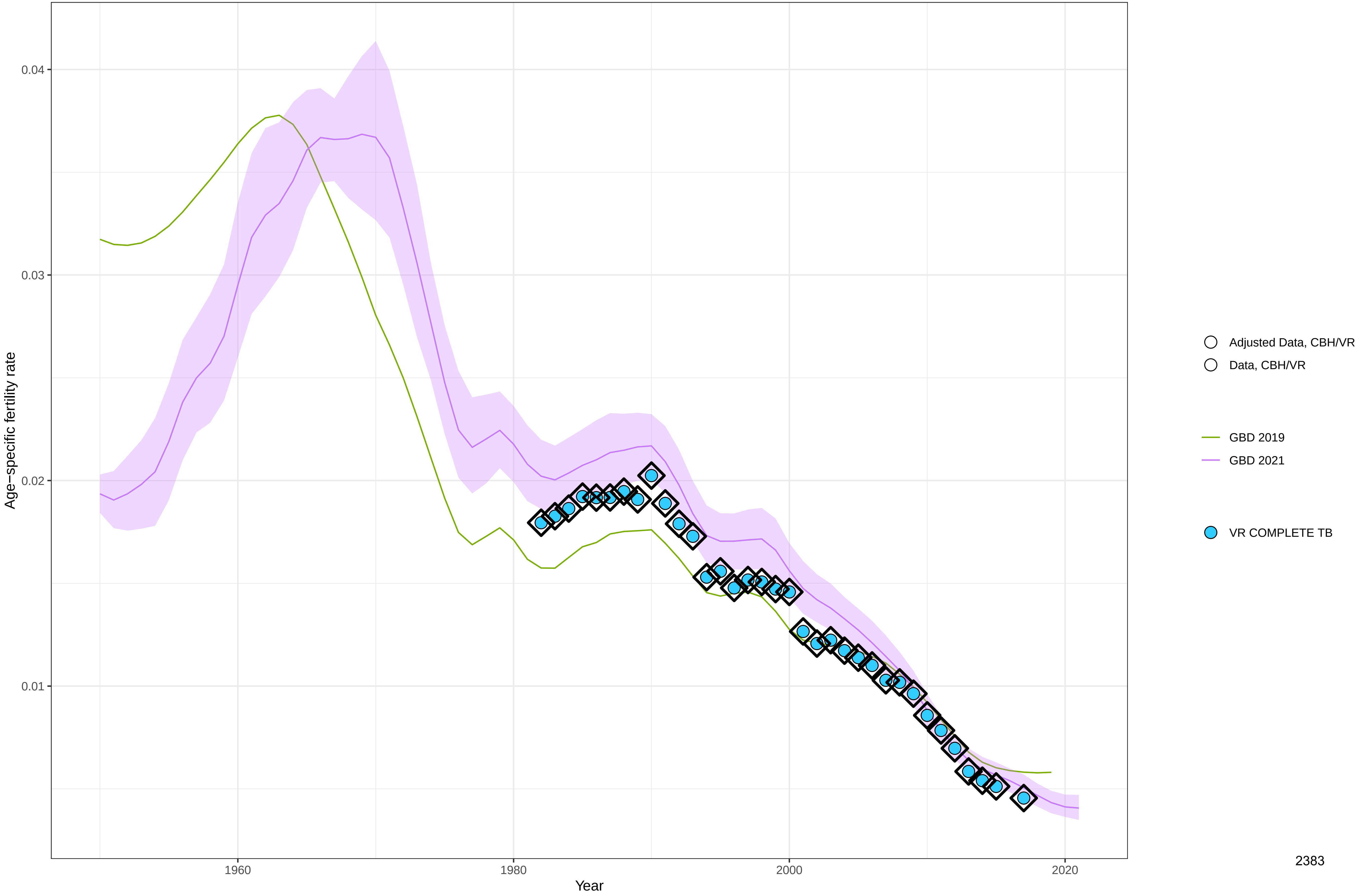

Age−specific fertility fitted trend lines, 1950−2021: Islington, Ages: 20−24
Data points enclosed with a diamond are reference sources

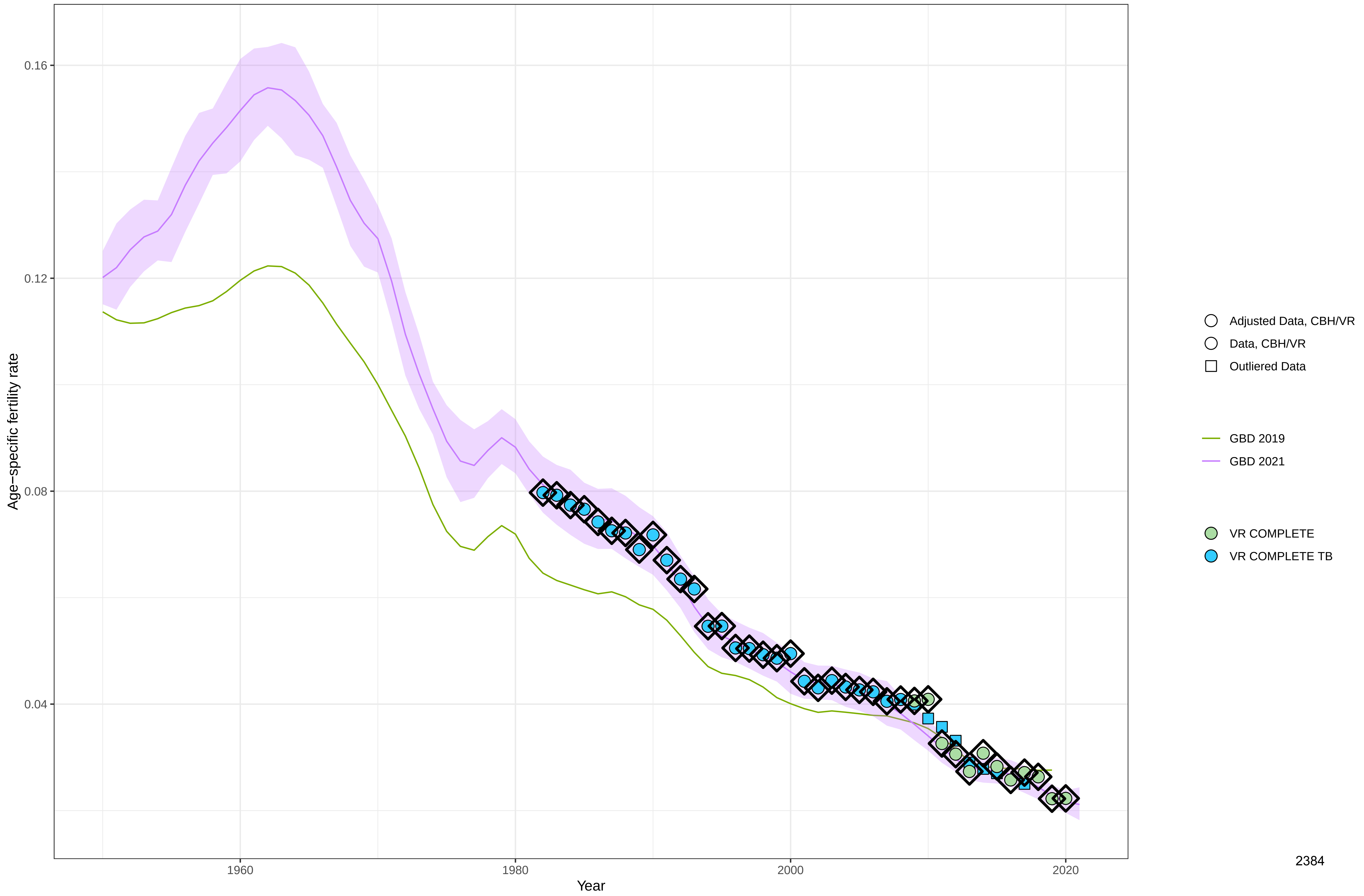

Age−specific fertility fitted trend lines, 1950−2021: Islington, Ages: 25−29
Data points enclosed with a diamond are reference sources

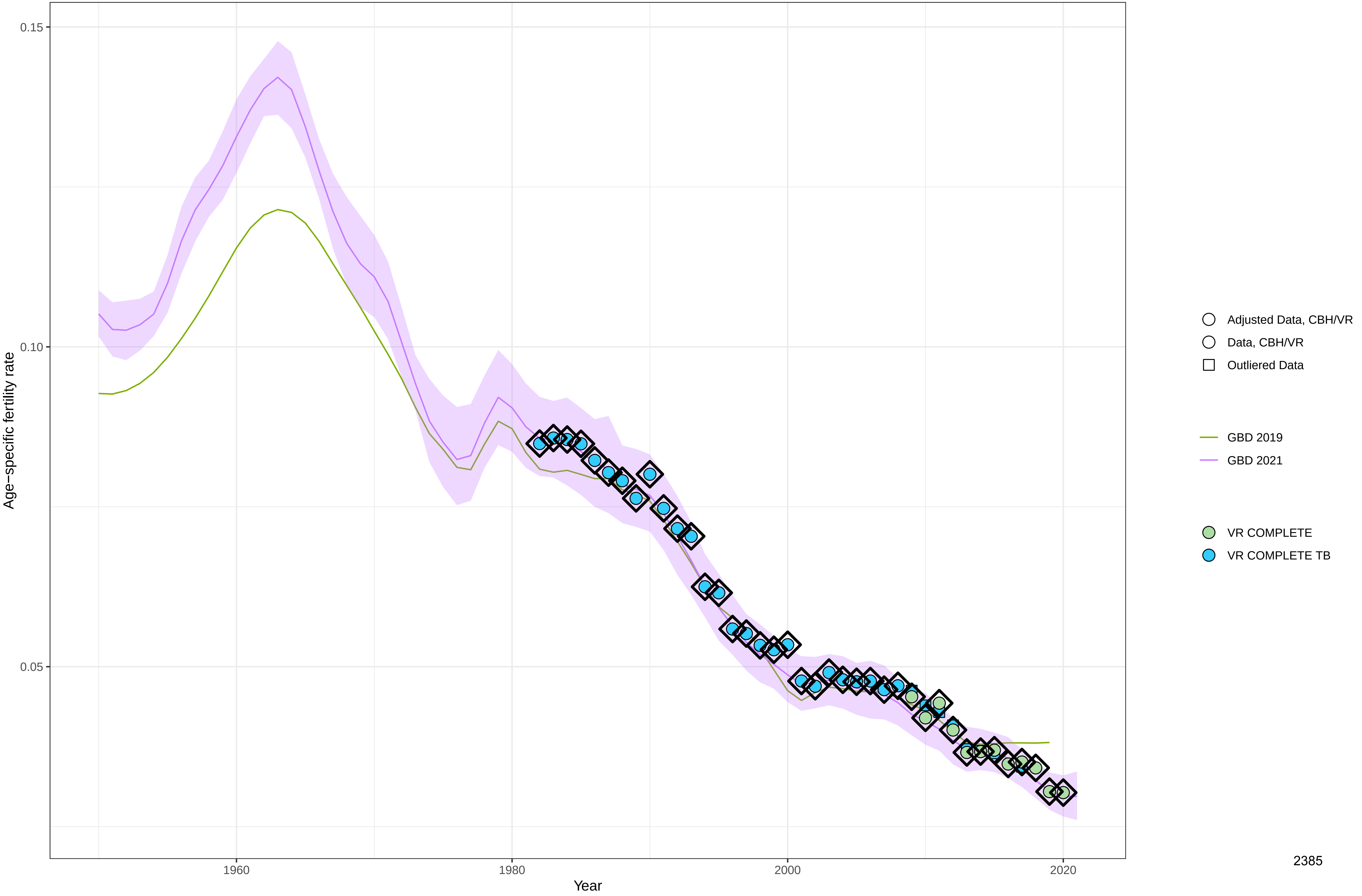

Age−specific fertility fitted trend lines, 1950−2021: Islington, Ages: 30−34
Data points enclosed with a diamond are reference sources

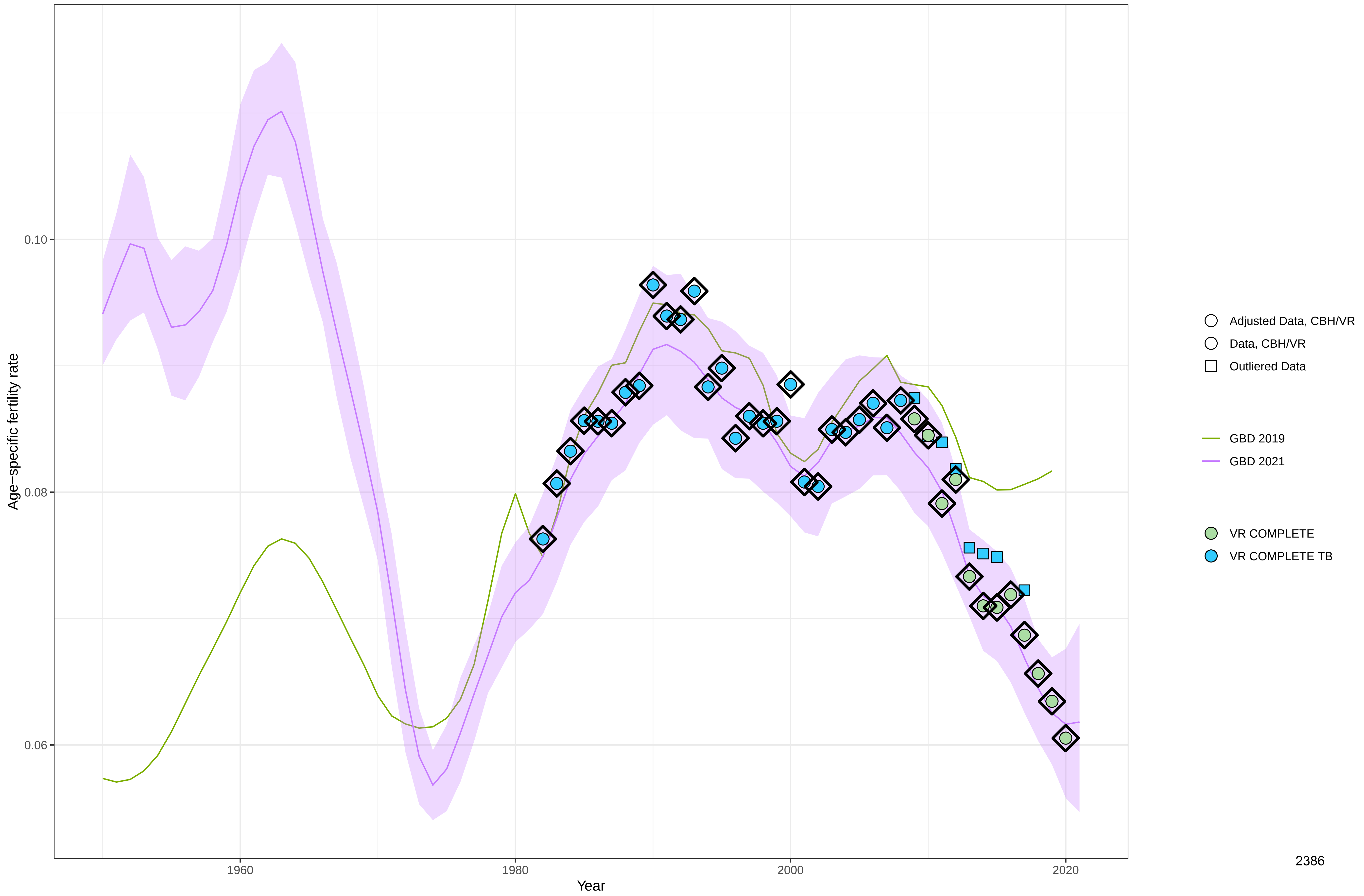

Age−specific fertility fitted trend lines, 1950−2021: Islington, Ages: 35−39
Data points enclosed with a diamond are reference sources

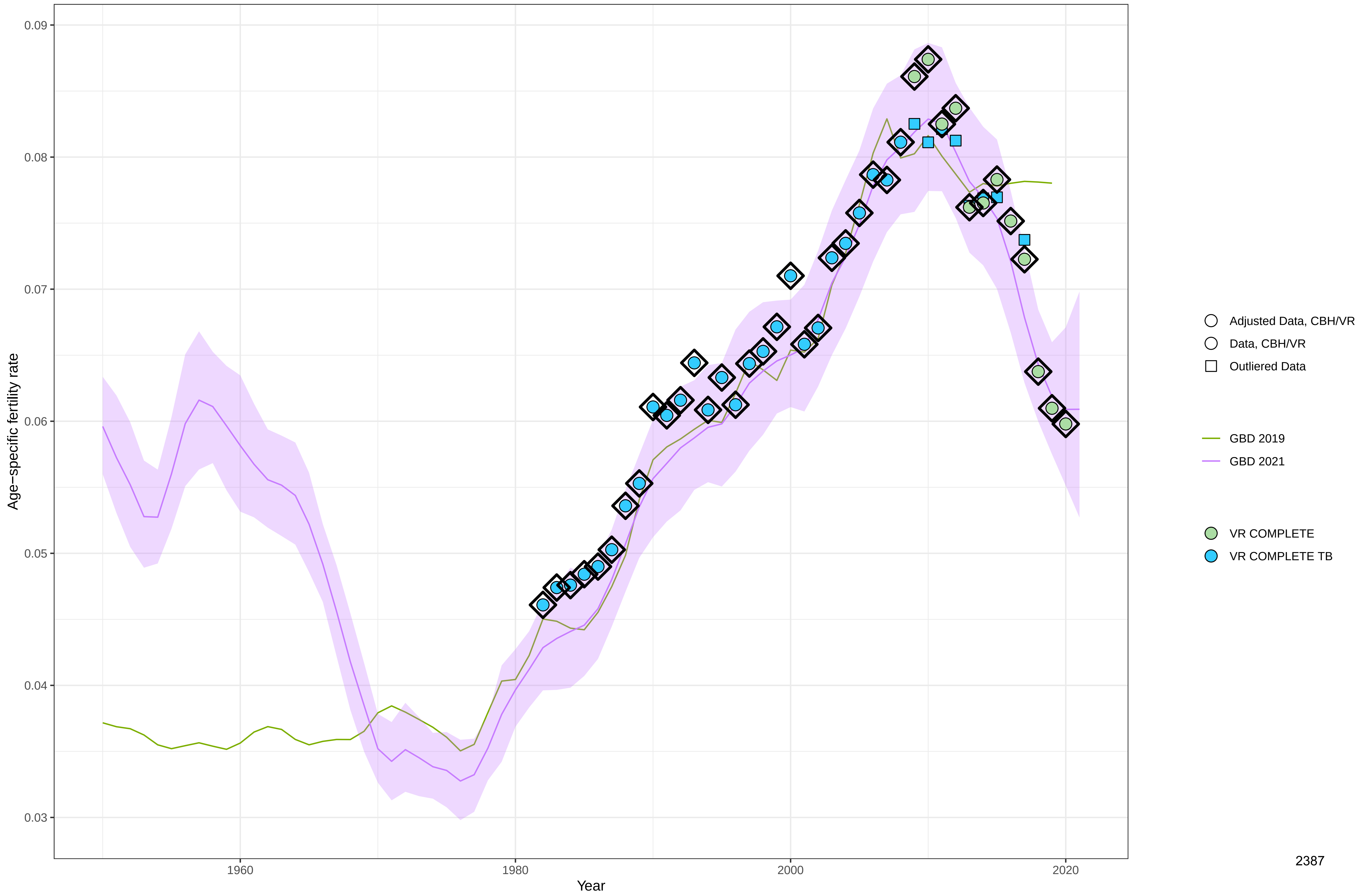

Age−specific fertility fitted trend lines, 1950−2021: Islington, Ages: 40−44
Data points enclosed with a diamond are reference sources

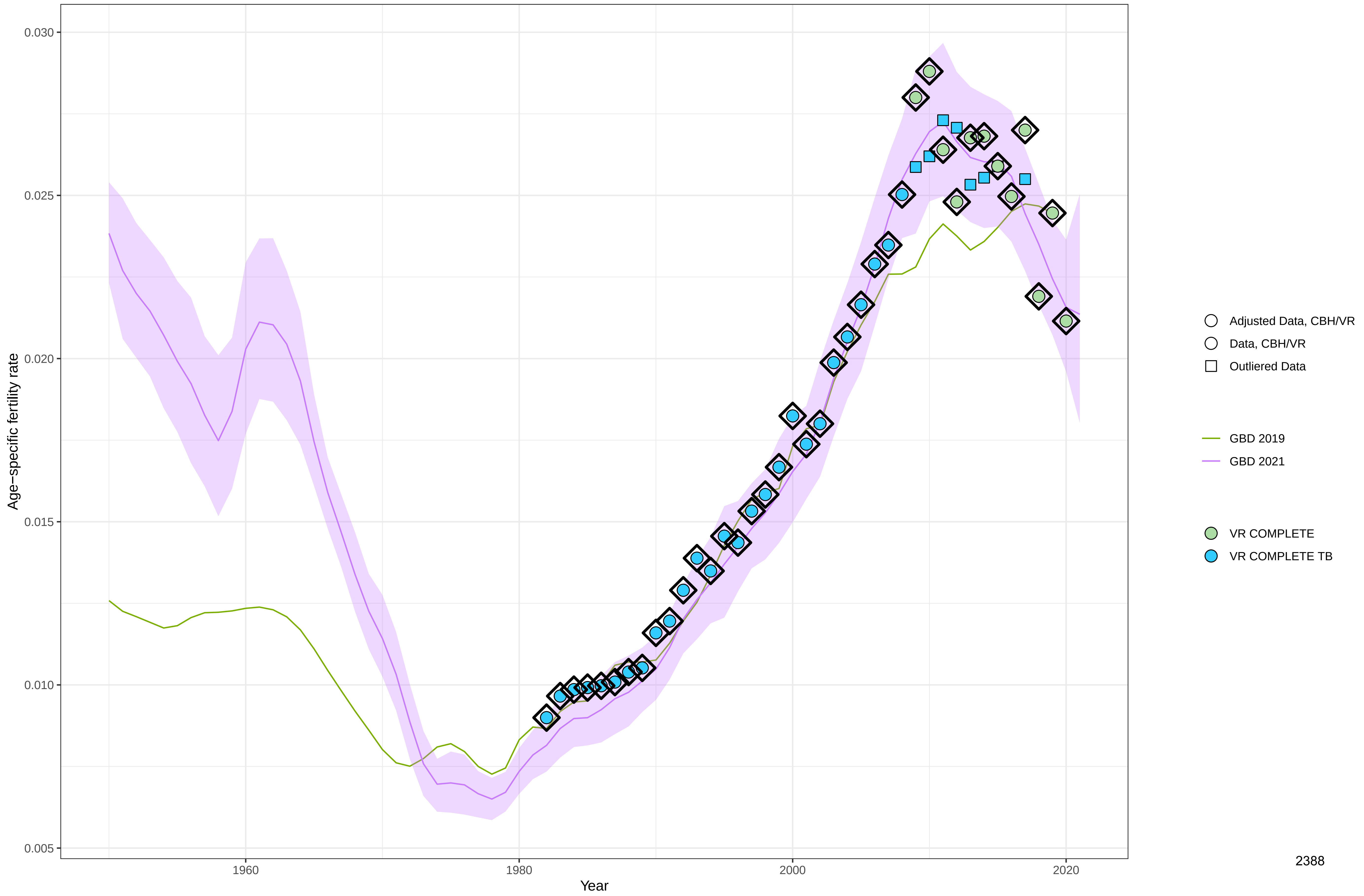

Age−specific fertility fitted trend lines, 1950−2021: Islington, Ages: 45−49
Data points enclosed with a diamond are reference sources

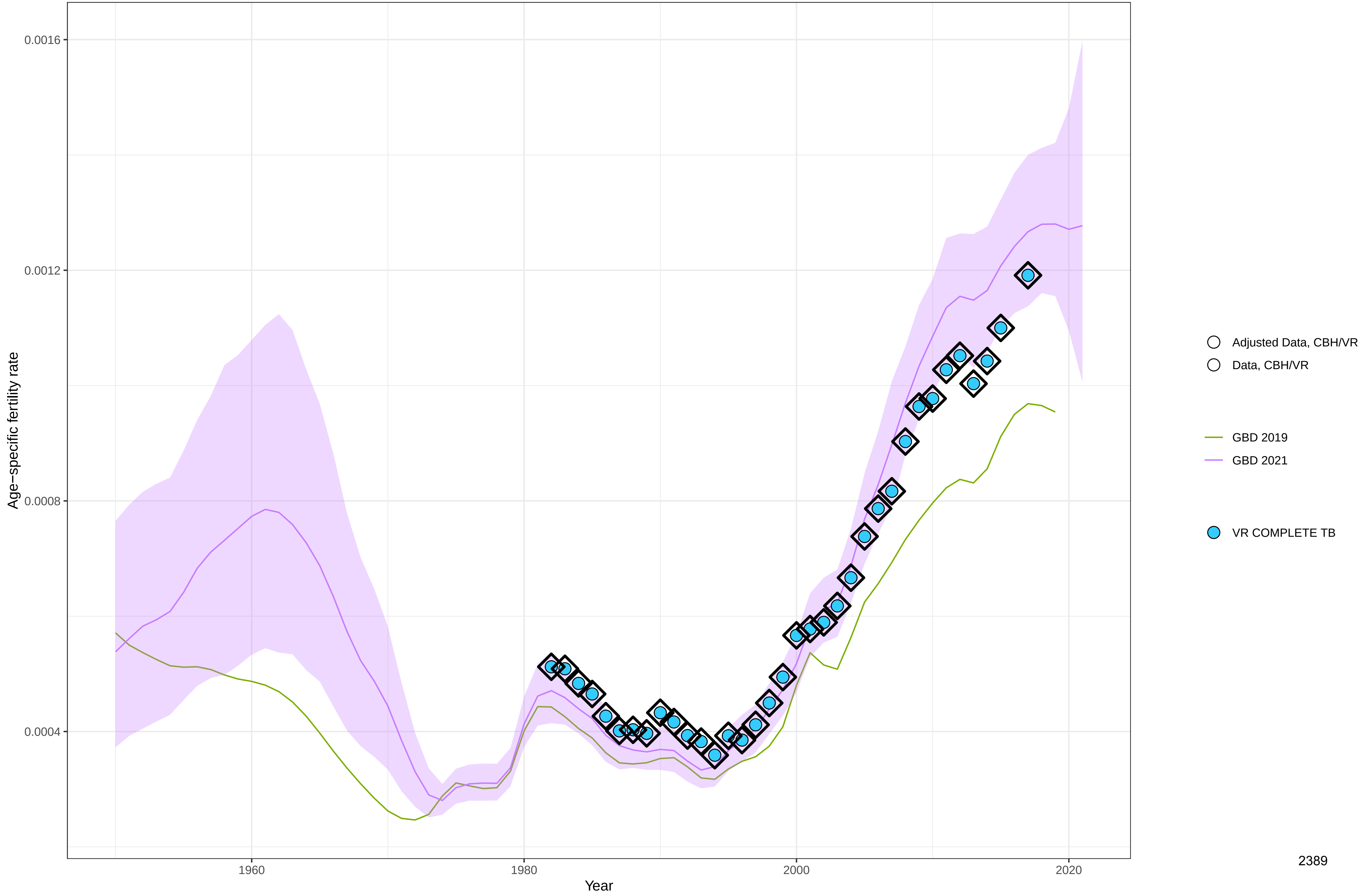

Age−specific fertility fitted trend lines, 1950−2021: Islington, Ages: 50−54
Data points enclosed with a diamond are reference sources

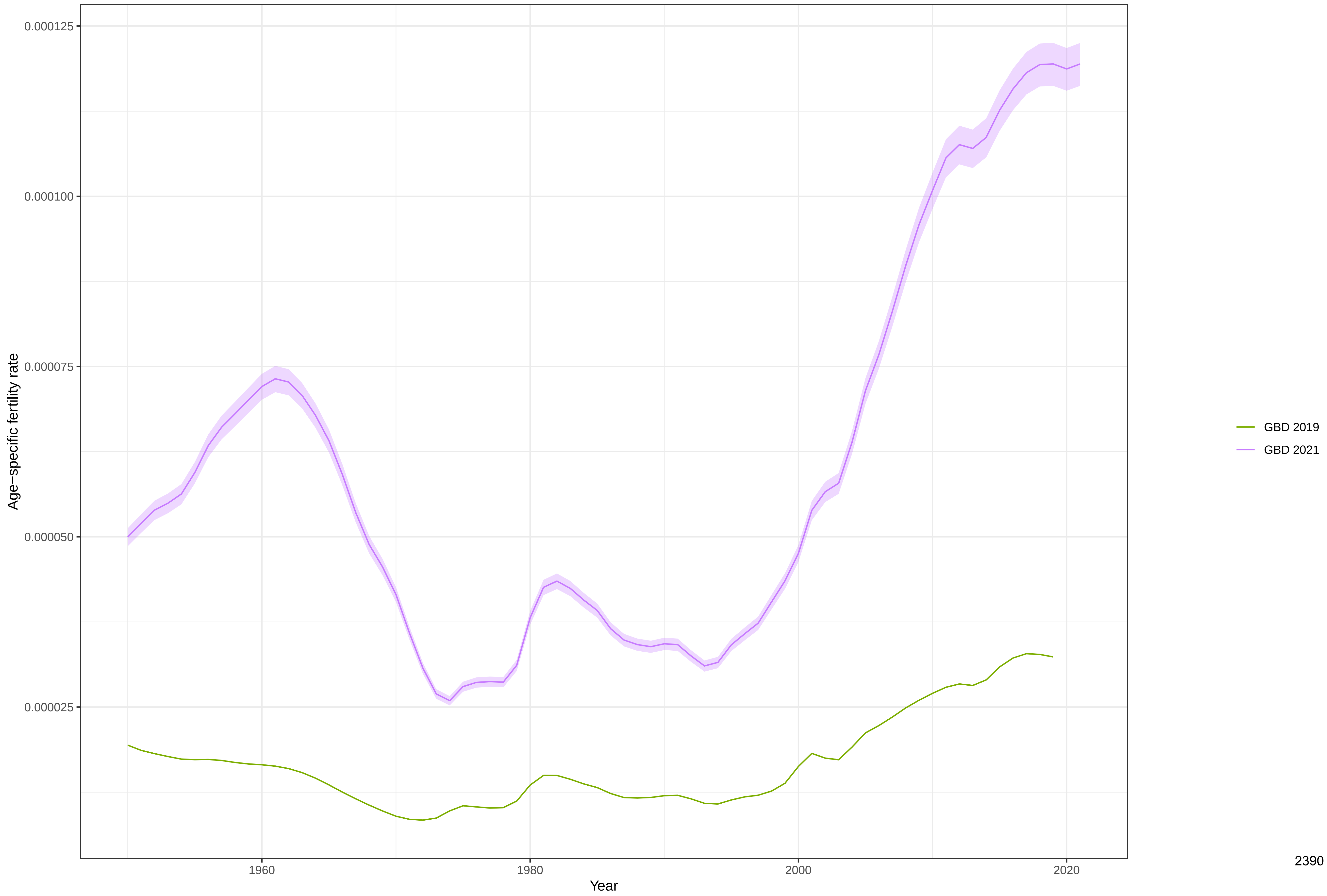

Total fertility rate fitted trend lines, 1950−2021: Islington TFR
Data points enclosed with a diamond are reference sources

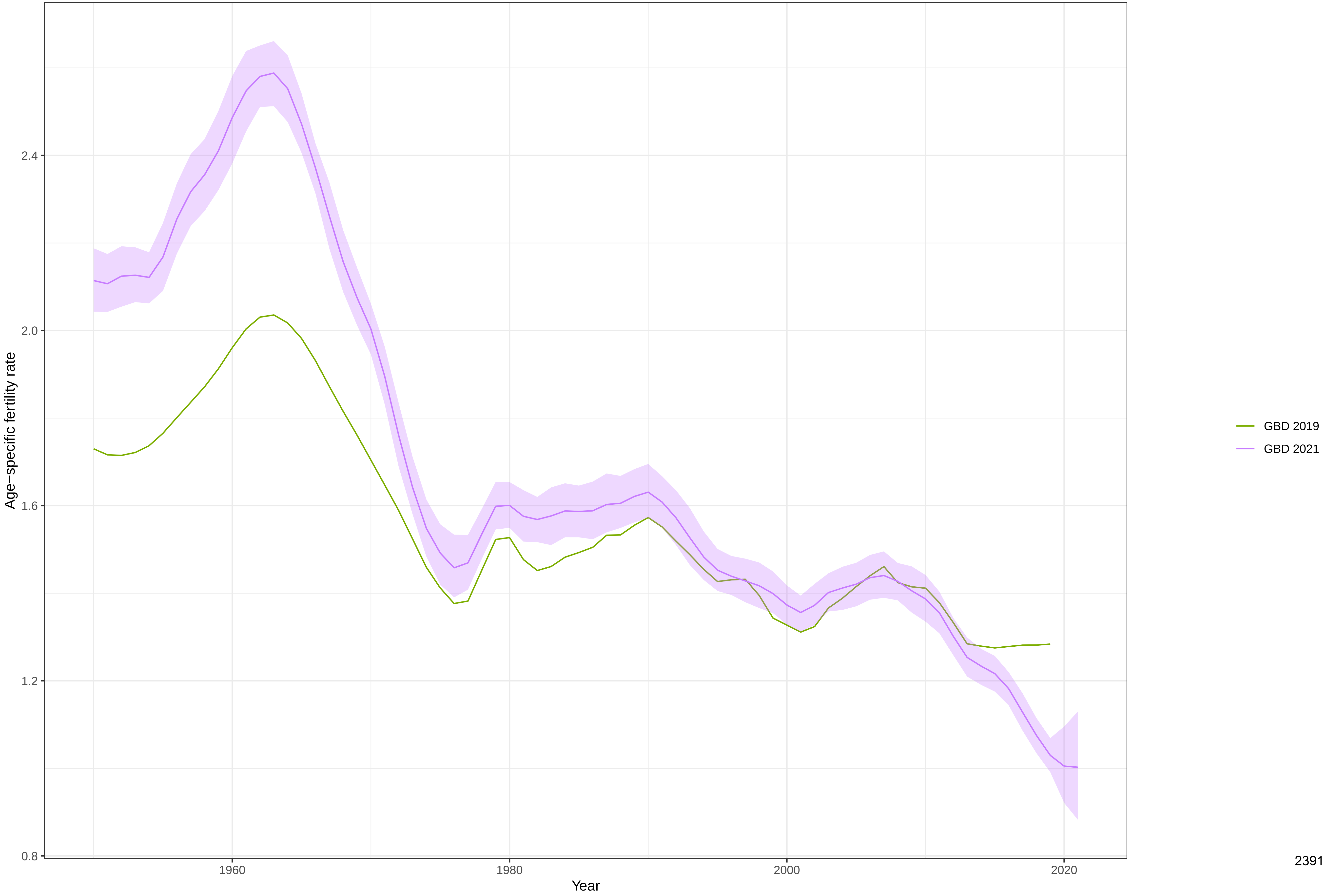

Age−specific fertility fitted trend lines, 1950−2021: Kensington and Chelsea, Ages: 10−14
Data points enclosed with a diamond are reference sources

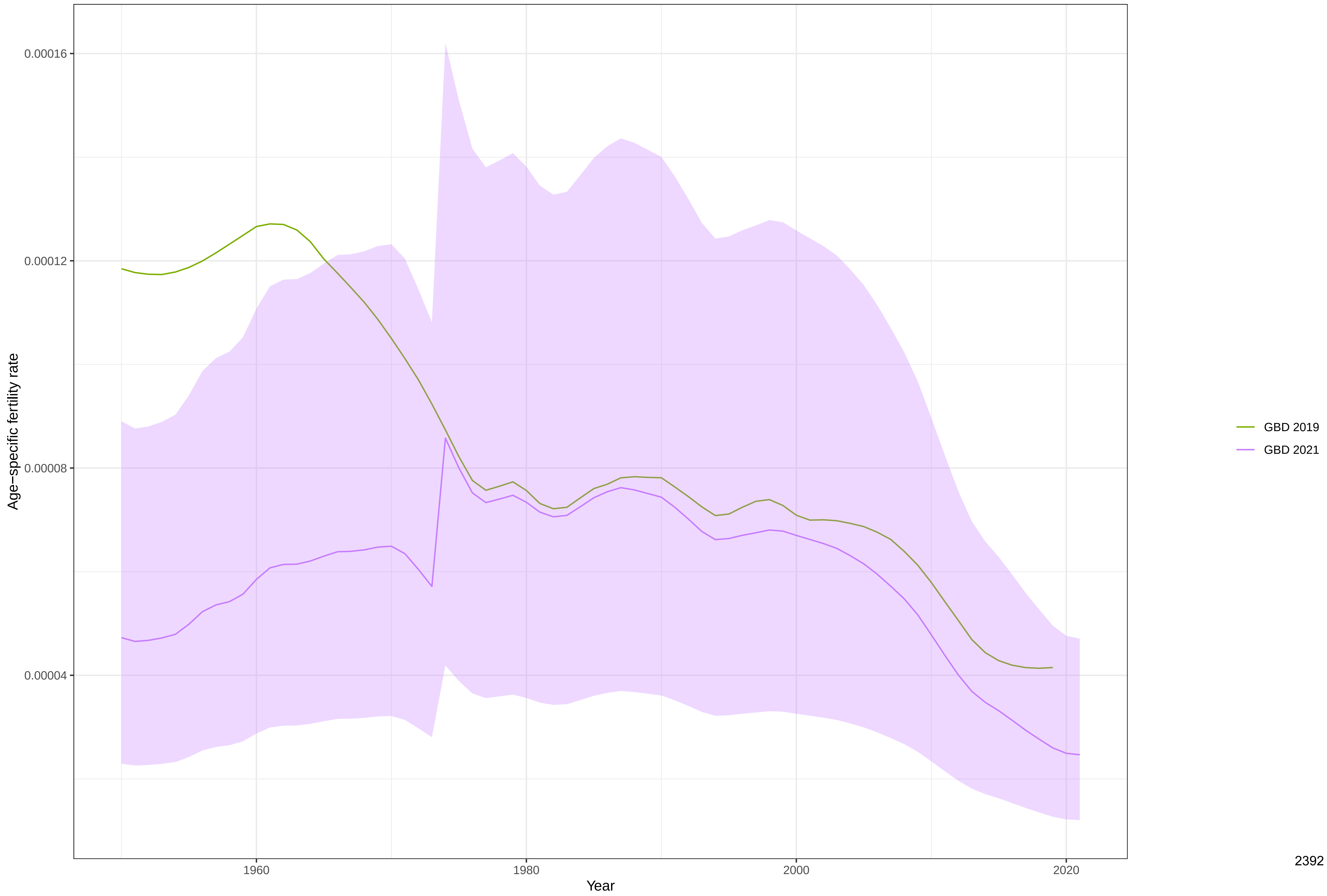

Age−specific fertility fitted trend lines, 1950−2021: Kensington and Chelsea, Ages: 15−19
Data points enclosed with a diamond are reference sources

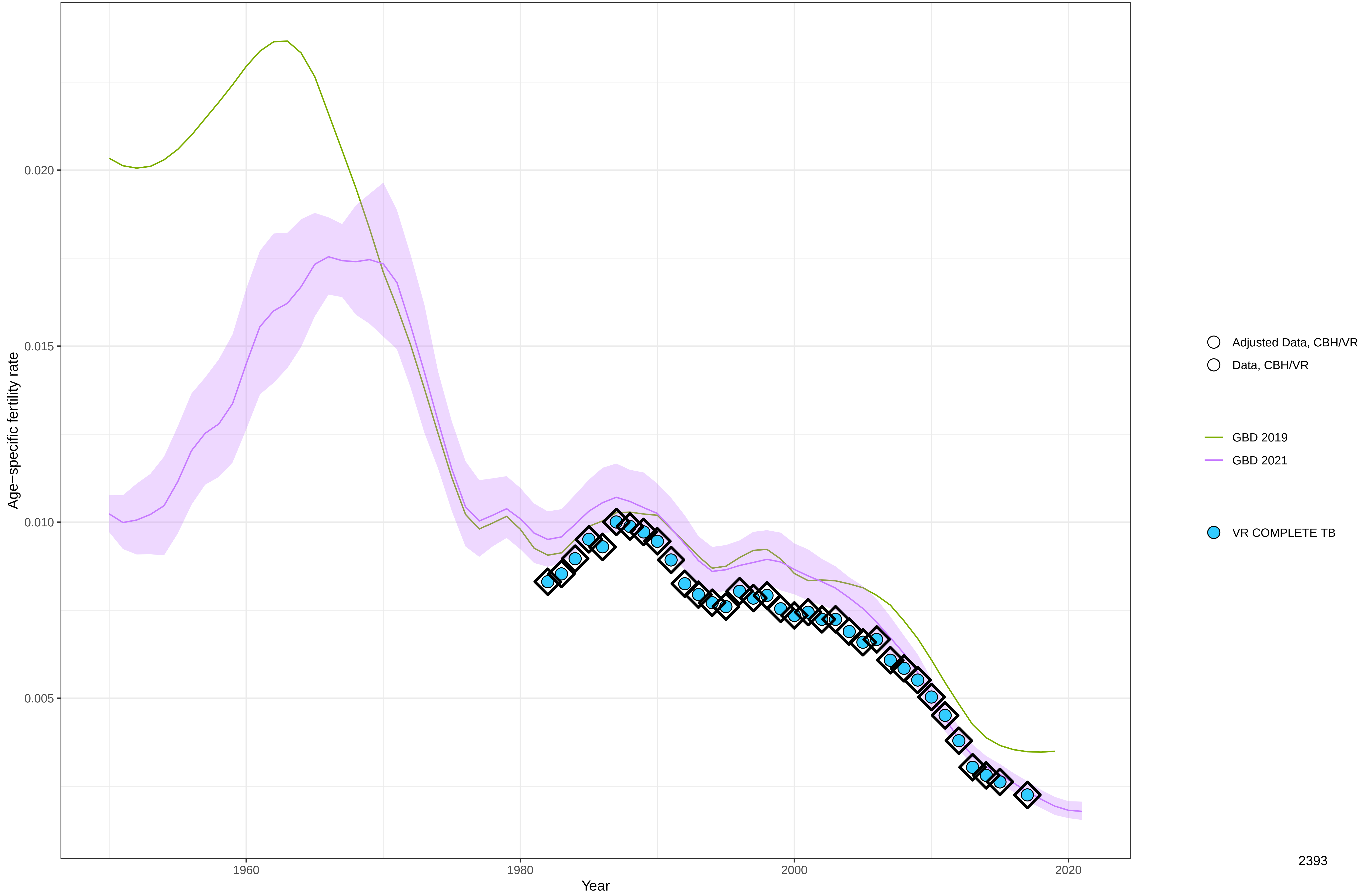

Age−specific fertility fitted trend lines, 1950−2021: Kensington and Chelsea, Ages: 20−24
Data points enclosed with a diamond are reference sources

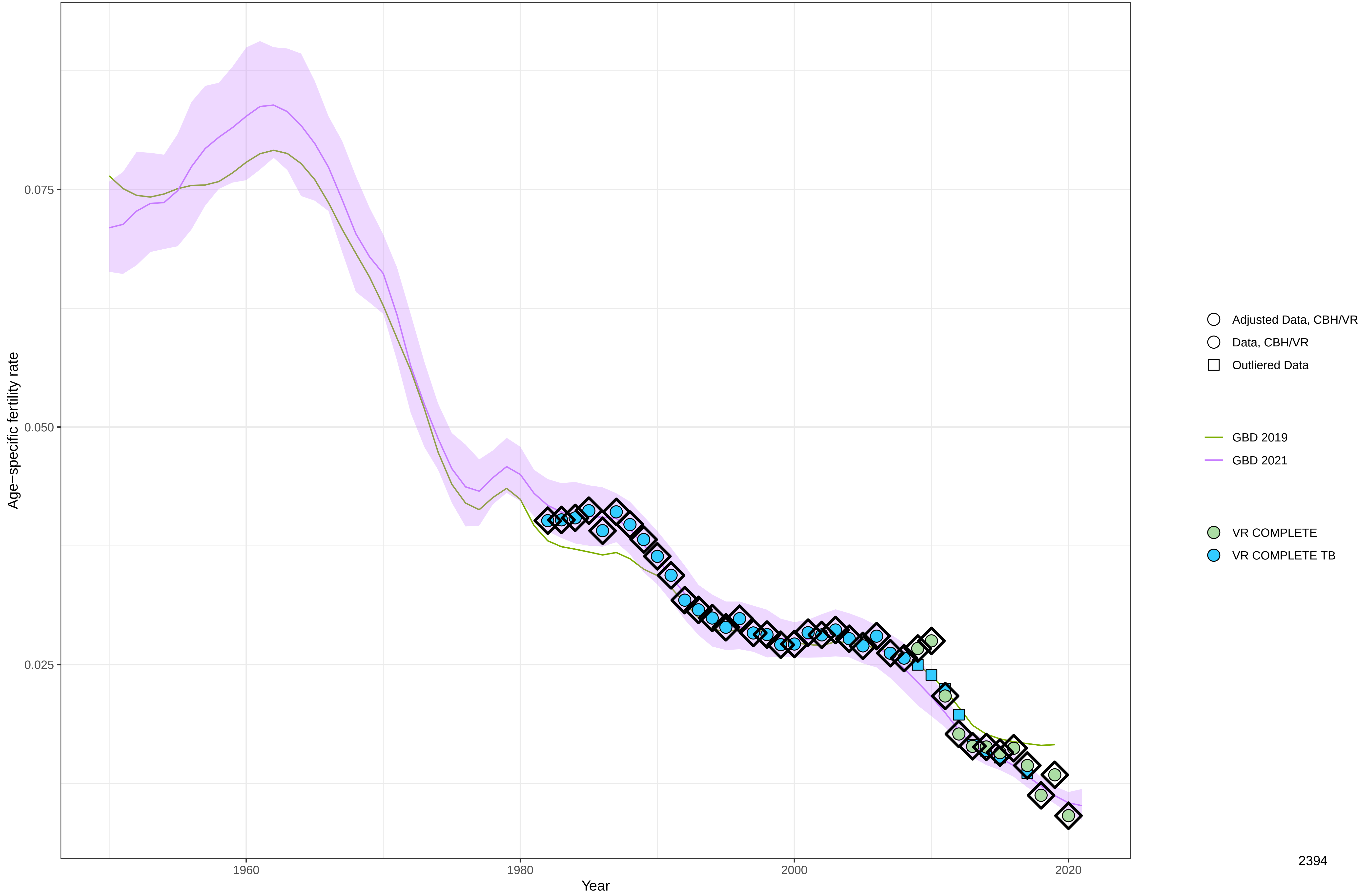

Age−specific fertility fitted trend lines, 1950−2021: Kensington and Chelsea, Ages: 25−29
Data points enclosed with a diamond are reference sources

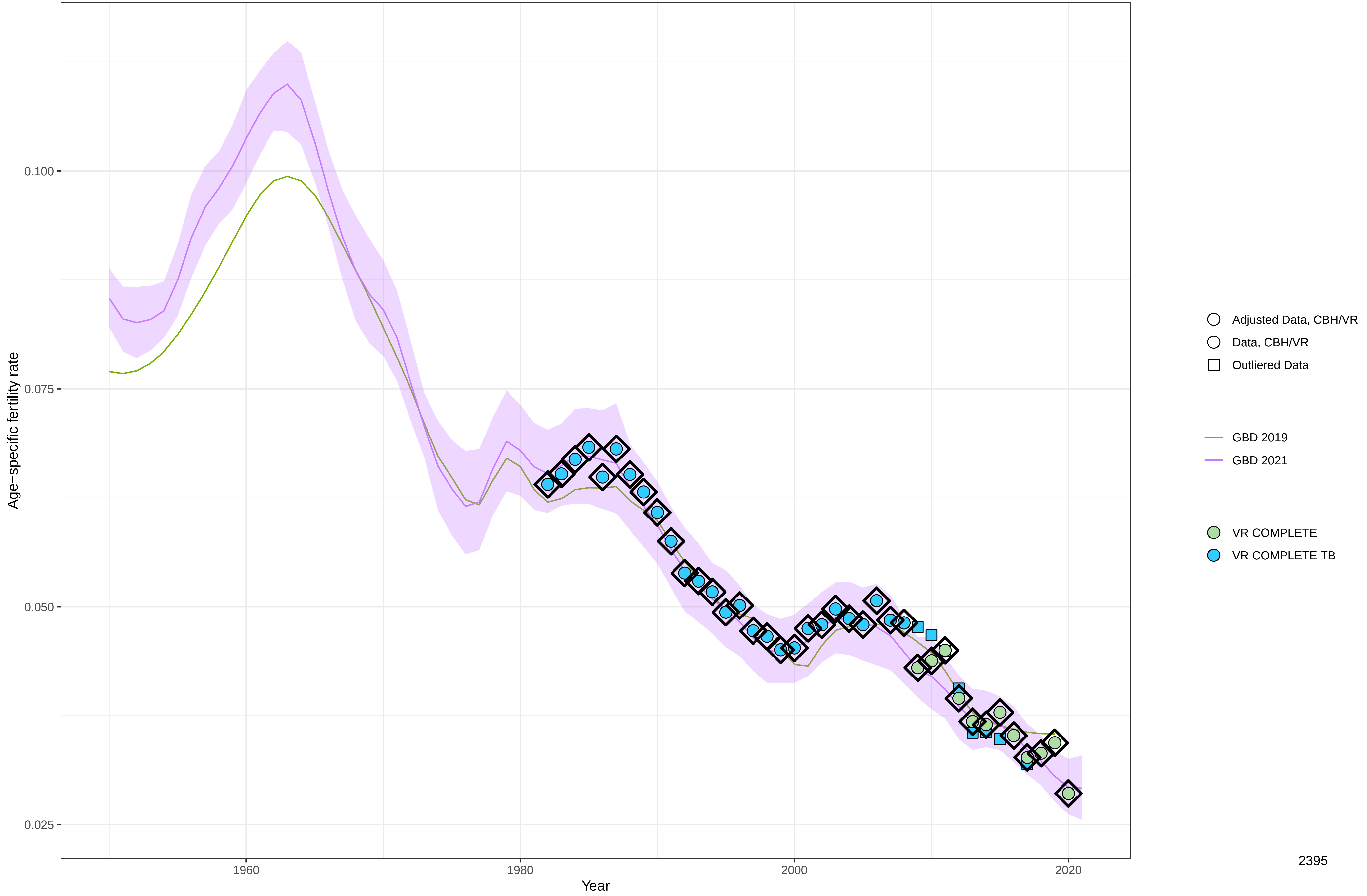

Age−specific fertility fitted trend lines, 1950−2021: Kensington and Chelsea, Ages: 30−34
Data points enclosed with a diamond are reference sources

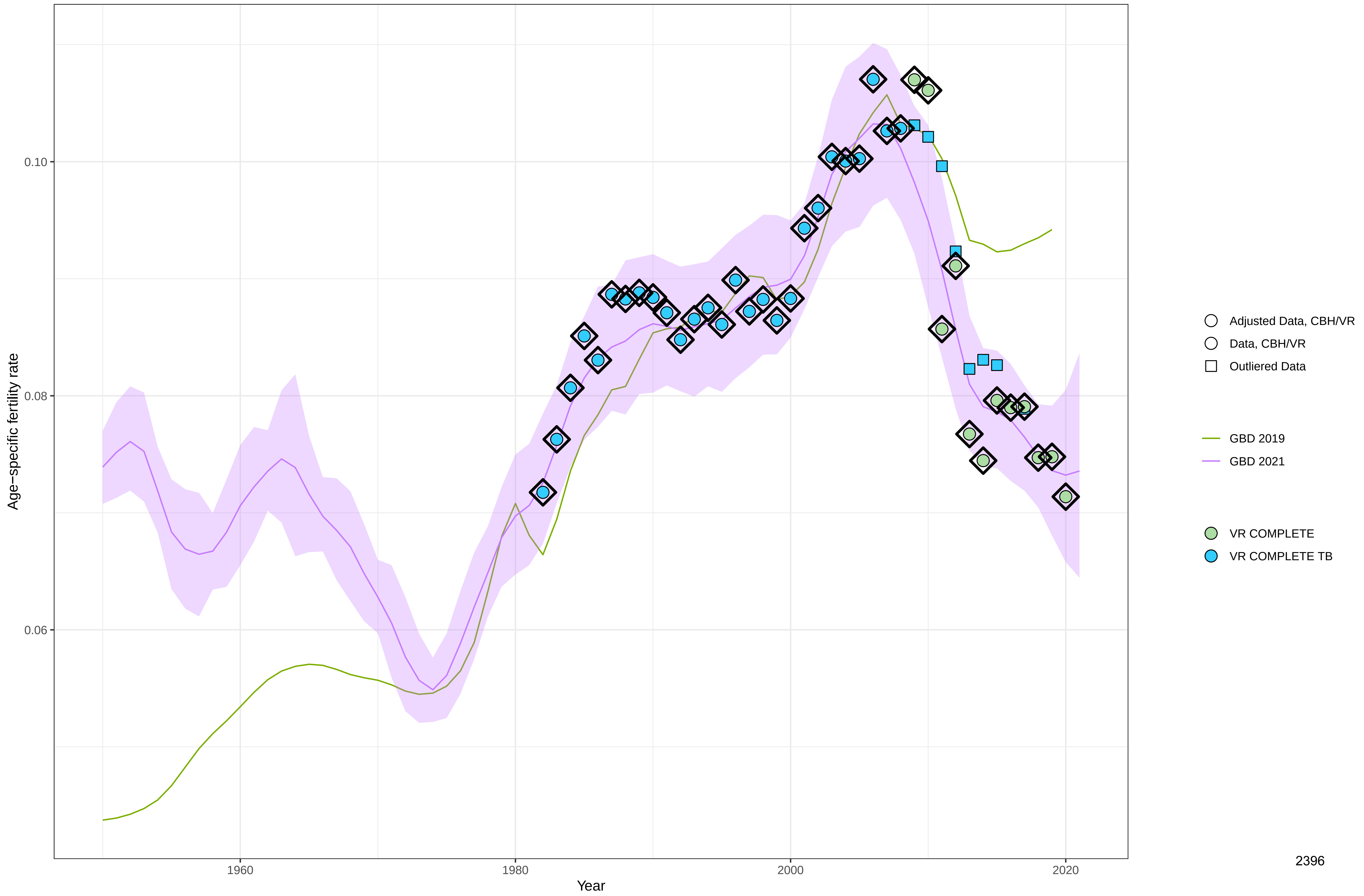

Age−specific fertility fitted trend lines, 1950−2021: Kensington and Chelsea, Ages: 35−39
Data points enclosed with a diamond are reference sources

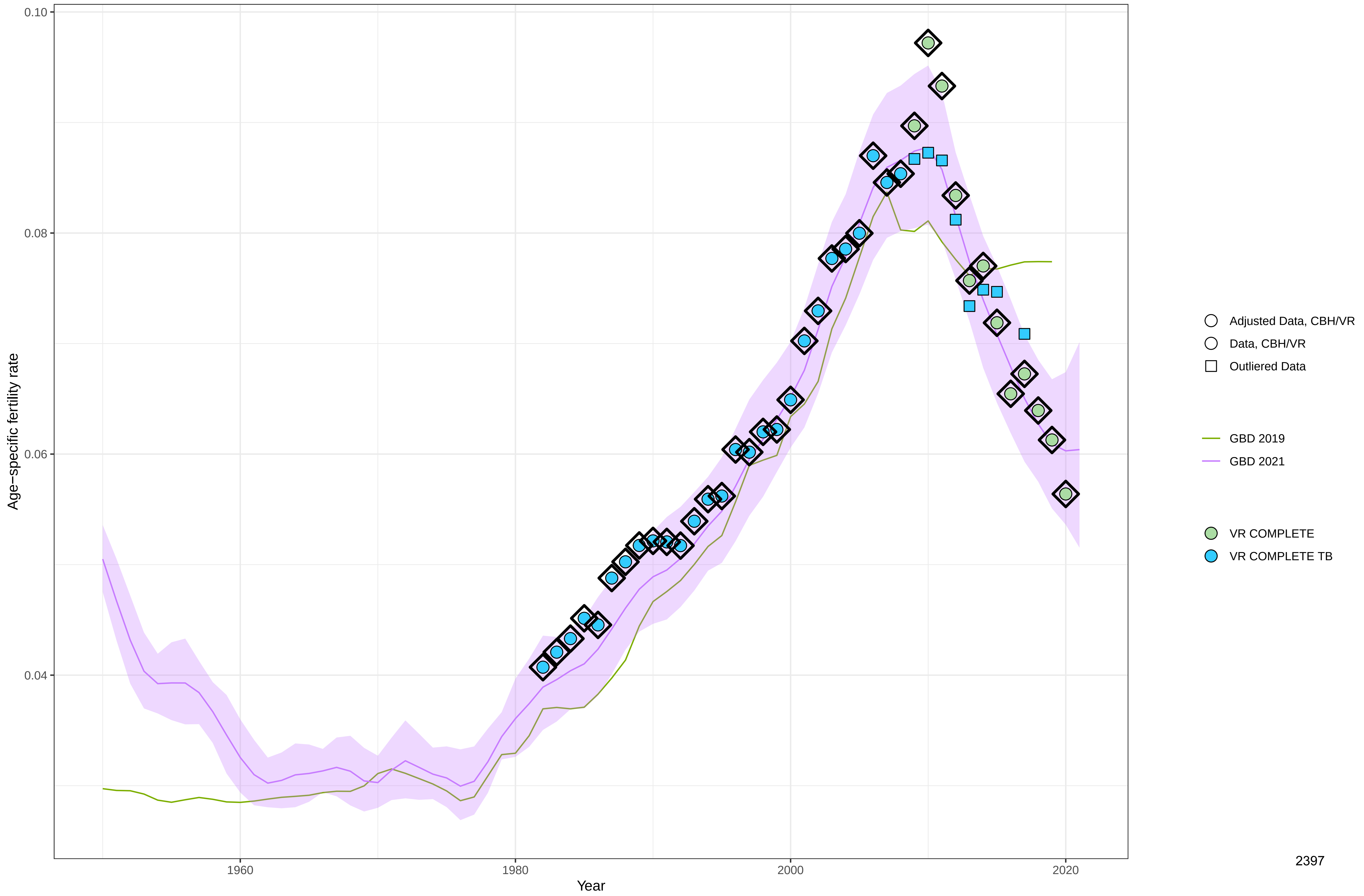

Age−specific fertility fitted trend lines, 1950−2021: Kensington and Chelsea, Ages: 40−44
Data points enclosed with a diamond are reference sources

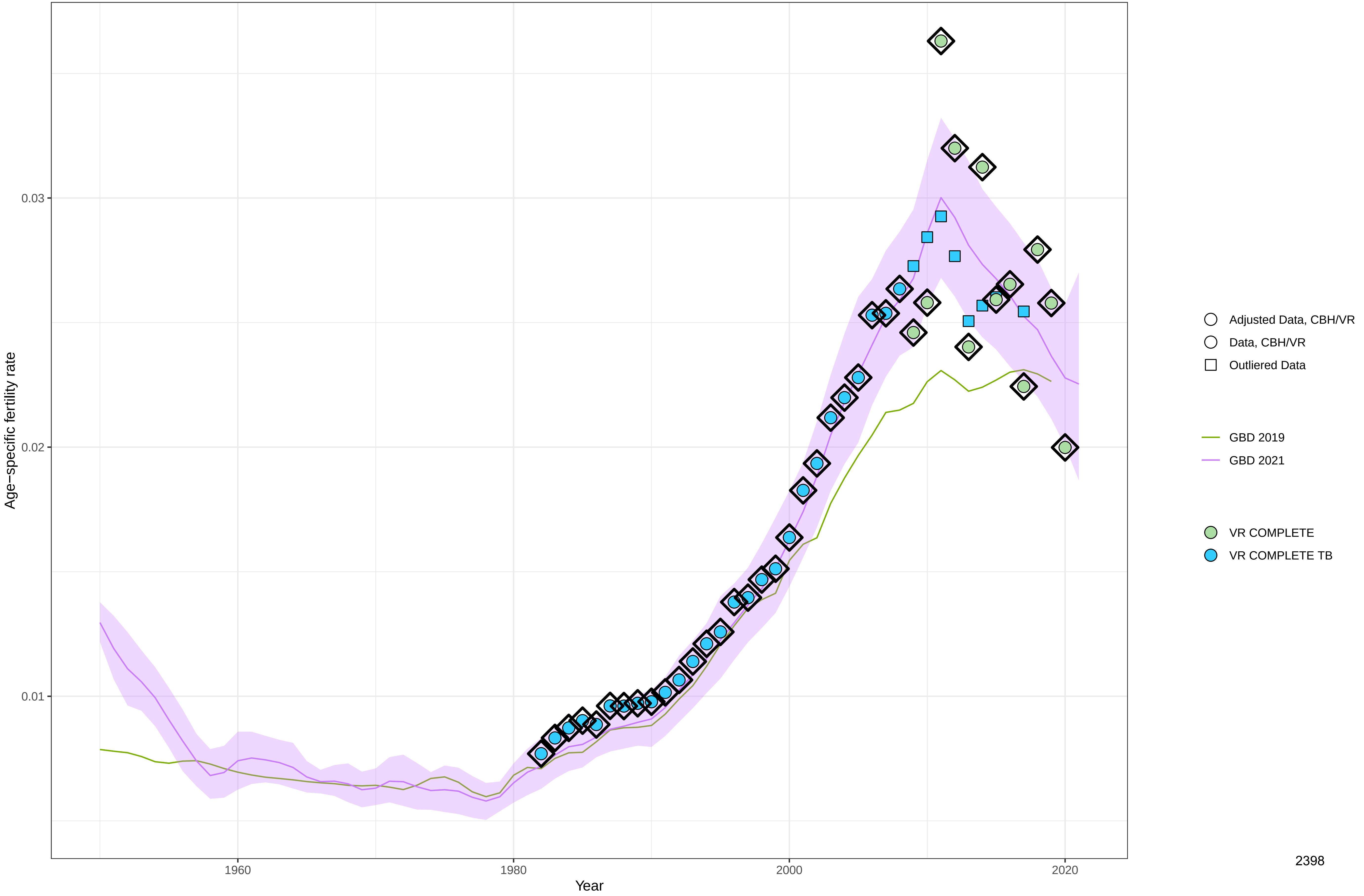

Age−specific fertility fitted trend lines, 1950−2021: Kensington and Chelsea, Ages: 45−49
Data points enclosed with a diamond are reference sources

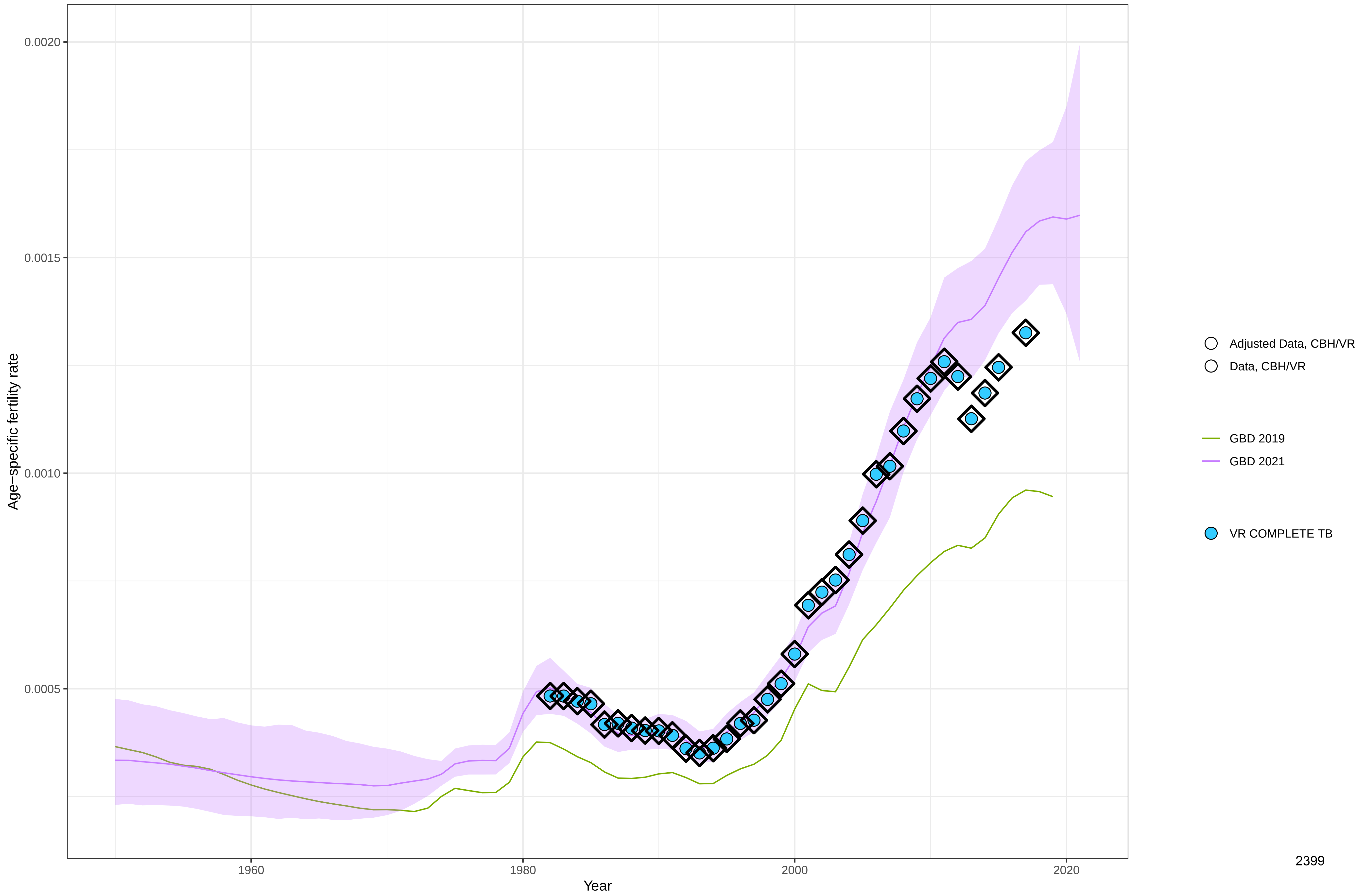

Age−specific fertility fitted trend lines, 1950−2021: Kensington and Chelsea, Ages: 50−54
Data points enclosed with a diamond are reference sources

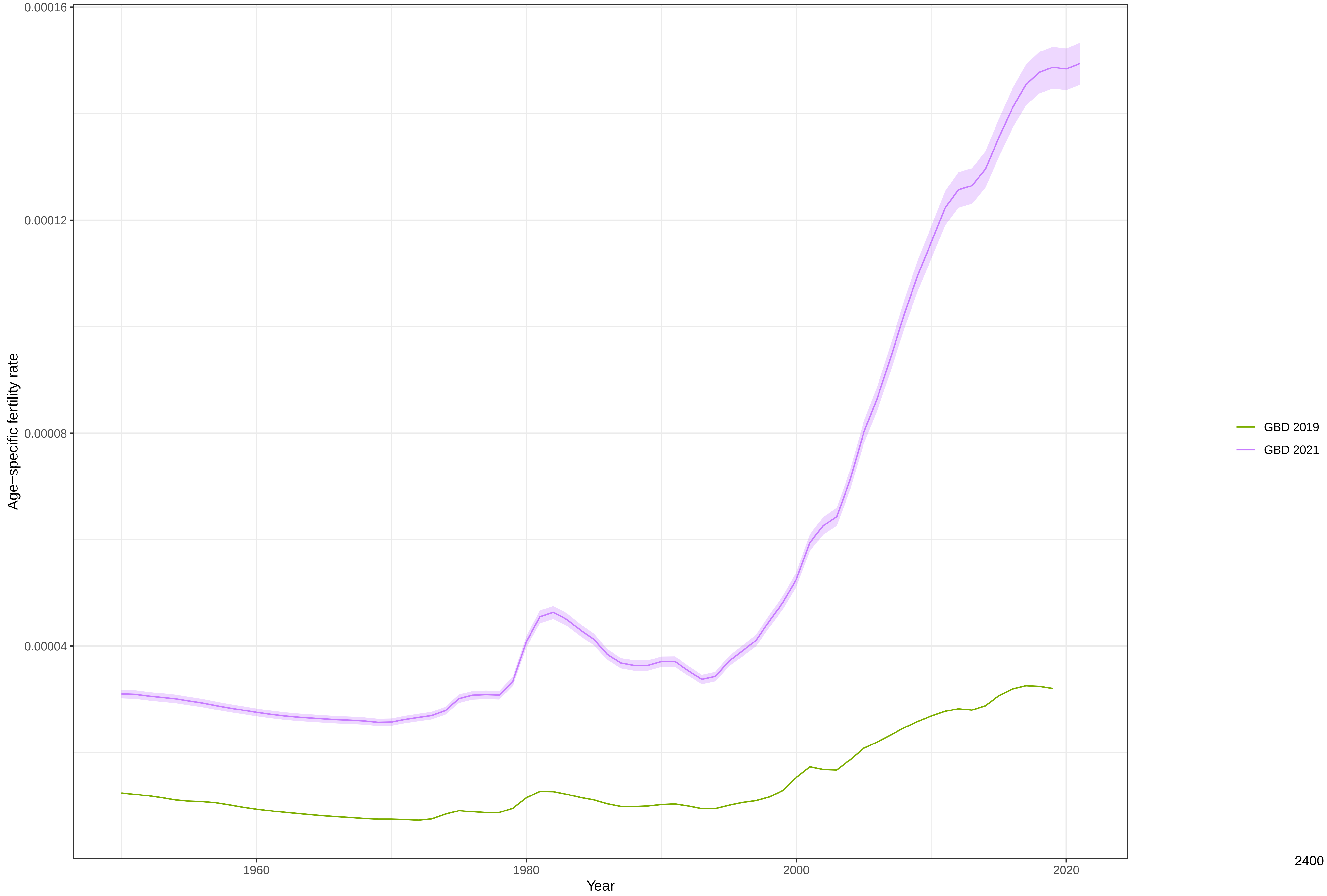

Total fertility rate fitted trend lines, 1950−2021: Kensington and Chelsea TFR
Data points enclosed with a diamond are reference sources

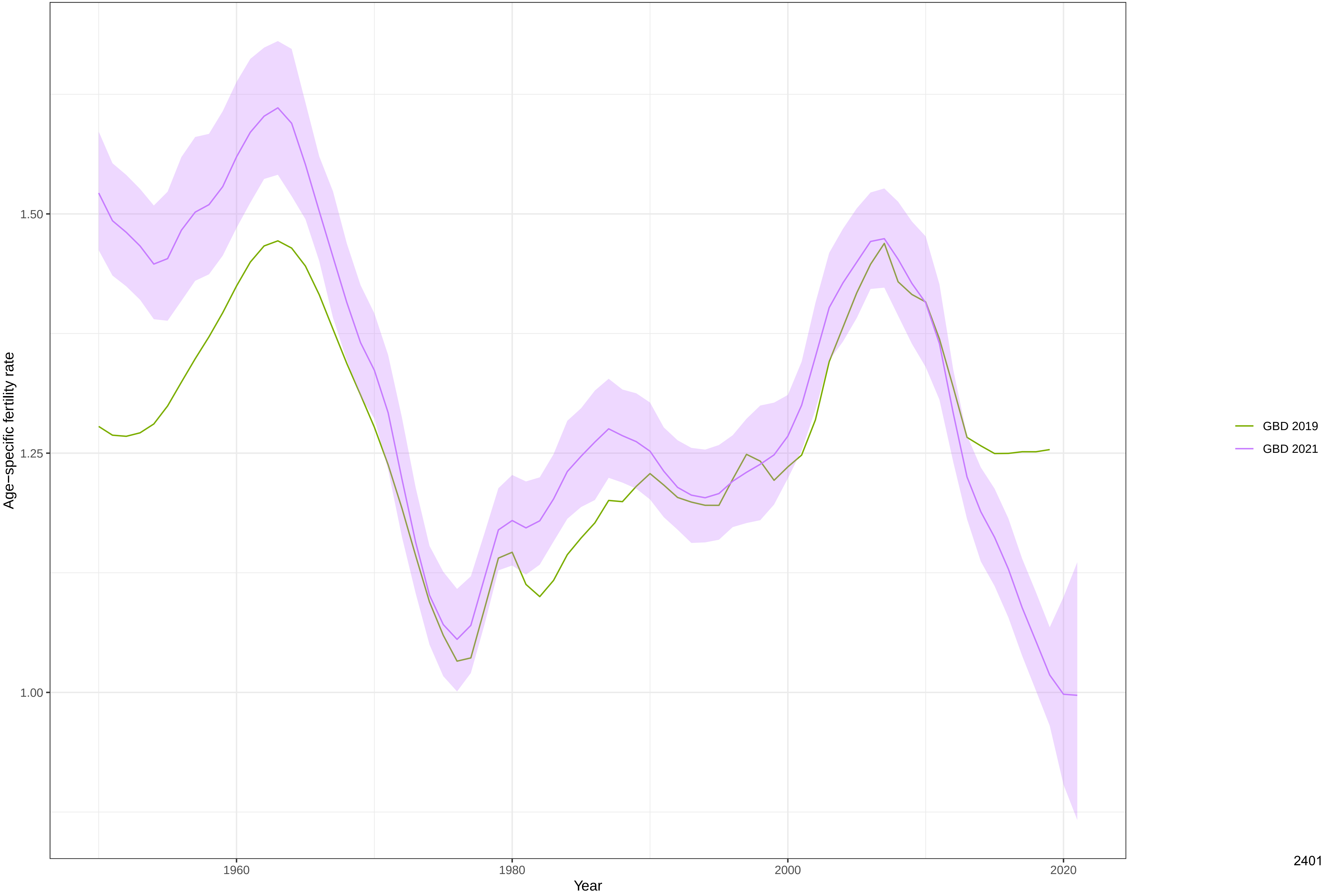

Age−specific fertility fitted trend lines, 1950−2021: Kingston upon Thames, Ages: 10−14
Data points enclosed with a diamond are reference sources

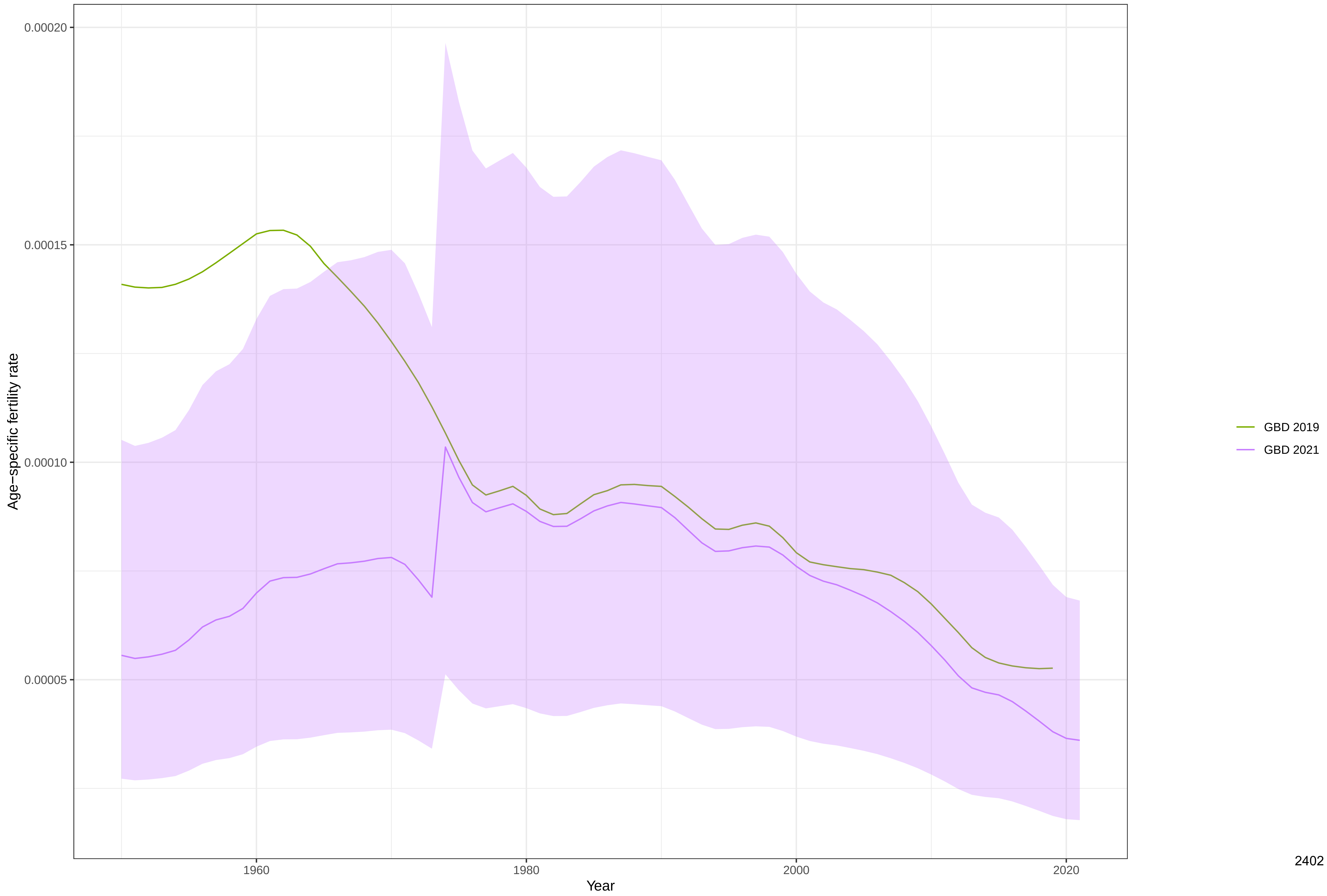

Age−specific fertility fitted trend lines, 1950−2021: Kingston upon Thames, Ages: 15−19
Data points enclosed with a diamond are reference sources

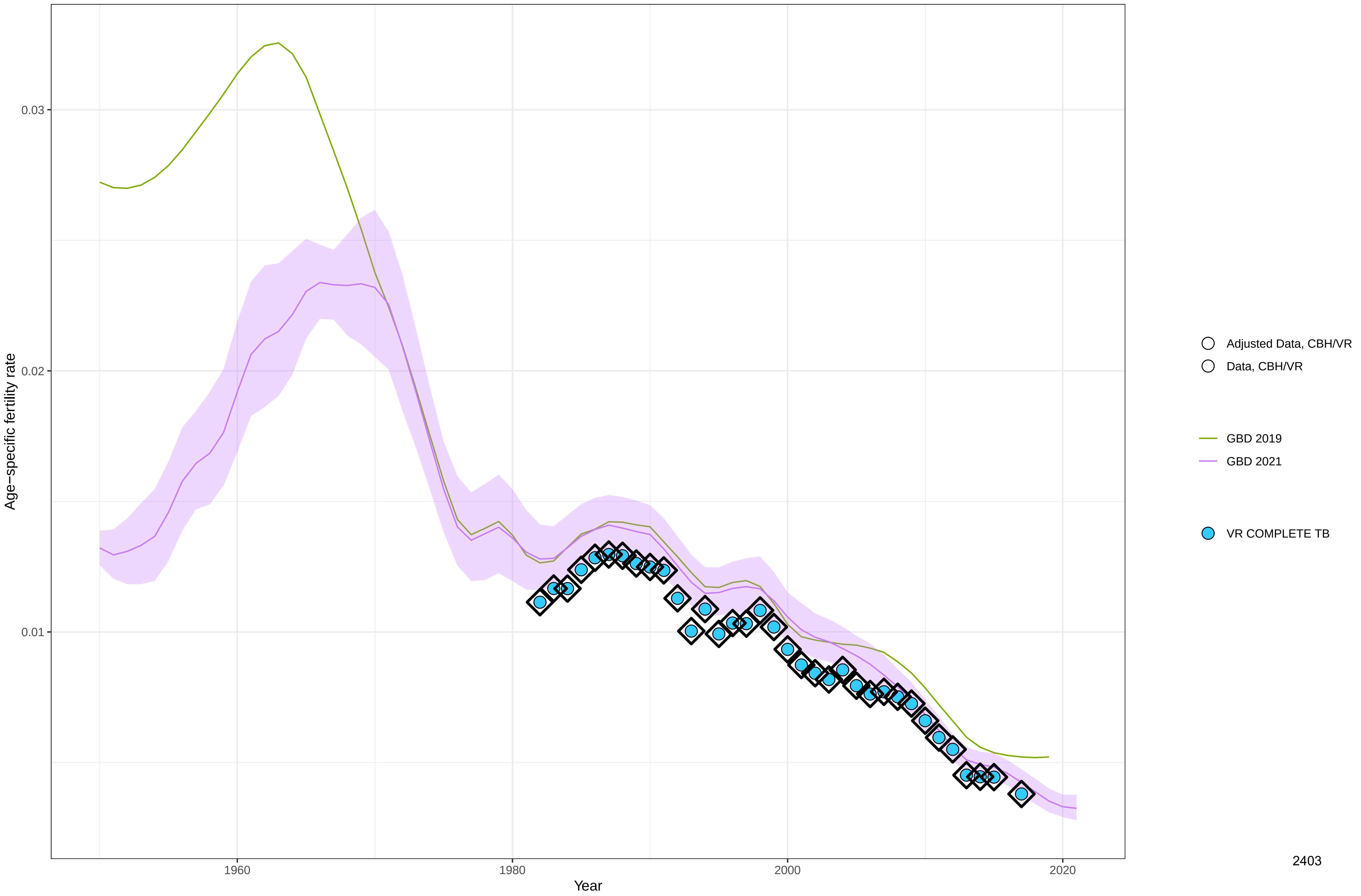

Age−specific fertility fitted trend lines, 1950−2021: Kingston upon Thames, Ages: 20−24
Data points enclosed with a diamond are reference sources

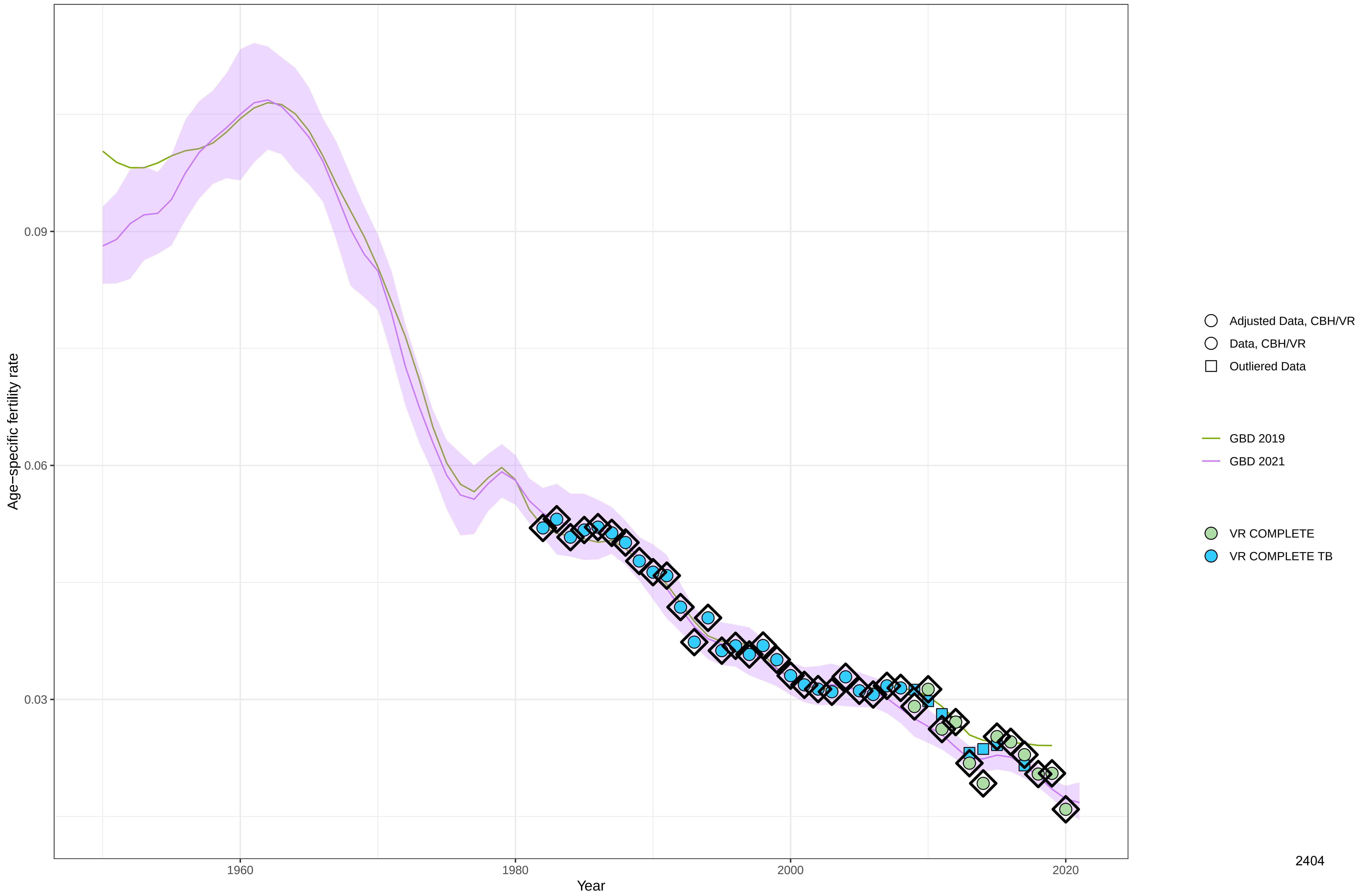

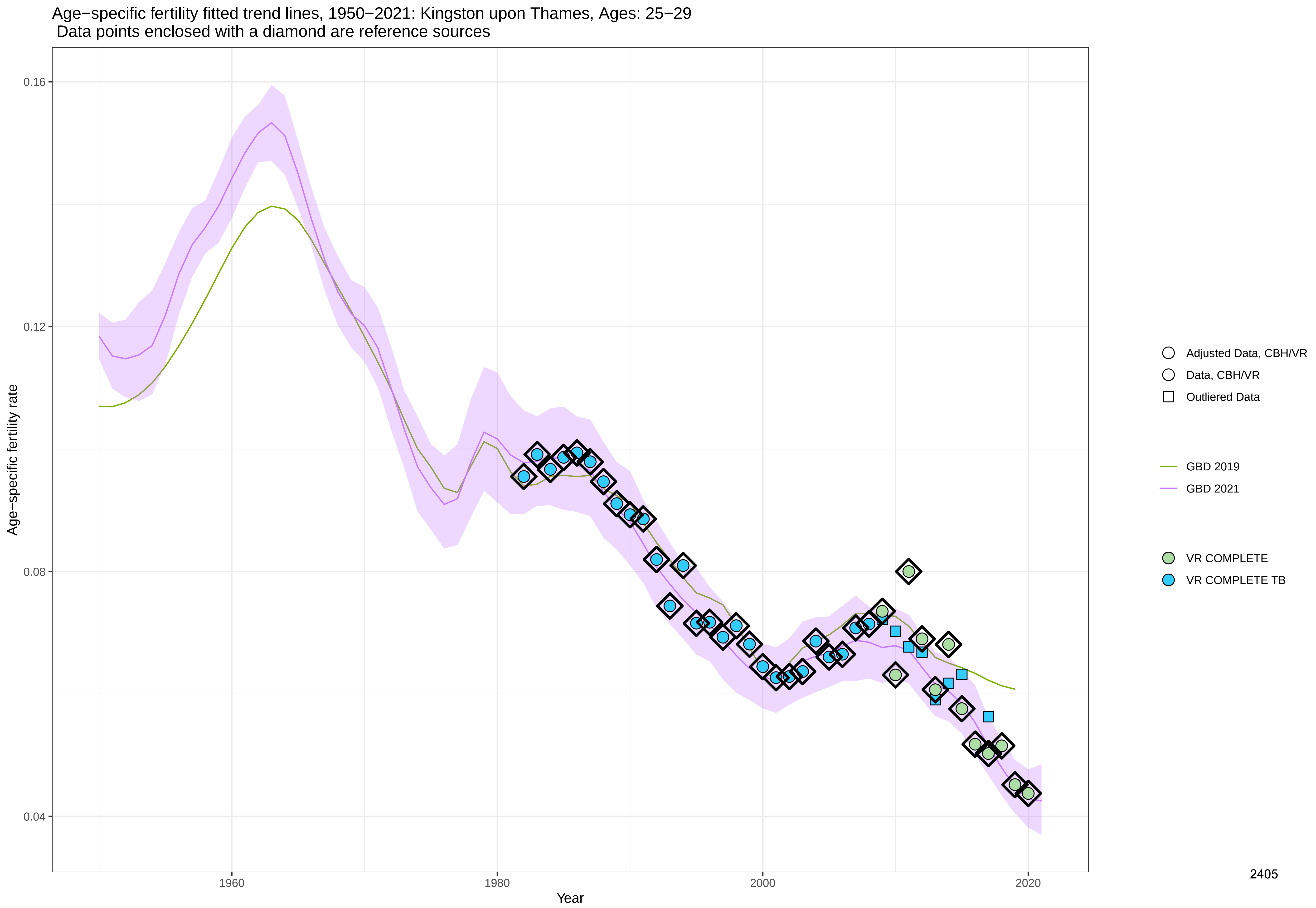

Age−specific fertility fitted trend lines, 1950−2021: Kingston upon Thames, Ages: 25−29
Data points enclosed with a diamond are reference sources
Age−specific fertility rate
0.16
0.12
0.08
0.04
1960
1980
2000
2020
Year
Adjusted Data, CBH/VR
Data, CBH/VR
Outliered Data
GBD 2019
GBD 2021
VR COMPLETE
VR COMPLETE TB

Age−specific fertility fitted trend lines, 1950−2021: Kingston upon Thames, Ages: 30−34
Data points enclosed with a diamond are reference sources

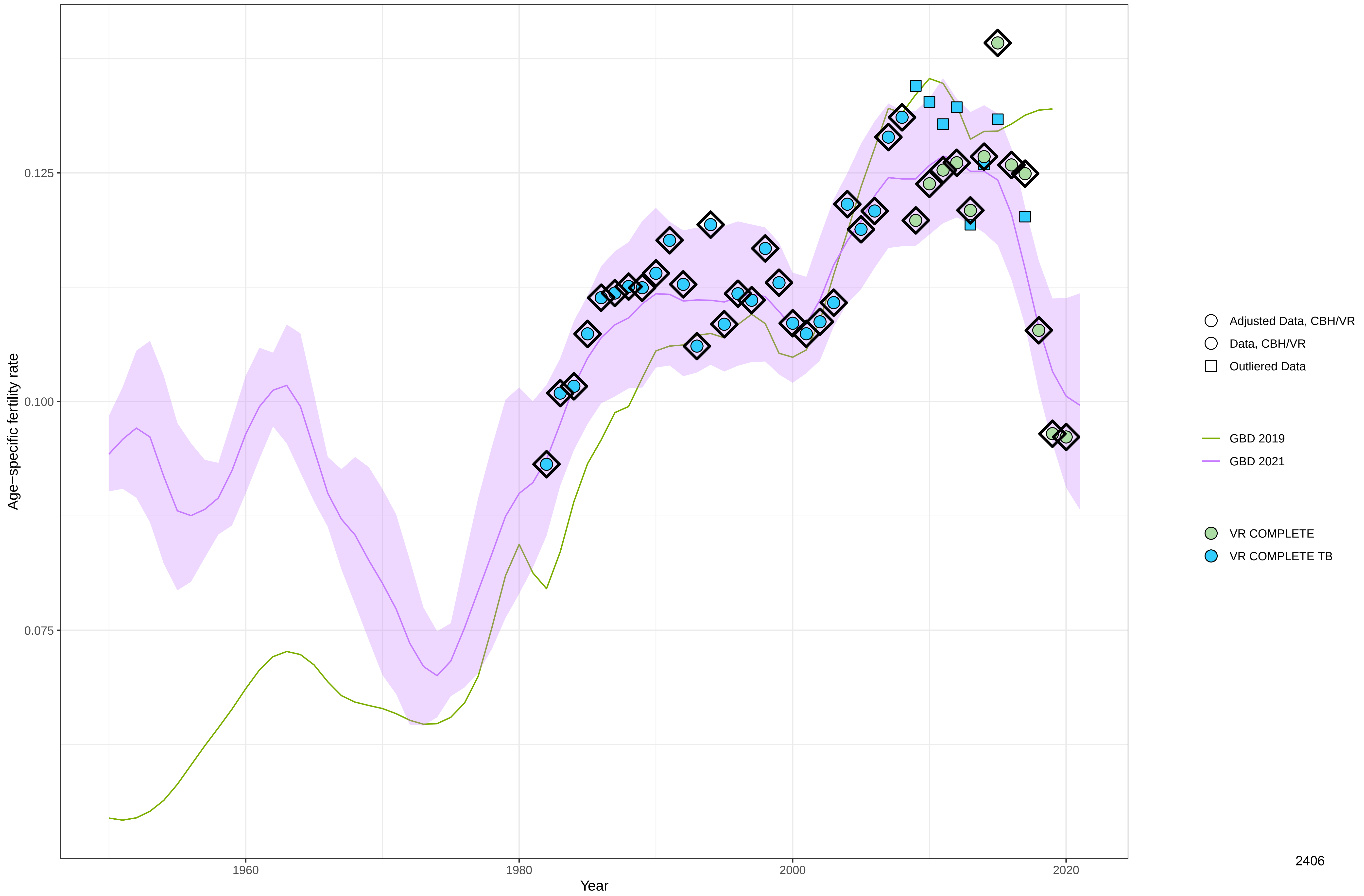

Age−specific fertility fitted trend lines, 1950−2021: Kingston upon Thames, Ages: 35−39
Data points enclosed with a diamond are reference sources

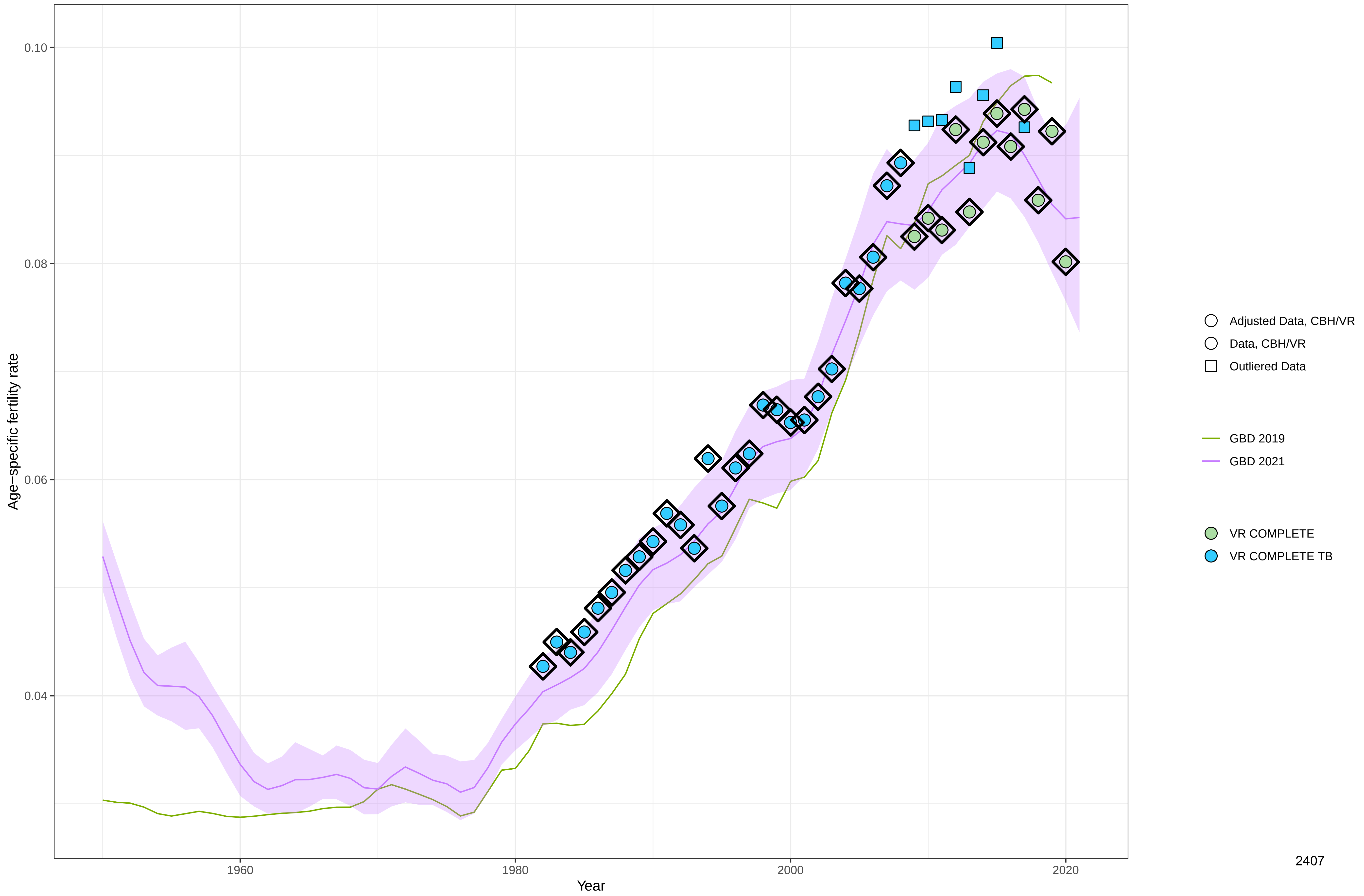

Age−specific fertility fitted trend lines, 1950−2021: Kingston upon Thames, Ages: 40−44
Data points enclosed with a diamond are reference sources

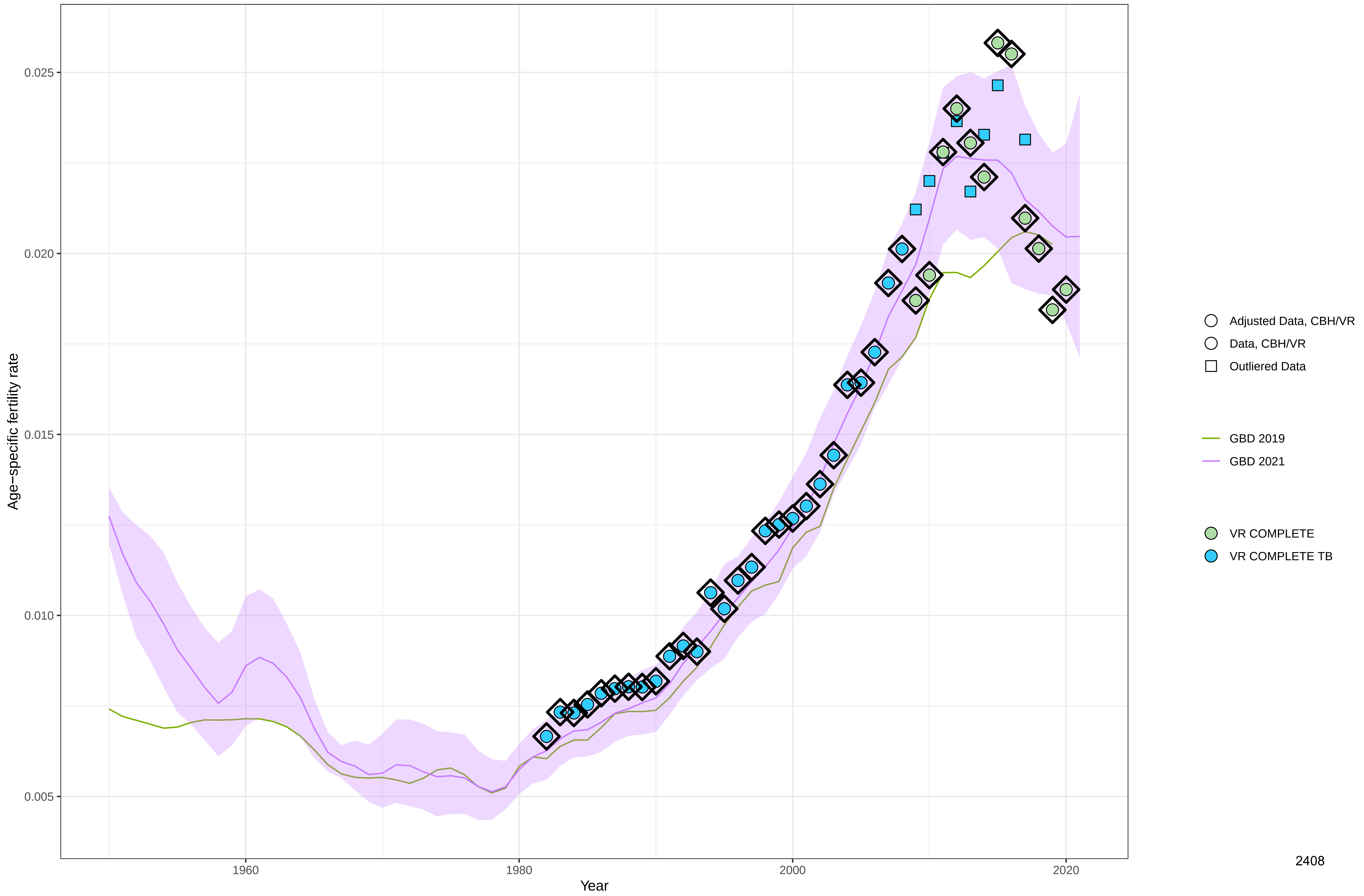

Age−specific fertility fitted trend lines, 1950−2021: Kingston upon Thames, Ages: 45−49
Data points enclosed with a diamond are reference sources

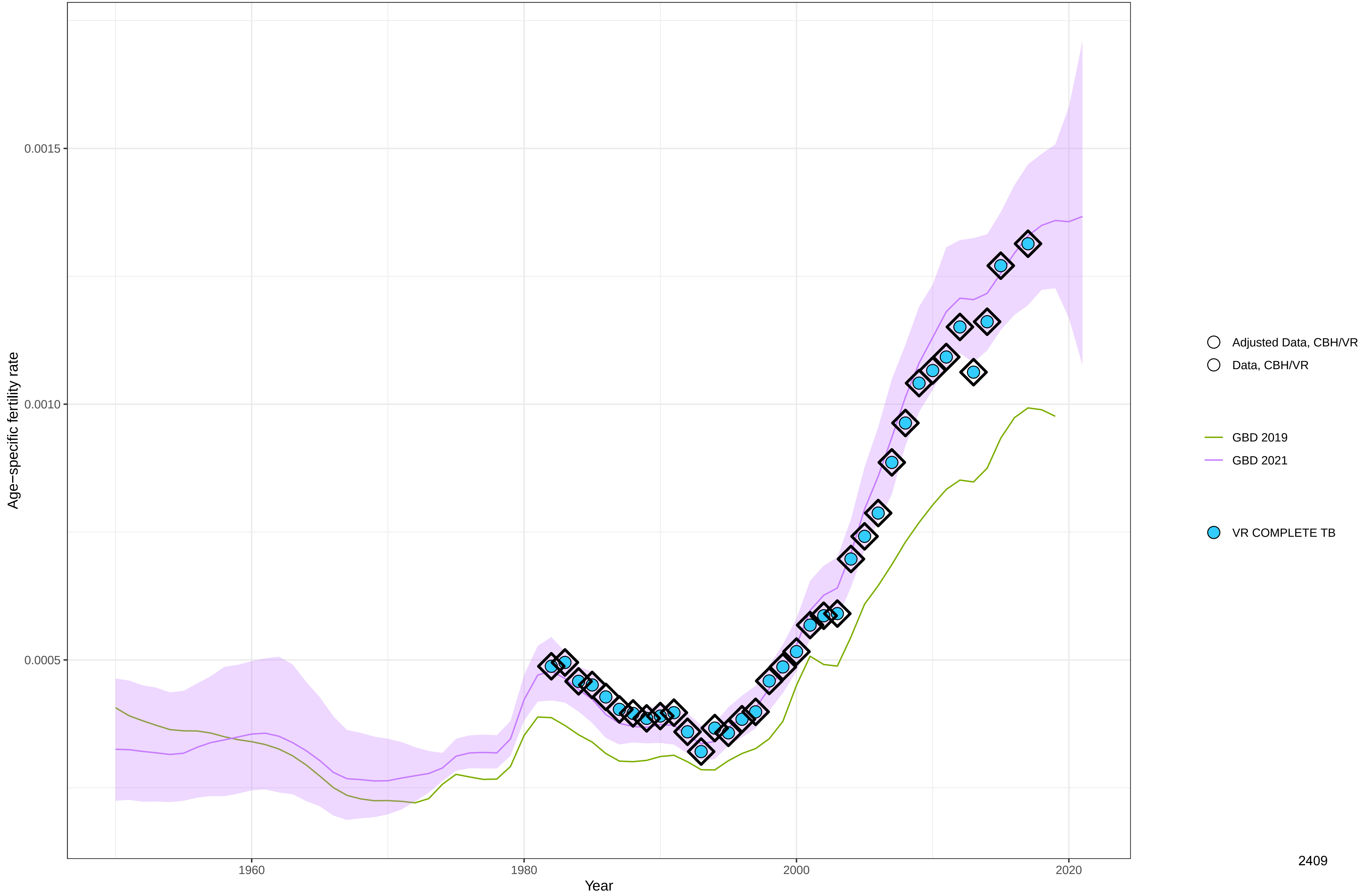

Age−specific fertility fitted trend lines, 1950−2021: Kingston upon Thames, Ages: 50−54
Data points enclosed with a diamond are reference sources

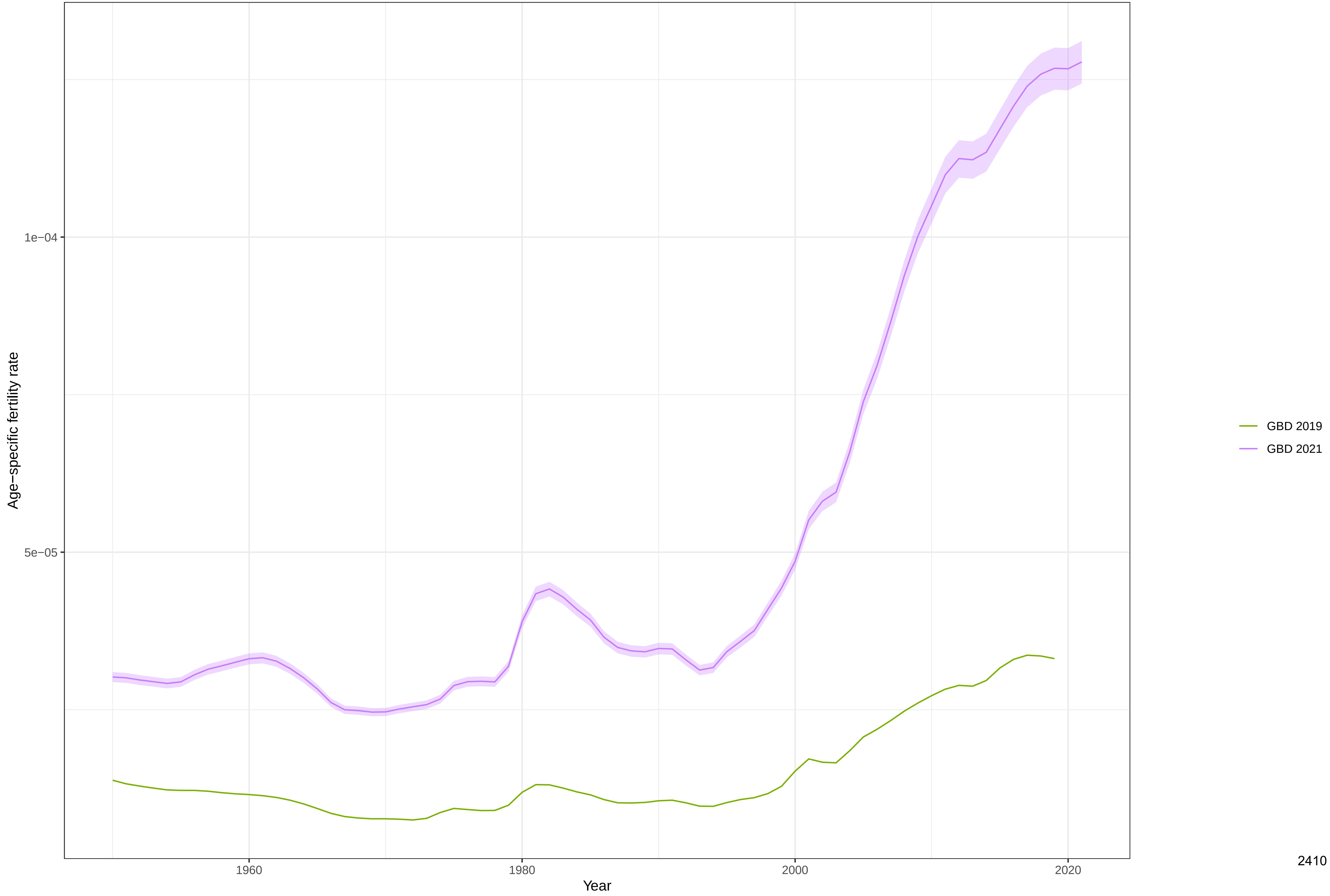

Total fertility rate fitted trend lines, 1950−2021: Kingston upon Thames TFR
Data points enclosed with a diamond are reference sources

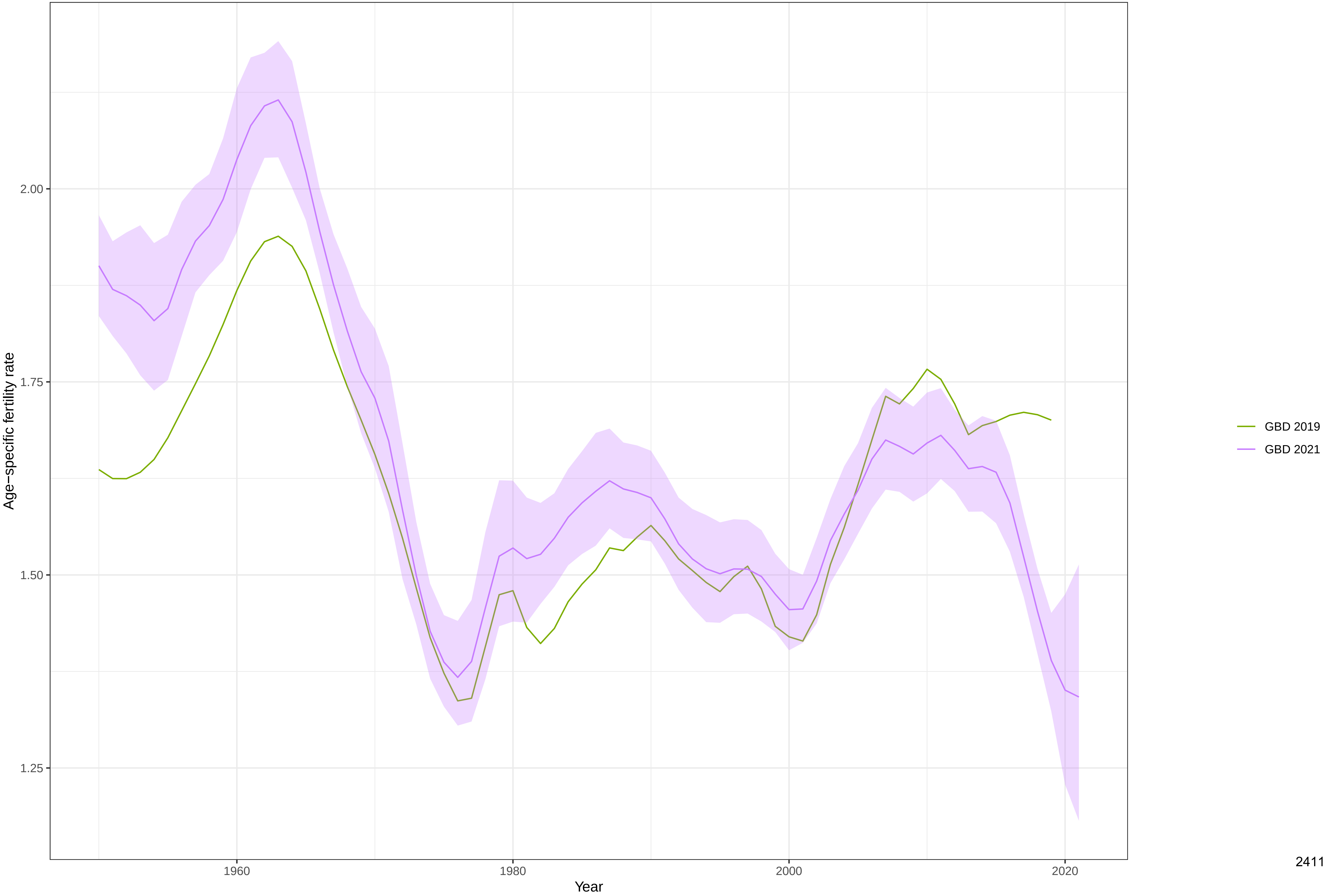

Age−specific fertility fitted trend lines, 1950−2021: Lambeth, Ages: 10−14
Data points enclosed with a diamond are reference sources

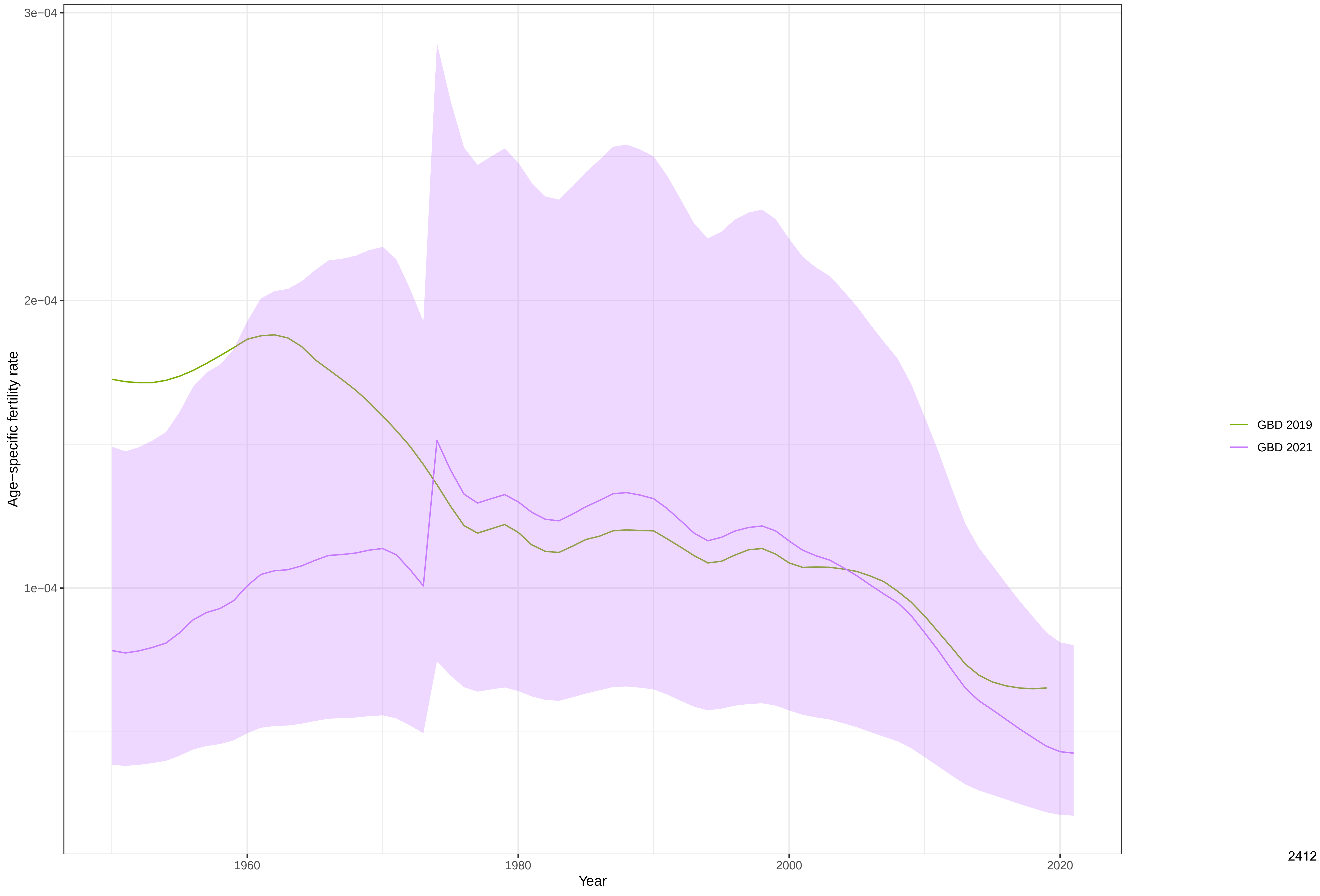

Age−specific fertility fitted trend lines, 1950−2021: Lambeth, Ages: 15−19
Data points enclosed with a diamond are reference sources

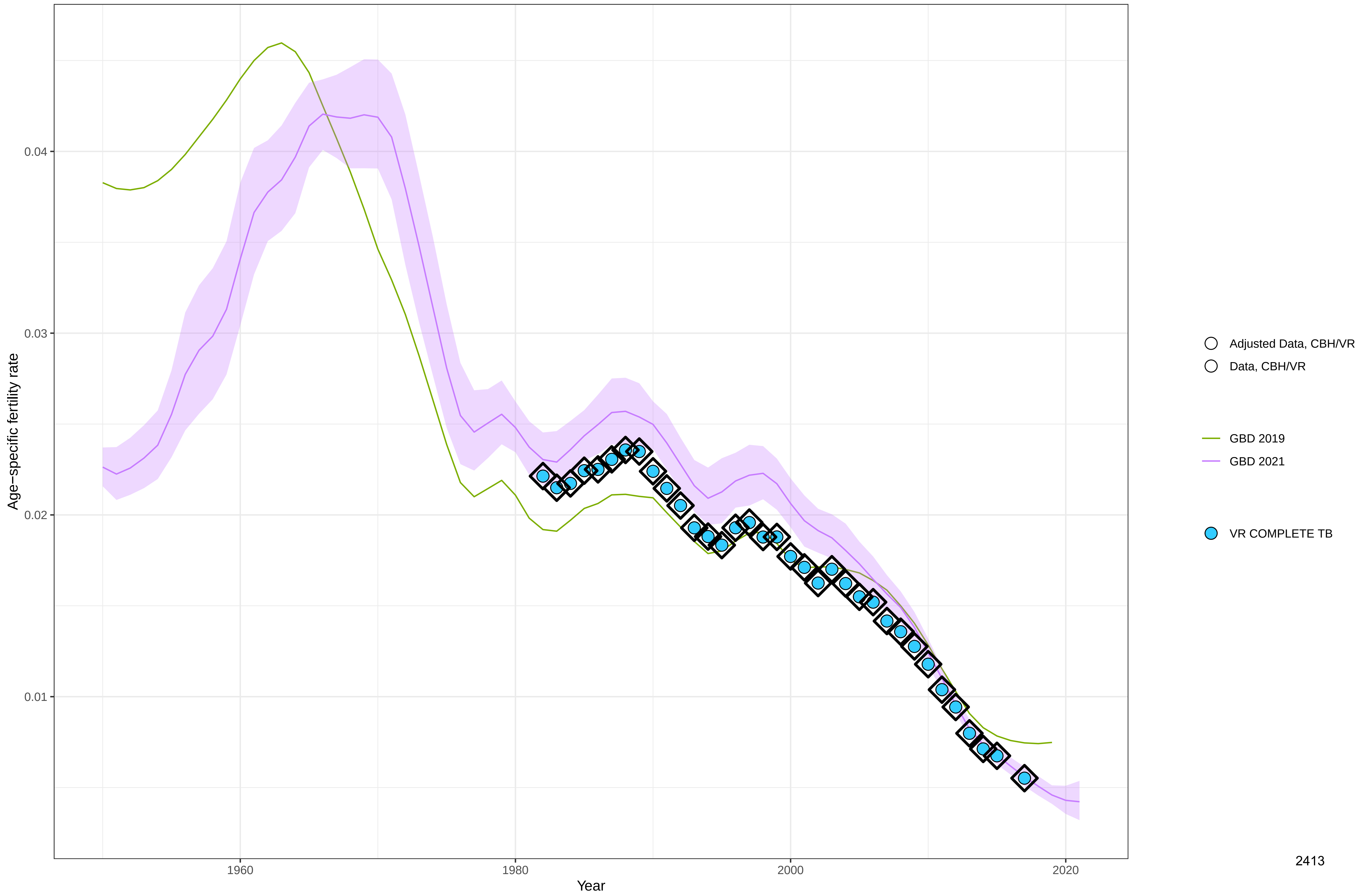

Age−specific fertility fitted trend lines, 1950−2021: Lambeth, Ages: 20−24
Data points enclosed with a diamond are reference sources

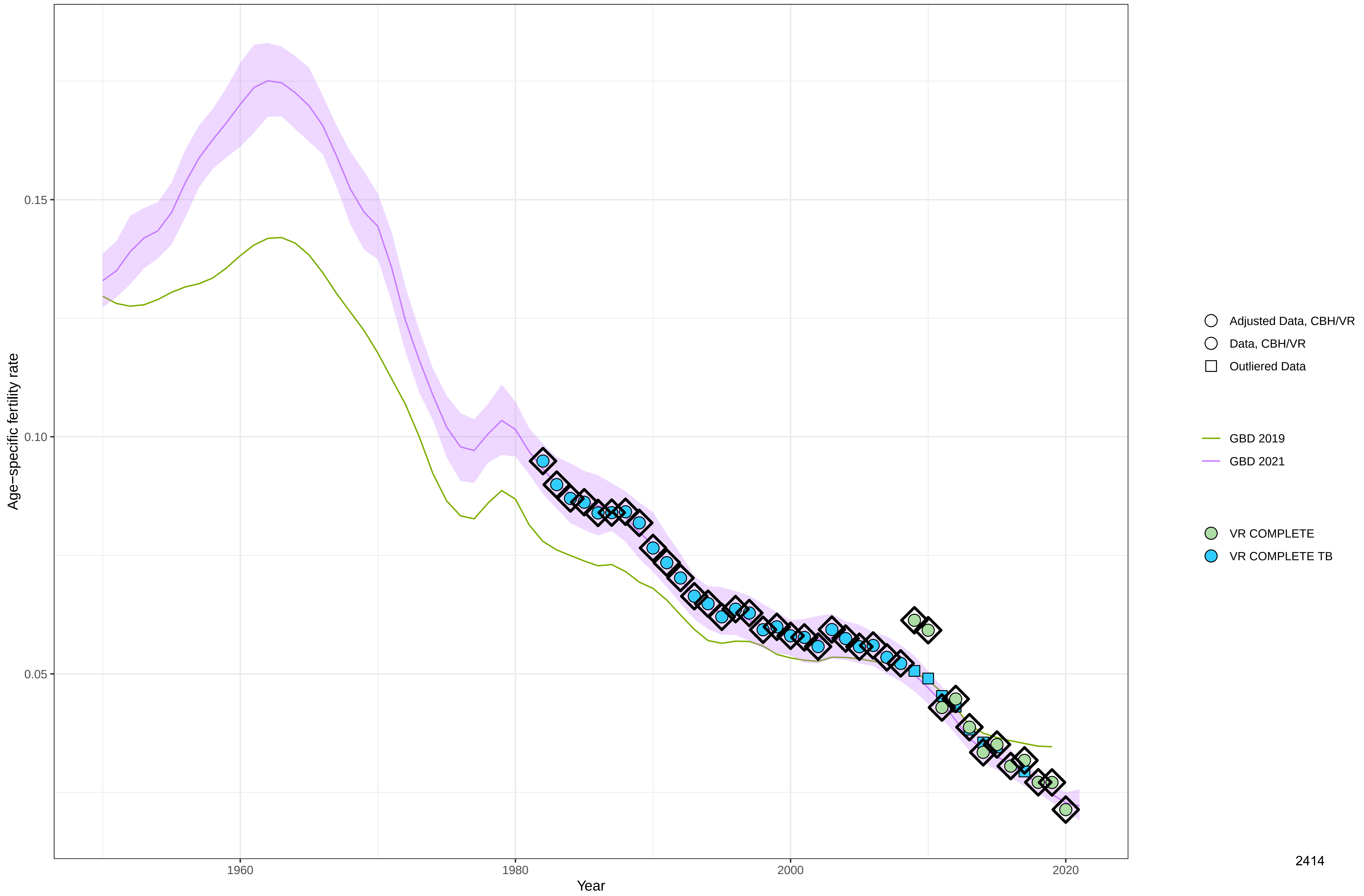

Age−specific fertility fitted trend lines, 1950−2021: Lambeth, Ages: 25−29
Data points enclosed with a diamond are reference sources

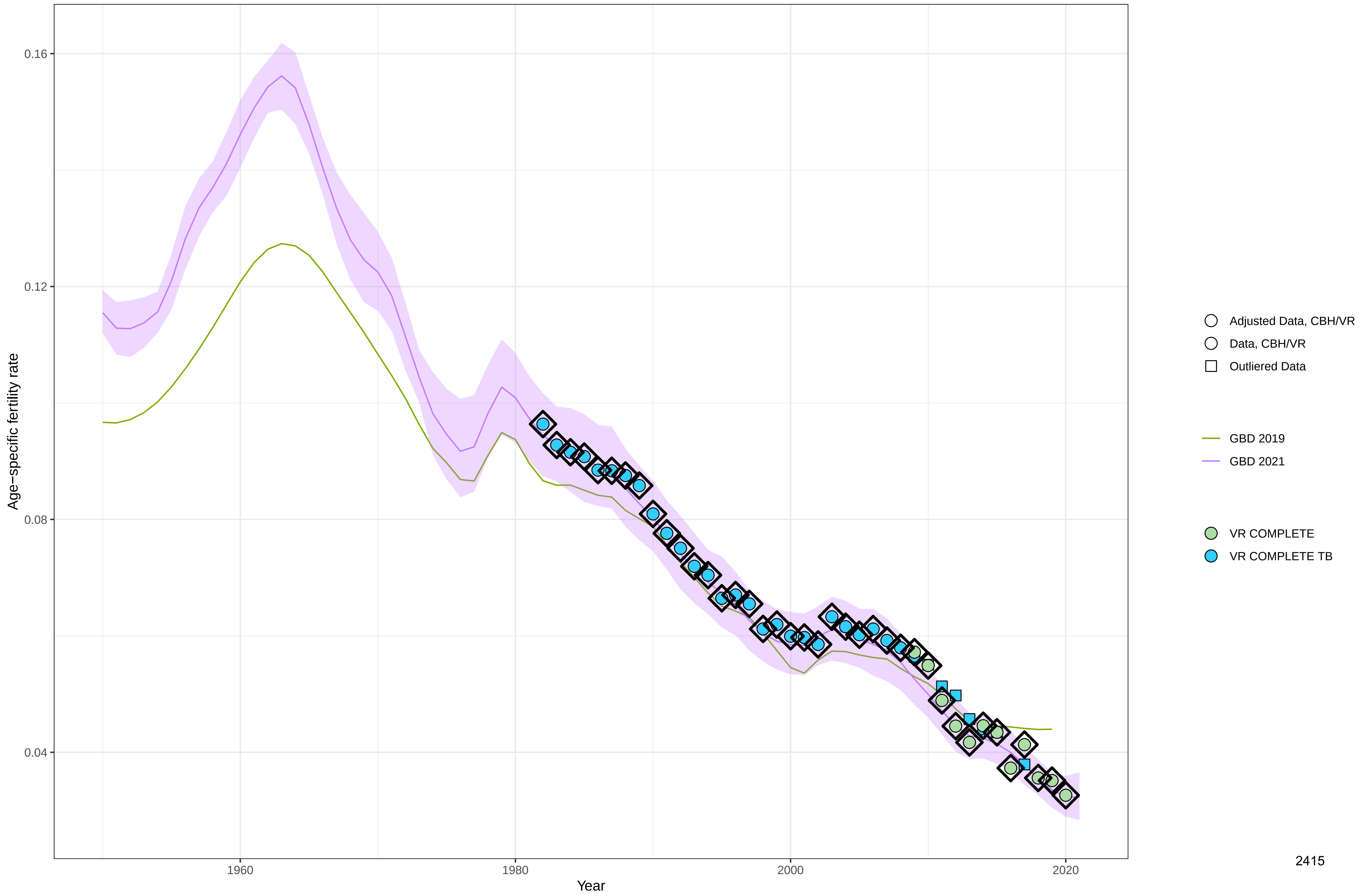

Age−specific fertility fitted trend lines, 1950−2021: Lambeth, Ages: 30−34
Data points enclosed with a diamond are reference sources

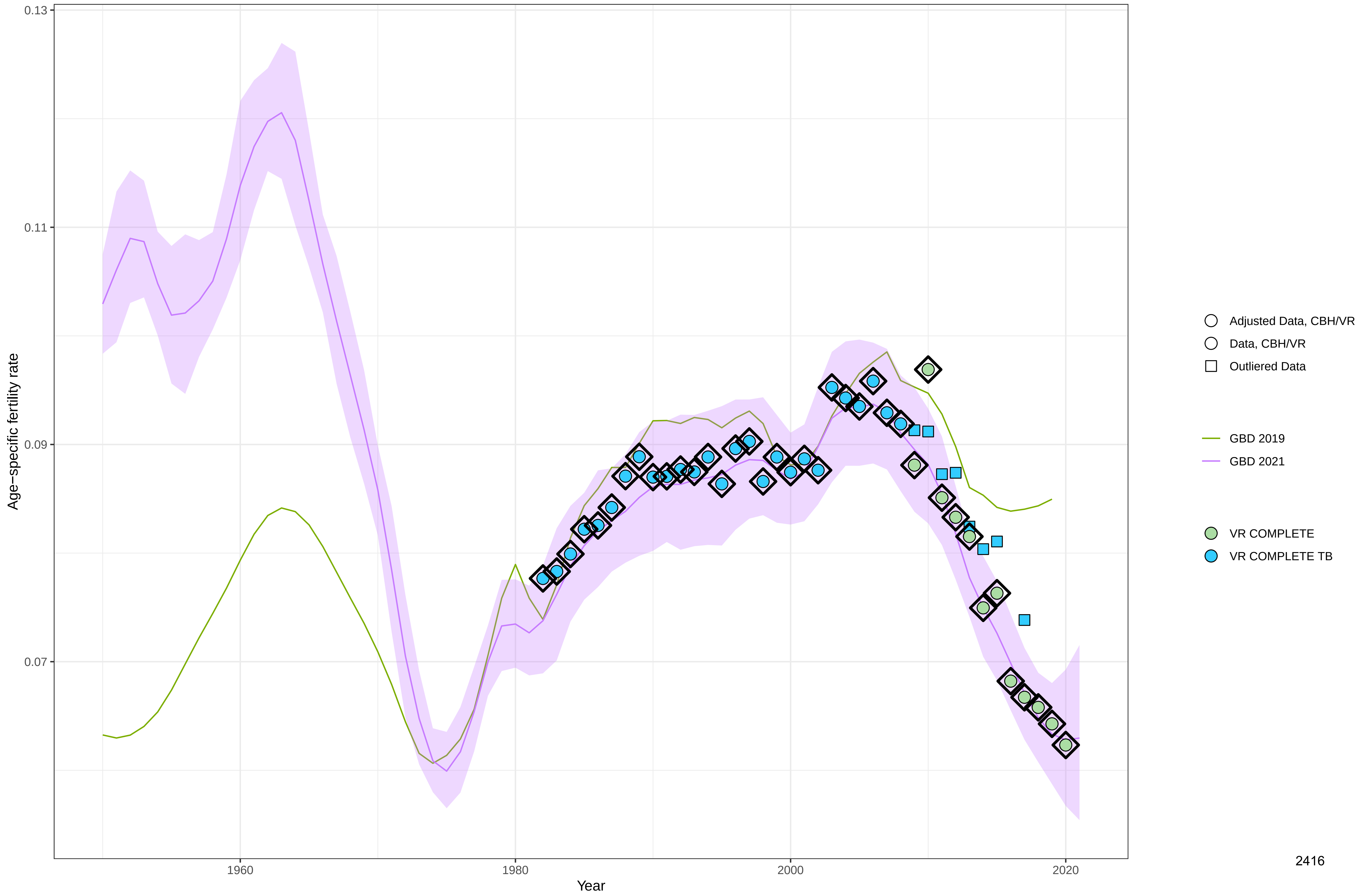

Age−specific fertility fitted trend lines, 1950−2021: Lambeth, Ages: 35−39
Data points enclosed with a diamond are reference sources

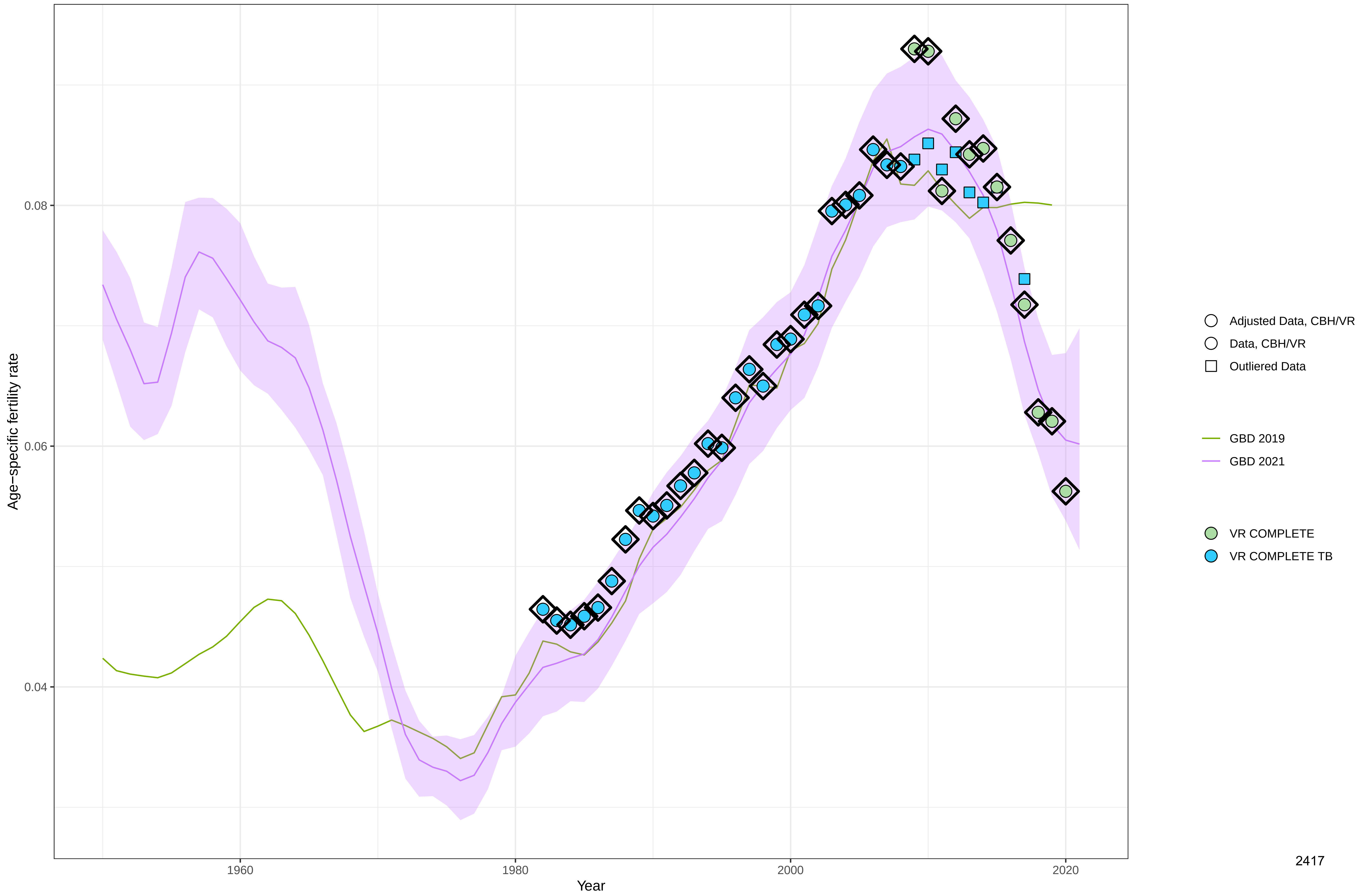

Age−specific fertility fitted trend lines, 1950−2021: Lambeth, Ages: 40−44
Data points enclosed with a diamond are reference sources

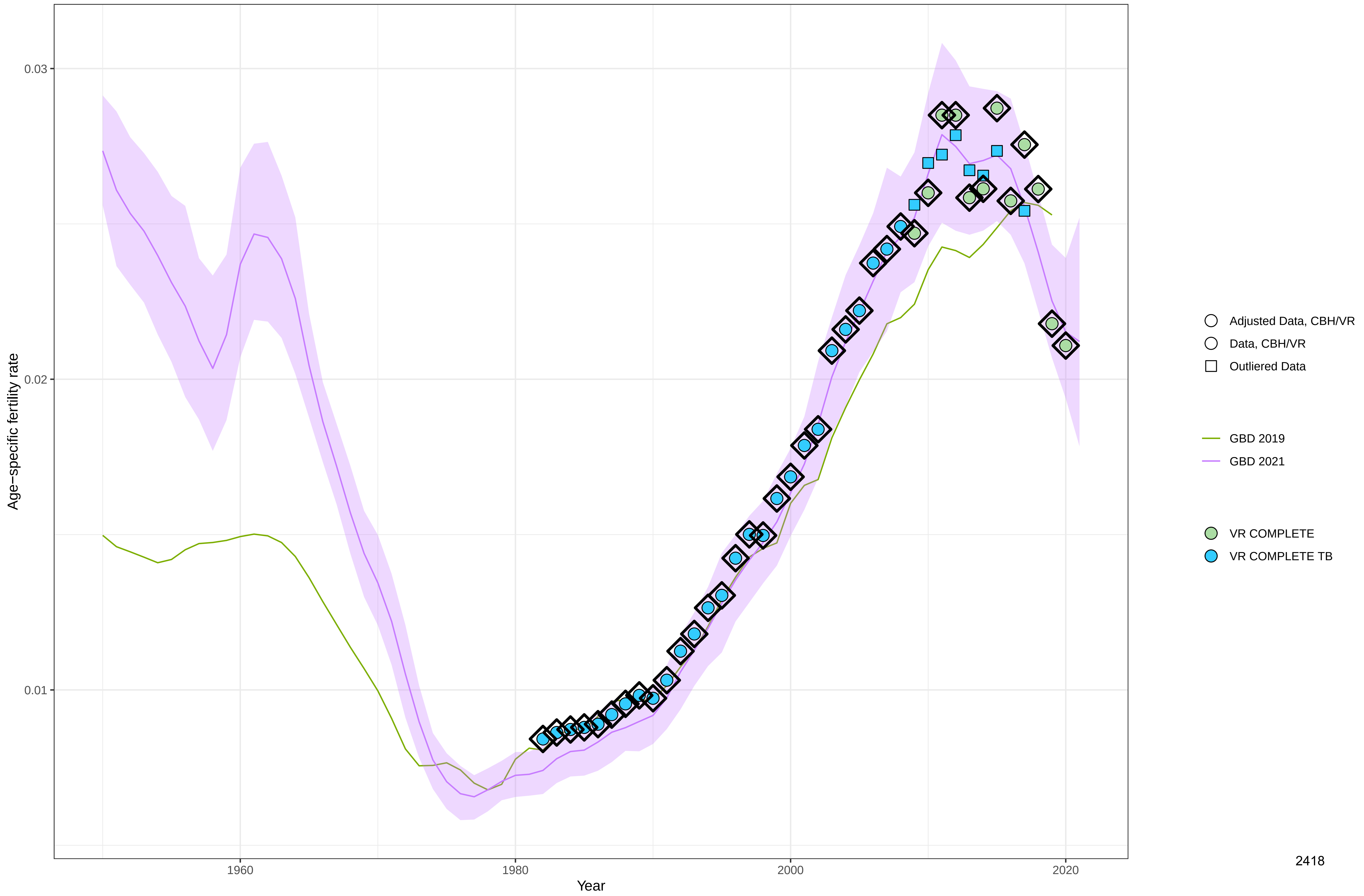

Age−specific fertility fitted trend lines, 1950−2021: Lambeth, Ages: 45−49
Data points enclosed with a diamond are reference sources

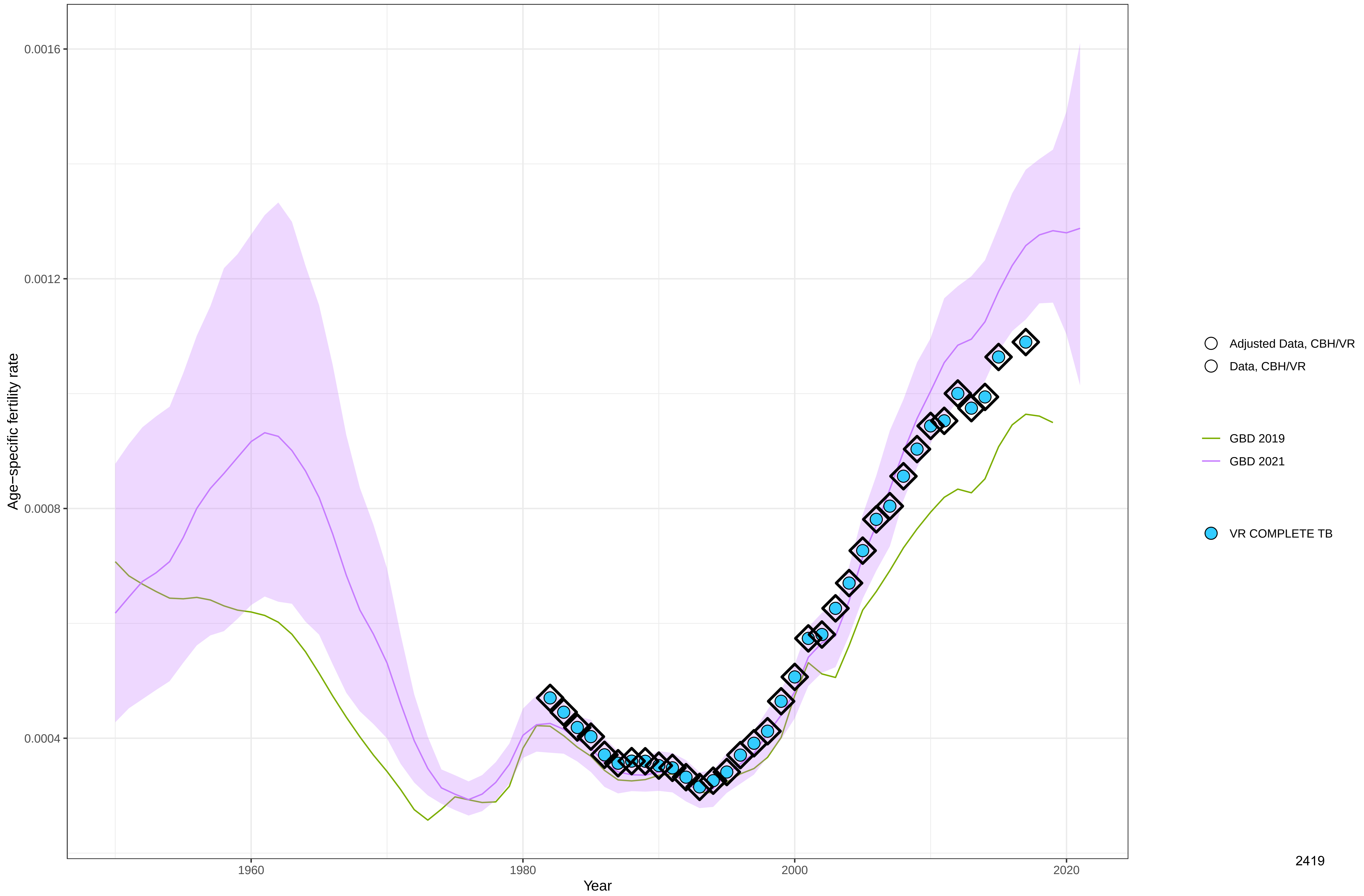

Age−specific fertility fitted trend lines, 1950−2021: Lambeth, Ages: 50−54
Data points enclosed with a diamond are reference sources

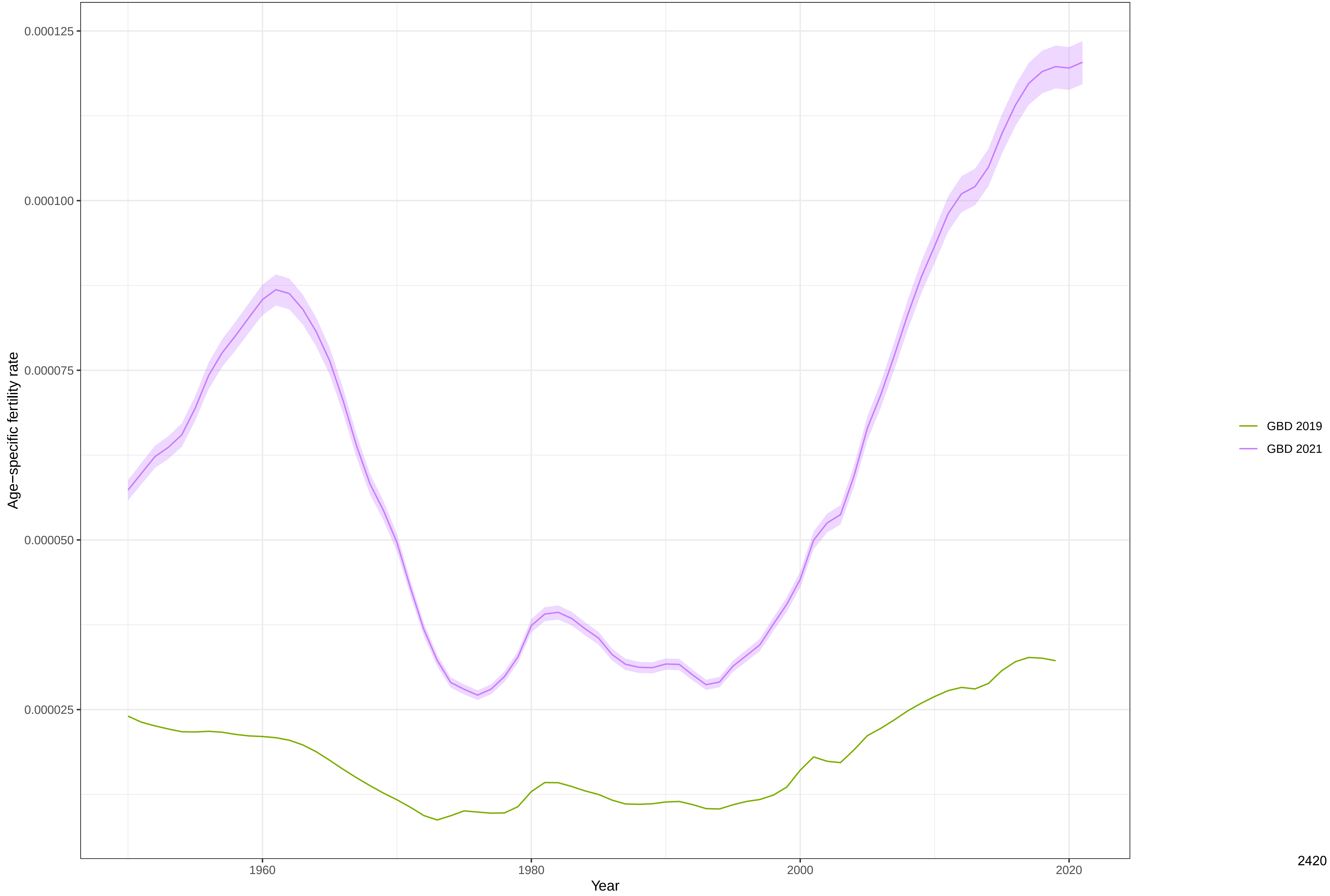

Total fertility rate fitted trend lines, 1950−2021: Lambeth TFR
Data points enclosed with a diamond are reference sources

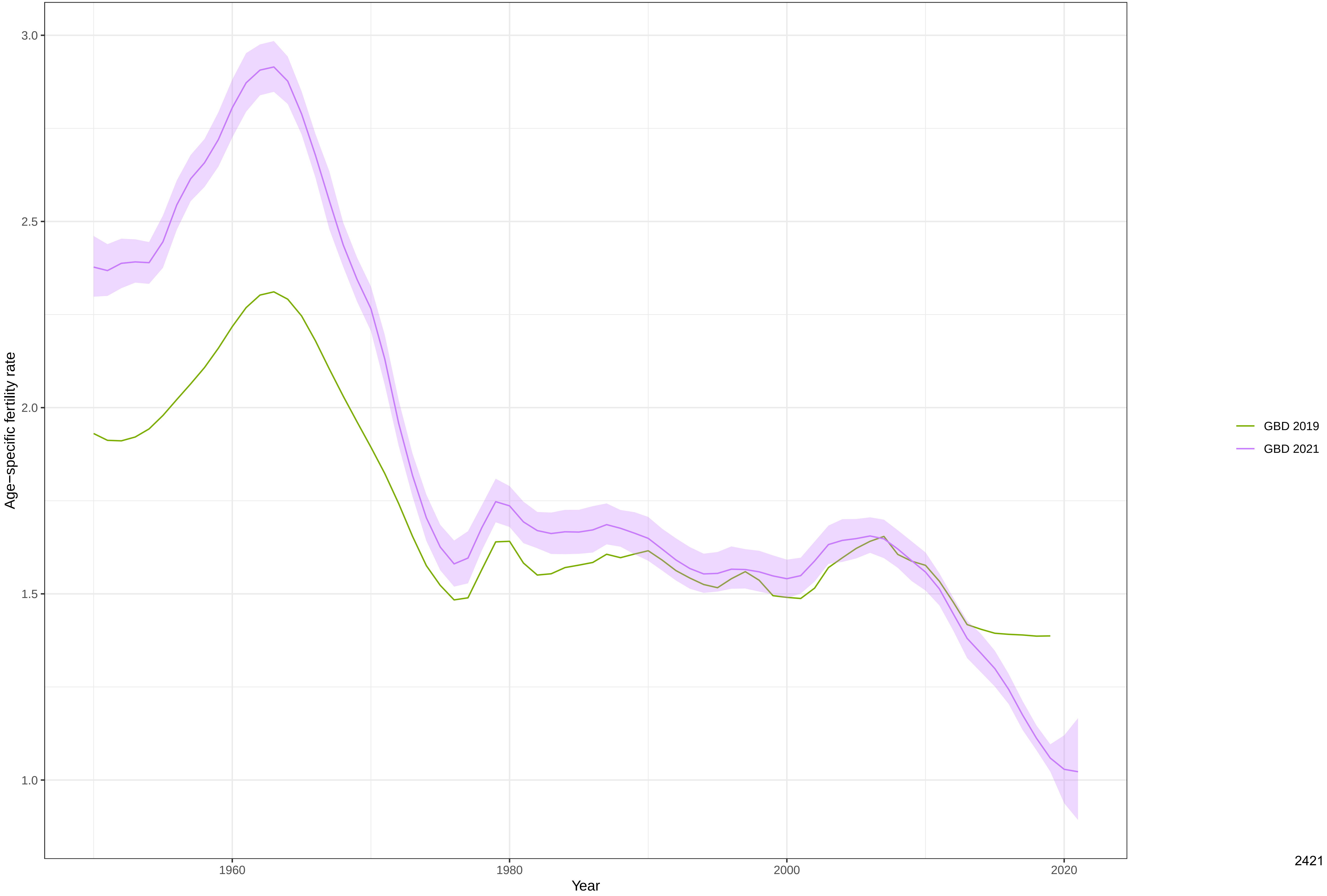

Age−specific fertility fitted trend lines, 1950−2021: Lewisham, Ages: 10−14
Data points enclosed with a diamond are reference sources

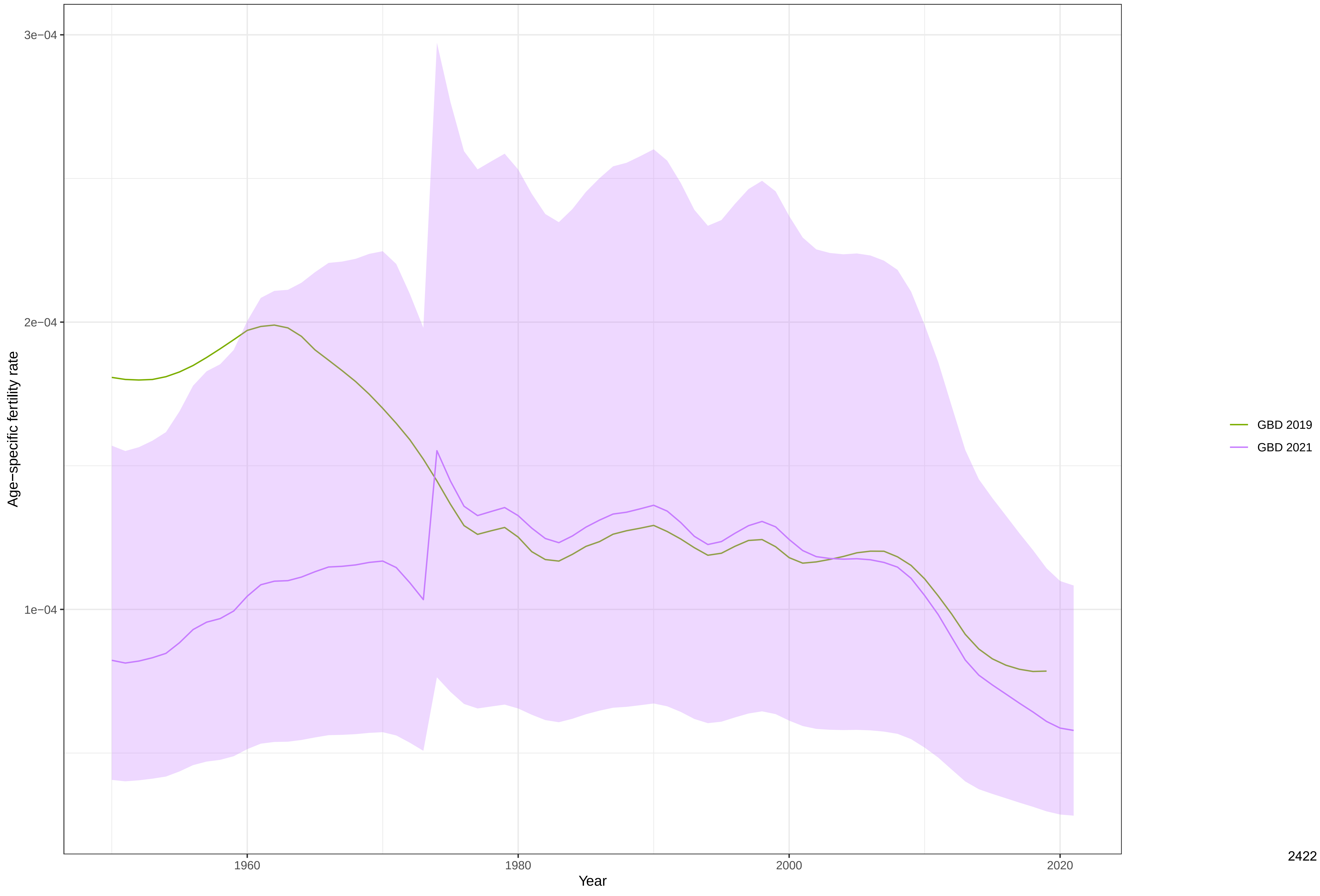

Age−specific fertility fitted trend lines, 1950−2021: Lewisham, Ages: 15−19
Data points enclosed with a diamond are reference sources

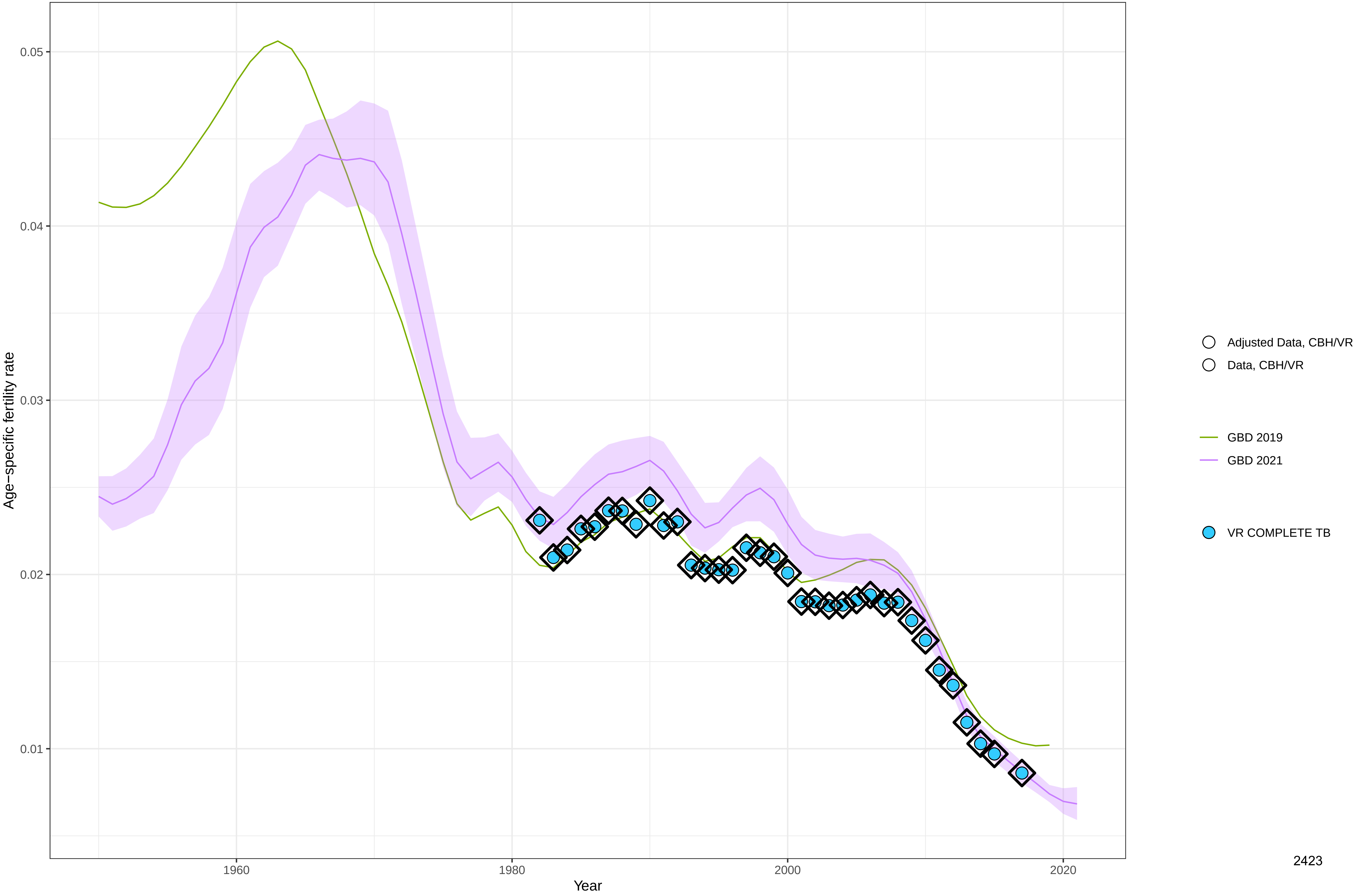

Age−specific fertility fitted trend lines, 1950−2021: Lewisham, Ages: 20−24
Data points enclosed with a diamond are reference sources

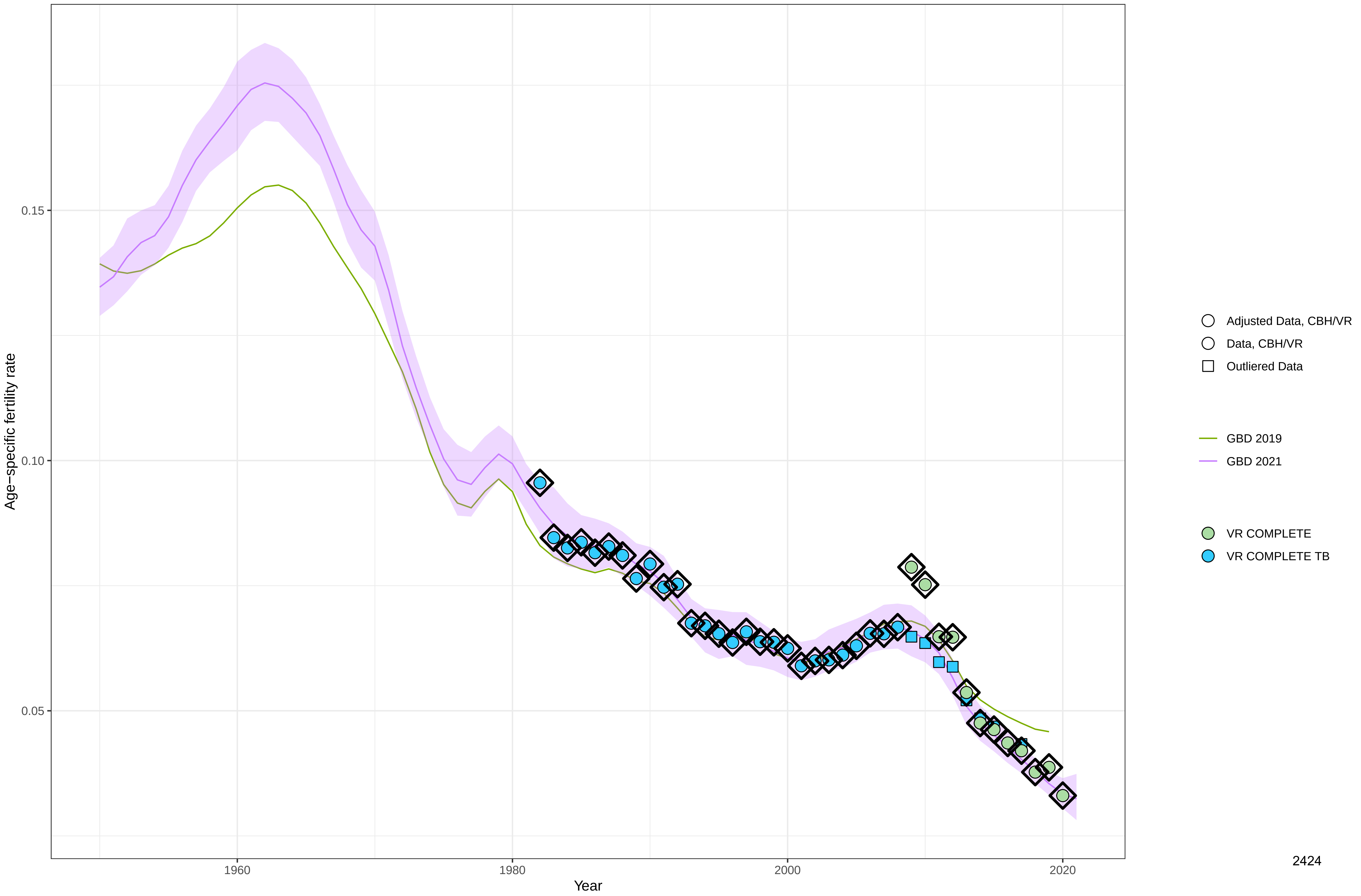

Age−specific fertility fitted trend lines, 1950−2021: Lewisham, Ages: 25−29
Data points enclosed with a diamond are reference sources

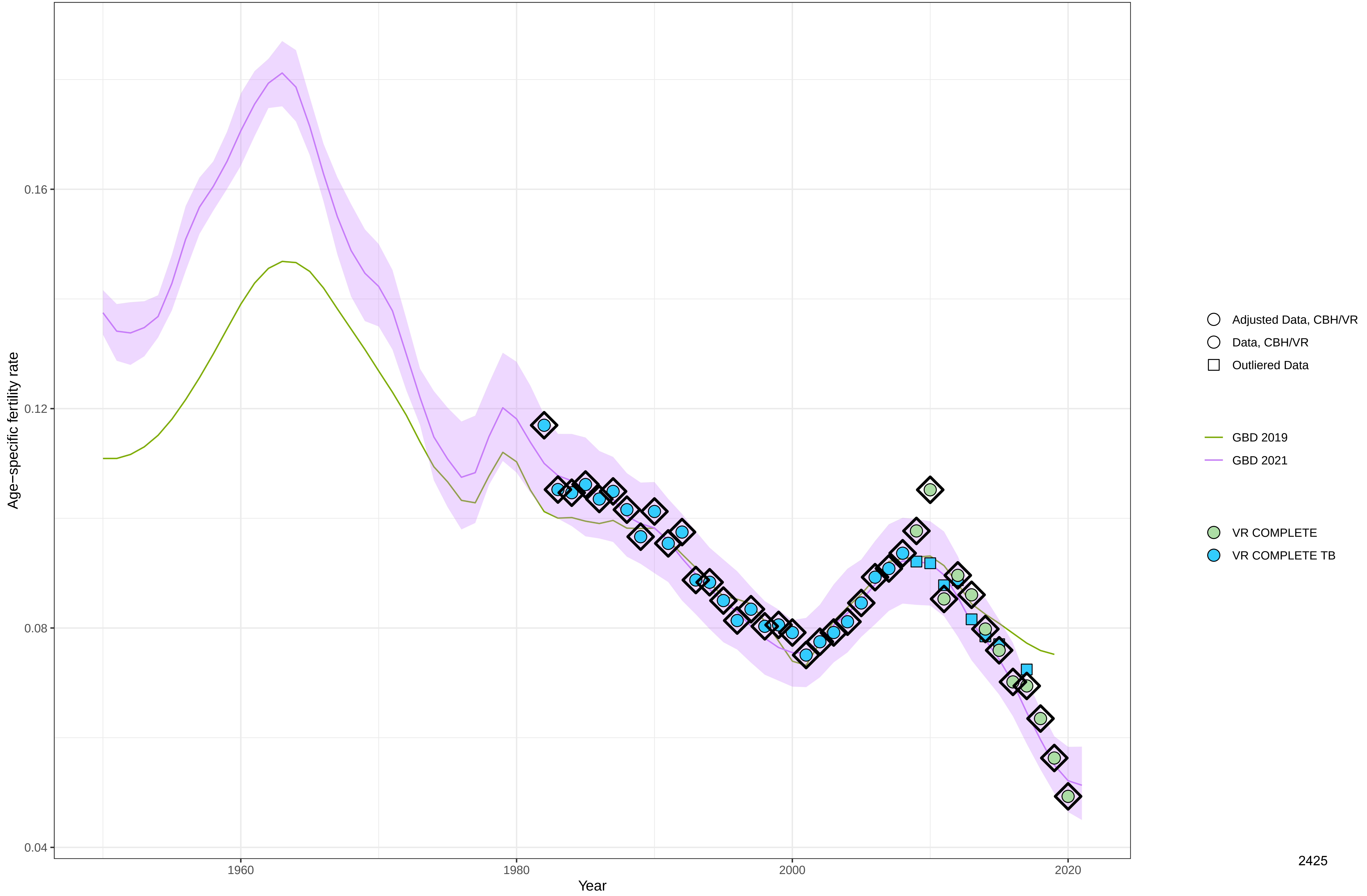

Age−specific fertility fitted trend lines, 1950−2021: Lewisham, Ages: 30−34
Data points enclosed with a diamond are reference sources

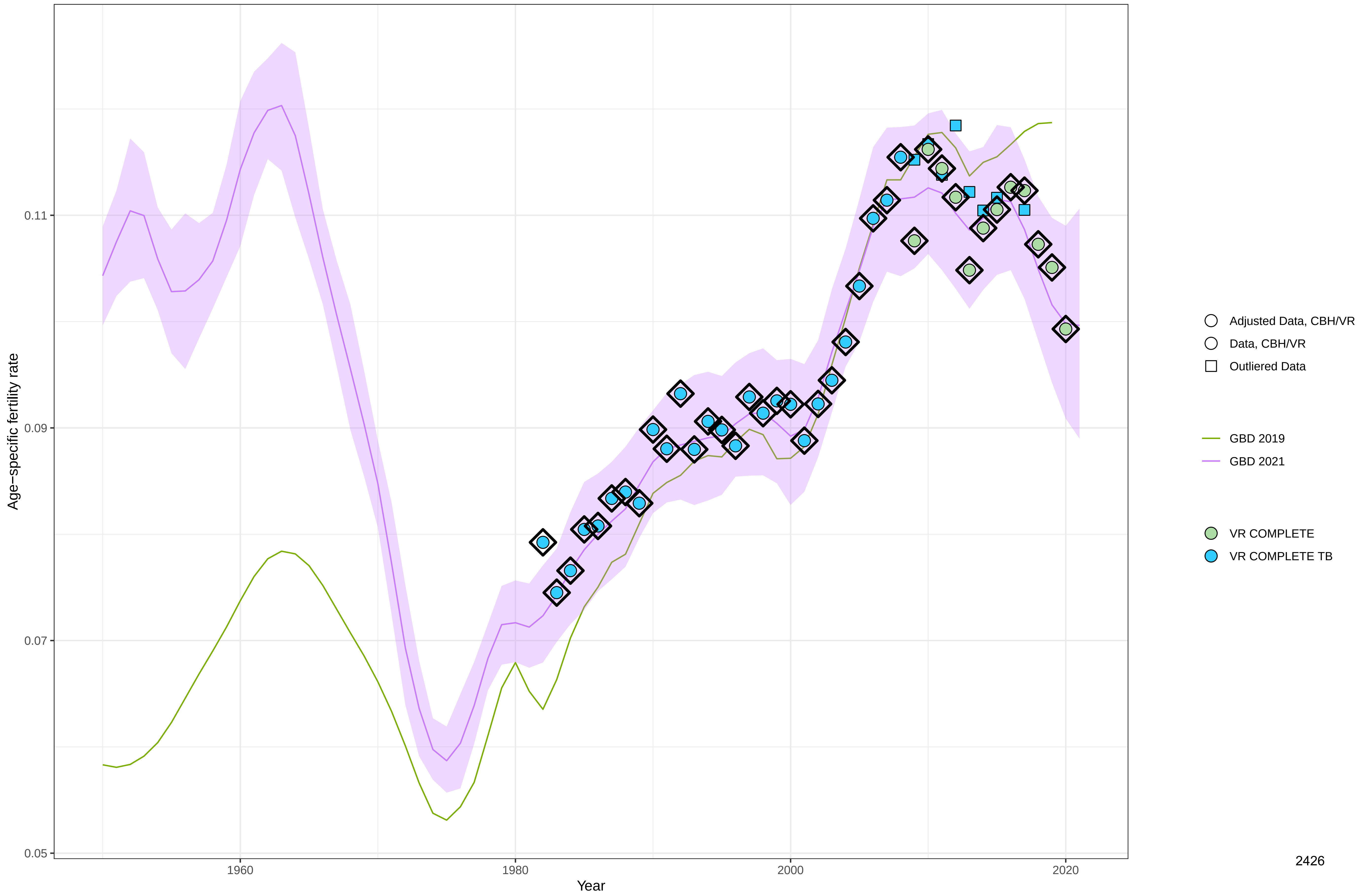

Age−specific fertility fitted trend lines, 1950−2021: Lewisham, Ages: 35−39
Data points enclosed with a diamond are reference sources

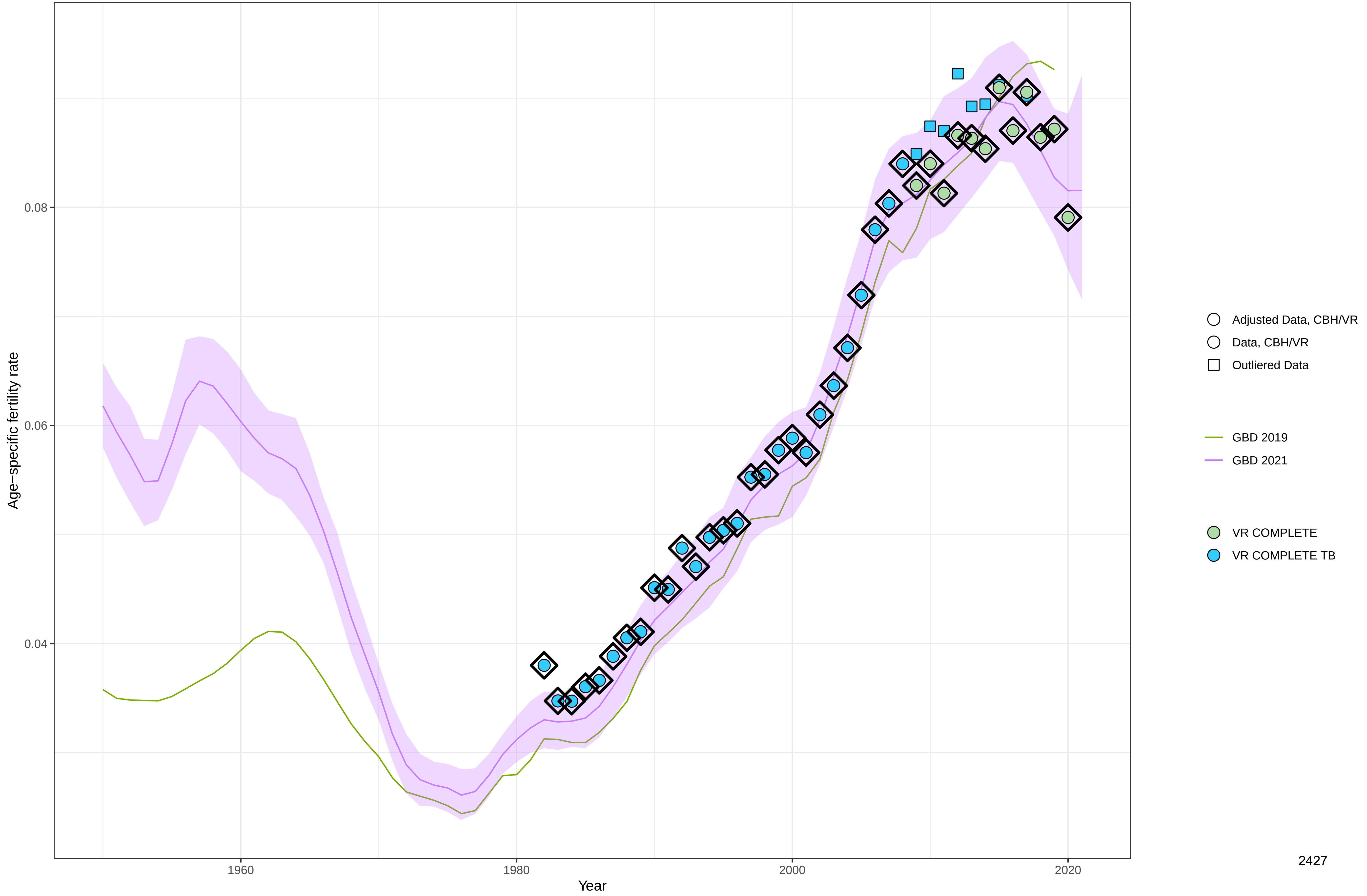

Age−specific fertility fitted trend lines, 1950−2021: Lewisham, Ages: 40−44
Data points enclosed with a diamond are reference sources

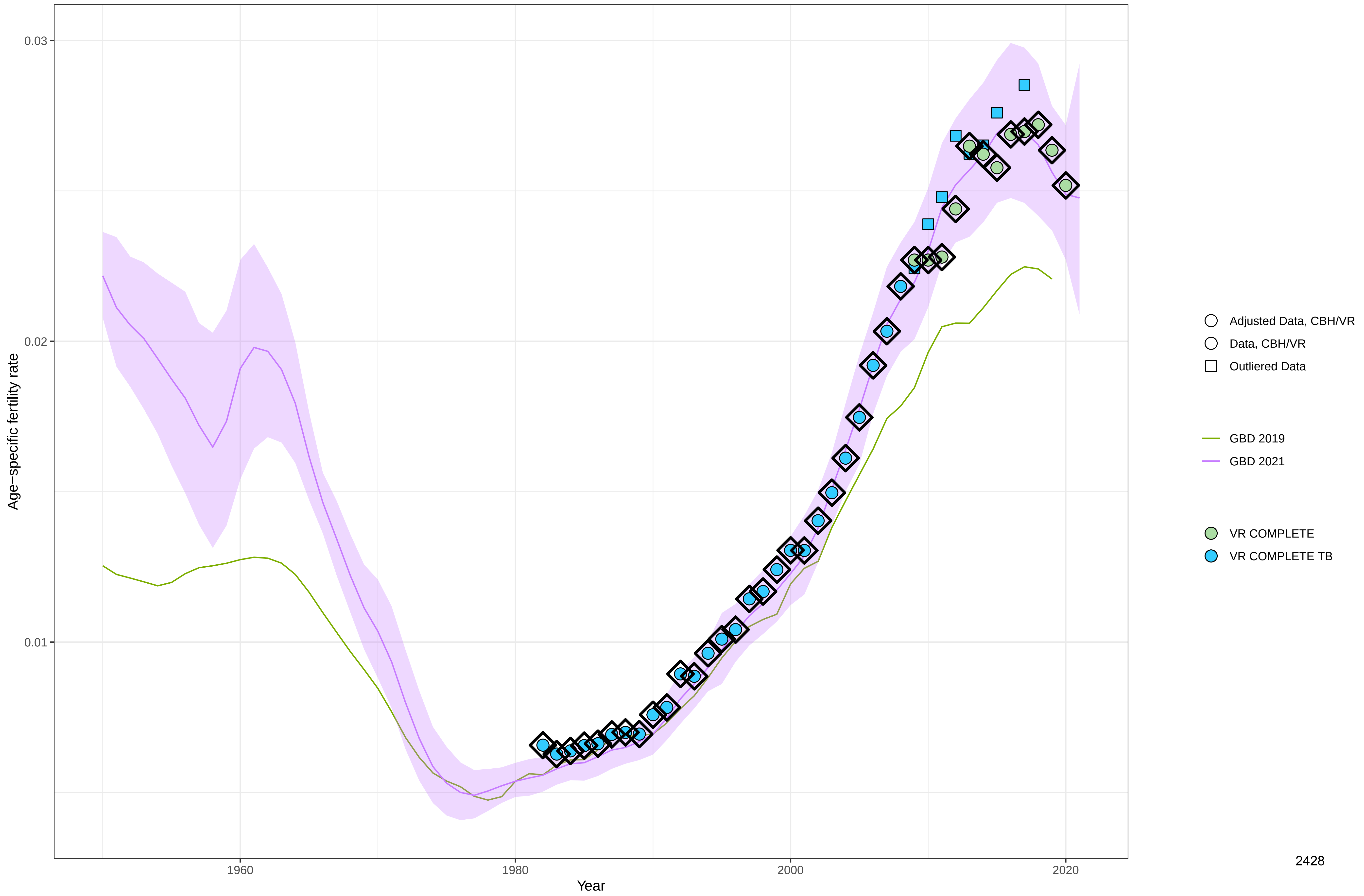

Age−specific fertility fitted trend lines, 1950−2021: Lewisham, Ages: 45−49
Data points enclosed with a diamond are reference sources

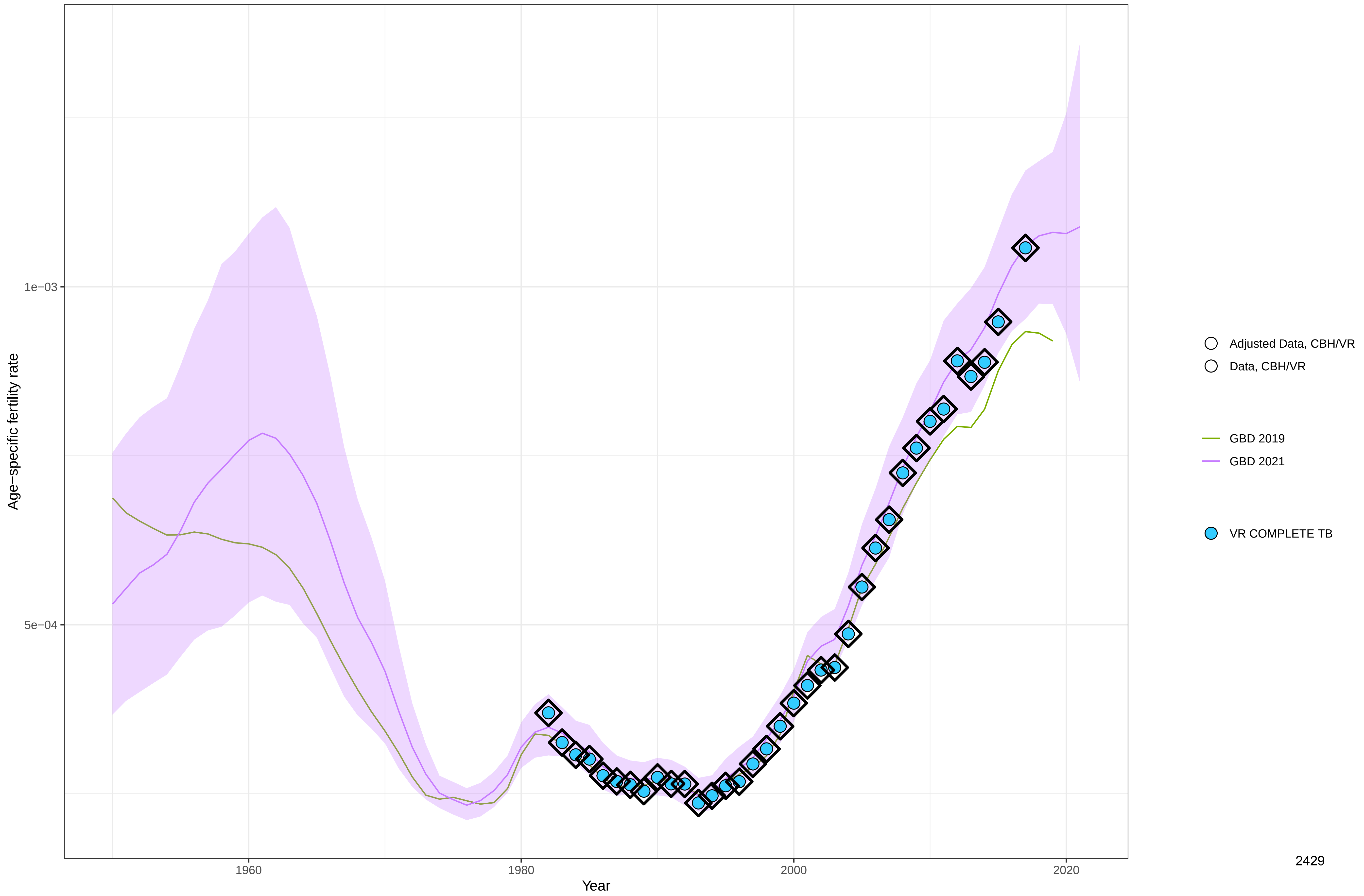

Age−specific fertility fitted trend lines, 1950−2021: Lewisham, Ages: 50−54
Data points enclosed with a diamond are reference sources

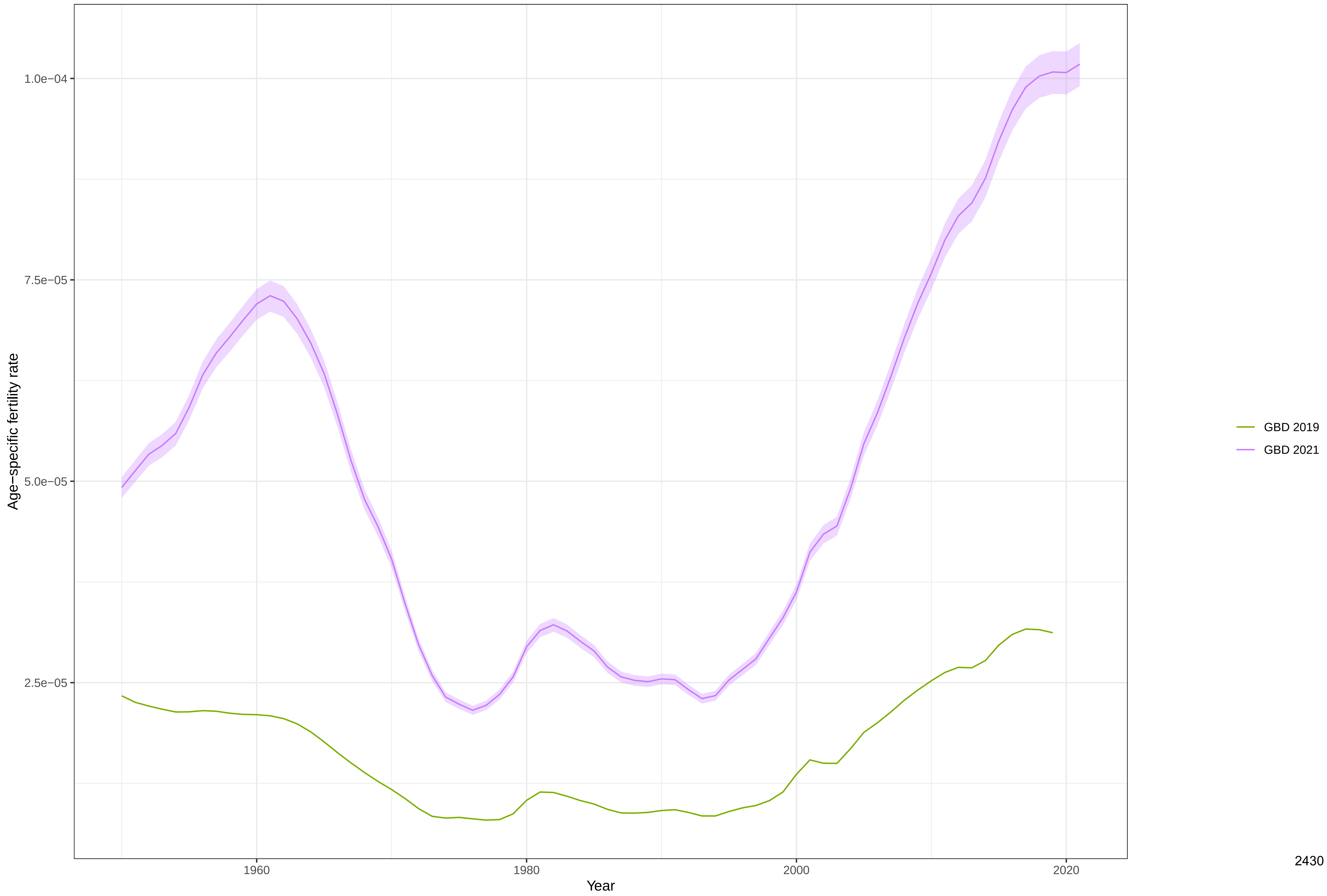

Total fertility rate fitted trend lines, 1950−2021: Lewisham TFR
Data points enclosed with a diamond are reference sources

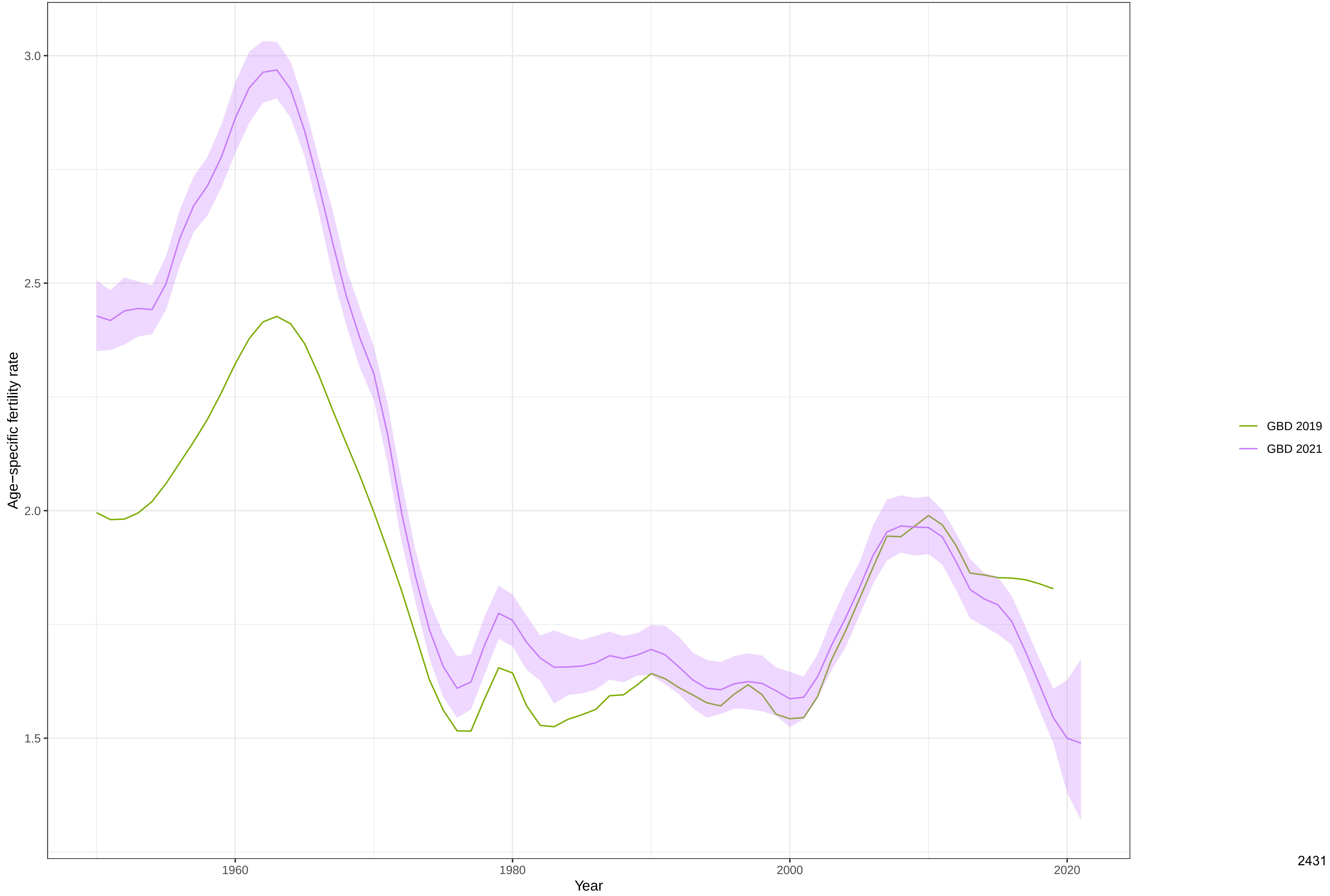

Age−specific fertility fitted trend lines, 1950−2021: Merton, Ages: 10−14
Data points enclosed with a diamond are reference sources

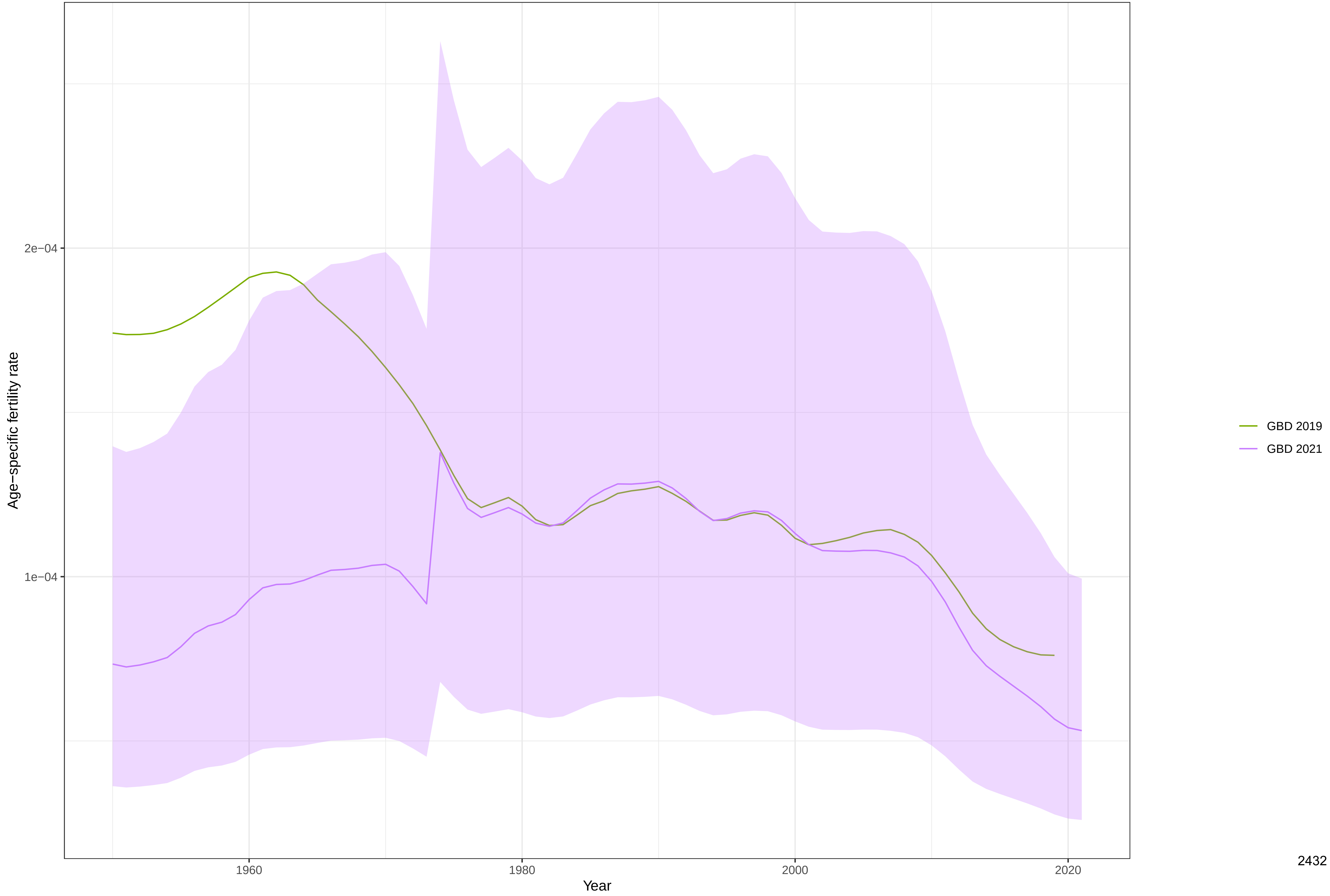

Age−specific fertility fitted trend lines, 1950−2021: Merton, Ages: 15−19
Data points enclosed with a diamond are reference sources

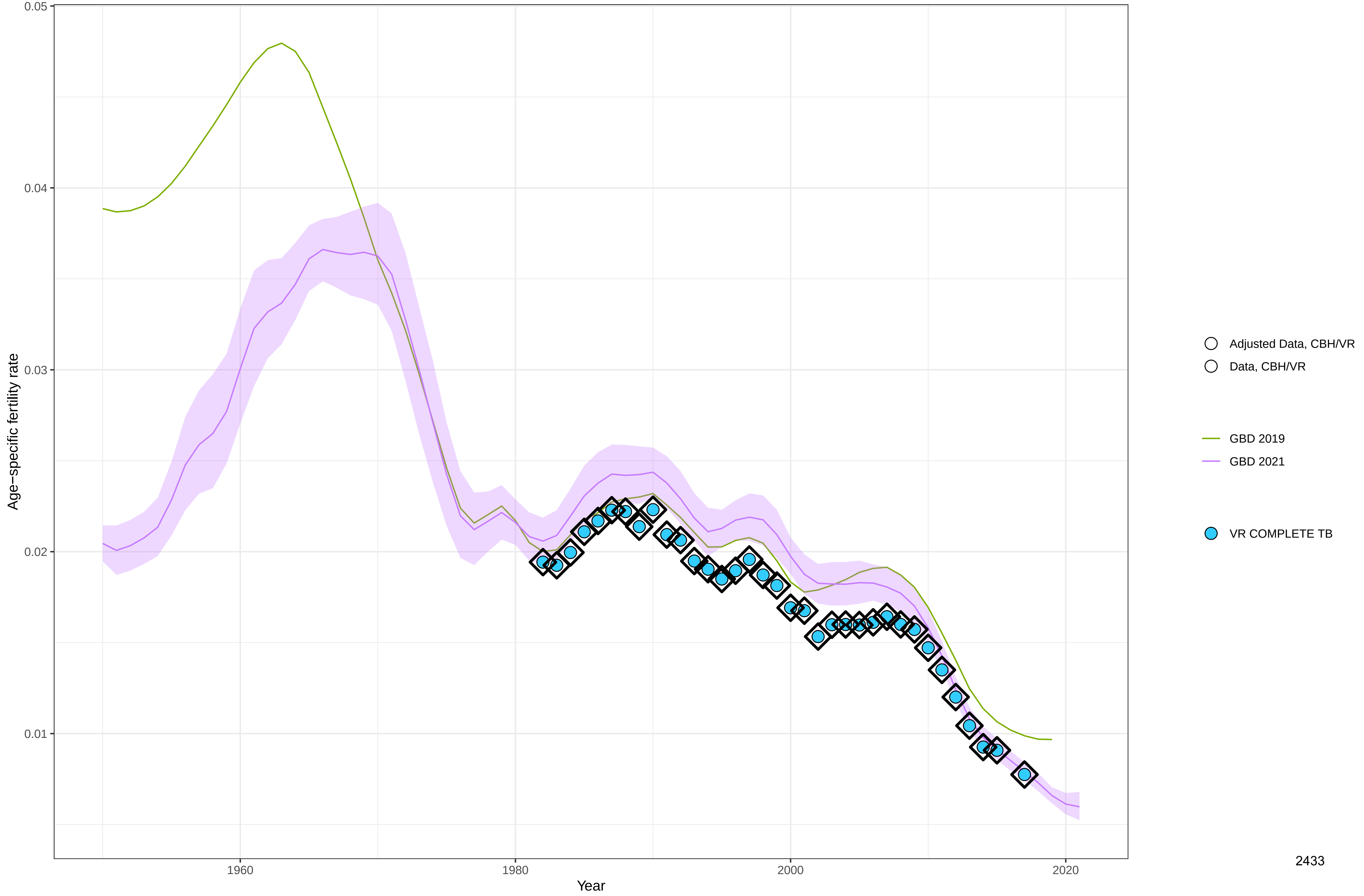

Age−specific fertility fitted trend lines, 1950−2021: Merton, Ages: 20−24
Data points enclosed with a diamond are reference sources

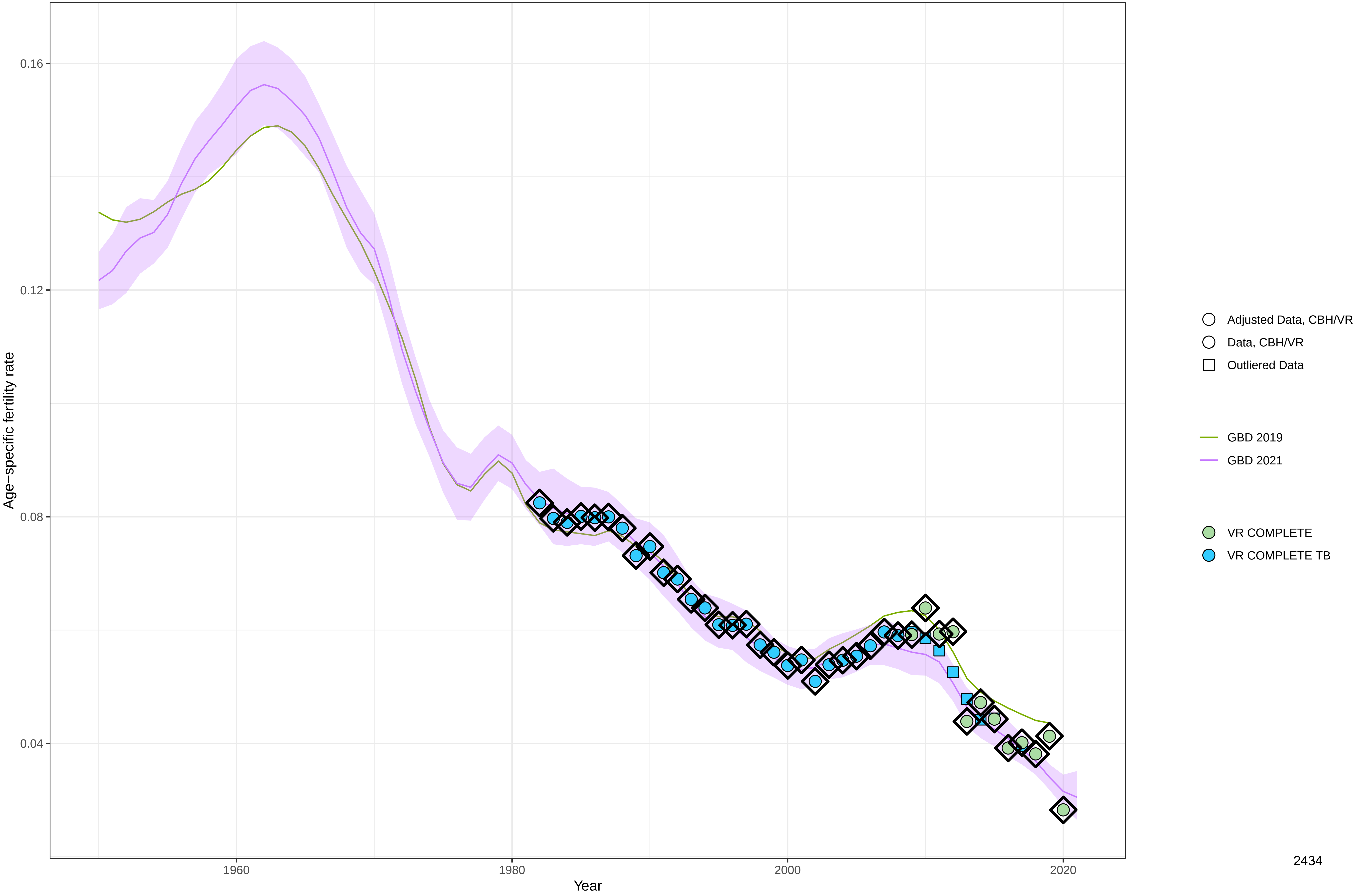

Age−specific fertility fitted trend lines, 1950−2021: Merton, Ages: 25−29
Data points enclosed with a diamond are reference sources

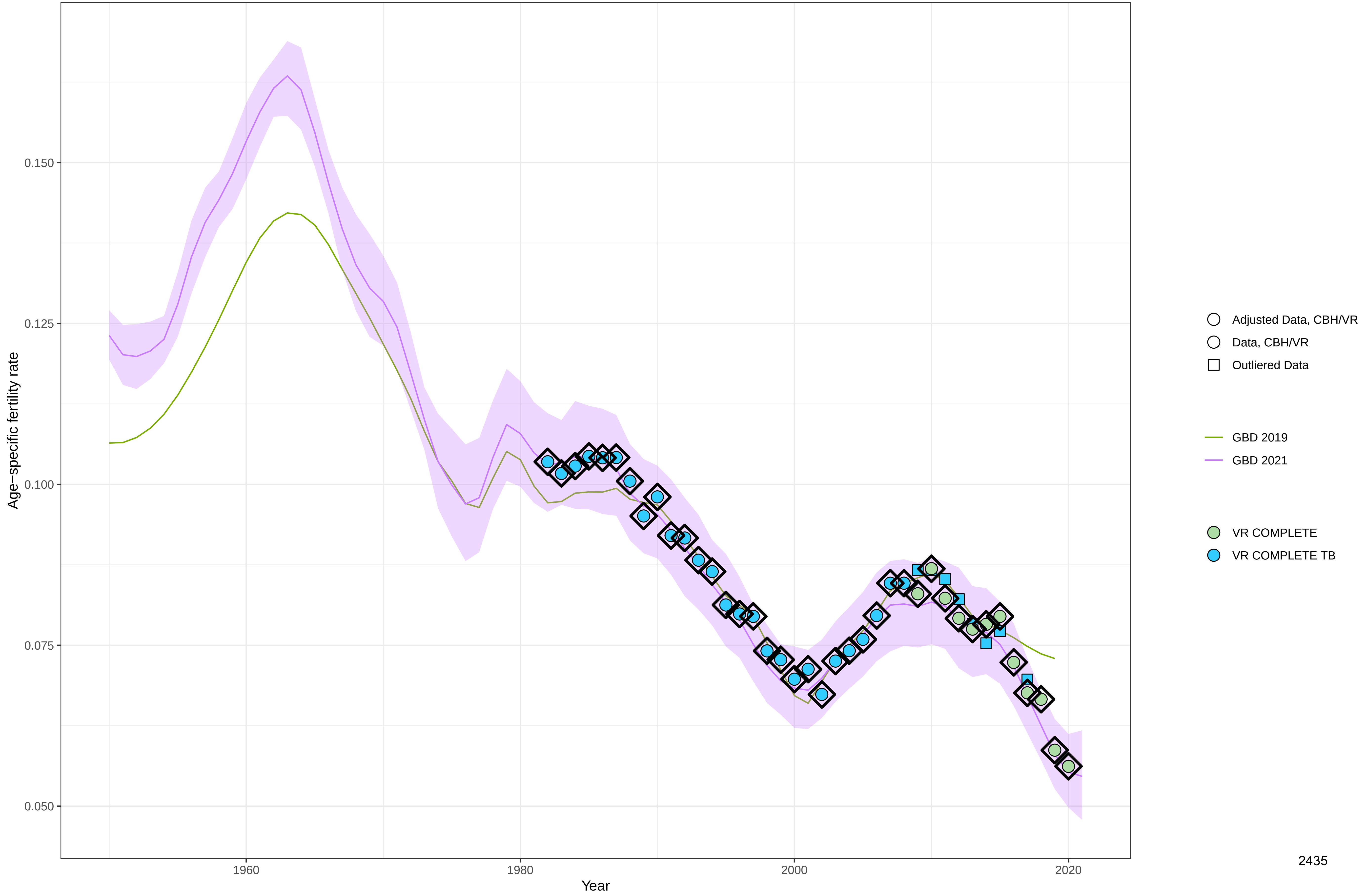

Age−specific fertility fitted trend lines, 1950−2021: Merton, Ages: 30−34
Data points enclosed with a diamond are reference sources

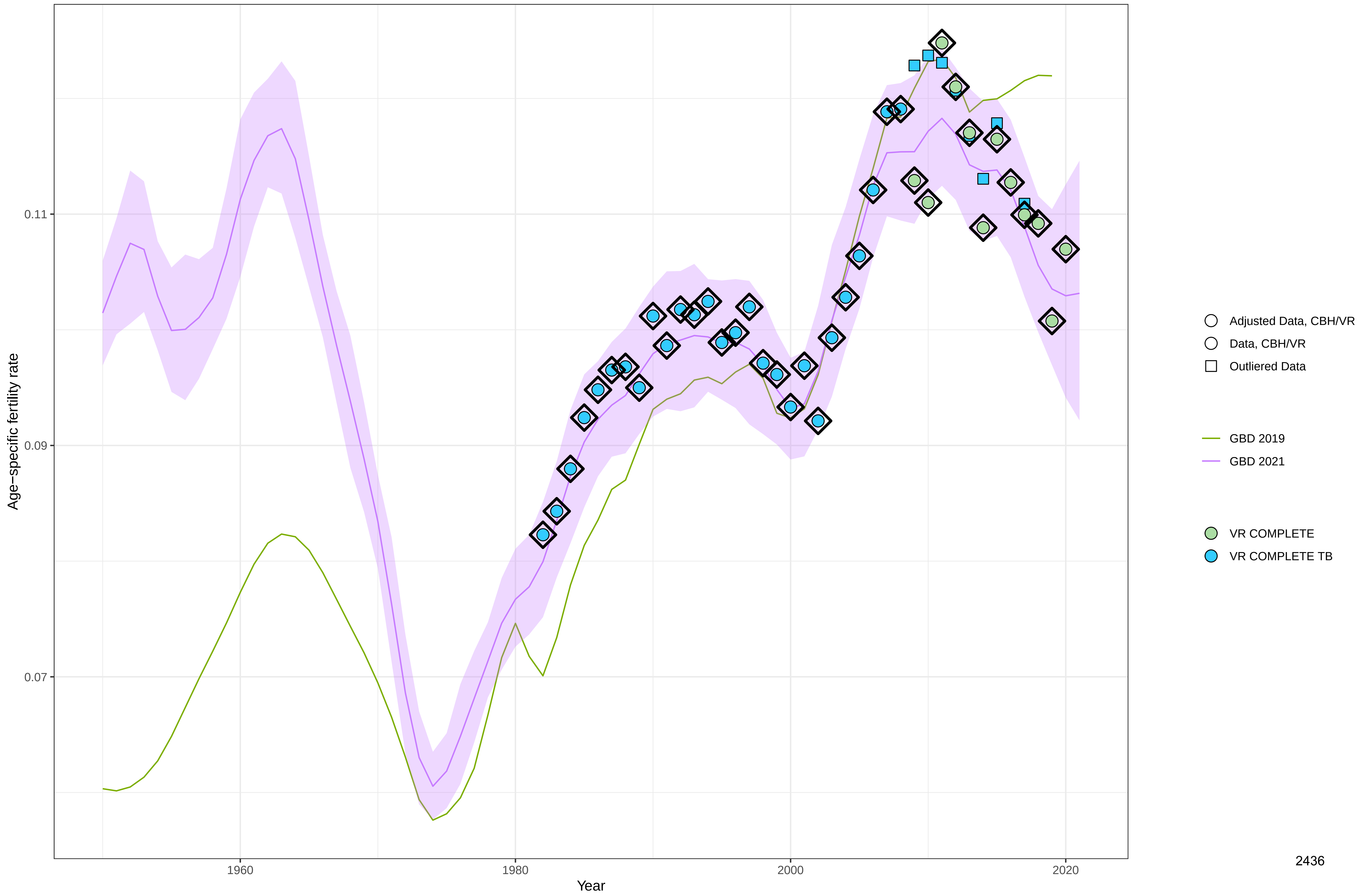

Age−specific fertility fitted trend lines, 1950−2021: Merton, Ages: 35−39
Data points enclosed with a diamond are reference sources

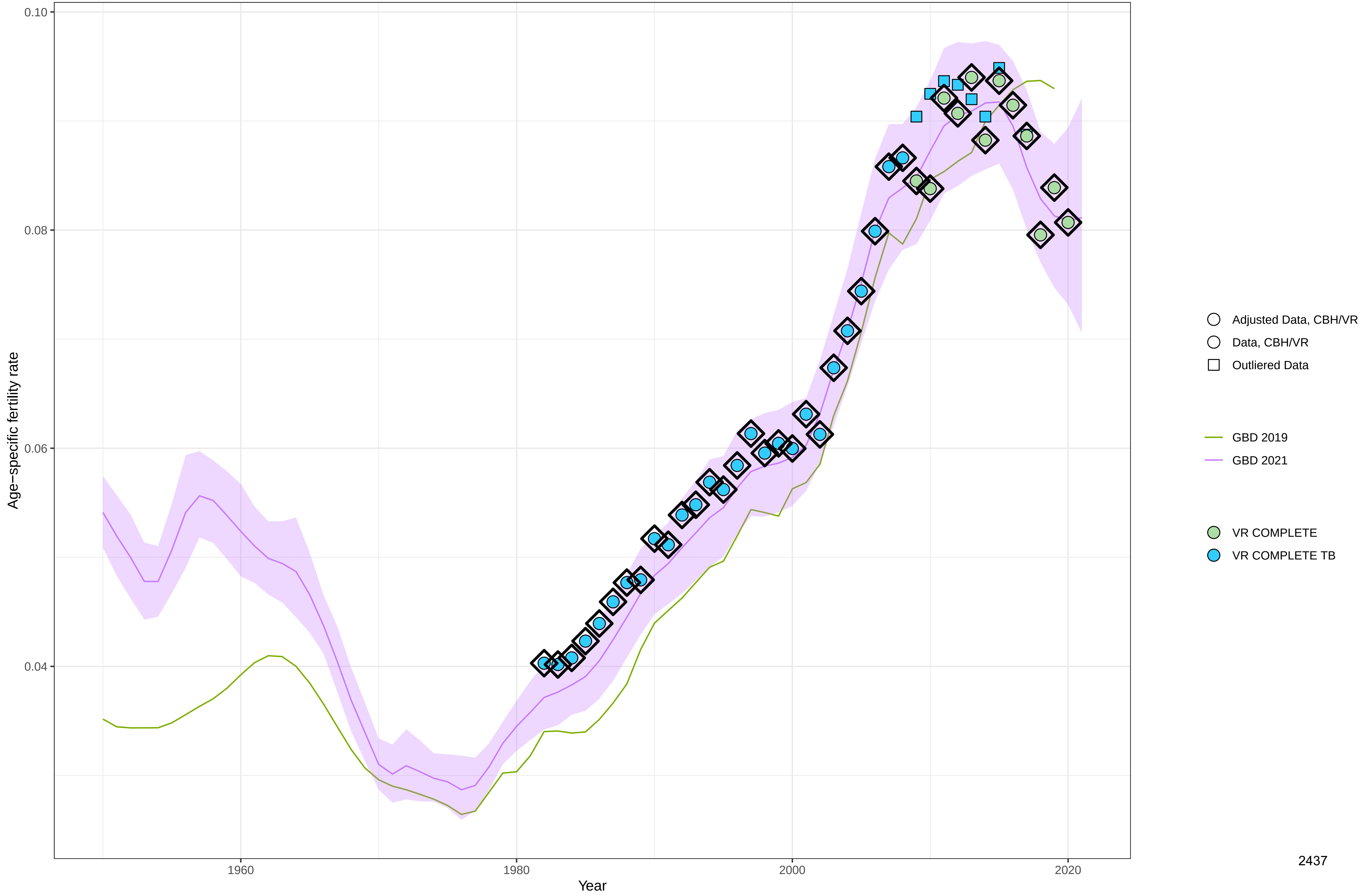

Age−specific fertility fitted trend lines, 1950−2021: Merton, Ages: 40−44
Data points enclosed with a diamond are reference sources

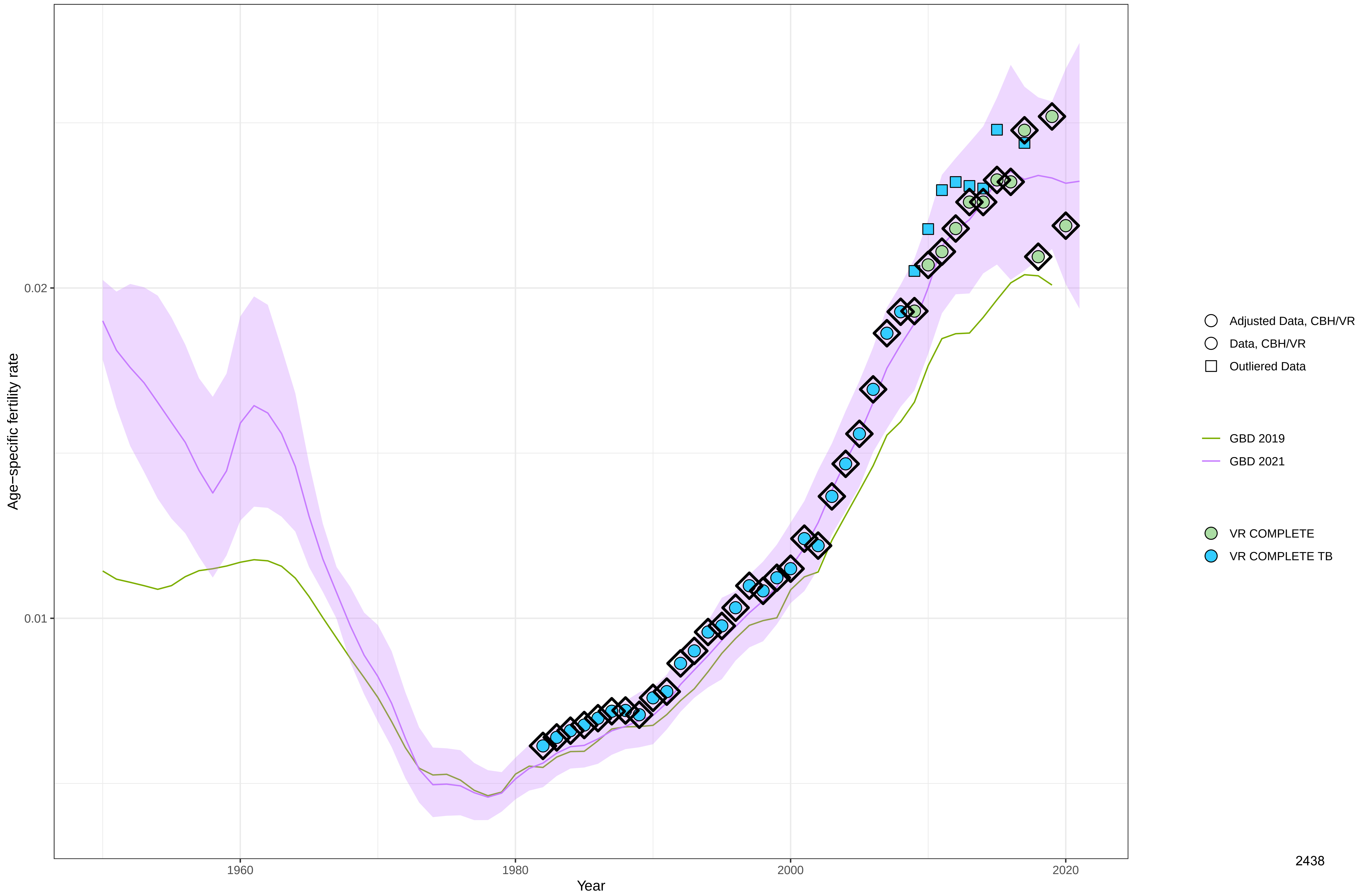

Age−specific fertility fitted trend lines, 1950−2021: Merton, Ages: 45−49
Data points enclosed with a diamond are reference sources

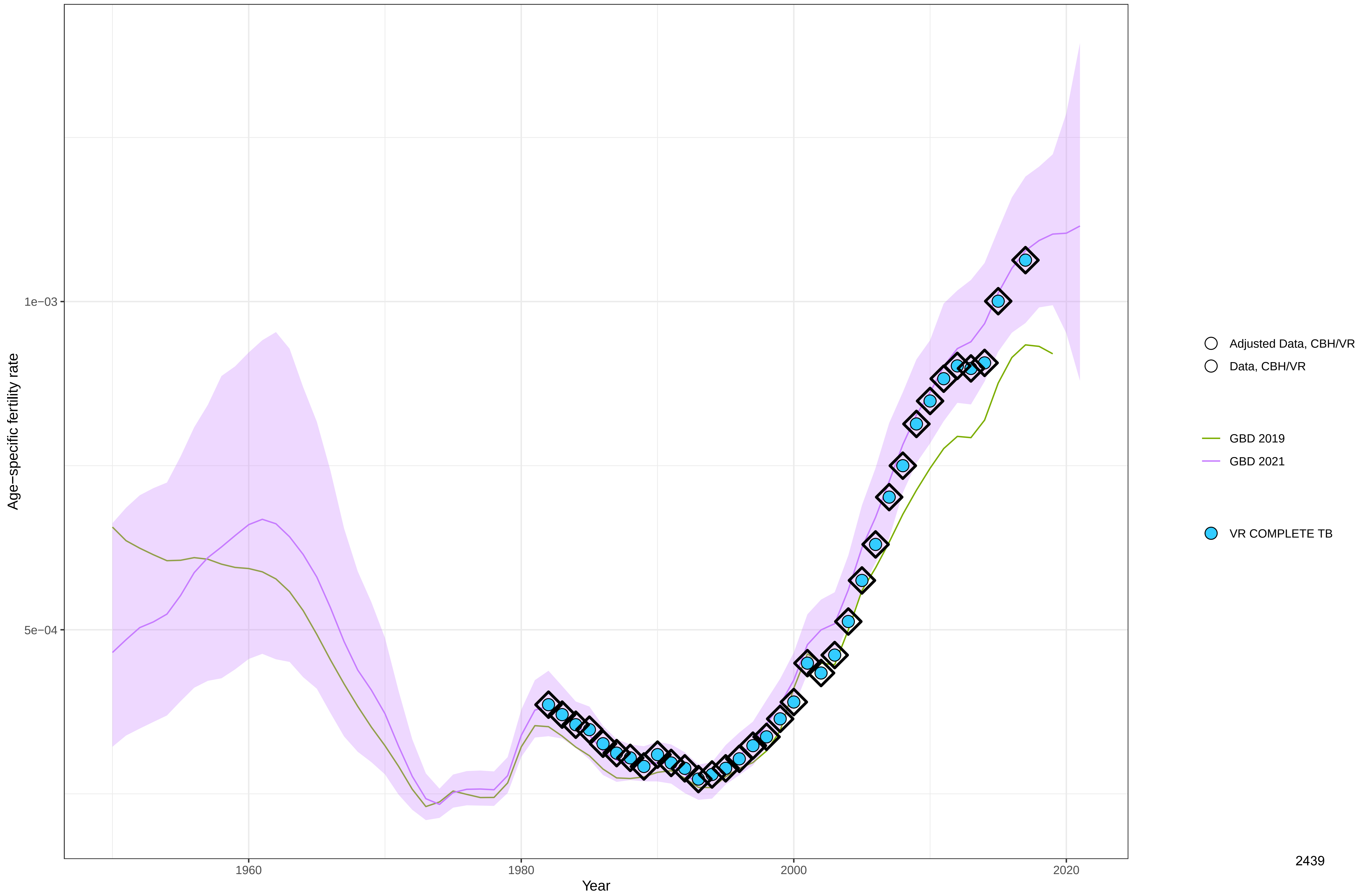

Age−specific fertility fitted trend lines, 1950−2021: Merton, Ages: 50−54
Data points enclosed with a diamond are reference sources

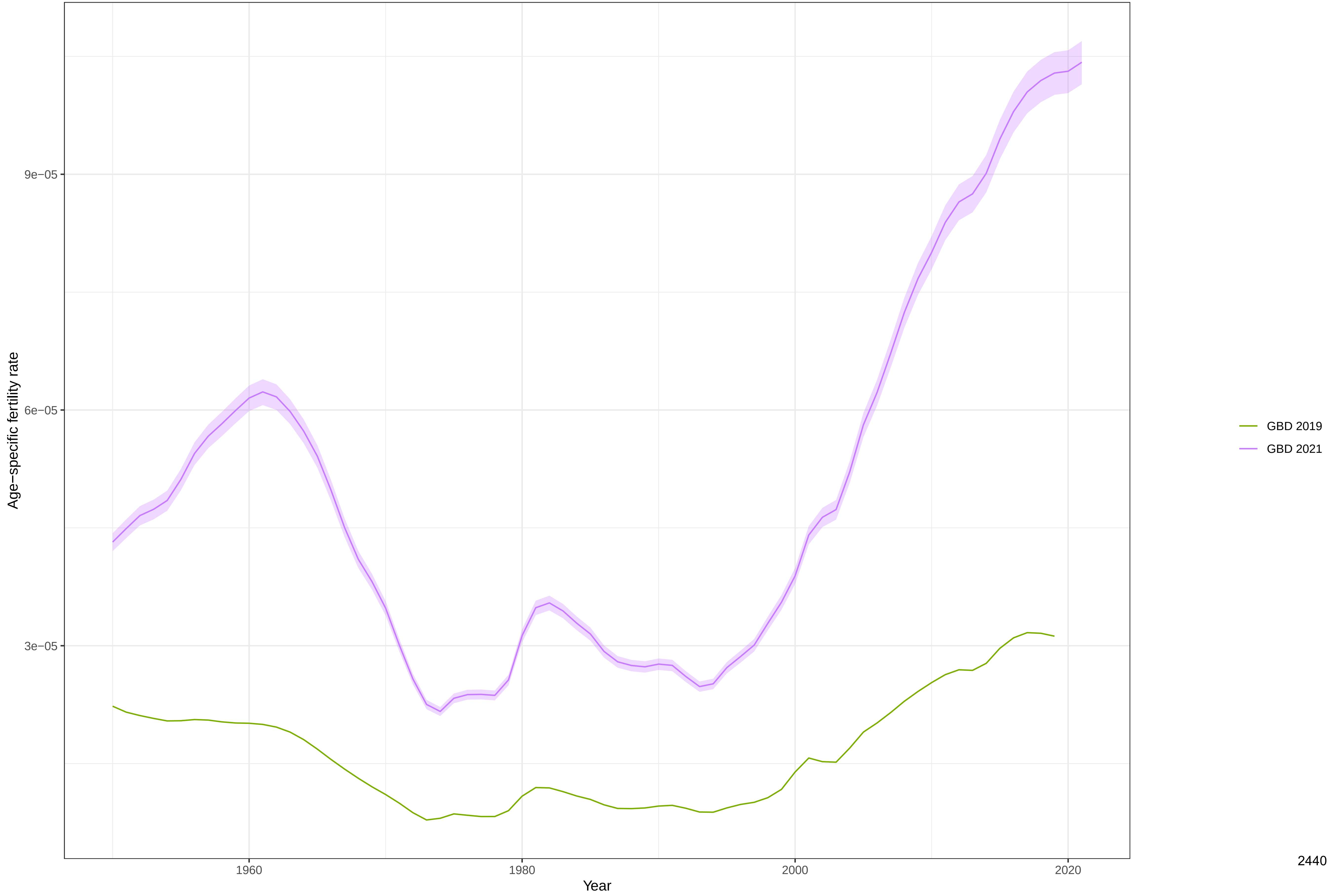

Total fertility rate fitted trend lines, 1950−2021: Merton TFR
Data points enclosed with a diamond are reference sources

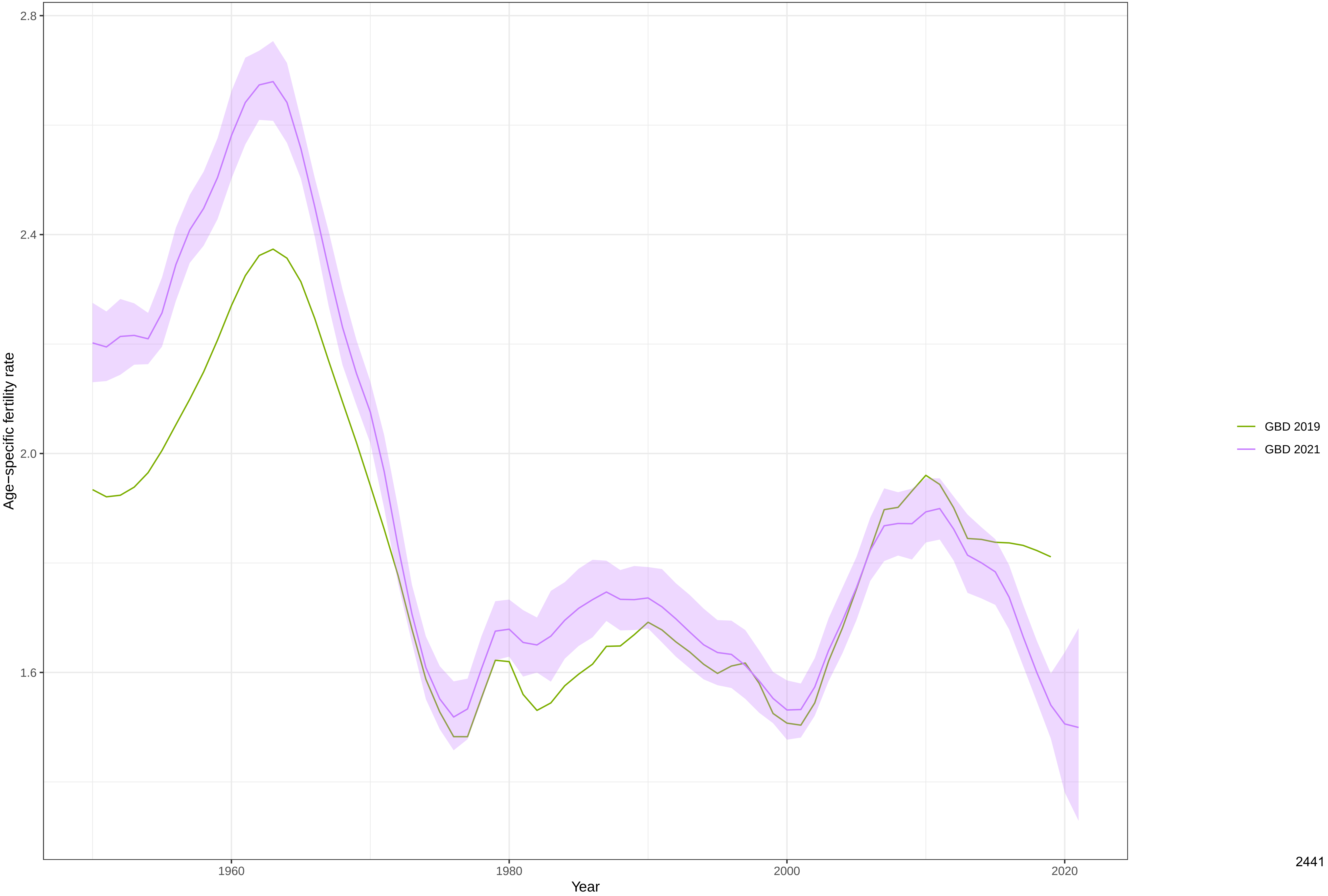

Age−specific fertility fitted trend lines, 1950−2021: Newham, Ages: 10−14
Data points enclosed with a diamond are reference sources

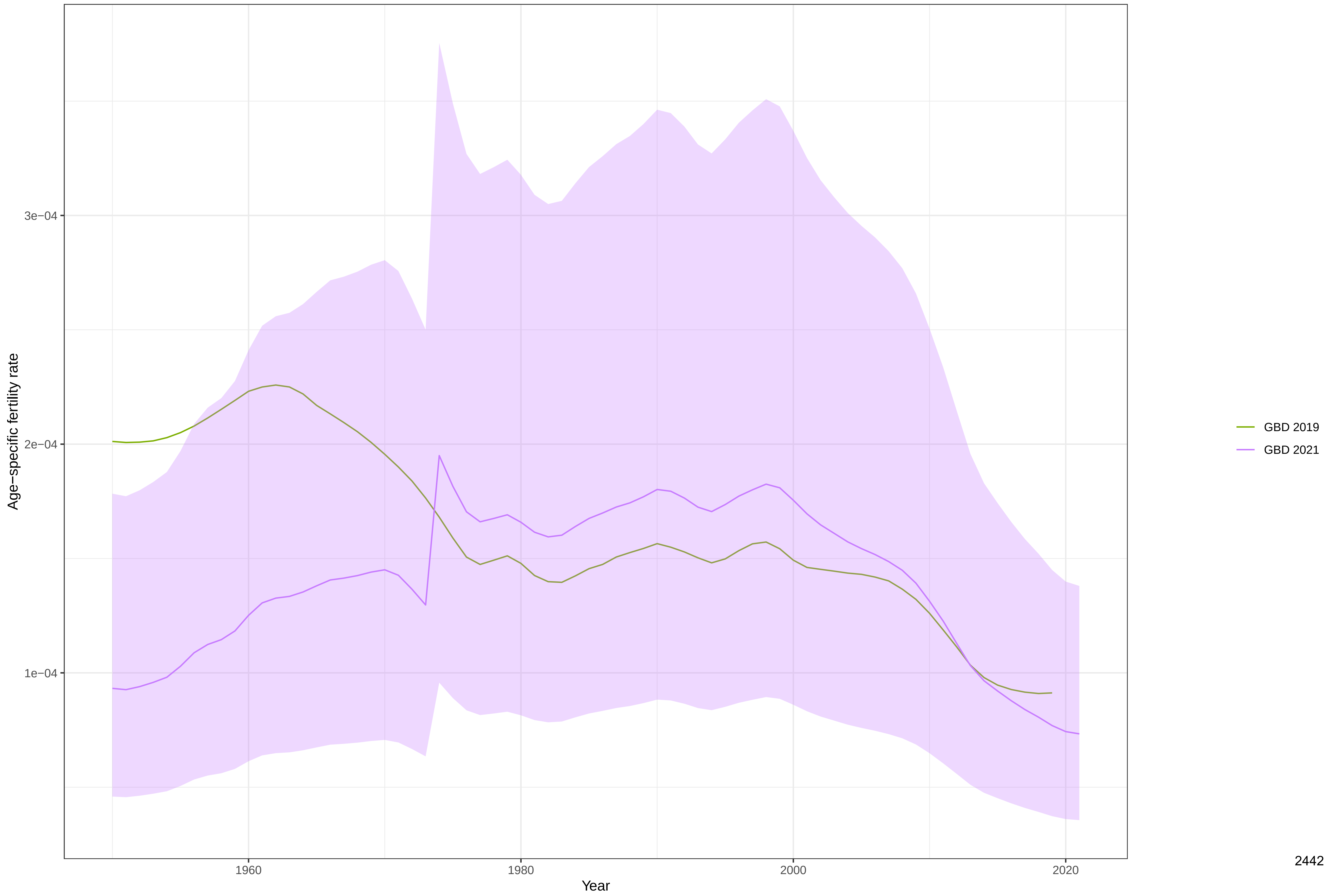

Age−specific fertility fitted trend lines, 1950−2021: Newham, Ages: 15−19
Data points enclosed with a diamond are reference sources

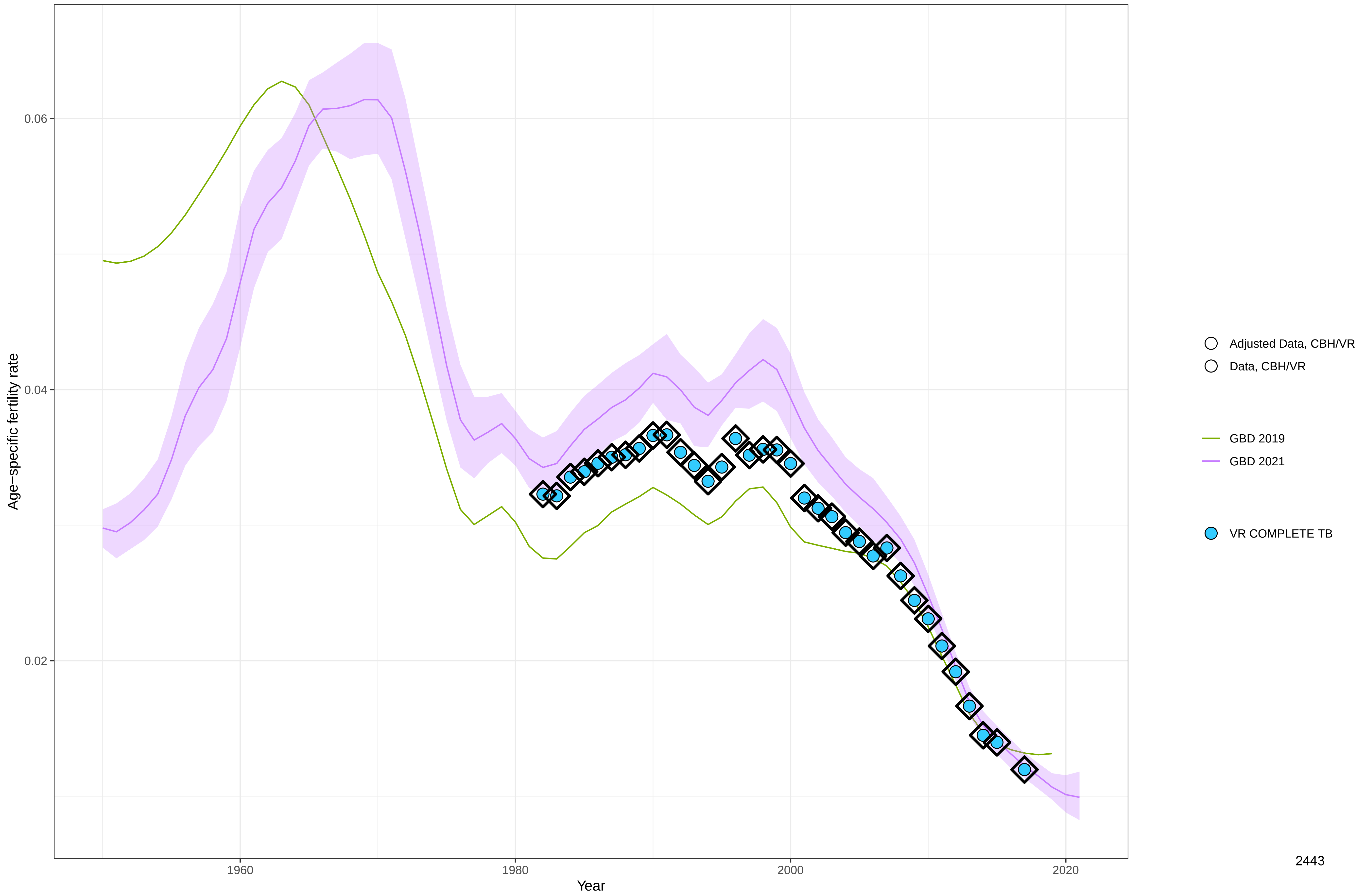

Age−specific fertility fitted trend lines, 1950−2021: Newham, Ages: 20−24
Data points enclosed with a diamond are reference sources

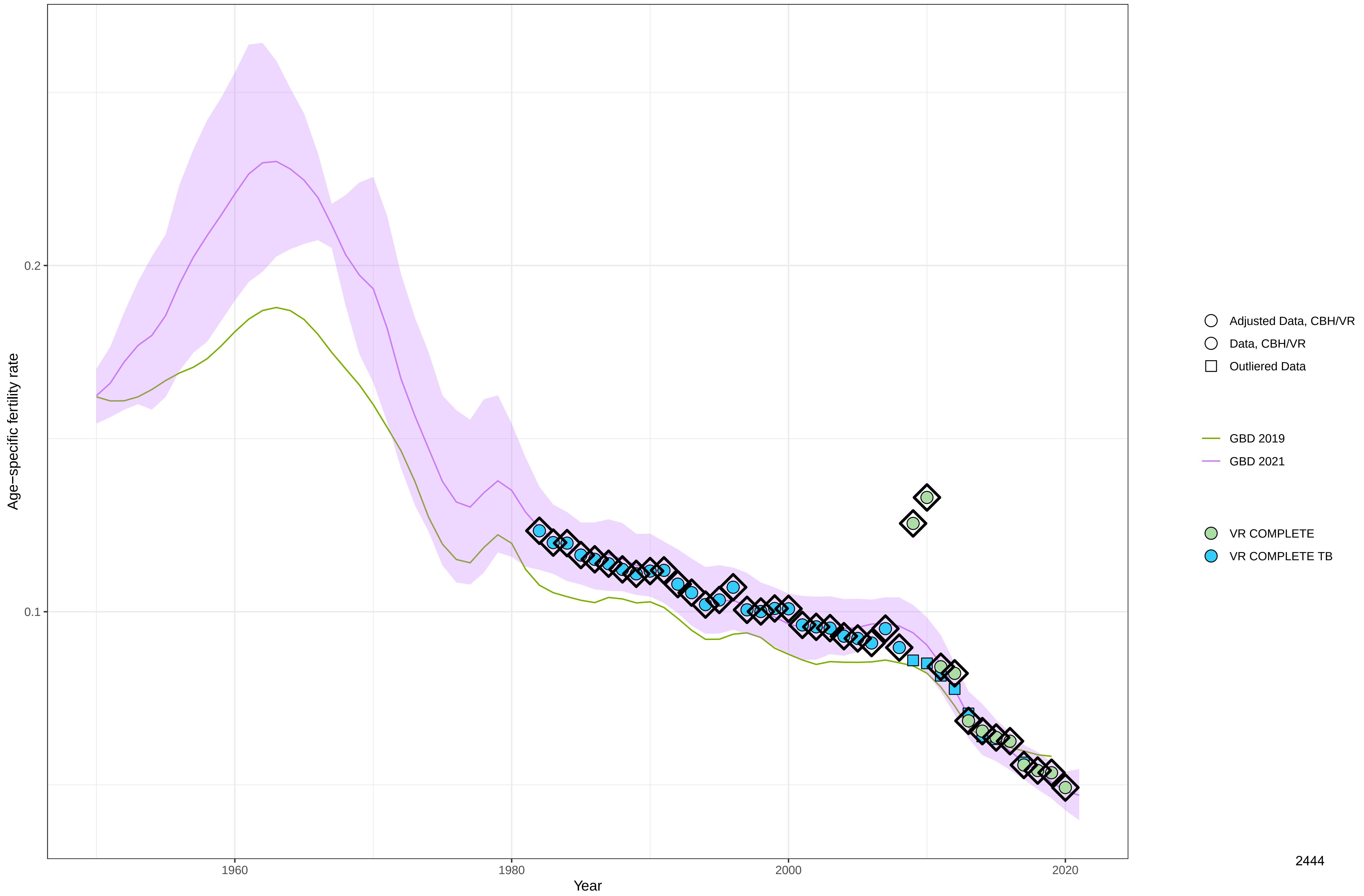

Age−specific fertility fitted trend lines, 1950−2021: Newham, Ages: 25−29
Data points enclosed with a diamond are reference sources

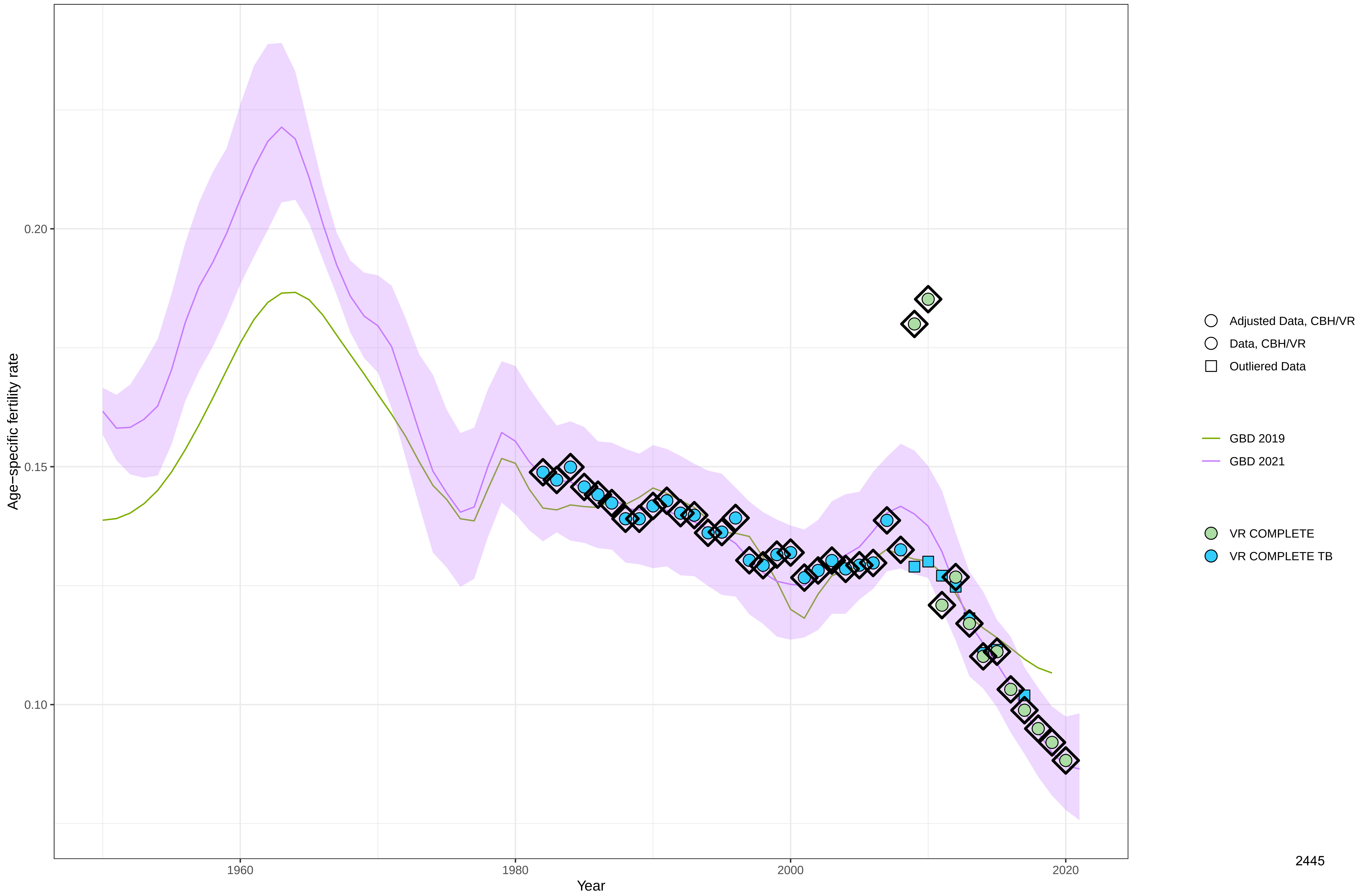

Age−specific fertility fitted trend lines, 1950−2021: Newham, Ages: 30−34
Data points enclosed with a diamond are reference sources

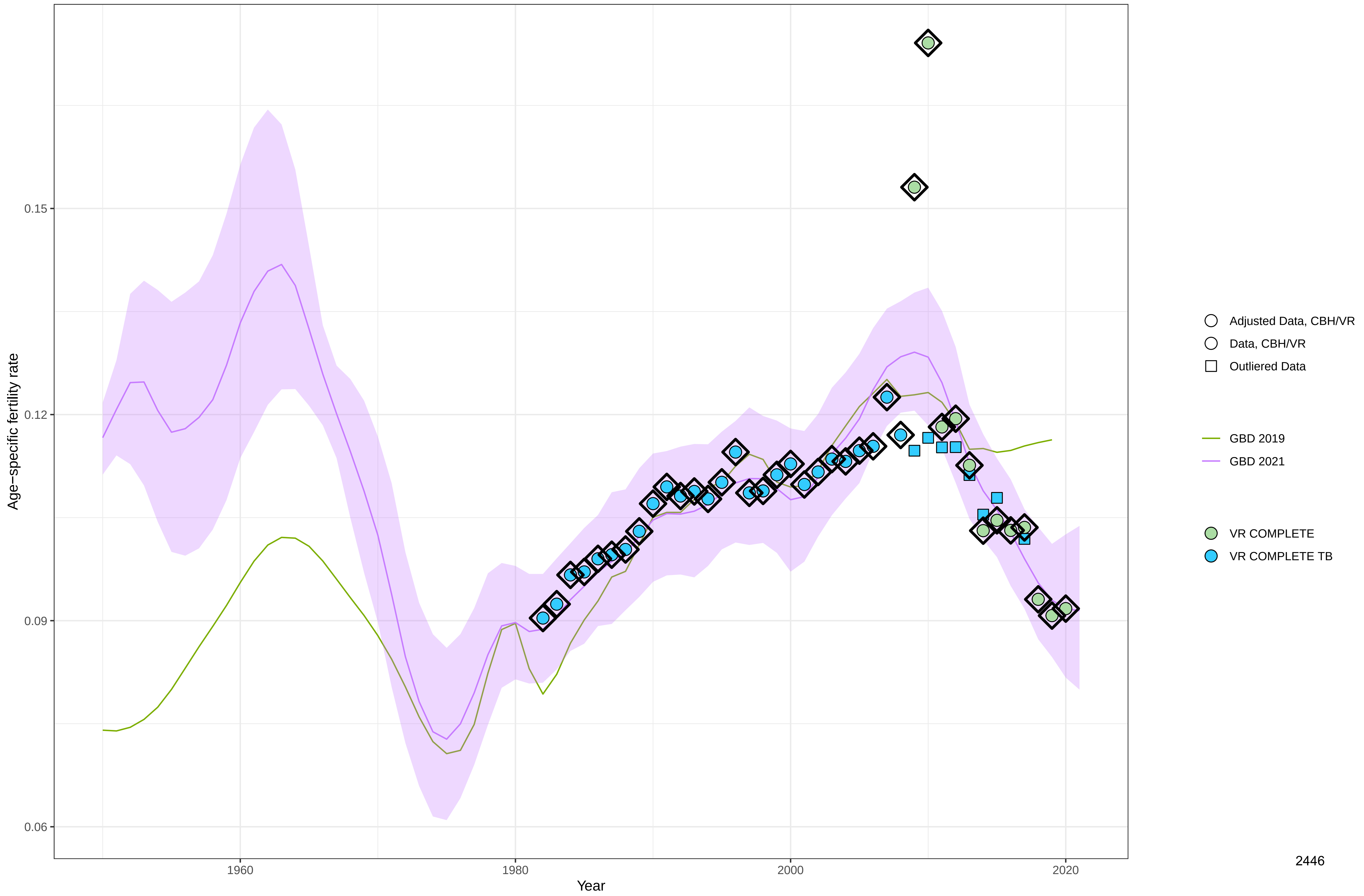

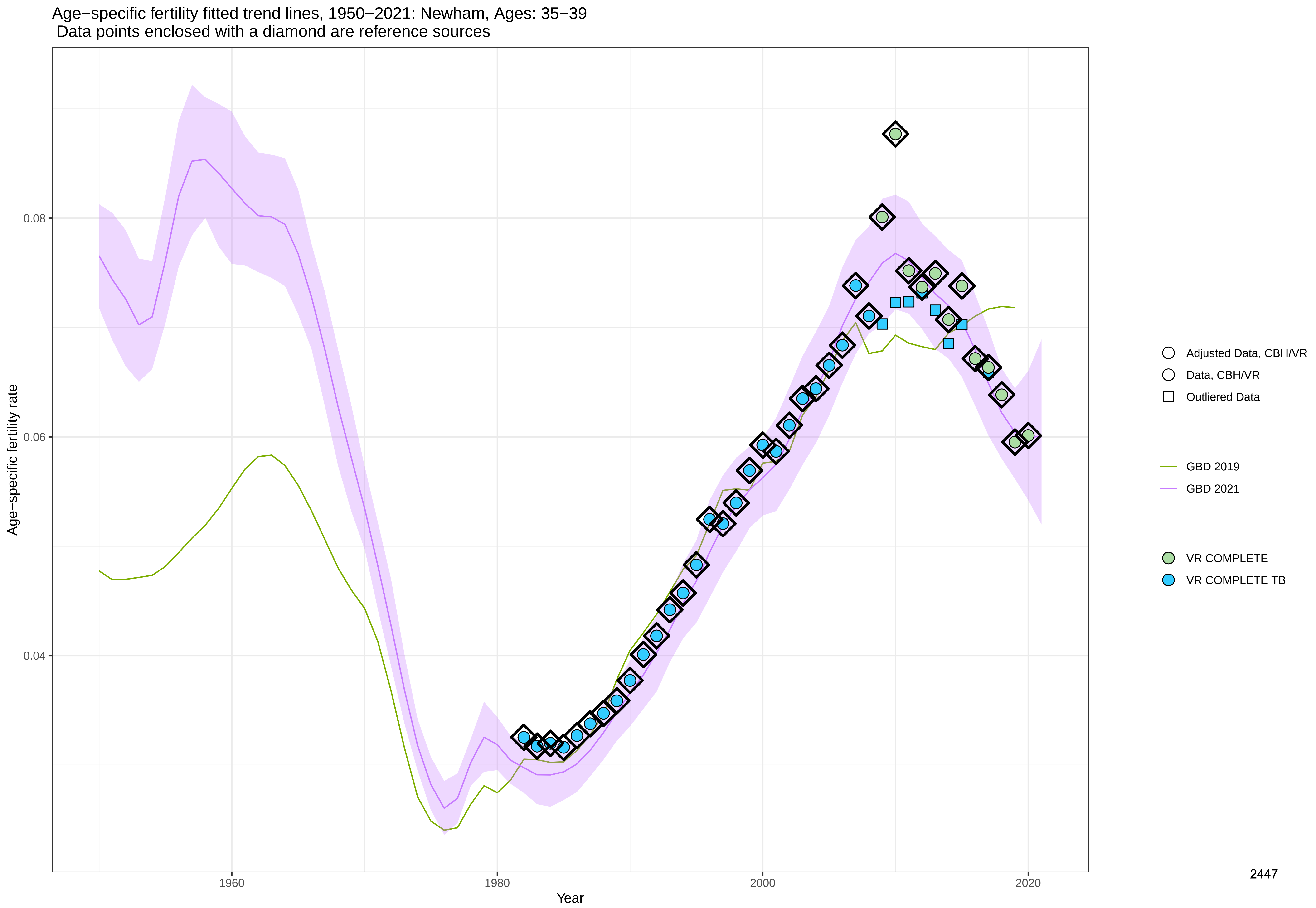
Age−specific fertility fitted trend lines, 1950−2021: Newham, Ages: 35−39
Data points enclosed with a diamond are reference sources
Age−specific fertility rate
0.04
0.06
0.08
Year
1960
1980
2000
2020
Adjusted Data, CBH/VR
Data, CBH/VR
Outliered Data
GBD 2019
GBD 2021
VR COMPLETE
VR COMPLETE TB

Age−specific fertility fitted trend lines, 1950−2021: Newham, Ages: 40−44
Data points enclosed with a diamond are reference sources

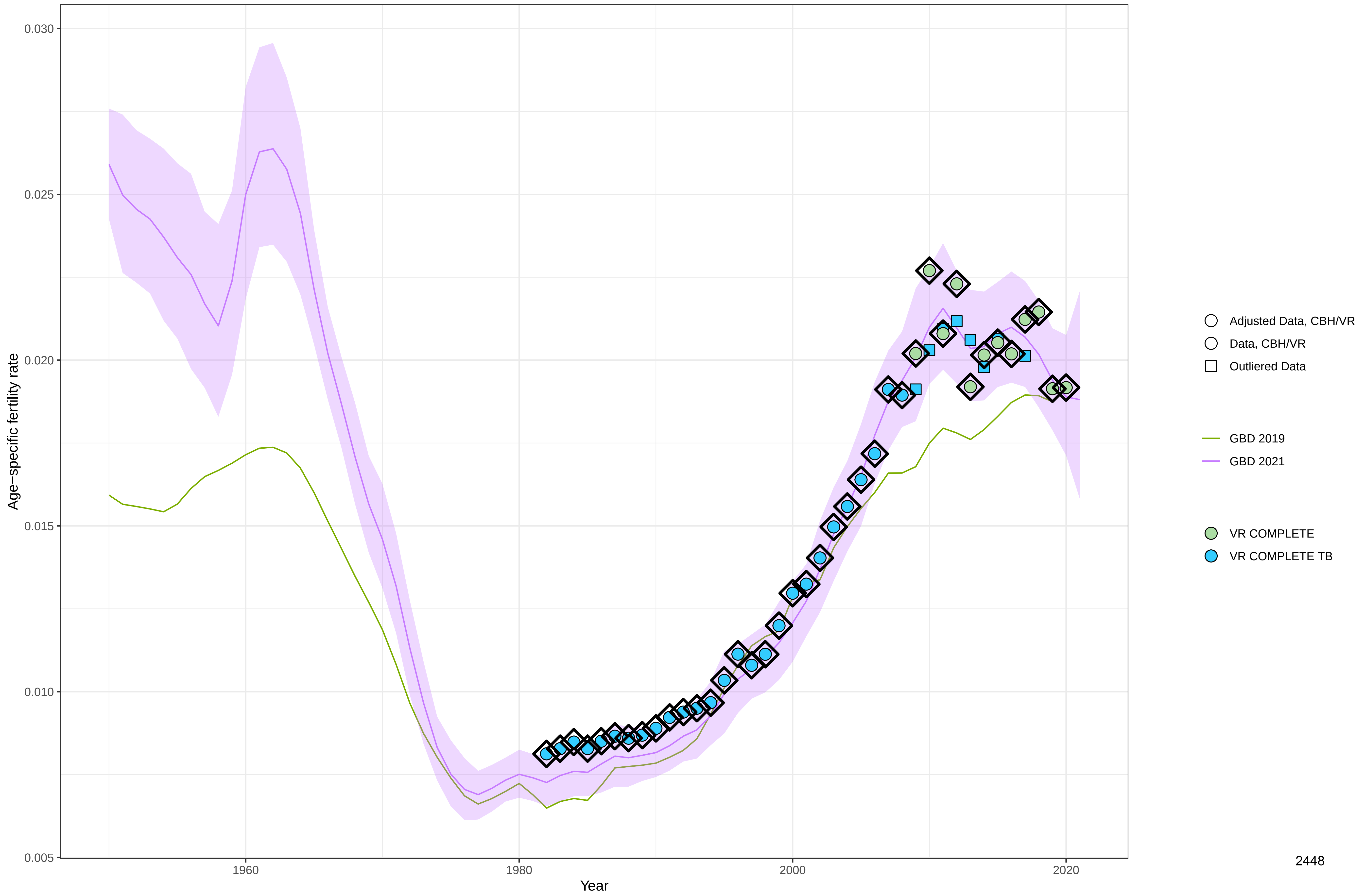

Age−specific fertility fitted trend lines, 1950−2021: Newham, Ages: 45−49
Data points enclosed with a diamond are reference sources

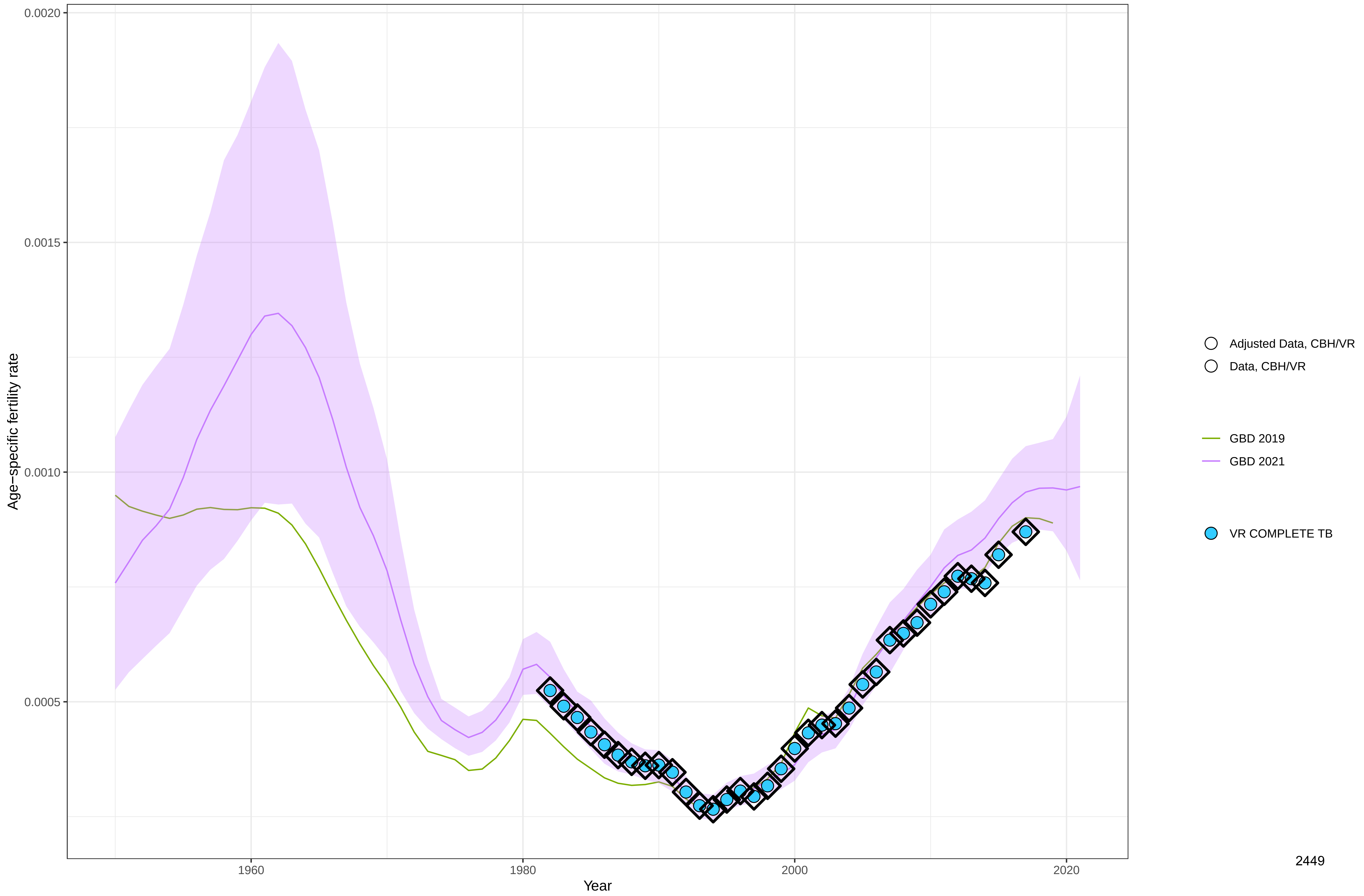

Age−specific fertility fitted trend lines, 1950−2021: Newham, Ages: 50−54
Data points enclosed with a diamond are reference sources

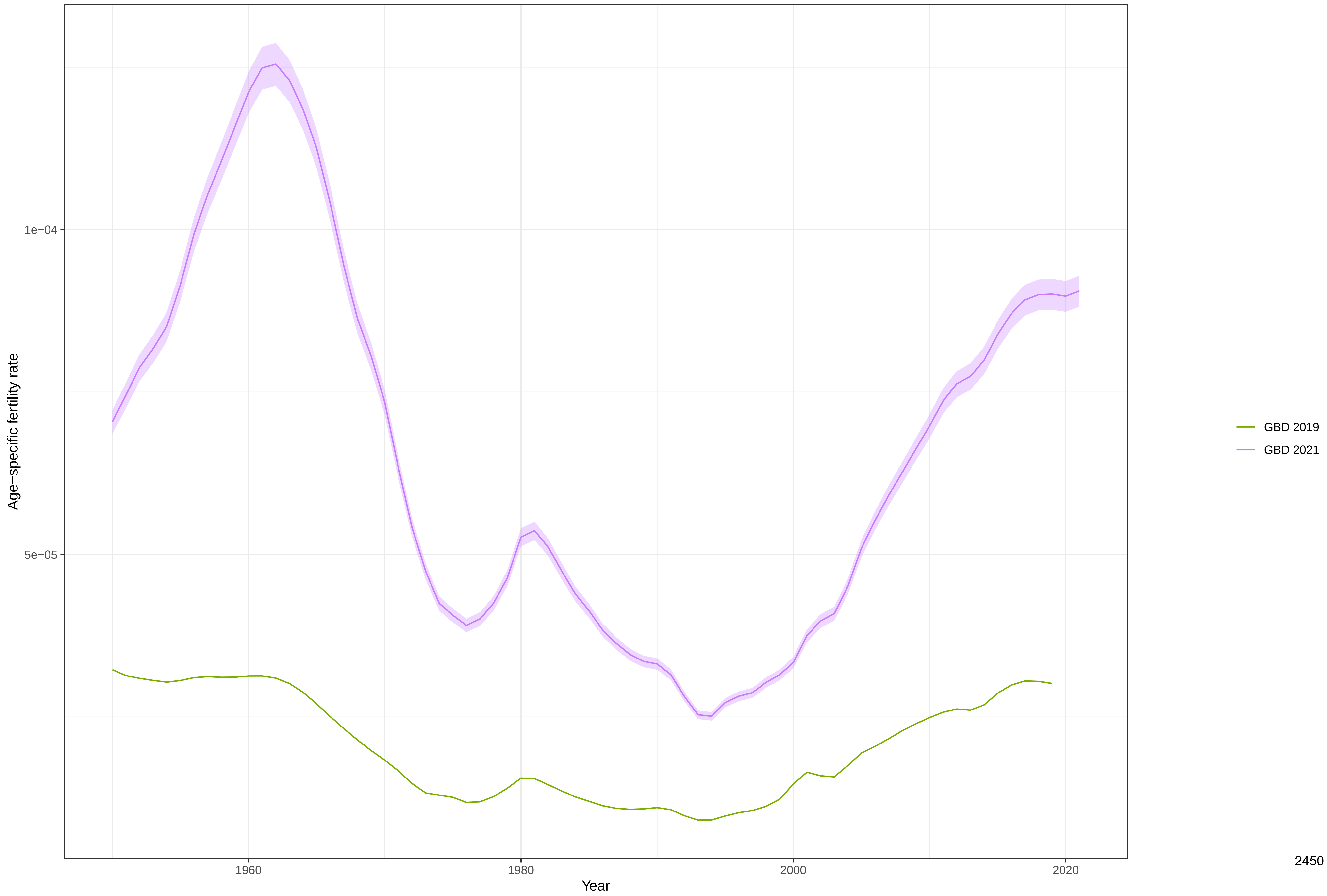

Total fertility rate fitted trend lines, 1950−2021: Newham TFR
Data points enclosed with a diamond are reference sources

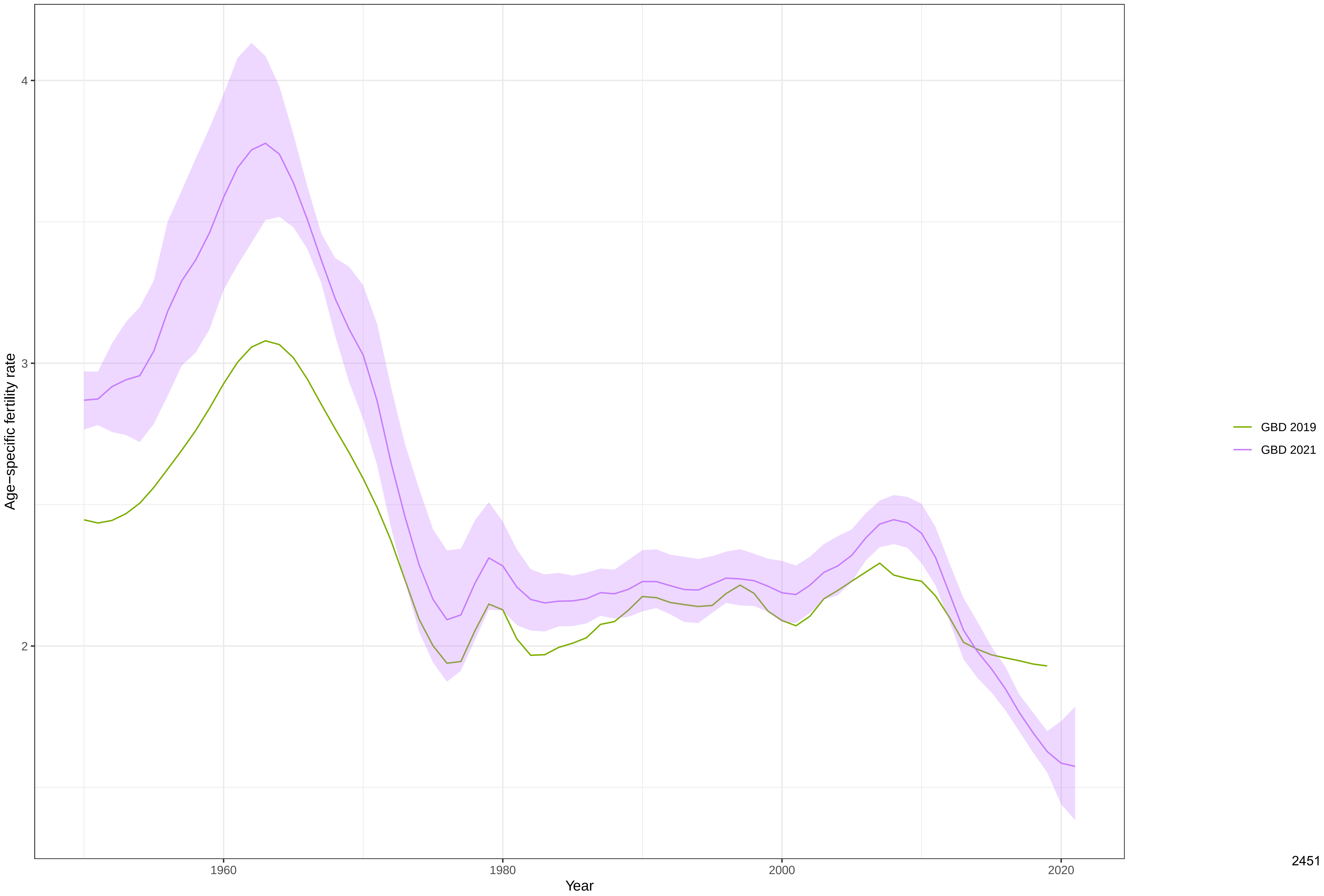

Age−specific fertility fitted trend lines, 1950−2021: Redbridge, Ages: 10−14
Data points enclosed with a diamond are reference sources

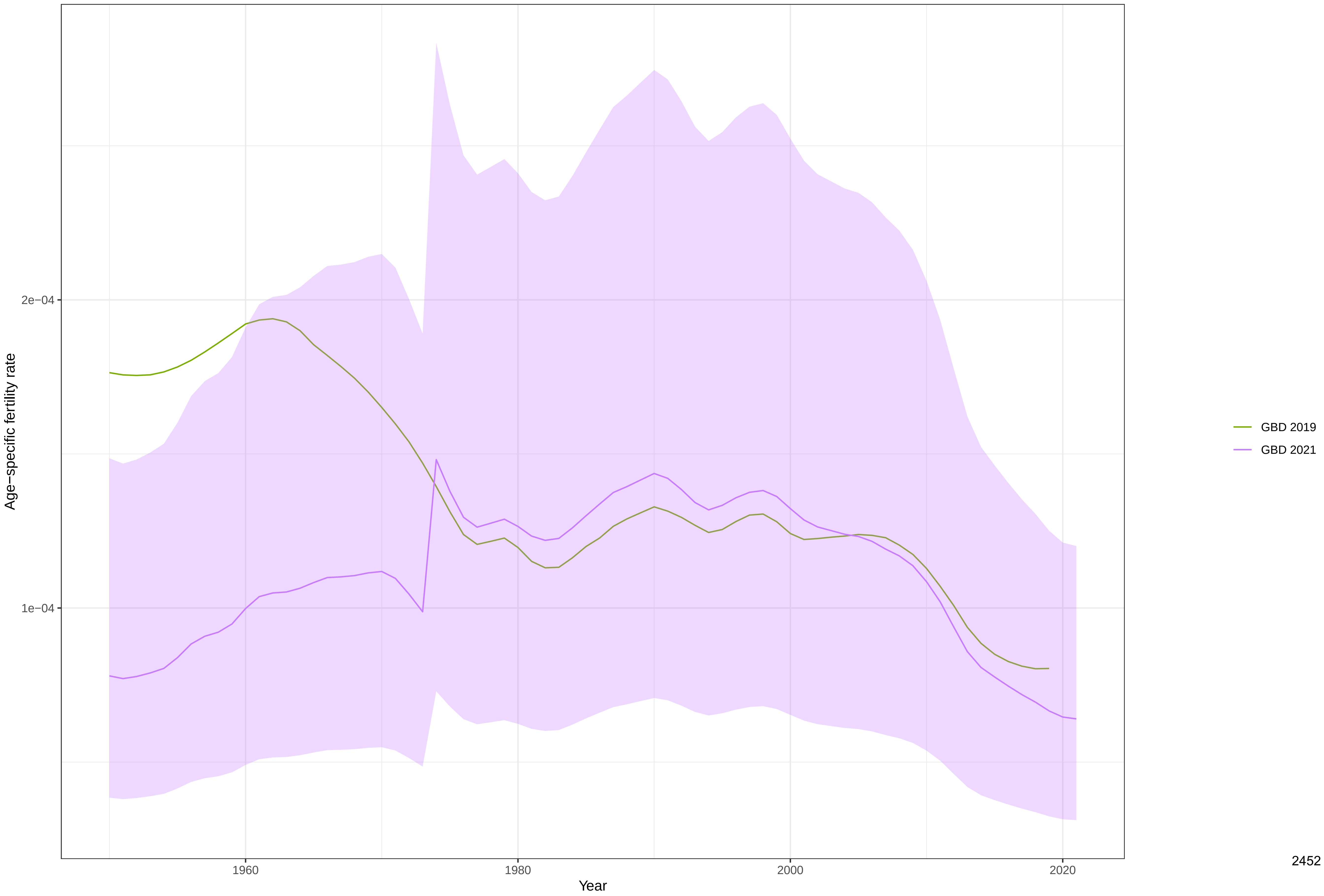

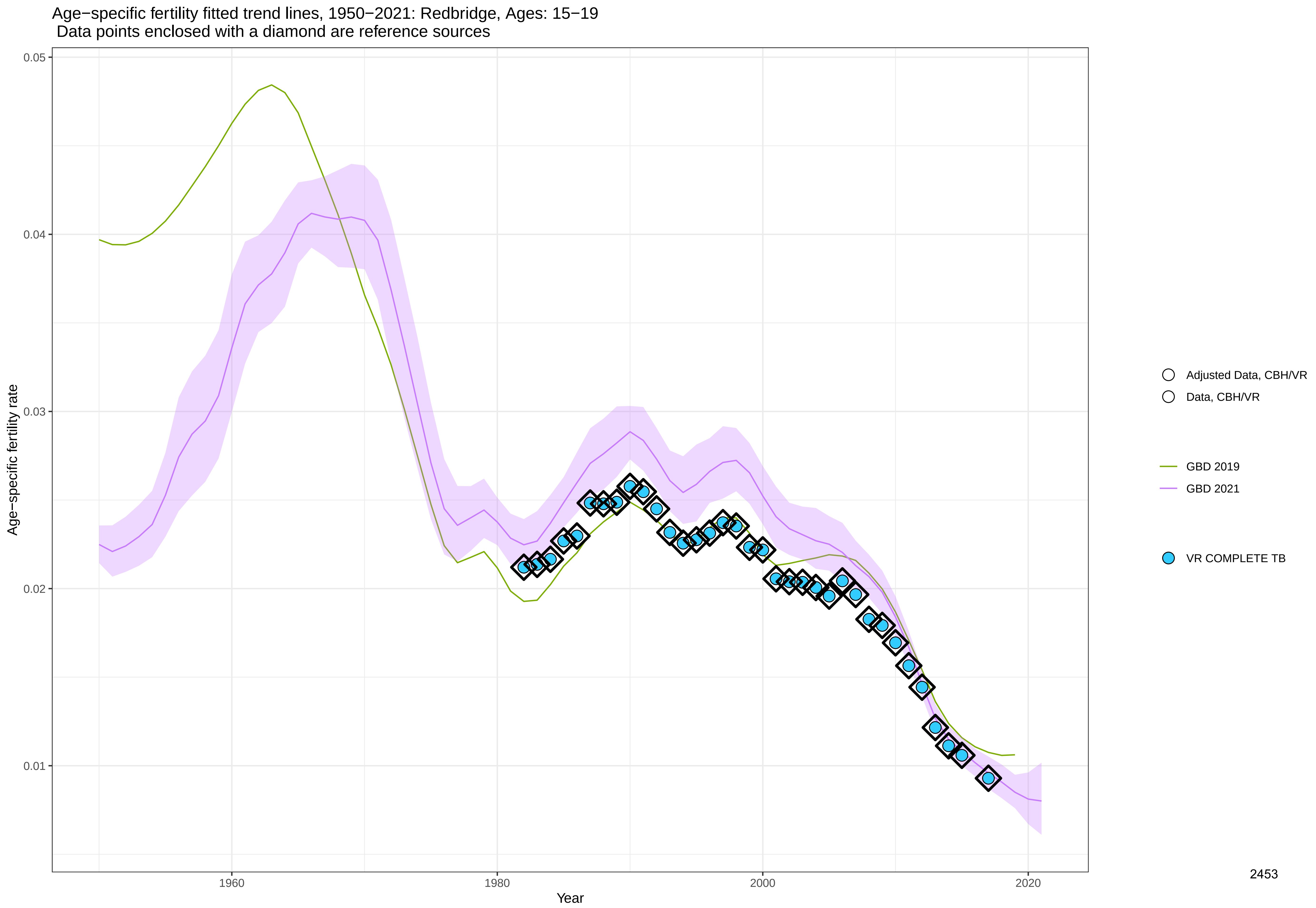

Age−specific fertility fitted trend lines, 1950−2021: Redbridge, Ages: 15−19
Data points enclosed with a diamond are reference sources
Age−specific fertility rate
Year
0.05
0.04
0.03
0.02
0.01
1960
1980
2000
2020
Adjusted Data, CBH/VR
Data, CBH/VR
GBD 2019
GBD 2021
VR COMPLETE TB

Age−specific fertility fitted trend lines, 1950−2021: Redbridge, Ages: 20−24
Data points enclosed with a diamond are reference sources

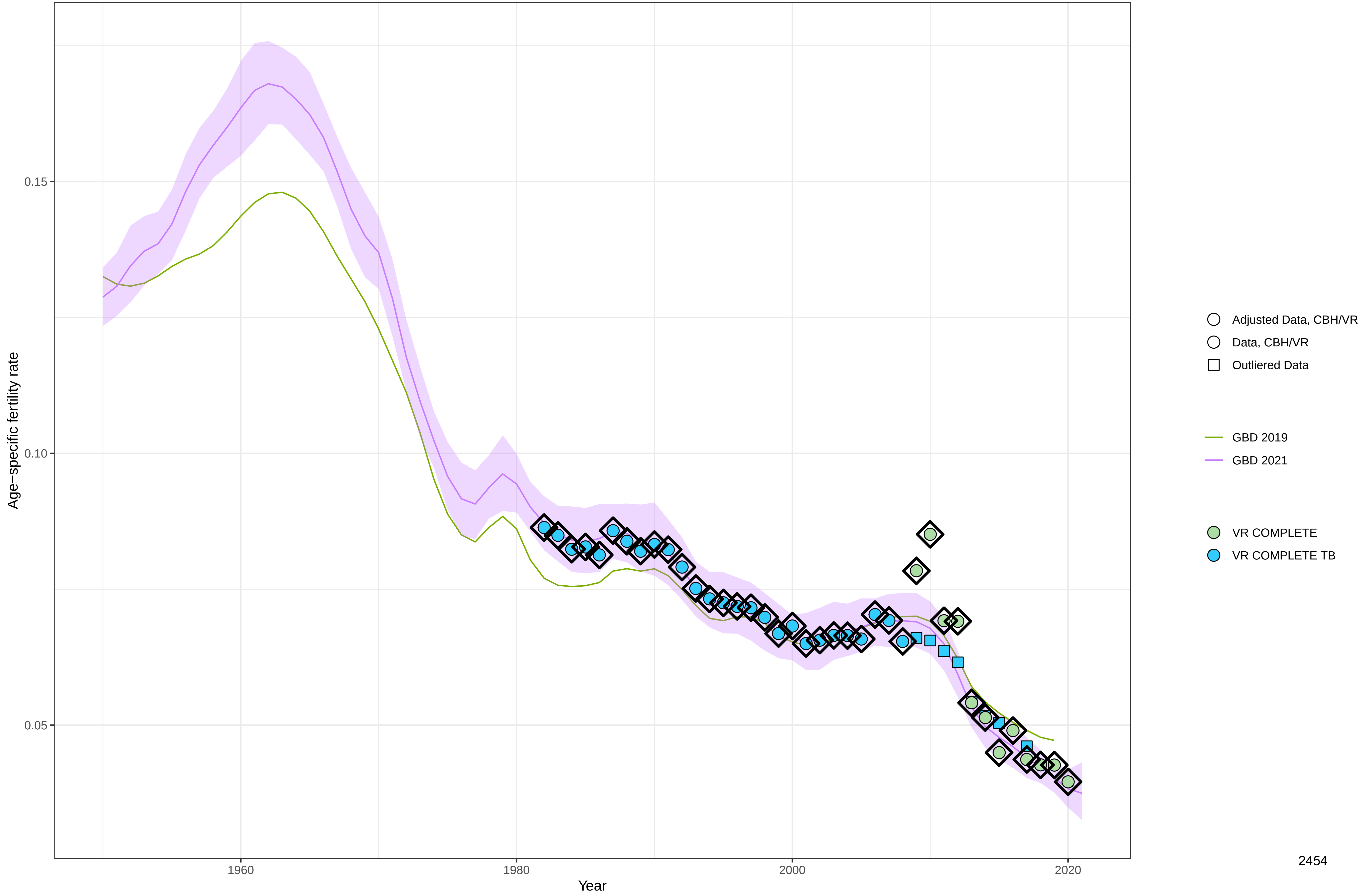

Age−specific fertility fitted trend lines, 1950−2021: Redbridge, Ages: 25−29
Data points enclosed with a diamond are reference sources

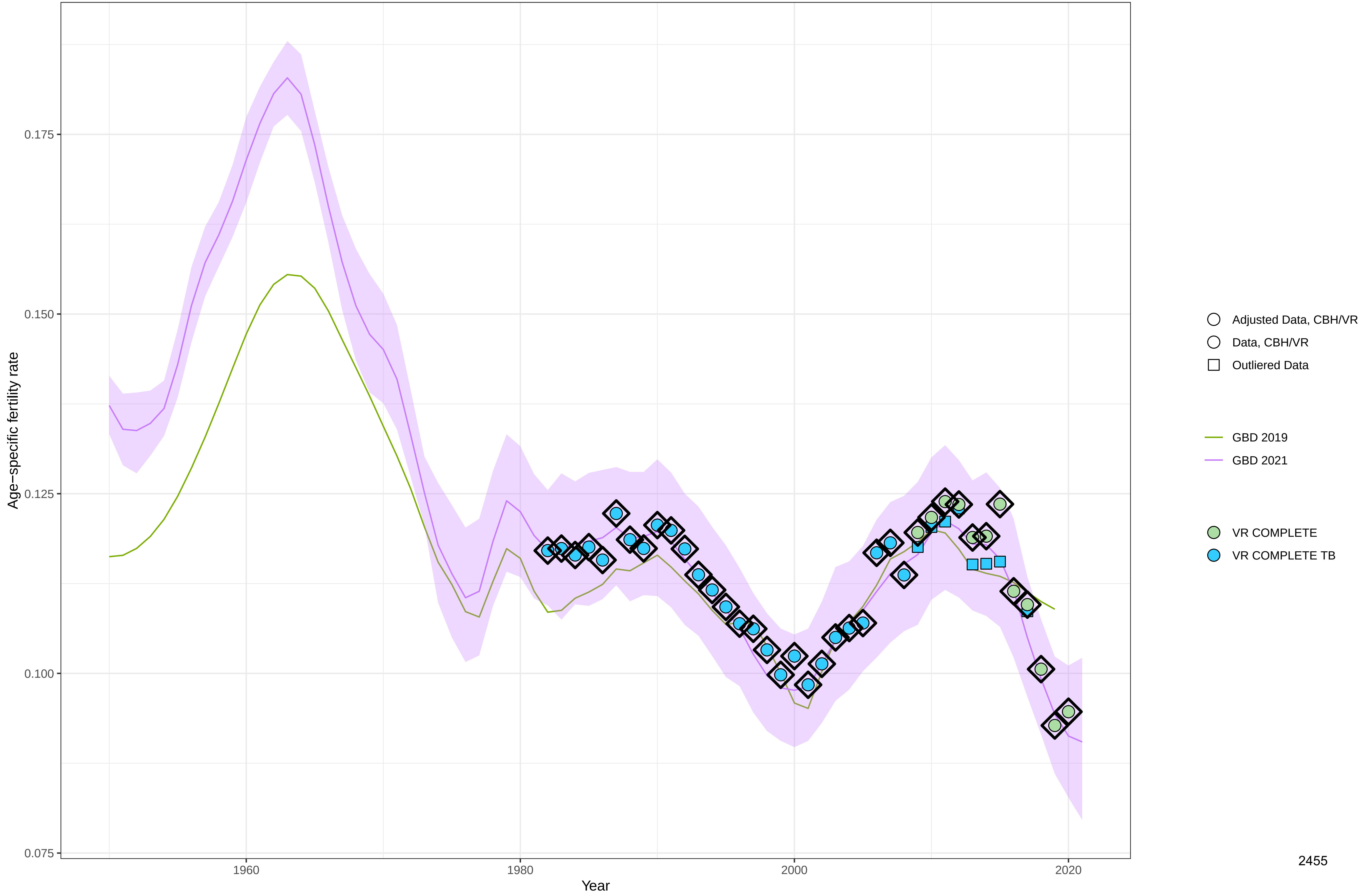

Age−specific fertility fitted trend lines, 1950−2021: Redbridge, Ages: 30−34
Data points enclosed with a diamond are reference sources

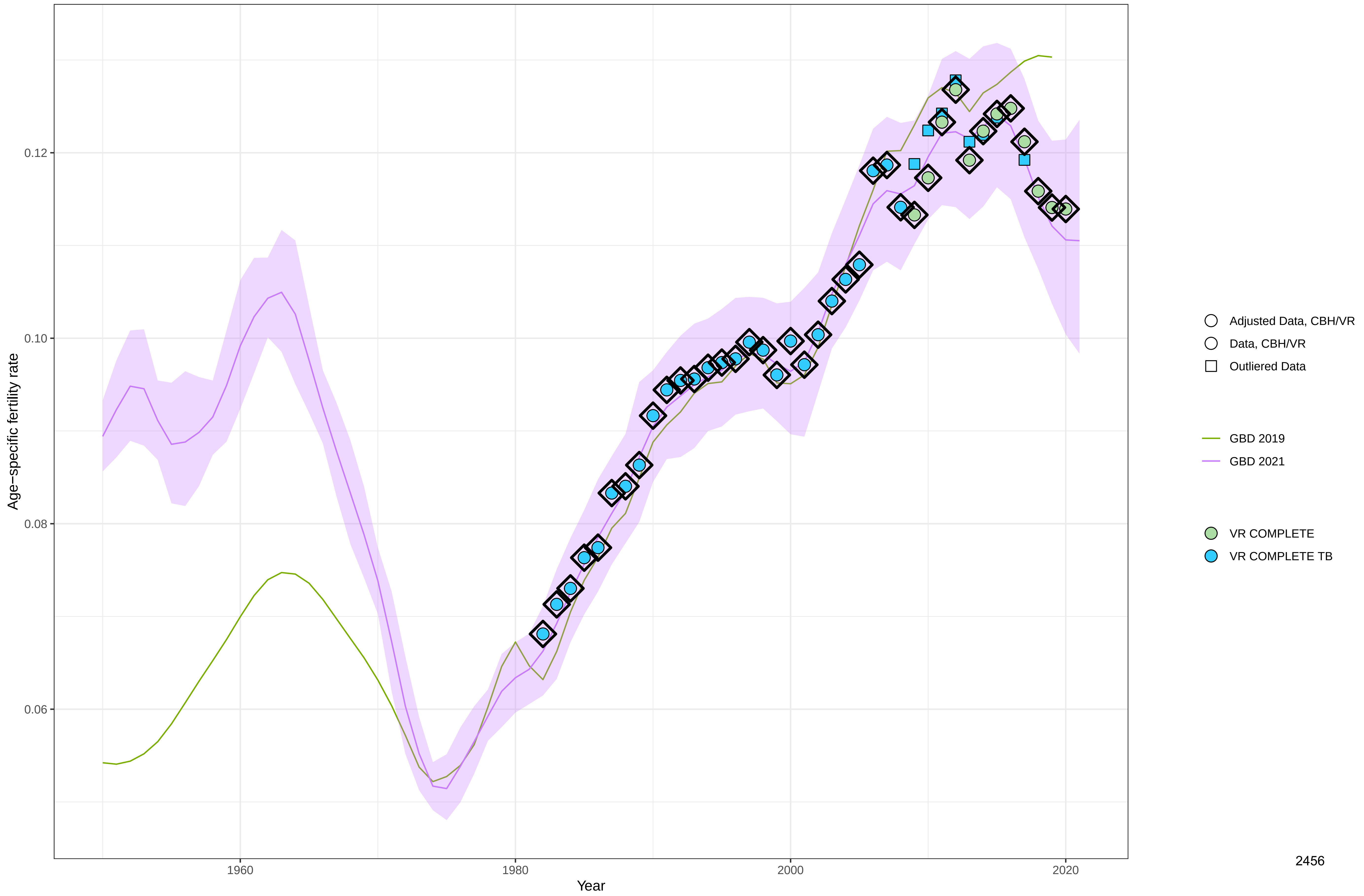

Age−specific fertility fitted trend lines, 1950−2021: Redbridge, Ages: 35−39
Data points enclosed with a diamond are reference sources

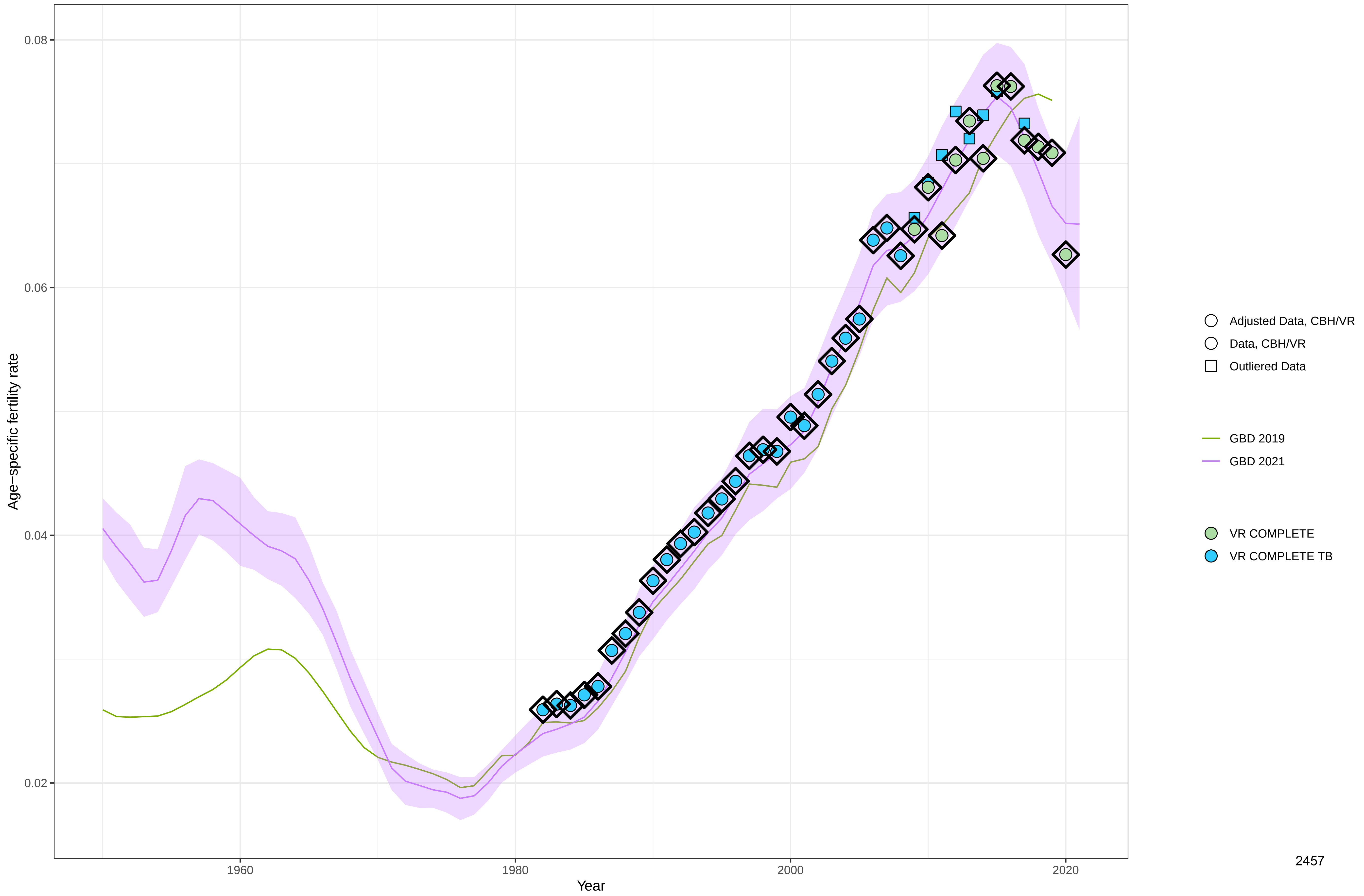

Age−specific fertility fitted trend lines, 1950−2021: Redbridge, Ages: 40−44
Data points enclosed with a diamond are reference sources

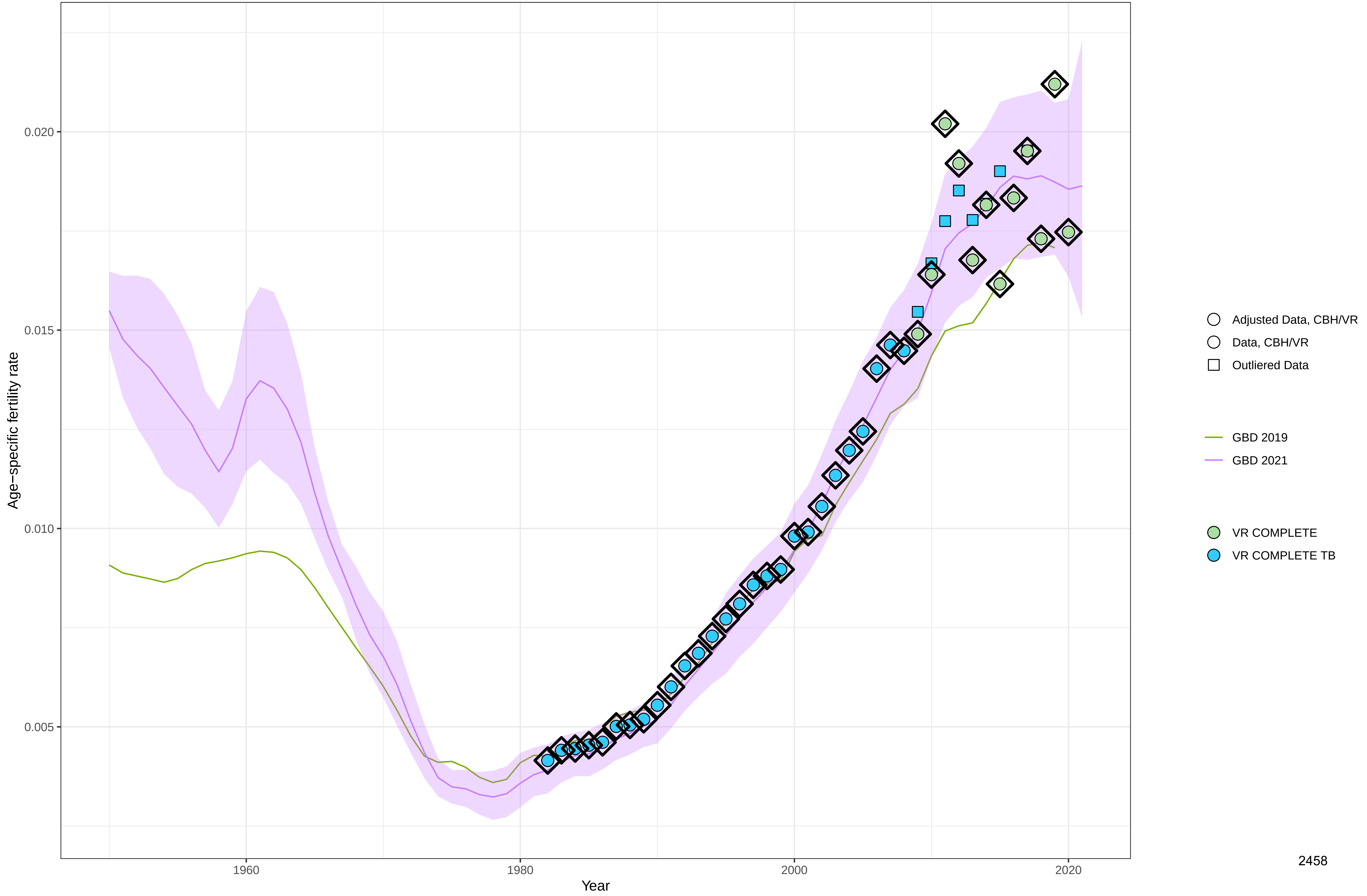

Age−specific fertility fitted trend lines, 1950−2021: Redbridge, Ages: 45−49
Data points enclosed with a diamond are reference sources

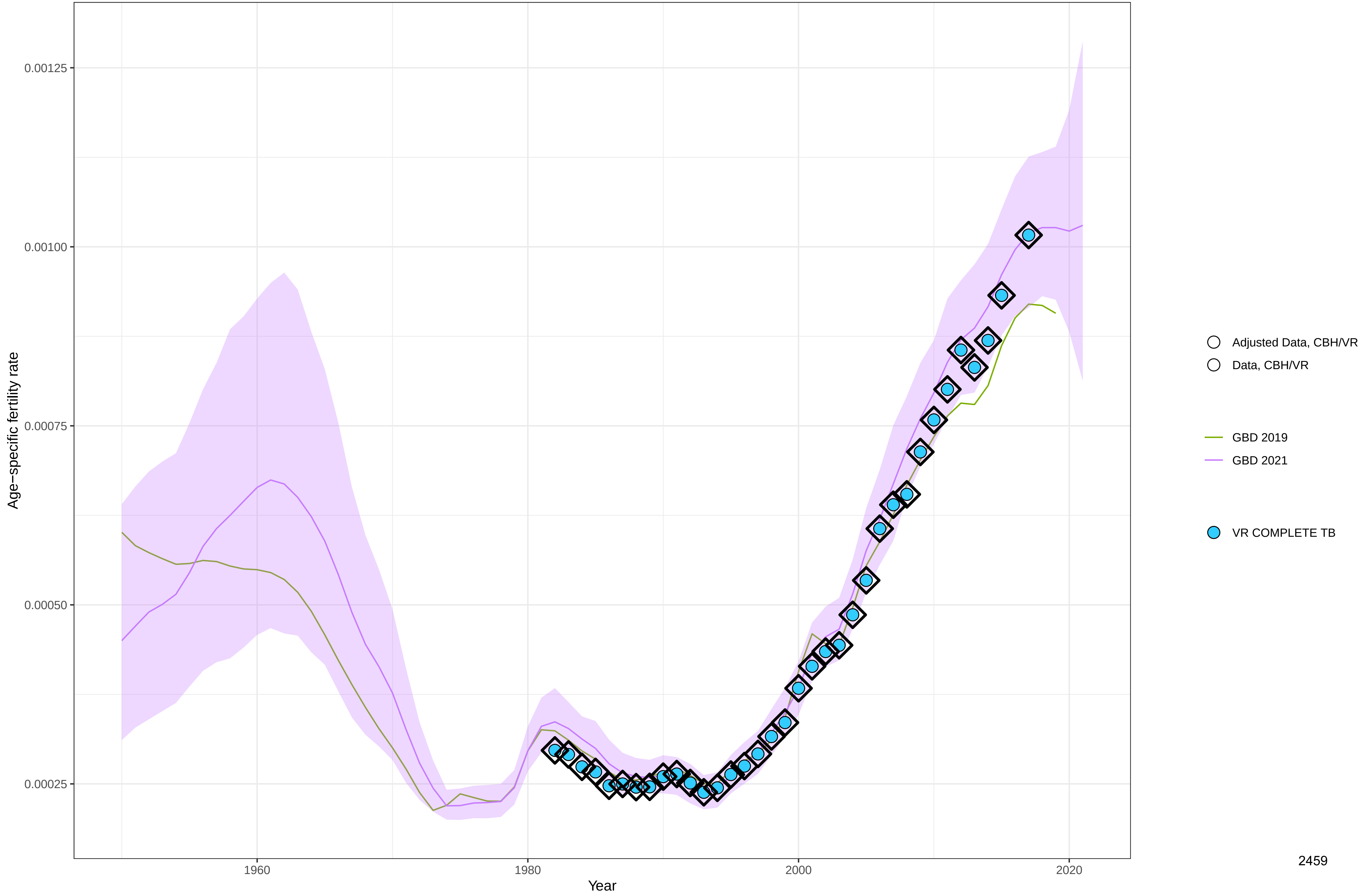

Age−specific fertility fitted trend lines, 1950−2021: Redbridge, Ages: 50−54
Data points enclosed with a diamond are reference sources

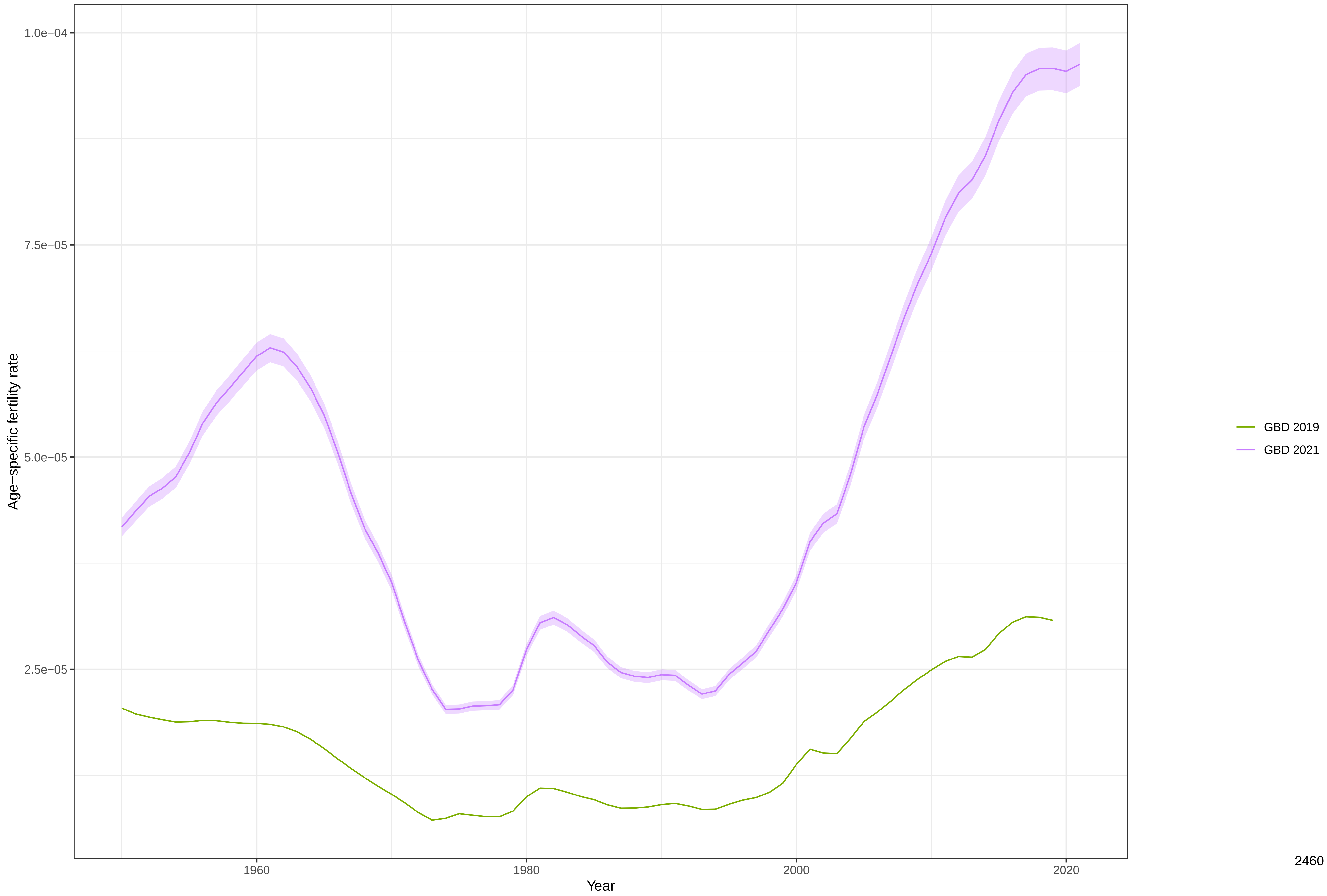

Total fertility rate fitted trend lines, 1950−2021: Redbridge TFR
Data points enclosed with a diamond are reference sources

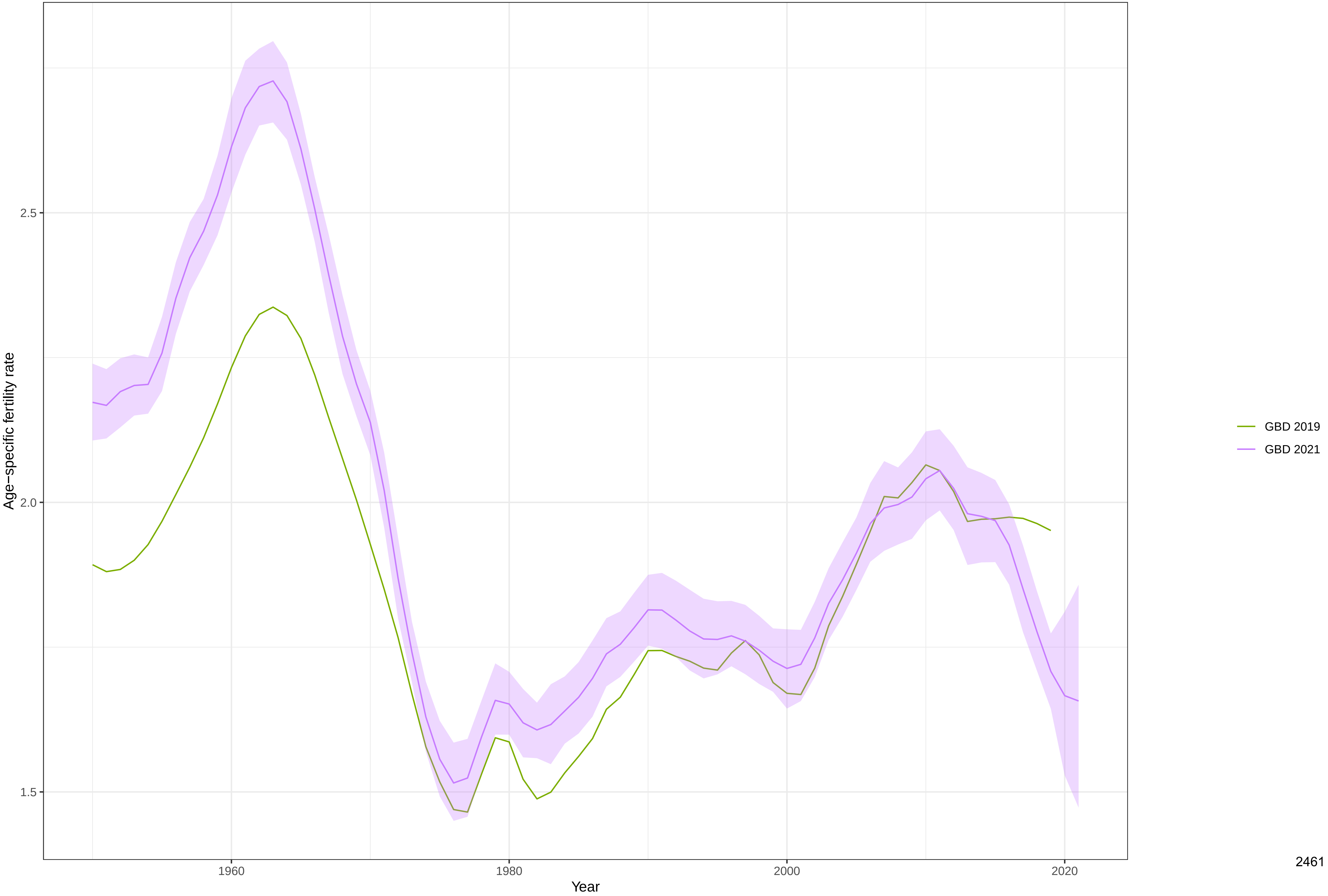

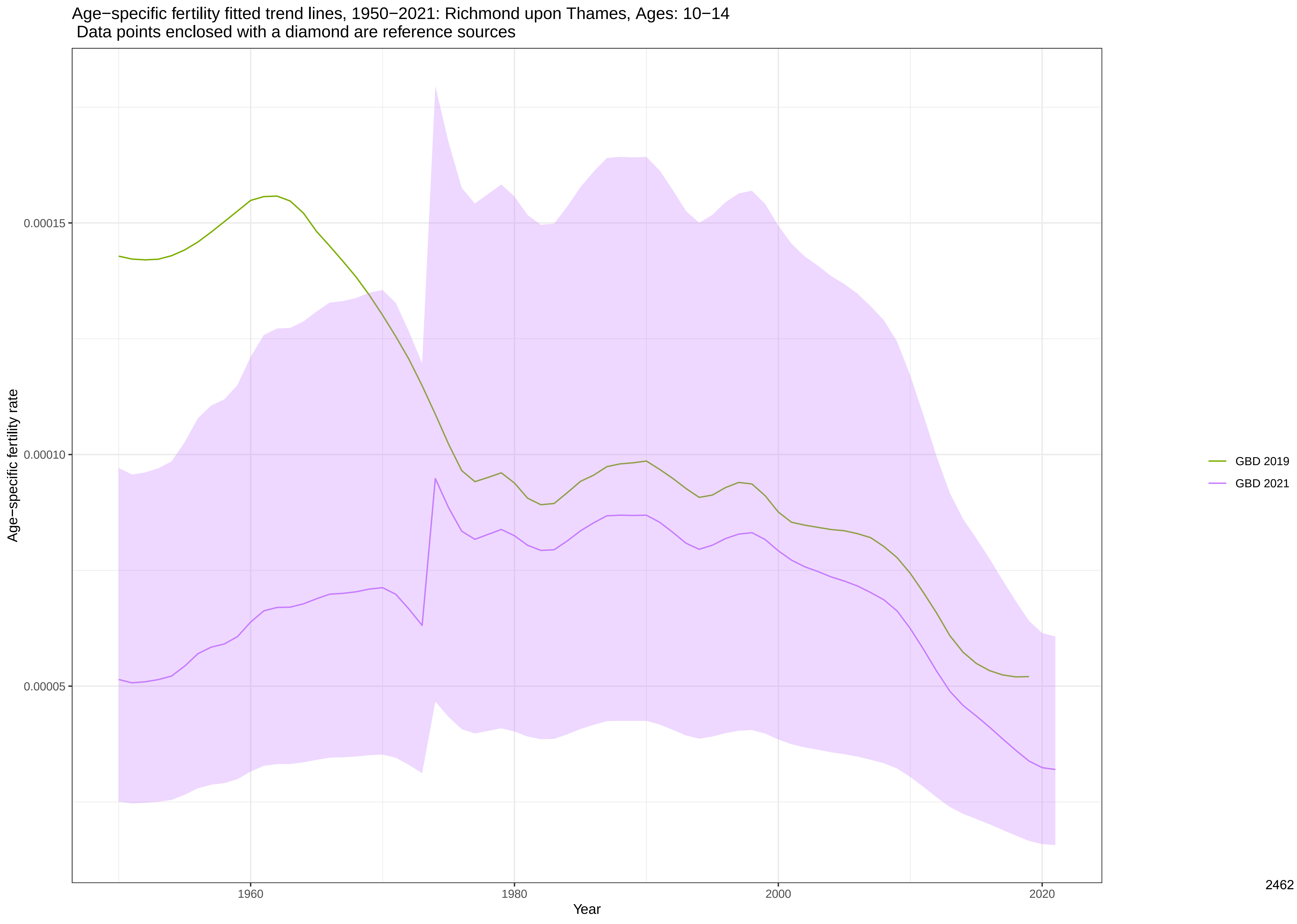

Age−specific fertility fitted trend lines, 1950−2021: Richmond upon Thames, Ages: 10−14
Data points enclosed with a diamond are reference sources
Age−specific fertility rate
0.00015
0.00010
0.00005
1960
1980
2000
2020
Year
GBD 2019
GBD 2021

Age−specific fertility fitted trend lines, 1950−2021: Richmond upon Thames, Ages: 15−19
Data points enclosed with a diamond are reference sources

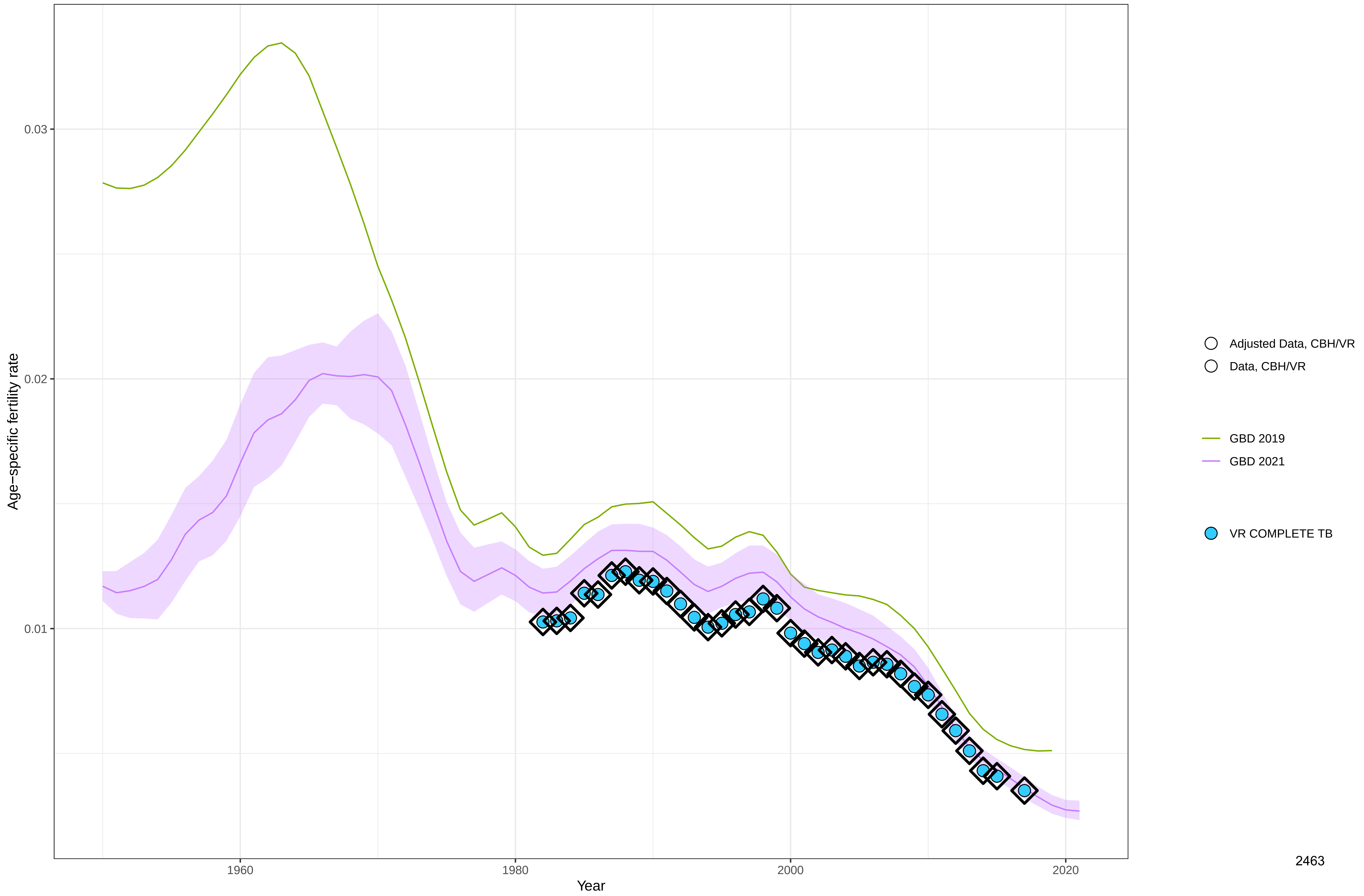

Age−specific fertility fitted trend lines, 1950−2021: Richmond upon Thames, Ages: 20−24
Data points enclosed with a diamond are reference sources

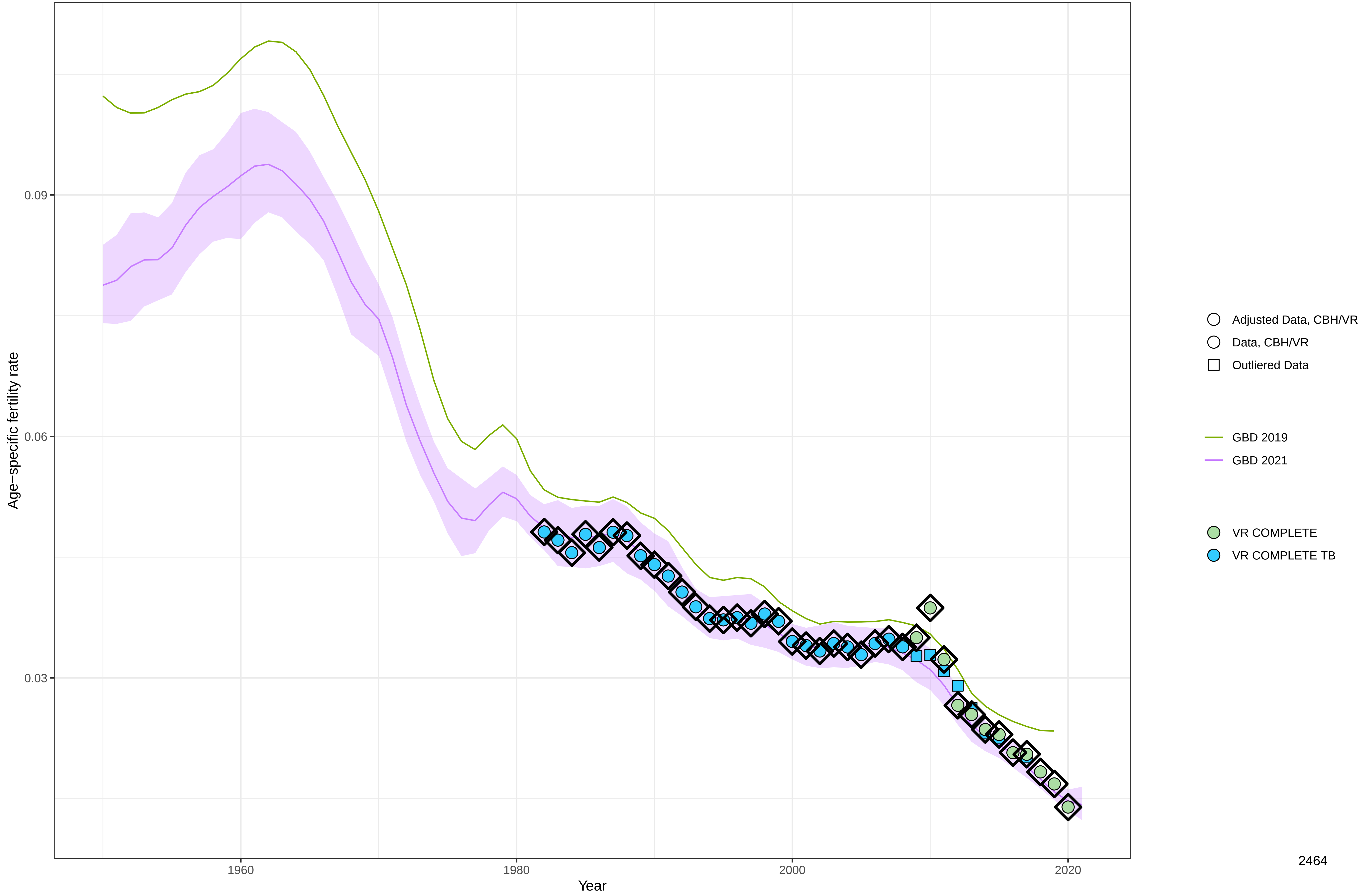

Age−specific fertility fitted trend lines, 1950−2021: Richmond upon Thames, Ages: 25−29
Data points enclosed with a diamond are reference sources

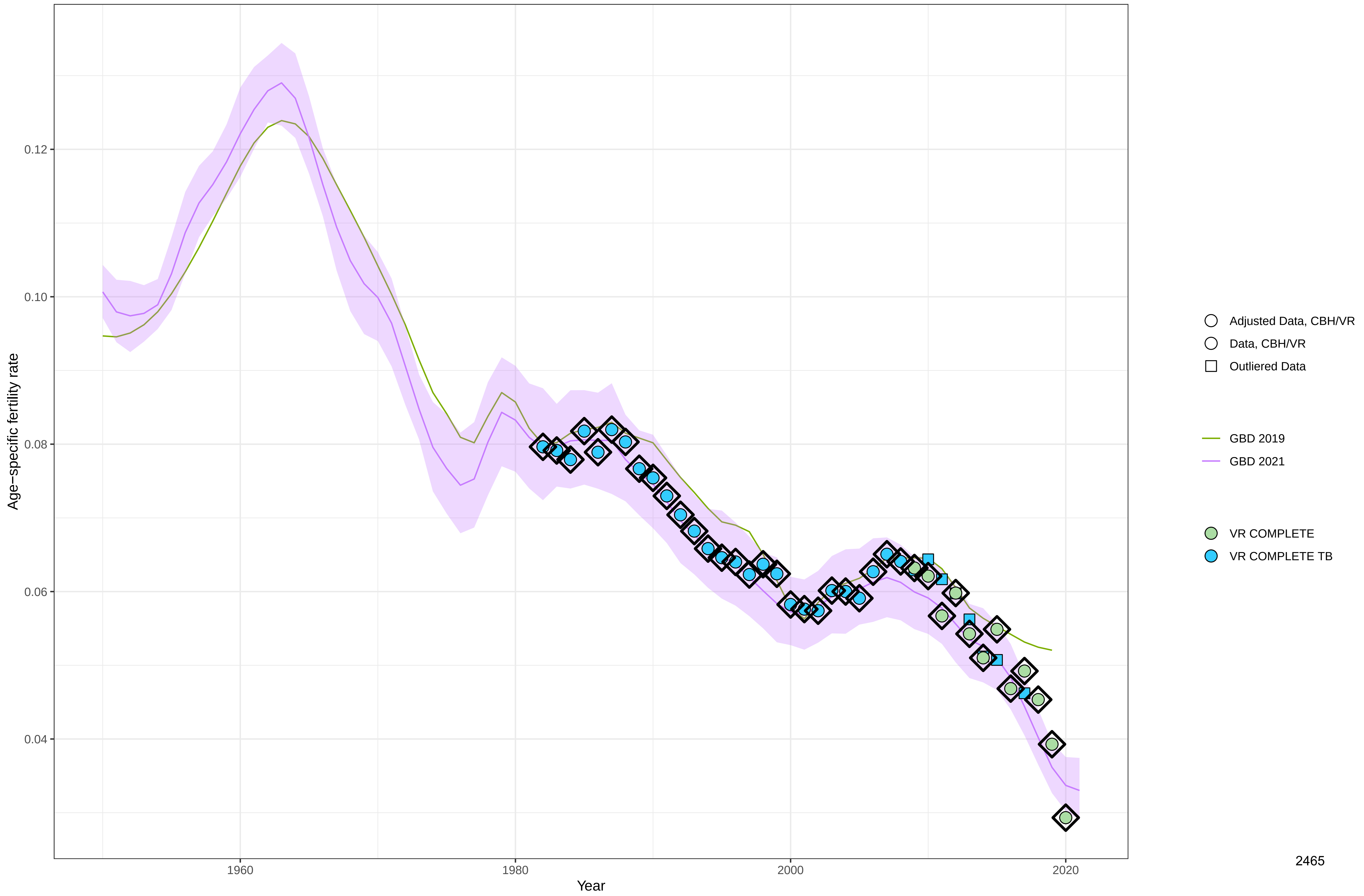

Age−specific fertility fitted trend lines, 1950−2021: Richmond upon Thames, Ages: 30−34
Data points enclosed with a diamond are reference sources

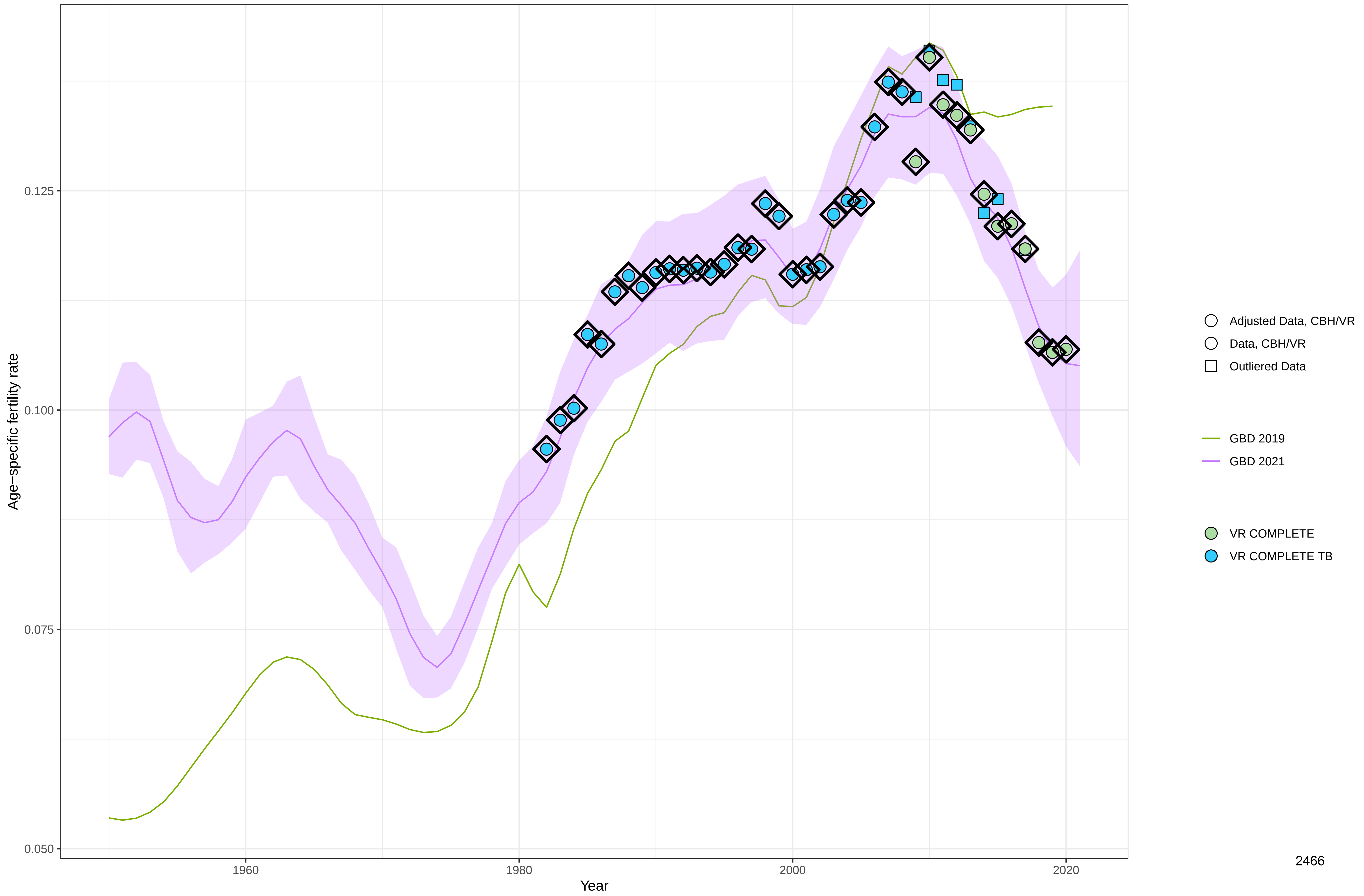

Age−specific fertility fitted trend lines, 1950−2021: Richmond upon Thames, Ages: 35−39
Data points enclosed with a diamond are reference sources

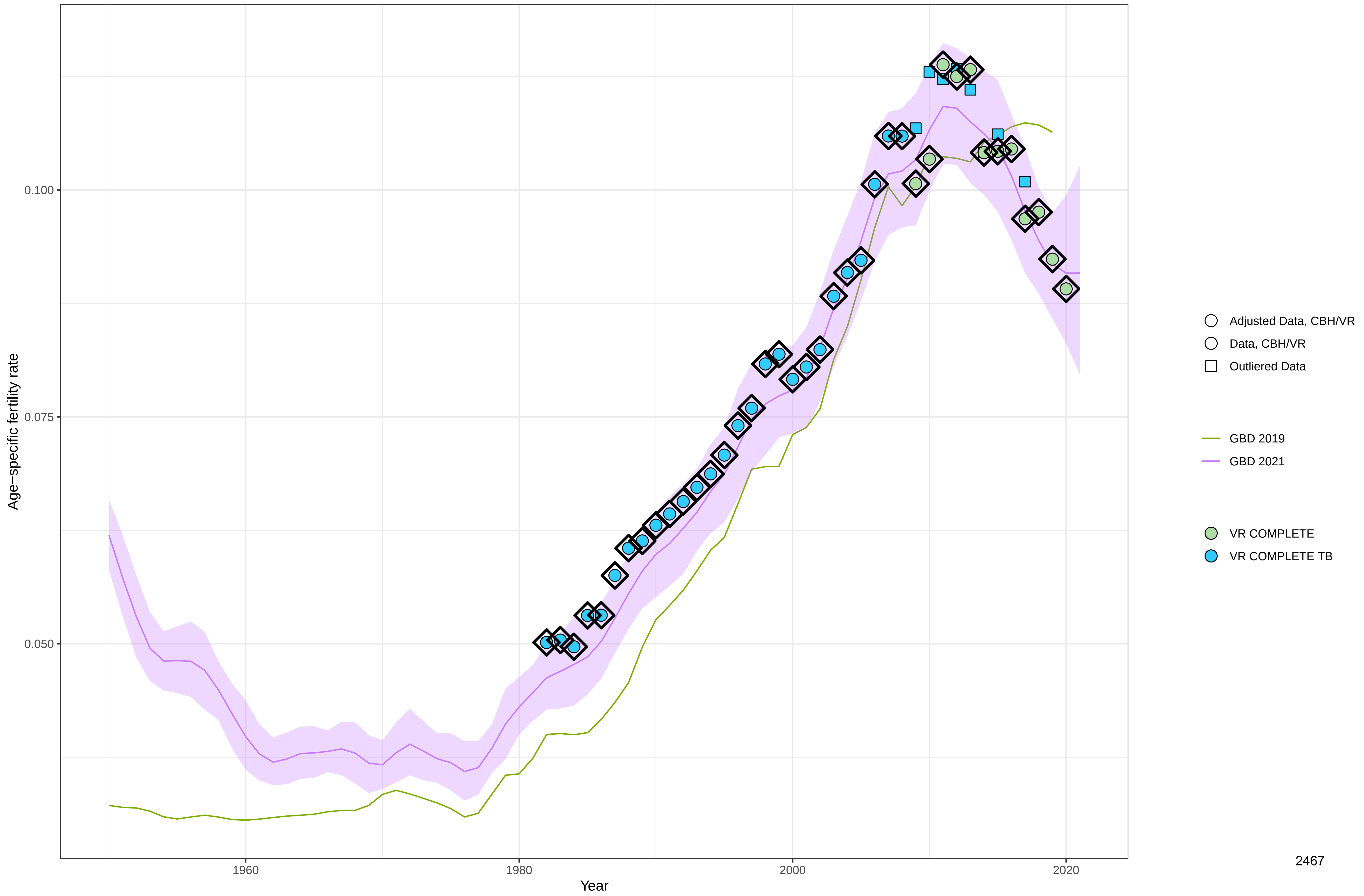

Age−specific fertility fitted trend lines, 1950−2021: Richmond upon Thames, Ages: 40−44
Data points enclosed with a diamond are reference sources

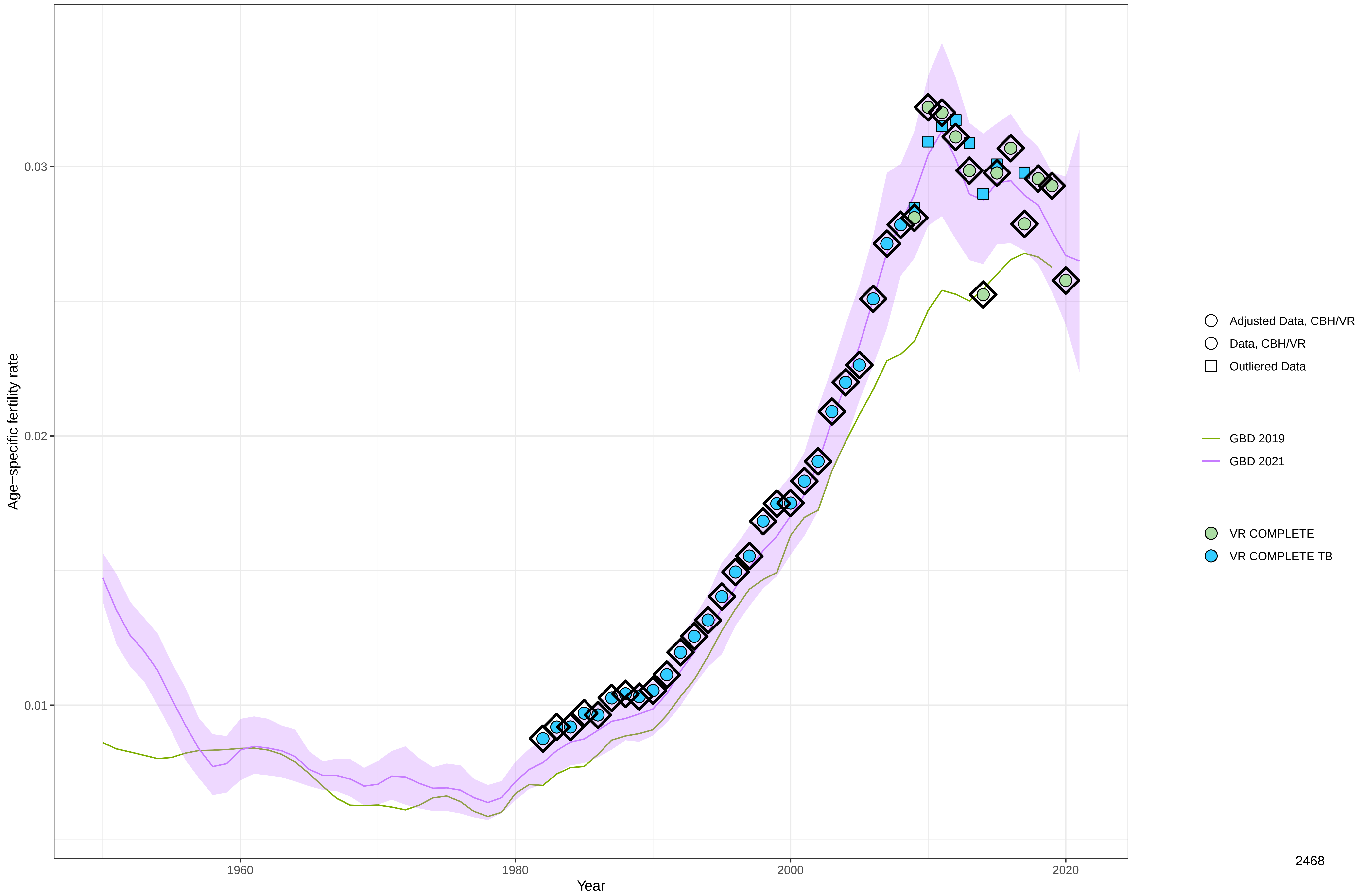

Age−specific fertility fitted trend lines, 1950−2021: Richmond upon Thames, Ages: 45−49
Data points enclosed with a diamond are reference sources

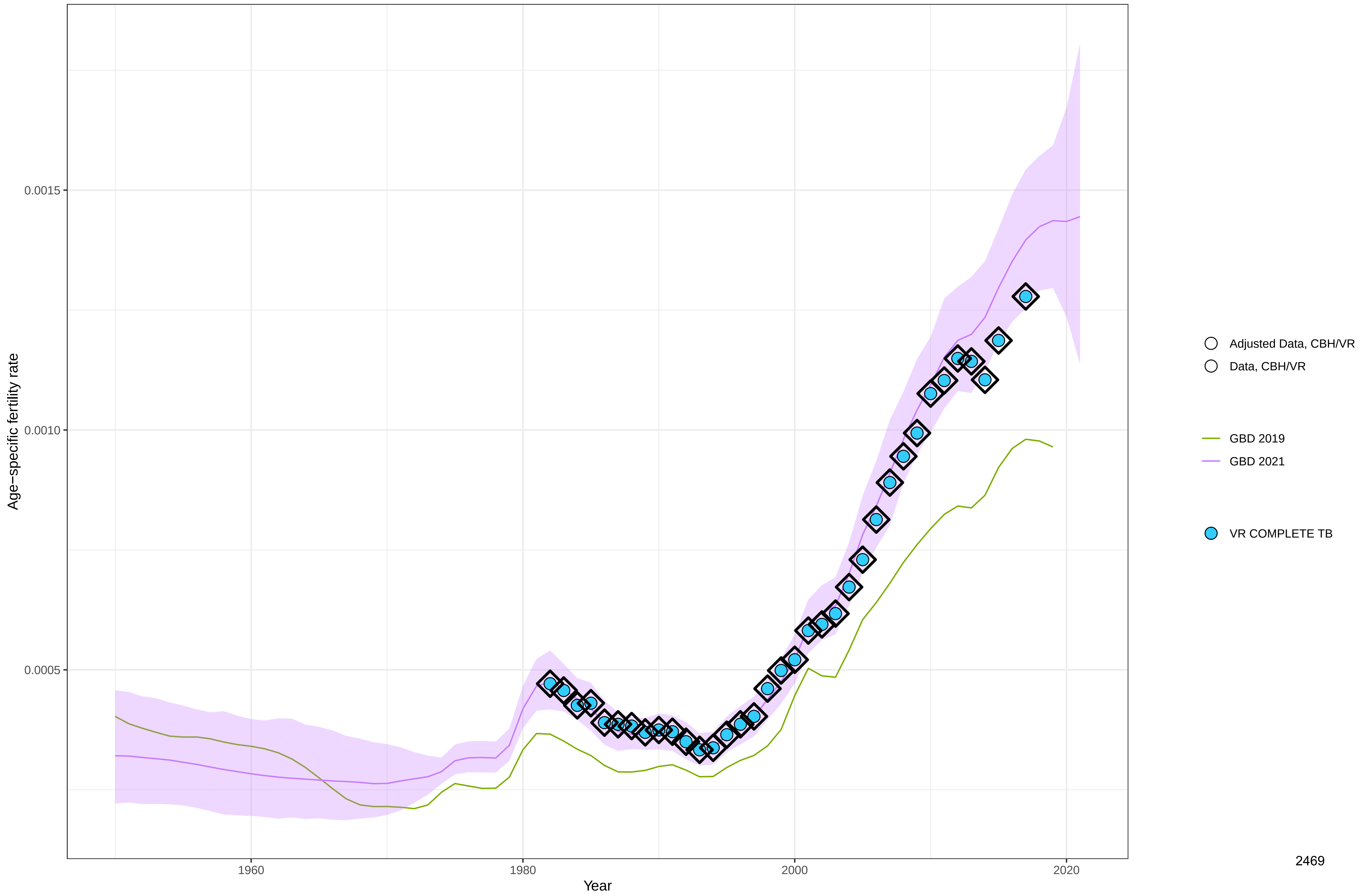

Age−specific fertility fitted trend lines, 1950−2021: Richmond upon Thames, Ages: 50−54
Data points enclosed with a diamond are reference sources

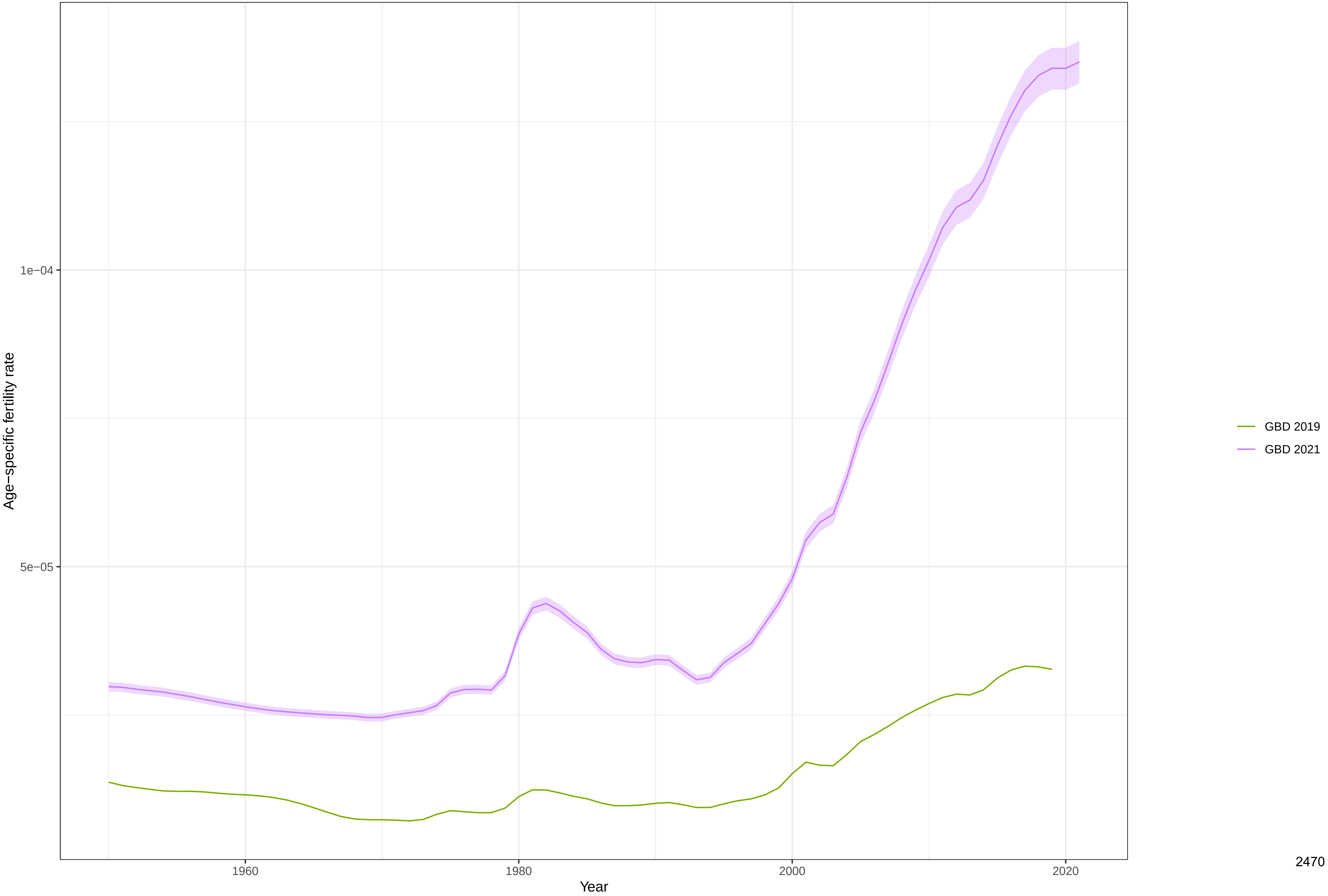

Total fertility rate fitted trend lines, 1950−2021: Richmond upon Thames TFR
Data points enclosed with a diamond are reference sources

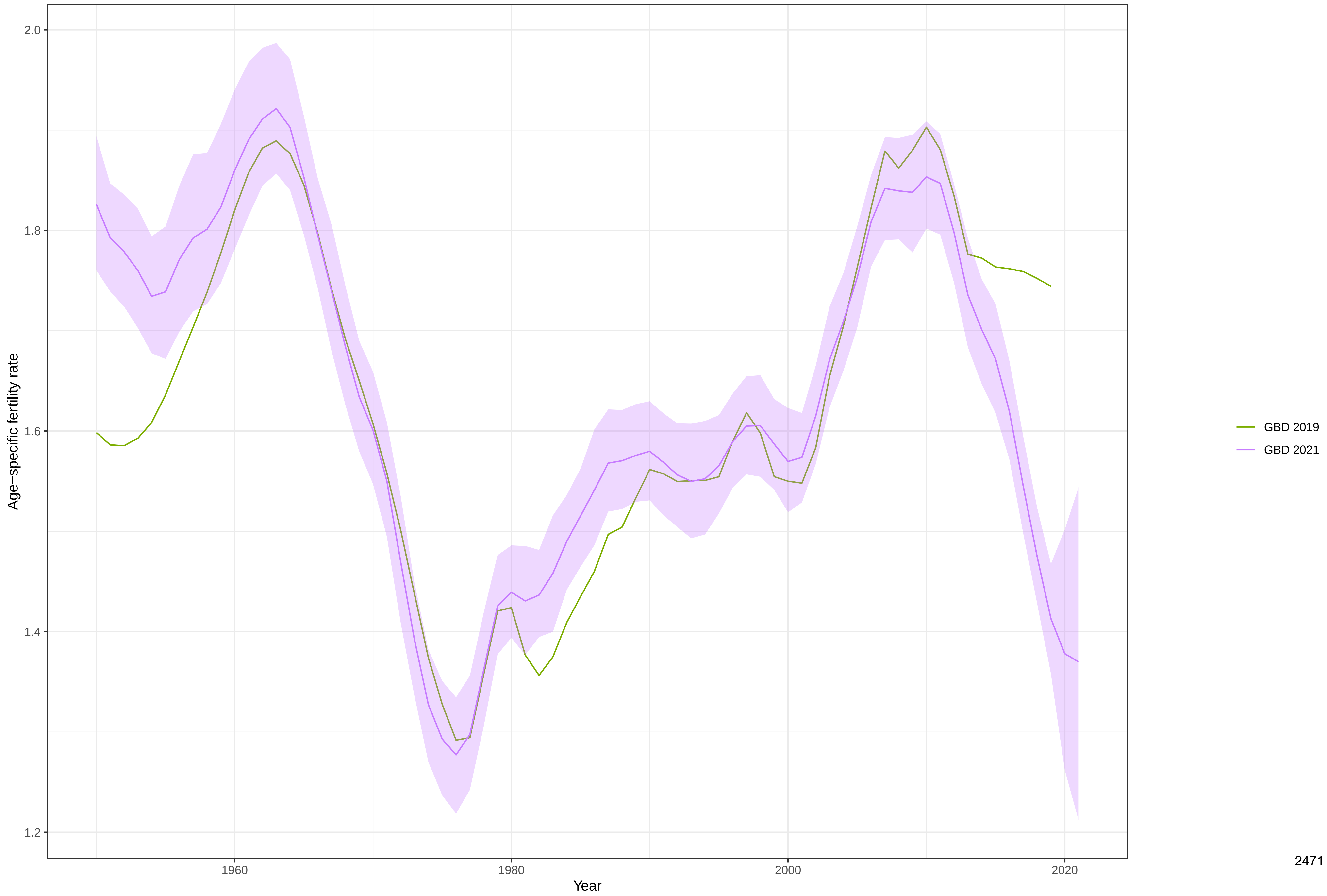

Age−specific fertility fitted trend lines, 1950−2021: Southwark, Ages: 10−14
Data points enclosed with a diamond are reference sources

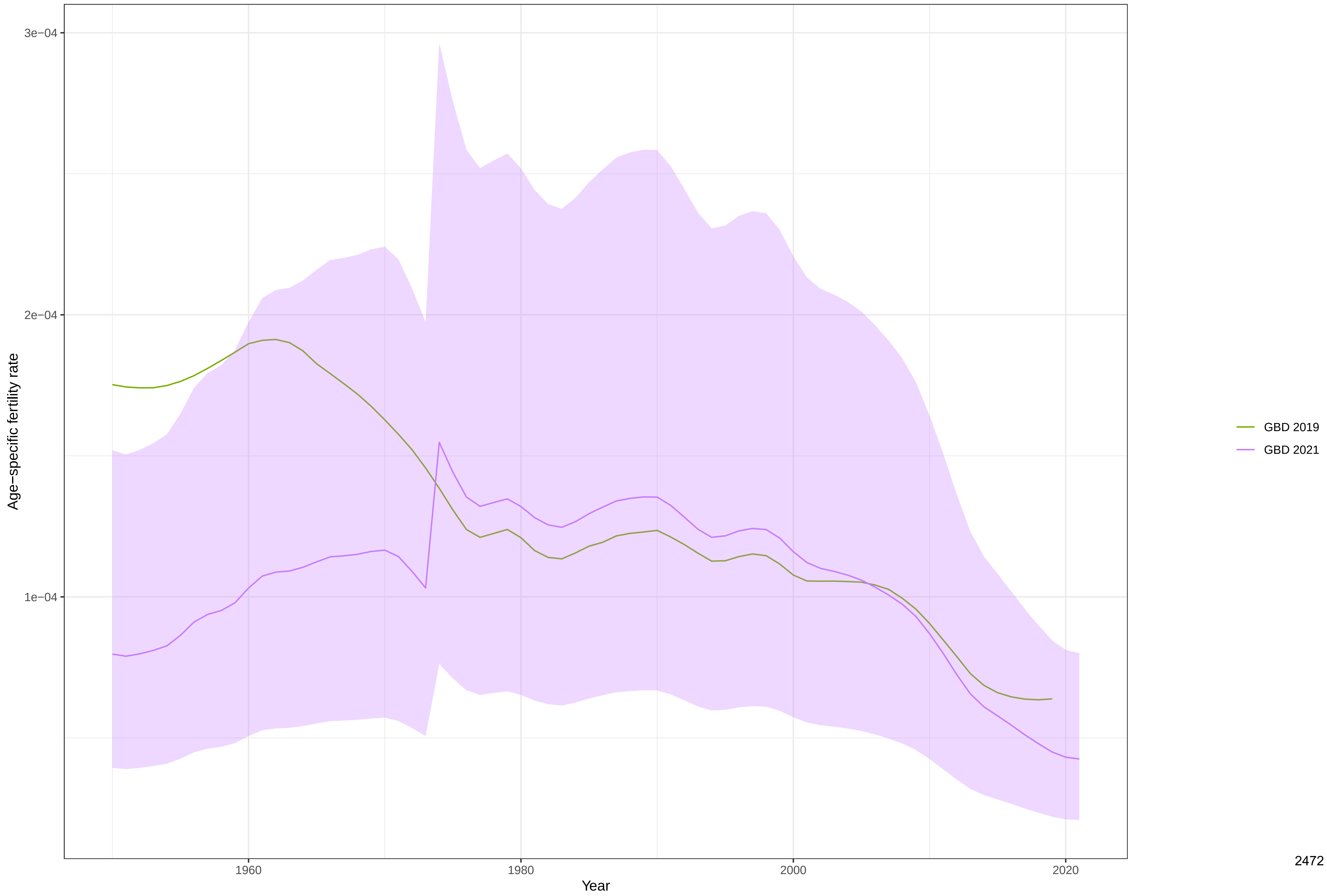

Age−specific fertility fitted trend lines, 1950−2021: Southwark, Ages: 15−19
Data points enclosed with a diamond are reference sources

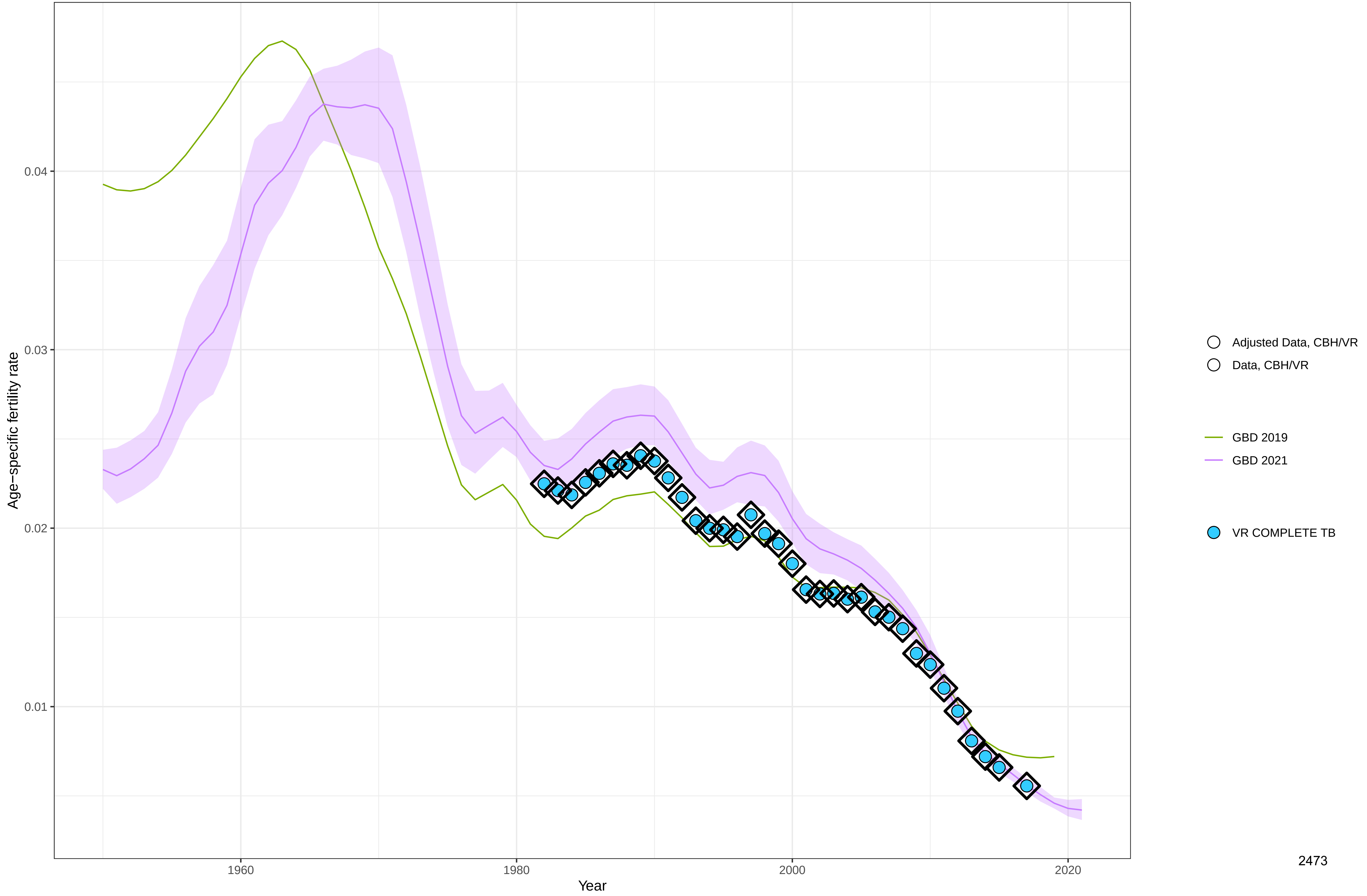

Age−specific fertility fitted trend lines, 1950−2021: Southwark, Ages: 20−24
Data points enclosed with a diamond are reference sources

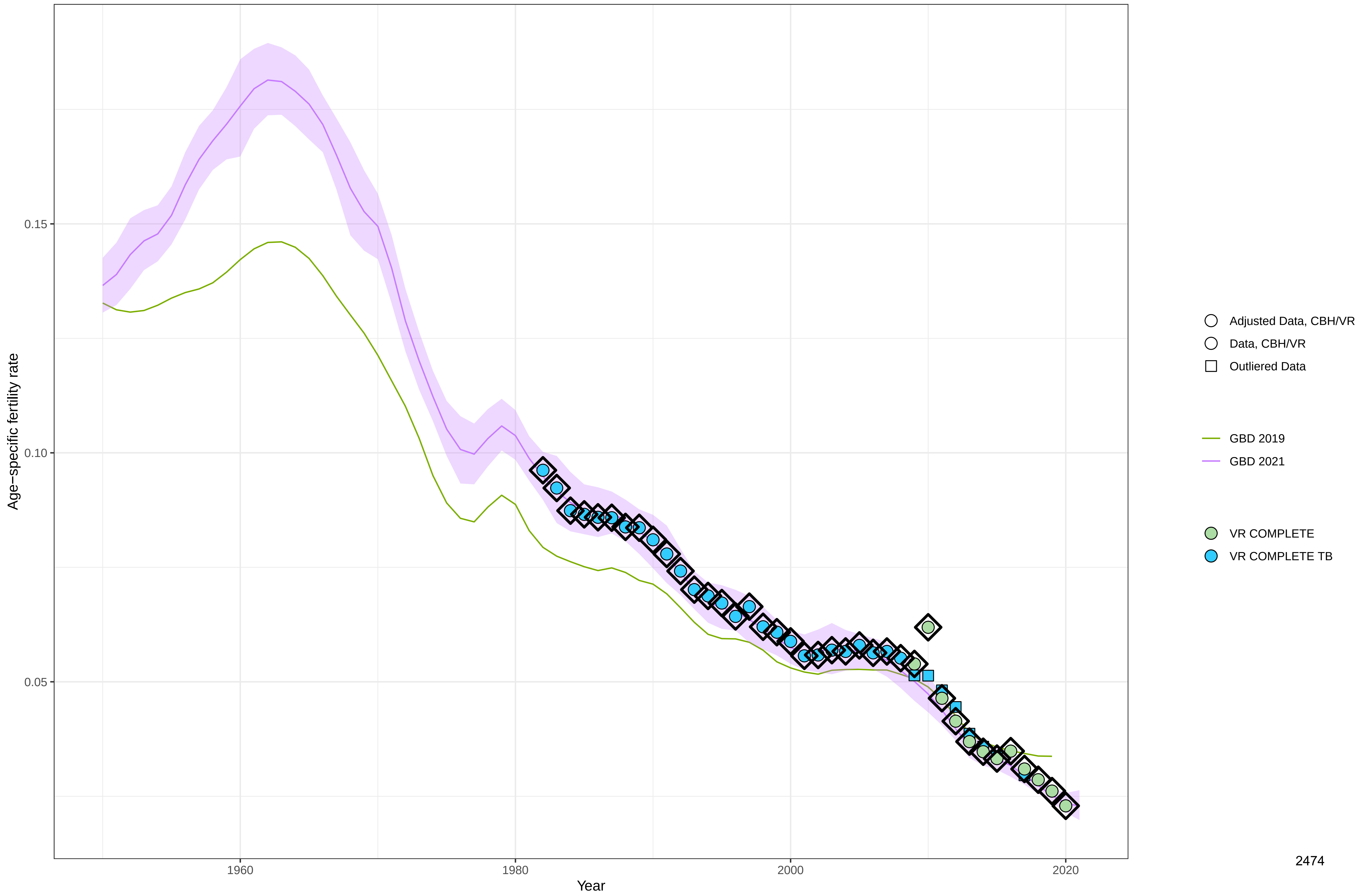

Age−specific fertility fitted trend lines, 1950−2021: Southwark, Ages: 25−29
Data points enclosed with a diamond are reference sources

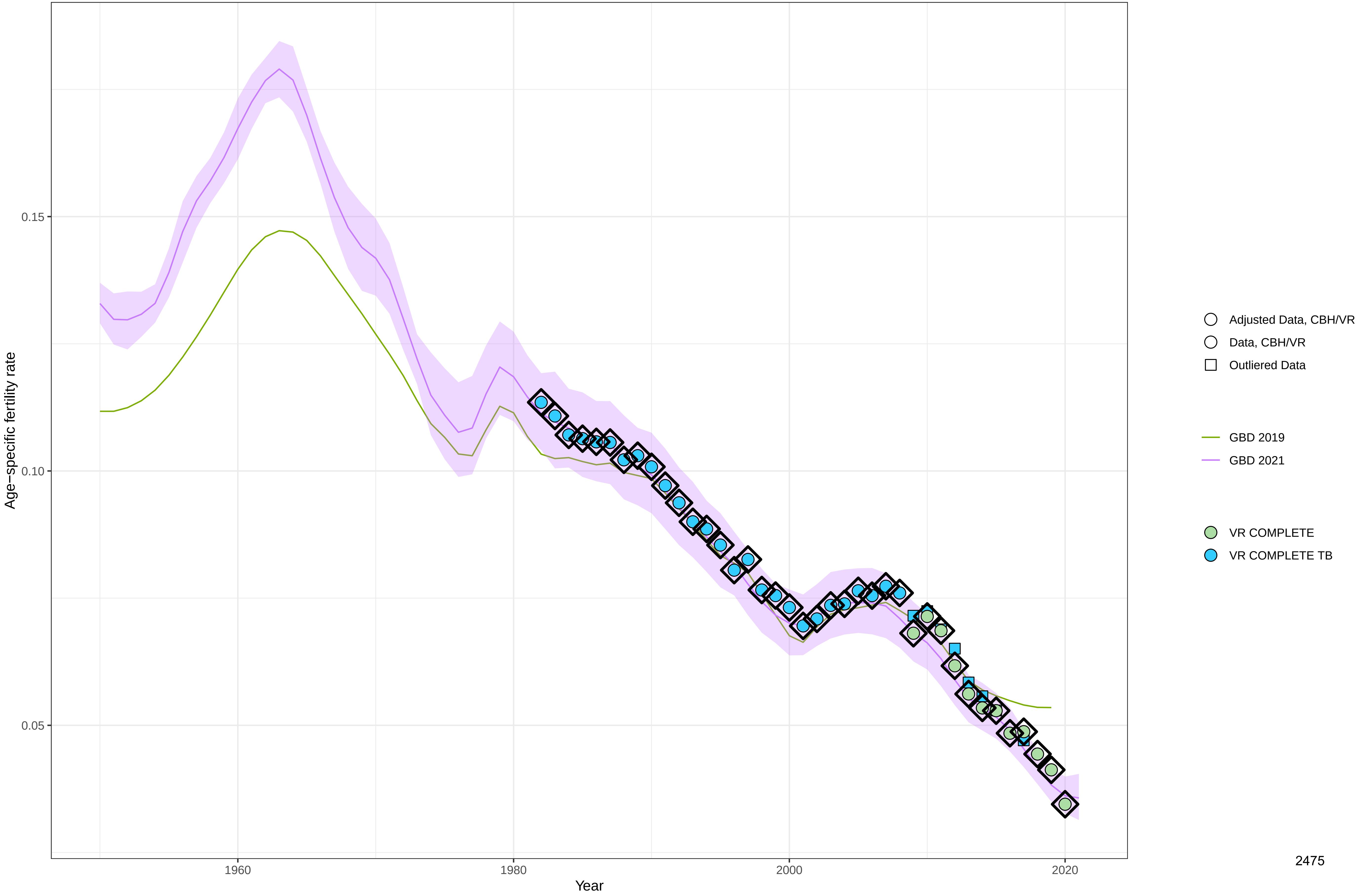

Age−specific fertility fitted trend lines, 1950−2021: Southwark, Ages: 30−34
Data points enclosed with a diamond are reference sources

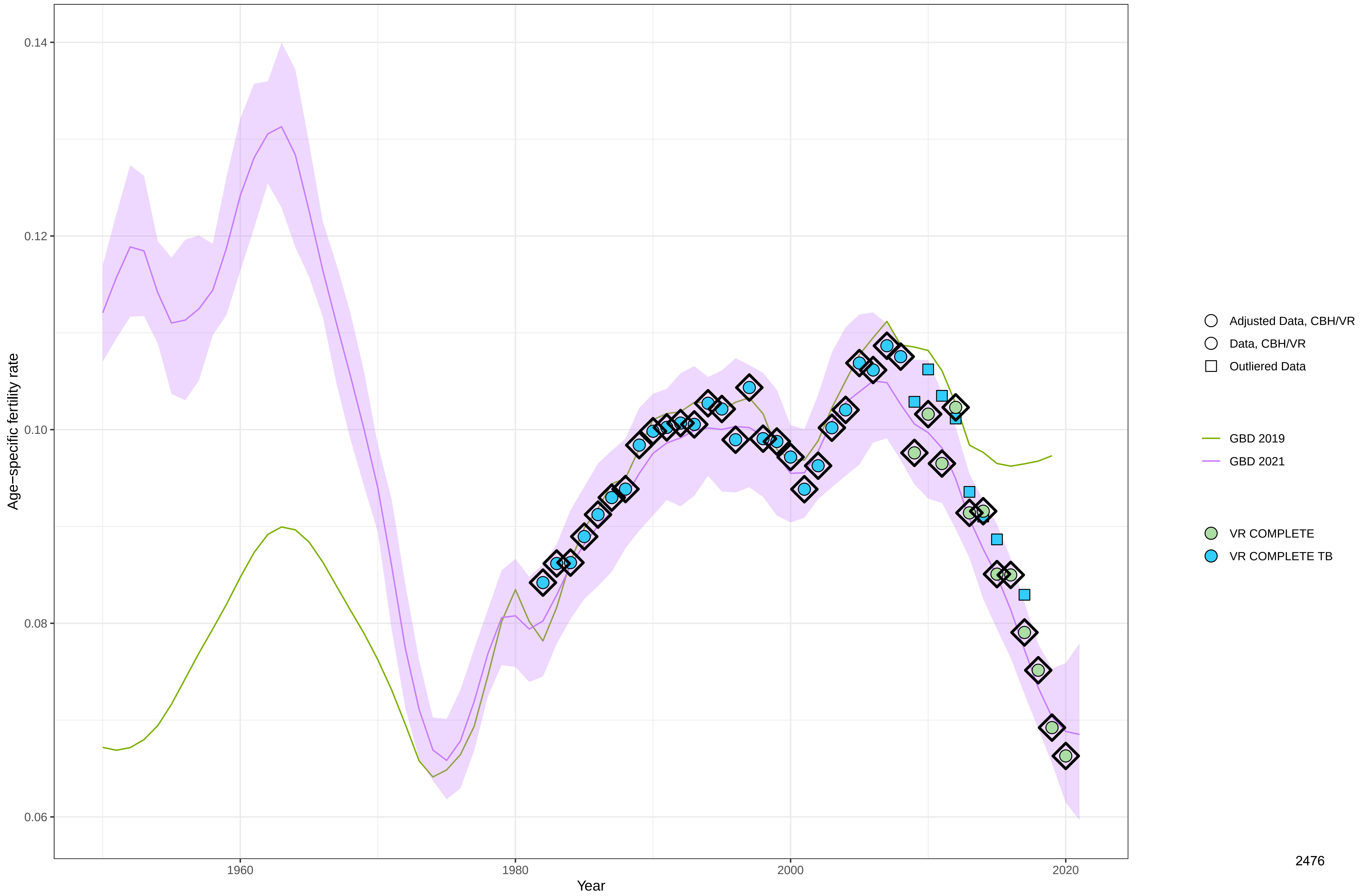

Age−specific fertility fitted trend lines, 1950−2021: Southwark, Ages: 35−39
Data points enclosed with a diamond are reference sources

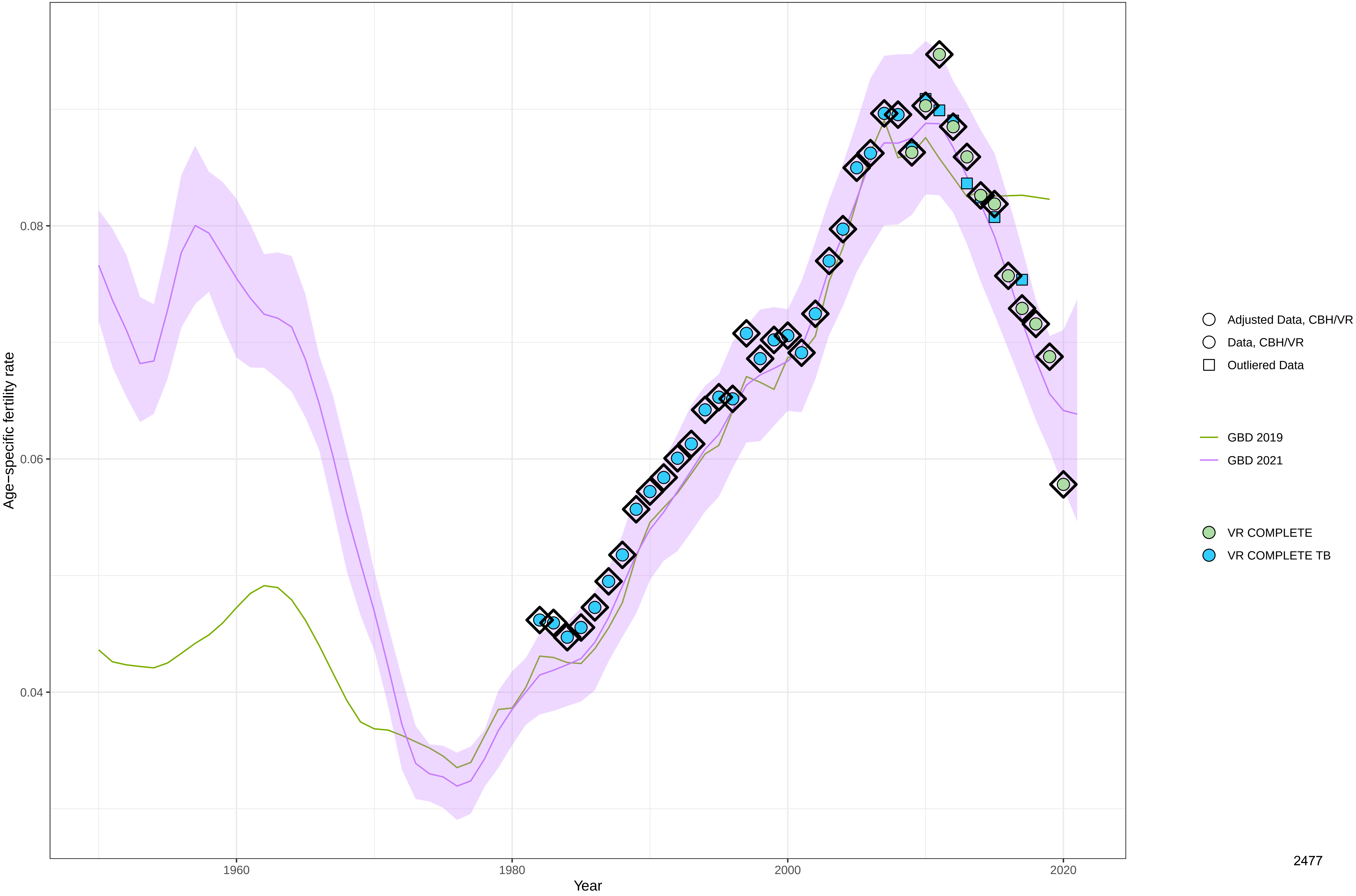

2477

Age−specific fertility fitted trend lines, 1950−2021: Southwark, Ages: 40−44
Data points enclosed with a diamond are reference sources

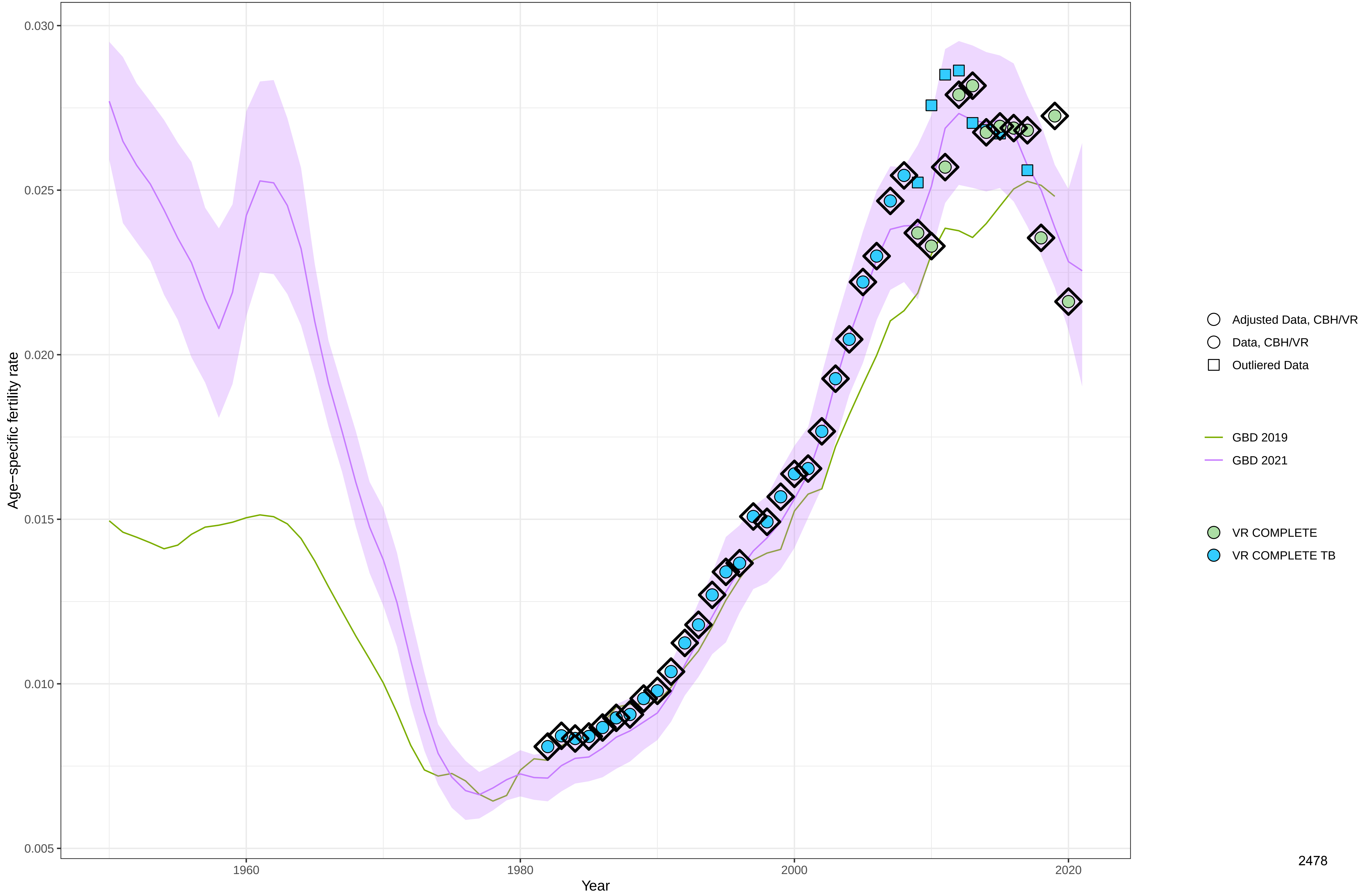

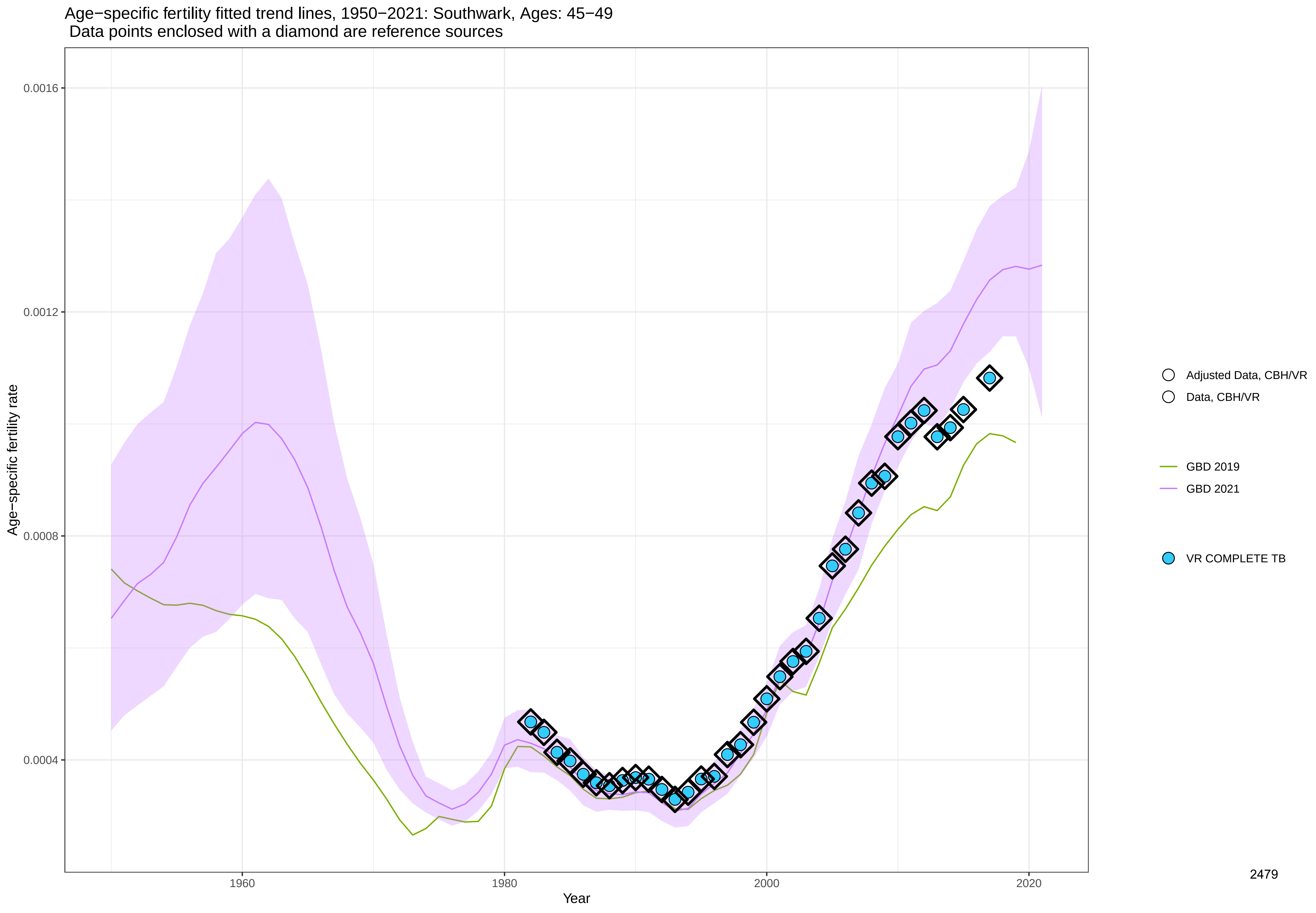

Age−specific fertility fitted trend lines, 1950−2021: Southwark, Ages: 45−49
Data points enclosed with a diamond are reference sources
Age−specific fertility rate
0.0016
0.0012
0.0008
0.0004
1960
1980
2000
2020
Year
Adjusted Data, CBH/VR
Data, CBH/VR
GBD 2019
GBD 2021
VR COMPLETE TB

Age−specific fertility fitted trend lines, 1950−2021: Southwark, Ages: 50−54
Data points enclosed with a diamond are reference sources

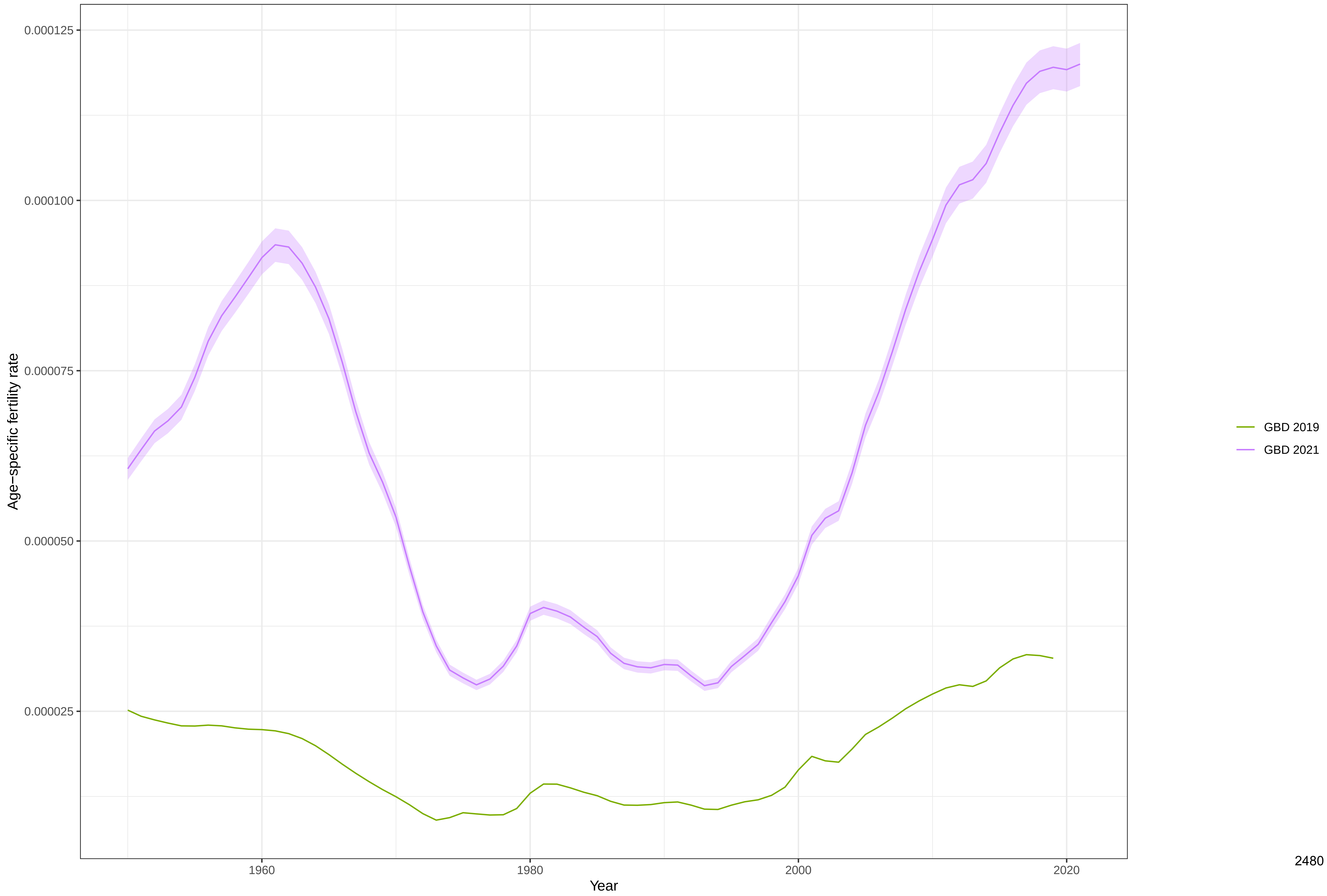

Total fertility rate fitted trend lines, 1950−2021: Southwark TFR
Data points enclosed with a diamond are reference sources

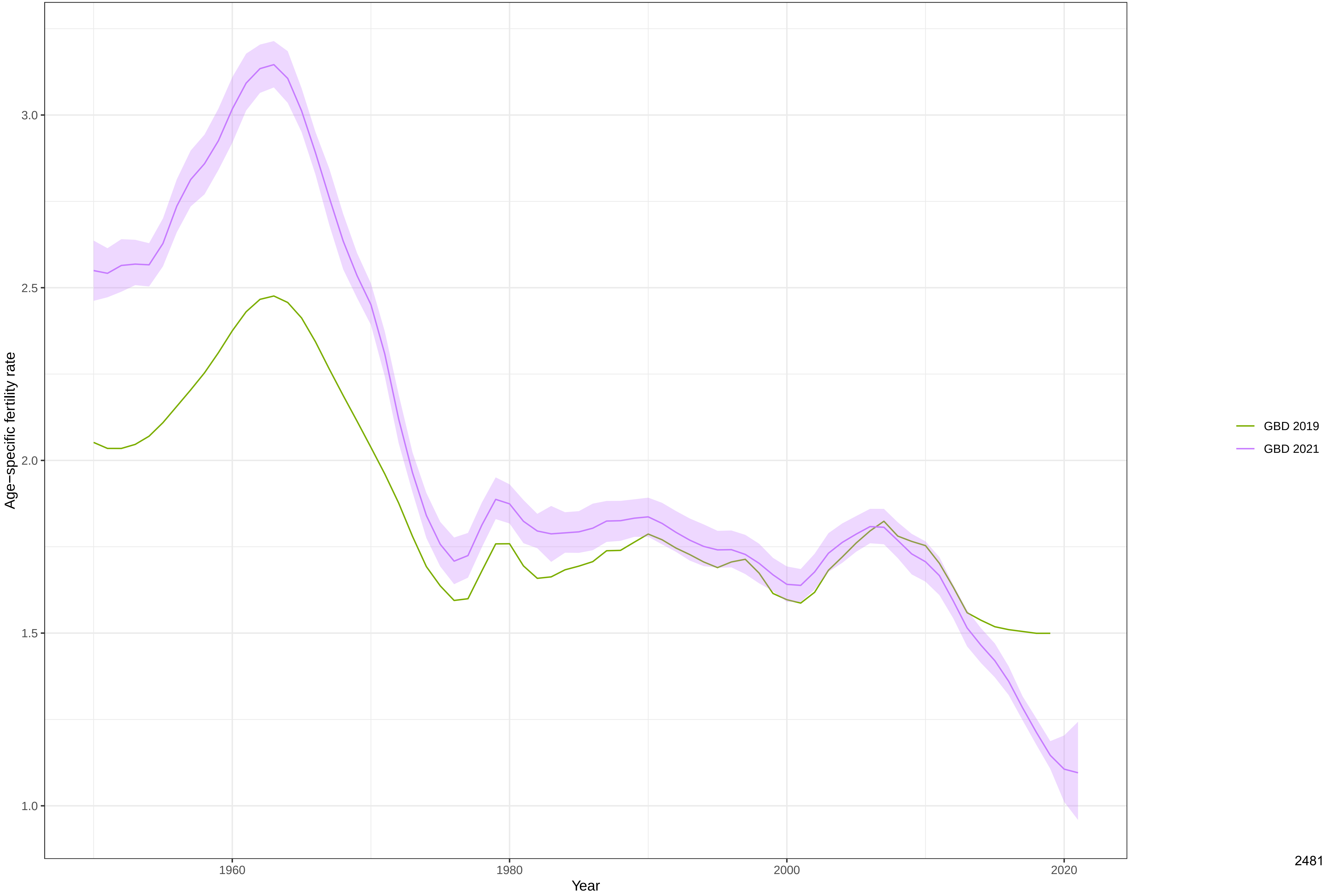

Age−specific fertility fitted trend lines, 1950−2021: Sutton, Ages: 10−14
Data points enclosed with a diamond are reference sources

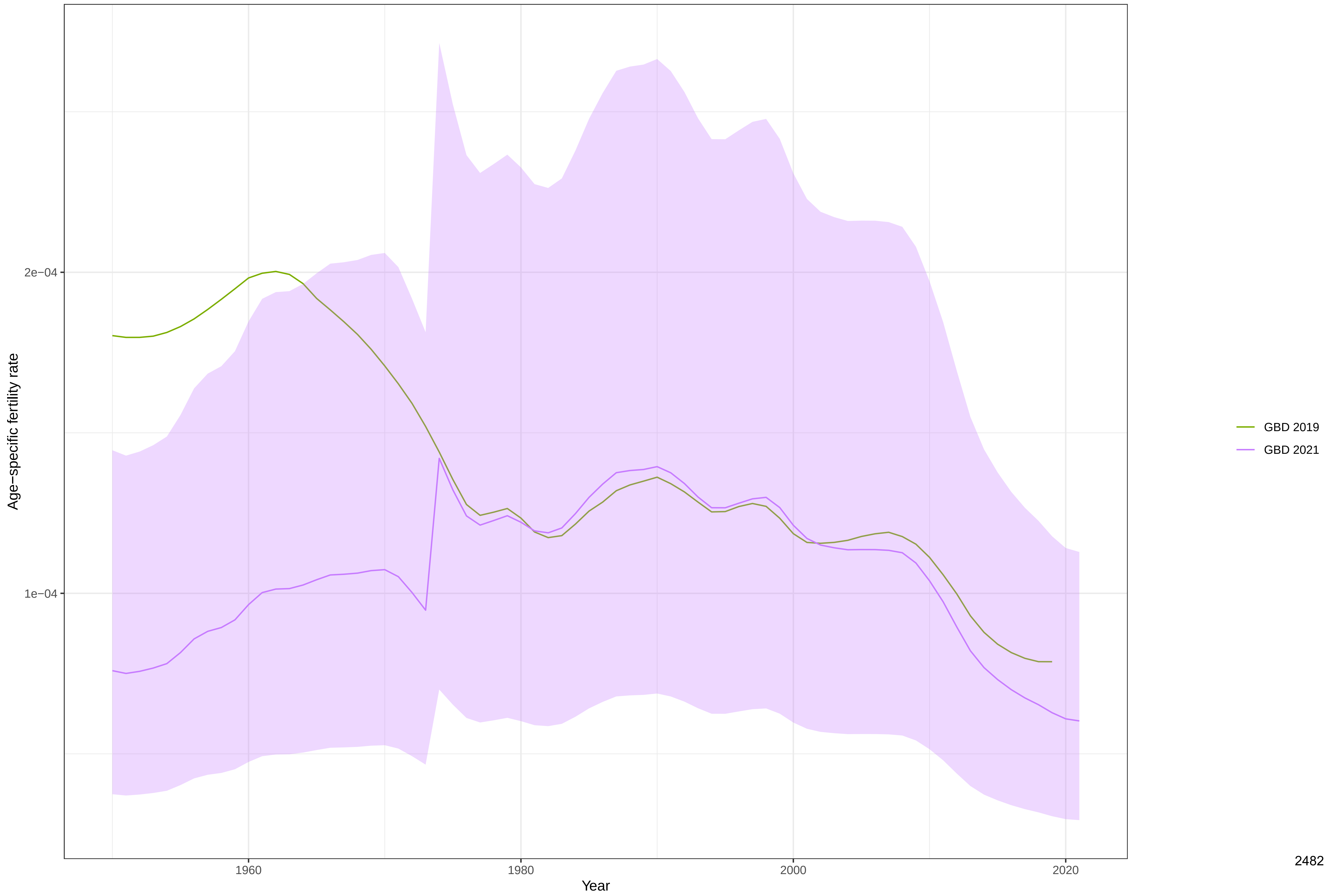

Age−specific fertility fitted trend lines, 1950−2021: Sutton, Ages: 15−19
Data points enclosed with a diamond are reference sources

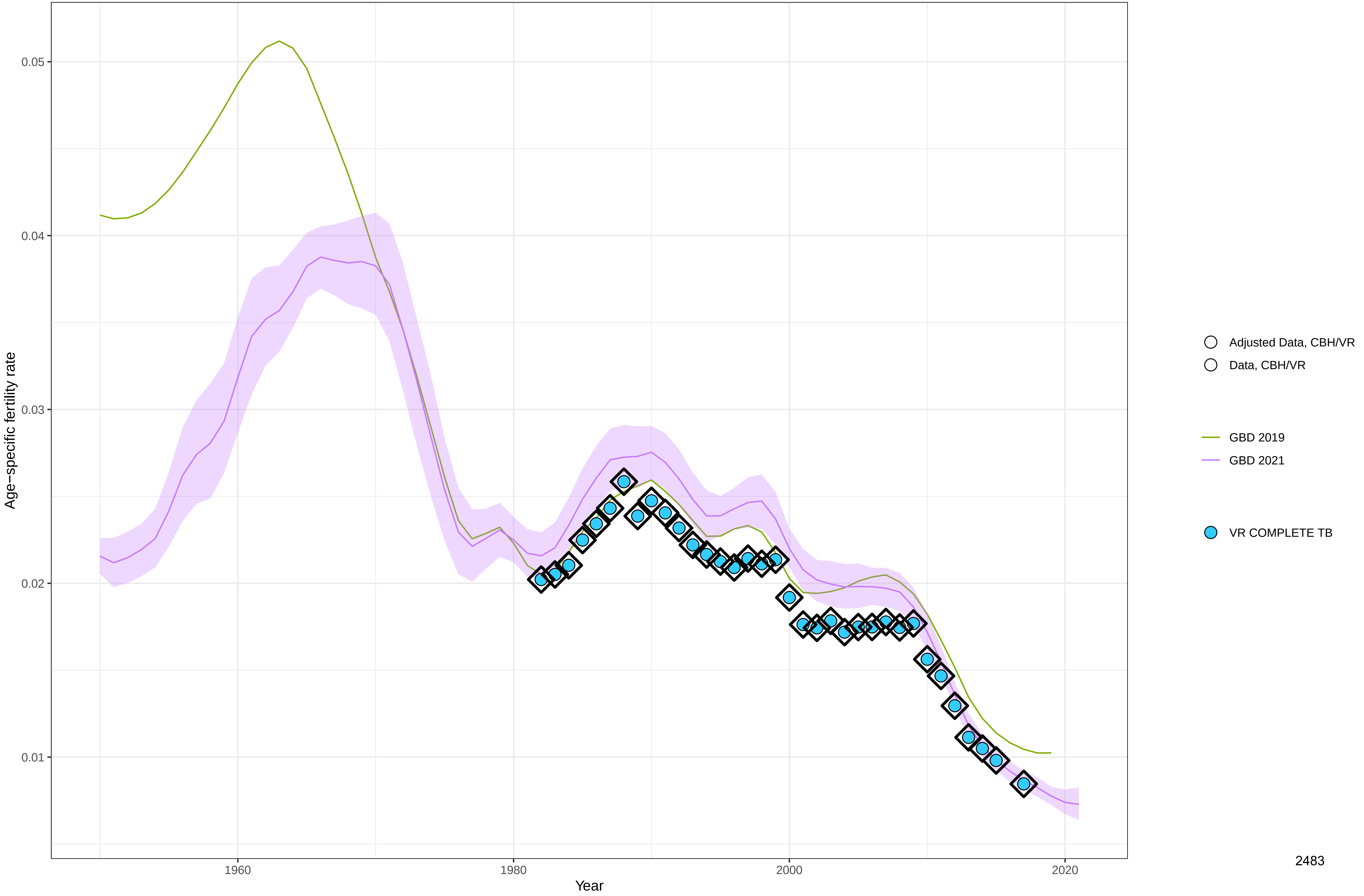

Age−specific fertility fitted trend lines, 1950−2021: Sutton, Ages: 20−24
Data points enclosed with a diamond are reference sources

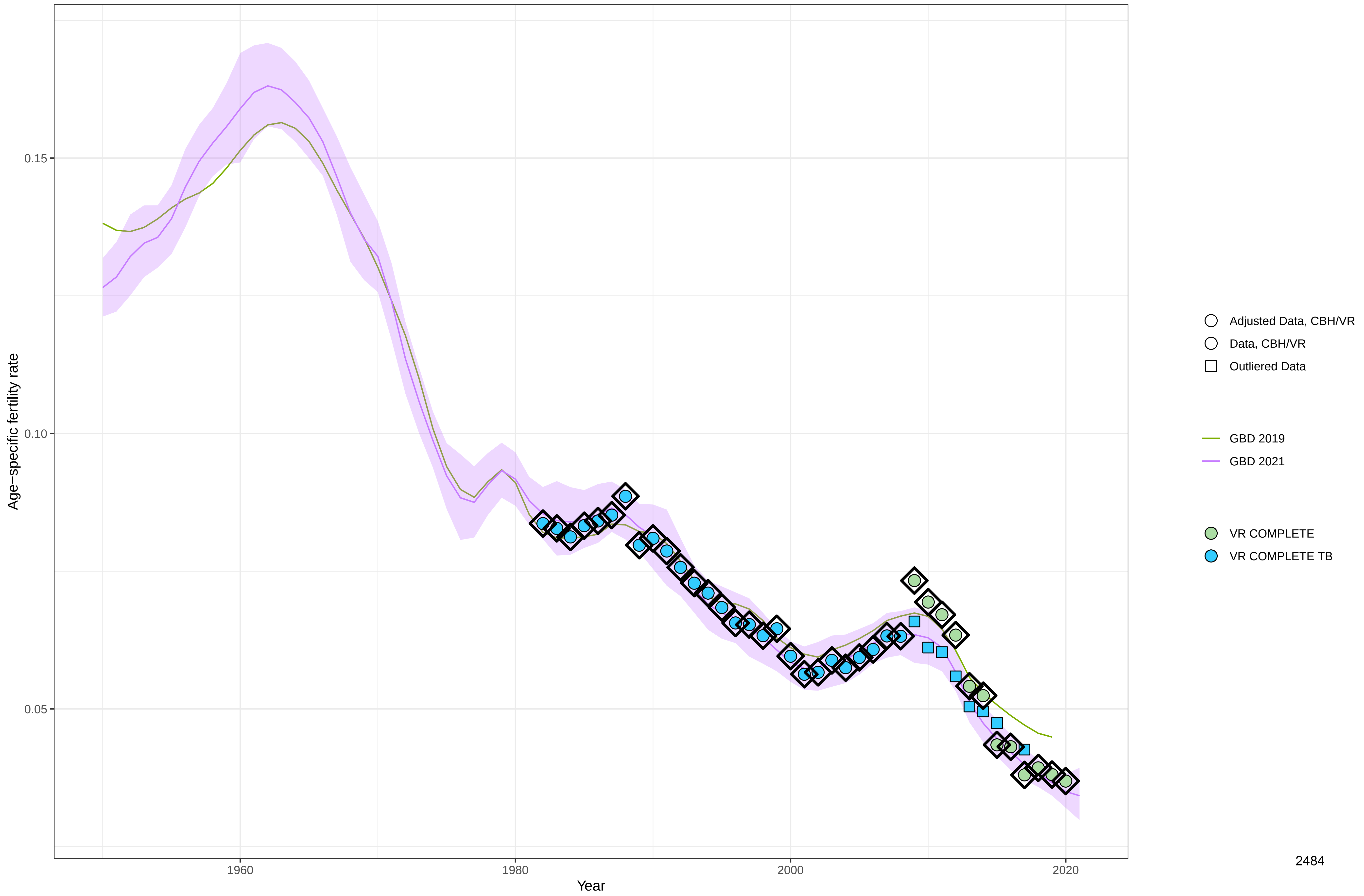

Age−specific fertility fitted trend lines, 1950−2021: Sutton, Ages: 25−29
Data points enclosed with a diamond are reference sources

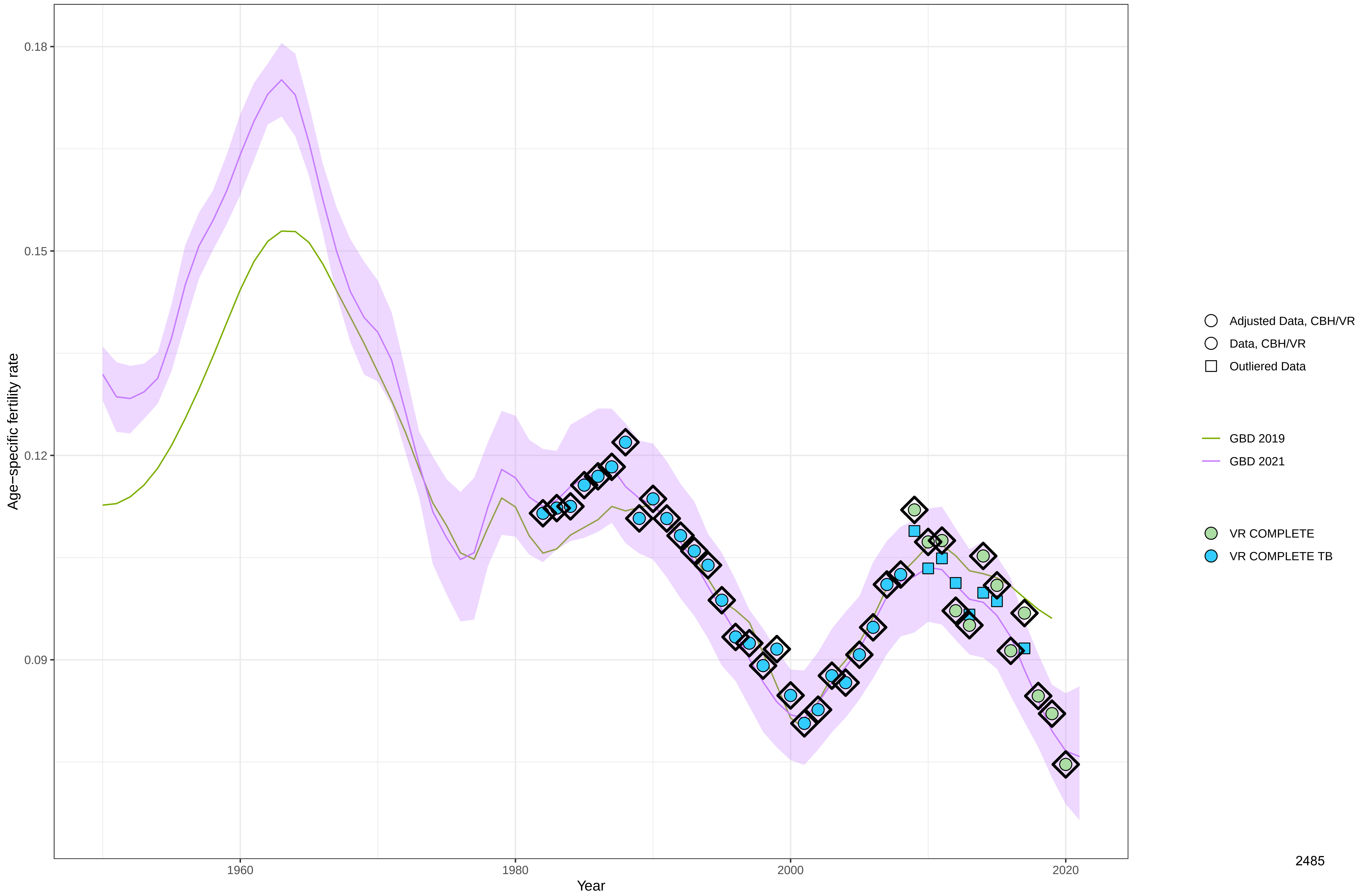

Age−specific fertility fitted trend lines, 1950−2021: Sutton, Ages: 30−34
Data points enclosed with a diamond are reference sources

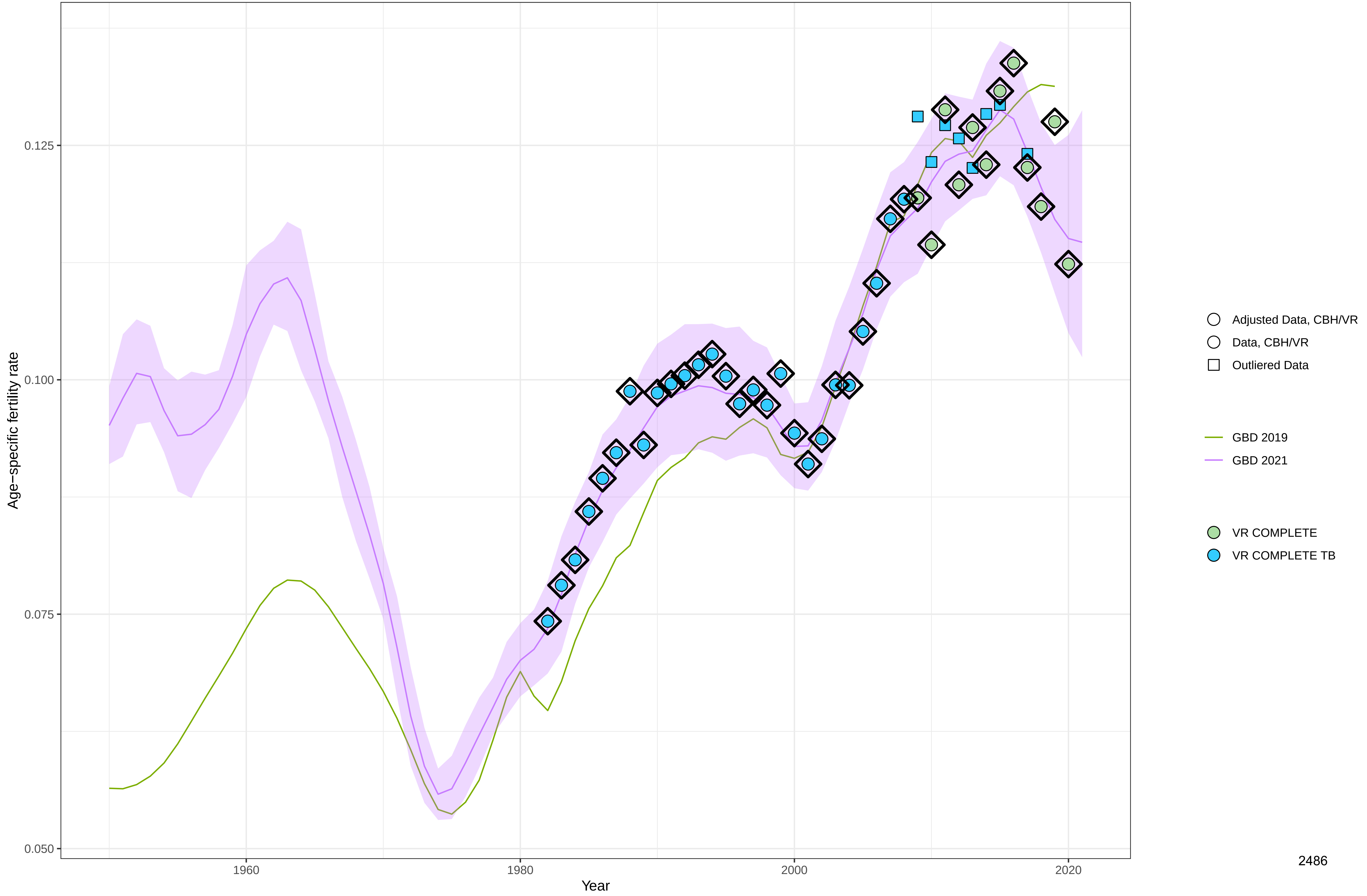

Age−specific fertility fitted trend lines, 1950−2021: Sutton, Ages: 35−39
Data points enclosed with a diamond are reference sources

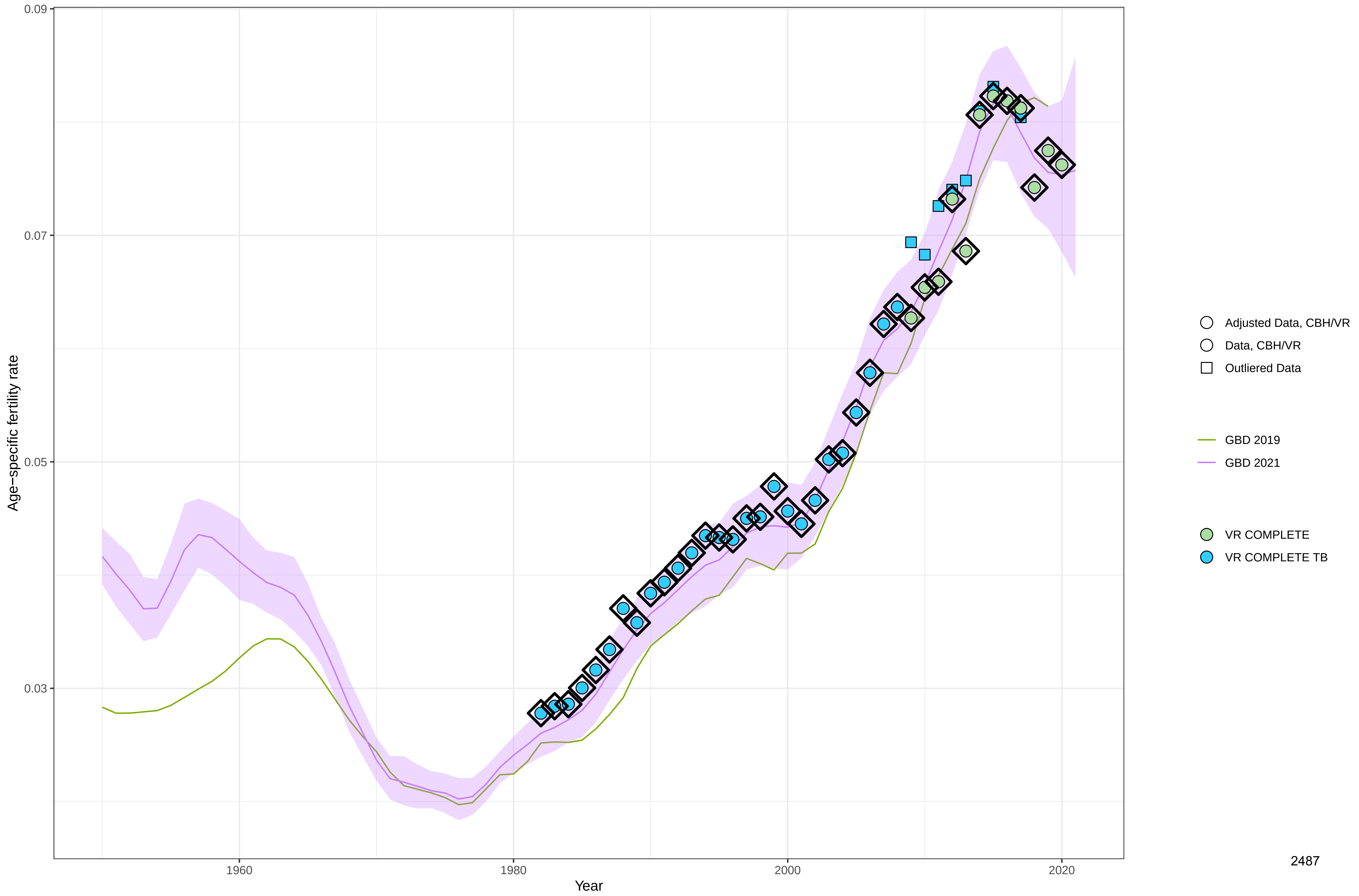

Age−specific fertility fitted trend lines, 1950−2021: Sutton, Ages: 40−44
Data points enclosed with a diamond are reference sources

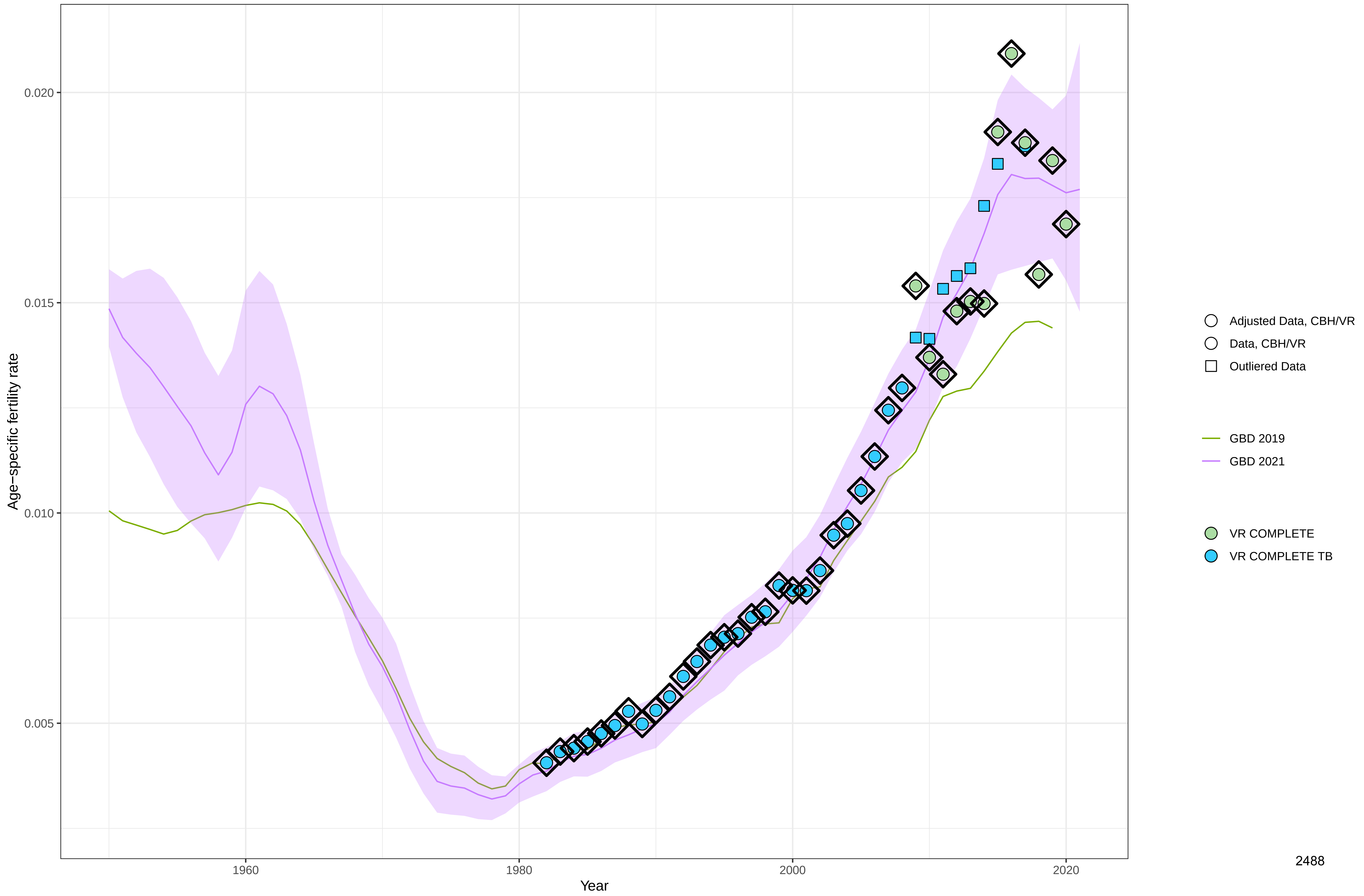

Age−specific fertility fitted trend lines, 1950−2021: Sutton, Ages: 45−49
Data points enclosed with a diamond are reference sources

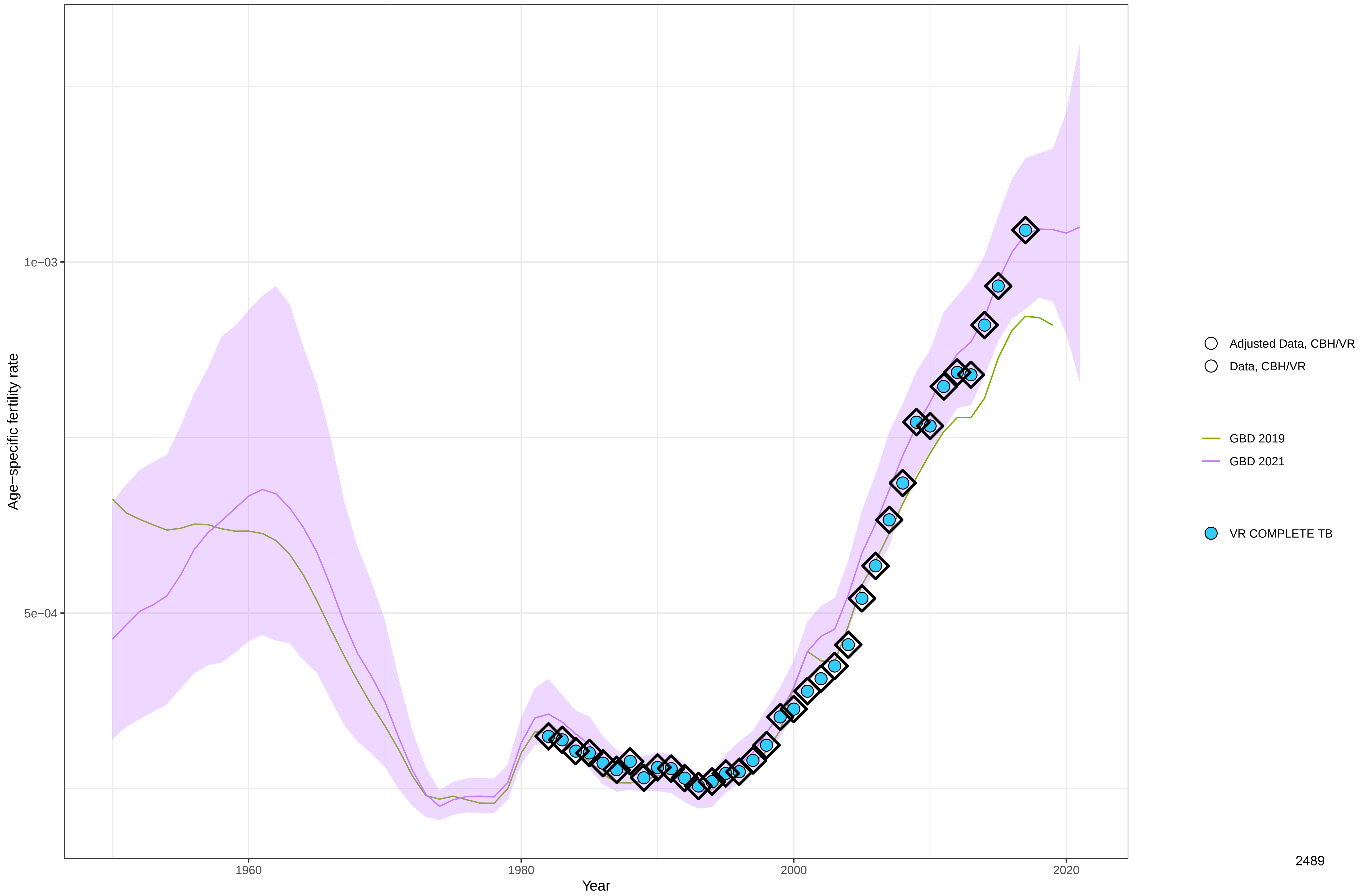

Age−specific fertility fitted trend lines, 1950−2021: Sutton, Ages: 50−54
Data points enclosed with a diamond are reference sources

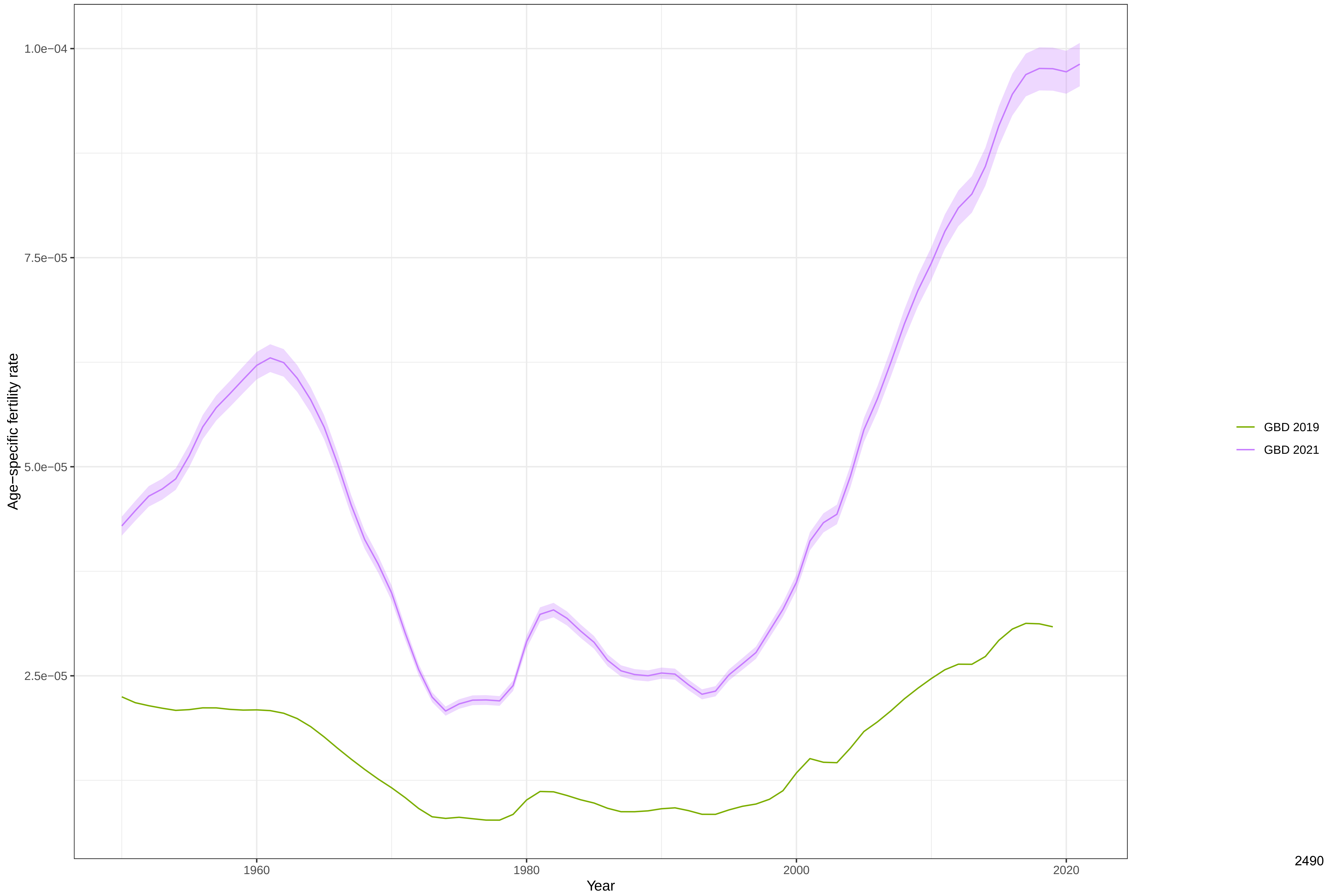

Total fertility rate fitted trend lines, 1950−2021: Sutton TFR
Data points enclosed with a diamond are reference sources

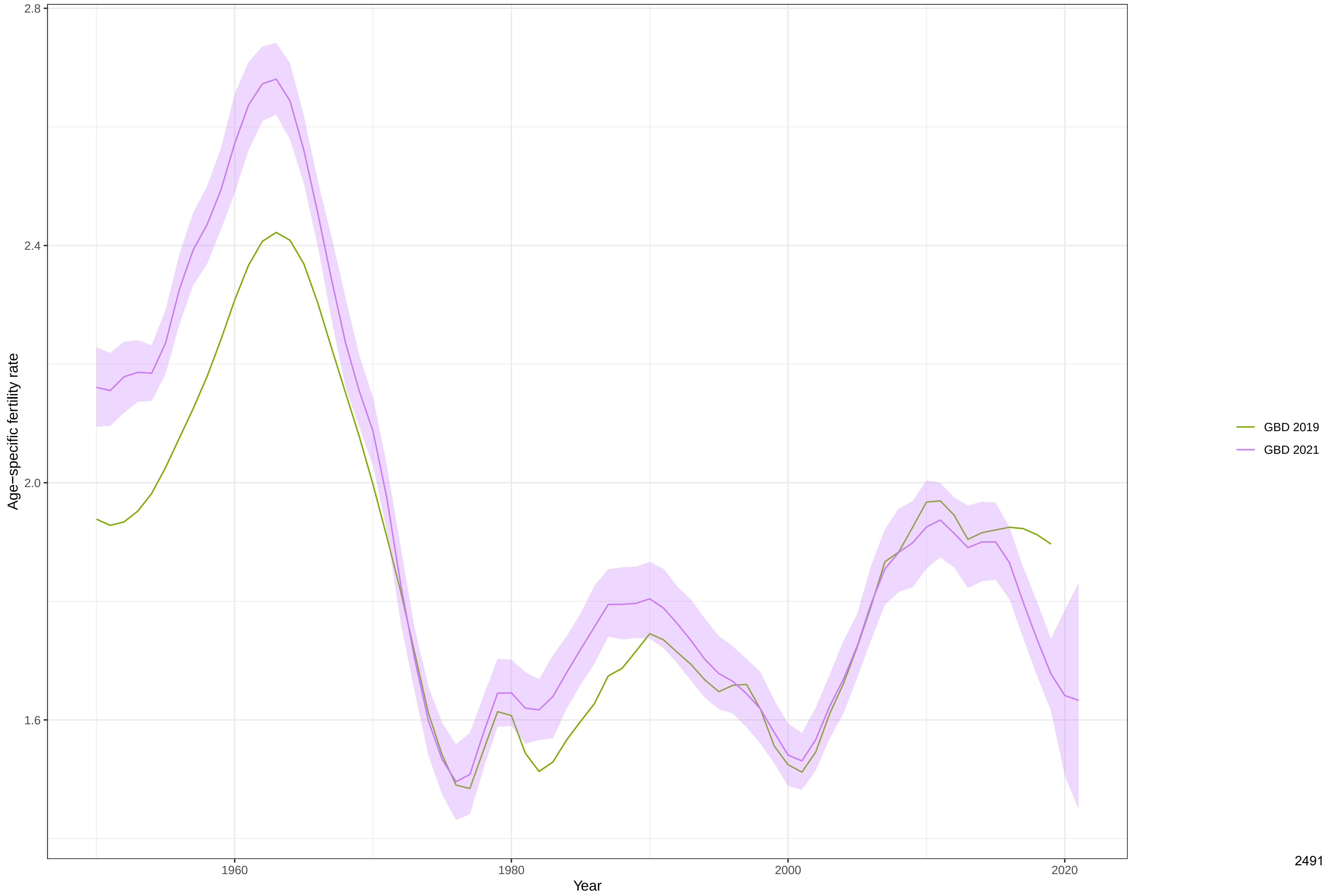

Age−specific fertility fitted trend lines, 1950−2021: Tower Hamlets, Ages: 10−14
Data points enclosed with a diamond are reference sources

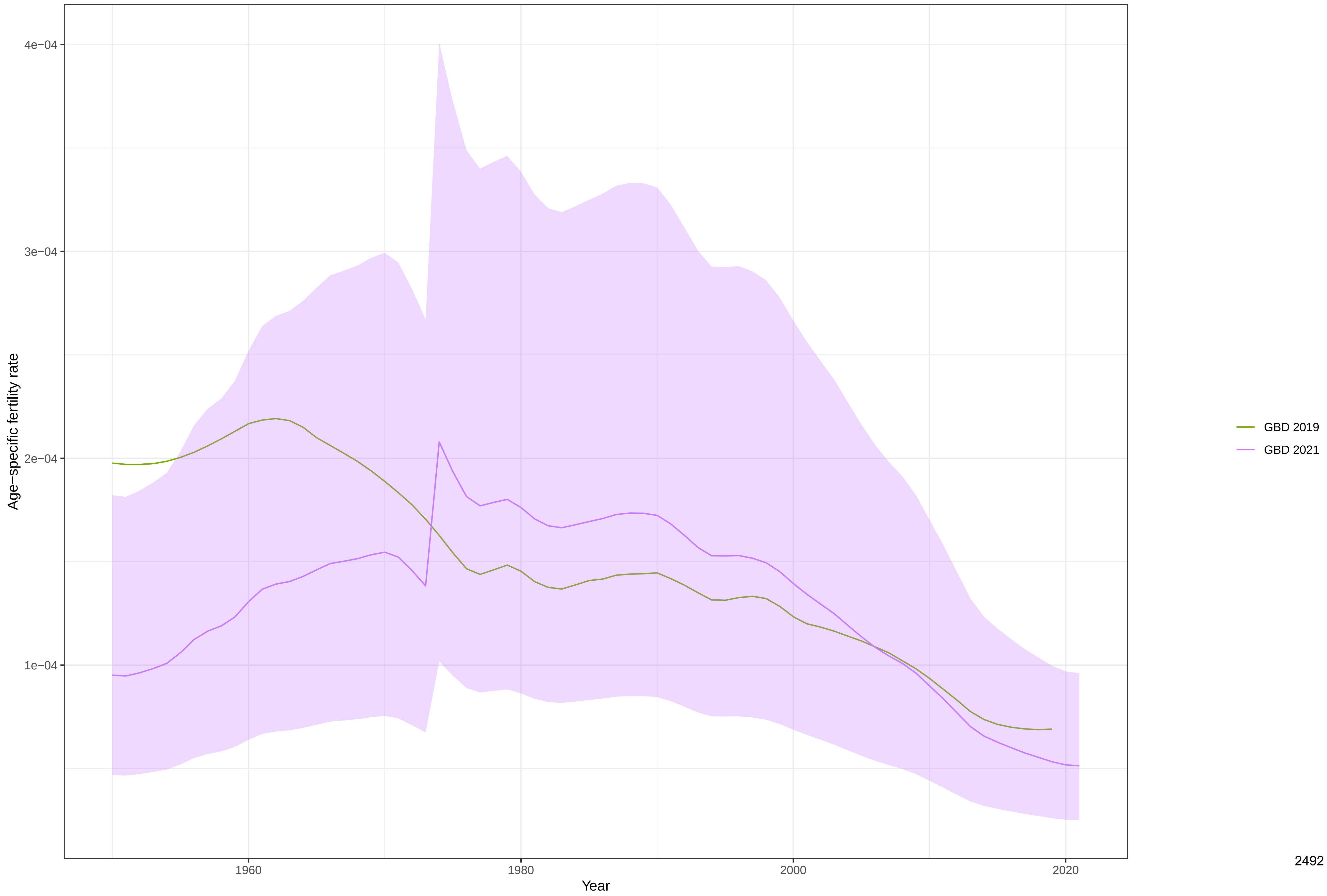

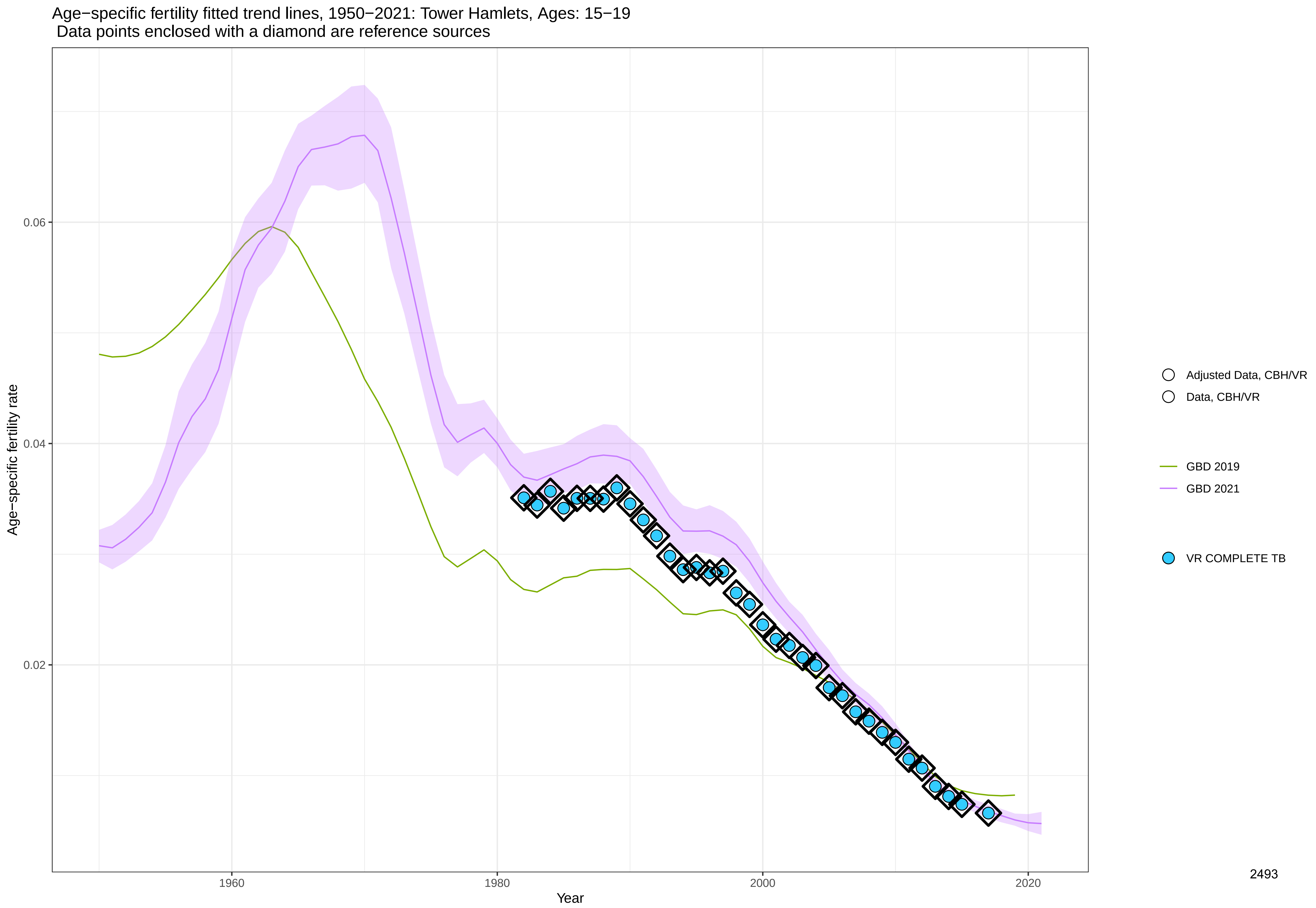

Age−specific fertility fitted trend lines, 1950−2021: Tower Hamlets, Ages: 15−19
Data points enclosed with a diamond are reference sources
Age−specific fertility rate
0.06
0.04
0.02
Year
1960
1980
2000
2020
Adjusted Data, CBH/VR
Data, CBH/VR
GBD 2019
GBD 2021
VR COMPLETE TB

Age−specific fertility fitted trend lines, 1950−2021: Tower Hamlets, Ages: 20−24
Data points enclosed with a diamond are reference sources

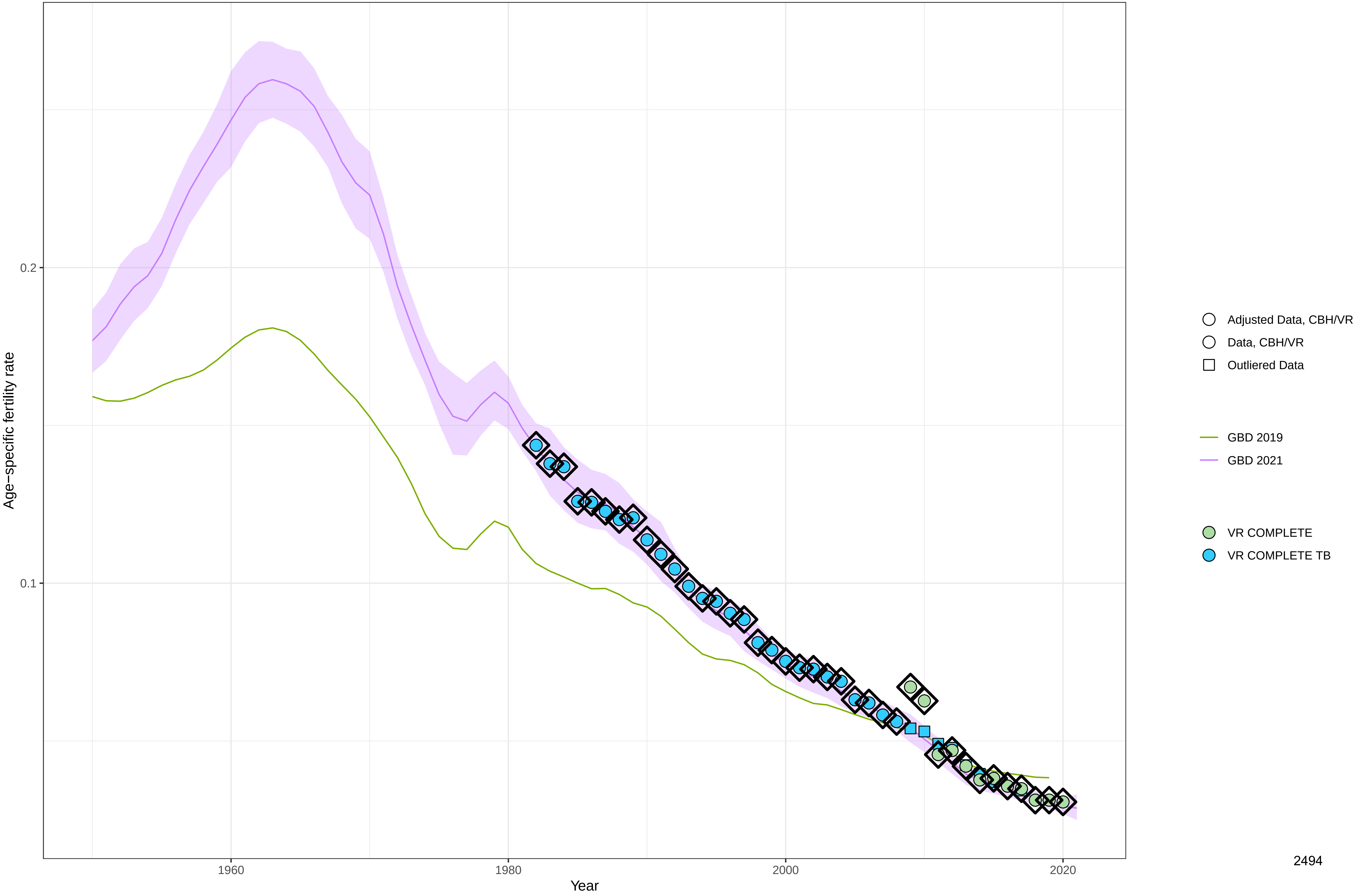

Age−specific fertility fitted trend lines, 1950−2021: Tower Hamlets, Ages: 25−29
Data points enclosed with a diamond are reference sources

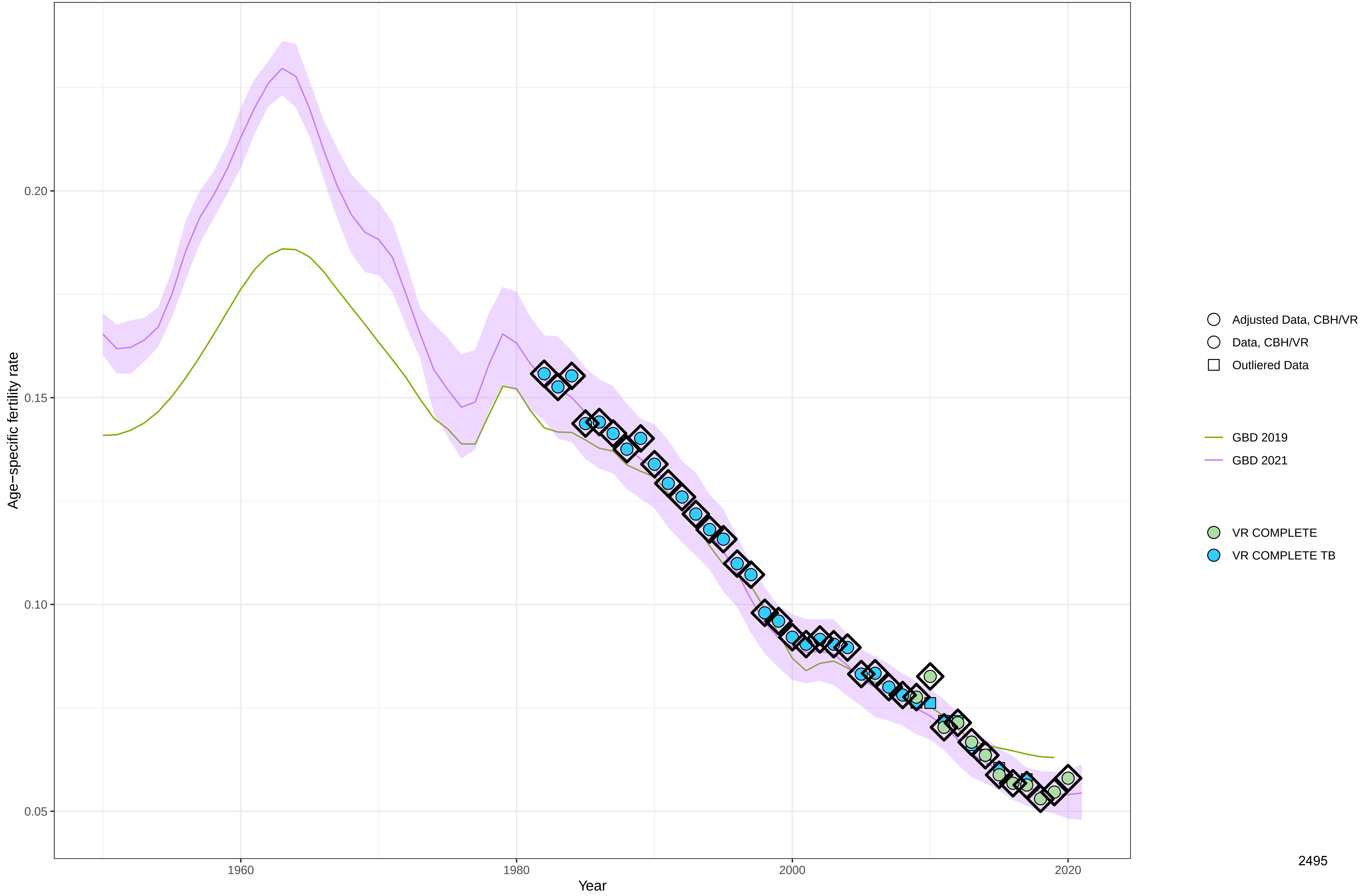

Age−specific fertility fitted trend lines, 1950−2021: Tower Hamlets, Ages: 30−34
Data points enclosed with a diamond are reference sources

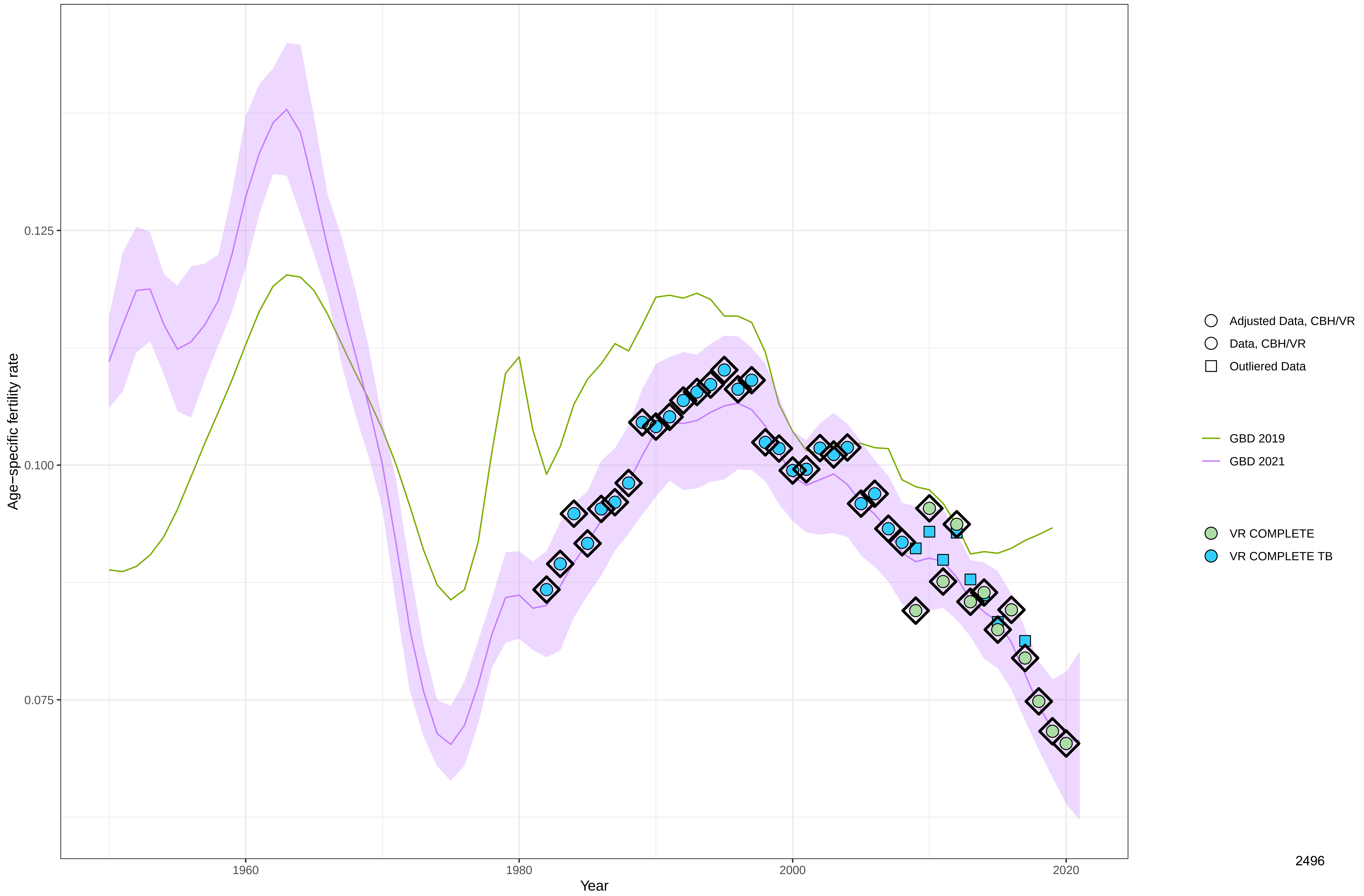

Age−specific fertility fitted trend lines, 1950−2021: Tower Hamlets, Ages: 35−39
Data points enclosed with a diamond are reference sources

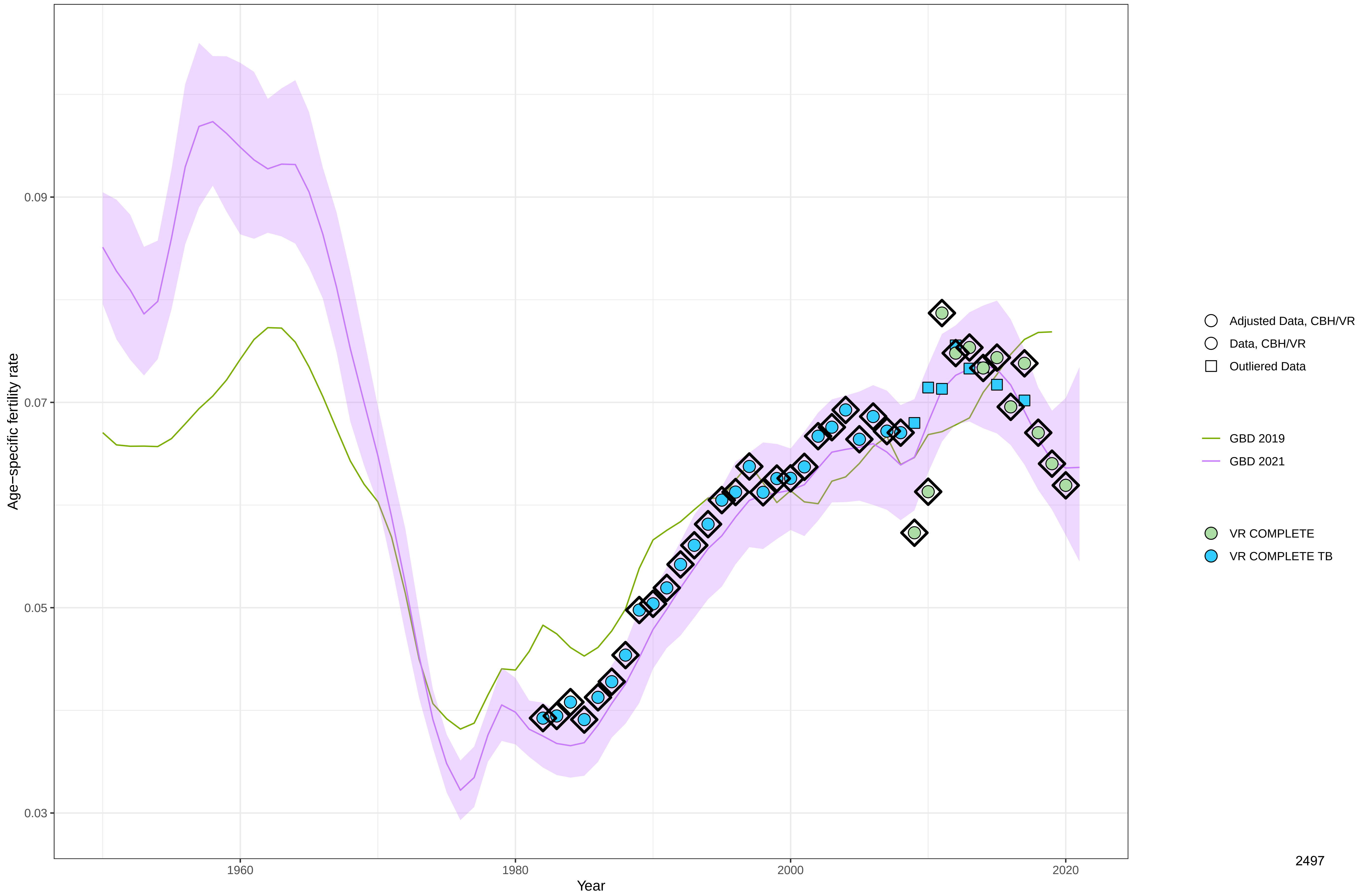

Age−specific fertility fitted trend lines, 1950−2021: Tower Hamlets, Ages: 40−44
Data points enclosed with a diamond are reference sources

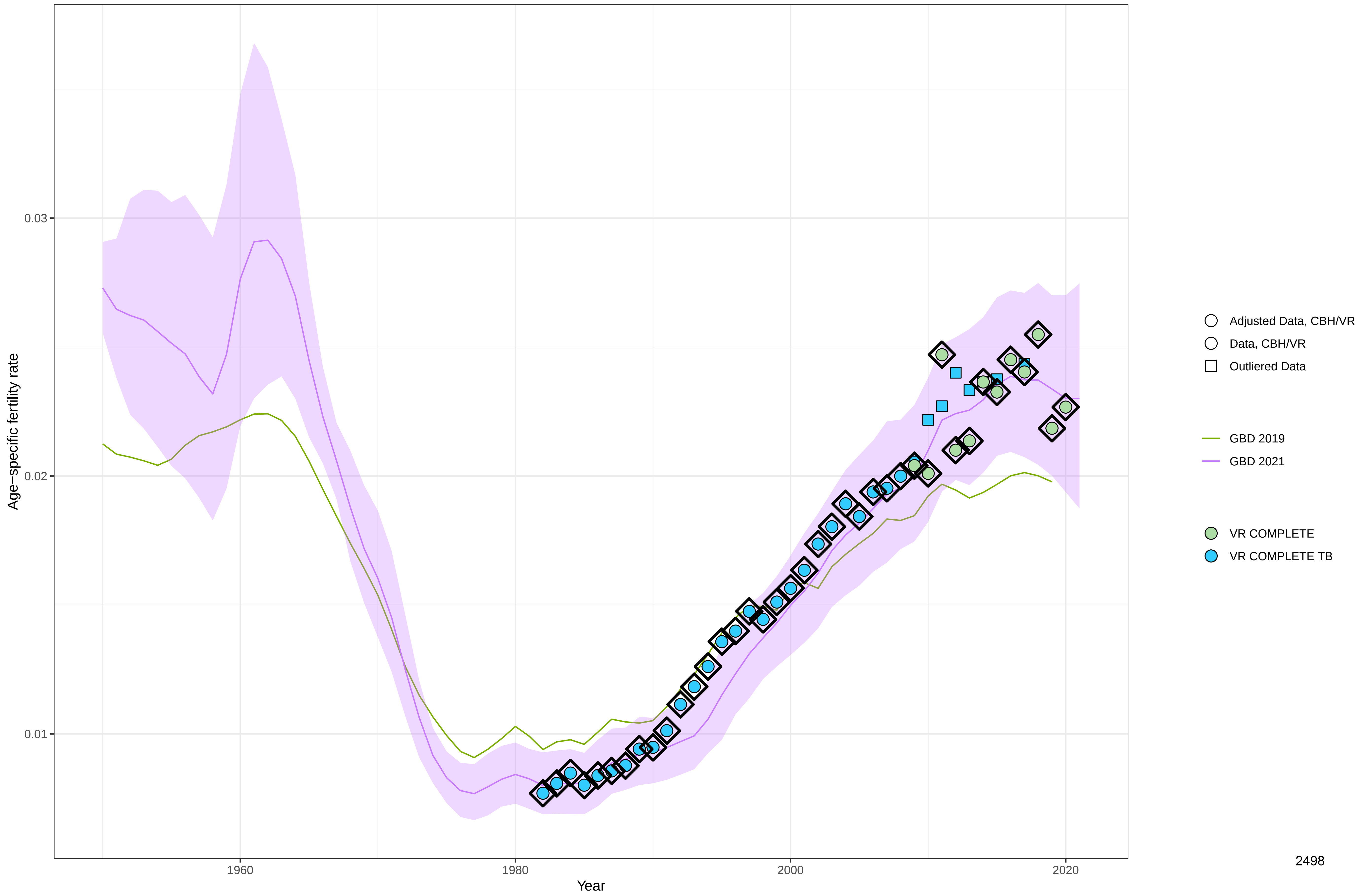

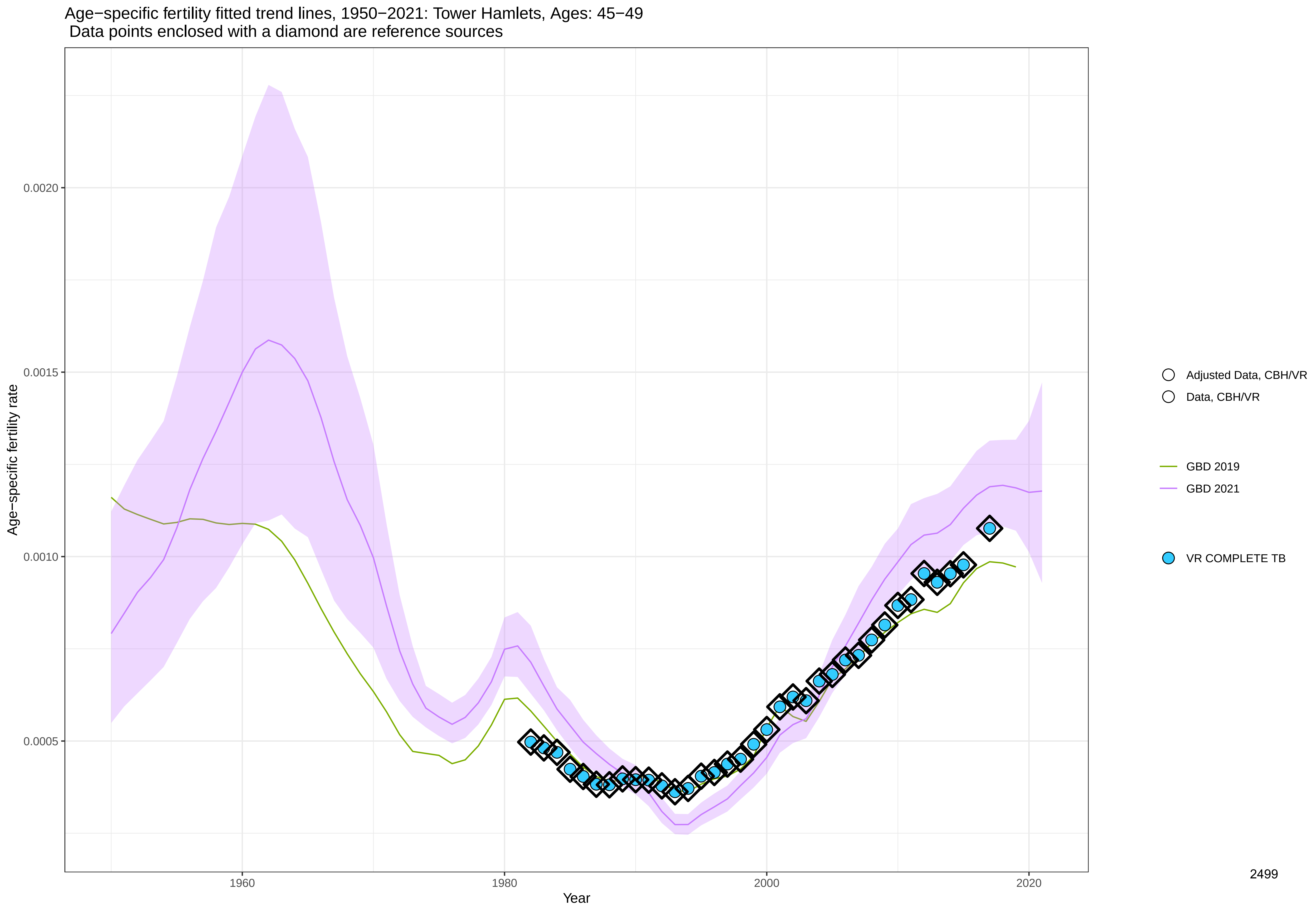

Age−specific fertility fitted trend lines, 1950−2021: Tower Hamlets, Ages: 45−49
Data points enclosed with a diamond are reference sources
Age−specific fertility rate
0.0020
0.0015
0.0010
0.0005
1960
1980
2000
2020
Year
Adjusted Data, CBH/VR
Data, CBH/VR
GBD 2019
GBD 2021
VR COMPLETE TB

Age−specific fertility fitted trend lines, 1950−2021: Tower Hamlets, Ages: 50−54
Data points enclosed with a diamond are reference sources

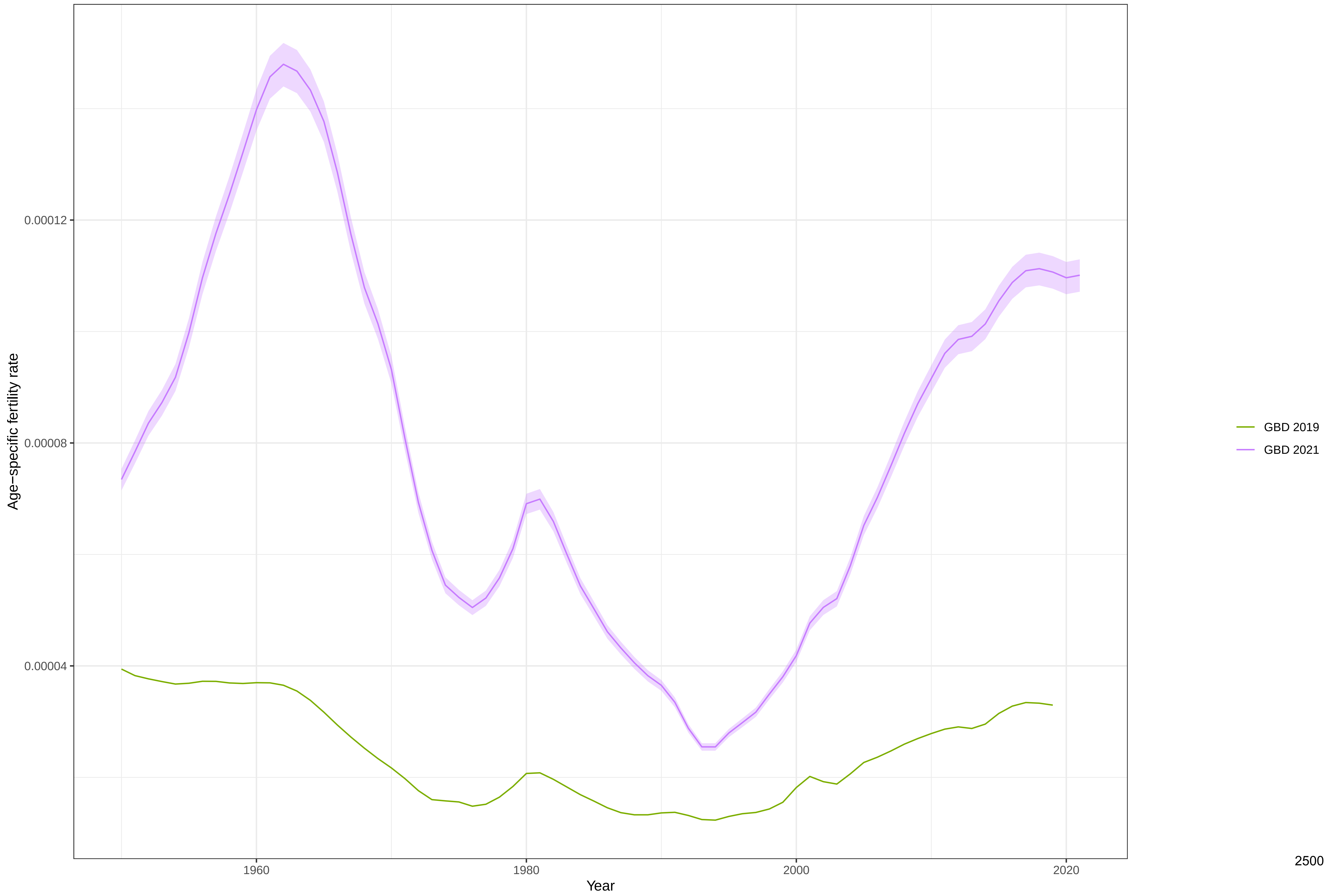

Total fertility rate fitted trend lines, 1950−2021: Tower Hamlets TFR
Data points enclosed with a diamond are reference sources

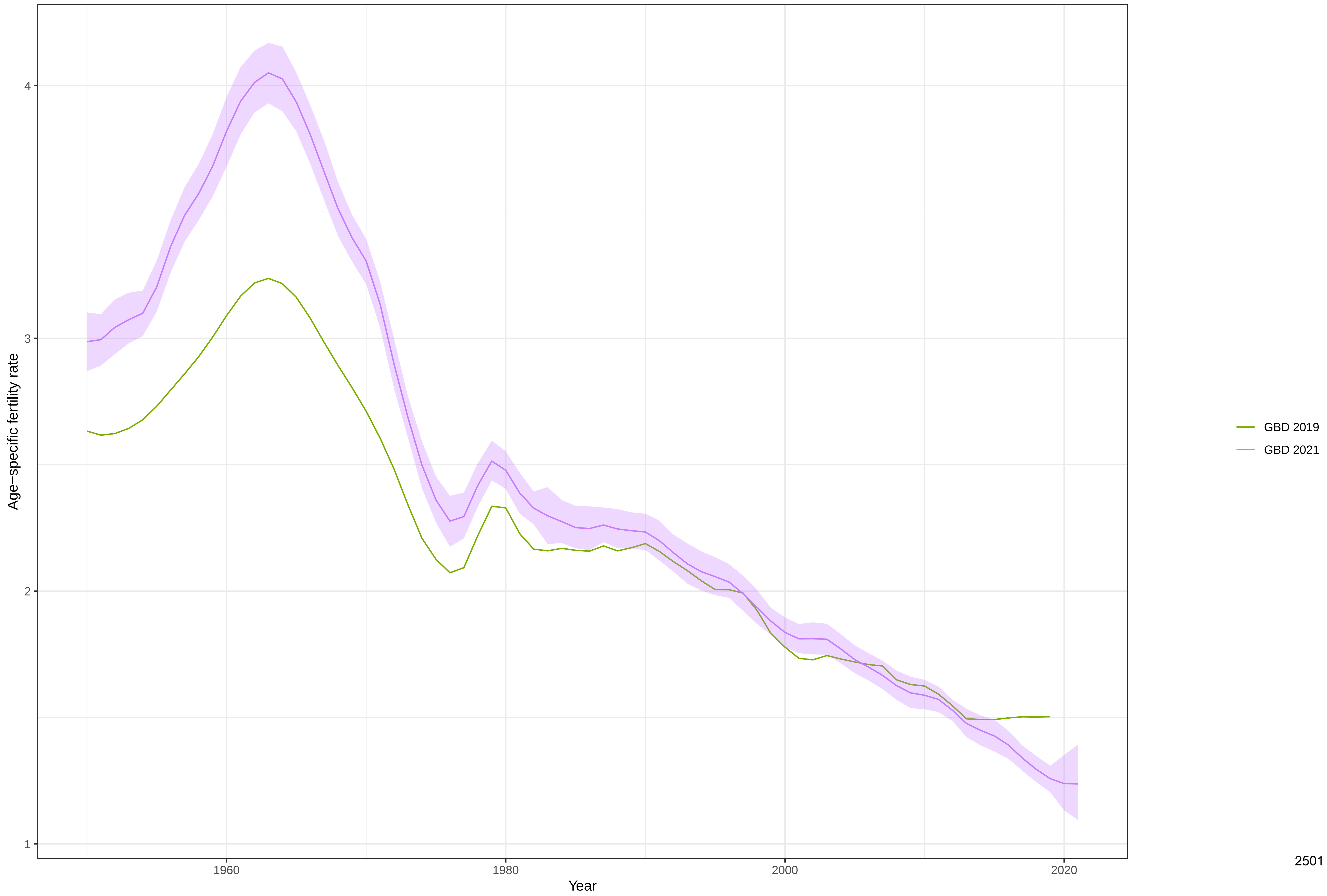

Age−specific fertility fitted trend lines, 1950−2021: Waltham Forest, Ages: 10−14
Data points enclosed with a diamond are reference sources

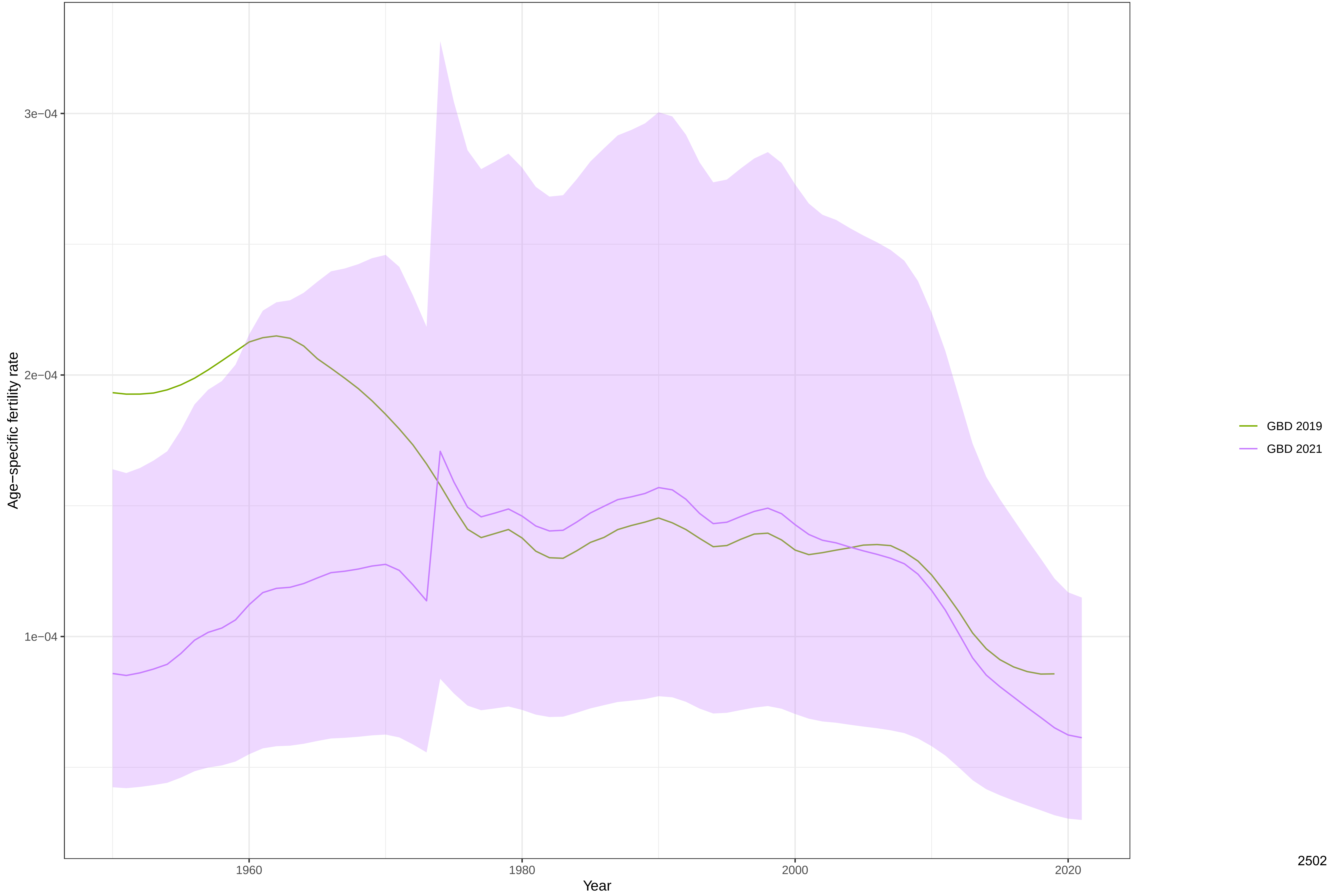

Age−specific fertility fitted trend lines, 1950−2021: Waltham Forest, Ages: 15−19
Data points enclosed with a diamond are reference sources

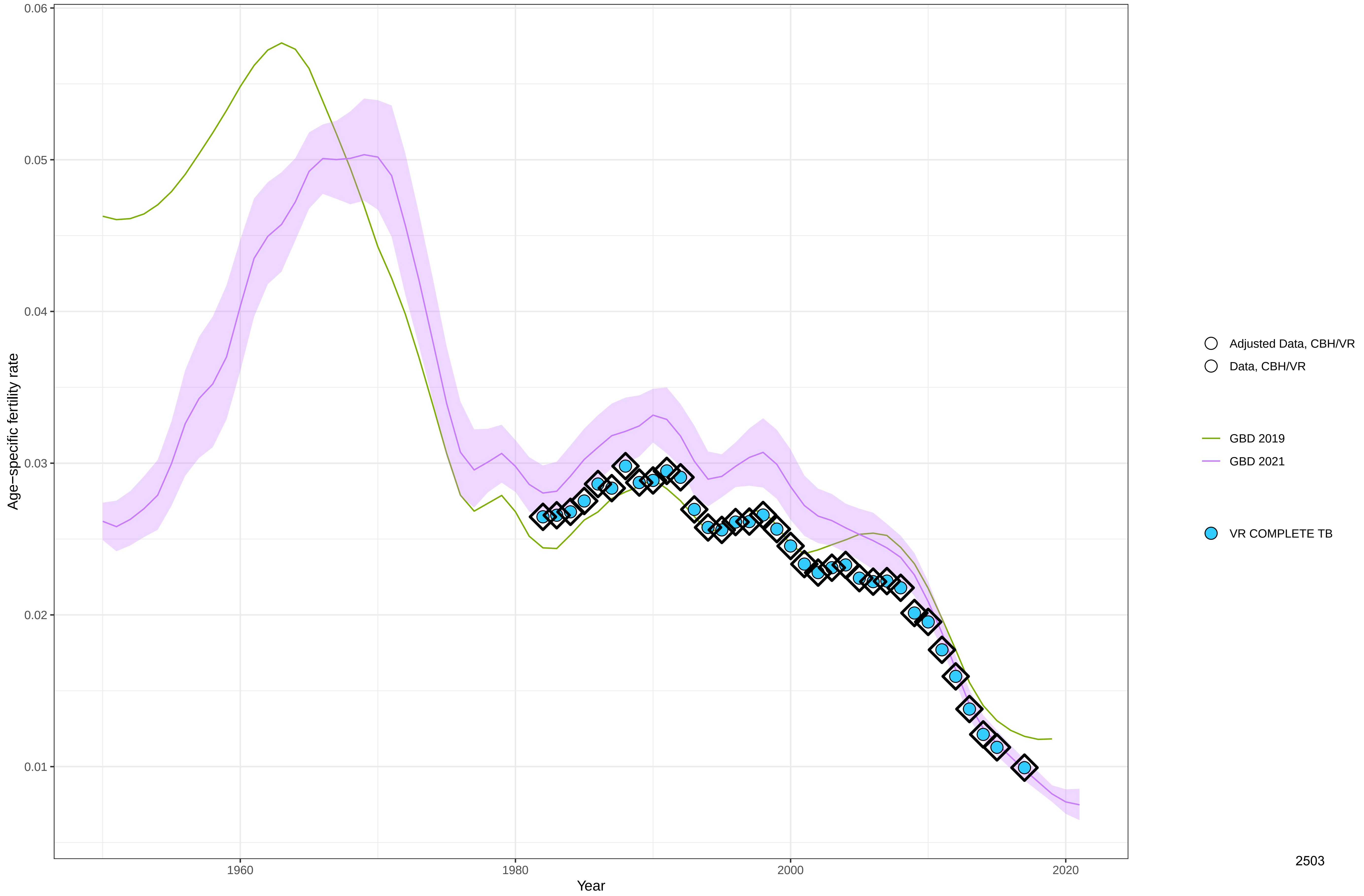

Age−specific fertility fitted trend lines, 1950−2021: Waltham Forest, Ages: 20−24
Data points enclosed with a diamond are reference sources

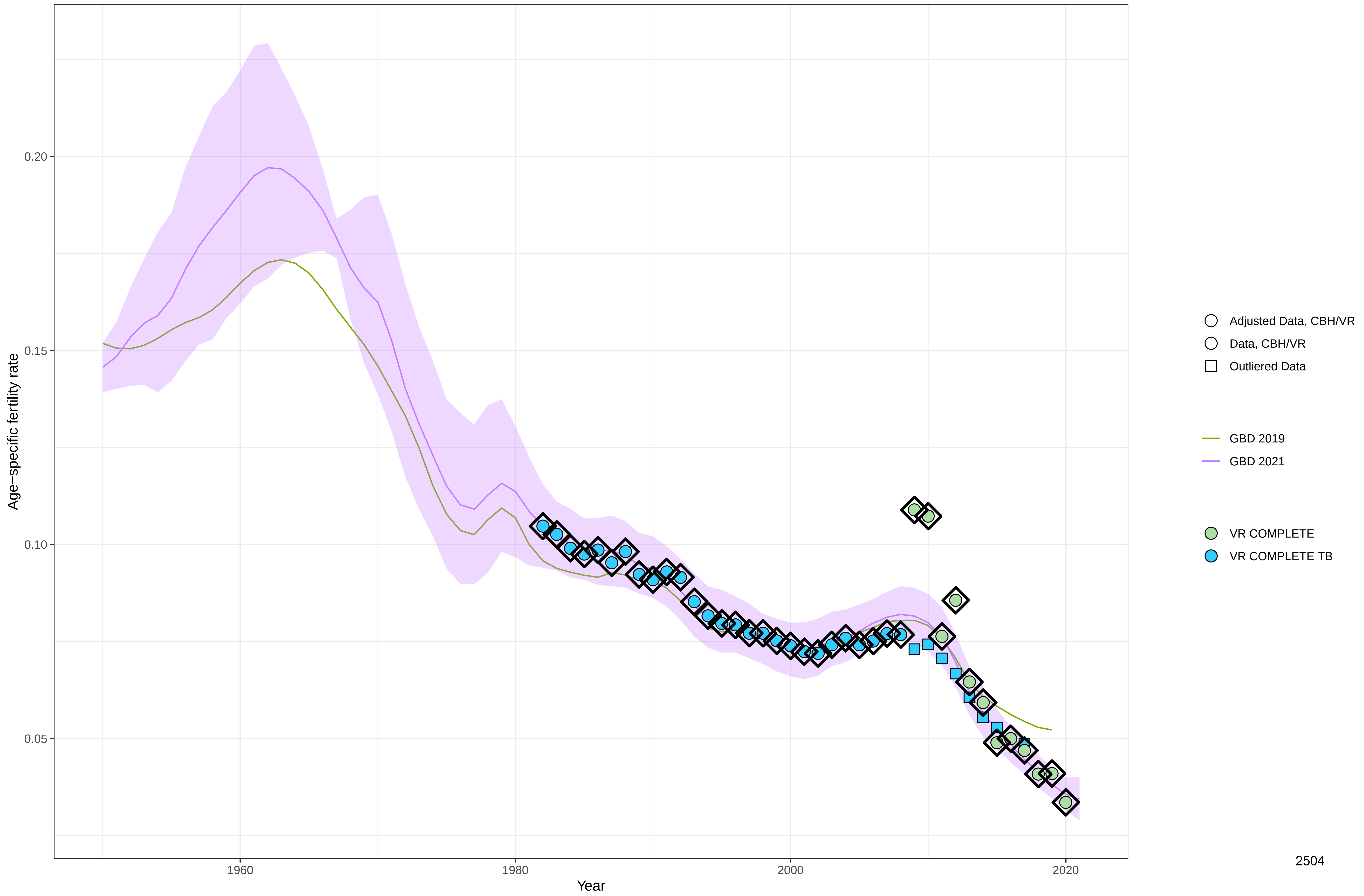

Age−specific fertility fitted trend lines, 1950−2021: Waltham Forest, Ages: 25−29
Data points enclosed with a diamond are reference sources

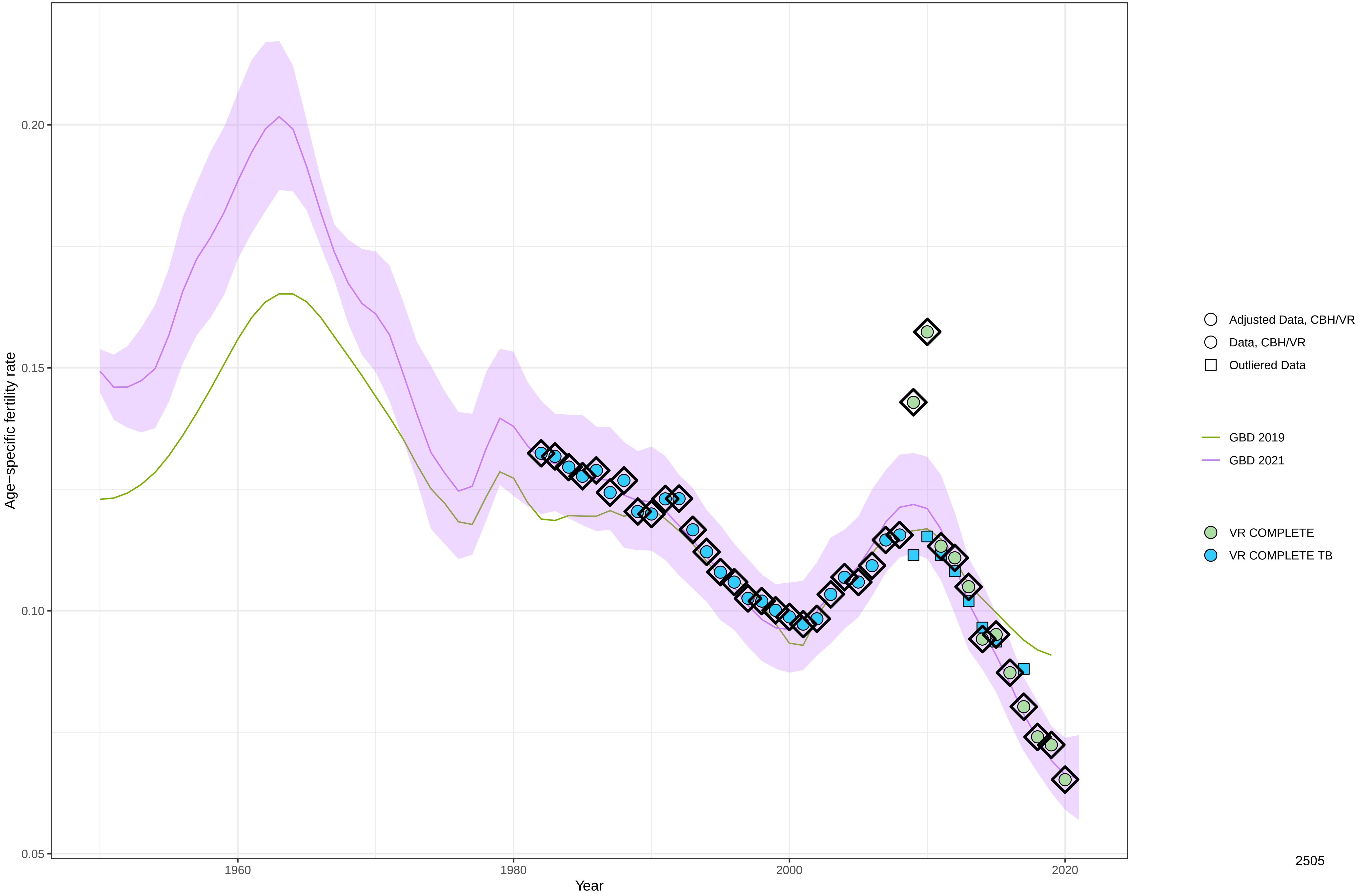

Age−specific fertility fitted trend lines, 1950−2021: Waltham Forest, Ages: 30−34
Data points enclosed with a diamond are reference sources

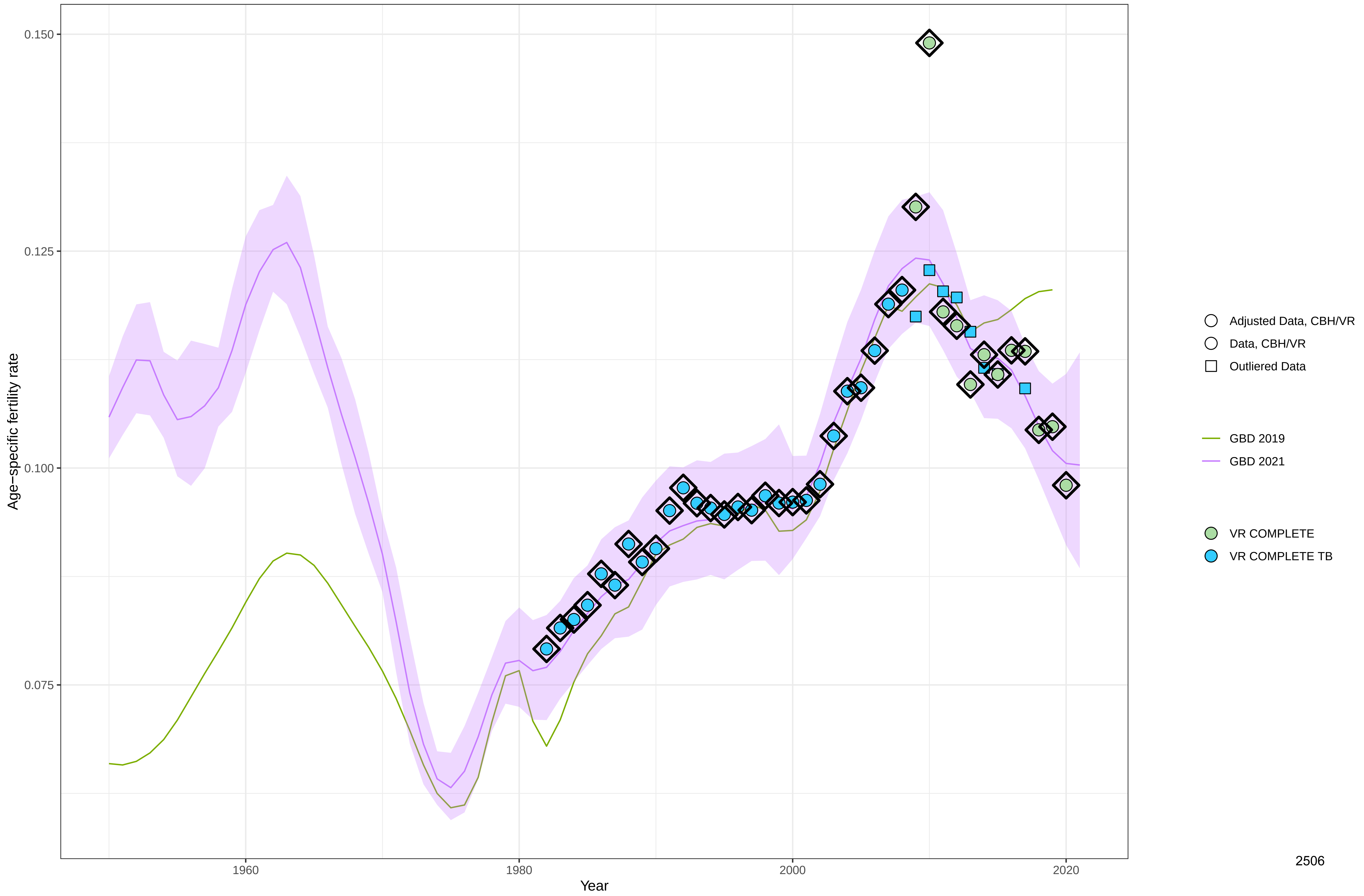

Age−specific fertility fitted trend lines, 1950−2021: Waltham Forest, Ages: 35−39
Data points enclosed with a diamond are reference sources

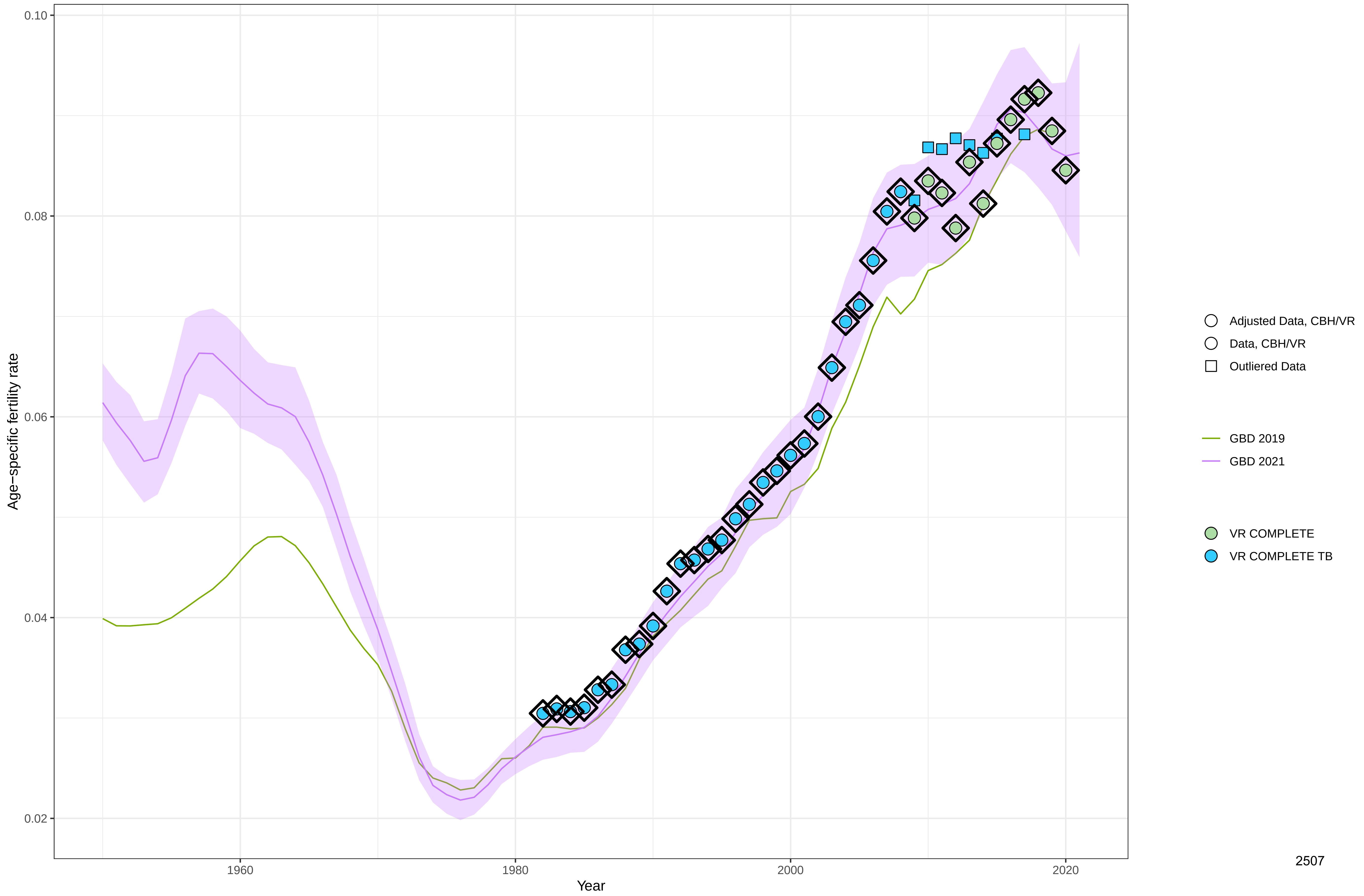

Age−specific fertility fitted trend lines, 1950−2021: Waltham Forest, Ages: 40−44
Data points enclosed with a diamond are reference sources

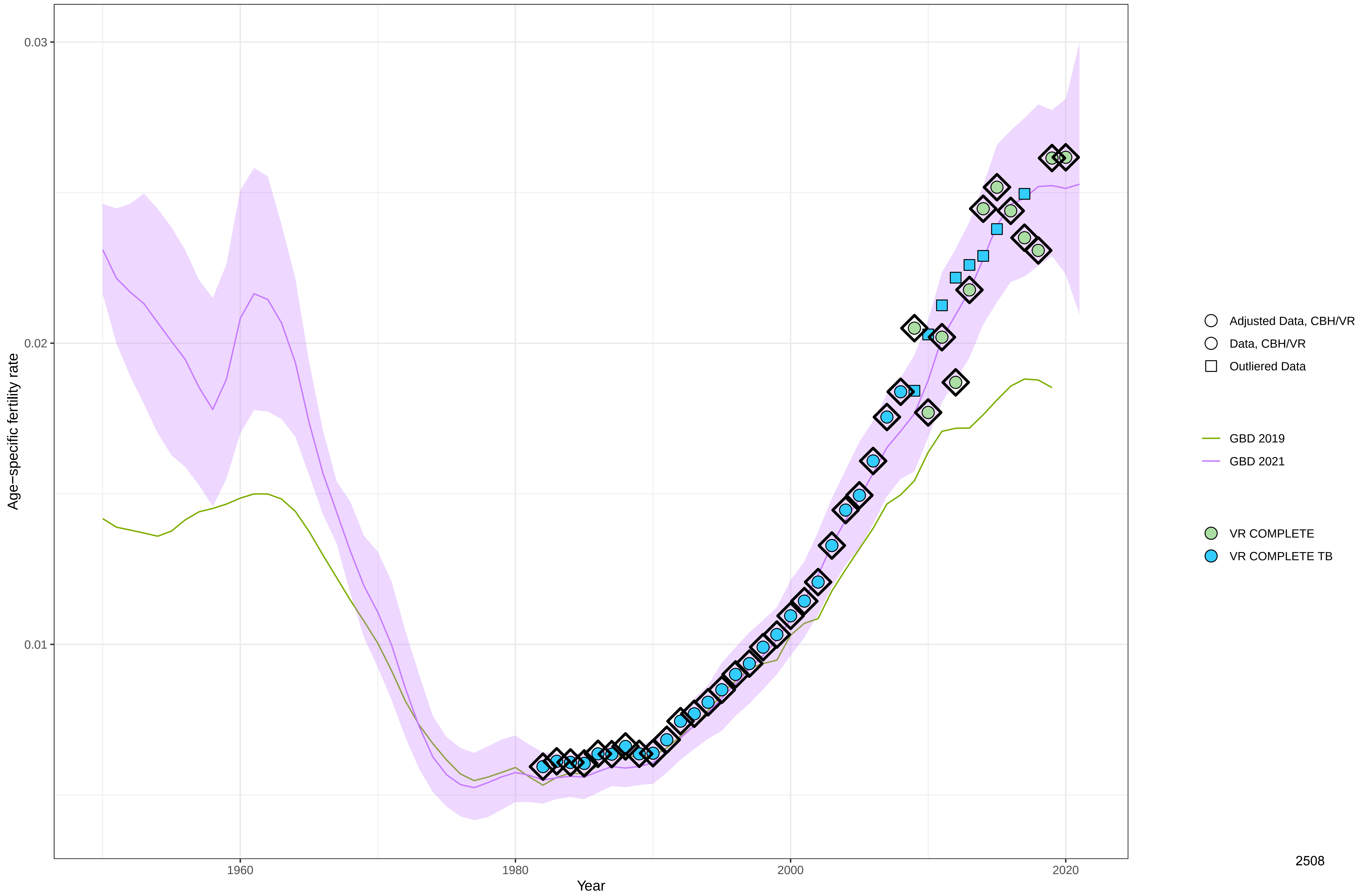

Age−specific fertility fitted trend lines, 1950−2021: Waltham Forest, Ages: 45−49
Data points enclosed with a diamond are reference sources

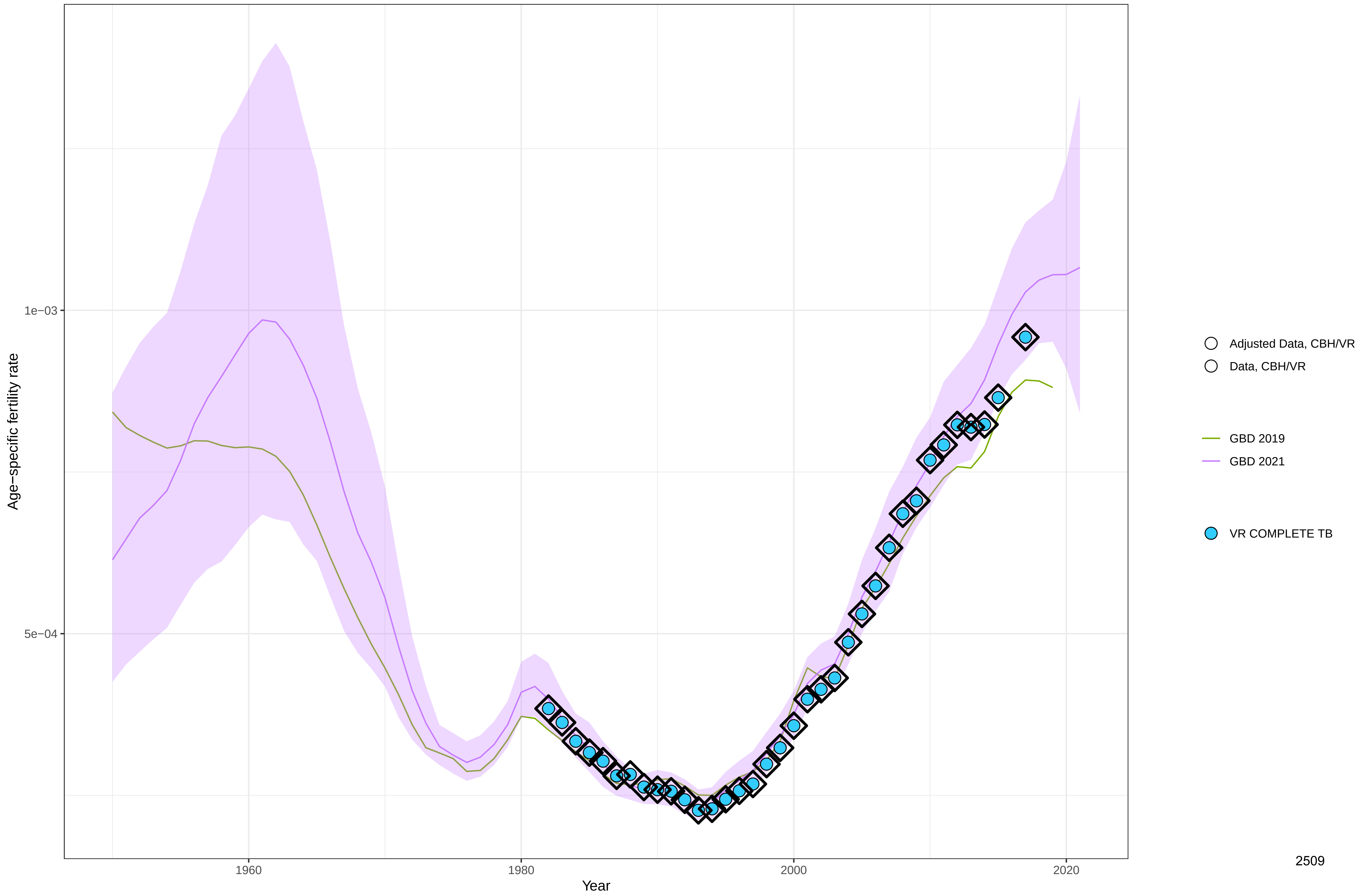

Age−specific fertility fitted trend lines, 1950−2021: Waltham Forest, Ages: 50−54
Data points enclosed with a diamond are reference sources

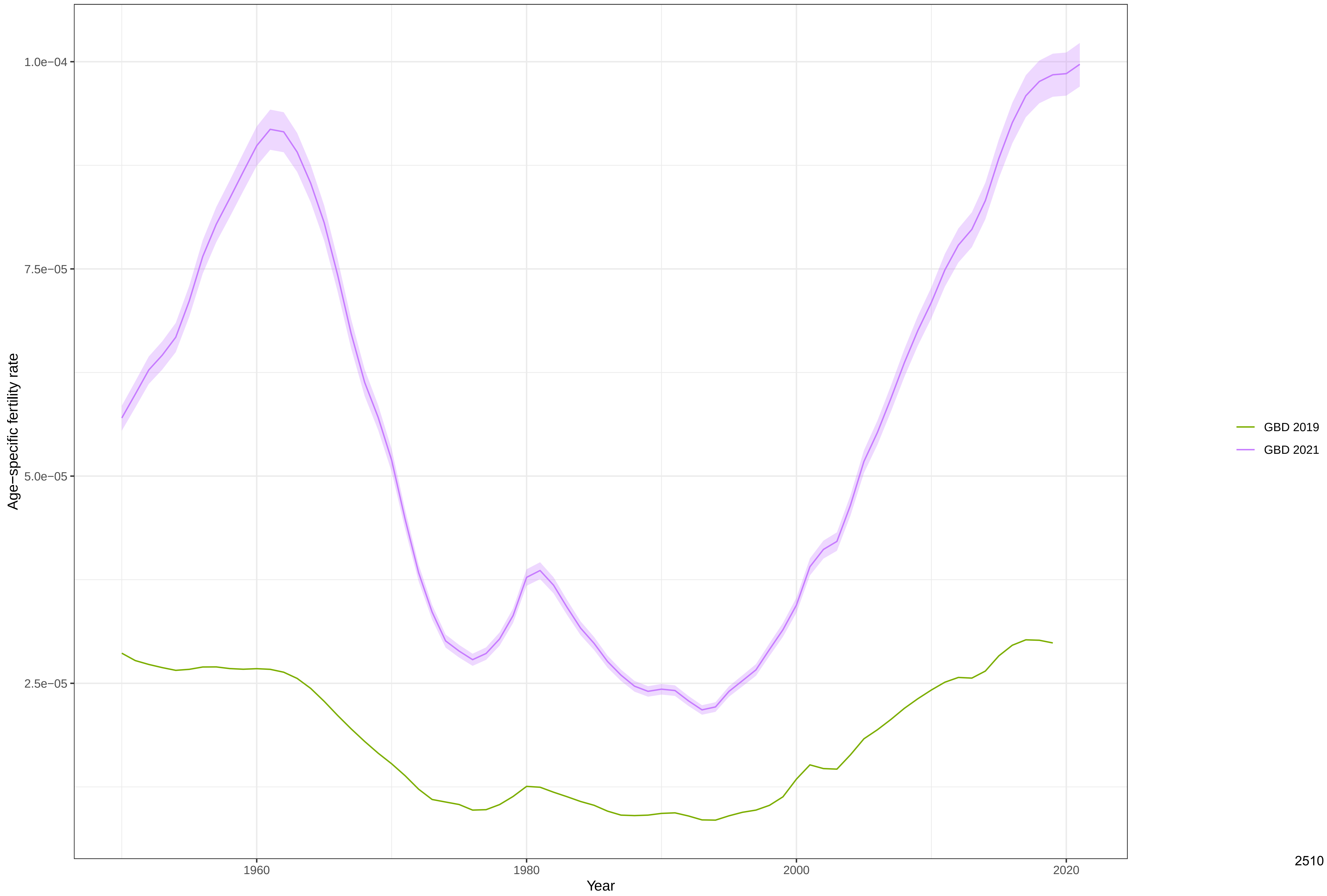

Total fertility rate fitted trend lines, 1950−2021: Waltham Forest TFR
 Data points enclosed with a diamond are reference sources

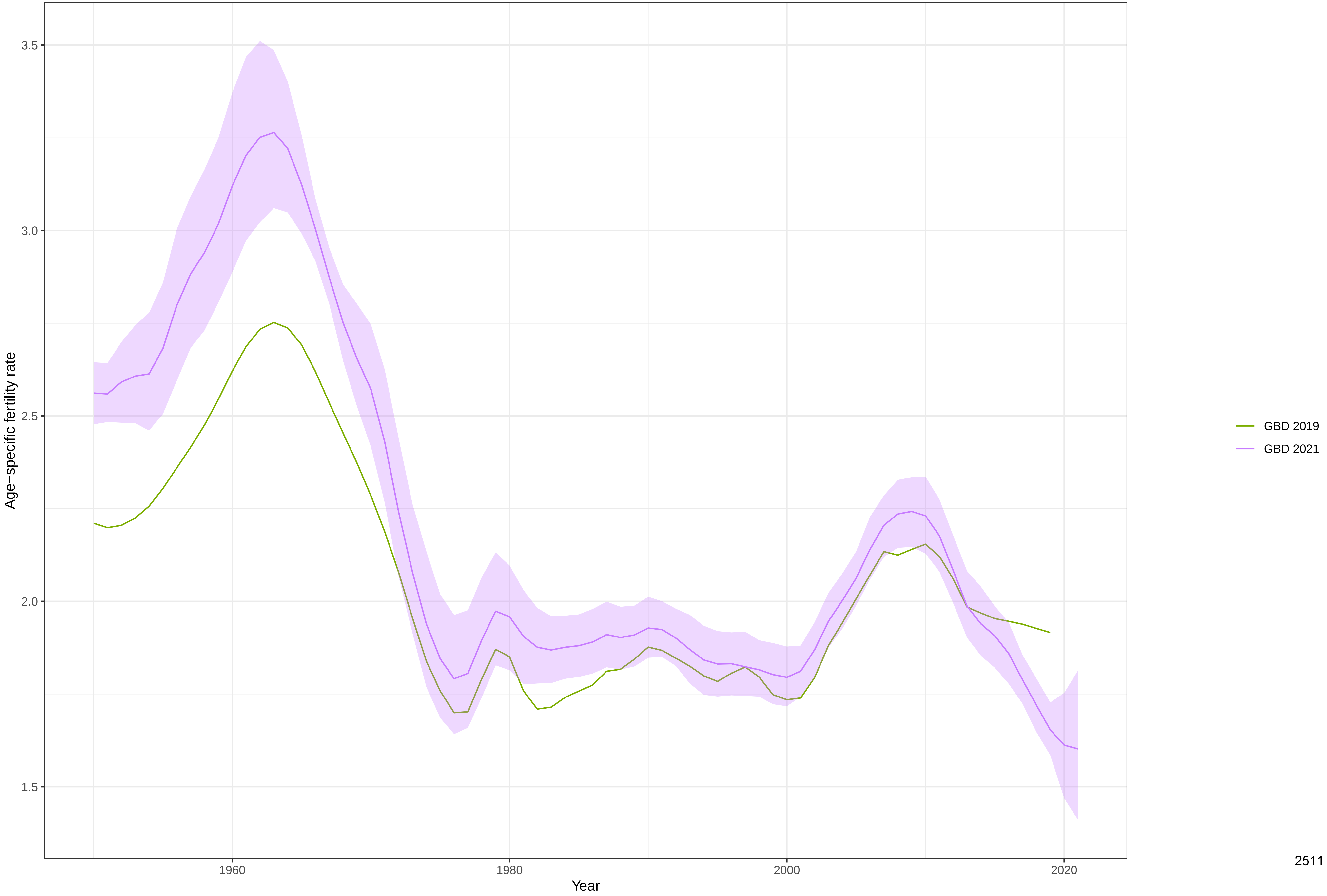

Age−specific fertility fitted trend lines, 1950−2021: Wandsworth, Ages: 10−14
Data points enclosed with a diamond are reference sources

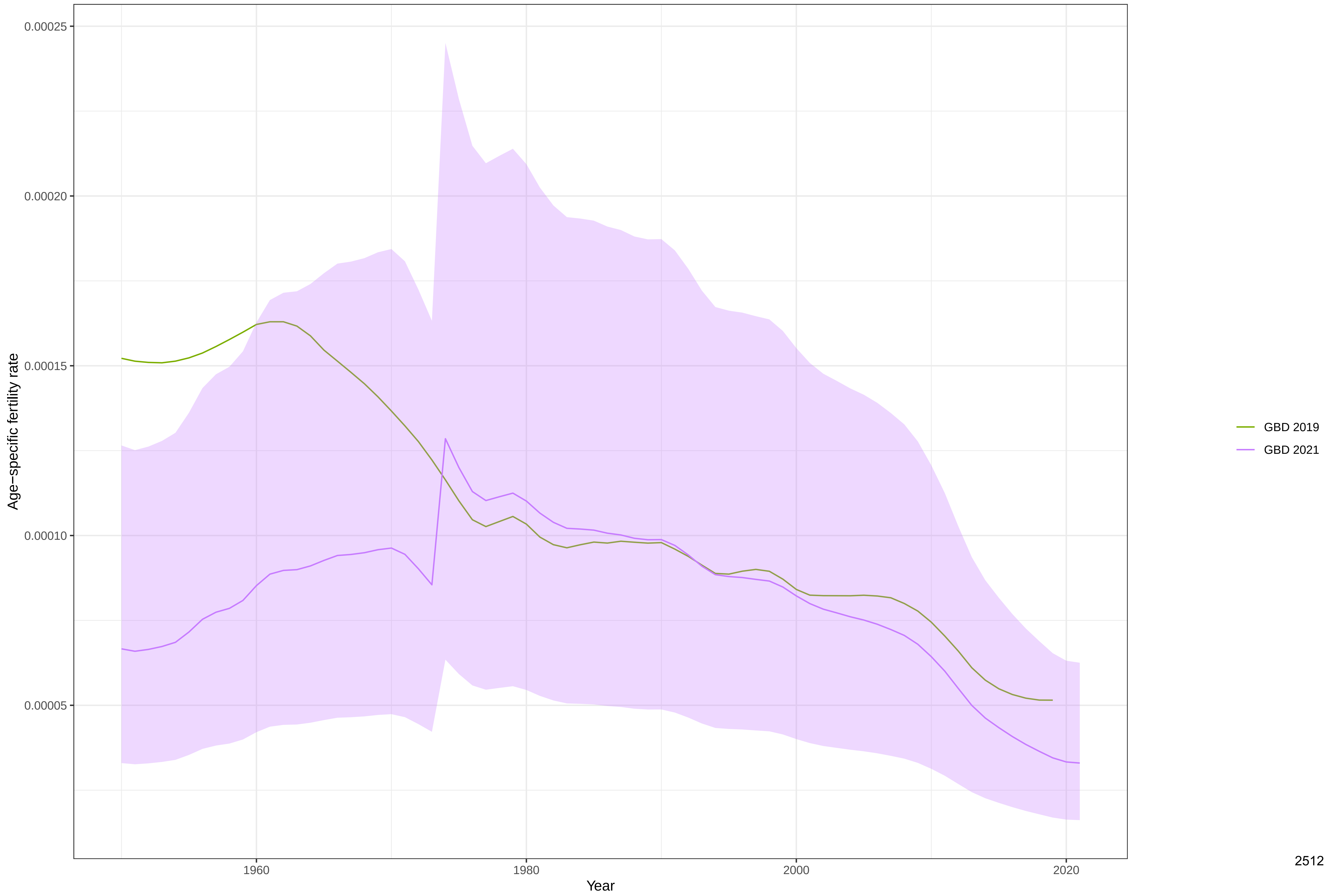

Age−specific fertility fitted trend lines, 1950−2021: Wandsworth, Ages: 15−19
Data points enclosed with a diamond are reference sources

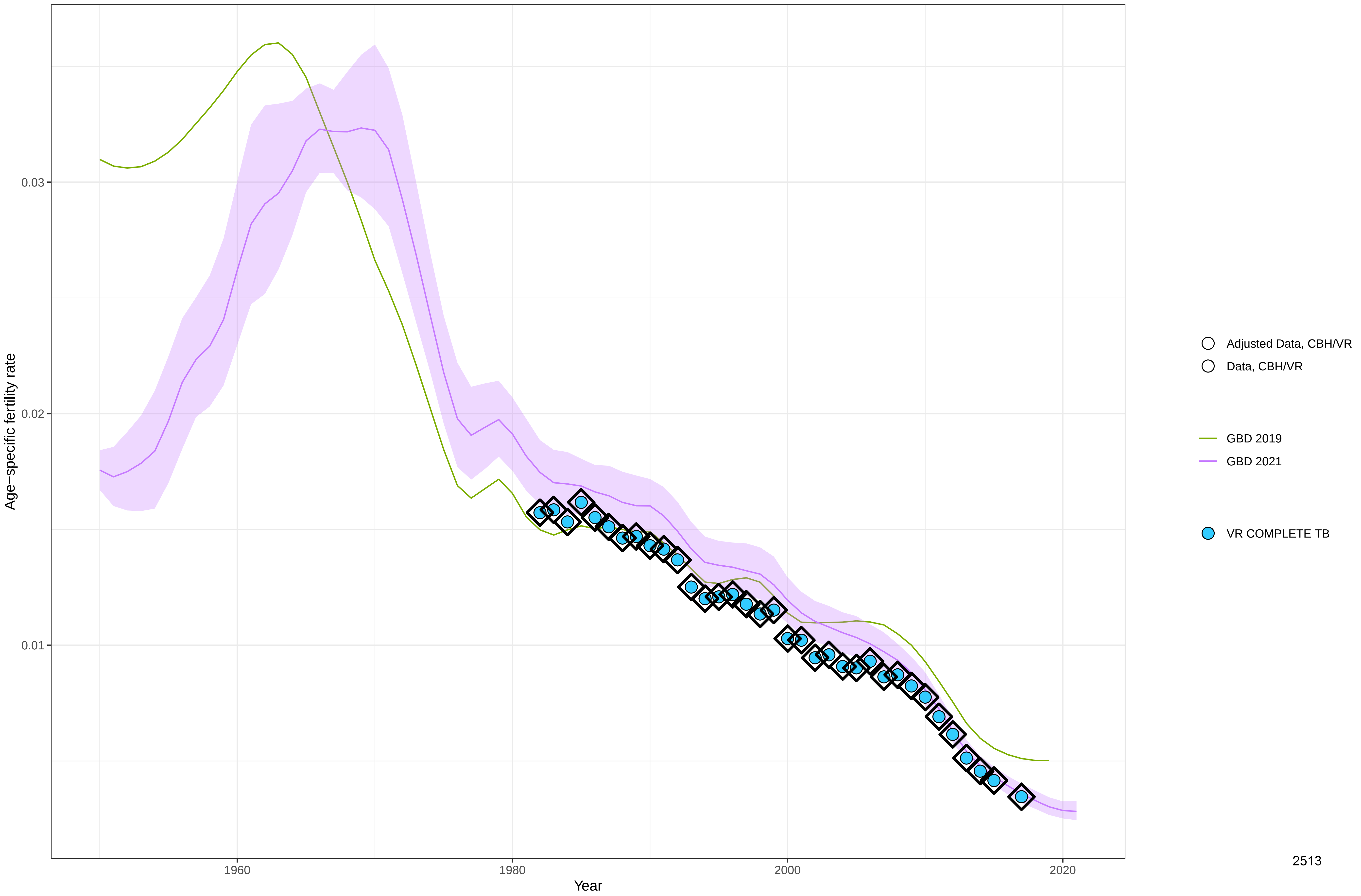

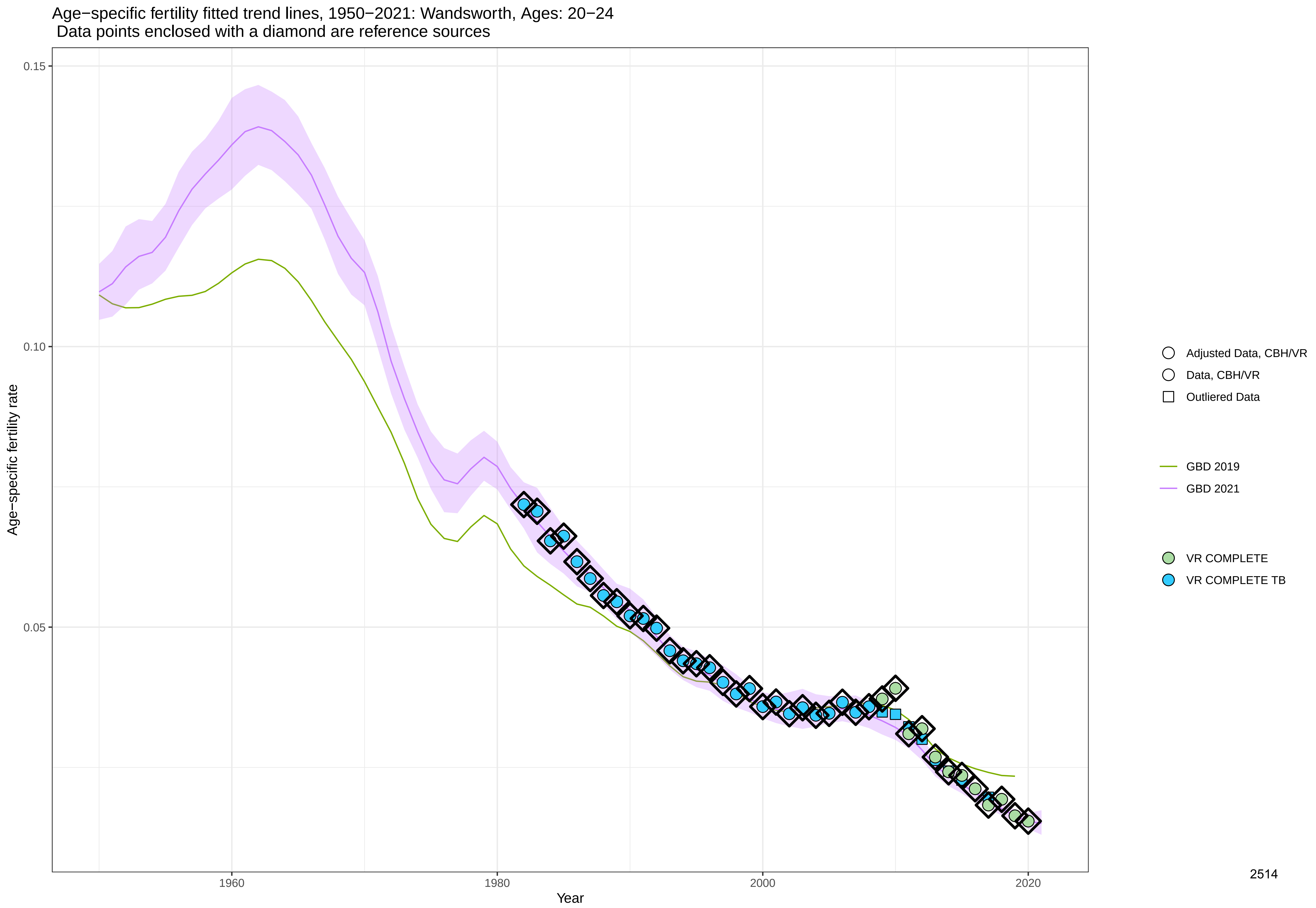

Age−specific fertility fitted trend lines, 1950−2021: Wandsworth, Ages: 20−24
Data points enclosed with a diamond are reference sources
0.15
0.10
0.05
Age−specific fertility rate
1960
1980
2000
2020
Year
Adjusted Data, CBH/VR
Data, CBH/VR
Outliered Data
GBD 2019
GBD 2021
VR COMPLETE
VR COMPLETE TB

Age−specific fertility fitted trend lines, 1950−2021: Wandsworth, Ages: 25−29
Data points enclosed with a diamond are reference sources

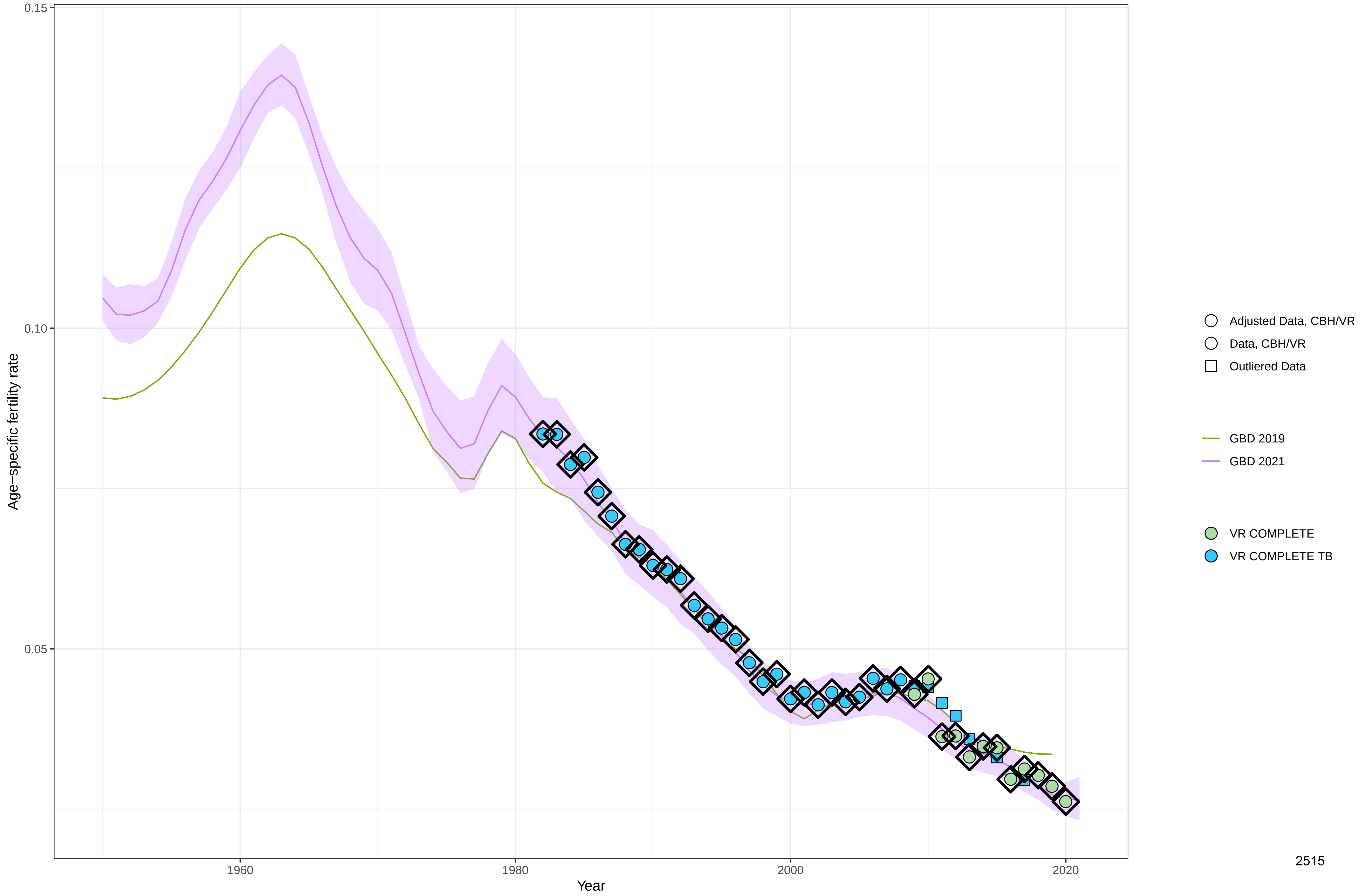

Age−specific fertility fitted trend lines, 1950−2021: Wandsworth, Ages: 30−34
Data points enclosed with a diamond are reference sources

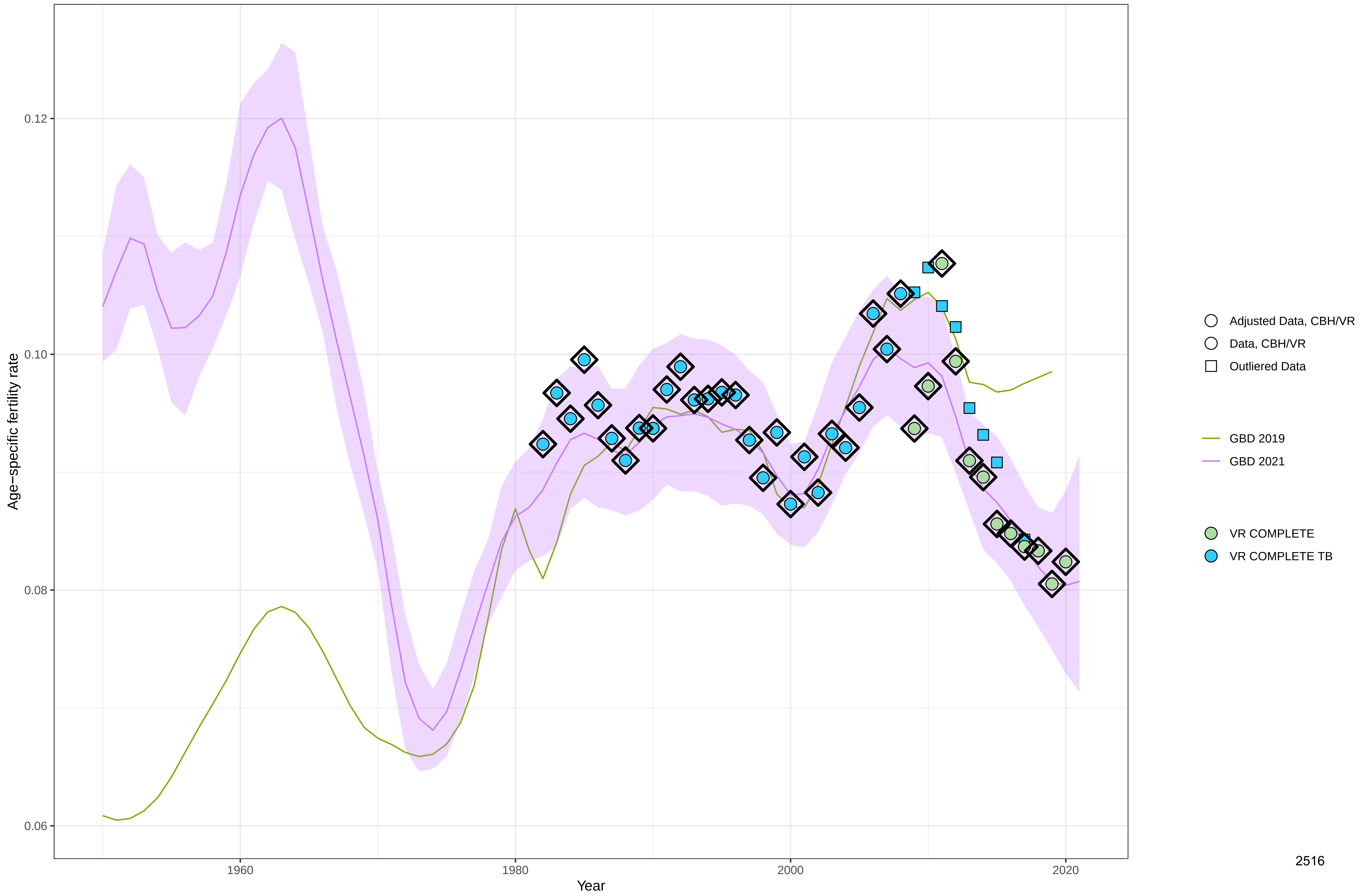

Age−specific fertility fitted trend lines, 1950−2021: Wandsworth, Ages: 35−39
Data points enclosed with a diamond are reference sources

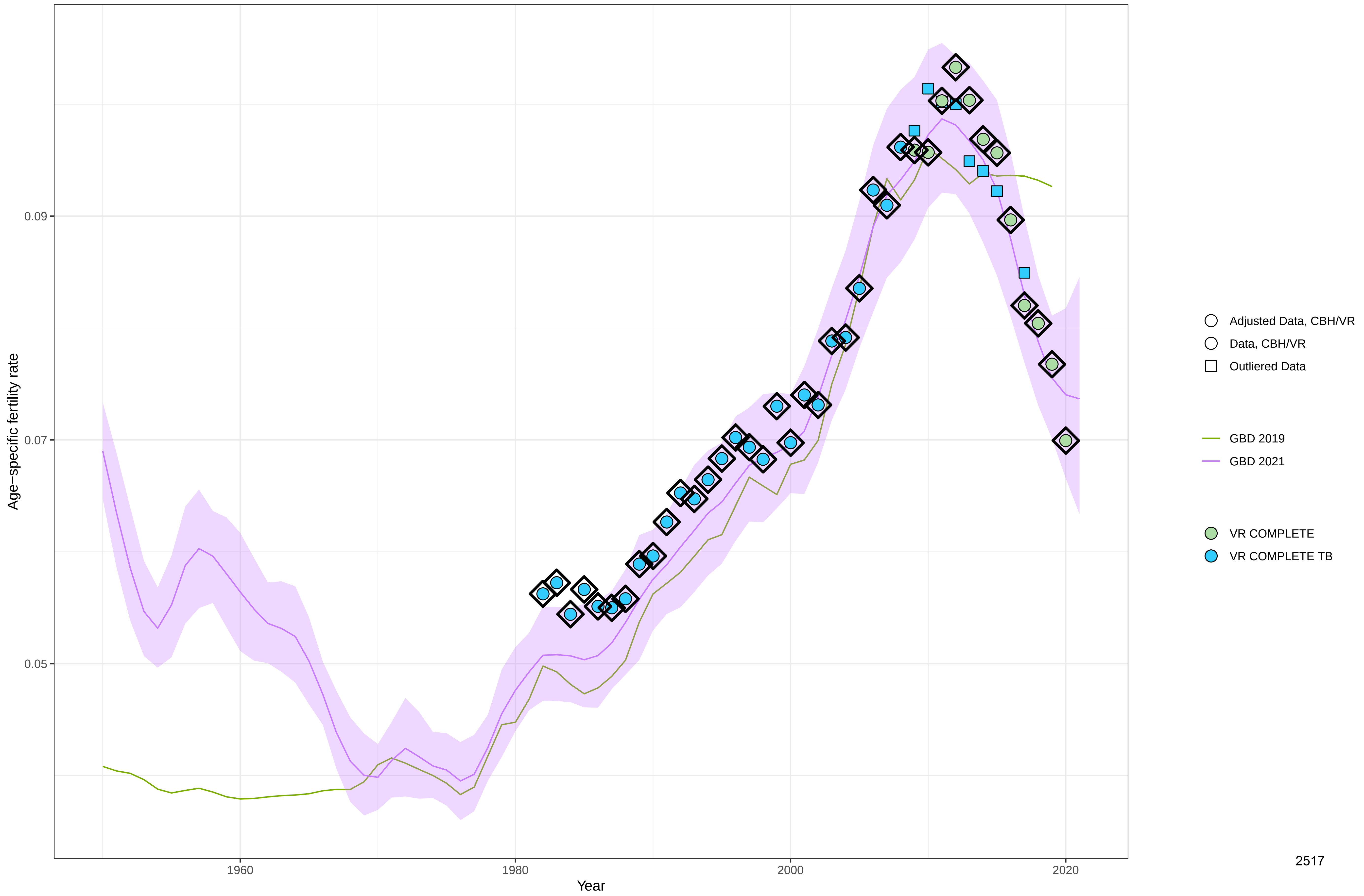

Age−specific fertility fitted trend lines, 1950−2021: Wandsworth, Ages: 40−44
Data points enclosed with a diamond are reference sources

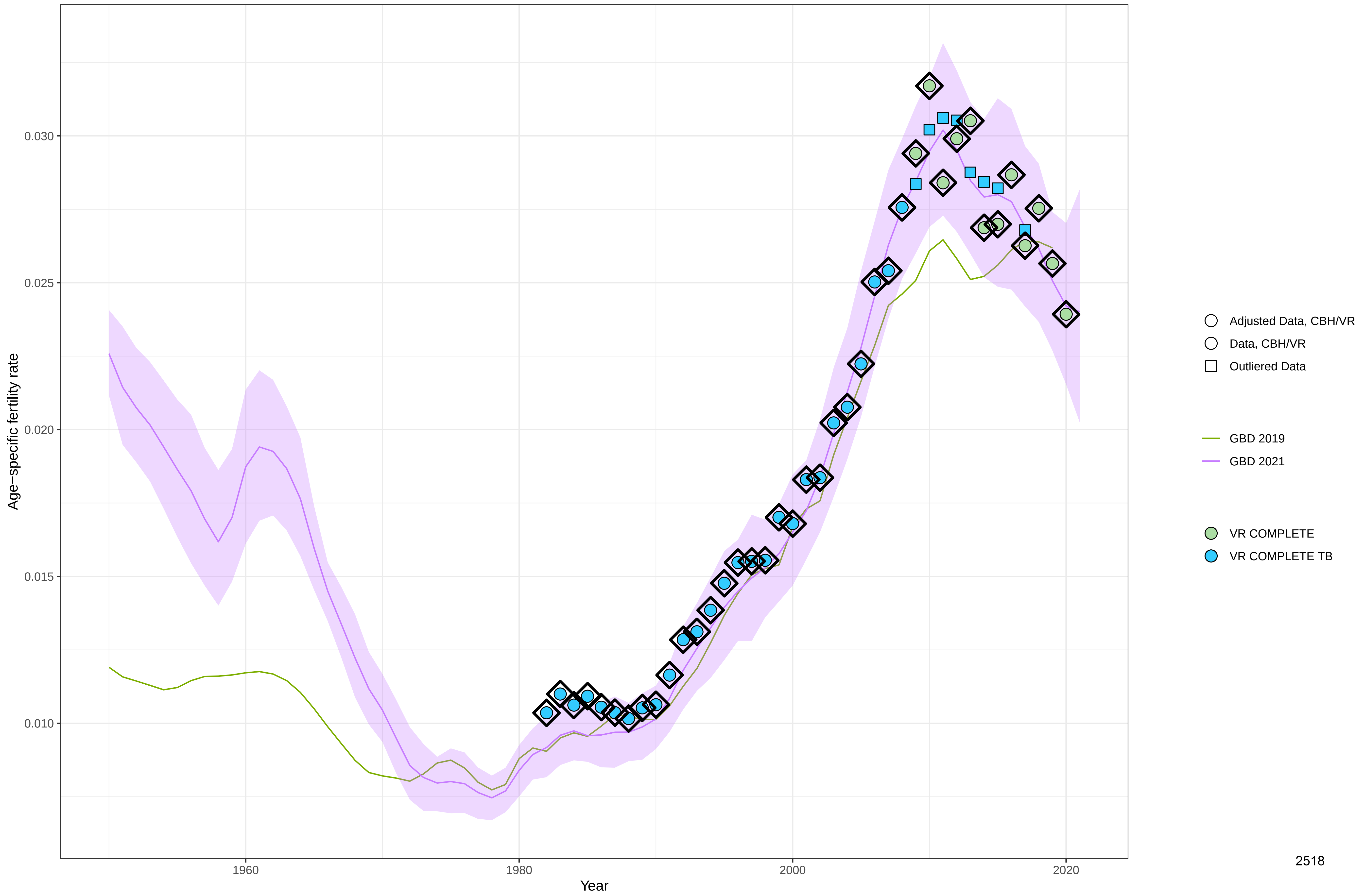

Age−specific fertility fitted trend lines, 1950−2021: Wandsworth, Ages: 45−49
Data points enclosed with a diamond are reference sources

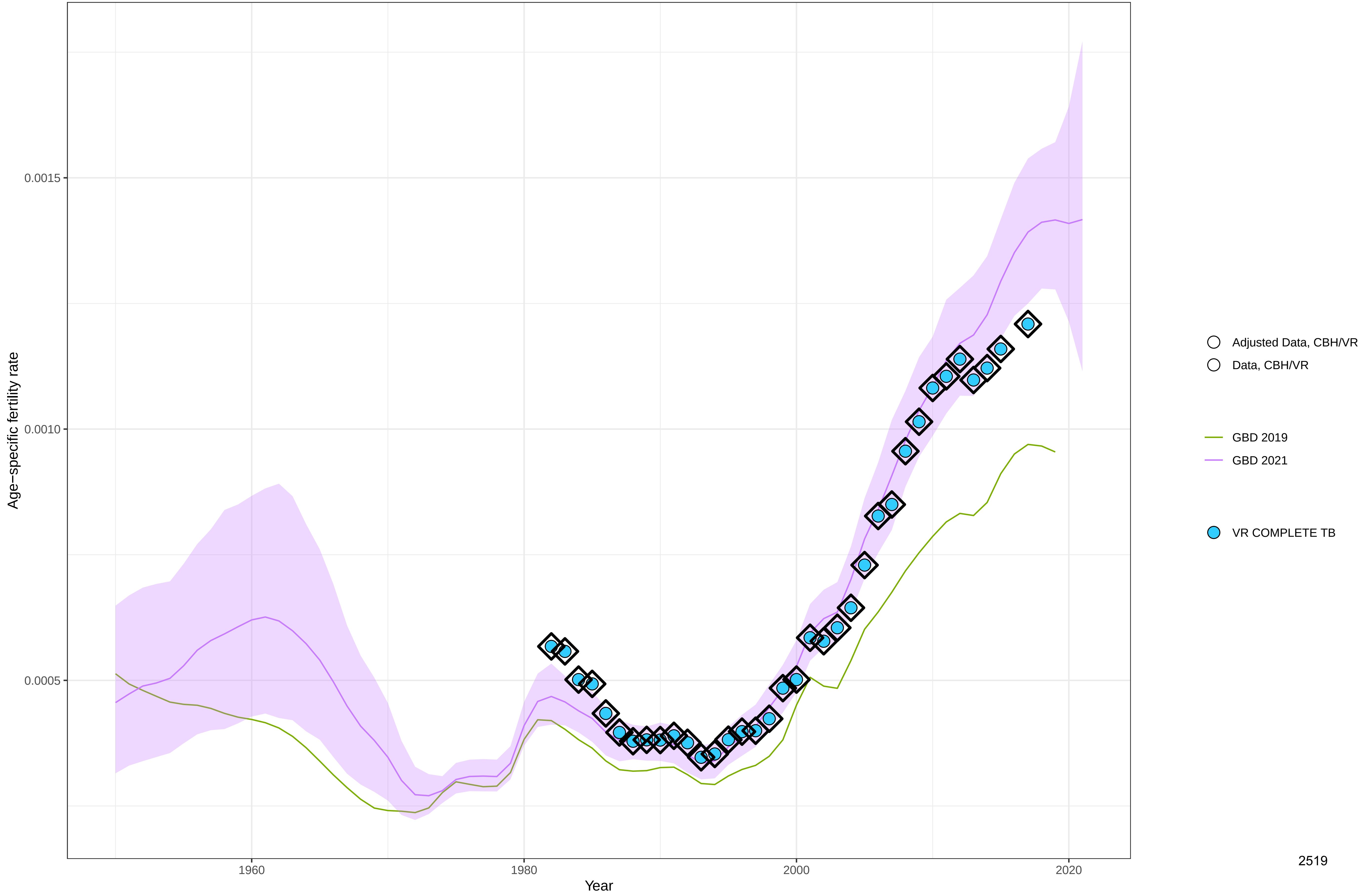

Age−specific fertility fitted trend lines, 1950−2021: Wandsworth, Ages: 50−54
Data points enclosed with a diamond are reference sources

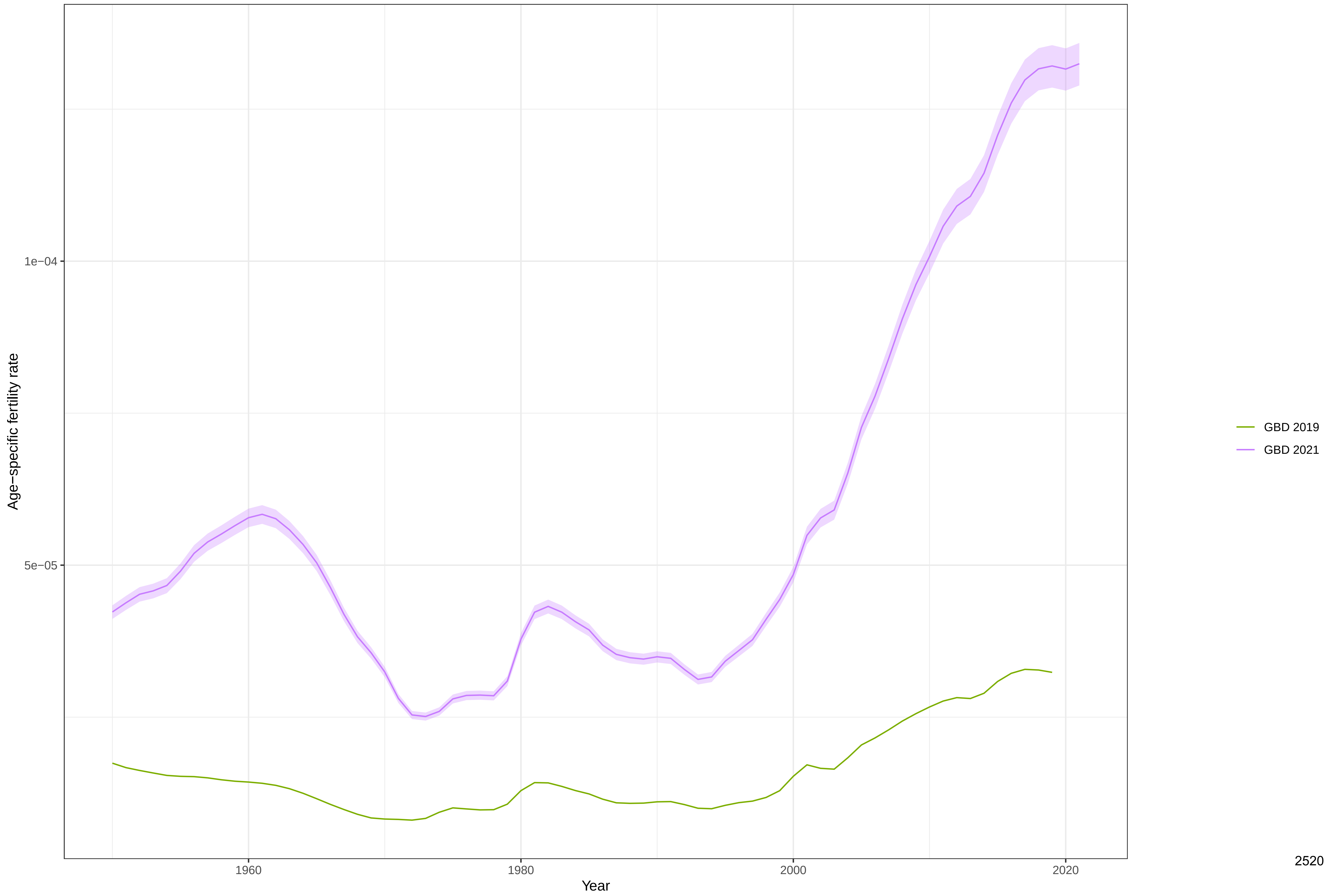

Total fertility rate fitted trend lines, 1950−2021: Wandsworth TFR
Data points enclosed with a diamond are reference sources

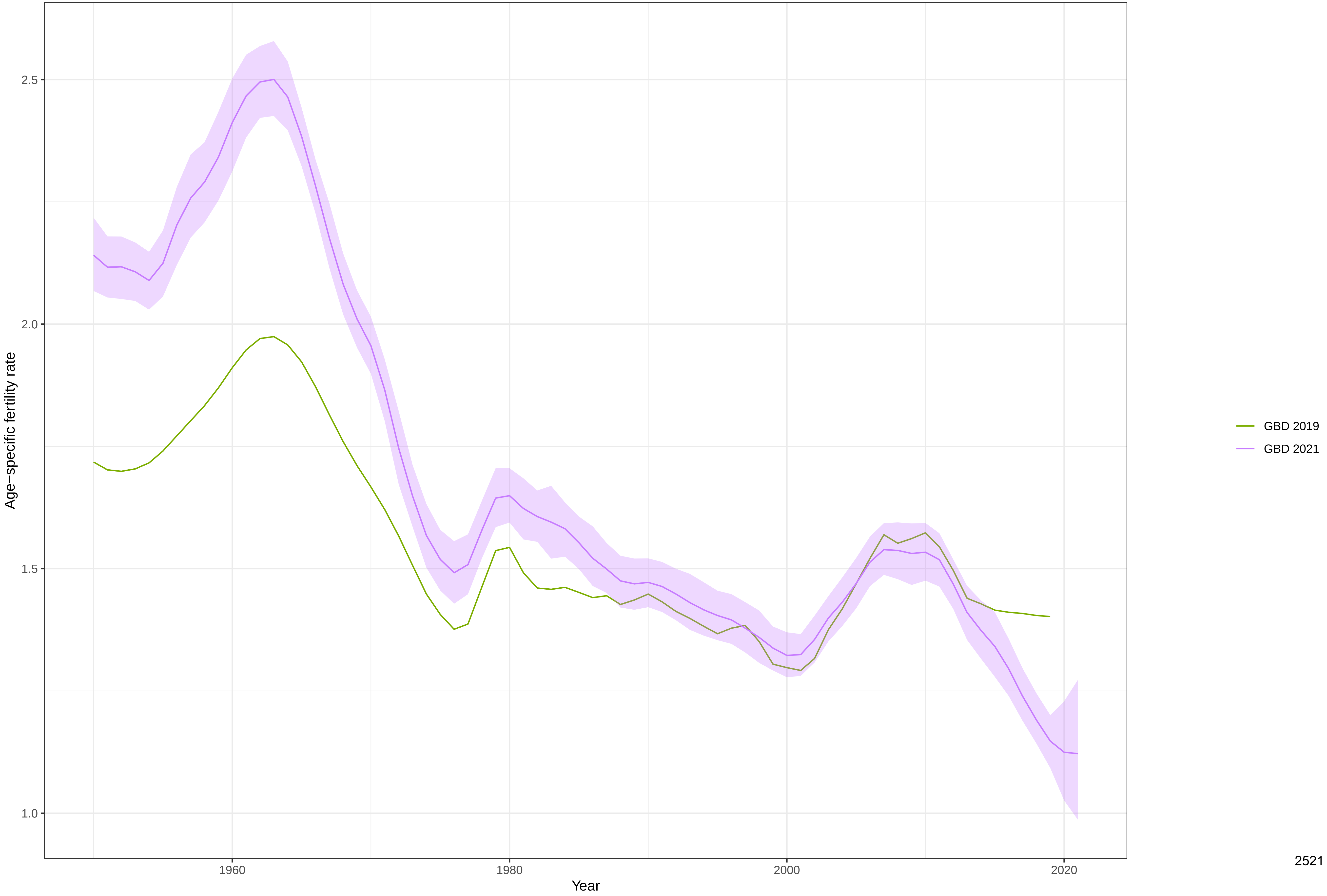

Age−specific fertility fitted trend lines, 1950−2021: Westminster, Ages: 10−14
Data points enclosed with a diamond are reference sources

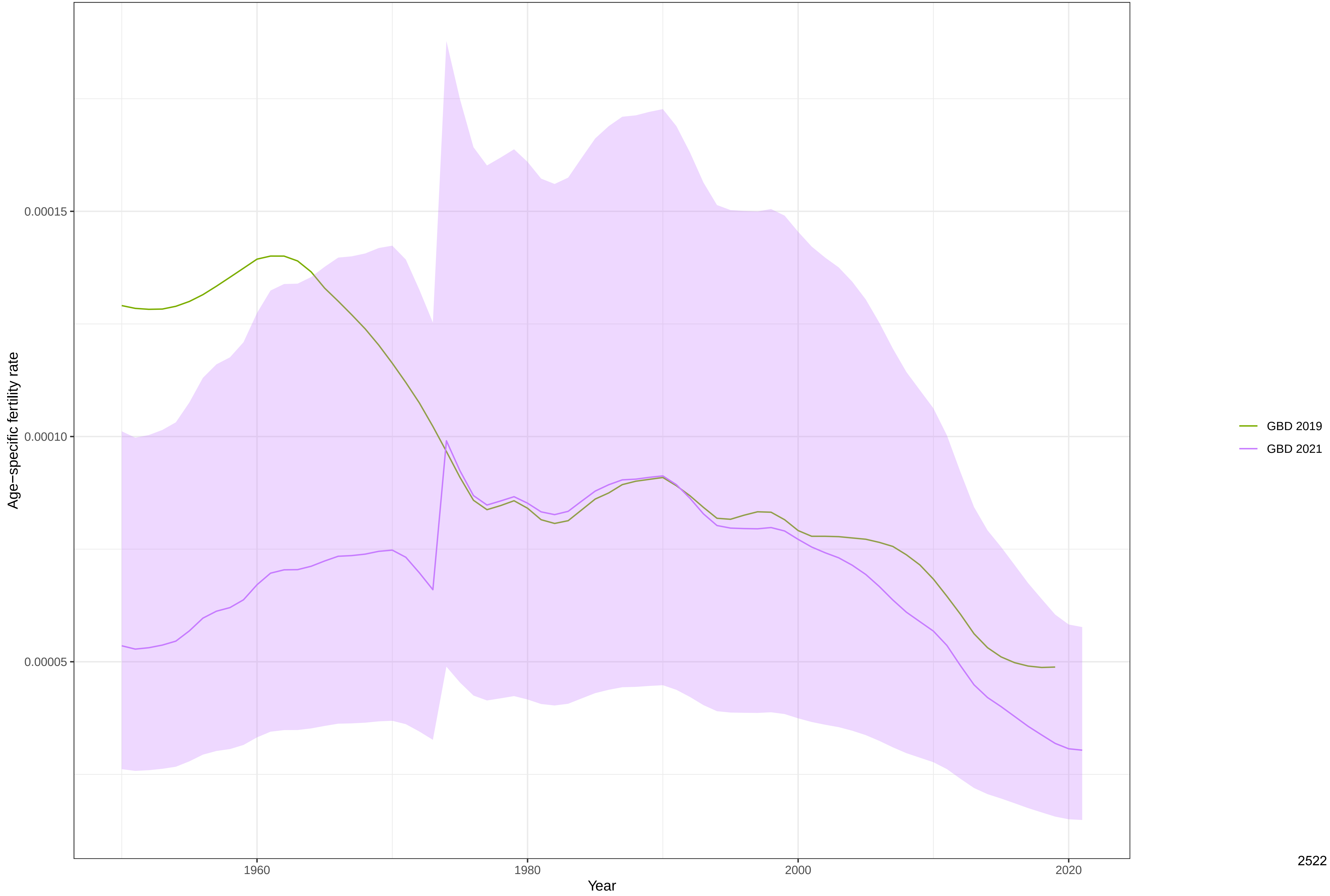

Age−specific fertility fitted trend lines, 1950−2021: Westminster, Ages: 15−19
Data points enclosed with a diamond are reference sources

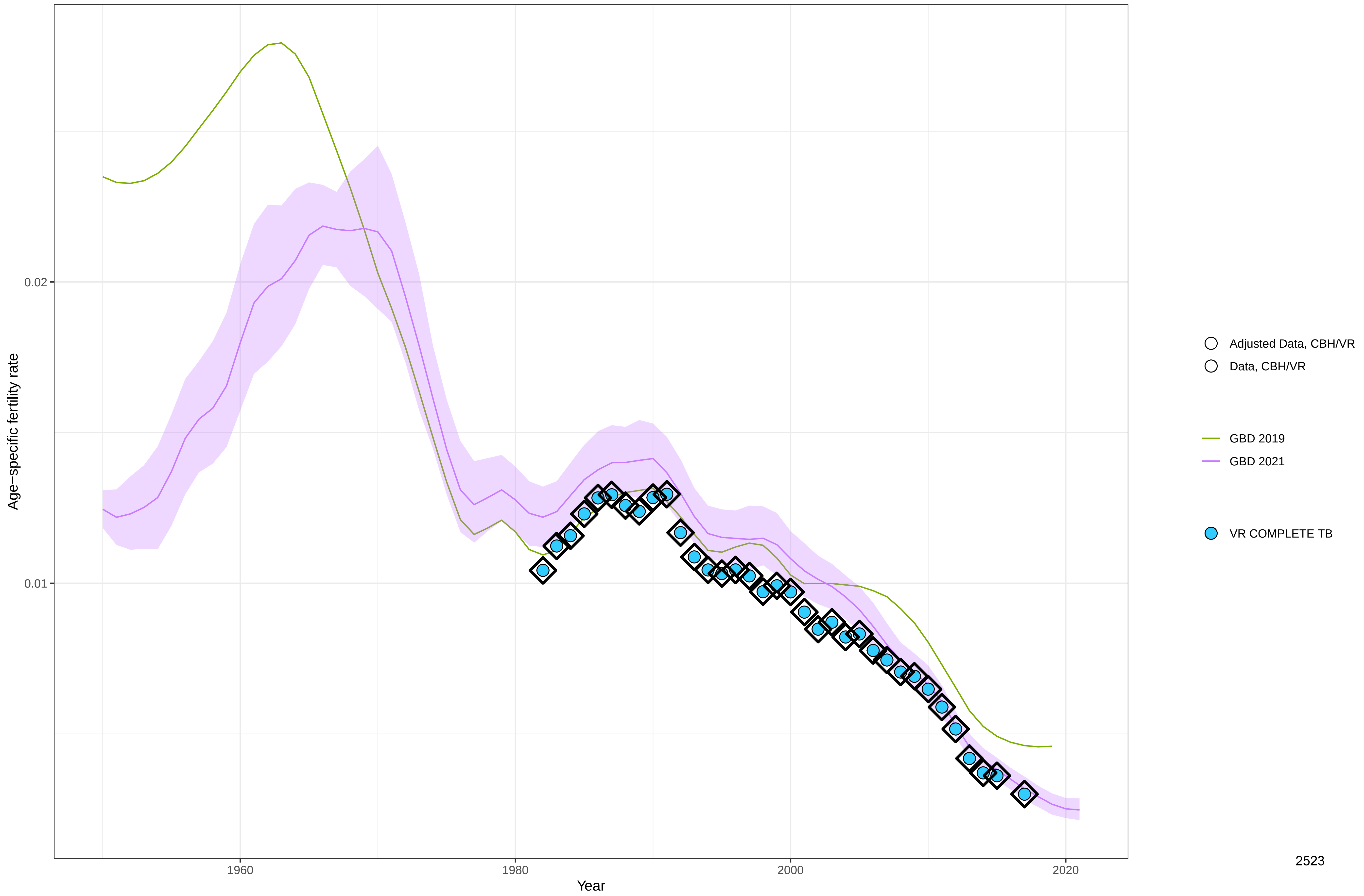

Age−specific fertility fitted trend lines, 1950−2021: Westminster, Ages: 20−24
Data points enclosed with a diamond are reference sources

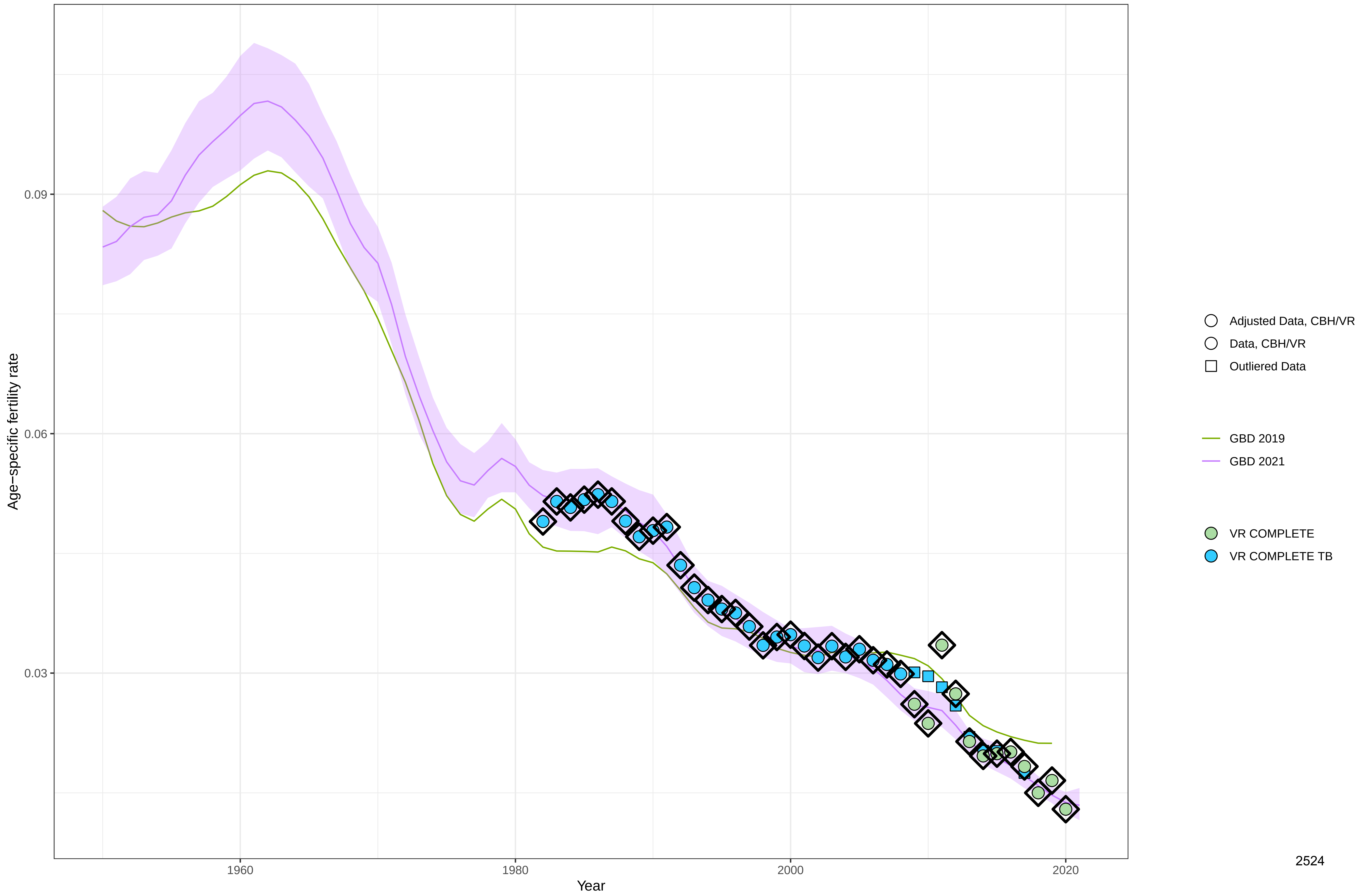

Age−specific fertility fitted trend lines, 1950−2021: Westminster, Ages: 25−29
Data points enclosed with a diamond are reference sources

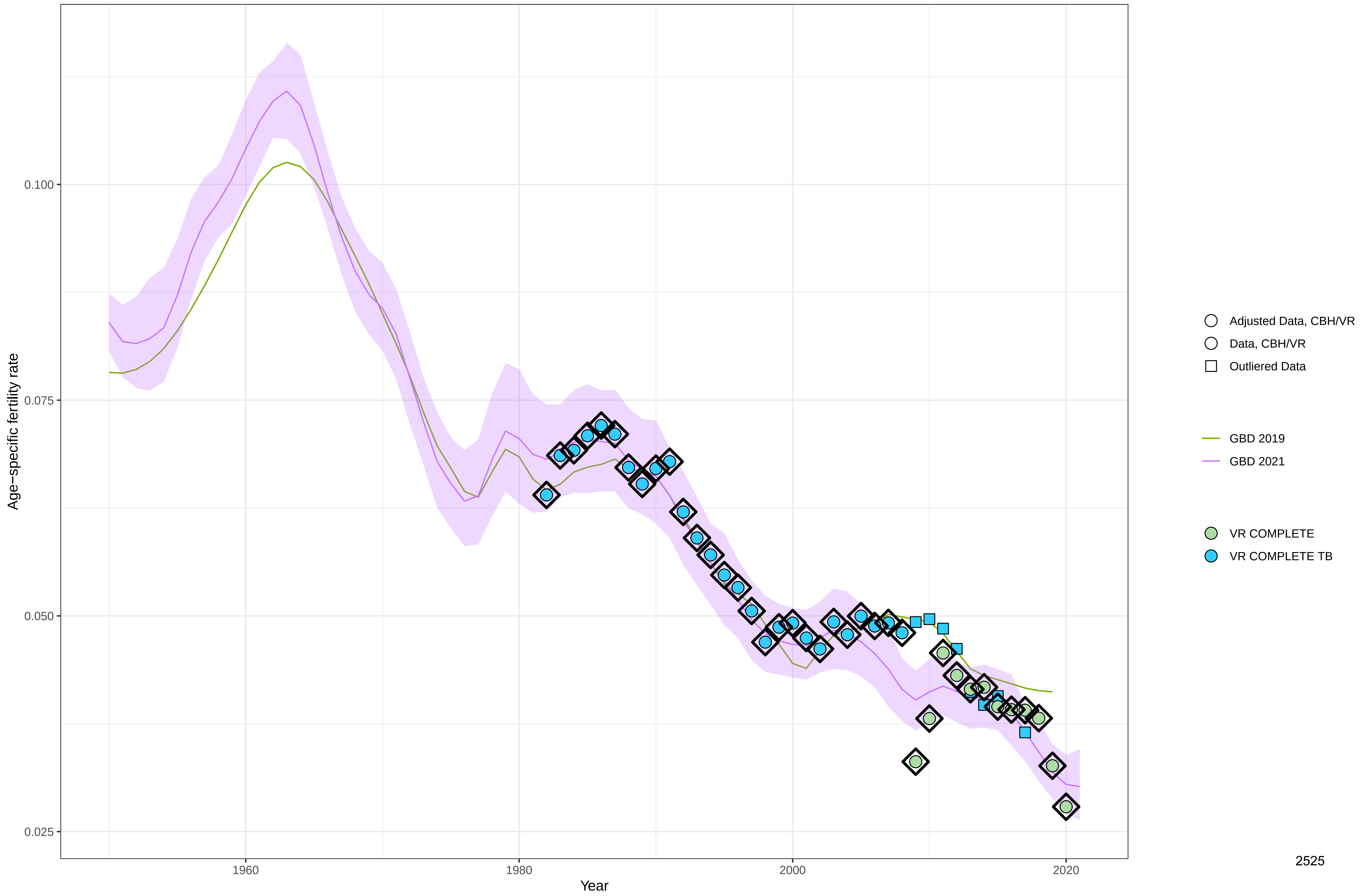

Age−specific fertility fitted trend lines, 1950−2021: Westminster, Ages: 30−34
Data points enclosed with a diamond are reference sources

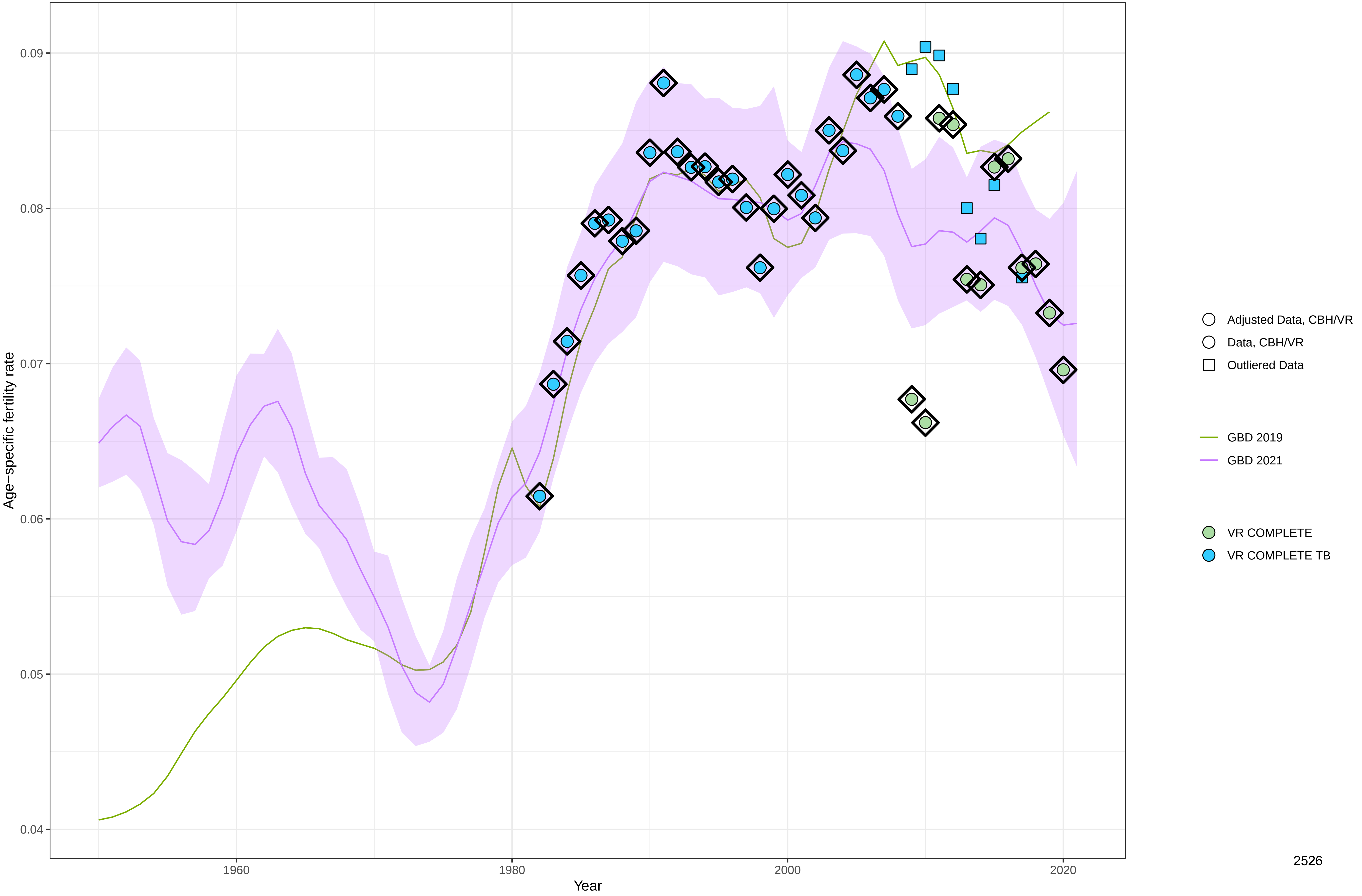

Age−specific fertility fitted trend lines, 1950−2021: Westminster, Ages: 35−39
Data points enclosed with a diamond are reference sources

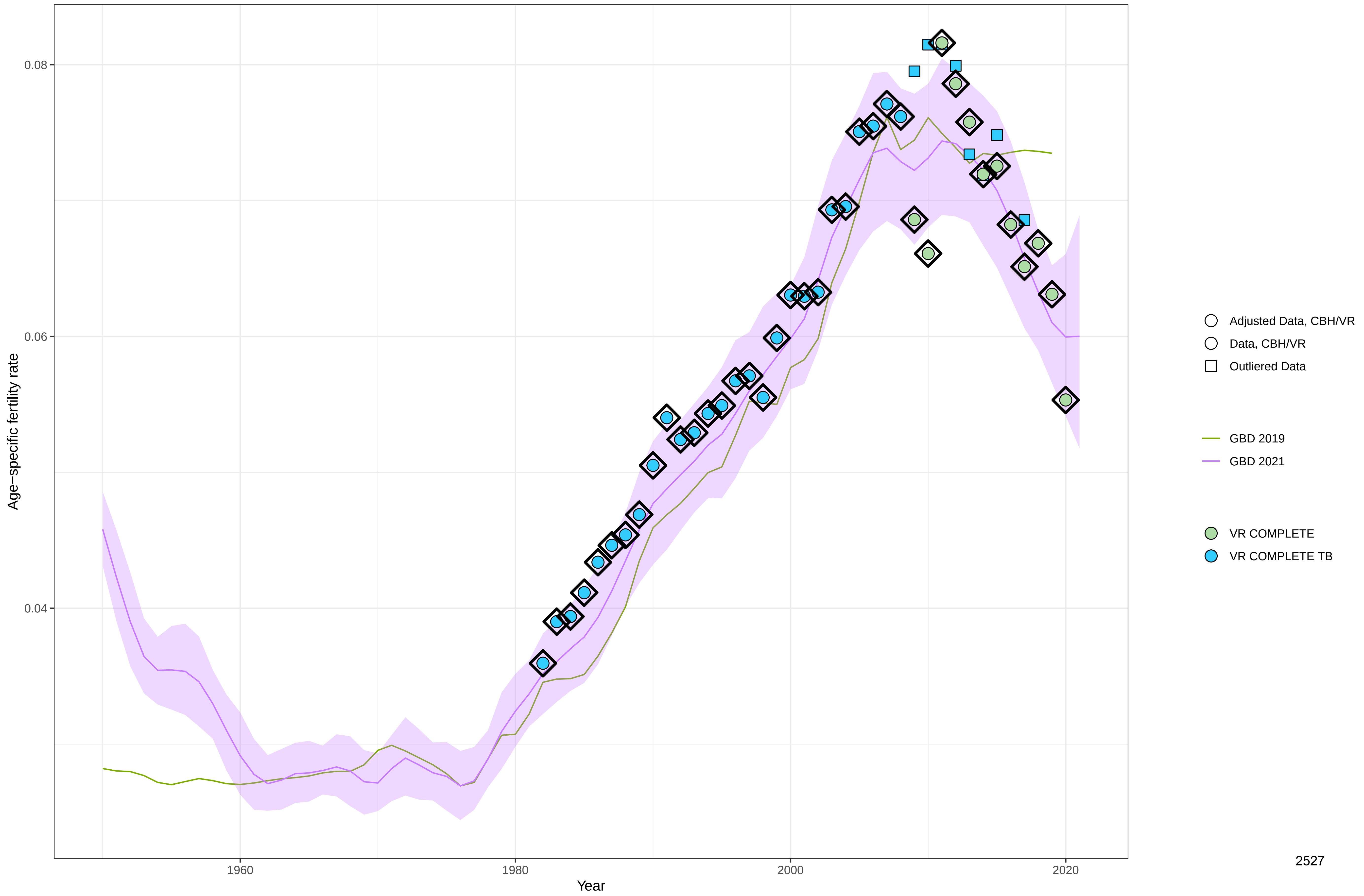

Age−specific fertility fitted trend lines, 1950−2021: Westminster, Ages: 40−44
Data points enclosed with a diamond are reference sources

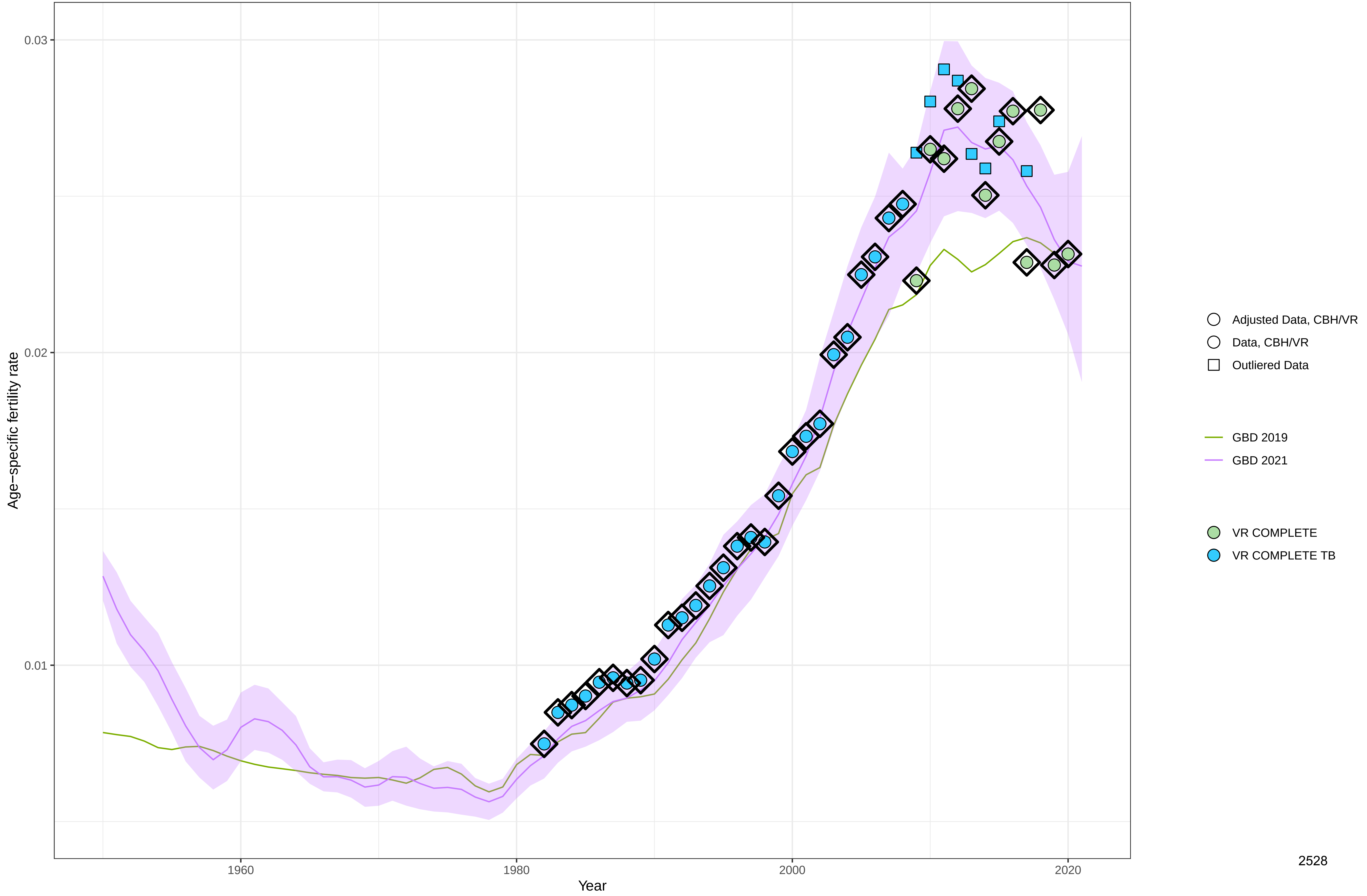

Age−specific fertility fitted trend lines, 1950−2021: Westminster, Ages: 45−49
Data points enclosed with a diamond are reference sources

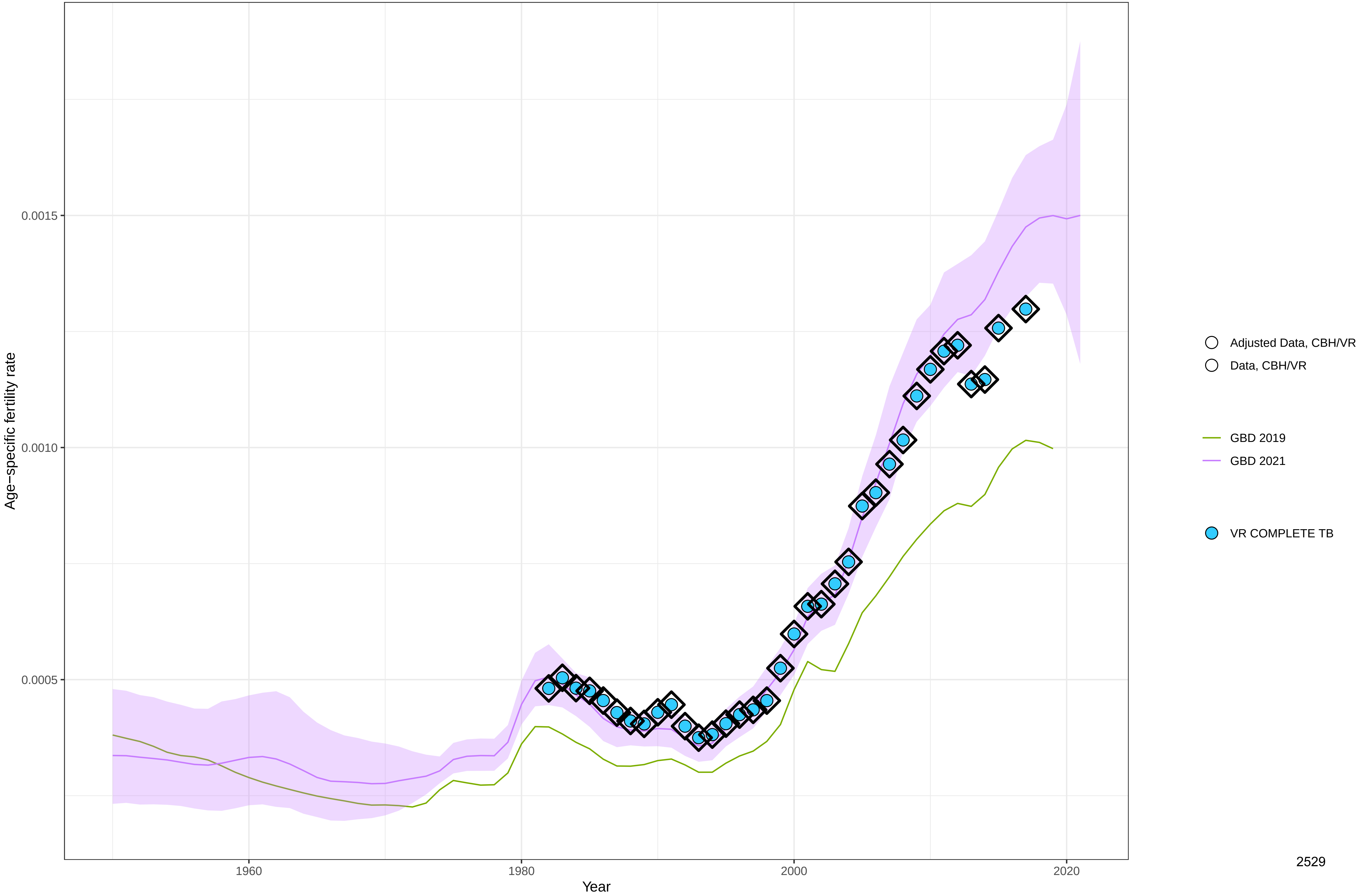

Age−specific fertility fitted trend lines, 1950−2021: Westminster, Ages: 50−54
Data points enclosed with a diamond are reference sources

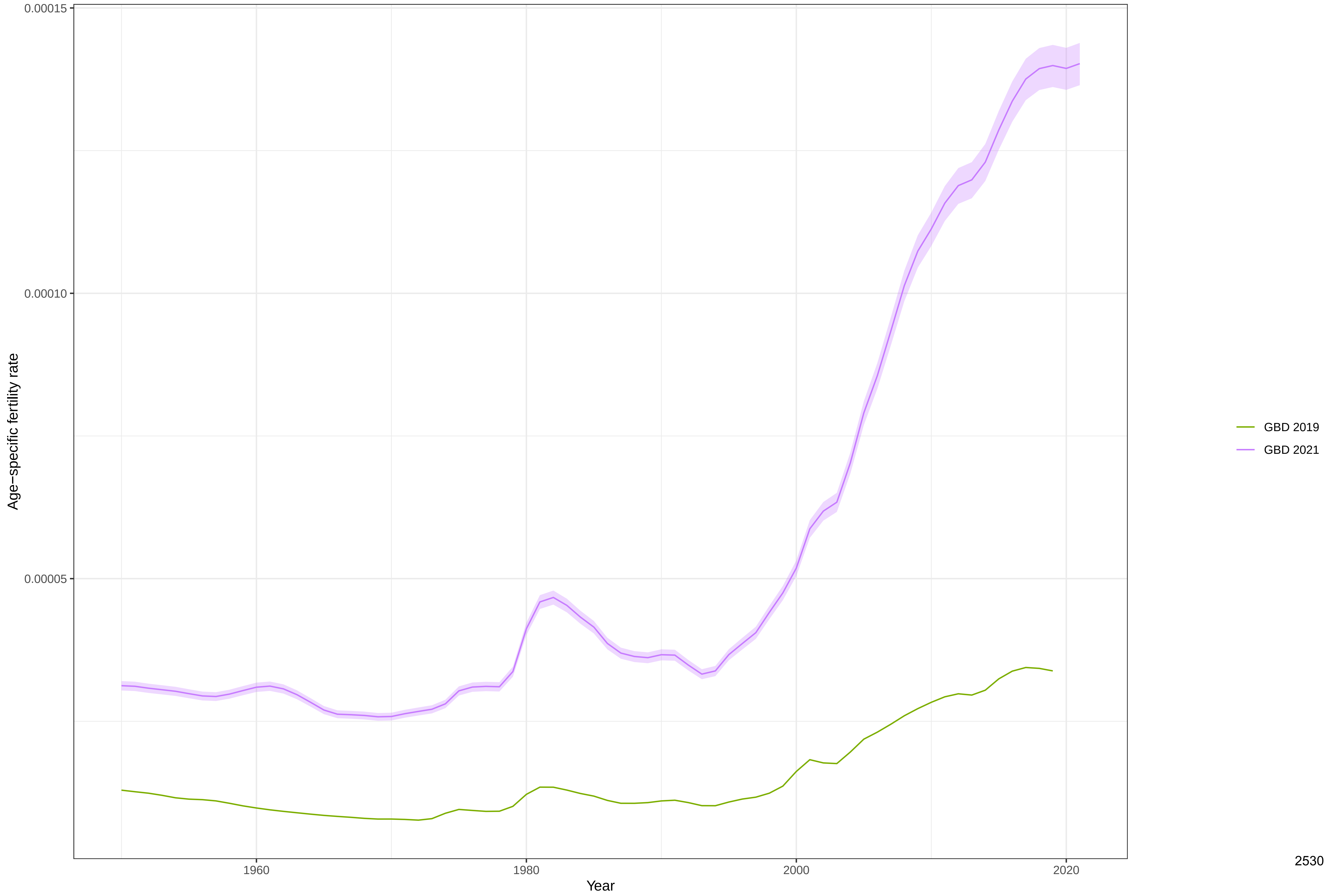

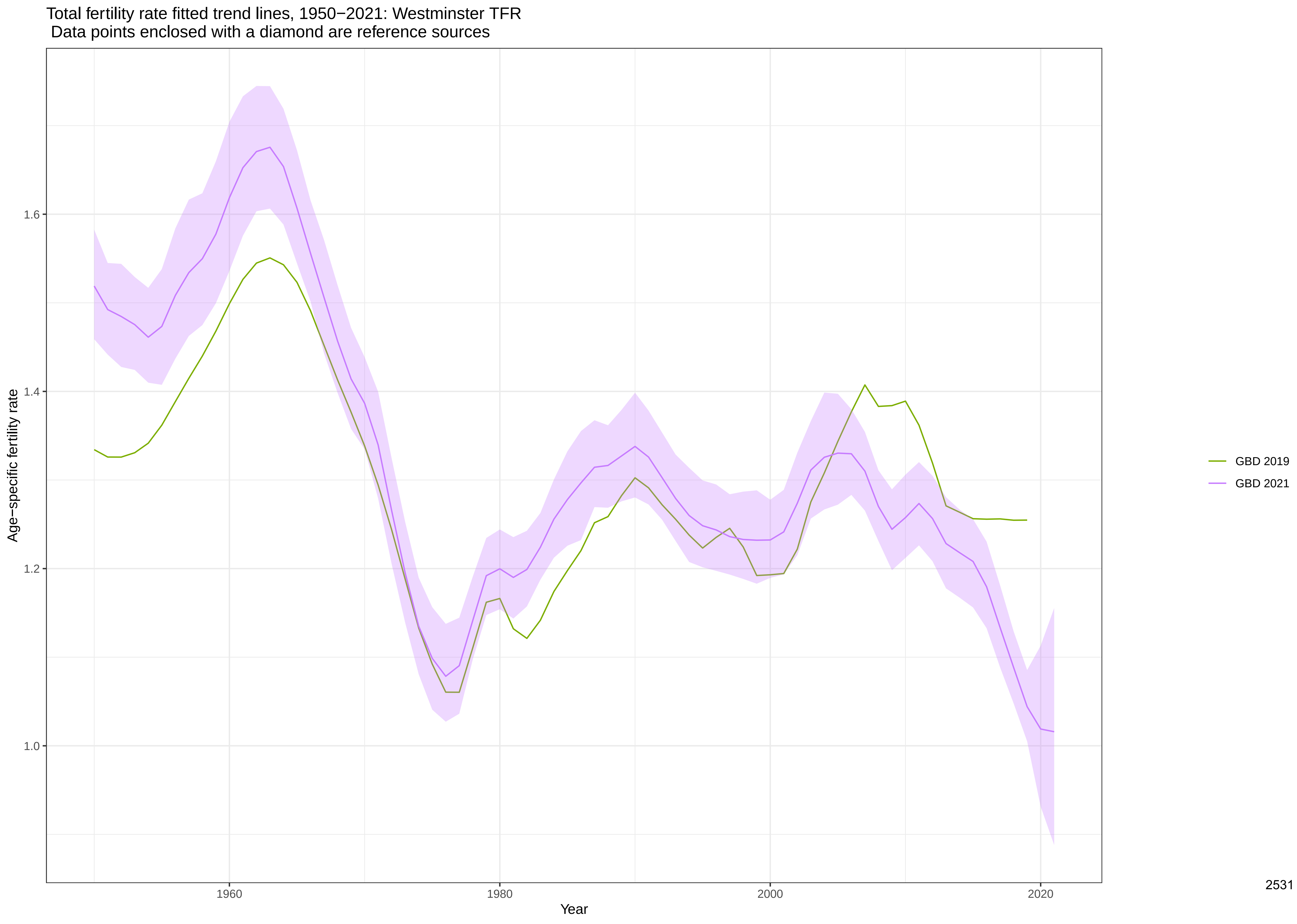
Total fertility rate fitted trend lines, 1950−2021: Westminster TFR
Data points enclosed with a diamond are reference sources
Age−specific fertility rate
1.0
1.2
1.4
1.6
1960
1980
2000
2020
Year
GBD 2019
GBD 2021

Age−specific fertility fitted trend lines, 1950−2021: North East England, Ages: 10−14
Data points enclosed with a diamond are reference sources

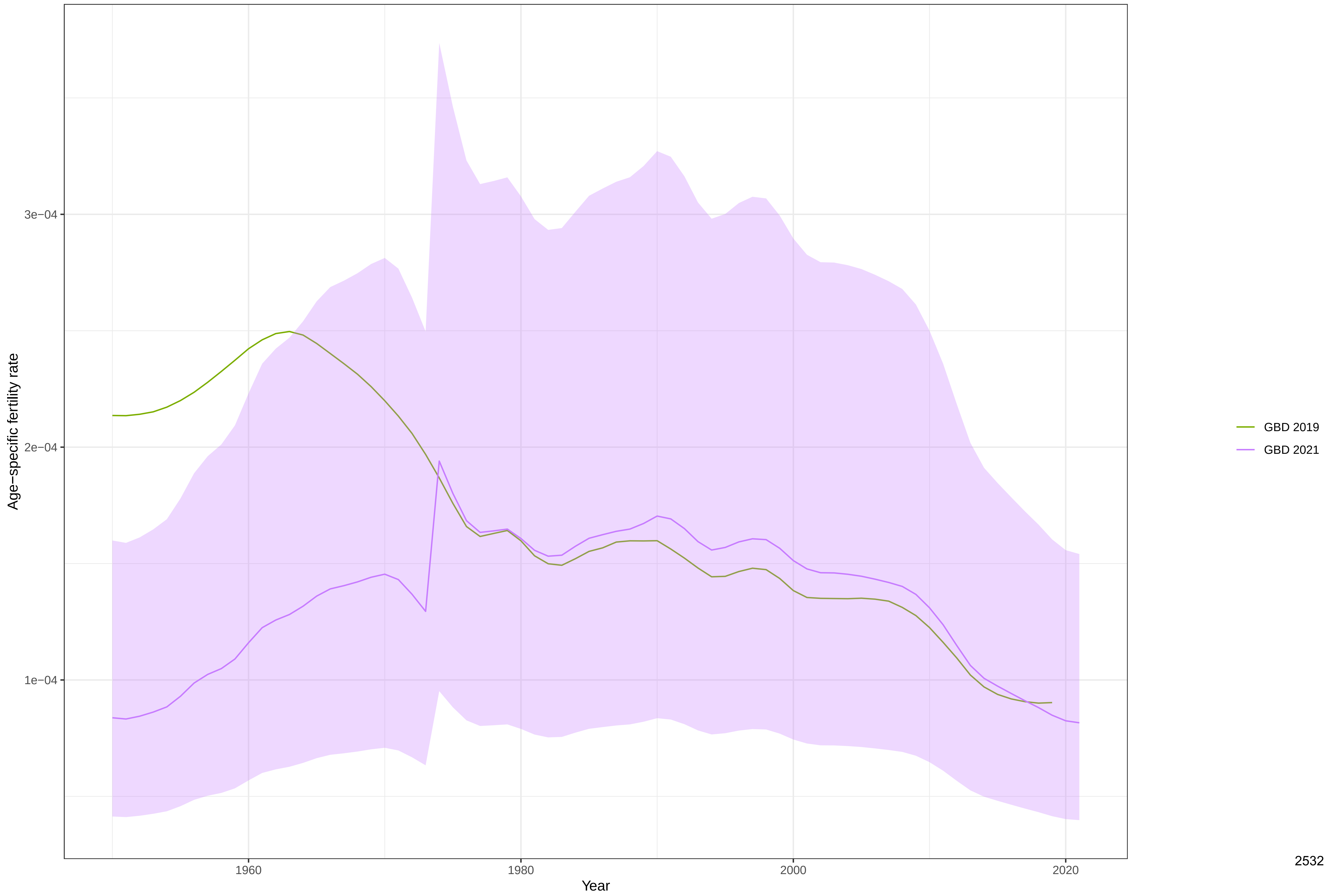

Age−specific fertility fitted trend lines, 1950−2021: North East England, Ages: 15−19
Data points enclosed with a diamond are reference sources

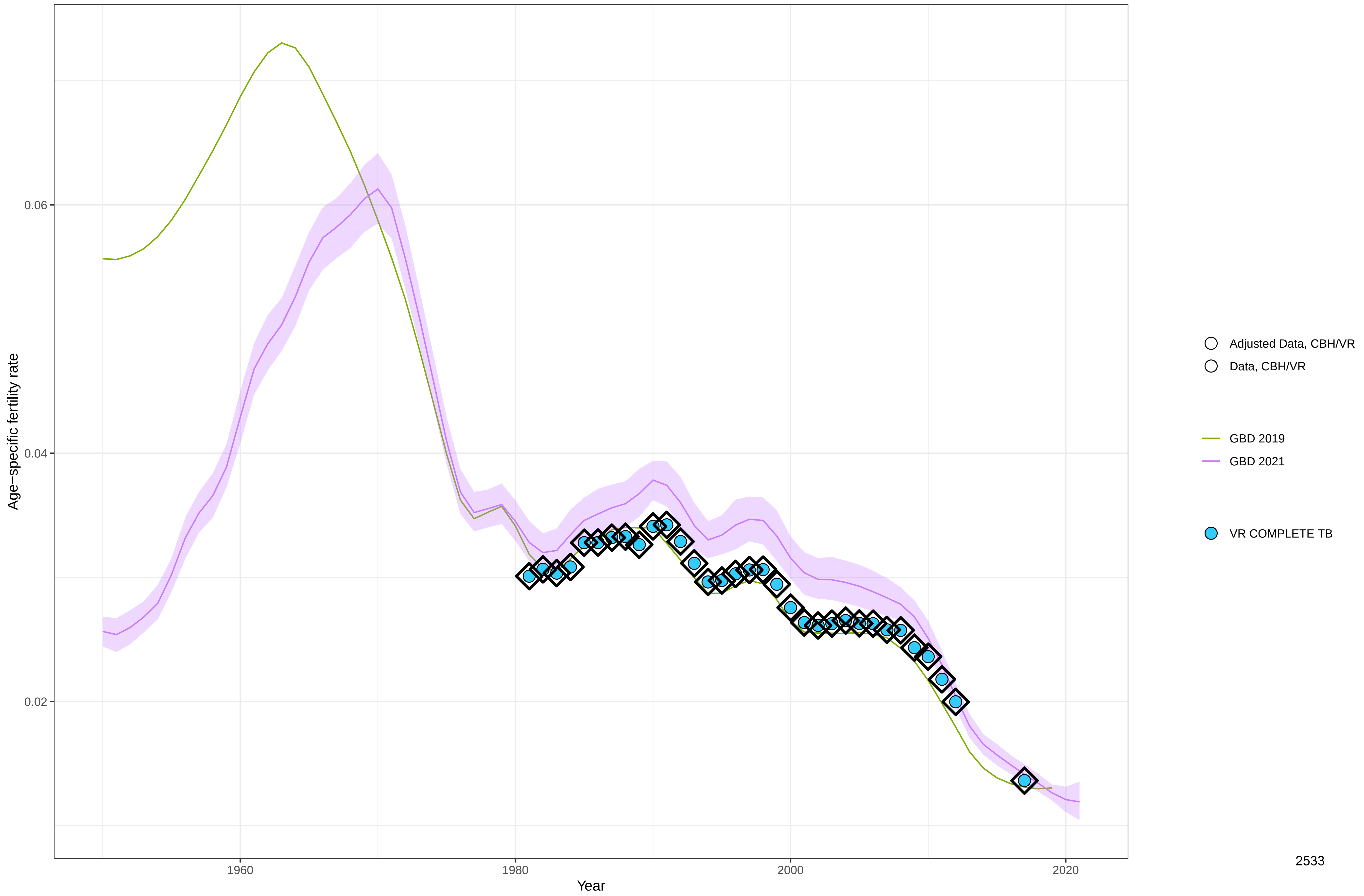

Age−specific fertility fitted trend lines, 1950−2021: North East England, Ages: 20−24
Data points enclosed with a diamond are reference sources

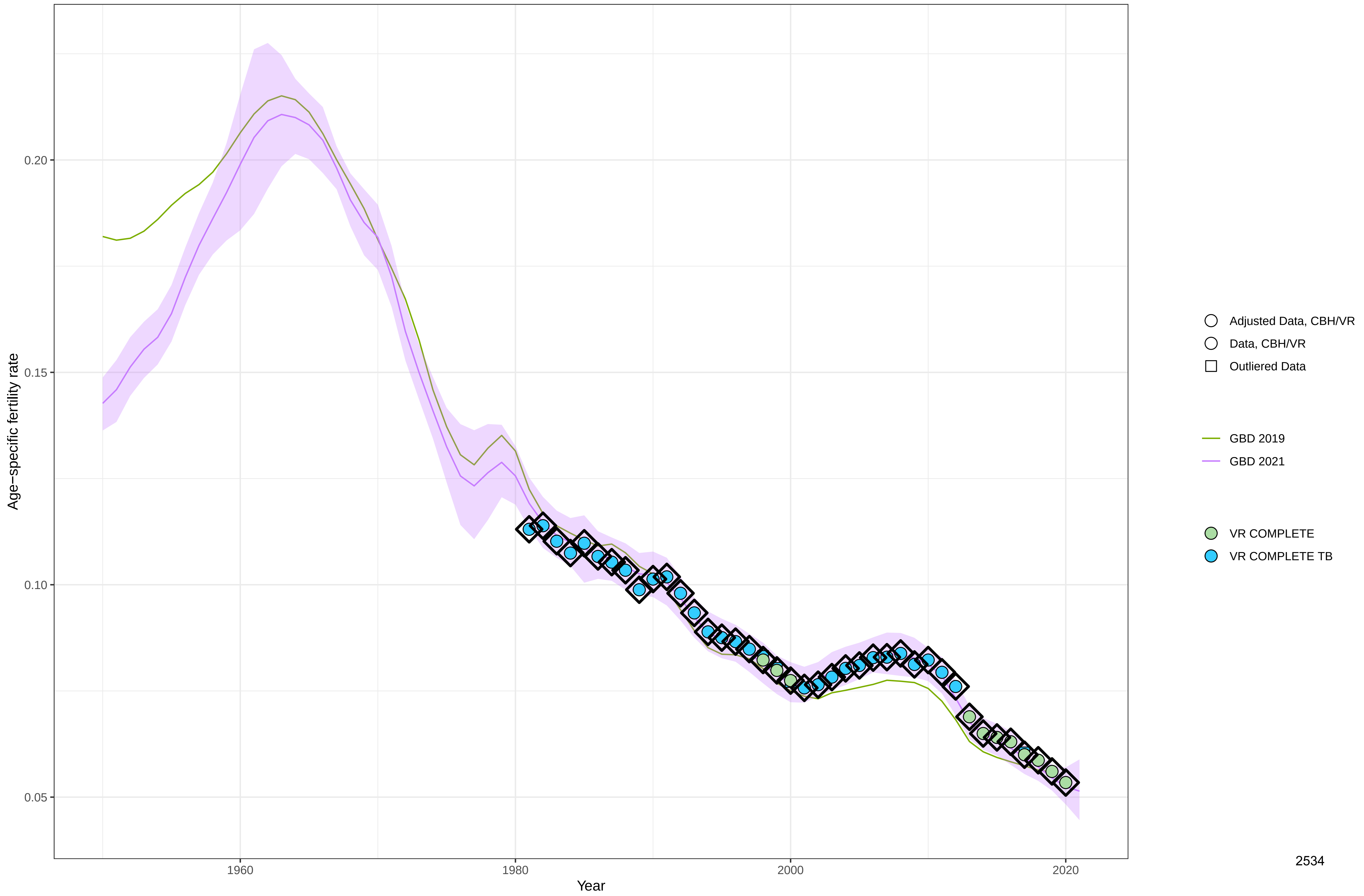

Age−specific fertility fitted trend lines, 1950−2021: North East England, Ages: 25−29
Data points enclosed with a diamond are reference sources

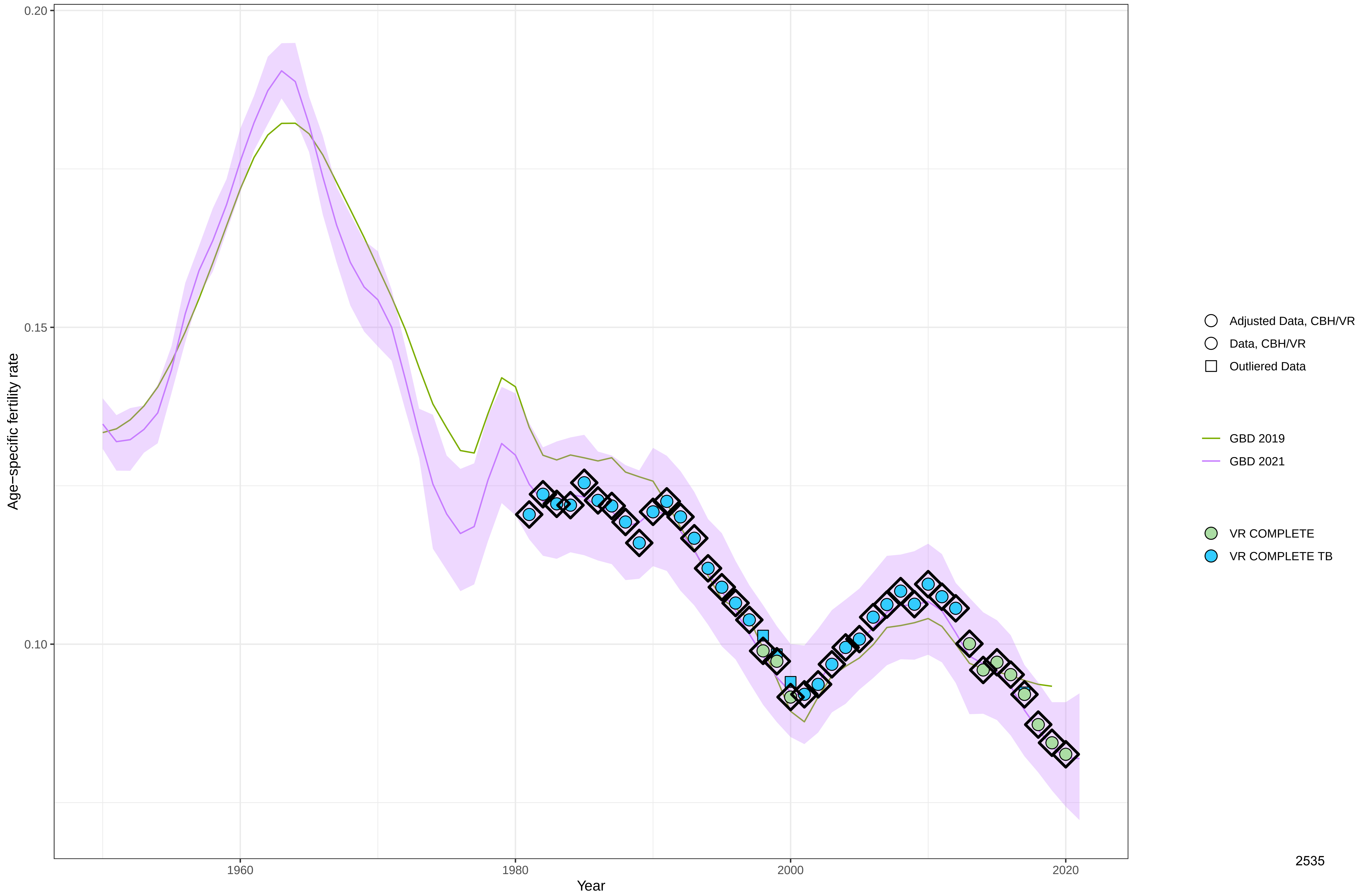

Age−specific fertility fitted trend lines, 1950−2021: North East England, Ages: 30−34
Data points enclosed with a diamond are reference sources

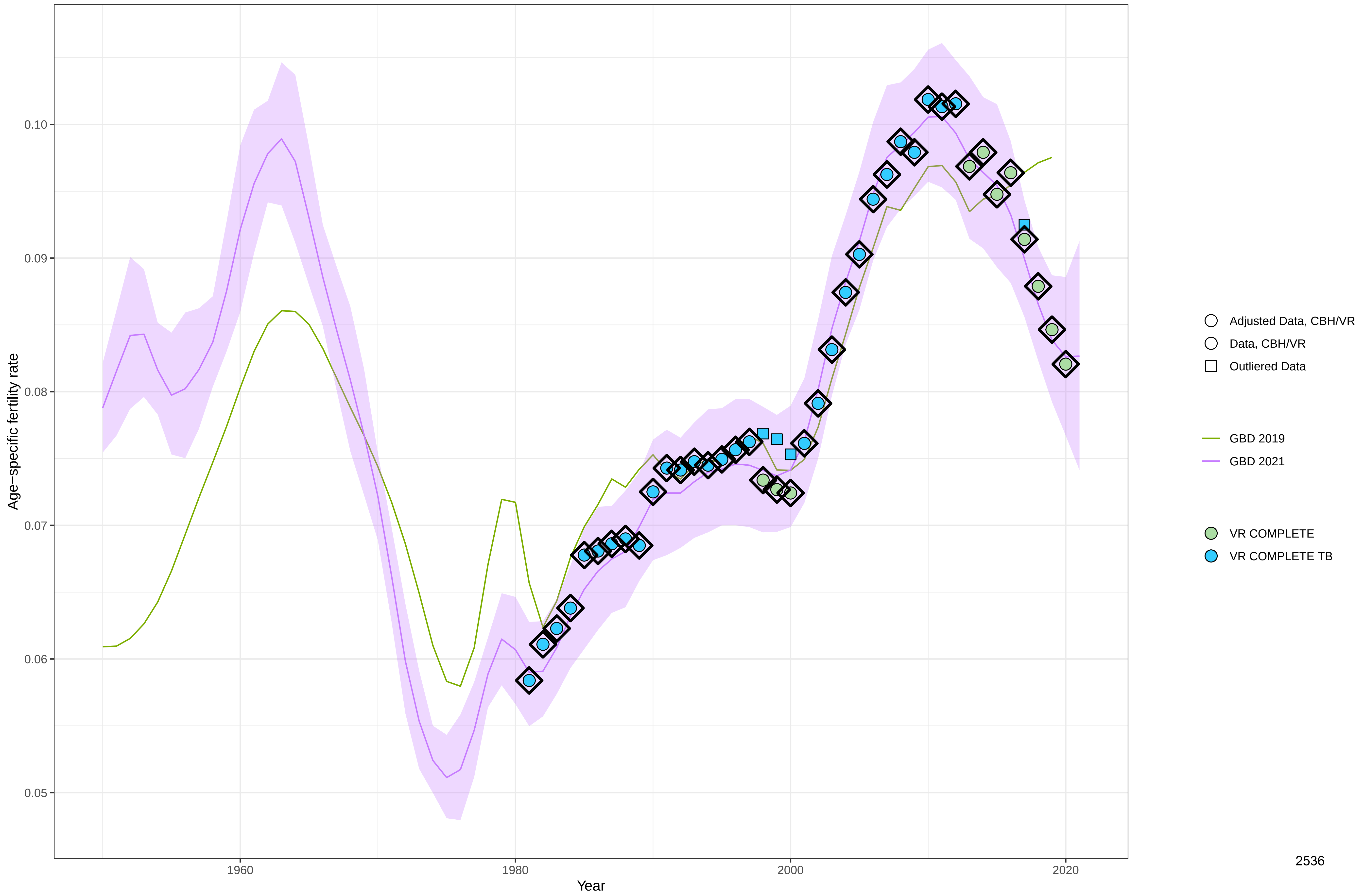

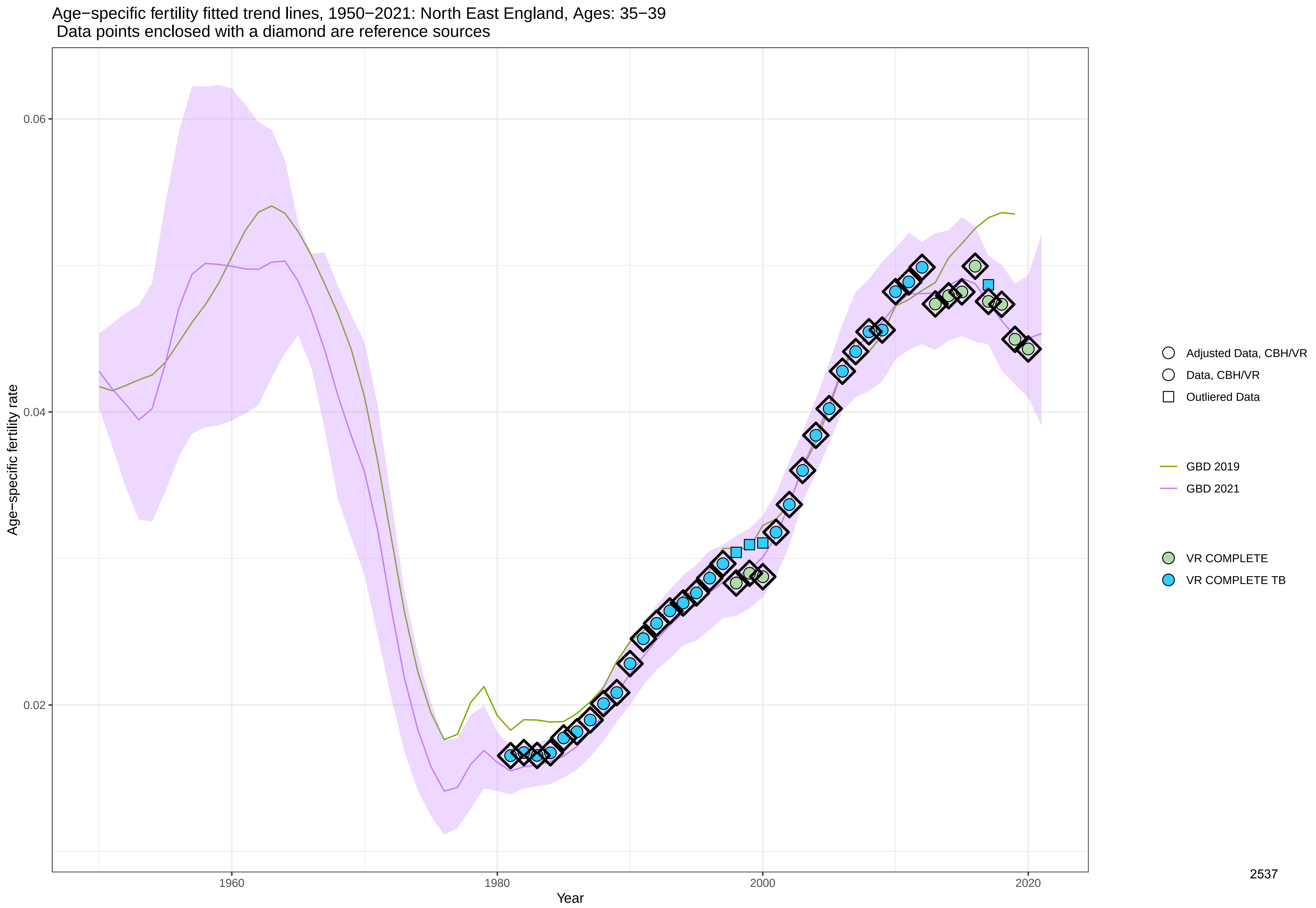

Age−specific fertility fitted trend lines, 1950−2021: North East England, Ages: 35−39
Data points enclosed with a diamond are reference sources
Age−specific fertility rate
0.06
0.04
0.02
1960
1980
2000
2020
Year
Adjusted Data, CBH/VR
Data, CBH/VR
Outliered Data
GBD 2019
GBD 2021
VR COMPLETE
VR COMPLETE TB

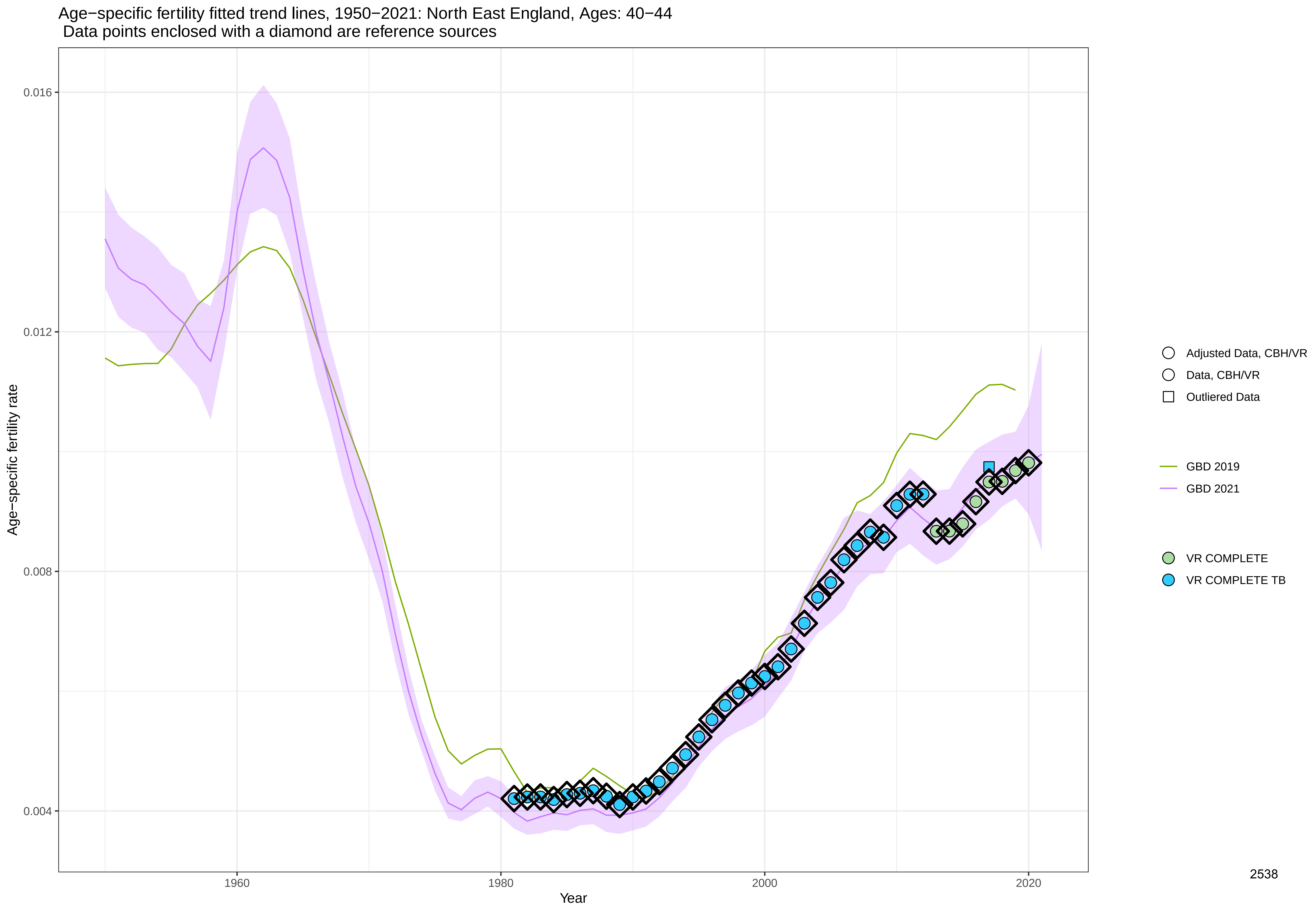

Age−specific fertility fitted trend lines, 1950−2021: North East England, Ages: 40−44
Data points enclosed with a diamond are reference sources
Age−specific fertility rate
0.016
0.012
0.008
0.004
1960
1980
2000
2020
Year
Adjusted Data, CBH/VR
Data, CBH/VR
Outliered Data
GBD 2019
GBD 2021
VR COMPLETE
VR COMPLETE TB

Age−specific fertility fitted trend lines, 1950−2021: North East England, Ages: 45−49
Data points enclosed with a diamond are reference sources

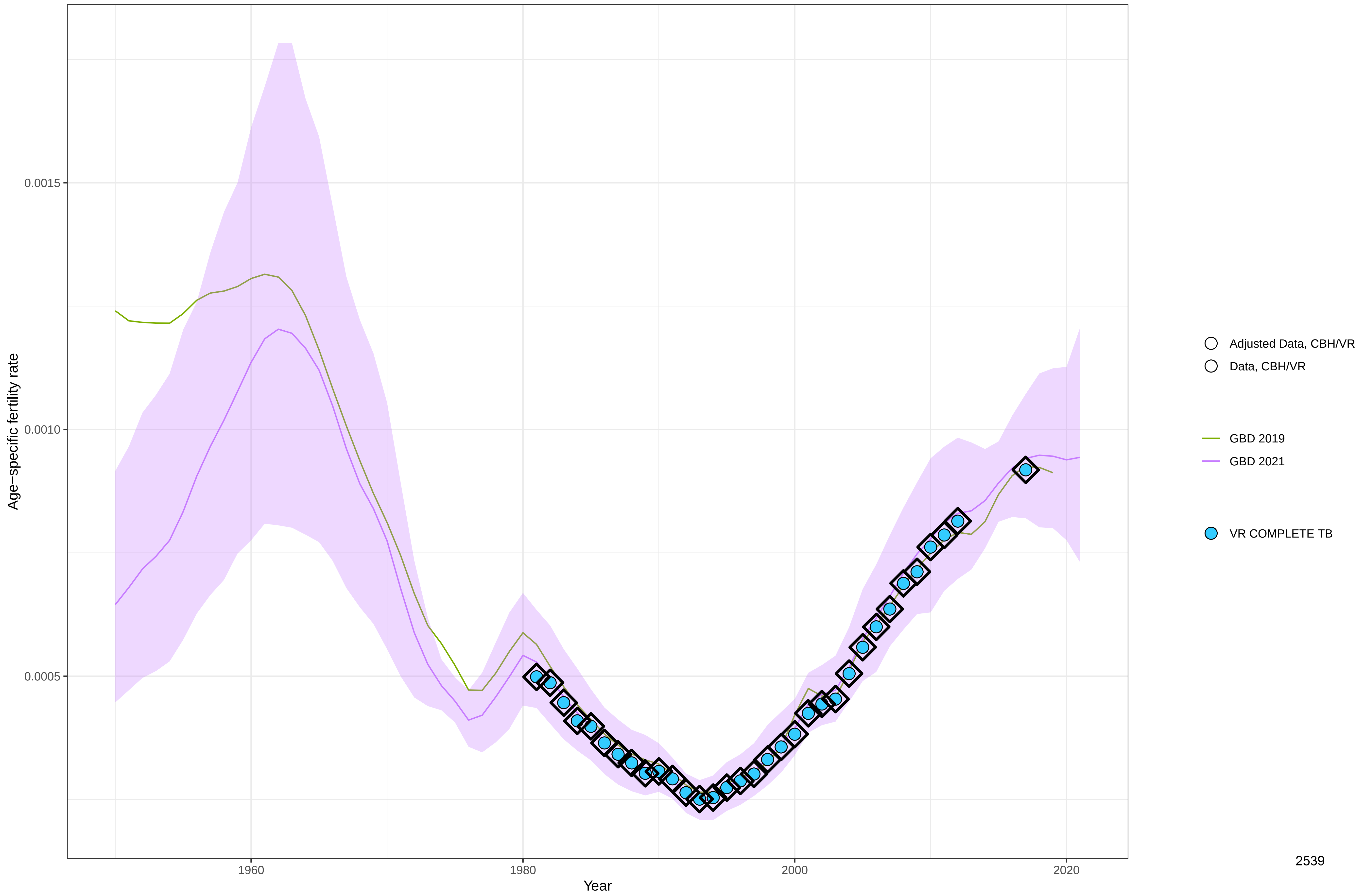

Age−specific fertility fitted trend lines, 1950−2021: North East England, Ages: 50−54
Data points enclosed with a diamond are reference sources

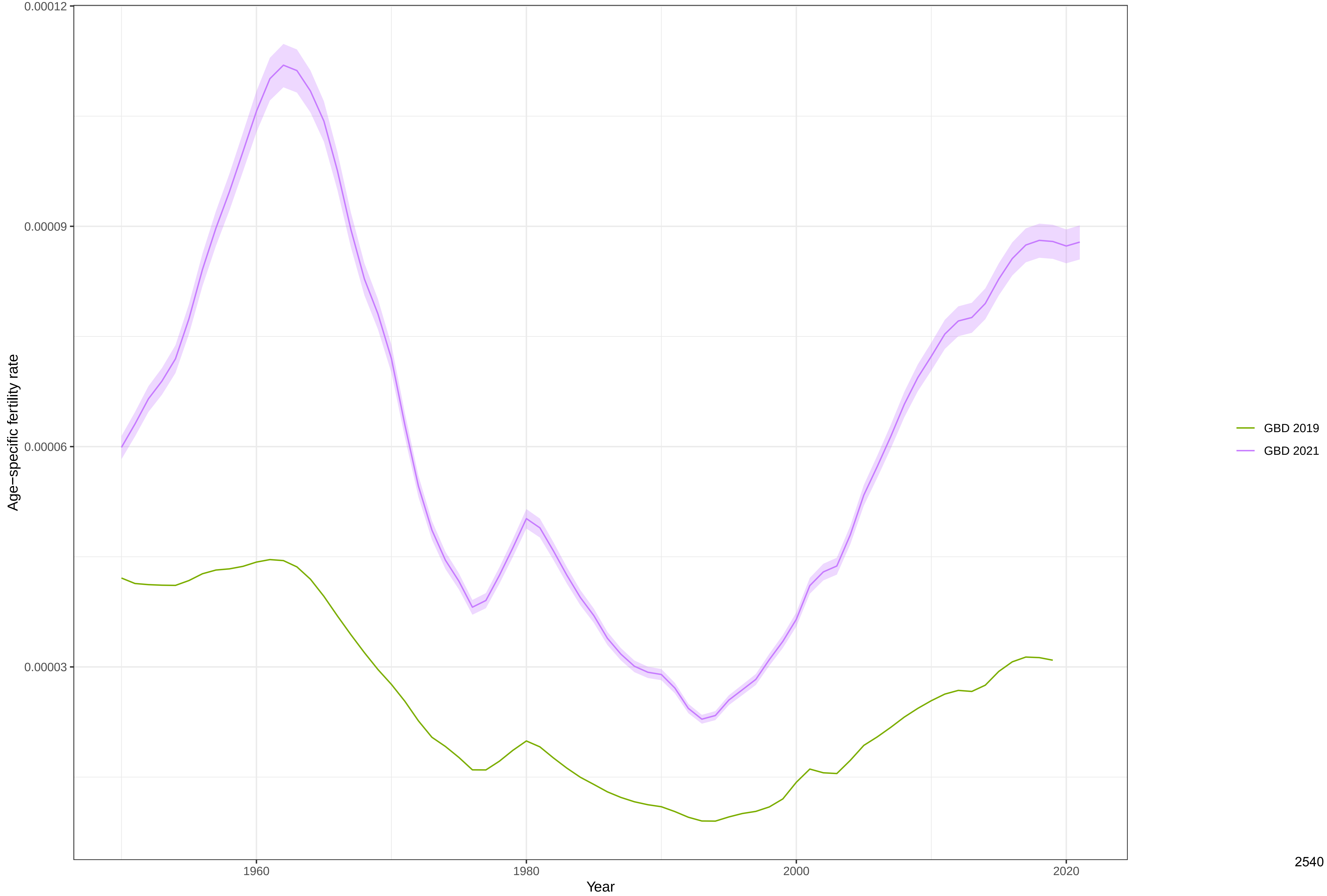

Total fertility rate fitted trend lines, 1950−2021: North East England TFR
Data points enclosed with a diamond are reference sources

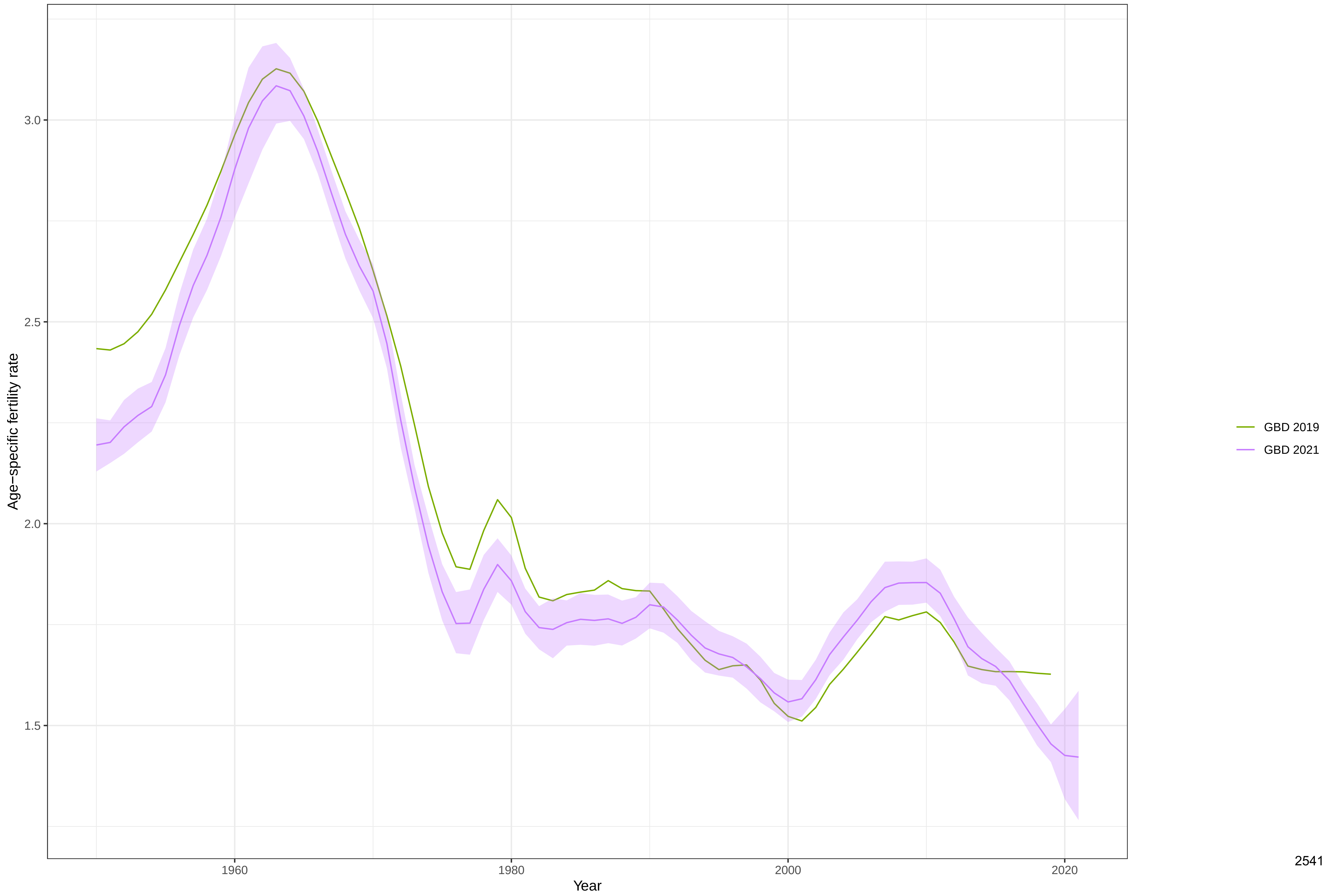

Age−specific fertility fitted trend lines, 1950−2021: County Durham, Ages: 10−14
Data points enclosed with a diamond are reference sources

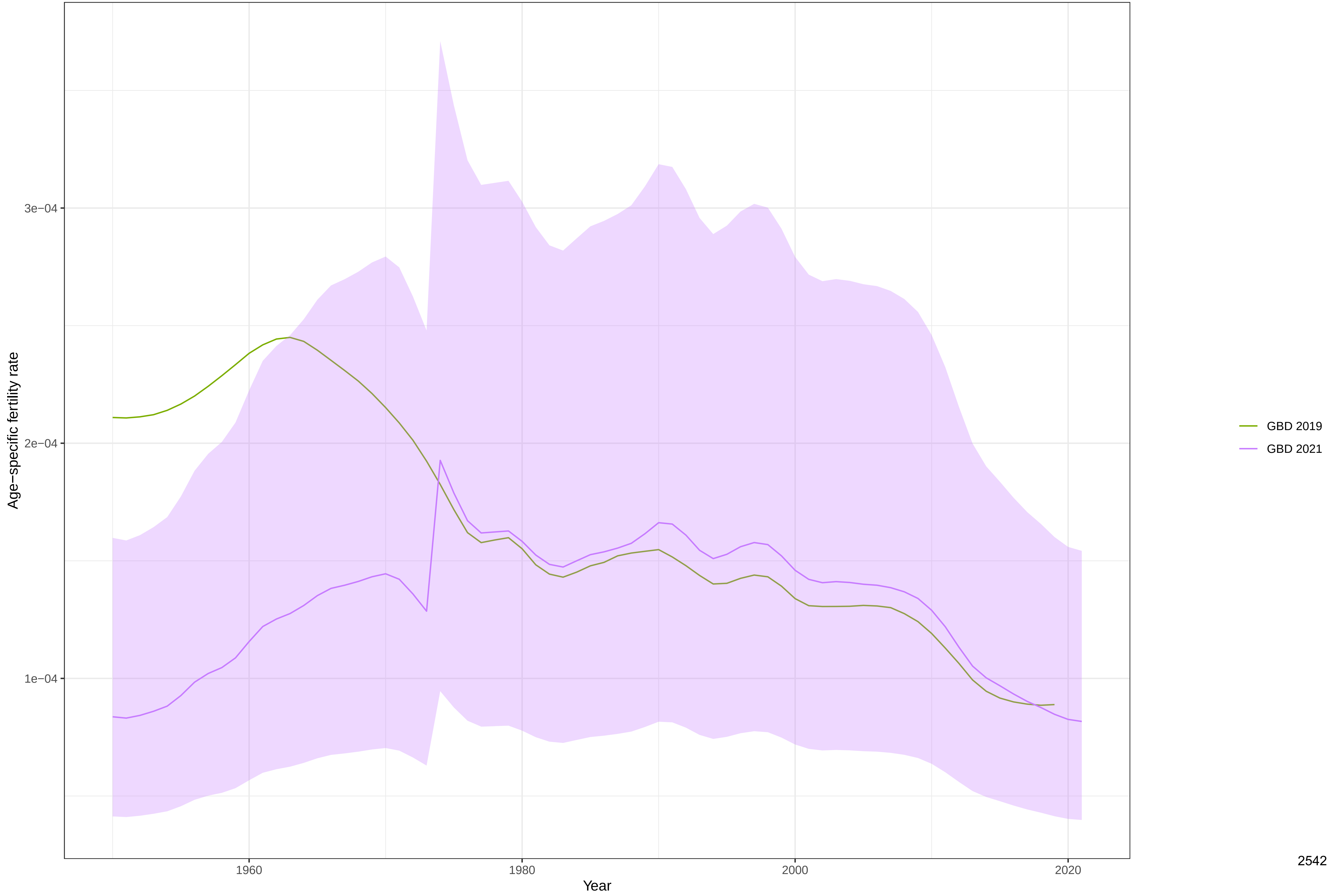

Age−specific fertility fitted trend lines, 1950−2021: County Durham, Ages: 15−19
Data points enclosed with a diamond are reference sources

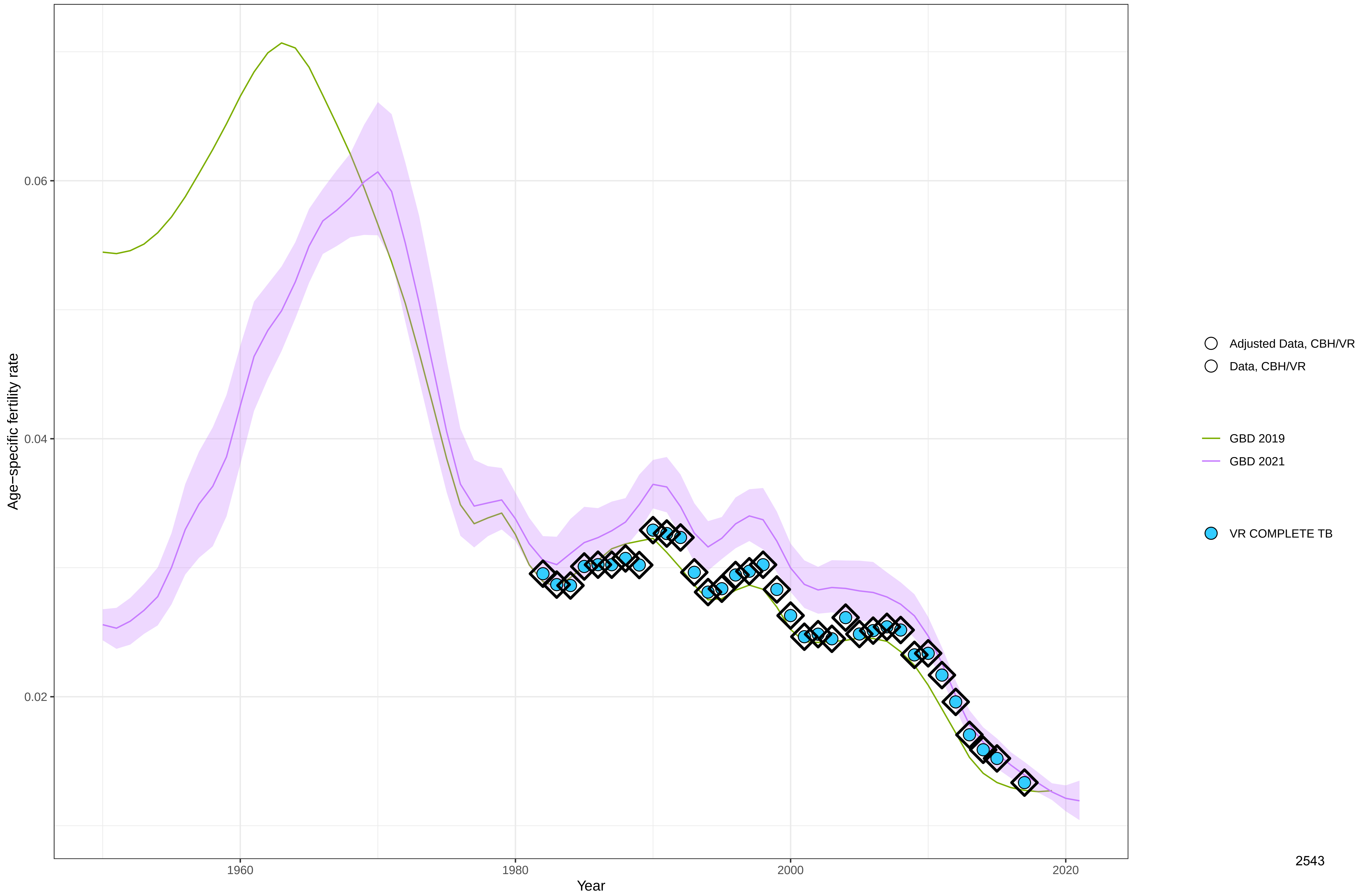

Age−specific fertility fitted trend lines, 1950−2021: County Durham, Ages: 20−24
Data points enclosed with a diamond are reference sources

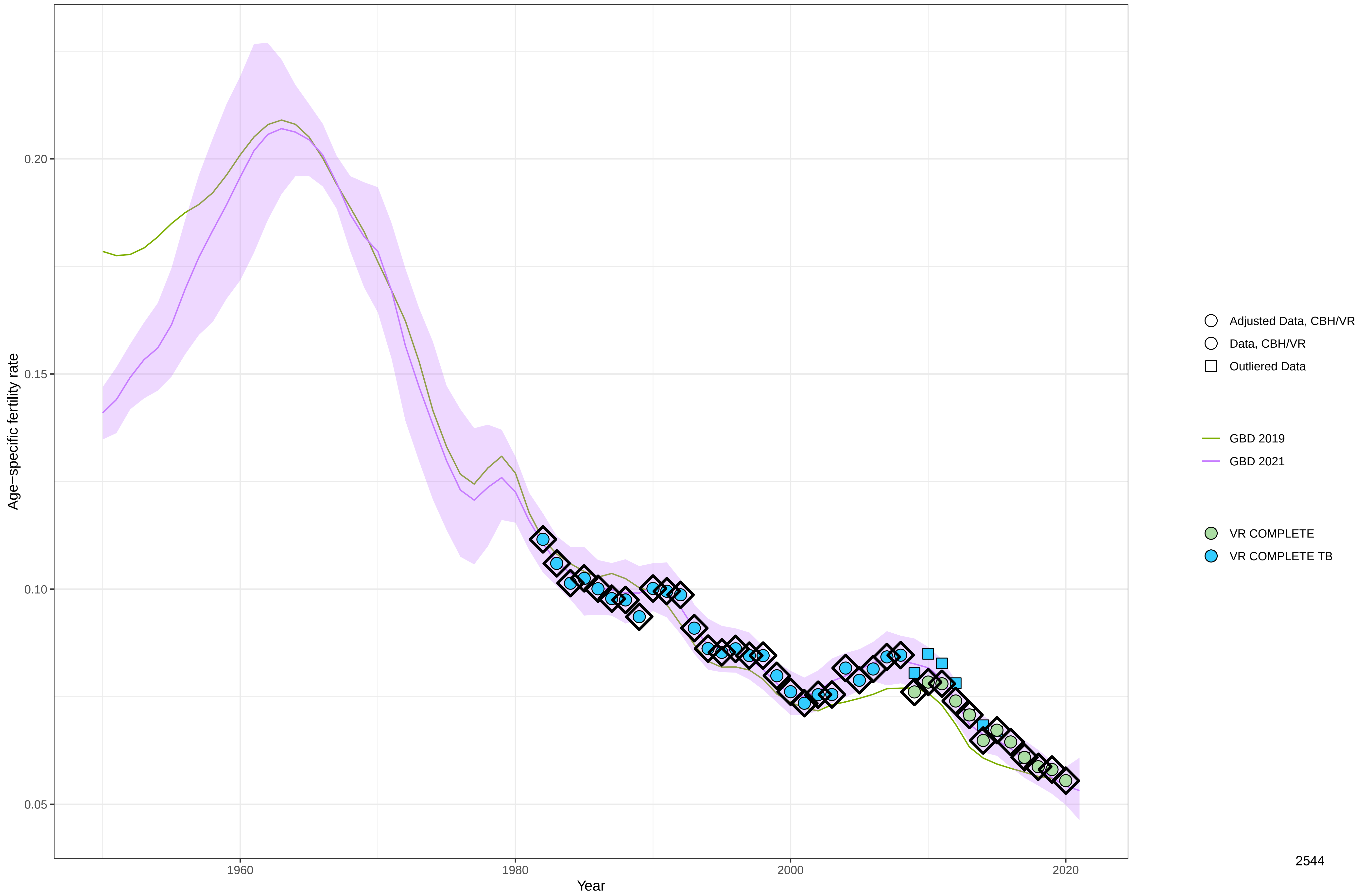

Age−specific fertility fitted trend lines, 1950−2021: County Durham, Ages: 25−29
Data points enclosed with a diamond are reference sources

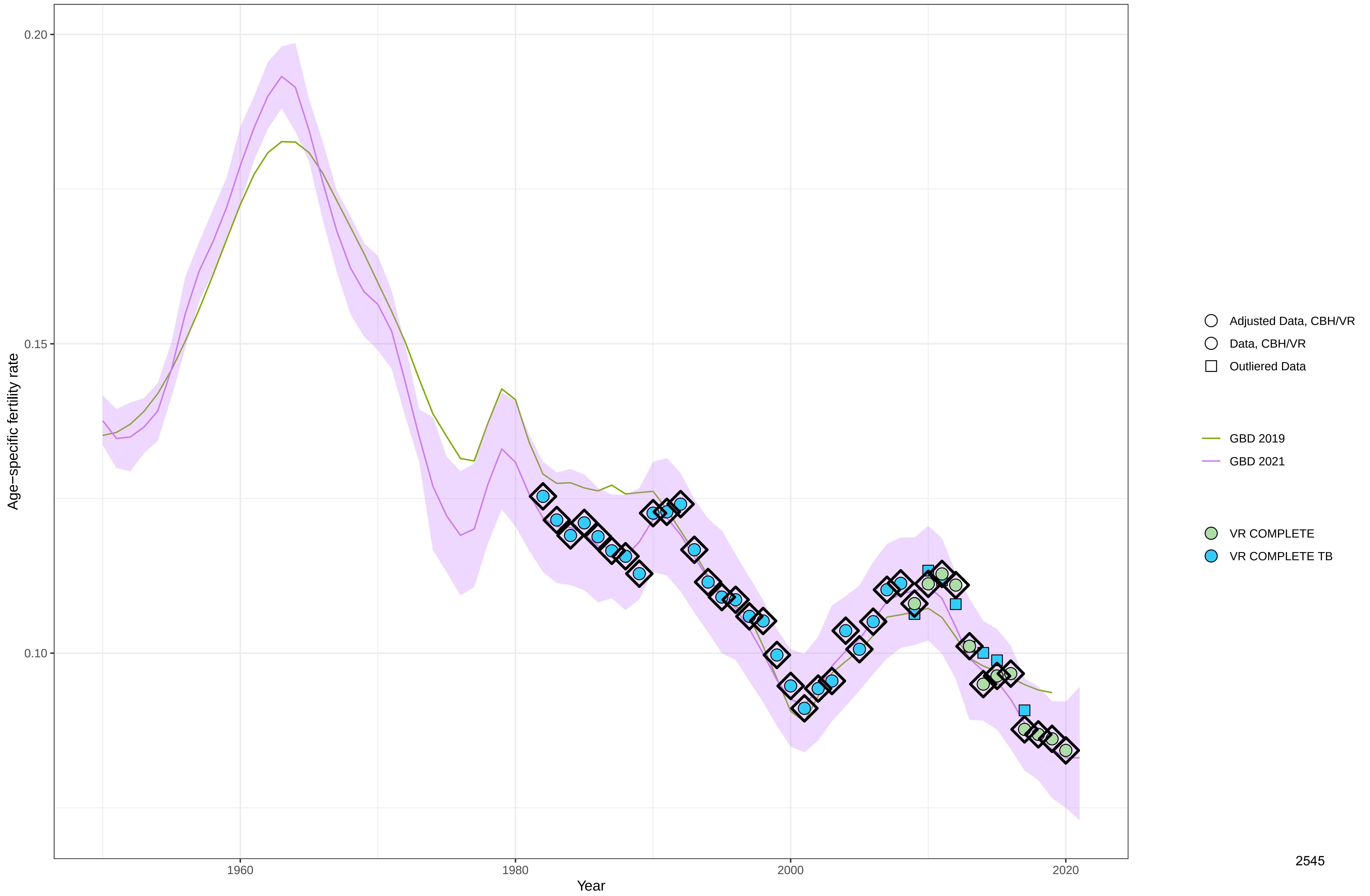

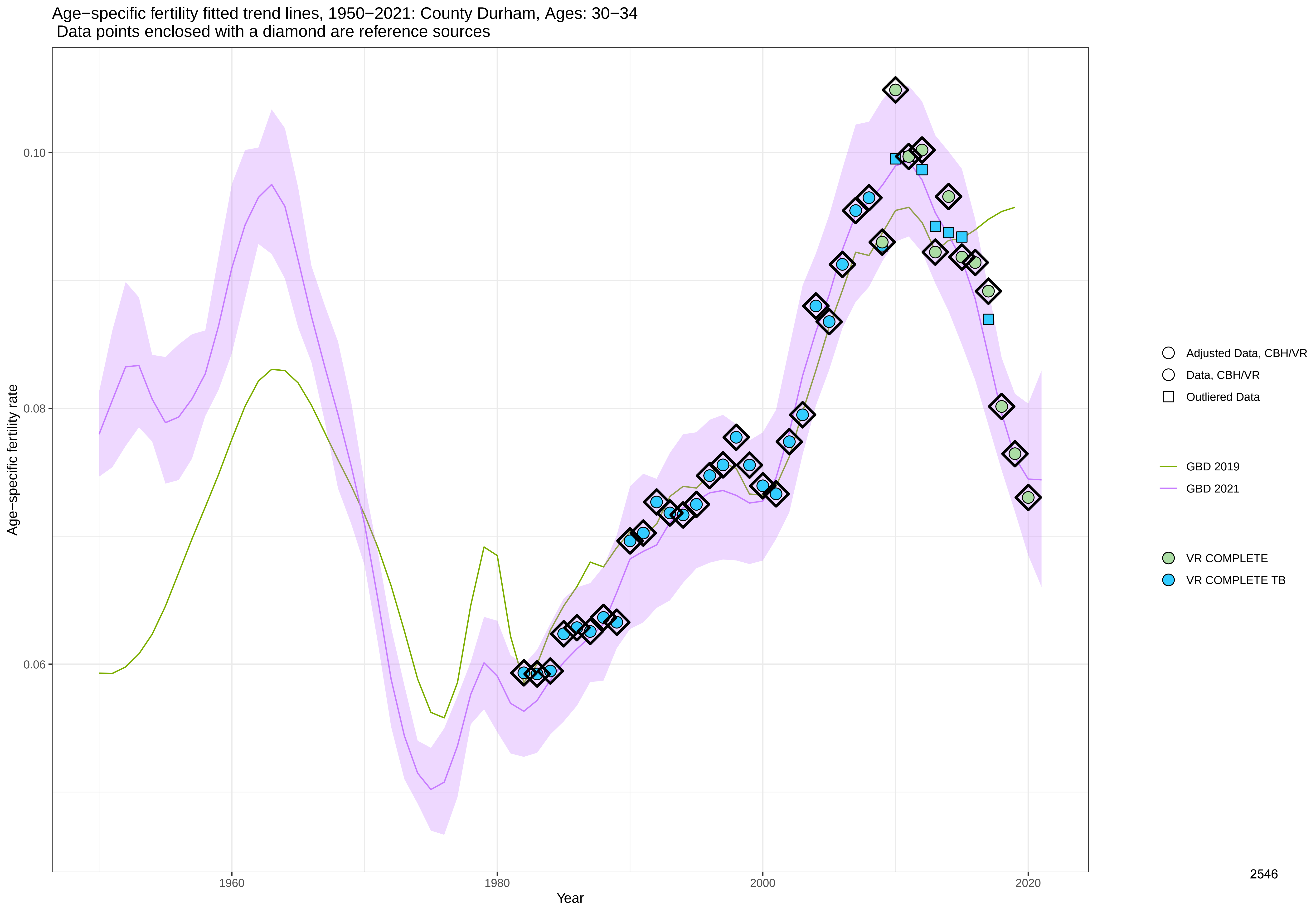

Age−specific fertility fitted trend lines, 1950−2021: County Durham, Ages: 30−34
Data points enclosed with a diamond are reference sources
Age−specific fertility rate
0.10
0.08
0.06
1960
1980
2000
2020
Year
Adjusted Data, CBH/VR
Data, CBH/VR
Outliered Data
GBD 2019
GBD 2021
VR COMPLETE
VR COMPLETE TB

Age−specific fertility fitted trend lines, 1950−2021: County Durham, Ages: 35−39
Data points enclosed with a diamond are reference sources

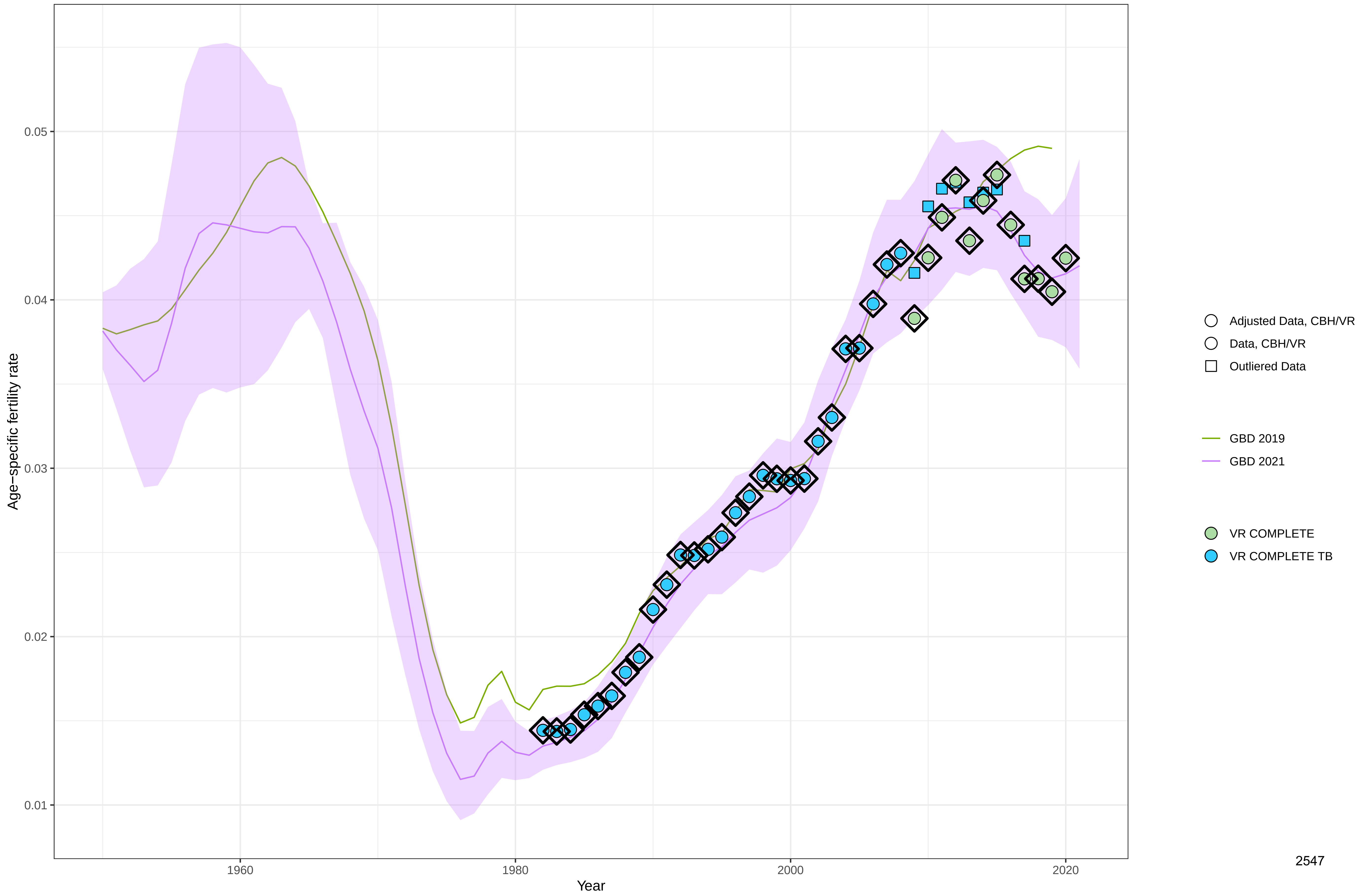

Age−specific fertility fitted trend lines, 1950−2021: County Durham, Ages: 40−44
Data points enclosed with a diamond are reference sources

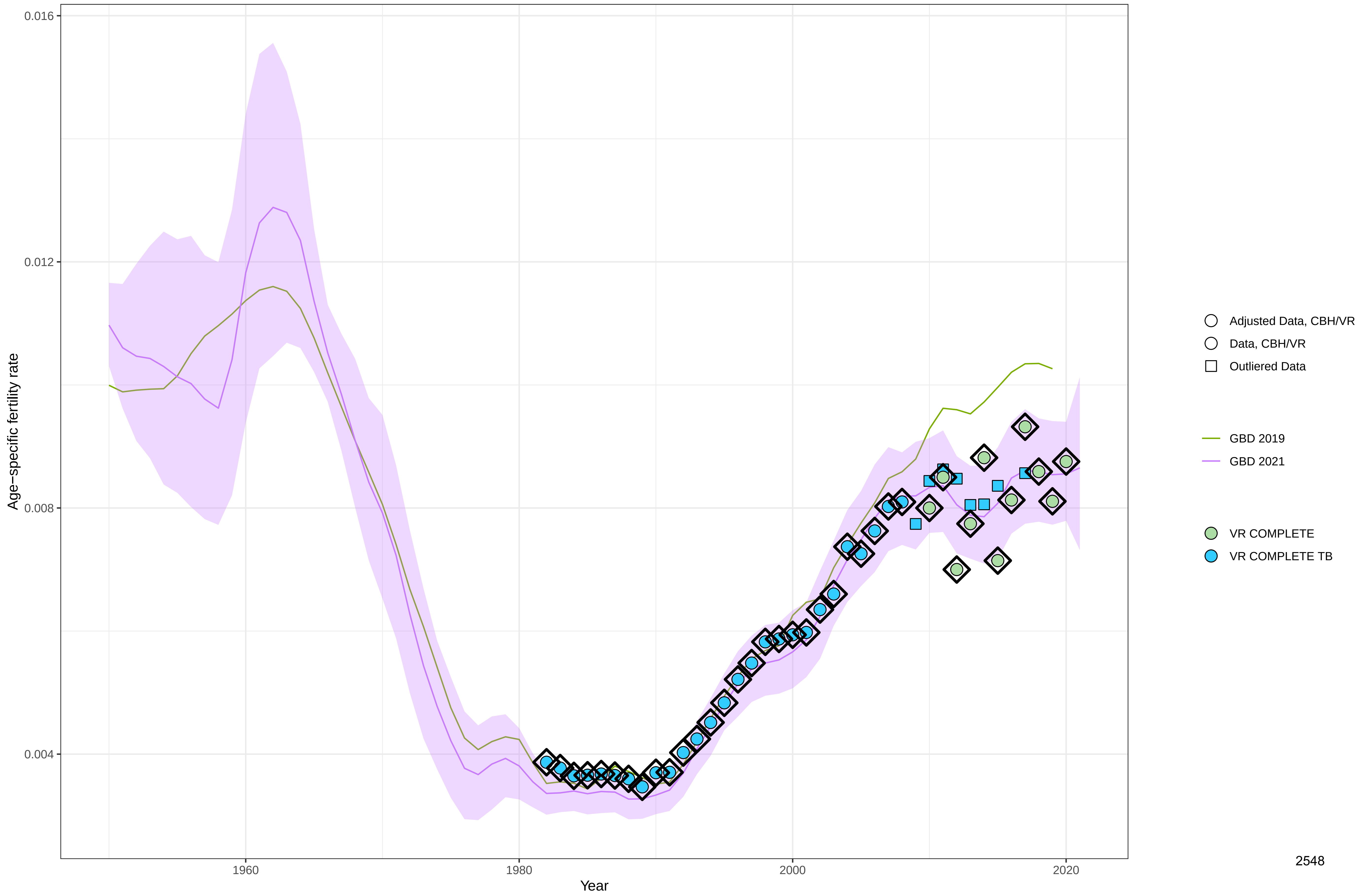

Age−specific fertility fitted trend lines, 1950−2021: County Durham, Ages: 45−49
Data points enclosed with a diamond are reference sources

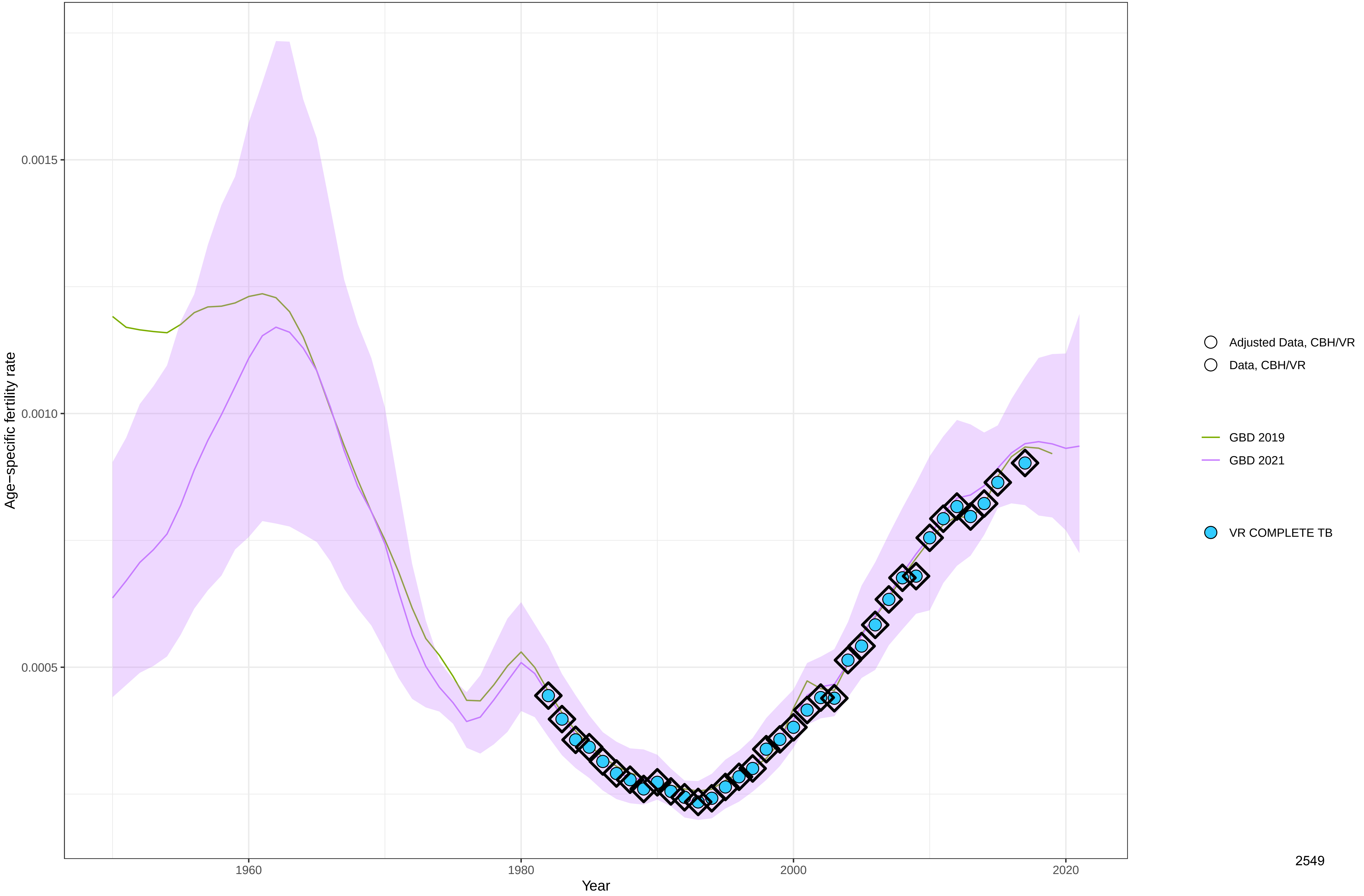

Age−specific fertility fitted trend lines, 1950−2021: County Durham, Ages: 50−54
Data points enclosed with a diamond are reference sources

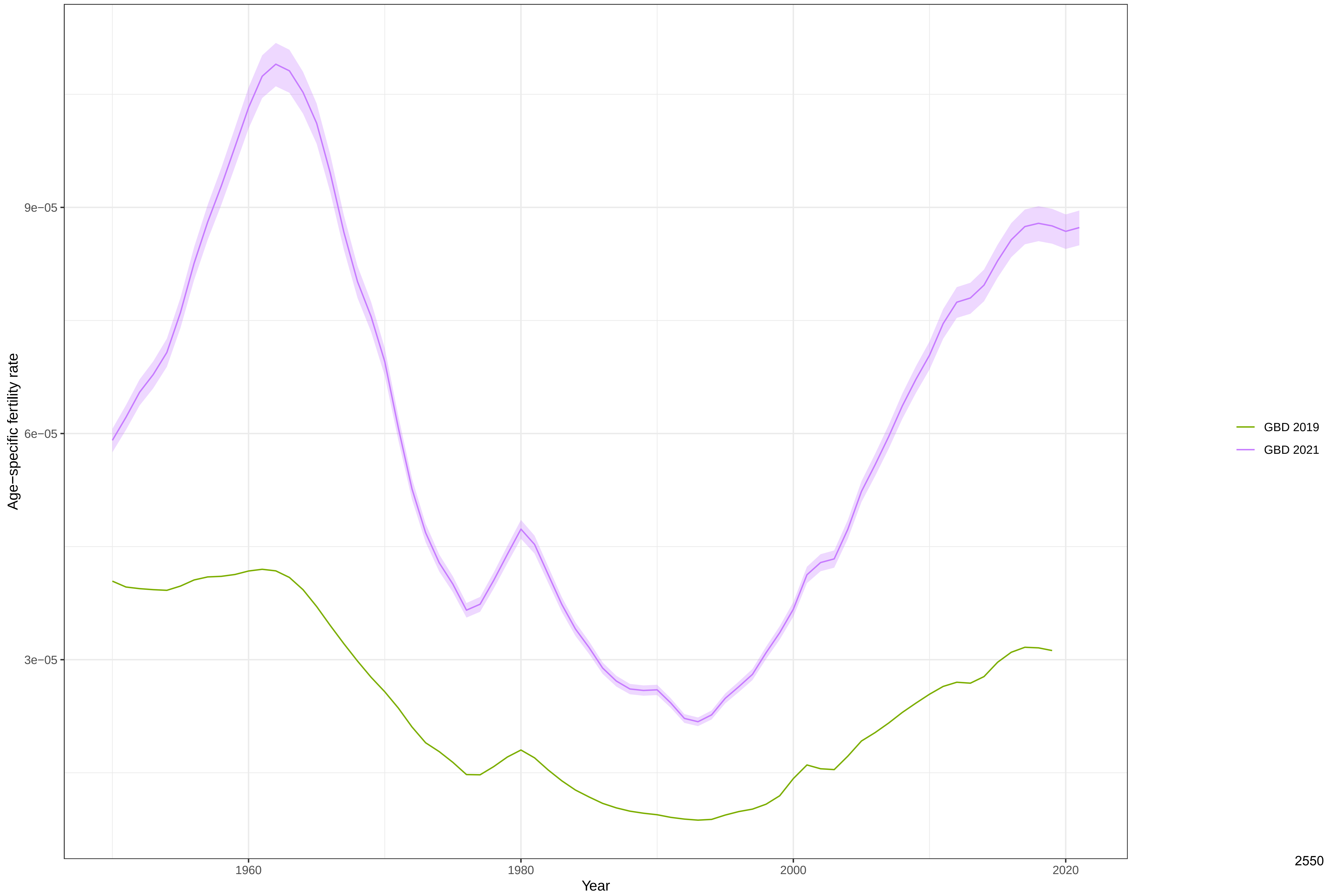

Total fertility rate fitted trend lines, 1950−2021: County Durham TFR
Data points enclosed with a diamond are reference sources

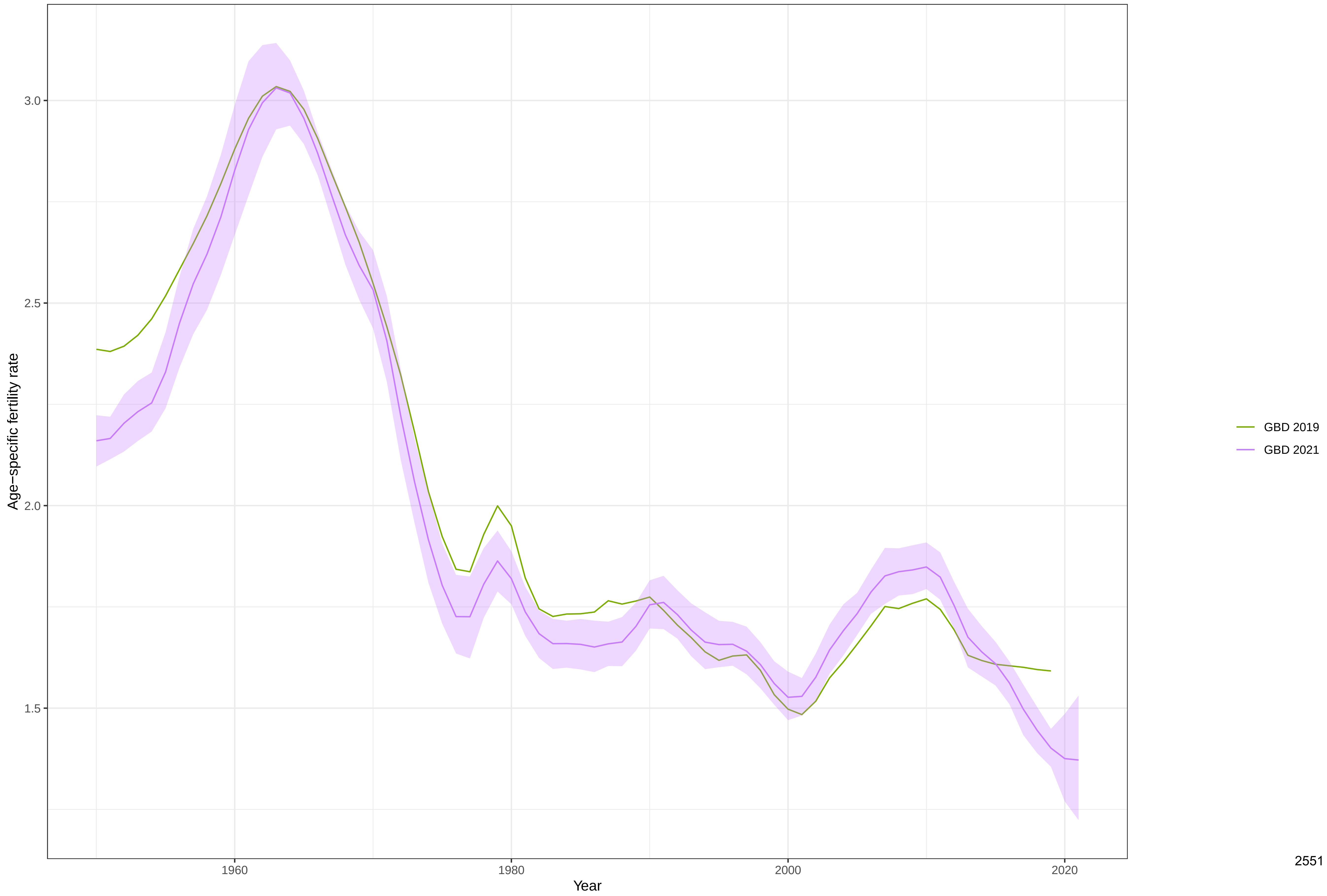

Age−specific fertility fitted trend lines, 1950−2021: Darlington, Ages: 10−14
Data points enclosed with a diamond are reference sources

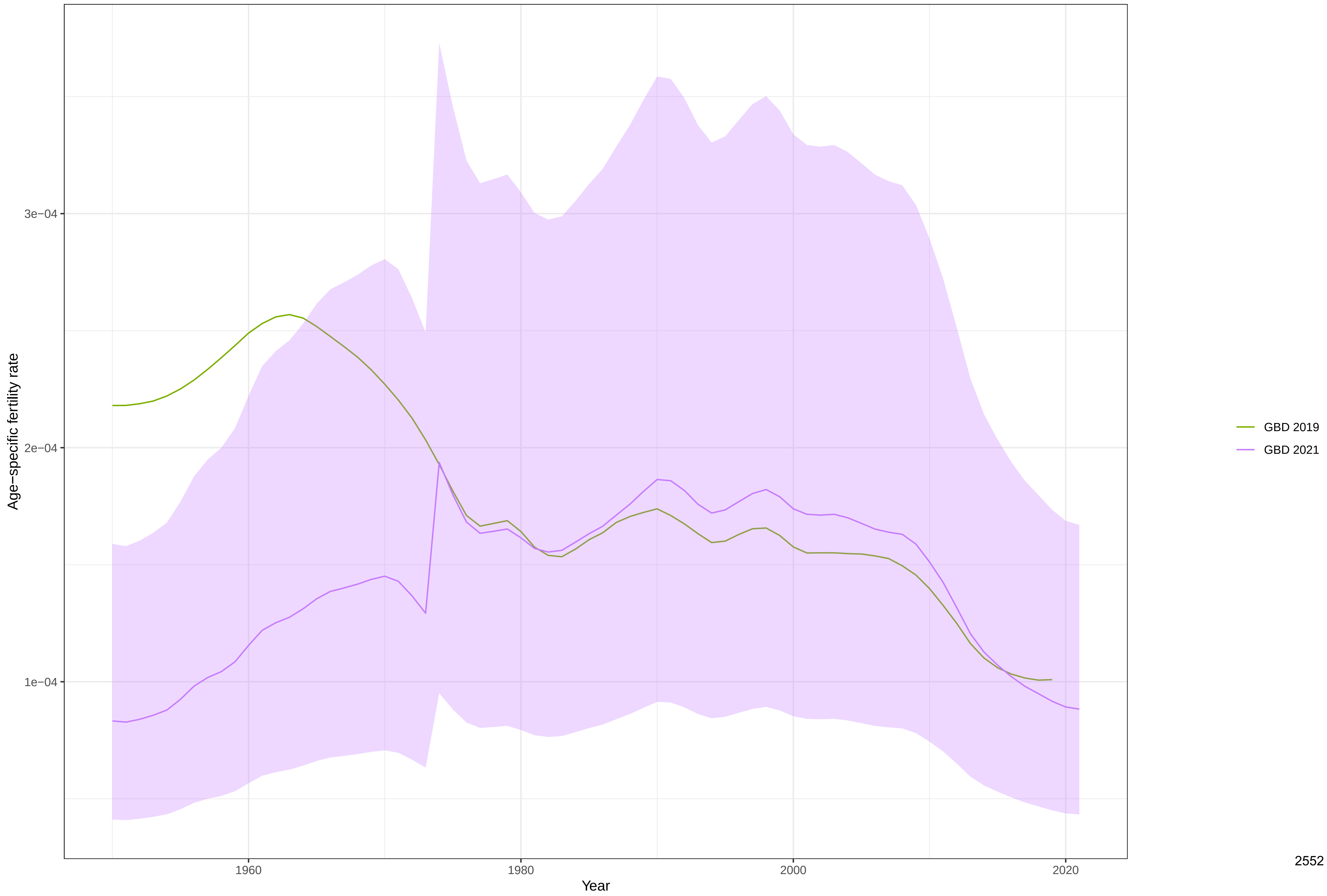

Age−specific fertility fitted trend lines, 1950−2021: Darlington, Ages: 15−19
Data points enclosed with a diamond are reference sources

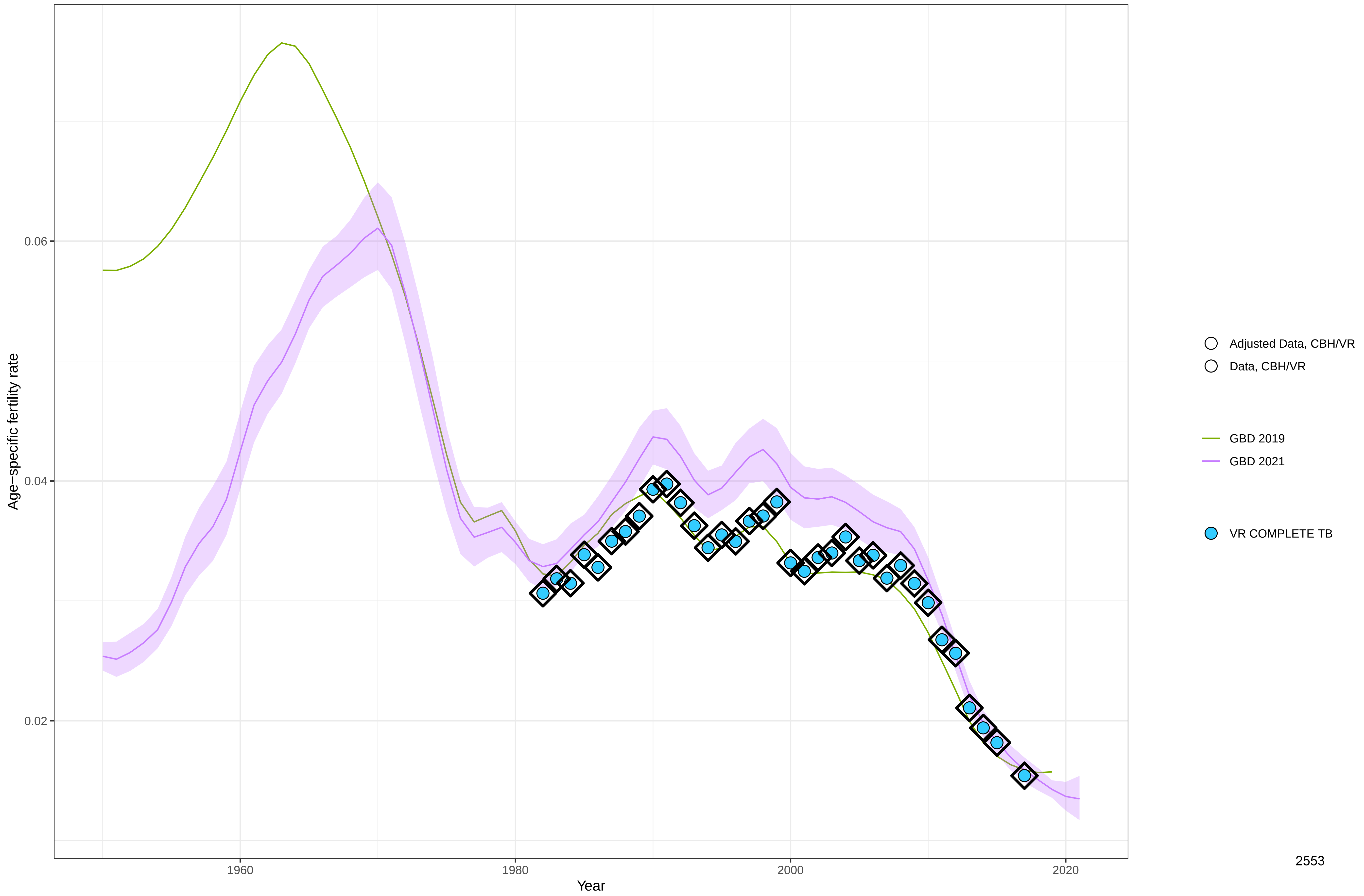

Age−specific fertility fitted trend lines, 1950−2021: Darlington, Ages: 20−24
Data points enclosed with a diamond are reference sources

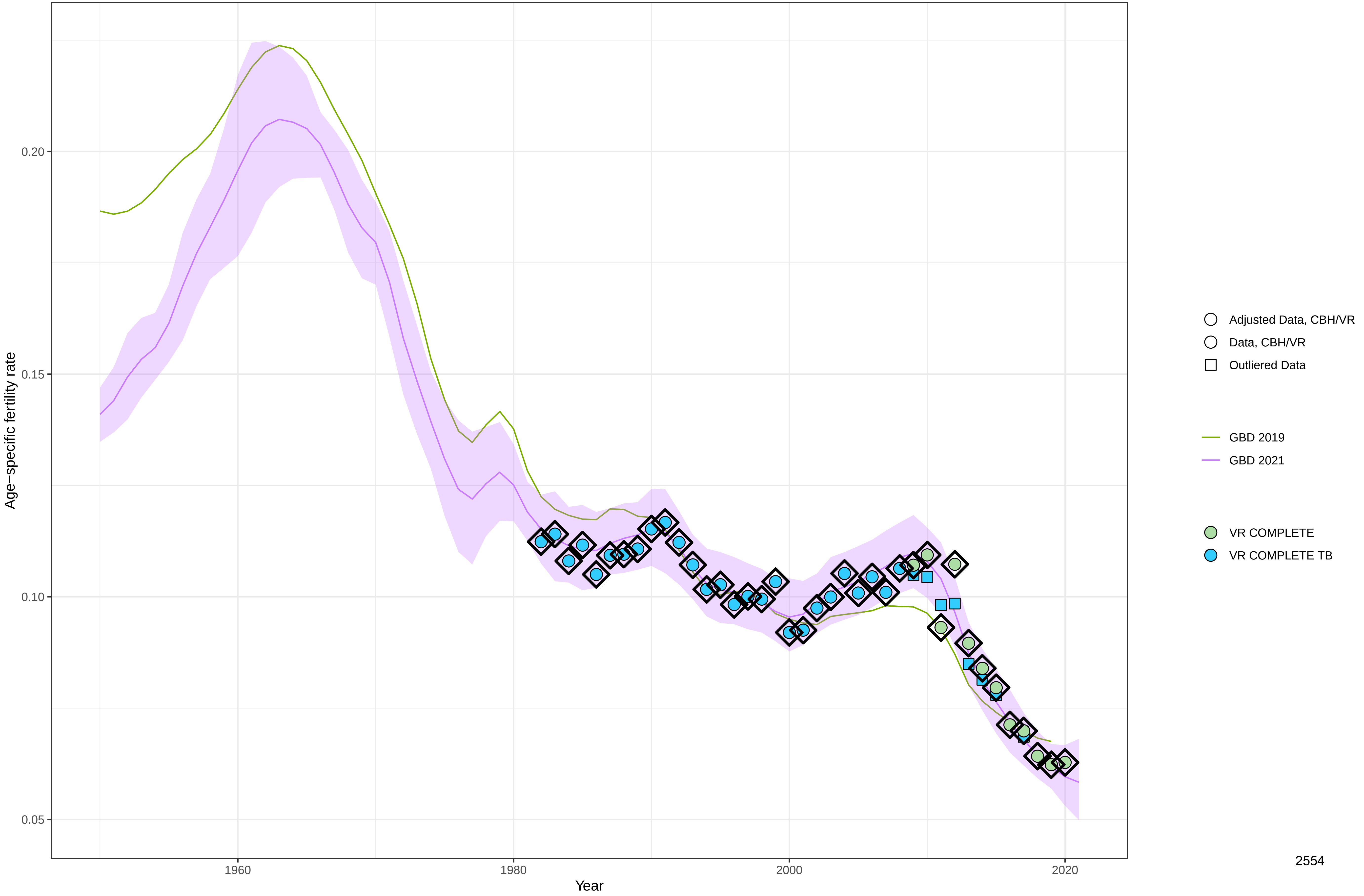

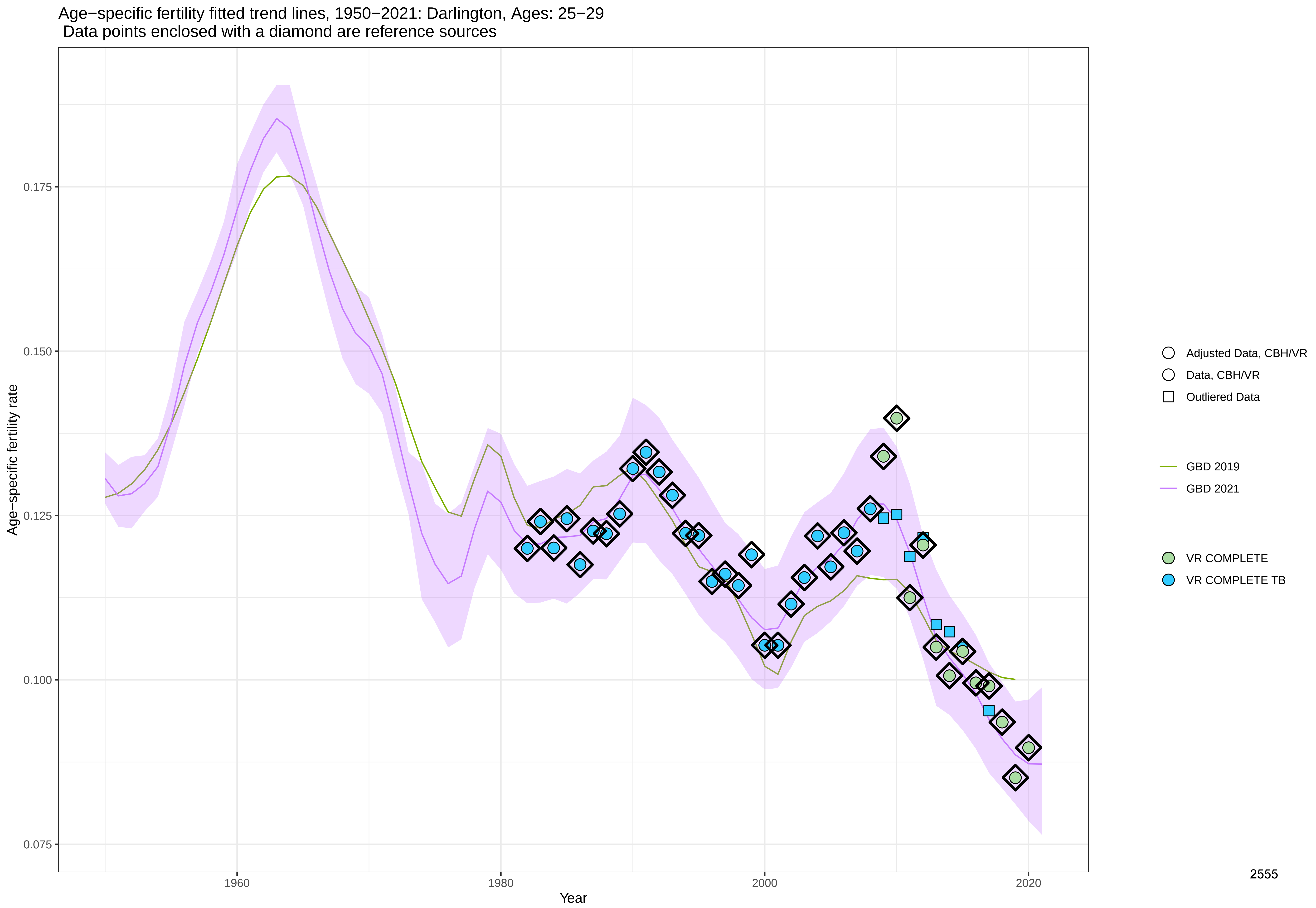

Age−specific fertility fitted trend lines, 1950−2021: Darlington, Ages: 25−29
Data points enclosed with a diamond are reference sources
Age−specific fertility rate
0.075
0.100
0.125
0.150
0.175
1960
1980
2000
2020
Year
Adjusted Data, CBH/VR
Data, CBH/VR
Outliered Data
GBD 2019
GBD 2021
VR COMPLETE
VR COMPLETE TB

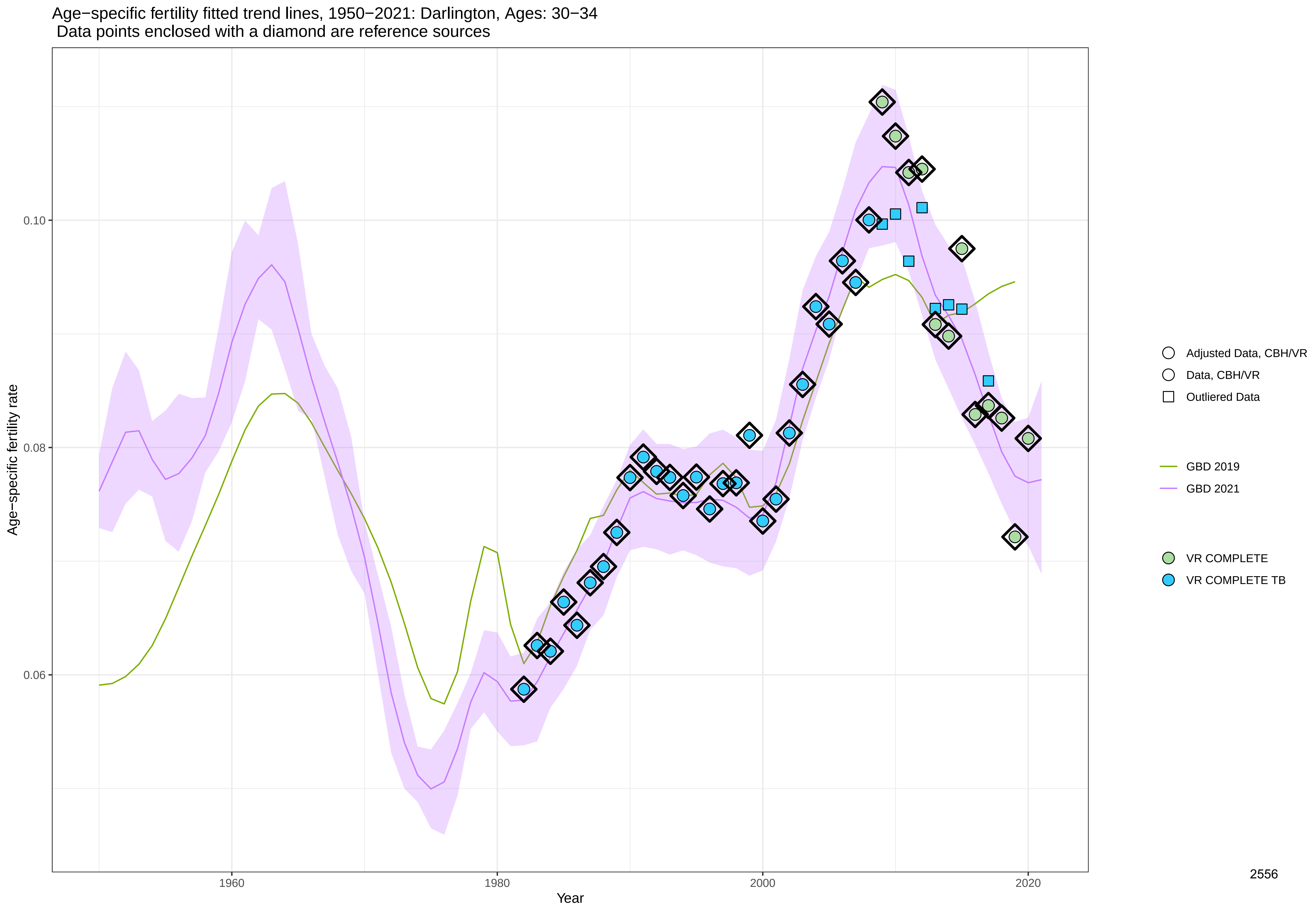

Age−specific fertility fitted trend lines, 1950−2021: Darlington, Ages: 30−34
Data points enclosed with a diamond are reference sources
Age−specific fertility rate
0.06
0.08
0.10
Year
1960
1980
2000
2020
Adjusted Data, CBH/VR
Data, CBH/VR
Outliered Data
GBD 2019
GBD 2021
VR COMPLETE
VR COMPLETE TB

Age−specific fertility fitted trend lines, 1950−2021: Darlington, Ages: 35−39
Data points enclosed with a diamond are reference sources

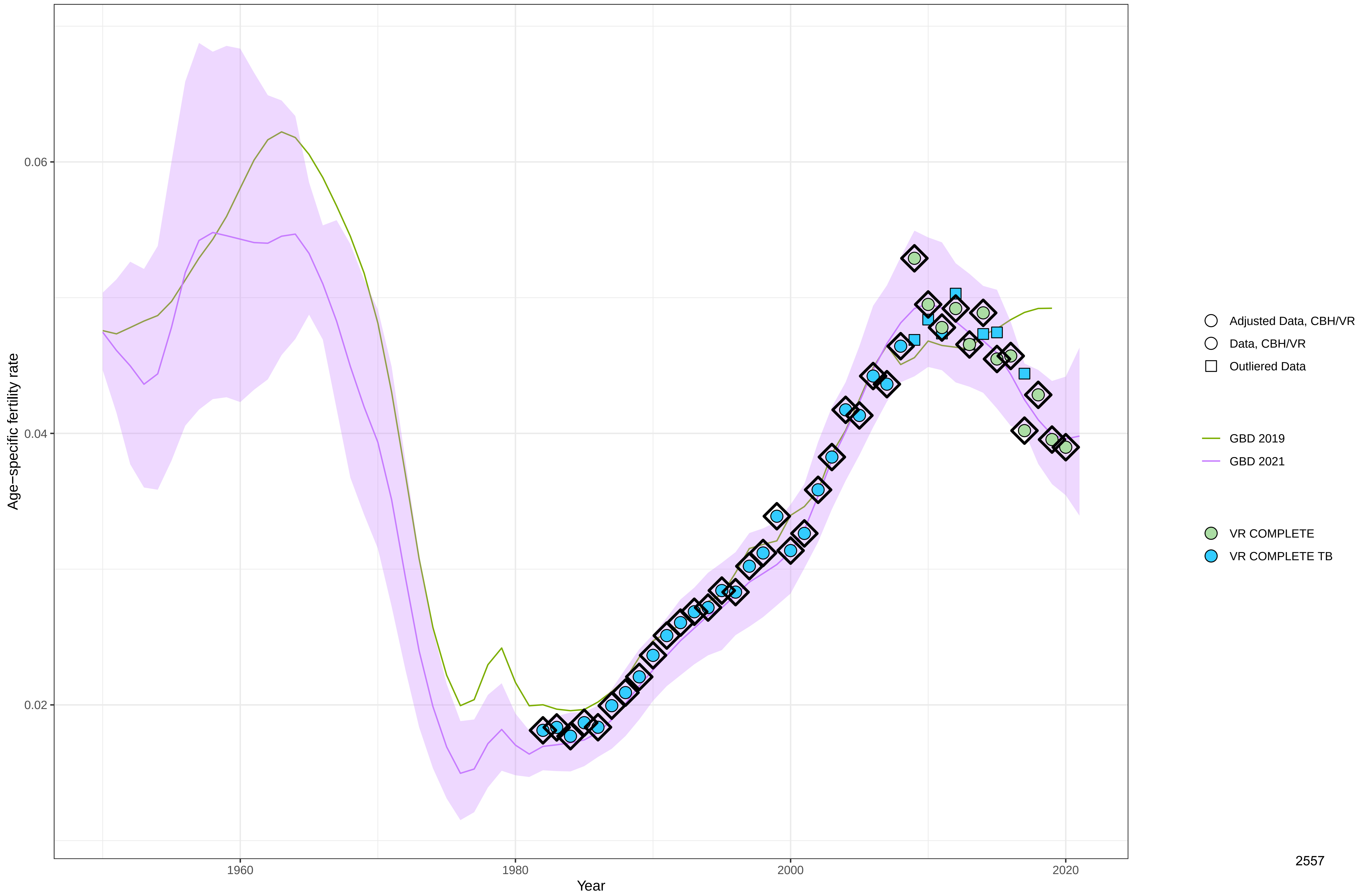

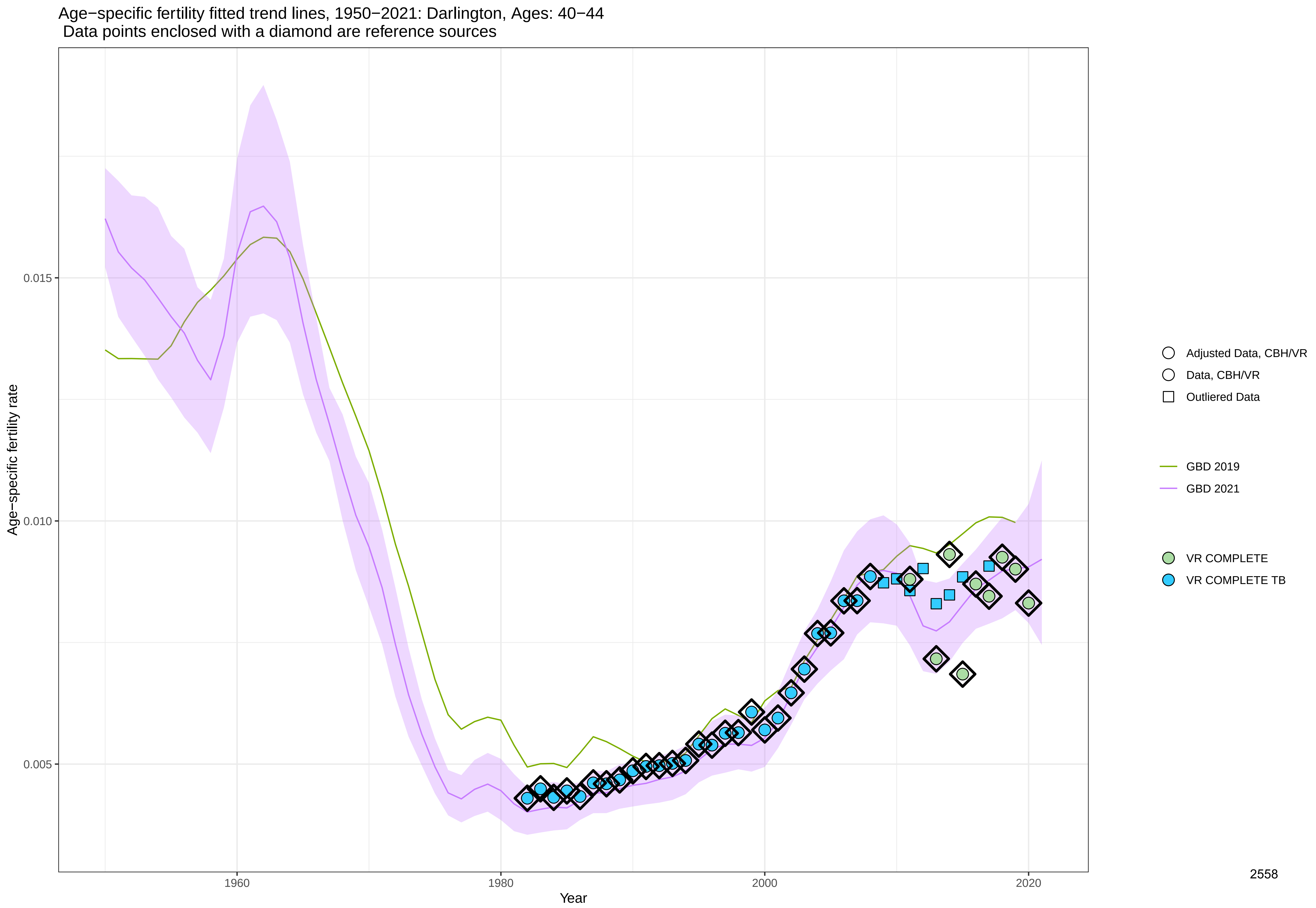
Age−specific fertility fitted trend lines, 1950−2021: Darlington, Ages: 40−44
Data points enclosed with a diamond are reference sources
Age−specific fertility rate
0.015
0.010
0.005
1960
1980
2000
2020
Year
Adjusted Data, CBH/VR
Data, CBH/VR
Outliered Data
GBD 2019
GBD 2021
VR COMPLETE
VR COMPLETE TB

Age−specific fertility fitted trend lines, 1950−2021: Darlington, Ages: 45−49
Data points enclosed with a diamond are reference sources

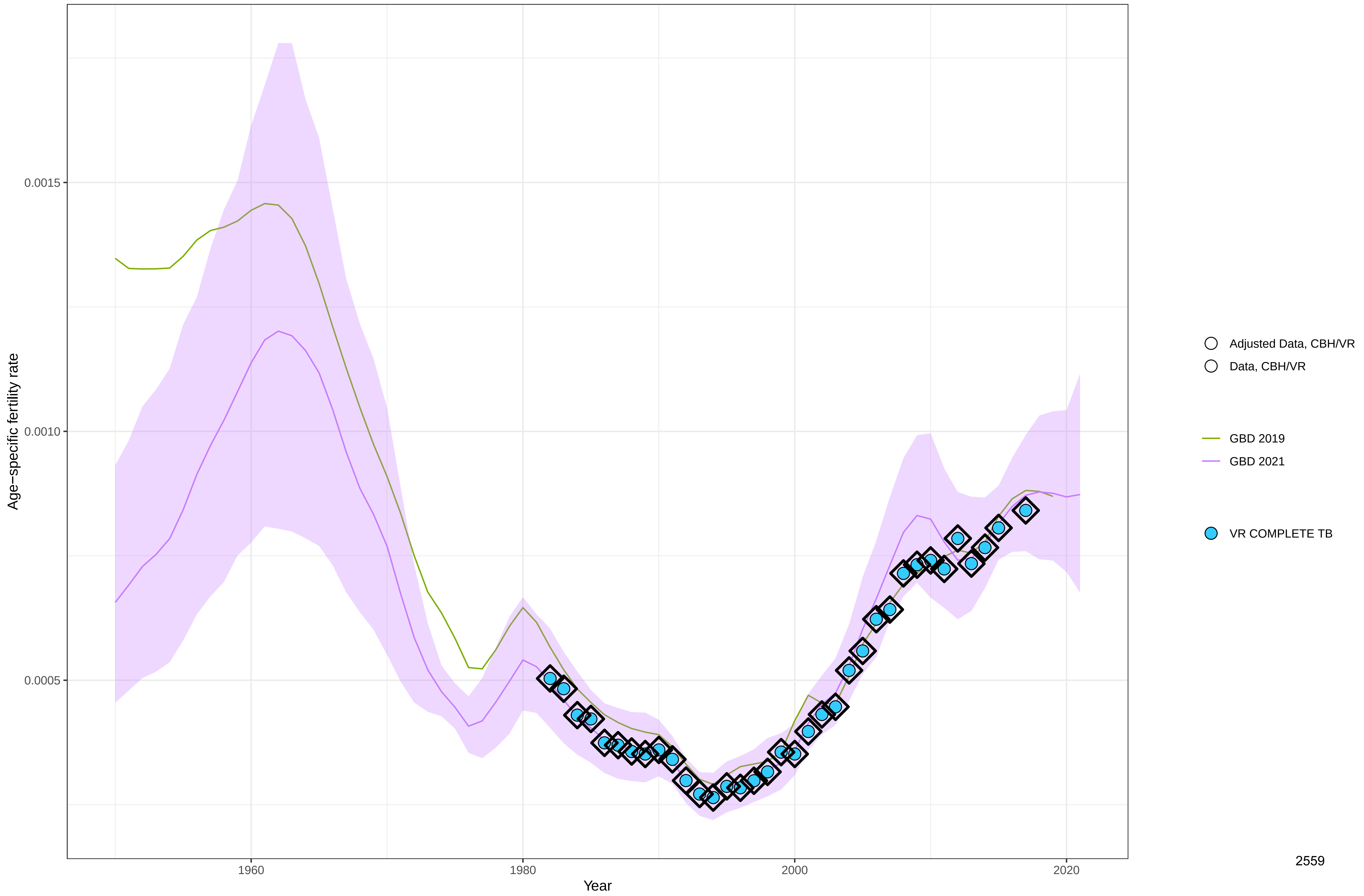

Age−specific fertility fitted trend lines, 1950−2021: Darlington, Ages: 50−54
Data points enclosed with a diamond are reference sources

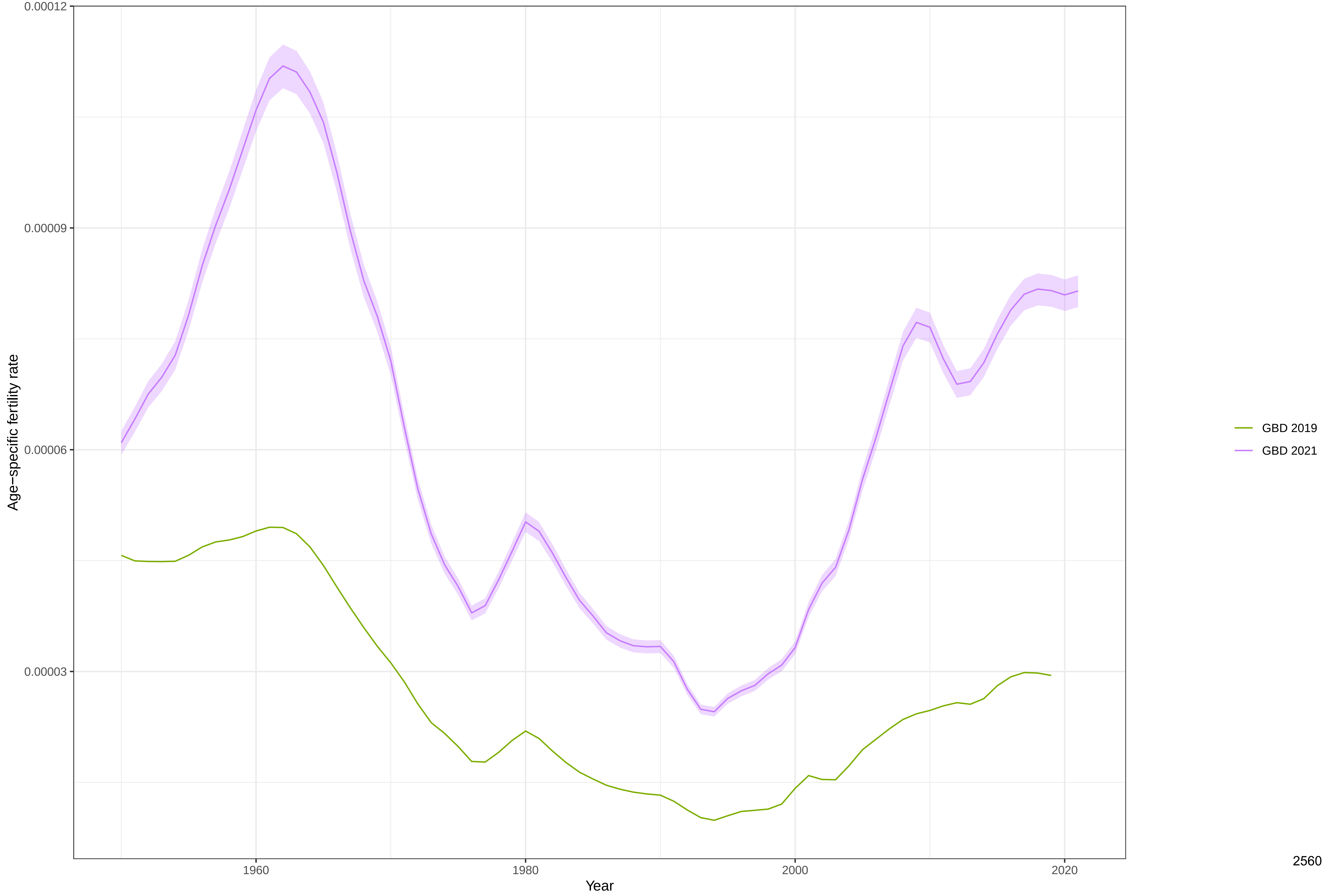

Total fertility rate fitted trend lines, 1950−2021: Darlington TFR
Data points enclosed with a diamond are reference sources

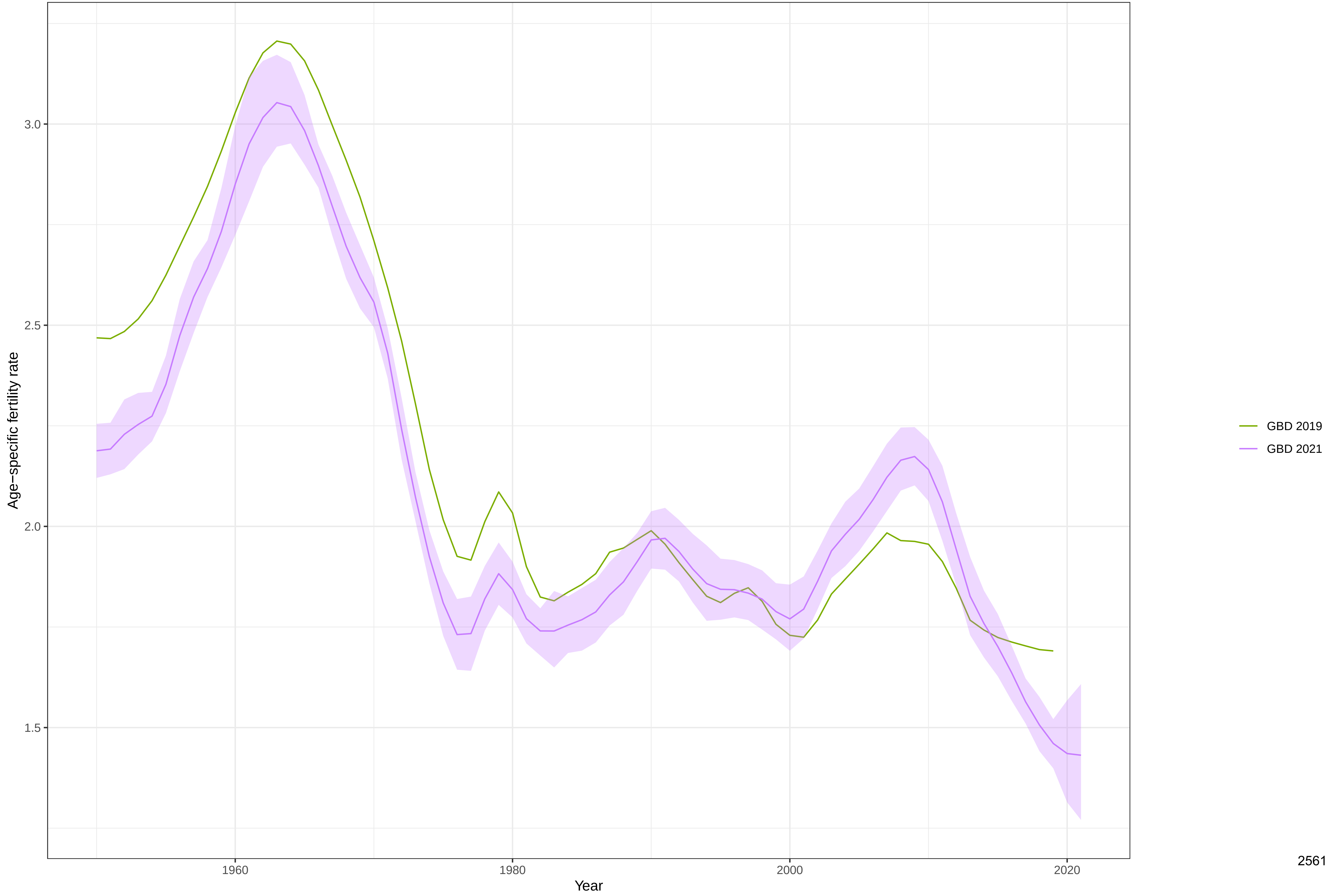

Age−specific fertility fitted trend lines, 1950−2021: Gateshead, Ages: 10−14
Data points enclosed with a diamond are reference sources

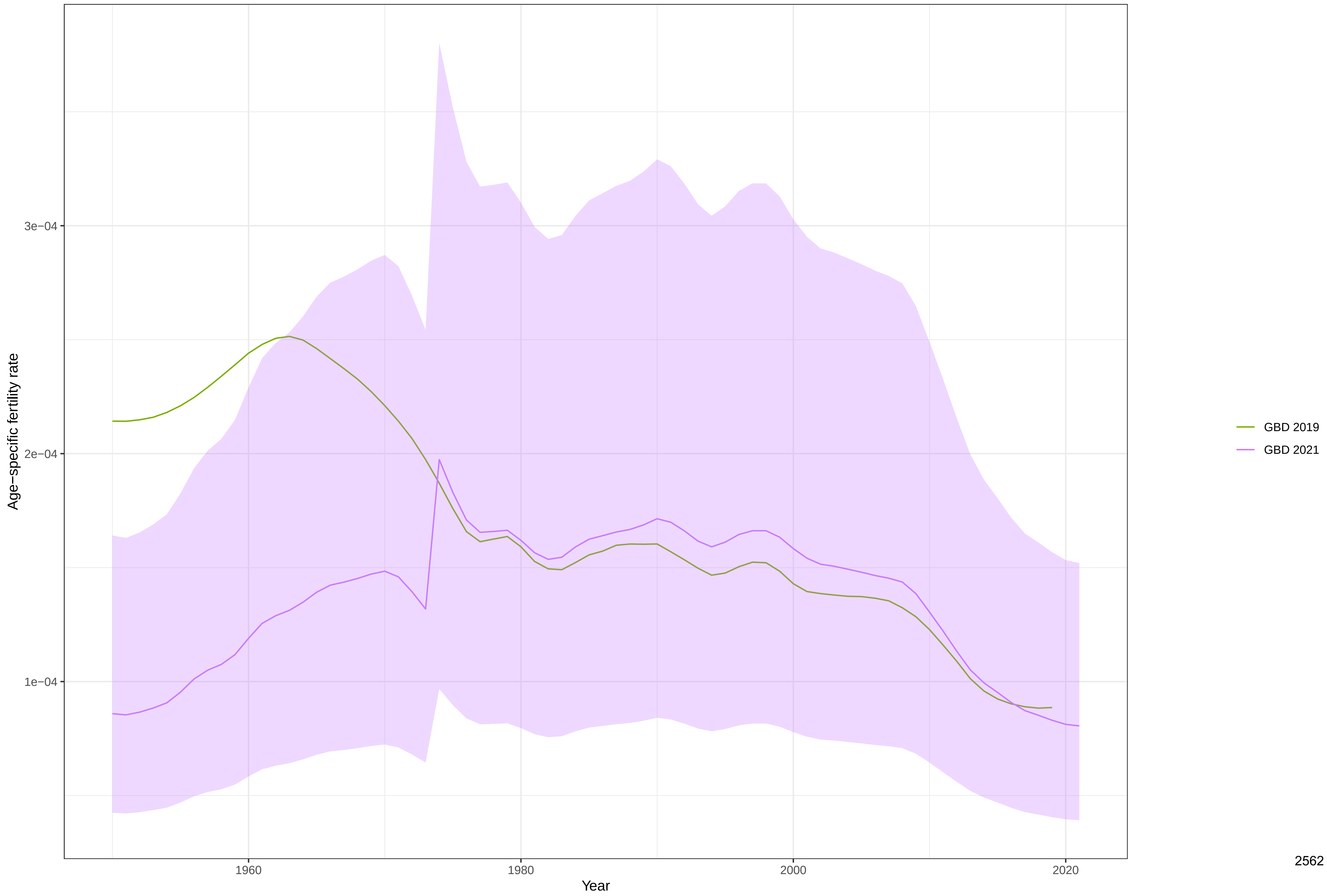

Age−specific fertility fitted trend lines, 1950−2021: Gateshead, Ages: 15−19
Data points enclosed with a diamond are reference sources

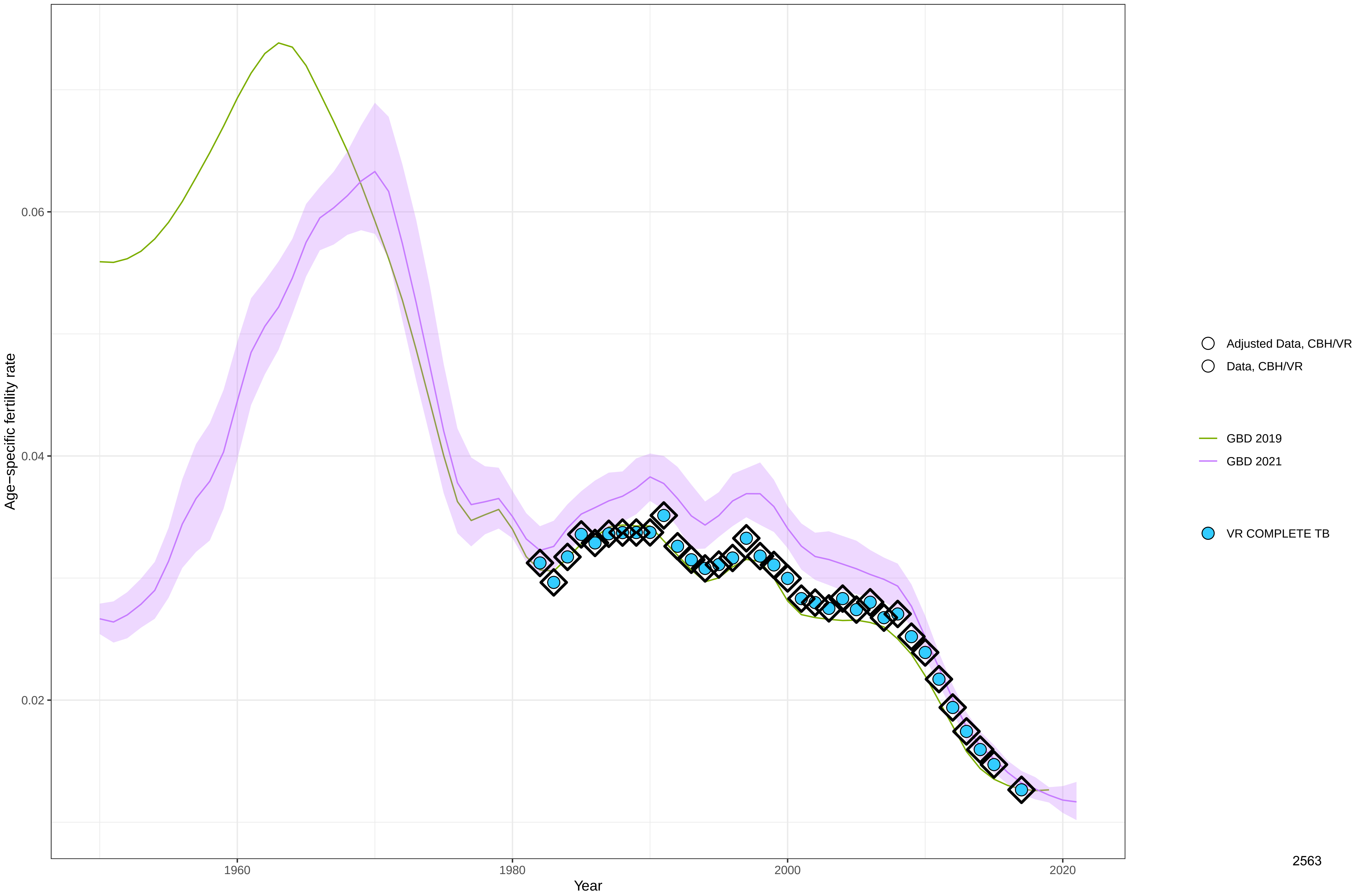

Age−specific fertility fitted trend lines, 1950−2021: Gateshead, Ages: 20−24
Data points enclosed with a diamond are reference sources

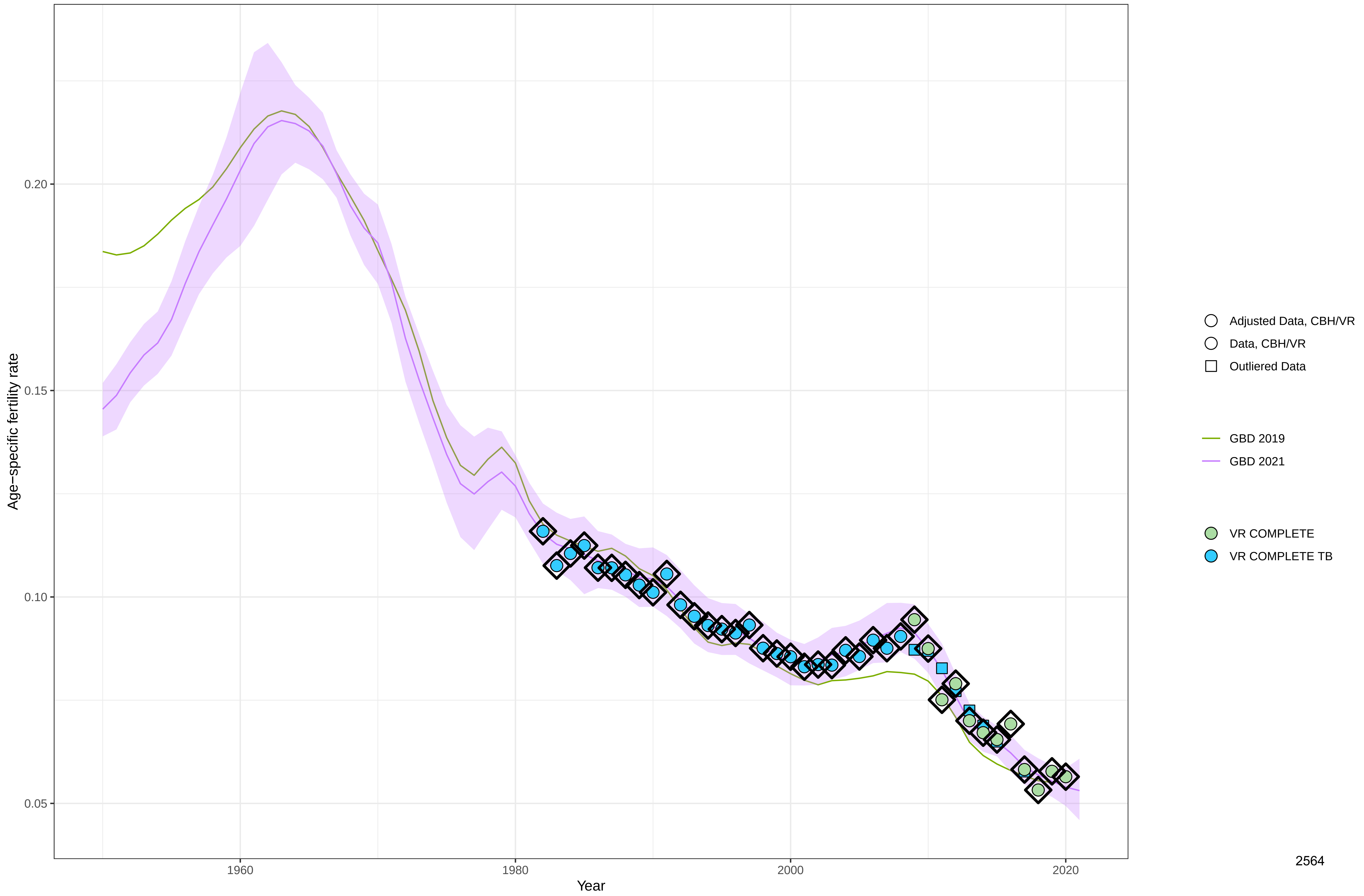

Age−specific fertility fitted trend lines, 1950−2021: Gateshead, Ages: 25−29
Data points enclosed with a diamond are reference sources

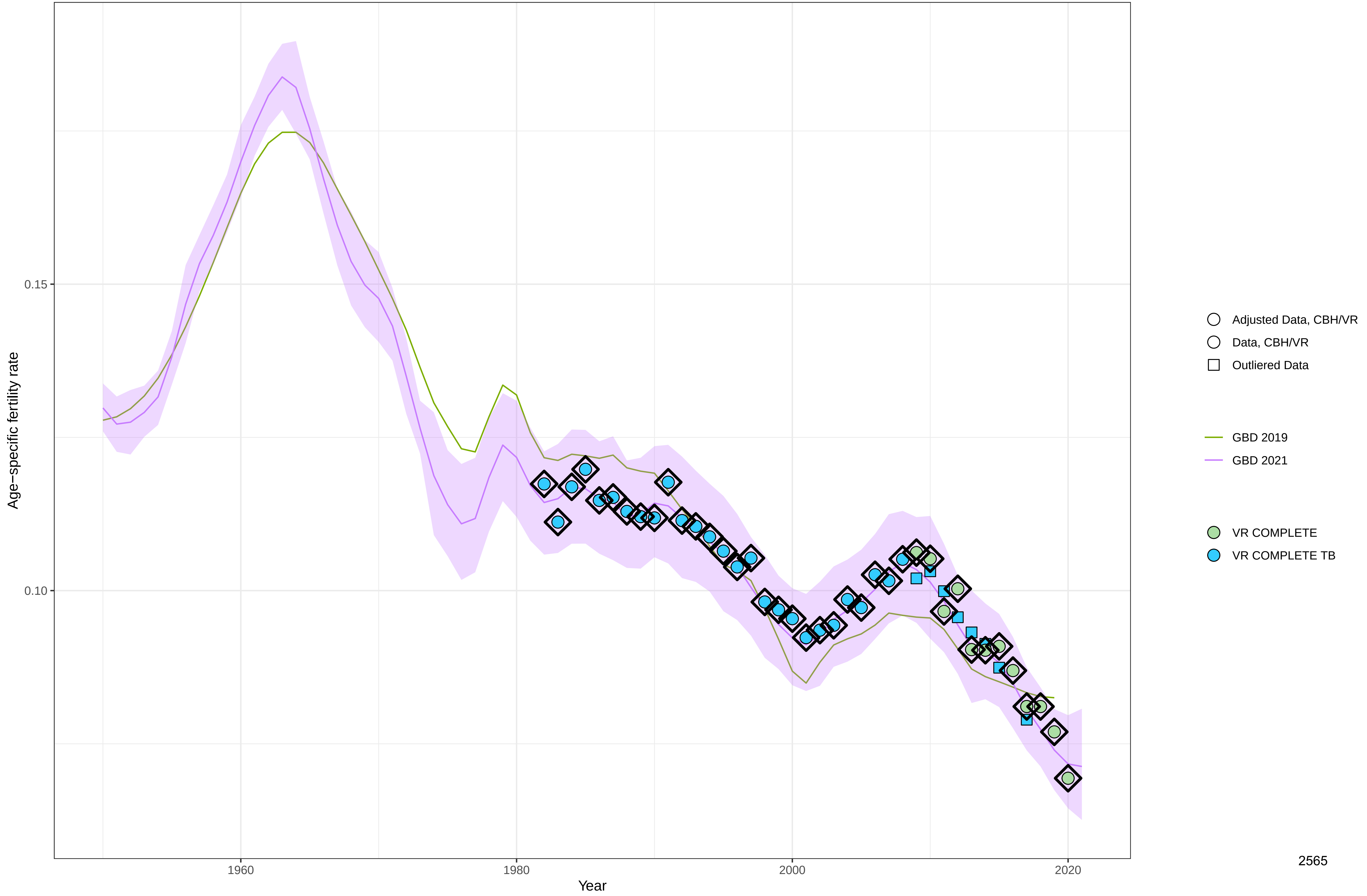

Age−specific fertility fitted trend lines, 1950−2021: Gateshead, Ages: 30−34
Data points enclosed with a diamond are reference sources

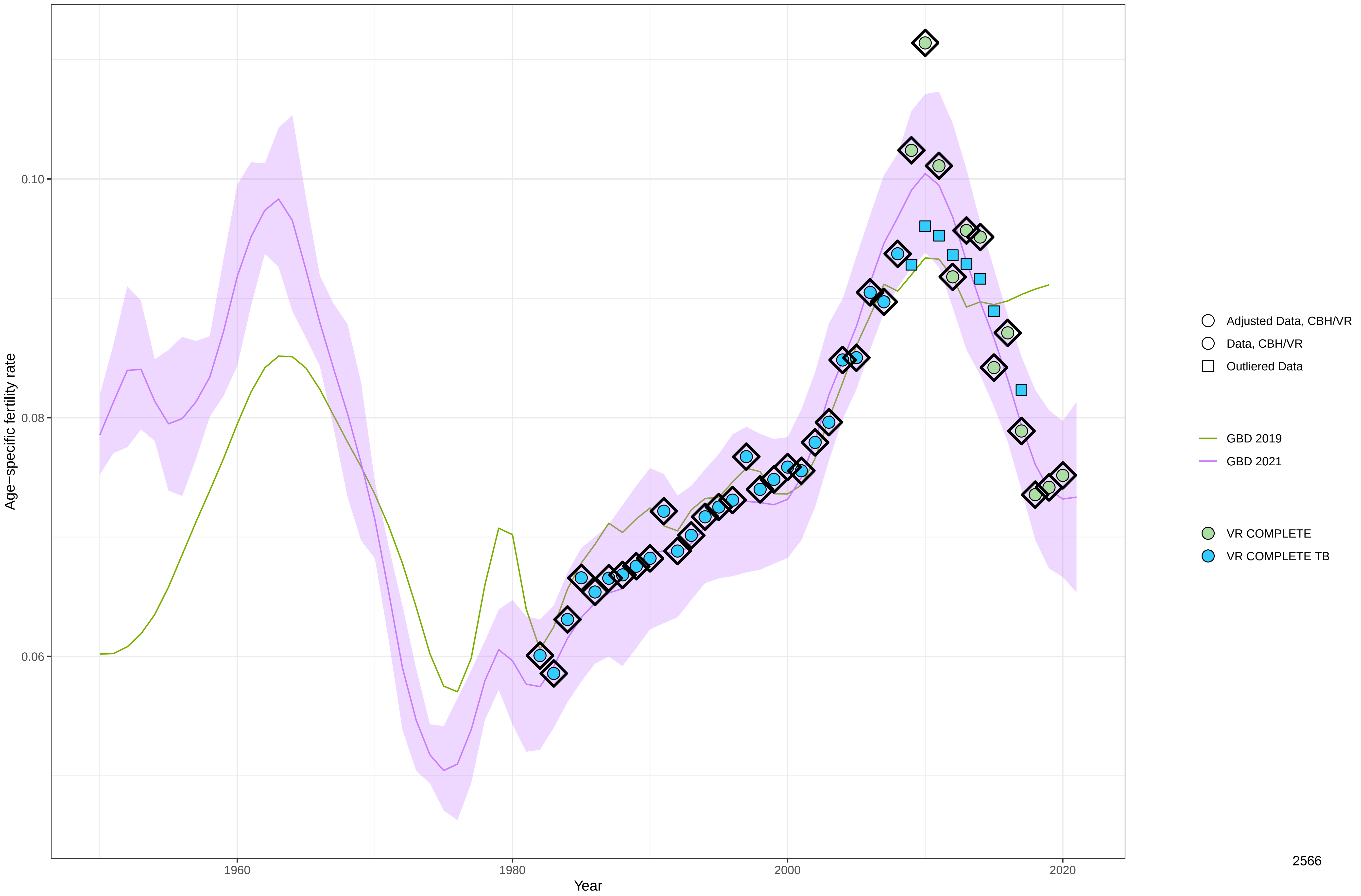

Age−specific fertility fitted trend lines, 1950−2021: Gateshead, Ages: 35−39
Data points enclosed with a diamond are reference sources

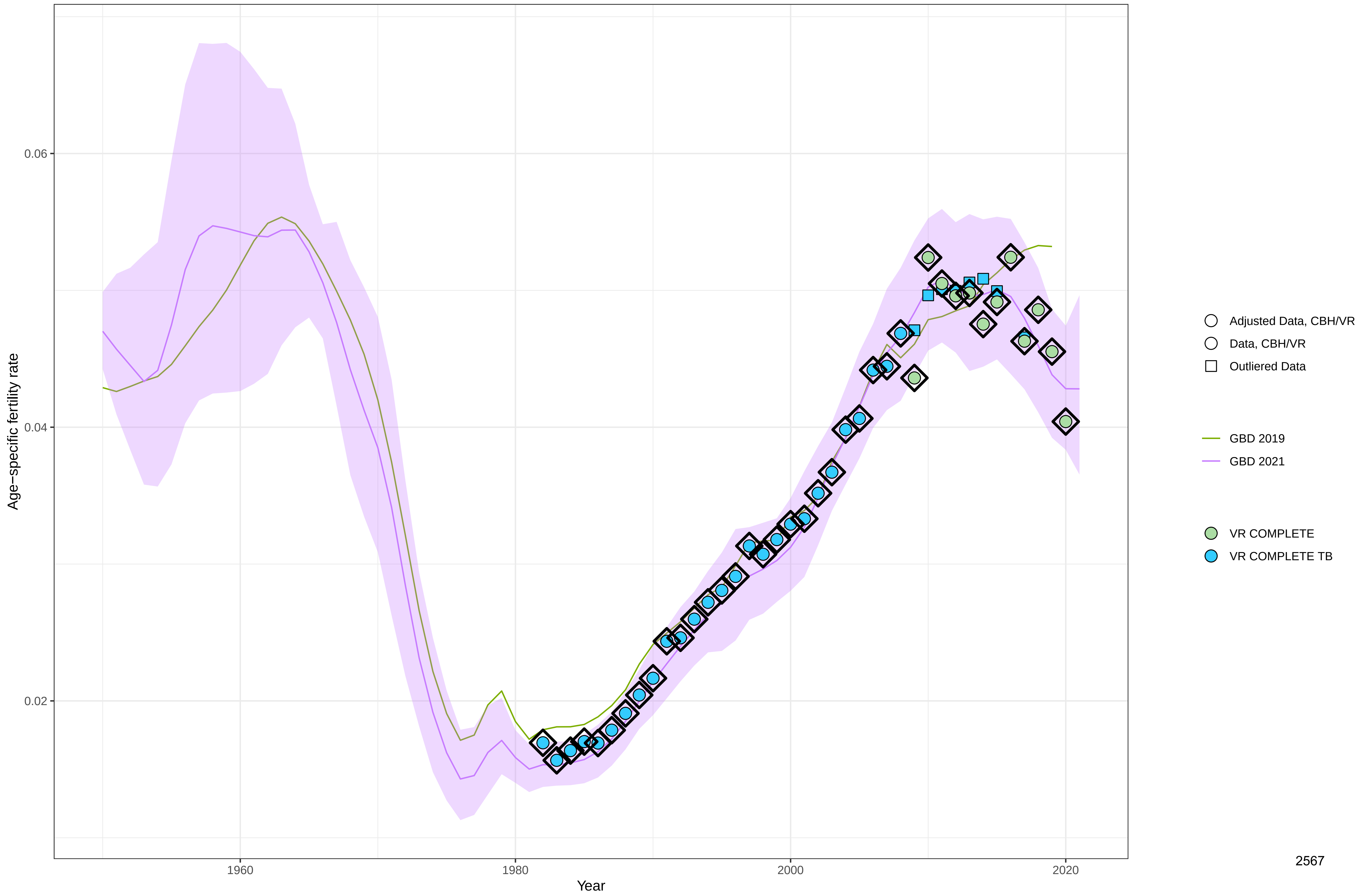

Age−specific fertility fitted trend lines, 1950−2021: Gateshead, Ages: 40−44
Data points enclosed with a diamond are reference sources

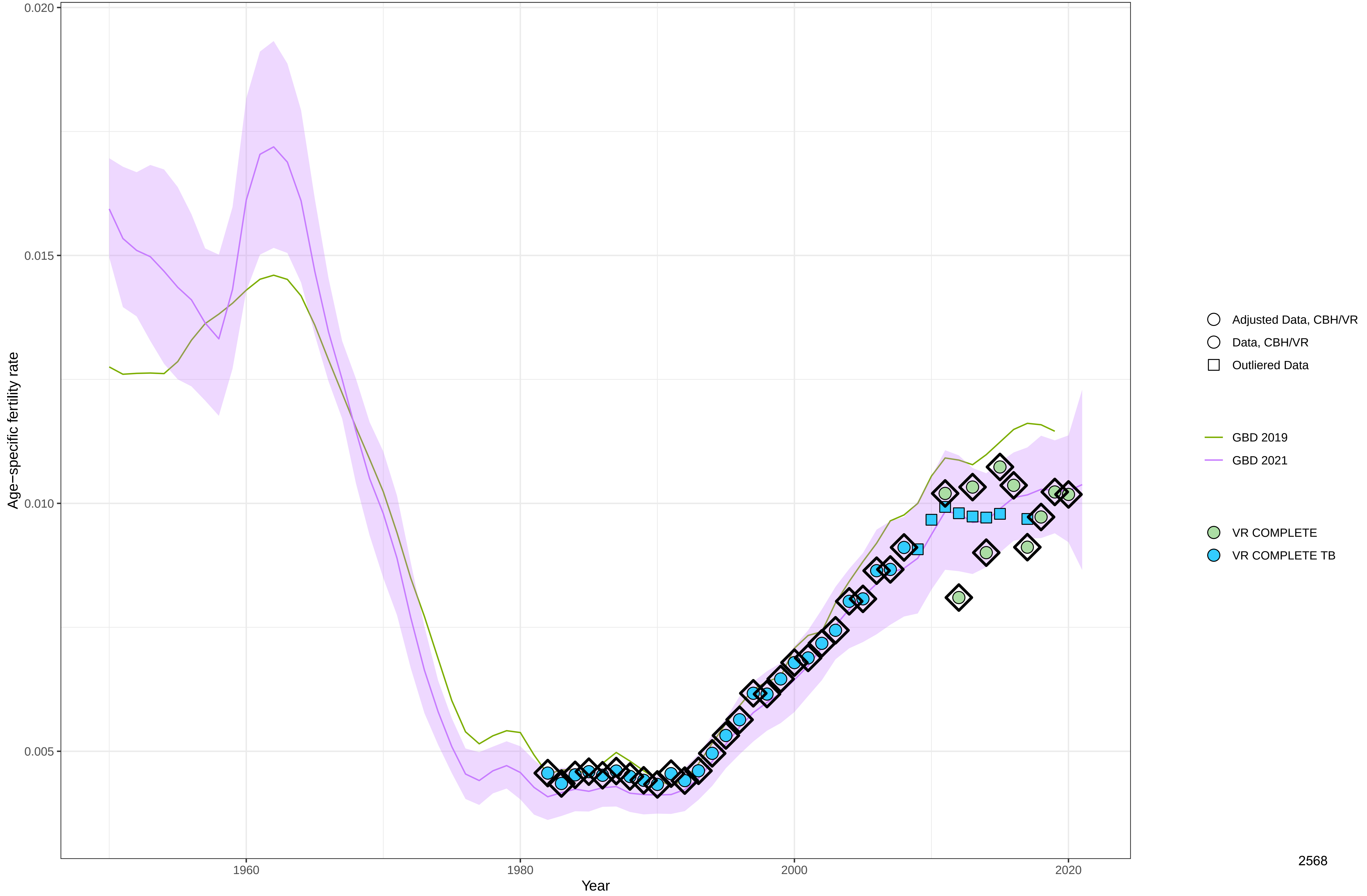

Age−specific fertility fitted trend lines, 1950−2021: Gateshead, Ages: 45−49
Data points enclosed with a diamond are reference sources

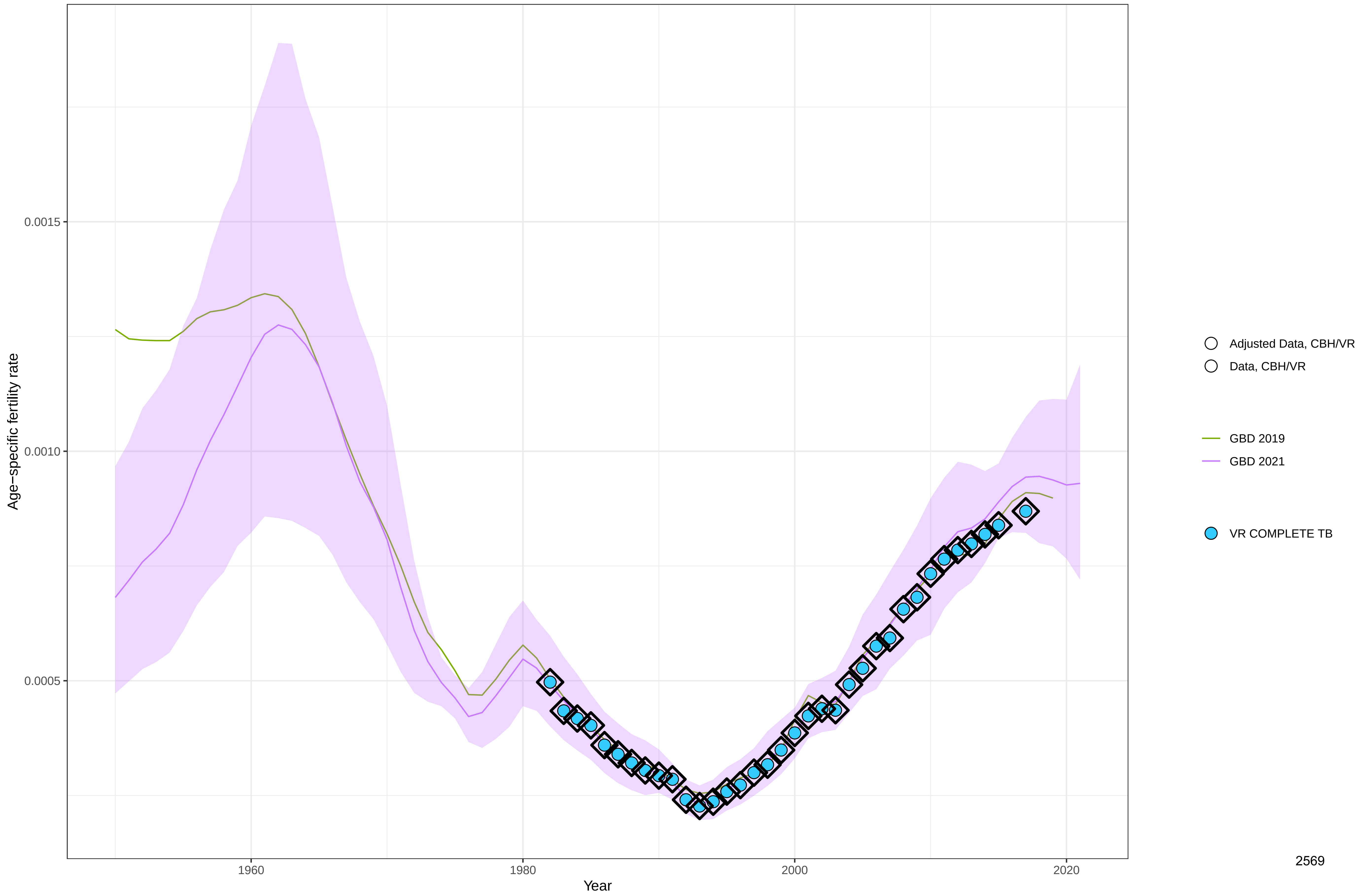

Age−specific fertility fitted trend lines, 1950−2021: Gateshead, Ages: 50−54
Data points enclosed with a diamond are reference sources

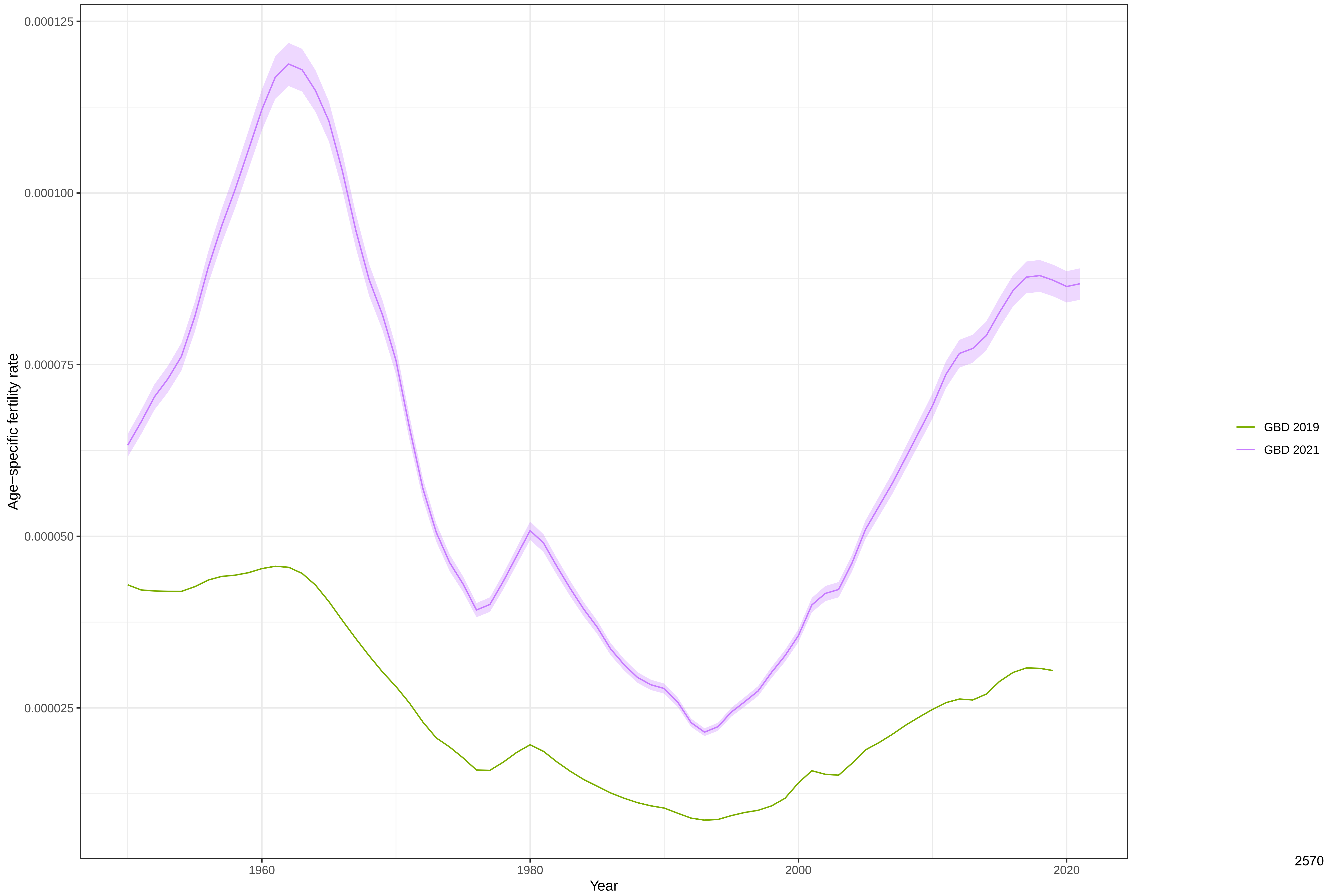

Total fertility rate fitted trend lines, 1950−2021: Gateshead TFR
Data points enclosed with a diamond are reference sources

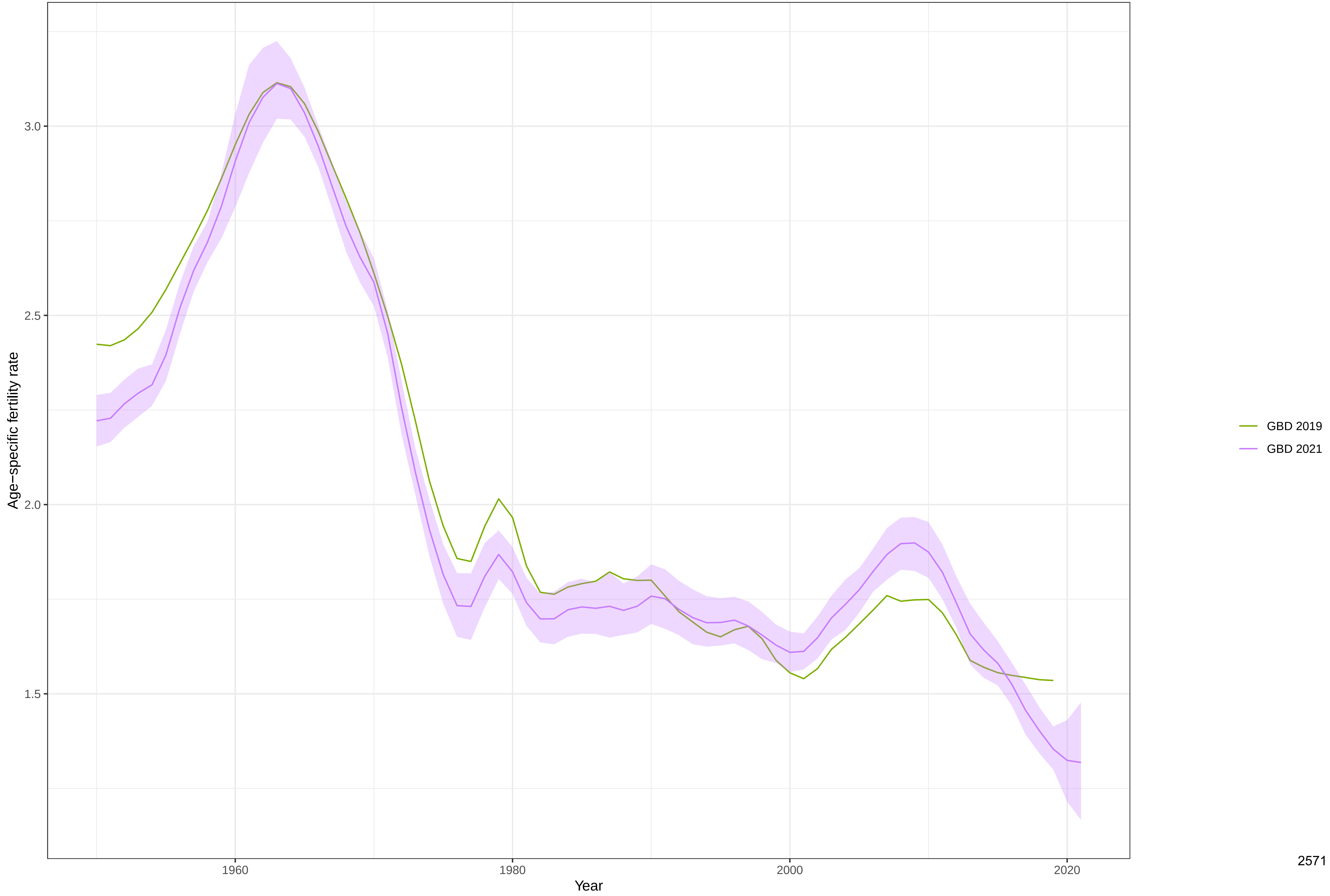

Age−specific fertility fitted trend lines, 1950−2021: Hartlepool, Ages: 10−14
Data points enclosed with a diamond are reference sources

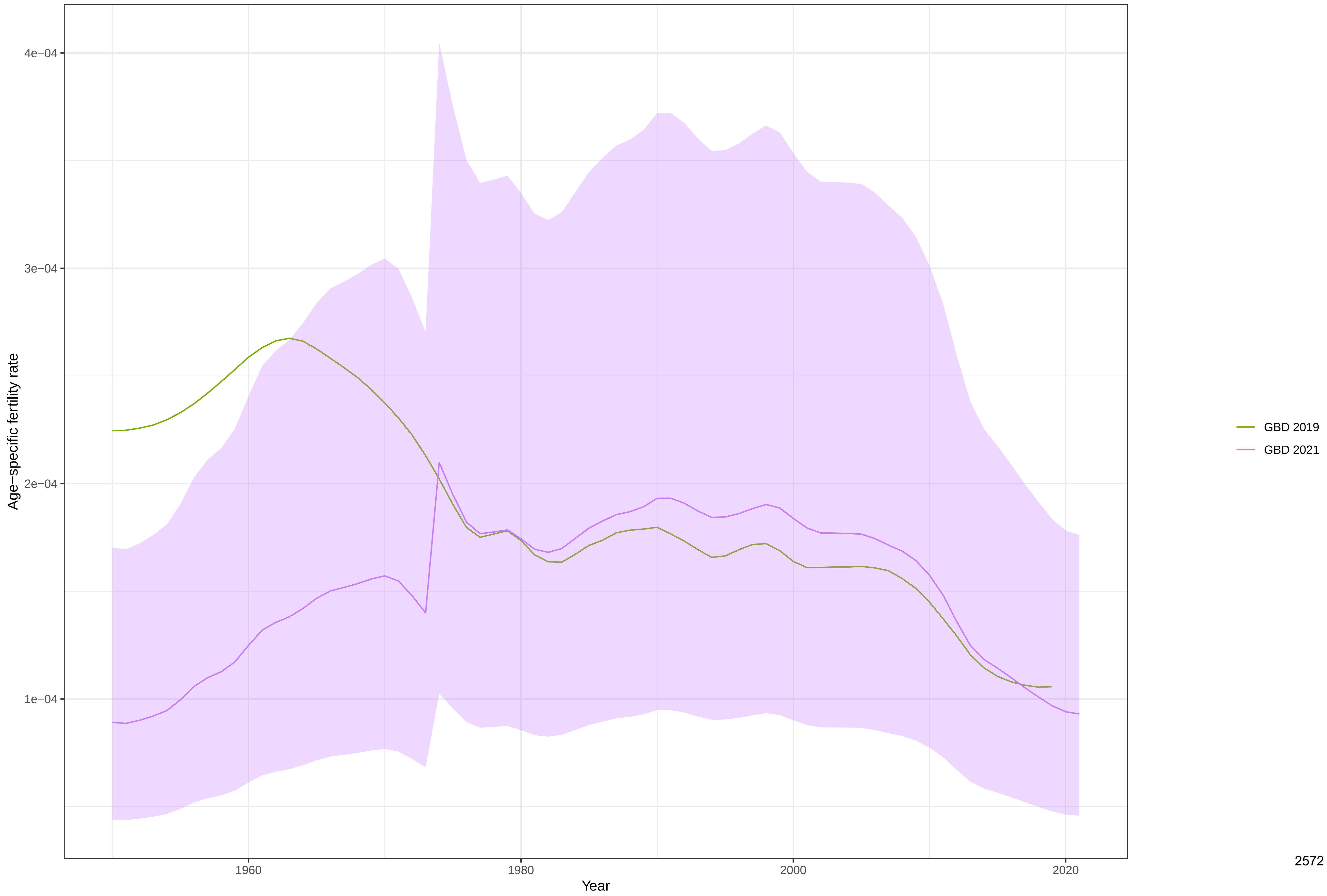

Age−specific fertility fitted trend lines, 1950−2021: Hartlepool, Ages: 15−19
Data points enclosed with a diamond are reference sources

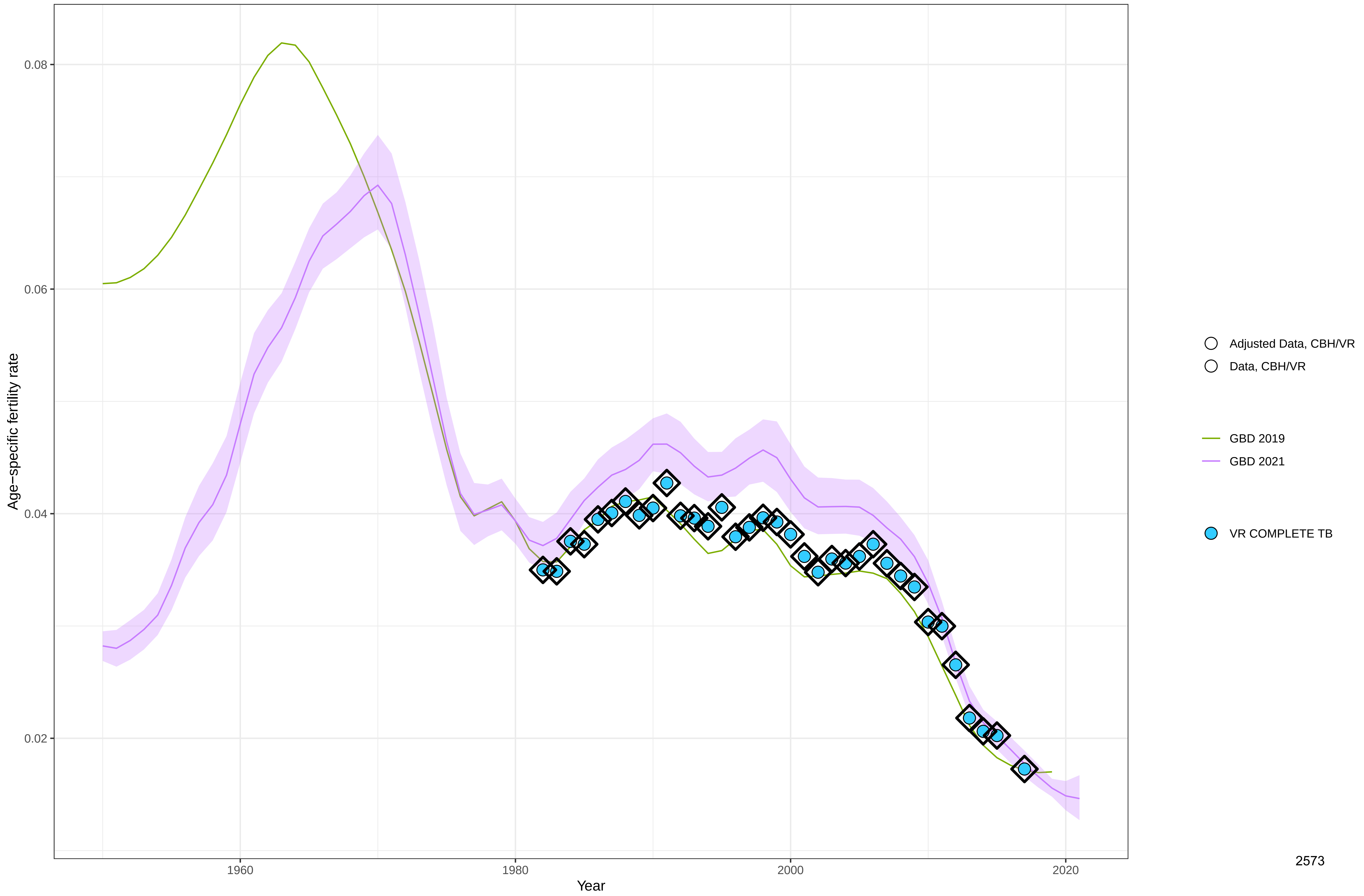

Age−specific fertility fitted trend lines, 1950−2021: Hartlepool, Ages: 20−24
Data points enclosed with a diamond are reference sources

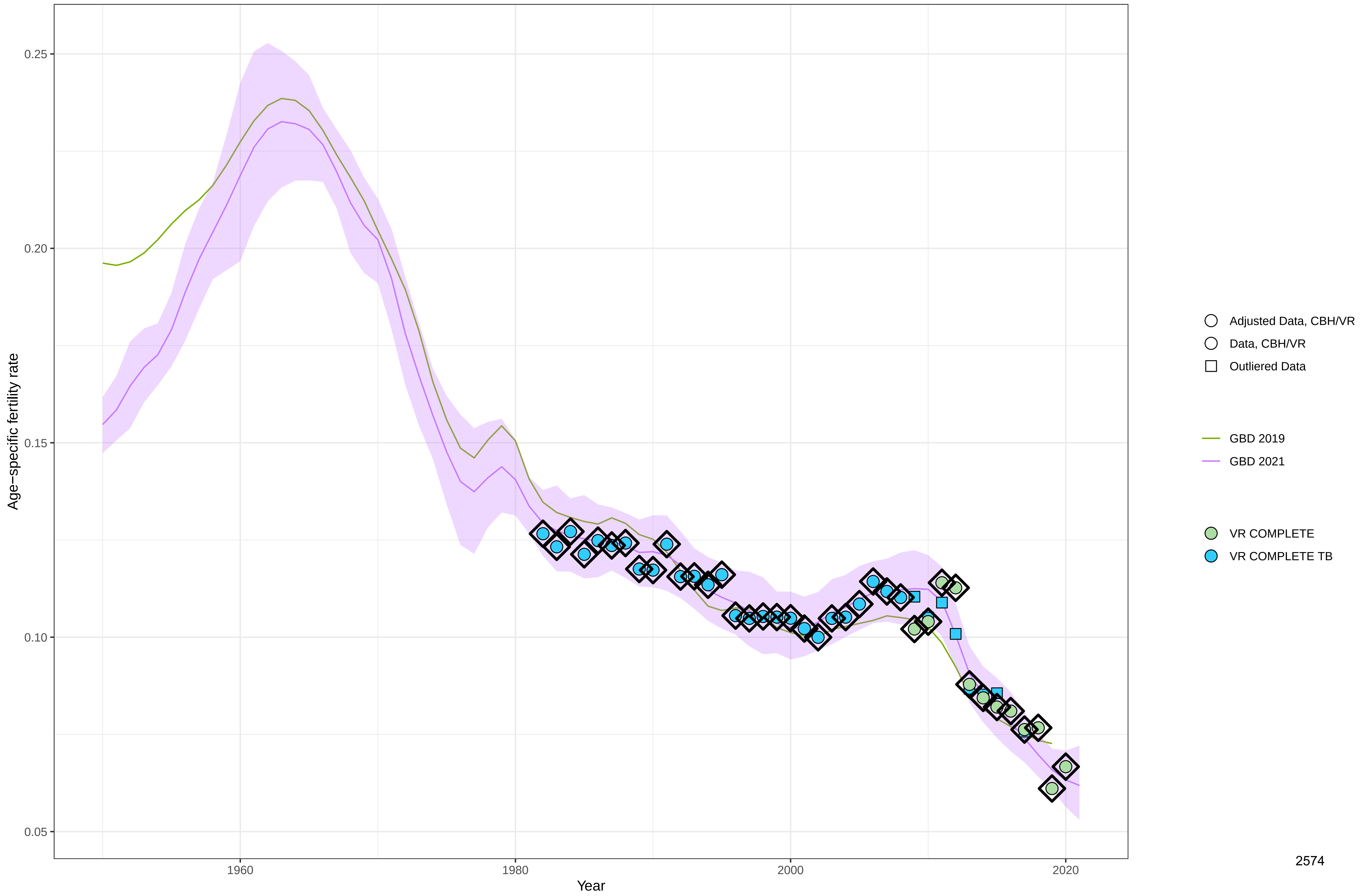

Age−specific fertility fitted trend lines, 1950−2021: Hartlepool, Ages: 25−29
Data points enclosed with a diamond are reference sources

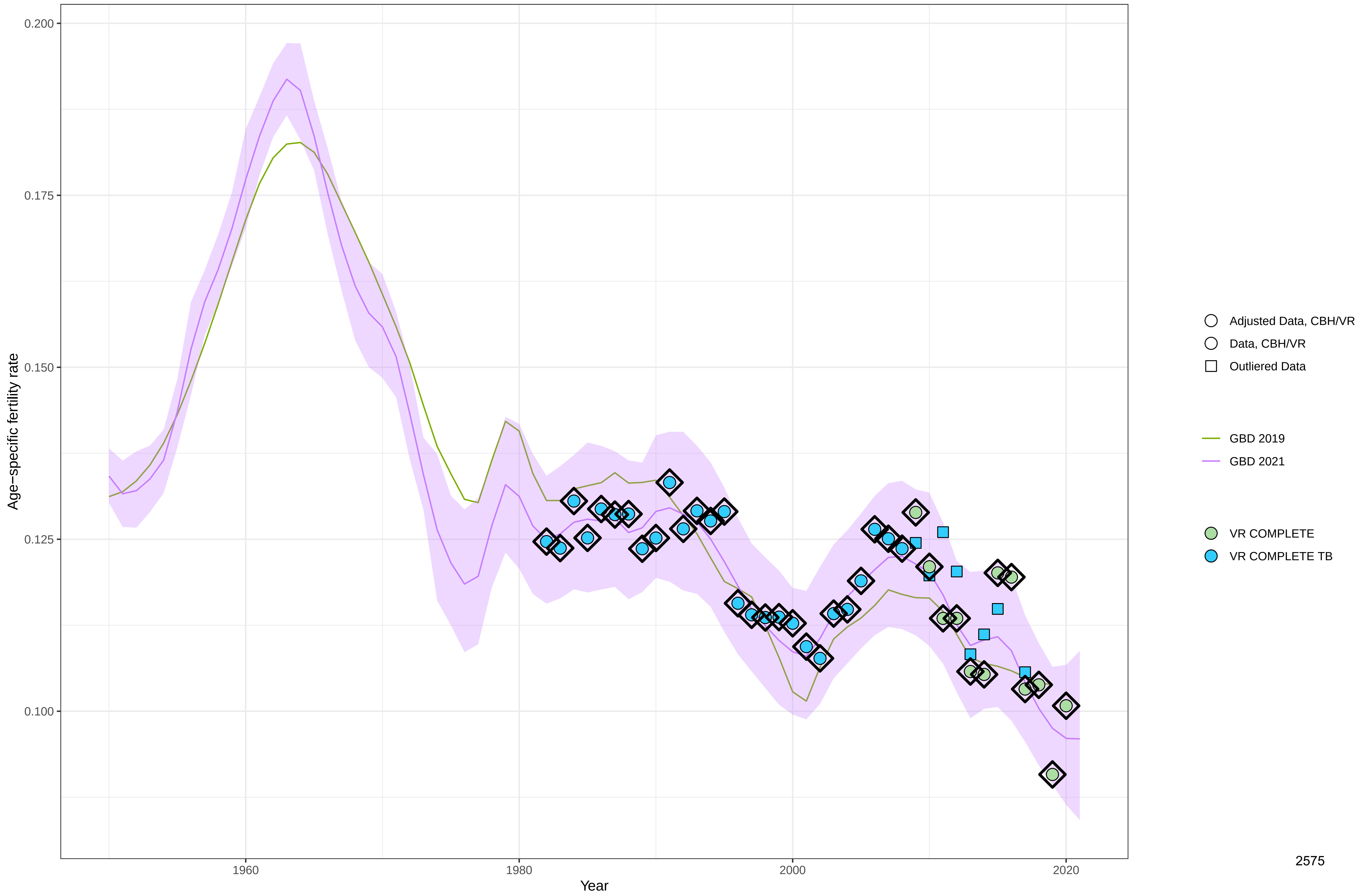

Age−specific fertility fitted trend lines, 1950−2021: Hartlepool, Ages: 30−34
Data points enclosed with a diamond are reference sources

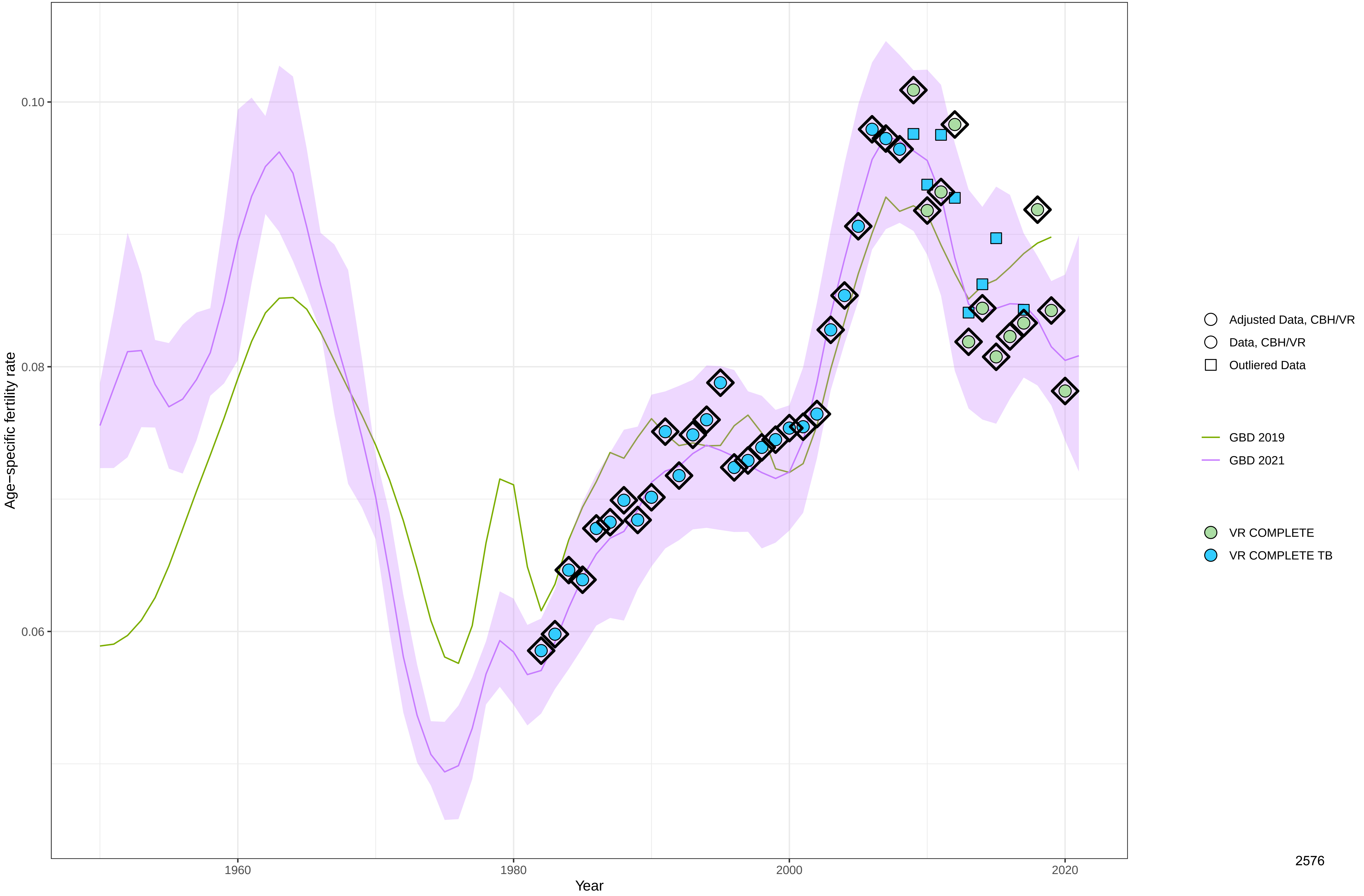

Age−specific fertility fitted trend lines, 1950−2021: Hartlepool, Ages: 35−39
Data points enclosed with a diamond are reference sources

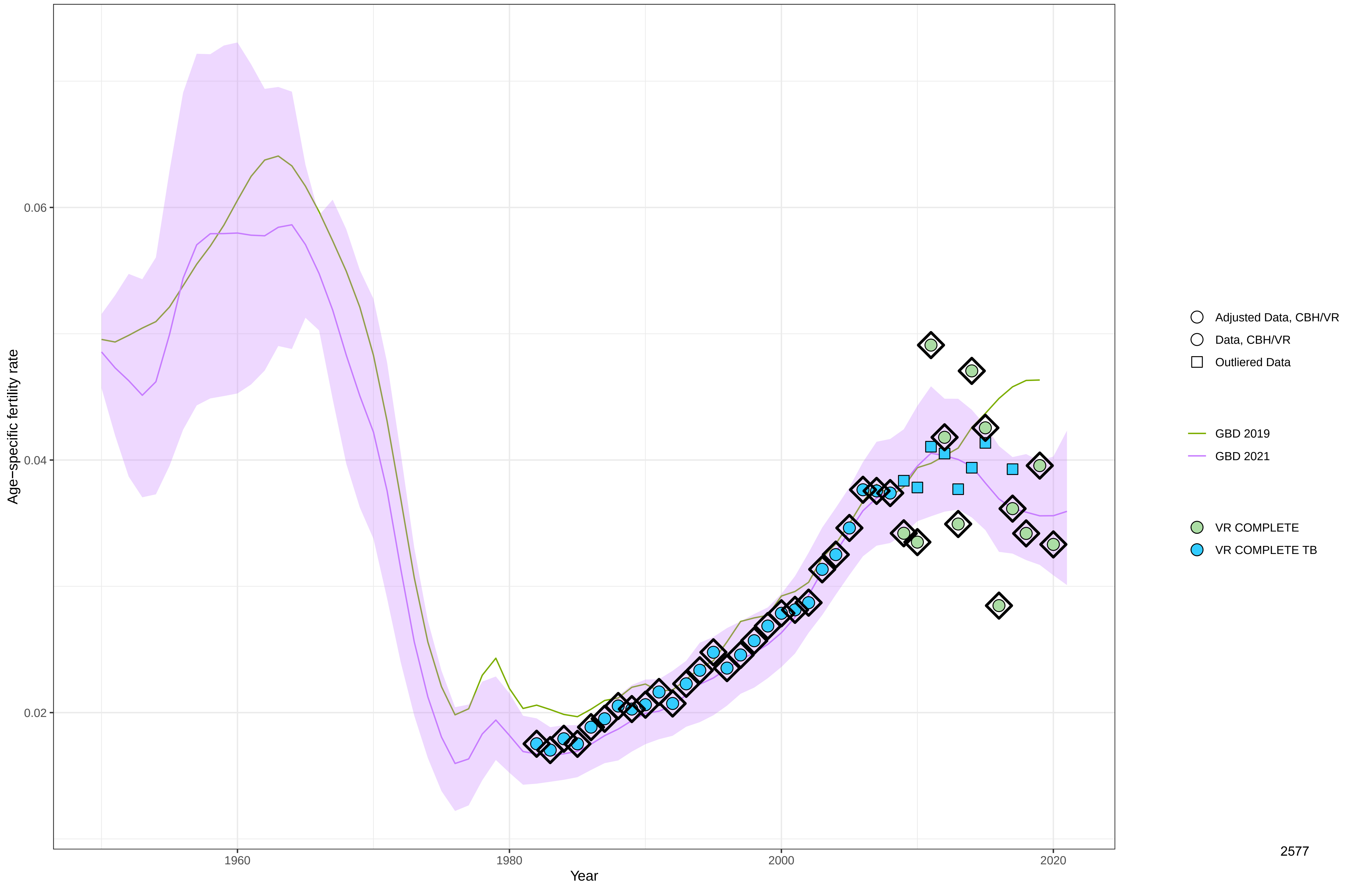

Age−specific fertility fitted trend lines, 1950−2021: Hartlepool, Ages: 40−44
Data points enclosed with a diamond are reference sources

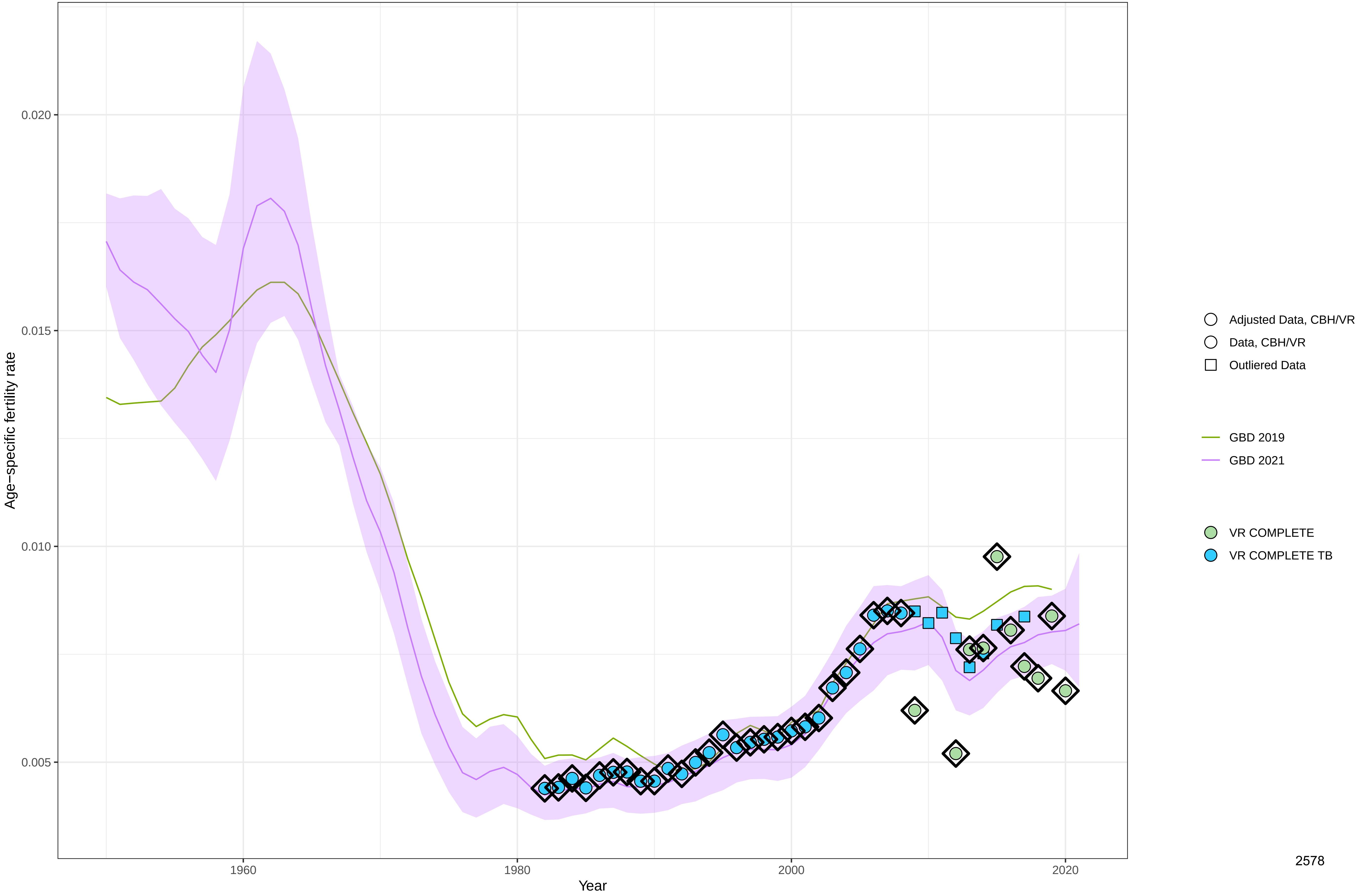

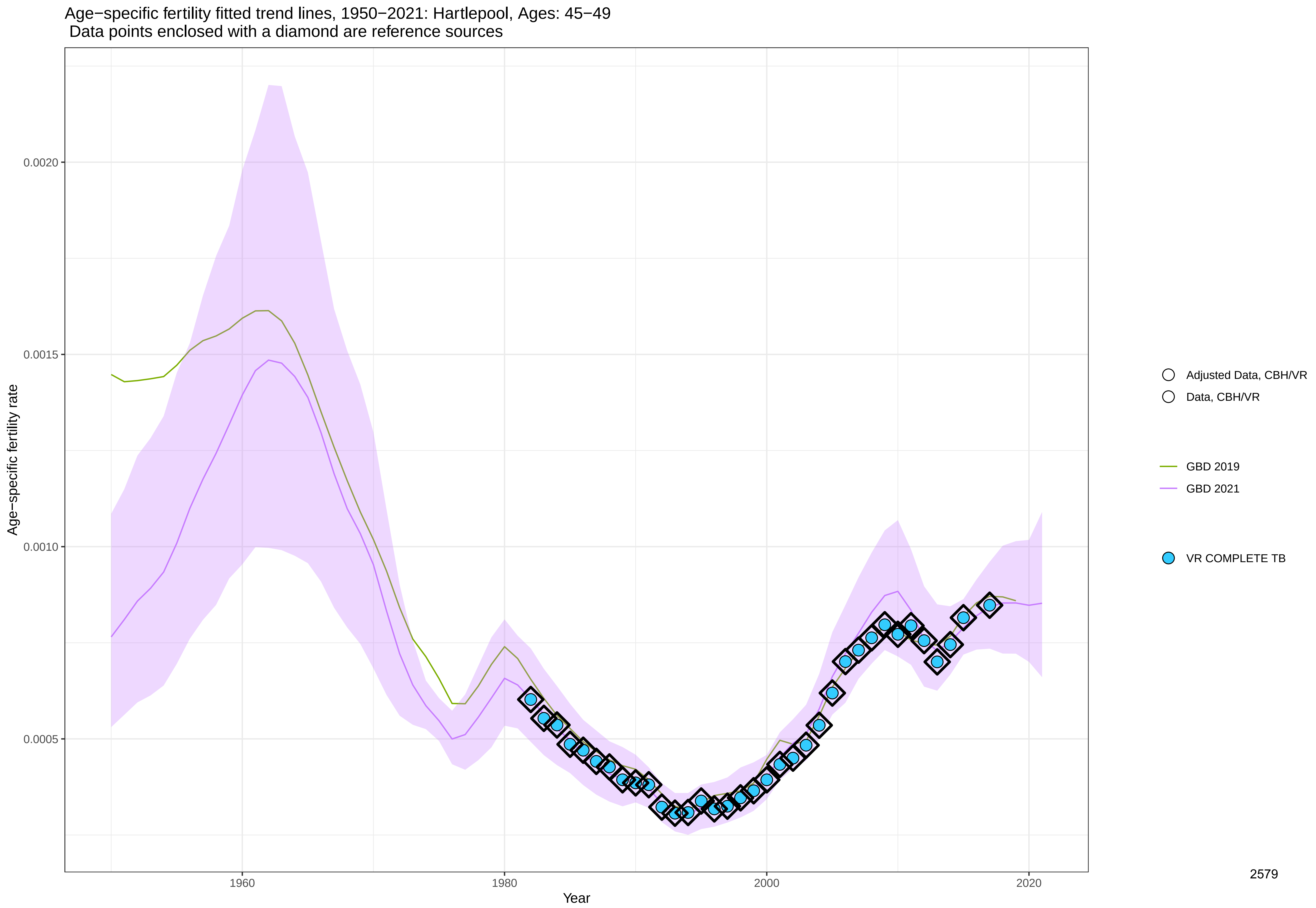

Age−specific fertility fitted trend lines, 1950−2021: Hartlepool, Ages: 45−49
Data points enclosed with a diamond are reference sources
Age−specific fertility rate
0.0020
0.0015
0.0010
0.0005
1960
1980
2000
2020
Year
Adjusted Data, CBH/VR
Data, CBH/VR
GBD 2019
GBD 2021
VR COMPLETE TB

Age−specific fertility fitted trend lines, 1950−2021: Hartlepool, Ages: 50−54
Data points enclosed with a diamond are reference sources

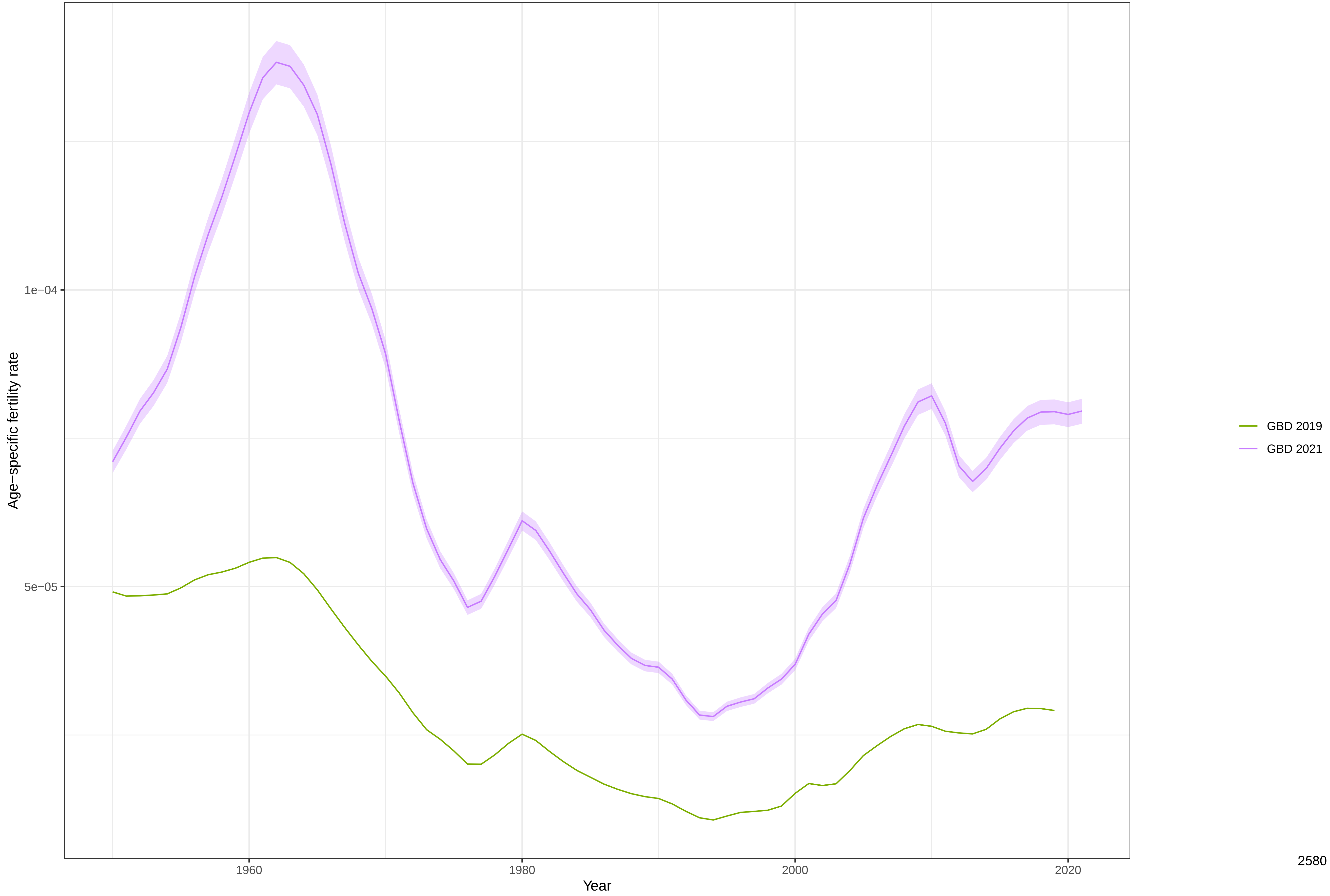

Total fertility rate fitted trend lines, 1950−2021: Hartlepool TFR
Data points enclosed with a diamond are reference sources

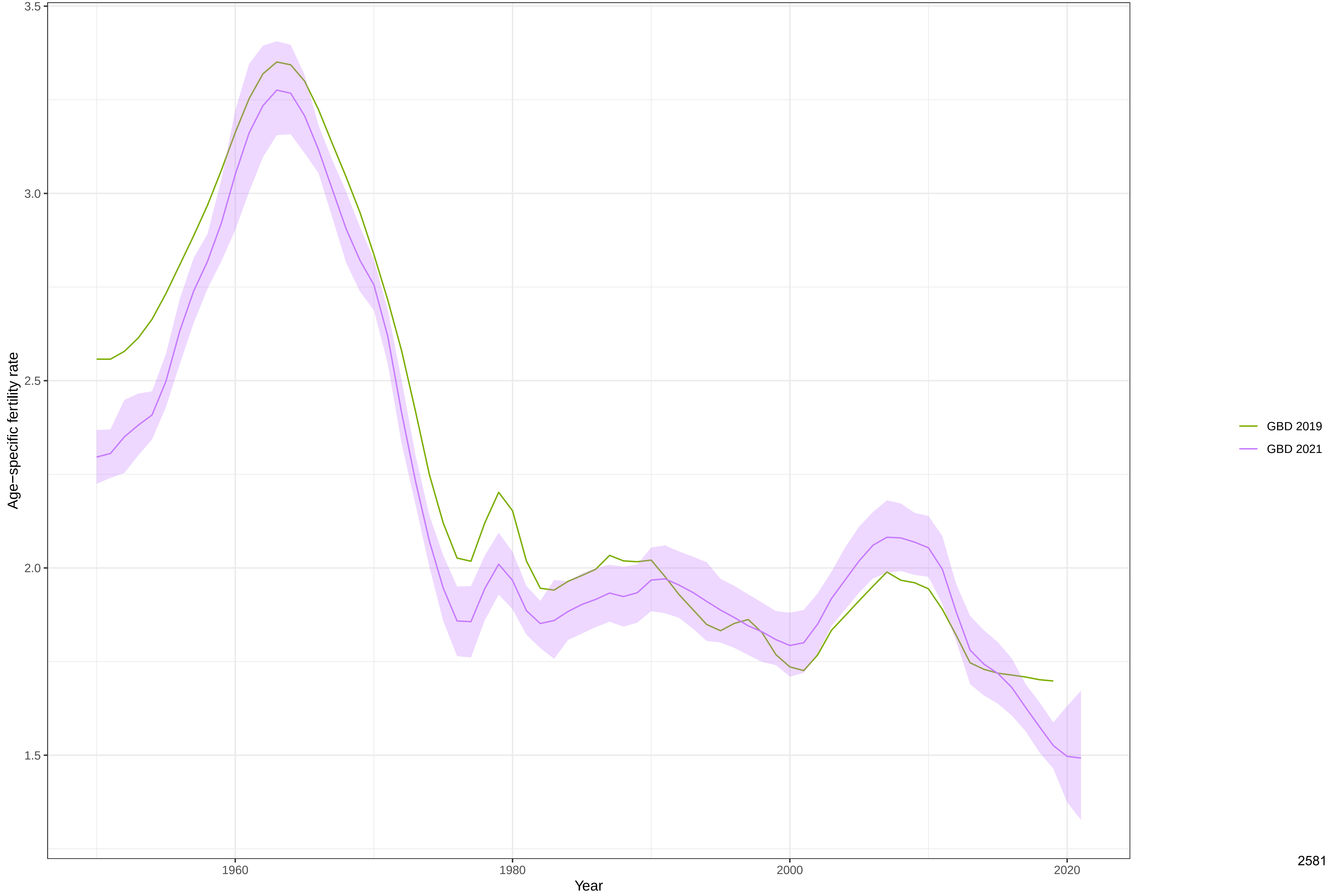

Age−specific fertility fitted trend lines, 1950−2021: Middlesbrough, Ages: 10−14
Data points enclosed with a diamond are reference sources

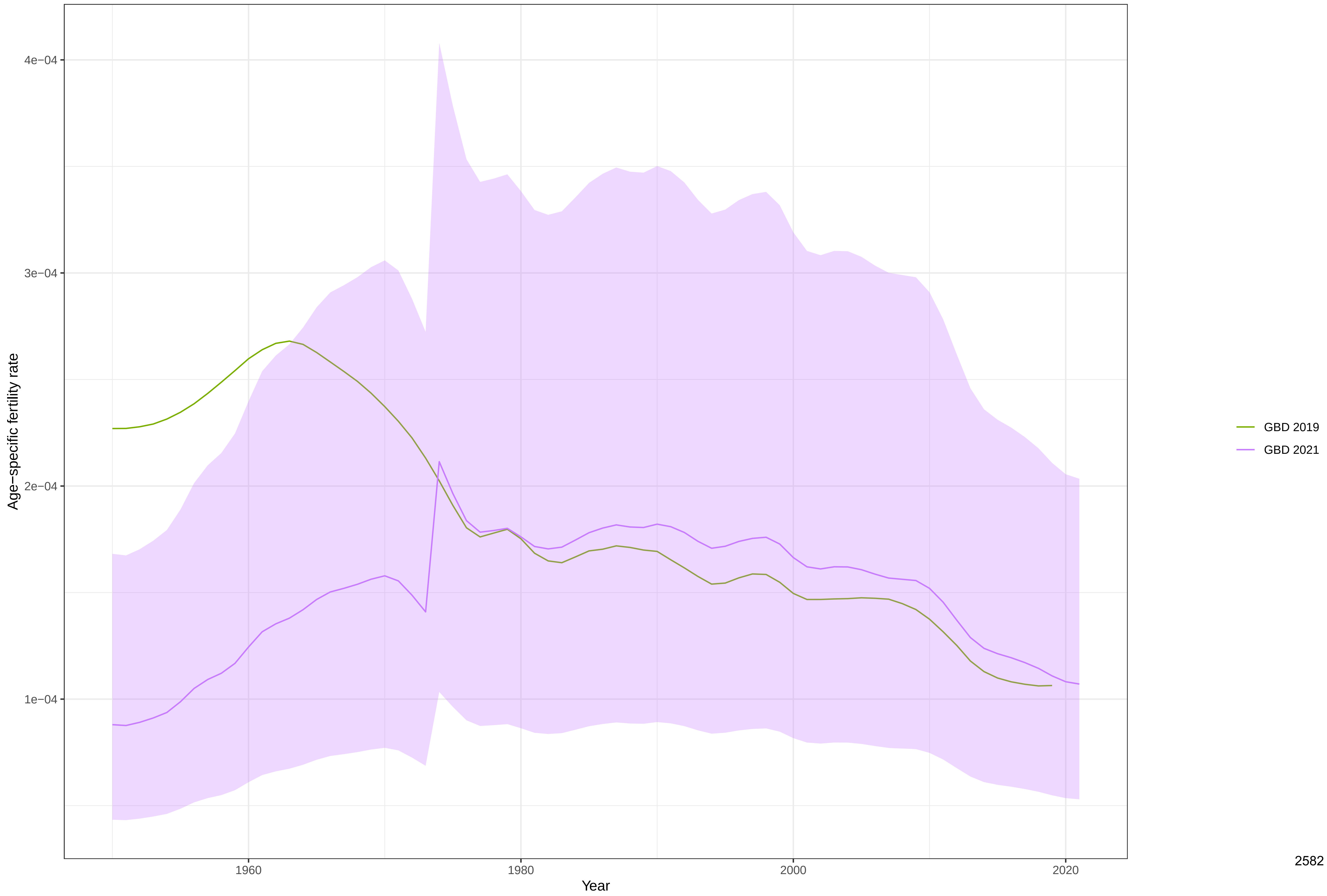

Age−specific fertility fitted trend lines, 1950−2021: Middlesbrough, Ages: 15−19
Data points enclosed with a diamond are reference sources

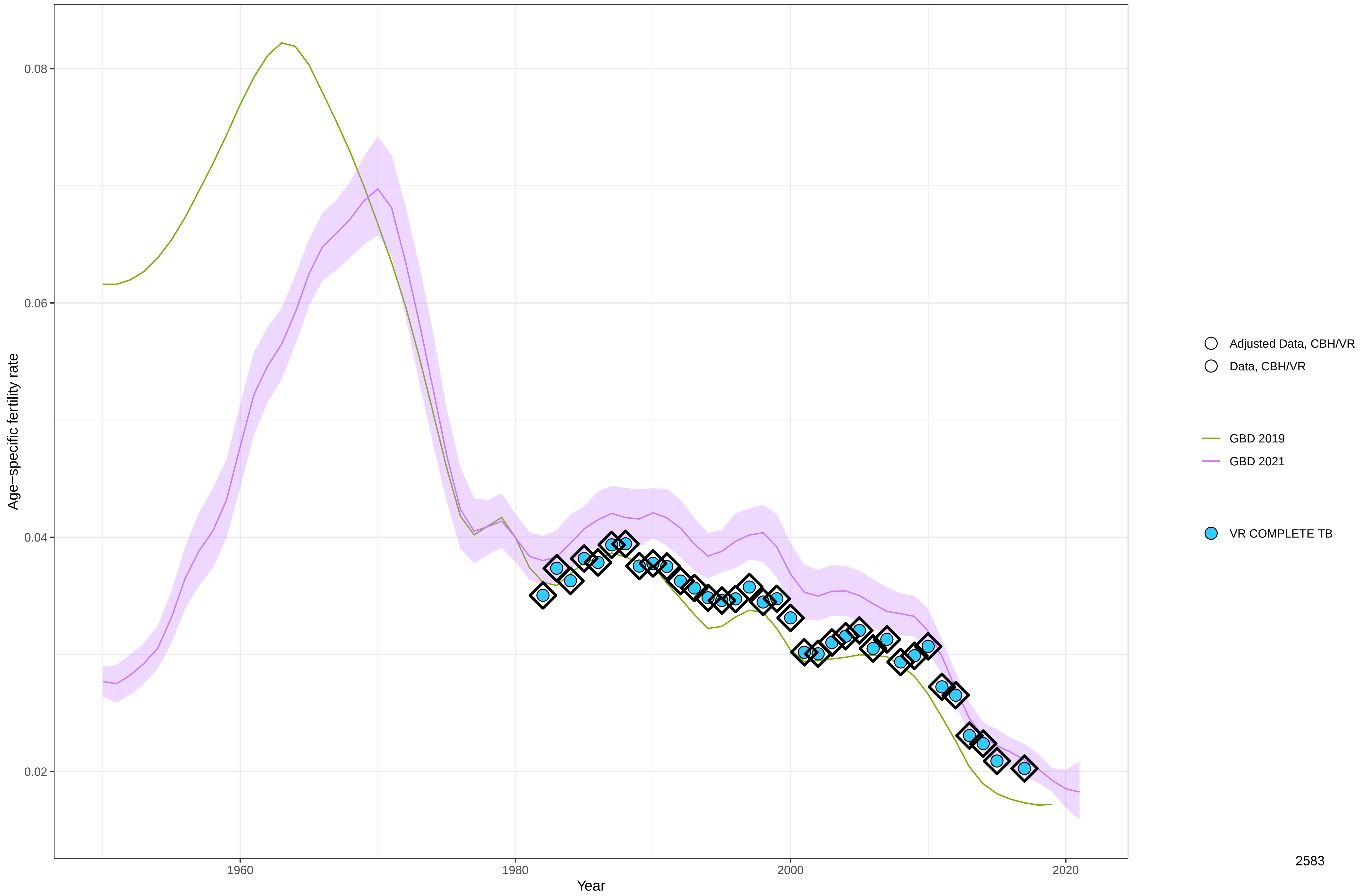

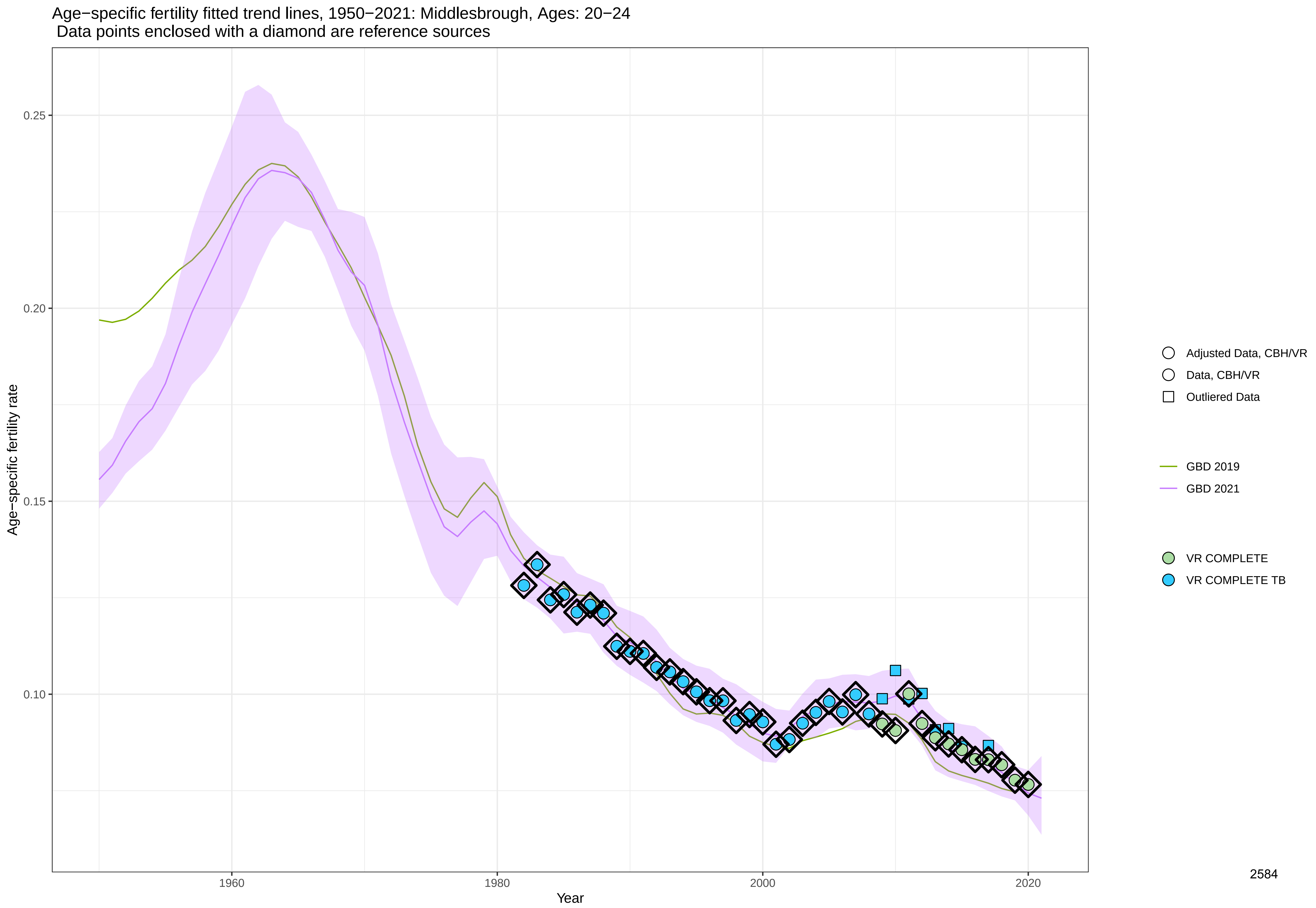

Age−specific fertility fitted trend lines, 1950−2021: Middlesbrough, Ages: 20−24
Data points enclosed with a diamond are reference sources
Age−specific fertility rate
Year
0.25
0.20
0.15
0.10
1960
1980
2000
2020
Adjusted Data, CBH/VR
Data, CBH/VR
Outliered Data
GBD 2019
GBD 2021
VR COMPLETE
VR COMPLETE TB

Age−specific fertility fitted trend lines, 1950−2021: Middlesbrough, Ages: 25−29
Data points enclosed with a diamond are reference sources

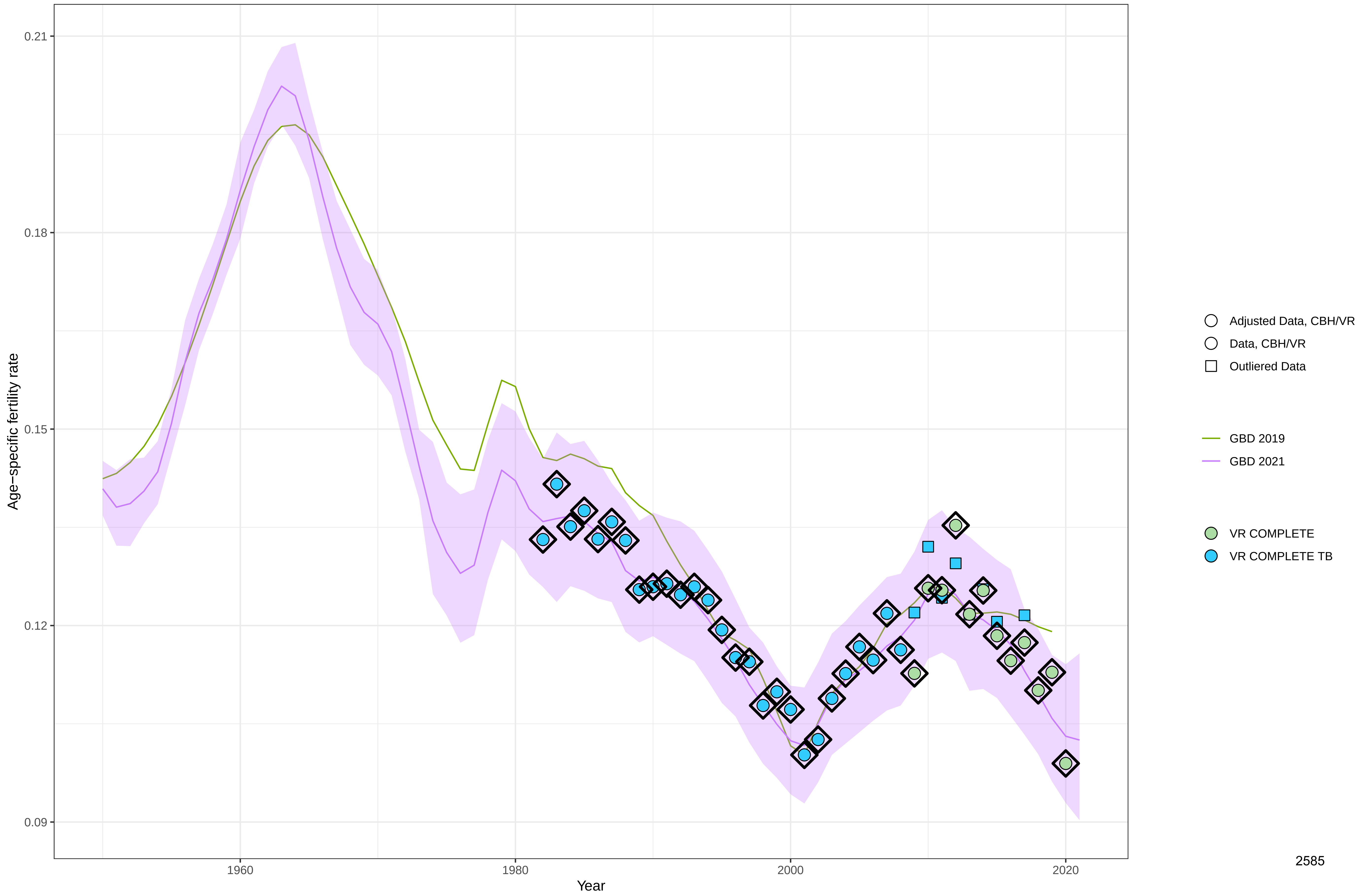

Age−specific fertility fitted trend lines, 1950−2021: Middlesbrough, Ages: 30−34
Data points enclosed with a diamond are reference sources

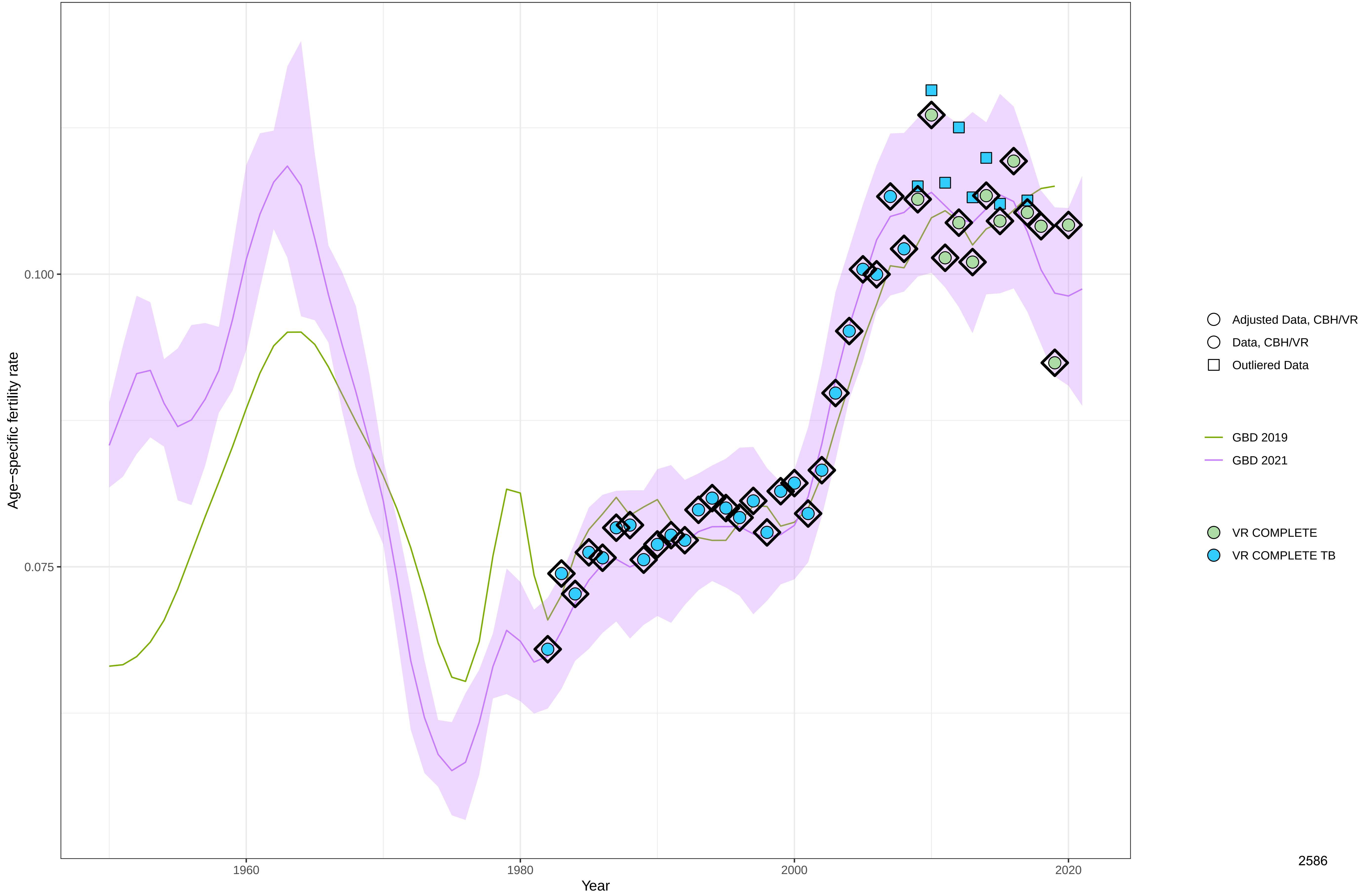

Age−specific fertility fitted trend lines, 1950−2021: Middlesbrough, Ages: 35−39
Data points enclosed with a diamond are reference sources

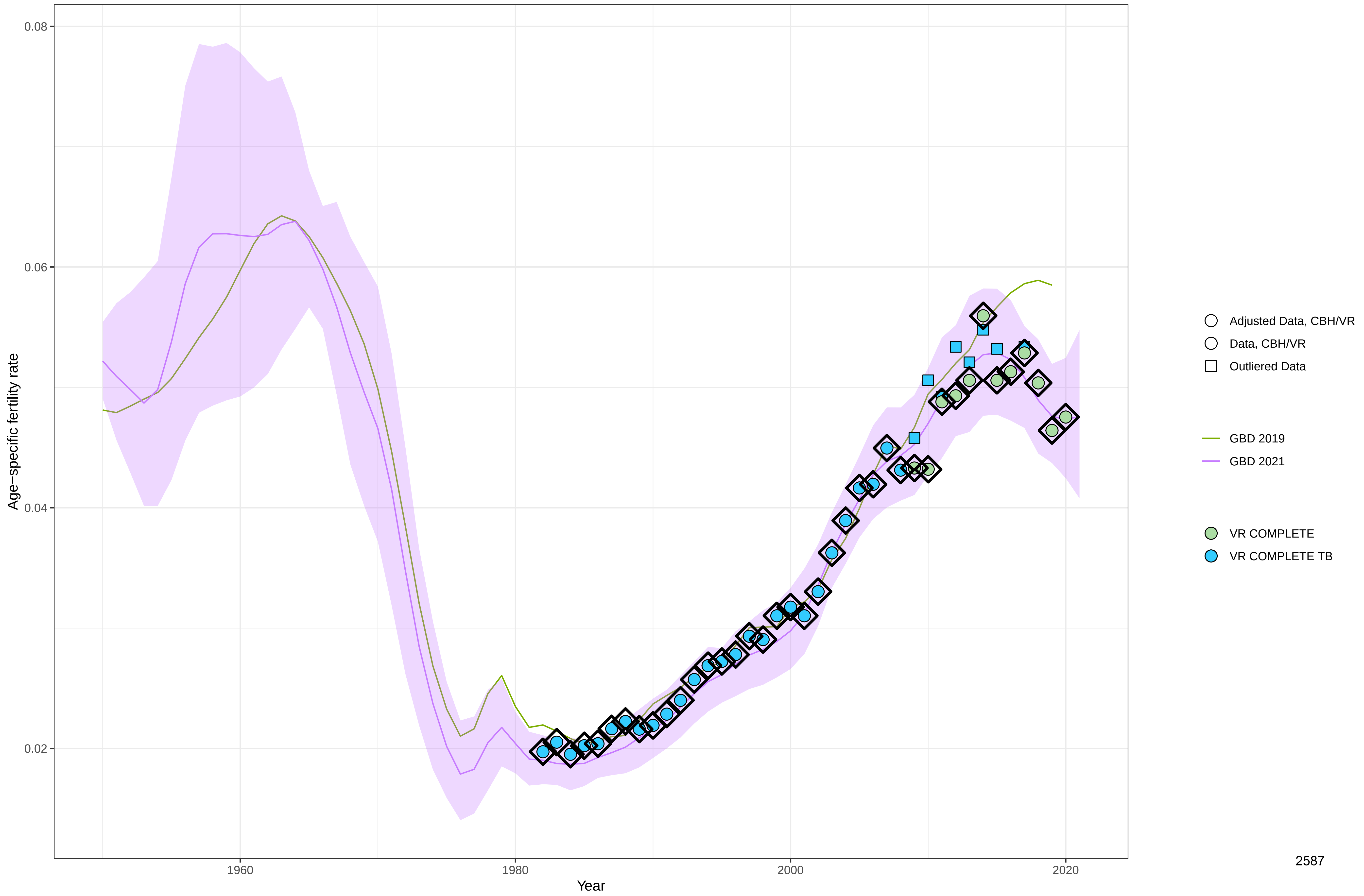

Age−specific fertility fitted trend lines, 1950−2021: Middlesbrough, Ages: 40−44
Data points enclosed with a diamond are reference sources

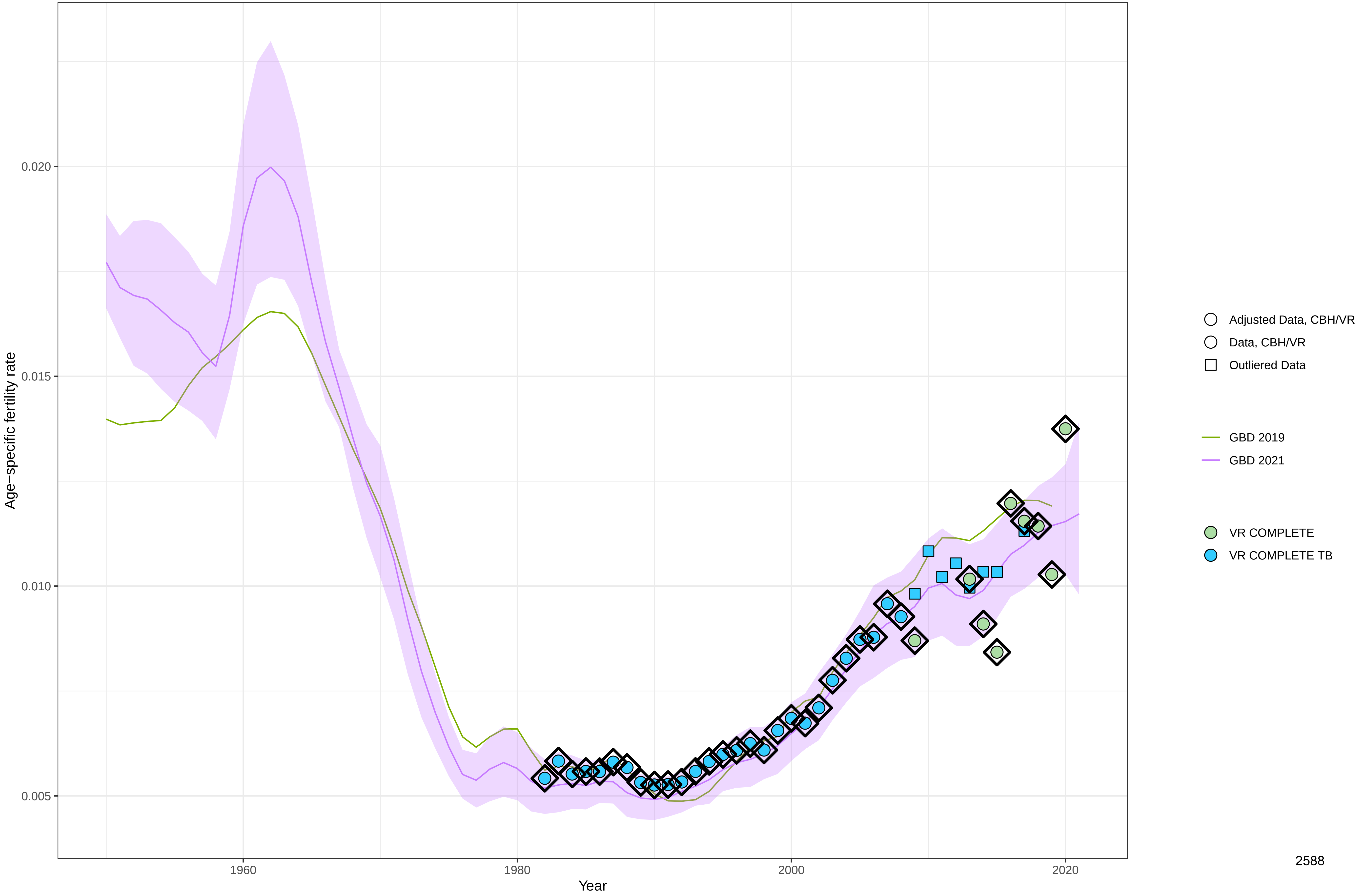

Age−specific fertility fitted trend lines, 1950−2021: Middlesbrough, Ages: 45−49
Data points enclosed with a diamond are reference sources

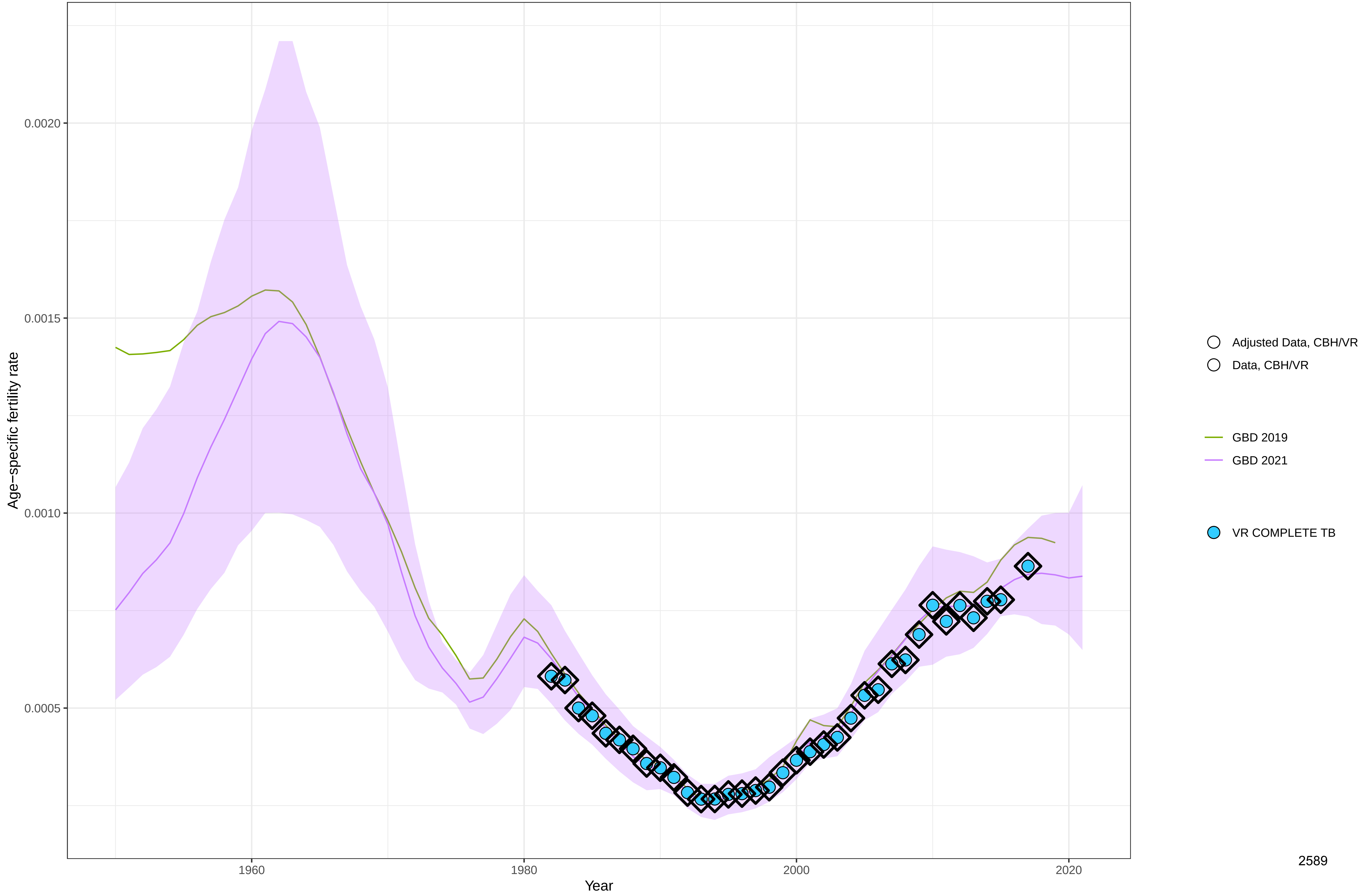

Age−specific fertility fitted trend lines, 1950−2021: Middlesbrough, Ages: 50−54
Data points enclosed with a diamond are reference sources

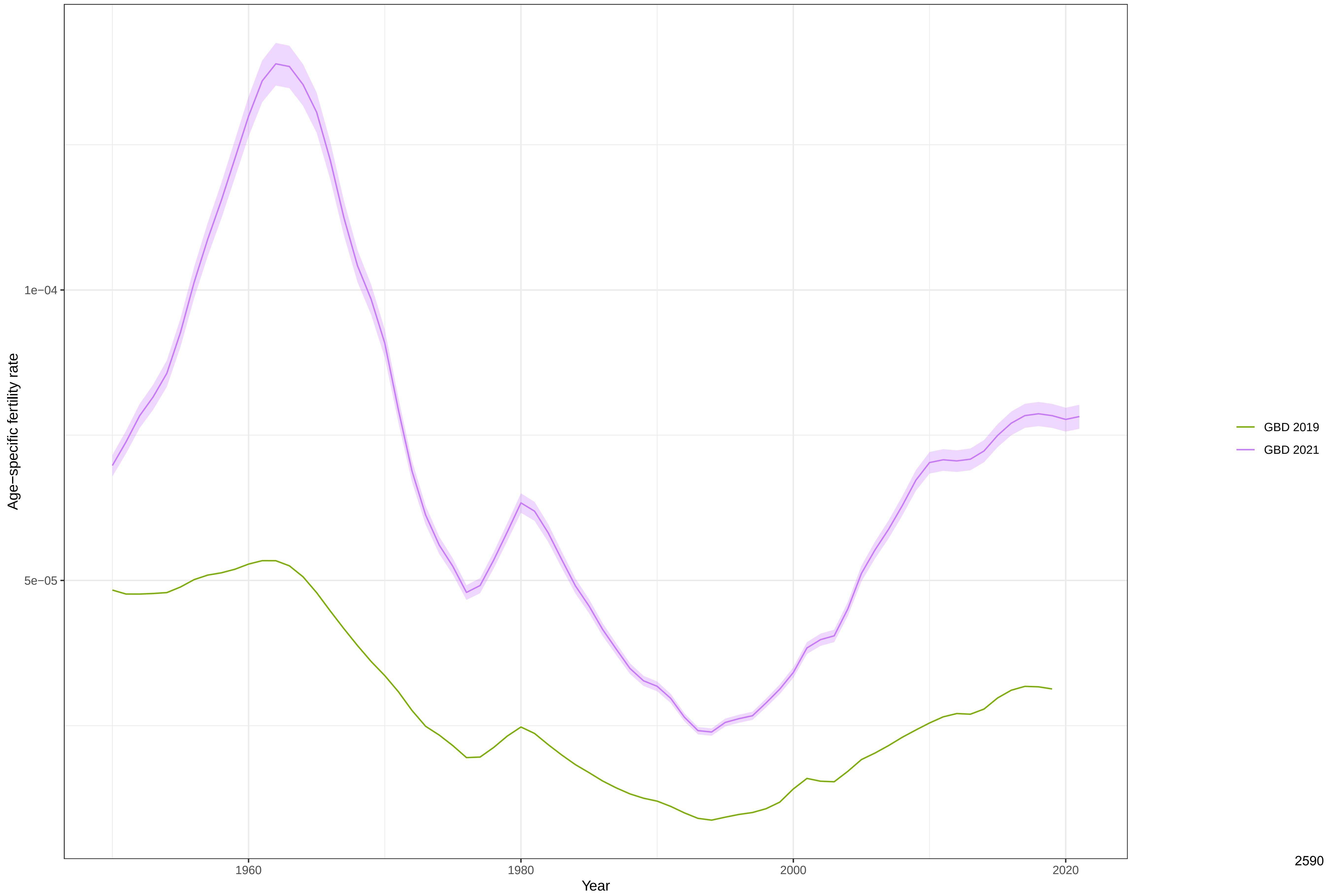

Total fertility rate fitted trend lines, 1950−2021: Middlesbrough TFR
Data points enclosed with a diamond are reference sources

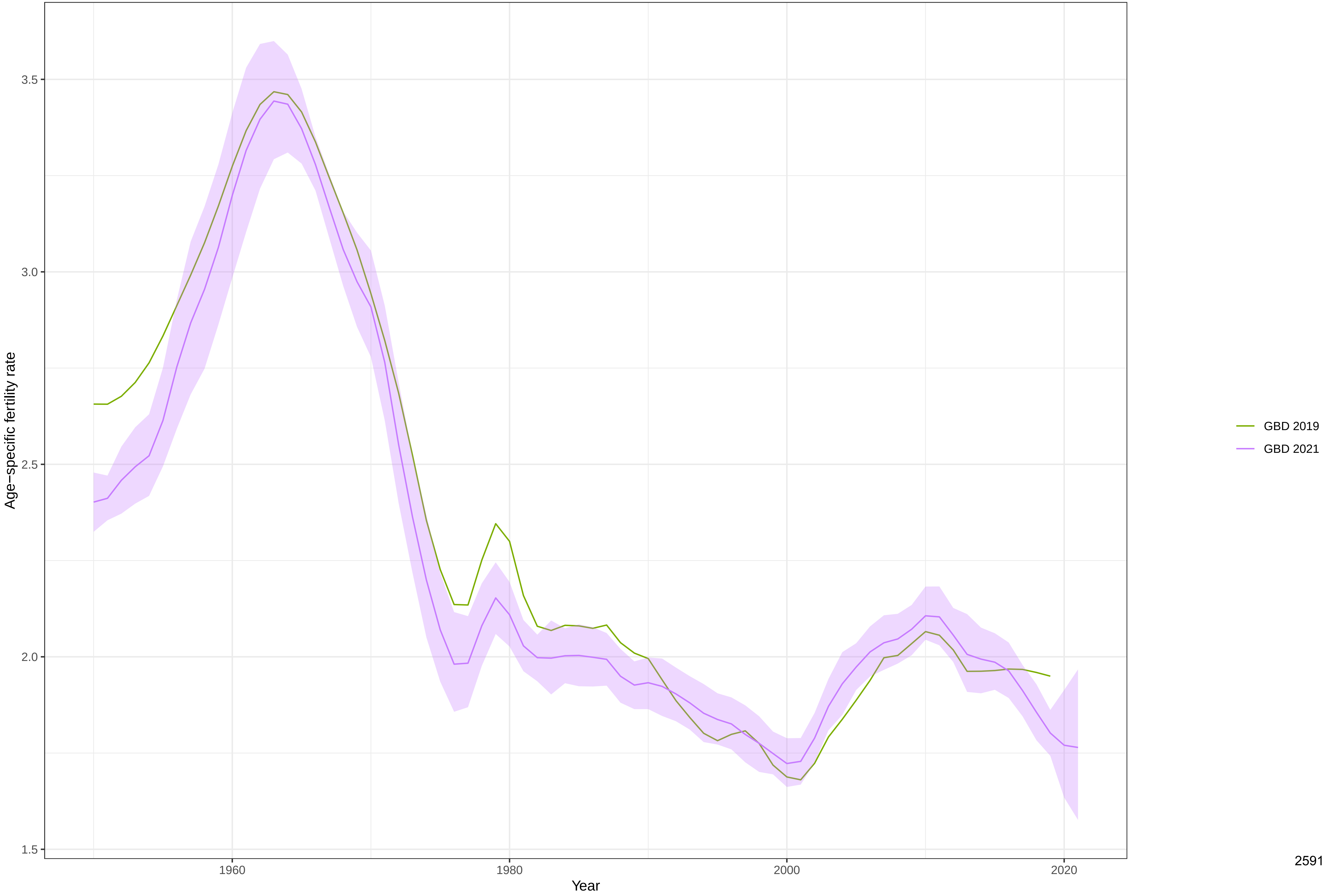

Age−specific fertility fitted trend lines, 1950−2021: Newcastle upon Tyne, Ages: 10−14
Data points enclosed with a diamond are reference sources

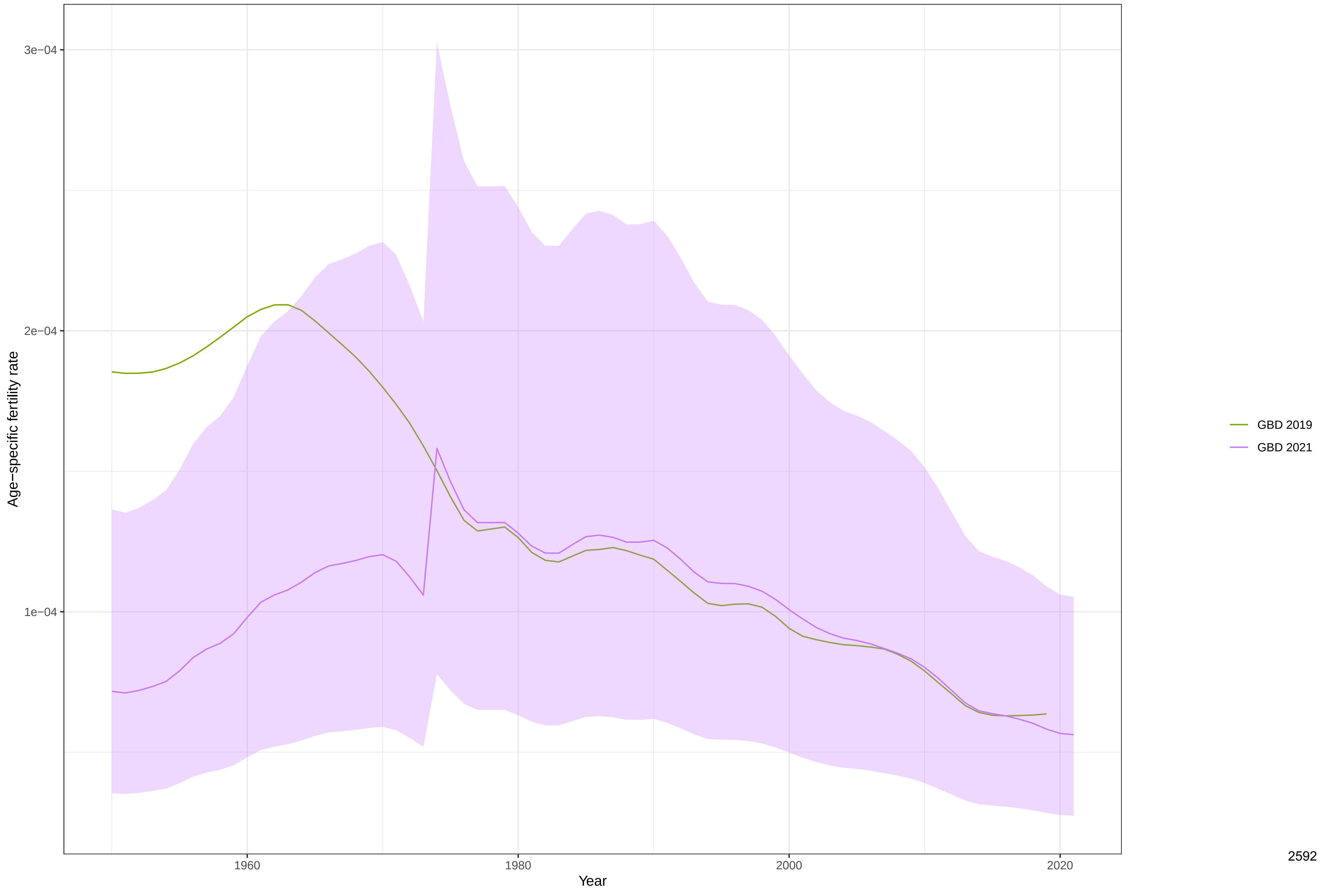

Age−specific fertility fitted trend lines, 1950−2021: Newcastle upon Tyne, Ages: 15−19
Data points enclosed with a diamond are reference sources

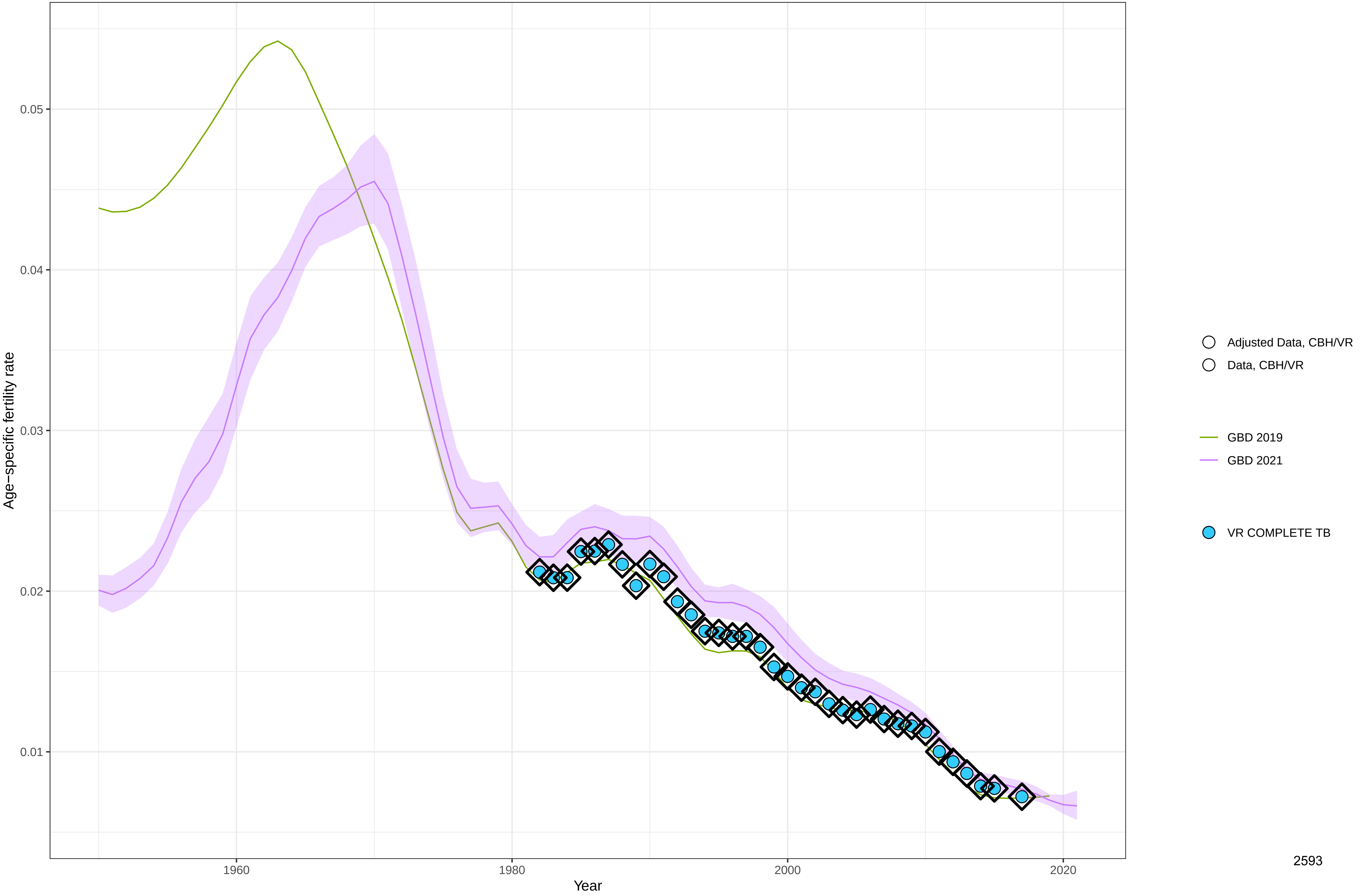

Age−specific fertility fitted trend lines, 1950−2021: Newcastle upon Tyne, Ages: 20−24
Data points enclosed with a diamond are reference sources

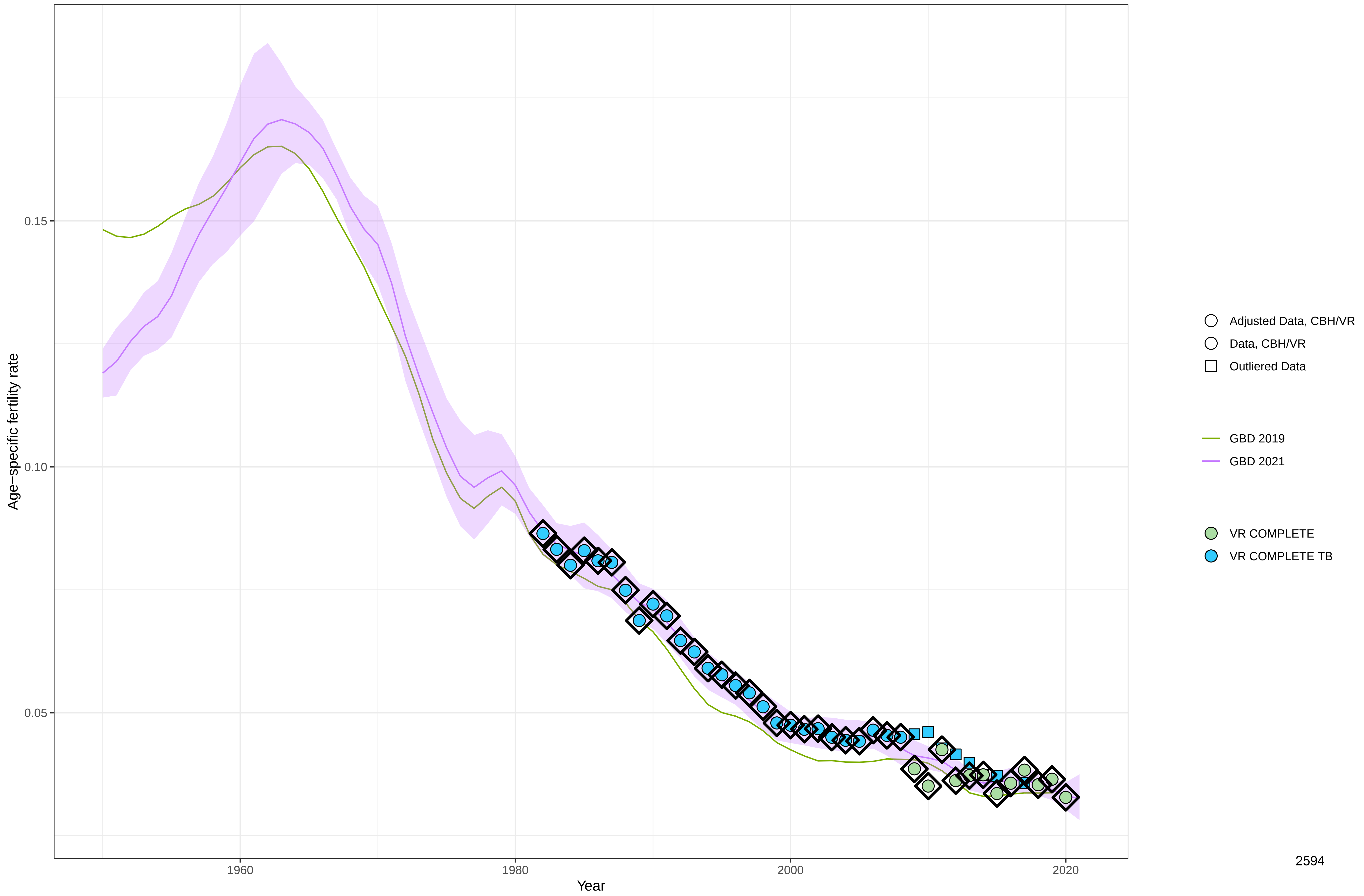

Age−specific fertility fitted trend lines, 1950−2021: Newcastle upon Tyne, Ages: 25−29
Data points enclosed with a diamond are reference sources

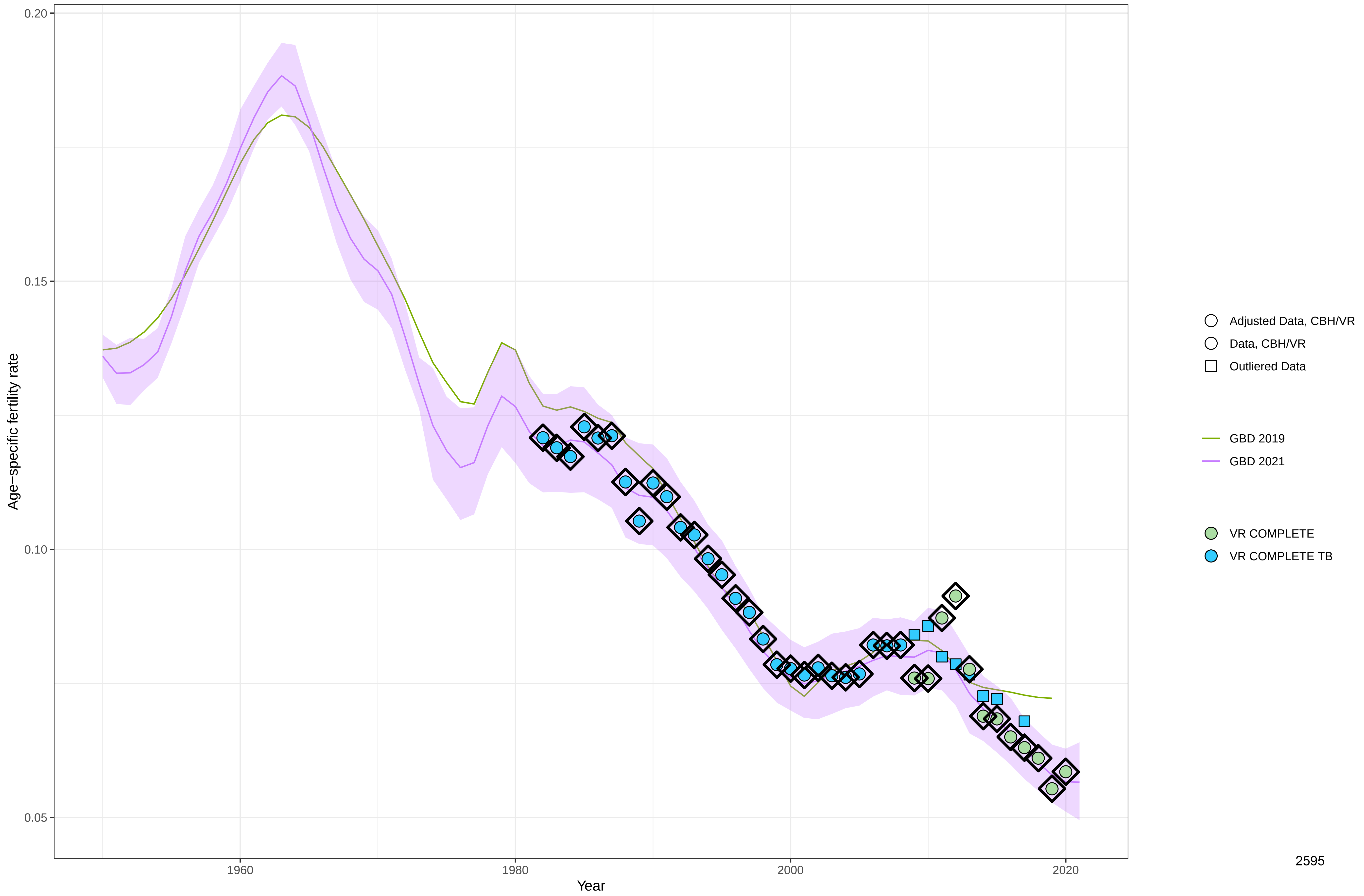

Age−specific fertility fitted trend lines, 1950−2021: Newcastle upon Tyne, Ages: 30−34
Data points enclosed with a diamond are reference sources

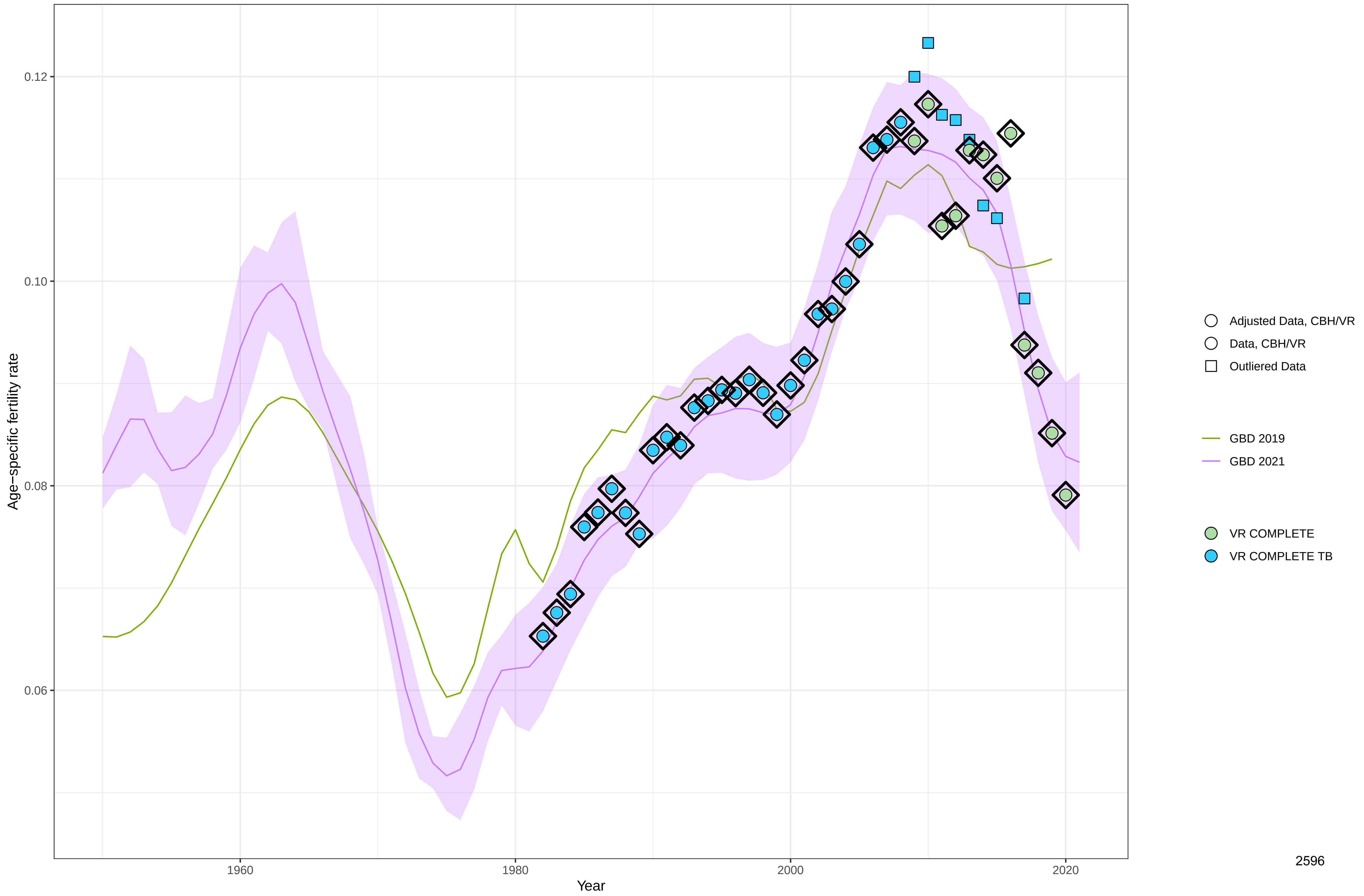

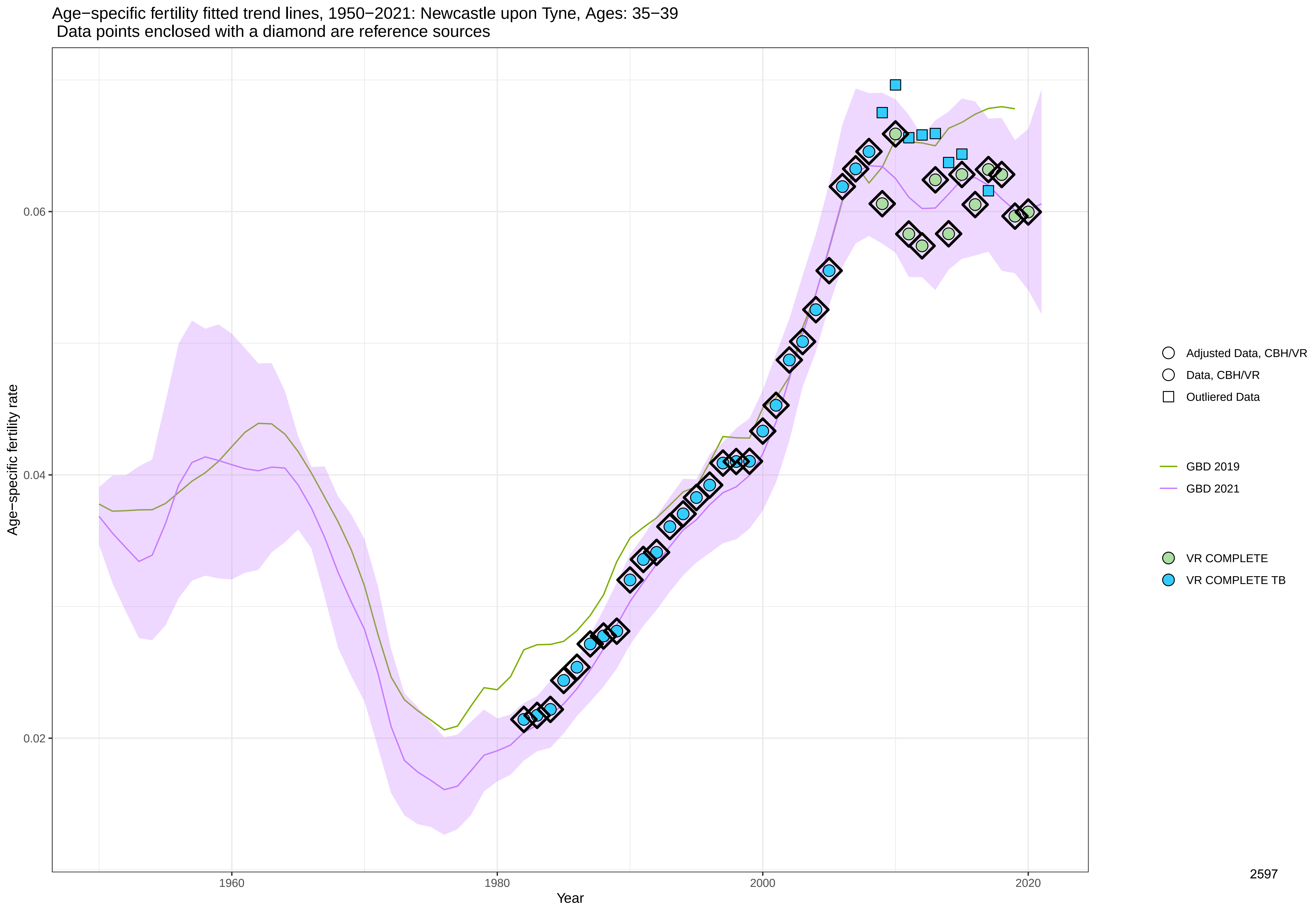

Age−specific fertility fitted trend lines, 1950−2021: Newcastle upon Tyne, Ages: 35−39
Data points enclosed with a diamond are reference sources
Age−specific fertility rate
Year
0.02
0.04
0.06
1960
1980
2000
2020
Adjusted Data, CBH/VR
Data, CBH/VR
Outliered Data
GBD 2019
GBD 2021
VR COMPLETE
VR COMPLETE TB

Age−specific fertility fitted trend lines, 1950−2021: Newcastle upon Tyne, Ages: 40−44
Data points enclosed with a diamond are reference sources

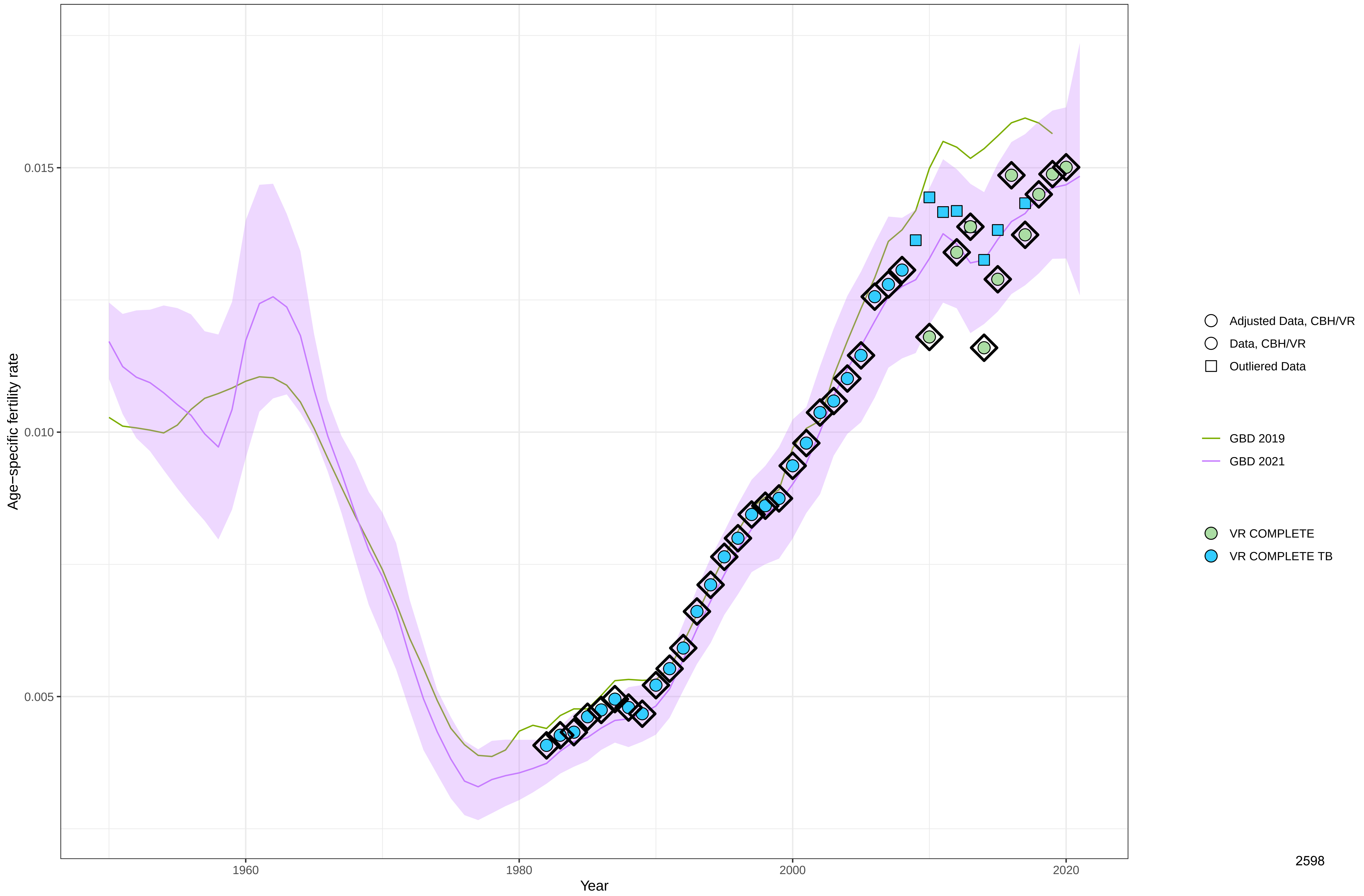

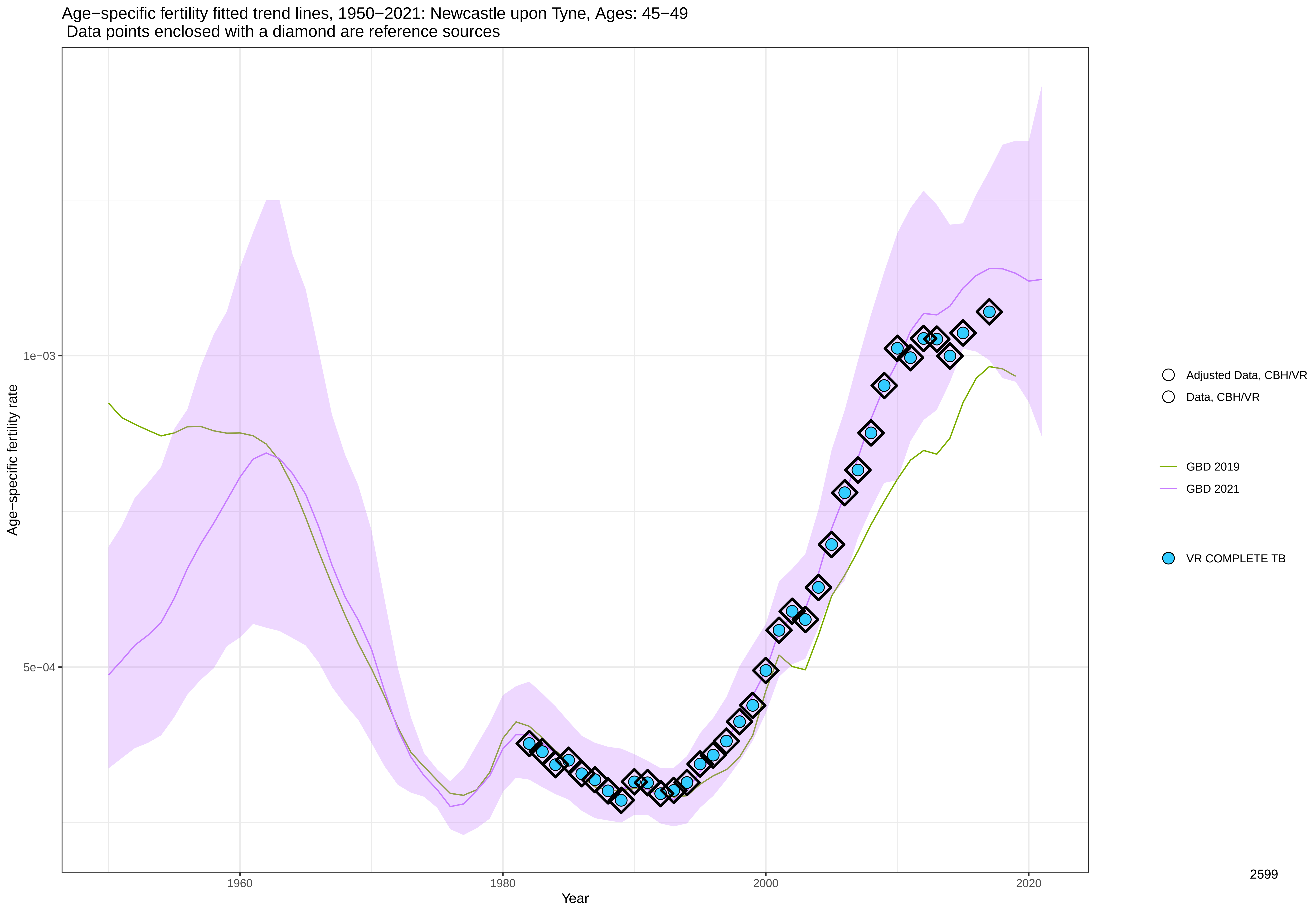
Age−specific fertility fitted trend lines, 1950−2021: Newcastle upon Tyne, Ages: 45−49
Data points enclosed with a diamond are reference sources
Age−specific fertility rate
1e−03
5e−04
1960
1980
2000
2020
Year
Adjusted Data, CBH/VR
Data, CBH/VR
GBD 2019
GBD 2021
VR COMPLETE TB

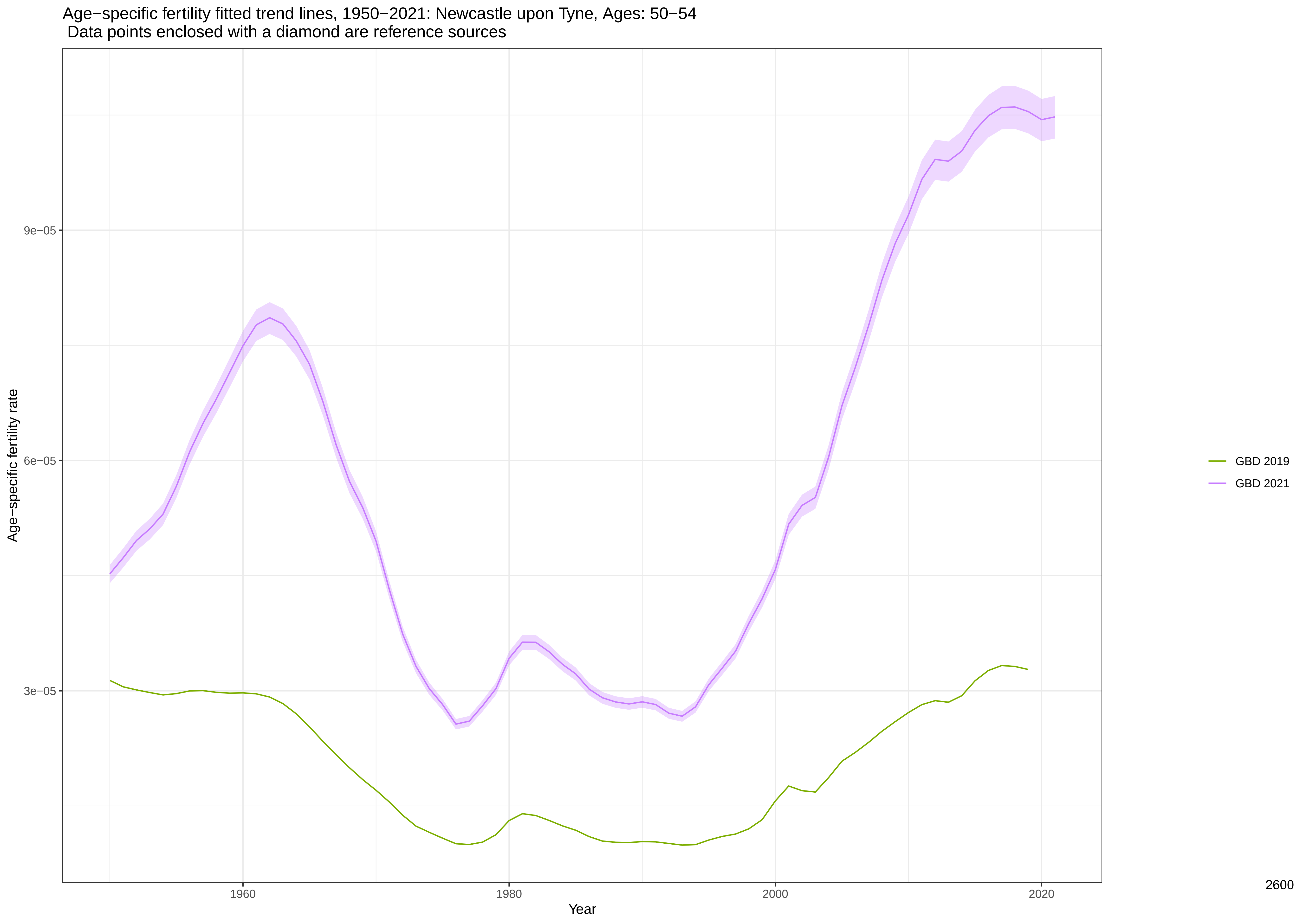
Age−specific fertility fitted trend lines, 1950−2021: Newcastle upon Tyne, Ages: 50−54
Data points enclosed with a diamond are reference sources
Age−specific fertility rate
9e−05
6e−05
3e−05
1960
1980
2000
2020
Year
GBD 2019
GBD 2021

Total fertility rate fitted trend lines, 1950−2021: Newcastle upon Tyne TFR
Data points enclosed with a diamond are reference sources

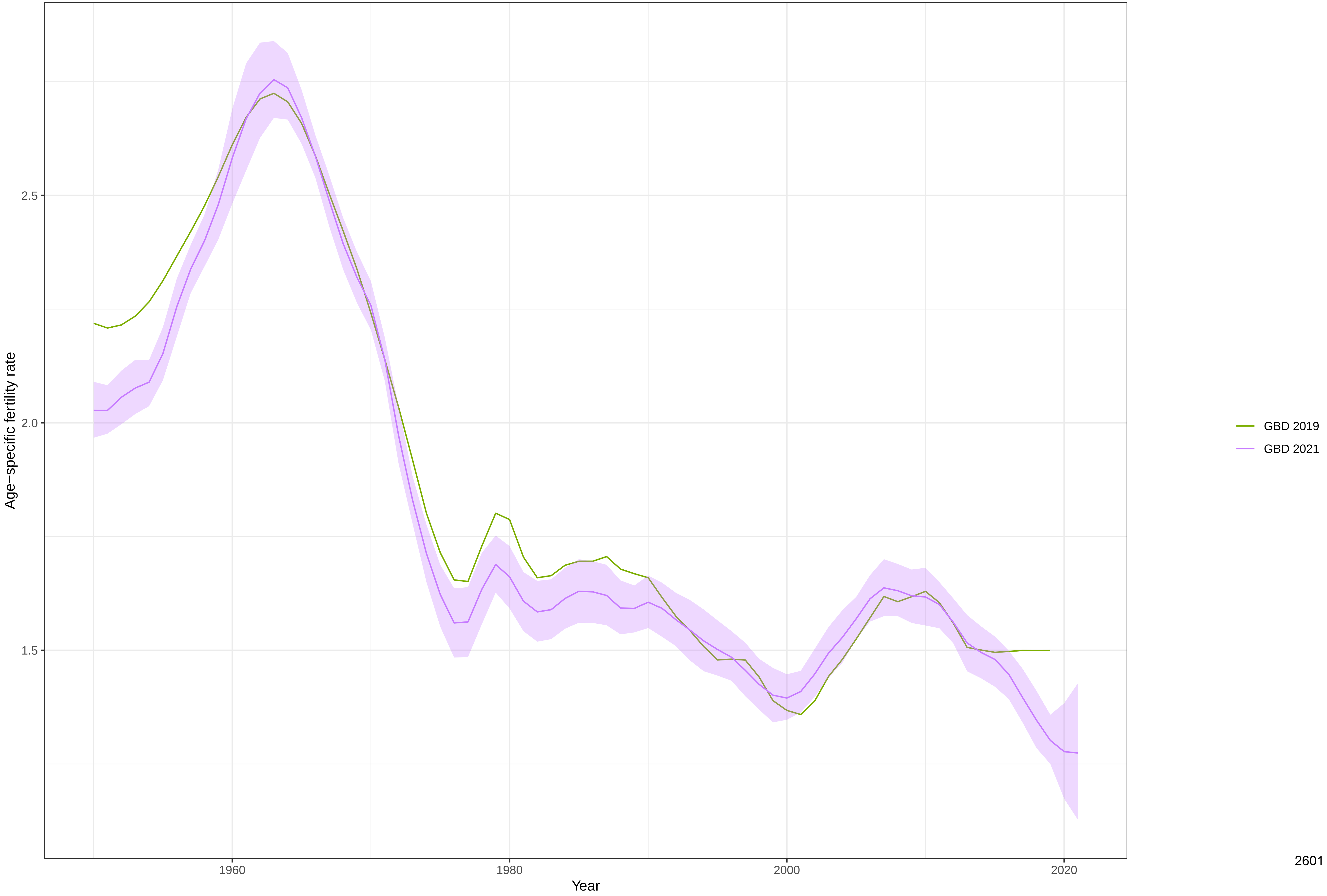

Age−specific fertility fitted trend lines, 1950−2021: North Tyneside, Ages: 10−14
Data points enclosed with a diamond are reference sources

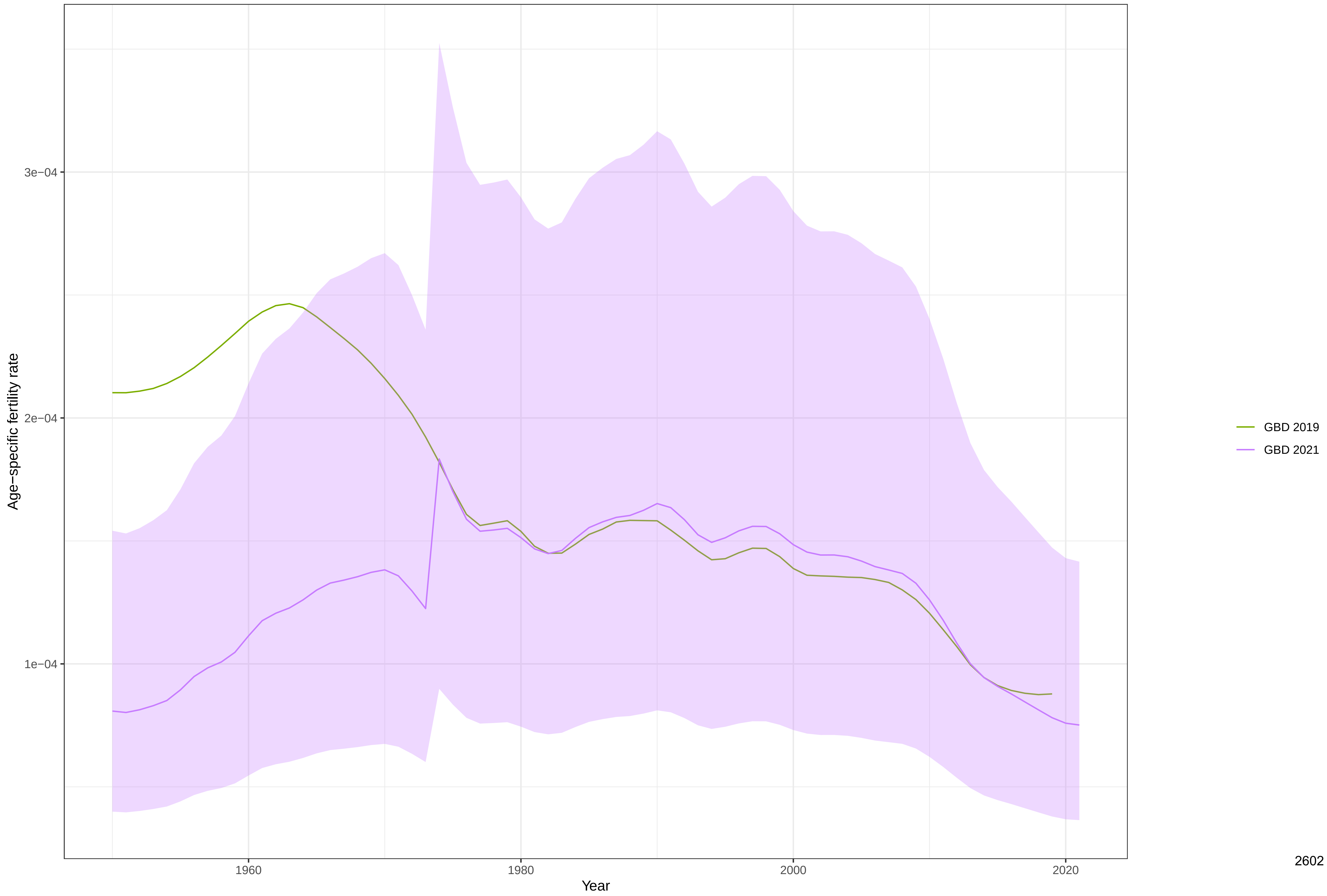

Age−specific fertility fitted trend lines, 1950−2021: North Tyneside, Ages: 15−19
Data points enclosed with a diamond are reference sources

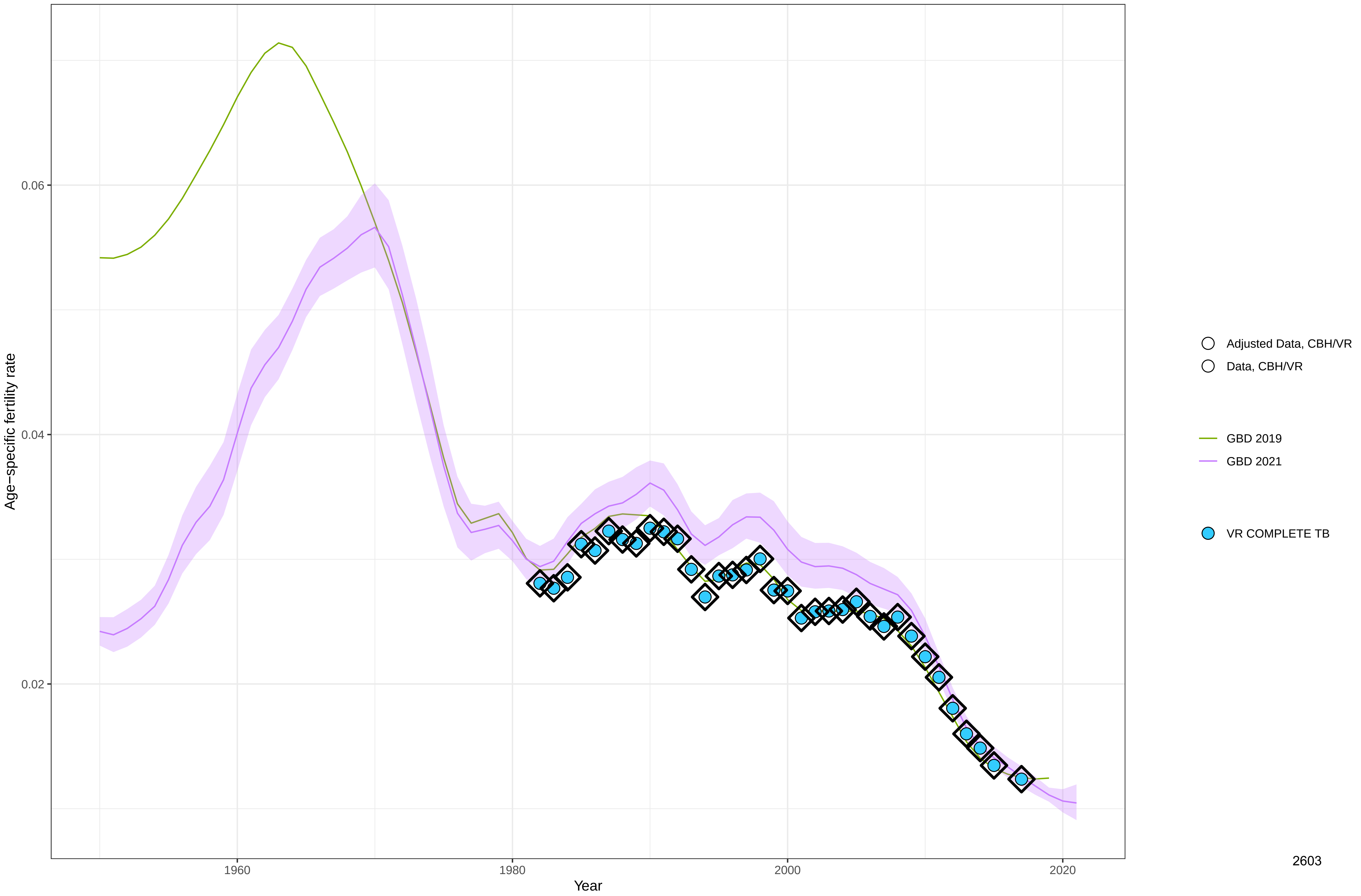

Age−specific fertility fitted trend lines, 1950−2021: North Tyneside, Ages: 20−24
Data points enclosed with a diamond are reference sources

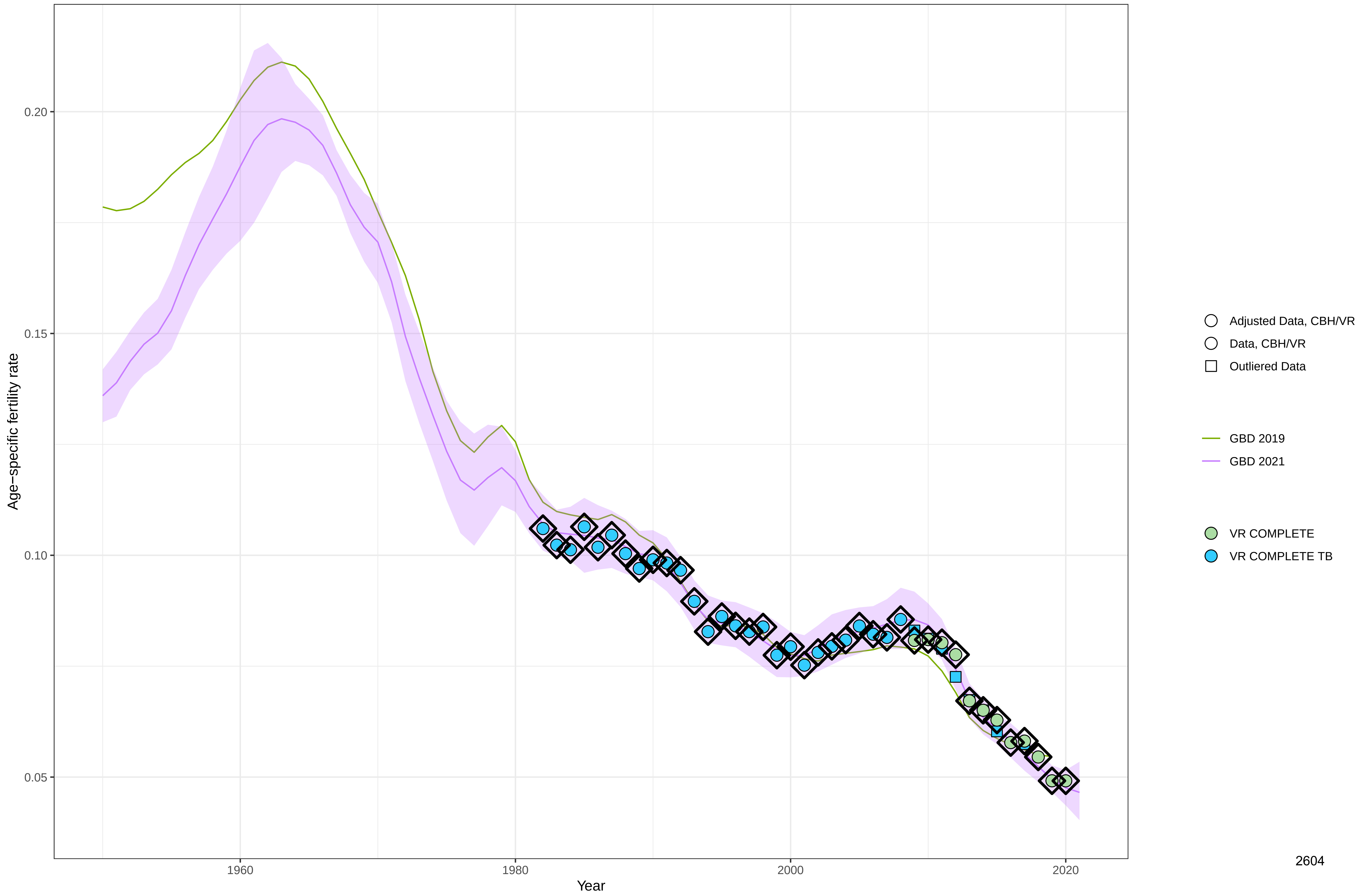

Age−specific fertility fitted trend lines, 1950−2021: North Tyneside, Ages: 25−29
Data points enclosed with a diamond are reference sources

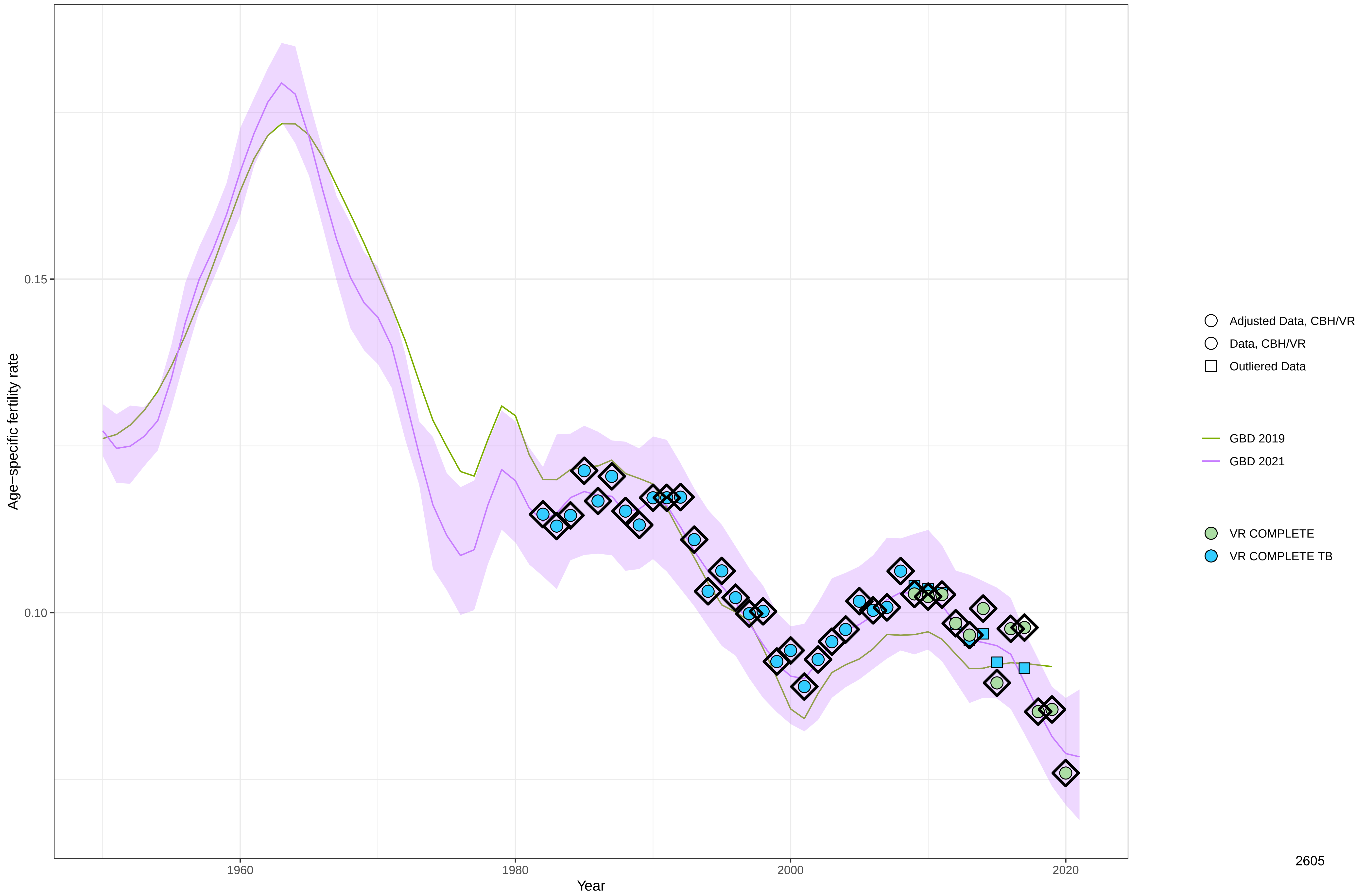

Age−specific fertility fitted trend lines, 1950−2021: North Tyneside, Ages: 30−34
Data points enclosed with a diamond are reference sources

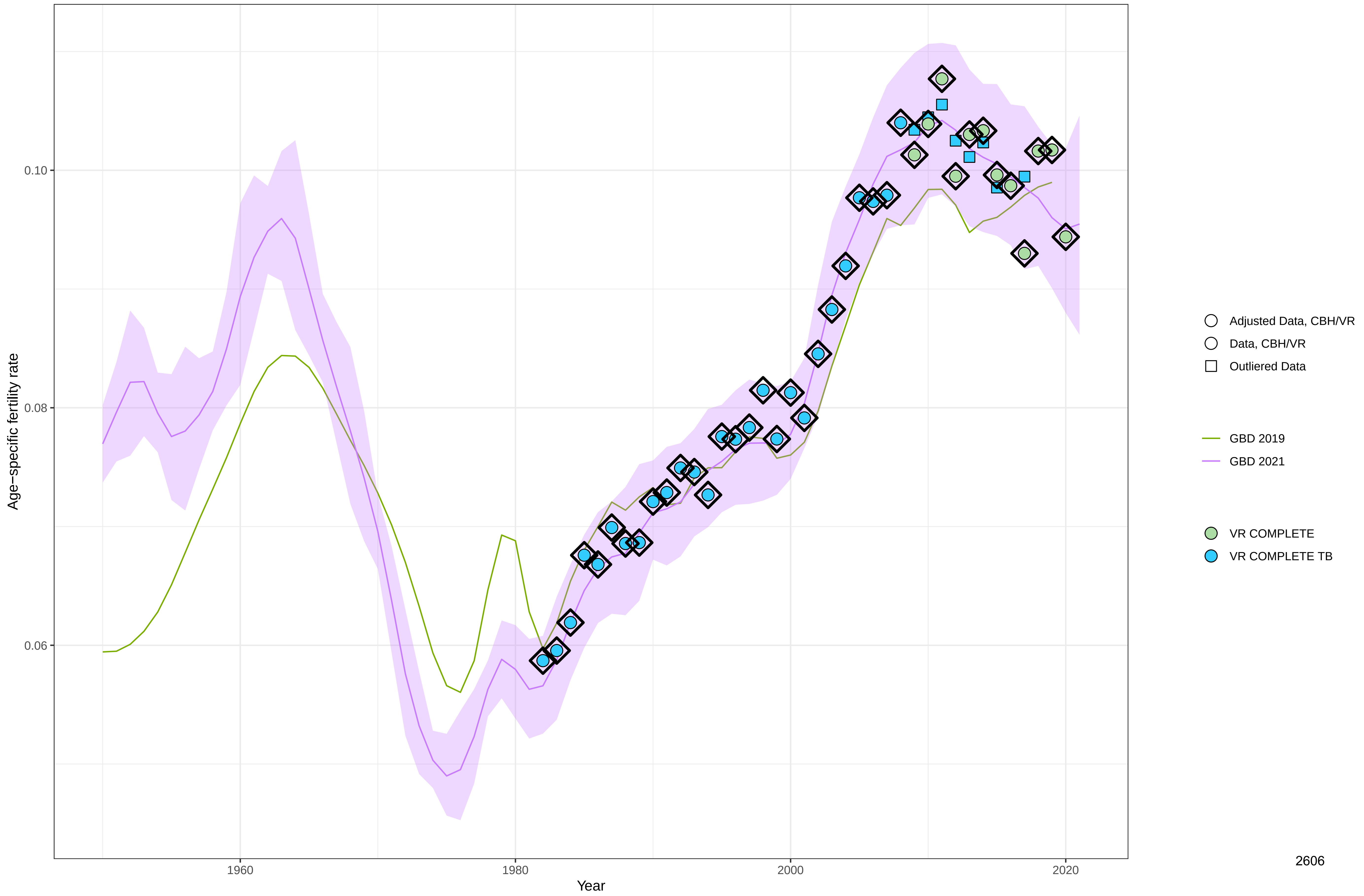

Age−specific fertility fitted trend lines, 1950−2021: North Tyneside, Ages: 35−39
Data points enclosed with a diamond are reference sources

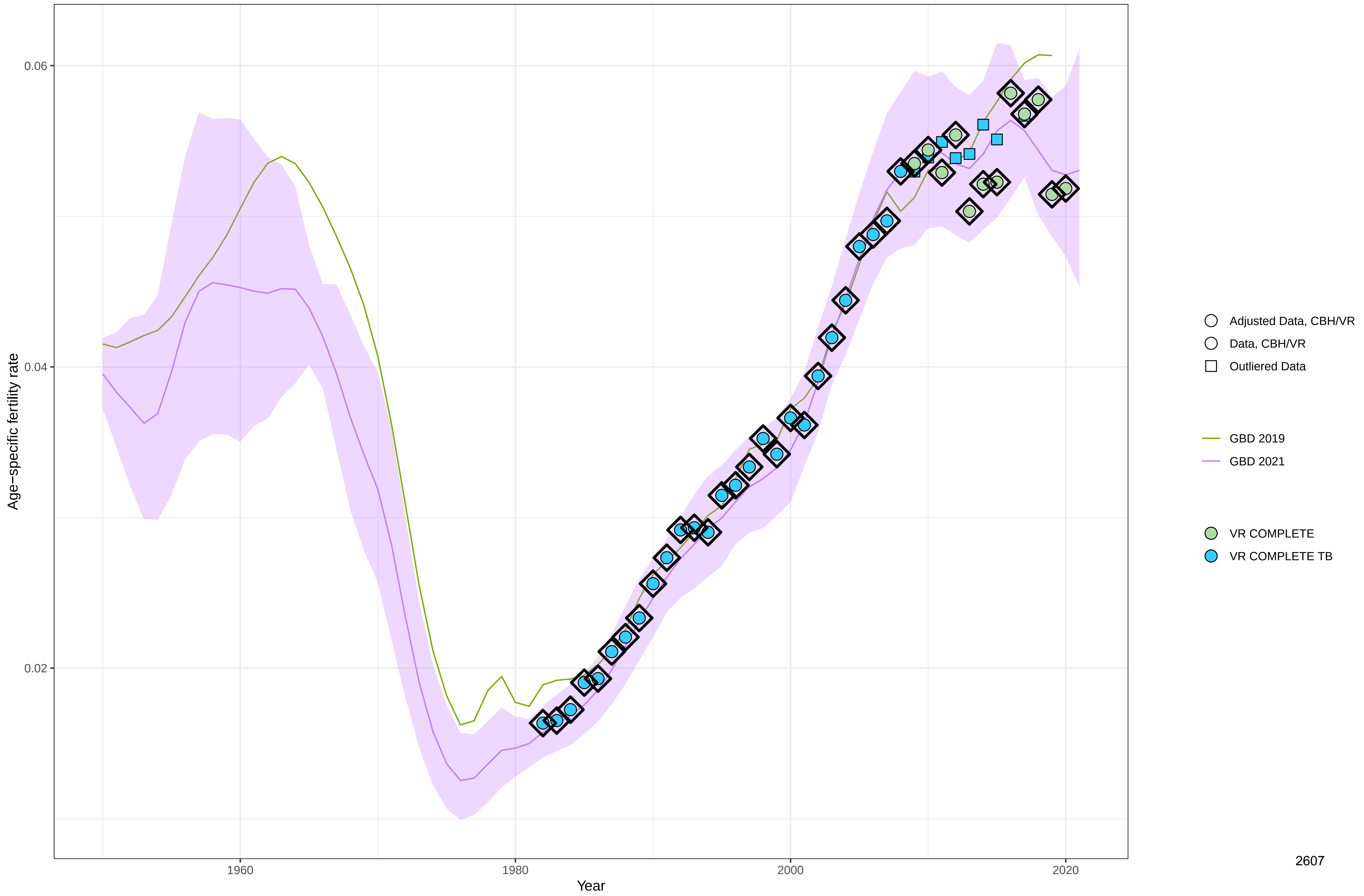

Age−specific fertility fitted trend lines, 1950−2021: North Tyneside, Ages: 40−44
Data points enclosed with a diamond are reference sources

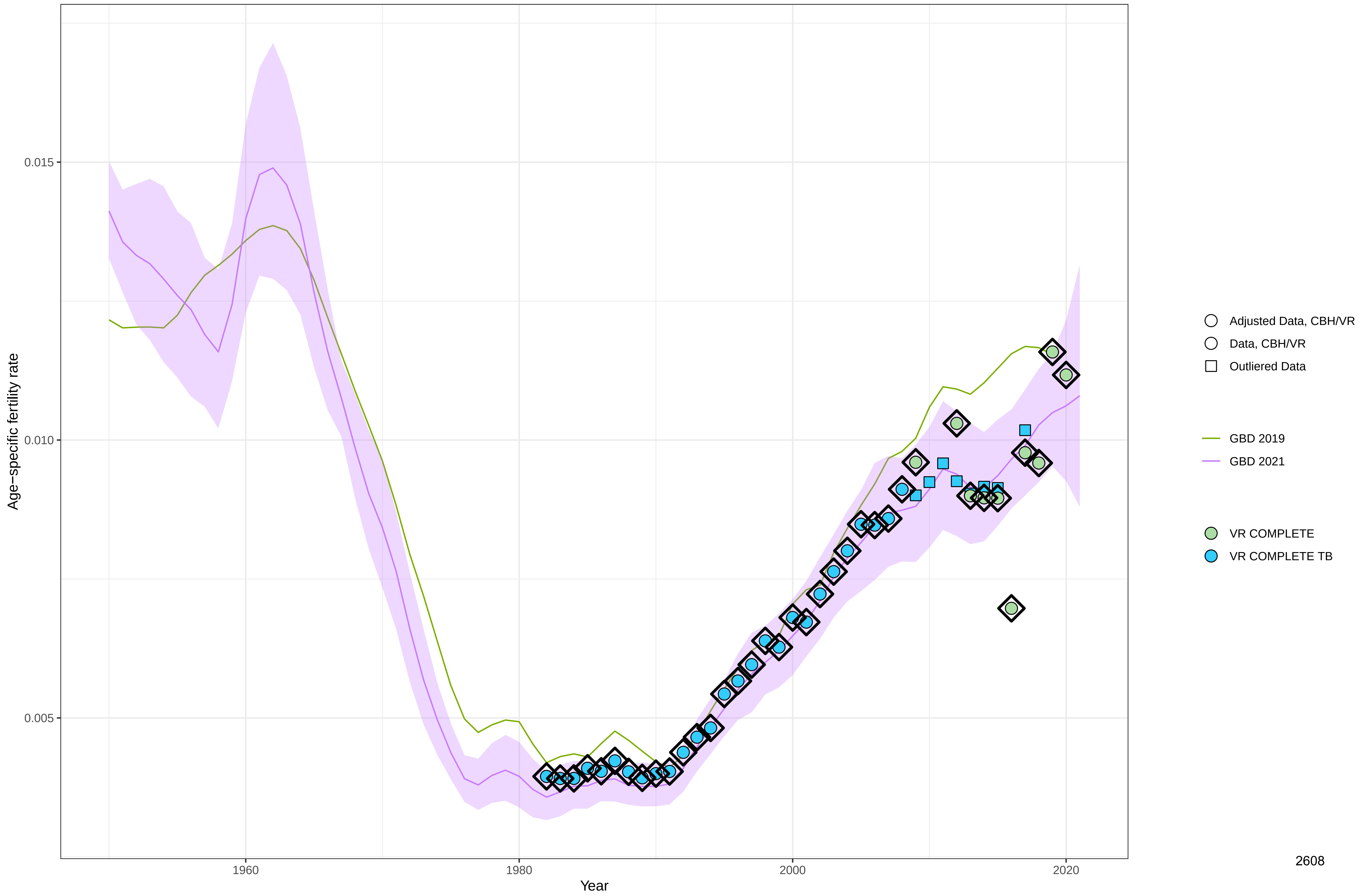

Age−specific fertility fitted trend lines, 1950−2021: North Tyneside, Ages: 45−49
Data points enclosed with a diamond are reference sources

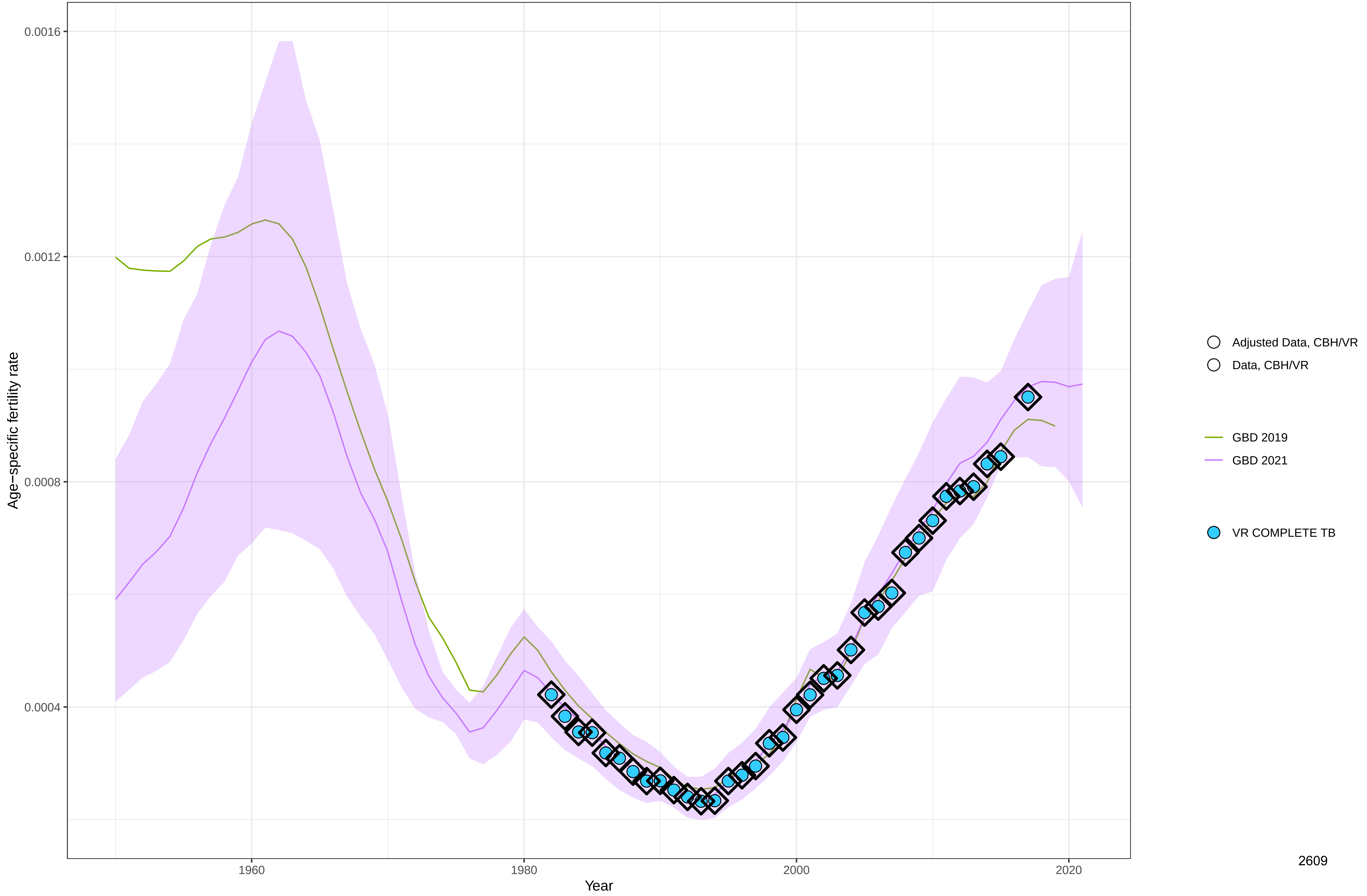

Age−specific fertility fitted trend lines, 1950−2021: North Tyneside, Ages: 50−54
Data points enclosed with a diamond are reference sources

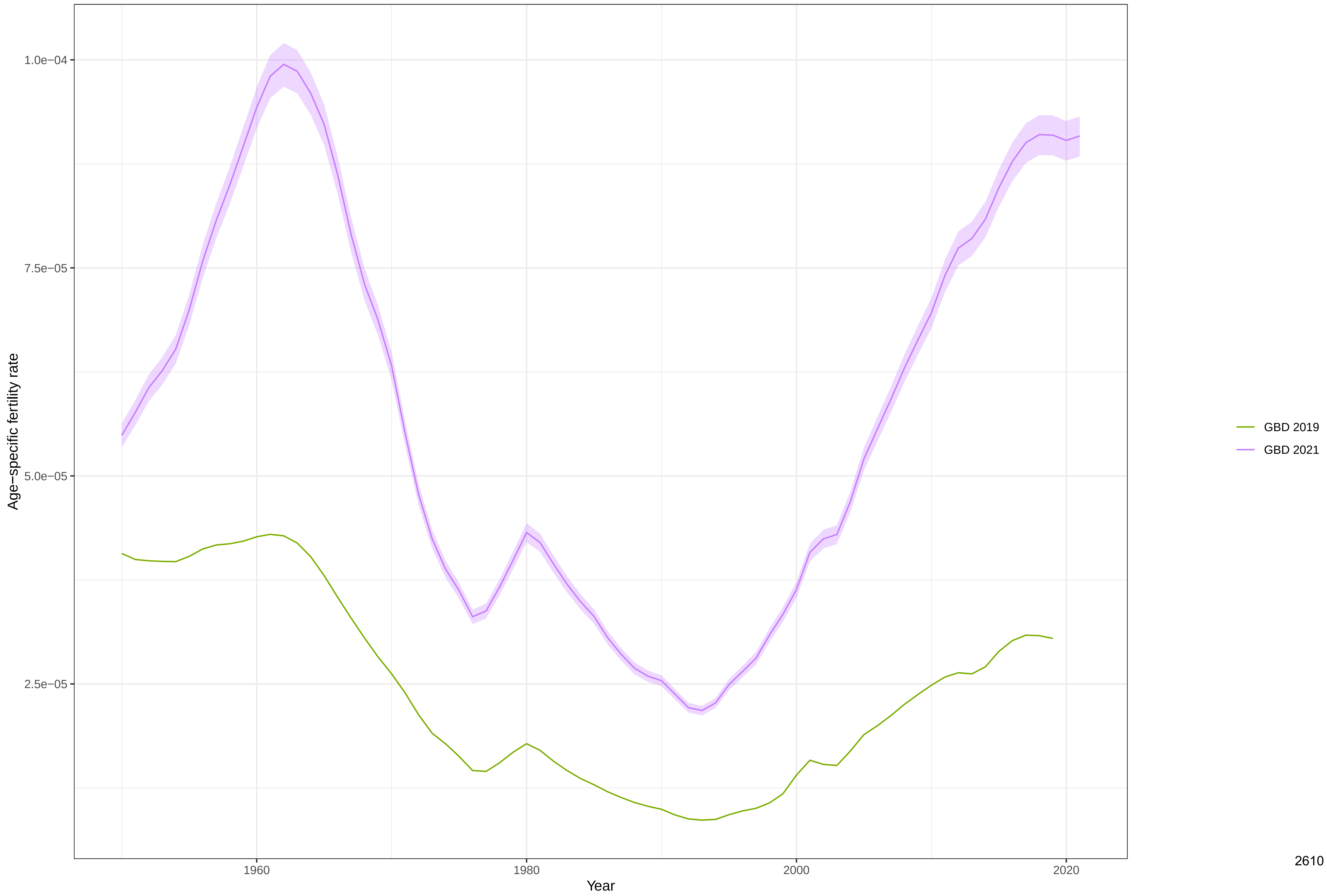

Total fertility rate fitted trend lines, 1950−2021: North Tyneside TFR
Data points enclosed with a diamond are reference sources

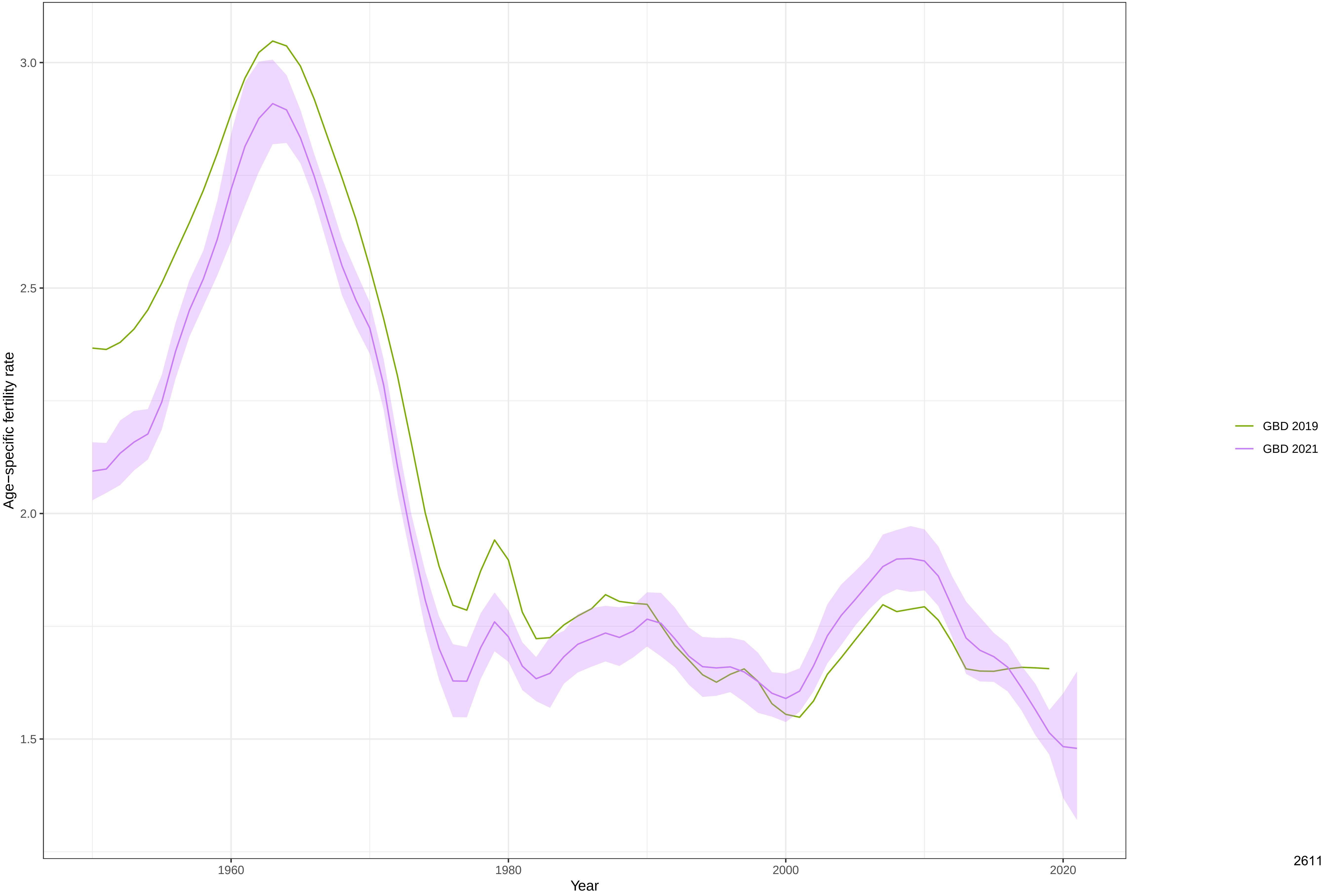

Age−specific fertility fitted trend lines, 1950−2021: Northumberland, Ages: 10−14
Data points enclosed with a diamond are reference sources

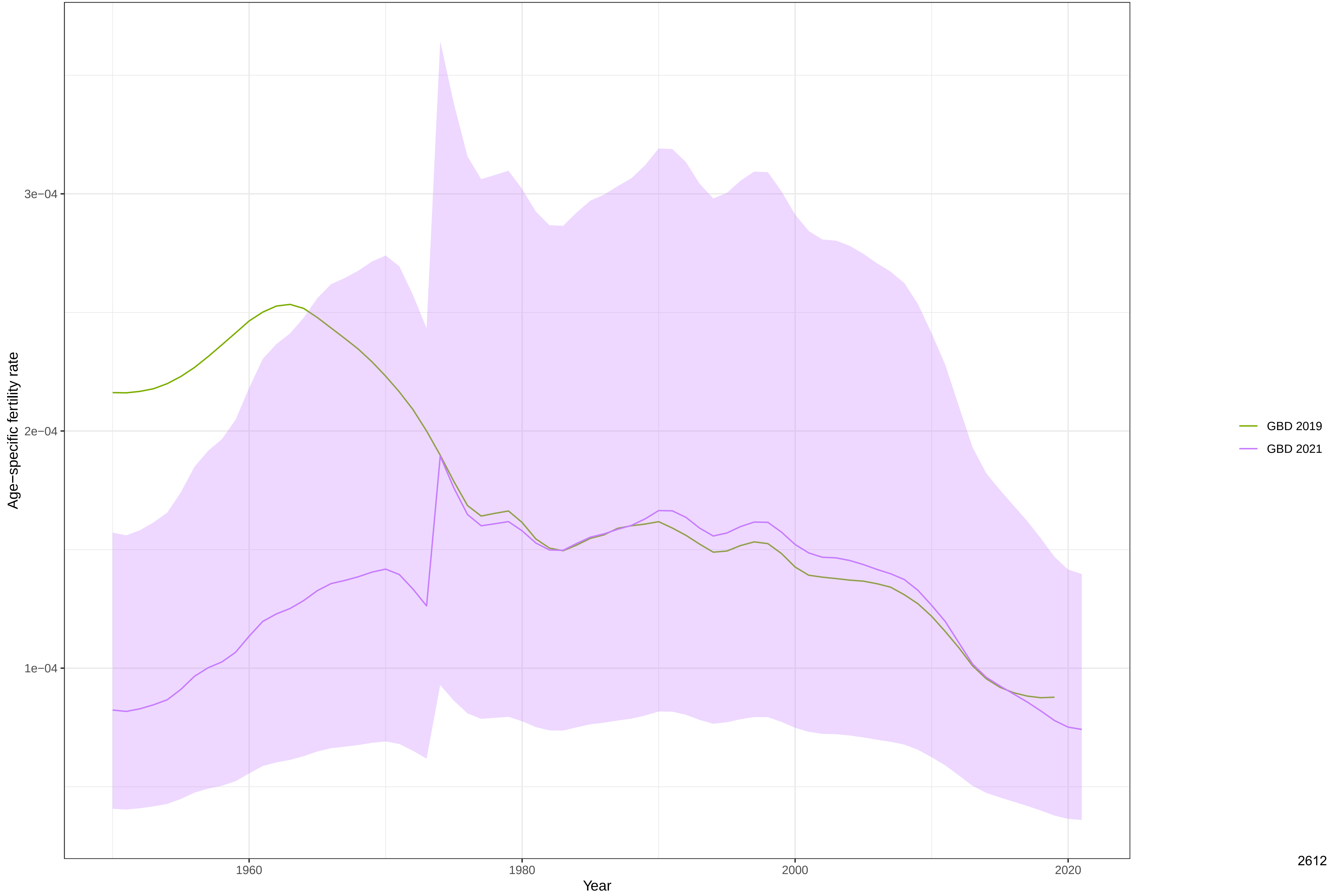

Age−specific fertility fitted trend lines, 1950−2021: Northumberland, Ages: 15−19
Data points enclosed with a diamond are reference sources

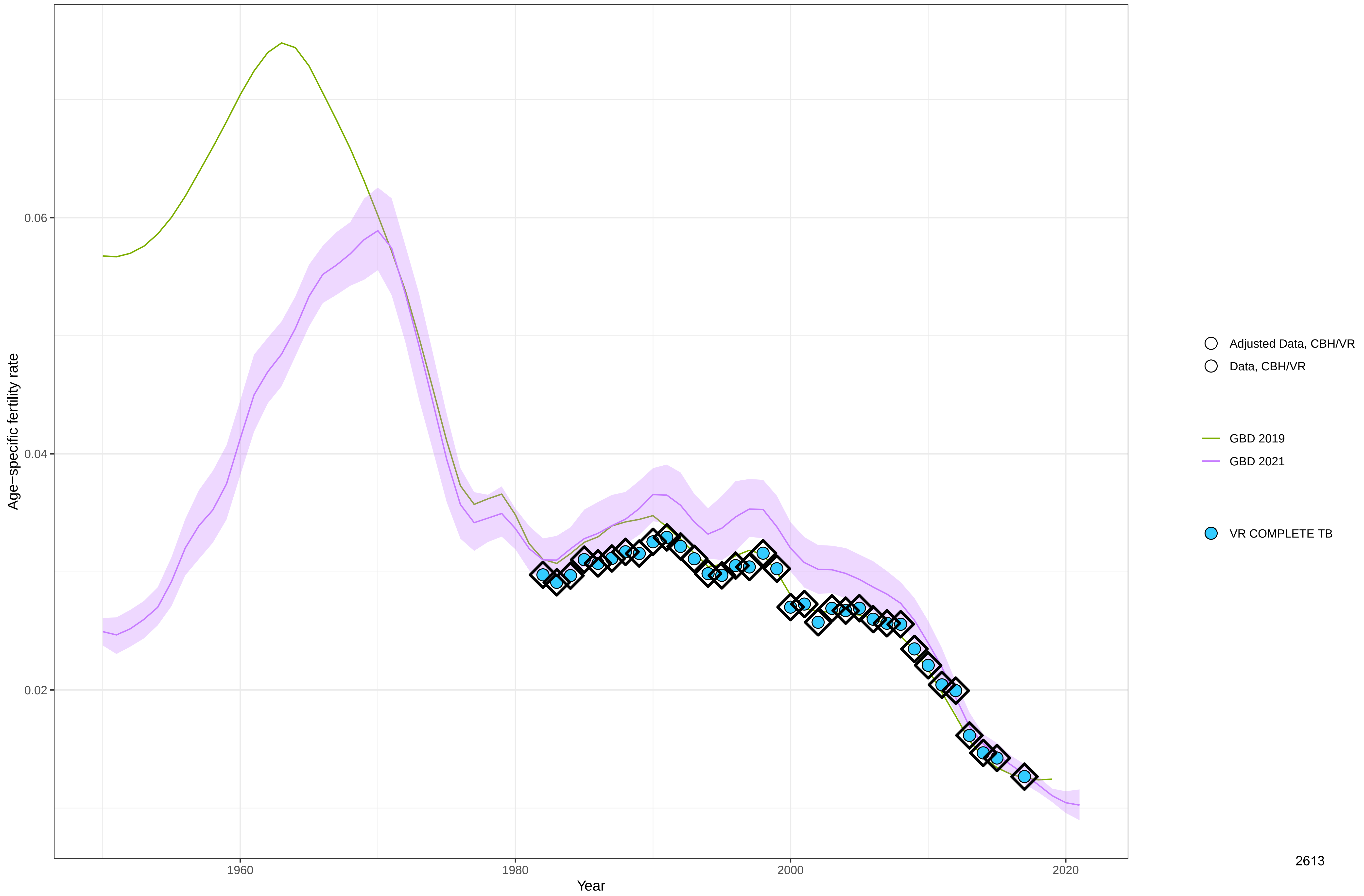

Age−specific fertility fitted trend lines, 1950−2021: Northumberland, Ages: 20−24
Data points enclosed with a diamond are reference sources

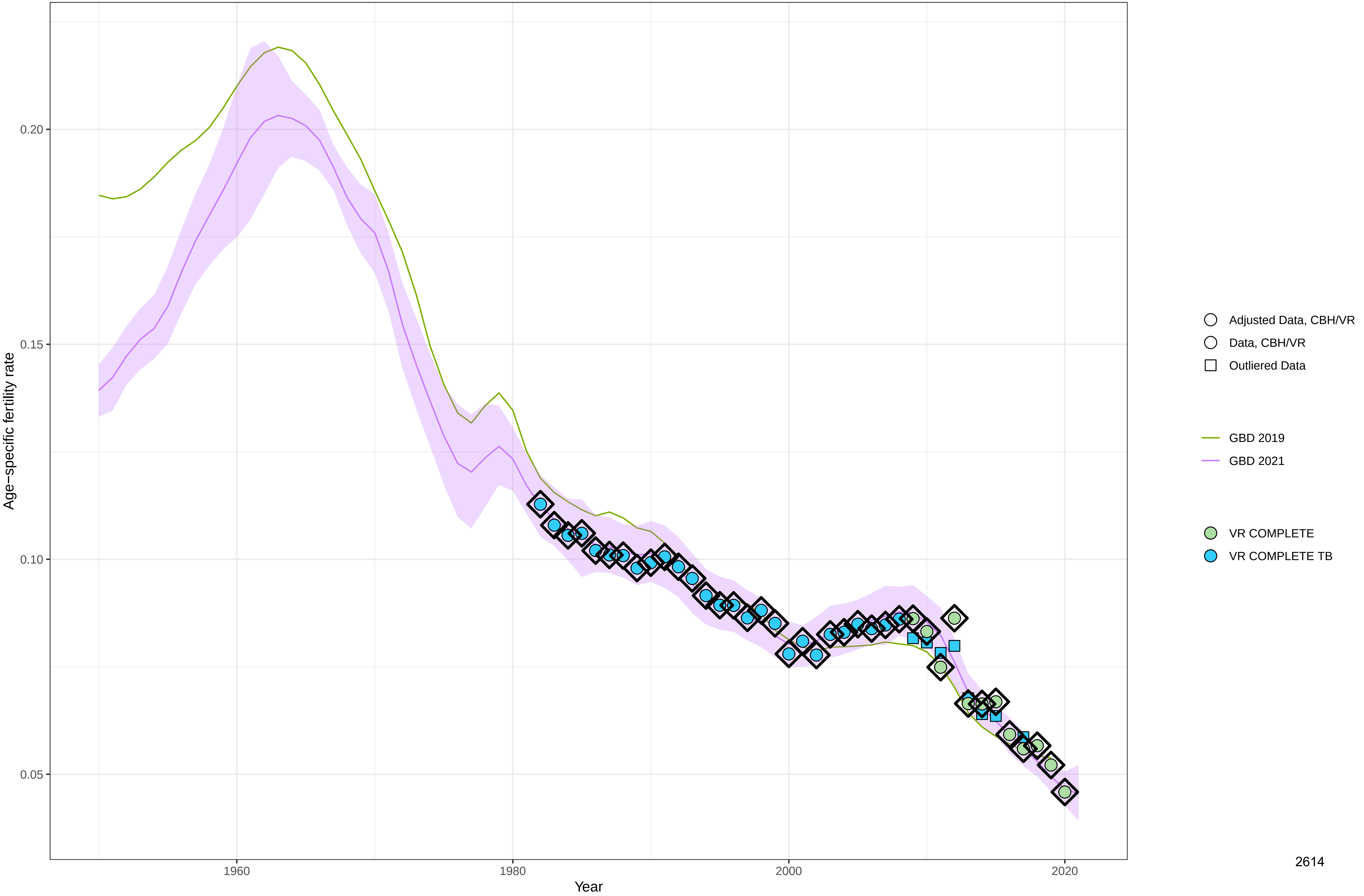

Age−specific fertility fitted trend lines, 1950−2021: Northumberland, Ages: 25−29
Data points enclosed with a diamond are reference sources

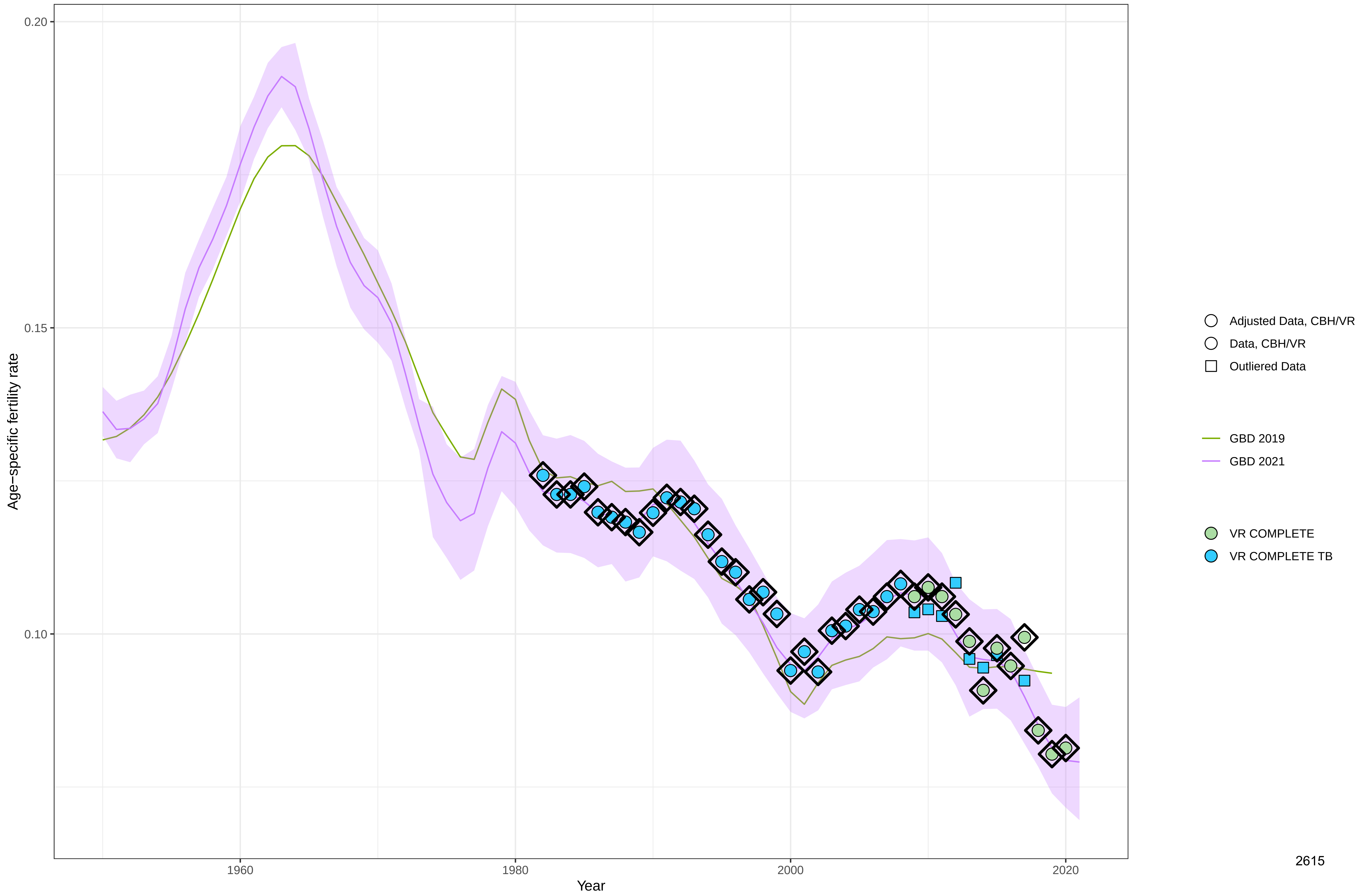

Age−specific fertility fitted trend lines, 1950−2021: Northumberland, Ages: 30−34
Data points enclosed with a diamond are reference sources

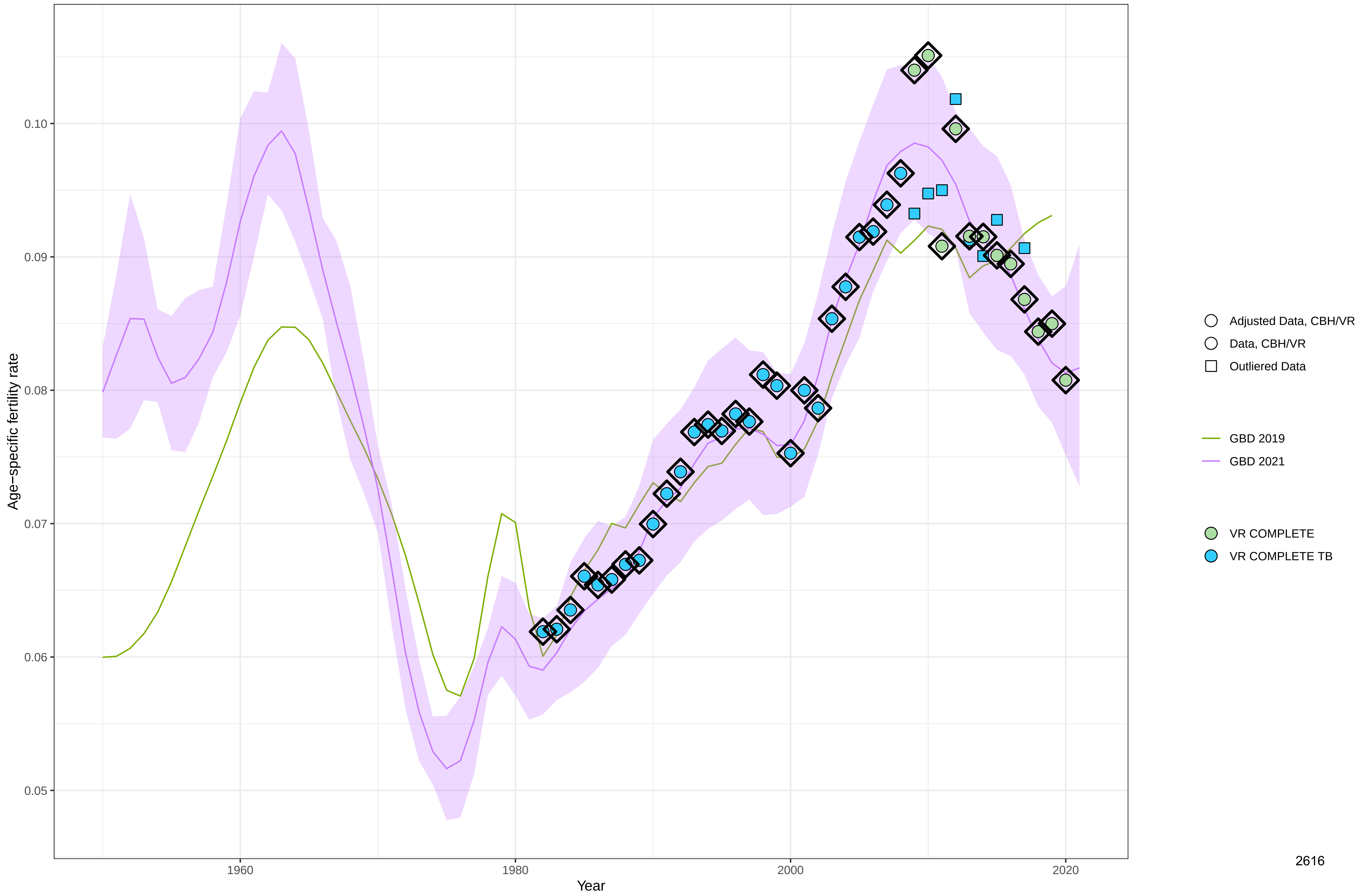

Age−specific fertility fitted trend lines, 1950−2021: Northumberland, Ages: 35−39
Data points enclosed with a diamond are reference sources

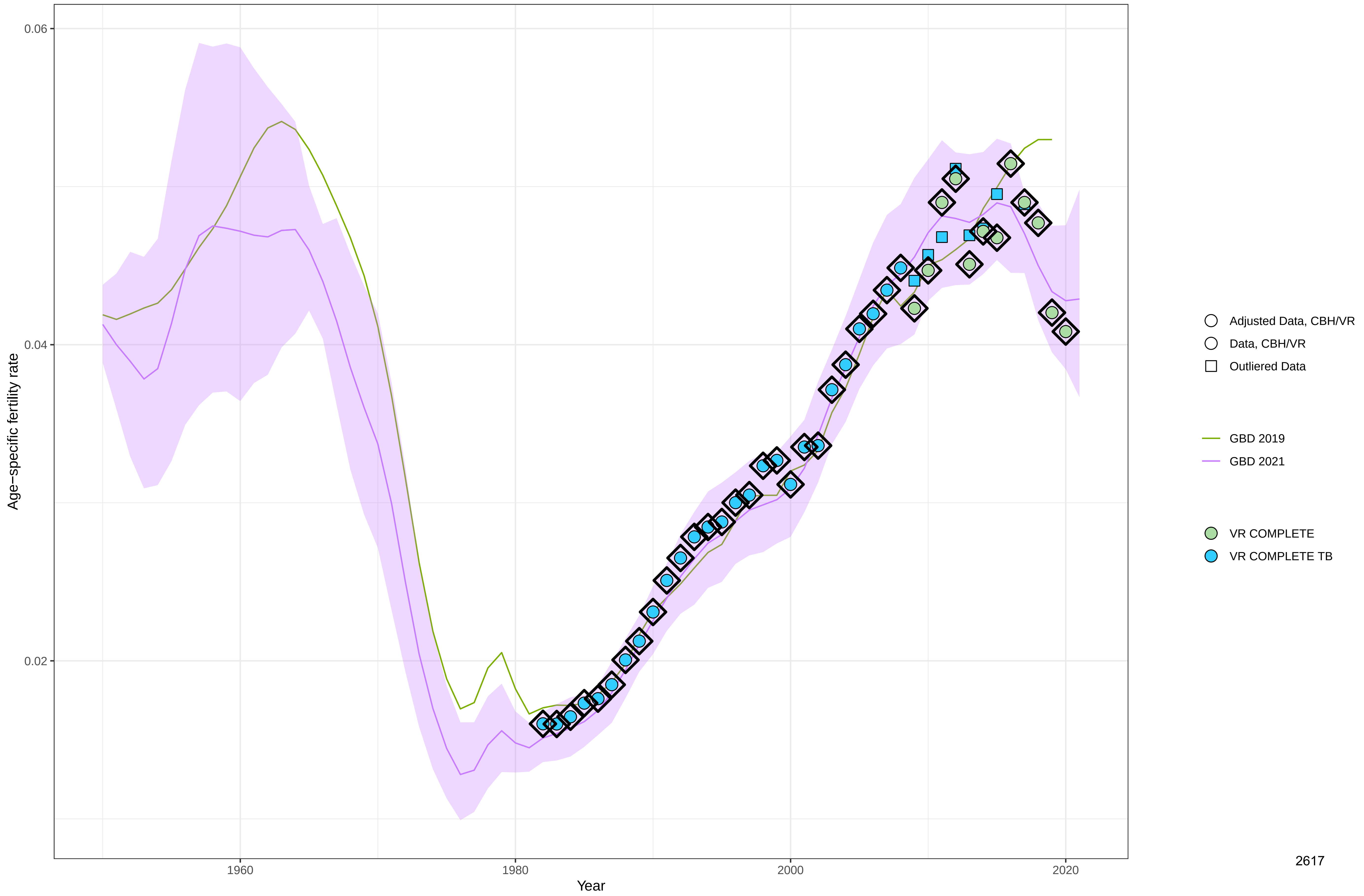

Age−specific fertility fitted trend lines, 1950−2021: Northumberland, Ages: 40−44
Data points enclosed with a diamond are reference sources

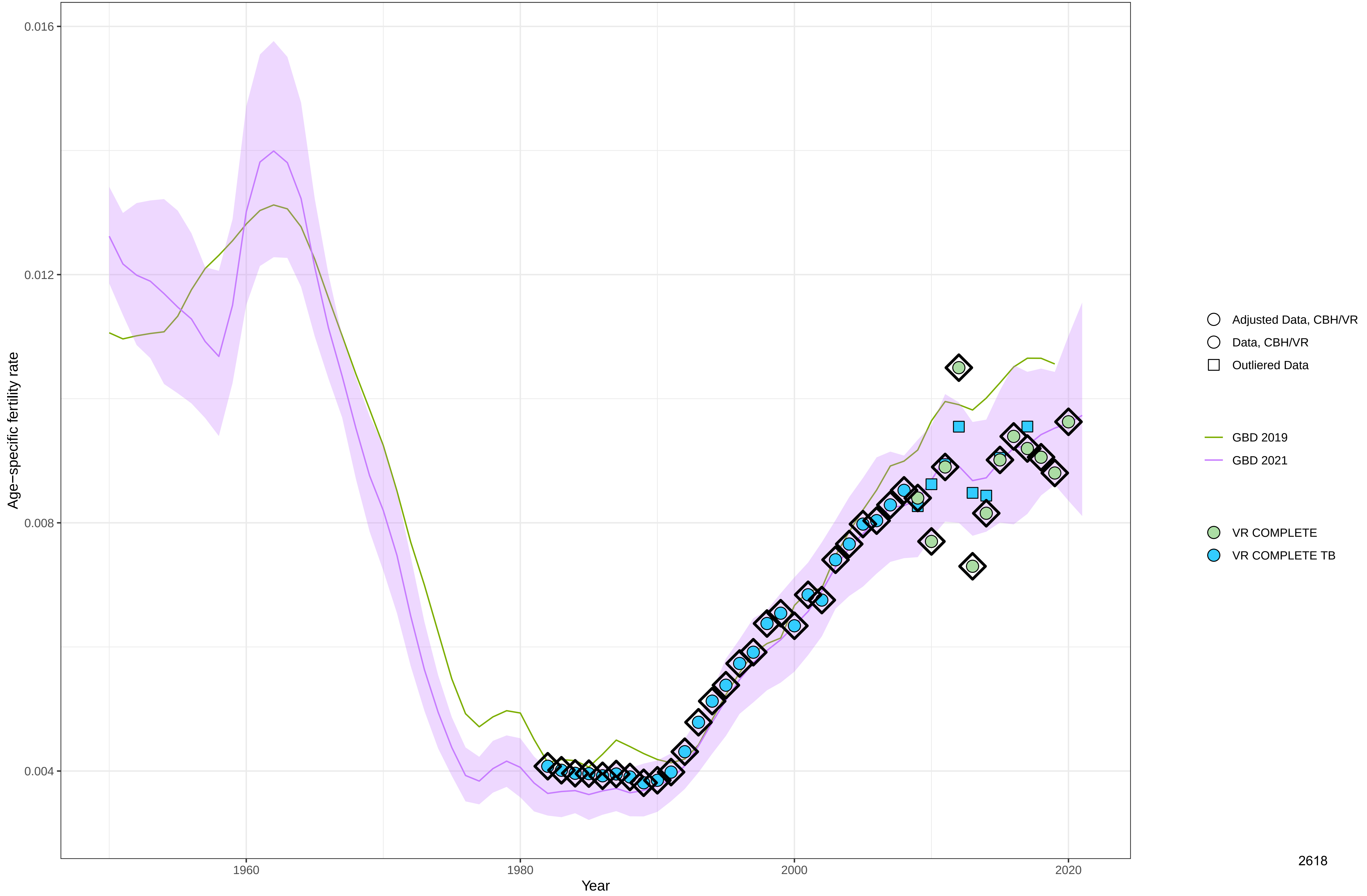

Age−specific fertility fitted trend lines, 1950−2021: Northumberland, Ages: 45−49
Data points enclosed with a diamond are reference sources

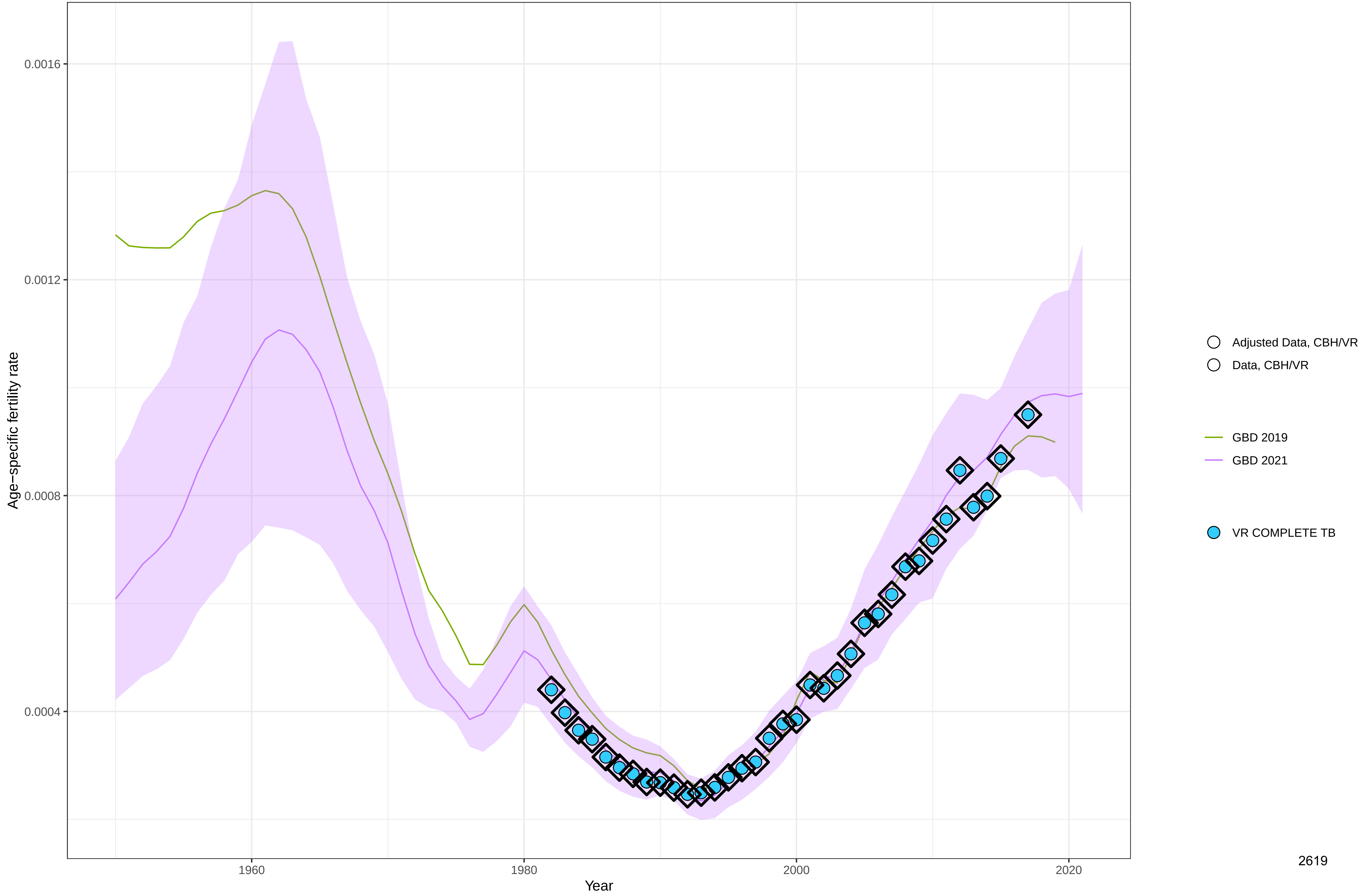

Age−specific fertility fitted trend lines, 1950−2021: Northumberland, Ages: 50−54
Data points enclosed with a diamond are reference sources

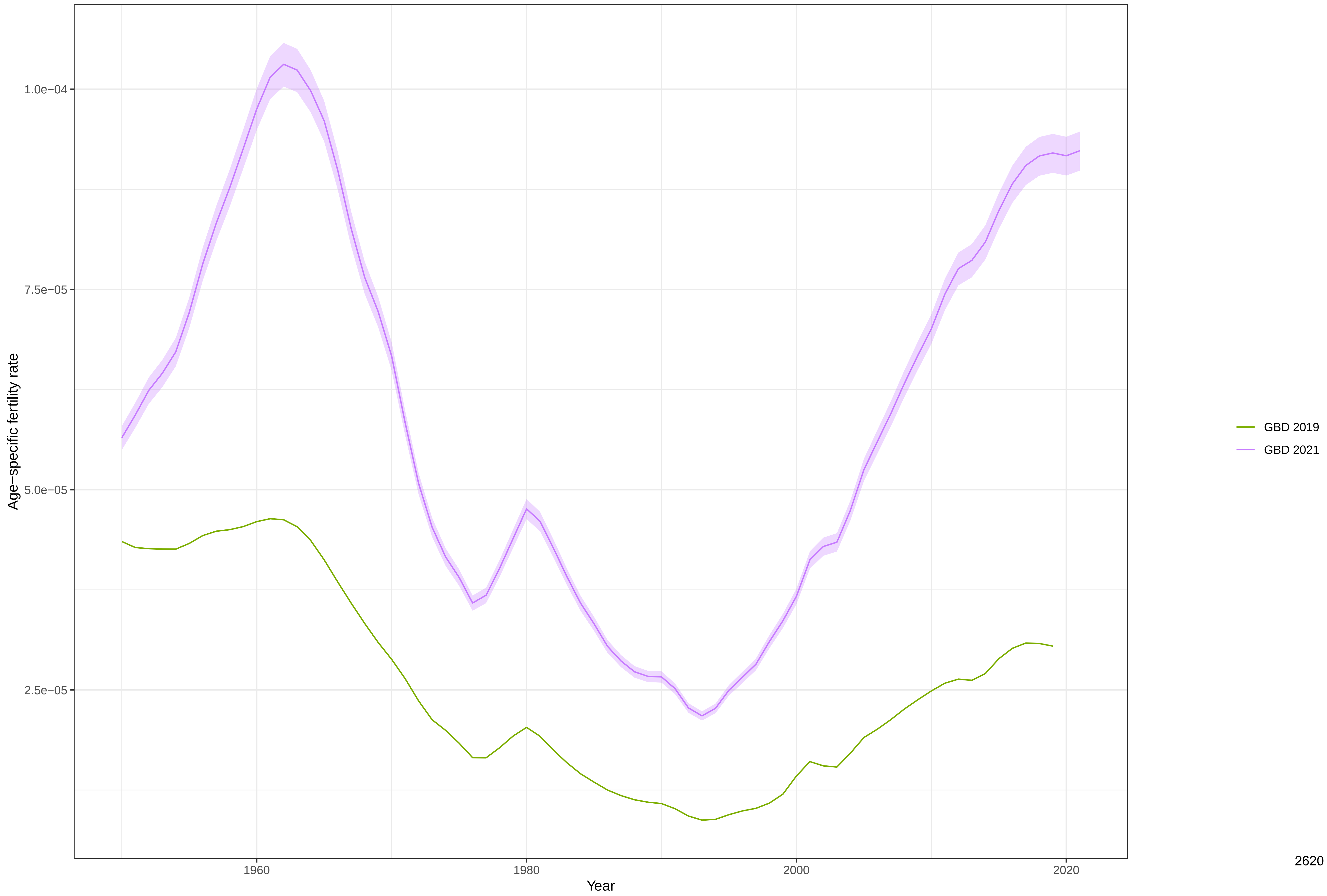

Total fertility rate fitted trend lines, 1950−2021: Northumberland TFR
Data points enclosed with a diamond are reference sources

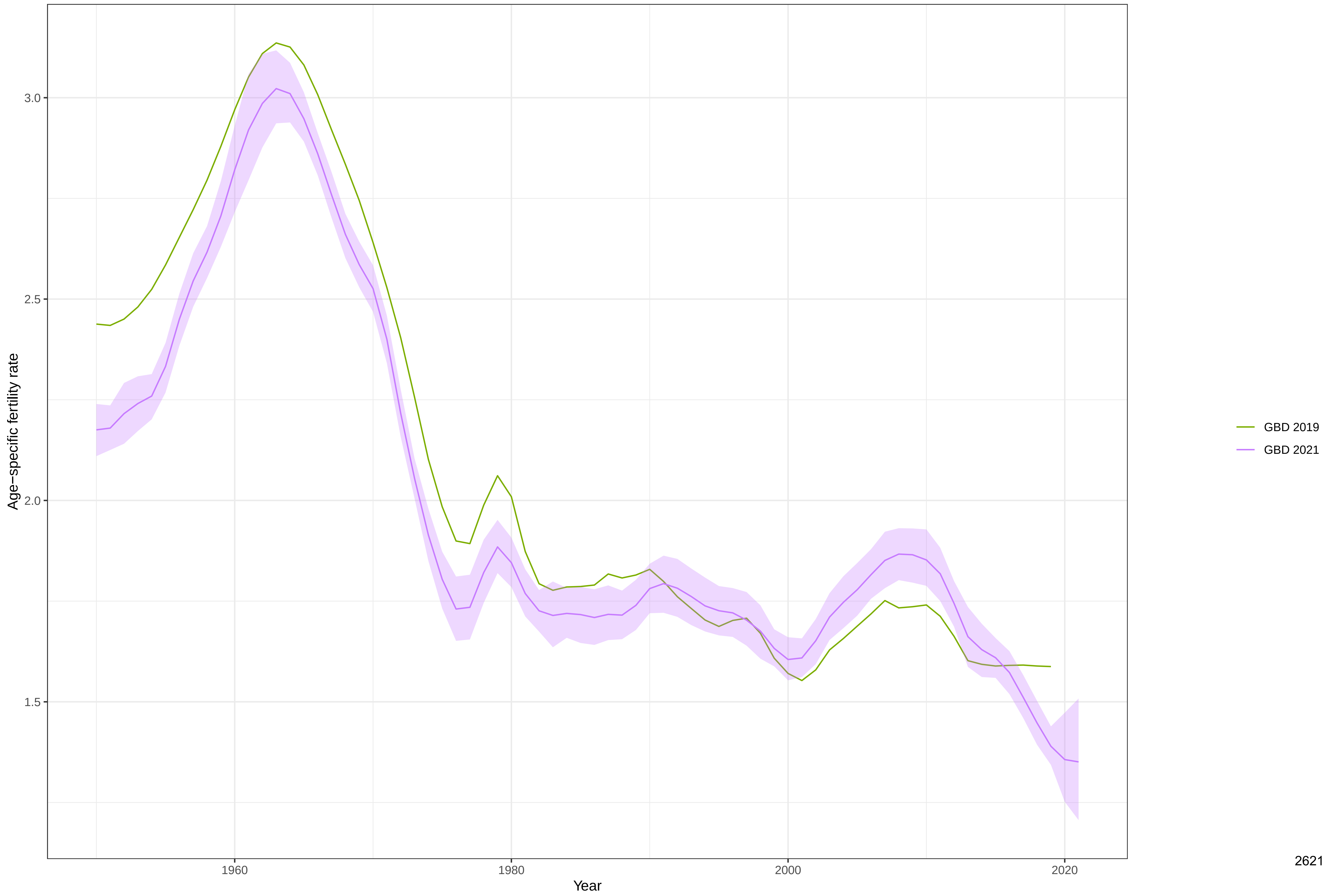

Age−specific fertility fitted trend lines, 1950−2021: Redcar and Cleveland, Ages: 10−14
Data points enclosed with a diamond are reference sources

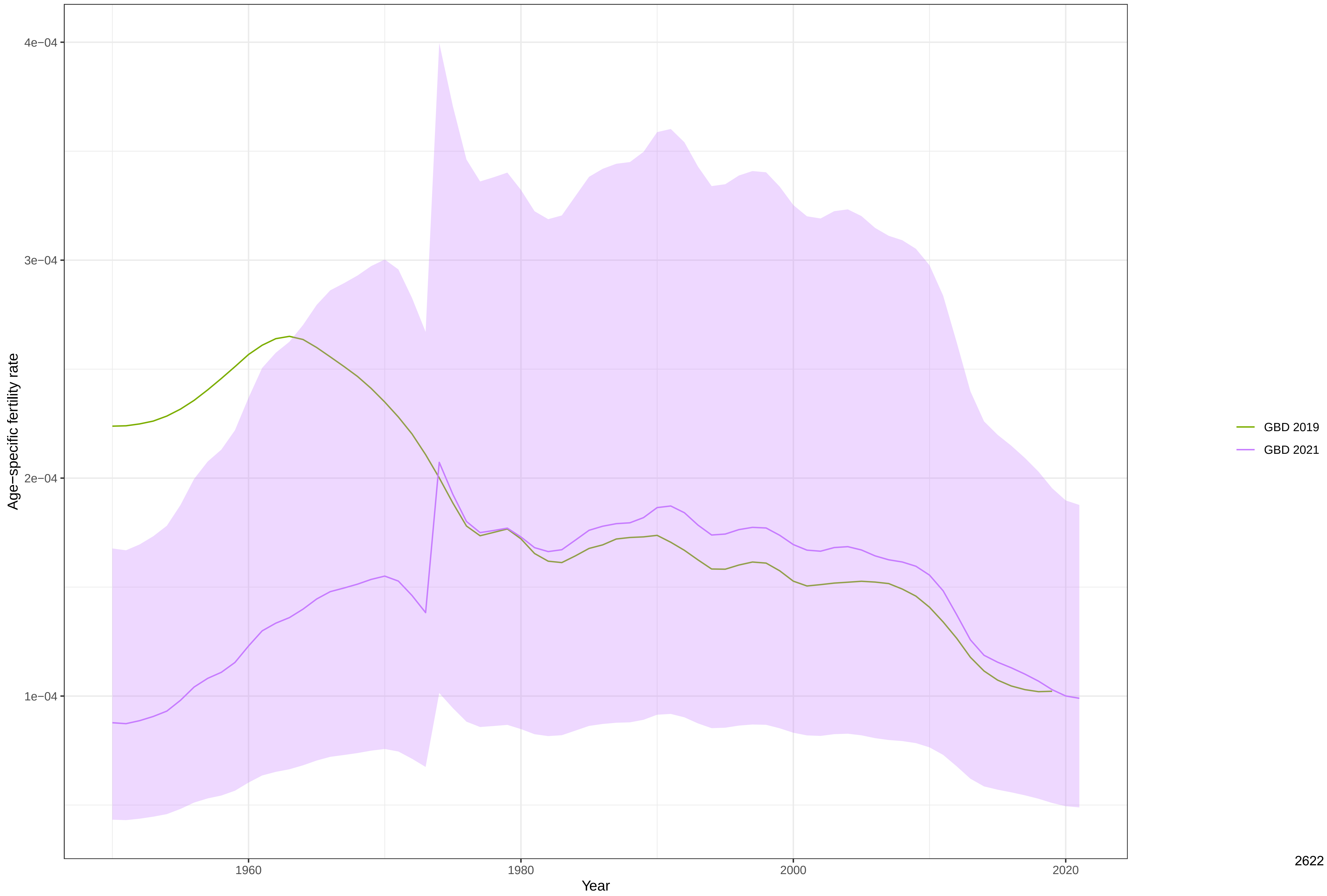

Age−specific fertility fitted trend lines, 1950−2021: Redcar and Cleveland, Ages: 15−19
Data points enclosed with a diamond are reference sources

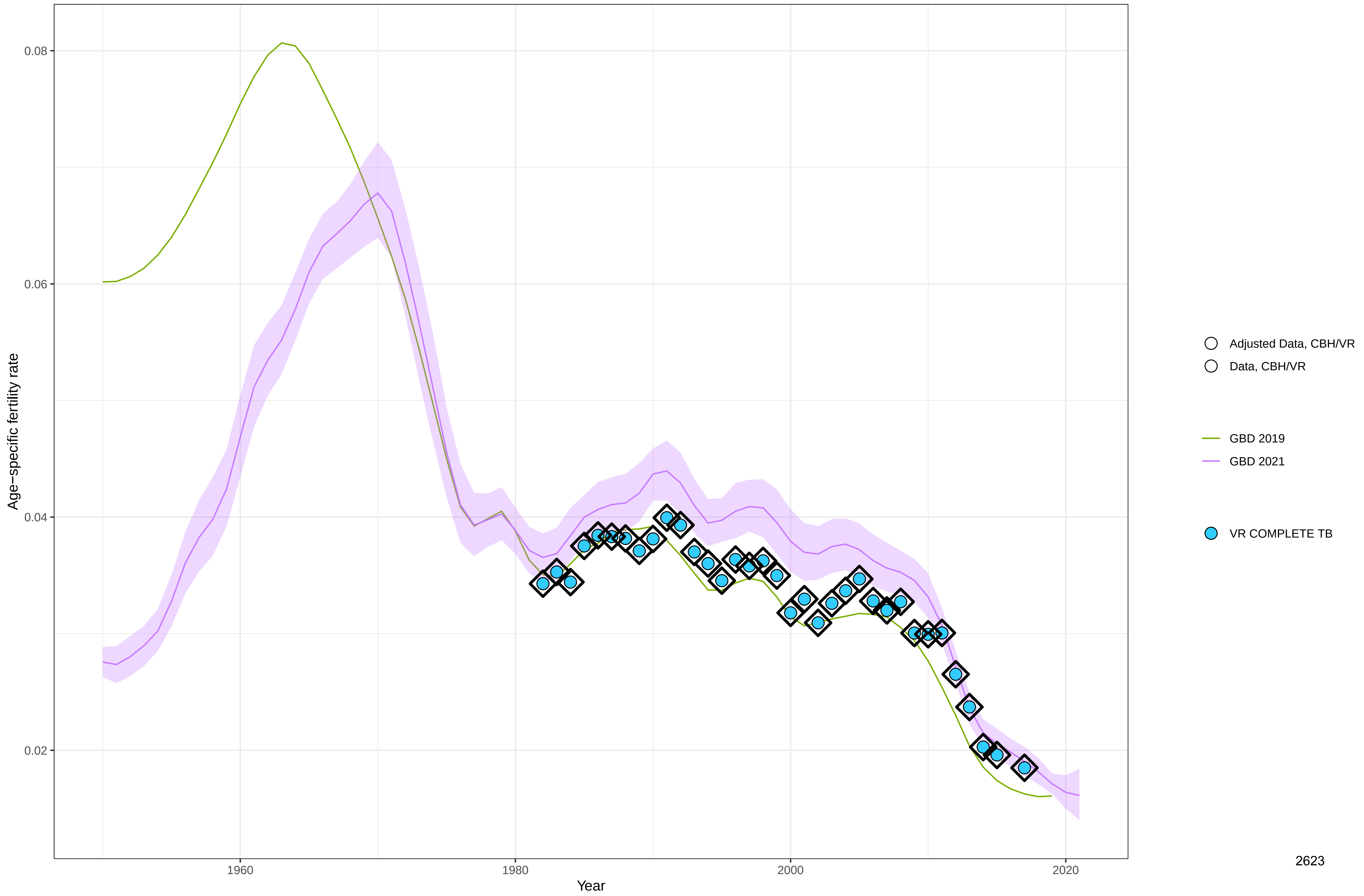

Age−specific fertility fitted trend lines, 1950−2021: Redcar and Cleveland, Ages: 20−24
Data points enclosed with a diamond are reference sources

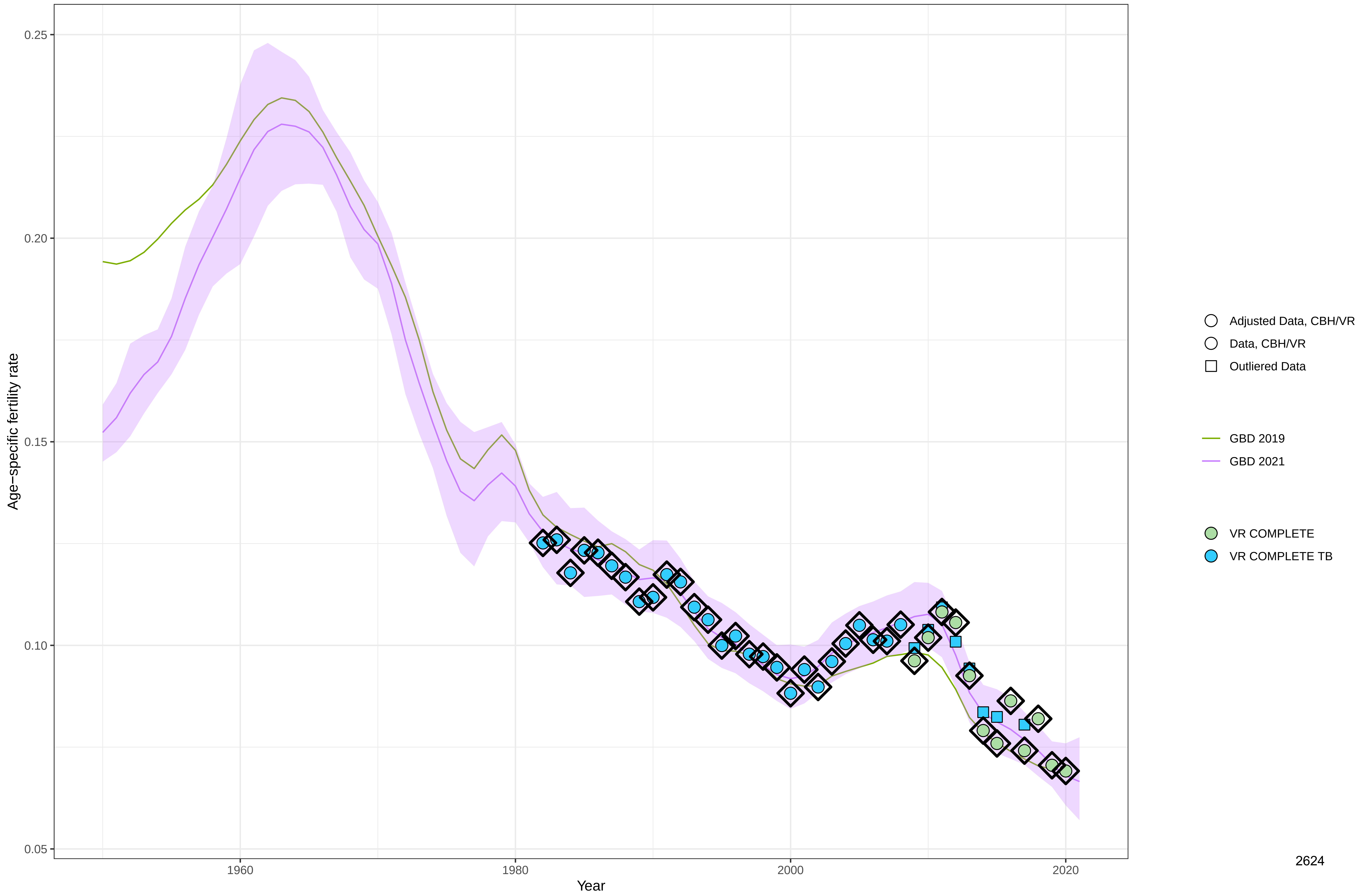

Age−specific fertility fitted trend lines, 1950−2021: Redcar and Cleveland, Ages: 25−29
Data points enclosed with a diamond are reference sources

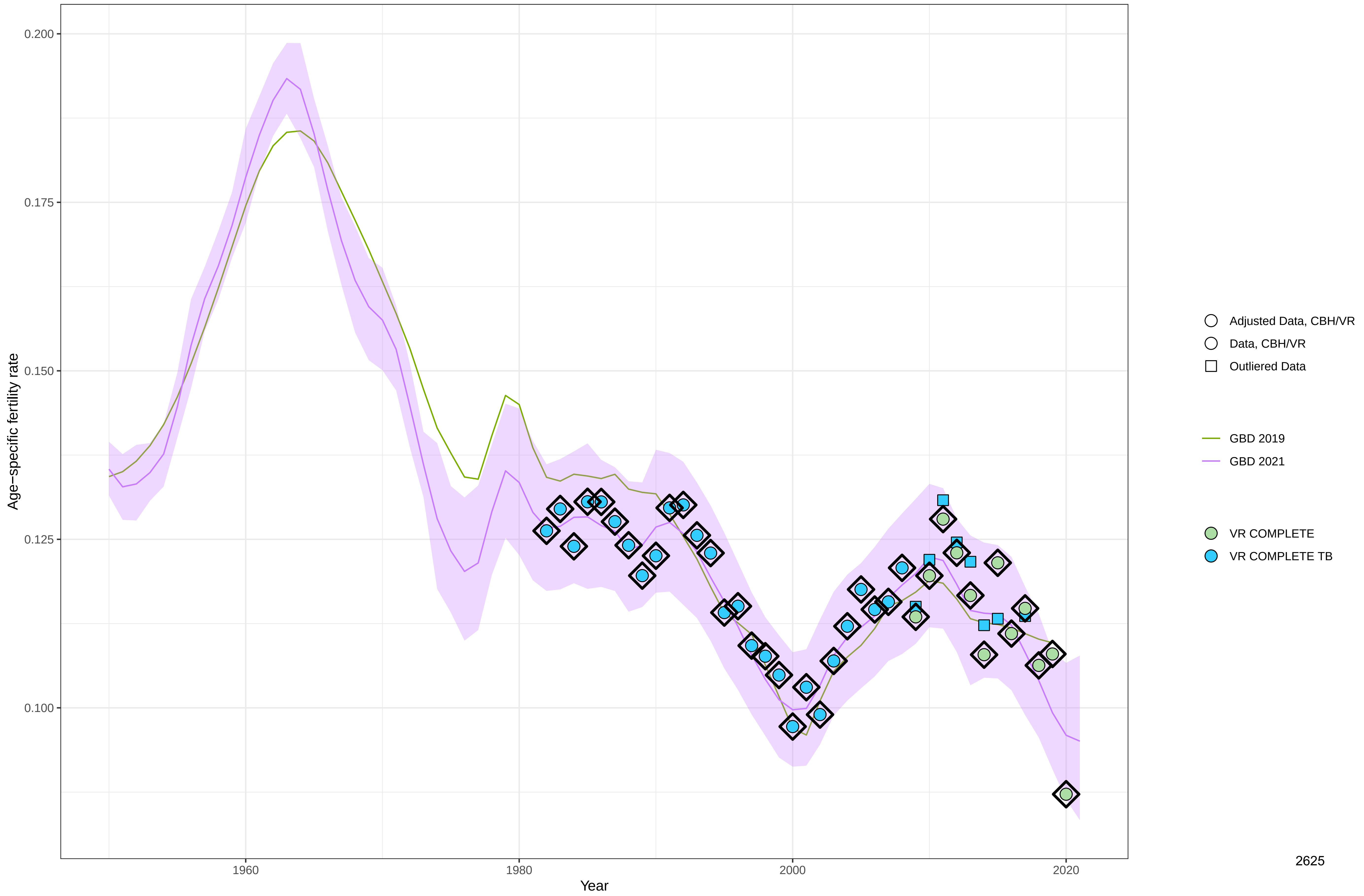

Age−specific fertility fitted trend lines, 1950−2021: Redcar and Cleveland, Ages: 30−34
Data points enclosed with a diamond are reference sources

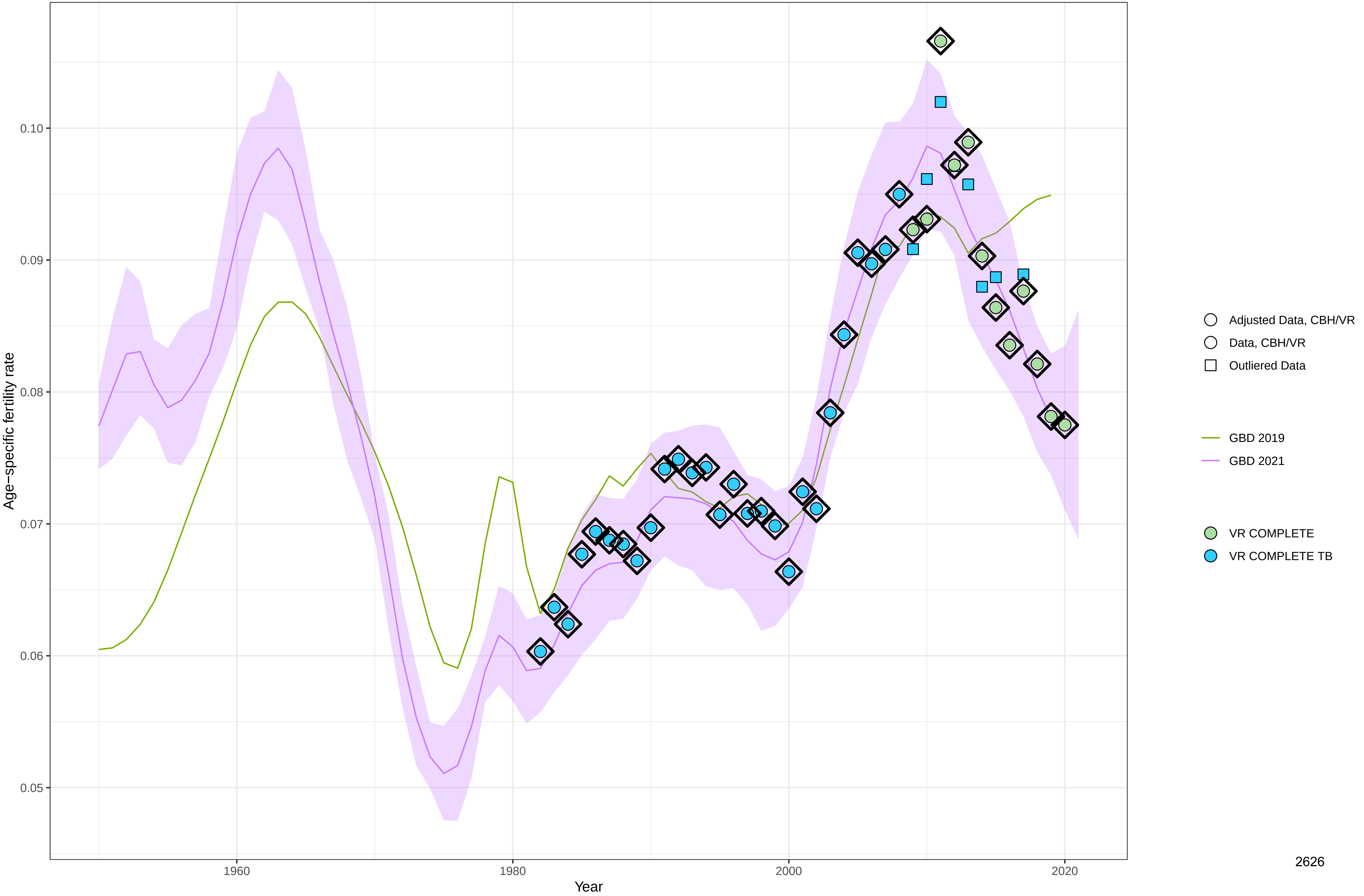

Age−specific fertility fitted trend lines, 1950−2021: Redcar and Cleveland, Ages: 35−39
Data points enclosed with a diamond are reference sources

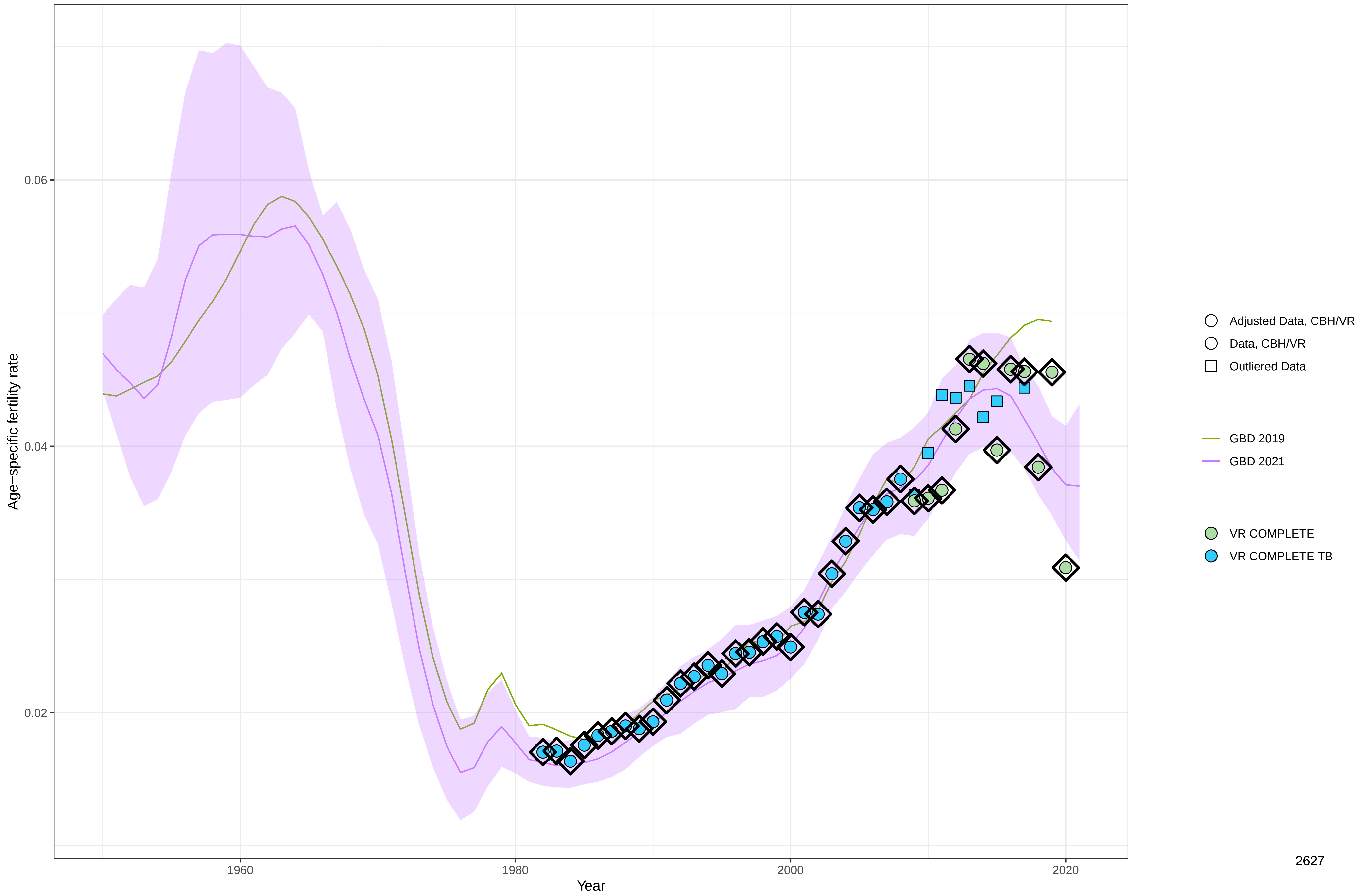

Age−specific fertility fitted trend lines, 1950−2021: Redcar and Cleveland, Ages: 40−44
Data points enclosed with a diamond are reference sources

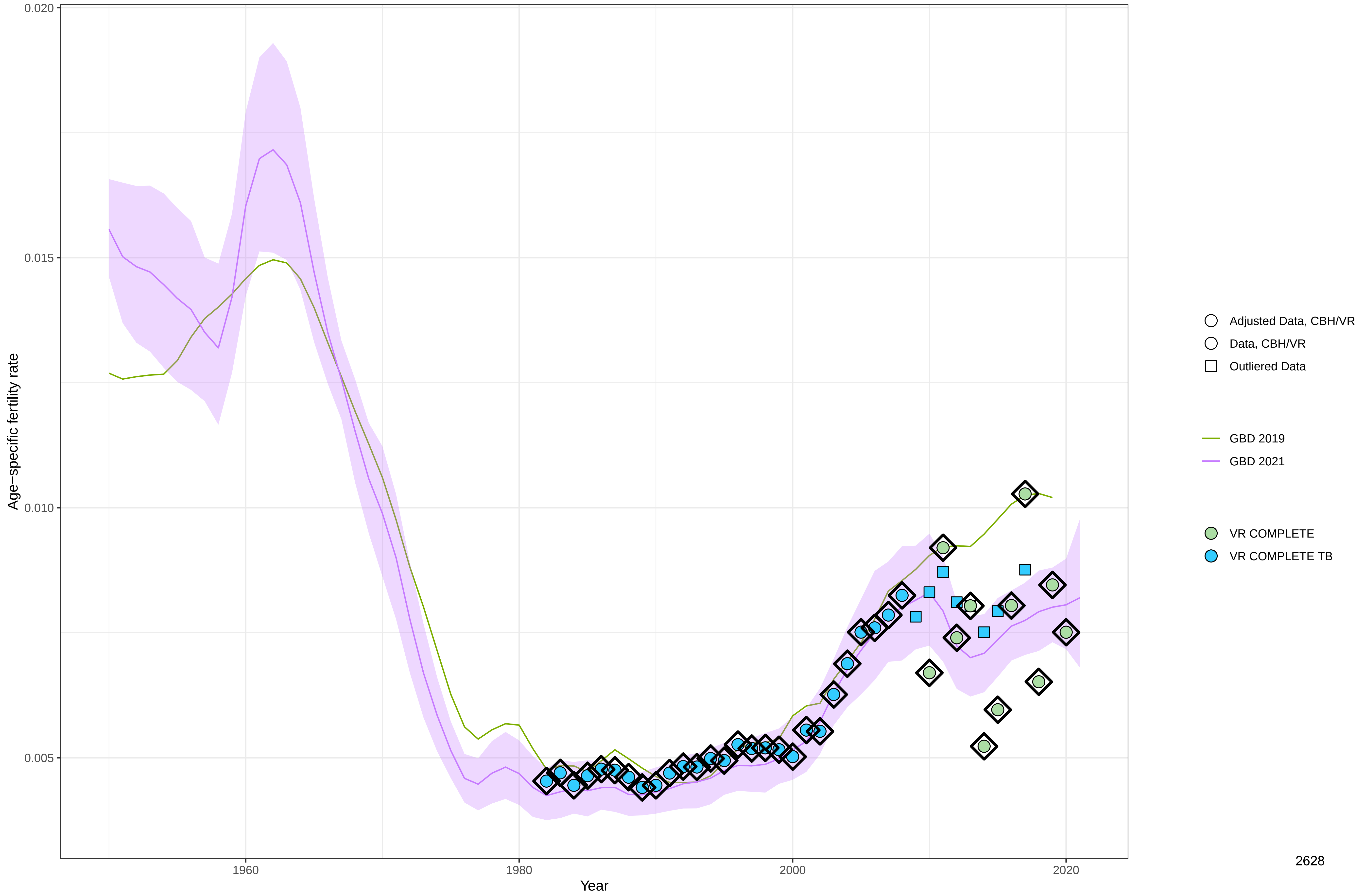

Age−specific fertility fitted trend lines, 1950−2021: Redcar and Cleveland, Ages: 45−49
Data points enclosed with a diamond are reference sources

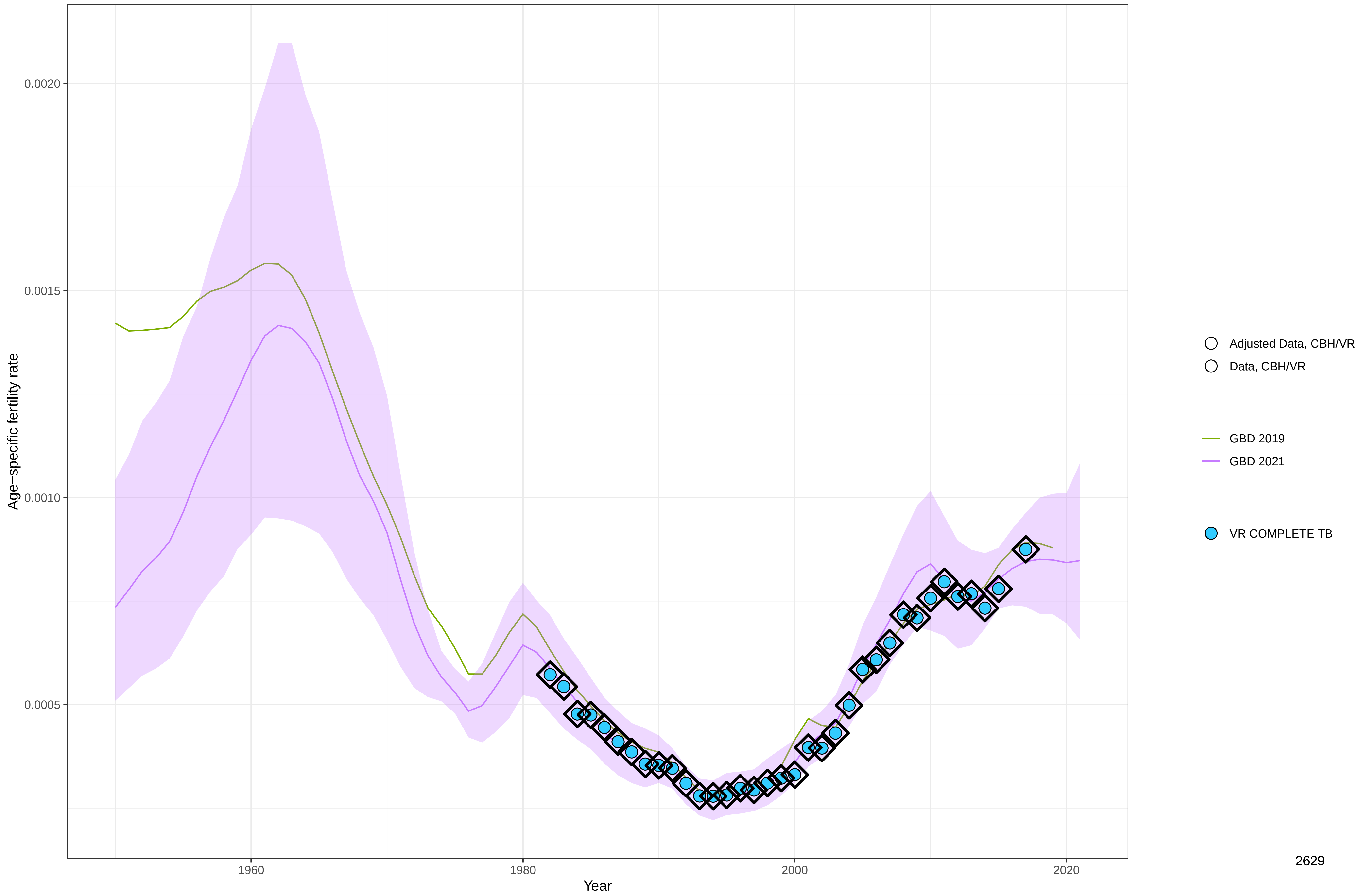

Age−specific fertility fitted trend lines, 1950−2021: Redcar and Cleveland, Ages: 50−54
Data points enclosed with a diamond are reference sources

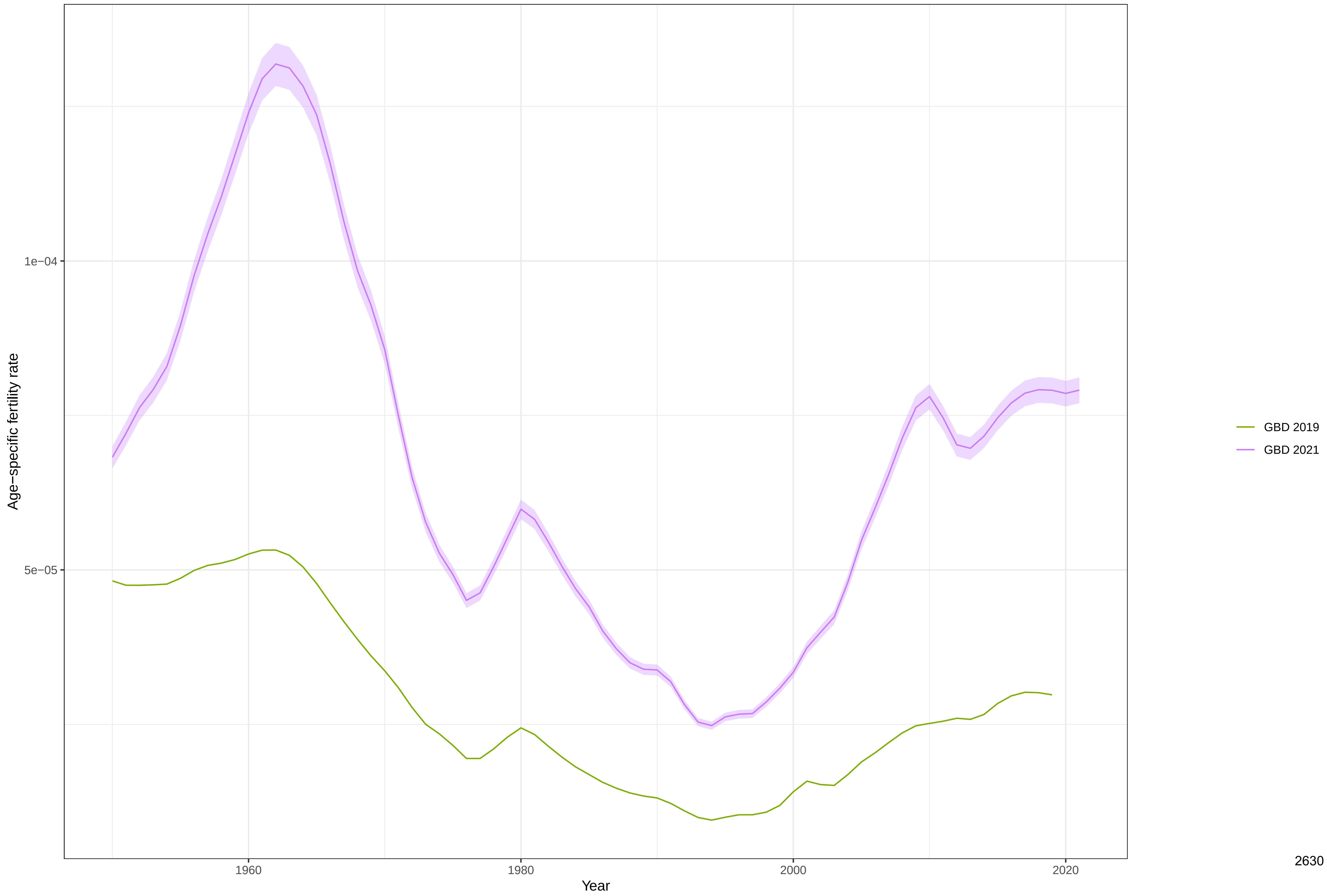

Total fertility rate fitted trend lines, 1950−2021: Redcar and Cleveland TFR
Data points enclosed with a diamond are reference sources

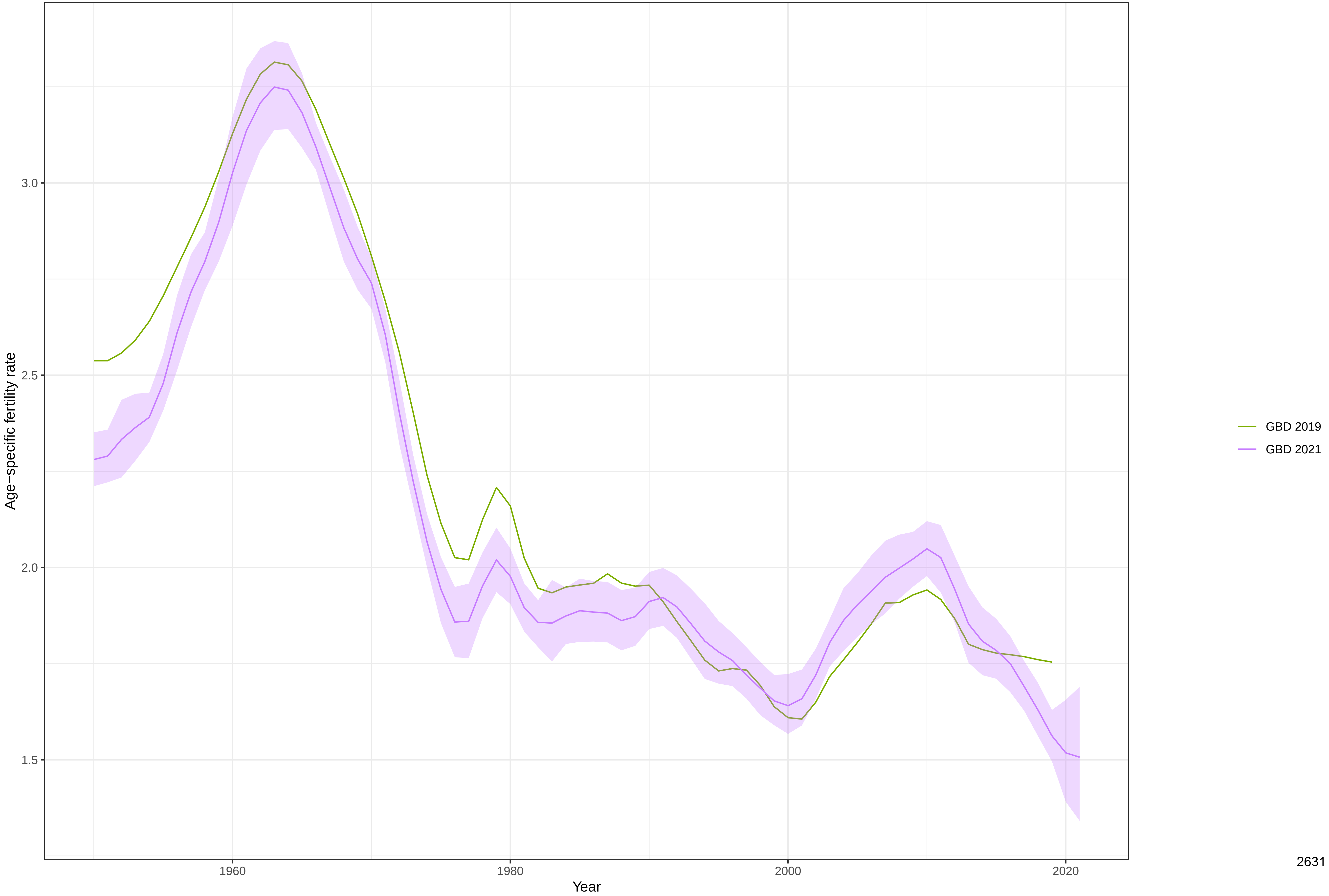

Age−specific fertility fitted trend lines, 1950−2021: South Tyneside, Ages: 10−14
Data points enclosed with a diamond are reference sources

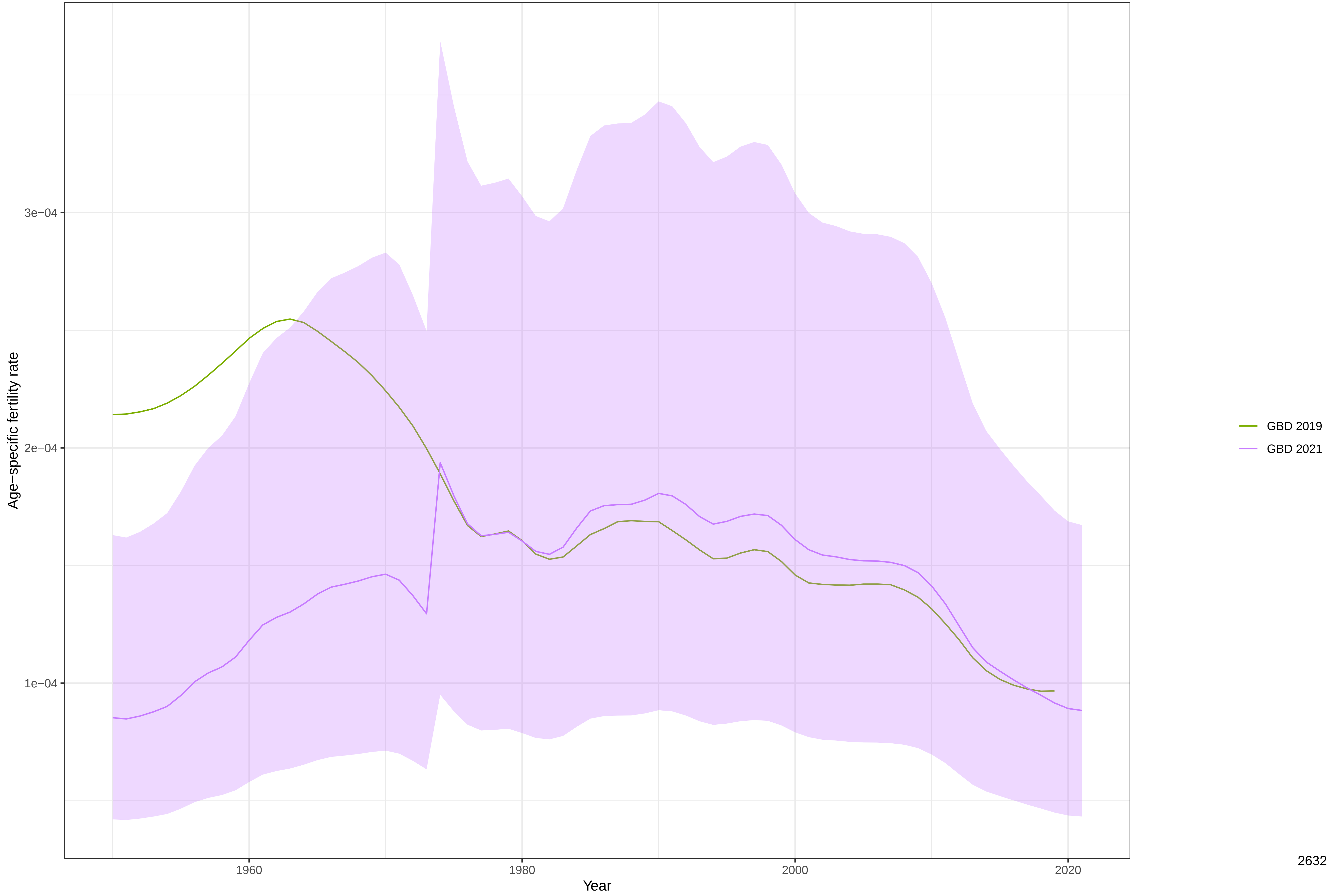

Age−specific fertility fitted trend lines, 1950−2021: South Tyneside, Ages: 15−19
Data points enclosed with a diamond are reference sources

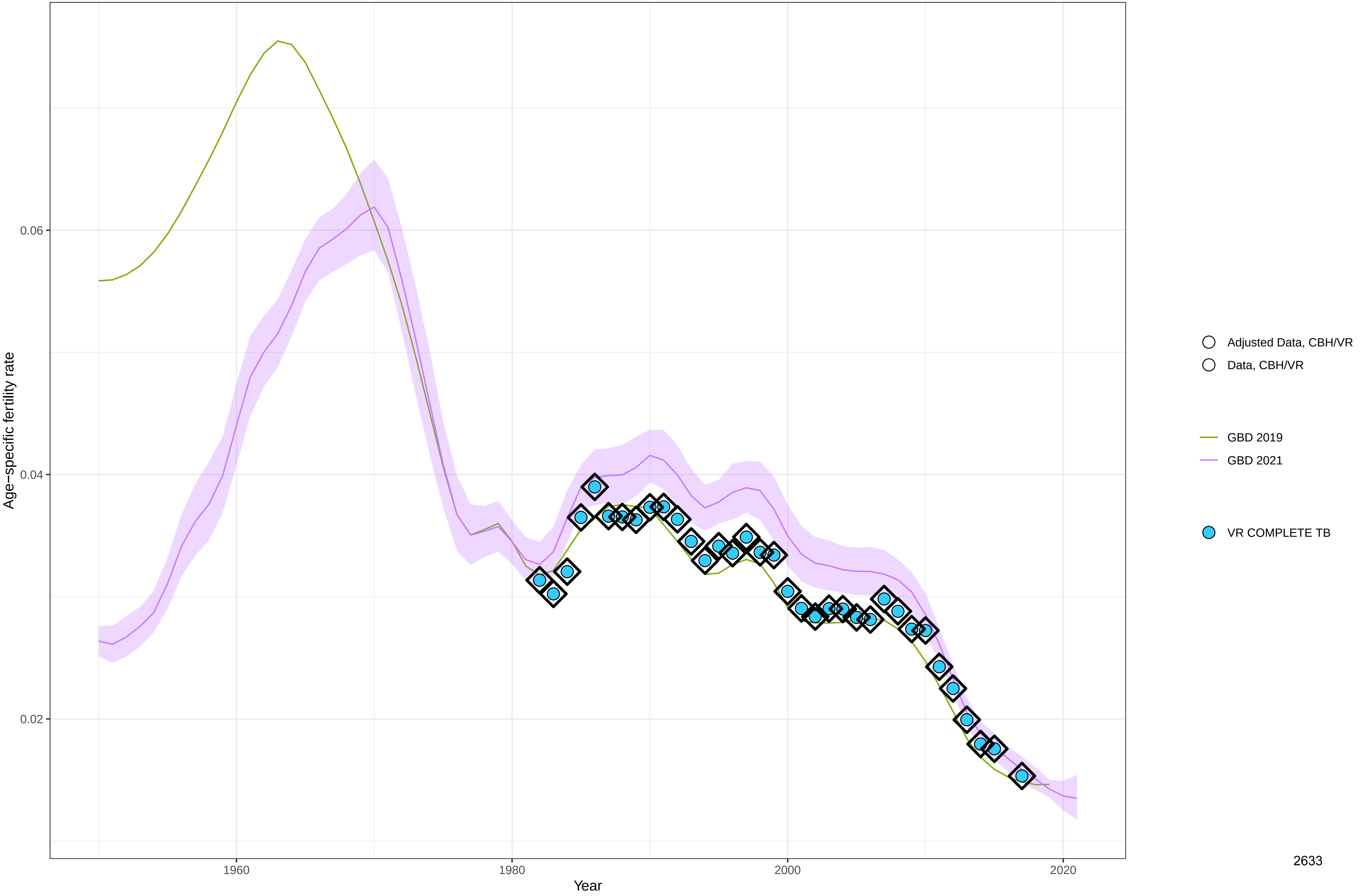

Age−specific fertility fitted trend lines, 1950−2021: South Tyneside, Ages: 20−24
Data points enclosed with a diamond are reference sources

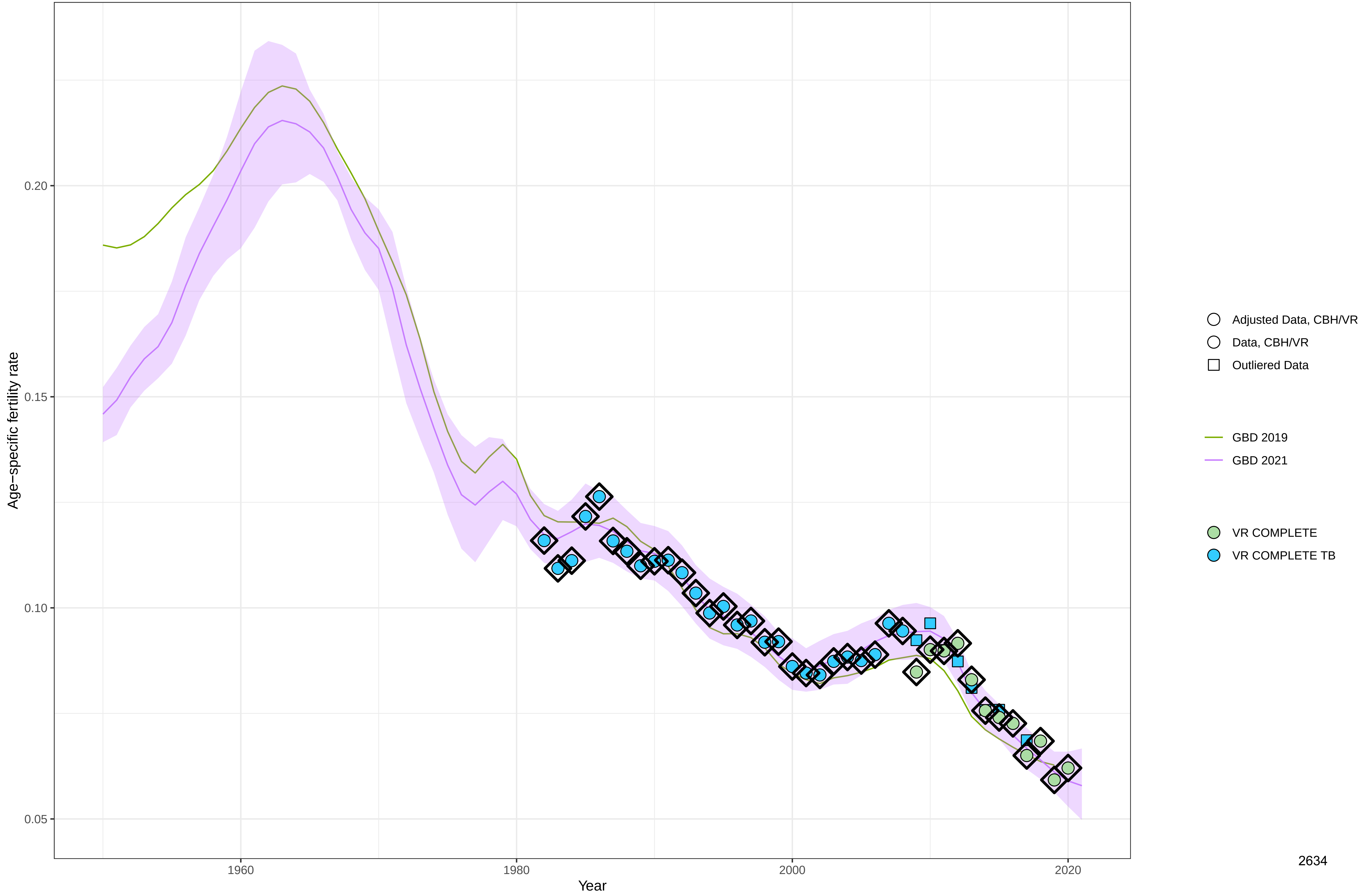

Age−specific fertility fitted trend lines, 1950−2021: South Tyneside, Ages: 25−29
Data points enclosed with a diamond are reference sources

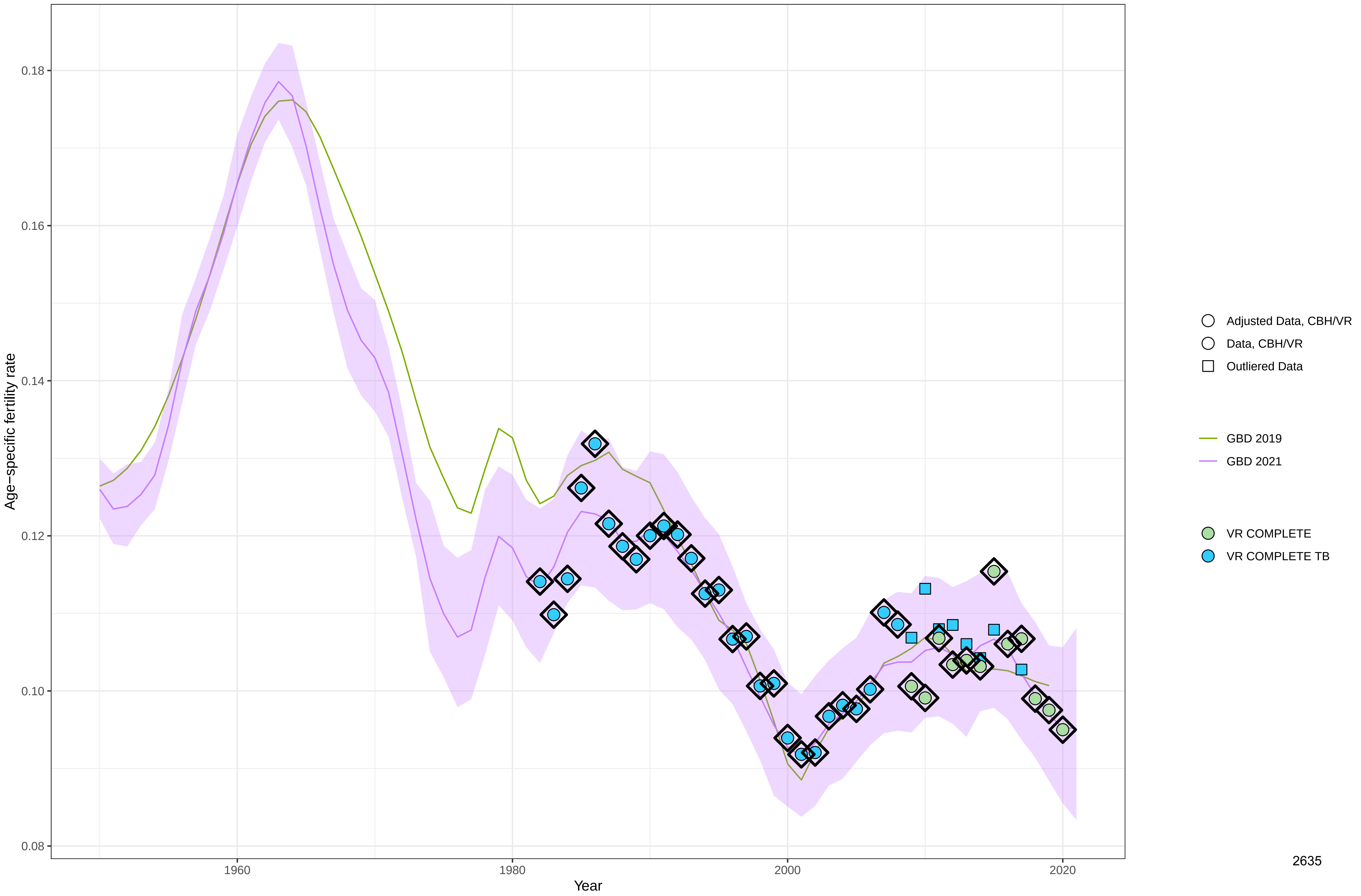

Age−specific fertility fitted trend lines, 1950−2021: South Tyneside, Ages: 30−34
Data points enclosed with a diamond are reference sources

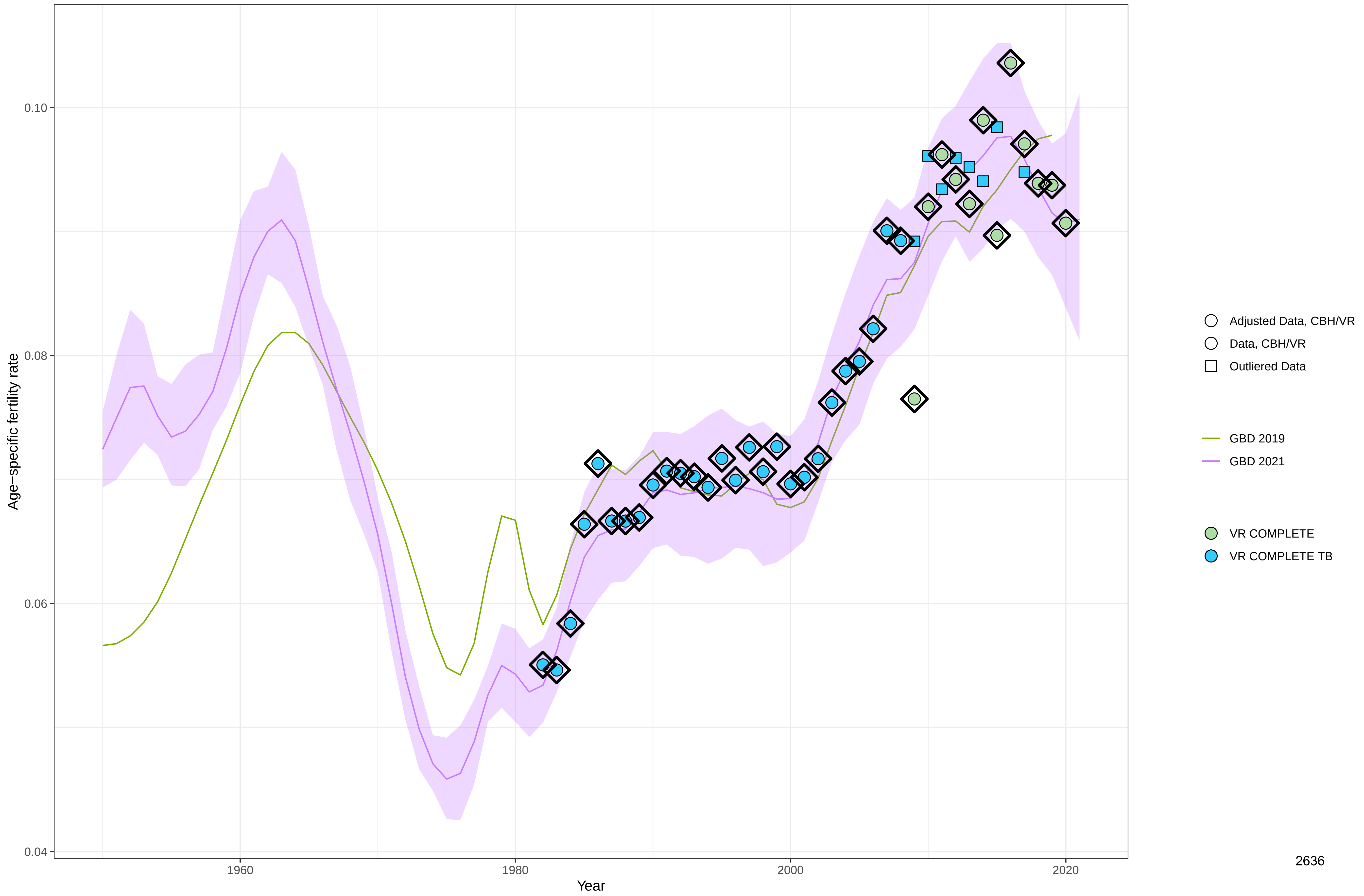

Age−specific fertility fitted trend lines, 1950−2021: South Tyneside, Ages: 35−39
Data points enclosed with a diamond are reference sources

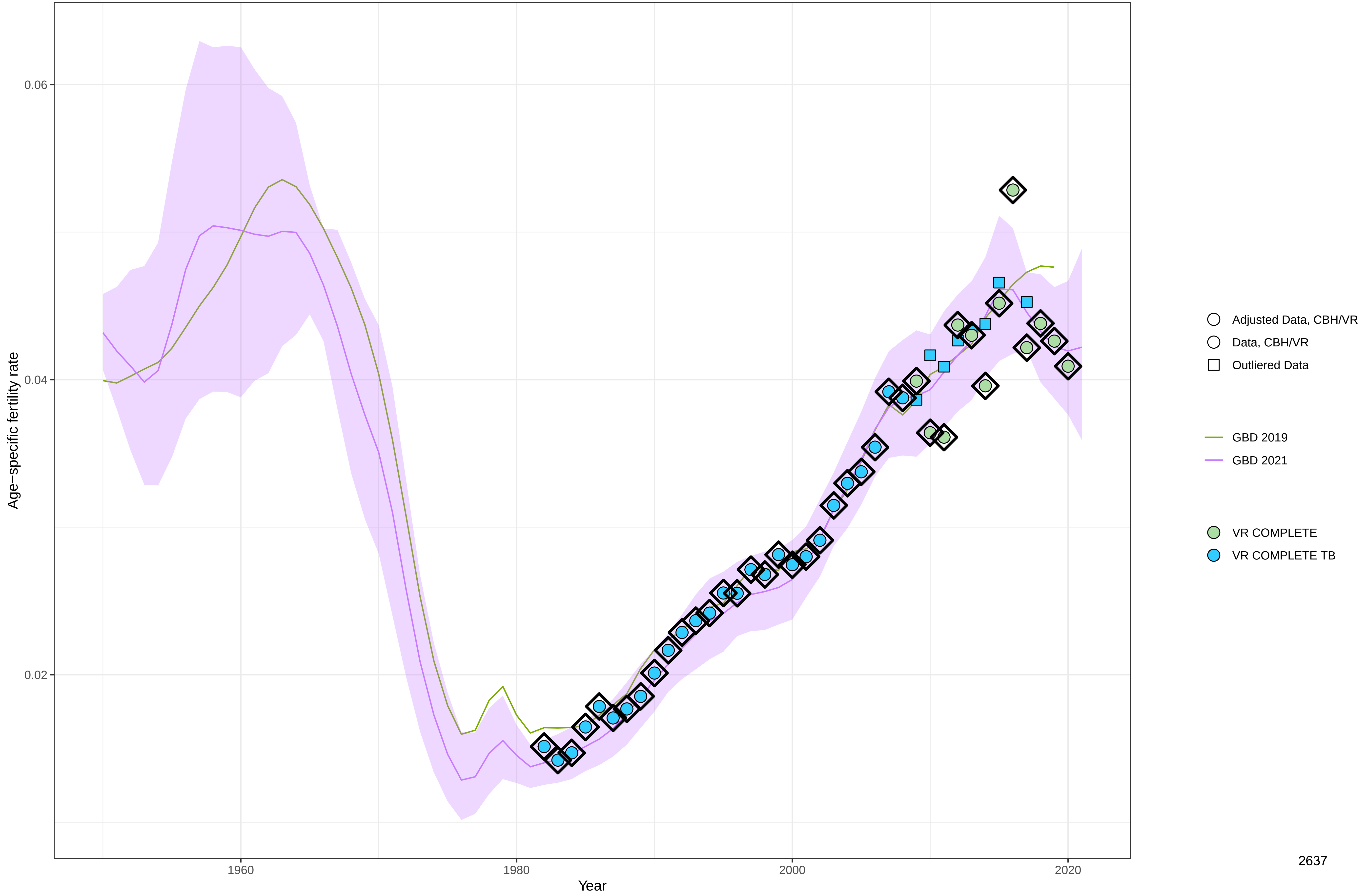

Age−specific fertility fitted trend lines, 1950−2021: South Tyneside, Ages: 40−44
Data points enclosed with a diamond are reference sources

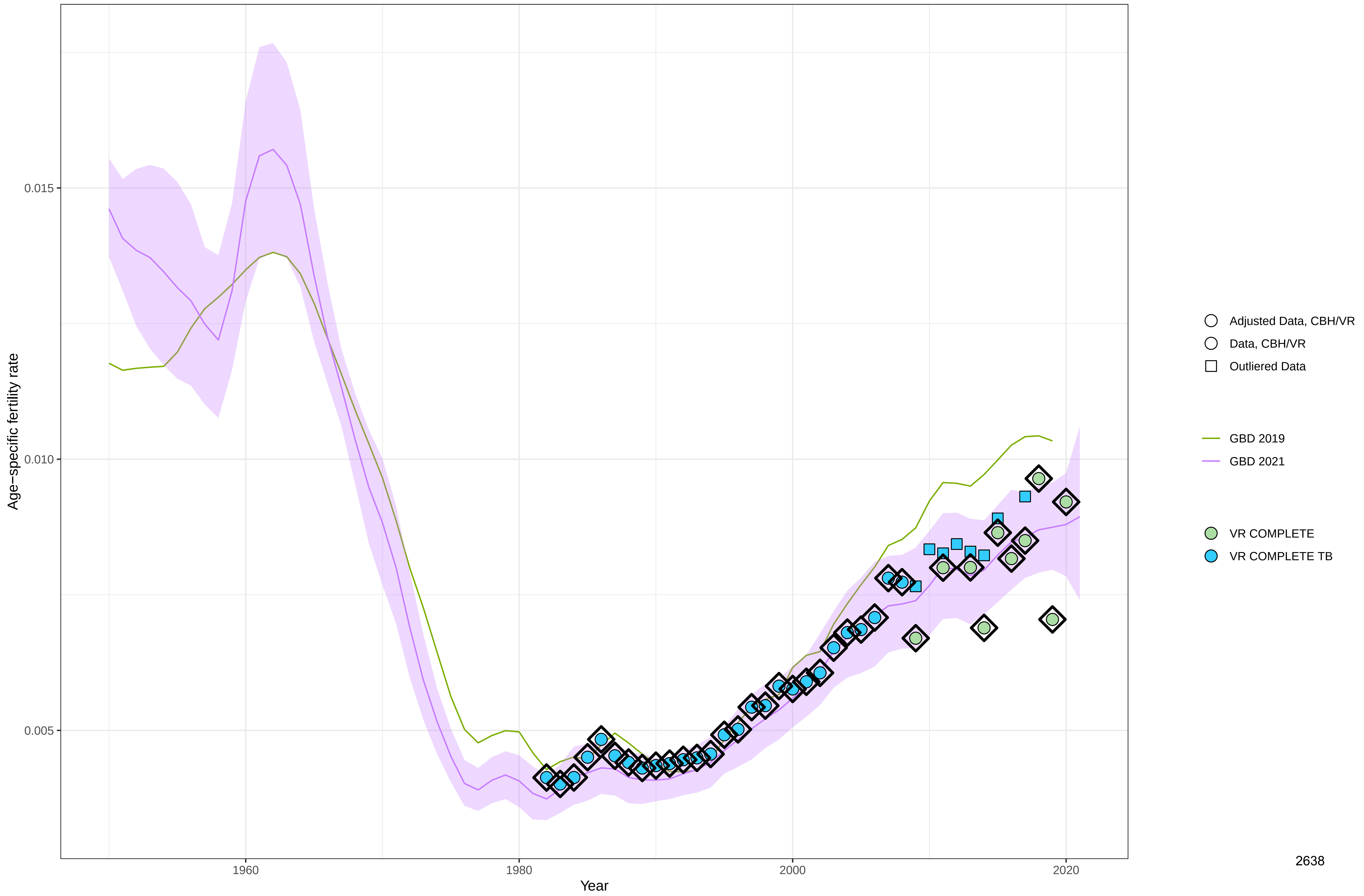

Age−specific fertility fitted trend lines, 1950−2021: South Tyneside, Ages: 45−49
Data points enclosed with a diamond are reference sources

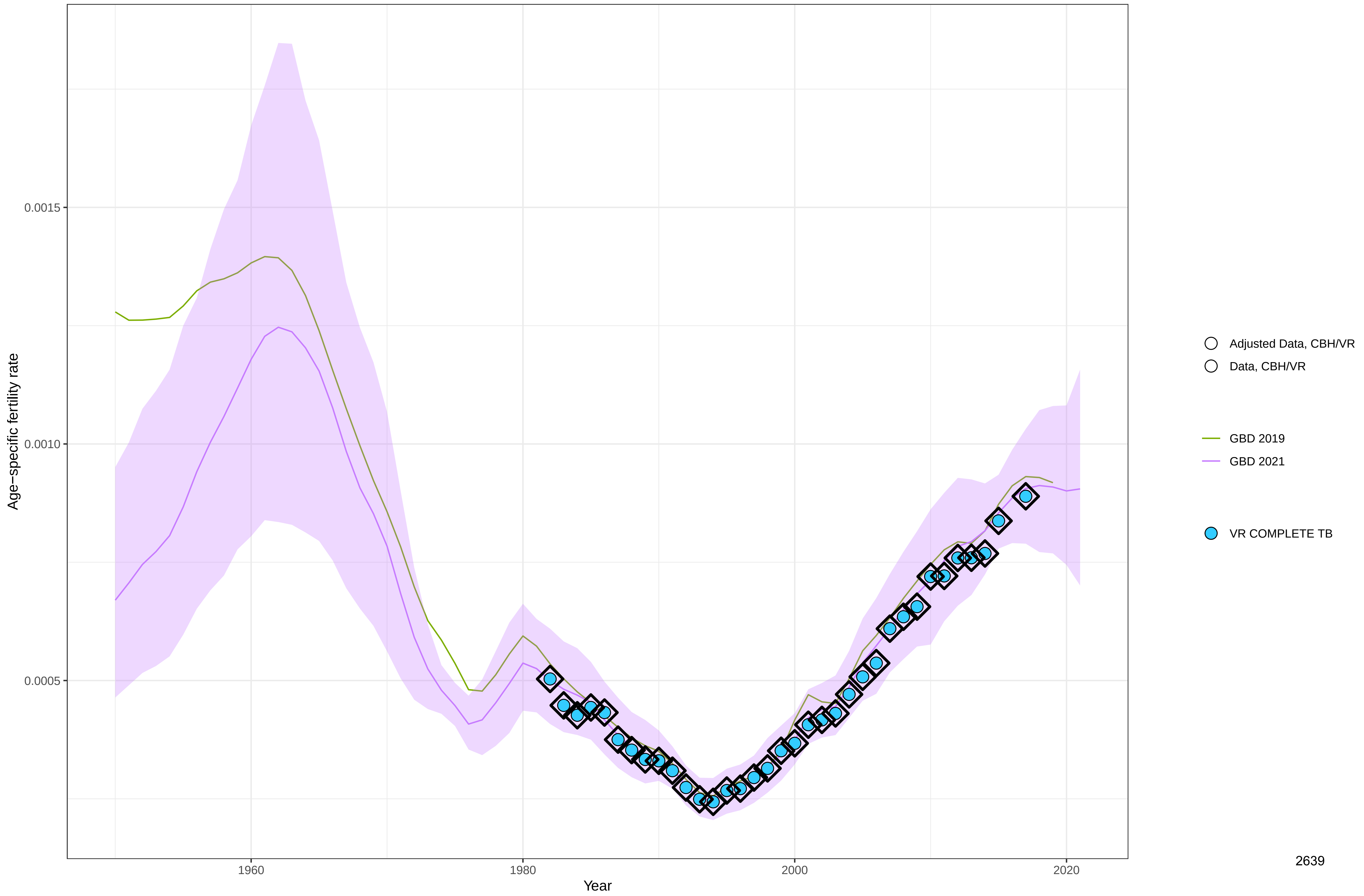

Age−specific fertility fitted trend lines, 1950−2021: South Tyneside, Ages: 50−54
Data points enclosed with a diamond are reference sources

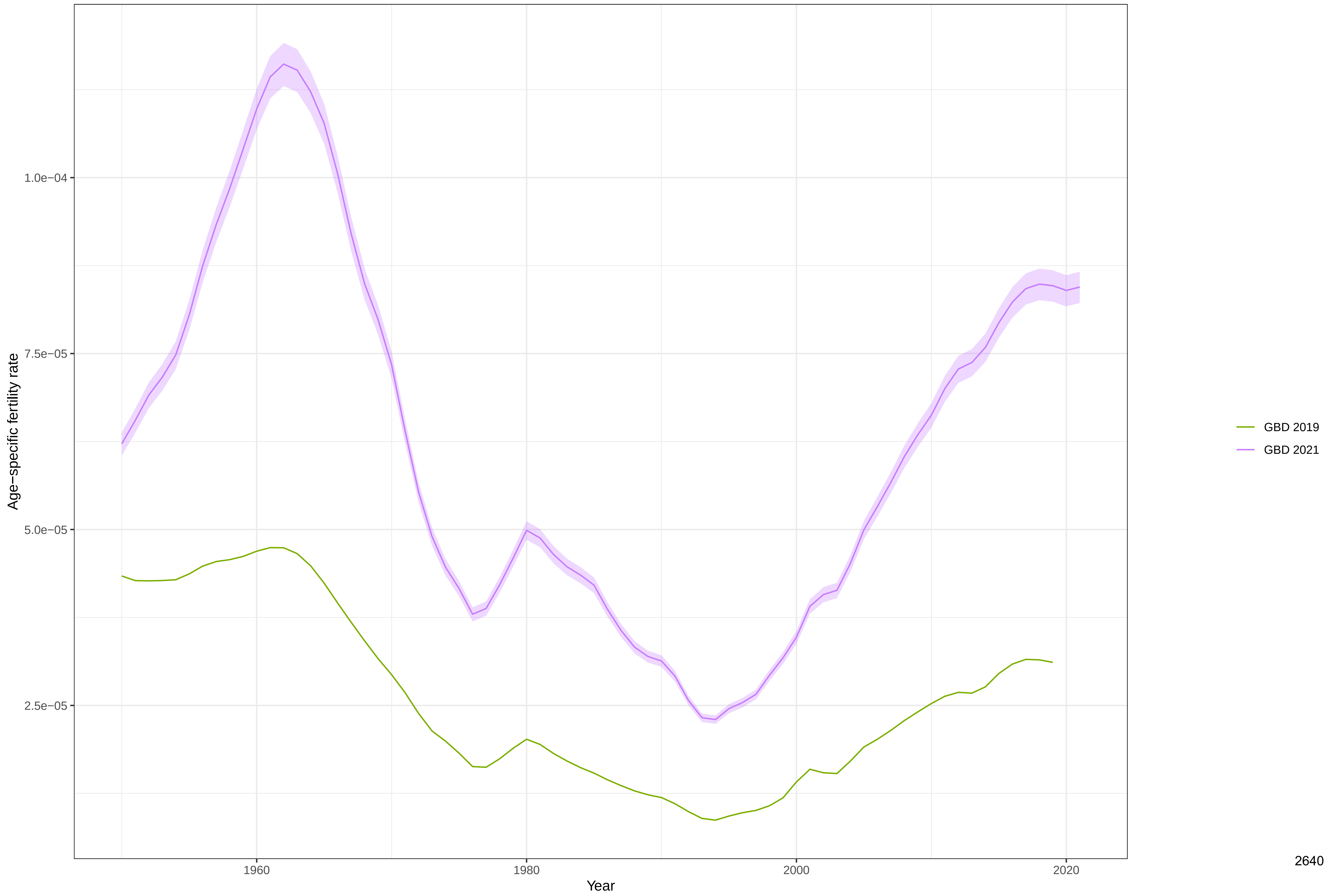

Total fertility rate fitted trend lines, 1950−2021: South Tyneside TFR
Data points enclosed with a diamond are reference sources

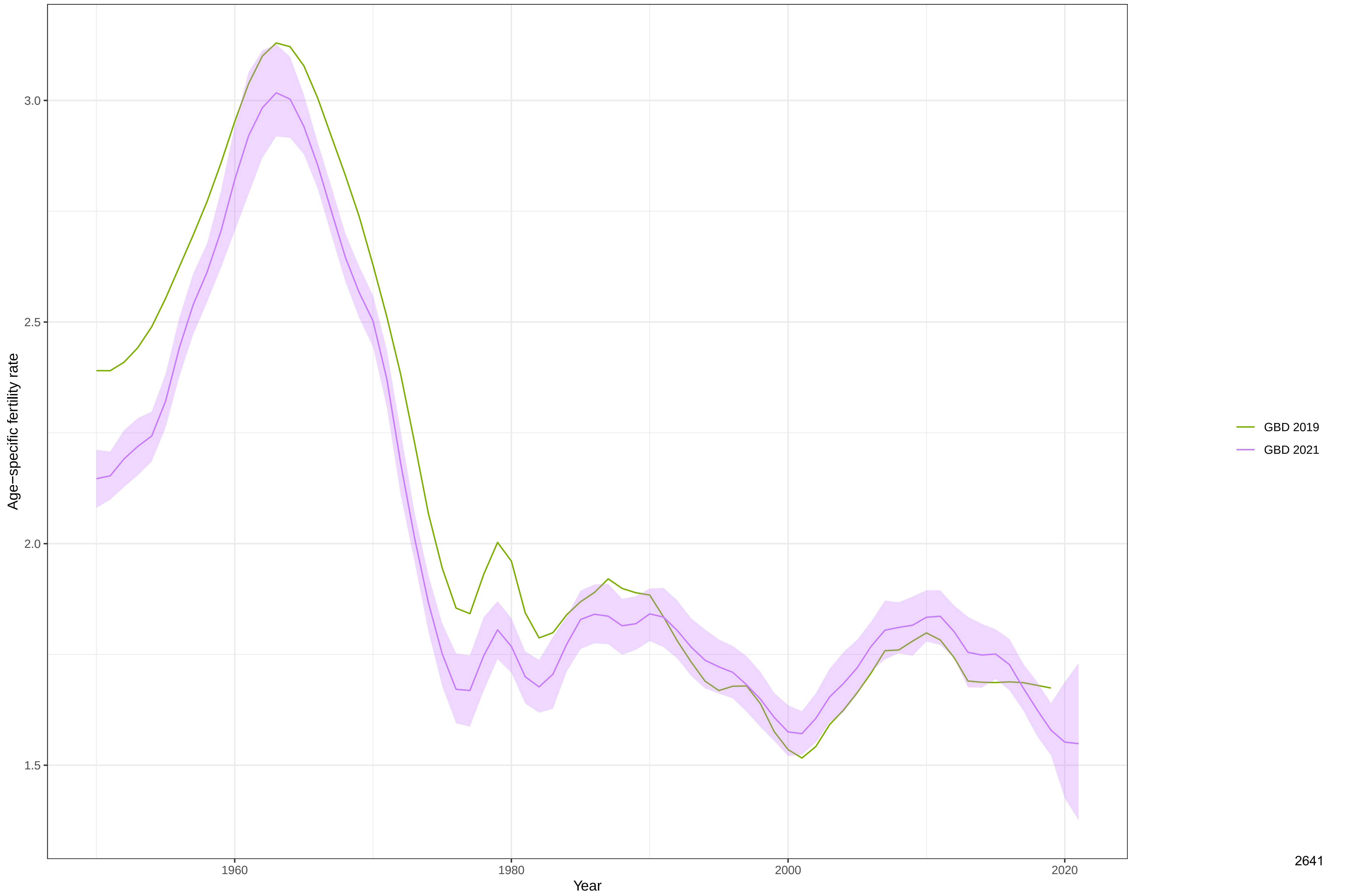

Age−specific fertility fitted trend lines, 1950−2021: Stockton−on−Tees, Ages: 10−14
Data points enclosed with a diamond are reference sources

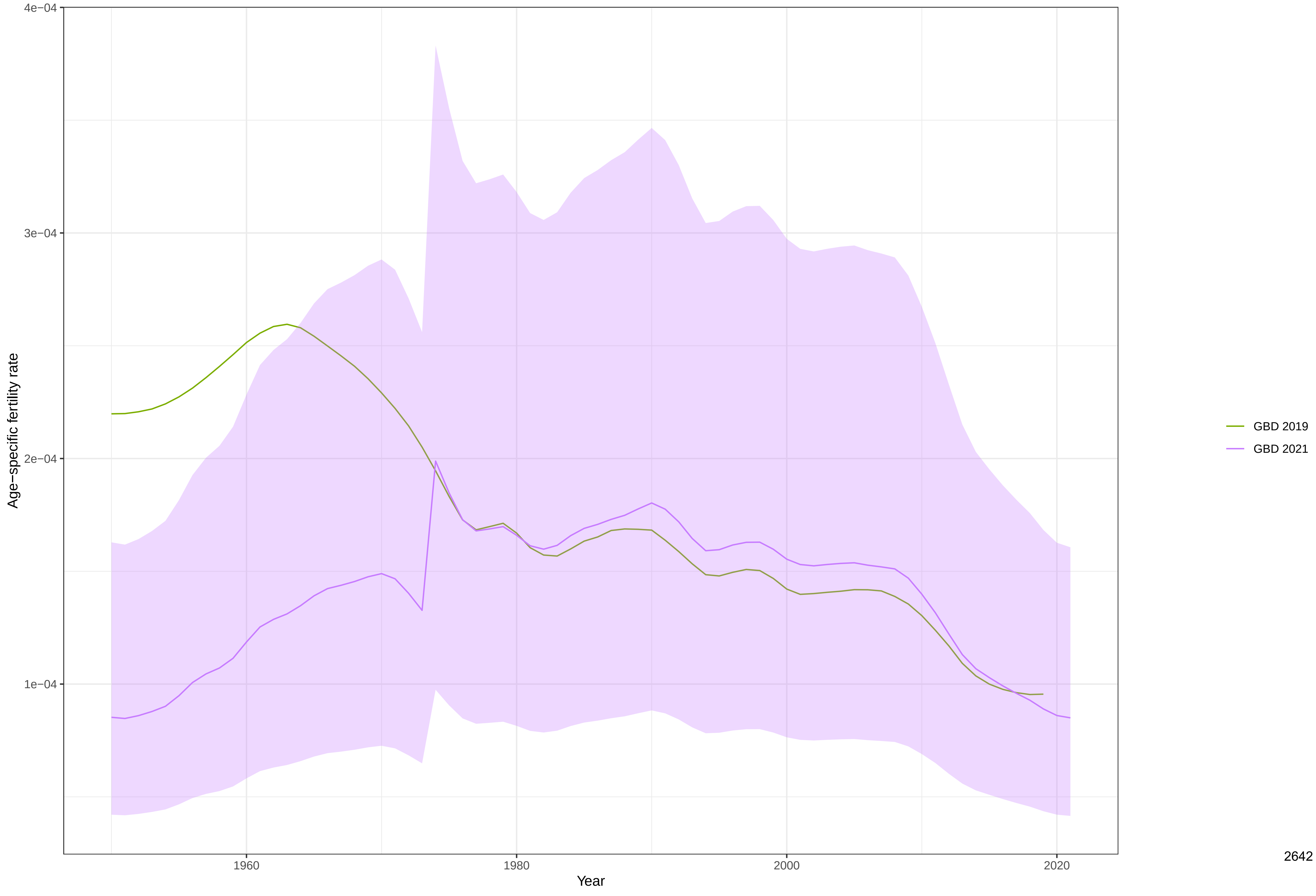

2642

Age−specific fertility fitted trend lines, 1950−2021: Stockton−on−Tees, Ages: 15−19
Data points enclosed with a diamond are reference sources

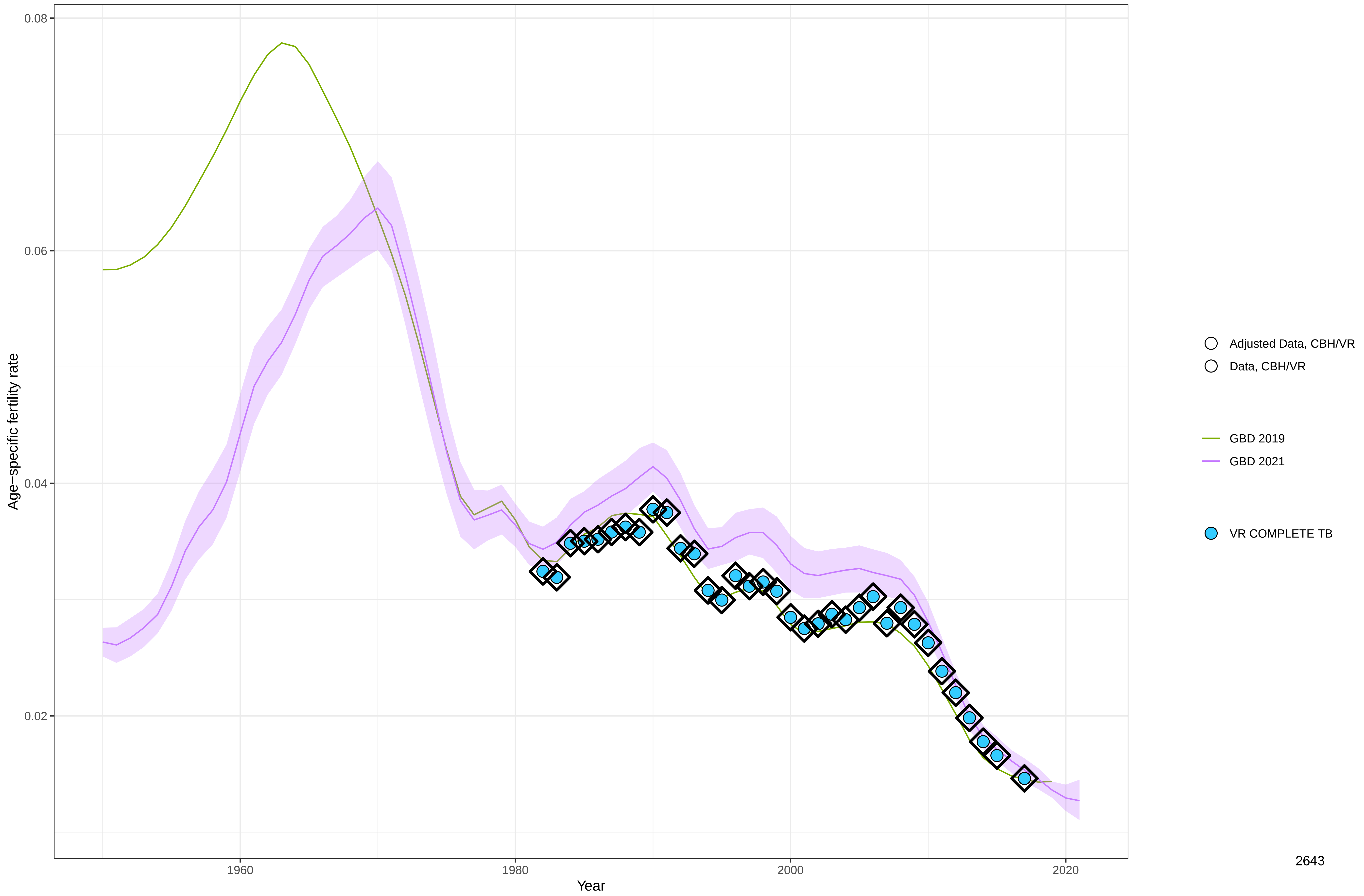

Age−specific fertility fitted trend lines, 1950−2021: Stockton−on−Tees, Ages: 20−24
Data points enclosed with a diamond are reference sources

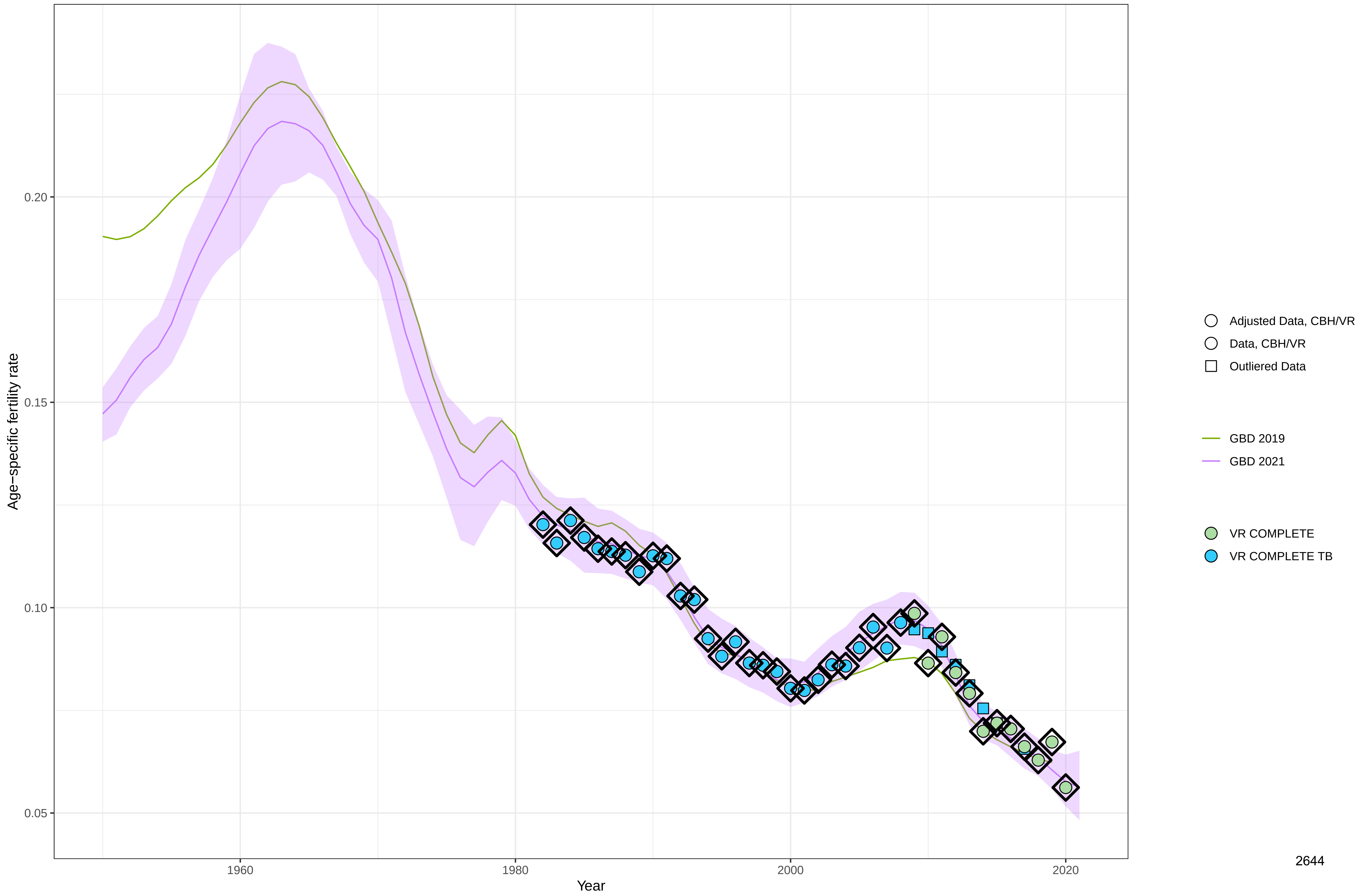

Age−specific fertility fitted trend lines, 1950−2021: Stockton−on−Tees, Ages: 25−29
Data points enclosed with a diamond are reference sources

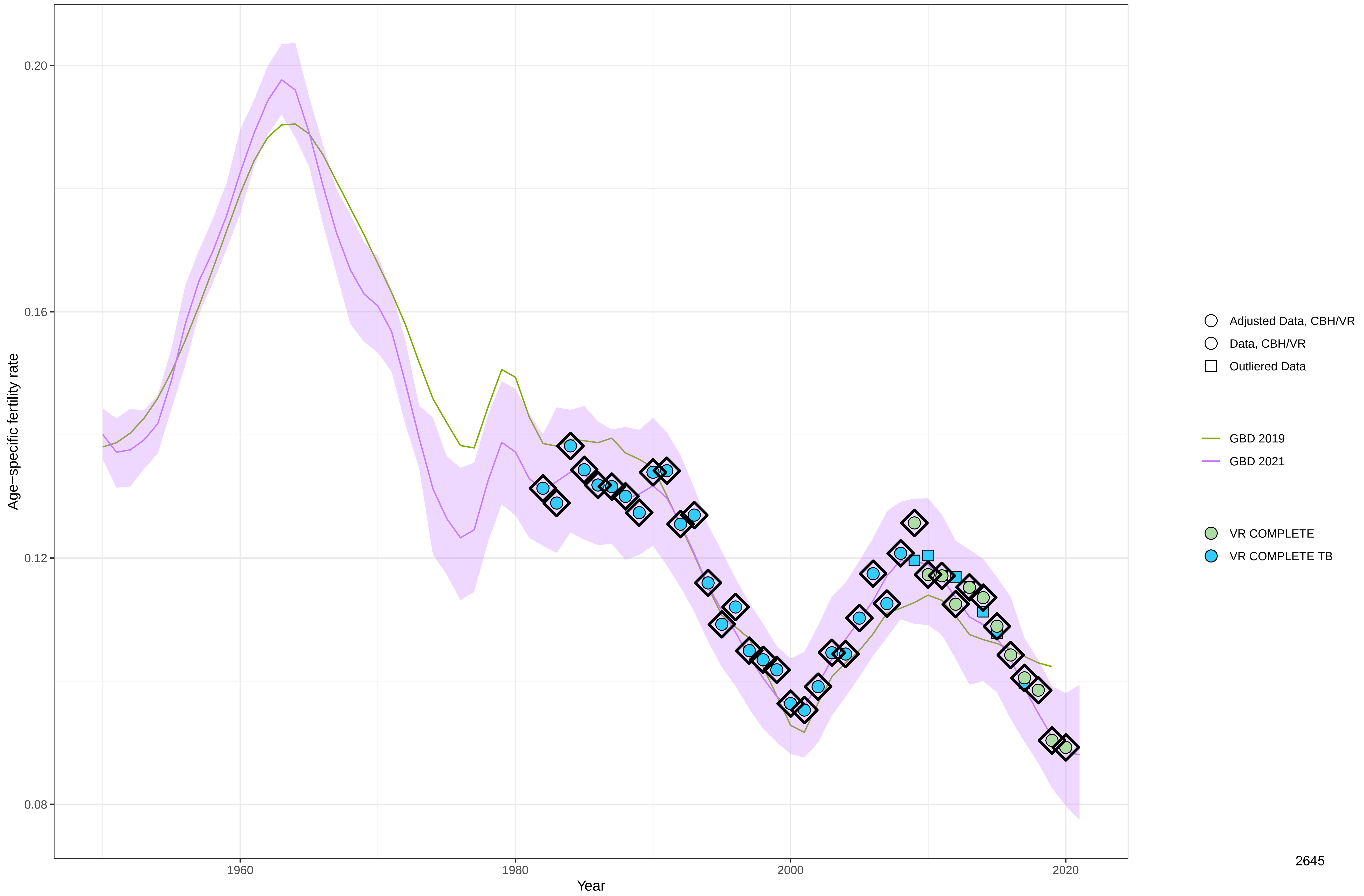

Age−specific fertility fitted trend lines, 1950−2021: Stockton−on−Tees, Ages: 30−34
Data points enclosed with a diamond are reference sources

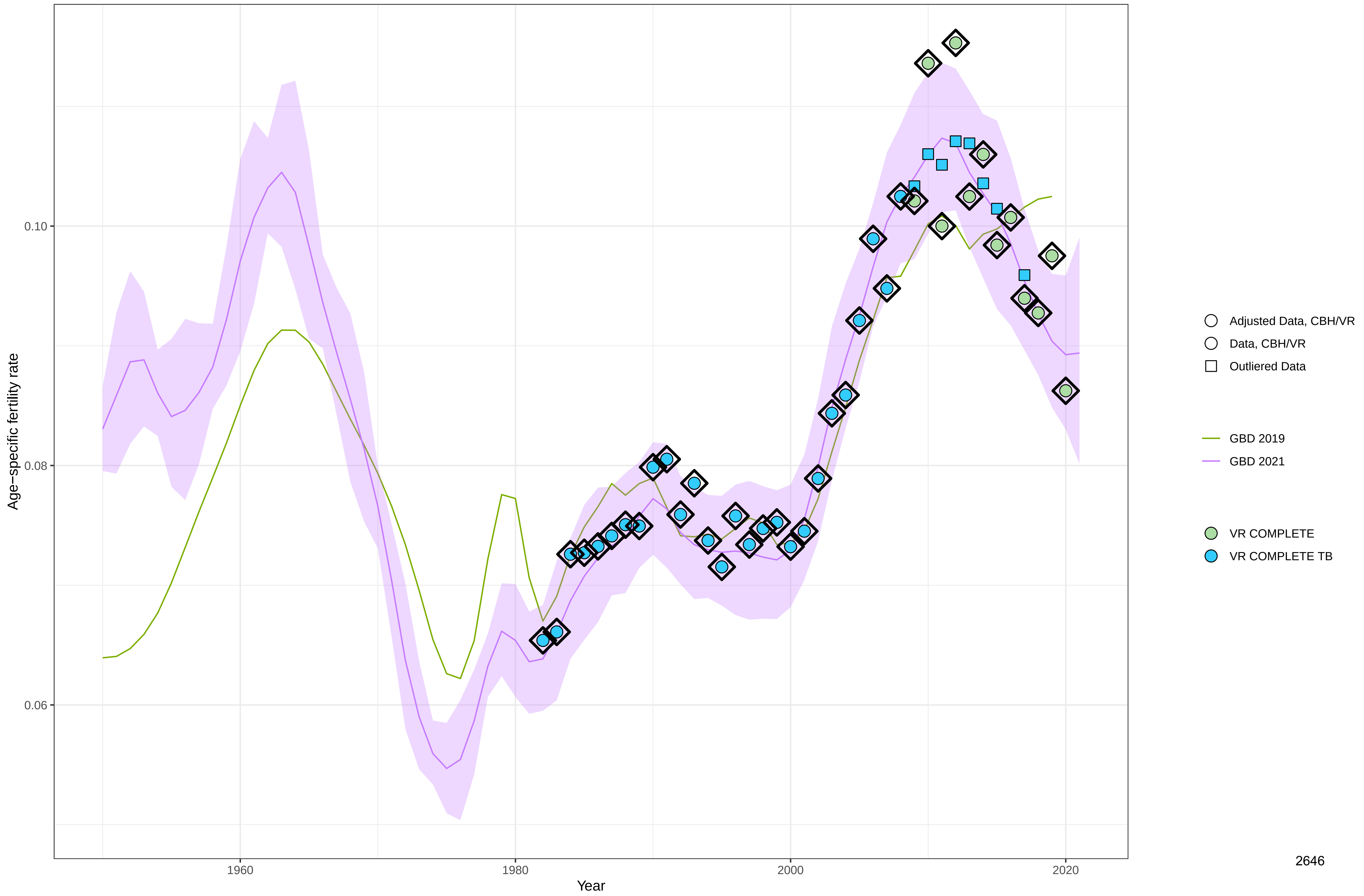

Age−specific fertility fitted trend lines, 1950−2021: Stockton−on−Tees, Ages: 35−39
Data points enclosed with a diamond are reference sources

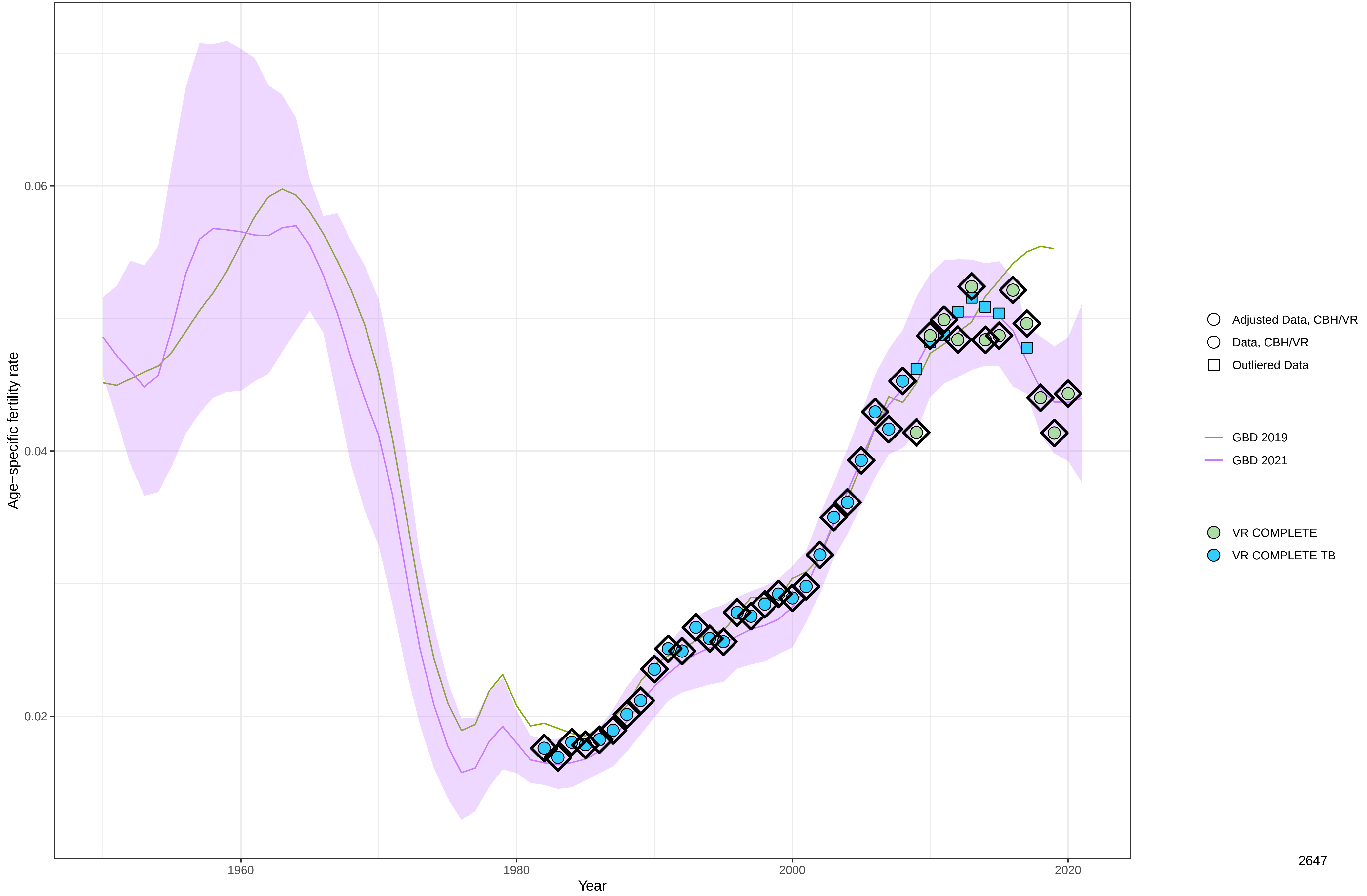

Age−specific fertility fitted trend lines, 1950−2021: Stockton−on−Tees, Ages: 40−44
Data points enclosed with a diamond are reference sources

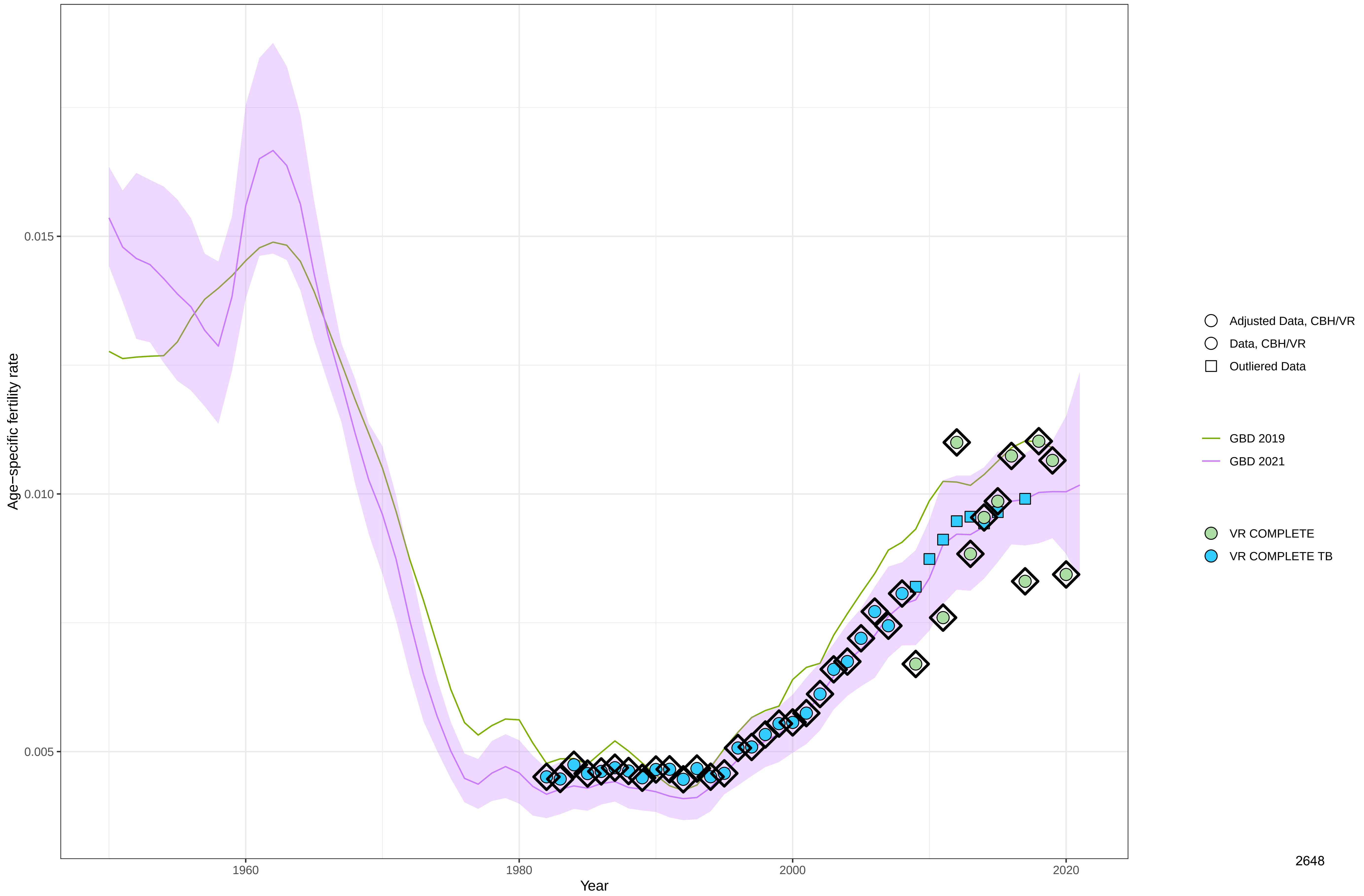

Age−specific fertility fitted trend lines, 1950−2021: Stockton−on−Tees, Ages: 45−49
Data points enclosed with a diamond are reference sources

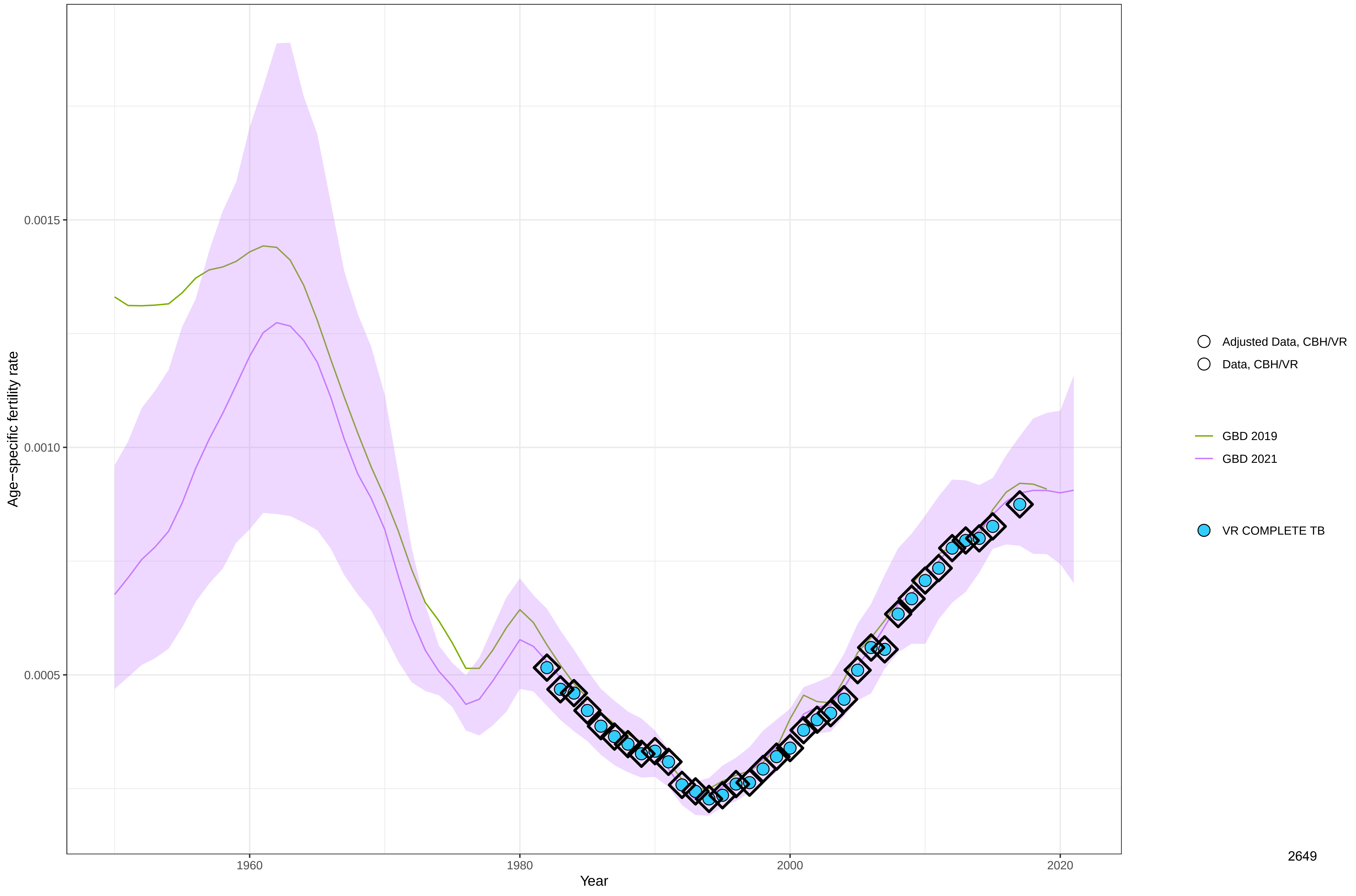

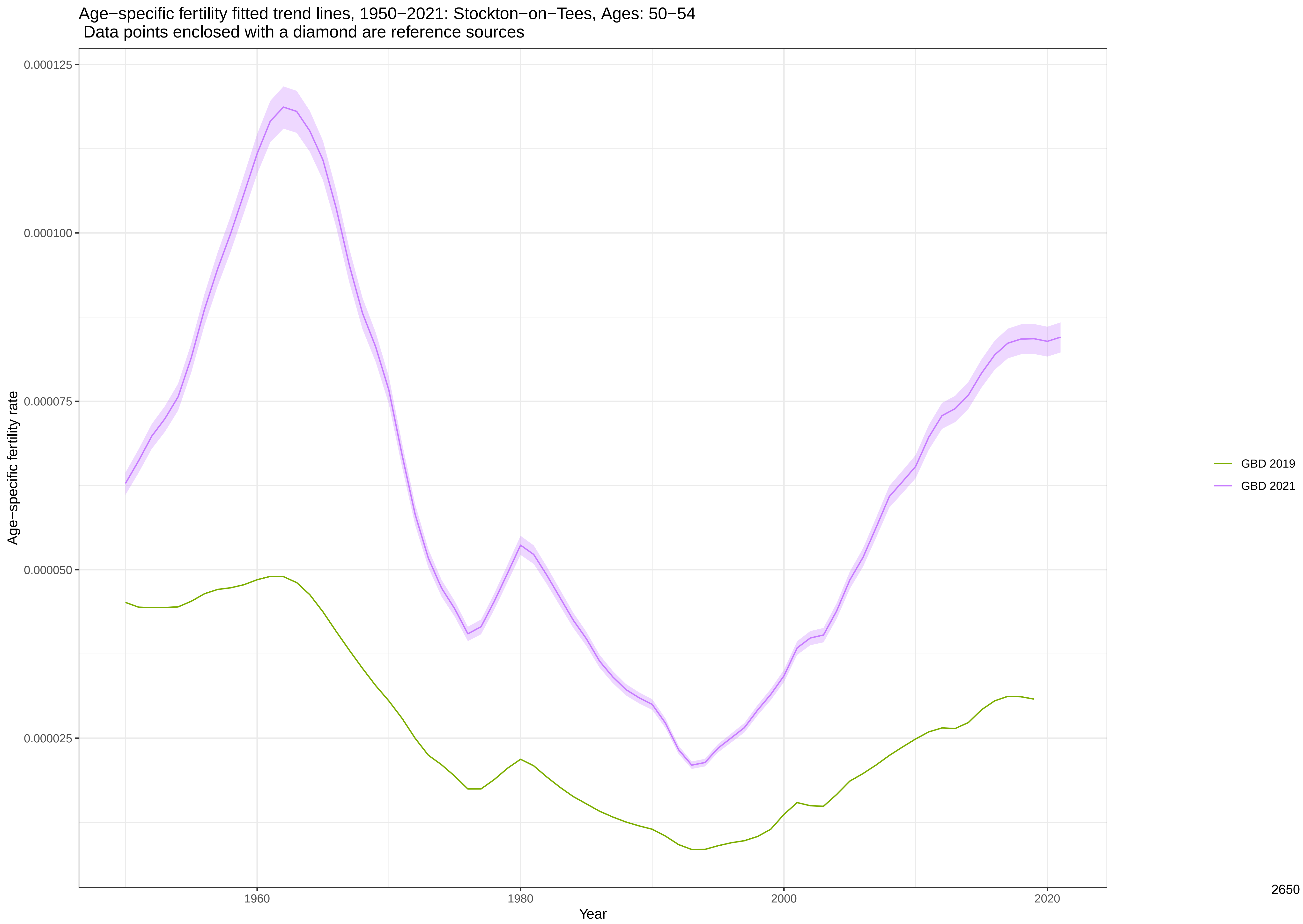
Age−specific fertility fitted trend lines, 1950−2021: Stockton−on−Tees, Ages: 50−54
Data points enclosed with a diamond are reference sources
Age−specific fertility rate
0.000125
0.000100
0.000075
0.000050
0.000025
1960
1980
2000
2020
Year
GBD 2019
GBD 2021

Total fertility rate fitted trend lines, 1950−2021: Stockton−on−Tees TFR
Data points enclosed with a diamond are reference sources

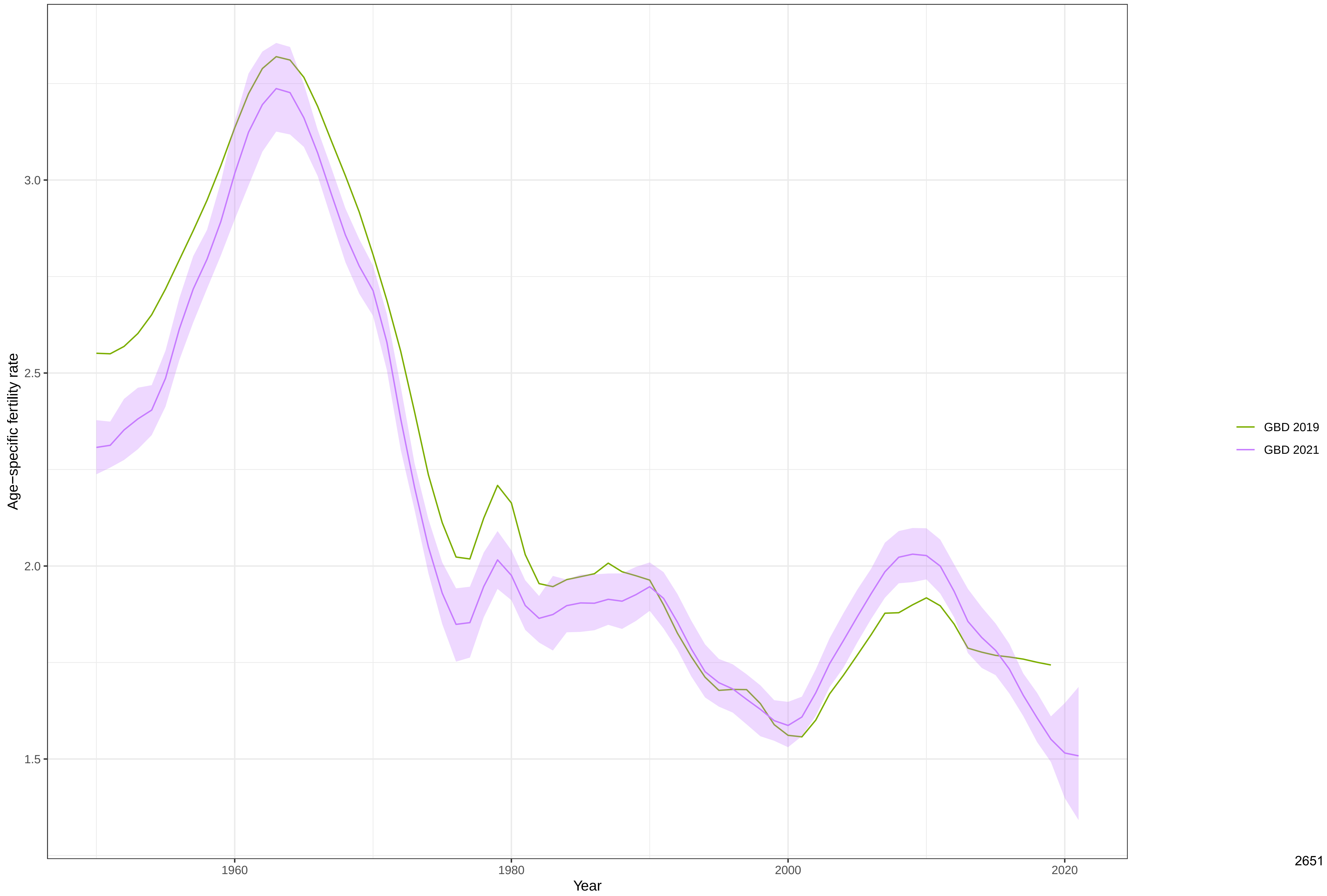

Age−specific fertility fitted trend lines, 1950−2021: Sunderland, Ages: 10−14
Data points enclosed with a diamond are reference sources

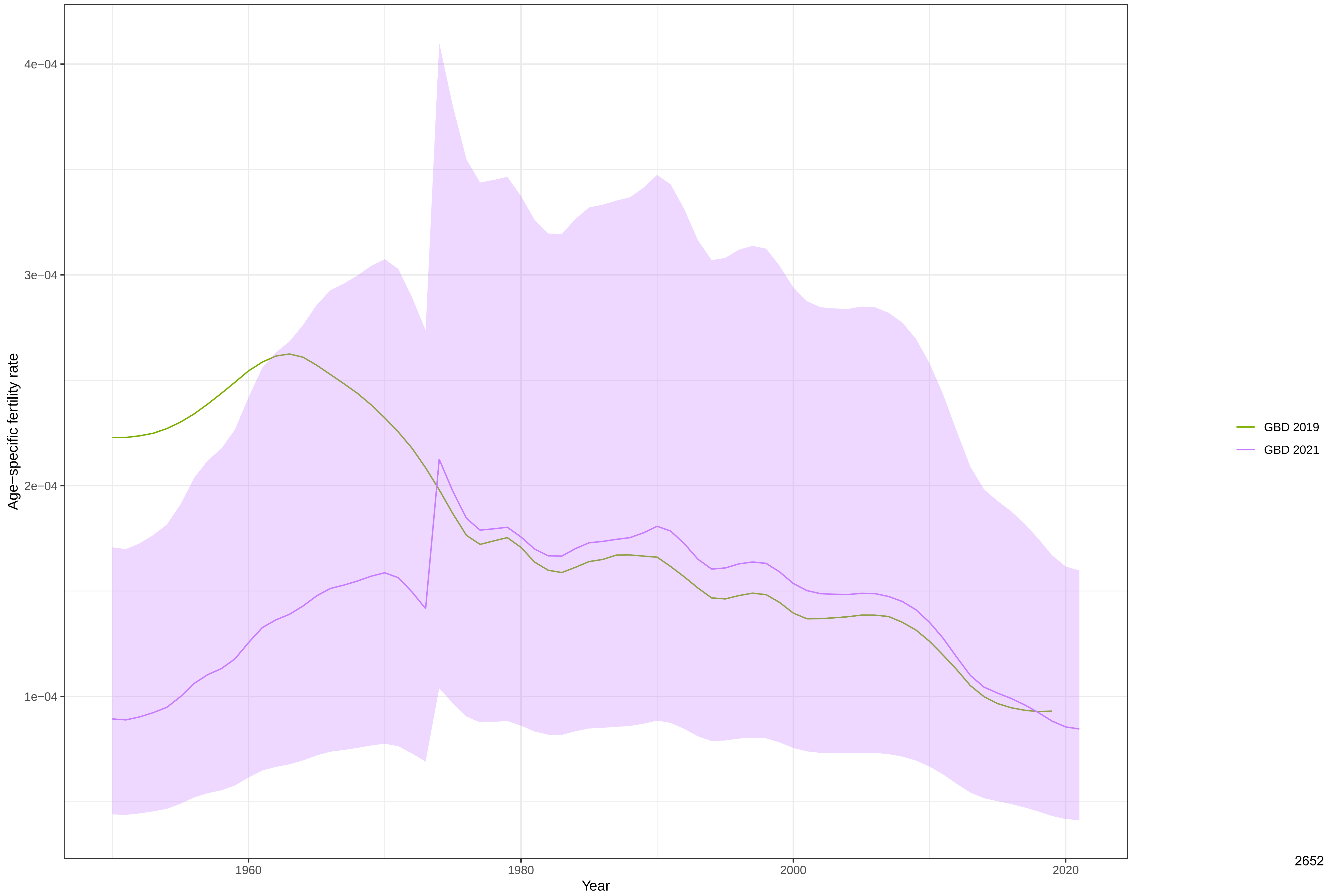

Age−specific fertility fitted trend lines, 1950−2021: Sunderland, Ages: 15−19
Data points enclosed with a diamond are reference sources

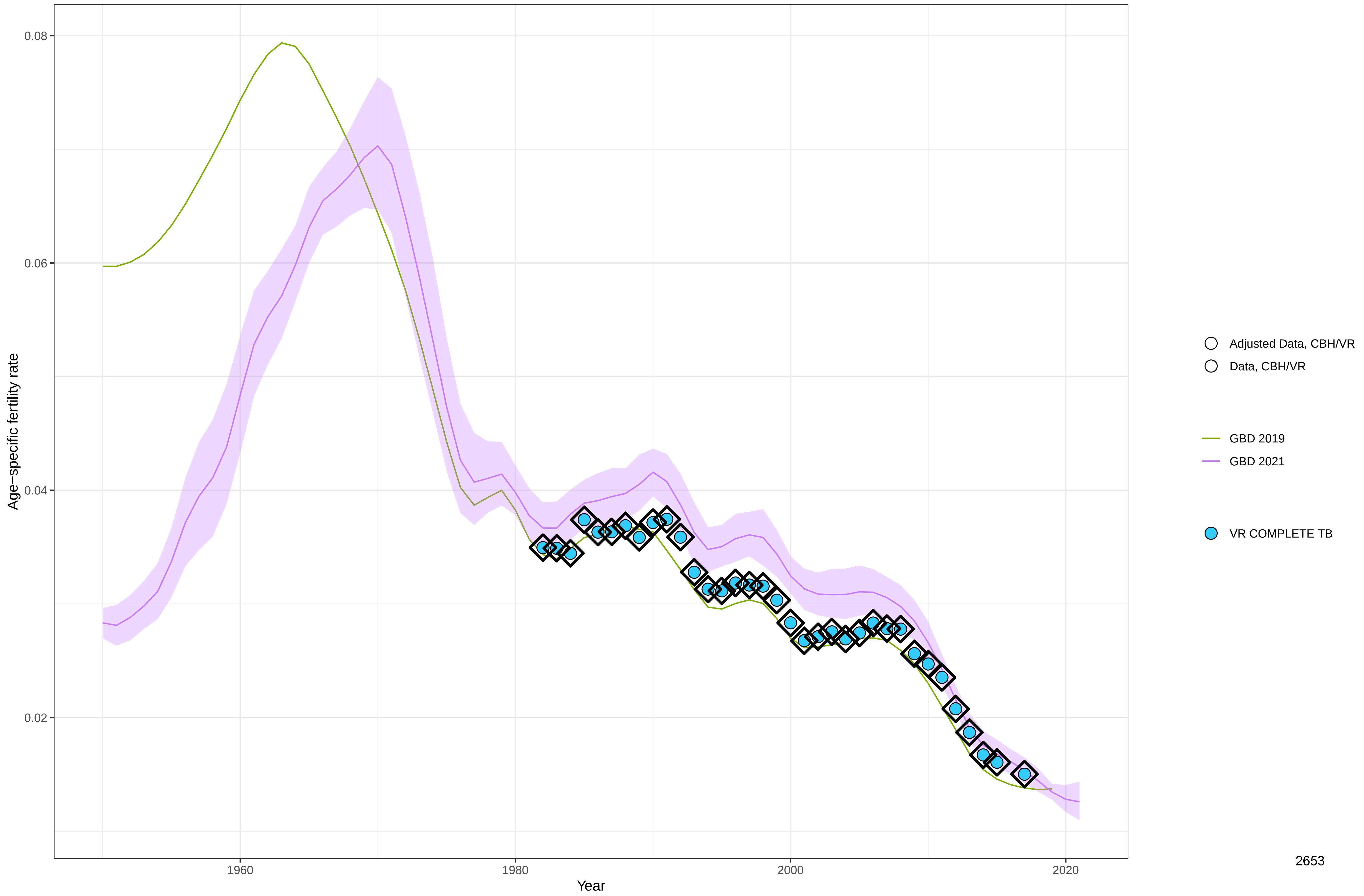

Age−specific fertility fitted trend lines, 1950−2021: Sunderland, Ages: 20−24
Data points enclosed with a diamond are reference sources

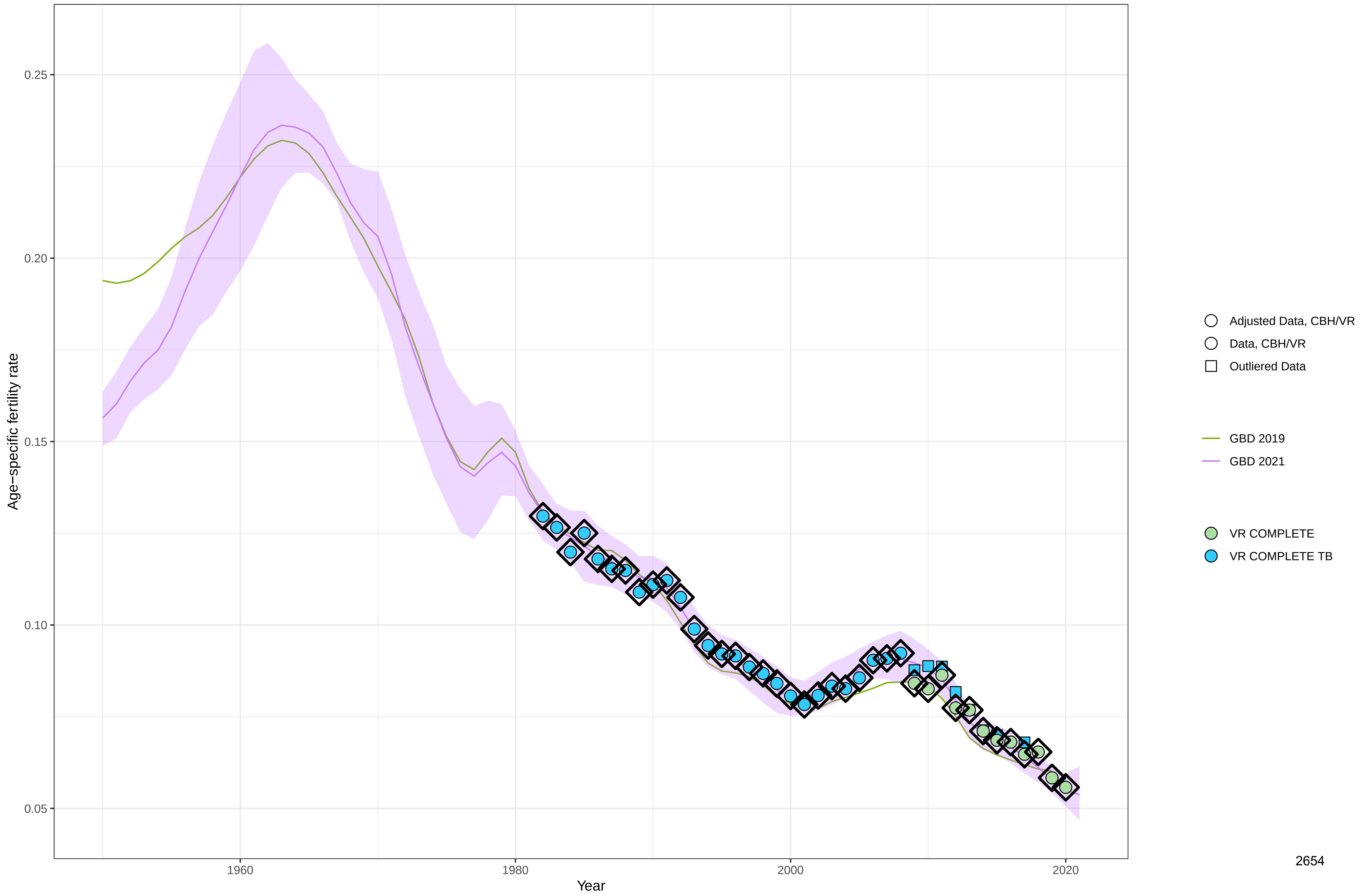

Age−specific fertility fitted trend lines, 1950−2021: Sunderland, Ages: 25−29
Data points enclosed with a diamond are reference sources

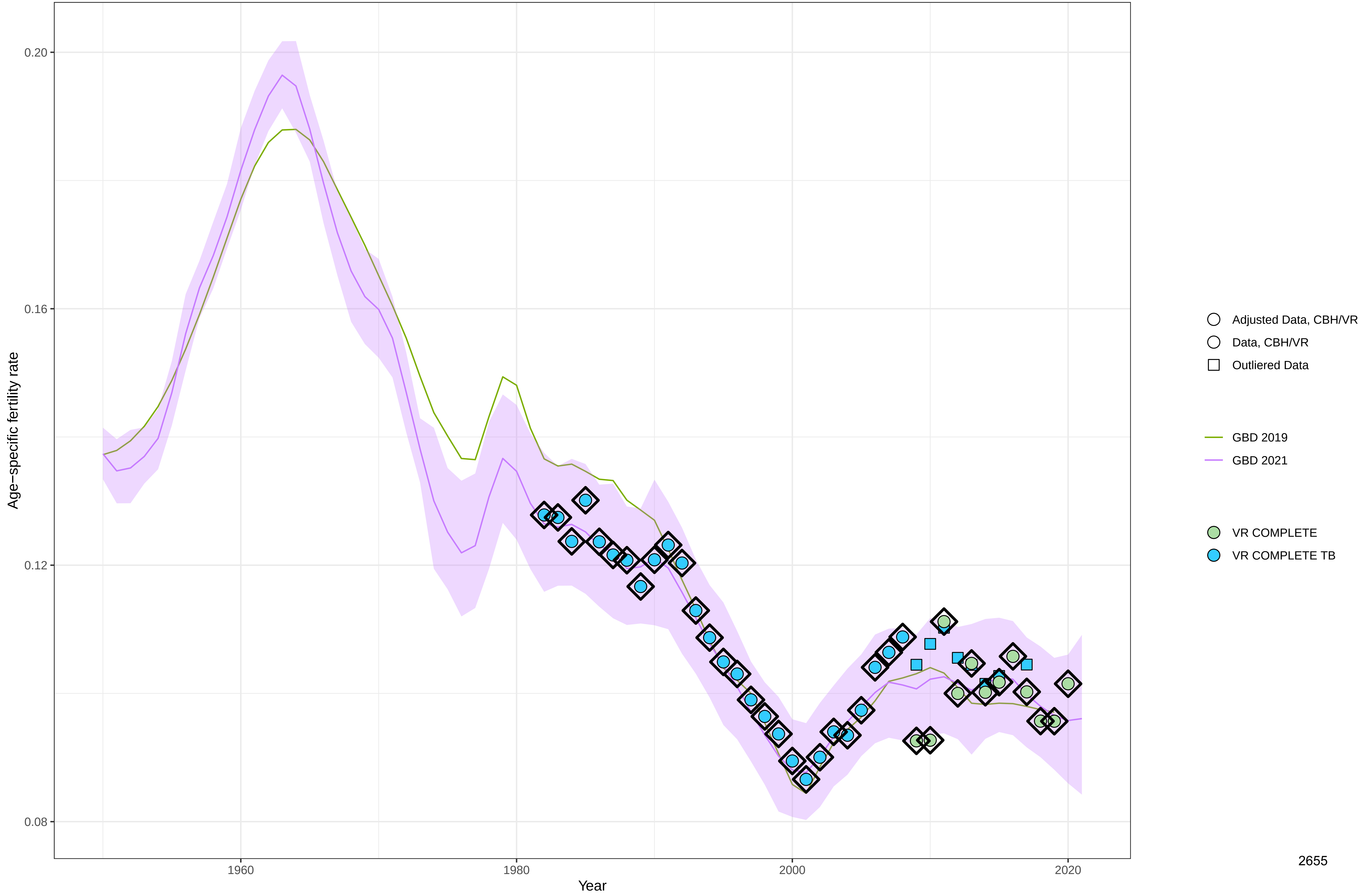

Age−specific fertility fitted trend lines, 1950−2021: Sunderland, Ages: 30−34
Data points enclosed with a diamond are reference sources

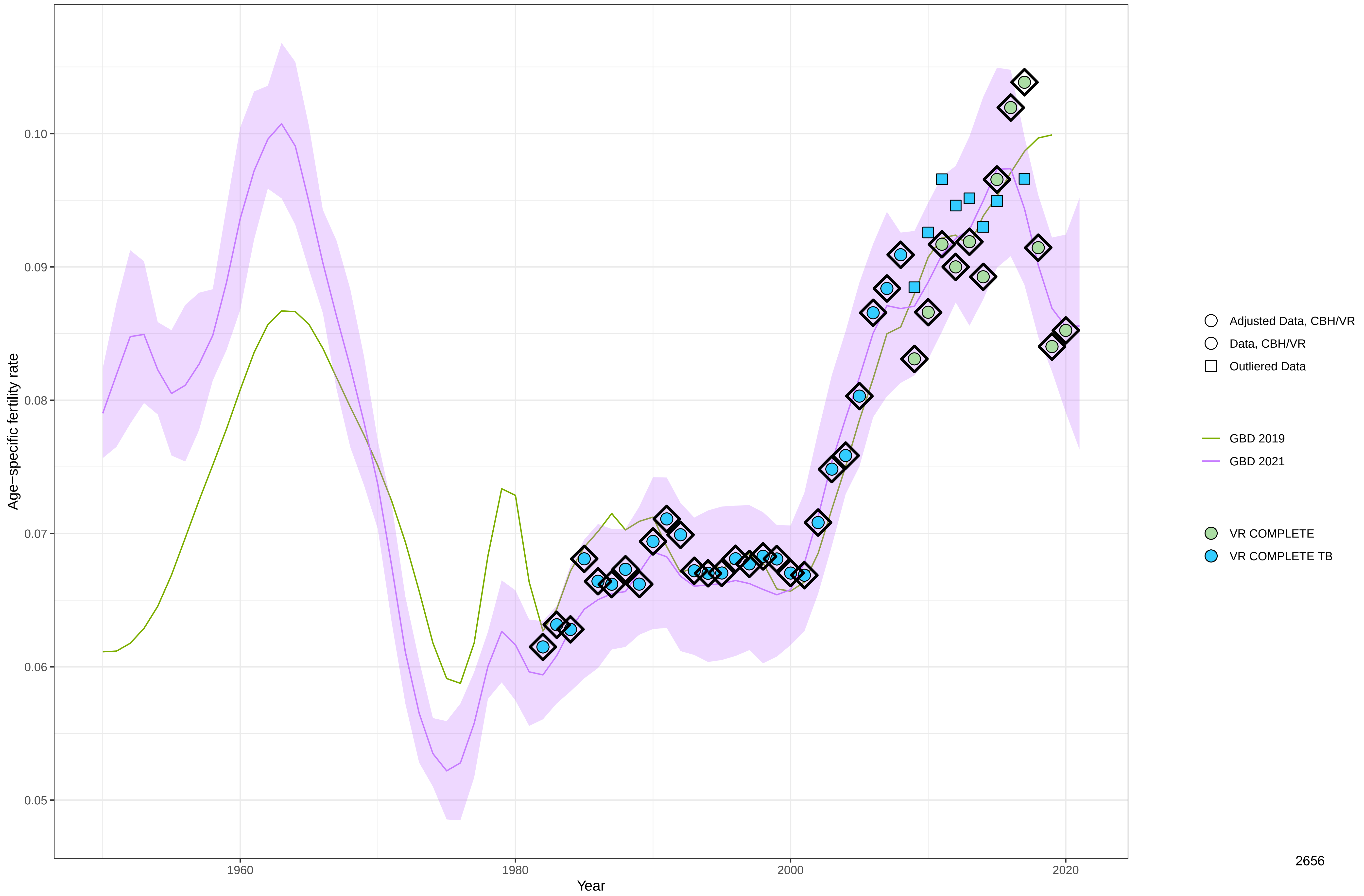

Age−specific fertility fitted trend lines, 1950−2021: Sunderland, Ages: 35−39
Data points enclosed with a diamond are reference sources

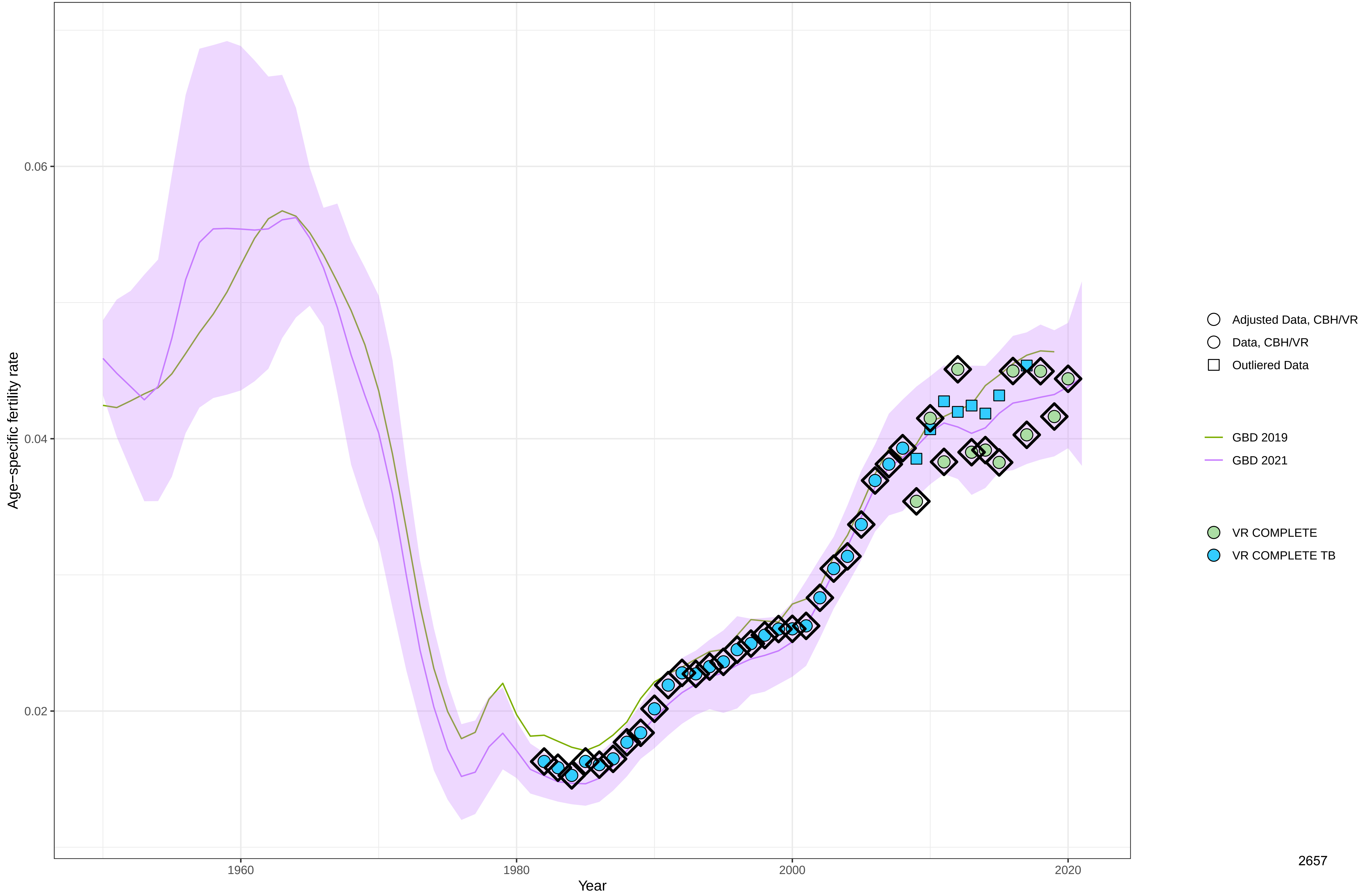

Age−specific fertility fitted trend lines, 1950−2021: Sunderland, Ages: 40−44
Data points enclosed with a diamond are reference sources

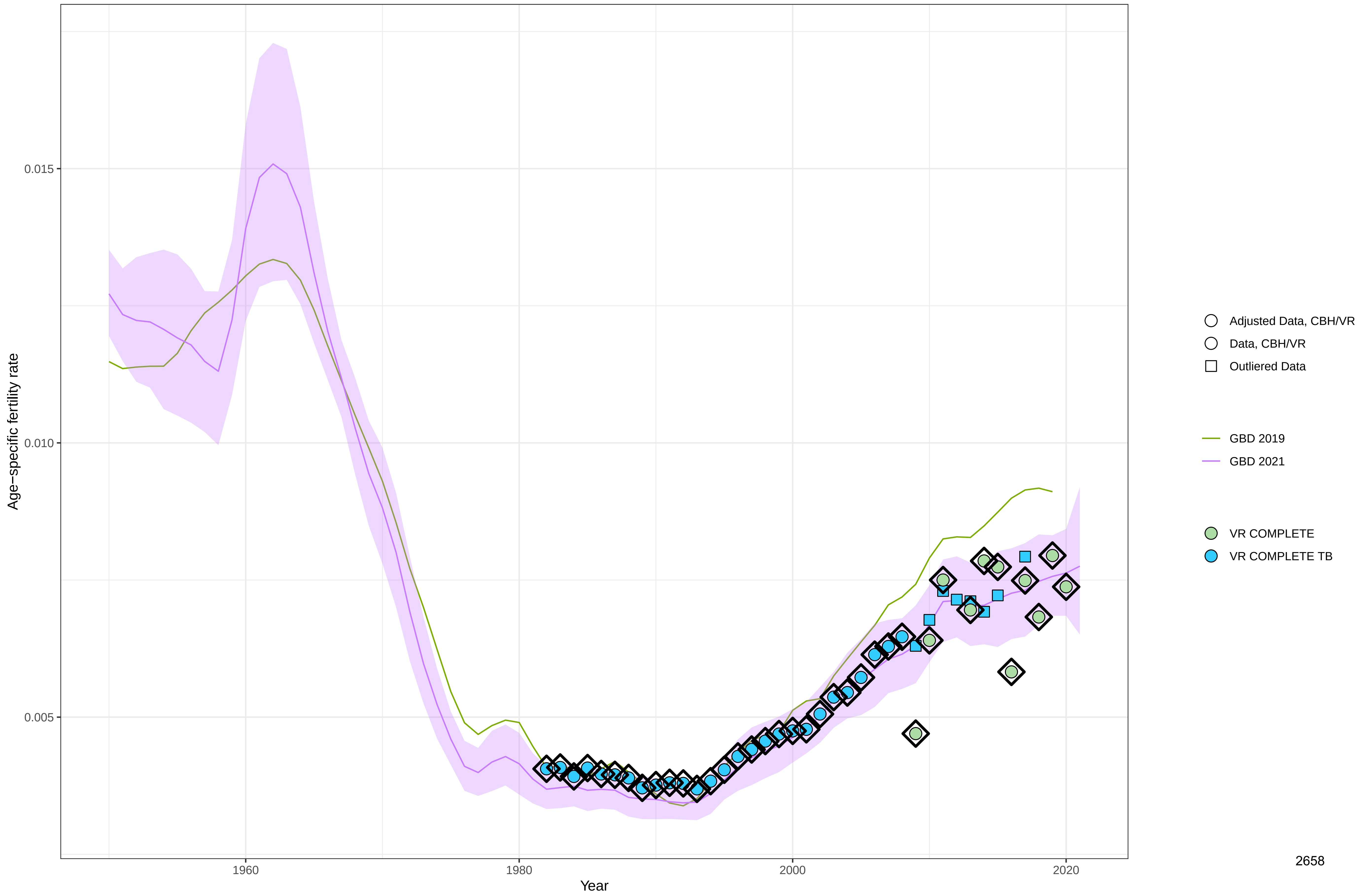

Age−specific fertility fitted trend lines, 1950−2021: Sunderland, Ages: 45−49
Data points enclosed with a diamond are reference sources

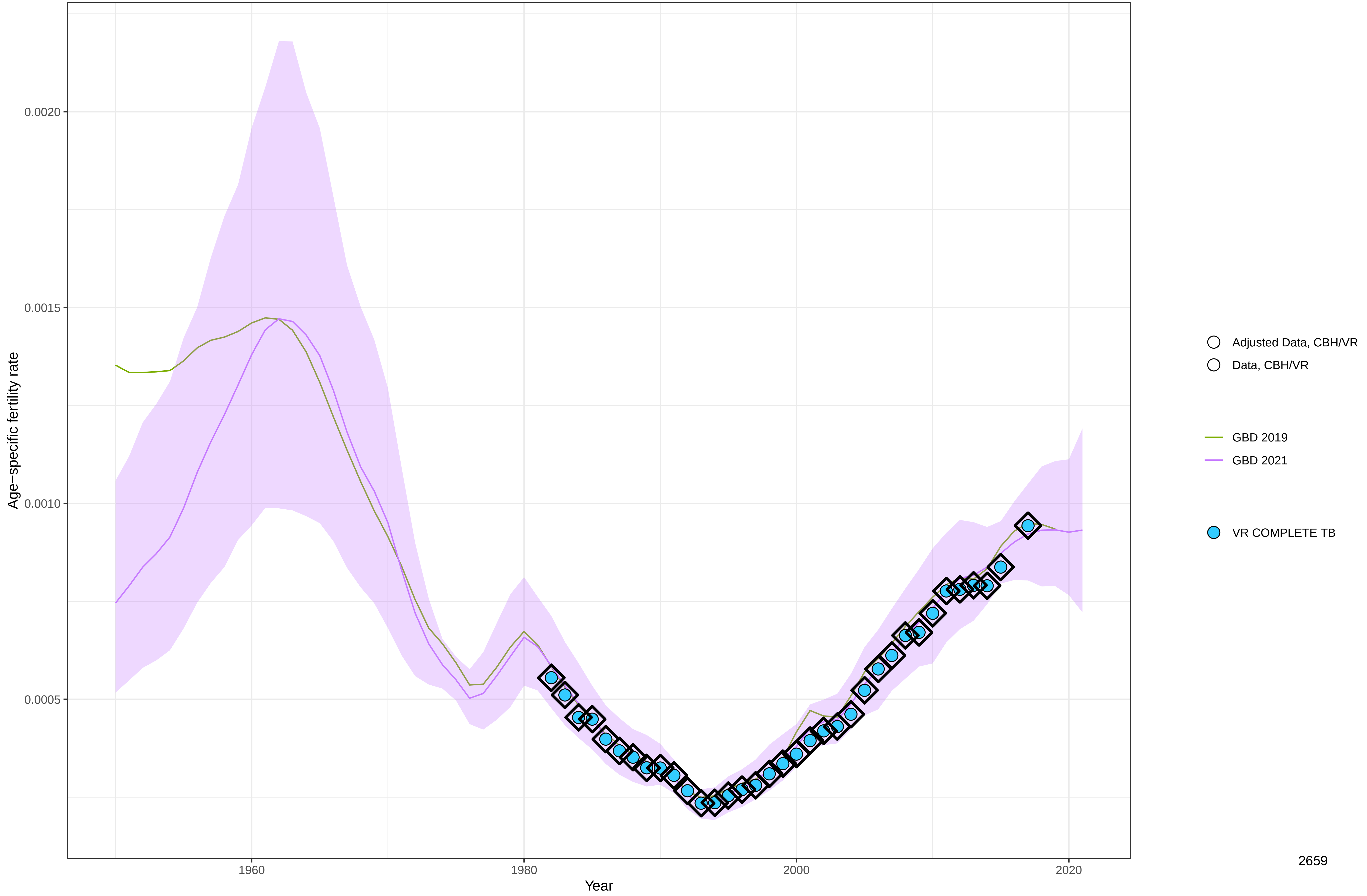

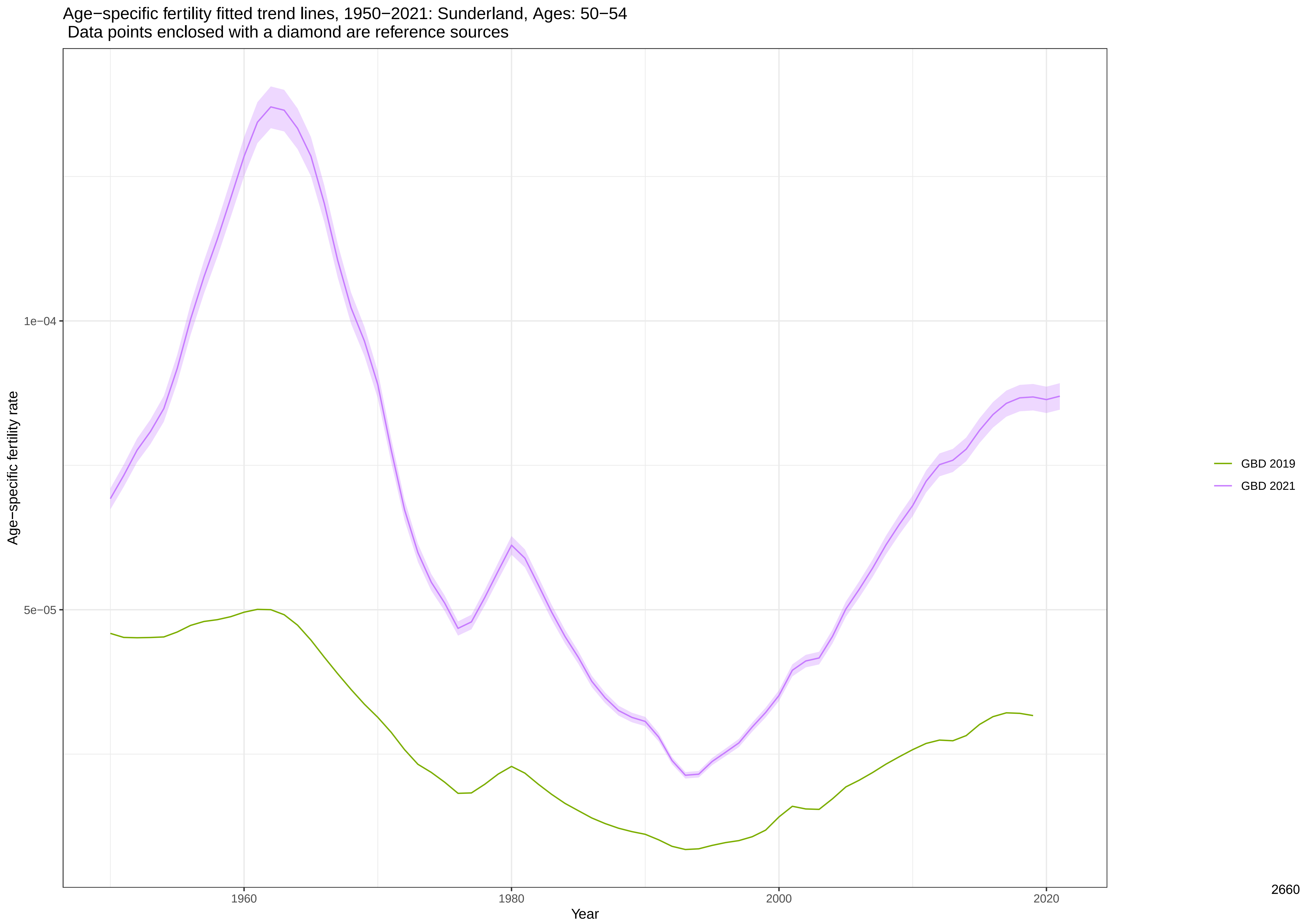

Age−specific fertility fitted trend lines, 1950−2021: Sunderland, Ages: 50−54
Data points enclosed with a diamond are reference sources
1e−04
5e−05
Age−specific fertility rate
1960
1980
2000
2020
Year
GBD 2019
GBD 2021

Total fertility rate fitted trend lines, 1950−2021: Sunderland TFR
Data points enclosed with a diamond are reference sources

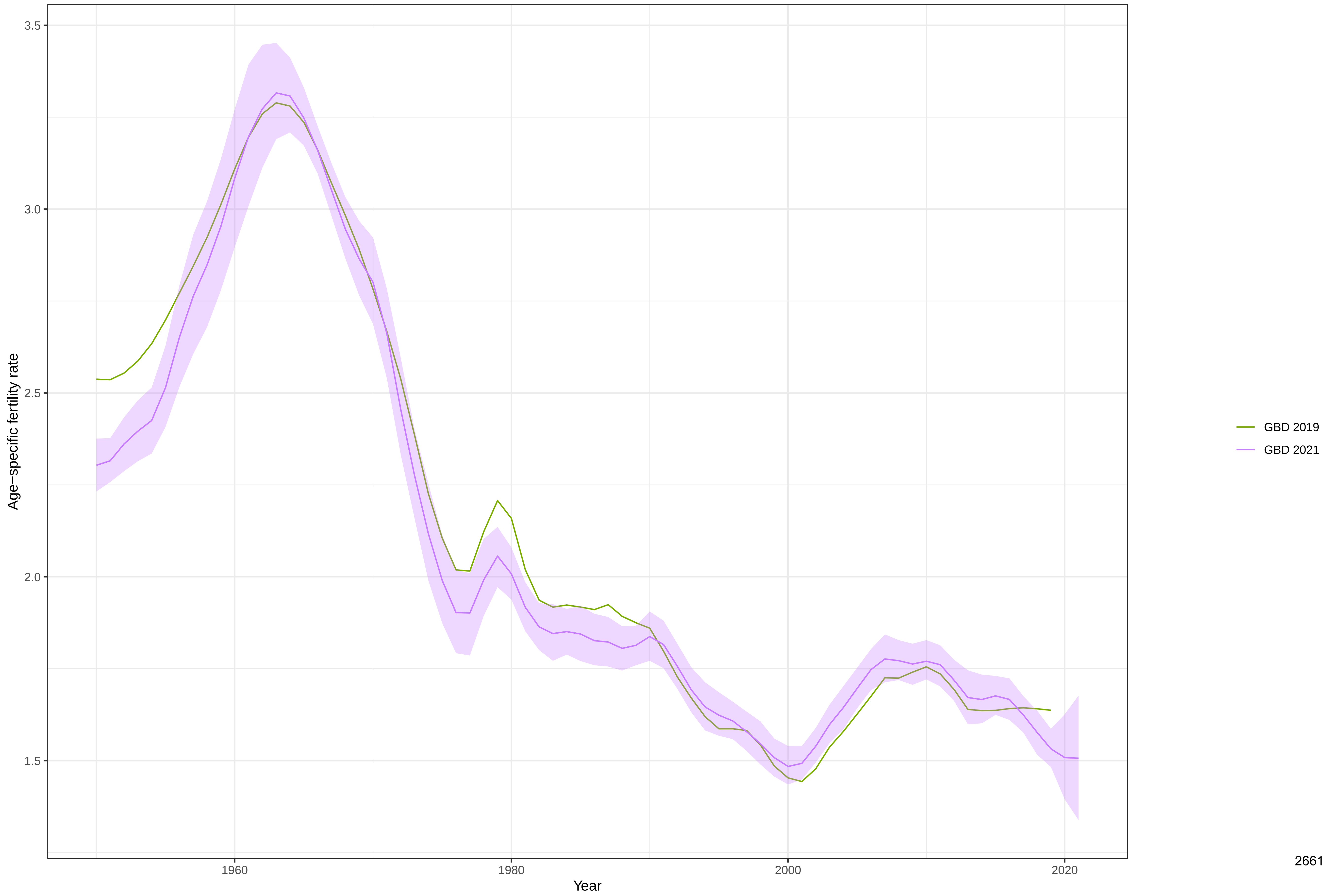

Age−specific fertility fitted trend lines, 1950−2021: North West England, Ages: 10−14
Data points enclosed with a diamond are reference sources

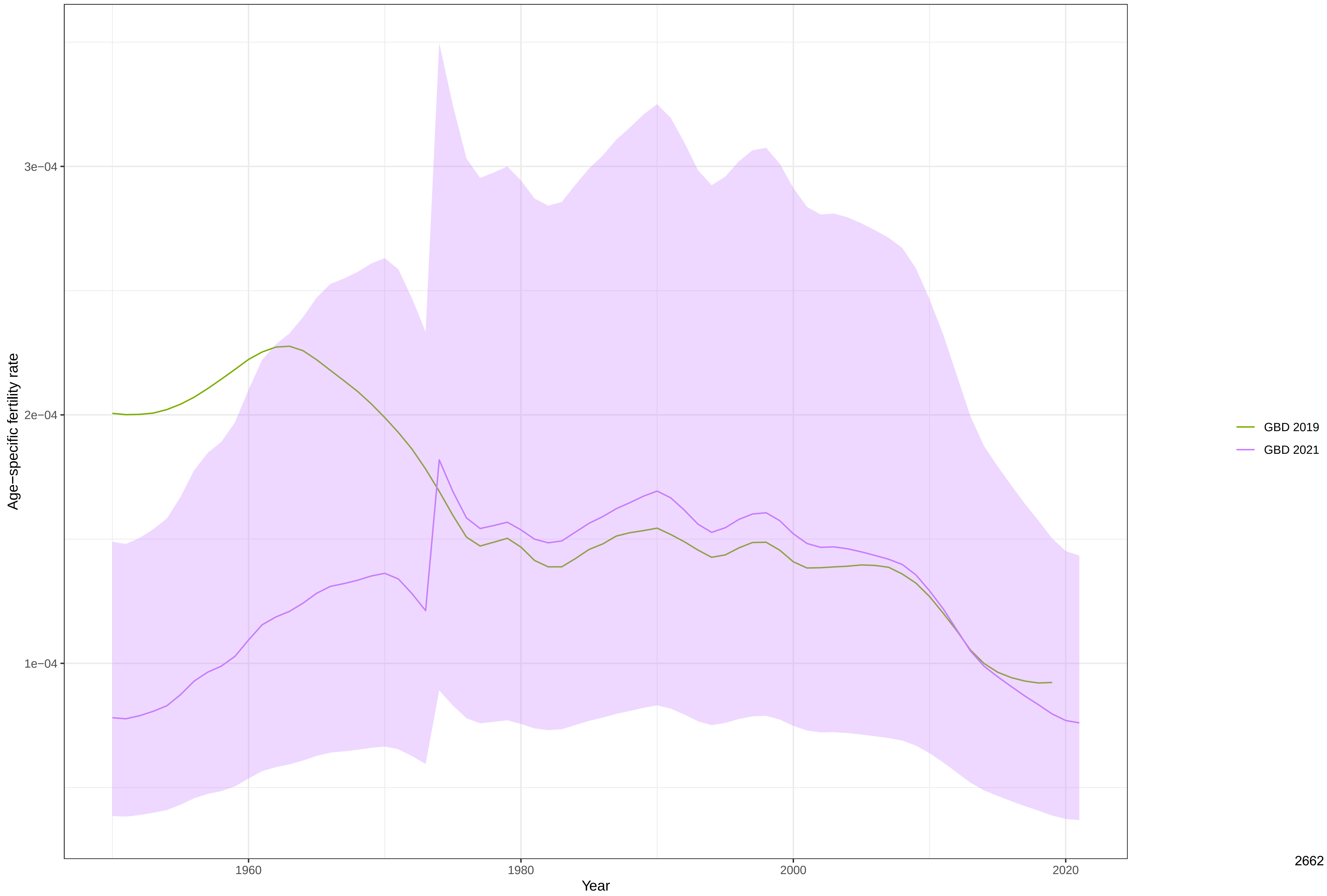

Age−specific fertility fitted trend lines, 1950−2021: North West England, Ages: 15−19
Data points enclosed with a diamond are reference sources

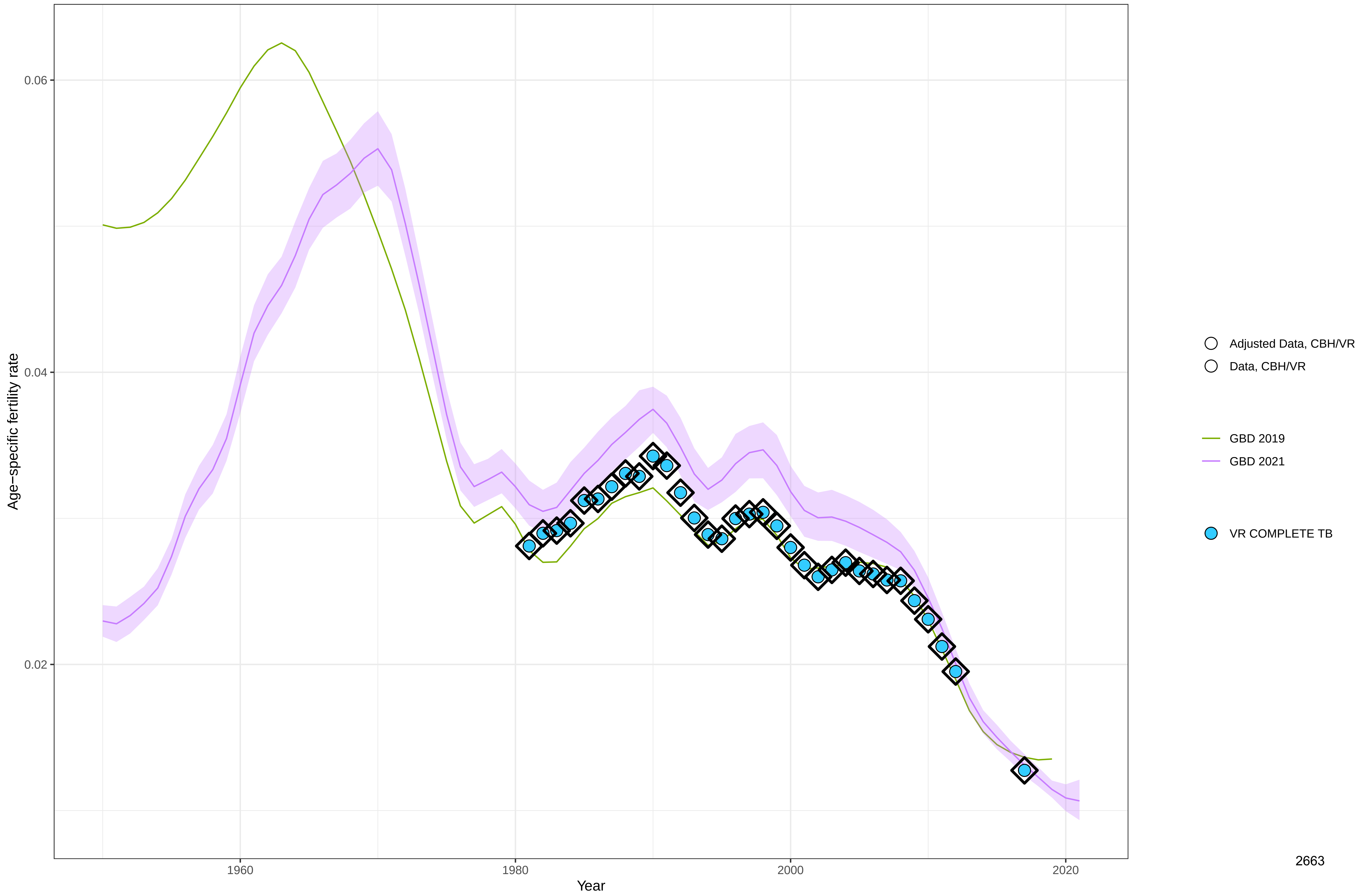

Age−specific fertility fitted trend lines, 1950−2021: North West England, Ages: 20−24
Data points enclosed with a diamond are reference sources

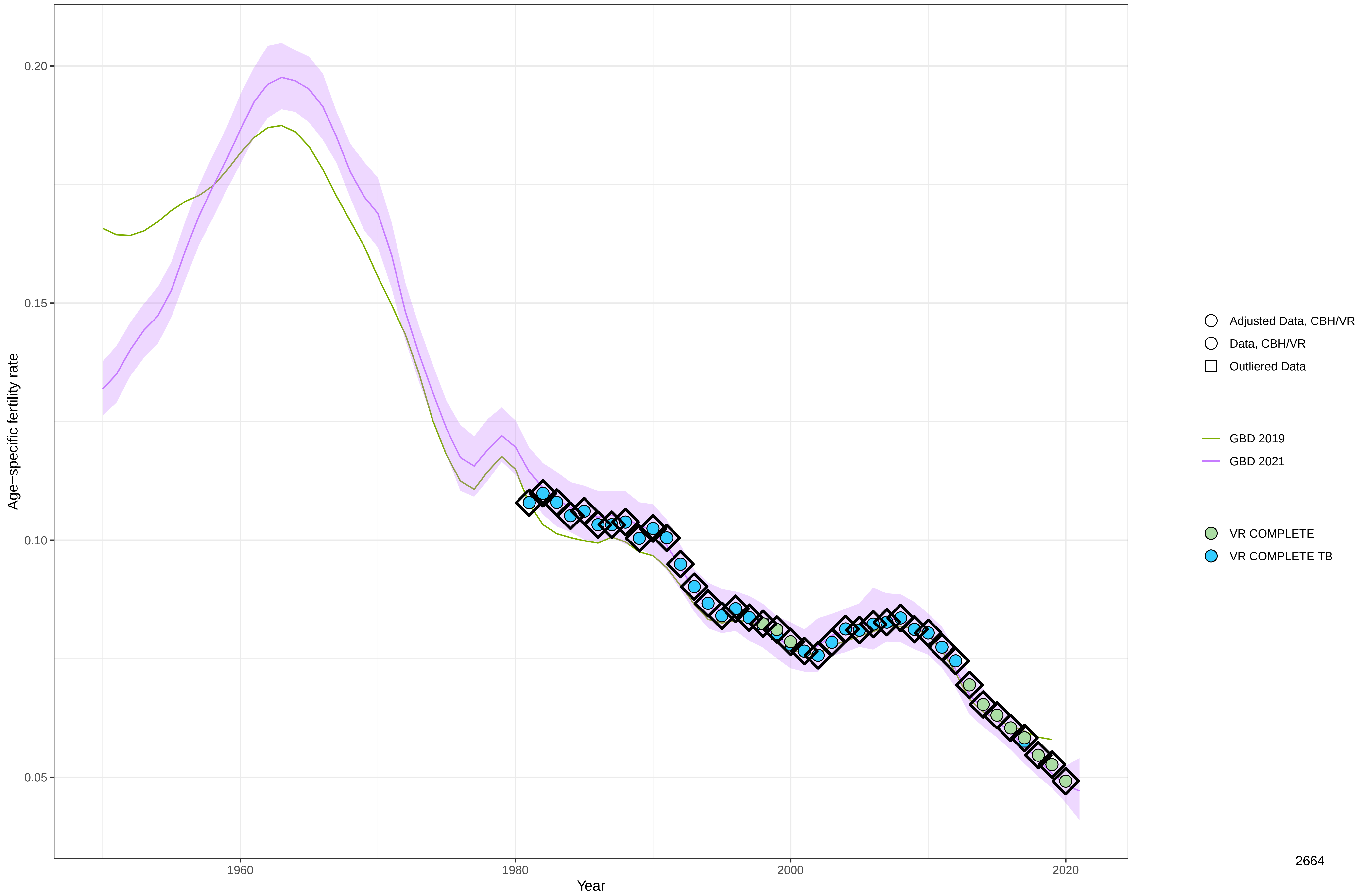

Age−specific fertility fitted trend lines, 1950−2021: North West England, Ages: 25−29
Data points enclosed with a diamond are reference sources

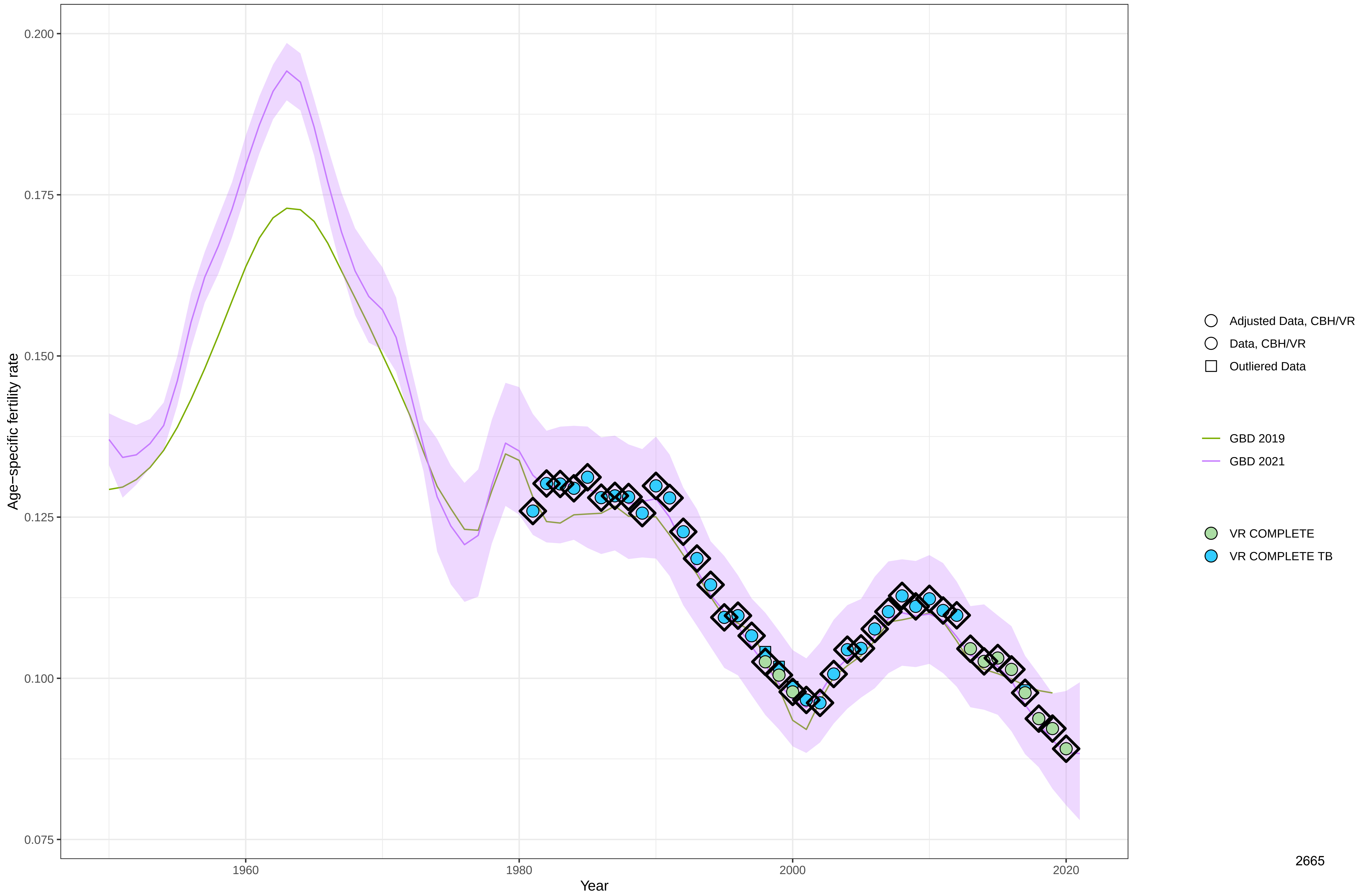

Age−specific fertility fitted trend lines, 1950−2021: North West England, Ages: 30−34
Data points enclosed with a diamond are reference sources

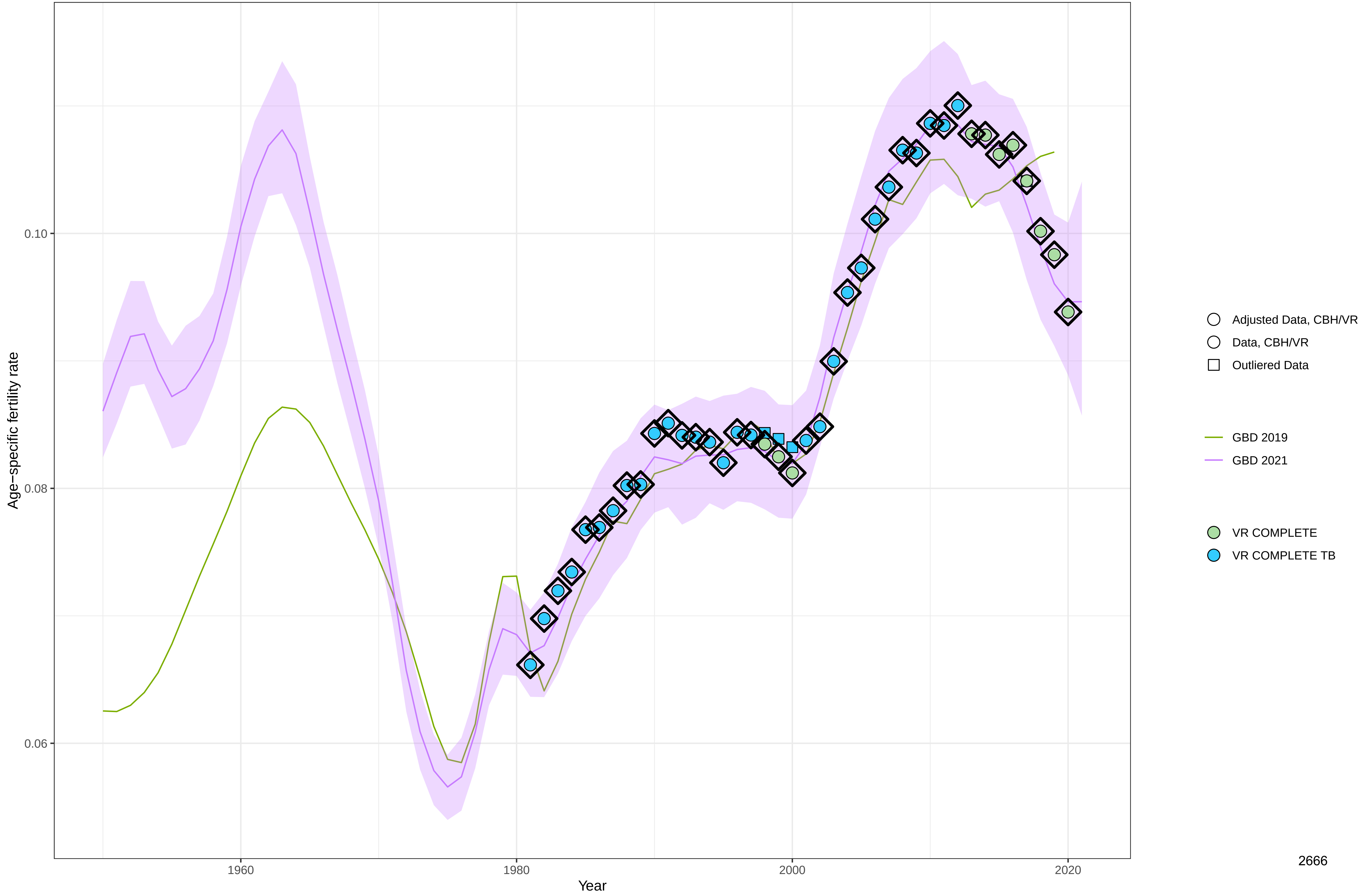

Age−specific fertility fitted trend lines, 1950−2021: North West England, Ages: 35−39
Data points enclosed with a diamond are reference sources

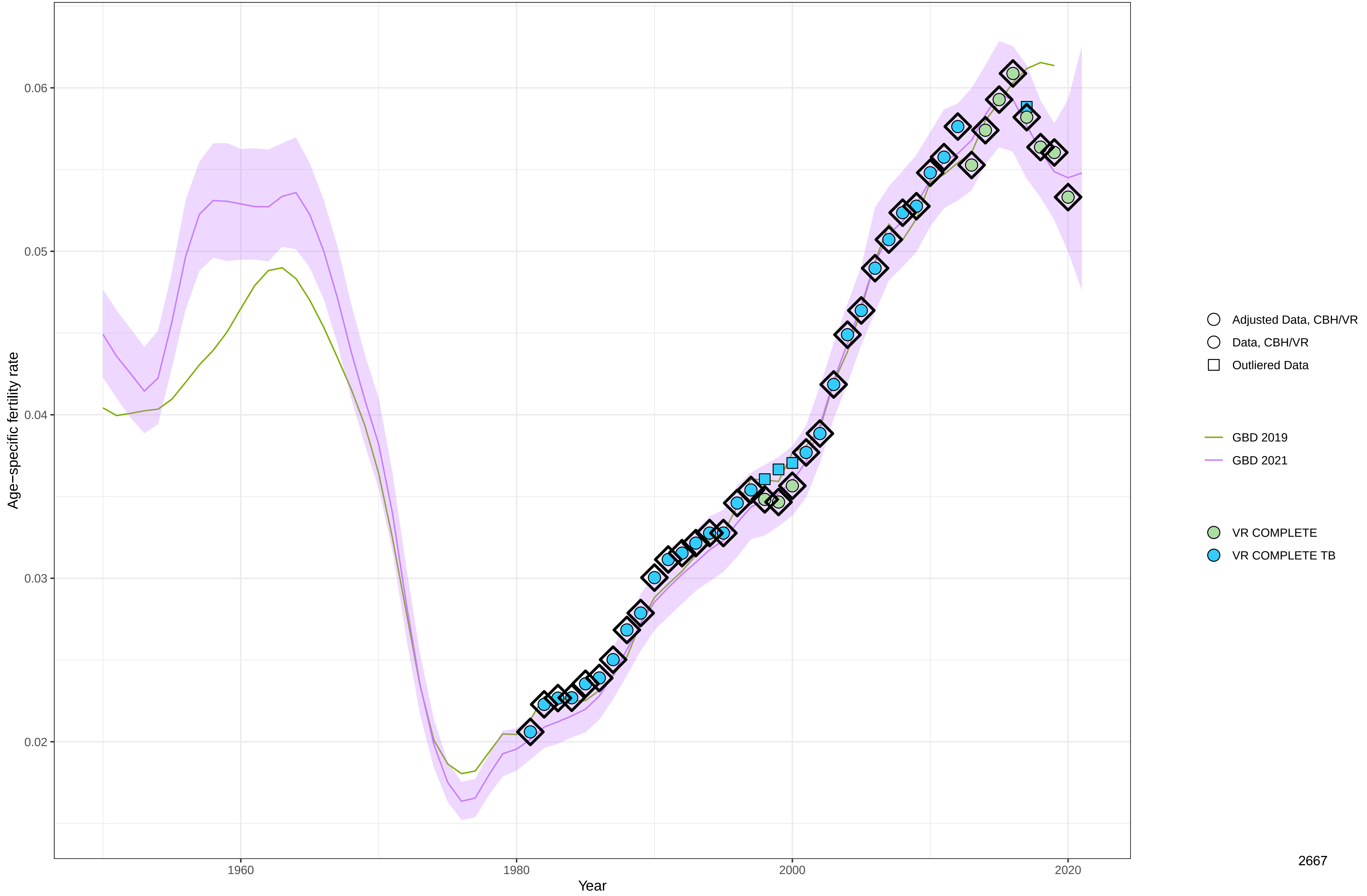

Age−specific fertility fitted trend lines, 1950−2021: North West England, Ages: 40−44
Data points enclosed with a diamond are reference sources

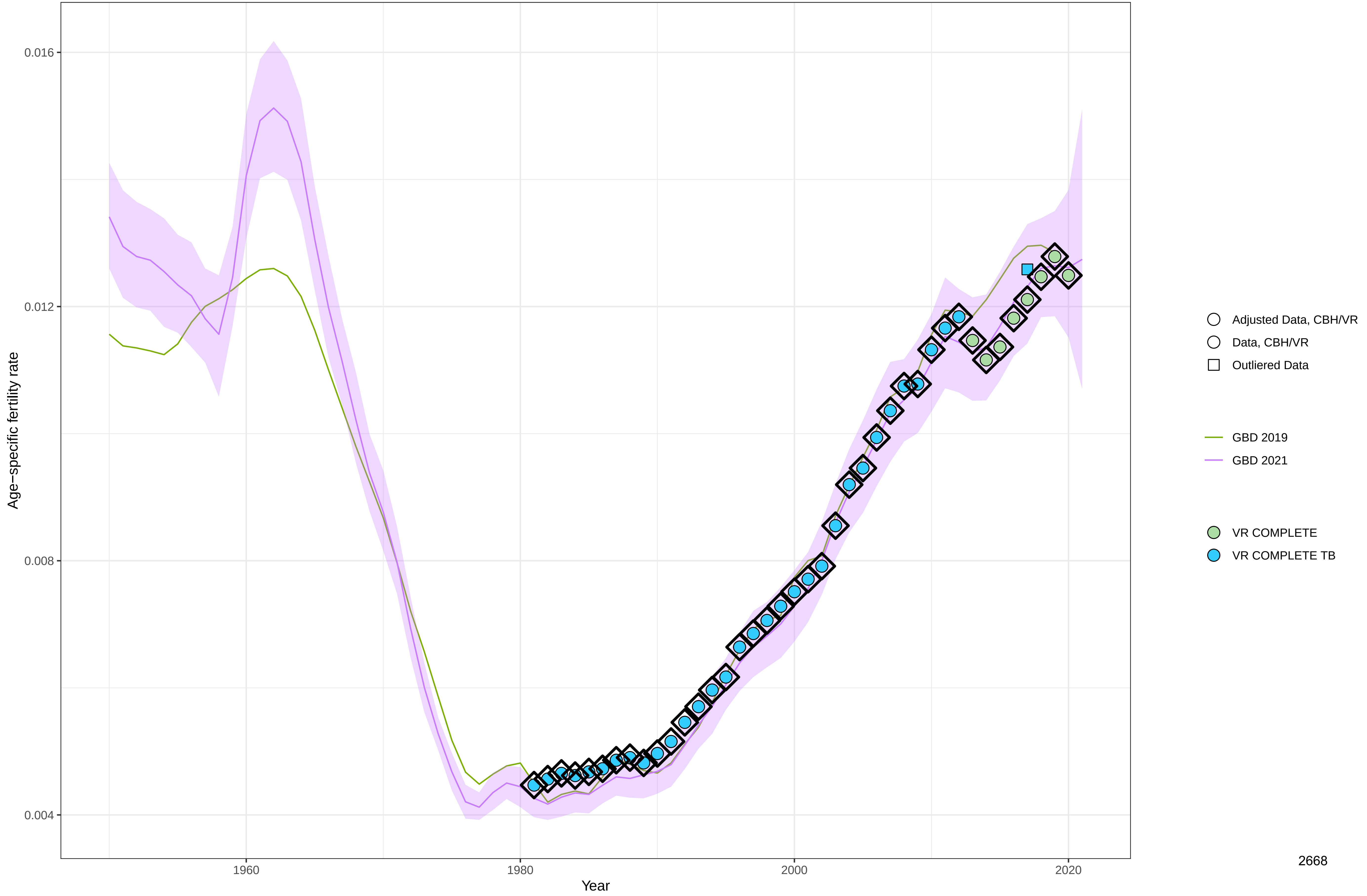

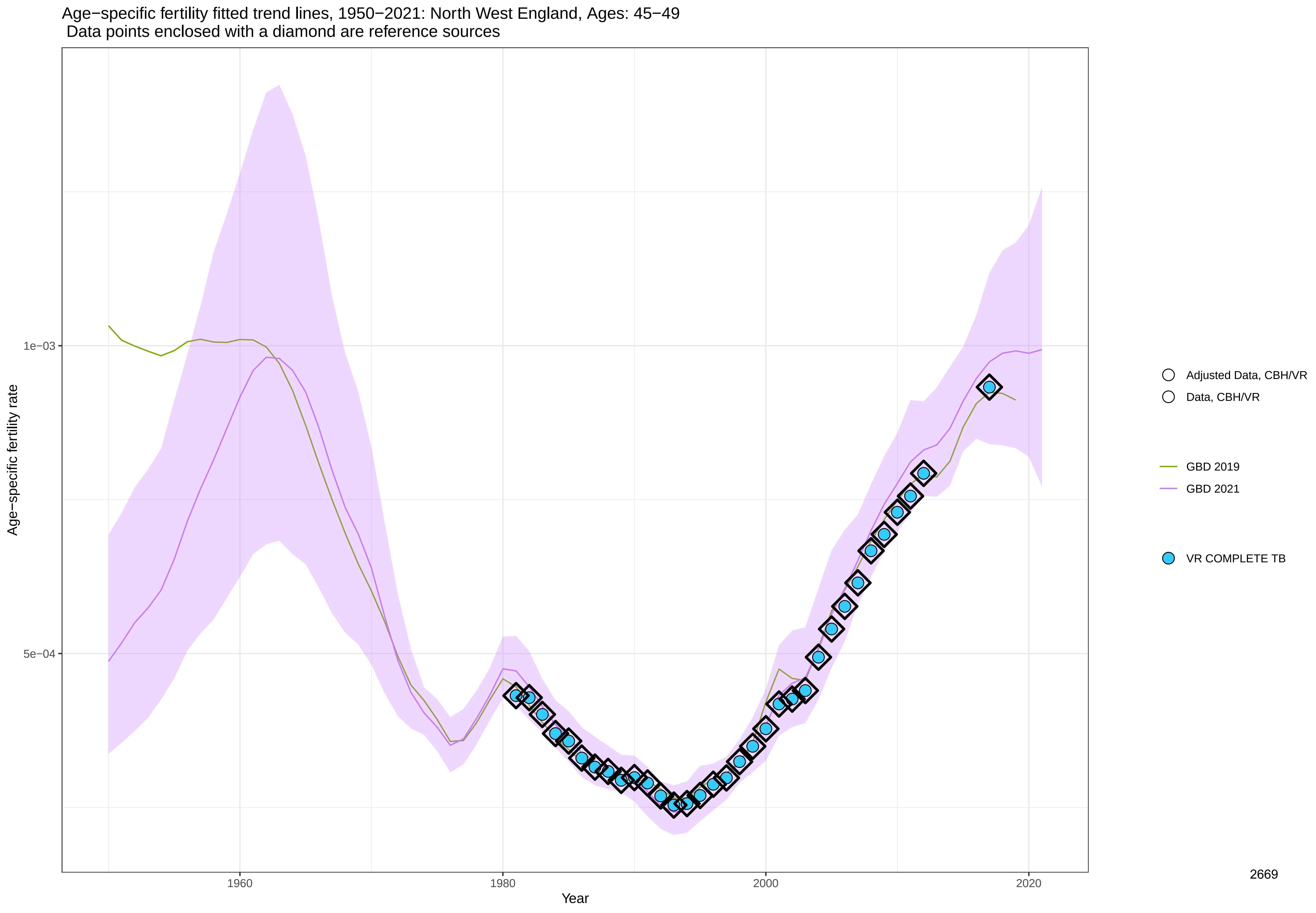
Age−specific fertility fitted trend lines, 1950−2021: North West England, Ages: 45−49
Data points enclosed with a diamond are reference sources
Age−specific fertility rate
1e−03
5e−04
1960
1980
2000
2020
Year
Adjusted Data, CBH/VR
Data, CBH/VR
GBD 2019
GBD 2021
VR COMPLETE TB

Age−specific fertility fitted trend lines, 1950−2021: North West England, Ages: 50−54
Data points enclosed with a diamond are reference sources

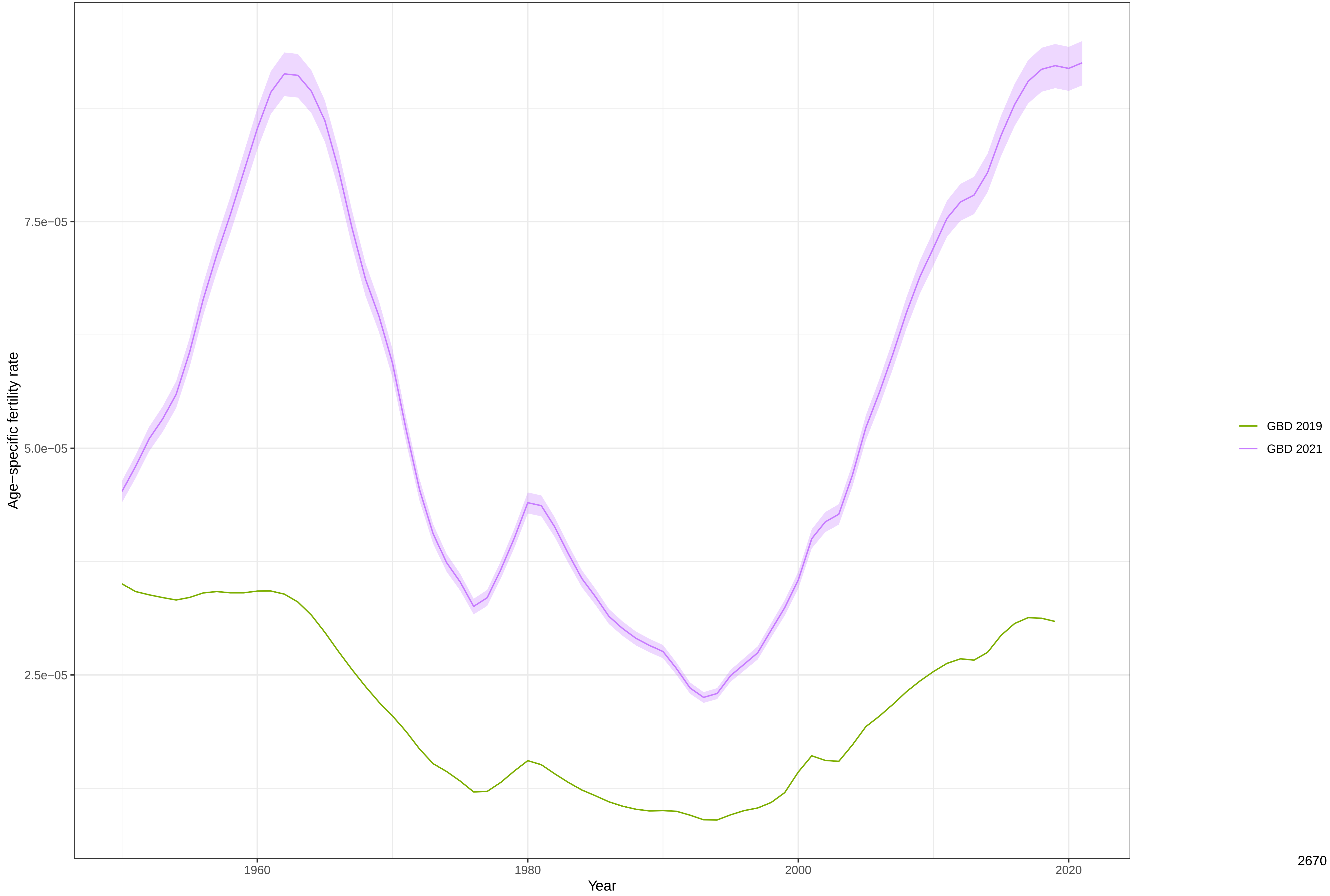

Total fertility rate fitted trend lines, 1950−2021: North West England TFR
Data points enclosed with a diamond are reference sources

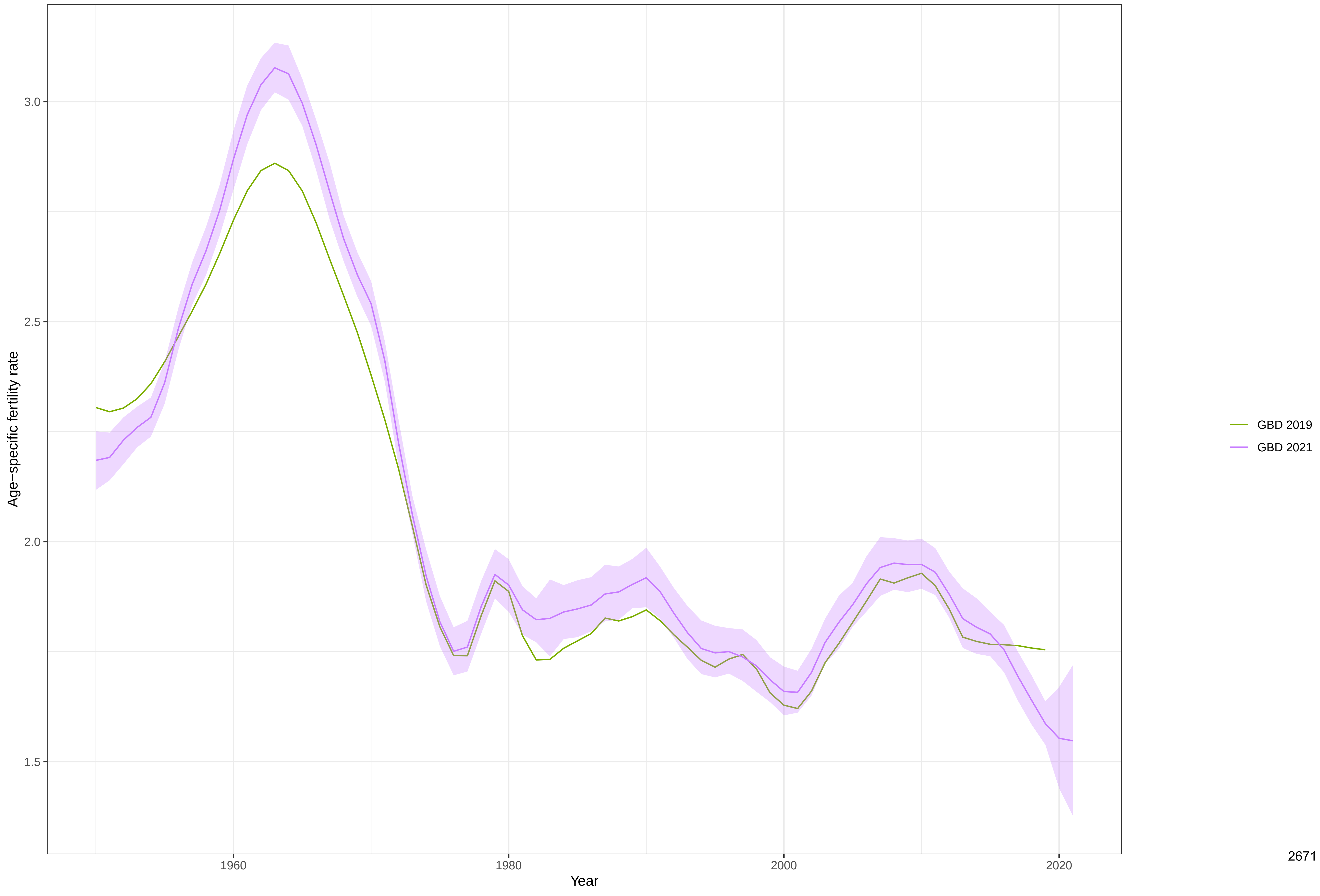

Age−specific fertility fitted trend lines, 1950−2021: Blackburn with Darwen, Ages: 10−14
Data points enclosed with a diamond are reference sources

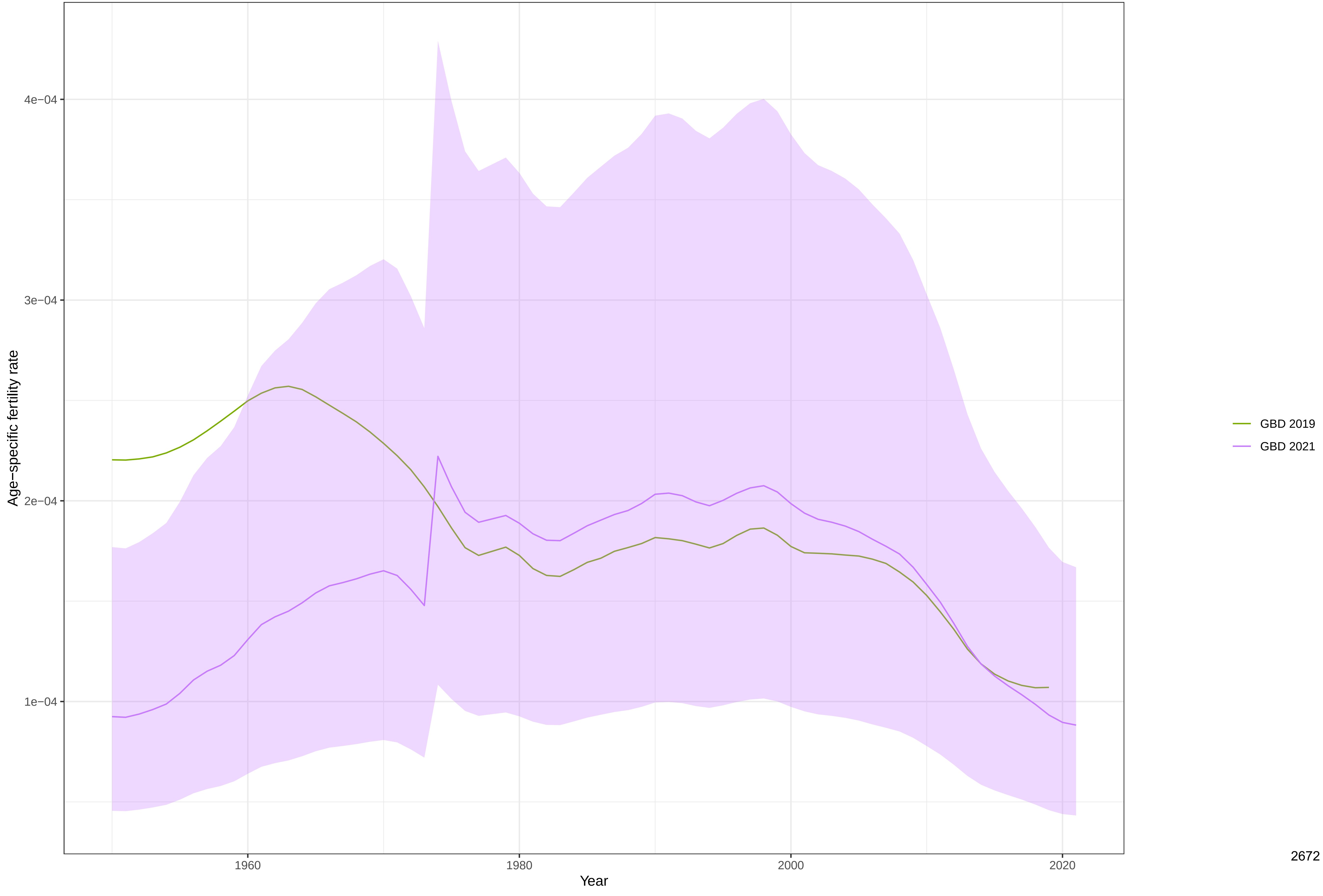

2672

Age−specific fertility fitted trend lines, 1950−2021: Blackburn with Darwen, Ages: 15−19
Data points enclosed with a diamond are reference sources

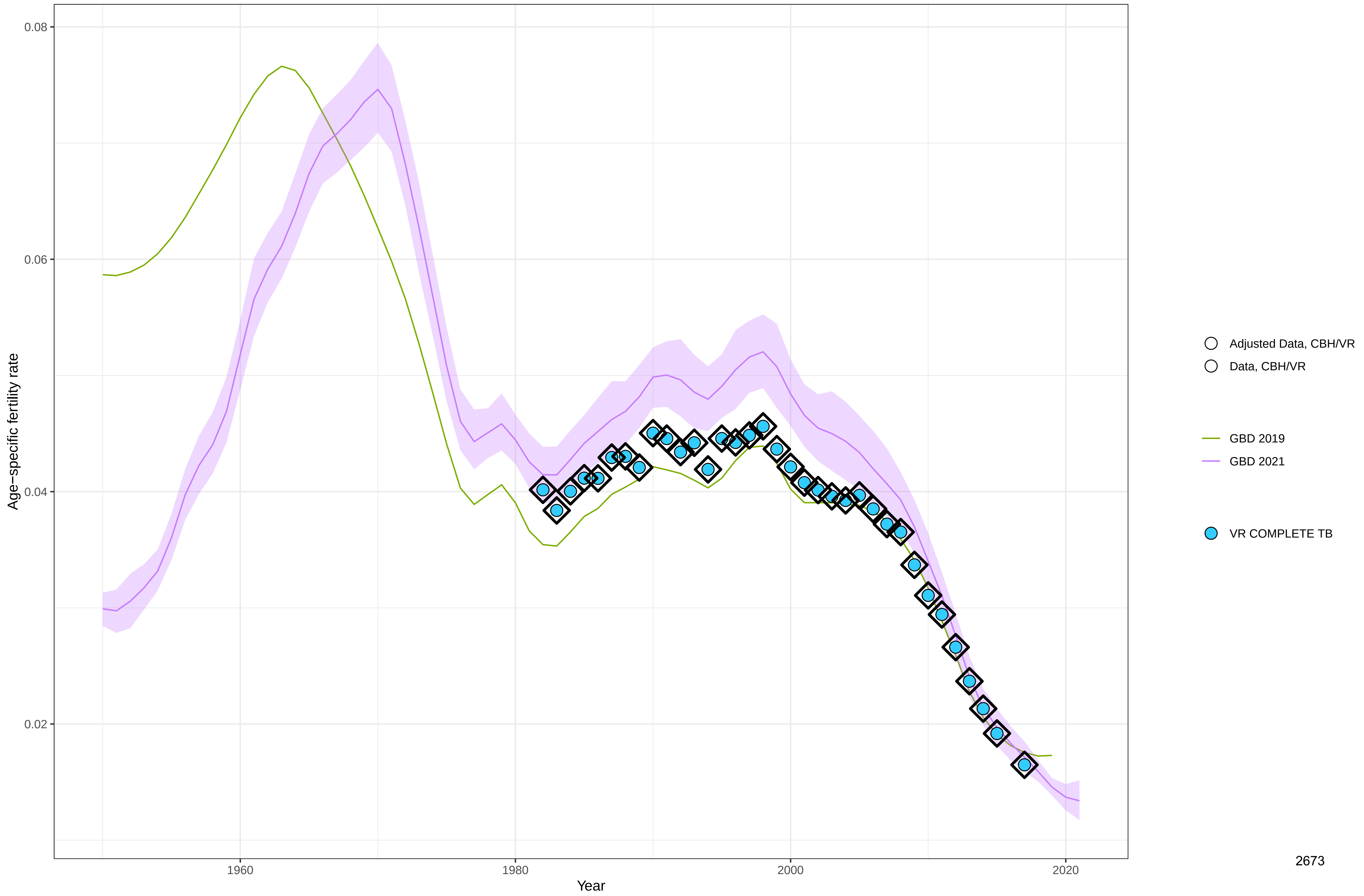

Age−specific fertility fitted trend lines, 1950−2021: Blackburn with Darwen, Ages: 20−24
Data points enclosed with a diamond are reference sources

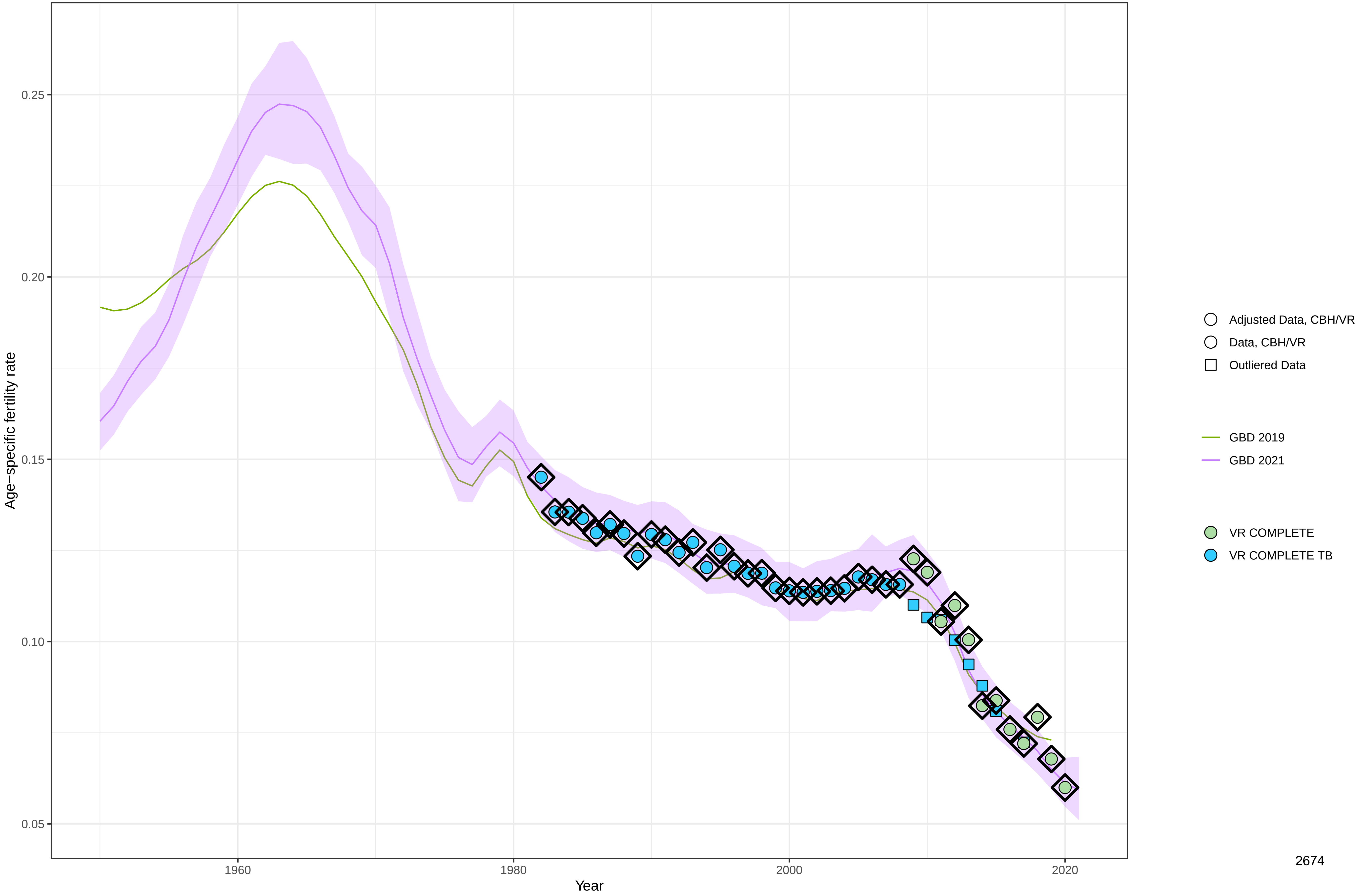

Age−specific fertility fitted trend lines, 1950−2021: Blackburn with Darwen, Ages: 25−29
Data points enclosed with a diamond are reference sources

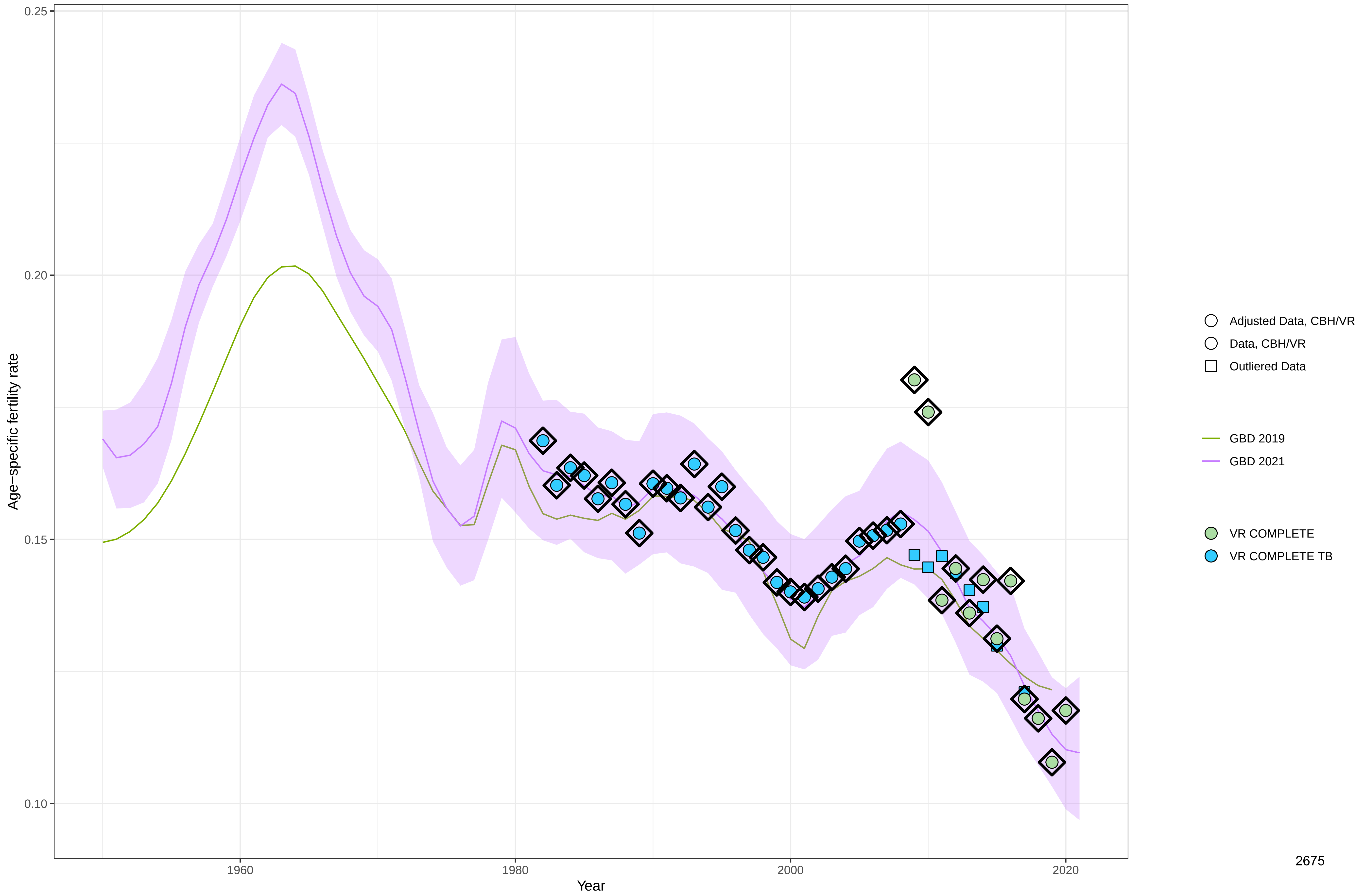

Age−specific fertility fitted trend lines, 1950−2021: Blackburn with Darwen, Ages: 30−34
Data points enclosed with a diamond are reference sources

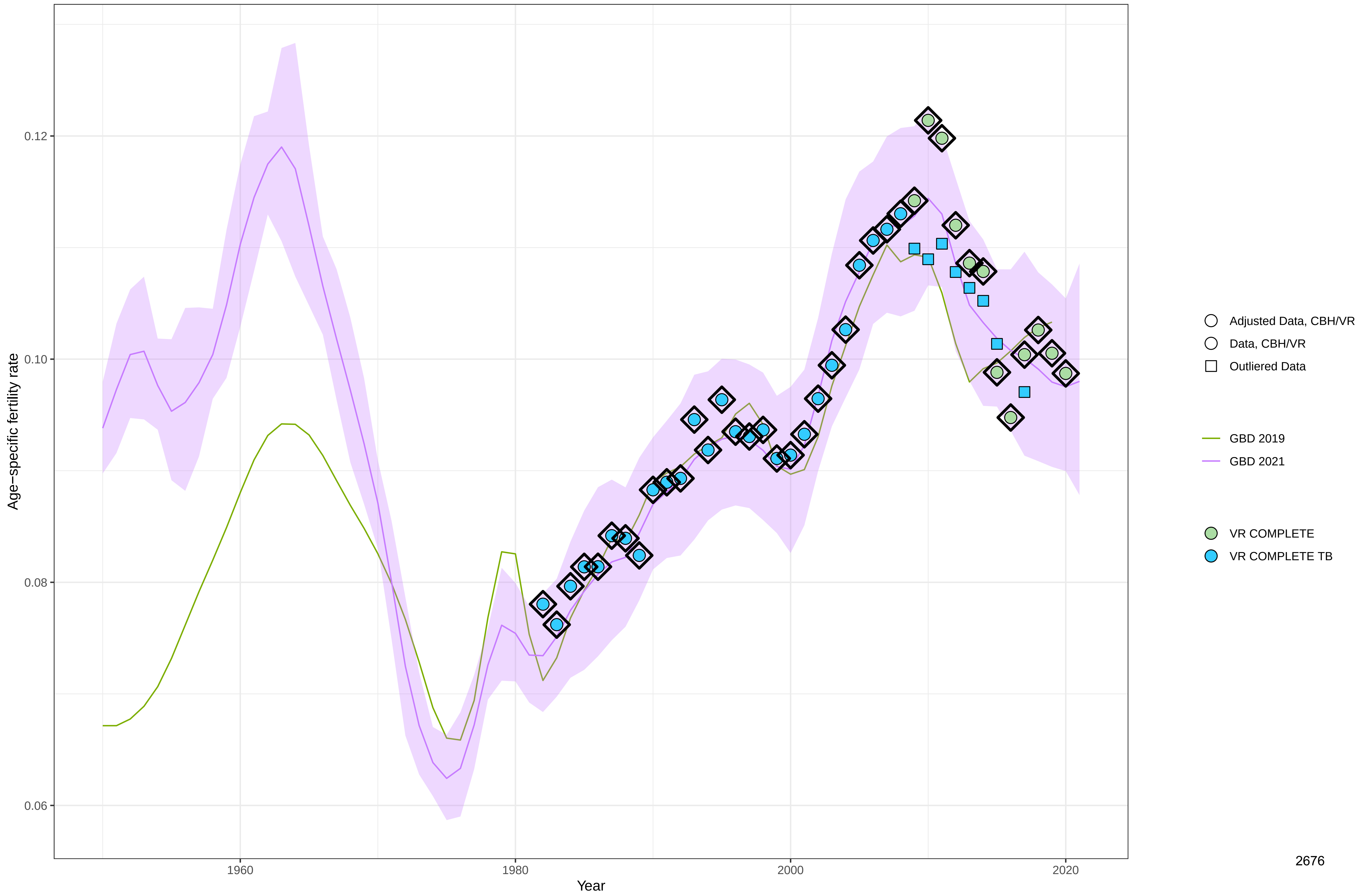

Age−specific fertility fitted trend lines, 1950−2021: Blackburn with Darwen, Ages: 35−39
Data points enclosed with a diamond are reference sources

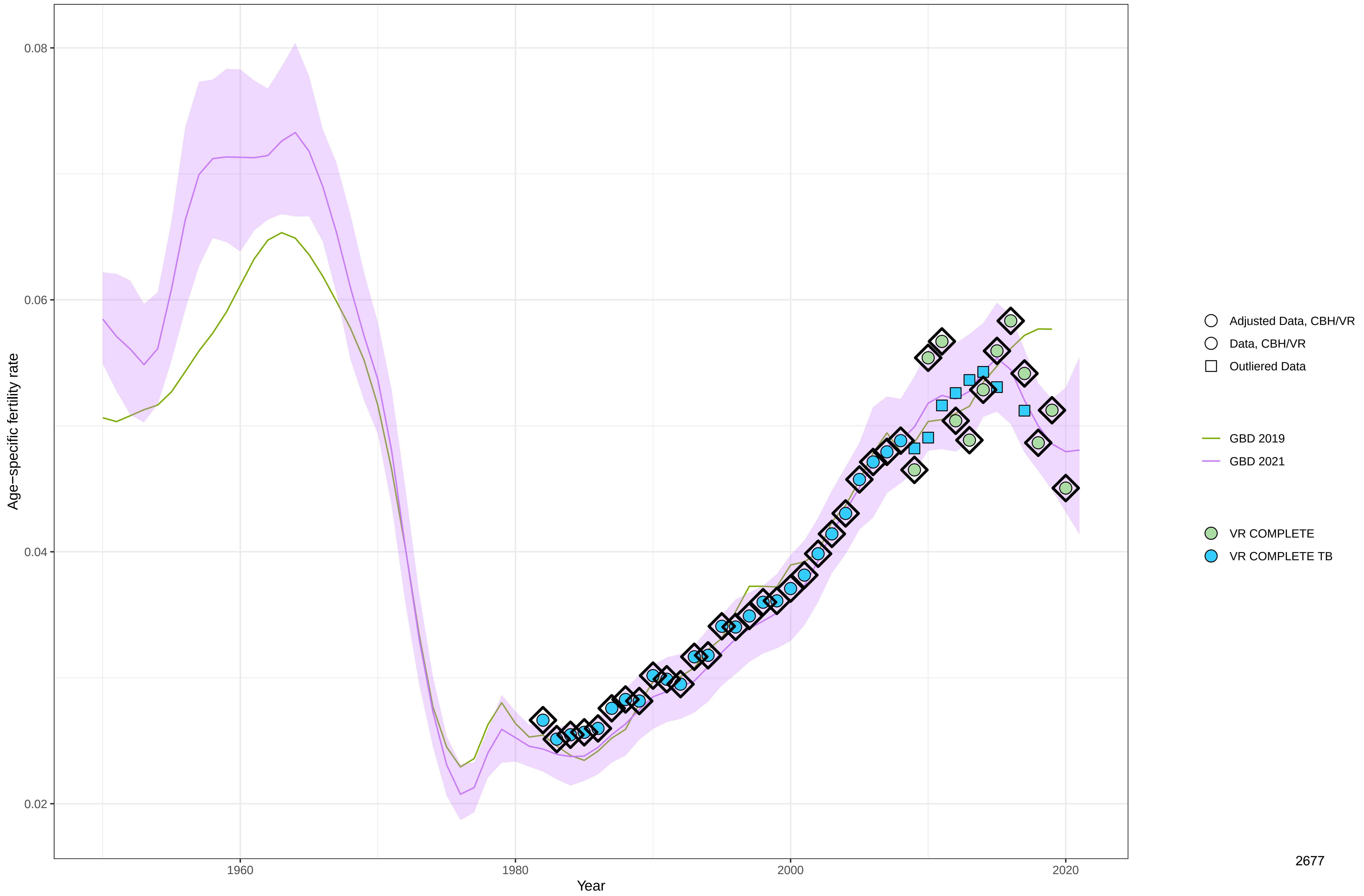

Age−specific fertility fitted trend lines, 1950−2021: Blackburn with Darwen, Ages: 40−44
Data points enclosed with a diamond are reference sources

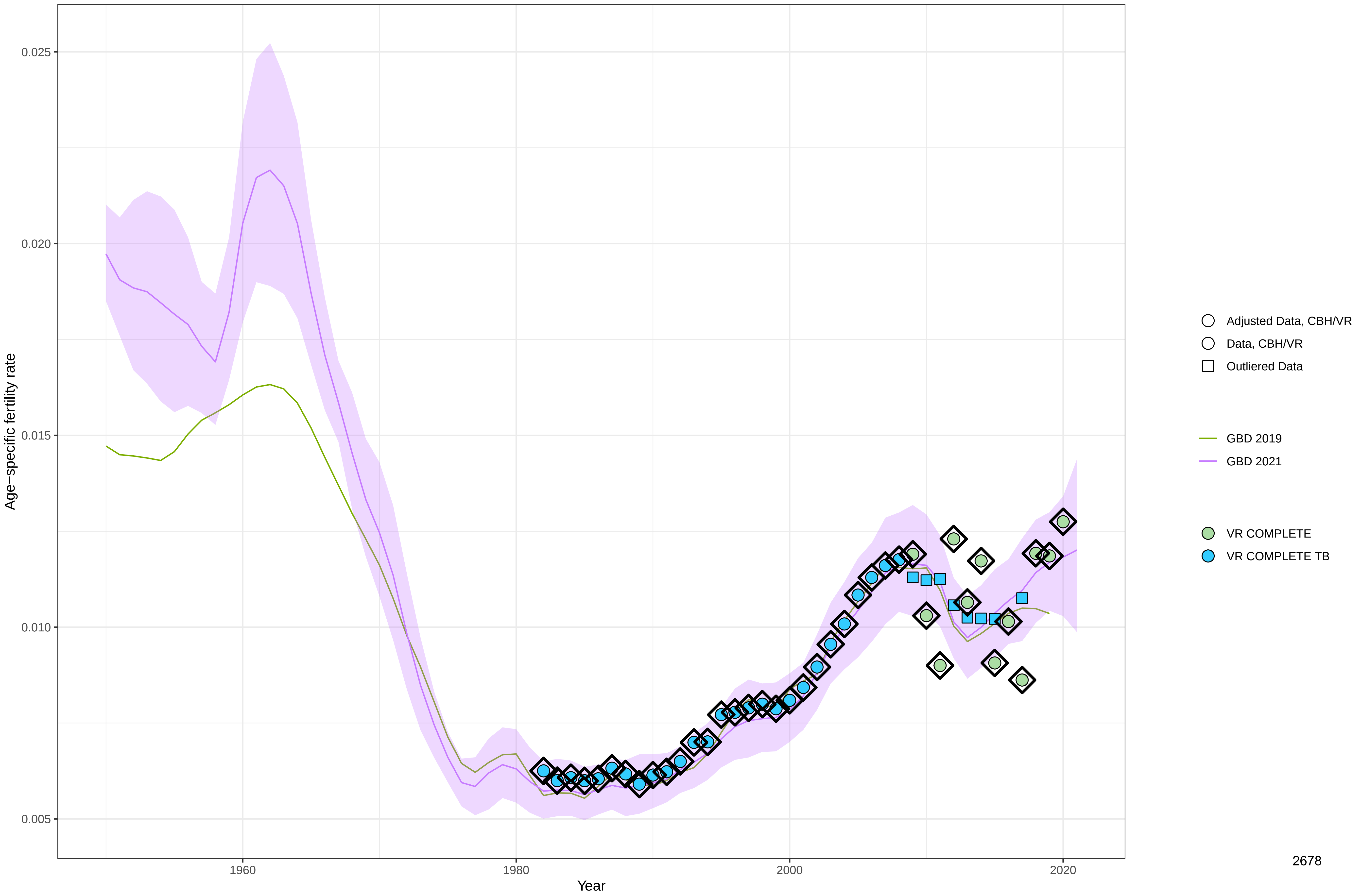

Age−specific fertility fitted trend lines, 1950−2021: Blackburn with Darwen, Ages: 45−49
Data points enclosed with a diamond are reference sources

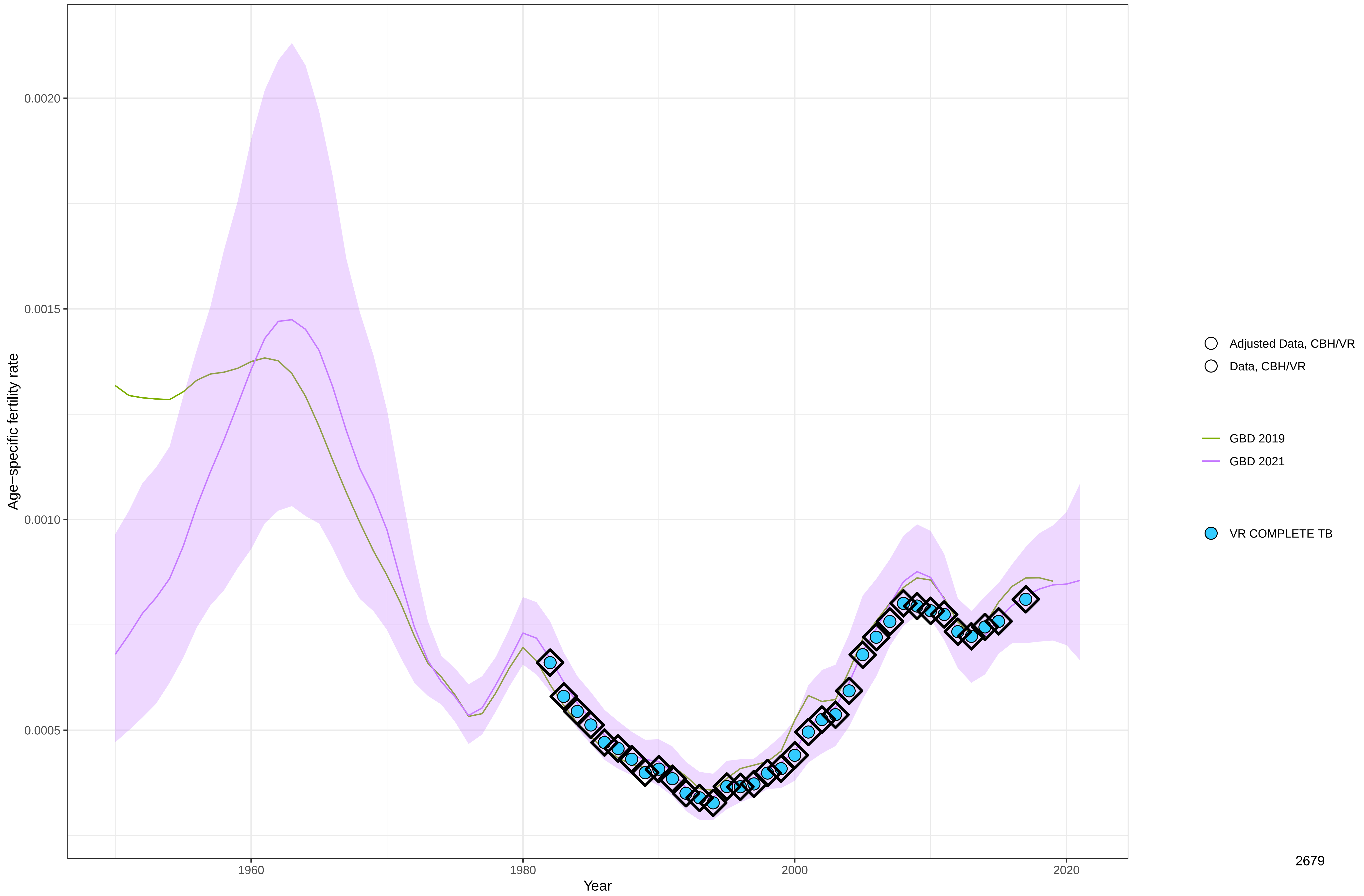

Age−specific fertility fitted trend lines, 1950−2021: Blackburn with Darwen, Ages: 50−54
Data points enclosed with a diamond are reference sources

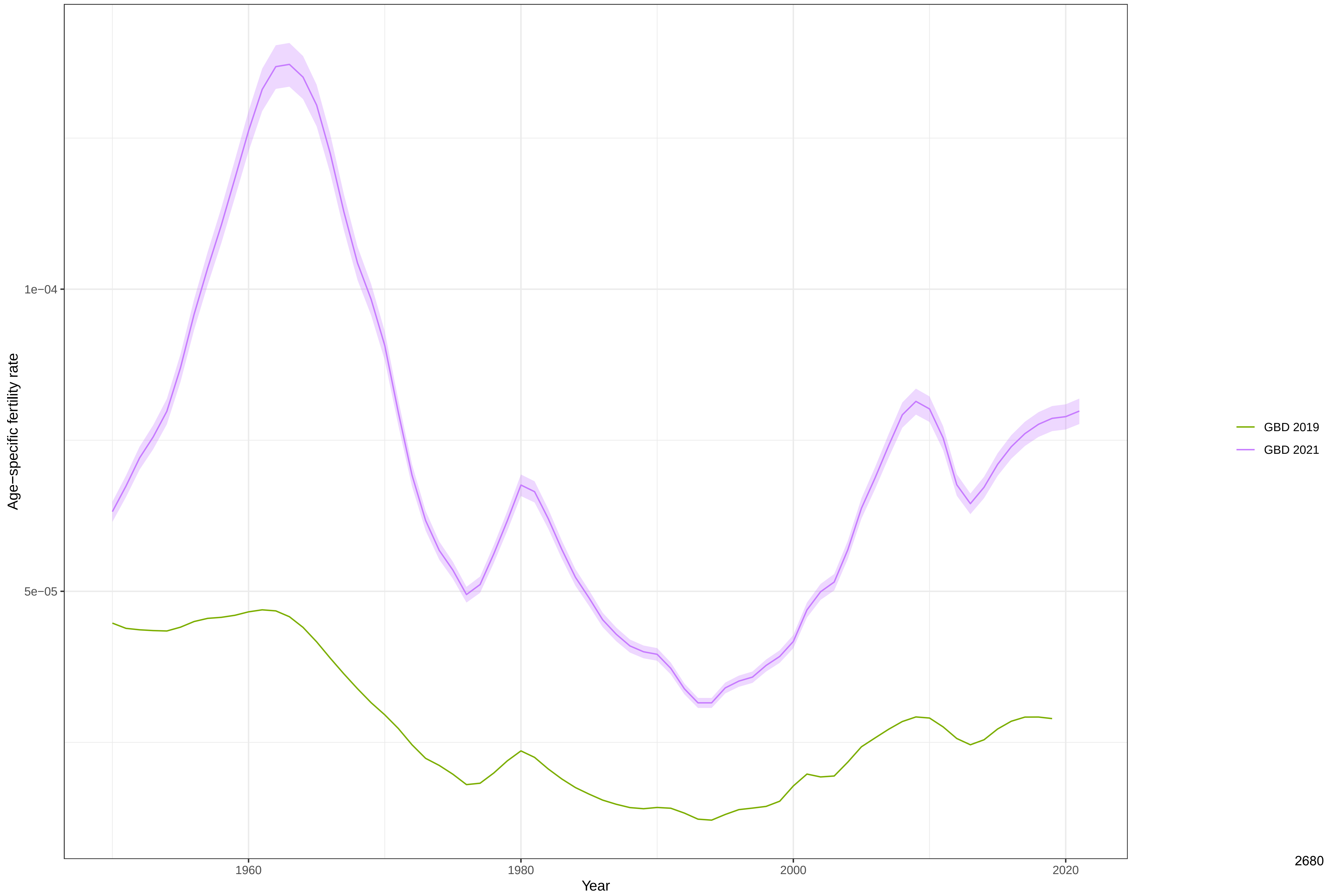

Total fertility rate fitted trend lines, 1950−2021: Blackburn with Darwen TFR
Data points enclosed with a diamond are reference sources

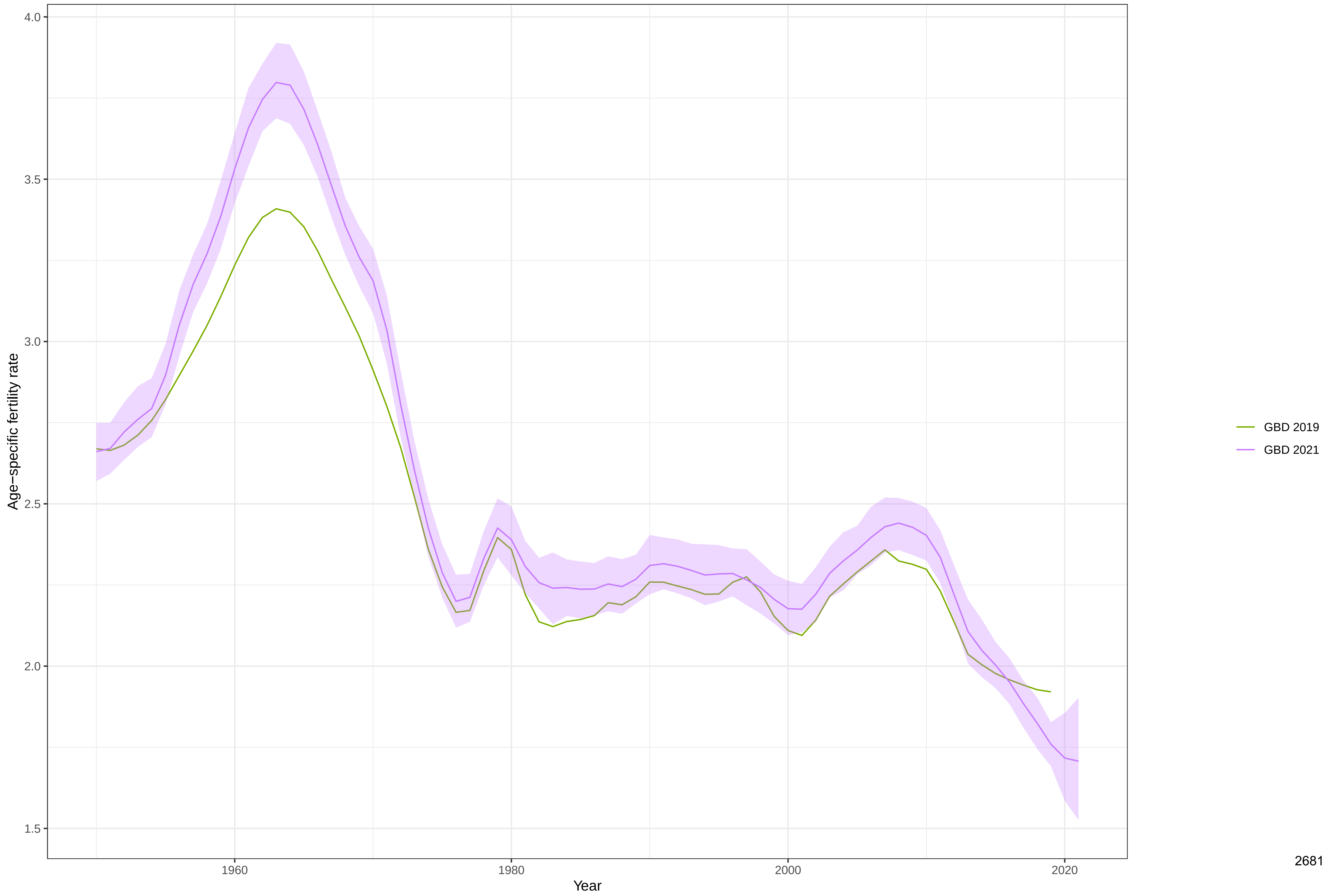

Age−specific fertility fitted trend lines, 1950−2021: Blackpool, Ages: 10−14
Data points enclosed with a diamond are reference sources

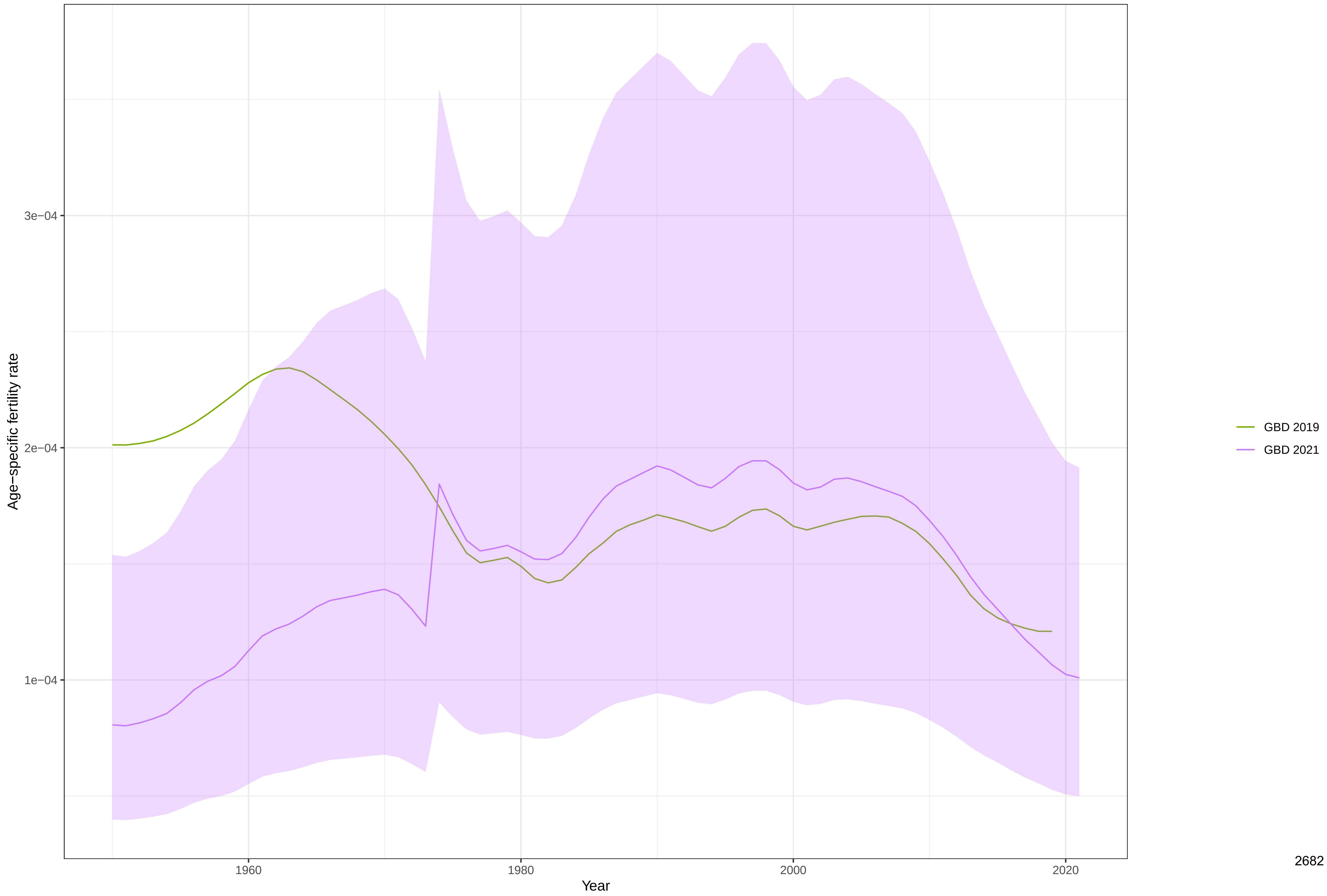

Age−specific fertility fitted trend lines, 1950−2021: Blackpool, Ages: 15−19
Data points enclosed with a diamond are reference sources

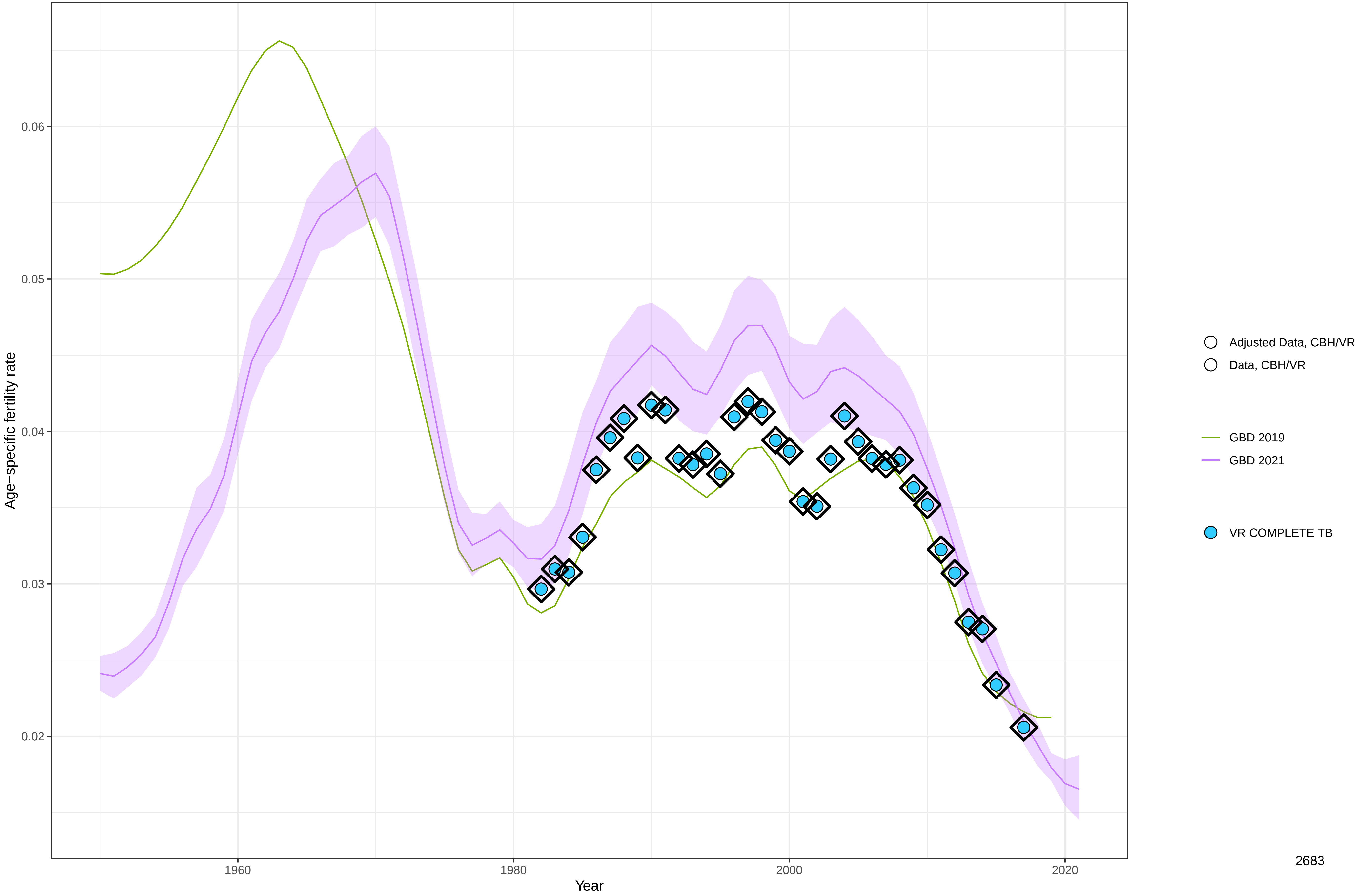

Age−specific fertility fitted trend lines, 1950−2021: Blackpool, Ages: 20−24
Data points enclosed with a diamond are reference sources

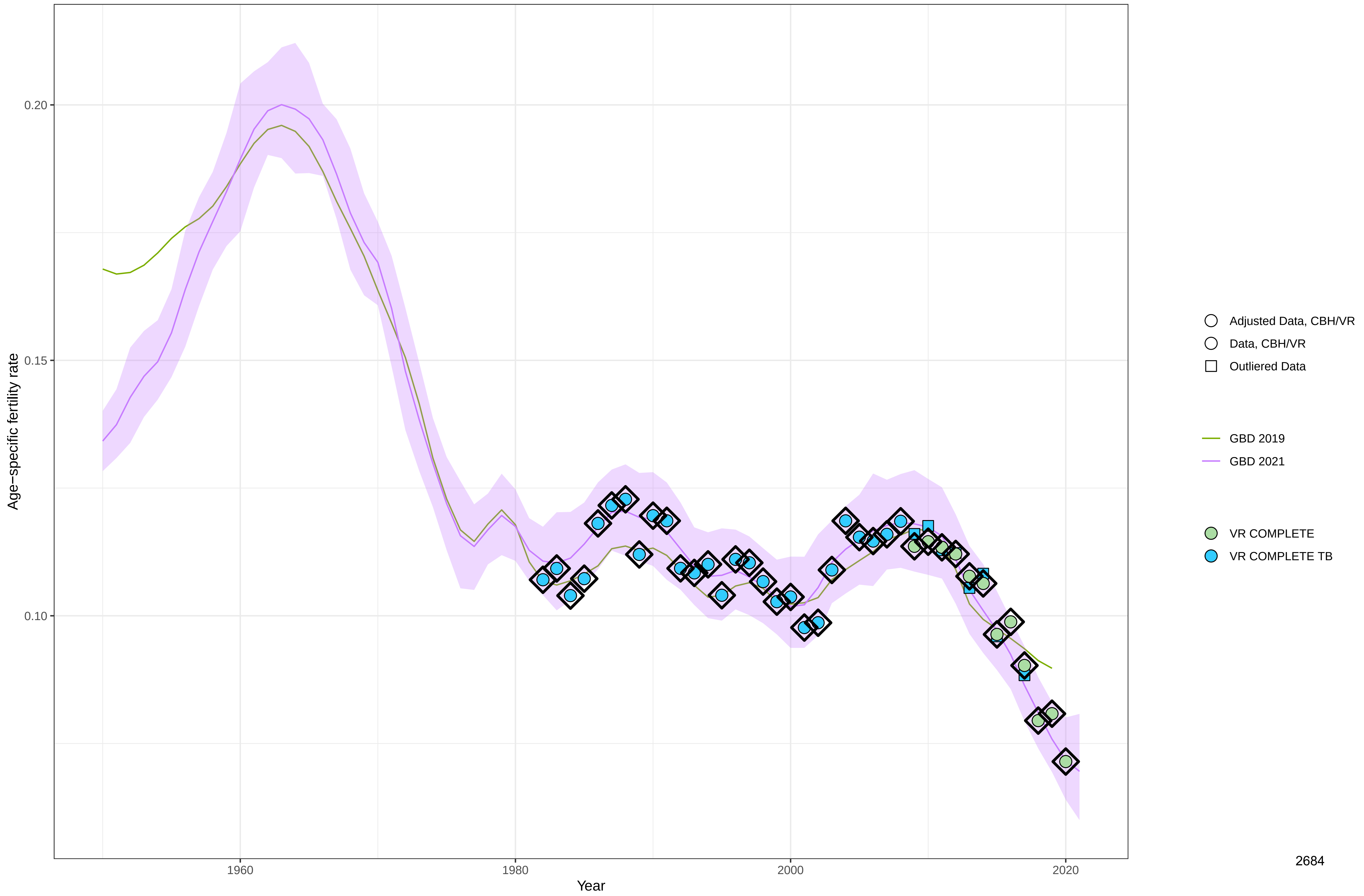

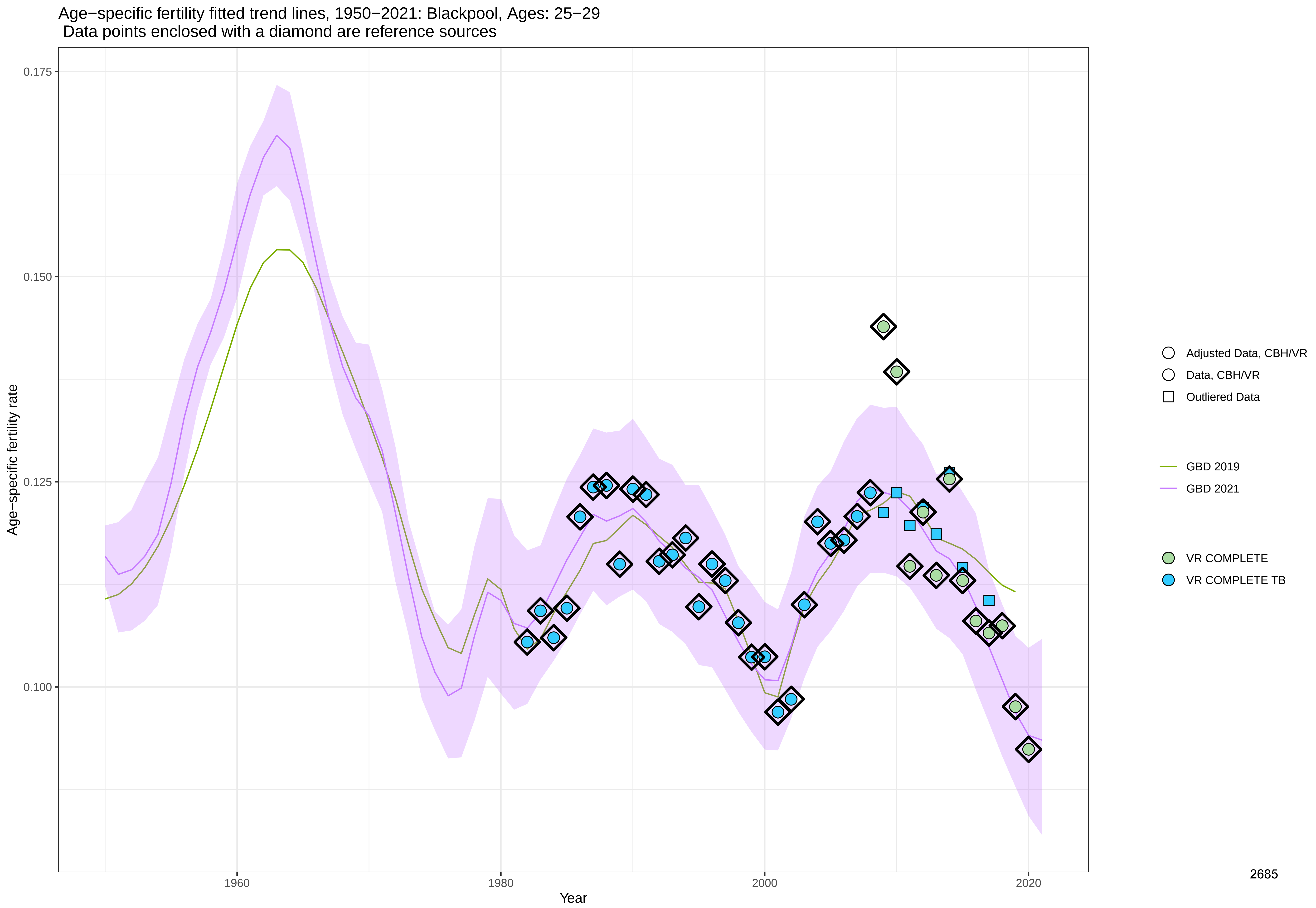
Age−specific fertility fitted trend lines, 1950−2021: Blackpool, Ages: 25−29
Data points enclosed with a diamond are reference sources
0.175
0.150
0.125
0.100
Age−specific fertility rate
1960
1980
2000
2020
Year
Adjusted Data, CBH/VR
Data, CBH/VR
Outliered Data
GBD 2019
GBD 2021
VR COMPLETE
VR COMPLETE TB

Age−specific fertility fitted trend lines, 1950−2021: Blackpool, Ages: 30−34
Data points enclosed with a diamond are reference sources

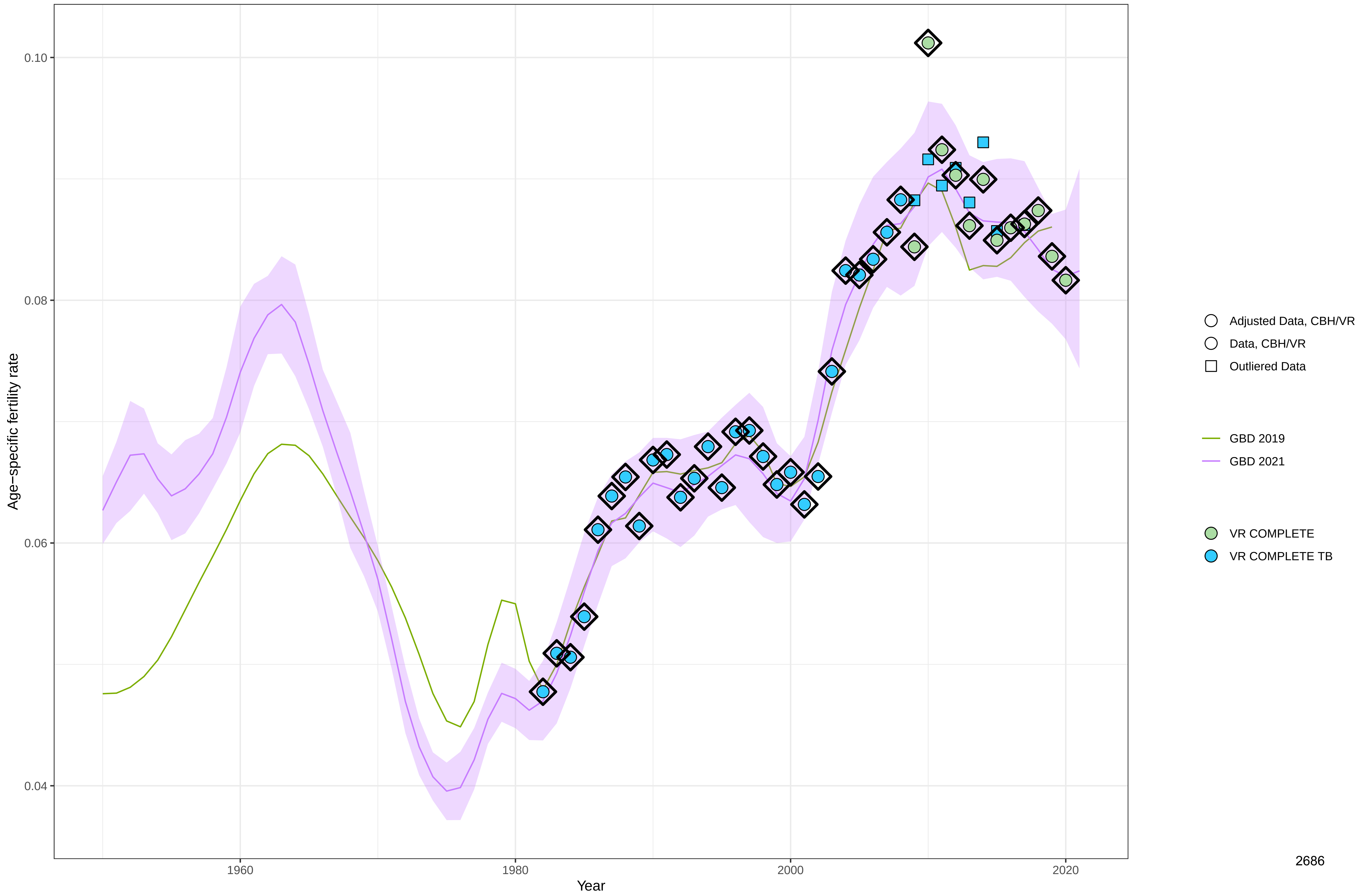

Age−specific fertility fitted trend lines, 1950−2021: Blackpool, Ages: 35−39
Data points enclosed with a diamond are reference sources

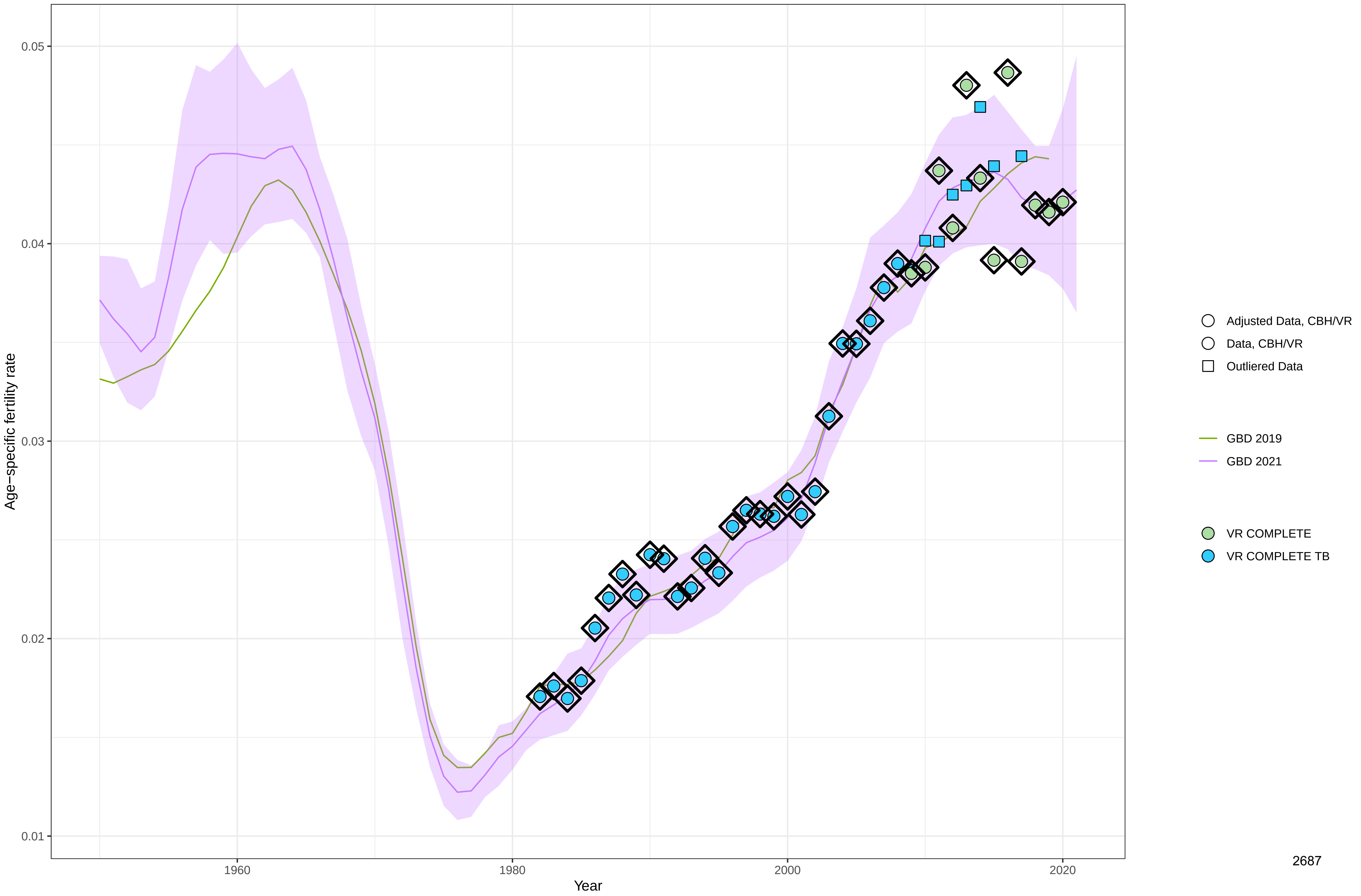

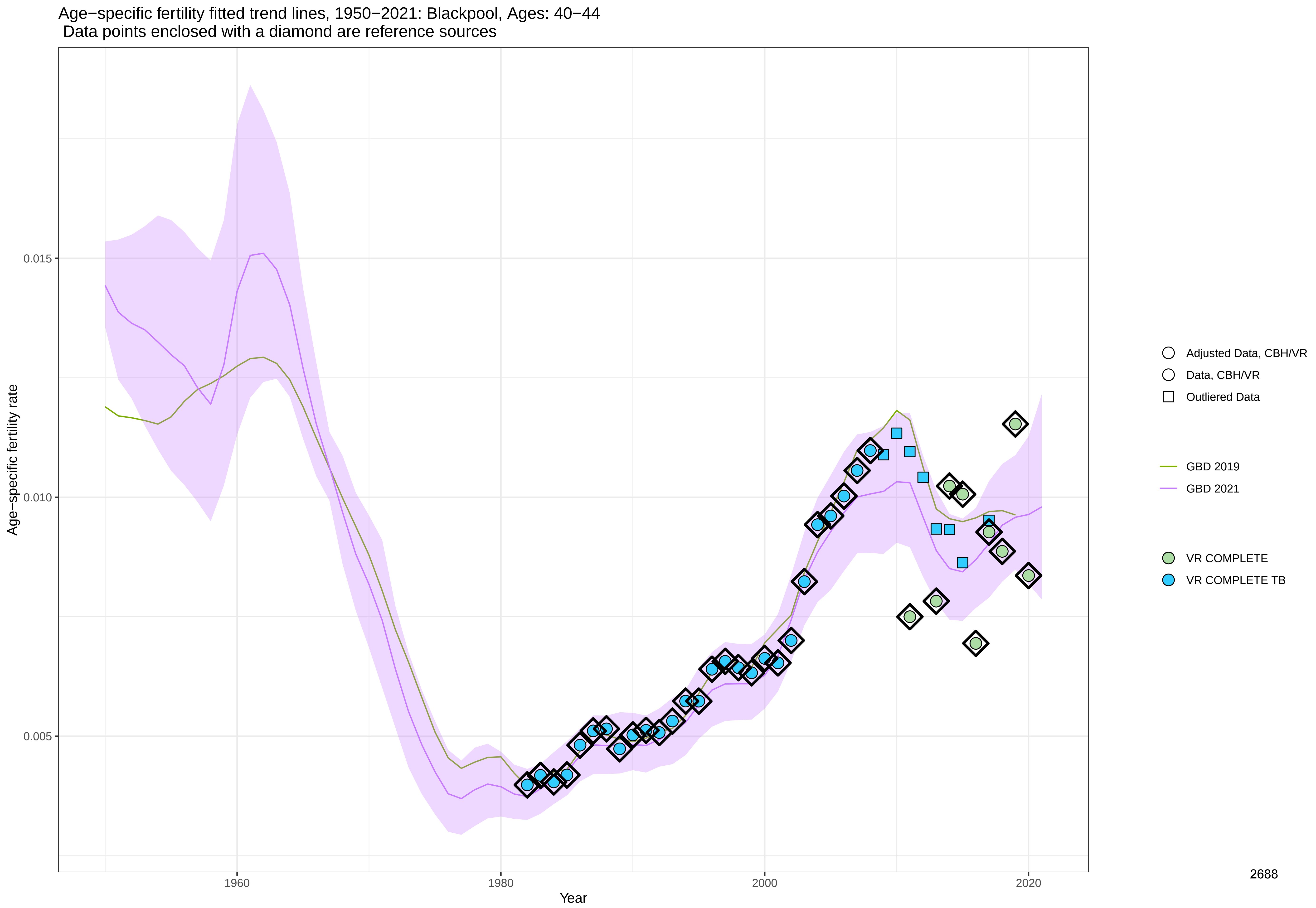

Age−specific fertility fitted trend lines, 1950−2021: Blackpool, Ages: 40−44
Data points enclosed with a diamond are reference sources
Age−specific fertility rate
0.015
0.010
0.005
1960
1980
2000
2020
Year
Adjusted Data, CBH/VR
Data, CBH/VR
Outliered Data
GBD 2019
GBD 2021
VR COMPLETE
VR COMPLETE TB

2688

Age−specific fertility fitted trend lines, 1950−2021: Blackpool, Ages: 45−49
Data points enclosed with a diamond are reference sources

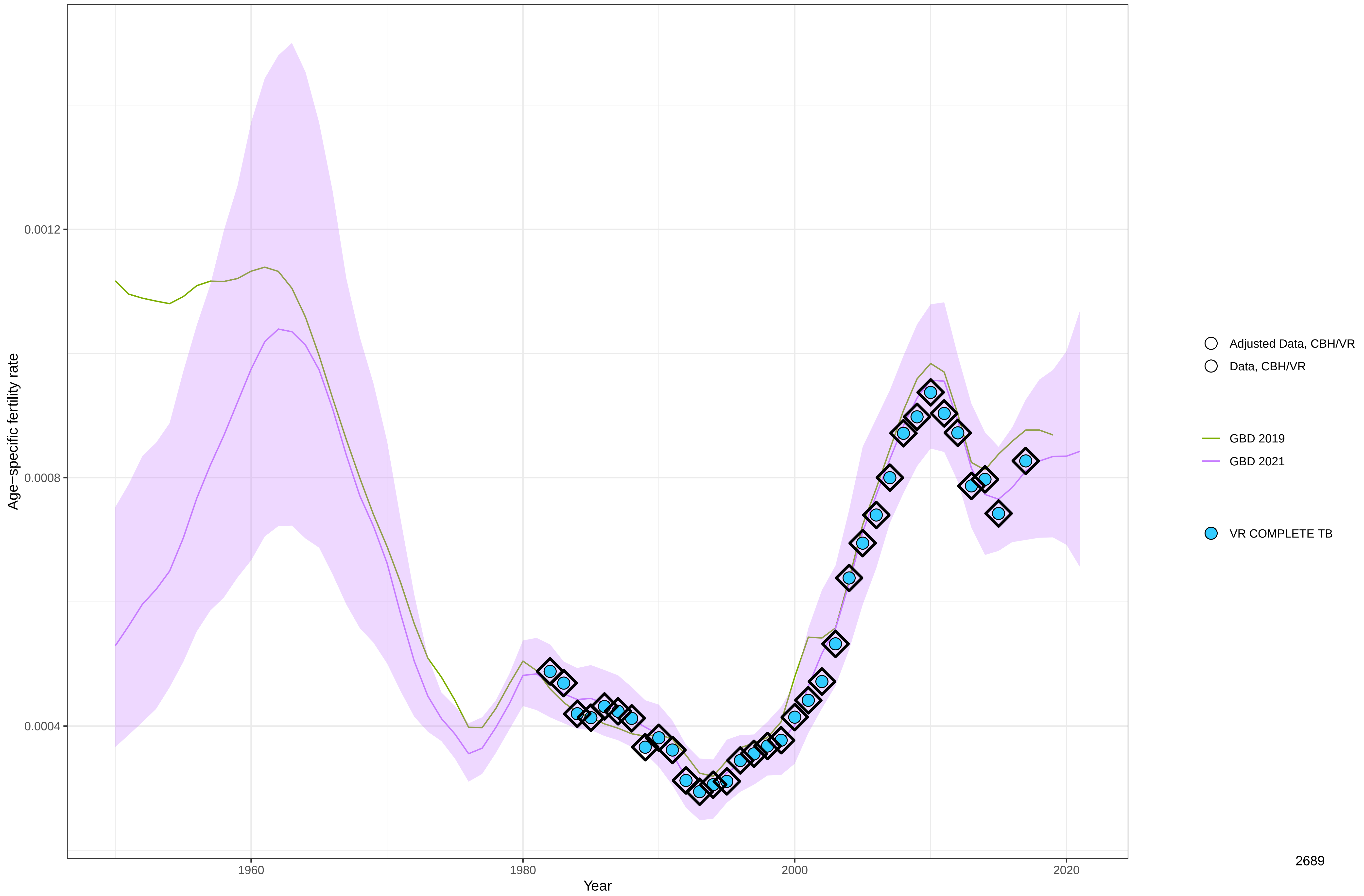

Age−specific fertility fitted trend lines, 1950−2021: Blackpool, Ages: 50−54
Data points enclosed with a diamond are reference sources

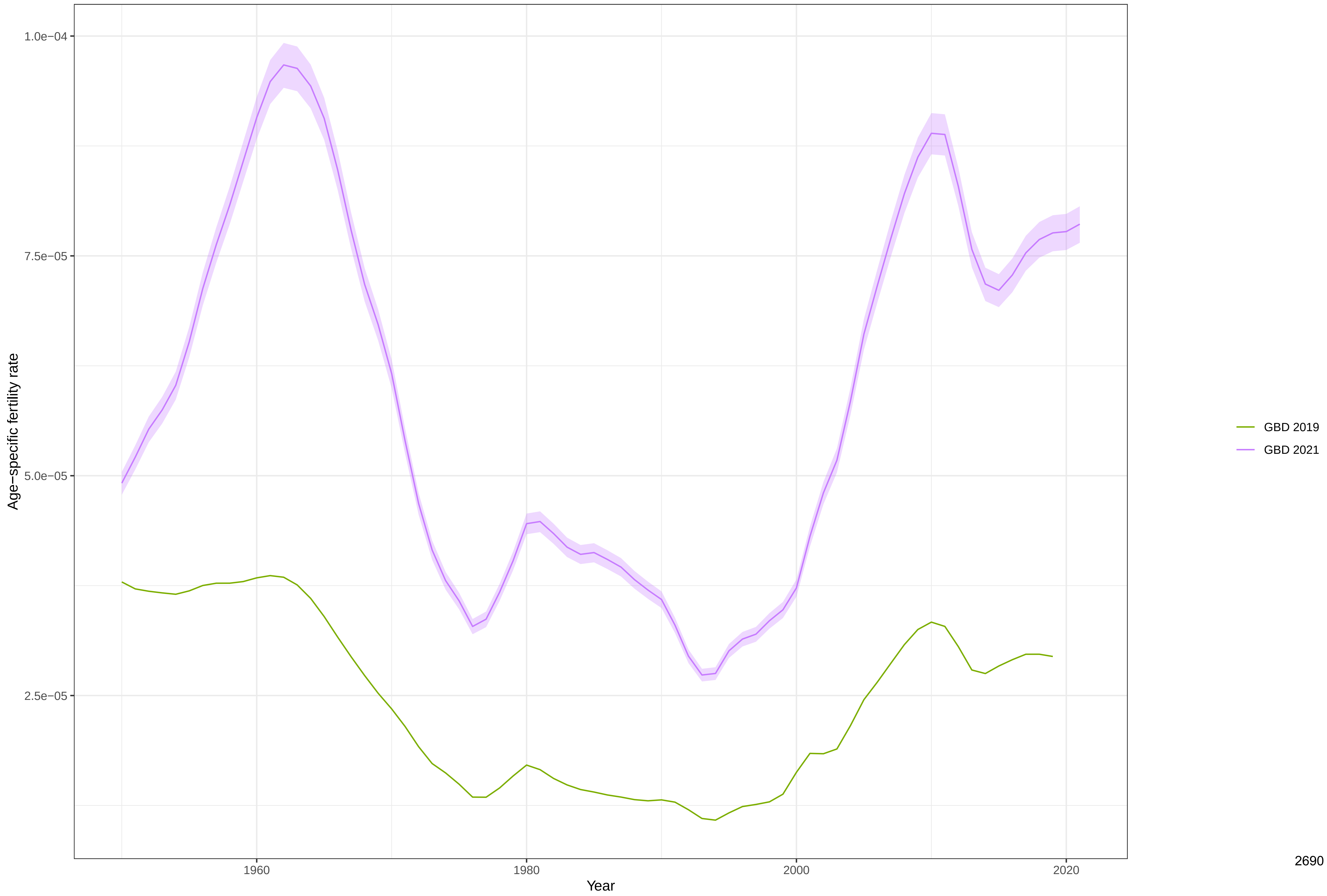

Total fertility rate fitted trend lines, 1950−2021: Blackpool TFR
Data points enclosed with a diamond are reference sources

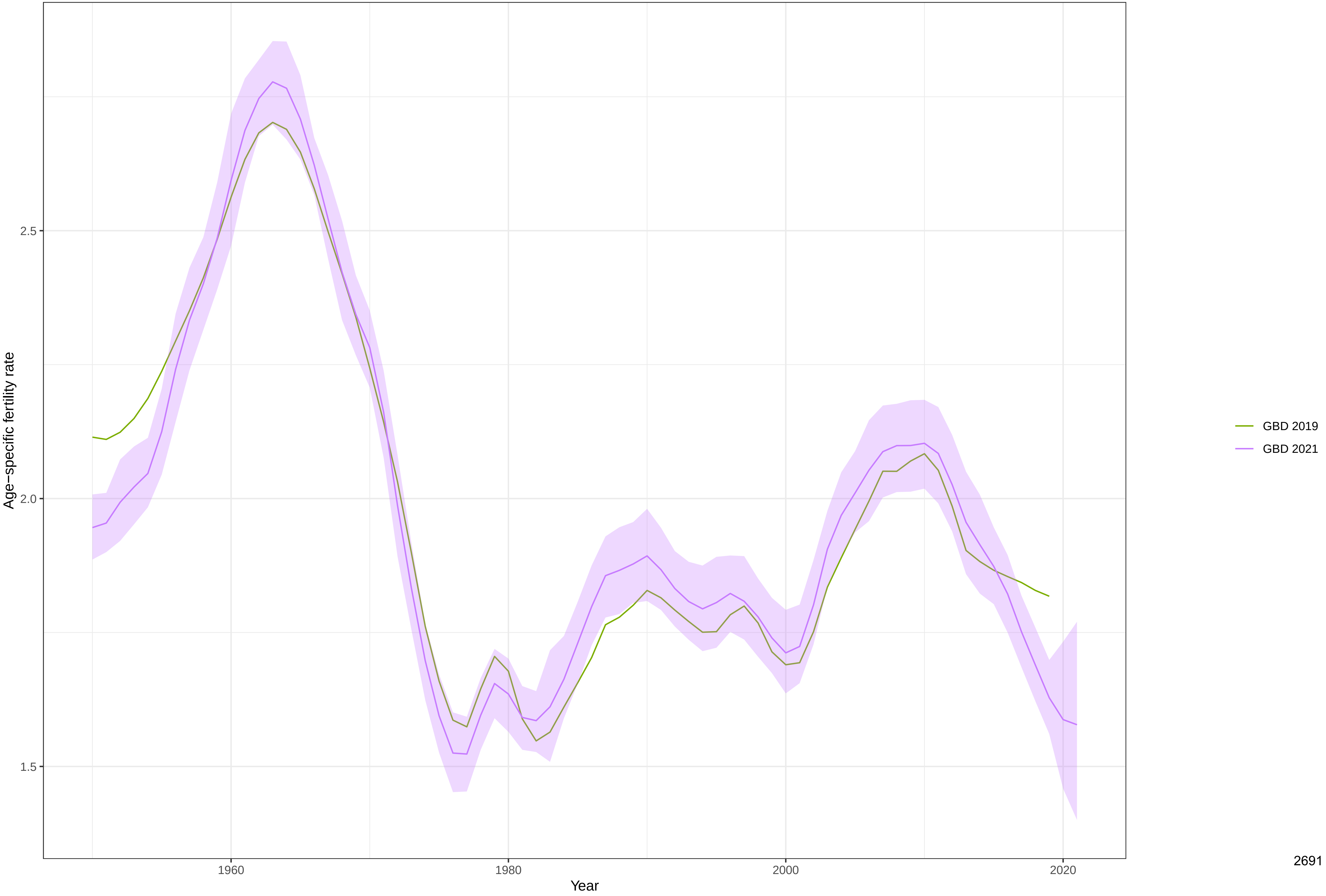

Age−specific fertility fitted trend lines, 1950−2021: Bolton, Ages: 10−14
Data points enclosed with a diamond are reference sources

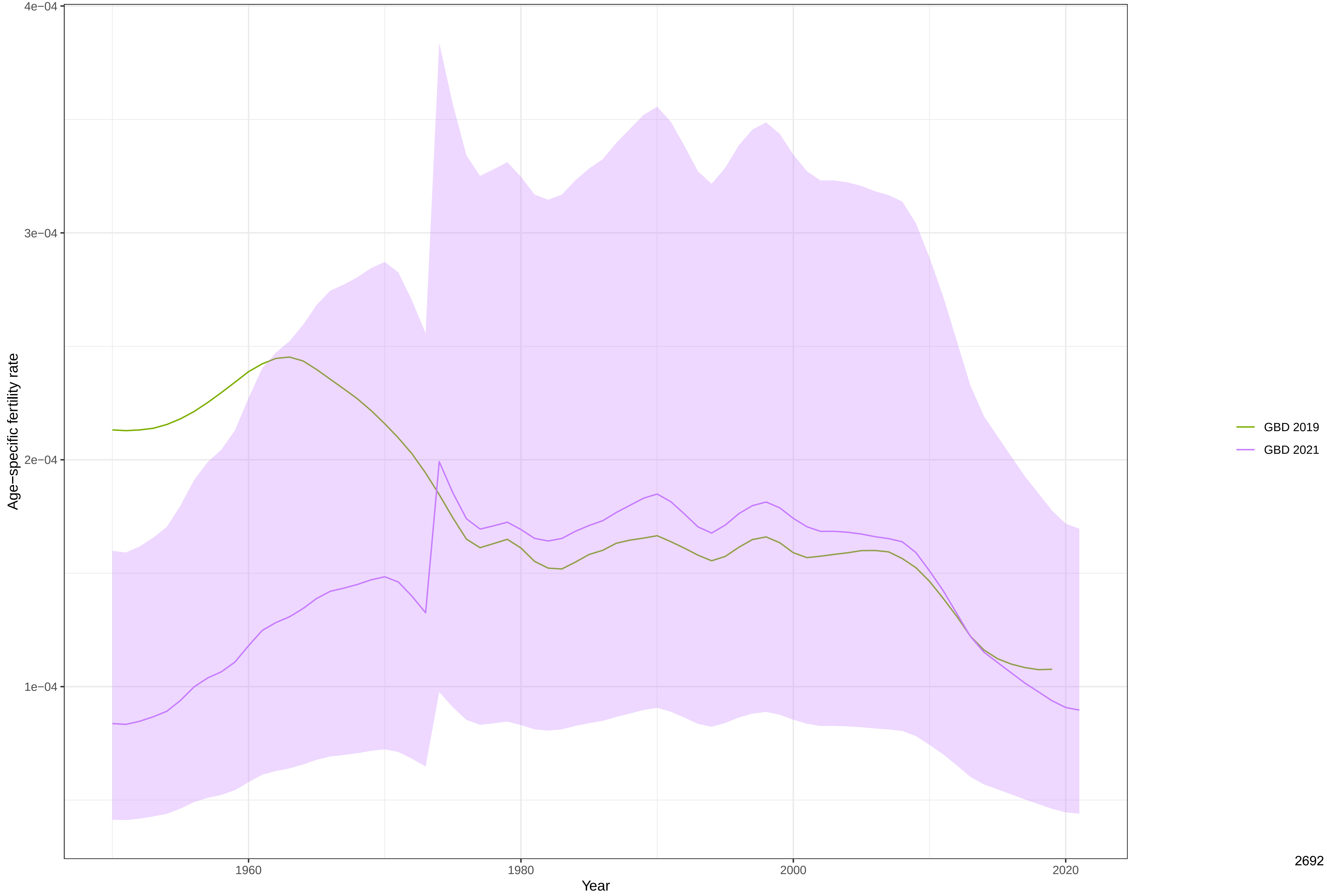

Age−specific fertility fitted trend lines, 1950−2021: Bolton, Ages: 15−19
Data points enclosed with a diamond are reference sources

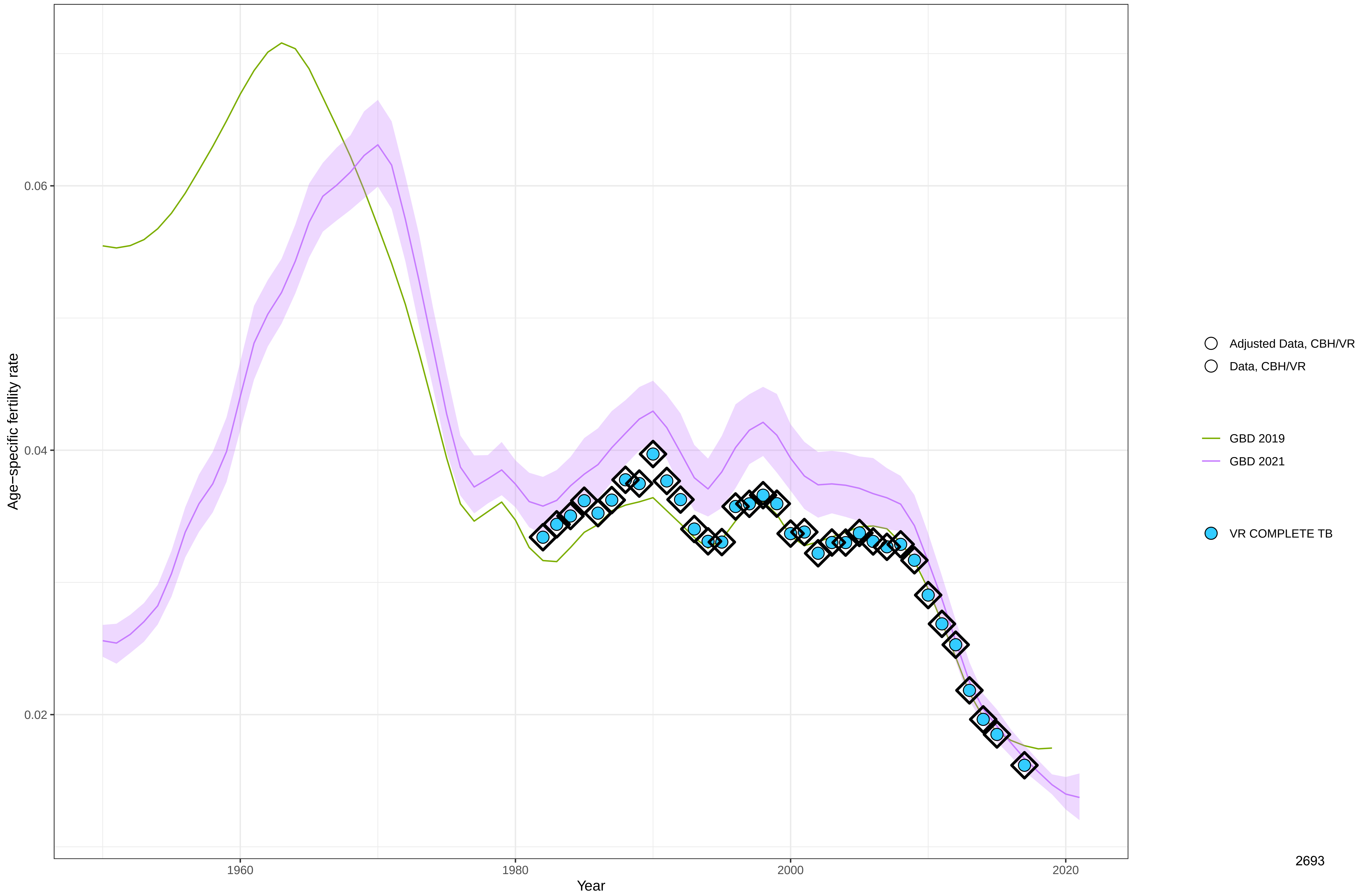

Age−specific fertility fitted trend lines, 1950−2021: Bolton, Ages: 20−24
Data points enclosed with a diamond are reference sources

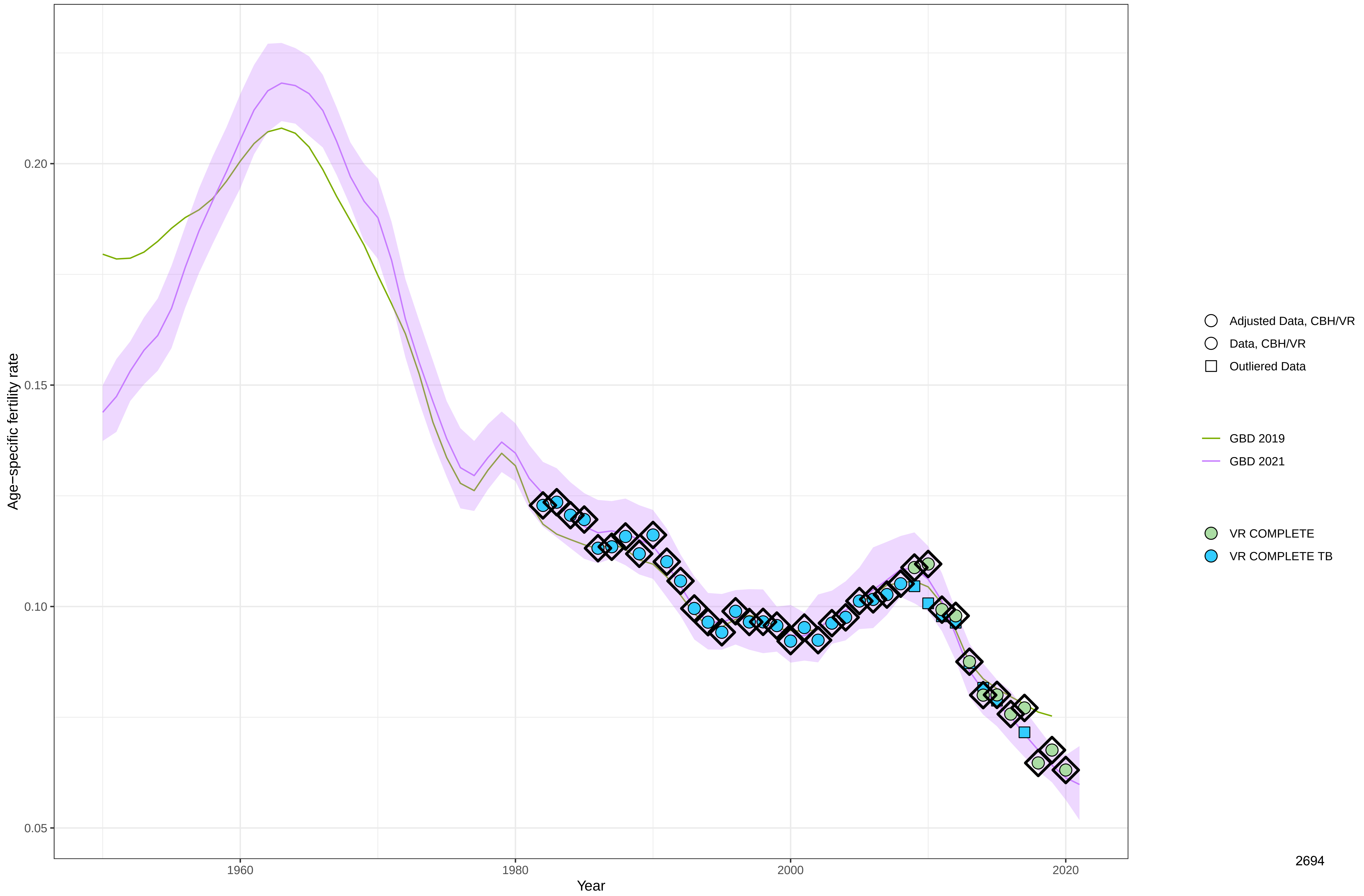

Age−specific fertility fitted trend lines, 1950−2021: Bolton, Ages: 25−29
Data points enclosed with a diamond are reference sources

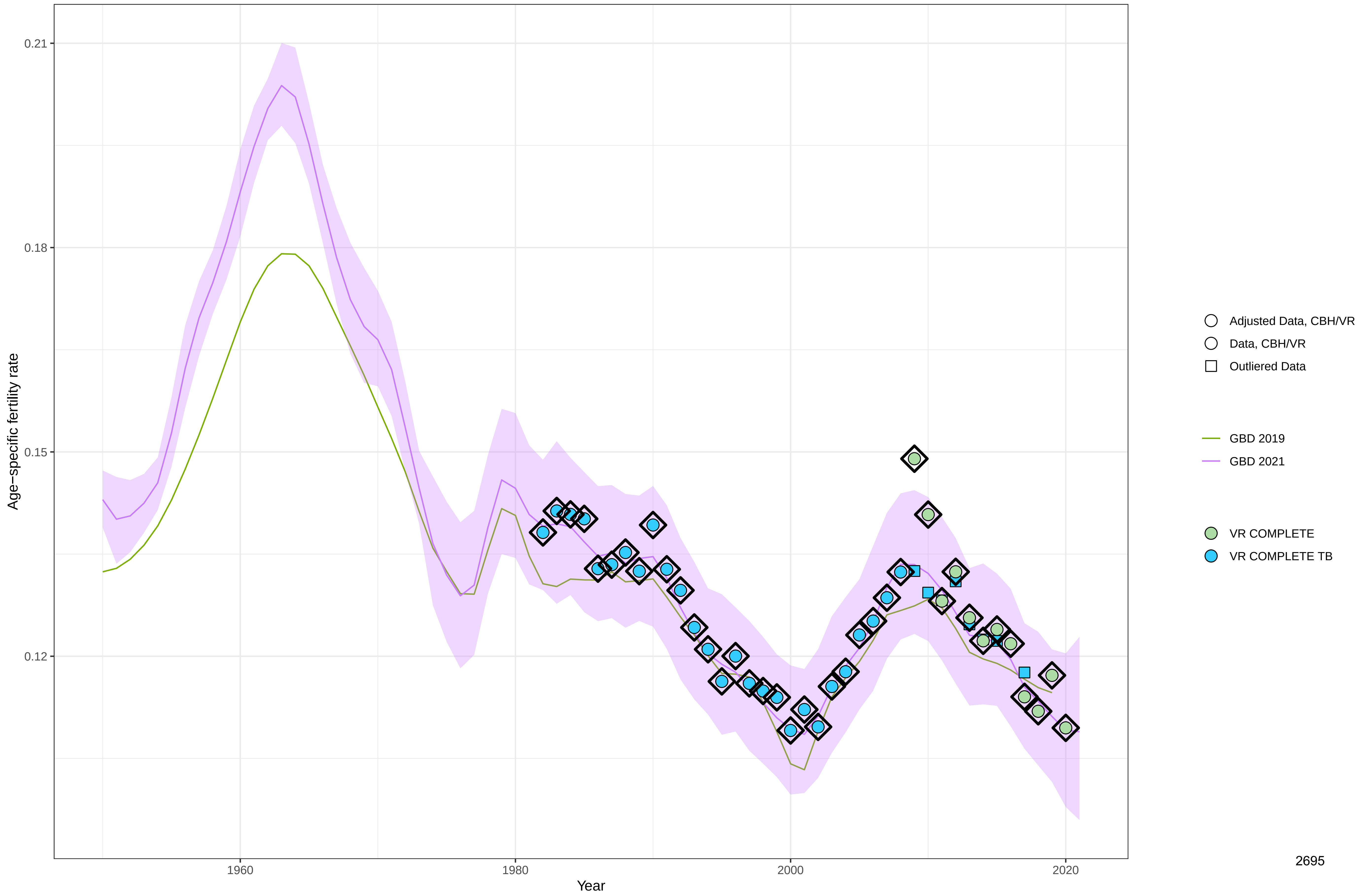

Age−specific fertility fitted trend lines, 1950−2021: Bolton, Ages: 30−34
Data points enclosed with a diamond are reference sources

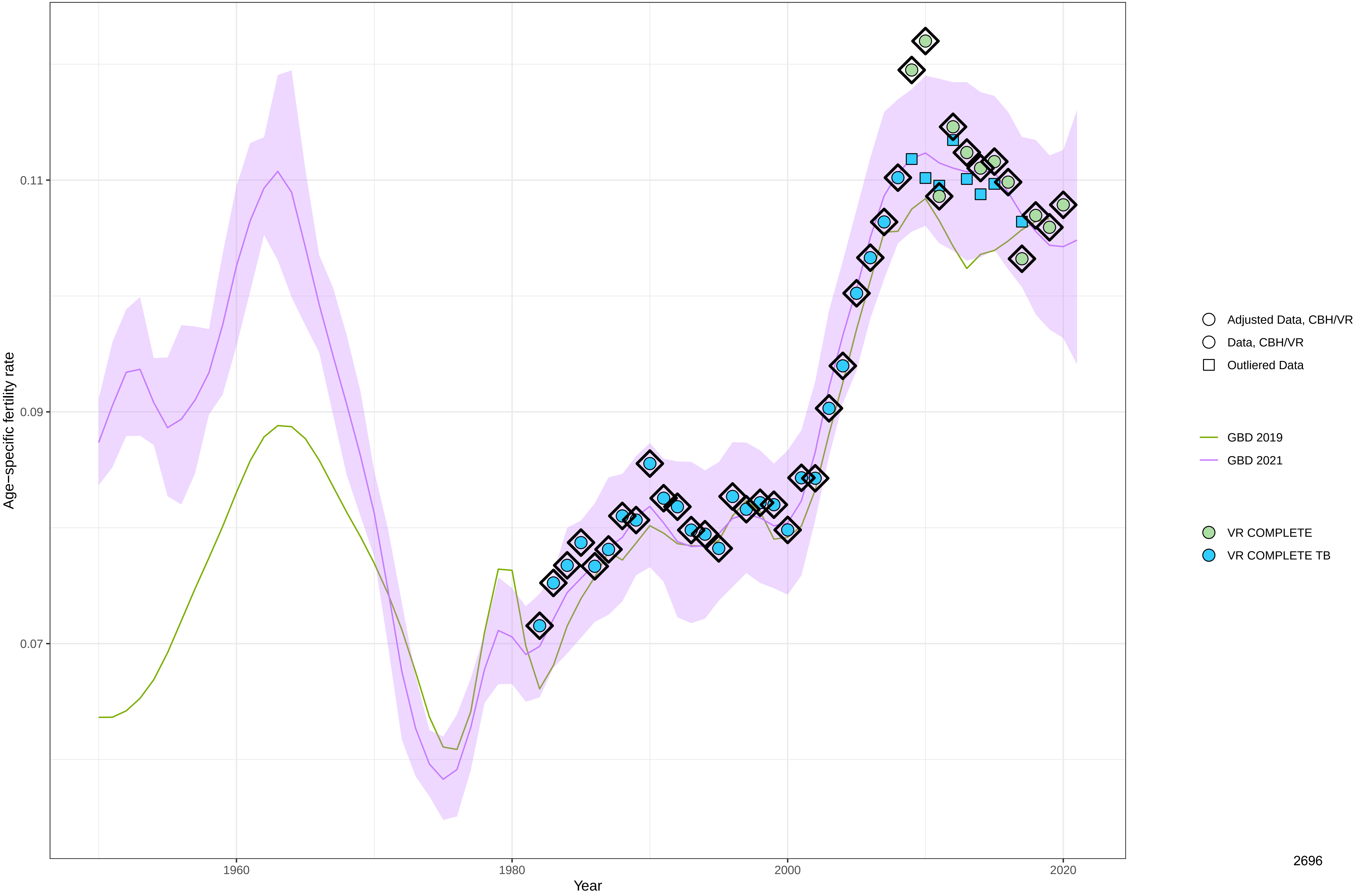

Age−specific fertility fitted trend lines, 1950−2021: Bolton, Ages: 35−39
Data points enclosed with a diamond are reference sources

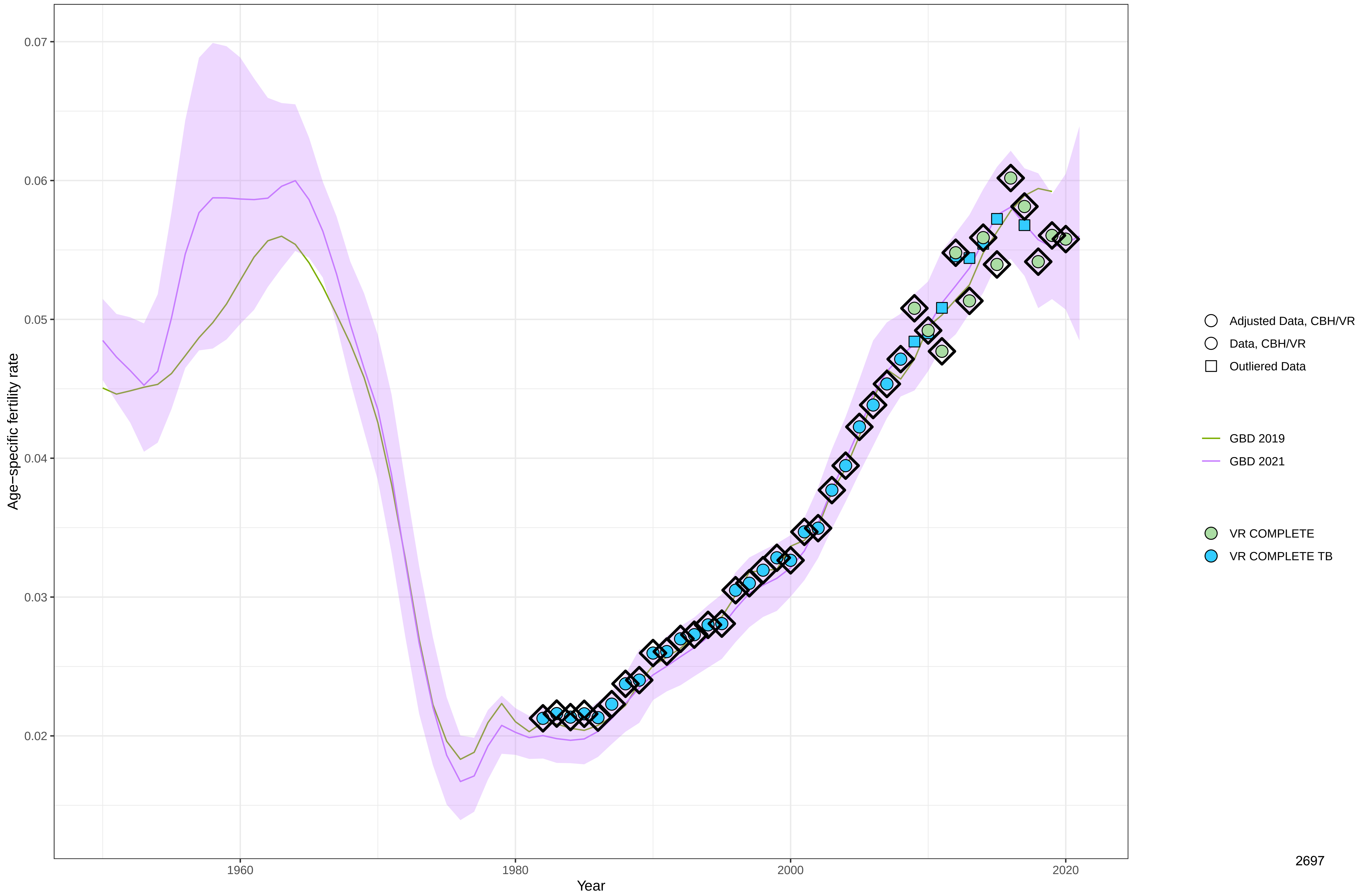

Age−specific fertility fitted trend lines, 1950−2021: Bolton, Ages: 40−44
Data points enclosed with a diamond are reference sources

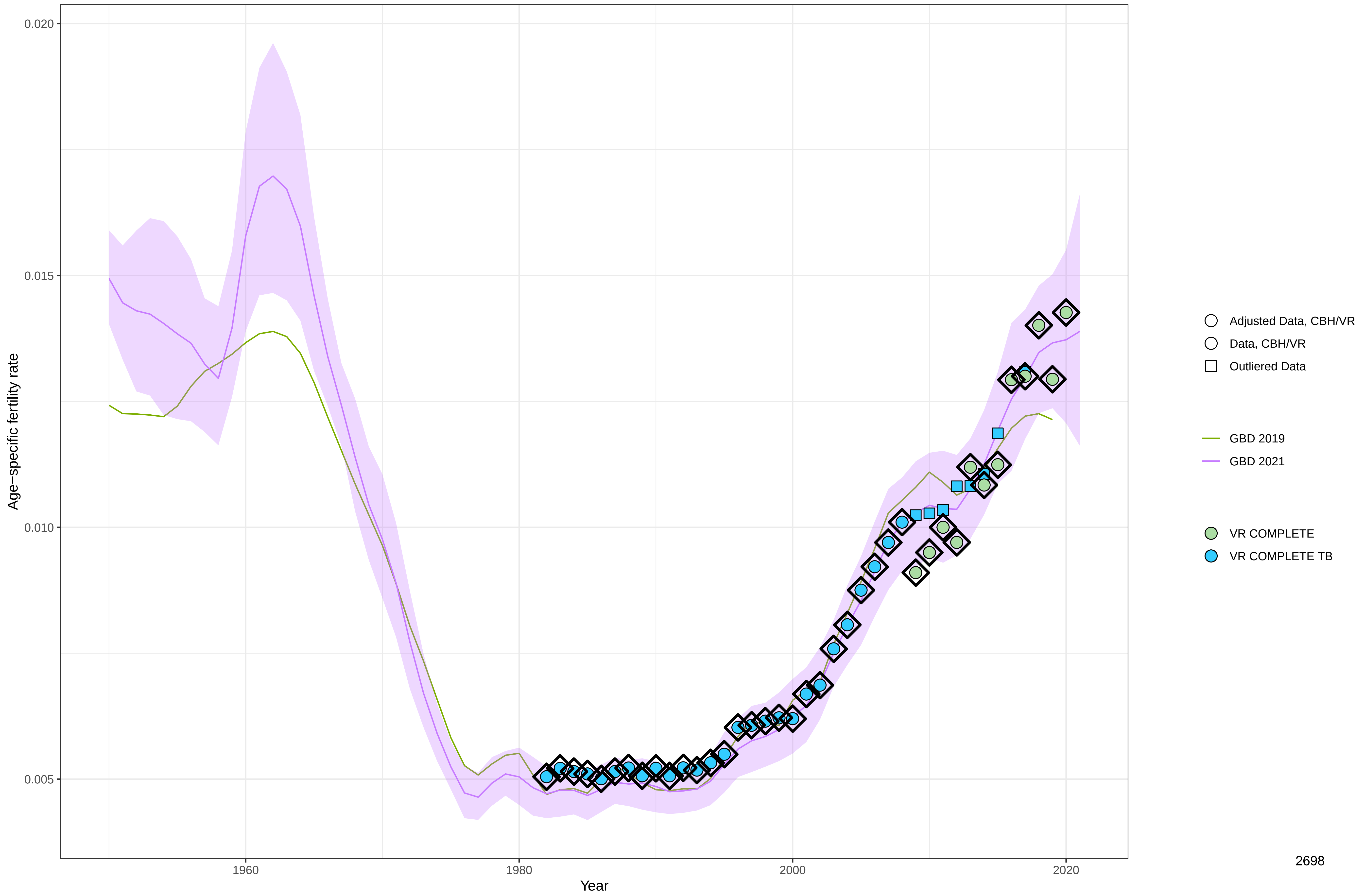

Age−specific fertility fitted trend lines, 1950−2021: Bolton, Ages: 45−49
Data points enclosed with a diamond are reference sources

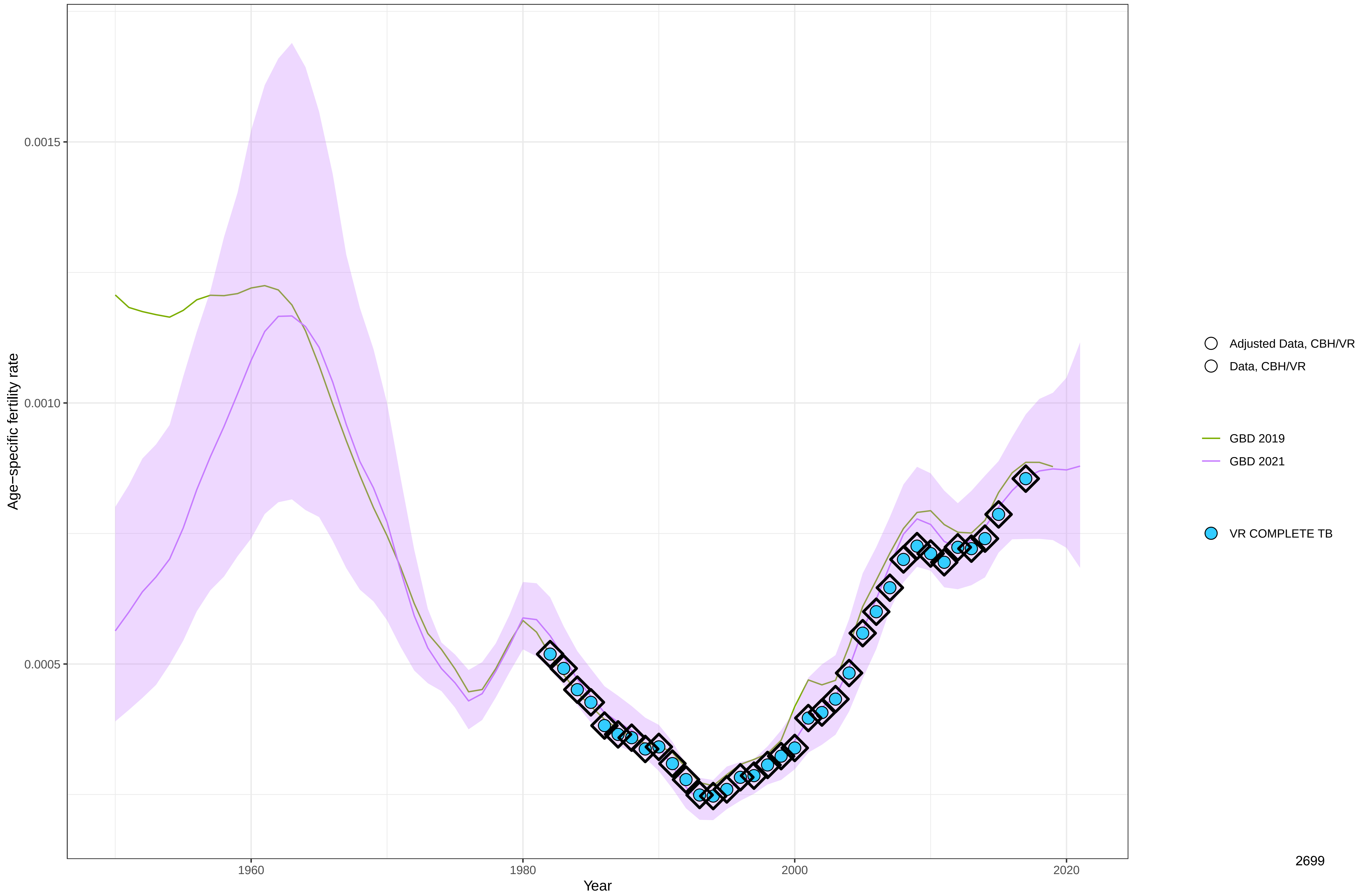

Age−specific fertility fitted trend lines, 1950−2021: Bolton, Ages: 50−54
Data points enclosed with a diamond are reference sources

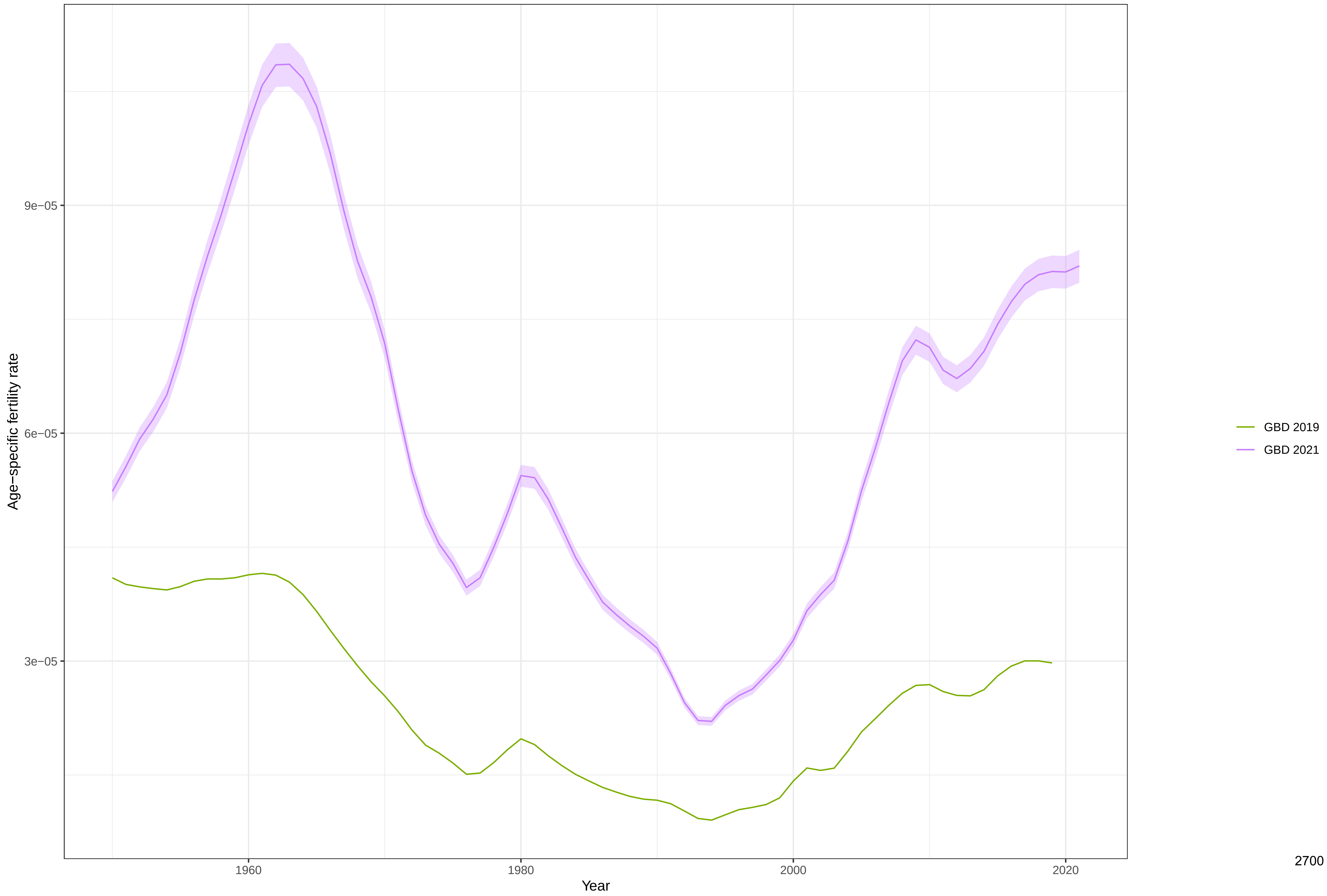

Total fertility rate fitted trend lines, 1950−2021: Bolton TFR
Data points enclosed with a diamond are reference sources

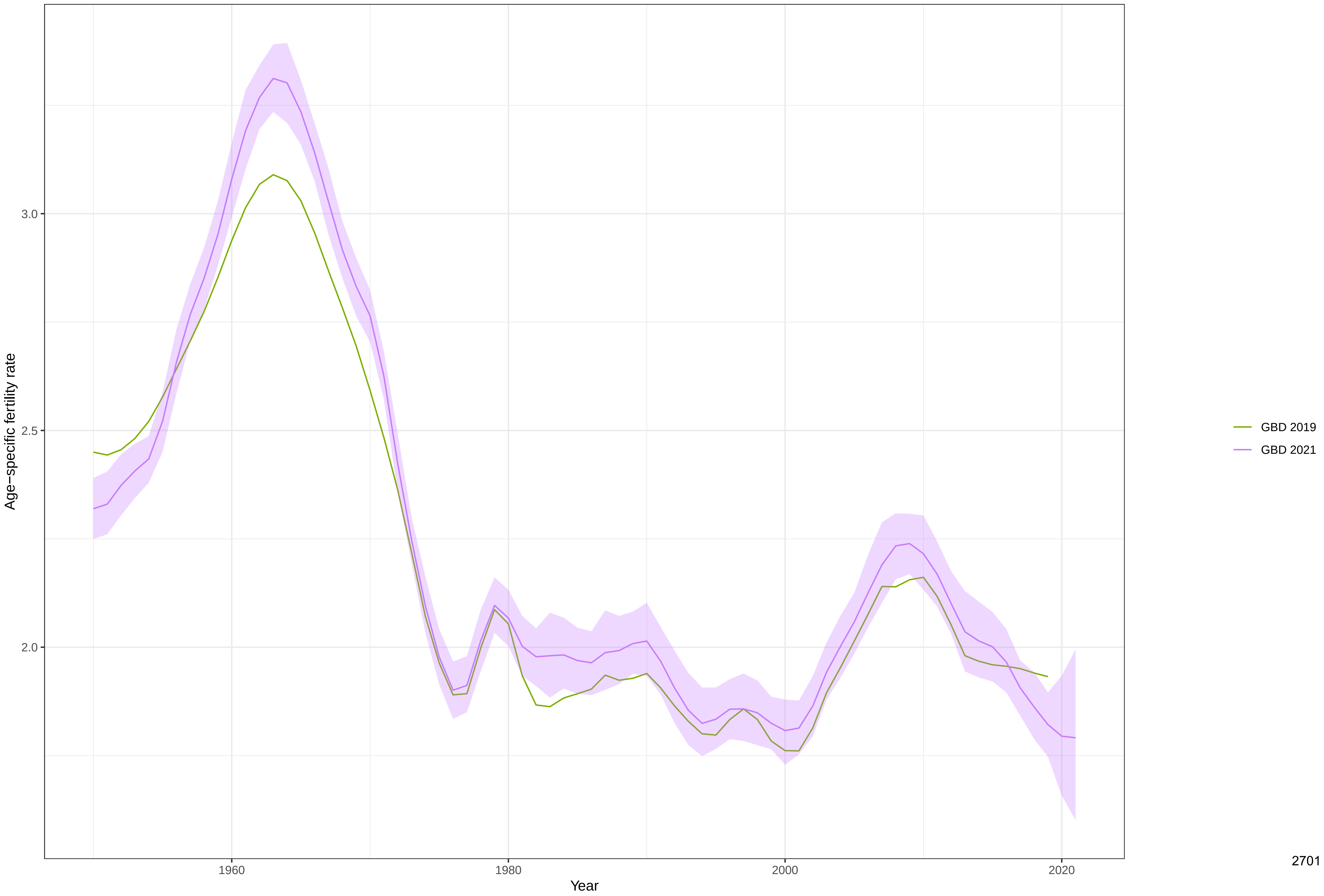

Age−specific fertility fitted trend lines, 1950−2021: Bury, Ages: 10−14
Data points enclosed with a diamond are reference sources

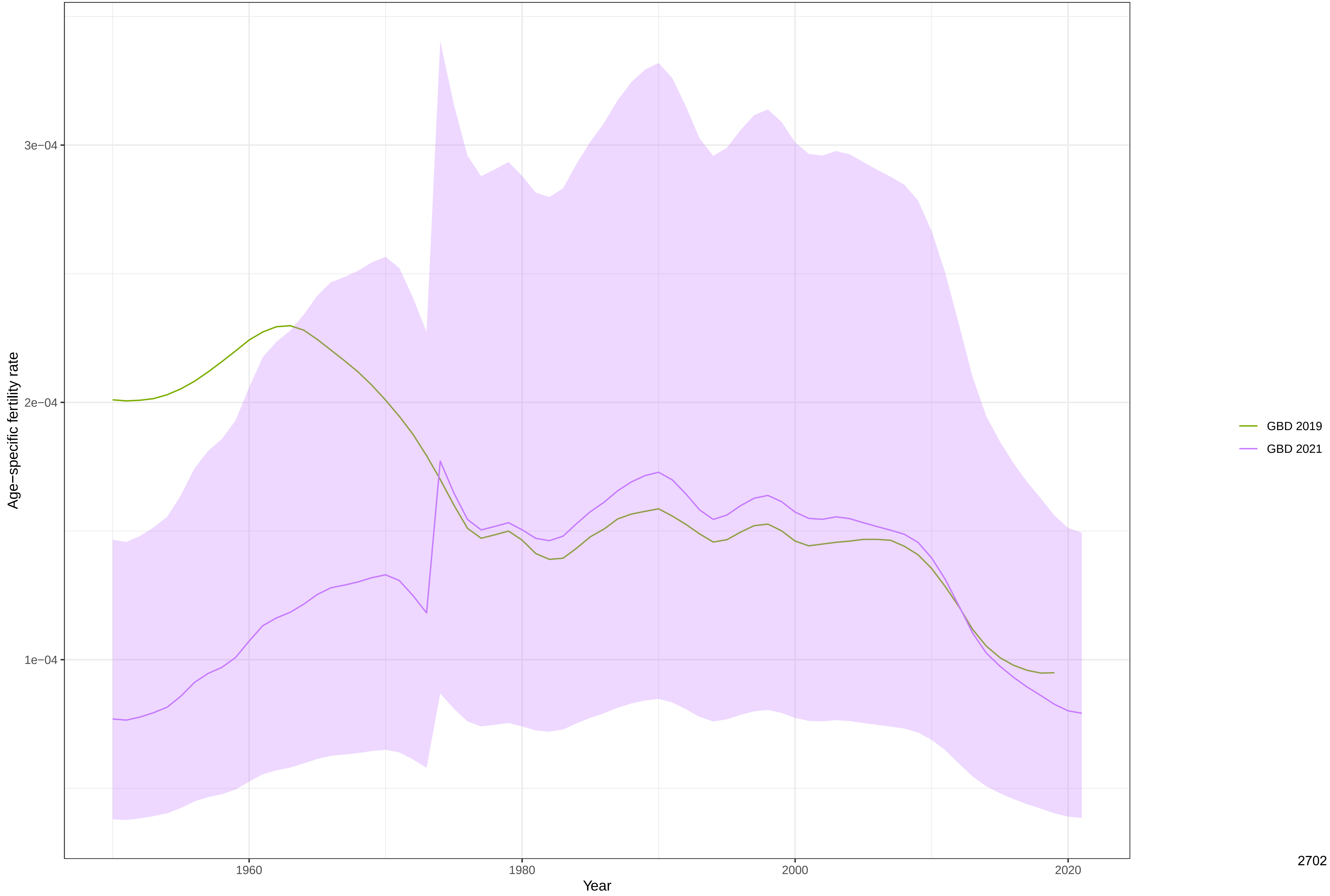

Age−specific fertility fitted trend lines, 1950−2021: Bury, Ages: 15−19
Data points enclosed with a diamond are reference sources

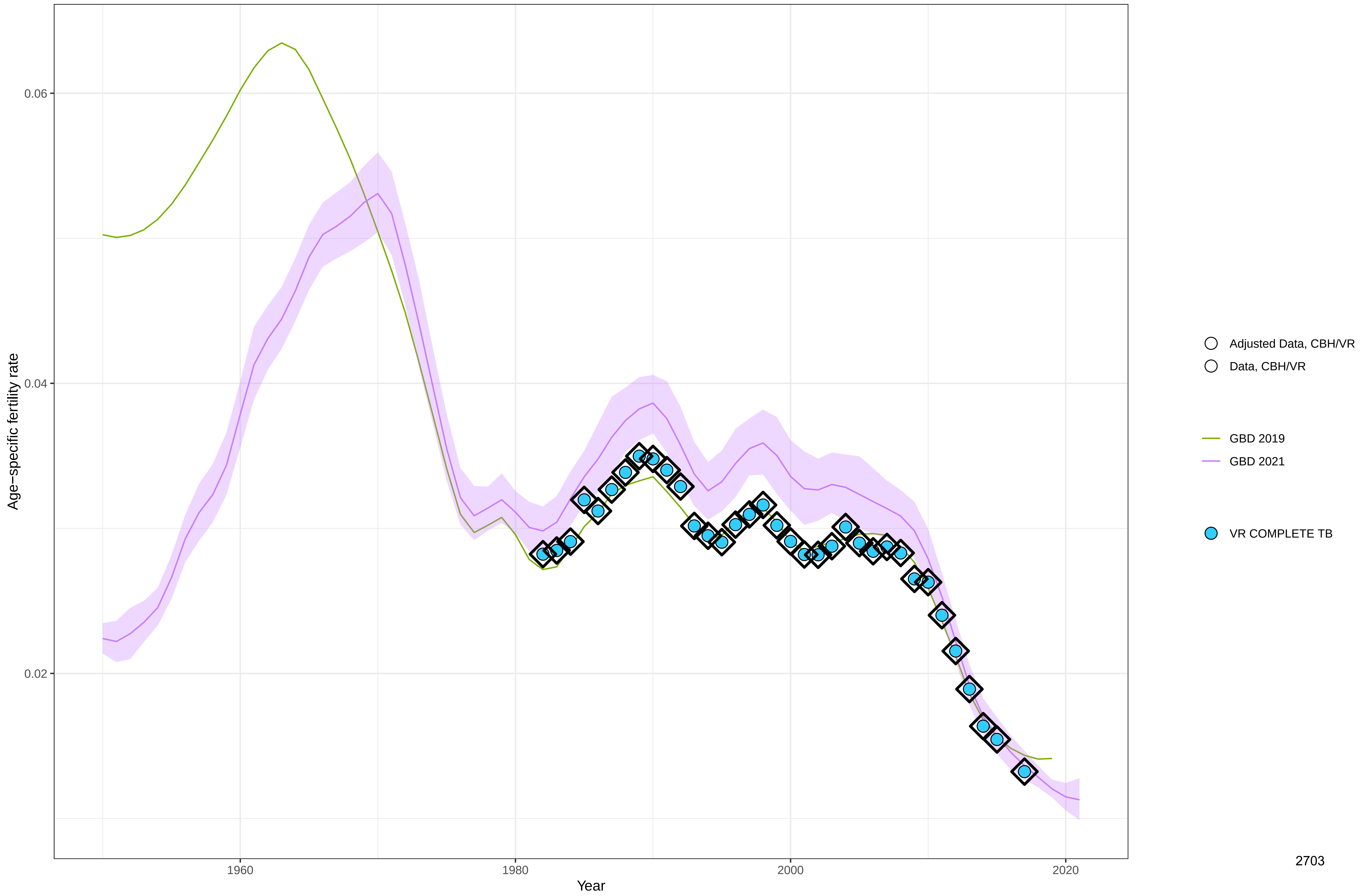

Age−specific fertility fitted trend lines, 1950−2021: Bury, Ages: 20−24
 Data points enclosed with a diamond are reference sources

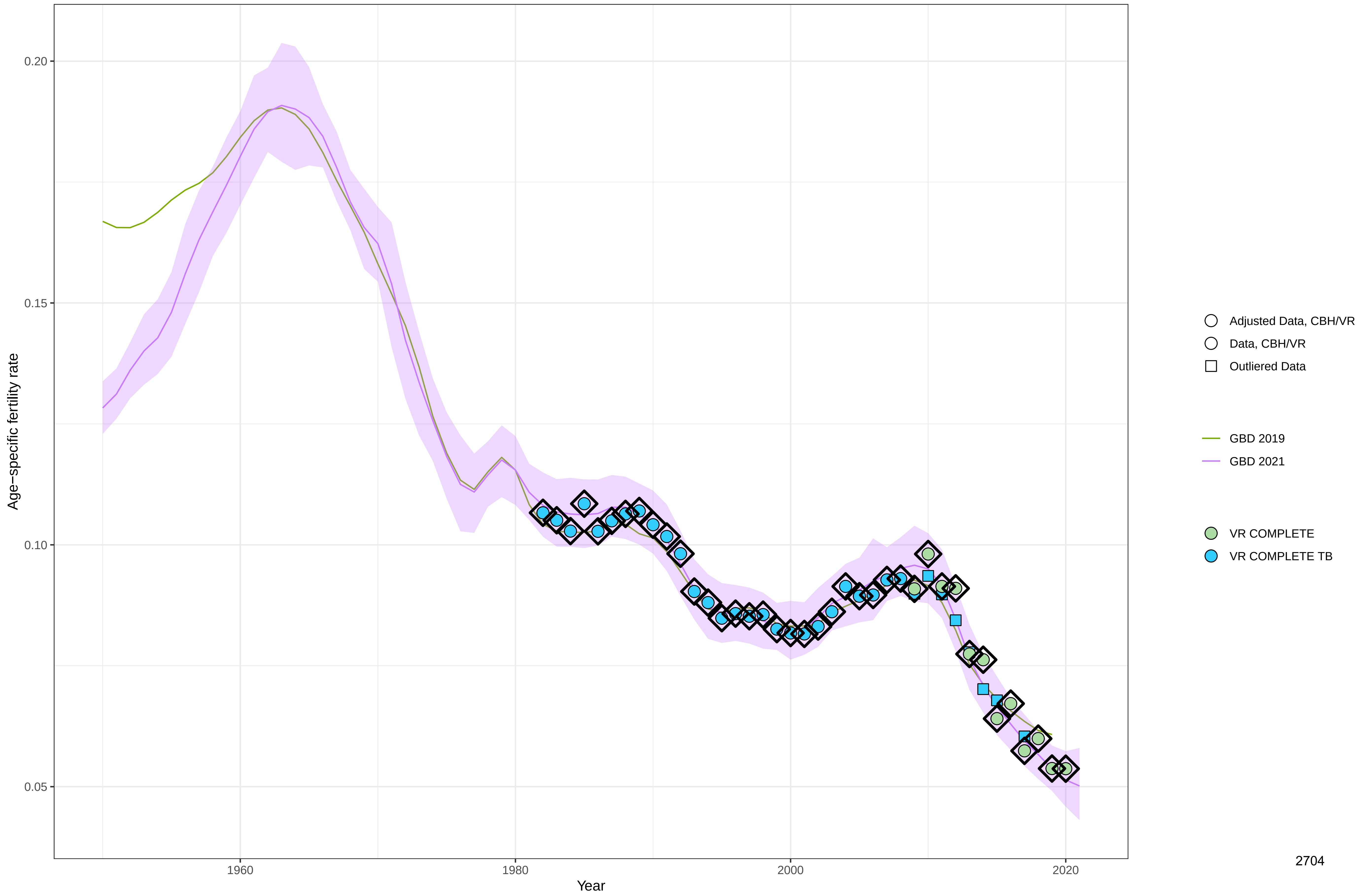

Age−specific fertility fitted trend lines, 1950−2021: Bury, Ages: 25−29
Data points enclosed with a diamond are reference sources

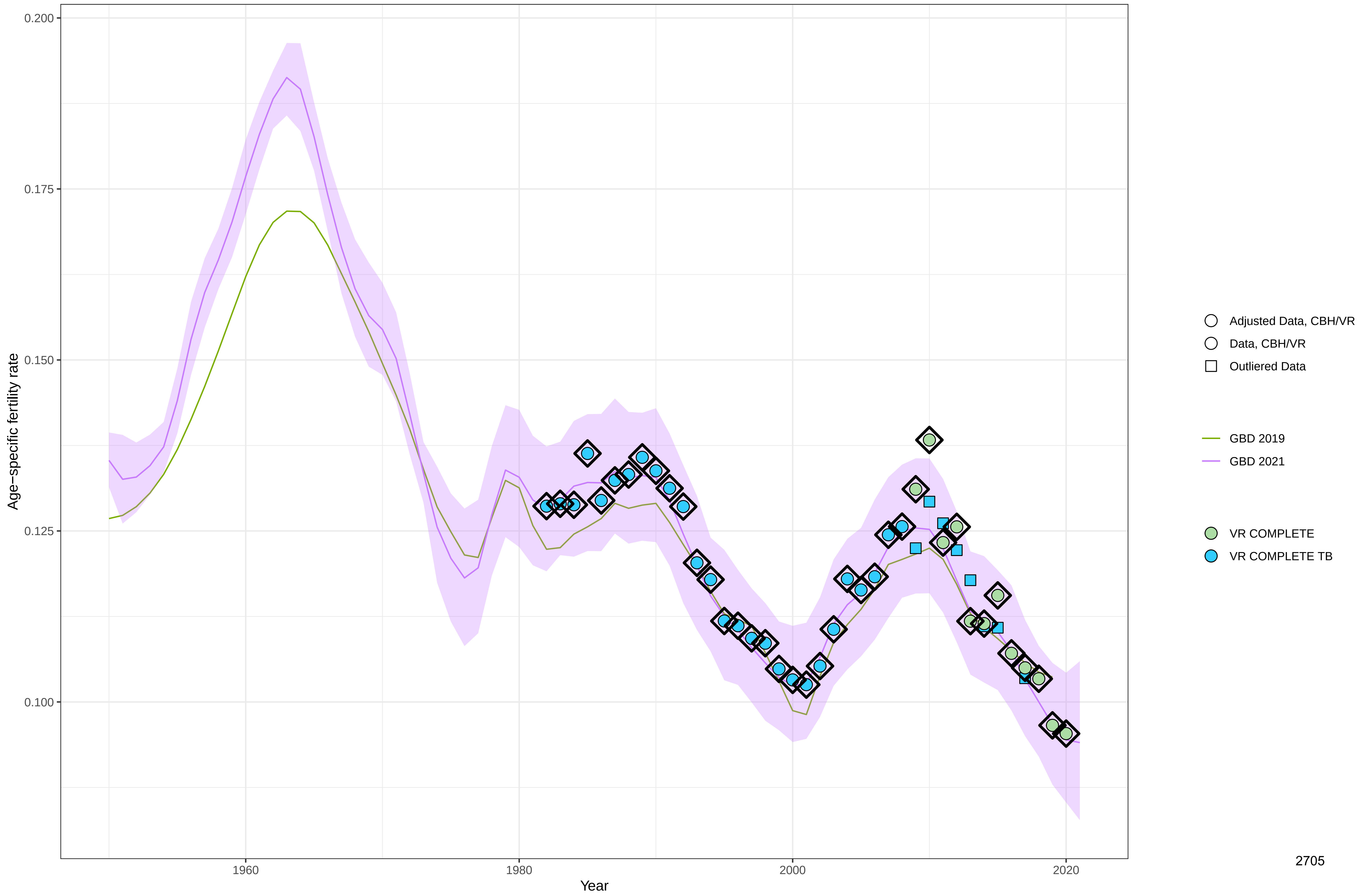

Age−specific fertility fitted trend lines, 1950−2021: Bury, Ages: 30−34
Data points enclosed with a diamond are reference sources

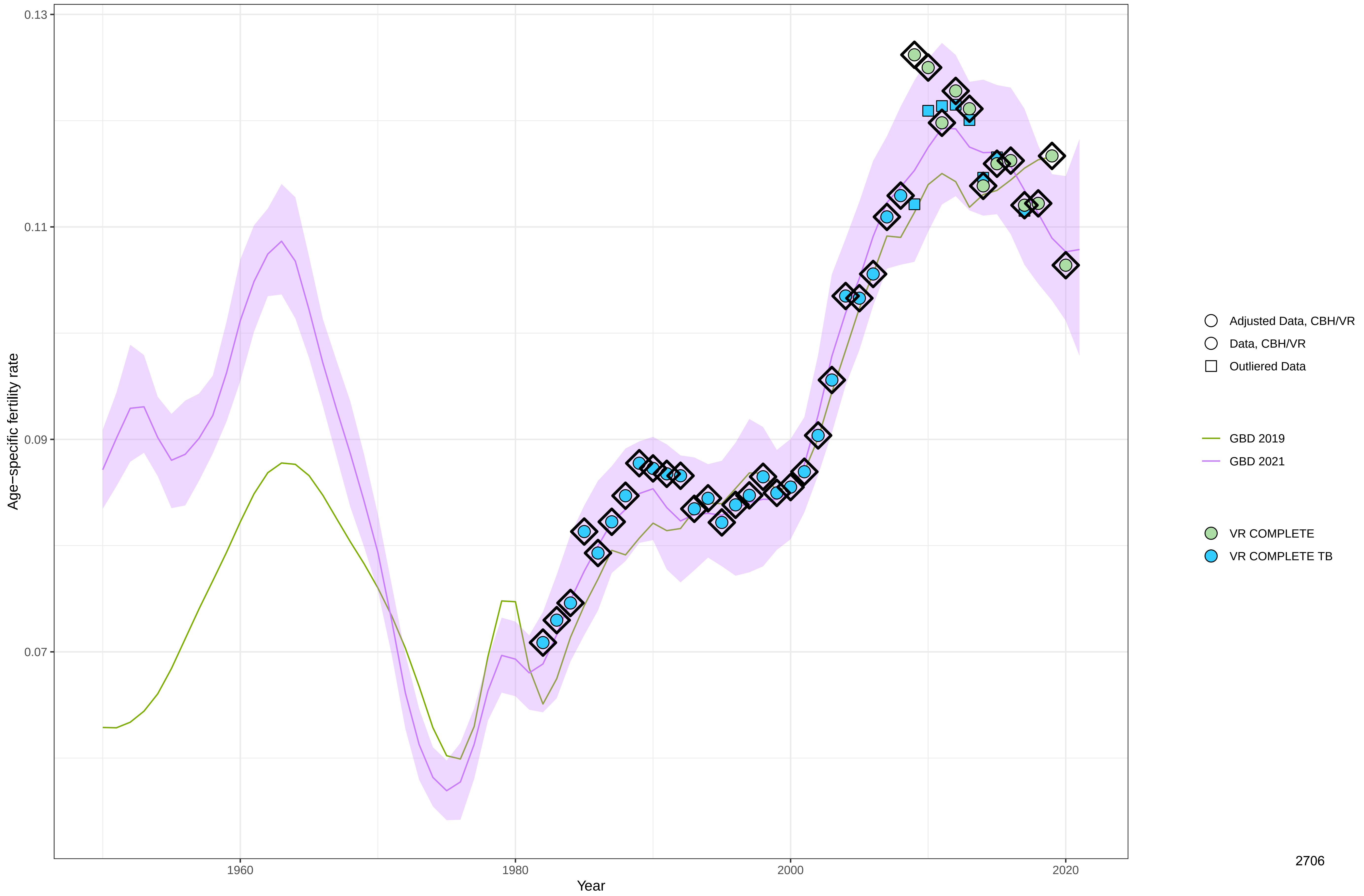

Age−specific fertility fitted trend lines, 1950−2021: Bury, Ages: 35−39
Data points enclosed with a diamond are reference sources

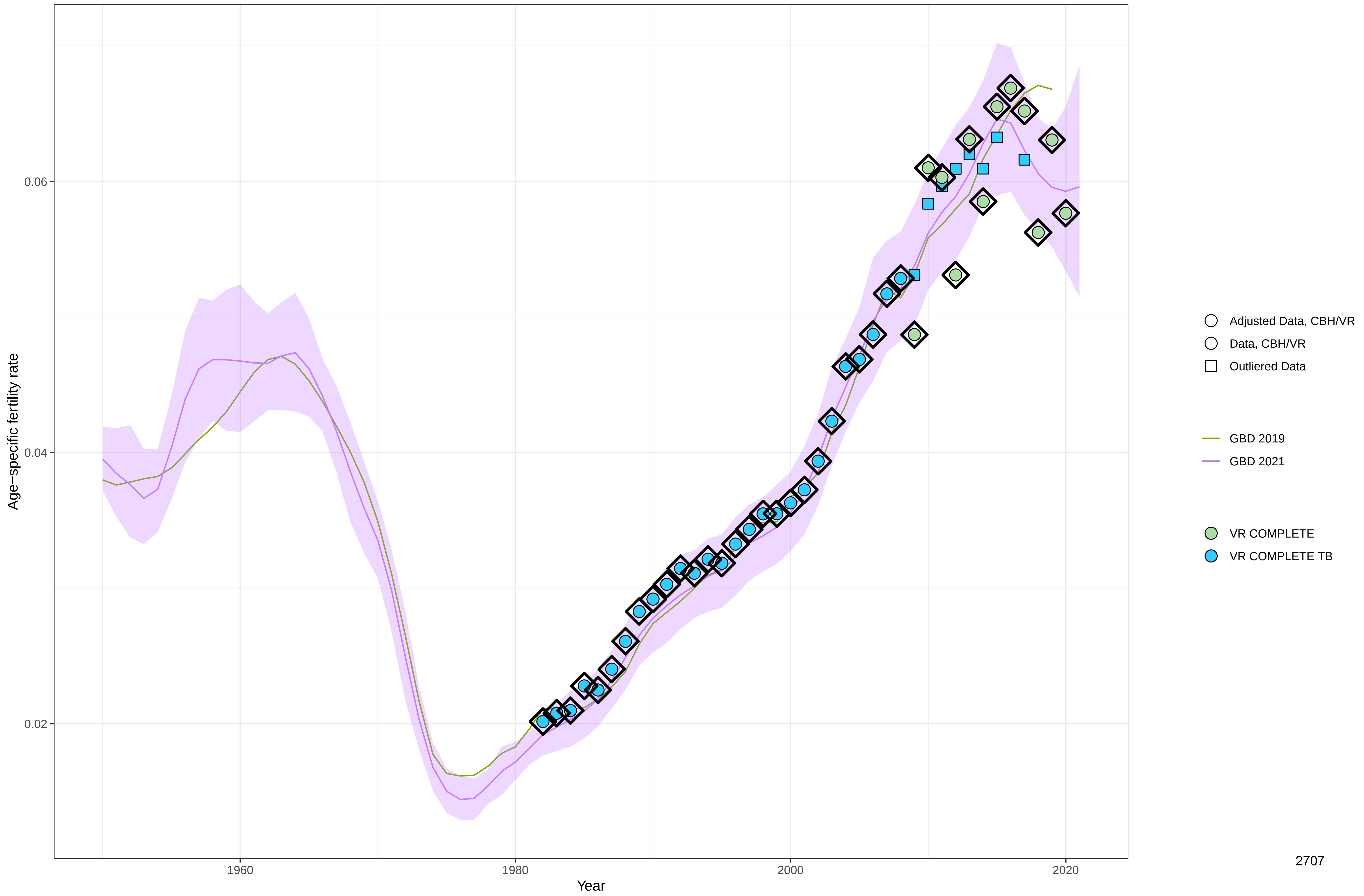

Age−specific fertility fitted trend lines, 1950−2021: Bury, Ages: 40−44
Data points enclosed with a diamond are reference sources

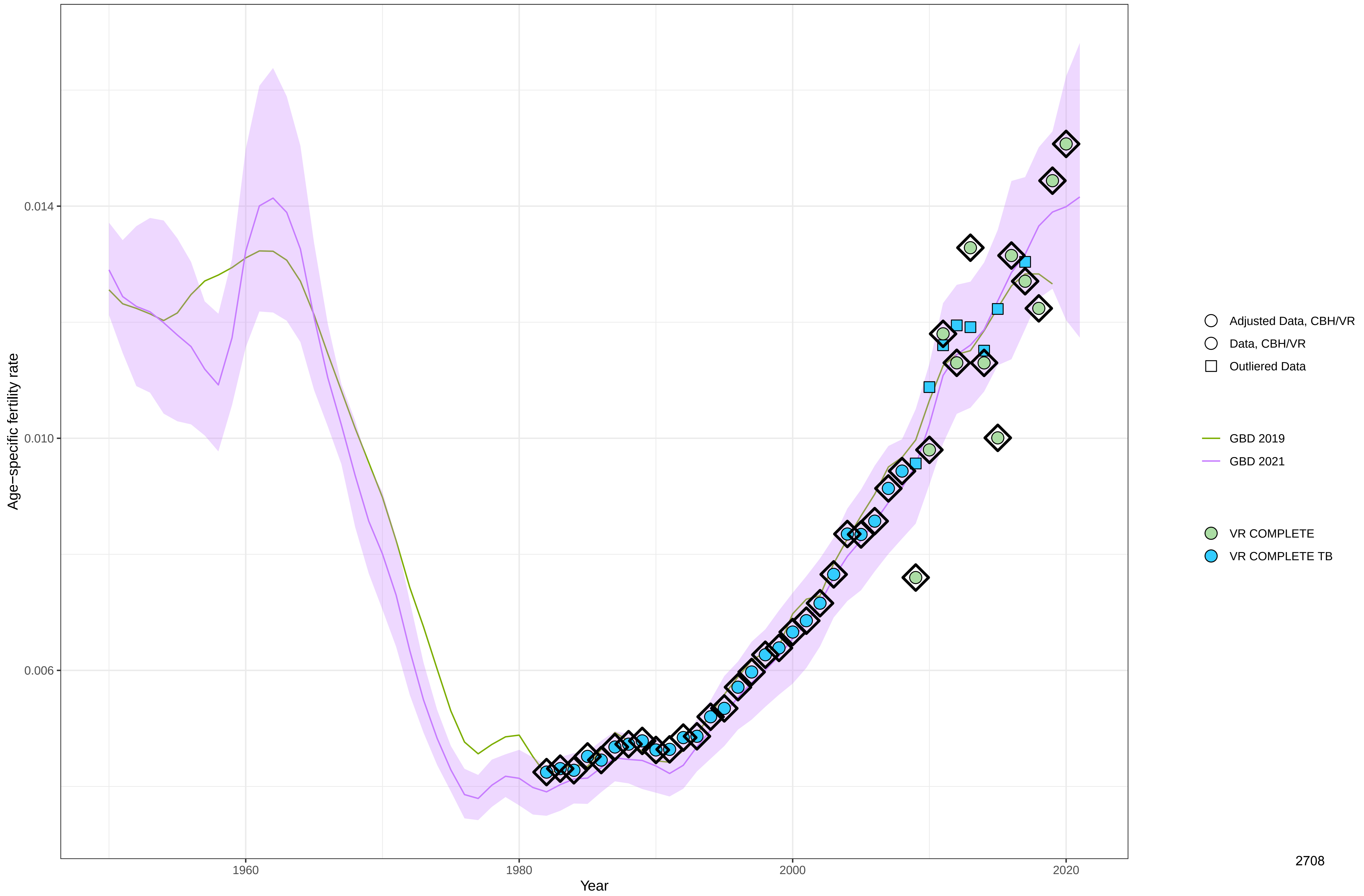

Age−specific fertility fitted trend lines, 1950−2021: Bury, Ages: 45−49
Data points enclosed with a diamond are reference sources

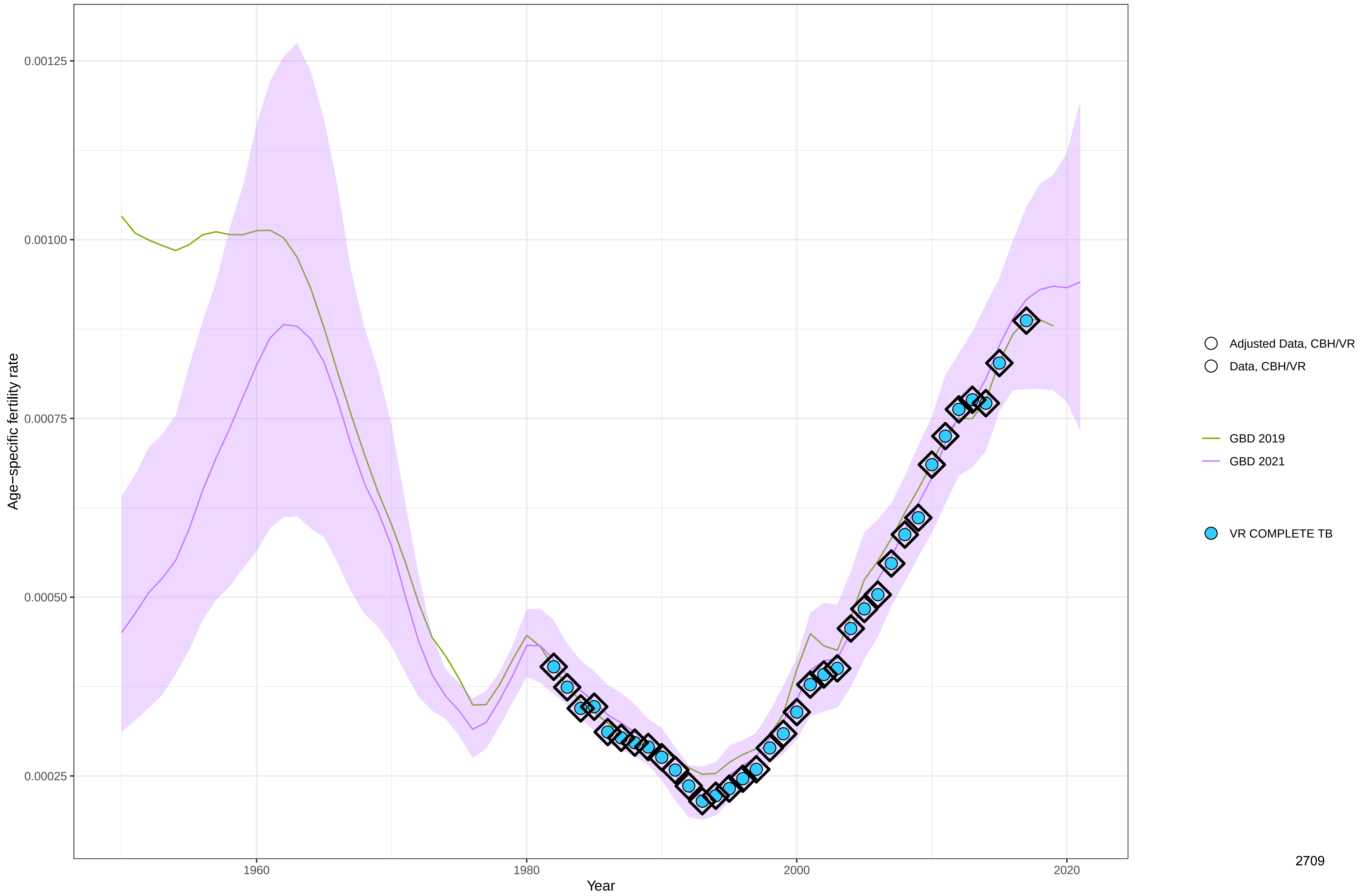

Age−specific fertility fitted trend lines, 1950−2021: Bury, Ages: 50−54
Data points enclosed with a diamond are reference sources

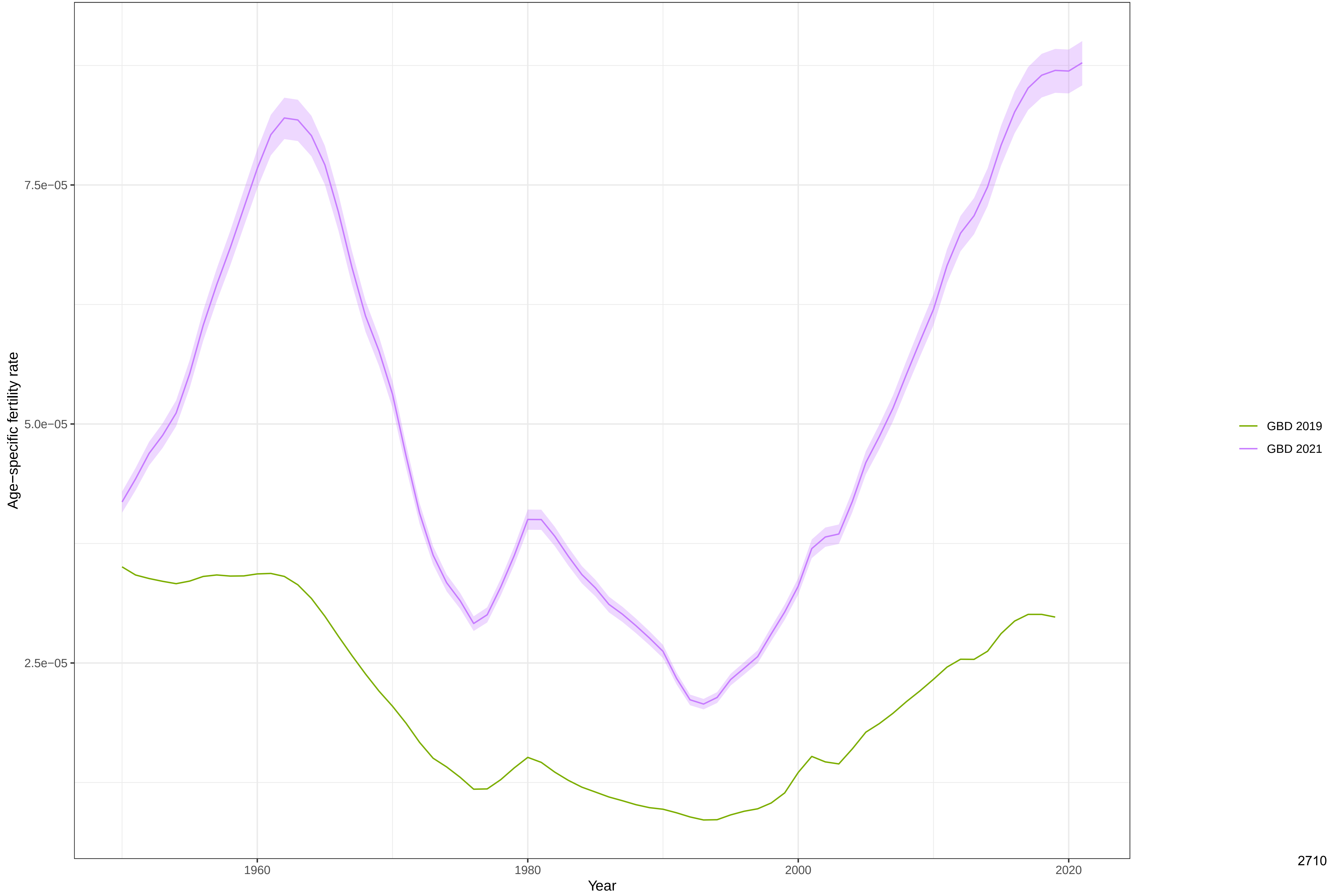

Total fertility rate fitted trend lines, 1950−2021: Bury TFR
Data points enclosed with a diamond are reference sources

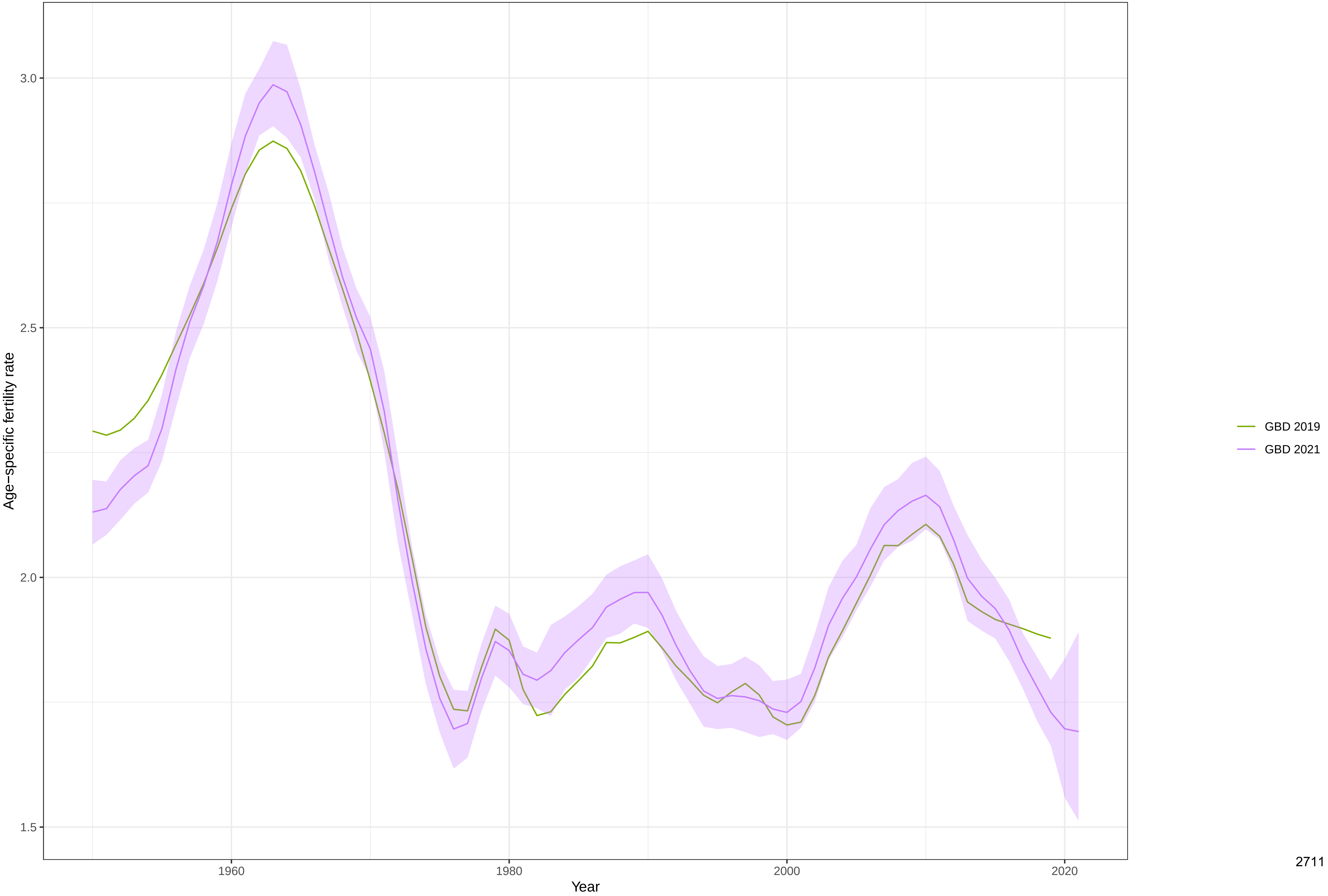

Age−specific fertility fitted trend lines, 1950−2021: Cheshire East, Ages: 10−14
Data points enclosed with a diamond are reference sources

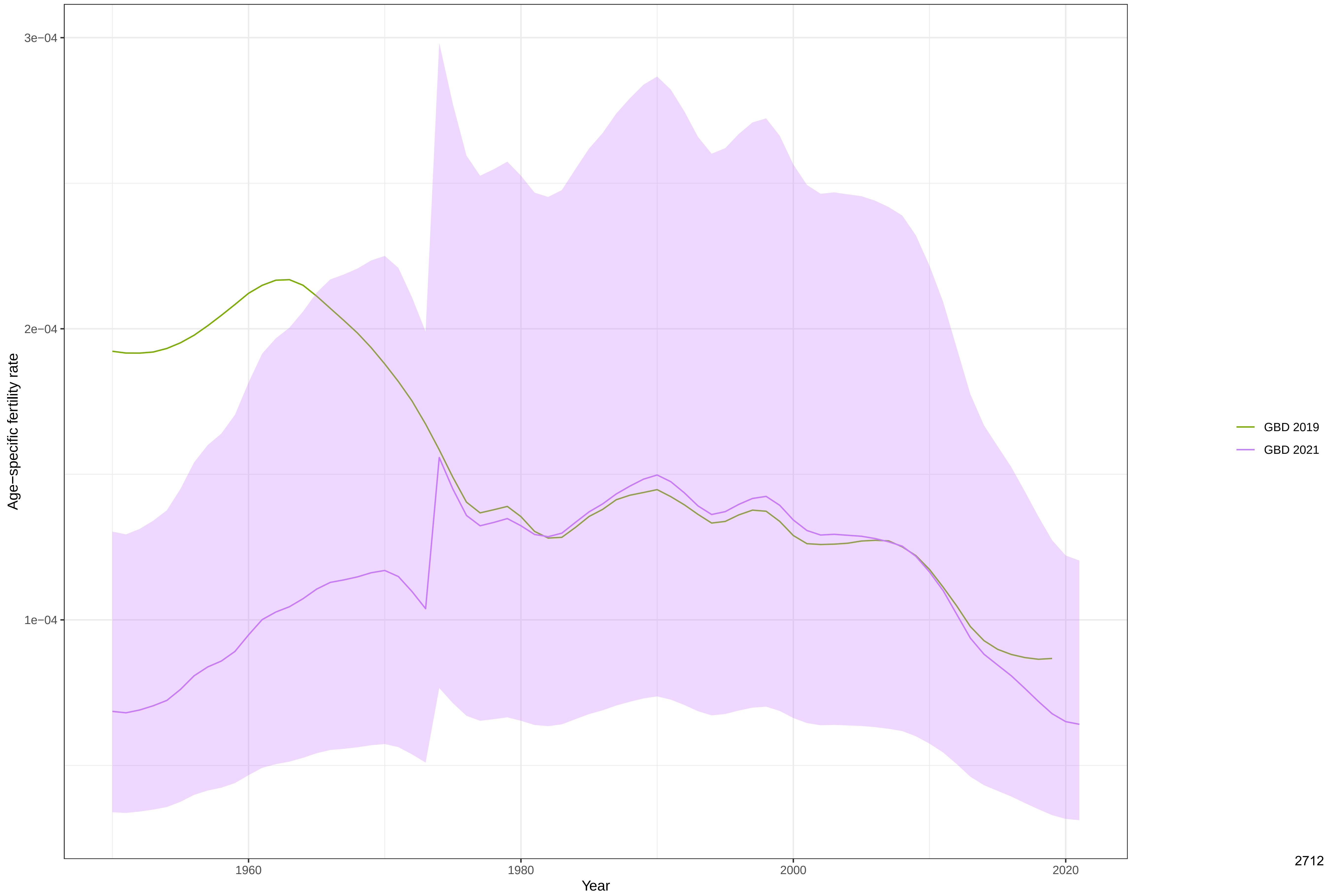

Age−specific fertility fitted trend lines, 1950−2021: Cheshire East, Ages: 15−19
Data points enclosed with a diamond are reference sources

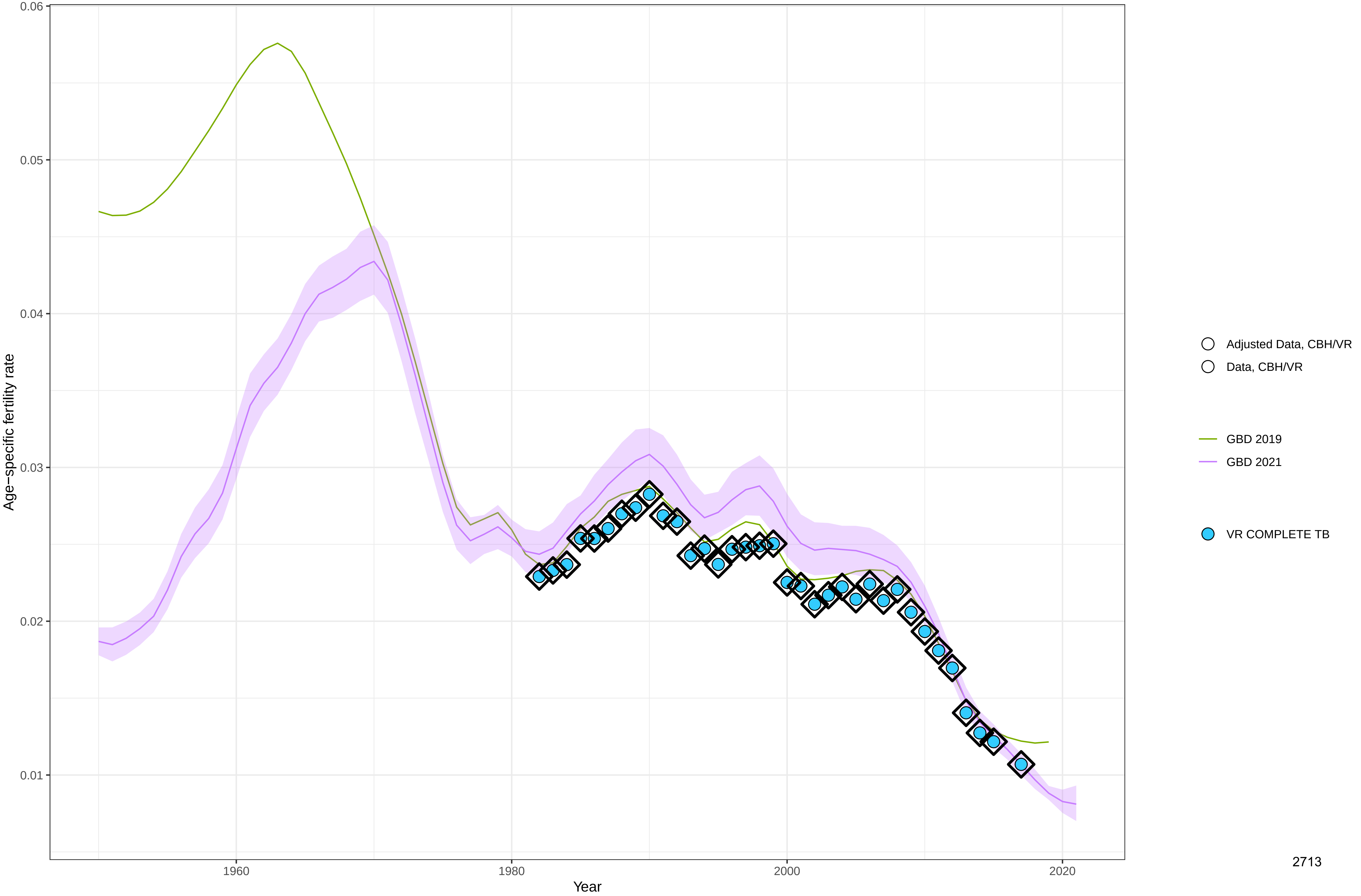

Age−specific fertility fitted trend lines, 1950−2021: Cheshire East, Ages: 20−24
Data points enclosed with a diamond are reference sources

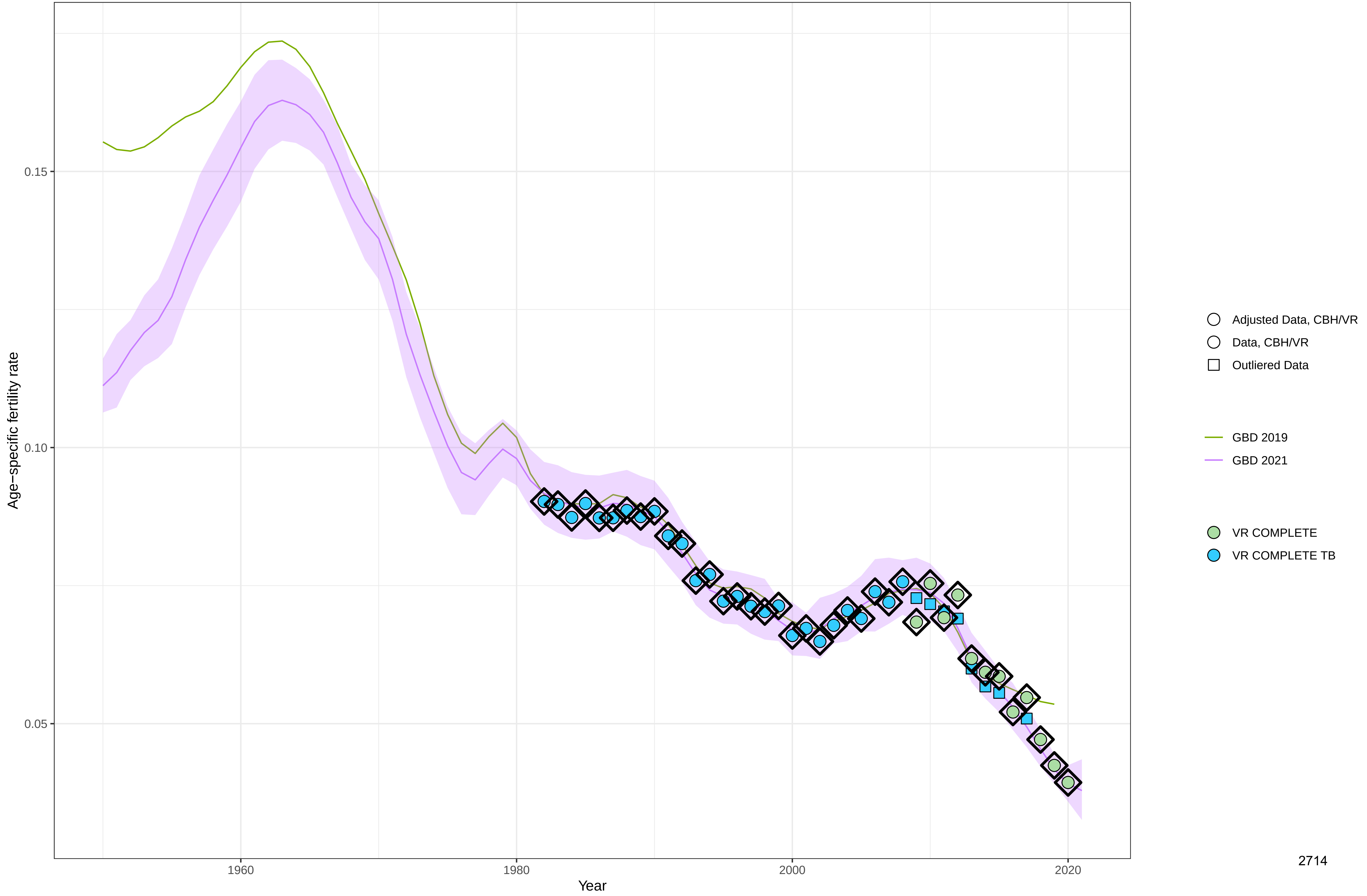

Age−specific fertility fitted trend lines, 1950−2021: Cheshire East, Ages: 25−29
Data points enclosed with a diamond are reference sources

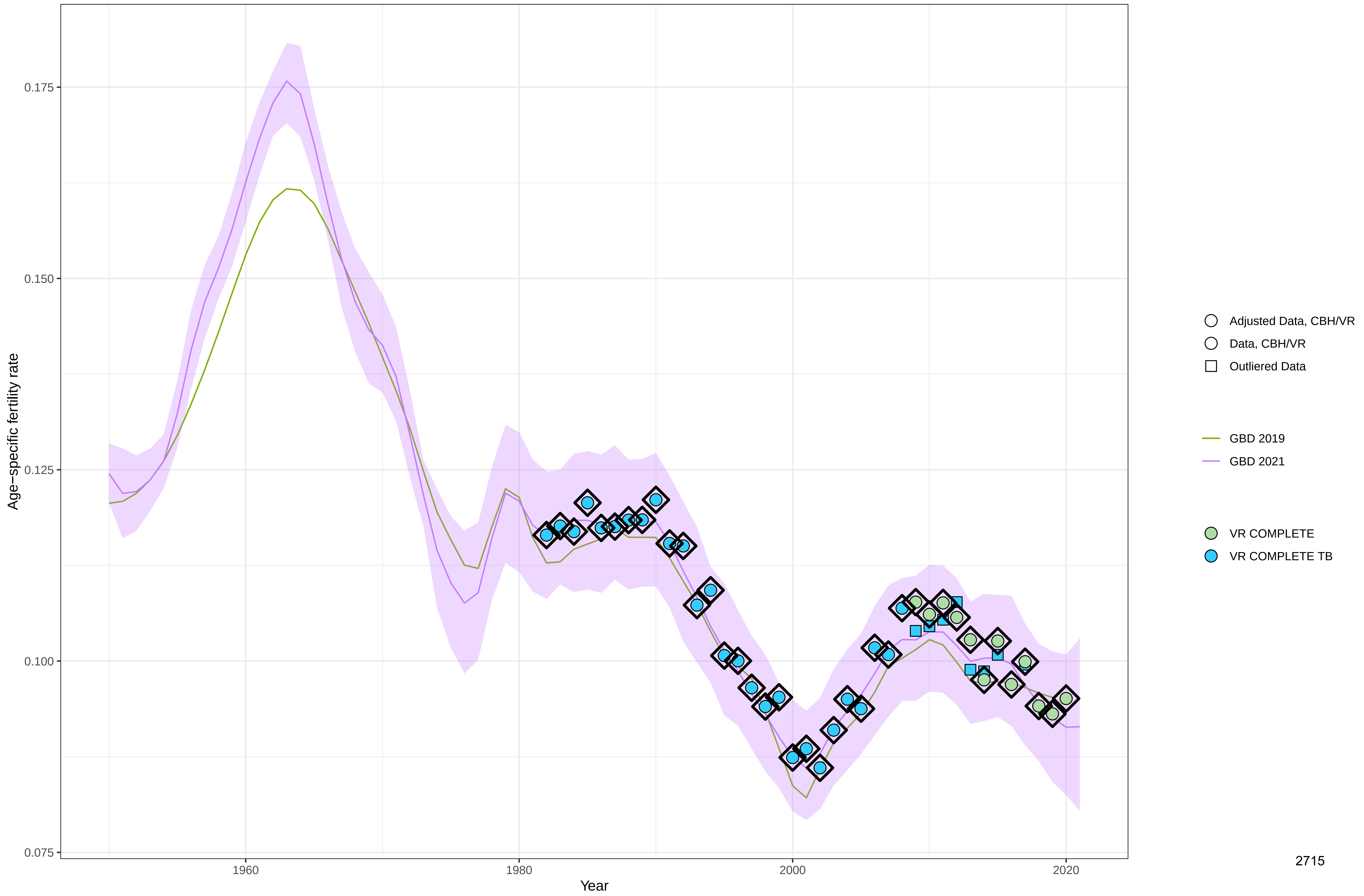

Age−specific fertility fitted trend lines, 1950−2021: Cheshire East, Ages: 30−34
Data points enclosed with a diamond are reference sources

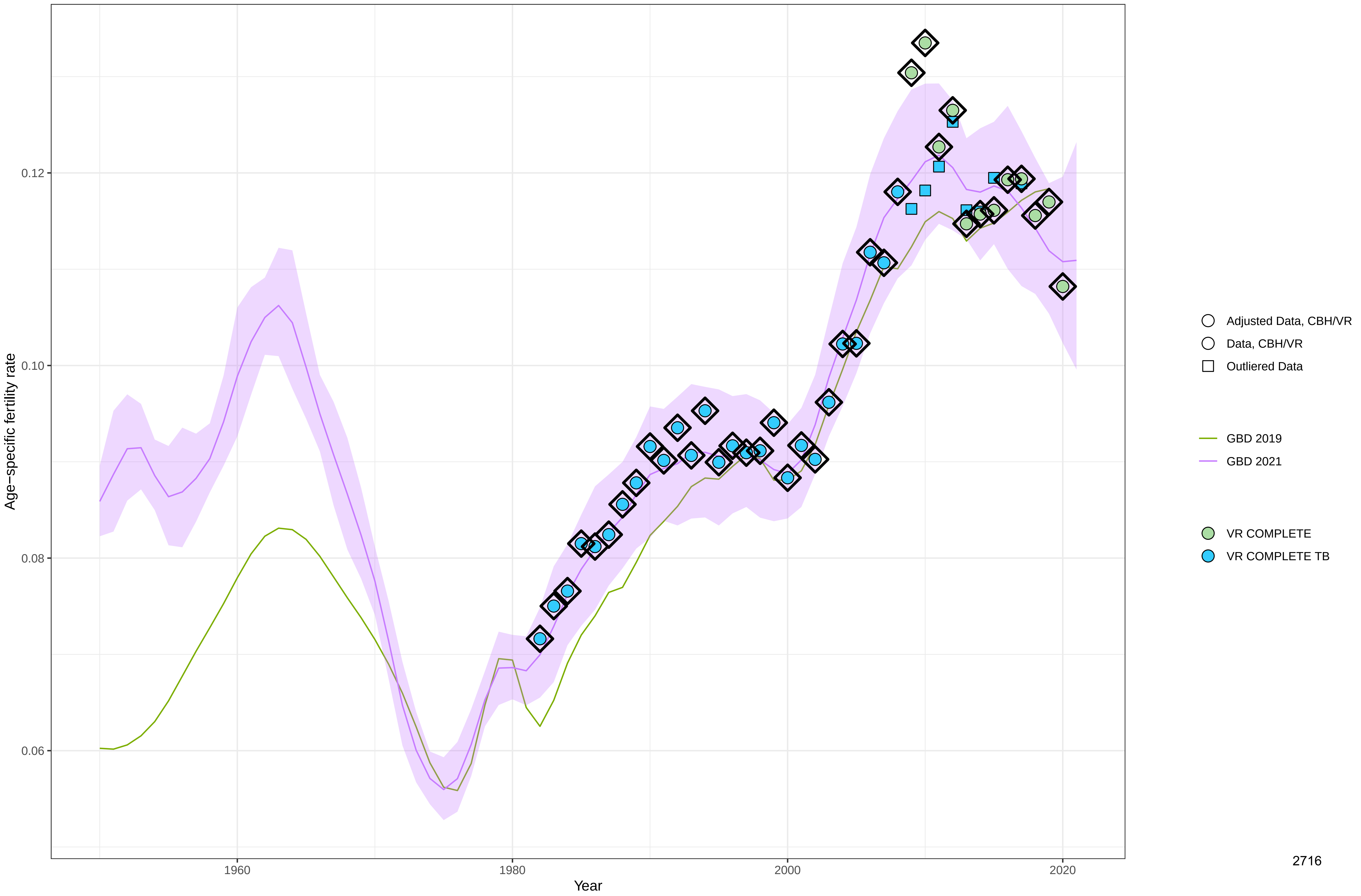

Age−specific fertility fitted trend lines, 1950−2021: Cheshire East, Ages: 35−39
Data points enclosed with a diamond are reference sources

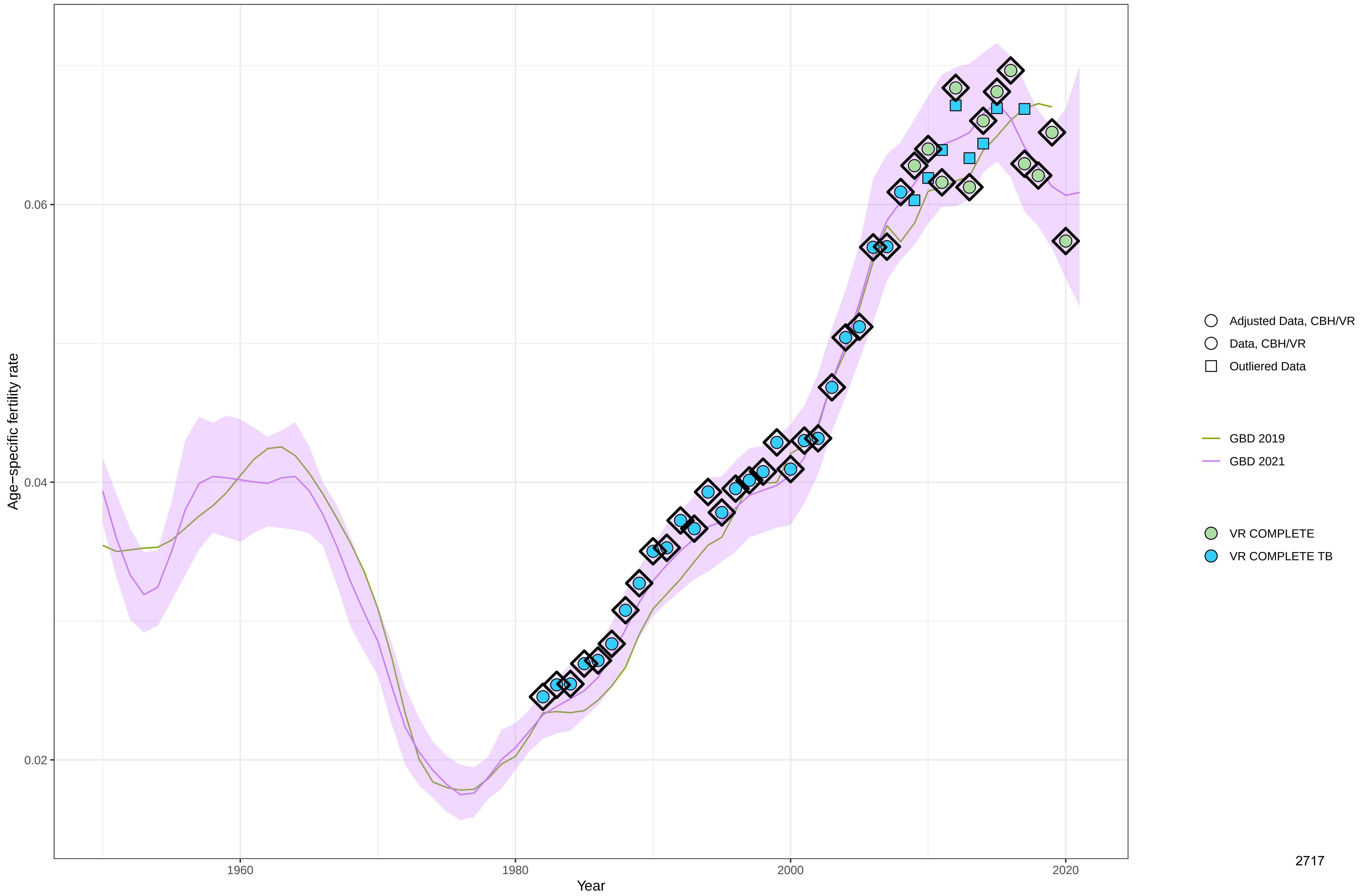

Age−specific fertility fitted trend lines, 1950−2021: Cheshire East, Ages: 40−44
Data points enclosed with a diamond are reference sources

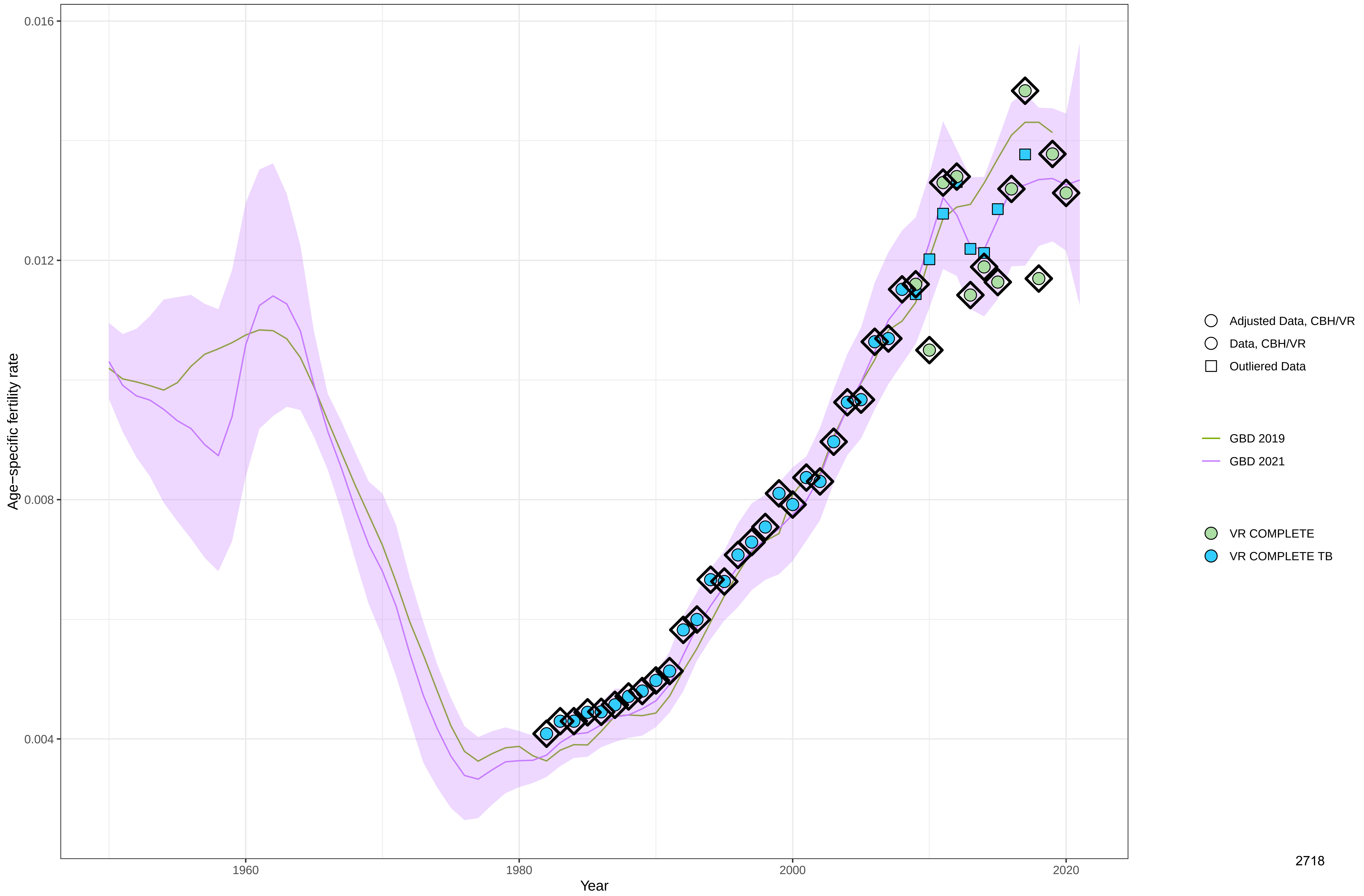

Age−specific fertility fitted trend lines, 1950−2021: Cheshire East, Ages: 45−49
Data points enclosed with a diamond are reference sources

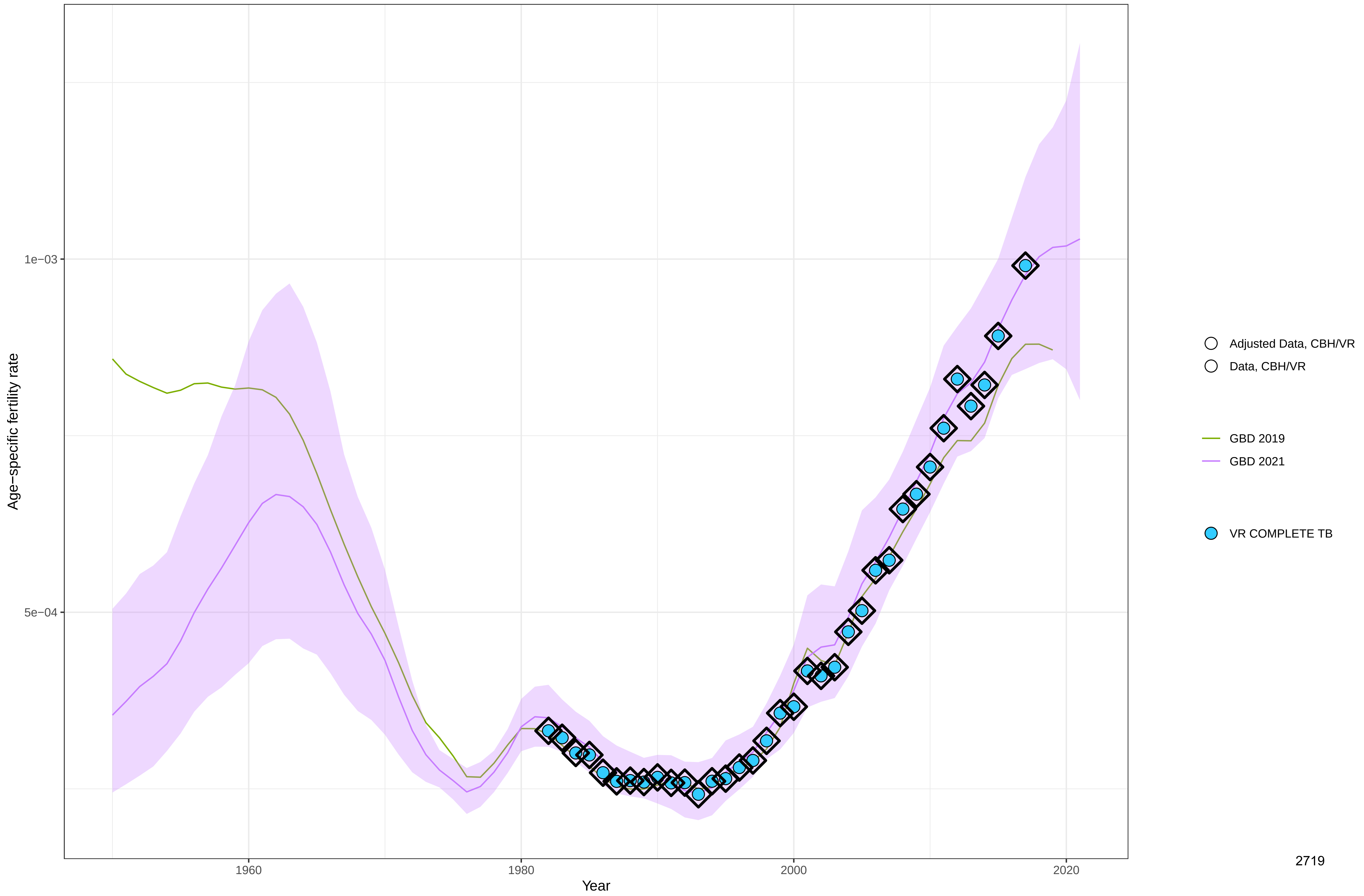

Age−specific fertility fitted trend lines, 1950−2021: Cheshire East, Ages: 50−54
Data points enclosed with a diamond are reference sources

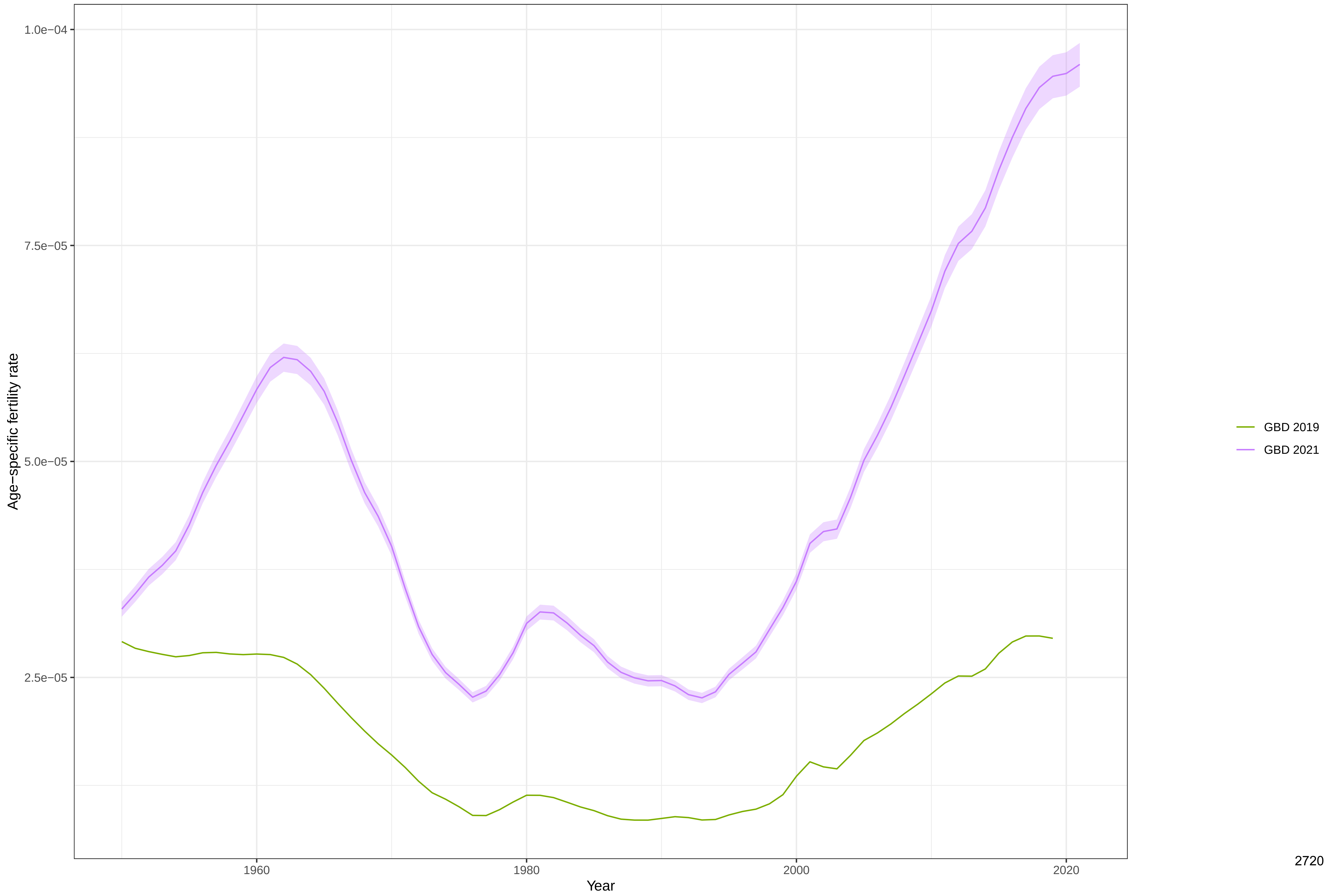

Total fertility rate fitted trend lines, 1950−2021: Cheshire East TFR
Data points enclosed with a diamond are reference sources

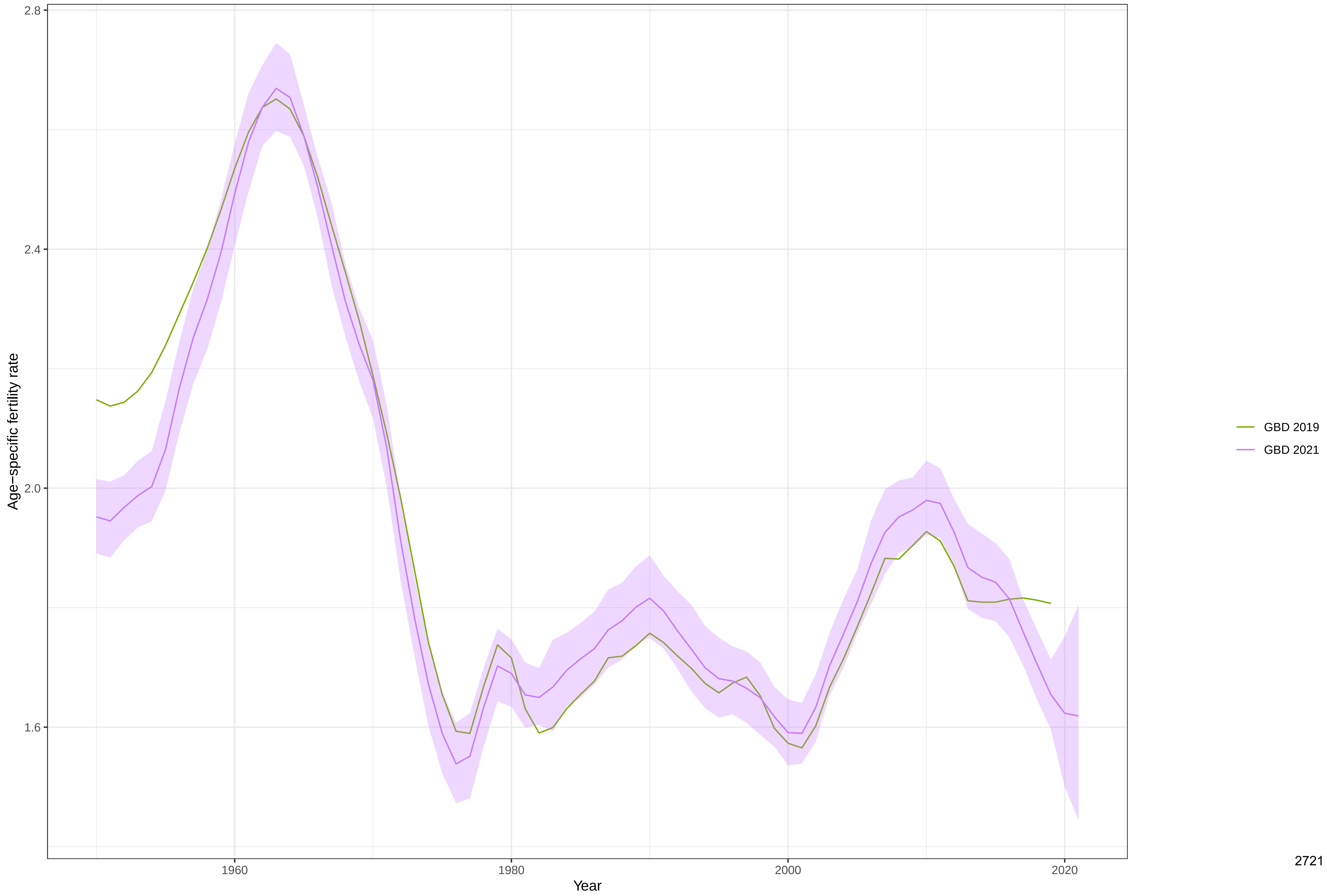

Age−specific fertility fitted trend lines, 1950−2021: Cheshire West and Chester, Ages: 10−14
Data points enclosed with a diamond are reference sources

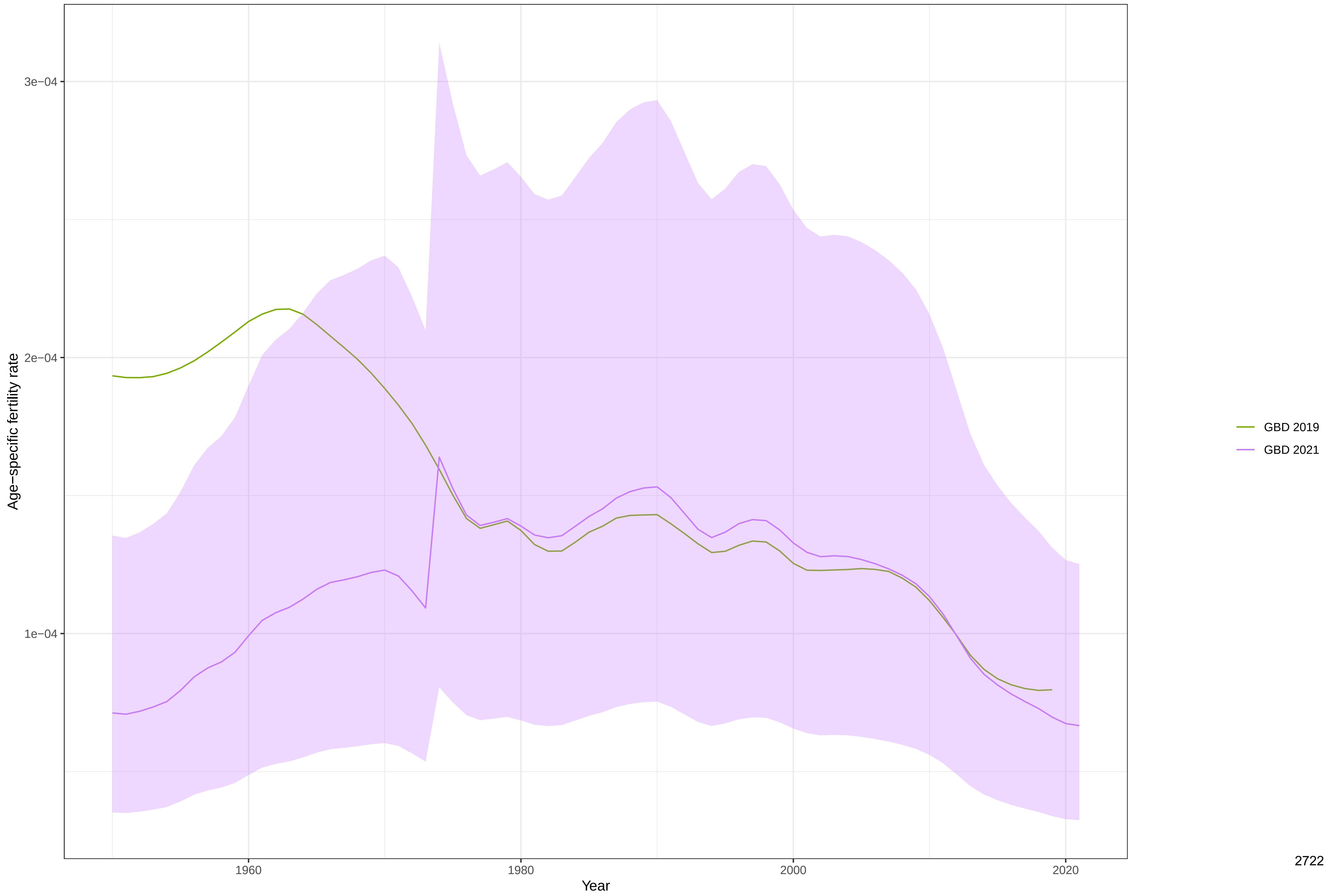

Age−specific fertility fitted trend lines, 1950−2021: Cheshire West and Chester, Ages: 15−19
Data points enclosed with a diamond are reference sources

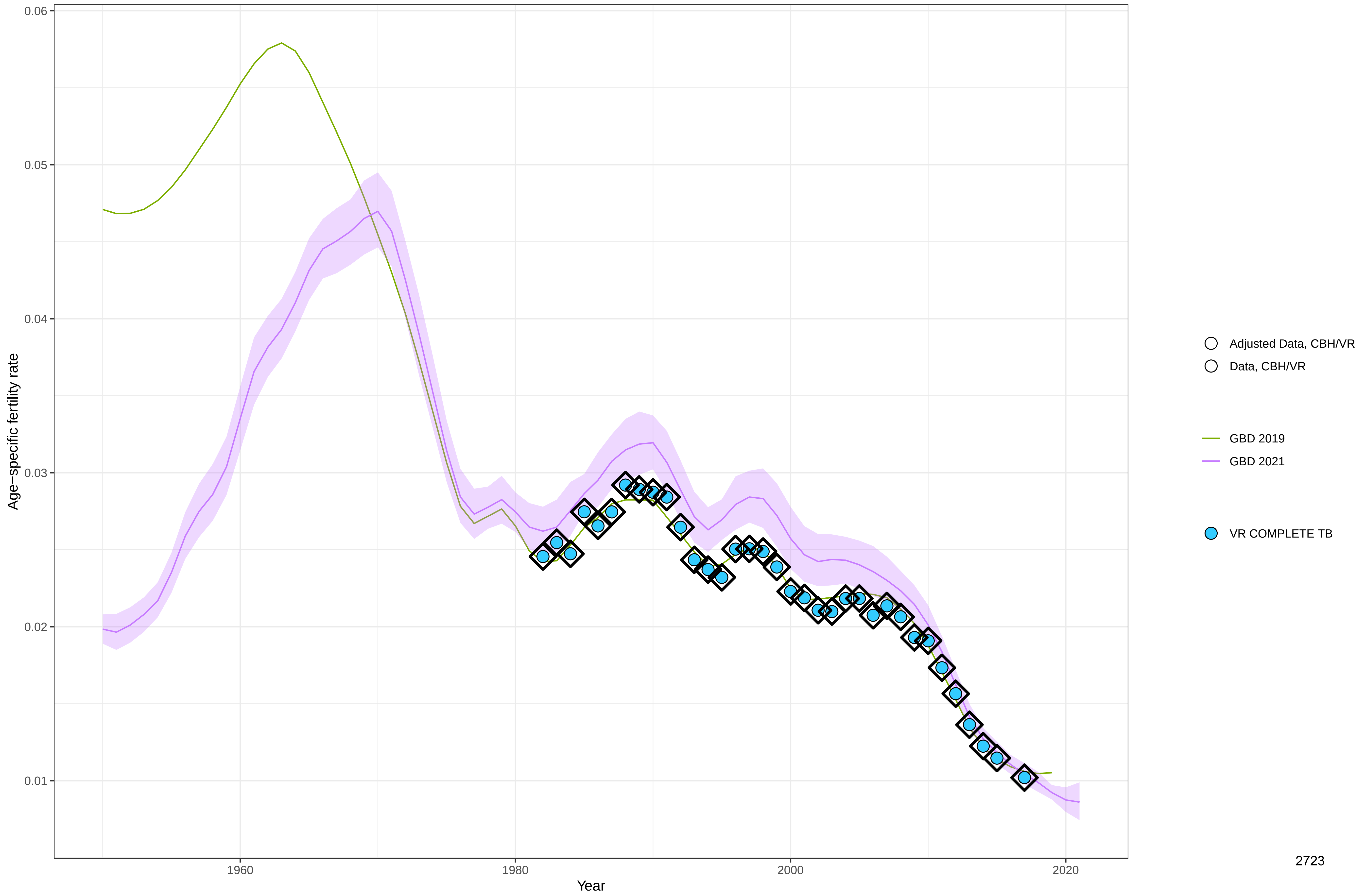

Age−specific fertility fitted trend lines, 1950−2021: Cheshire West and Chester, Ages: 20−24
Data points enclosed with a diamond are reference sources

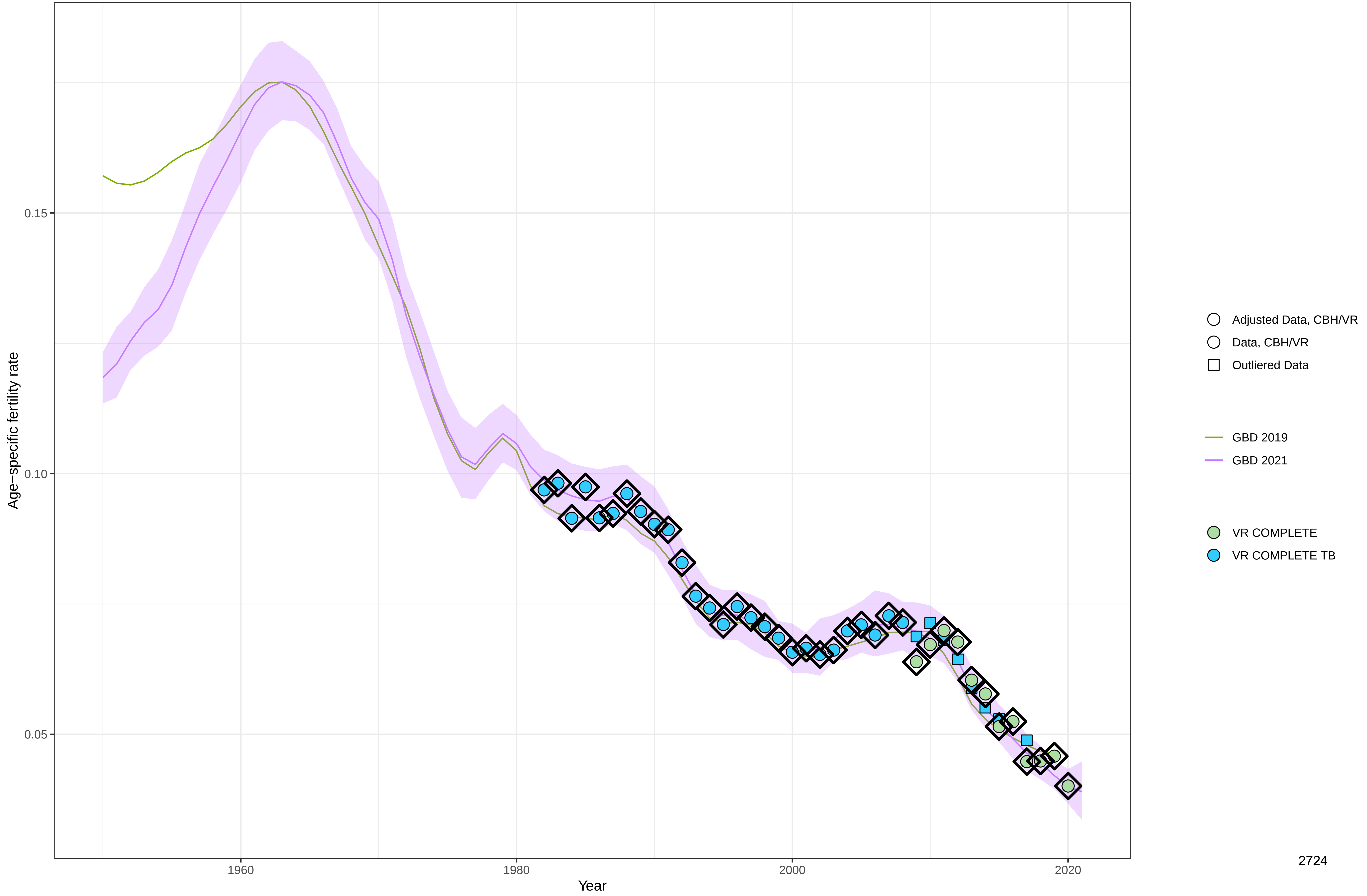

Age−specific fertility fitted trend lines, 1950−2021: Cheshire West and Chester, Ages: 25−29
Data points enclosed with a diamond are reference sources

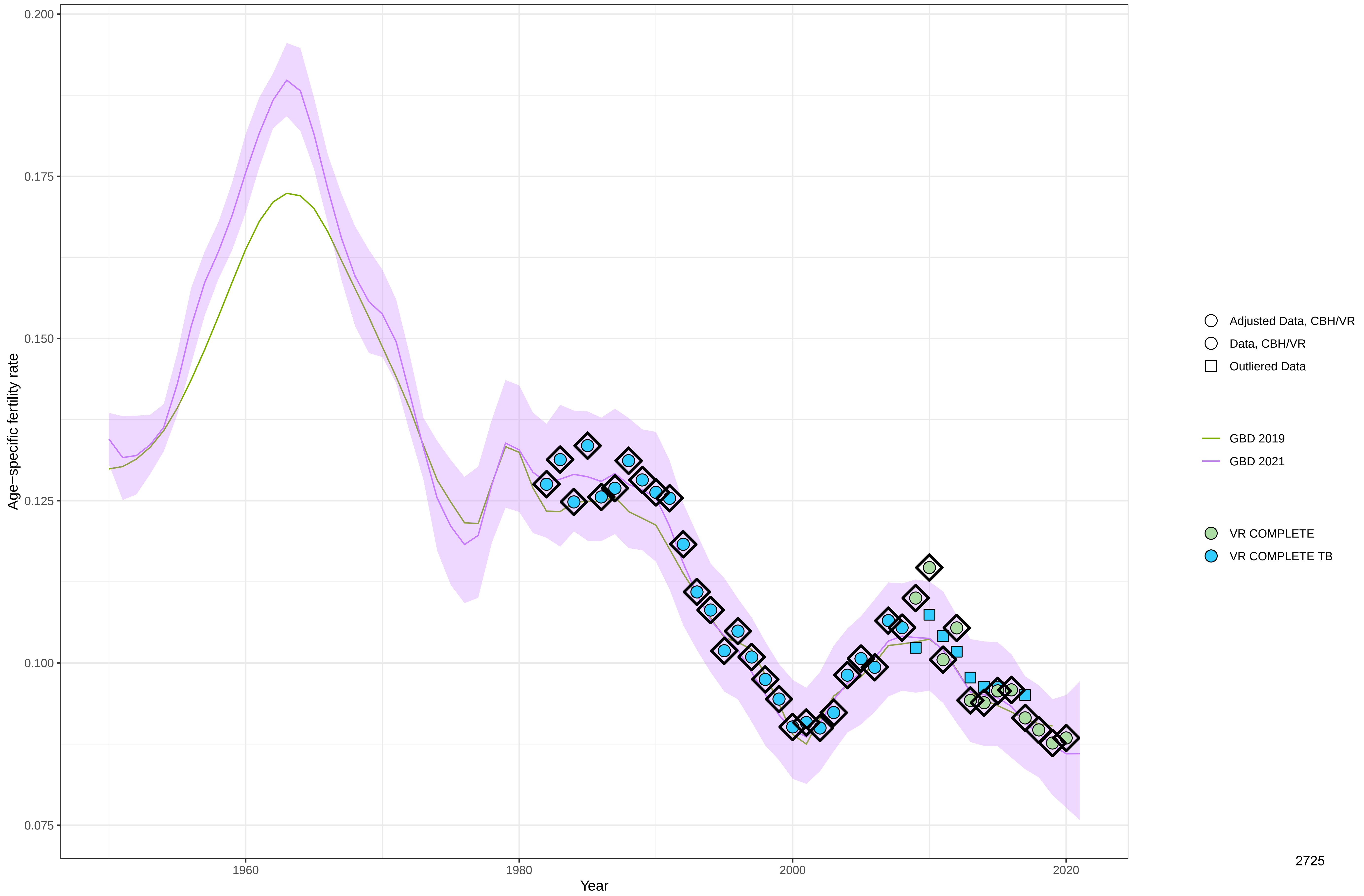

Age−specific fertility fitted trend lines, 1950−2021: Cheshire West and Chester, Ages: 30−34
Data points enclosed with a diamond are reference sources

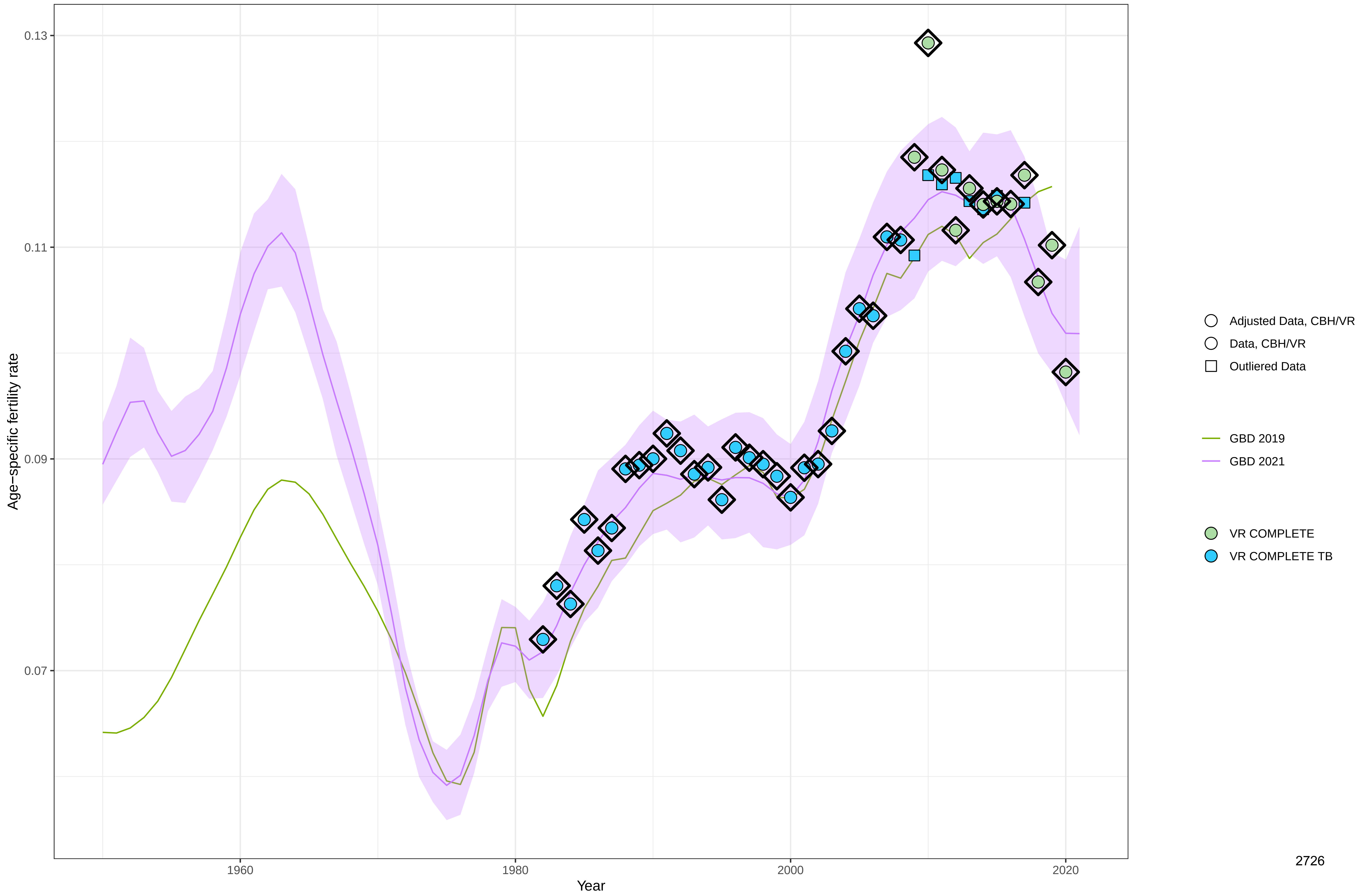

Age−specific fertility fitted trend lines, 1950−2021: Cheshire West and Chester, Ages: 35−39
Data points enclosed with a diamond are reference sources

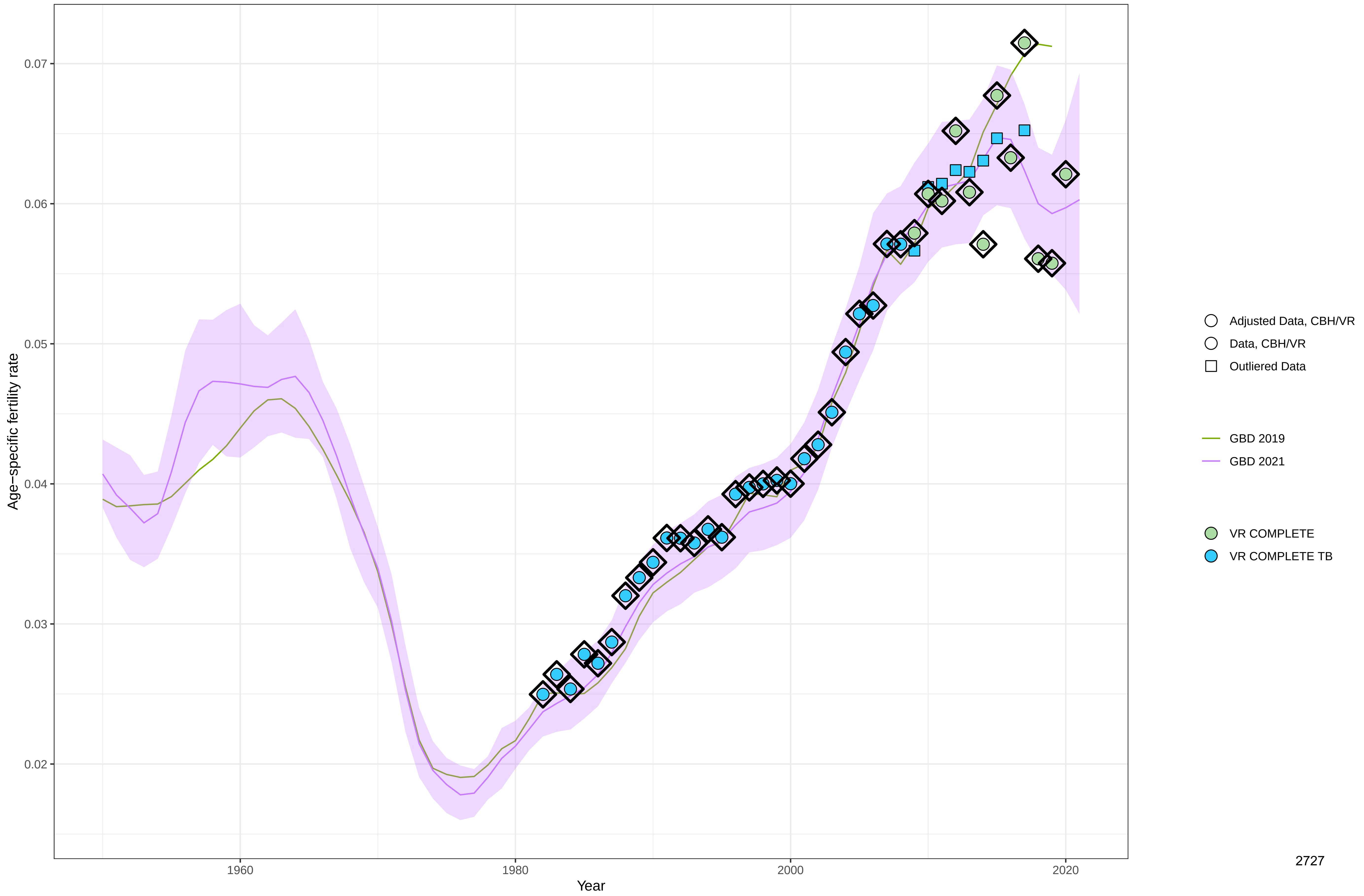

Age−specific fertility fitted trend lines, 1950−2021: Cheshire West and Chester, Ages: 40−44
Data points enclosed with a diamond are reference sources

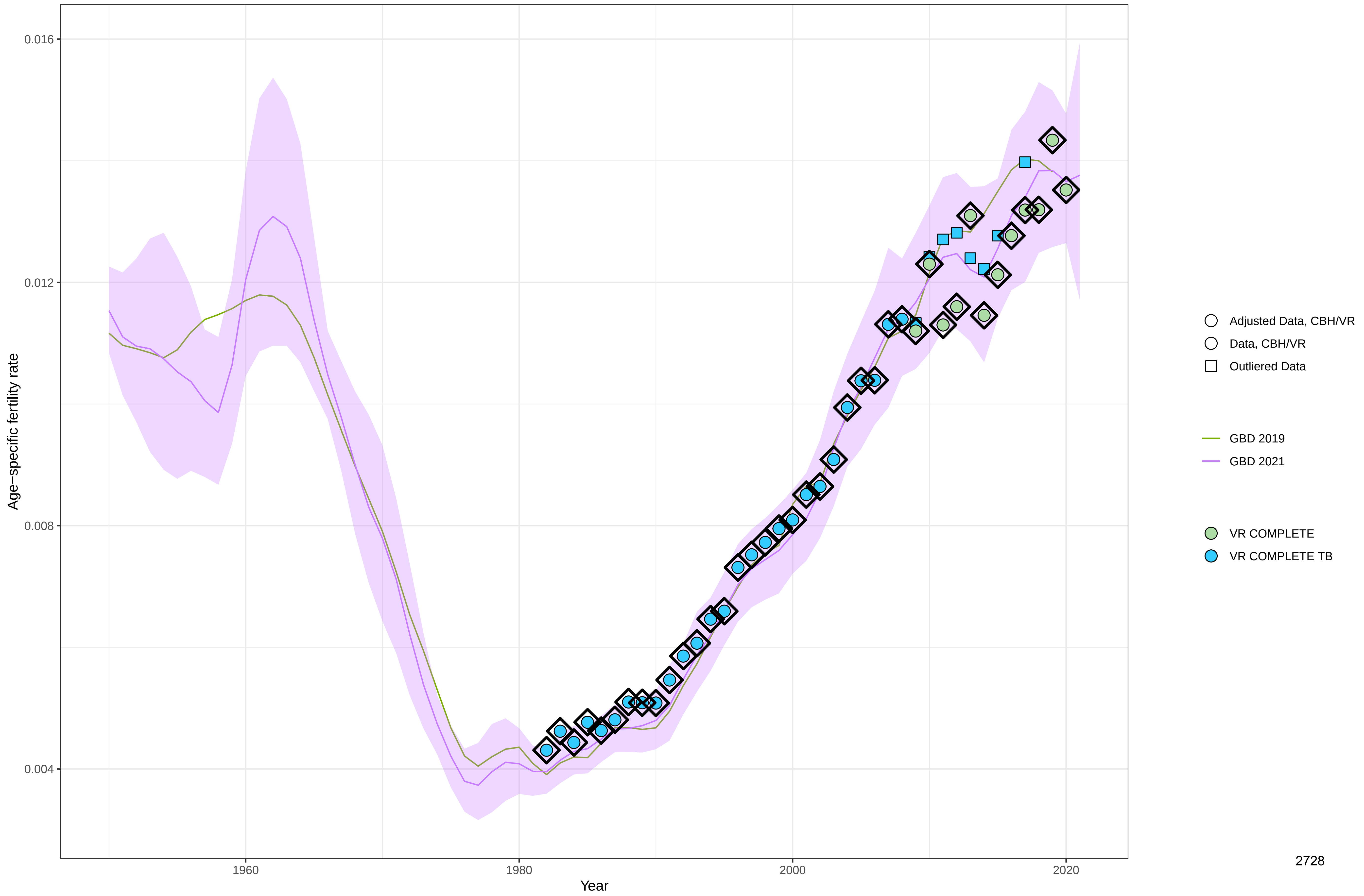

Age−specific fertility fitted trend lines, 1950−2021: Cheshire West and Chester, Ages: 45−49
Data points enclosed with a diamond are reference sources

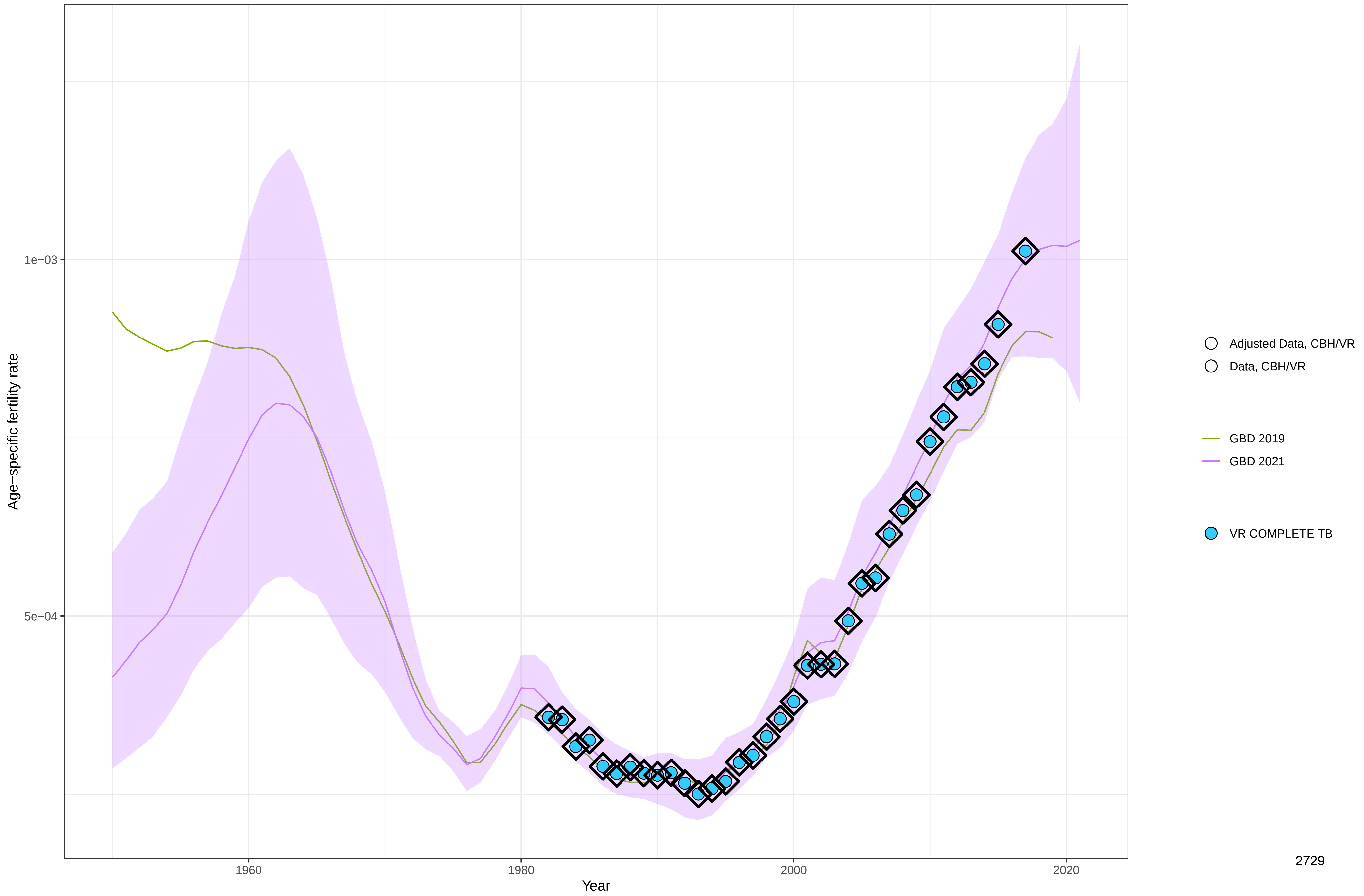

Age−specific fertility fitted trend lines, 1950−2021: Cheshire West and Chester, Ages: 50−54
Data points enclosed with a diamond are reference sources

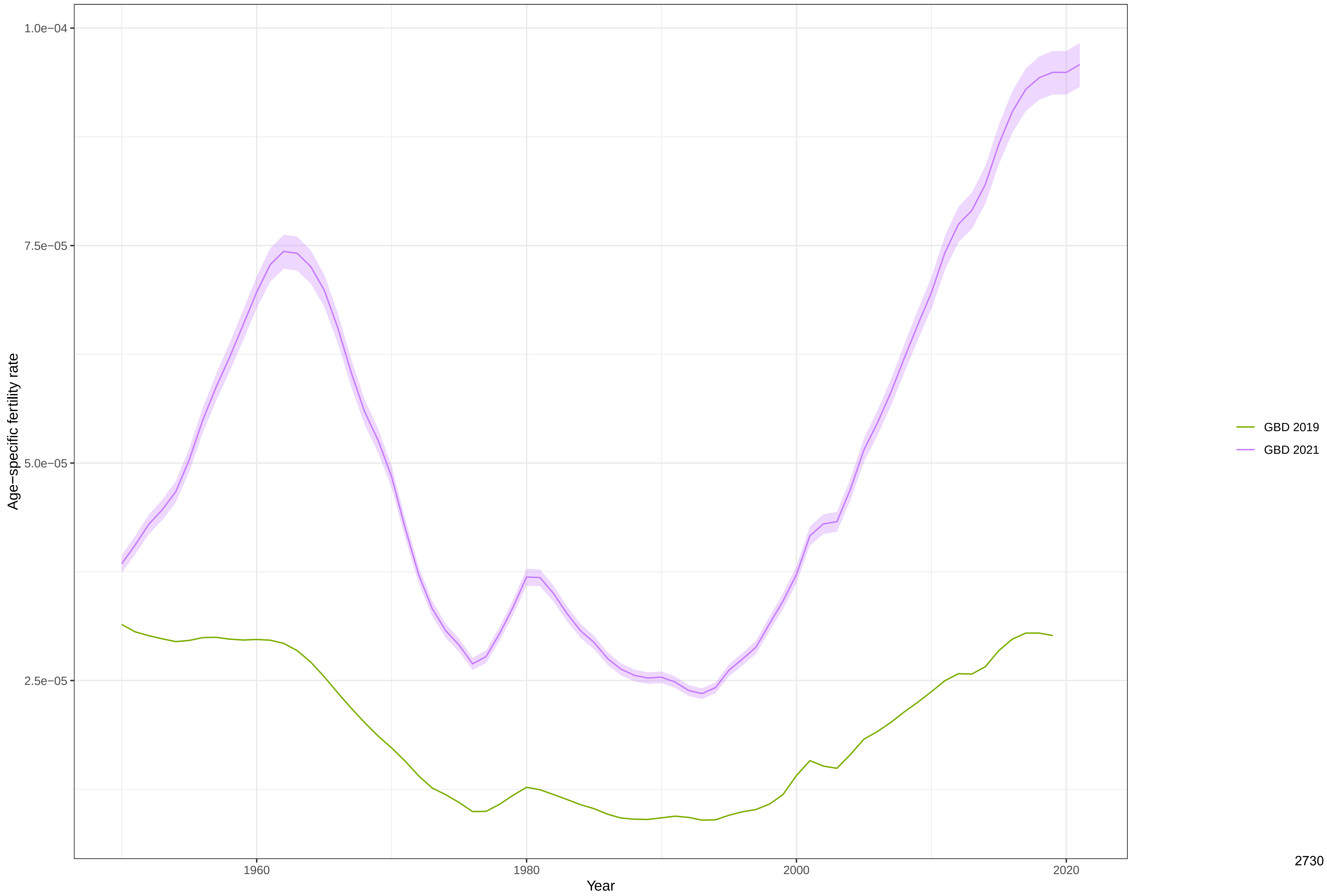

Total fertility rate fitted trend lines, 1950−2021: Cheshire West and Chester TFR
Data points enclosed with a diamond are reference sources

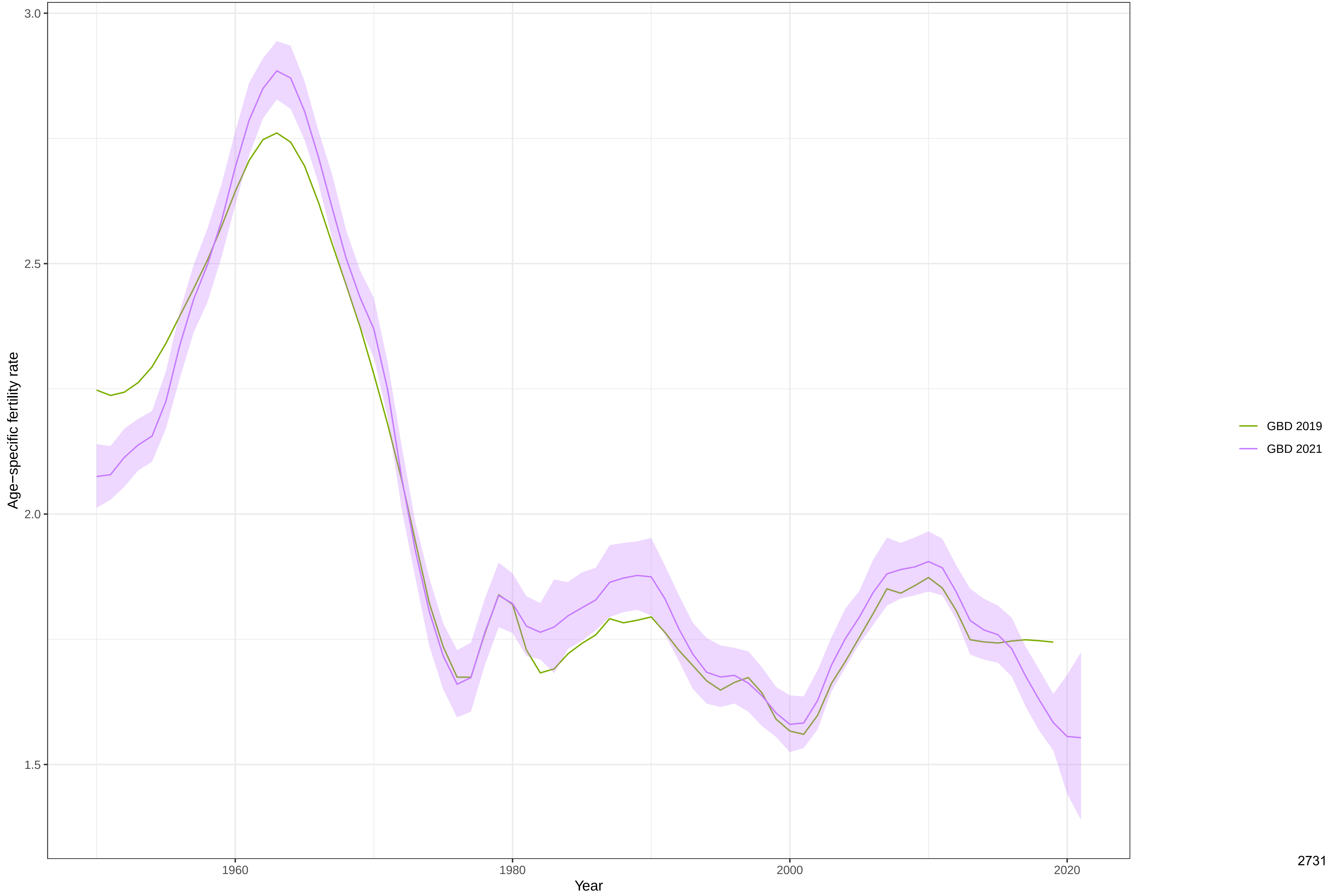

Age−specific fertility fitted trend lines, 1950−2021: Cumbria, Ages: 10−14
Data points enclosed with a diamond are reference sources

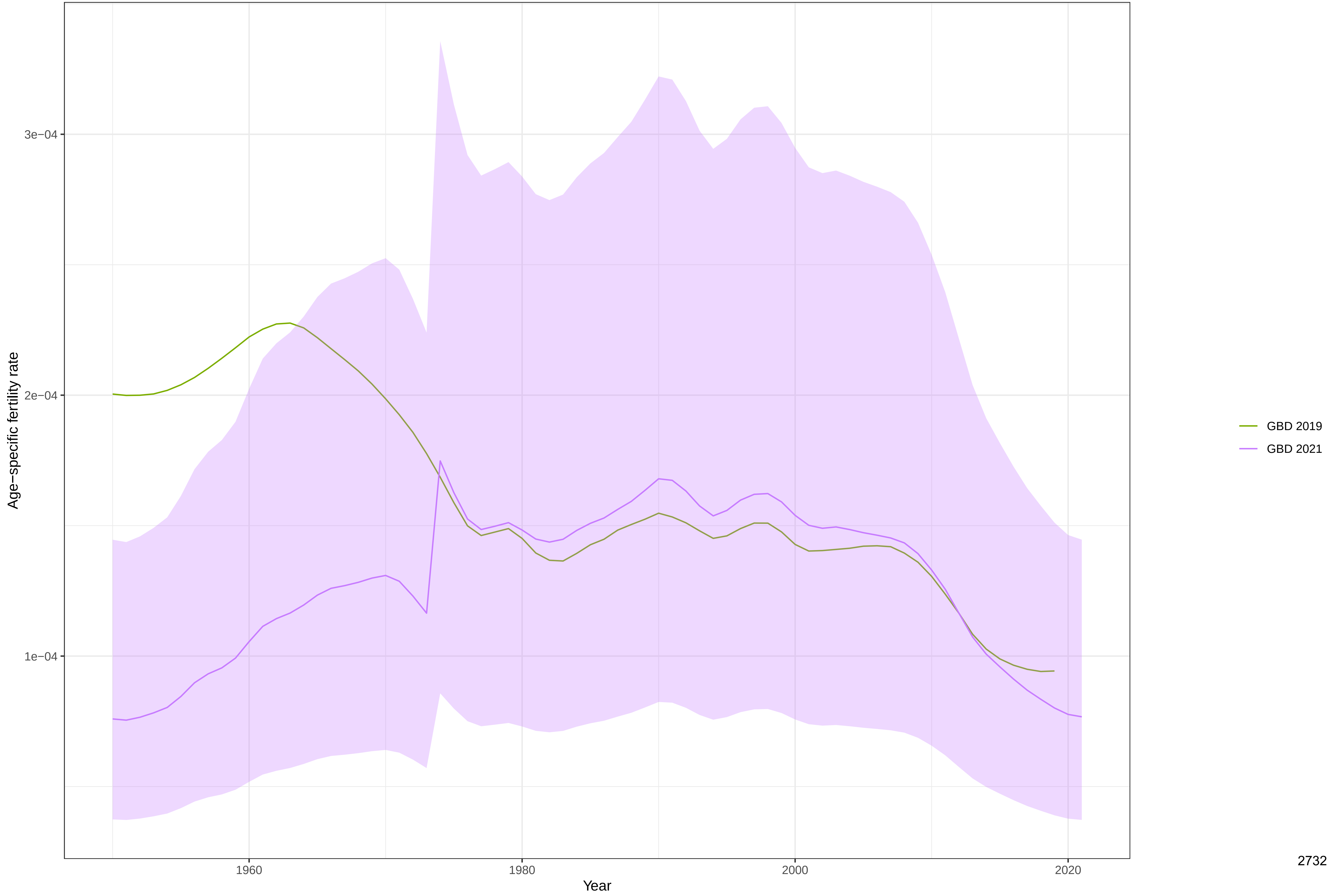

Age−specific fertility fitted trend lines, 1950−2021: Cumbria, Ages: 15−19
Data points enclosed with a diamond are reference sources

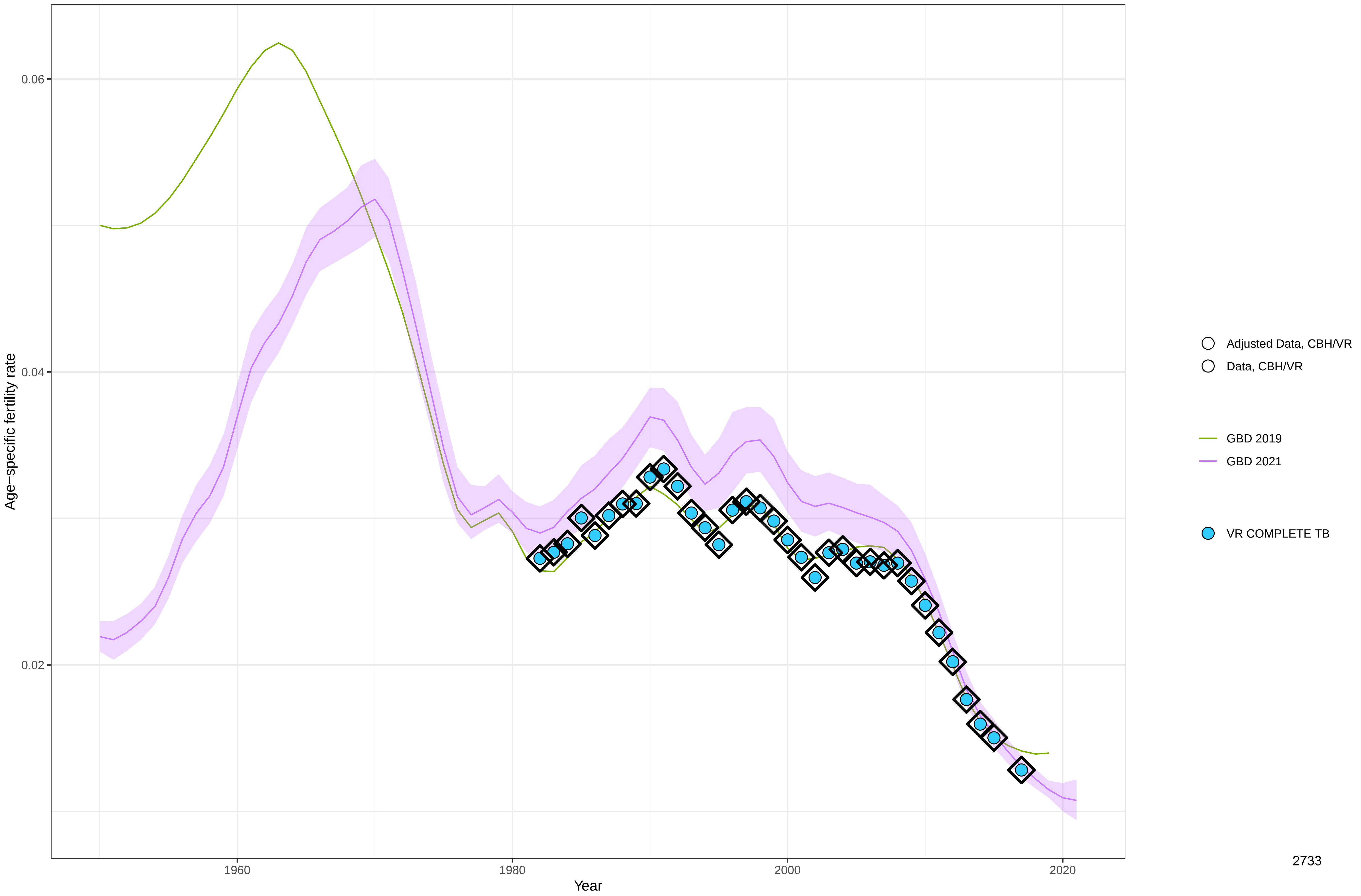

Age−specific fertility fitted trend lines, 1950−2021: Cumbria, Ages: 20−24
Data points enclosed with a diamond are reference sources

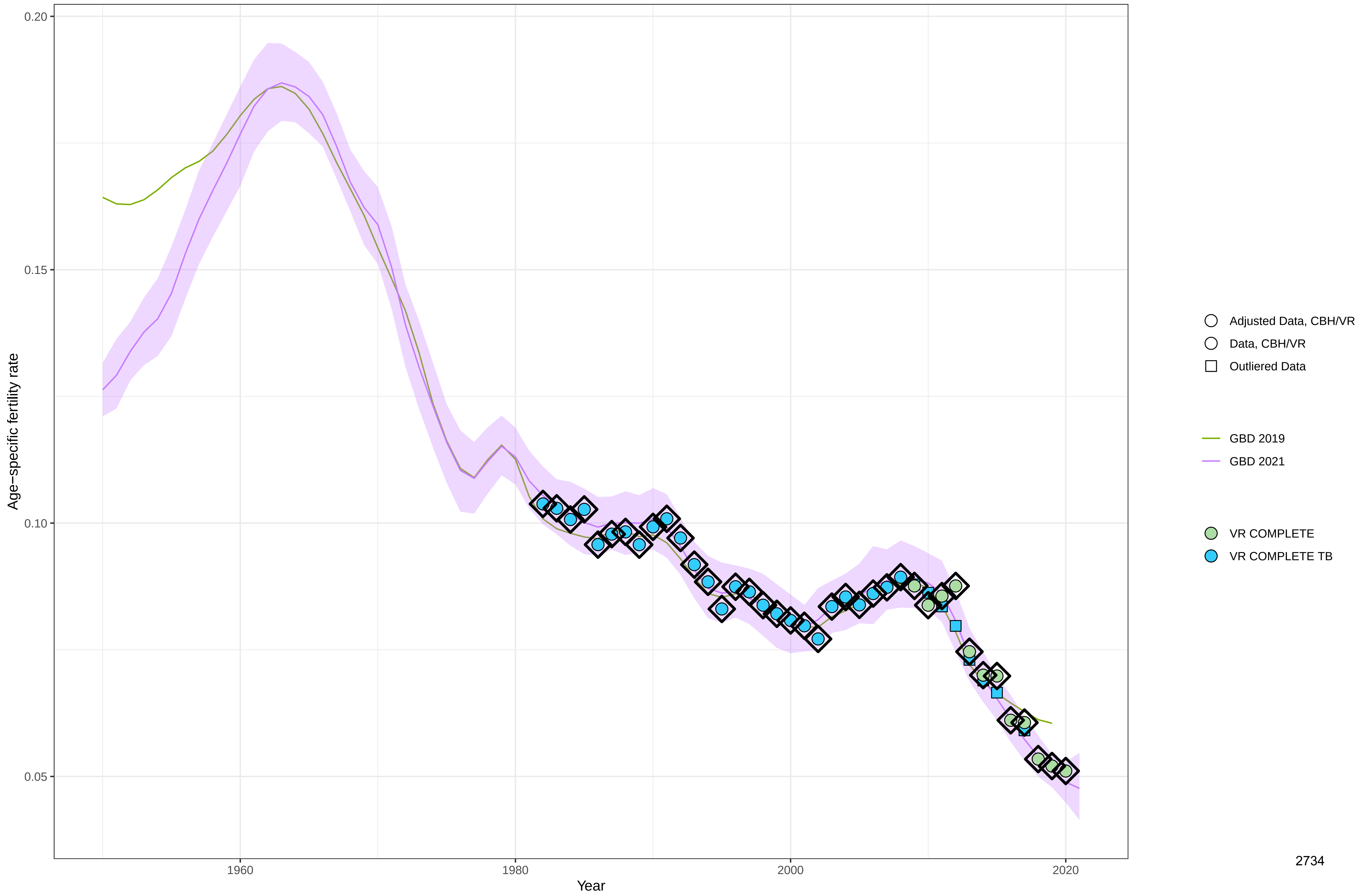

Age−specific fertility fitted trend lines, 1950−2021: Cumbria, Ages: 25−29
Data points enclosed with a diamond are reference sources

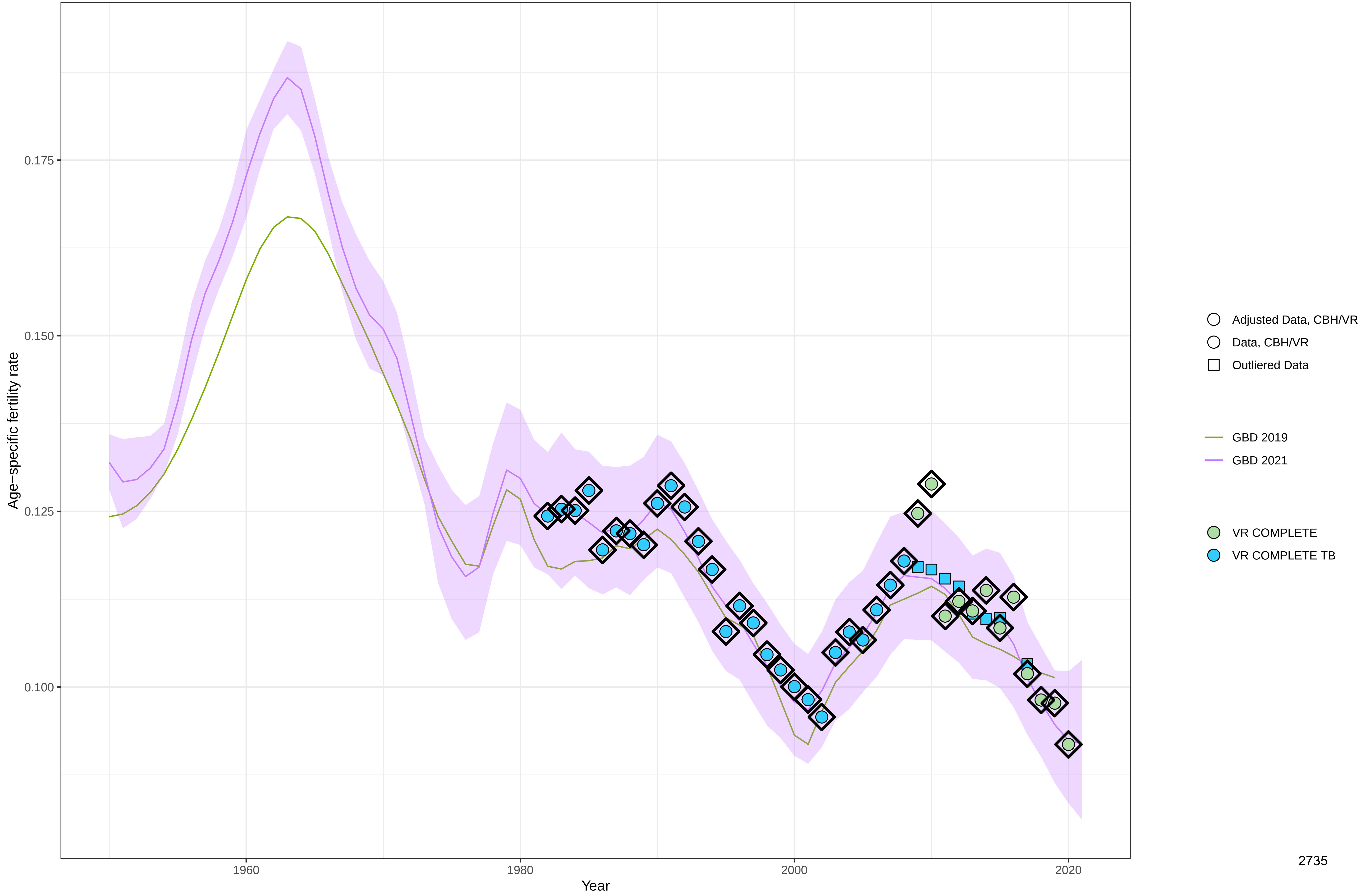

Age−specific fertility fitted trend lines, 1950−2021: Cumbria, Ages: 30−34
Data points enclosed with a diamond are reference sources

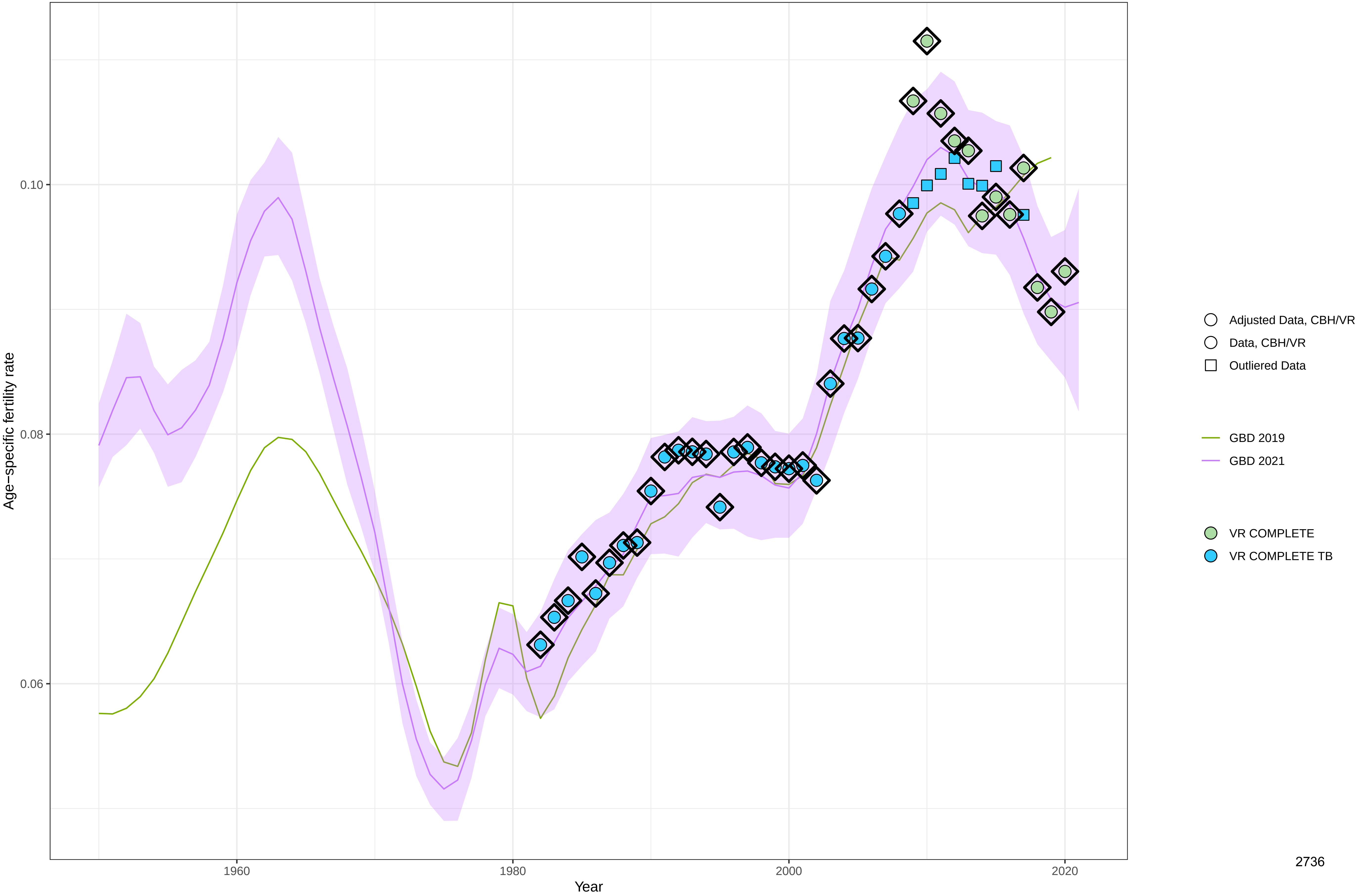

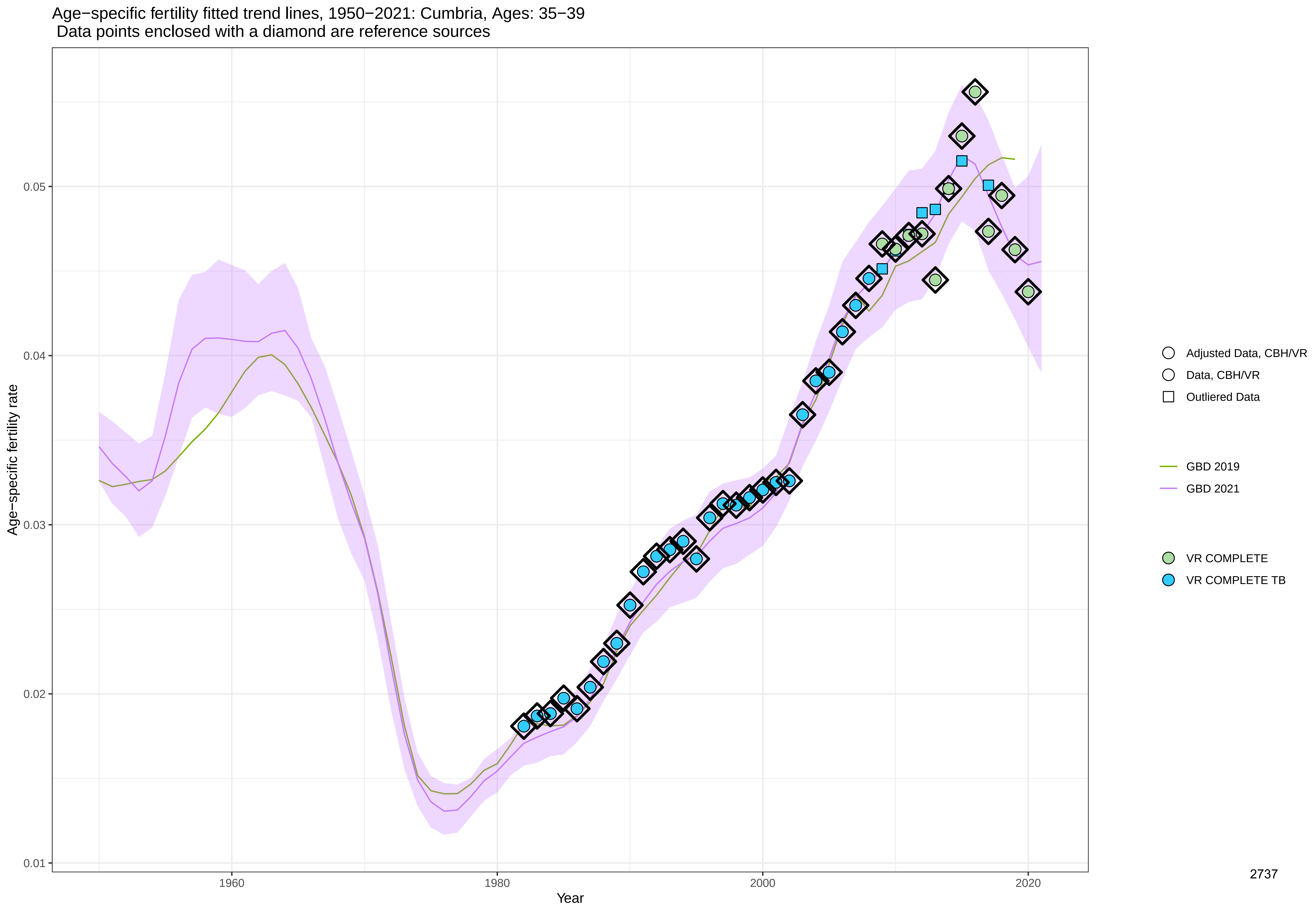

Age−specific fertility fitted trend lines, 1950−2021: Cumbria, Ages: 35−39
Data points enclosed with a diamond are reference sources
Age−specific fertility rate
0.01
0.02
0.03
0.04
0.05
1960
1980
2000
2020
Year
Adjusted Data, CBH/VR
Data, CBH/VR
Outliered Data
GBD 2019
GBD 2021
VR COMPLETE
VR COMPLETE TB

Age−specific fertility fitted trend lines, 1950−2021: Cumbria, Ages: 40−44
Data points enclosed with a diamond are reference sources

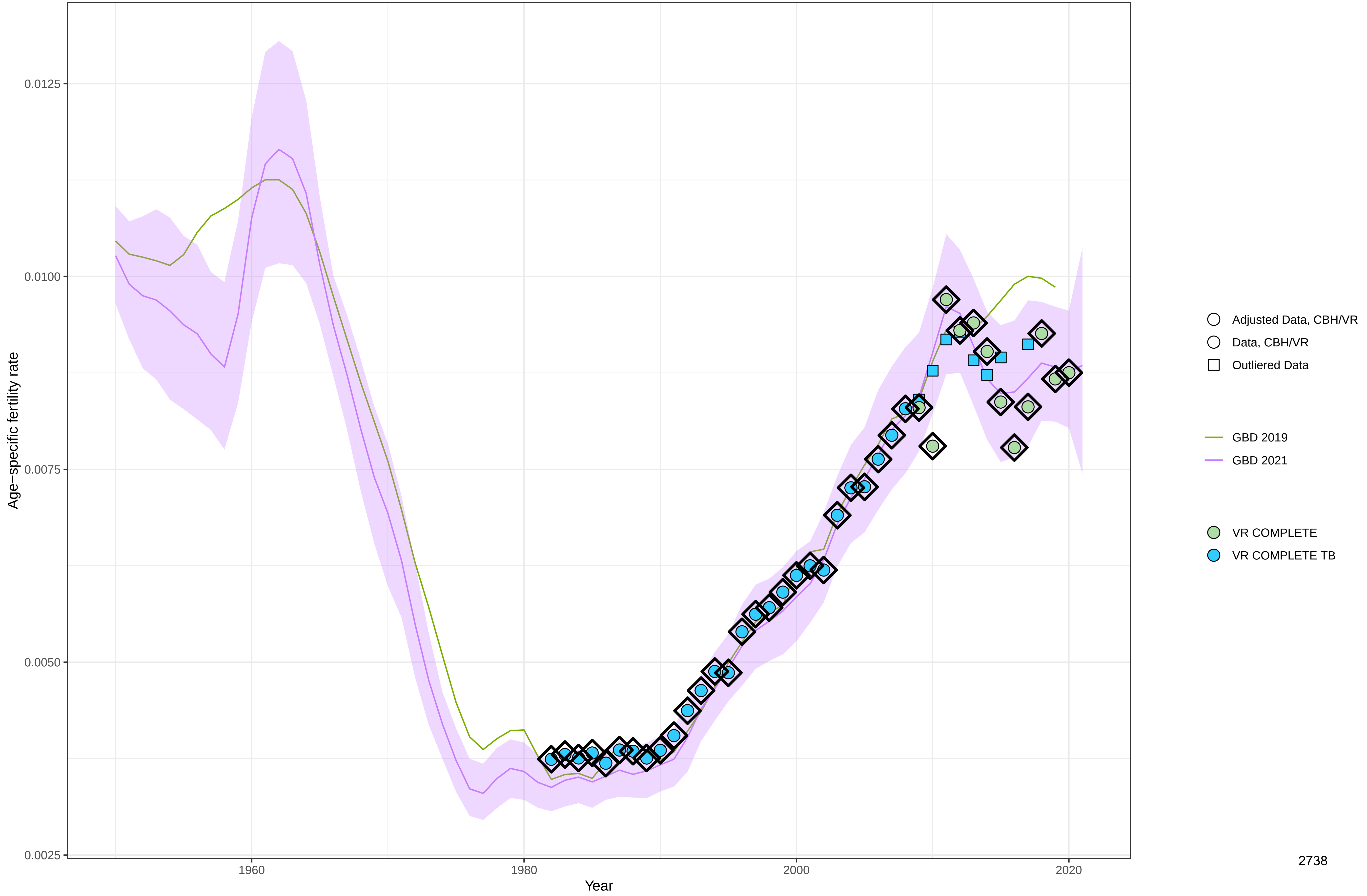

Age−specific fertility fitted trend lines, 1950−2021: Cumbria, Ages: 45−49
Data points enclosed with a diamond are reference sources

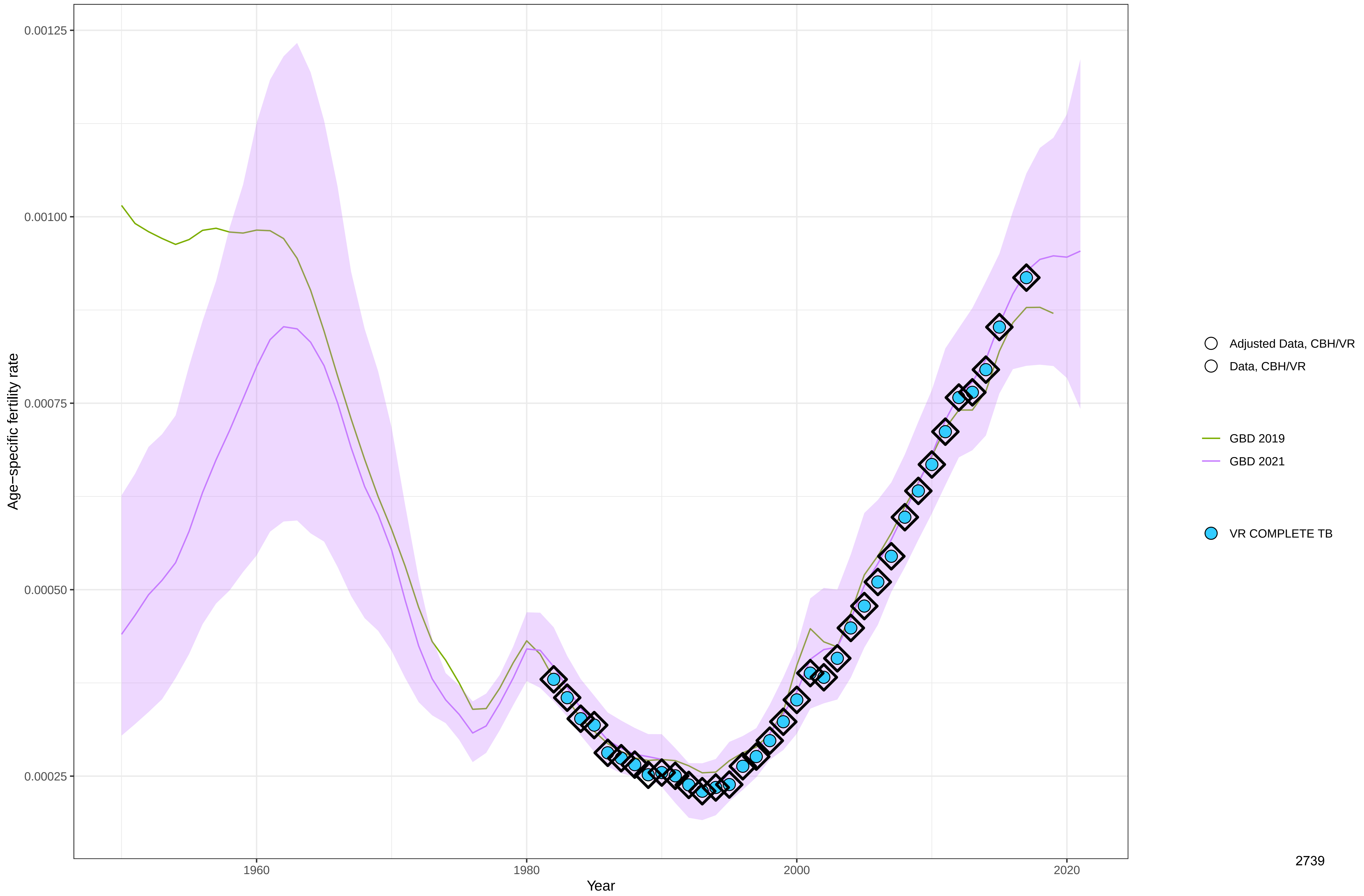

Age−specific fertility fitted trend lines, 1950−2021: Cumbria, Ages: 50−54
Data points enclosed with a diamond are reference sources

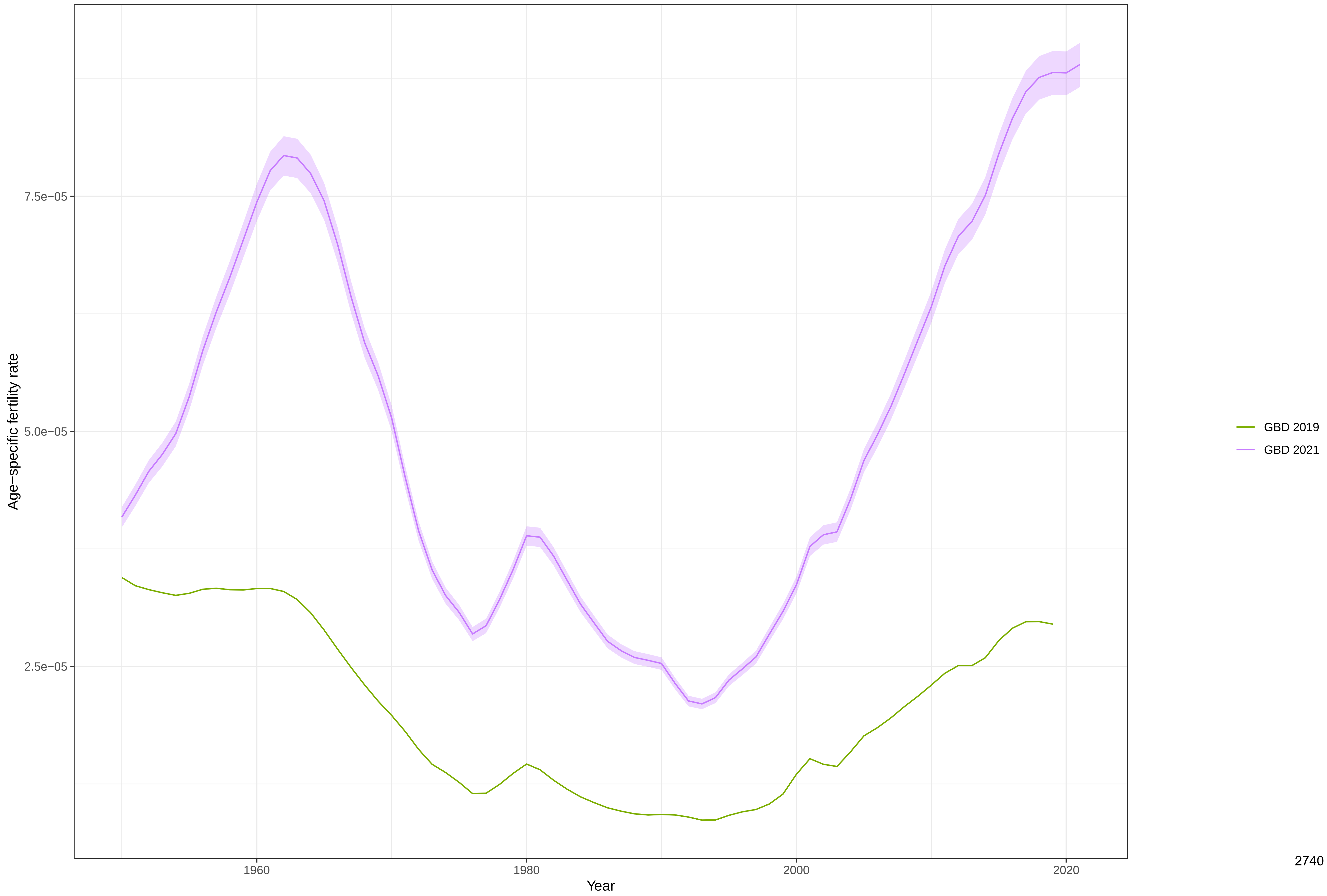

Total fertility rate fitted trend lines, 1950−2021: Cumbria TFR
Data points enclosed with a diamond are reference sources

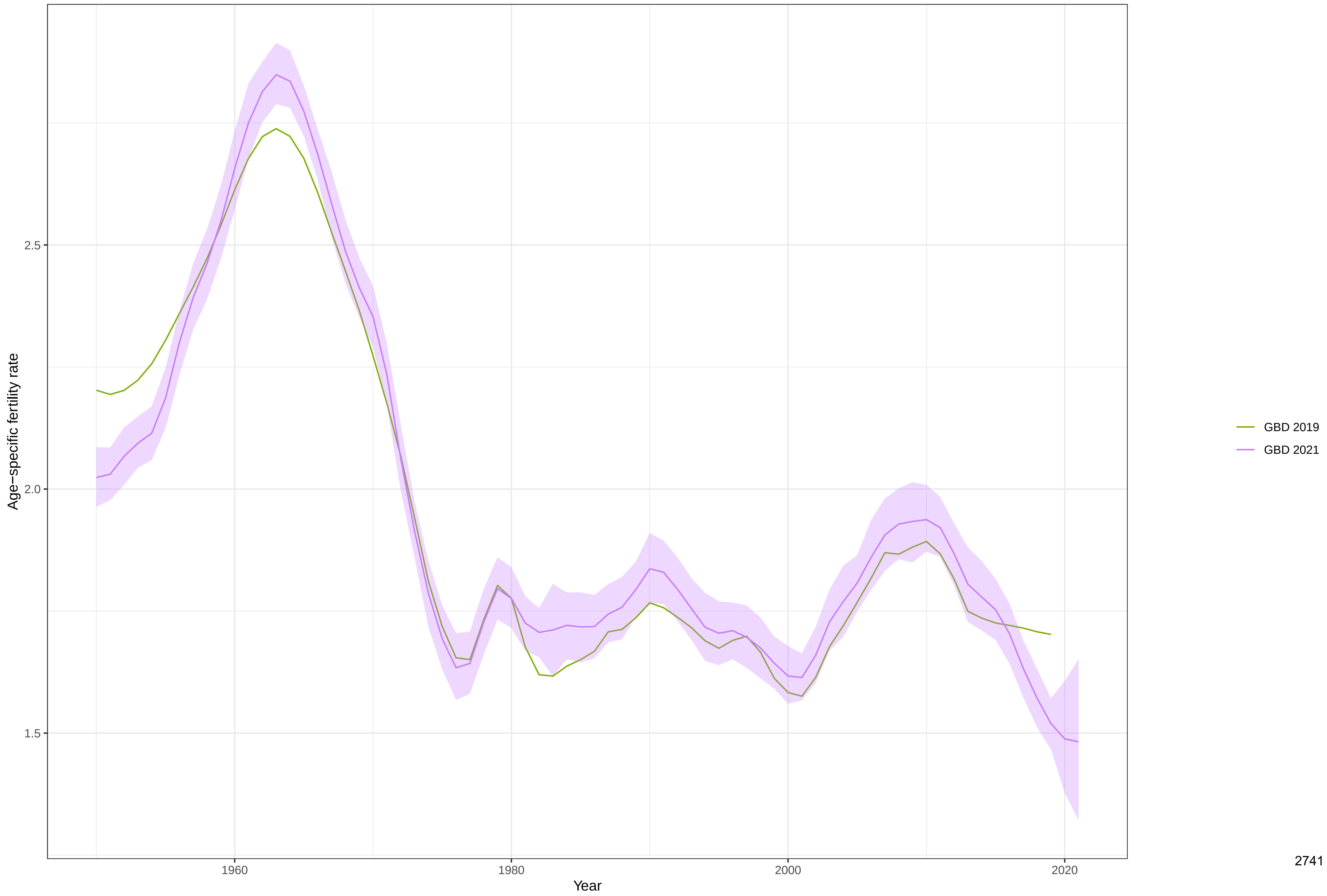

Age−specific fertility fitted trend lines, 1950−2021: Halton, Ages: 10−14
Data points enclosed with a diamond are reference sources

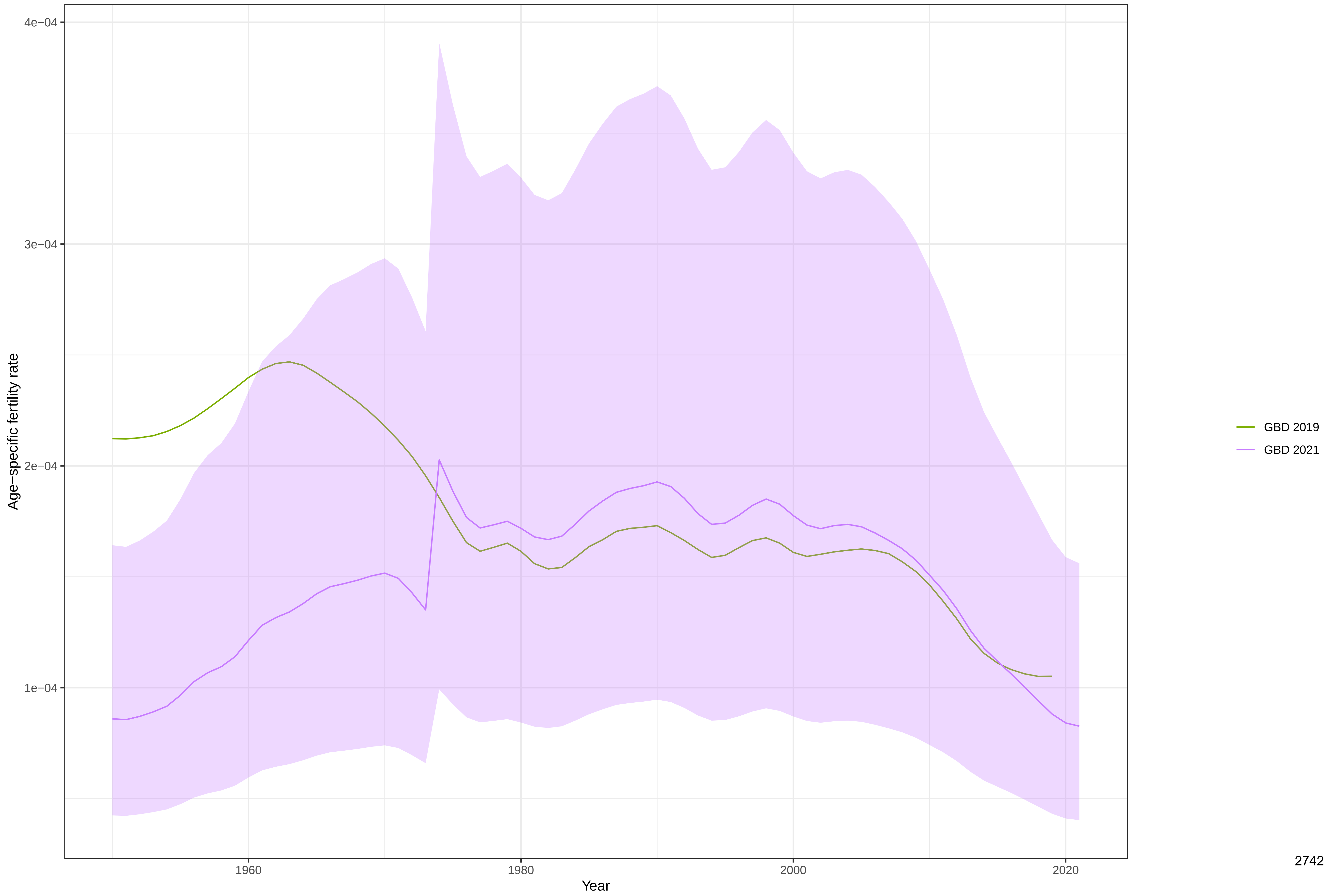

Age−specific fertility fitted trend lines, 1950−2021: Halton, Ages: 15−19
Data points enclosed with a diamond are reference sources

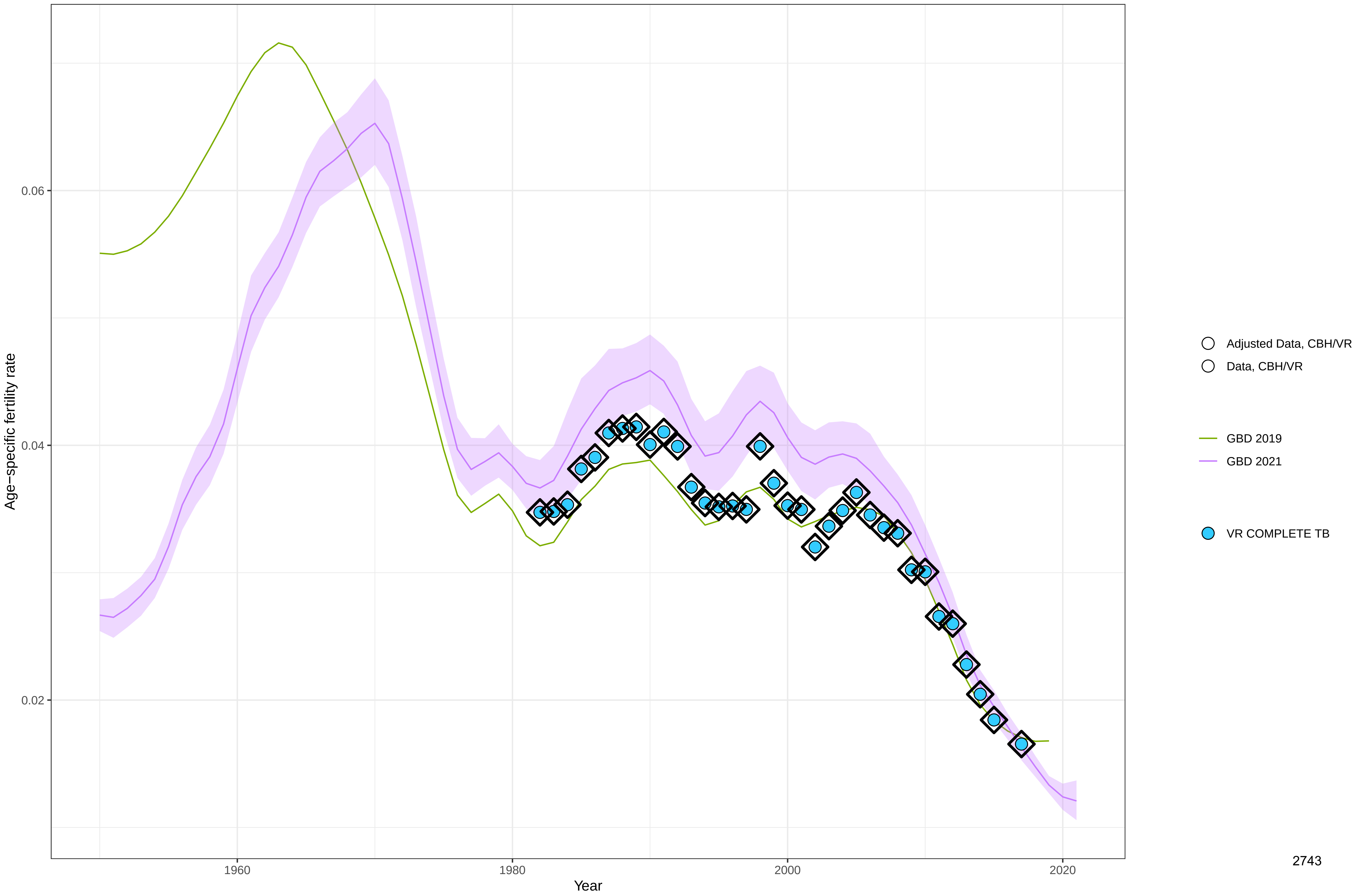

Age−specific fertility fitted trend lines, 1950−2021: Halton, Ages: 20−24
Data points enclosed with a diamond are reference sources

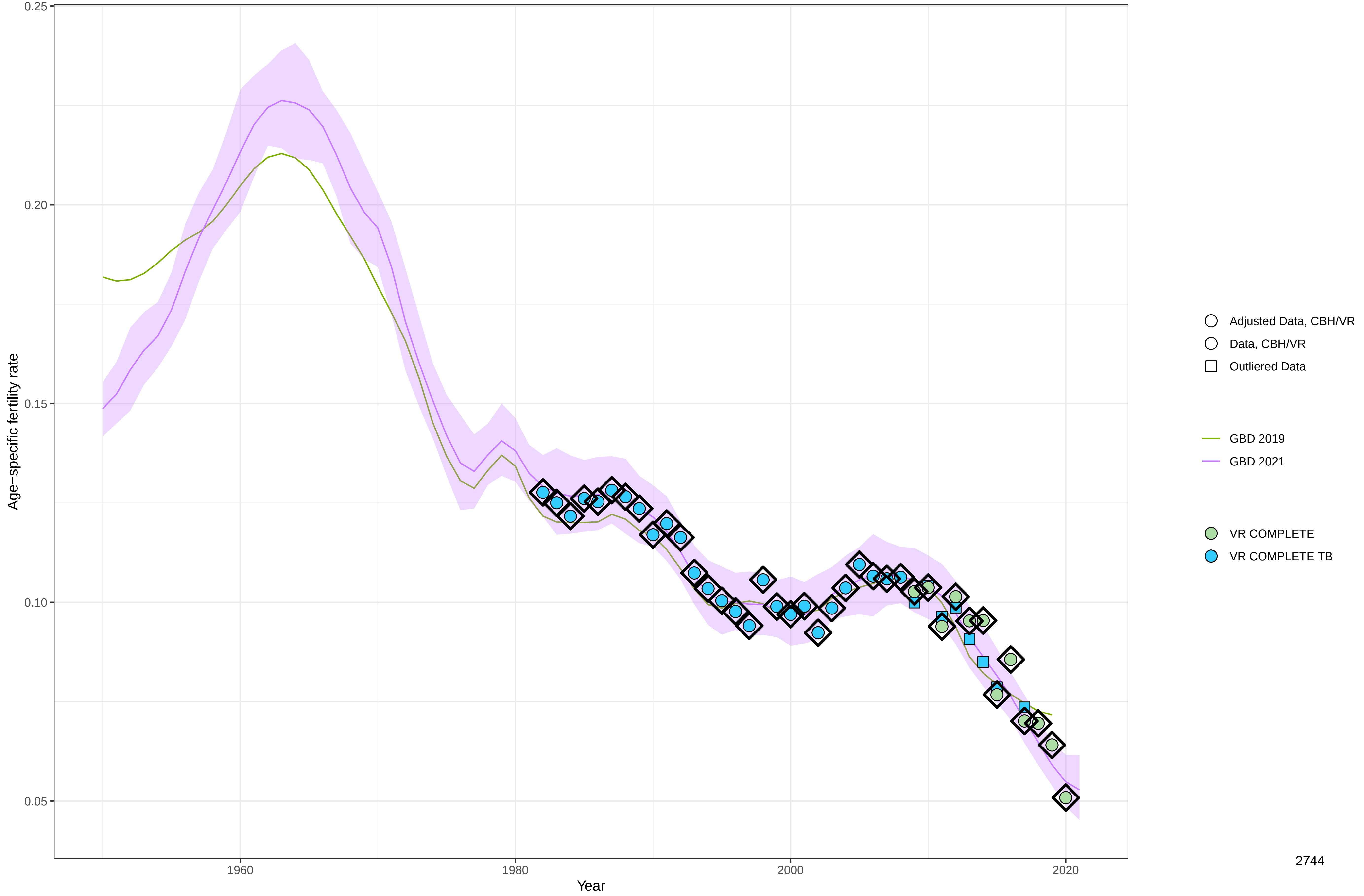

Age−specific fertility fitted trend lines, 1950−2021: Halton, Ages: 25−29
Data points enclosed with a diamond are reference sources

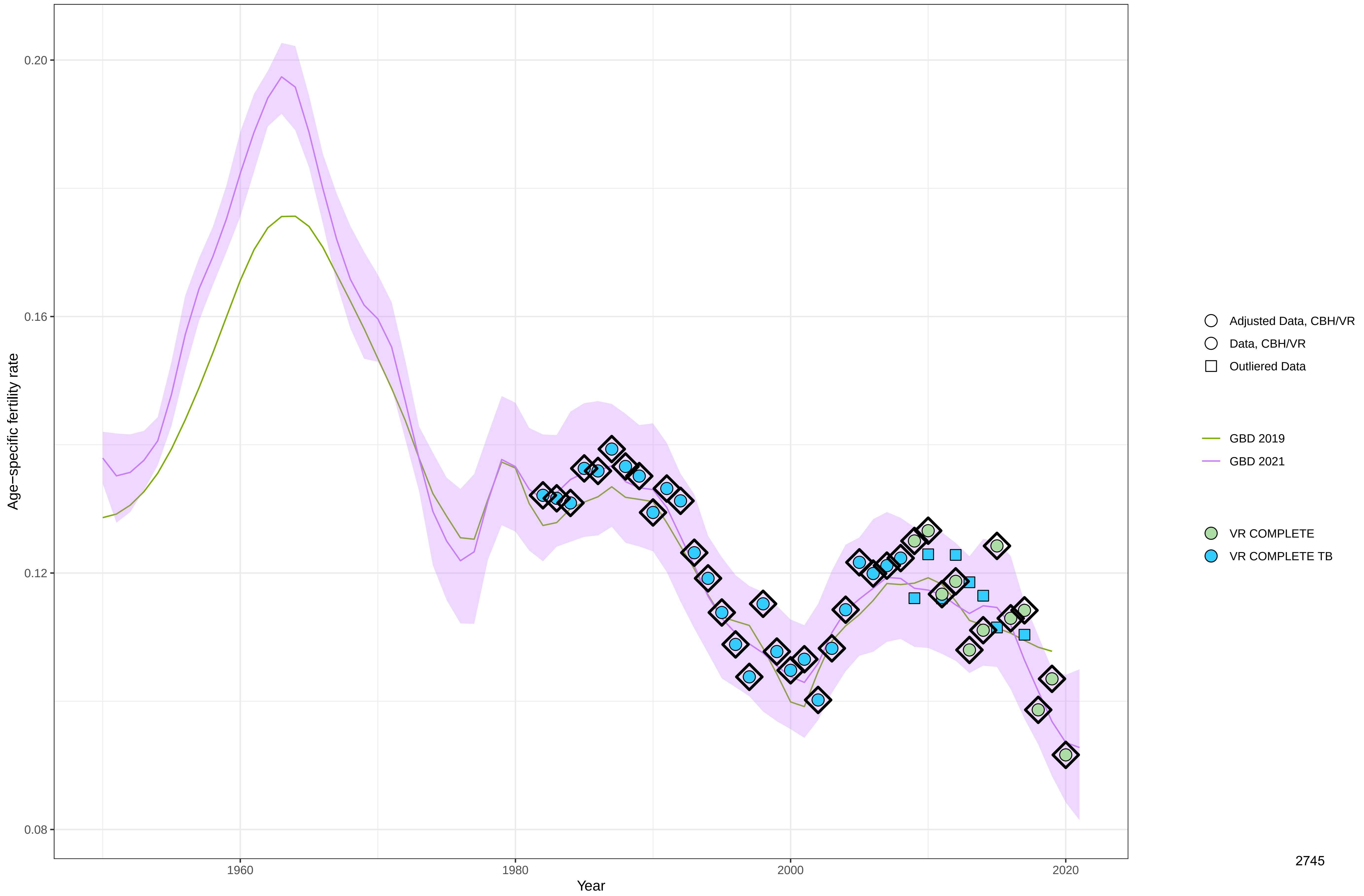

Age−specific fertility fitted trend lines, 1950−2021: Halton, Ages: 30−34
Data points enclosed with a diamond are reference sources

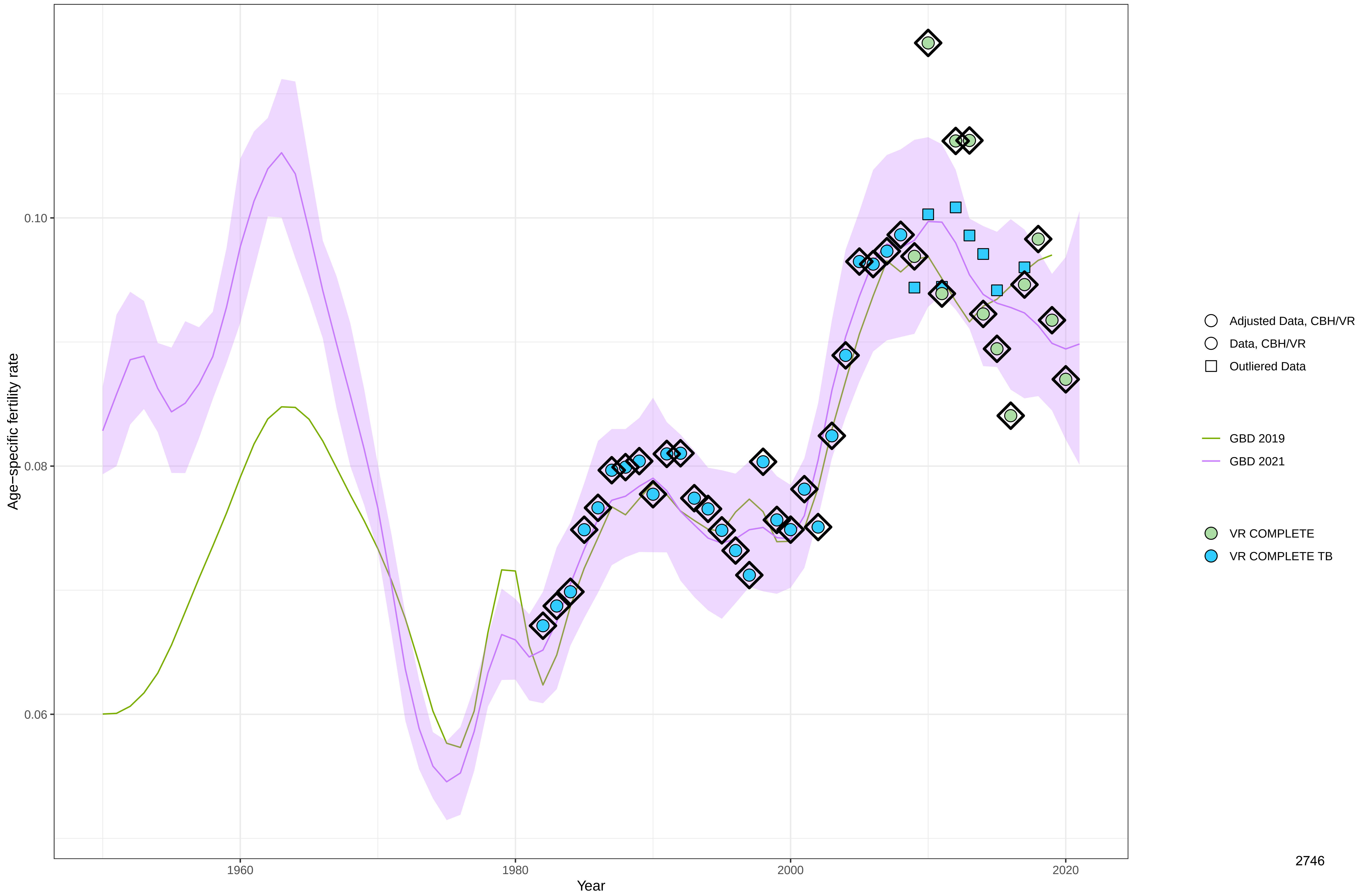

Age−specific fertility fitted trend lines, 1950−2021: Halton, Ages: 35−39
Data points enclosed with a diamond are reference sources

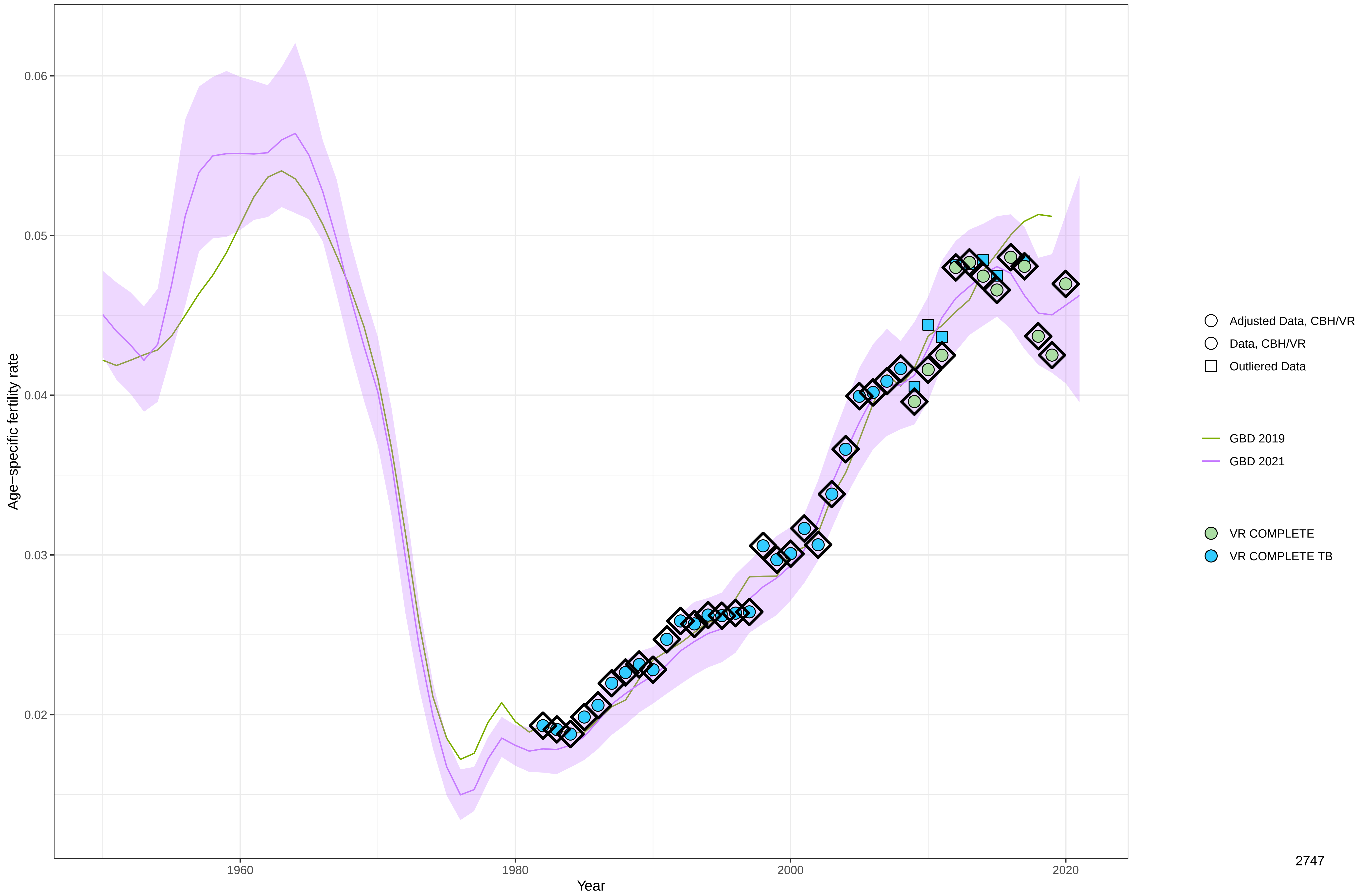

2747

Age−specific fertility fitted trend lines, 1950−2021: Halton, Ages: 40−44
Data points enclosed with a diamond are reference sources

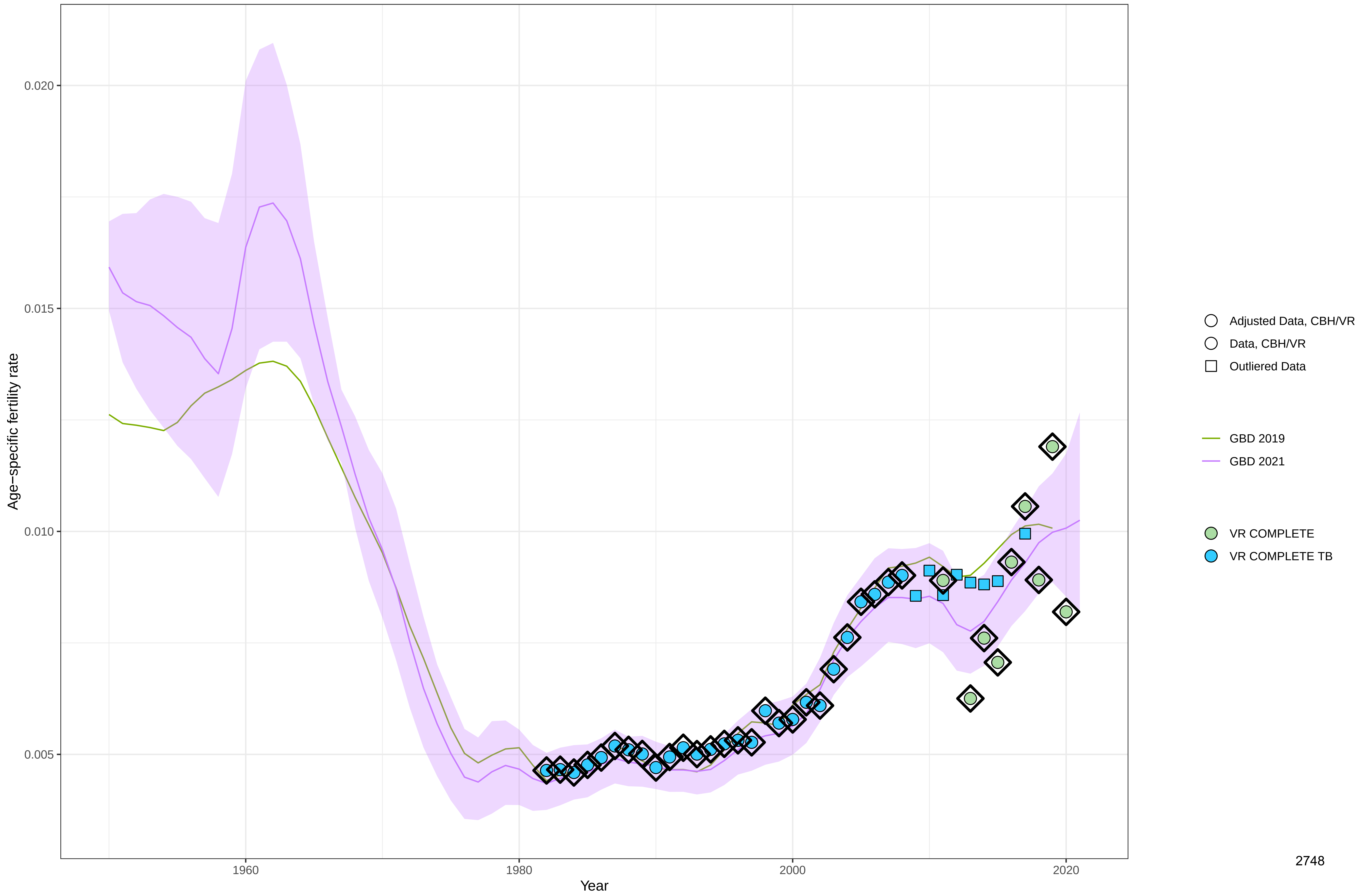

Age−specific fertility fitted trend lines, 1950−2021: Halton, Ages: 45−49
Data points enclosed with a diamond are reference sources

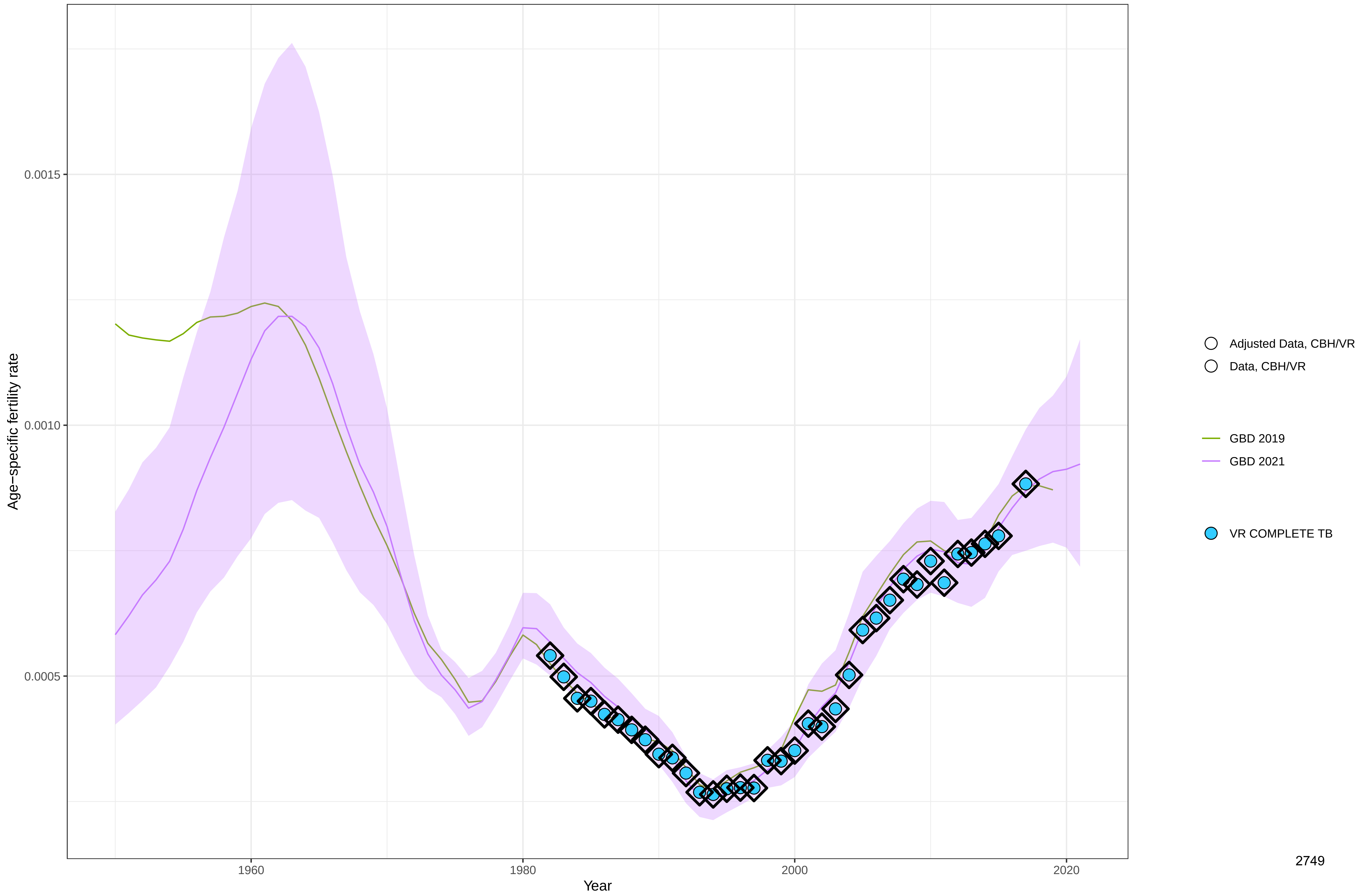

Age−specific fertility fitted trend lines, 1950−2021: Halton, Ages: 50−54
Data points enclosed with a diamond are reference sources

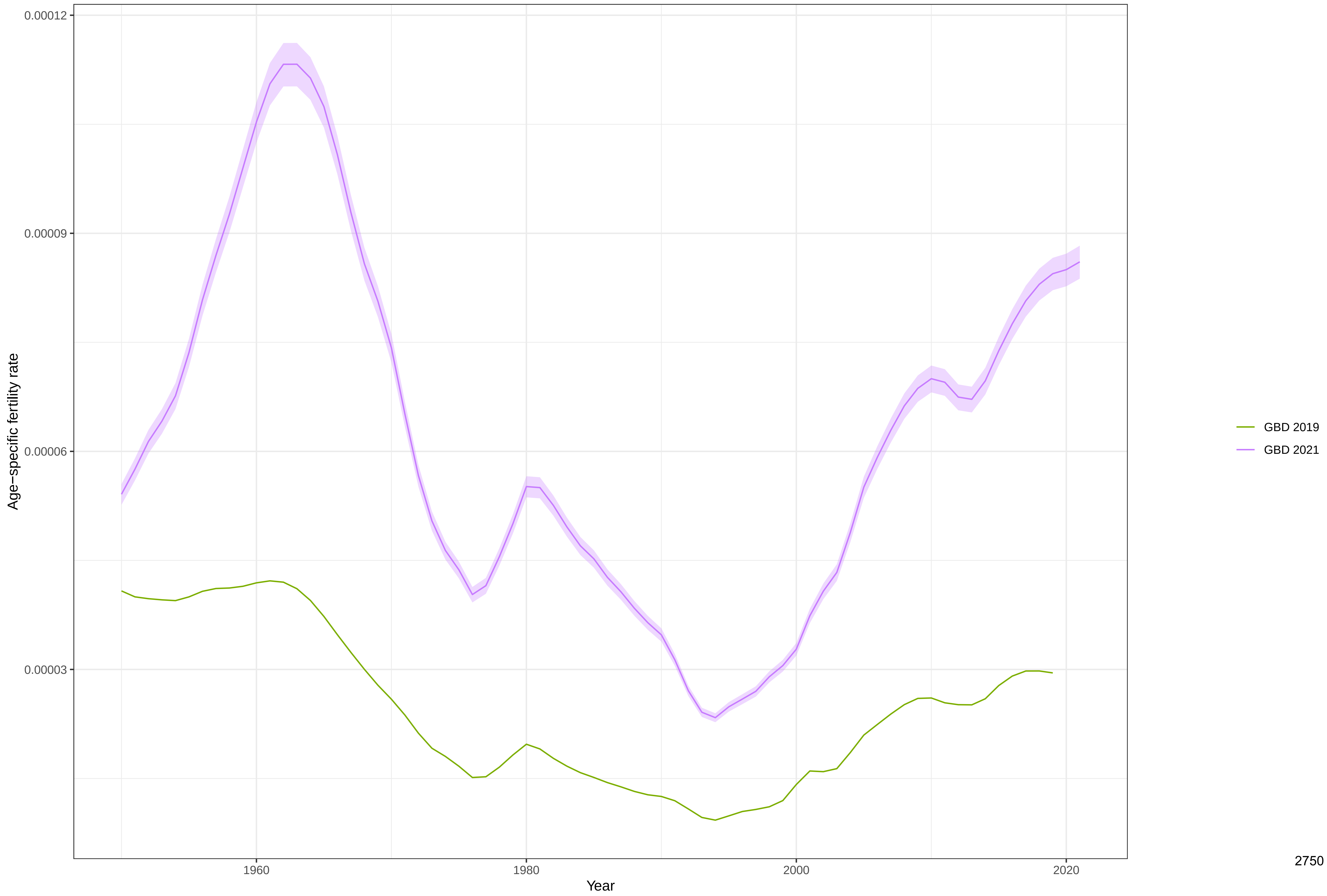

Total fertility rate fitted trend lines, 1950−2021: Halton TFR
Data points enclosed with a diamond are reference sources

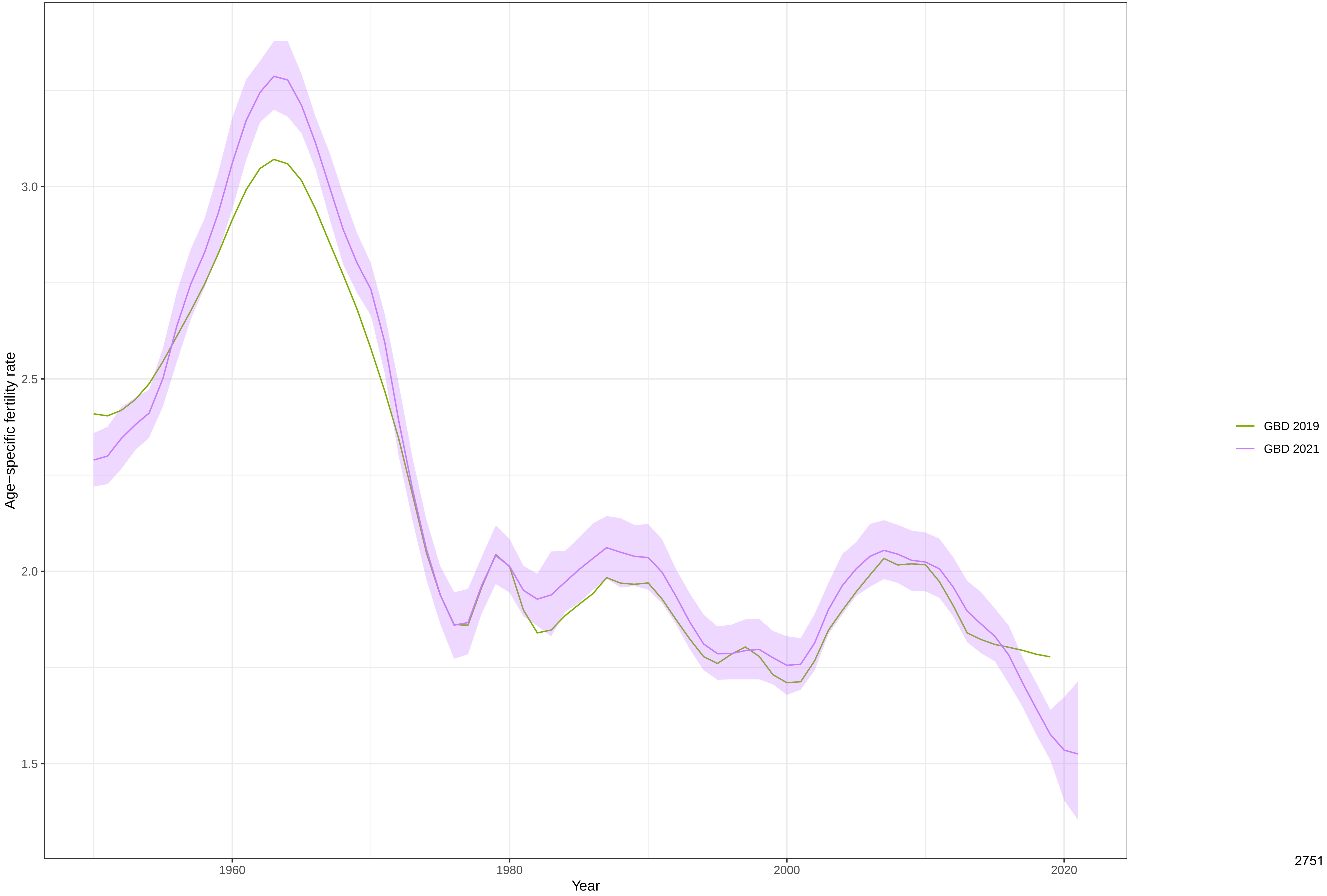

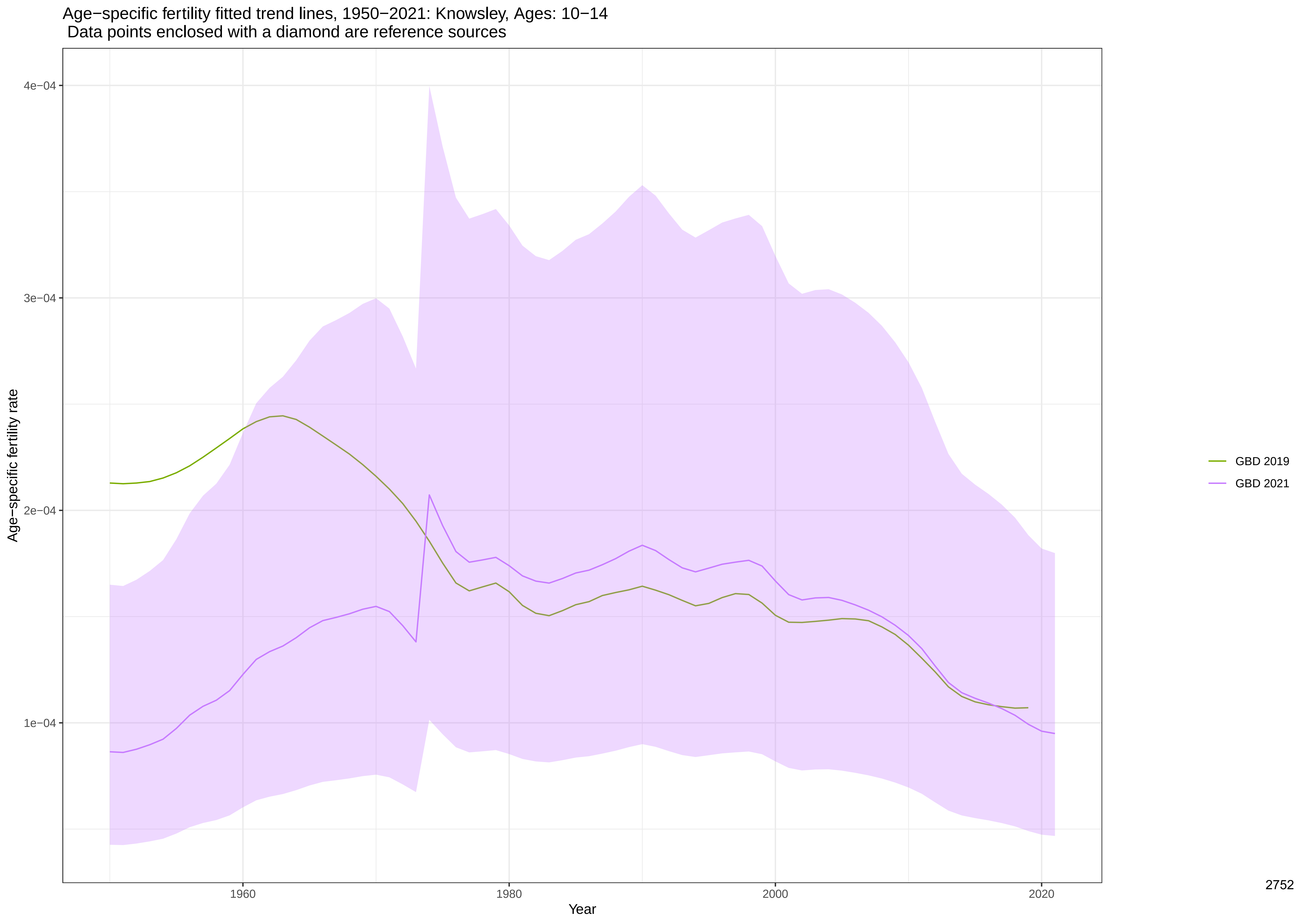

Age−specific fertility fitted trend lines, 1950−2021: Knowsley, Ages: 10−14
Data points enclosed with a diamond are reference sources
Age−specific fertility rate
4e−04
3e−04
2e−04
1e−04
1960
1980
2000
2020
Year
GBD 2019
GBD 2021

Age−specific fertility fitted trend lines, 1950−2021: Knowsley, Ages: 15−19
Data points enclosed with a diamond are reference sources

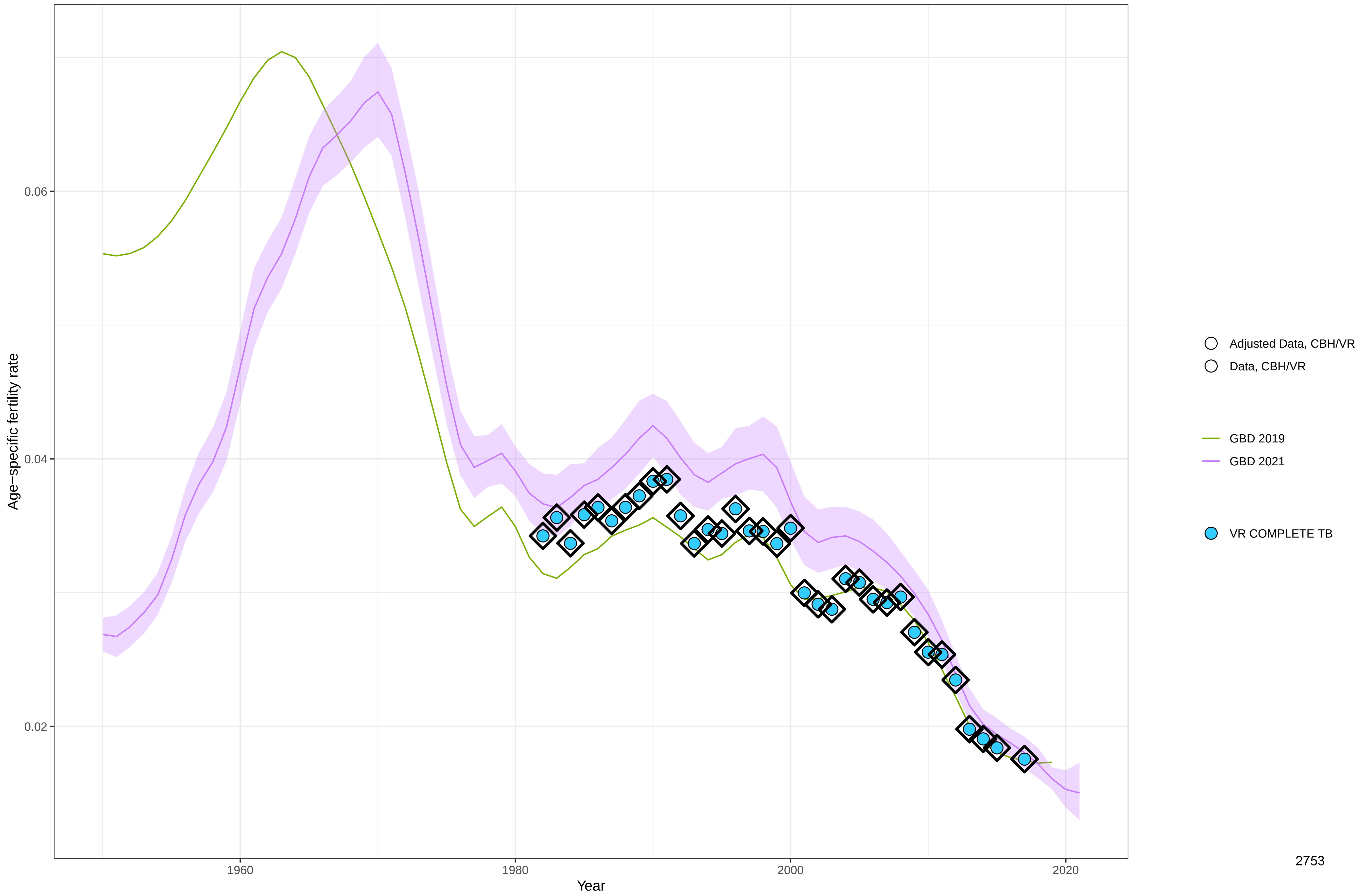

Age−specific fertility fitted trend lines, 1950−2021: Knowsley, Ages: 20−24
Data points enclosed with a diamond are reference sources

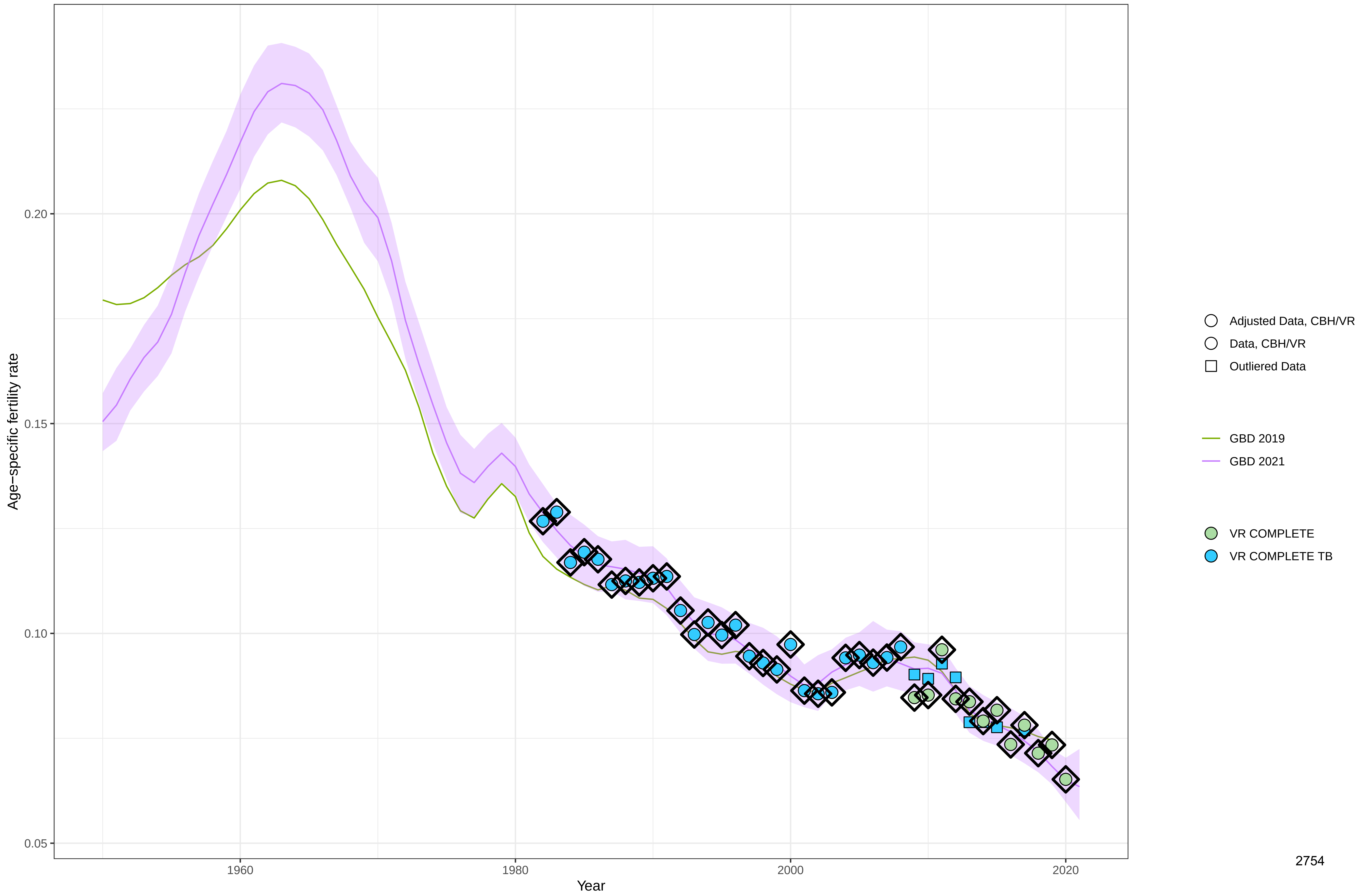

Age−specific fertility fitted trend lines, 1950−2021: Knowsley, Ages: 25−29
Data points enclosed with a diamond are reference sources

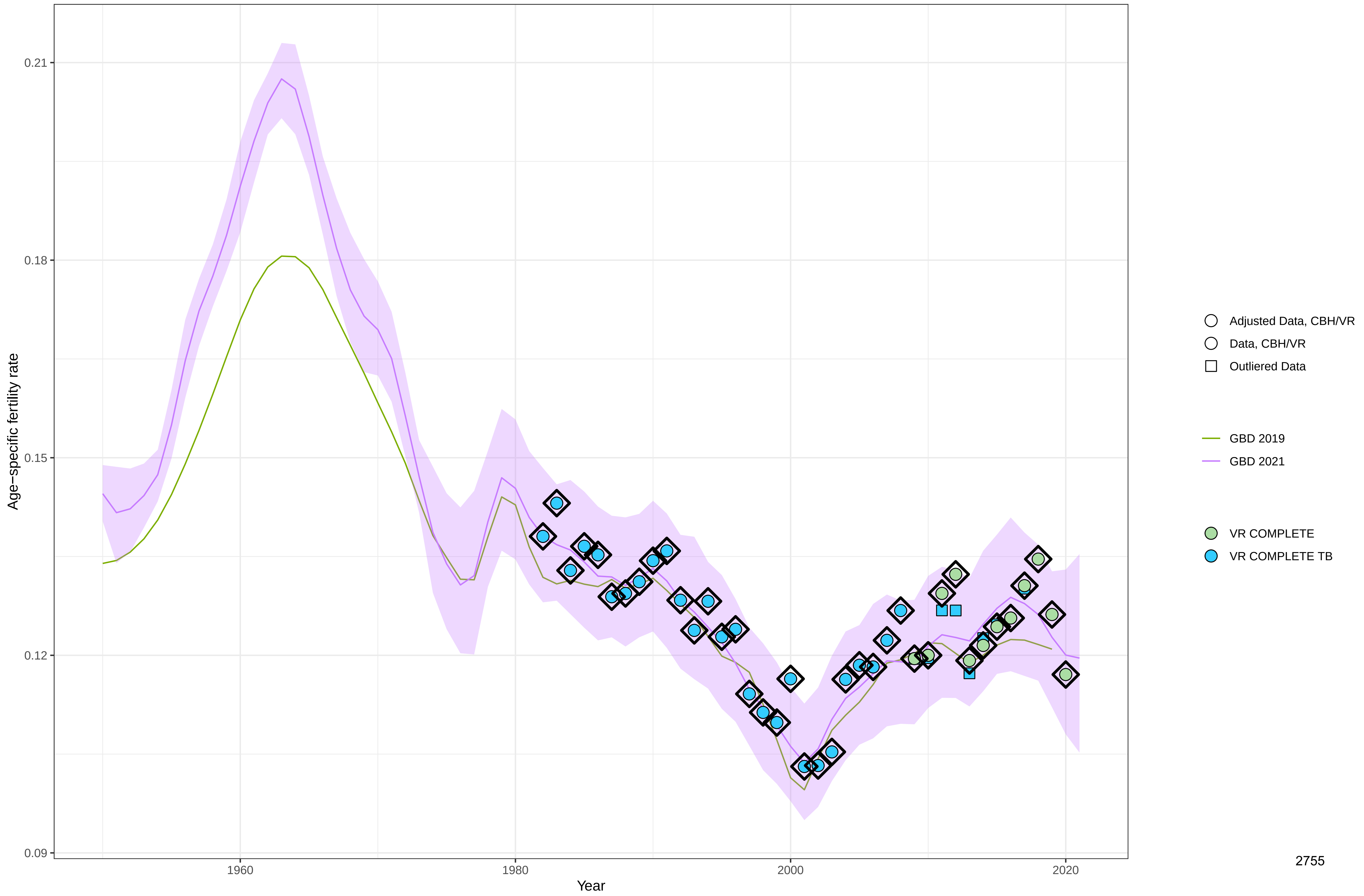

Age−specific fertility fitted trend lines, 1950−2021: Knowsley, Ages: 30−34
Data points enclosed with a diamond are reference sources

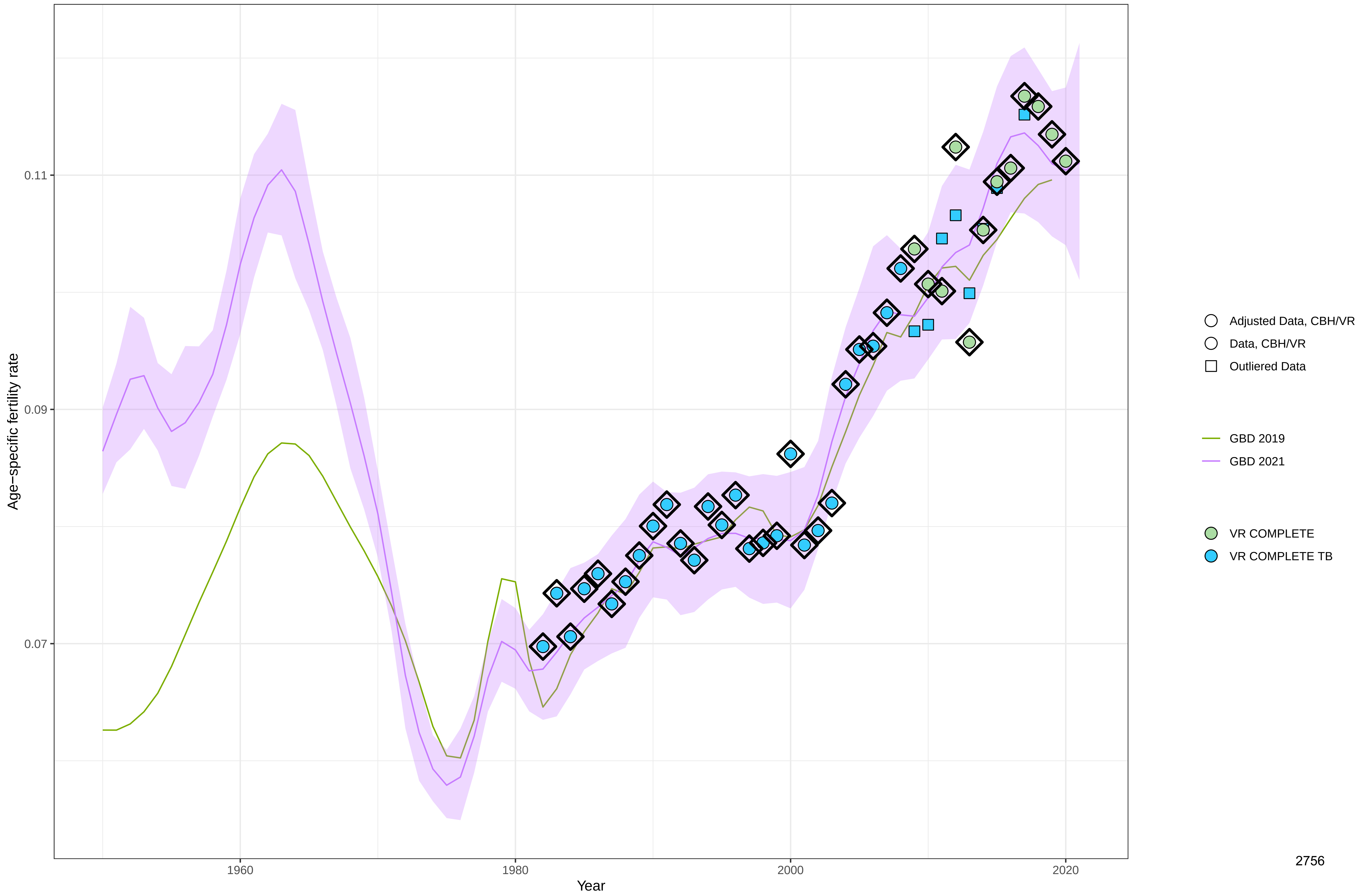

Age−specific fertility fitted trend lines, 1950−2021: Knowsley, Ages: 35−39
Data points enclosed with a diamond are reference sources

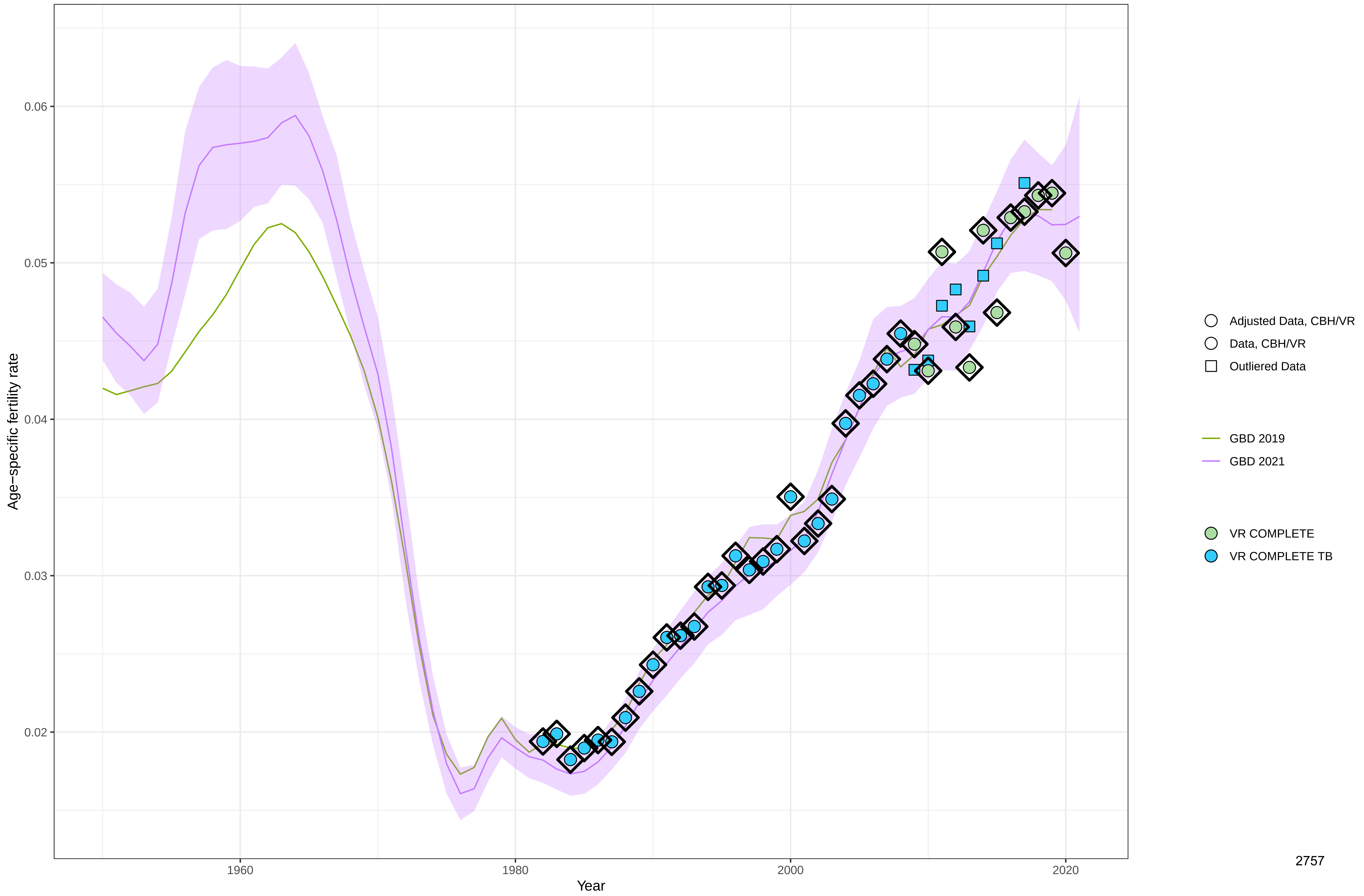

Age−specific fertility fitted trend lines, 1950−2021: Knowsley, Ages: 40−44
Data points enclosed with a diamond are reference sources

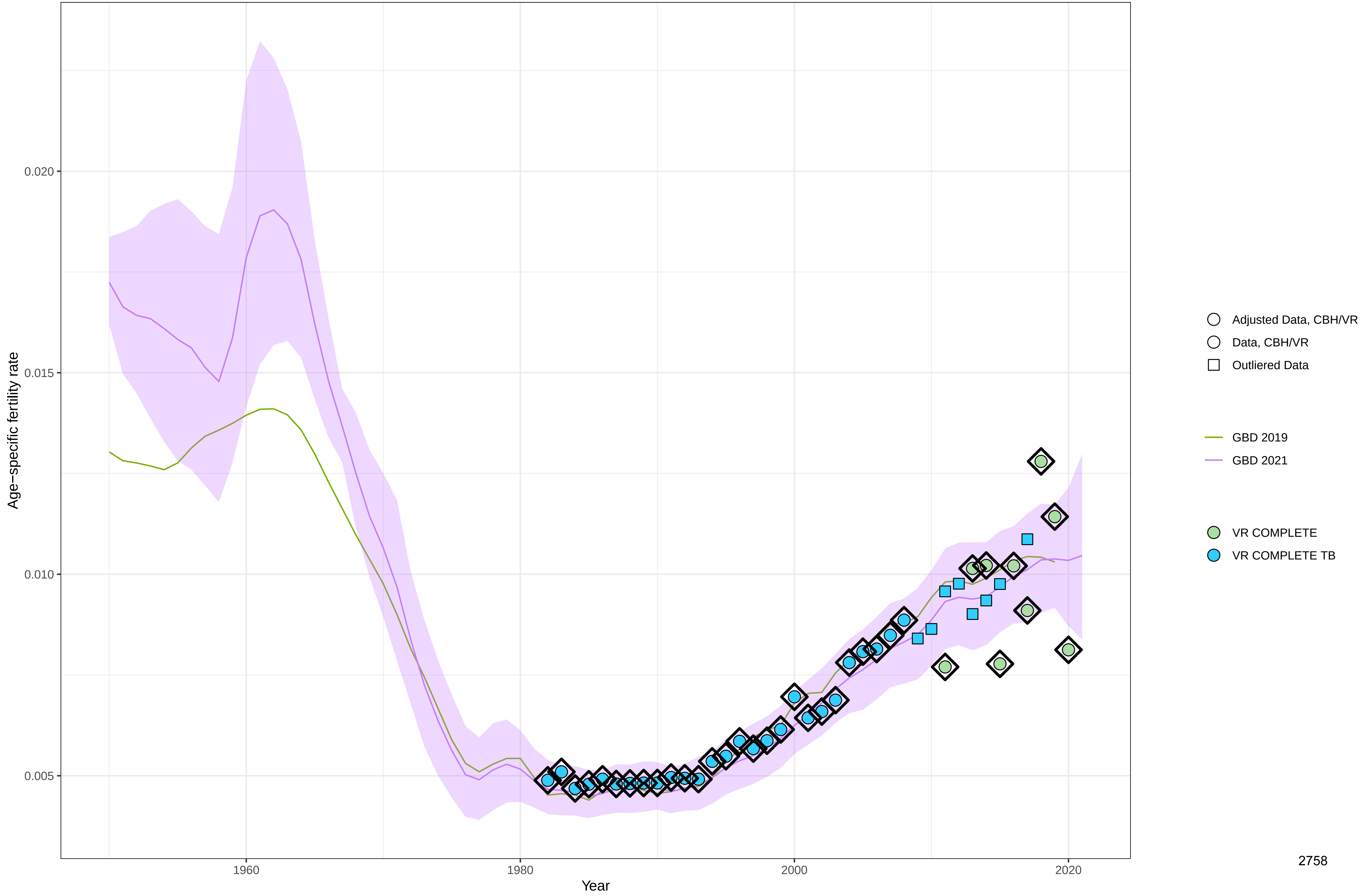

Age−specific fertility fitted trend lines, 1950−2021: Knowsley, Ages: 45−49
Data points enclosed with a diamond are reference sources

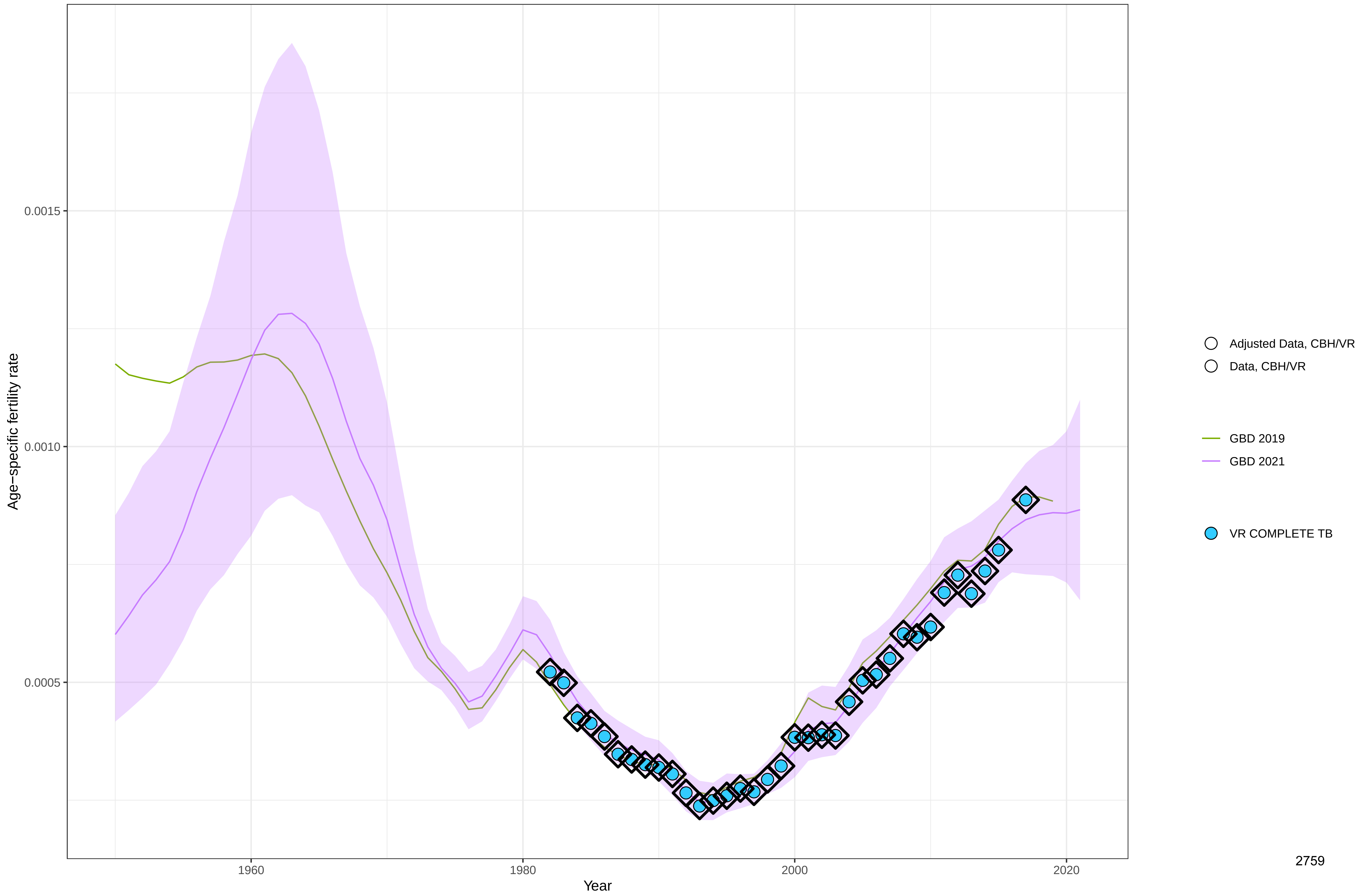

Age−specific fertility fitted trend lines, 1950−2021: Knowsley, Ages: 50−54
Data points enclosed with a diamond are reference sources

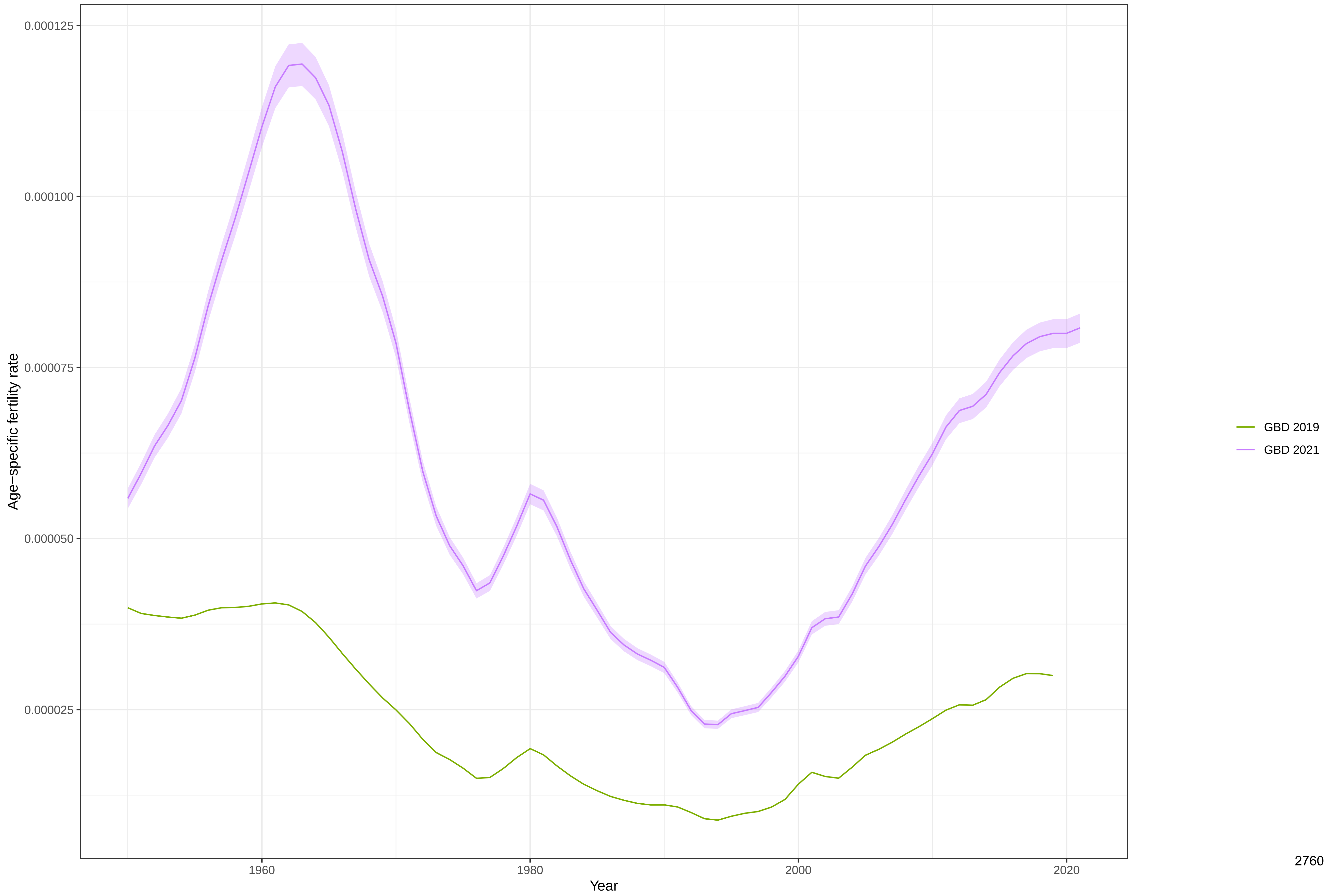

Total fertility rate fitted trend lines, 1950−2021: Knowsley TFR
Data points enclosed with a diamond are reference sources

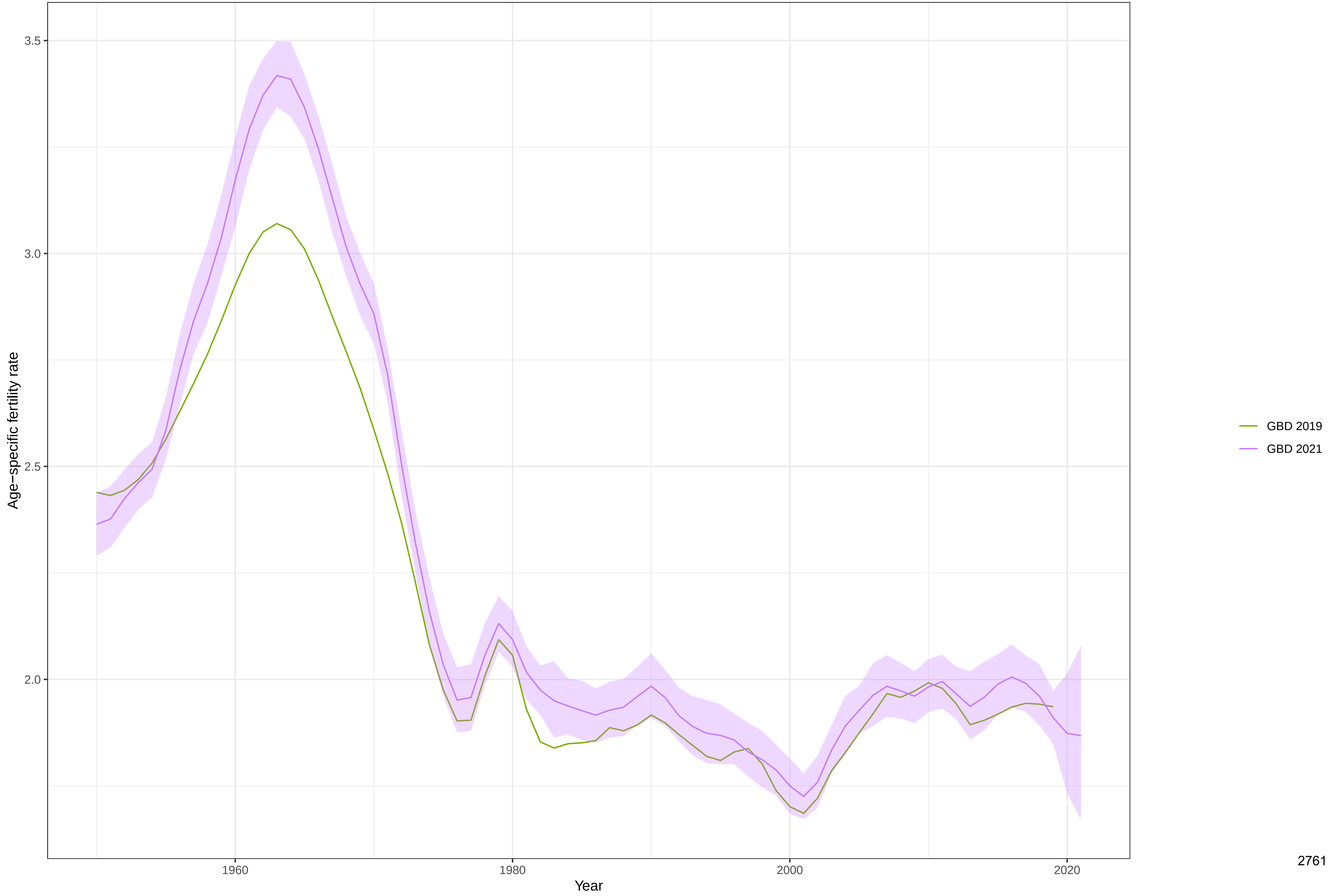

Age−specific fertility fitted trend lines, 1950−2021: Lancashire, Ages: 10−14
Data points enclosed with a diamond are reference sources

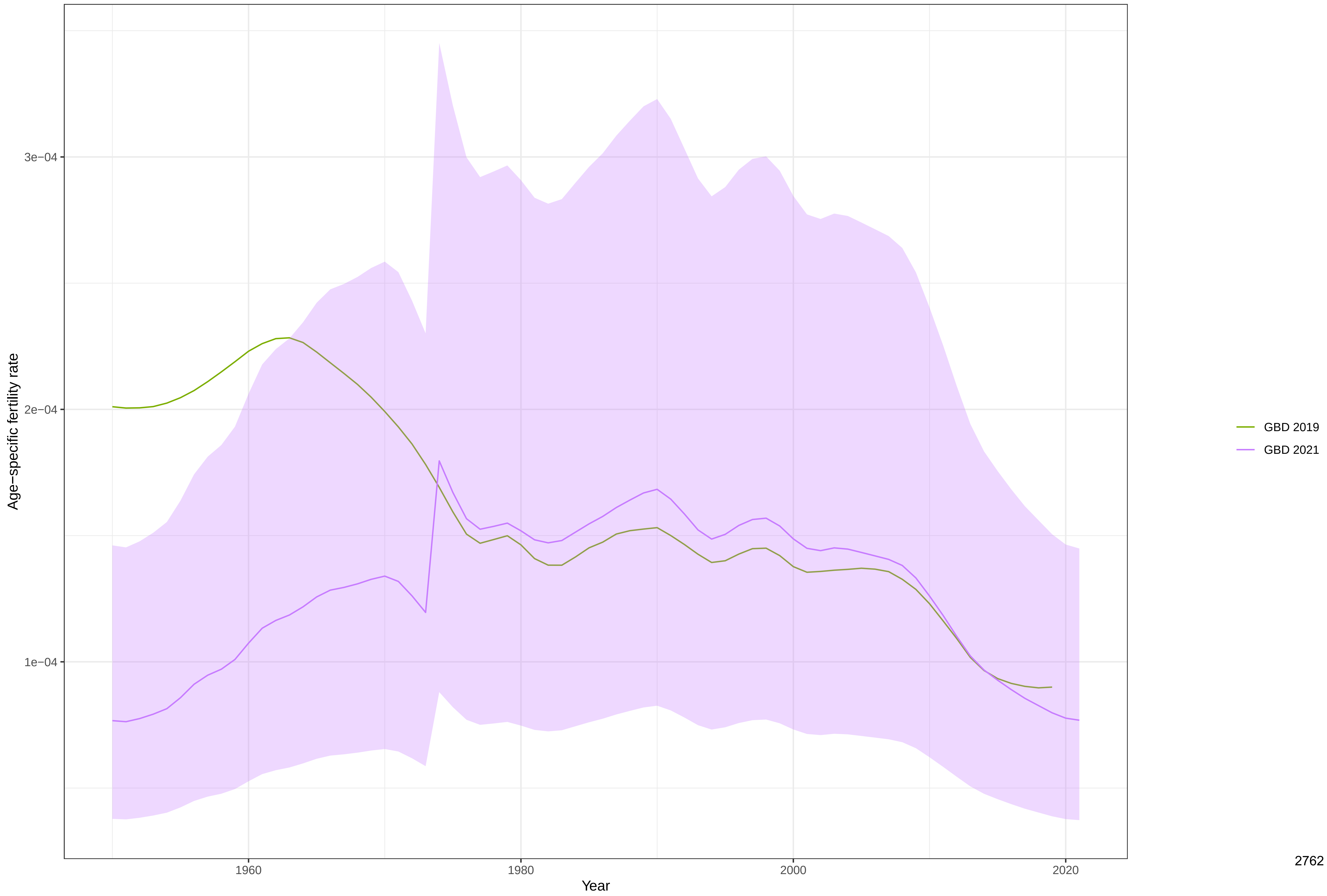

Age−specific fertility fitted trend lines, 1950−2021: Lancashire, Ages: 15−19
Data points enclosed with a diamond are reference sources

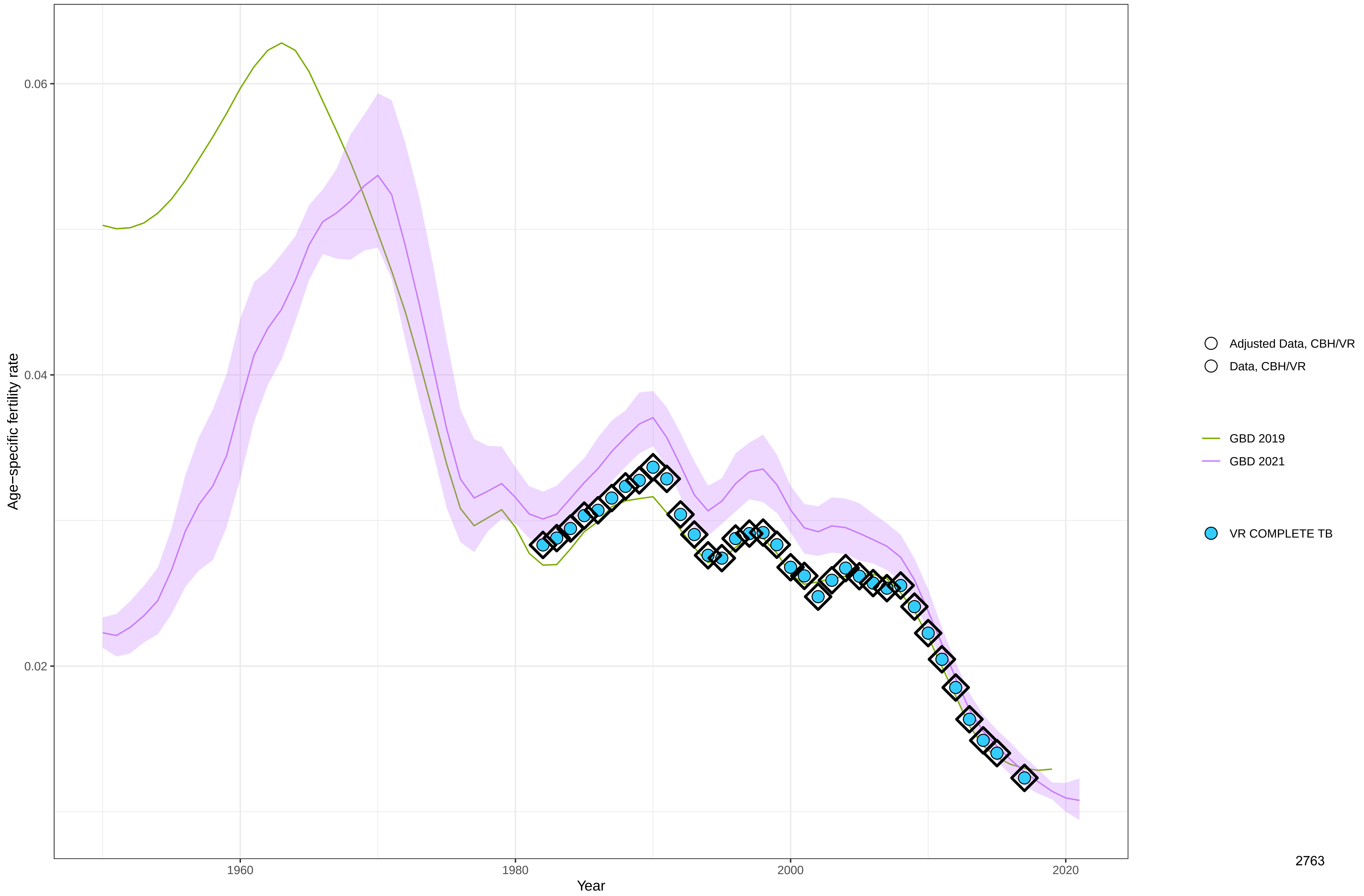

Age−specific fertility fitted trend lines, 1950−2021: Lancashire, Ages: 20−24
Data points enclosed with a diamond are reference sources

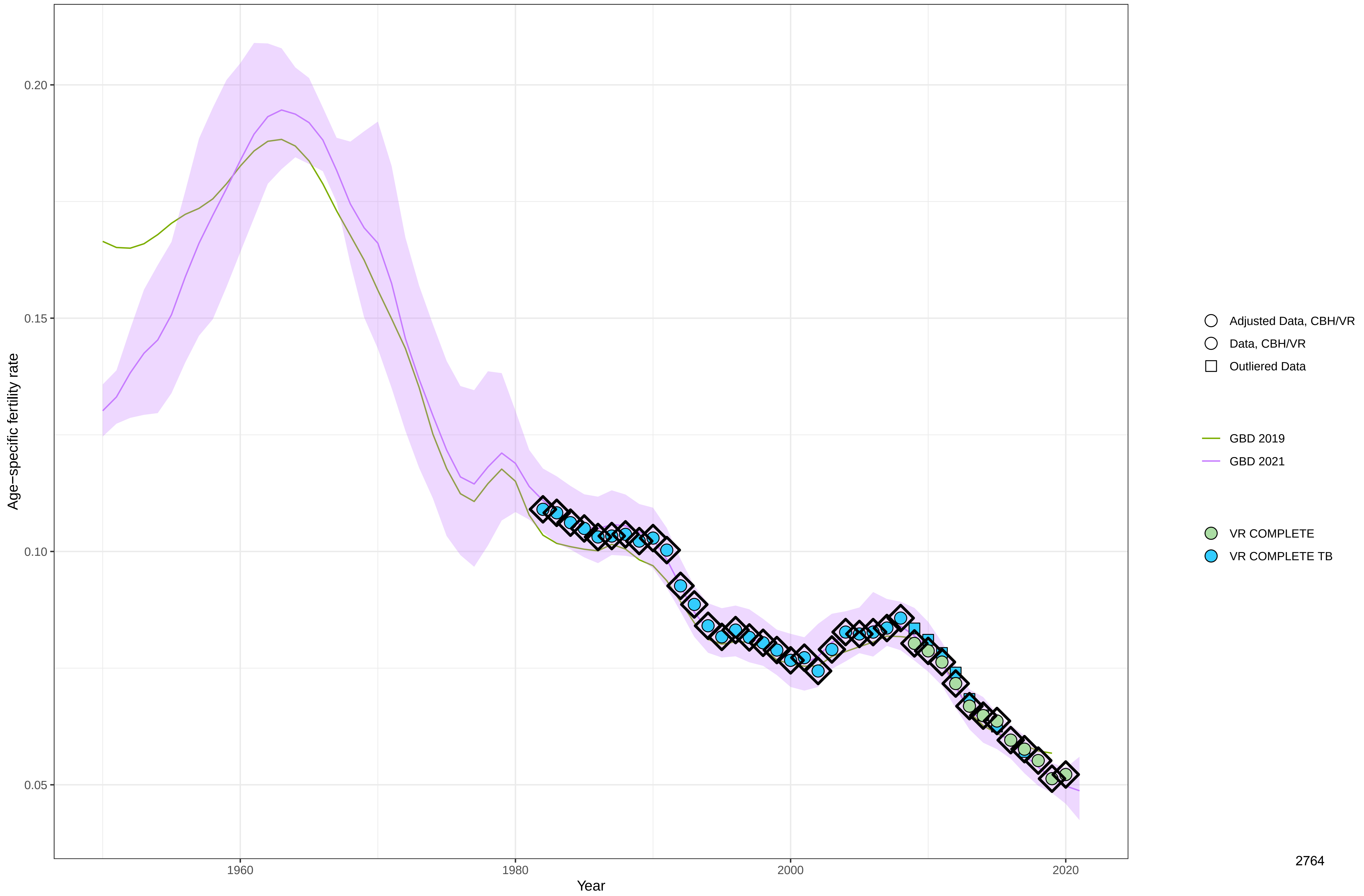

Age−specific fertility fitted trend lines, 1950−2021: Lancashire, Ages: 25−29
Data points enclosed with a diamond are reference sources

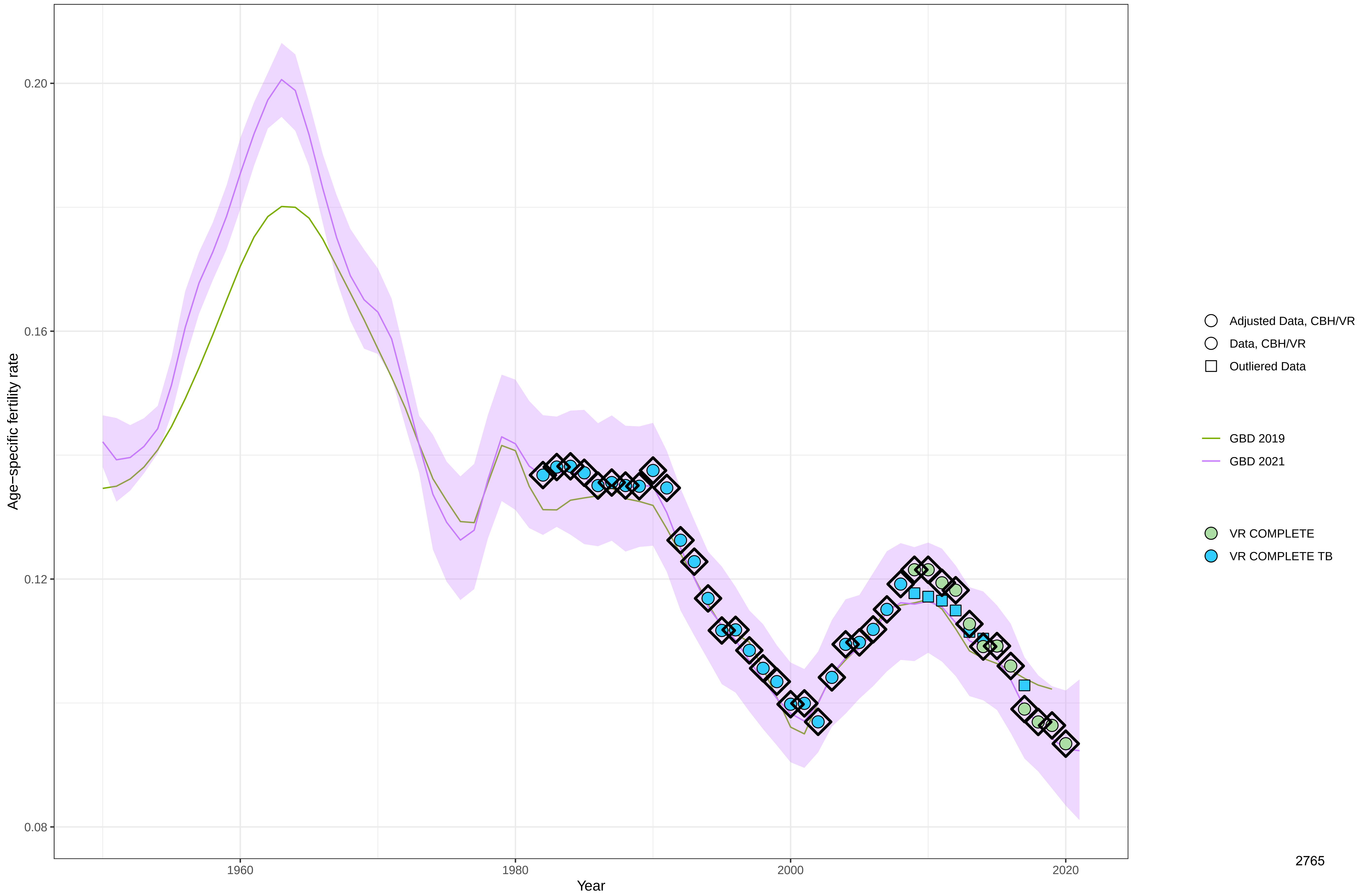

Age−specific fertility fitted trend lines, 1950−2021: Lancashire, Ages: 30−34
Data points enclosed with a diamond are reference sources

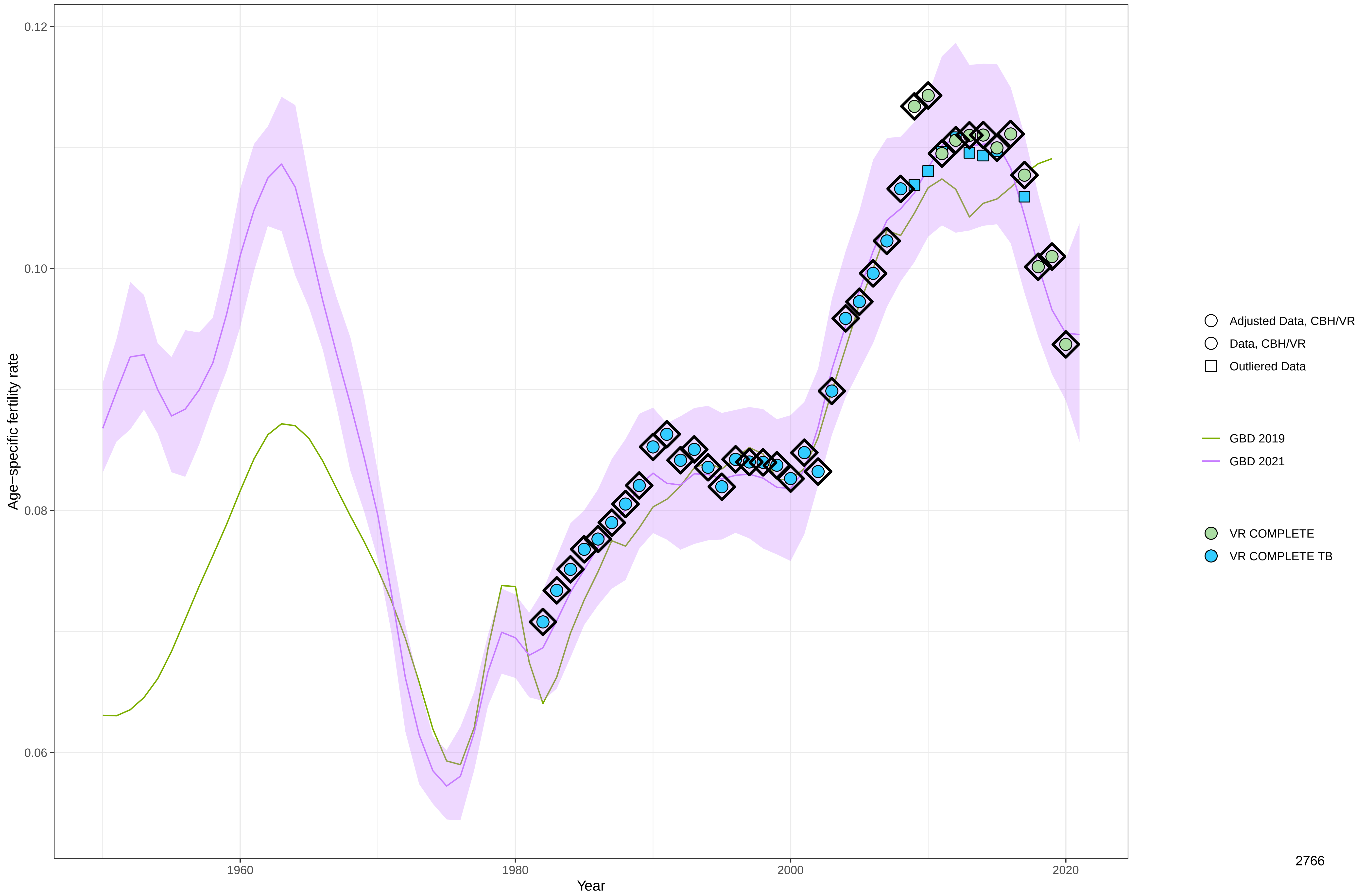

Age−specific fertility fitted trend lines, 1950−2021: Lancashire, Ages: 35−39
Data points enclosed with a diamond are reference sources

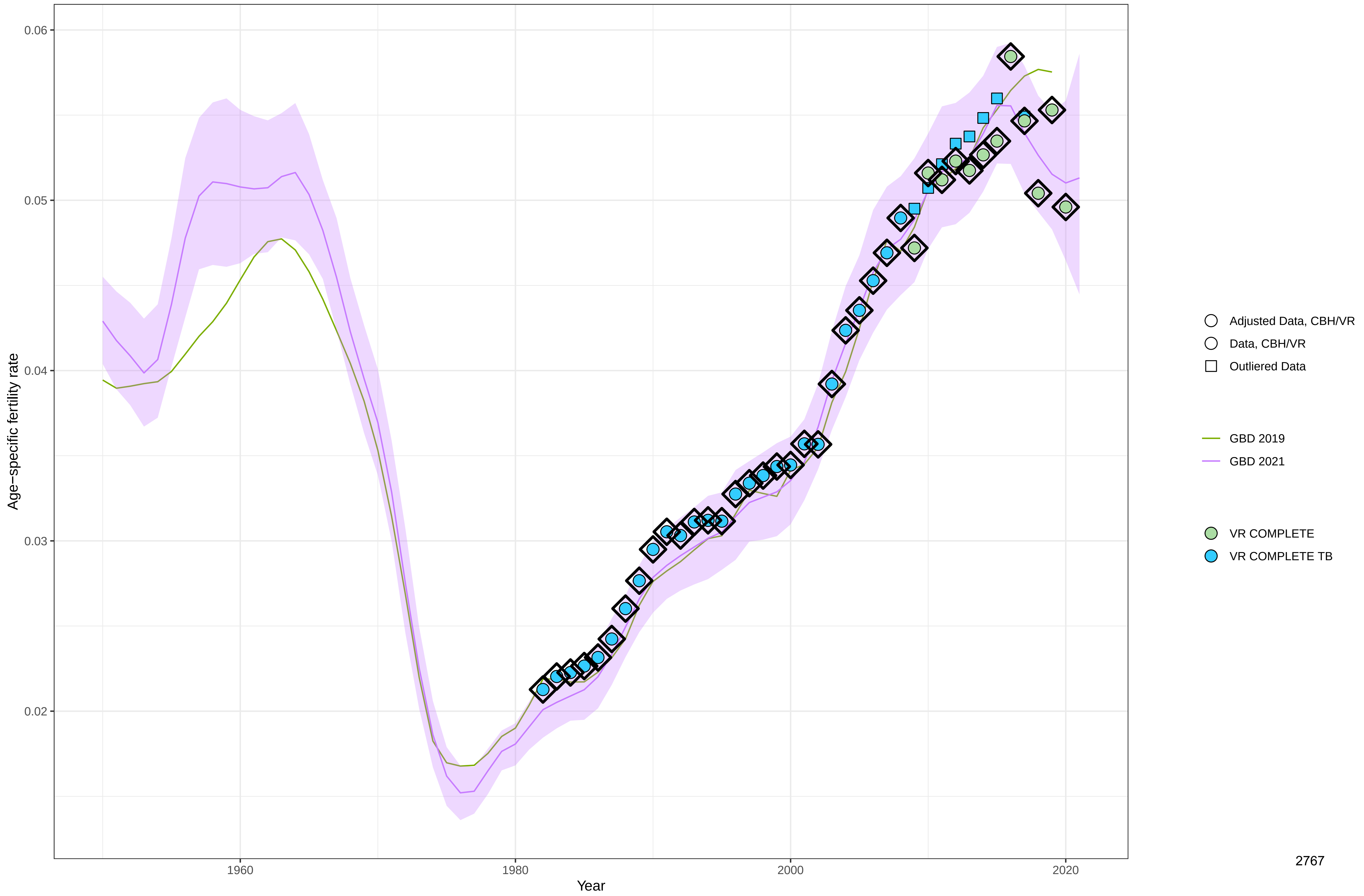

Age−specific fertility fitted trend lines, 1950−2021: Lancashire, Ages: 40−44
Data points enclosed with a diamond are reference sources

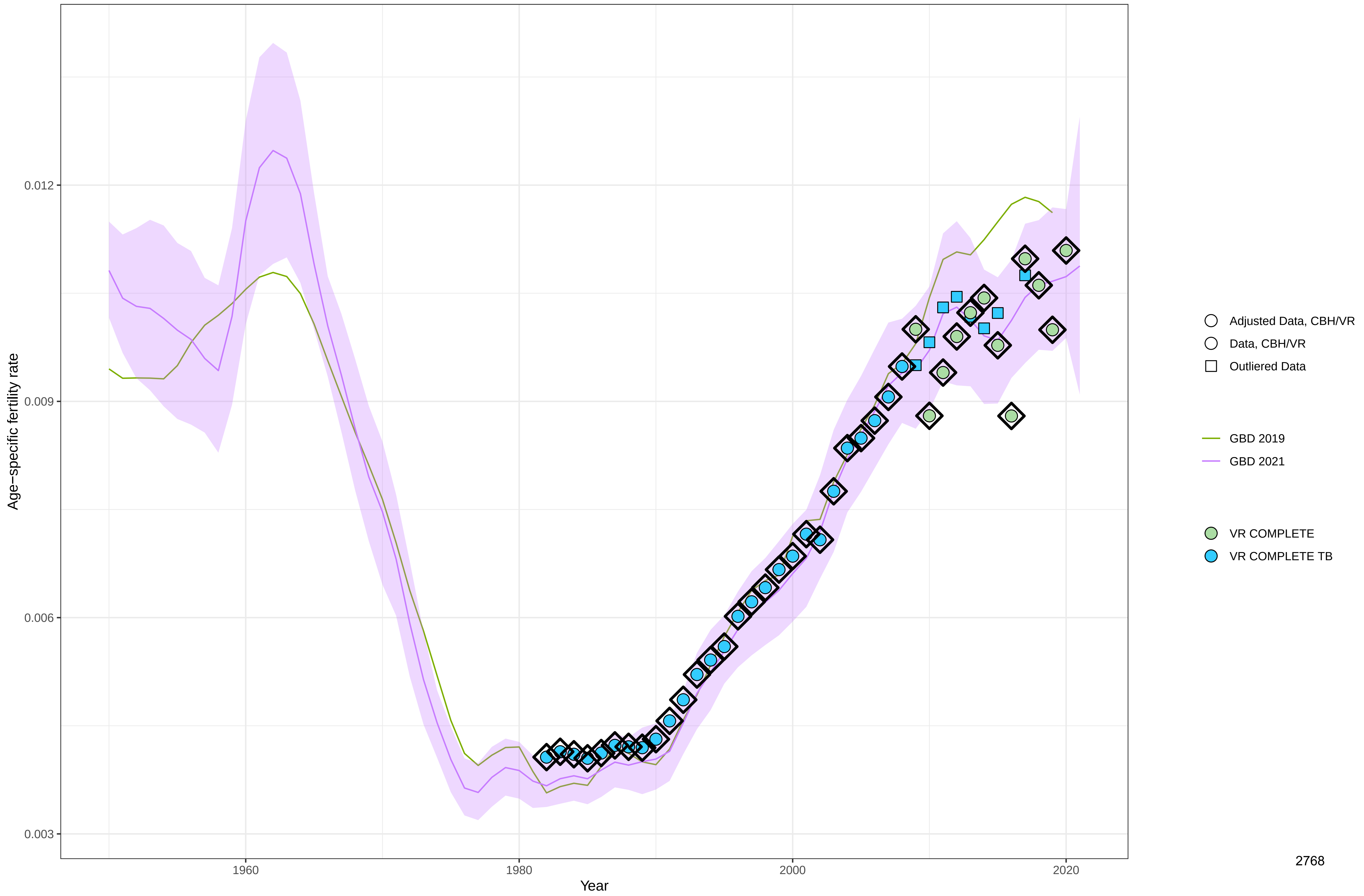

Age−specific fertility fitted trend lines, 1950−2021: Lancashire, Ages: 45−49
Data points enclosed with a diamond are reference sources

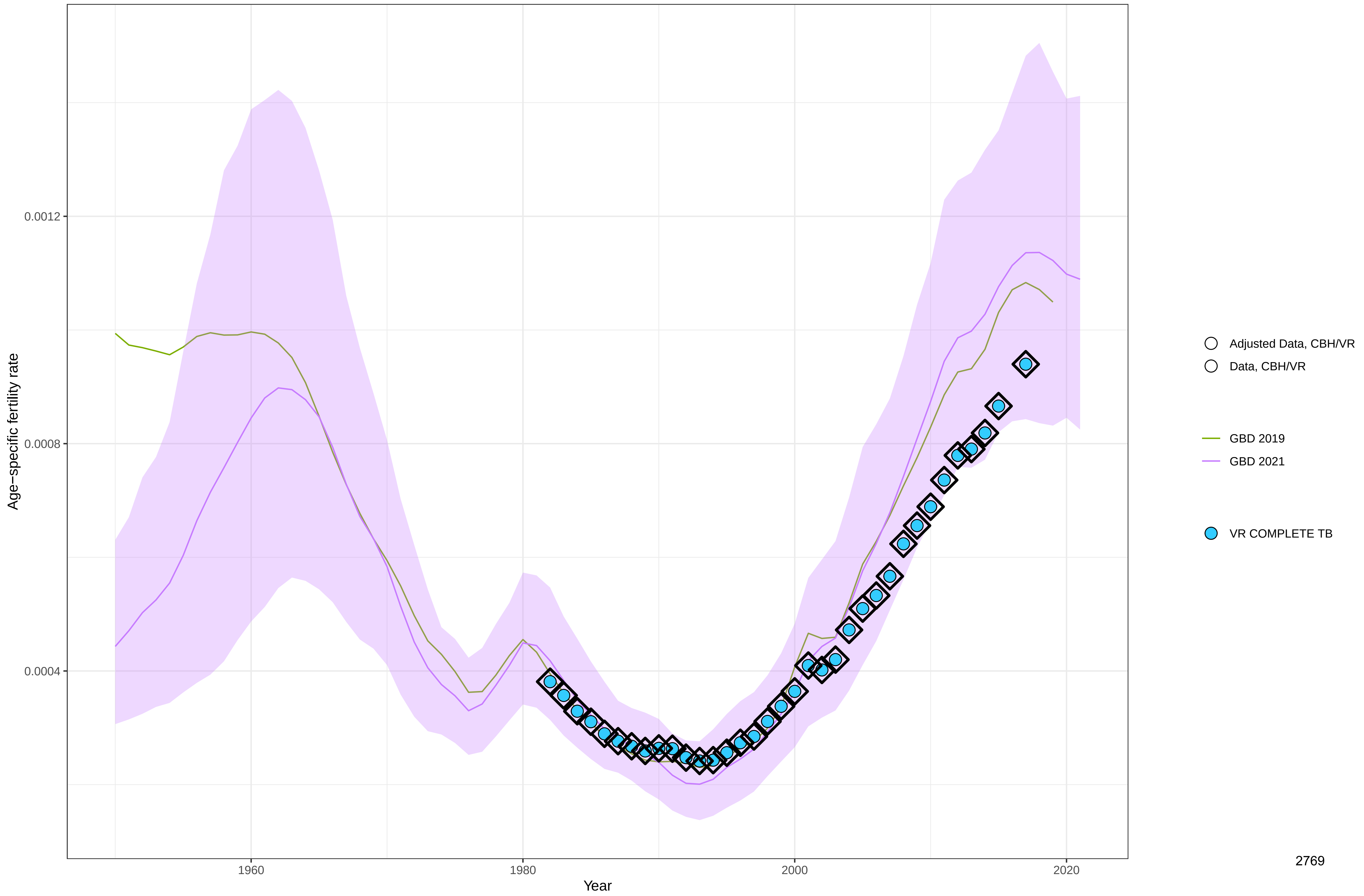

Age−specific fertility fitted trend lines, 1950−2021: Lancashire, Ages: 50−54
Data points enclosed with a diamond are reference sources

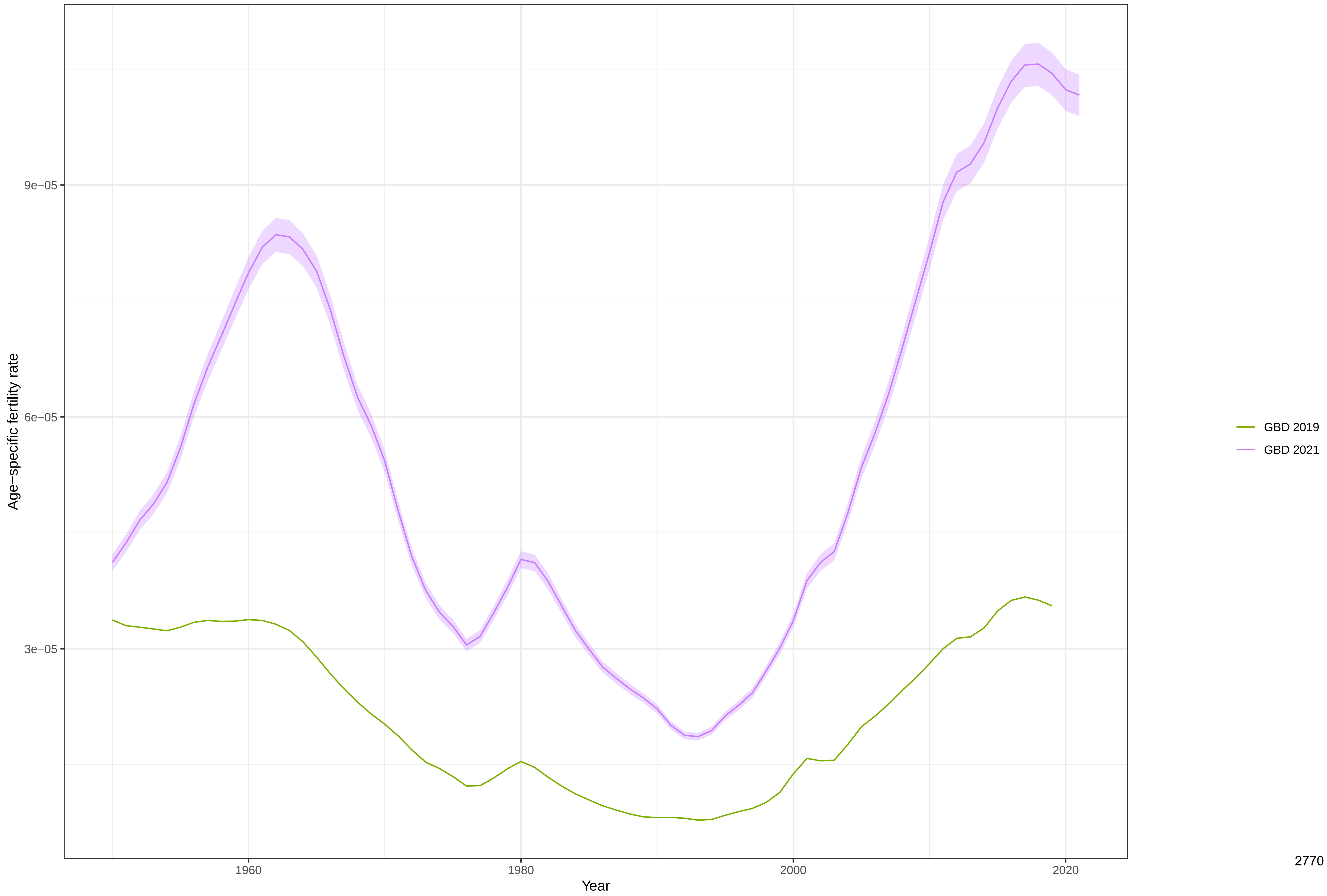

Total fertility rate fitted trend lines, 1950−2021: Lancashire TFR
Data points enclosed with a diamond are reference sources

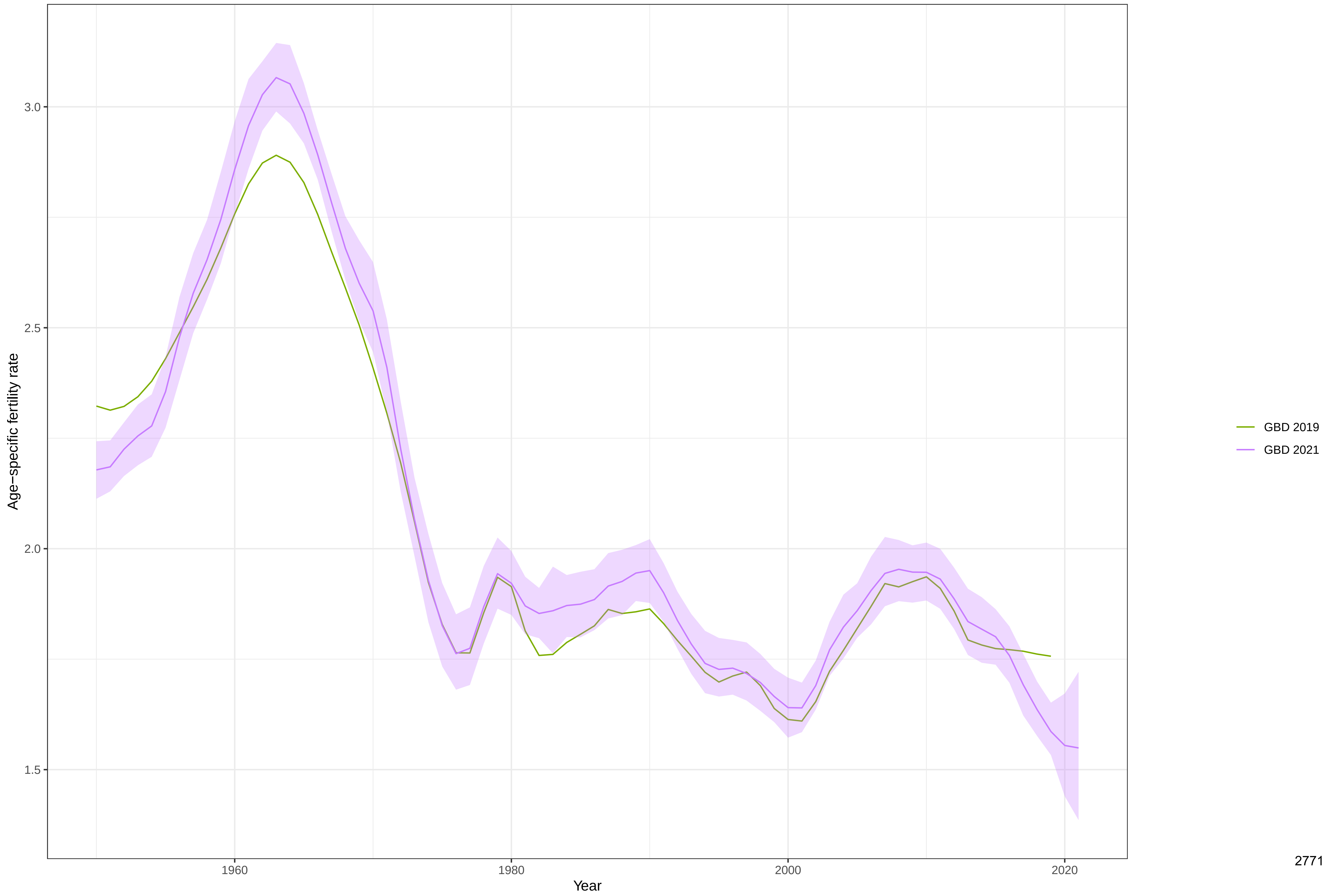

Age−specific fertility fitted trend lines, 1950−2021: Liverpool, Ages: 10−14
Data points enclosed with a diamond are reference sources

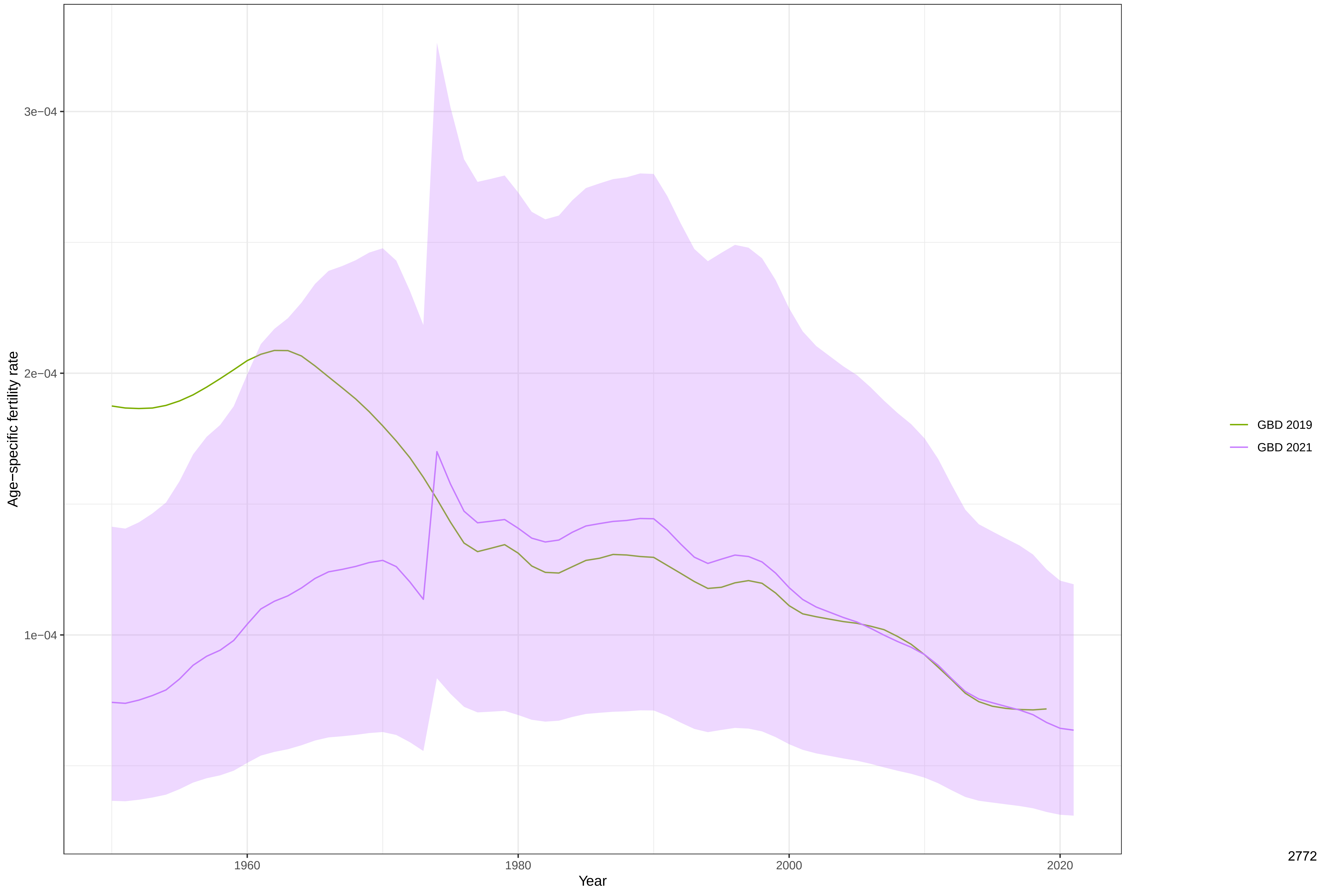

Age−specific fertility fitted trend lines, 1950−2021: Liverpool, Ages: 15−19
Data points enclosed with a diamond are reference sources

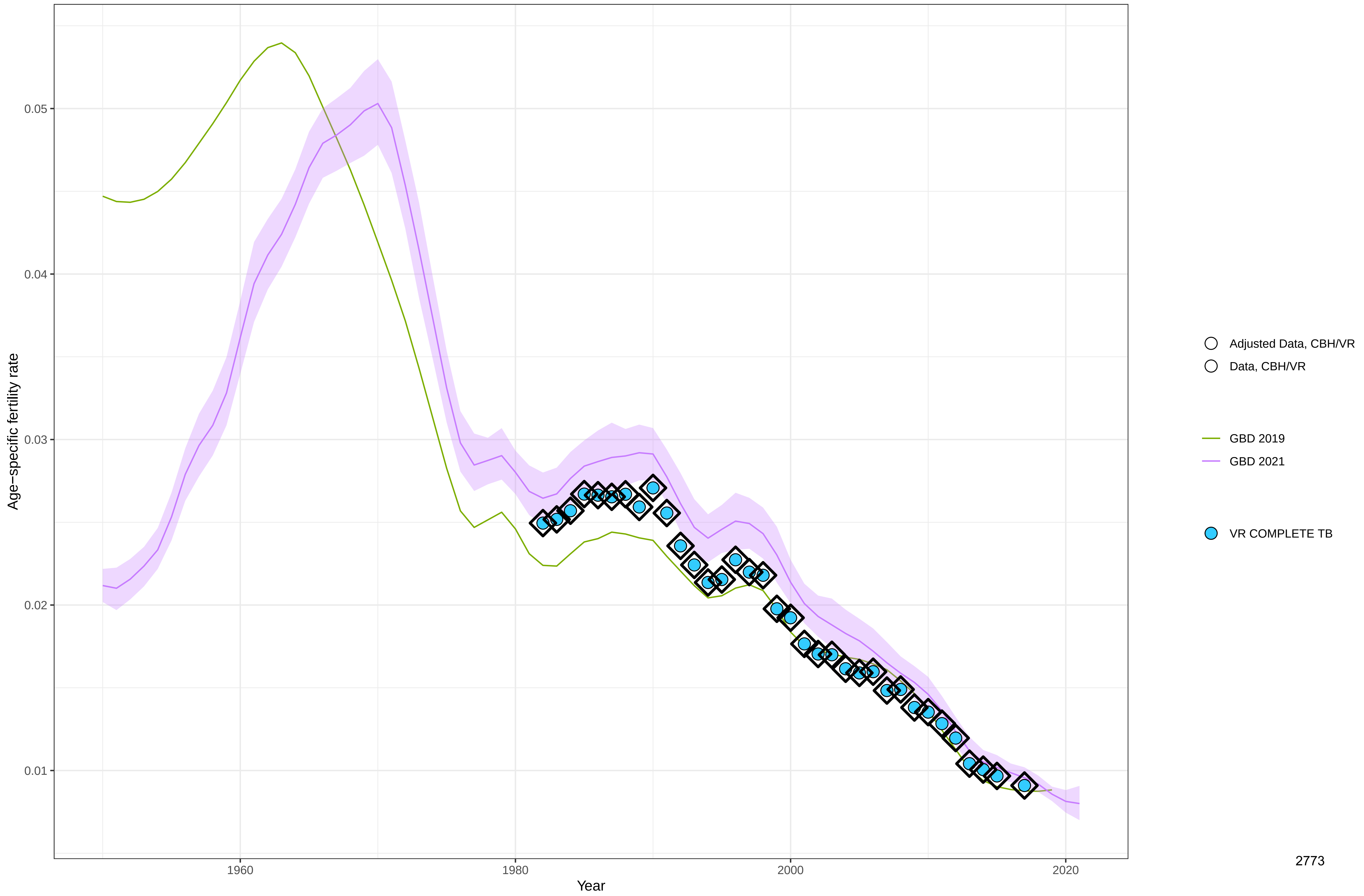

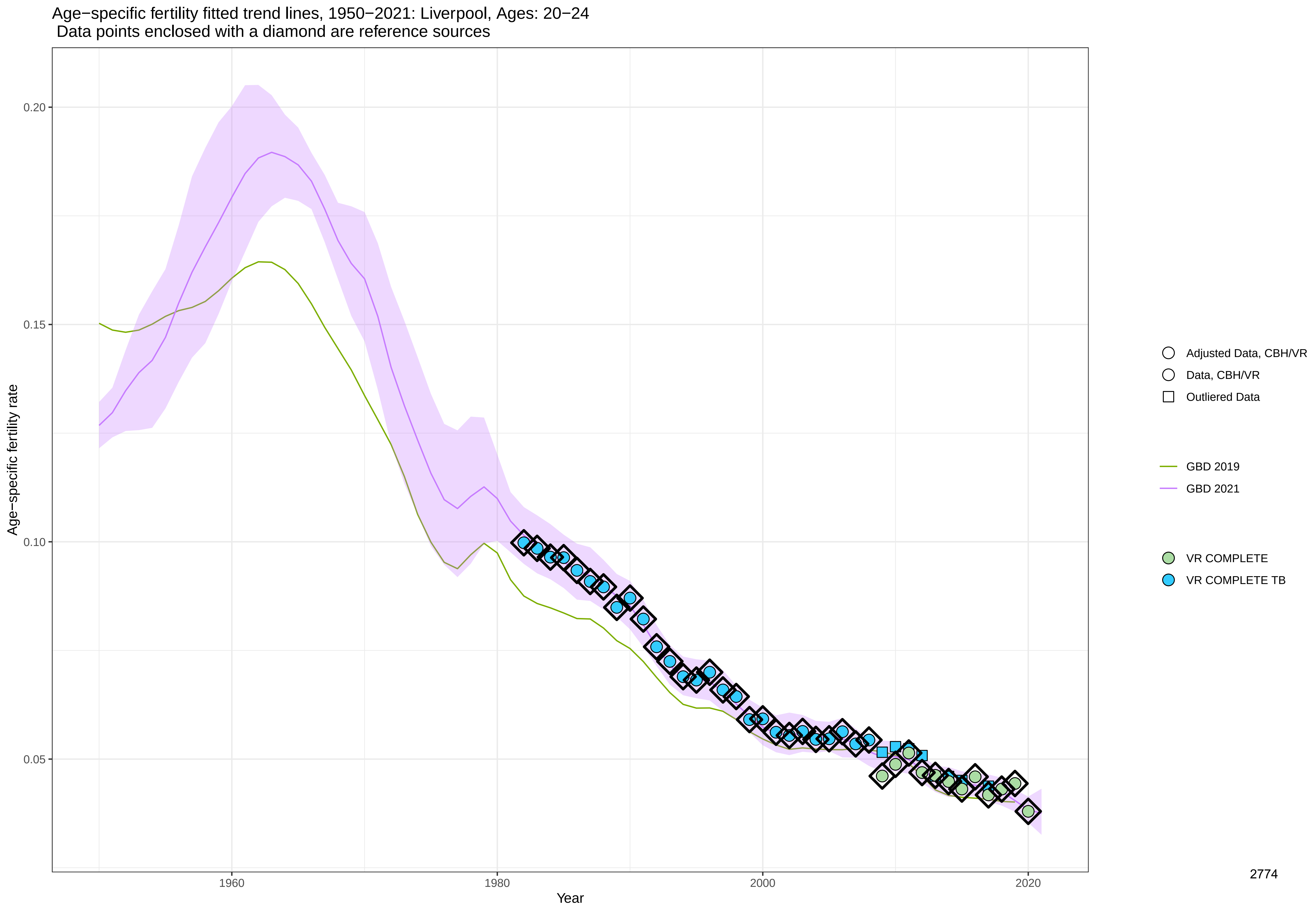

Age−specific fertility fitted trend lines, 1950−2021: Liverpool, Ages: 20−24
Data points enclosed with a diamond are reference sources
Age−specific fertility rate
0.20
0.15
0.10
0.05
1960
1980
2000
2020
Year
Adjusted Data, CBH/VR
Data, CBH/VR
Outliered Data
GBD 2019
GBD 2021
VR COMPLETE
VR COMPLETE TB

Age−specific fertility fitted trend lines, 1950−2021: Liverpool, Ages: 25−29
Data points enclosed with a diamond are reference sources

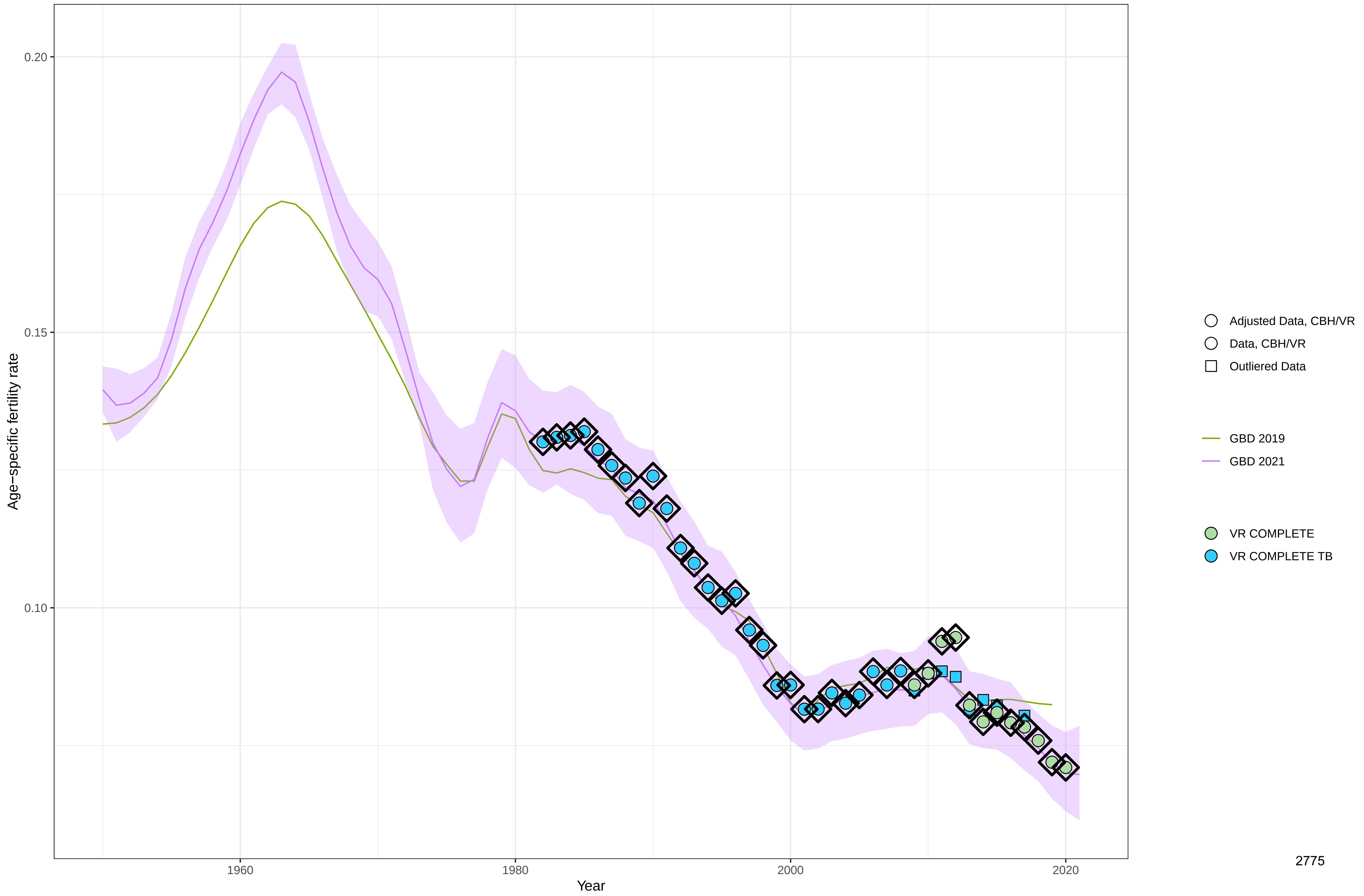

Age−specific fertility fitted trend lines, 1950−2021: Liverpool, Ages: 30−34
Data points enclosed with a diamond are reference sources

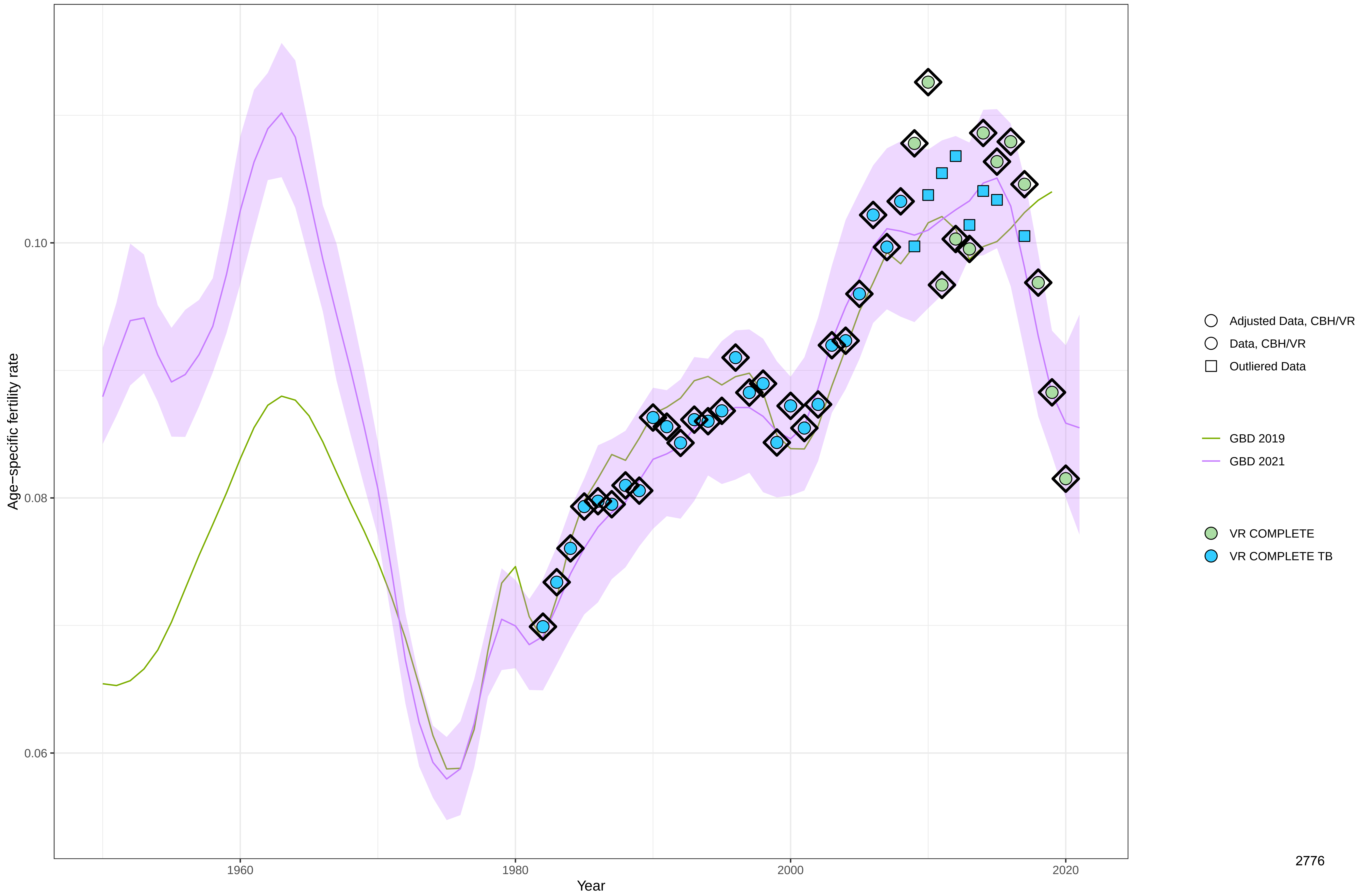

Age−specific fertility fitted trend lines, 1950−2021: Liverpool, Ages: 35−39
Data points enclosed with a diamond are reference sources

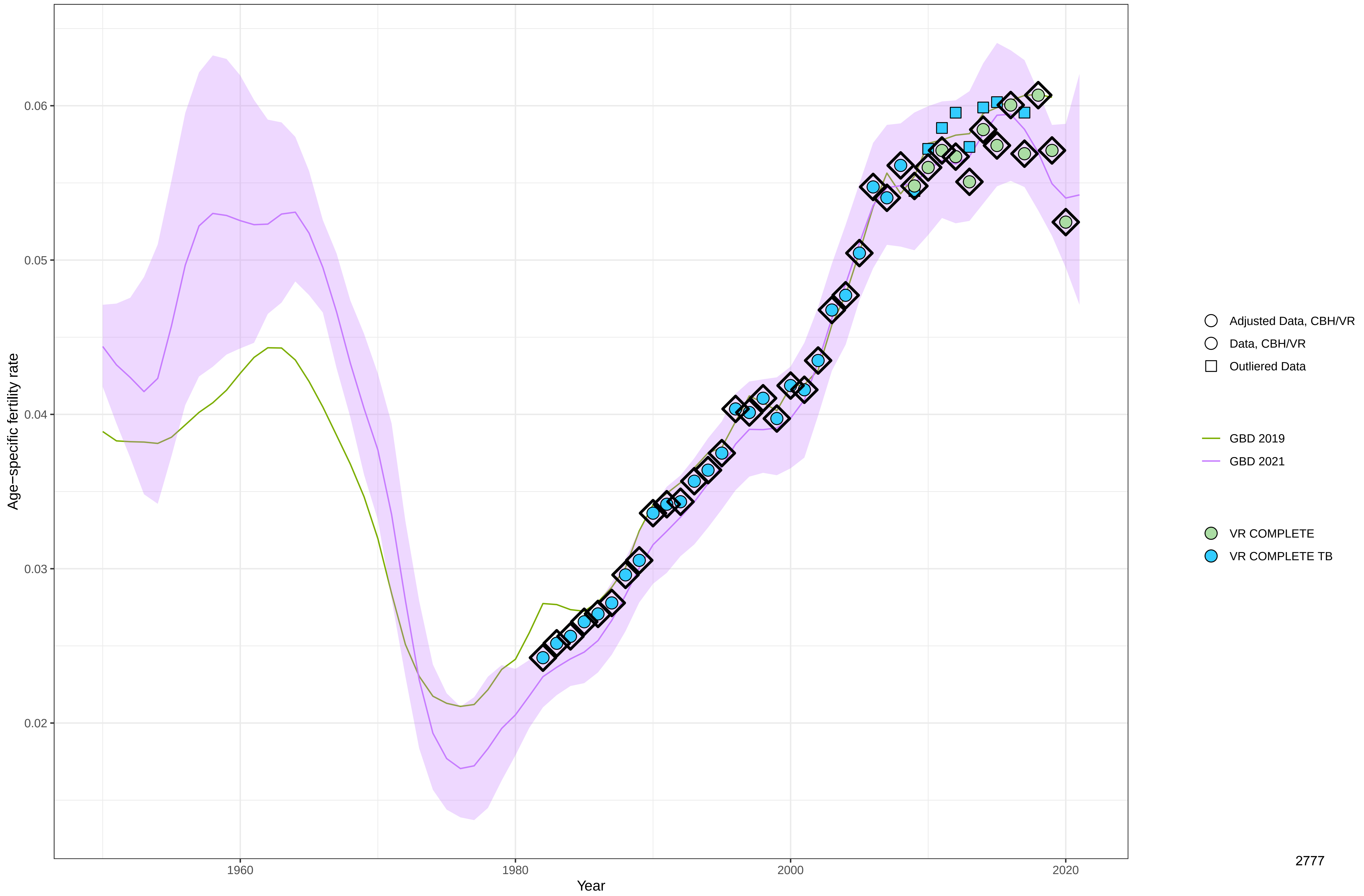

Age−specific fertility fitted trend lines, 1950−2021: Liverpool, Ages: 40−44
Data points enclosed with a diamond are reference sources

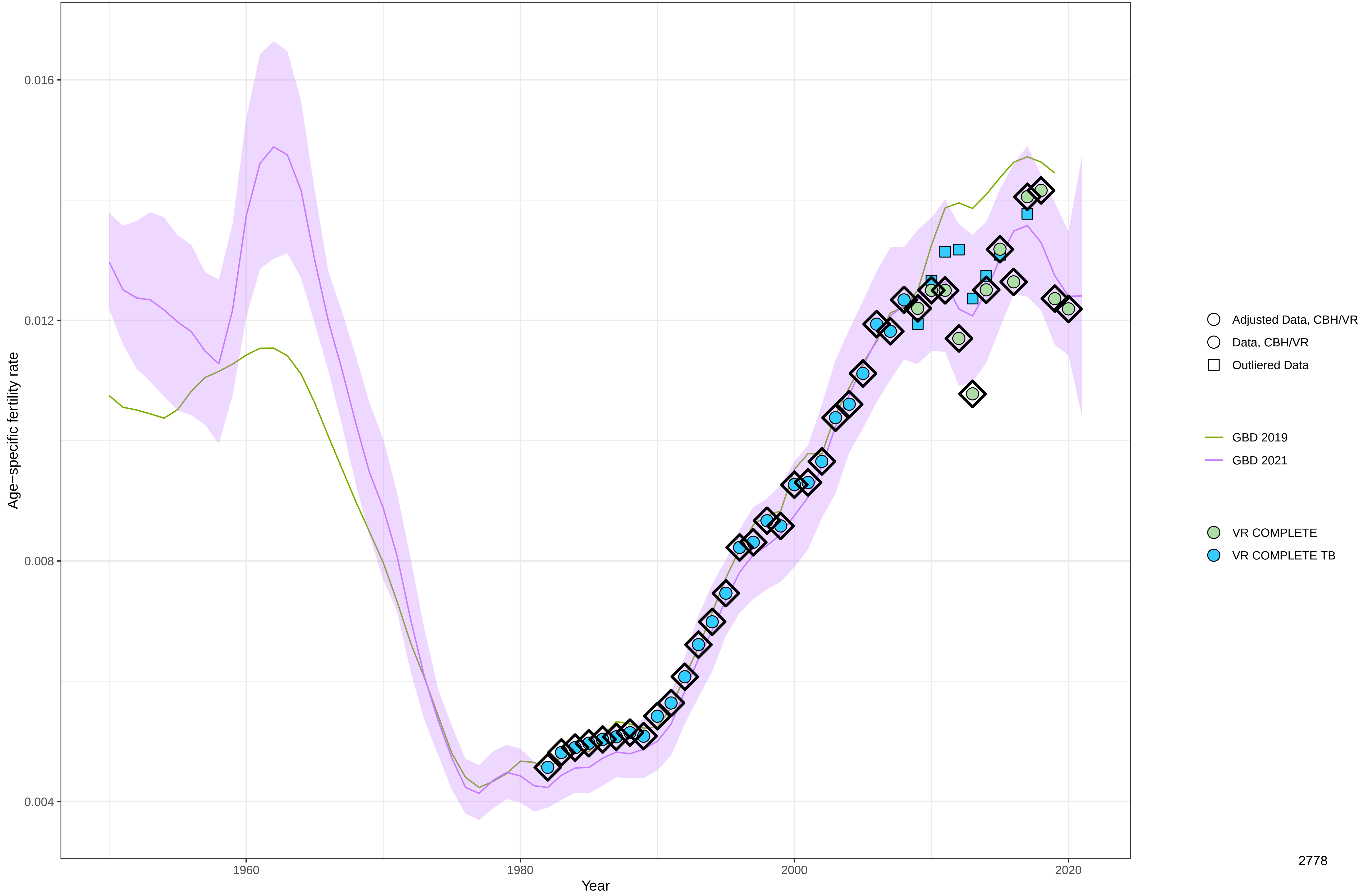

Age−specific fertility fitted trend lines, 1950−2021: Liverpool, Ages: 45−49
Data points enclosed with a diamond are reference sources

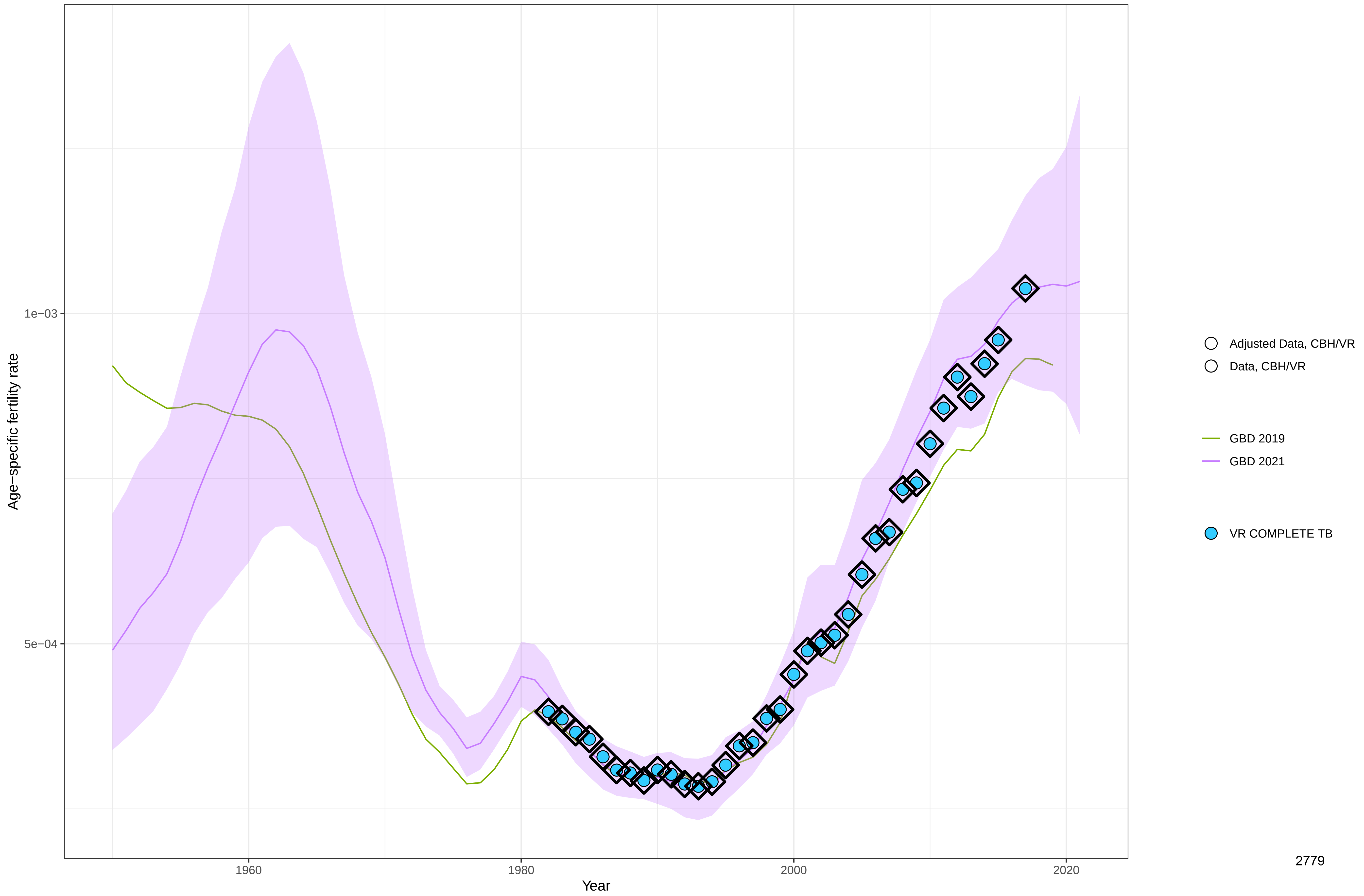

Age−specific fertility fitted trend lines, 1950−2021: Liverpool, Ages: 50−54
Data points enclosed with a diamond are reference sources

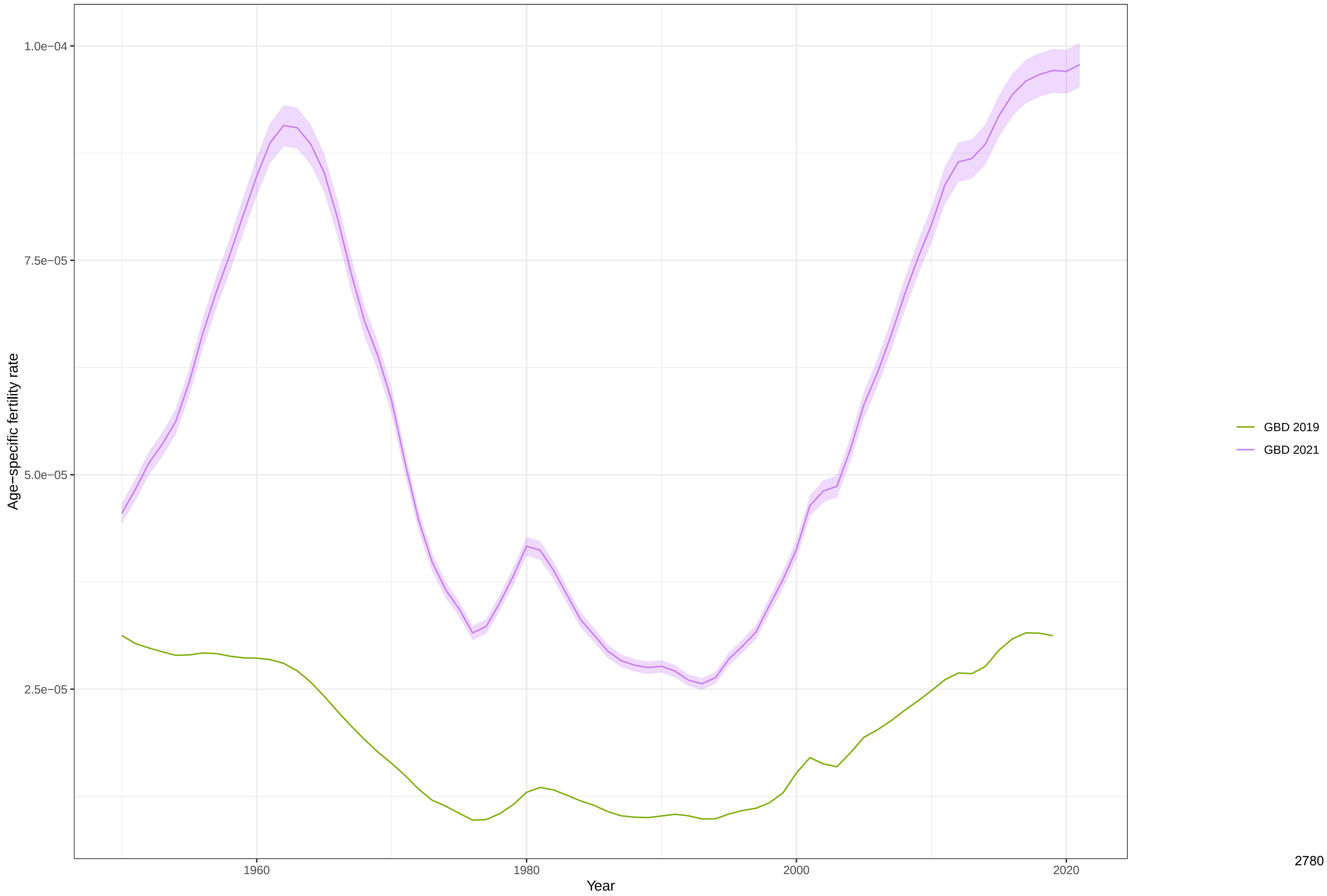

Total fertility rate fitted trend lines, 1950−2021: Liverpool TFR
Data points enclosed with a diamond are reference sources

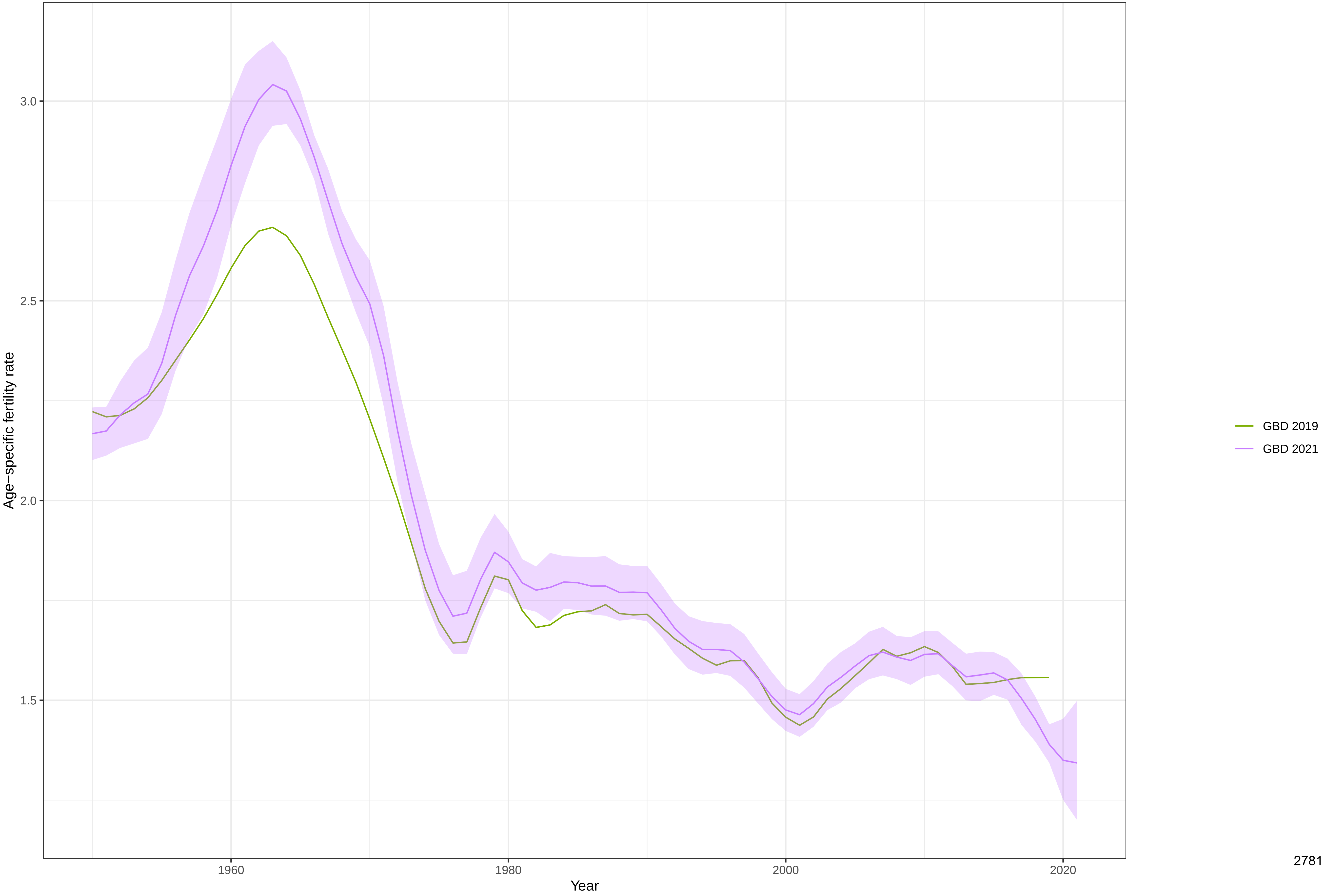

Age−specific fertility fitted trend lines, 1950−2021: Manchester, Ages: 10−14
Data points enclosed with a diamond are reference sources

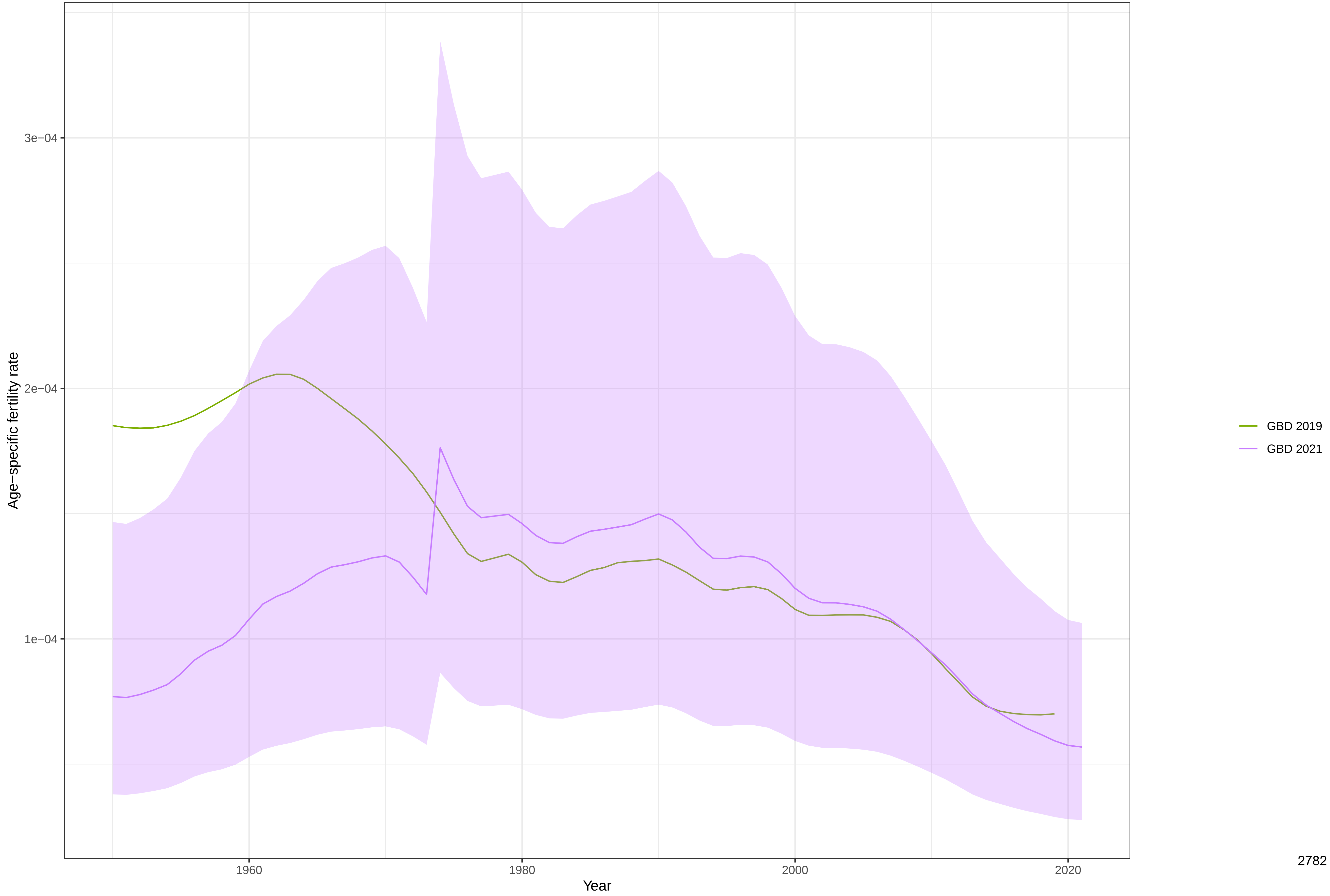

Age−specific fertility fitted trend lines, 1950−2021: Manchester, Ages: 15−19
Data points enclosed with a diamond are reference sources

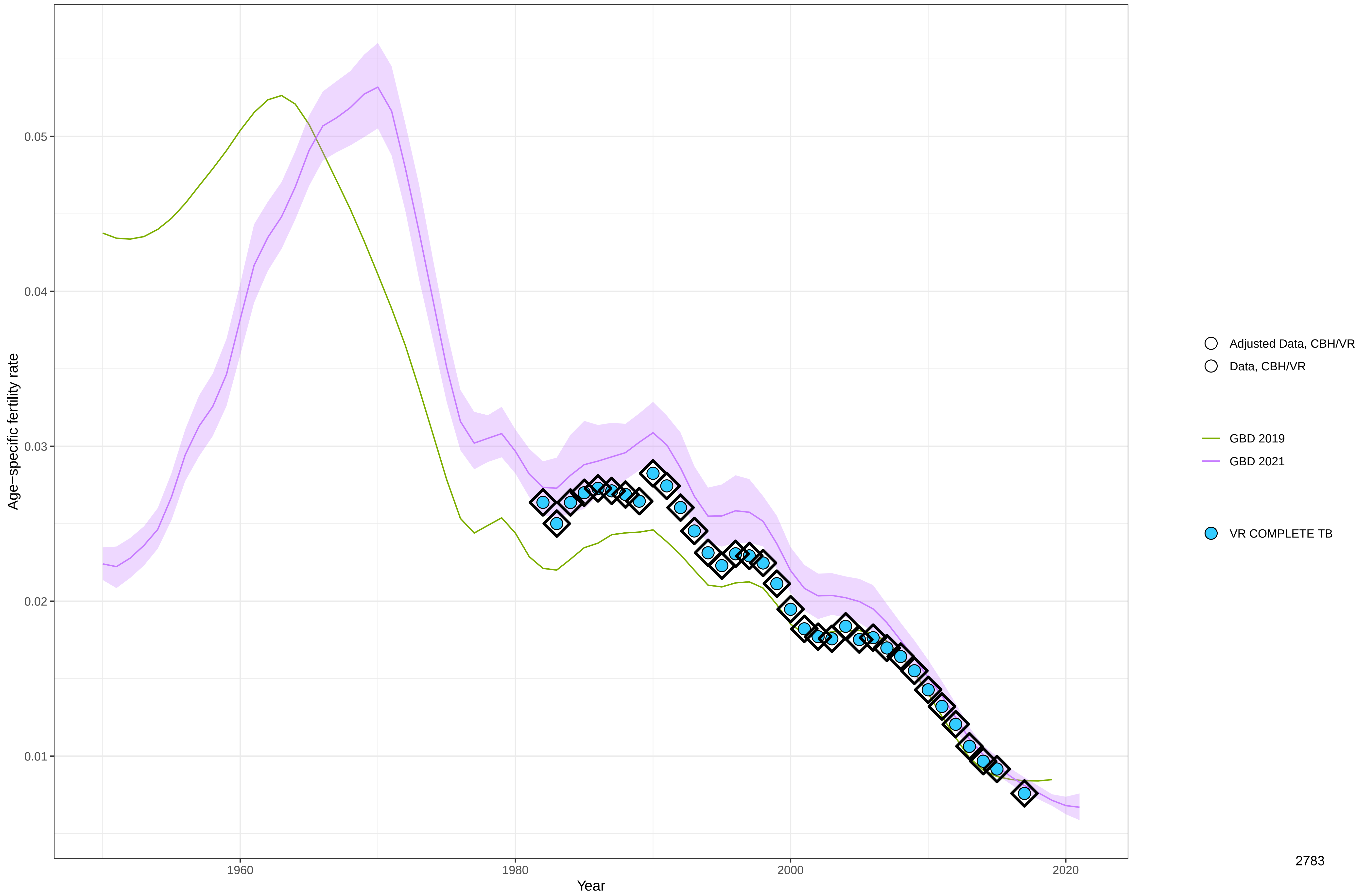

Age−specific fertility fitted trend lines, 1950−2021: Manchester, Ages: 20−24
Data points enclosed with a diamond are reference sources

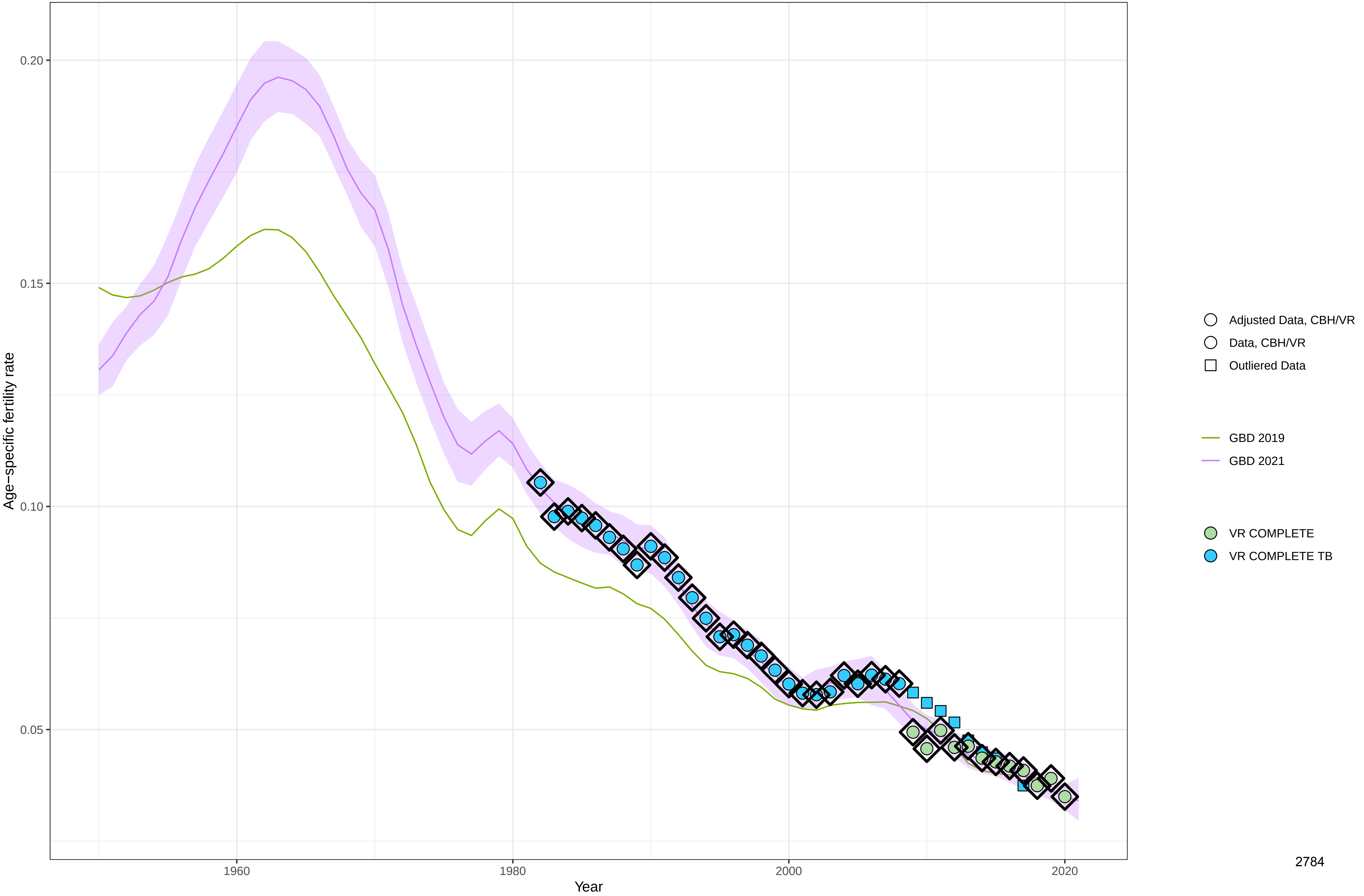

Age−specific fertility fitted trend lines, 1950−2021: Manchester, Ages: 25−29
Data points enclosed with a diamond are reference sources

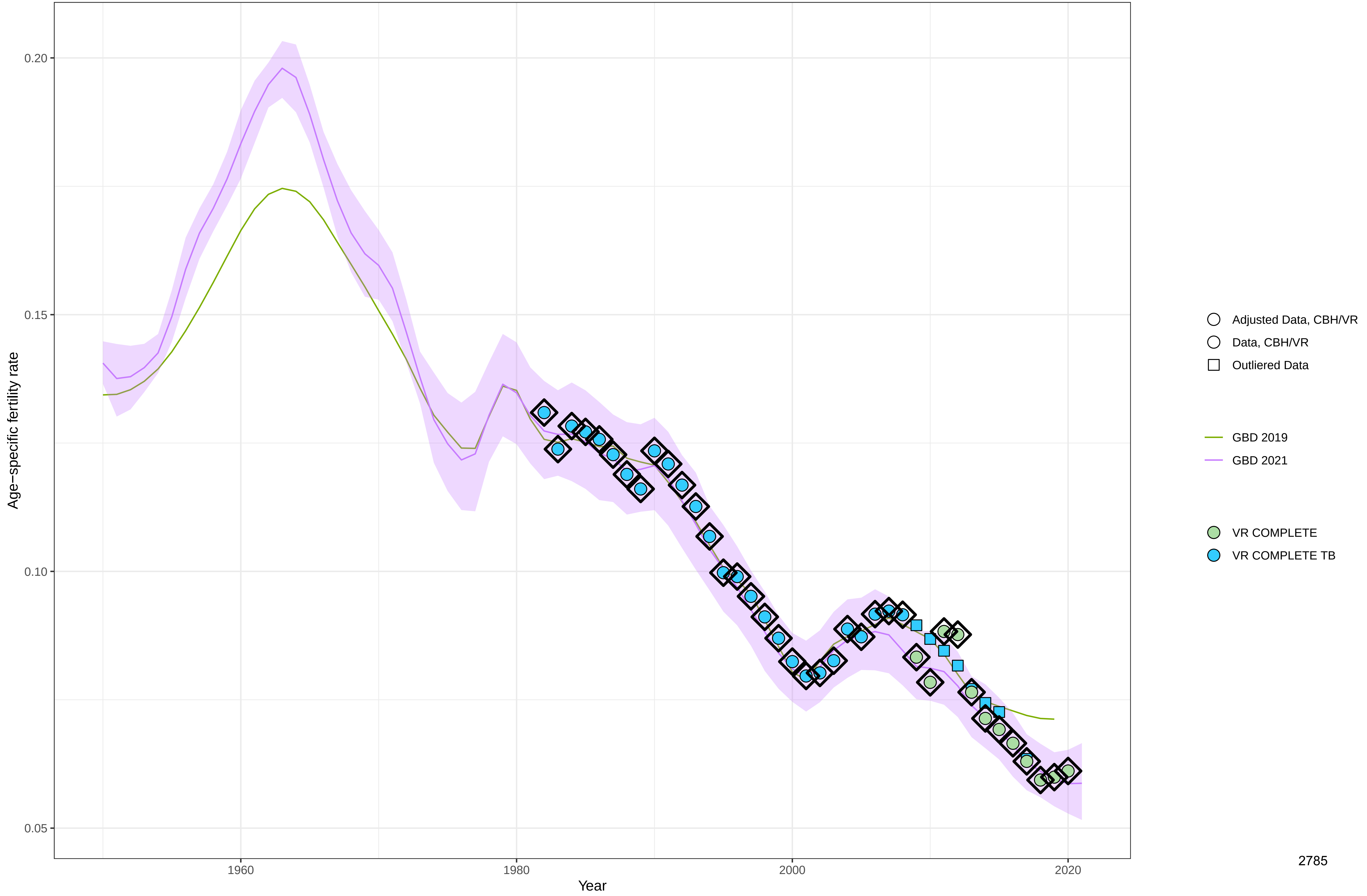

Age−specific fertility fitted trend lines, 1950−2021: Manchester, Ages: 30−34
Data points enclosed with a diamond are reference sources

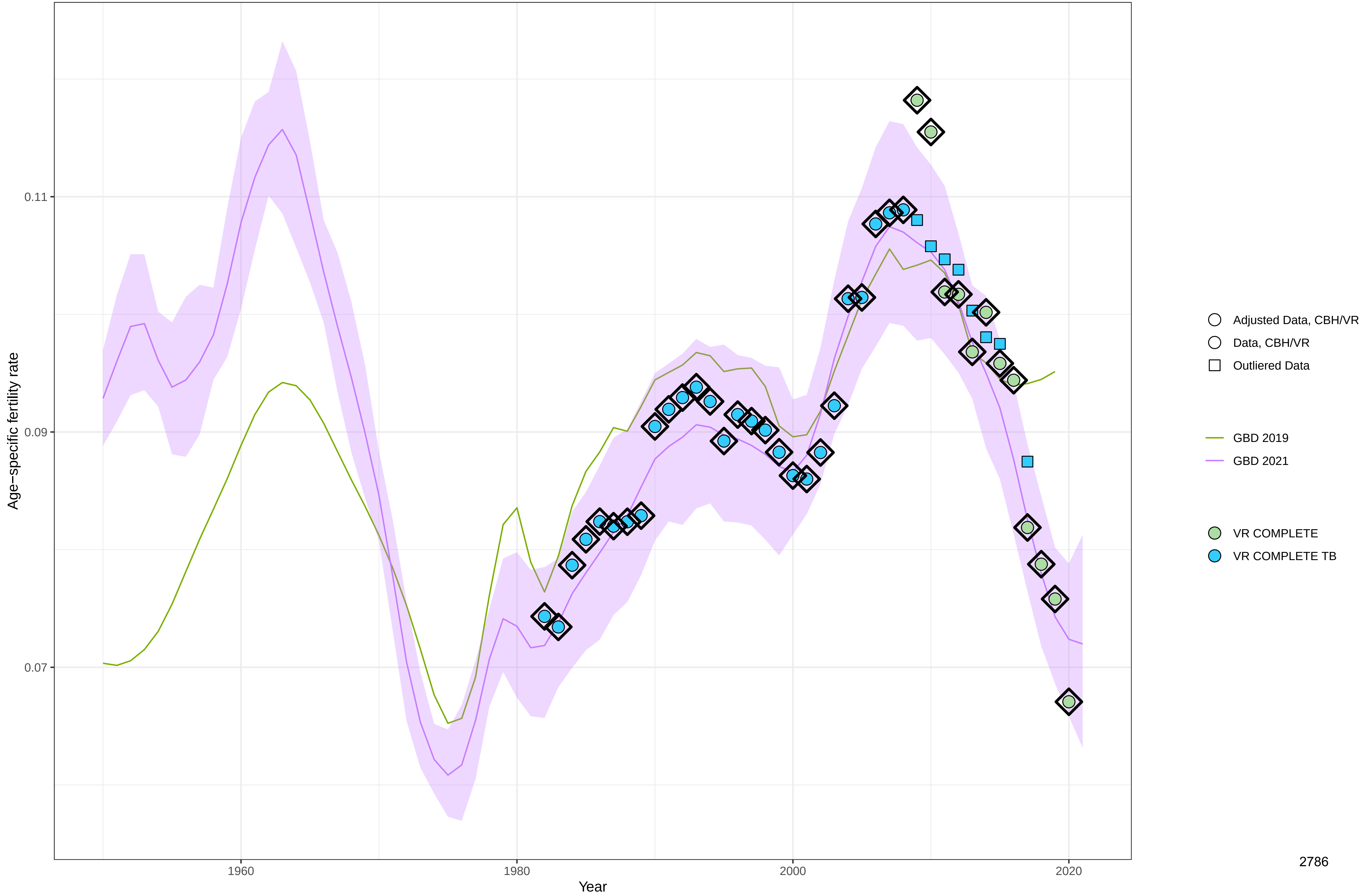

2786

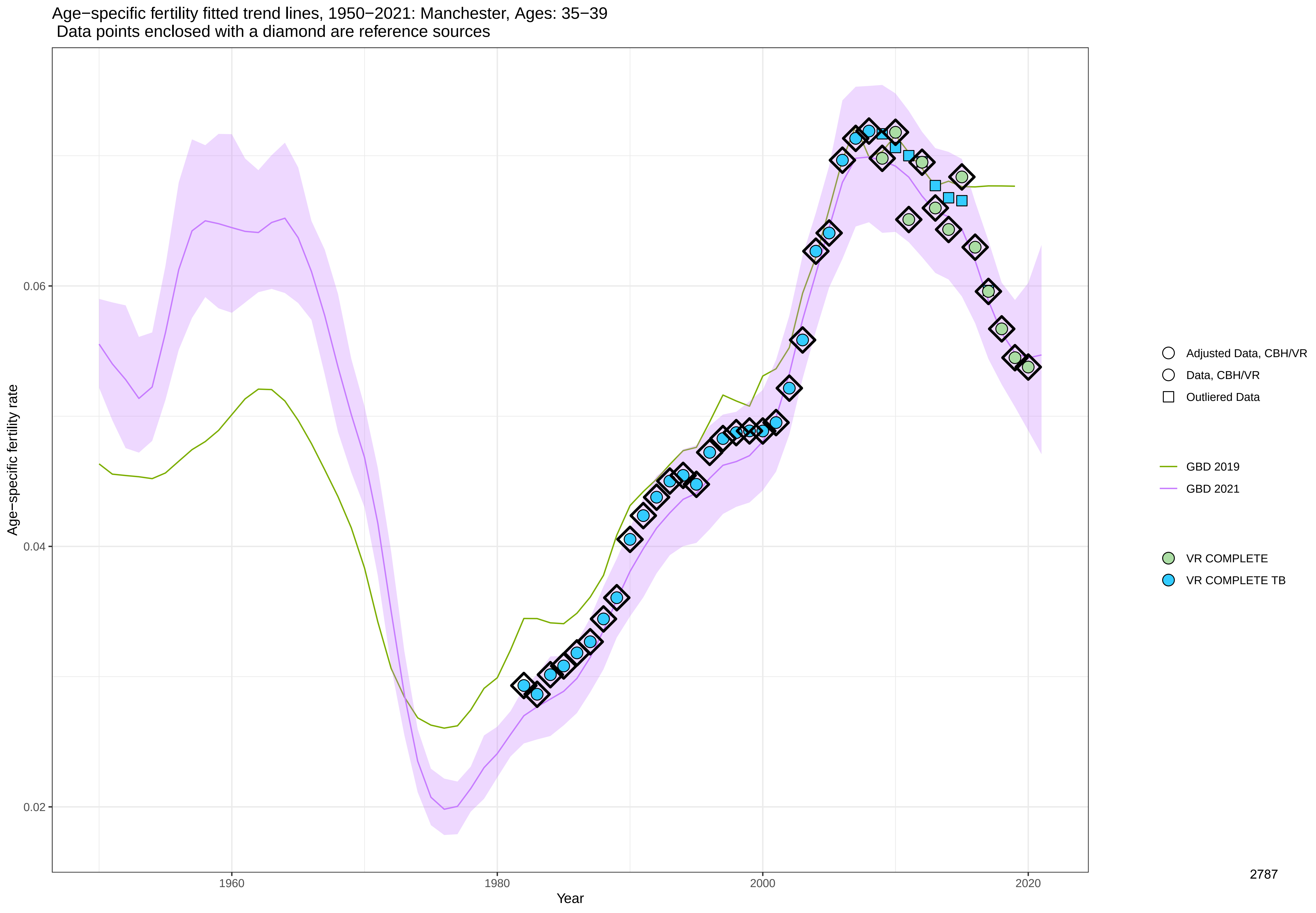

Age−specific fertility fitted trend lines, 1950−2021: Manchester, Ages: 35−39
Data points enclosed with a diamond are reference sources
Age−specific fertility rate
0.06
0.04
0.02
1960
1980
2000
2020
Year
Adjusted Data, CBH/VR
Data, CBH/VR
Outliered Data
GBD 2019
GBD 2021
VR COMPLETE
VR COMPLETE TB

2787

Age−specific fertility fitted trend lines, 1950−2021: Manchester, Ages: 40−44
Data points enclosed with a diamond are reference sources

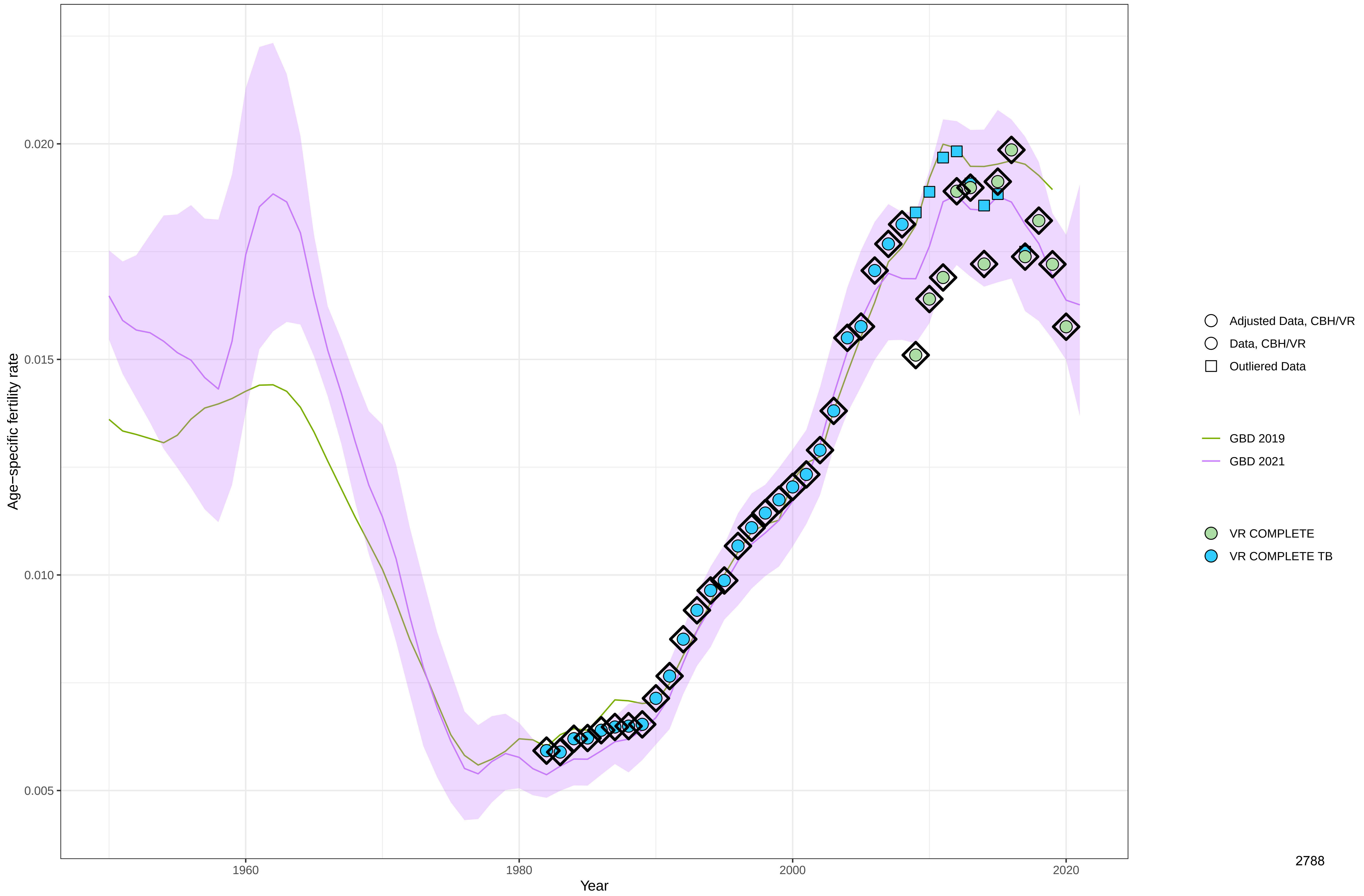

Age−specific fertility fitted trend lines, 1950−2021: Manchester, Ages: 45−49
Data points enclosed with a diamond are reference sources

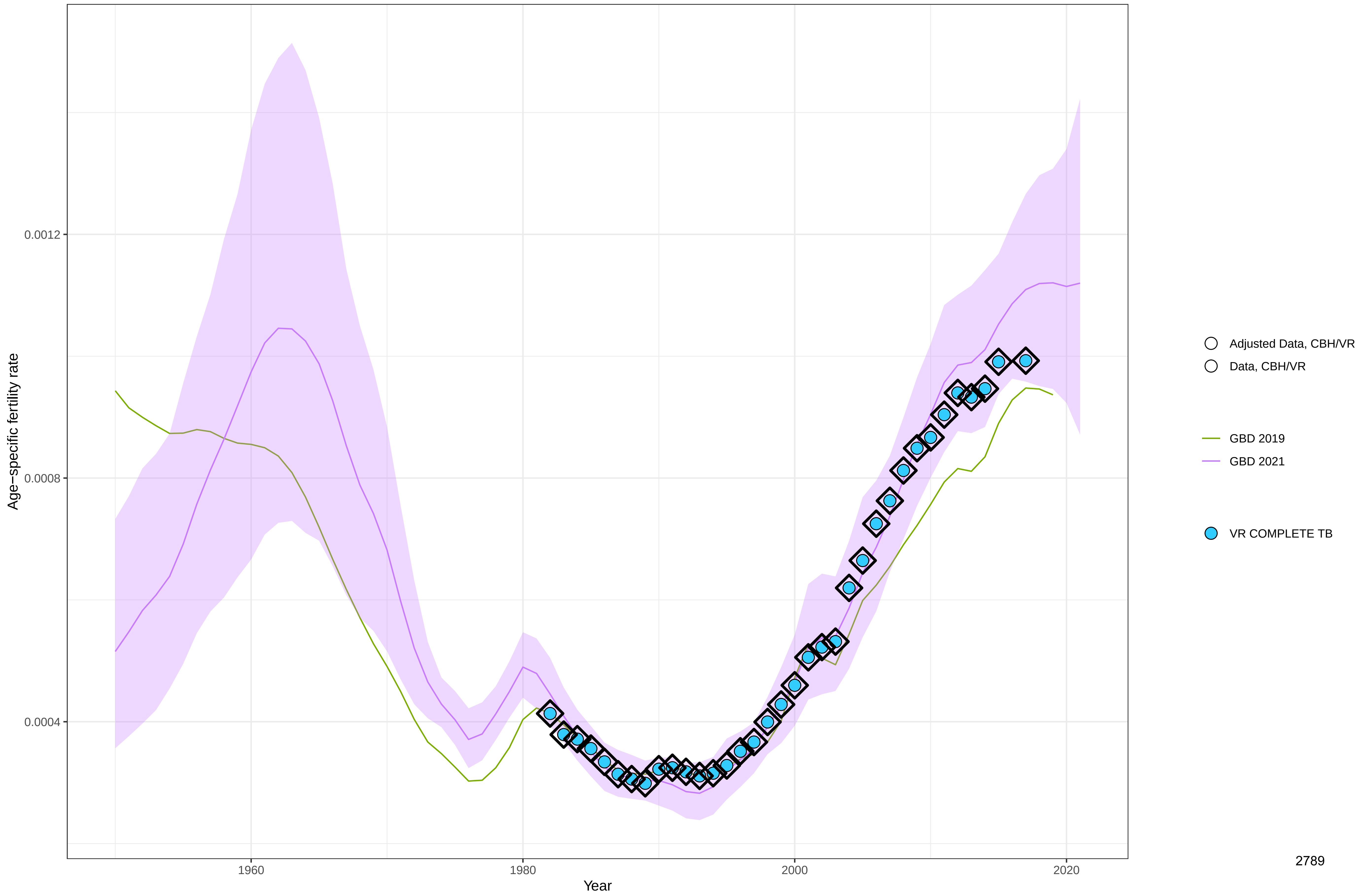

Age−specific fertility fitted trend lines, 1950−2021: Manchester, Ages: 50−54
Data points enclosed with a diamond are reference sources

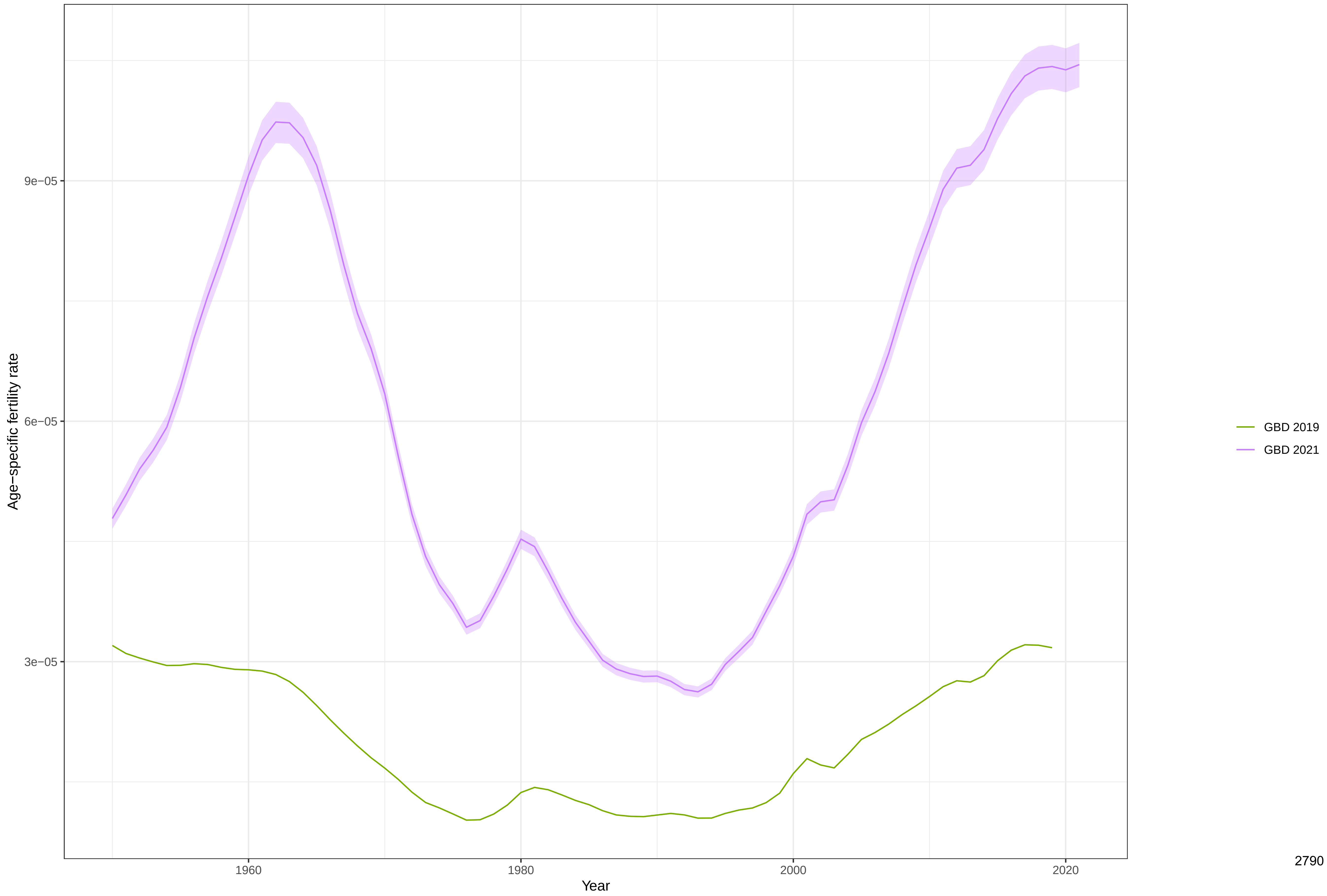

Total fertility rate fitted trend lines, 1950−2021: Manchester TFR
Data points enclosed with a diamond are reference sources

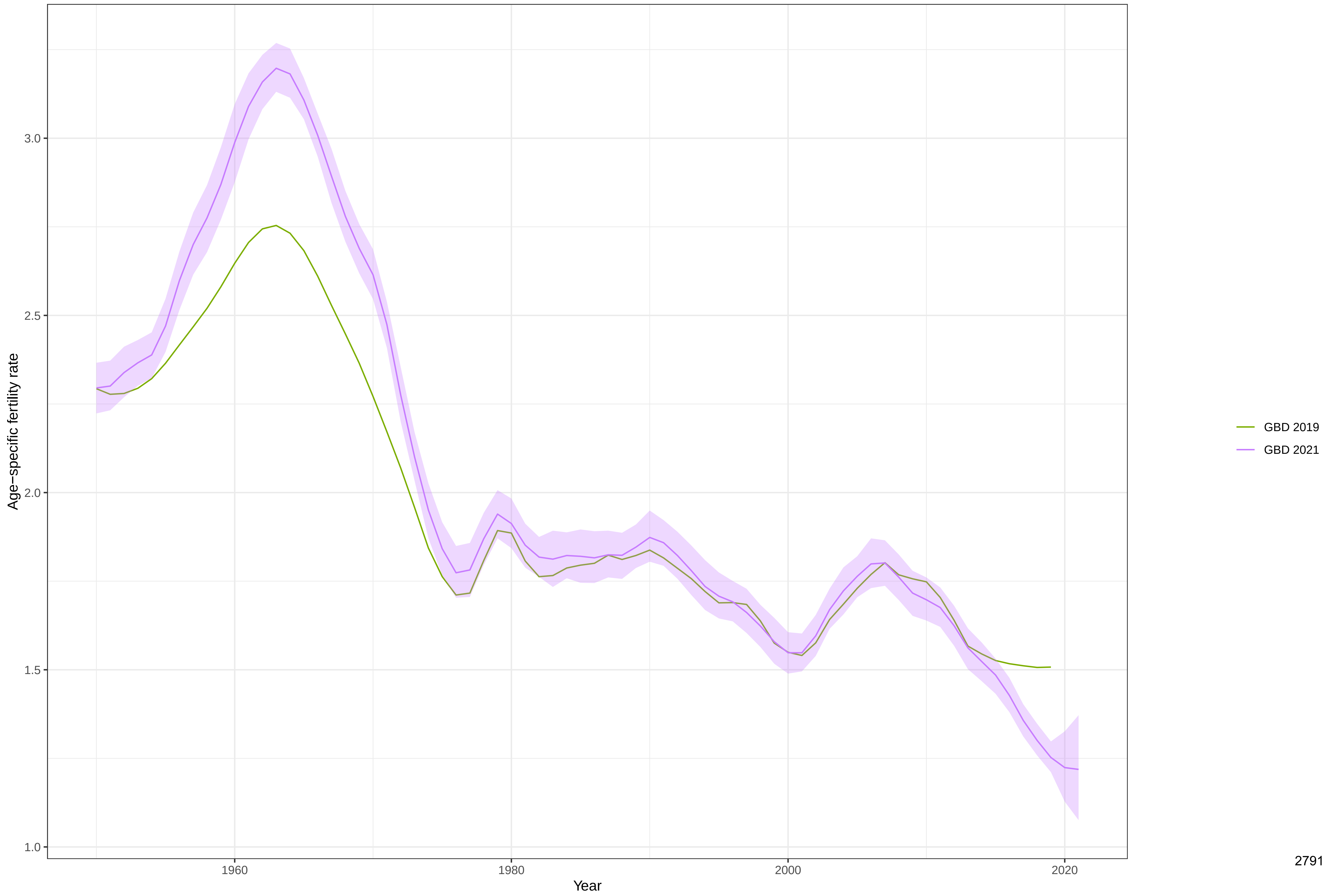

Age−specific fertility fitted trend lines, 1950−2021: Oldham, Ages: 10−14
 Data points enclosed with a diamond are reference sources

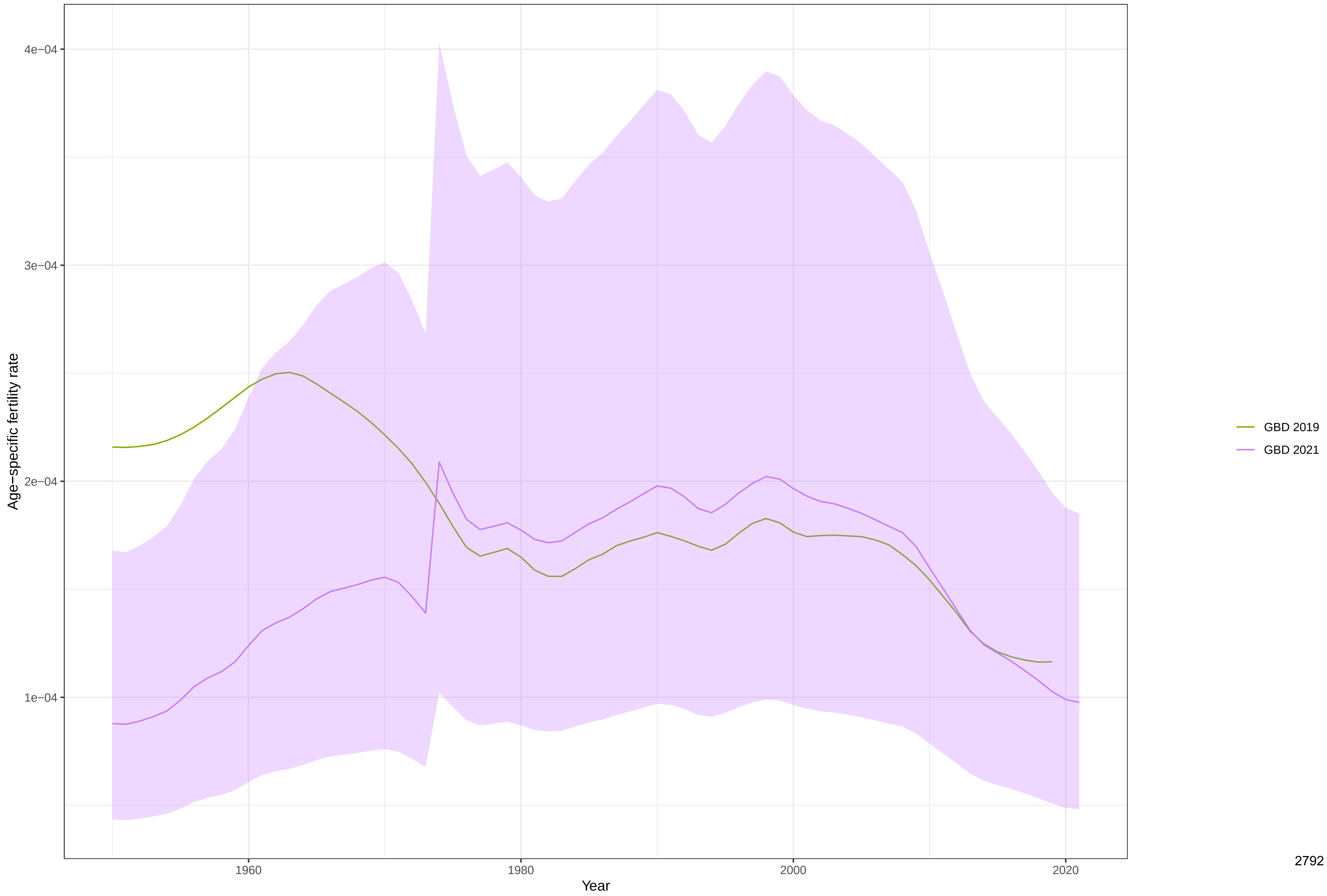

Age−specific fertility fitted trend lines, 1950−2021: Oldham, Ages: 15−19
Data points enclosed with a diamond are reference sources

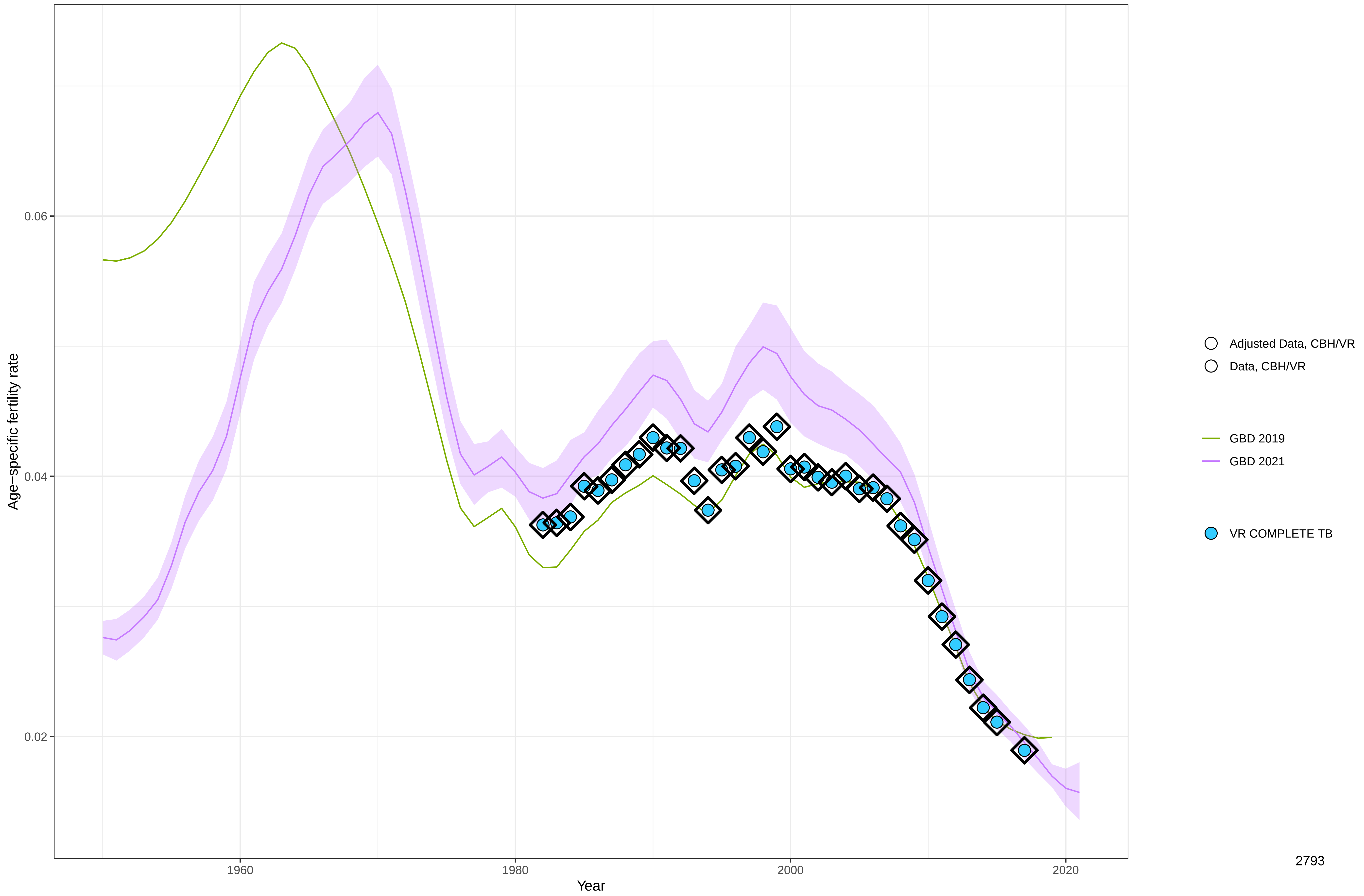

Age−specific fertility fitted trend lines, 1950−2021: Oldham, Ages: 20−24
Data points enclosed with a diamond are reference sources

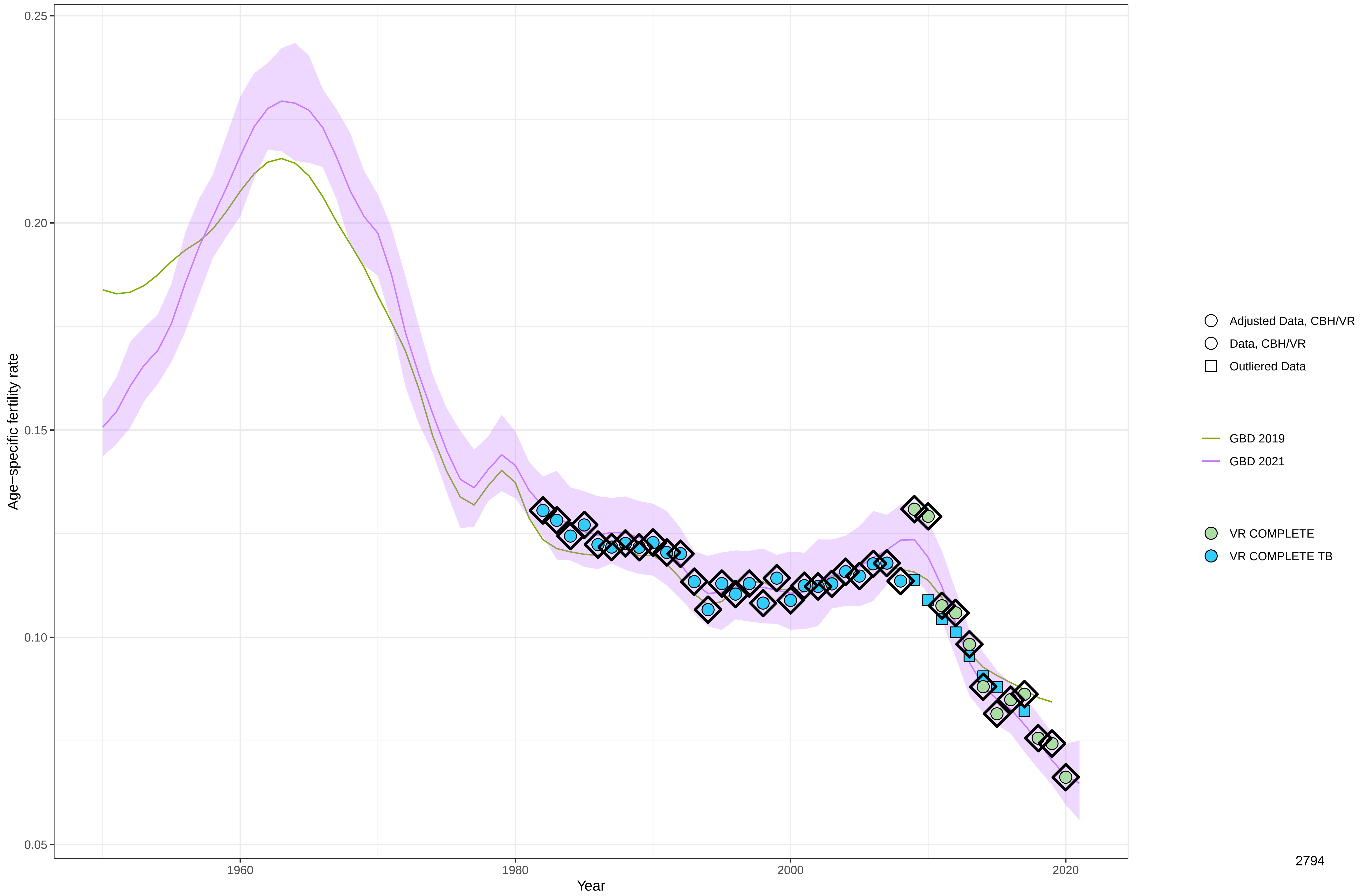

Age−specific fertility fitted trend lines, 1950−2021: Oldham, Ages: 25−29
Data points enclosed with a diamond are reference sources

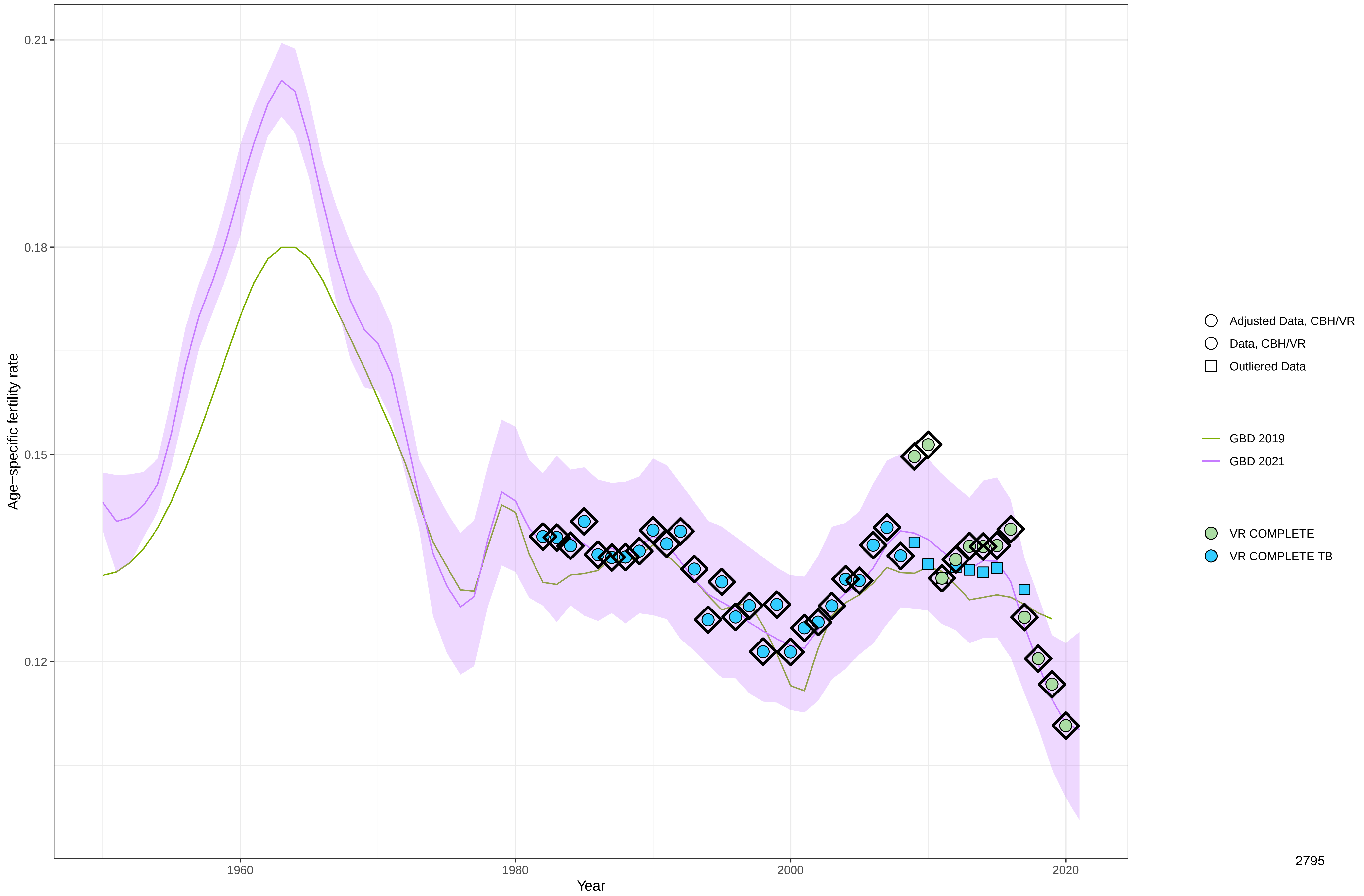

Age−specific fertility fitted trend lines, 1950−2021: Oldham, Ages: 30−34
Data points enclosed with a diamond are reference sources

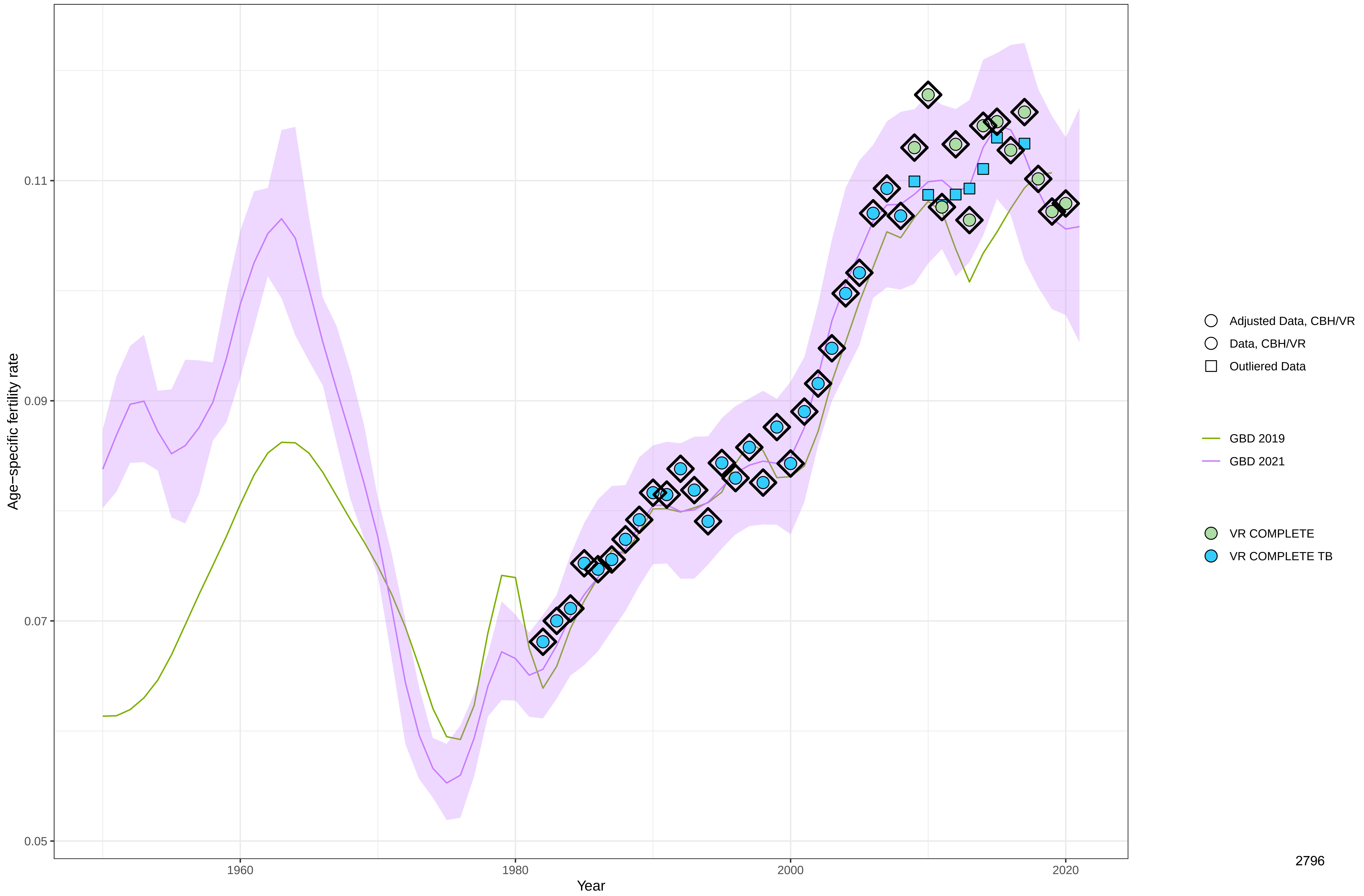

Age−specific fertility fitted trend lines, 1950−2021: Oldham, Ages: 35−39
Data points enclosed with a diamond are reference sources

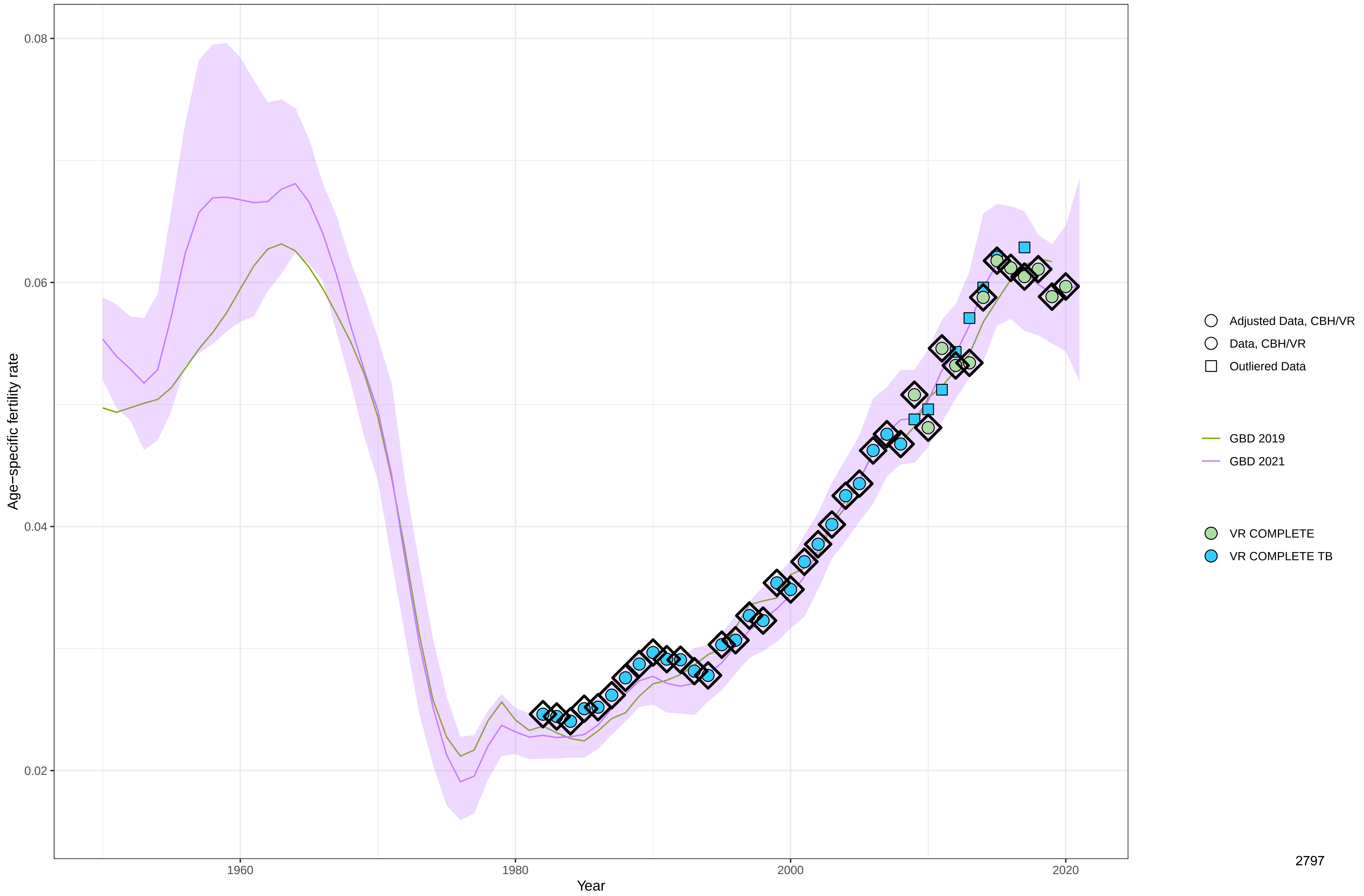

Age−specific fertility fitted trend lines, 1950−2021: Oldham, Ages: 40−44
Data points enclosed with a diamond are reference sources

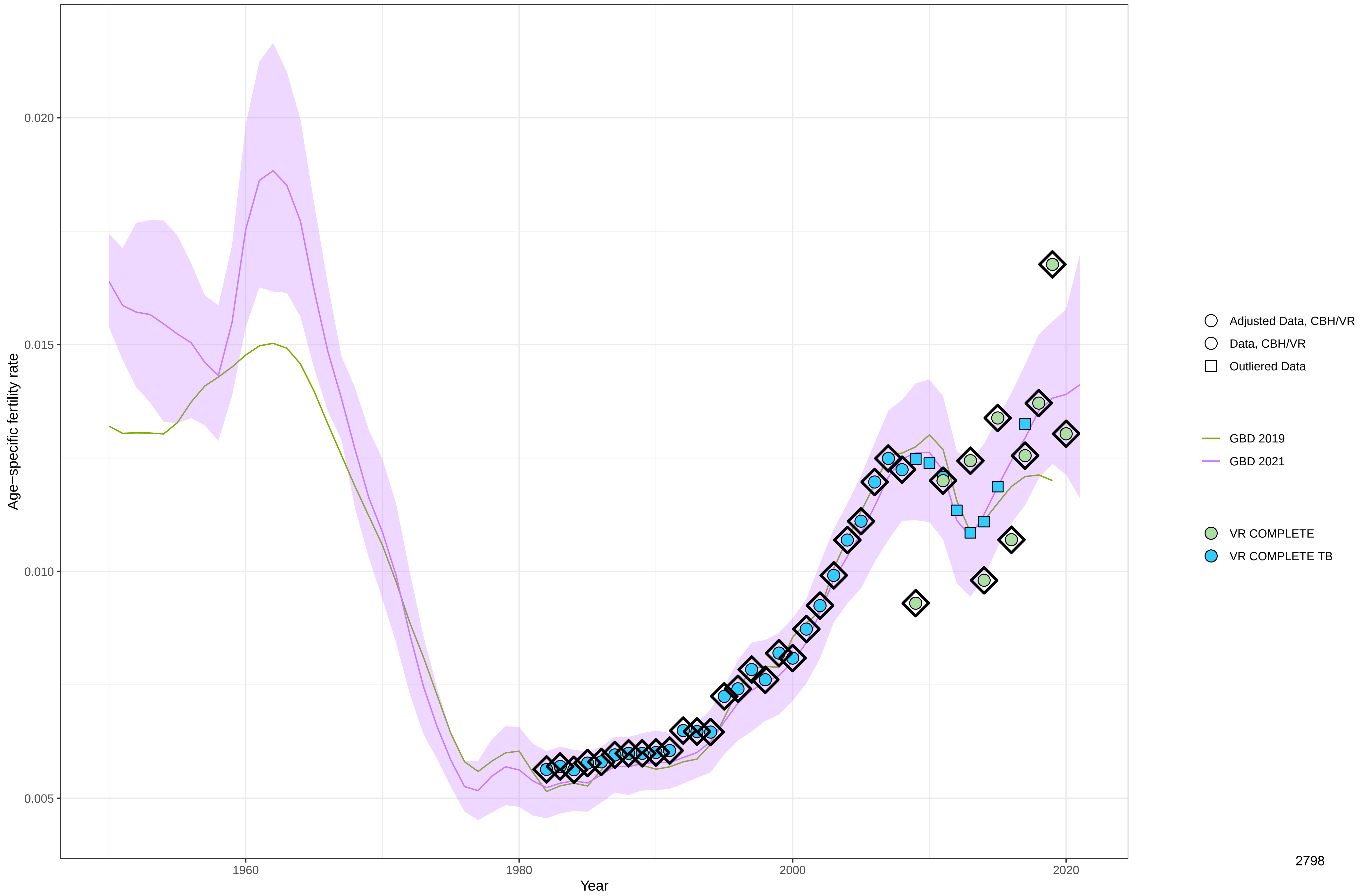

Age−specific fertility fitted trend lines, 1950−2021: Oldham, Ages: 45−49
Data points enclosed with a diamond are reference sources

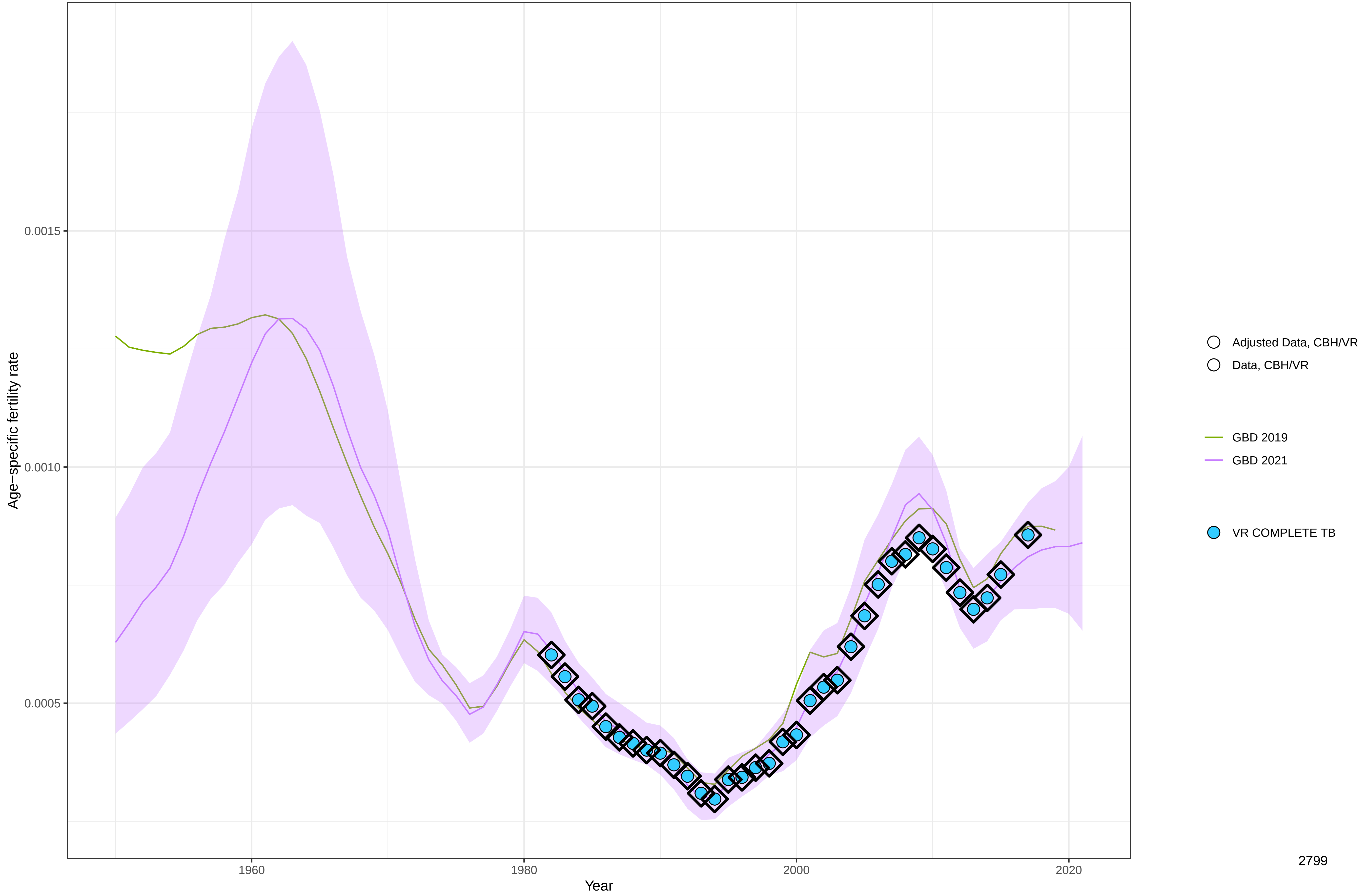

Age−specific fertility fitted trend lines, 1950−2021: Oldham, Ages: 50−54
Data points enclosed with a diamond are reference sources

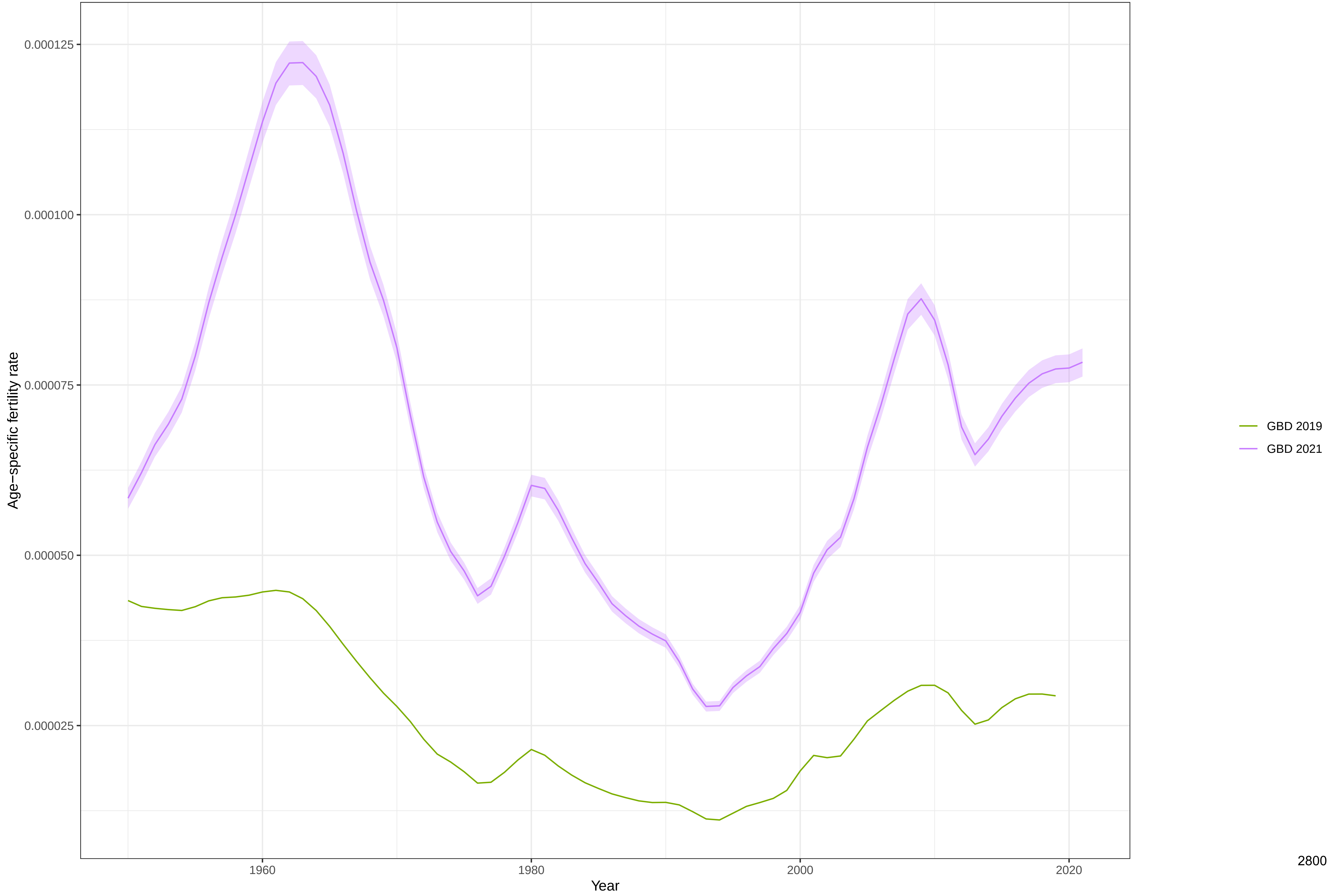

Total fertility rate fitted trend lines, 1950−2021: Oldham TFR
Data points enclosed with a diamond are reference sources

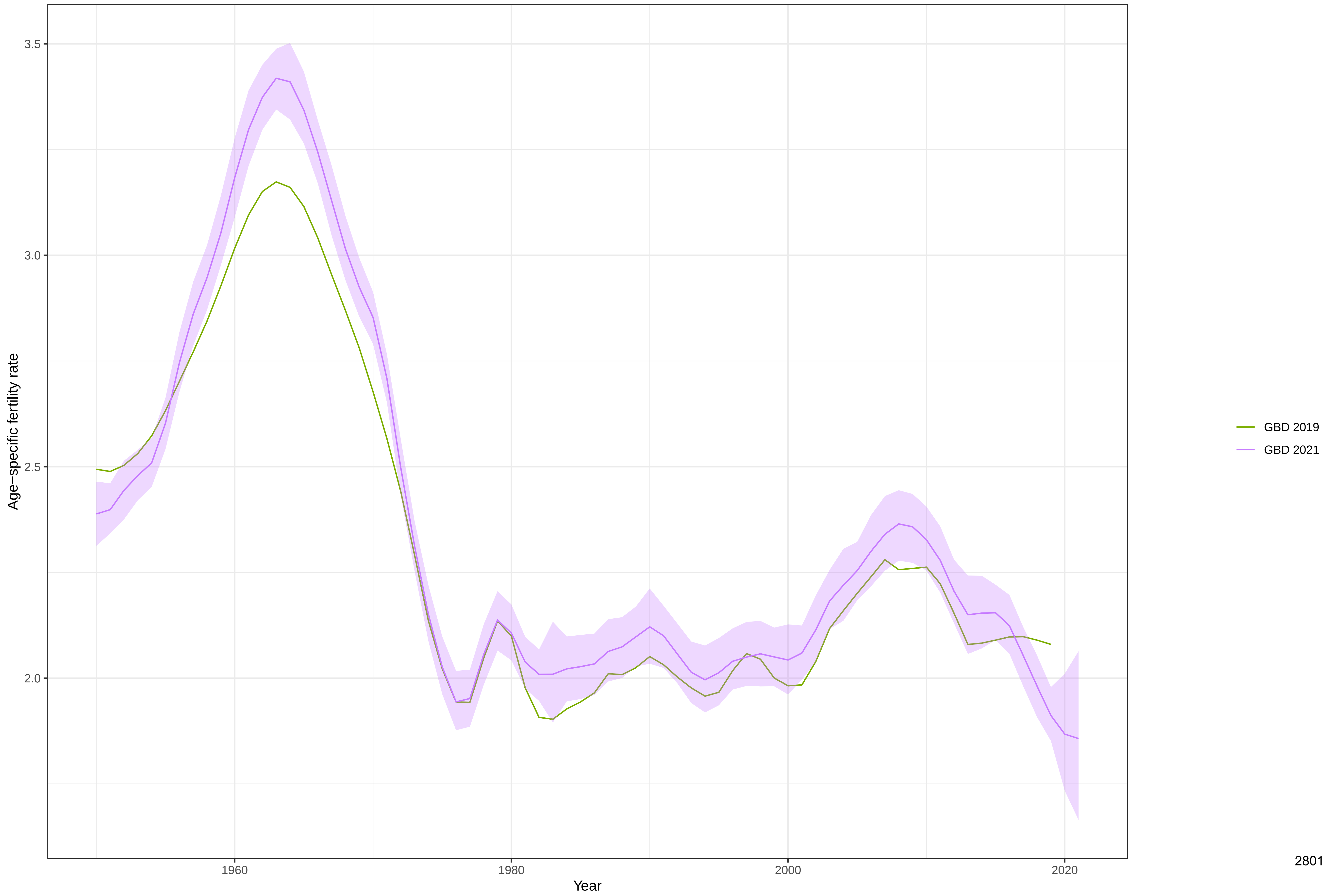

Age−specific fertility fitted trend lines, 1950−2021: Rochdale, Ages: 10−14
Data points enclosed with a diamond are reference sources

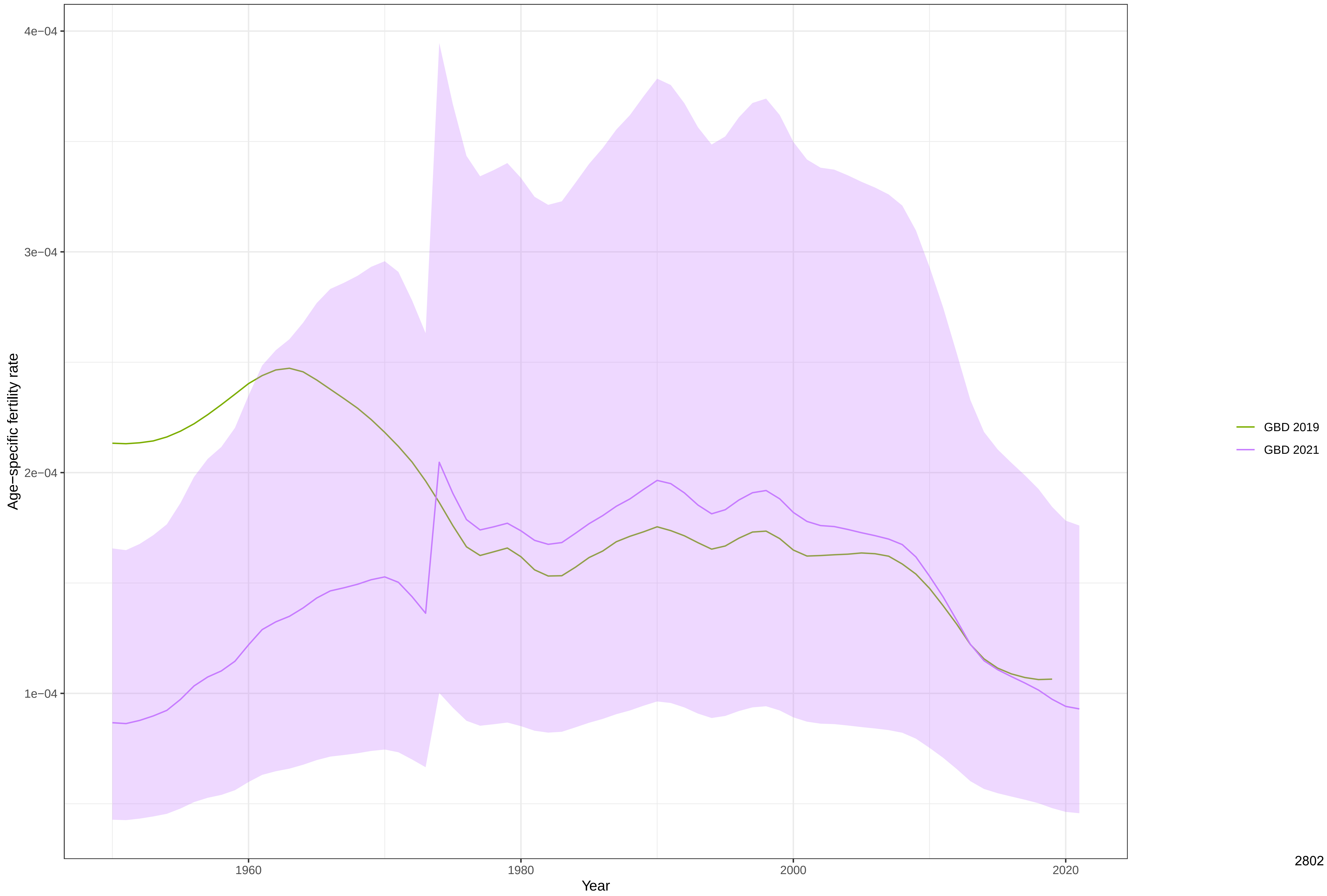

Age−specific fertility fitted trend lines, 1950−2021: Rochdale, Ages: 15−19
Data points enclosed with a diamond are reference sources

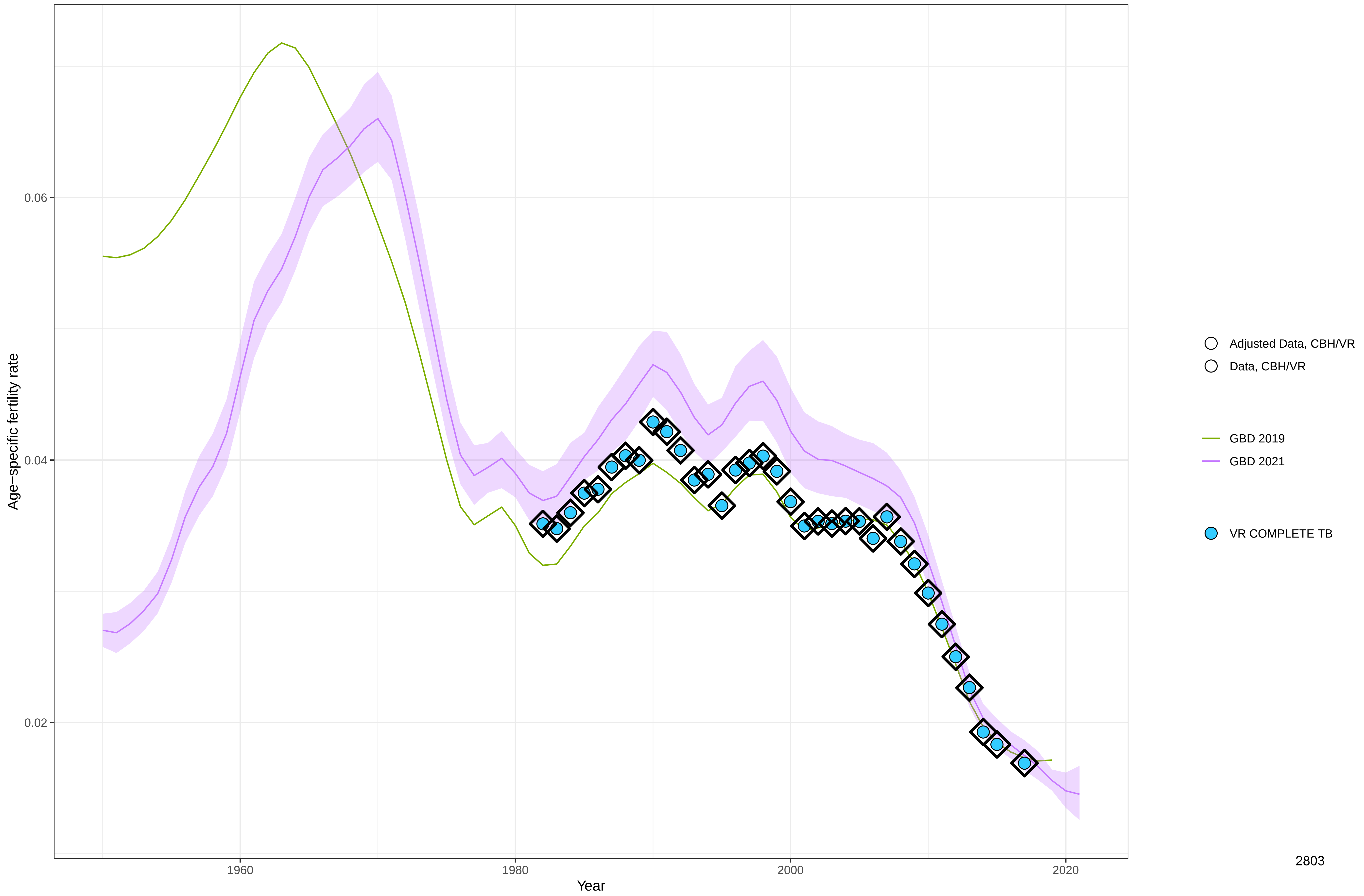

Age−specific fertility fitted trend lines, 1950−2021: Rochdale, Ages: 20−24
Data points enclosed with a diamond are reference sources

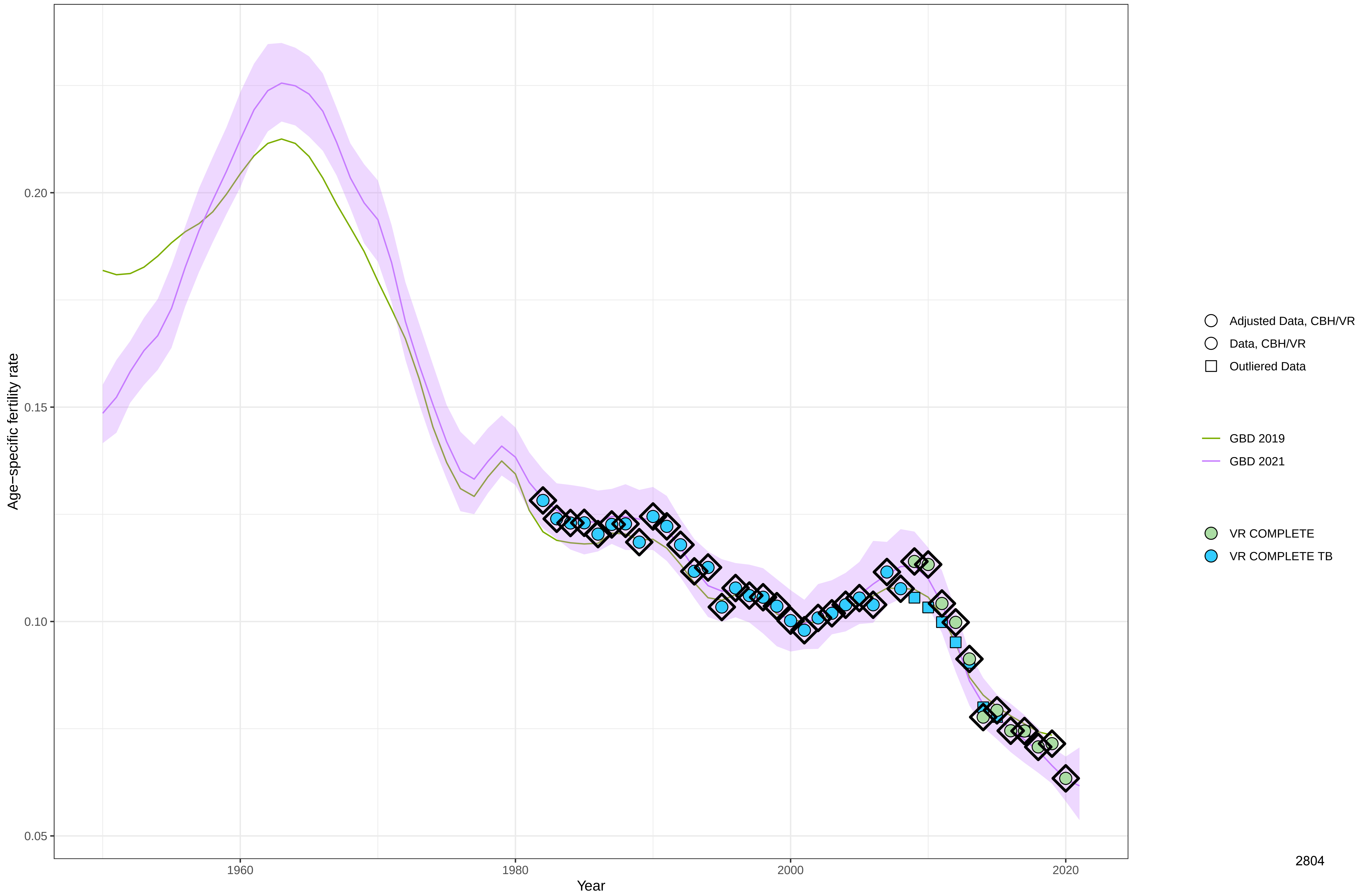

Age−specific fertility fitted trend lines, 1950−2021: Rochdale, Ages: 25−29
Data points enclosed with a diamond are reference sources

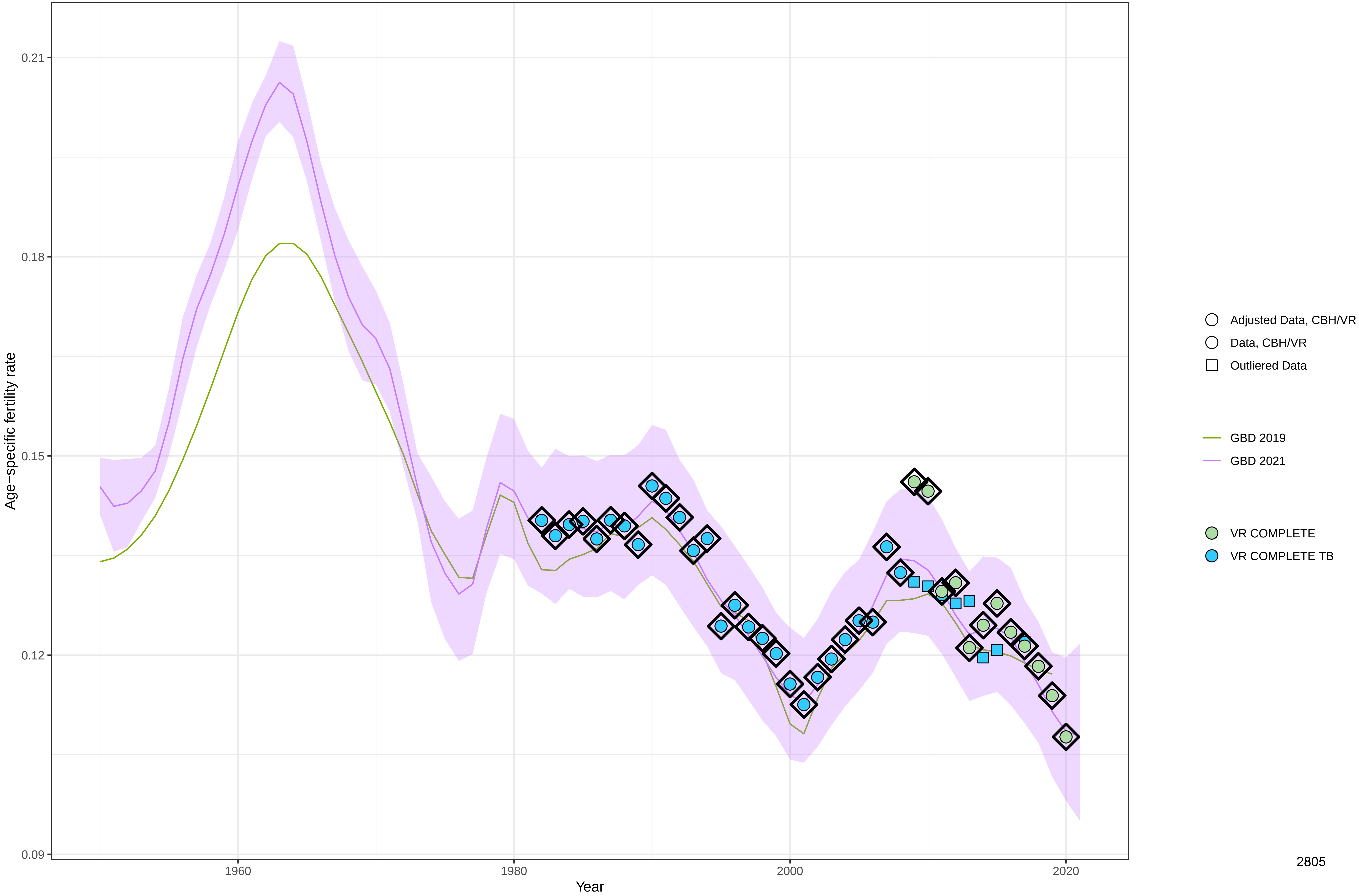

Age−specific fertility fitted trend lines, 1950−2021: Rochdale, Ages: 30−34
Data points enclosed with a diamond are reference sources

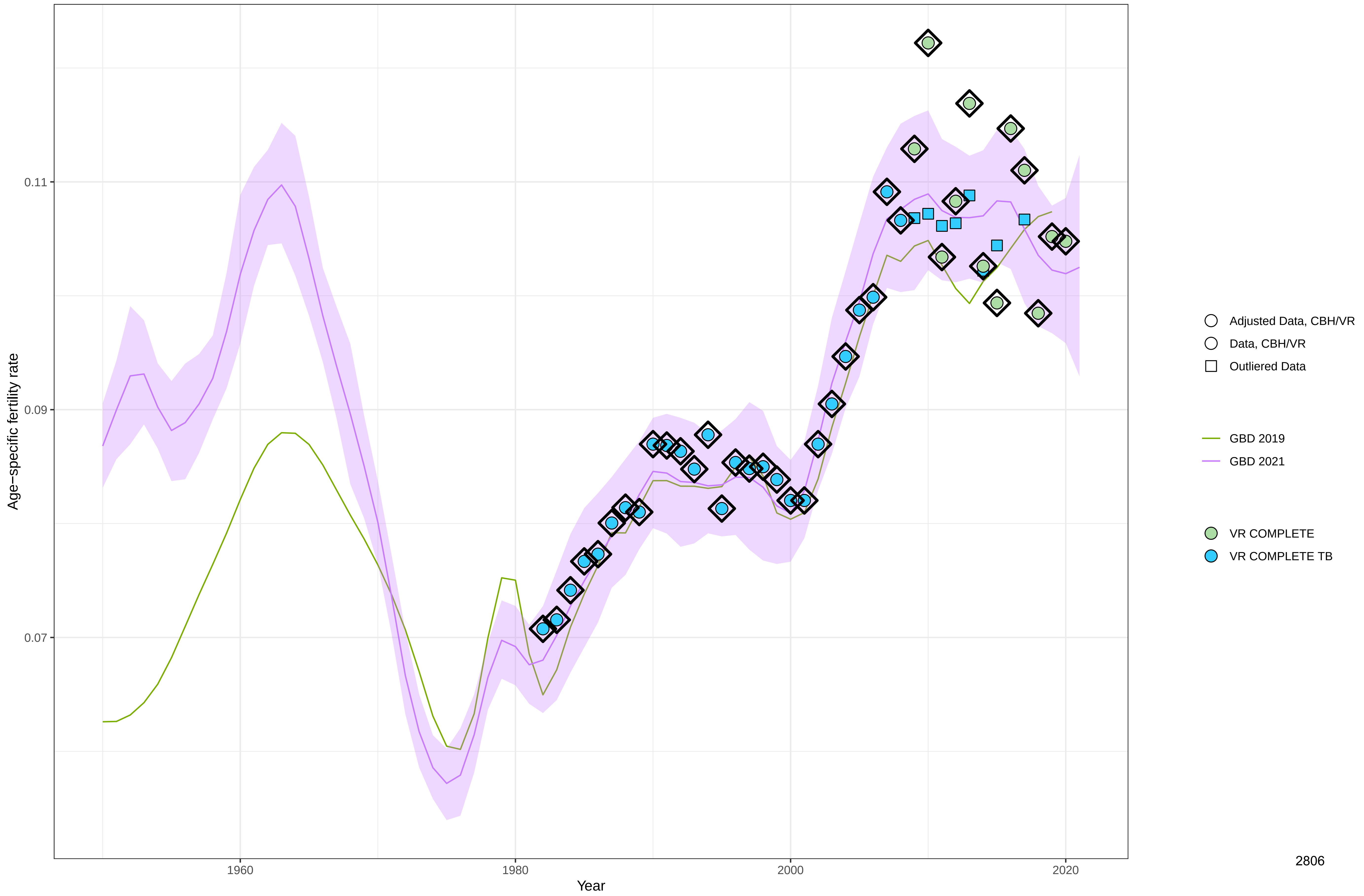

Age−specific fertility fitted trend lines, 1950−2021: Rochdale, Ages: 35−39
Data points enclosed with a diamond are reference sources

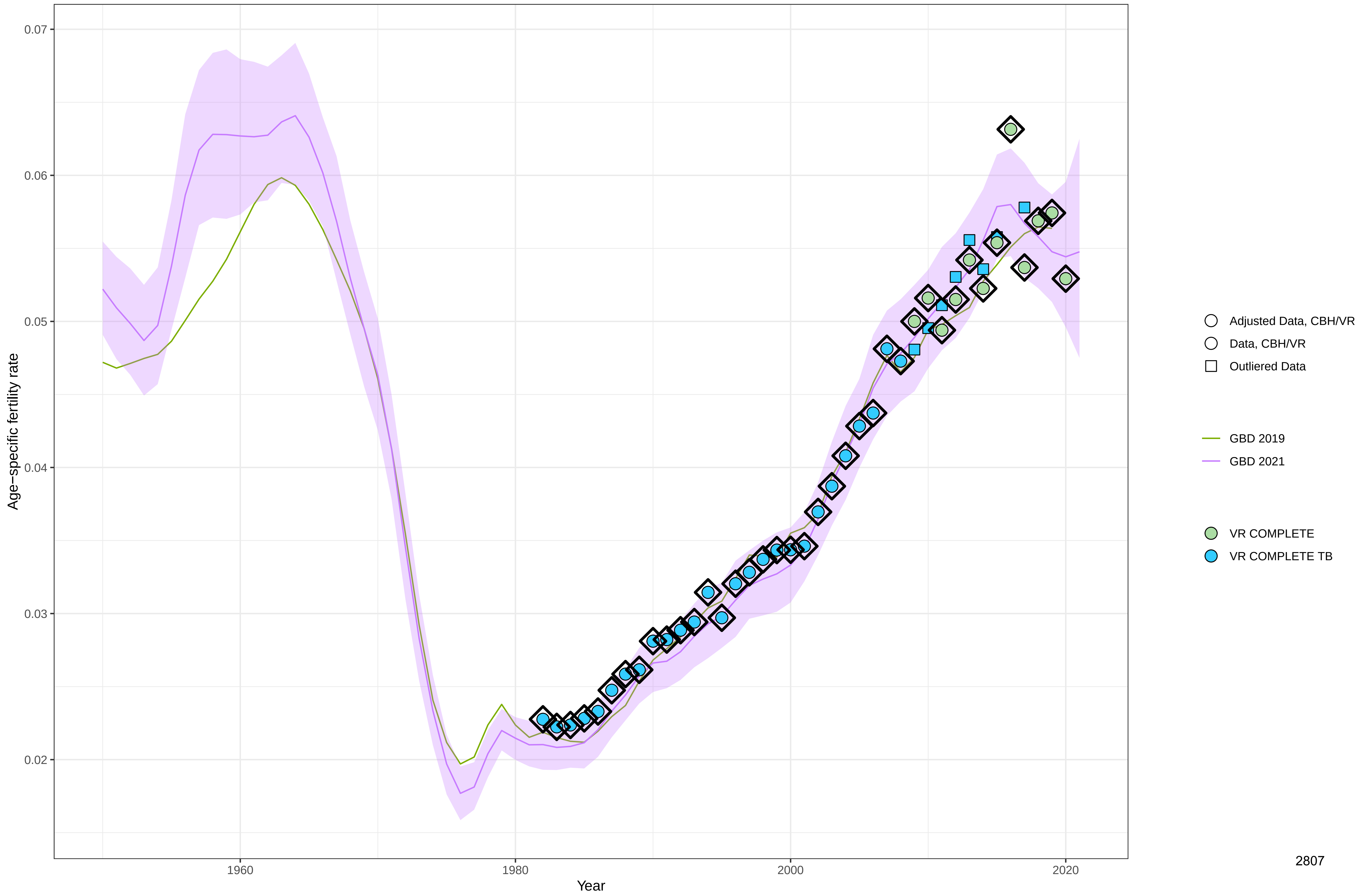

Age−specific fertility fitted trend lines, 1950−2021: Rochdale, Ages: 40−44
Data points enclosed with a diamond are reference sources

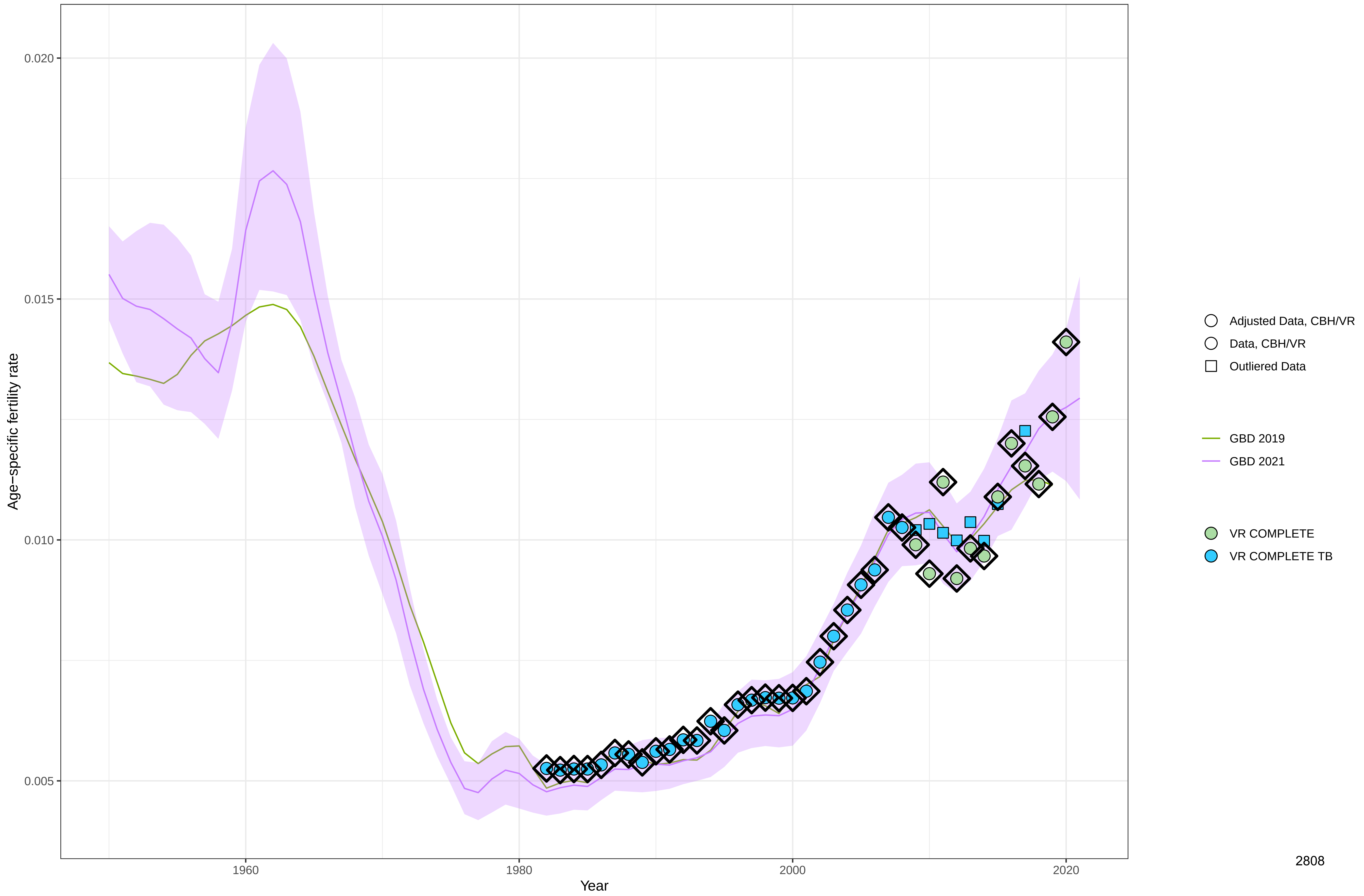

Age−specific fertility fitted trend lines, 1950−2021: Rochdale, Ages: 45−49
Data points enclosed with a diamond are reference sources

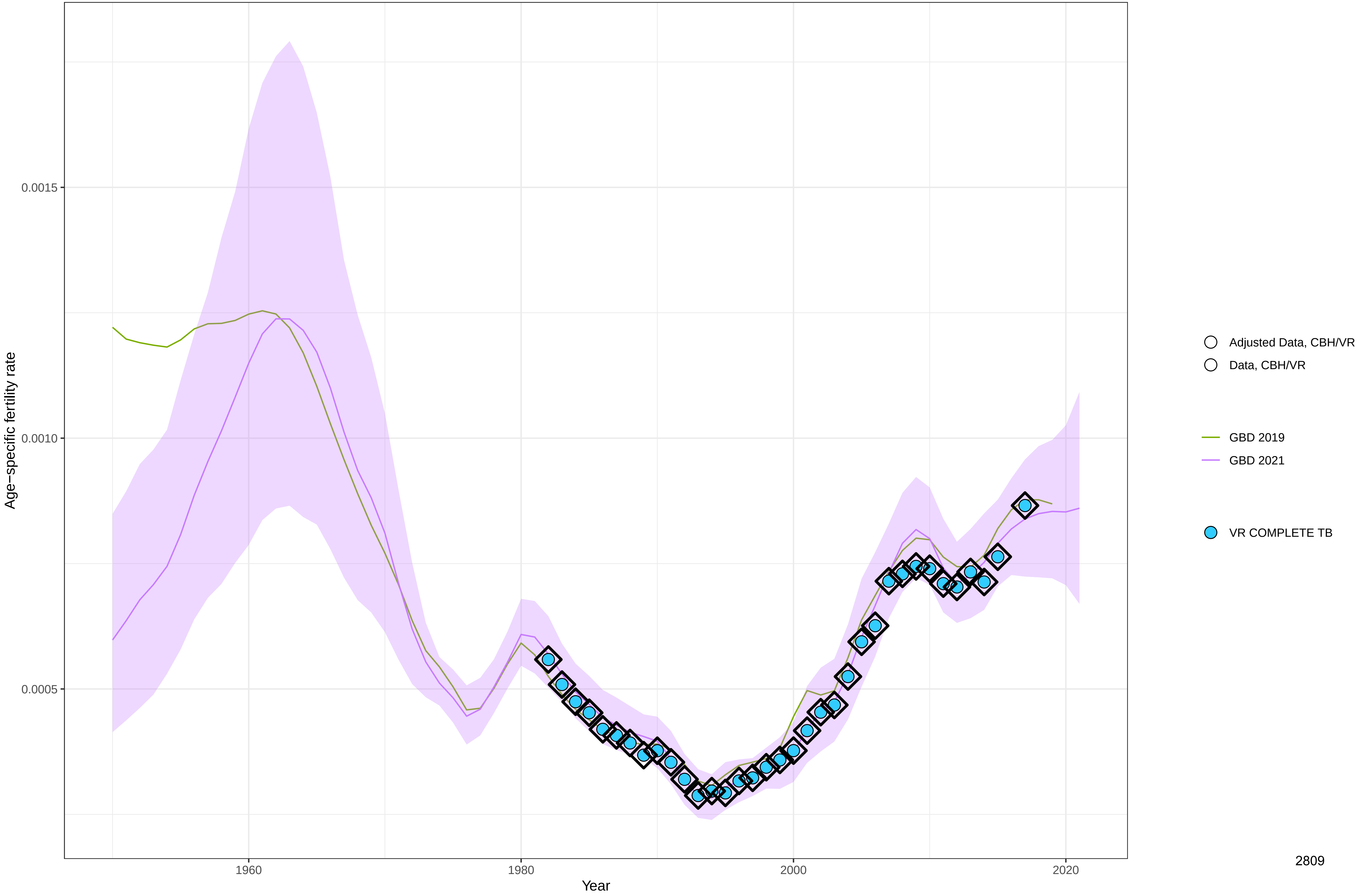

Age−specific fertility fitted trend lines, 1950−2021: Rochdale, Ages: 50−54
Data points enclosed with a diamond are reference sources

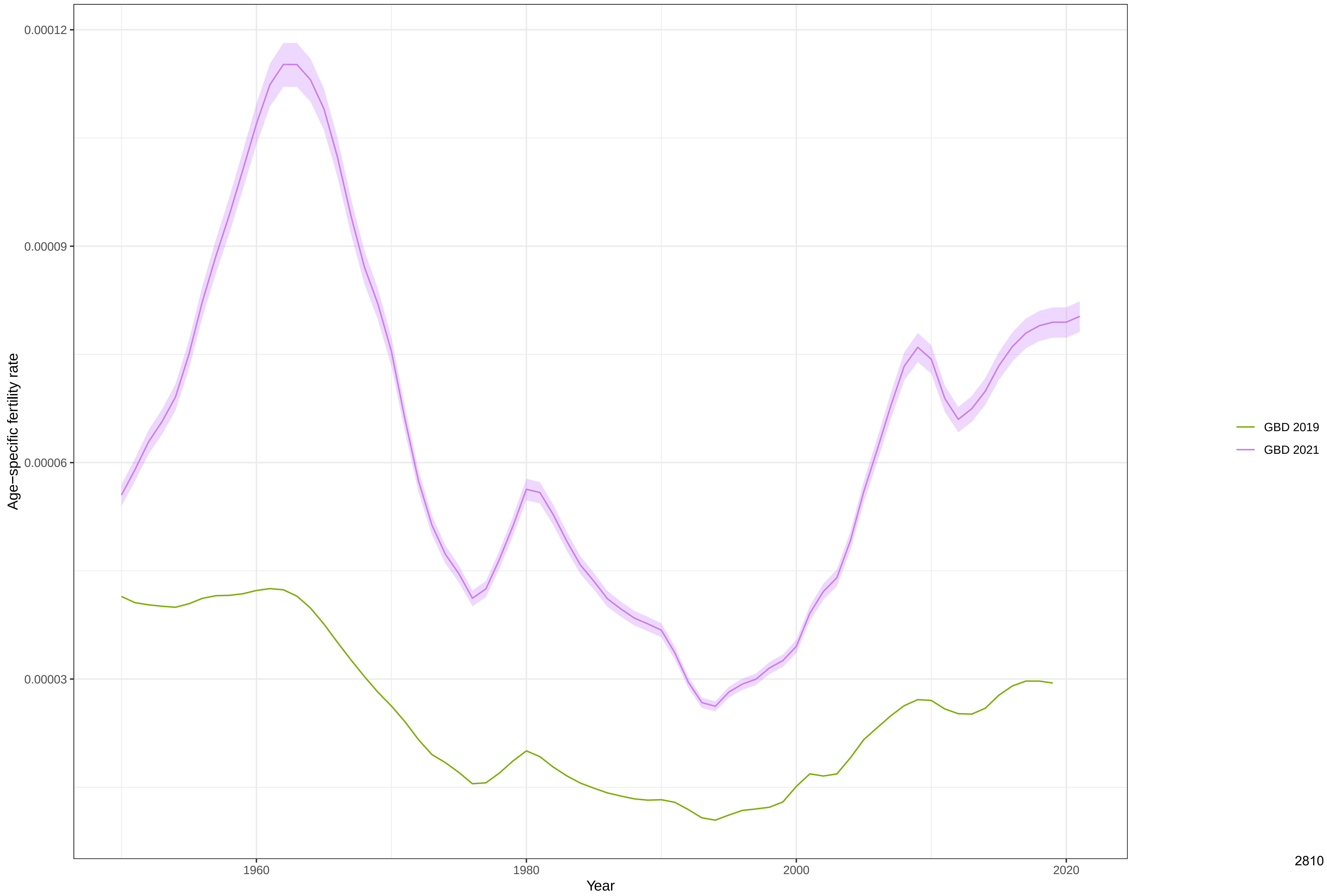

Total fertility rate fitted trend lines, 1950−2021: Rochdale TFR
Data points enclosed with a diamond are reference sources

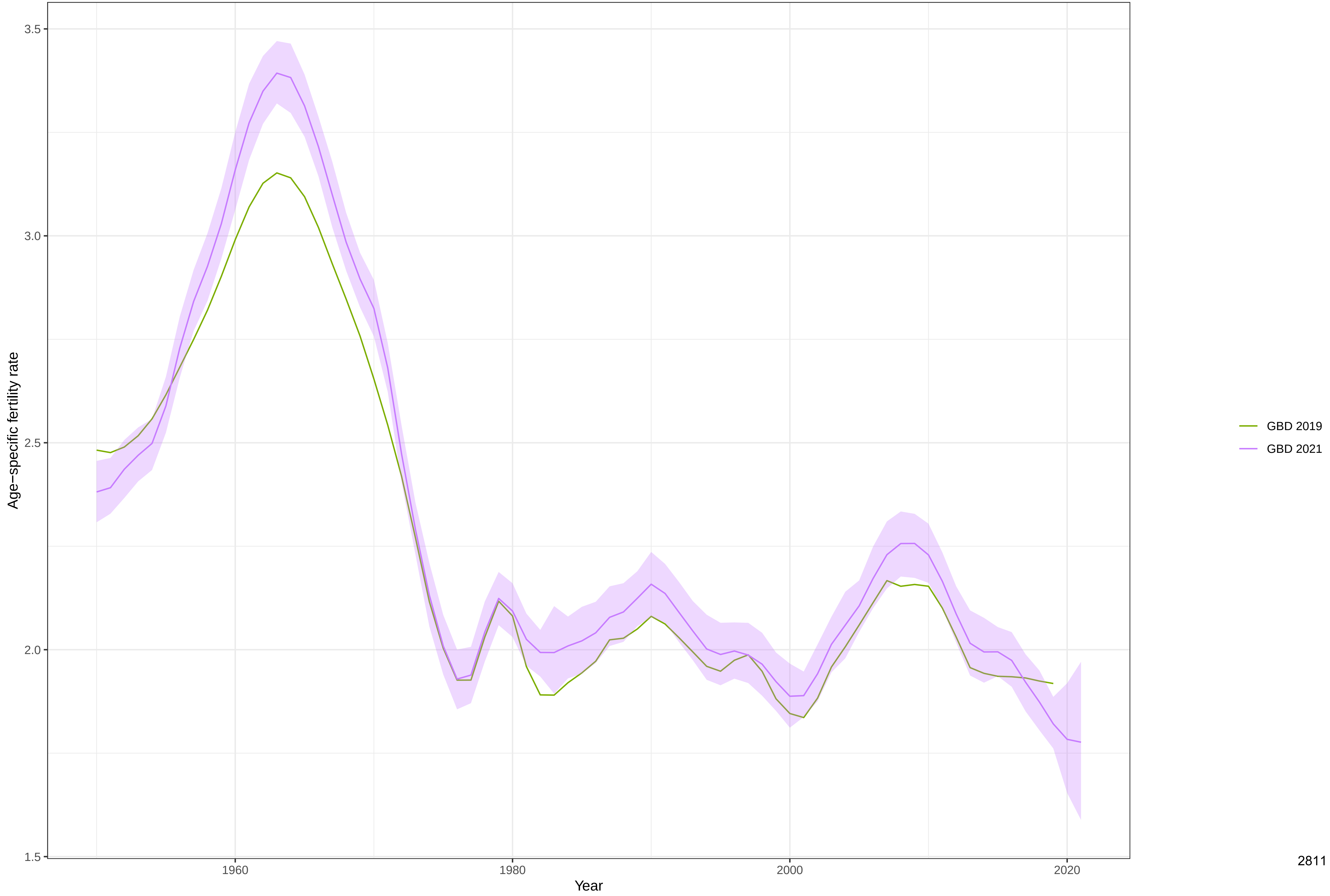

Age−specific fertility fitted trend lines, 1950−2021: Salford, Ages: 10−14
Data points enclosed with a diamond are reference sources

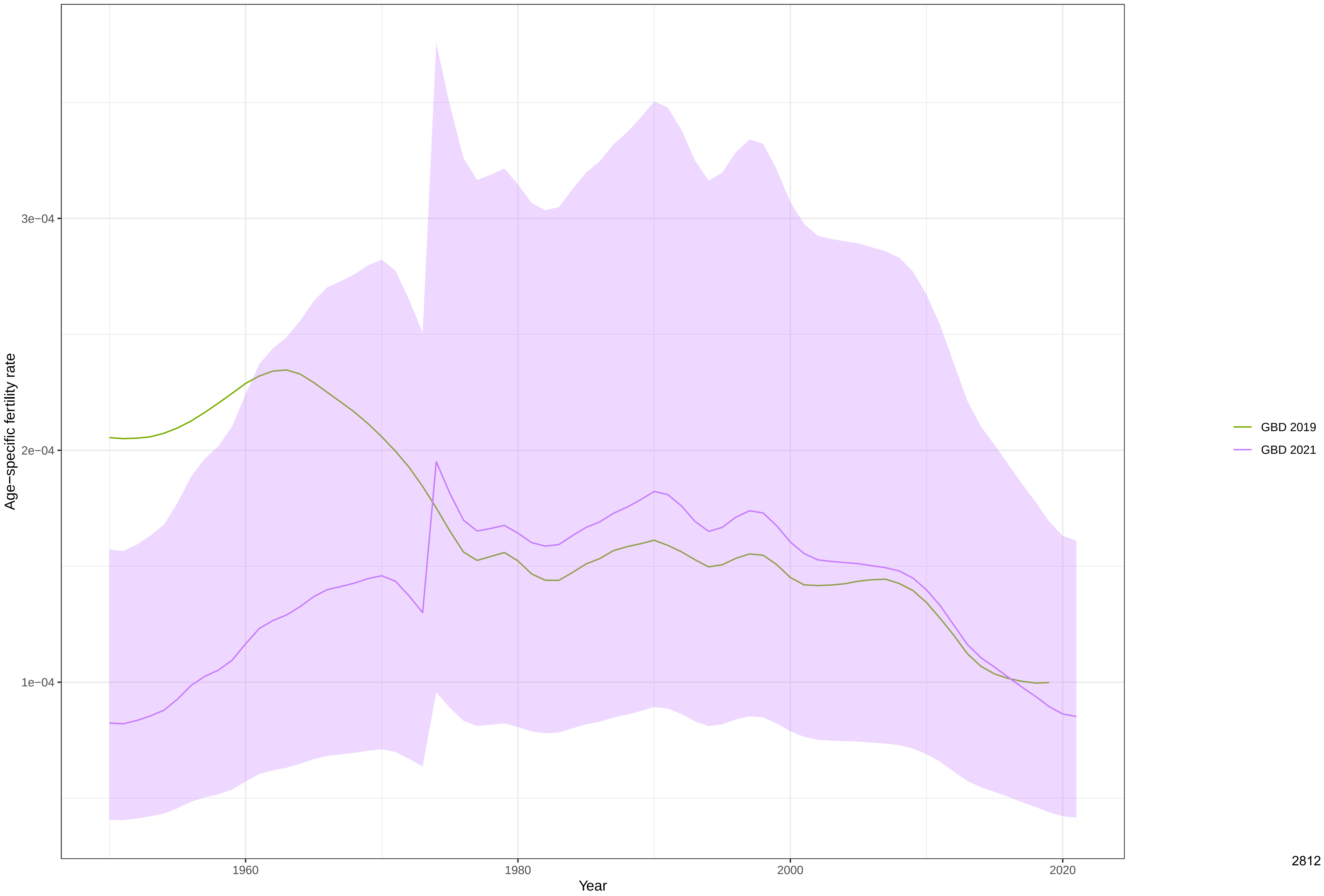

Age−specific fertility fitted trend lines, 1950−2021: Salford, Ages: 15−19
Data points enclosed with a diamond are reference sources

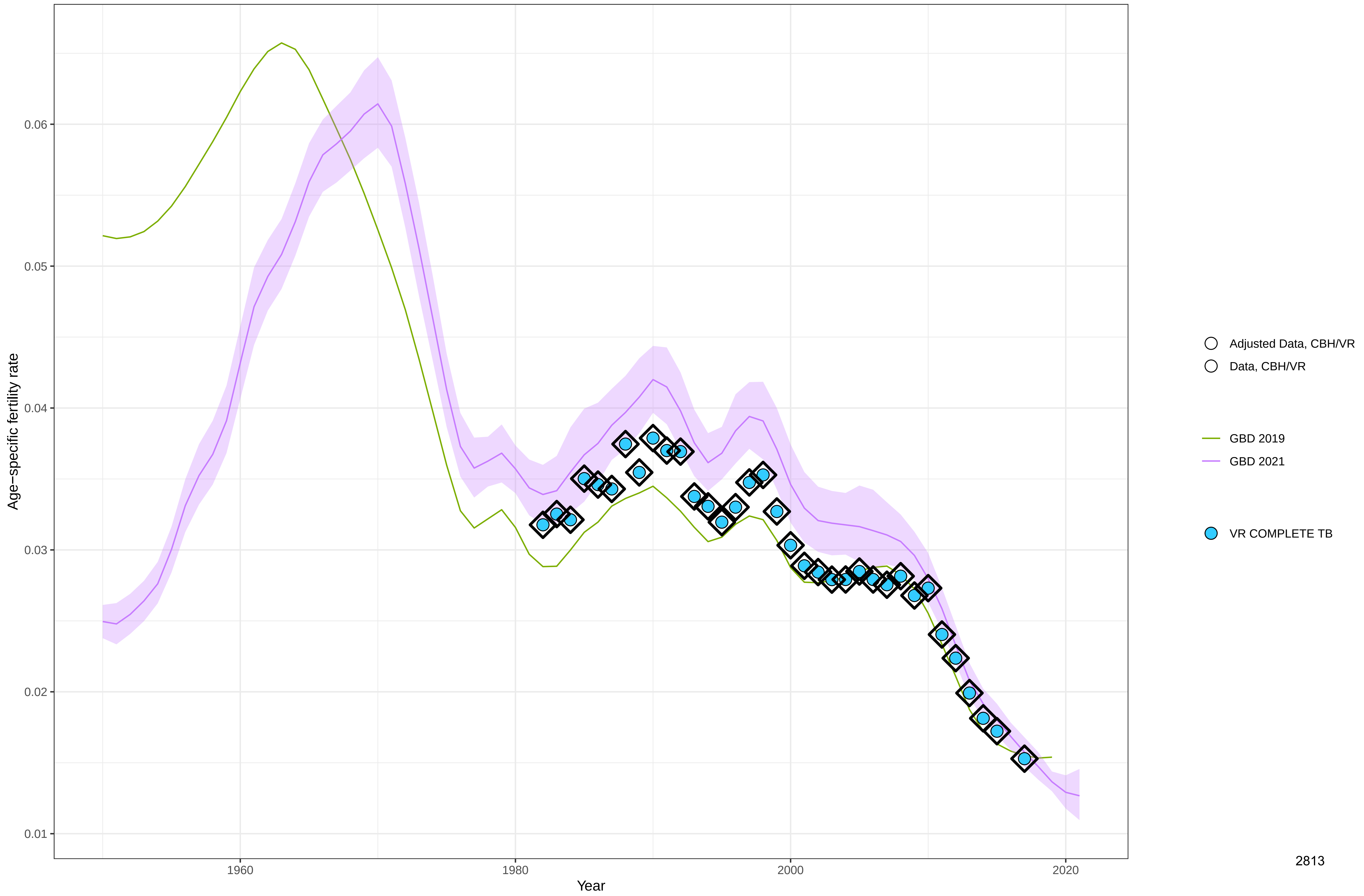

Age−specific fertility fitted trend lines, 1950−2021: Salford, Ages: 20−24
Data points enclosed with a diamond are reference sources

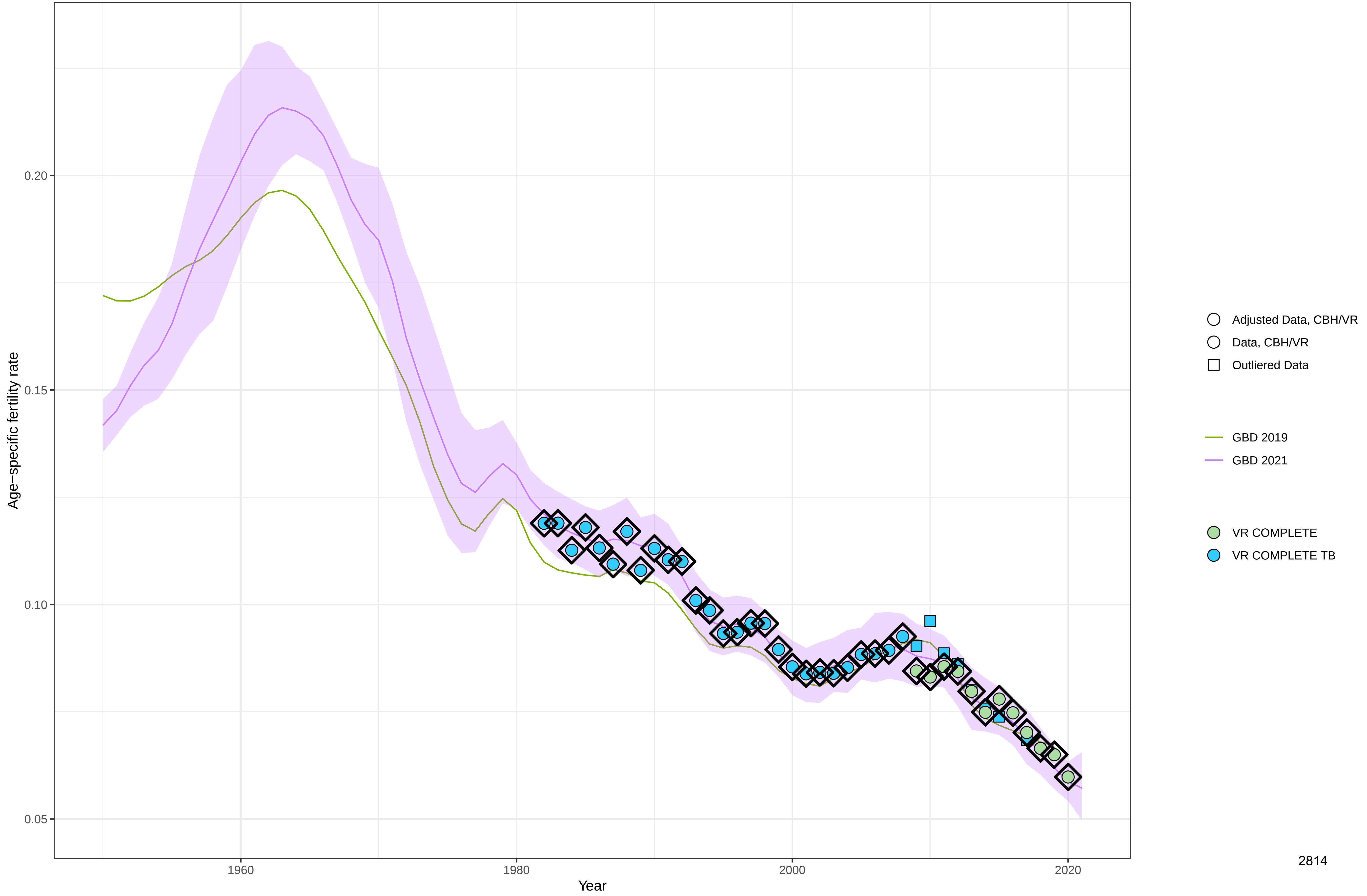

Age−specific fertility fitted trend lines, 1950−2021: Salford, Ages: 25−29
Data points enclosed with a diamond are reference sources

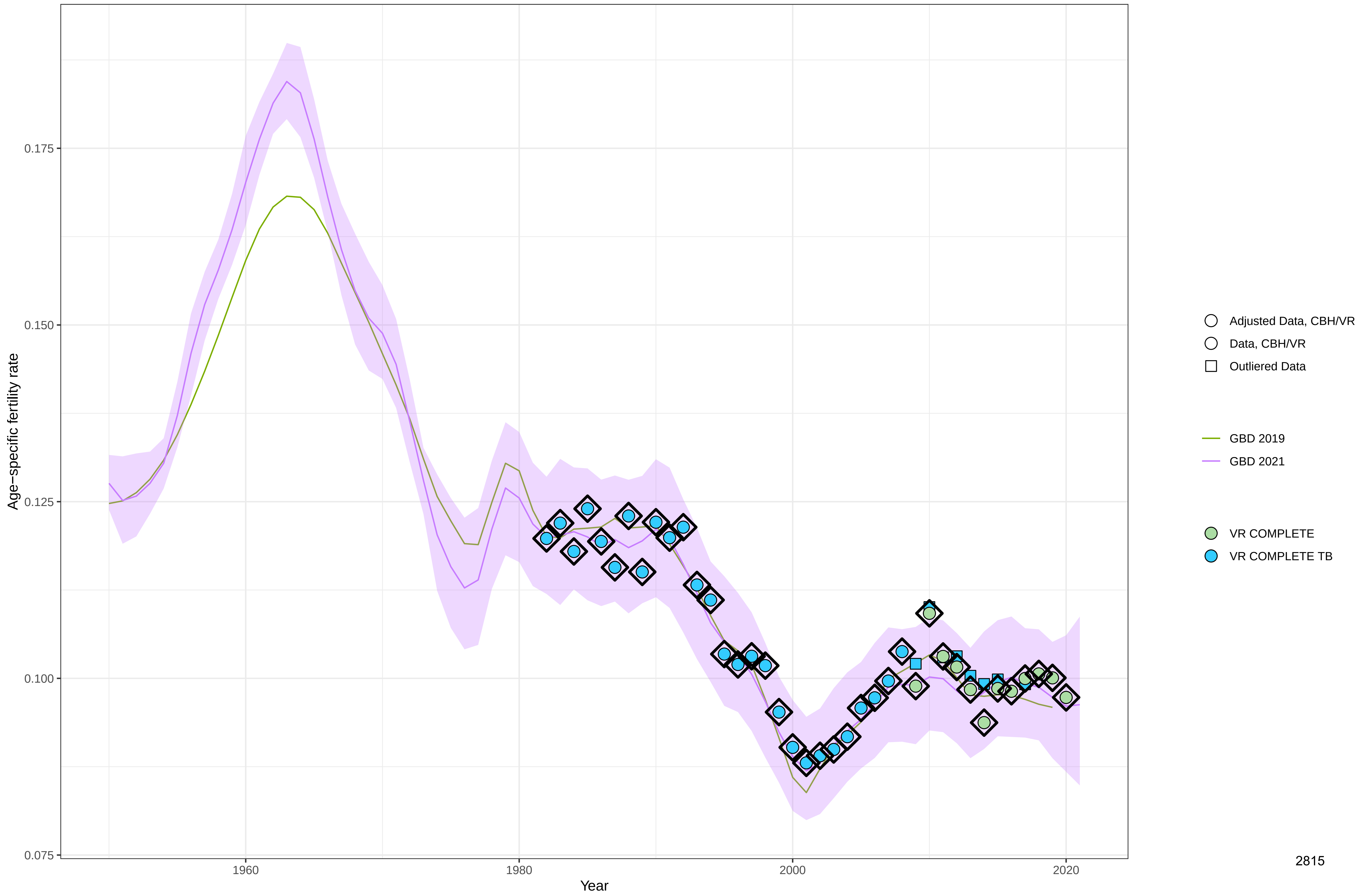

Age−specific fertility fitted trend lines, 1950−2021: Salford, Ages: 30−34
Data points enclosed with a diamond are reference sources

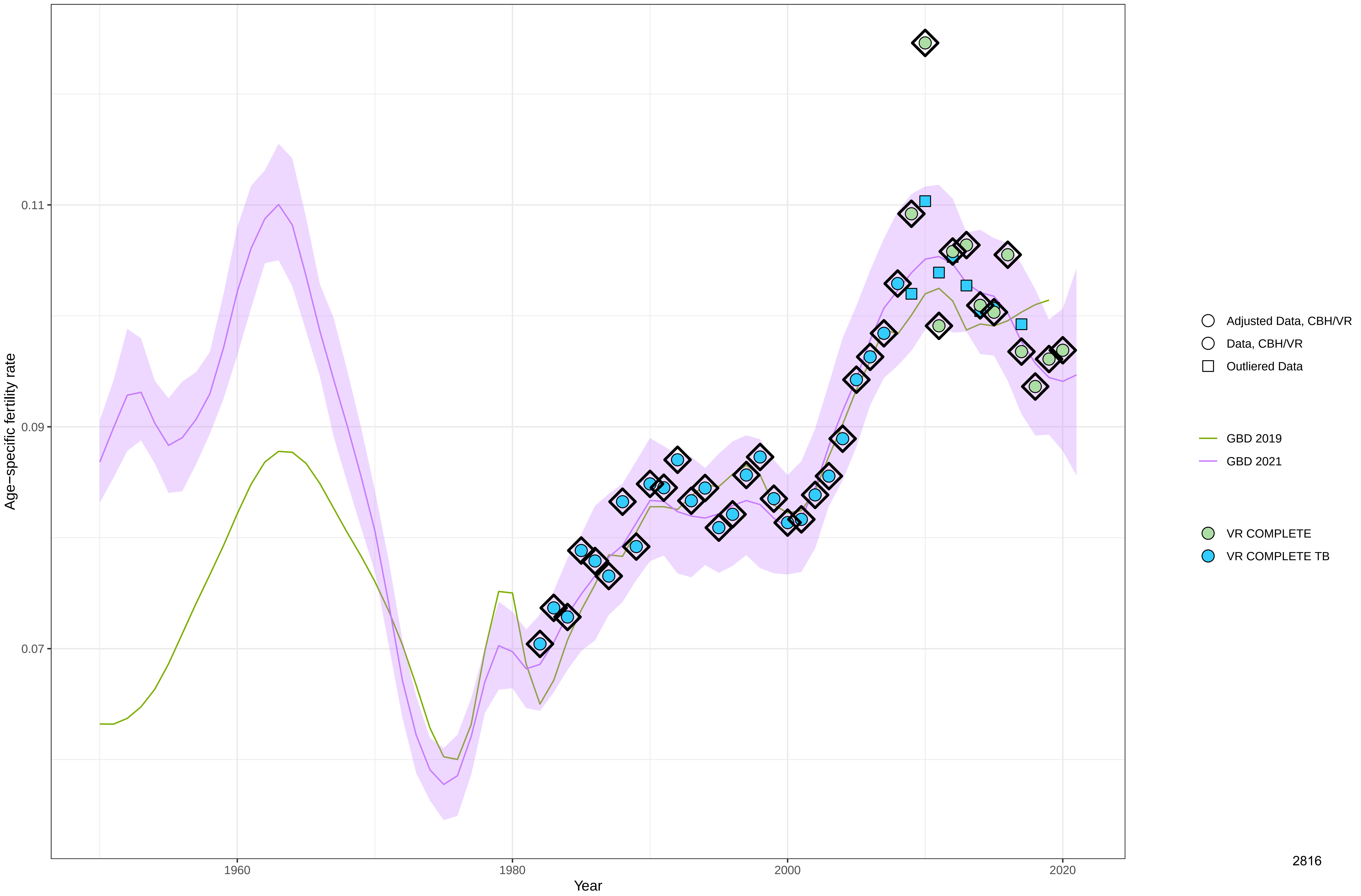

Age−specific fertility fitted trend lines, 1950−2021: Salford, Ages: 35−39
Data points enclosed with a diamond are reference sources

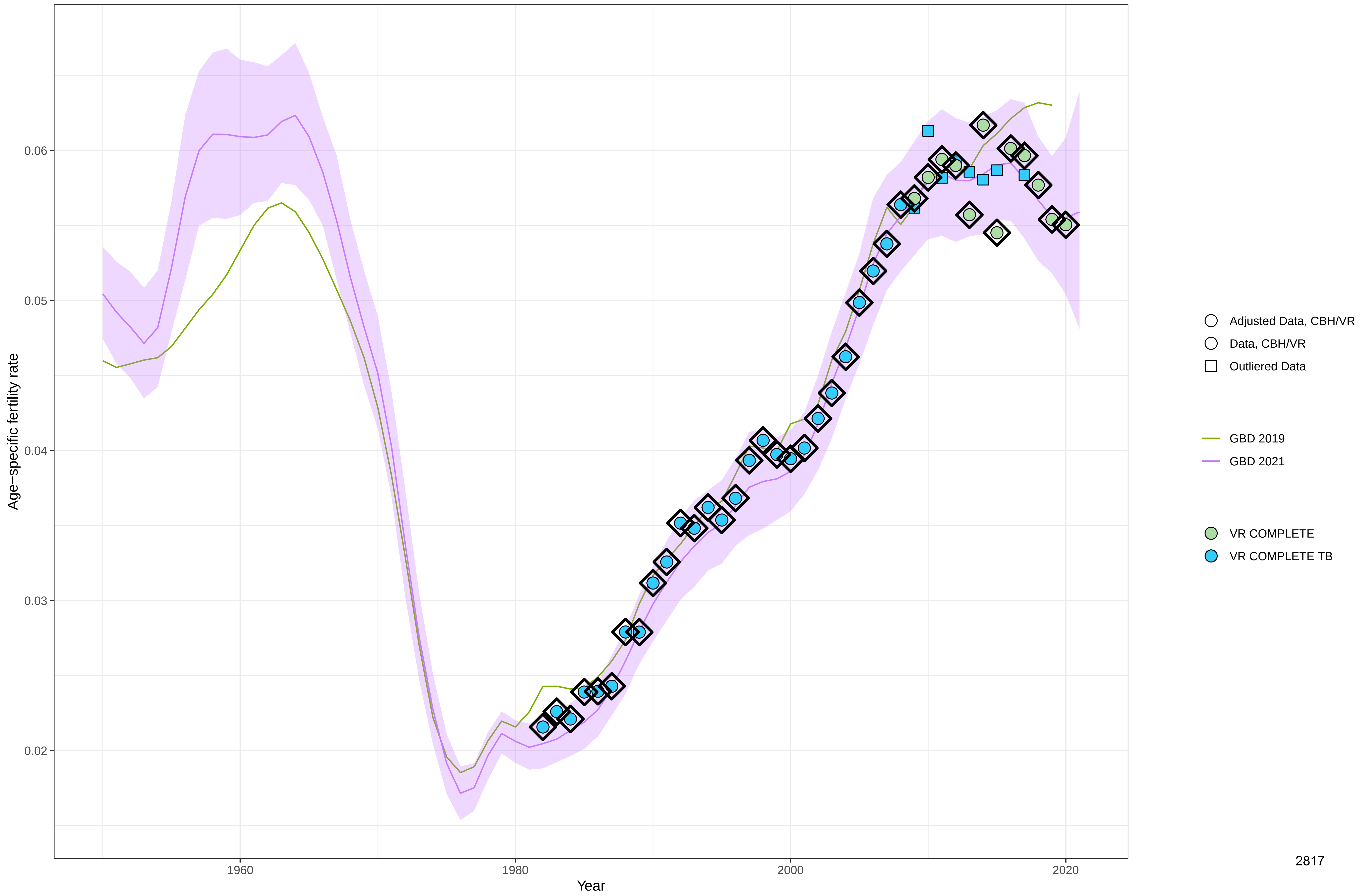

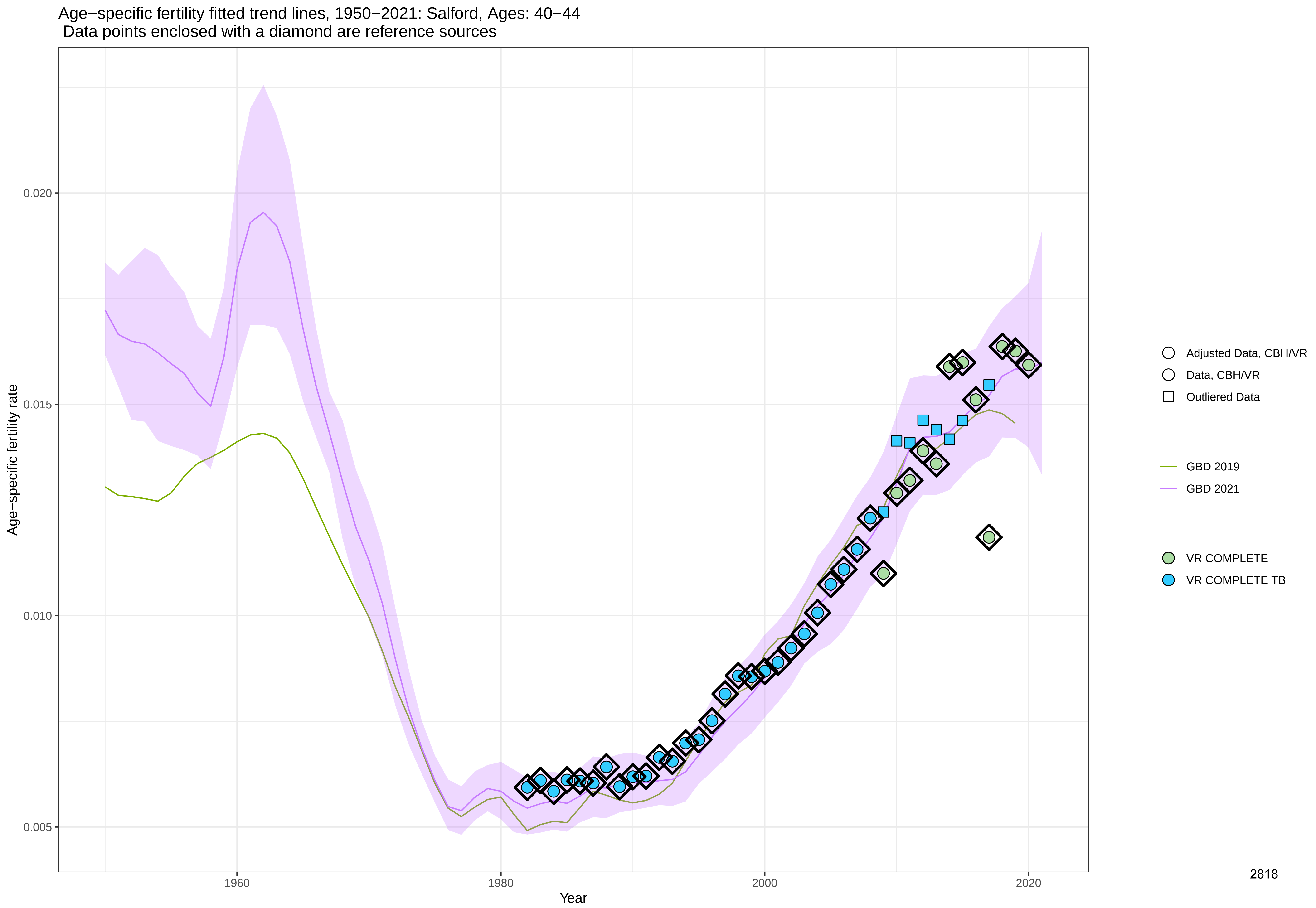
Age−specific fertility fitted trend lines, 1950−2021: Salford, Ages: 40−44
Data points enclosed with a diamond are reference sources
Age−specific fertility rate
0.020
0.015
0.010
0.005
1960
1980
2000
2020
Year
Adjusted Data, CBH/VR
Data, CBH/VR
Outliered Data
GBD 2019
GBD 2021
VR COMPLETE
VR COMPLETE TB

Age−specific fertility fitted trend lines, 1950−2021: Salford, Ages: 45−49
Data points enclosed with a diamond are reference sources

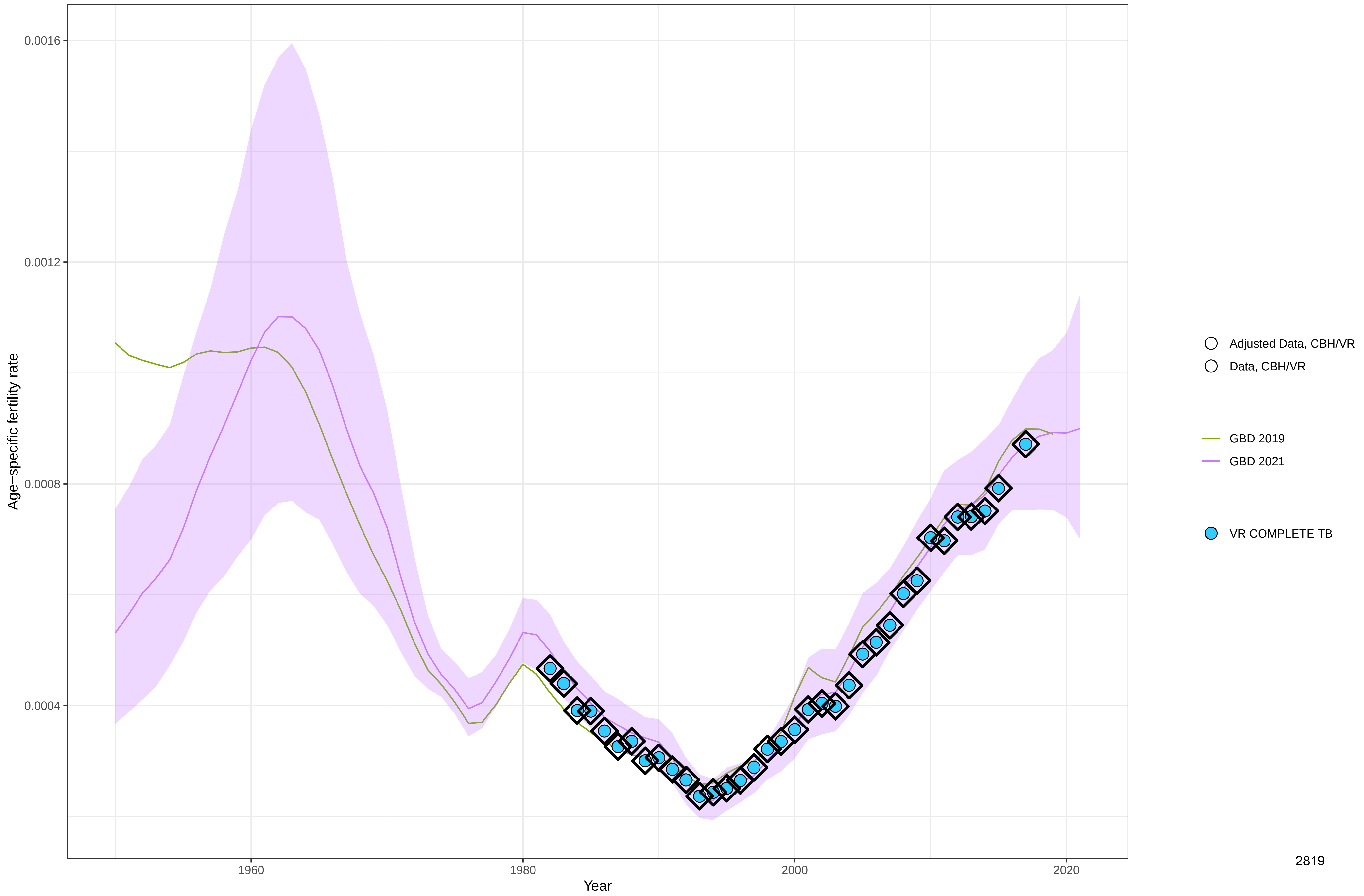

Age−specific fertility fitted trend lines, 1950−2021: Salford, Ages: 50−54
Data points enclosed with a diamond are reference sources

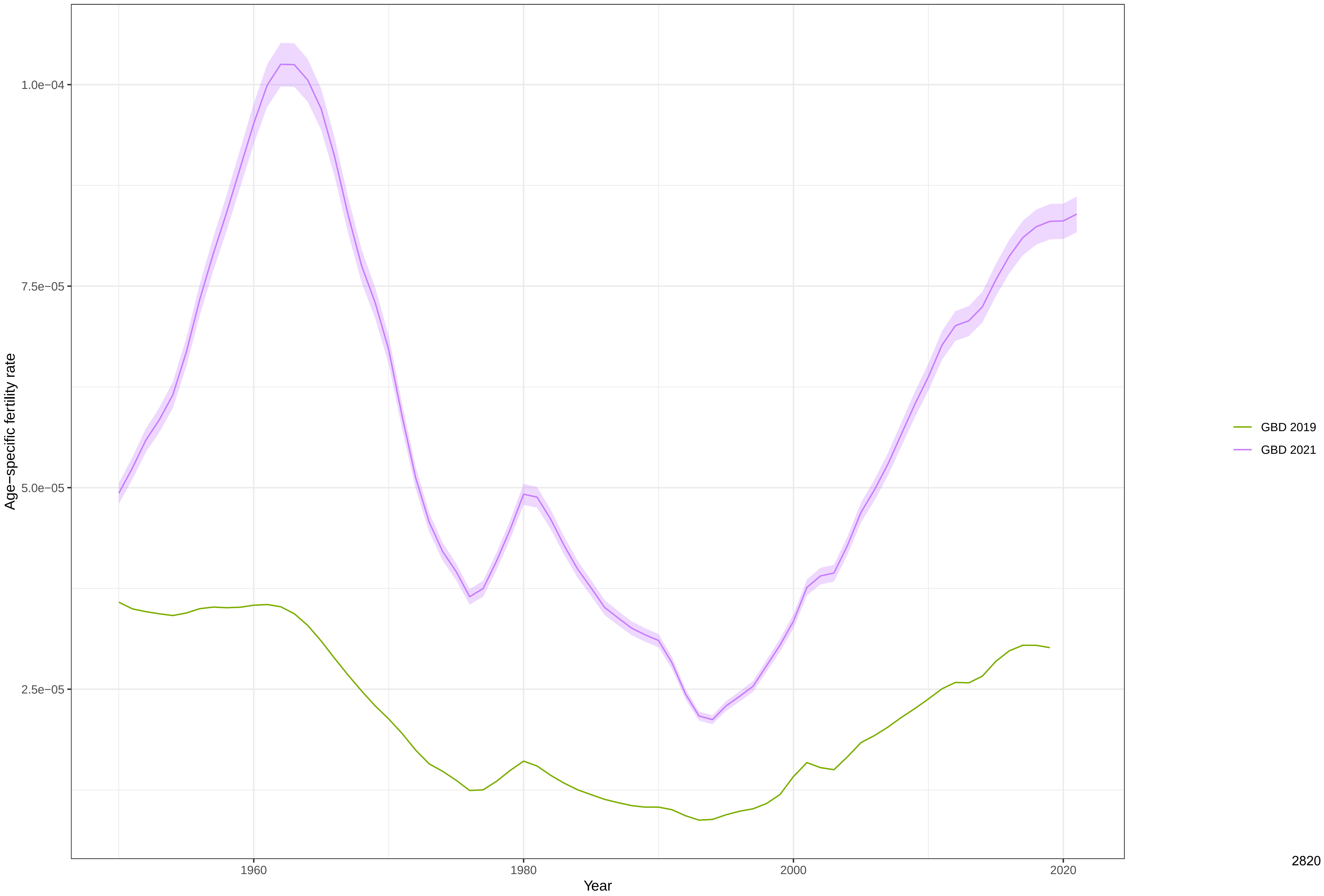

Total fertility rate fitted trend lines, 1950−2021: Salford TFR
Data points enclosed with a diamond are reference sources

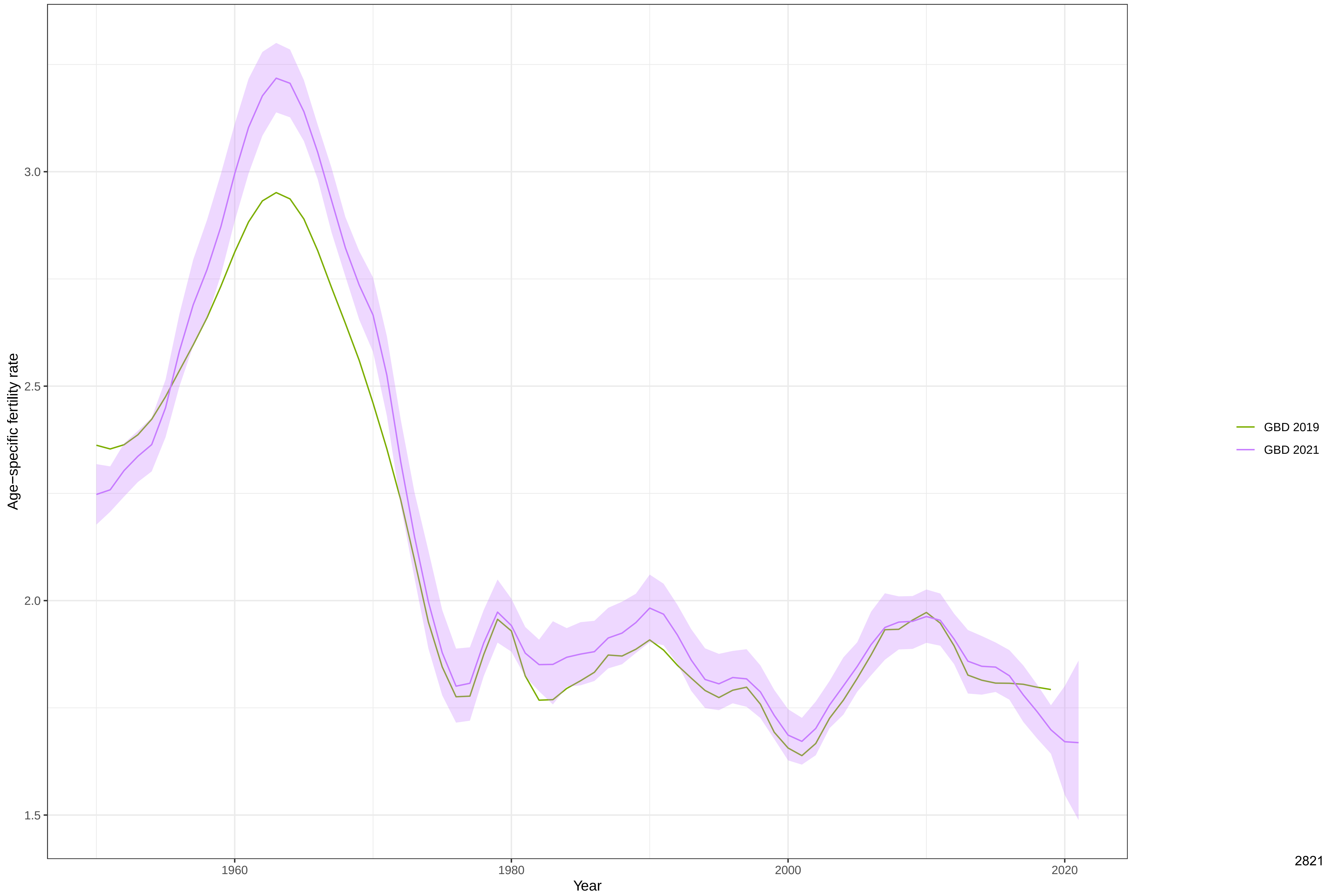

Age−specific fertility fitted trend lines, 1950−2021: Sefton, Ages: 10−14
Data points enclosed with a diamond are reference sources

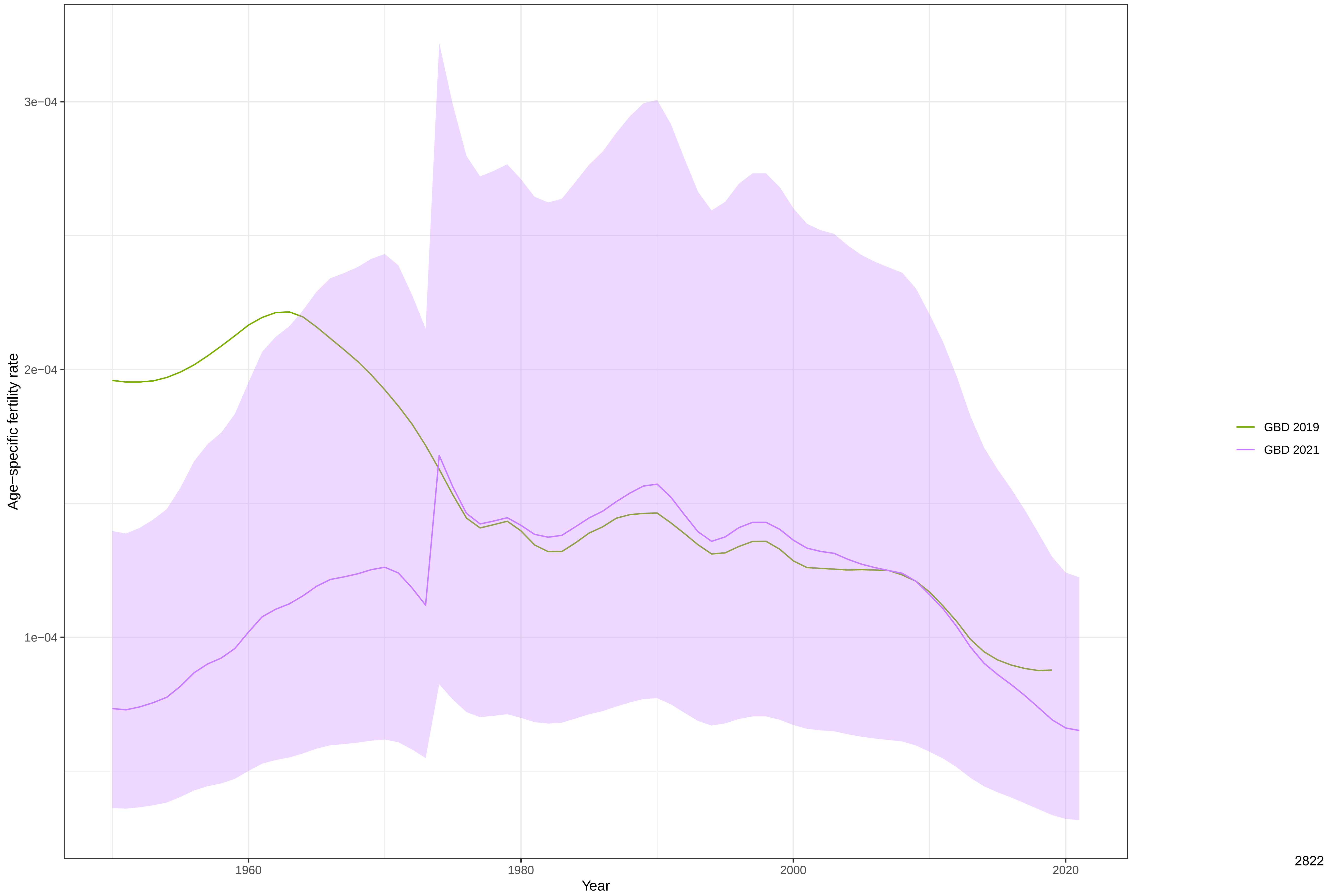

Age−specific fertility fitted trend lines, 1950−2021: Sefton, Ages: 15−19
Data points enclosed with a diamond are reference sources

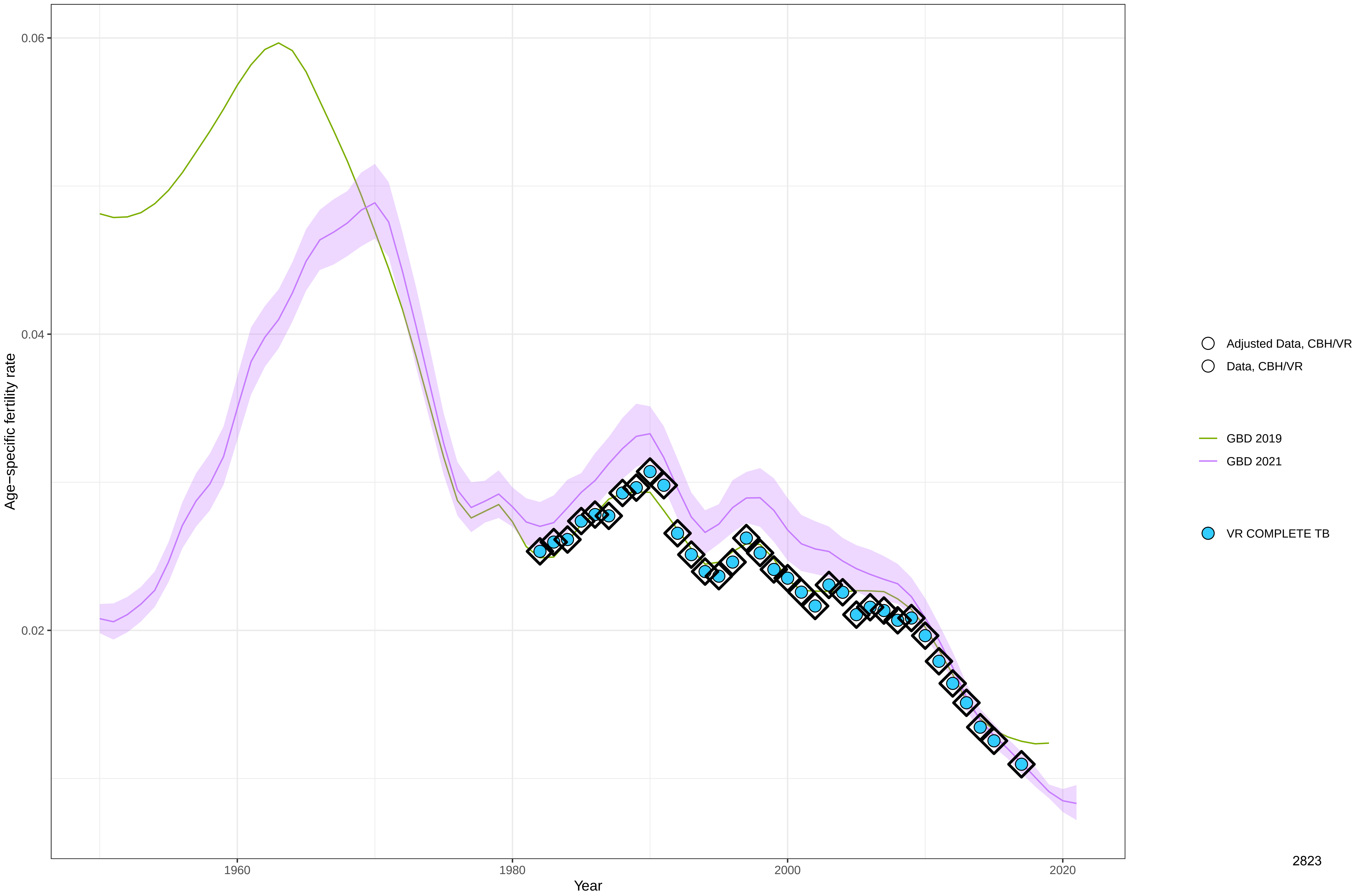

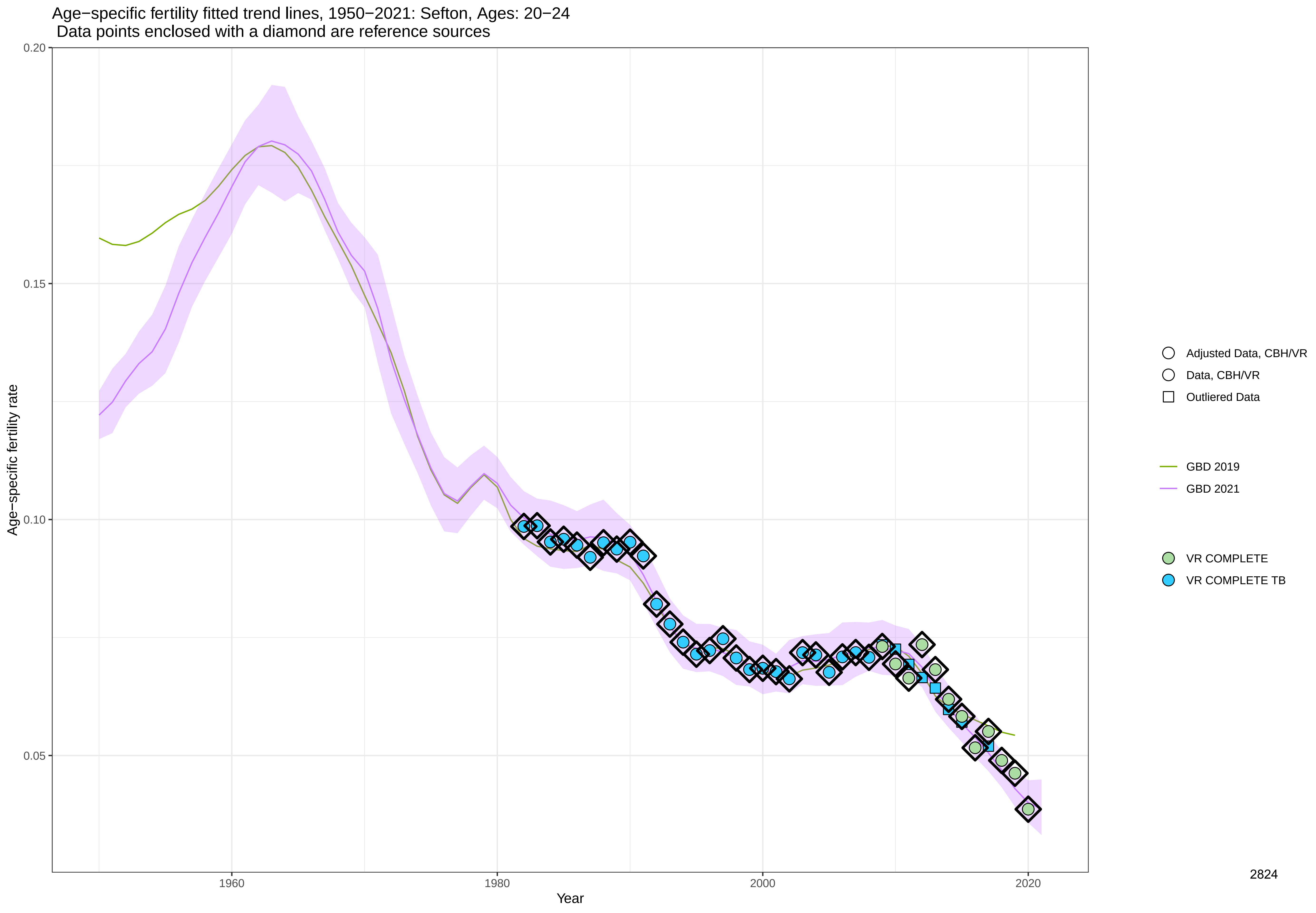

Age−specific fertility fitted trend lines, 1950−2021: Sefton, Ages: 20−24
Data points enclosed with a diamond are reference sources
Age−specific fertility rate
0.20
0.15
0.10
0.05
1960
1980
2000
2020
Year
Adjusted Data, CBH/VR
Data, CBH/VR
Outliered Data
GBD 2019
GBD 2021
VR COMPLETE
VR COMPLETE TB

Age−specific fertility fitted trend lines, 1950−2021: Sefton, Ages: 25−29
Data points enclosed with a diamond are reference sources

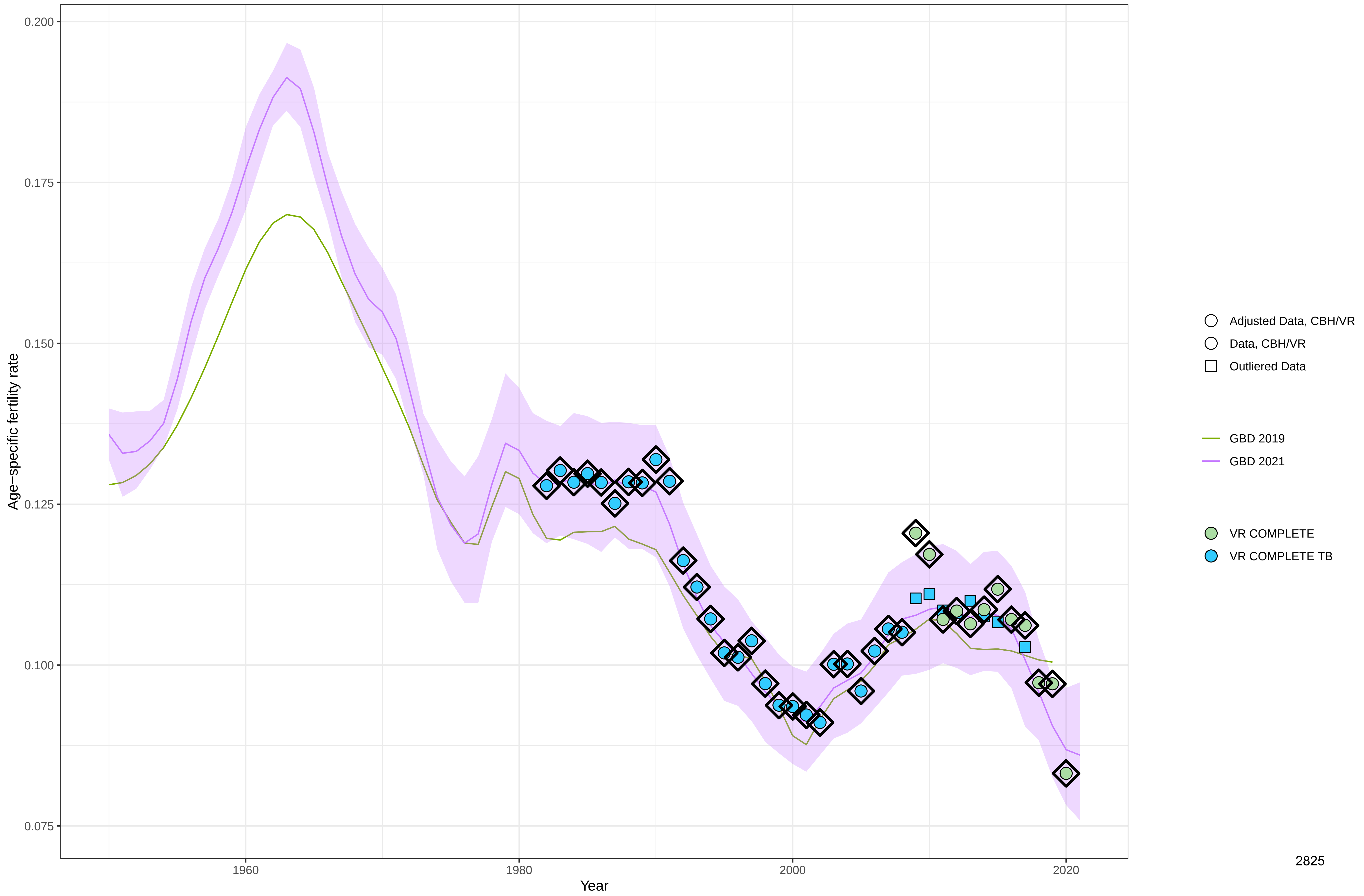

Age−specific fertility fitted trend lines, 1950−2021: Sefton, Ages: 30−34
Data points enclosed with a diamond are reference sources

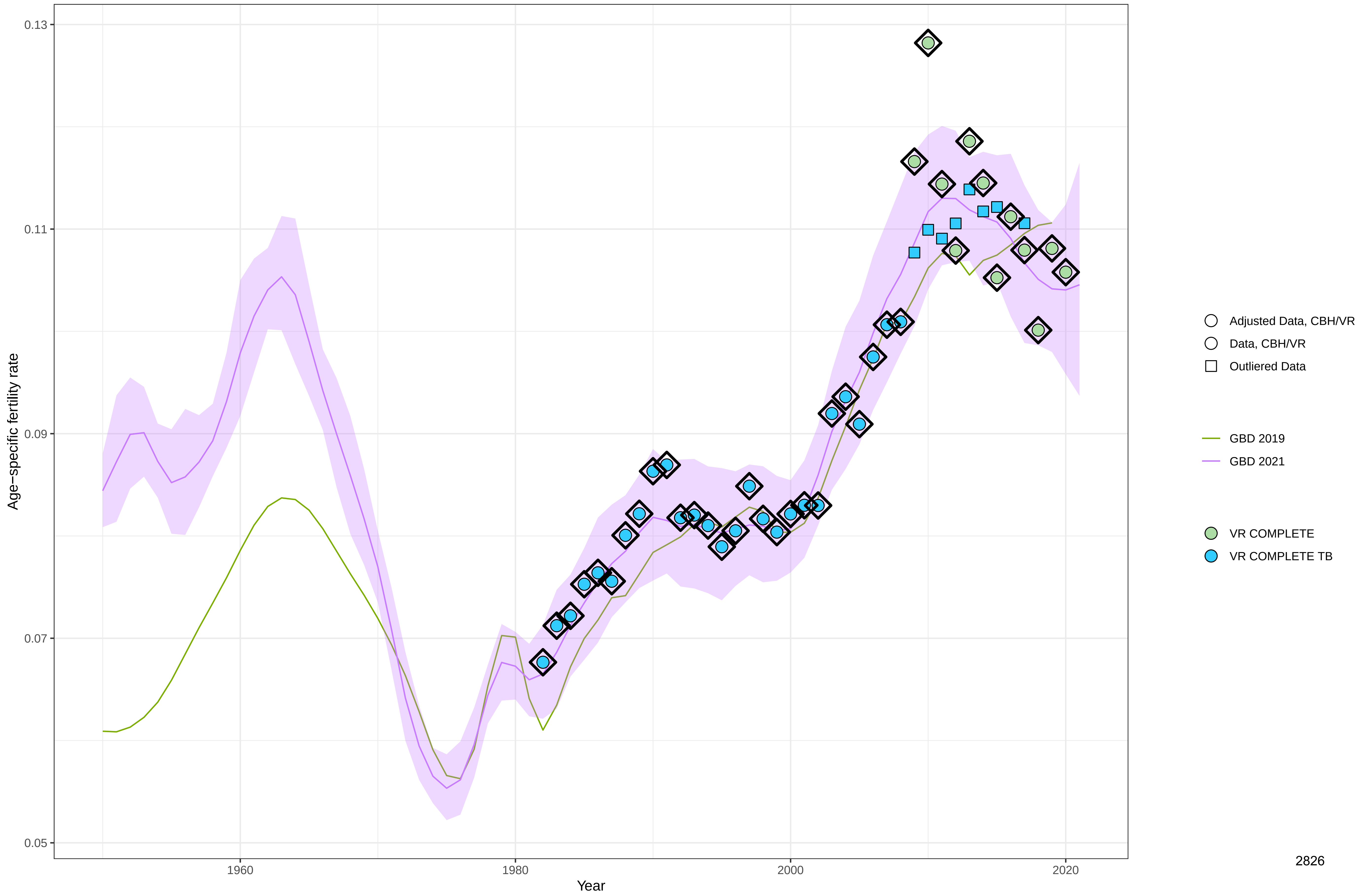

Age−specific fertility fitted trend lines, 1950−2021: Sefton, Ages: 35−39
Data points enclosed with a diamond are reference sources

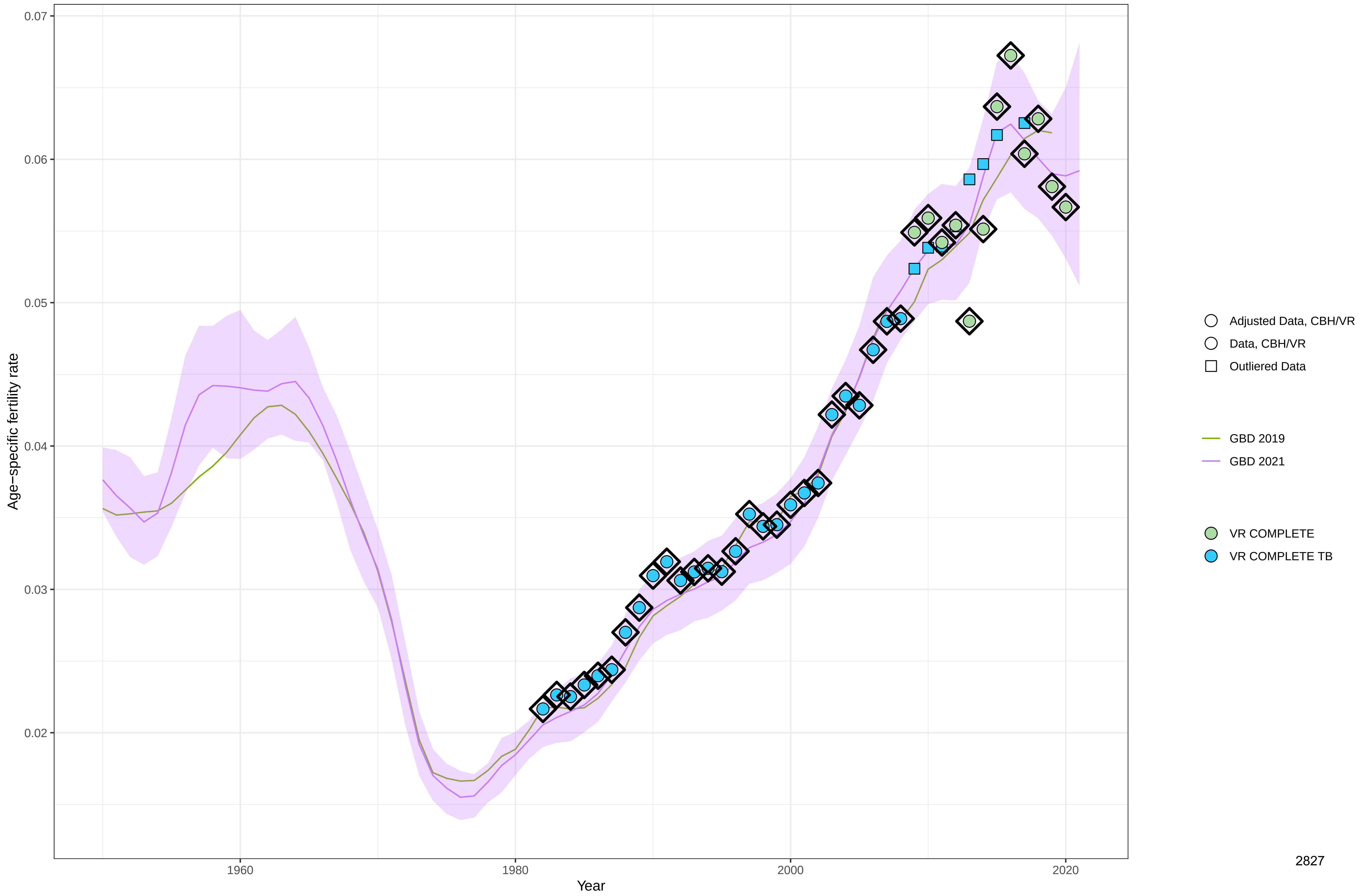

Age−specific fertility fitted trend lines, 1950−2021: Sefton, Ages: 40−44
Data points enclosed with a diamond are reference sources

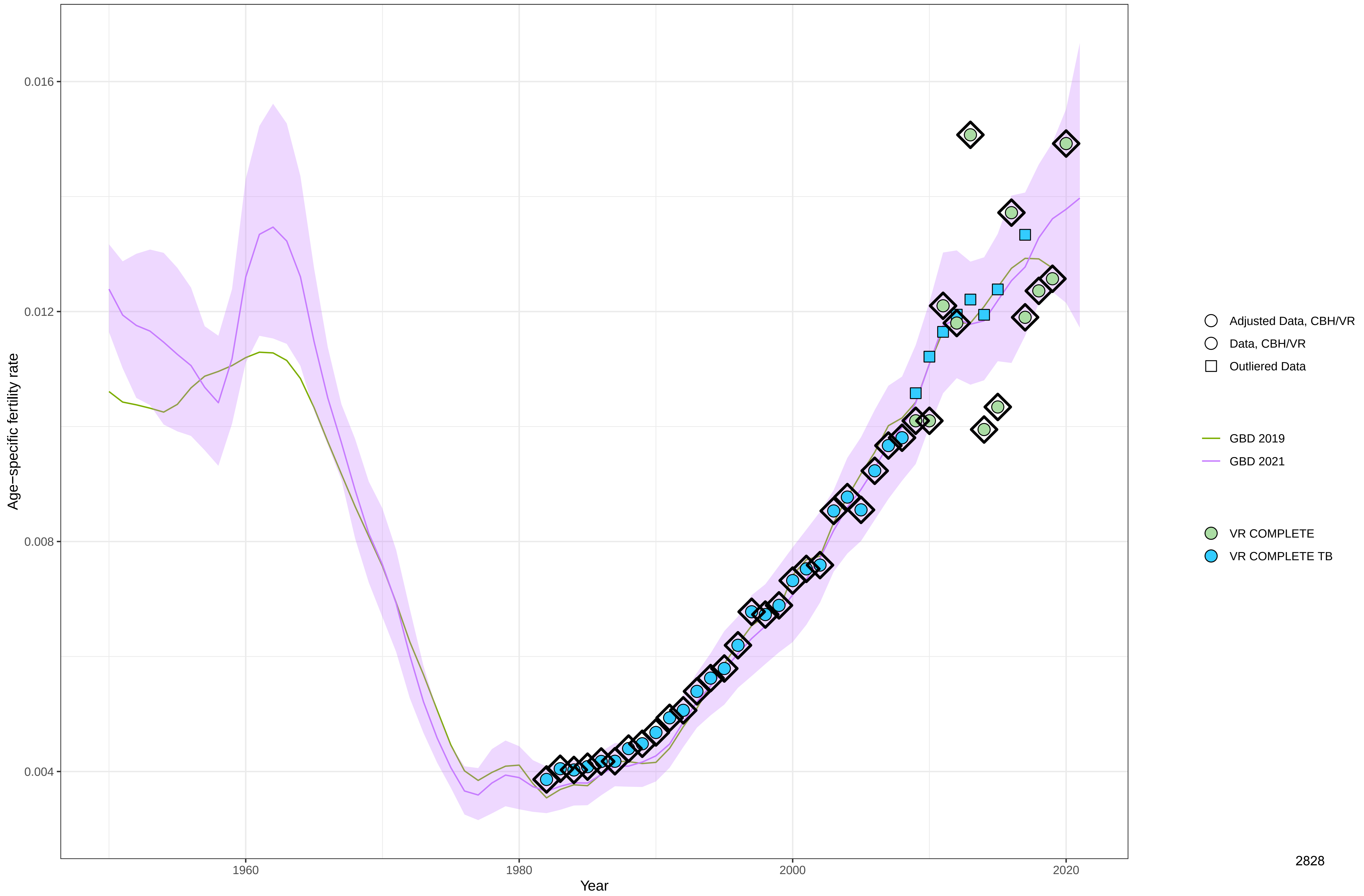

Age−specific fertility fitted trend lines, 1950−2021: Sefton, Ages: 45−49
Data points enclosed with a diamond are reference sources

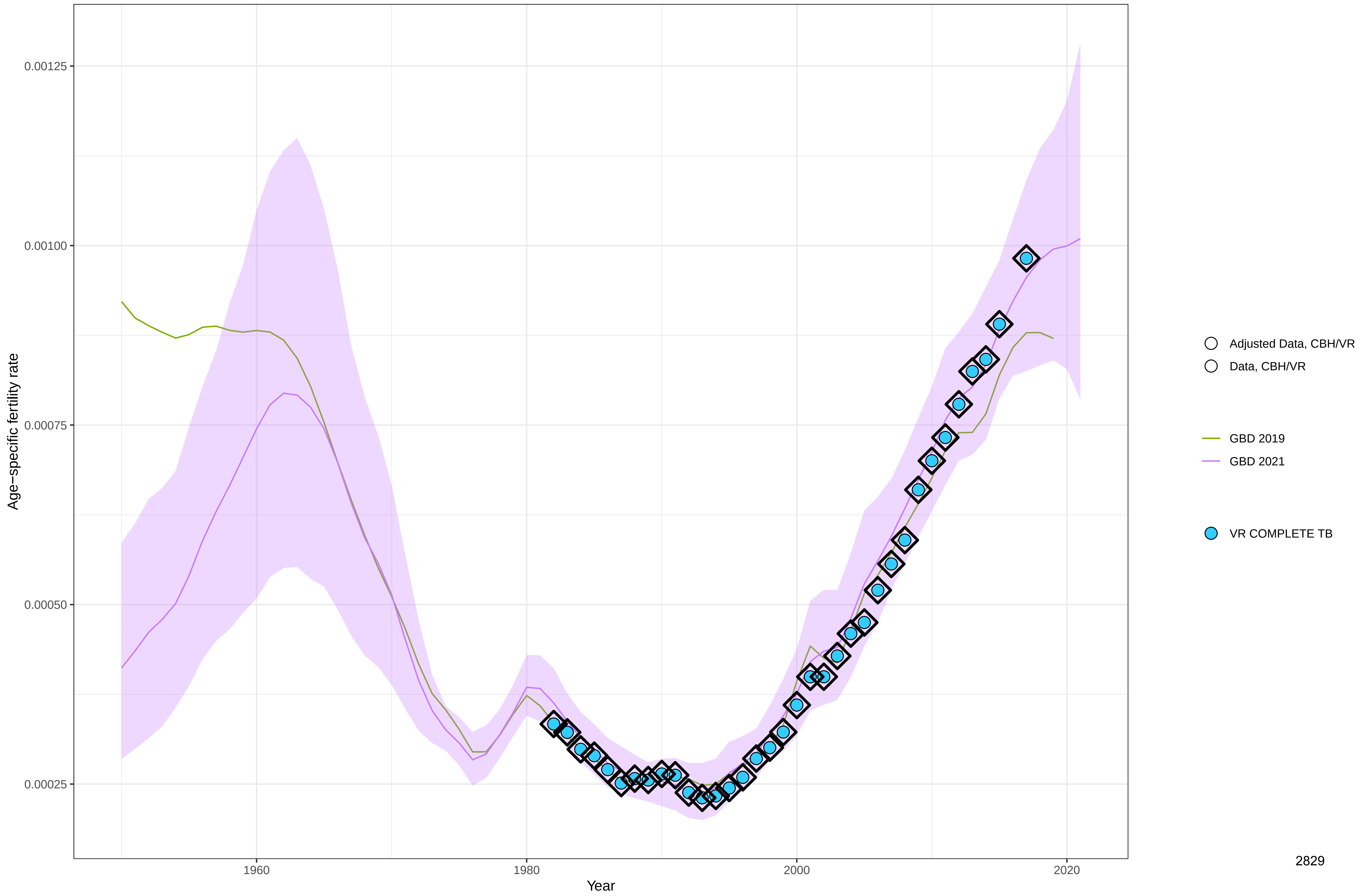

Age−specific fertility fitted trend lines, 1950−2021: Sefton, Ages: 50−54
Data points enclosed with a diamond are reference sources

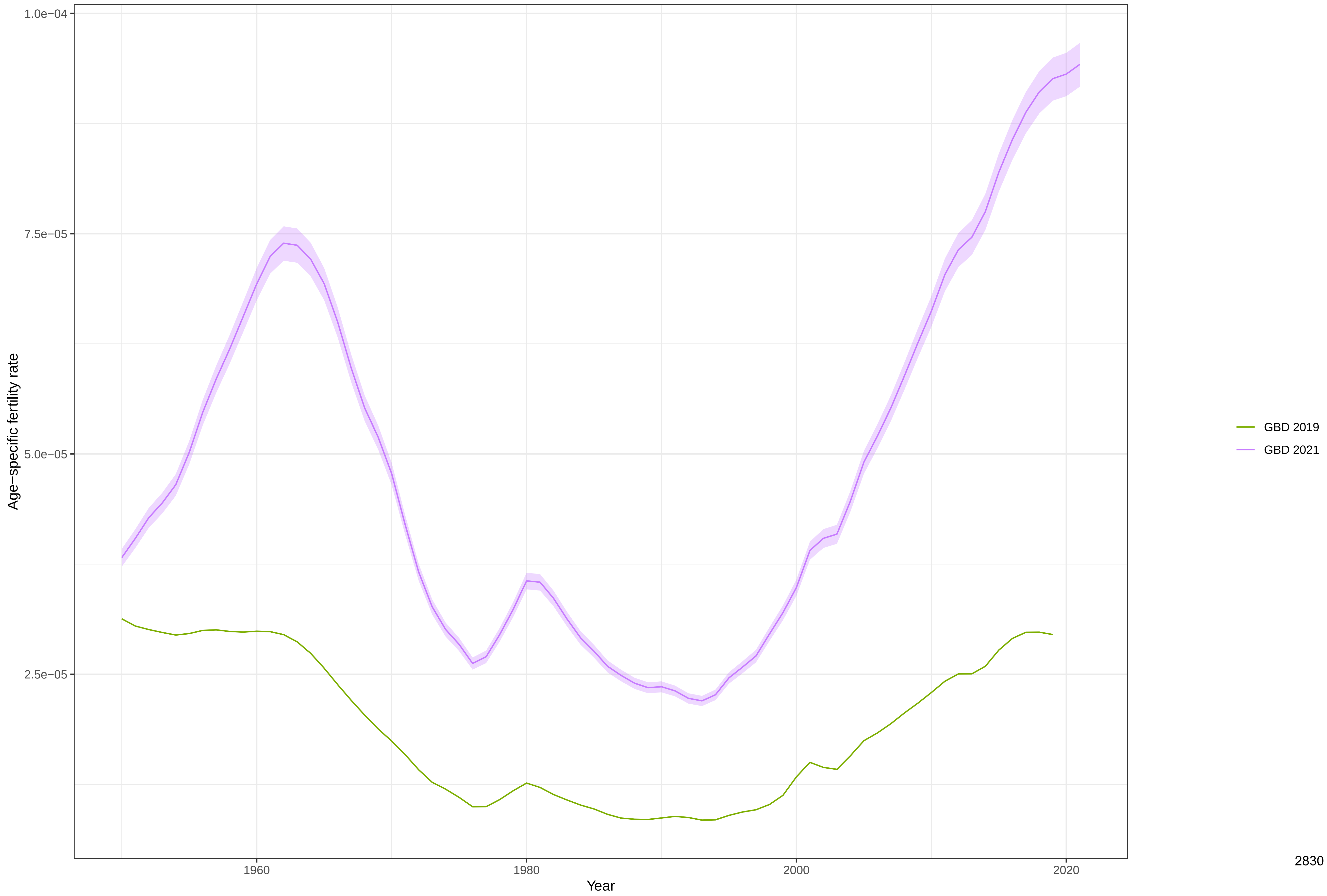

Total fertility rate fitted trend lines, 1950−2021: Sefton TFR
Data points enclosed with a diamond are reference sources

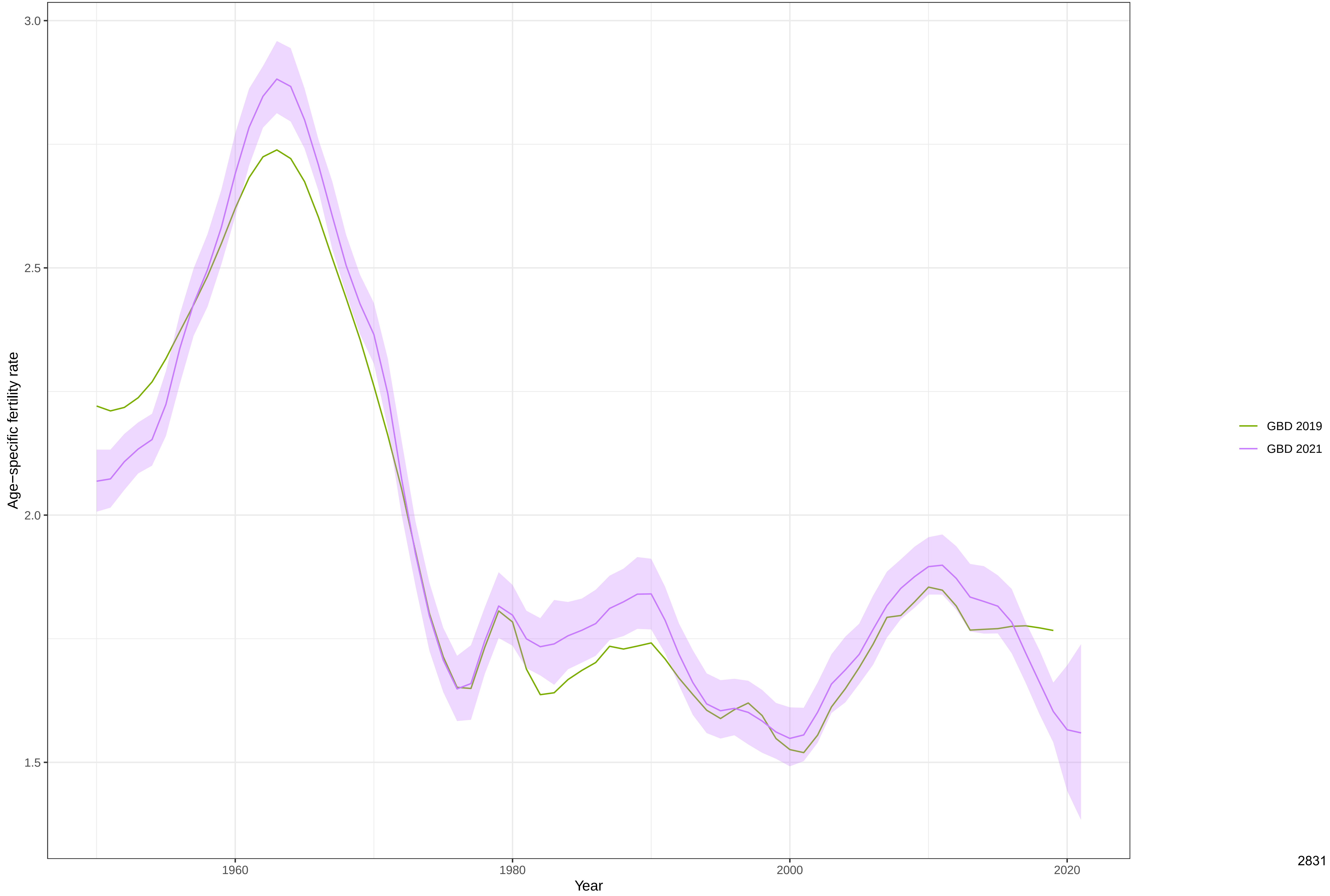

Age−specific fertility fitted trend lines, 1950−2021: St Helens, Ages: 10−14
Data points enclosed with a diamond are reference sources

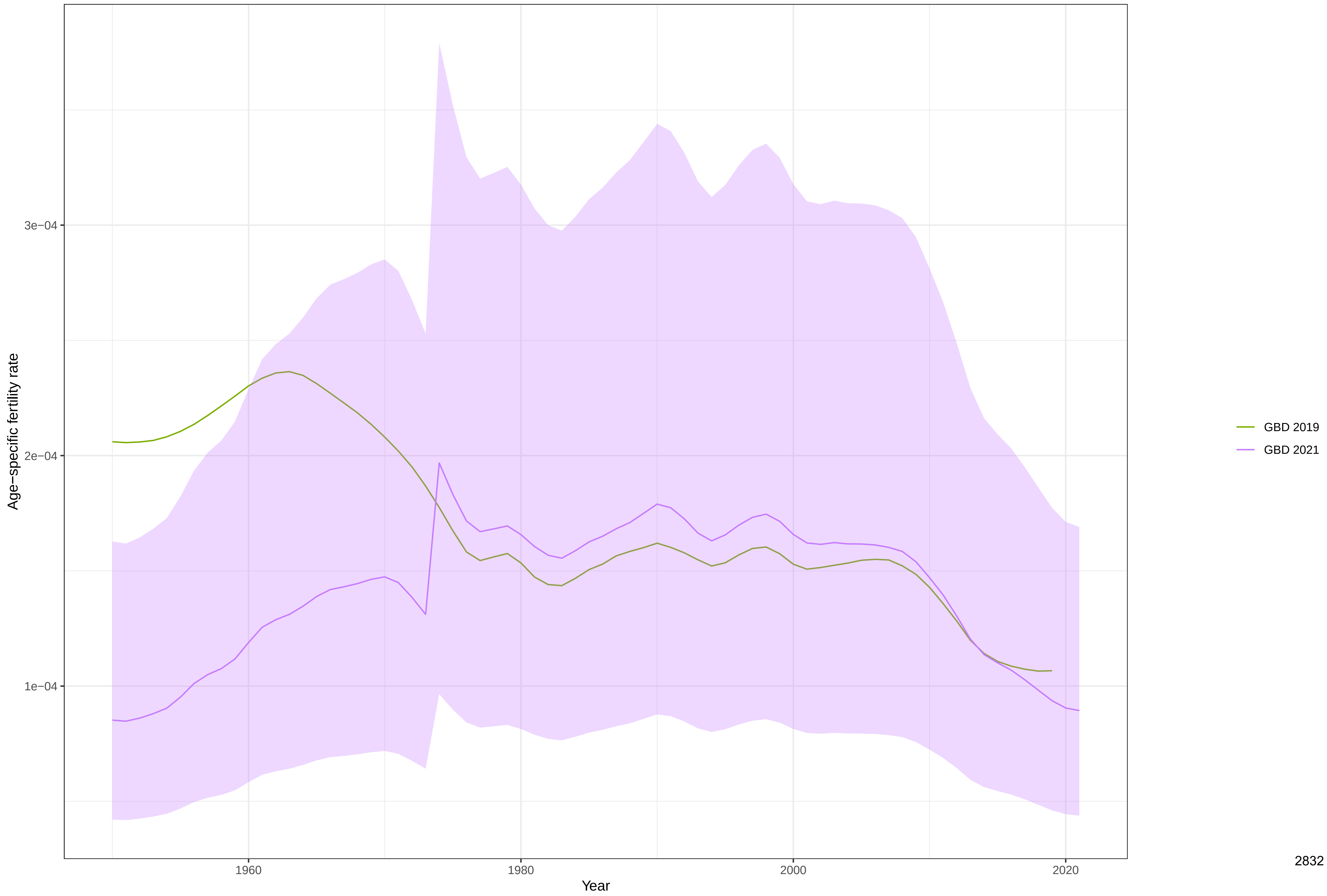

Age−specific fertility fitted trend lines, 1950−2021: St Helens, Ages: 15−19
Data points enclosed with a diamond are reference sources

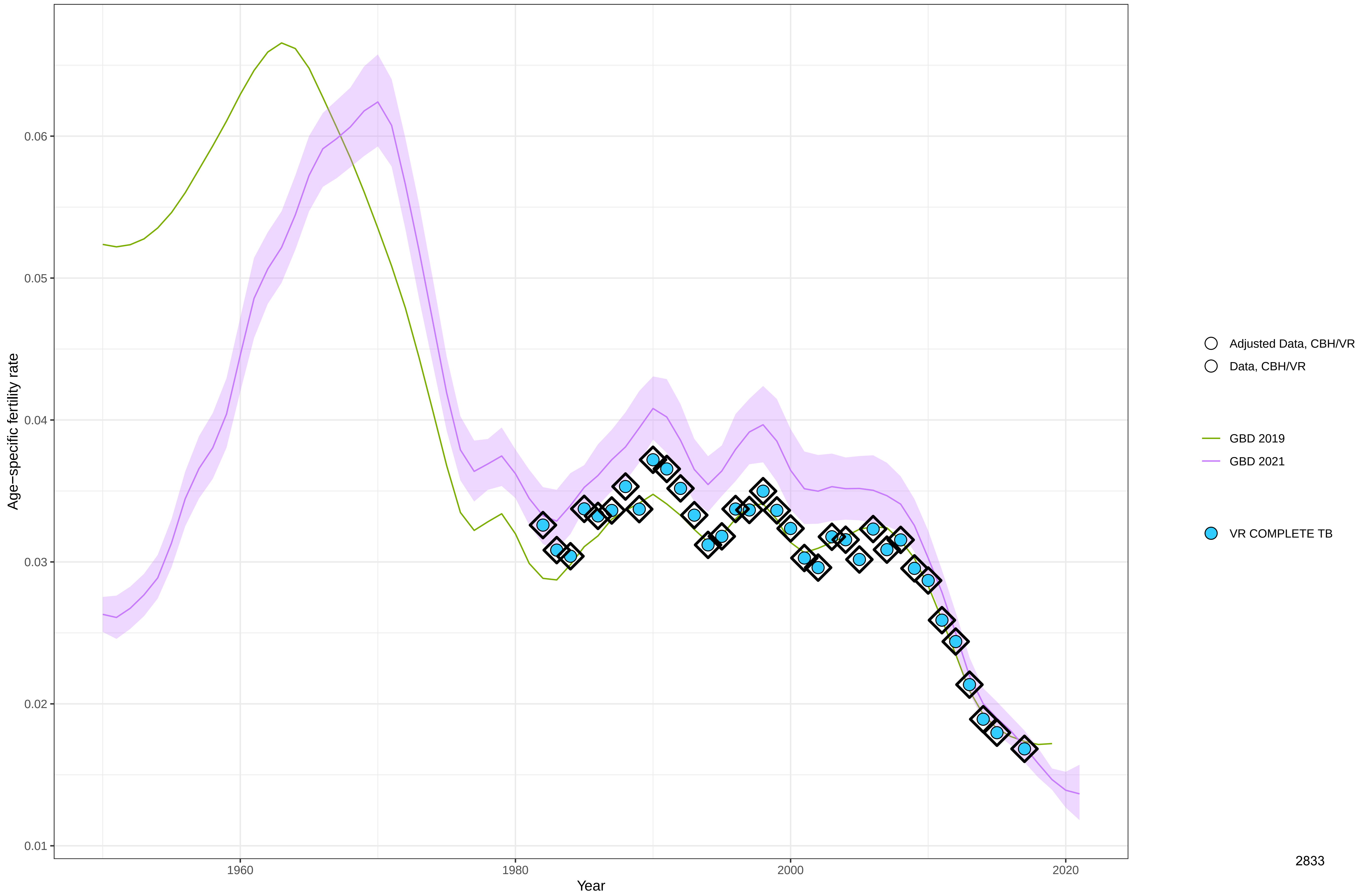

Age−specific fertility fitted trend lines, 1950−2021: St Helens, Ages: 20−24
Data points enclosed with a diamond are reference sources

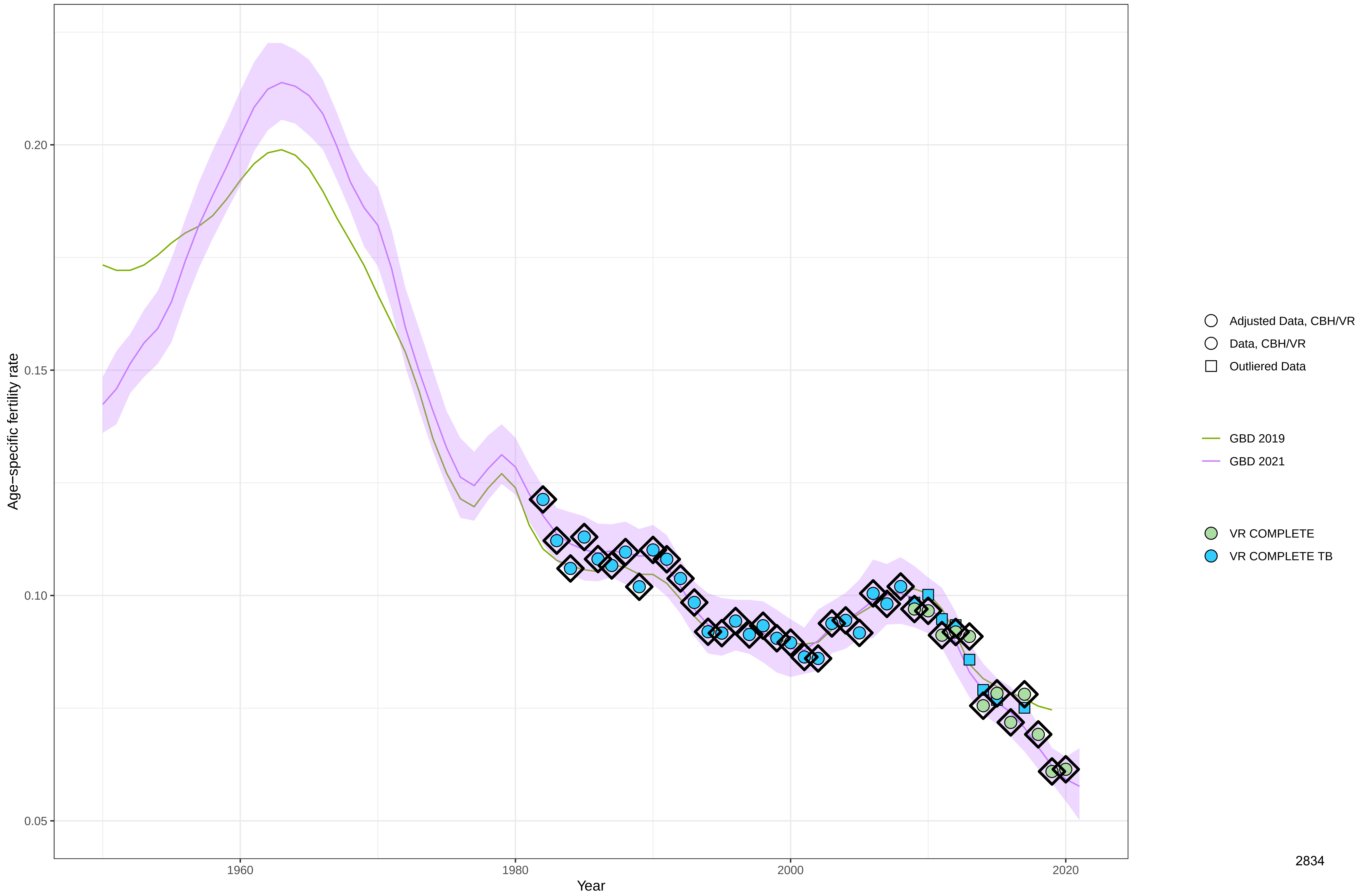

Age−specific fertility fitted trend lines, 1950−2021: St Helens, Ages: 25−29
Data points enclosed with a diamond are reference sources

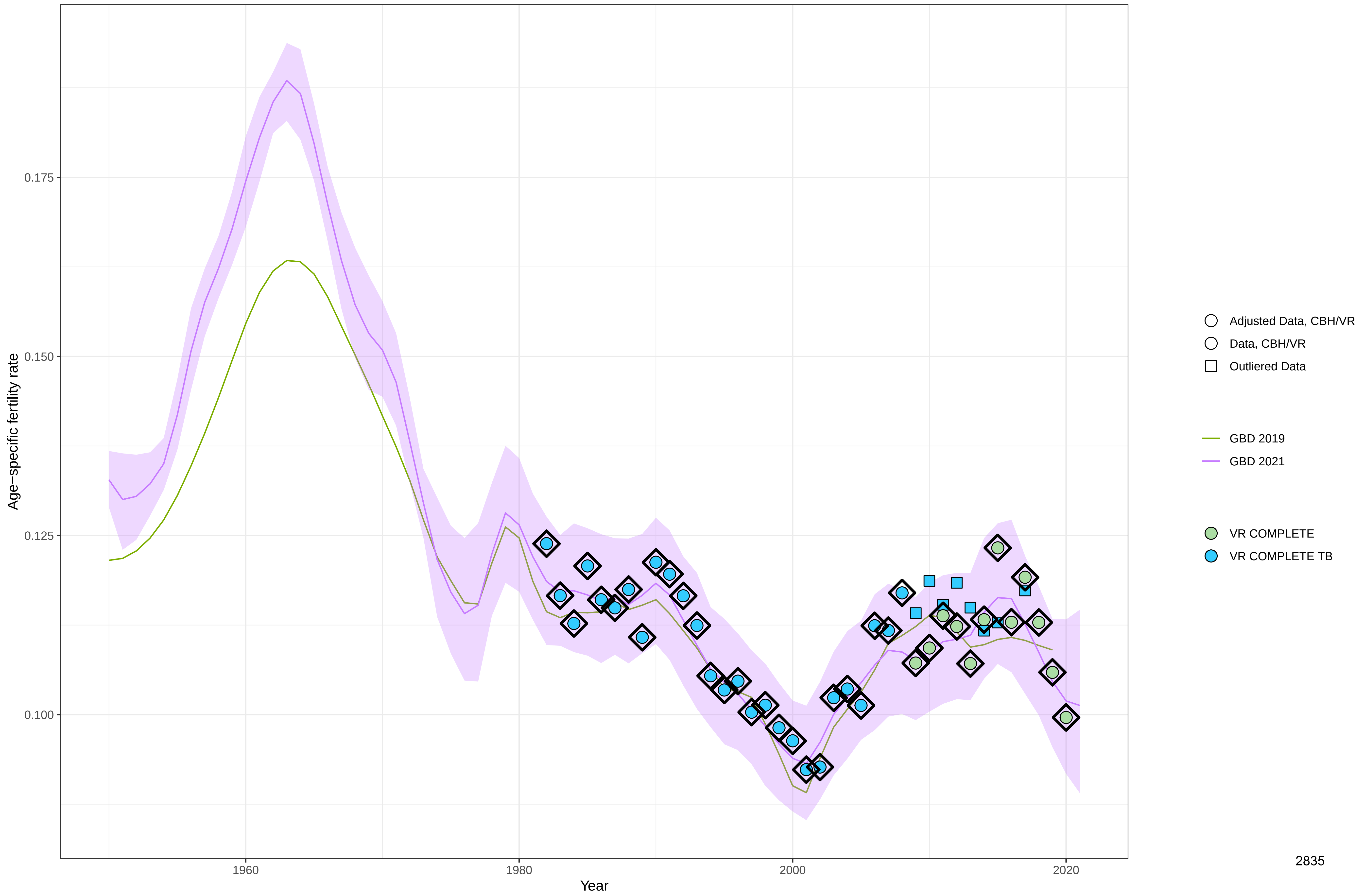

Age−specific fertility fitted trend lines, 1950−2021: St Helens, Ages: 30−34
Data points enclosed with a diamond are reference sources

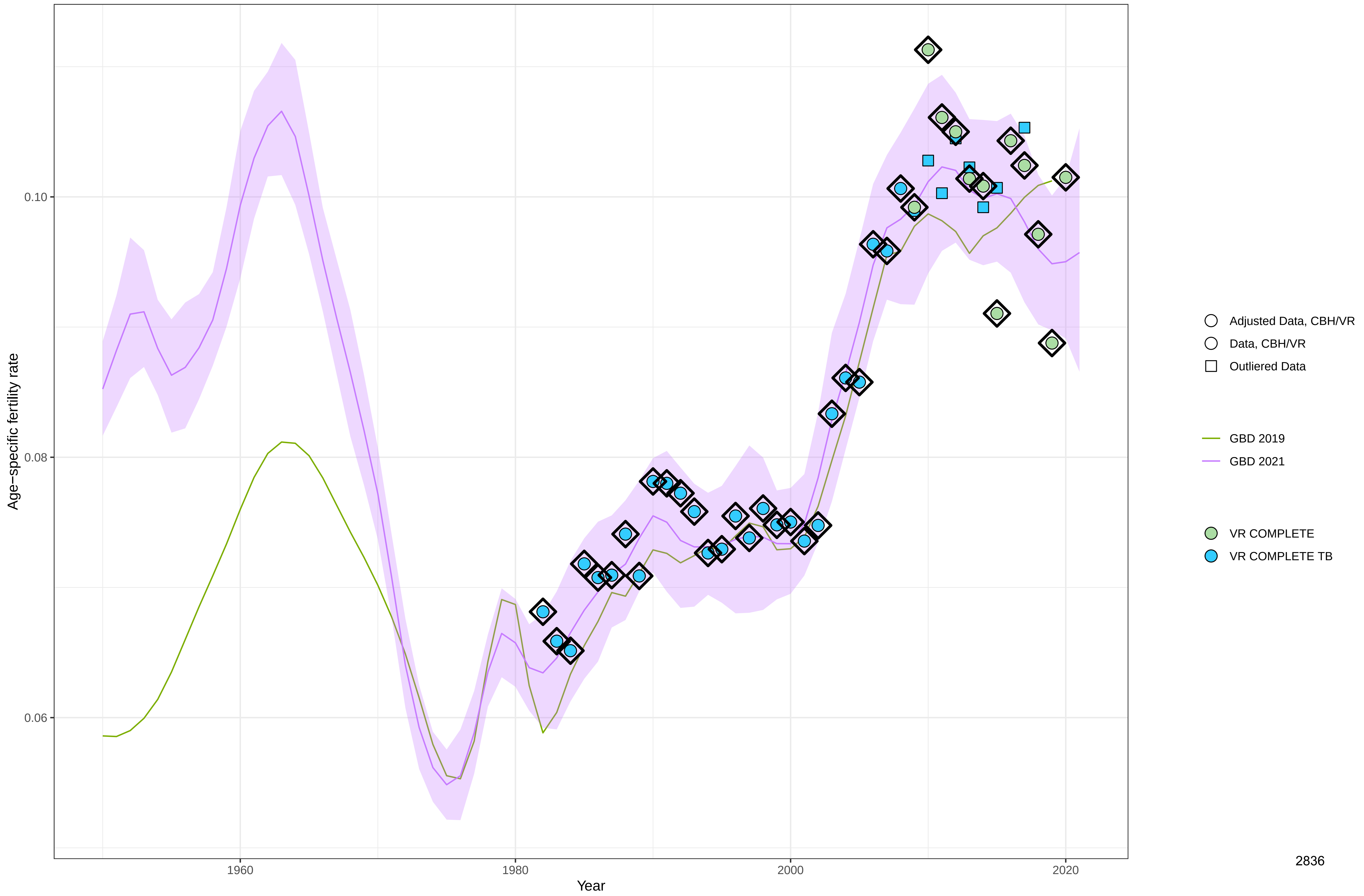

Age−specific fertility fitted trend lines, 1950−2021: St Helens, Ages: 35−39
Data points enclosed with a diamond are reference sources

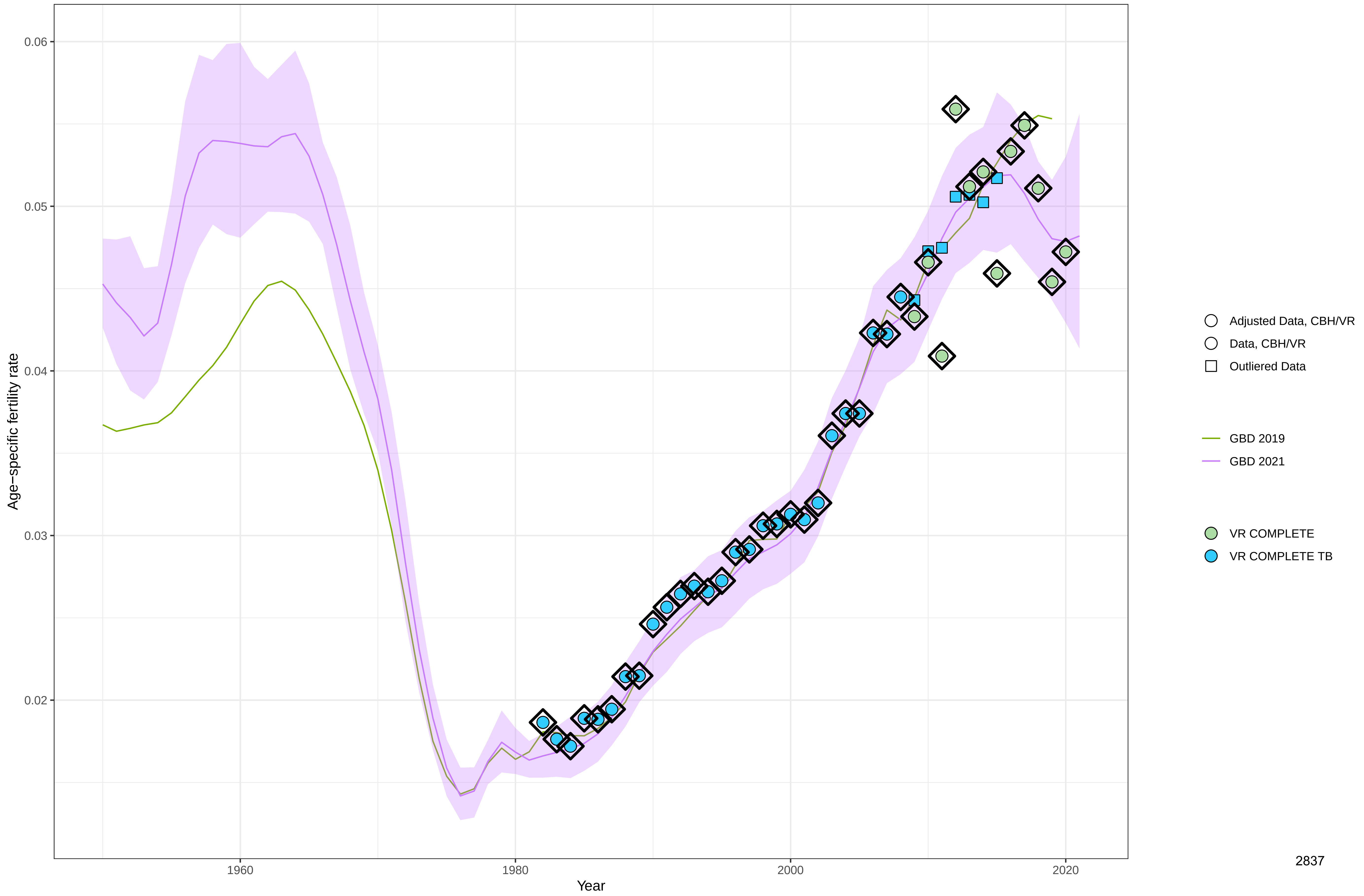

Age−specific fertility fitted trend lines, 1950−2021: St Helens, Ages: 40−44
Data points enclosed with a diamond are reference sources

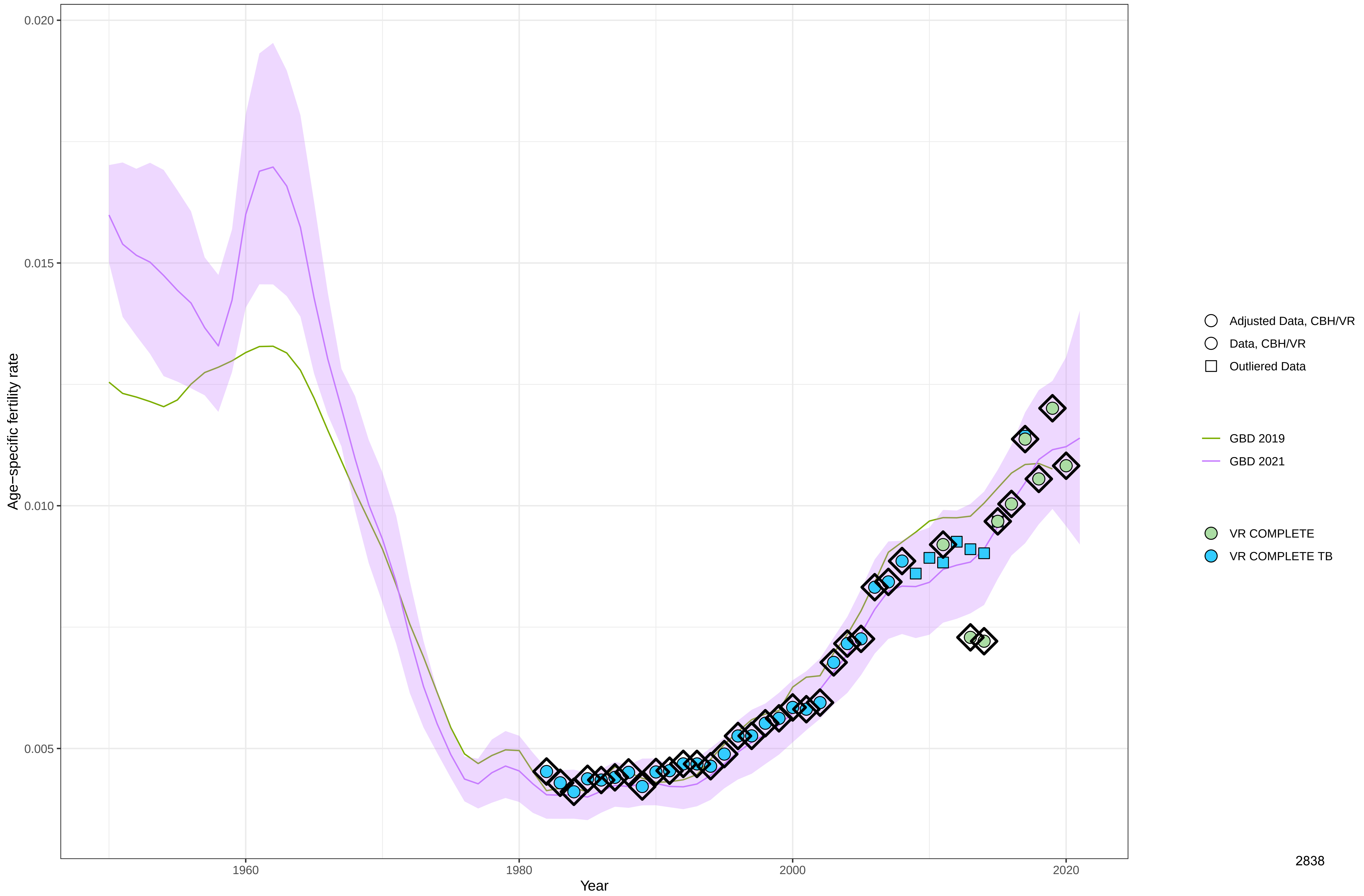

Age−specific fertility fitted trend lines, 1950−2021: St Helens, Ages: 45−49
Data points enclosed with a diamond are reference sources

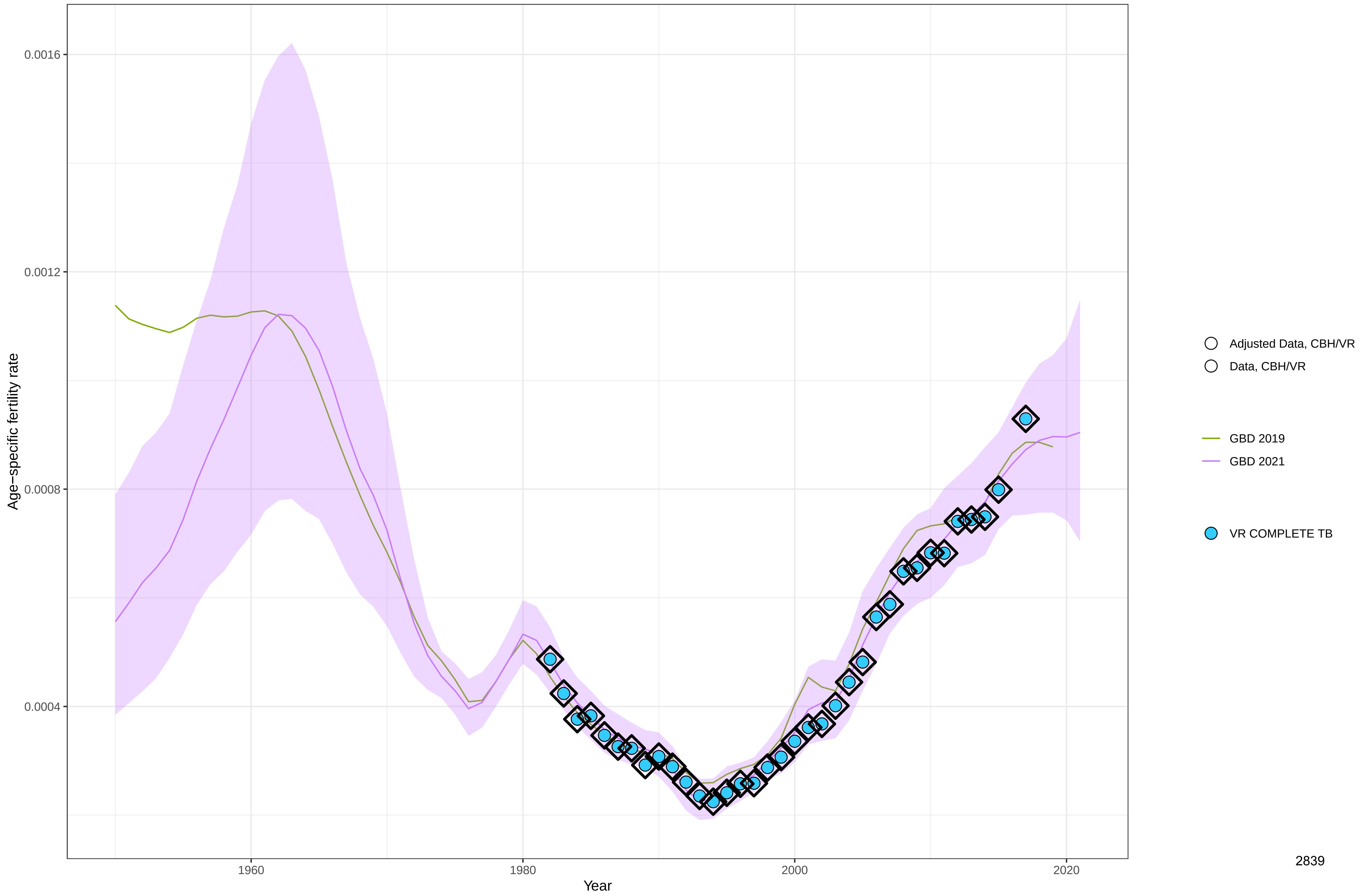

Age−specific fertility fitted trend lines, 1950−2021: St Helens, Ages: 50−54
Data points enclosed with a diamond are reference sources

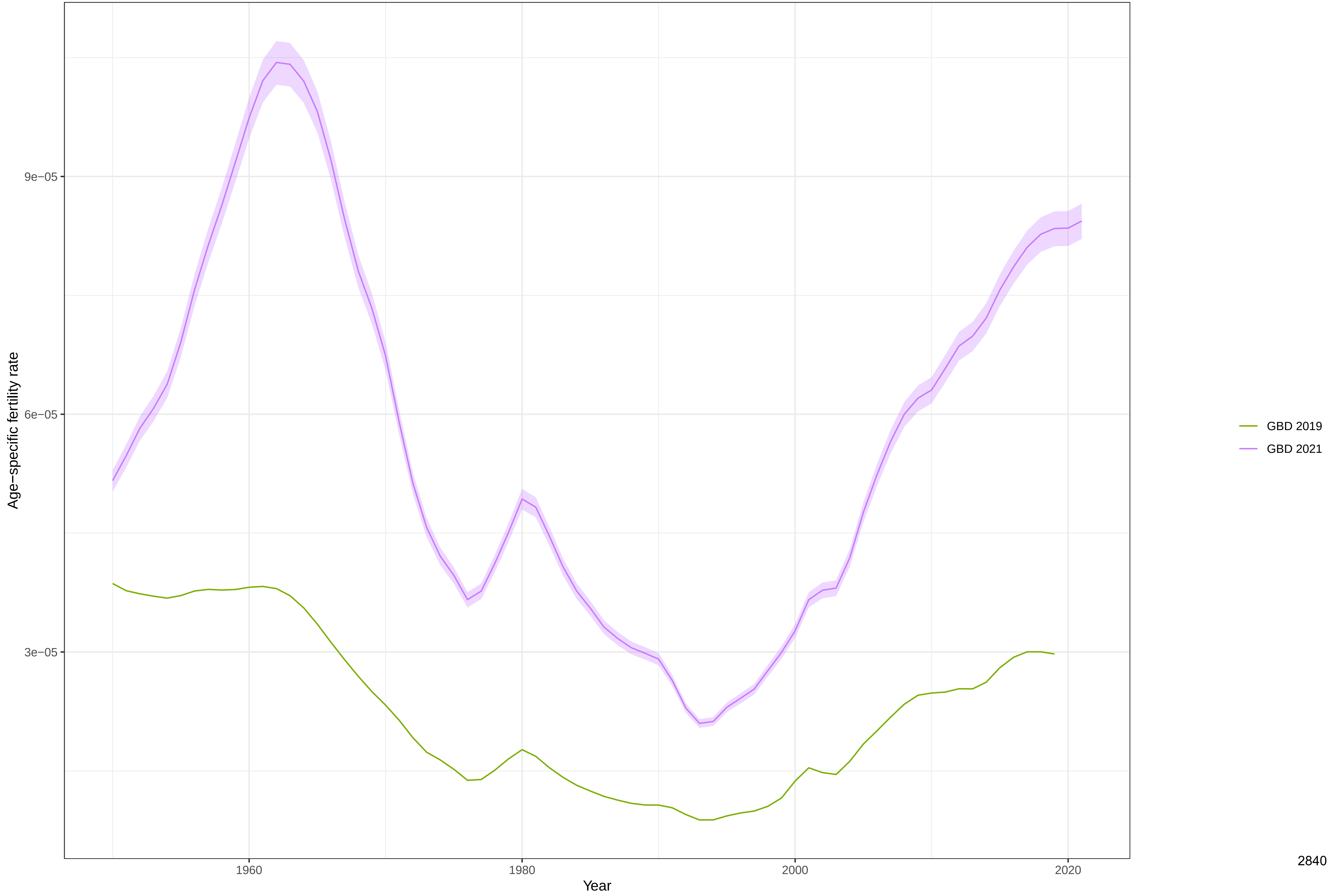

Total fertility rate fitted trend lines, 1950−2021: St Helens TFR
Data points enclosed with a diamond are reference sources

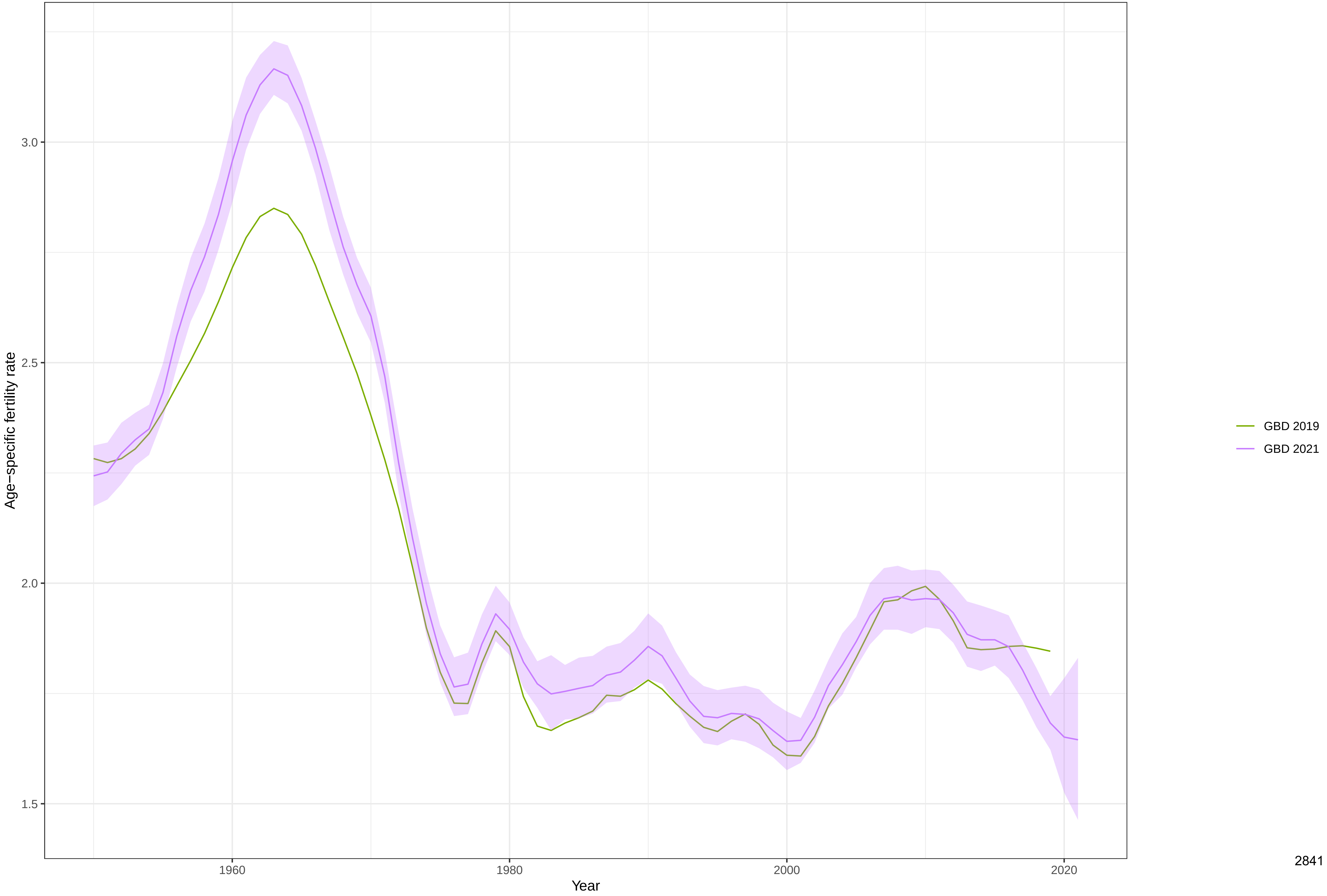

Age−specific fertility fitted trend lines, 1950−2021: Stockport, Ages: 10−14
Data points enclosed with a diamond are reference sources

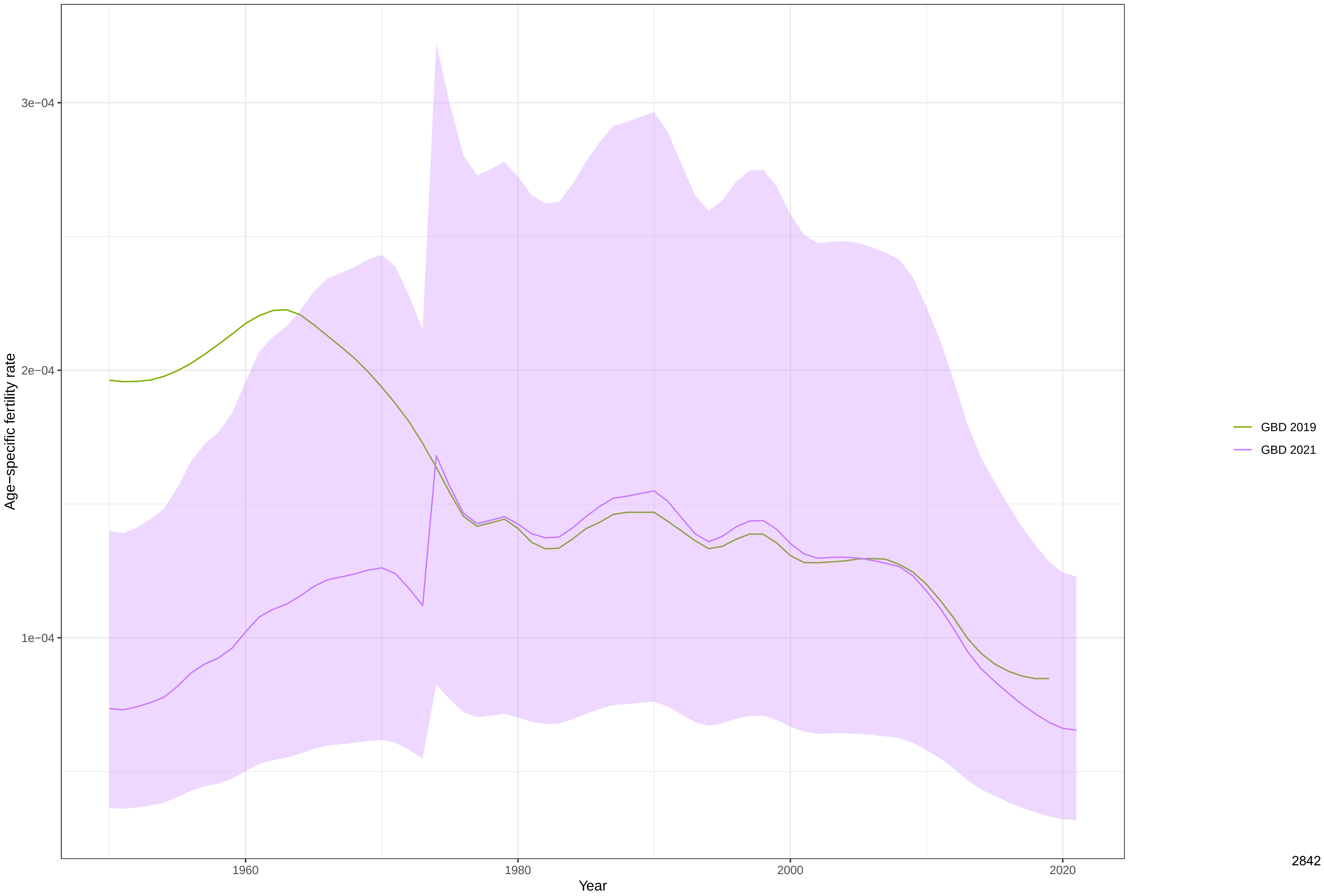

Age−specific fertility fitted trend lines, 1950−2021: Stockport, Ages: 15−19
Data points enclosed with a diamond are reference sources

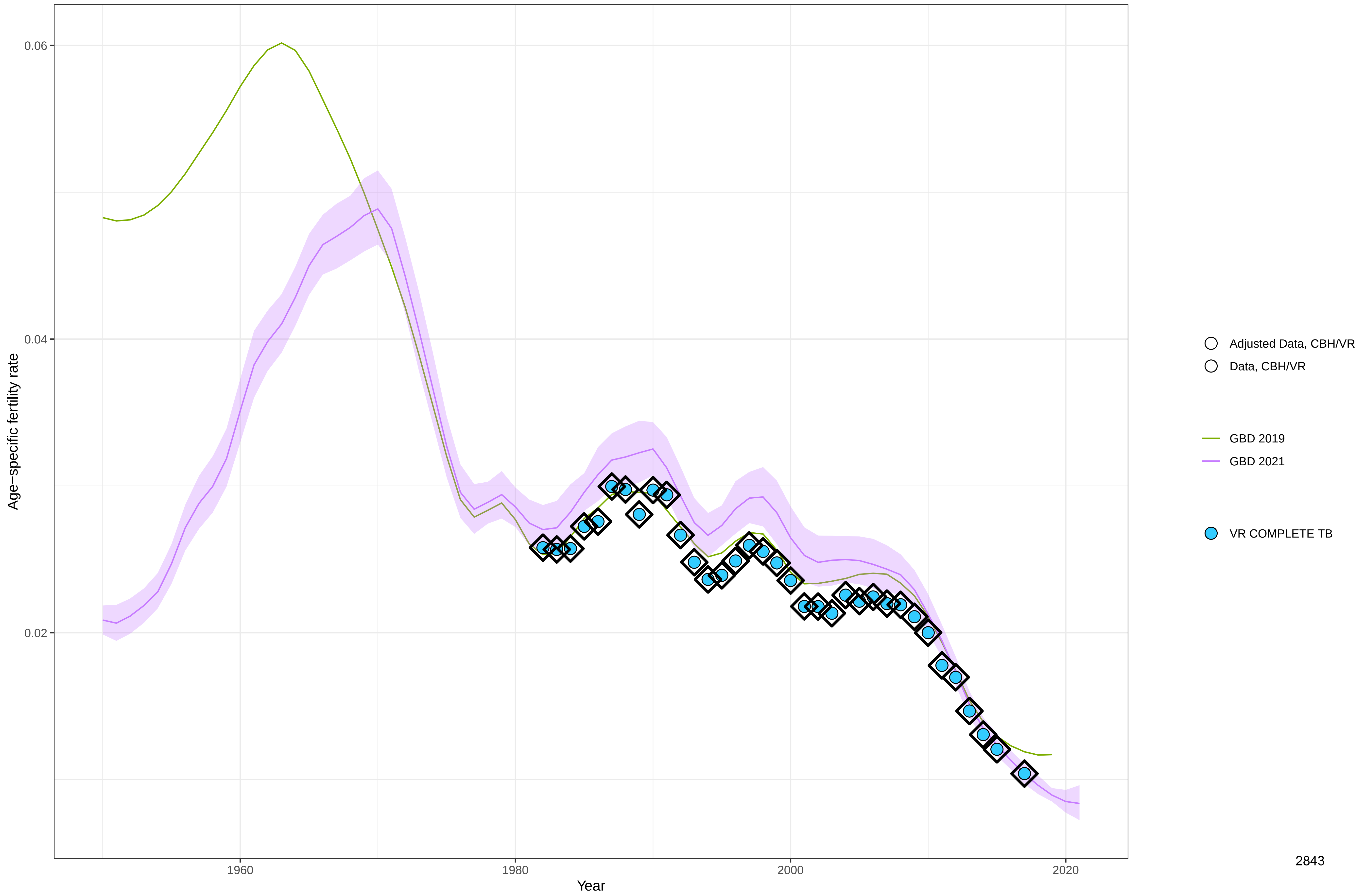

Age−specific fertility fitted trend lines, 1950−2021: Stockport, Ages: 20−24
Data points enclosed with a diamond are reference sources

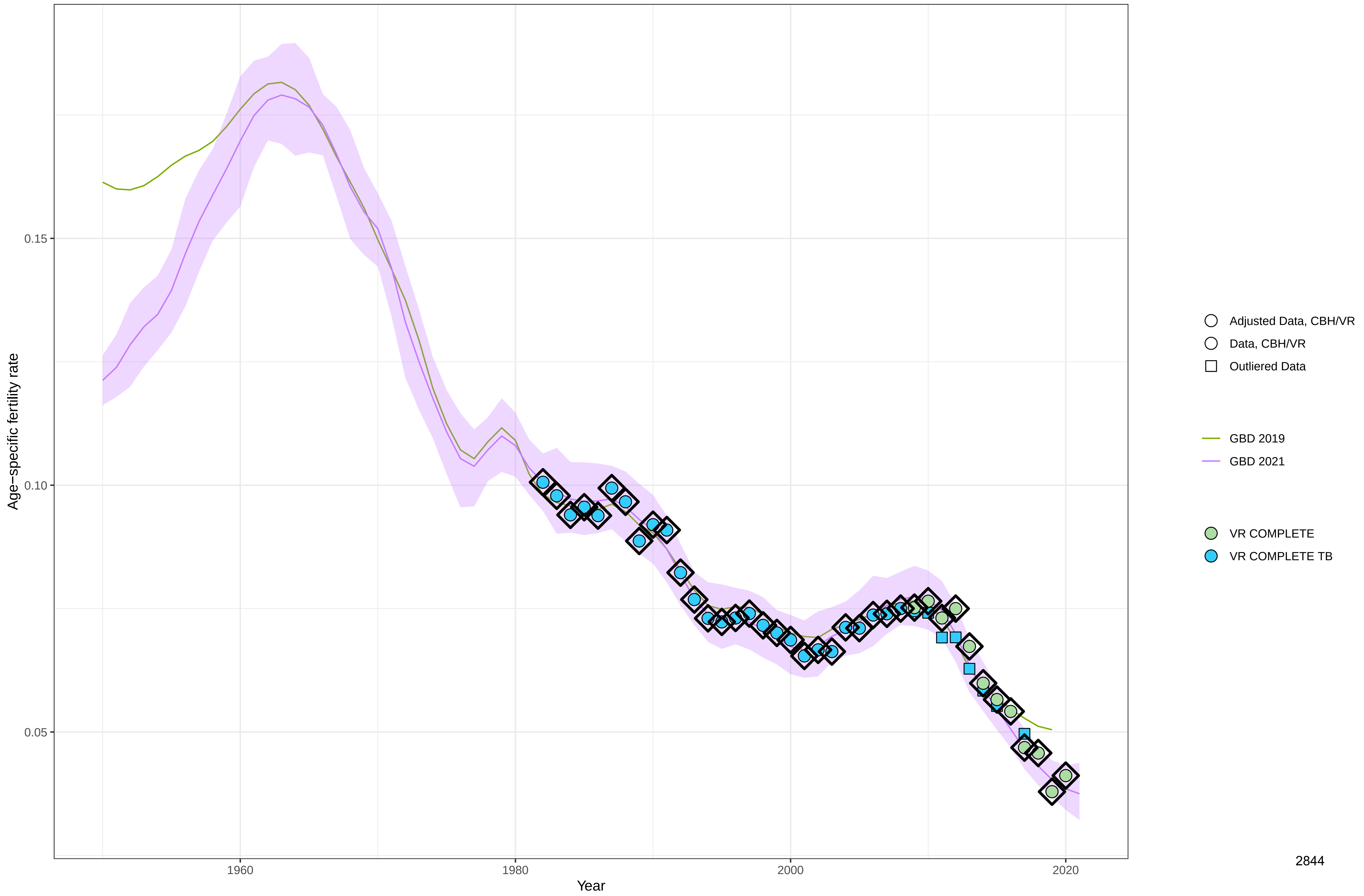

2844

Age−specific fertility fitted trend lines, 1950−2021: Stockport, Ages: 25−29
Data points enclosed with a diamond are reference sources

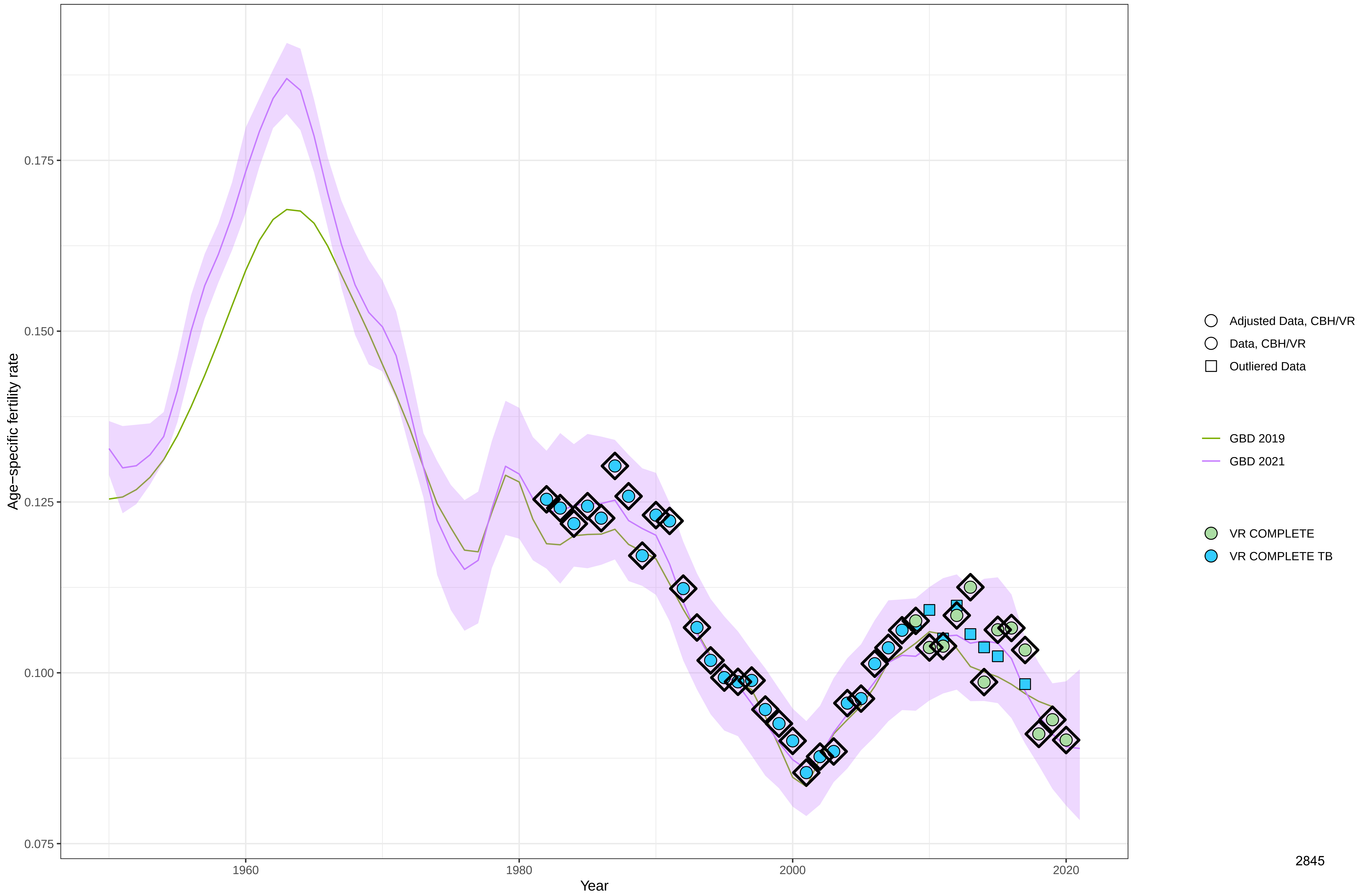

2845

Age−specific fertility fitted trend lines, 1950−2021: Stockport, Ages: 30−34
Data points enclosed with a diamond are reference sources

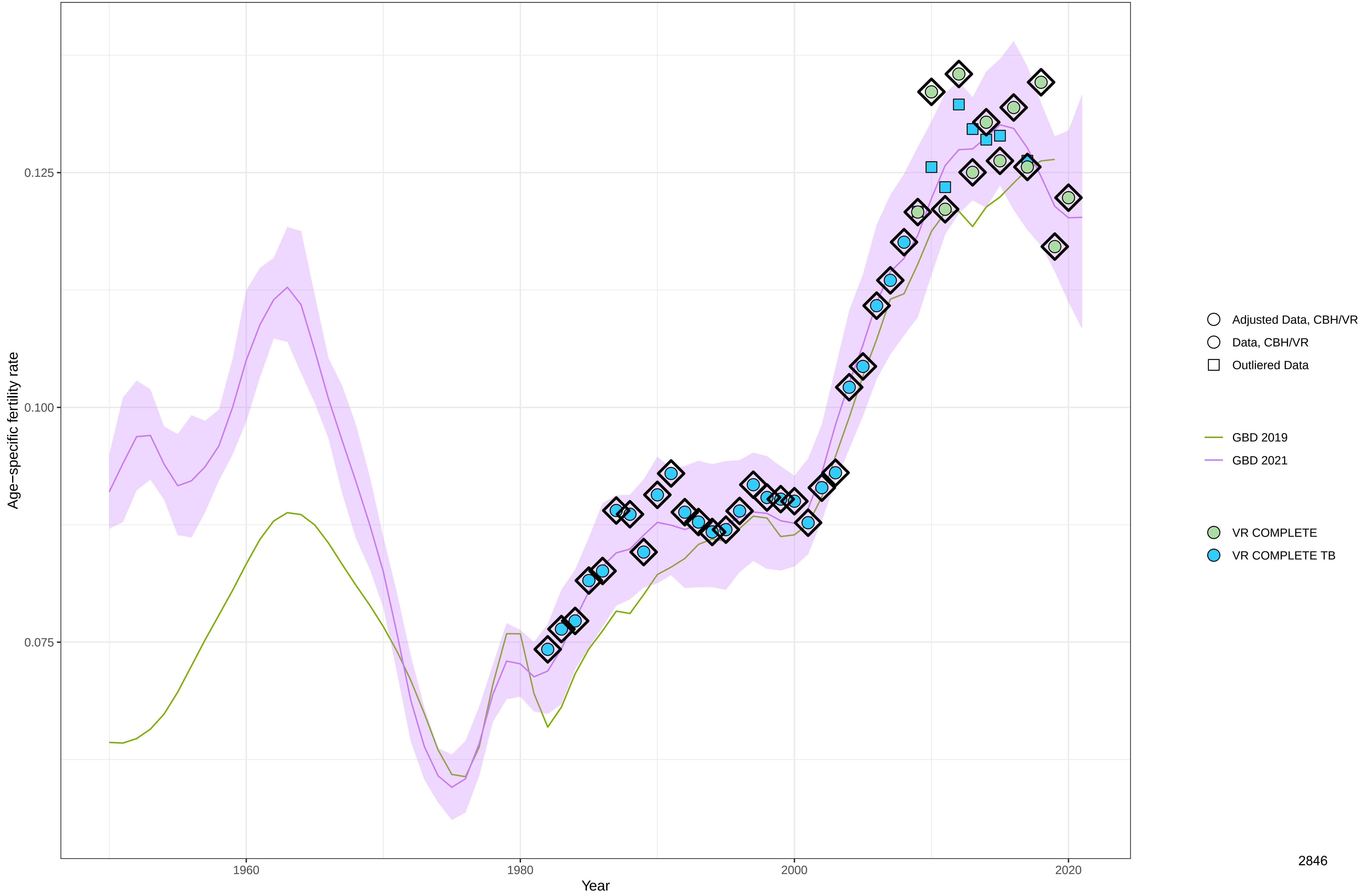

Age−specific fertility fitted trend lines, 1950−2021: Stockport, Ages: 35−39
Data points enclosed with a diamond are reference sources

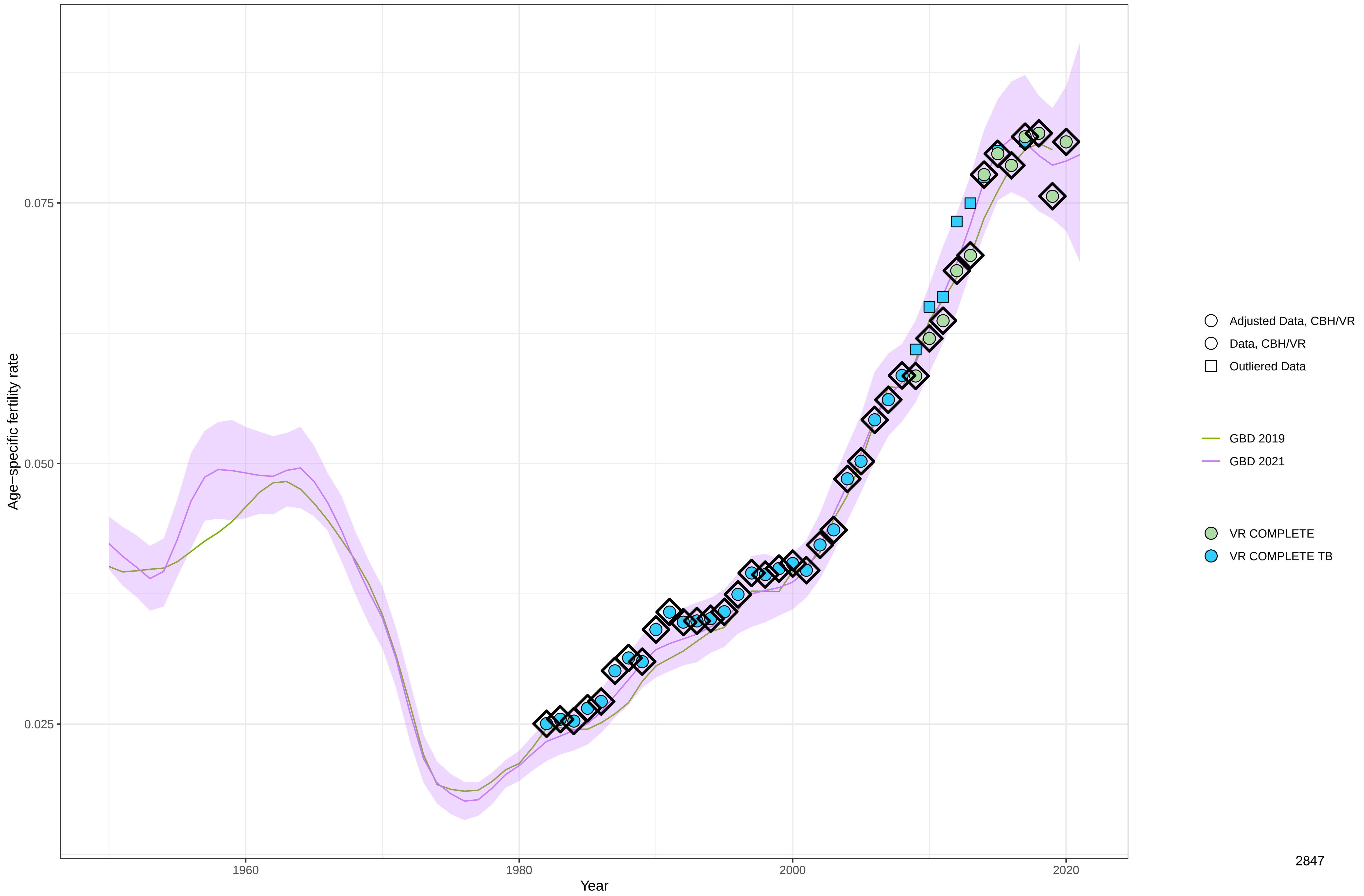

Age−specific fertility fitted trend lines, 1950−2021: Stockport, Ages: 40−44
Data points enclosed with a diamond are reference sources

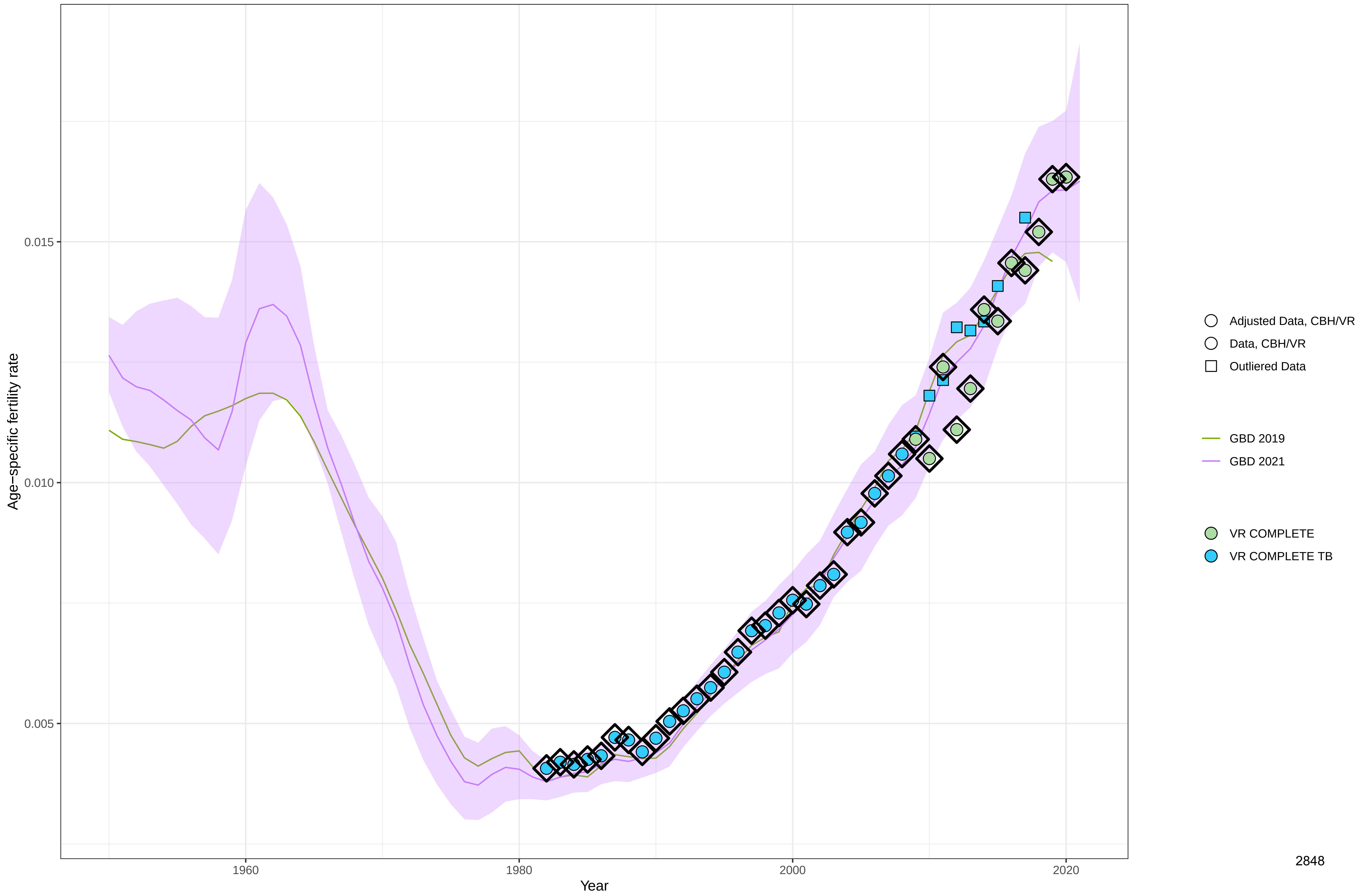

Age−specific fertility fitted trend lines, 1950−2021: Stockport, Ages: 45−49
Data points enclosed with a diamond are reference sources

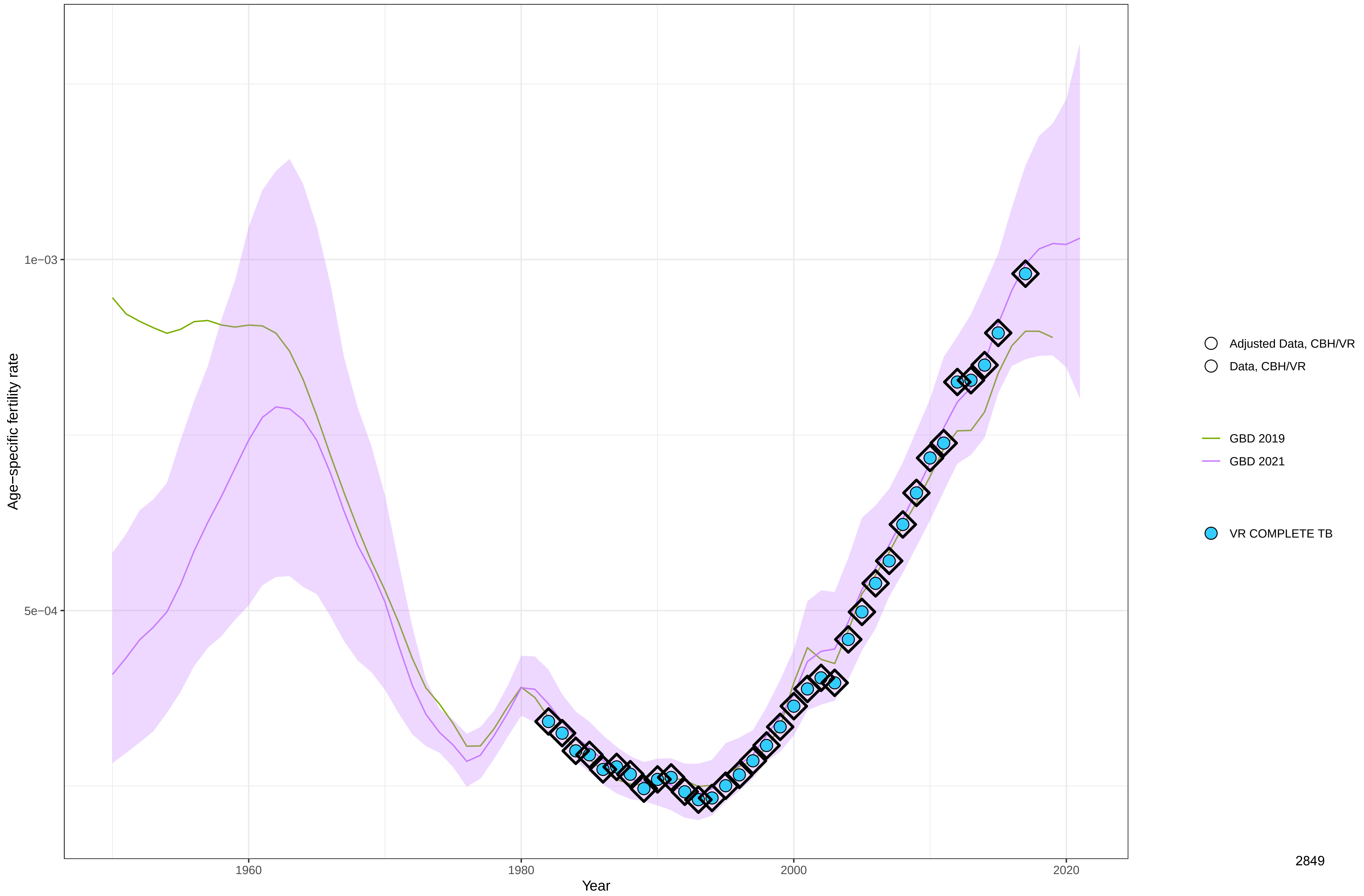

Age−specific fertility fitted trend lines, 1950−2021: Stockport, Ages: 50−54
Data points enclosed with a diamond are reference sources

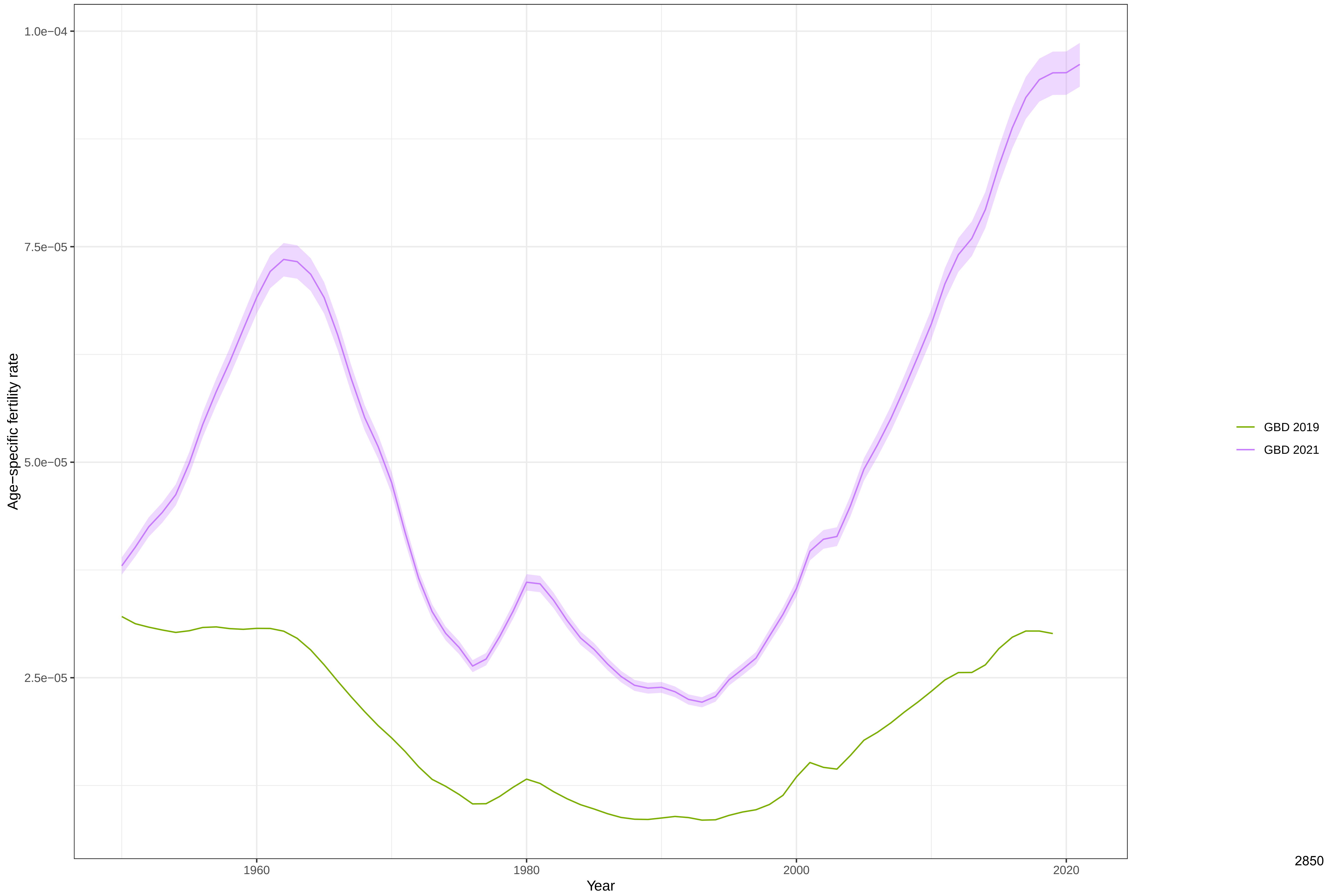

Total fertility rate fitted trend lines, 1950−2021: Stockport TFR
Data points enclosed with a diamond are reference sources

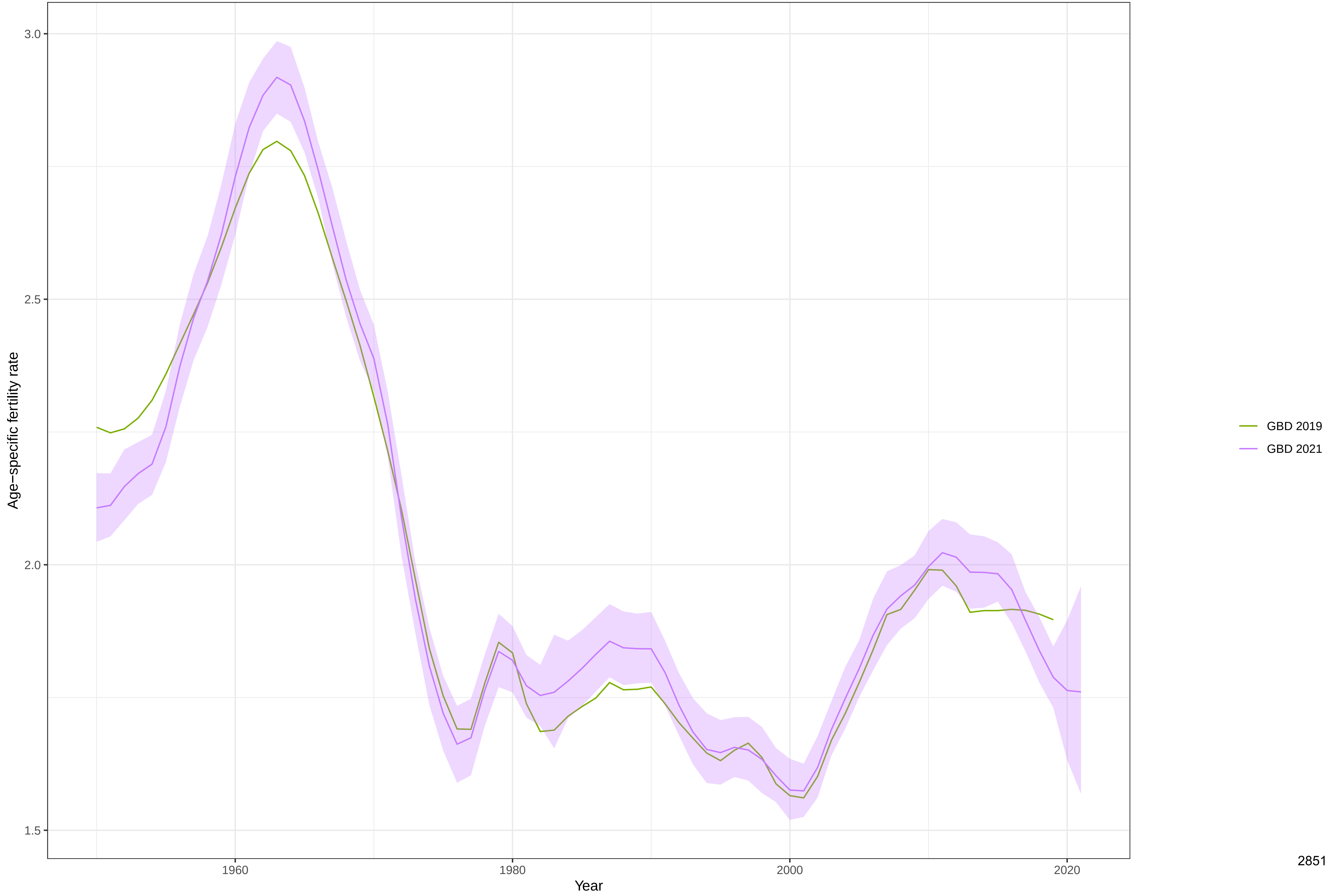

Age−specific fertility fitted trend lines, 1950−2021: Tameside, Ages: 10−14
Data points enclosed with a diamond are reference sources

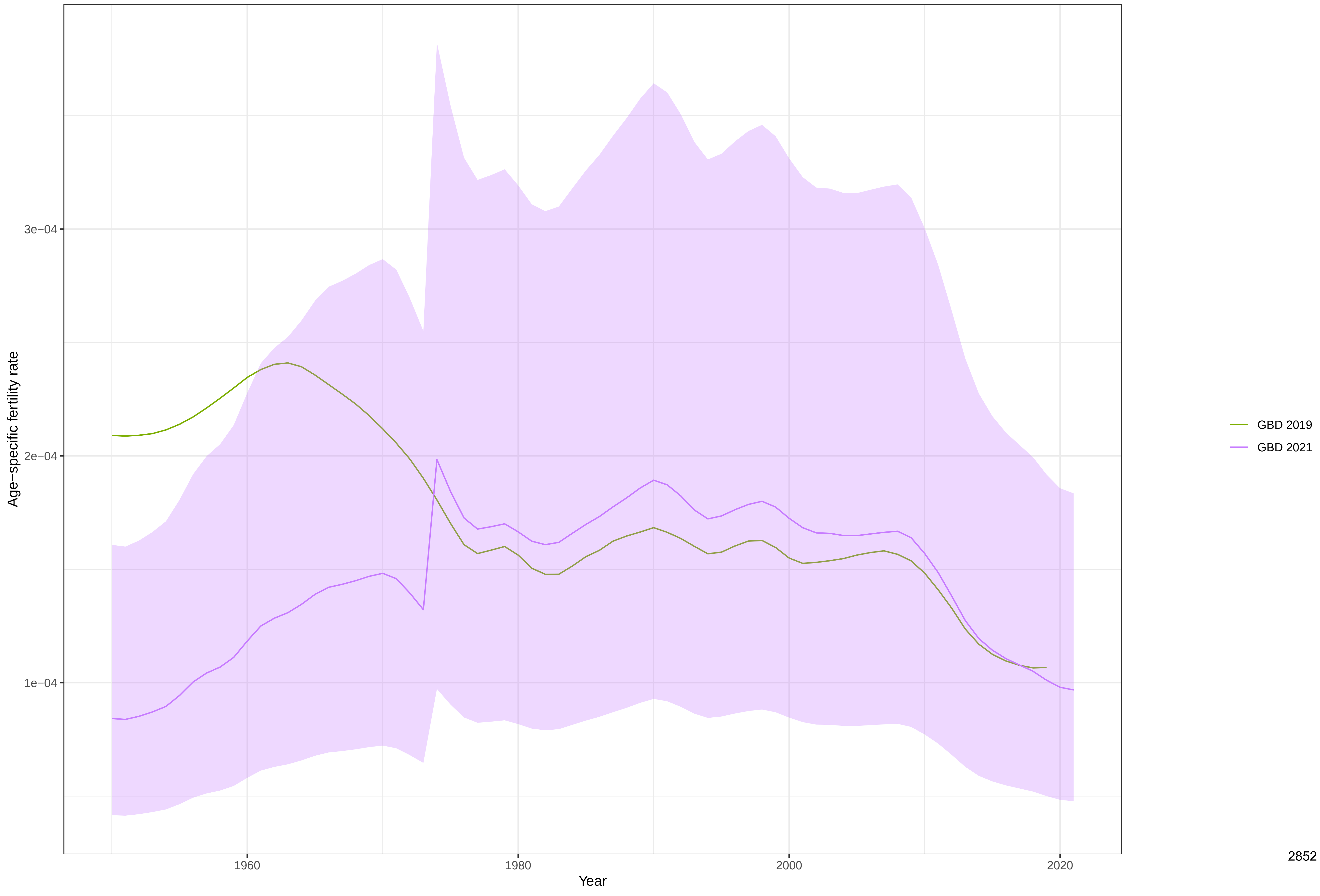

Age−specific fertility fitted trend lines, 1950−2021: Tameside, Ages: 15−19
Data points enclosed with a diamond are reference sources

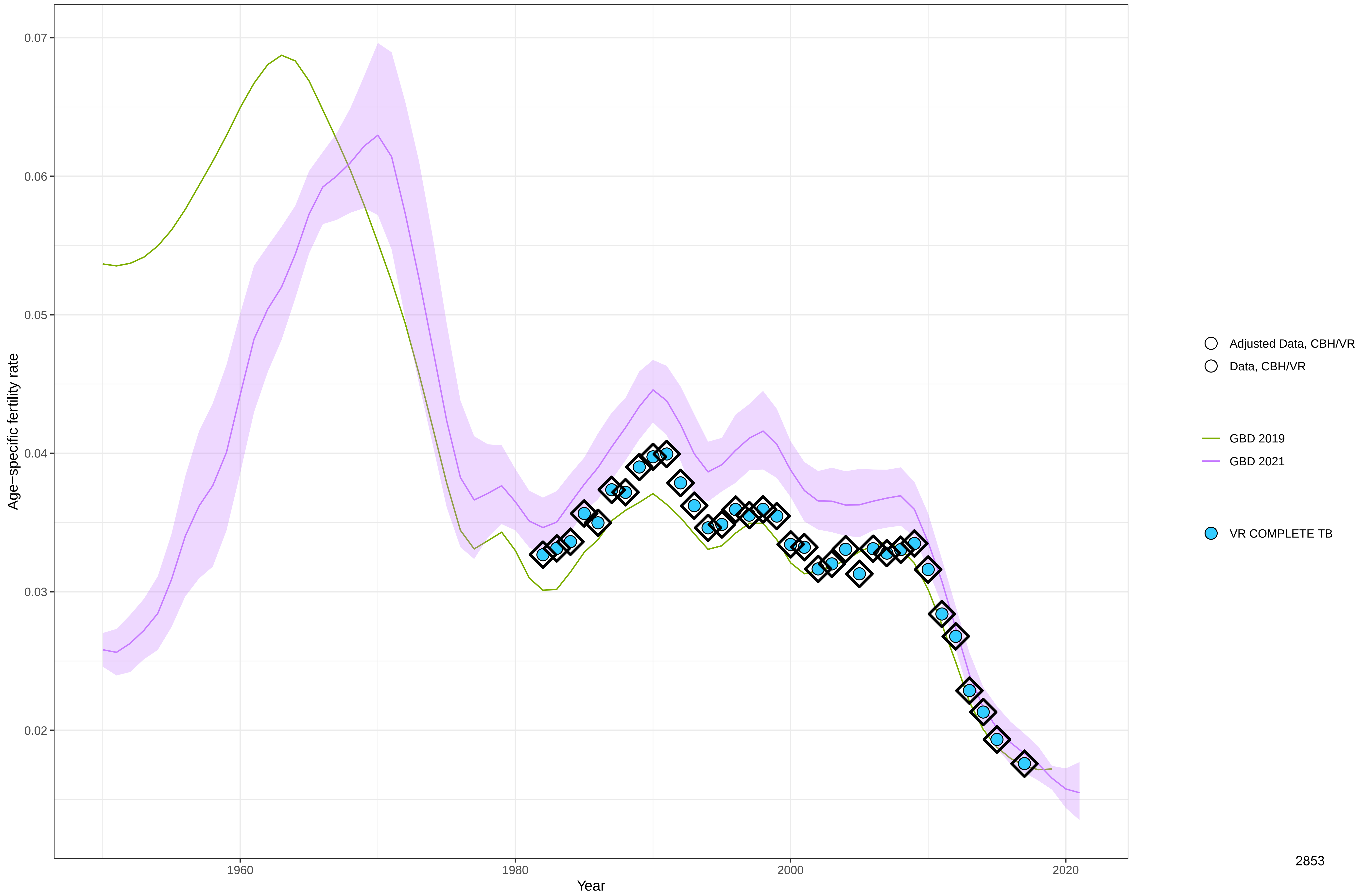

Age−specific fertility fitted trend lines, 1950−2021: Tameside, Ages: 20−24
Data points enclosed with a diamond are reference sources

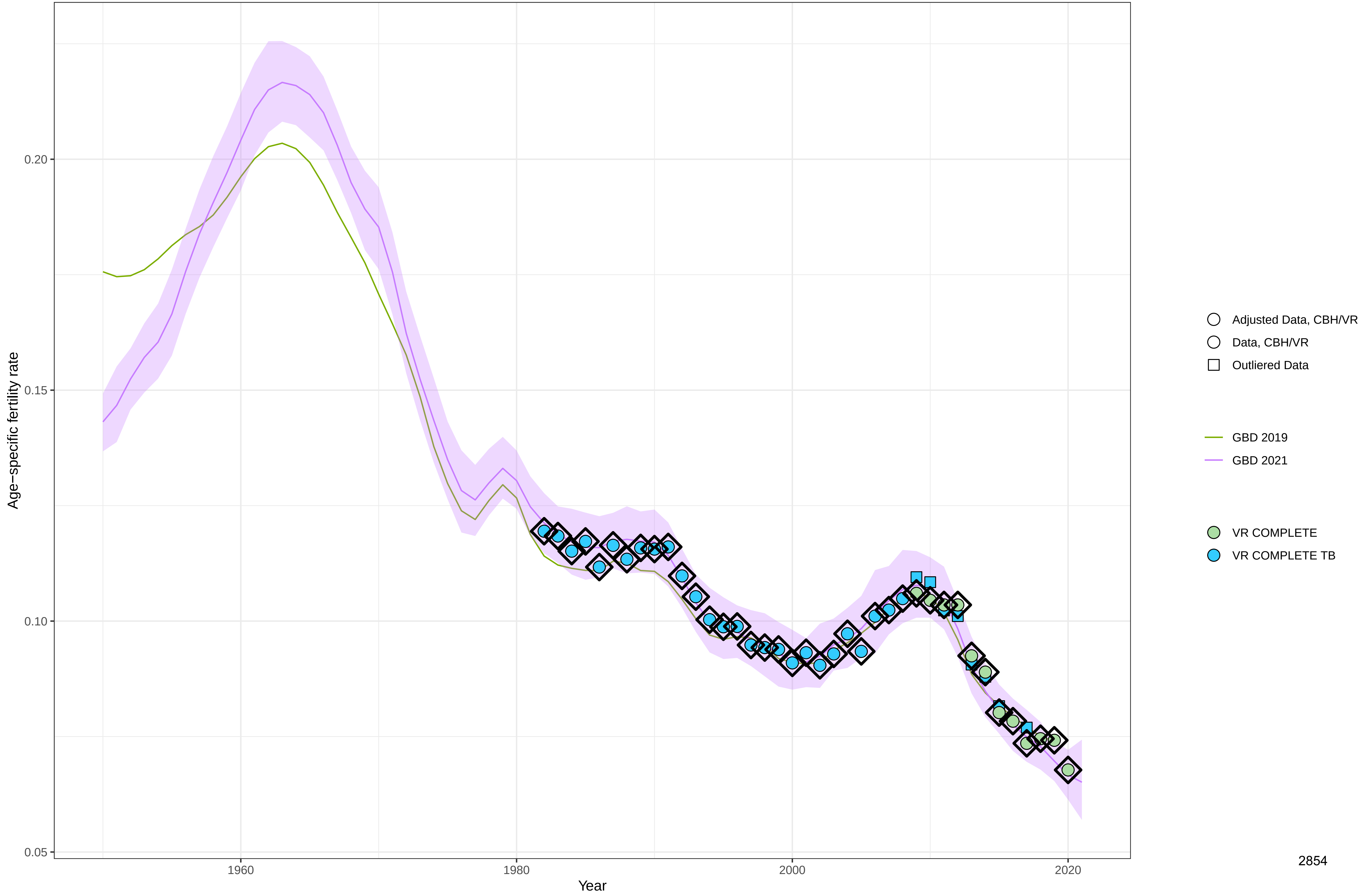

Age−specific fertility fitted trend lines, 1950−2021: Tameside, Ages: 25−29
Data points enclosed with a diamond are reference sources

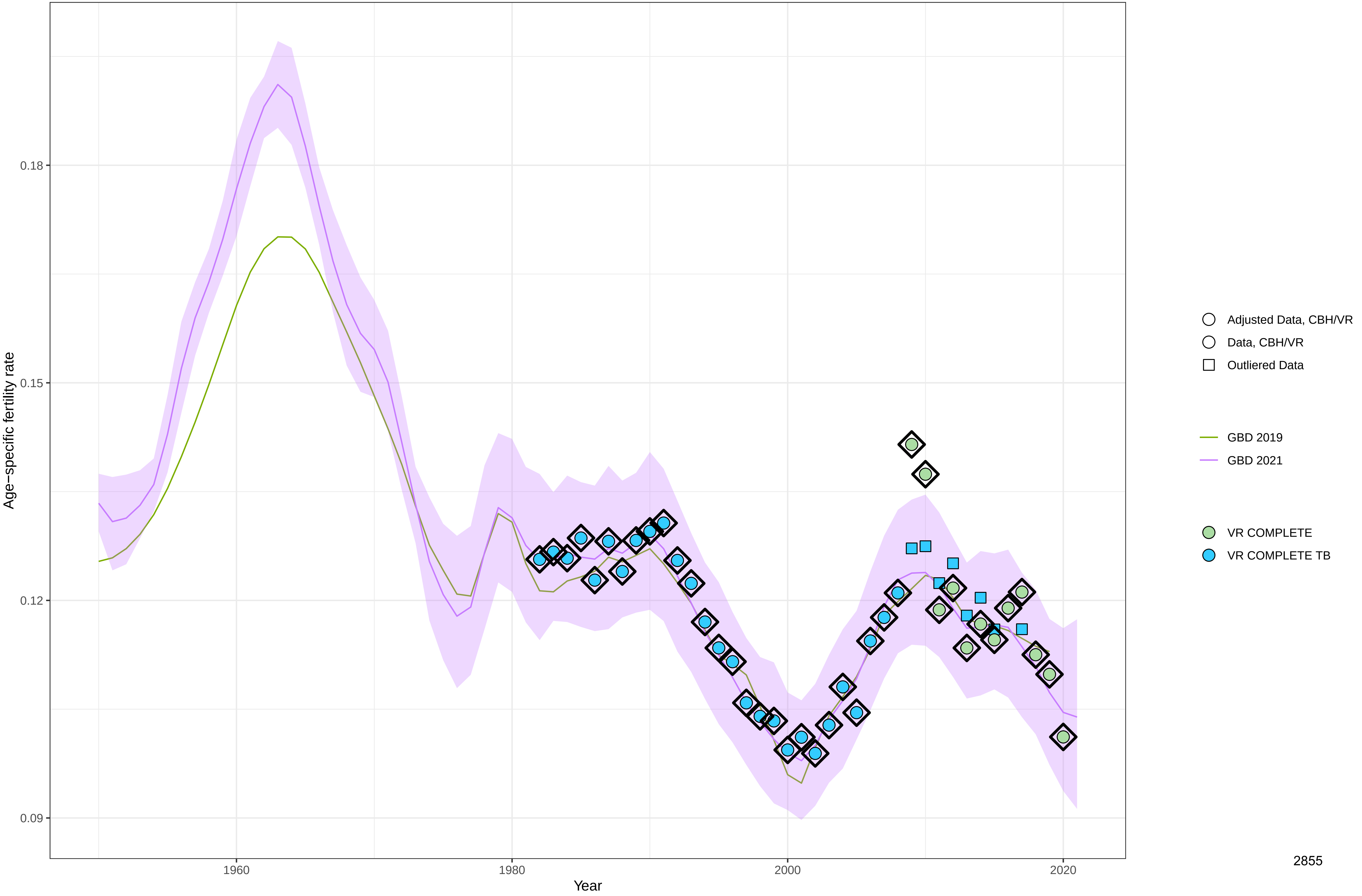

Age−specific fertility fitted trend lines, 1950−2021: Tameside, Ages: 30−34
Data points enclosed with a diamond are reference sources

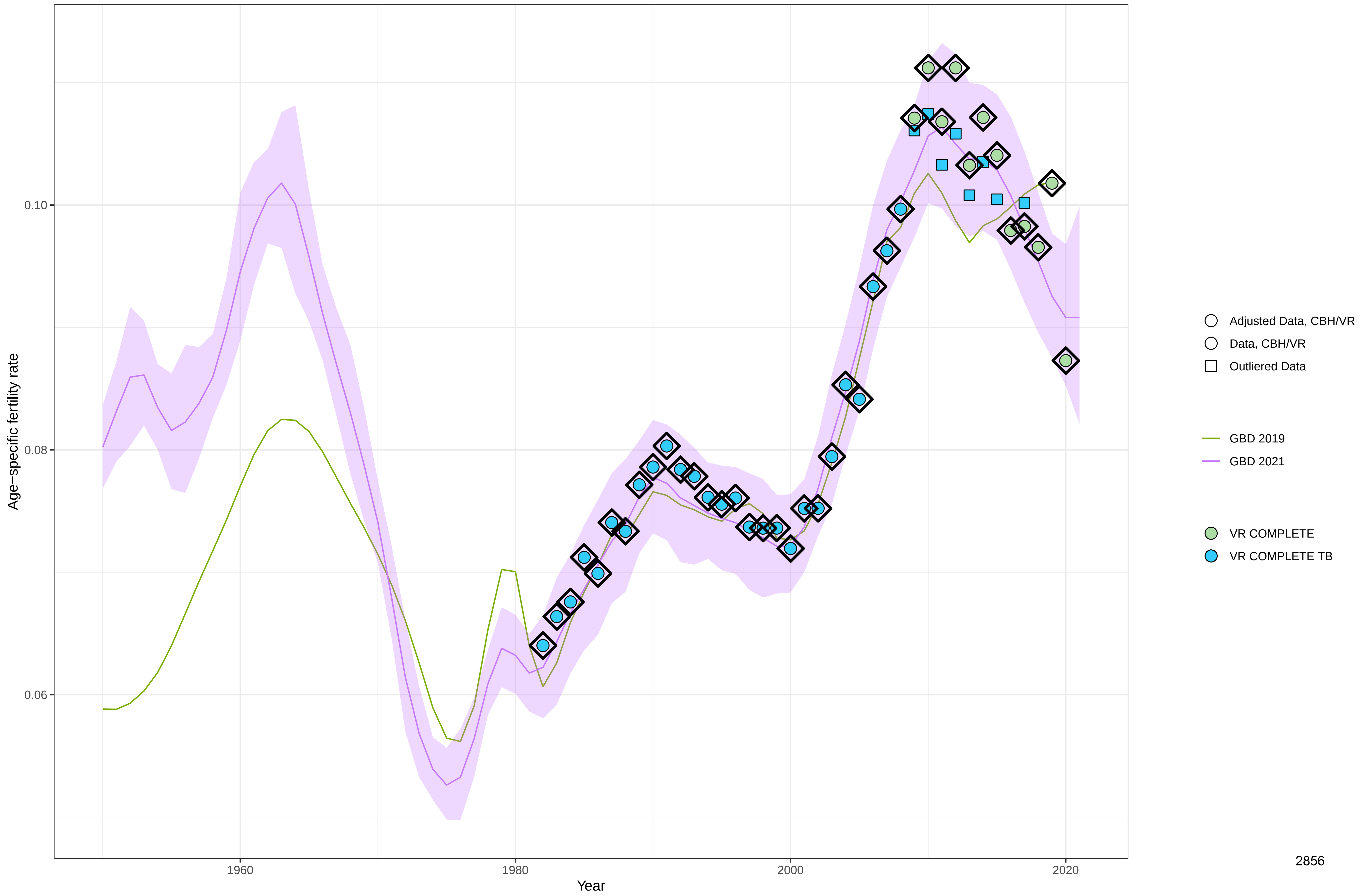

Age−specific fertility fitted trend lines, 1950−2021: Tameside, Ages: 35−39
Data points enclosed with a diamond are reference sources

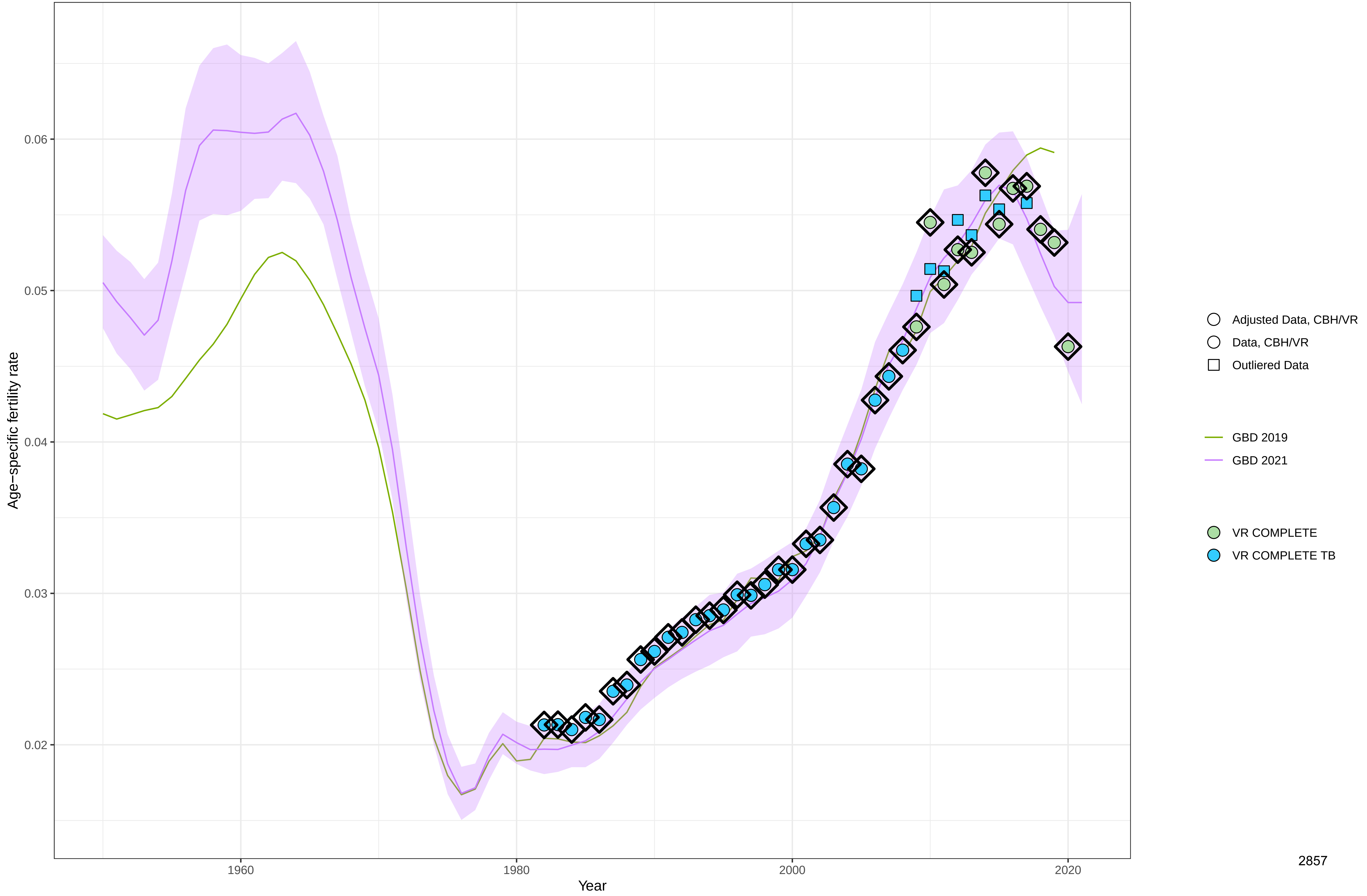

Age−specific fertility fitted trend lines, 1950−2021: Tameside, Ages: 40−44
Data points enclosed with a diamond are reference sources

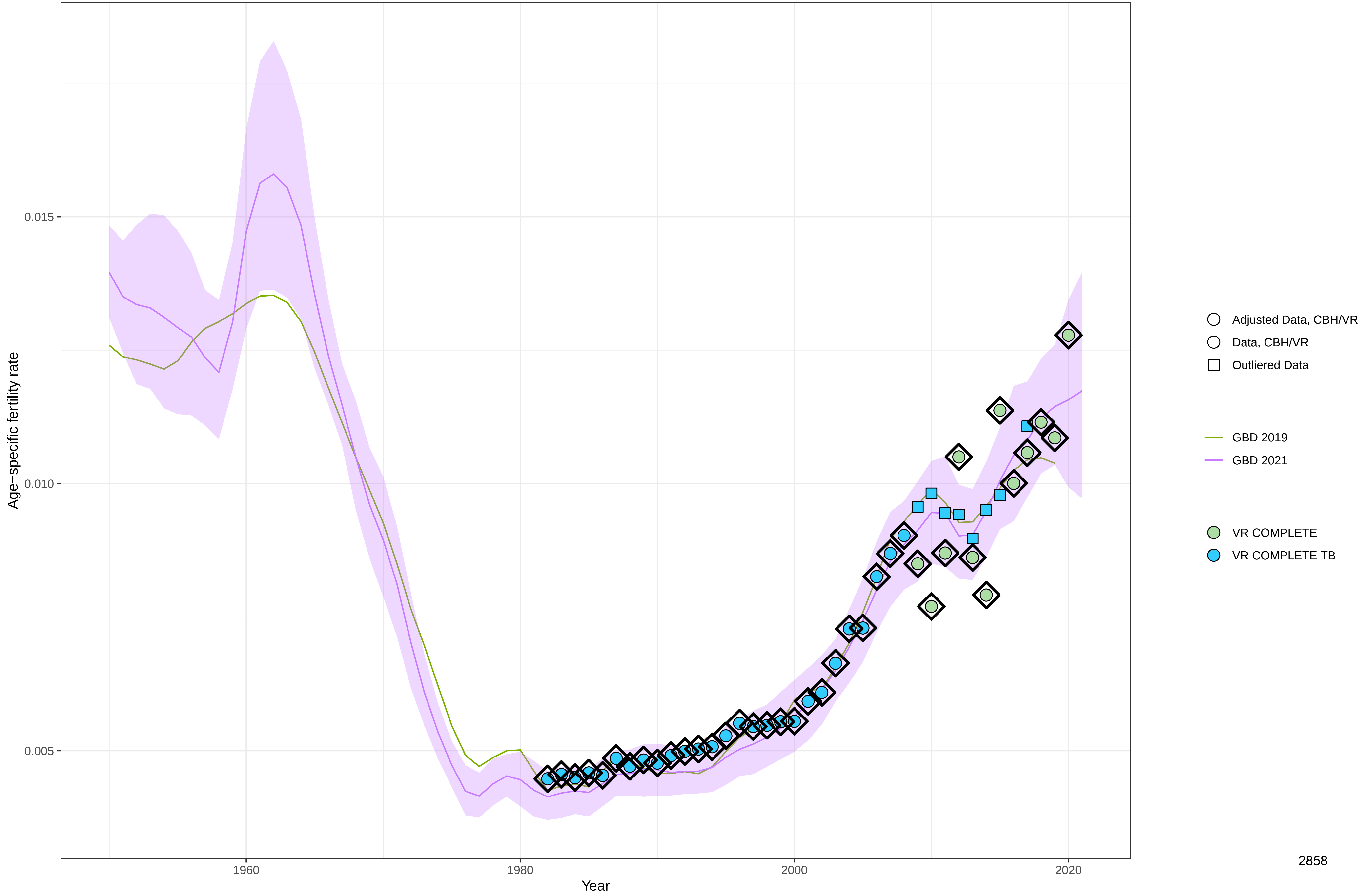

Age−specific fertility fitted trend lines, 1950−2021: Tameside, Ages: 45−49
Data points enclosed with a diamond are reference sources

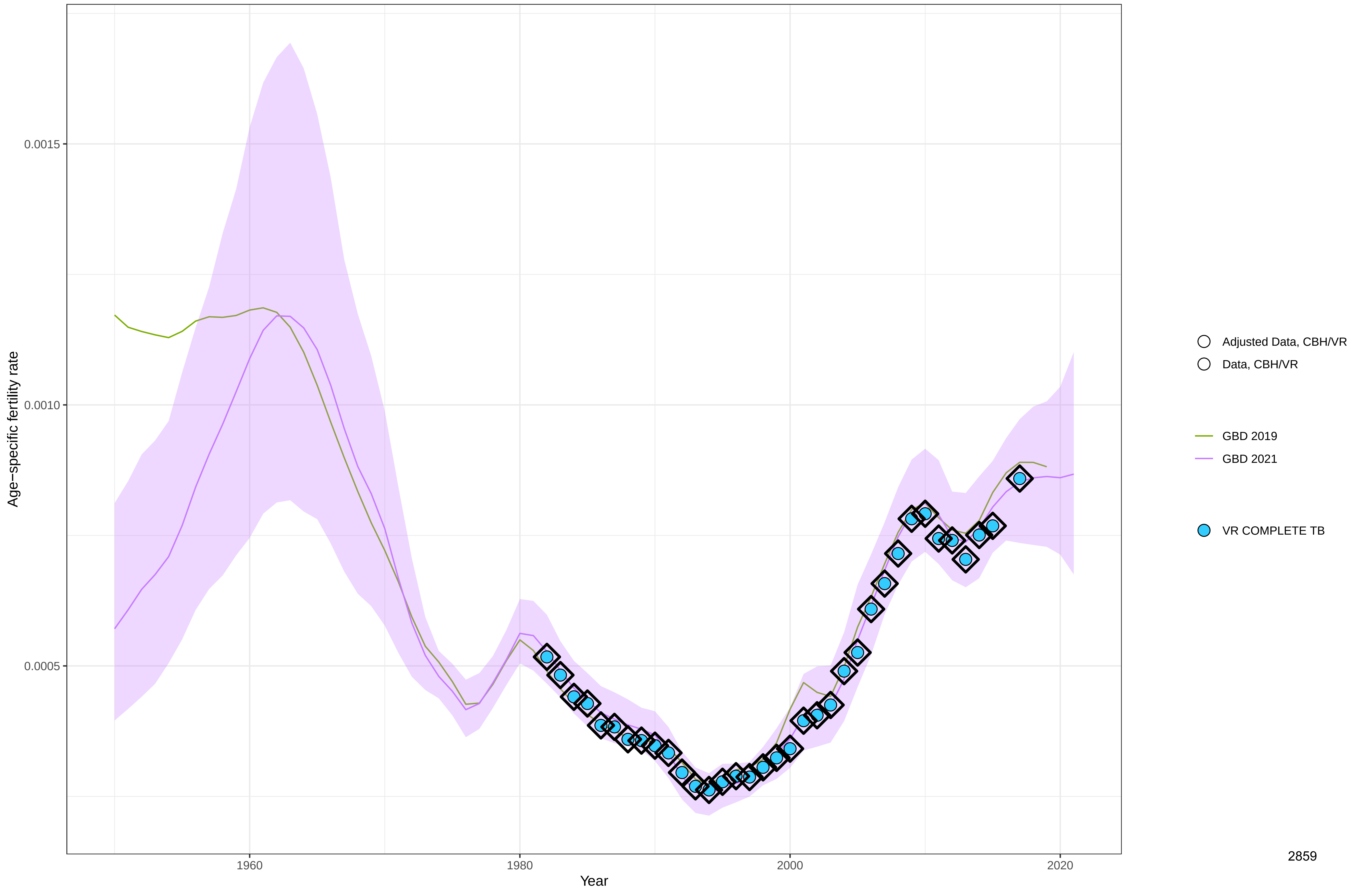

Age−specific fertility fitted trend lines, 1950−2021: Tameside, Ages: 50−54
Data points enclosed with a diamond are reference sources

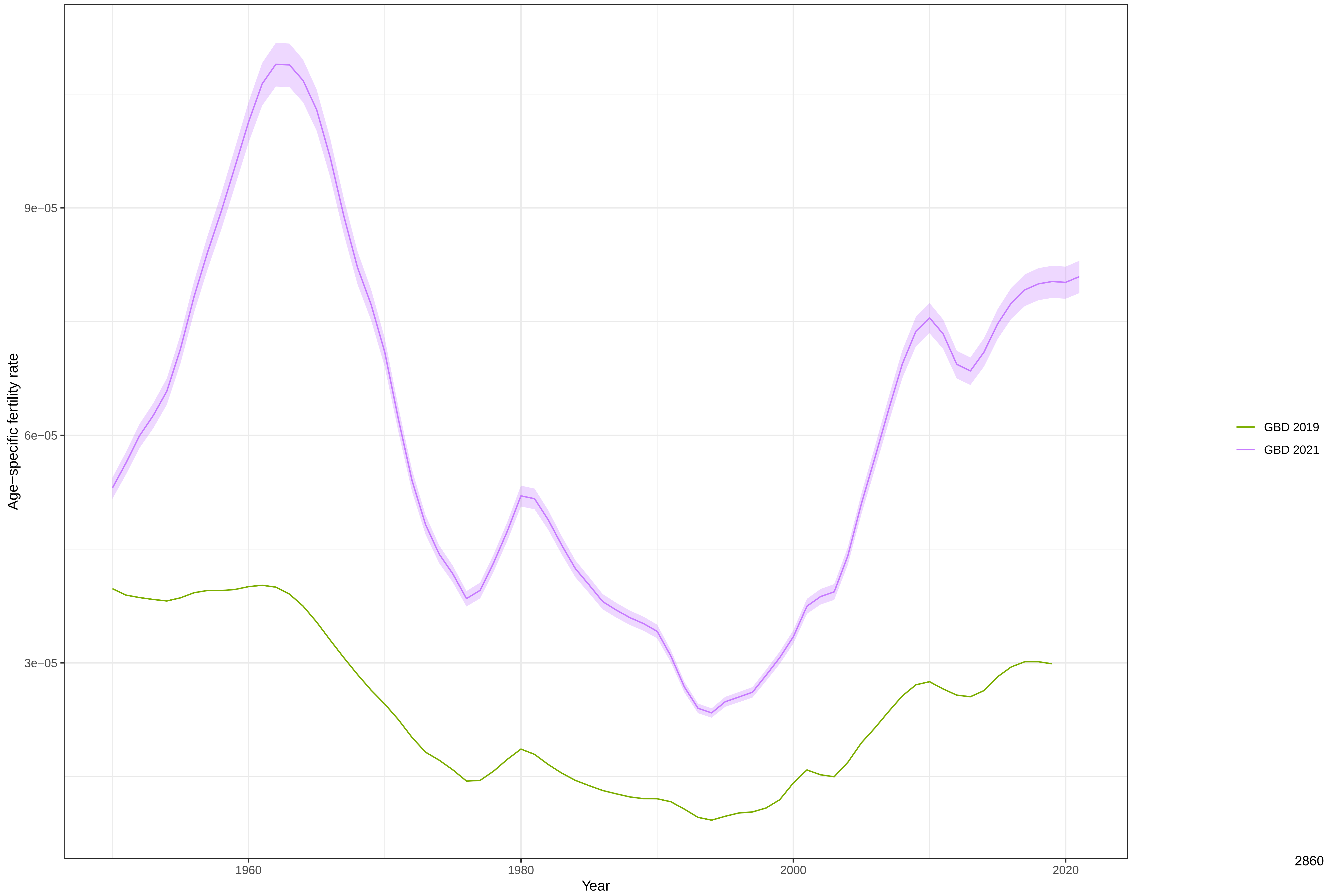

Total fertility rate fitted trend lines, 1950−2021: Tameside TFR
Data points enclosed with a diamond are reference sources

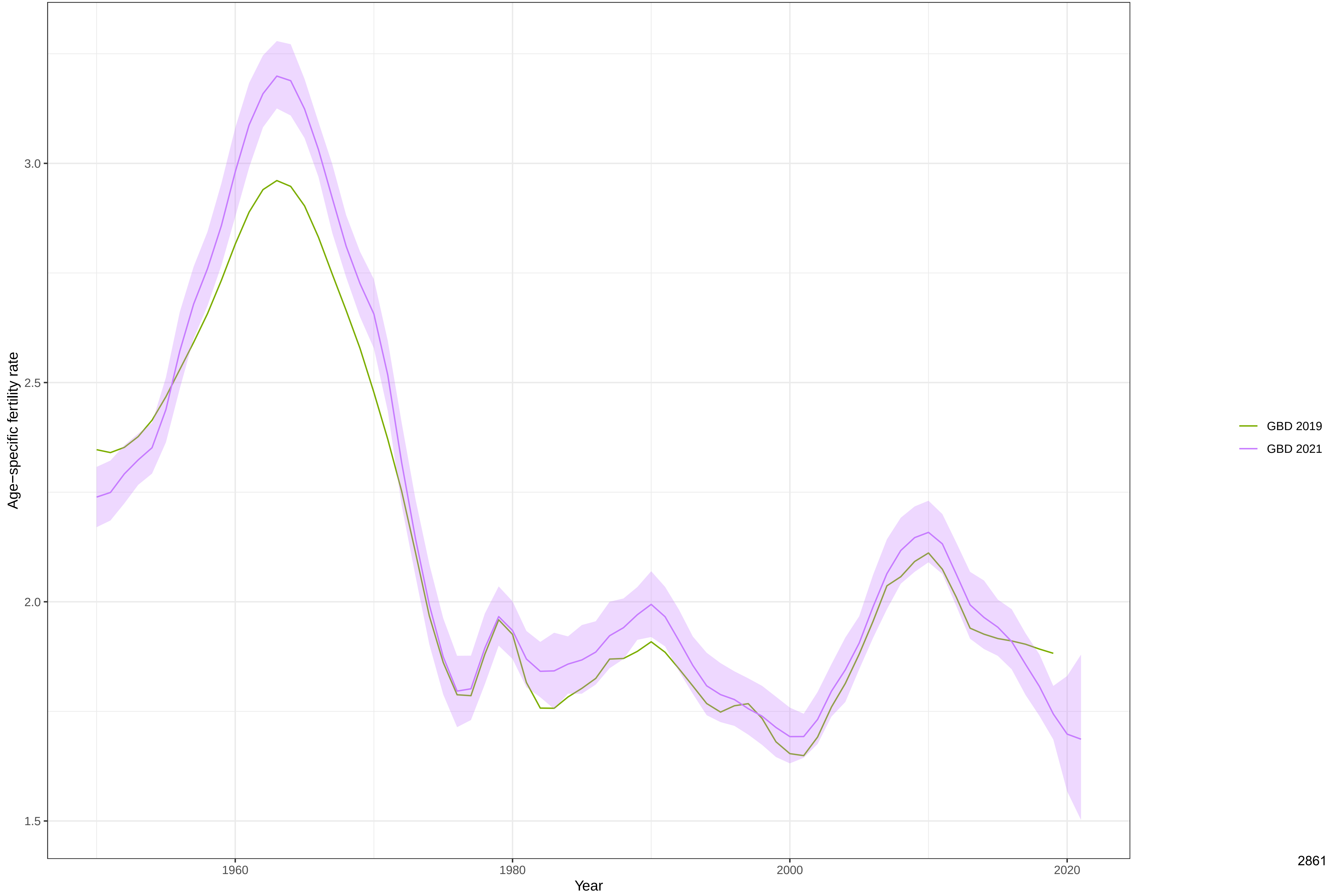

Age−specific fertility fitted trend lines, 1950−2021: Trafford, Ages: 10−14
Data points enclosed with a diamond are reference sources

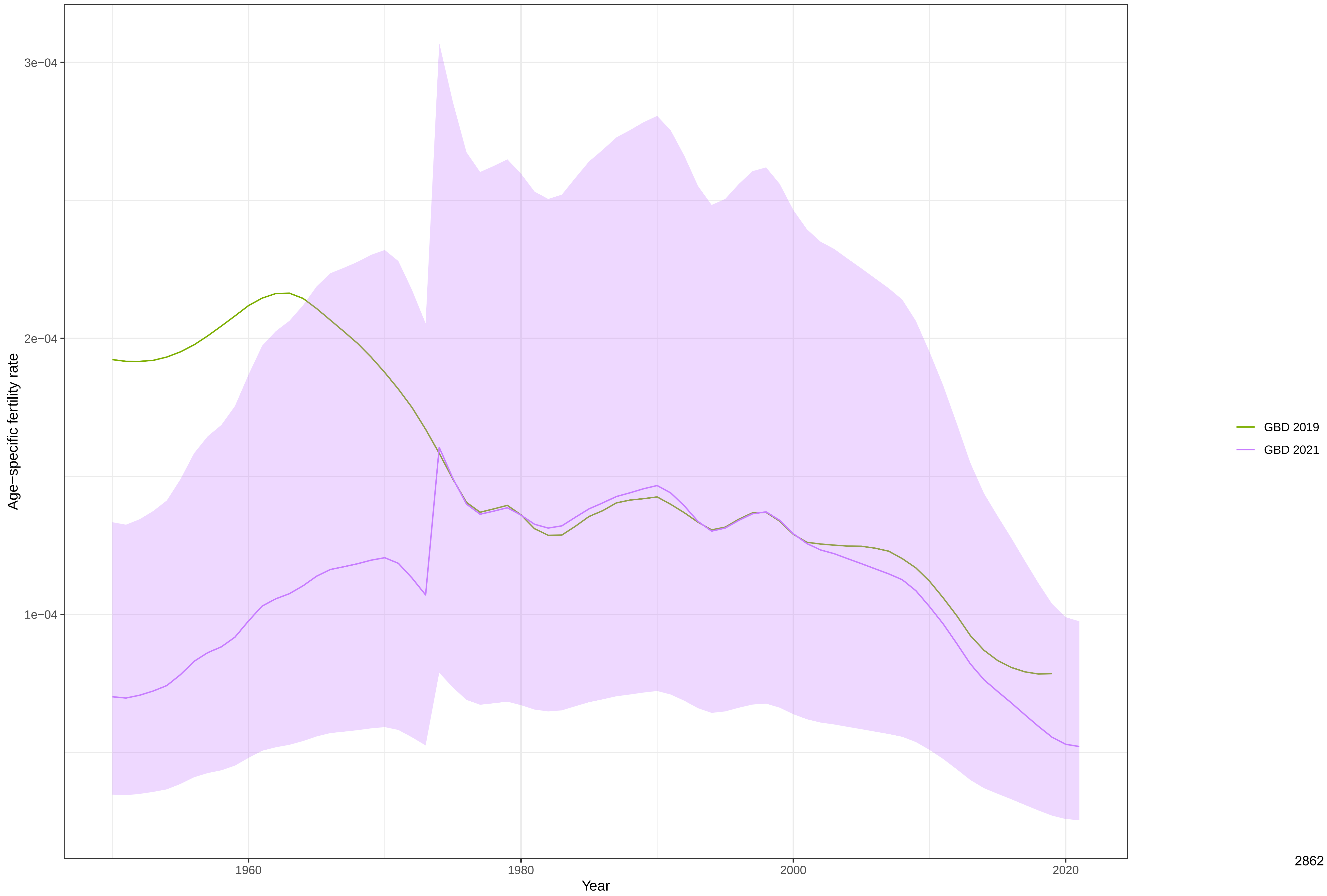

Age−specific fertility fitted trend lines, 1950−2021: Trafford, Ages: 15−19
Data points enclosed with a diamond are reference sources

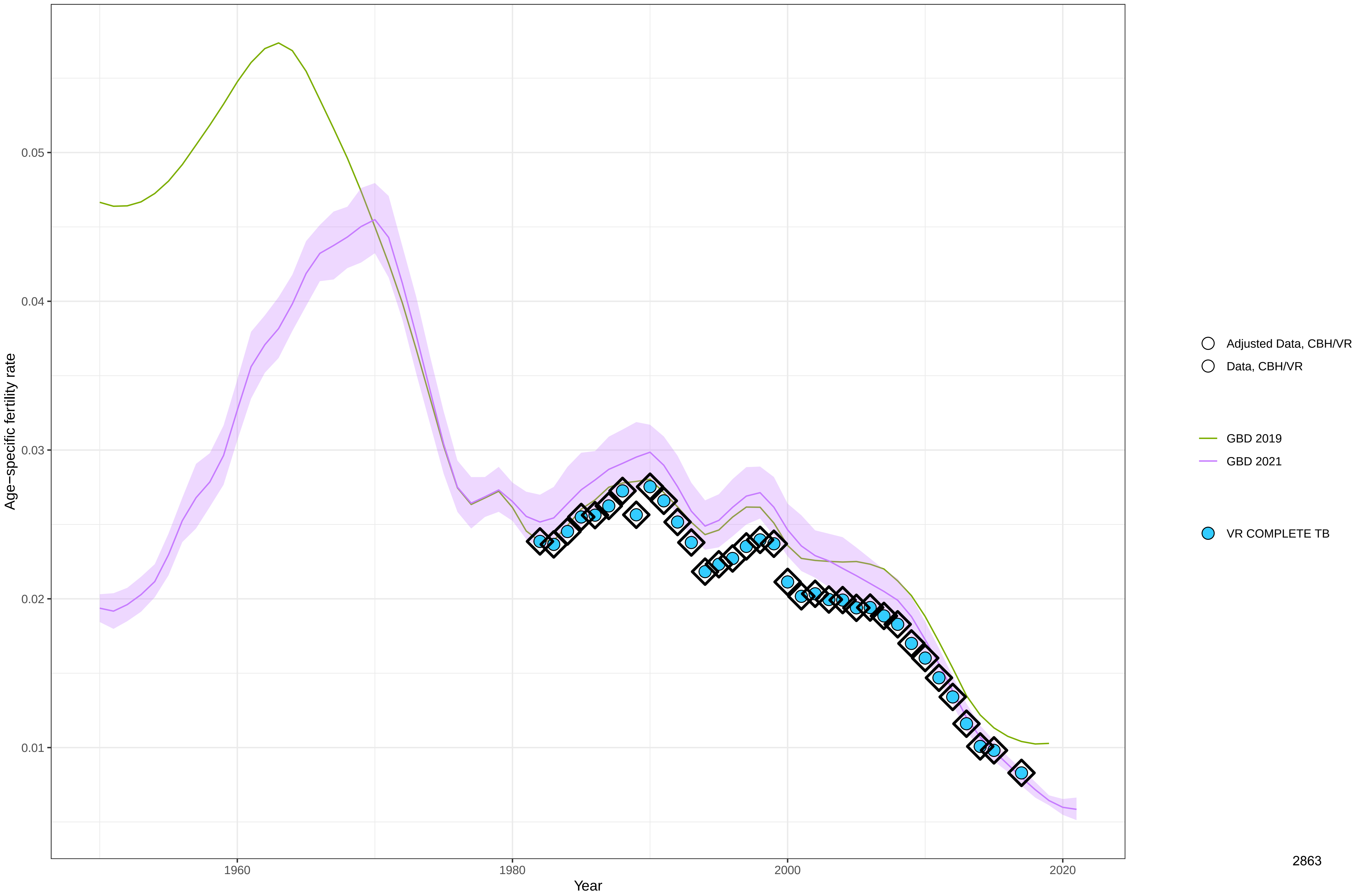

2863

Age−specific fertility fitted trend lines, 1950−2021: Trafford, Ages: 20−24
Data points enclosed with a diamond are reference sources

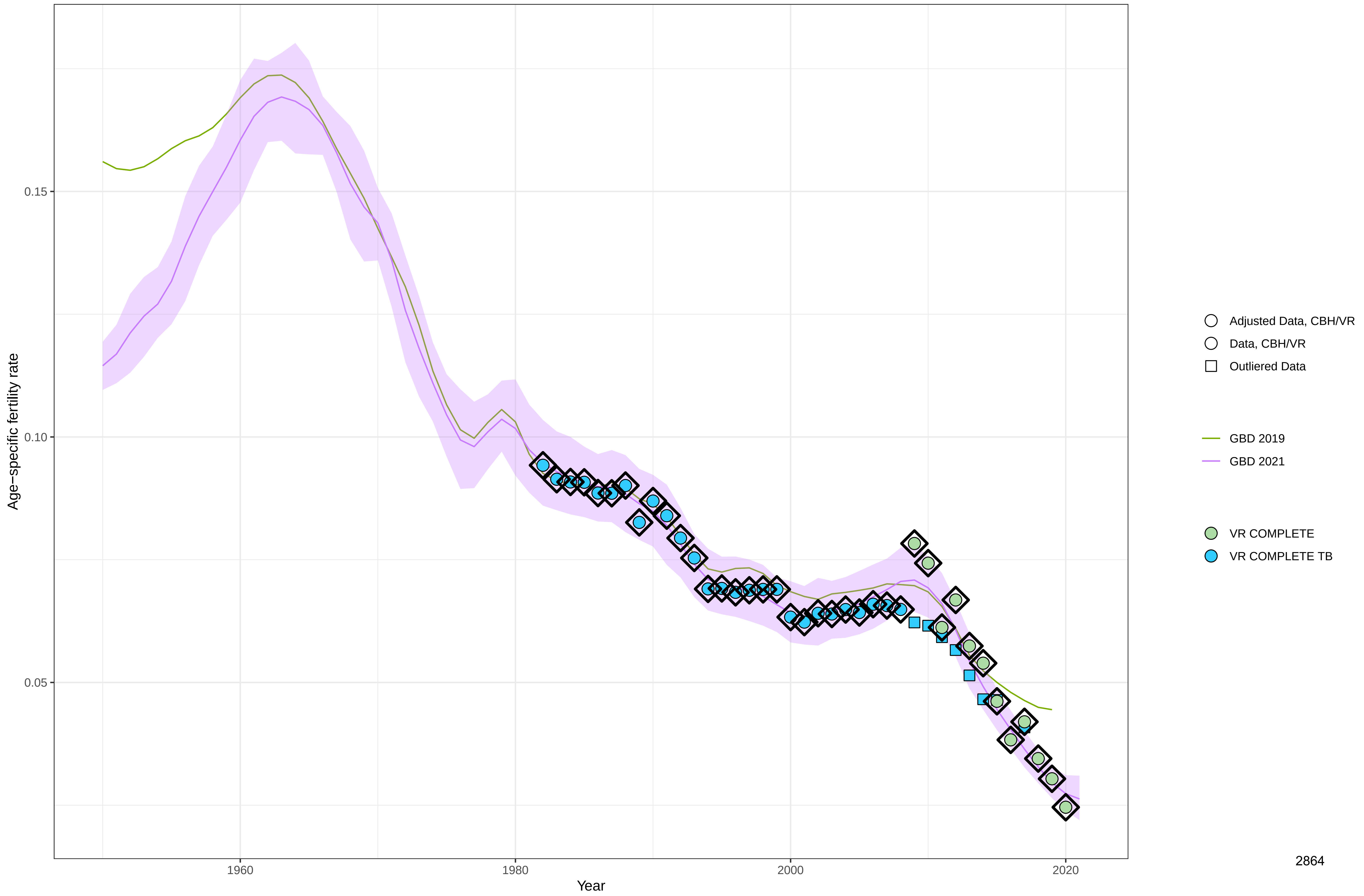

Age−specific fertility fitted trend lines, 1950−2021: Trafford, Ages: 25−29
Data points enclosed with a diamond are reference sources

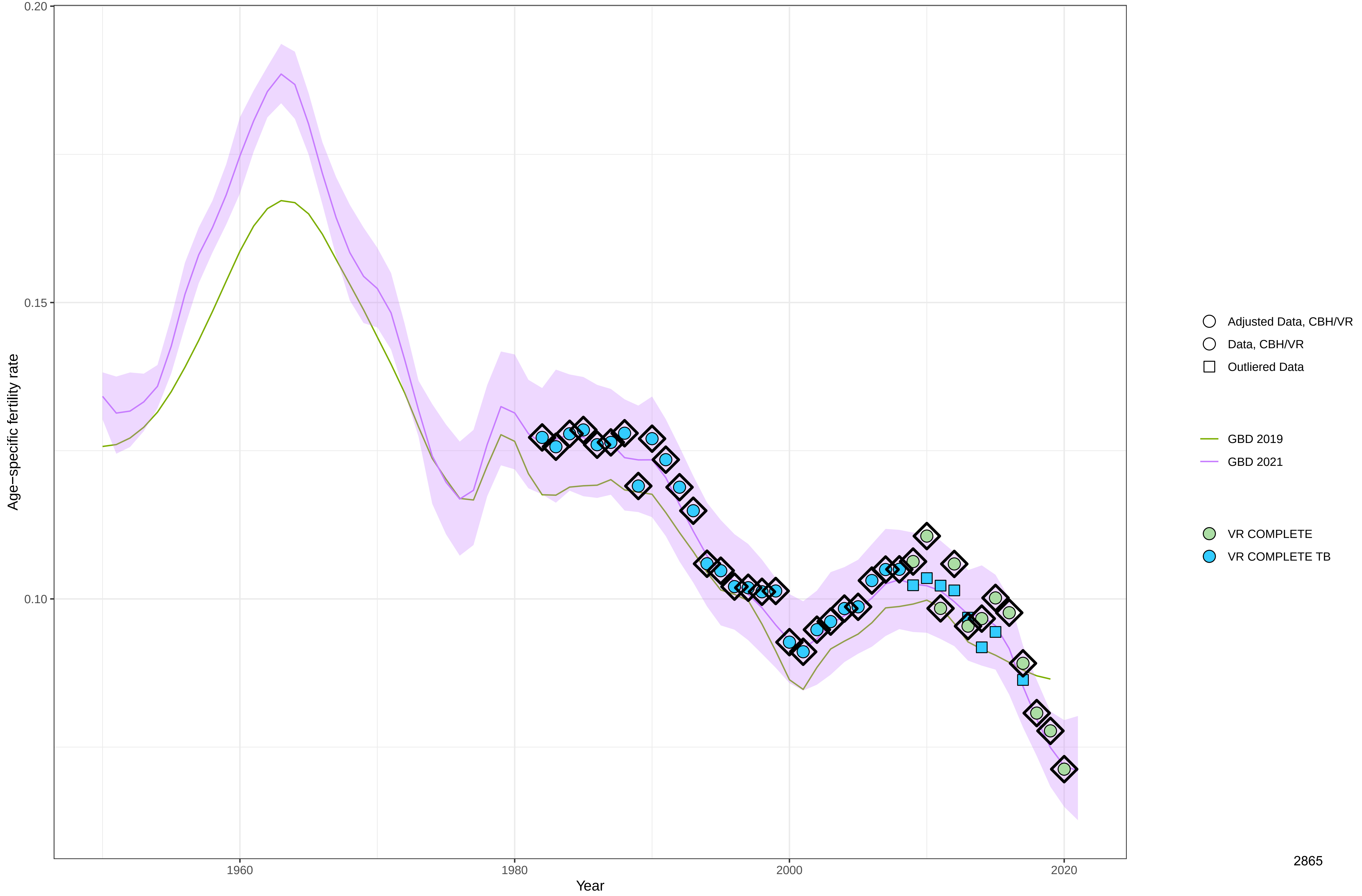

Age−specific fertility fitted trend lines, 1950−2021: Trafford, Ages: 30−34
Data points enclosed with a diamond are reference sources

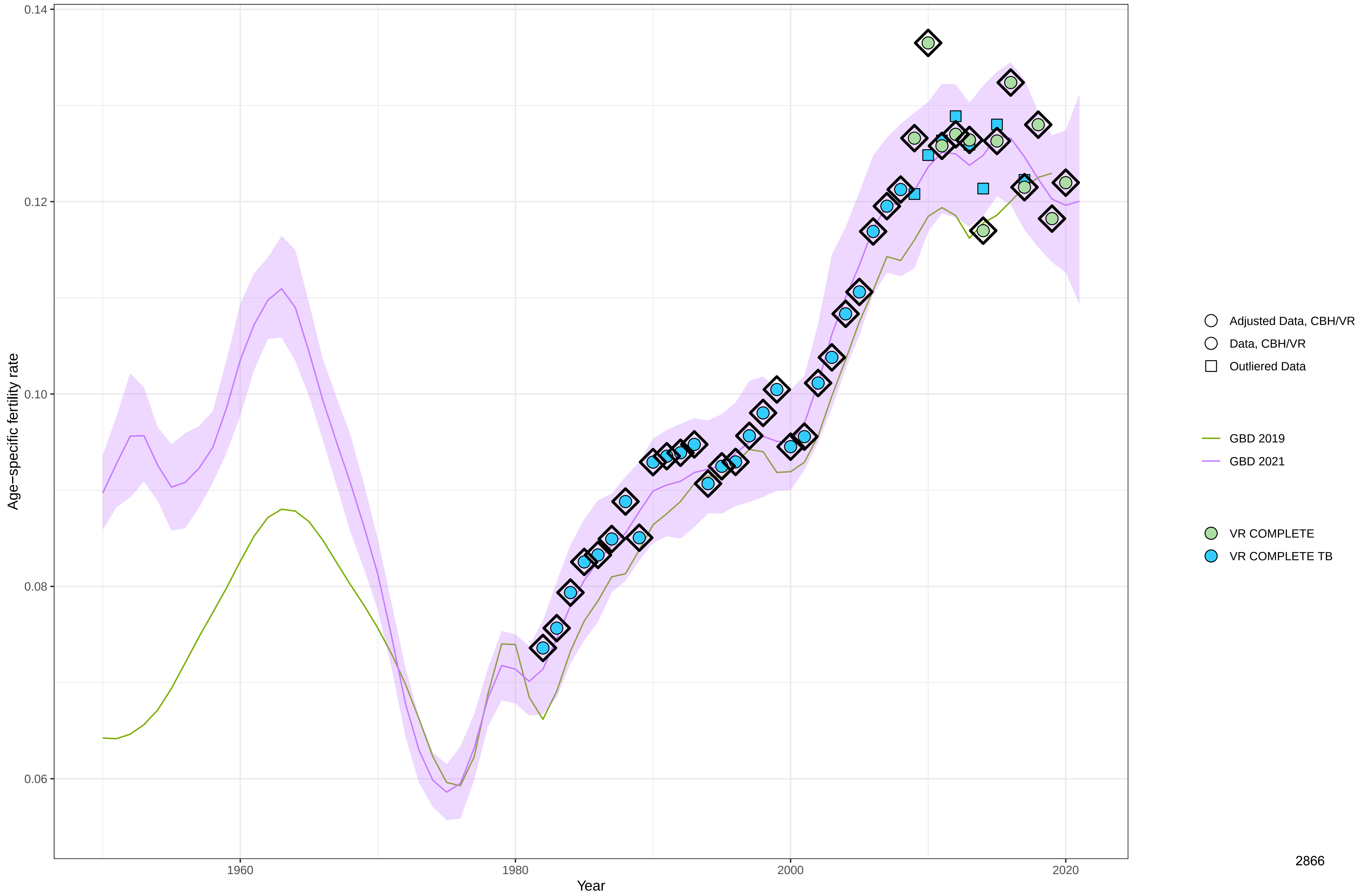

Age−specific fertility fitted trend lines, 1950−2021: Trafford, Ages: 35−39
Data points enclosed with a diamond are reference sources

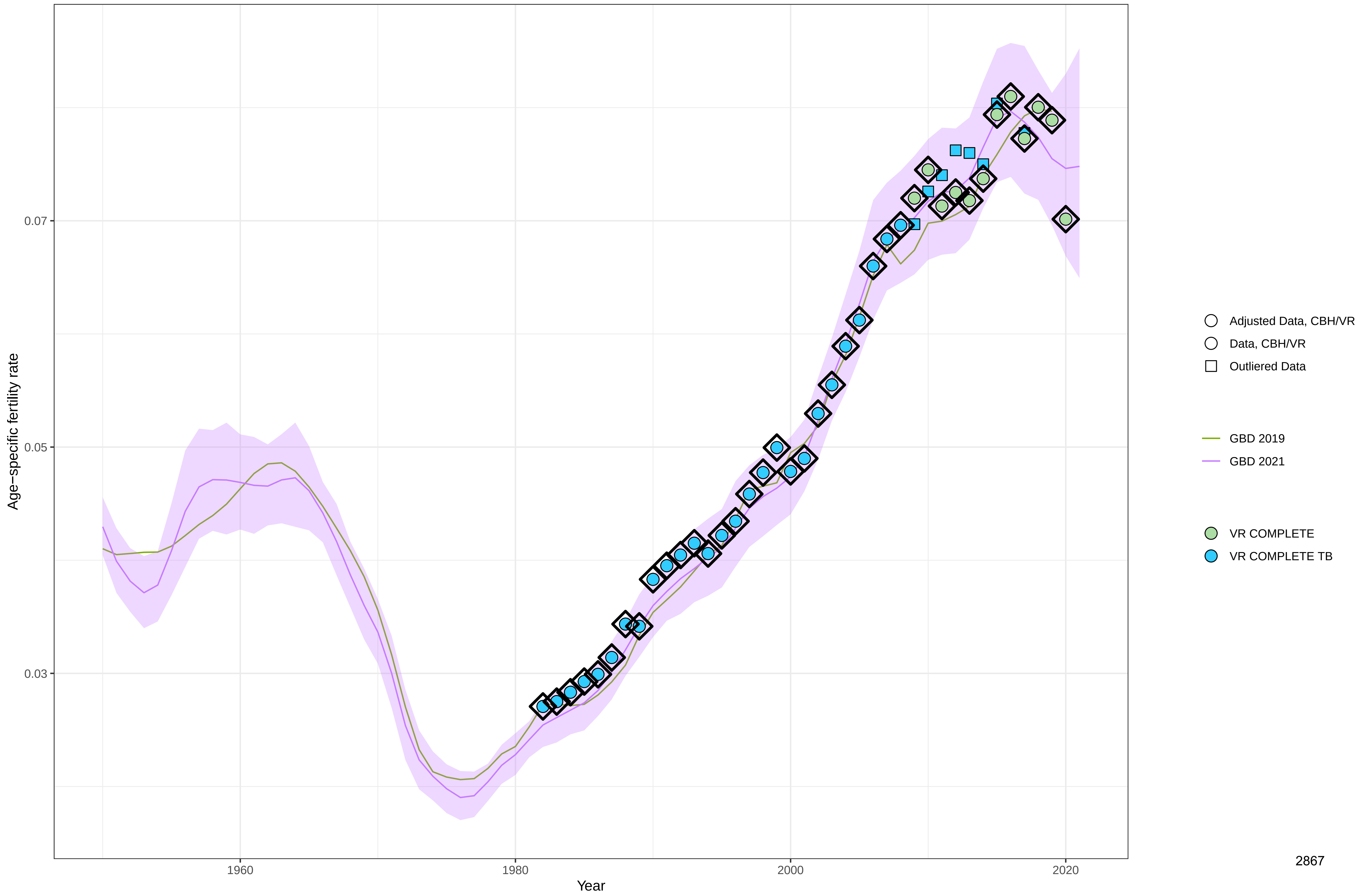

Age−specific fertility fitted trend lines, 1950−2021: Trafford, Ages: 40−44
Data points enclosed with a diamond are reference sources

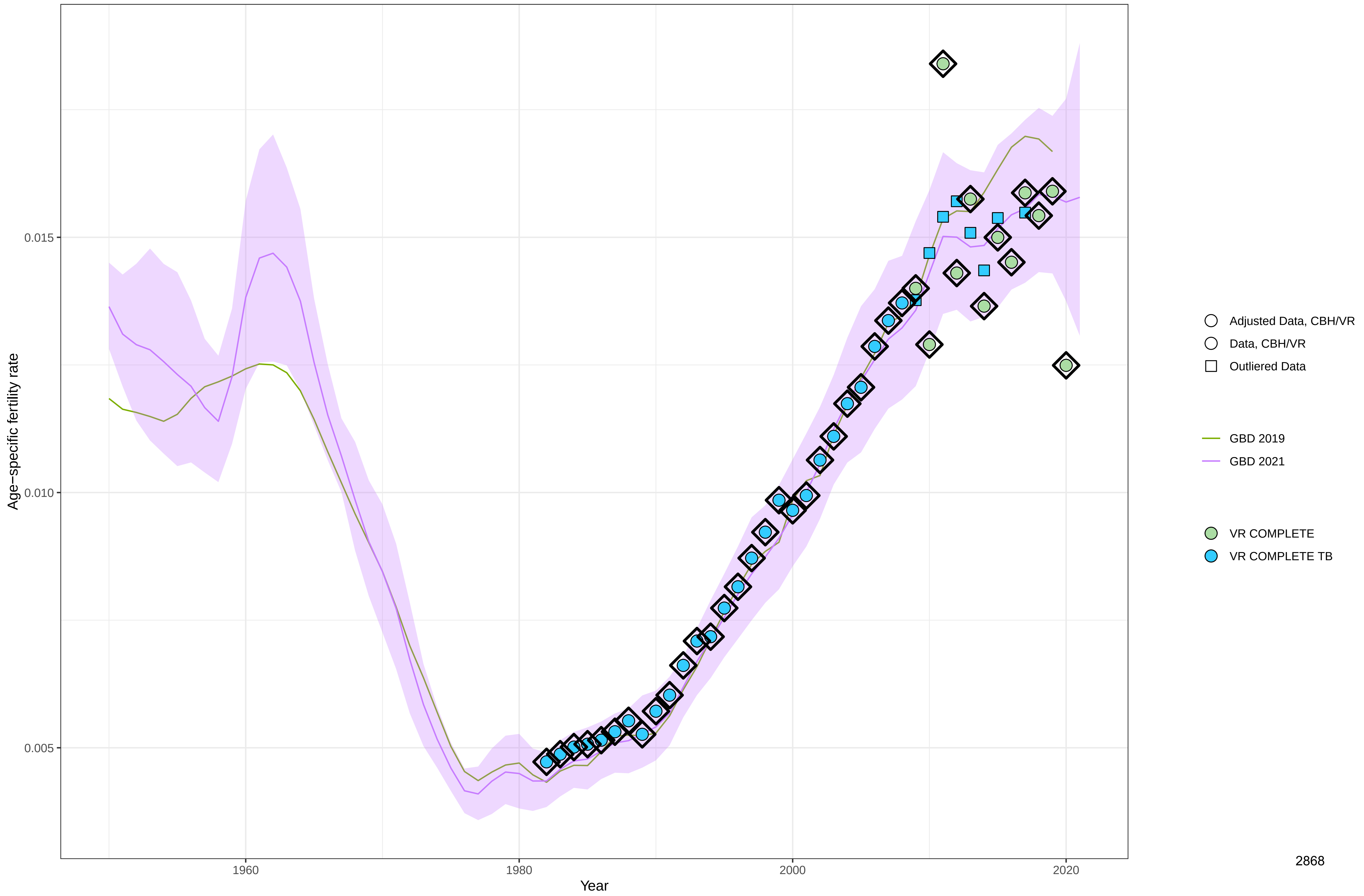

Age−specific fertility fitted trend lines, 1950−2021: Trafford, Ages: 45−49
Data points enclosed with a diamond are reference sources

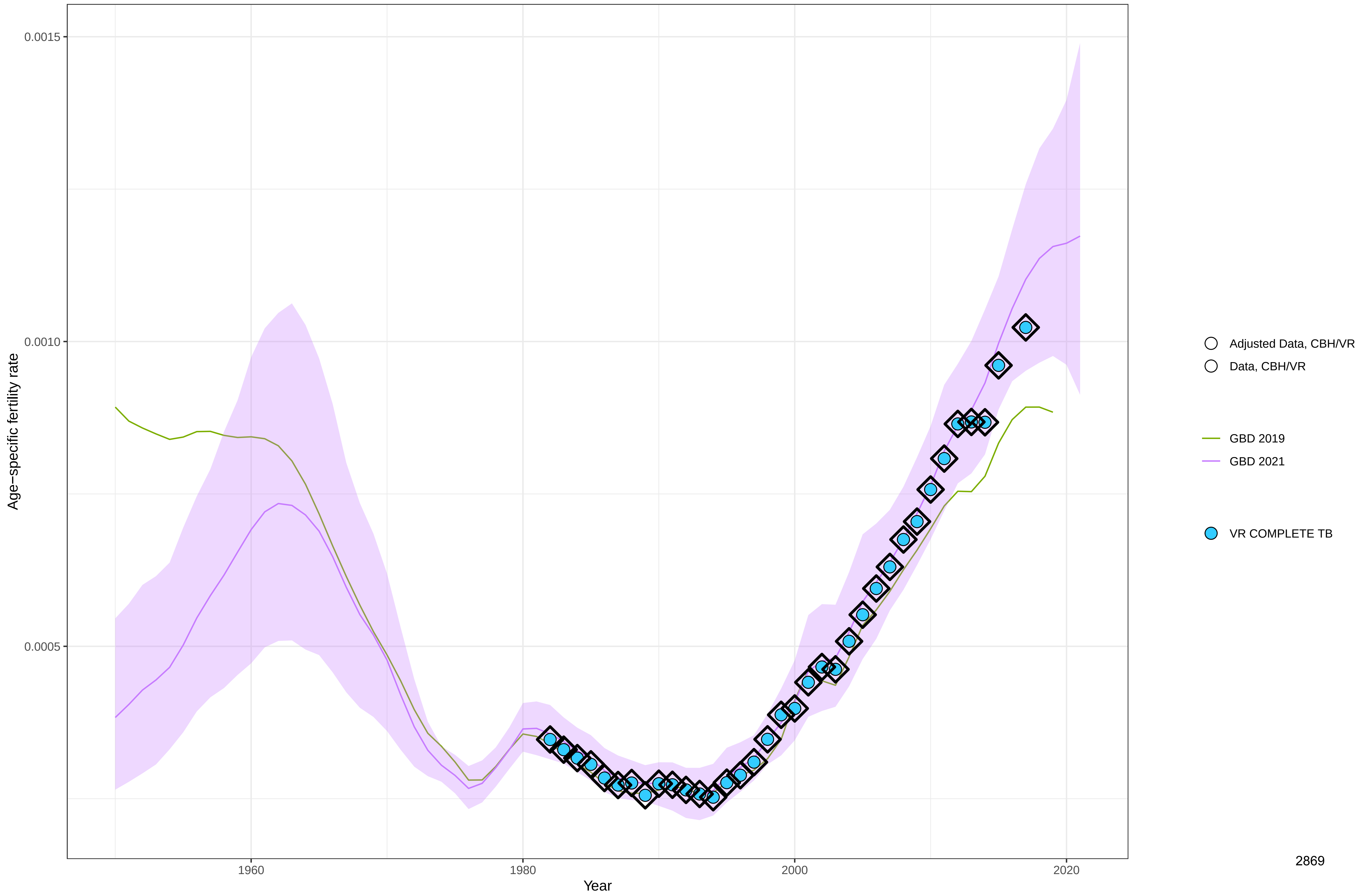

Age−specific fertility fitted trend lines, 1950−2021: Trafford, Ages: 50−54
Data points enclosed with a diamond are reference sources

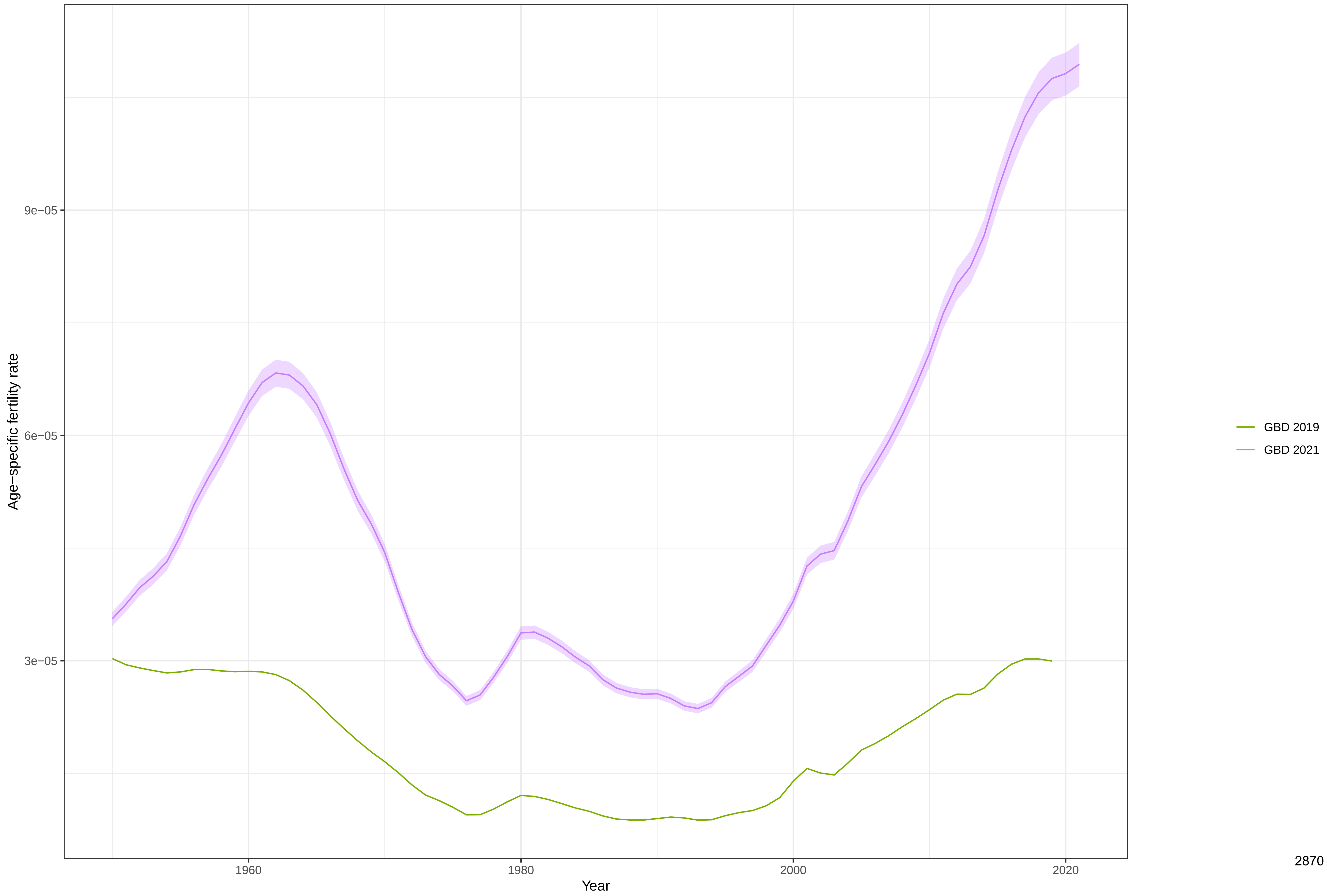

Total fertility rate fitted trend lines, 1950−2021: Trafford TFR
Data points enclosed with a diamond are reference sources

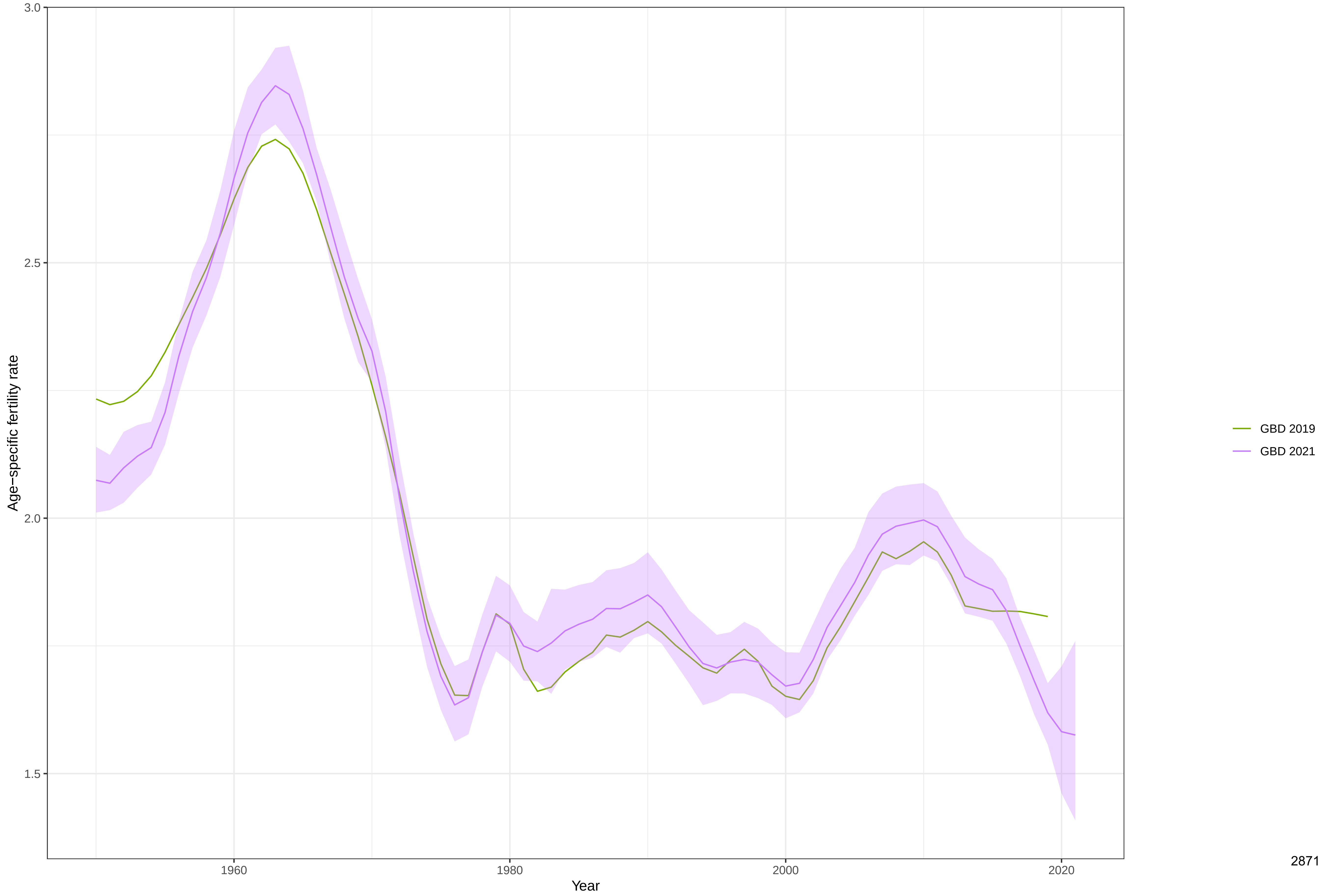

Age−specific fertility fitted trend lines, 1950−2021: Warrington, Ages: 10−14
Data points enclosed with a diamond are reference sources

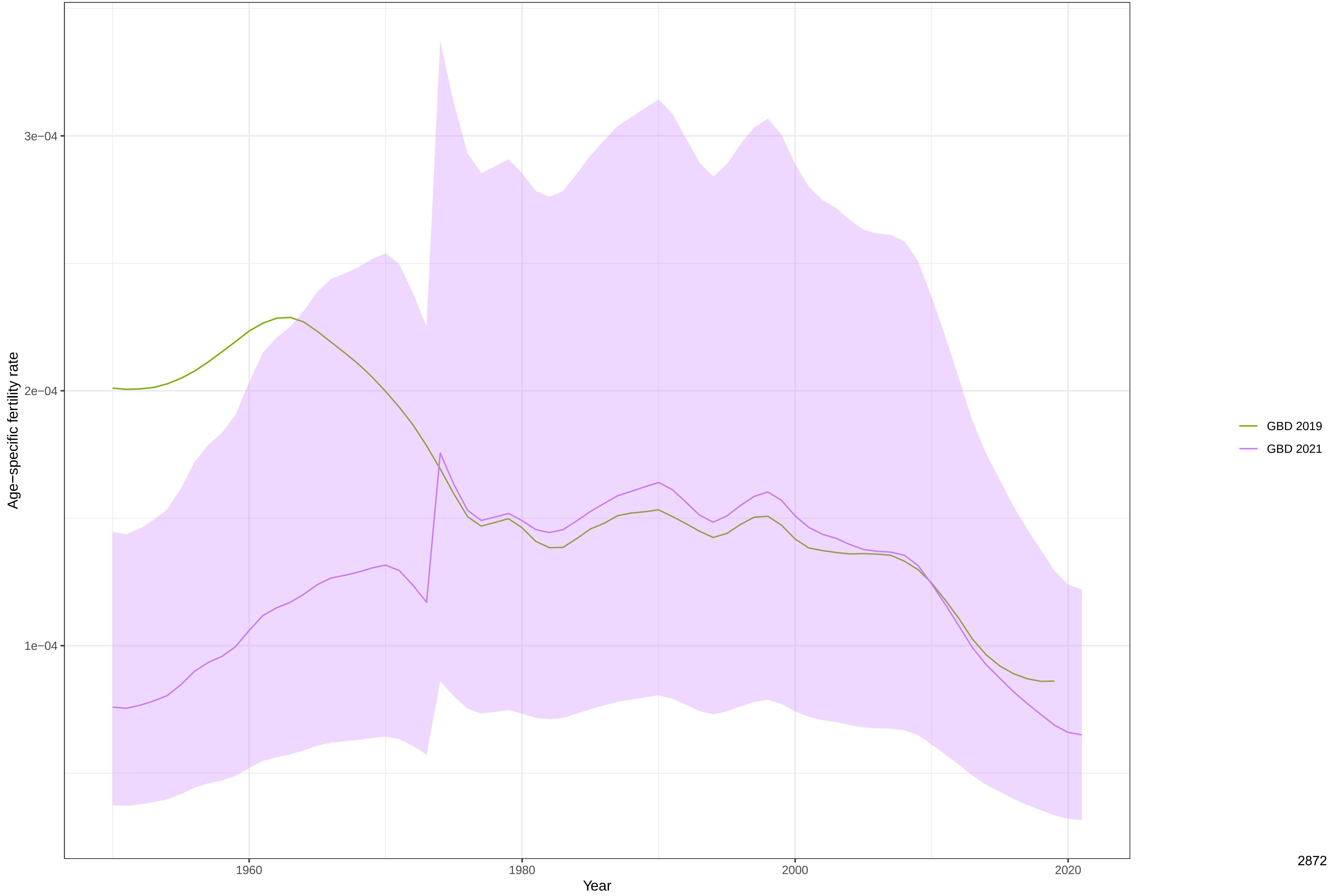

Age−specific fertility fitted trend lines, 1950−2021: Warrington, Ages: 15−19
Data points enclosed with a diamond are reference sources

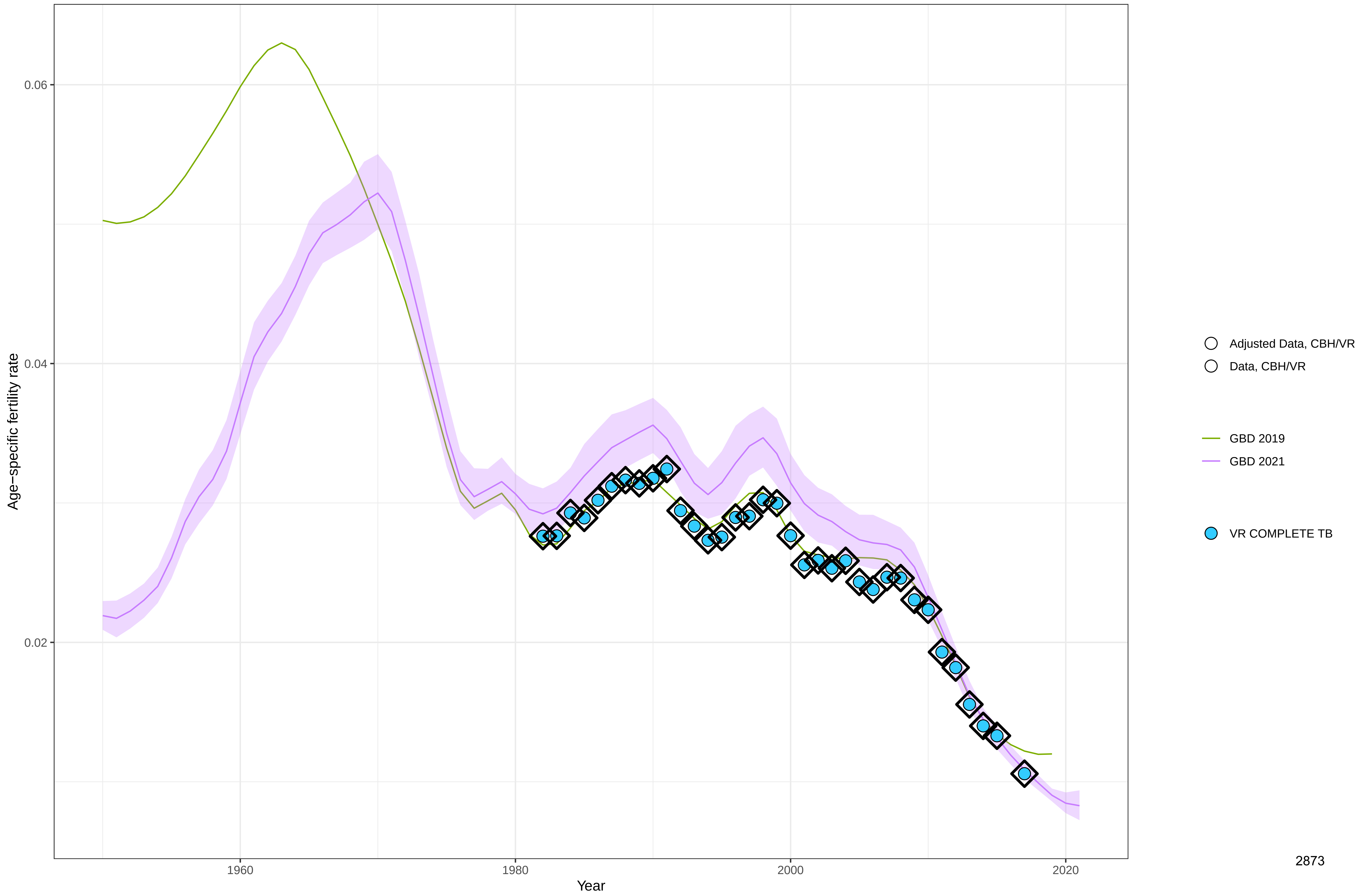

Age−specific fertility fitted trend lines, 1950−2021: Warrington, Ages: 20−24
Data points enclosed with a diamond are reference sources

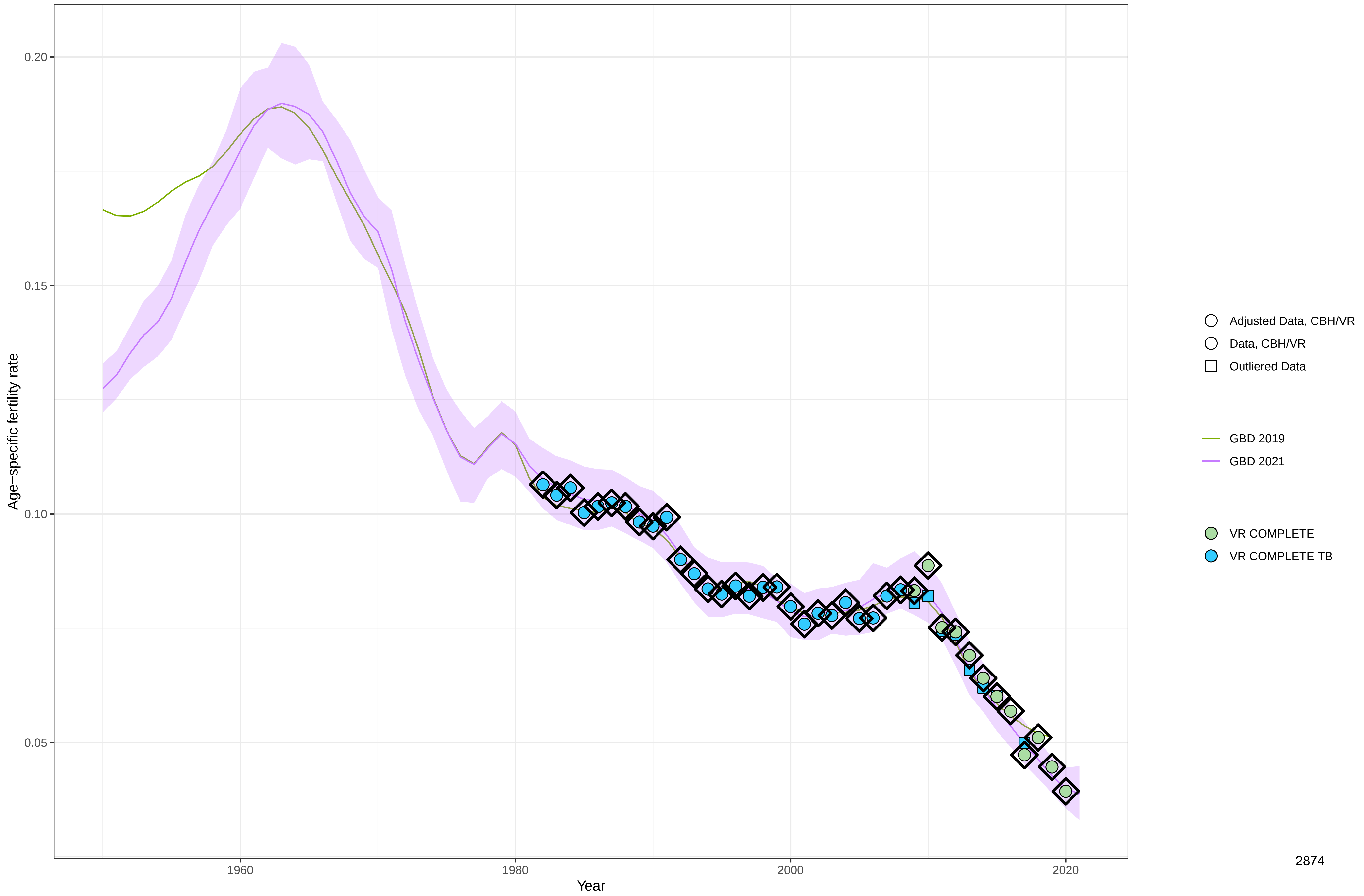

Age−specific fertility fitted trend lines, 1950−2021: Warrington, Ages: 25−29
Data points enclosed with a diamond are reference sources

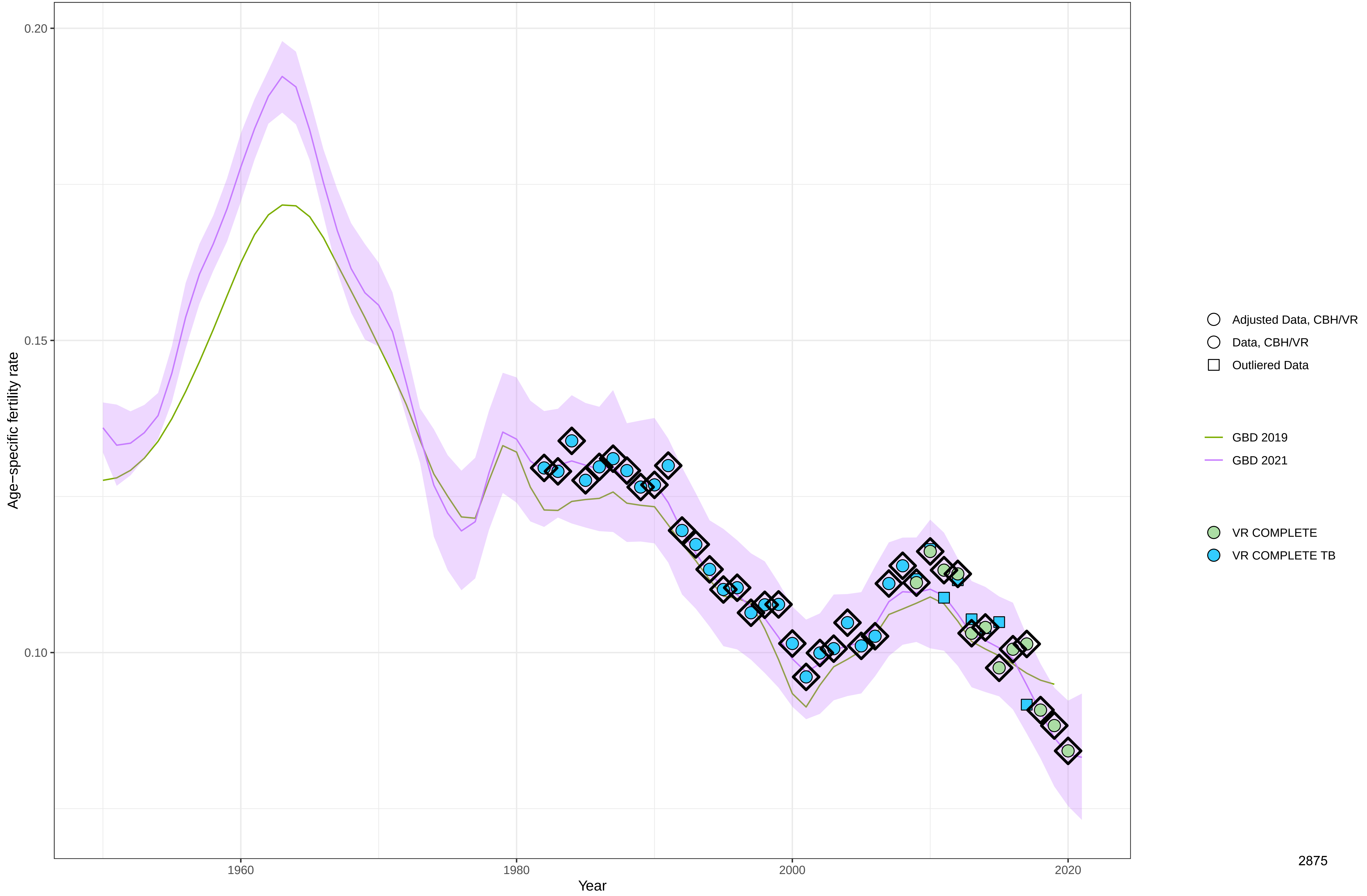

Age−specific fertility fitted trend lines, 1950−2021: Warrington, Ages: 30−34
Data points enclosed with a diamond are reference sources

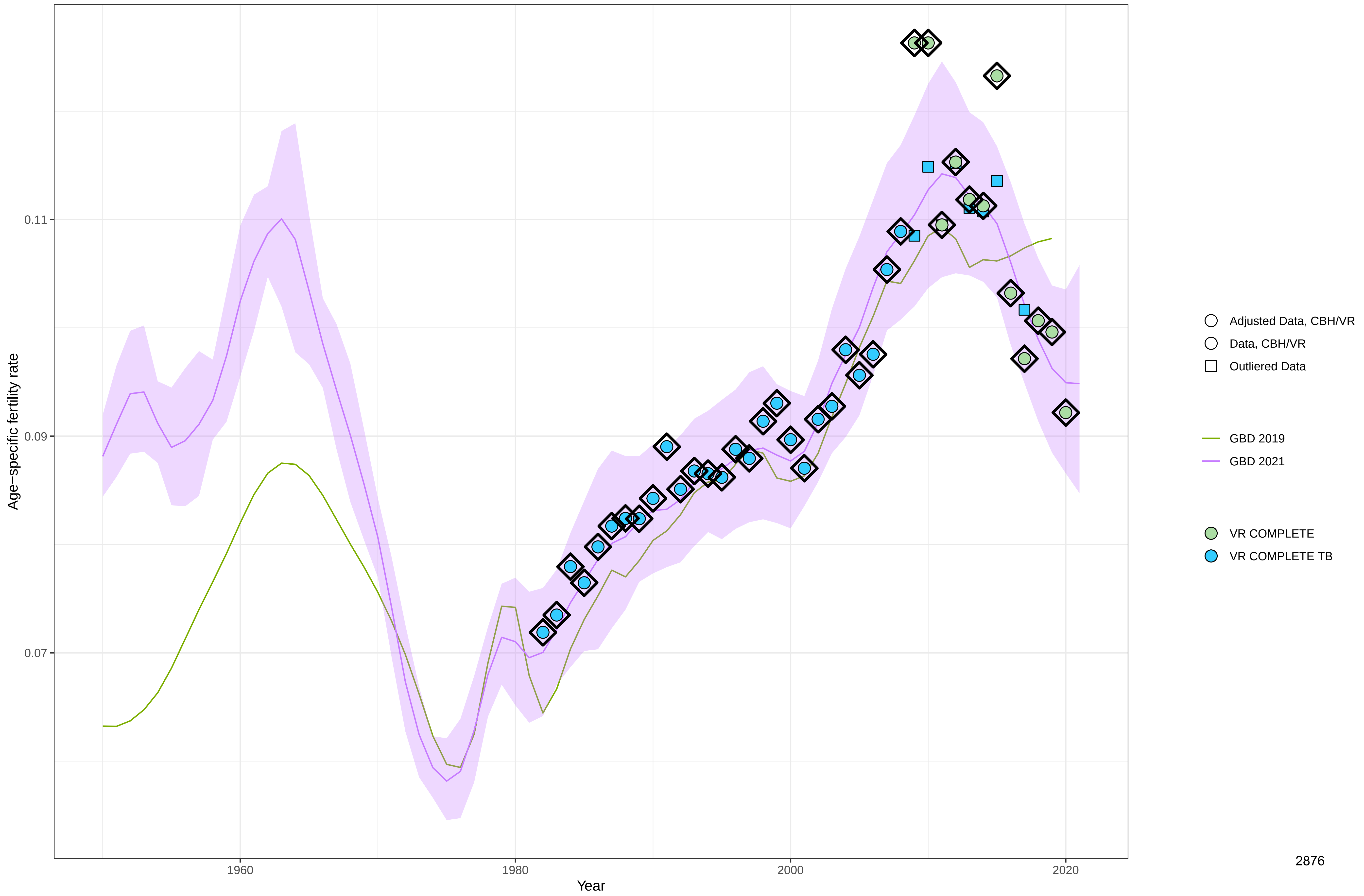

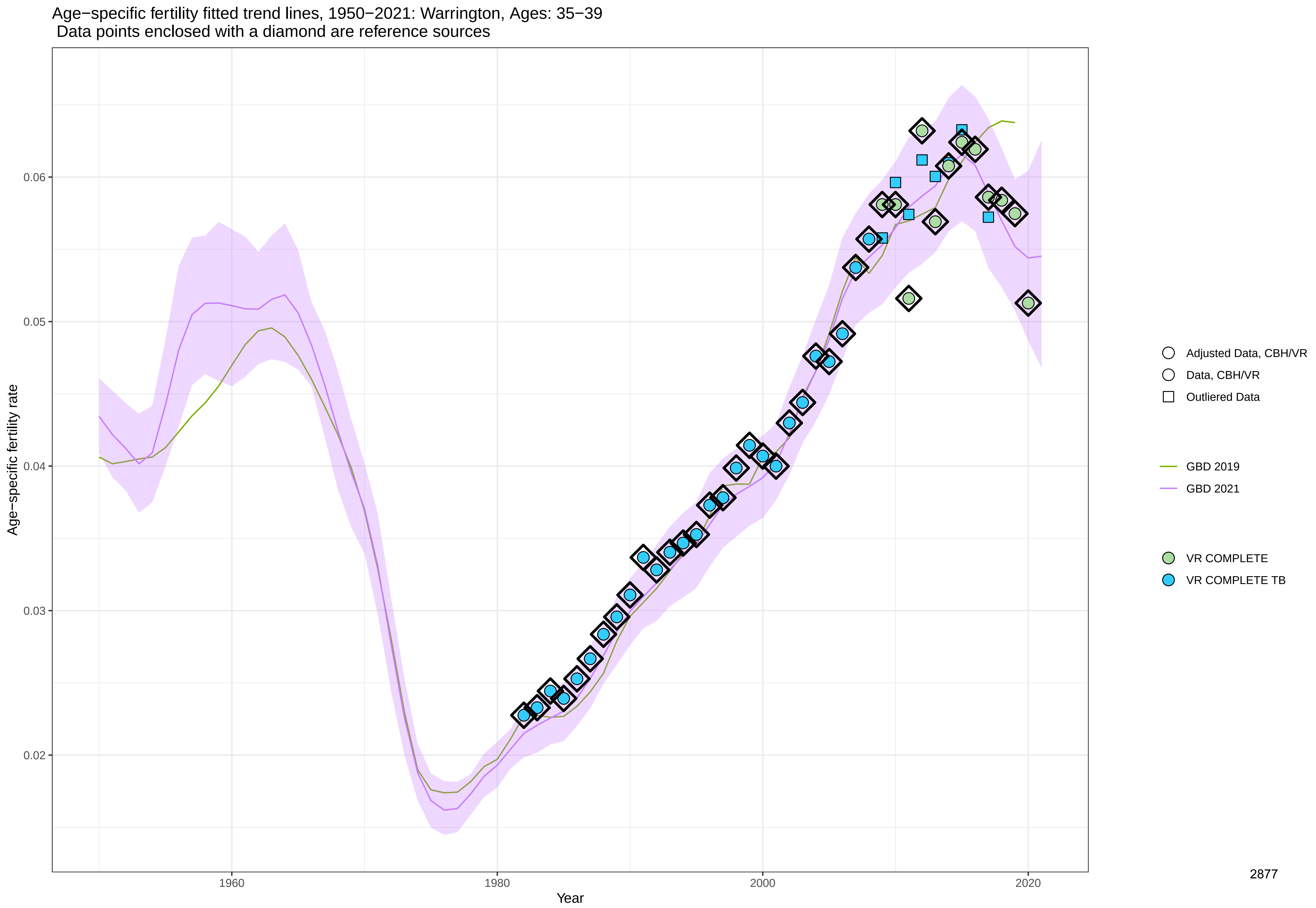

Age−specific fertility fitted trend lines, 1950−2021: Warrington, Ages: 35−39
Data points enclosed with a diamond are reference sources
Age−specific fertility rate
0.06
0.05
0.04
0.03
0.02
1960
1980
2000
2020
Year
Adjusted Data, CBH/VR
Data, CBH/VR
Outliered Data
GBD 2019
GBD 2021
VR COMPLETE
VR COMPLETE TB

Age−specific fertility fitted trend lines, 1950−2021: Warrington, Ages: 40−44
Data points enclosed with a diamond are reference sources

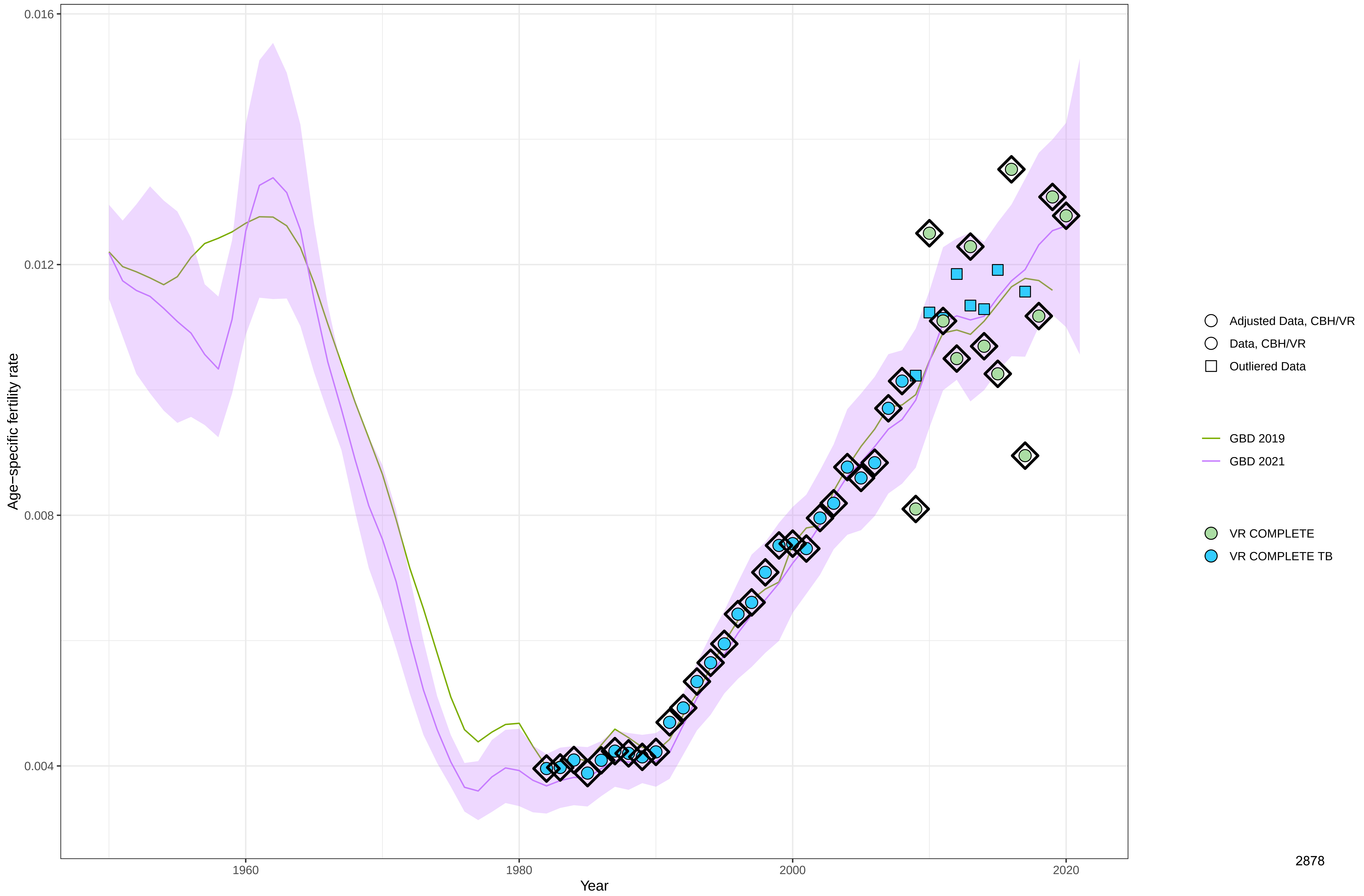

Age−specific fertility fitted trend lines, 1950−2021: Warrington, Ages: 45−49
Data points enclosed with a diamond are reference sources

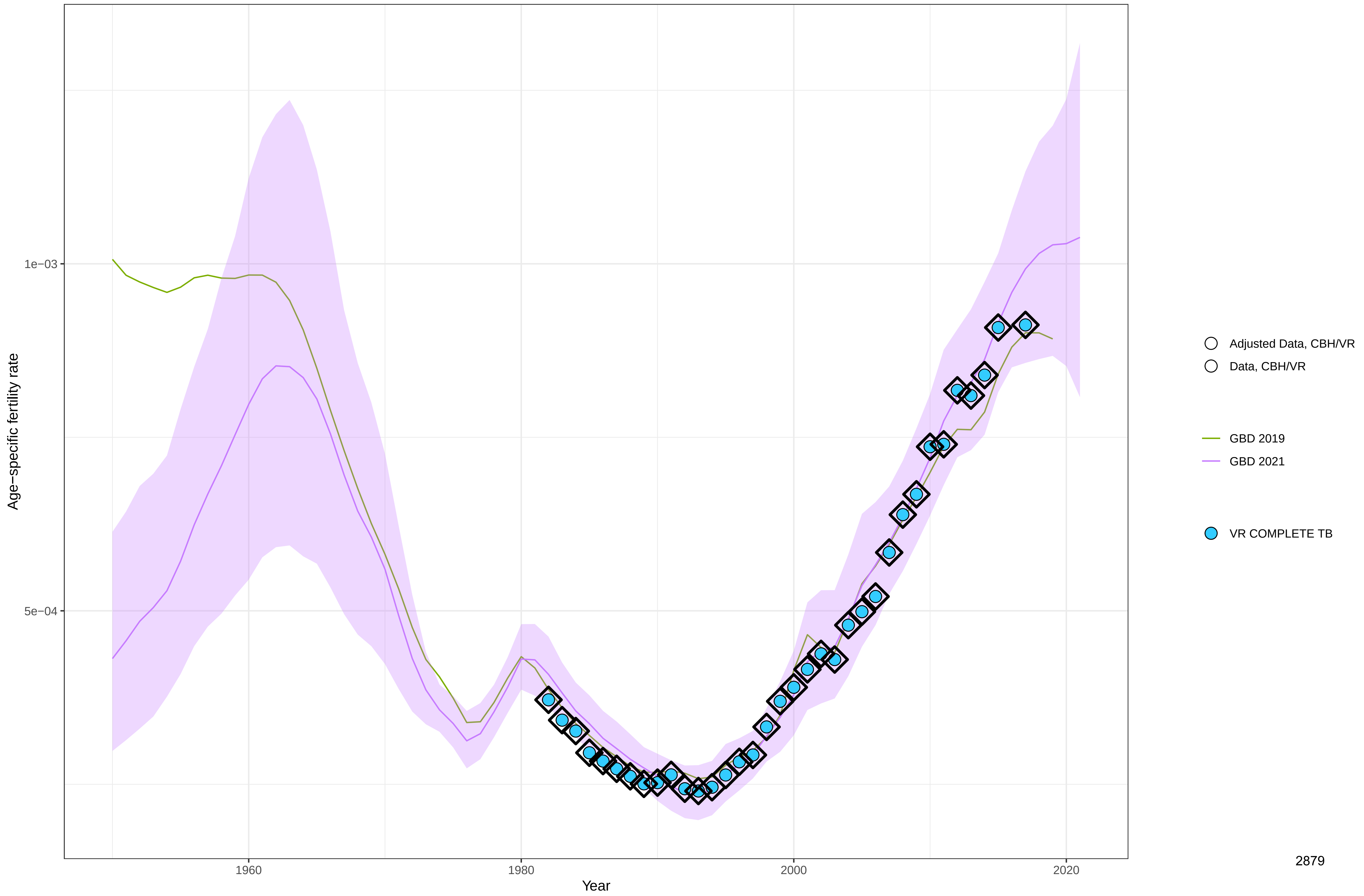

Age−specific fertility fitted trend lines, 1950−2021: Warrington, Ages: 50−54
Data points enclosed with a diamond are reference sources

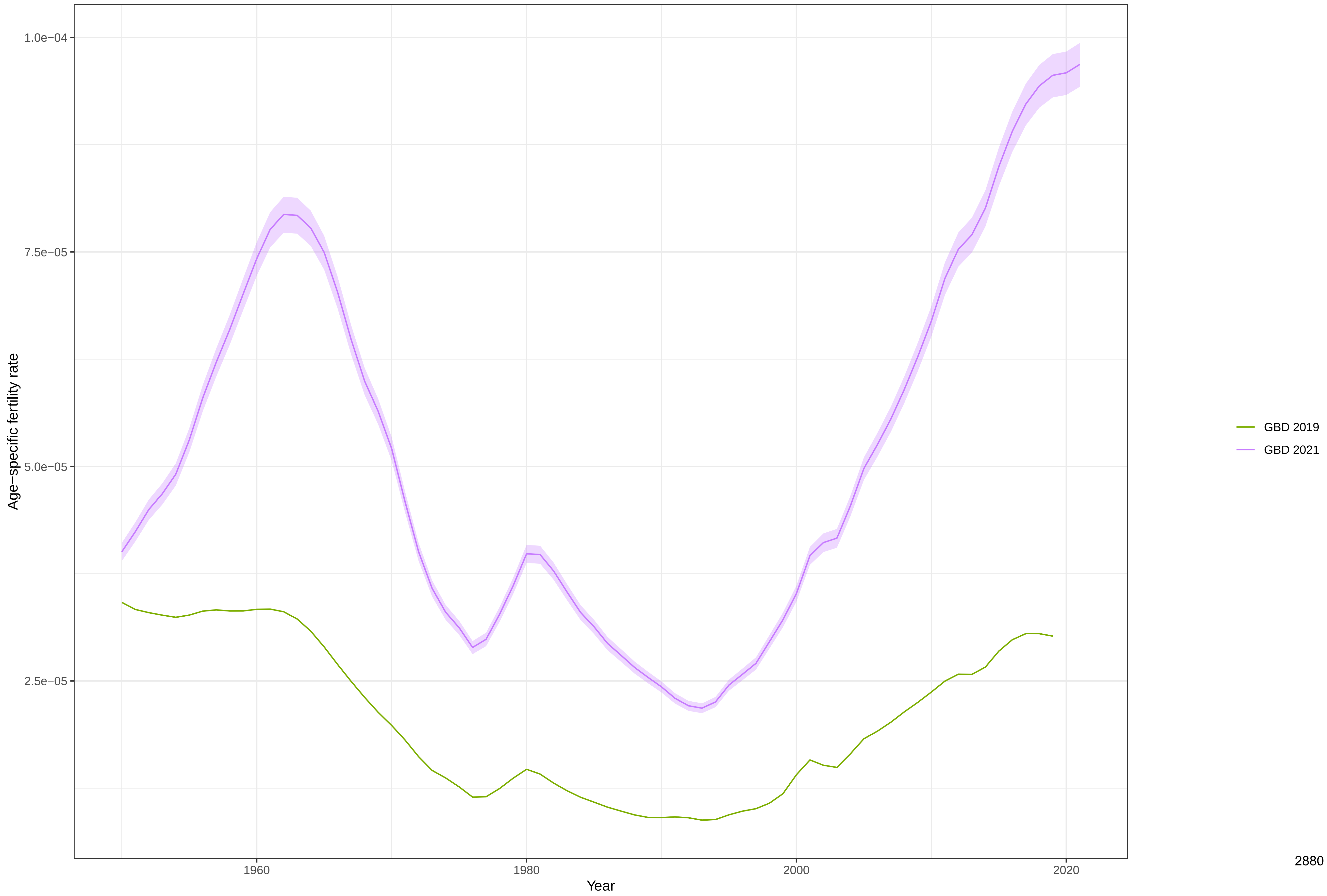

Total fertility rate fitted trend lines, 1950−2021: Warrington TFR
Data points enclosed with a diamond are reference sources

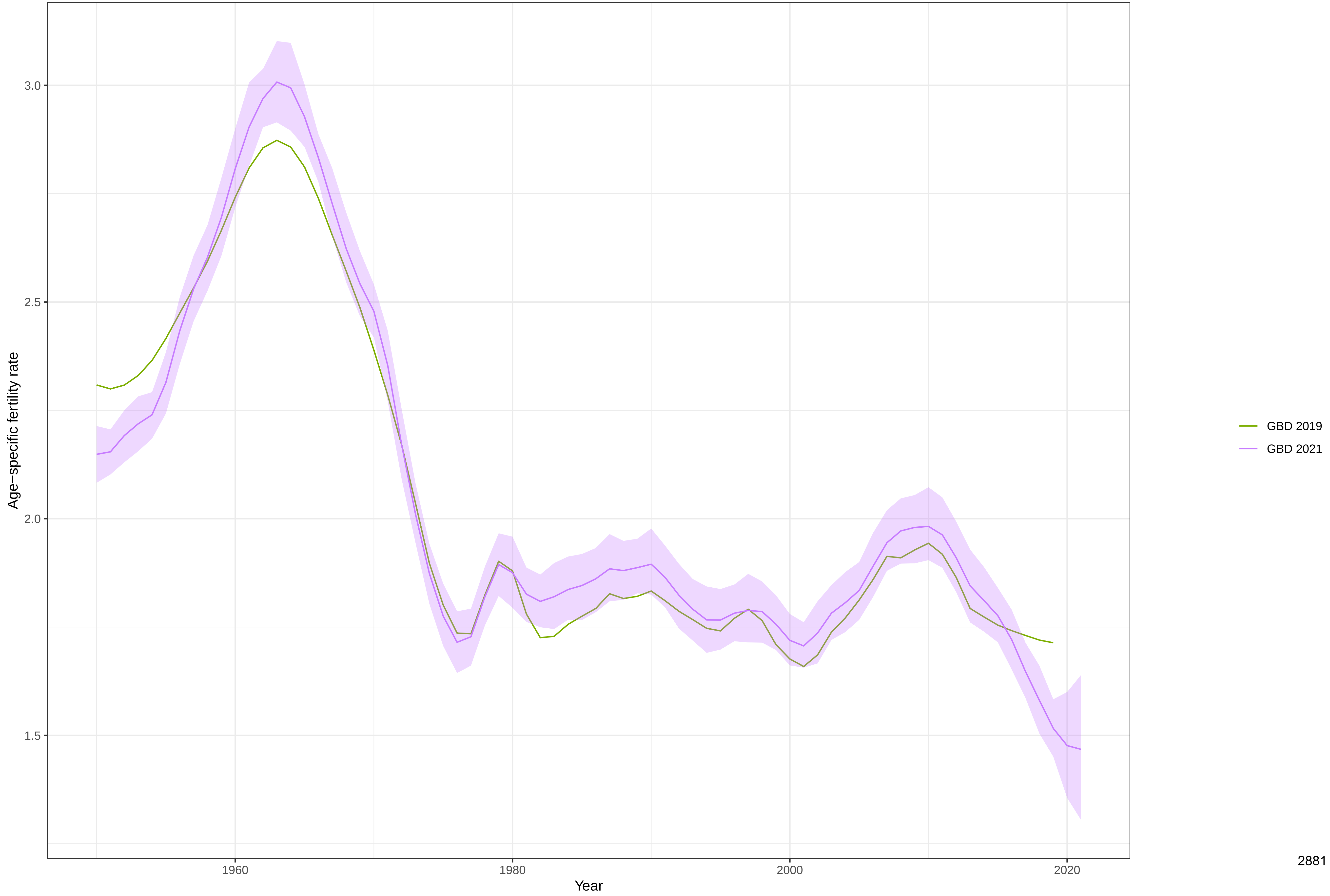

Age−specific fertility fitted trend lines, 1950−2021: Wigan, Ages: 10−14
Data points enclosed with a diamond are reference sources

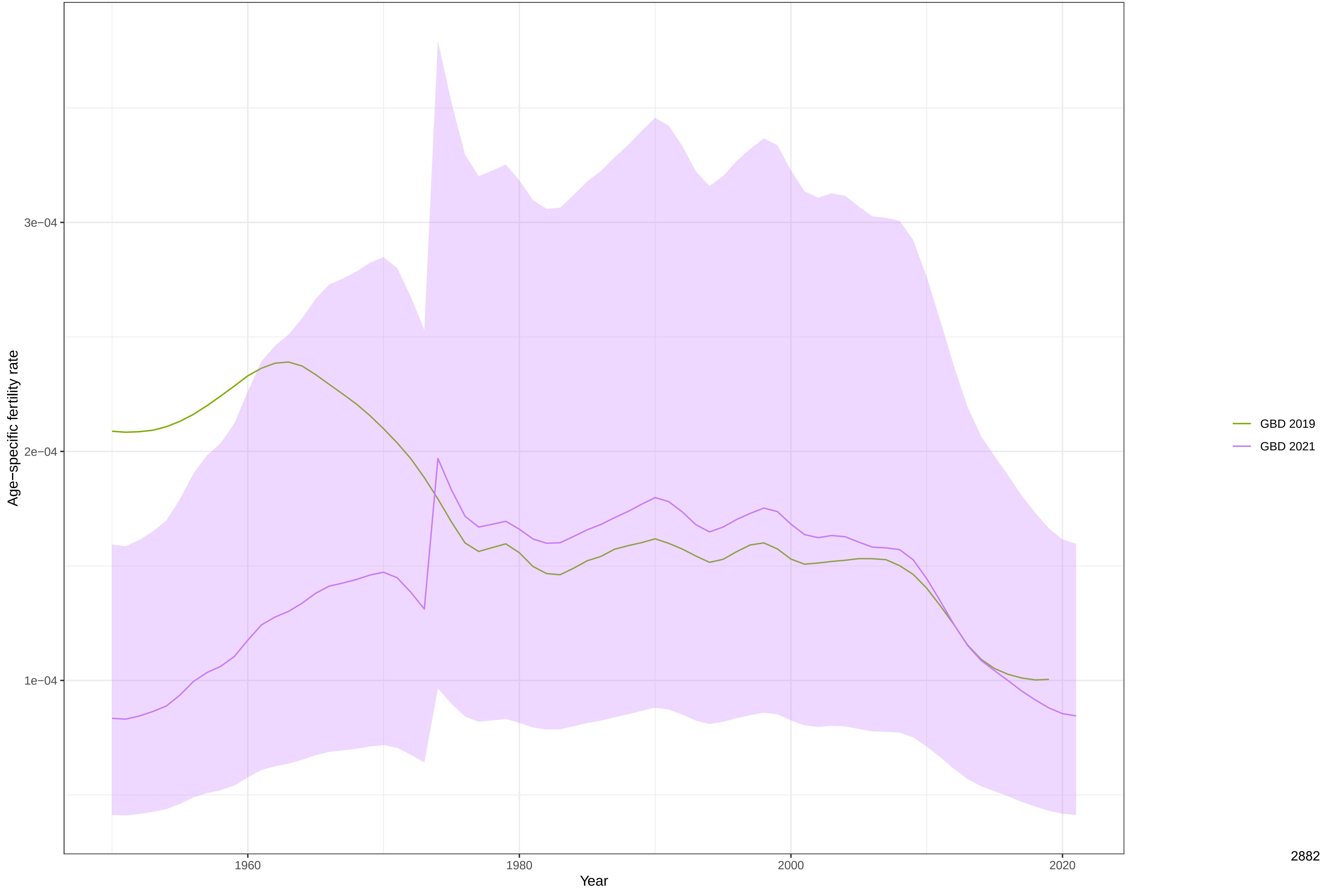

Age−specific fertility fitted trend lines, 1950−2021: Wigan, Ages: 15−19
Data points enclosed with a diamond are reference sources

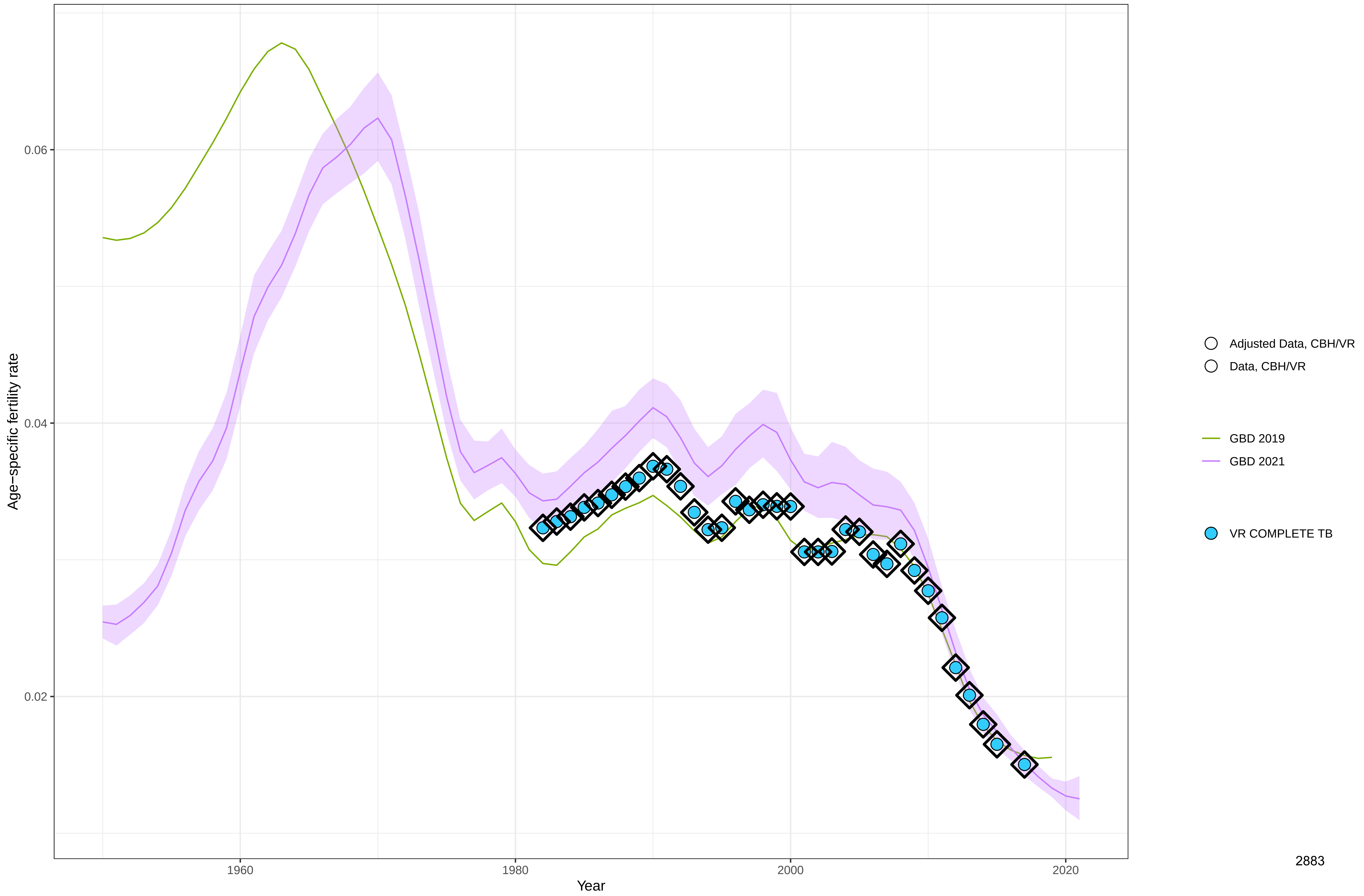

Age−specific fertility fitted trend lines, 1950−2021: Wigan, Ages: 20−24
Data points enclosed with a diamond are reference sources

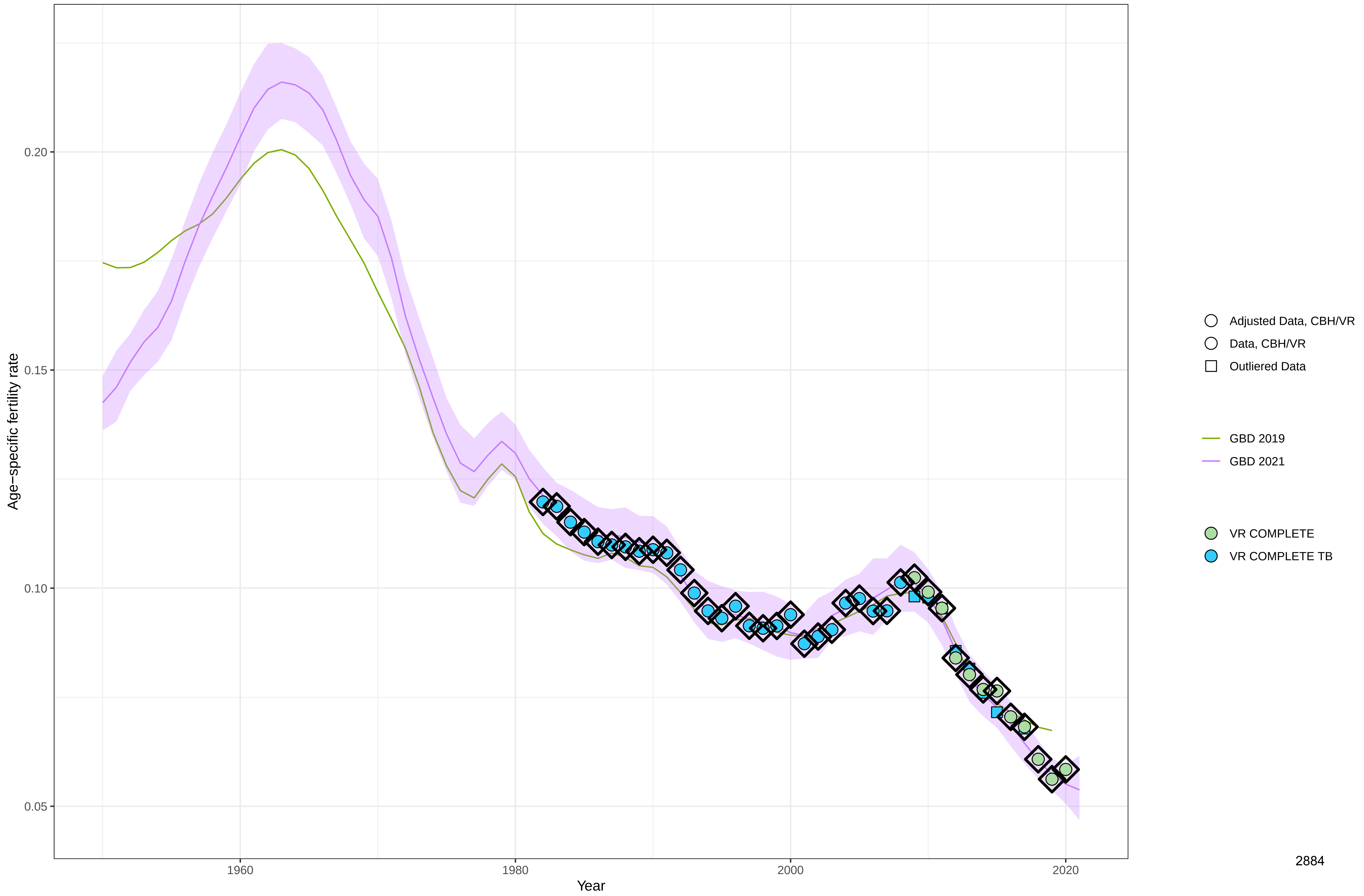

Age−specific fertility fitted trend lines, 1950−2021: Wigan, Ages: 25−29
Data points enclosed with a diamond are reference sources

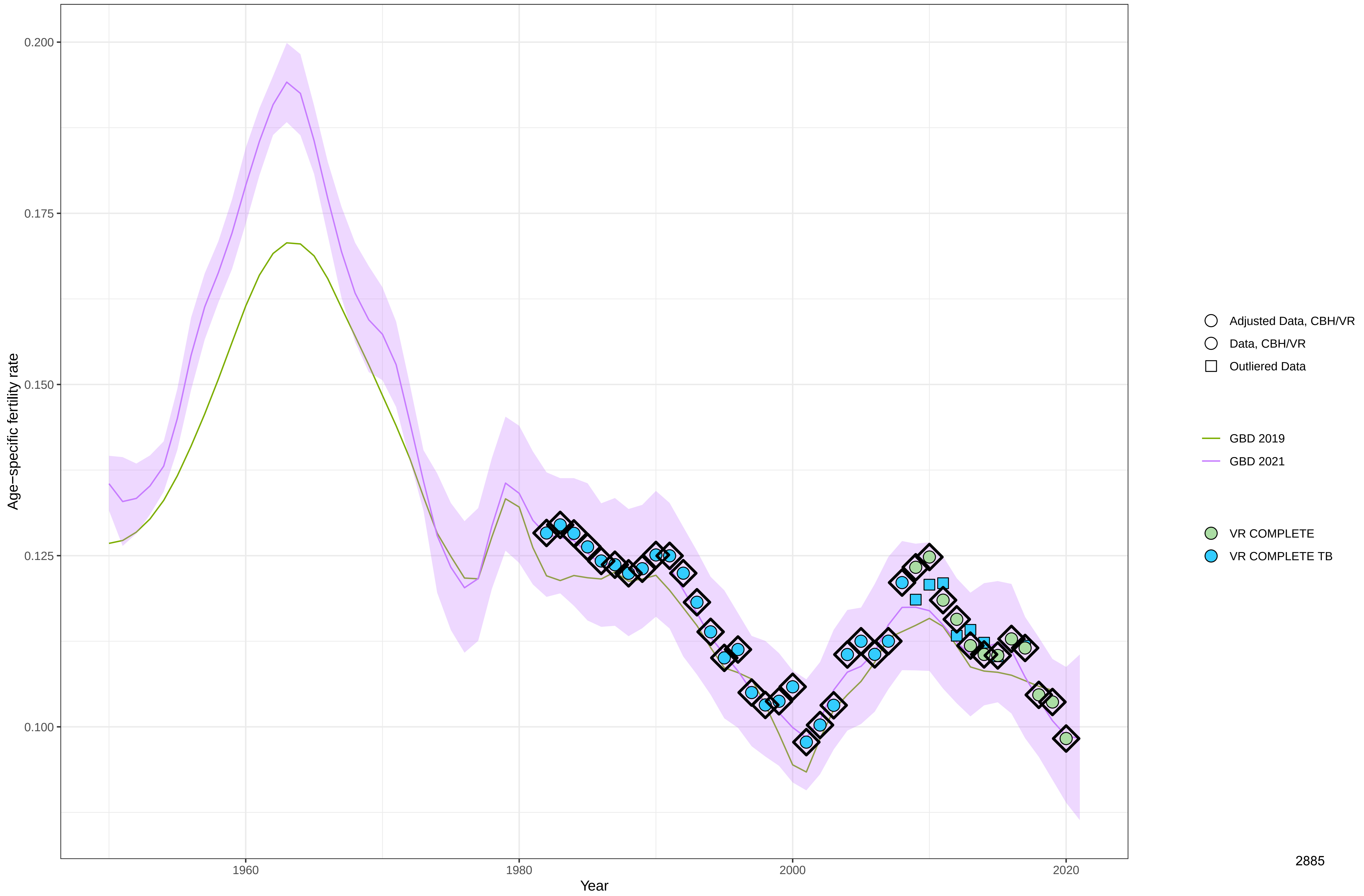

Age−specific fertility fitted trend lines, 1950−2021: Wigan, Ages: 30−34
Data points enclosed with a diamond are reference sources

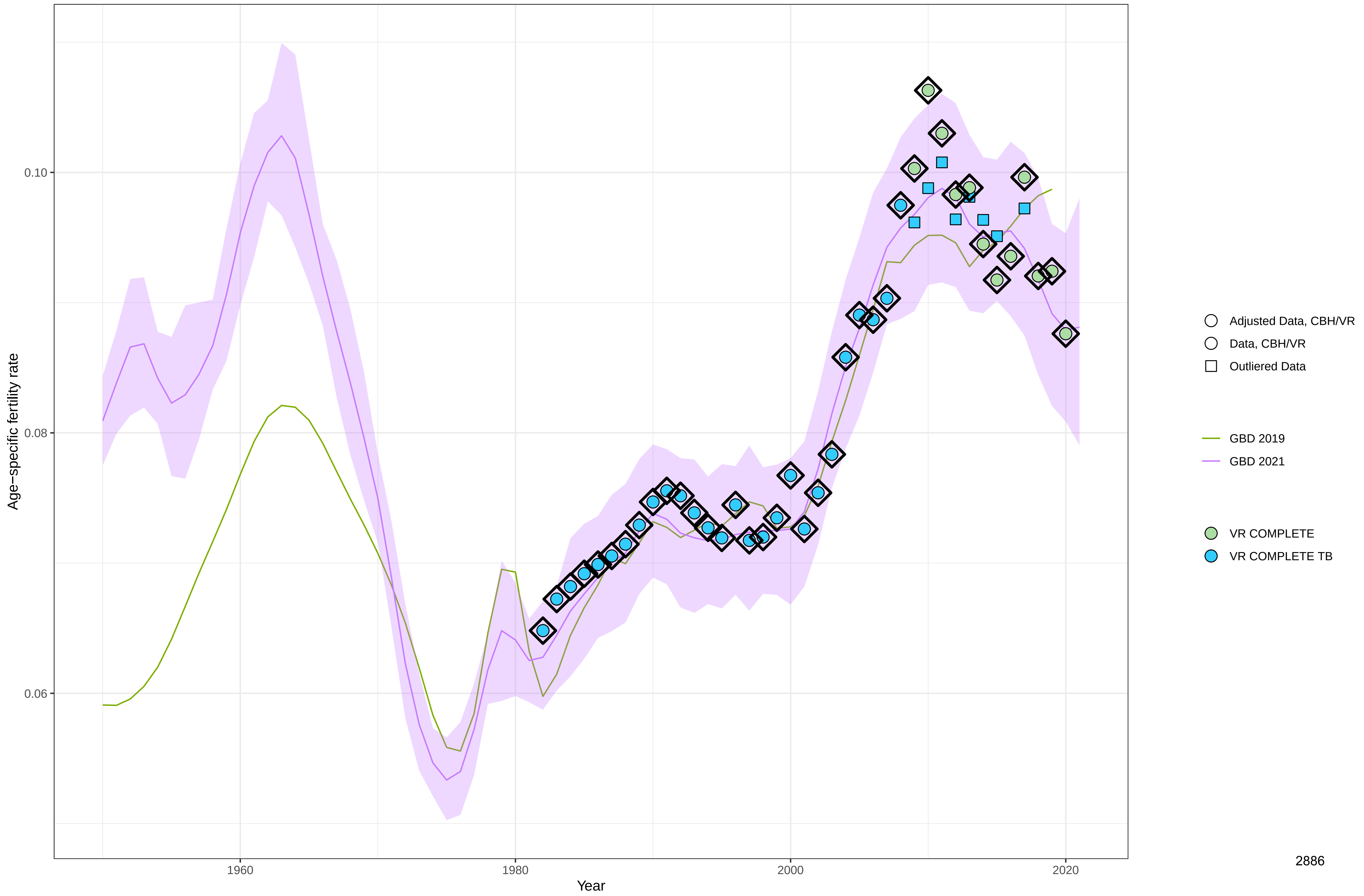

Age−specific fertility fitted trend lines, 1950−2021: Wigan, Ages: 35−39
Data points enclosed with a diamond are reference sources

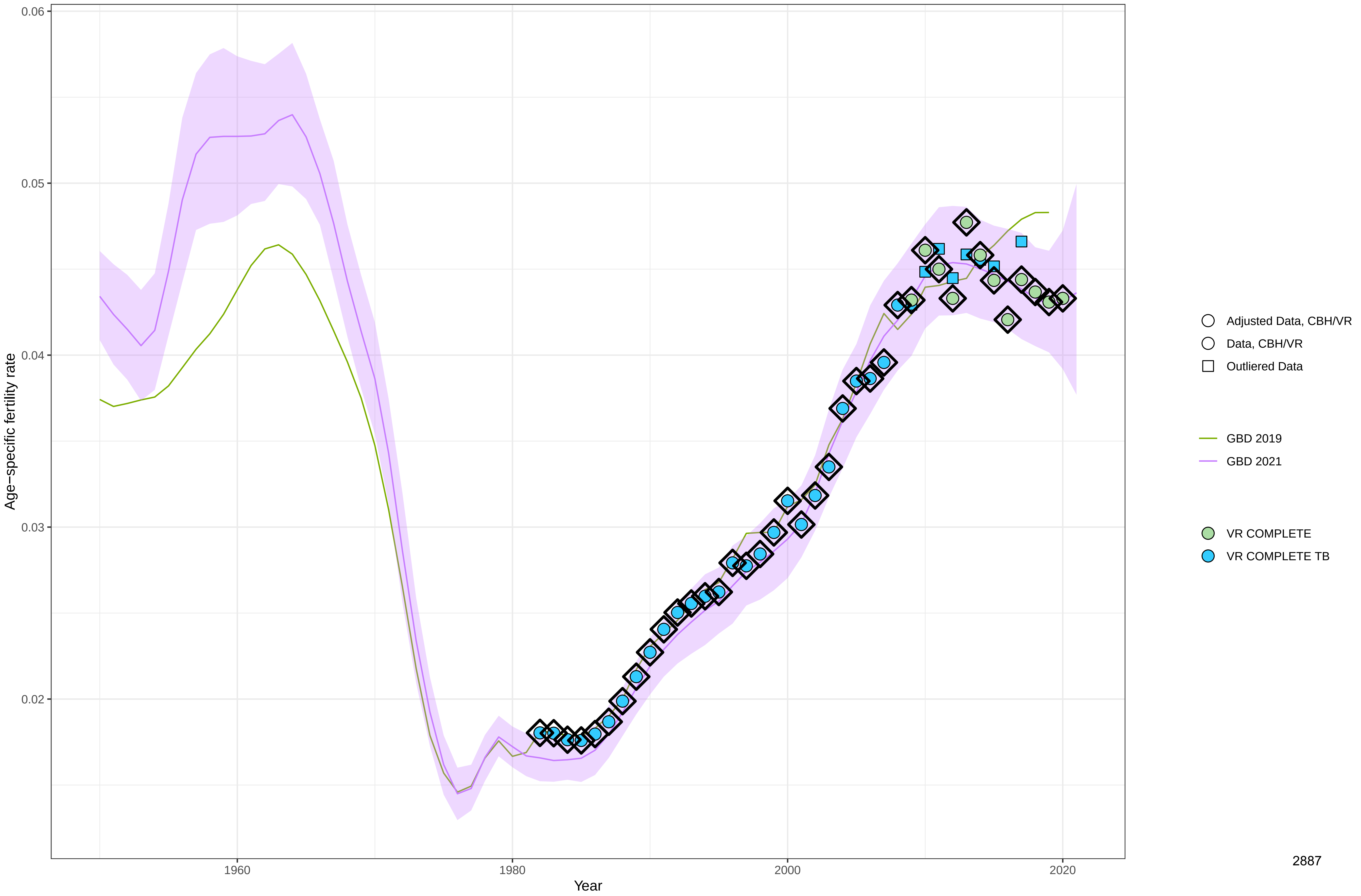

Age−specific fertility fitted trend lines, 1950−2021: Wigan, Ages: 40−44
Data points enclosed with a diamond are reference sources

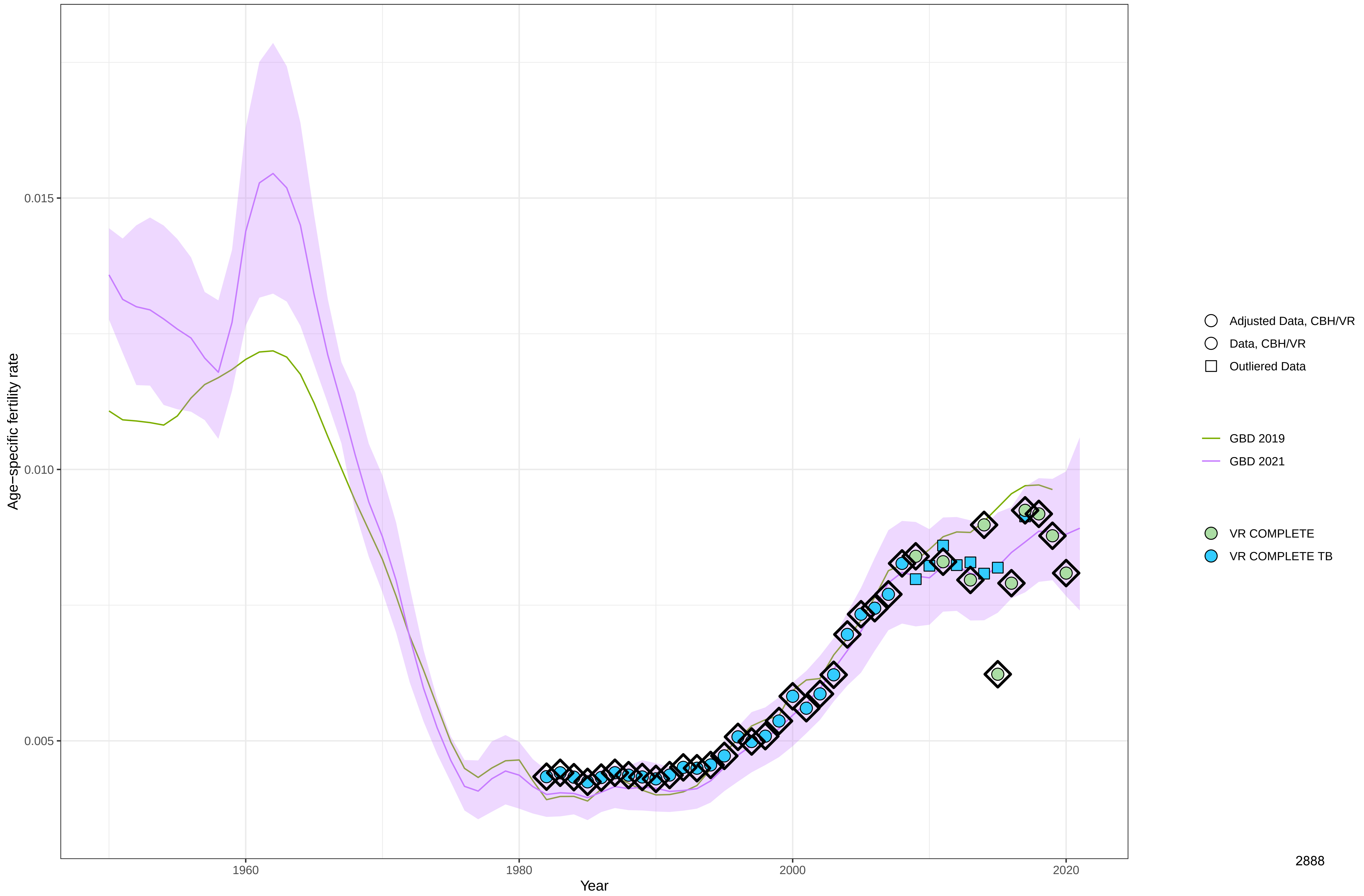

Age−specific fertility fitted trend lines, 1950−2021: Wigan, Ages: 45−49
Data points enclosed with a diamond are reference sources

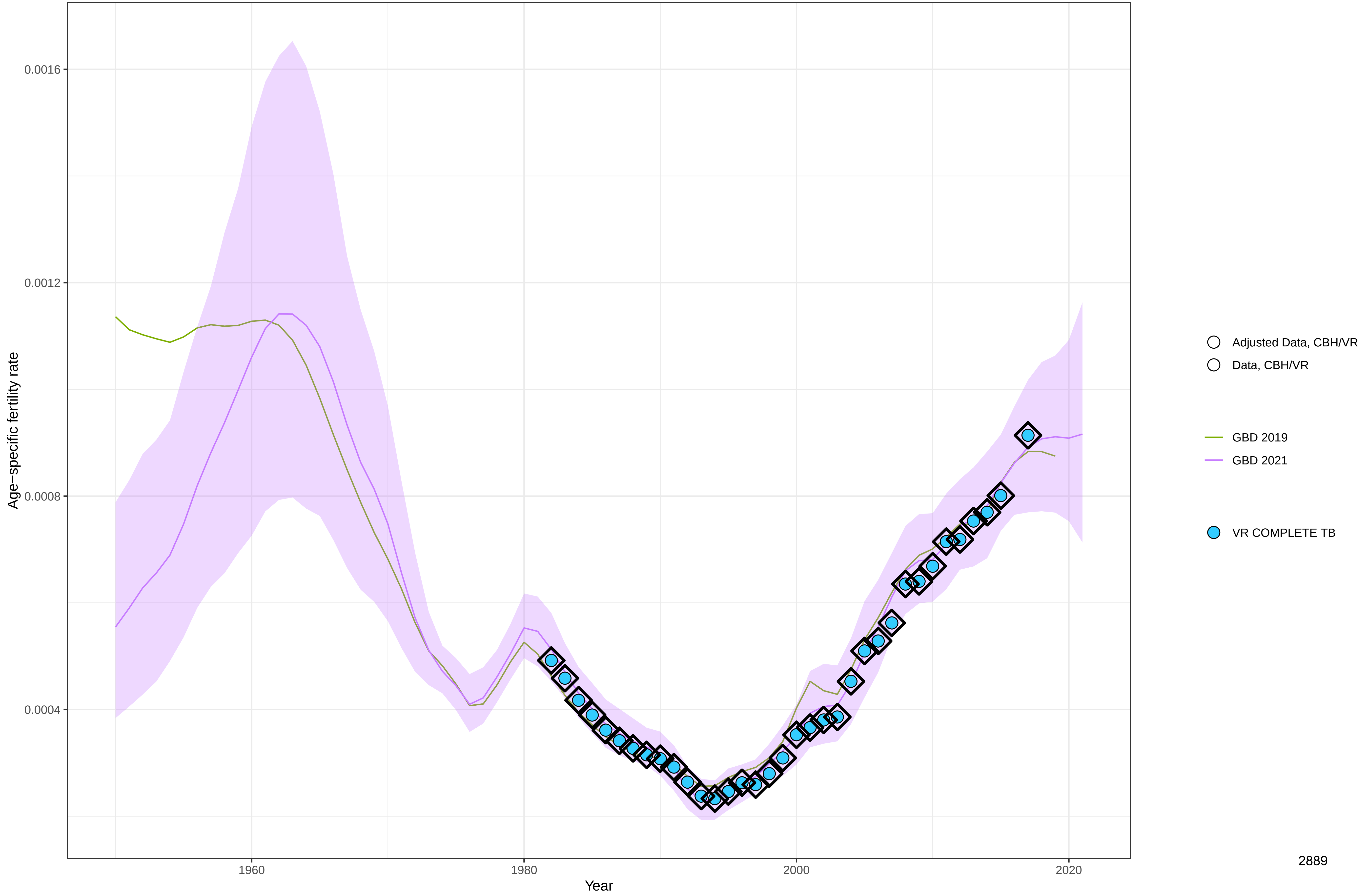

Age−specific fertility fitted trend lines, 1950−2021: Wigan, Ages: 50−54
Data points enclosed with a diamond are reference sources

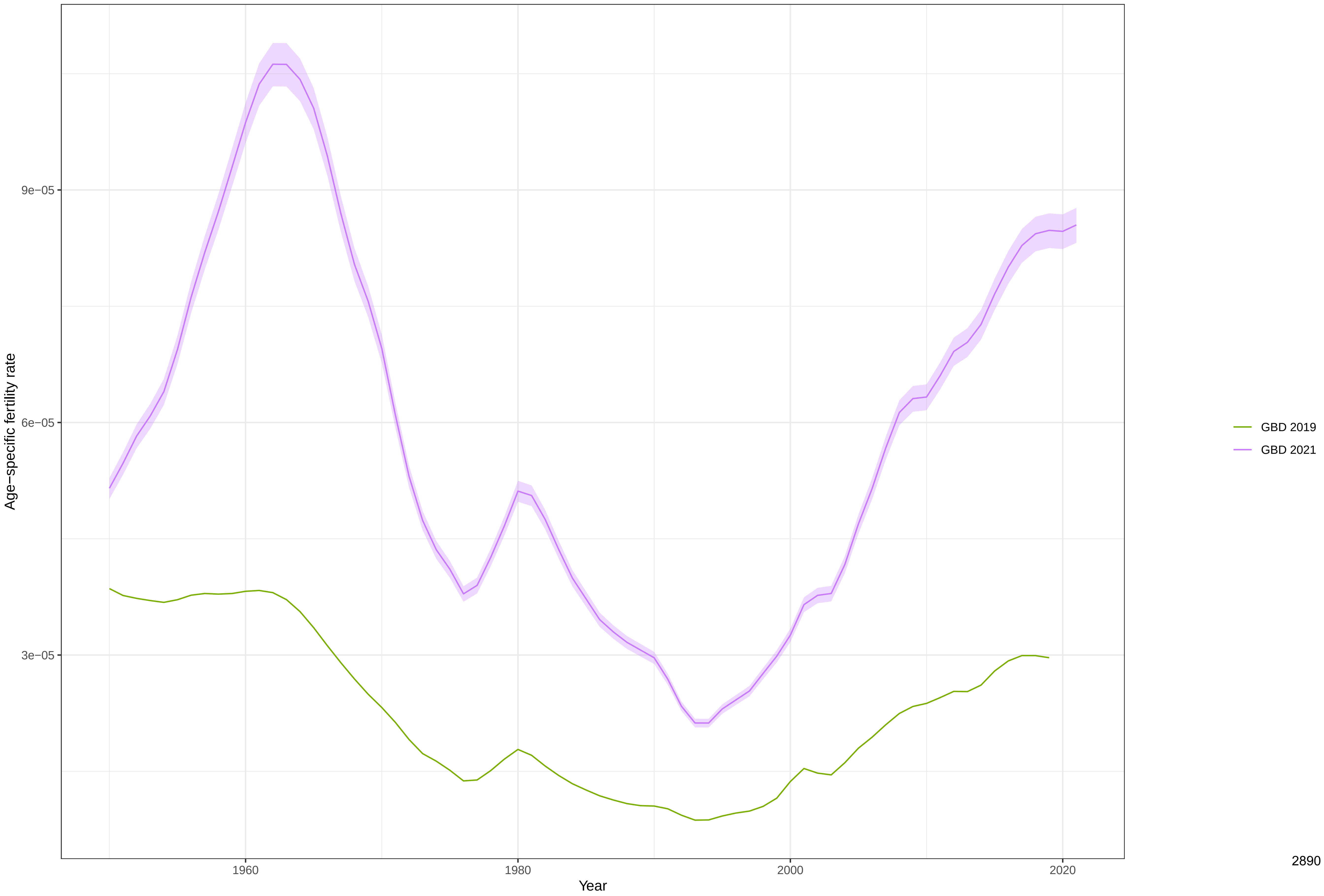

2890

Total fertility rate fitted trend lines, 1950−2021: Wigan TFR
Data points enclosed with a diamond are reference sources

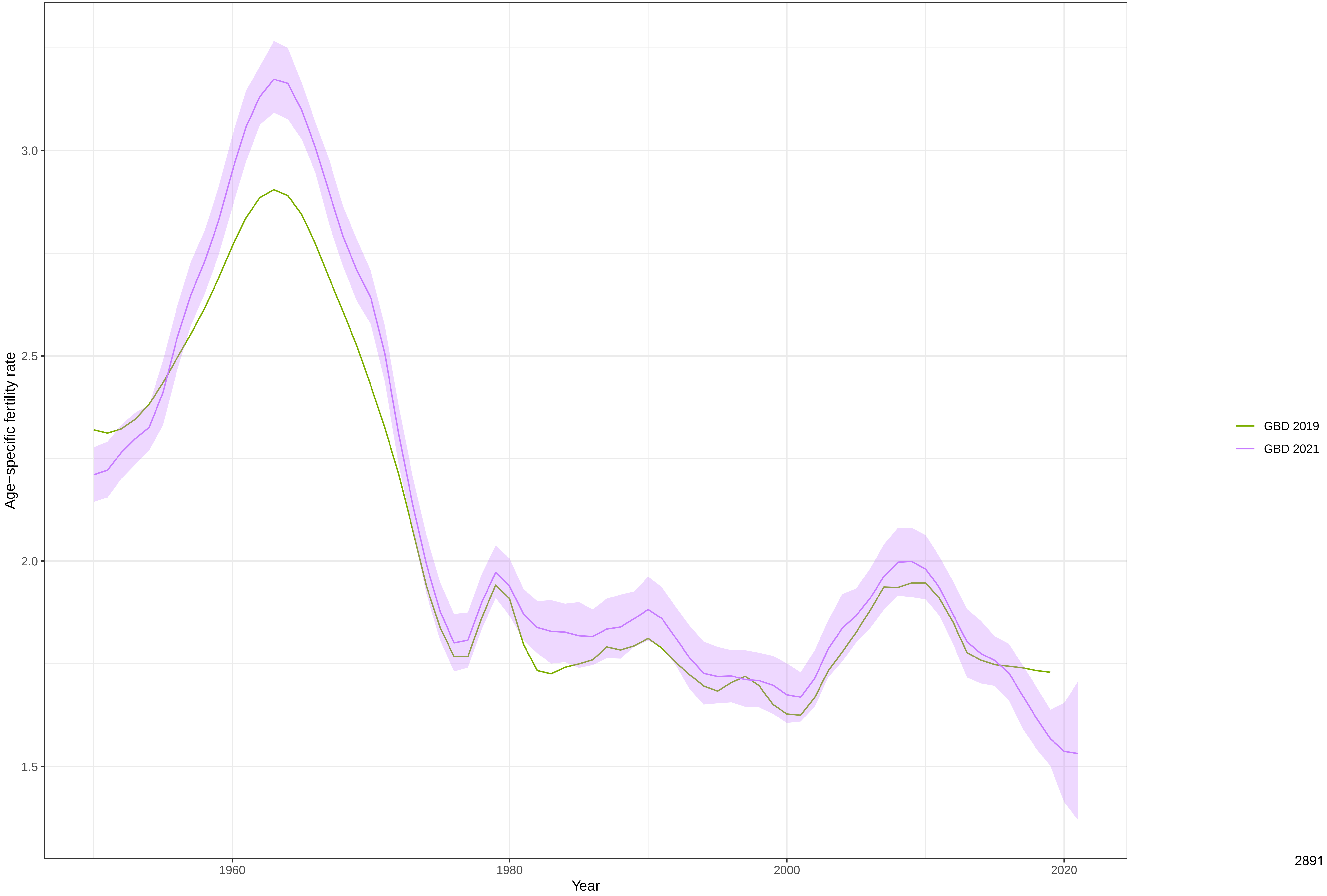

Age−specific fertility fitted trend lines, 1950−2021: Wirral, Ages: 10−14
Data points enclosed with a diamond are reference sources

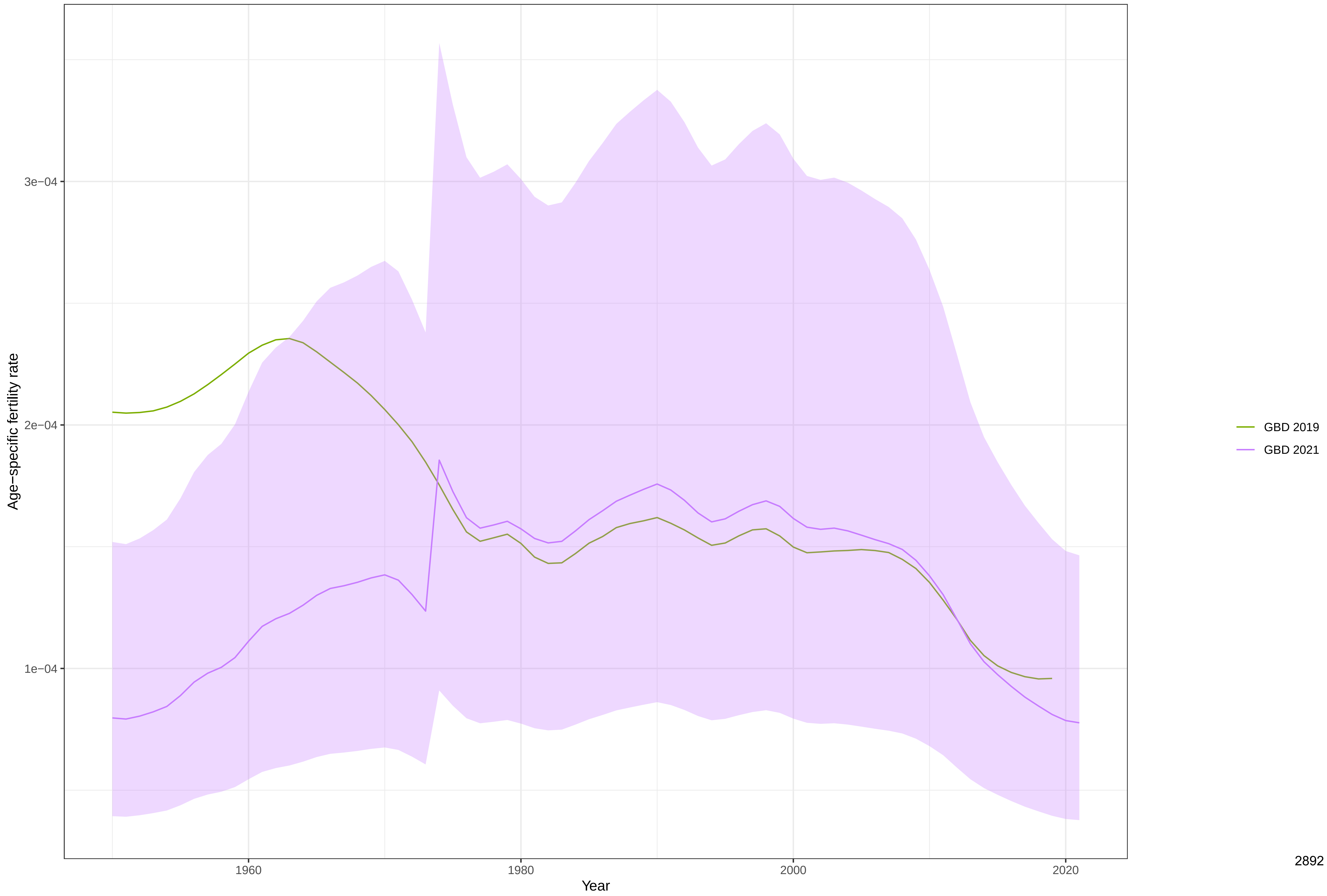

2892

Age−specific fertility fitted trend lines, 1950−2021: Wirral, Ages: 15−19
Data points enclosed with a diamond are reference sources

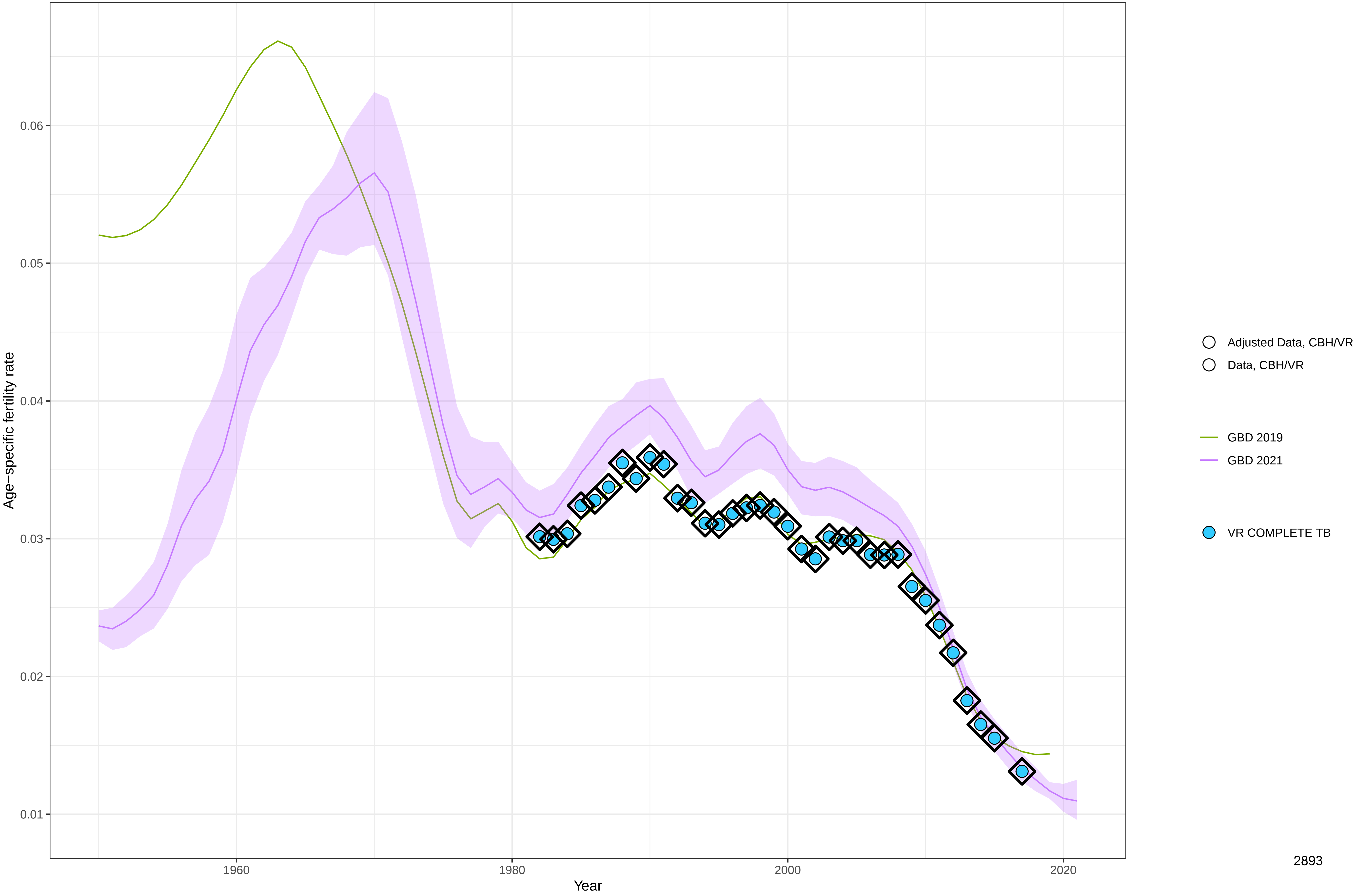

Age−specific fertility fitted trend lines, 1950−2021: Wirral, Ages: 20−24
Data points enclosed with a diamond are reference sources

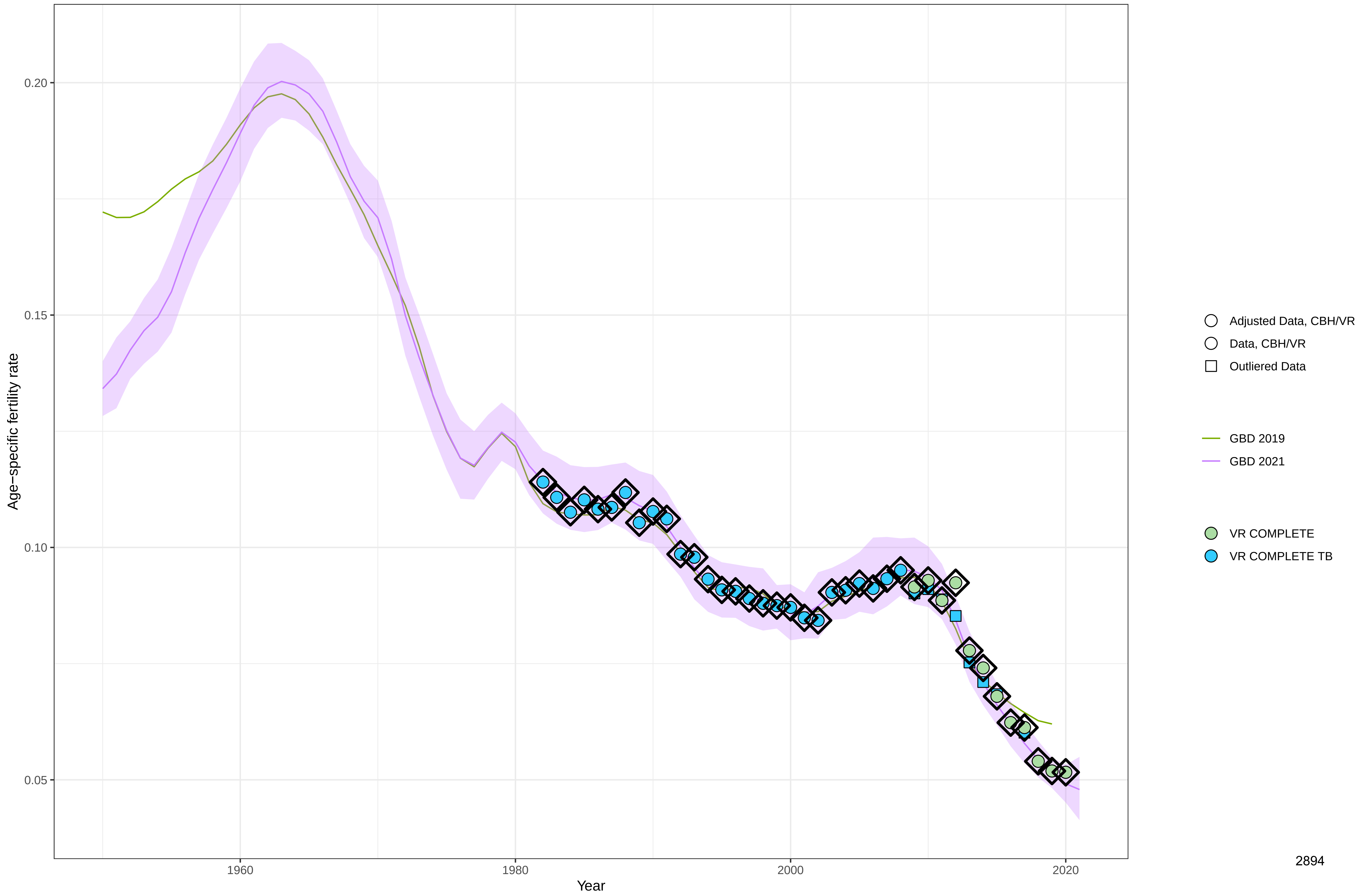

Age−specific fertility fitted trend lines, 1950−2021: Wirral, Ages: 25−29
Data points enclosed with a diamond are reference sources

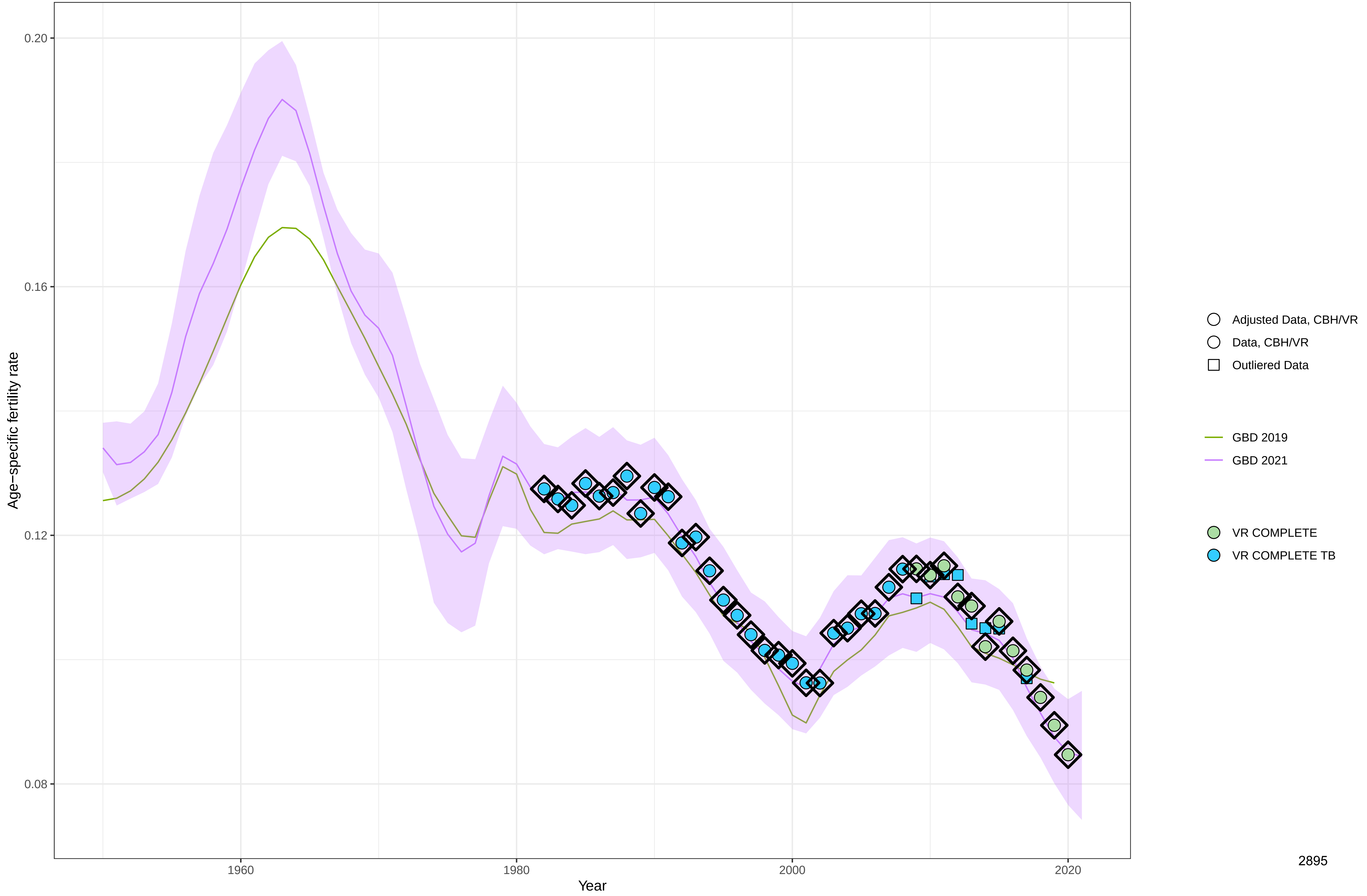

Age−specific fertility fitted trend lines, 1950−2021: Wirral, Ages: 30−34
Data points enclosed with a diamond are reference sources

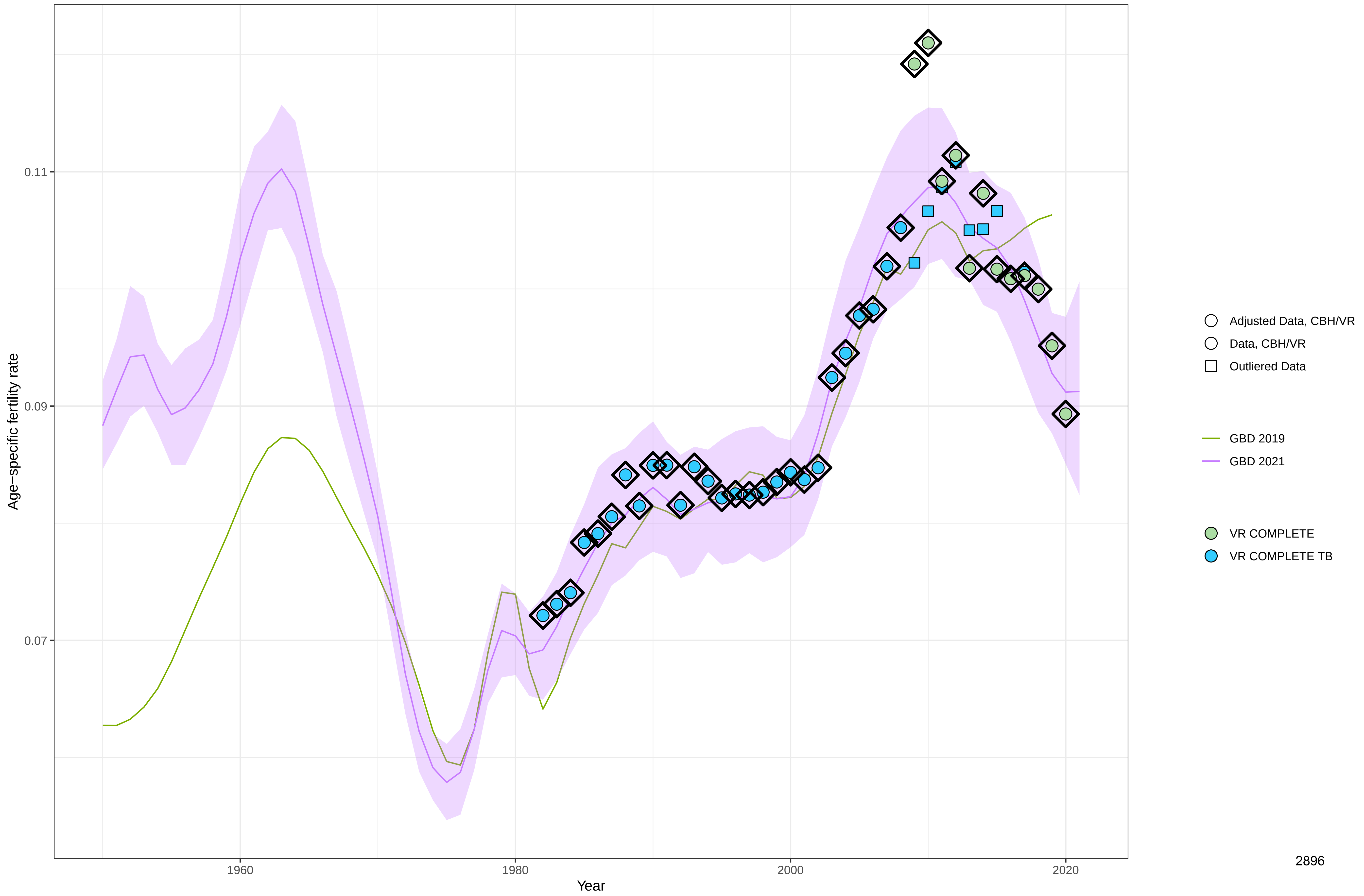

Age−specific fertility fitted trend lines, 1950−2021: Wirral, Ages: 35−39
Data points enclosed with a diamond are reference sources

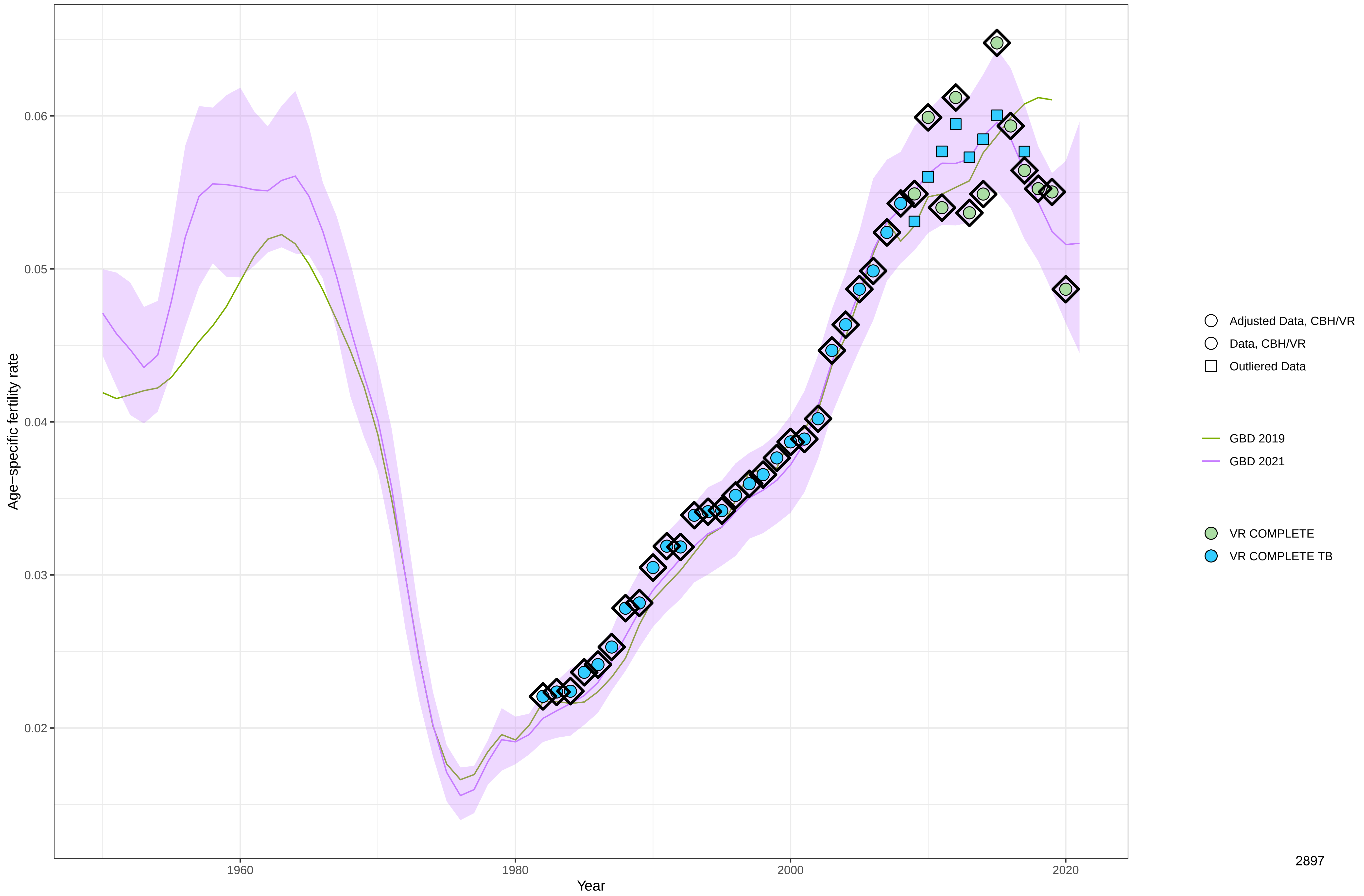

Age−specific fertility fitted trend lines, 1950−2021: Wirral, Ages: 40−44
Data points enclosed with a diamond are reference sources

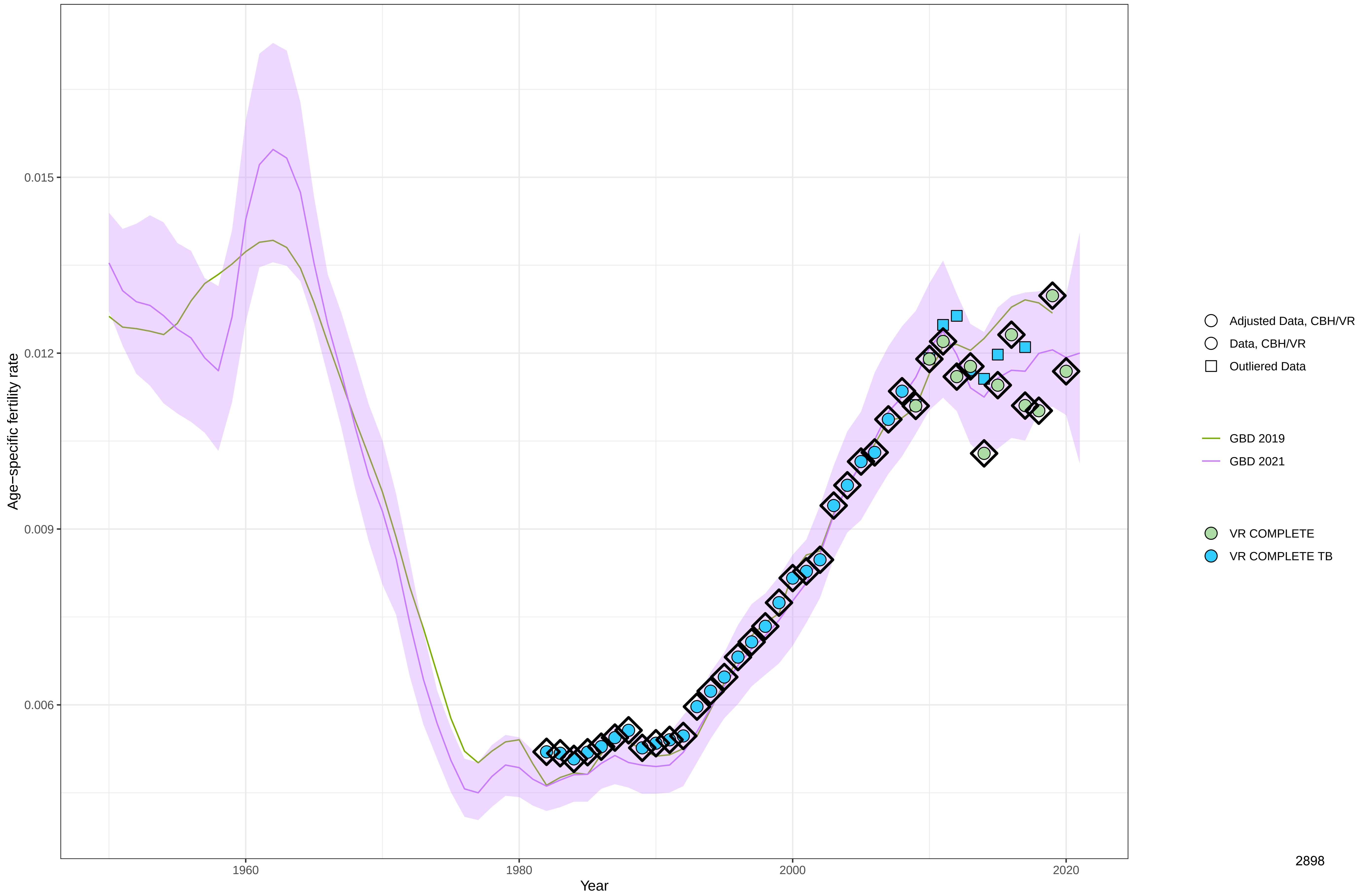

Age−specific fertility fitted trend lines, 1950−2021: Wirral, Ages: 45−49
Data points enclosed with a diamond are reference sources

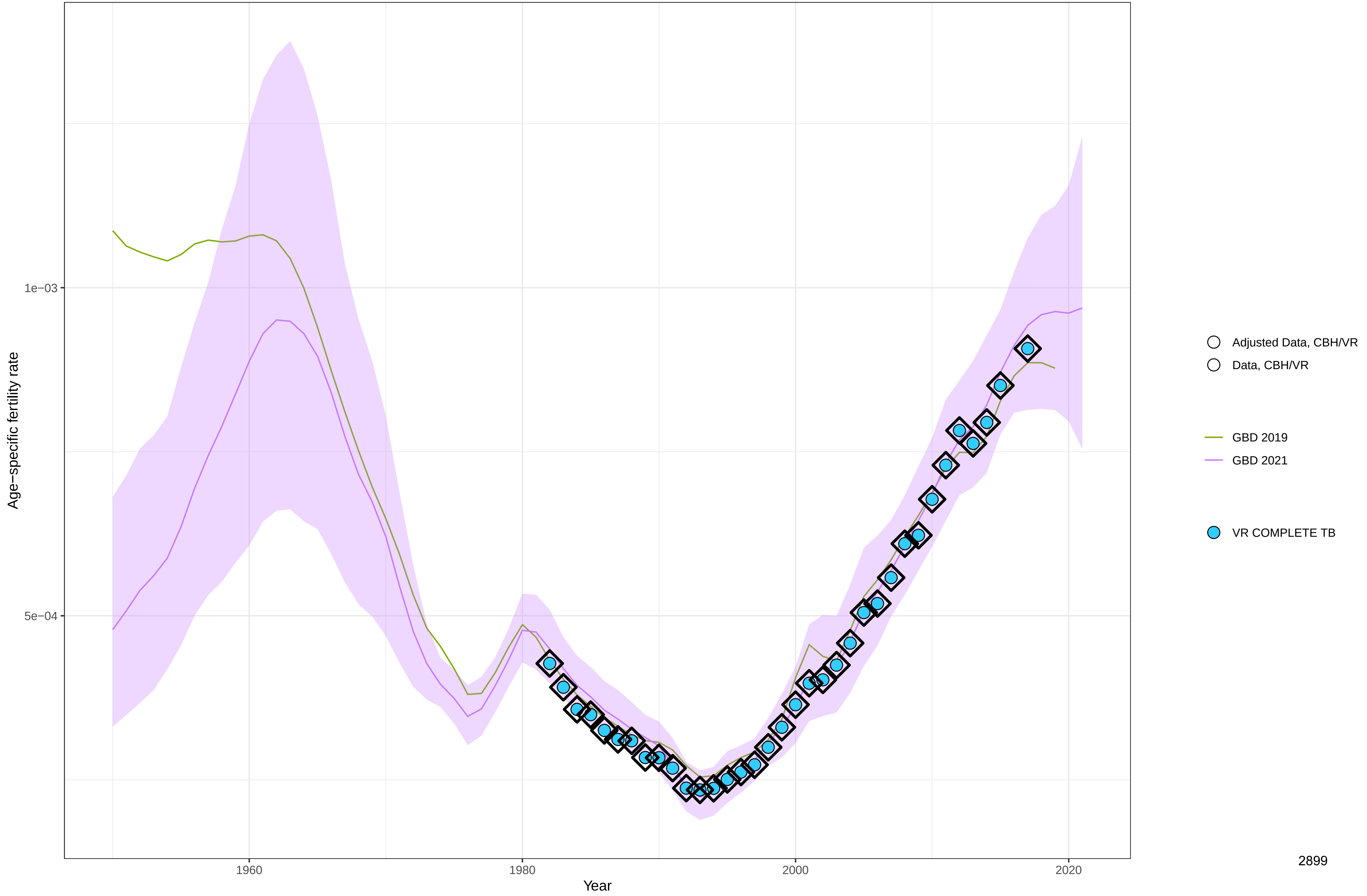

Age−specific fertility fitted trend lines, 1950−2021: Wirral, Ages: 50−54
Data points enclosed with a diamond are reference sources

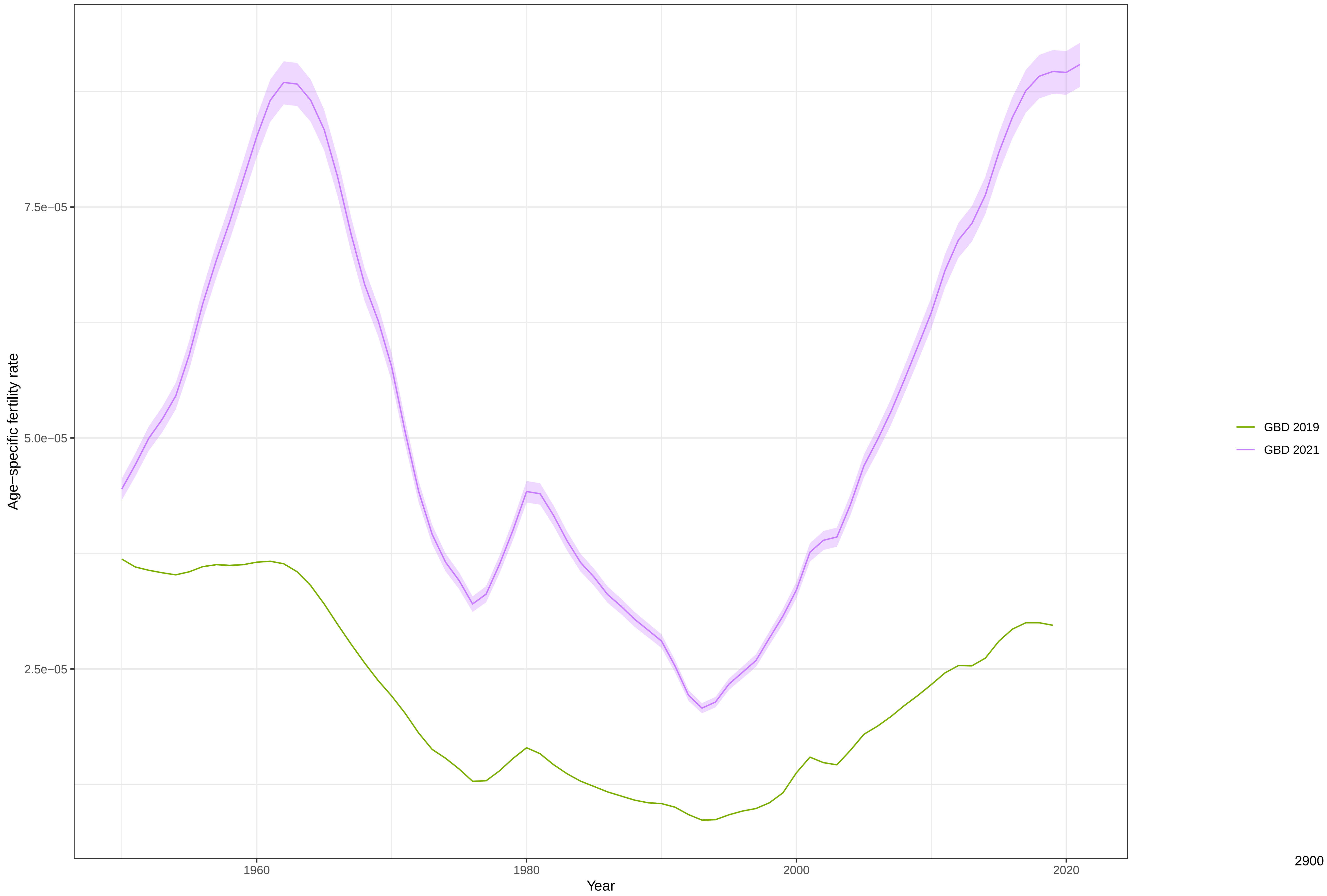

Total fertility rate fitted trend lines, 1950−2021: Wirral TFR
Data points enclosed with a diamond are reference sources

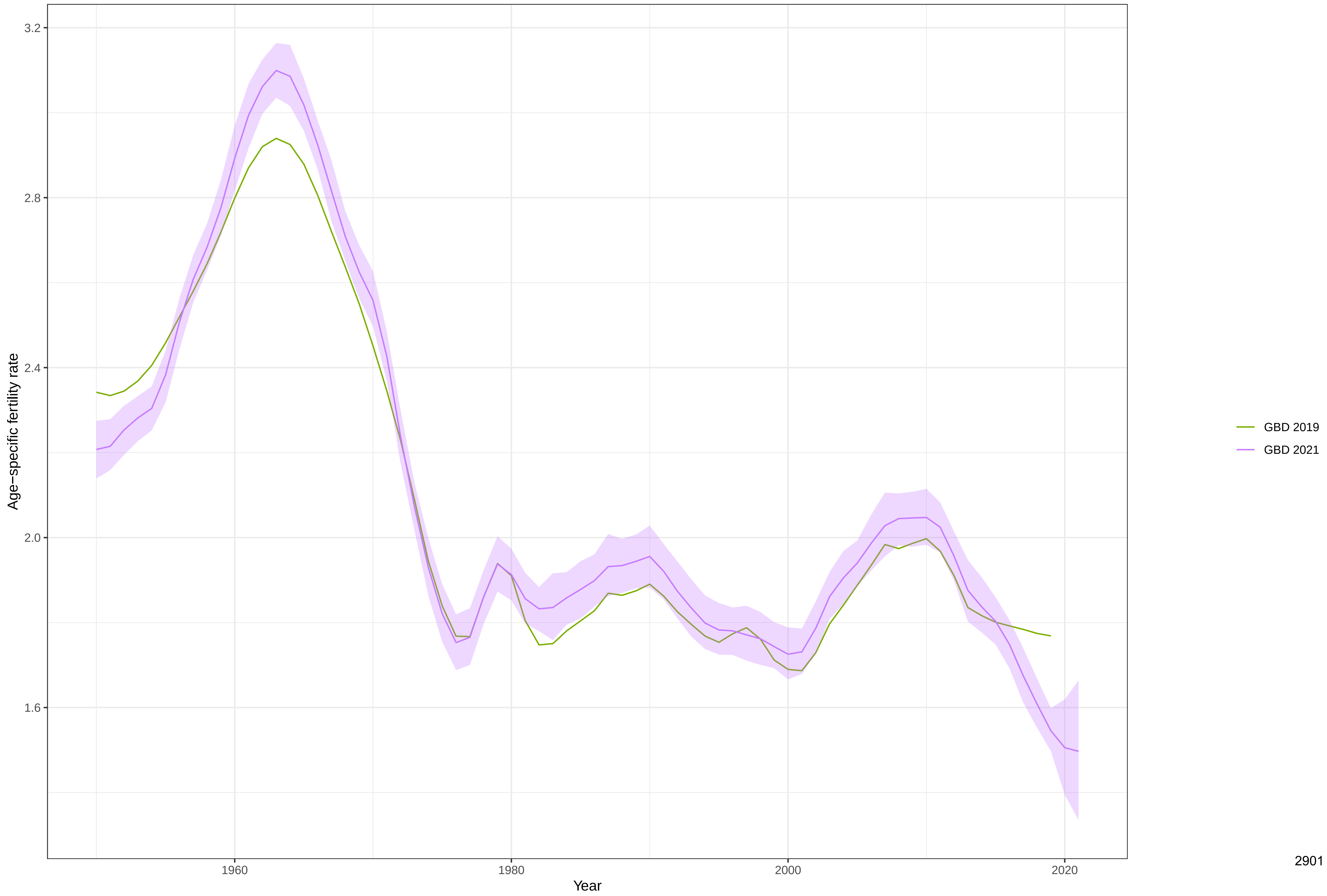

Age−specific fertility fitted trend lines, 1950−2021: South East England, Ages: 10−14
Data points enclosed with a diamond are reference sources

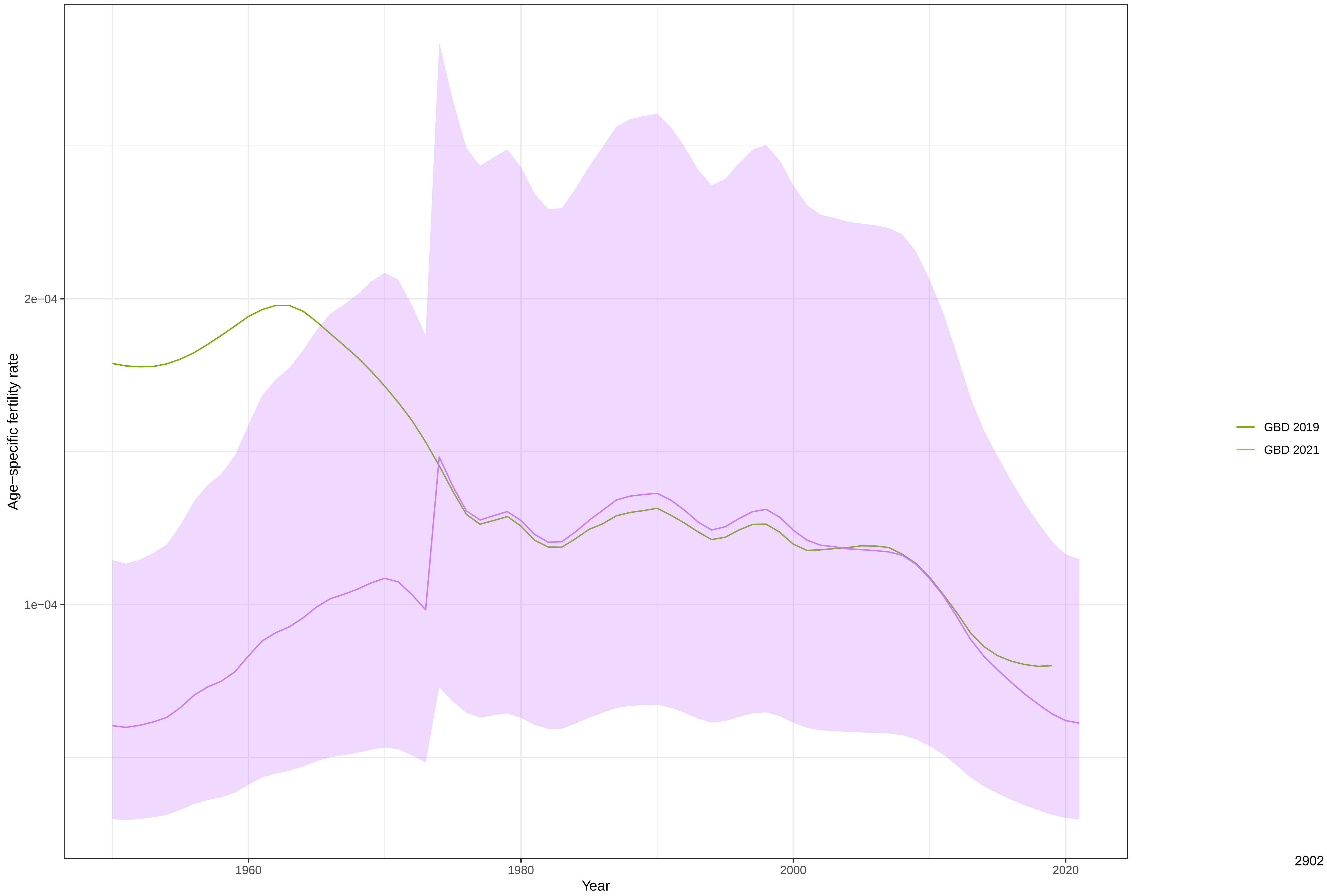

Age−specific fertility fitted trend lines, 1950−2021: South East England, Ages: 15−19
Data points enclosed with a diamond are reference sources

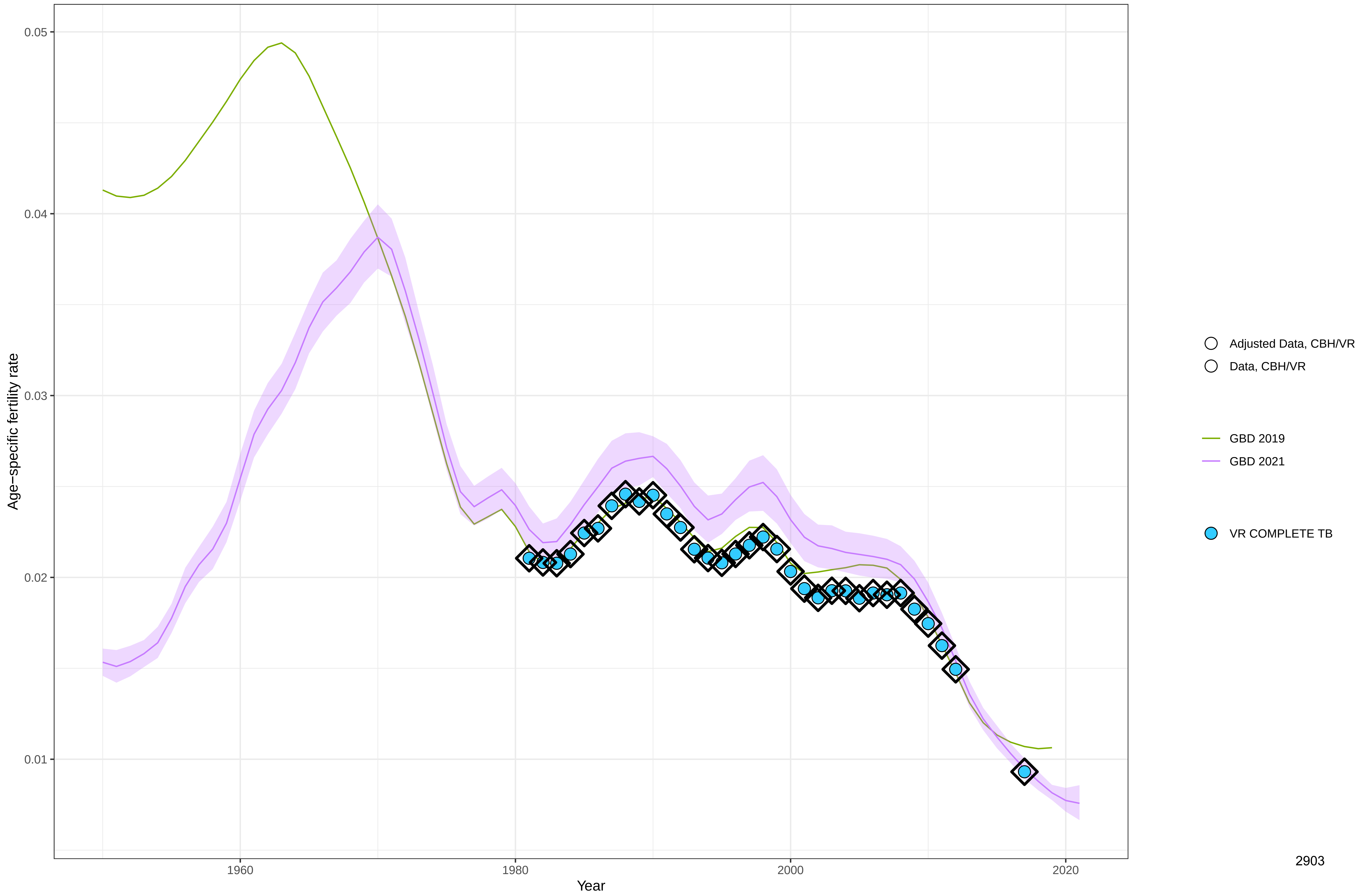

2903

Age−specific fertility fitted trend lines, 1950−2021: South East England, Ages: 20−24
Data points enclosed with a diamond are reference sources

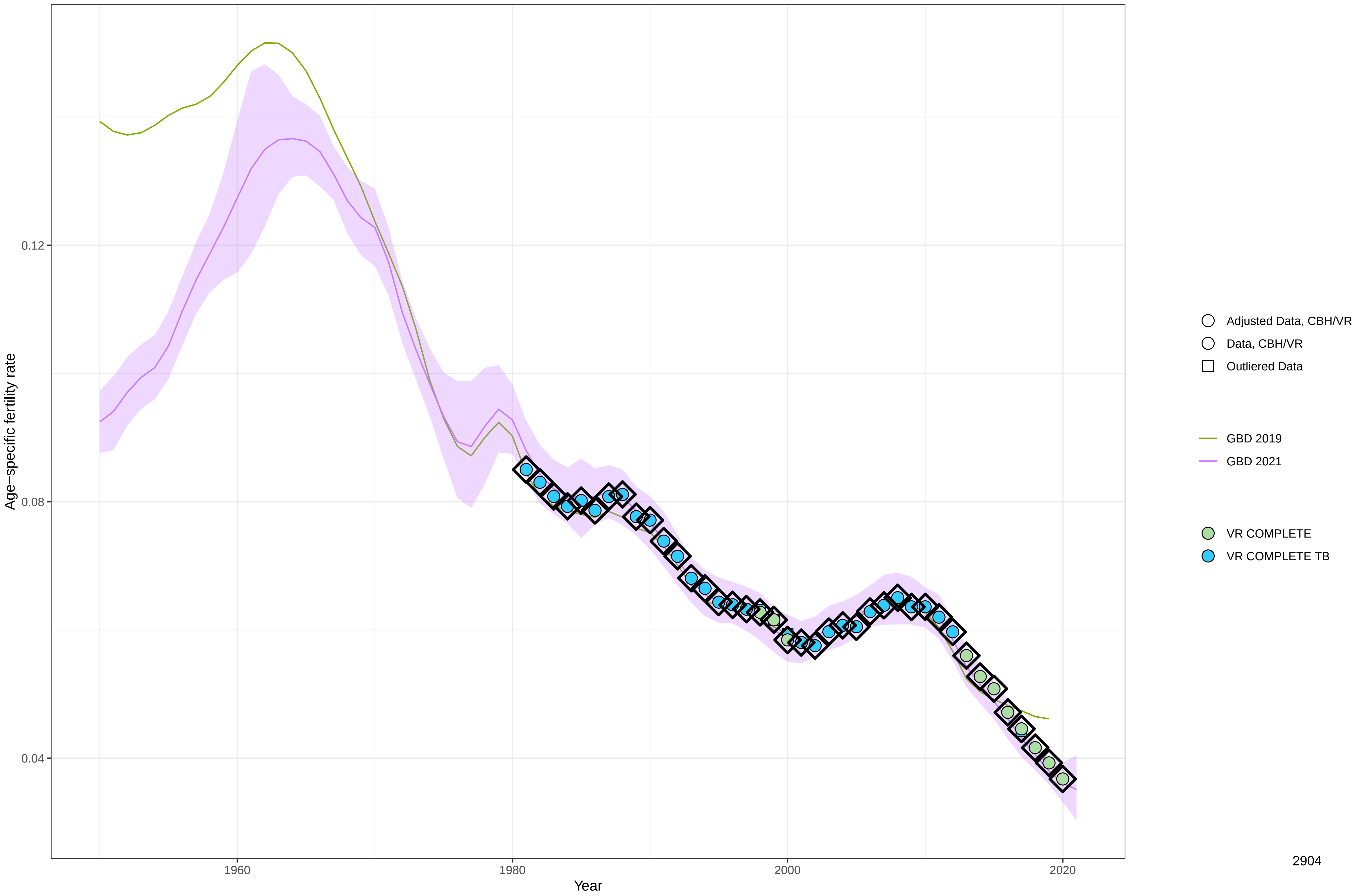

Age−specific fertility fitted trend lines, 1950−2021: South East England, Ages: 25−29
Data points enclosed with a diamond are reference sources

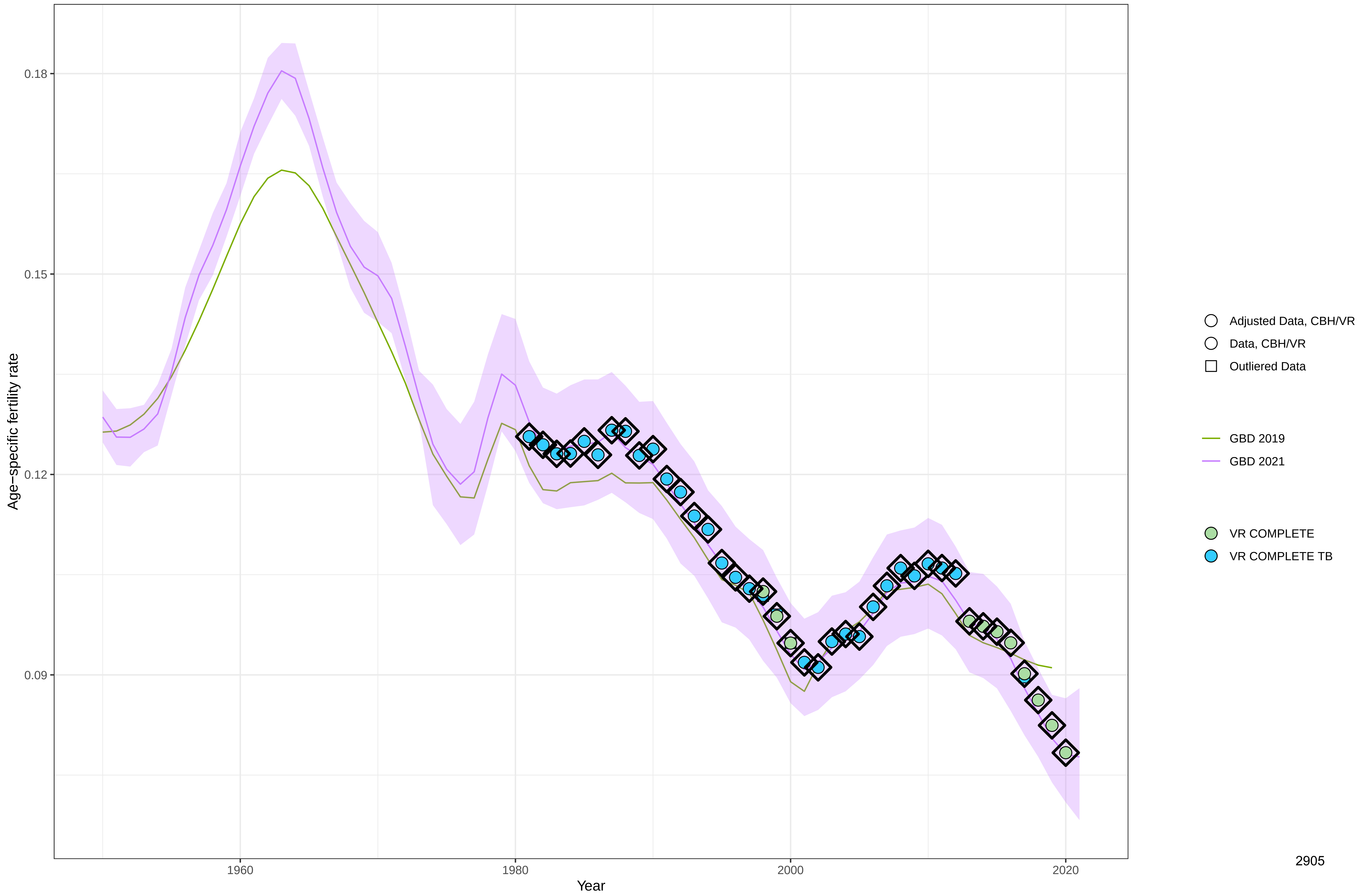

Age−specific fertility fitted trend lines, 1950−2021: South East England, Ages: 30−34
Data points enclosed with a diamond are reference sources

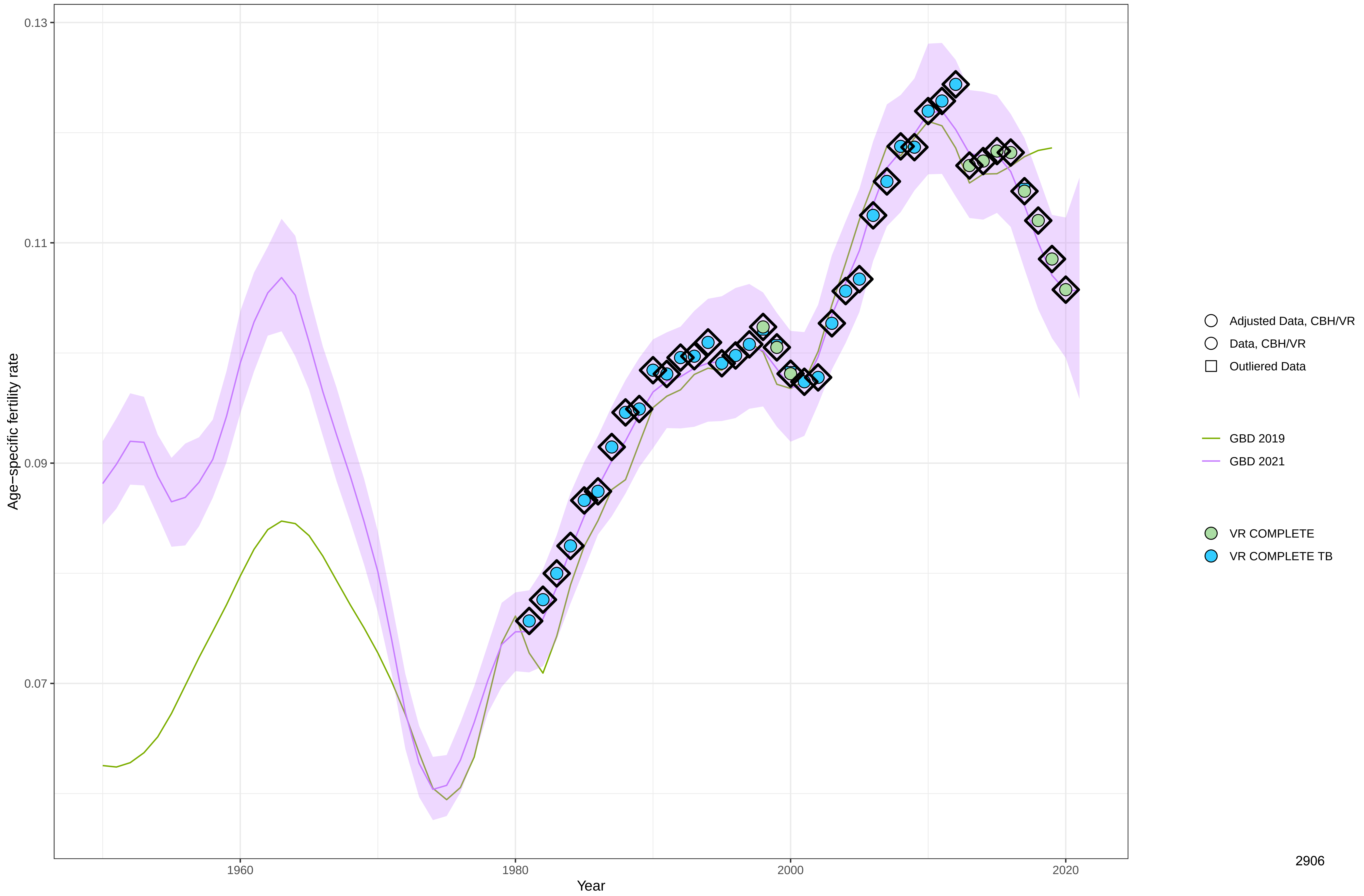

Age−specific fertility fitted trend lines, 1950−2021: South East England, Ages: 35−39
Data points enclosed with a diamond are reference sources

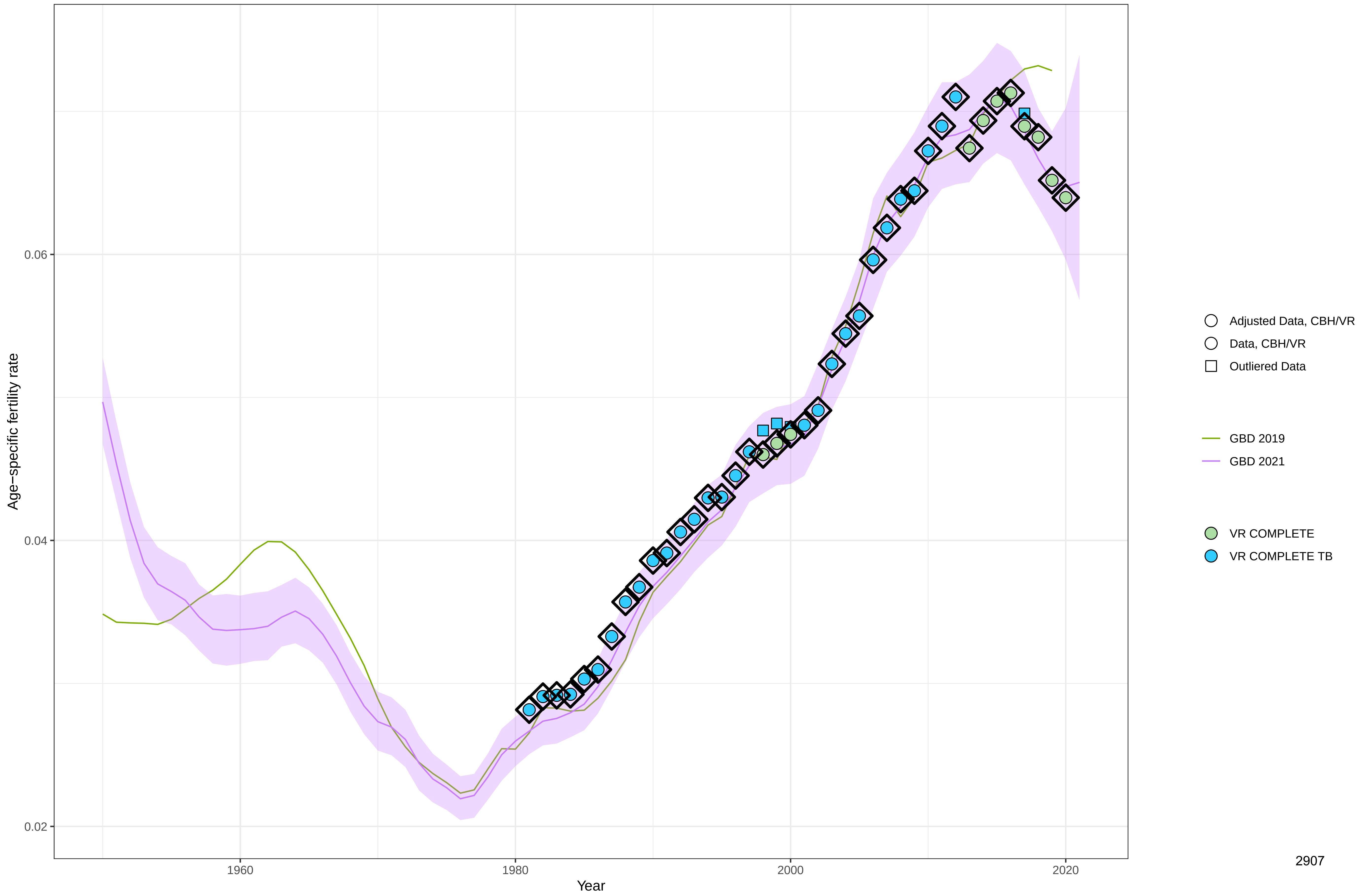

Age−specific fertility fitted trend lines, 1950−2021: South East England, Ages: 40−44
Data points enclosed with a diamond are reference sources

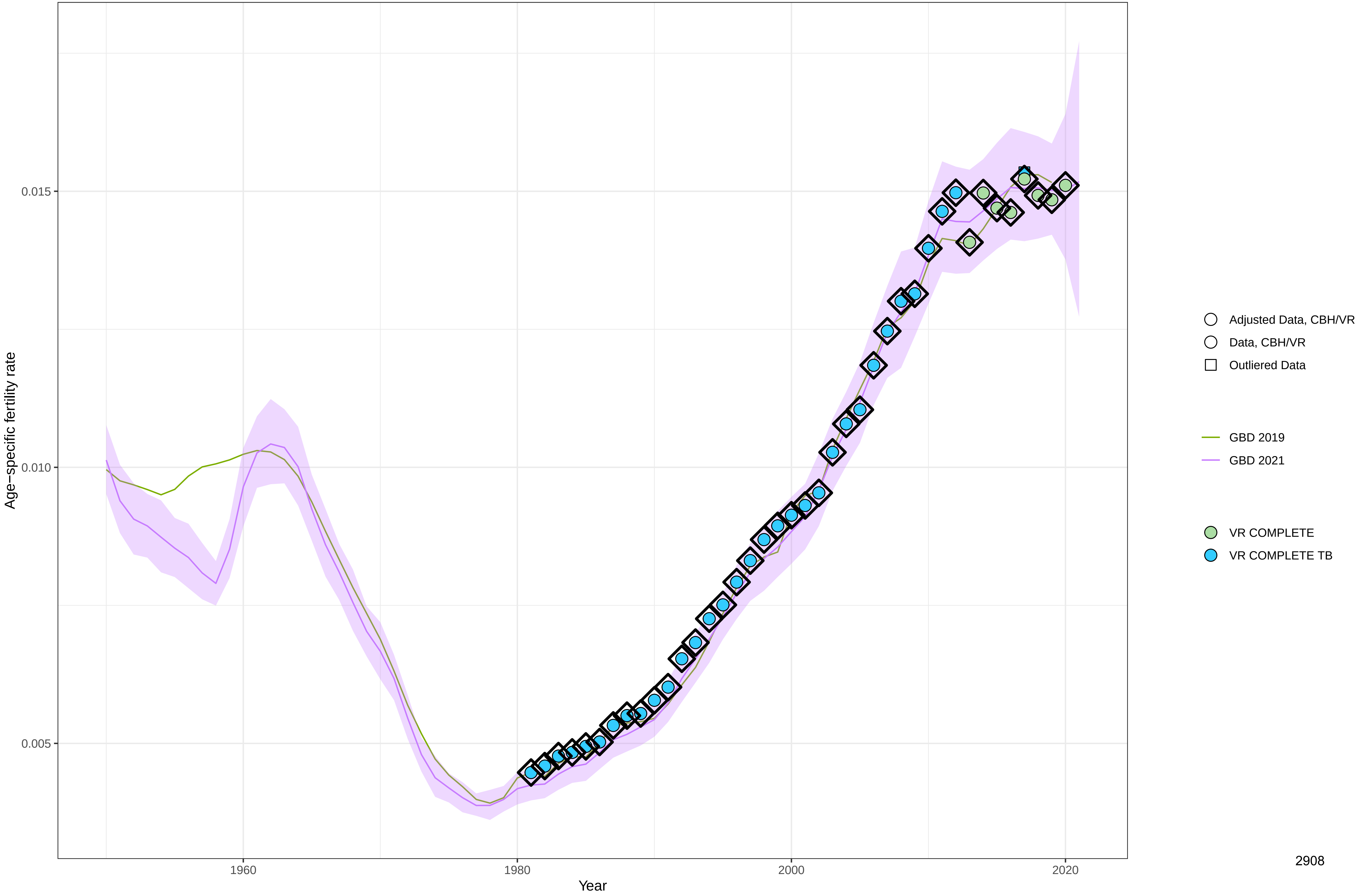

Age−specific fertility fitted trend lines, 1950−2021: South East England, Ages: 45−49
Data points enclosed with a diamond are reference sources

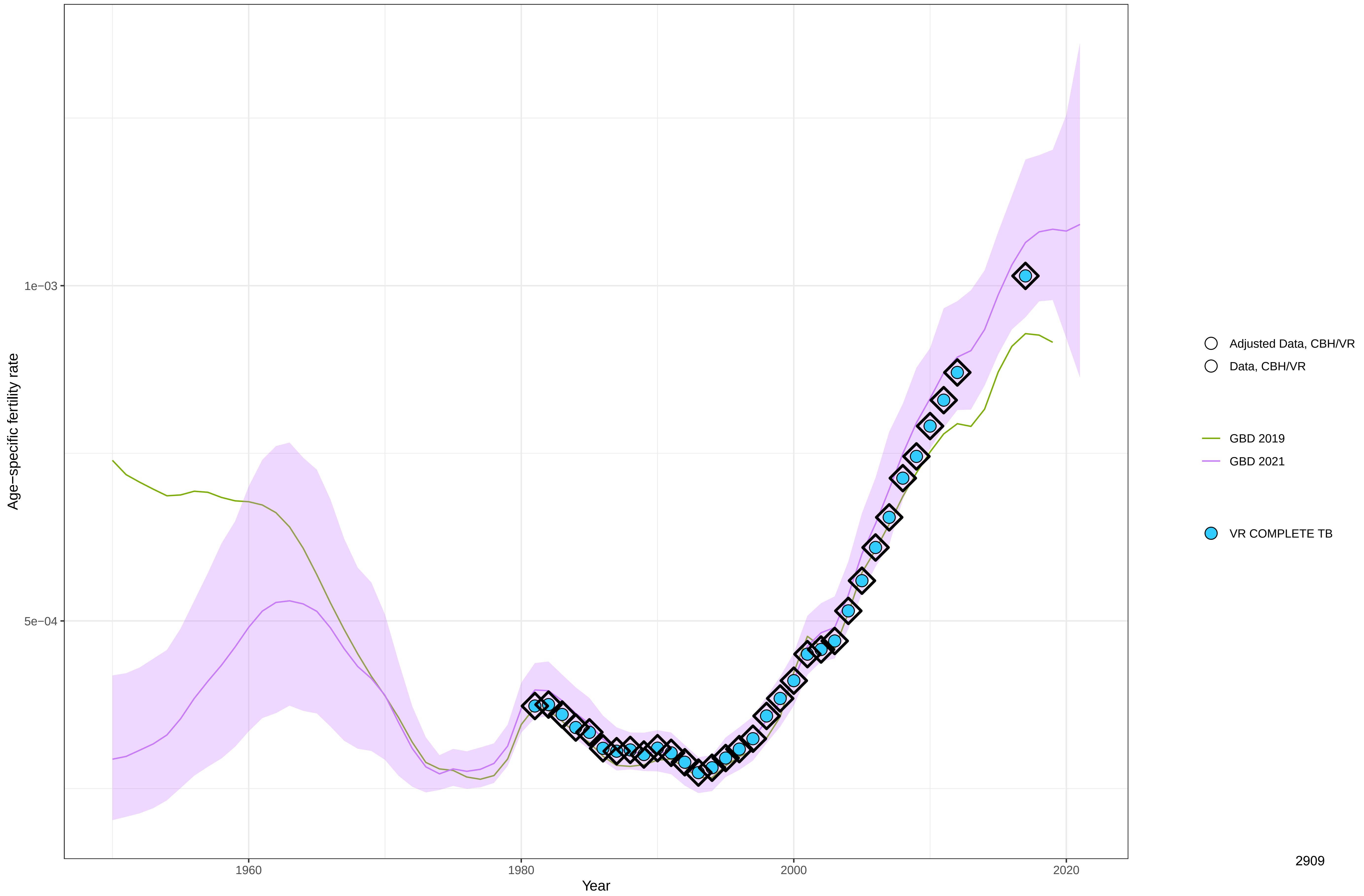

Age−specific fertility fitted trend lines, 1950−2021: South East England, Ages: 50−54
Data points enclosed with a diamond are reference sources

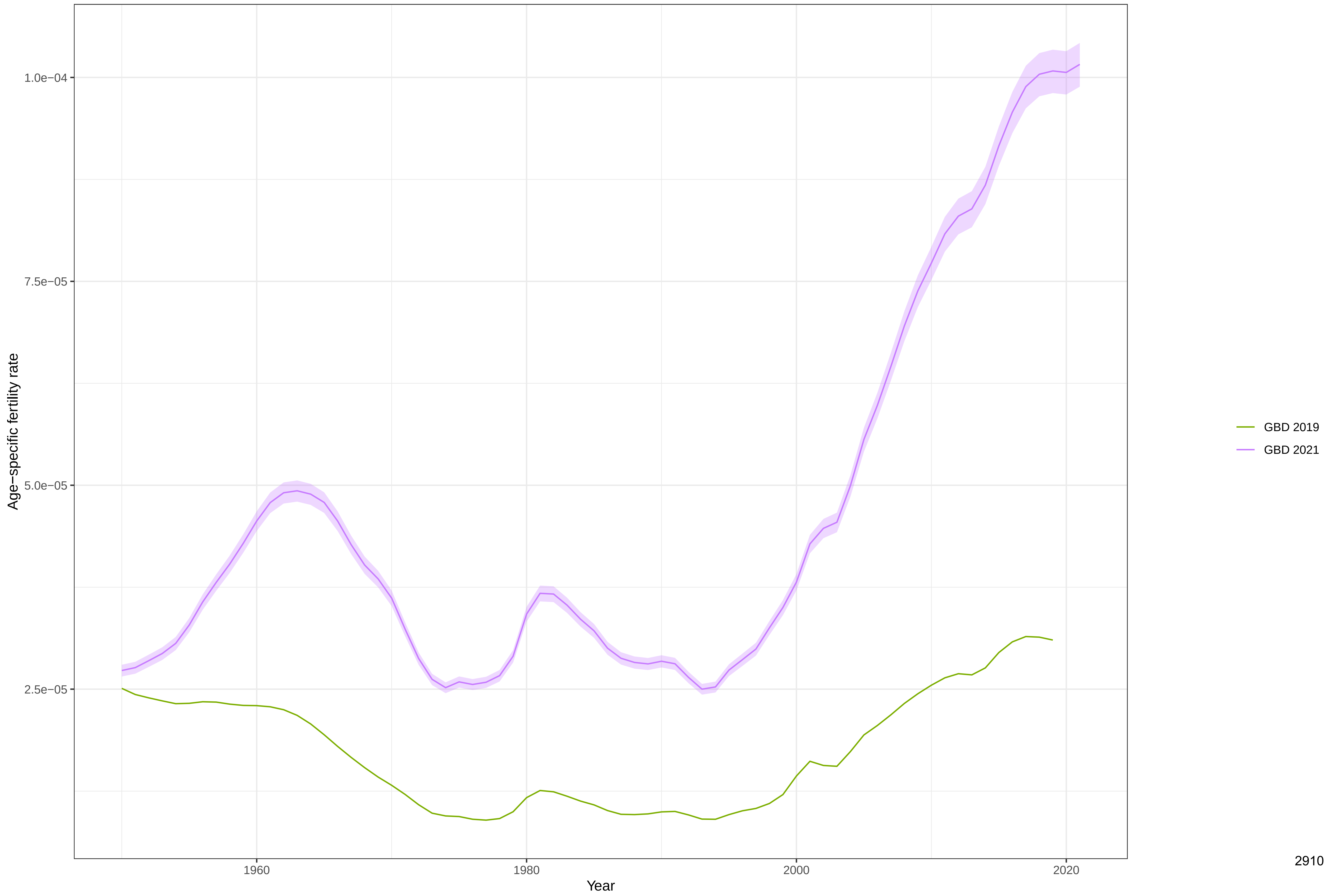

Total fertility rate fitted trend lines, 1950−2021: South East England TFR
Data points enclosed with a diamond are reference sources

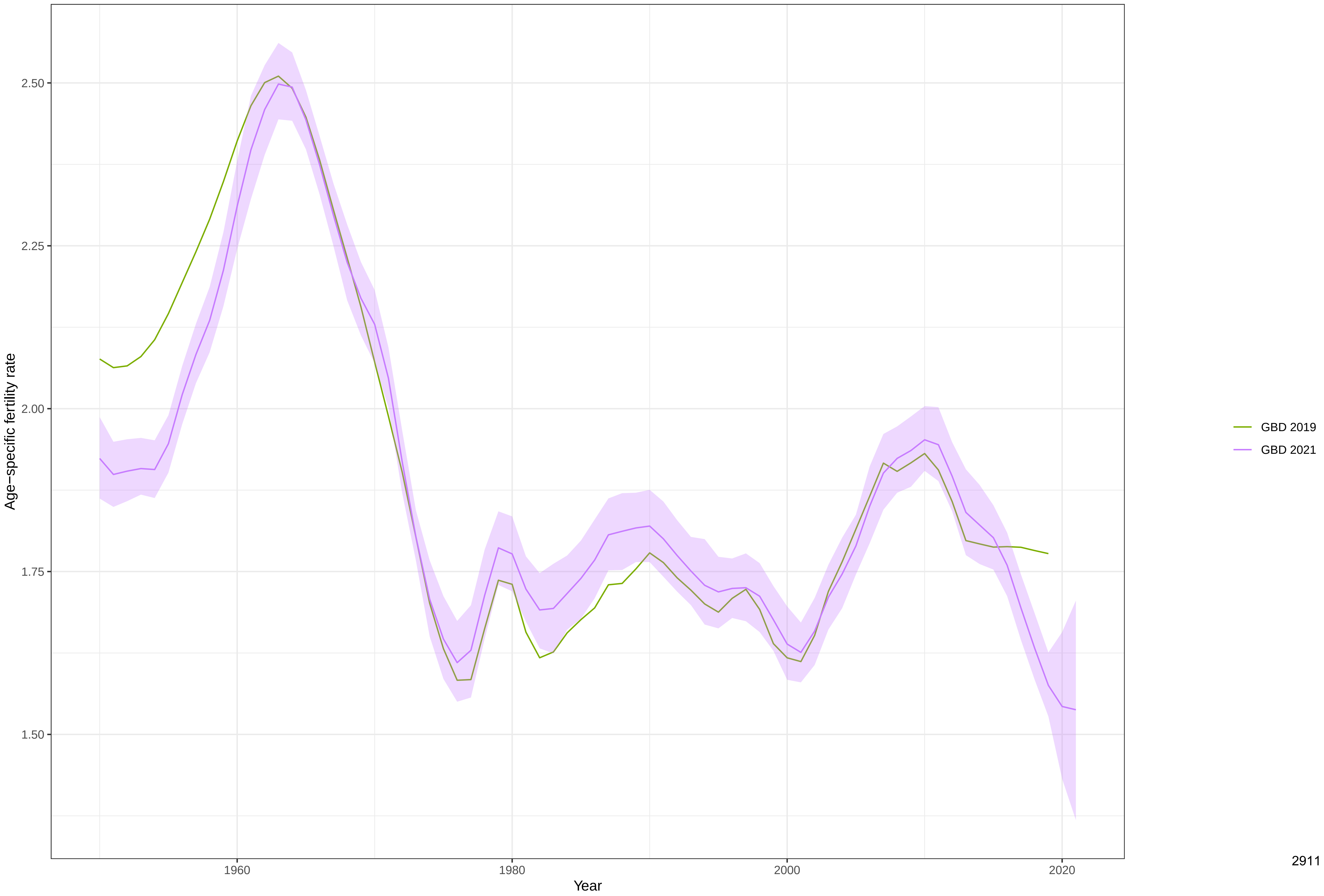

Age−specific fertility fitted trend lines, 1950−2021: Bracknell Forest, Ages: 10−14
Data points enclosed with a diamond are reference sources

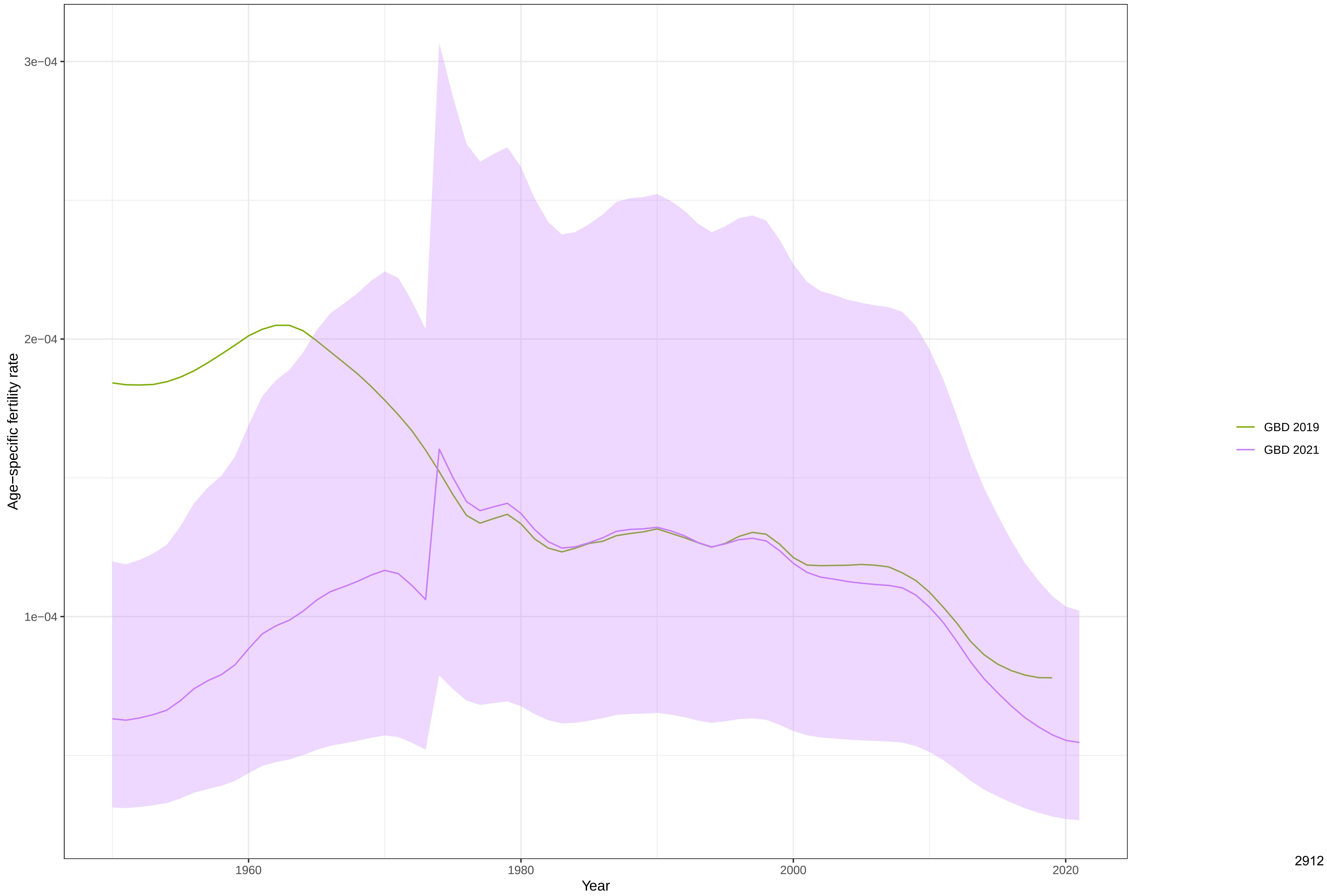

Age−specific fertility fitted trend lines, 1950−2021: Bracknell Forest, Ages: 15−19
Data points enclosed with a diamond are reference sources

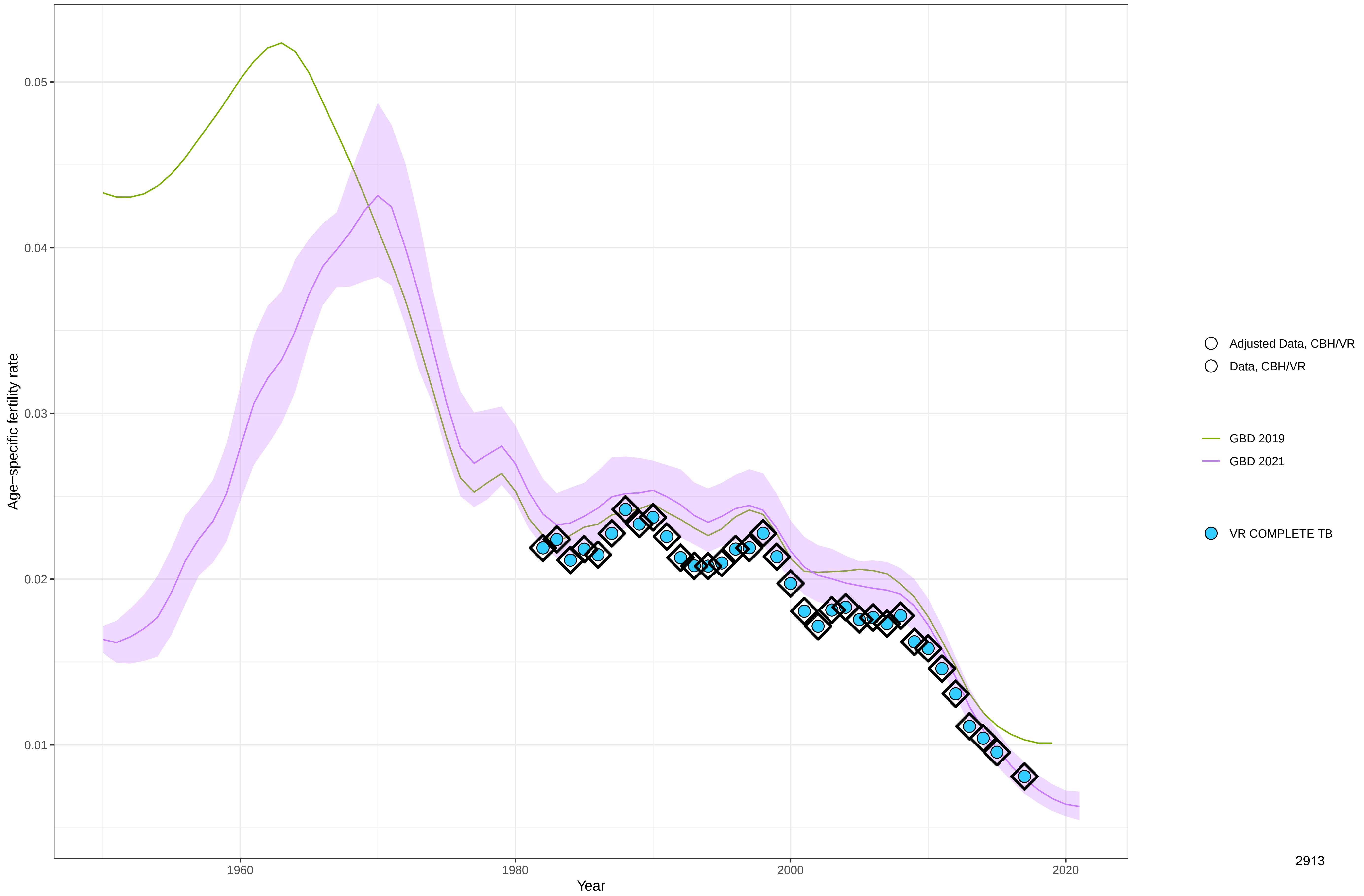

Age−specific fertility fitted trend lines, 1950−2021: Bracknell Forest, Ages: 20−24
Data points enclosed with a diamond are reference sources

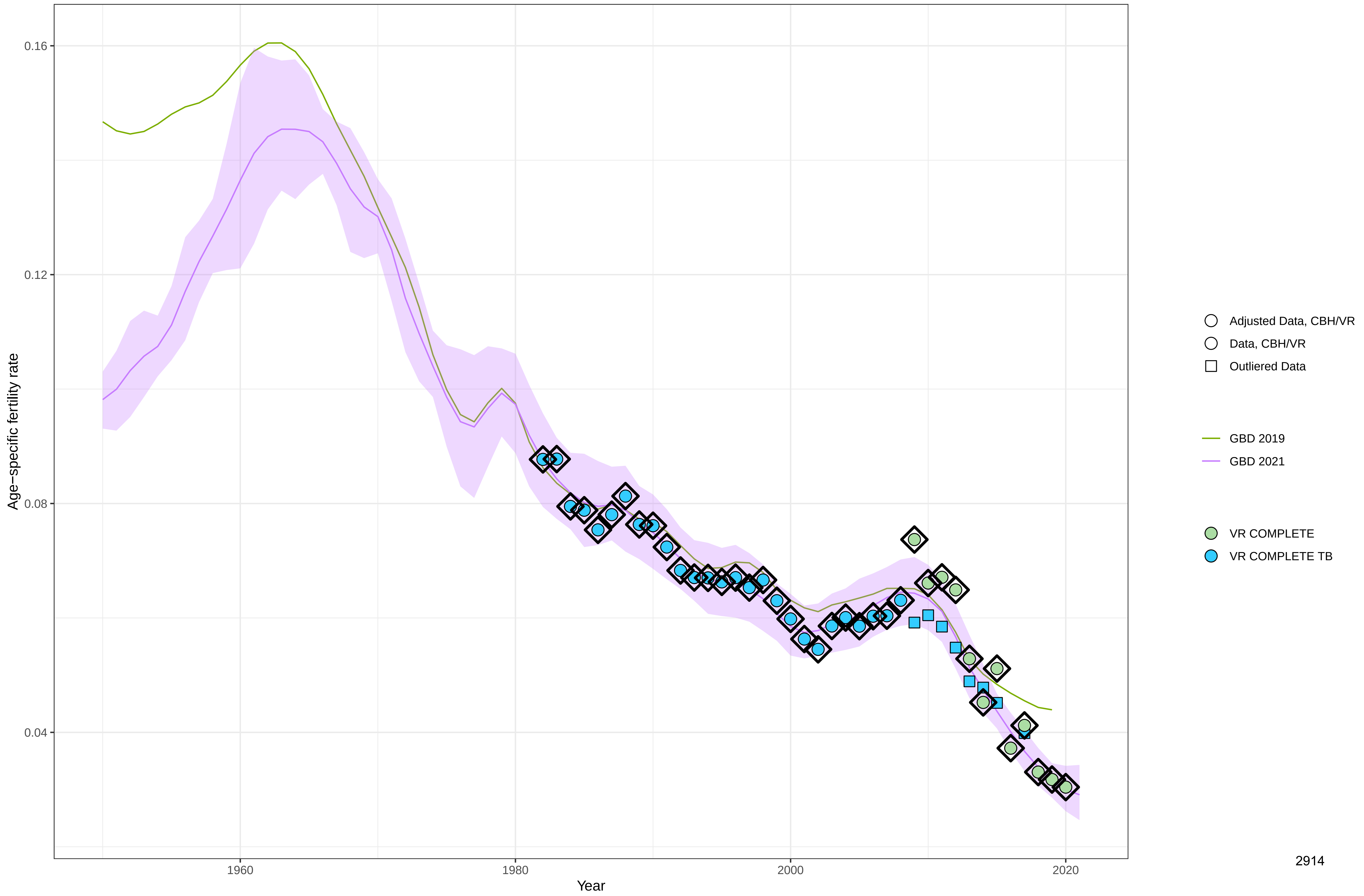

Age−specific fertility fitted trend lines, 1950−2021: Bracknell Forest, Ages: 25−29
Data points enclosed with a diamond are reference sources

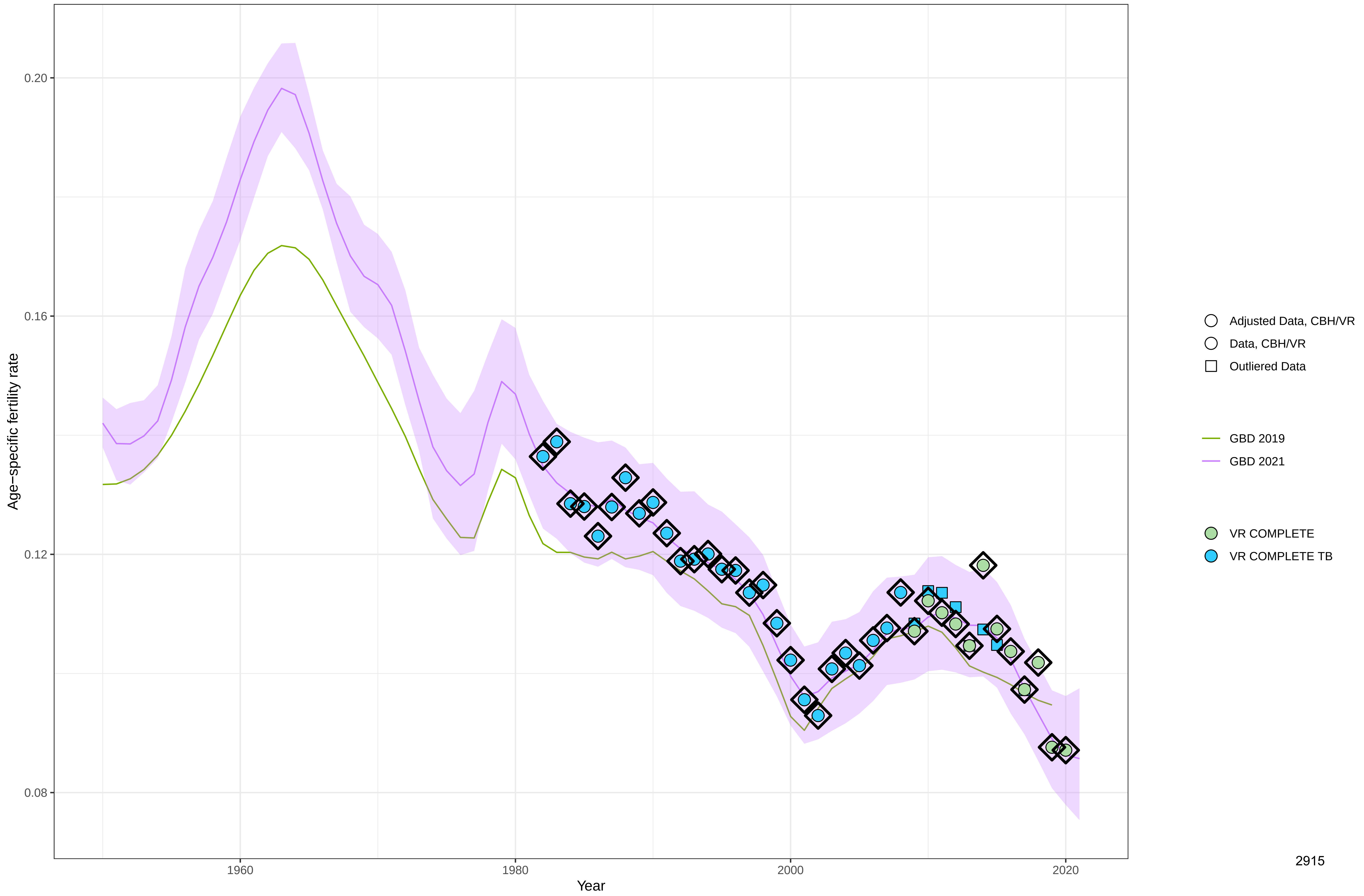

Age−specific fertility fitted trend lines, 1950−2021: Bracknell Forest, Ages: 30−34
Data points enclosed with a diamond are reference sources

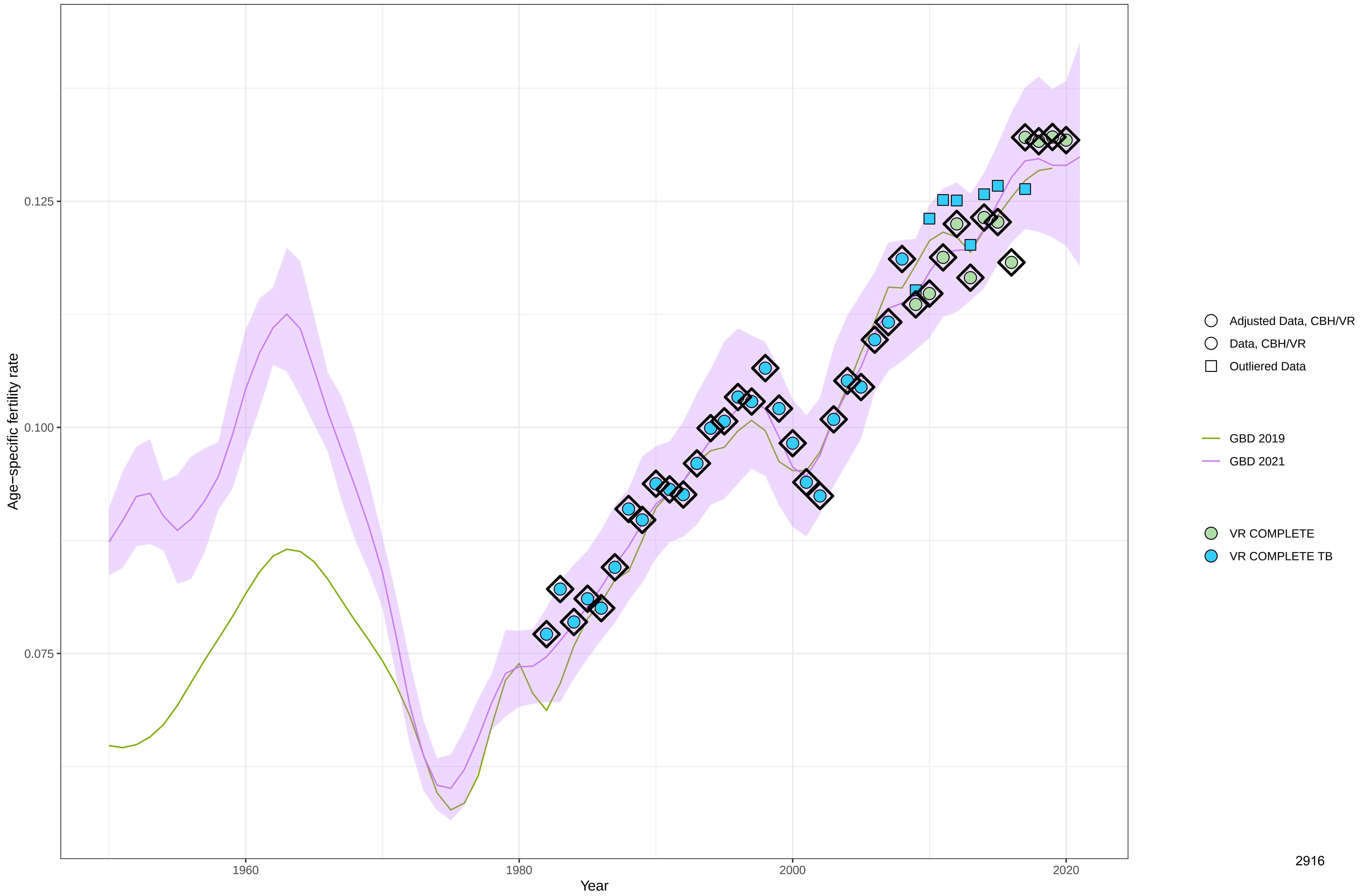

Age−specific fertility fitted trend lines, 1950−2021: Bracknell Forest, Ages: 35−39
Data points enclosed with a diamond are reference sources

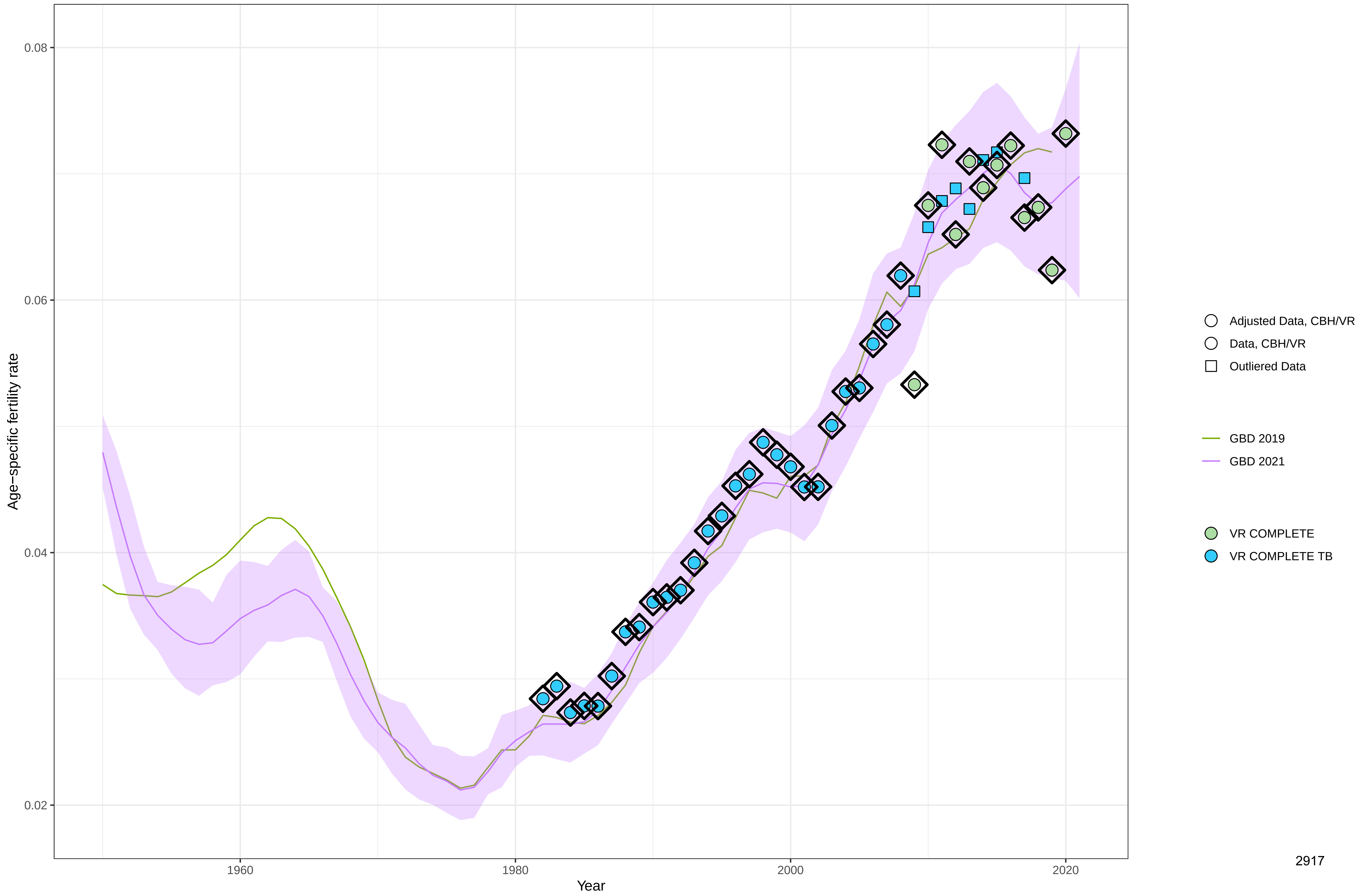

Age−specific fertility fitted trend lines, 1950−2021: Bracknell Forest, Ages: 40−44
Data points enclosed with a diamond are reference sources

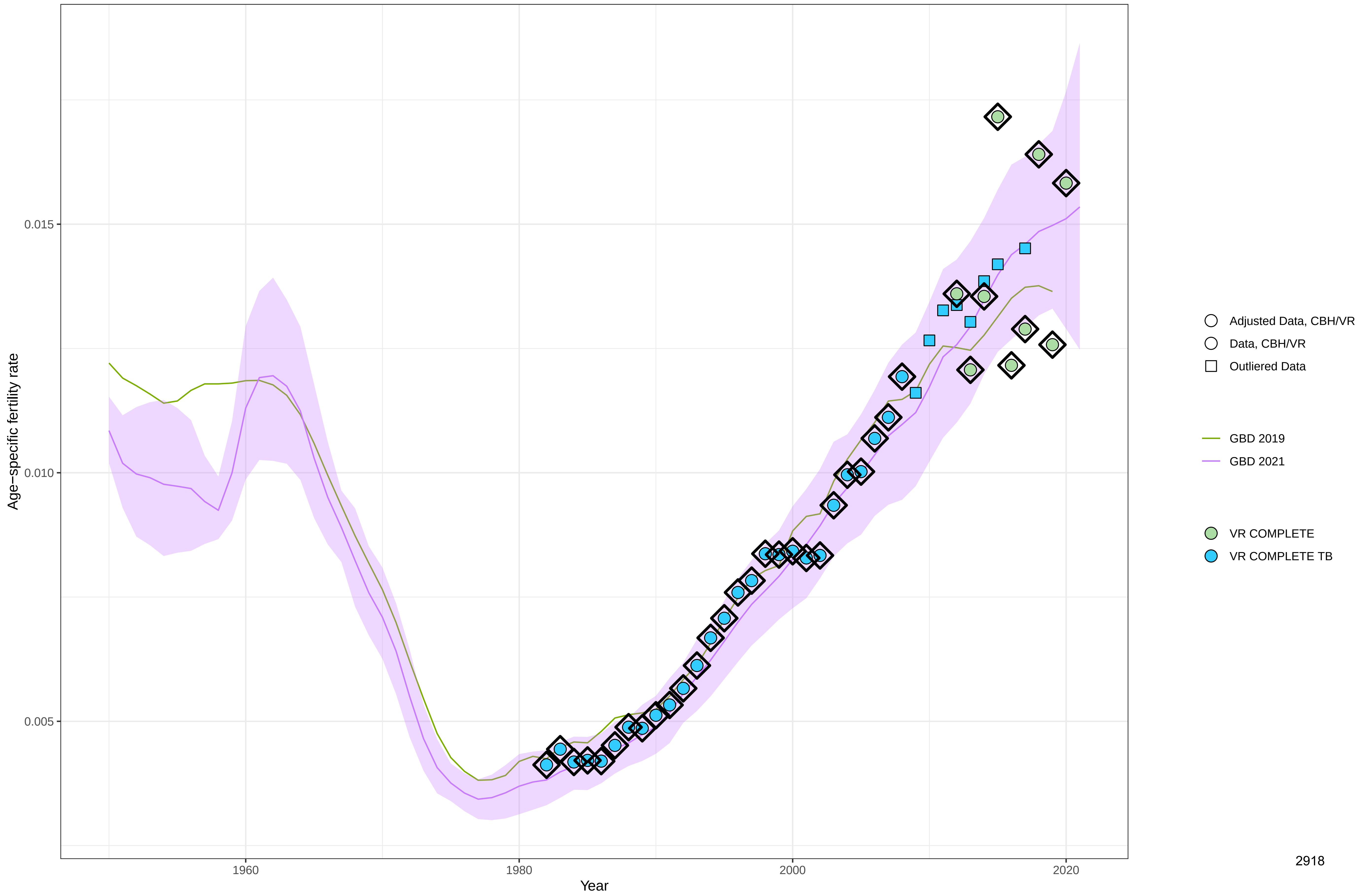

Age−specific fertility fitted trend lines, 1950−2021: Bracknell Forest, Ages: 45−49
Data points enclosed with a diamond are reference sources

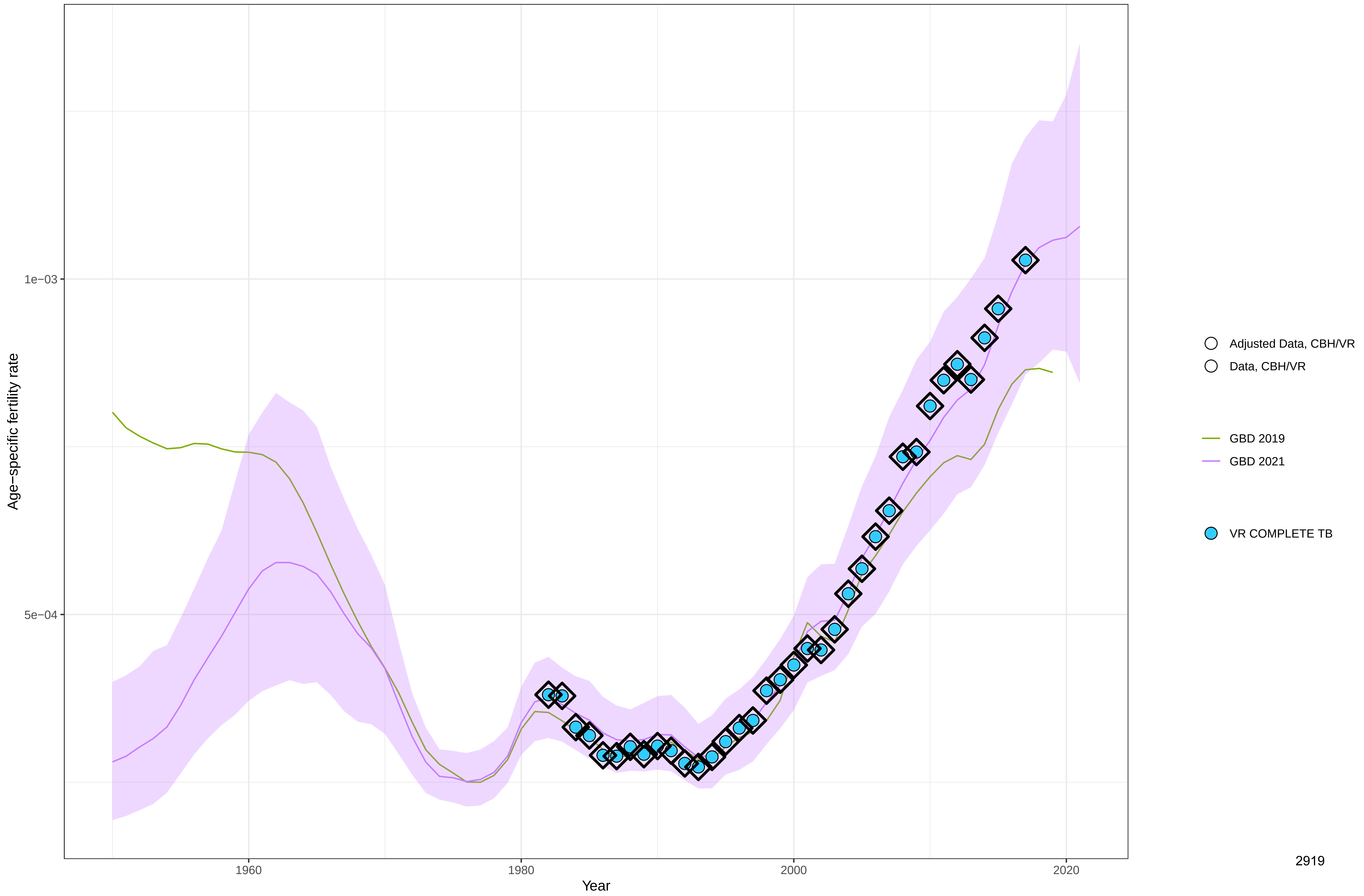

Age−specific fertility fitted trend lines, 1950−2021: Bracknell Forest, Ages: 50−54
Data points enclosed with a diamond are reference sources

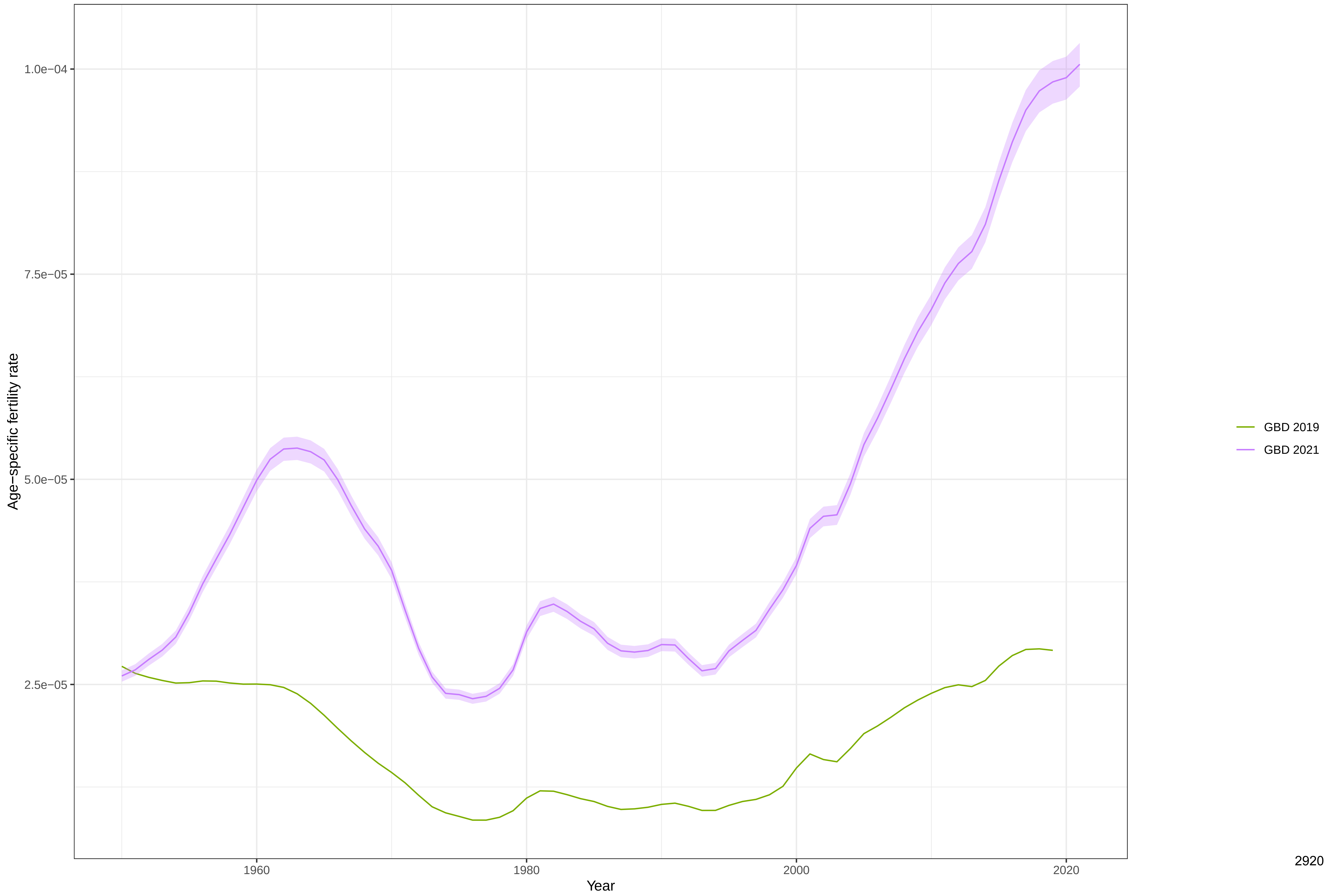

Total fertility rate fitted trend lines, 1950−2021: Bracknell Forest TFR
Data points enclosed with a diamond are reference sources

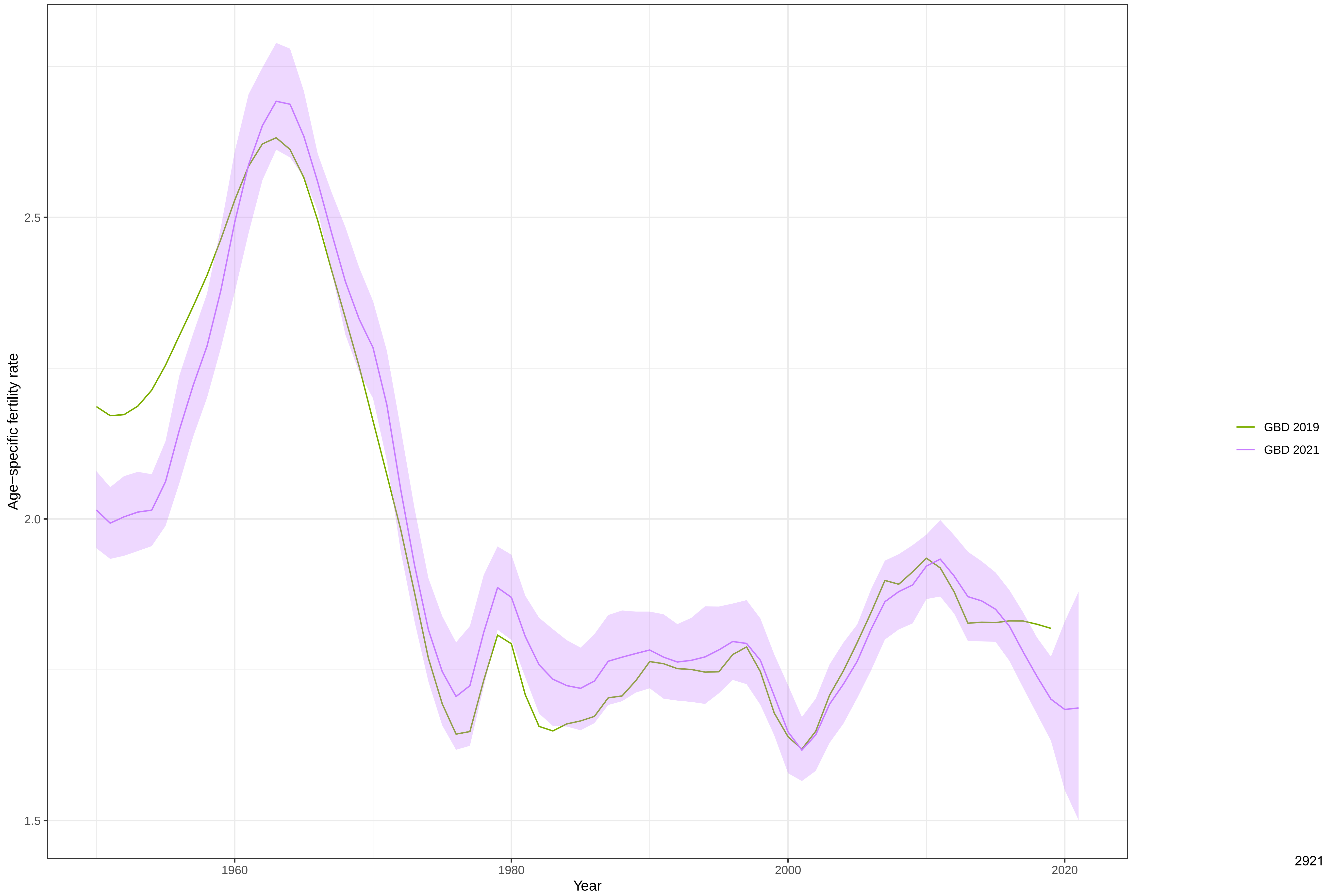

Age−specific fertility fitted trend lines, 1950−2021: Brighton and Hove, Ages: 10−14
Data points enclosed with a diamond are reference sources

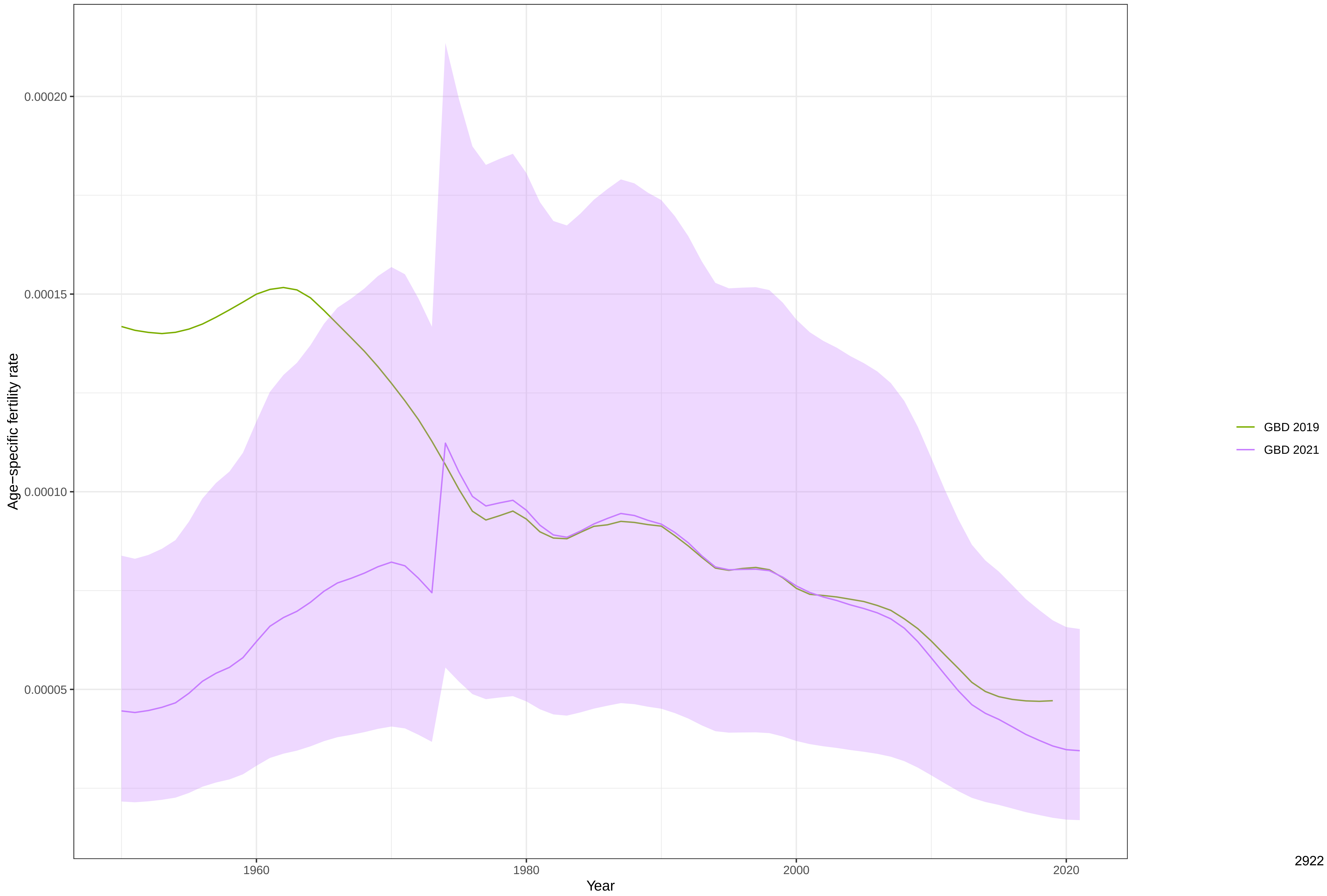

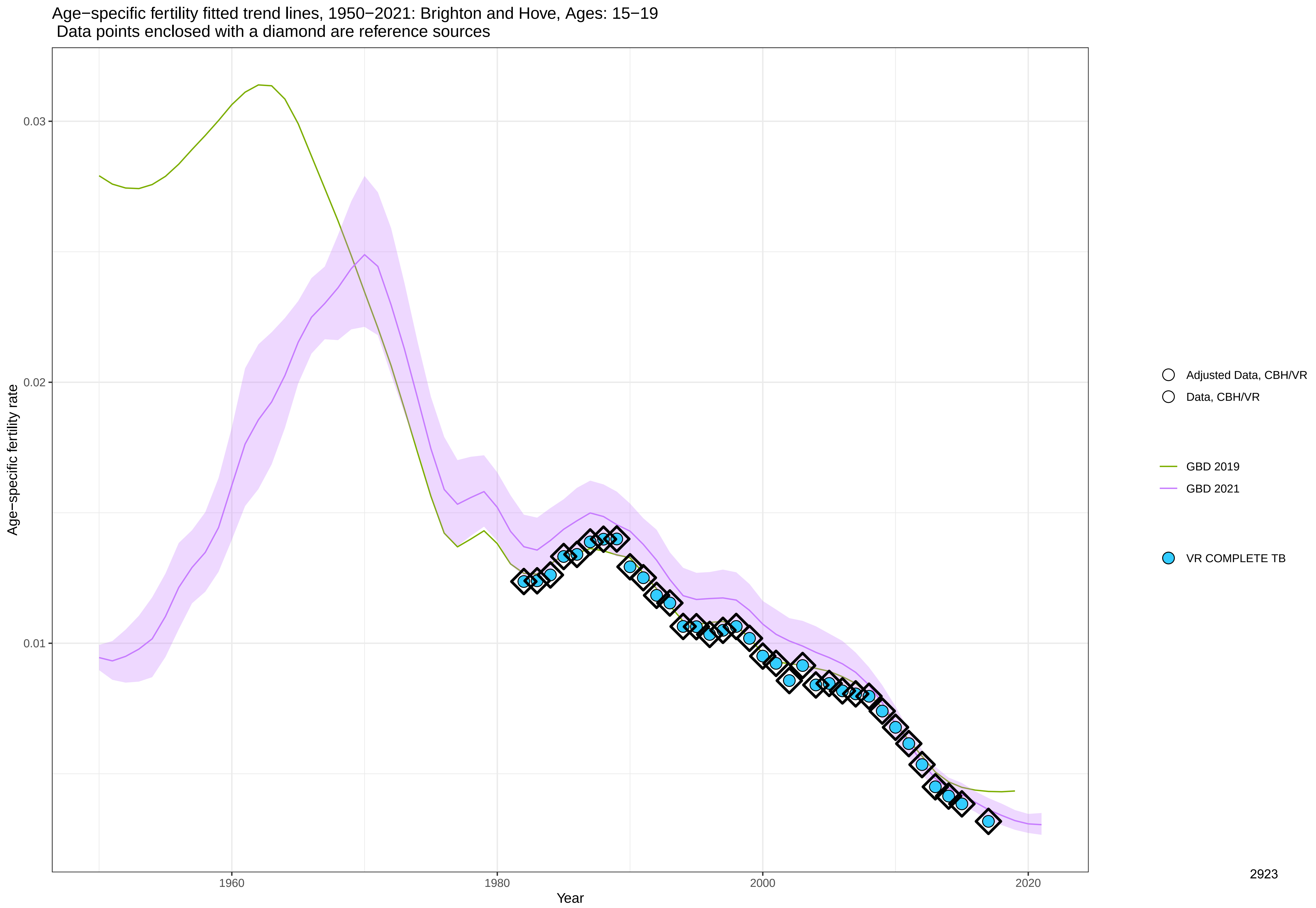

Age−specific fertility fitted trend lines, 1950−2021: Brighton and Hove, Ages: 15−19
Data points enclosed with a diamond are reference sources
Age−specific fertility rate
0.03
0.02
0.01
1960
1980
2000
2020
Year
Adjusted Data, CBH/VR
Data, CBH/VR
GBD 2019
GBD 2021
VR COMPLETE TB

Age−specific fertility fitted trend lines, 1950−2021: Brighton and Hove, Ages: 20−24
Data points enclosed with a diamond are reference sources

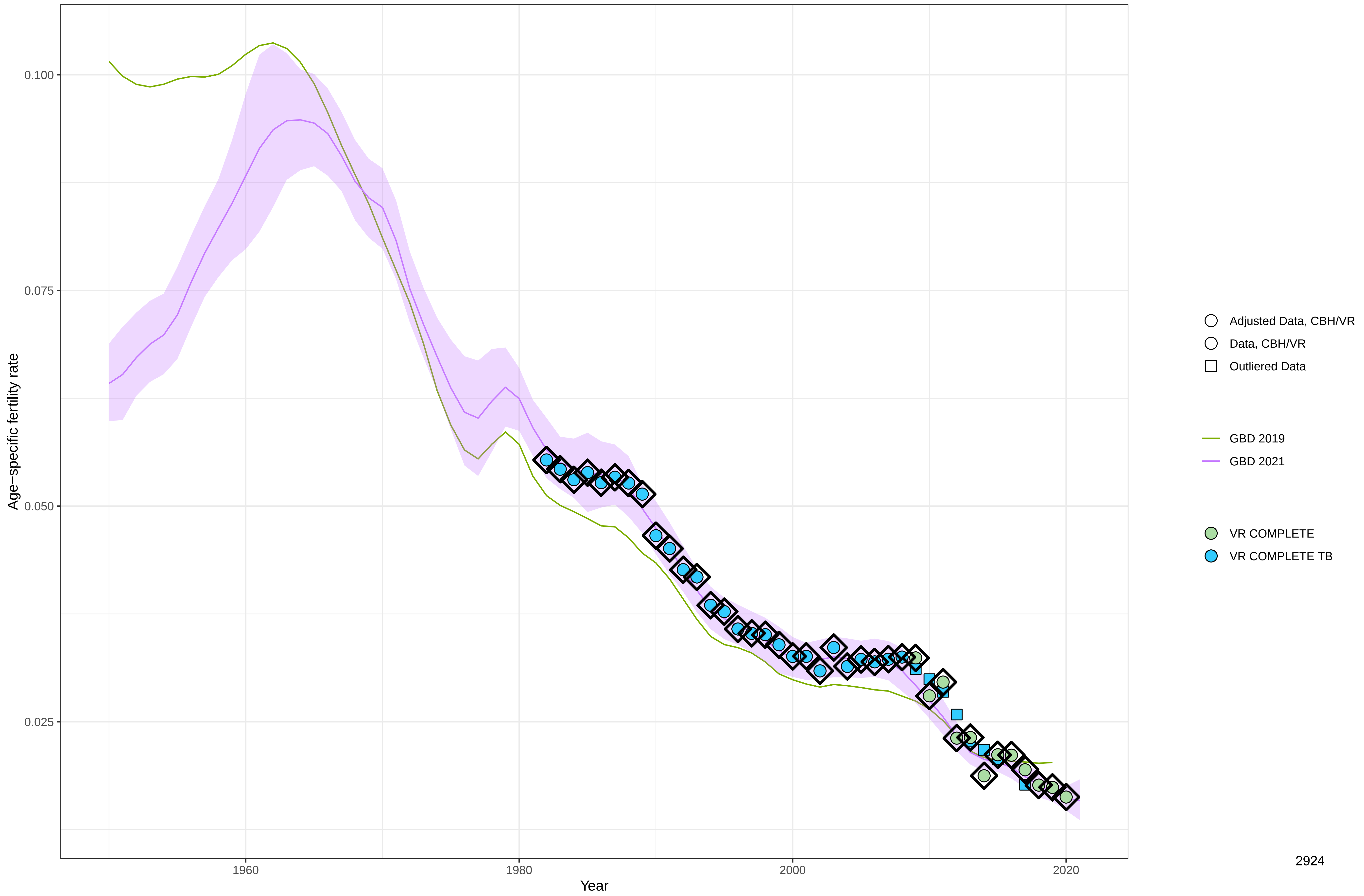

Age−specific fertility fitted trend lines, 1950−2021: Brighton and Hove, Ages: 25−29
Data points enclosed with a diamond are reference sources

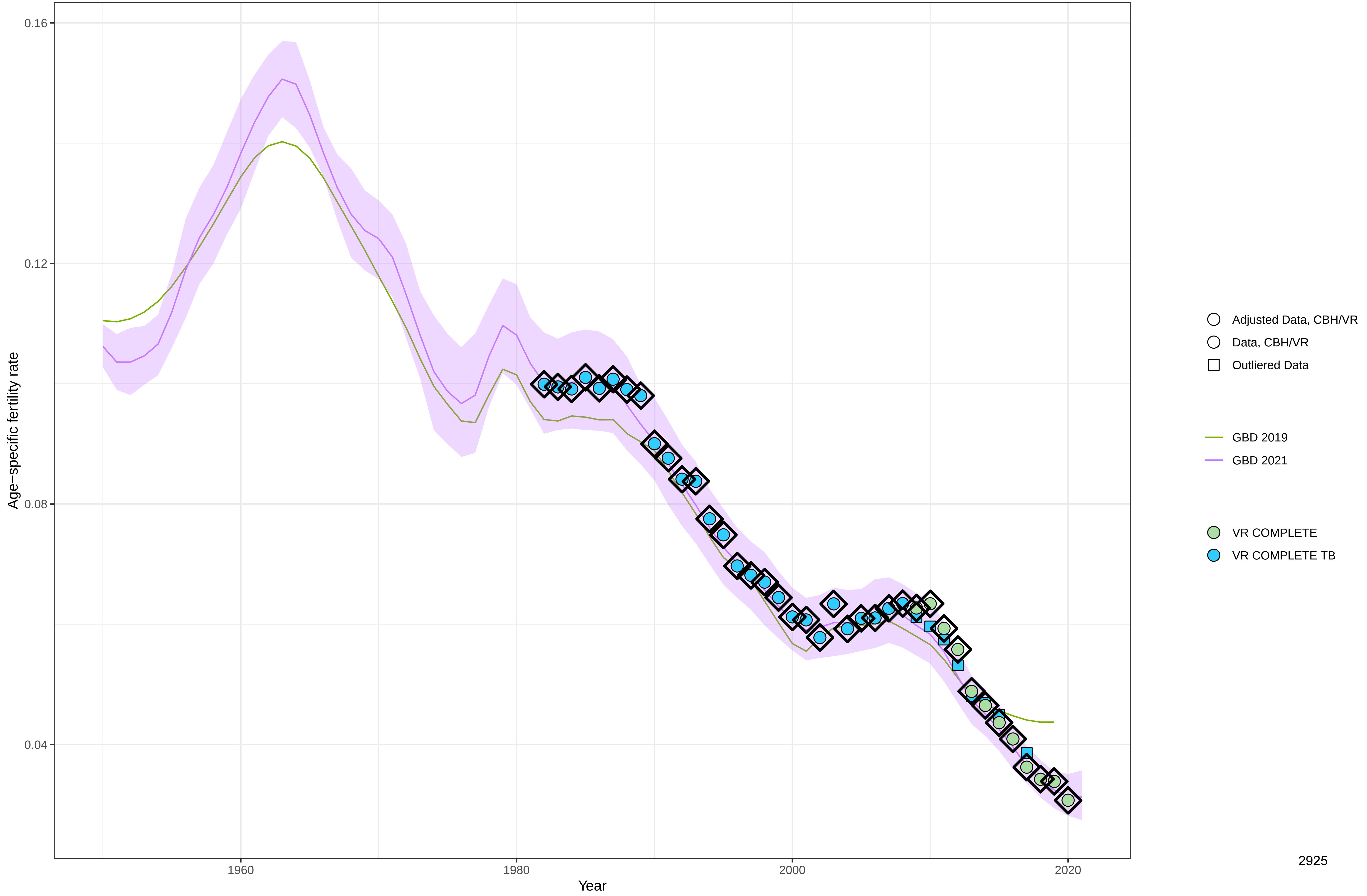

Age−specific fertility fitted trend lines, 1950−2021: Brighton and Hove, Ages: 30−34
Data points enclosed with a diamond are reference sources

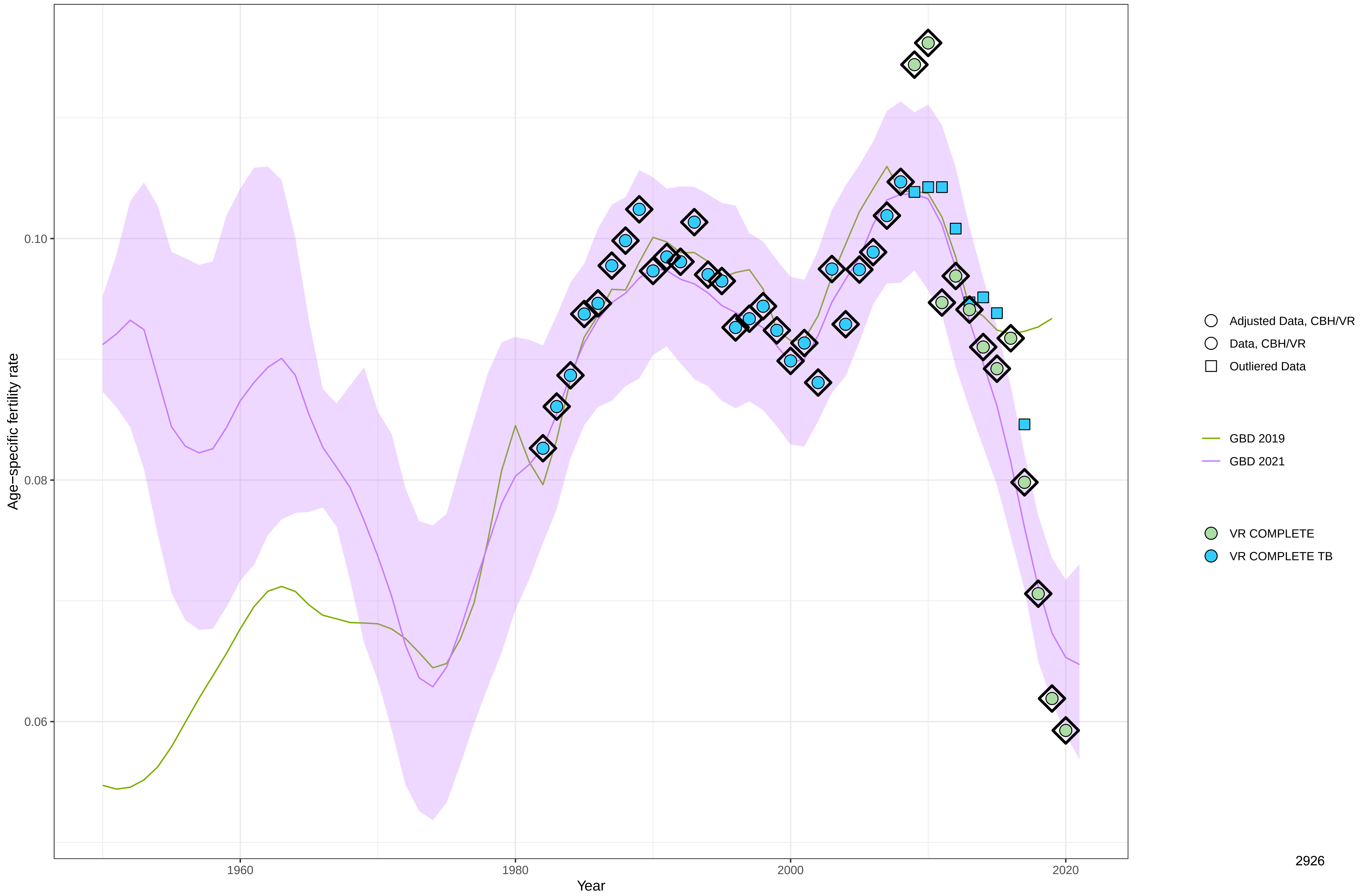

Age−specific fertility fitted trend lines, 1950−2021: Brighton and Hove, Ages: 35−39
Data points enclosed with a diamond are reference sources

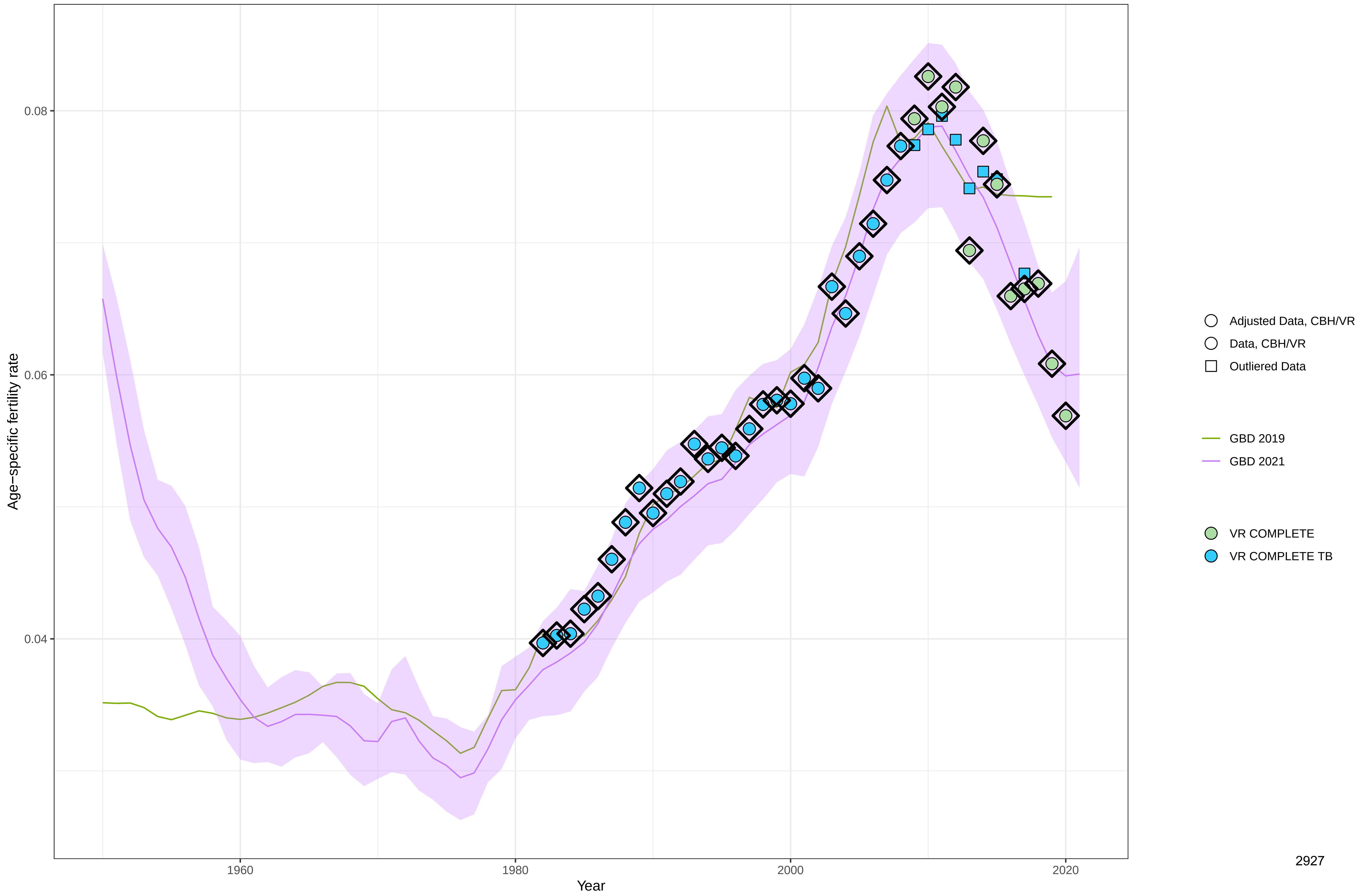

Age−specific fertility fitted trend lines, 1950−2021: Brighton and Hove, Ages: 40−44
Data points enclosed with a diamond are reference sources

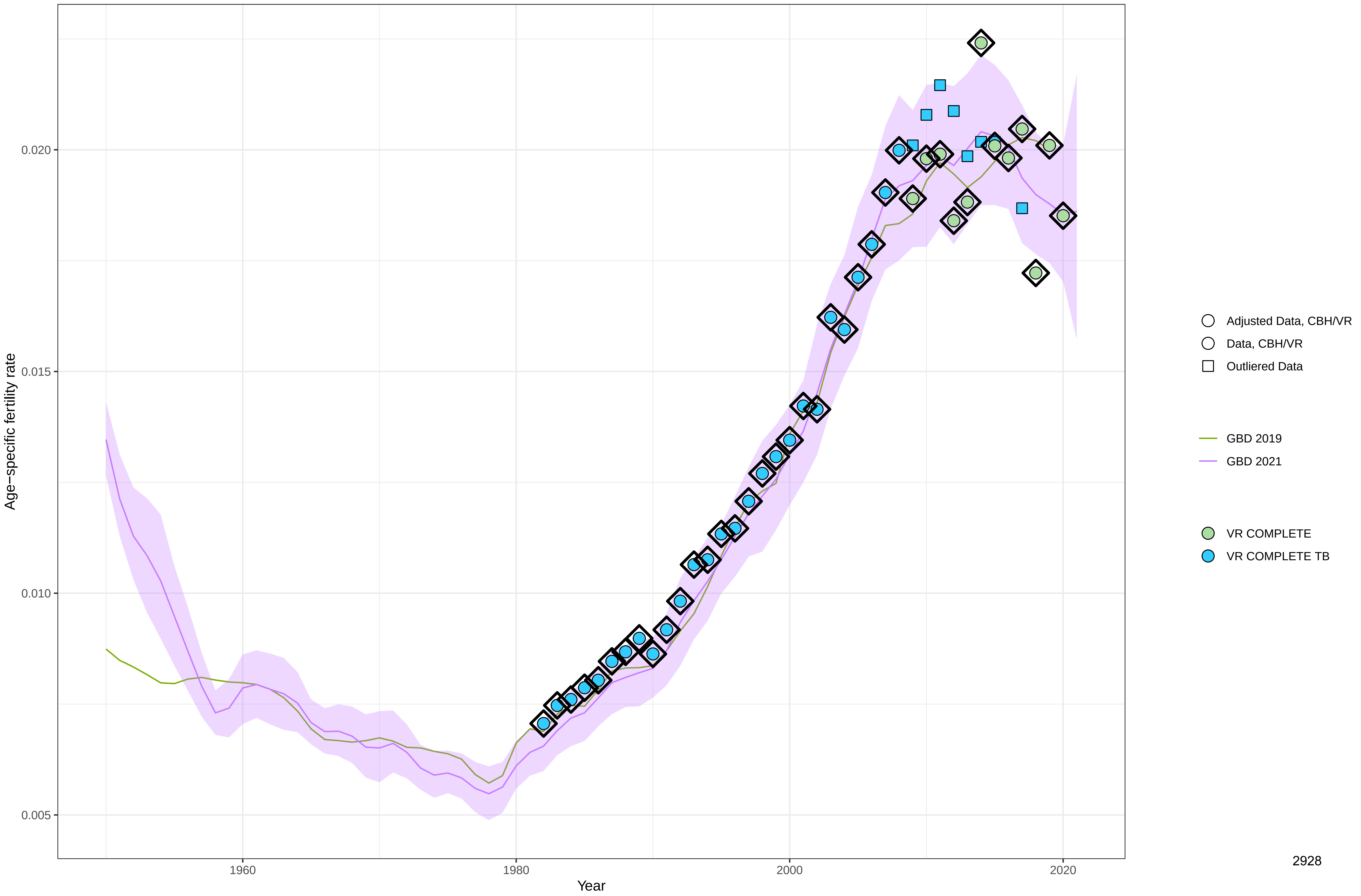

Age−specific fertility fitted trend lines, 1950−2021: Brighton and Hove, Ages: 45−49
Data points enclosed with a diamond are reference sources

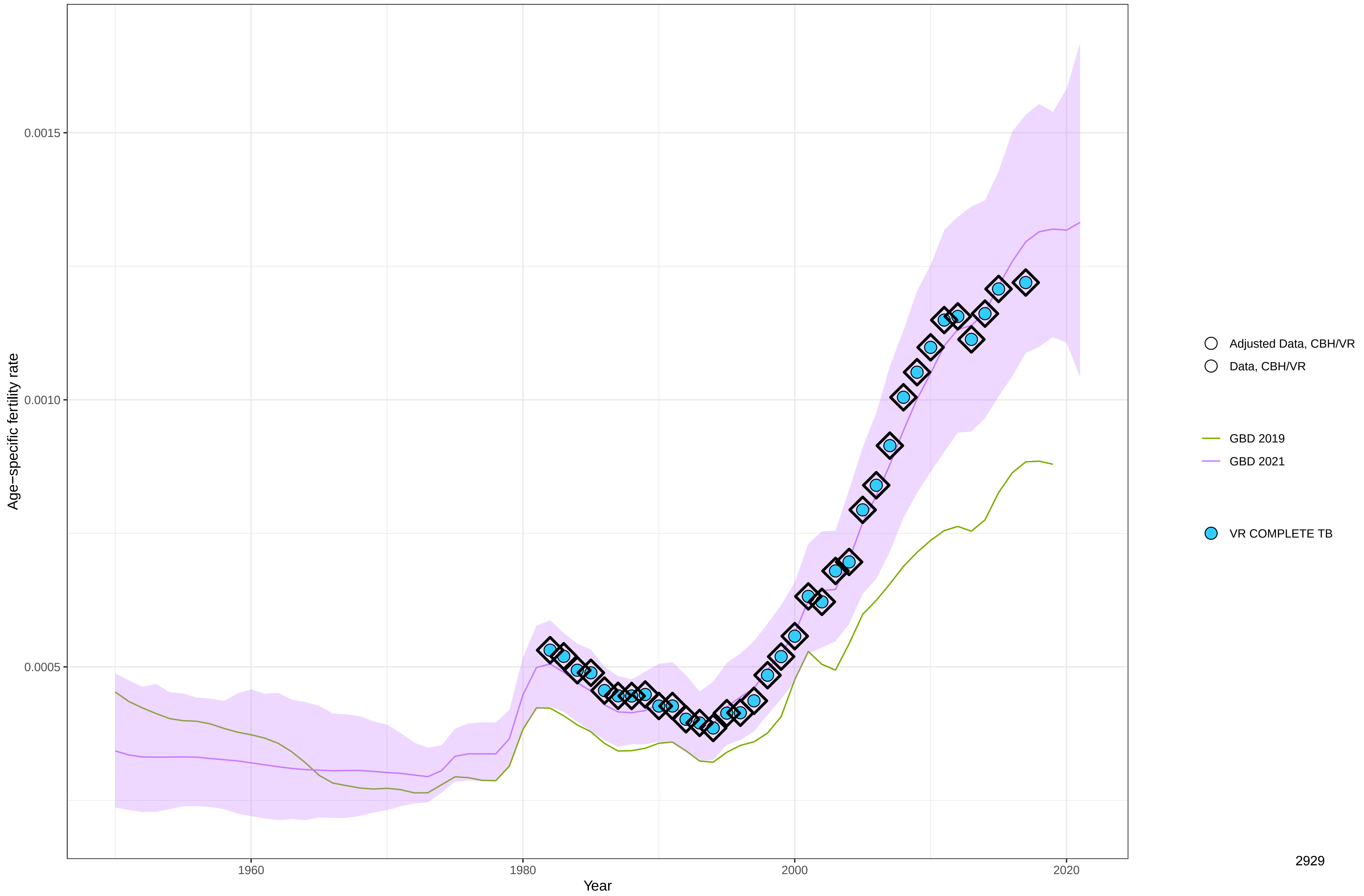

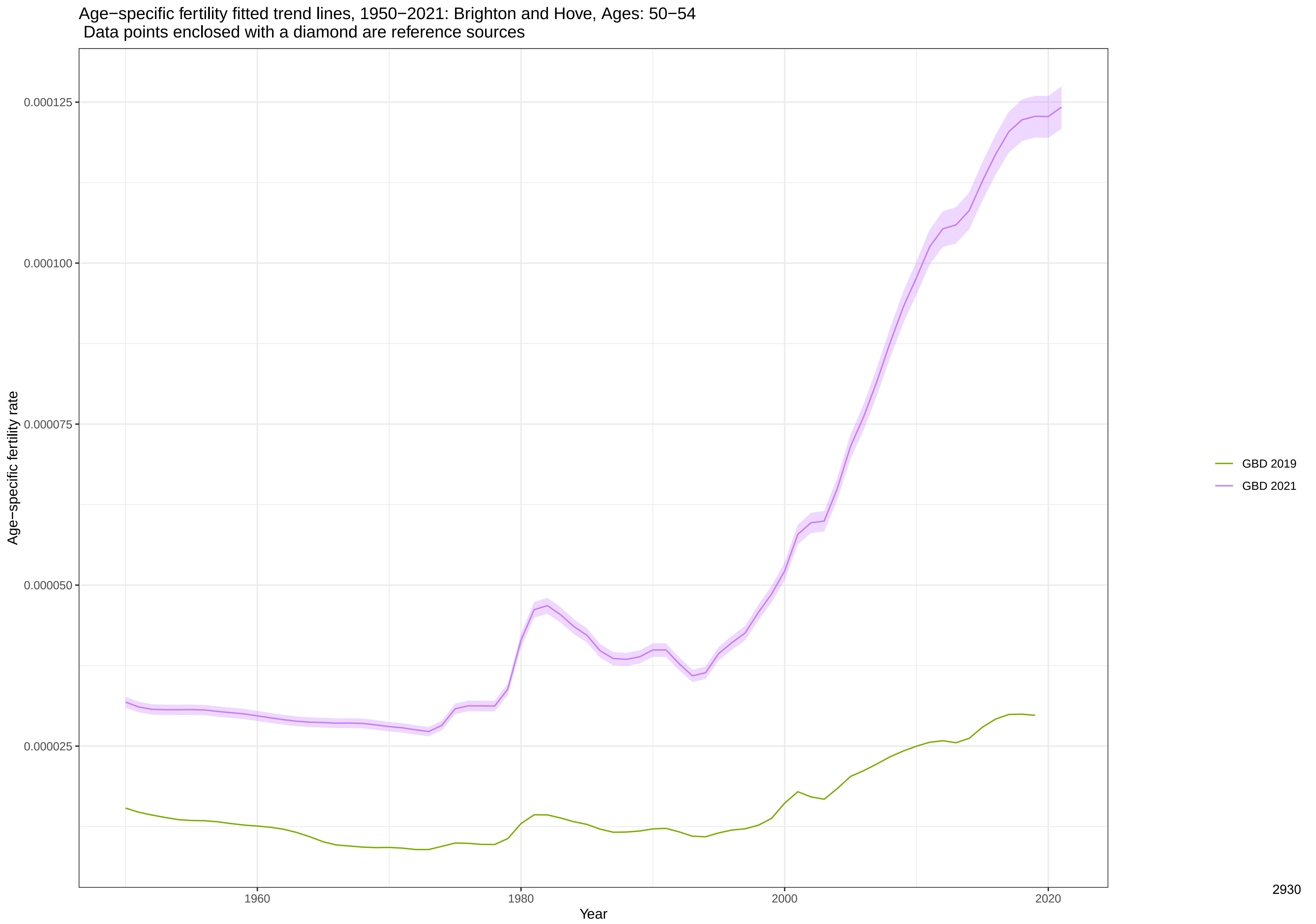

Age−specific fertility fitted trend lines, 1950−2021: Brighton and Hove, Ages: 50−54
Data points enclosed with a diamond are reference sources
Age−specific fertility rate
0.000125
0.000100
0.000075
0.000050
0.000025
1960
1980
2000
2020
Year
GBD 2019
GBD 2021

Total fertility rate fitted trend lines, 1950−2021: Brighton and Hove TFR
Data points enclosed with a diamond are reference sources

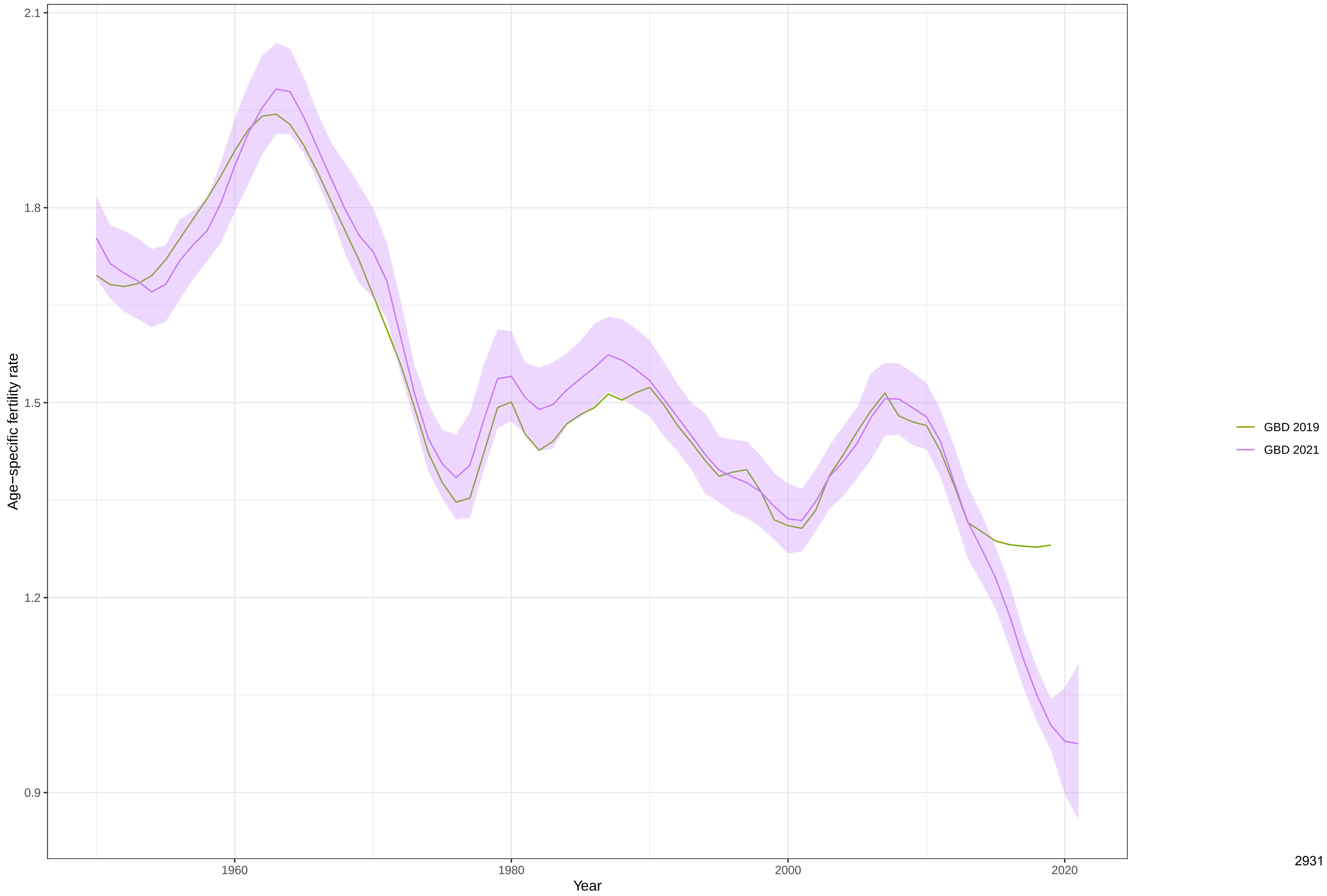

Age−specific fertility fitted trend lines, 1950−2021: Buckinghamshire, Ages: 10−14
Data points enclosed with a diamond are reference sources

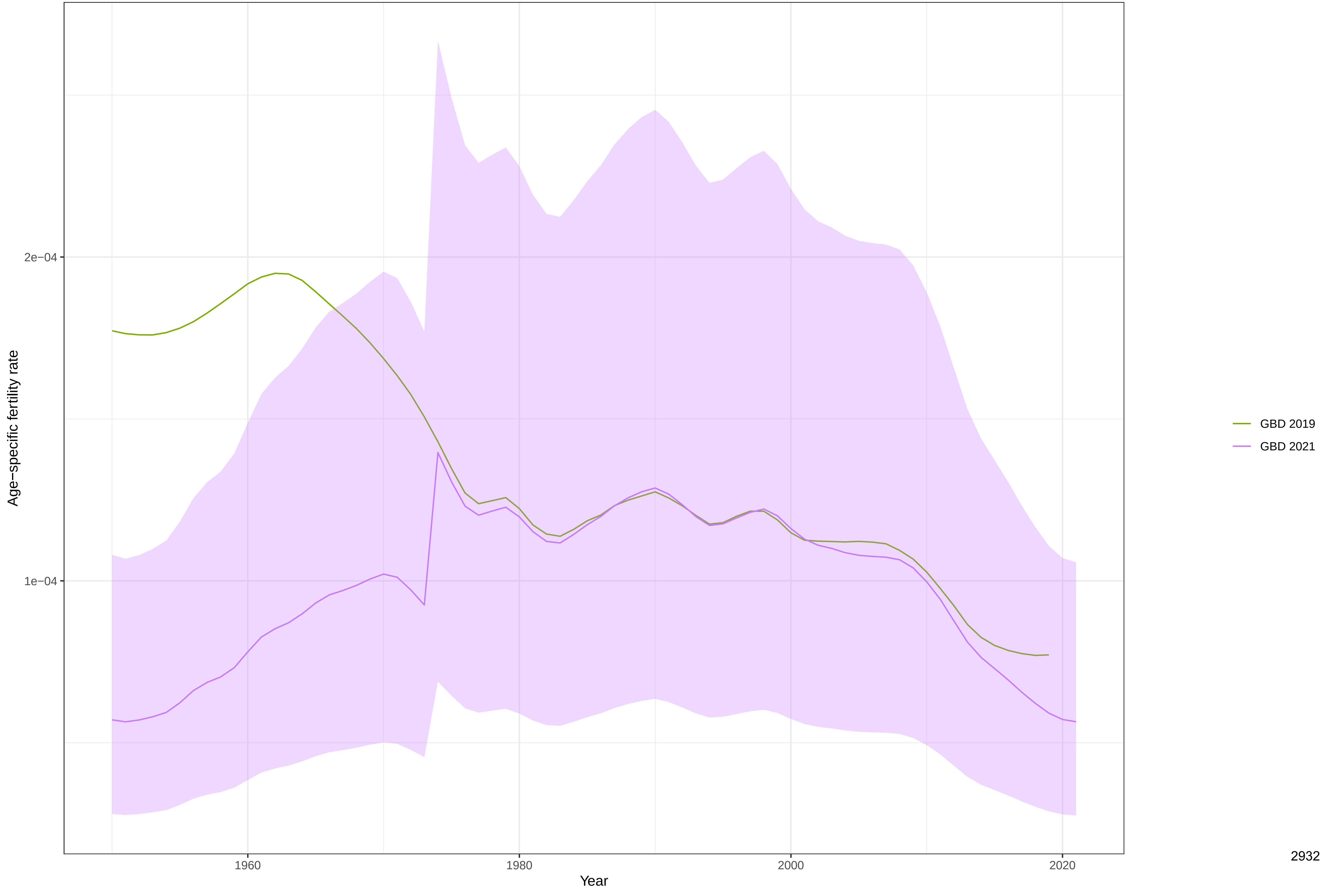

Age−specific fertility fitted trend lines, 1950−2021: Buckinghamshire, Ages: 15−19
Data points enclosed with a diamond are reference sources

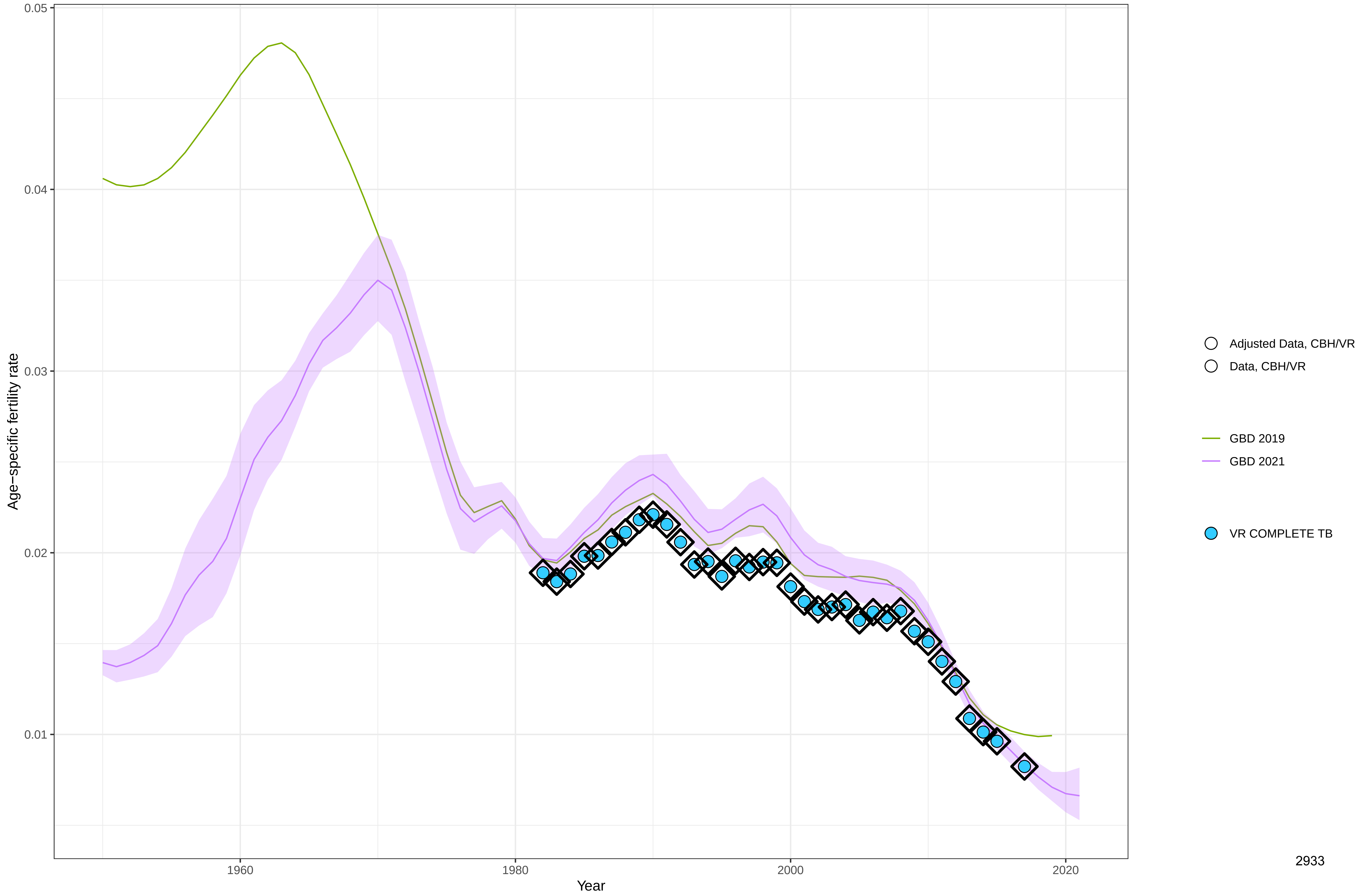

Age−specific fertility fitted trend lines, 1950−2021: Buckinghamshire, Ages: 20−24
Data points enclosed with a diamond are reference sources

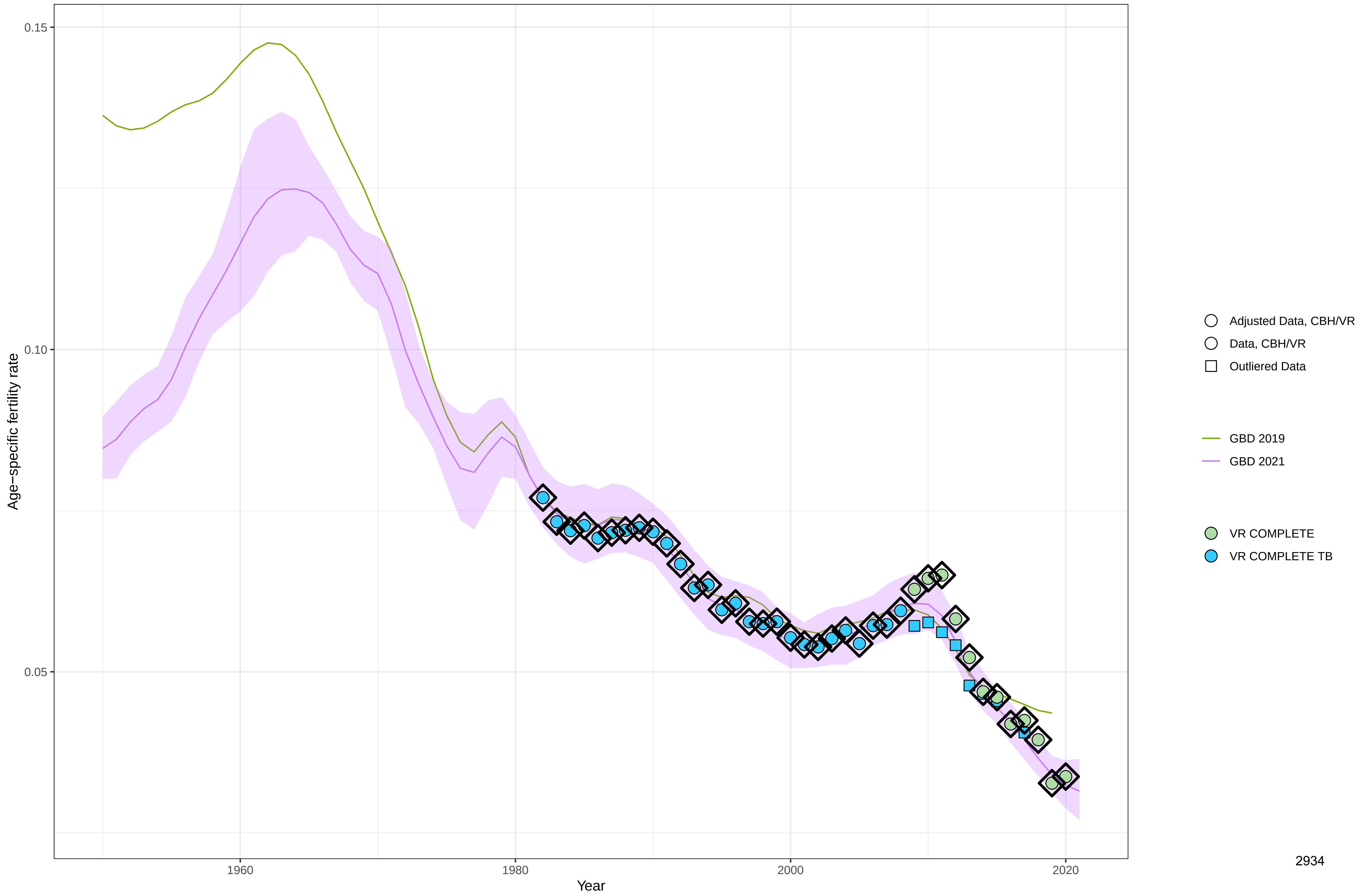

Age−specific fertility fitted trend lines, 1950−2021: Buckinghamshire, Ages: 25−29
Data points enclosed with a diamond are reference sources

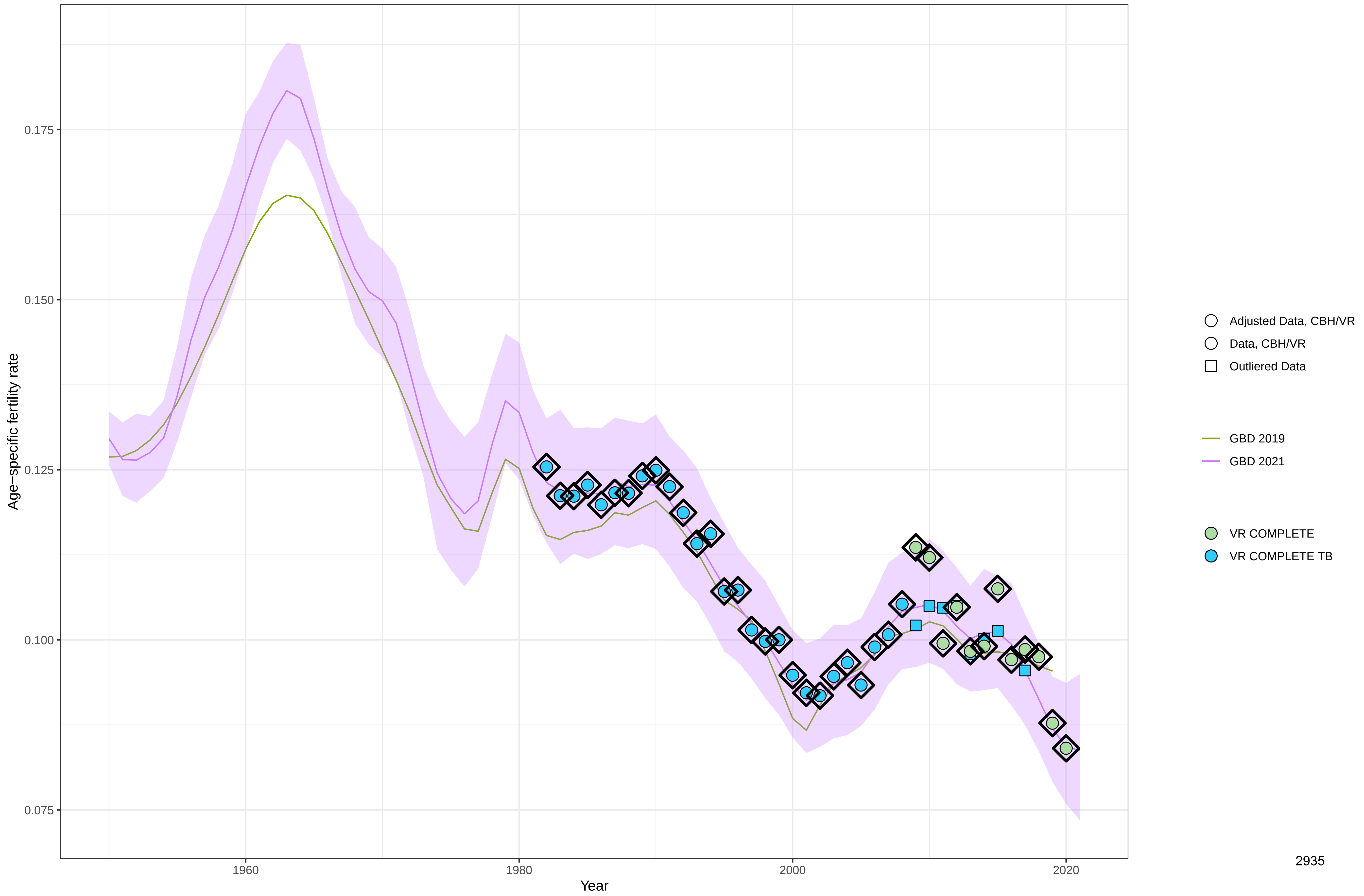

Age−specific fertility fitted trend lines, 1950−2021: Buckinghamshire, Ages: 30−34
Data points enclosed with a diamond are reference sources

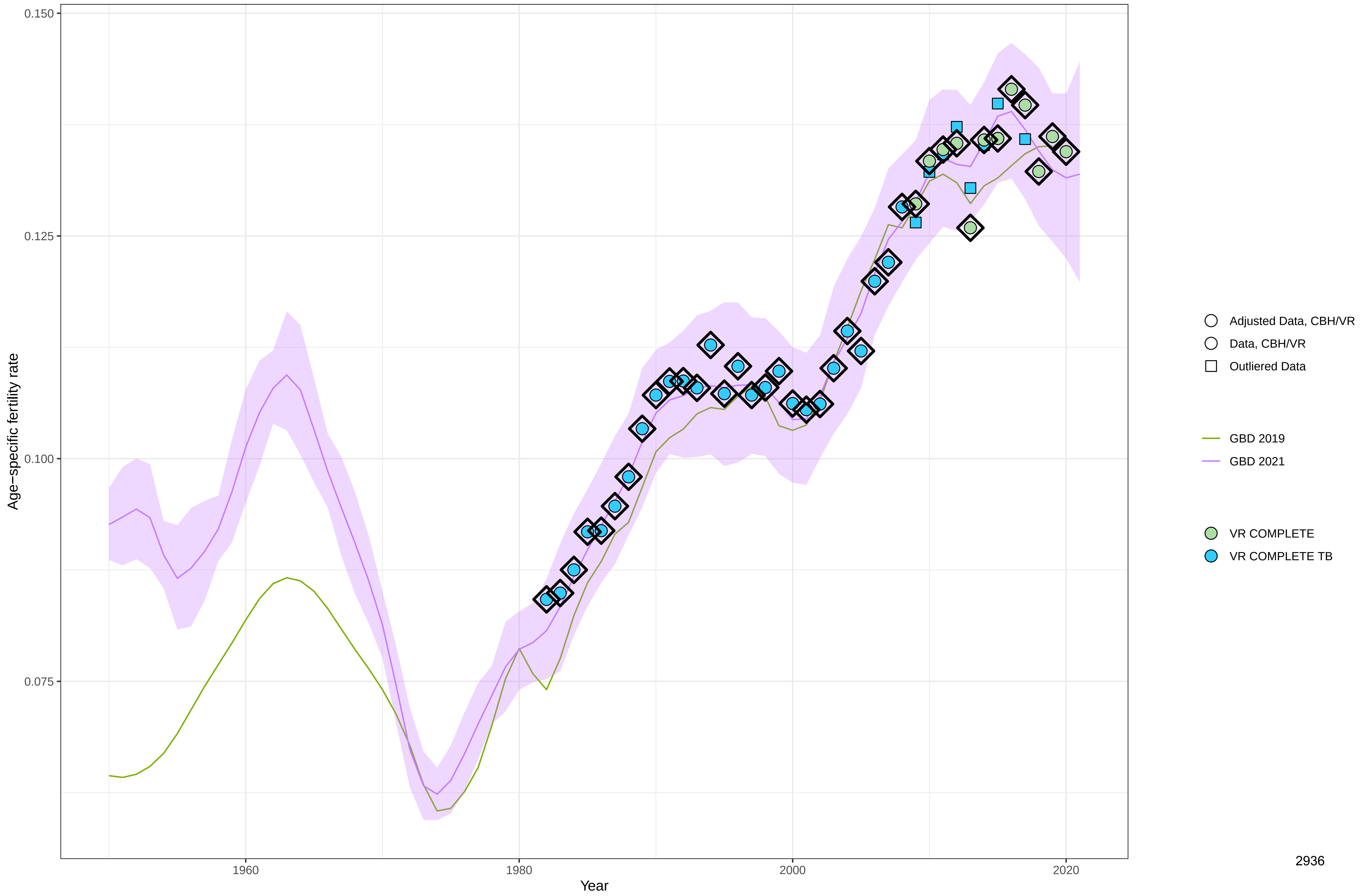

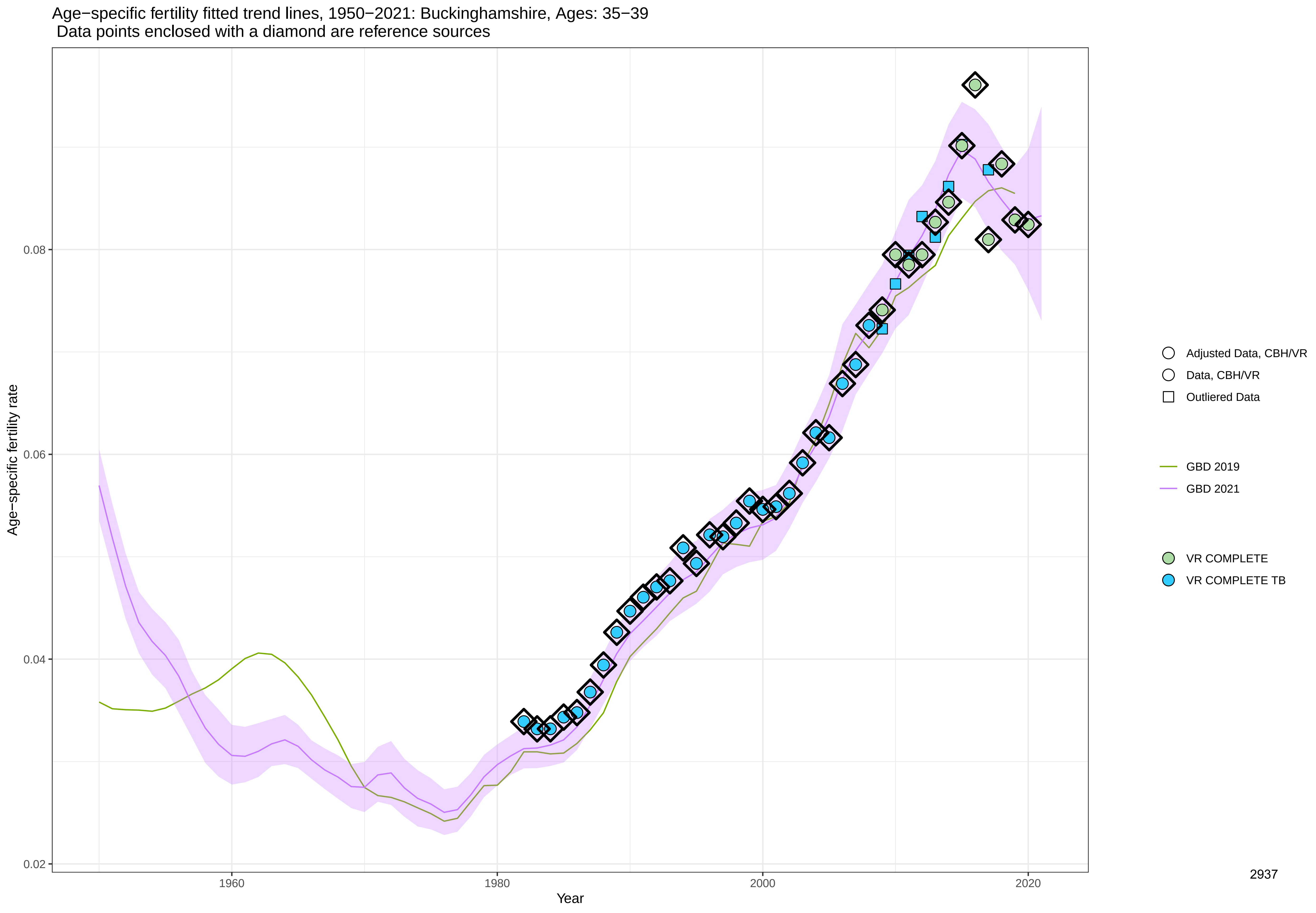

Age−specific fertility fitted trend lines, 1950−2021: Buckinghamshire, Ages: 35−39
Data points enclosed with a diamond are reference sources
Age−specific fertility rate
0.02
0.04
0.06
0.08
1960
1980
2000
2020
Year
Adjusted Data, CBH/VR
Data, CBH/VR
Outliered Data
GBD 2019
GBD 2021
VR COMPLETE
VR COMPLETE TB

2937

Age−specific fertility fitted trend lines, 1950−2021: Buckinghamshire, Ages: 40−44
Data points enclosed with a diamond are reference sources

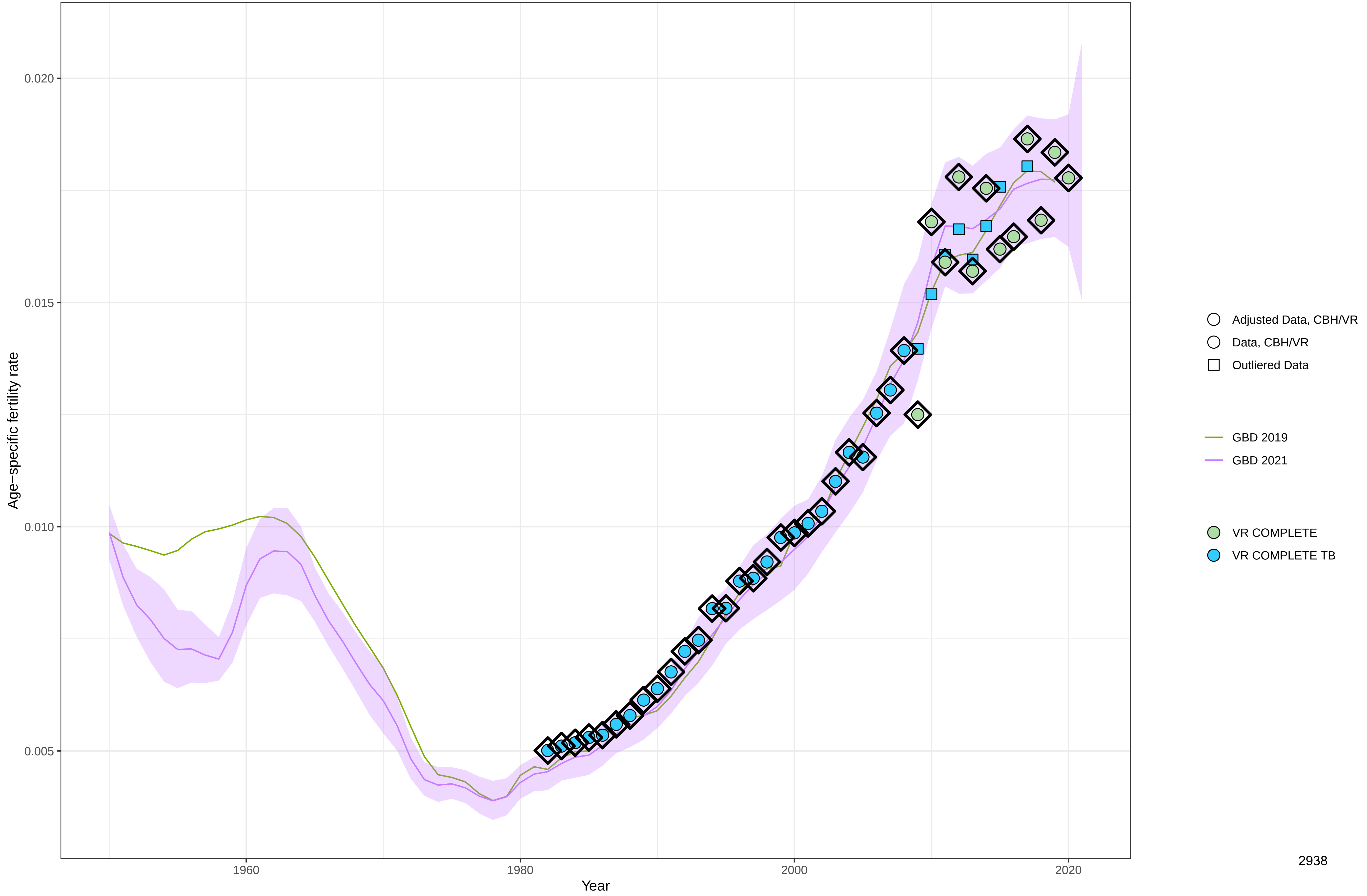

Age−specific fertility fitted trend lines, 1950−2021: Buckinghamshire, Ages: 45−49
Data points enclosed with a diamond are reference sources

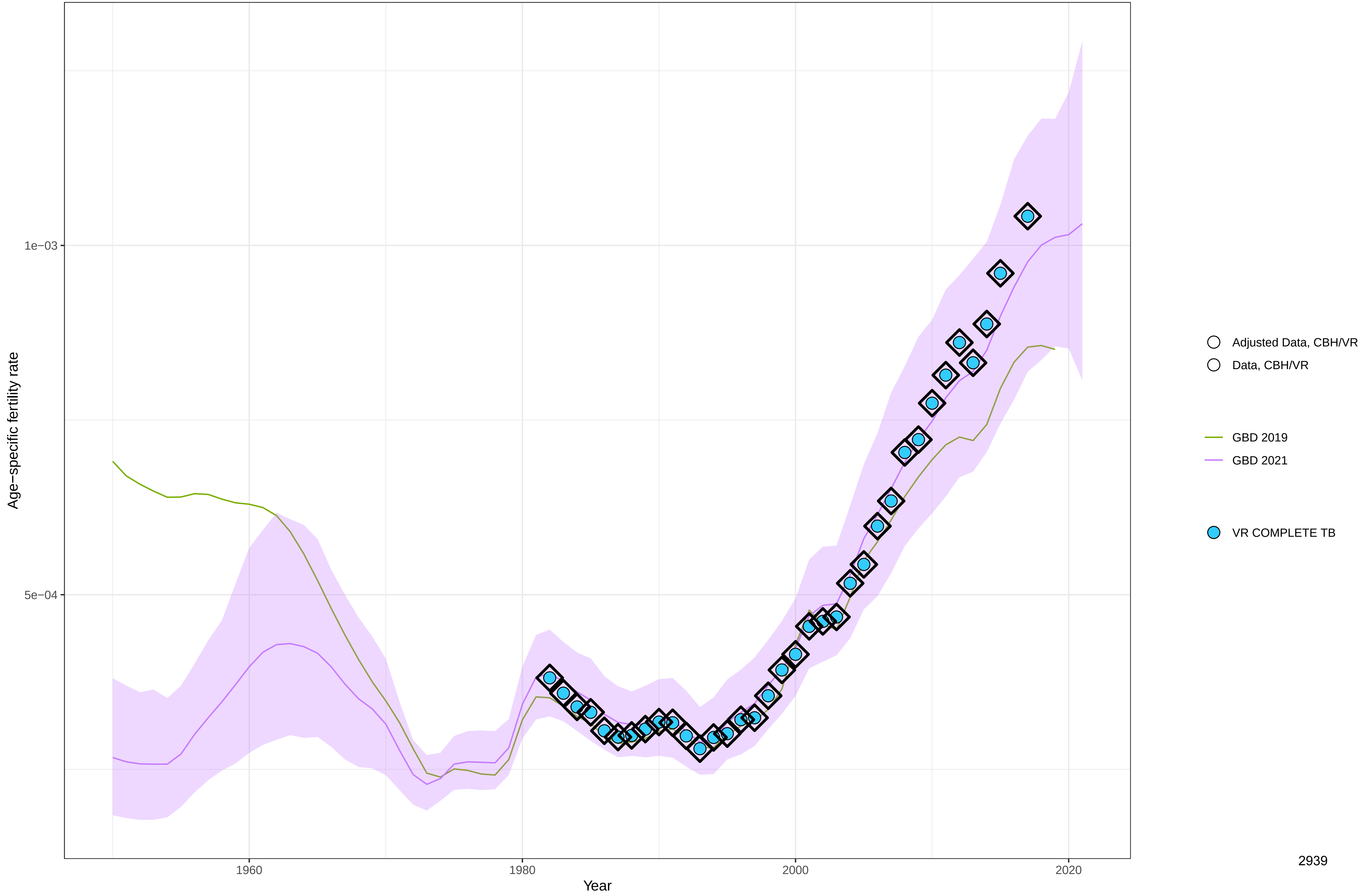

Age−specific fertility fitted trend lines, 1950−2021: Buckinghamshire, Ages: 50−54
Data points enclosed with a diamond are reference sources

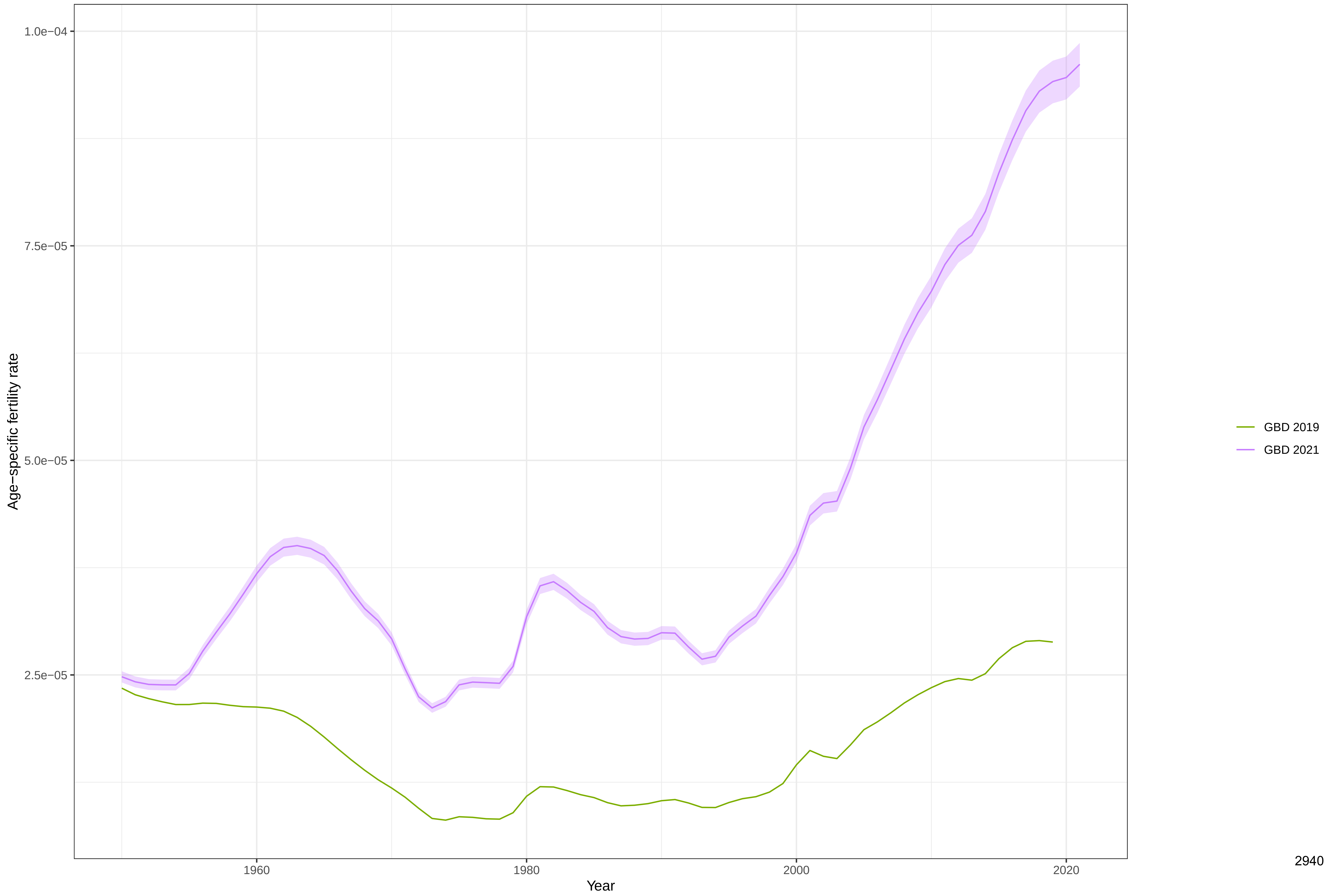

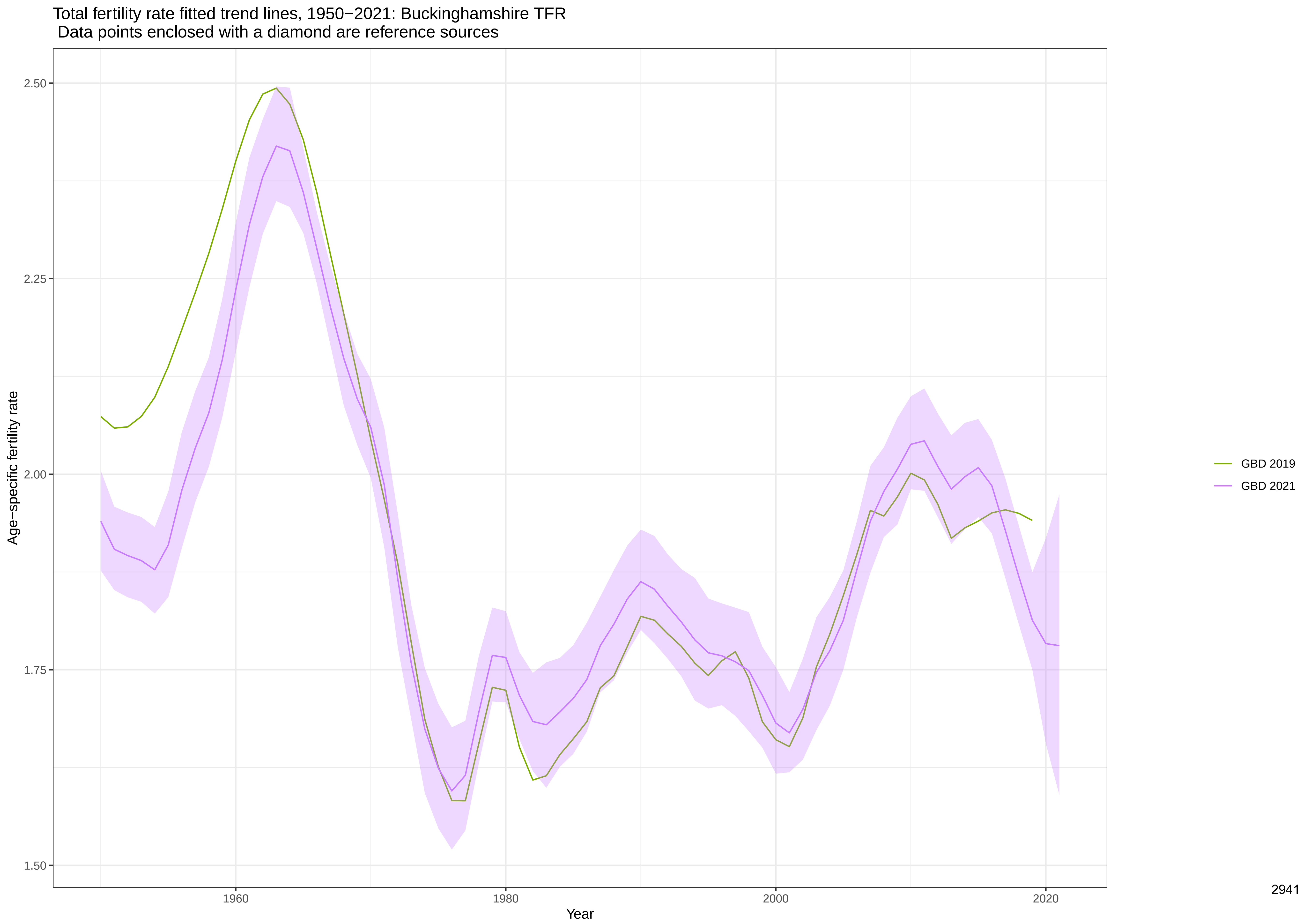

Total fertility rate fitted trend lines, 1950−2021: Buckinghamshire TFR
Data points enclosed with a diamond are reference sources
2.50
2.25
2.00
1.75
1.50
Age−specific fertility rate
1960
1980
2000
2020
Year
GBD 2019
GBD 2021

Age−specific fertility fitted trend lines, 1950−2021: East Sussex, Ages: 10−14
Data points enclosed with a diamond are reference sources

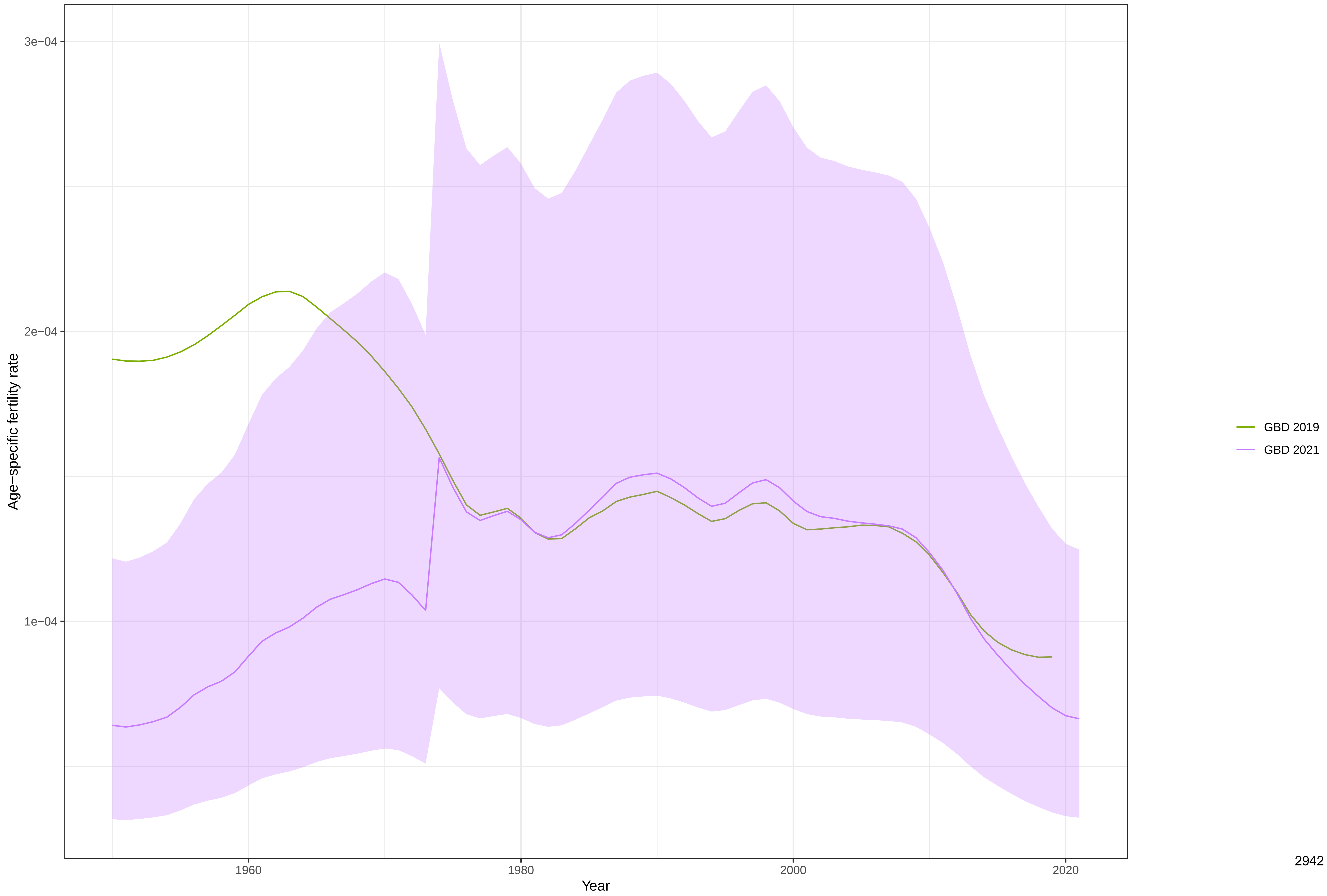

Age−specific fertility fitted trend lines, 1950−2021: East Sussex, Ages: 15−19
Data points enclosed with a diamond are reference sources

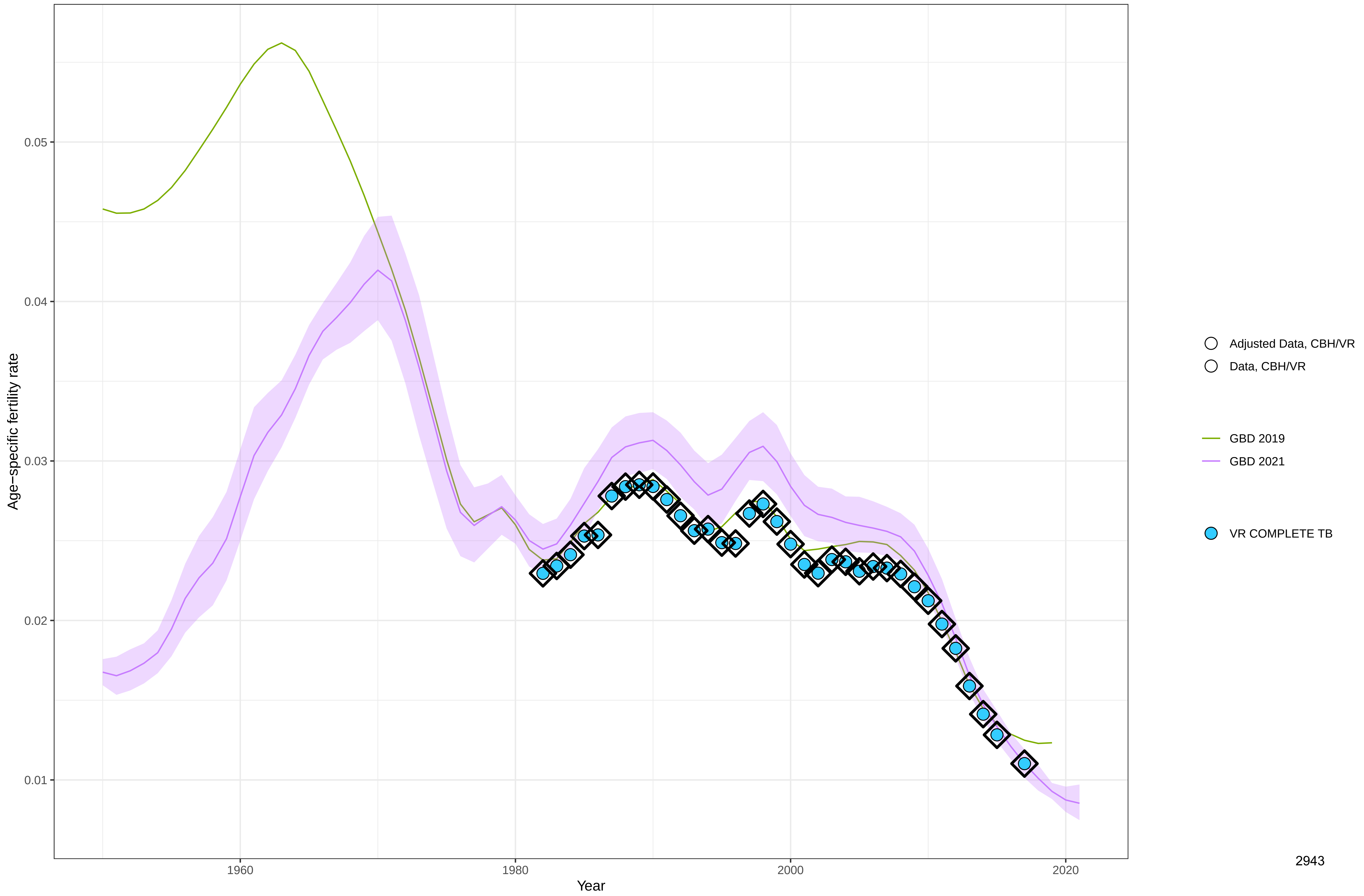

Age−specific fertility fitted trend lines, 1950−2021: East Sussex, Ages: 20−24
Data points enclosed with a diamond are reference sources

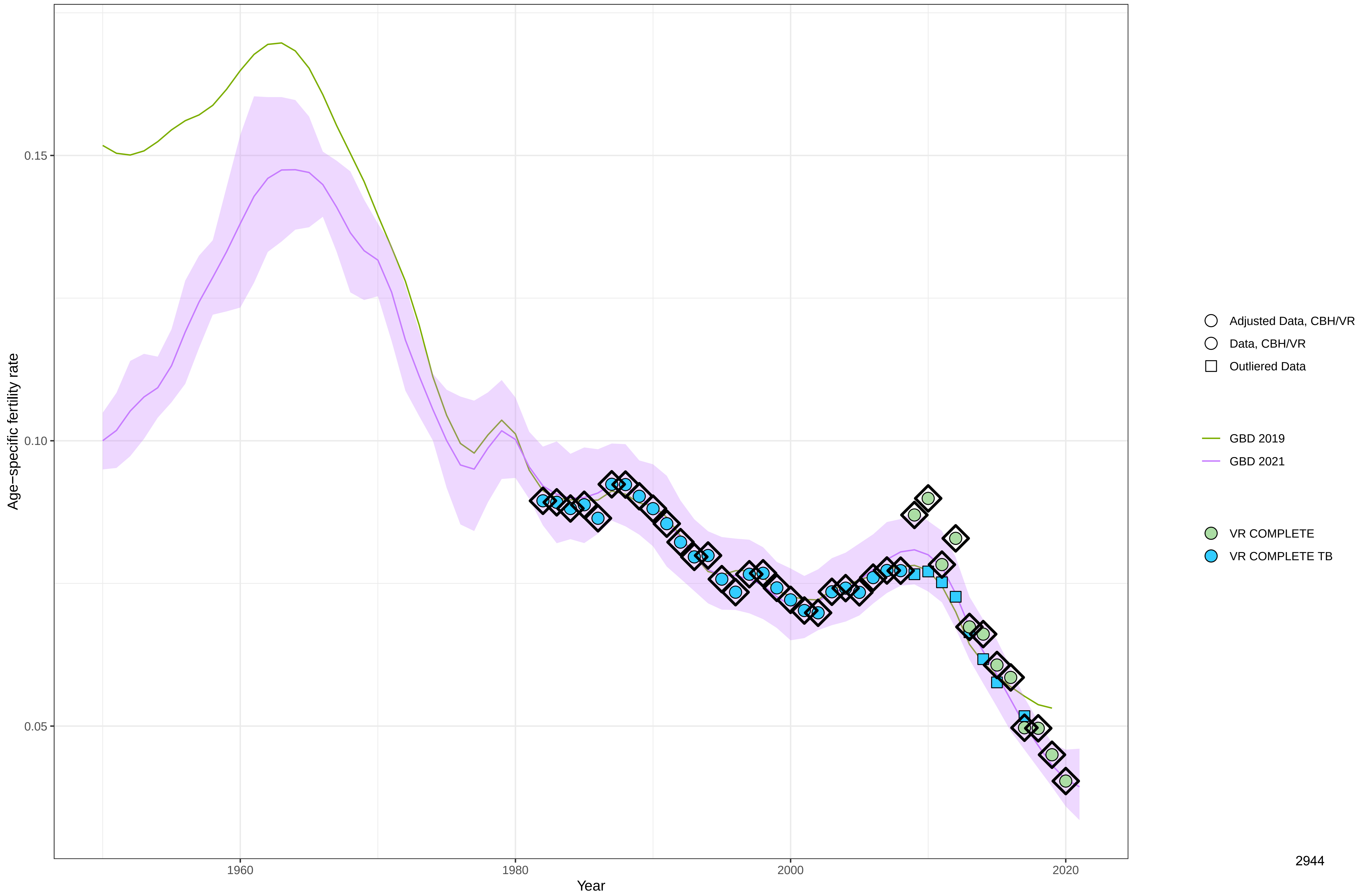

Age−specific fertility fitted trend lines, 1950−2021: East Sussex, Ages: 25−29
Data points enclosed with a diamond are reference sources

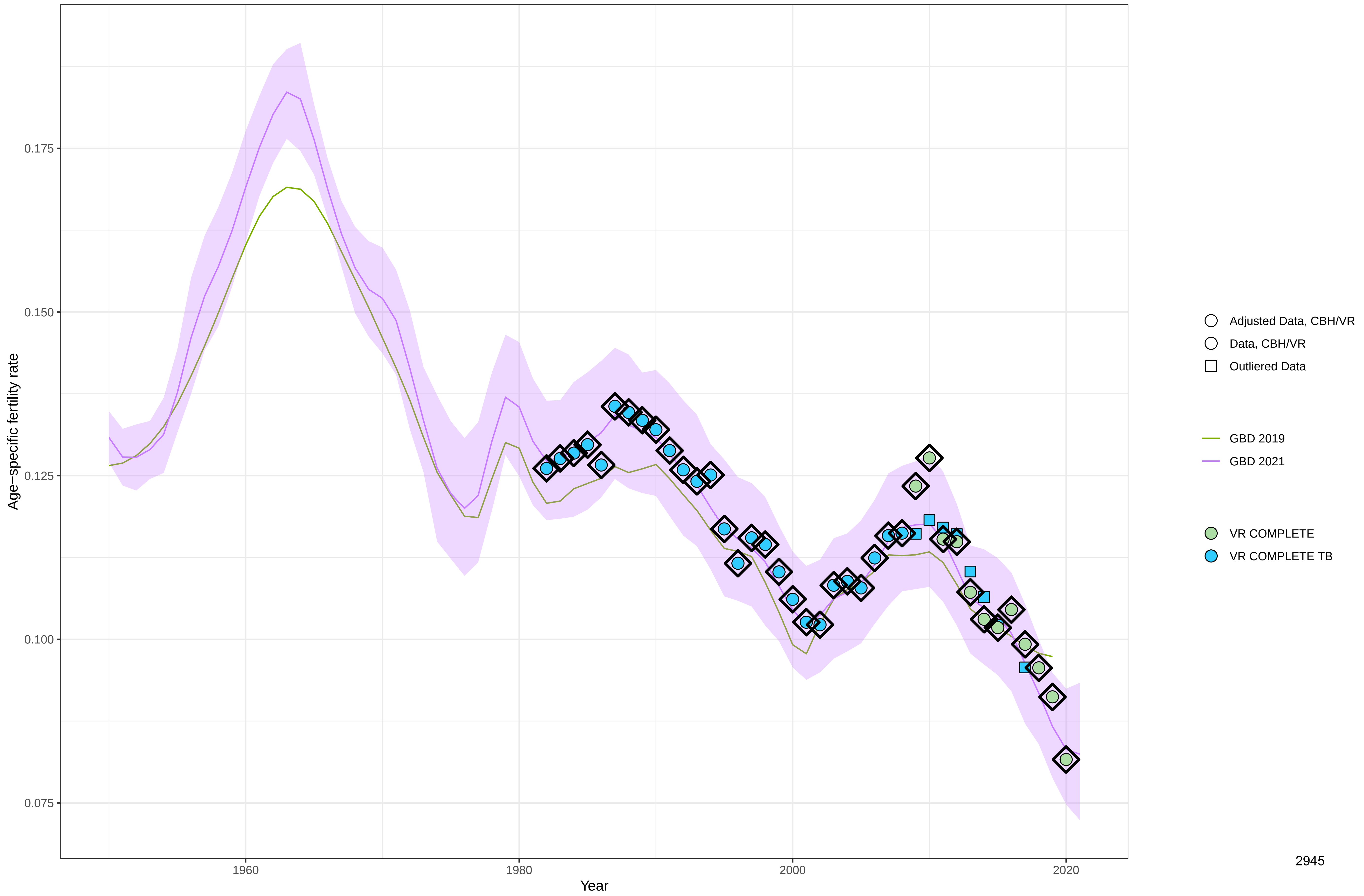

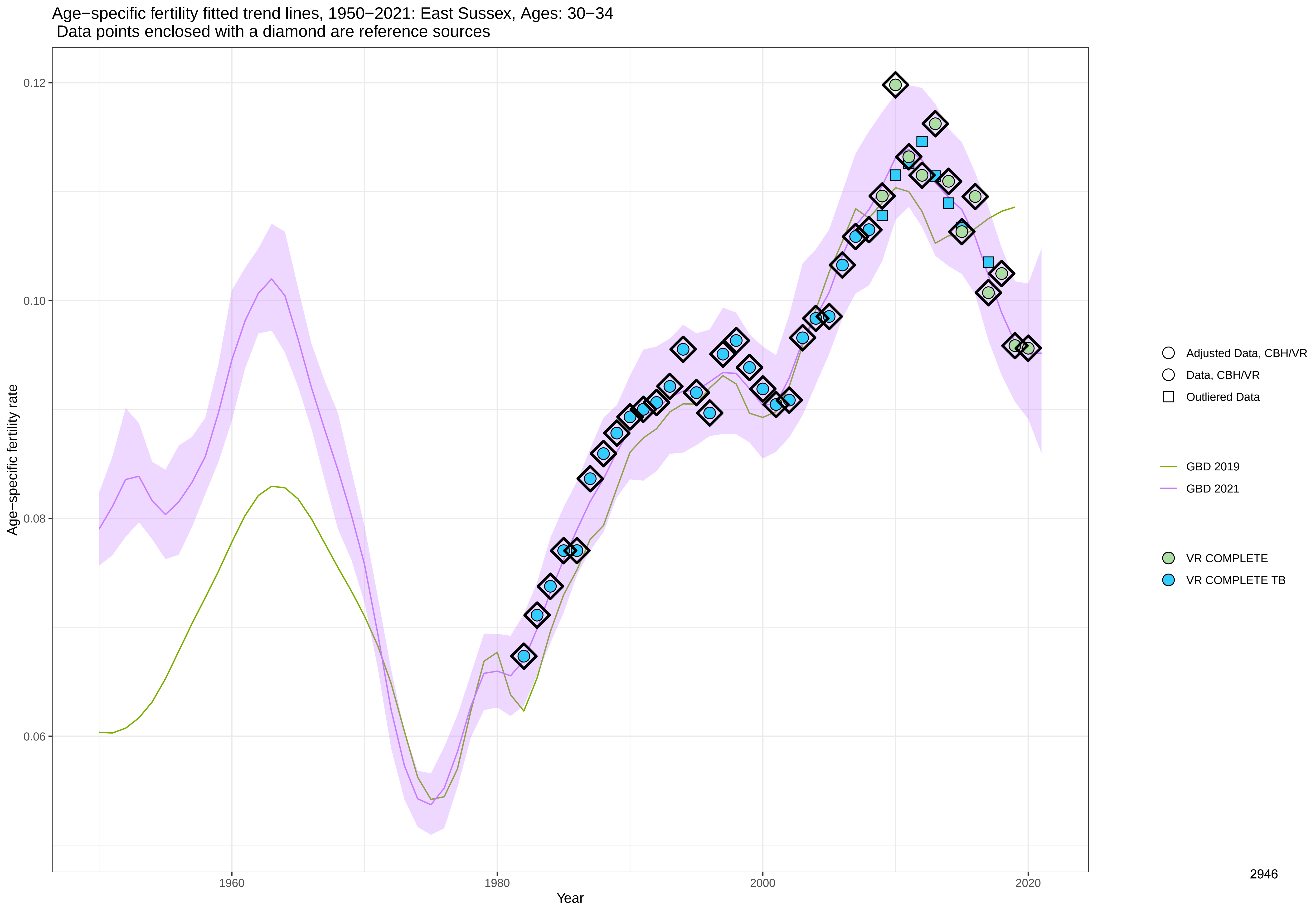

Age−specific fertility fitted trend lines, 1950−2021: East Sussex, Ages: 30−34
Data points enclosed with a diamond are reference sources
Age−specific fertility rate
0.12
0.10
0.08
0.06
Year
1960
1980
2000
2020
Adjusted Data, CBH/VR
Data, CBH/VR
Outliered Data
GBD 2019
GBD 2021
VR COMPLETE
VR COMPLETE TB

Age−specific fertility fitted trend lines, 1950−2021: East Sussex, Ages: 35−39
Data points enclosed with a diamond are reference sources

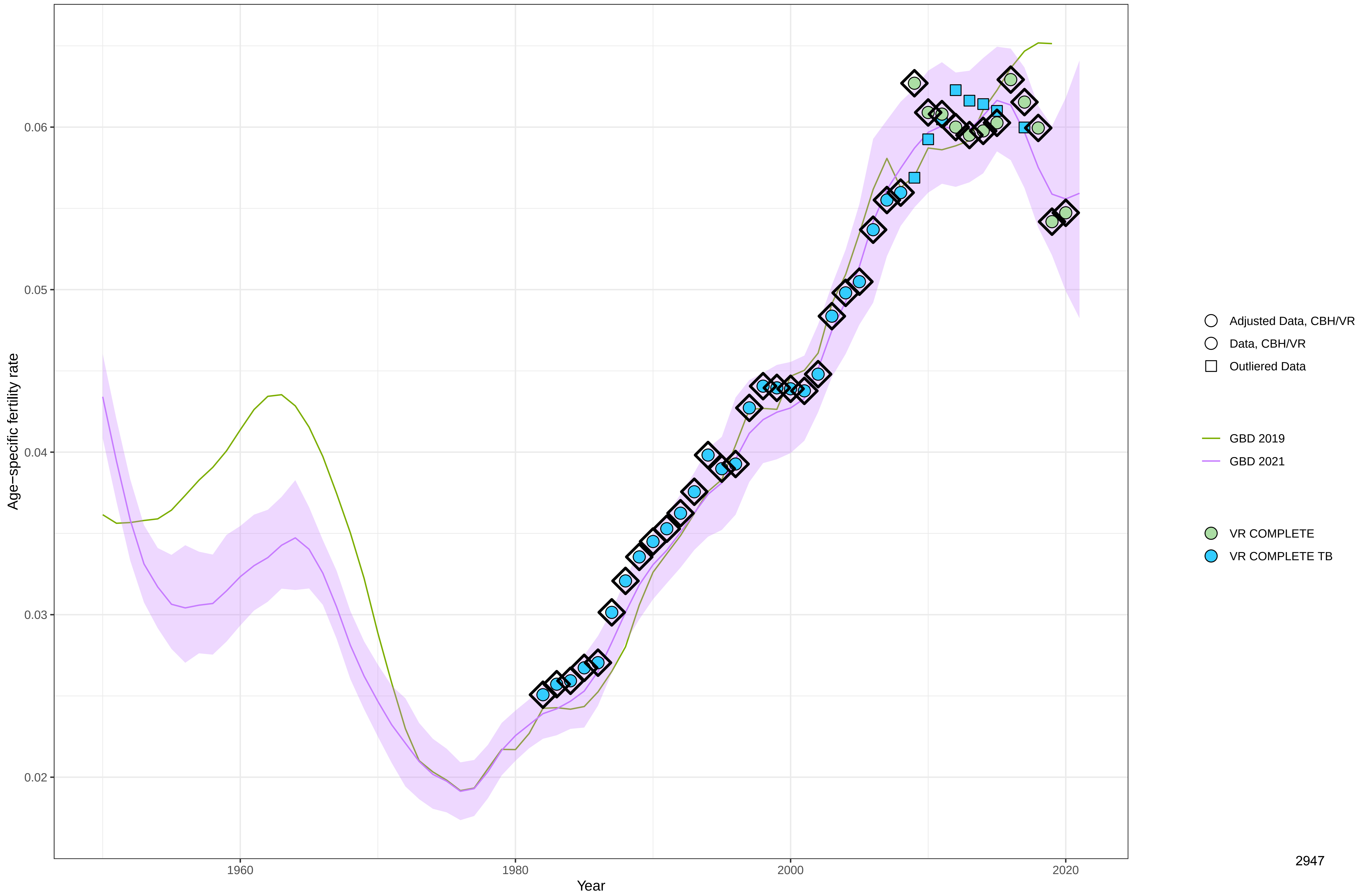

Age−specific fertility fitted trend lines, 1950−2021: East Sussex, Ages: 40−44
Data points enclosed with a diamond are reference sources

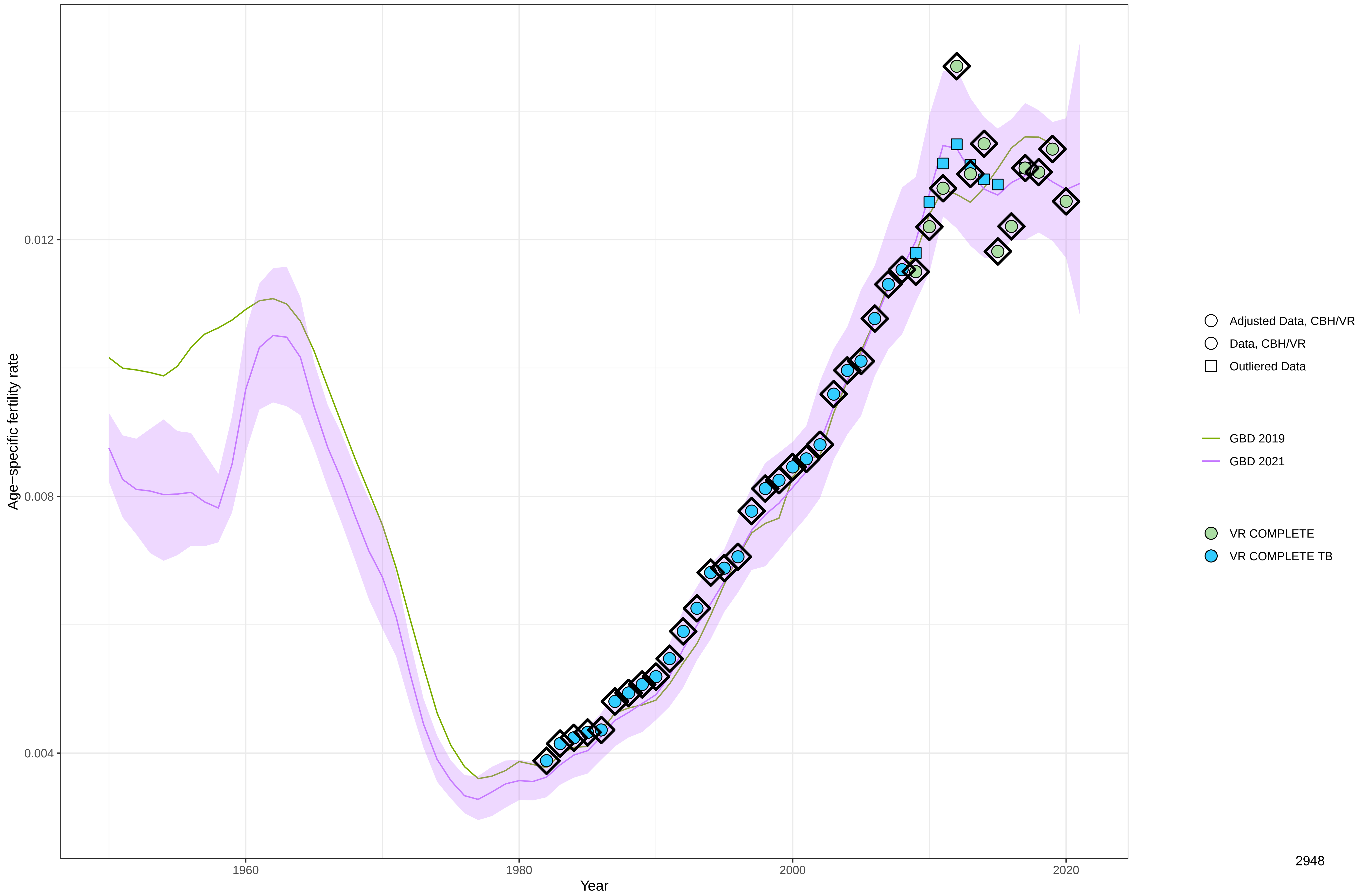

Age−specific fertility fitted trend lines, 1950−2021: East Sussex, Ages: 45−49
Data points enclosed with a diamond are reference sources

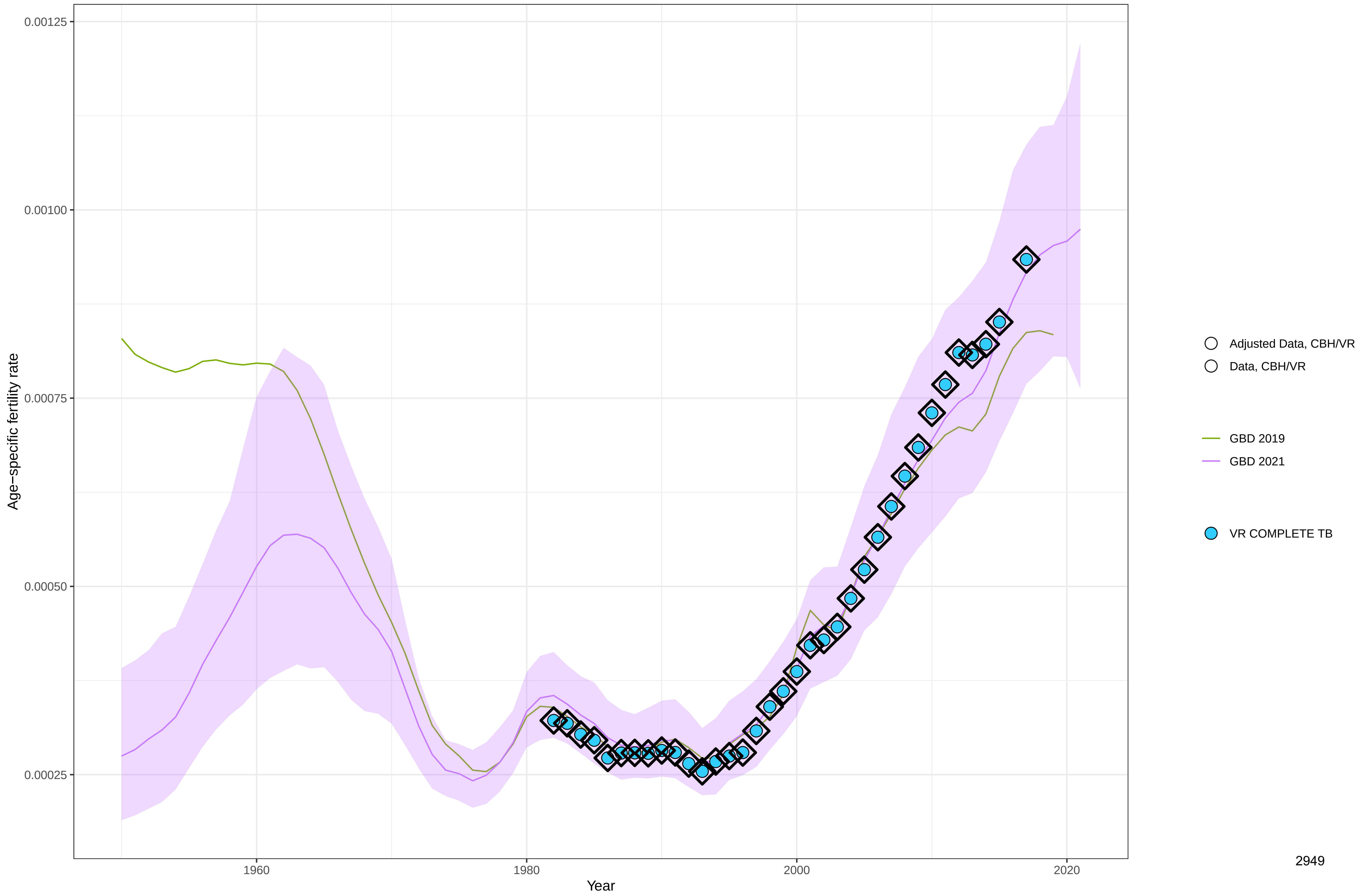

Age−specific fertility fitted trend lines, 1950−2021: East Sussex, Ages: 50−54
Data points enclosed with a diamond are reference sources

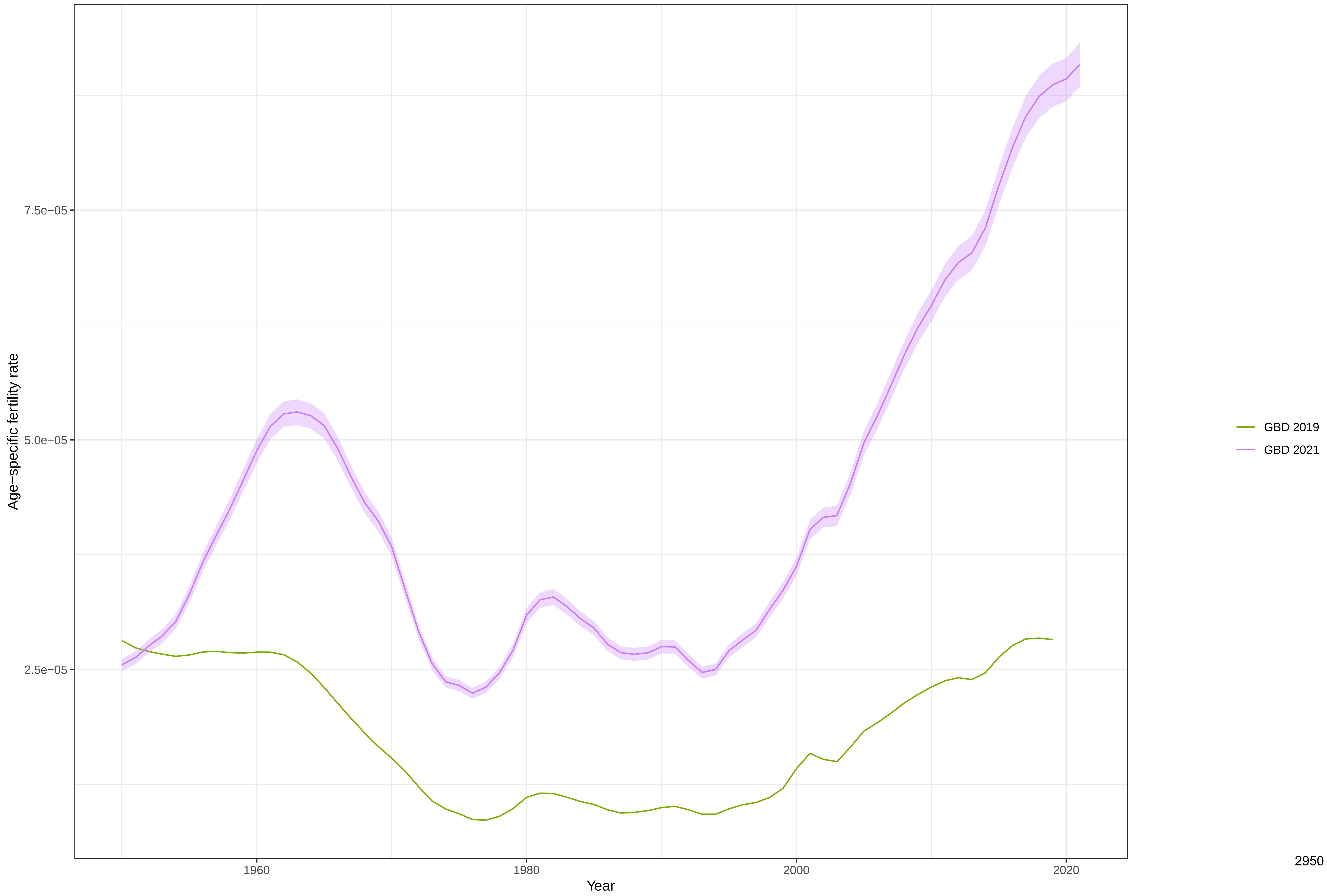

Total fertility rate fitted trend lines, 1950−2021: East Sussex TFR
Data points enclosed with a diamond are reference sources

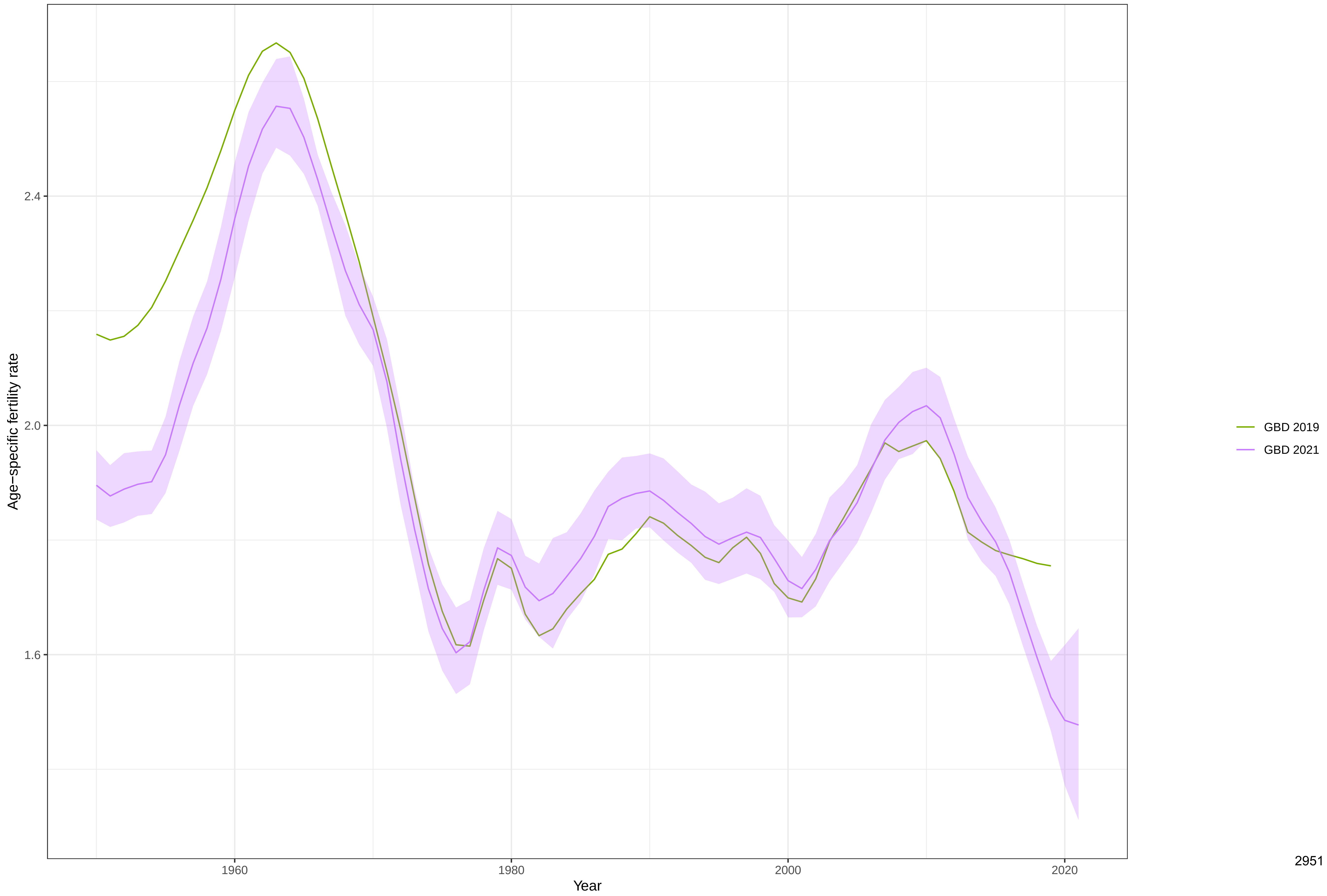

Age−specific fertility fitted trend lines, 1950−2021: Hampshire, Ages: 10−14
Data points enclosed with a diamond are reference sources

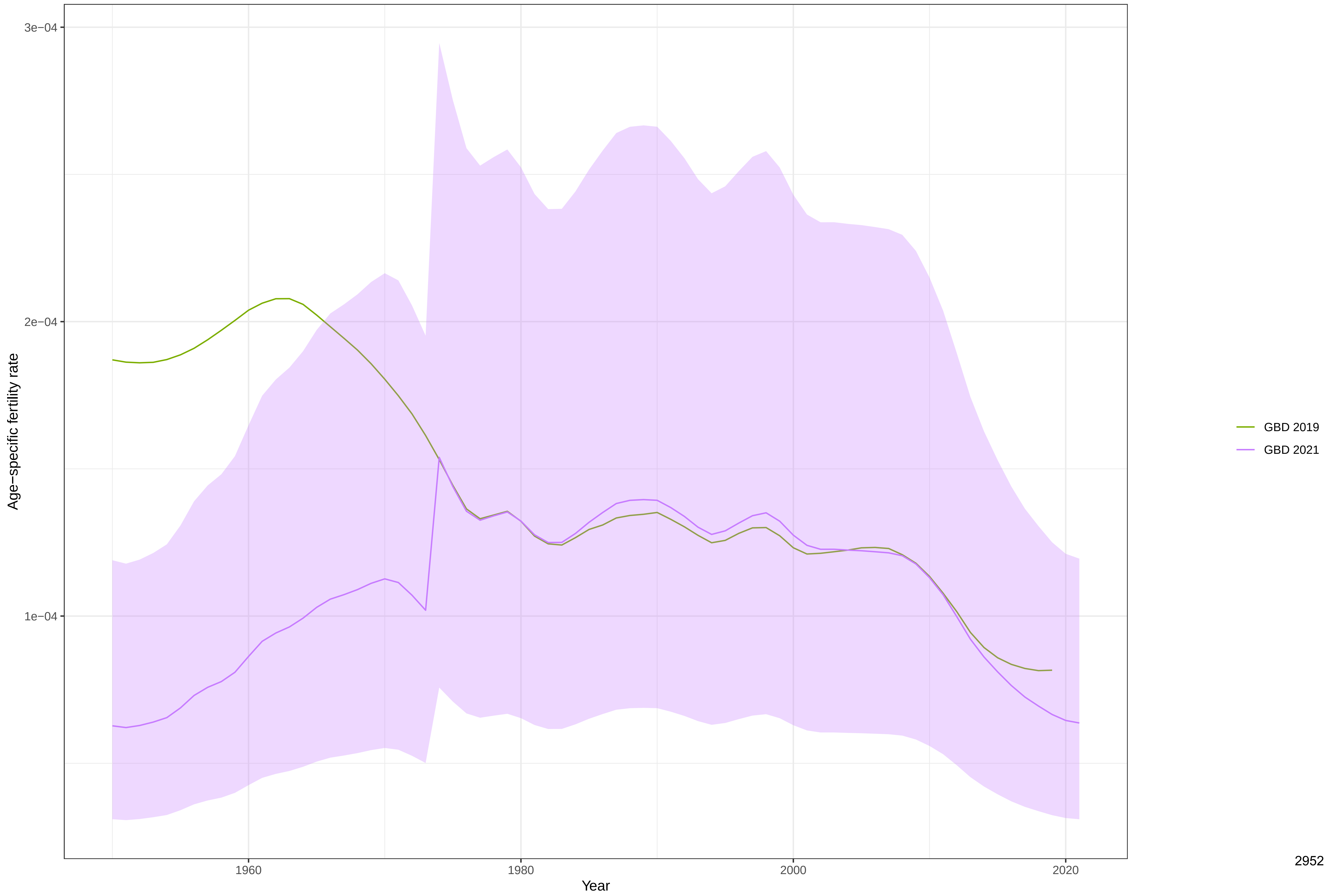

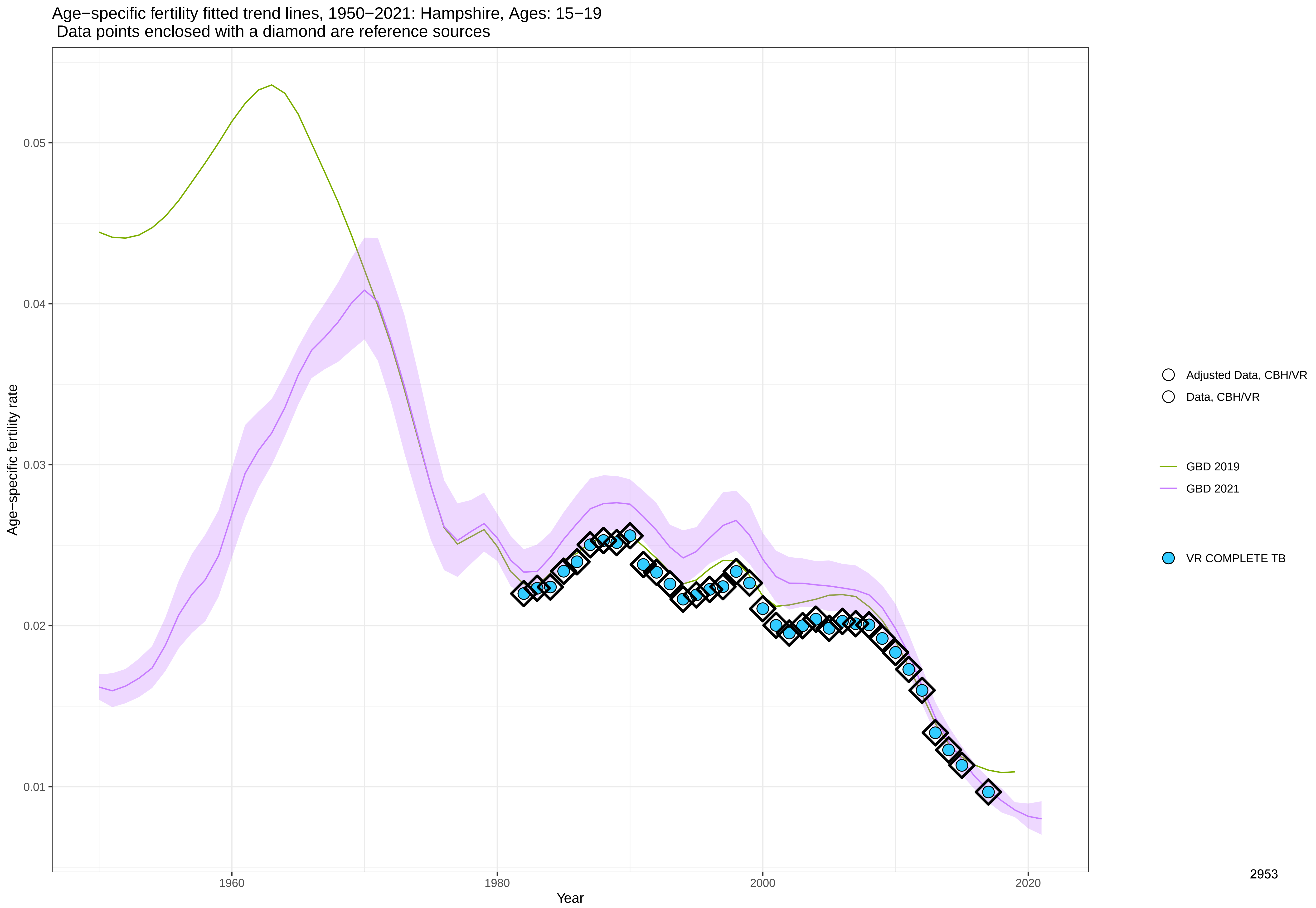

Age−specific fertility fitted trend lines, 1950−2021: Hampshire, Ages: 15−19
Data points enclosed with a diamond are reference sources
Age−specific fertility rate
0.05
0.04
0.03
0.02
0.01
1960
1980
2000
2020
Year
Adjusted Data, CBH/VR
Data, CBH/VR
GBD 2019
GBD 2021
VR COMPLETE TB

2953

Age−specific fertility fitted trend lines, 1950−2021: Hampshire, Ages: 20−24
Data points enclosed with a diamond are reference sources

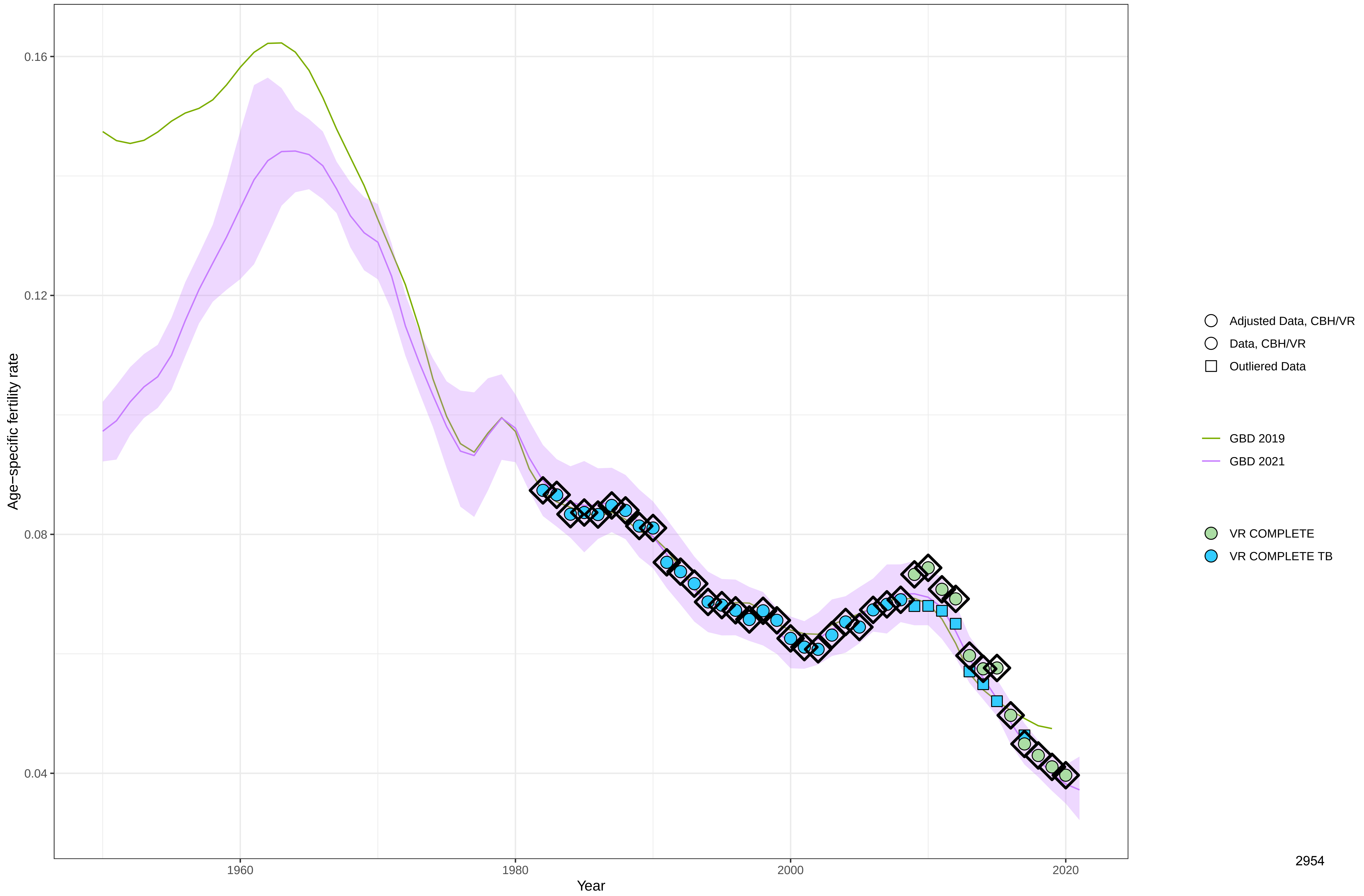

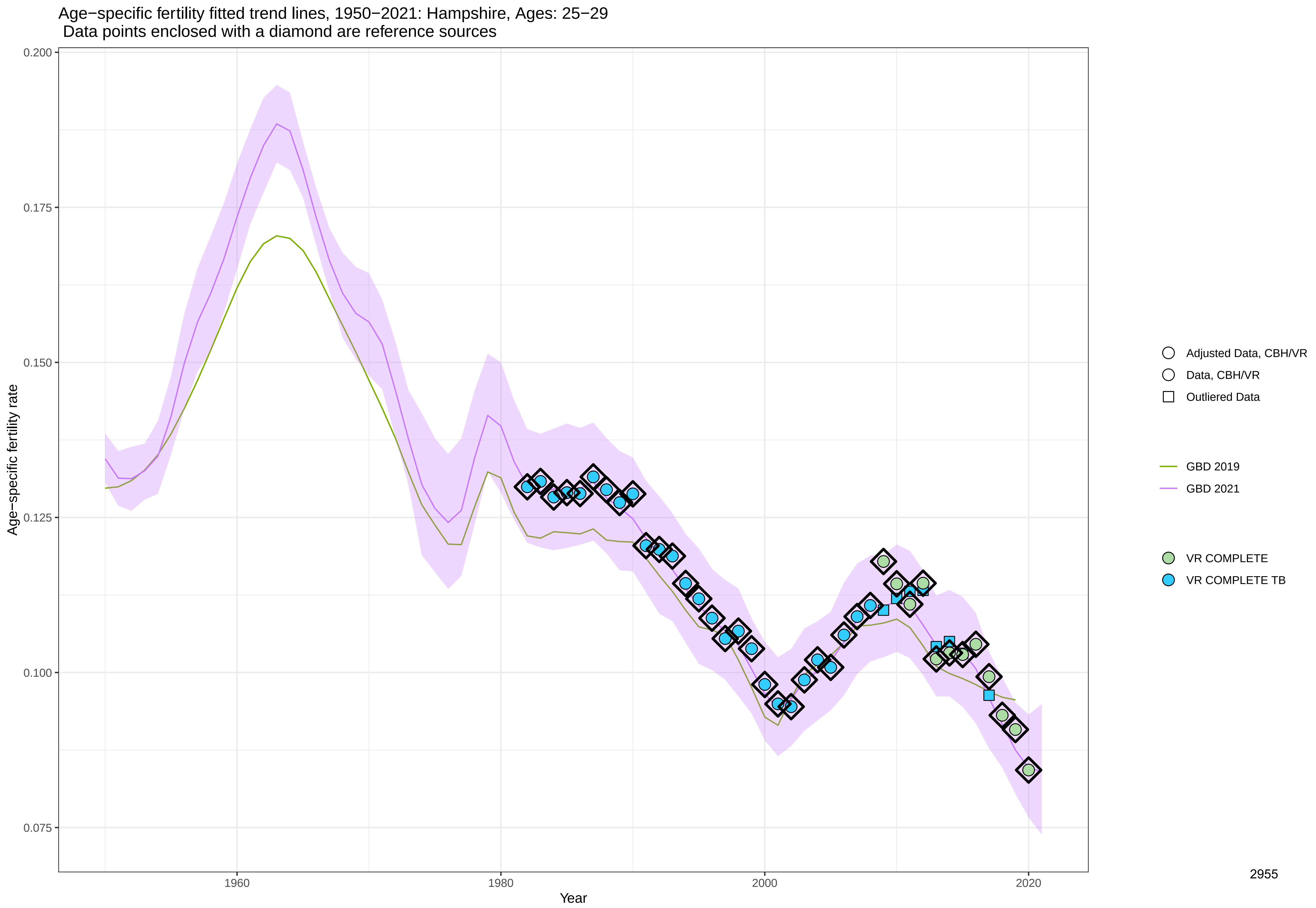

Age−specific fertility fitted trend lines, 1950−2021: Hampshire, Ages: 25−29
Data points enclosed with a diamond are reference sources
0.200
0.175
0.150
0.125
0.100
0.075
Age−specific fertility rate
1960
1980
2000
2020
Year
Adjusted Data, CBH/VR
Data, CBH/VR
Outliered Data
GBD 2019
GBD 2021
VR COMPLETE
VR COMPLETE TB

Age−specific fertility fitted trend lines, 1950−2021: Hampshire, Ages: 30−34
Data points enclosed with a diamond are reference sources

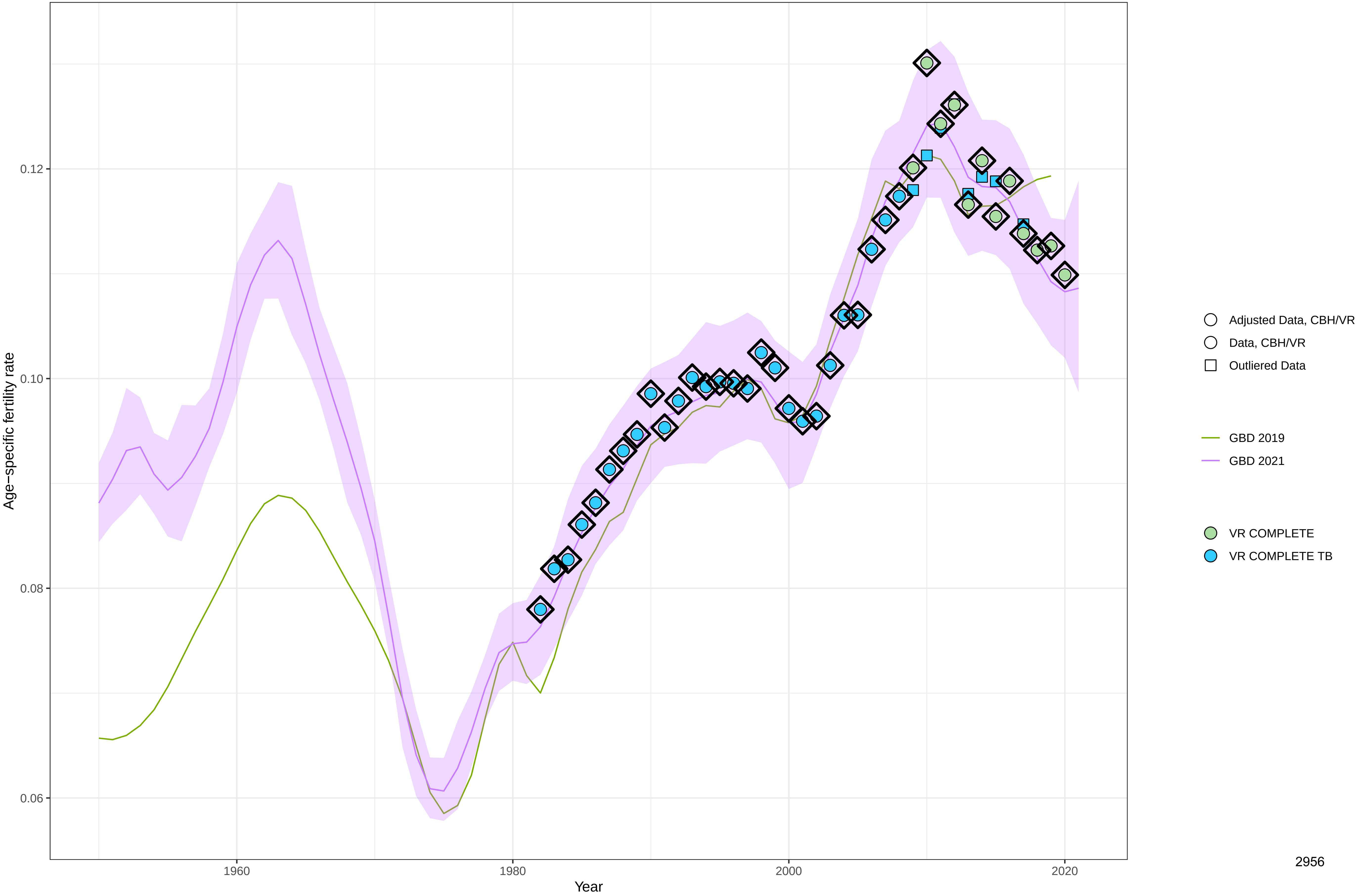

Age−specific fertility fitted trend lines, 1950−2021: Hampshire, Ages: 35−39
Data points enclosed with a diamond are reference sources

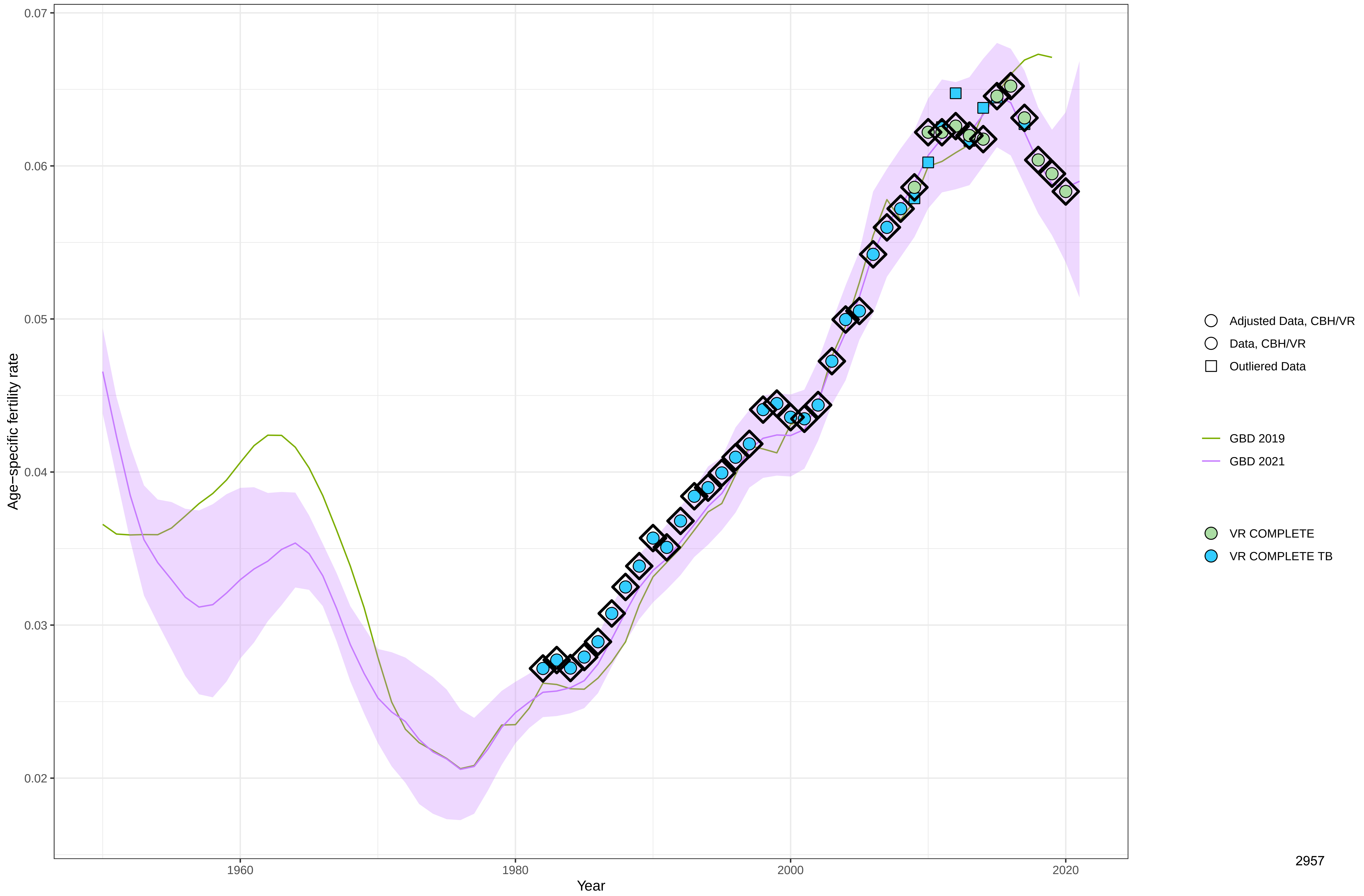

Age−specific fertility fitted trend lines, 1950−2021: Hampshire, Ages: 40−44
Data points enclosed with a diamond are reference sources

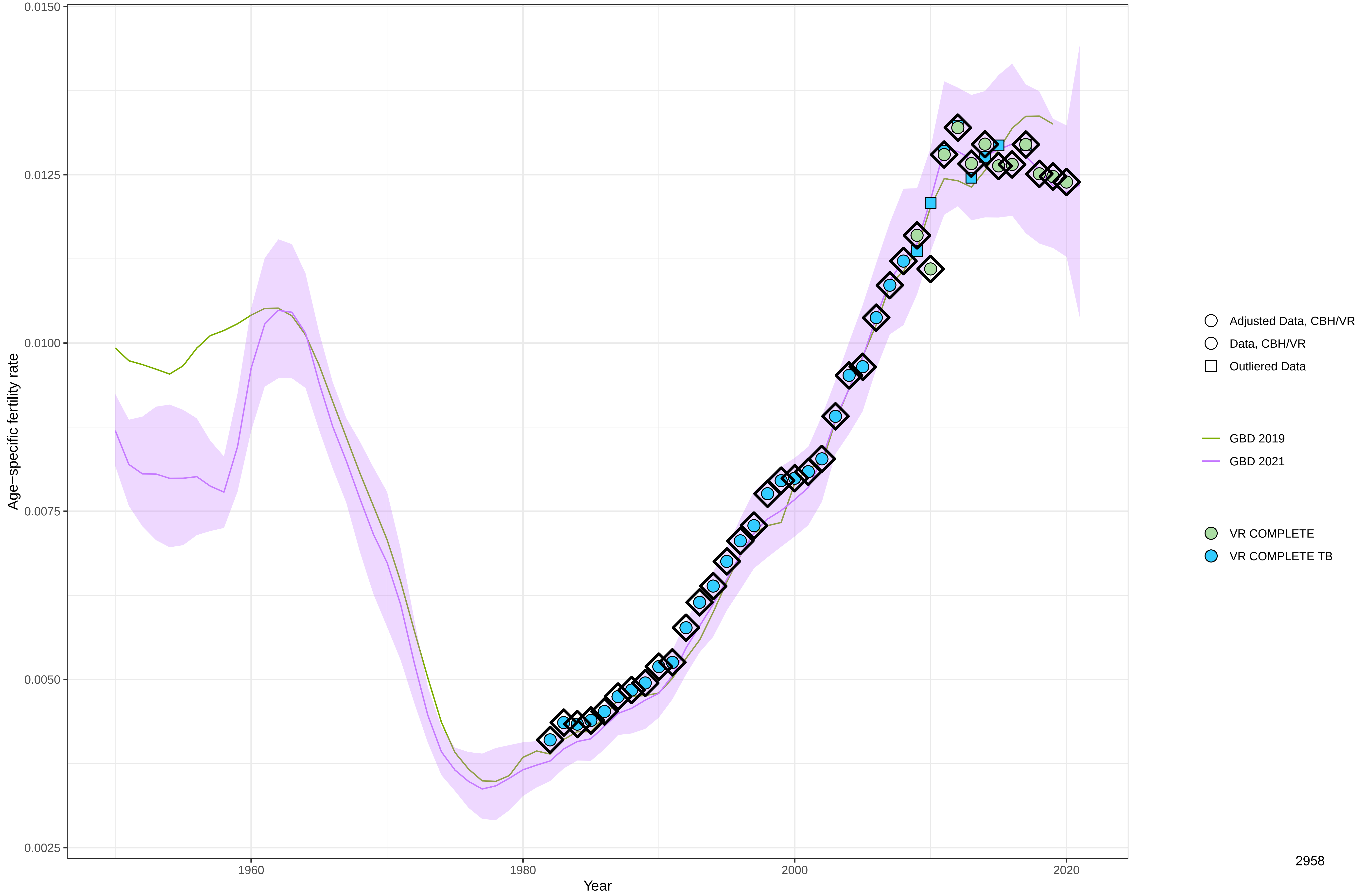

Age−specific fertility fitted trend lines, 1950−2021: Hampshire, Ages: 45−49
Data points enclosed with a diamond are reference sources

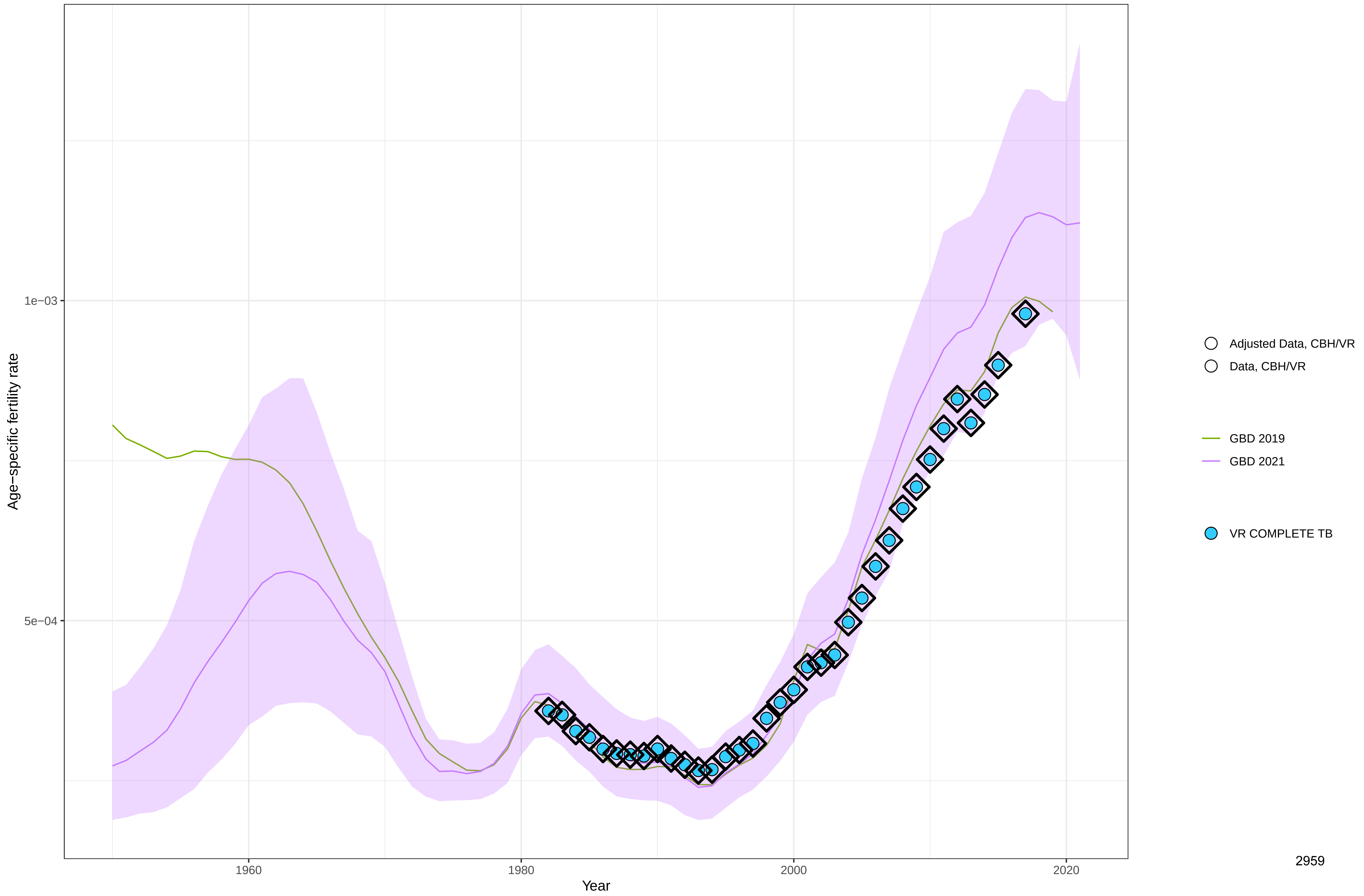

Age−specific fertility fitted trend lines, 1950−2021: Hampshire, Ages: 50−54
Data points enclosed with a diamond are reference sources

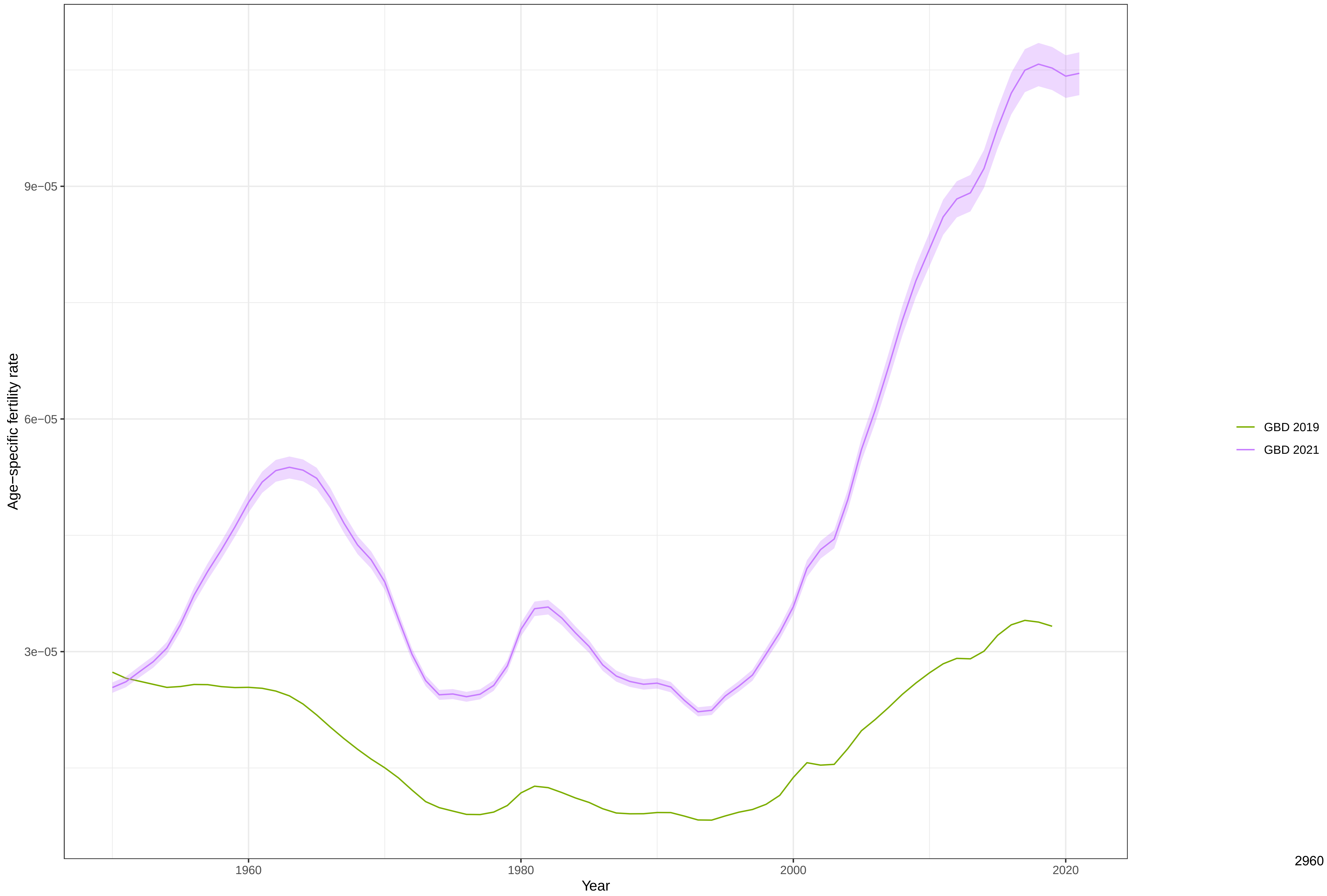

Total fertility rate fitted trend lines, 1950−2021: Hampshire TFR
Data points enclosed with a diamond are reference sources

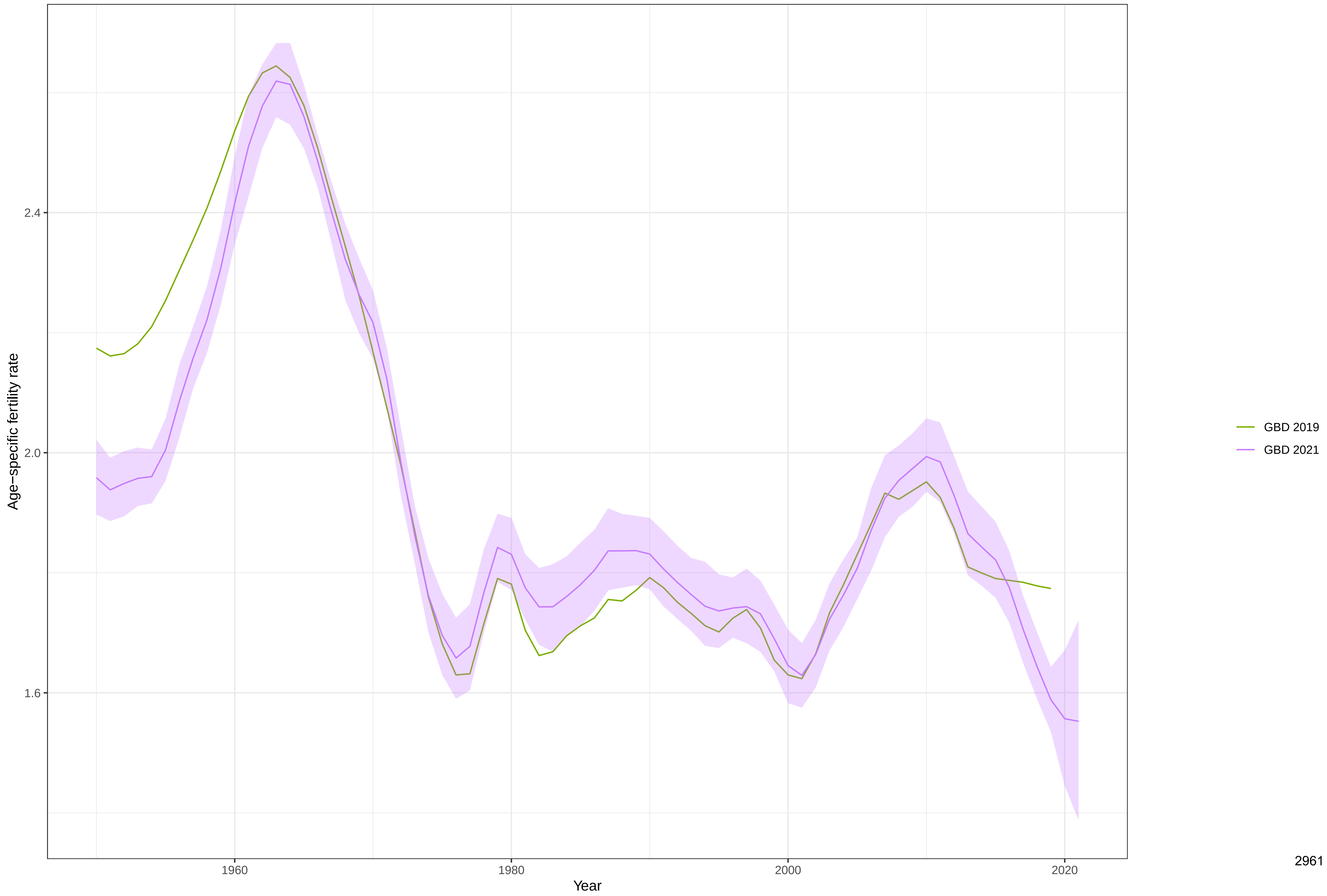

Age−specific fertility fitted trend lines, 1950−2021: Isle of Wight, Ages: 10−14
 Data points enclosed with a diamond are reference sources

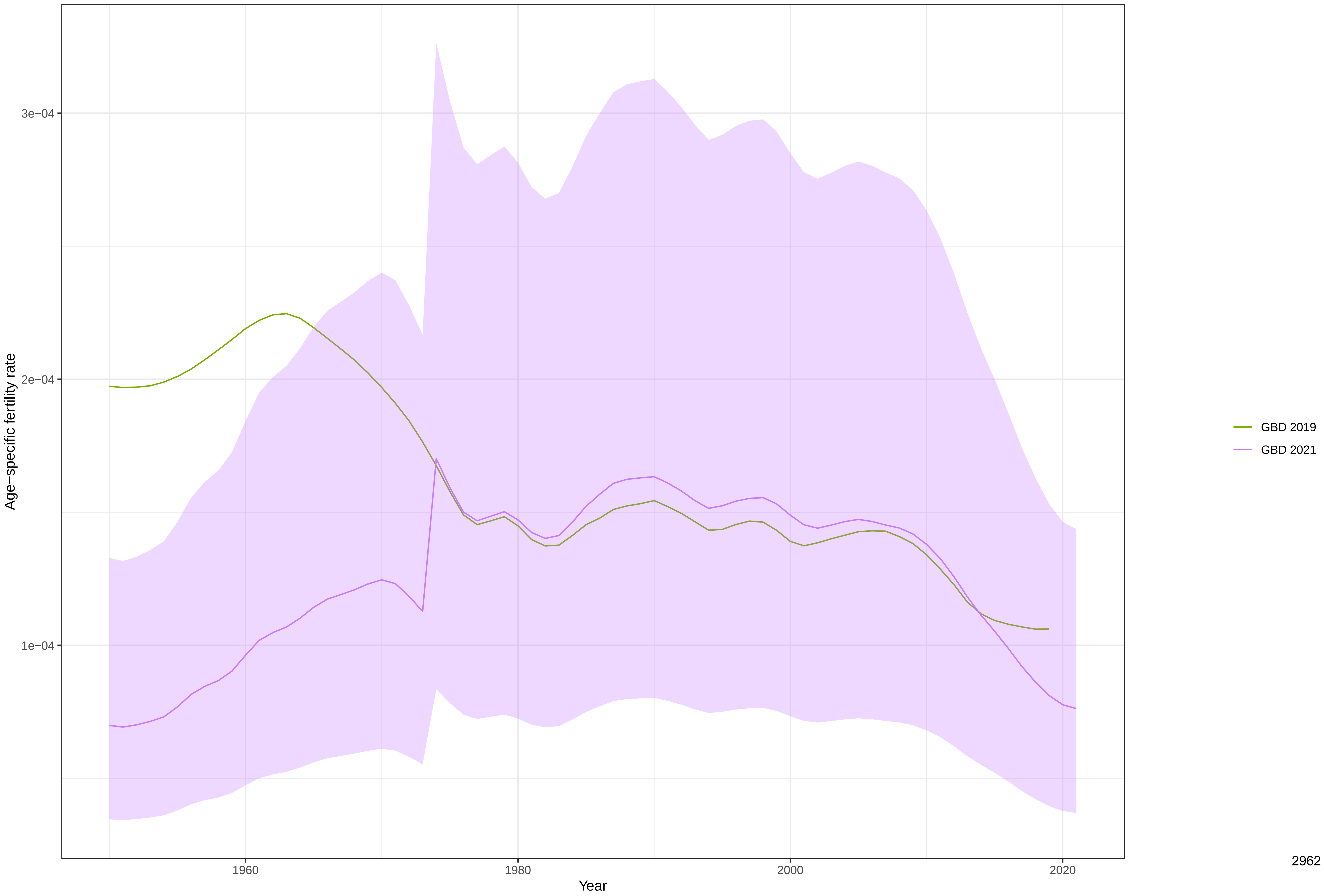

Age−specific fertility fitted trend lines, 1950−2021: Isle of Wight, Ages: 15−19
Data points enclosed with a diamond are reference sources

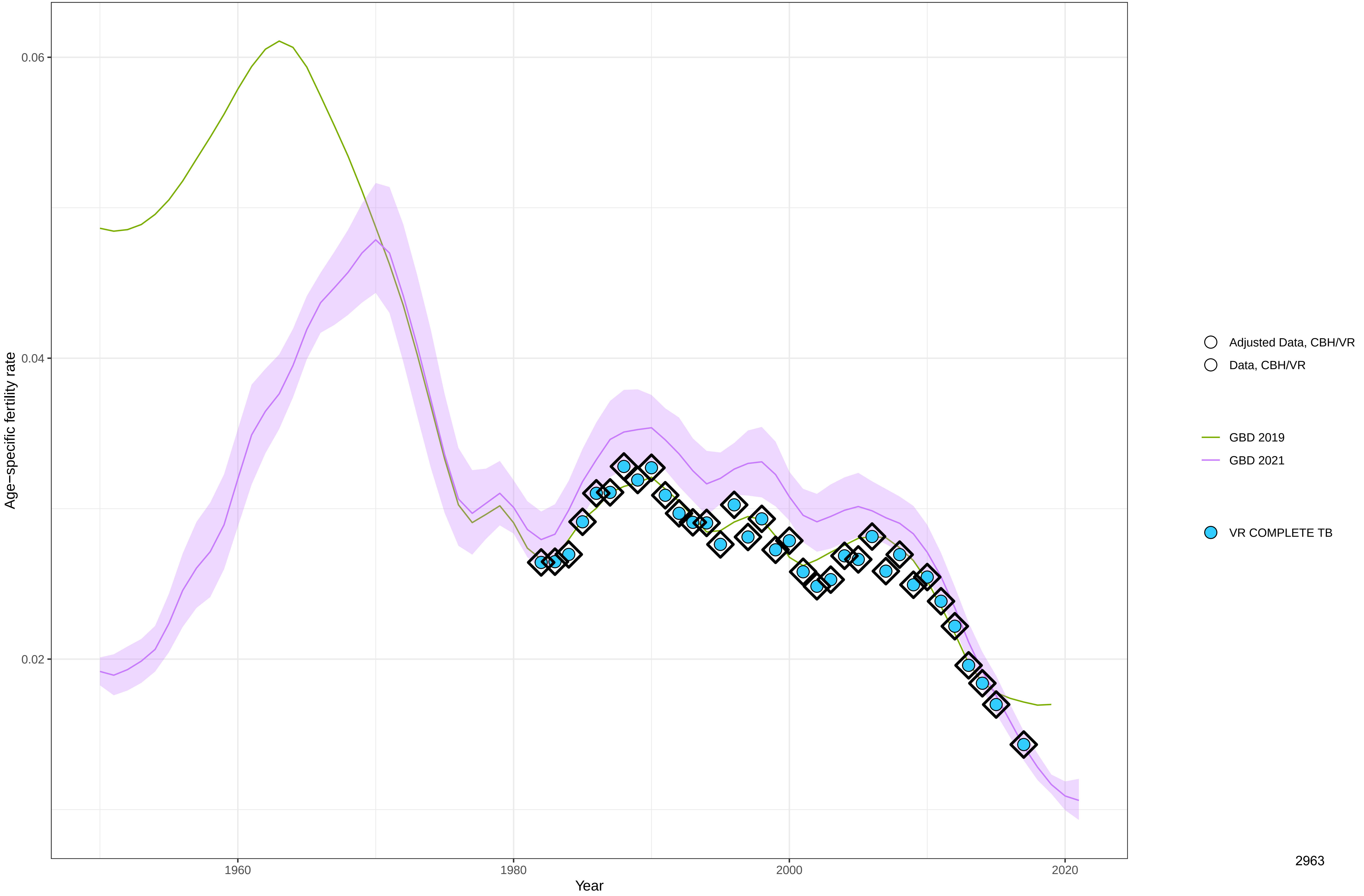

Age−specific fertility fitted trend lines, 1950−2021: Isle of Wight, Ages: 20−24
Data points enclosed with a diamond are reference sources

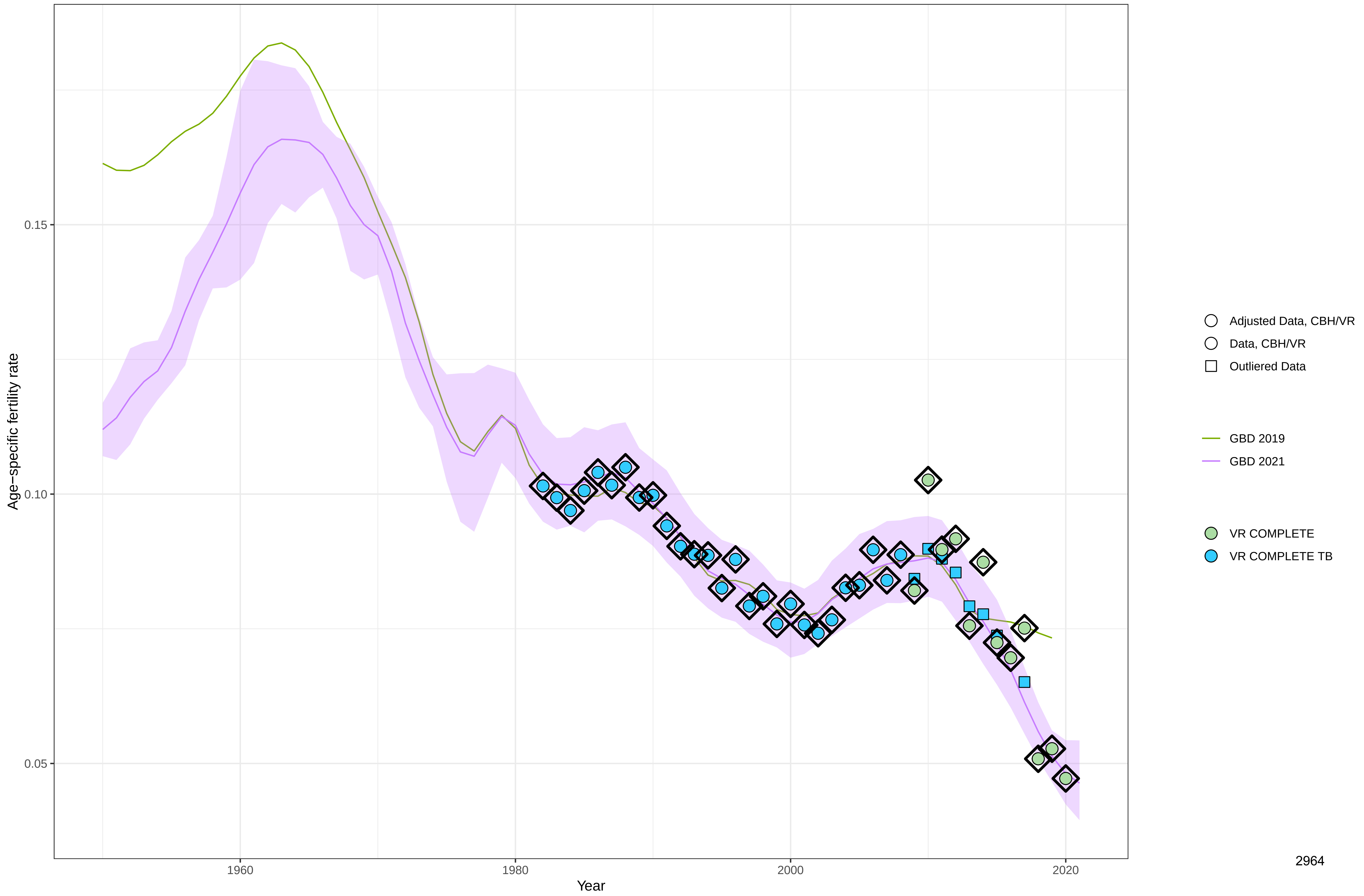

Age−specific fertility fitted trend lines, 1950−2021: Isle of Wight, Ages: 25−29
Data points enclosed with a diamond are reference sources

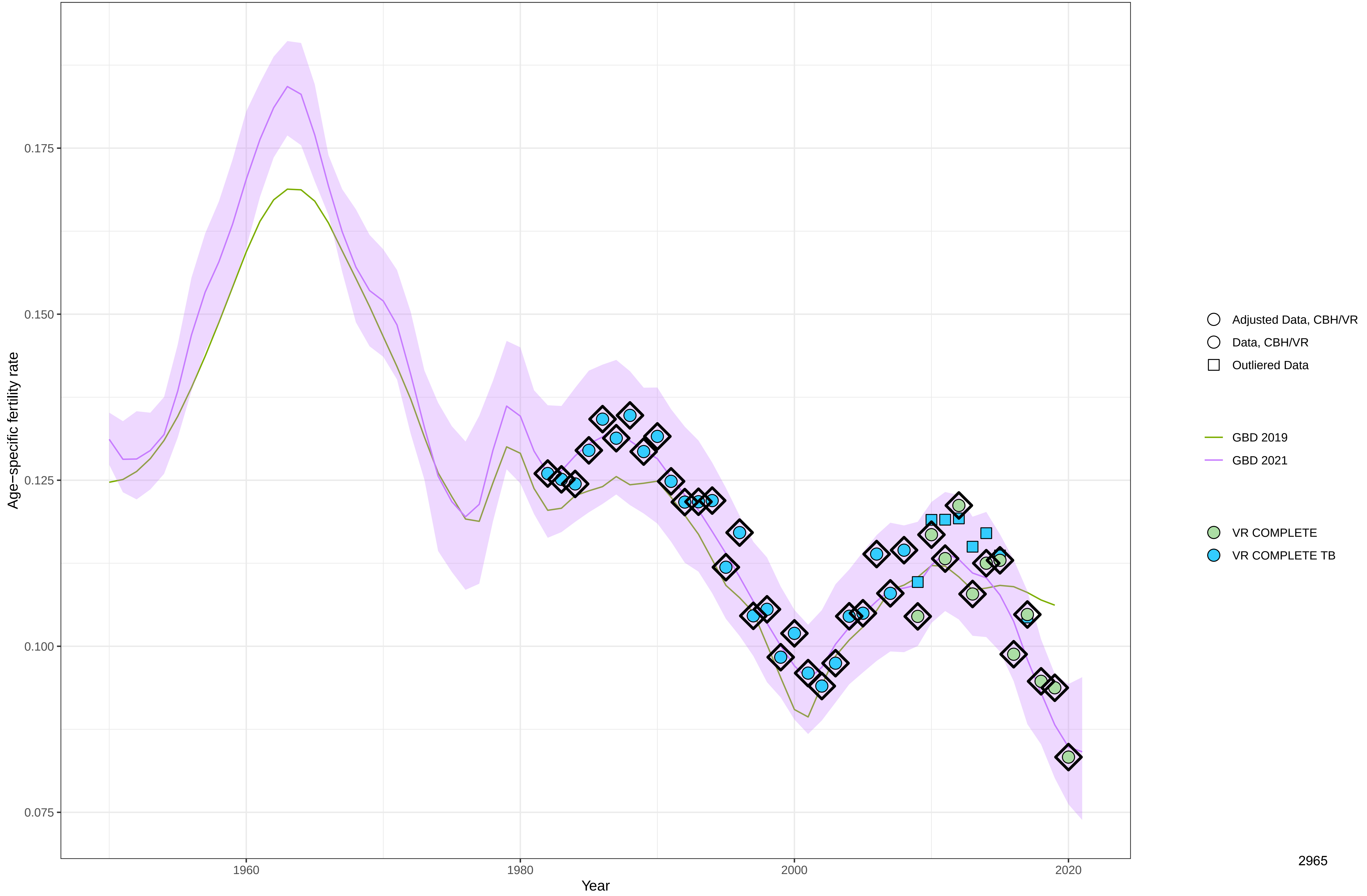

Age−specific fertility fitted trend lines, 1950−2021: Isle of Wight, Ages: 30−34
Data points enclosed with a diamond are reference sources

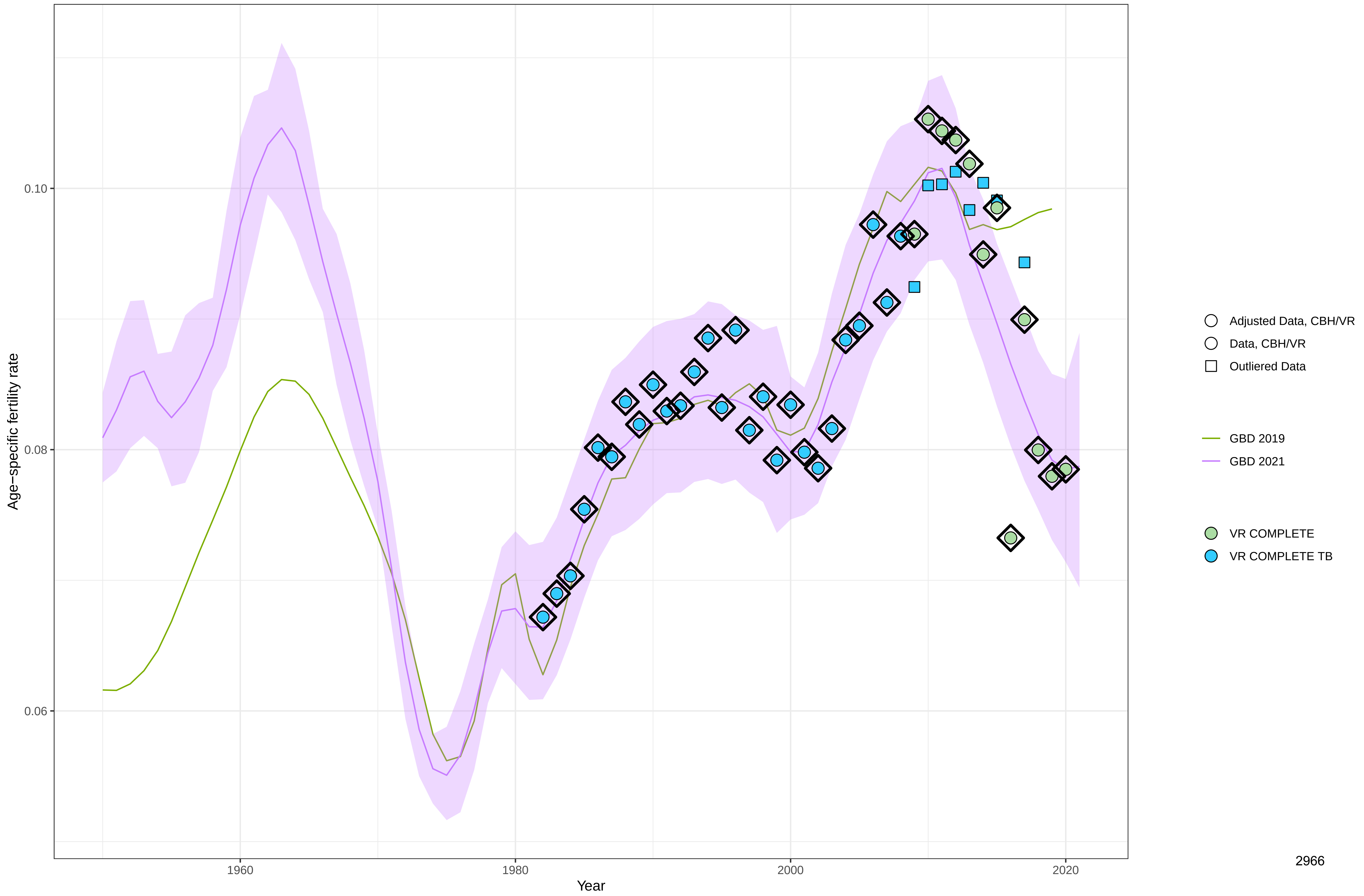

Age−specific fertility fitted trend lines, 1950−2021: Isle of Wight, Ages: 35−39
Data points enclosed with a diamond are reference sources

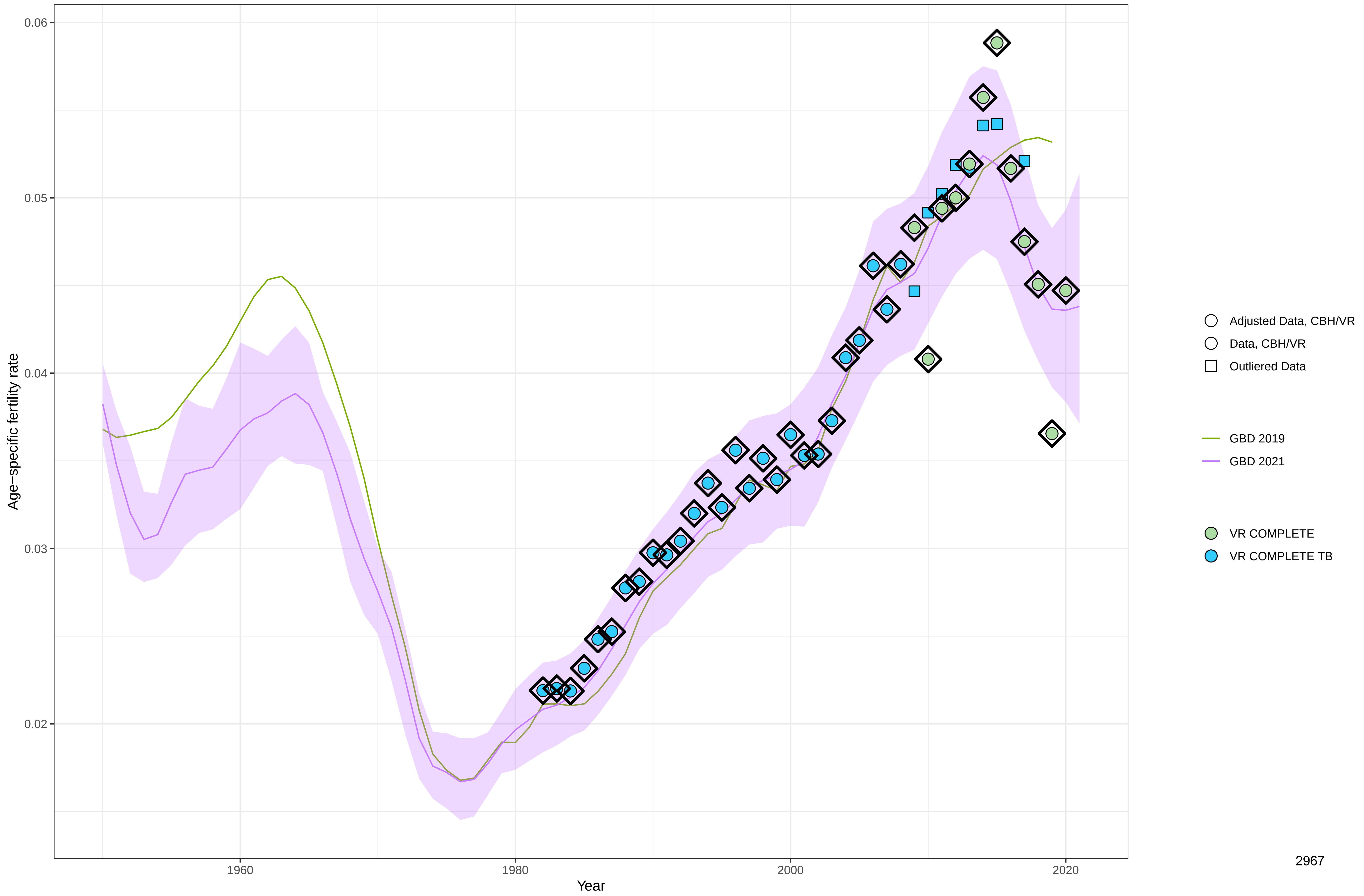

Age−specific fertility fitted trend lines, 1950−2021: Isle of Wight, Ages: 40−44
Data points enclosed with a diamond are reference sources

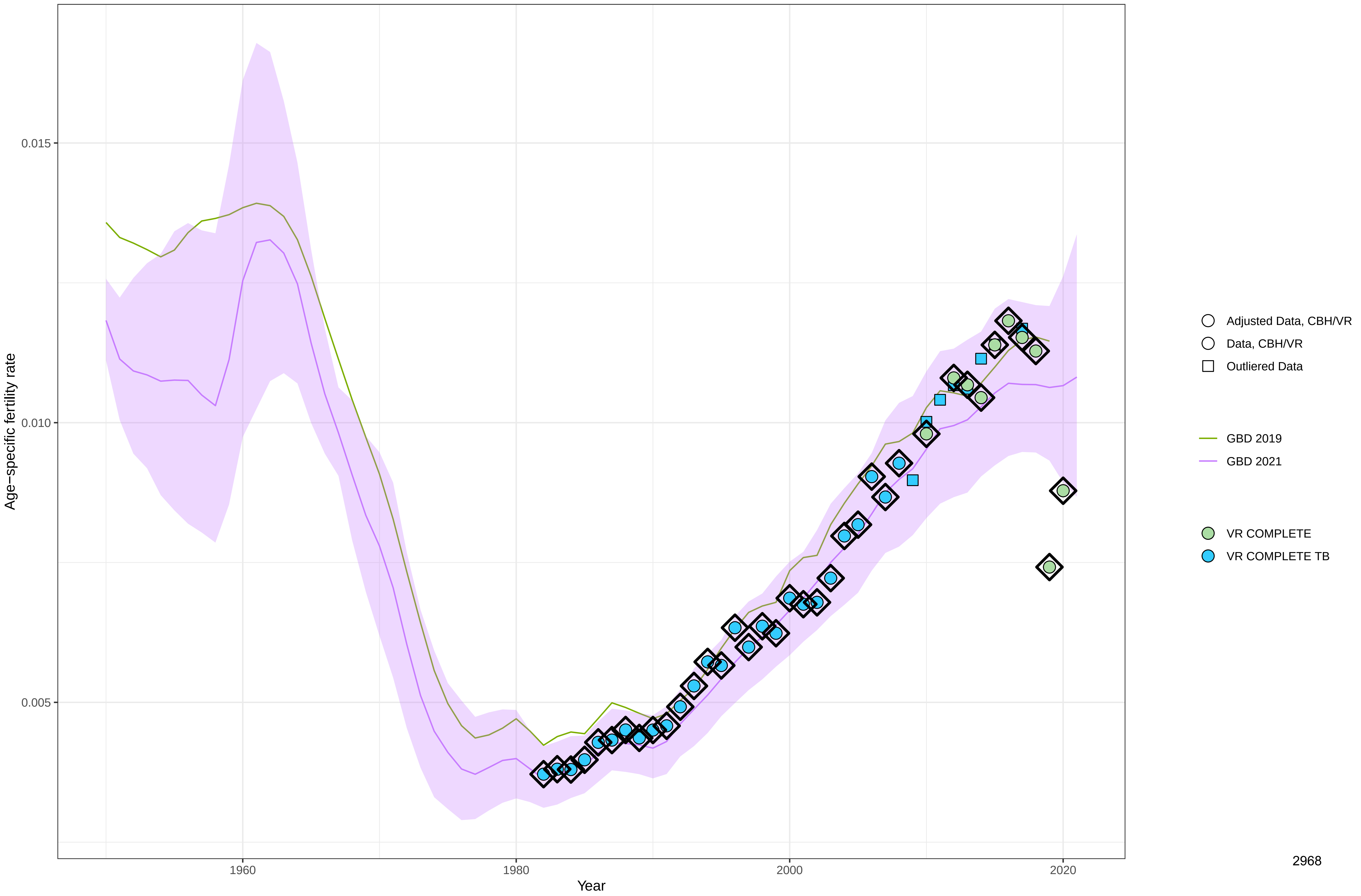

Age−specific fertility fitted trend lines, 1950−2021: Isle of Wight, Ages: 45−49
Data points enclosed with a diamond are reference sources

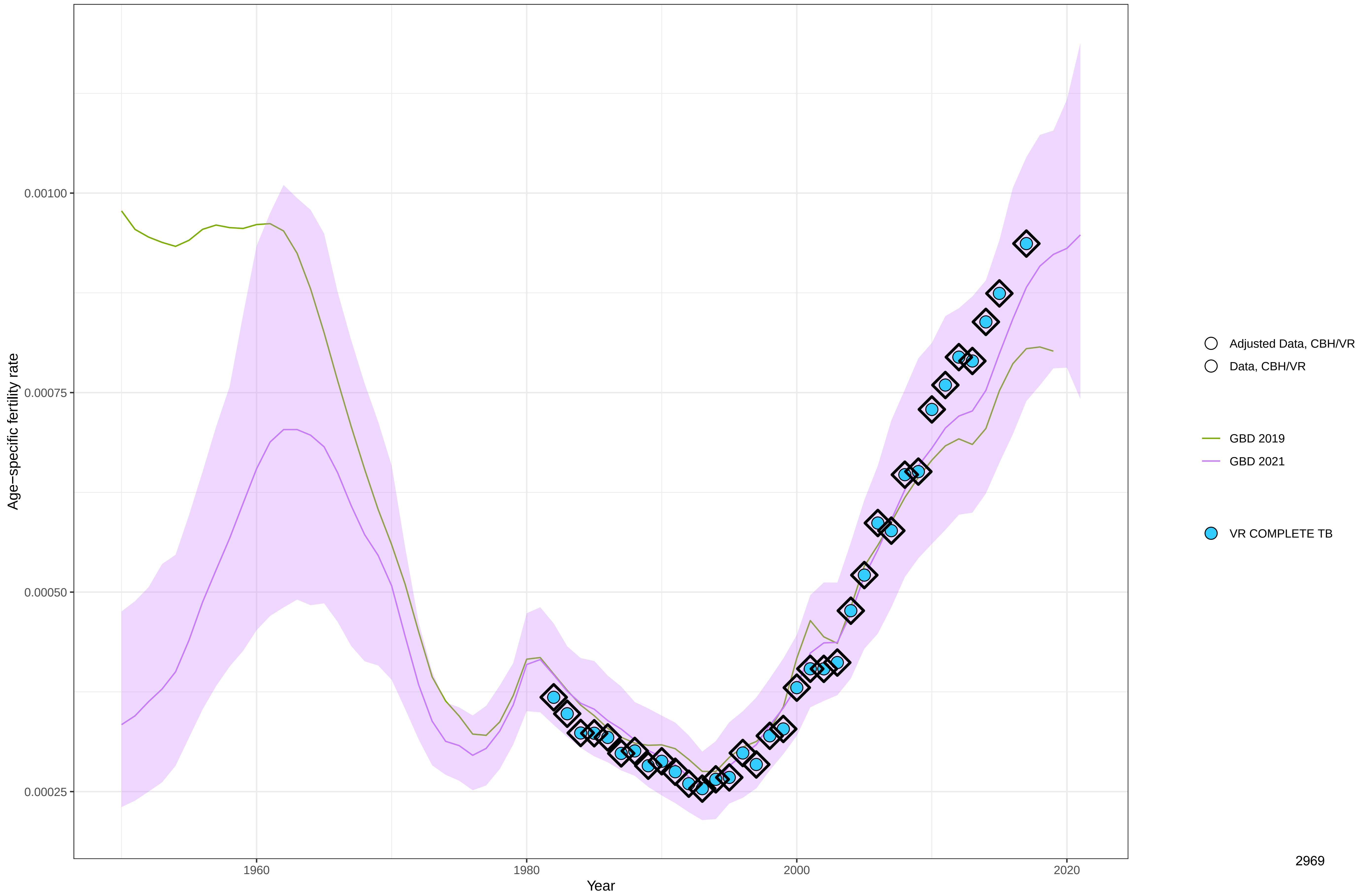

Age−specific fertility fitted trend lines, 1950−2021: Isle of Wight, Ages: 50−54
Data points enclosed with a diamond are reference sources

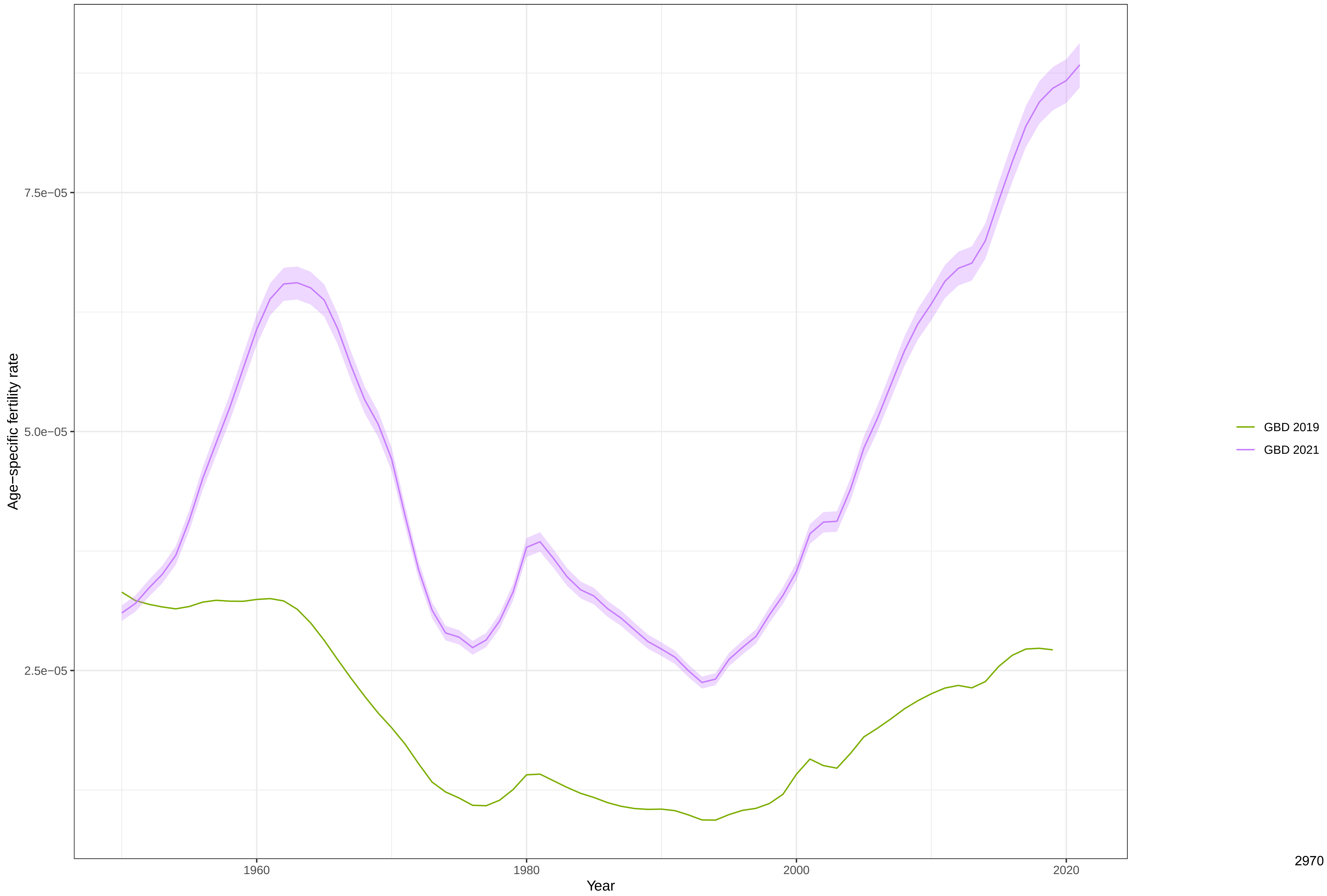

Total fertility rate fitted trend lines, 1950−2021: Isle of Wight TFR
Data points enclosed with a diamond are reference sources

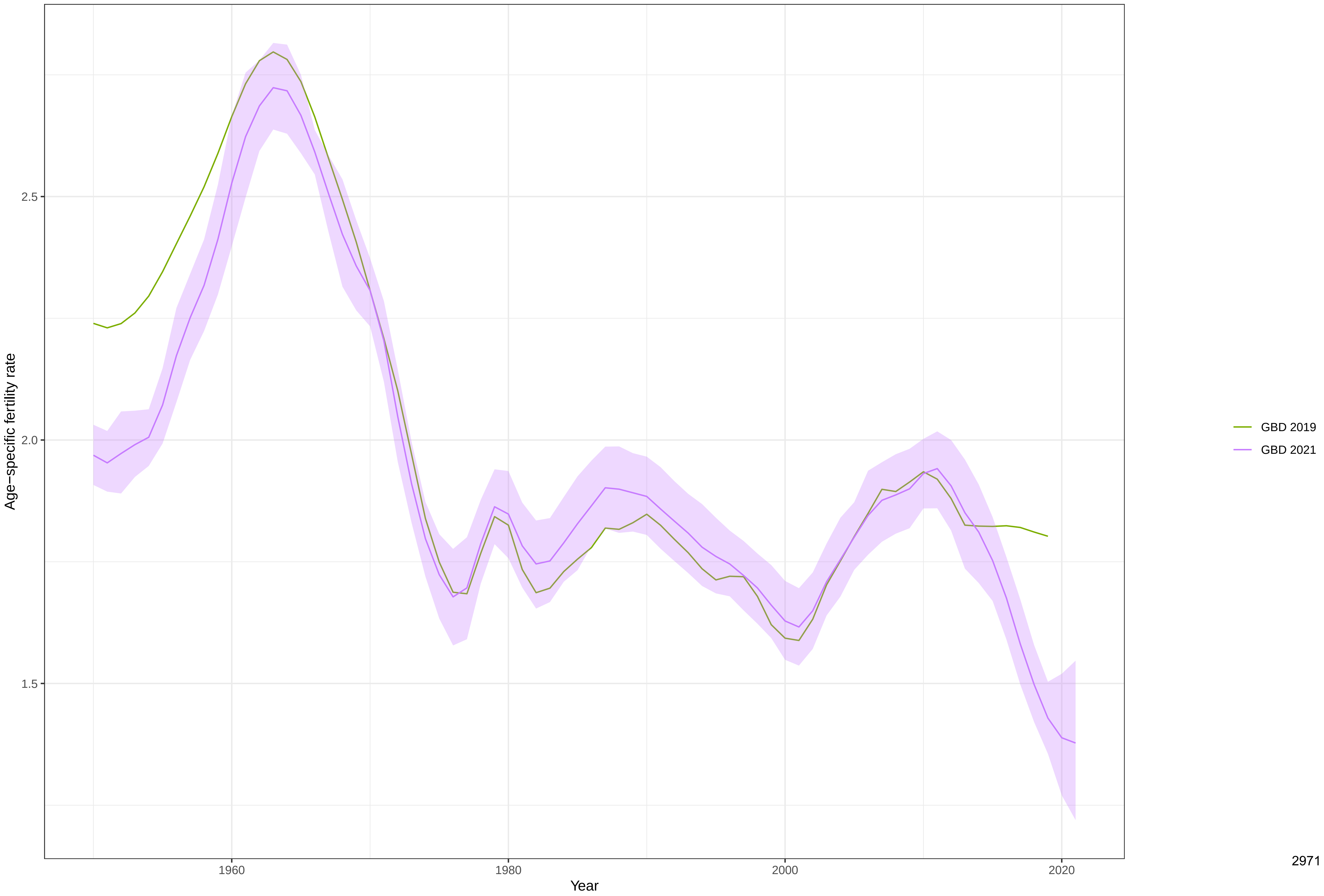

Age−specific fertility fitted trend lines, 1950−2021: Kent, Ages: 10−14
Data points enclosed with a diamond are reference sources

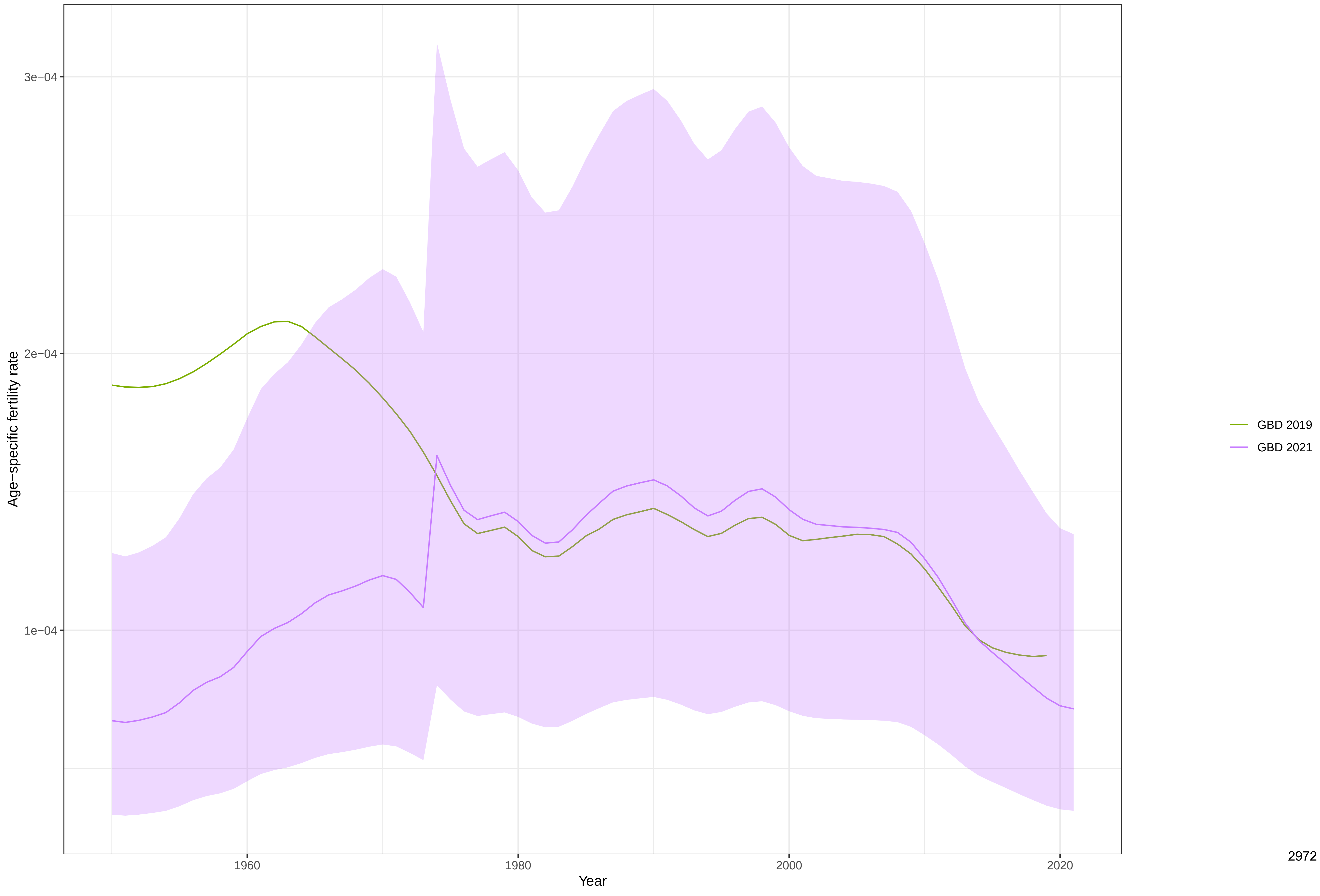

Age−specific fertility fitted trend lines, 1950−2021: Kent, Ages: 15−19
Data points enclosed with a diamond are reference sources

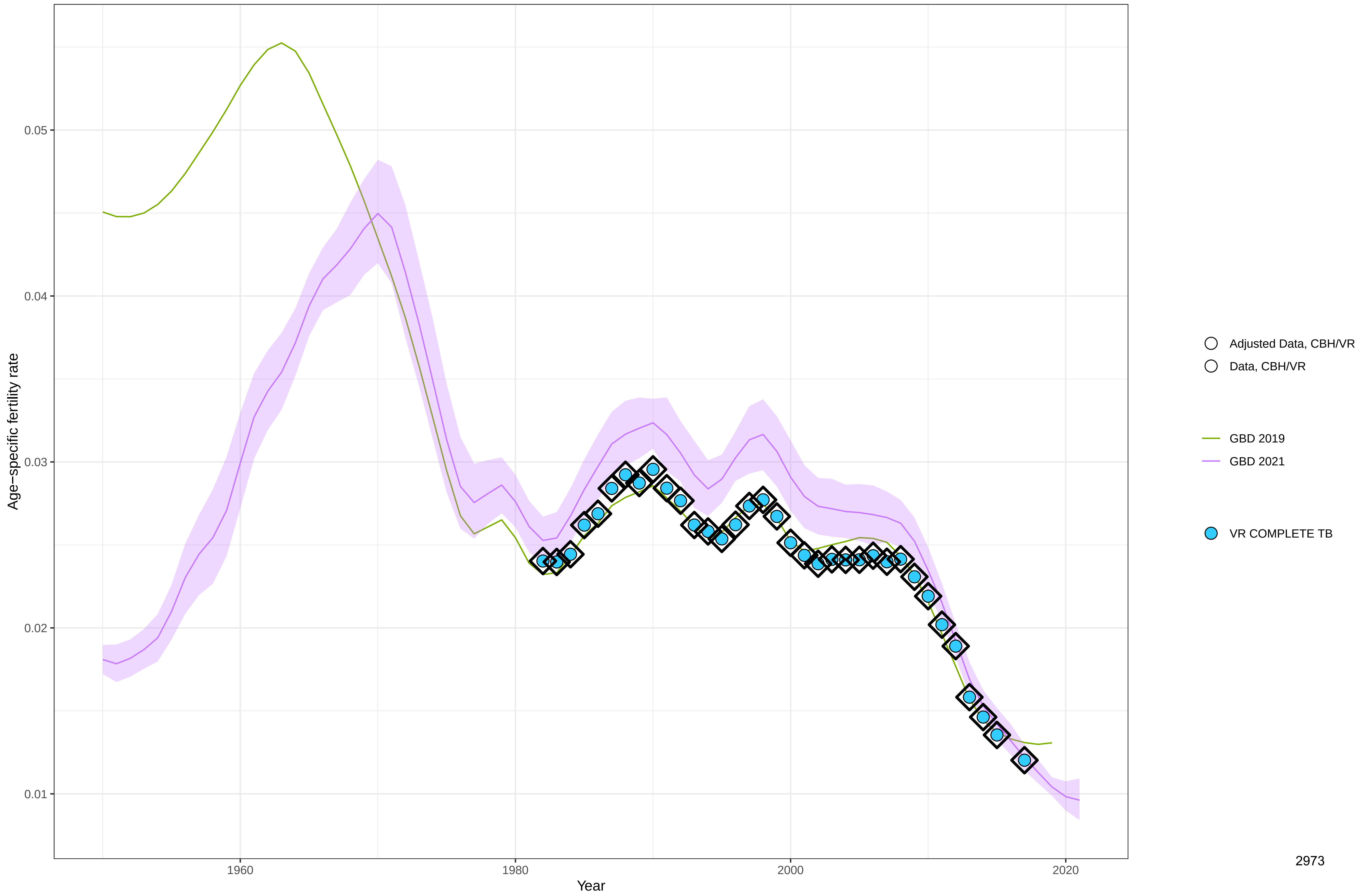

Age−specific fertility fitted trend lines, 1950−2021: Kent, Ages: 20−24
Data points enclosed with a diamond are reference sources

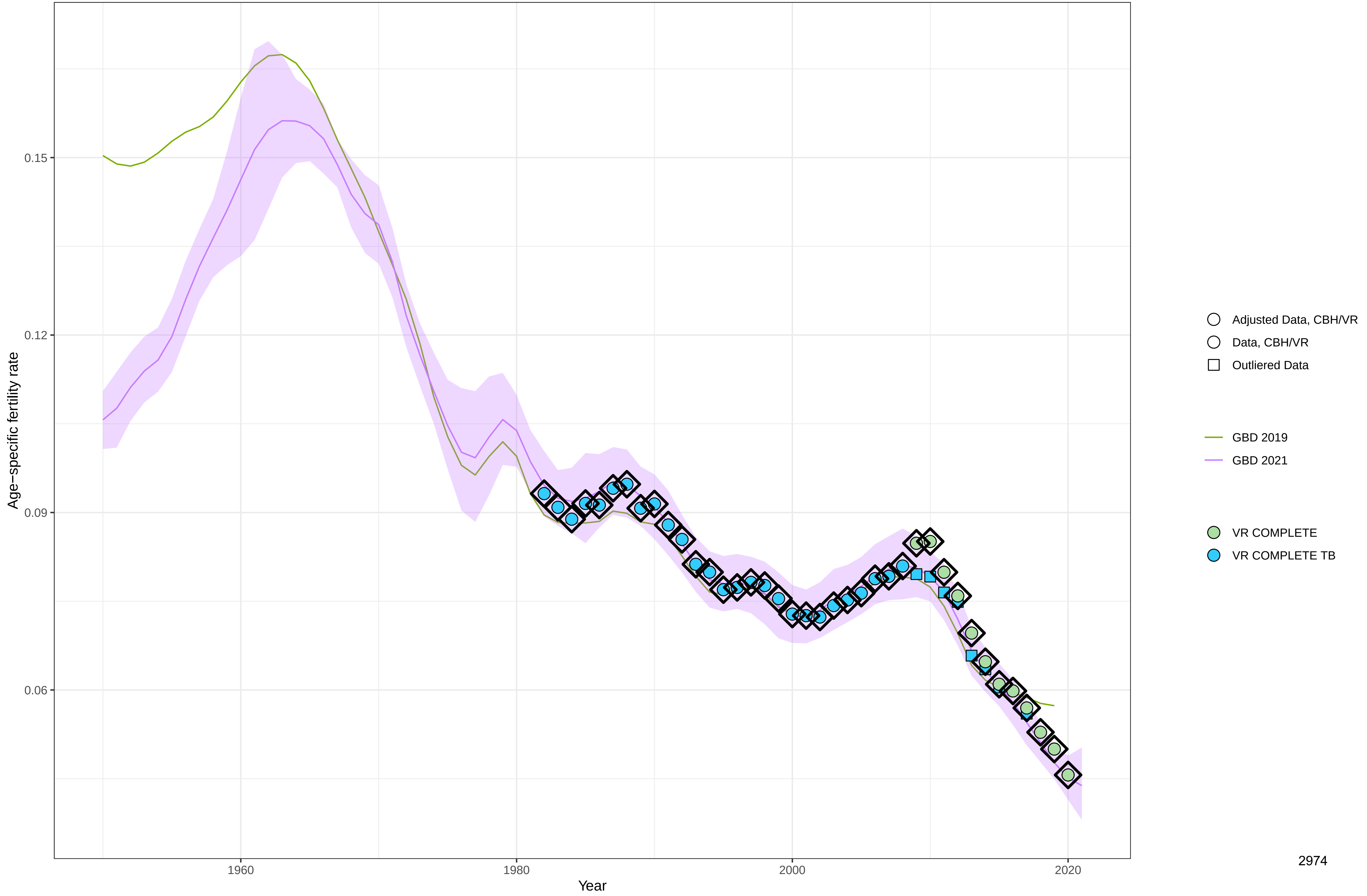

Age−specific fertility fitted trend lines, 1950−2021: Kent, Ages: 25−29
Data points enclosed with a diamond are reference sources

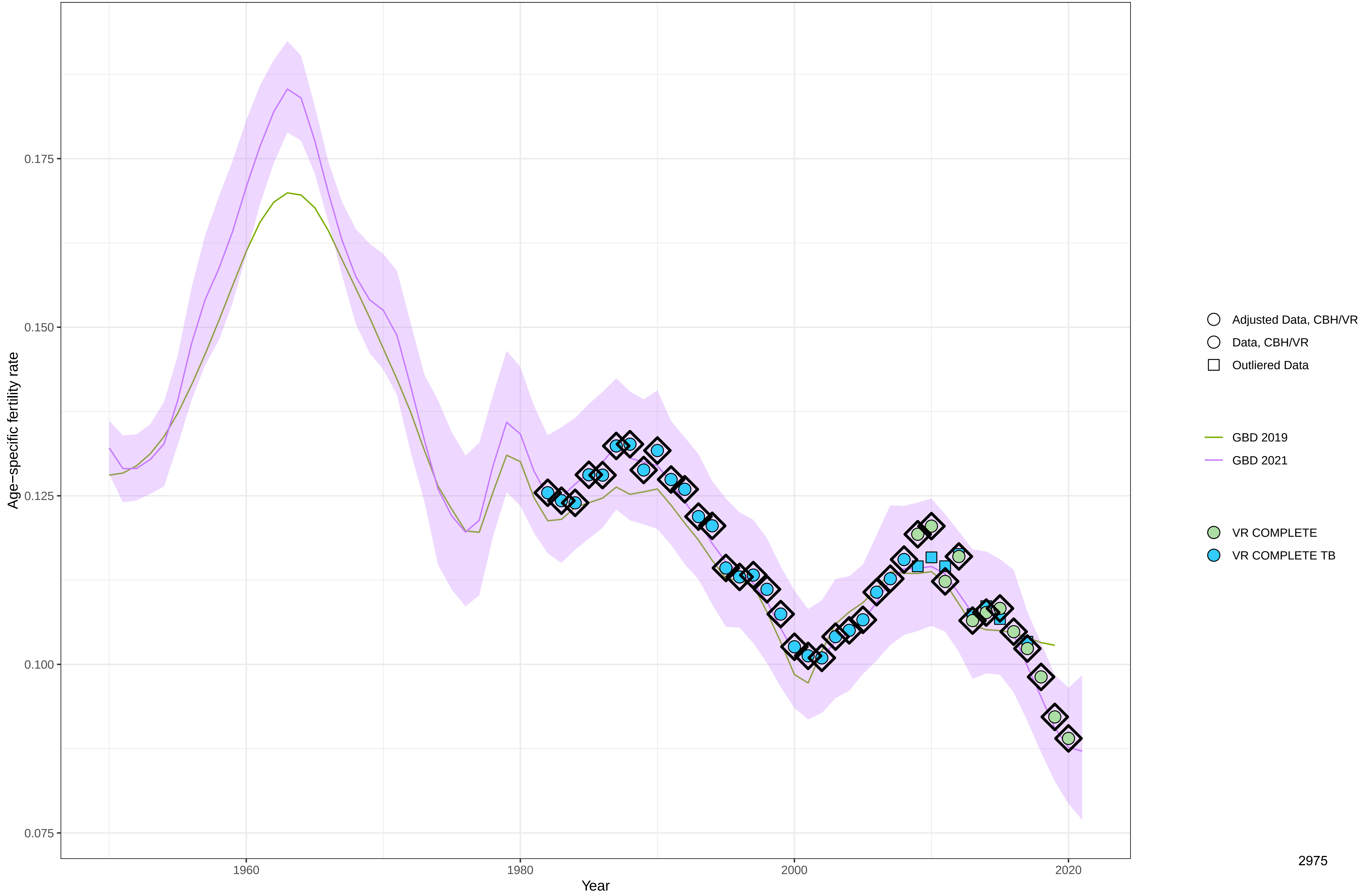

Age−specific fertility fitted trend lines, 1950−2021: Kent, Ages: 30−34
Data points enclosed with a diamond are reference sources

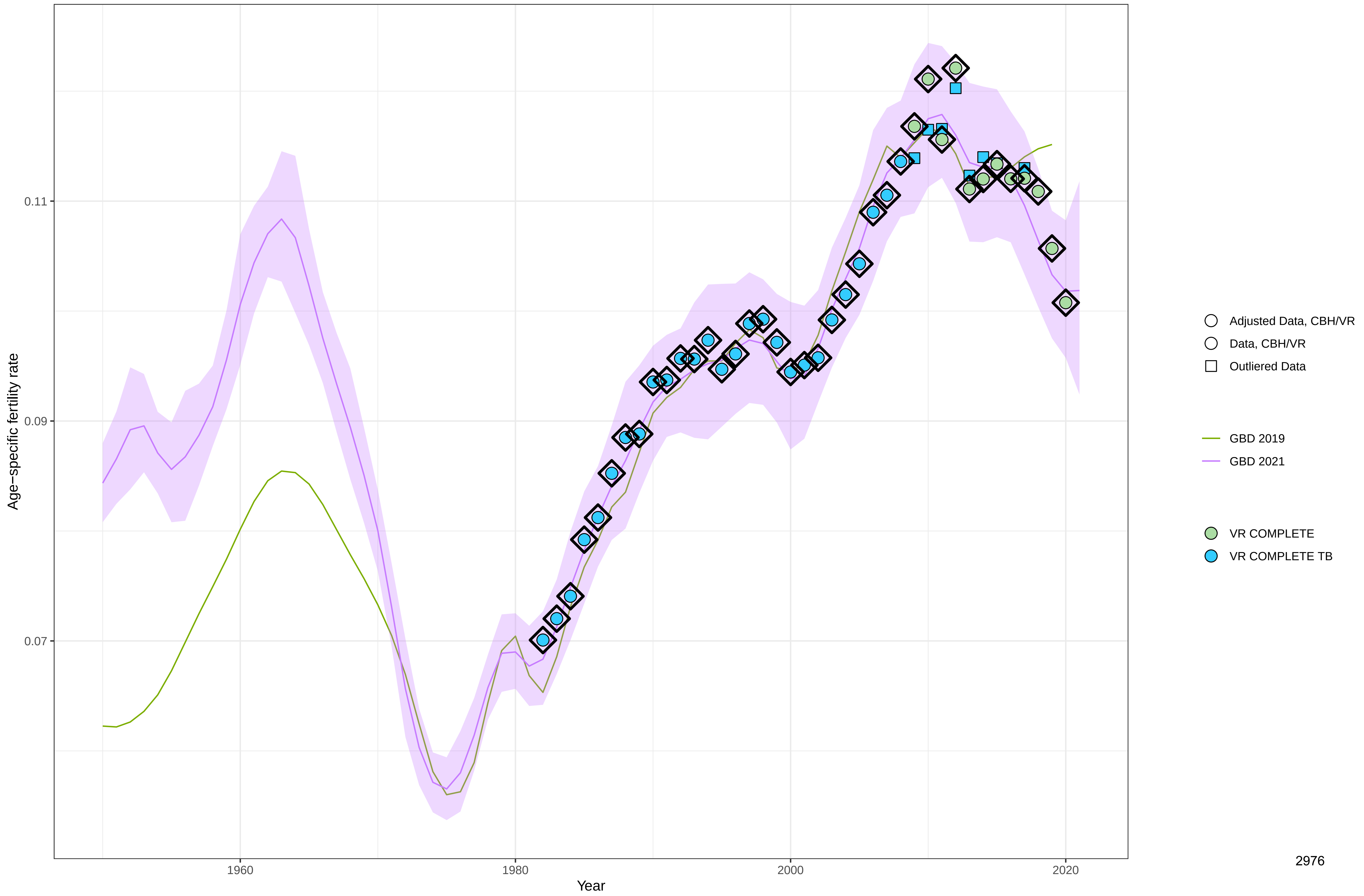

Age−specific fertility fitted trend lines, 1950−2021: Kent, Ages: 35−39
Data points enclosed with a diamond are reference sources

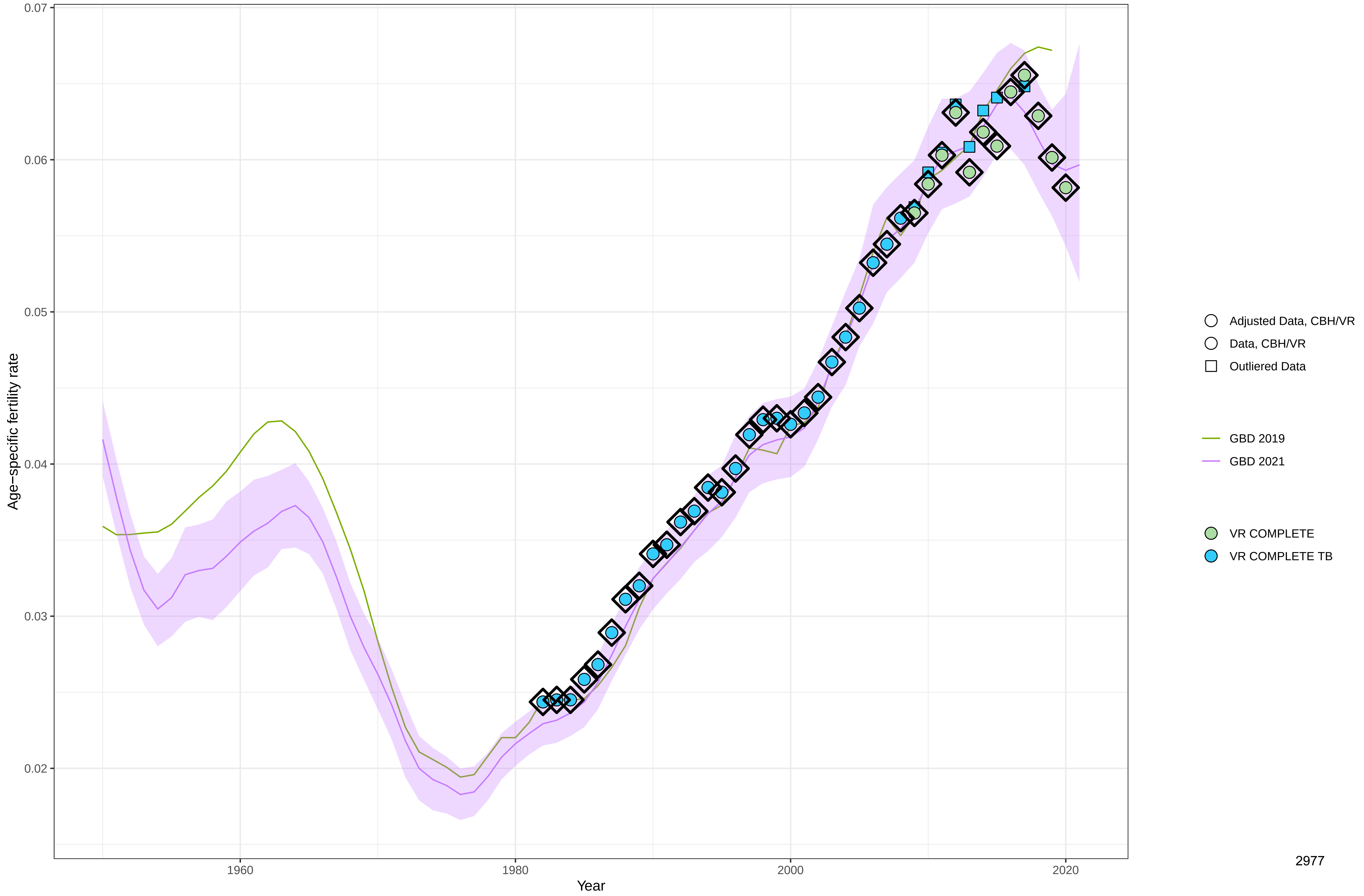

Age−specific fertility fitted trend lines, 1950−2021: Kent, Ages: 40−44
Data points enclosed with a diamond are reference sources

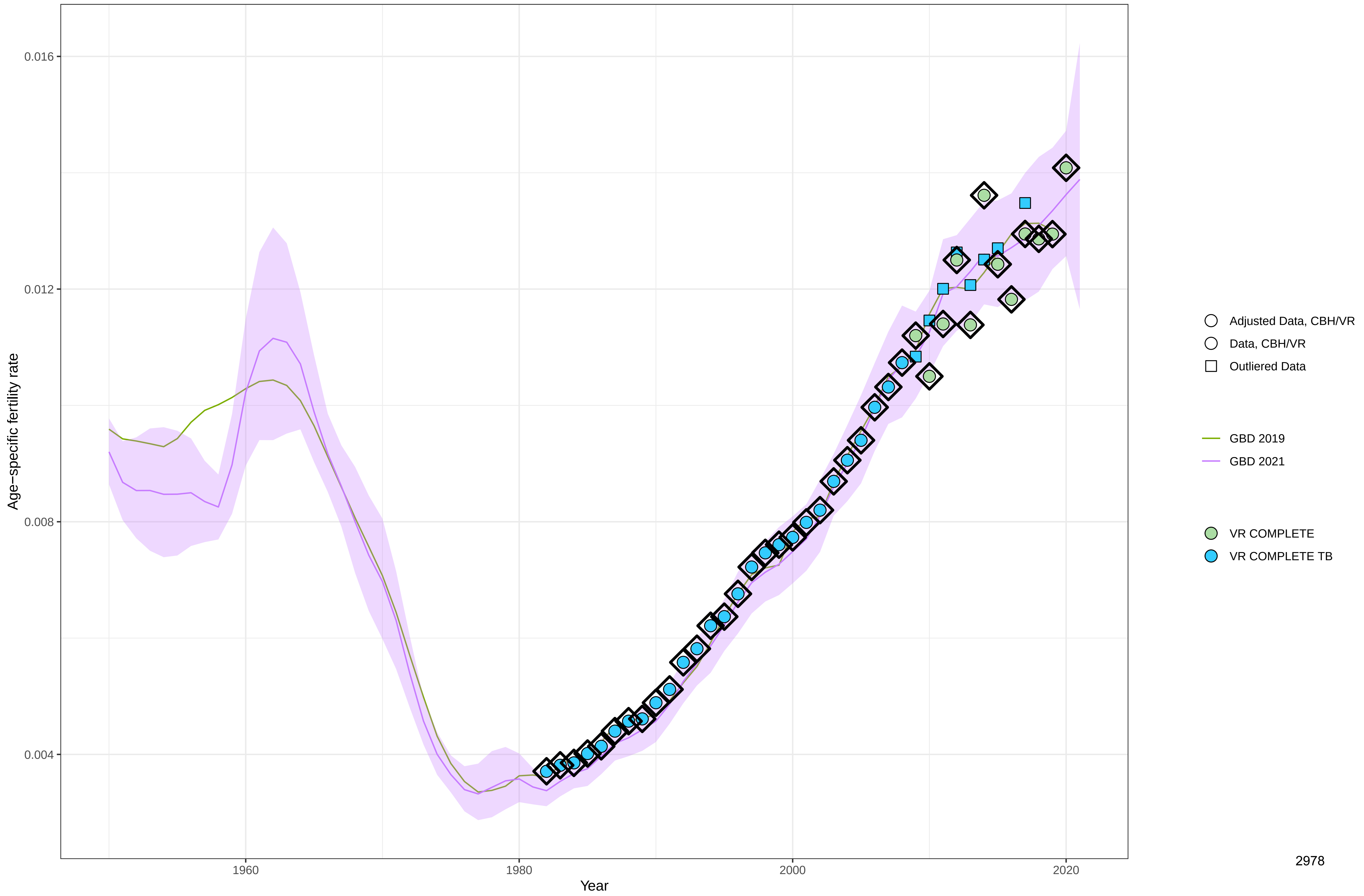

Age−specific fertility fitted trend lines, 1950−2021: Kent, Ages: 45−49
Data points enclosed with a diamond are reference sources

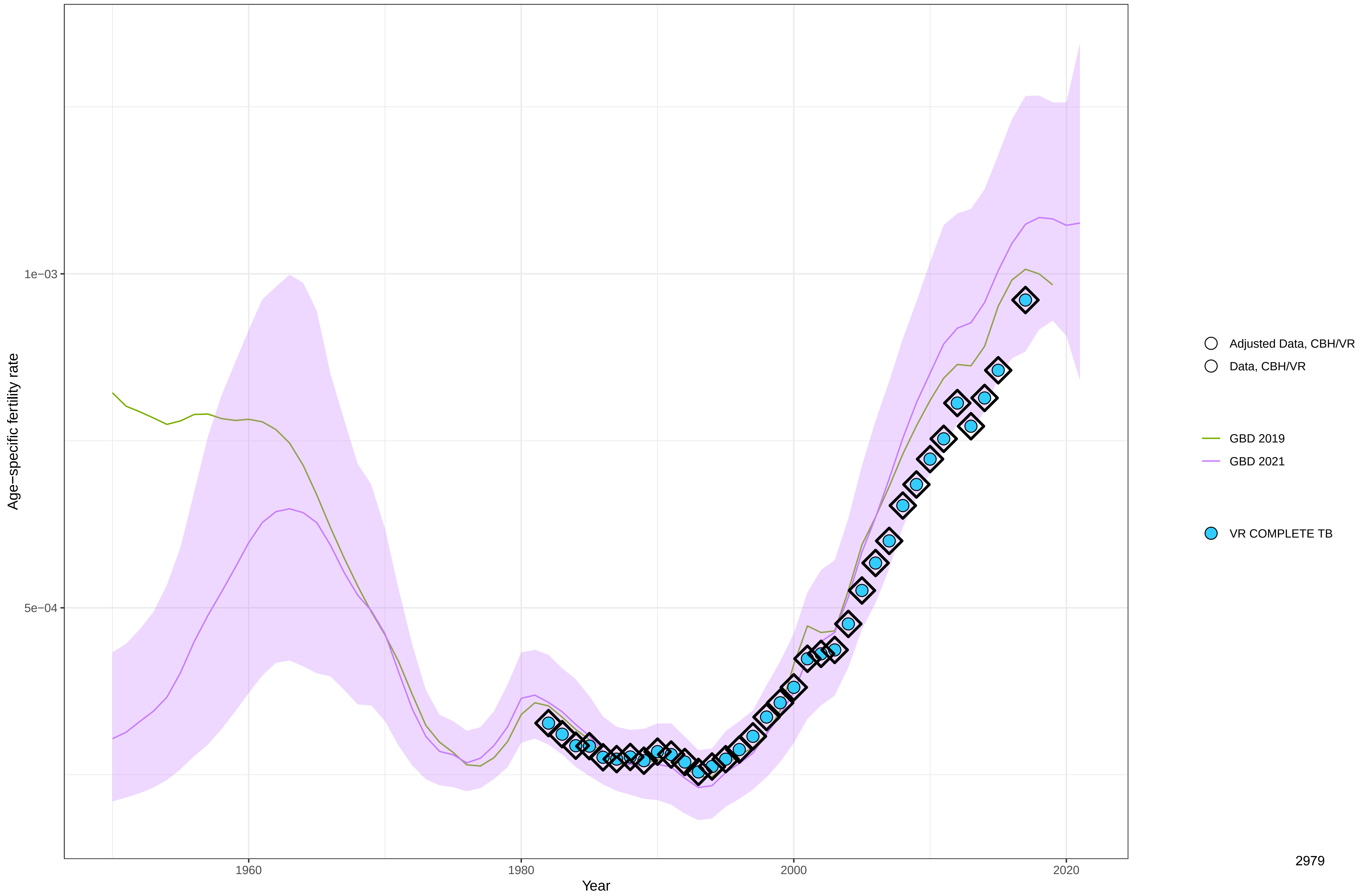

Age−specific fertility fitted trend lines, 1950−2021: Kent, Ages: 50−54
Data points enclosed with a diamond are reference sources

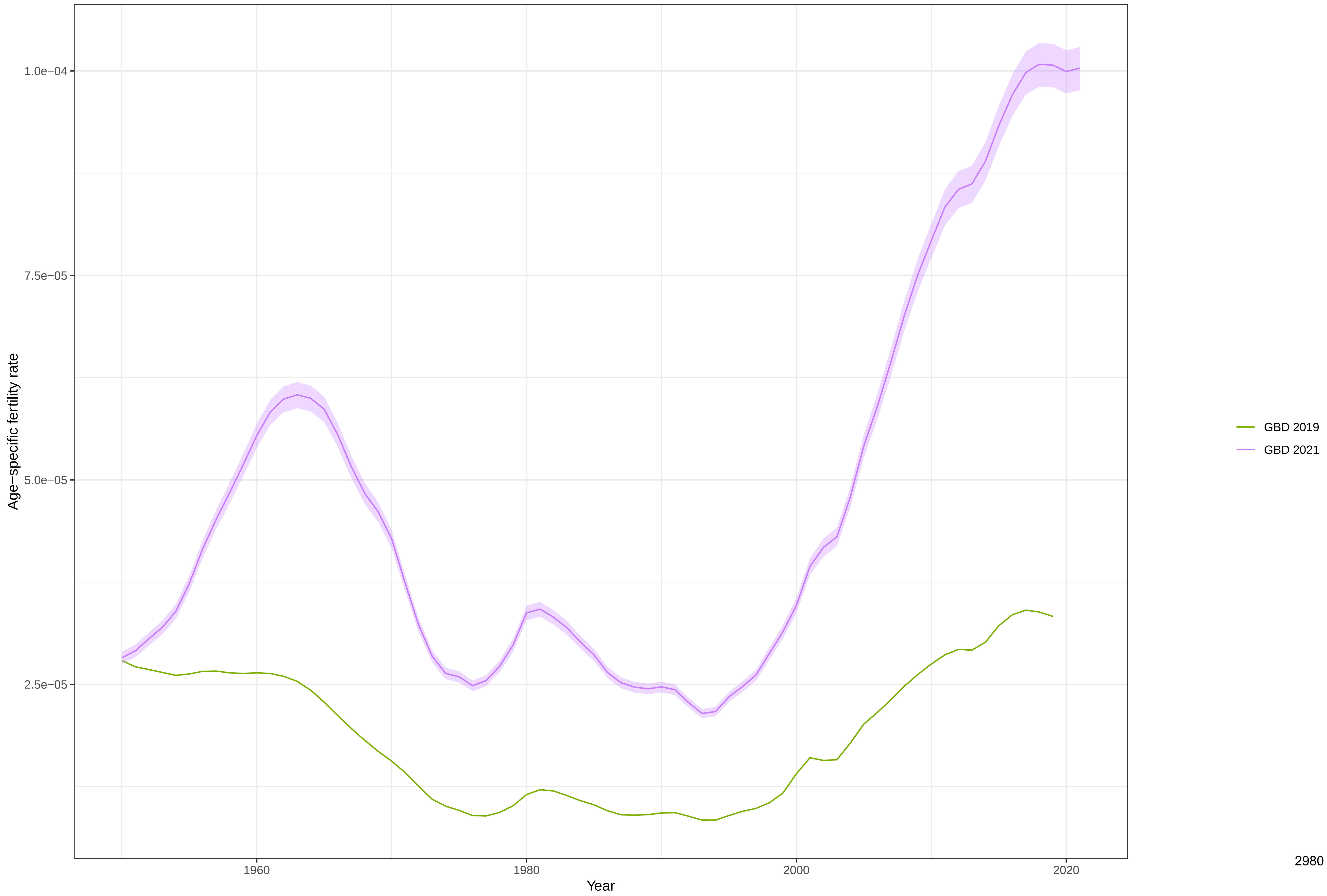

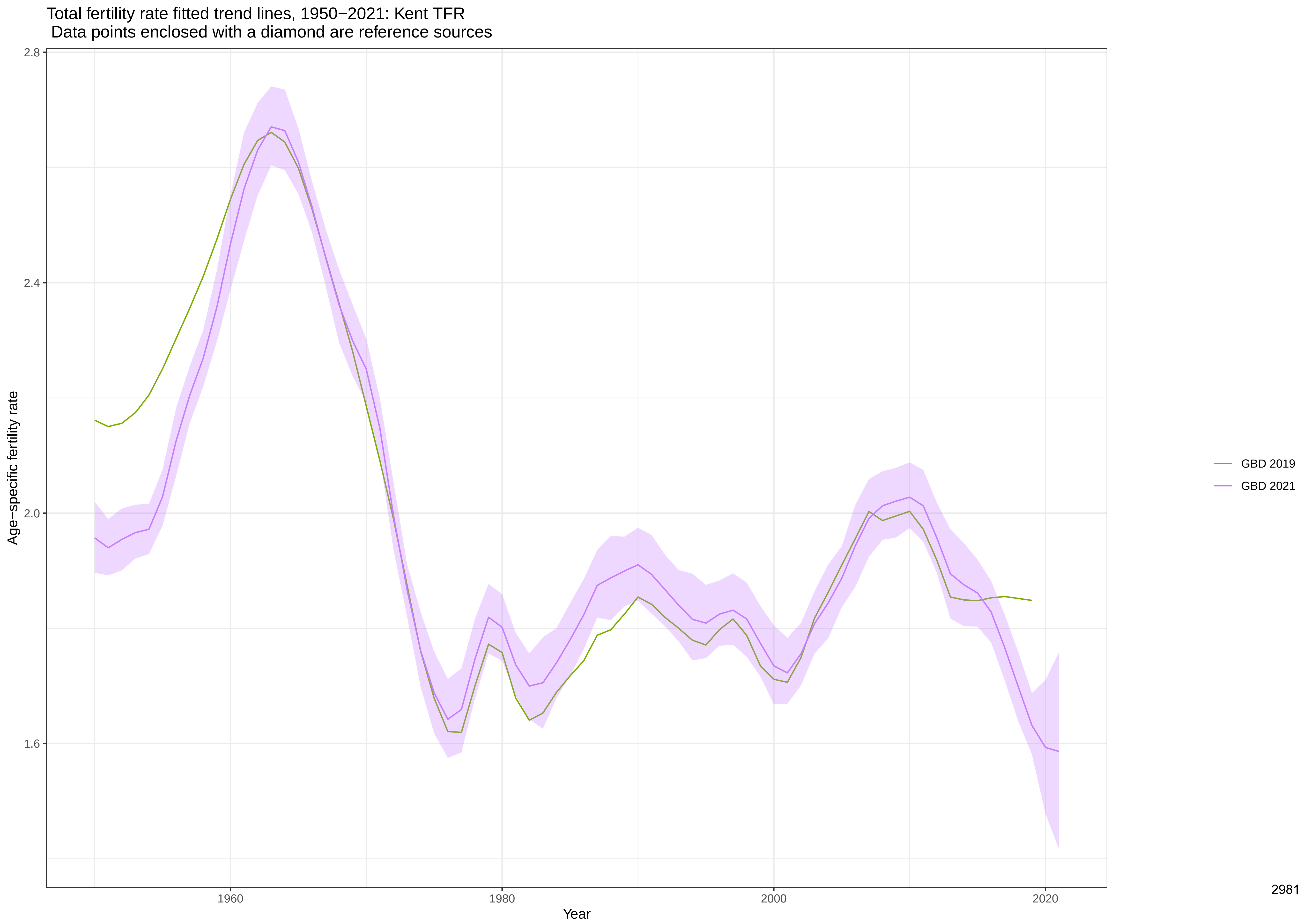

Total fertility rate fitted trend lines, 1950−2021: Kent TFR
Data points enclosed with a diamond are reference sources
2.8
2.4
2.0
1.6
Age−specific fertility rate
1960
1980
2000
2020
Year
GBD 2019
GBD 2021

Age−specific fertility fitted trend lines, 1950−2021: Medway, Ages: 10−14
Data points enclosed with a diamond are reference sources

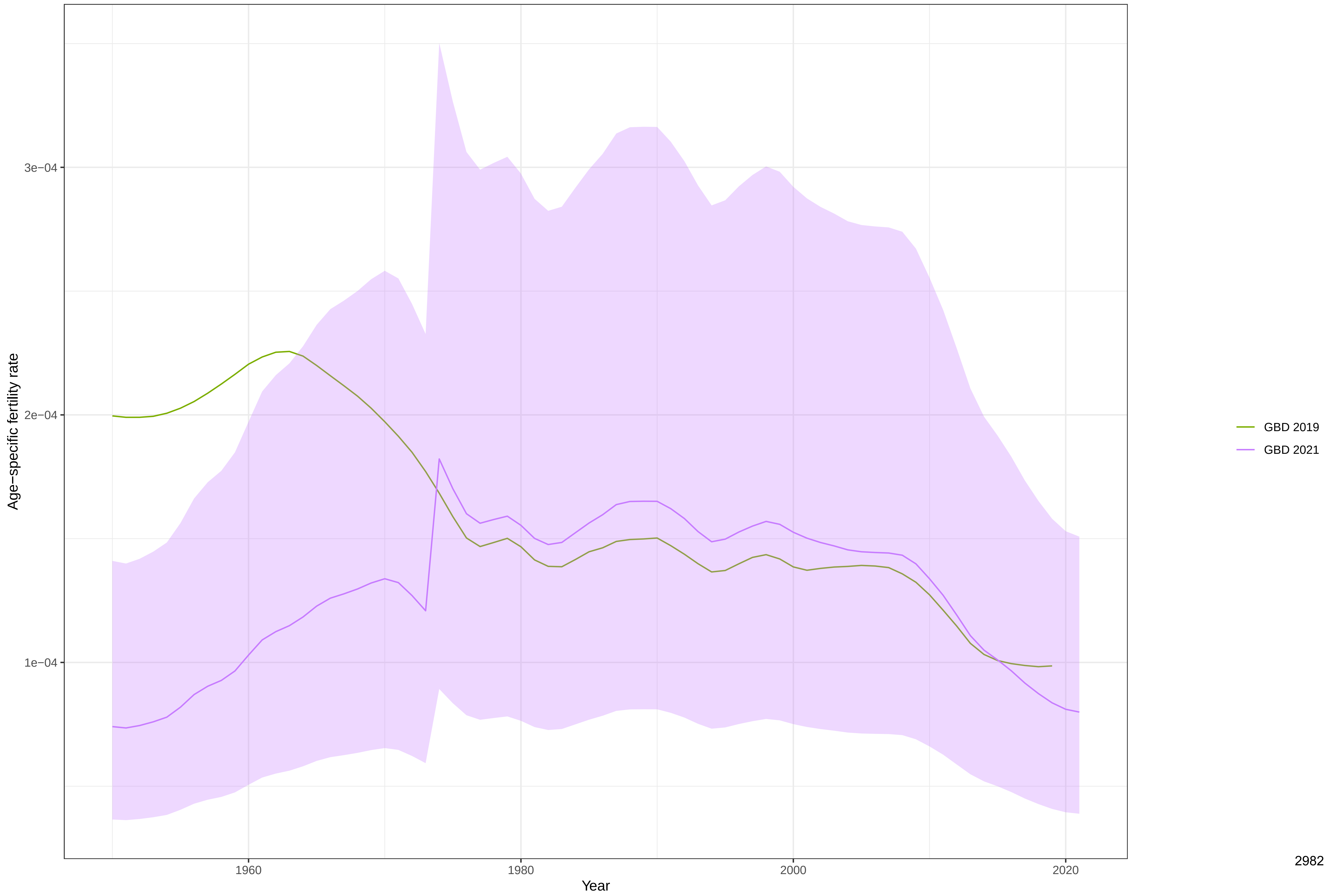

Age−specific fertility fitted trend lines, 1950−2021: Medway, Ages: 15−19
Data points enclosed with a diamond are reference sources

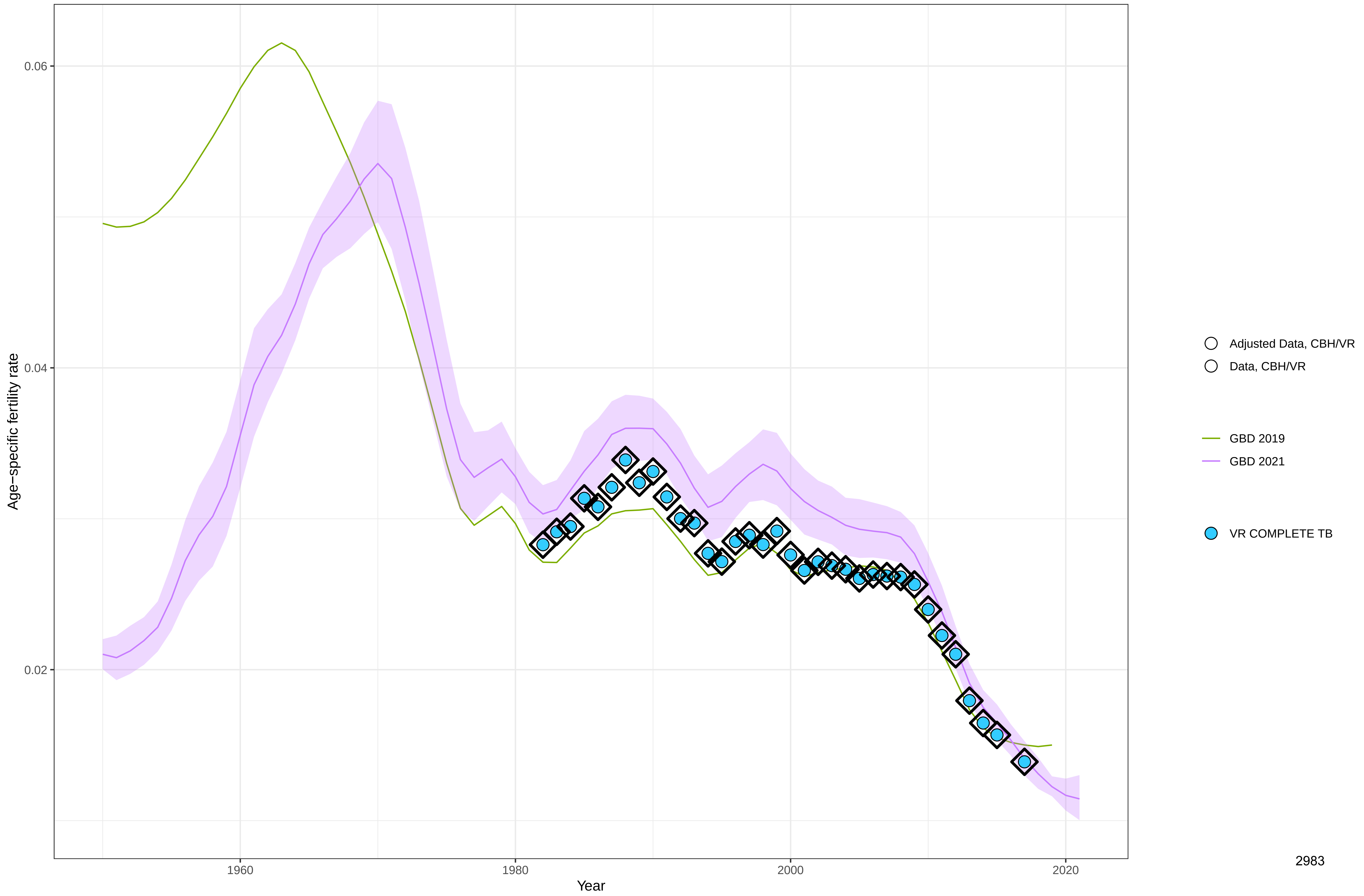

Age−specific fertility fitted trend lines, 1950−2021: Medway, Ages: 20−24
Data points enclosed with a diamond are reference sources

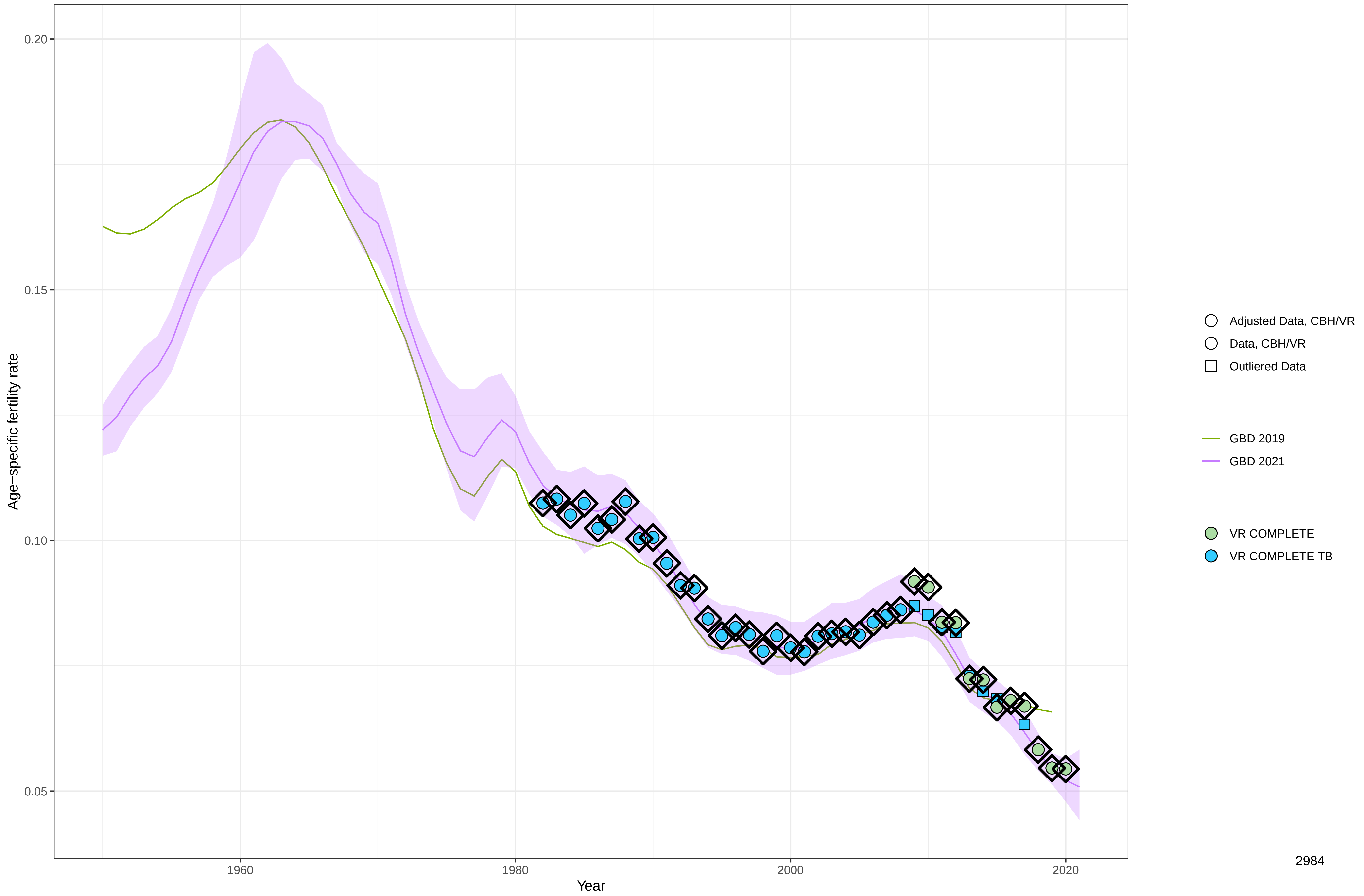

Age−specific fertility fitted trend lines, 1950−2021: Medway, Ages: 25−29
Data points enclosed with a diamond are reference sources

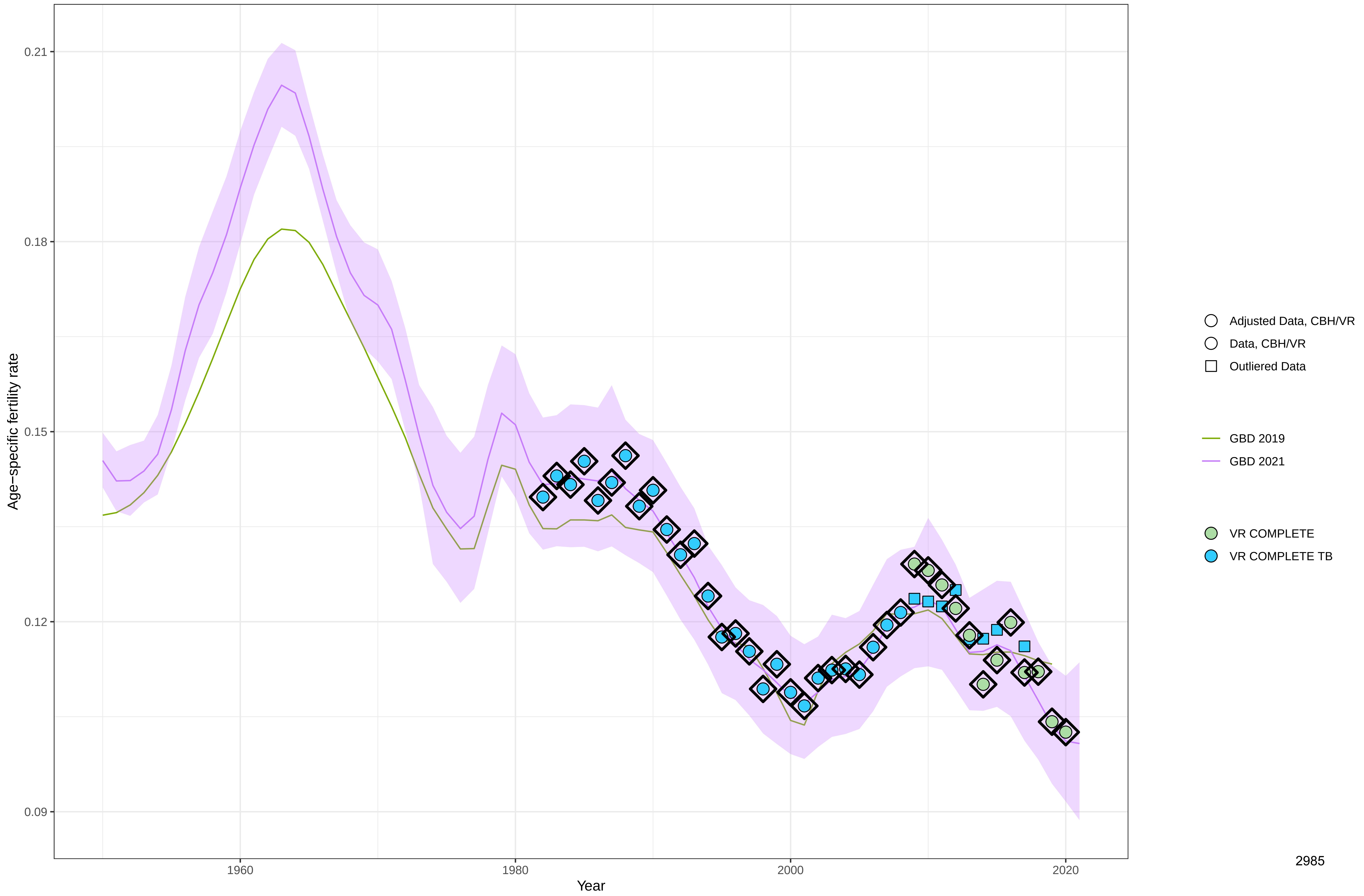

Age−specific fertility fitted trend lines, 1950−2021: Medway, Ages: 30−34
Data points enclosed with a diamond are reference sources

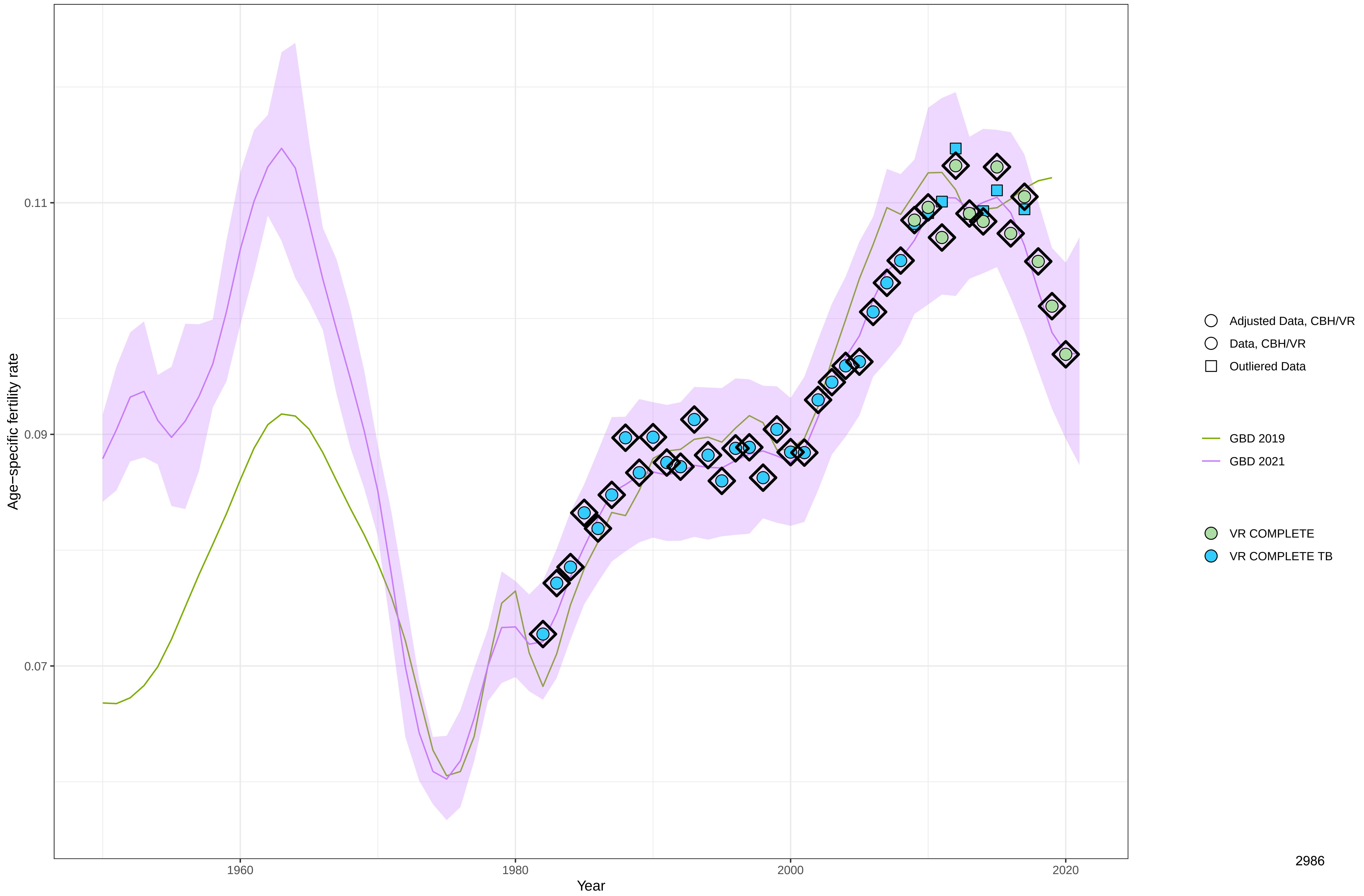

Age−specific fertility fitted trend lines, 1950−2021: Medway, Ages: 35−39
Data points enclosed with a diamond are reference sources

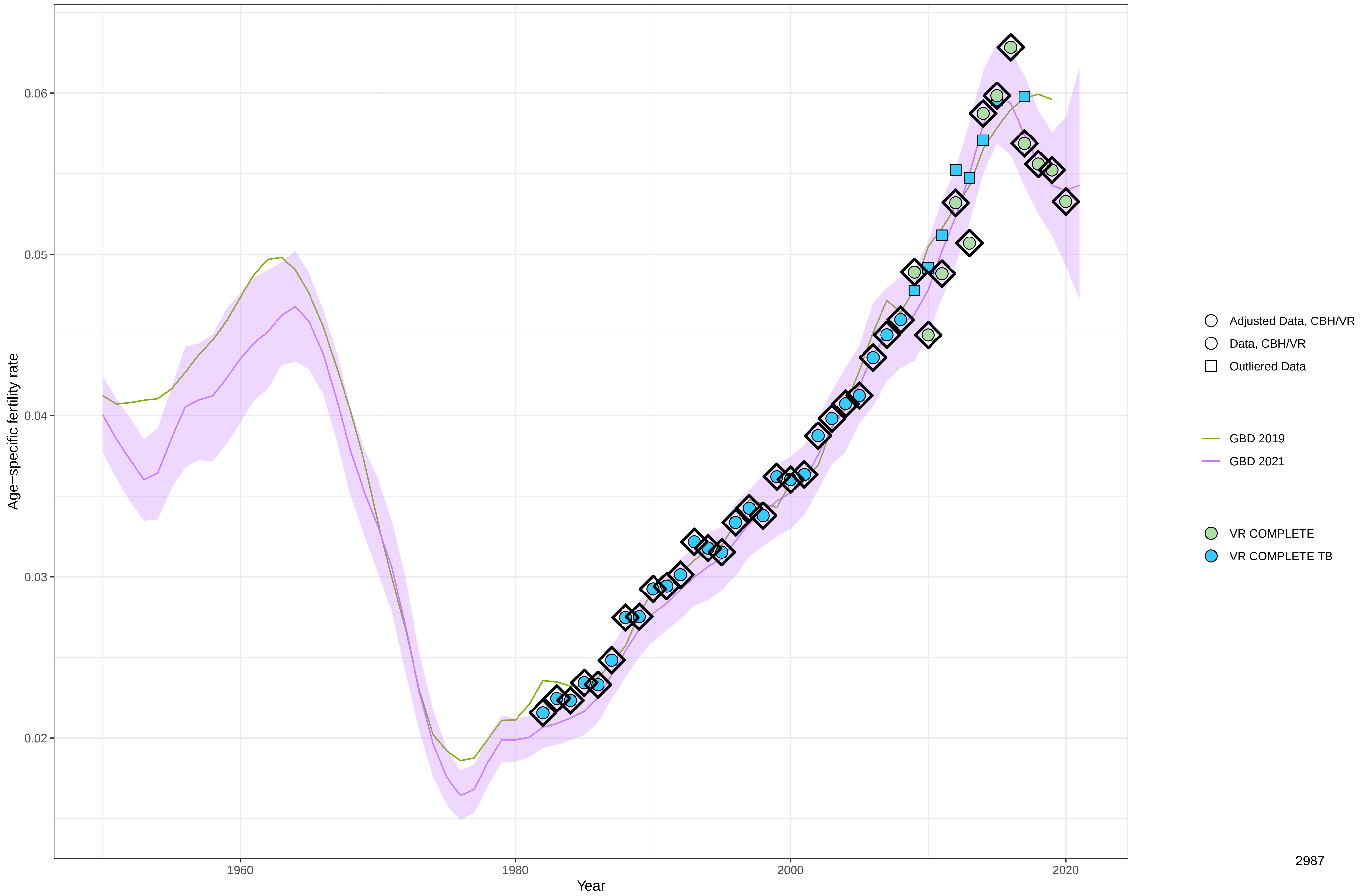

Age−specific fertility fitted trend lines, 1950−2021: Medway, Ages: 40−44
Data points enclosed with a diamond are reference sources

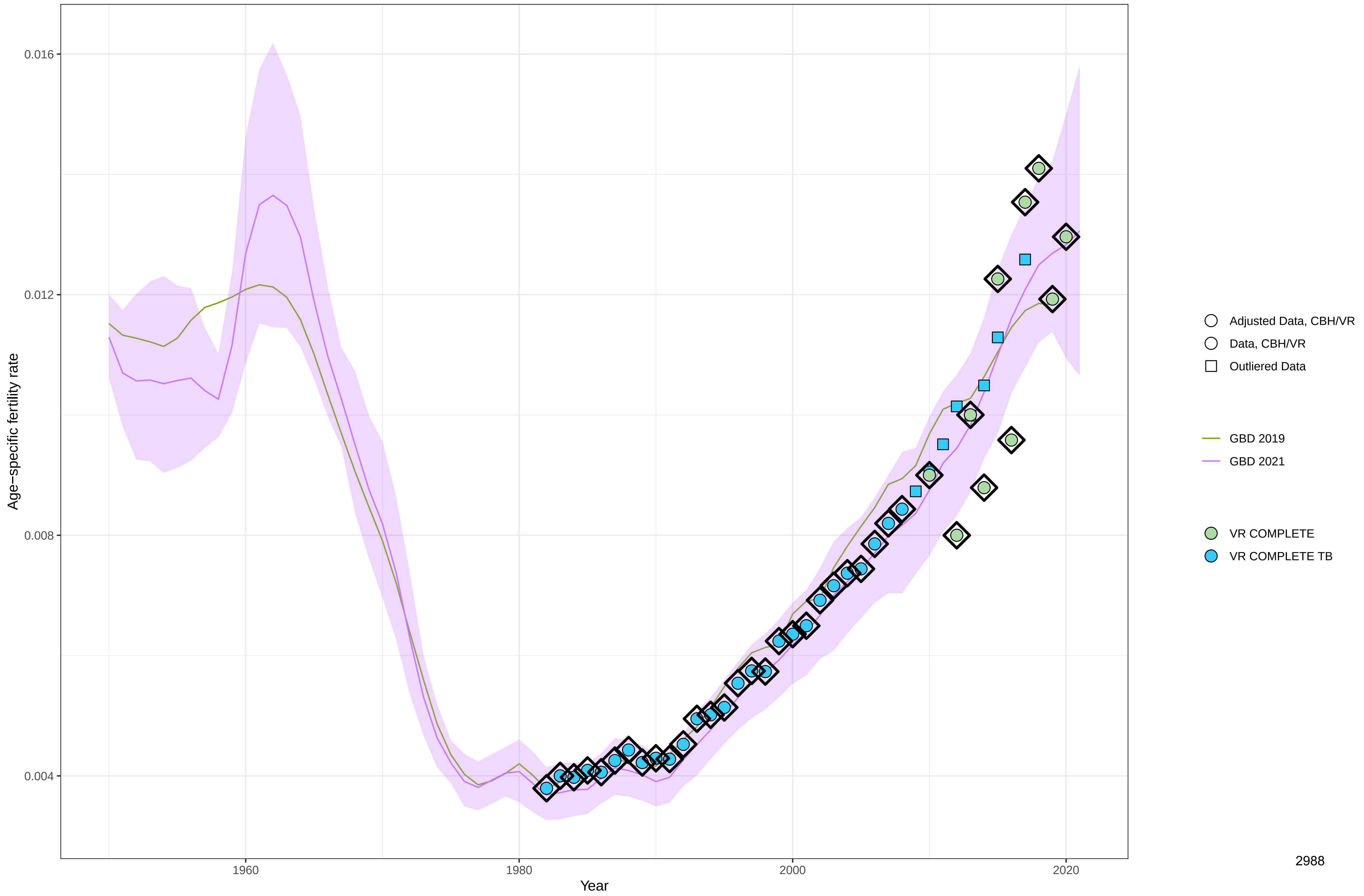

Age−specific fertility fitted trend lines, 1950−2021: Medway, Ages: 45−49
Data points enclosed with a diamond are reference sources

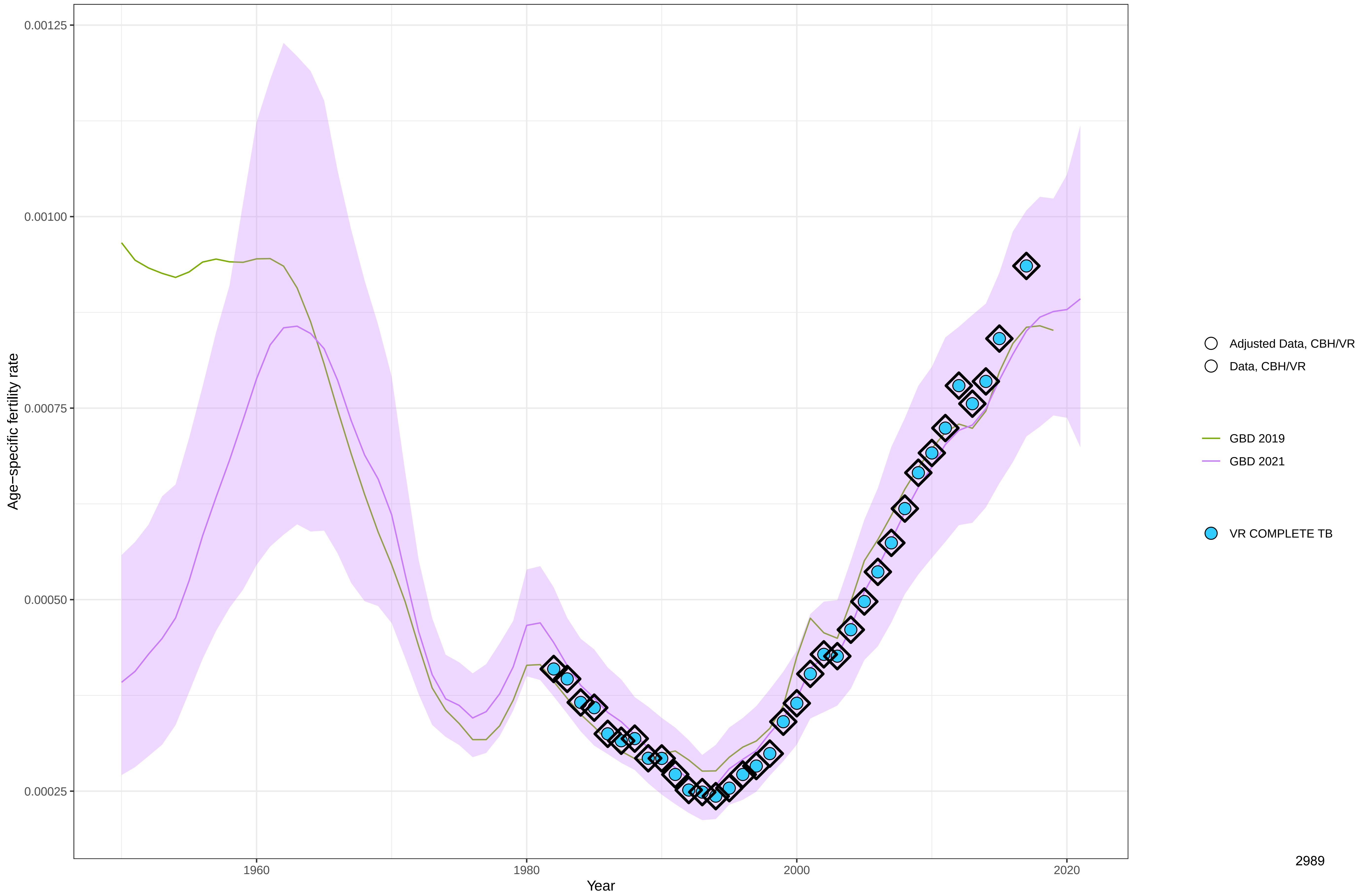

Age−specific fertility fitted trend lines, 1950−2021: Medway, Ages: 50−54
Data points enclosed with a diamond are reference sources

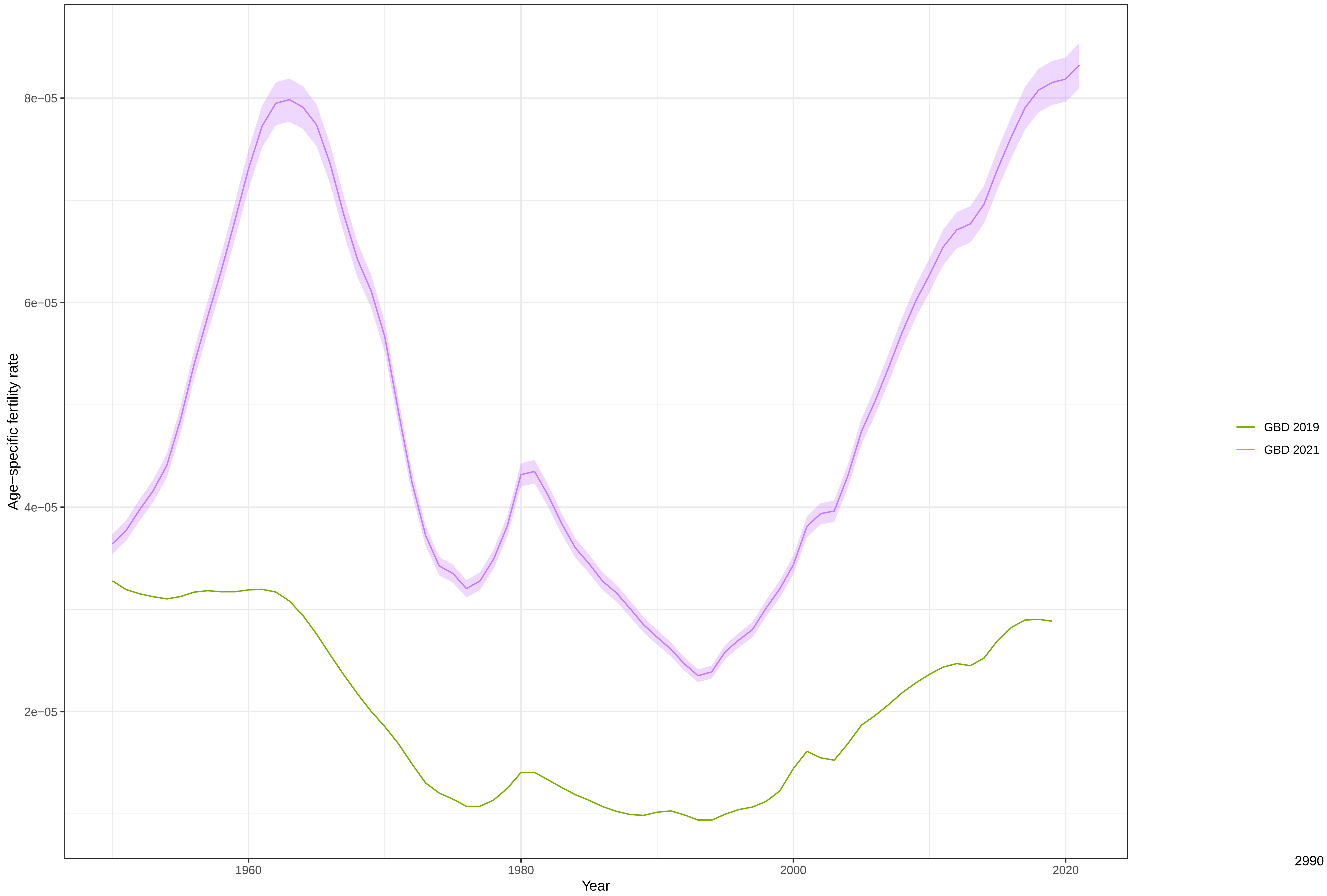

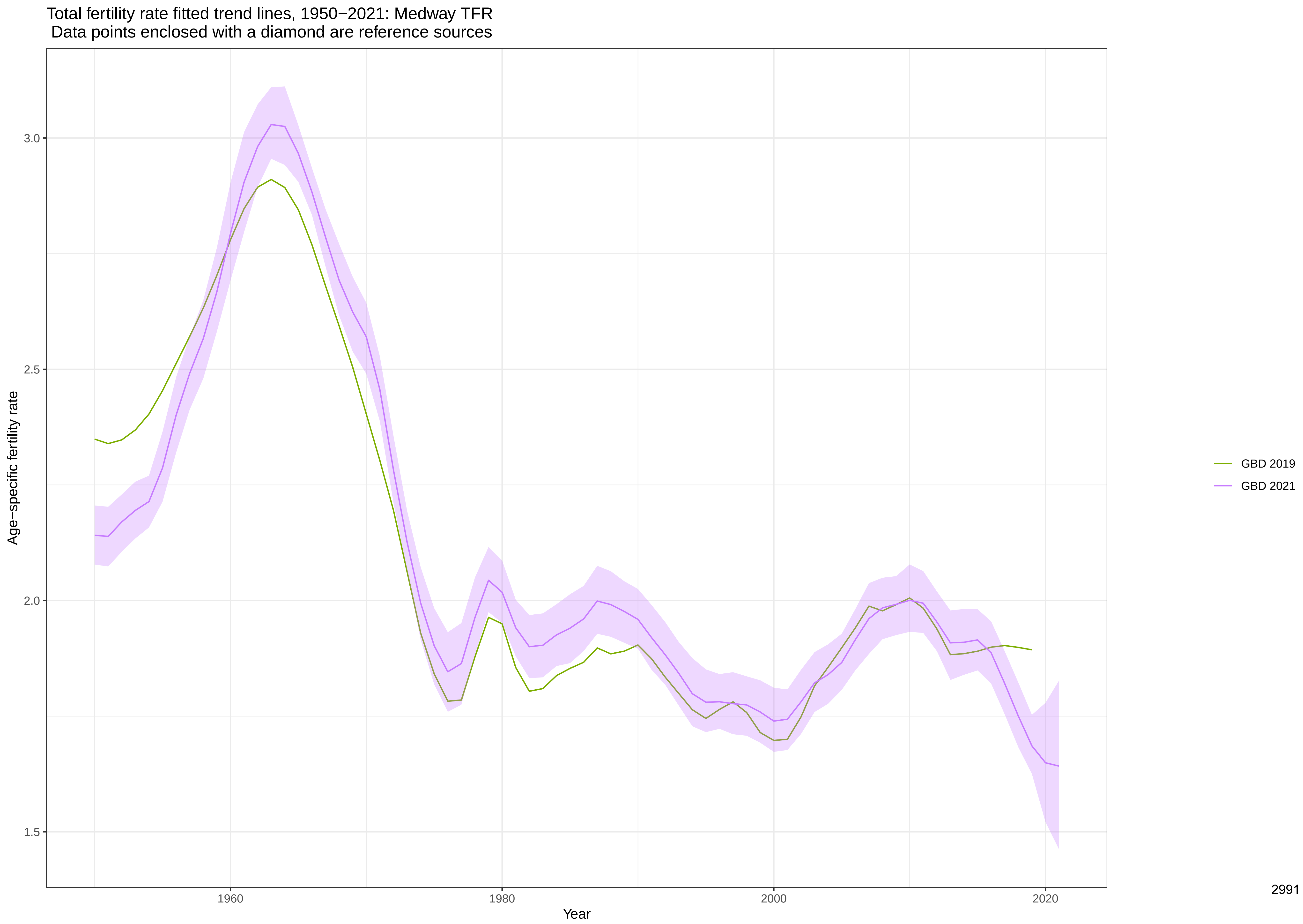
Total fertility rate fitted trend lines, 1950−2021: Medway TFR
Data points enclosed with a diamond are reference sources
Age−specific fertility rate
Year
3.0
2.5
2.0
1.5
1960
1980
2000
2020
GBD 2019
GBD 2021

Age−specific fertility fitted trend lines, 1950−2021: Milton Keynes, Ages: 10−14
Data points enclosed with a diamond are reference sources

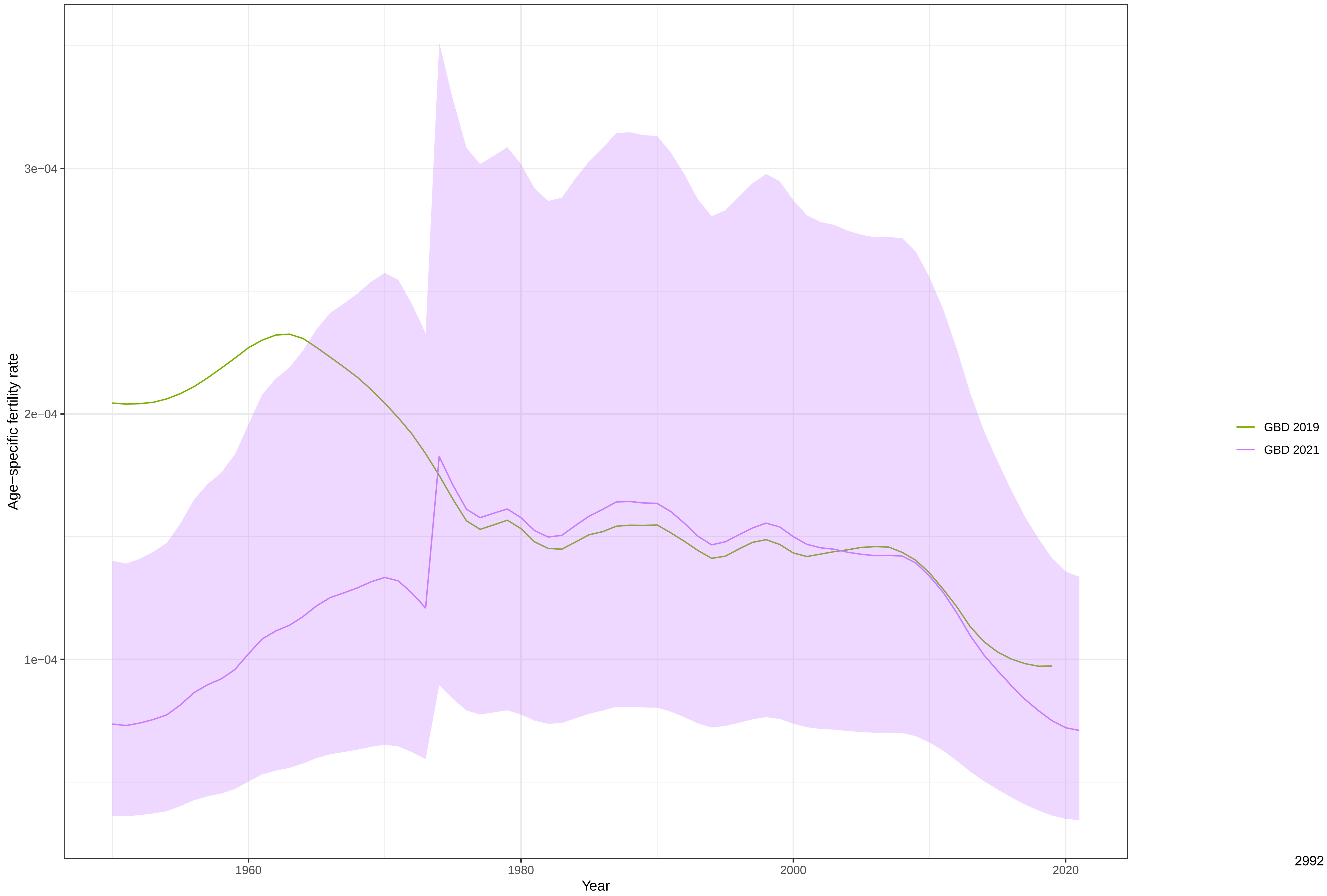

Age−specific fertility fitted trend lines, 1950−2021: Milton Keynes, Ages: 15−19
Data points enclosed with a diamond are reference sources

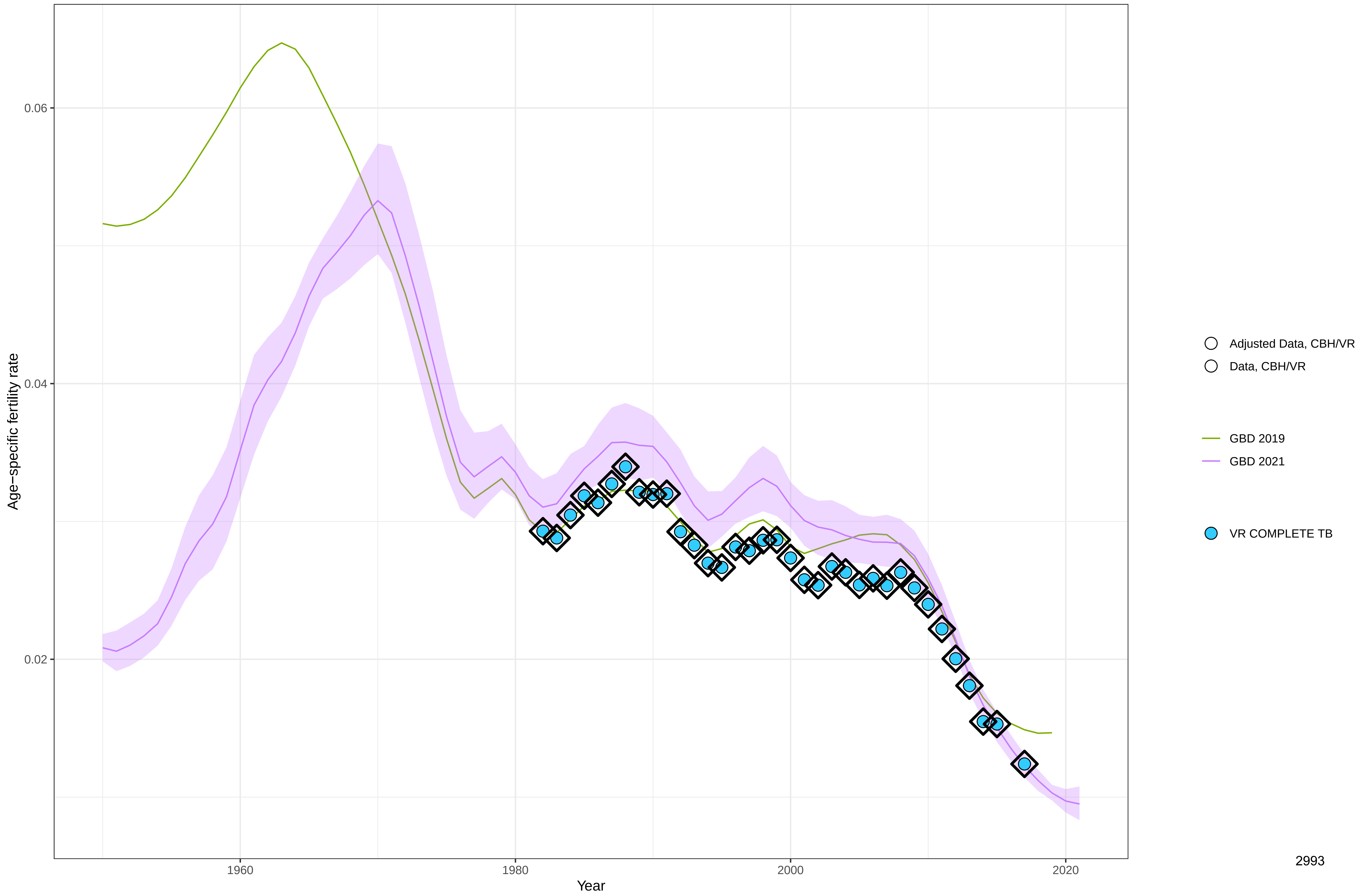

Age−specific fertility fitted trend lines, 1950−2021: Milton Keynes, Ages: 20−24
Data points enclosed with a diamond are reference sources

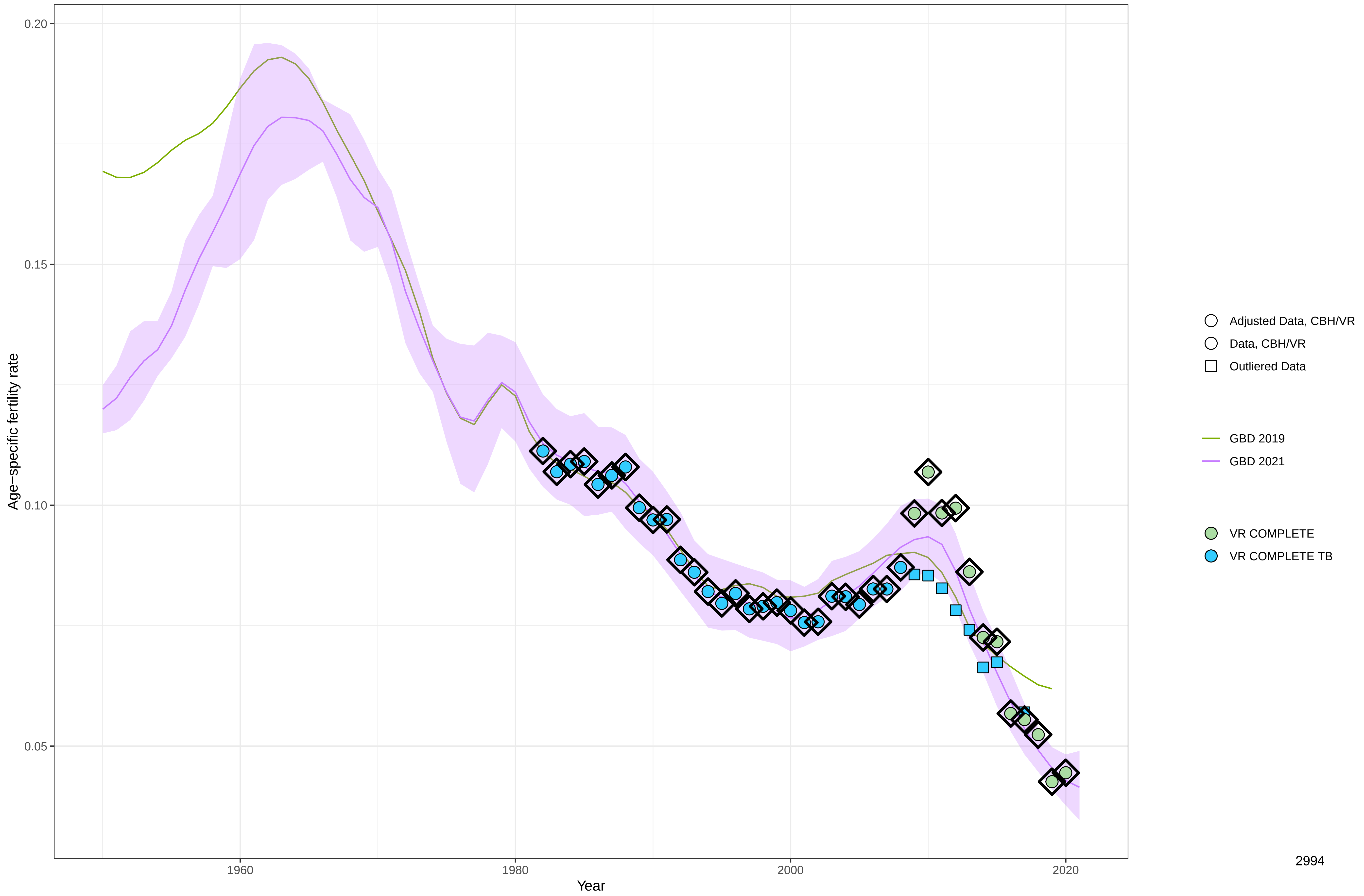

Age−specific fertility fitted trend lines, 1950−2021: Milton Keynes, Ages: 25−29
Data points enclosed with a diamond are reference sources

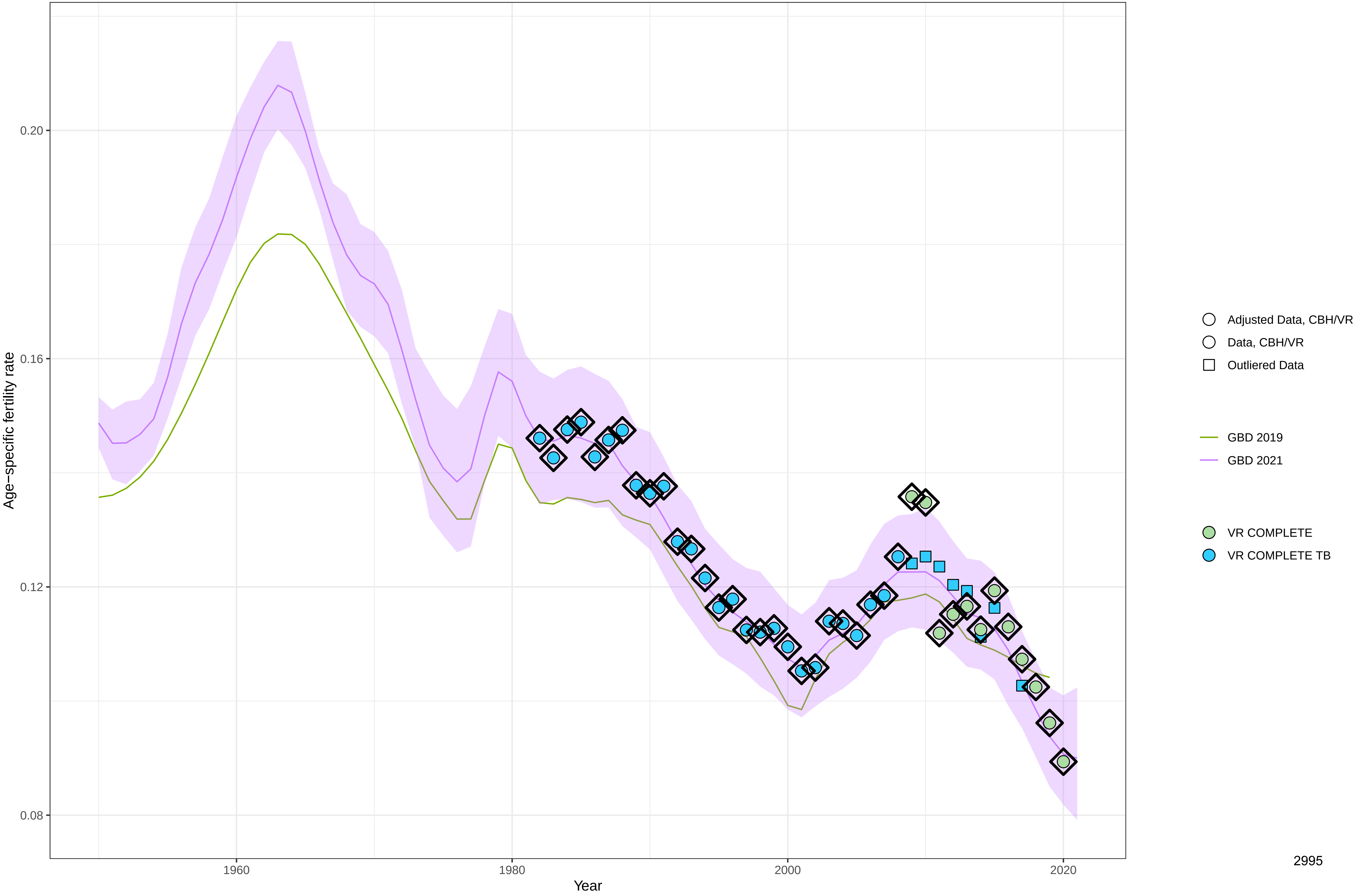

Age−specific fertility fitted trend lines, 1950−2021: Milton Keynes, Ages: 30−34
Data points enclosed with a diamond are reference sources

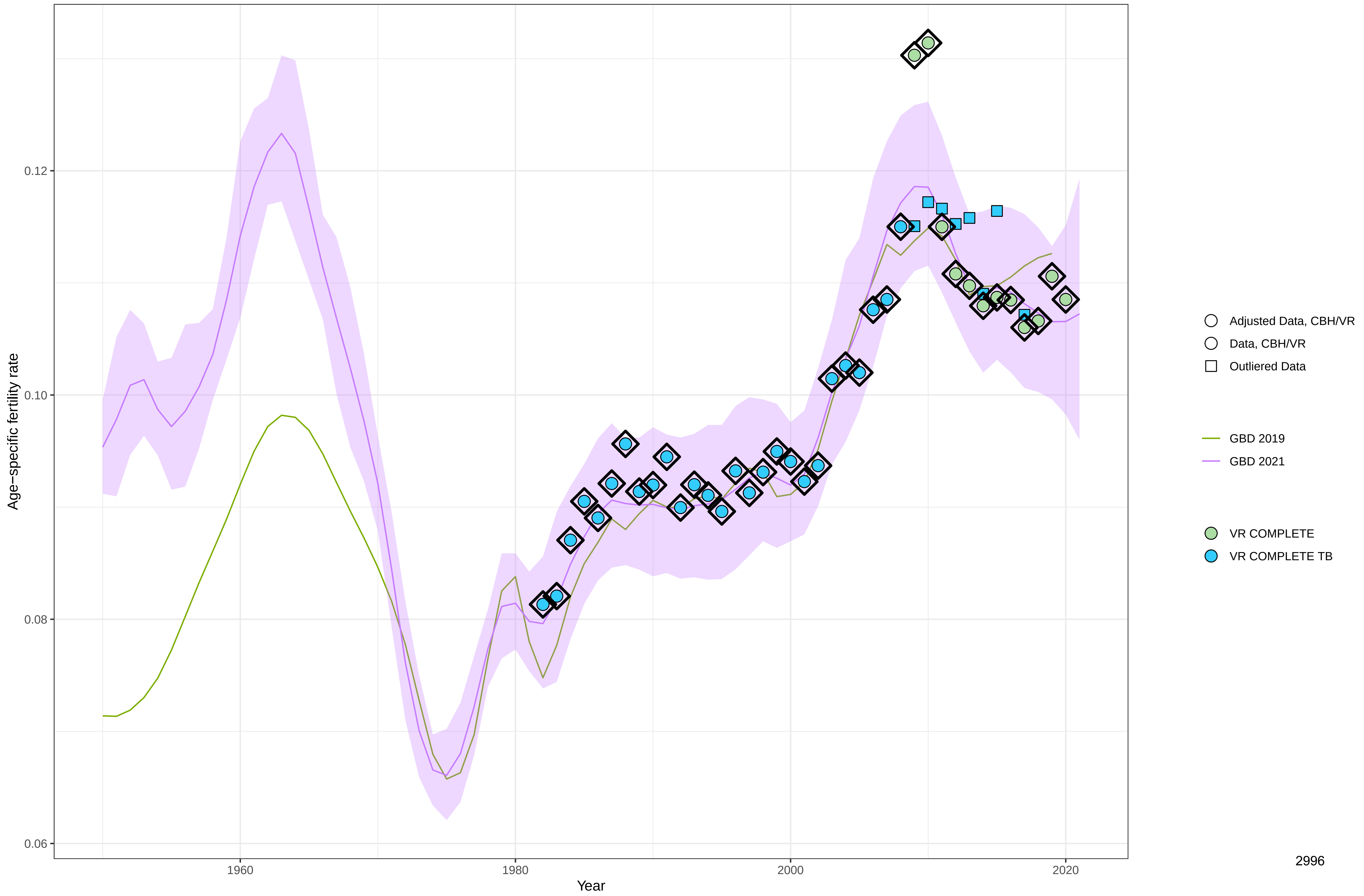

Age−specific fertility fitted trend lines, 1950−2021: Milton Keynes, Ages: 35−39
Data points enclosed with a diamond are reference sources

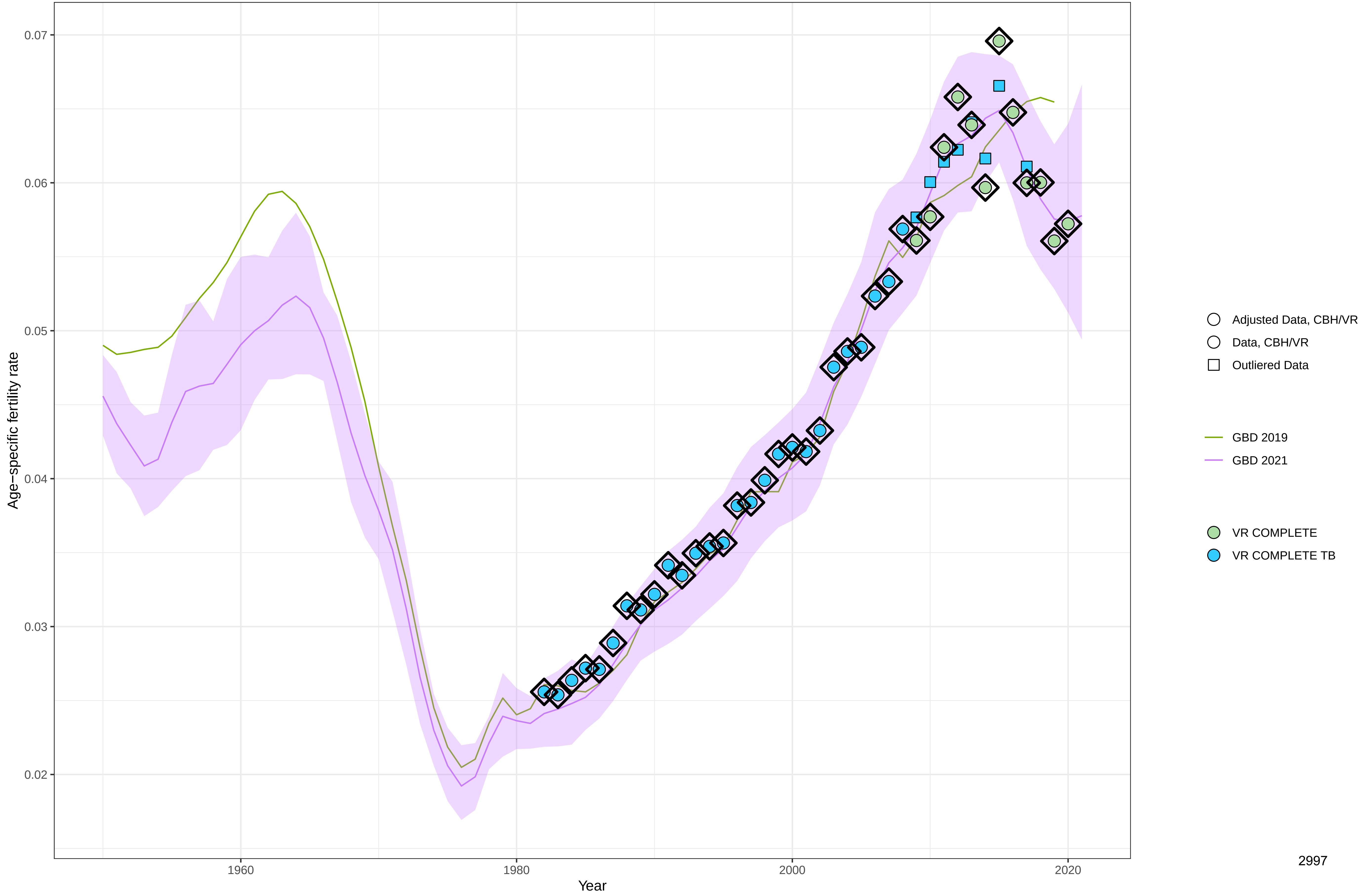

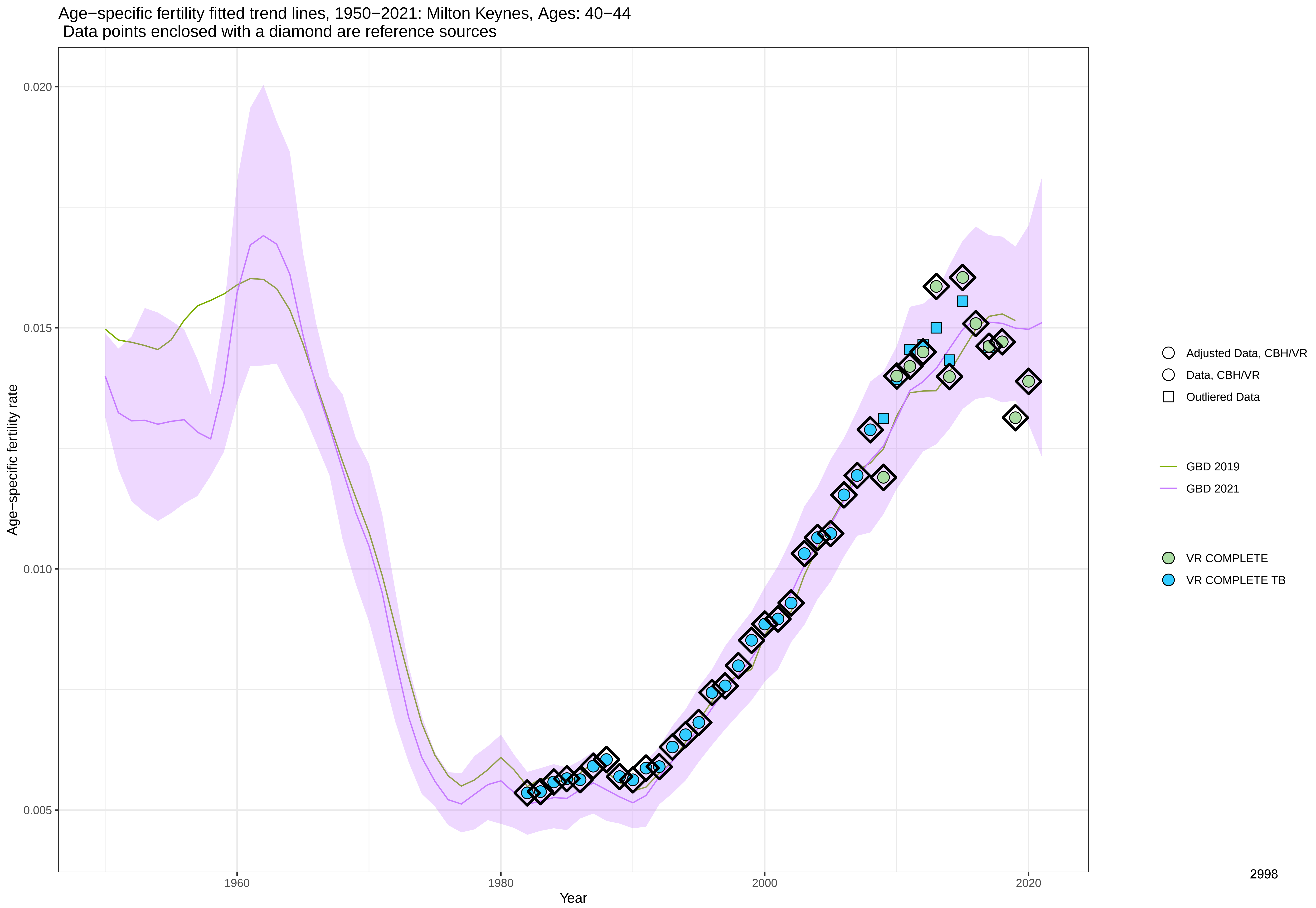
Age−specific fertility fitted trend lines, 1950−2021: Milton Keynes, Ages: 40−44
Data points enclosed with a diamond are reference sources
Age−specific fertility rate
0.020
0.015
0.010
0.005
1960
1980
2000
2020
Year
Adjusted Data, CBH/VR
Data, CBH/VR
Outliered Data
GBD 2019
GBD 2021
VR COMPLETE
VR COMPLETE TB

Age−specific fertility fitted trend lines, 1950−2021: Milton Keynes, Ages: 45−49
Data points enclosed with a diamond are reference sources

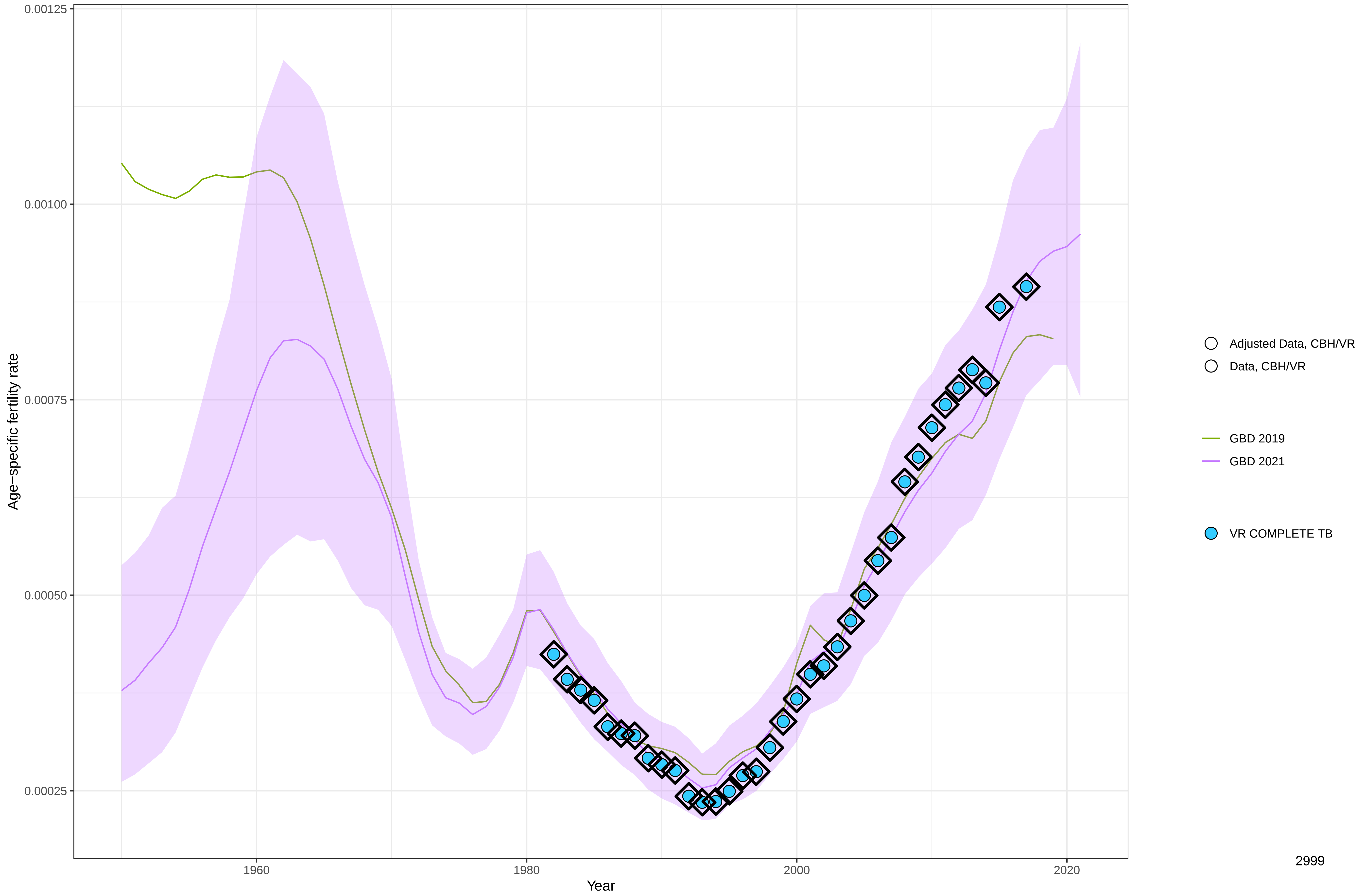

Age−specific fertility fitted trend lines, 1950−2021: Milton Keynes, Ages: 50−54
Data points enclosed with a diamond are reference sources

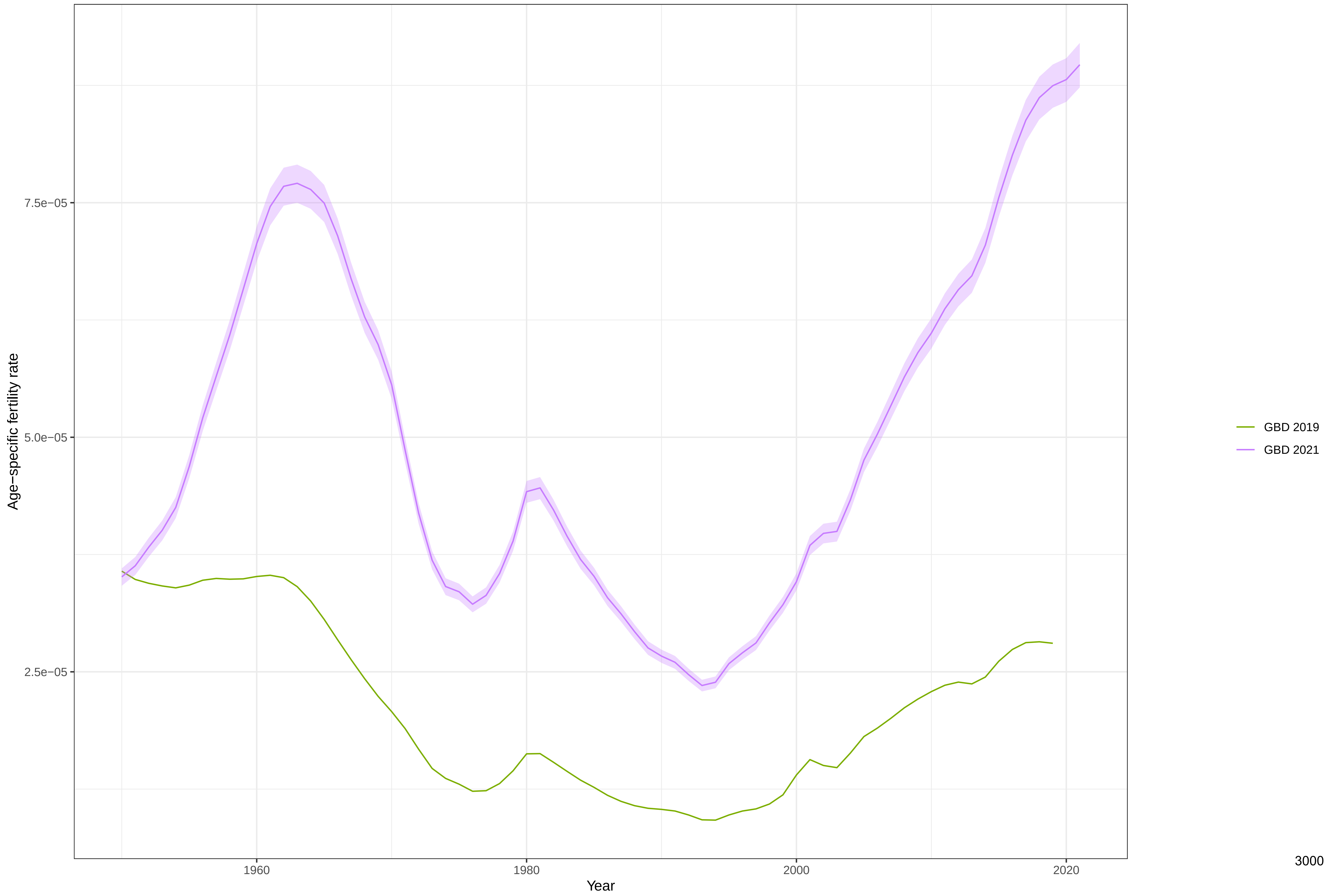

Total fertility rate fitted trend lines, 1950−2021: Milton Keynes TFR
Data points enclosed with a diamond are reference sources

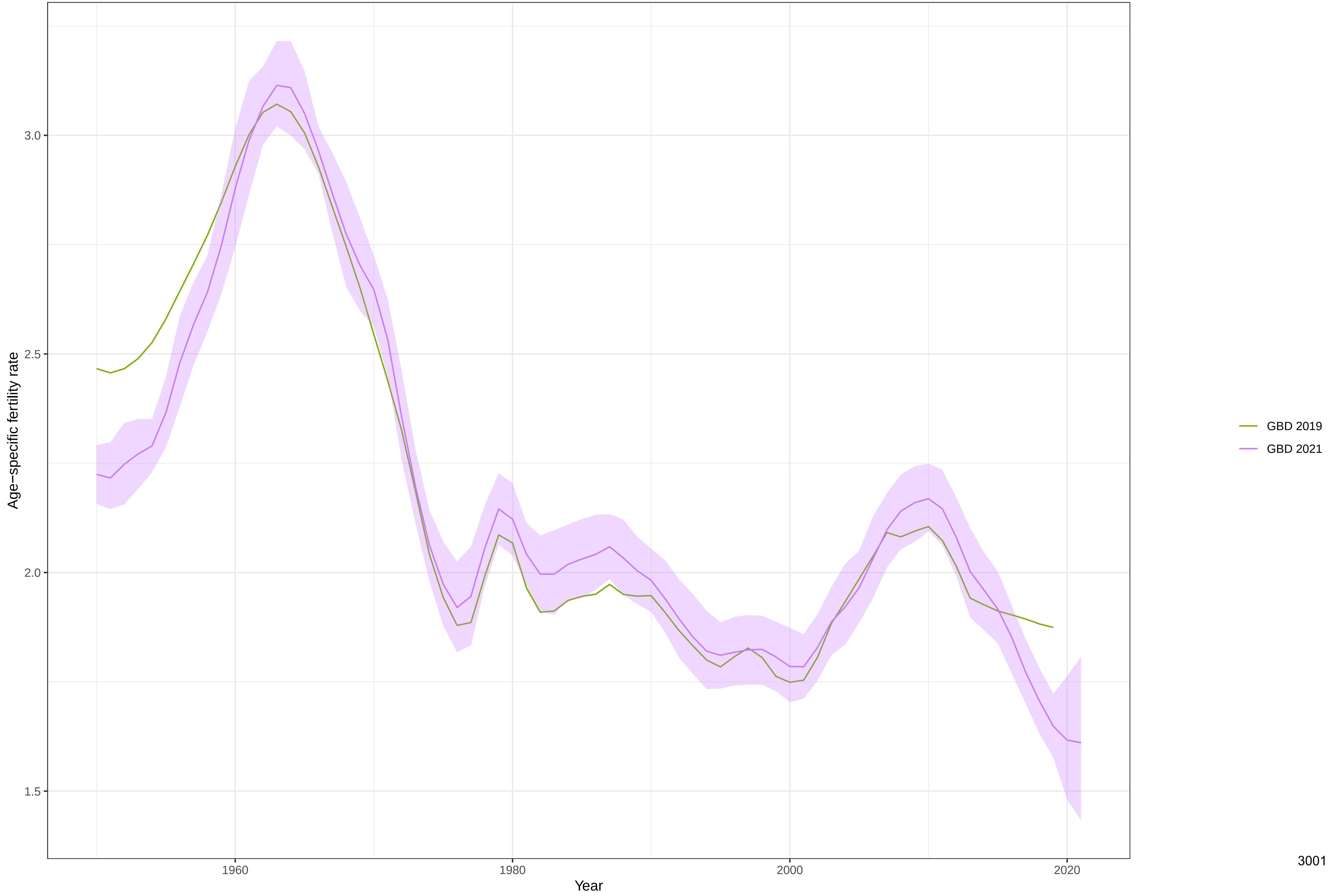

Age−specific fertility fitted trend lines, 1950−2021: Oxfordshire, Ages: 10−14
Data points enclosed with a diamond are reference sources

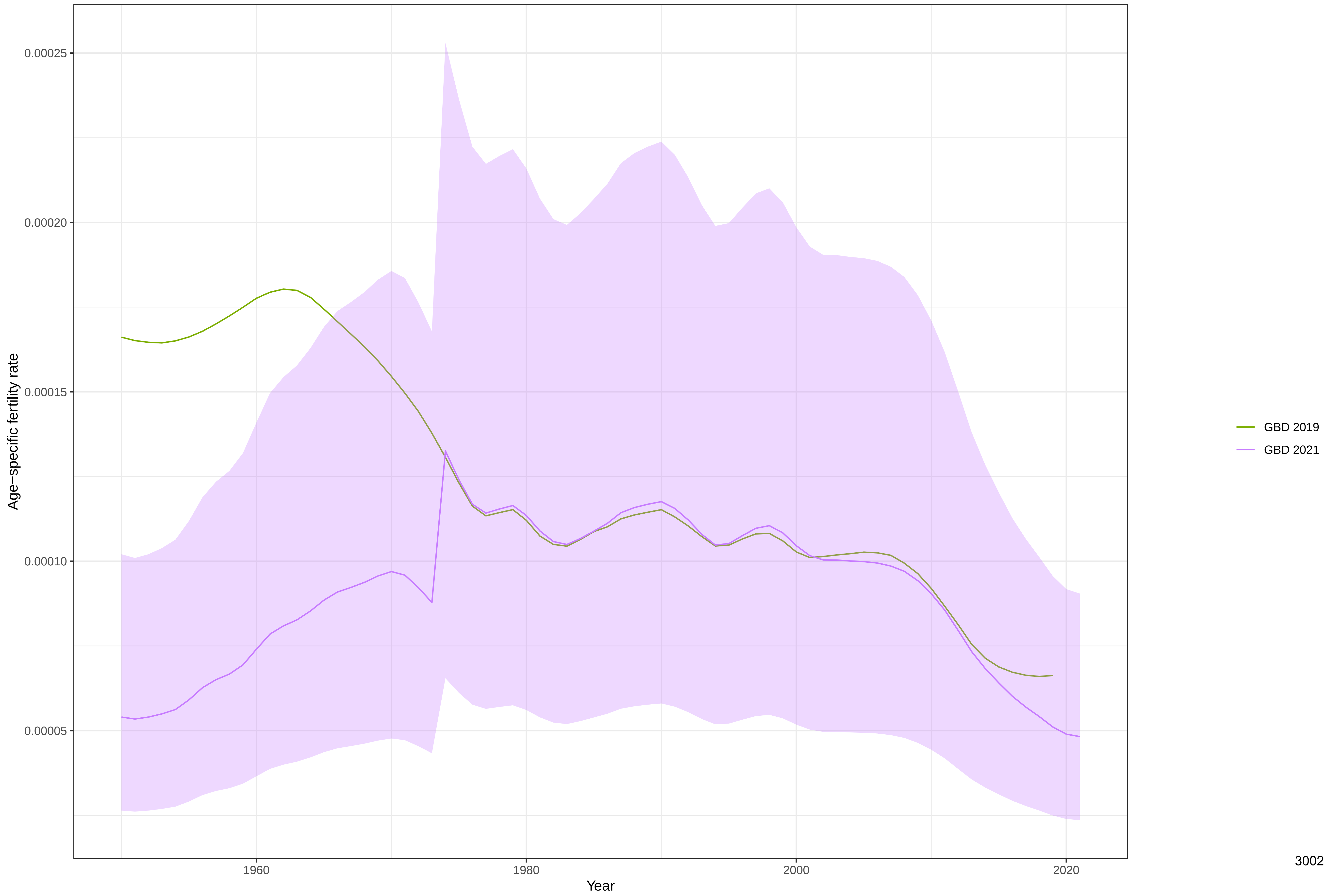

Age−specific fertility fitted trend lines, 1950−2021: Oxfordshire, Ages: 15−19
Data points enclosed with a diamond are reference sources

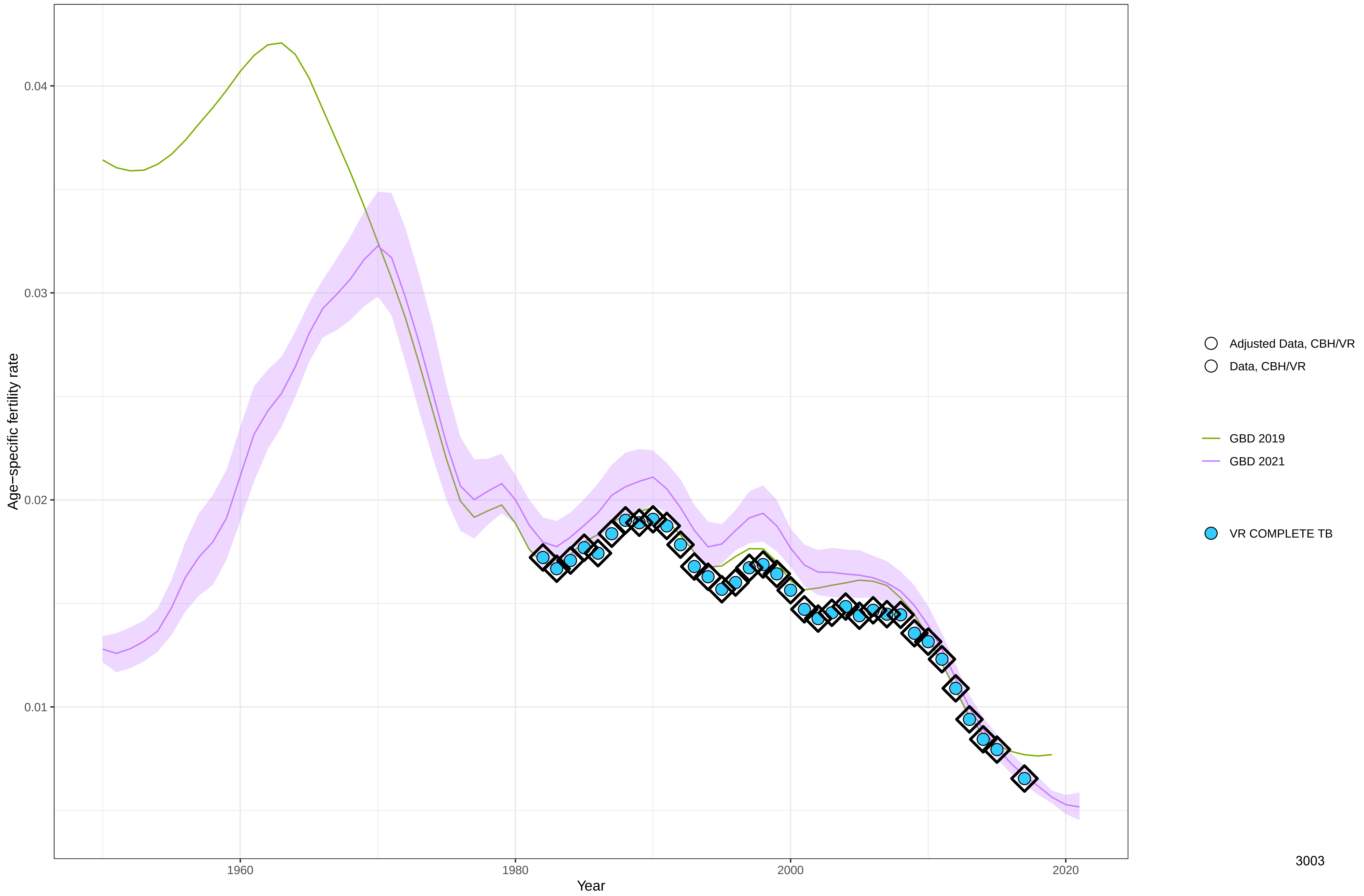

Age−specific fertility fitted trend lines, 1950−2021: Oxfordshire, Ages: 20−24
Data points enclosed with a diamond are reference sources

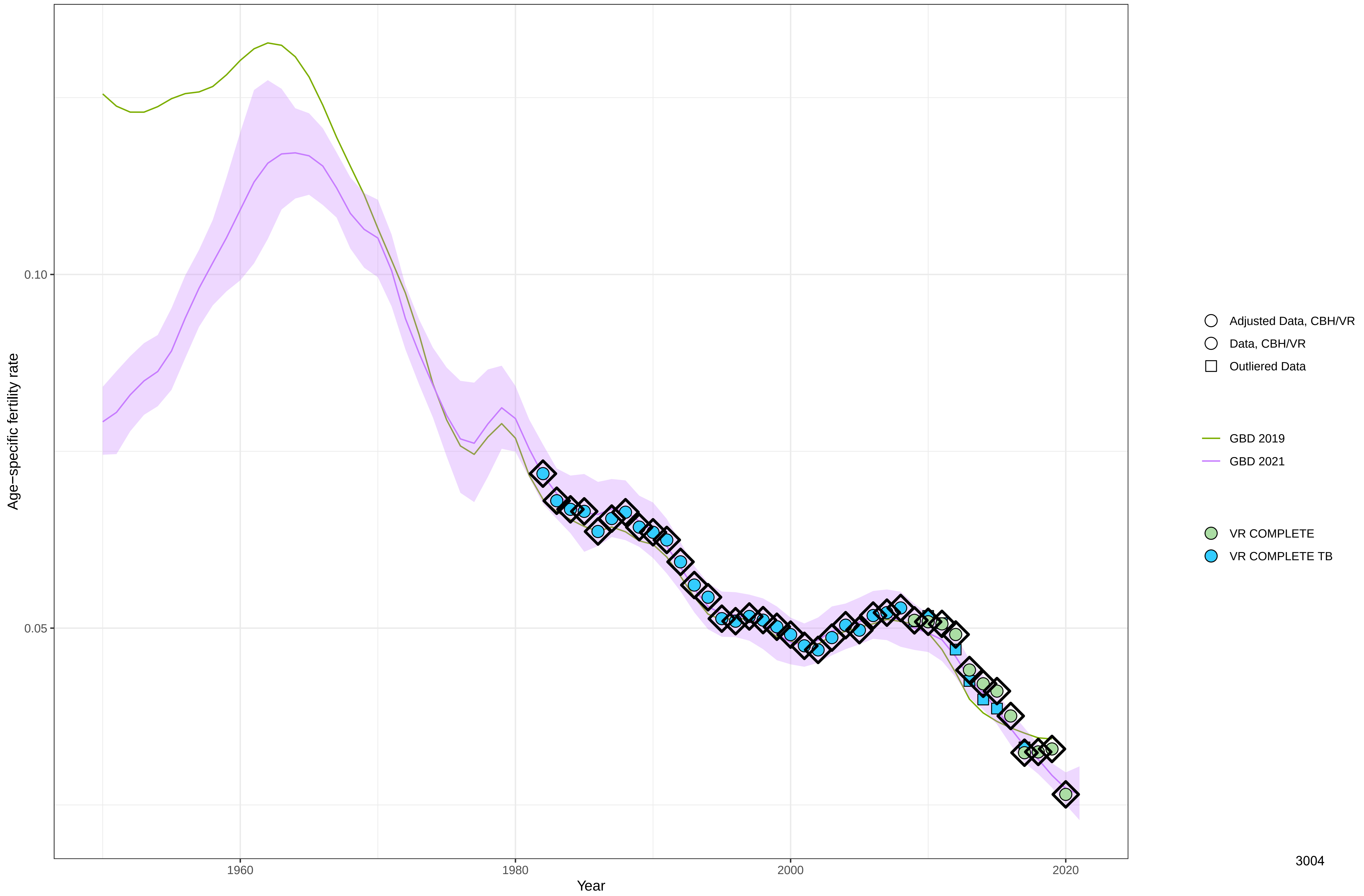

Age−specific fertility fitted trend lines, 1950−2021: Oxfordshire, Ages: 25−29
Data points enclosed with a diamond are reference sources

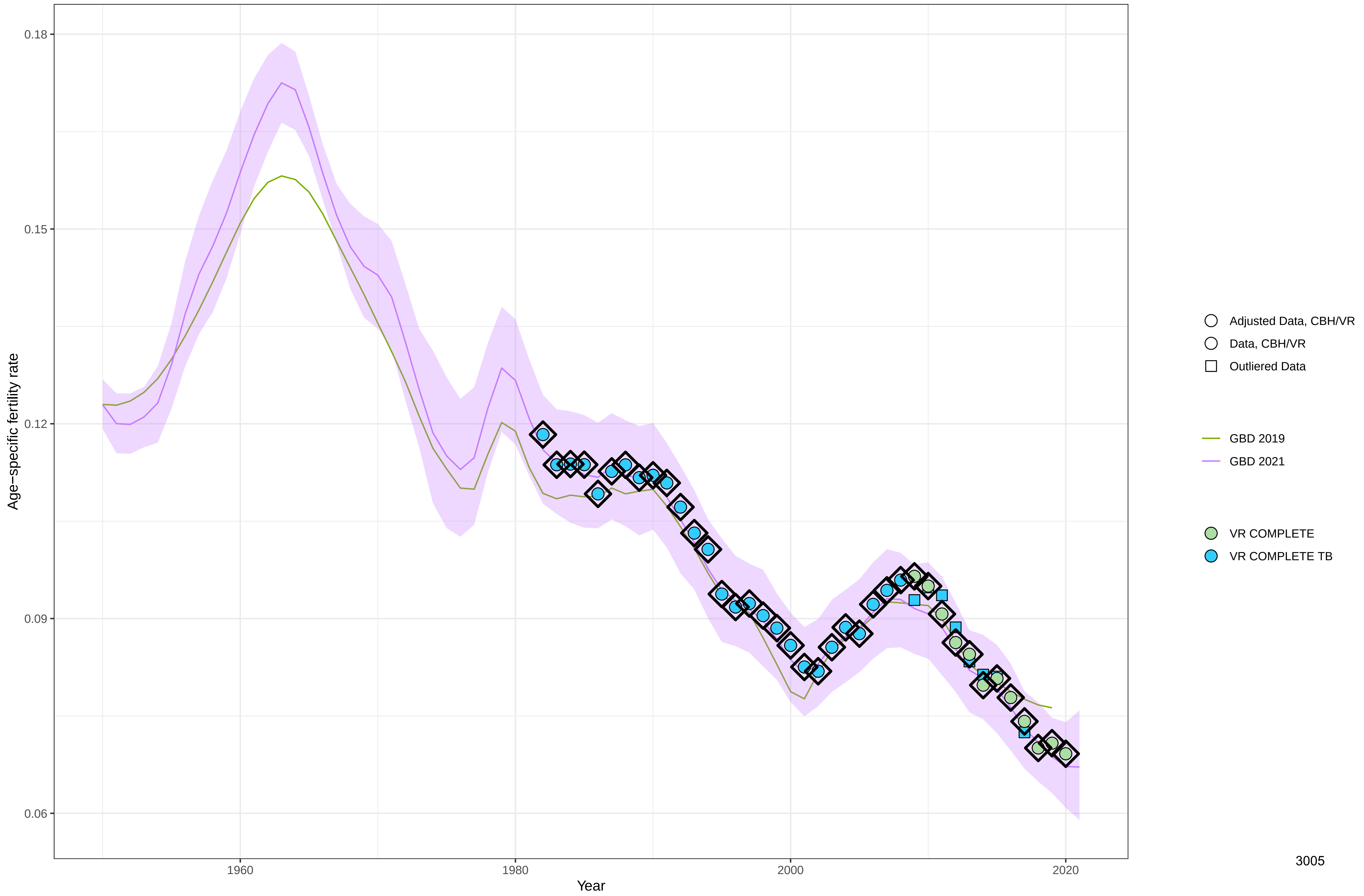

Age−specific fertility fitted trend lines, 1950−2021: Oxfordshire, Ages: 30−34
Data points enclosed with a diamond are reference sources

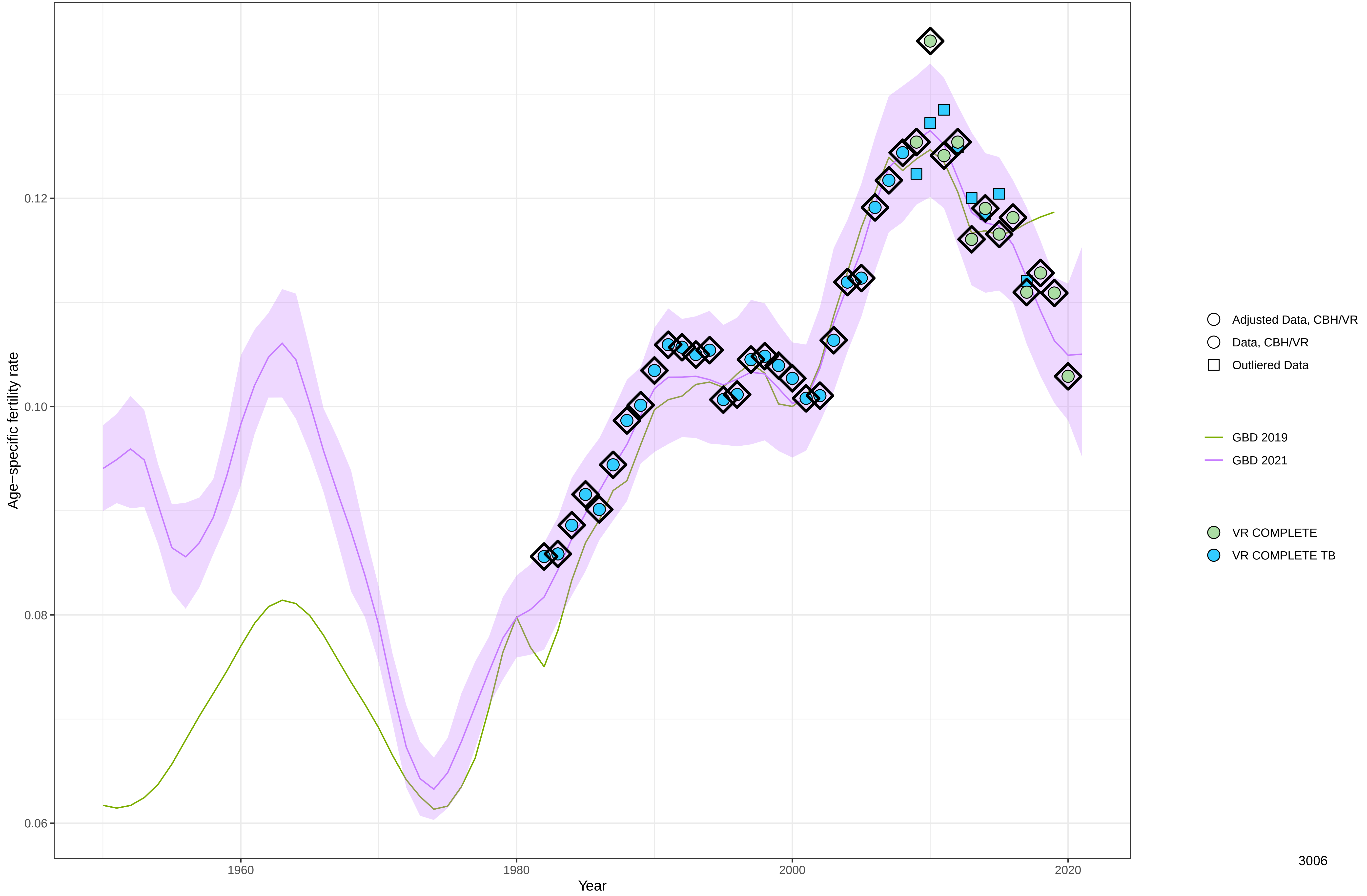

Age−specific fertility fitted trend lines, 1950−2021: Oxfordshire, Ages: 35−39
Data points enclosed with a diamond are reference sources

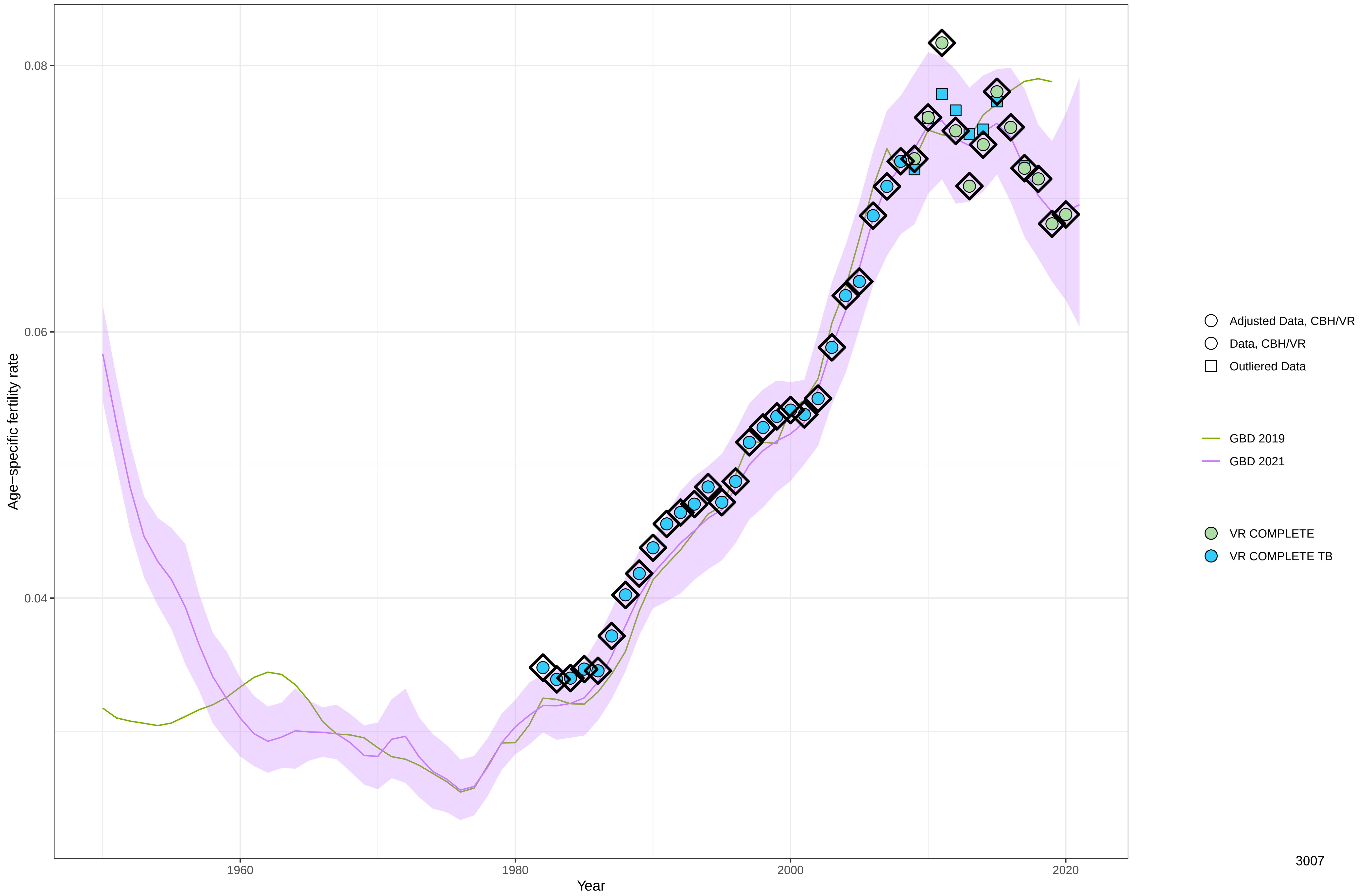

Age−specific fertility fitted trend lines, 1950−2021: Oxfordshire, Ages: 40−44
Data points enclosed with a diamond are reference sources

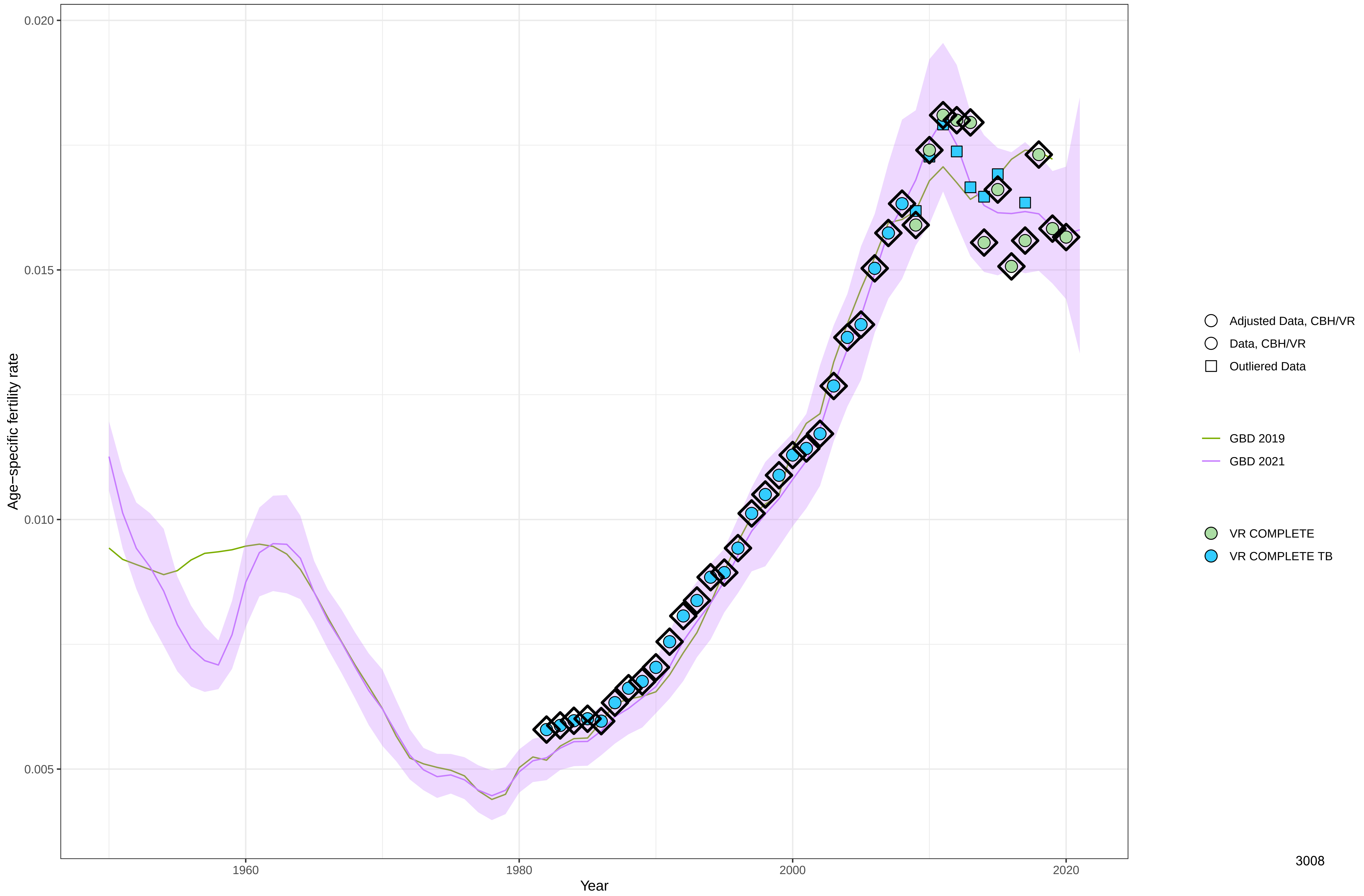

Age−specific fertility fitted trend lines, 1950−2021: Oxfordshire, Ages: 45−49
Data points enclosed with a diamond are reference sources

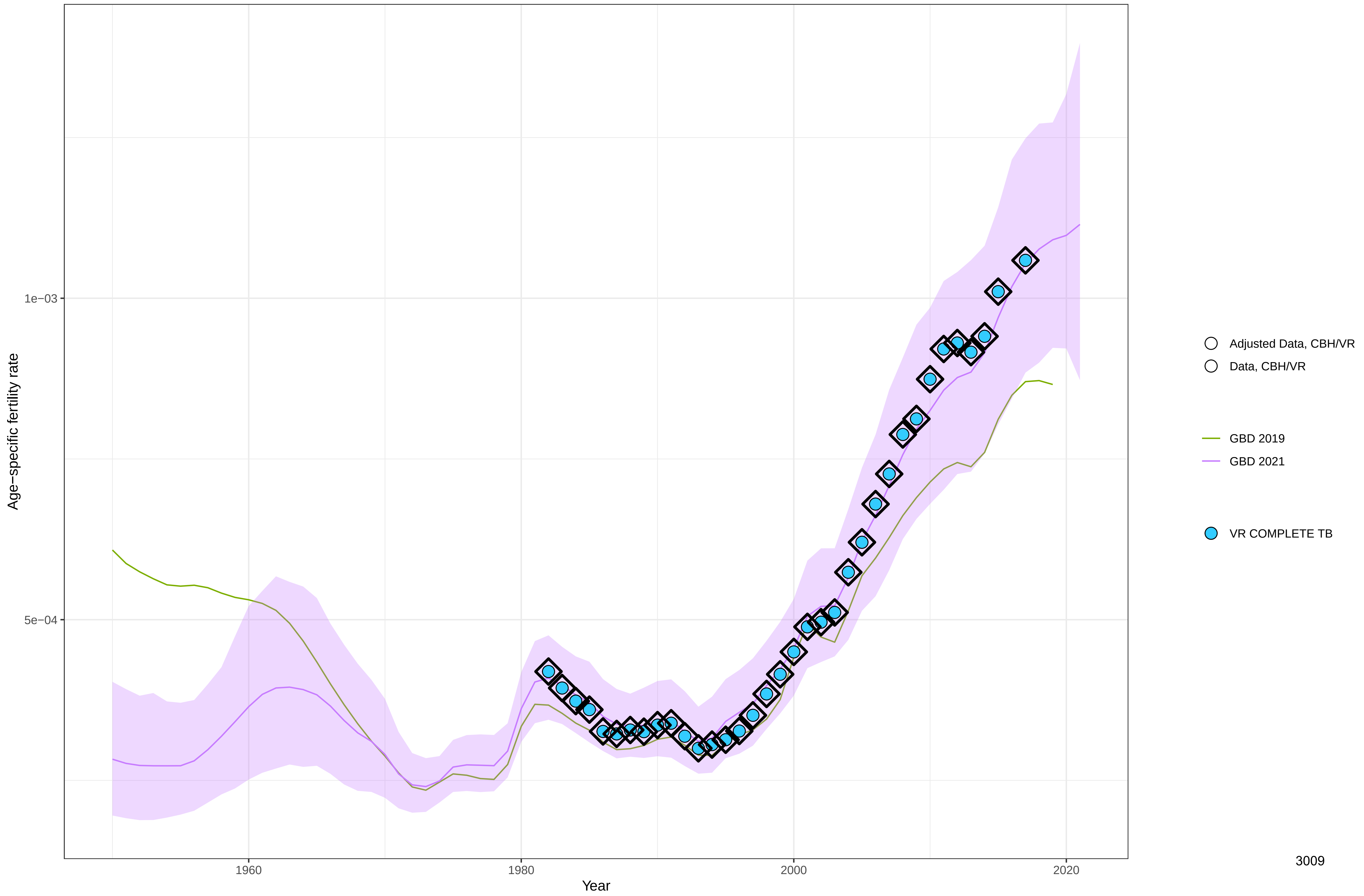

Age−specific fertility fitted trend lines, 1950−2021: Oxfordshire, Ages: 50−54
Data points enclosed with a diamond are reference sources

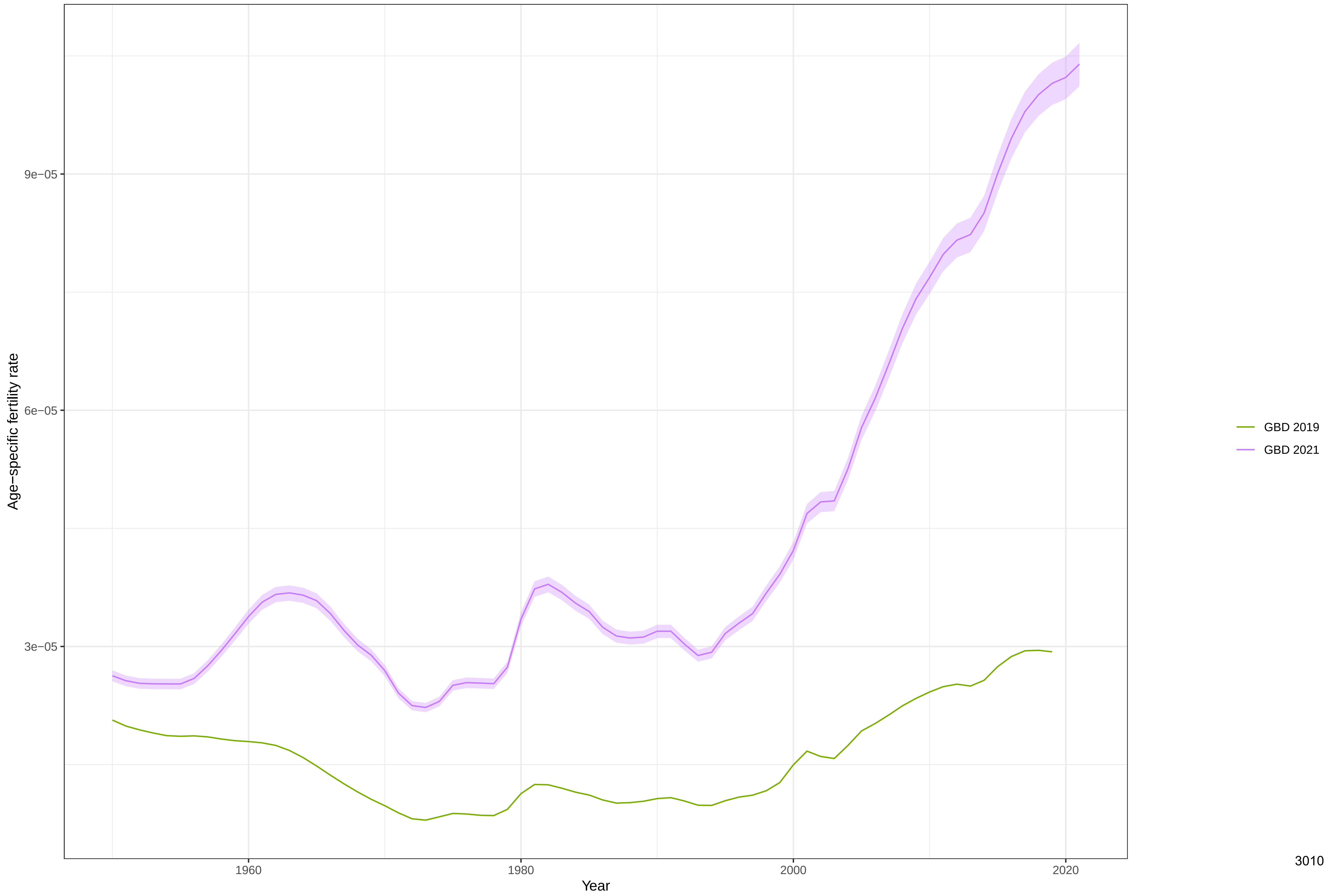

Total fertility rate fitted trend lines, 1950−2021: Oxfordshire TFR
Data points enclosed with a diamond are reference sources

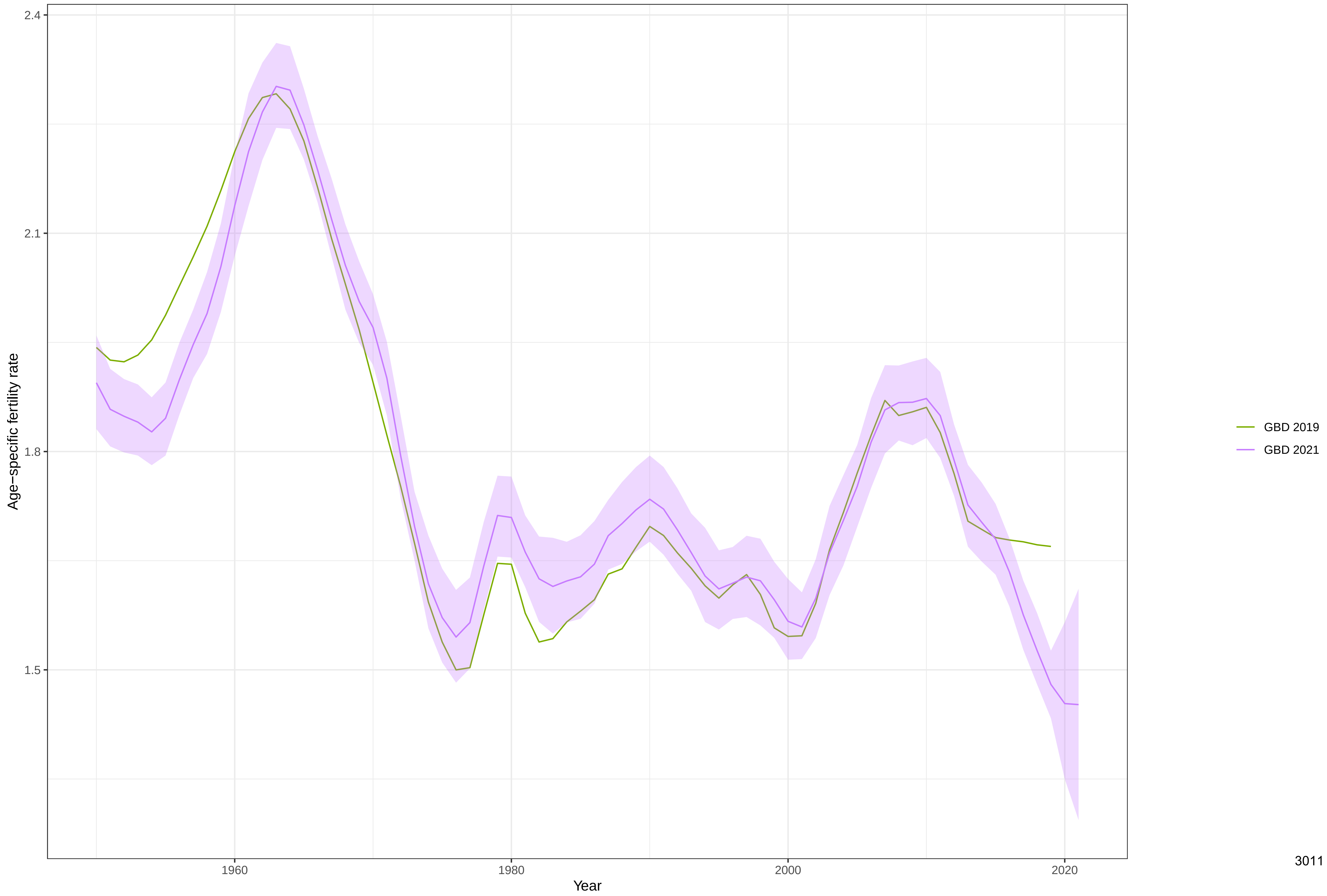

Age−specific fertility fitted trend lines, 1950−2021: Portsmouth, Ages: 10−14
Data points enclosed with a diamond are reference sources

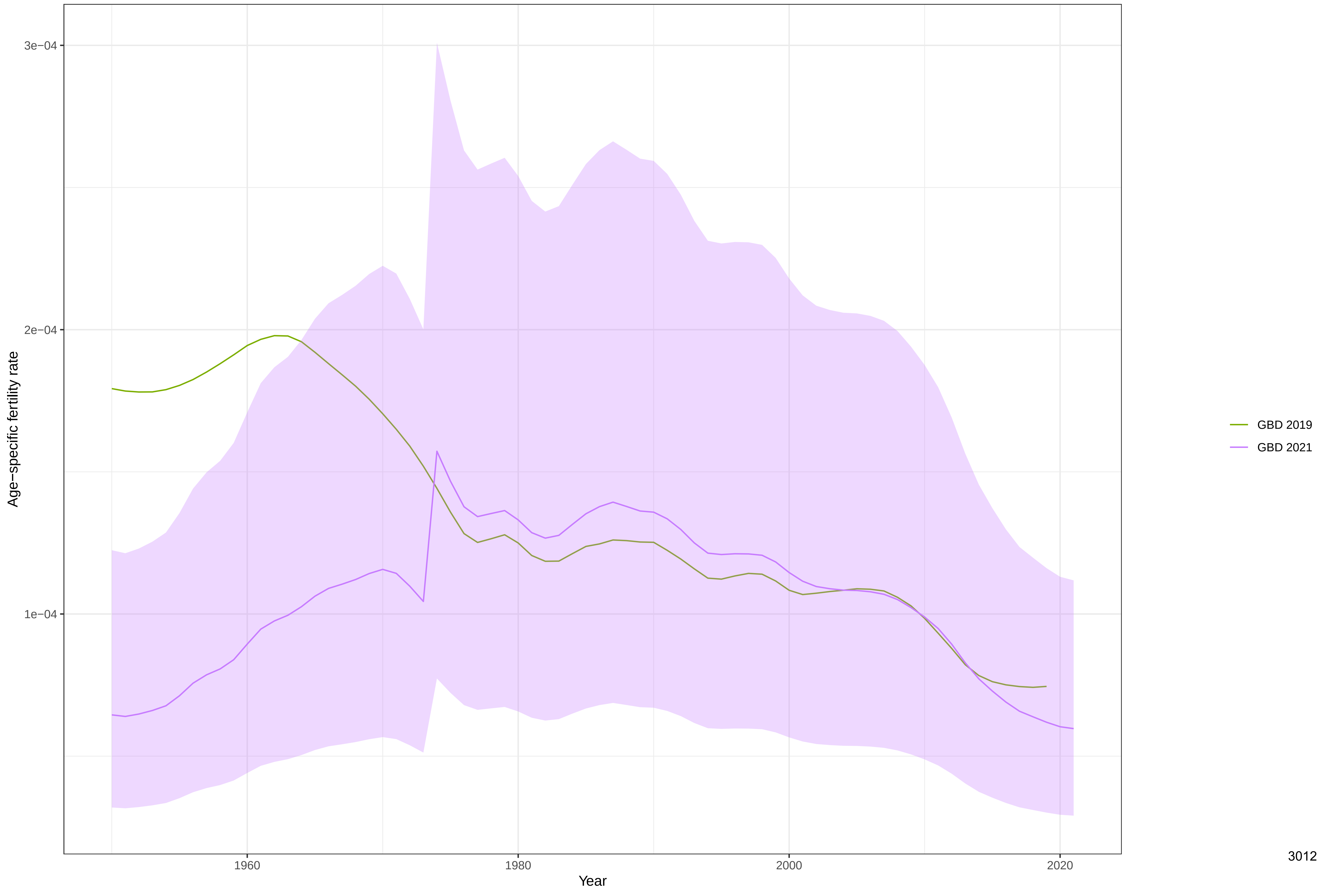

Age−specific fertility fitted trend lines, 1950−2021: Portsmouth, Ages: 15−19
Data points enclosed with a diamond are reference sources

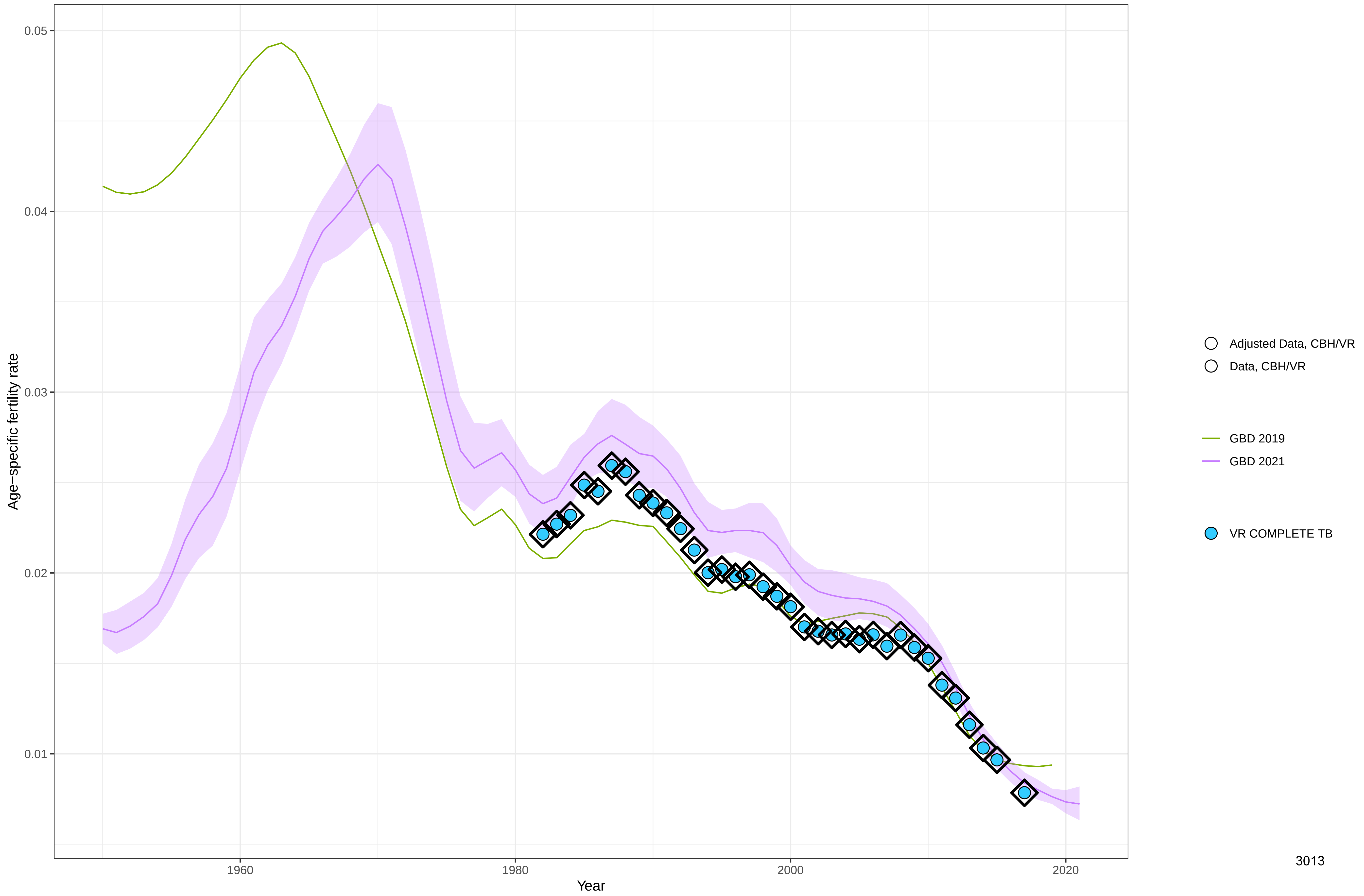

Age−specific fertility fitted trend lines, 1950−2021: Portsmouth, Ages: 20−24
Data points enclosed with a diamond are reference sources

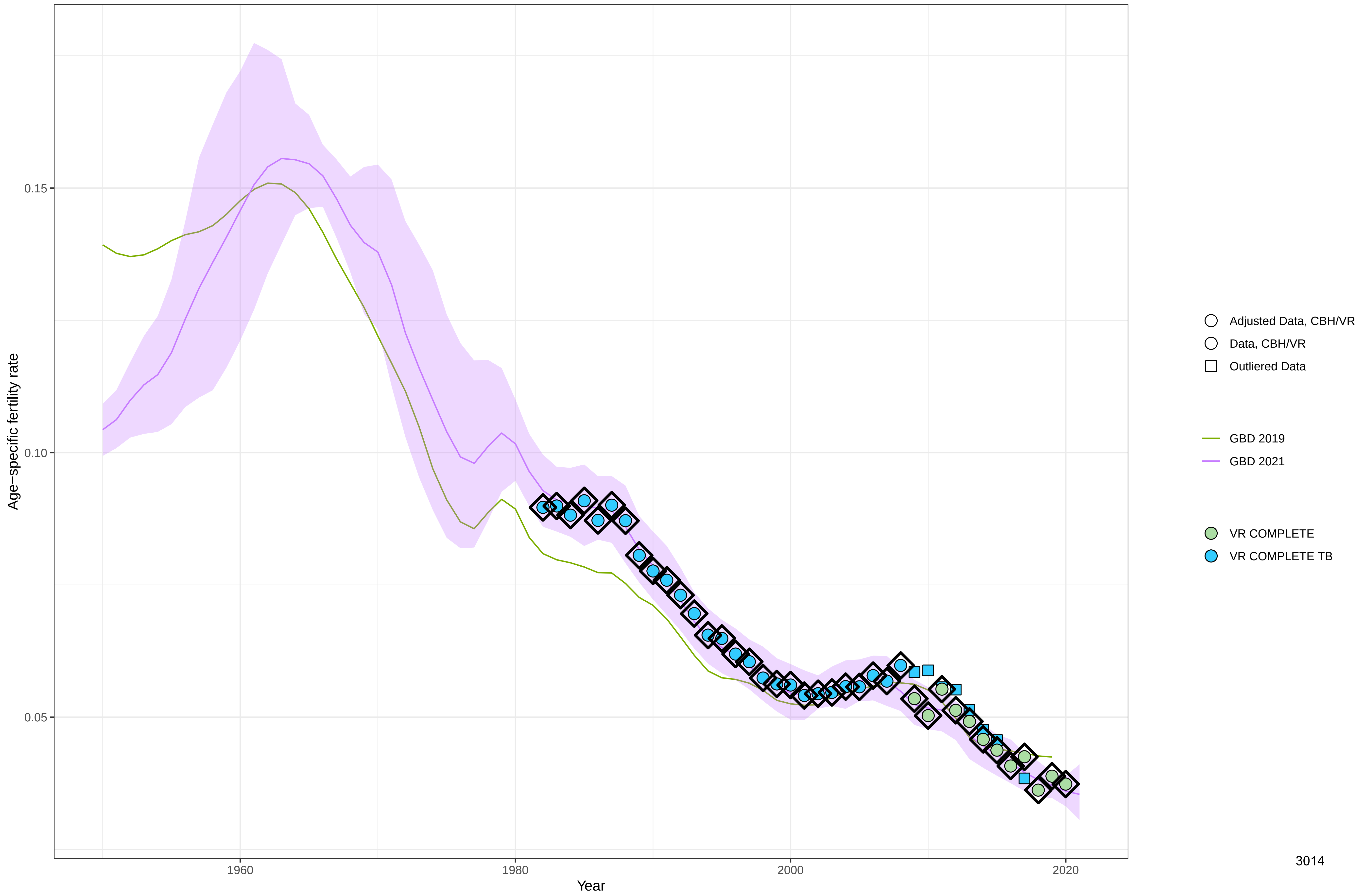

Age−specific fertility fitted trend lines, 1950−2021: Portsmouth, Ages: 25−29
Data points enclosed with a diamond are reference sources

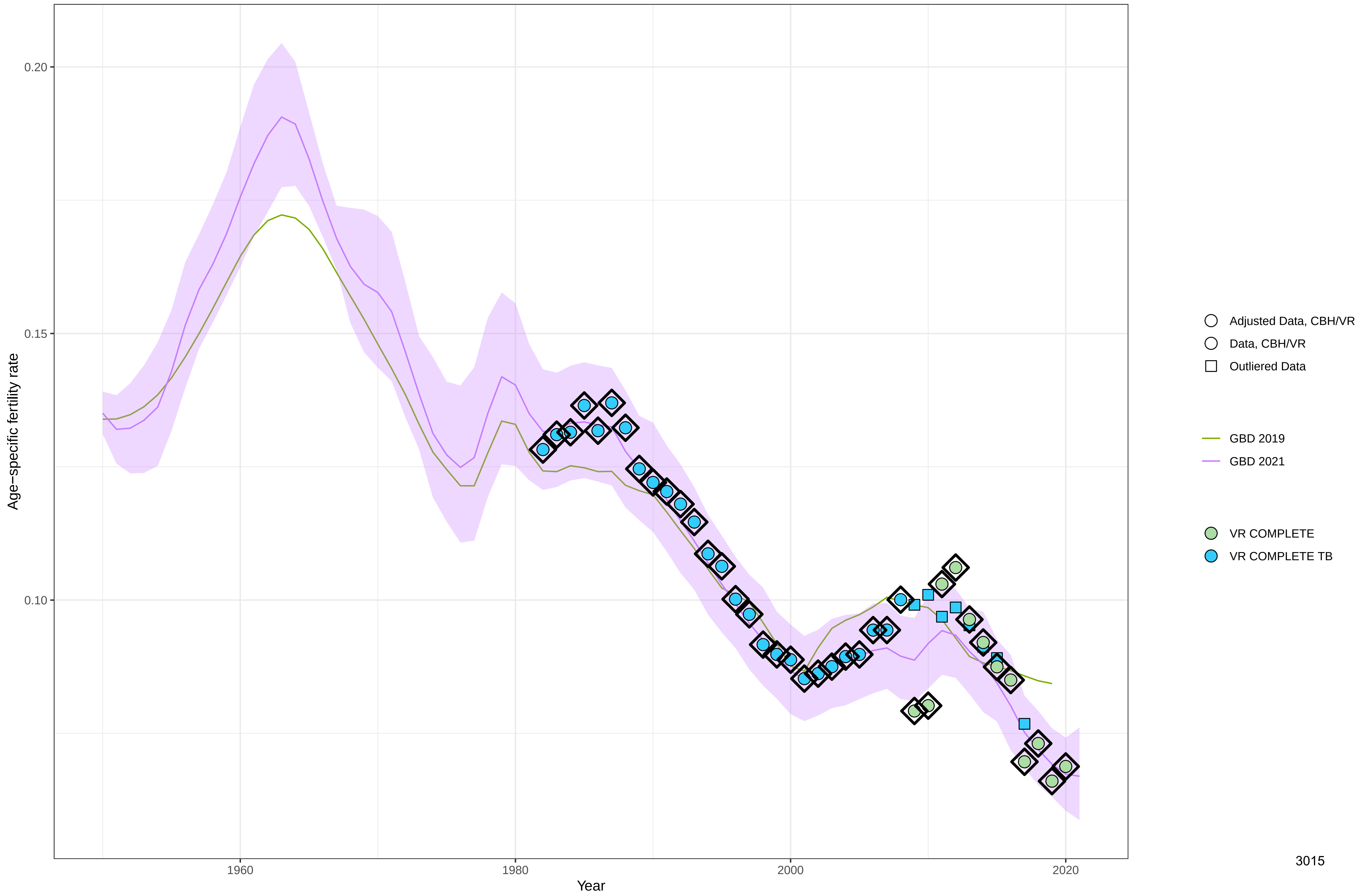

Age−specific fertility fitted trend lines, 1950−2021: Portsmouth, Ages: 30−34
Data points enclosed with a diamond are reference sources

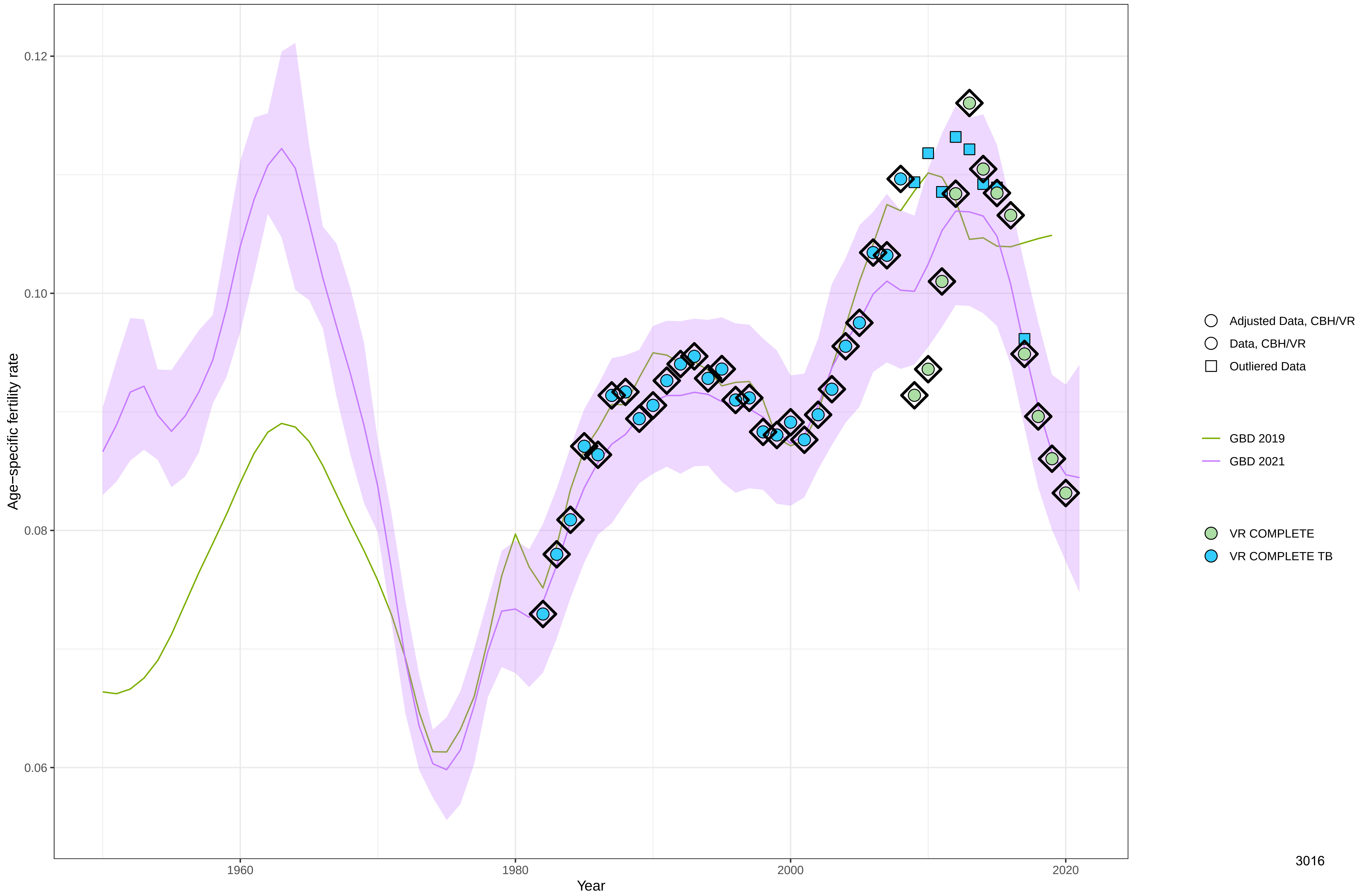

Age−specific fertility fitted trend lines, 1950−2021: Portsmouth, Ages: 35−39
Data points enclosed with a diamond are reference sources

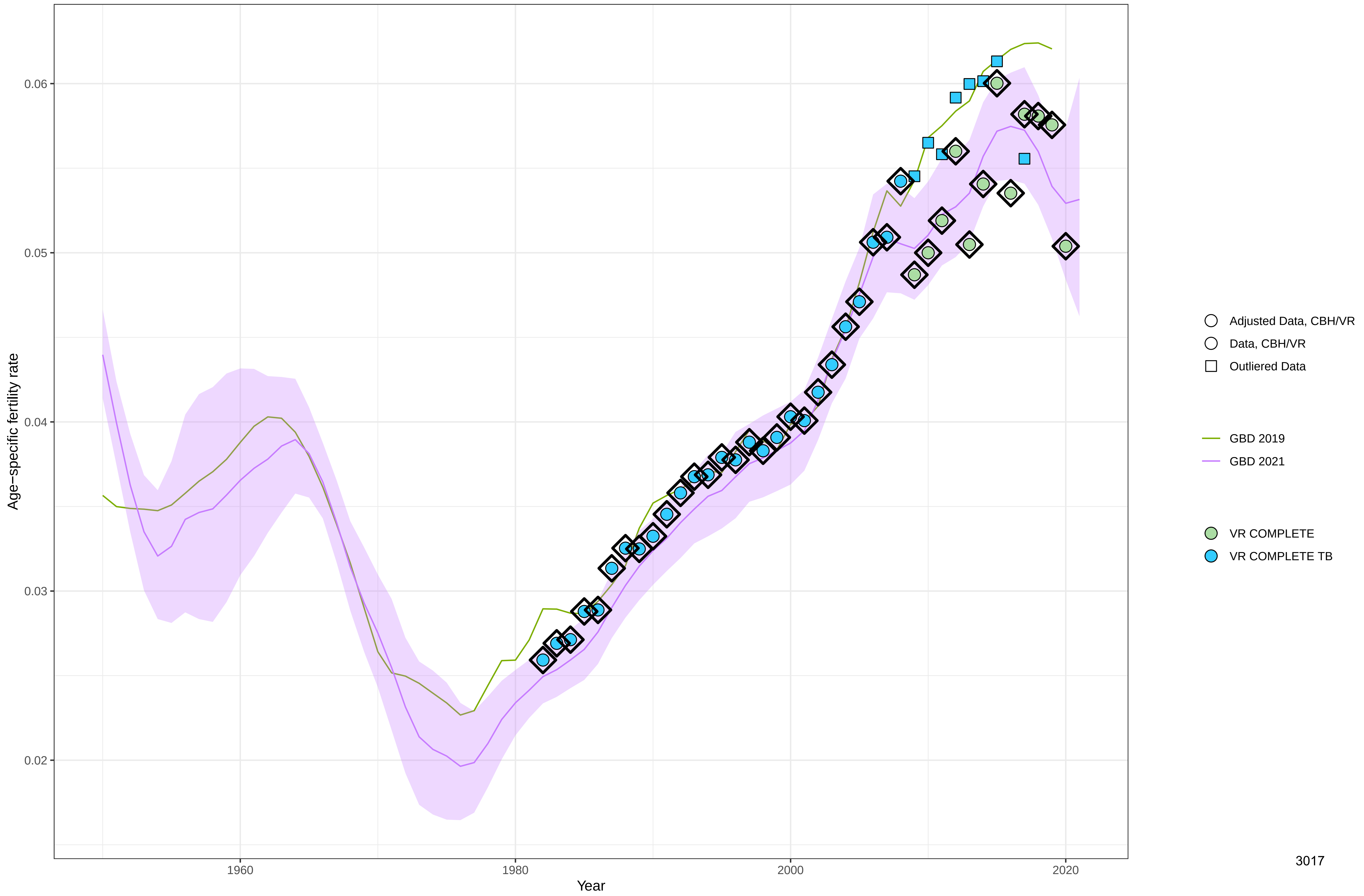

Age−specific fertility fitted trend lines, 1950−2021: Portsmouth, Ages: 40−44
Data points enclosed with a diamond are reference sources

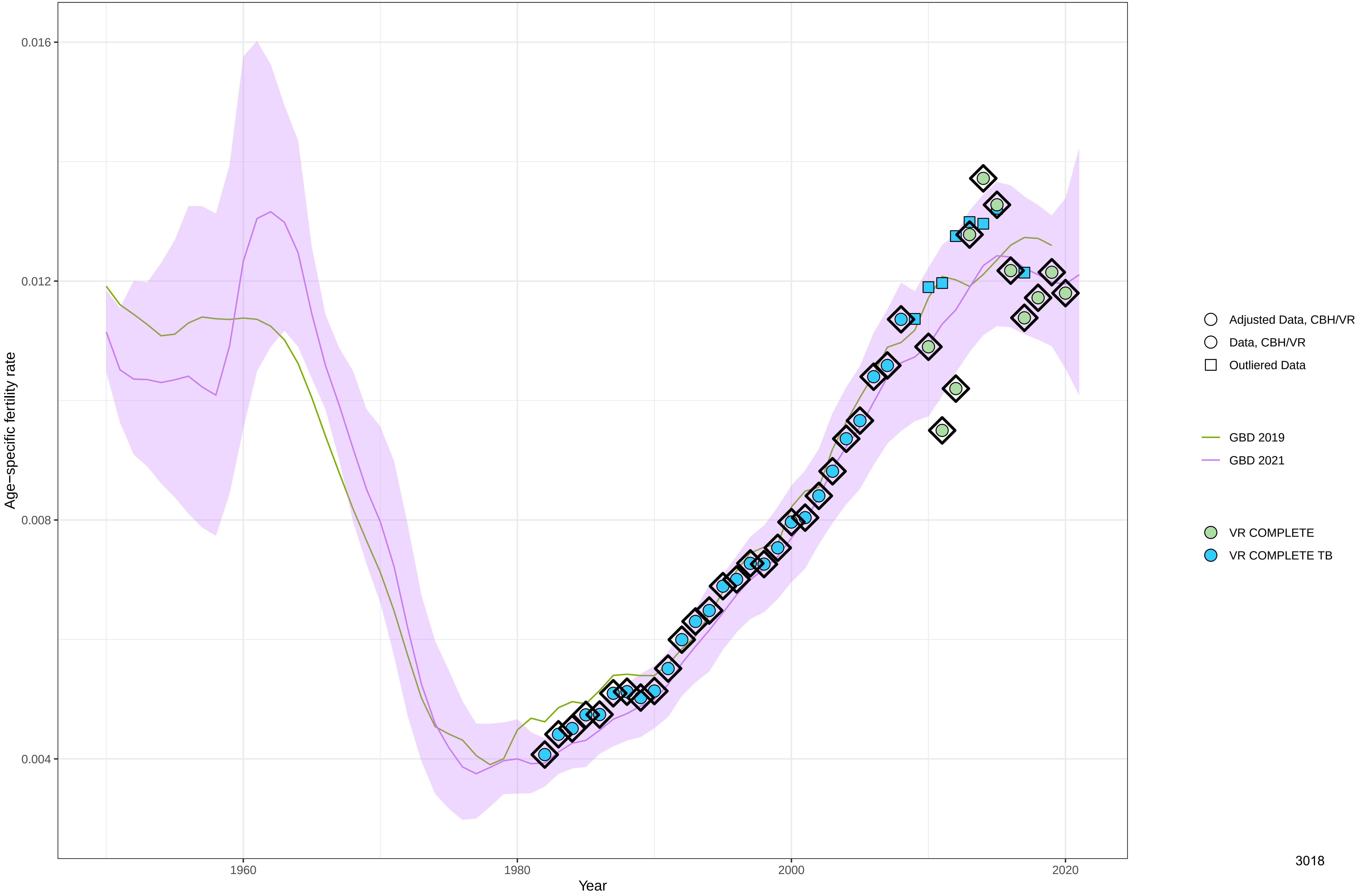

Age−specific fertility fitted trend lines, 1950−2021: Portsmouth, Ages: 45−49
Data points enclosed with a diamond are reference sources

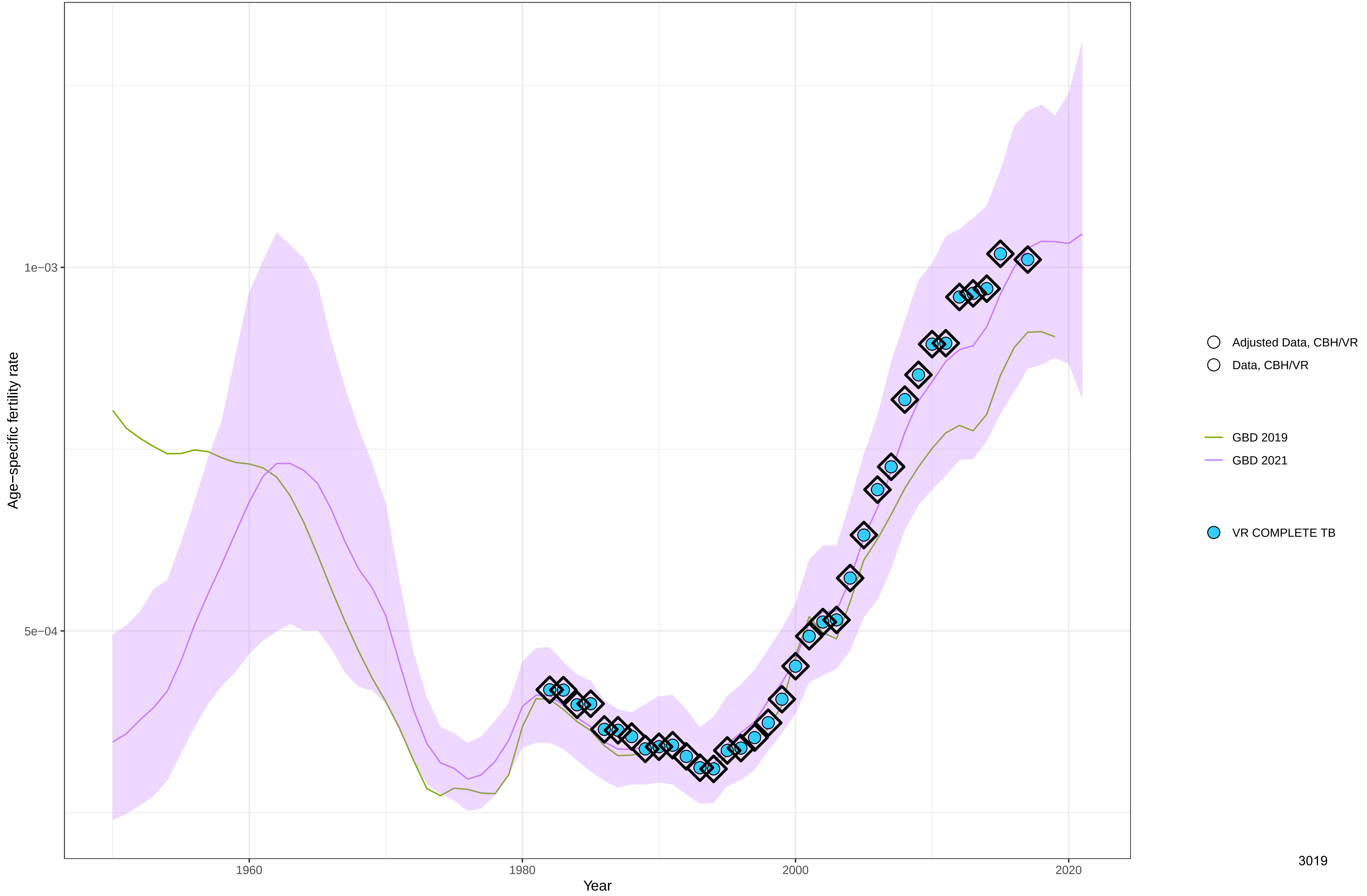

Age−specific fertility fitted trend lines, 1950−2021: Portsmouth, Ages: 50−54
Data points enclosed with a diamond are reference sources

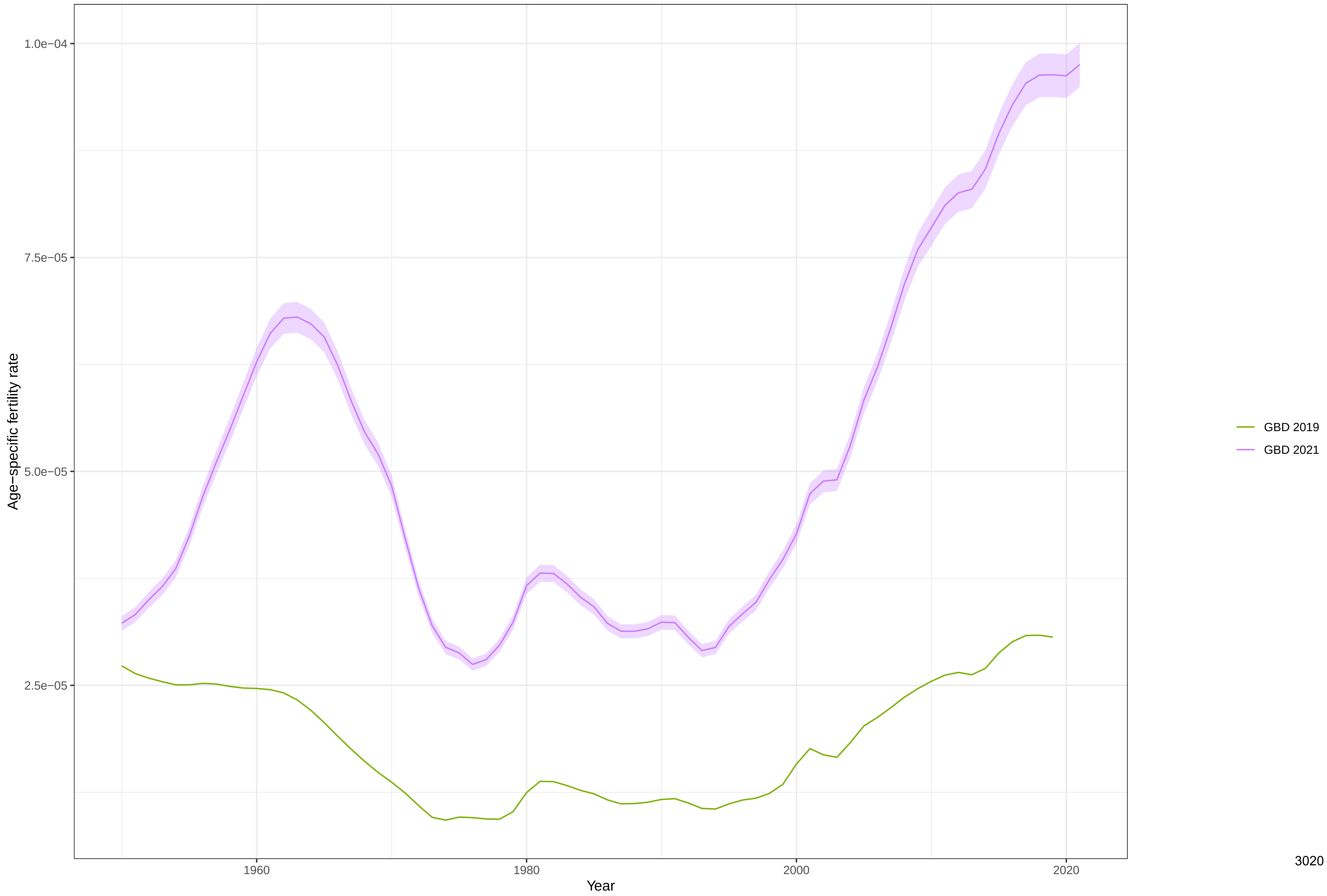

Total fertility rate fitted trend lines, 1950−2021: Portsmouth TFR
Data points enclosed with a diamond are reference sources

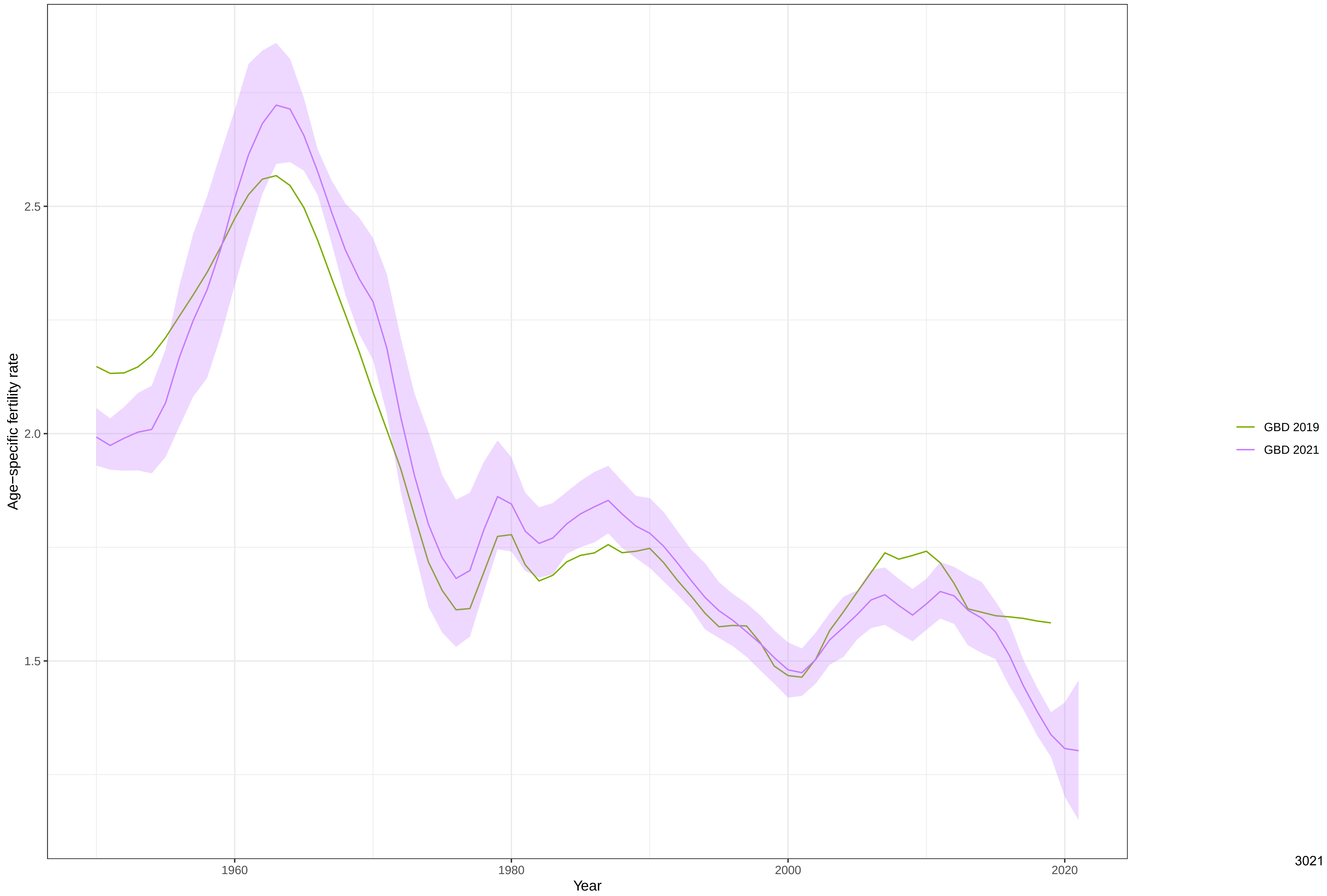

Age−specific fertility fitted trend lines, 1950−2021: Reading, Ages: 10−14
Data points enclosed with a diamond are reference sources

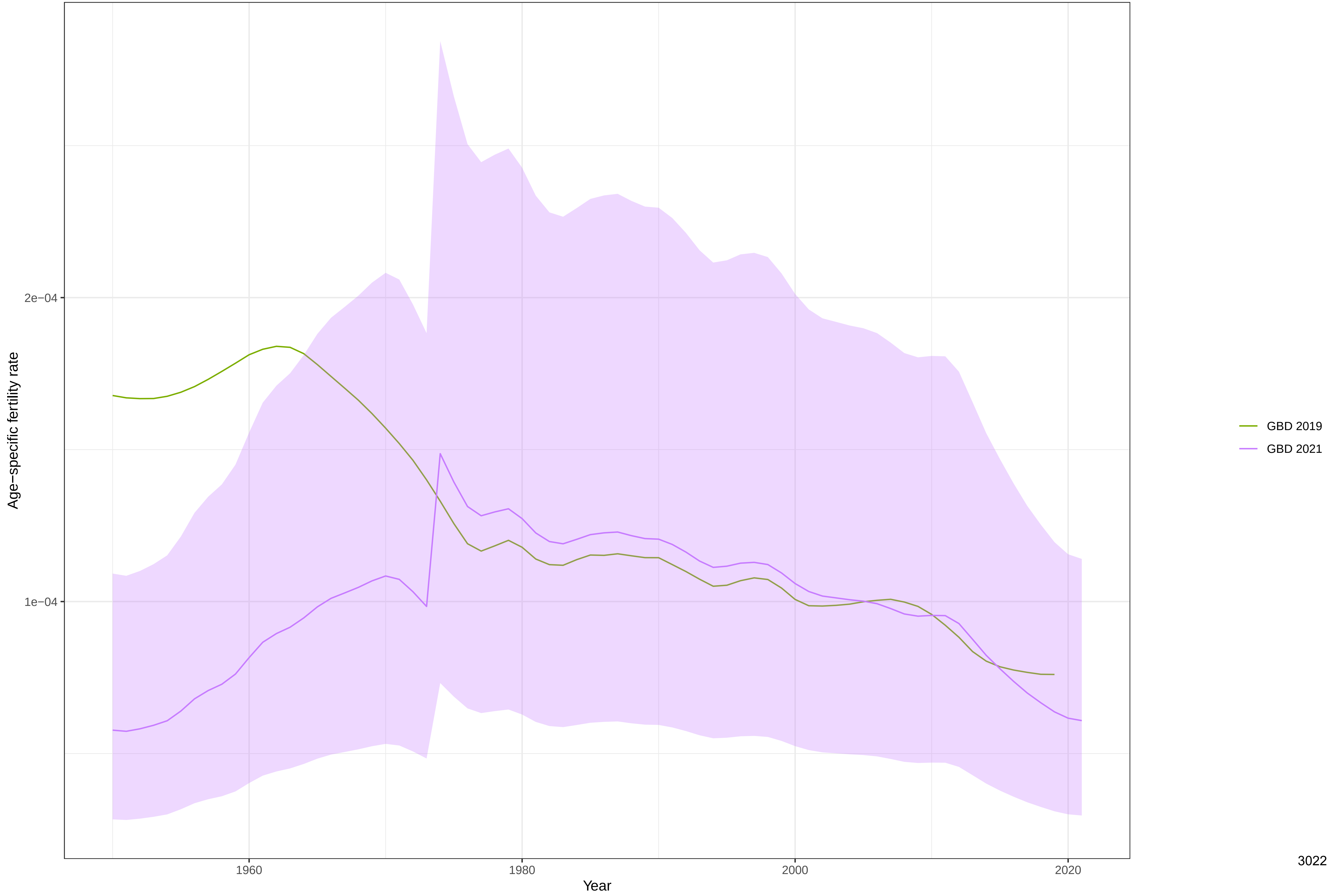

Age−specific fertility fitted trend lines, 1950−2021: Reading, Ages: 15−19
Data points enclosed with a diamond are reference sources

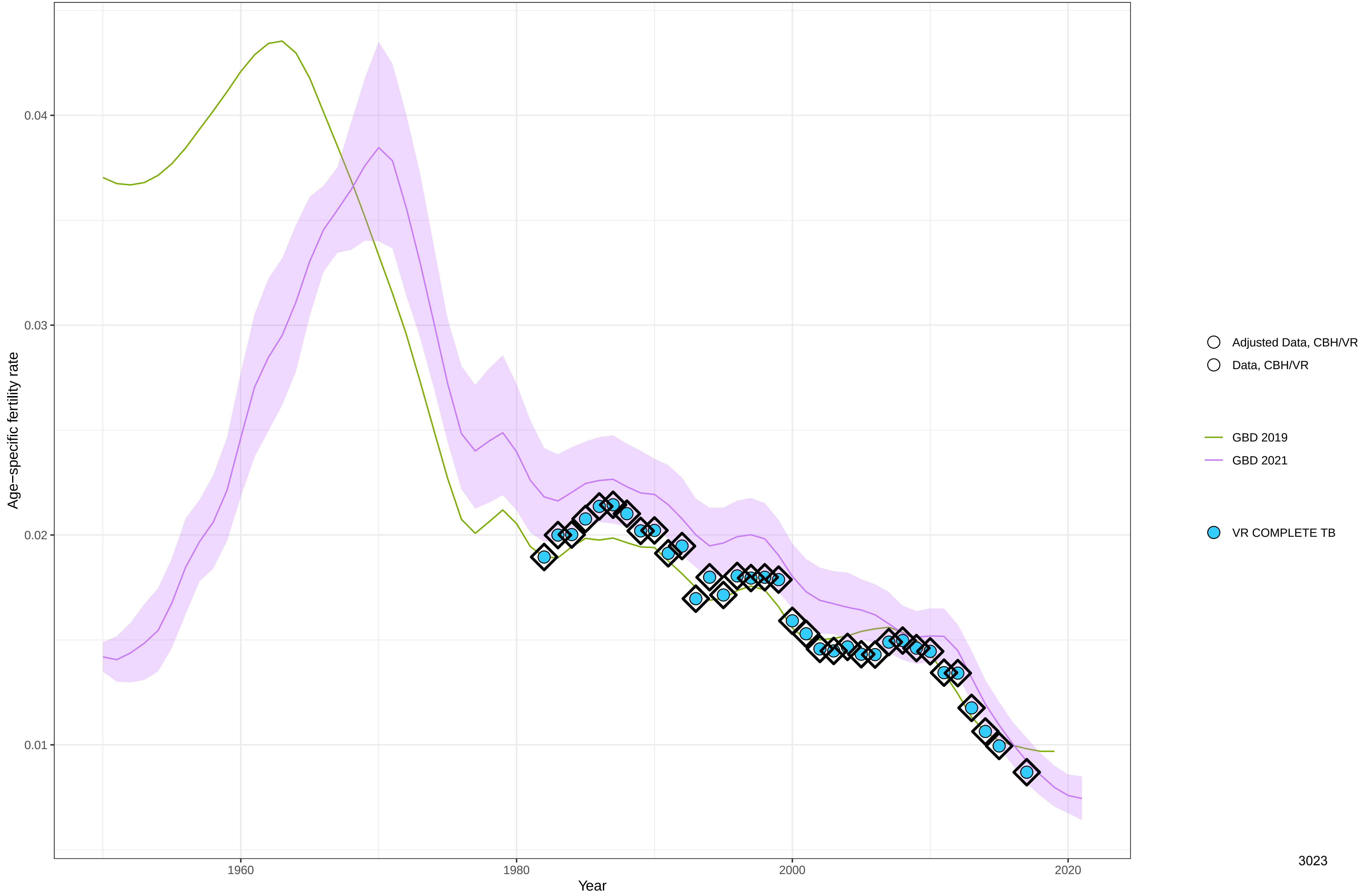

Age−specific fertility fitted trend lines, 1950−2021: Reading, Ages: 20−24
Data points enclosed with a diamond are reference sources

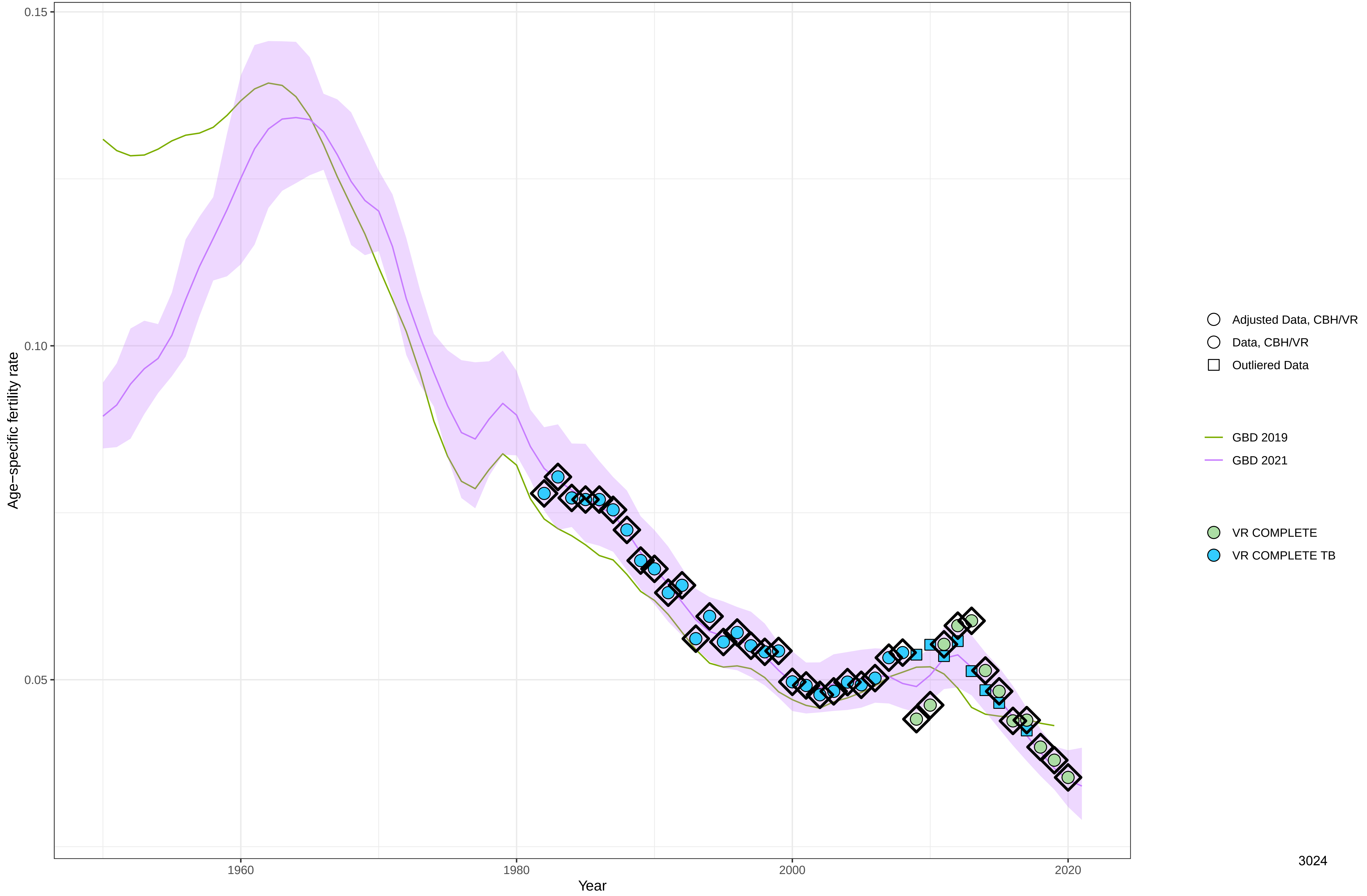

Age−specific fertility fitted trend lines, 1950−2021: Reading, Ages: 25−29
 Data points enclosed with a diamond are reference sources

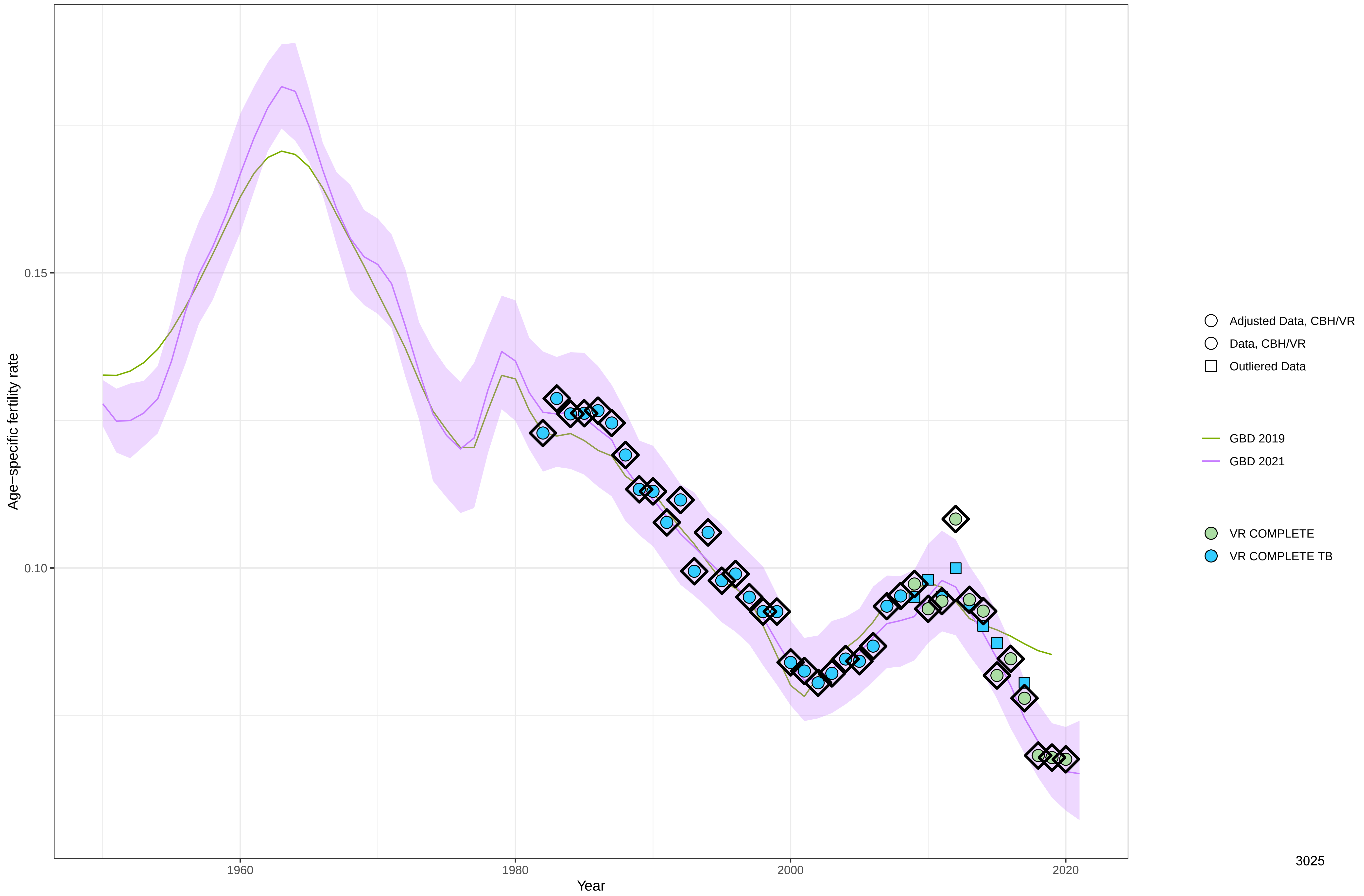

Age−specific fertility fitted trend lines, 1950−2021: Reading, Ages: 30−34
Data points enclosed with a diamond are reference sources

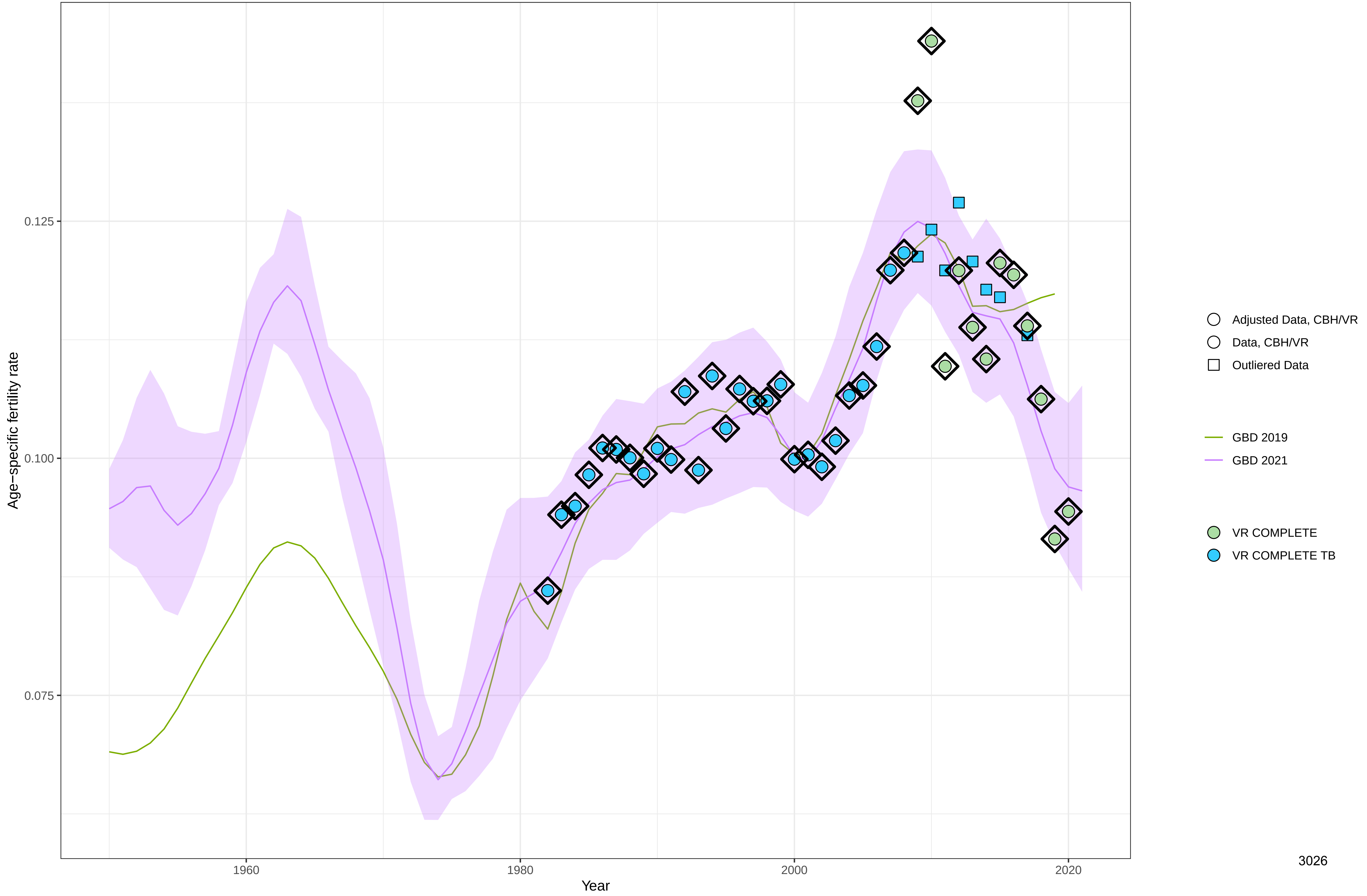

Age−specific fertility fitted trend lines, 1950−2021: Reading, Ages: 35−39
Data points enclosed with a diamond are reference sources

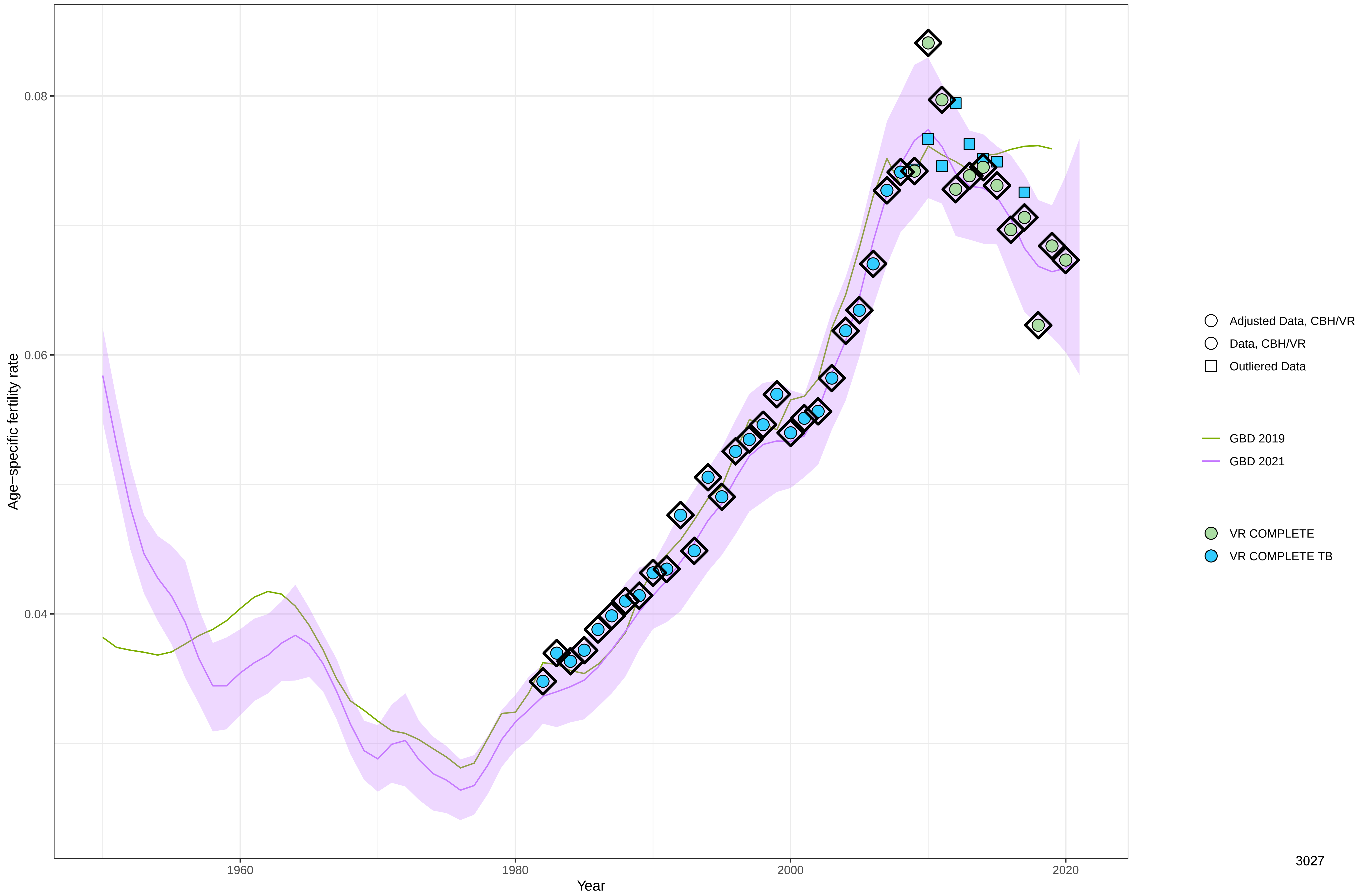

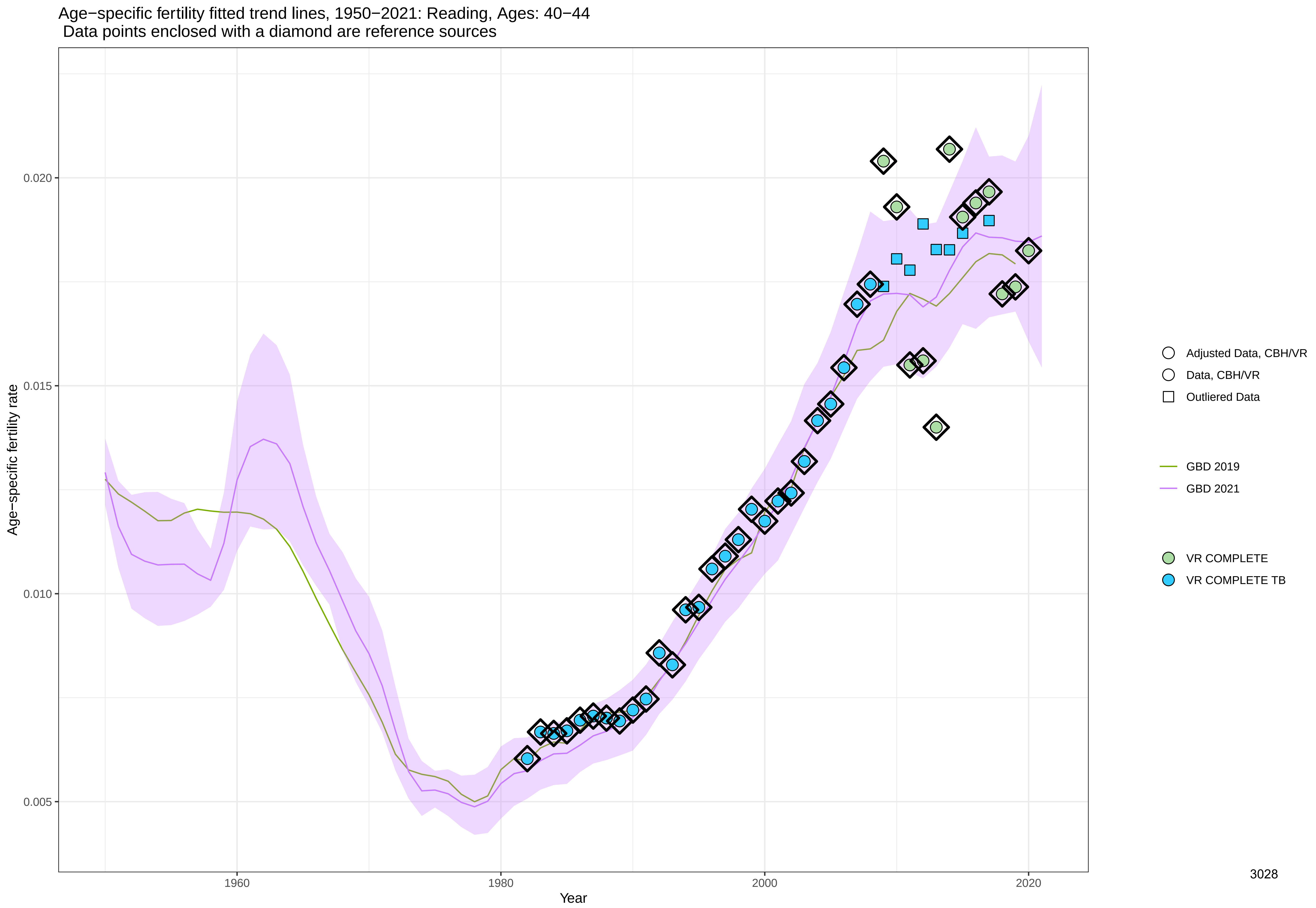

Age−specific fertility fitted trend lines, 1950−2021: Reading, Ages: 40−44
Data points enclosed with a diamond are reference sources
Age−specific fertility rate
0.020
0.015
0.010
0.005
1960
1980
2000
2020
Year
Adjusted Data, CBH/VR
Data, CBH/VR
Outliered Data
GBD 2019
GBD 2021
VR COMPLETE
VR COMPLETE TB

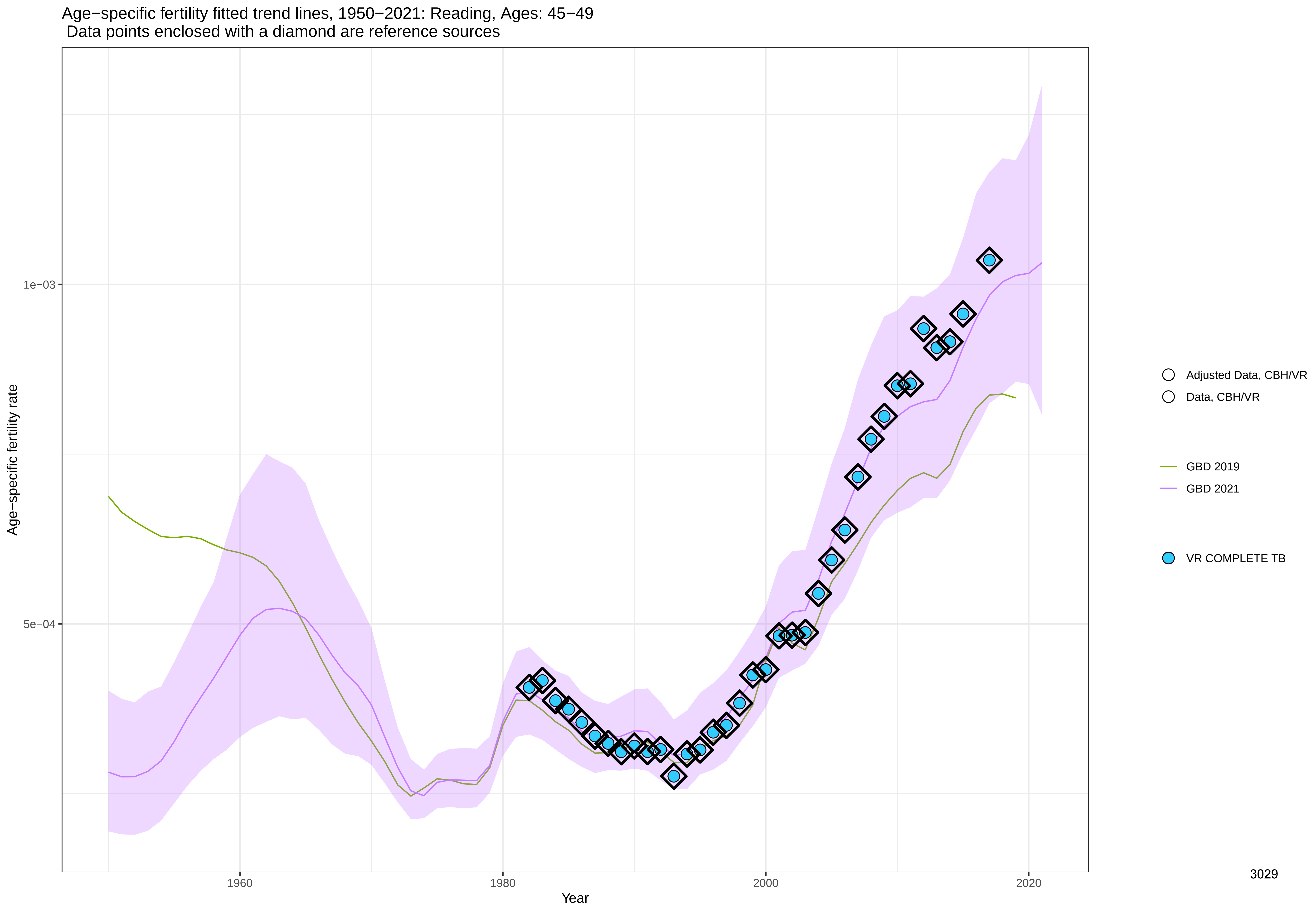
Age−specific fertility fitted trend lines, 1950−2021: Reading, Ages: 45−49
Data points enclosed with a diamond are reference sources
Age−specific fertility rate
1e−03
5e−04
1960
1980
2000
2020
Year
Adjusted Data, CBH/VR
Data, CBH/VR
GBD 2019
GBD 2021
VR COMPLETE TB

Age−specific fertility fitted trend lines, 1950−2021: Reading, Ages: 50−54
Data points enclosed with a diamond are reference sources

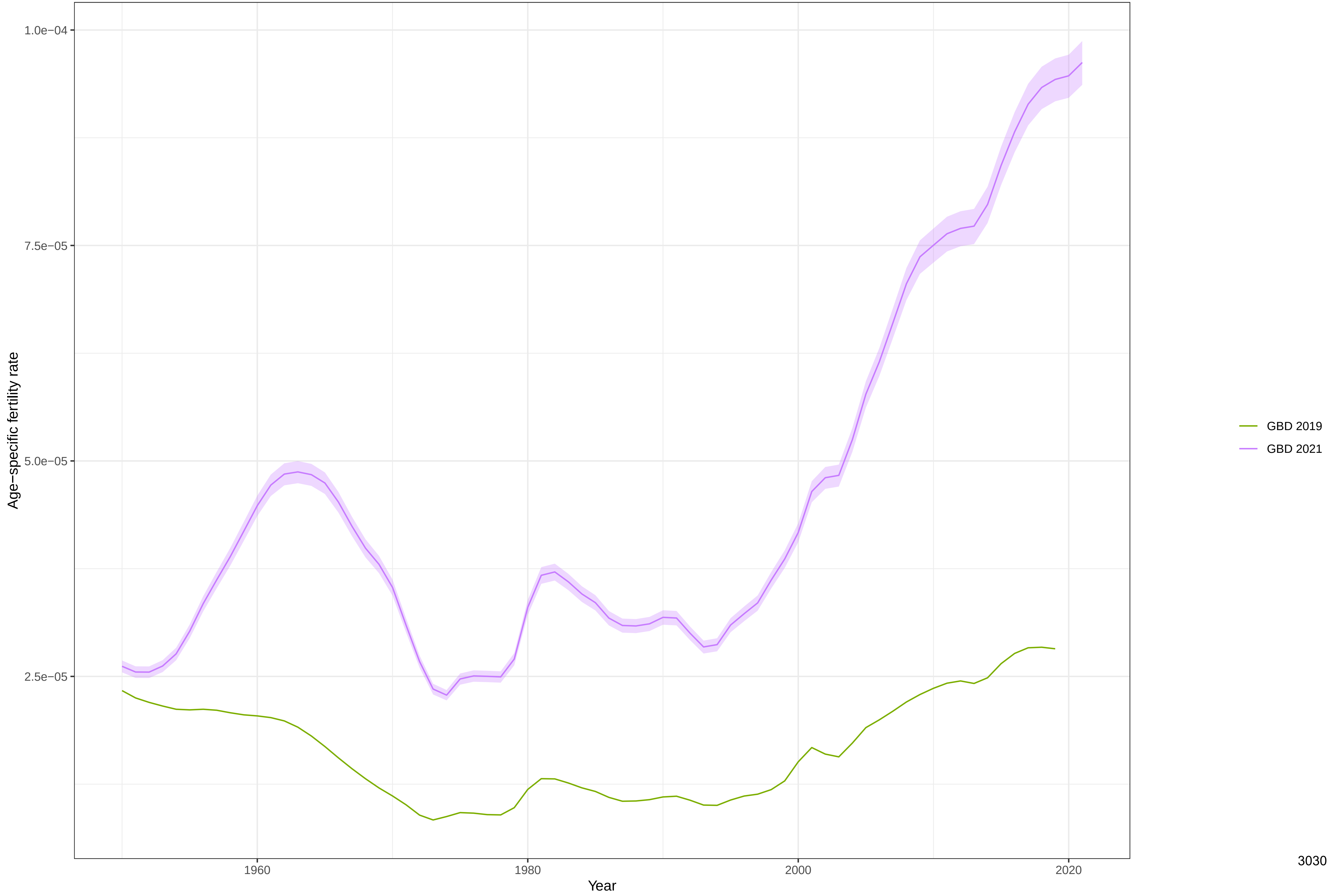

Total fertility rate fitted trend lines, 1950−2021: Reading TFR
Data points enclosed with a diamond are reference sources

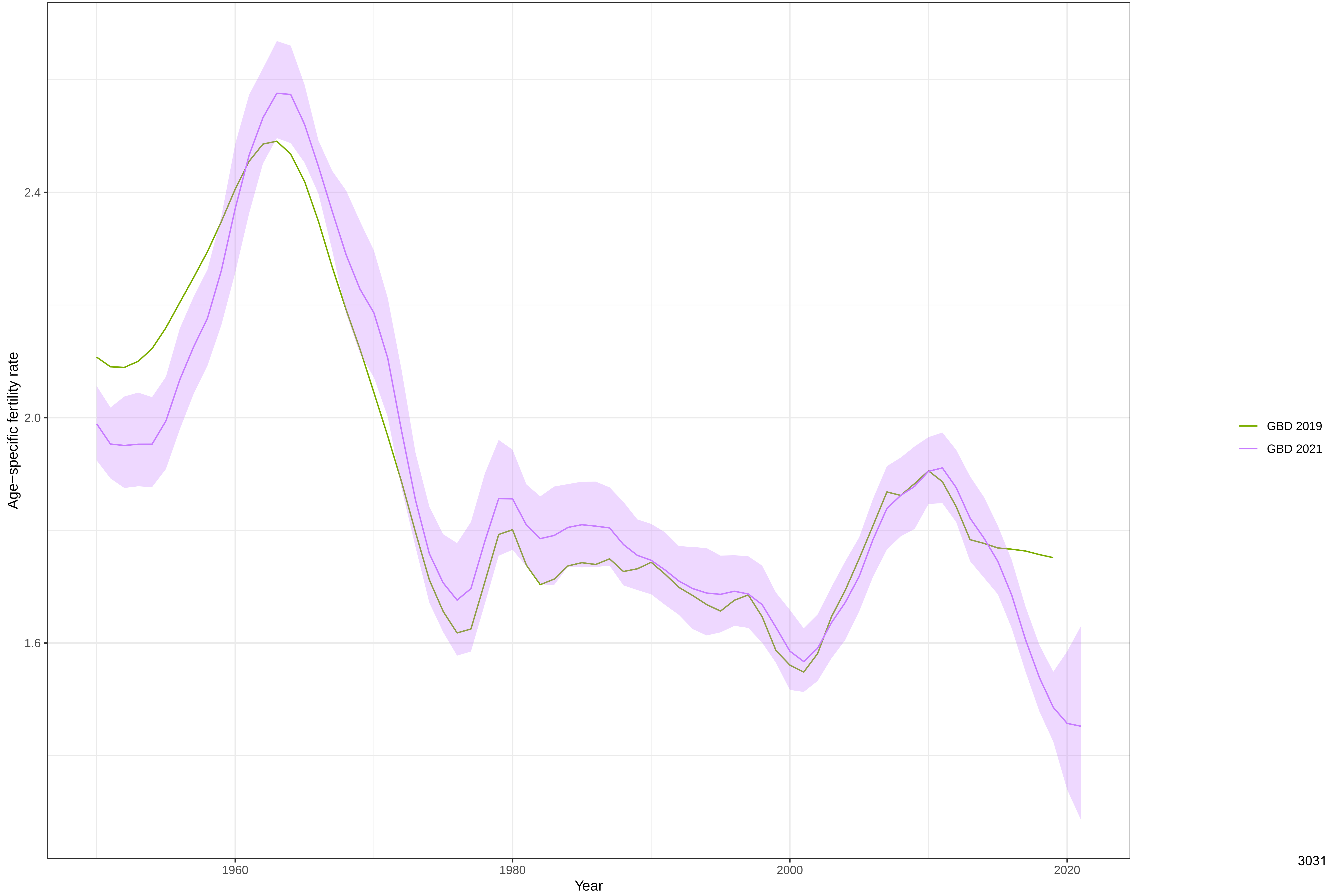

Age−specific fertility fitted trend lines, 1950−2021: Slough, Ages: 10−14
Data points enclosed with a diamond are reference sources

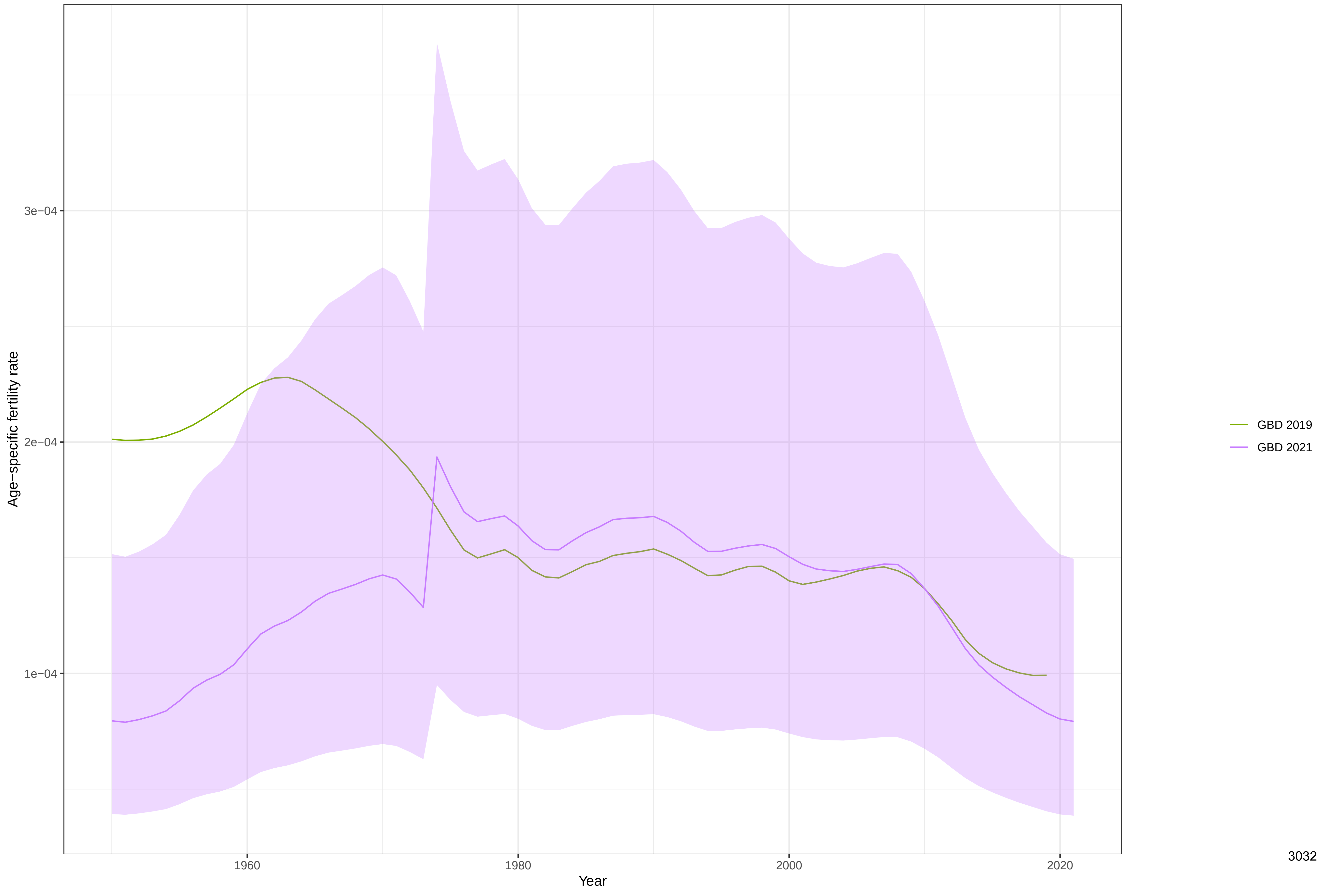

3032

Age−specific fertility fitted trend lines, 1950−2021: Slough, Ages: 15−19
Data points enclosed with a diamond are reference sources

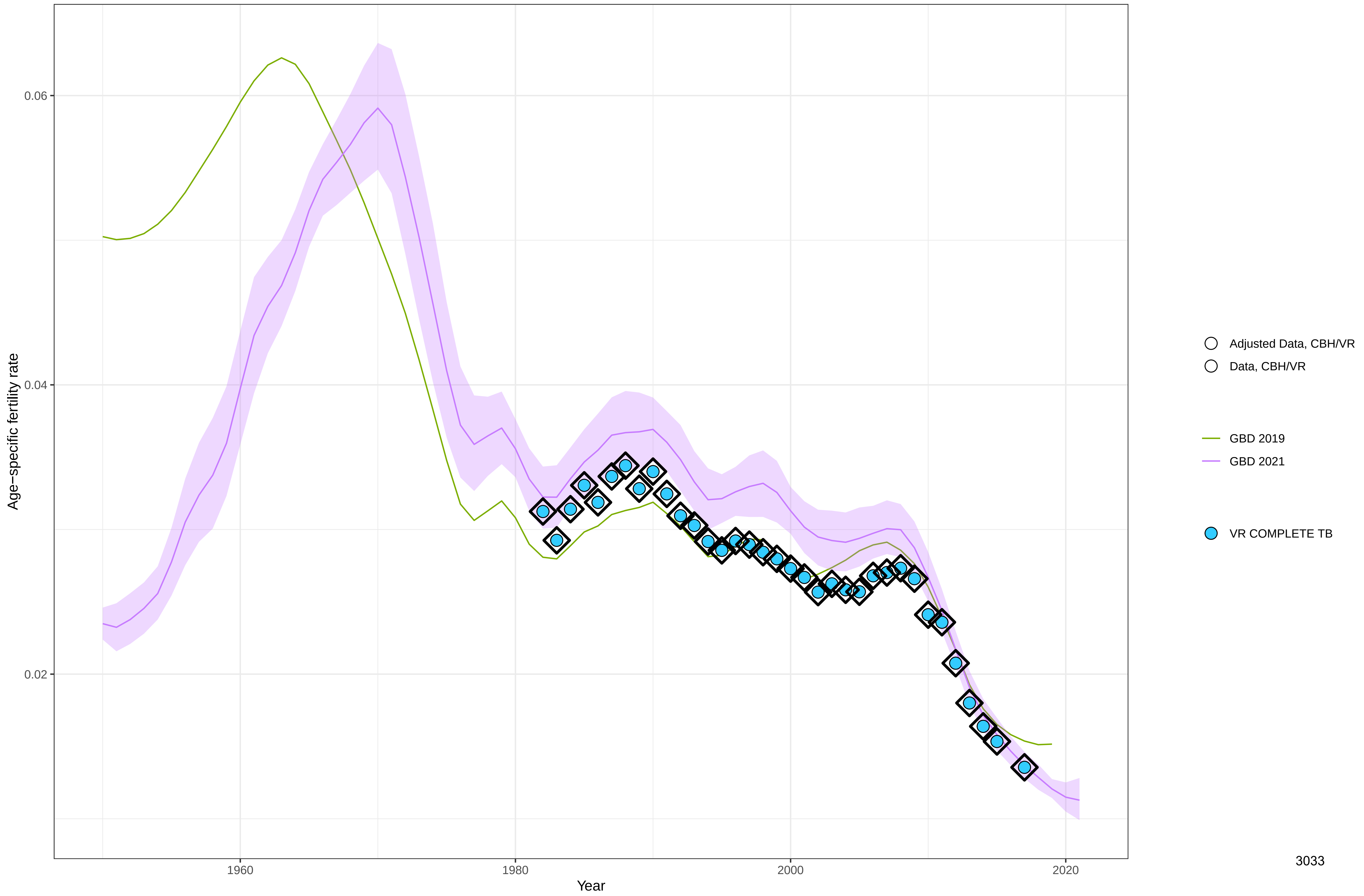

Age−specific fertility fitted trend lines, 1950−2021: Slough, Ages: 20−24
Data points enclosed with a diamond are reference sources

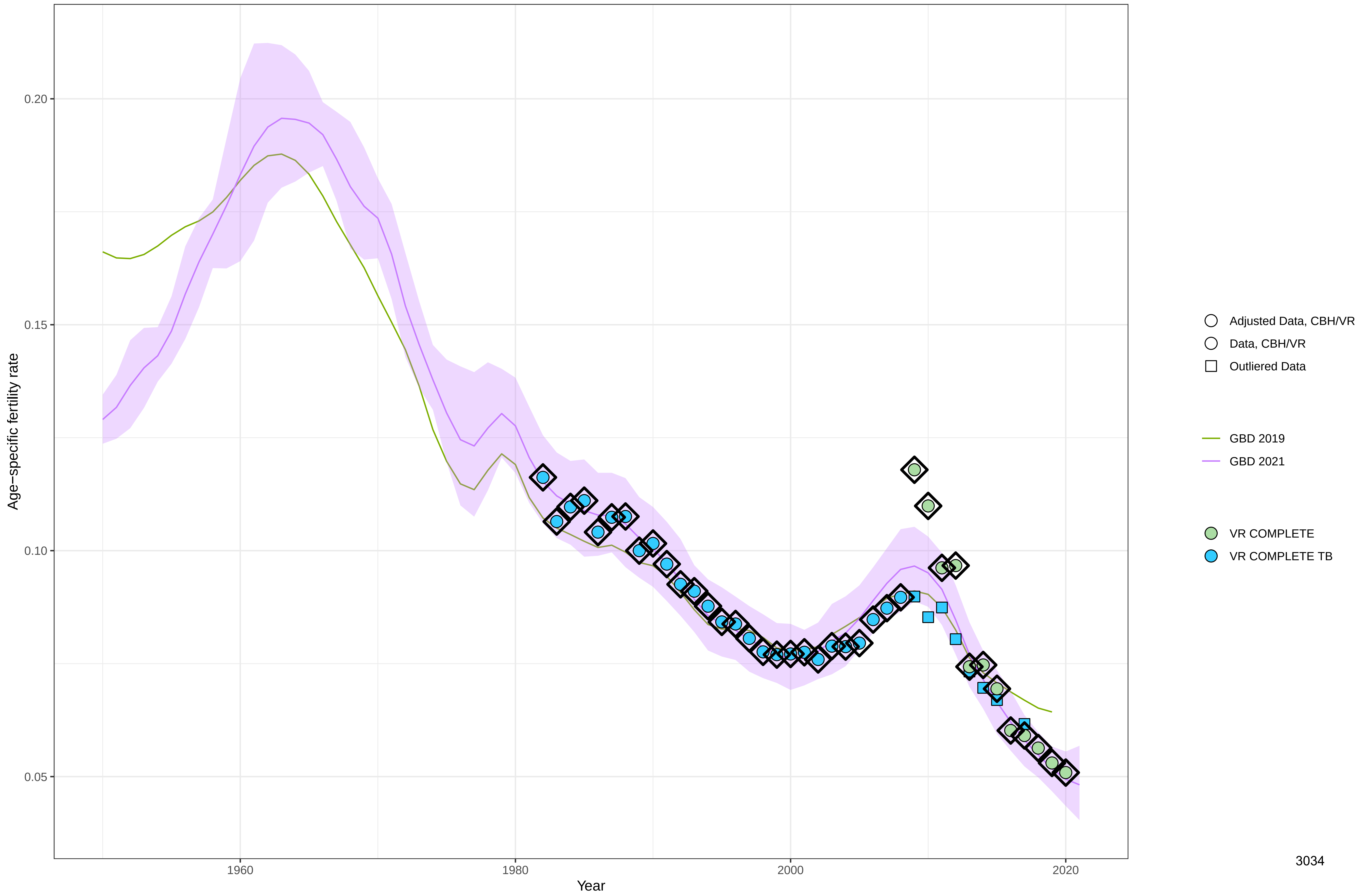

Age−specific fertility fitted trend lines, 1950−2021: Slough, Ages: 25−29
Data points enclosed with a diamond are reference sources

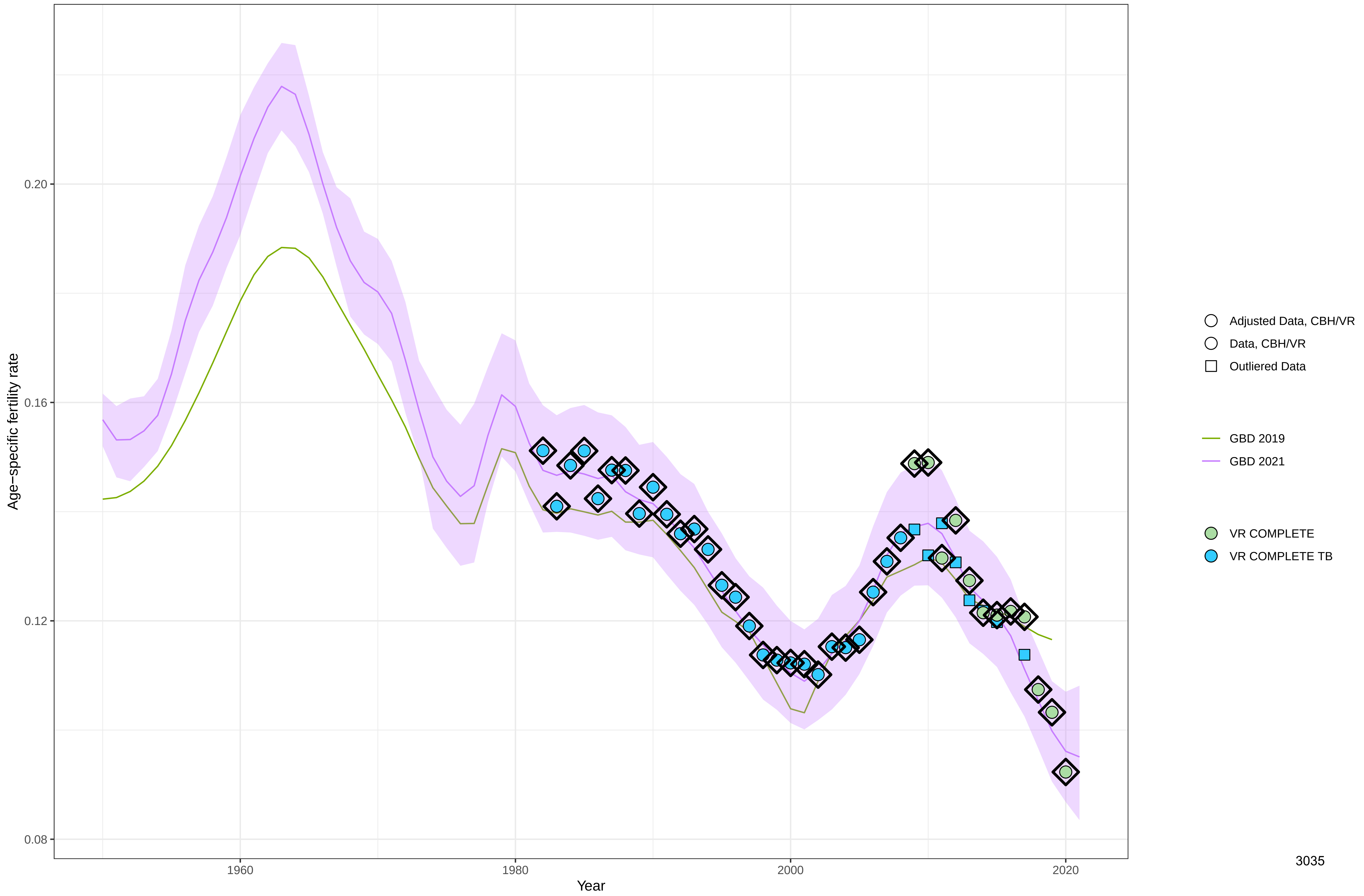

Age−specific fertility fitted trend lines, 1950−2021: Slough, Ages: 30−34
Data points enclosed with a diamond are reference sources

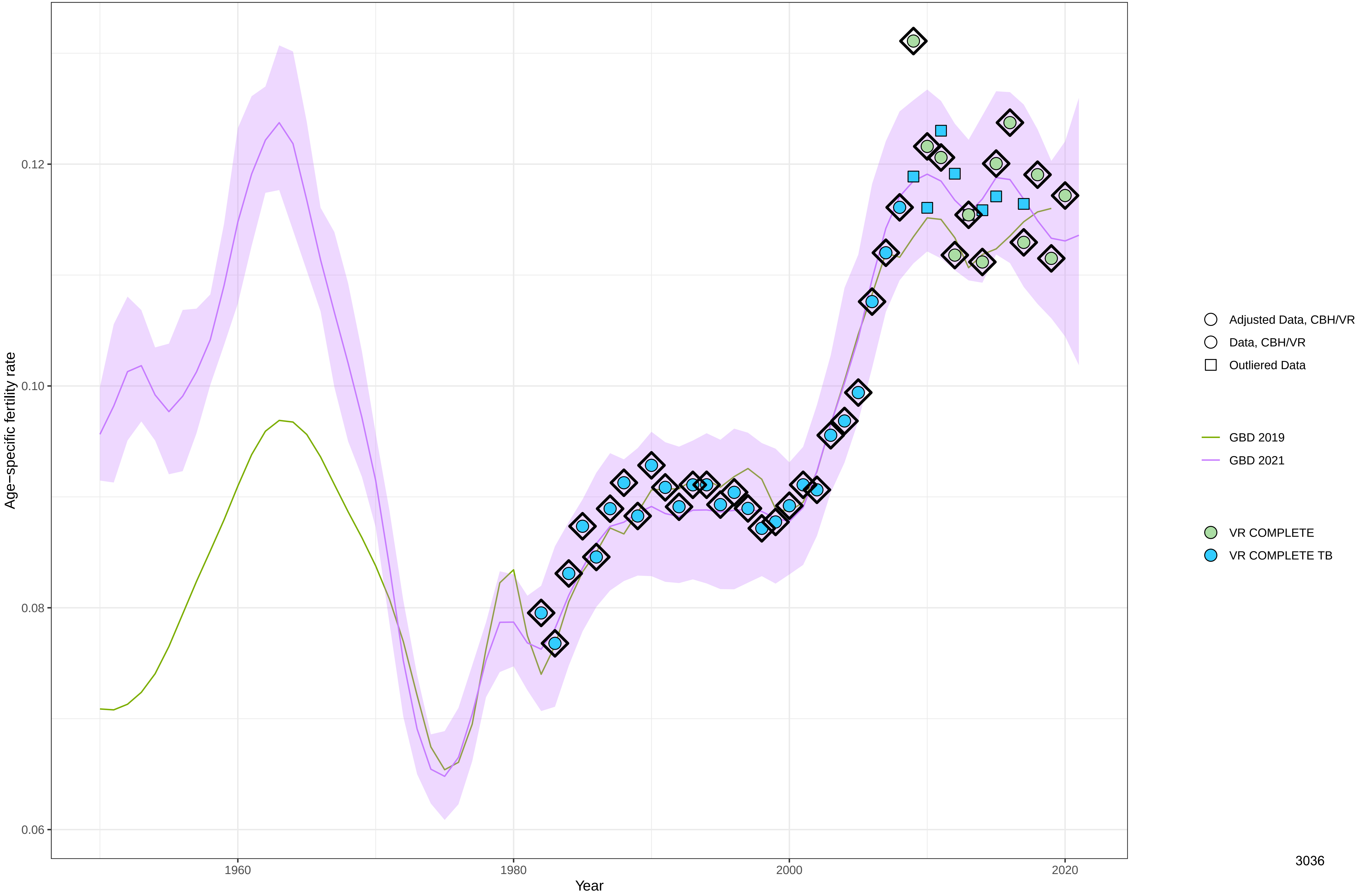

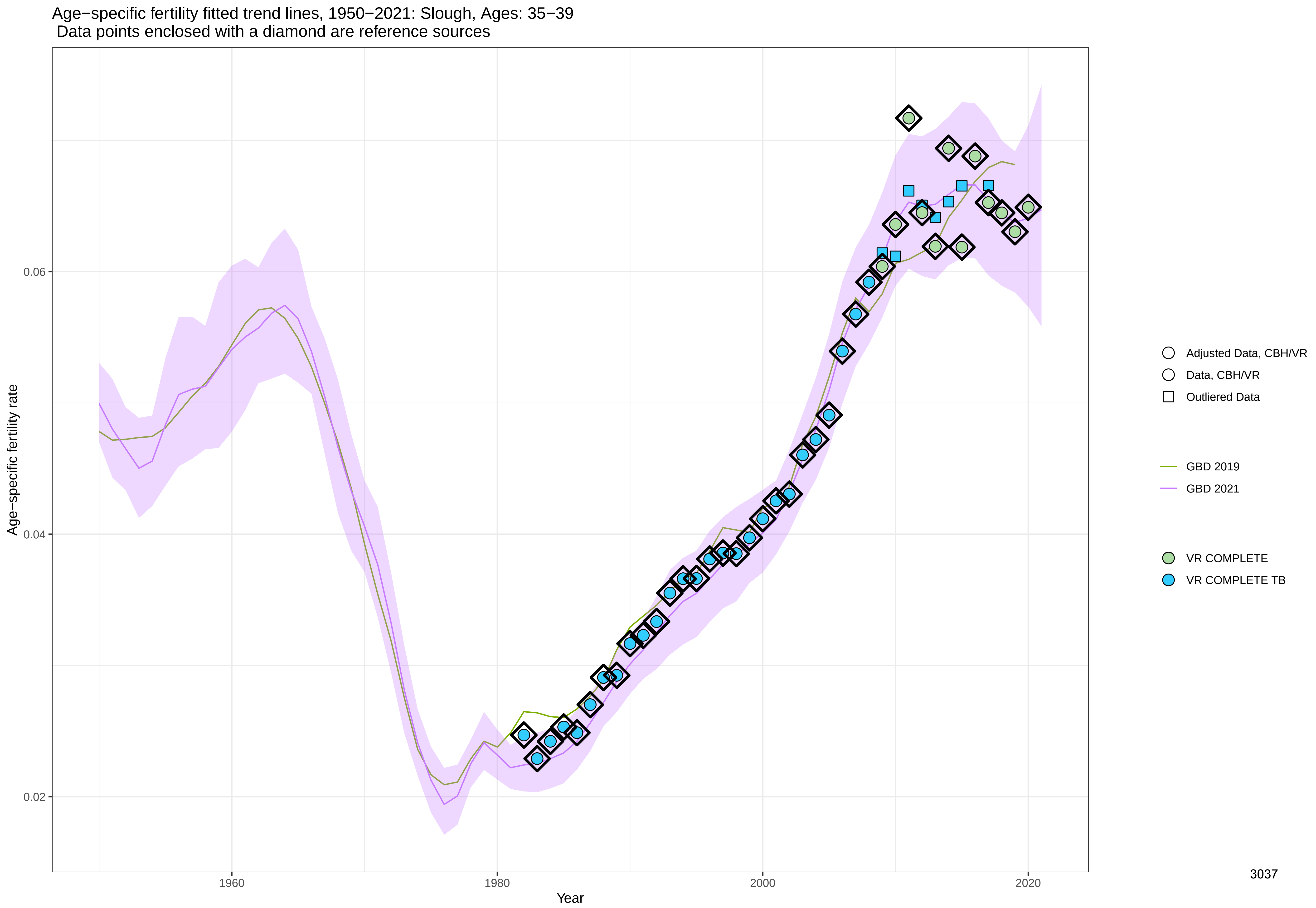
Age−specific fertility fitted trend lines, 1950−2021: Slough, Ages: 35−39
Data points enclosed with a diamond are reference sources
Age−specific fertility rate
0.06
0.04
0.02
1960
1980
2000
2020
Year
Adjusted Data, CBH/VR
Data, CBH/VR
Outliered Data
GBD 2019
GBD 2021
VR COMPLETE
VR COMPLETE TB

Age−specific fertility fitted trend lines, 1950−2021: Slough, Ages: 40−44
Data points enclosed with a diamond are reference sources

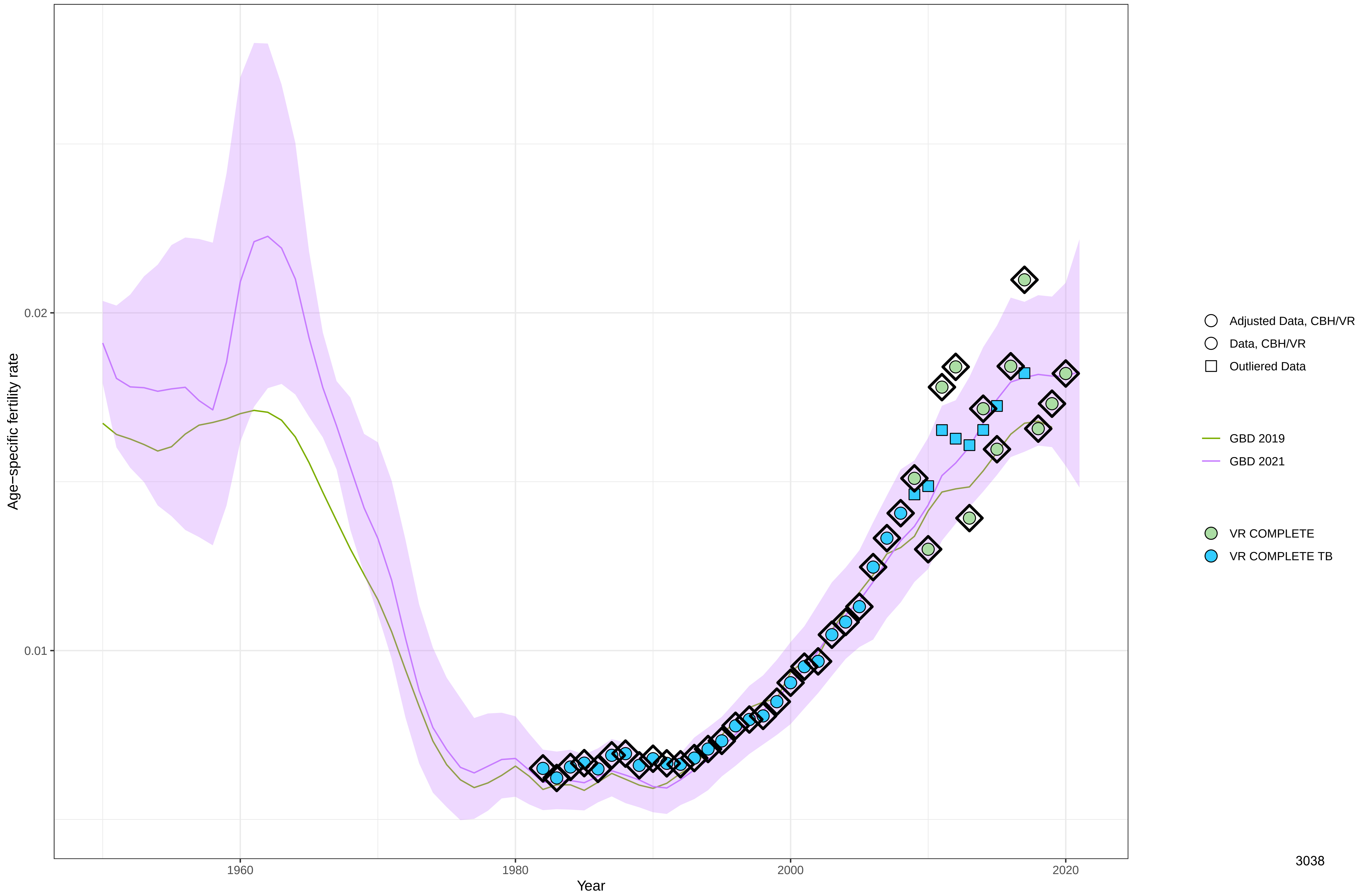

Age−specific fertility fitted trend lines, 1950−2021: Slough, Ages: 45−49
Data points enclosed with a diamond are reference sources

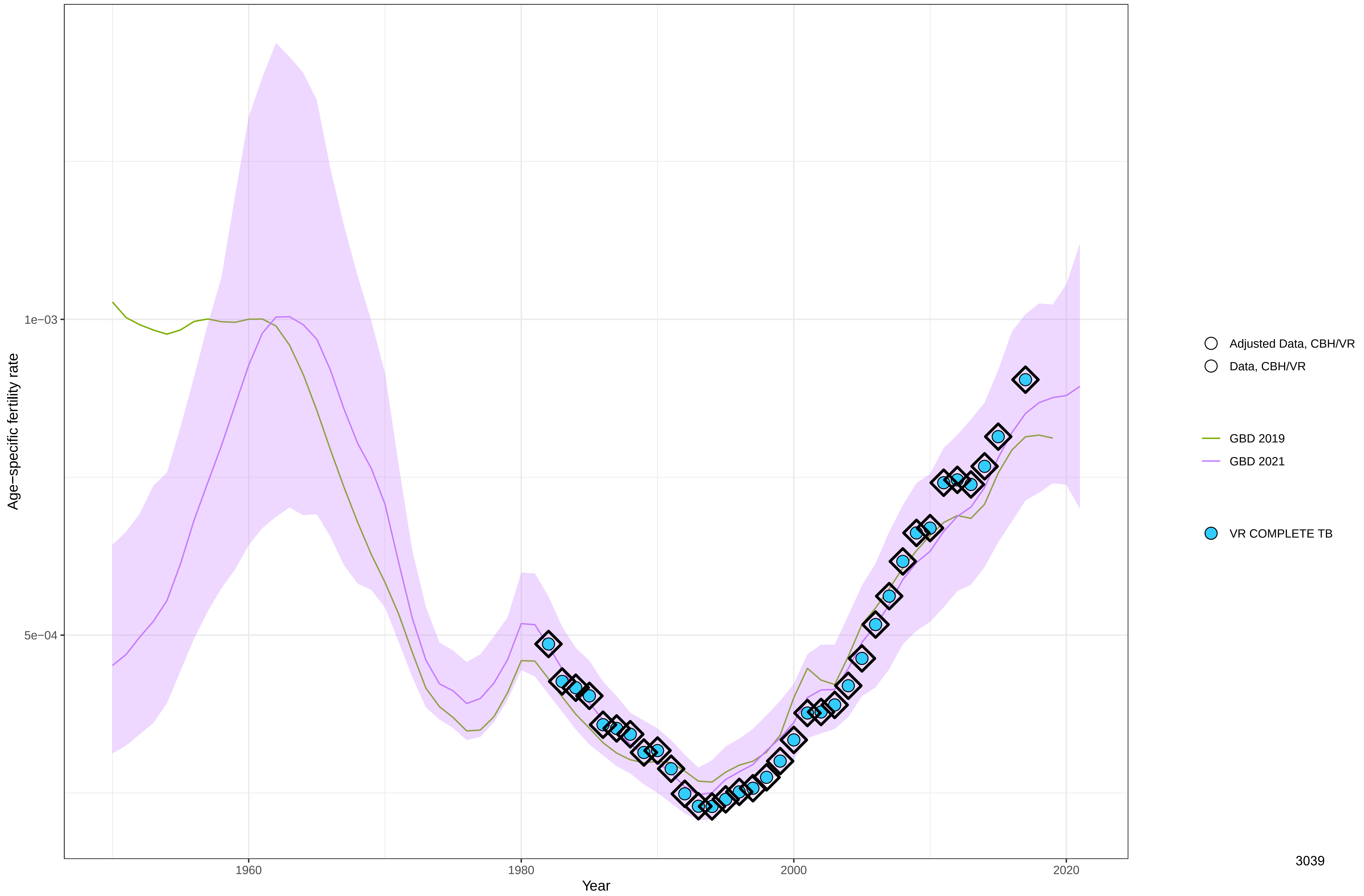

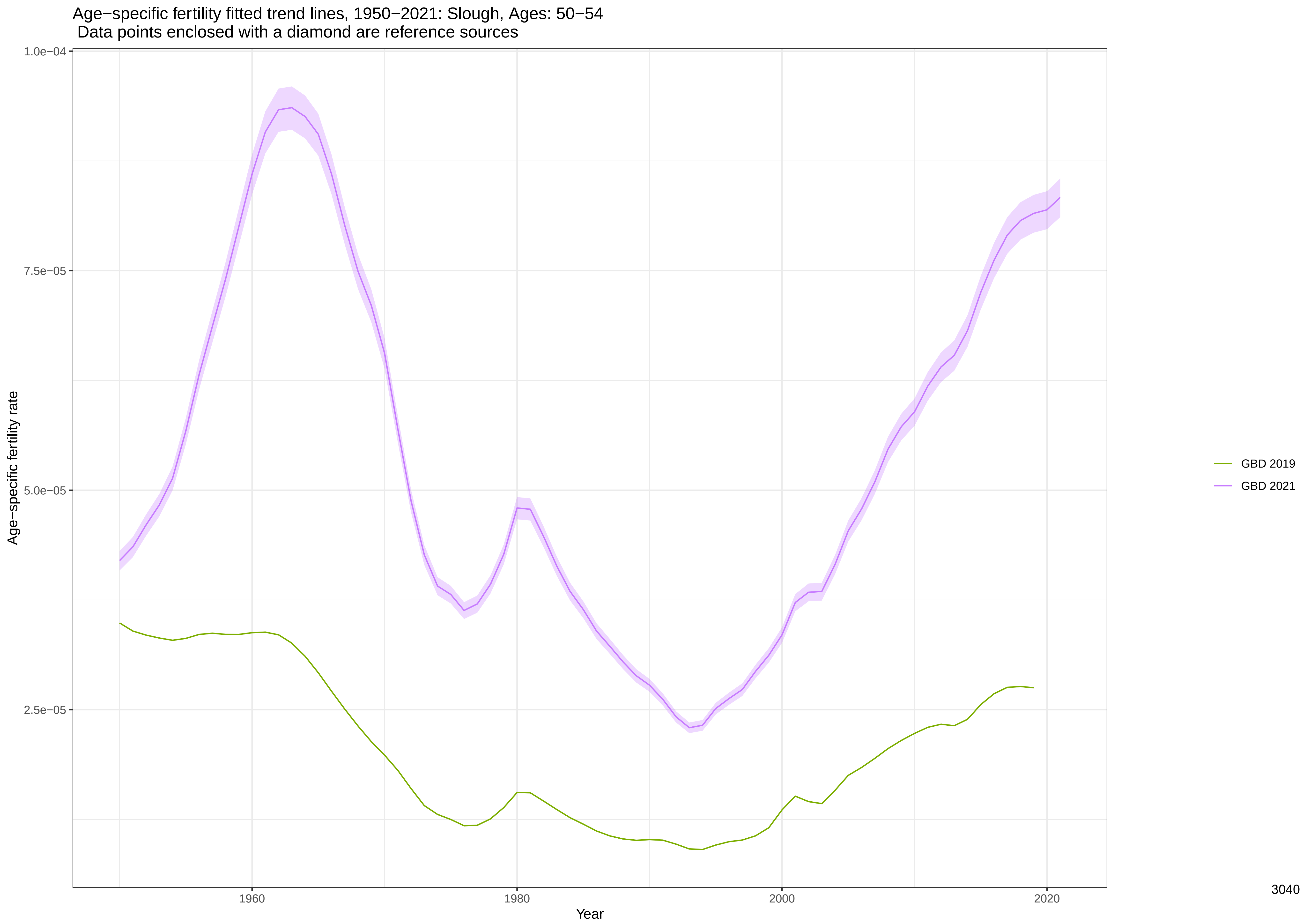

Age−specific fertility fitted trend lines, 1950−2021: Slough, Ages: 50−54
Data points enclosed with a diamond are reference sources
1.0e−04
7.5e−05
5.0e−05
2.5e−05
Age−specific fertility rate
1960
1980
2000
2020
Year
GBD 2019
GBD 2021

Total fertility rate fitted trend lines, 1950−2021: Slough TFR
Data points enclosed with a diamond are reference sources

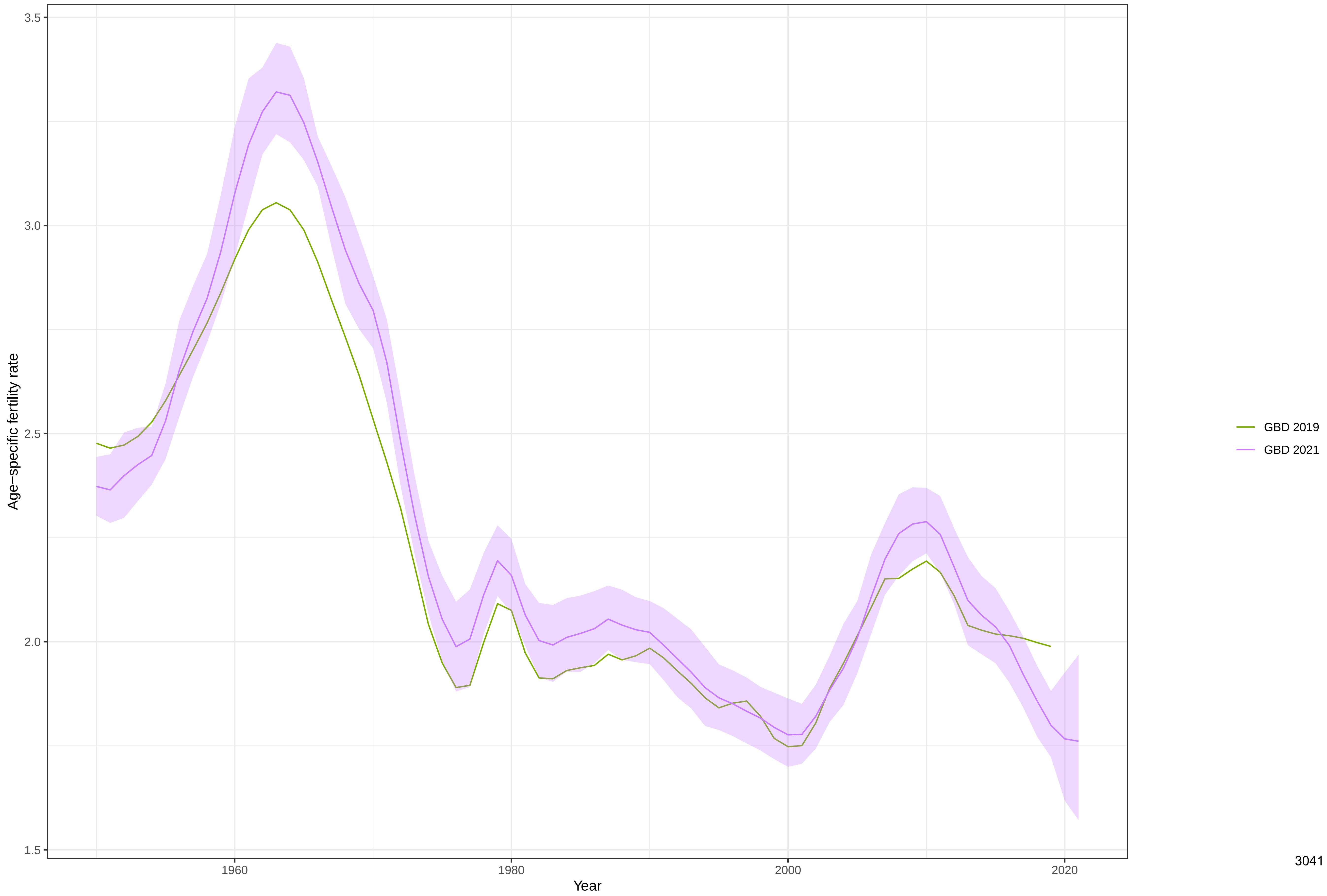

Age−specific fertility fitted trend lines, 1950−2021: Southampton, Ages: 10−14
Data points enclosed with a diamond are reference sources

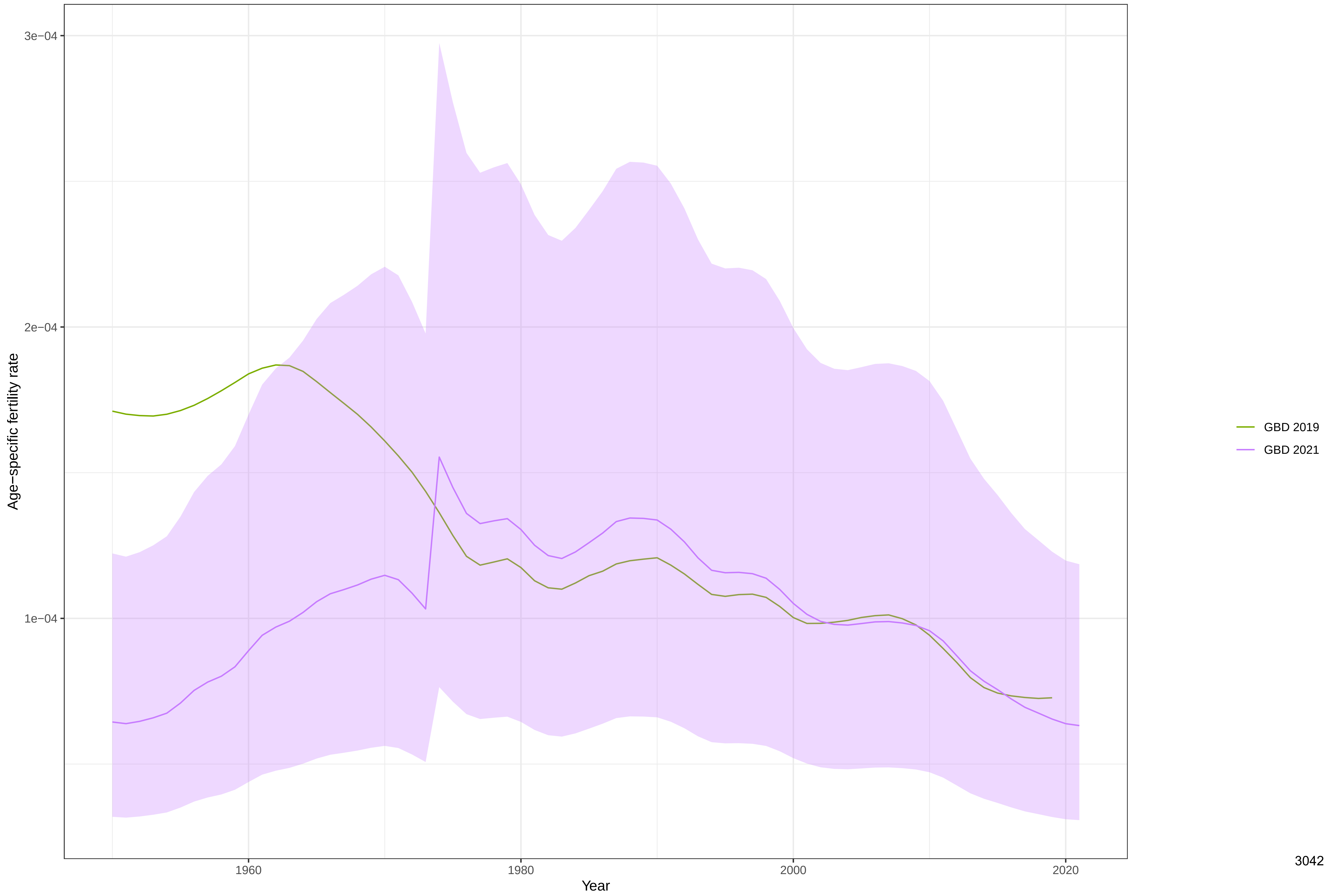

Age−specific fertility fitted trend lines, 1950−2021: Southampton, Ages: 15−19
Data points enclosed with a diamond are reference sources

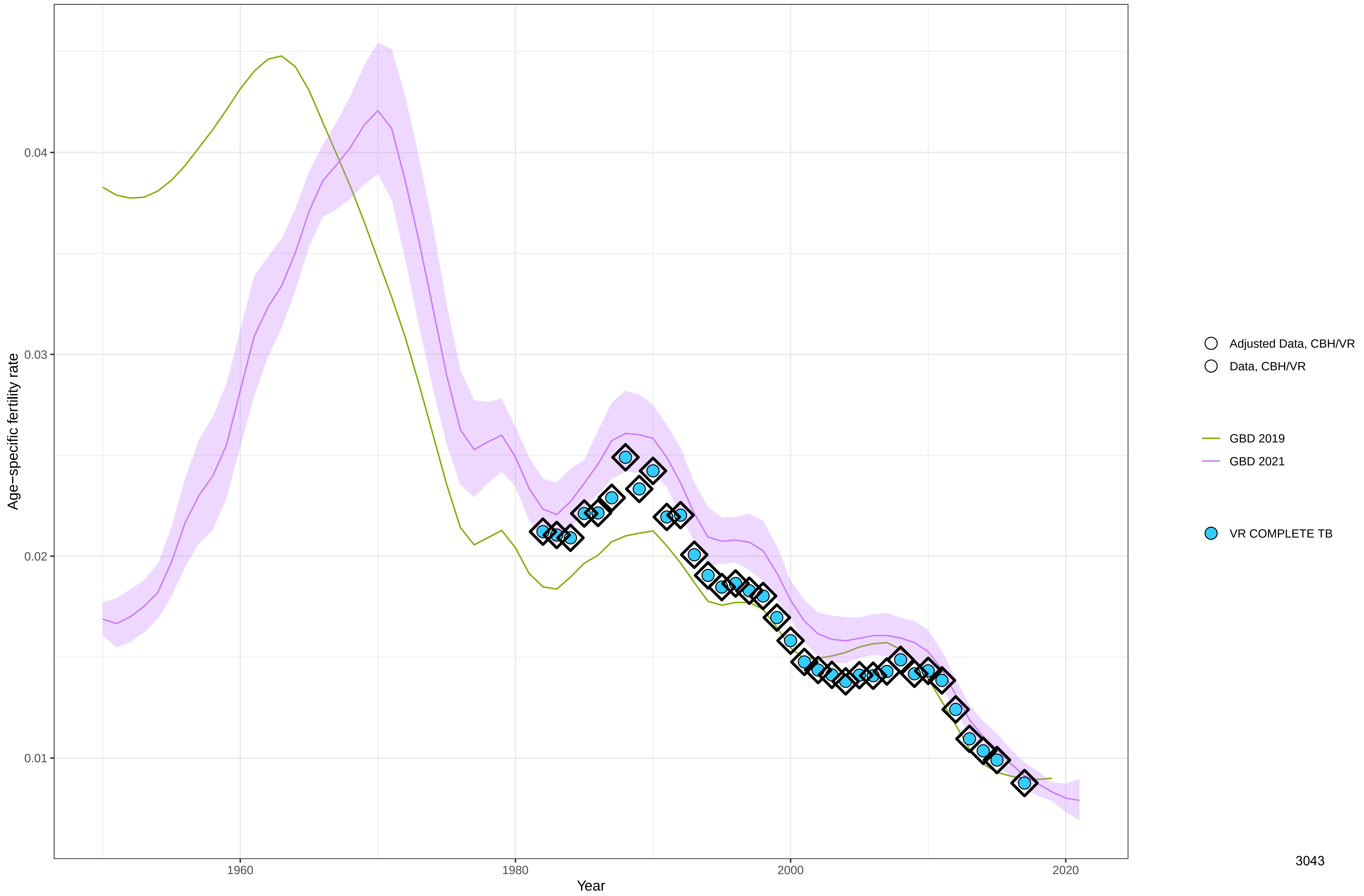

Age−specific fertility fitted trend lines, 1950−2021: Southampton, Ages: 20−24
Data points enclosed with a diamond are reference sources

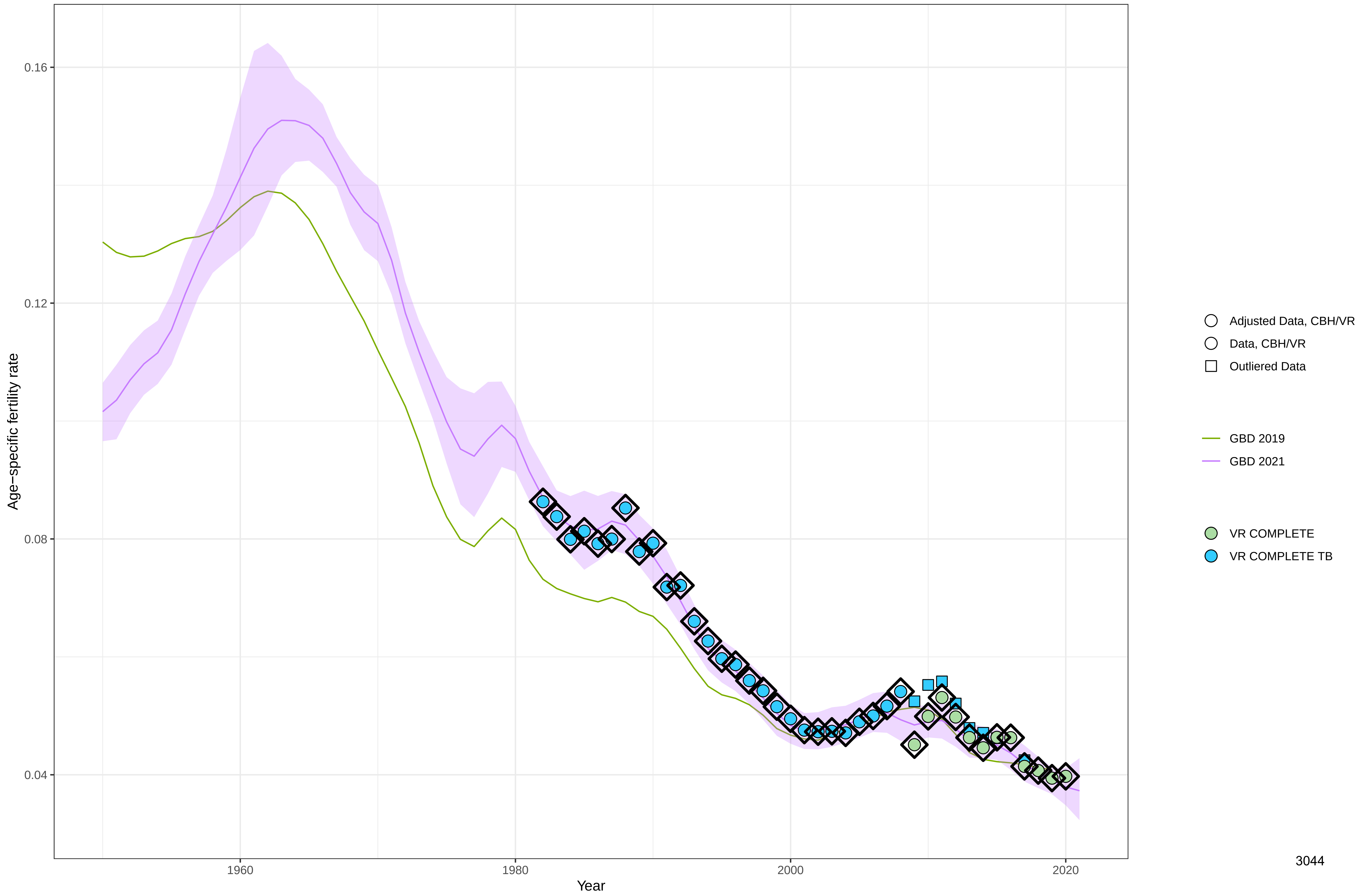

Age−specific fertility fitted trend lines, 1950−2021: Southampton, Ages: 25−29
Data points enclosed with a diamond are reference sources

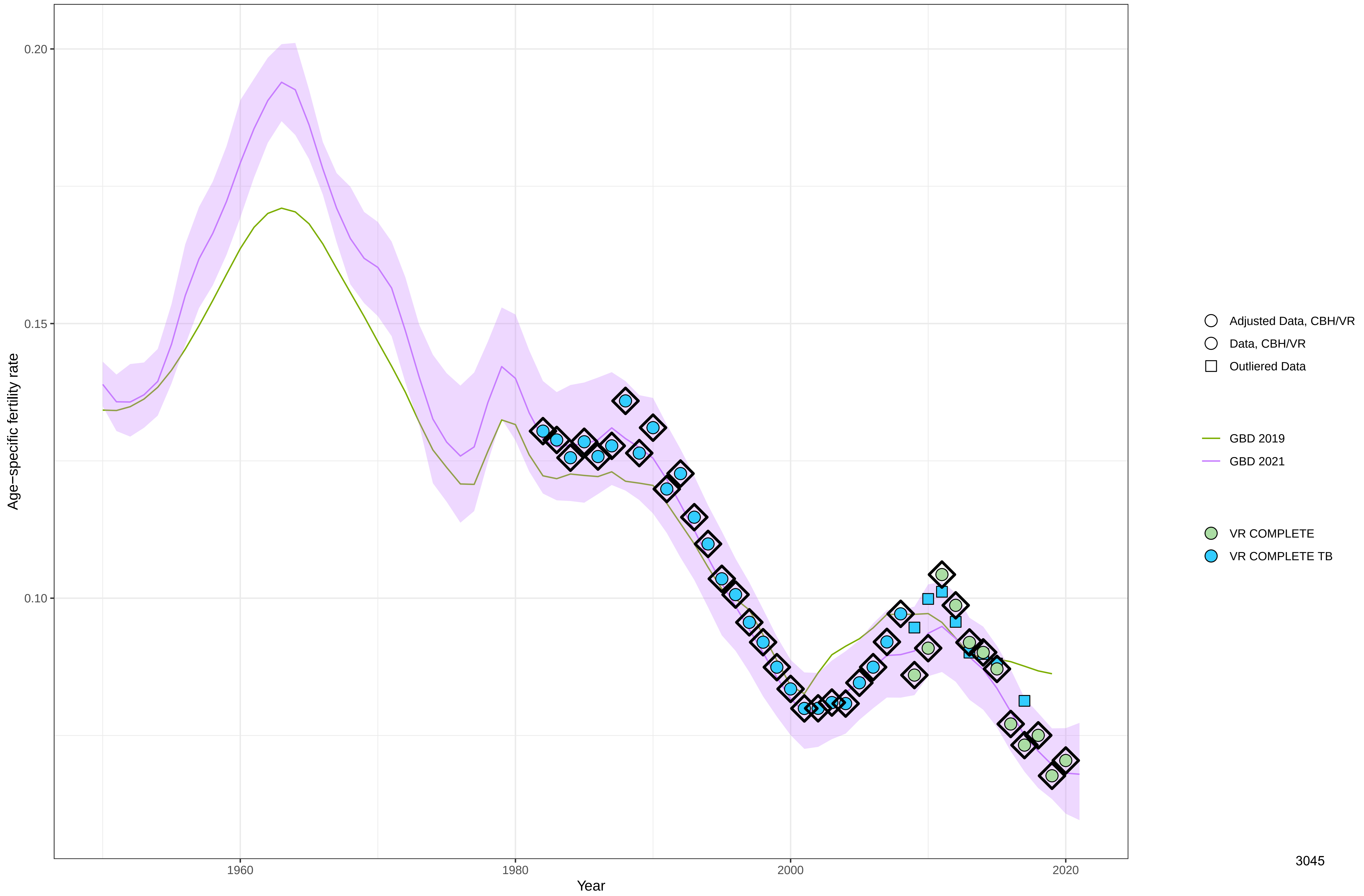

Age−specific fertility fitted trend lines, 1950−2021: Southampton, Ages: 30−34
Data points enclosed with a diamond are reference sources

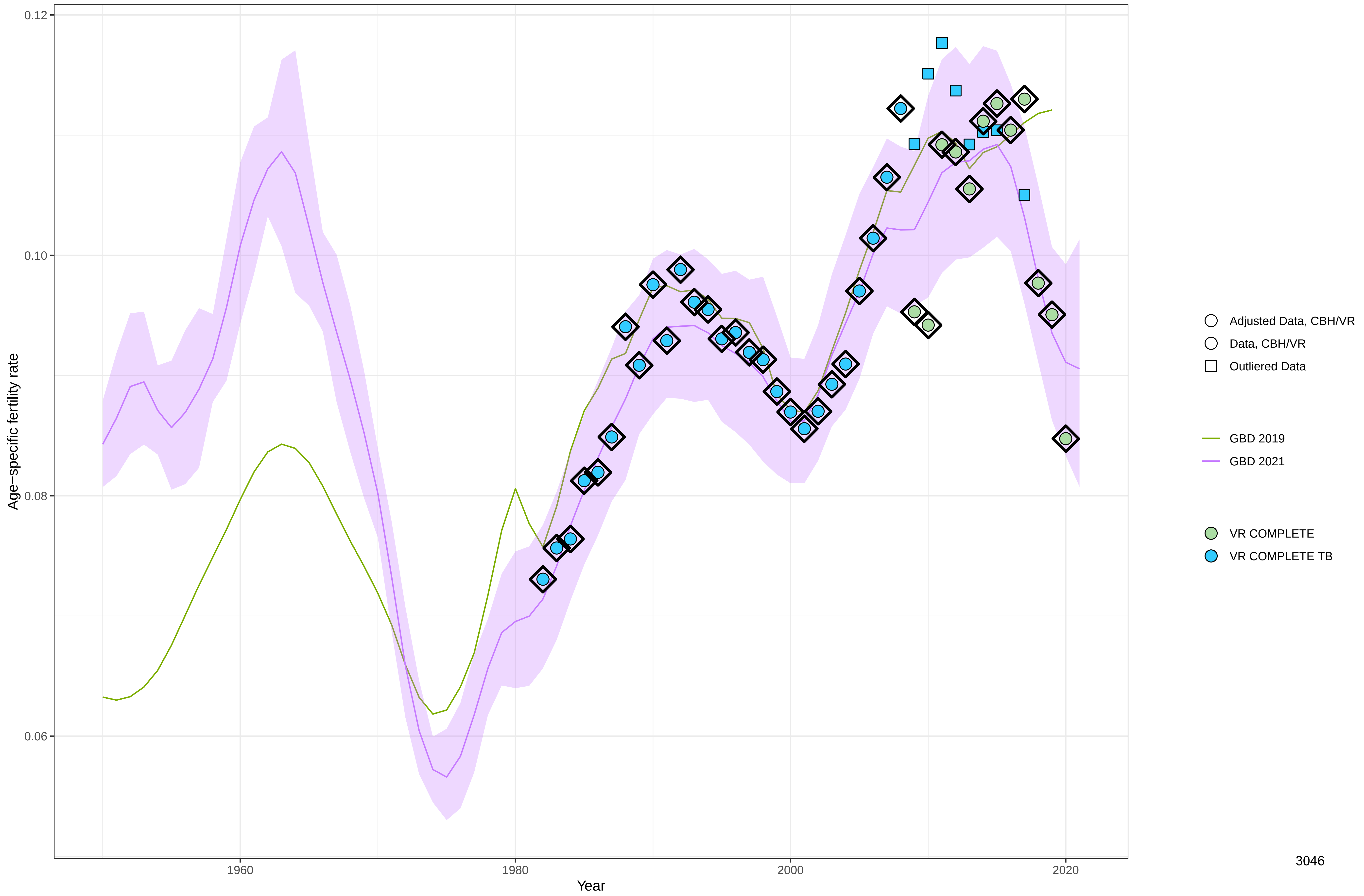

Age−specific fertility fitted trend lines, 1950−2021: Southampton, Ages: 35−39
Data points enclosed with a diamond are reference sources

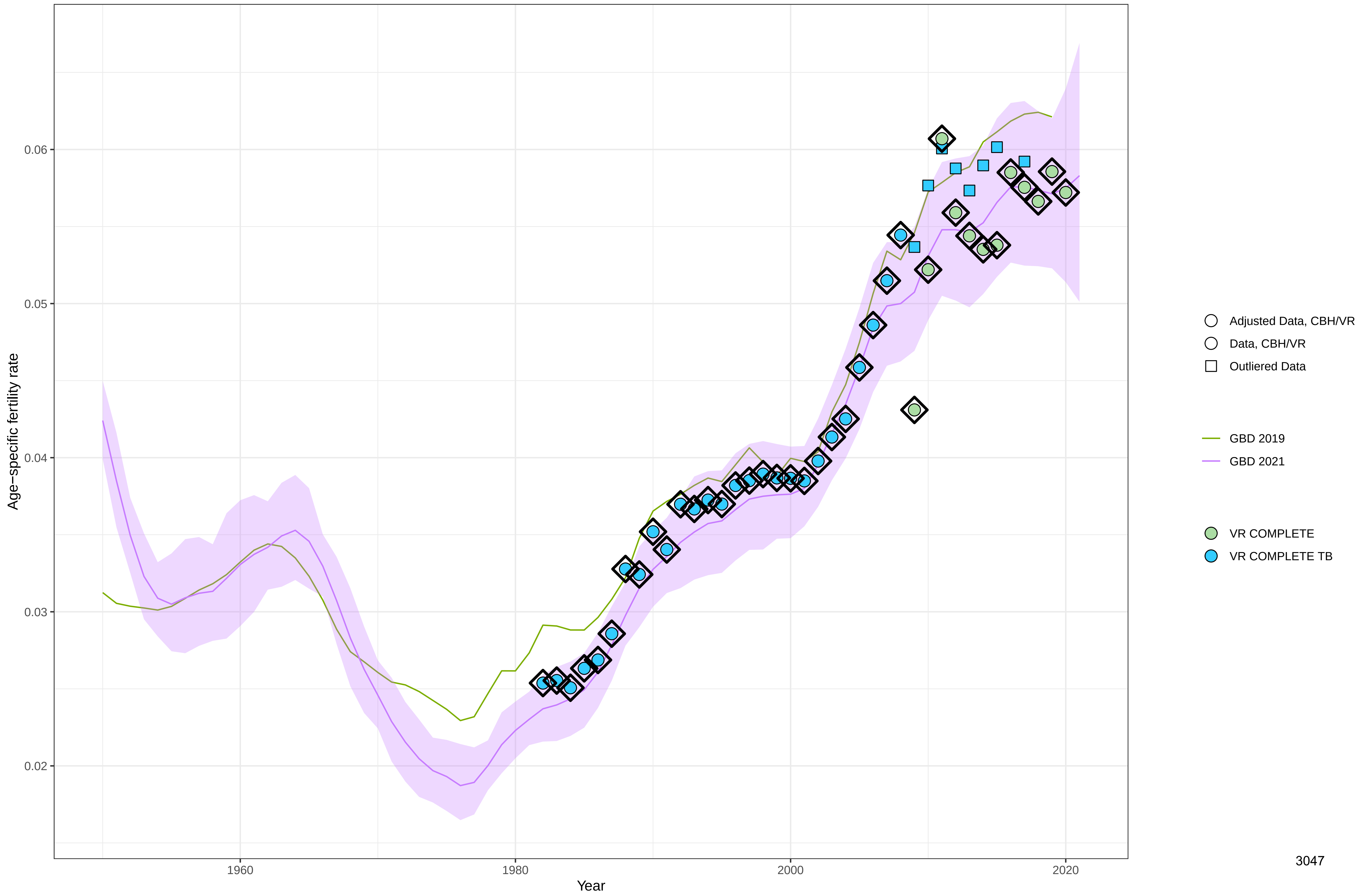

Age−specific fertility fitted trend lines, 1950−2021: Southampton, Ages: 40−44
Data points enclosed with a diamond are reference sources

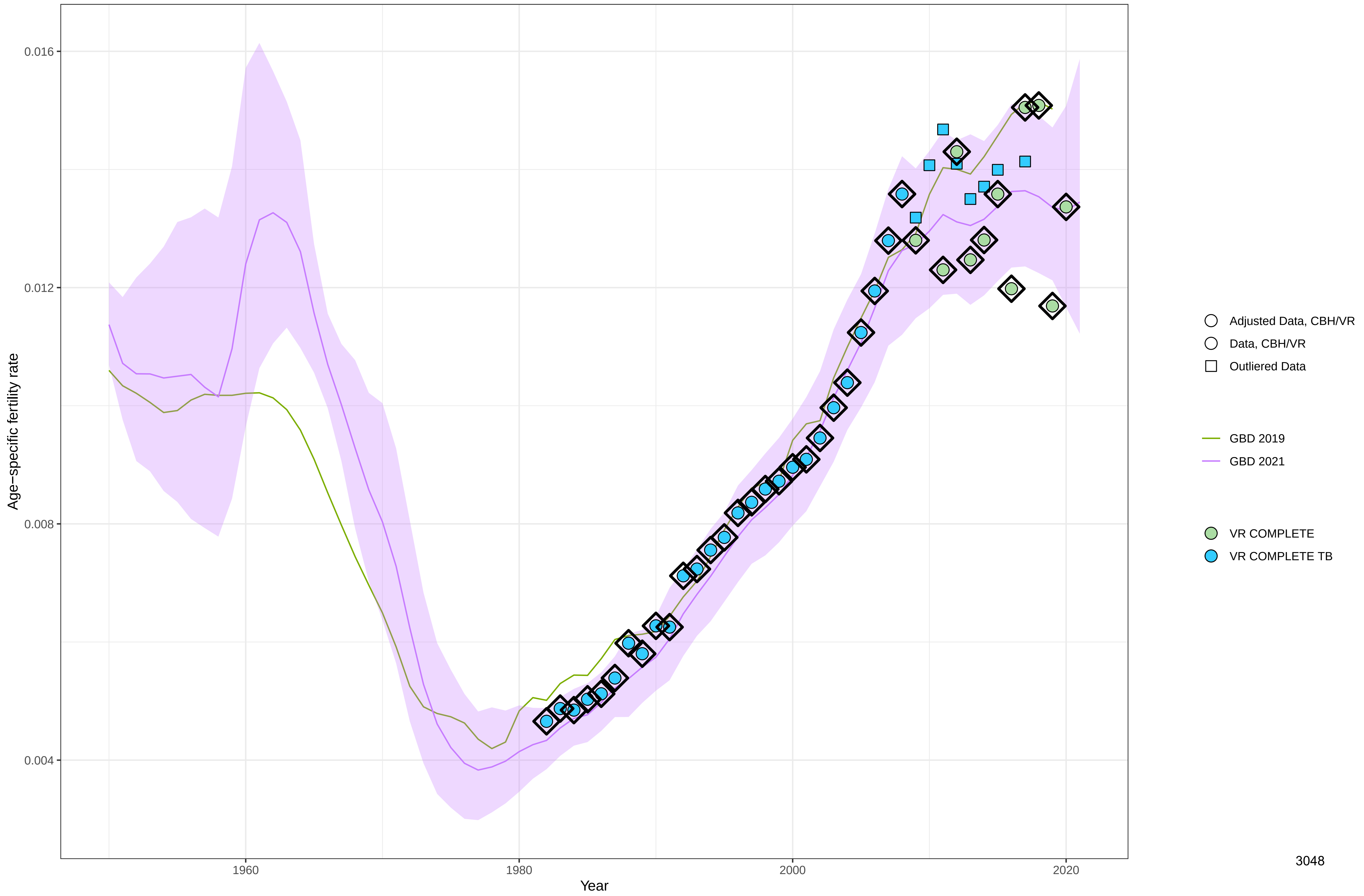

Age−specific fertility fitted trend lines, 1950−2021: Southampton, Ages: 45−49
Data points enclosed with a diamond are reference sources

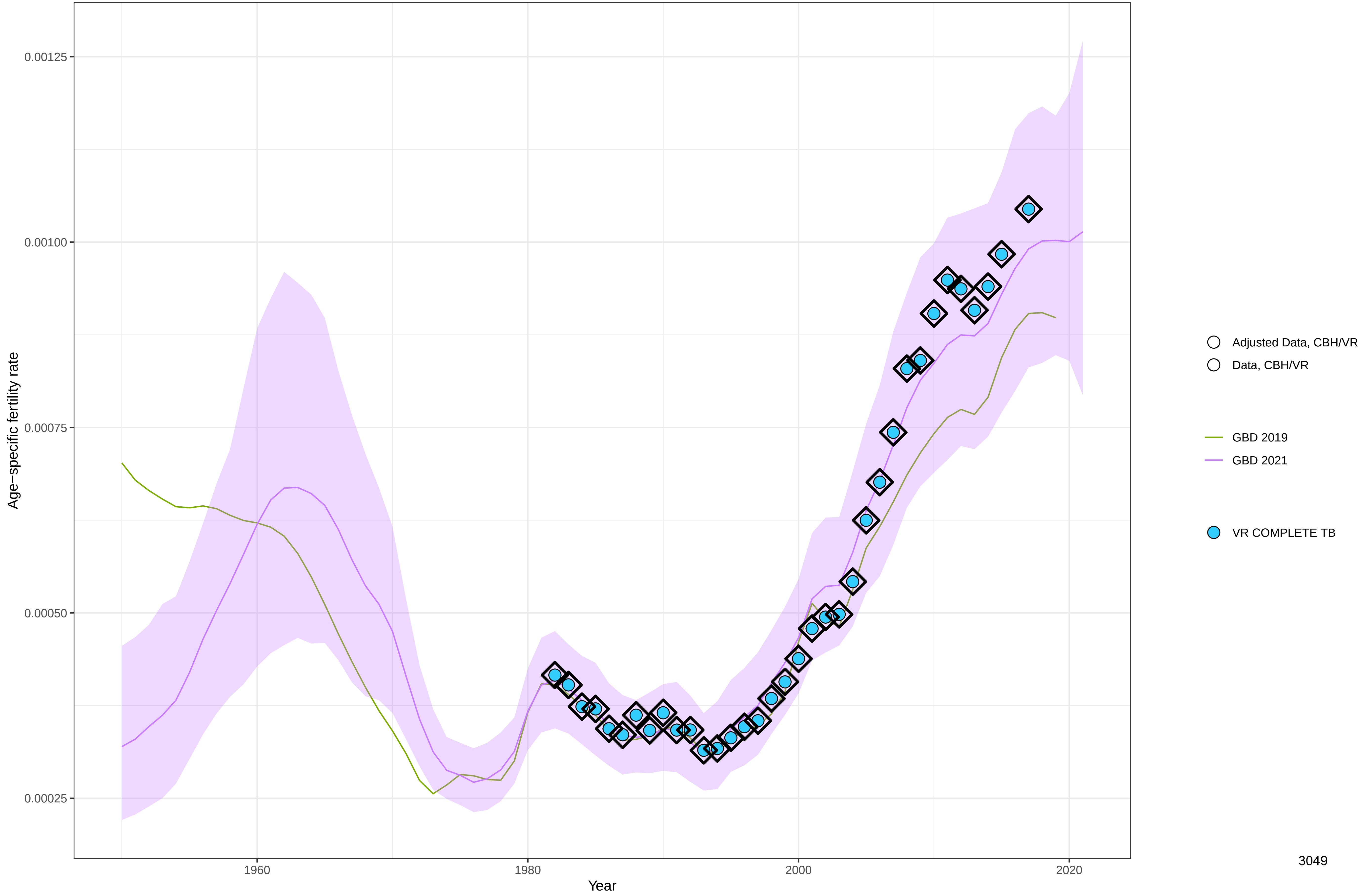

Age−specific fertility fitted trend lines, 1950−2021: Southampton, Ages: 50−54
Data points enclosed with a diamond are reference sources

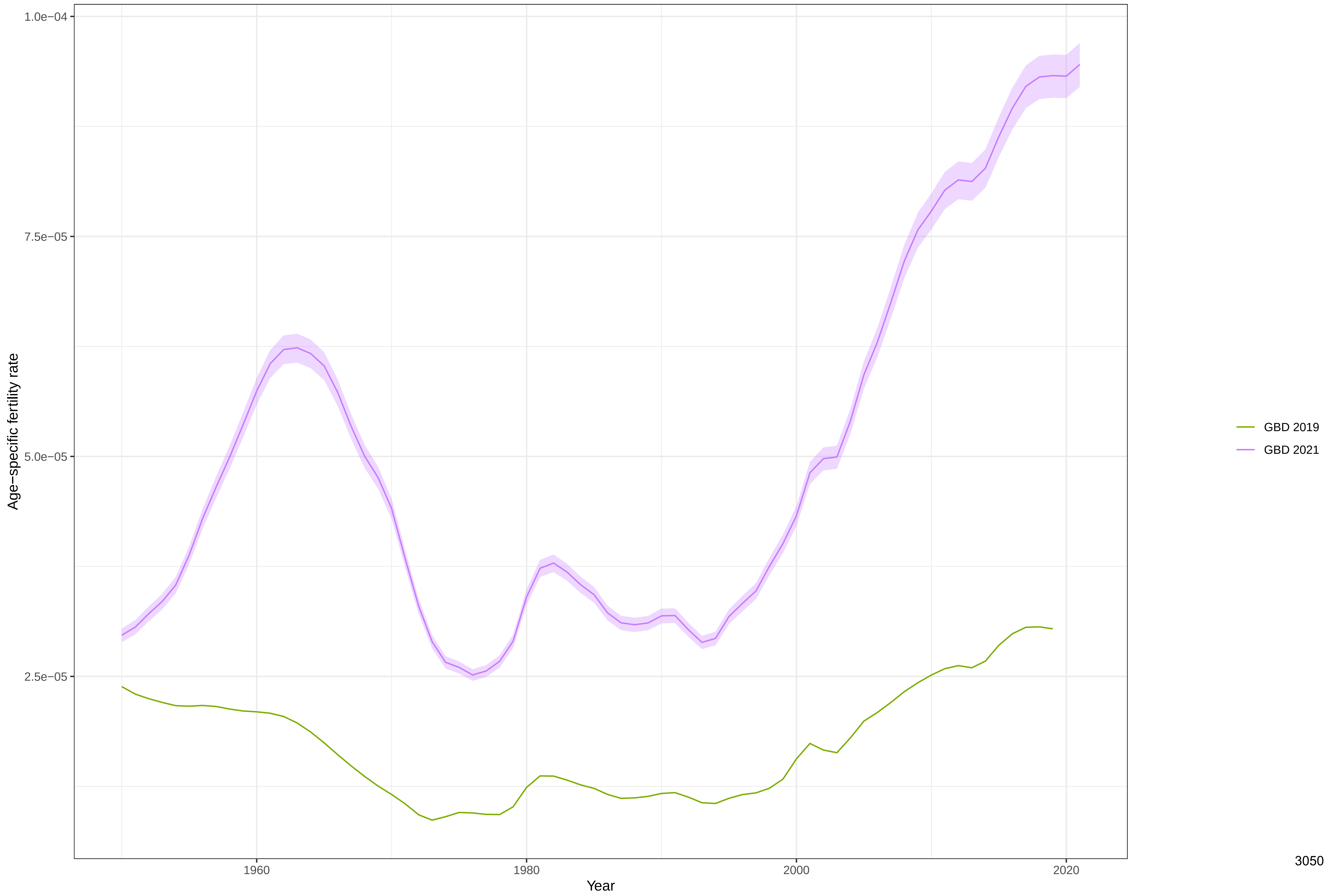

Total fertility rate fitted trend lines, 1950−2021: Southampton TFR
Data points enclosed with a diamond are reference sources

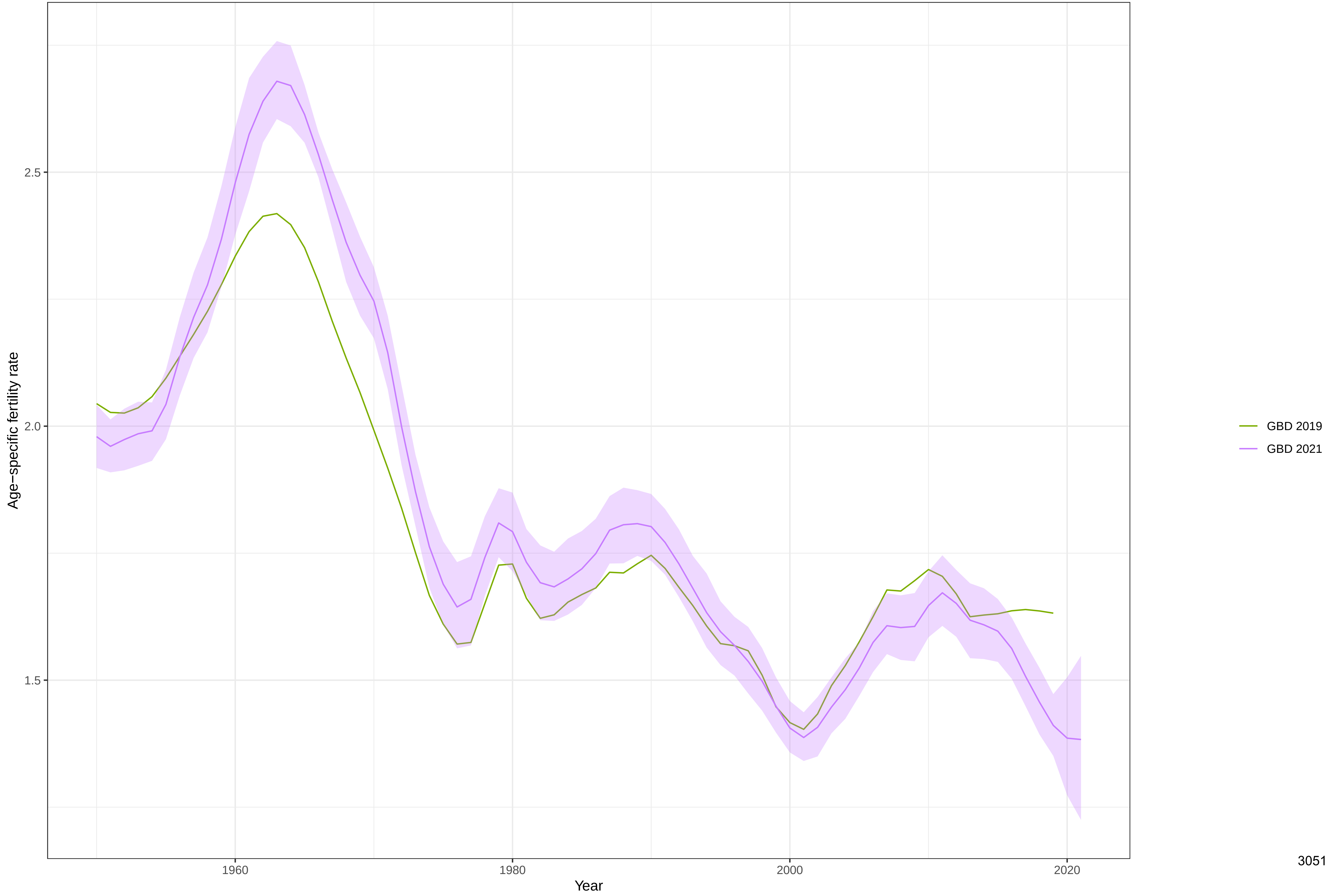

Age−specific fertility fitted trend lines, 1950−2021: Surrey, Ages: 10−14
Data points enclosed with a diamond are reference sources

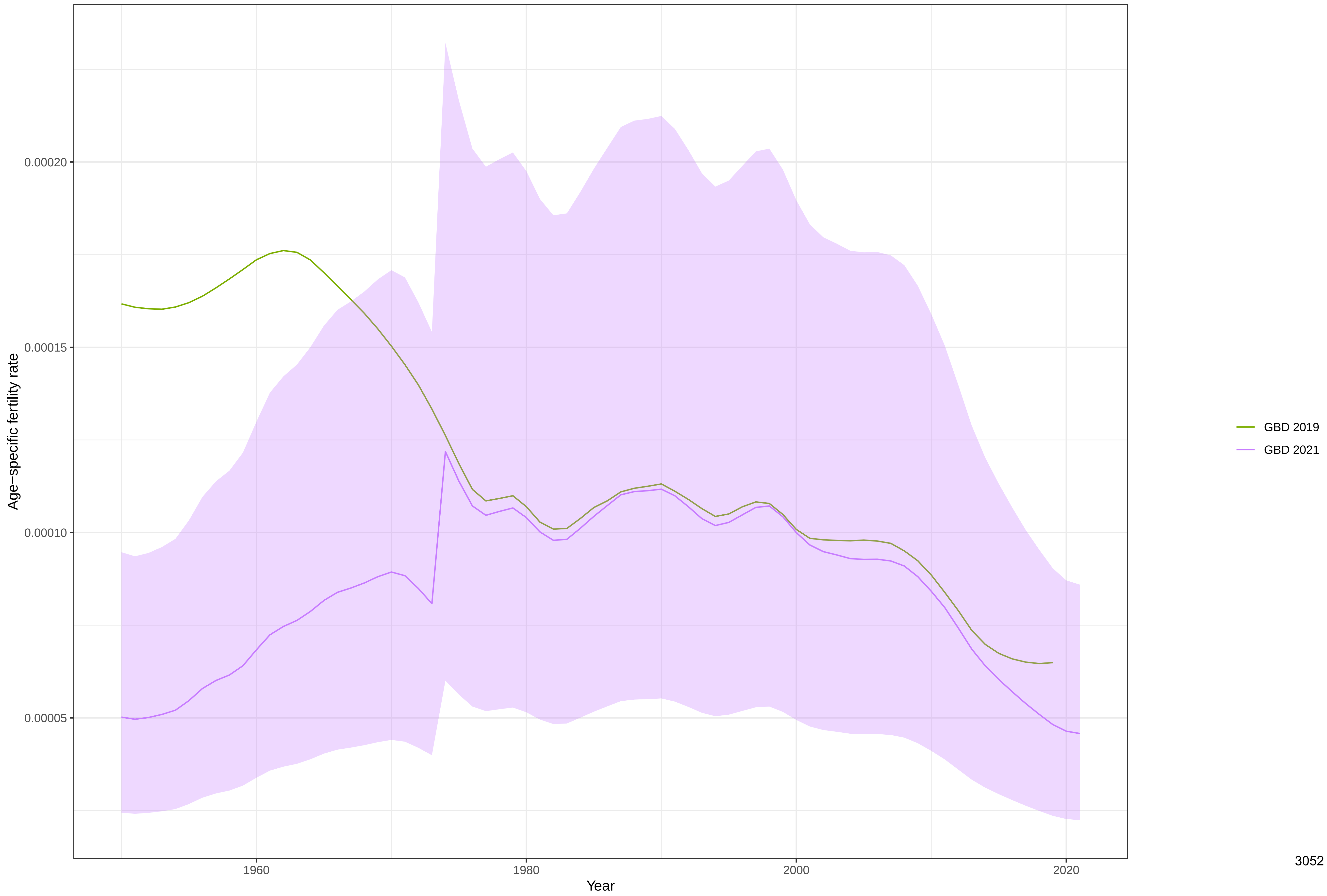

Age−specific fertility fitted trend lines, 1950−2021: Surrey, Ages: 15−19
Data points enclosed with a diamond are reference sources

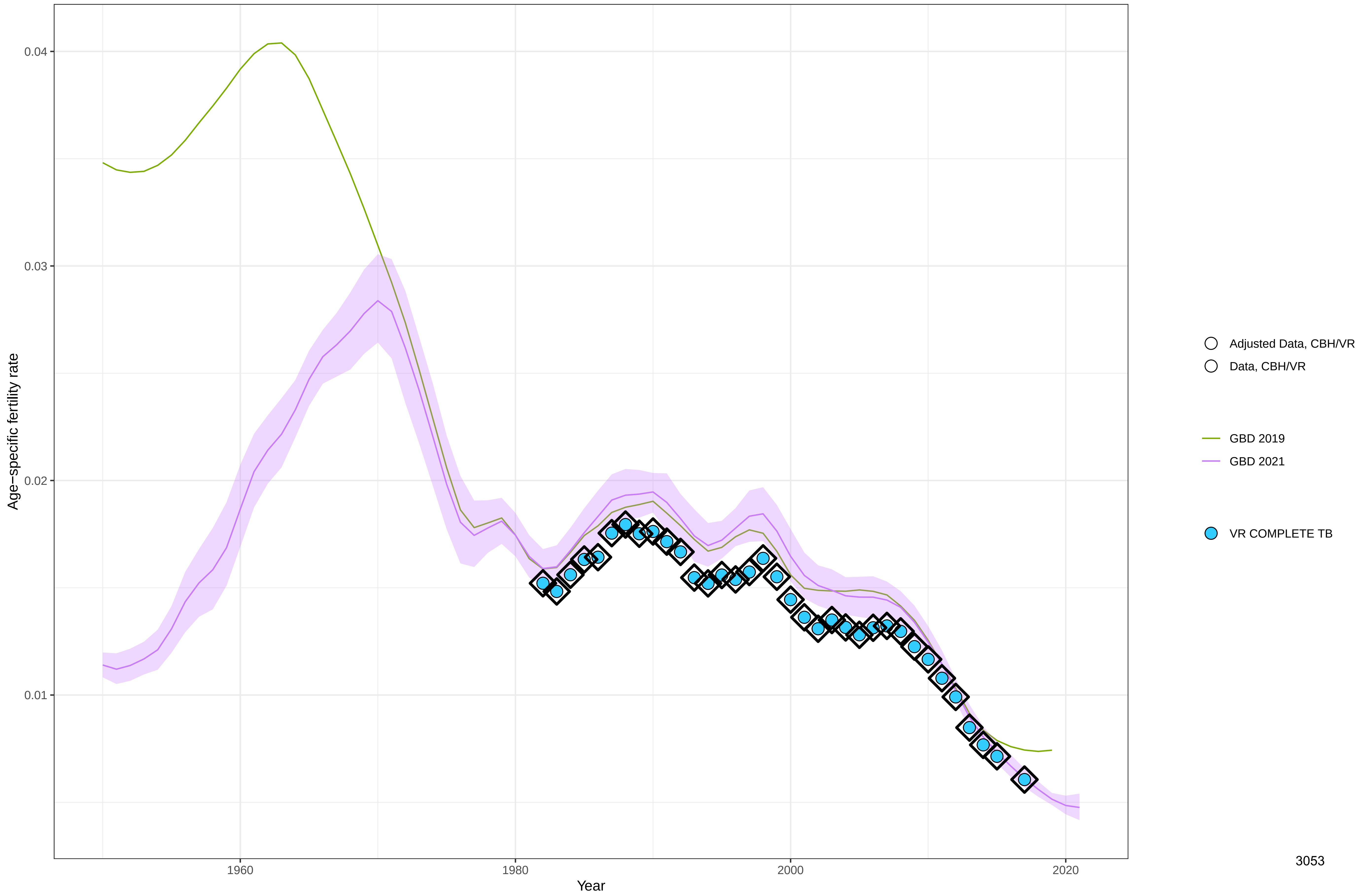

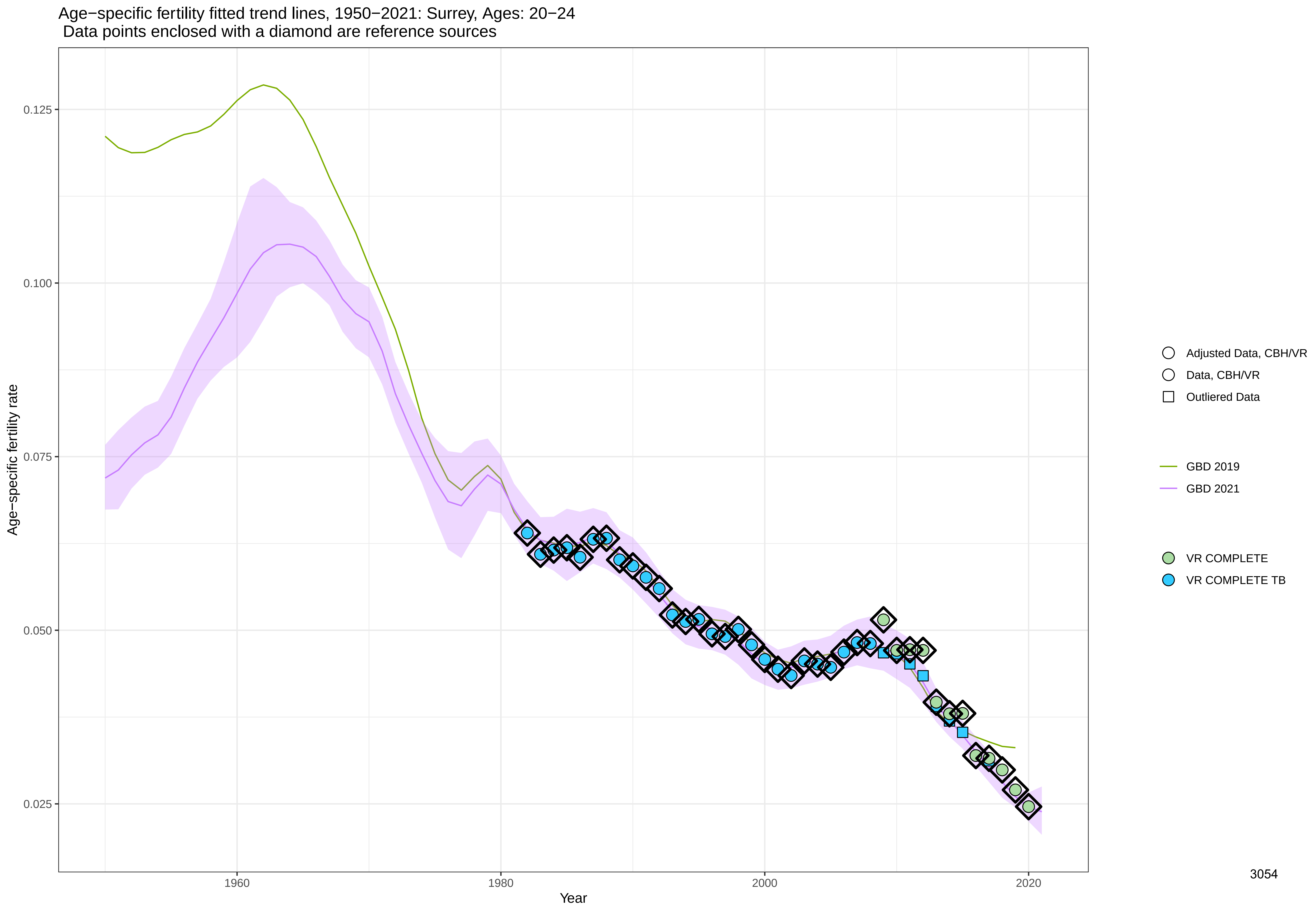

Age−specific fertility fitted trend lines, 1950−2021: Surrey, Ages: 20−24
Data points enclosed with a diamond are reference sources
Age−specific fertility rate
0.125
0.100
0.075
0.050
0.025
1960
1980
2000
2020
Year
Adjusted Data, CBH/VR
Data, CBH/VR
Outliered Data
GBD 2019
GBD 2021
VR COMPLETE
VR COMPLETE TB

Age−specific fertility fitted trend lines, 1950−2021: Surrey, Ages: 25−29
Data points enclosed with a diamond are reference sources

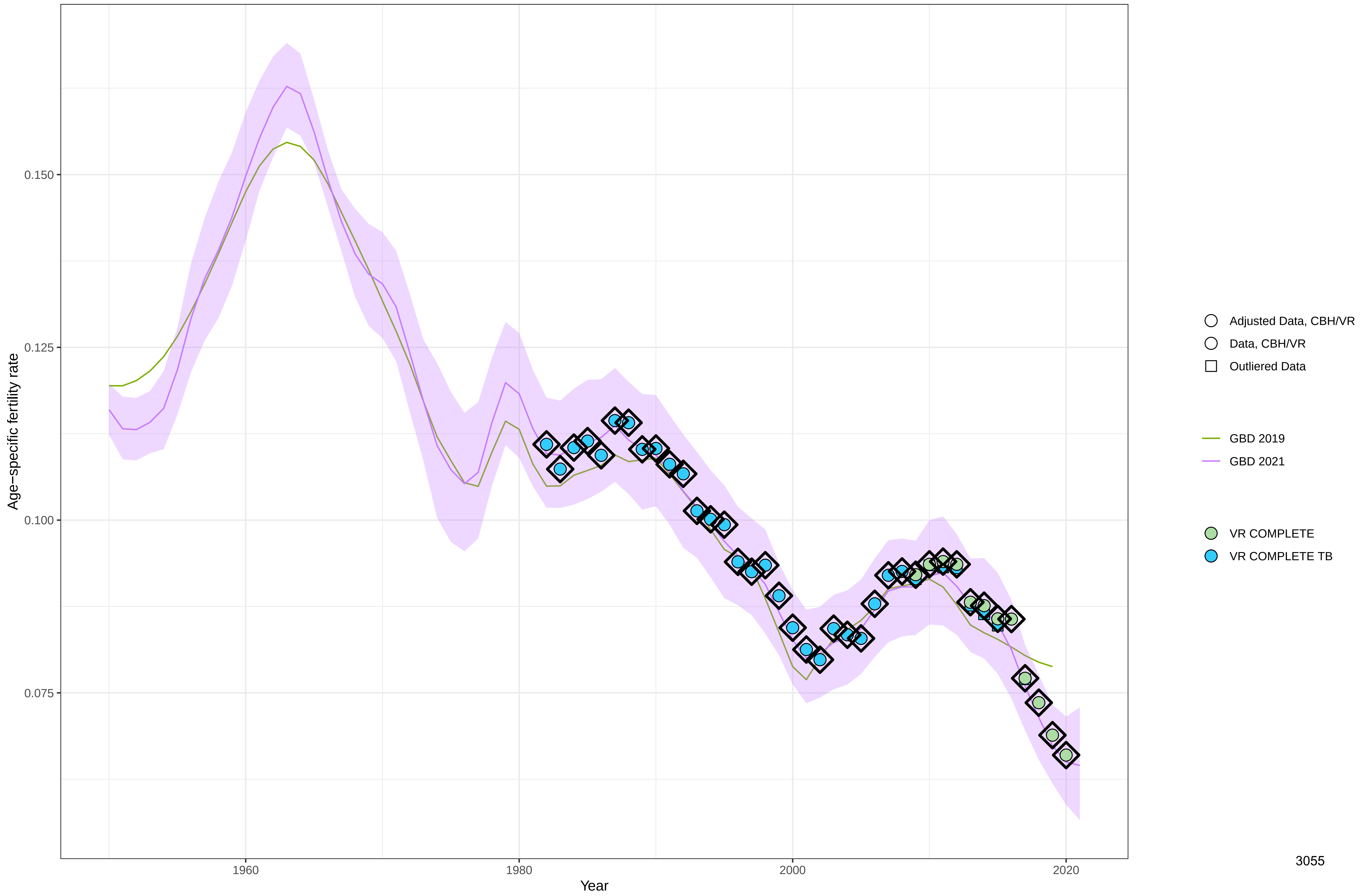

Age−specific fertility fitted trend lines, 1950−2021: Surrey, Ages: 30−34
Data points enclosed with a diamond are reference sources

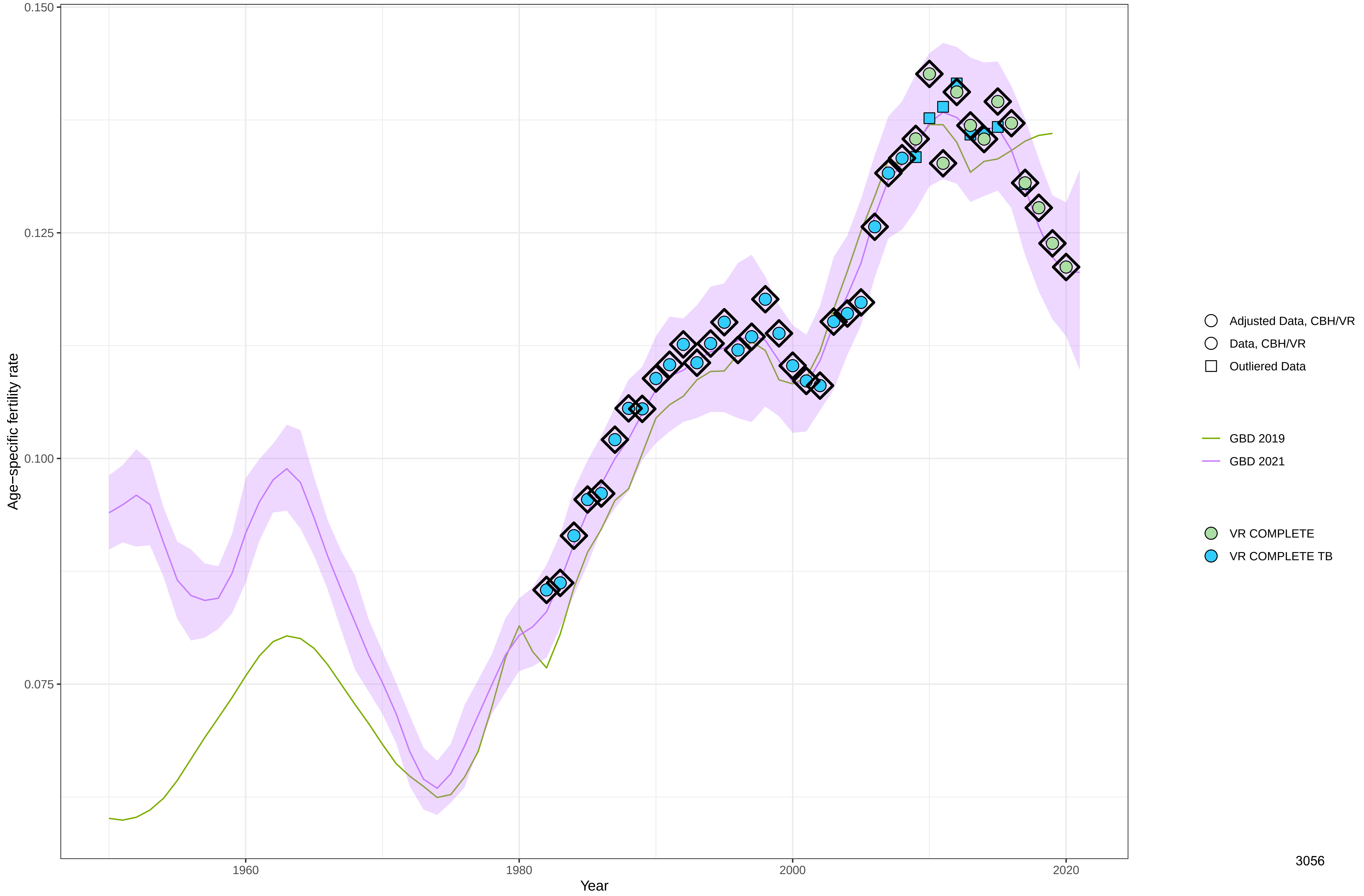

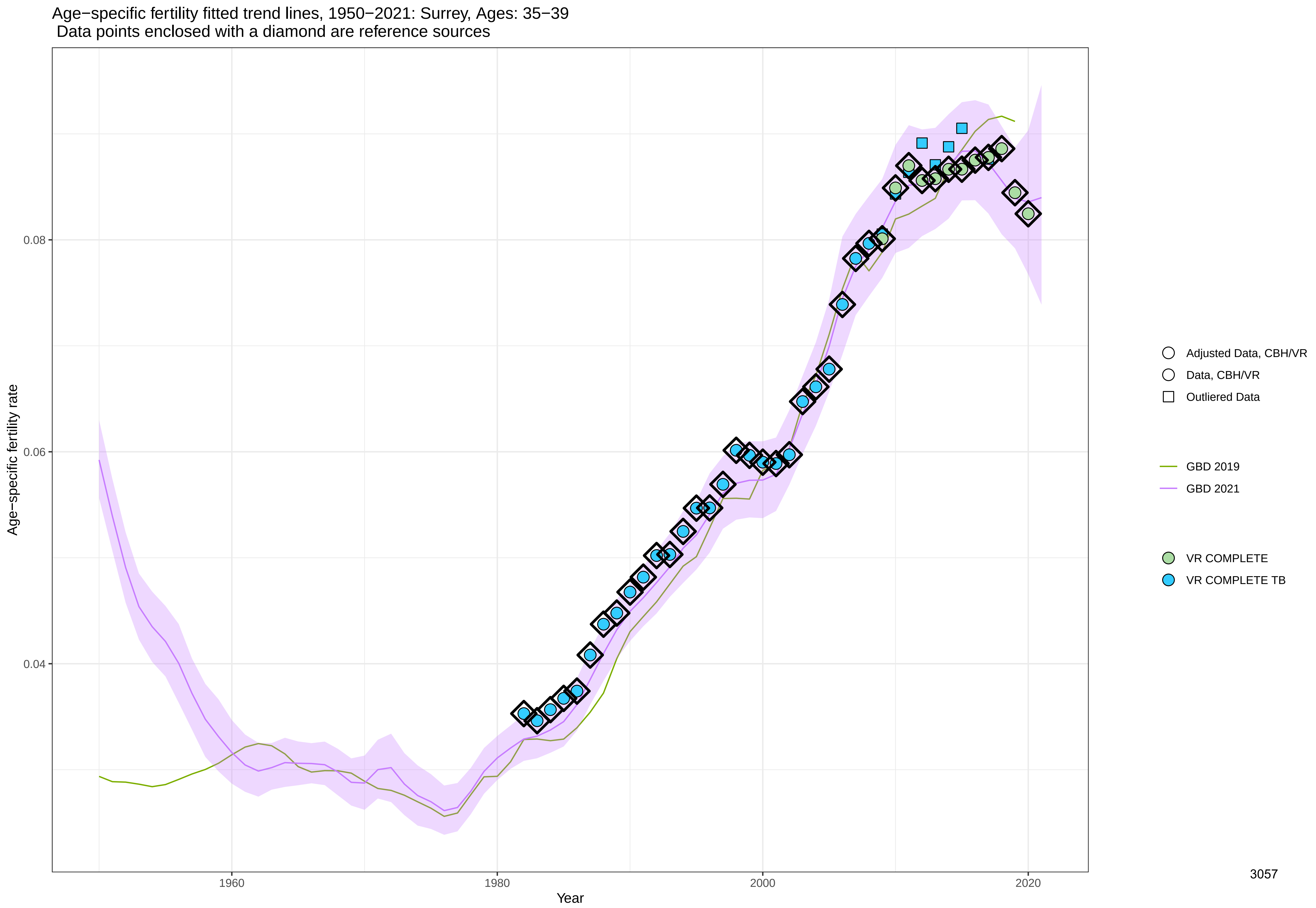

Age−specific fertility fitted trend lines, 1950−2021: Surrey, Ages: 35−39
Data points enclosed with a diamond are reference sources
Age−specific fertility rate
0.04
0.06
0.08
1960
1980
2000
2020
Year
Adjusted Data, CBH/VR
Data, CBH/VR
Outliered Data
GBD 2019
GBD 2021
VR COMPLETE
VR COMPLETE TB

Age−specific fertility fitted trend lines, 1950−2021: Surrey, Ages: 40−44
Data points enclosed with a diamond are reference sources

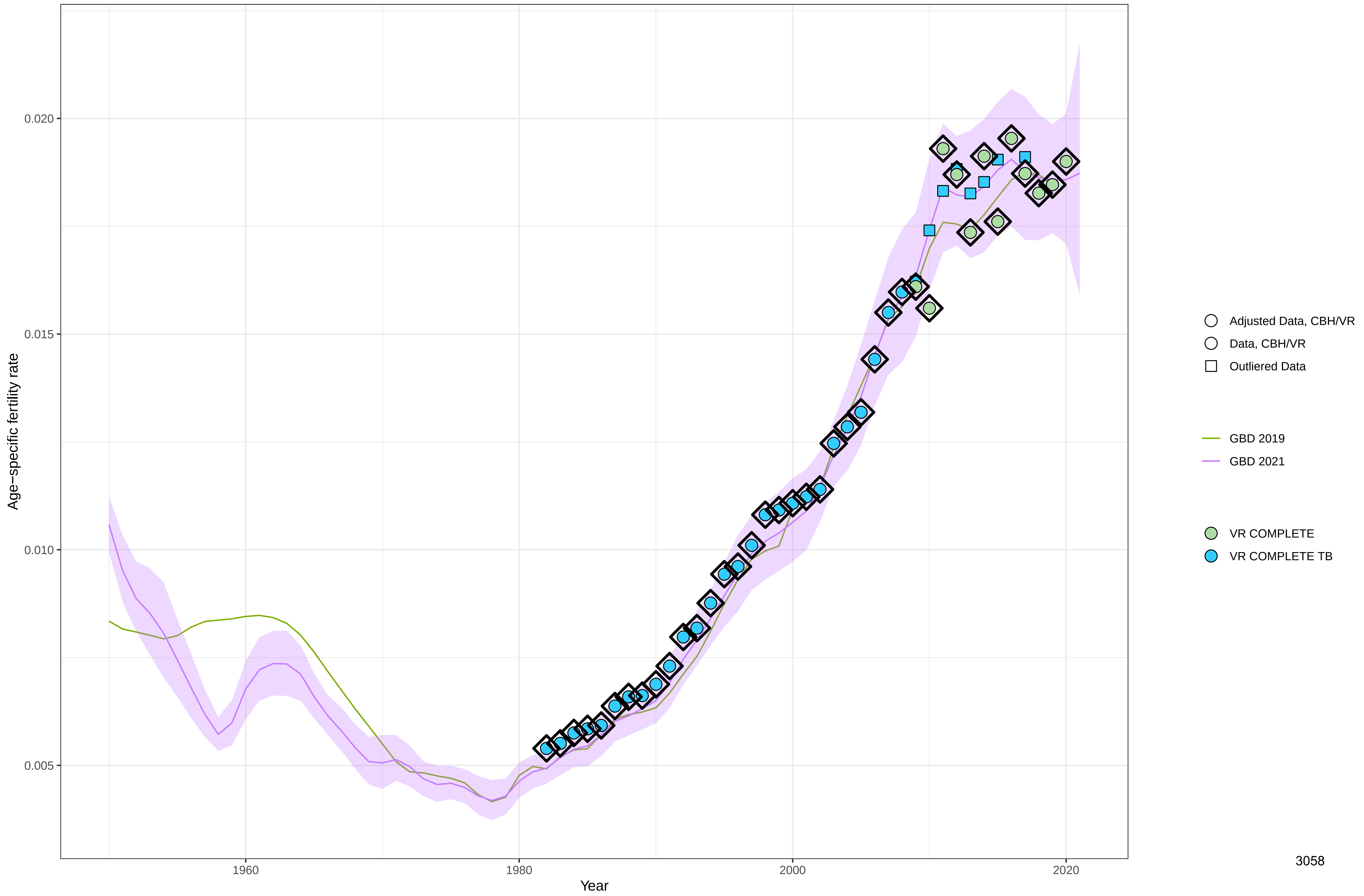

Age−specific fertility fitted trend lines, 1950−2021: Surrey, Ages: 45−49
Data points enclosed with a diamond are reference sources

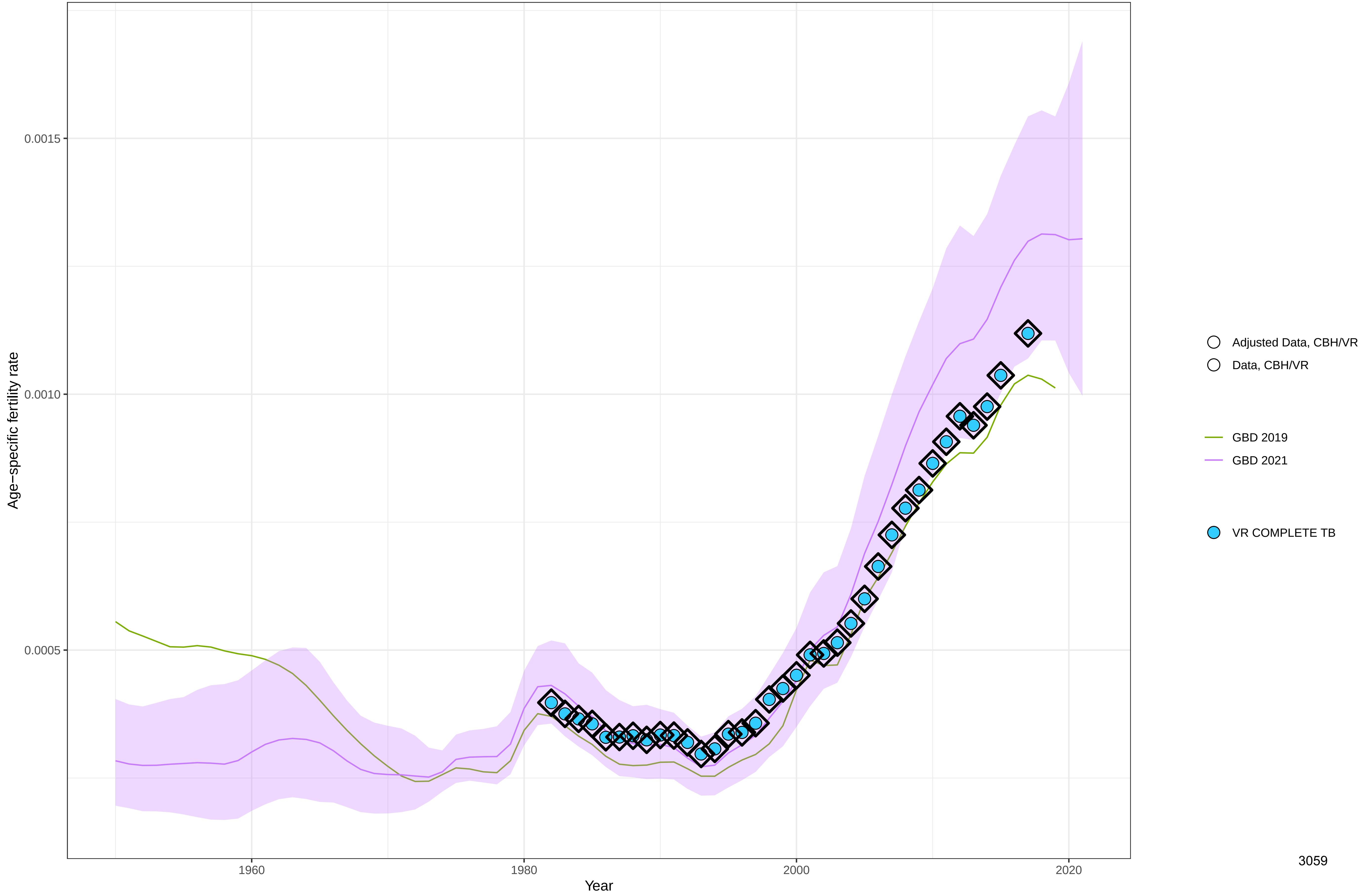

Age−specific fertility fitted trend lines, 1950−2021: Surrey, Ages: 50−54
Data points enclosed with a diamond are reference sources

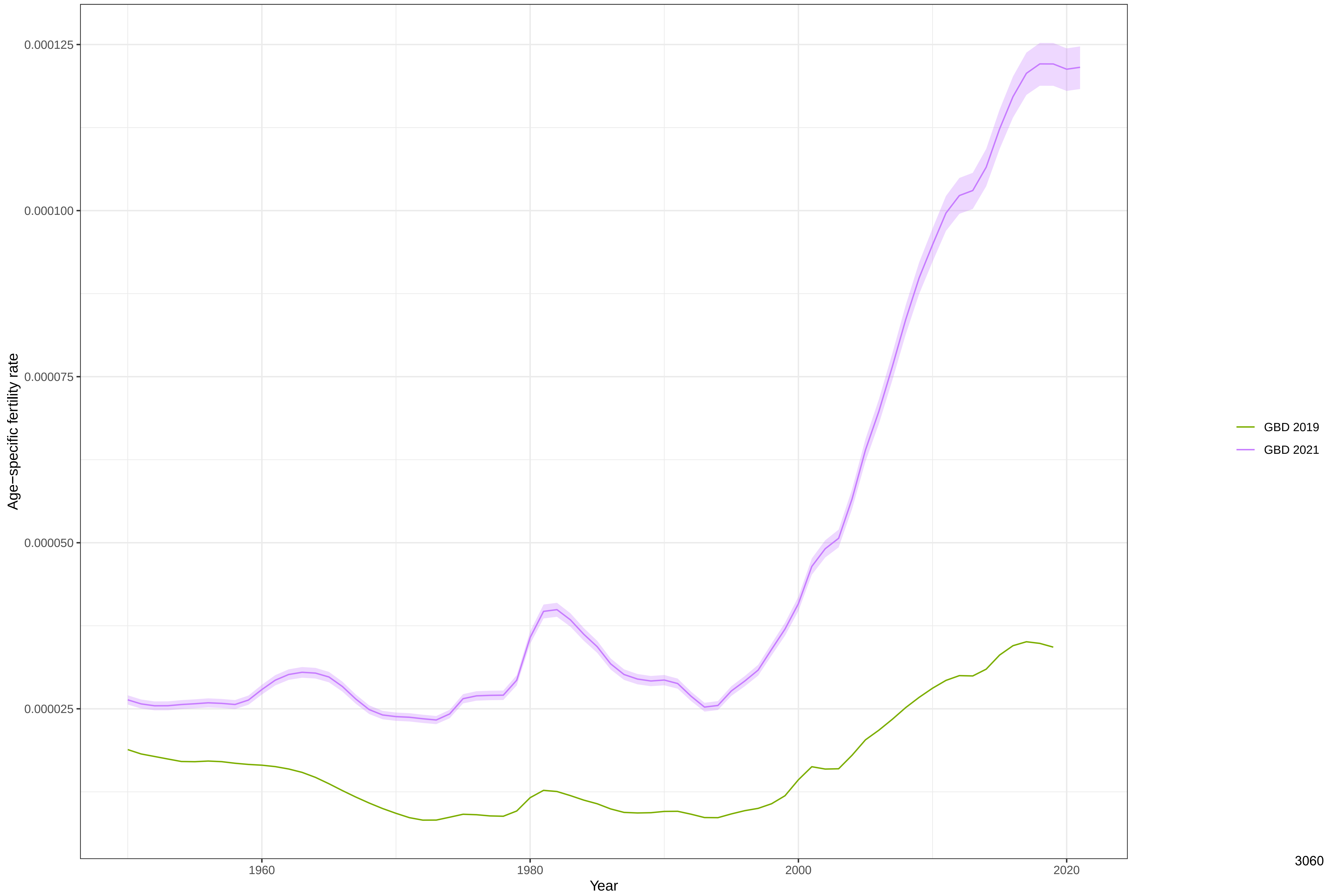

Total fertility rate fitted trend lines, 1950−2021: Surrey TFR
Data points enclosed with a diamond are reference sources

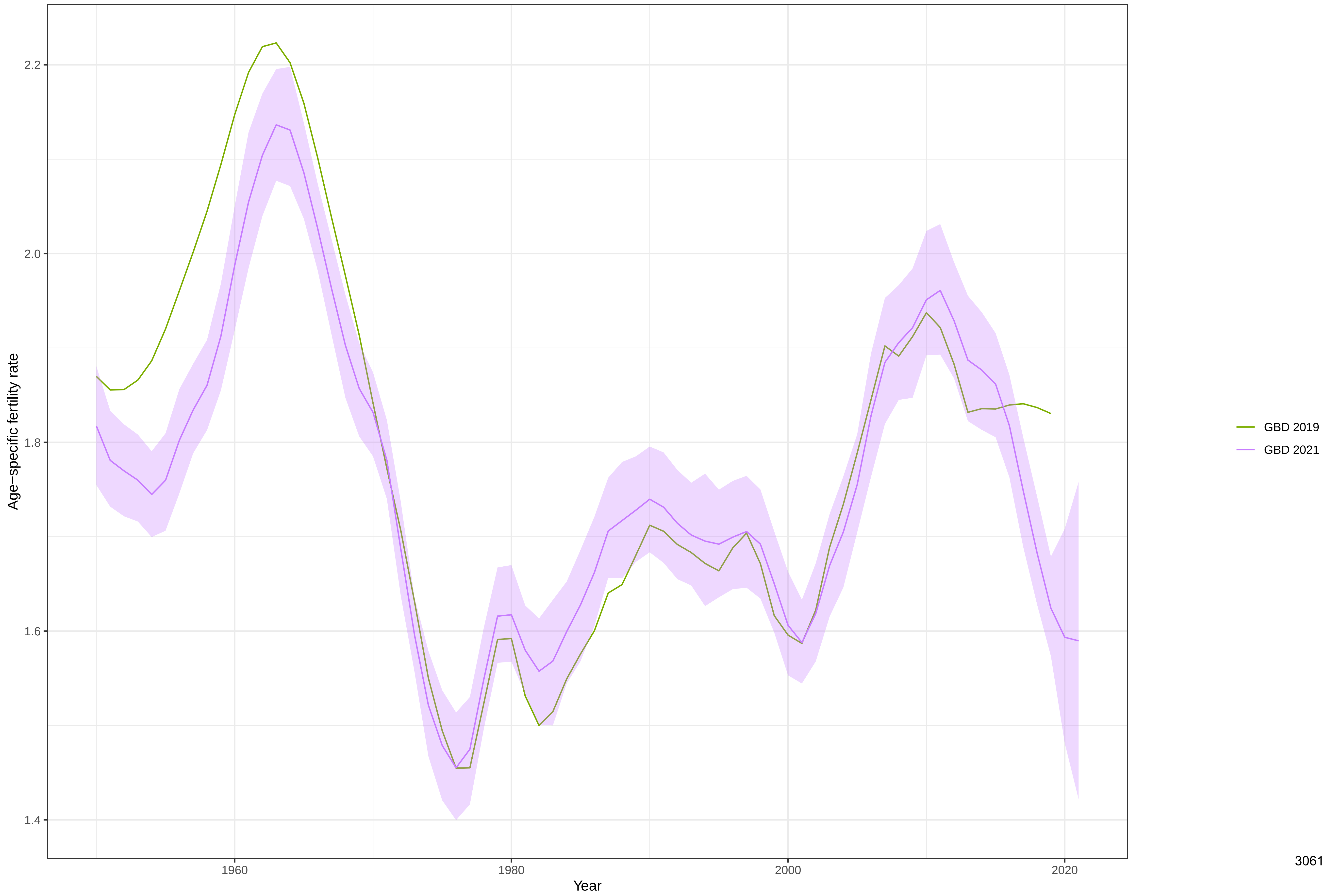

Age−specific fertility fitted trend lines, 1950−2021: West Berkshire, Ages: 10−14
Data points enclosed with a diamond are reference sources

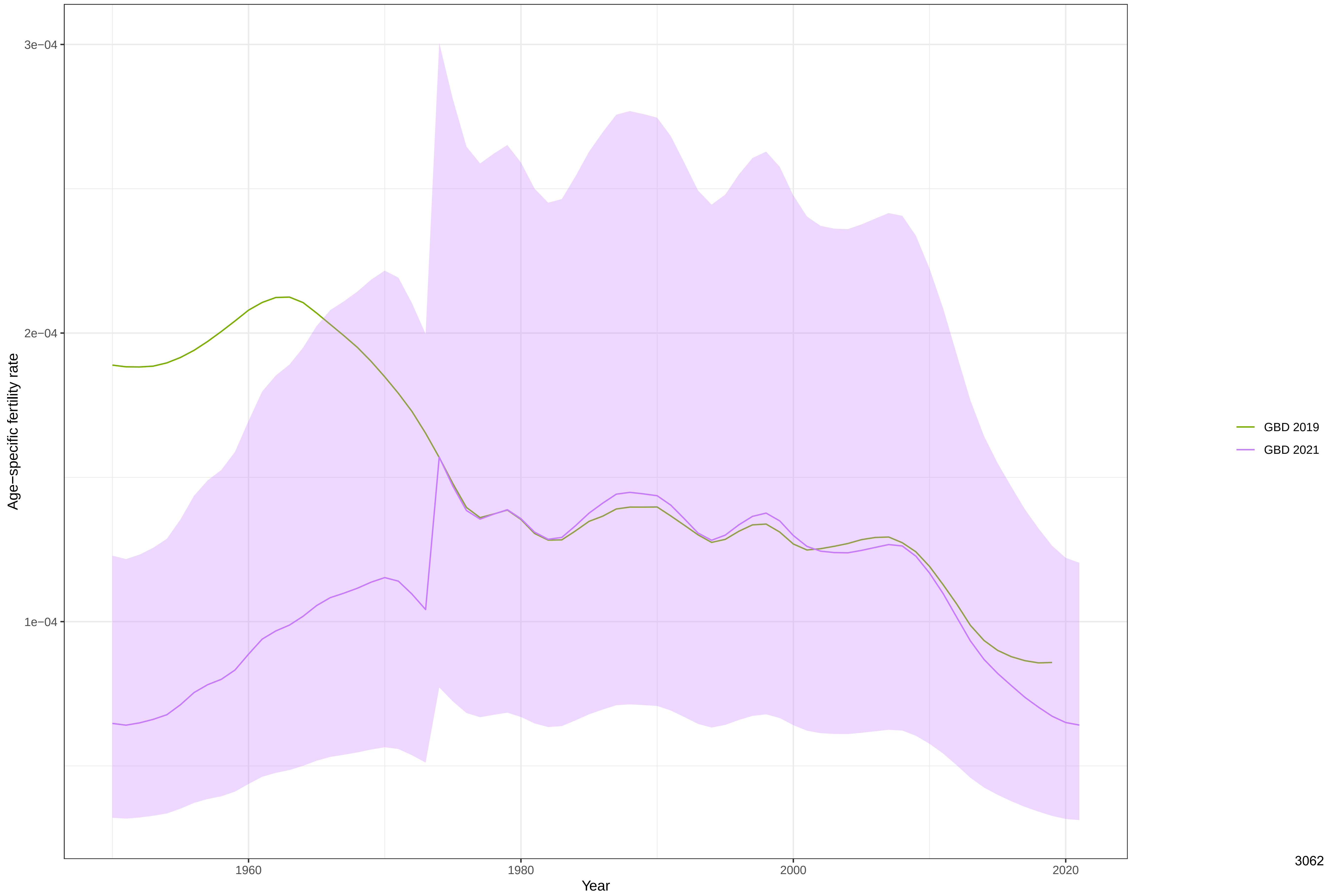

Age−specific fertility fitted trend lines, 1950−2021: West Berkshire, Ages: 15−19
Data points enclosed with a diamond are reference sources

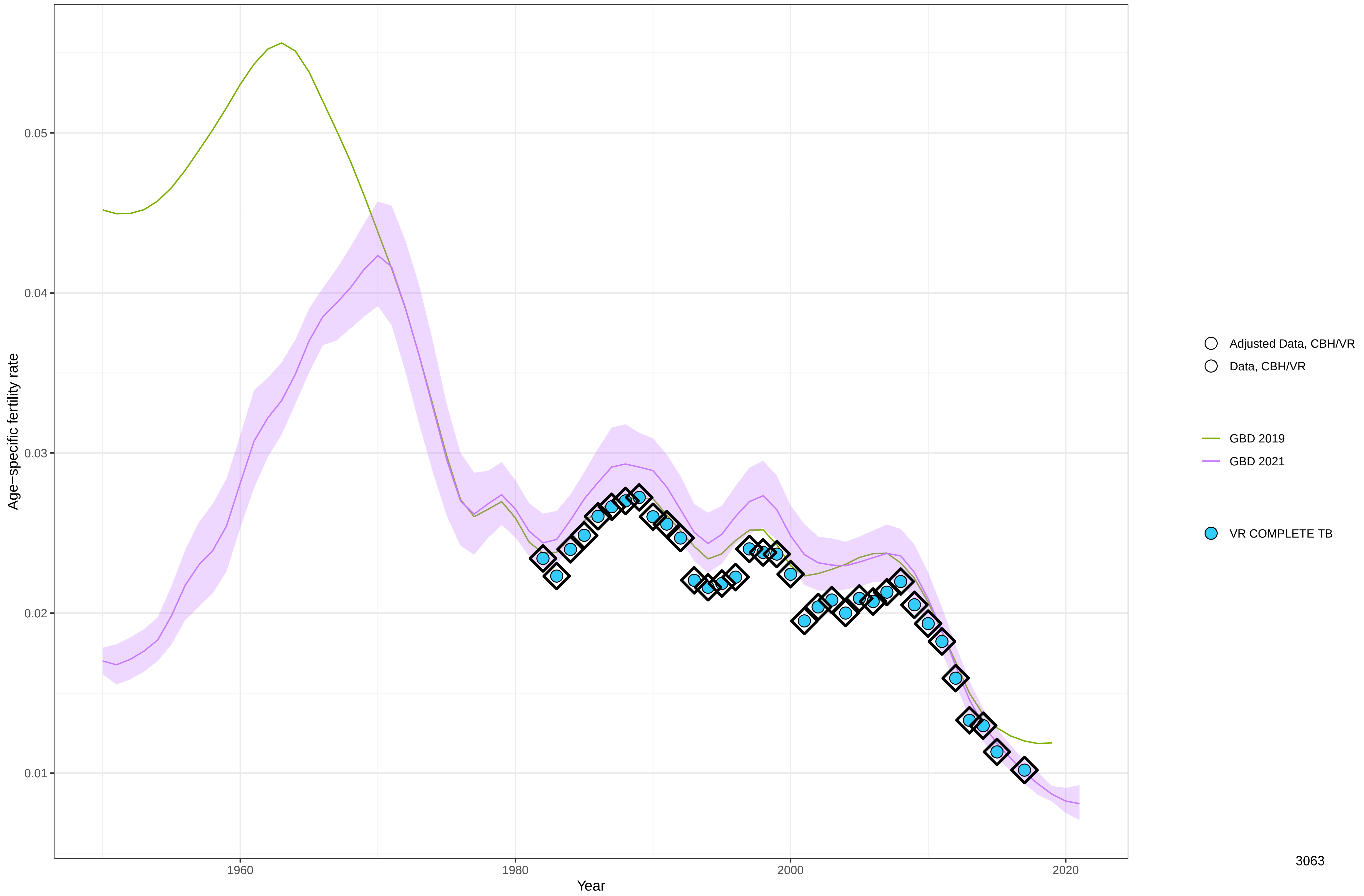

3063

Age−specific fertility fitted trend lines, 1950−2021: West Berkshire, Ages: 20−24
Data points enclosed with a diamond are reference sources

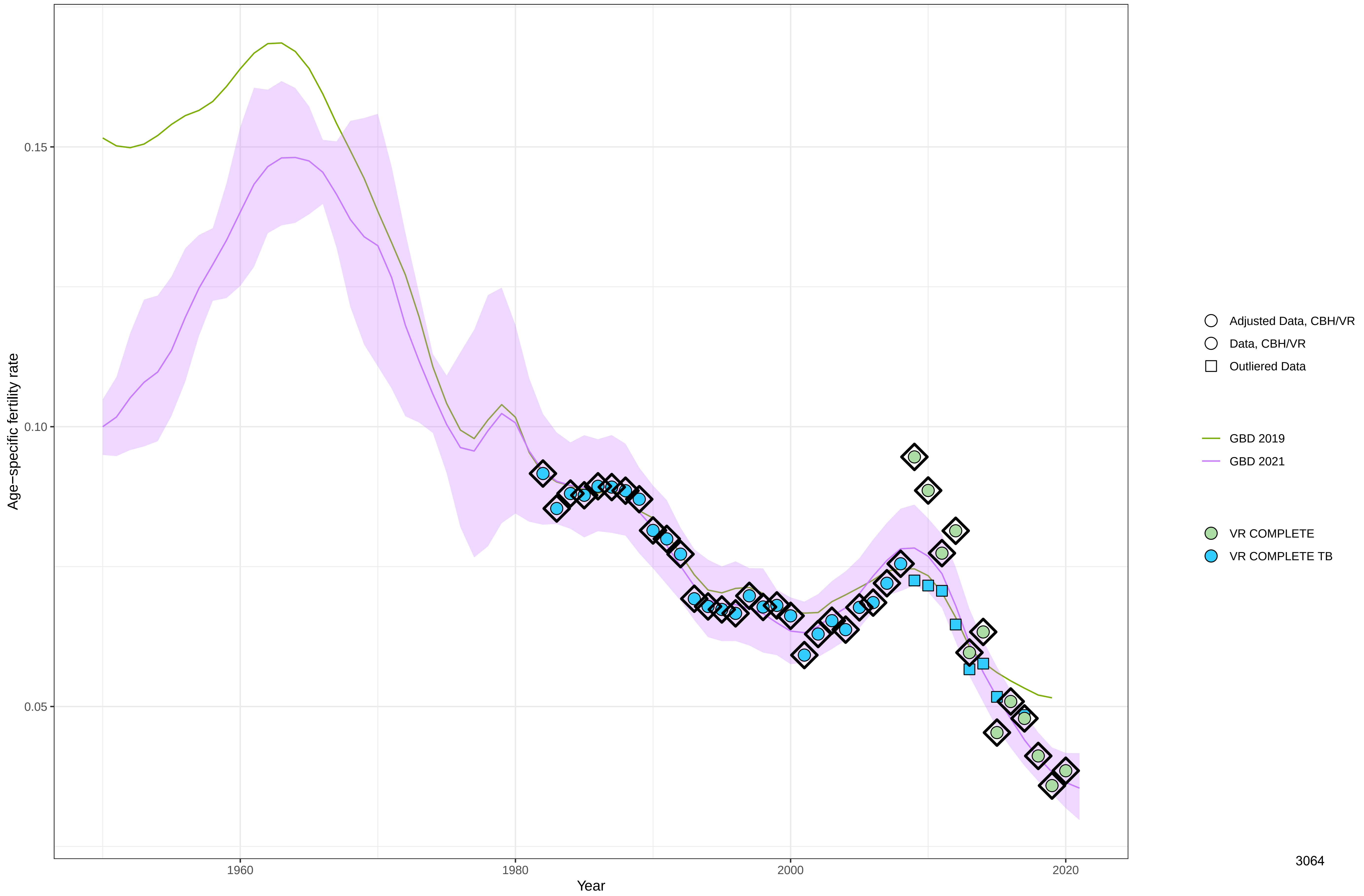

Age−specific fertility fitted trend lines, 1950−2021: West Berkshire, Ages: 25−29
Data points enclosed with a diamond are reference sources

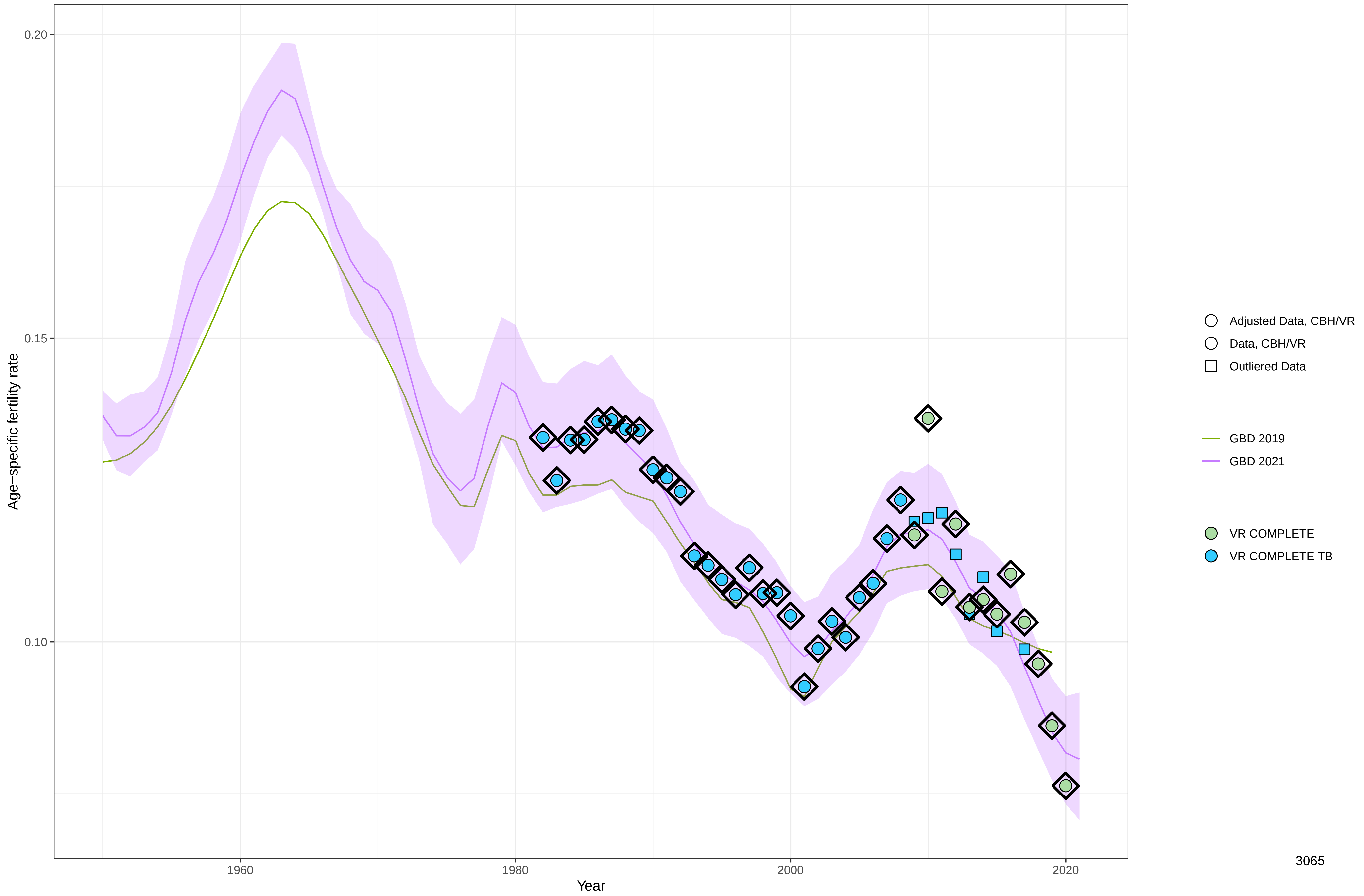

Age−specific fertility fitted trend lines, 1950−2021: West Berkshire, Ages: 30−34
Data points enclosed with a diamond are reference sources

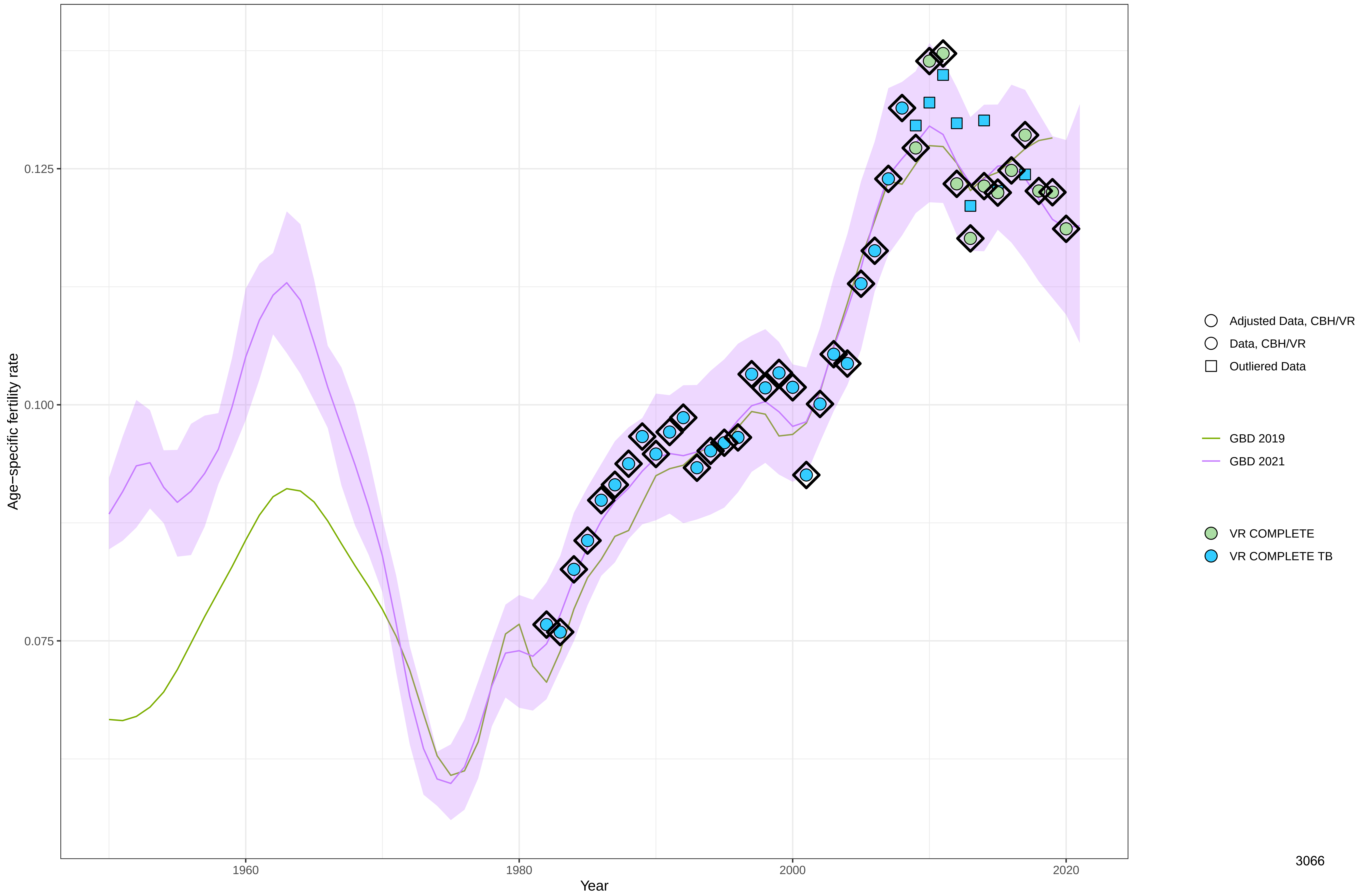

3066

Age−specific fertility fitted trend lines, 1950−2021: West Berkshire, Ages: 35−39
Data points enclosed with a diamond are reference sources

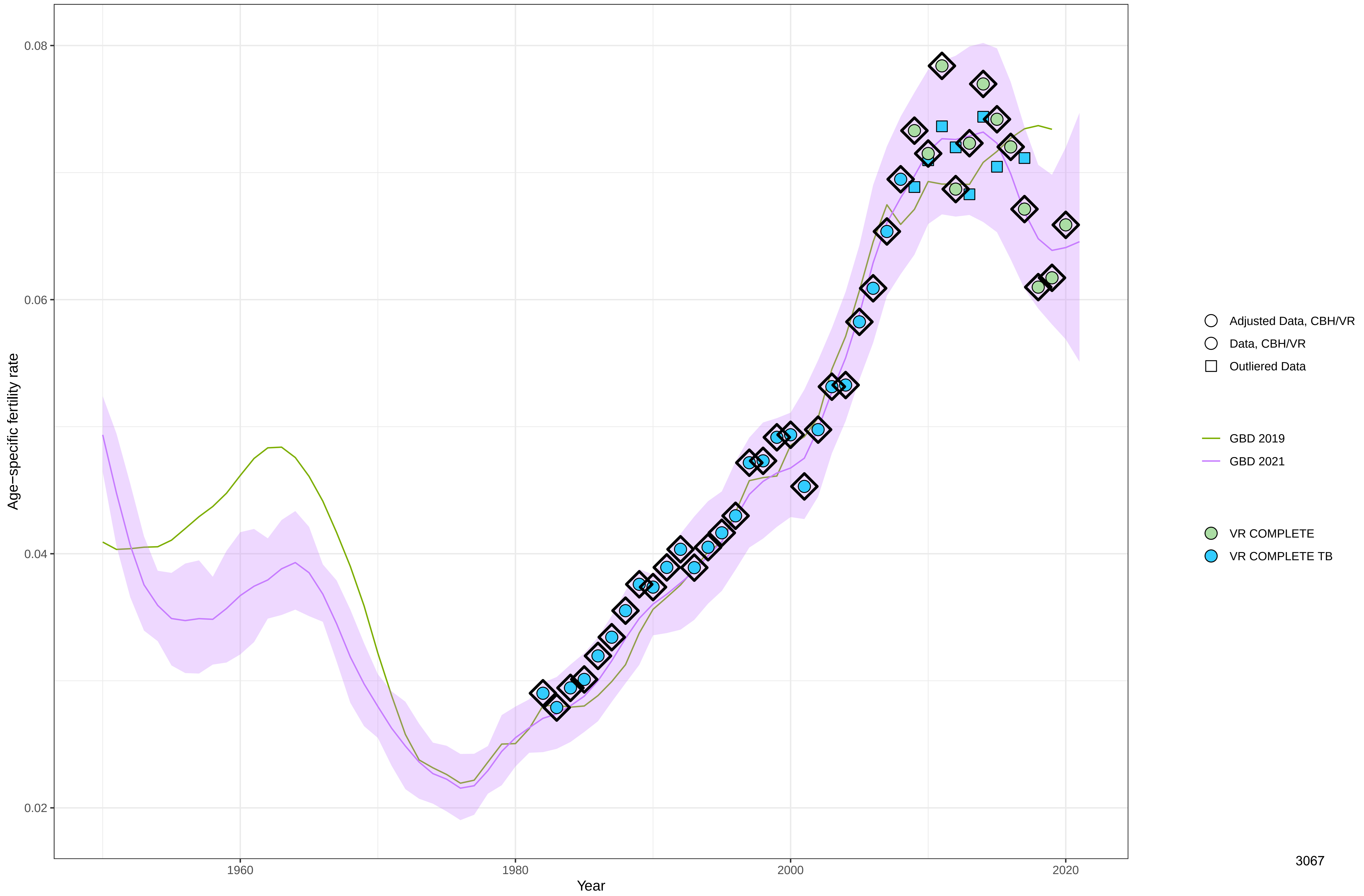

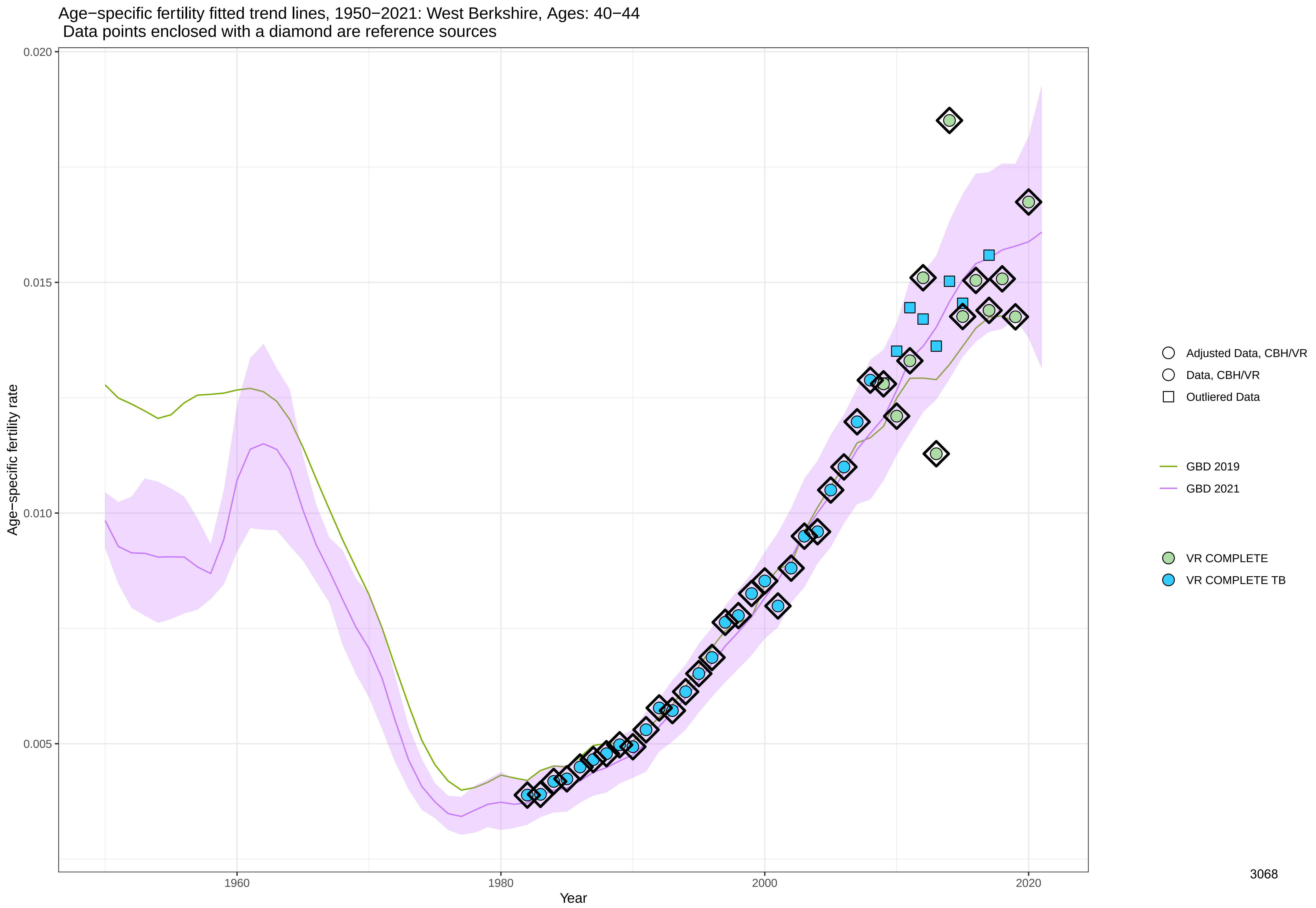
Age−specific fertility fitted trend lines, 1950−2021: West Berkshire, Ages: 40−44
Data points enclosed with a diamond are reference sources
Age−specific fertility rate
0.020
0.015
0.010
0.005
1960
1980
2000
2020
Year
Adjusted Data, CBH/VR
Data, CBH/VR
Outliered Data
GBD 2019
GBD 2021
VR COMPLETE
VR COMPLETE TB

Age−specific fertility fitted trend lines, 1950−2021: West Berkshire, Ages: 45−49
Data points enclosed with a diamond are reference sources

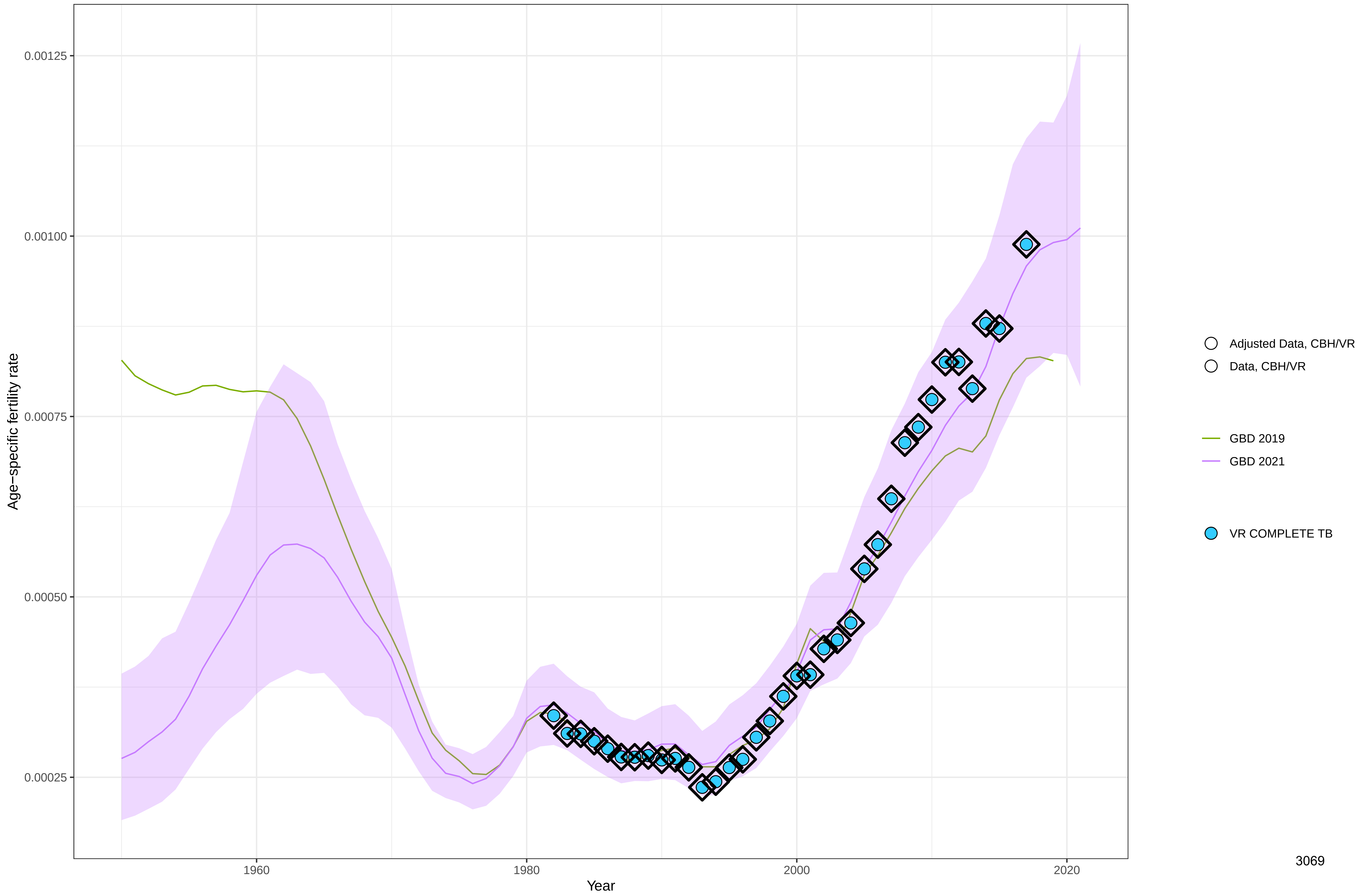

Age−specific fertility fitted trend lines, 1950−2021: West Berkshire, Ages: 50−54
Data points enclosed with a diamond are reference sources

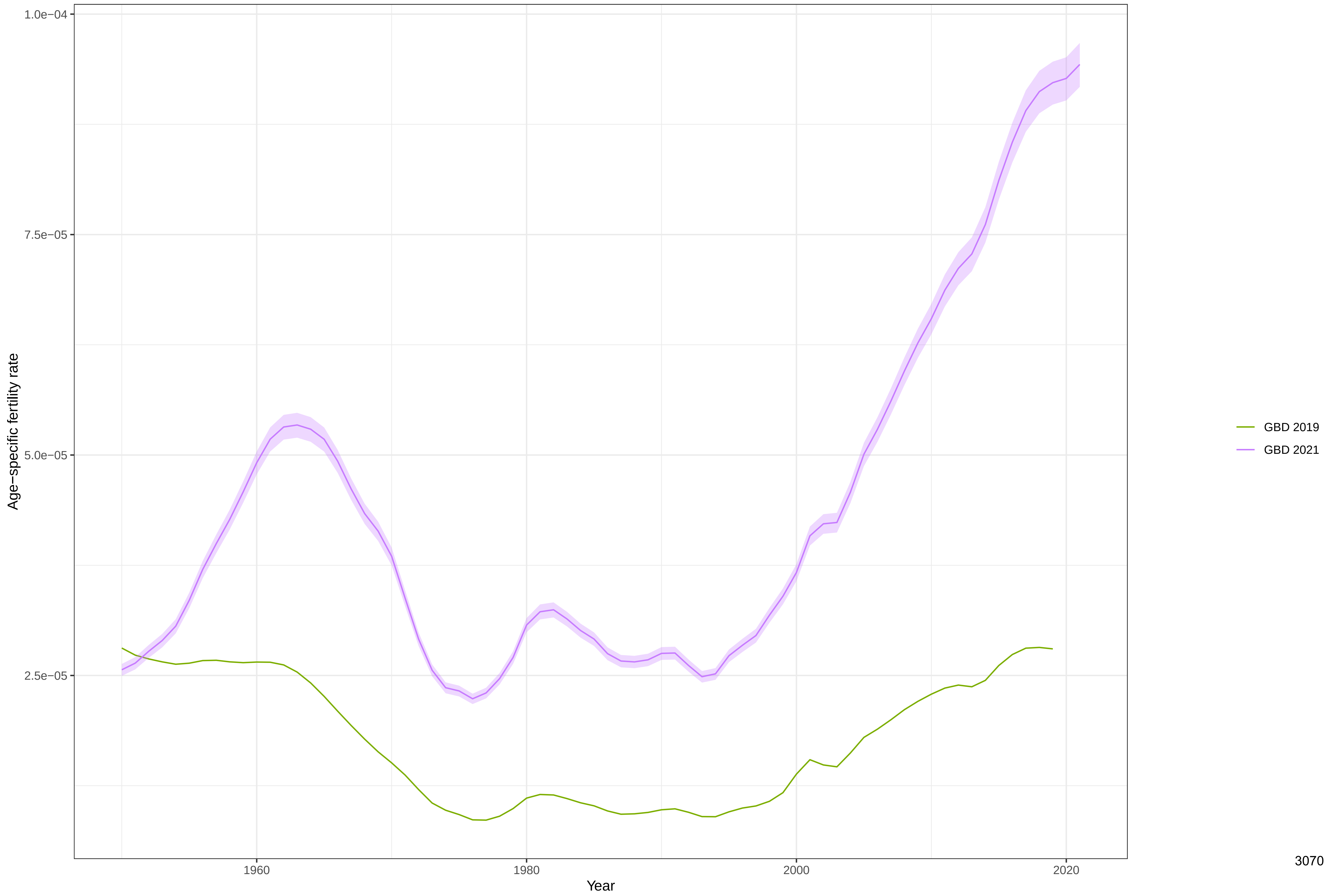

Total fertility rate fitted trend lines, 1950−2021: West Berkshire TFR
Data points enclosed with a diamond are reference sources

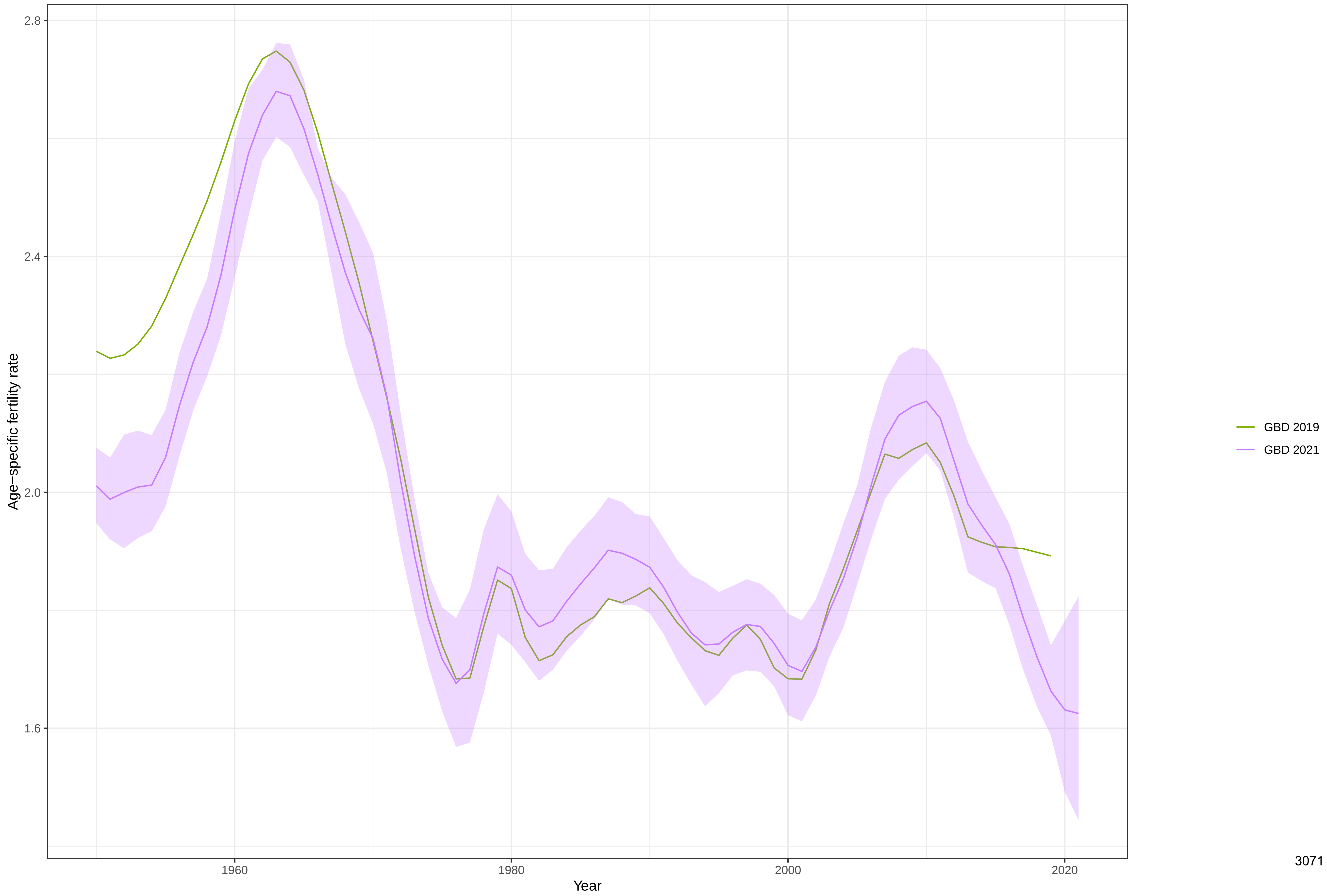

Age−specific fertility fitted trend lines, 1950−2021: West Sussex, Ages: 10−14
Data points enclosed with a diamond are reference sources

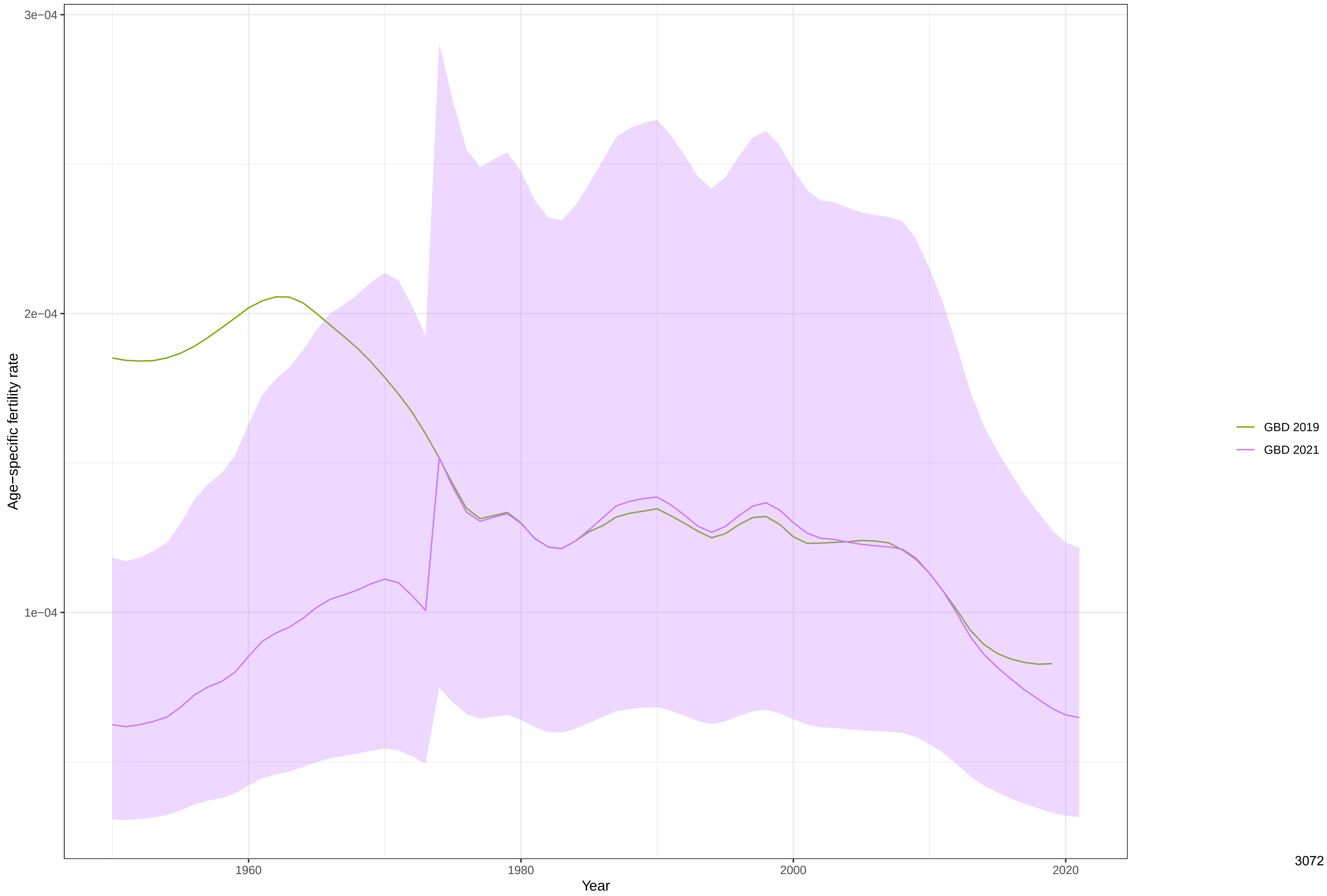

Age−specific fertility fitted trend lines, 1950−2021: West Sussex, Ages: 15−19
Data points enclosed with a diamond are reference sources

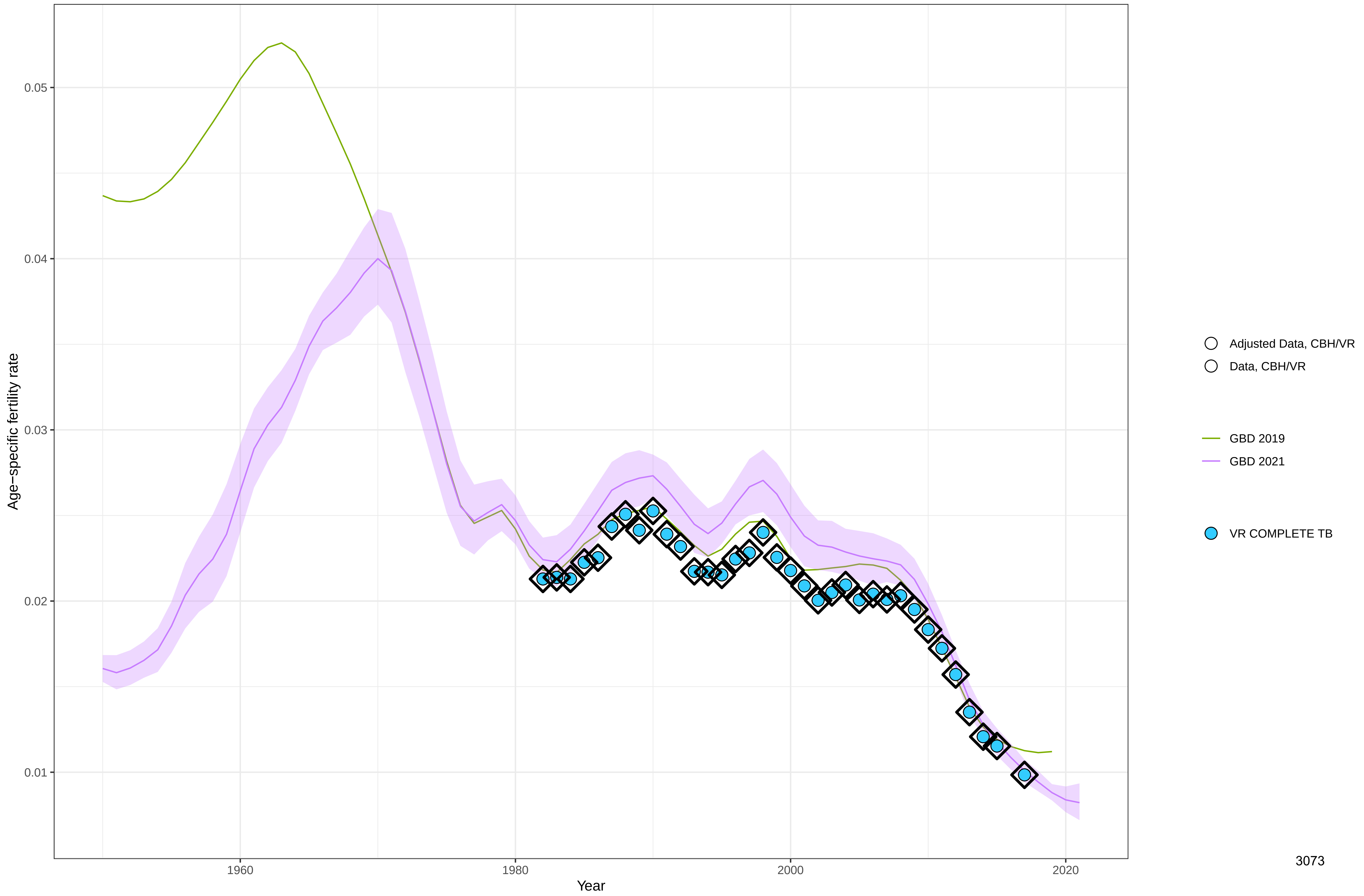

Age−specific fertility fitted trend lines, 1950−2021: West Sussex, Ages: 20−24
Data points enclosed with a diamond are reference sources

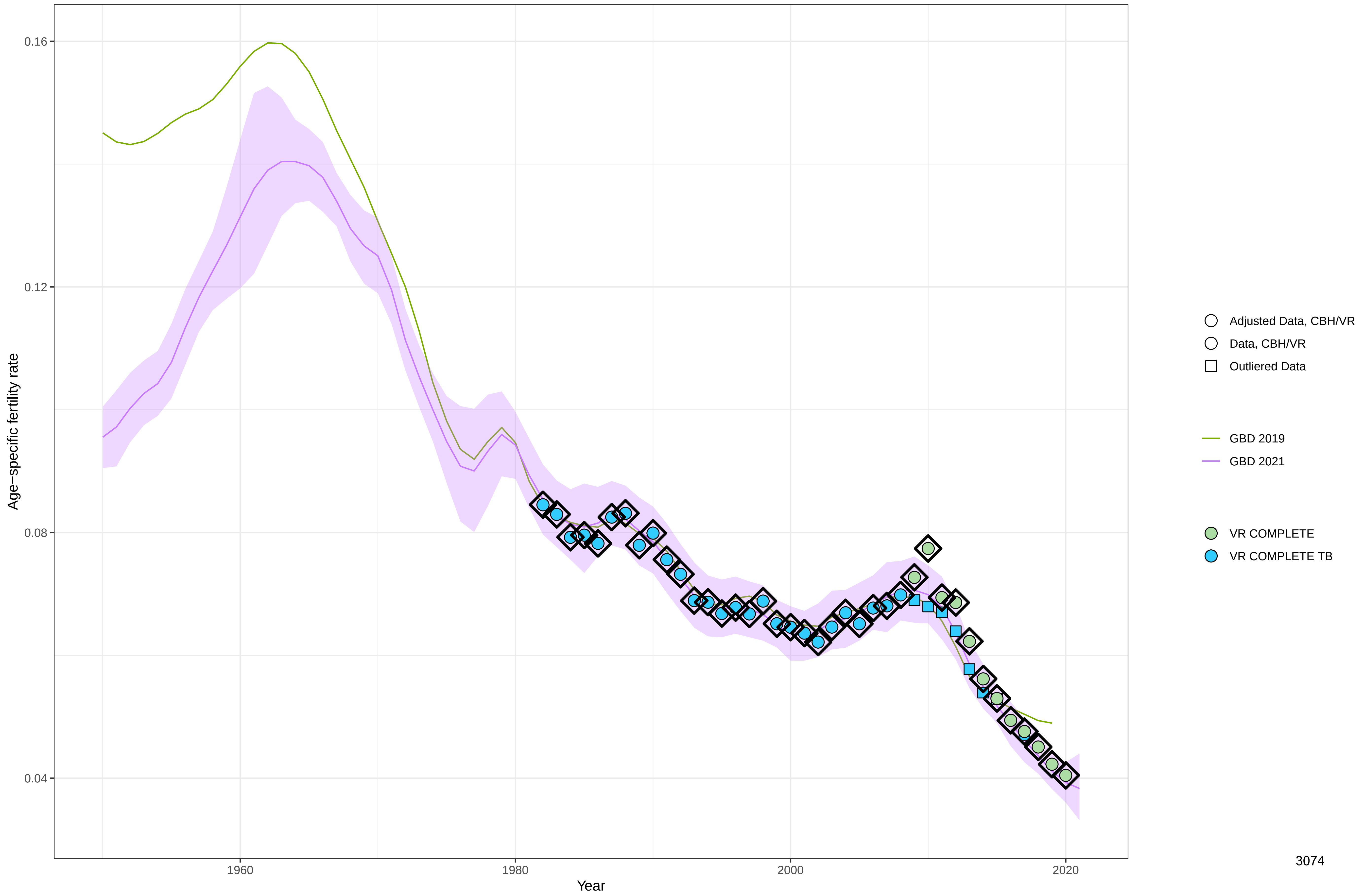

Age−specific fertility fitted trend lines, 1950−2021: West Sussex, Ages: 25−29
Data points enclosed with a diamond are reference sources

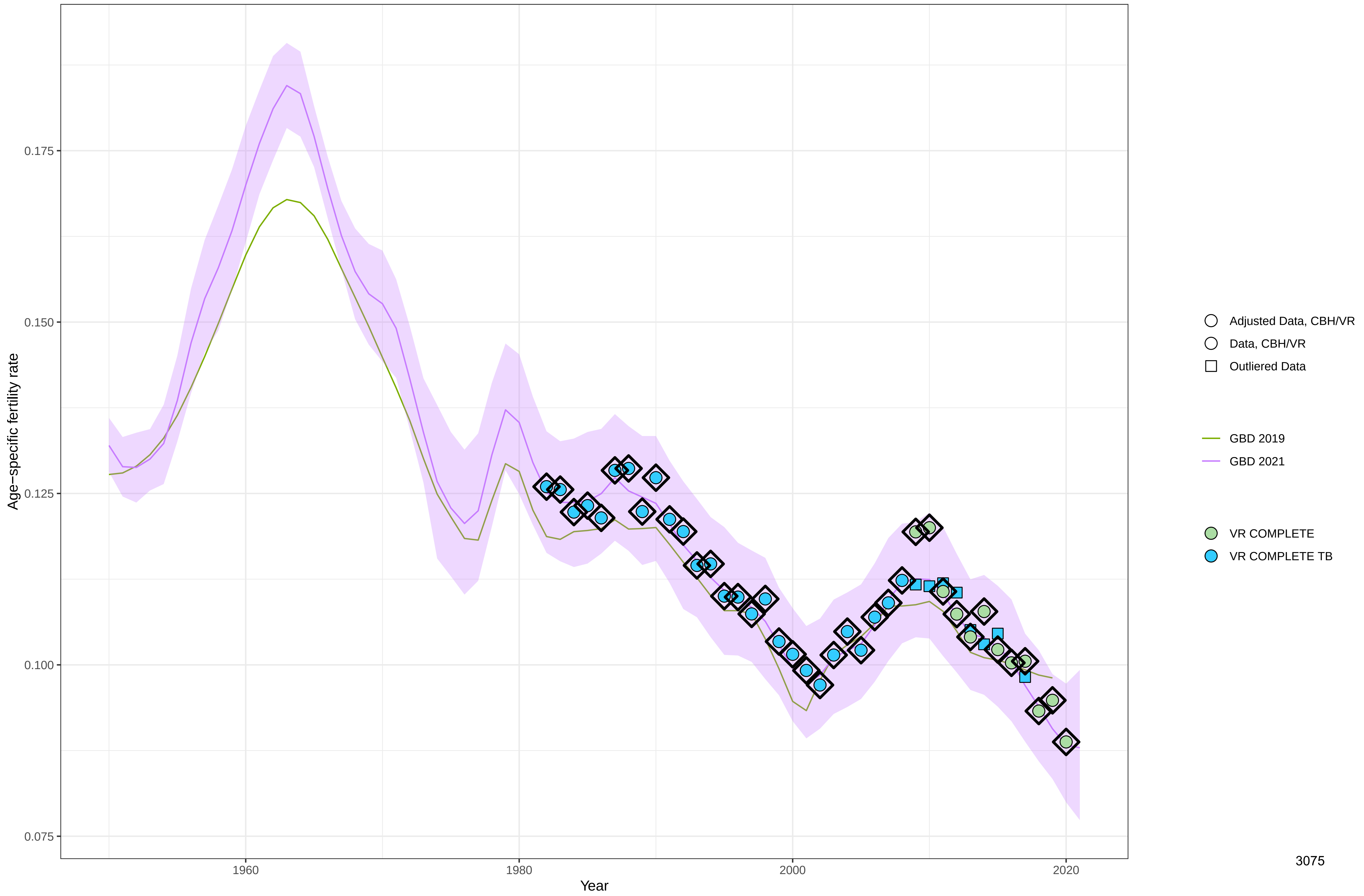

Age−specific fertility fitted trend lines, 1950−2021: West Sussex, Ages: 30−34
Data points enclosed with a diamond are reference sources

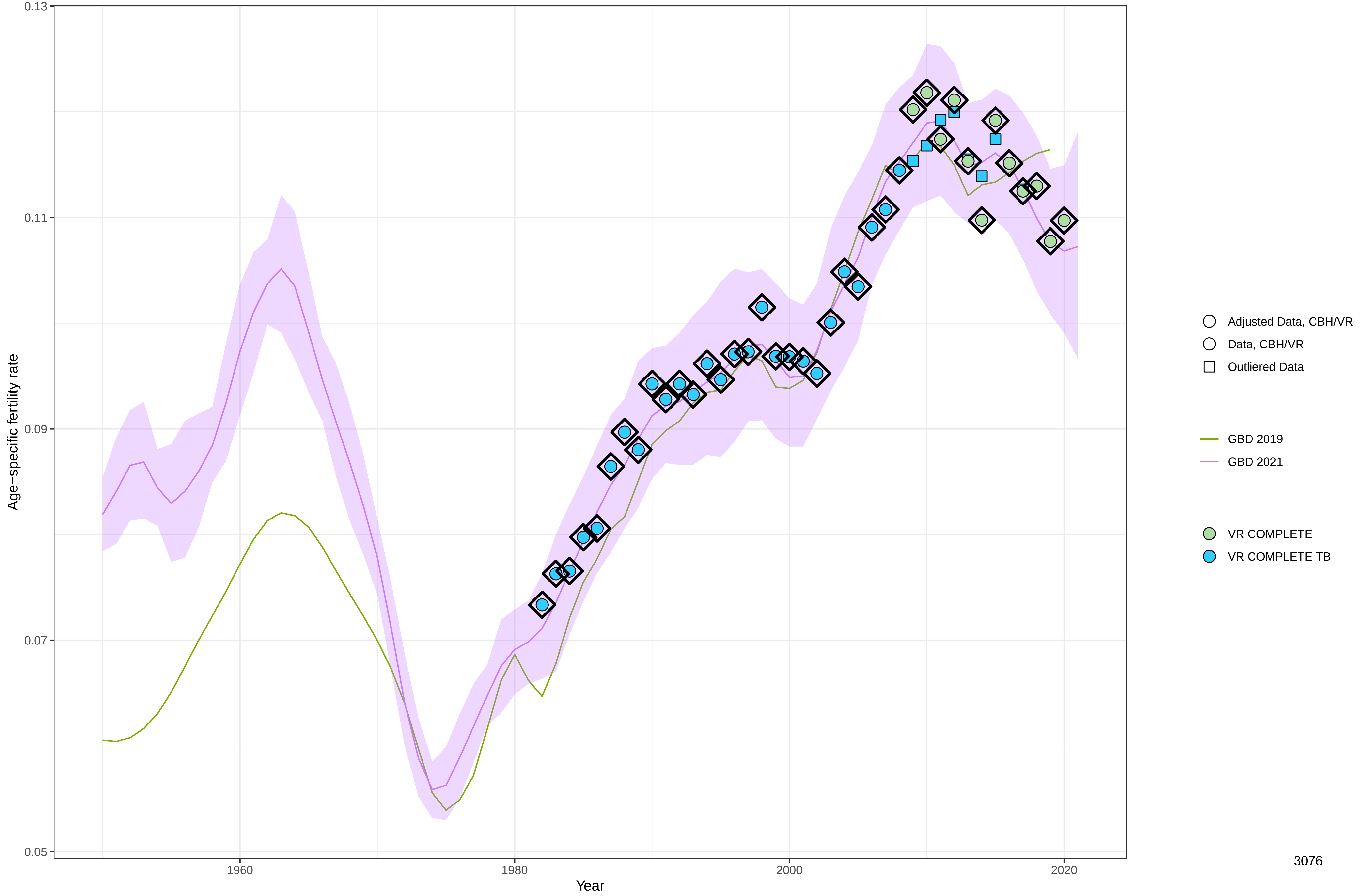

Age−specific fertility fitted trend lines, 1950−2021: West Sussex, Ages: 35−39
Data points enclosed with a diamond are reference sources

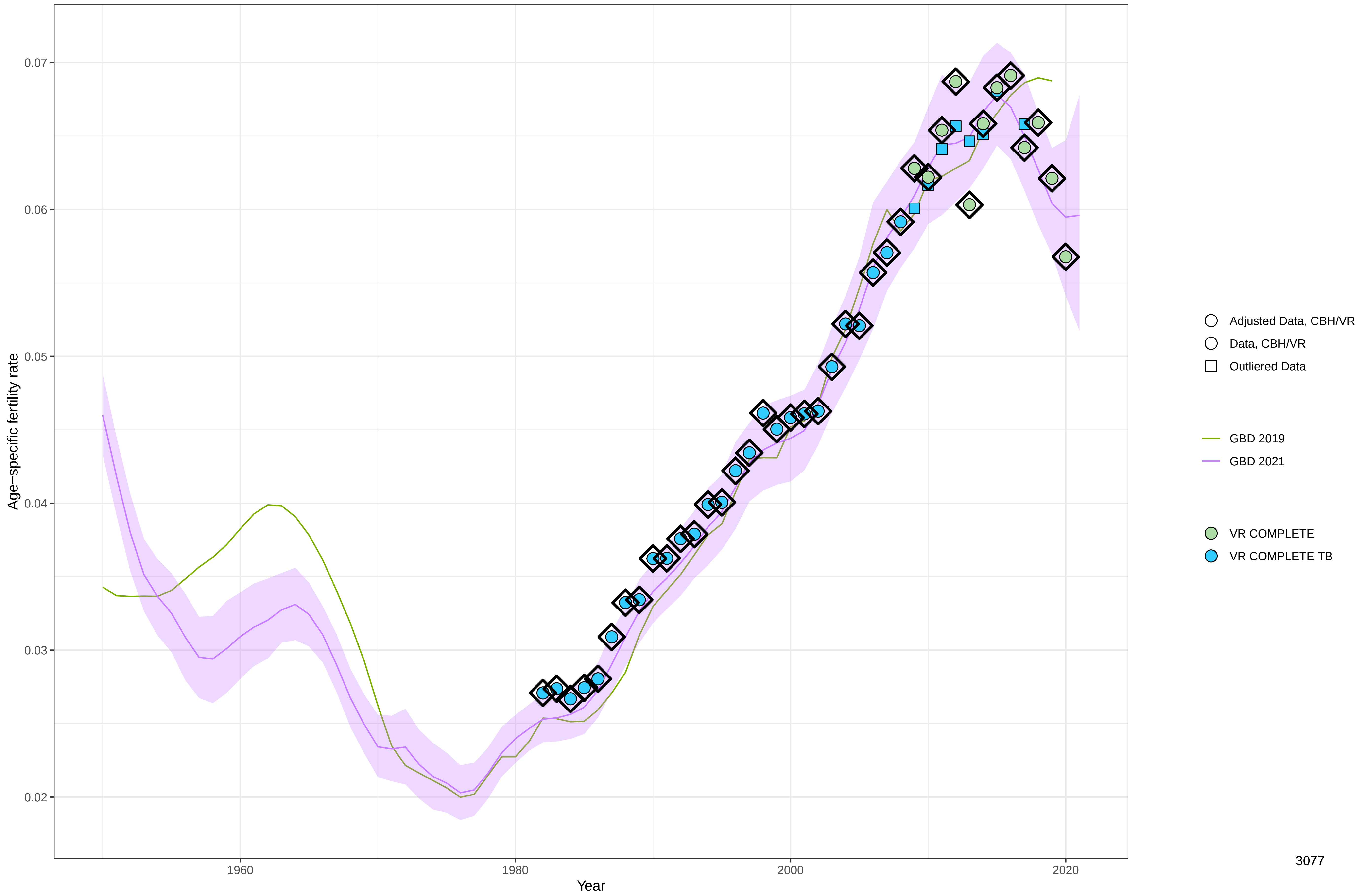

Age−specific fertility fitted trend lines, 1950−2021: West Sussex, Ages: 40−44
Data points enclosed with a diamond are reference sources

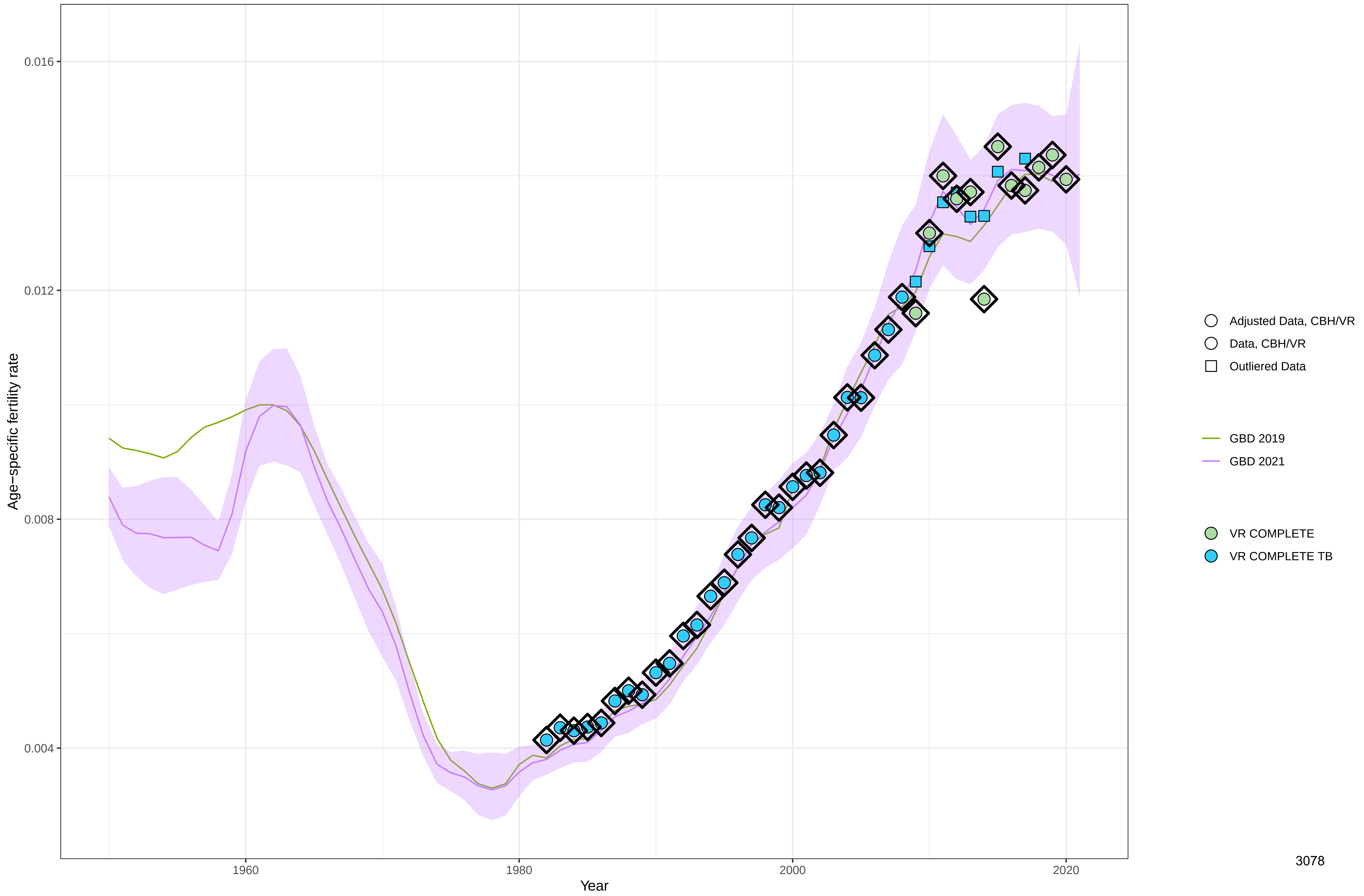

Age−specific fertility fitted trend lines, 1950−2021: West Sussex, Ages: 45−49
Data points enclosed with a diamond are reference sources

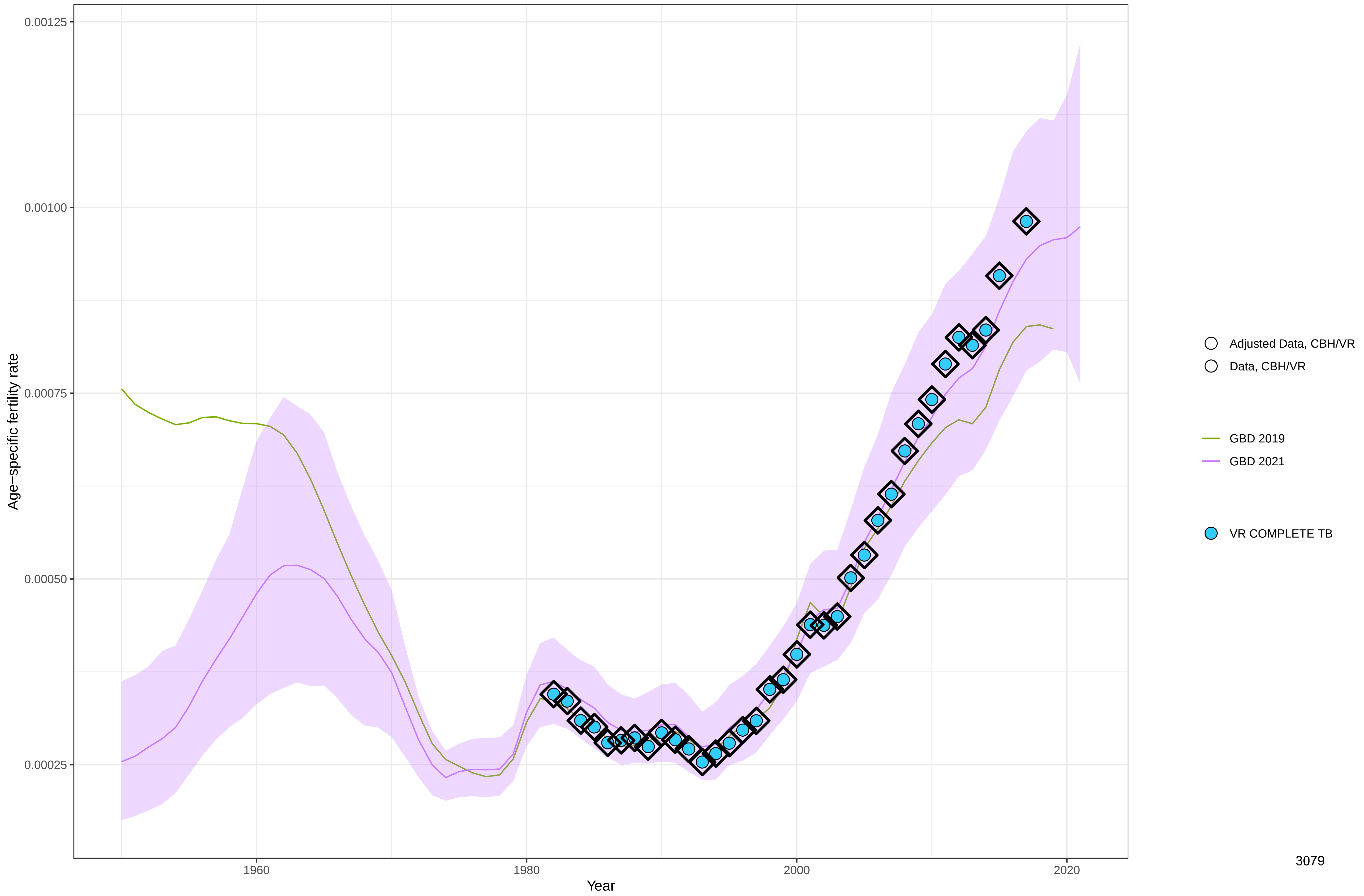

Age−specific fertility fitted trend lines, 1950−2021: West Sussex, Ages: 50−54
Data points enclosed with a diamond are reference sources

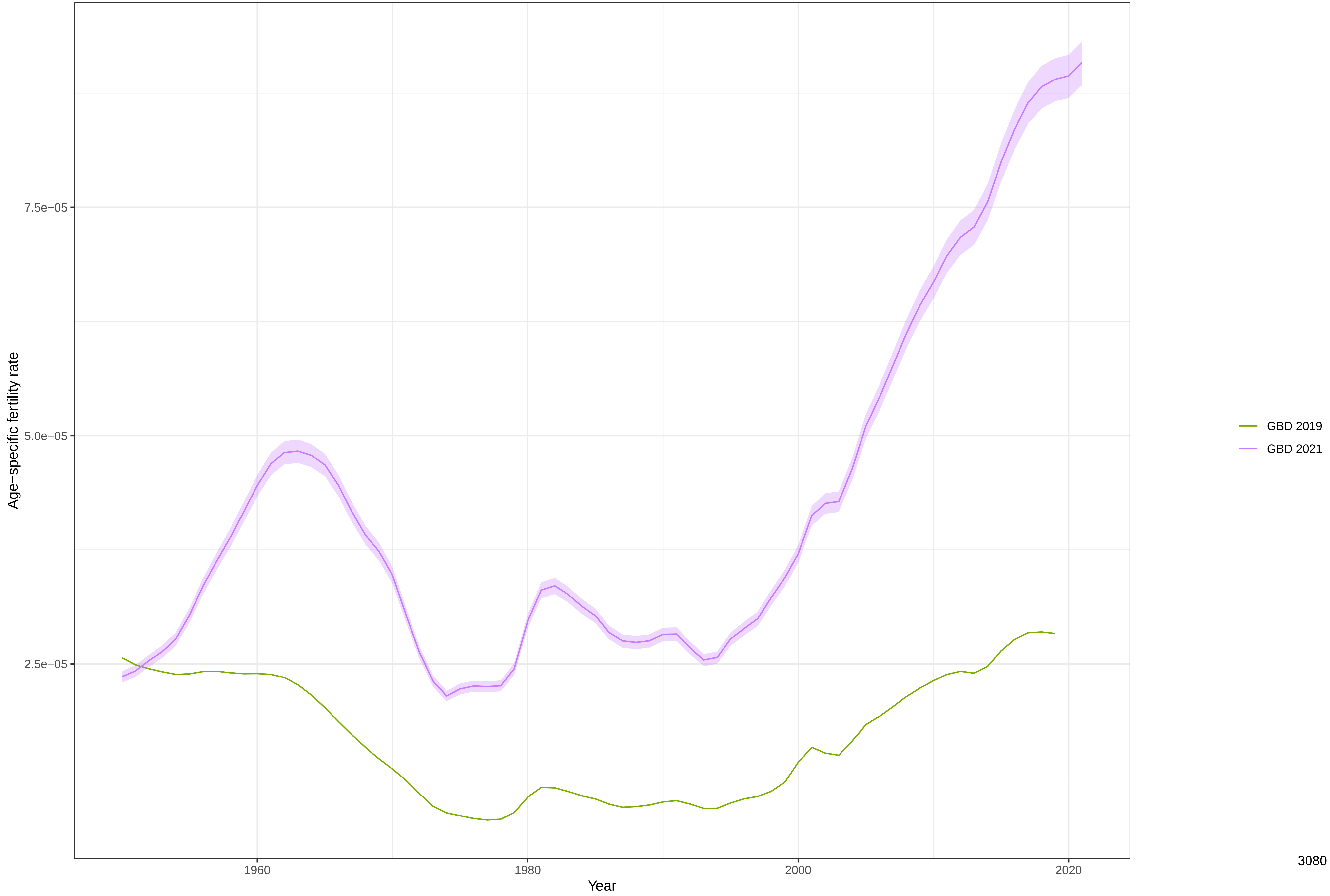

Total fertility rate fitted trend lines, 1950−2021: West Sussex TFR
Data points enclosed with a diamond are reference sources

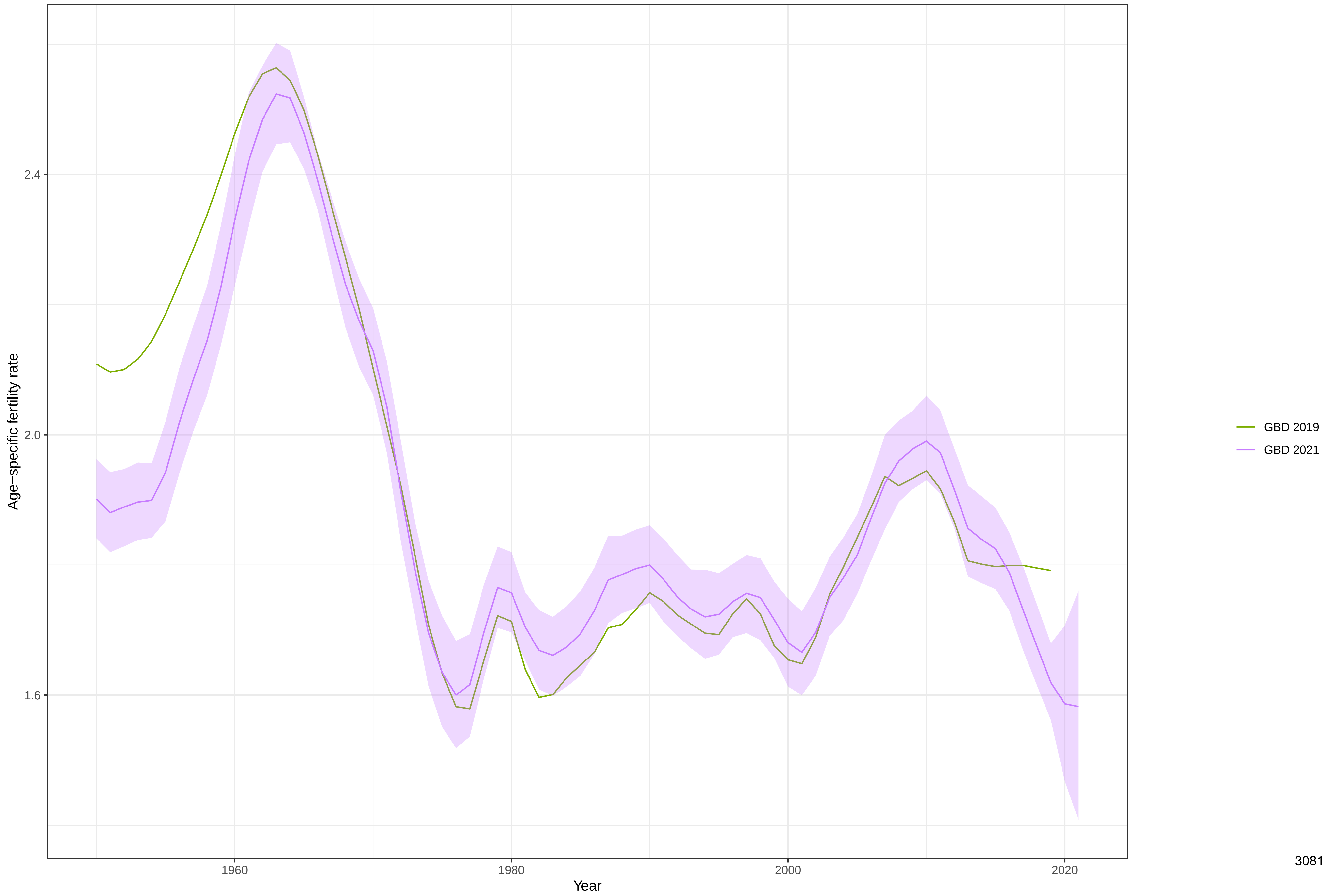

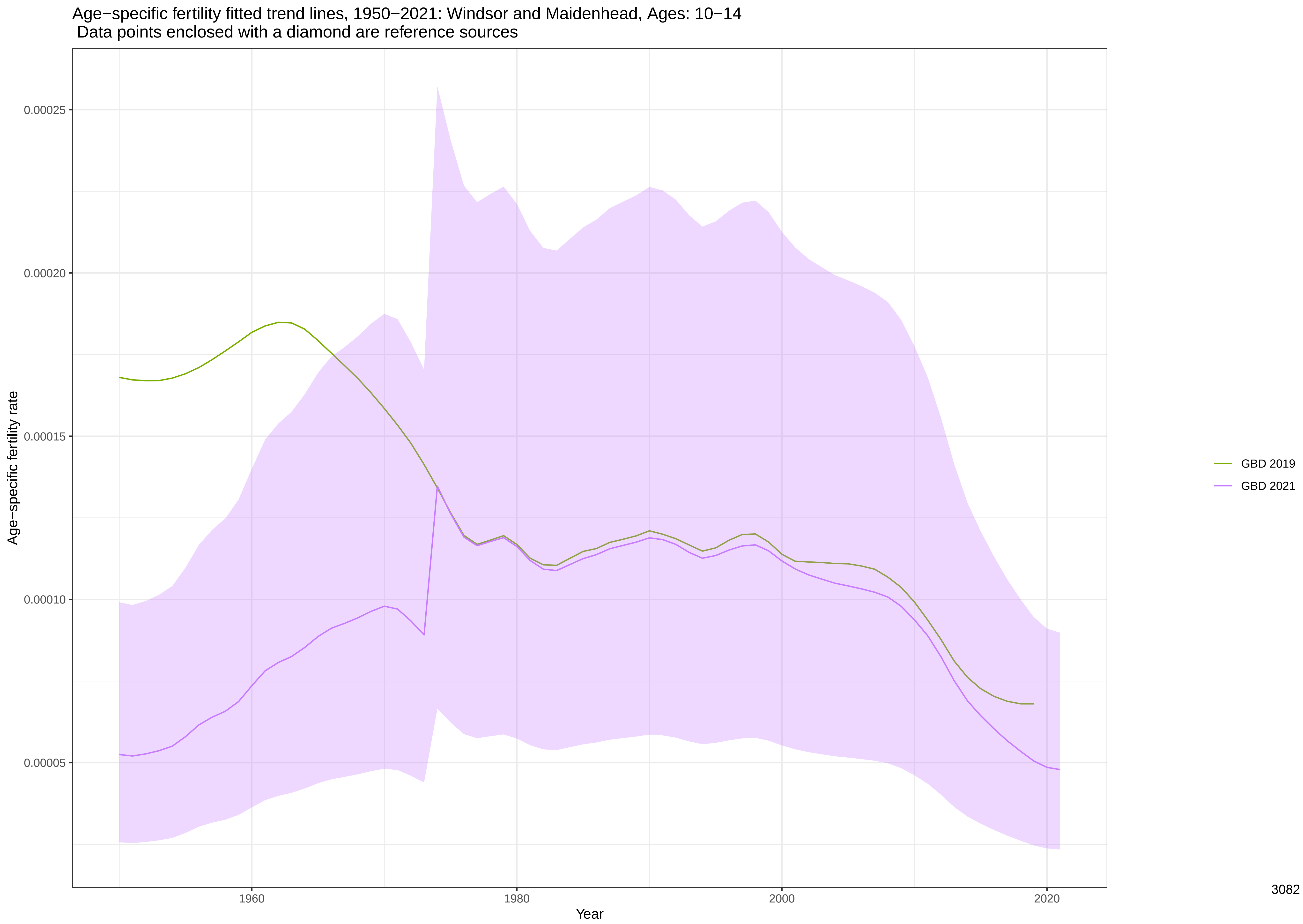
Age−specific fertility fitted trend lines, 1950−2021: Windsor and Maidenhead, Ages: 10−14
Data points enclosed with a diamond are reference sources
Age−specific fertility rate
0.00025
0.00020
0.00015
0.00010
0.00005
1960
1980
2000
2020
Year
GBD 2019
GBD 2021

Age−specific fertility fitted trend lines, 1950−2021: Windsor and Maidenhead, Ages: 15−19
Data points enclosed with a diamond are reference sources

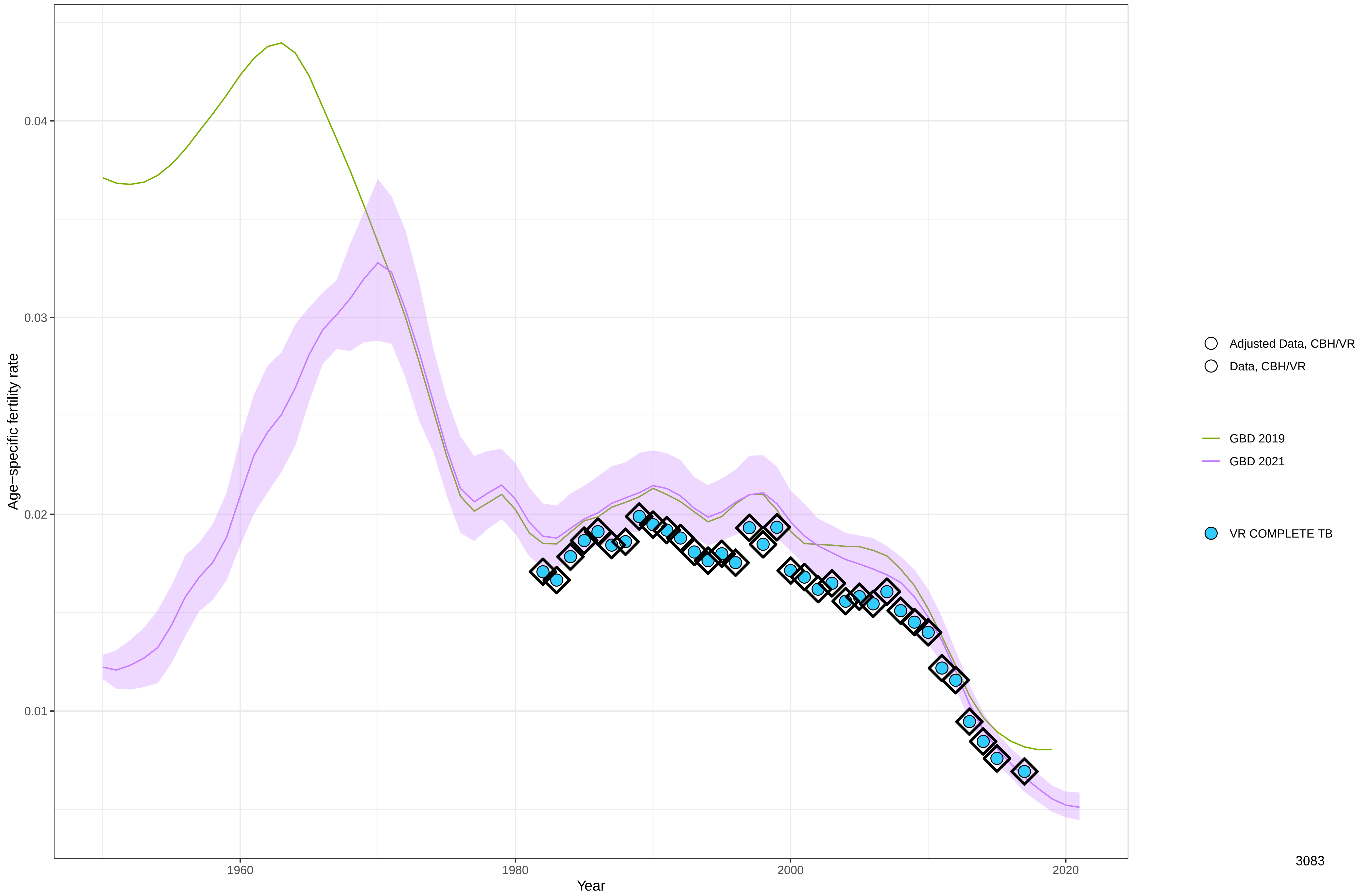

Age−specific fertility fitted trend lines, 1950−2021: Windsor and Maidenhead, Ages: 20−24
Data points enclosed with a diamond are reference sources

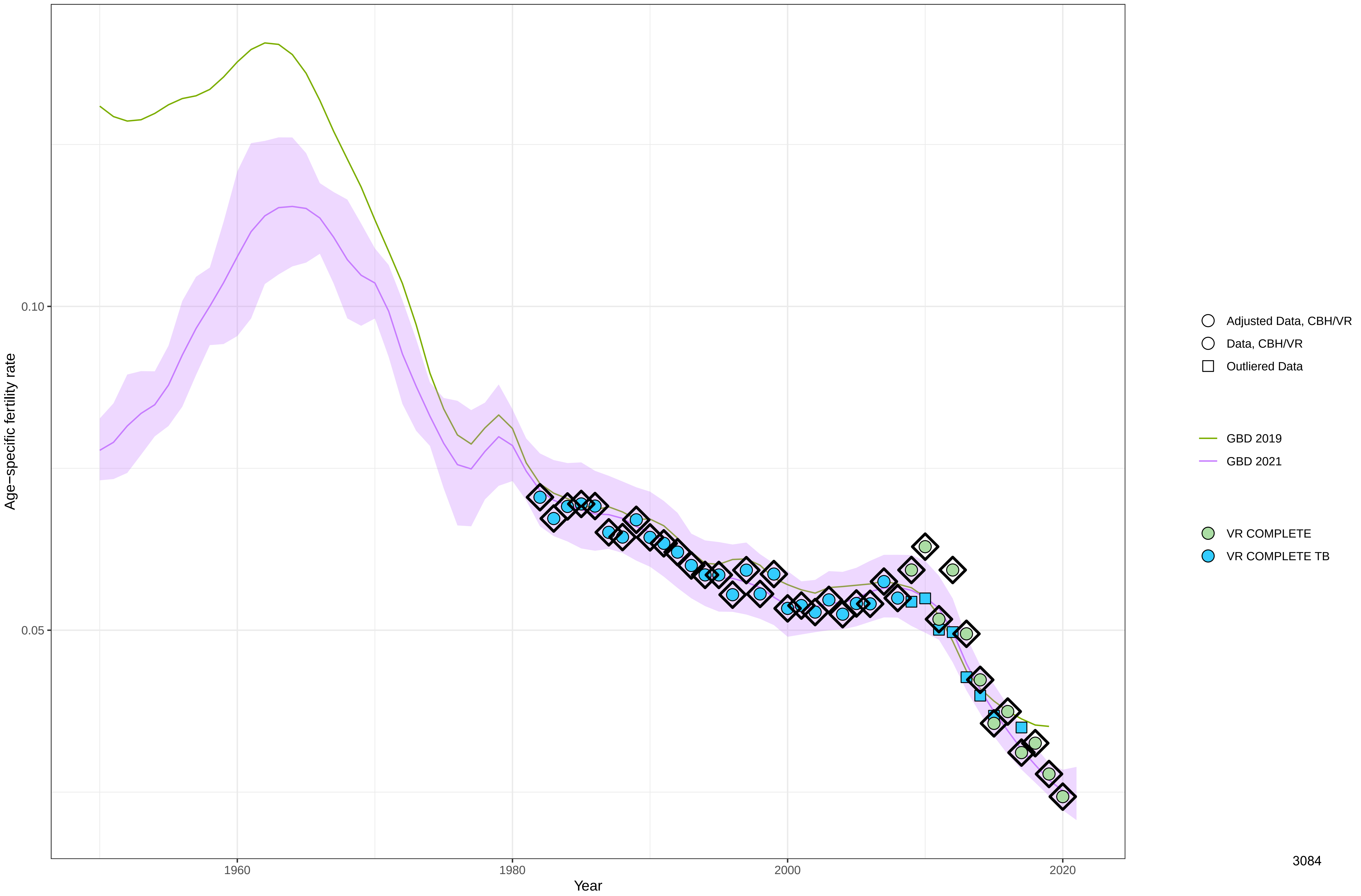

Age−specific fertility fitted trend lines, 1950−2021: Windsor and Maidenhead, Ages: 25−29
Data points enclosed with a diamond are reference sources

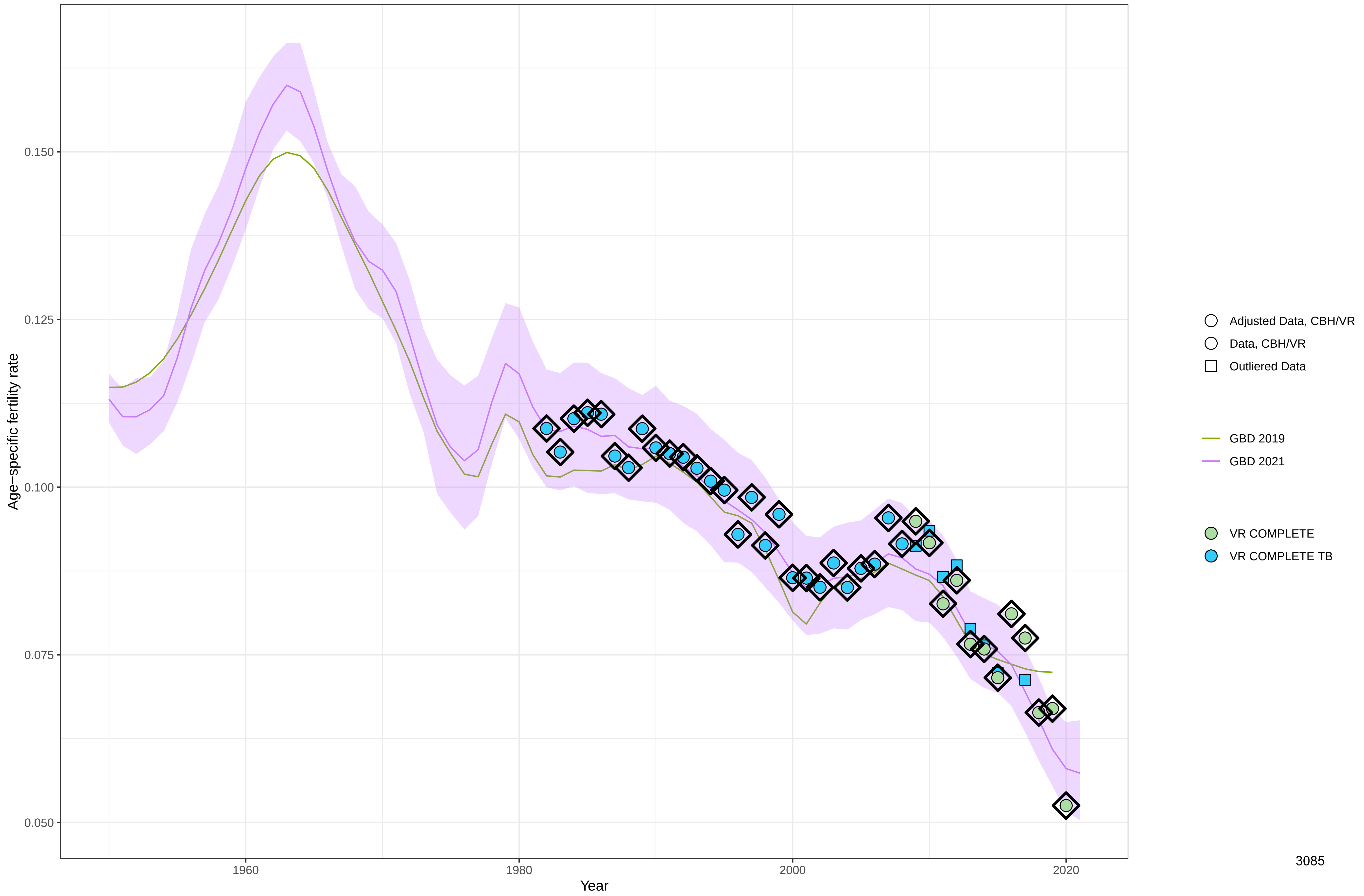

Age−specific fertility fitted trend lines, 1950−2021: Windsor and Maidenhead, Ages: 30−34
Data points enclosed with a diamond are reference sources

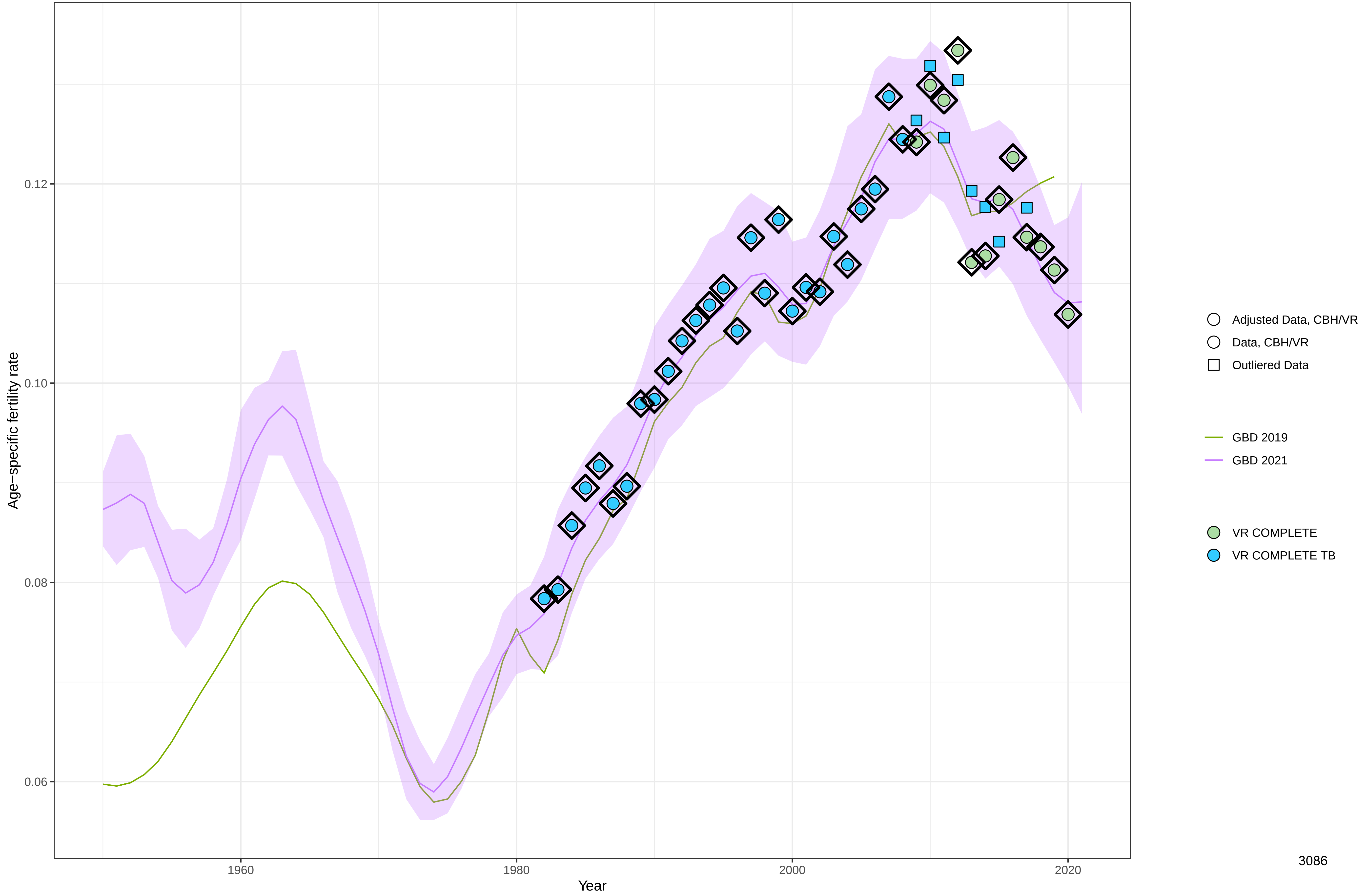

Age−specific fertility fitted trend lines, 1950−2021: Windsor and Maidenhead, Ages: 35−39
Data points enclosed with a diamond are reference sources

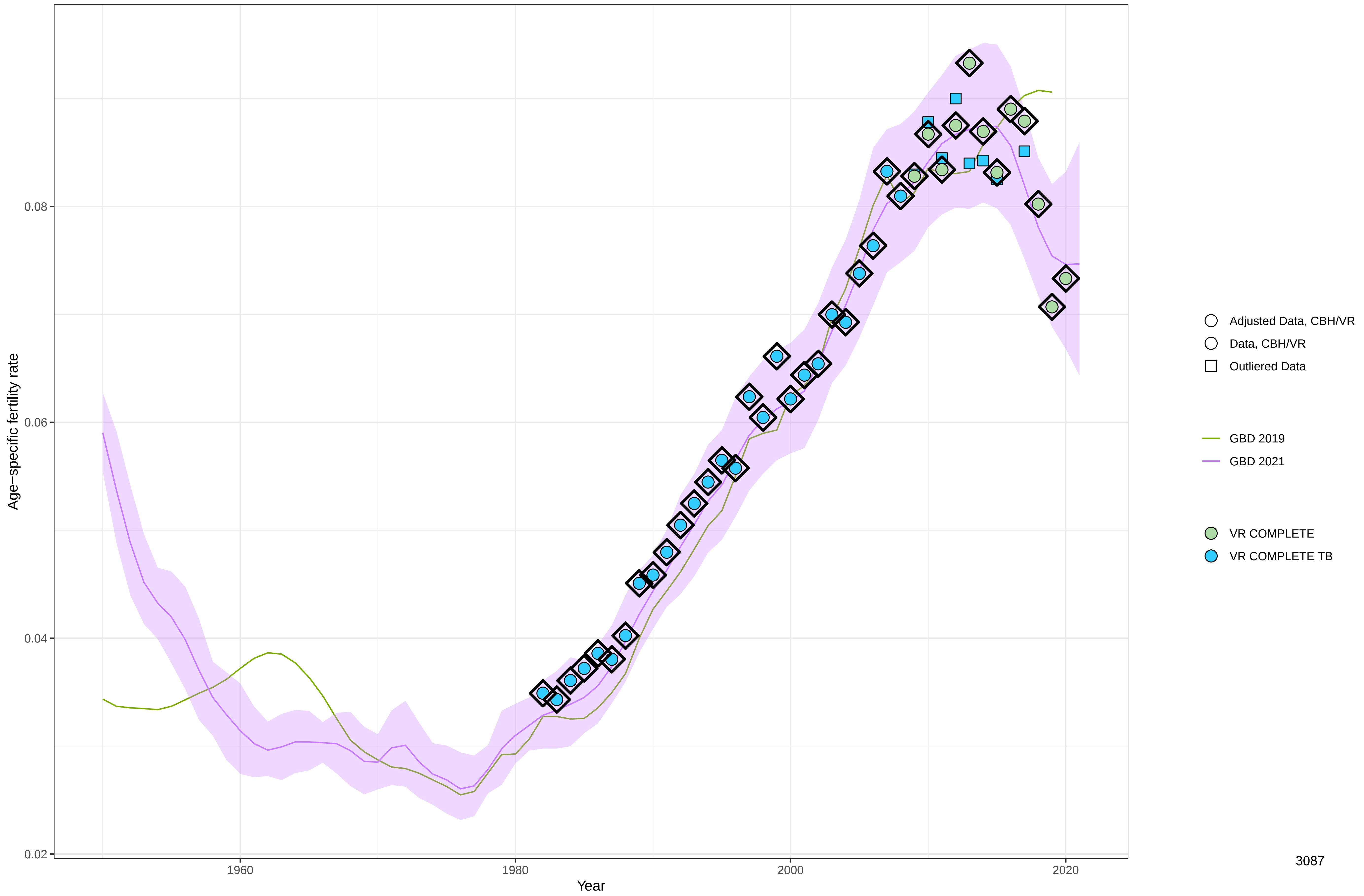

Age−specific fertility fitted trend lines, 1950−2021: Windsor and Maidenhead, Ages: 40−44
Data points enclosed with a diamond are reference sources

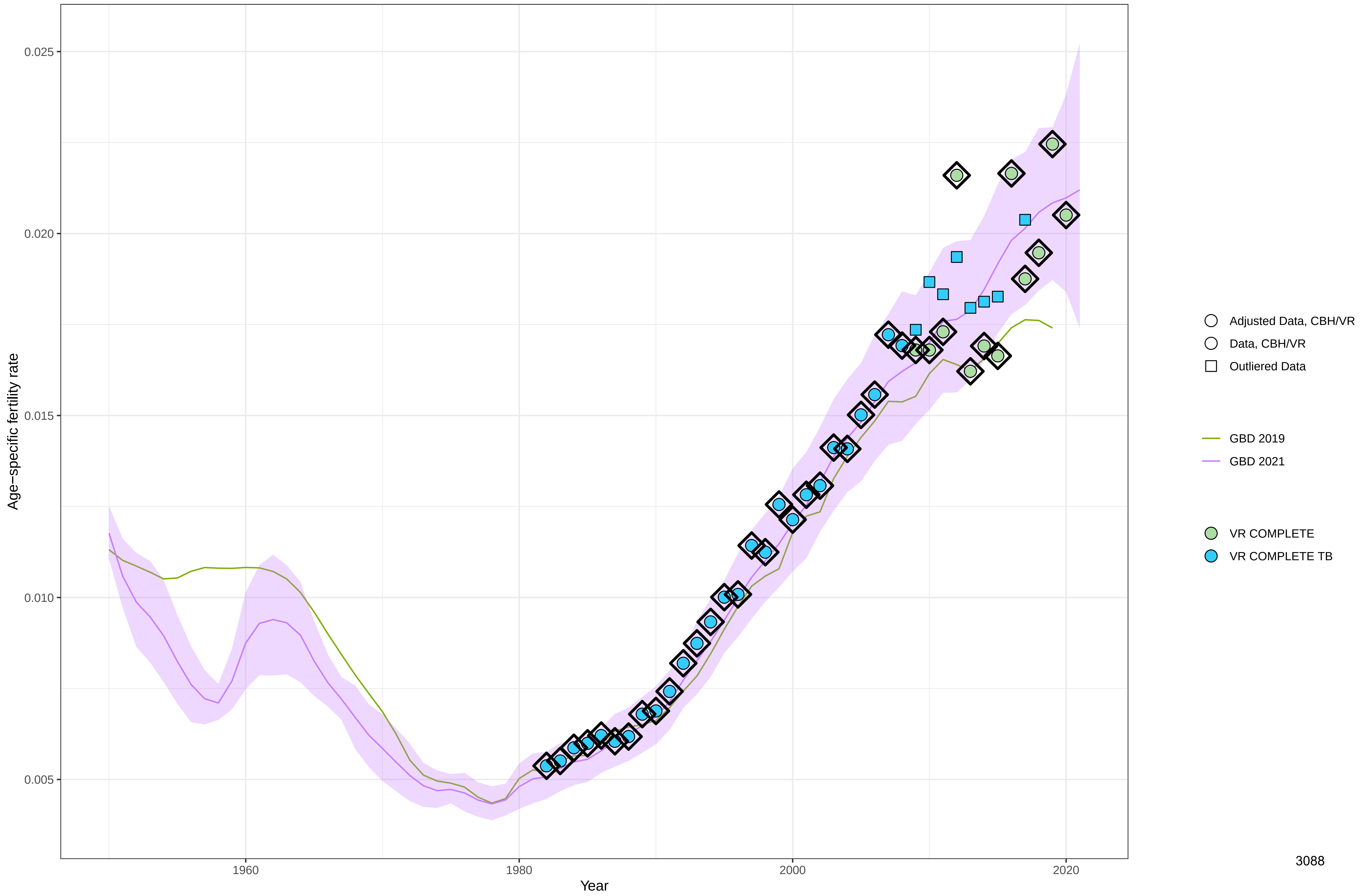

Age−specific fertility fitted trend lines, 1950−2021: Windsor and Maidenhead, Ages: 45−49
Data points enclosed with a diamond are reference sources

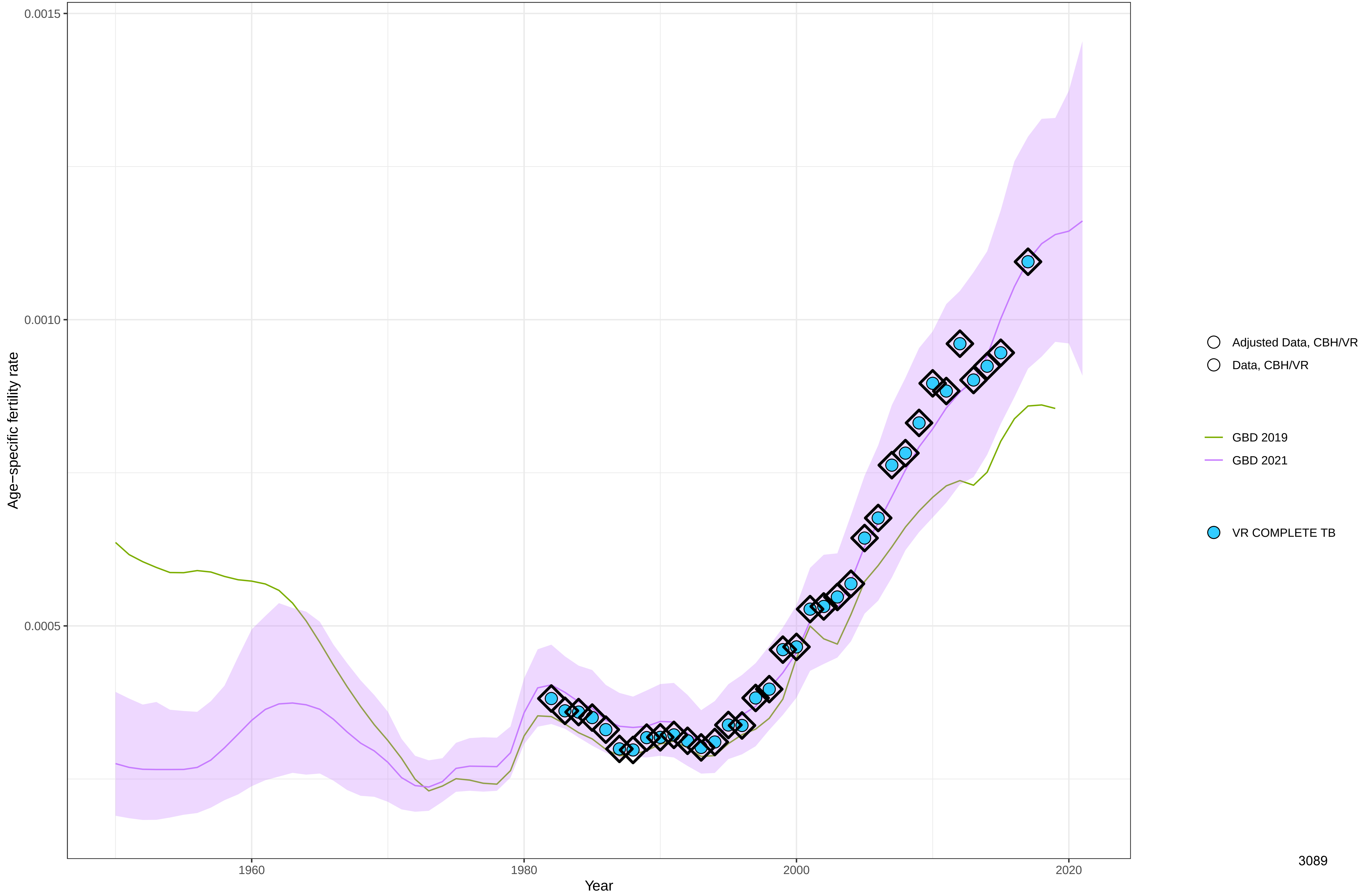

Age−specific fertility fitted trend lines, 1950−2021: Windsor and Maidenhead, Ages: 50−54
Data points enclosed with a diamond are reference sources

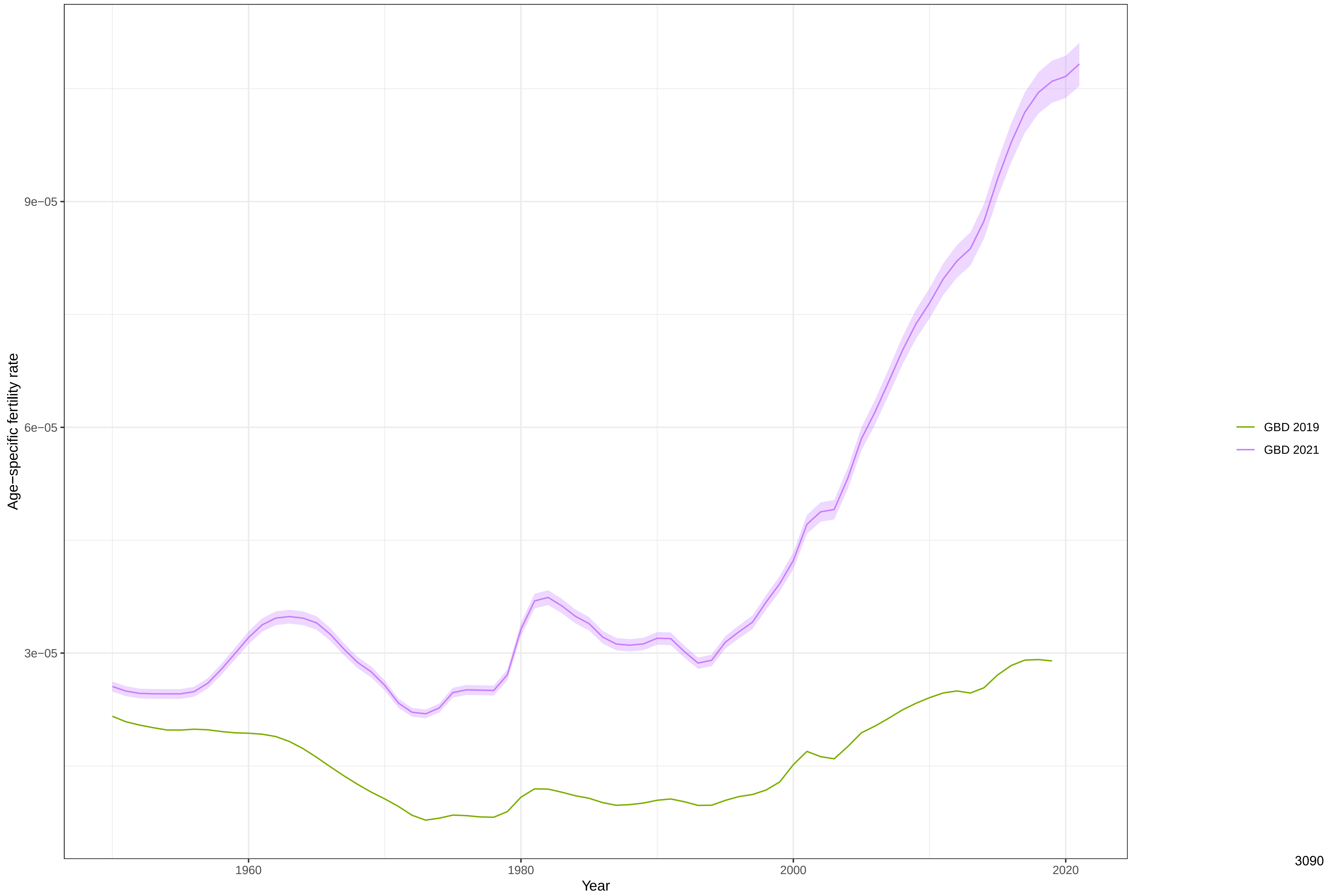

Total fertility rate fitted trend lines, 1950−2021: Windsor and Maidenhead TFR
Data points enclosed with a diamond are reference sources

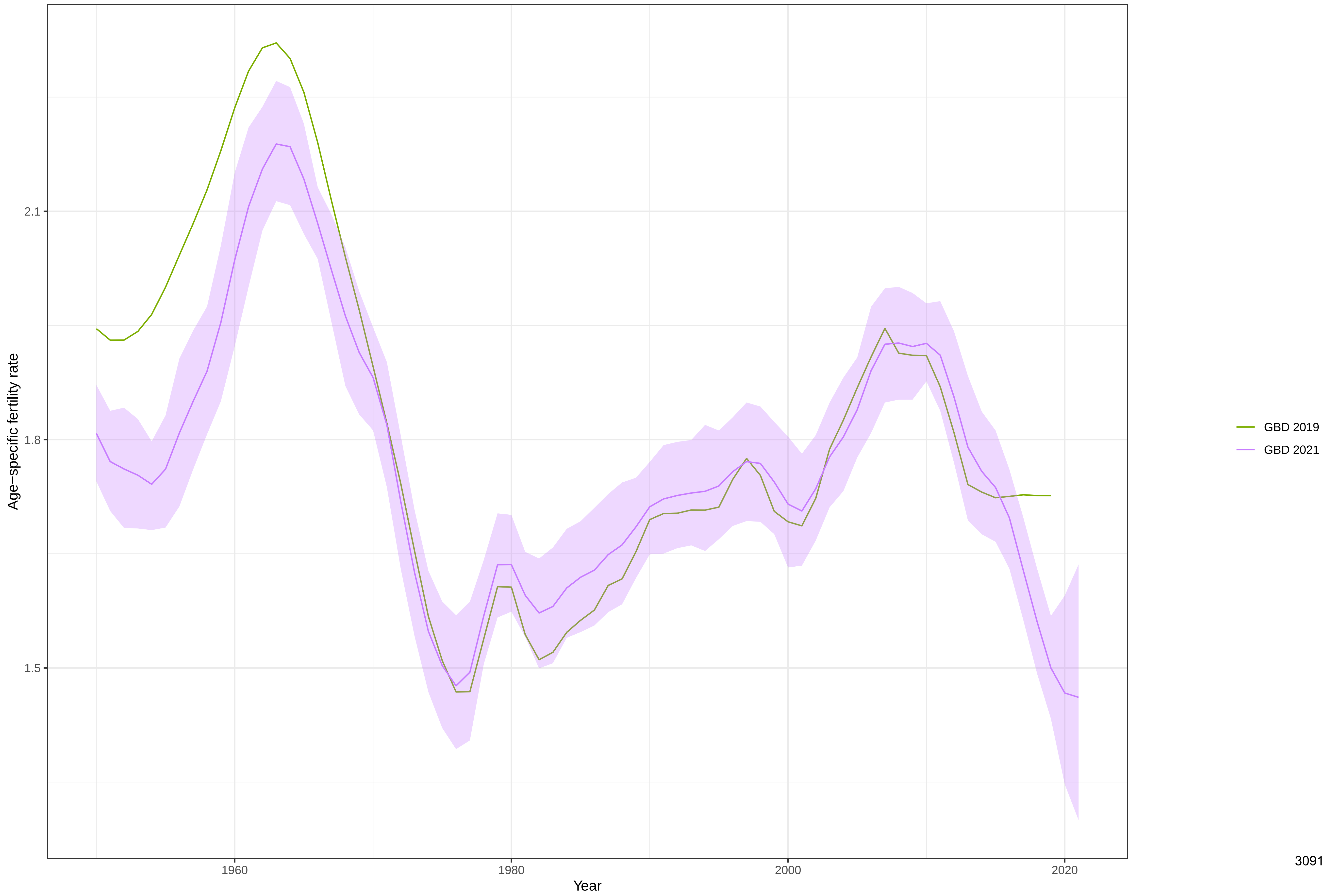

Age−specific fertility fitted trend lines, 1950−2021: Wokingham, Ages: 10−14
Data points enclosed with a diamond are reference sources

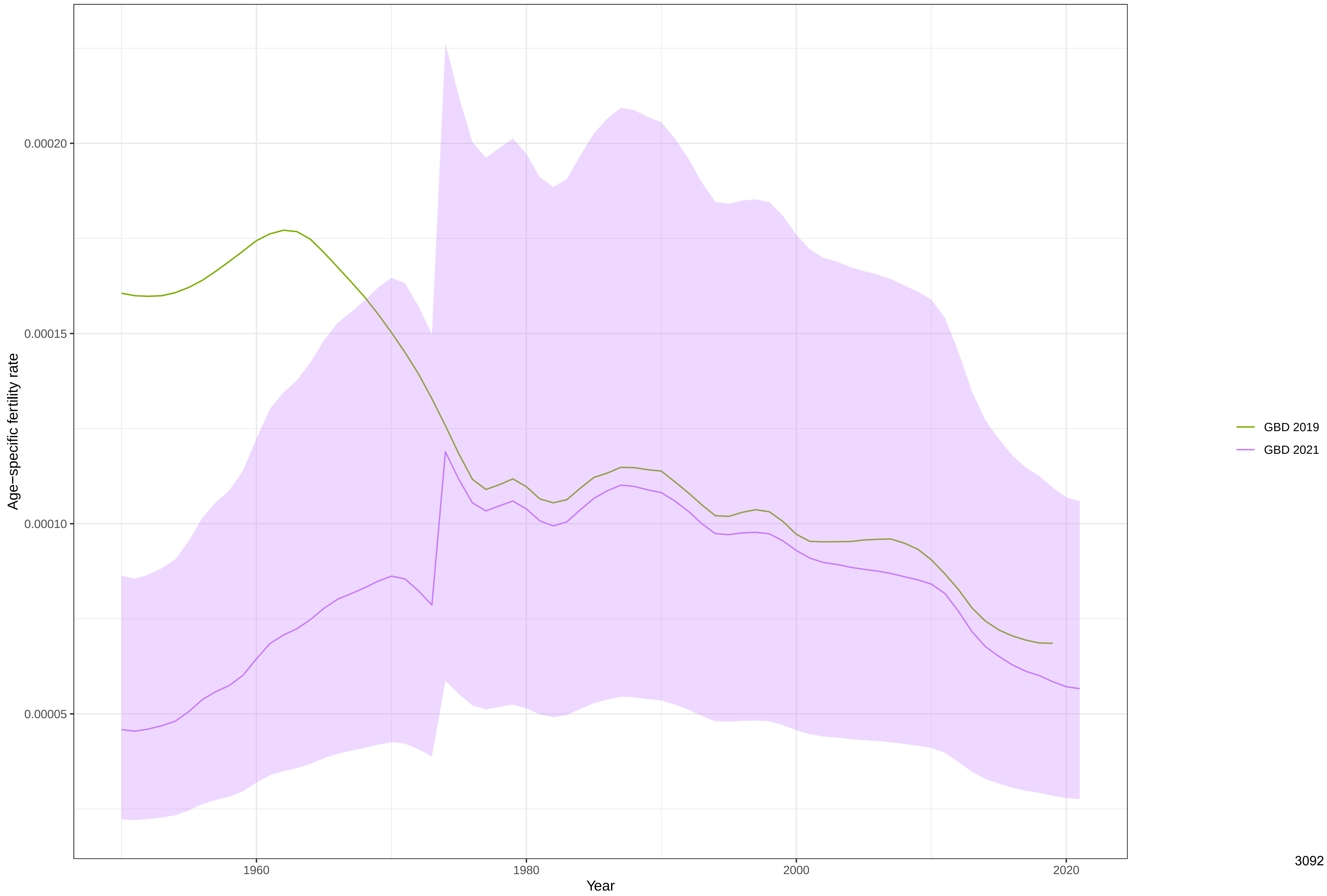

Age−specific fertility fitted trend lines, 1950−2021: Wokingham, Ages: 15−19
Data points enclosed with a diamond are reference sources

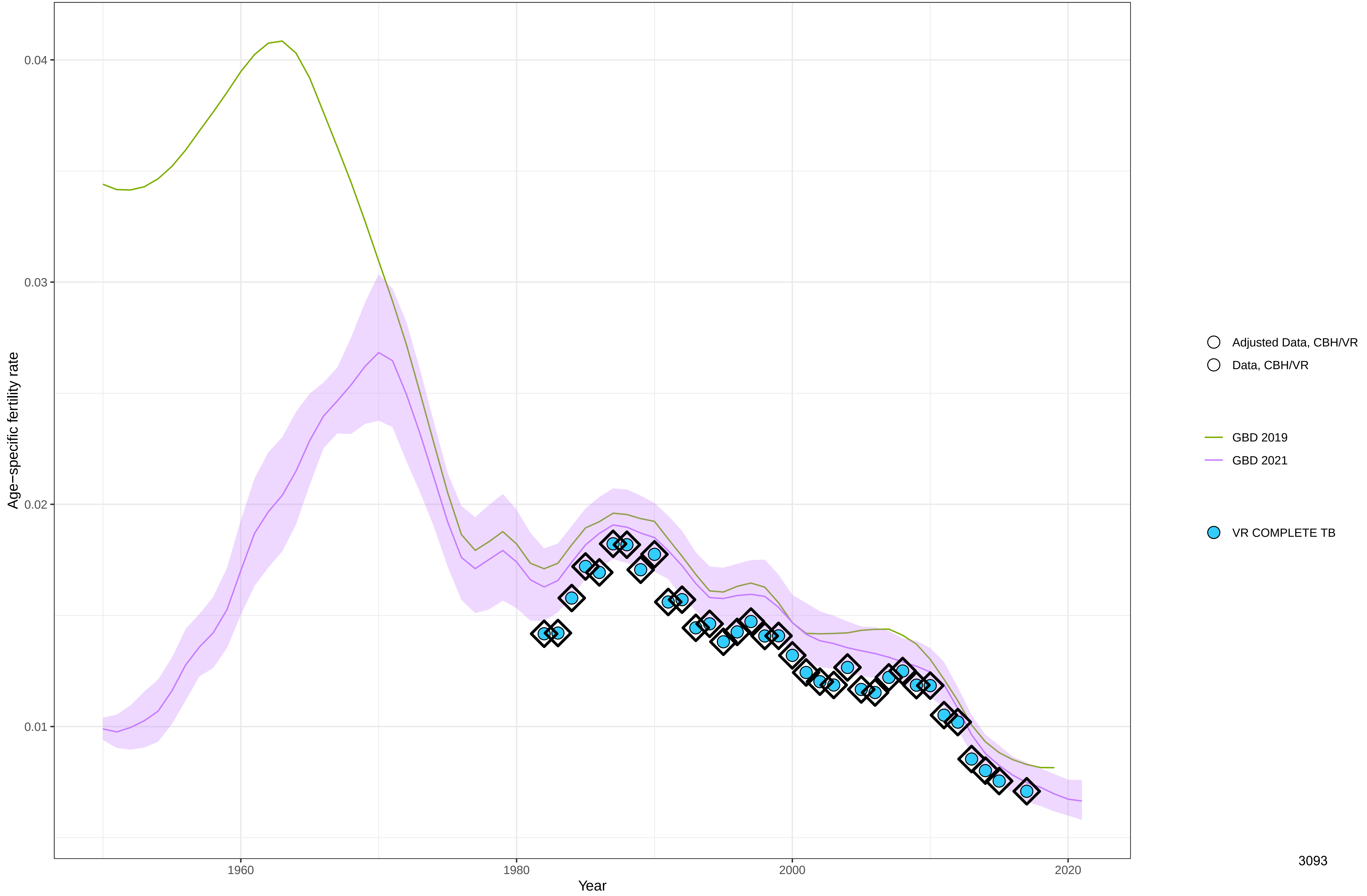

Age−specific fertility fitted trend lines, 1950−2021: Wokingham, Ages: 20−24
Data points enclosed with a diamond are reference sources

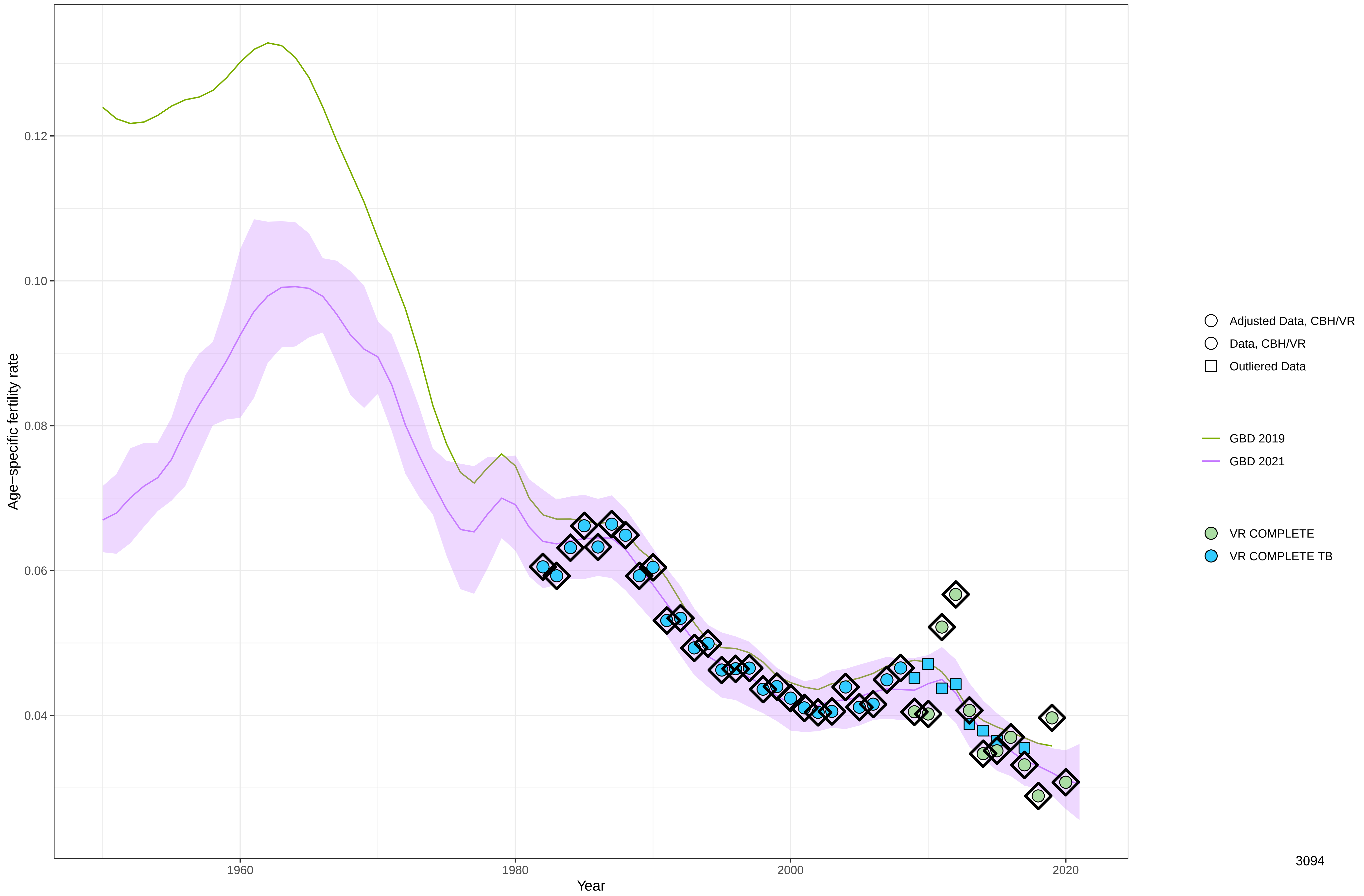

Age−specific fertility fitted trend lines, 1950−2021: Wokingham, Ages: 25−29
Data points enclosed with a diamond are reference sources

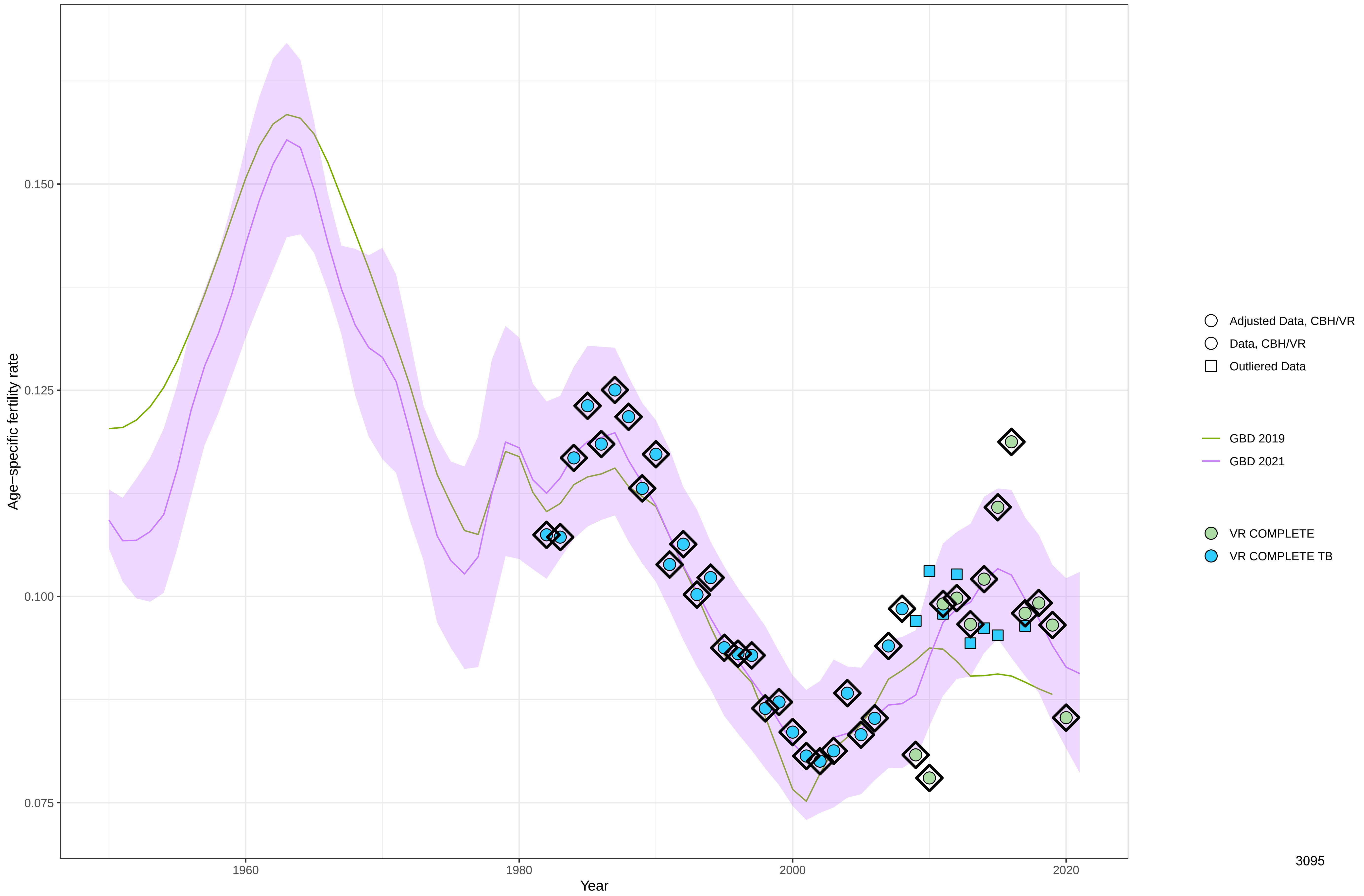

Age−specific fertility fitted trend lines, 1950−2021: Wokingham, Ages: 30−34
Data points enclosed with a diamond are reference sources

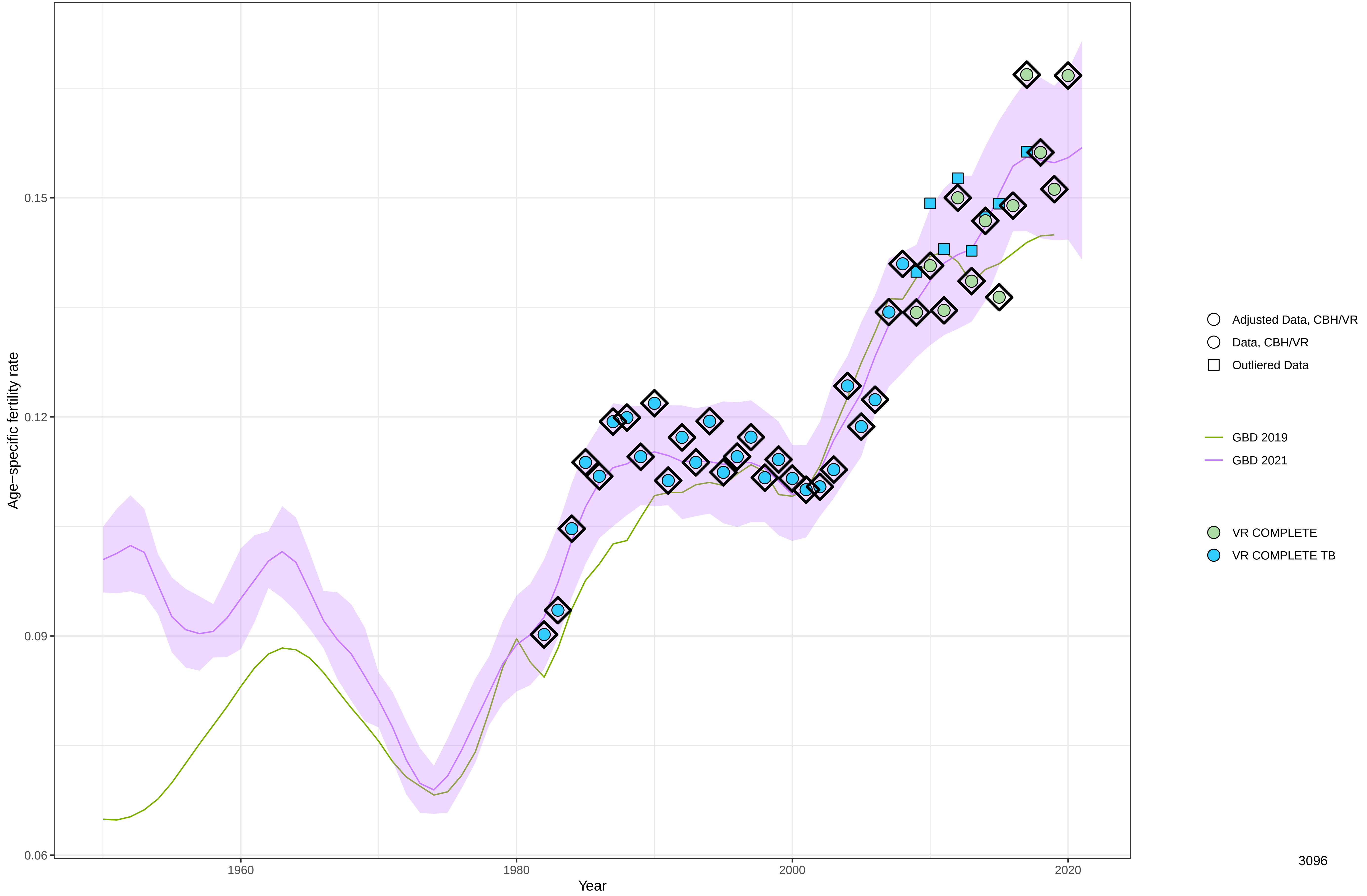

Age−specific fertility fitted trend lines, 1950−2021: Wokingham, Ages: 35−39
Data points enclosed with a diamond are reference sources

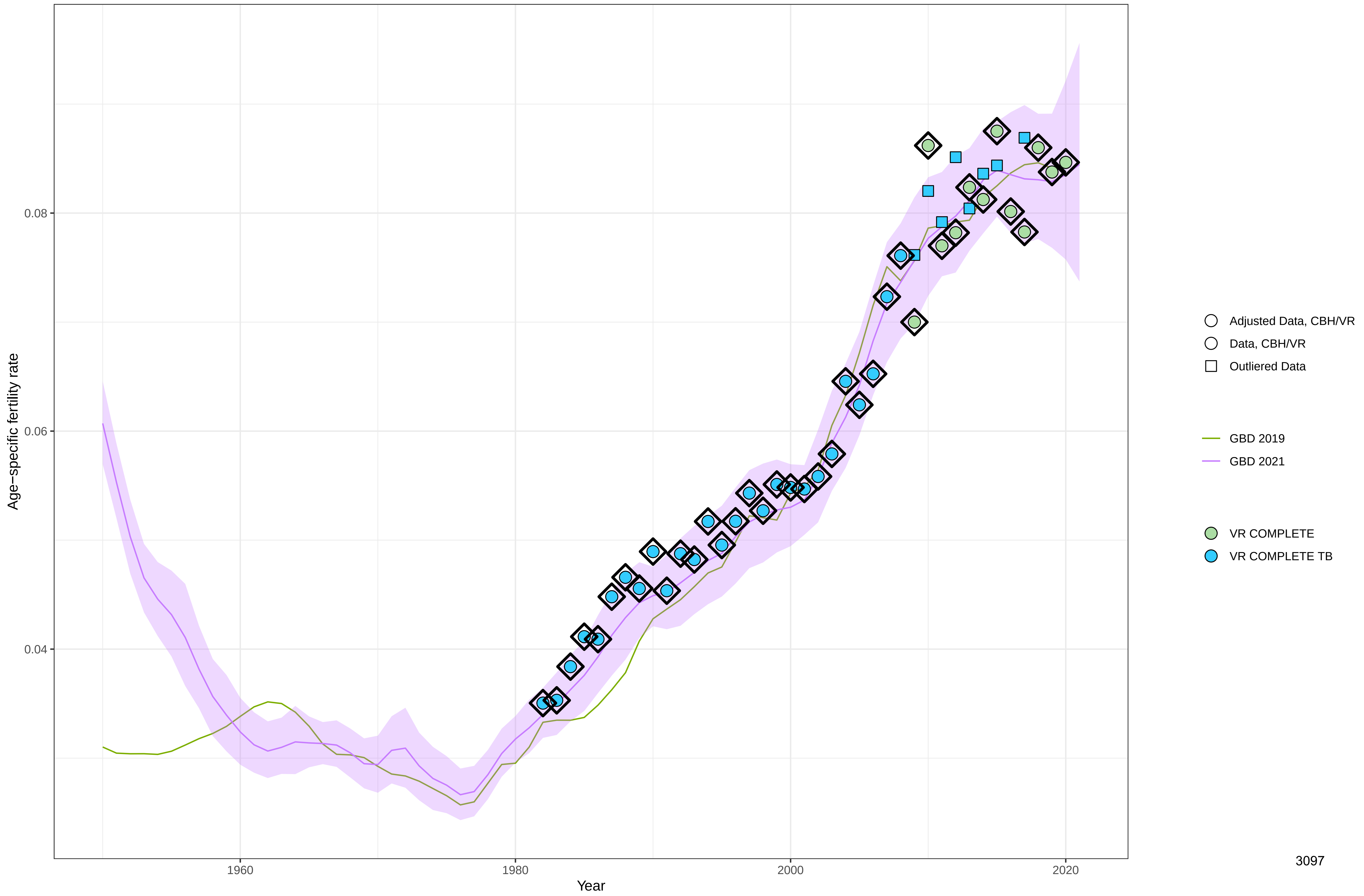

Age−specific fertility fitted trend lines, 1950−2021: Wokingham, Ages: 40−44
Data points enclosed with a diamond are reference sources

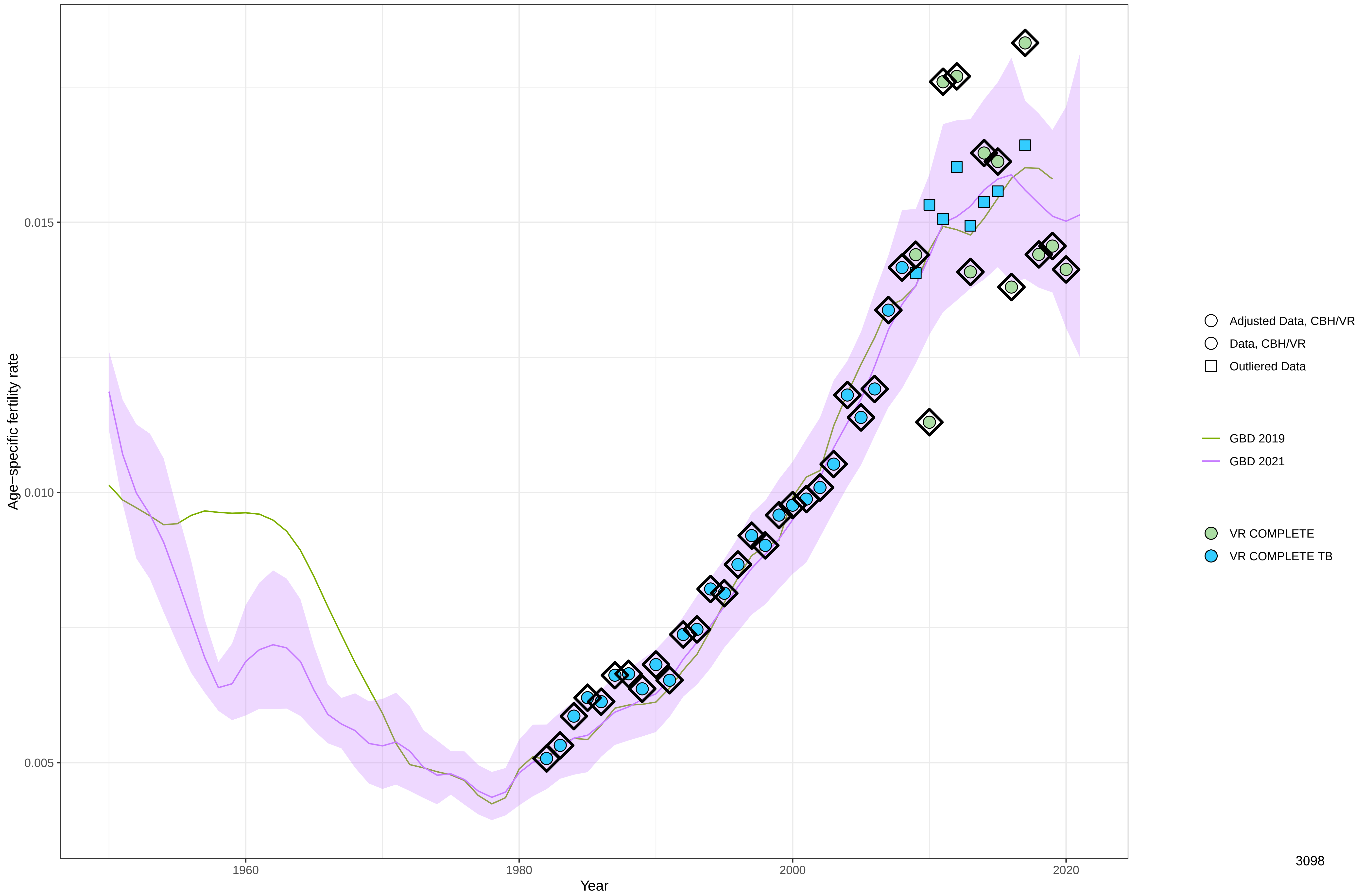

Age−specific fertility fitted trend lines, 1950−2021: Wokingham, Ages: 45−49
Data points enclosed with a diamond are reference sources

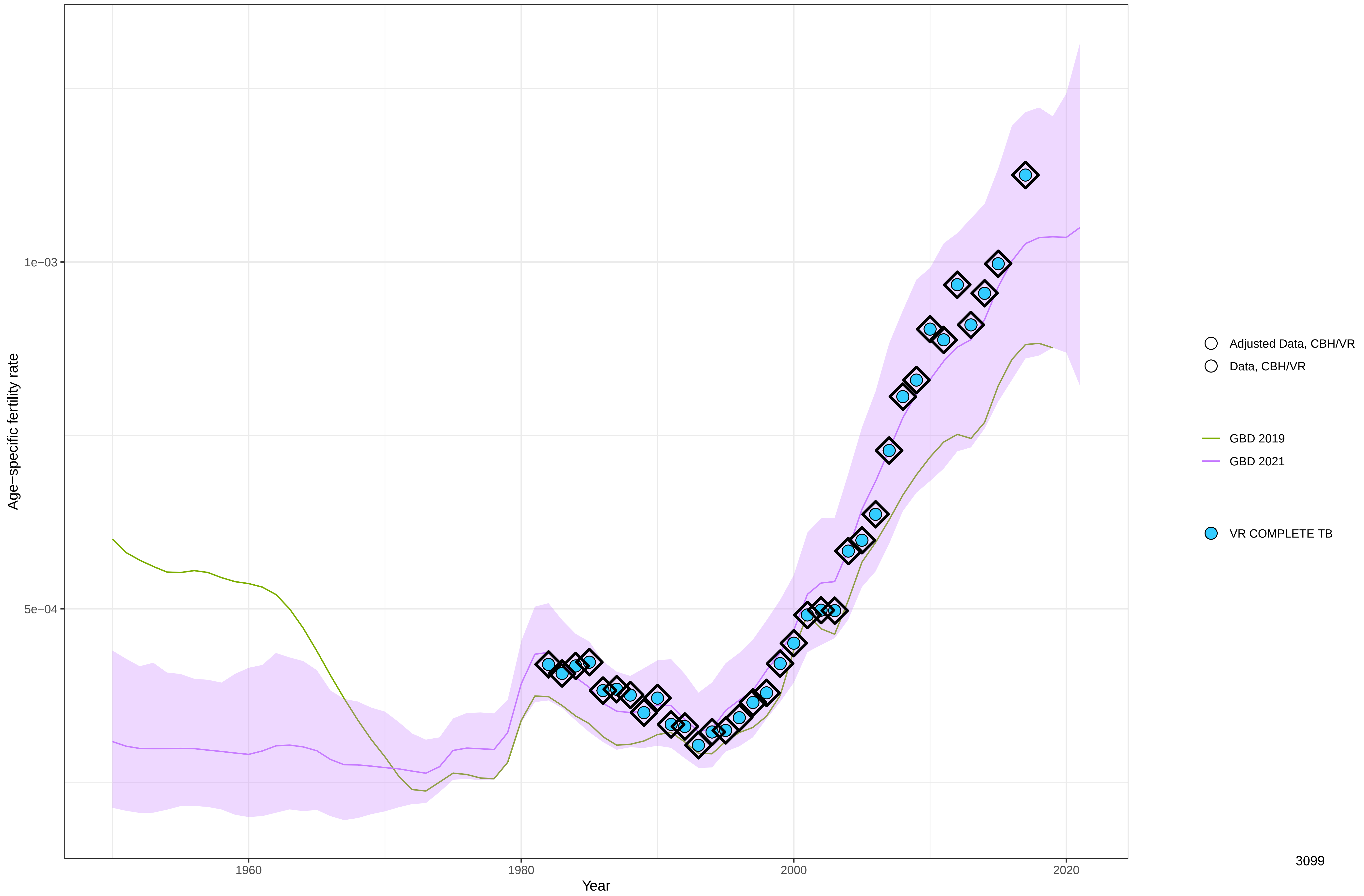

Age−specific fertility fitted trend lines, 1950−2021: Wokingham, Ages: 50−54
Data points enclosed with a diamond are reference sources

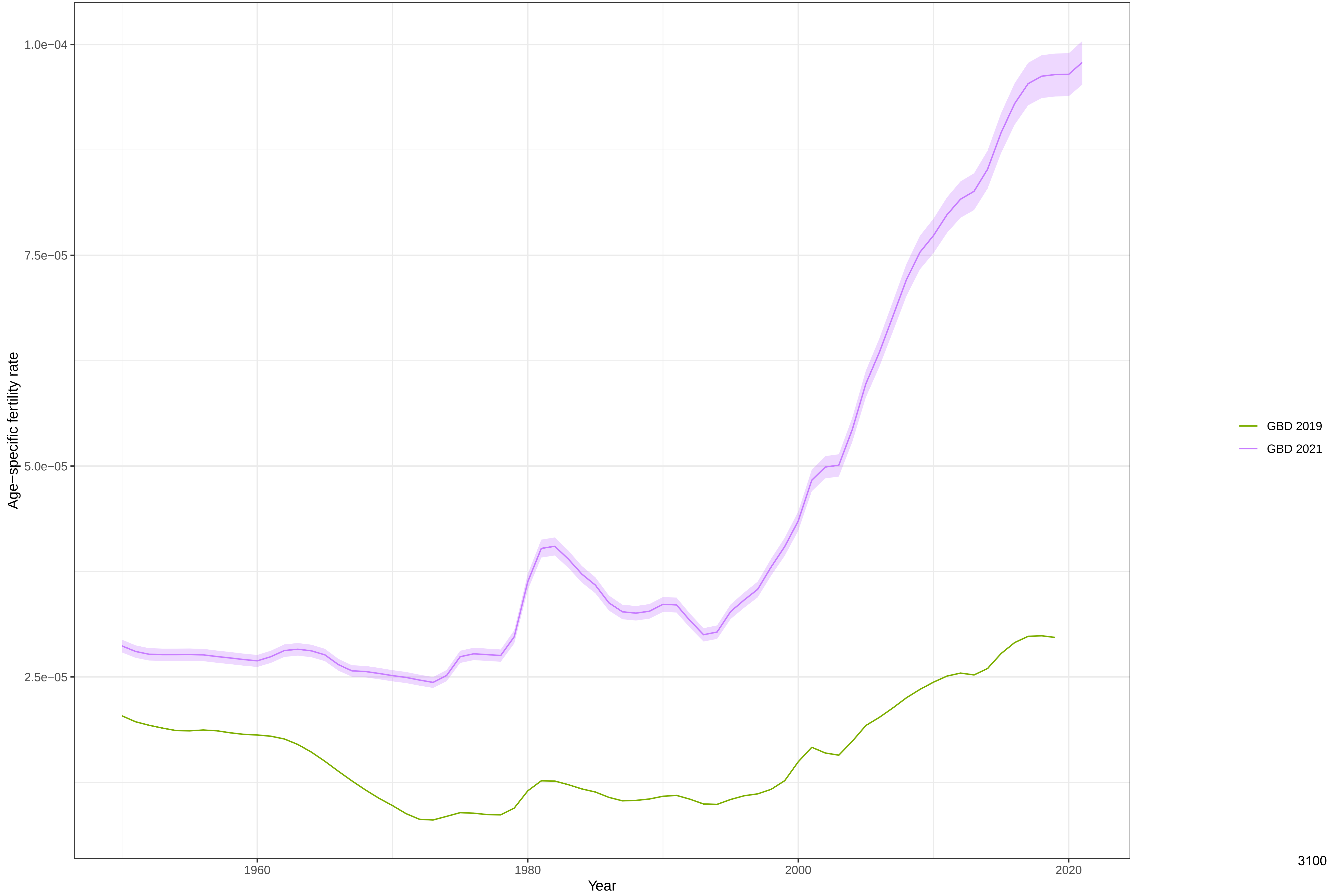

Total fertility rate fitted trend lines, 1950−2021: Wokingham TFR
Data points enclosed with a diamond are reference sources

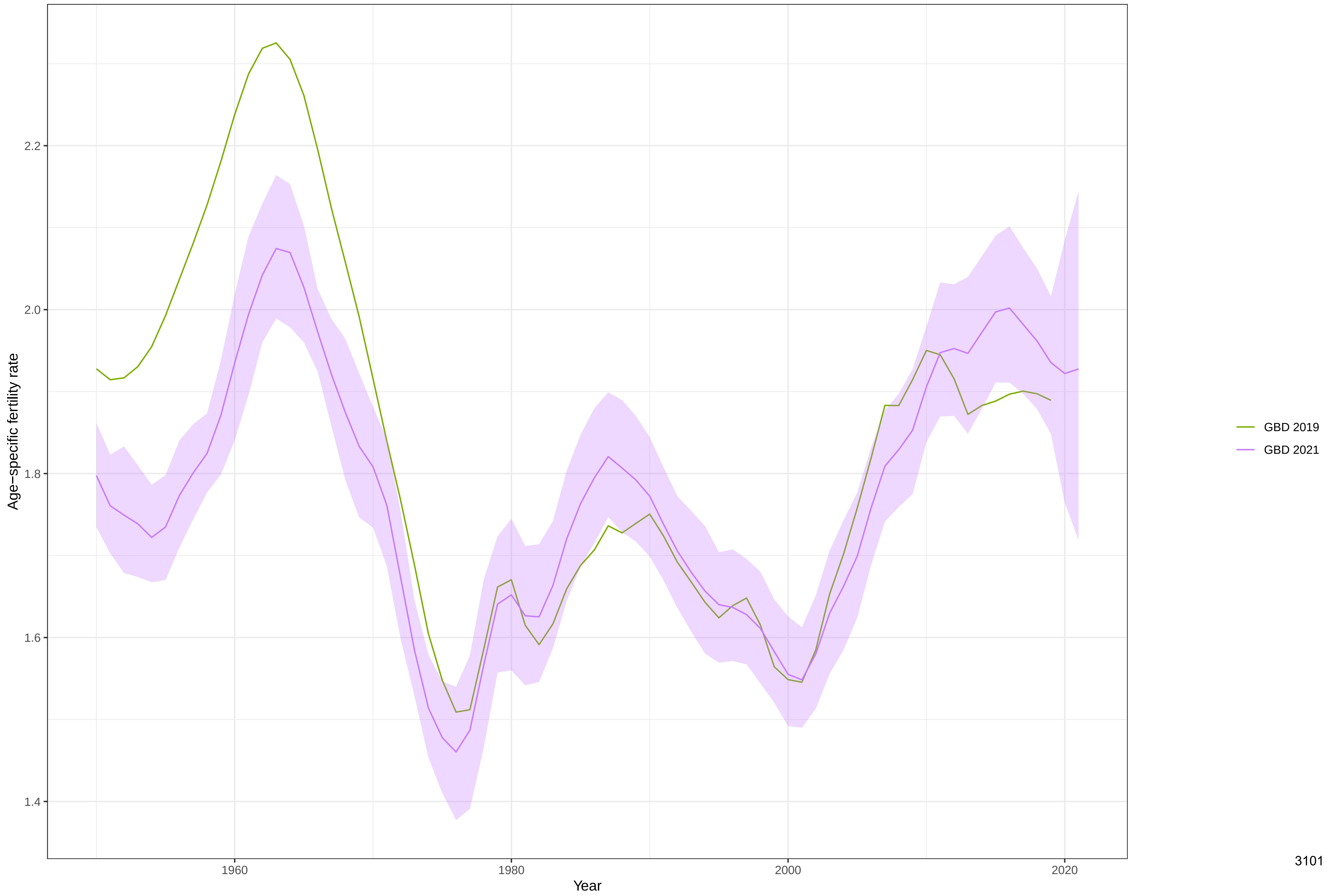

Age−specific fertility fitted trend lines, 1950−2021: South West England, Ages: 10−14
 Data points enclosed with a diamond are reference sources

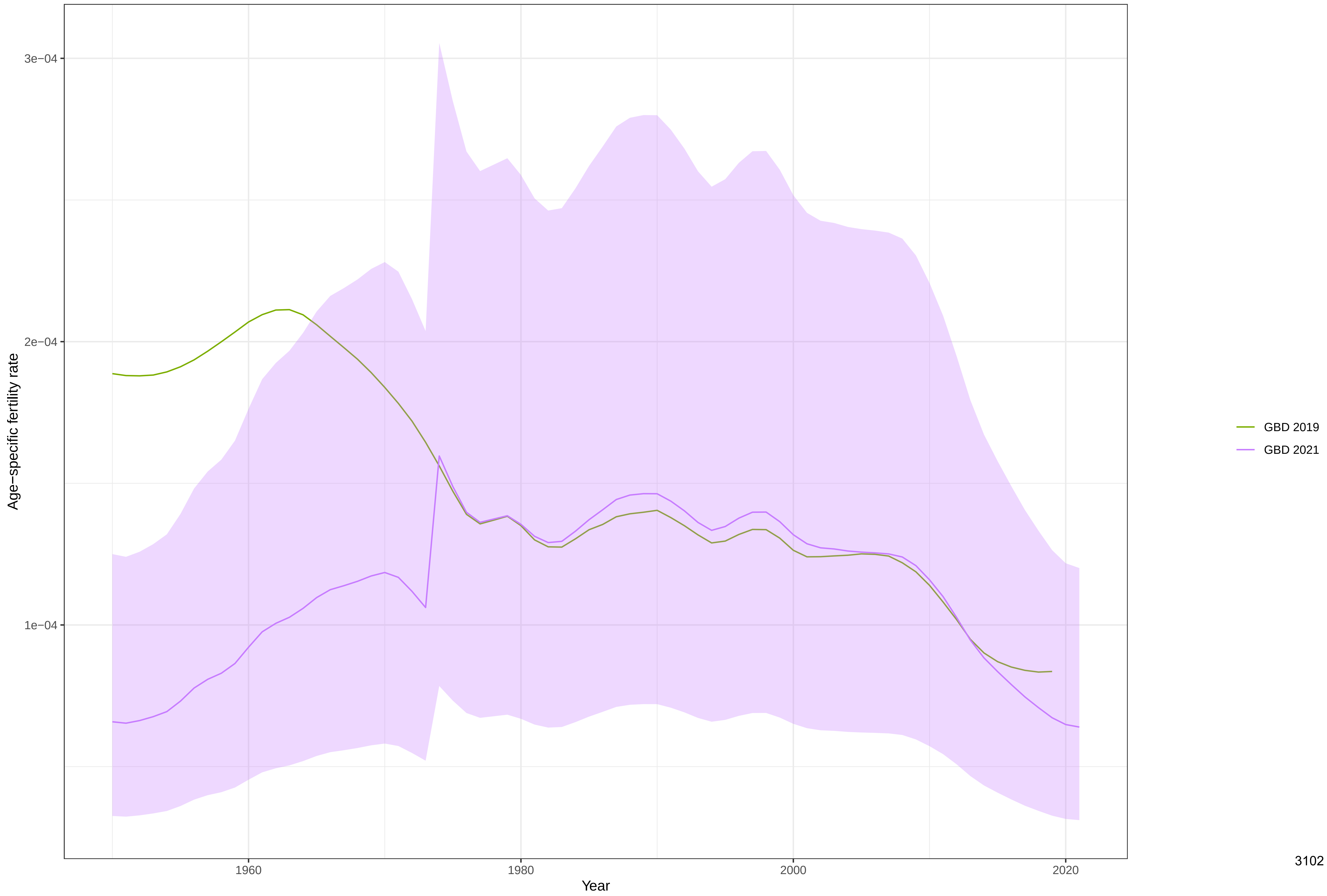

Age−specific fertility fitted trend lines, 1950−2021: South West England, Ages: 15−19
Data points enclosed with a diamond are reference sources

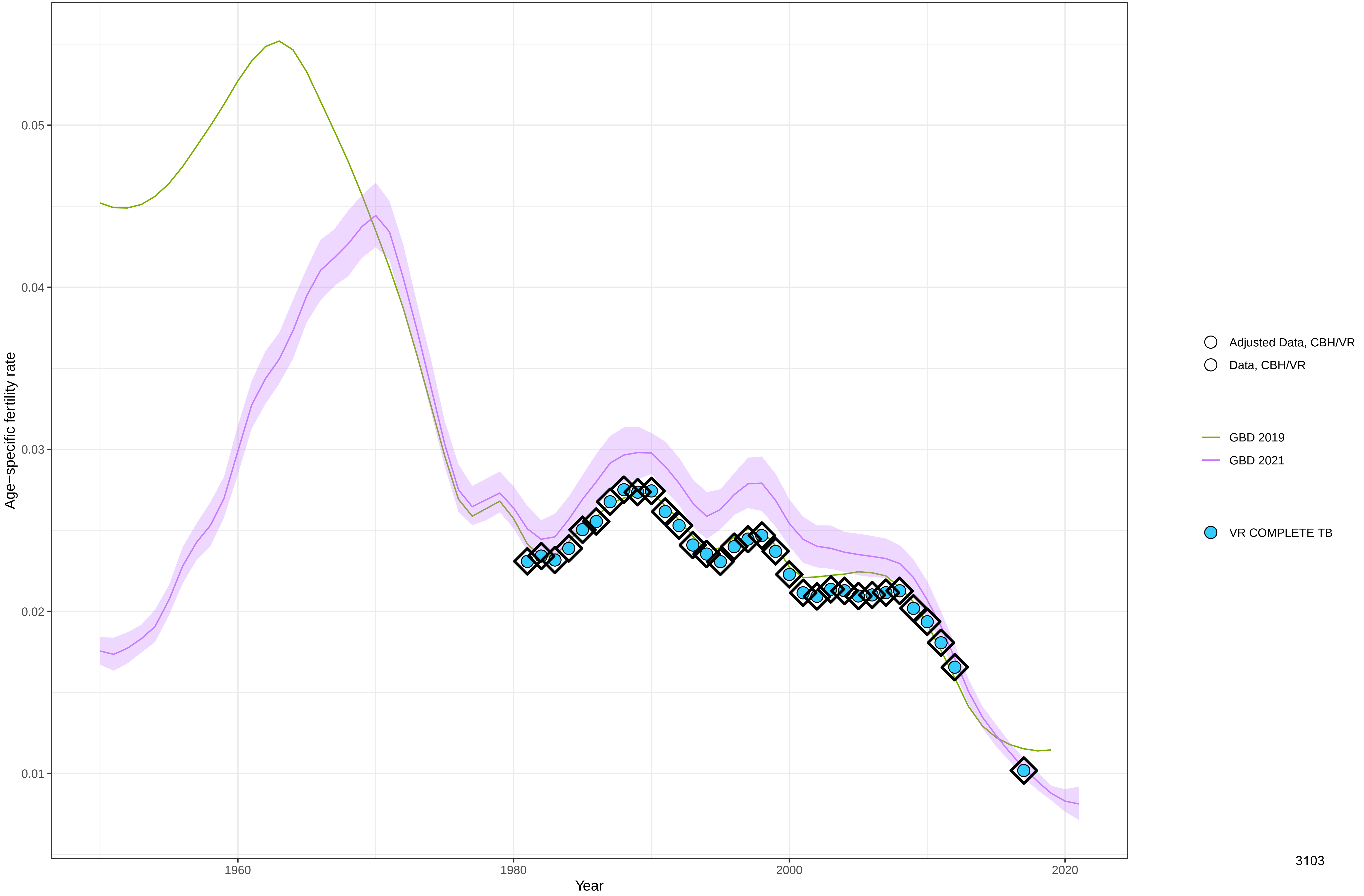

Age−specific fertility fitted trend lines, 1950−2021: South West England, Ages: 20−24
Data points enclosed with a diamond are reference sources

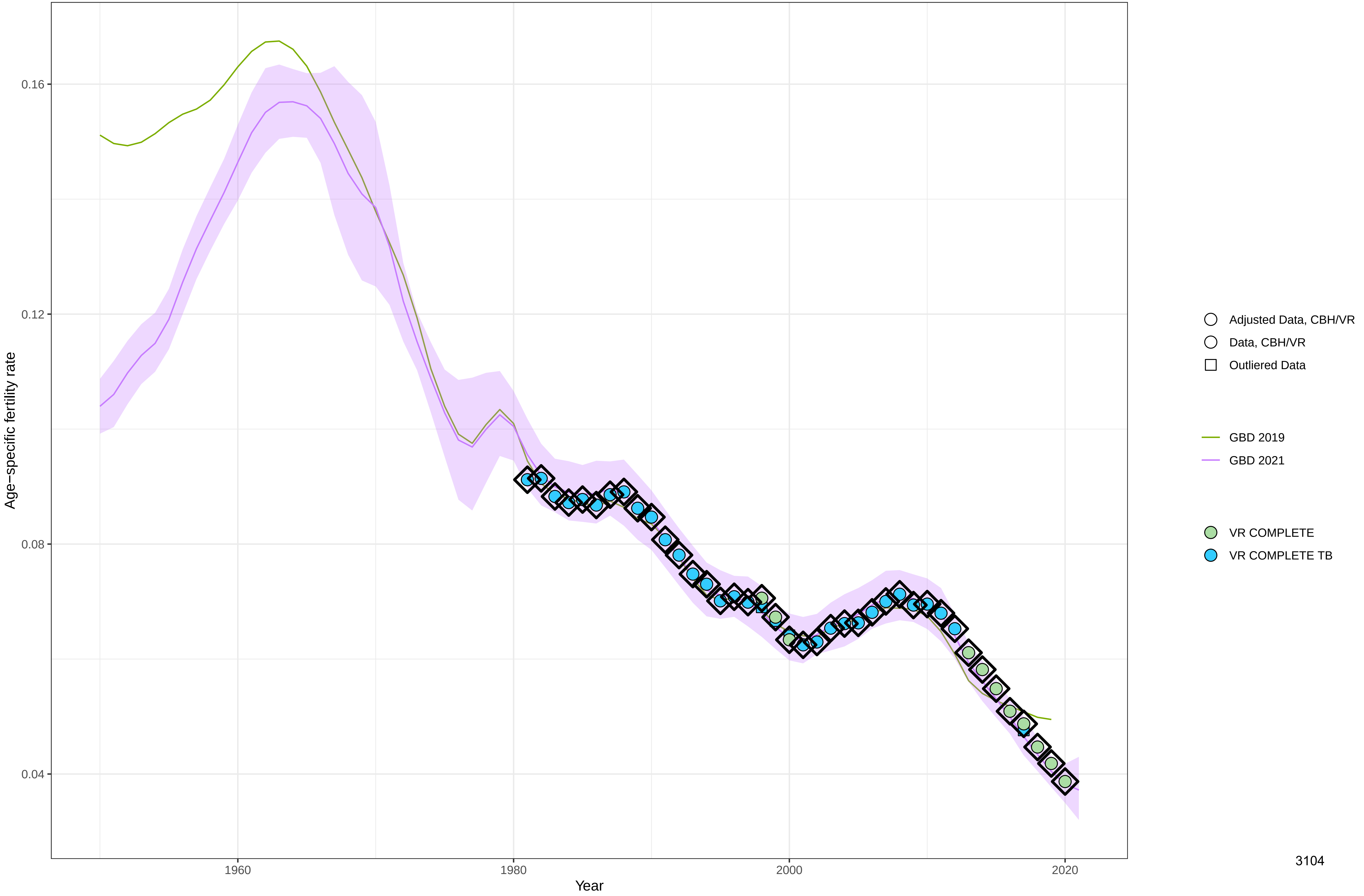

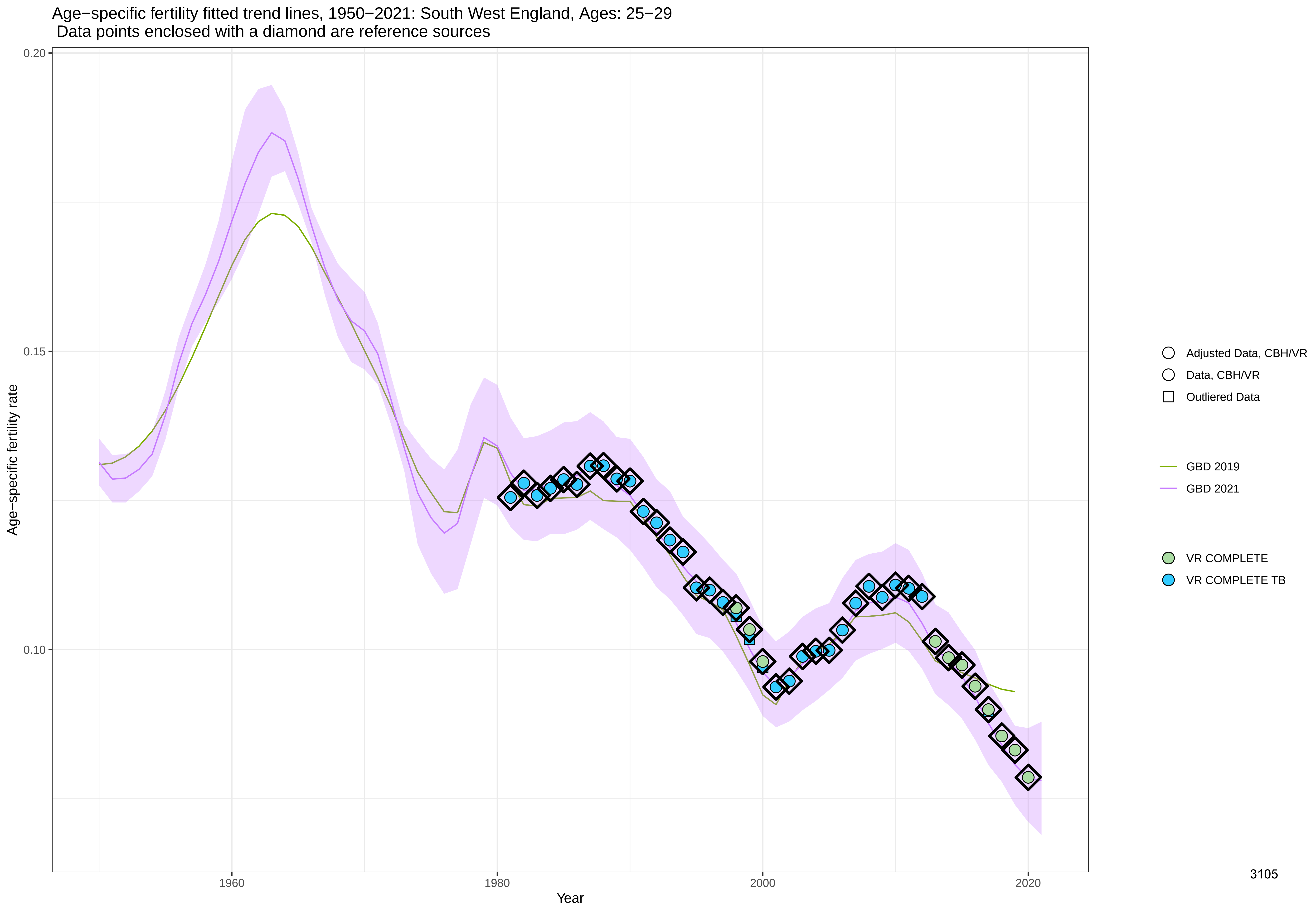

Age−specific fertility fitted trend lines, 1950−2021: South West England, Ages: 25−29
Data points enclosed with a diamond are reference sources
0.20
0.15
0.10
Age−specific fertility rate
1960
1980
2000
2020
Year
Adjusted Data, CBH/VR
Data, CBH/VR
Outliered Data
GBD 2019
GBD 2021
VR COMPLETE
VR COMPLETE TB

Age−specific fertility fitted trend lines, 1950−2021: South West England, Ages: 30−34
Data points enclosed with a diamond are reference sources

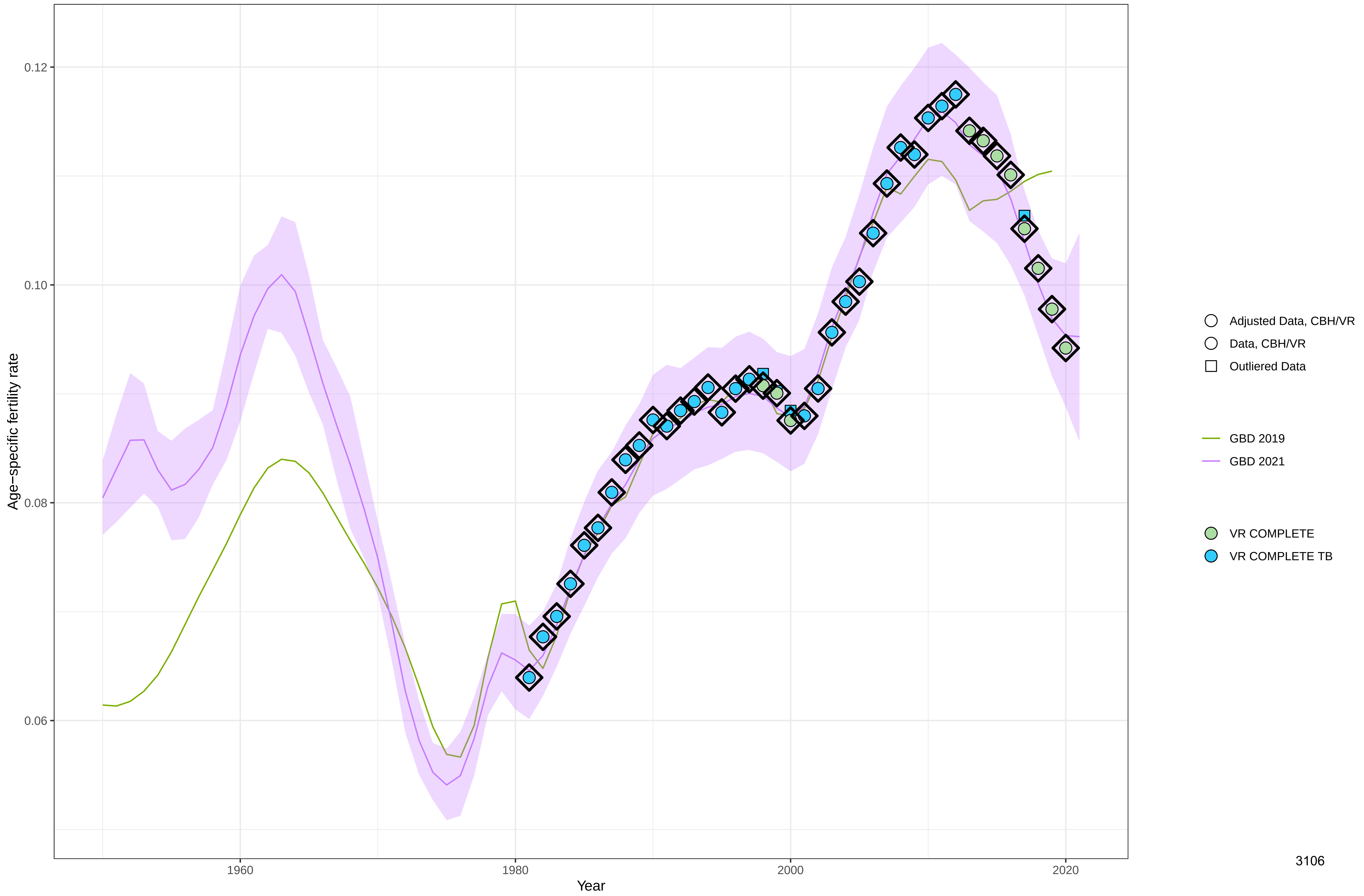

Age−specific fertility fitted trend lines, 1950−2021: South West England, Ages: 35−39
Data points enclosed with a diamond are reference sources

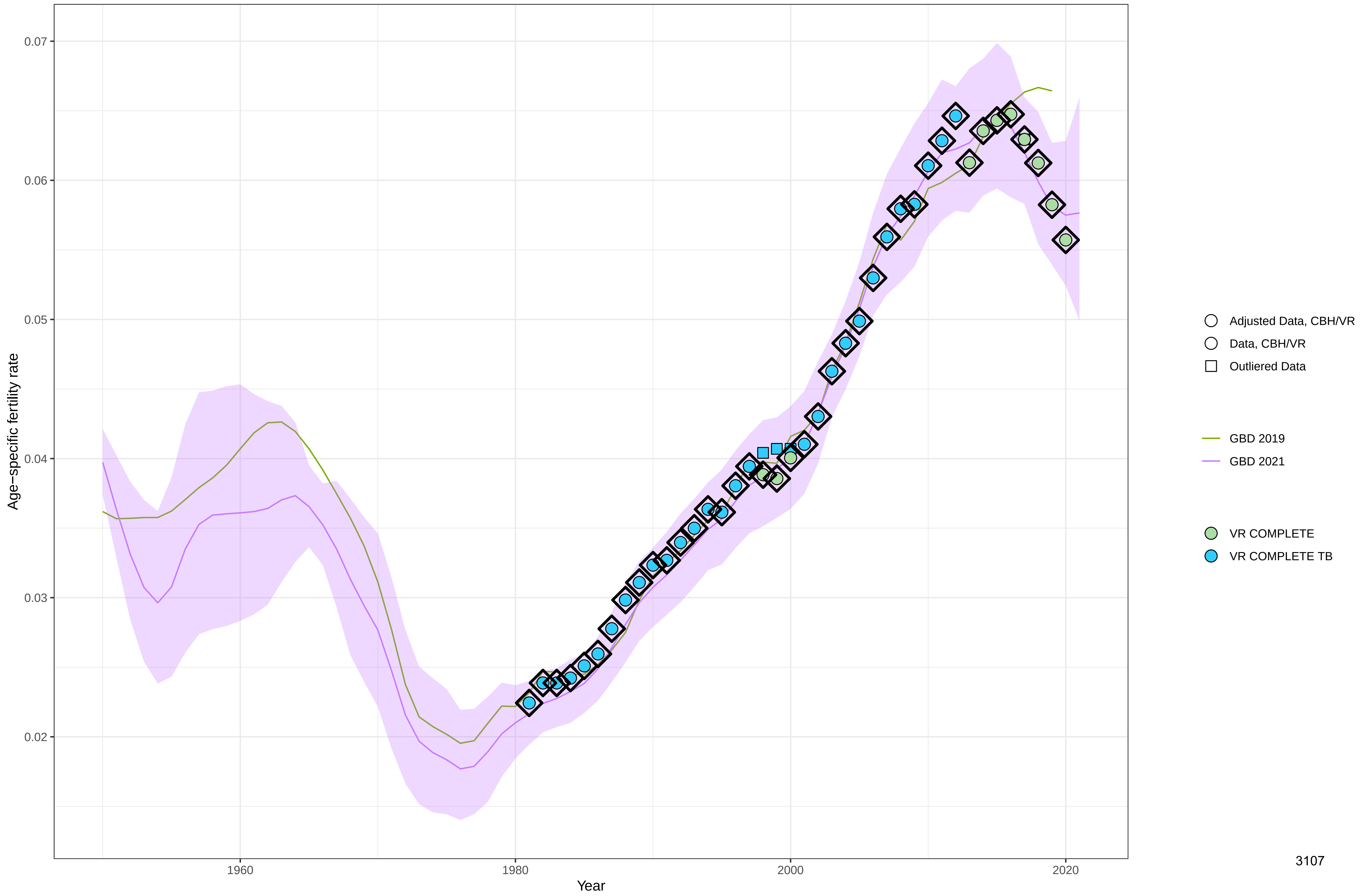

Age−specific fertility fitted trend lines, 1950−2021: South West England, Ages: 40−44
Data points enclosed with a diamond are reference sources

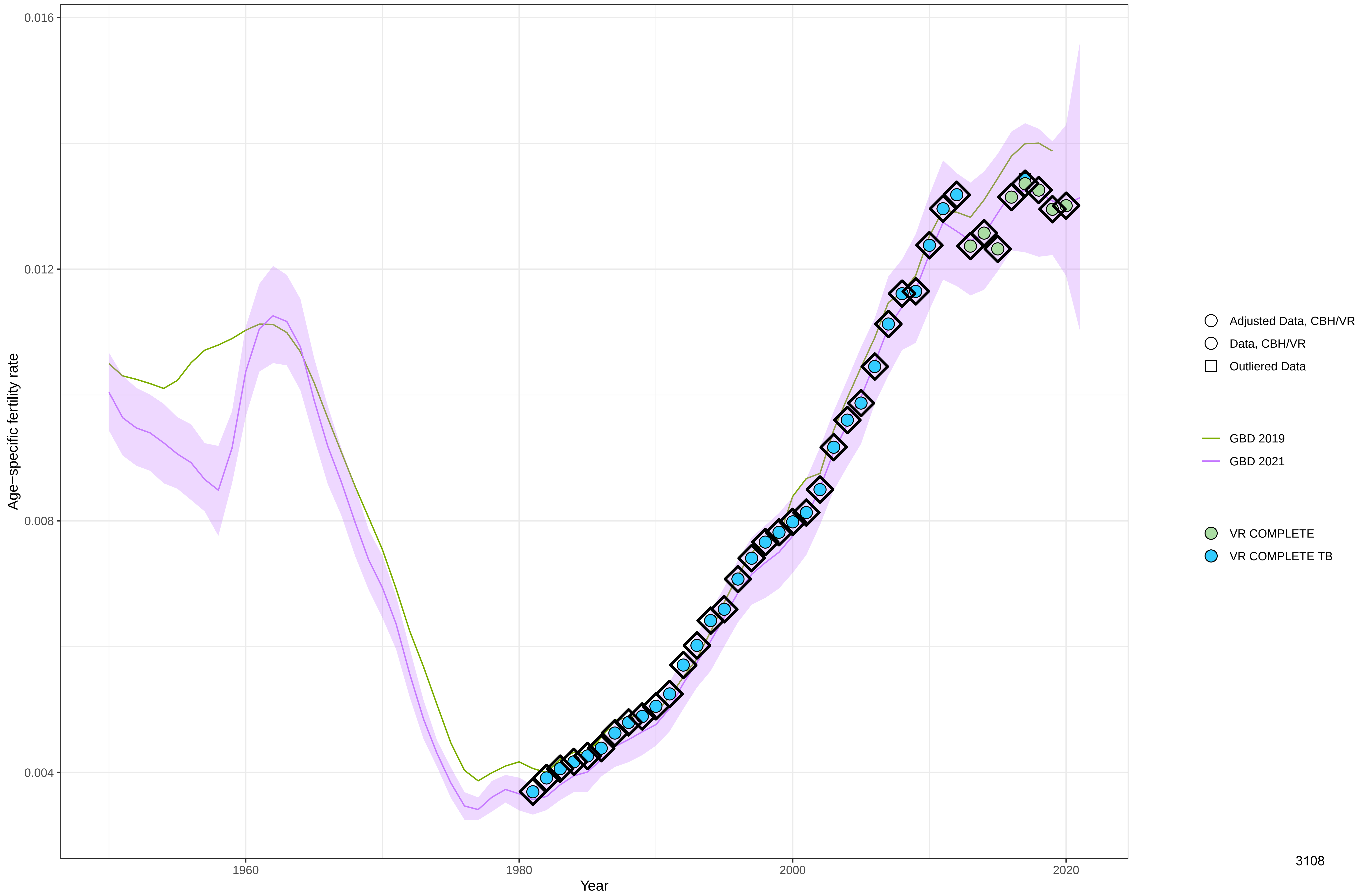

Age−specific fertility fitted trend lines, 1950−2021: South West England, Ages: 45−49
Data points enclosed with a diamond are reference sources

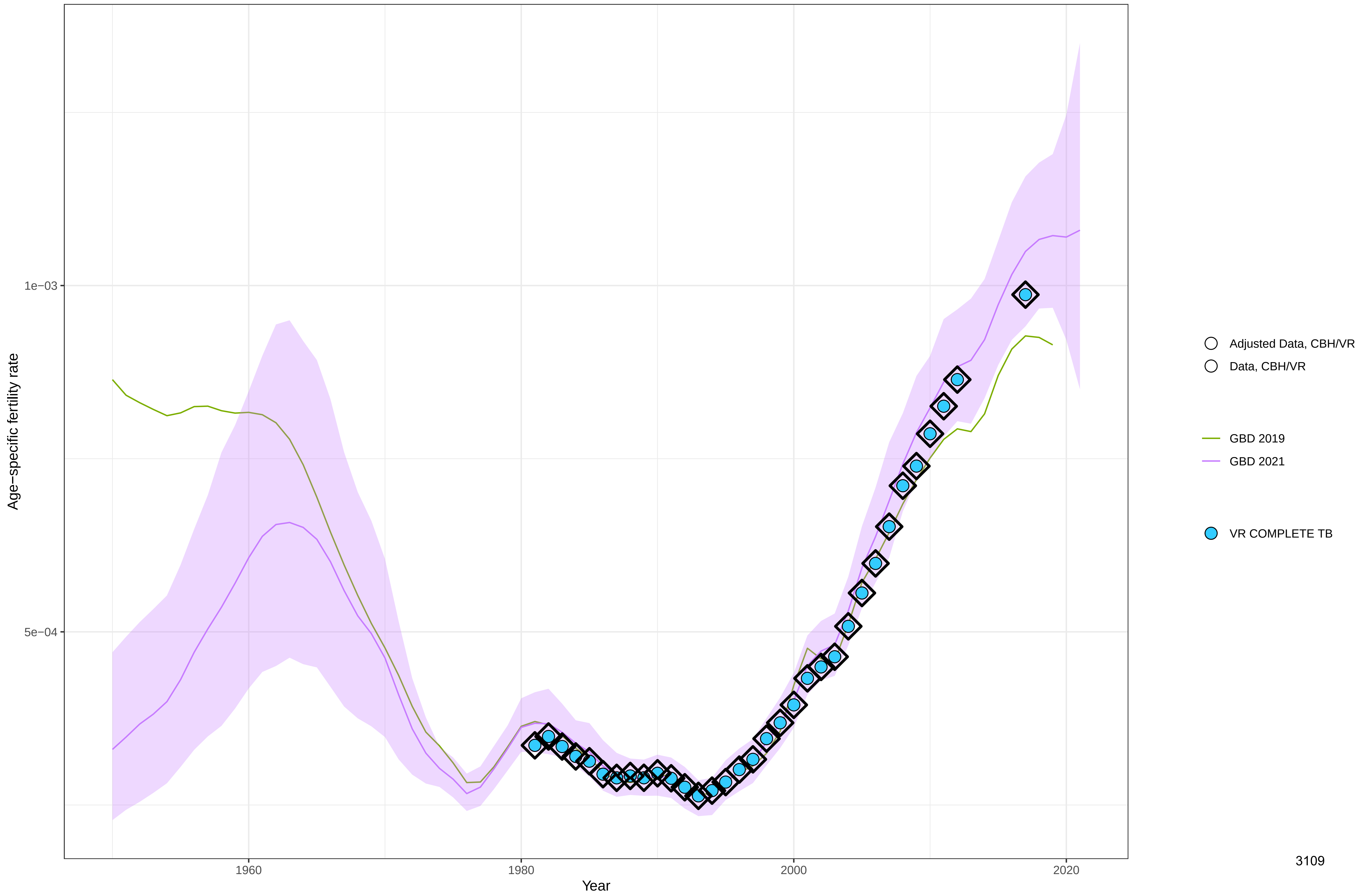

Age−specific fertility fitted trend lines, 1950−2021: South West England, Ages: 50−54
Data points enclosed with a diamond are reference sources

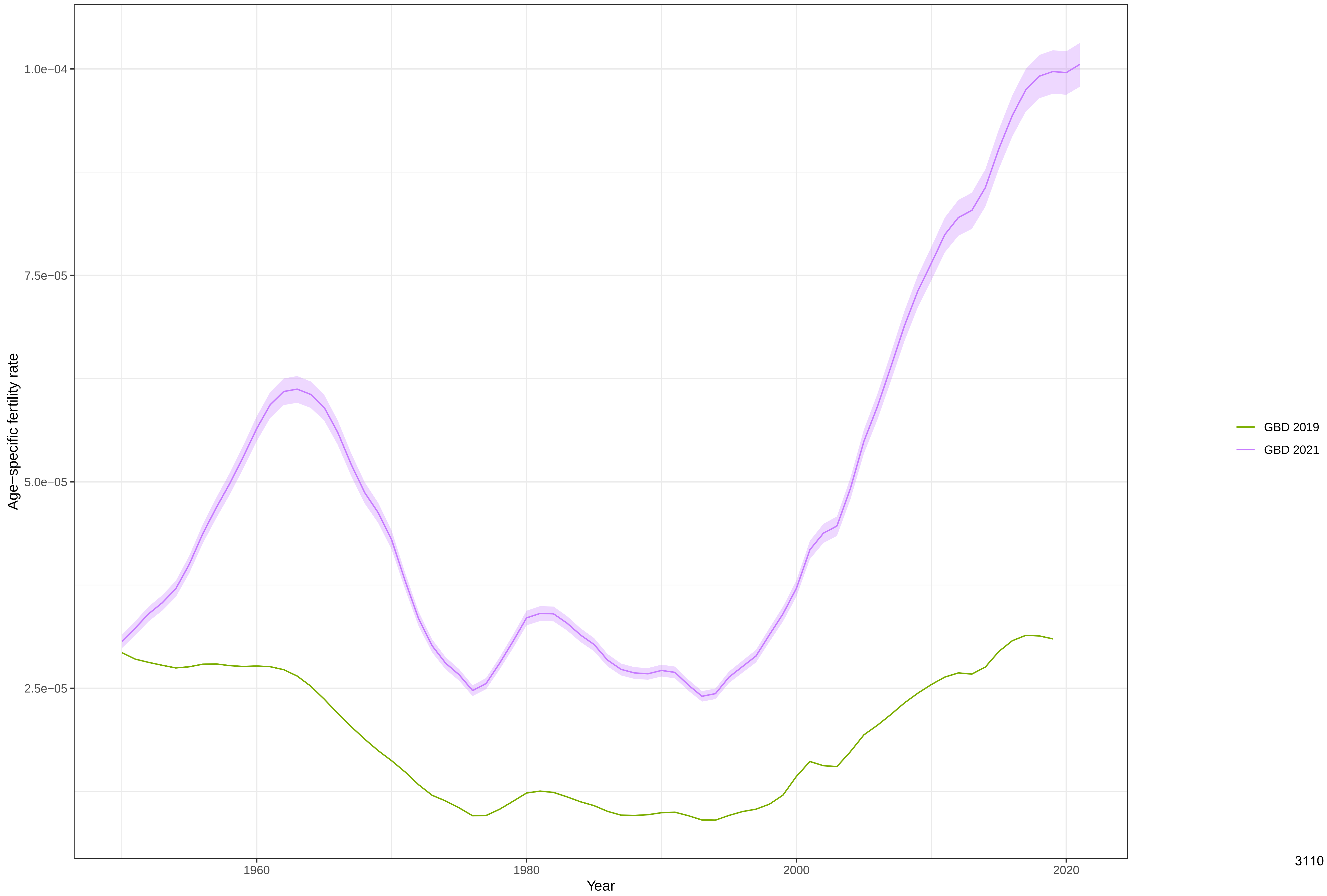

Total fertility rate fitted trend lines, 1950−2021: South West England TFR
Data points enclosed with a diamond are reference sources

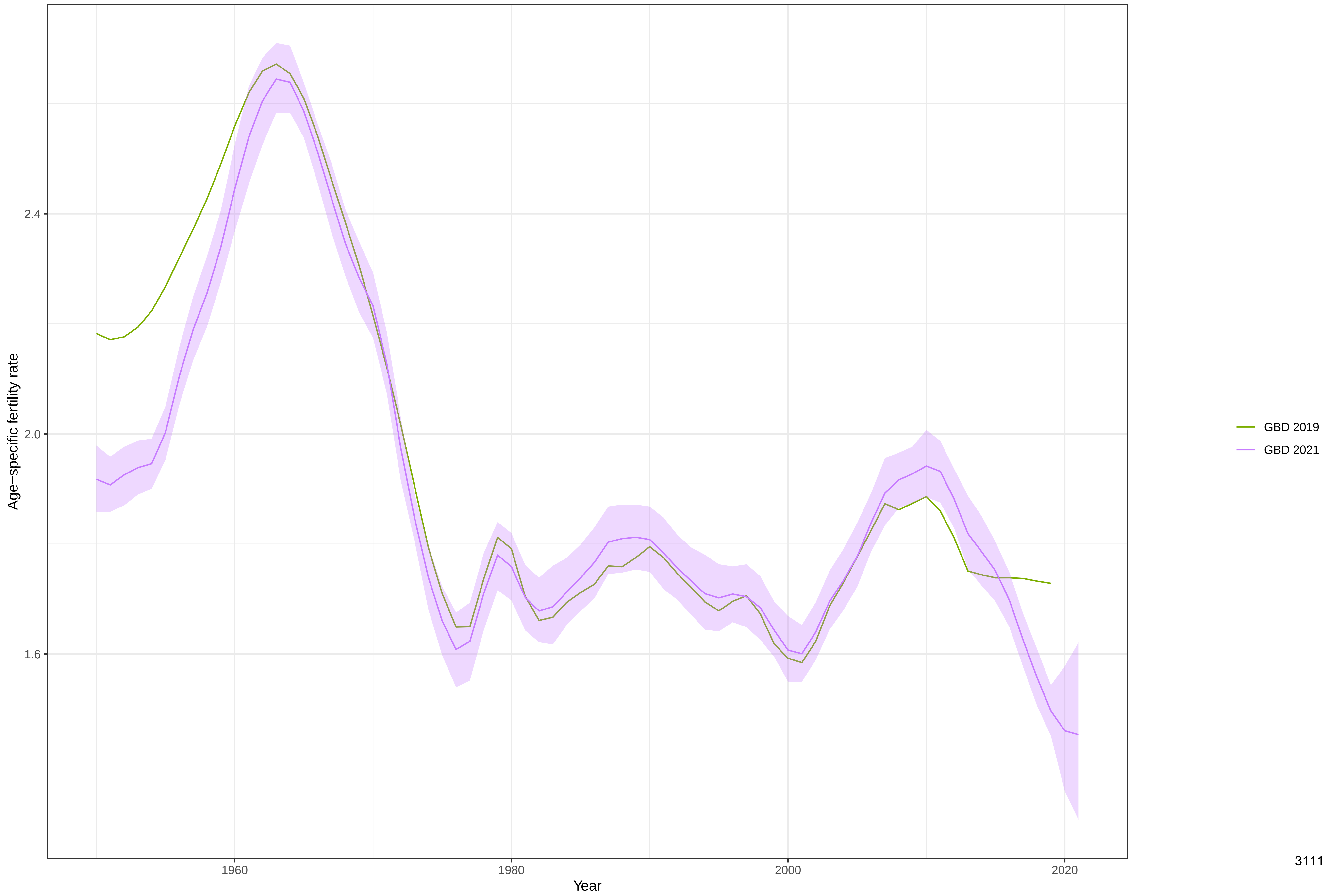

Age−specific fertility fitted trend lines, 1950−2021: Bath and North East Somerset, Ages: 10−14
Data points enclosed with a diamond are reference sources

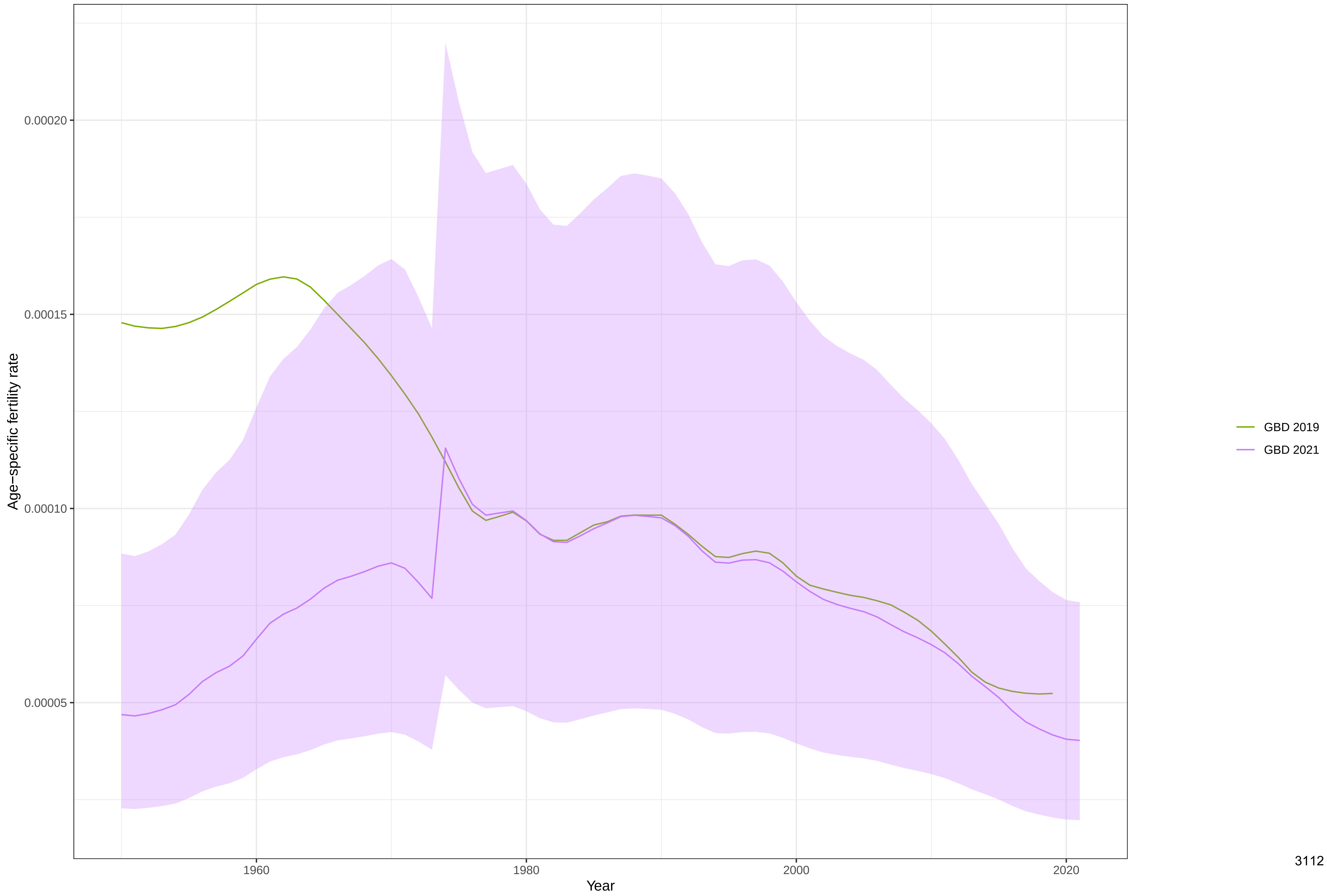

Age−specific fertility fitted trend lines, 1950−2021: Bath and North East Somerset, Ages: 15−19
Data points enclosed with a diamond are reference sources

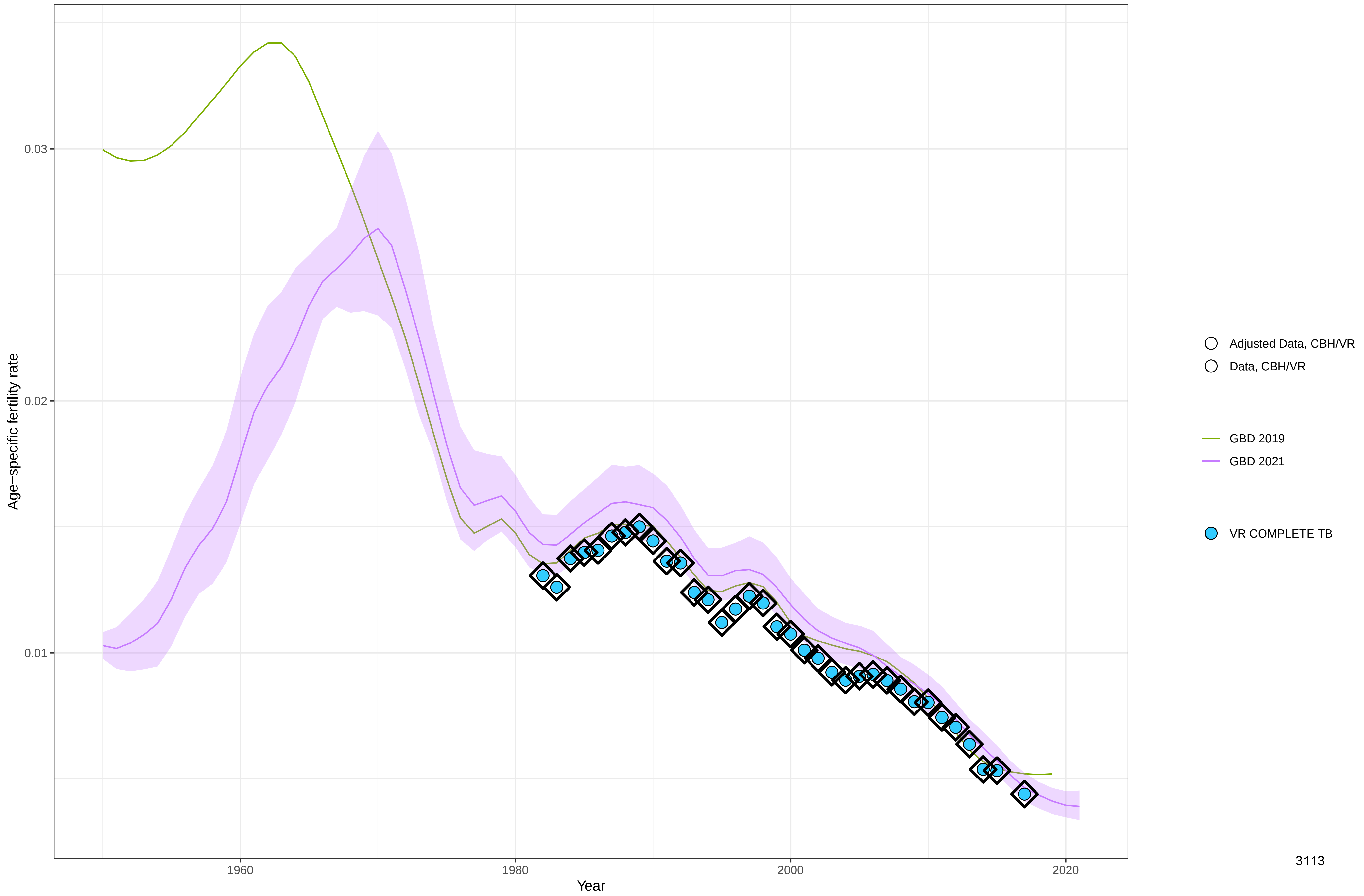

Age−specific fertility fitted trend lines, 1950−2021: Bath and North East Somerset, Ages: 20−24
Data points enclosed with a diamond are reference sources

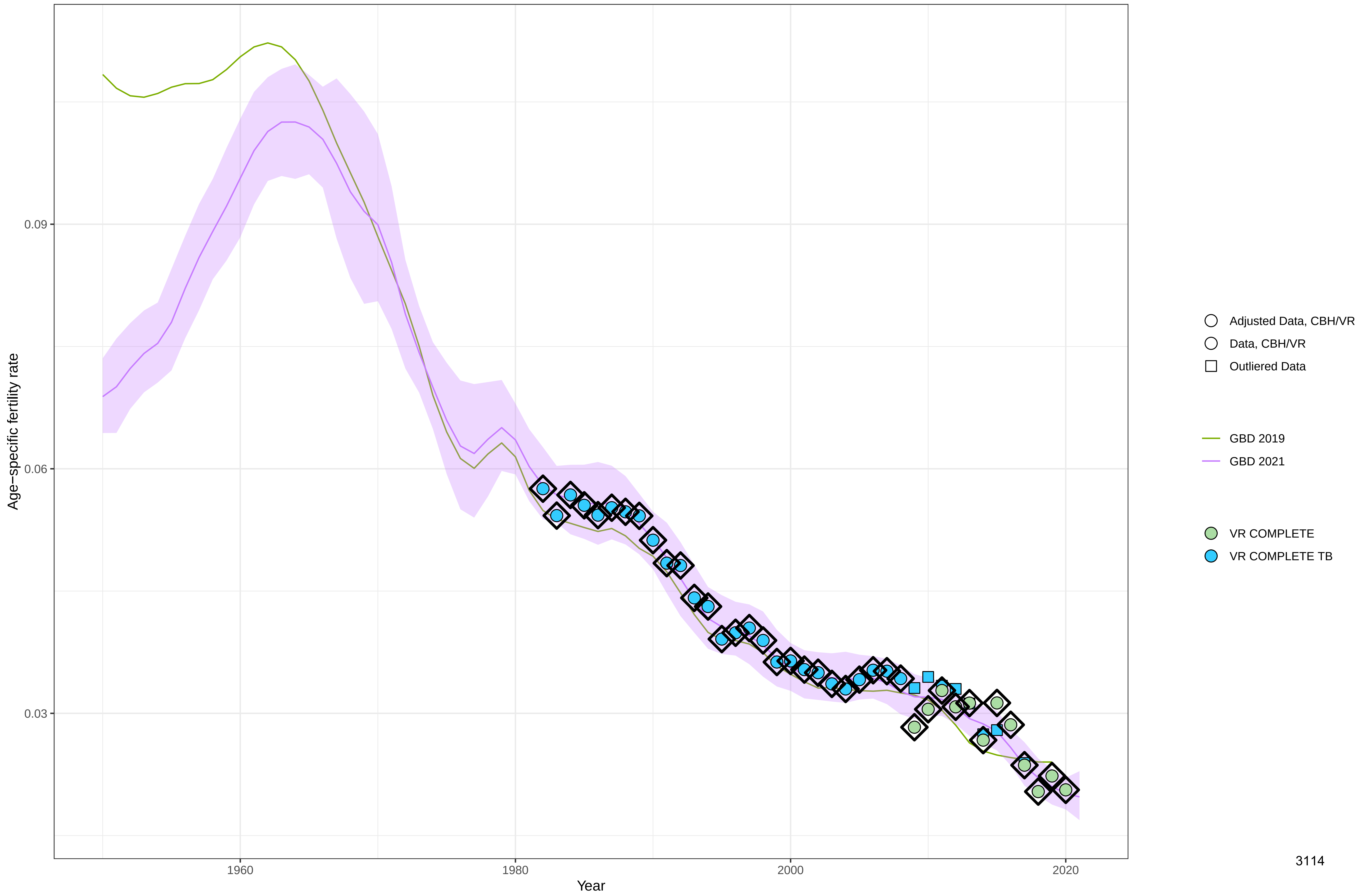

Age−specific fertility fitted trend lines, 1950−2021: Bath and North East Somerset, Ages: 25−29
Data points enclosed with a diamond are reference sources

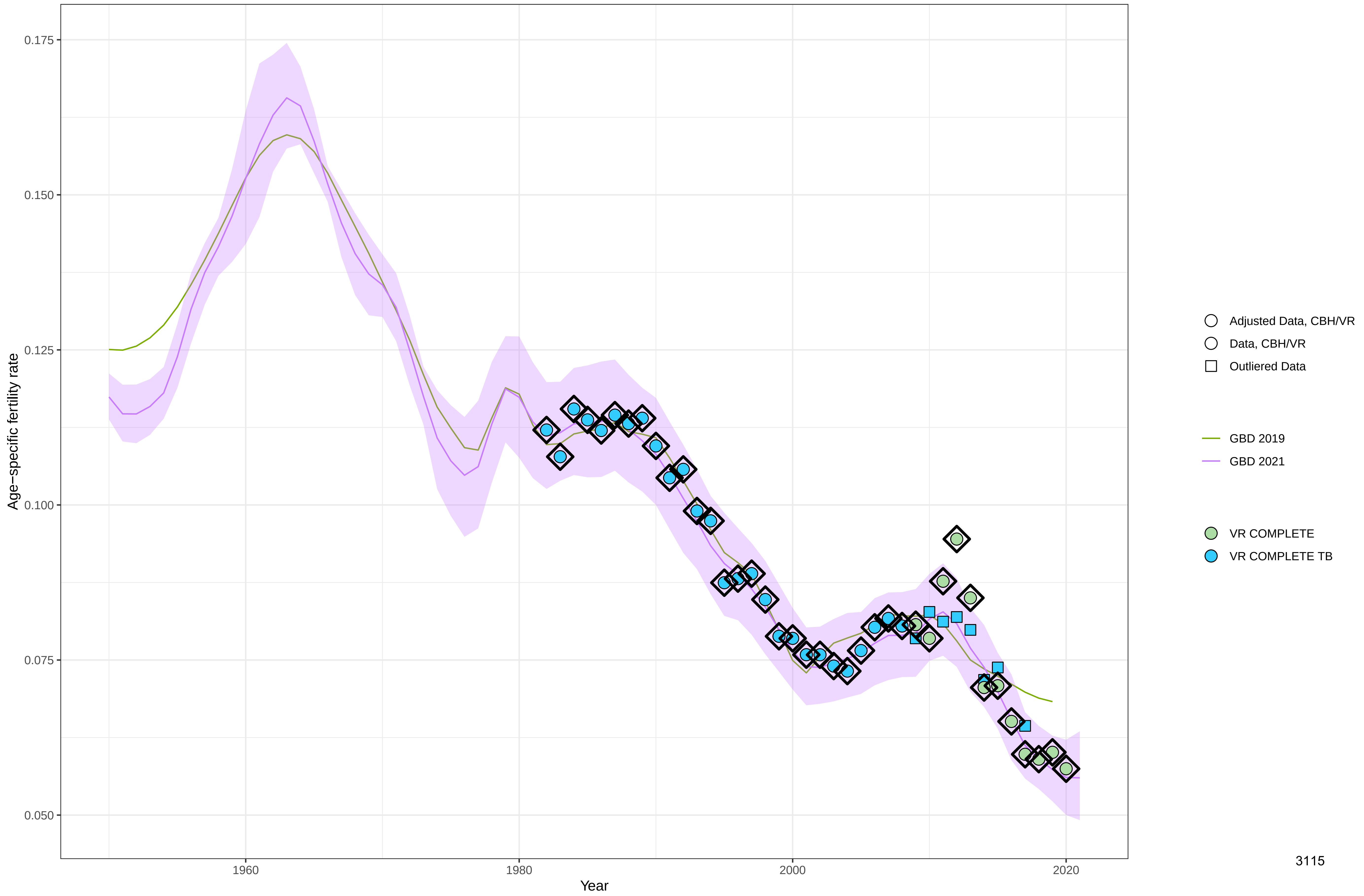

Age−specific fertility fitted trend lines, 1950−2021: Bath and North East Somerset, Ages: 30−34
Data points enclosed with a diamond are reference sources

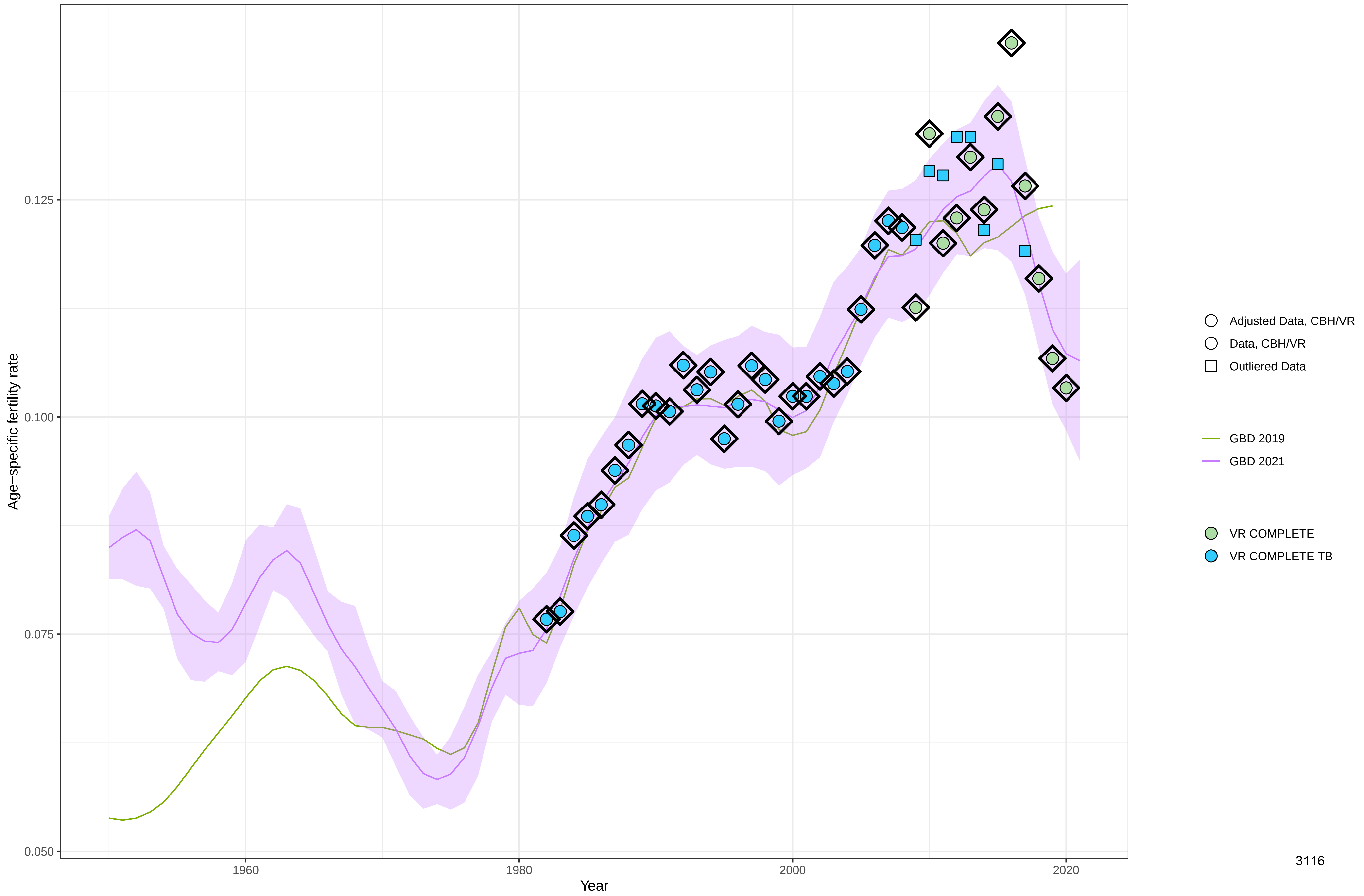

Age−specific fertility fitted trend lines, 1950−2021: Bath and North East Somerset, Ages: 35−39
Data points enclosed with a diamond are reference sources

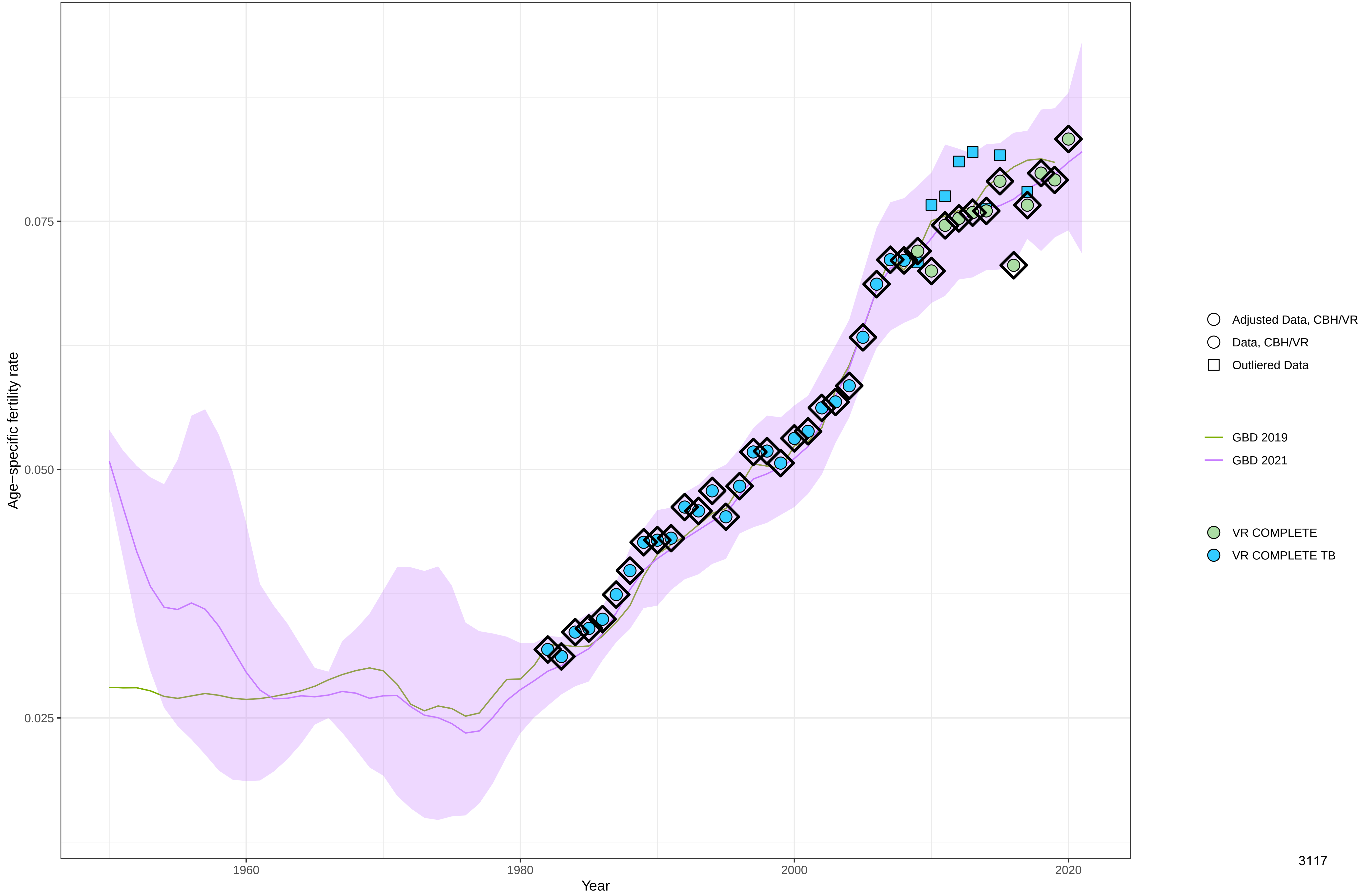

Age−specific fertility fitted trend lines, 1950−2021: Bath and North East Somerset, Ages: 40−44
Data points enclosed with a diamond are reference sources

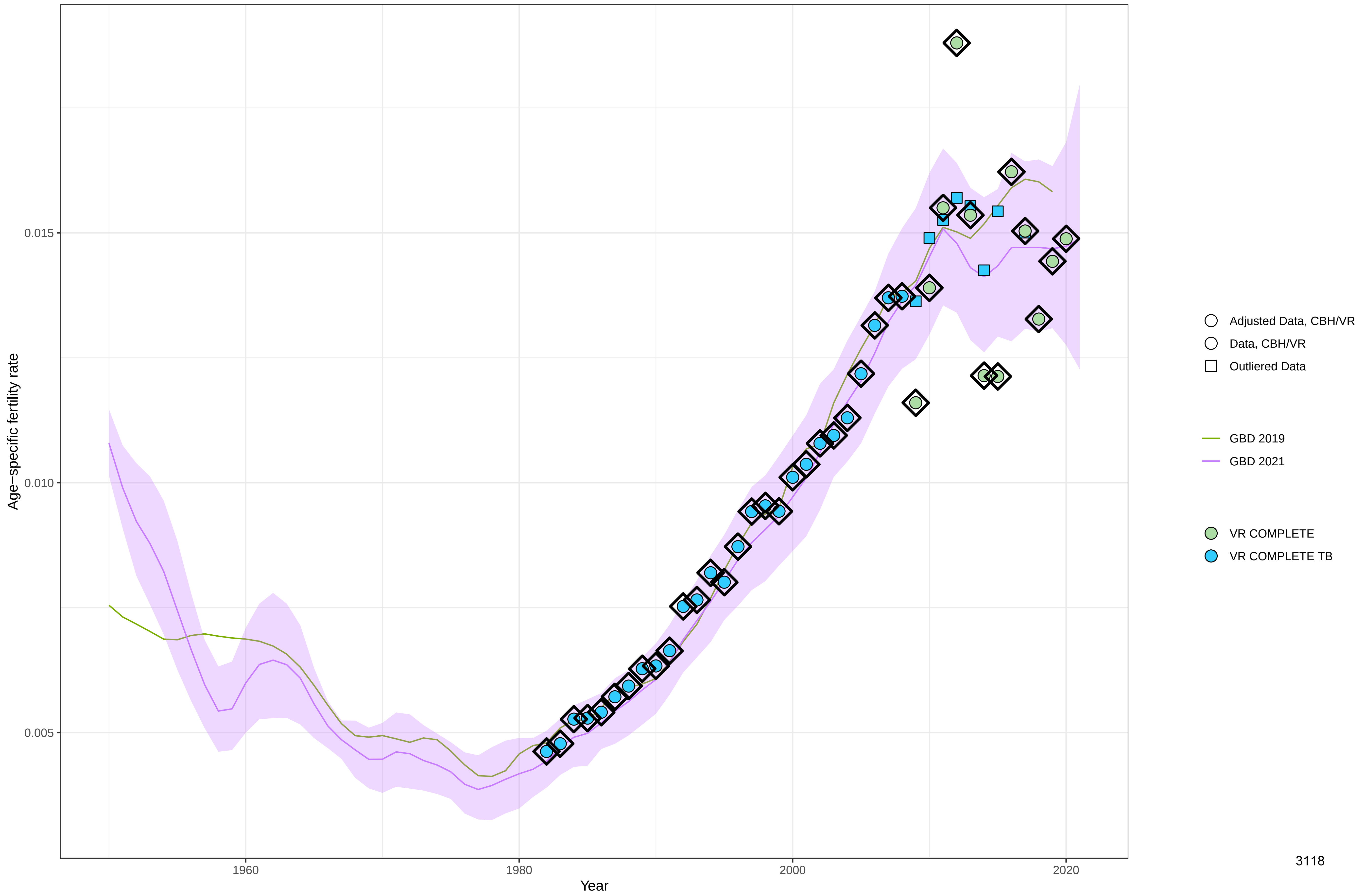

Age−specific fertility fitted trend lines, 1950−2021: Bath and North East Somerset, Ages: 45−49
Data points enclosed with a diamond are reference sources

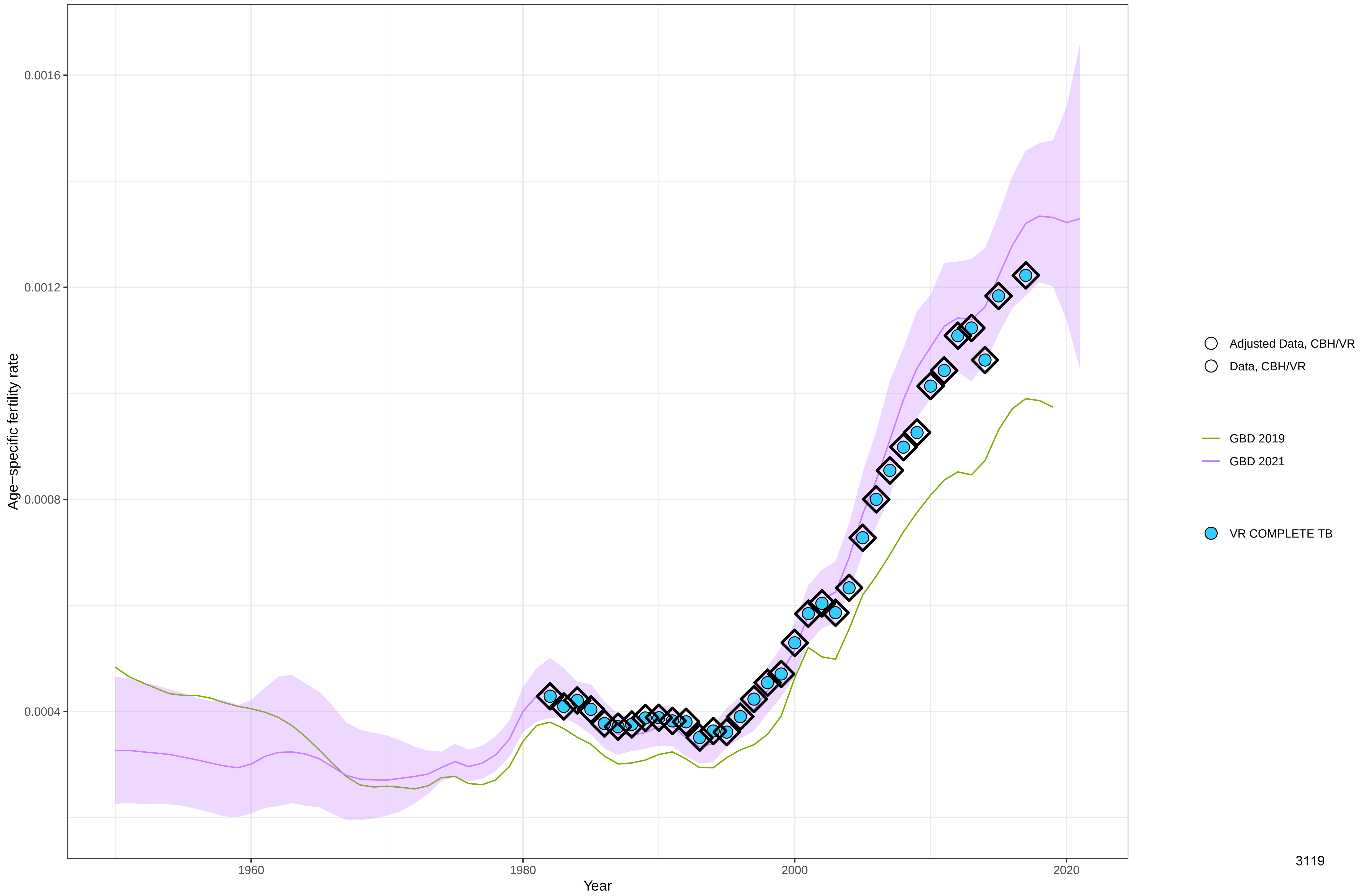

Age−specific fertility fitted trend lines, 1950−2021: Bath and North East Somerset, Ages: 50−54
Data points enclosed with a diamond are reference sources

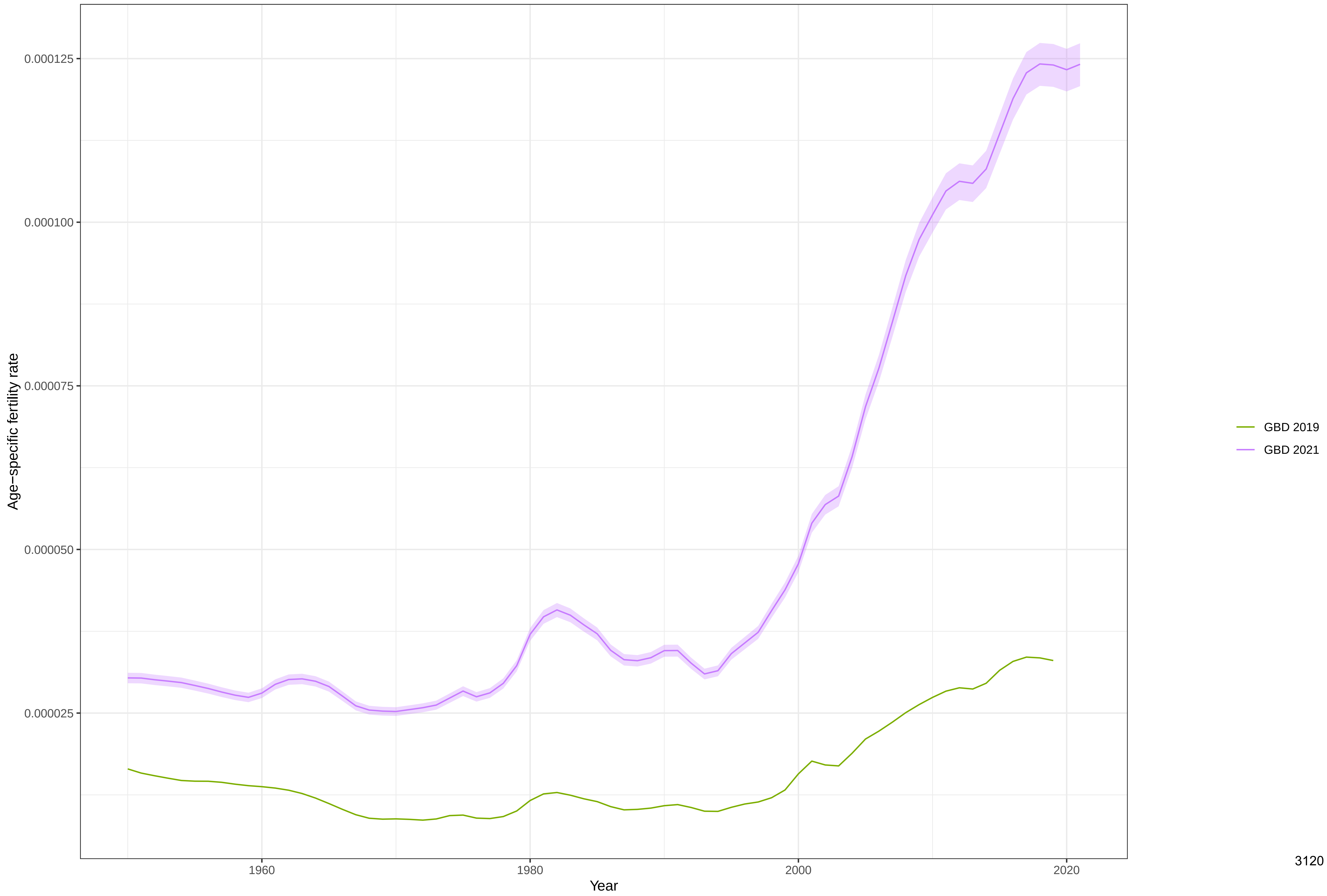

Total fertility rate fitted trend lines, 1950−2021: Bath and North East Somerset TFR
Data points enclosed with a diamond are reference sources

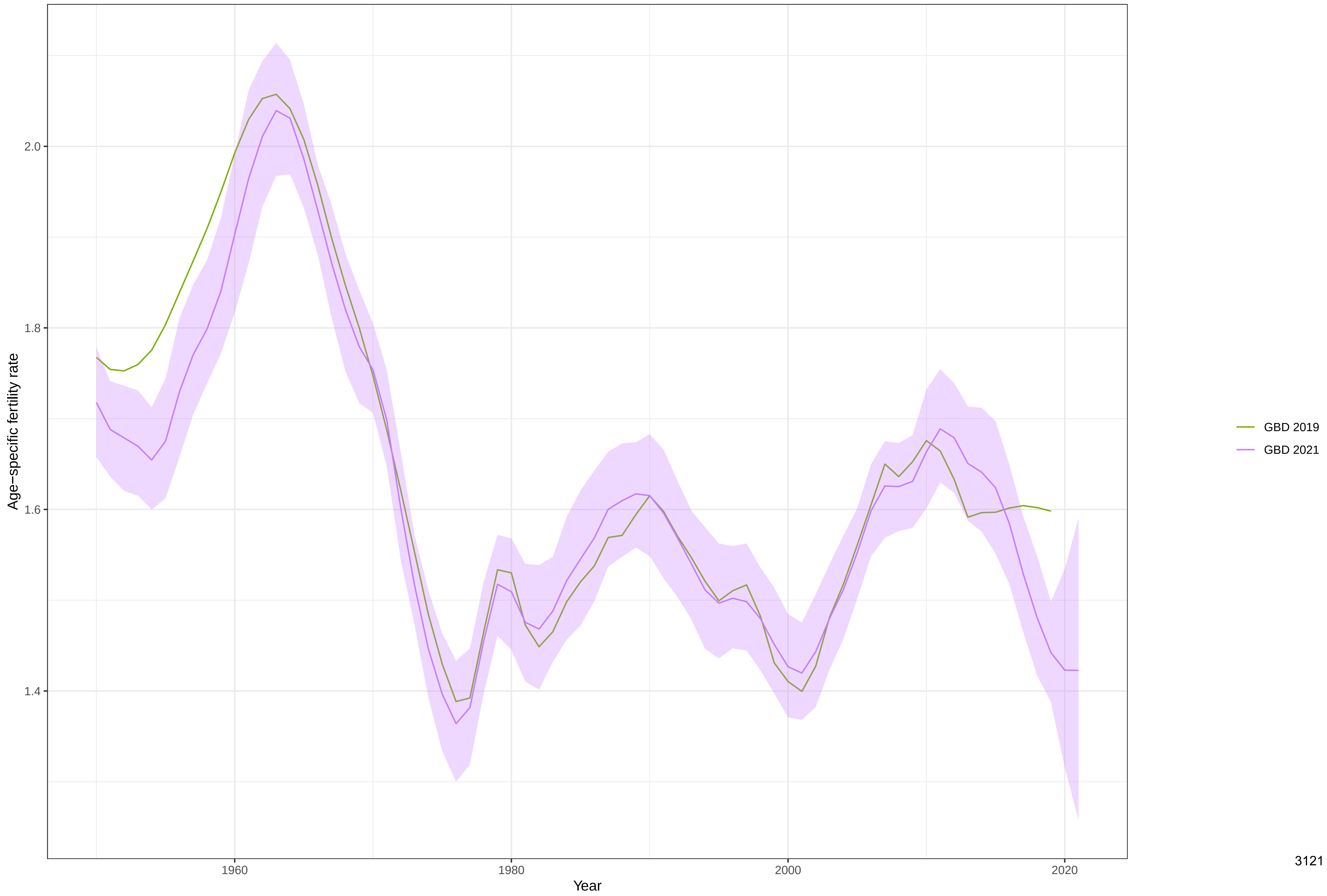

Age−specific fertility fitted trend lines, 1950−2021: Bournemouth, Ages: 10−14
Data points enclosed with a diamond are reference sources

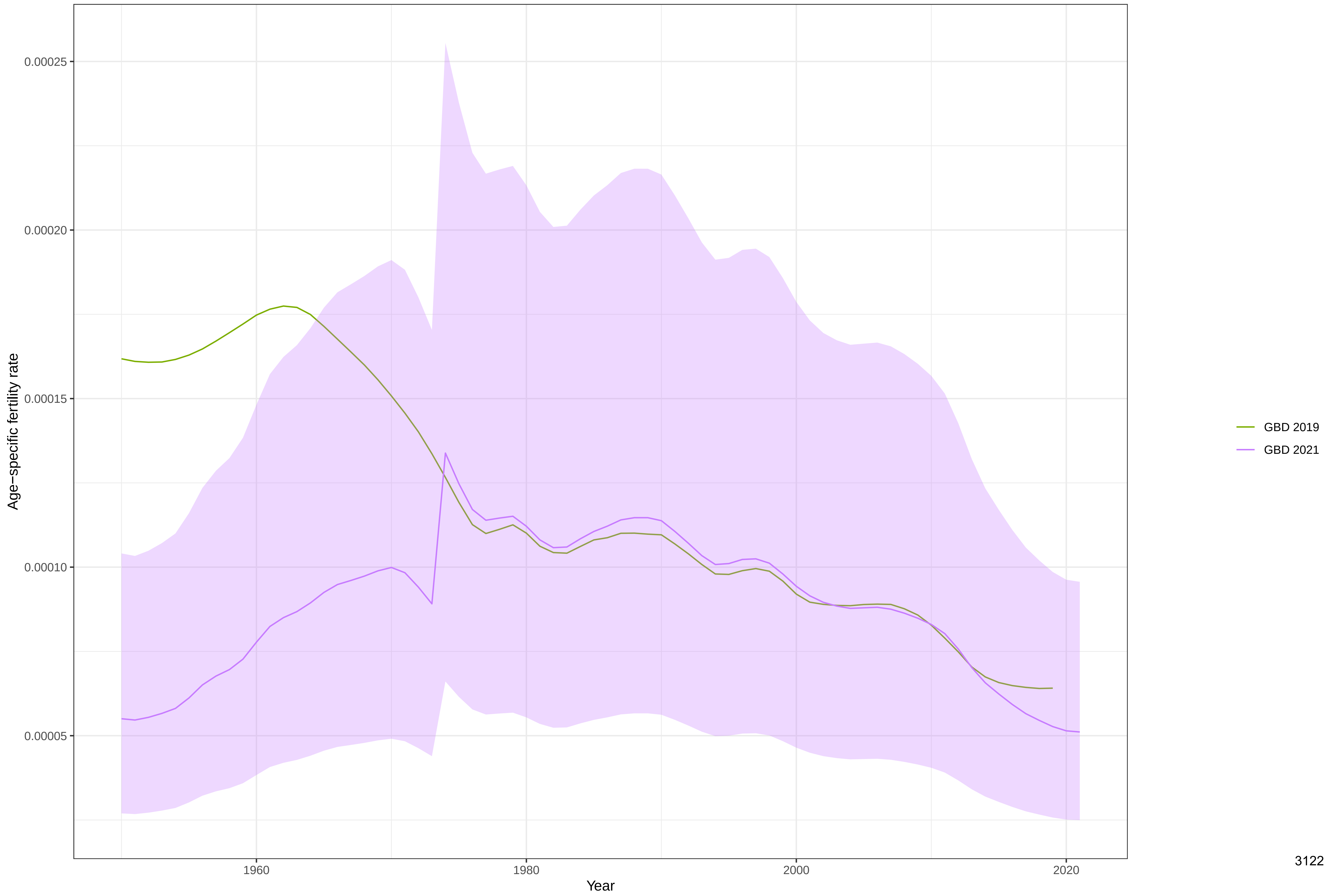

Age−specific fertility fitted trend lines, 1950−2021: Bournemouth, Ages: 15−19
Data points enclosed with a diamond are reference sources

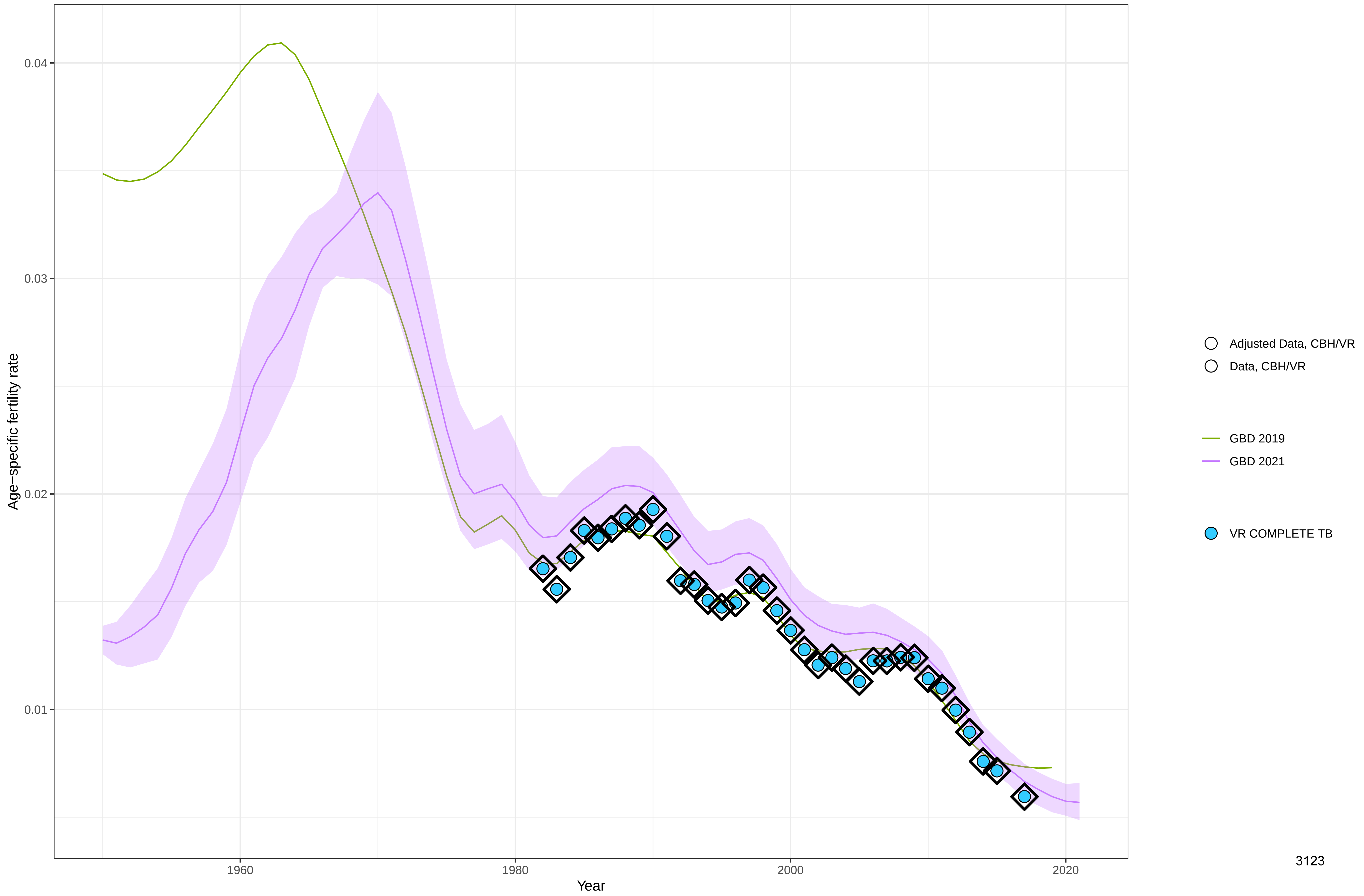

Age−specific fertility fitted trend lines, 1950−2021: Bournemouth, Ages: 20−24
Data points enclosed with a diamond are reference sources

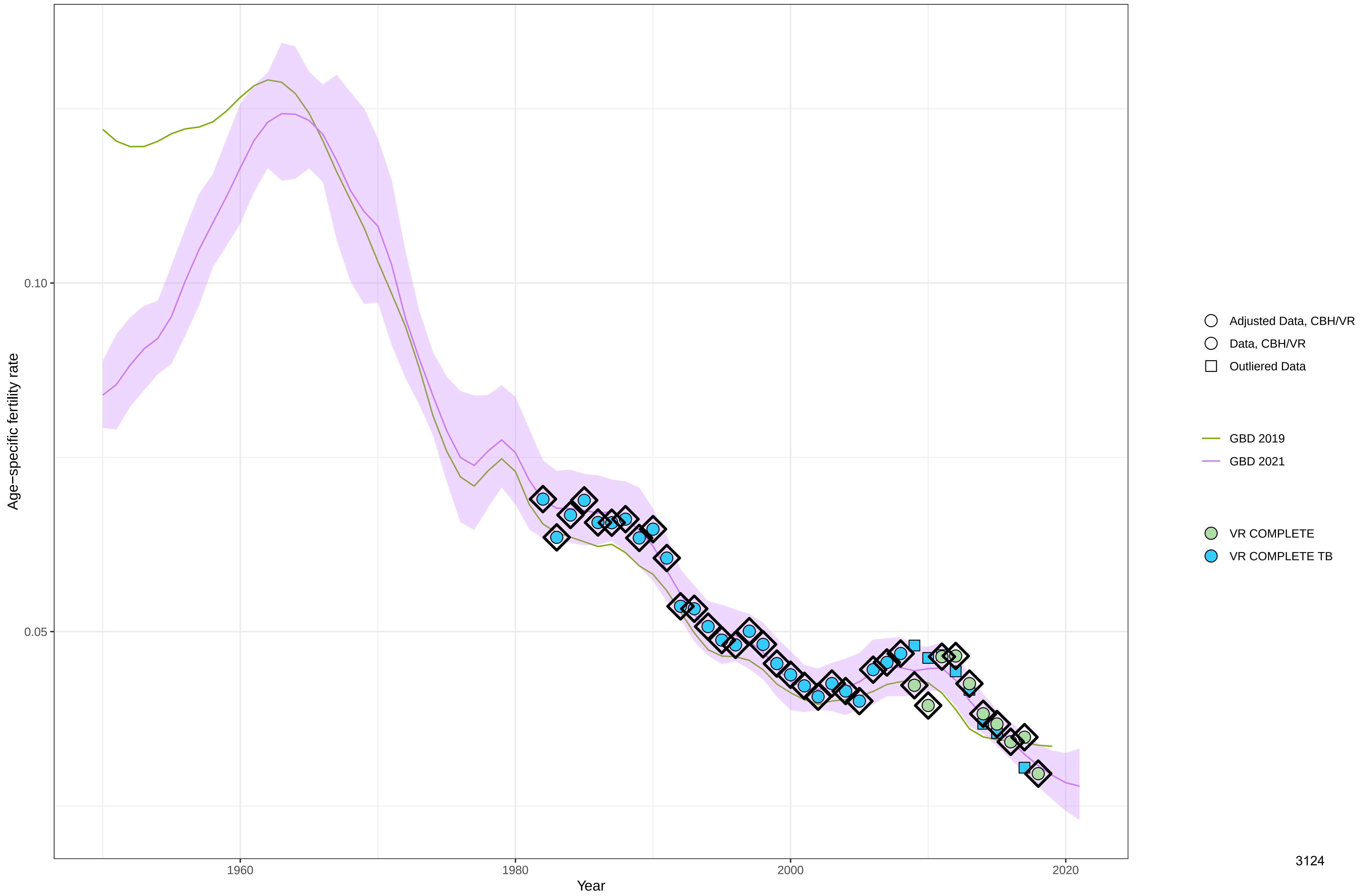

Age−specific fertility fitted trend lines, 1950−2021: Bournemouth, Ages: 25−29
Data points enclosed with a diamond are reference sources

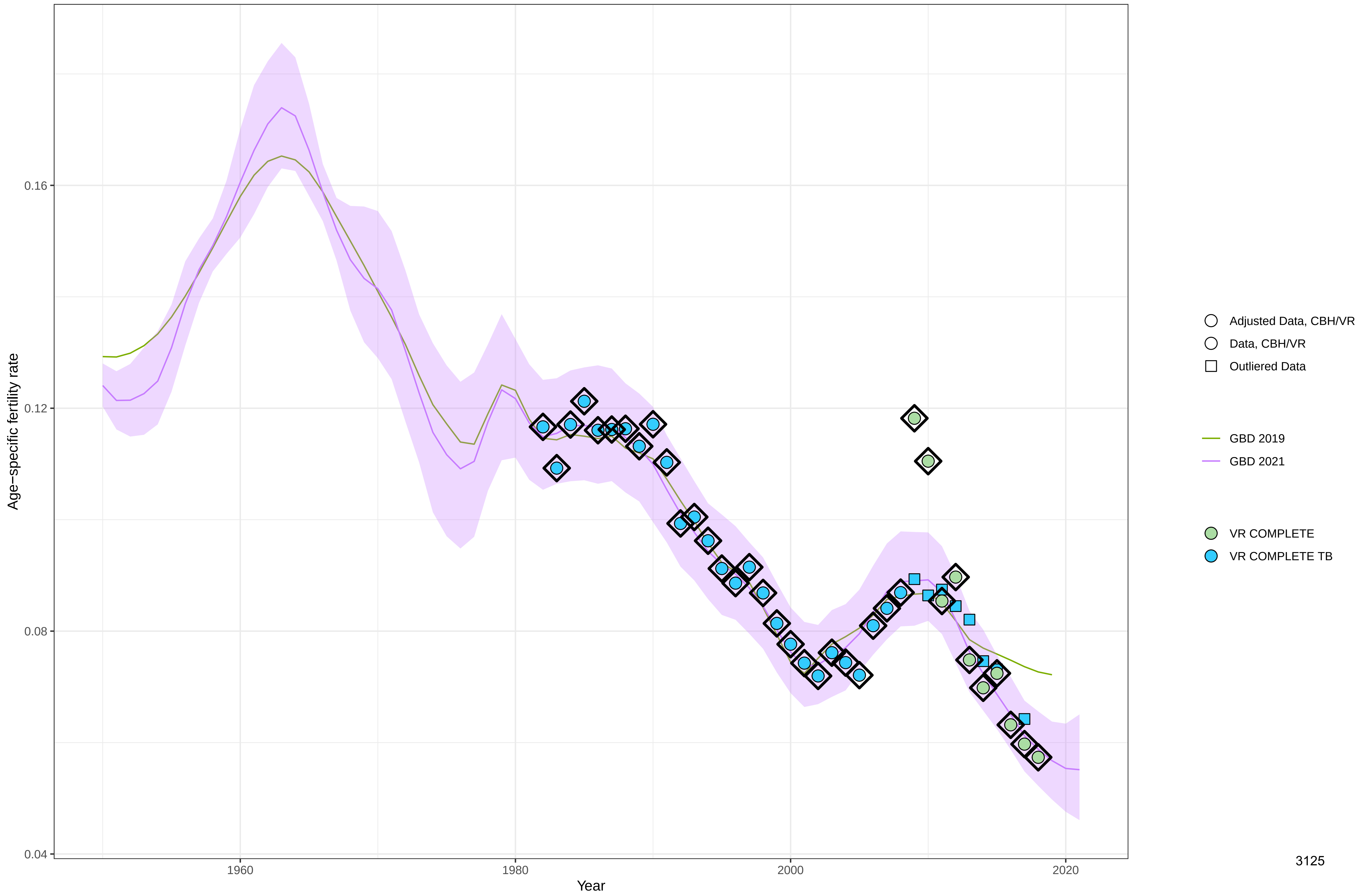

Age−specific fertility fitted trend lines, 1950−2021: Bournemouth, Ages: 30−34
Data points enclosed with a diamond are reference sources

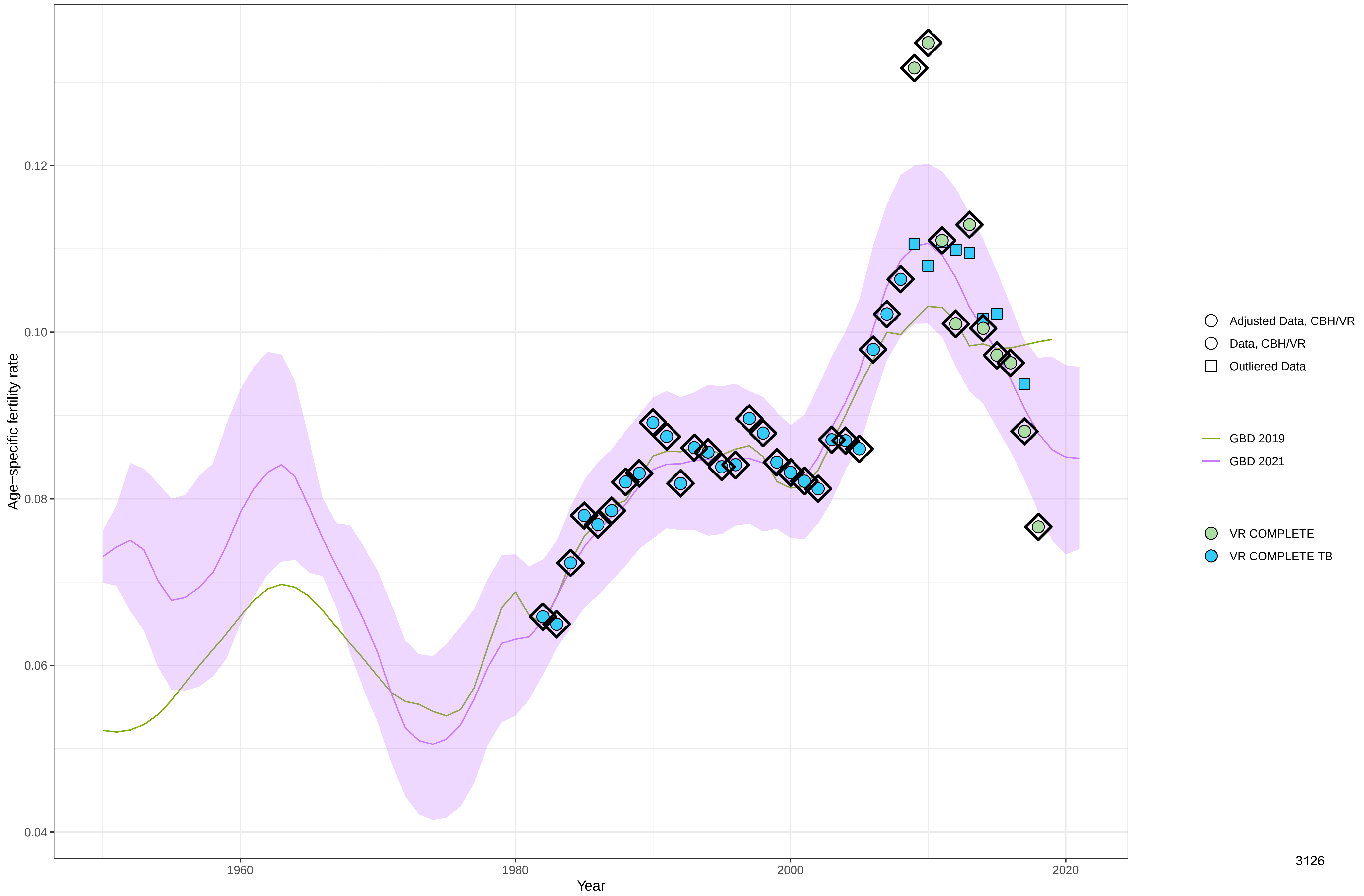

Age−specific fertility fitted trend lines, 1950−2021: Bournemouth, Ages: 35−39
Data points enclosed with a diamond are reference sources

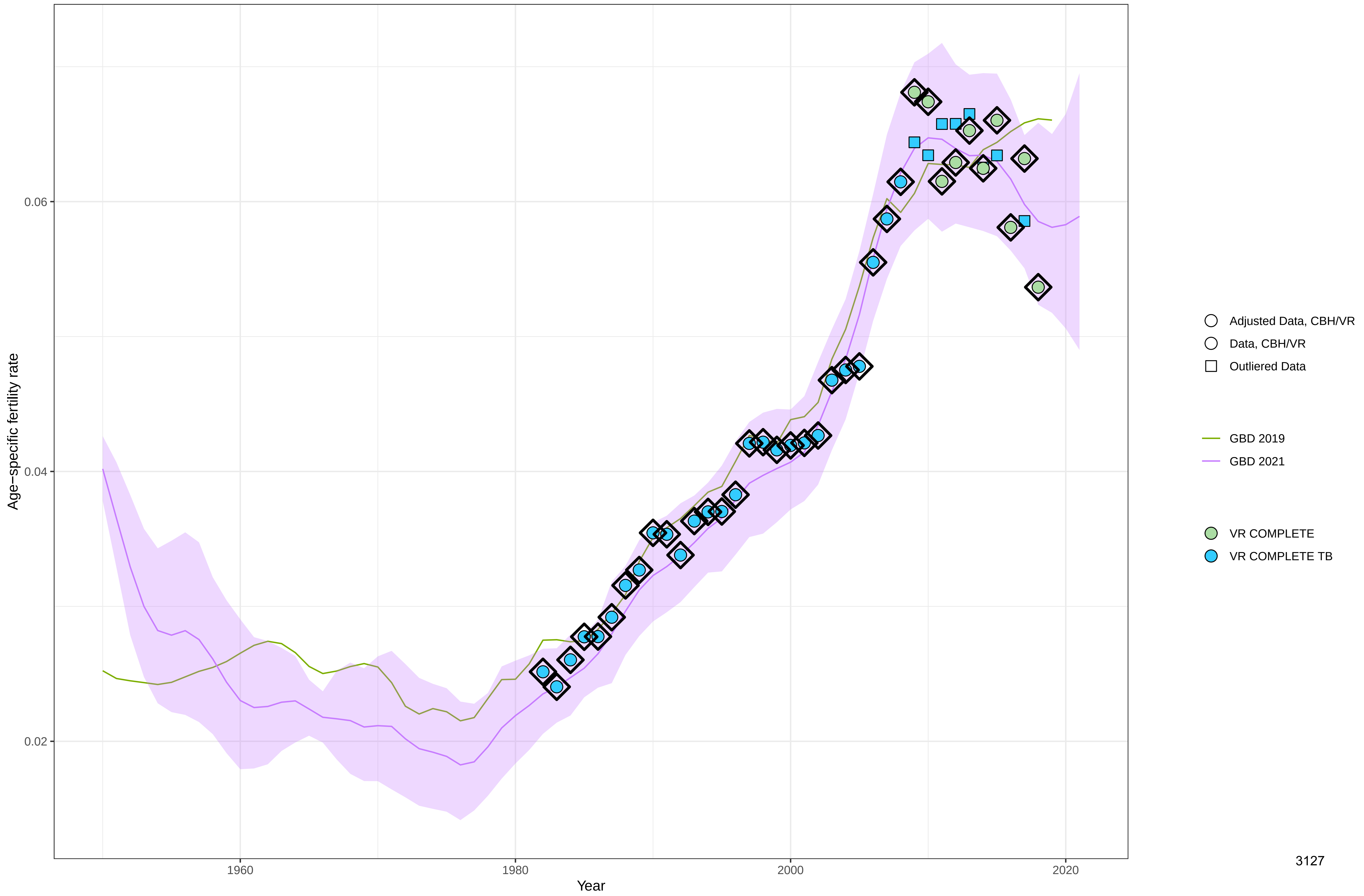

Age−specific fertility fitted trend lines, 1950−2021: Bournemouth, Ages: 40−44
Data points enclosed with a diamond are reference sources

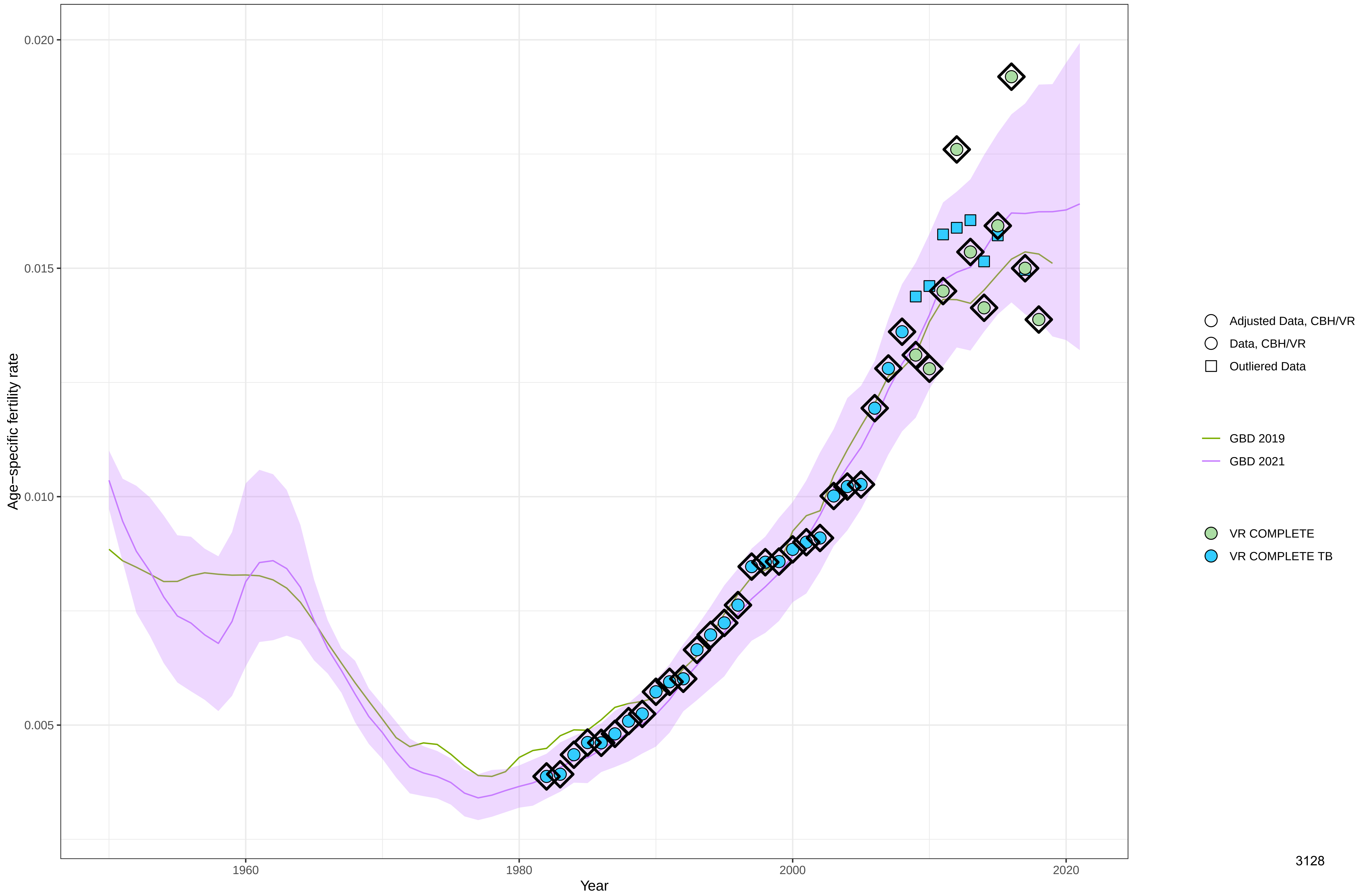

Age−specific fertility fitted trend lines, 1950−2021: Bournemouth, Ages: 45−49
Data points enclosed with a diamond are reference sources

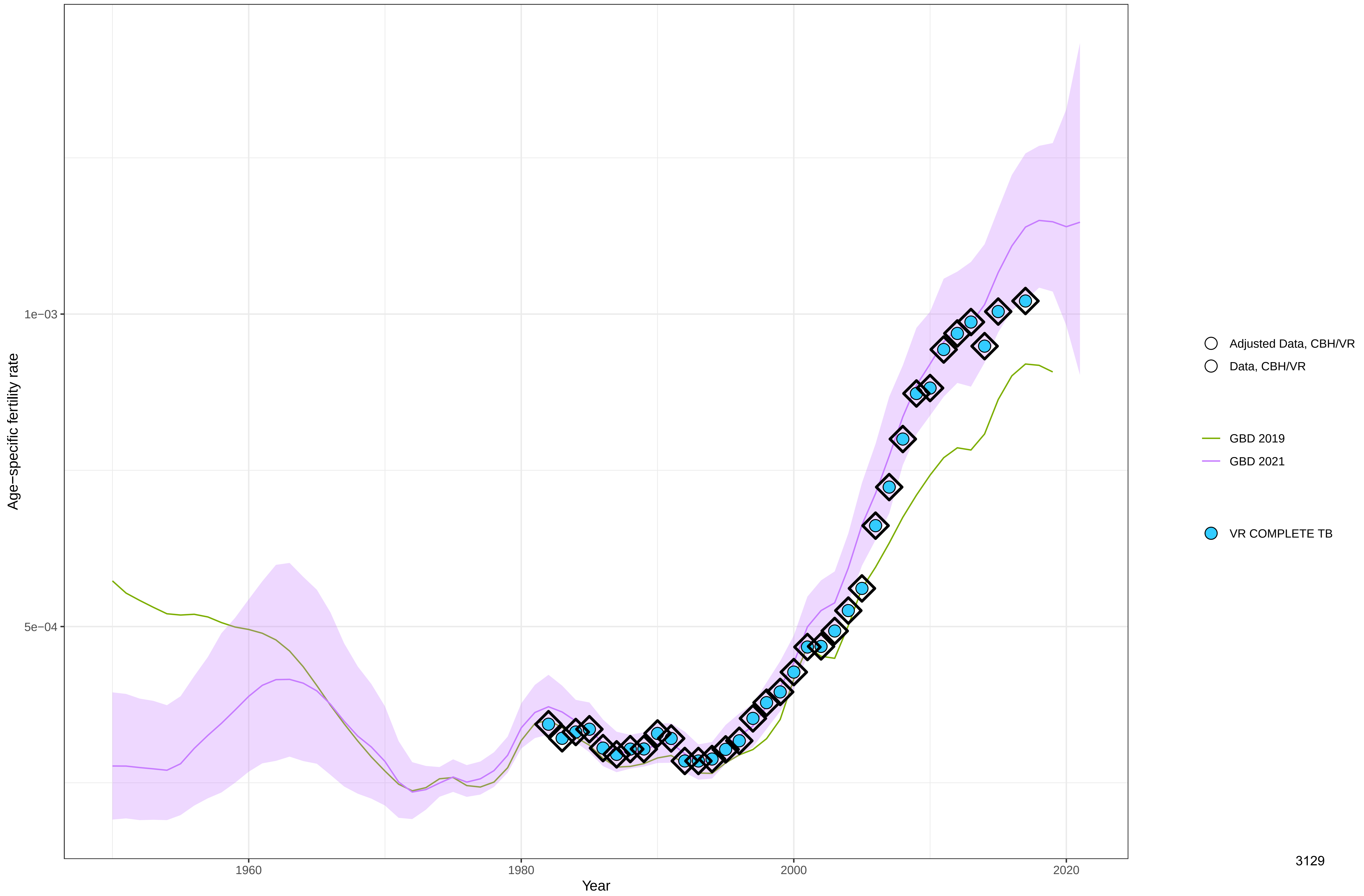

Age−specific fertility fitted trend lines, 1950−2021: Bournemouth, Ages: 50−54
Data points enclosed with a diamond are reference sources

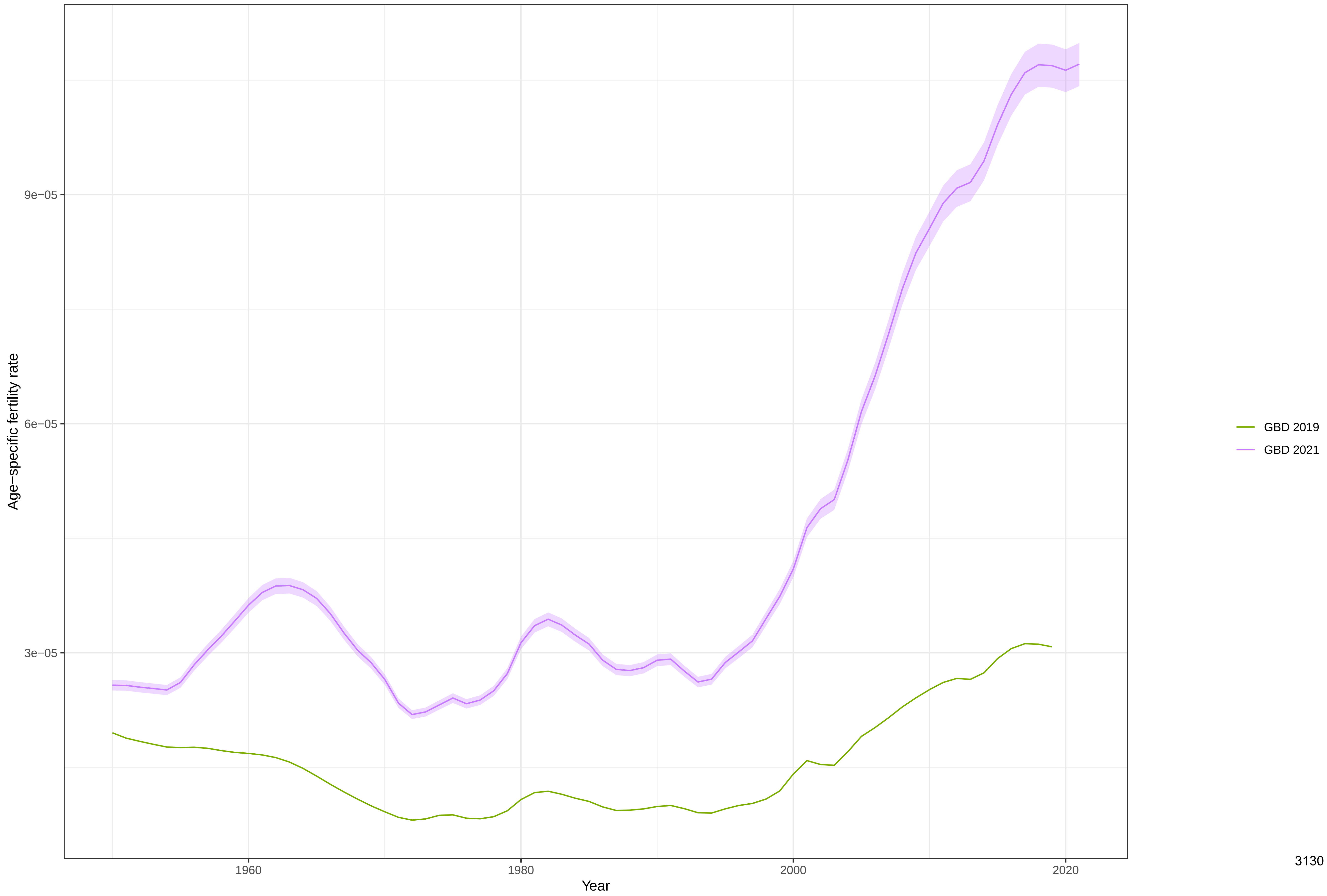

Total fertility rate fitted trend lines, 1950−2021: Bournemouth TFR
Data points enclosed with a diamond are reference sources

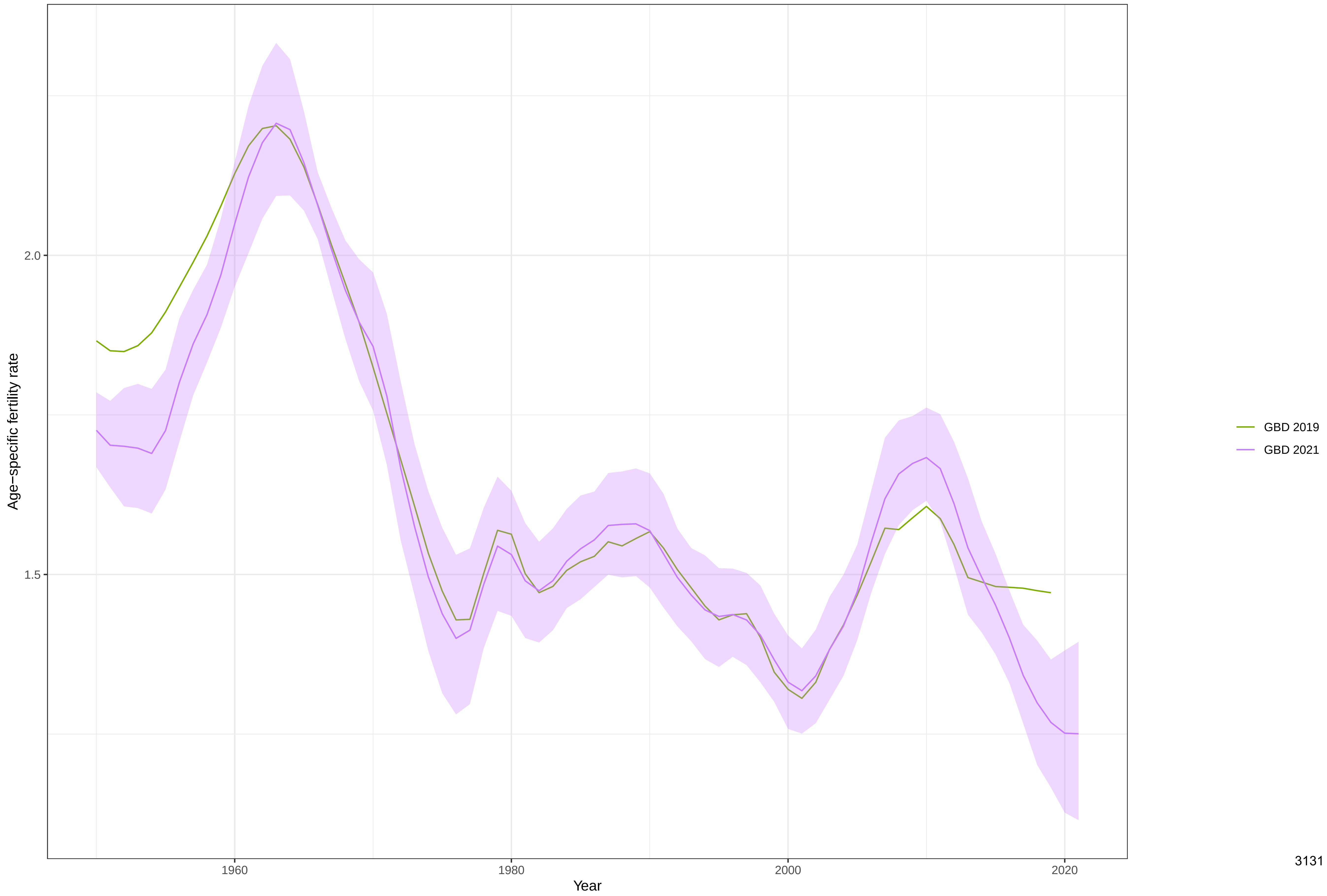

Age−specific fertility fitted trend lines, 1950−2021: Bristol, City of, Ages: 10−14
Data points enclosed with a diamond are reference sources

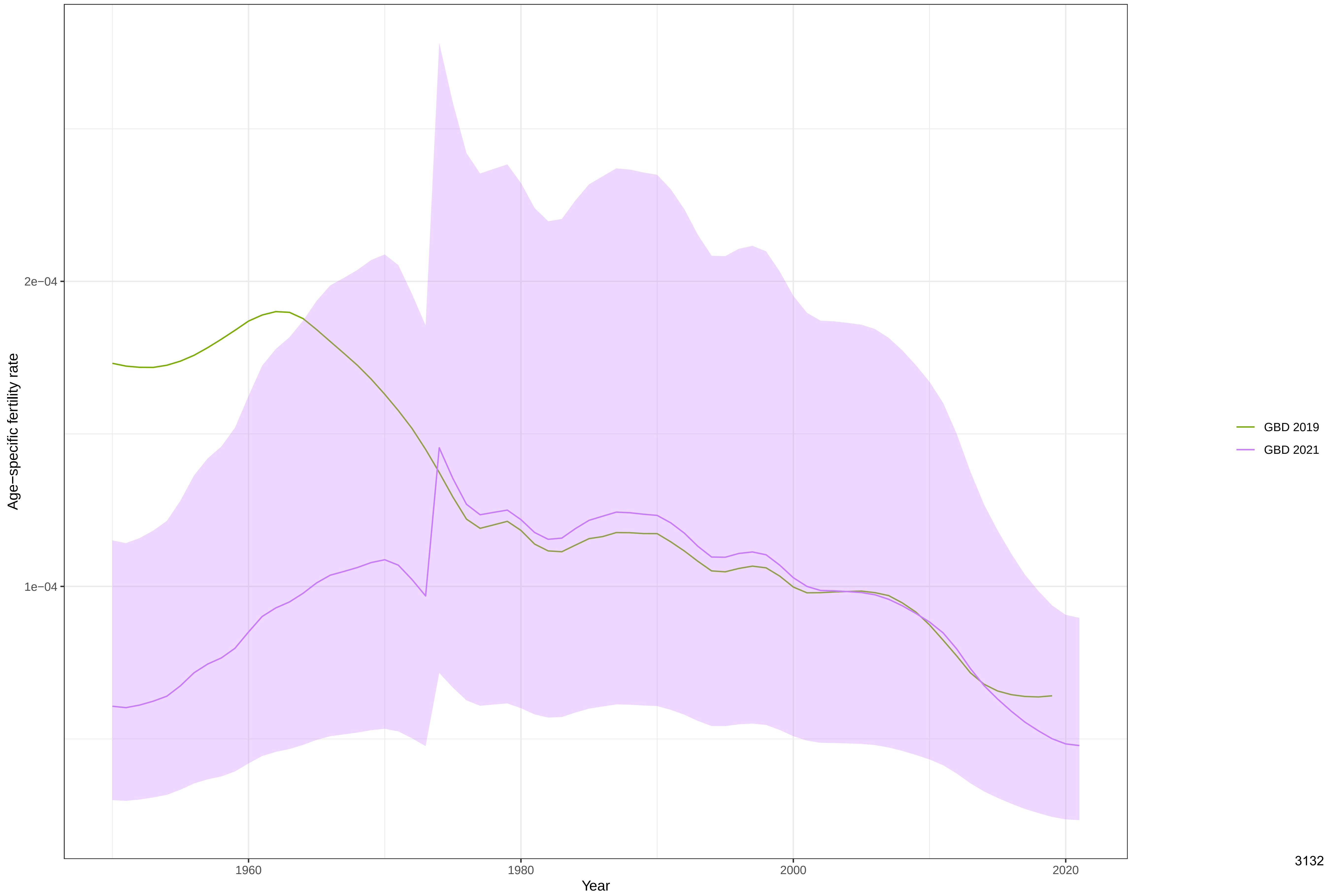

Age−specific fertility fitted trend lines, 1950−2021: Bristol, City of, Ages: 15−19
Data points enclosed with a diamond are reference sources

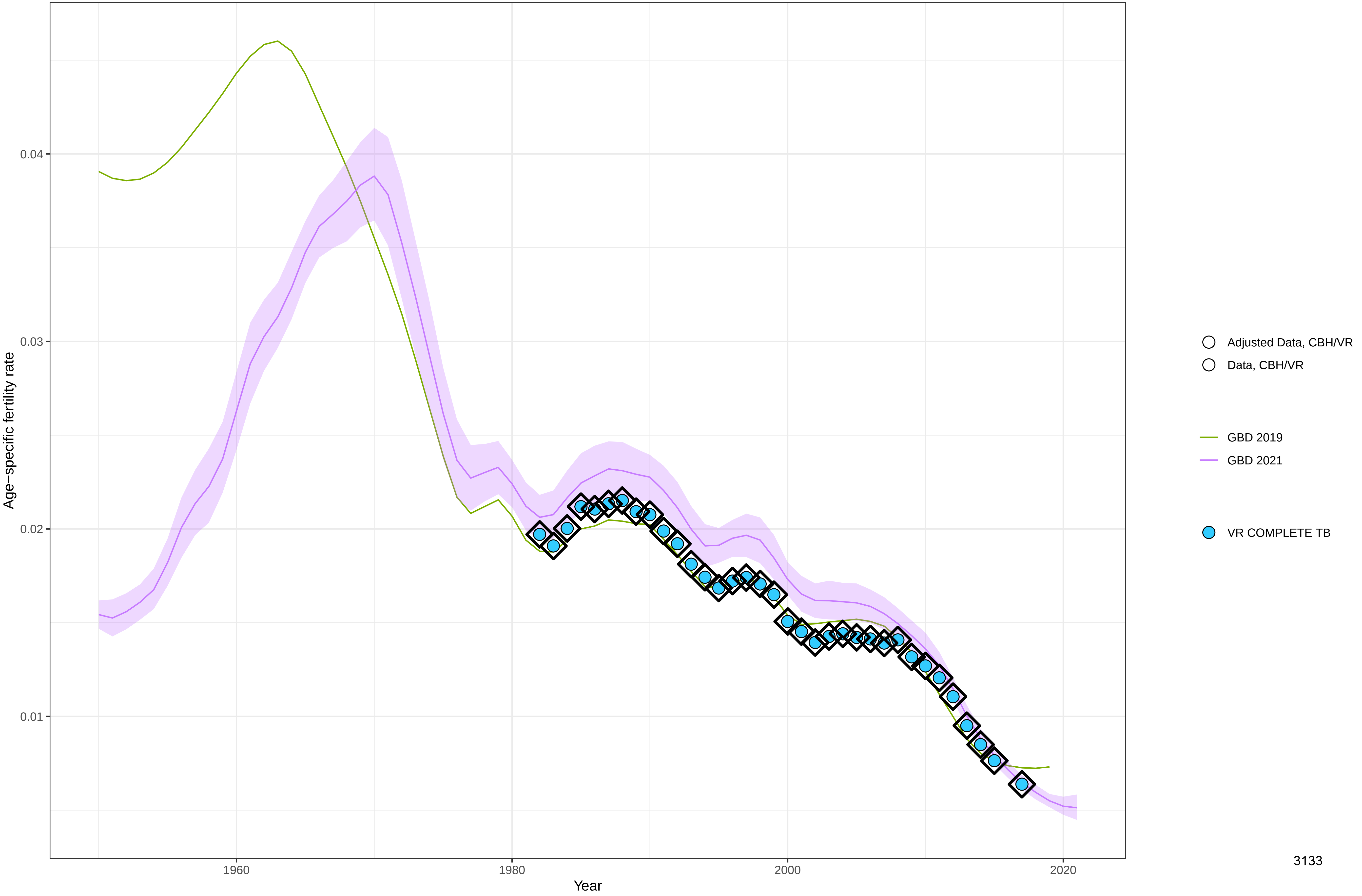

Age−specific fertility fitted trend lines, 1950−2021: Bristol, City of, Ages: 20−24
Data points enclosed with a diamond are reference sources

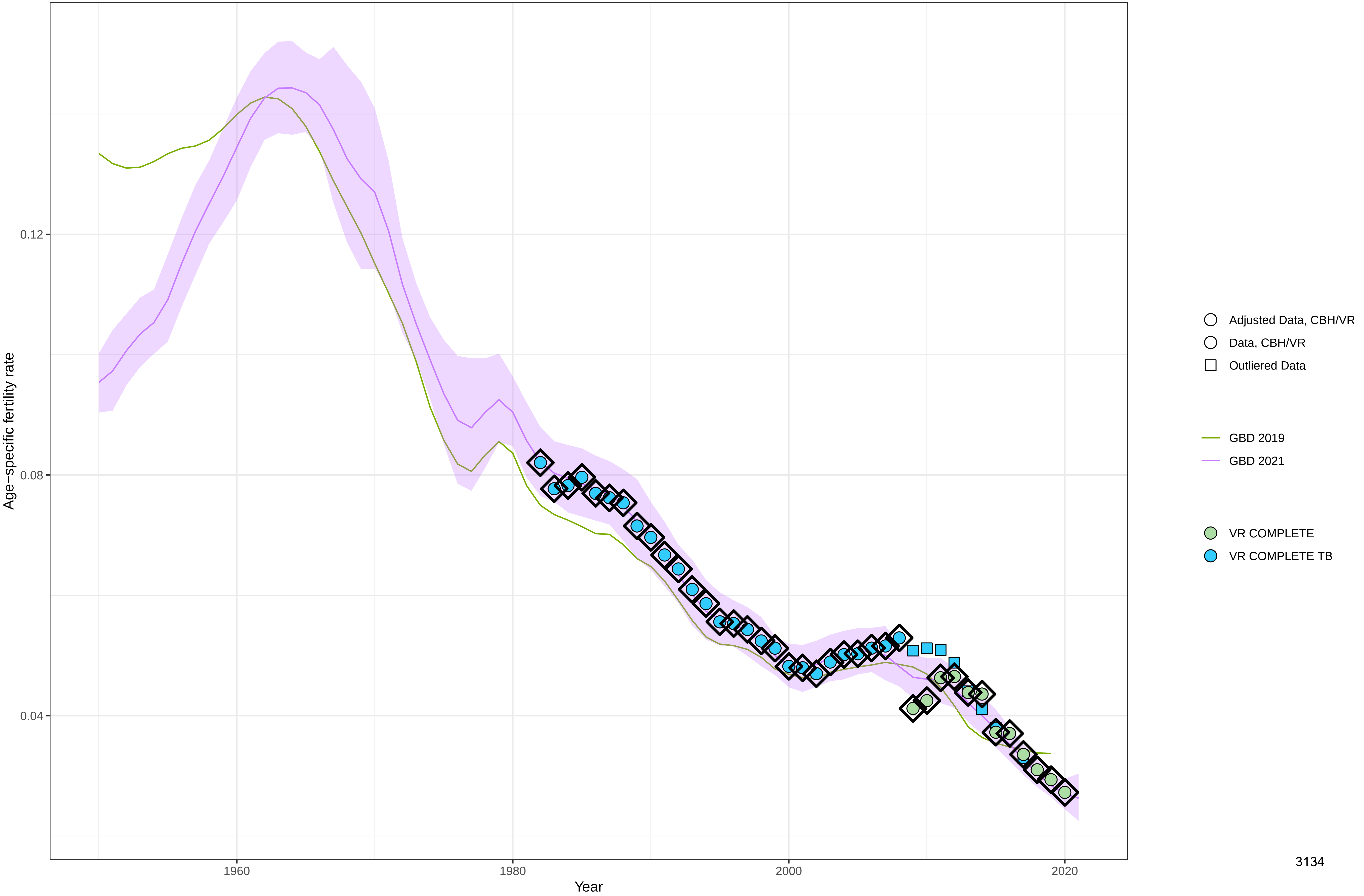

Age−specific fertility fitted trend lines, 1950−2021: Bristol, City of, Ages: 25−29
Data points enclosed with a diamond are reference sources

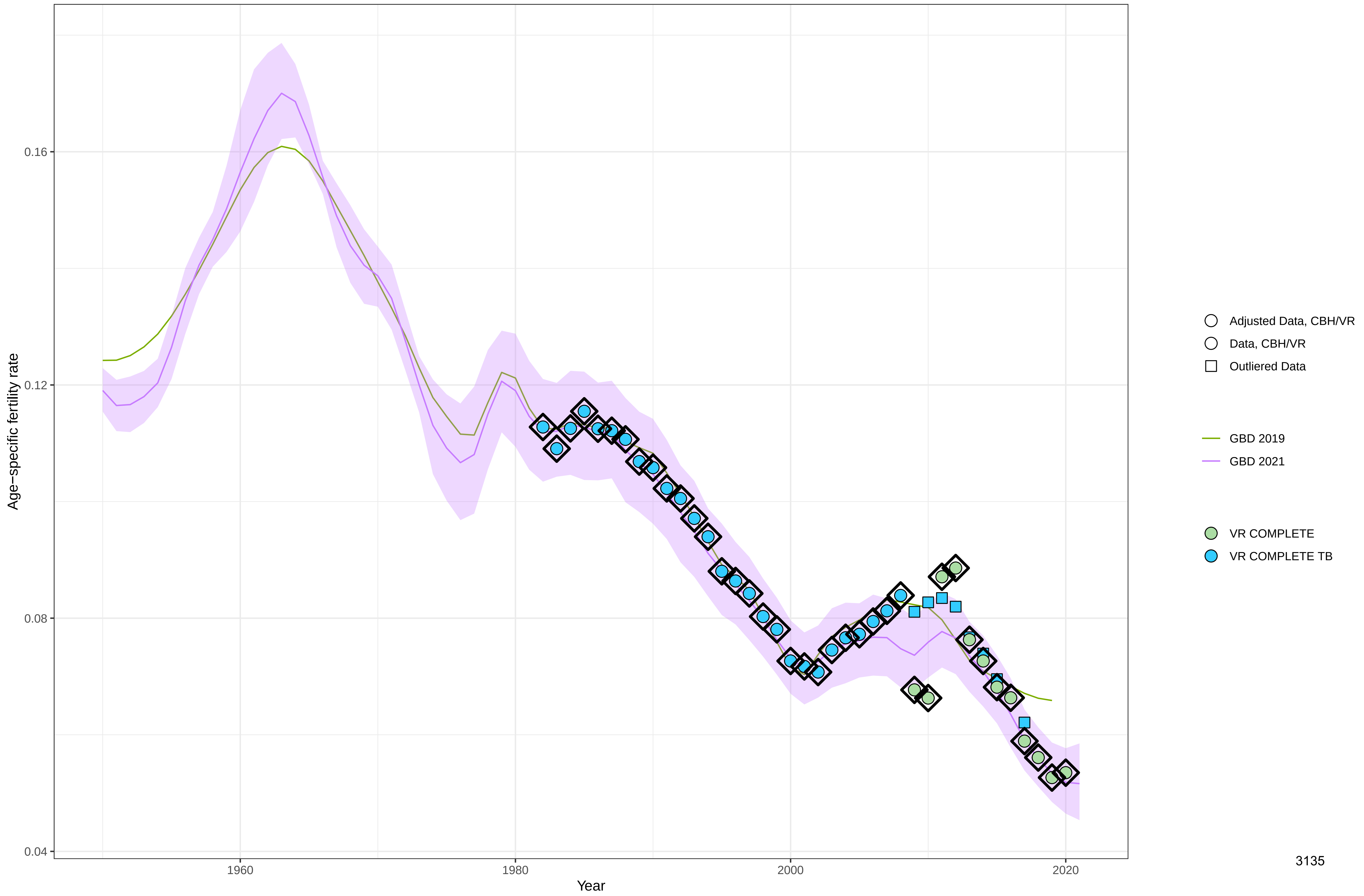

Age−specific fertility fitted trend lines, 1950−2021: Bristol, City of, Ages: 30−34
Data points enclosed with a diamond are reference sources

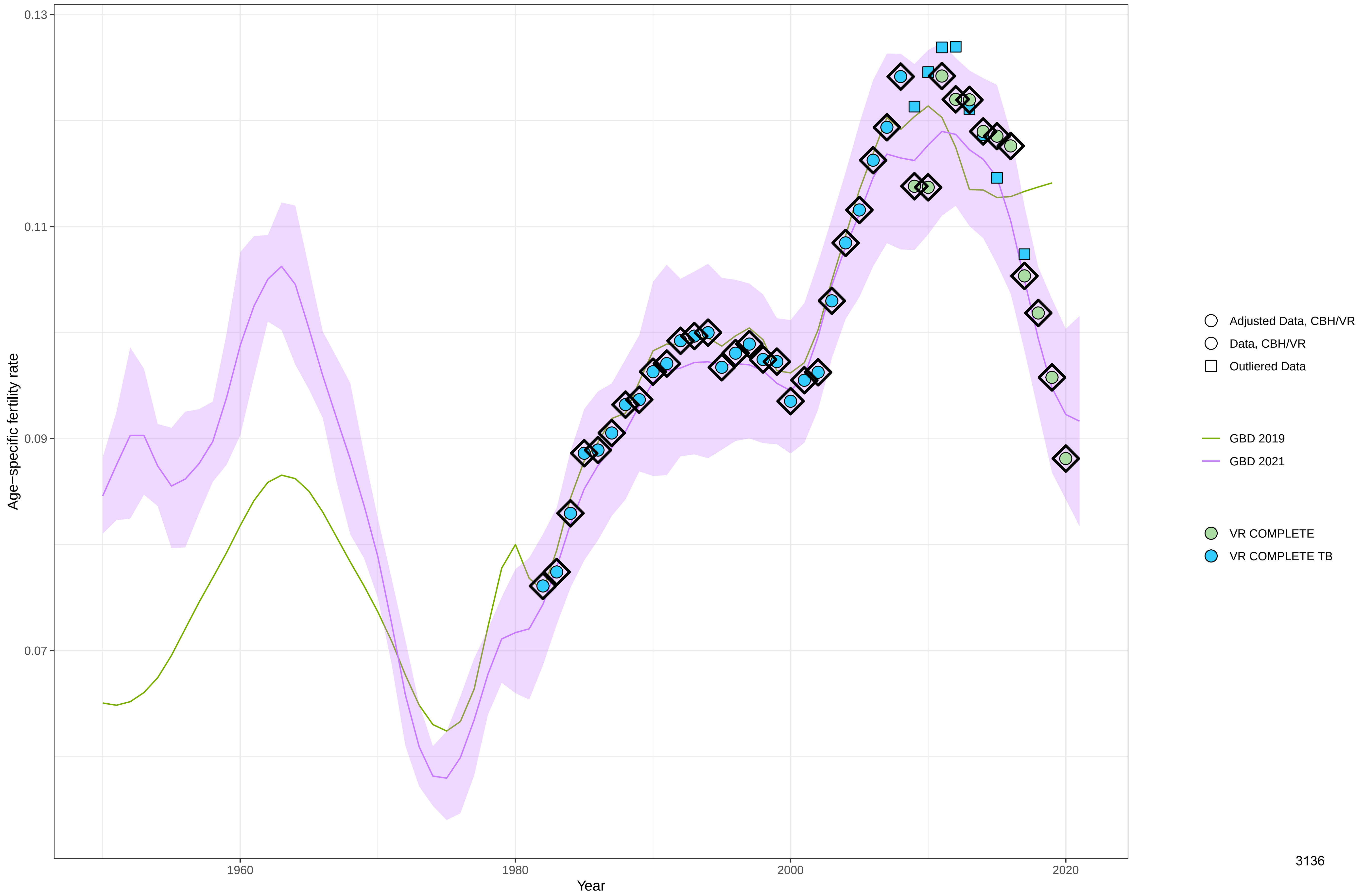

Age−specific fertility fitted trend lines, 1950−2021: Bristol, City of, Ages: 35−39
Data points enclosed with a diamond are reference sources

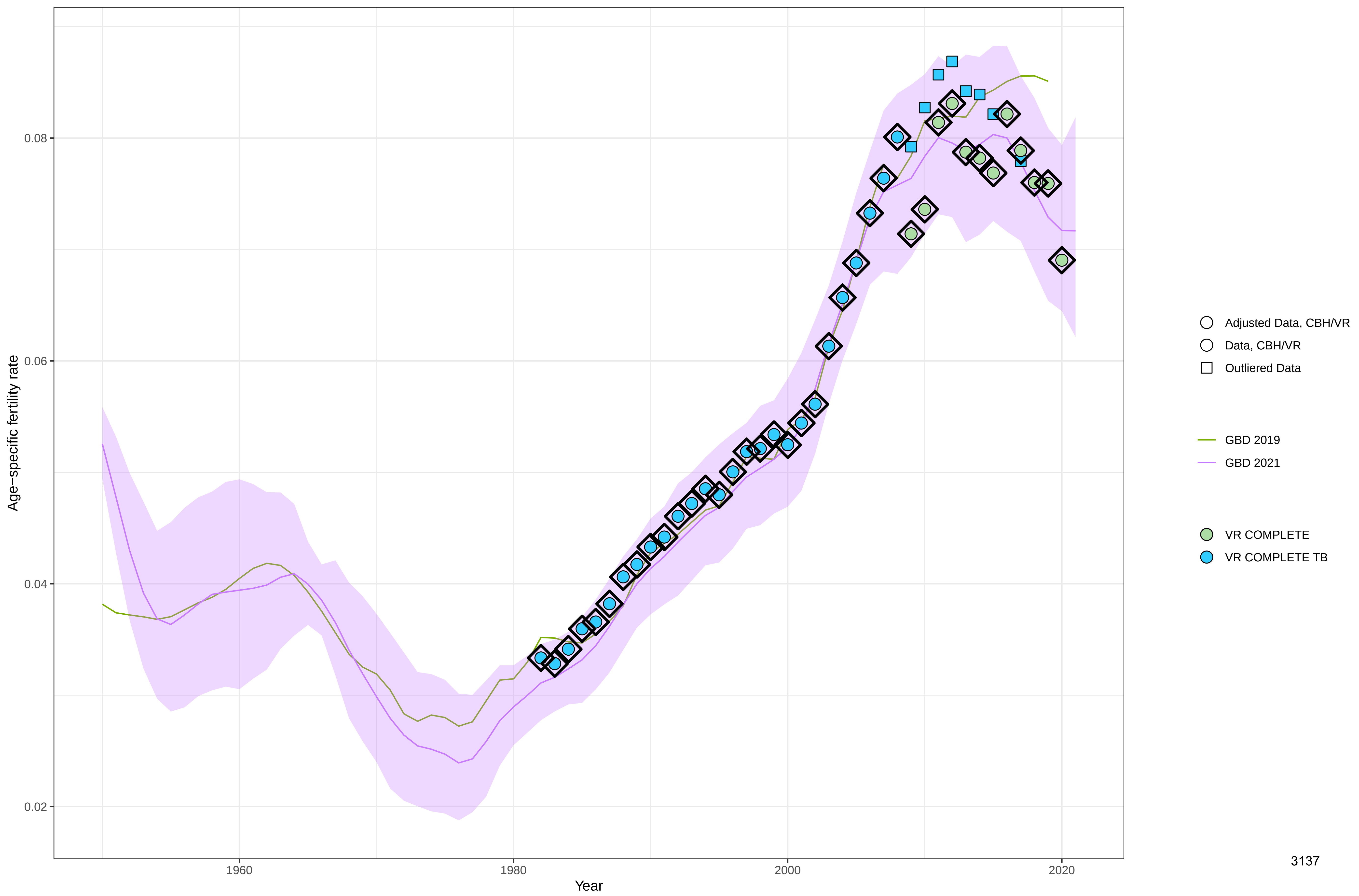

Age−specific fertility fitted trend lines, 1950−2021: Bristol, City of, Ages: 40−44
Data points enclosed with a diamond are reference sources

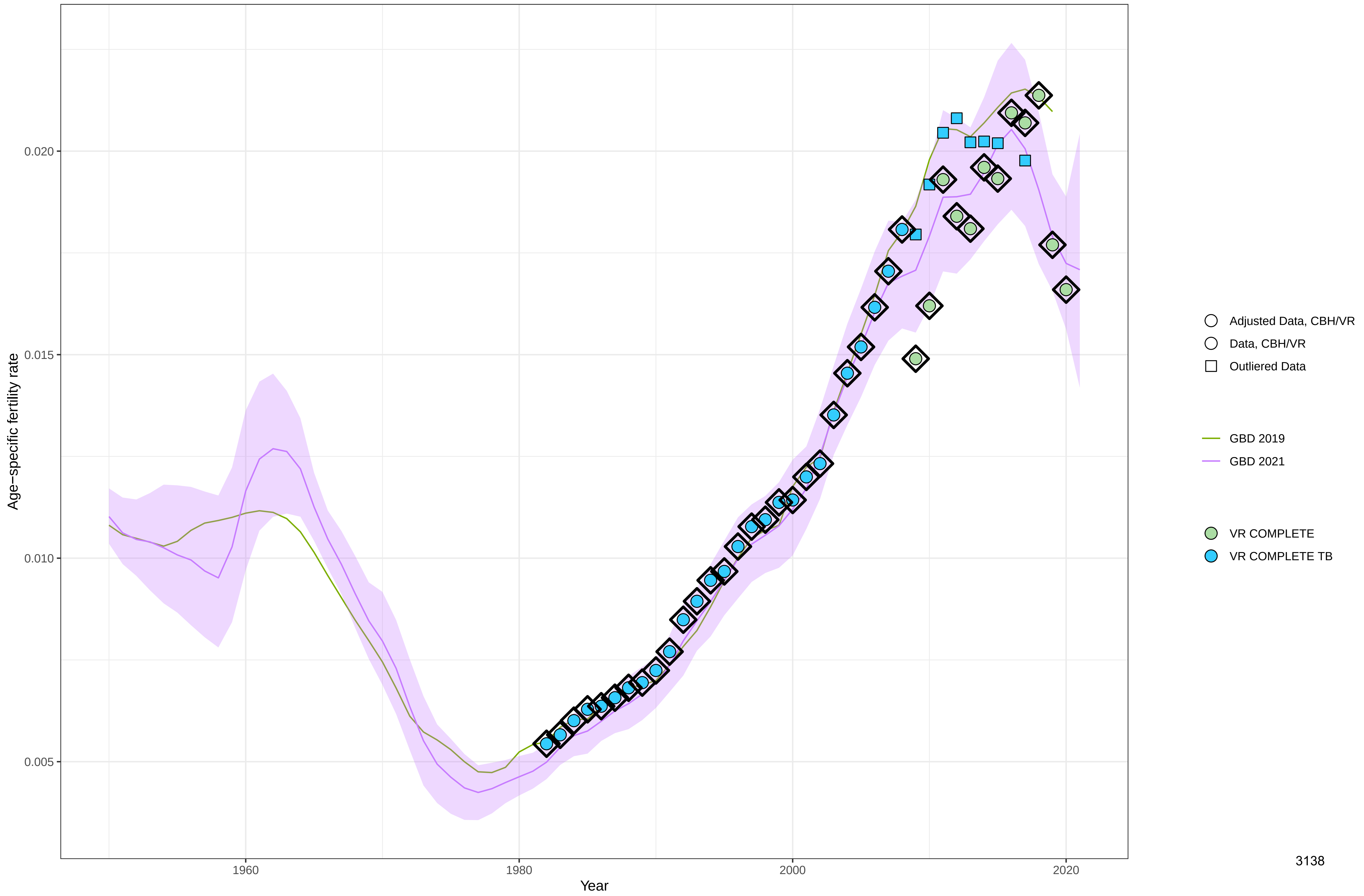

Age−specific fertility fitted trend lines, 1950−2021: Bristol, City of, Ages: 45−49
Data points enclosed with a diamond are reference sources

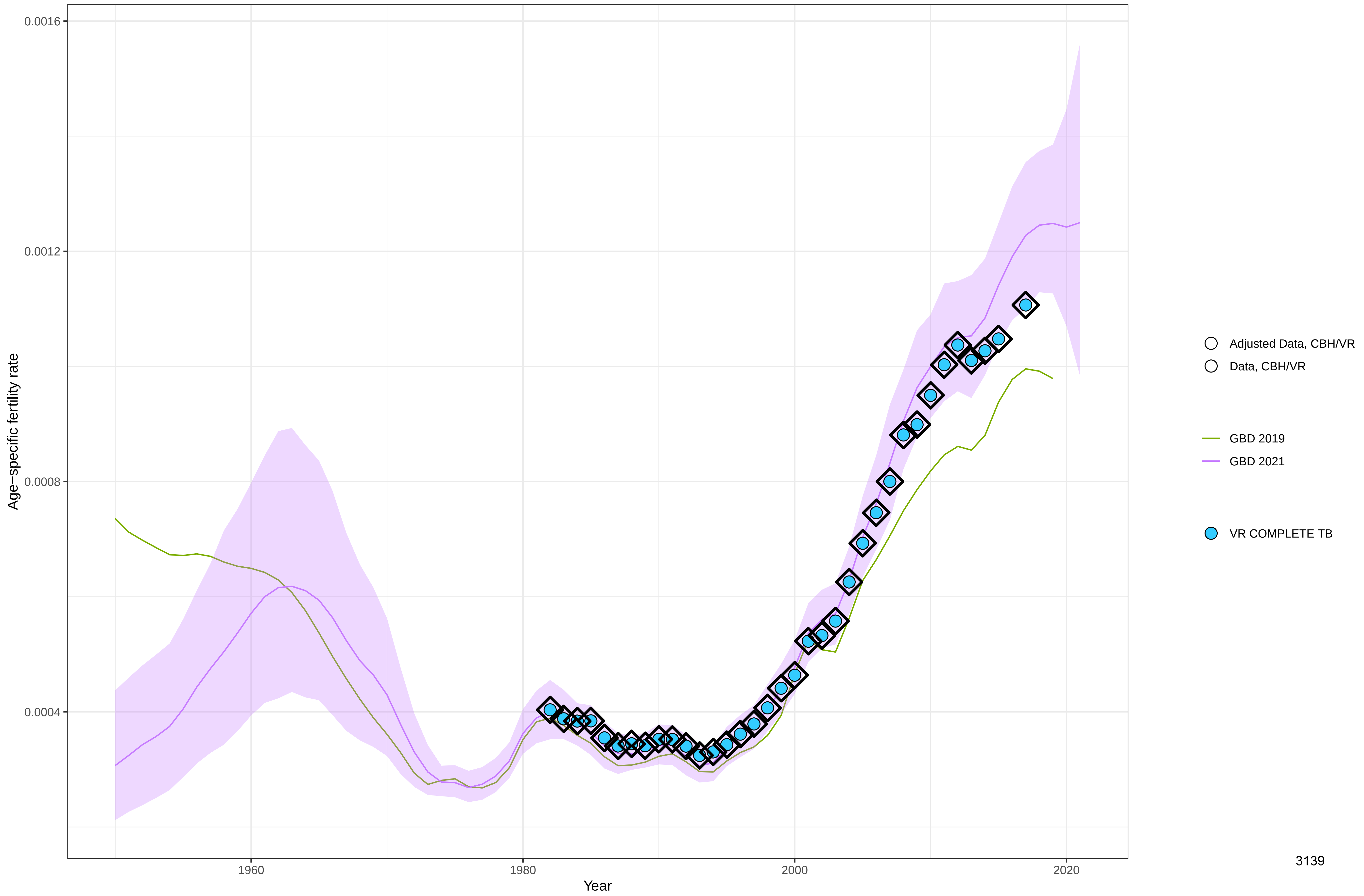

Age−specific fertility fitted trend lines, 1950−2021: Bristol, City of, Ages: 50−54
Data points enclosed with a diamond are reference sources

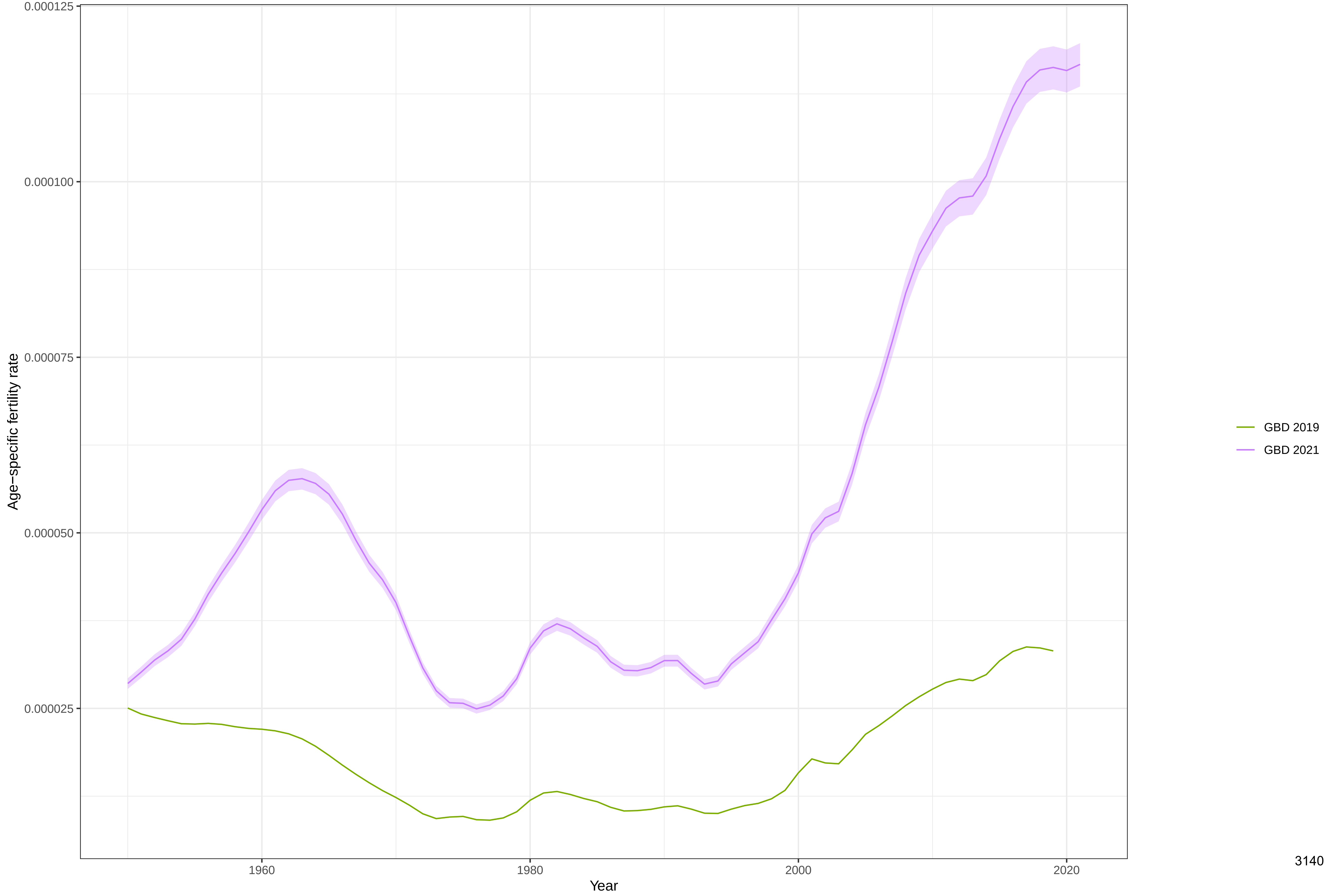

Total fertility rate fitted trend lines, 1950−2021: Bristol, City of TFR
Data points enclosed with a diamond are reference sources

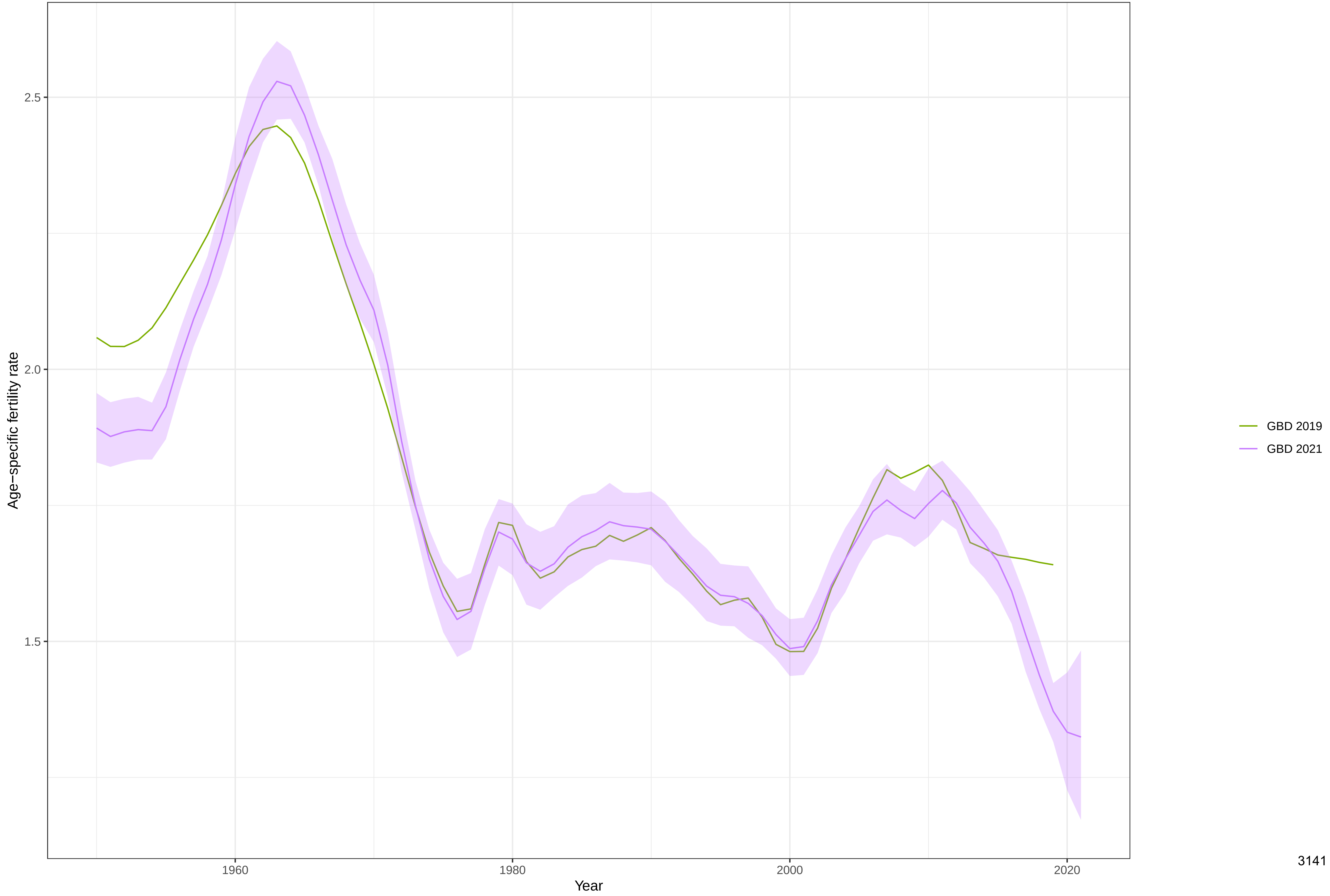

Age−specific fertility fitted trend lines, 1950−2021: Cornwall, Ages: 10−14
Data points enclosed with a diamond are reference sources

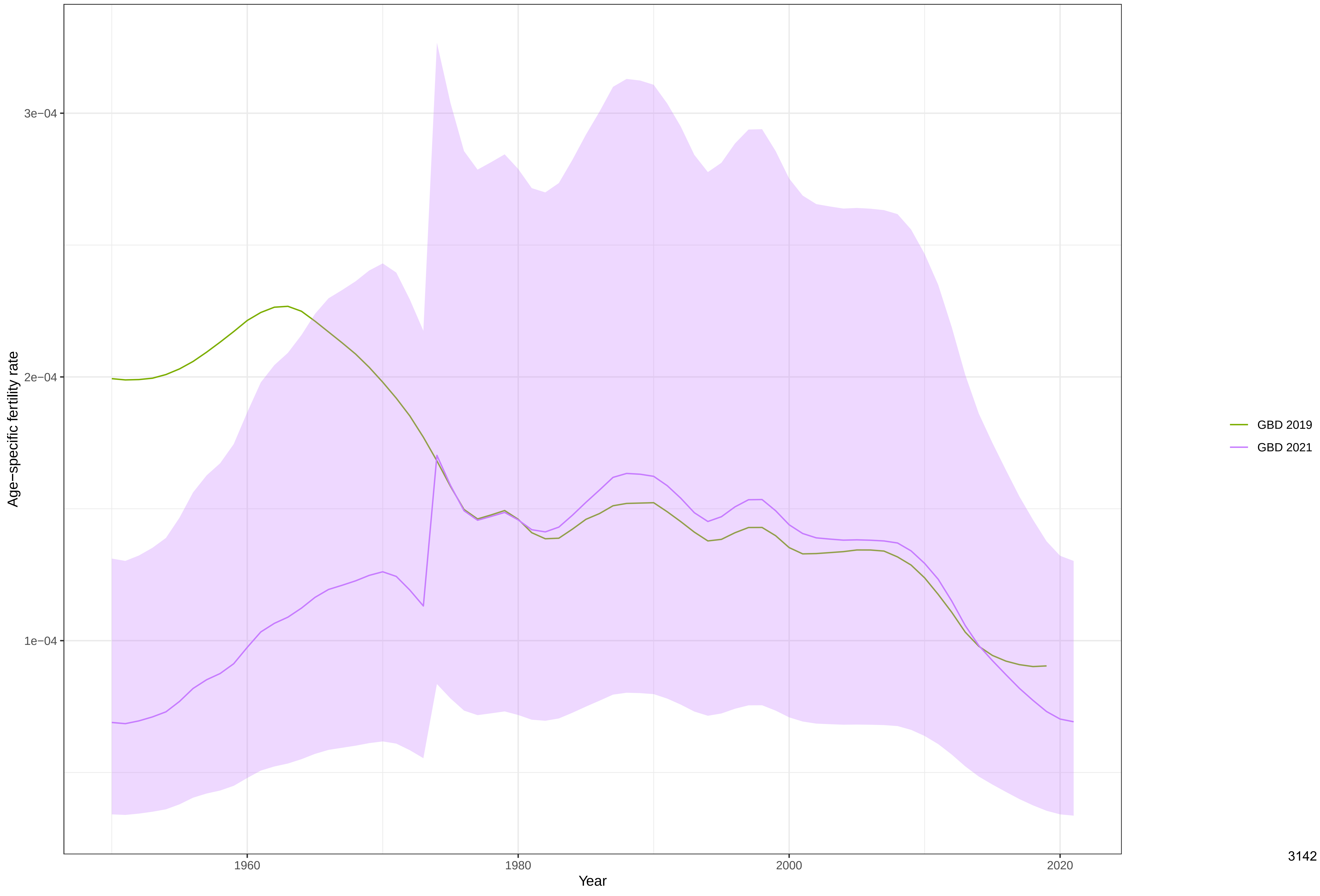

Age−specific fertility fitted trend lines, 1950−2021: Cornwall, Ages: 15−19
Data points enclosed with a diamond are reference sources

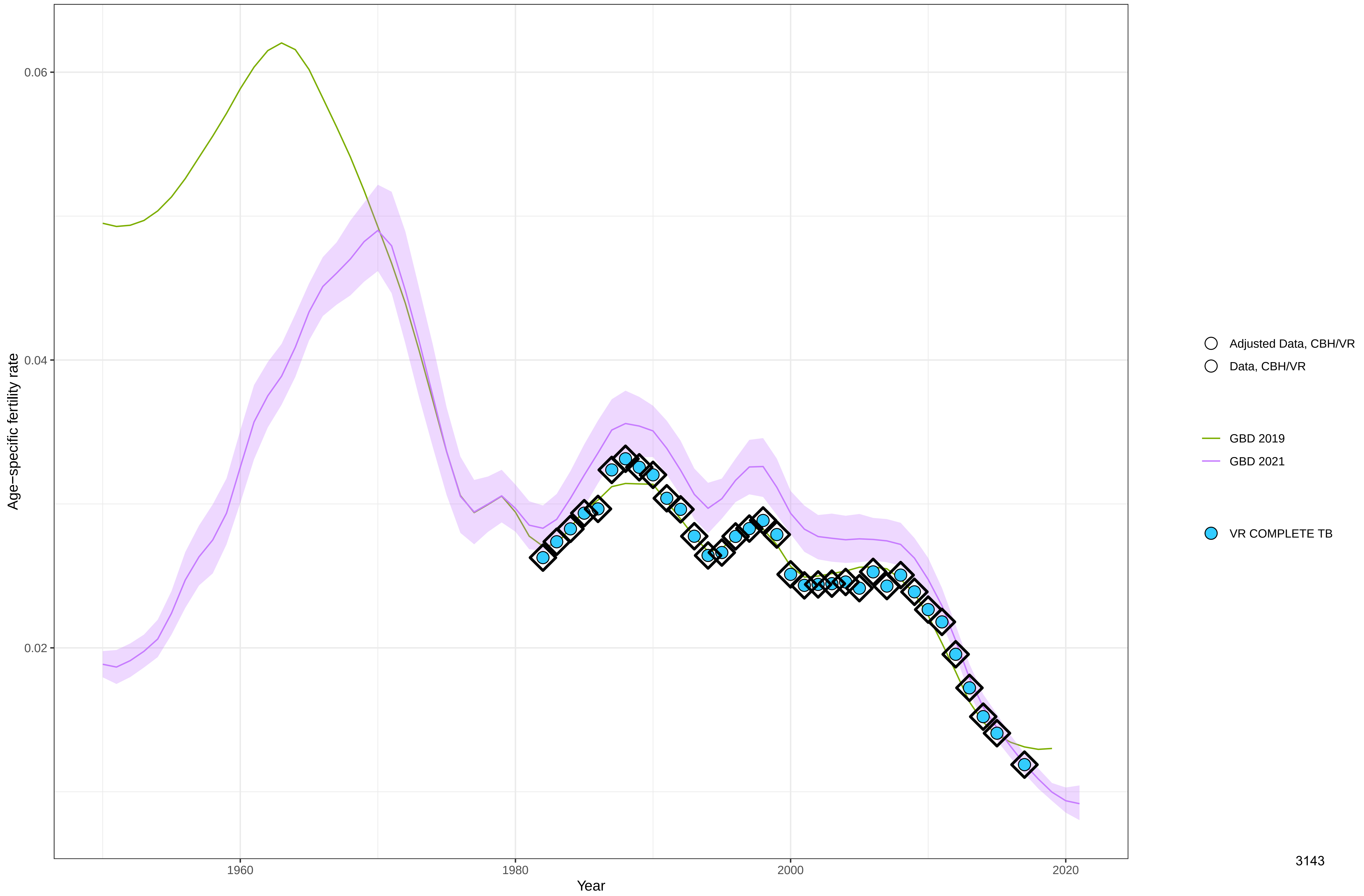

Age−specific fertility fitted trend lines, 1950−2021: Cornwall, Ages: 20−24
Data points enclosed with a diamond are reference sources

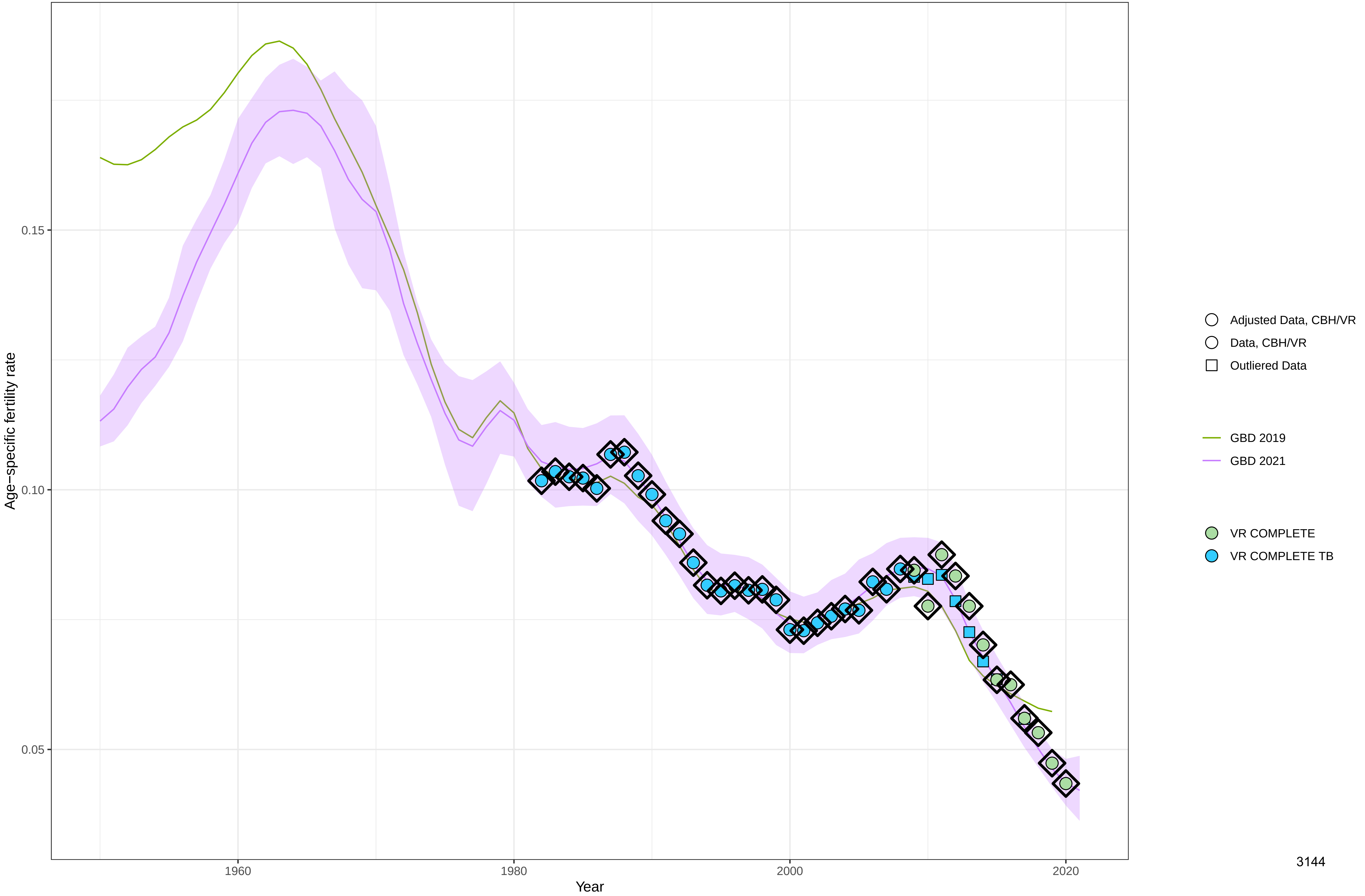

Age−specific fertility fitted trend lines, 1950−2021: Cornwall, Ages: 25−29
Data points enclosed with a diamond are reference sources

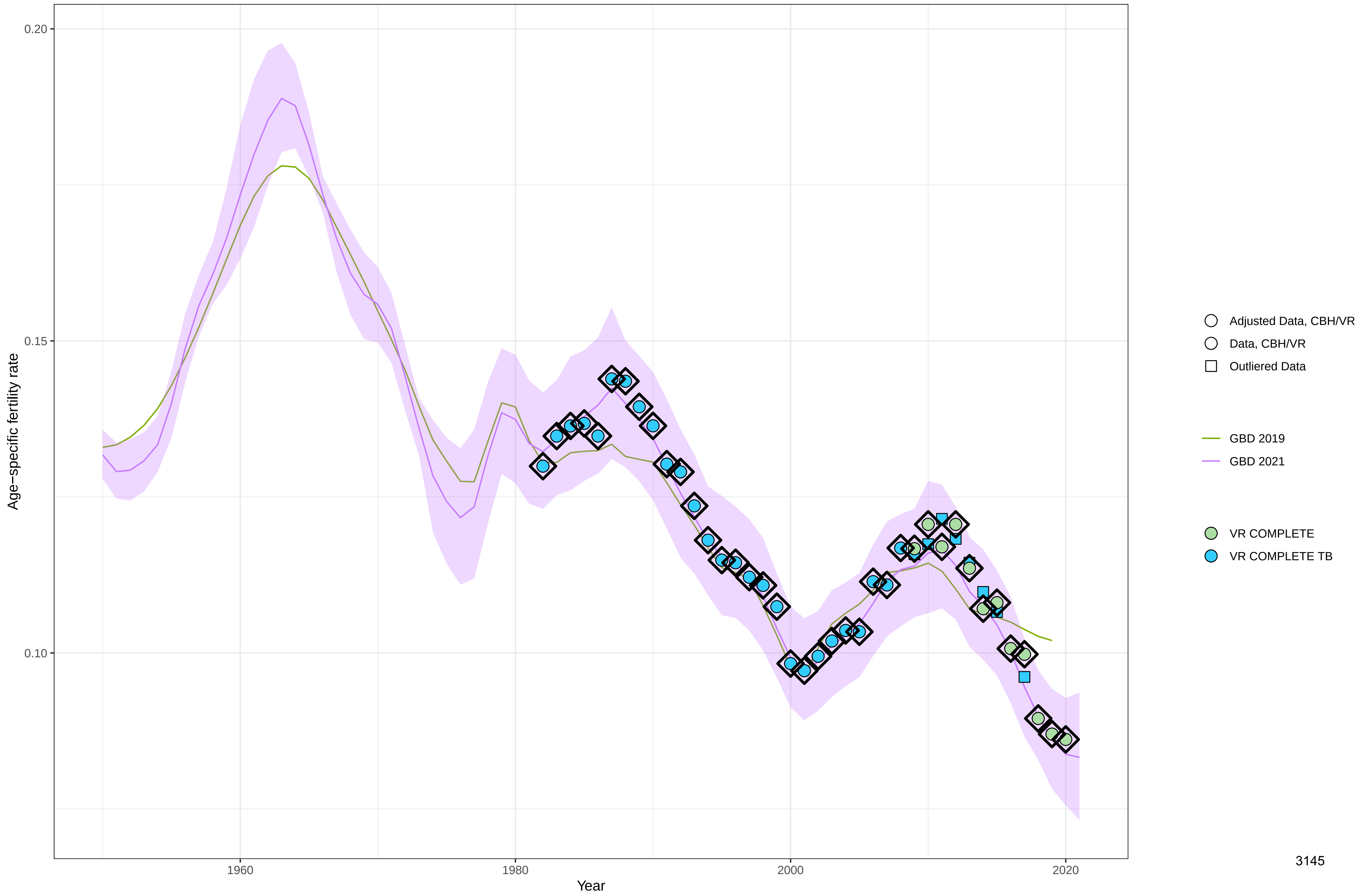

Age−specific fertility fitted trend lines, 1950−2021: Cornwall, Ages: 30−34
Data points enclosed with a diamond are reference sources

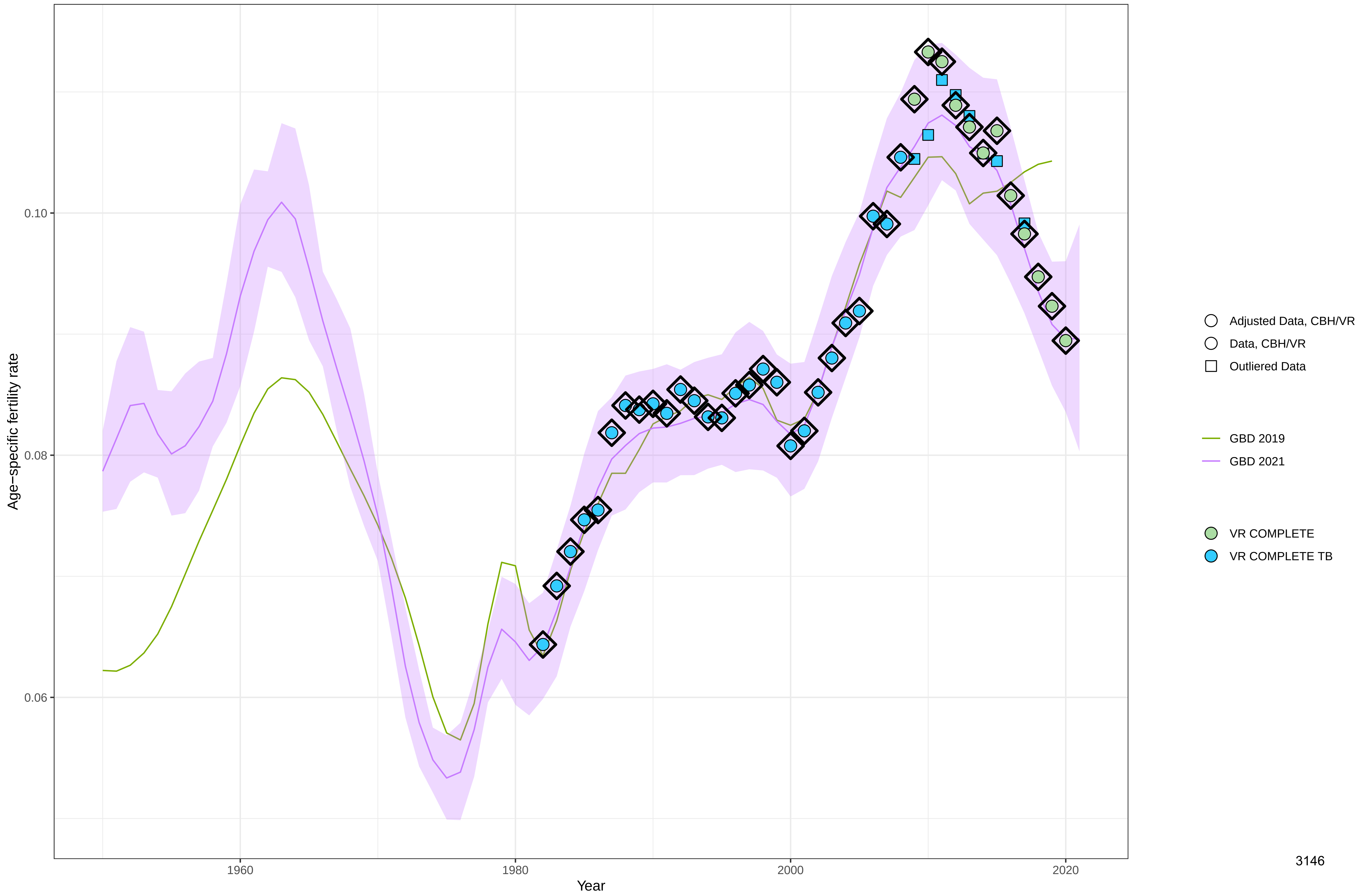

Age−specific fertility fitted trend lines, 1950−2021: Cornwall, Ages: 35−39
Data points enclosed with a diamond are reference sources

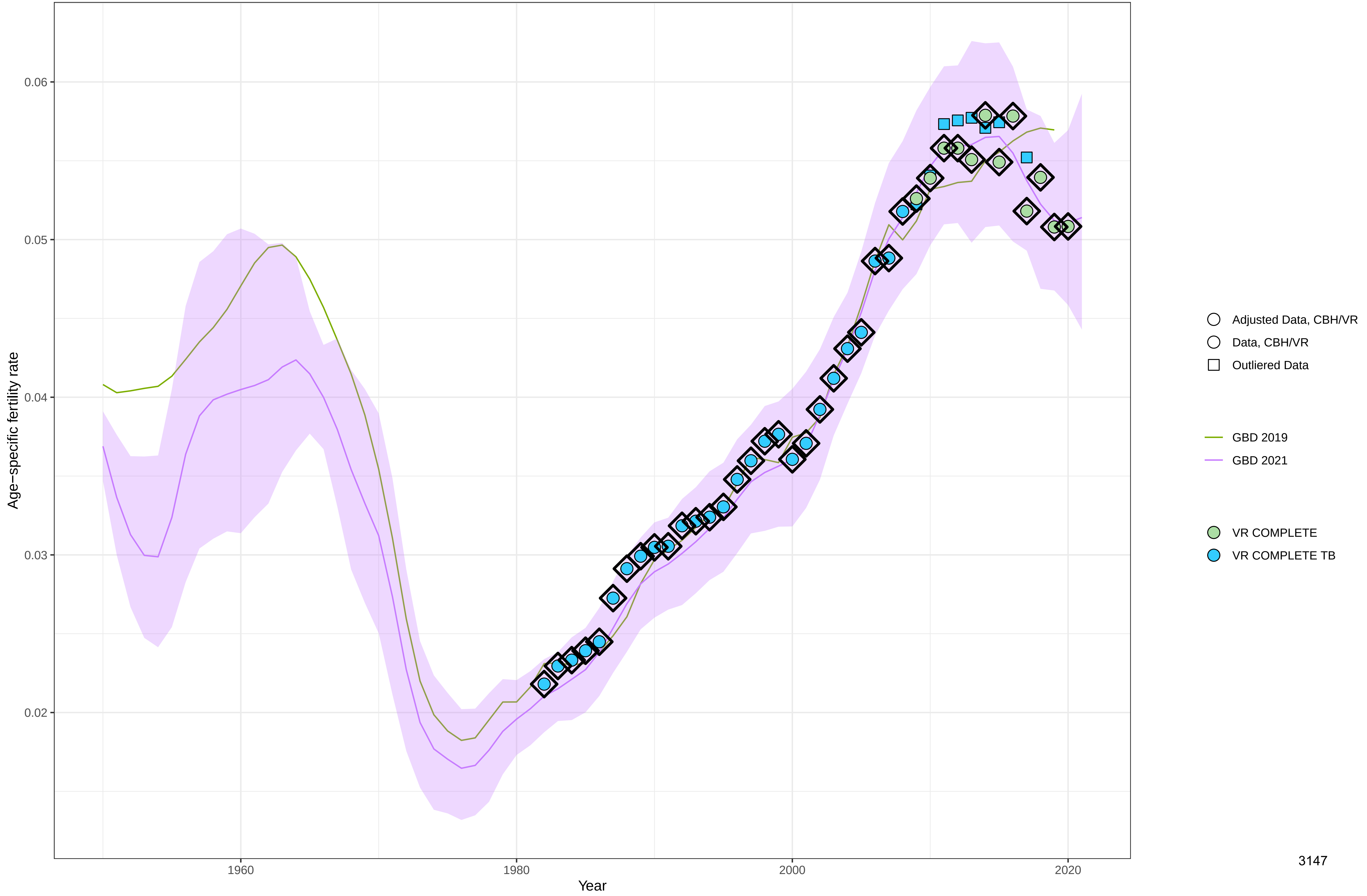

Age−specific fertility fitted trend lines, 1950−2021: Cornwall, Ages: 40−44
Data points enclosed with a diamond are reference sources

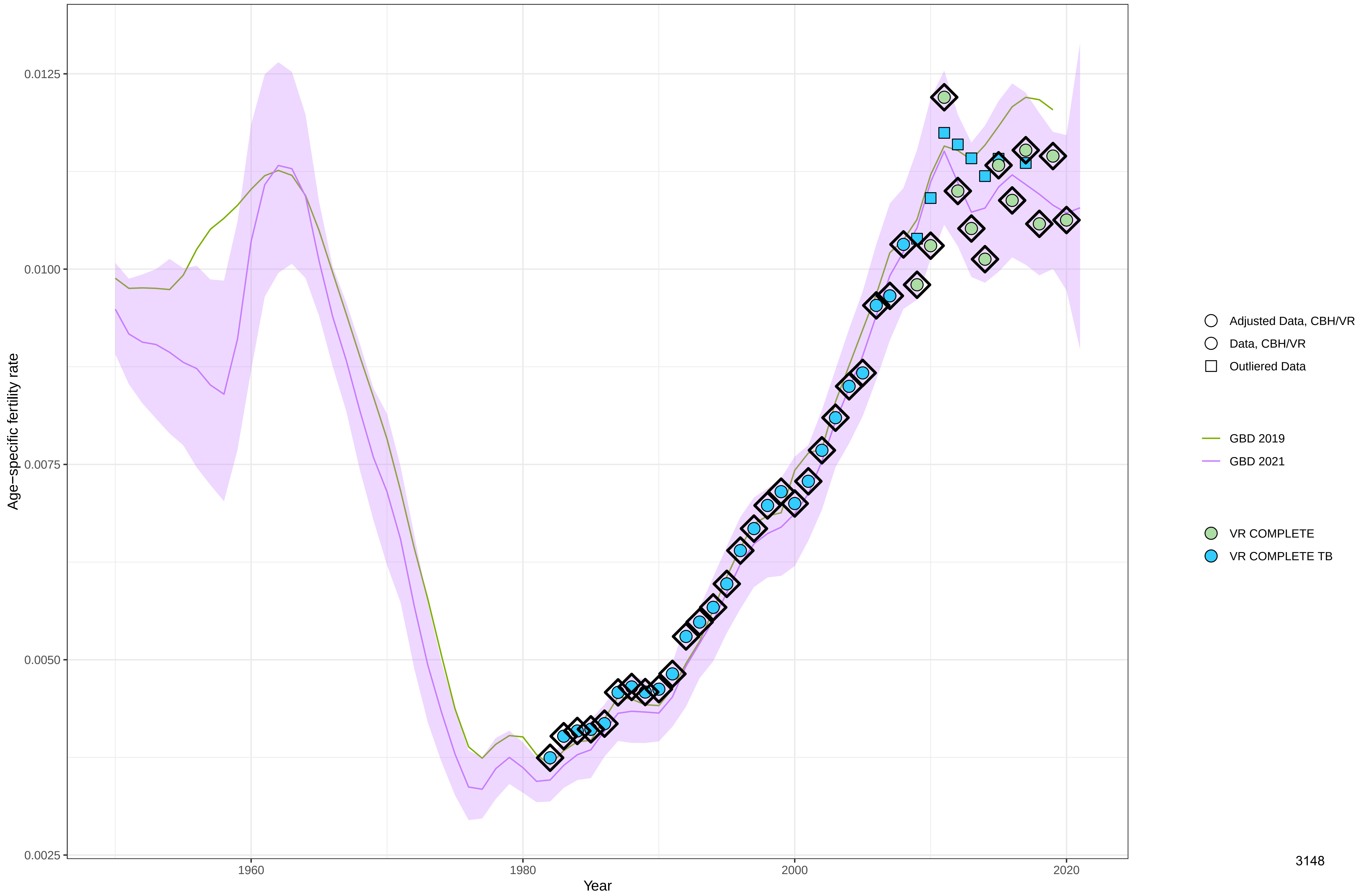

Age−specific fertility fitted trend lines, 1950−2021: Cornwall, Ages: 45−49
Data points enclosed with a diamond are reference sources

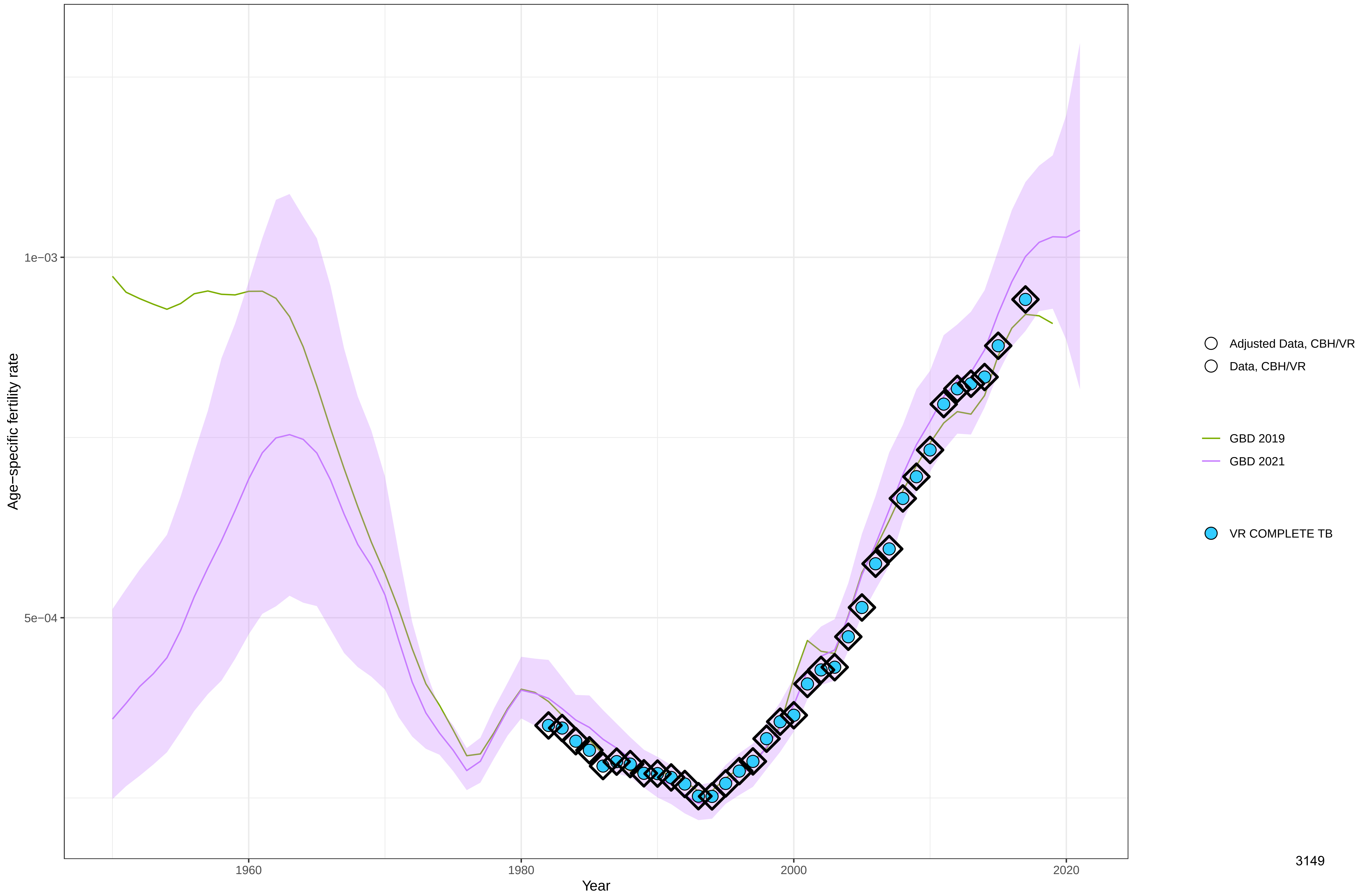

Age−specific fertility fitted trend lines, 1950−2021: Cornwall, Ages: 50−54
Data points enclosed with a diamond are reference sources

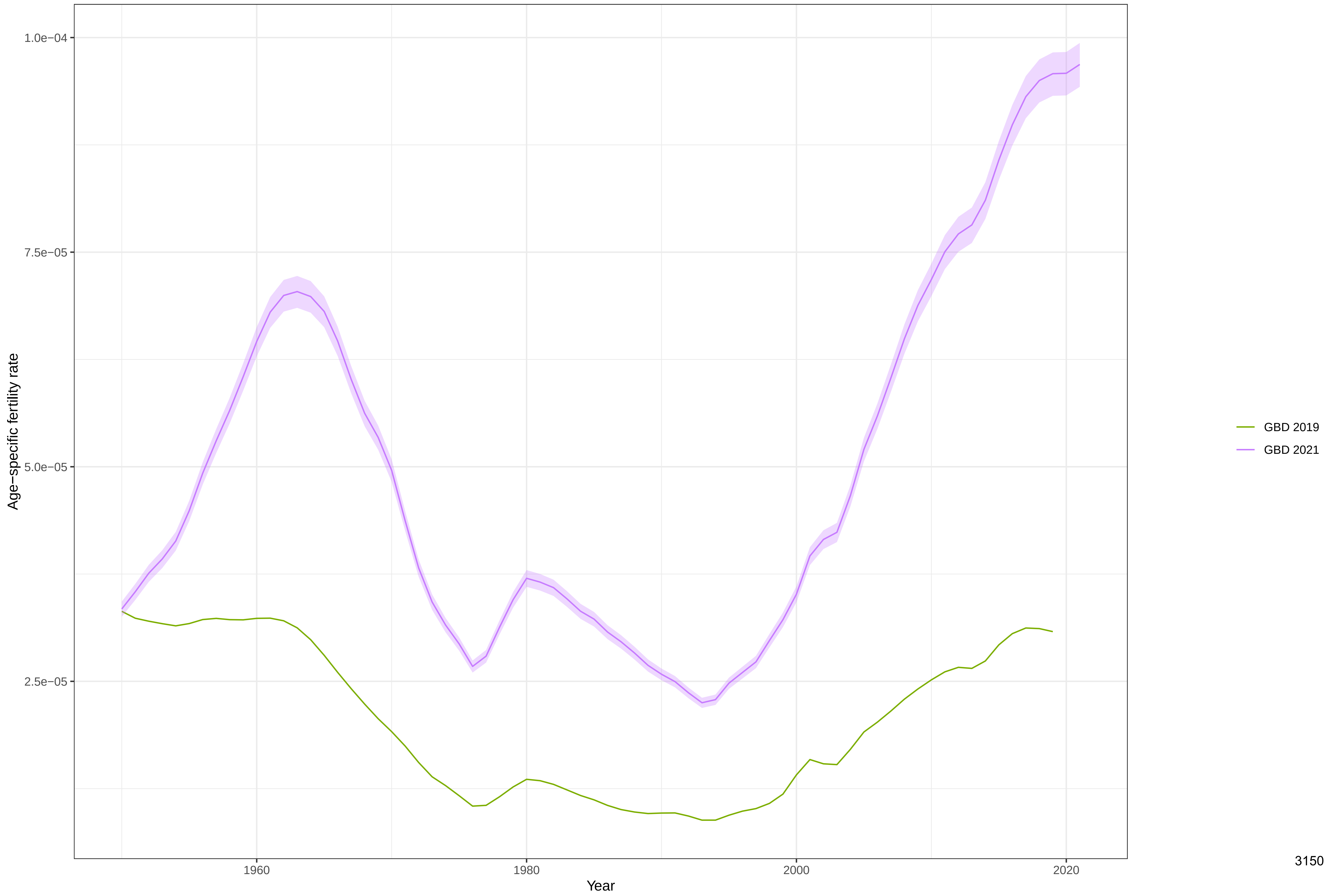

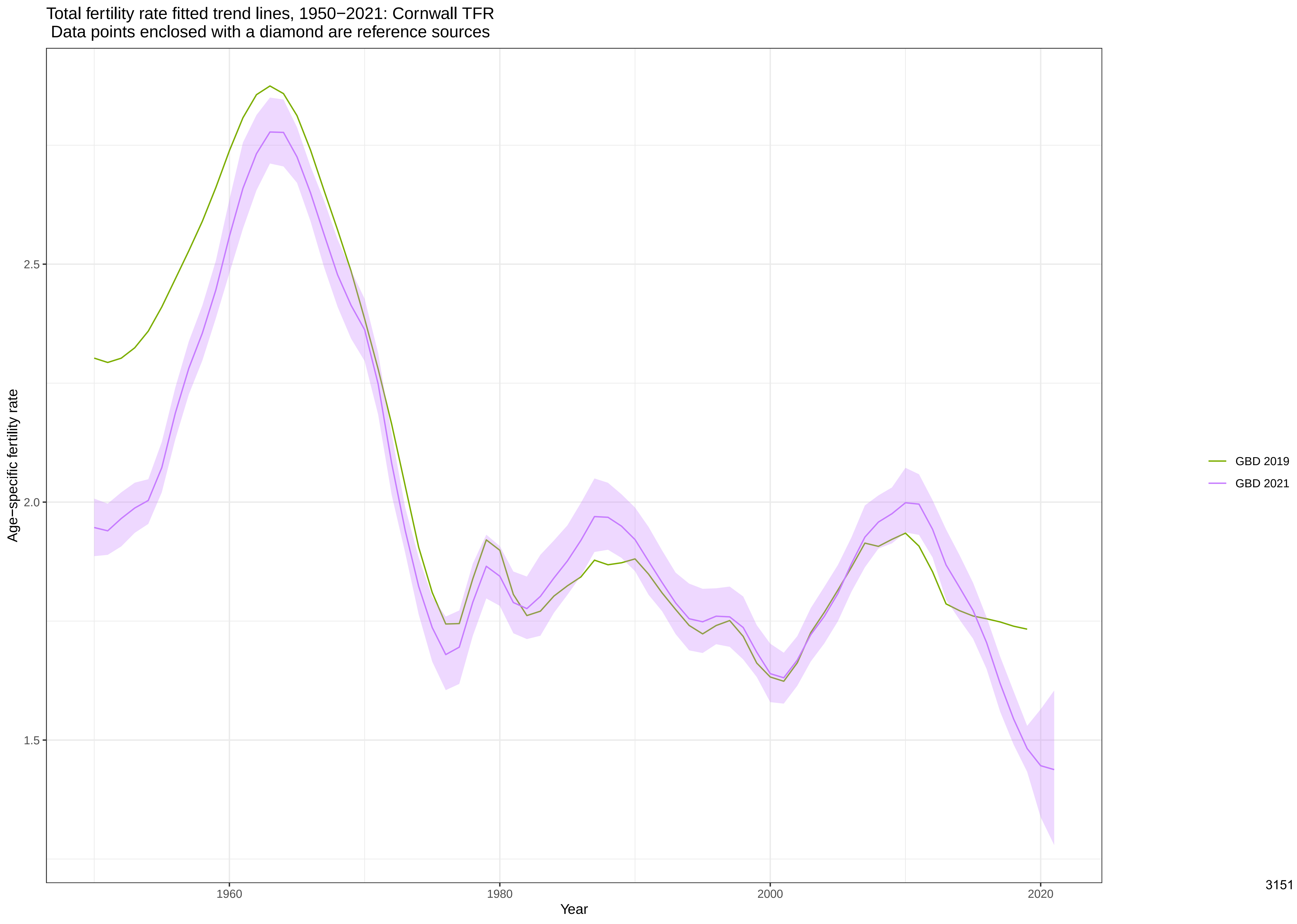

Total fertility rate fitted trend lines, 1950−2021: Cornwall TFR
Data points enclosed with a diamond are reference sources
Age−specific fertility rate
2.5
2.0
1.5
Year
1960
1980
2000
2020
GBD 2019
GBD 2021

Age−specific fertility fitted trend lines, 1950−2021: Devon, Ages: 10−14
Data points enclosed with a diamond are reference sources

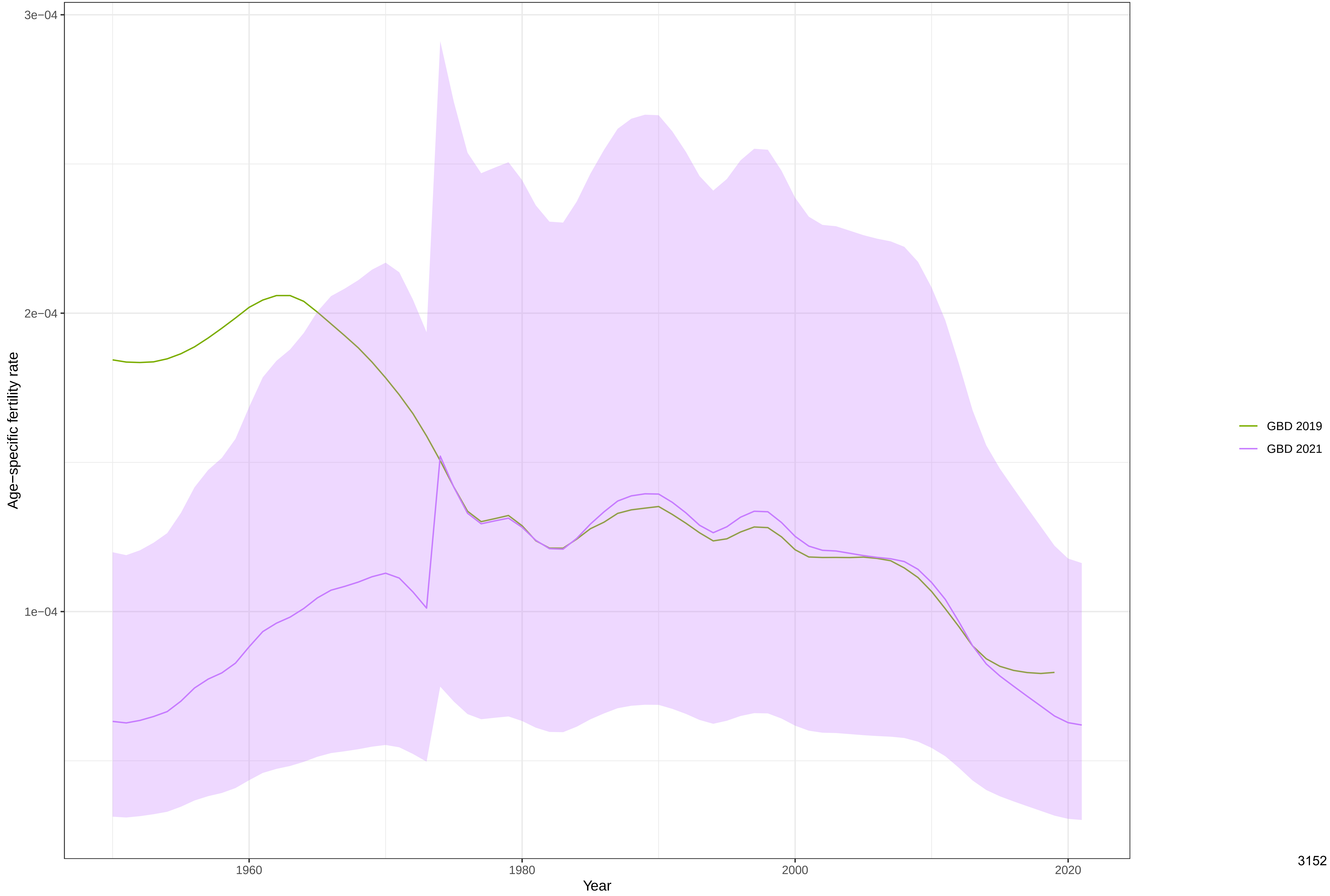

Age−specific fertility fitted trend lines, 1950−2021: Devon, Ages: 15−19
Data points enclosed with a diamond are reference sources

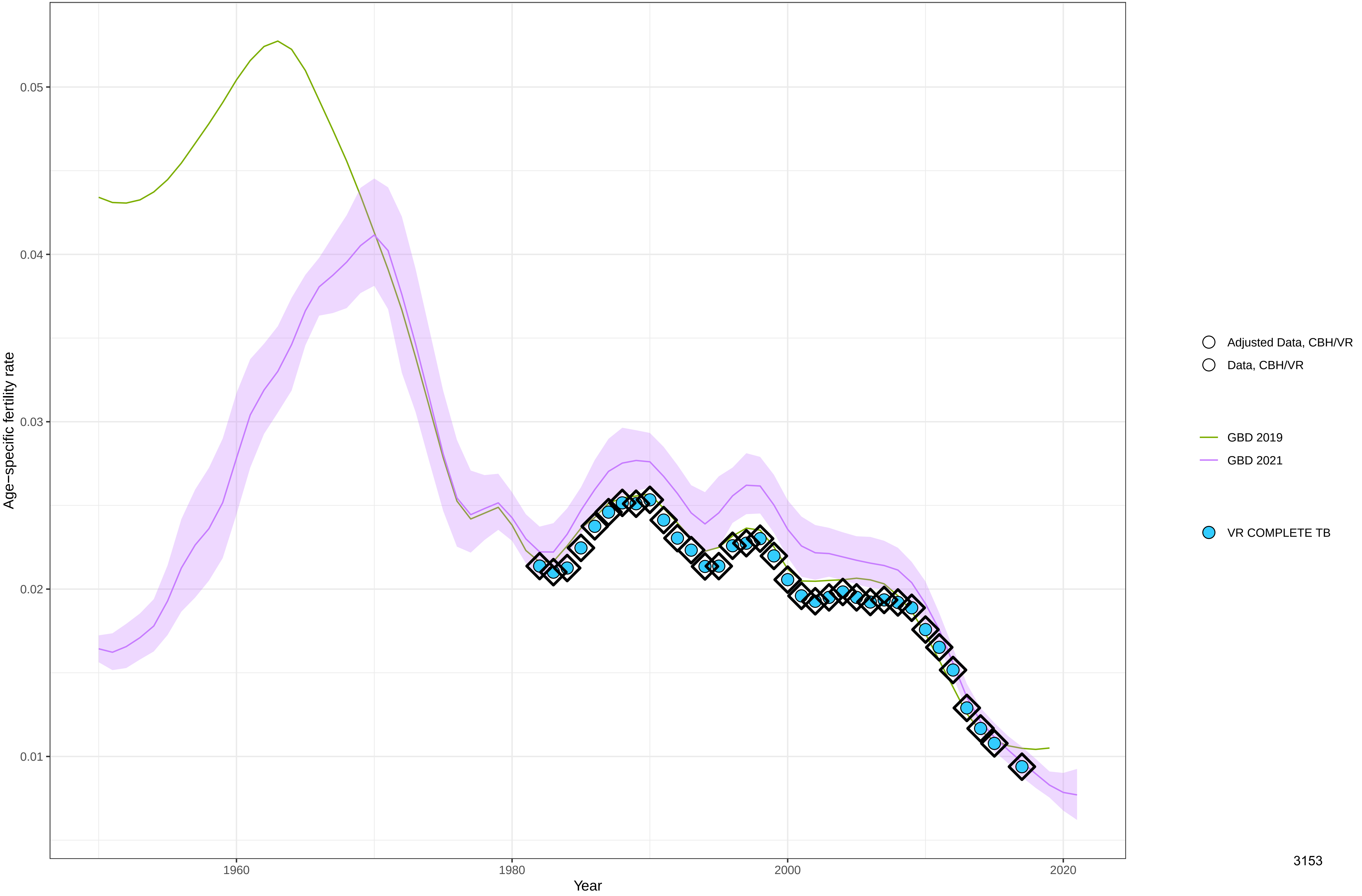

Age−specific fertility fitted trend lines, 1950−2021: Devon, Ages: 20−24
Data points enclosed with a diamond are reference sources

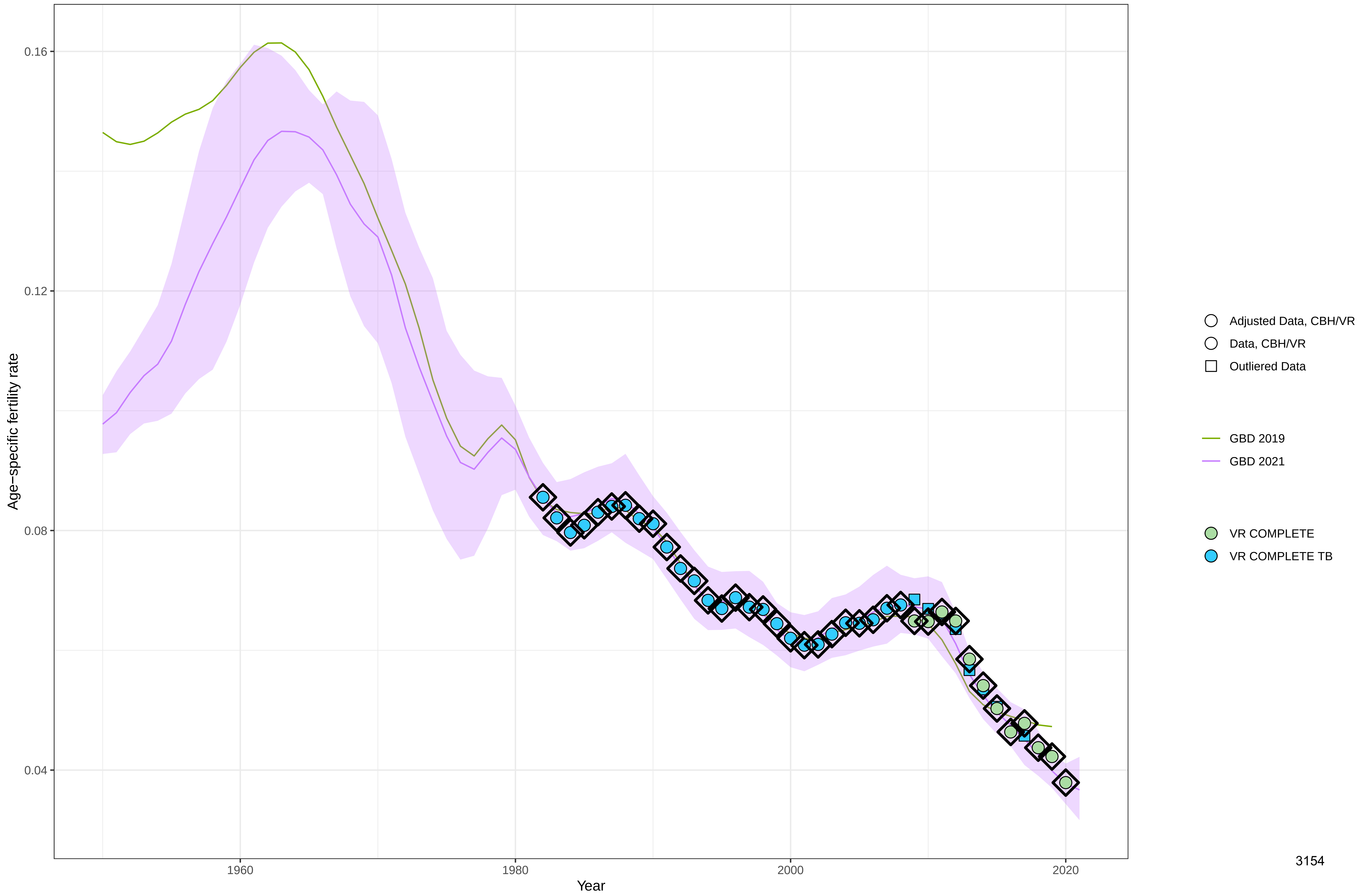

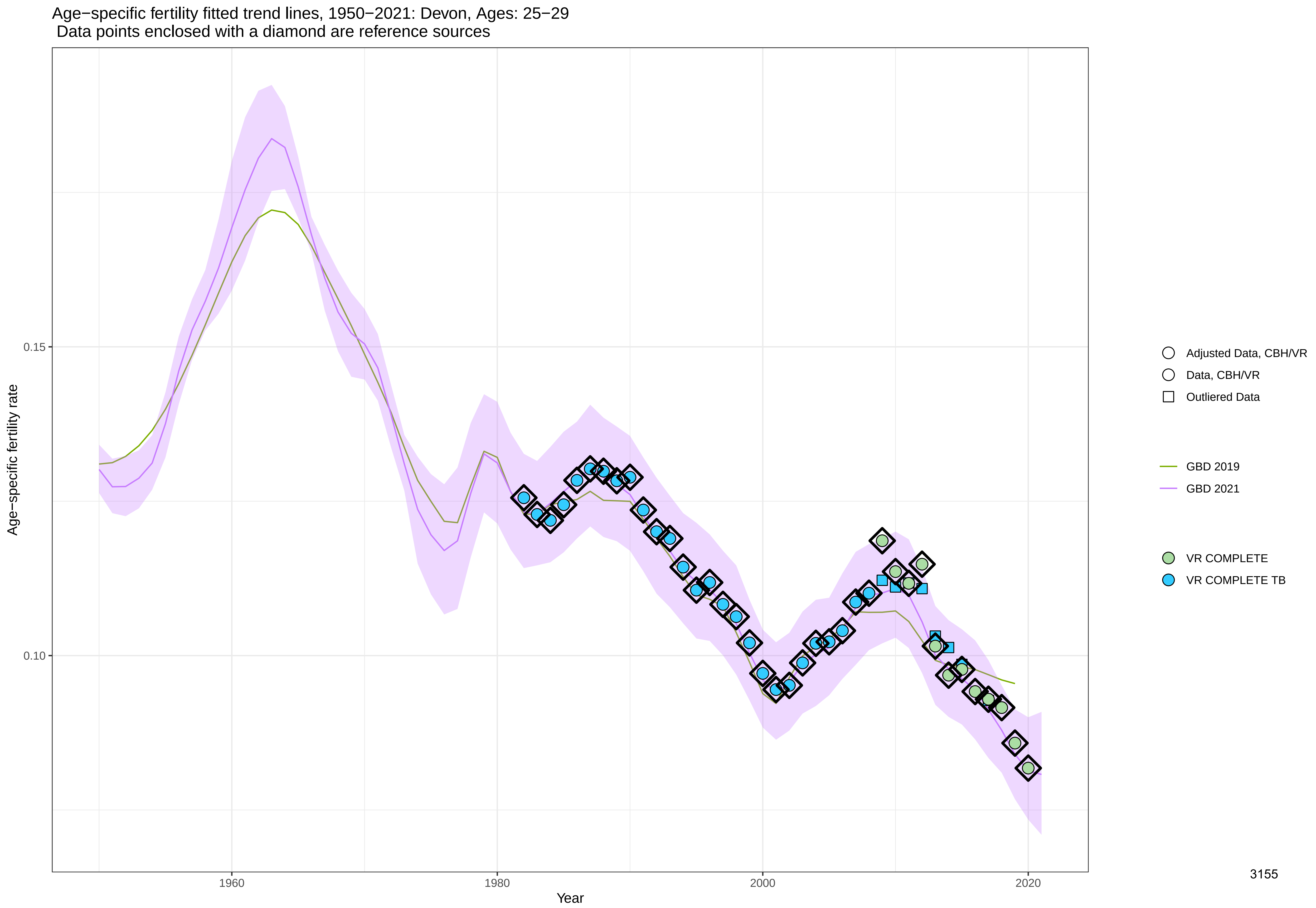

Age−specific fertility fitted trend lines, 1950−2021: Devon, Ages: 25−29
Data points enclosed with a diamond are reference sources
Age−specific fertility rate
0.15
0.10
1960
1980
2000
2020
Year
Adjusted Data, CBH/VR
Data, CBH/VR
Outliered Data
GBD 2019
GBD 2021
VR COMPLETE
VR COMPLETE TB

Age−specific fertility fitted trend lines, 1950−2021: Devon, Ages: 30−34
Data points enclosed with a diamond are reference sources

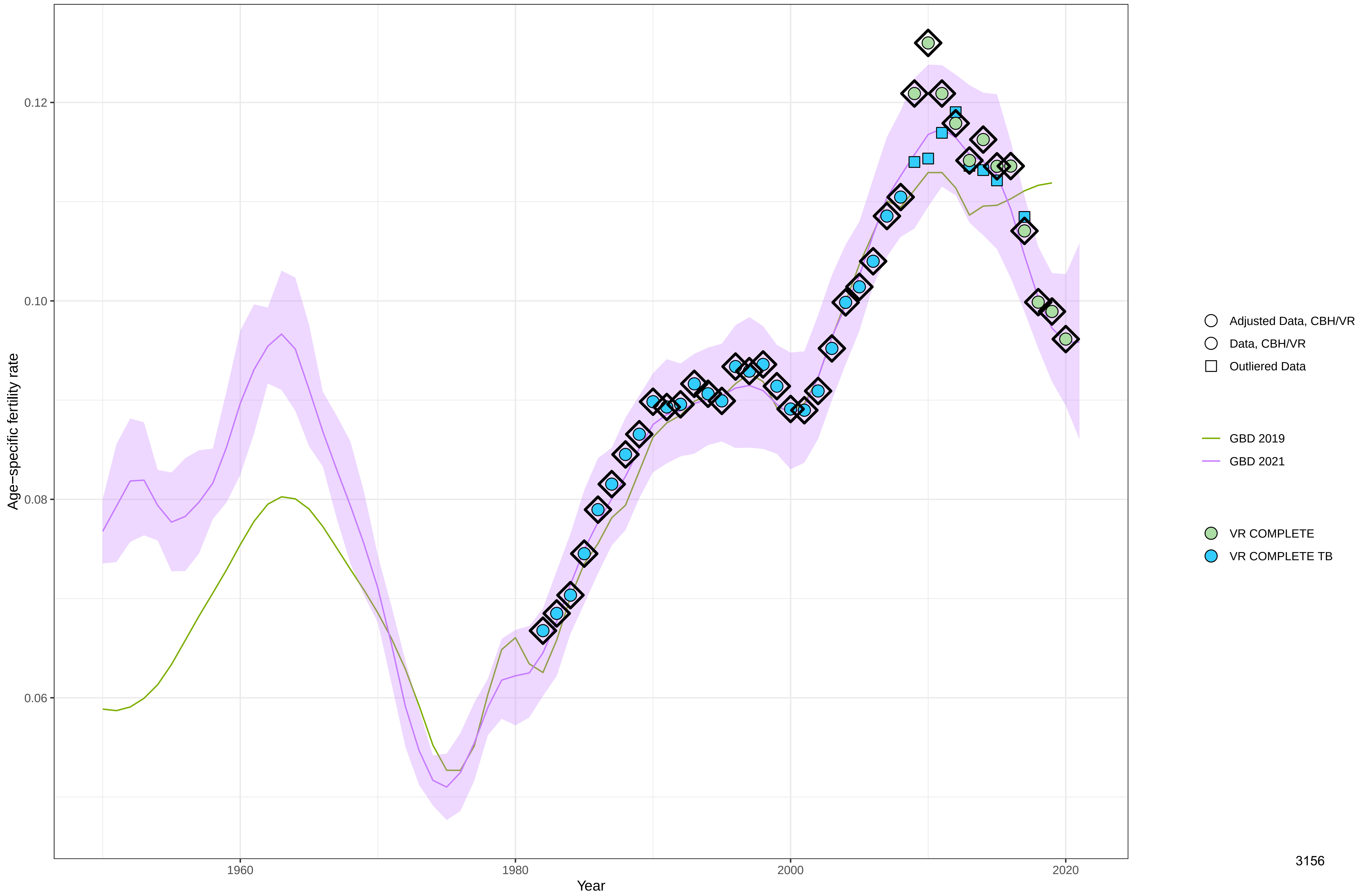

Age−specific fertility fitted trend lines, 1950−2021: Devon, Ages: 35−39
Data points enclosed with a diamond are reference sources

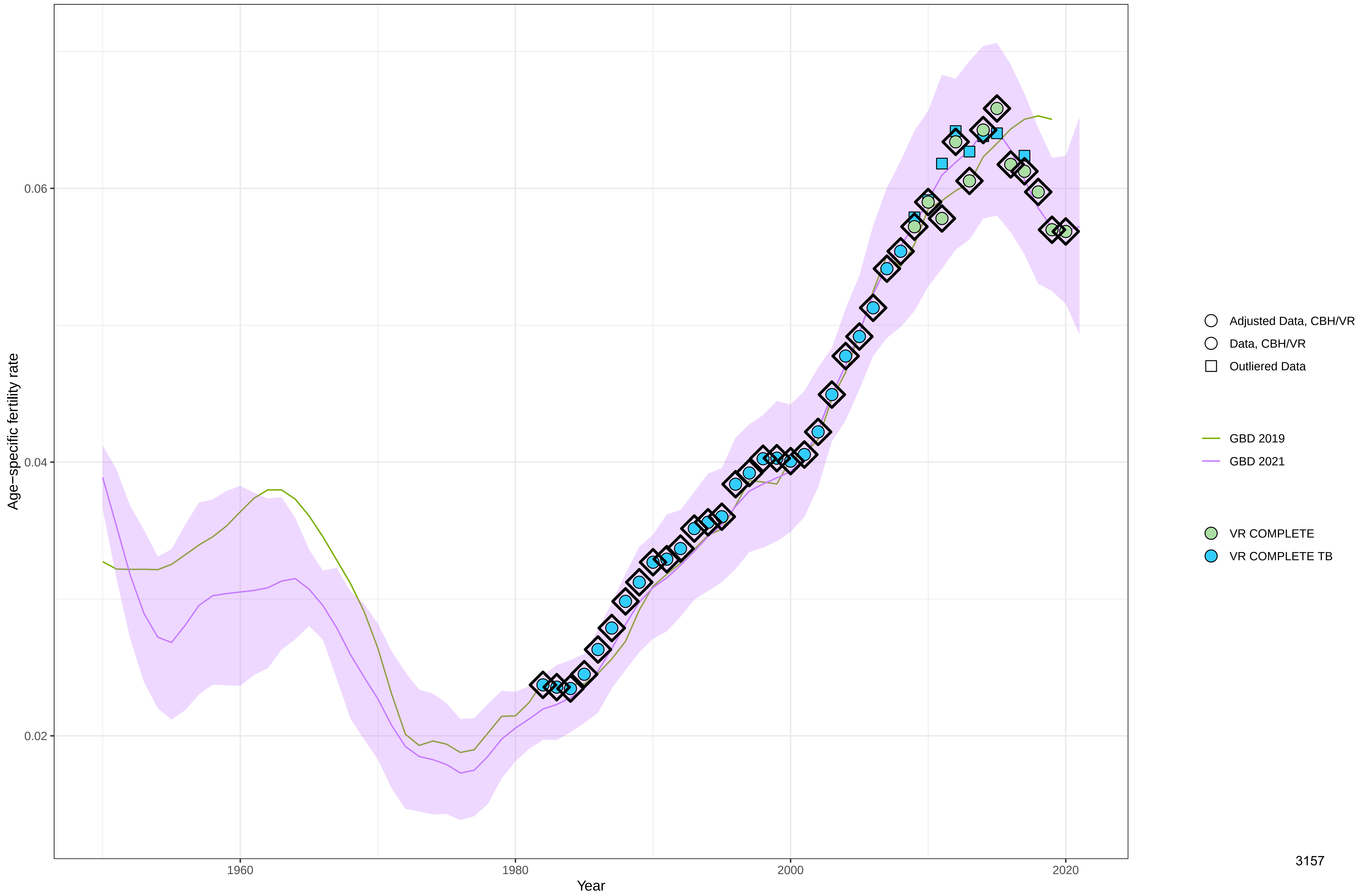

Age−specific fertility fitted trend lines, 1950−2021: Devon, Ages: 40−44
Data points enclosed with a diamond are reference sources

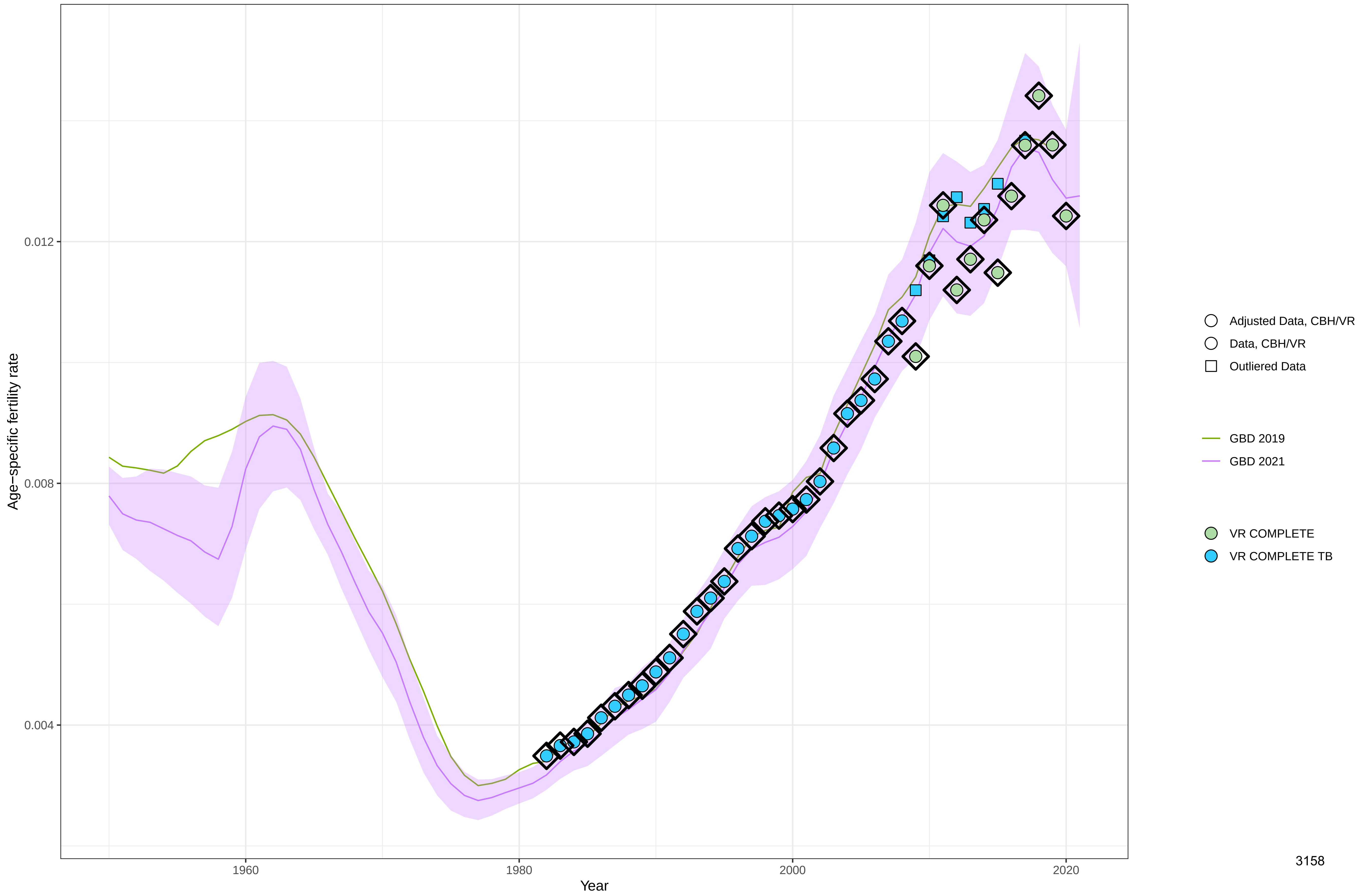

Age−specific fertility fitted trend lines, 1950−2021: Devon, Ages: 45−49
Data points enclosed with a diamond are reference sources

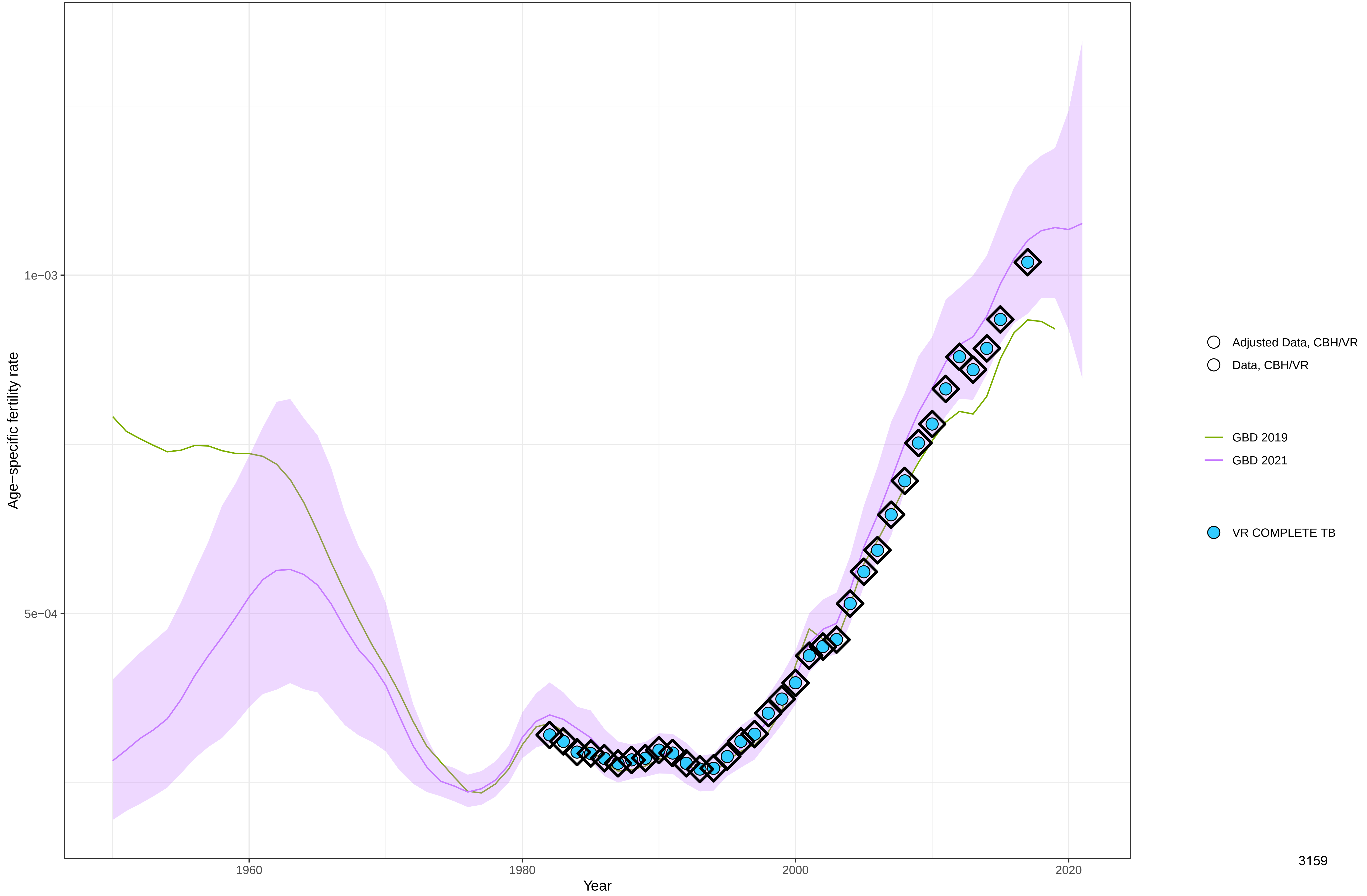

Age−specific fertility fitted trend lines, 1950−2021: Devon, Ages: 50−54
Data points enclosed with a diamond are reference sources

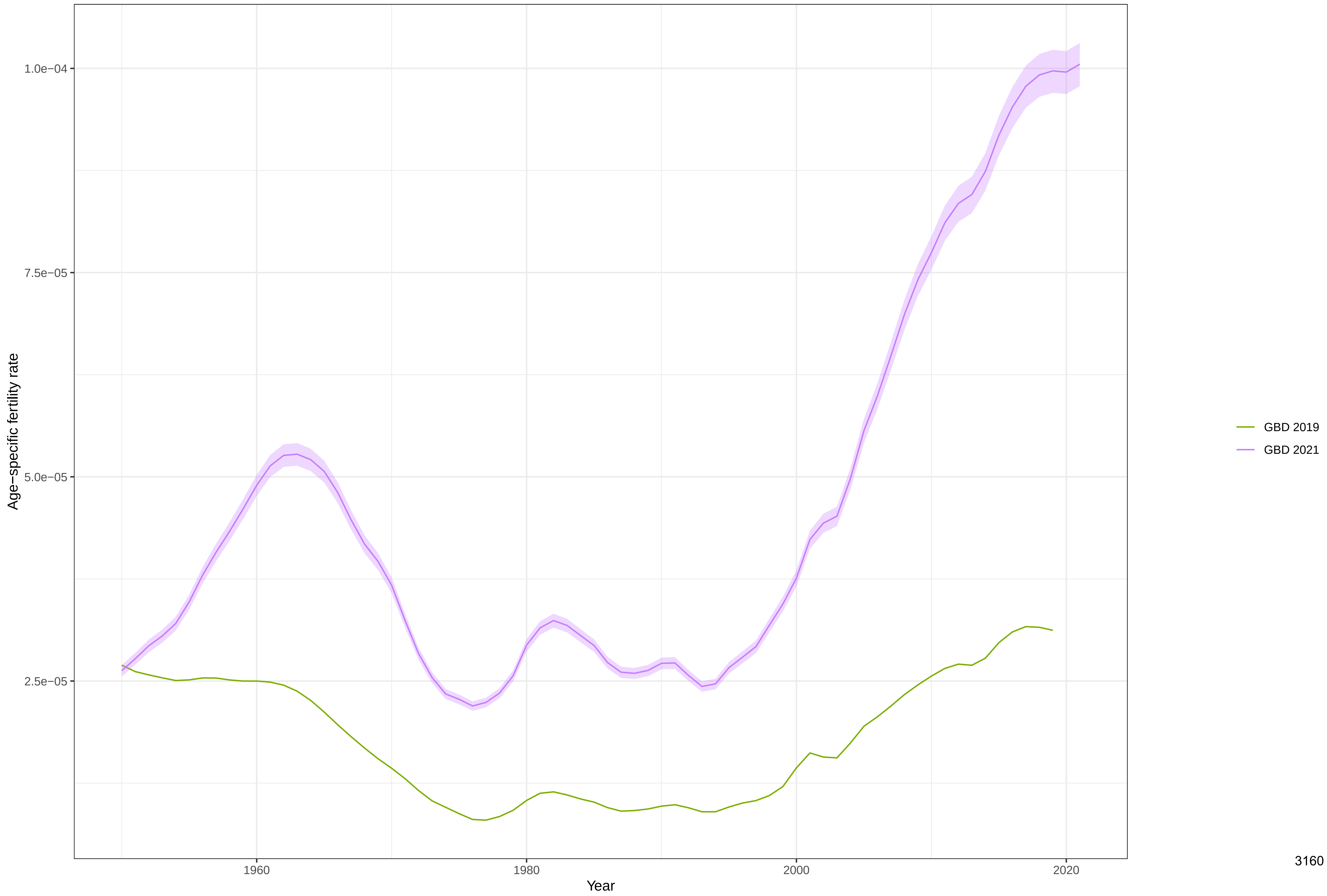

Total fertility rate fitted trend lines, 1950−2021: Devon TFR
Data points enclosed with a diamond are reference sources

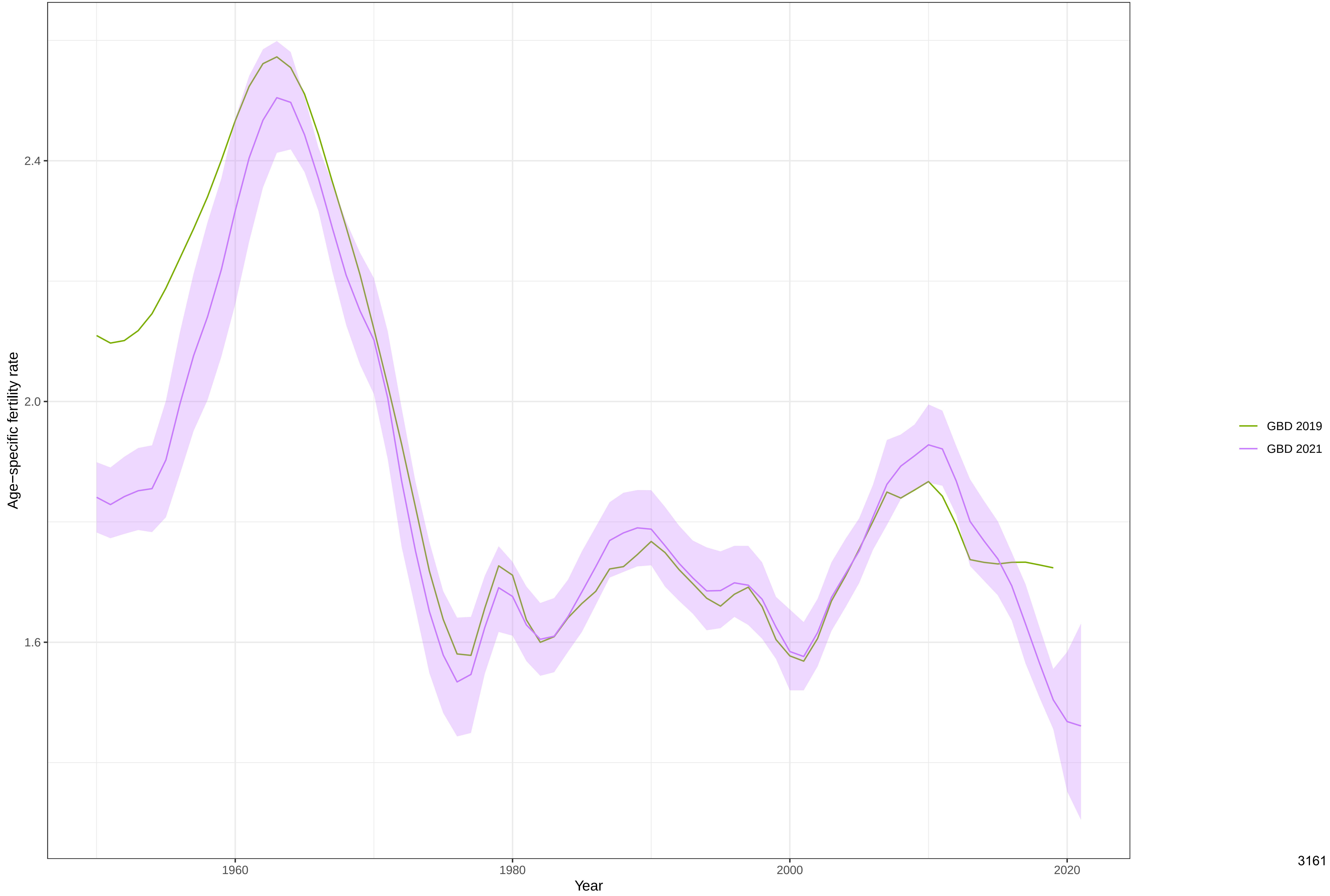

Age−specific fertility fitted trend lines, 1950−2021: Dorset, Ages: 10−14
Data points enclosed with a diamond are reference sources

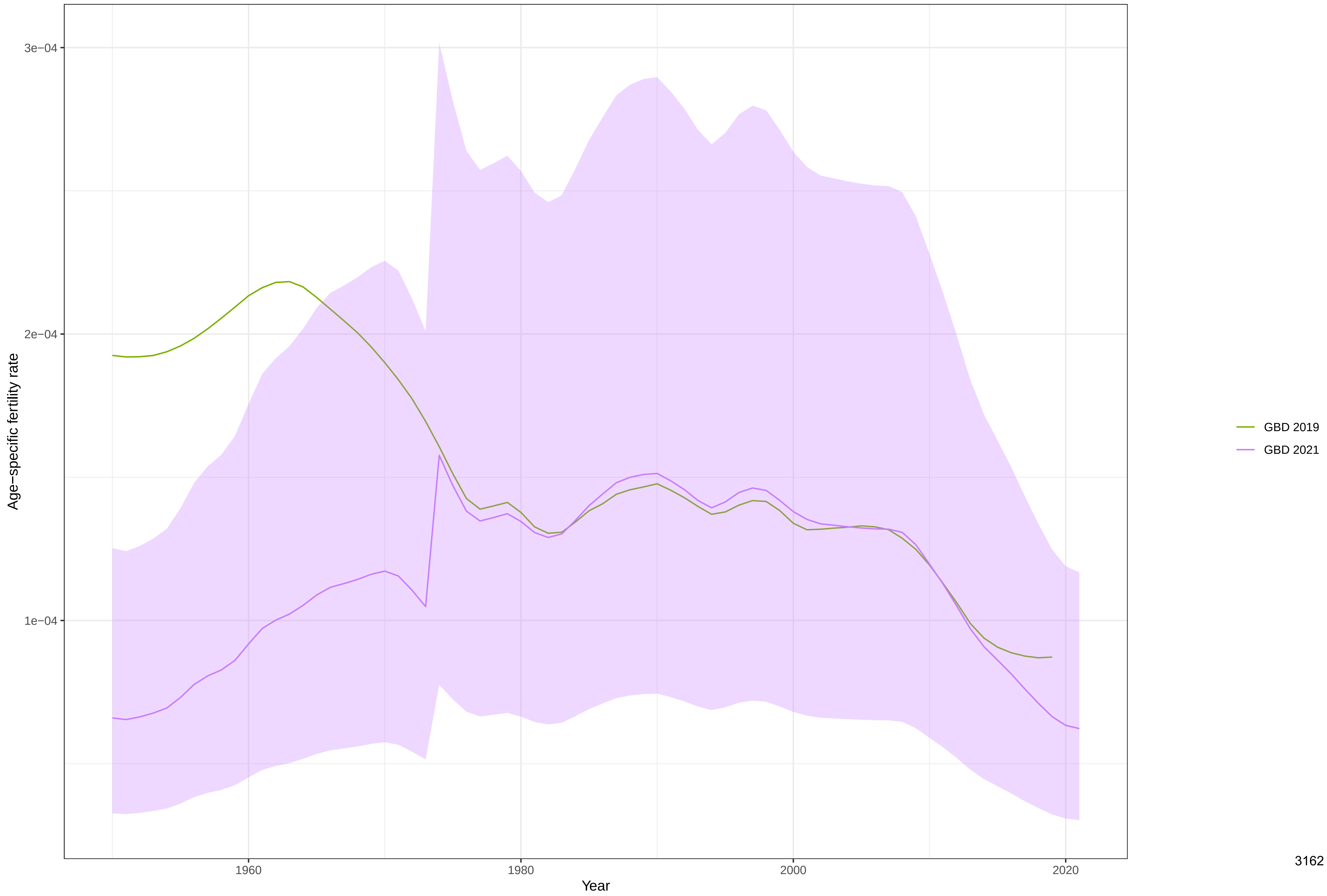

Age−specific fertility fitted trend lines, 1950−2021: Dorset, Ages: 15−19
Data points enclosed with a diamond are reference sources

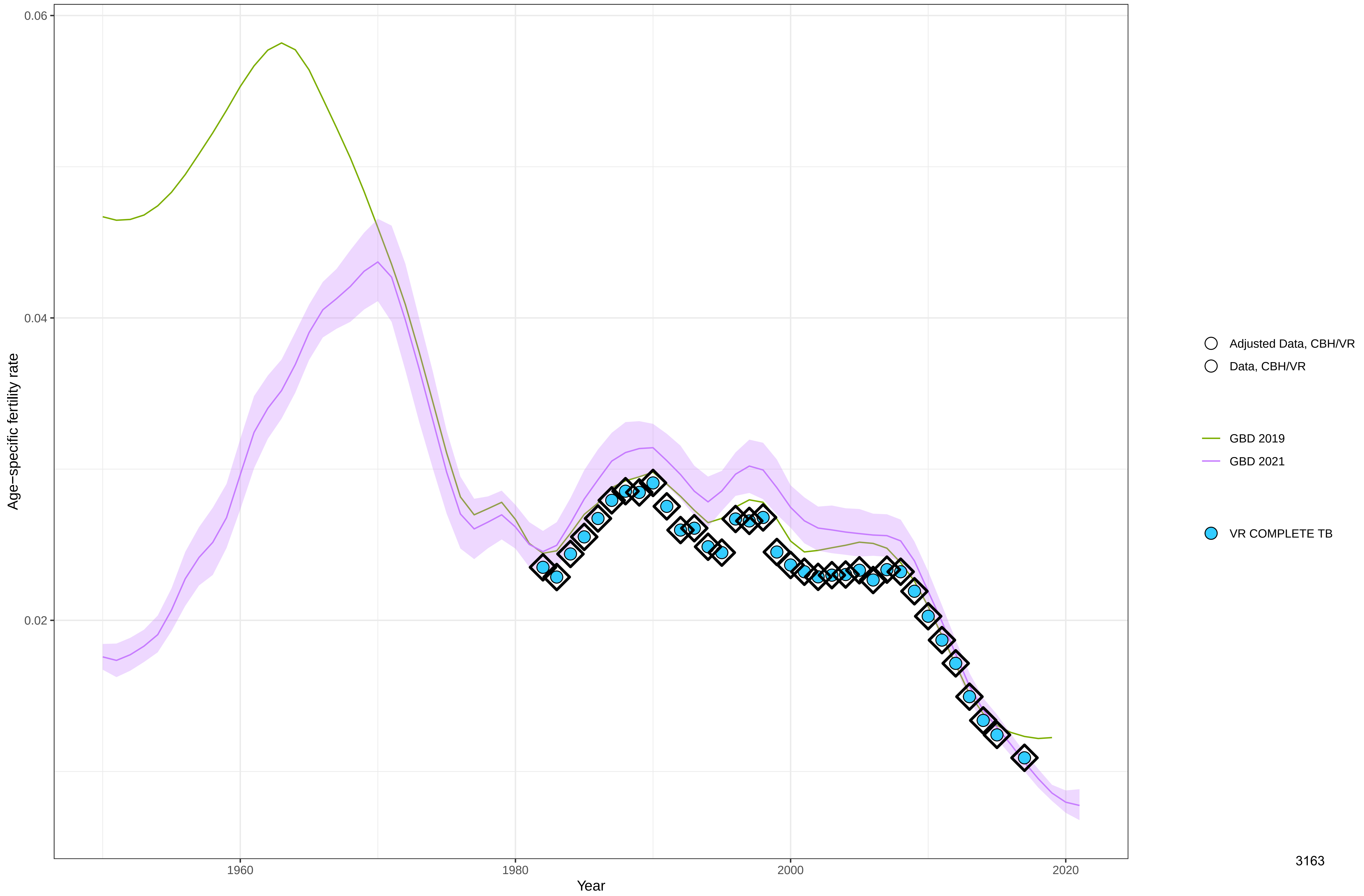

Age−specific fertility fitted trend lines, 1950−2021: Dorset, Ages: 20−24
Data points enclosed with a diamond are reference sources

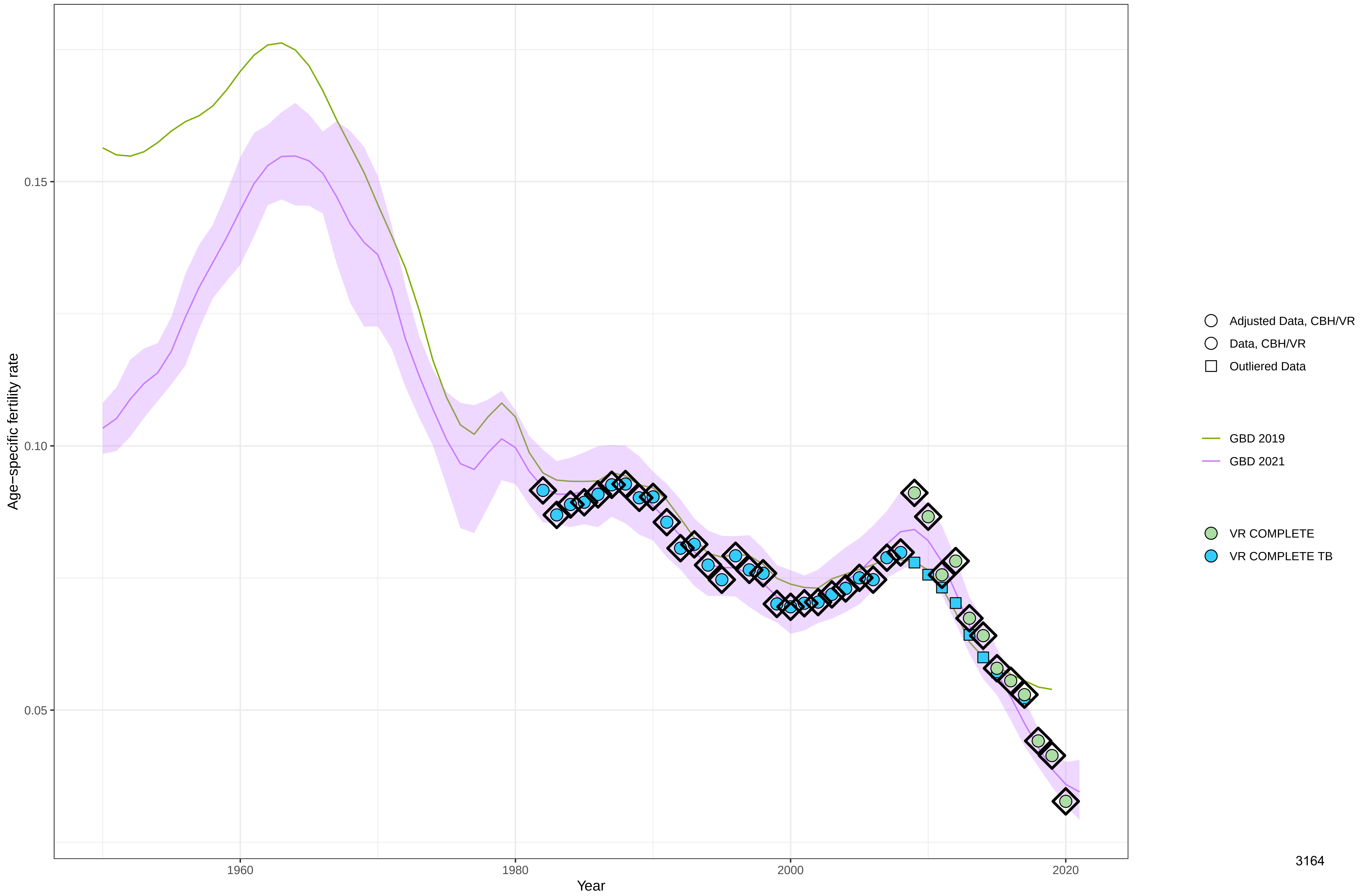

Age−specific fertility fitted trend lines, 1950−2021: Dorset, Ages: 25−29
Data points enclosed with a diamond are reference sources

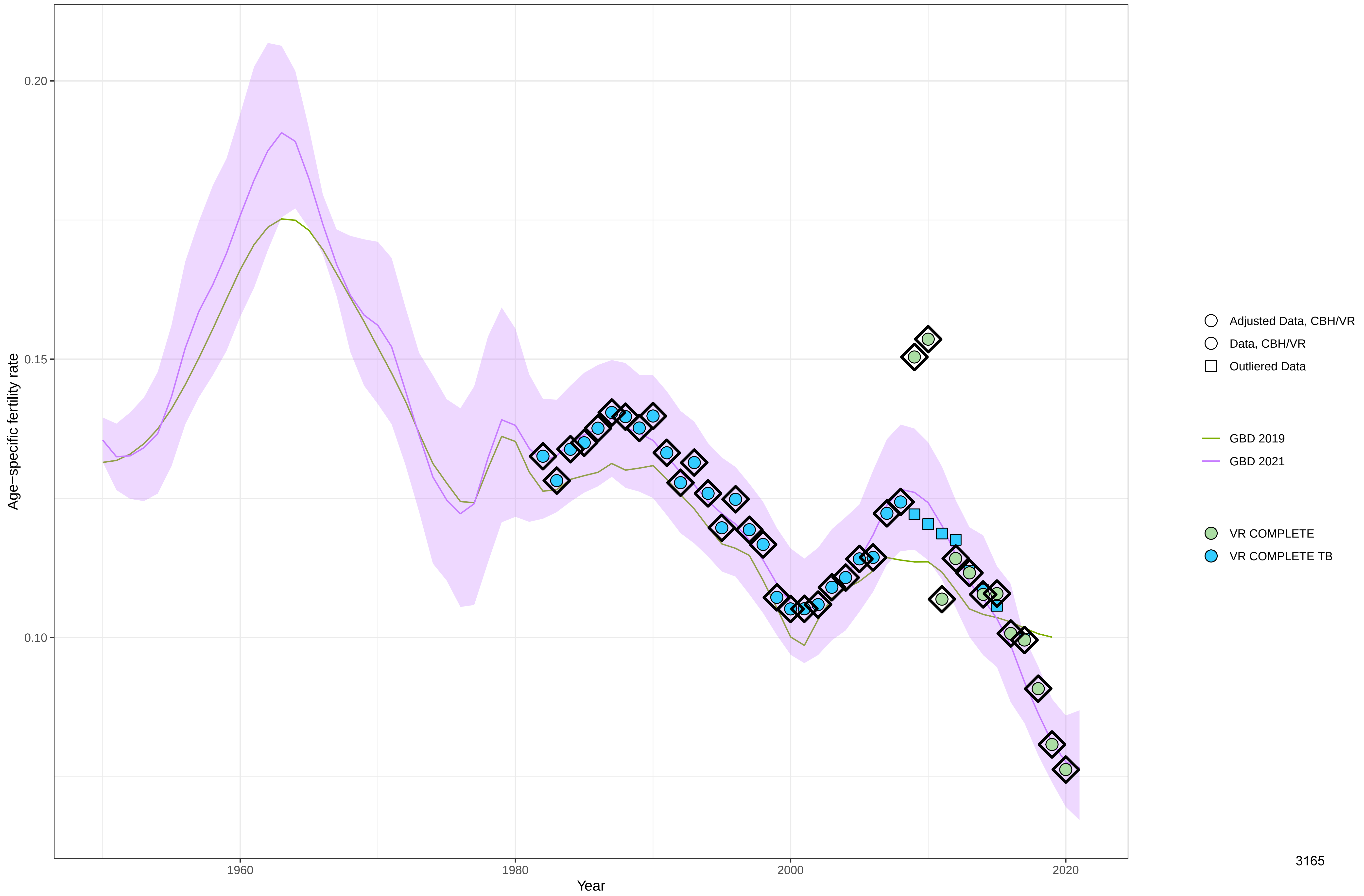

Age−specific fertility fitted trend lines, 1950−2021: Dorset, Ages: 30−34
Data points enclosed with a diamond are reference sources

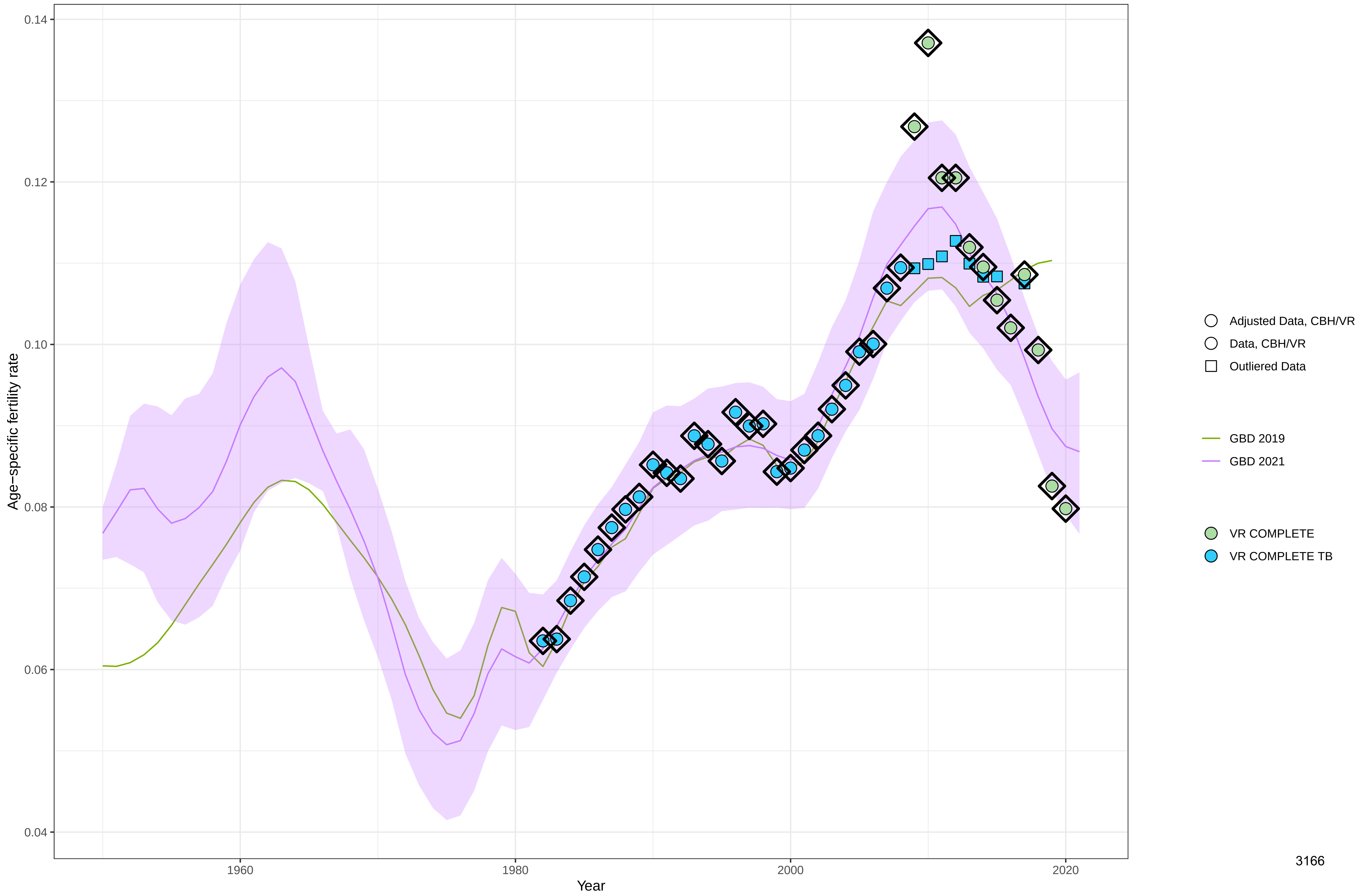

Age−specific fertility fitted trend lines, 1950−2021: Dorset, Ages: 35−39
Data points enclosed with a diamond are reference sources

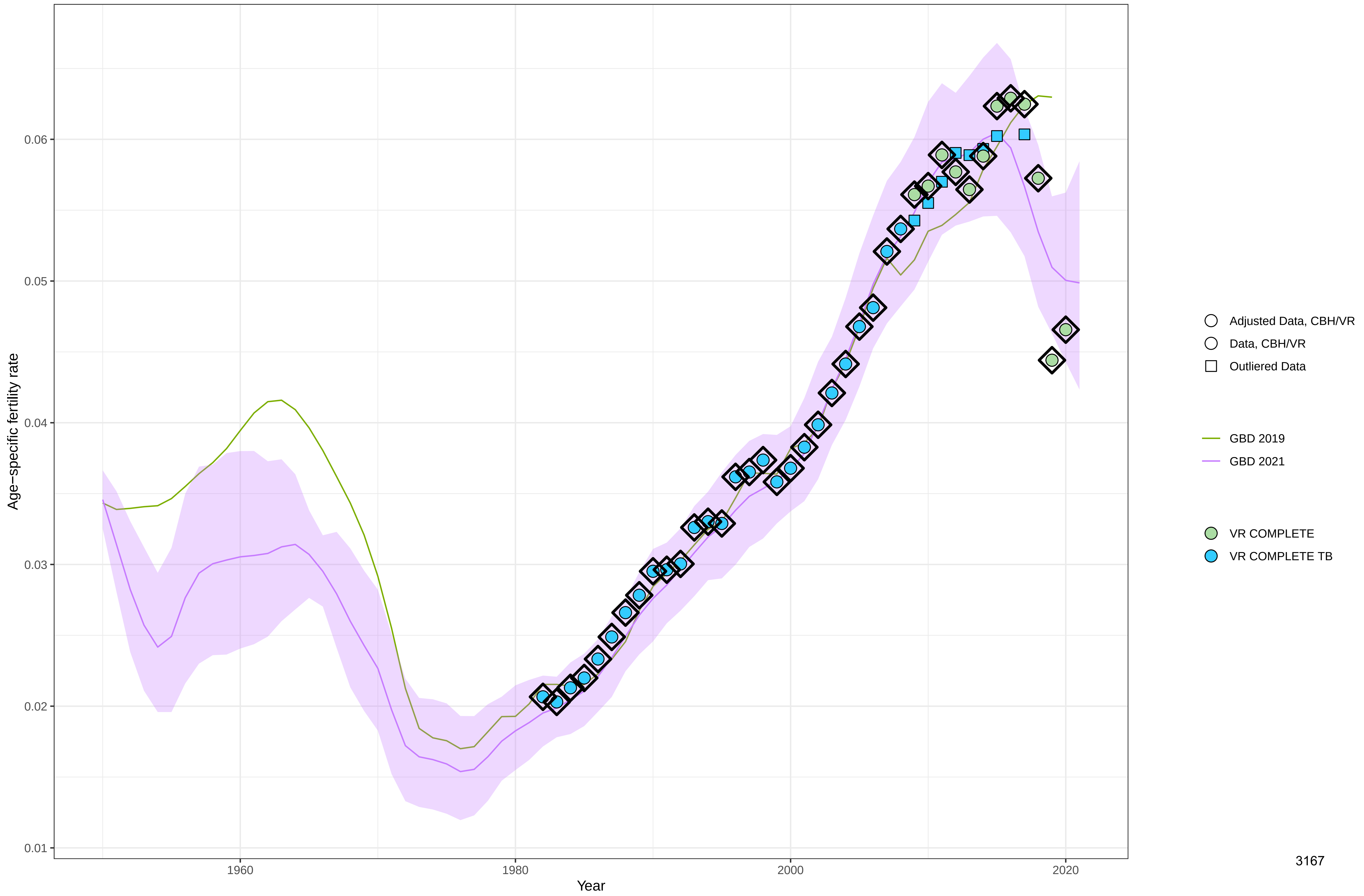

Age−specific fertility fitted trend lines, 1950−2021: Dorset, Ages: 40−44
Data points enclosed with a diamond are reference sources

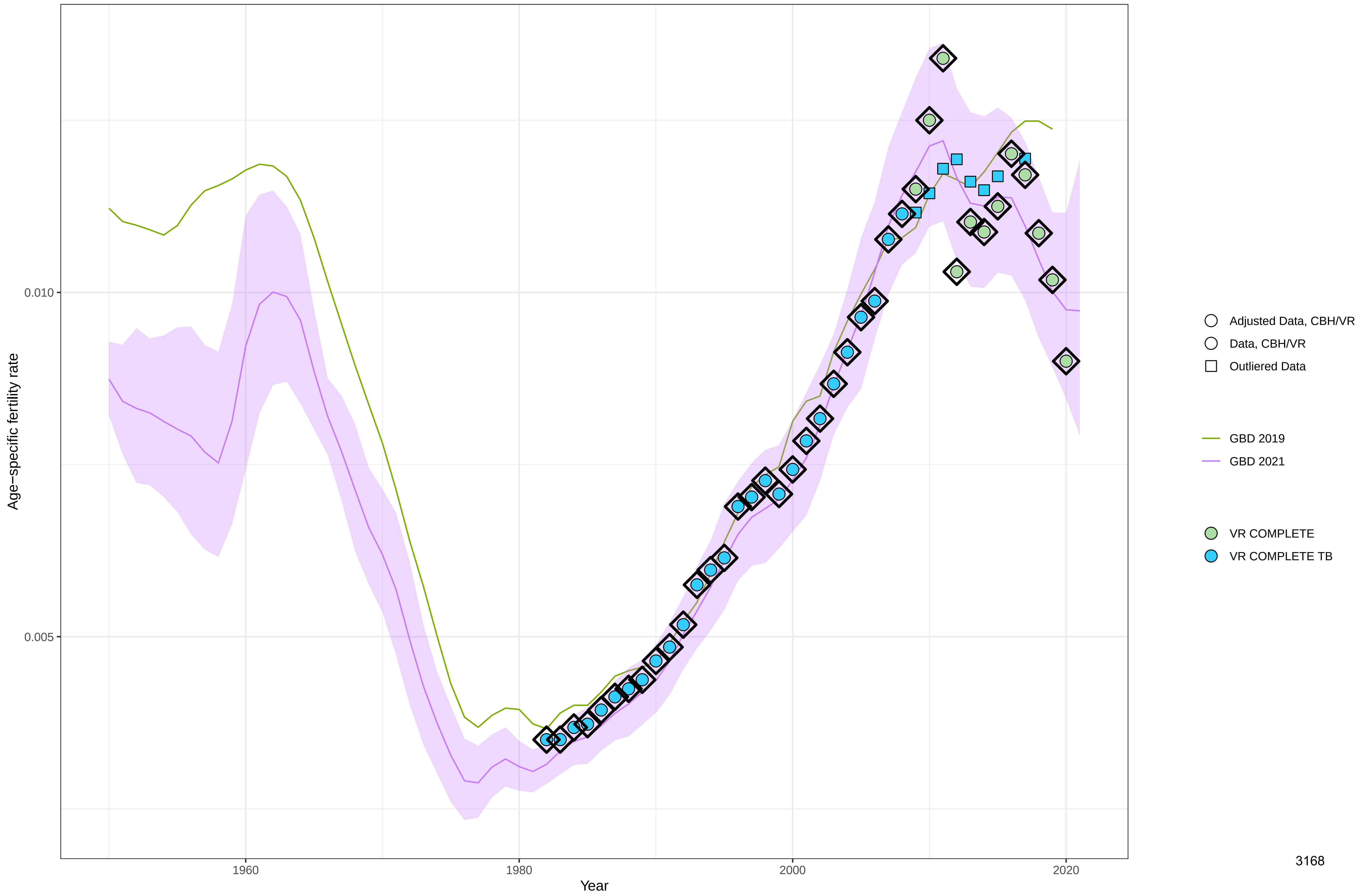

Age−specific fertility fitted trend lines, 1950−2021: Dorset, Ages: 45−49
Data points enclosed with a diamond are reference sources

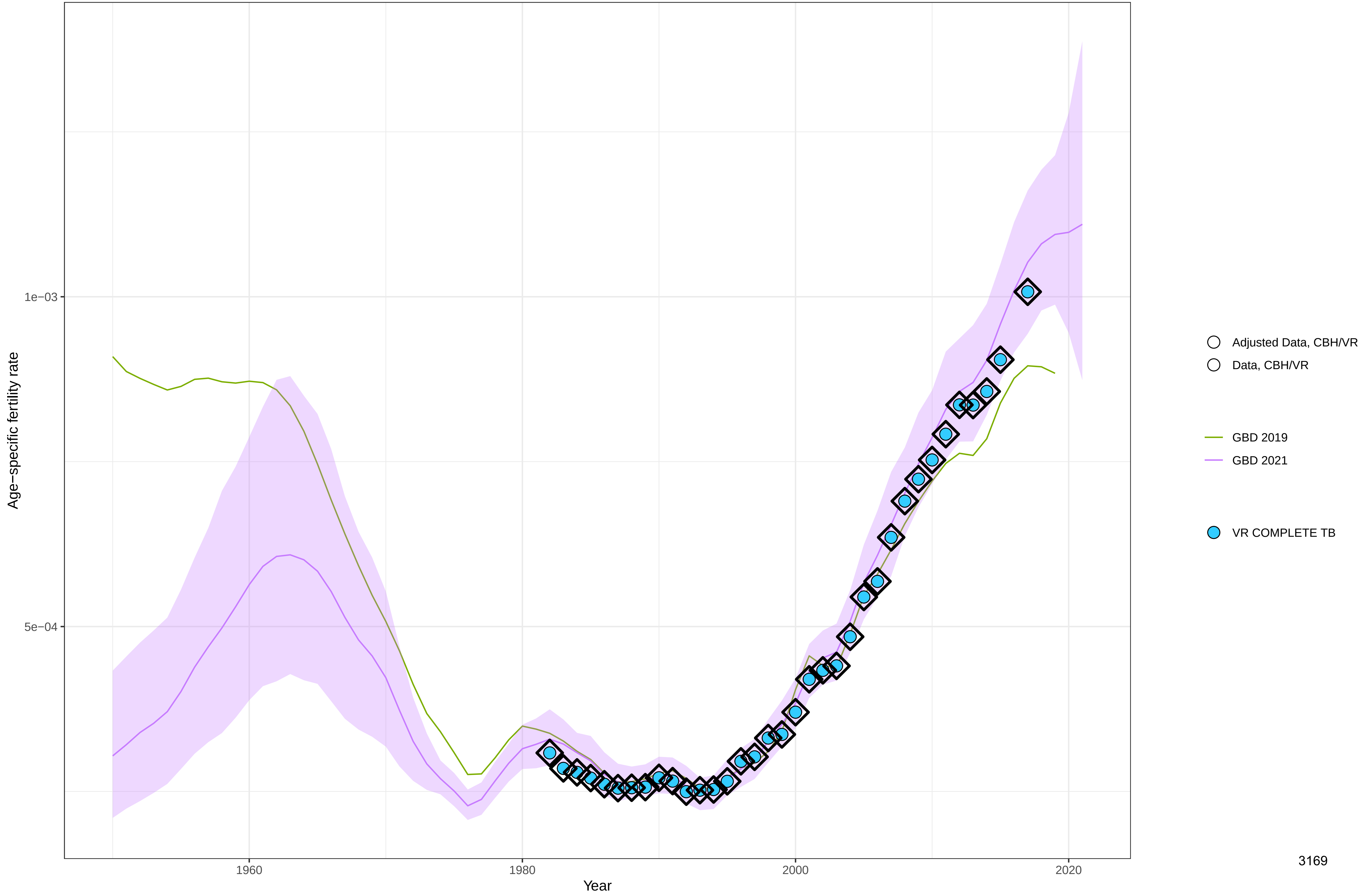

Age−specific fertility fitted trend lines, 1950−2021: Dorset, Ages: 50−54
Data points enclosed with a diamond are reference sources

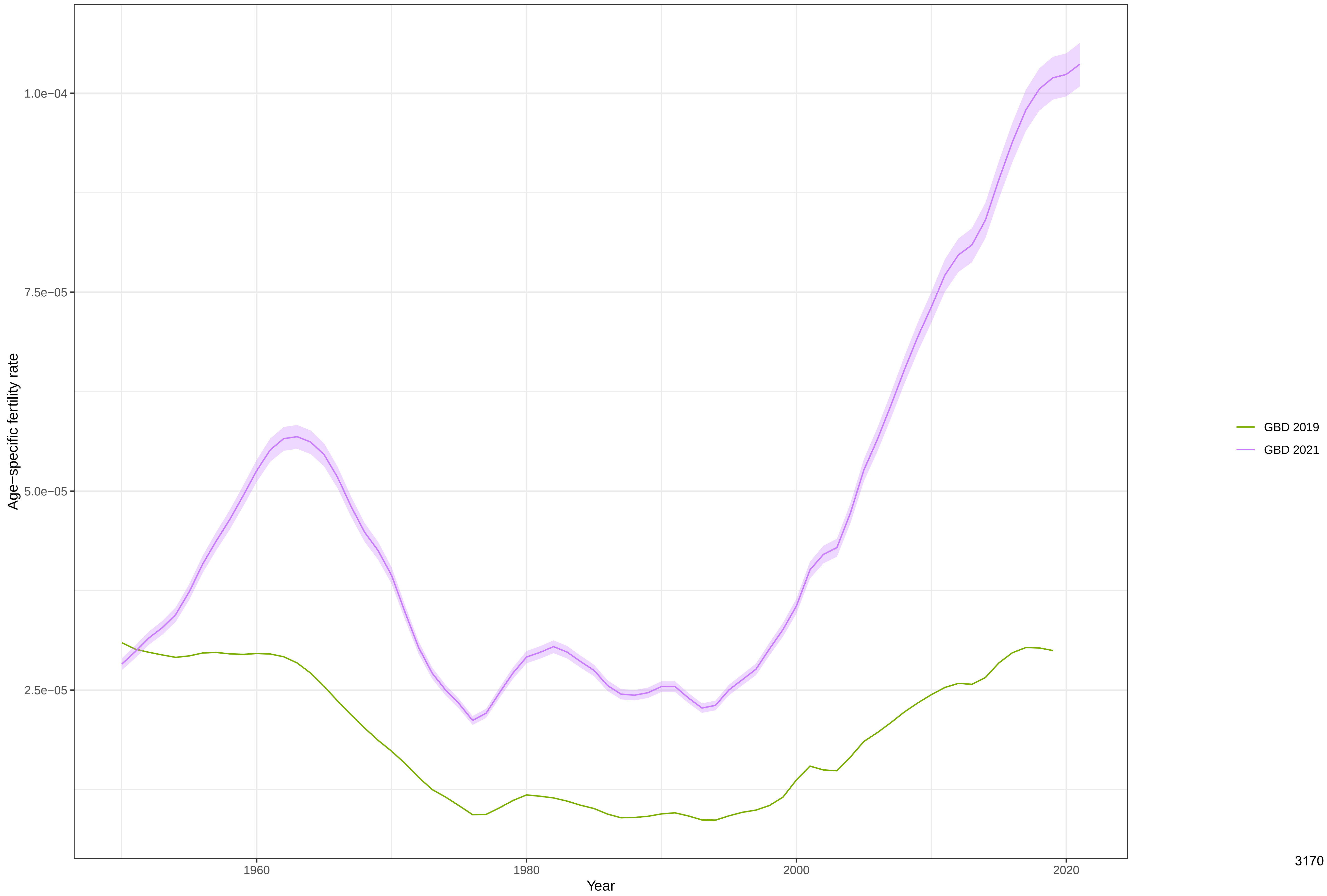

Total fertility rate fitted trend lines, 1950−2021: Dorset TFR
Data points enclosed with a diamond are reference sources

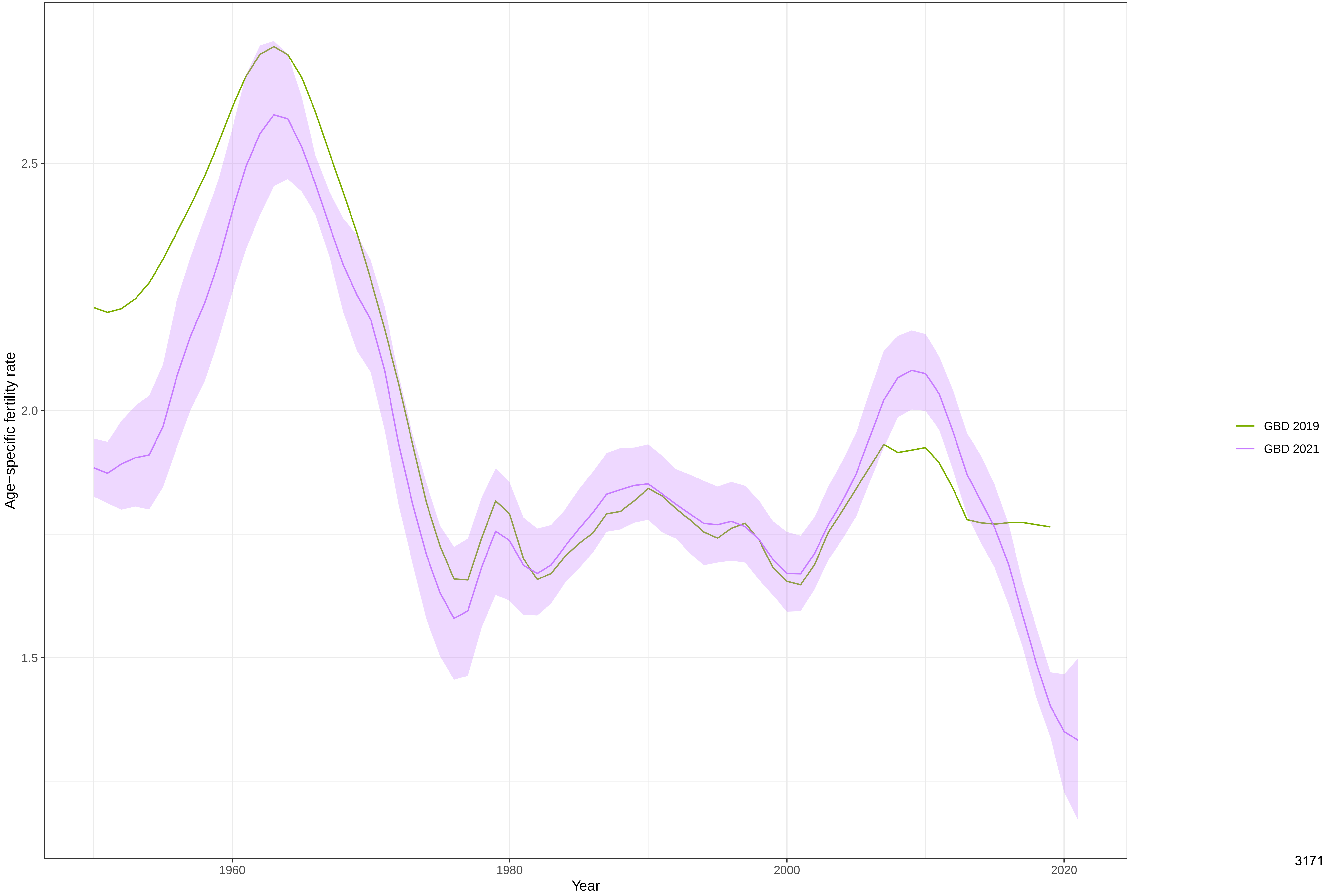

Age−specific fertility fitted trend lines, 1950−2021: Gloucestershire, Ages: 10−14
Data points enclosed with a diamond are reference sources

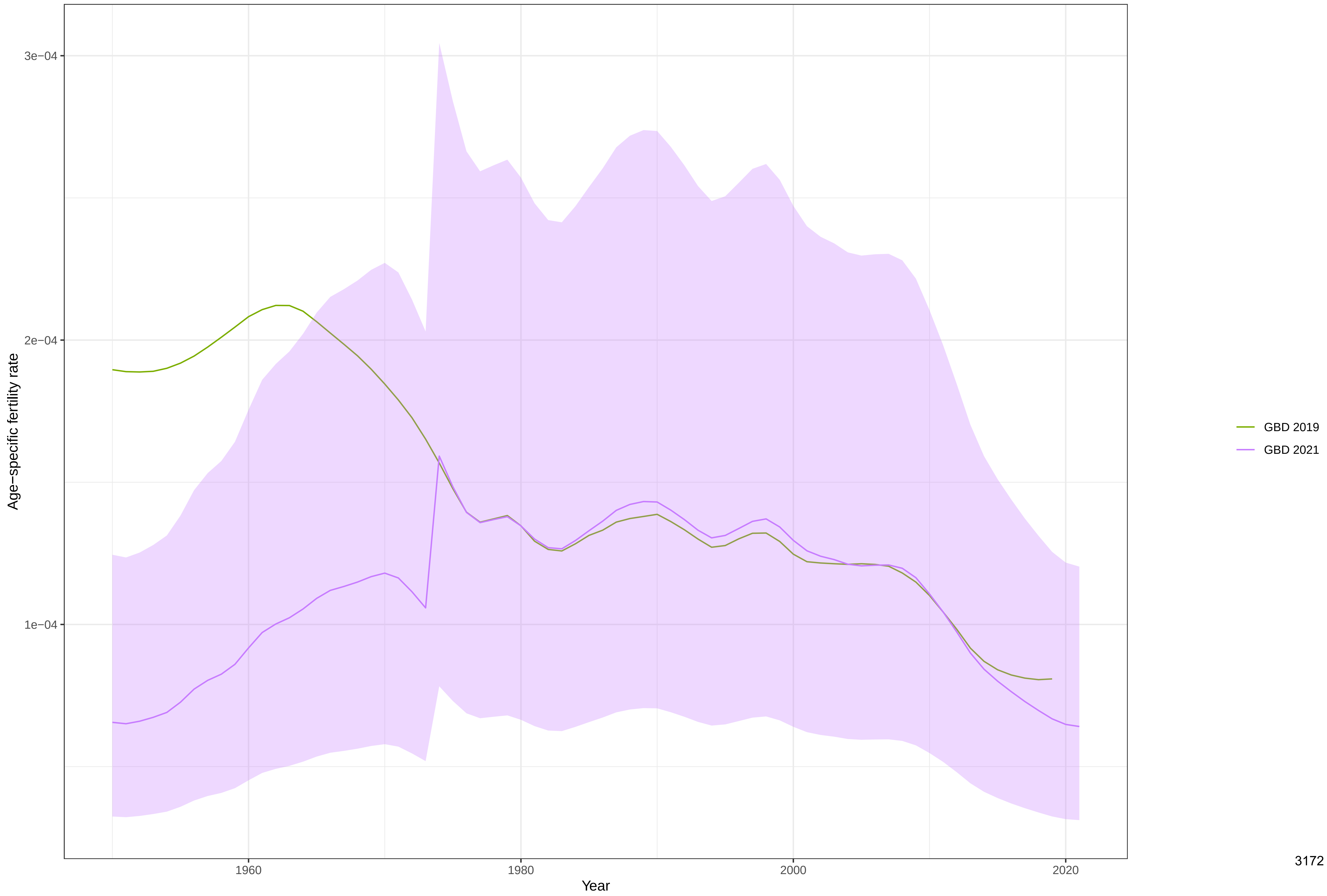

Age−specific fertility fitted trend lines, 1950−2021: Gloucestershire, Ages: 15−19
Data points enclosed with a diamond are reference sources

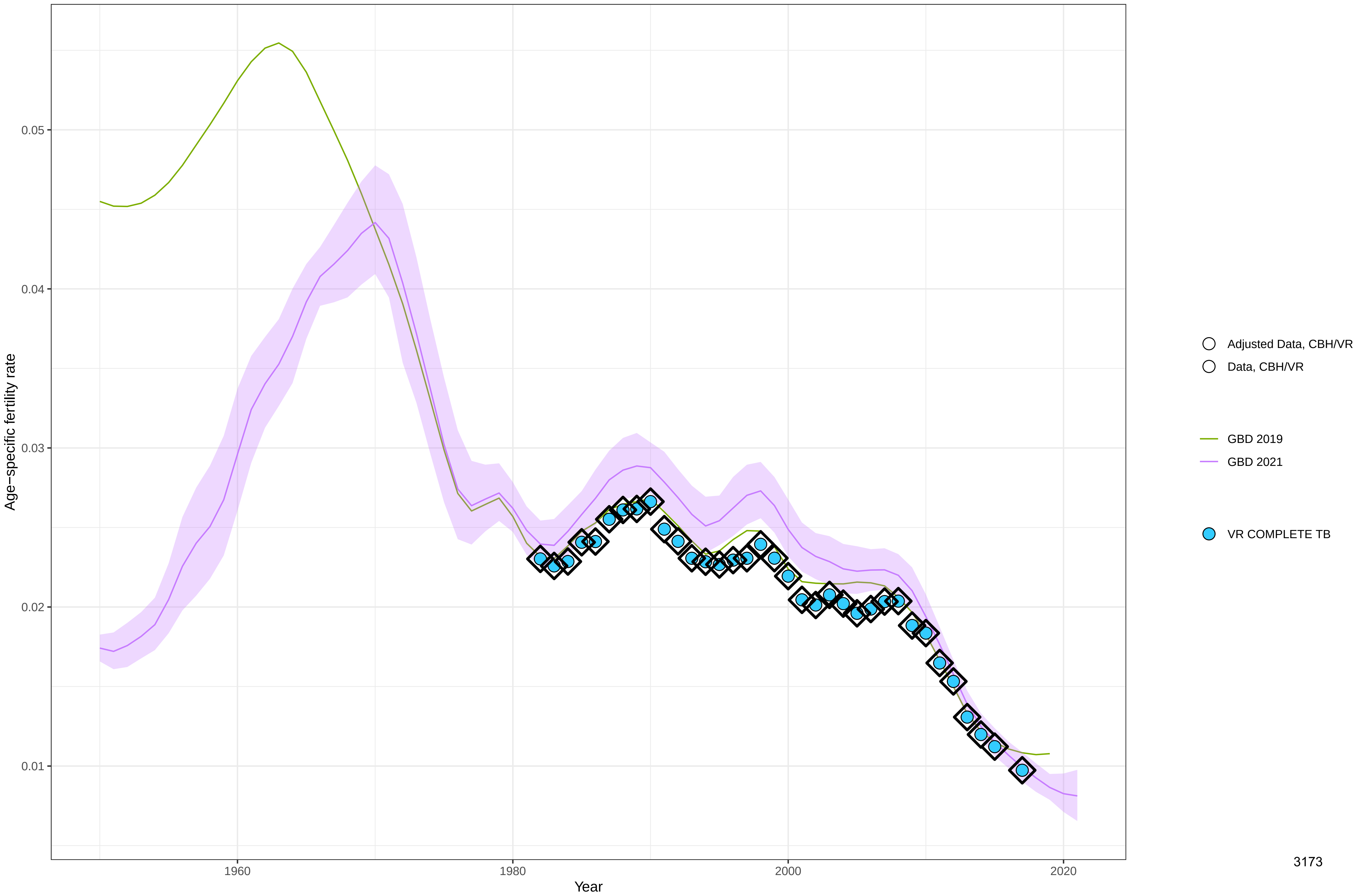

Age−specific fertility fitted trend lines, 1950−2021: Gloucestershire, Ages: 20−24
Data points enclosed with a diamond are reference sources

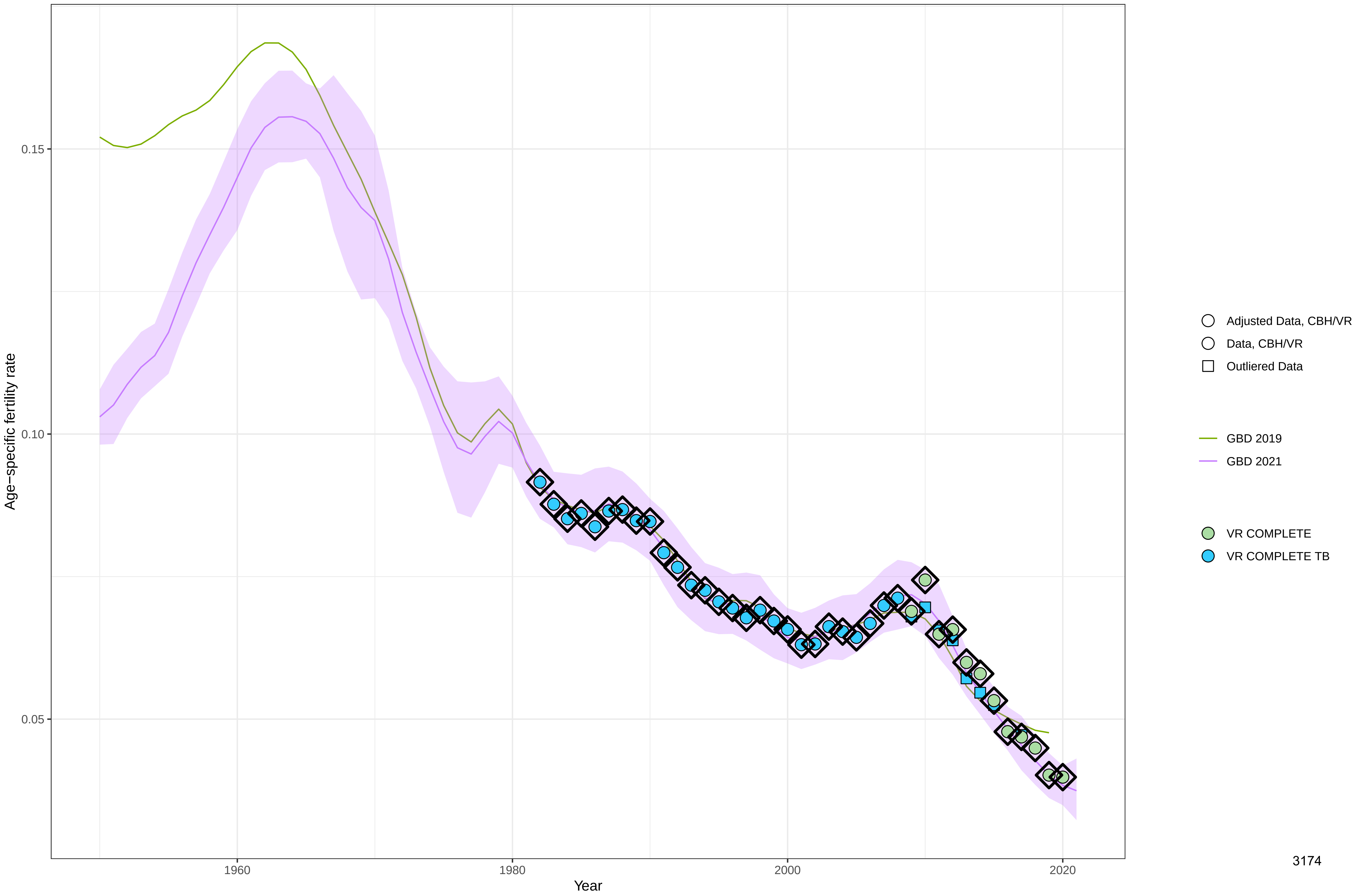

Age−specific fertility fitted trend lines, 1950−2021: Gloucestershire, Ages: 25−29
Data points enclosed with a diamond are reference sources

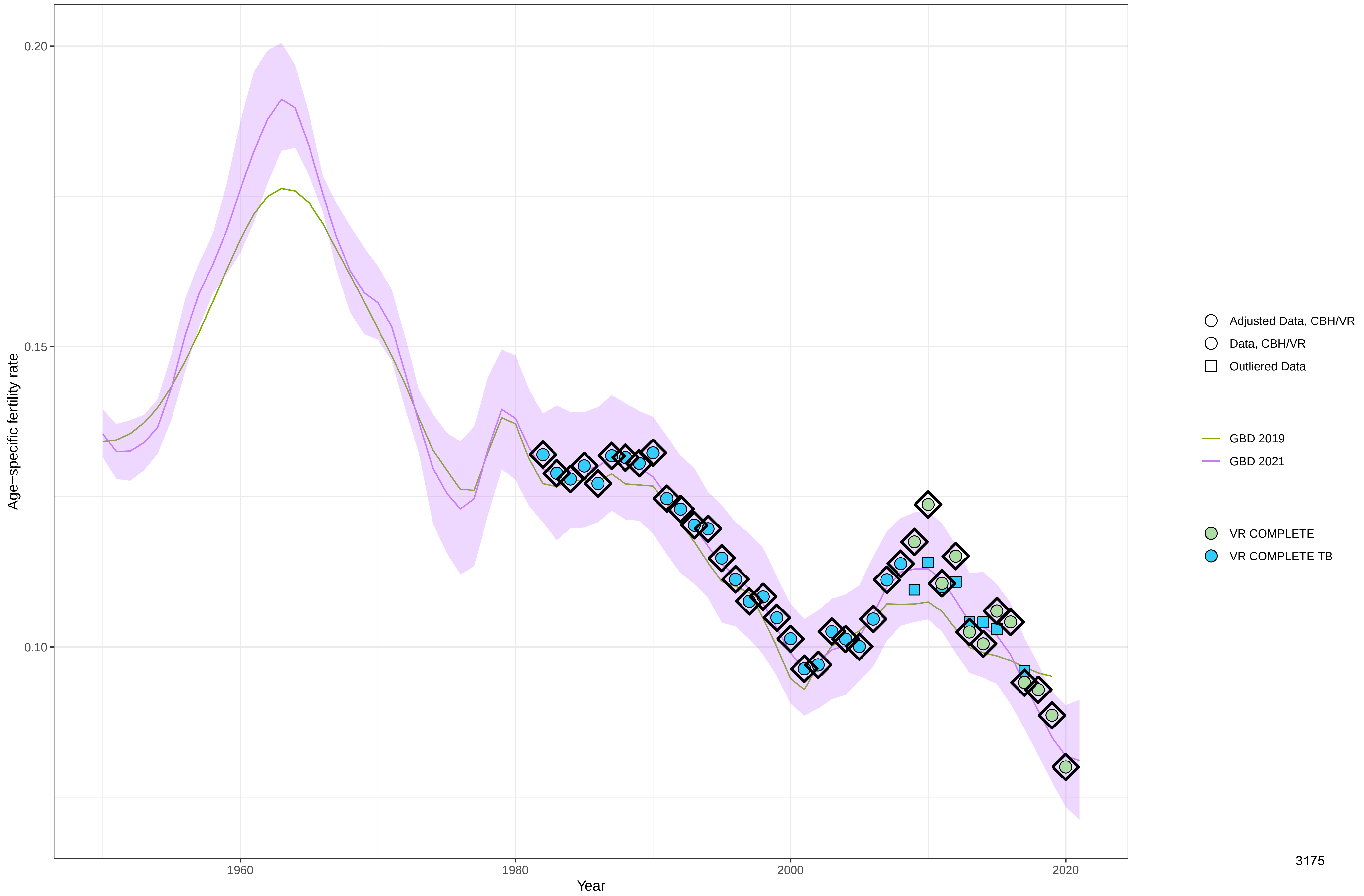

Age−specific fertility fitted trend lines, 1950−2021: Gloucestershire, Ages: 30−34
Data points enclosed with a diamond are reference sources

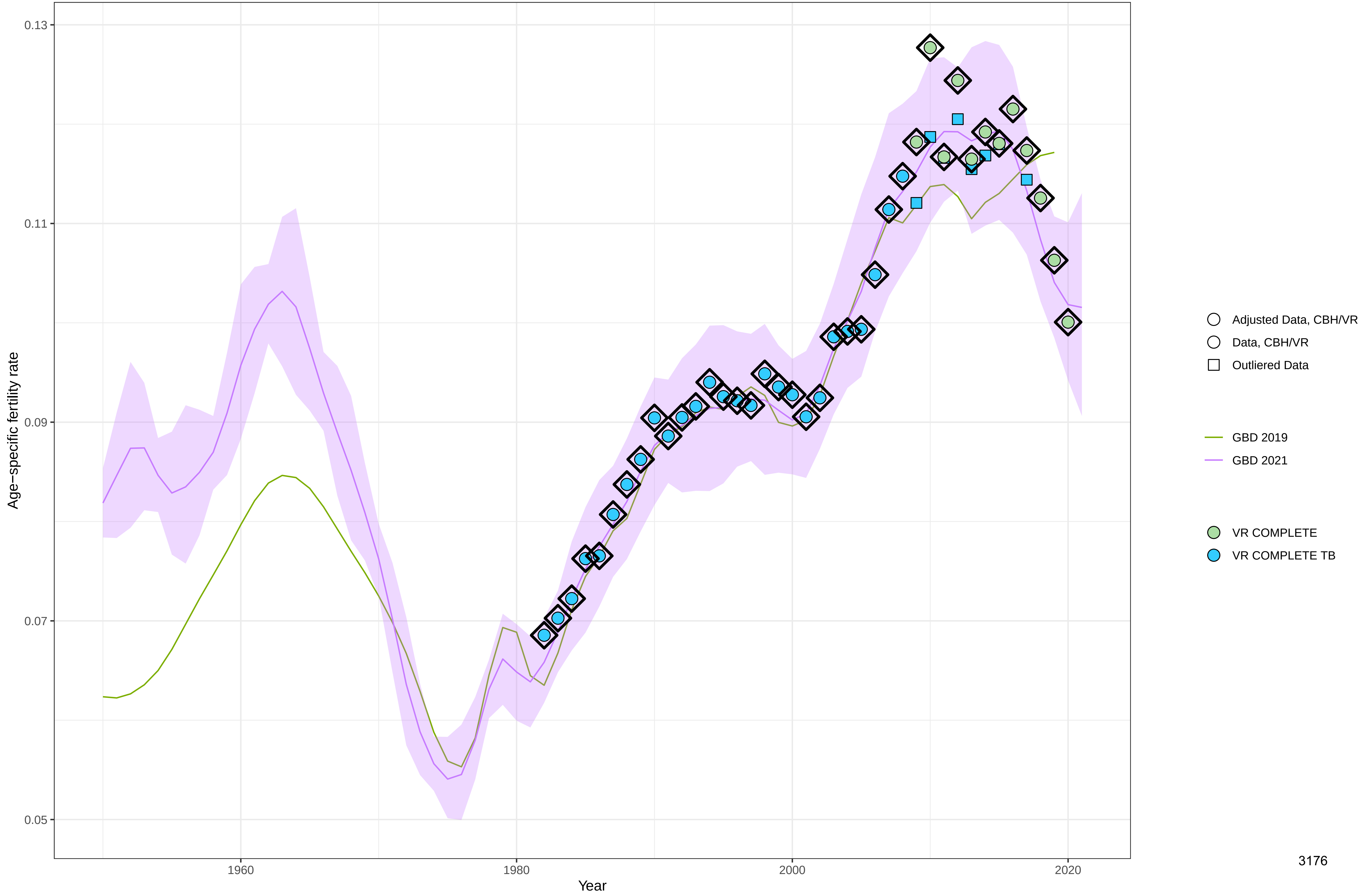

Age−specific fertility fitted trend lines, 1950−2021: Gloucestershire, Ages: 35−39
Data points enclosed with a diamond are reference sources

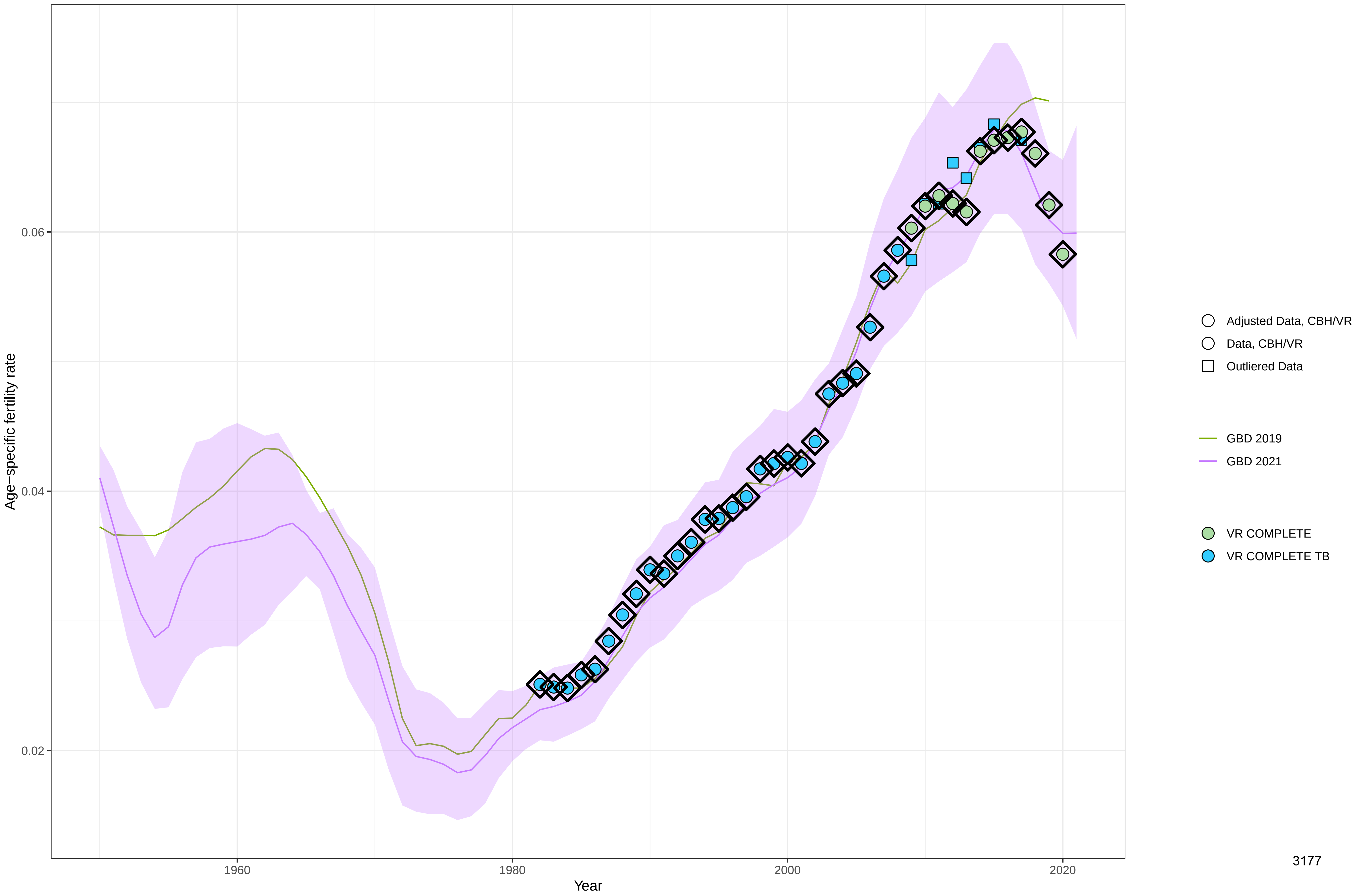

Age−specific fertility fitted trend lines, 1950−2021: Gloucestershire, Ages: 40−44
Data points enclosed with a diamond are reference sources

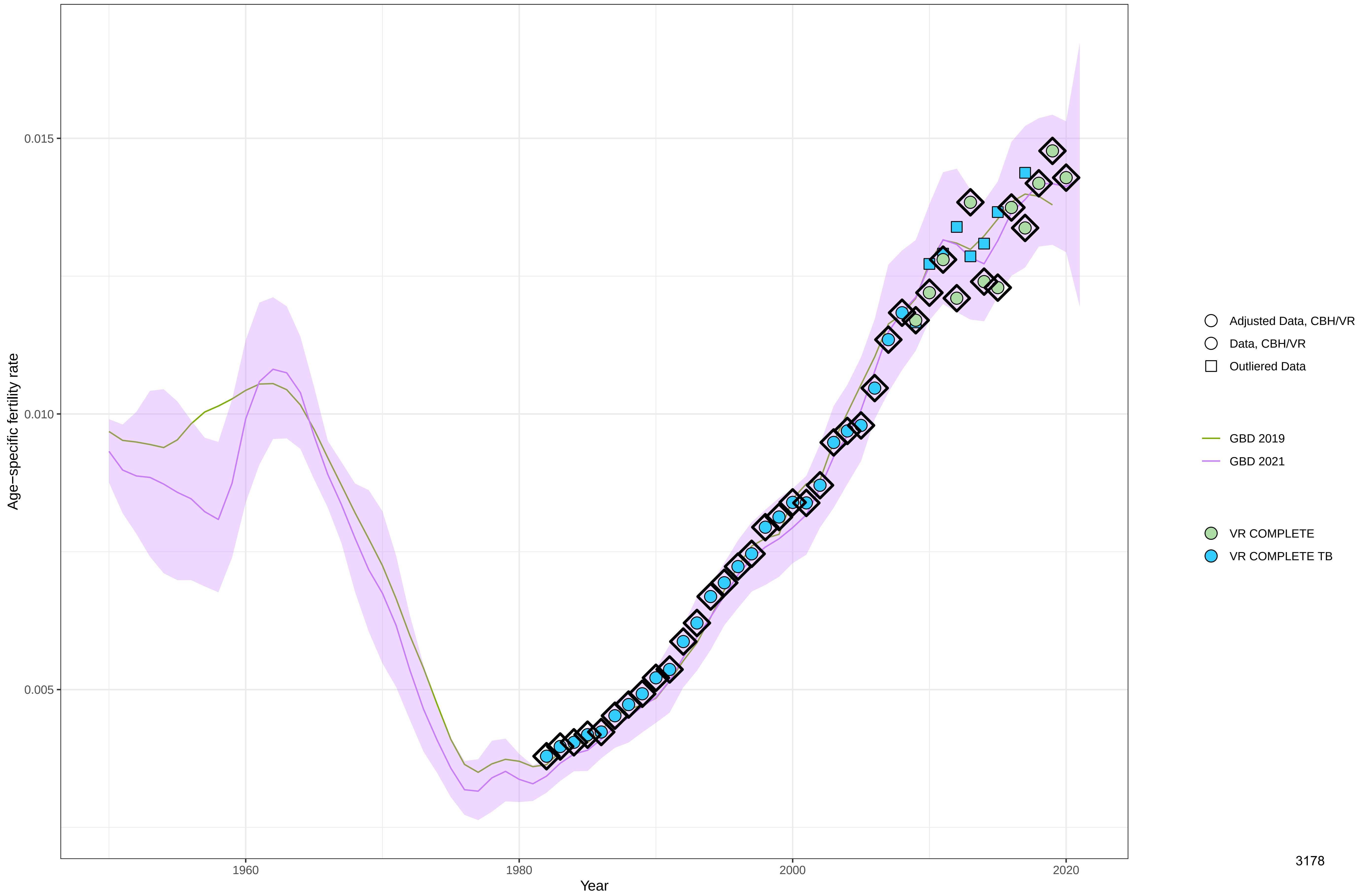

Age−specific fertility fitted trend lines, 1950−2021: Gloucestershire, Ages: 45−49
Data points enclosed with a diamond are reference sources

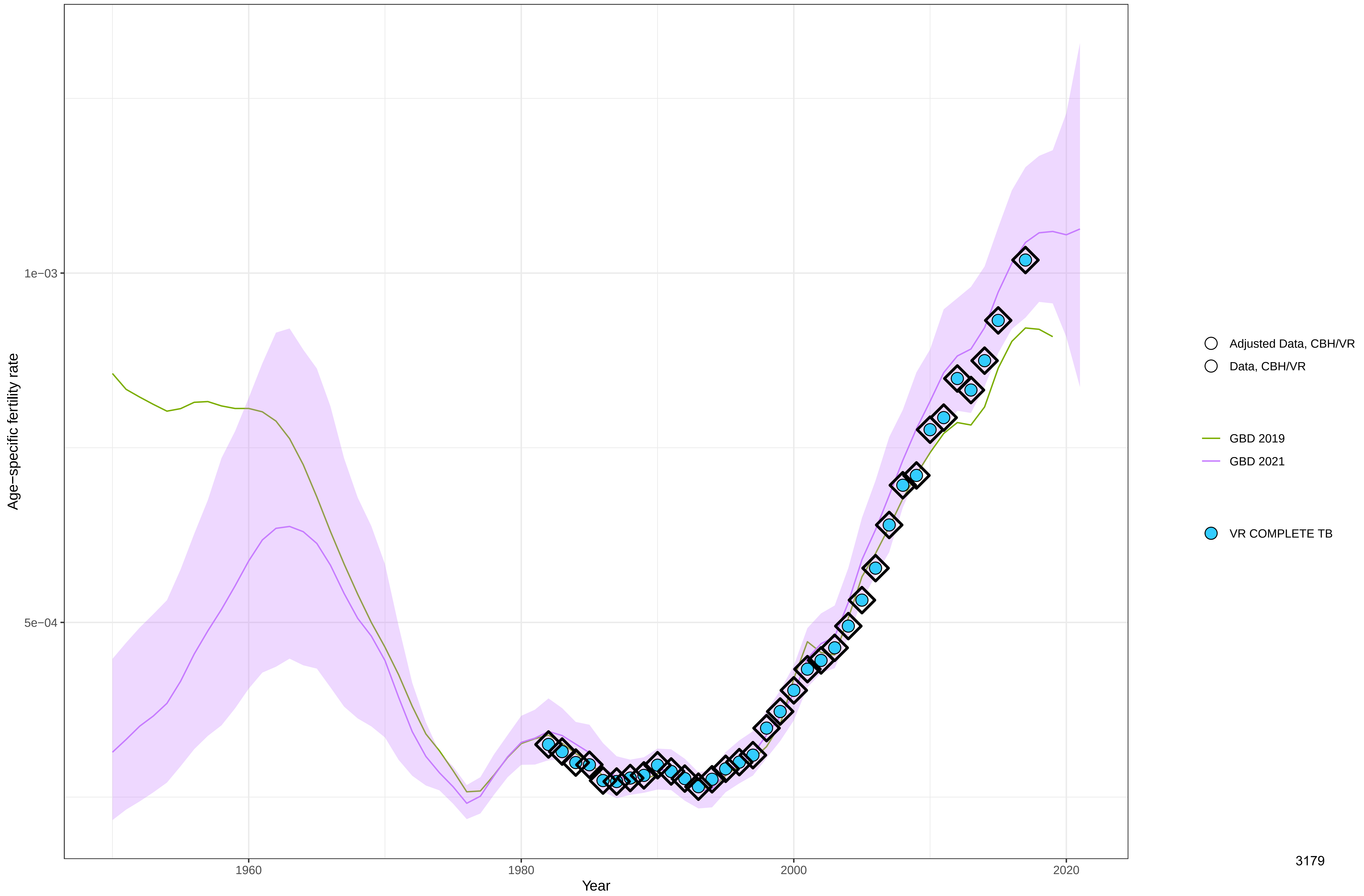

Age−specific fertility fitted trend lines, 1950−2021: Gloucestershire, Ages: 50−54
Data points enclosed with a diamond are reference sources

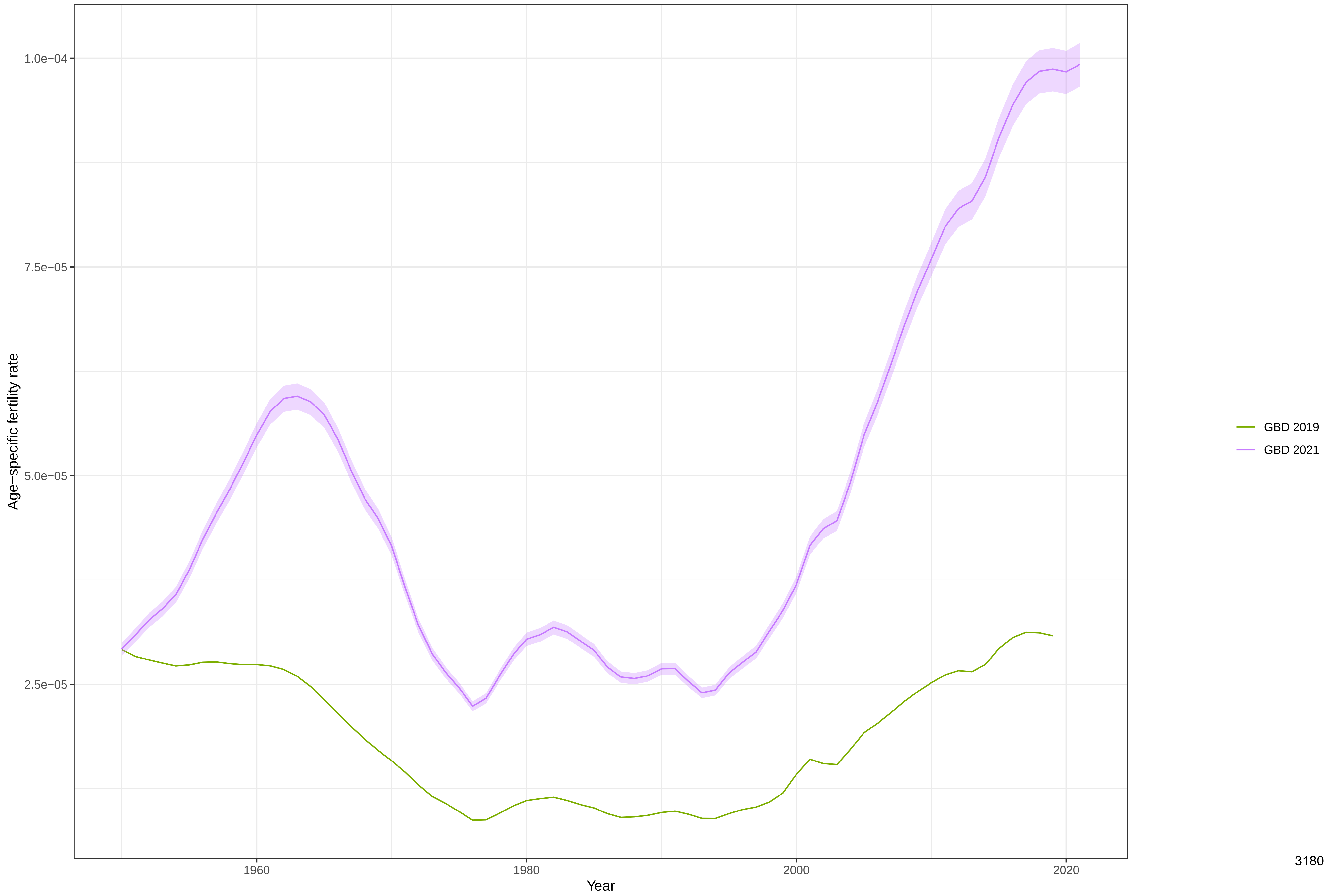

3180

Total fertility rate fitted trend lines, 1950−2021: Gloucestershire TFR
Data points enclosed with a diamond are reference sources

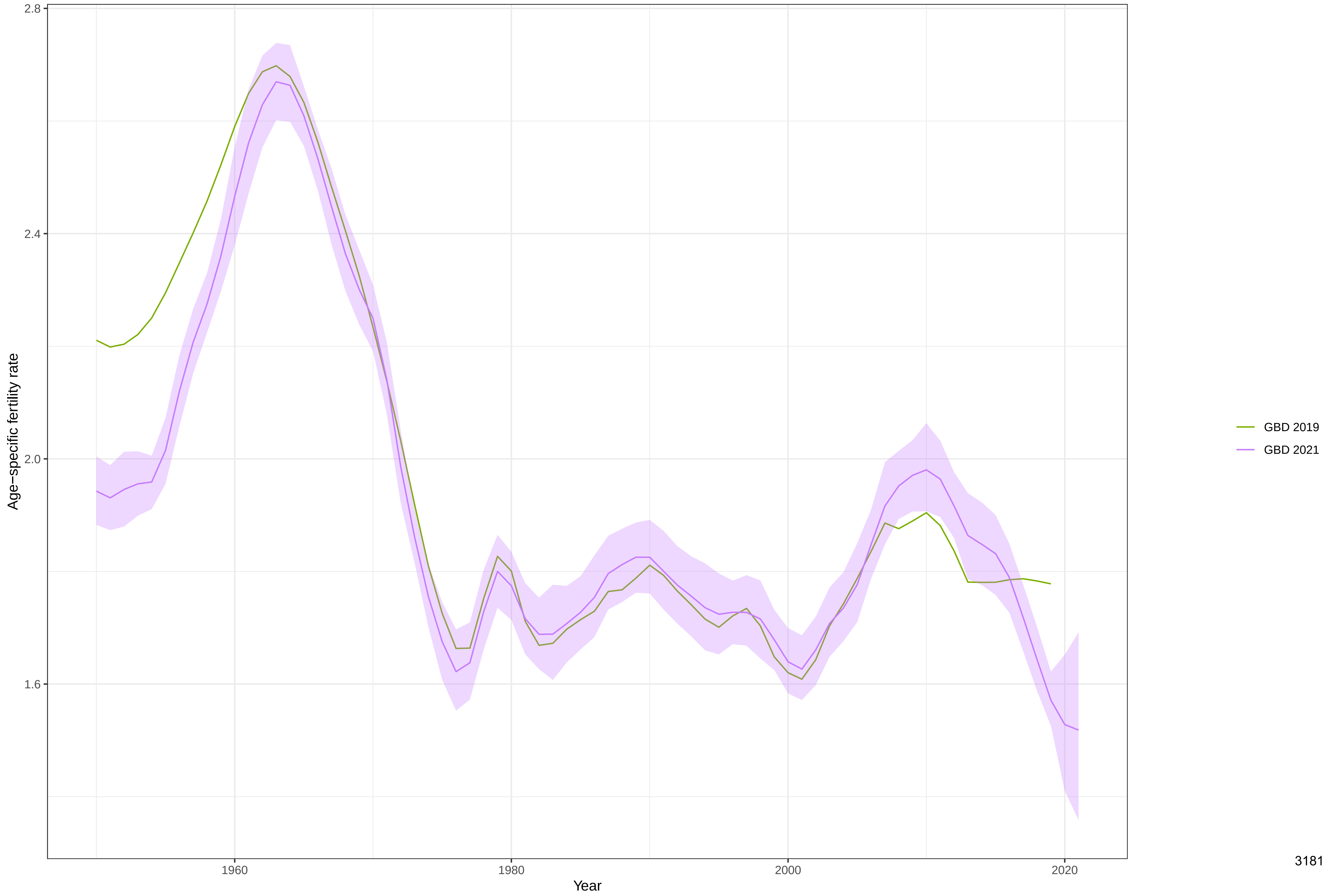

Age−specific fertility fitted trend lines, 1950−2021: North Somerset, Ages: 10−14
Data points enclosed with a diamond are reference sources

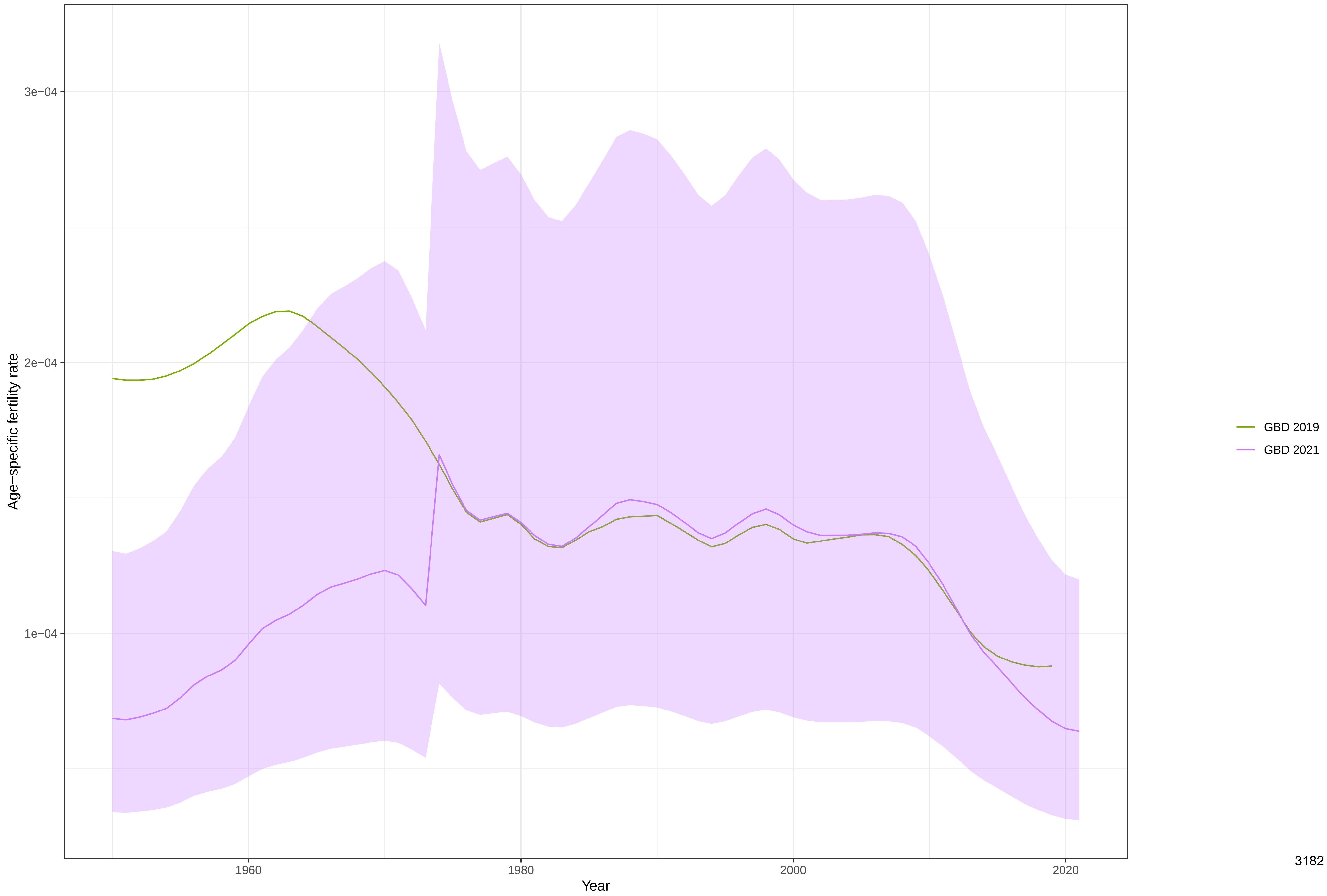

Age−specific fertility fitted trend lines, 1950−2021: North Somerset, Ages: 15−19
Data points enclosed with a diamond are reference sources

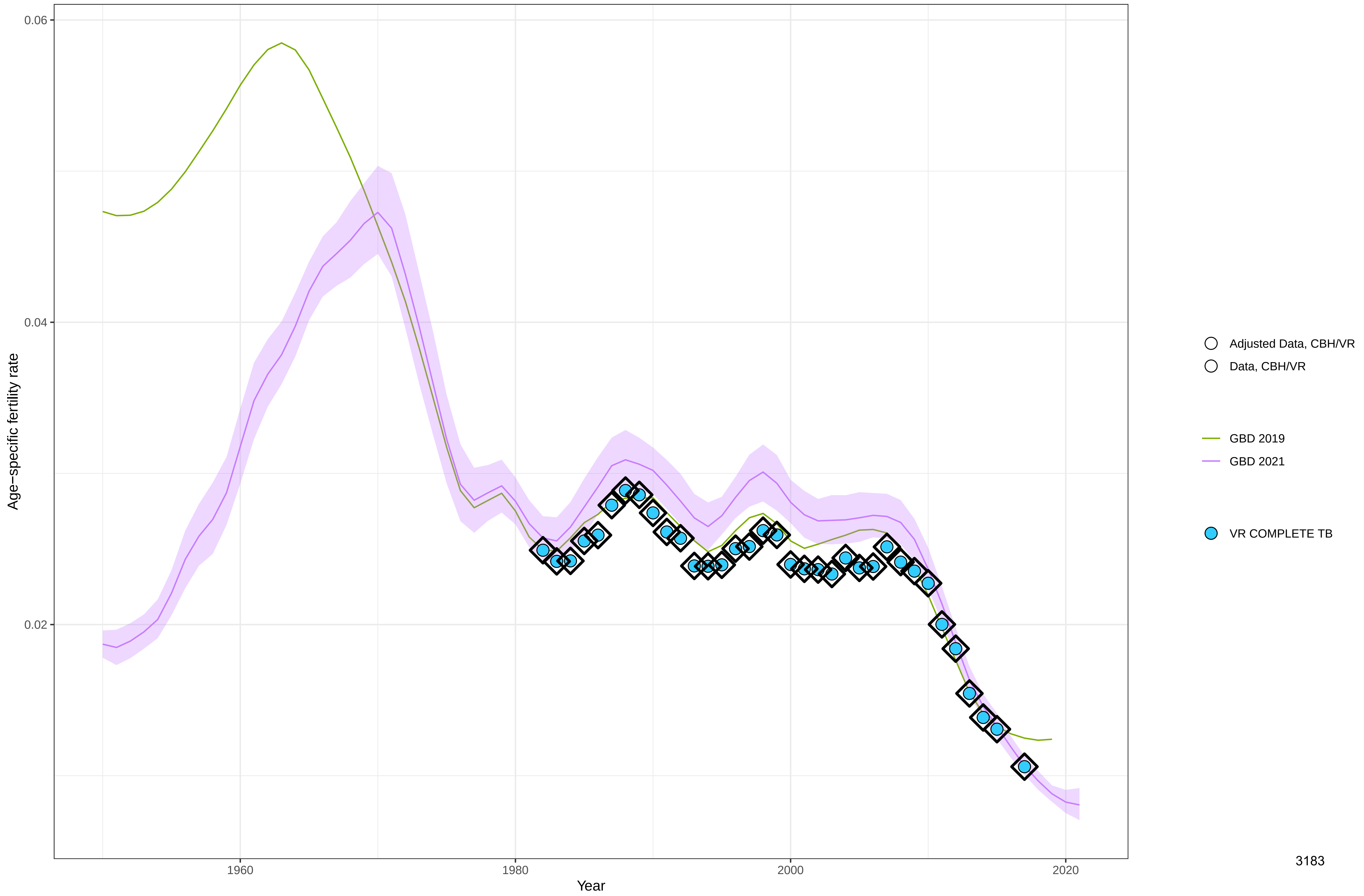

Age−specific fertility fitted trend lines, 1950−2021: North Somerset, Ages: 20−24
Data points enclosed with a diamond are reference sources

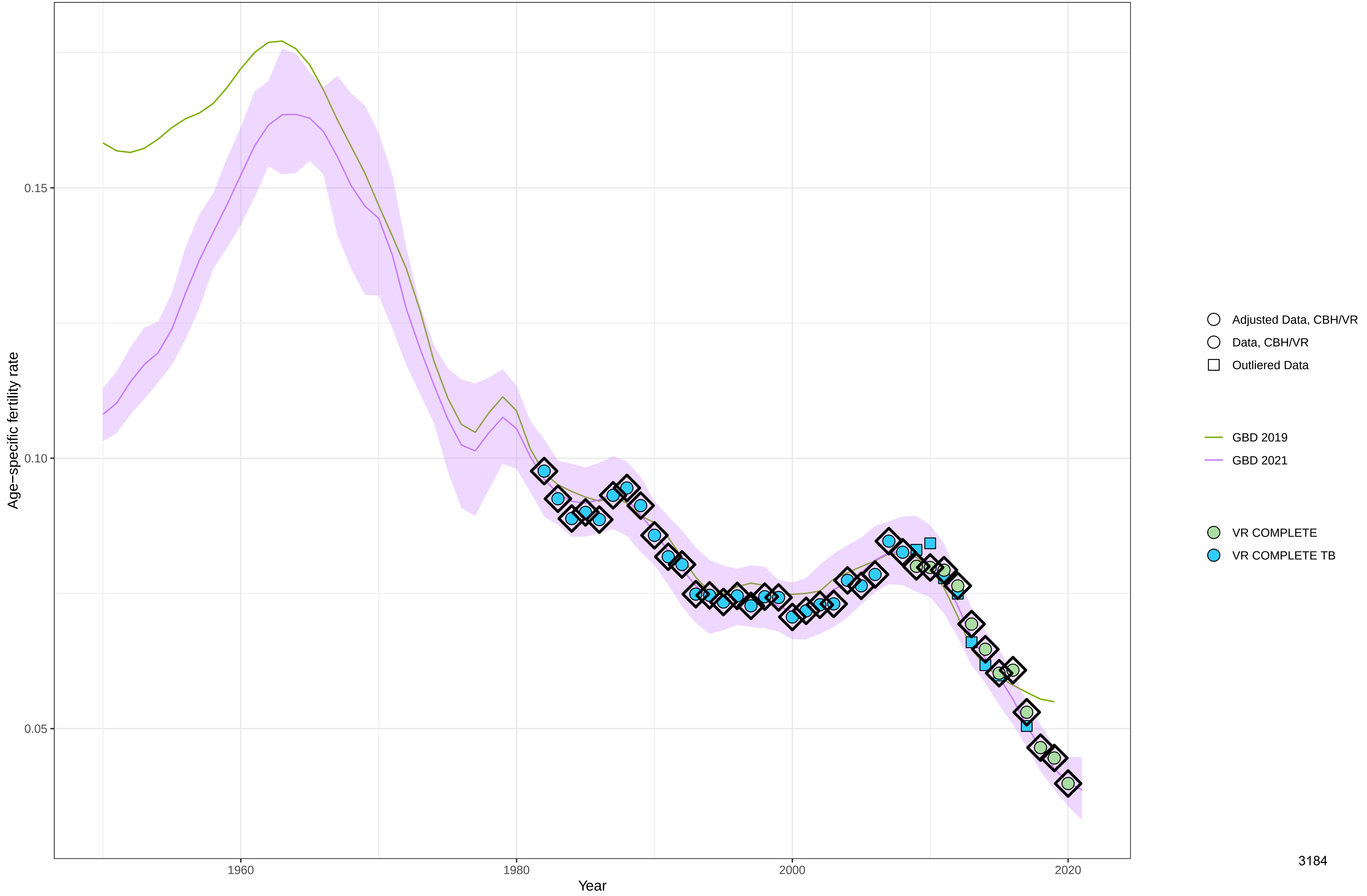

Age−specific fertility fitted trend lines, 1950−2021: North Somerset, Ages: 25−29
Data points enclosed with a diamond are reference sources

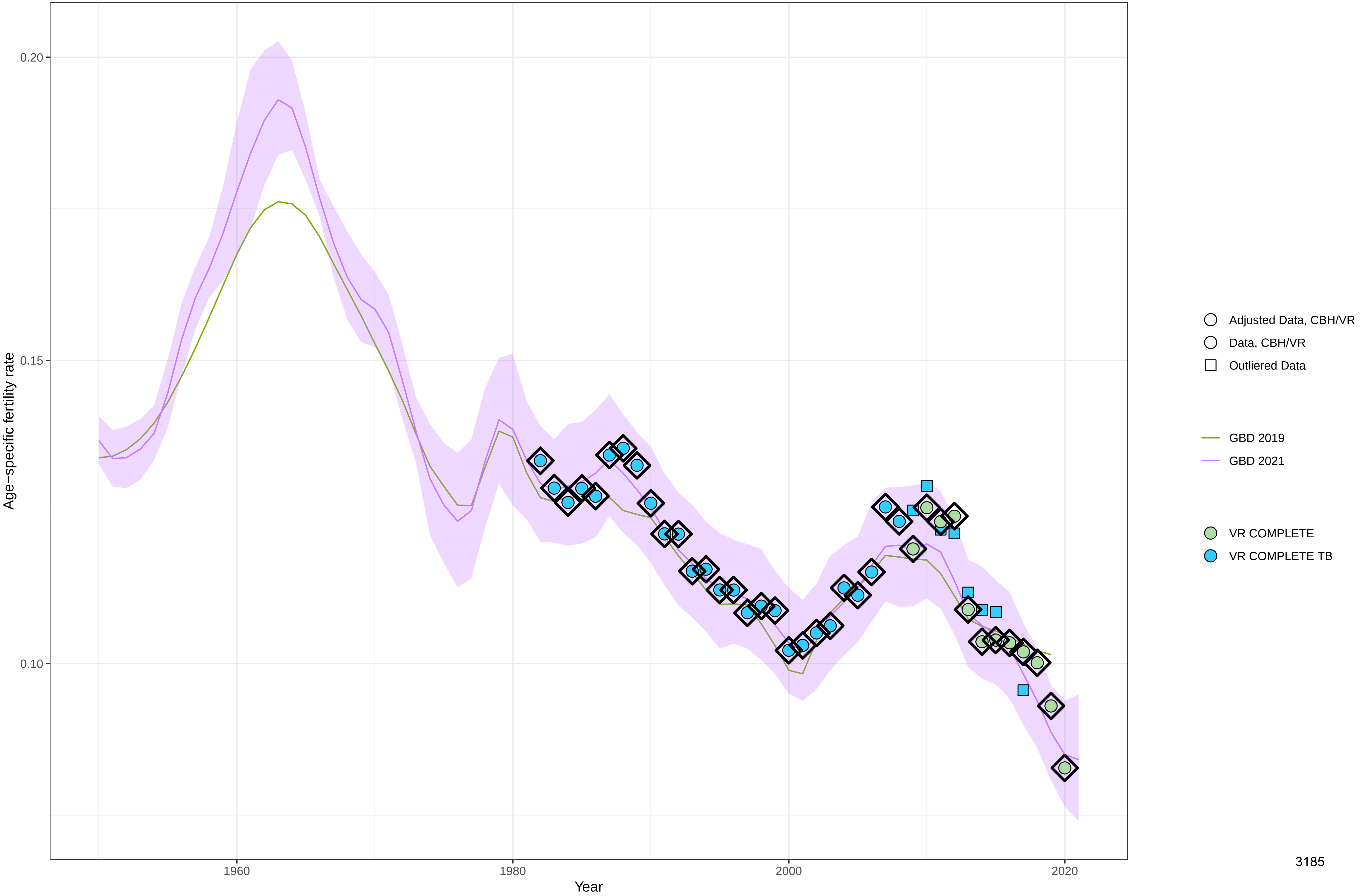

Age−specific fertility fitted trend lines, 1950−2021: North Somerset, Ages: 30−34
Data points enclosed with a diamond are reference sources

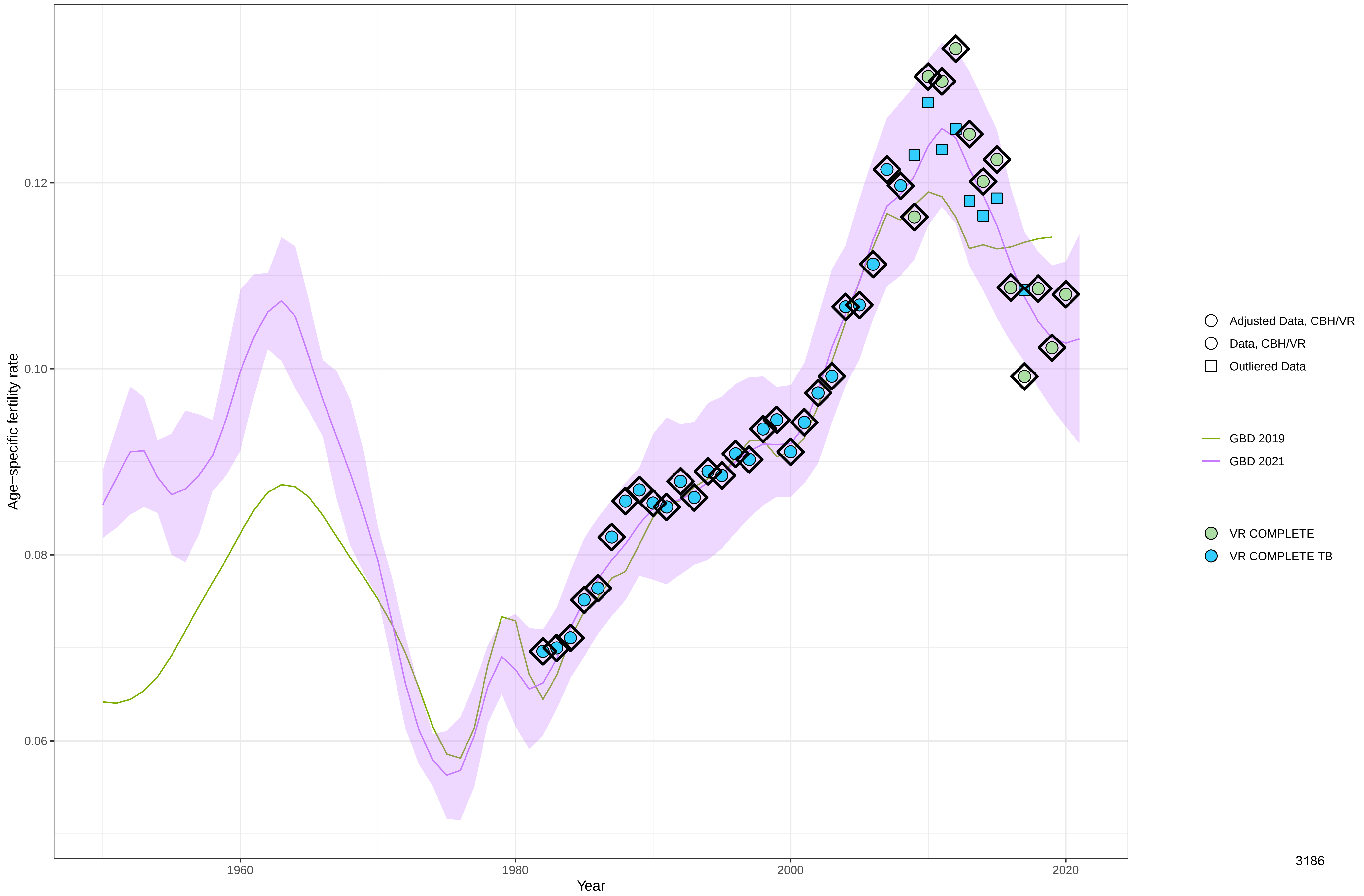

Age−specific fertility fitted trend lines, 1950−2021: North Somerset, Ages: 35−39
Data points enclosed with a diamond are reference sources

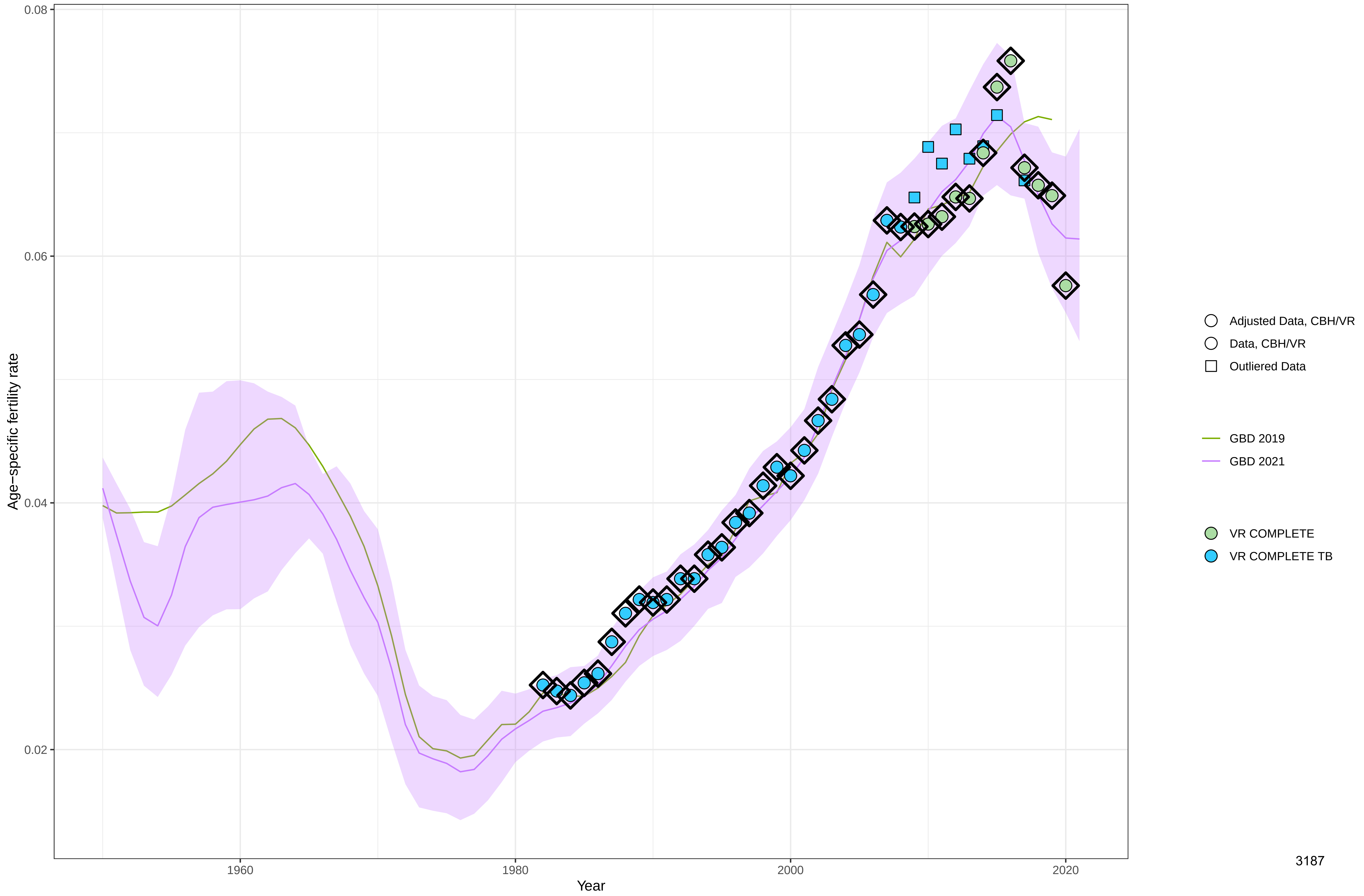

Age−specific fertility fitted trend lines, 1950−2021: North Somerset, Ages: 40−44
Data points enclosed with a diamond are reference sources

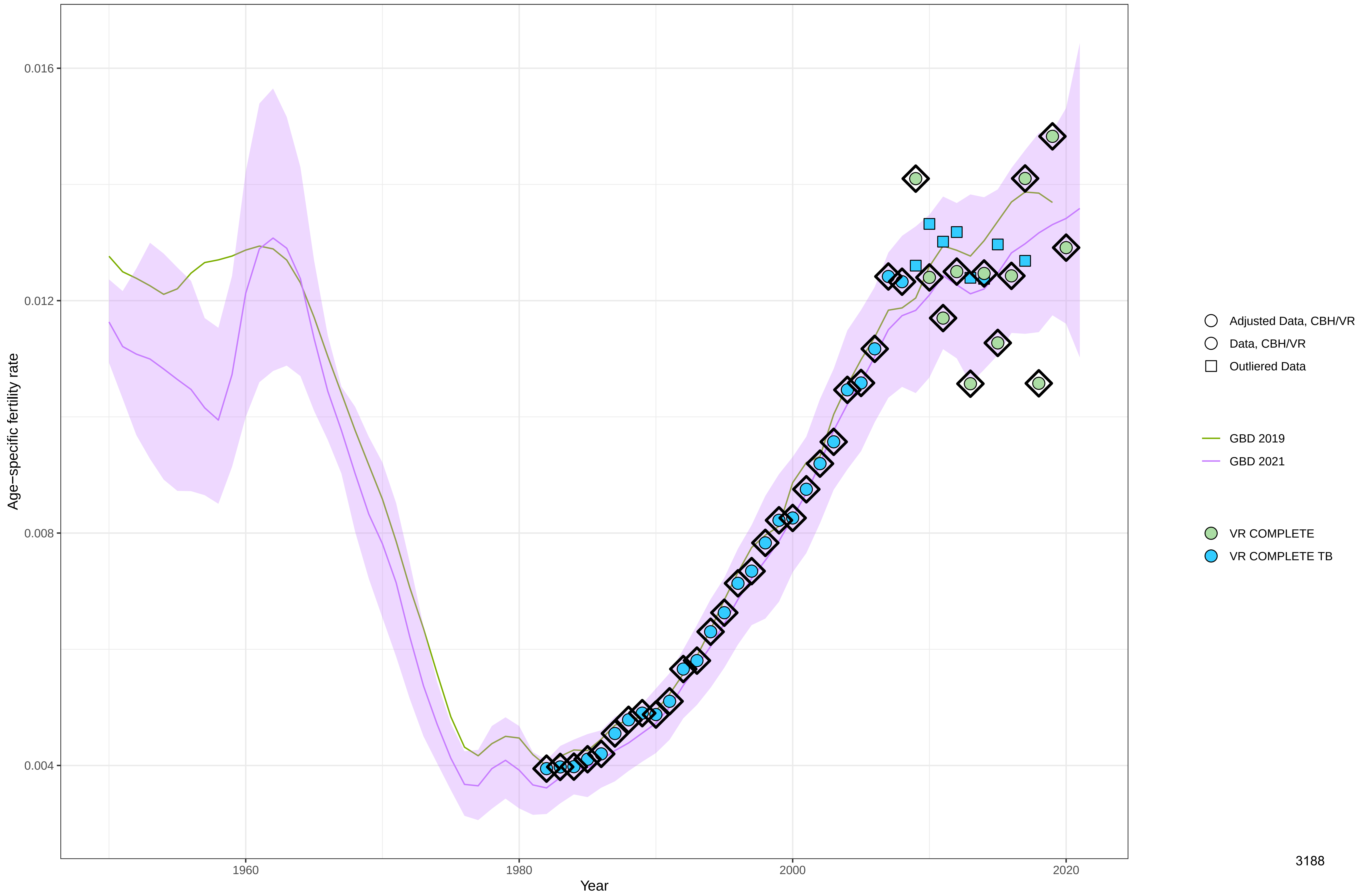

Age−specific fertility fitted trend lines, 1950−2021: North Somerset, Ages: 45−49
Data points enclosed with a diamond are reference sources

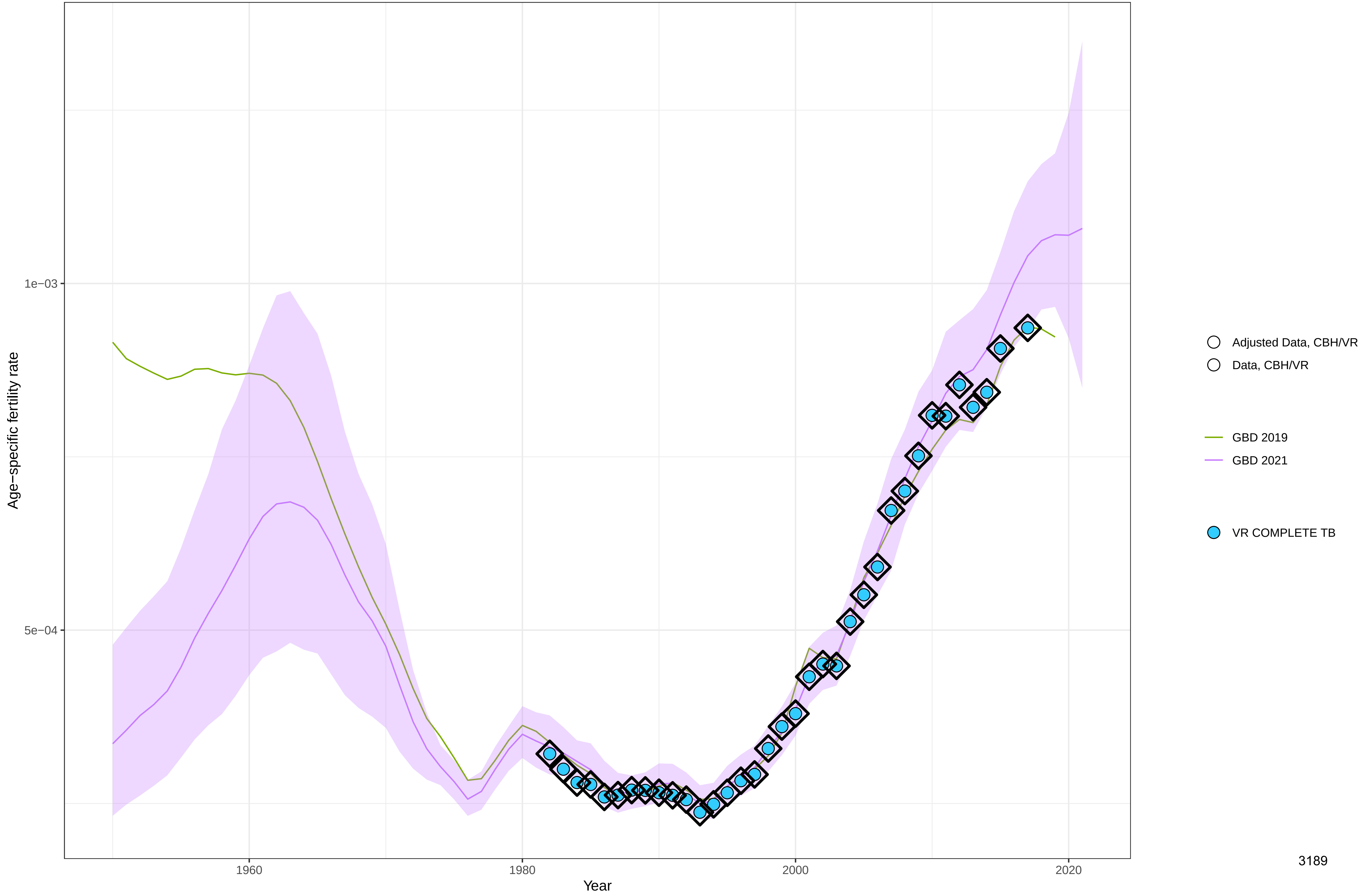

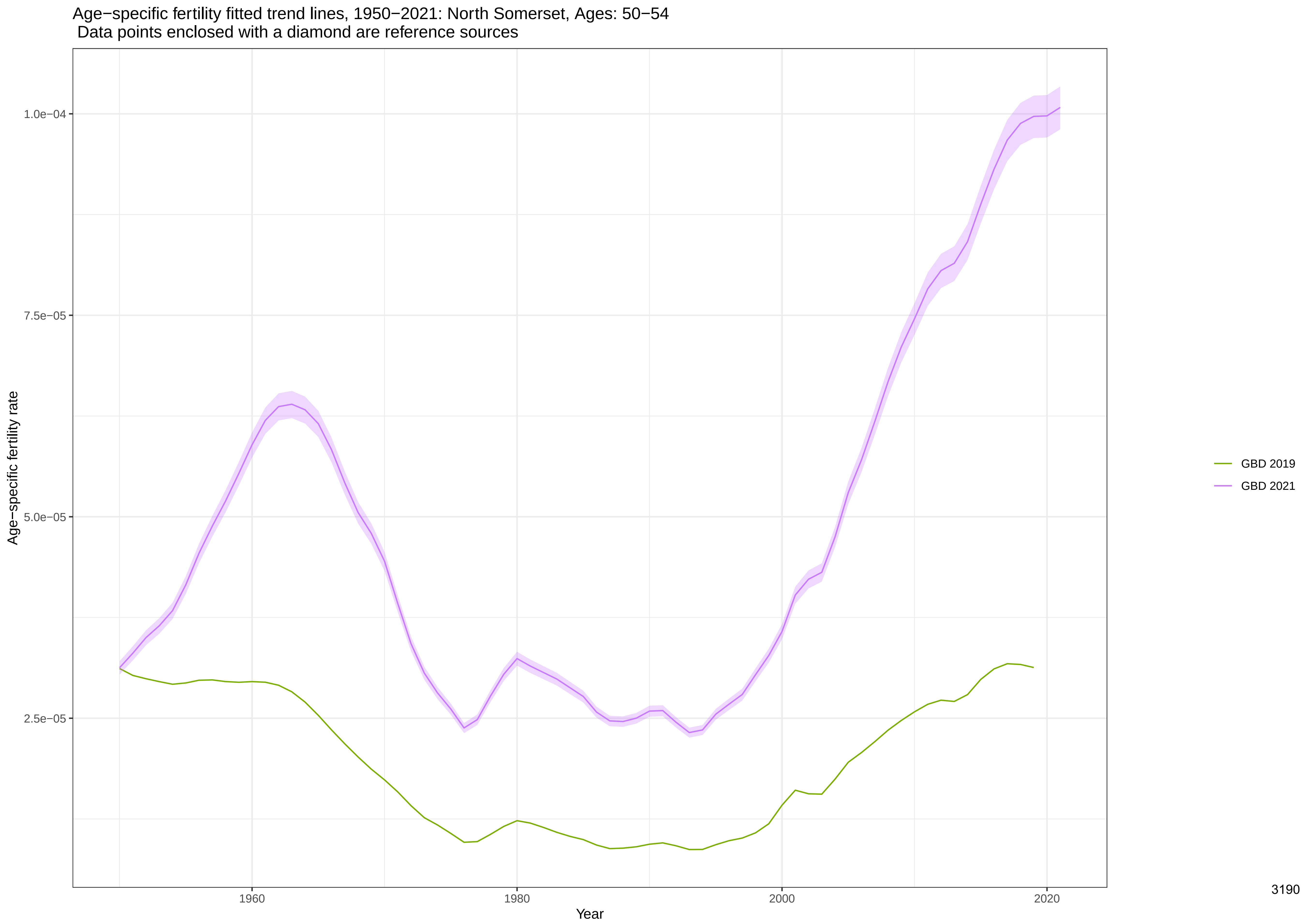

Age−specific fertility fitted trend lines, 1950−2021: North Somerset, Ages: 50−54
Data points enclosed with a diamond are reference sources
Age−specific fertility rate
1.0e−04
7.5e−05
5.0e−05
2.5e−05
1960
1980
2000
2020
Year
GBD 2019
GBD 2021

3190

Total fertility rate fitted trend lines, 1950−2021: North Somerset TFR
Data points enclosed with a diamond are reference sources

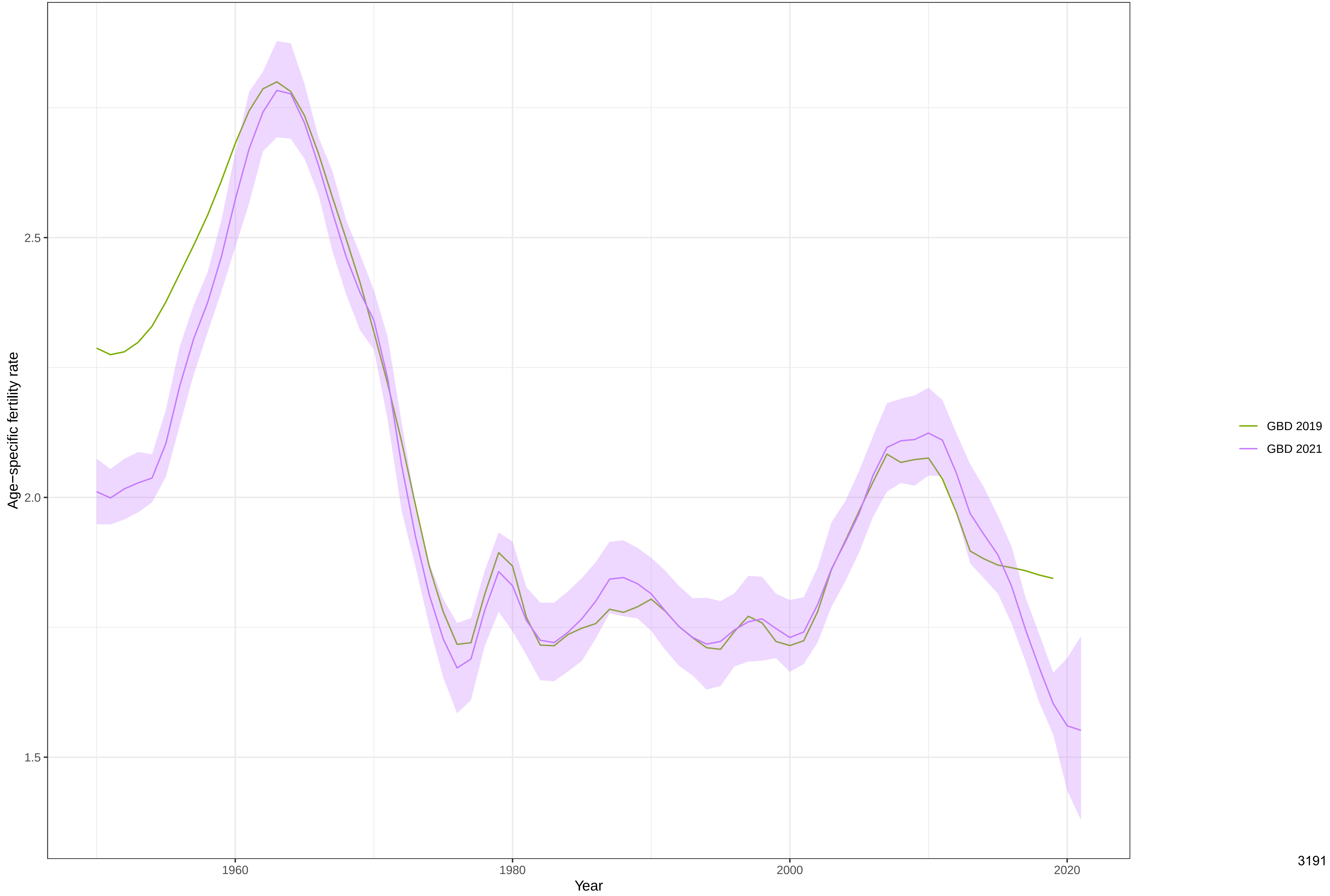

Age−specific fertility fitted trend lines, 1950−2021: Plymouth, Ages: 10−14
Data points enclosed with a diamond are reference sources

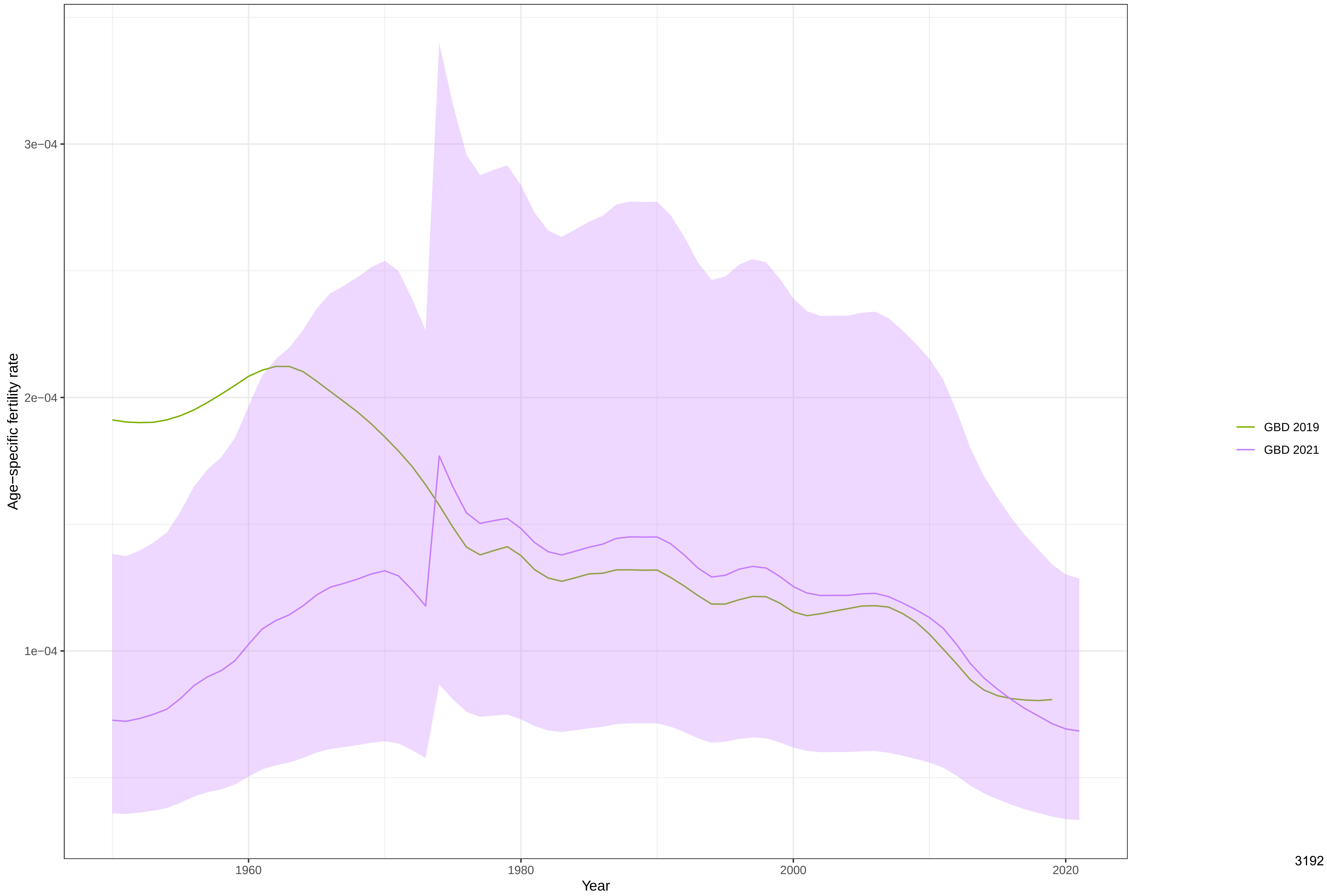

Age−specific fertility fitted trend lines, 1950−2021: Plymouth, Ages: 15−19
Data points enclosed with a diamond are reference sources

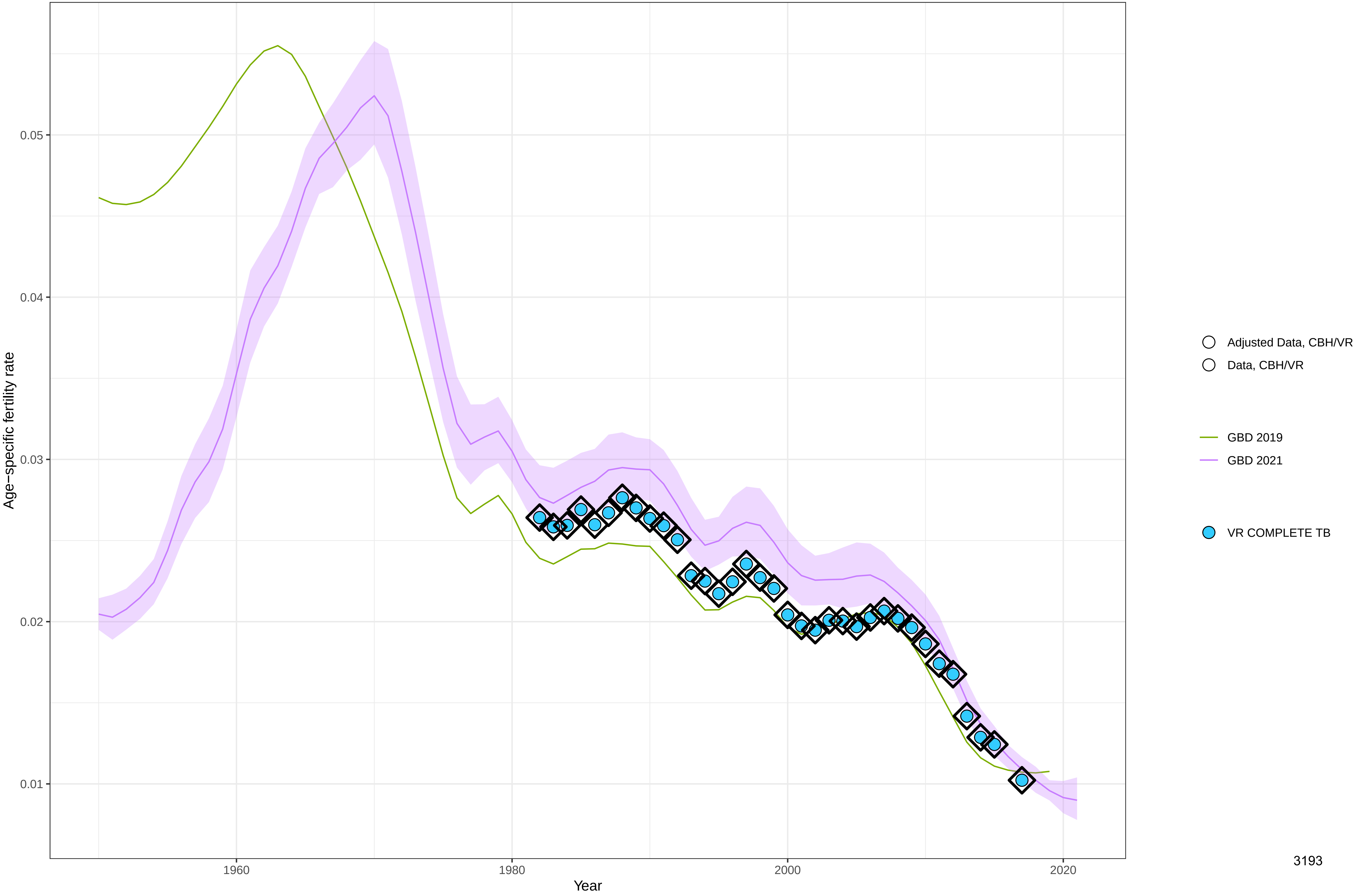

Age−specific fertility fitted trend lines, 1950−2021: Plymouth, Ages: 20−24
Data points enclosed with a diamond are reference sources

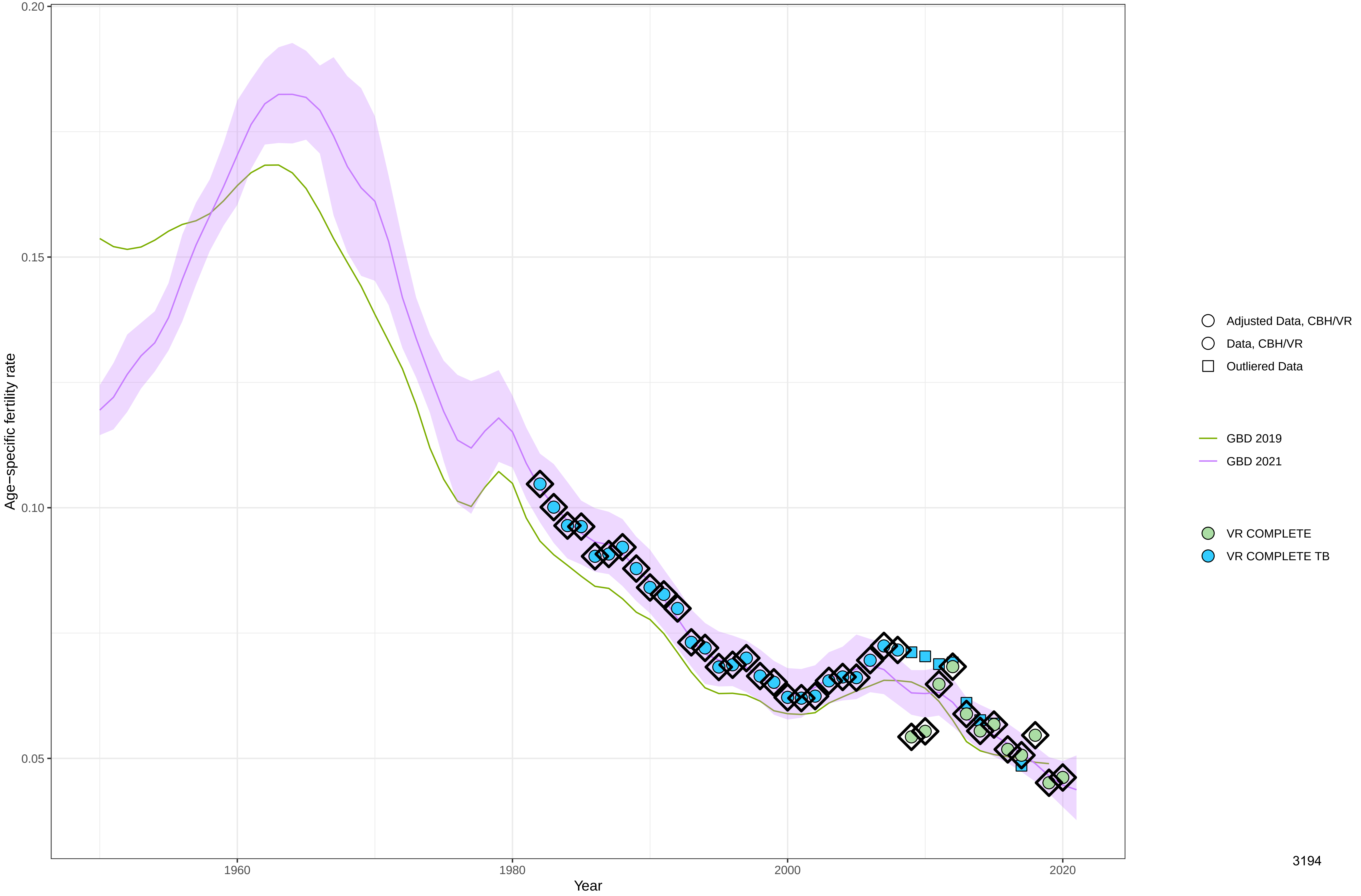

Age−specific fertility fitted trend lines, 1950−2021: Plymouth, Ages: 25−29
Data points enclosed with a diamond are reference sources

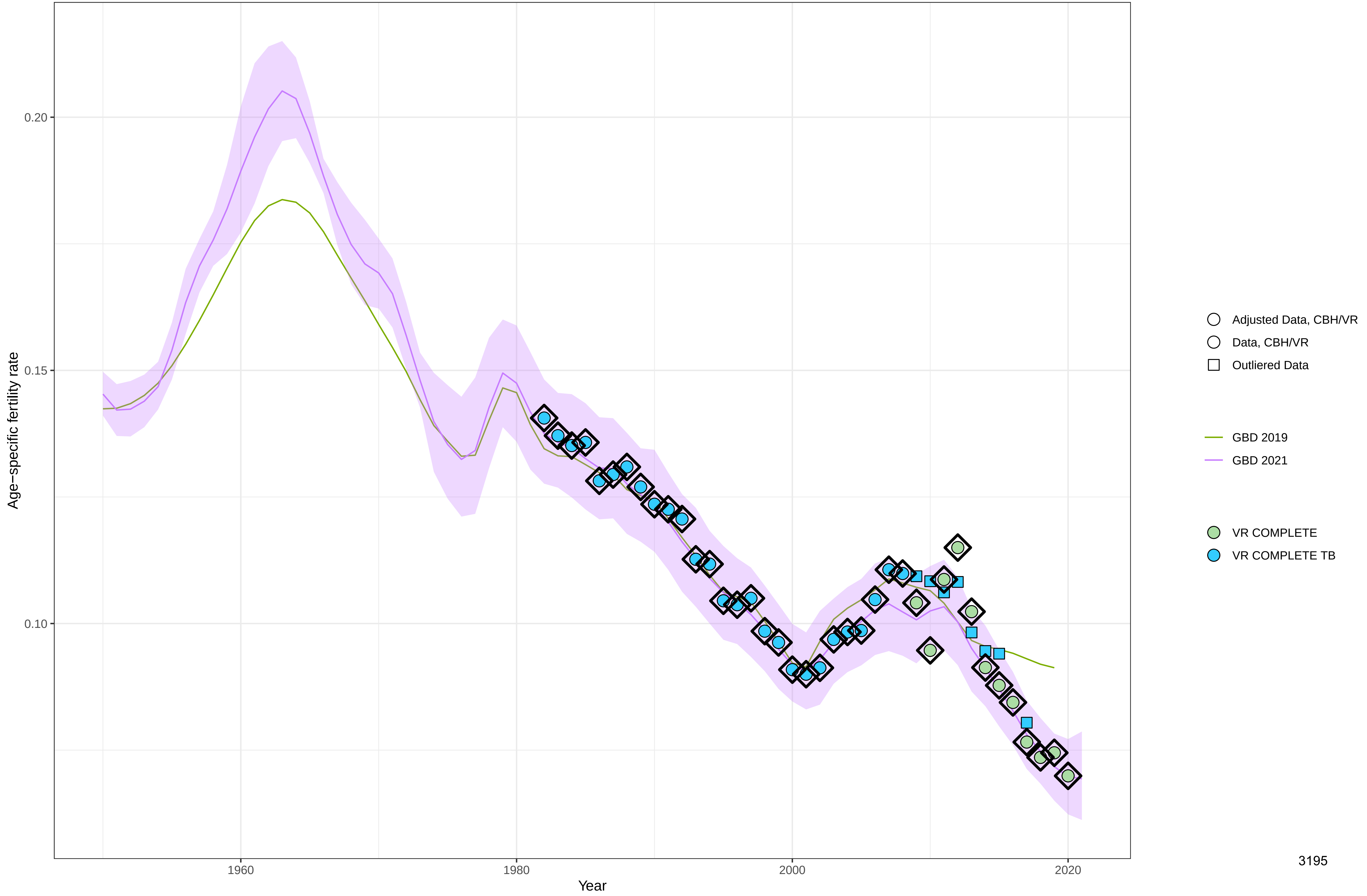

3195

Age−specific fertility fitted trend lines, 1950−2021: Plymouth, Ages: 30−34
Data points enclosed with a diamond are reference sources

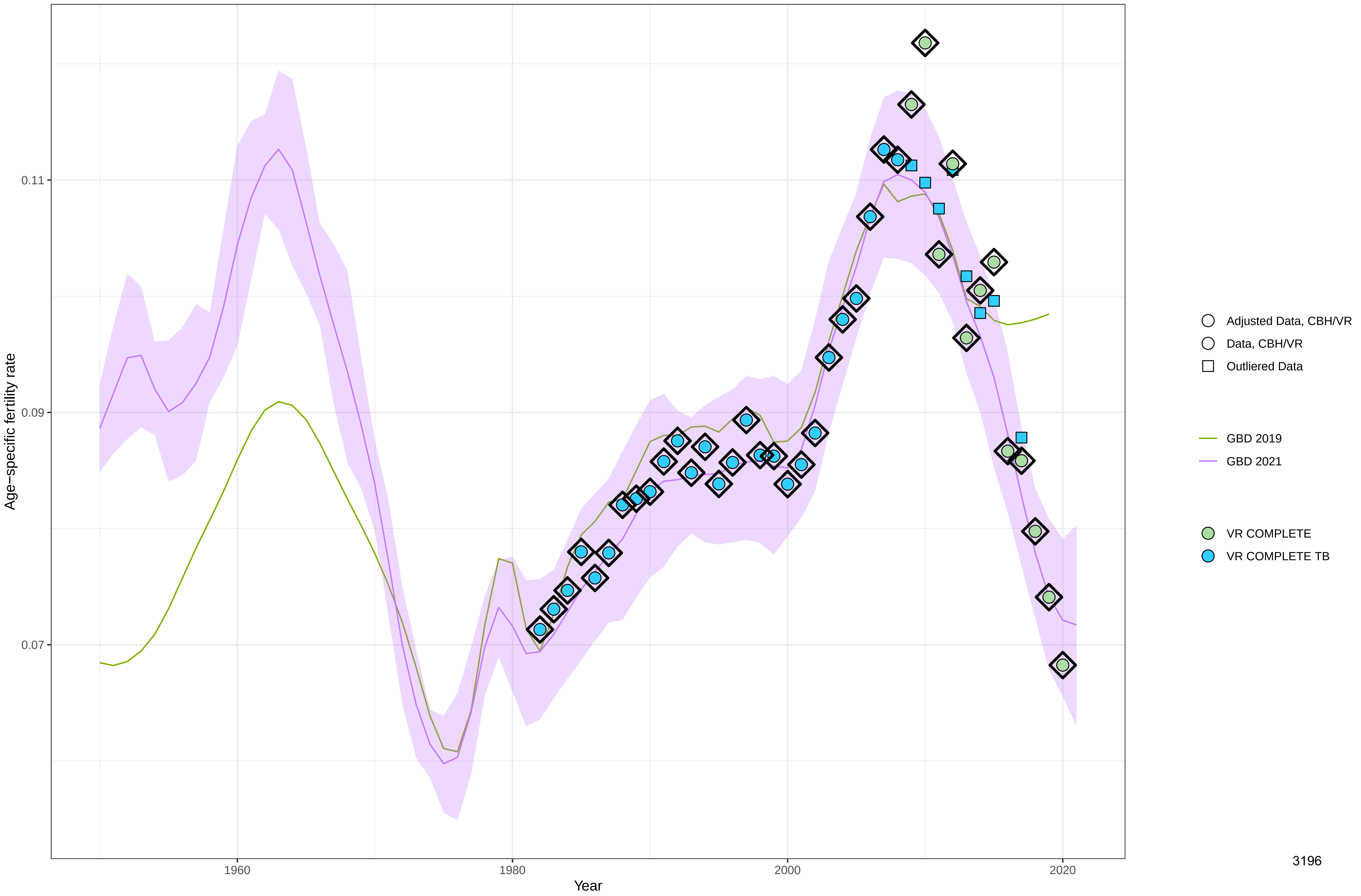

Age−specific fertility fitted trend lines, 1950−2021: Plymouth, Ages: 35−39
Data points enclosed with a diamond are reference sources

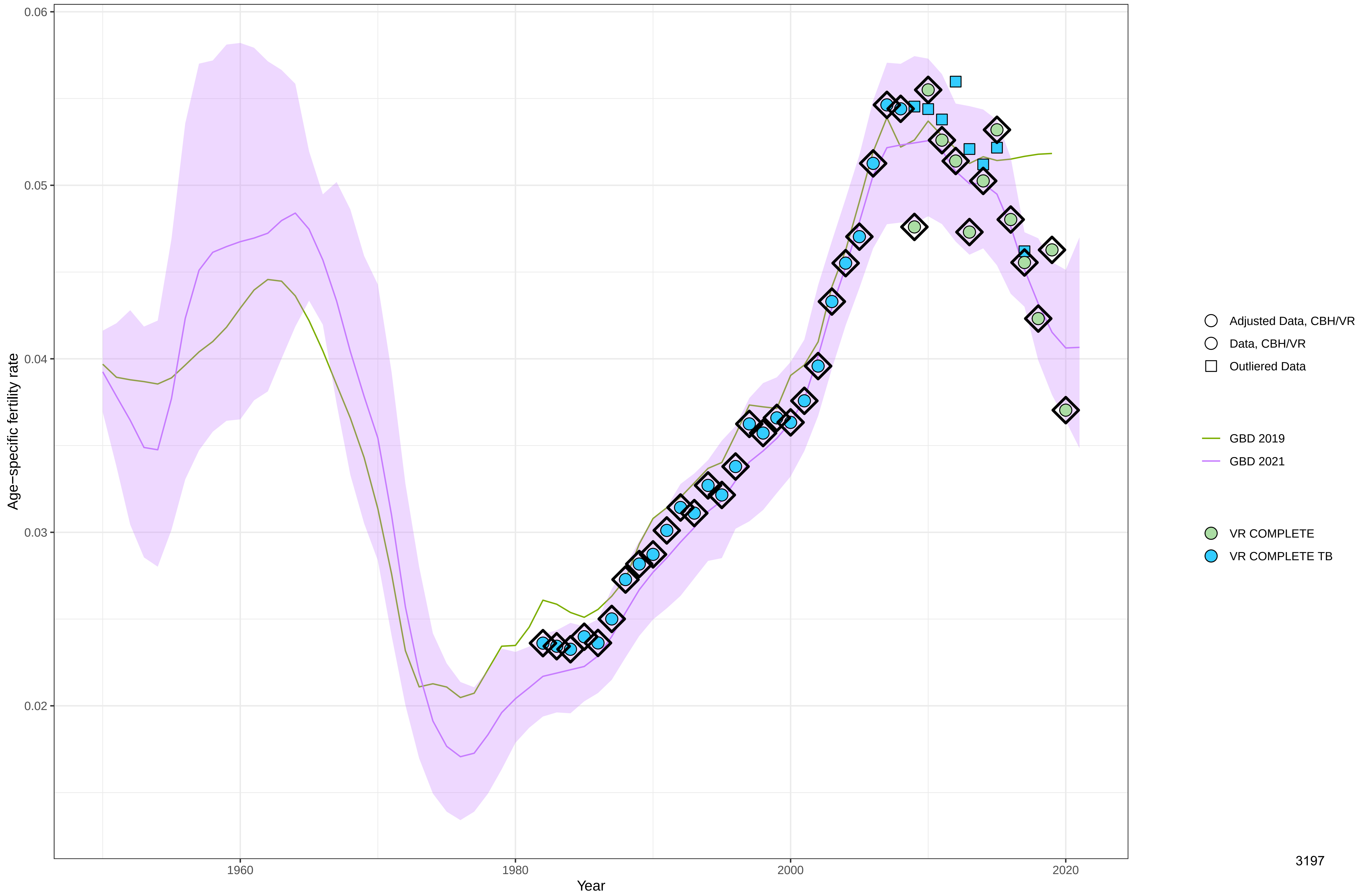

Age−specific fertility fitted trend lines, 1950−2021: Plymouth, Ages: 40−44
Data points enclosed with a diamond are reference sources

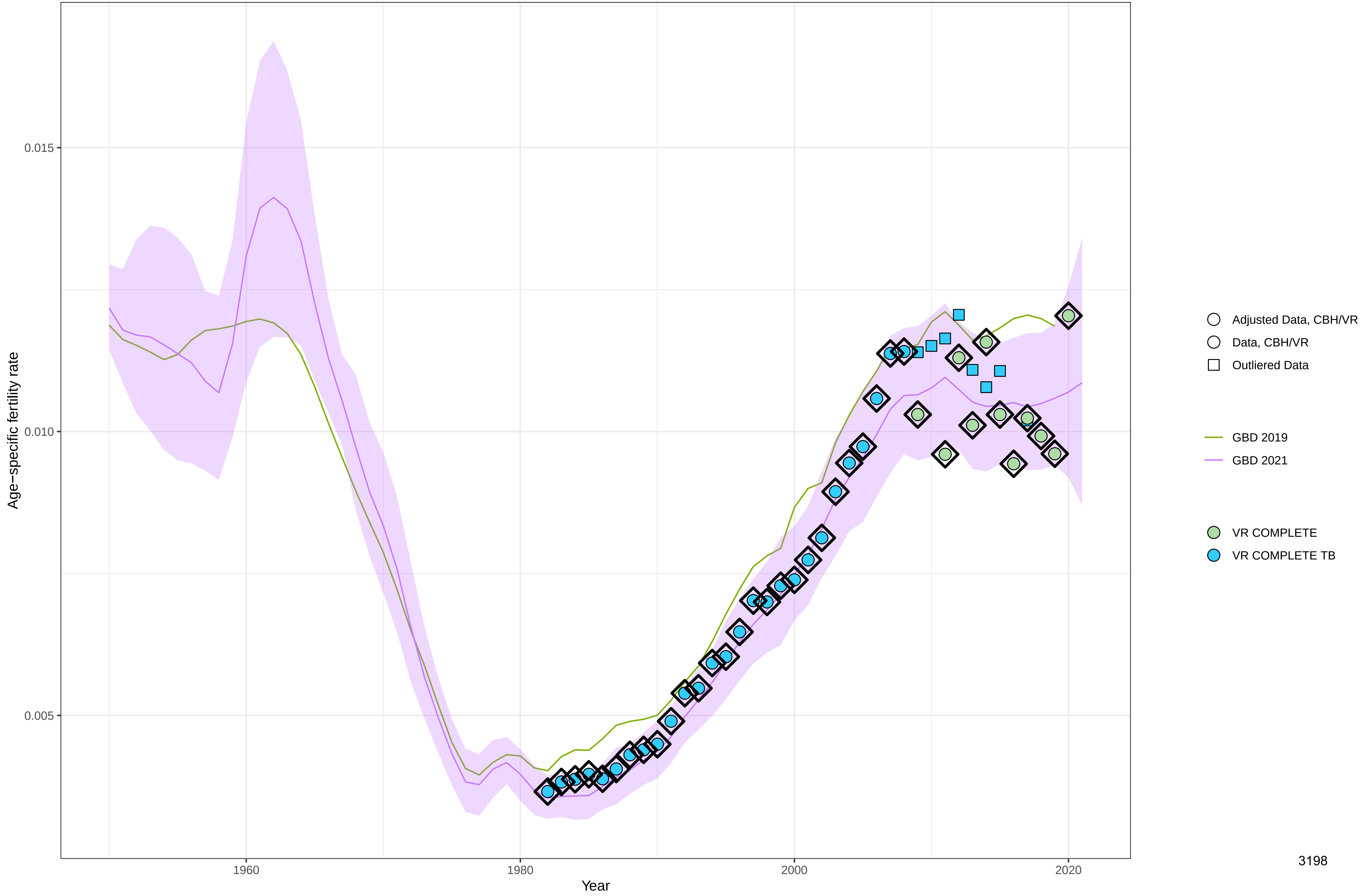

Age−specific fertility fitted trend lines, 1950−2021: Plymouth, Ages: 45−49
Data points enclosed with a diamond are reference sources

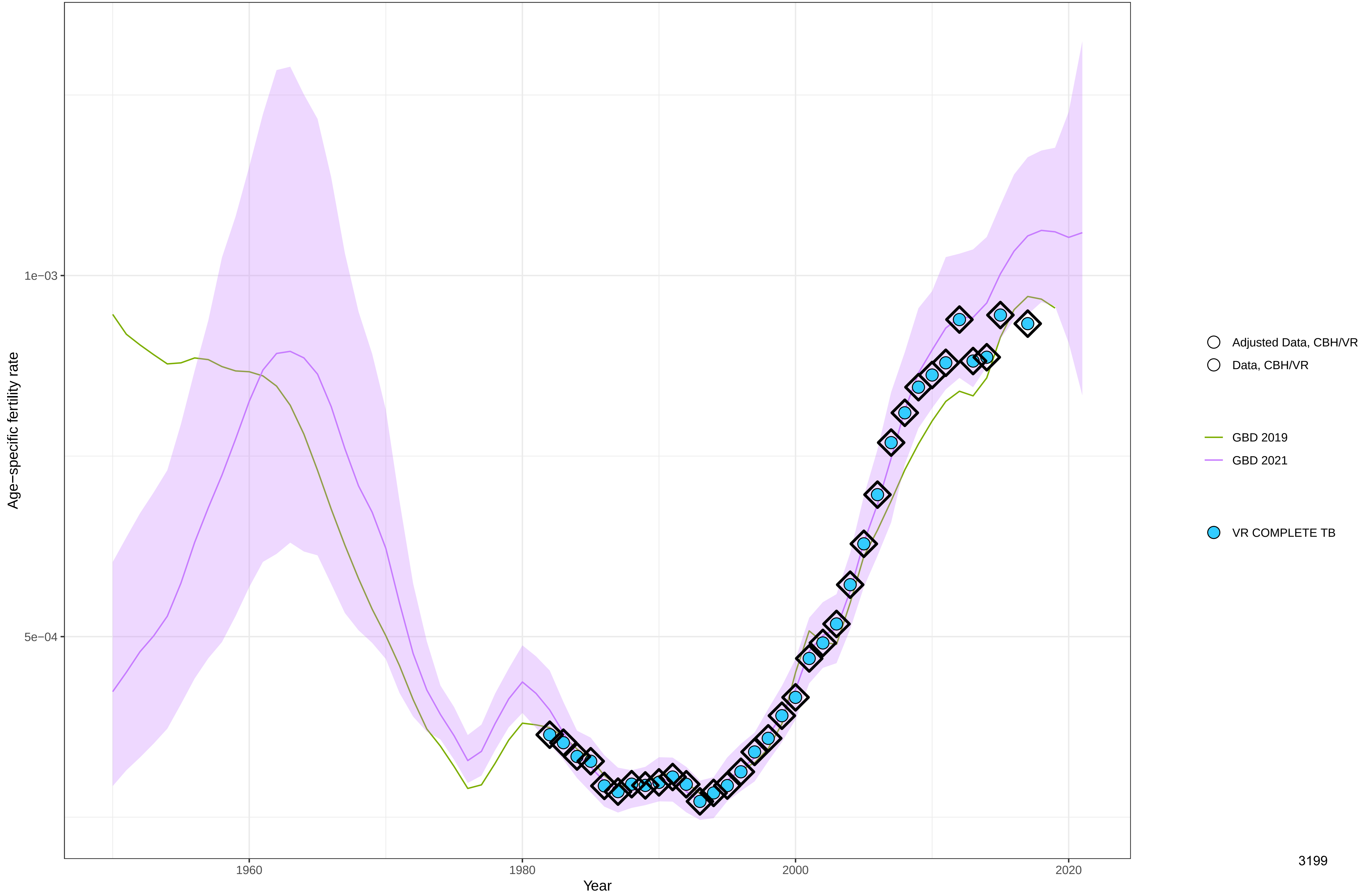

Age−specific fertility fitted trend lines, 1950−2021: Plymouth, Ages: 50−54
Data points enclosed with a diamond are reference sources

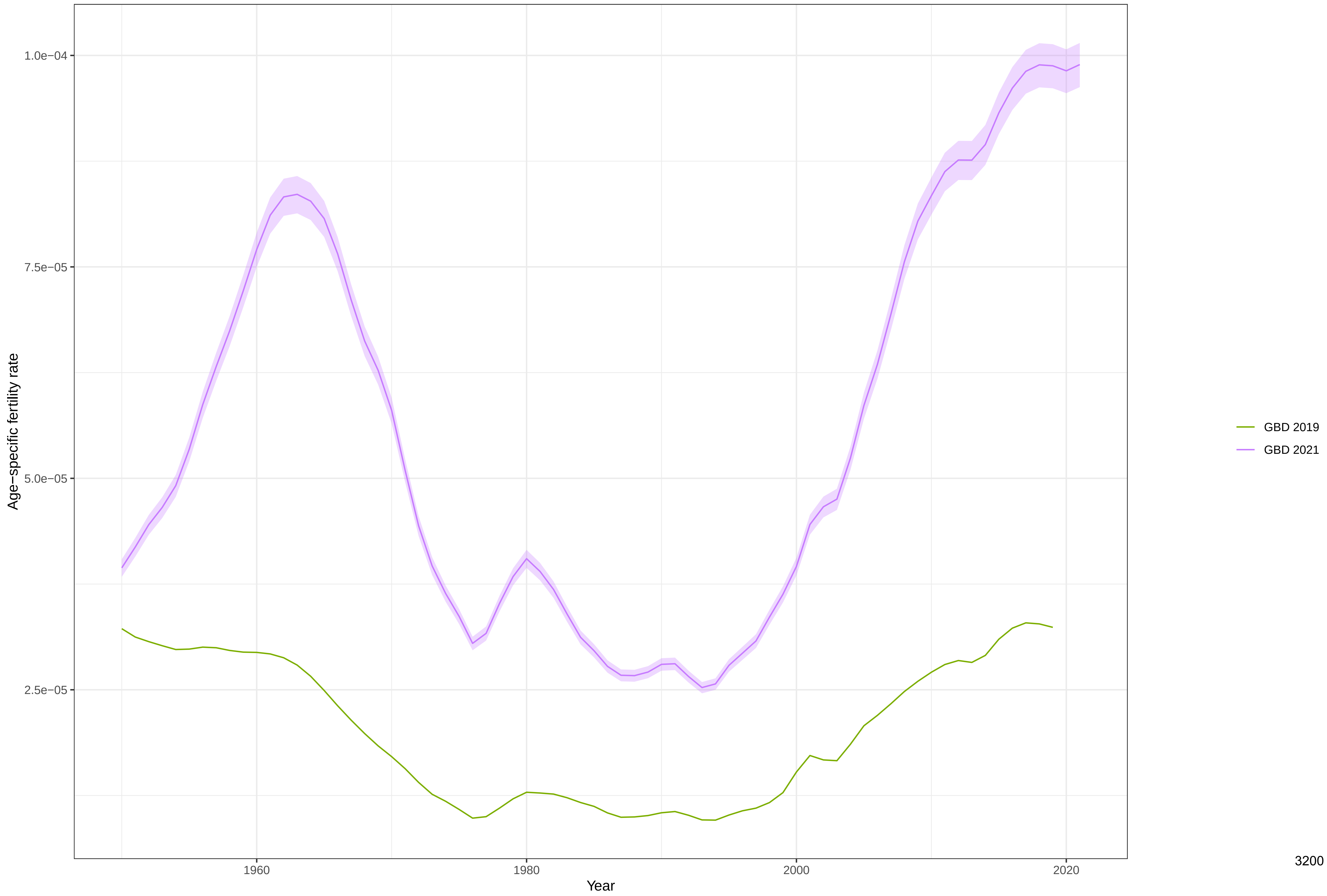

Total fertility rate fitted trend lines, 1950−2021: Plymouth TFR
Data points enclosed with a diamond are reference sources

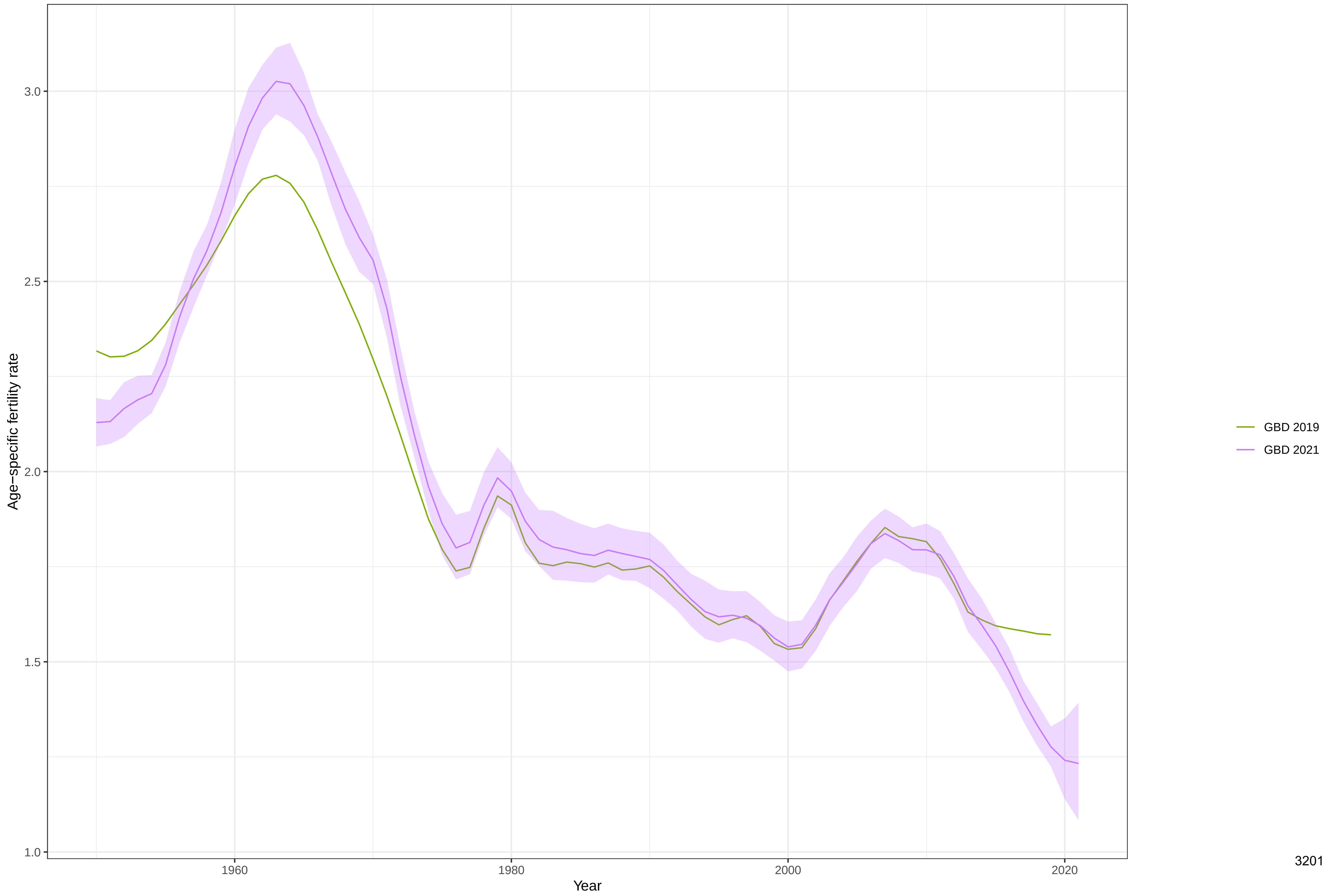

Age−specific fertility fitted trend lines, 1950−2021: Poole, Ages: 10−14
Data points enclosed with a diamond are reference sources

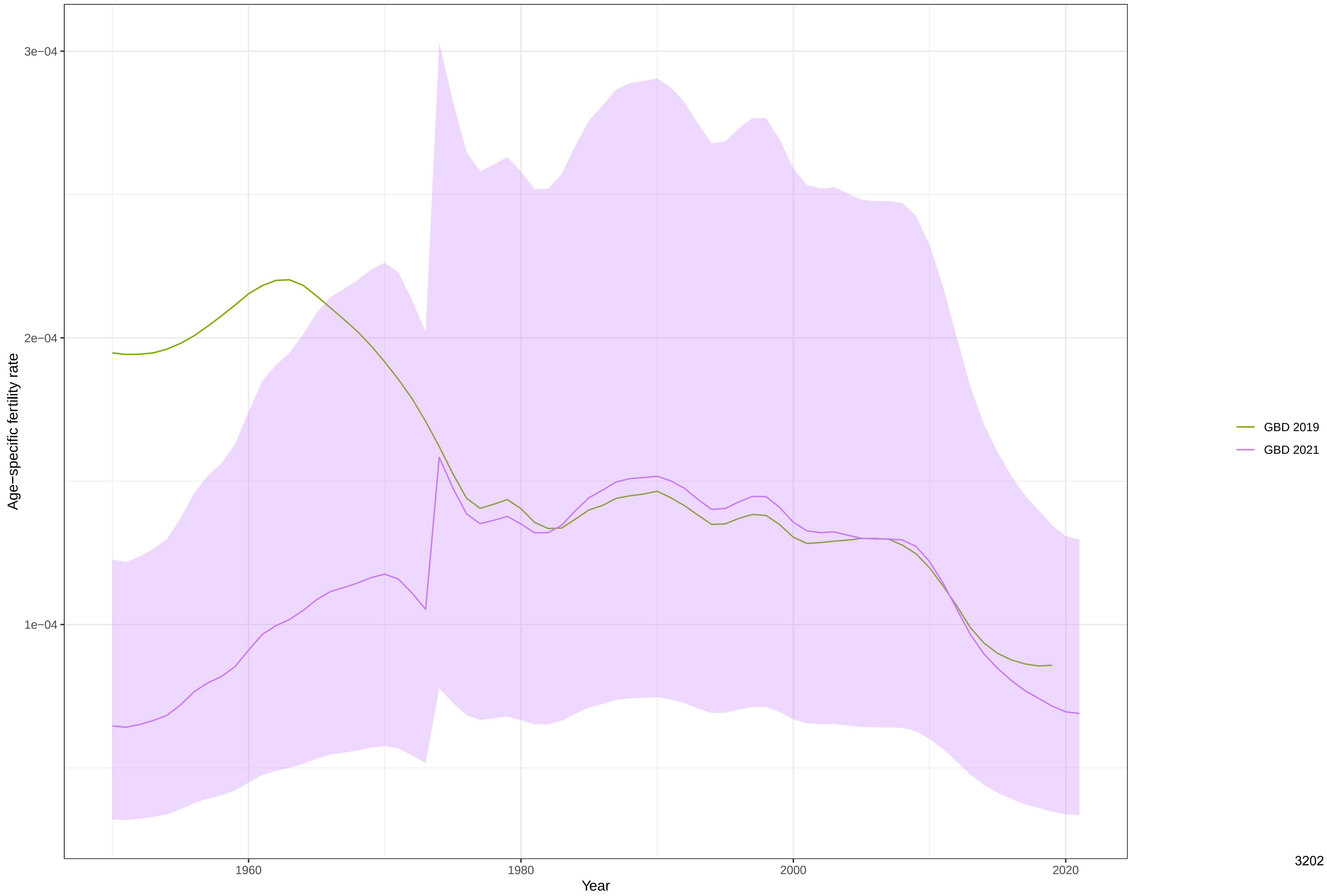

Age−specific fertility fitted trend lines, 1950−2021: Poole, Ages: 15−19
Data points enclosed with a diamond are reference sources

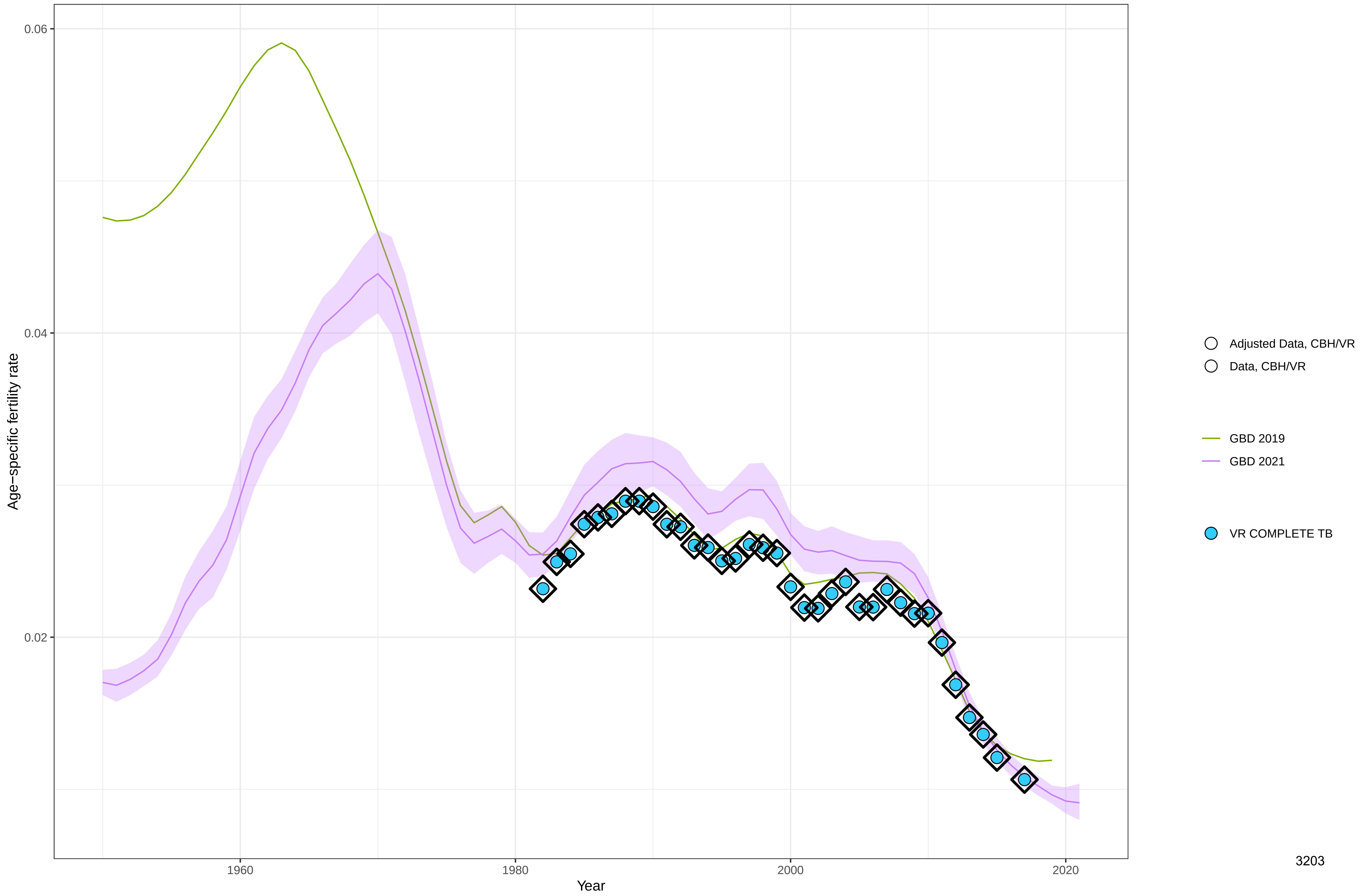

Age−specific fertility fitted trend lines, 1950−2021: Poole, Ages: 20−24
Data points enclosed with a diamond are reference sources

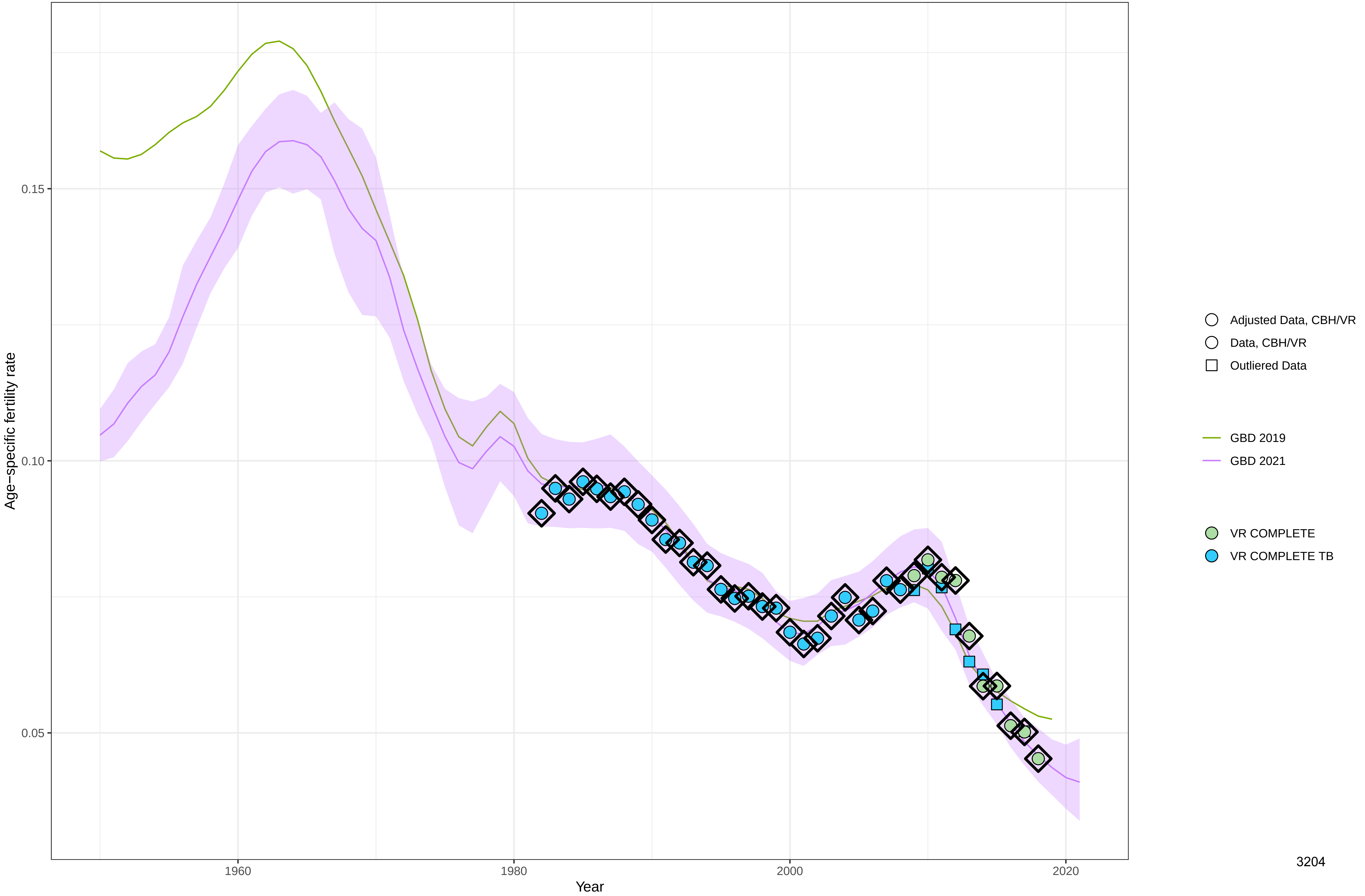

Age−specific fertility fitted trend lines, 1950−2021: Poole, Ages: 25−29
Data points enclosed with a diamond are reference sources

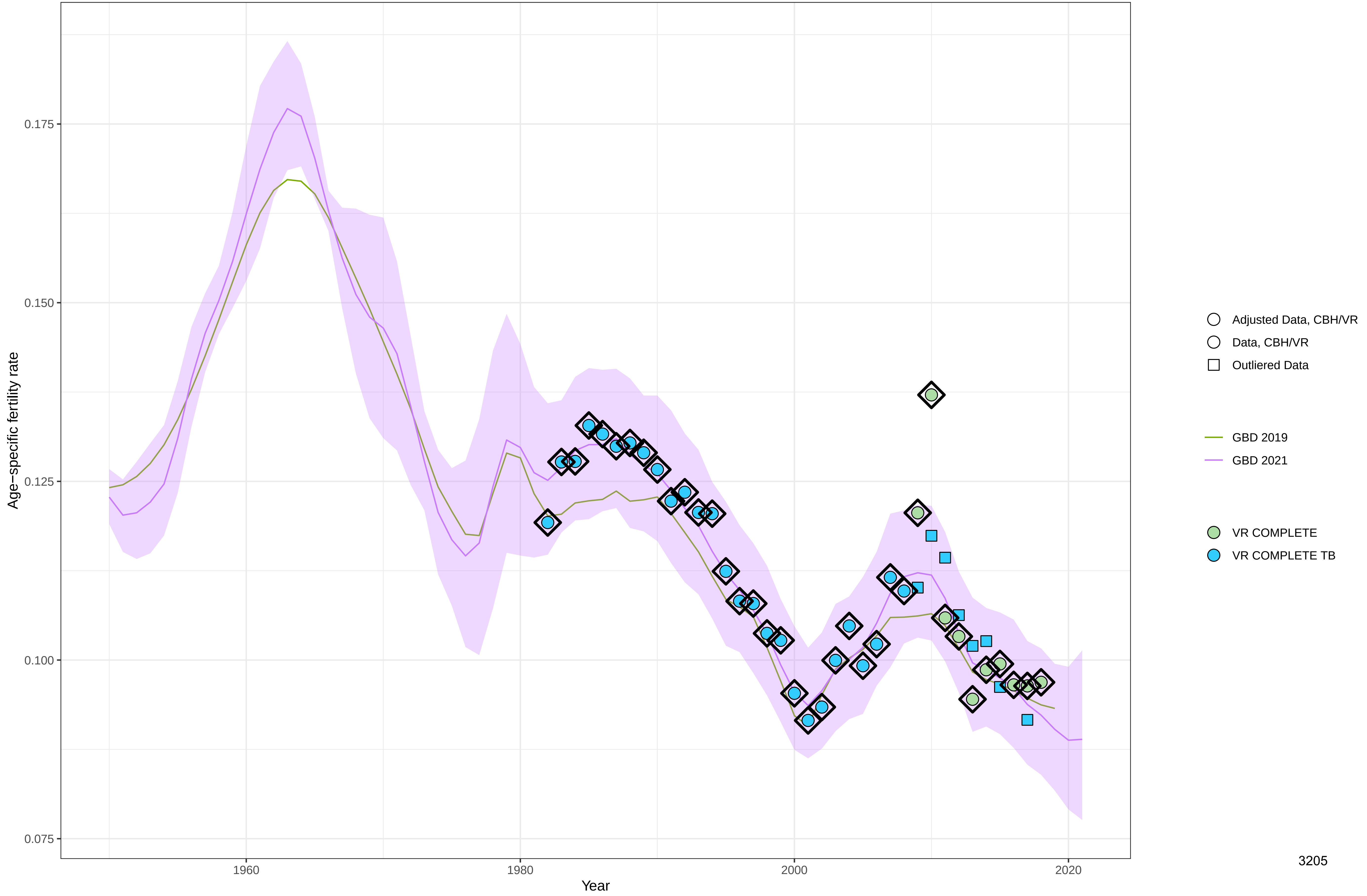

Age−specific fertility fitted trend lines, 1950−2021: Poole, Ages: 30−34
Data points enclosed with a diamond are reference sources

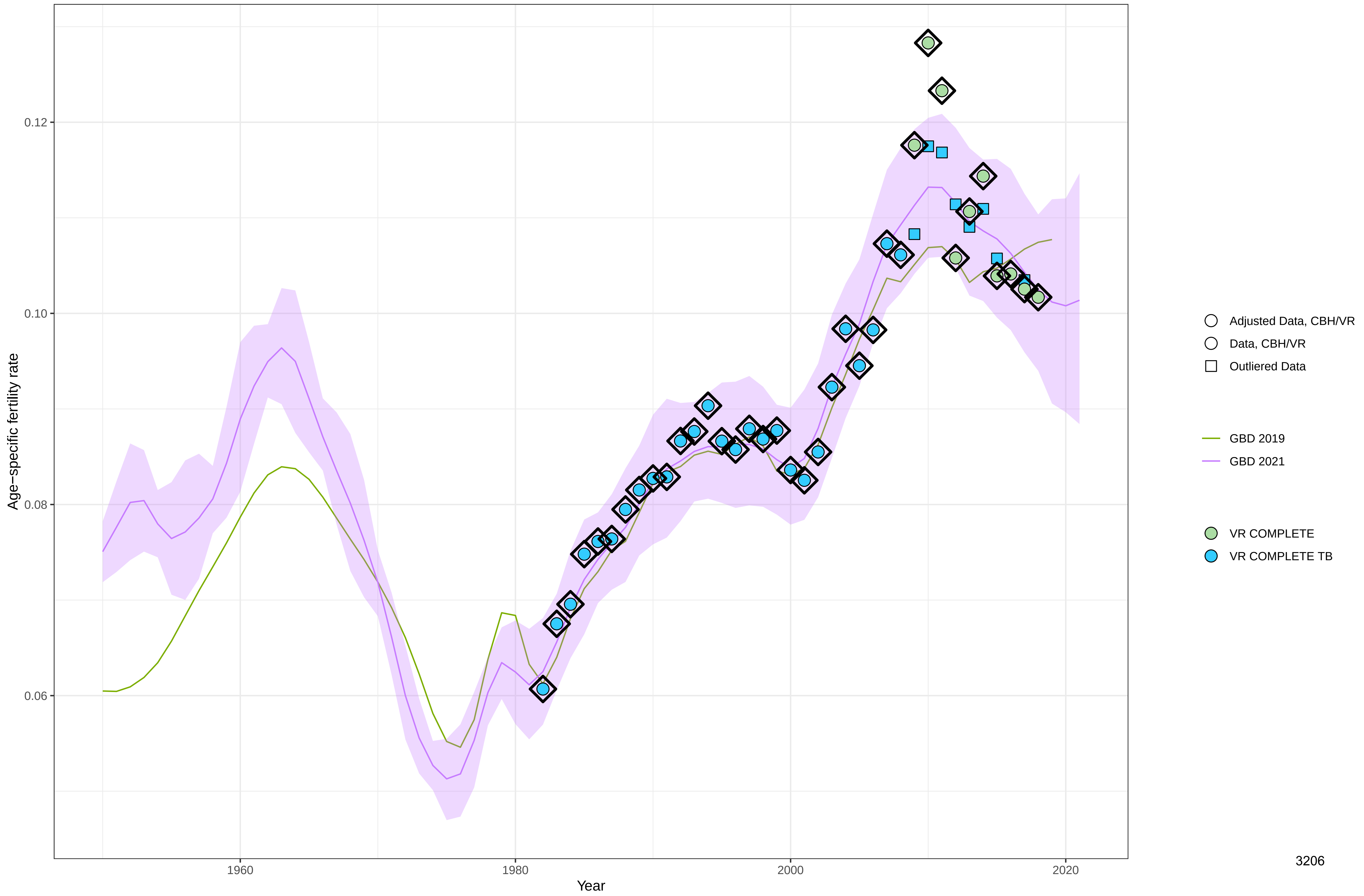

Age−specific fertility fitted trend lines, 1950−2021: Poole, Ages: 35−39
Data points enclosed with a diamond are reference sources

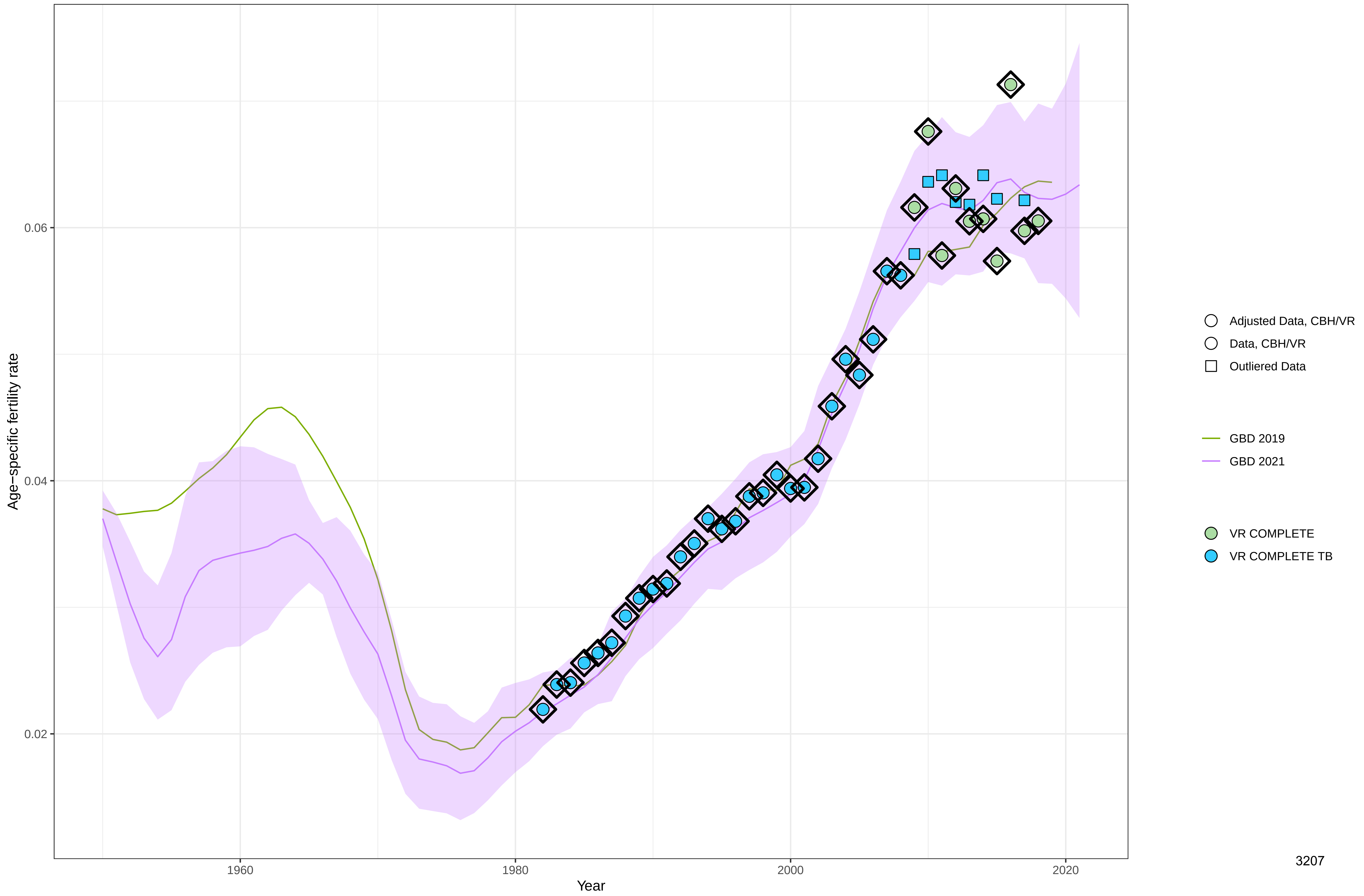

Age−specific fertility fitted trend lines, 1950−2021: Poole, Ages: 40−44
Data points enclosed with a diamond are reference sources

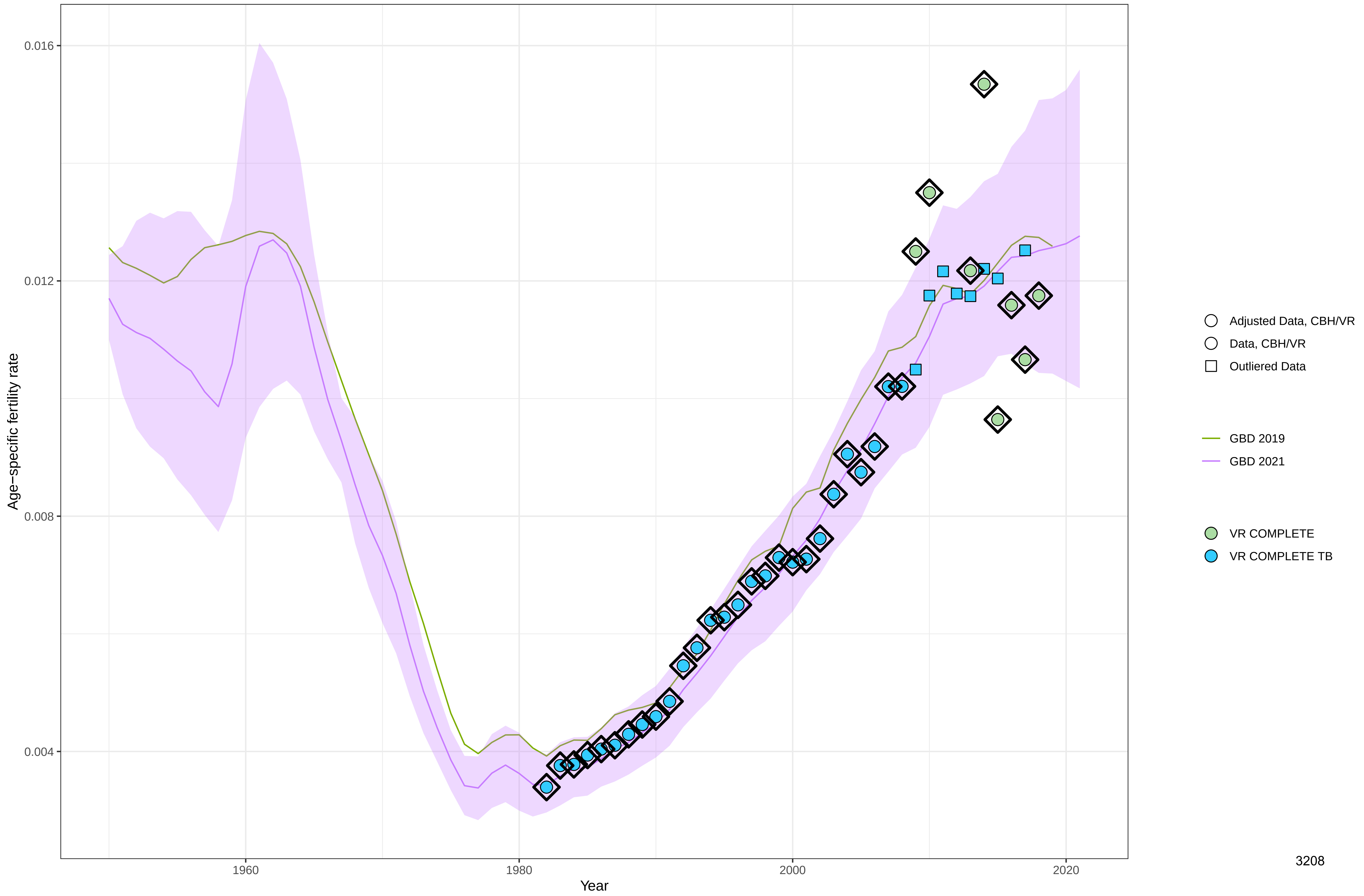

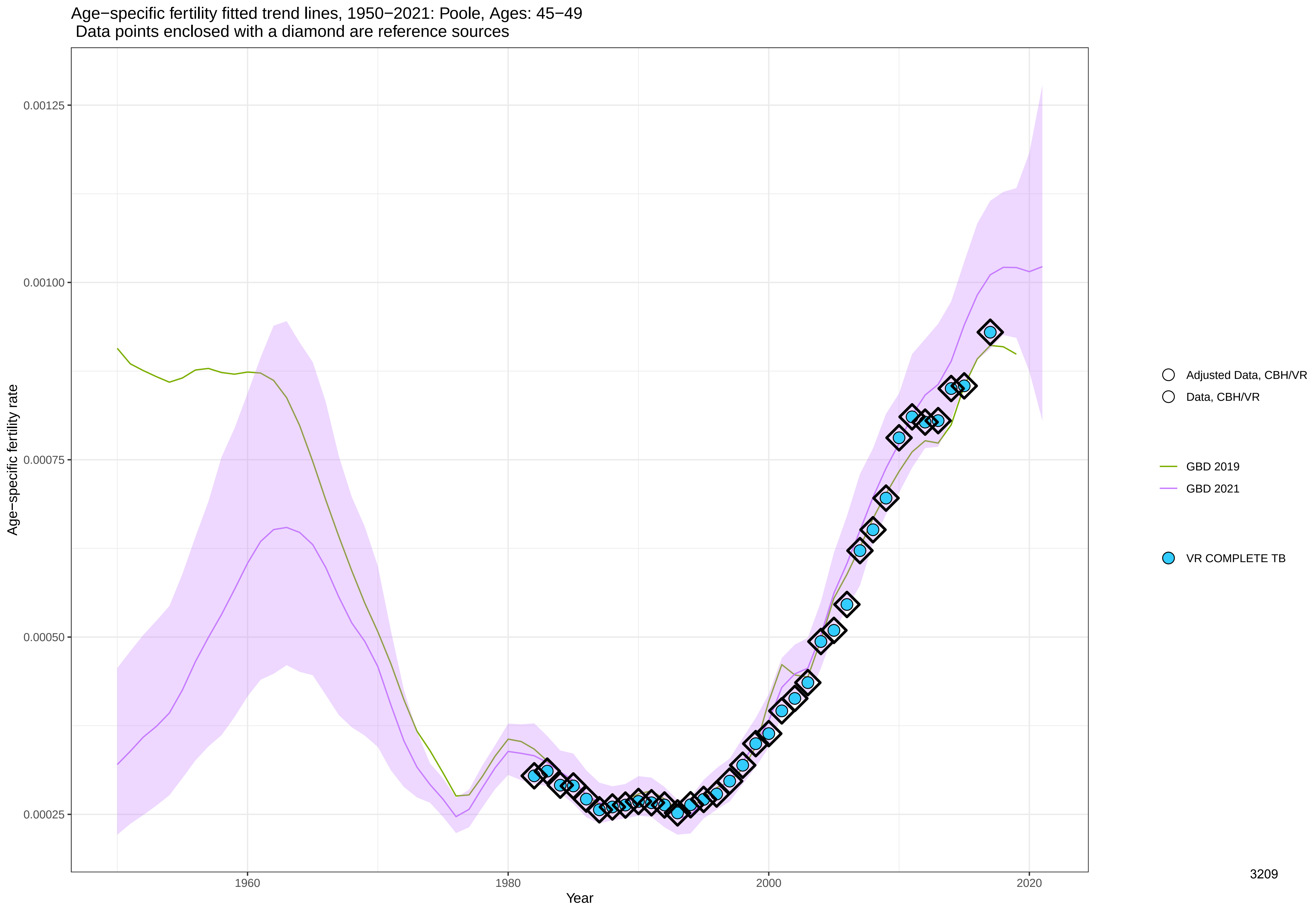

Age−specific fertility fitted trend lines, 1950−2021: Poole, Ages: 45−49
Data points enclosed with a diamond are reference sources
Age−specific fertility rate
0.00125
0.00100
0.00075
0.00050
0.00025
1960
1980
2000
2020
Year
Adjusted Data, CBH/VR
Data, CBH/VR
GBD 2019
GBD 2021
VR COMPLETE TB

Age−specific fertility fitted trend lines, 1950−2021: Poole, Ages: 50−54
Data points enclosed with a diamond are reference sources

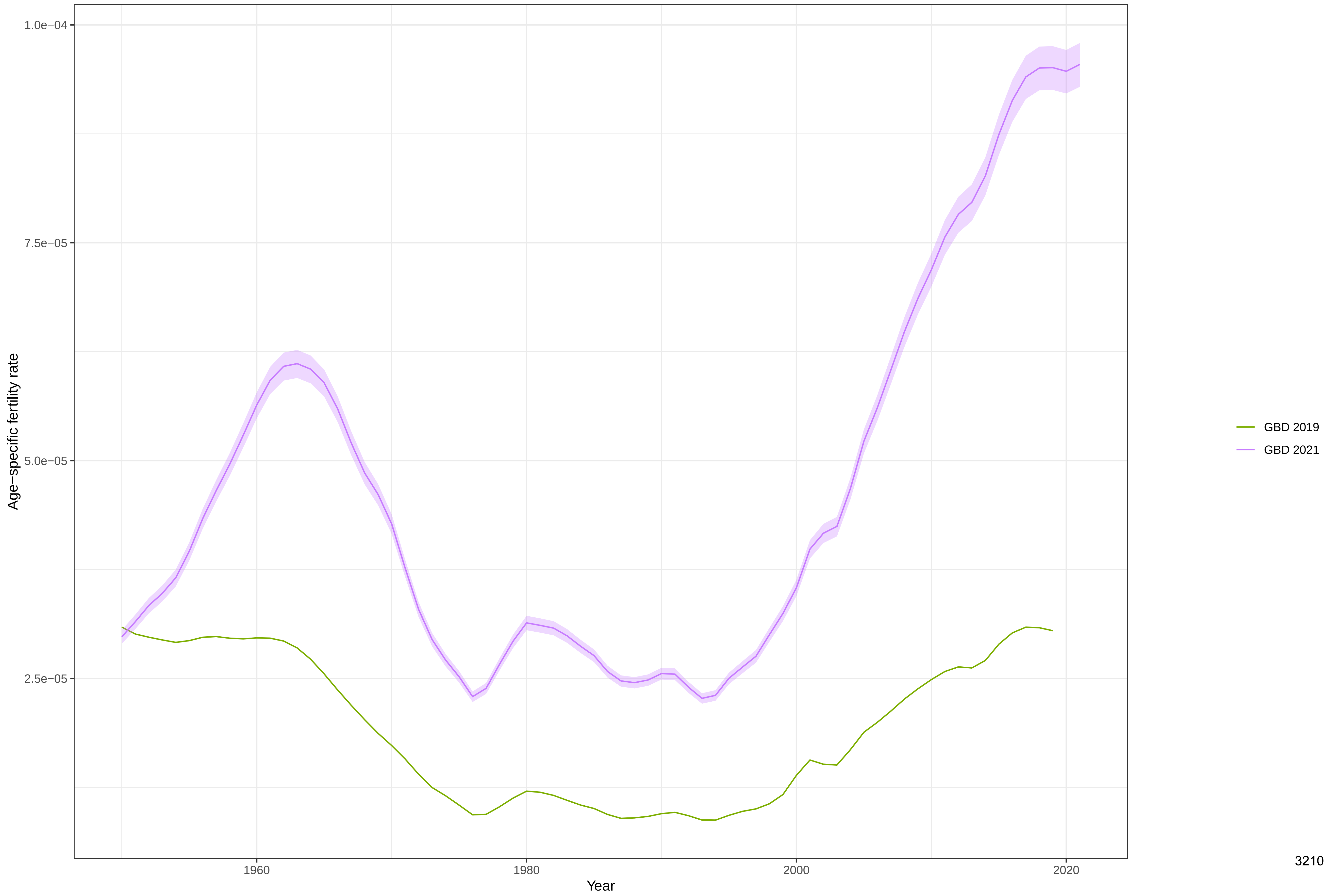

Total fertility rate fitted trend lines, 1950−2021: Poole TFR
Data points enclosed with a diamond are reference sources

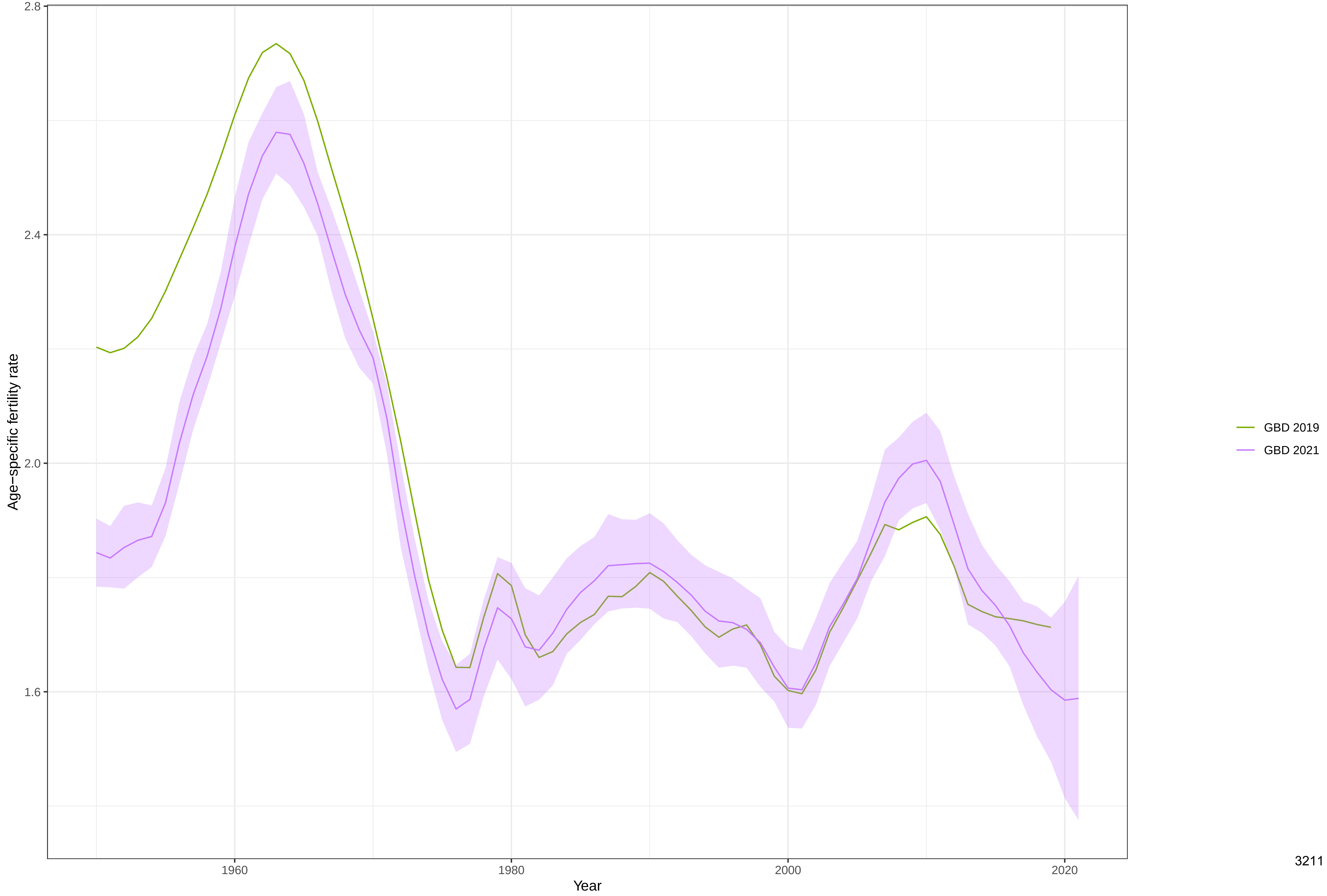

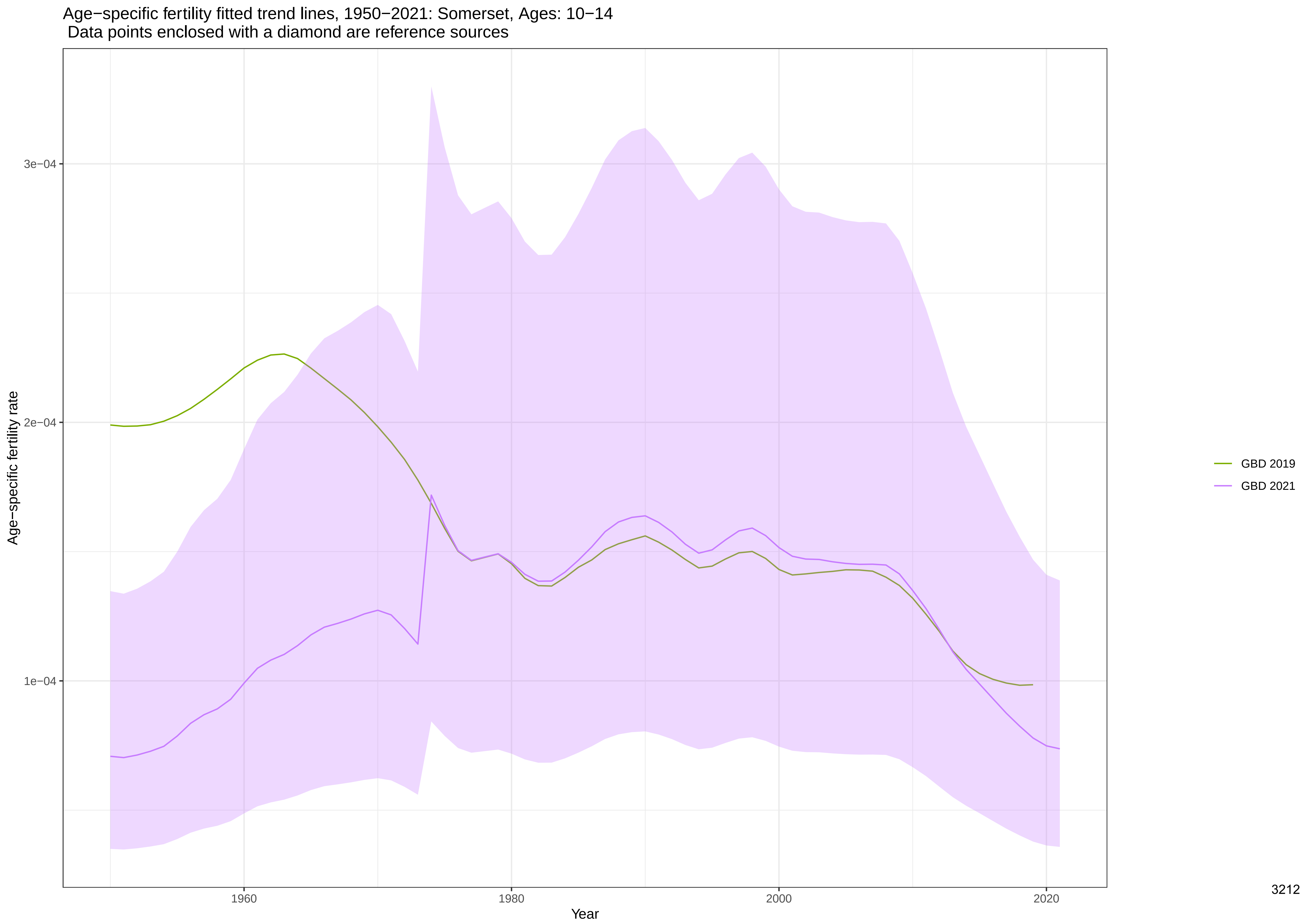

Age−specific fertility fitted trend lines, 1950−2021: Somerset, Ages: 10−14
Data points enclosed with a diamond are reference sources
Age−specific fertility rate
3e−04
2e−04
1e−04
1960
1980
2000
2020
Year
GBD 2019
GBD 2021

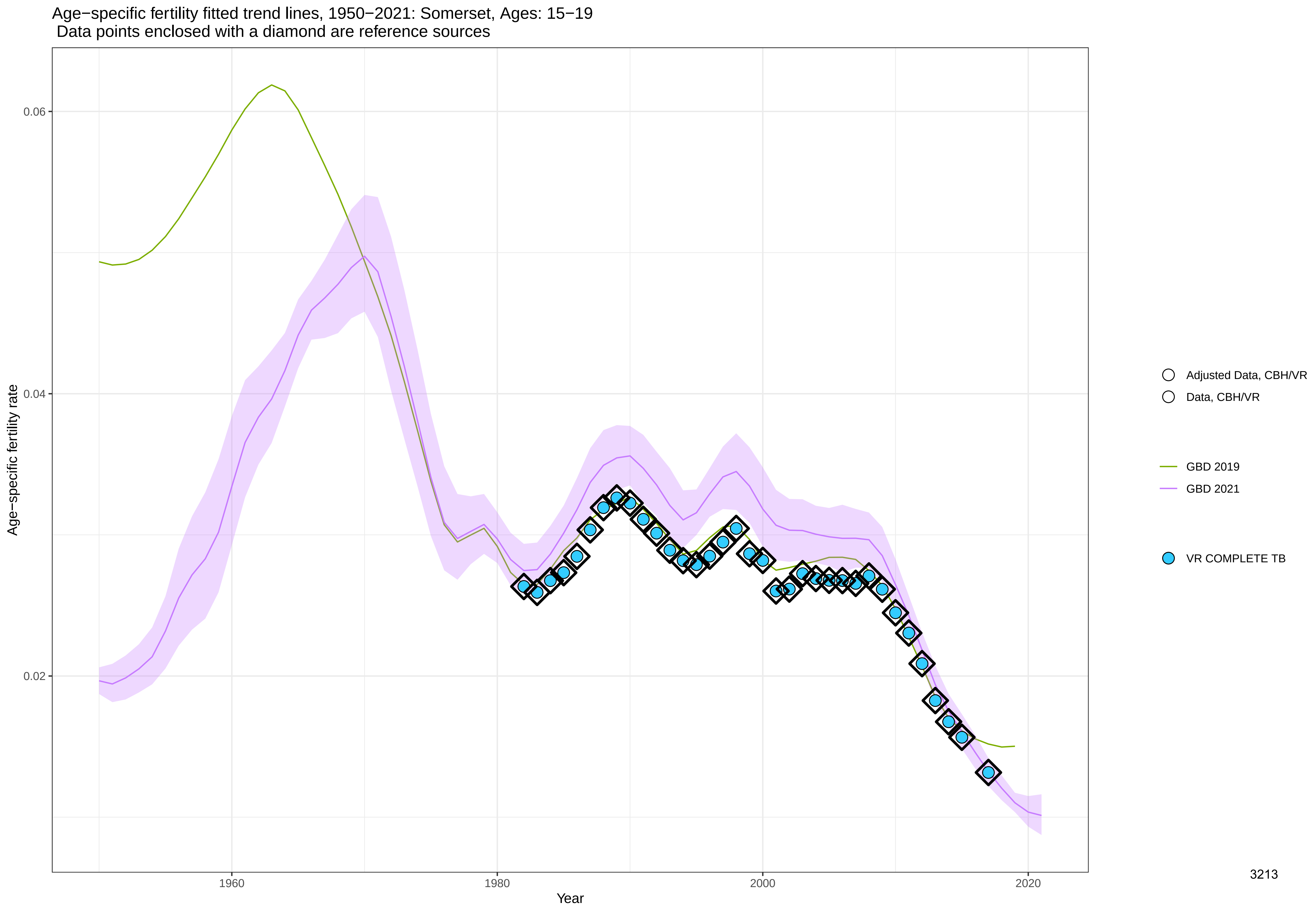

Age−specific fertility fitted trend lines, 1950−2021: Somerset, Ages: 15−19
Data points enclosed with a diamond are reference sources
Age−specific fertility rate
0.06
0.04
0.02
1960
1980
2000
2020
Year
Adjusted Data, CBH/VR
Data, CBH/VR
GBD 2019
GBD 2021
VR COMPLETE TB

3213

Age−specific fertility fitted trend lines, 1950−2021: Somerset, Ages: 20−24
Data points enclosed with a diamond are reference sources

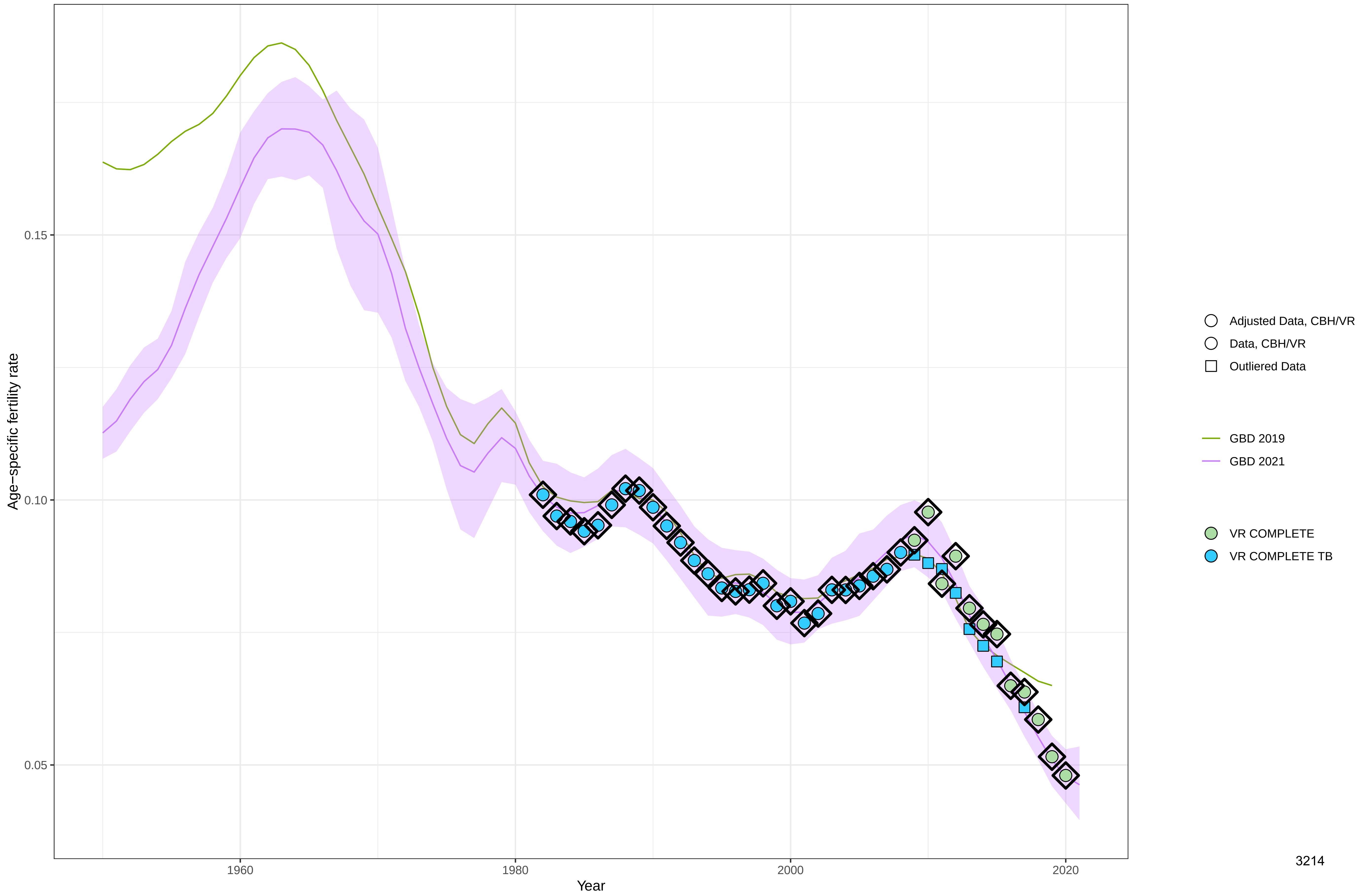

Age−specific fertility fitted trend lines, 1950−2021: Somerset, Ages: 25−29
Data points enclosed with a diamond are reference sources

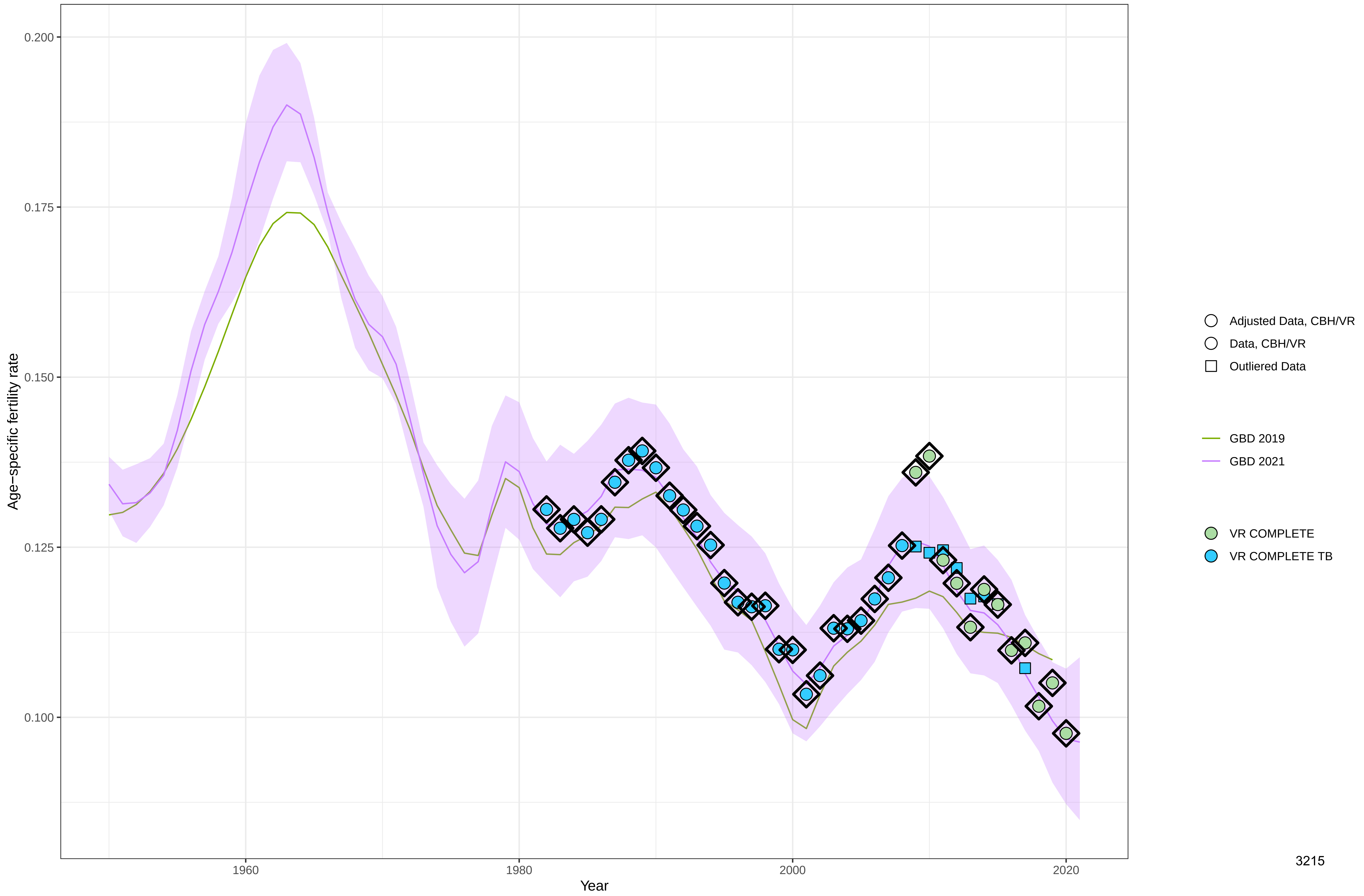

Age−specific fertility fitted trend lines, 1950−2021: Somerset, Ages: 30−34
Data points enclosed with a diamond are reference sources

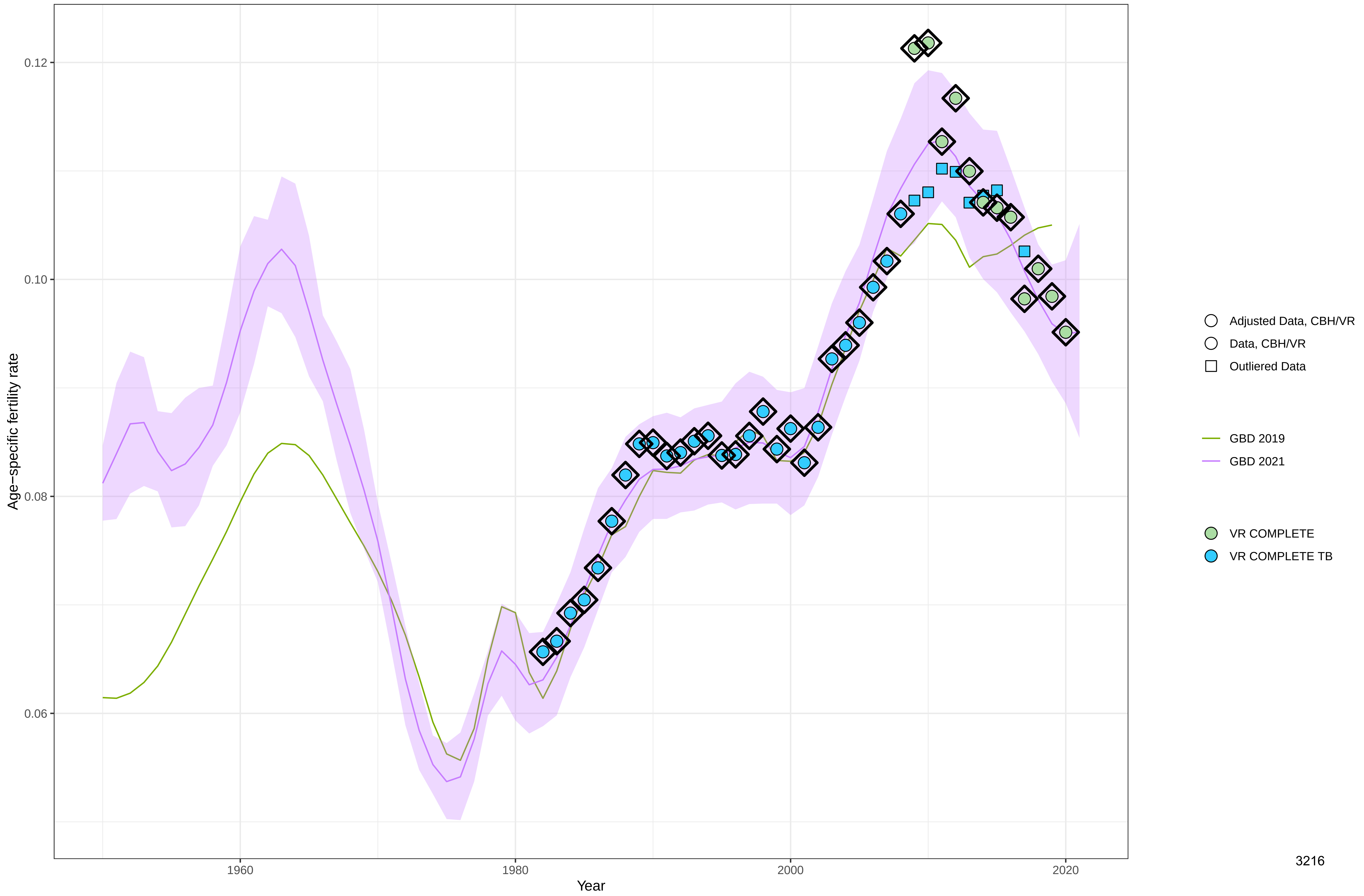

Age−specific fertility fitted trend lines, 1950−2021: Somerset, Ages: 35−39
Data points enclosed with a diamond are reference sources

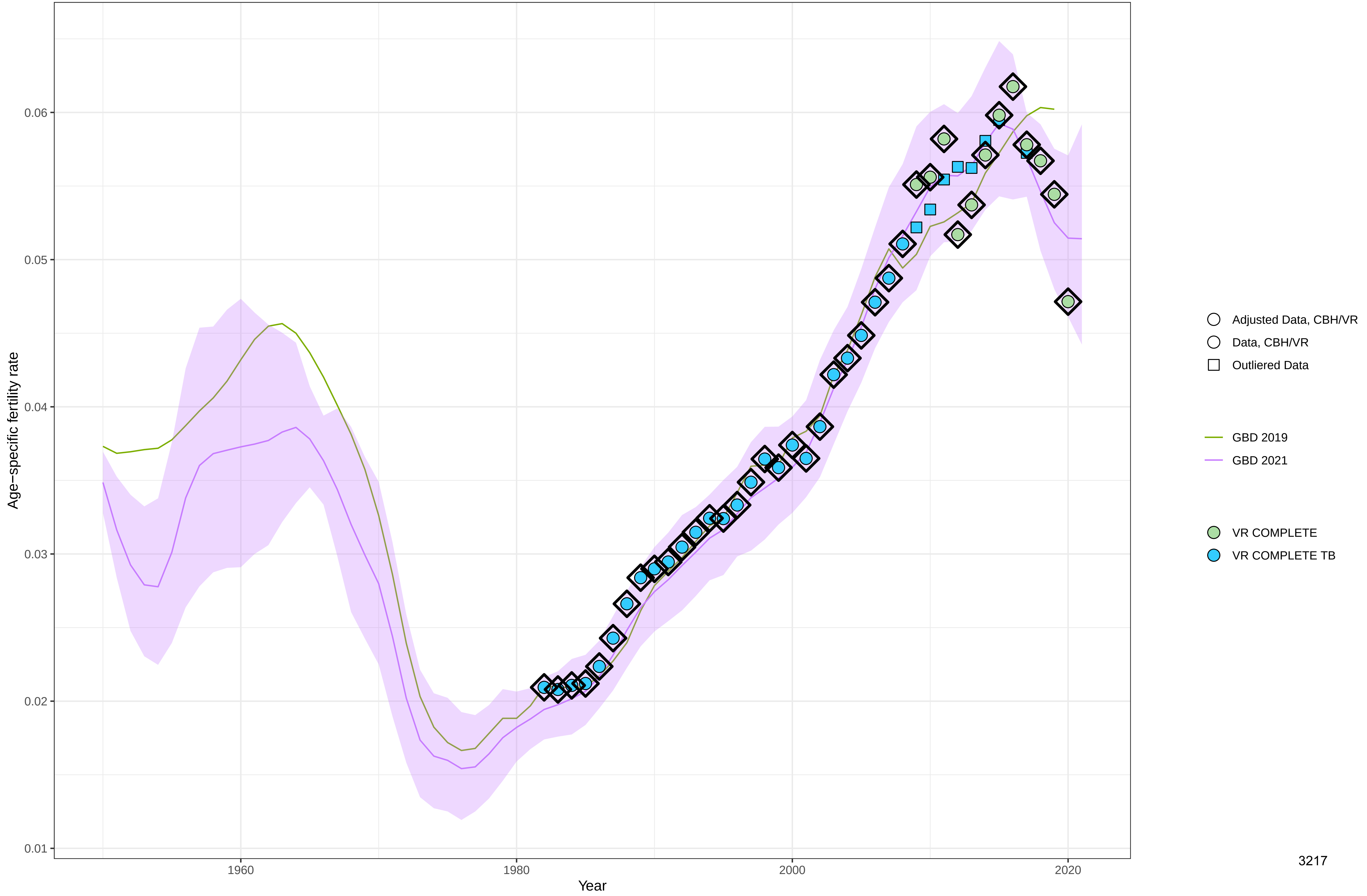

Age−specific fertility fitted trend lines, 1950−2021: Somerset, Ages: 40−44
Data points enclosed with a diamond are reference sources

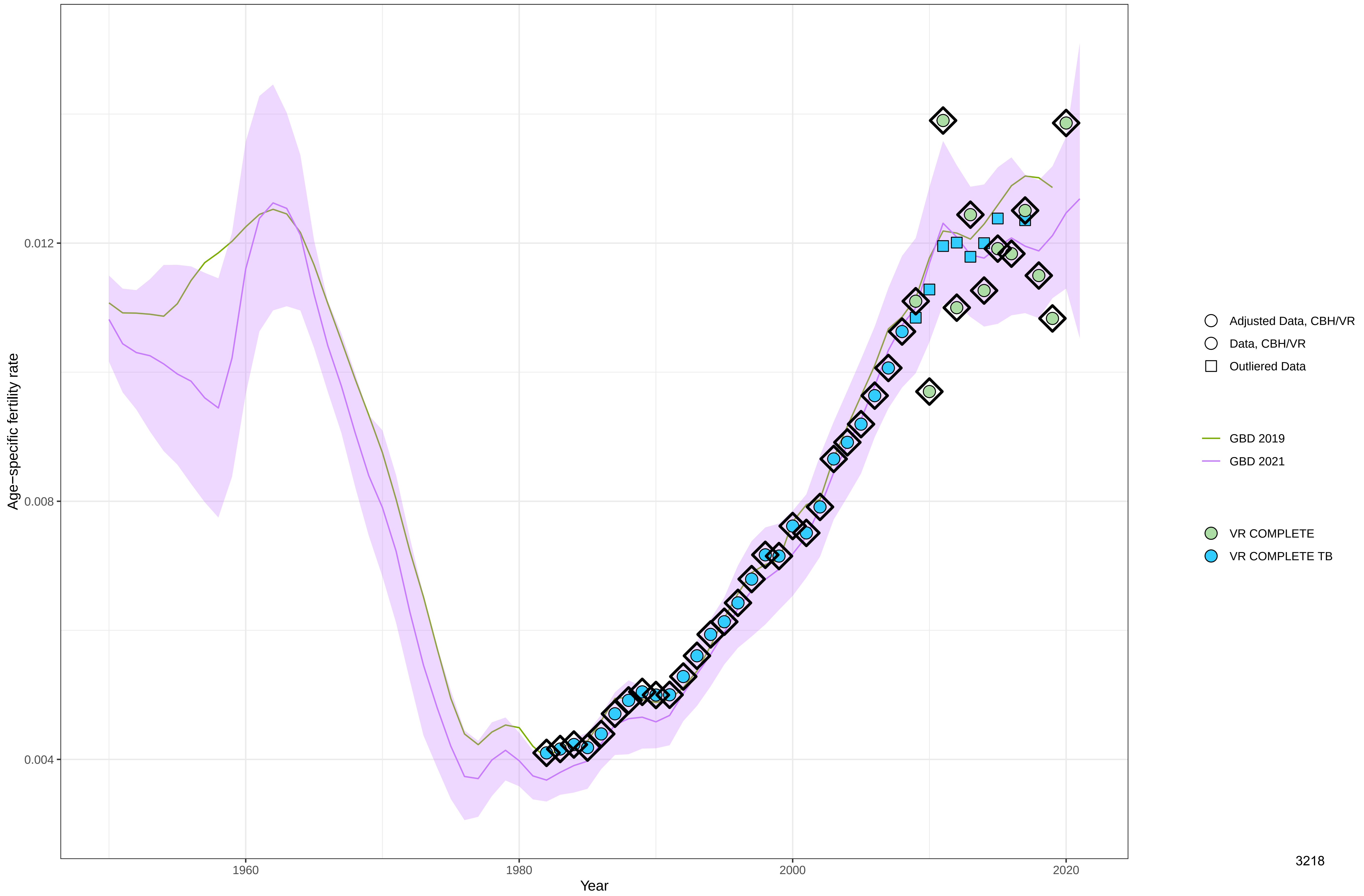

Age−specific fertility fitted trend lines, 1950−2021: Somerset, Ages: 45−49
Data points enclosed with a diamond are reference sources

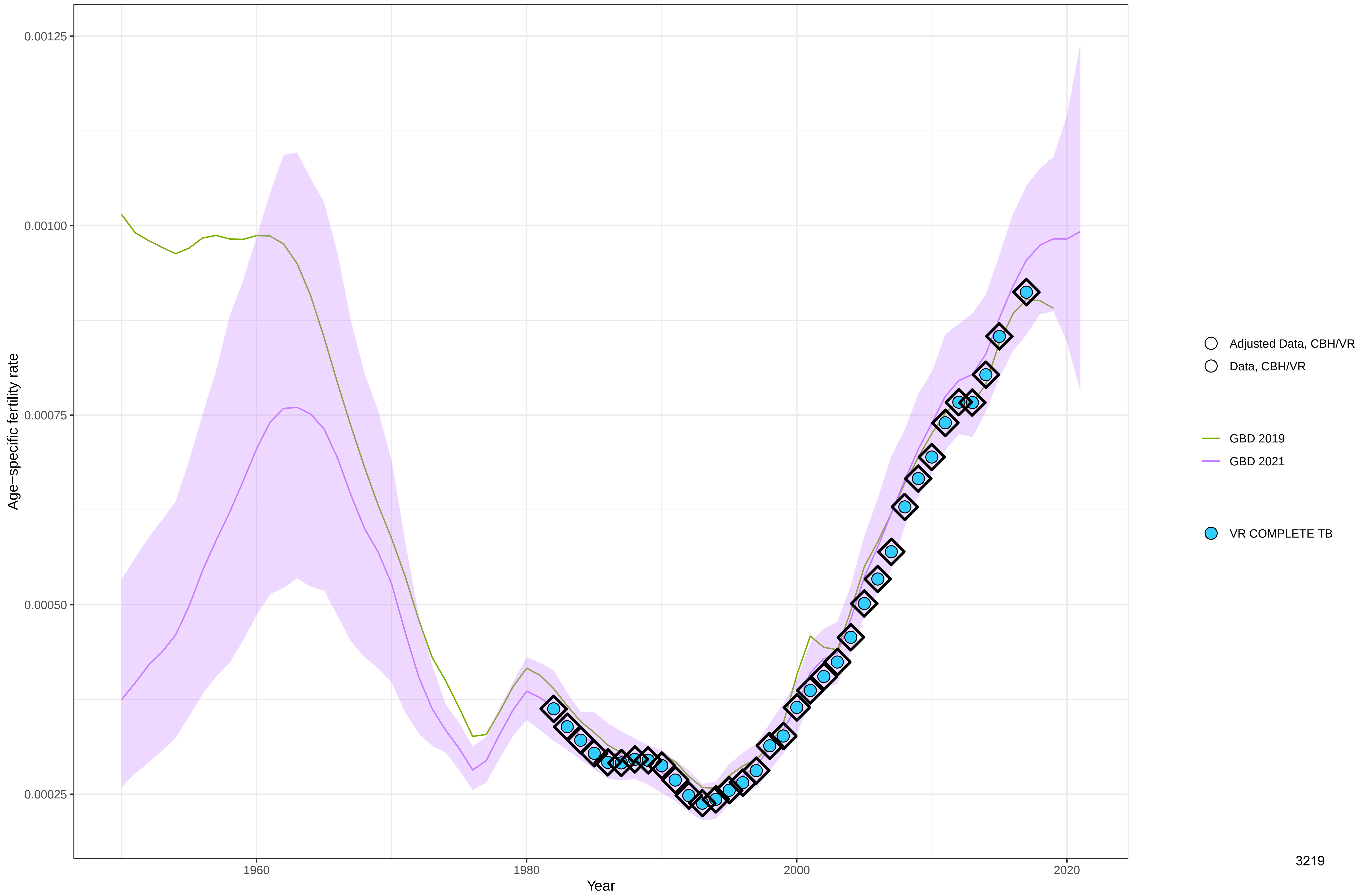

Age−specific fertility fitted trend lines, 1950−2021: Somerset, Ages: 50−54
Data points enclosed with a diamond are reference sources

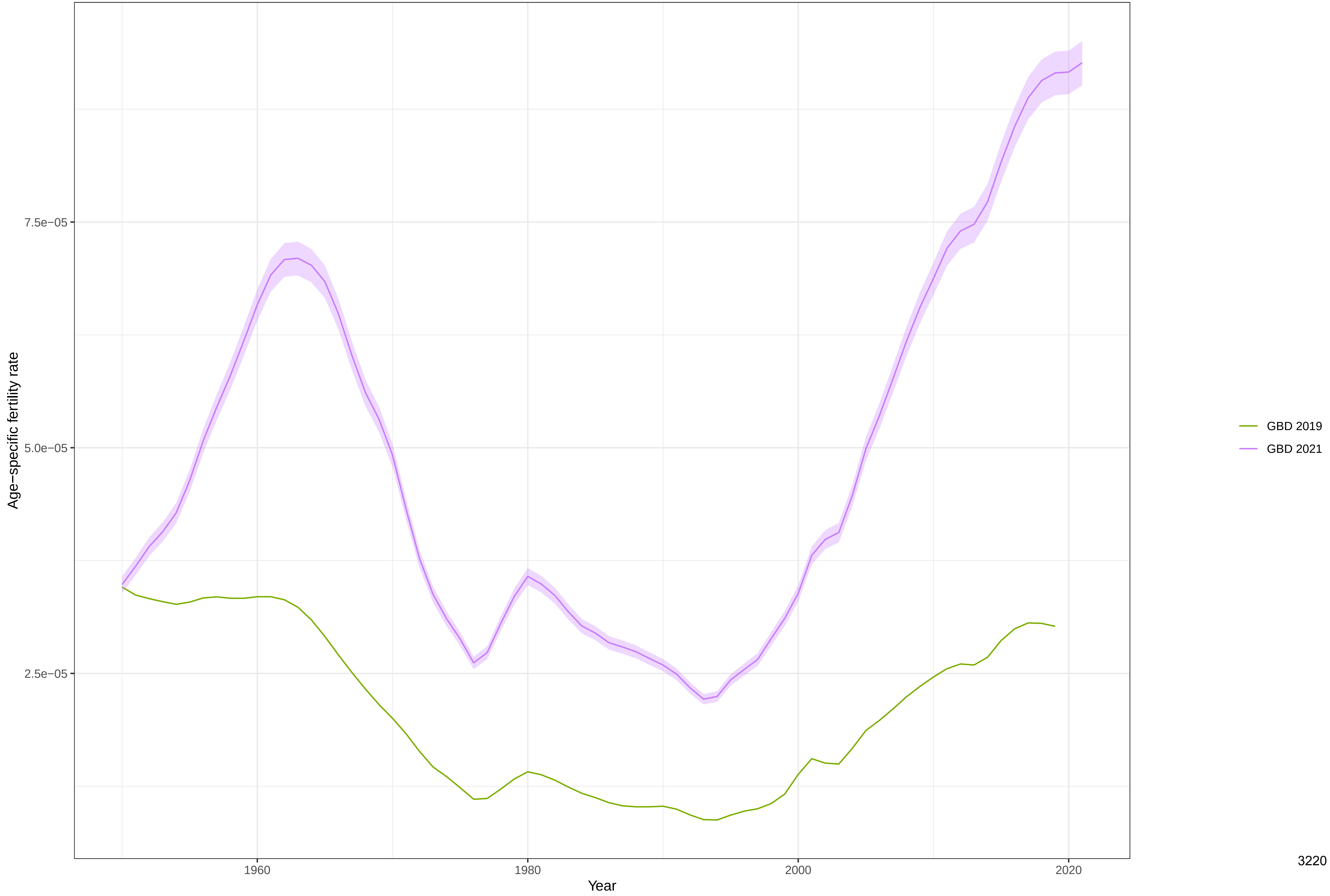

Total fertility rate fitted trend lines, 1950−2021: Somerset TFR
Data points enclosed with a diamond are reference sources

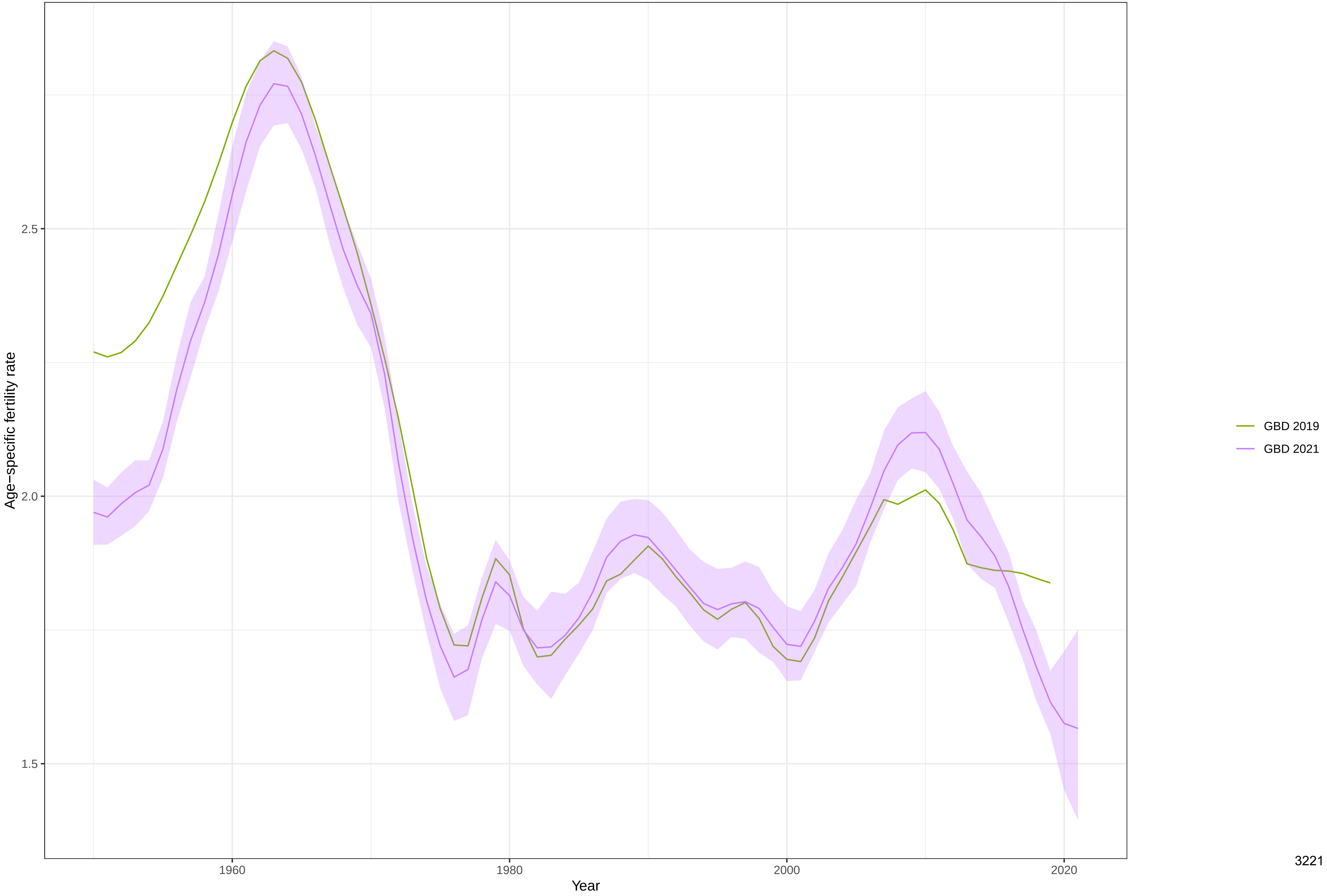

Age−specific fertility fitted trend lines, 1950−2021: South Gloucestershire, Ages: 10−14
Data points enclosed with a diamond are reference sources

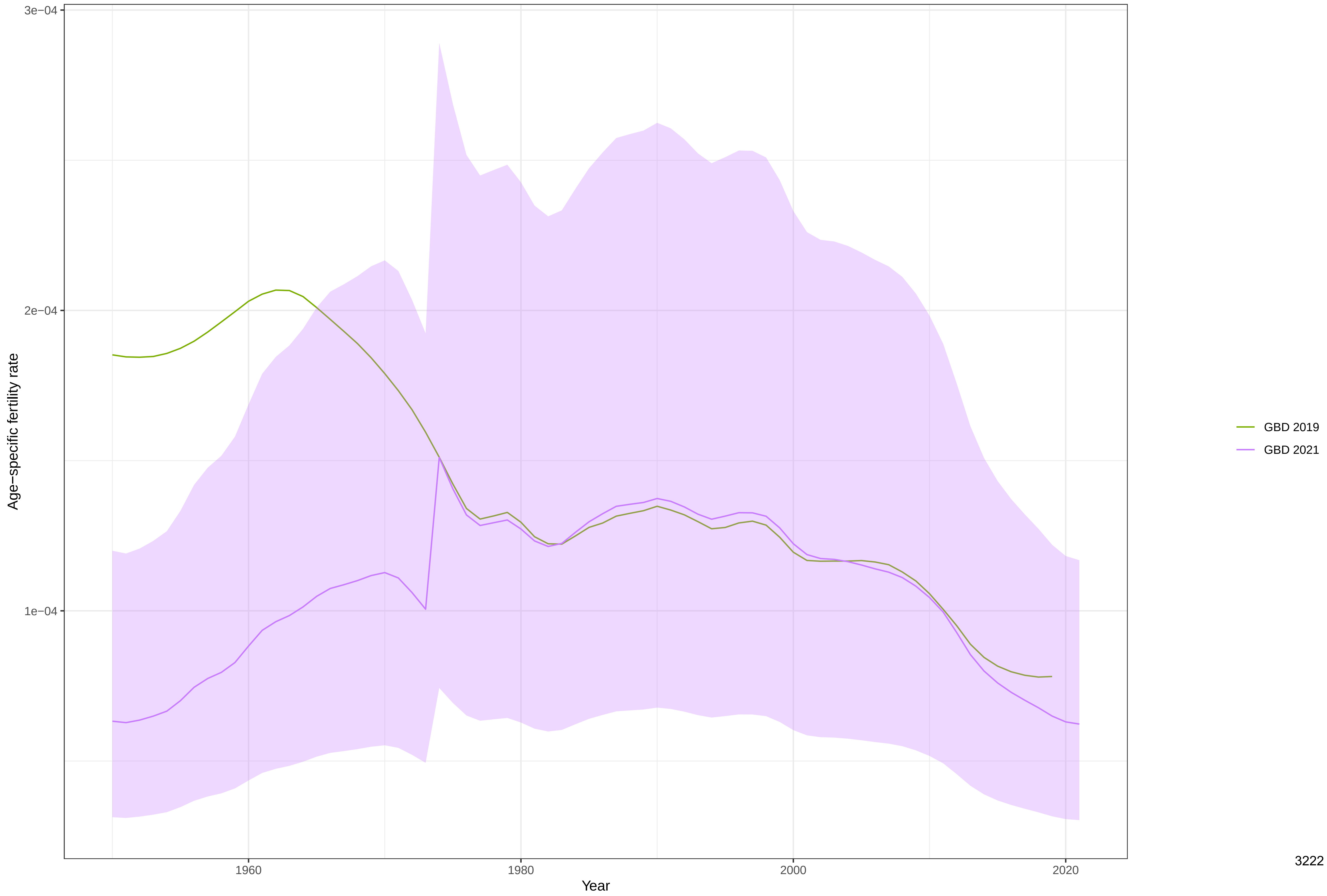

3222

Age−specific fertility fitted trend lines, 1950−2021: South Gloucestershire, Ages: 15−19
Data points enclosed with a diamond are reference sources

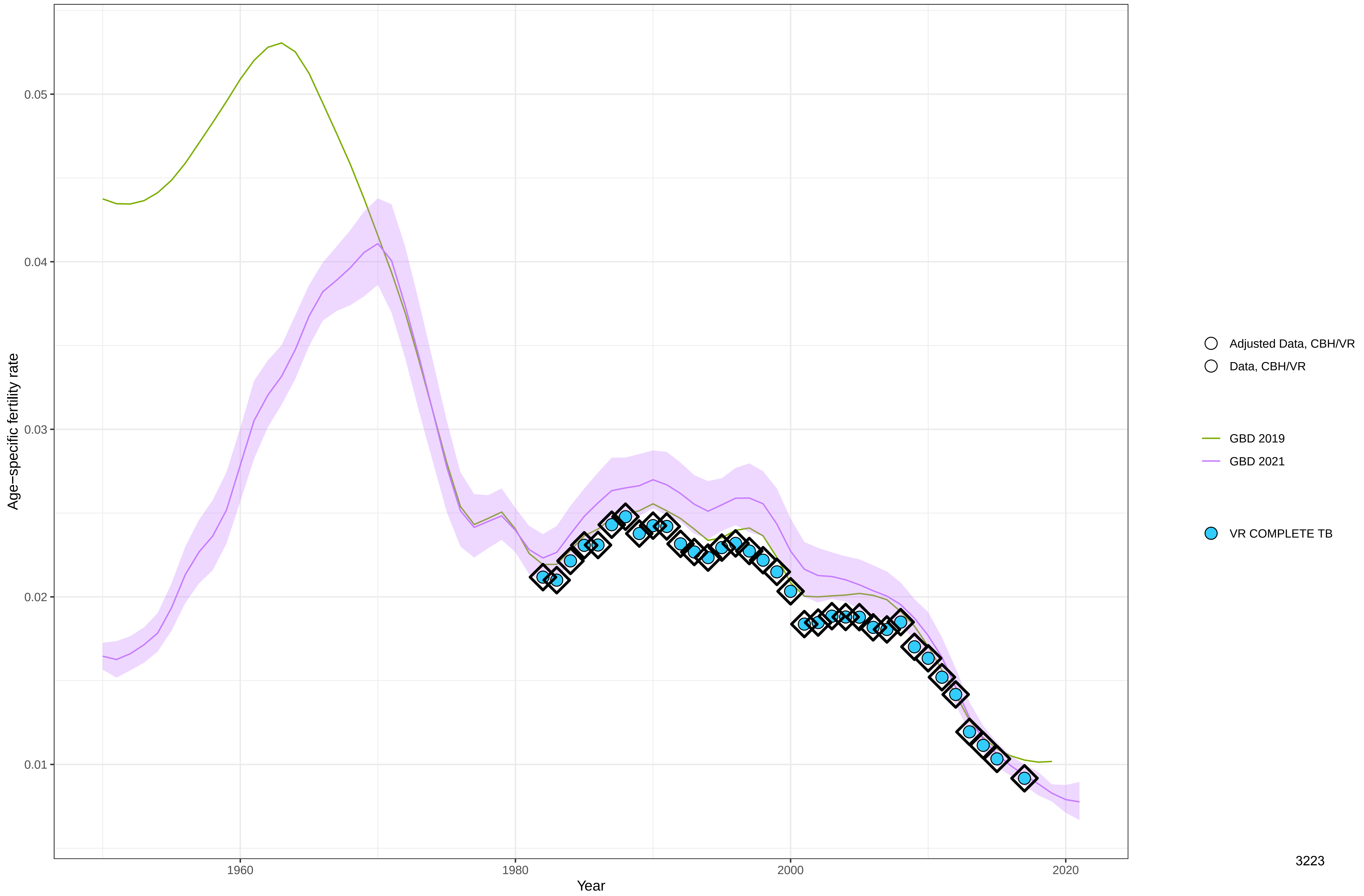

Age−specific fertility fitted trend lines, 1950−2021: South Gloucestershire, Ages: 20−24
Data points enclosed with a diamond are reference sources

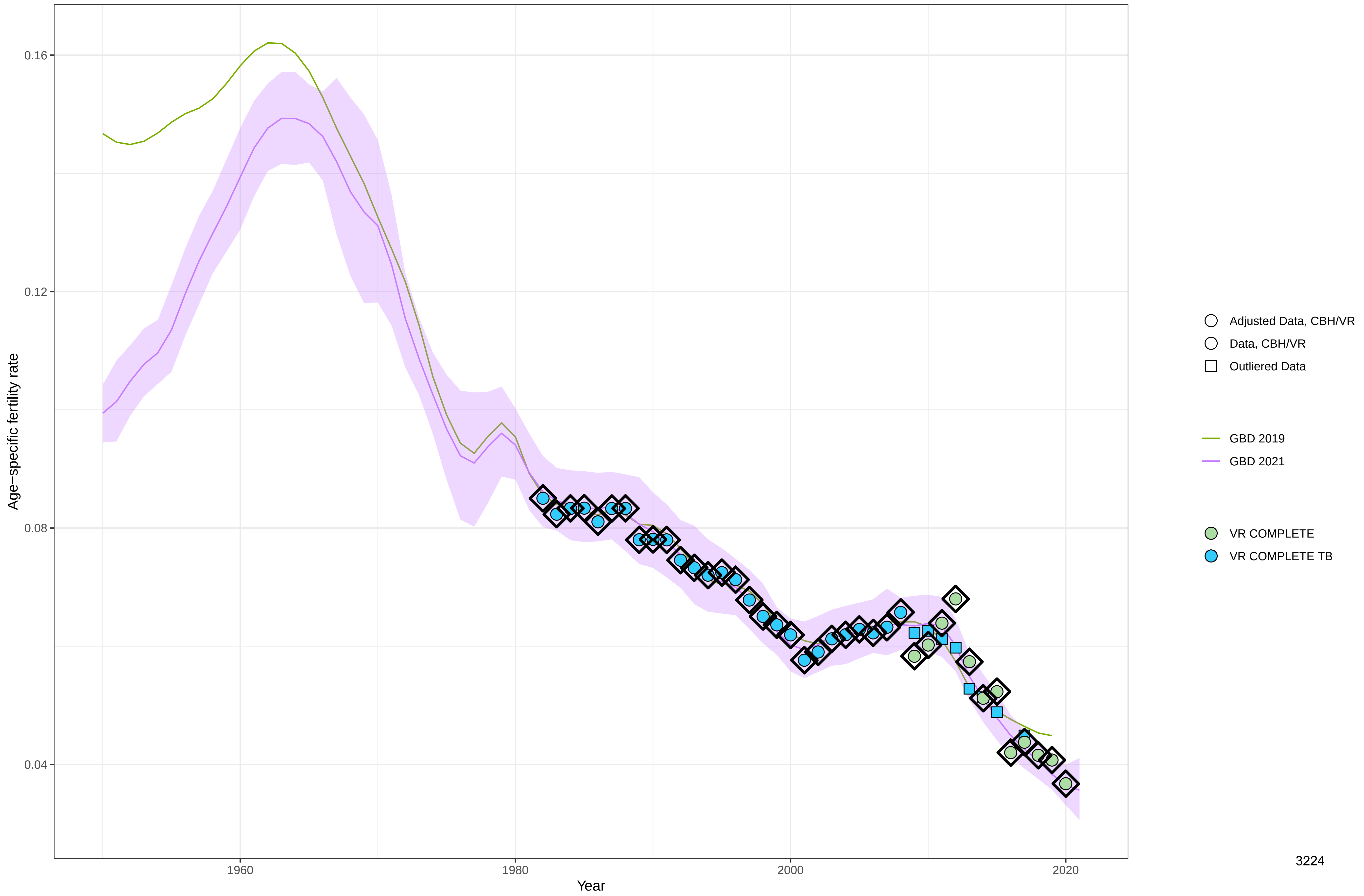

3224

Age−specific fertility fitted trend lines, 1950−2021: South Gloucestershire, Ages: 25−29
Data points enclosed with a diamond are reference sources

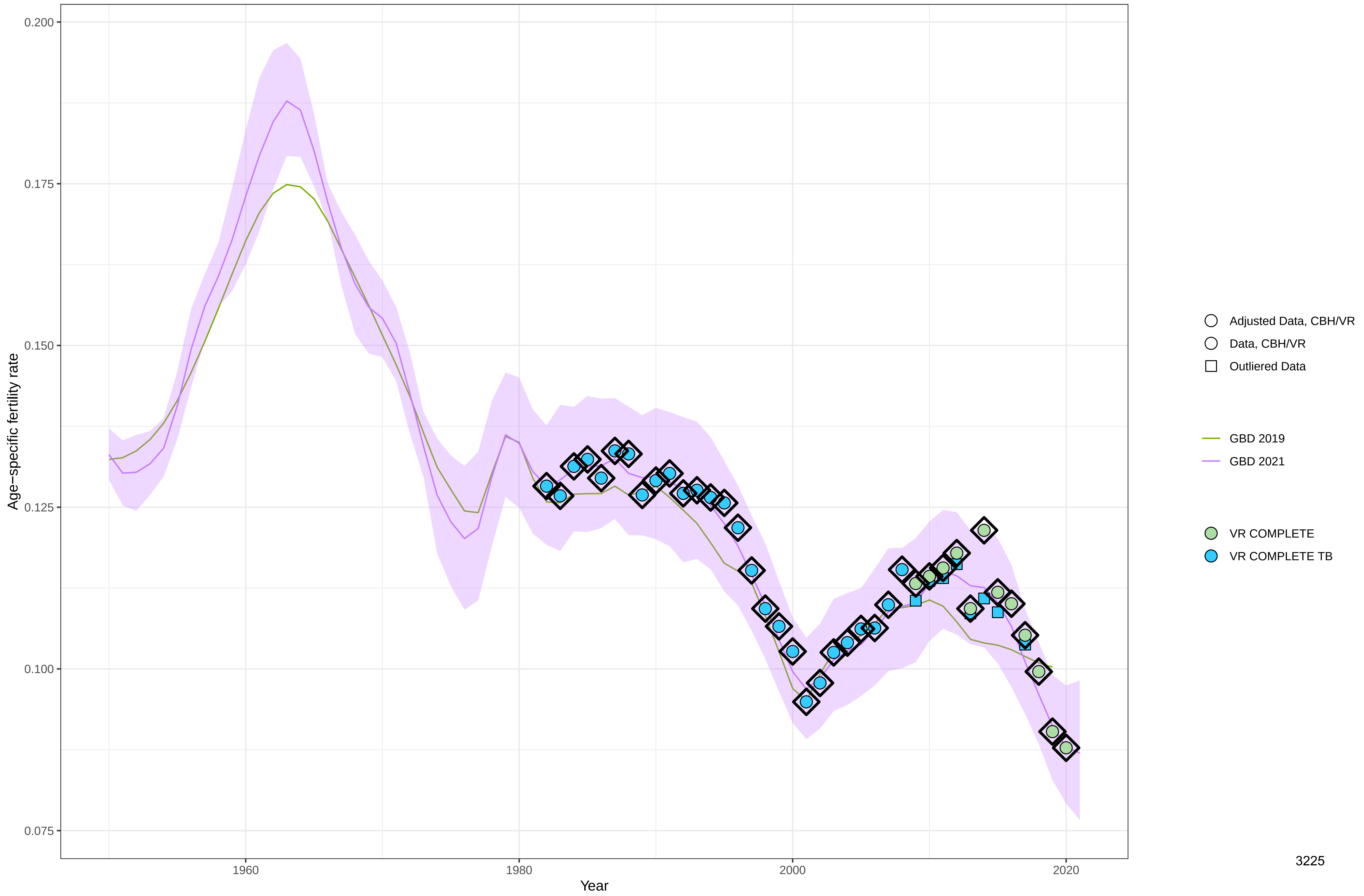

Age−specific fertility fitted trend lines, 1950−2021: South Gloucestershire, Ages: 30−34
Data points enclosed with a diamond are reference sources

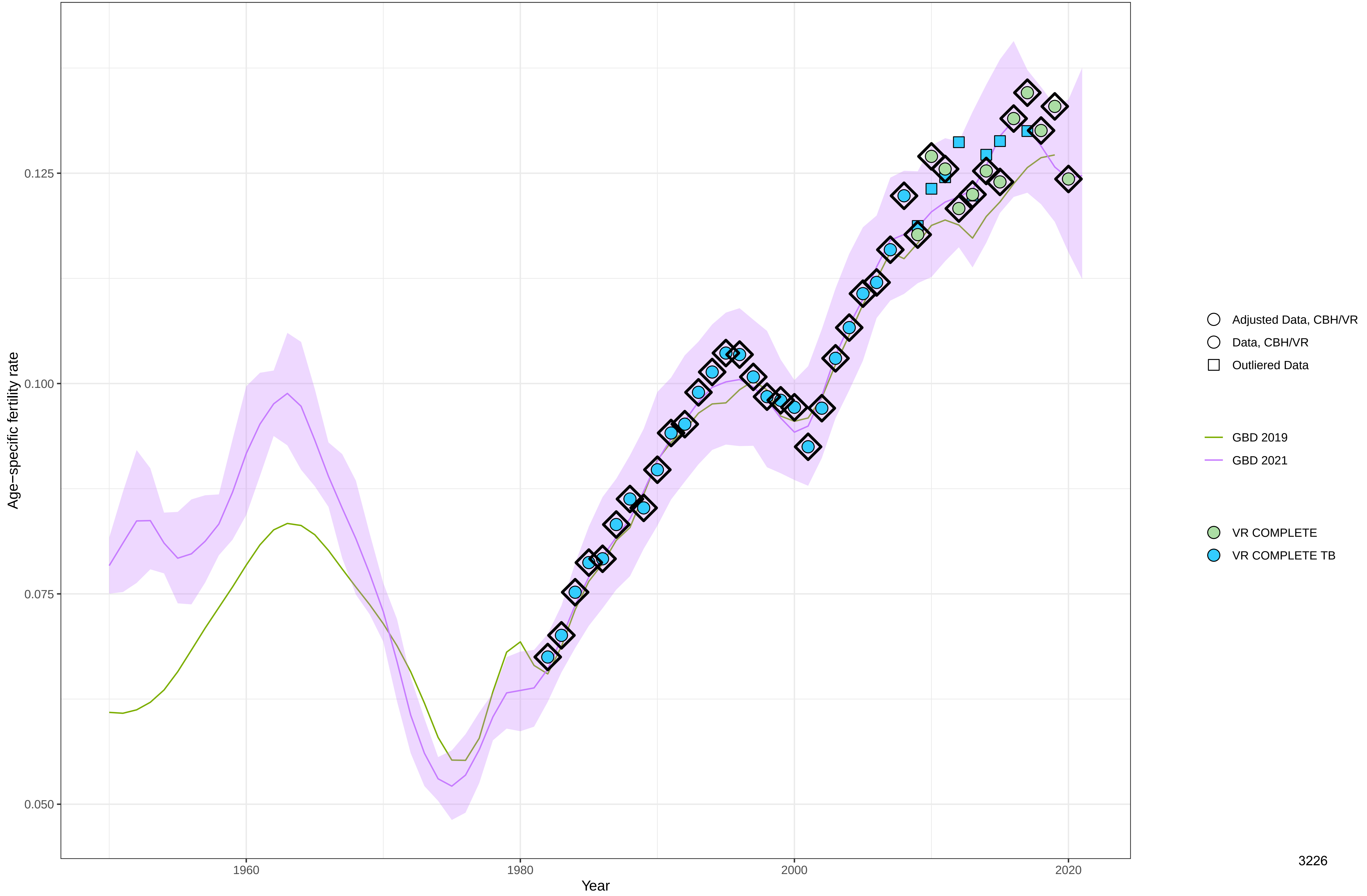

Age−specific fertility fitted trend lines, 1950−2021: South Gloucestershire, Ages: 35−39
Data points enclosed with a diamond are reference sources

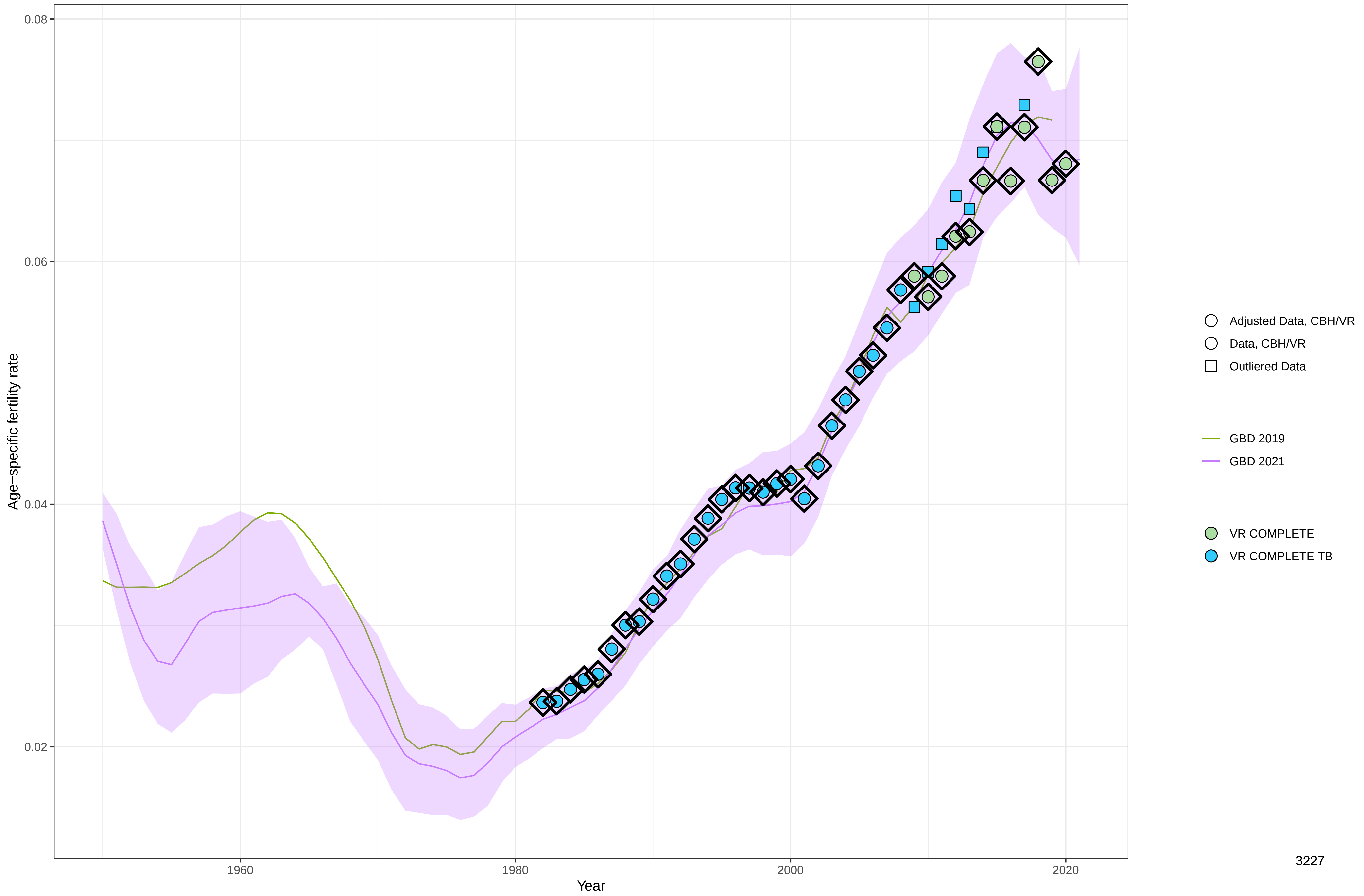

3227

Age−specific fertility fitted trend lines, 1950−2021: South Gloucestershire, Ages: 40−44
Data points enclosed with a diamond are reference sources

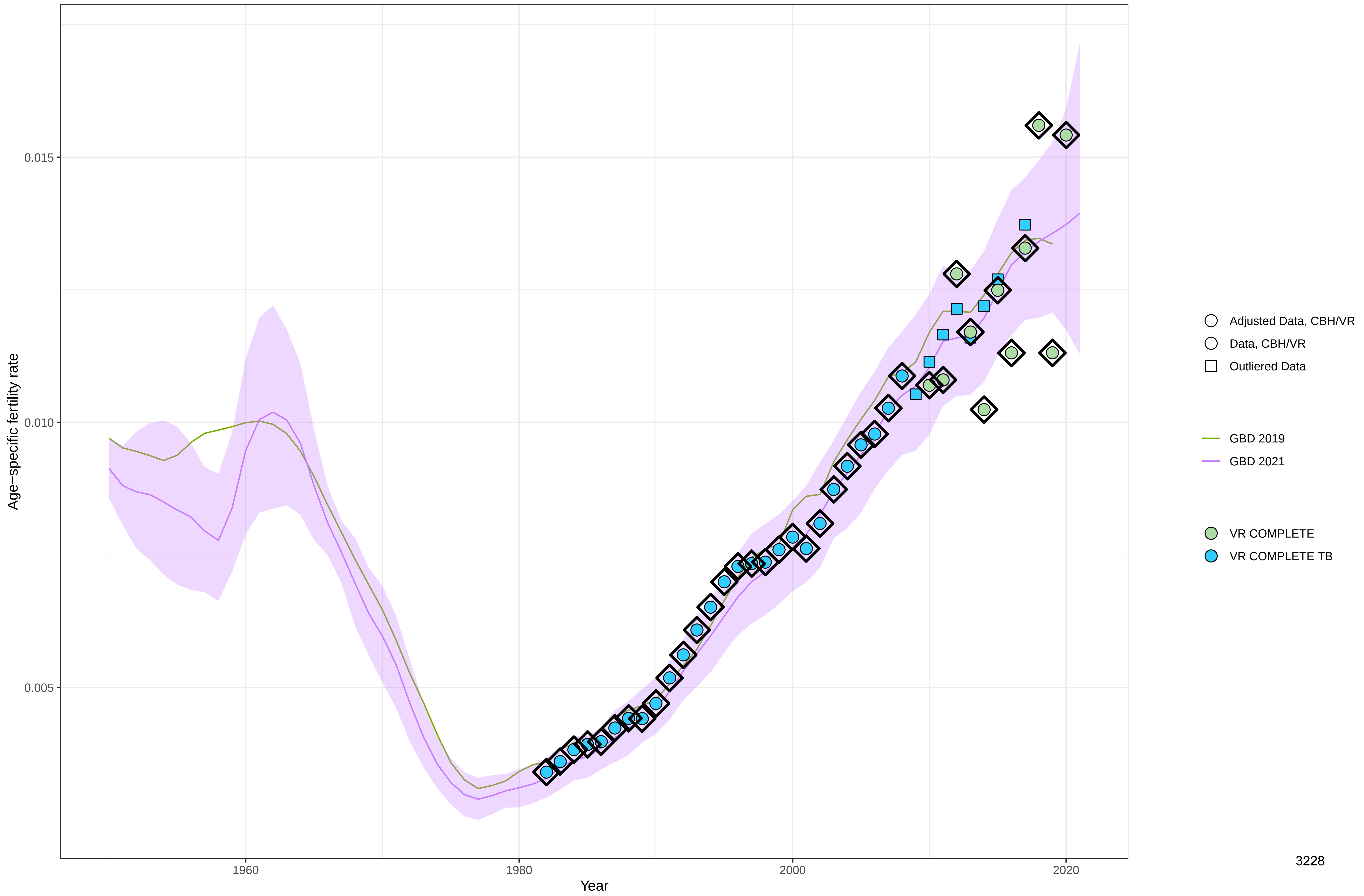

Age−specific fertility fitted trend lines, 1950−2021: South Gloucestershire, Ages: 45−49
Data points enclosed with a diamond are reference sources

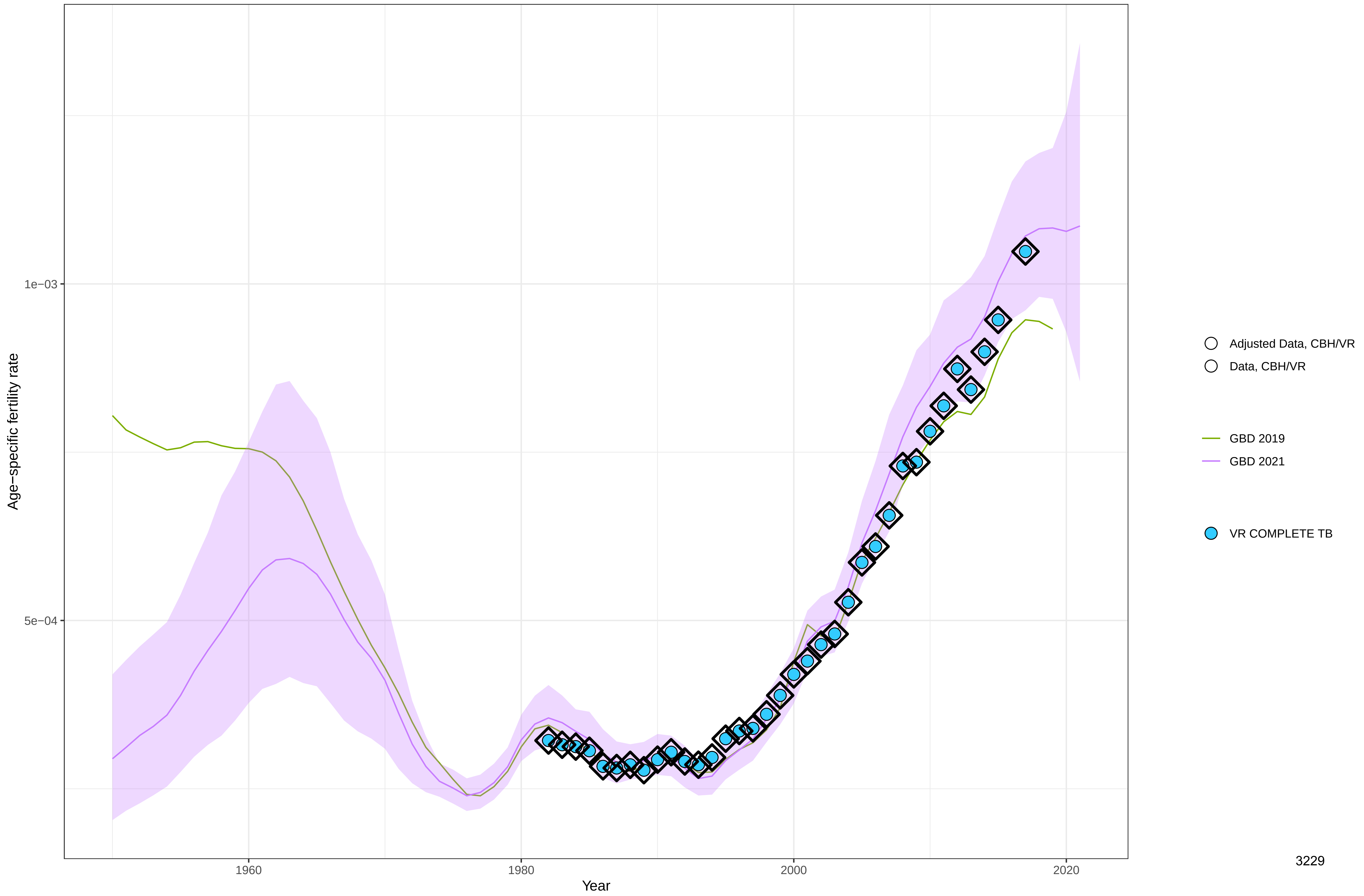

Age−specific fertility fitted trend lines, 1950−2021: South Gloucestershire, Ages: 50−54
Data points enclosed with a diamond are reference sources

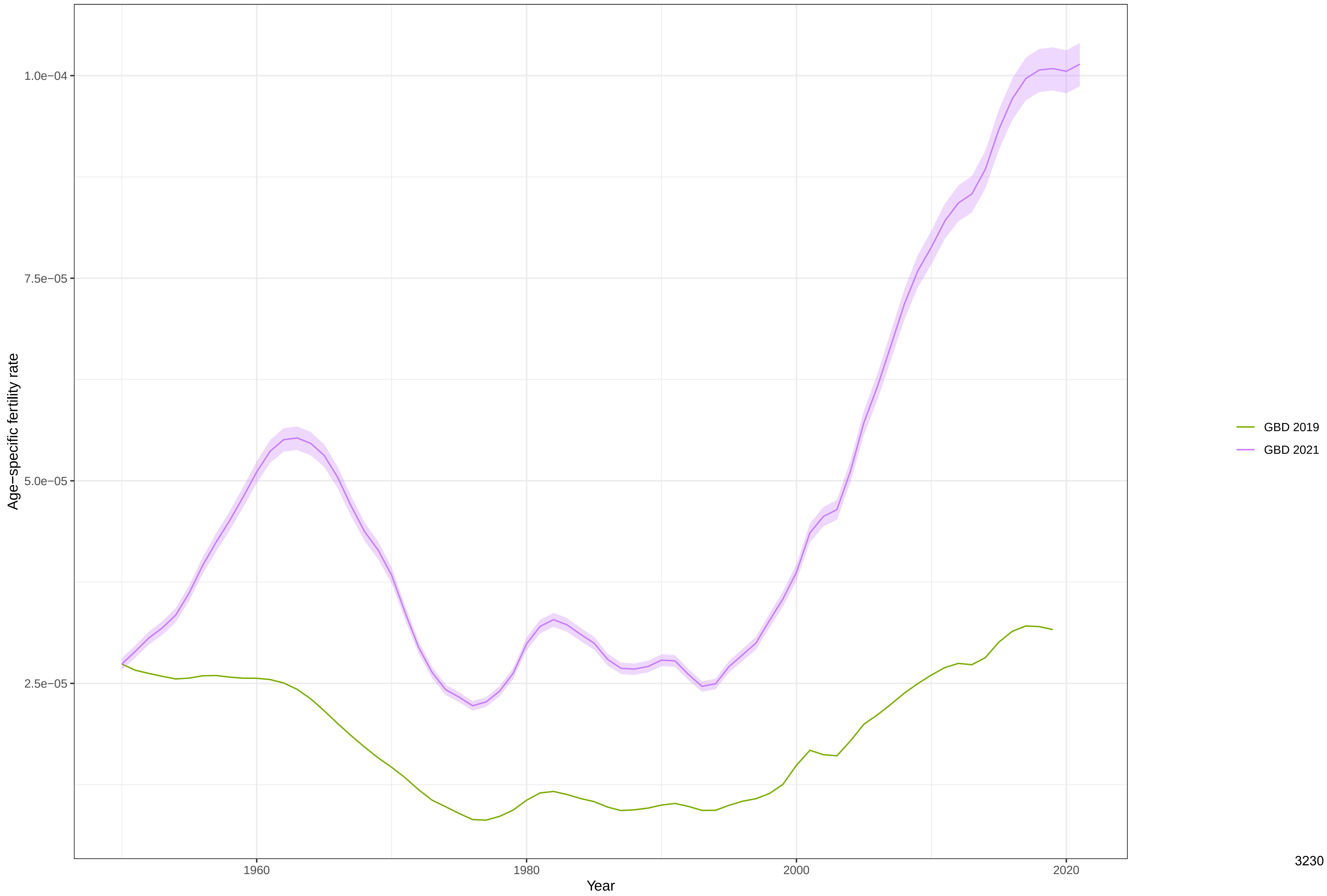

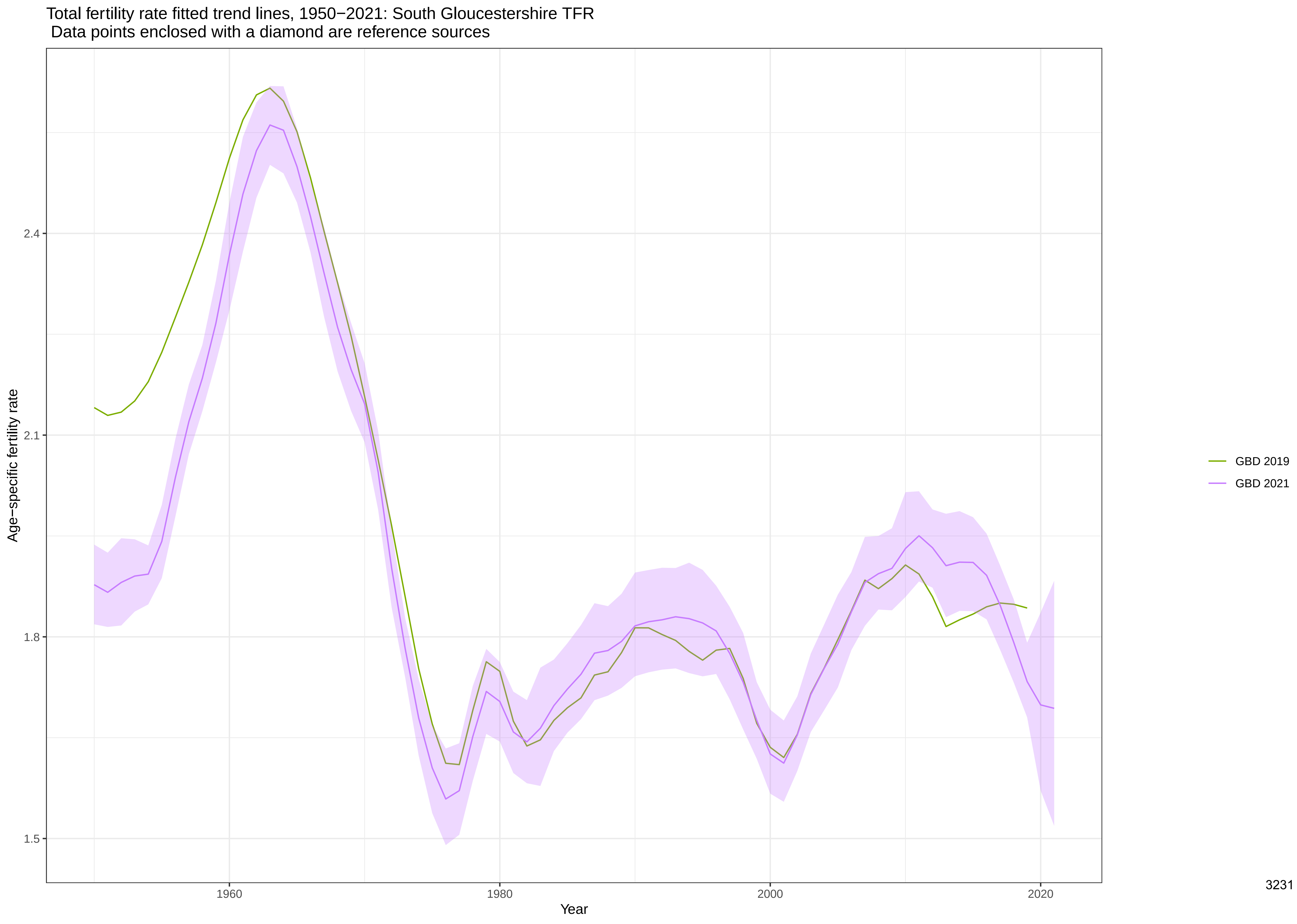
Total fertility rate fitted trend lines, 1950−2021: South Gloucestershire TFR
Data points enclosed with a diamond are reference sources
Age−specific fertility rate
2.4
2.1
1.8
1.5
1960
1980
2000
2020
Year
GBD 2019
GBD 2021

Age−specific fertility fitted trend lines, 1950−2021: Swindon, Ages: 10−14
Data points enclosed with a diamond are reference sources

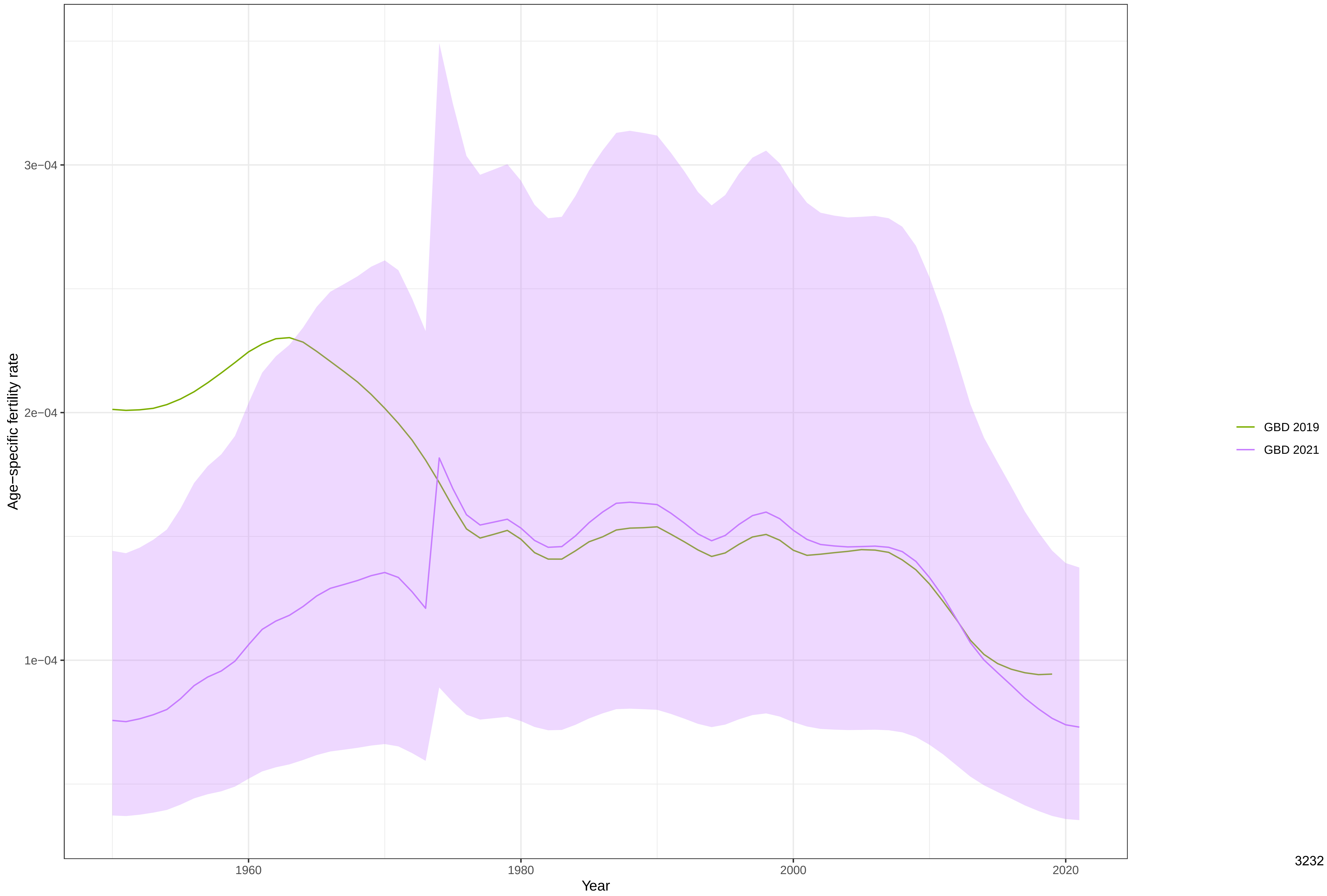

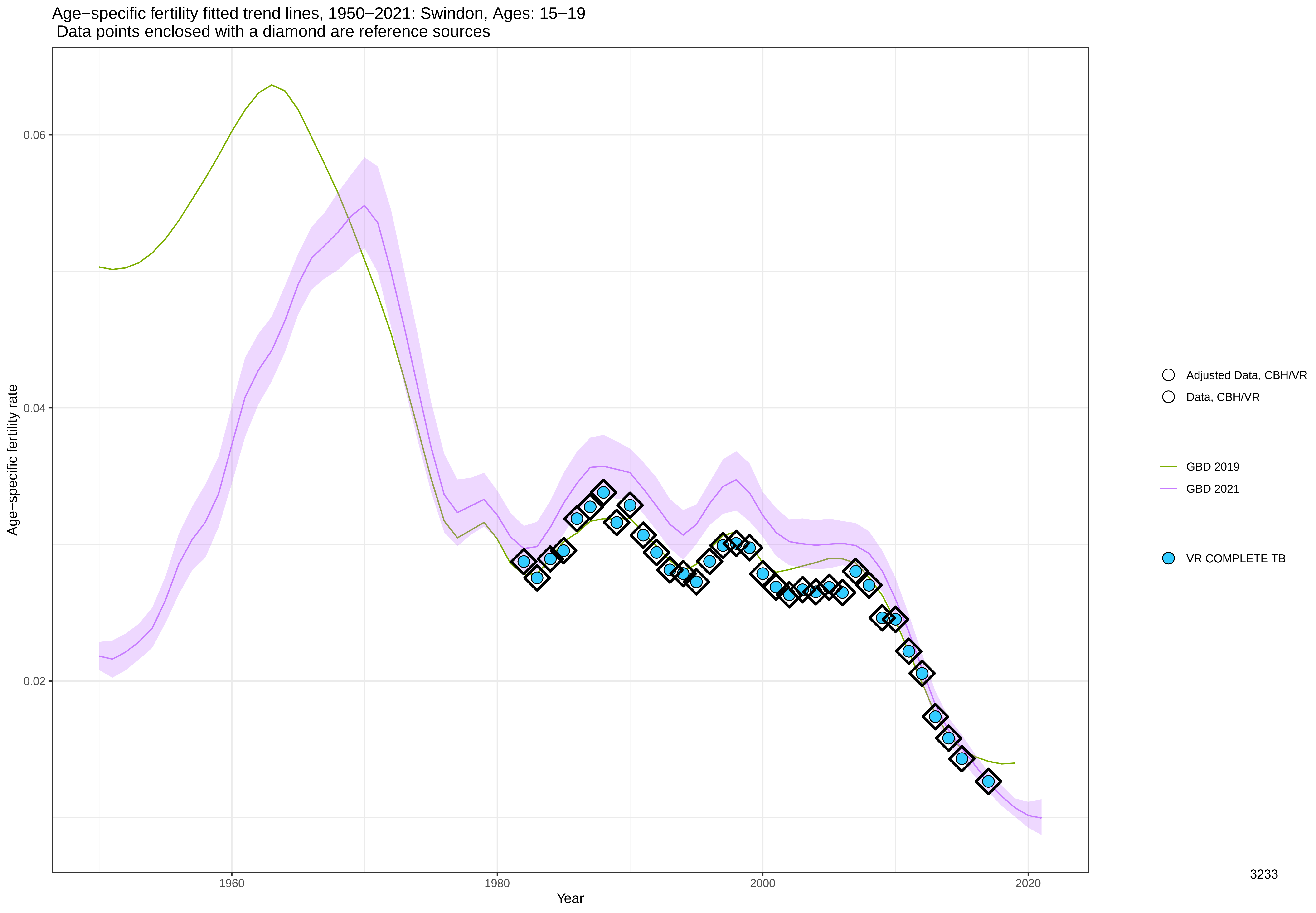

Age−specific fertility fitted trend lines, 1950−2021: Swindon, Ages: 15−19
Data points enclosed with a diamond are reference sources
0.06
0.04
0.02
Age−specific fertility rate
1960
1980
2000
2020
Year
Adjusted Data, CBH/VR
Data, CBH/VR
GBD 2019
GBD 2021
VR COMPLETE TB

3233

Age−specific fertility fitted trend lines, 1950−2021: Swindon, Ages: 20−24
Data points enclosed with a diamond are reference sources

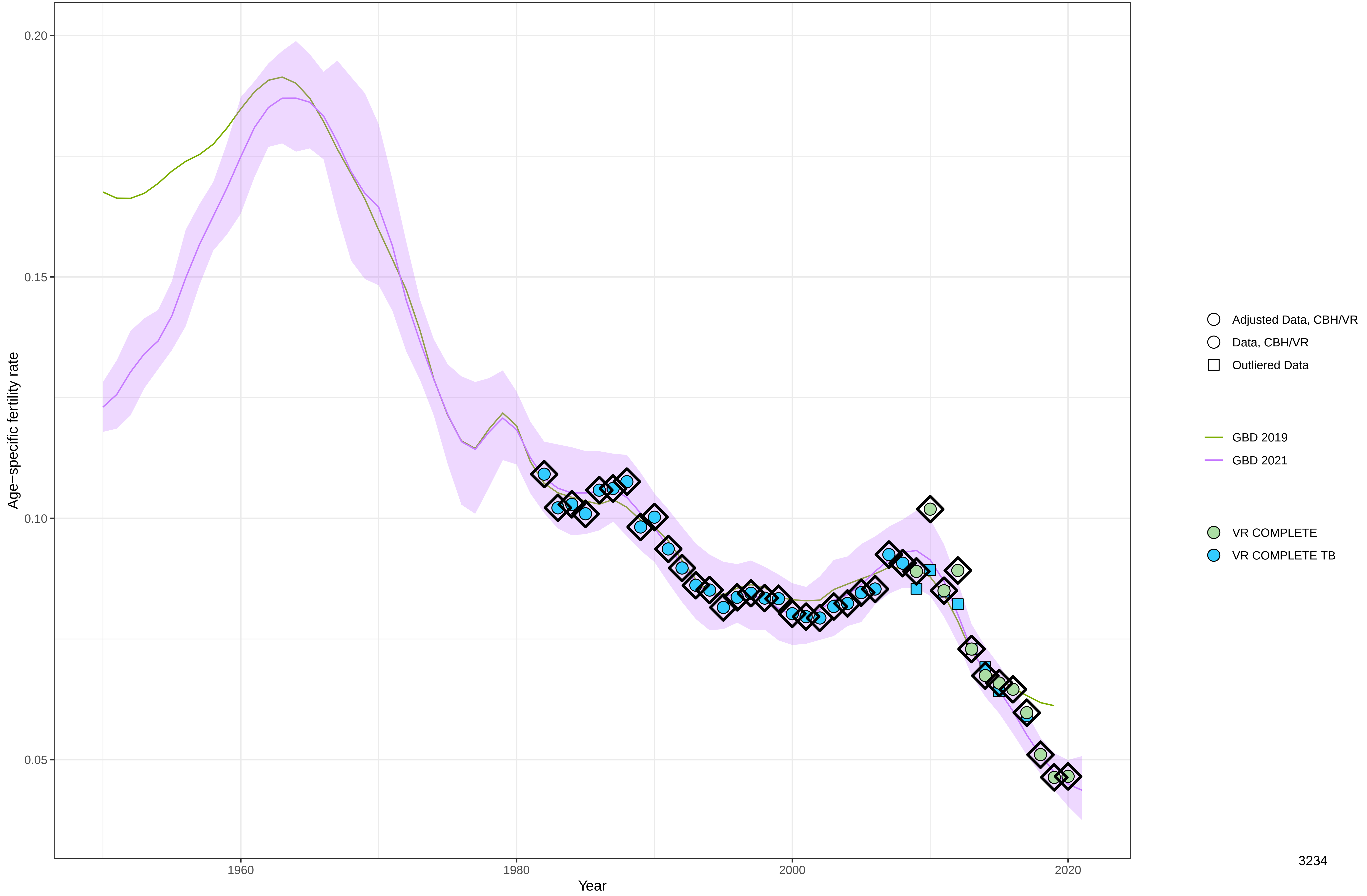

Age−specific fertility fitted trend lines, 1950−2021: Swindon, Ages: 25−29
Data points enclosed with a diamond are reference sources

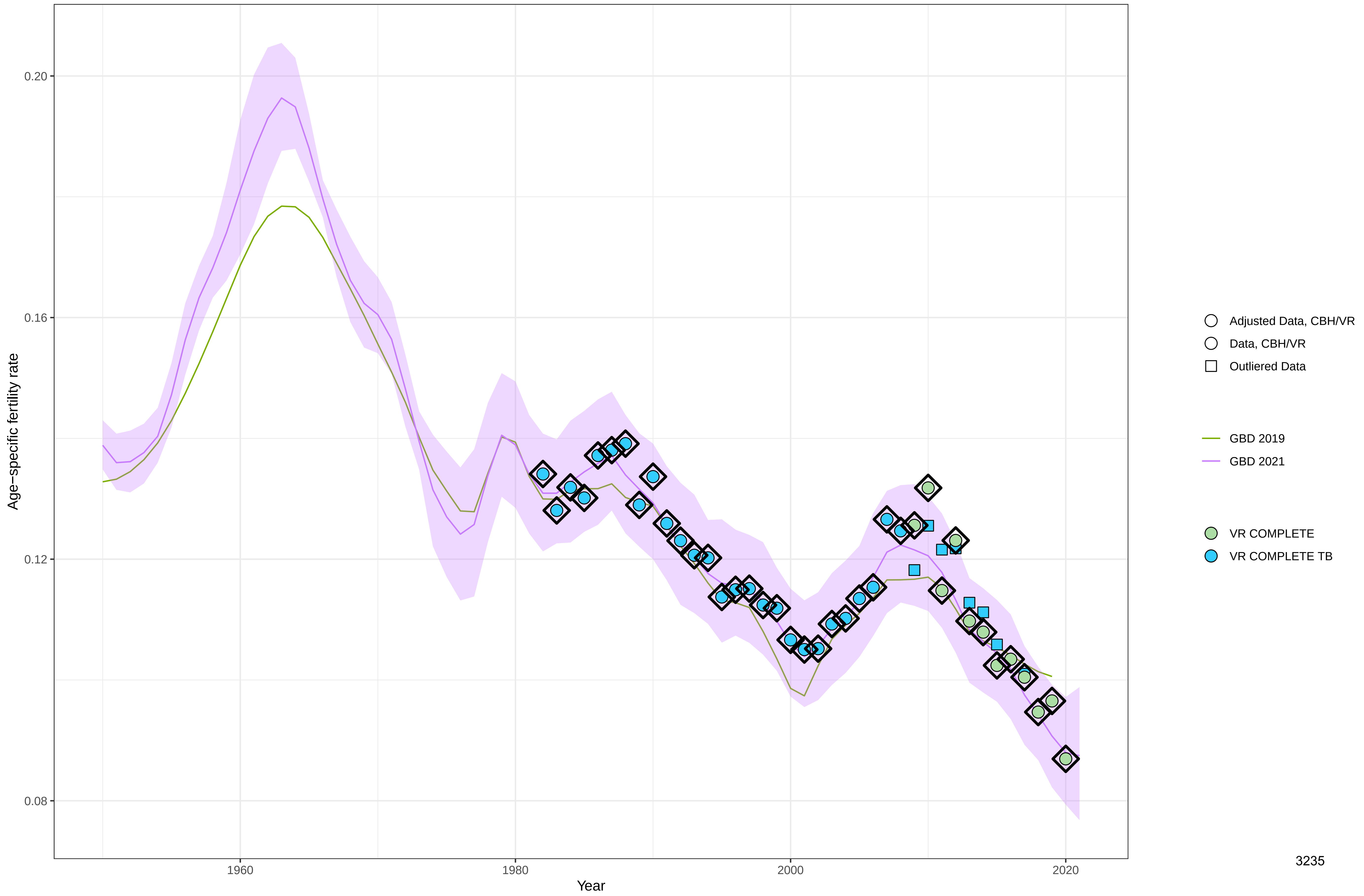

Age−specific fertility fitted trend lines, 1950−2021: Swindon, Ages: 30−34
Data points enclosed with a diamond are reference sources

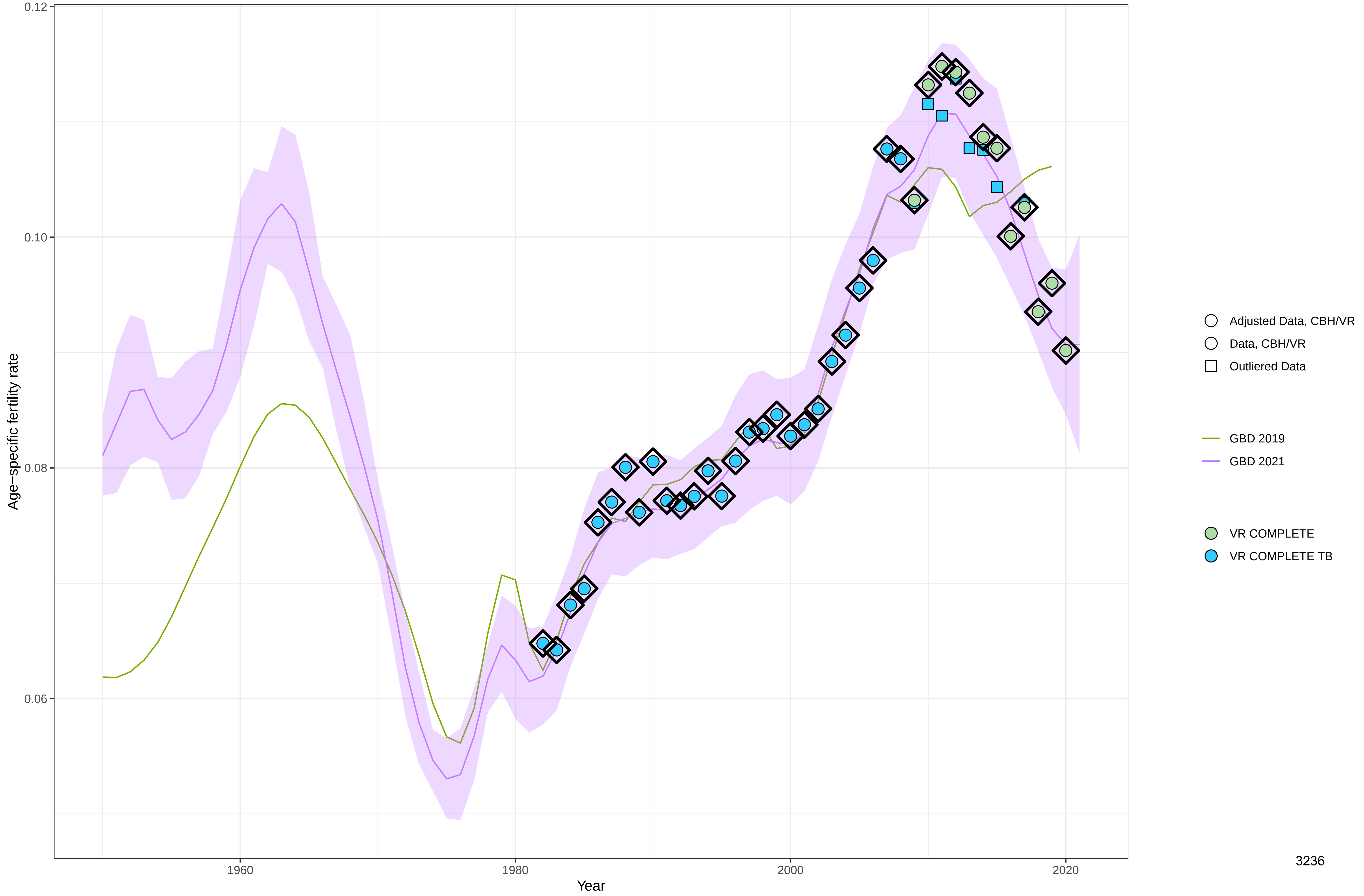

3236

Age−specific fertility fitted trend lines, 1950−2021: Swindon, Ages: 35−39
Data points enclosed with a diamond are reference sources

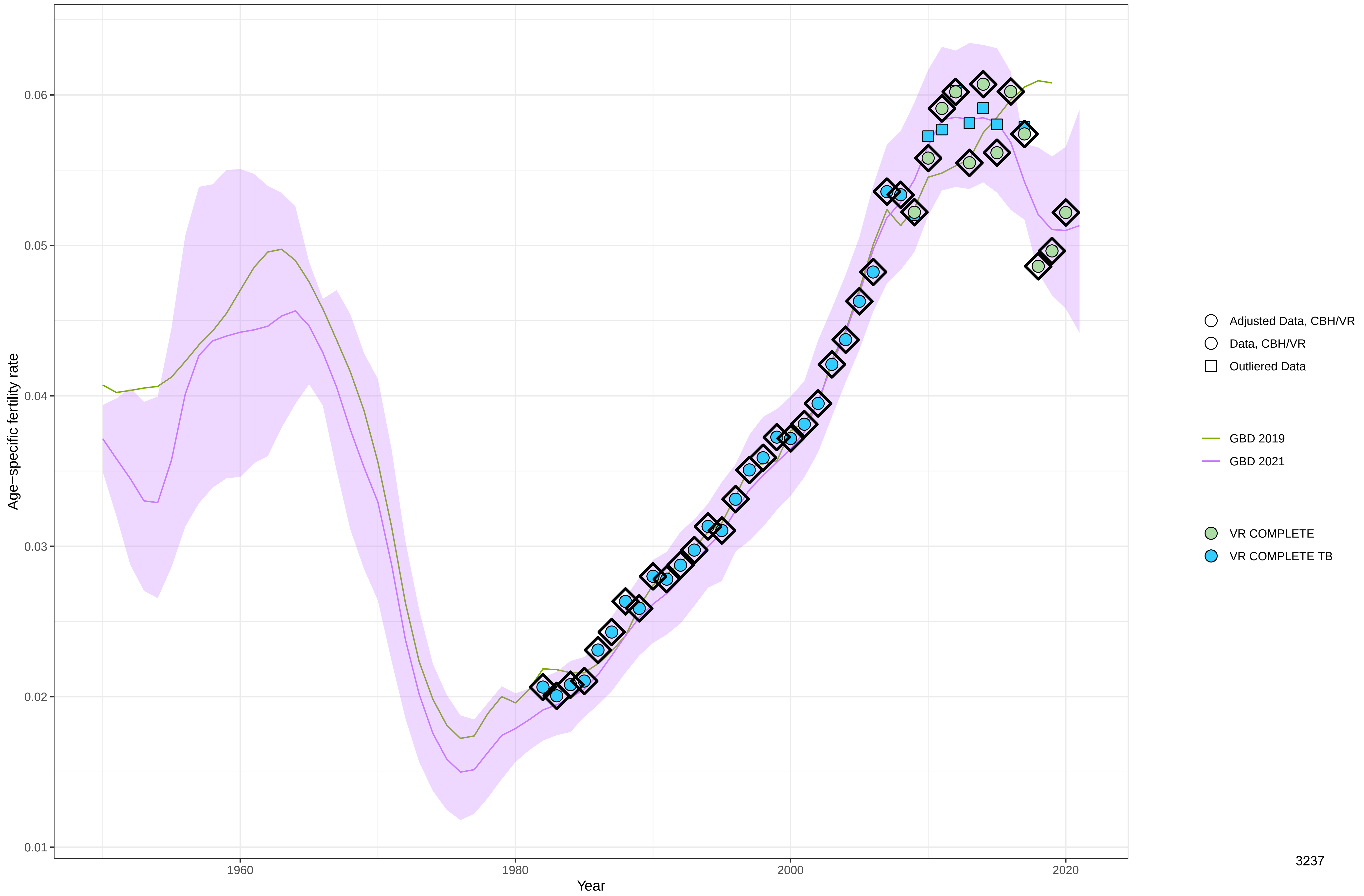

Age−specific fertility fitted trend lines, 1950−2021: Swindon, Ages: 40−44
Data points enclosed with a diamond are reference sources

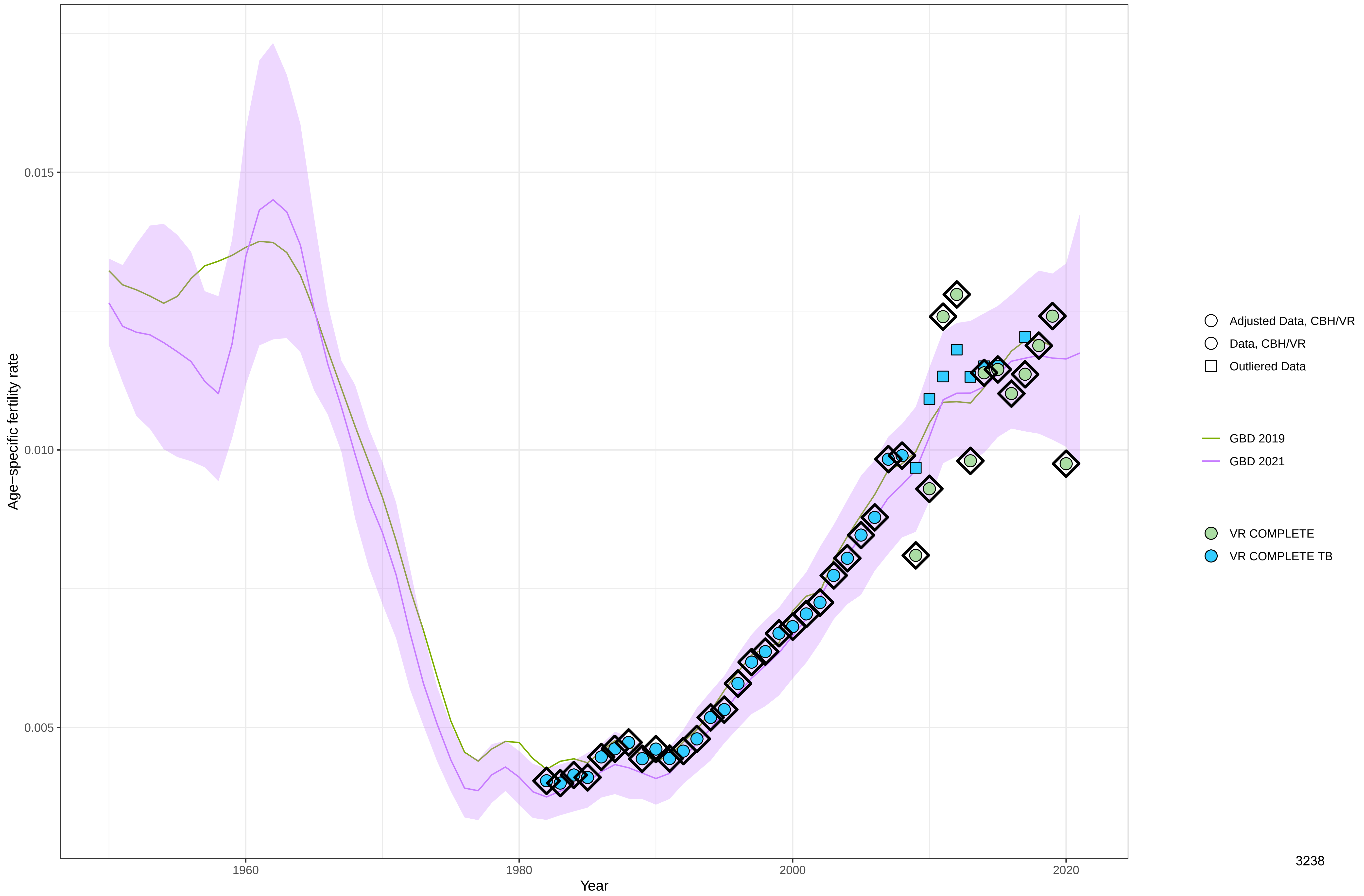

Age−specific fertility fitted trend lines, 1950−2021: Swindon, Ages: 45−49
Data points enclosed with a diamond are reference sources

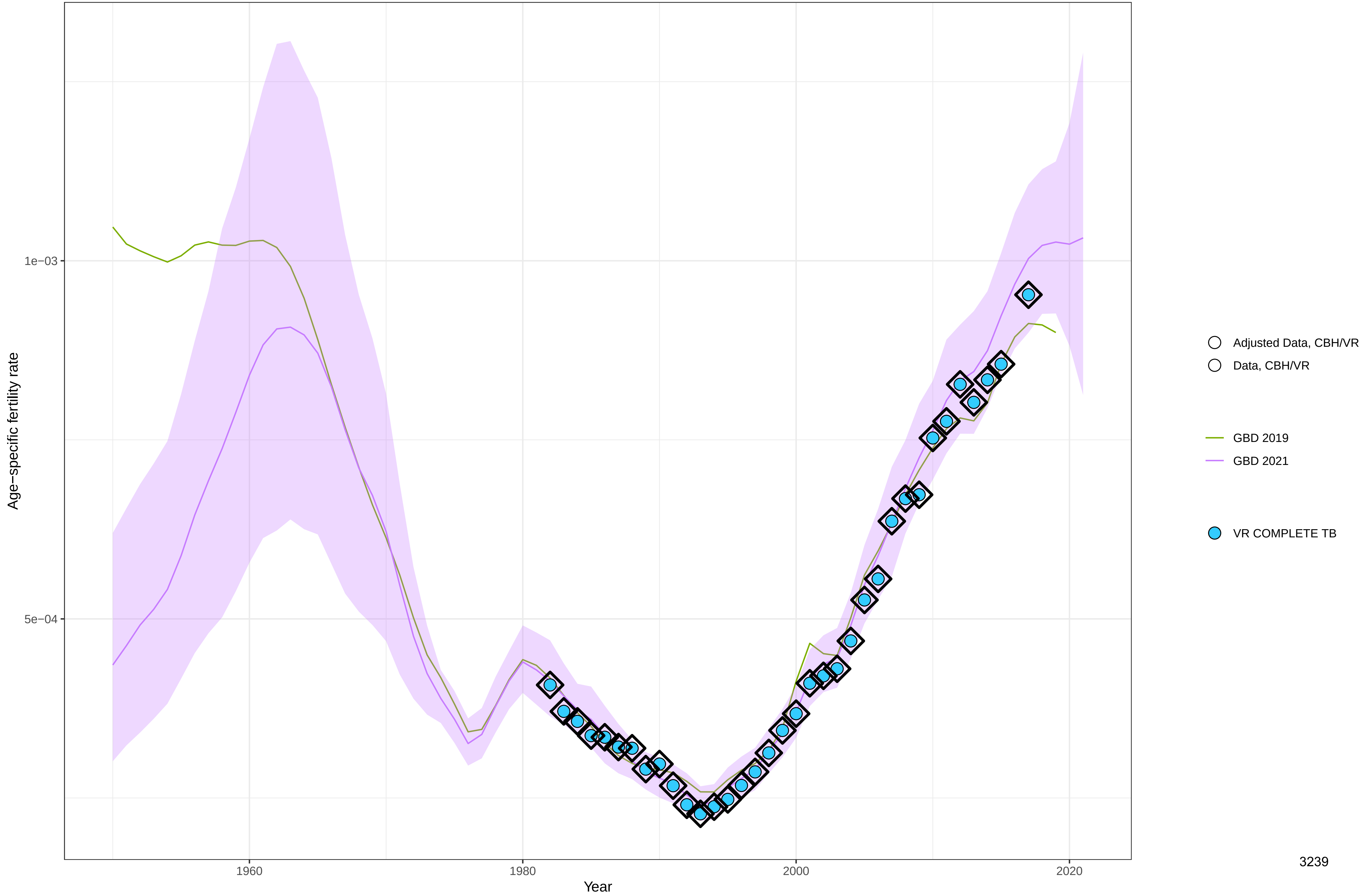

Age−specific fertility fitted trend lines, 1950−2021: Swindon, Ages: 50−54
Data points enclosed with a diamond are reference sources

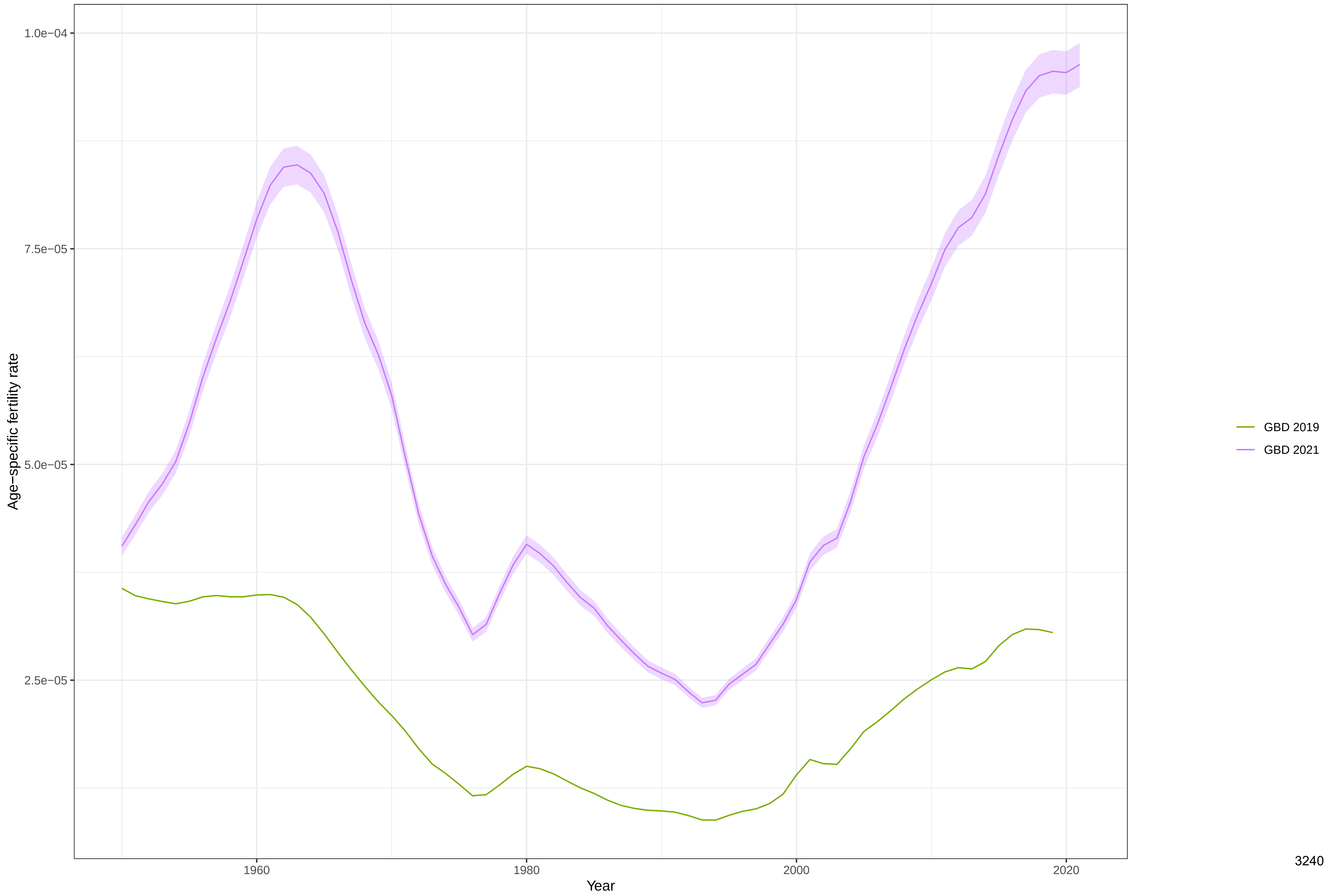

Total fertility rate fitted trend lines, 1950−2021: Swindon TFR
Data points enclosed with a diamond are reference sources

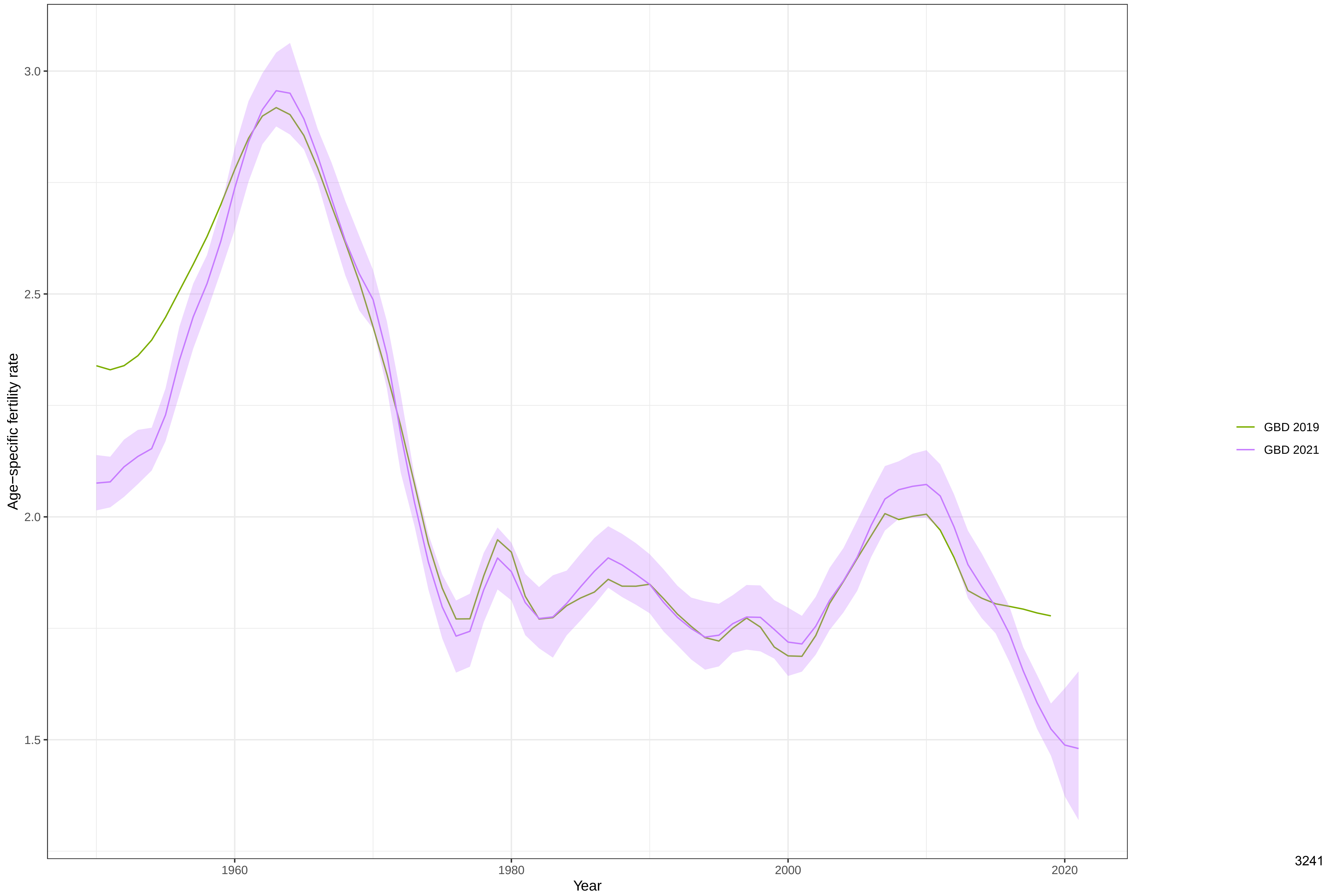

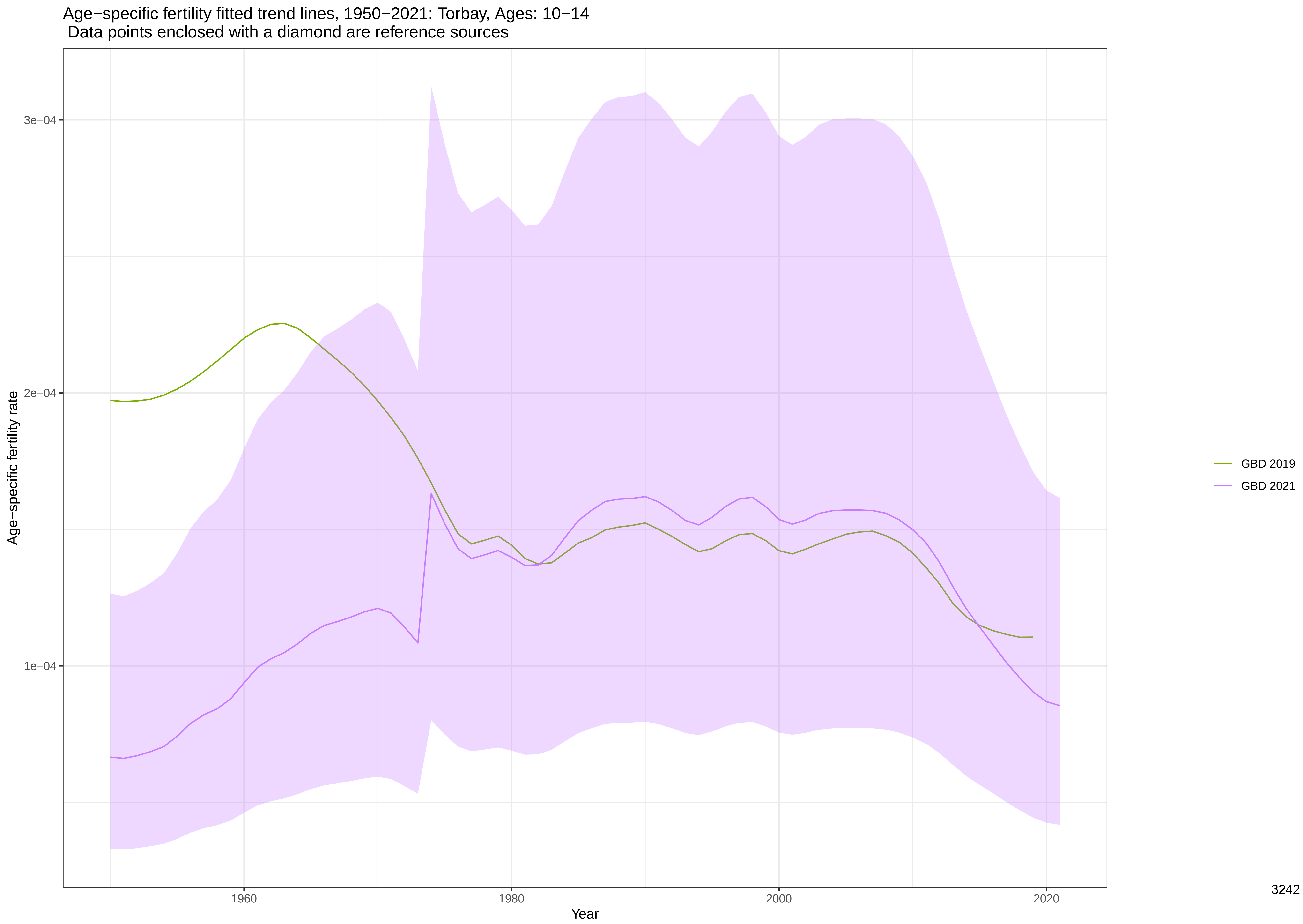
Age−specific fertility fitted trend lines, 1950−2021: Torbay, Ages: 10−14
Data points enclosed with a diamond are reference sources
3e−04
2e−04
1e−04
Age−specific fertility rate
1960
1980
2000
2020
Year
GBD 2019
GBD 2021

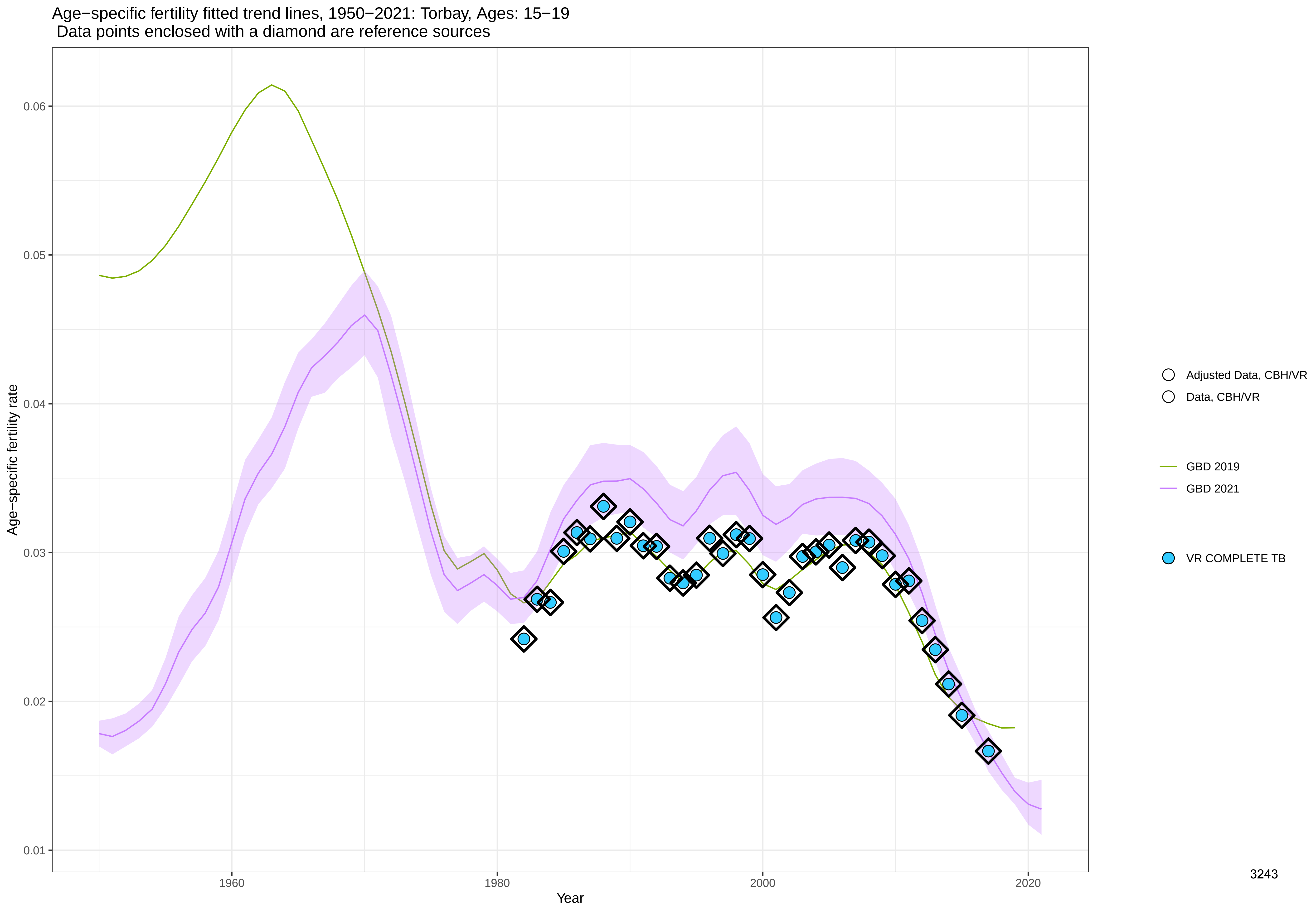

Age−specific fertility fitted trend lines, 1950−2021: Torbay, Ages: 15−19
Data points enclosed with a diamond are reference sources
Age−specific fertility rate
0.06
0.05
0.04
0.03
0.02
0.01
1960
1980
2000
2020
Year
Adjusted Data, CBH/VR
Data, CBH/VR
GBD 2019
GBD 2021
VR COMPLETE TB

3243

Age−specific fertility fitted trend lines, 1950−2021: Torbay, Ages: 20−24
Data points enclosed with a diamond are reference sources

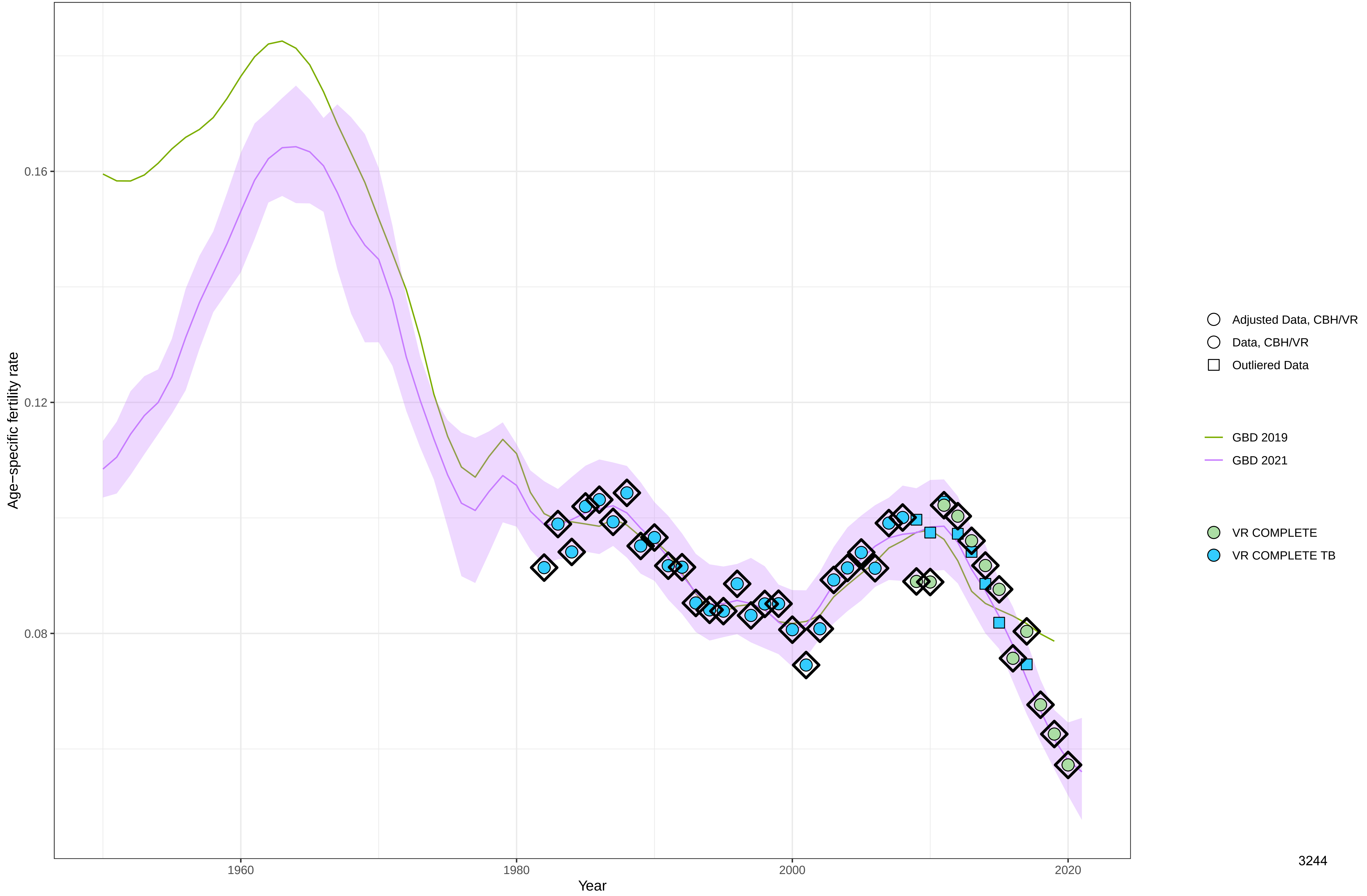

Age−specific fertility fitted trend lines, 1950−2021: Torbay, Ages: 25−29
Data points enclosed with a diamond are reference sources

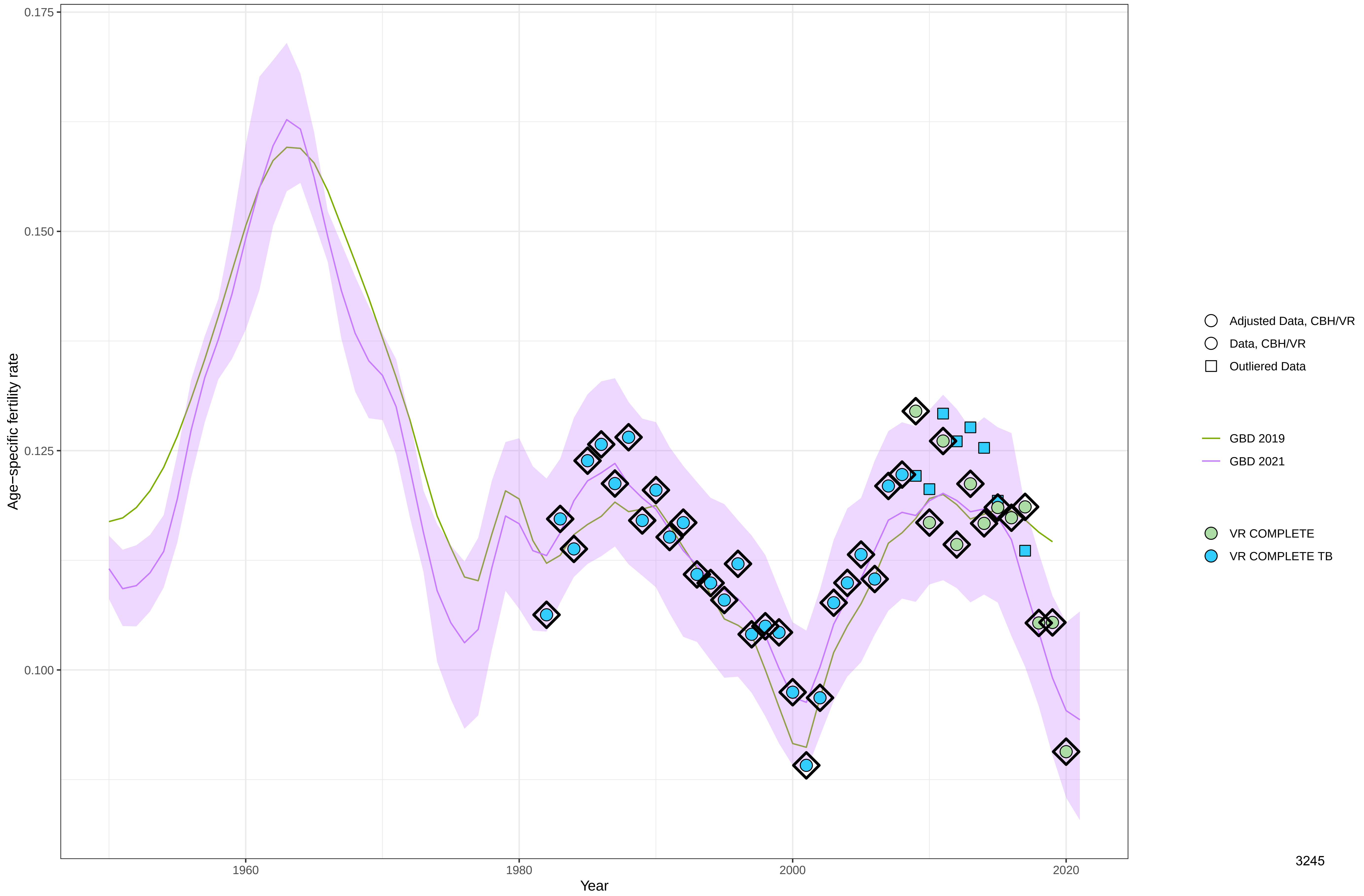

Age−specific fertility fitted trend lines, 1950−2021: Torbay, Ages: 30−34
Data points enclosed with a diamond are reference sources

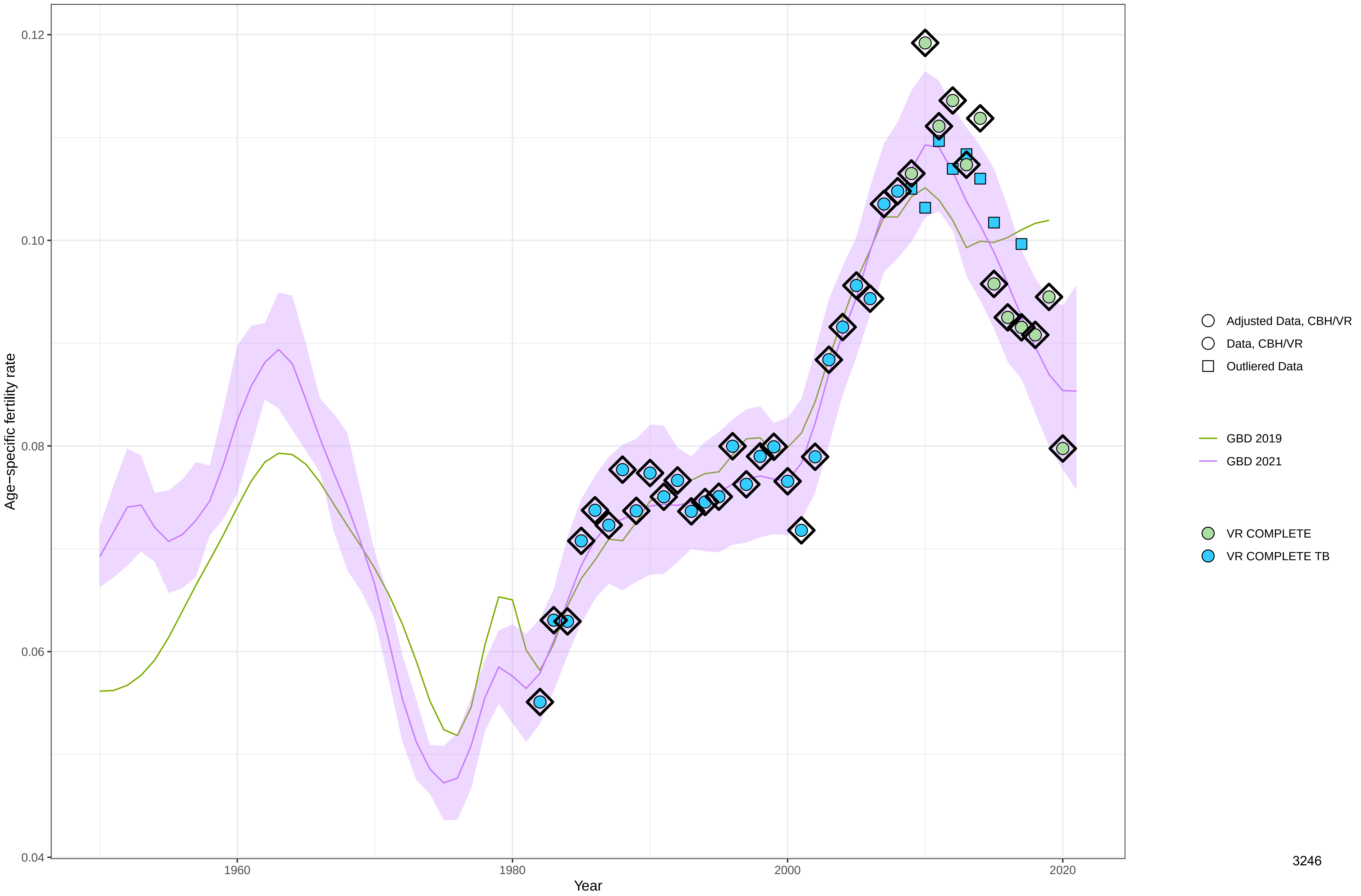

Age−specific fertility fitted trend lines, 1950−2021: Torbay, Ages: 35−39
Data points enclosed with a diamond are reference sources

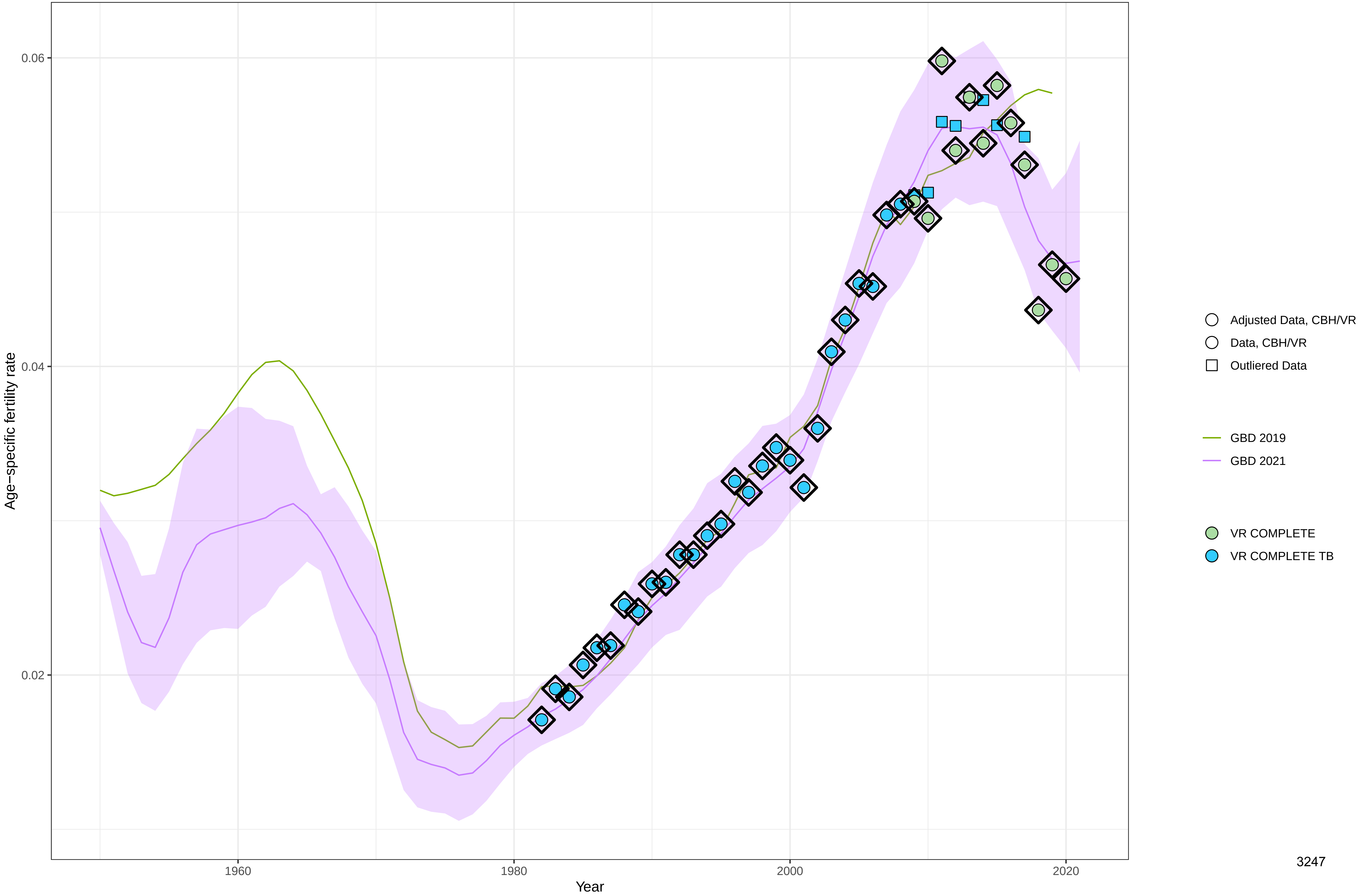

Age−specific fertility fitted trend lines, 1950−2021: Torbay, Ages: 40−44
Data points enclosed with a diamond are reference sources

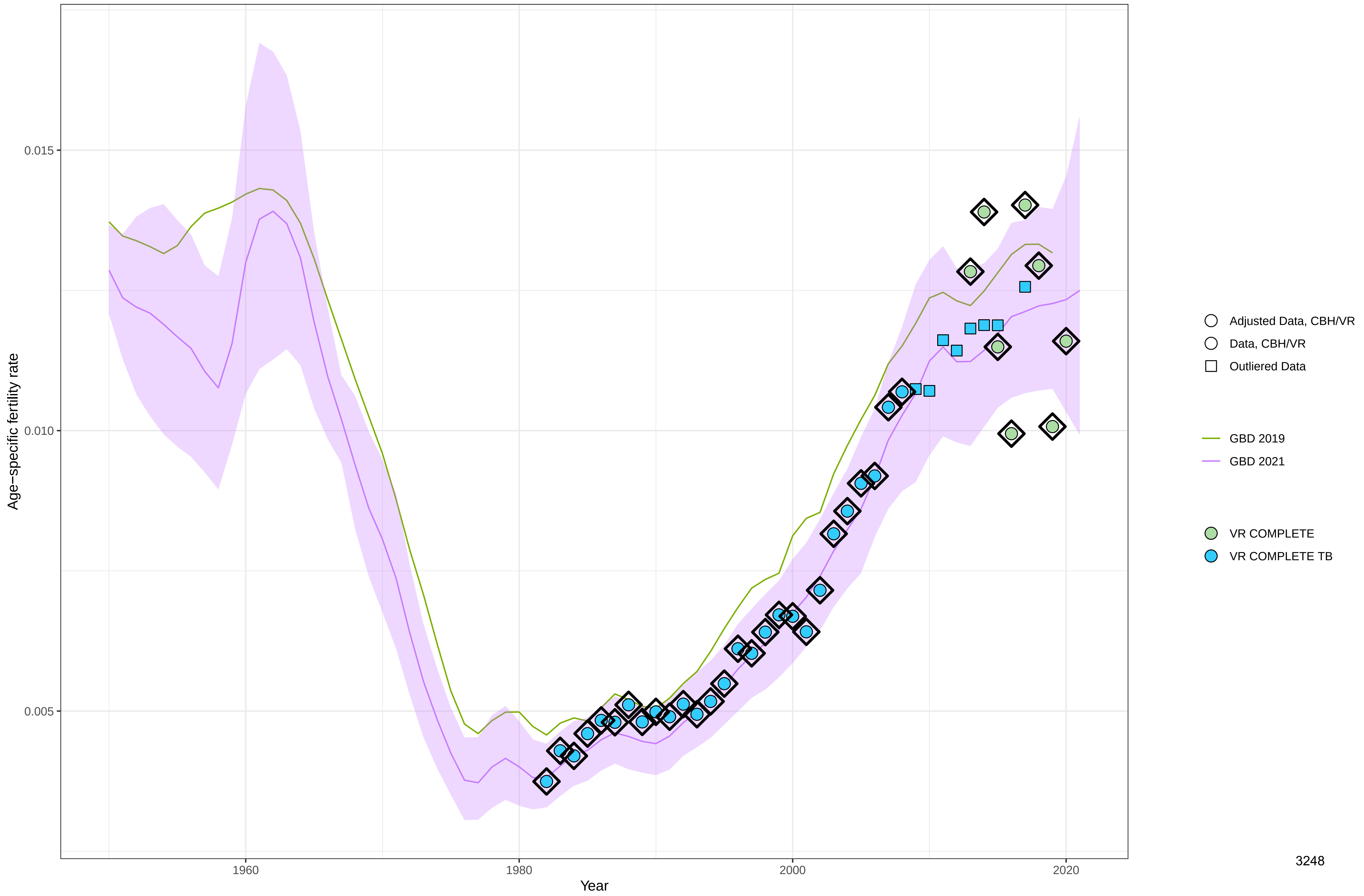

Age−specific fertility fitted trend lines, 1950−2021: Torbay, Ages: 45−49
Data points enclosed with a diamond are reference sources

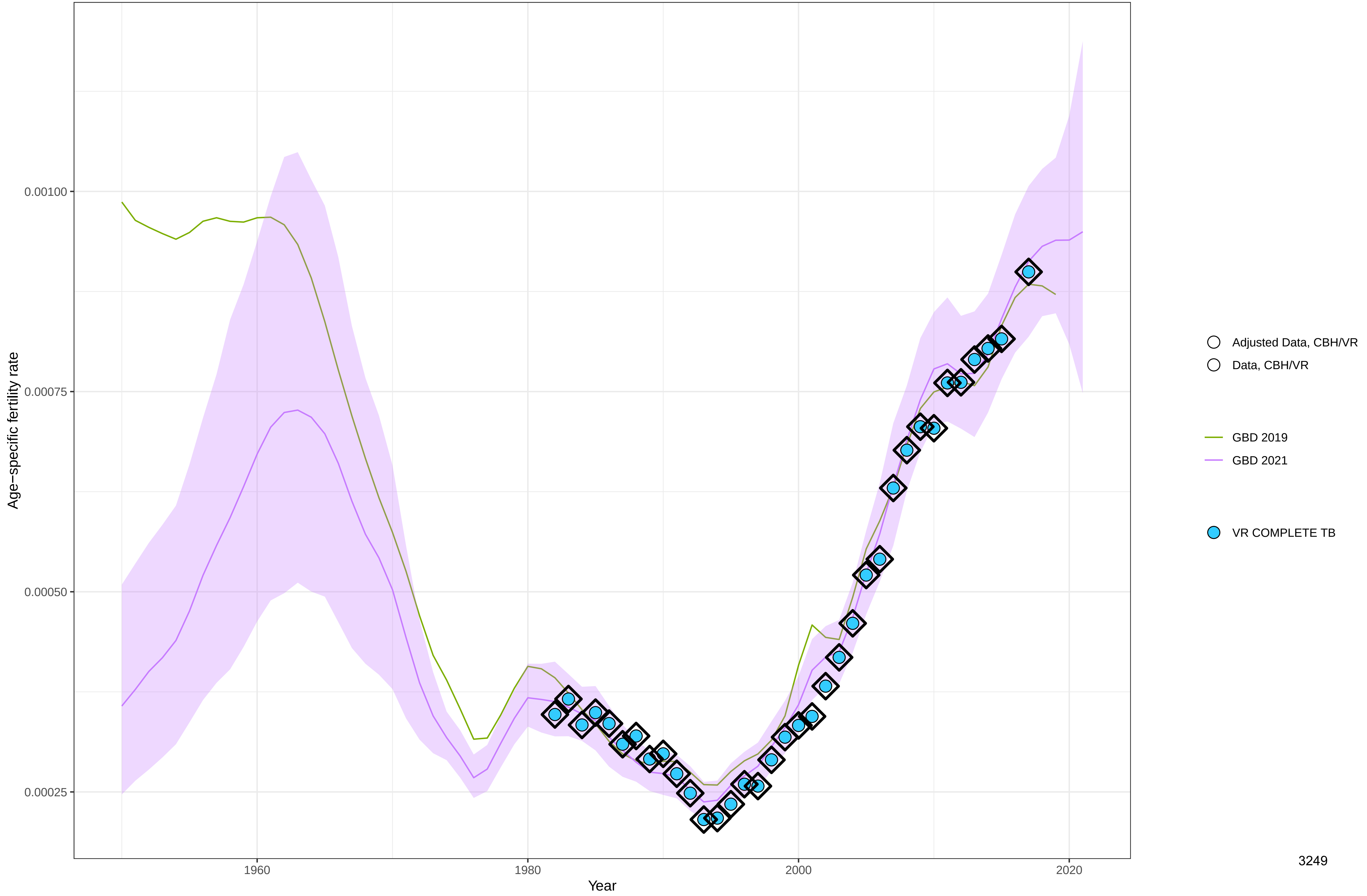

Age−specific fertility fitted trend lines, 1950−2021: Torbay, Ages: 50−54
Data points enclosed with a diamond are reference sources

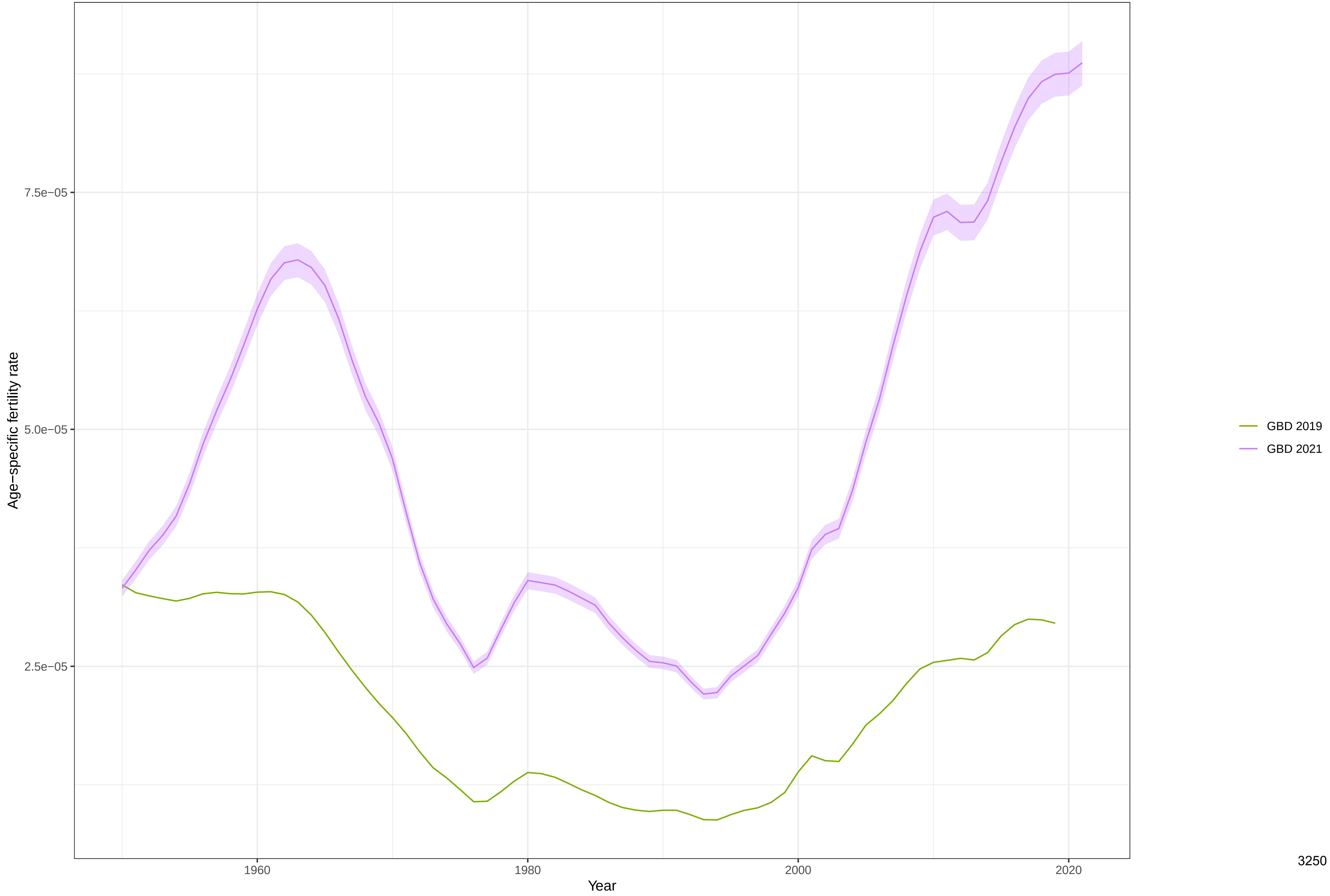

Total fertility rate fitted trend lines, 1950−2021: Torbay TFR
Data points enclosed with a diamond are reference sources

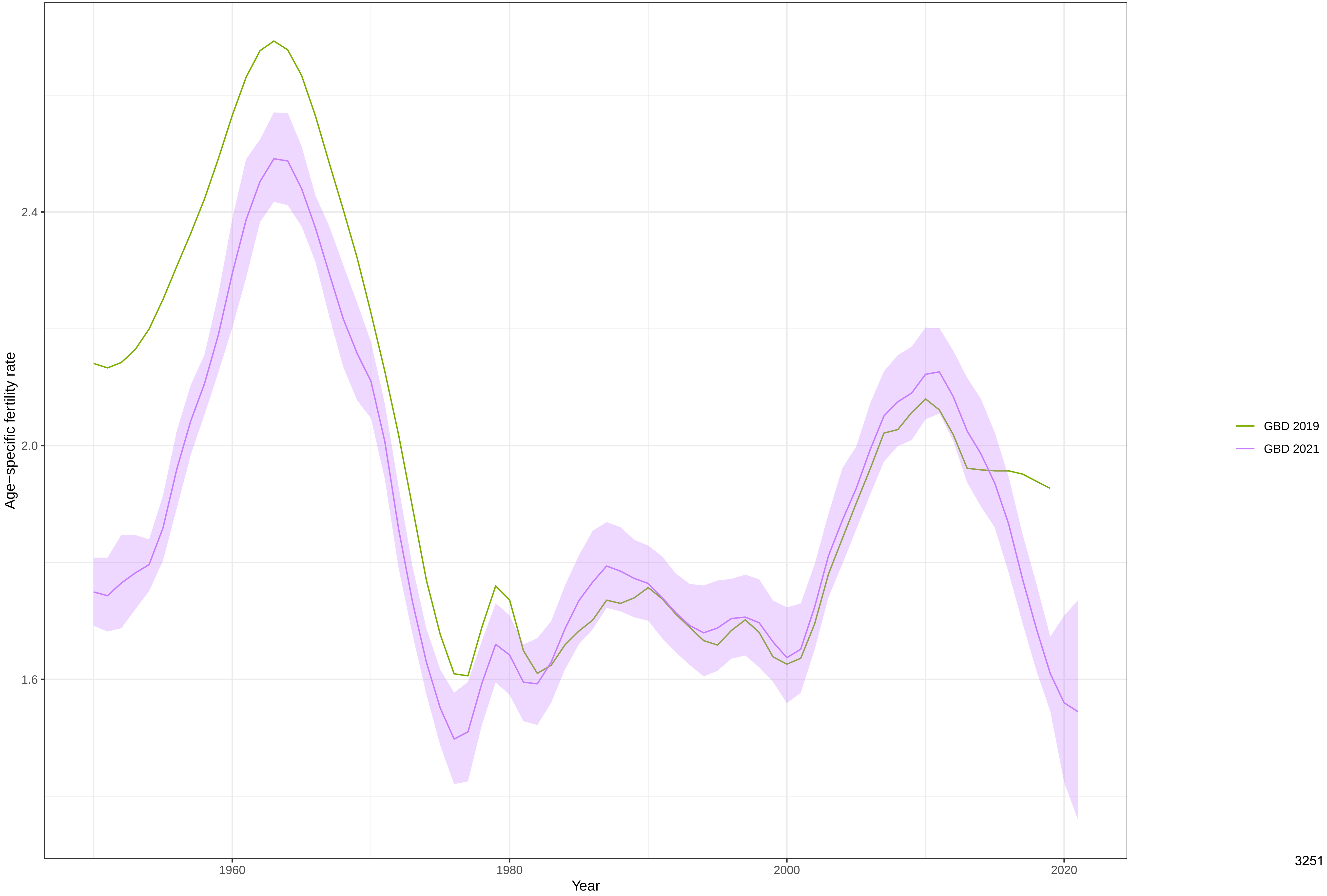

Age−specific fertility fitted trend lines, 1950−2021: Wiltshire, Ages: 10−14
Data points enclosed with a diamond are reference sources

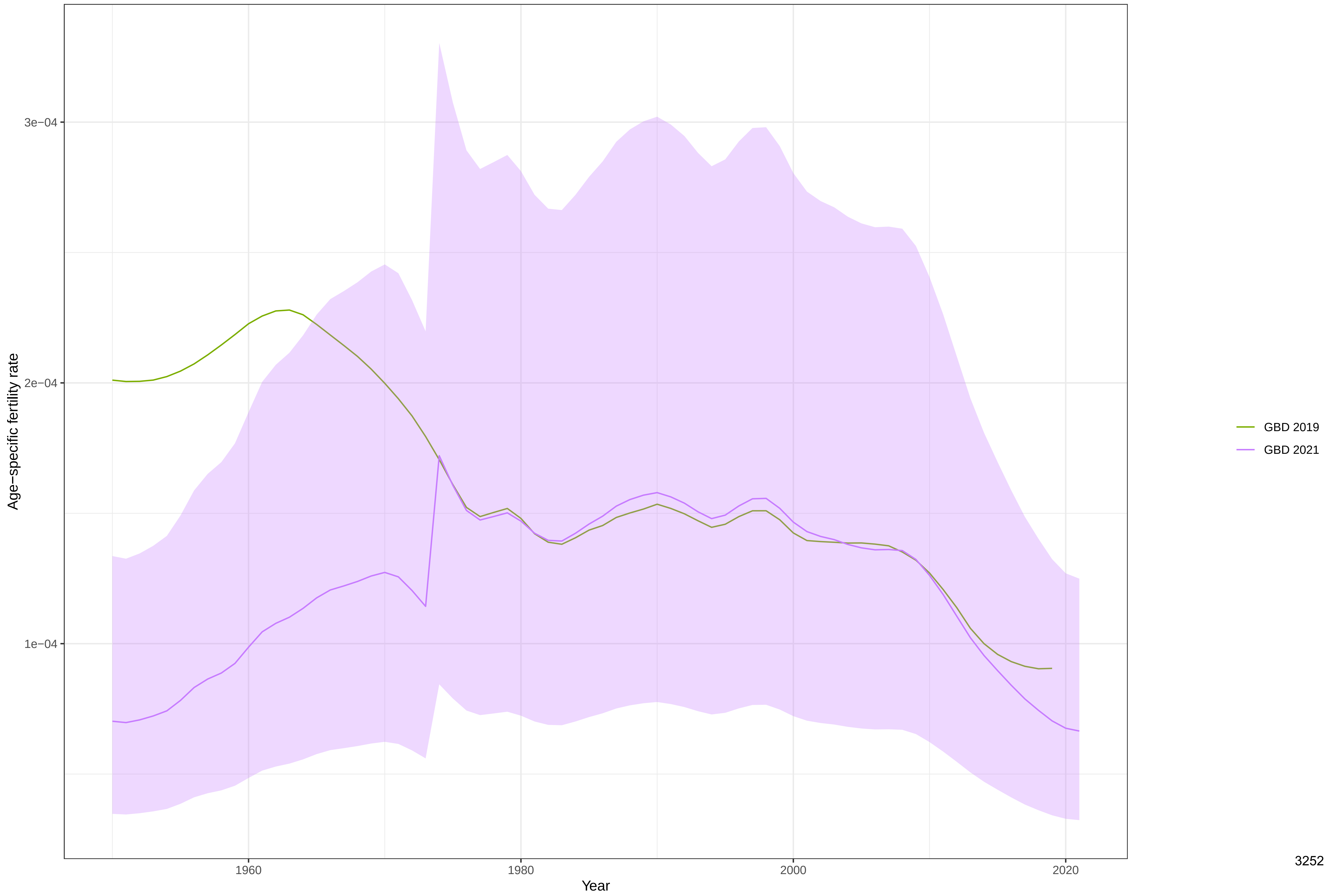

Age−specific fertility fitted trend lines, 1950−2021: Wiltshire, Ages: 15−19
Data points enclosed with a diamond are reference sources

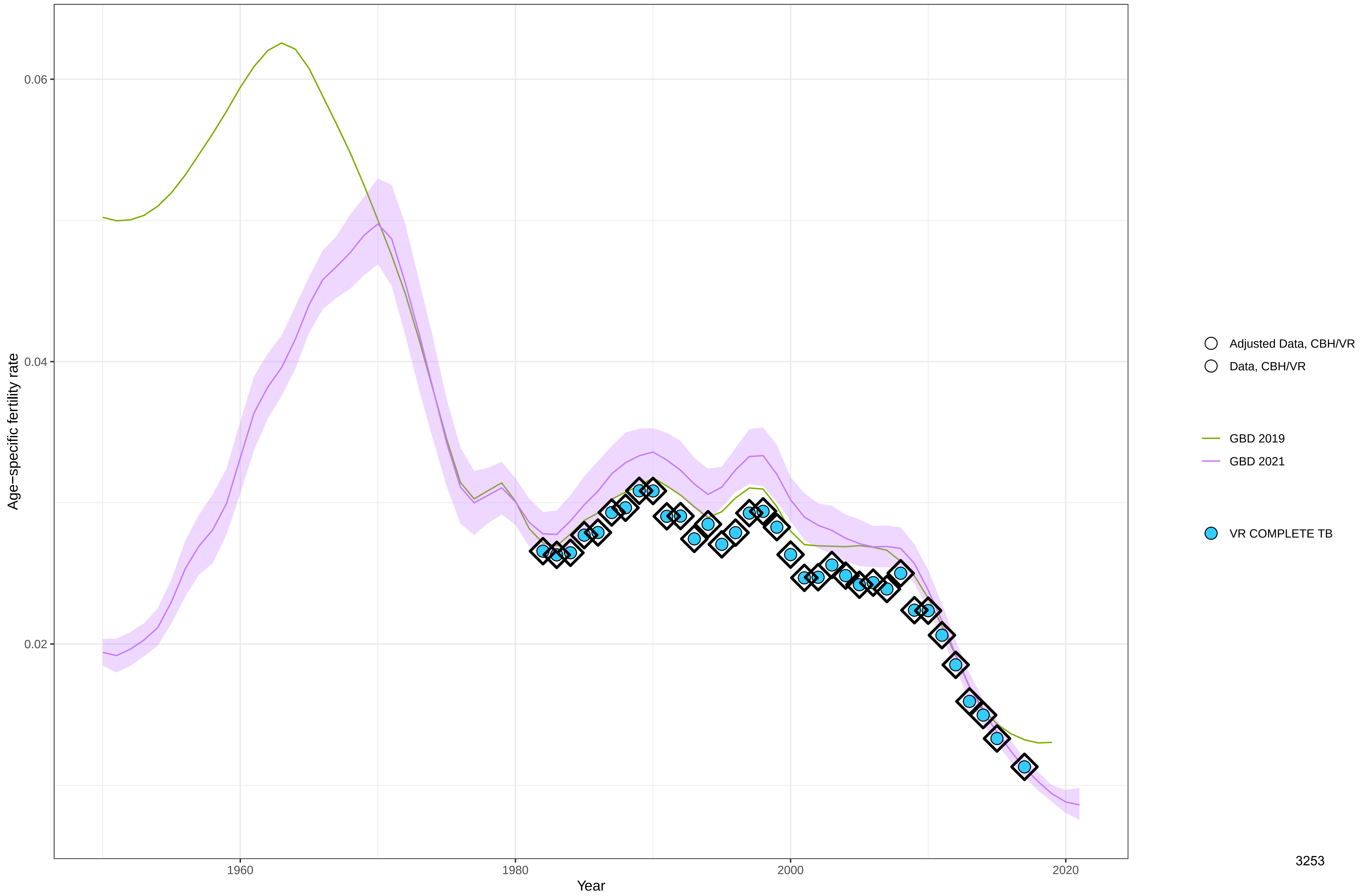

3253

Age−specific fertility fitted trend lines, 1950−2021: Wiltshire, Ages: 20−24
Data points enclosed with a diamond are reference sources

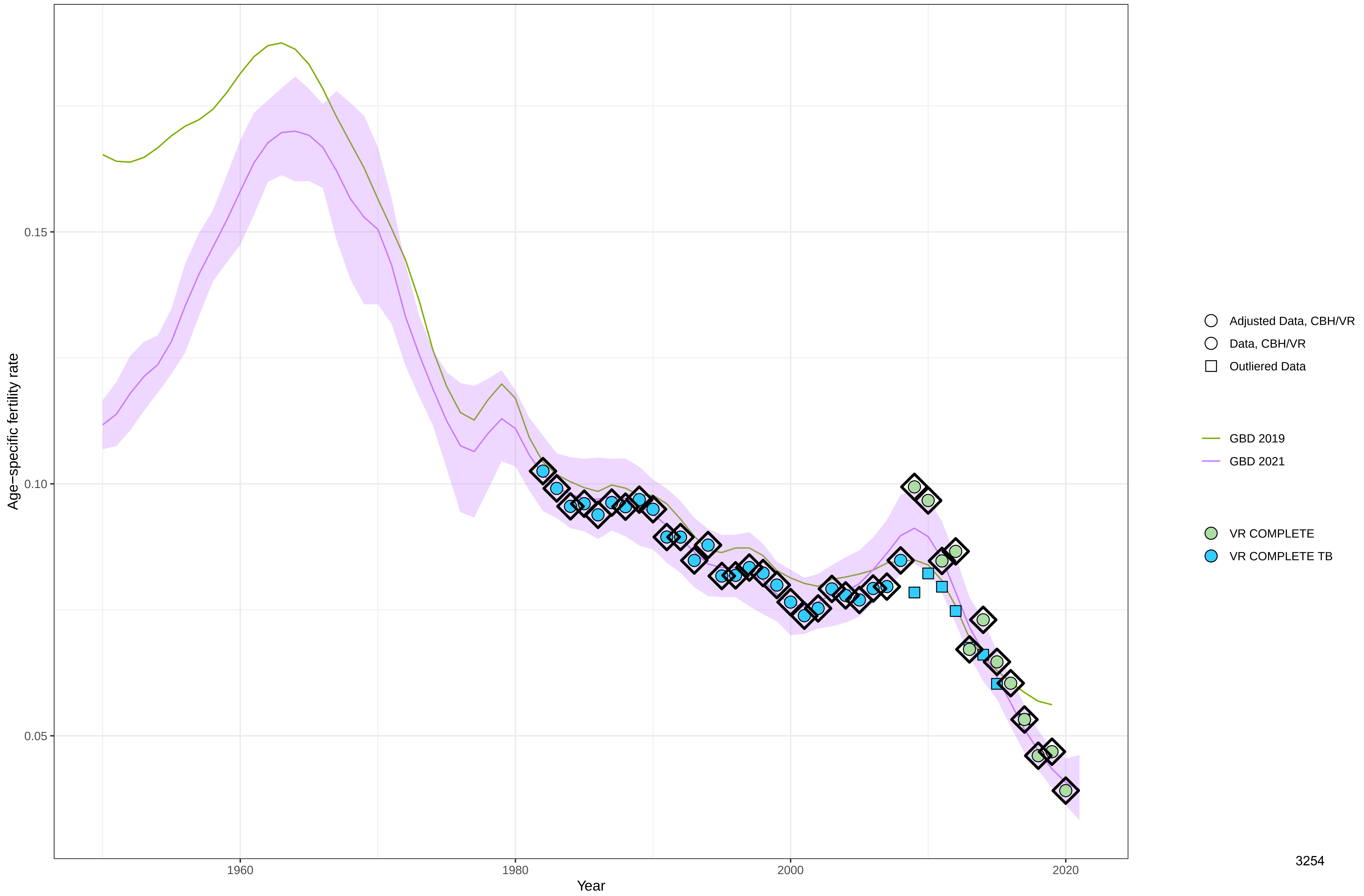

Age−specific fertility fitted trend lines, 1950−2021: Wiltshire, Ages: 25−29
Data points enclosed with a diamond are reference sources

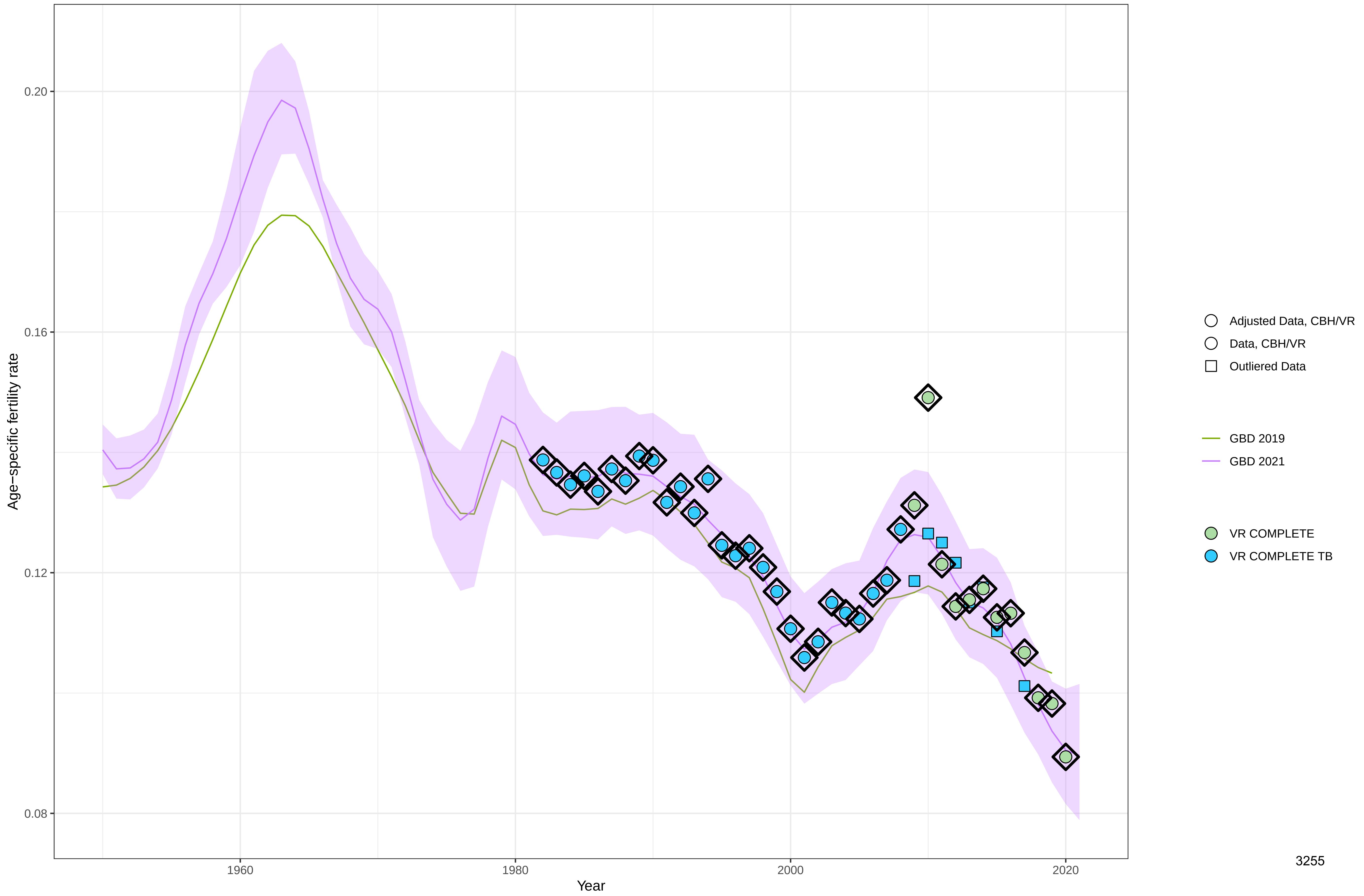

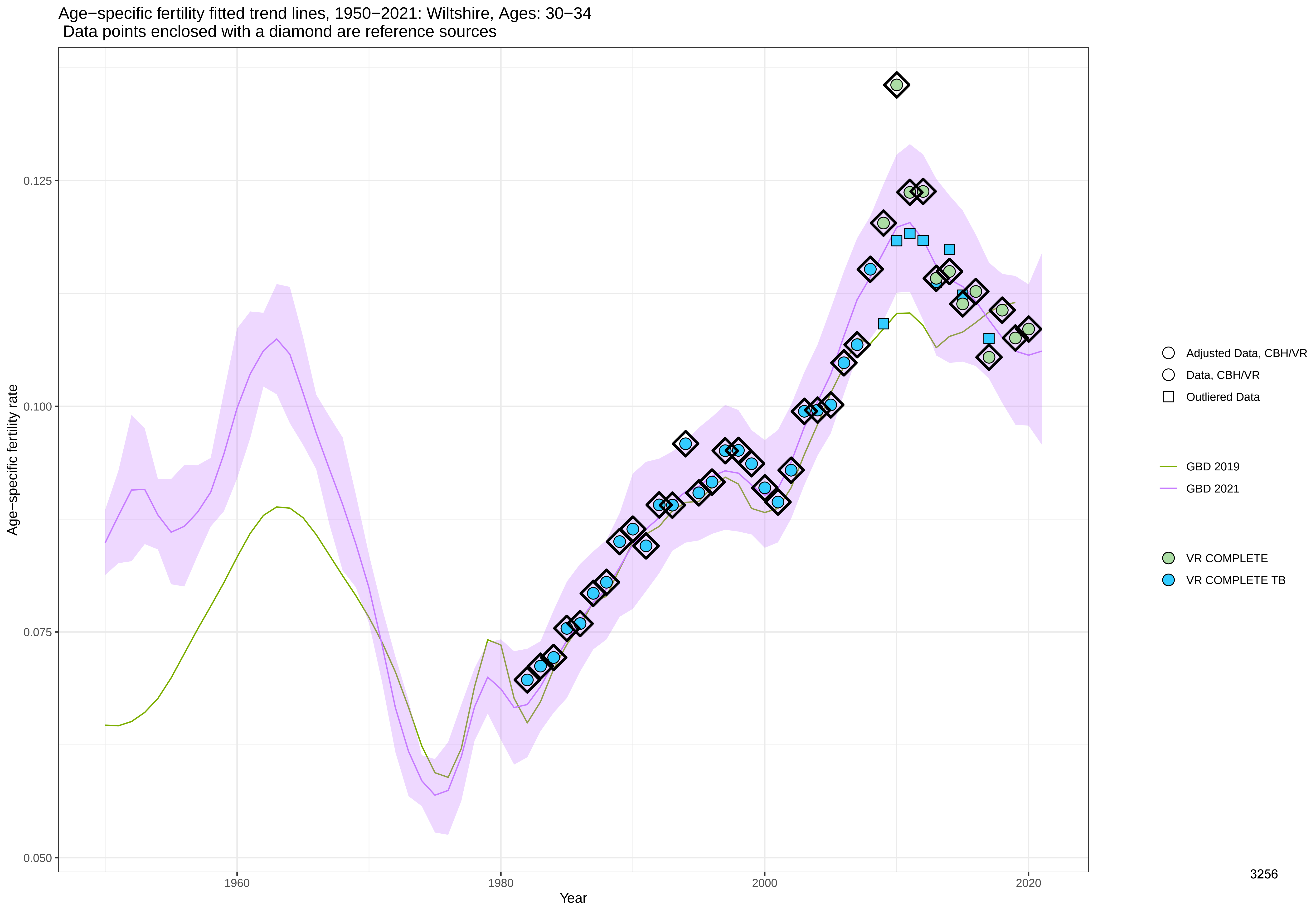
Age−specific fertility fitted trend lines, 1950−2021: Wiltshire, Ages: 30−34
Data points enclosed with a diamond are reference sources
Age−specific fertility rate
0.125
0.100
0.075
0.050
1960
1980
2000
2020
Year
Adjusted Data, CBH/VR
Data, CBH/VR
Outliered Data
GBD 2019
GBD 2021
VR COMPLETE
VR COMPLETE TB

Age−specific fertility fitted trend lines, 1950−2021: Wiltshire, Ages: 35−39
Data points enclosed with a diamond are reference sources

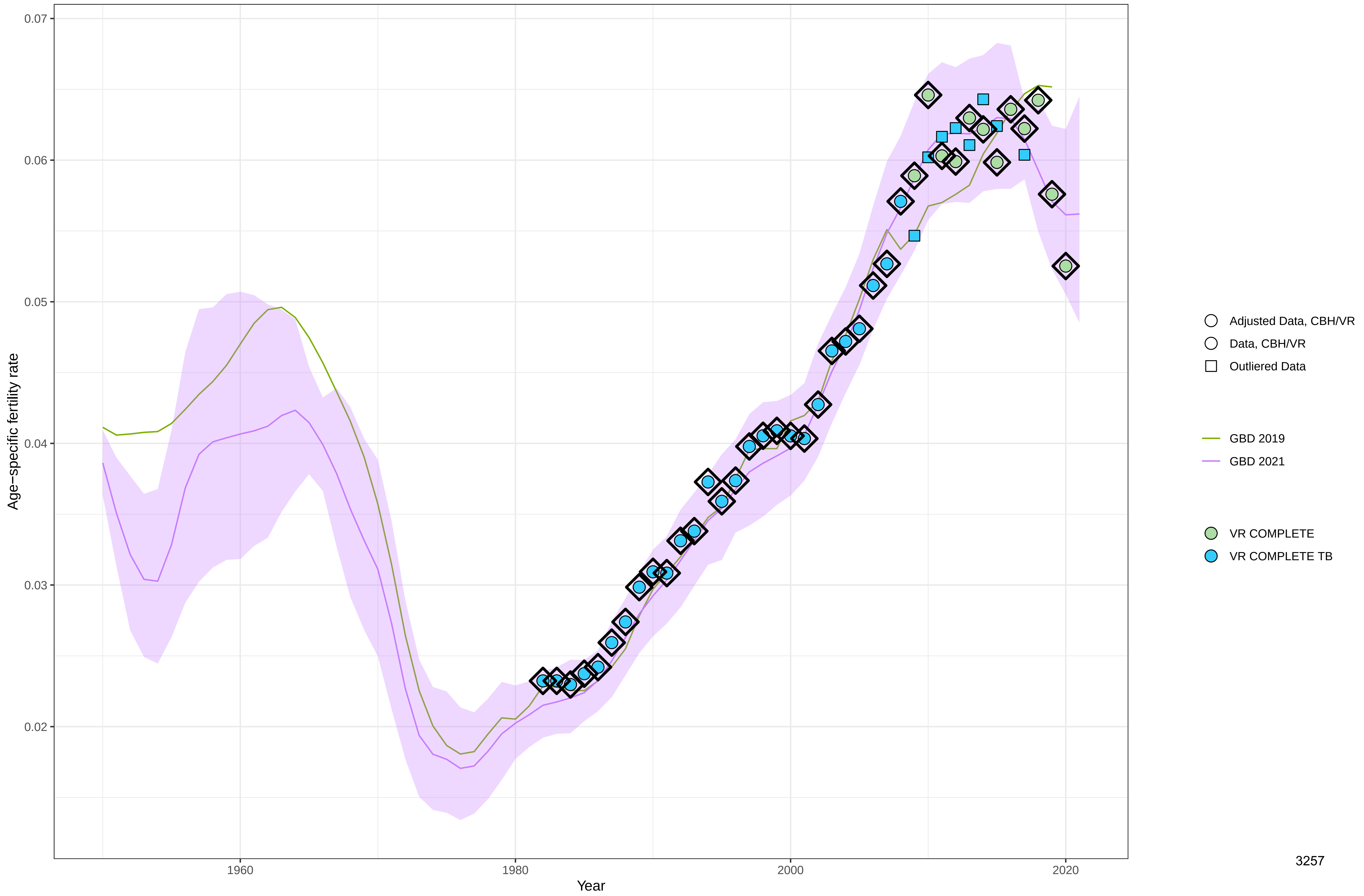

Age−specific fertility fitted trend lines, 1950−2021: Wiltshire, Ages: 40−44
Data points enclosed with a diamond are reference sources

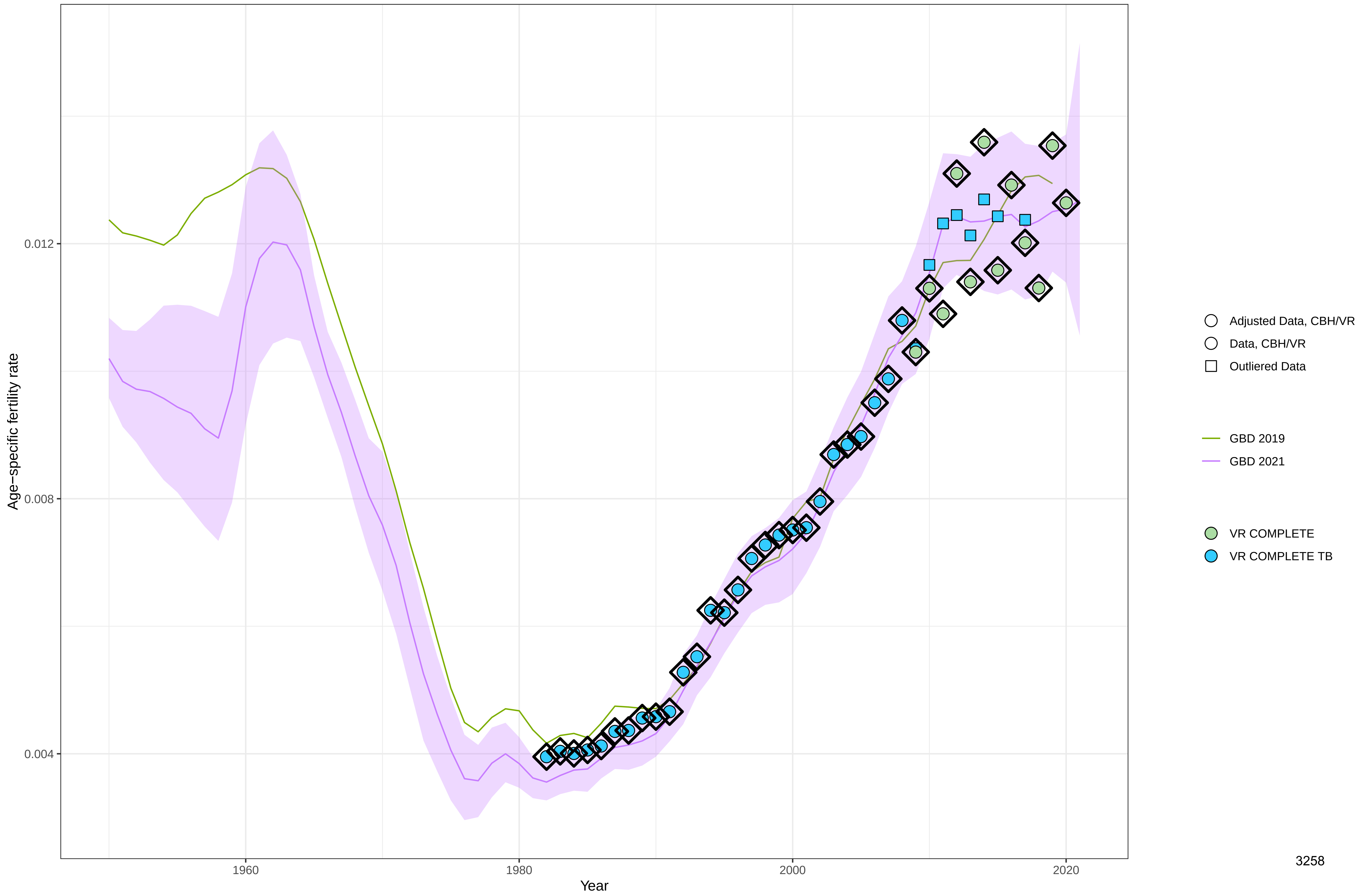

Age−specific fertility fitted trend lines, 1950−2021: Wiltshire, Ages: 45−49
Data points enclosed with a diamond are reference sources

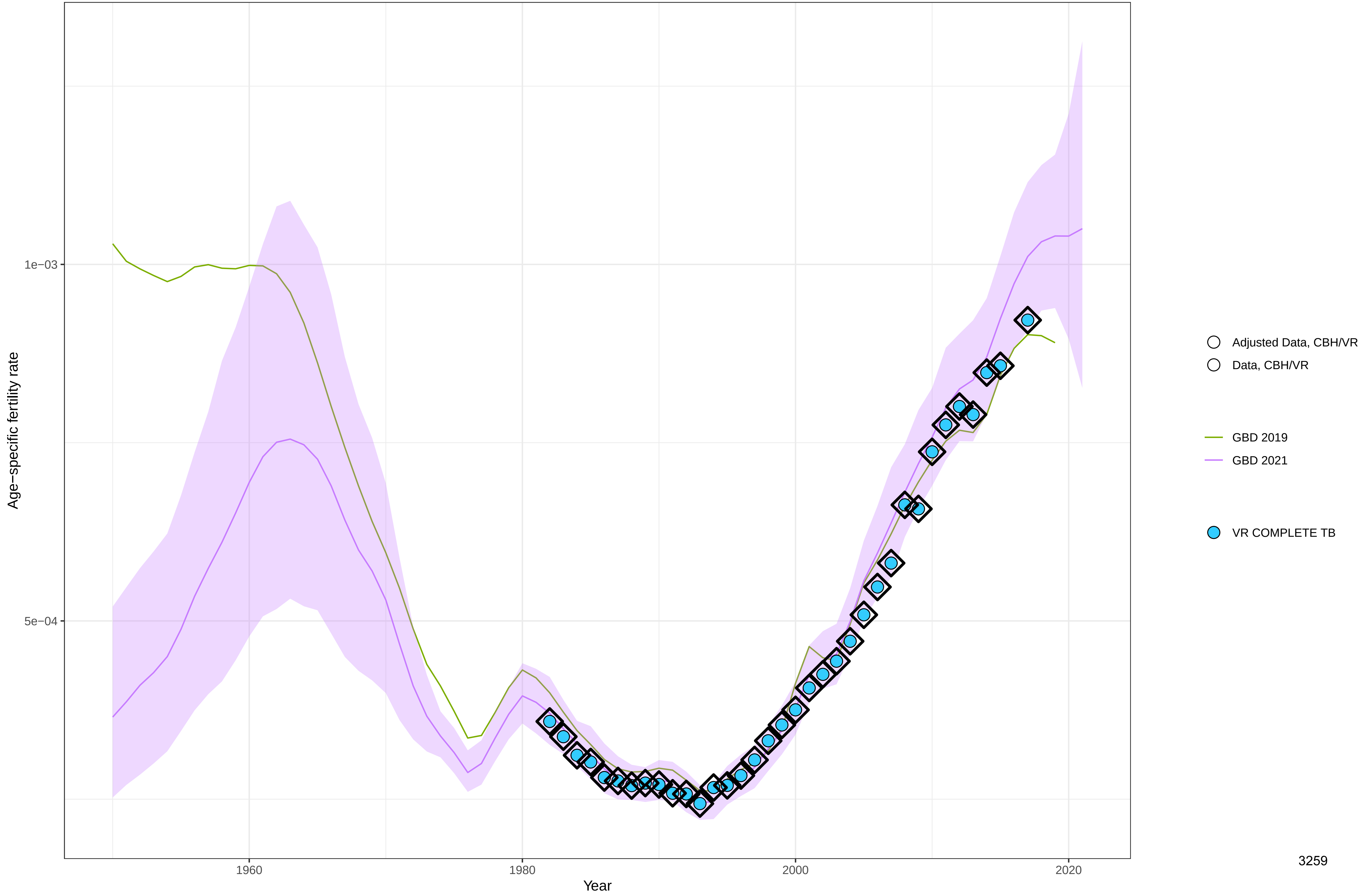

Age−specific fertility fitted trend lines, 1950−2021: Wiltshire, Ages: 50−54
Data points enclosed with a diamond are reference sources

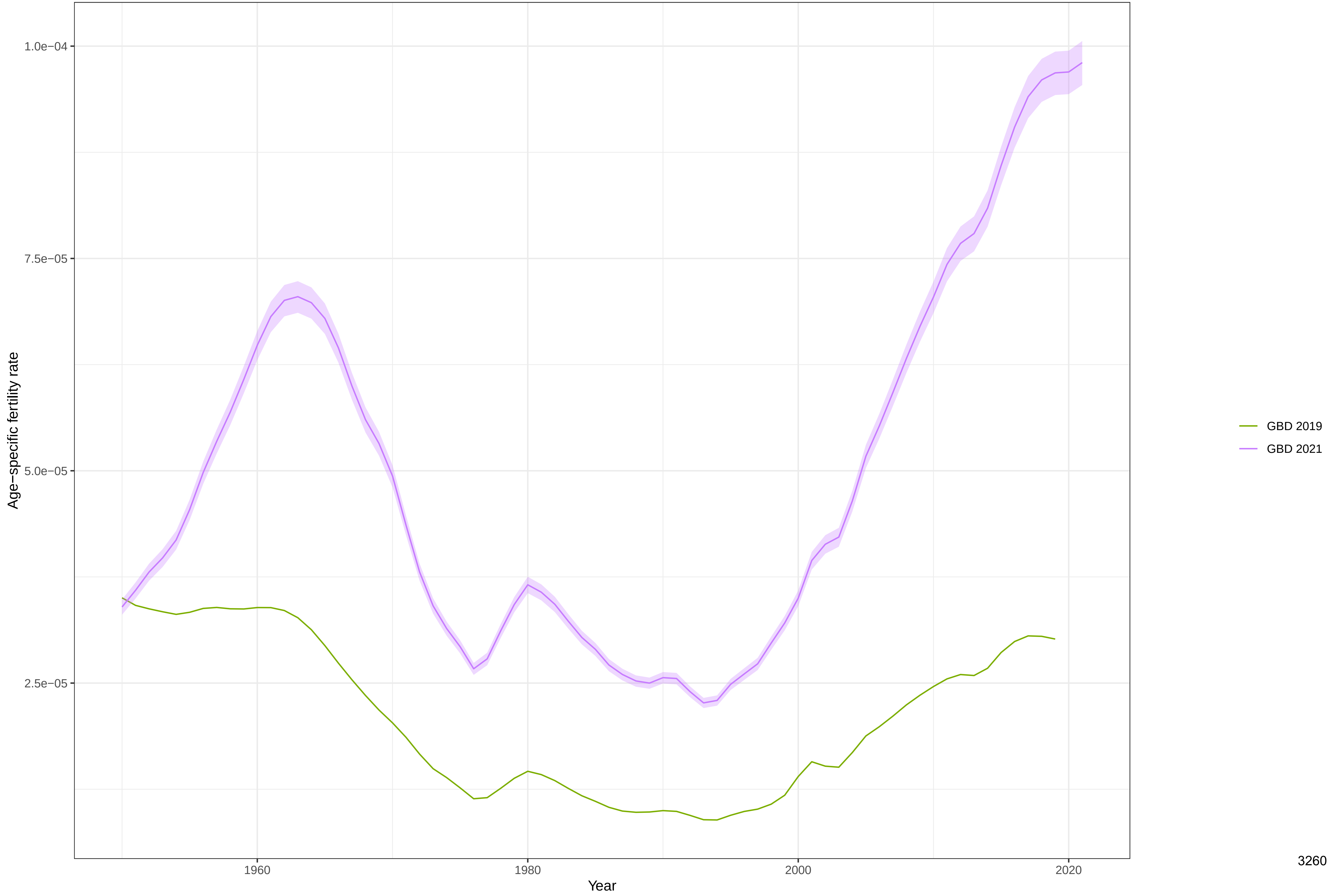

Total fertility rate fitted trend lines, 1950−2021: Wiltshire TFR
Data points enclosed with a diamond are reference sources

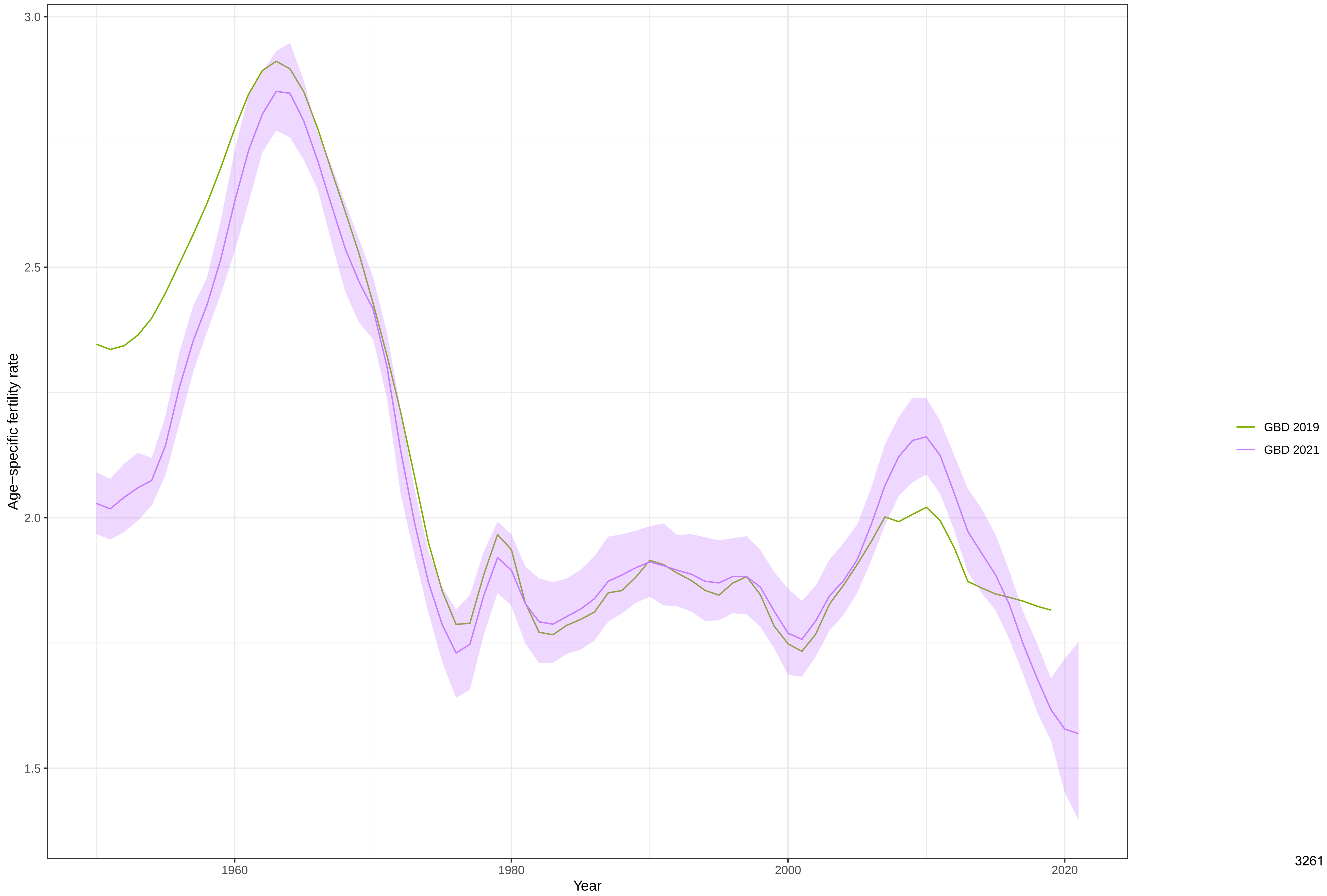

Age−specific fertility fitted trend lines, 1950−2021: West Midlands, Ages: 10−14
Data points enclosed with a diamond are reference sources

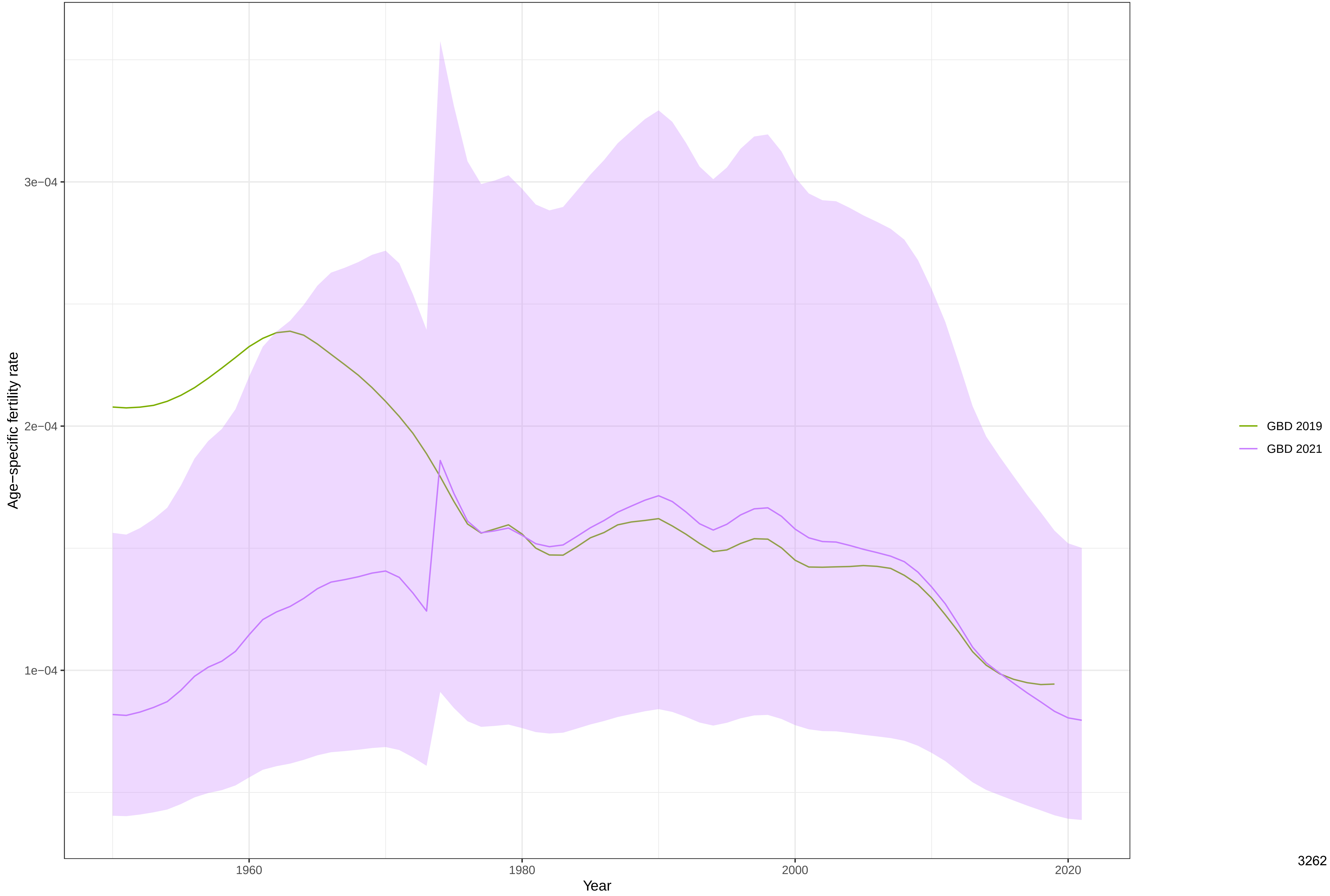

Age−specific fertility fitted trend lines, 1950−2021: West Midlands, Ages: 15−19
Data points enclosed with a diamond are reference sources

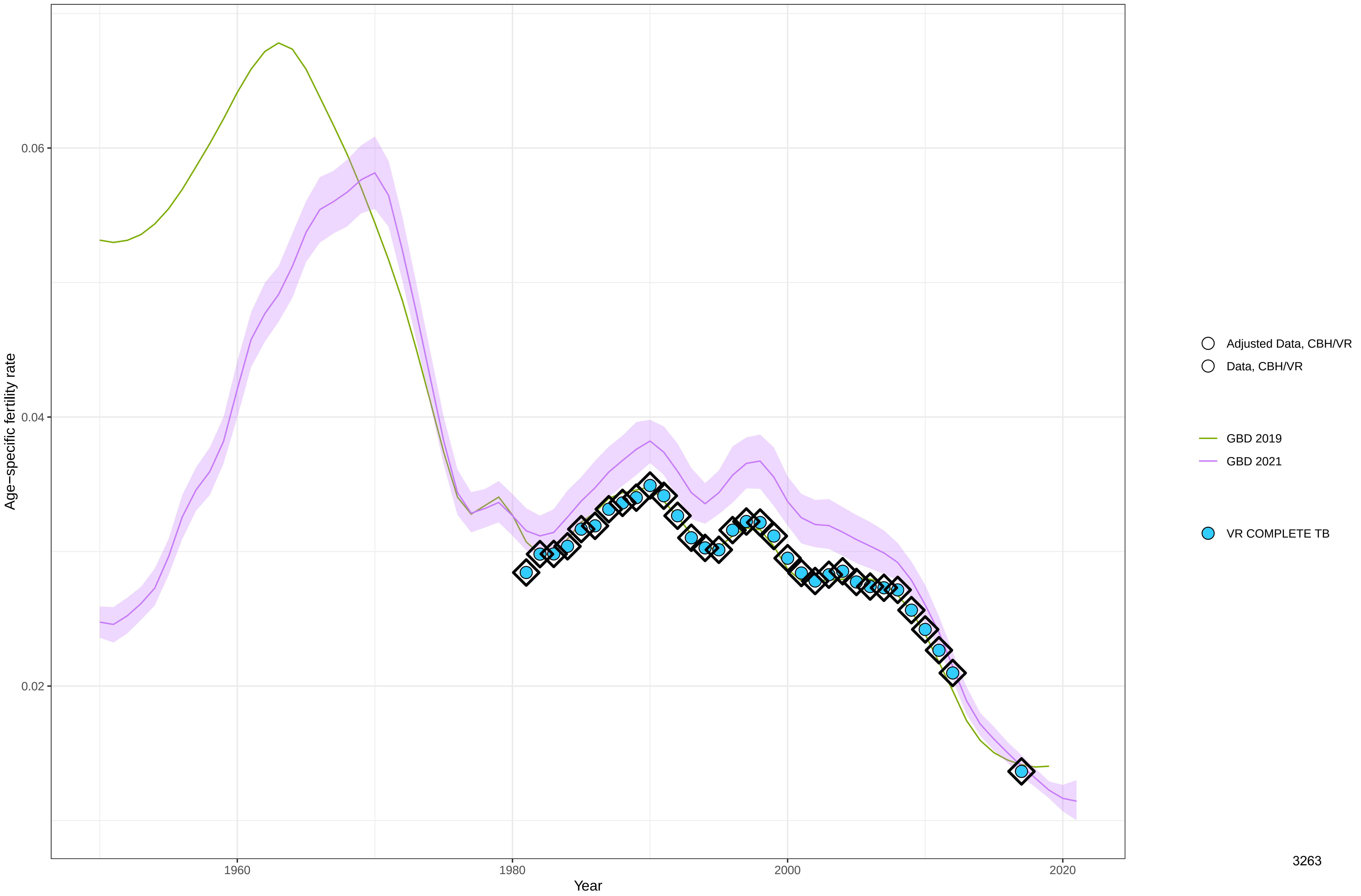

Age−specific fertility fitted trend lines, 1950−2021: West Midlands, Ages: 20−24
Data points enclosed with a diamond are reference sources

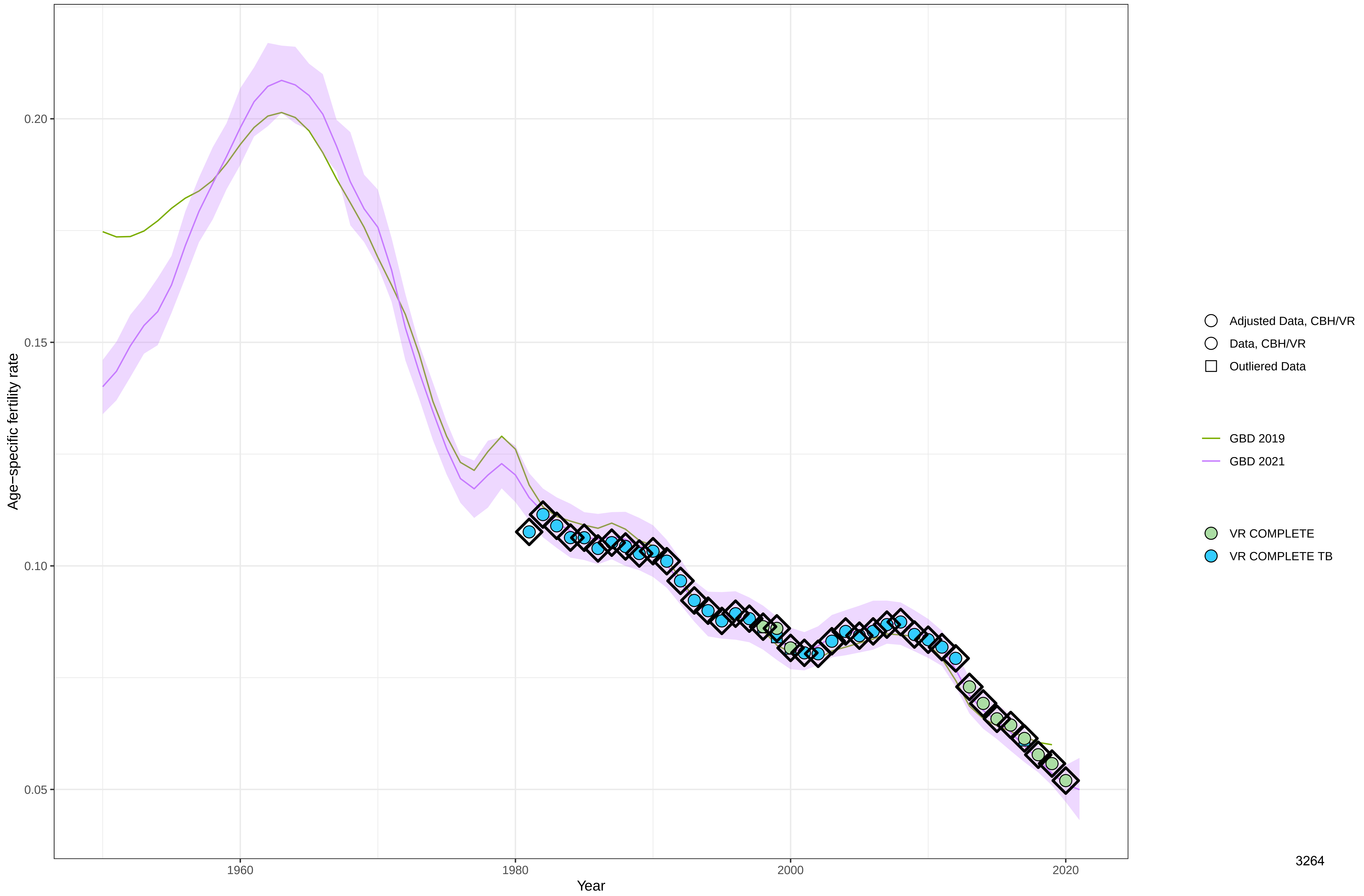

Age−specific fertility fitted trend lines, 1950−2021: West Midlands, Ages: 25−29
Data points enclosed with a diamond are reference sources

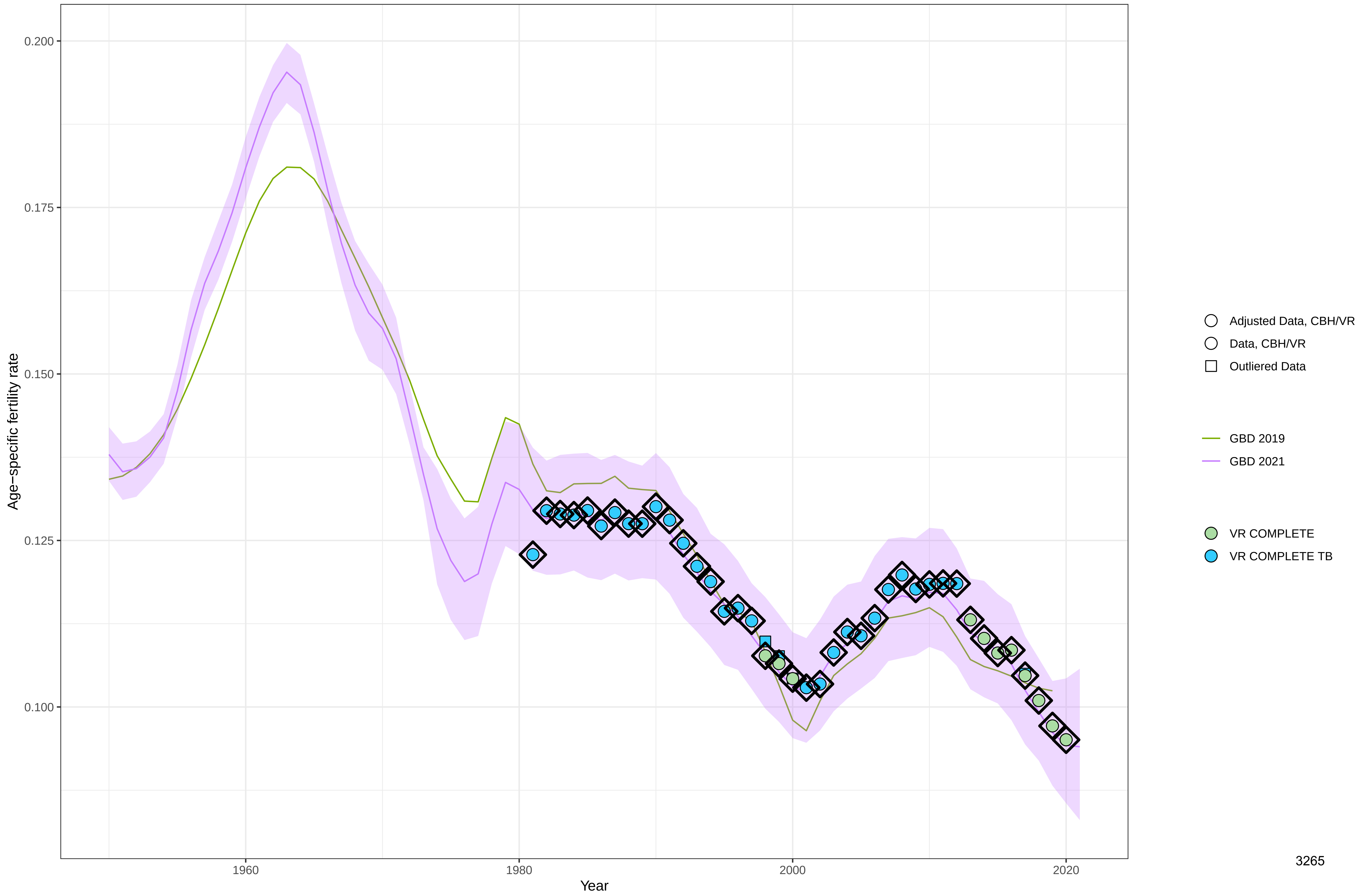

Age−specific fertility fitted trend lines, 1950−2021: West Midlands, Ages: 30−34
Data points enclosed with a diamond are reference sources

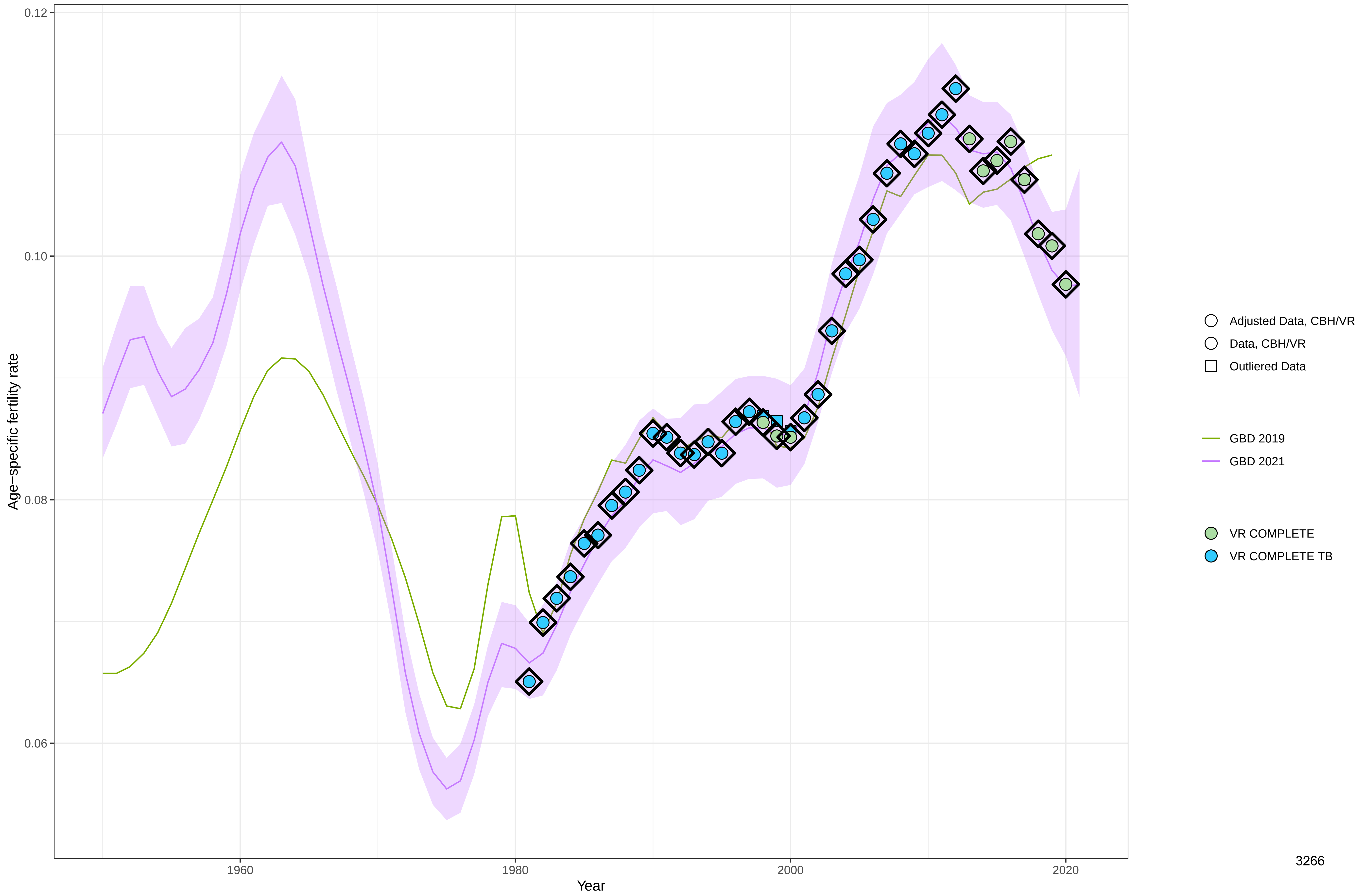

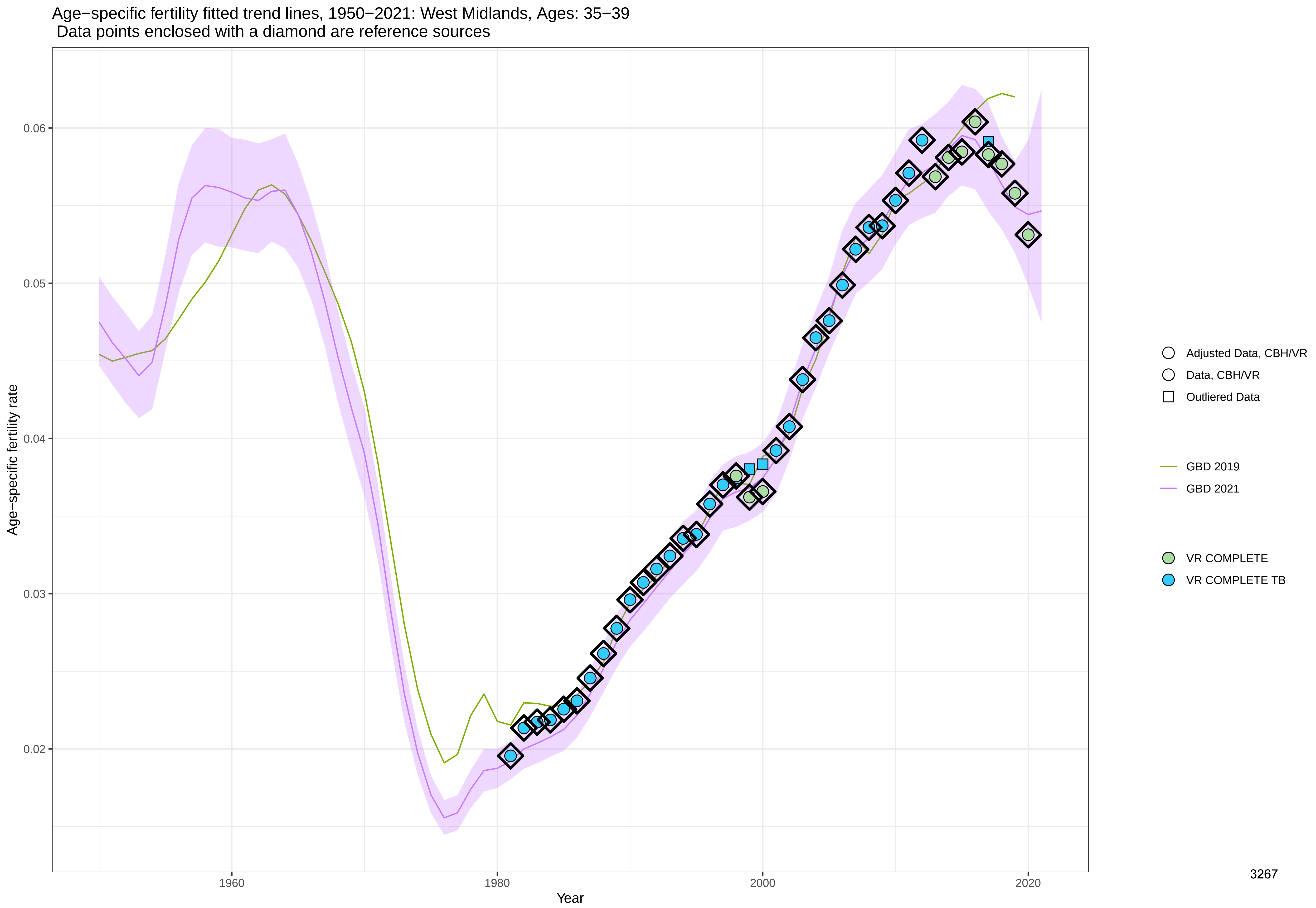
Age−specific fertility fitted trend lines, 1950−2021: West Midlands, Ages: 35−39
Data points enclosed with a diamond are reference sources
Age−specific fertility rate
0.06
0.05
0.04
0.03
0.02
1960
1980
2000
2020
Year
Adjusted Data, CBH/VR
Data, CBH/VR
Outliered Data
GBD 2019
GBD 2021
VR COMPLETE
VR COMPLETE TB

Age−specific fertility fitted trend lines, 1950−2021: West Midlands, Ages: 40−44
Data points enclosed with a diamond are reference sources

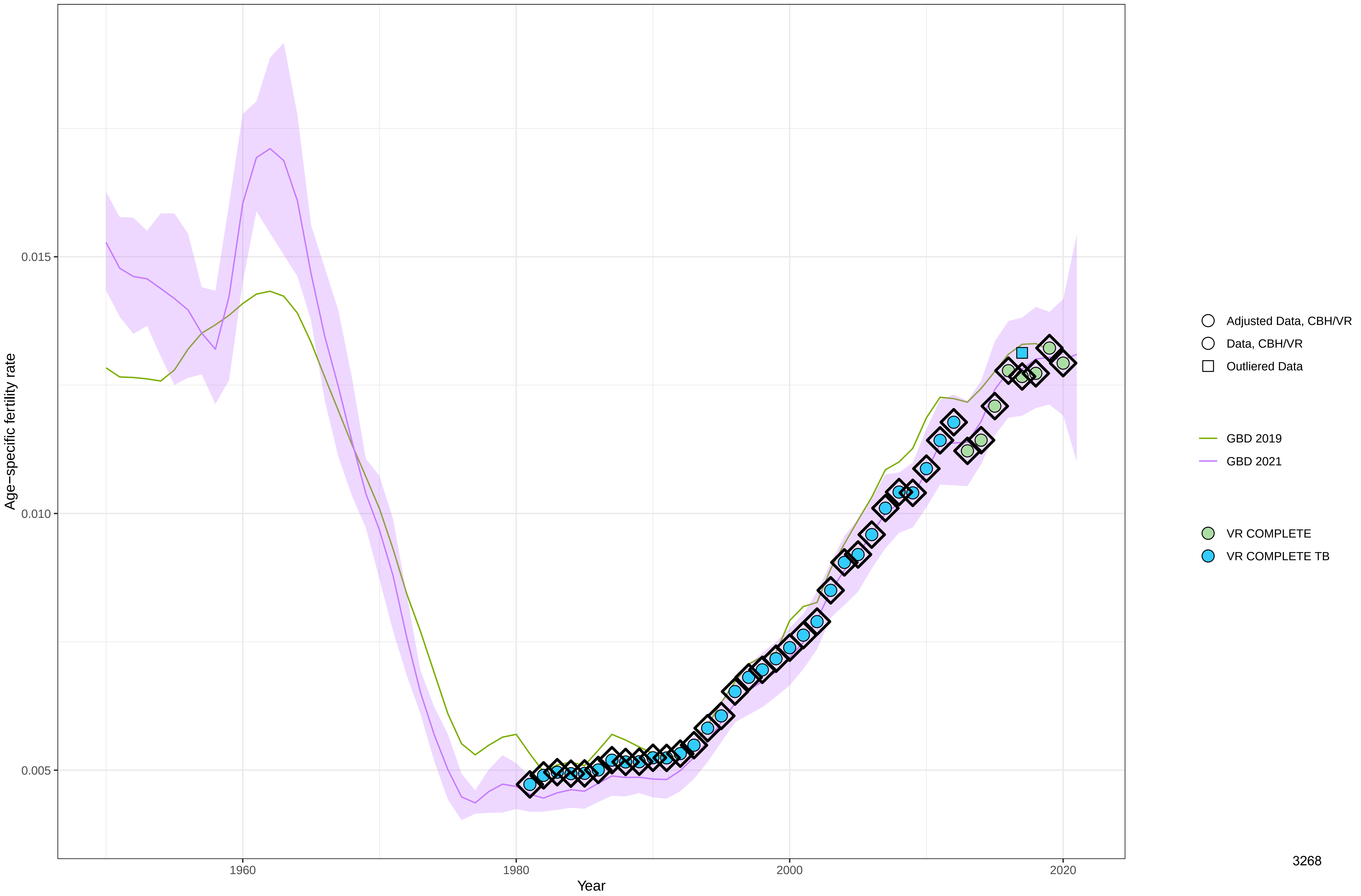

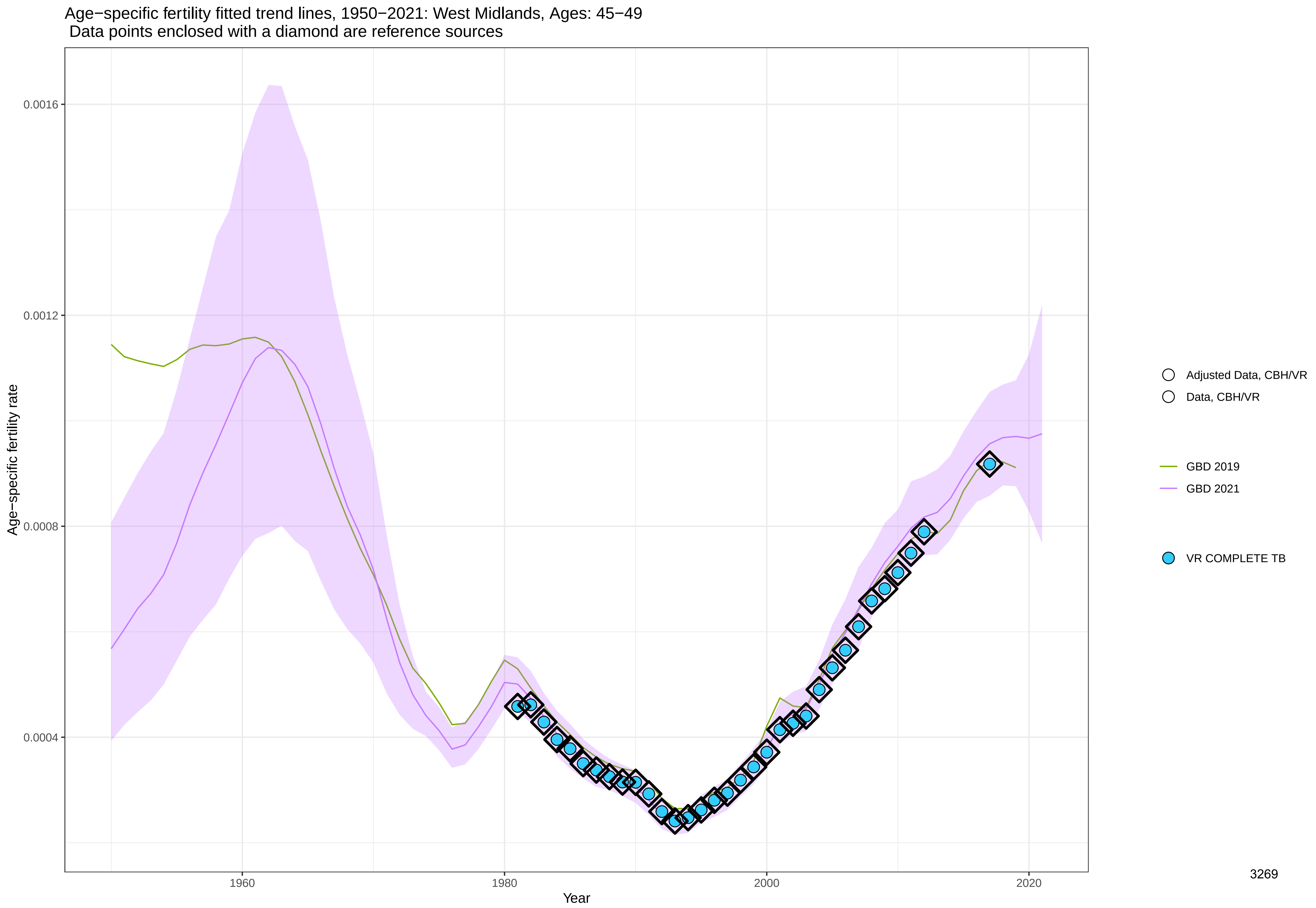

Age−specific fertility fitted trend lines, 1950−2021: West Midlands, Ages: 45−49
Data points enclosed with a diamond are reference sources
0.0016
0.0012
0.0008
0.0004
Age−specific fertility rate
1960
1980
2000
2020
Year
Adjusted Data, CBH/VR
Data, CBH/VR
GBD 2019
GBD 2021
VR COMPLETE TB

Age−specific fertility fitted trend lines, 1950−2021: West Midlands, Ages: 50−54
Data points enclosed with a diamond are reference sources

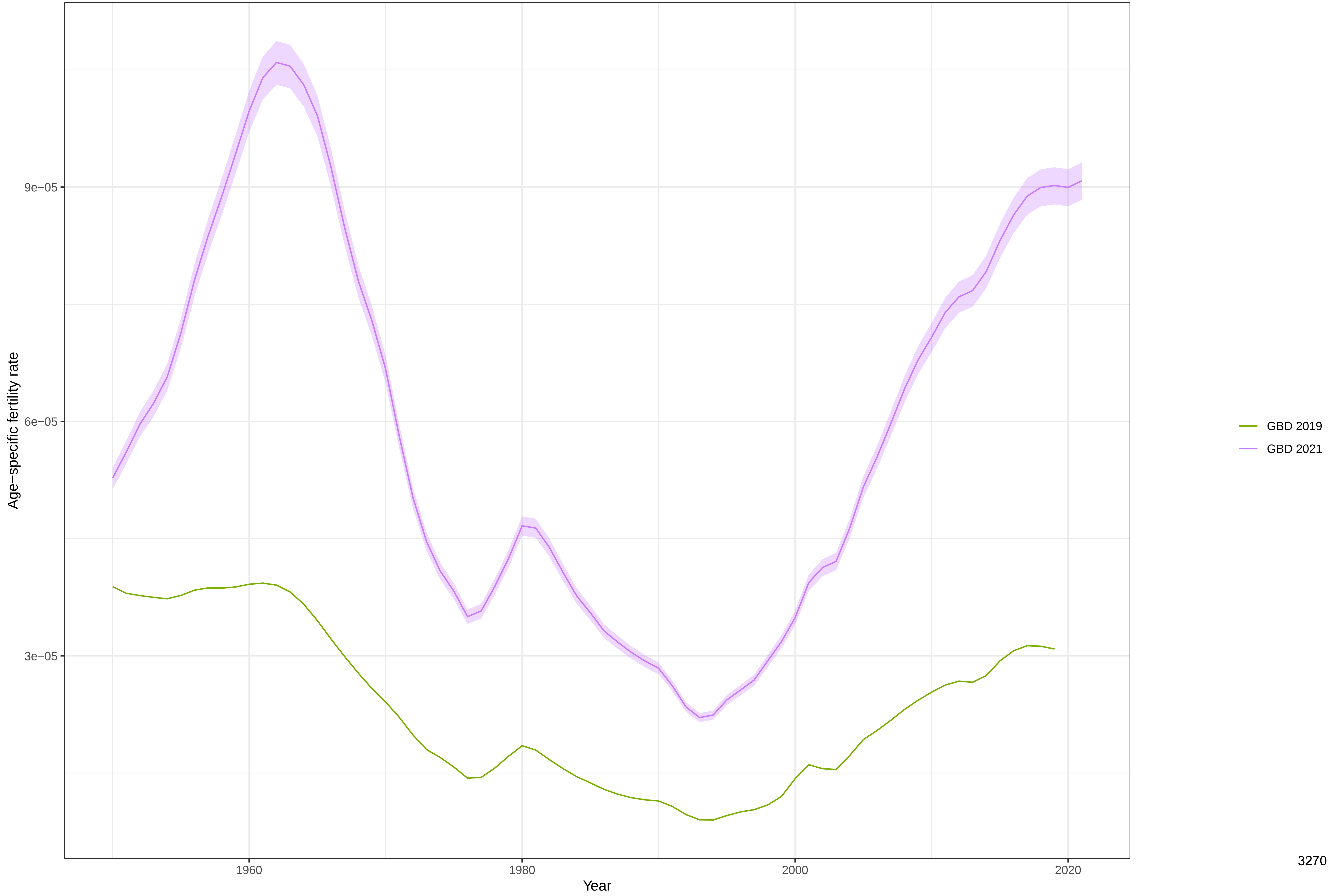

Total fertility rate fitted trend lines, 1950−2021: West Midlands TFR
Data points enclosed with a diamond are reference sources

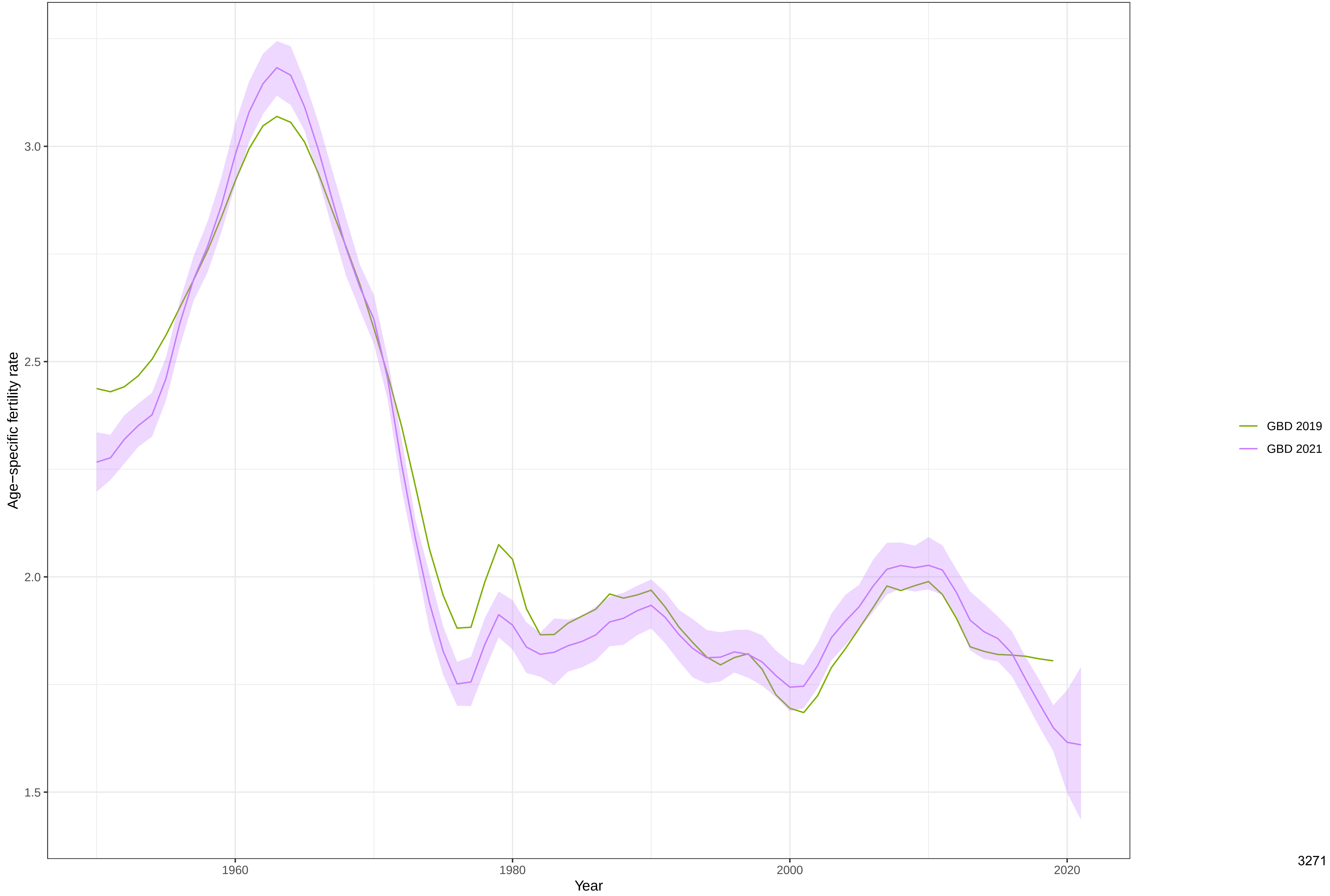

Age−specific fertility fitted trend lines, 1950−2021: Birmingham, Ages: 10−14
Data points enclosed with a diamond are reference sources

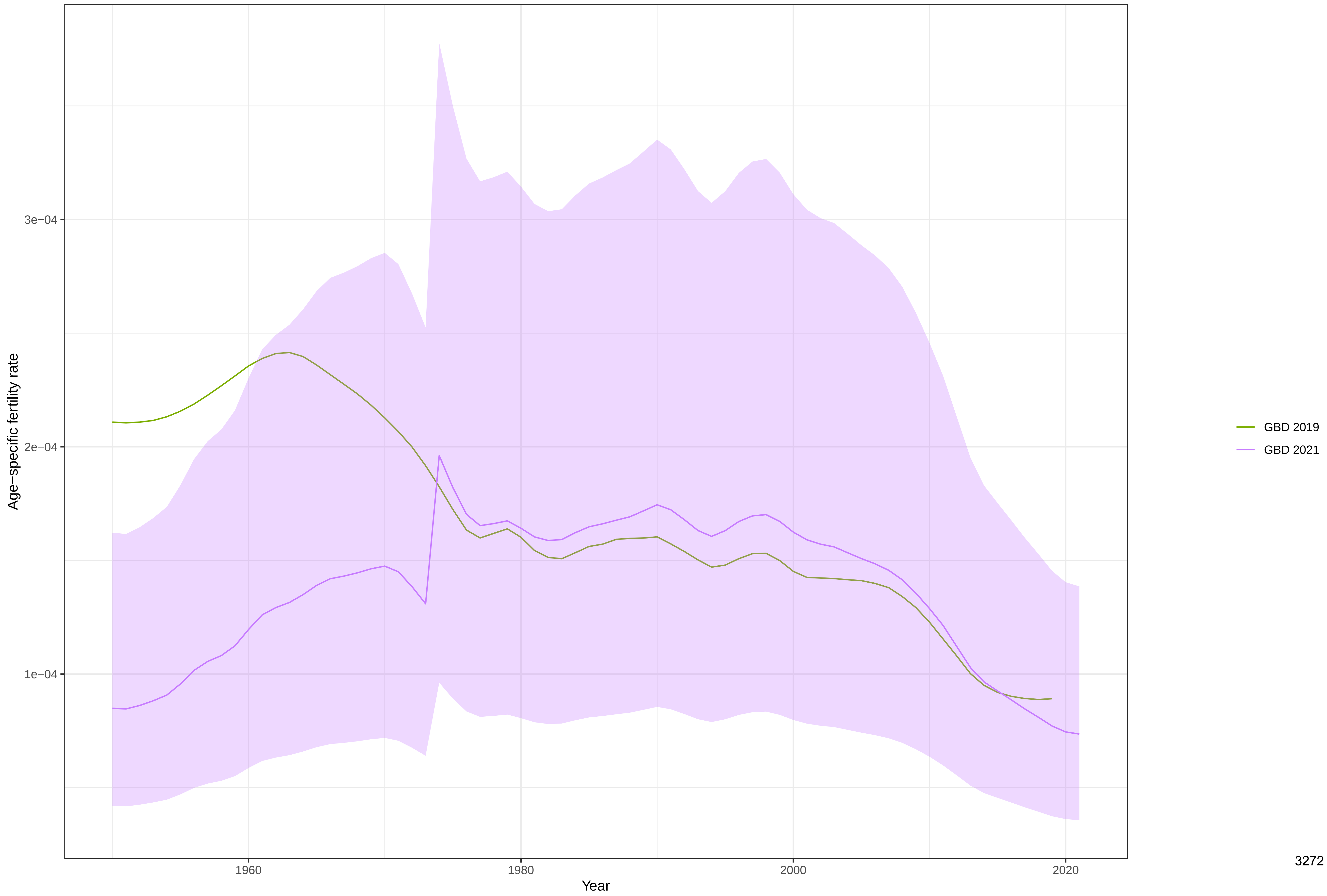

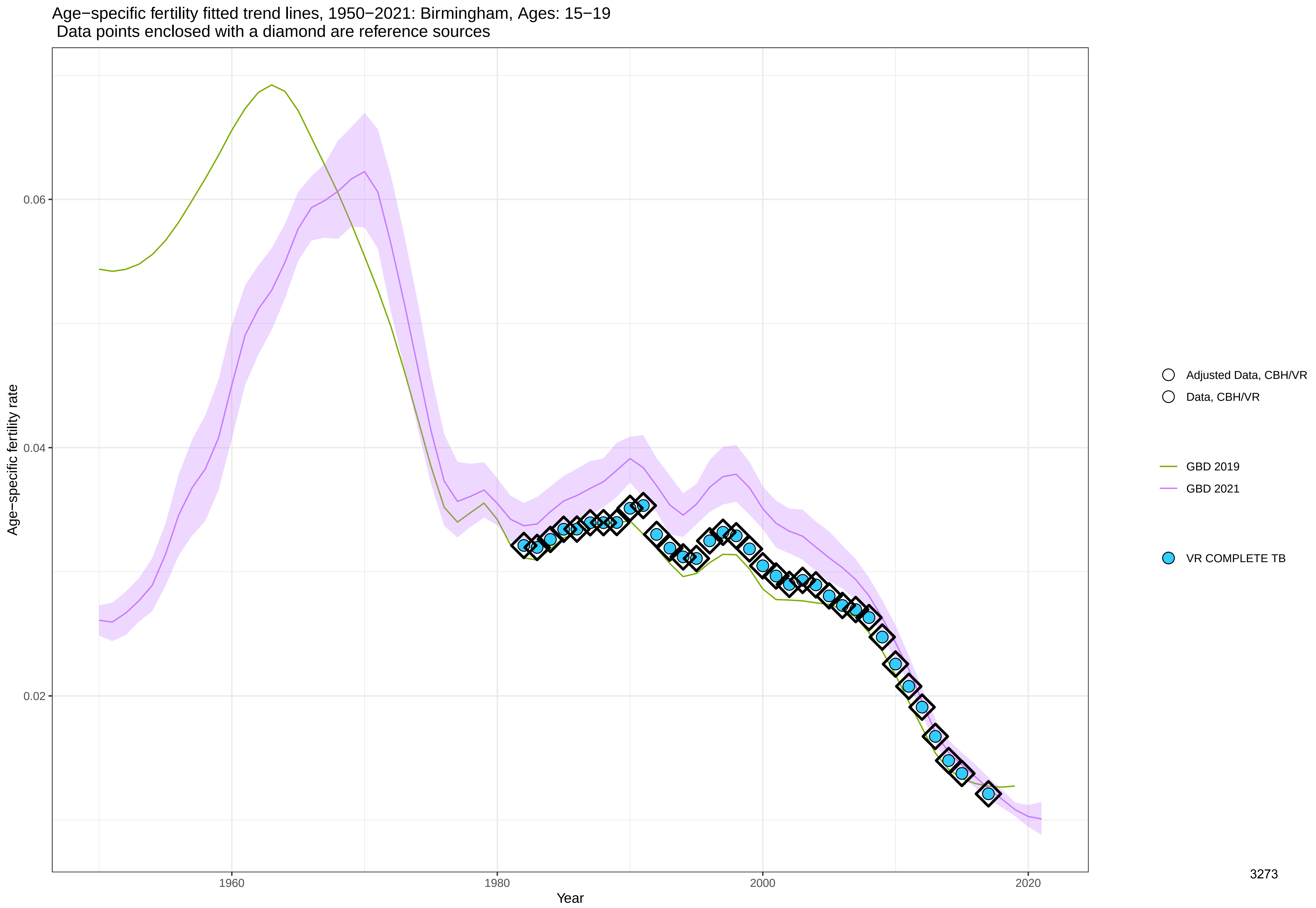

Age−specific fertility fitted trend lines, 1950−2021: Birmingham, Ages: 15−19
Data points enclosed with a diamond are reference sources
0.06
0.04
0.02
Age−specific fertility rate
1960
1980
2000
2020
Year
Adjusted Data, CBH/VR
Data, CBH/VR
GBD 2019
GBD 2021
VR COMPLETE TB

Age−specific fertility fitted trend lines, 1950−2021: Birmingham, Ages: 20−24
Data points enclosed with a diamond are reference sources

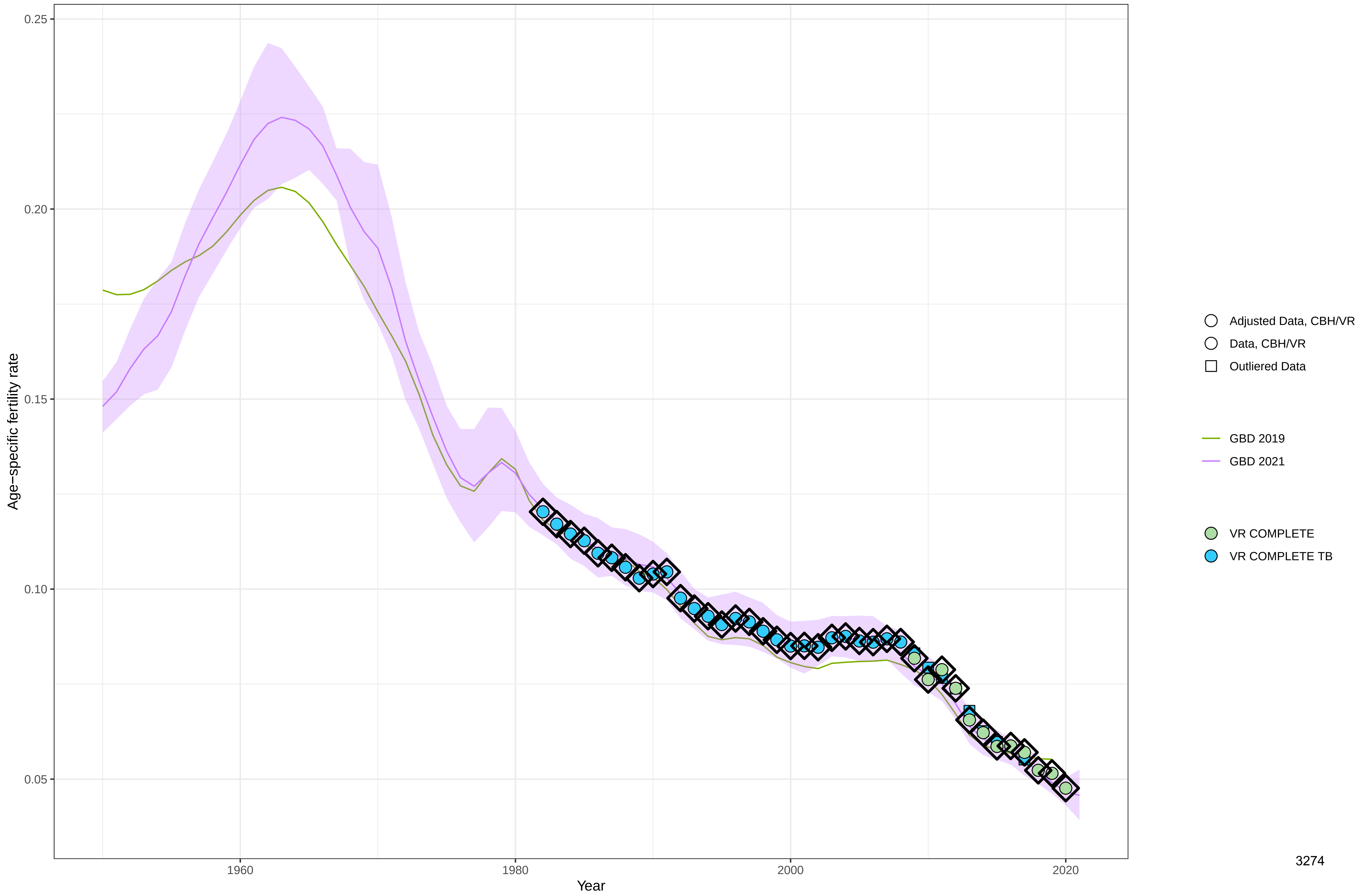

Age−specific fertility fitted trend lines, 1950−2021: Birmingham, Ages: 25−29
Data points enclosed with a diamond are reference sources

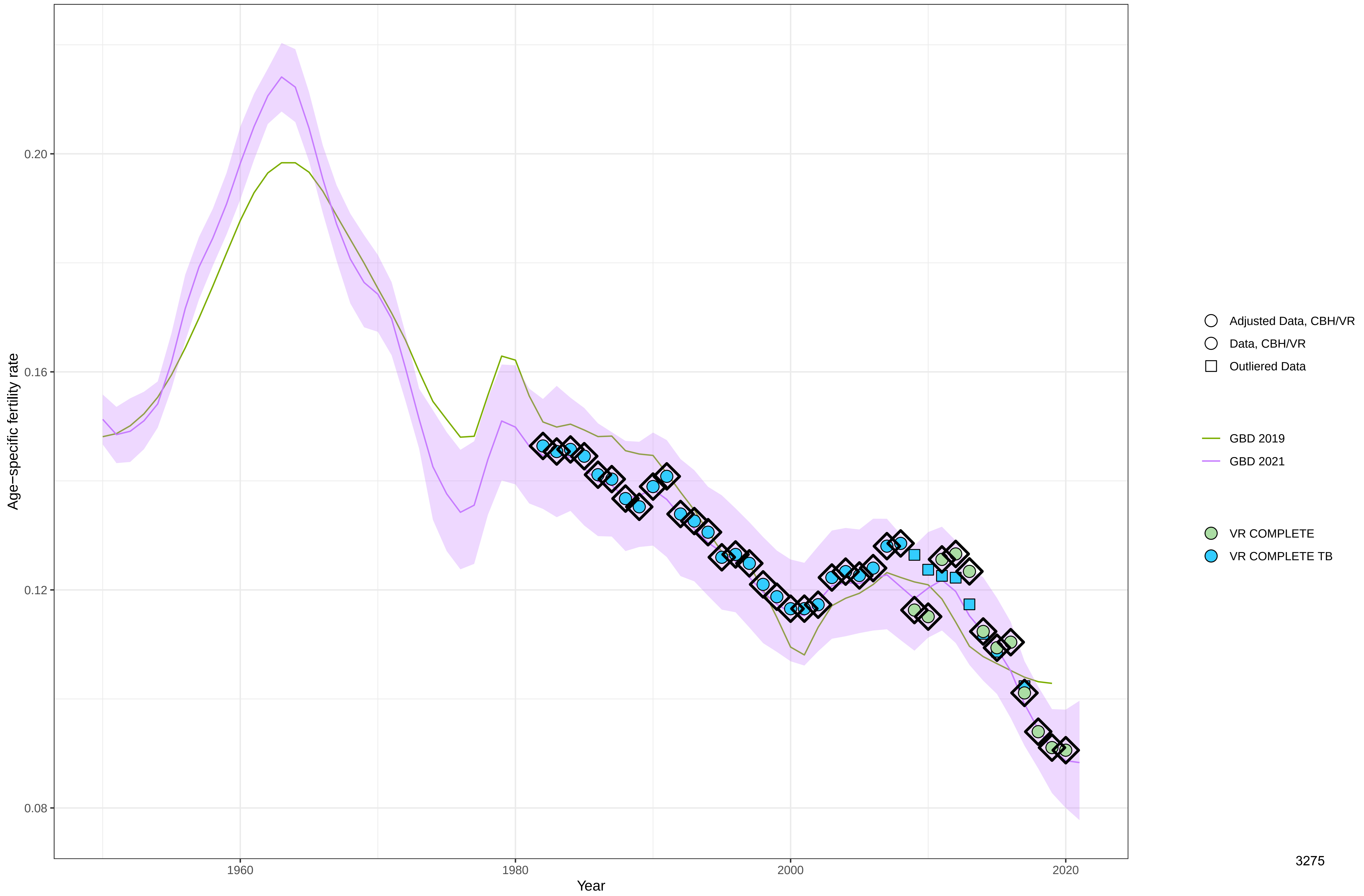

Age−specific fertility fitted trend lines, 1950−2021: Birmingham, Ages: 30−34
Data points enclosed with a diamond are reference sources

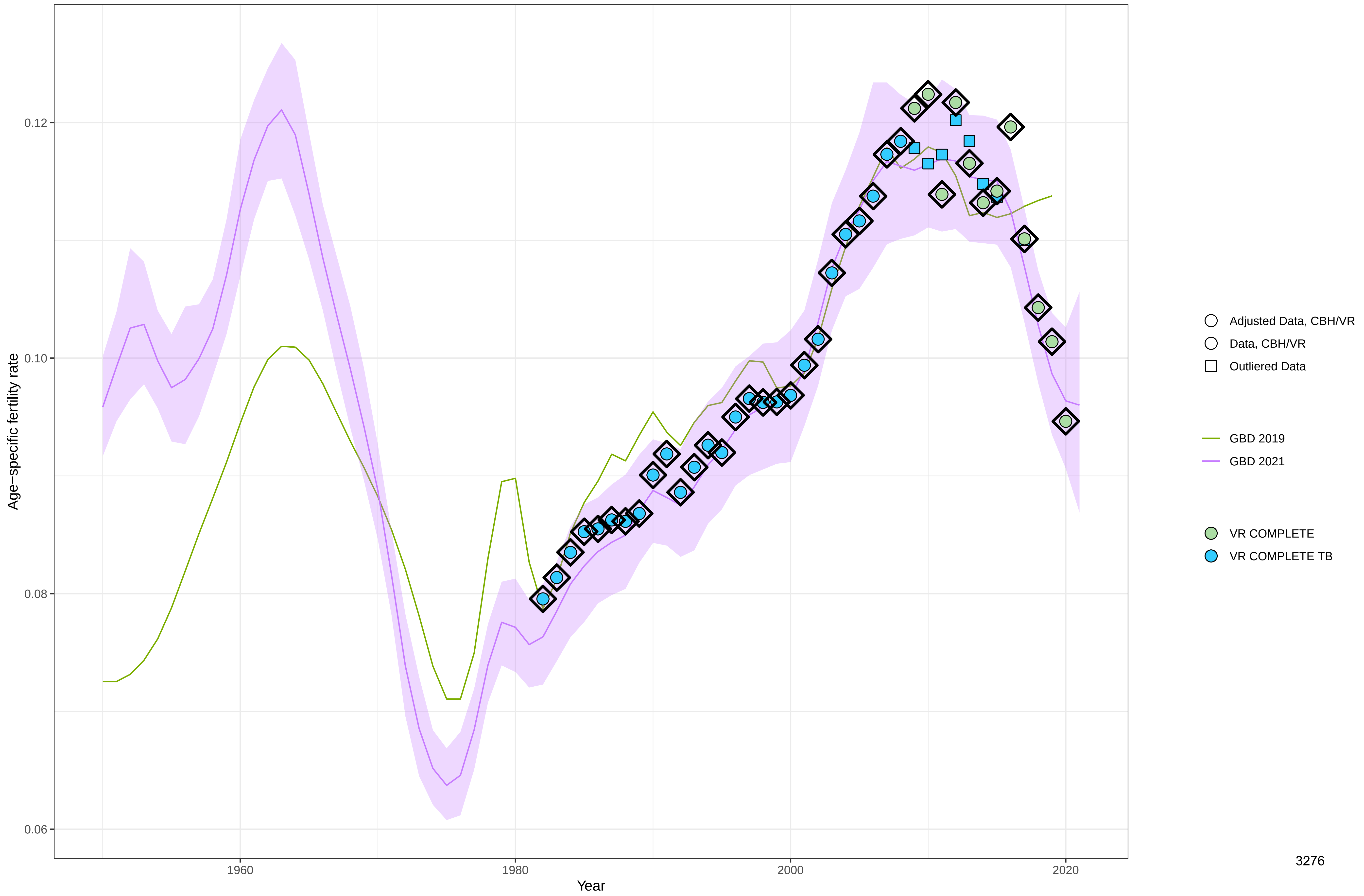

Age−specific fertility fitted trend lines, 1950−2021: Birmingham, Ages: 35−39
Data points enclosed with a diamond are reference sources

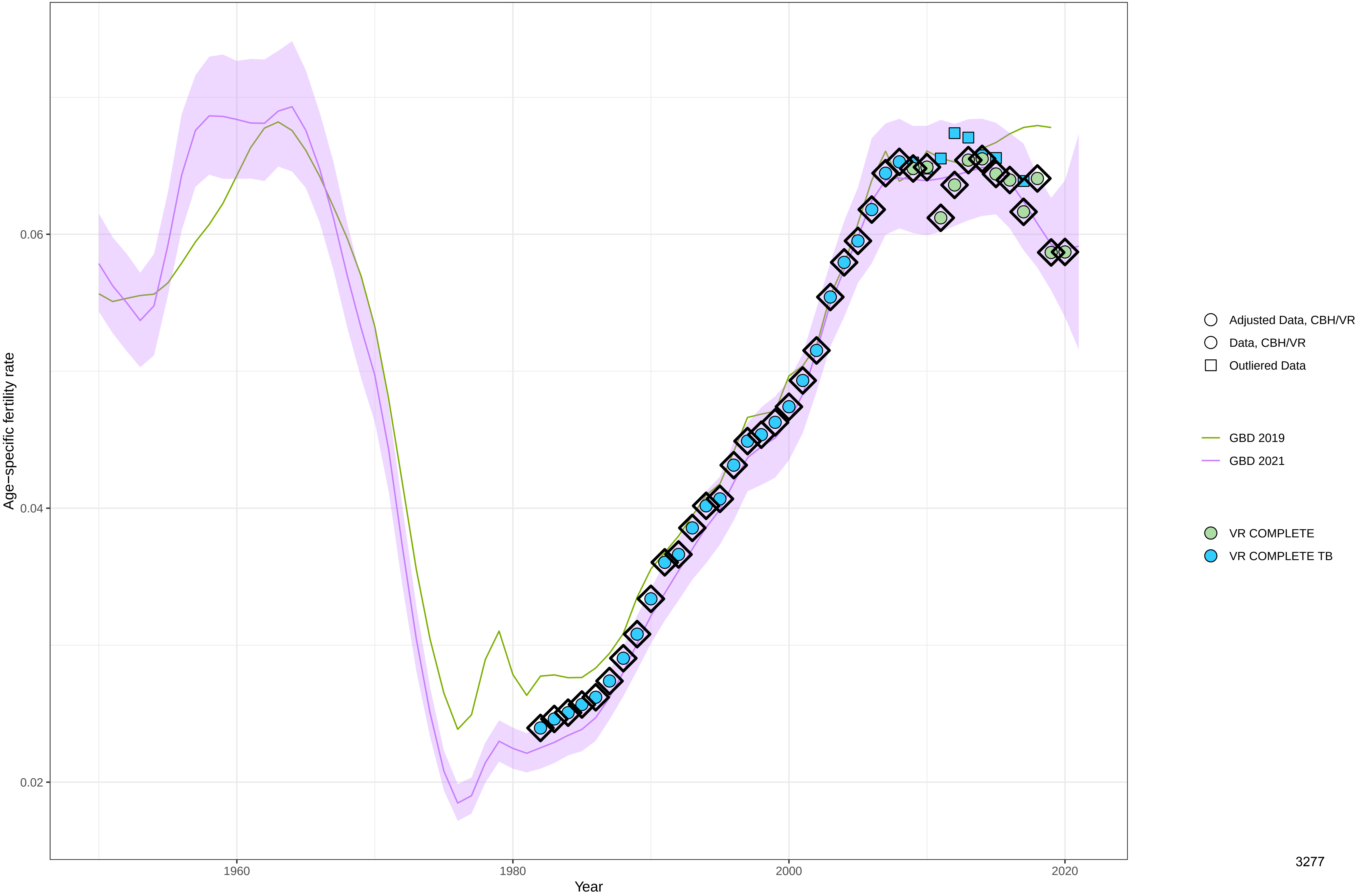

Age−specific fertility fitted trend lines, 1950−2021: Birmingham, Ages: 40−44
Data points enclosed with a diamond are reference sources

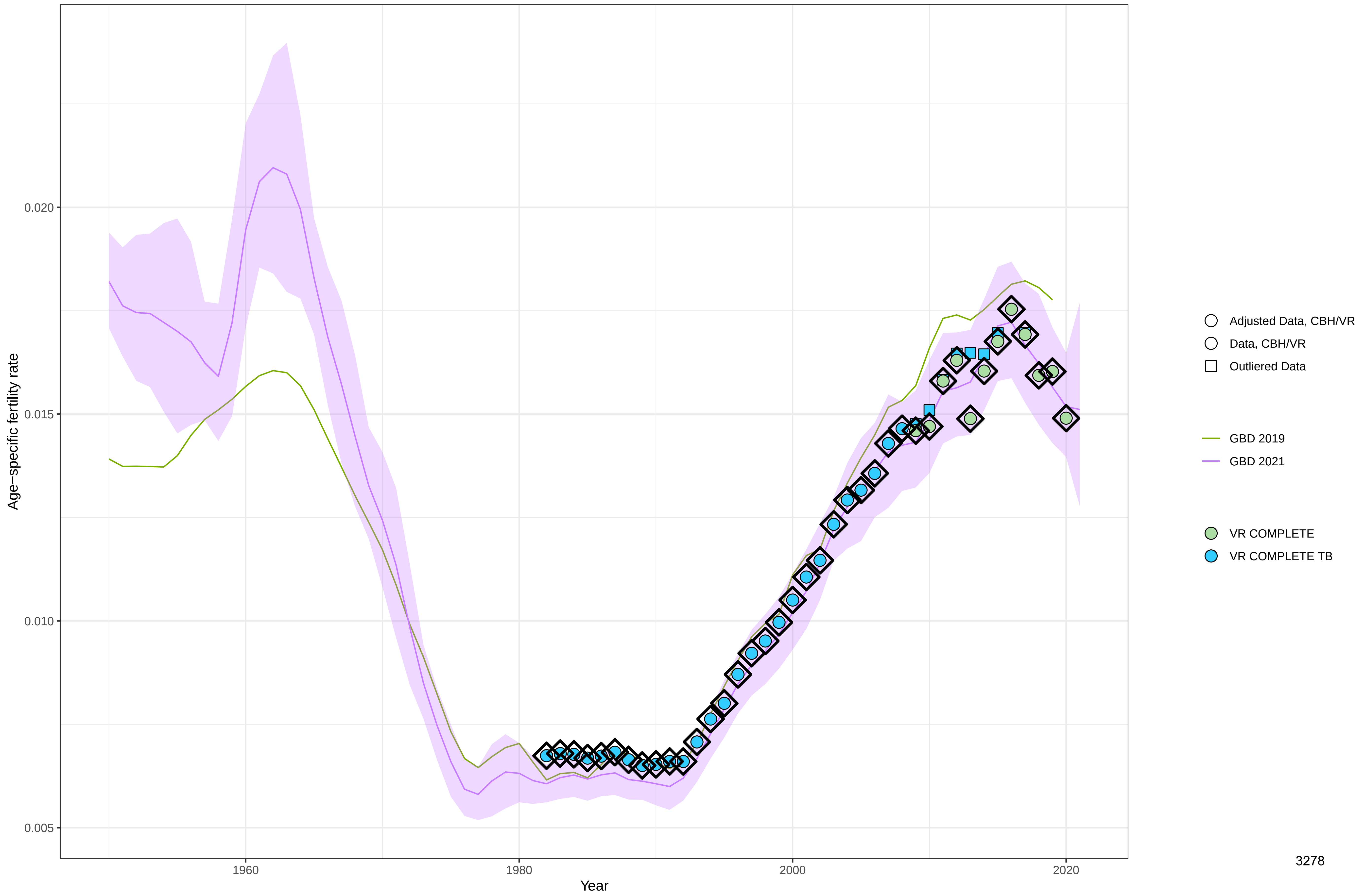

Age−specific fertility fitted trend lines, 1950−2021: Birmingham, Ages: 45−49
Data points enclosed with a diamond are reference sources

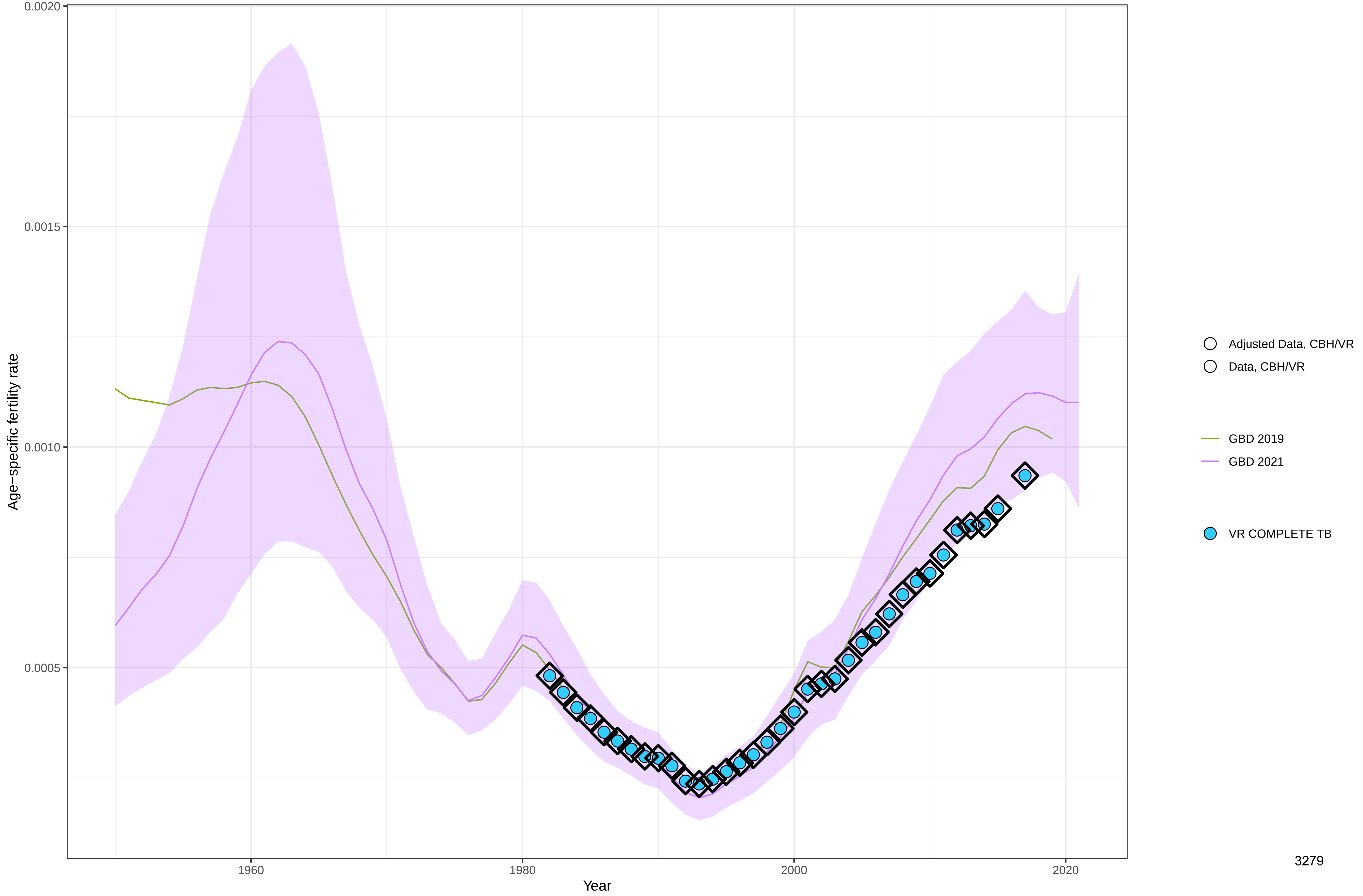

Age−specific fertility fitted trend lines, 1950−2021: Birmingham, Ages: 50−54
Data points enclosed with a diamond are reference sources

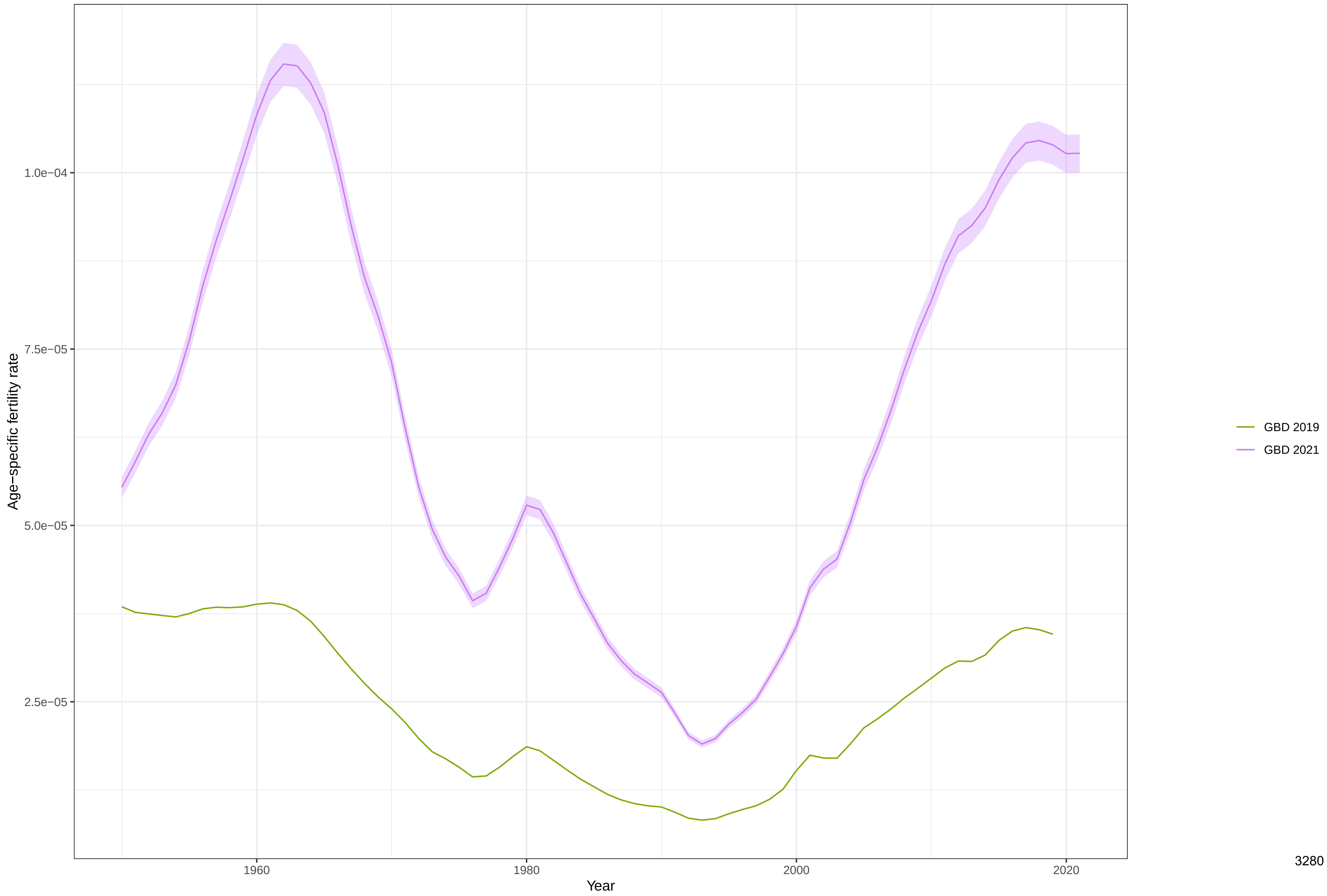

Total fertility rate fitted trend lines, 1950−2021: Birmingham TFR
Data points enclosed with a diamond are reference sources

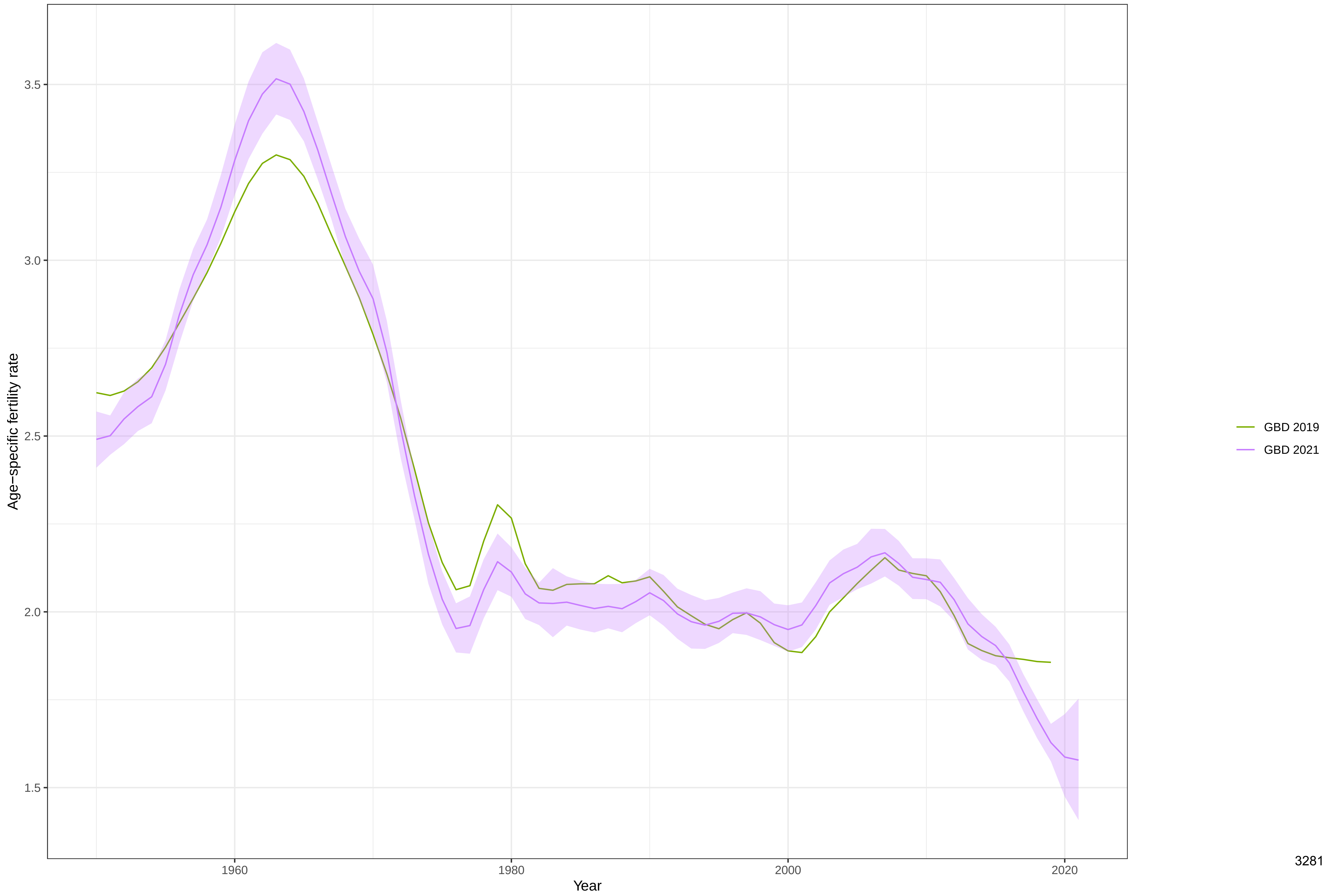

Age−specific fertility fitted trend lines, 1950−2021: Coventry, Ages: 10−14
Data points enclosed with a diamond are reference sources

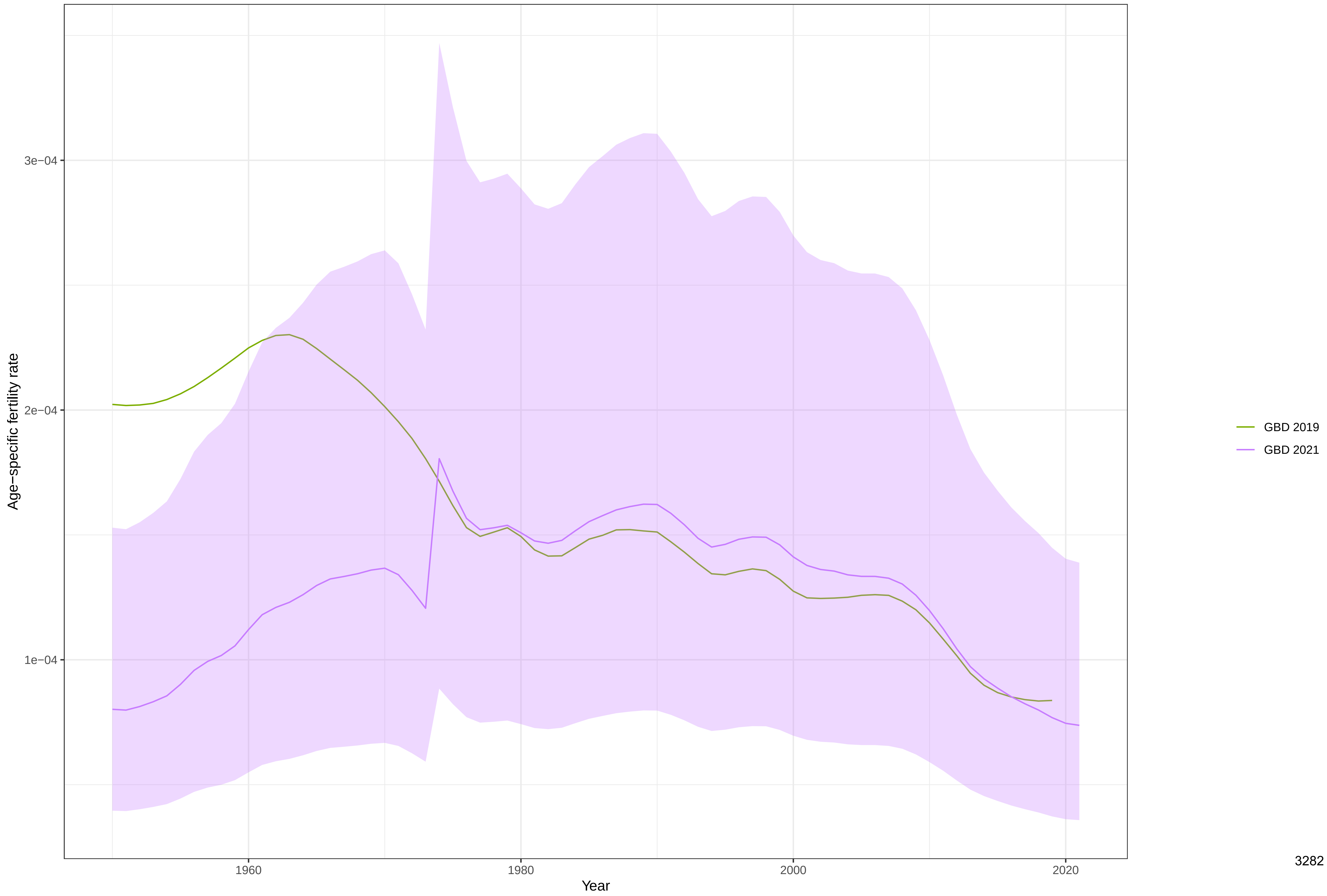

Age−specific fertility fitted trend lines, 1950−2021: Coventry, Ages: 15−19
Data points enclosed with a diamond are reference sources

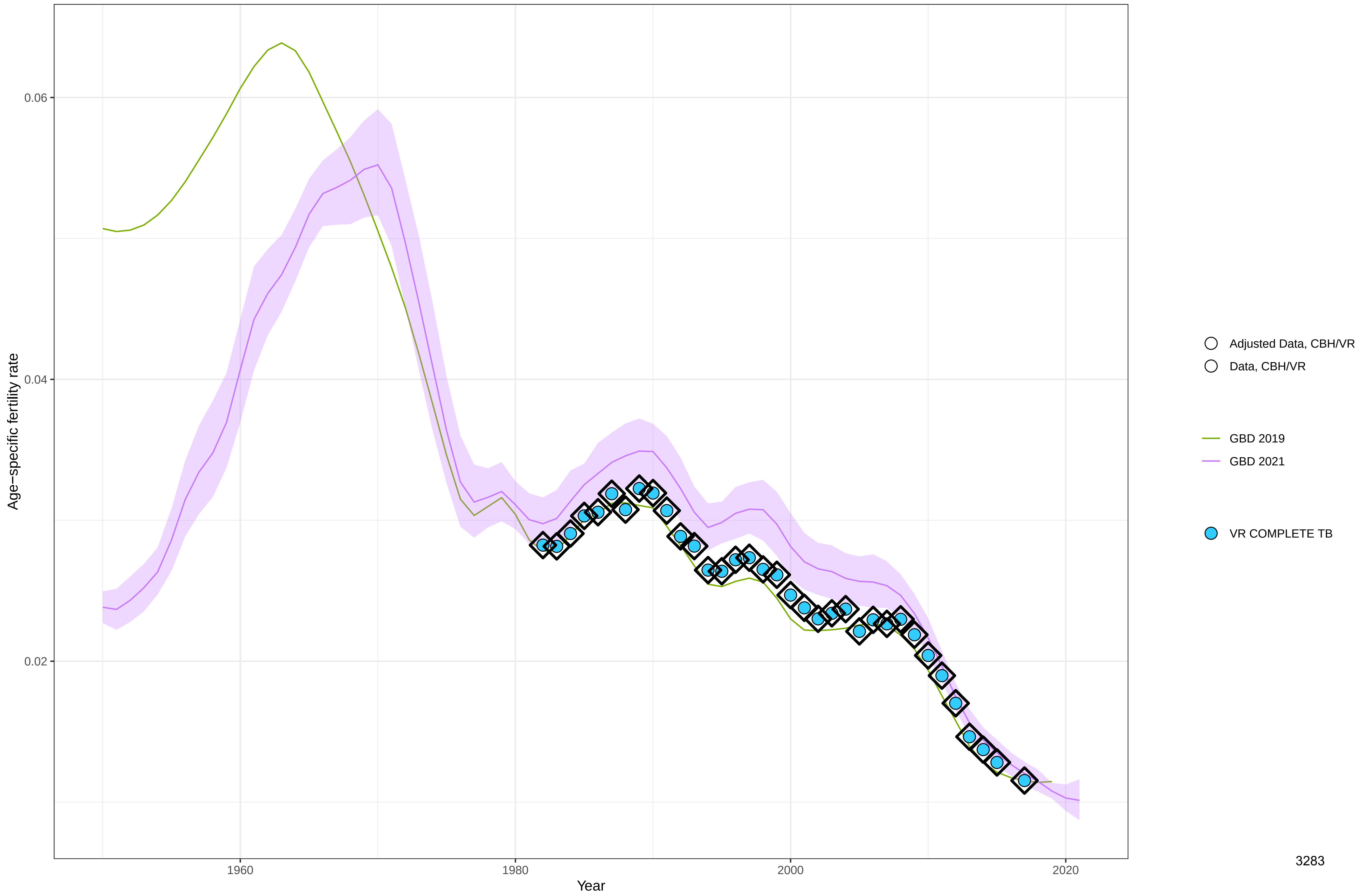

Age−specific fertility fitted trend lines, 1950−2021: Coventry, Ages: 20−24
Data points enclosed with a diamond are reference sources

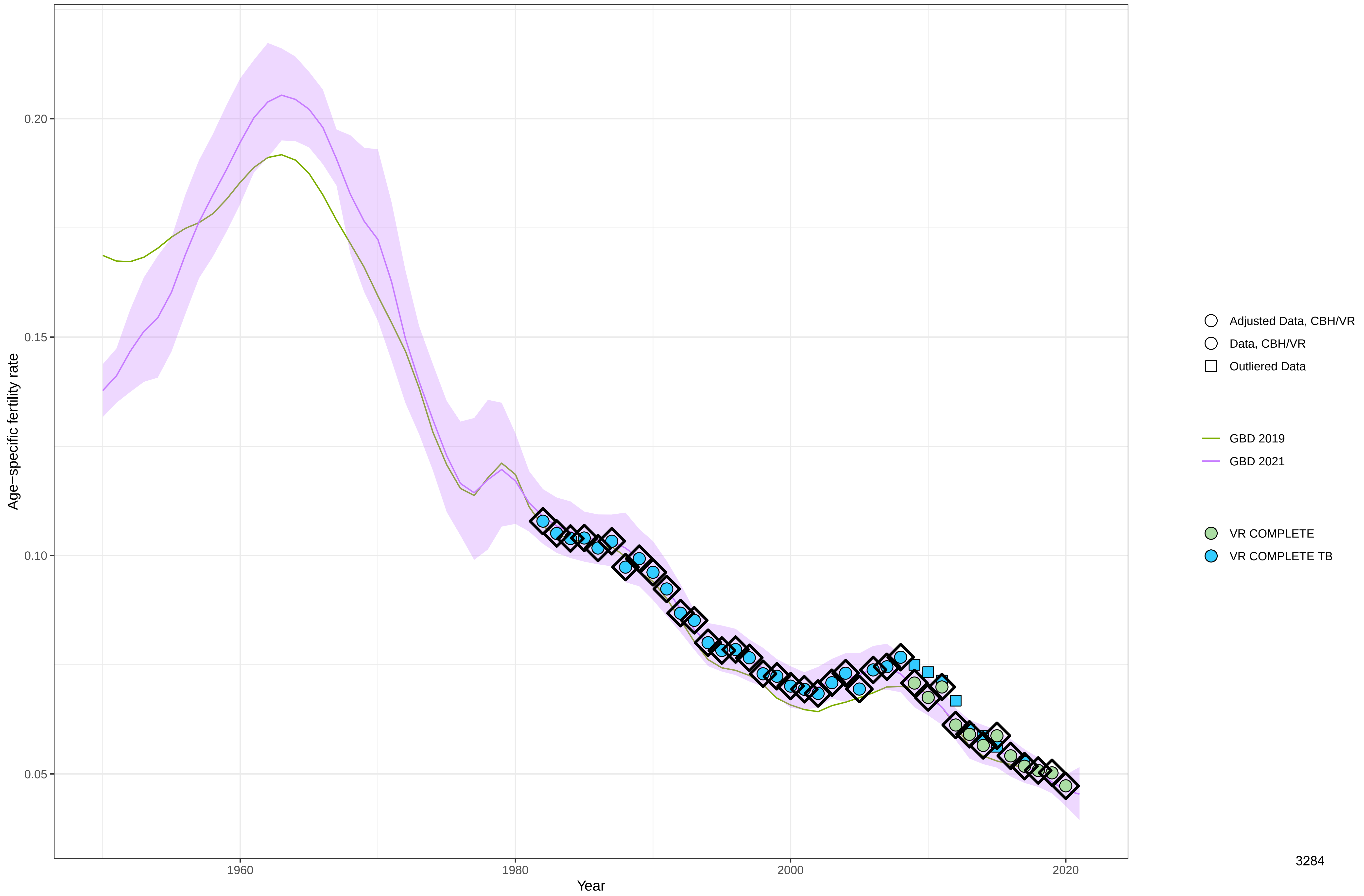

Age−specific fertility fitted trend lines, 1950−2021: Coventry, Ages: 25−29
Data points enclosed with a diamond are reference sources

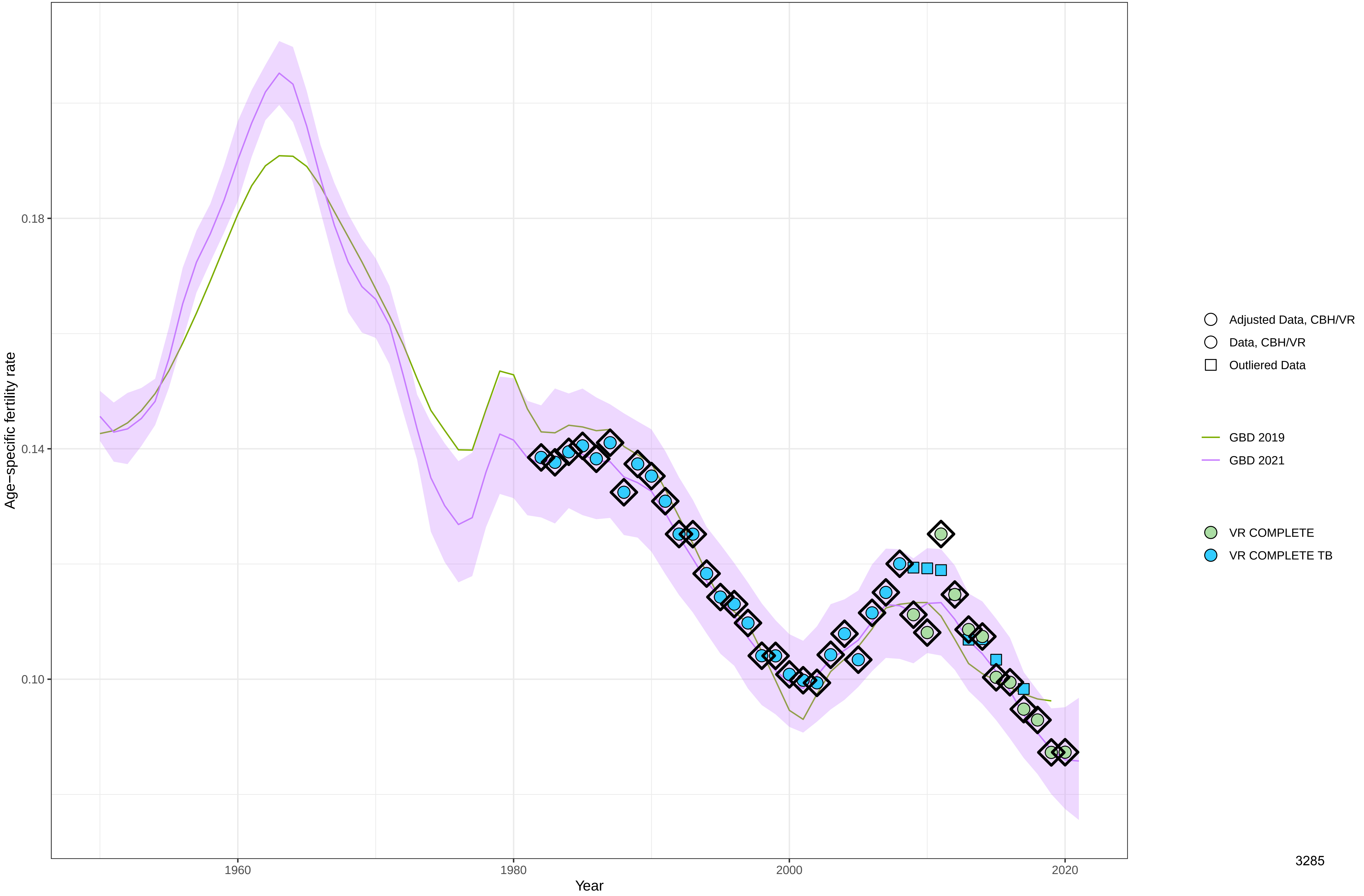

Age−specific fertility fitted trend lines, 1950−2021: Coventry, Ages: 30−34
Data points enclosed with a diamond are reference sources

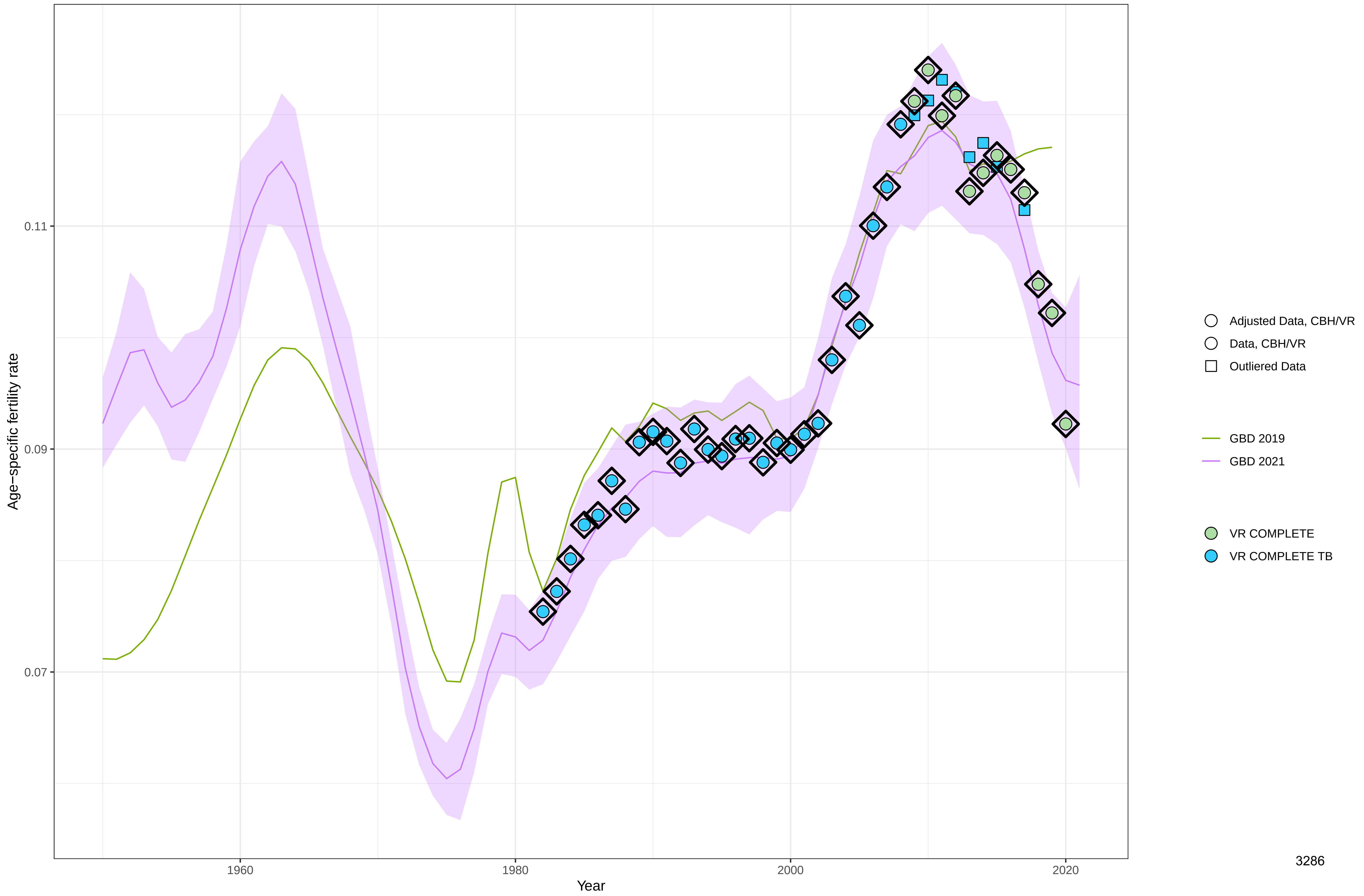

Age−specific fertility fitted trend lines, 1950−2021: Coventry, Ages: 35−39
Data points enclosed with a diamond are reference sources

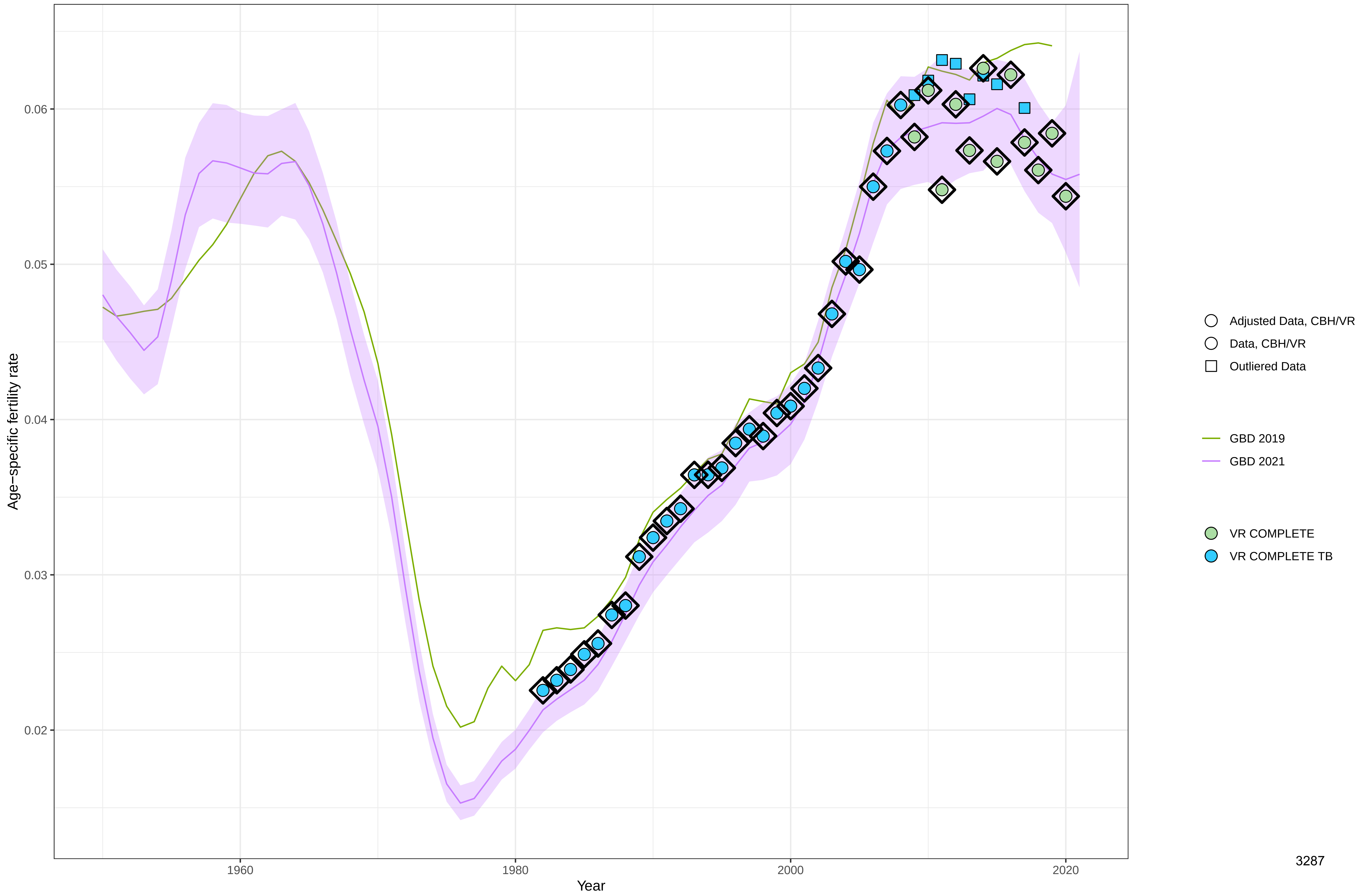

Age−specific fertility fitted trend lines, 1950−2021: Coventry, Ages: 40−44
Data points enclosed with a diamond are reference sources

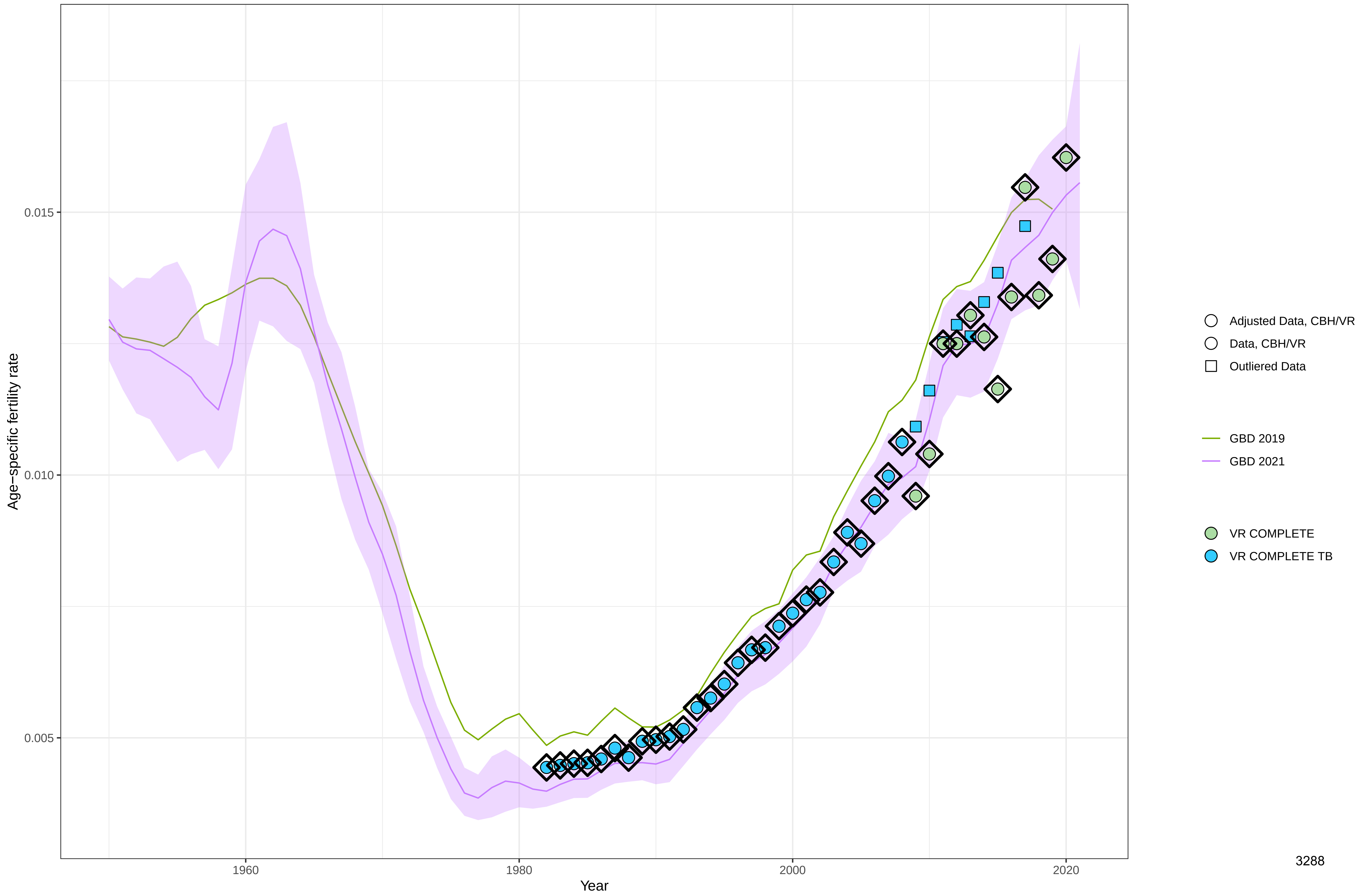

Age−specific fertility fitted trend lines, 1950−2021: Coventry, Ages: 45−49
Data points enclosed with a diamond are reference sources

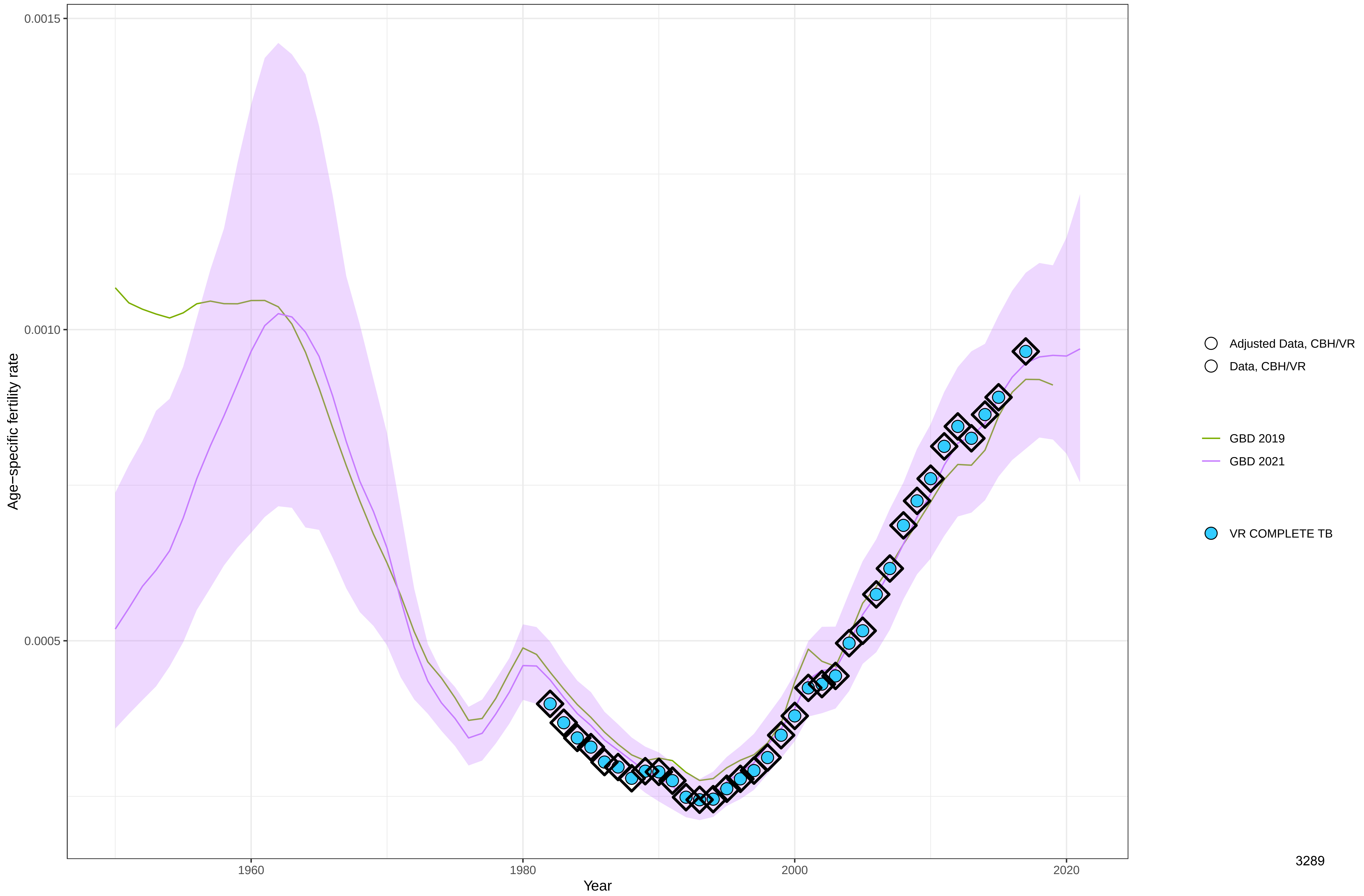

Age−specific fertility fitted trend lines, 1950−2021: Coventry, Ages: 50−54
Data points enclosed with a diamond are reference sources

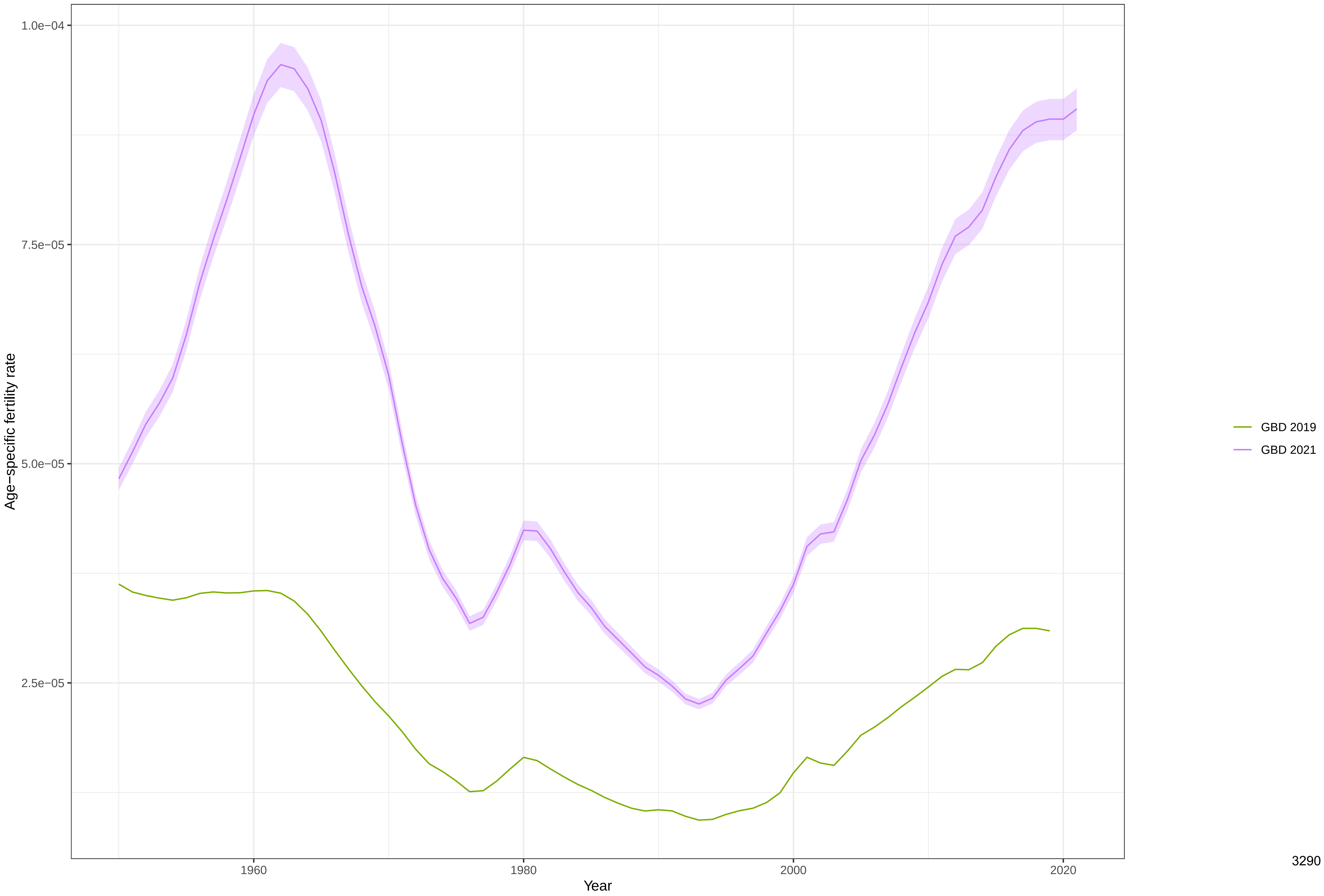

Total fertility rate fitted trend lines, 1950−2021: Coventry TFR
Data points enclosed with a diamond are reference sources

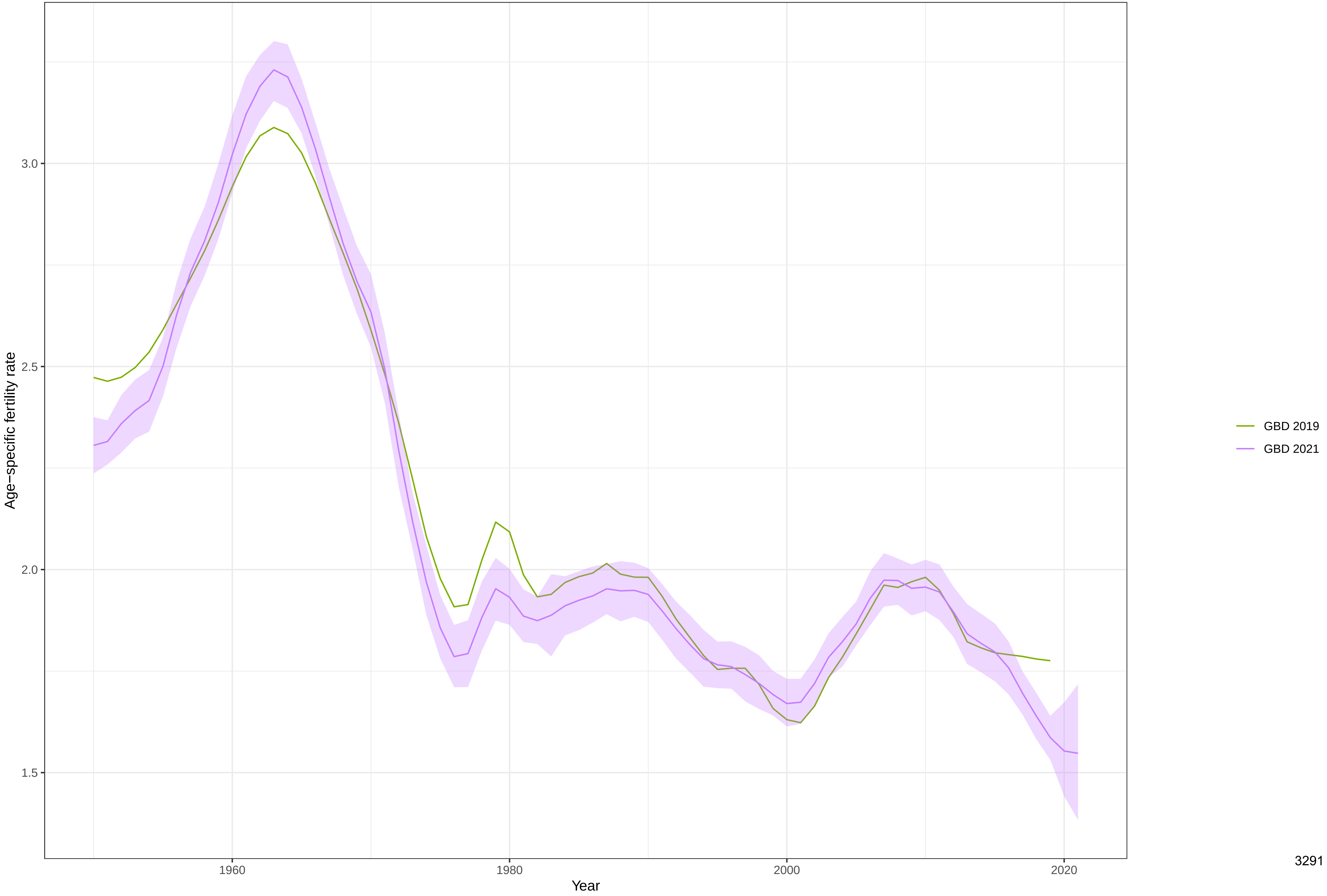

Age−specific fertility fitted trend lines, 1950−2021: Dudley, Ages: 10−14
Data points enclosed with a diamond are reference sources

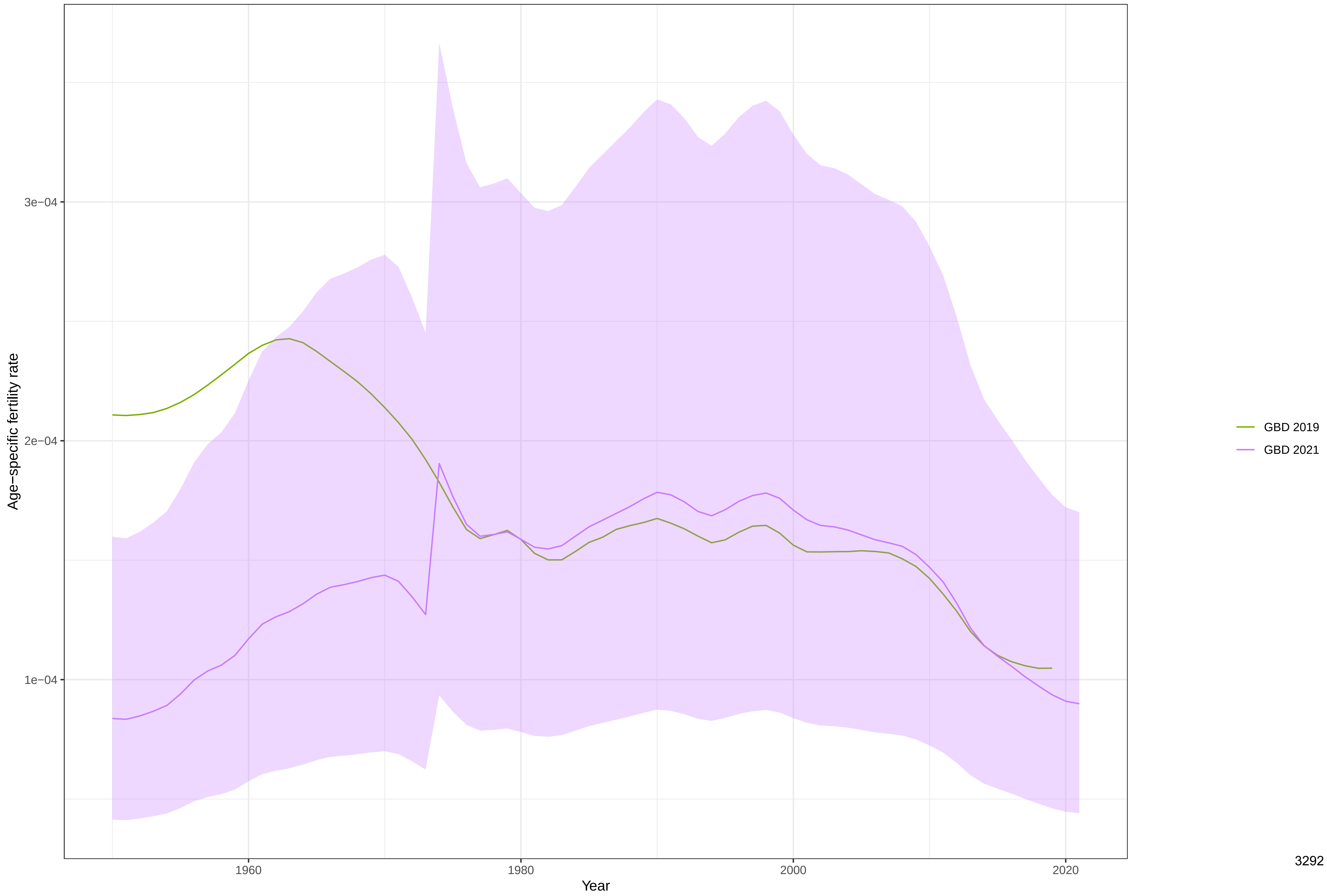

3292

Age−specific fertility fitted trend lines, 1950−2021: Dudley, Ages: 15−19
Data points enclosed with a diamond are reference sources

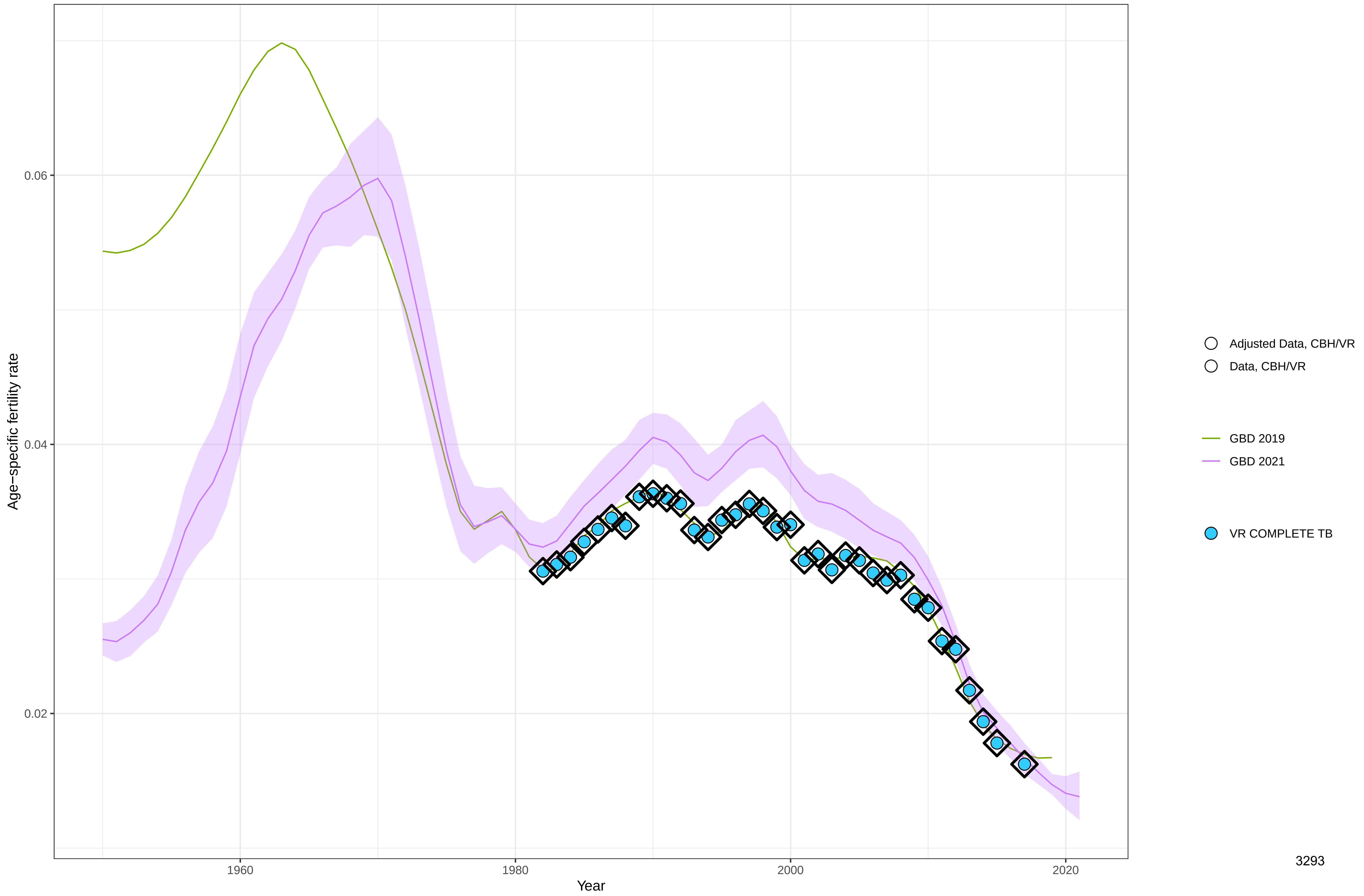

Age−specific fertility fitted trend lines, 1950−2021: Dudley, Ages: 20−24
Data points enclosed with a diamond are reference sources

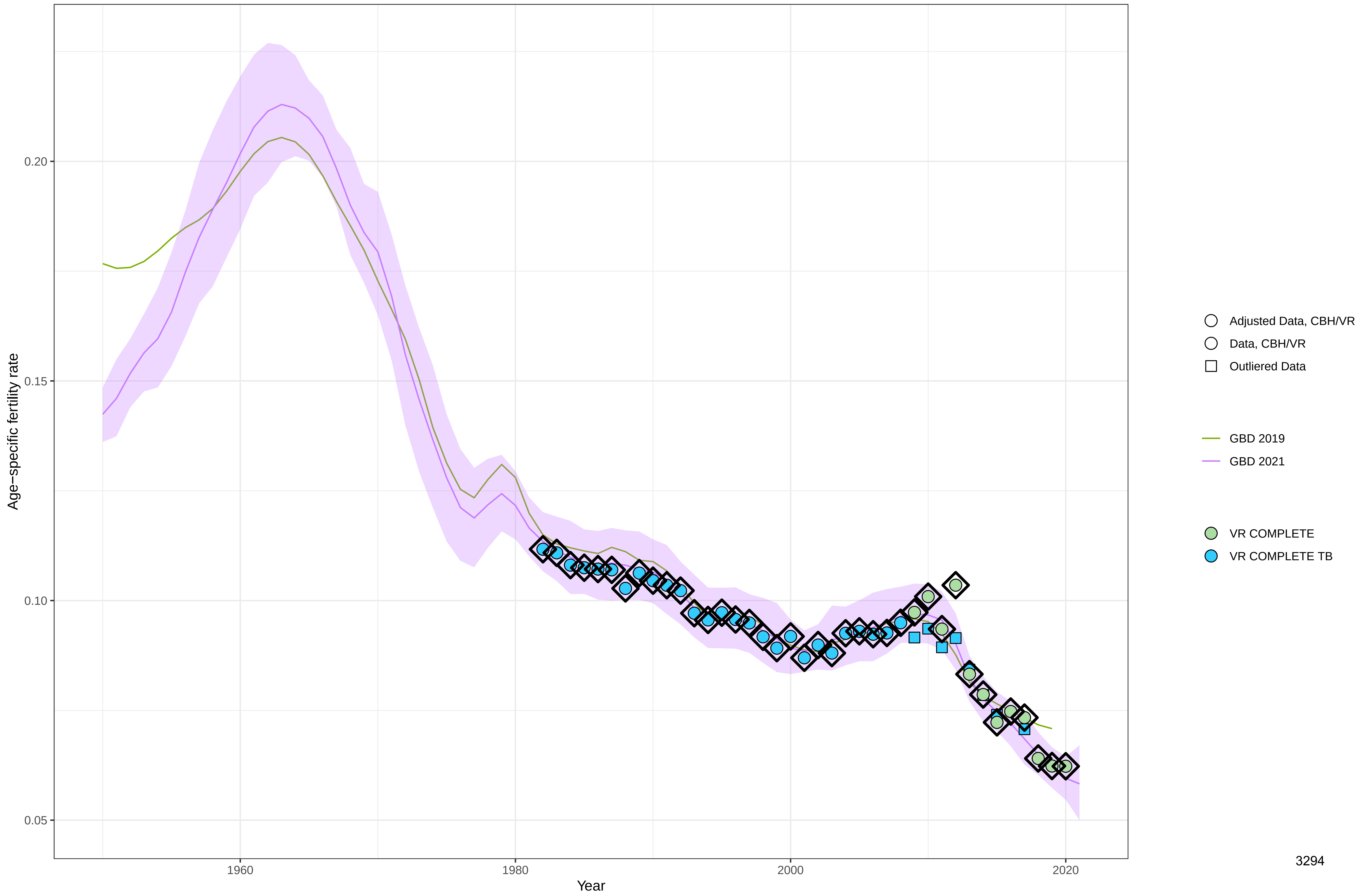

Age−specific fertility fitted trend lines, 1950−2021: Dudley, Ages: 25−29
Data points enclosed with a diamond are reference sources

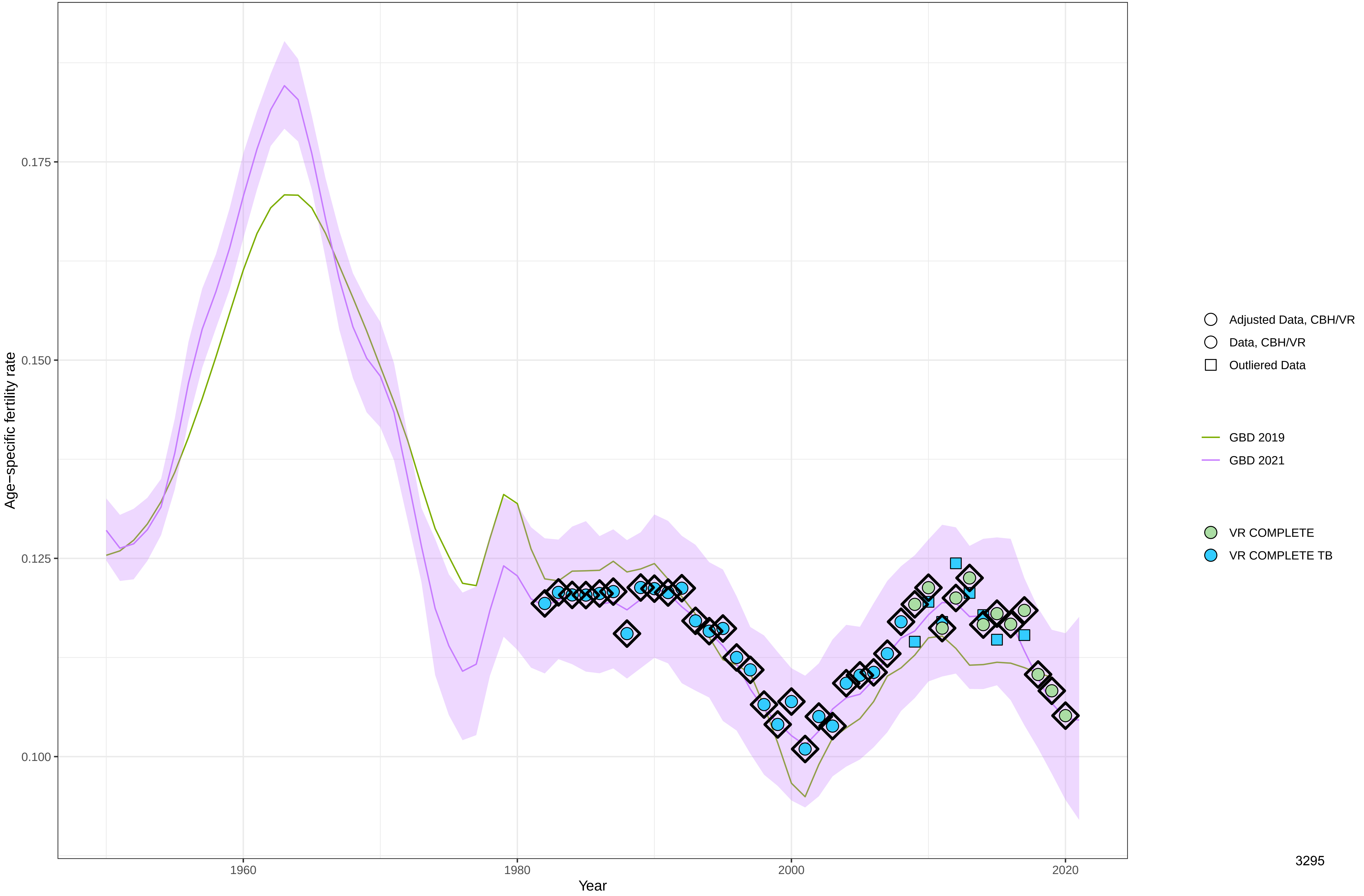

Age−specific fertility fitted trend lines, 1950−2021: Dudley, Ages: 30−34
Data points enclosed with a diamond are reference sources

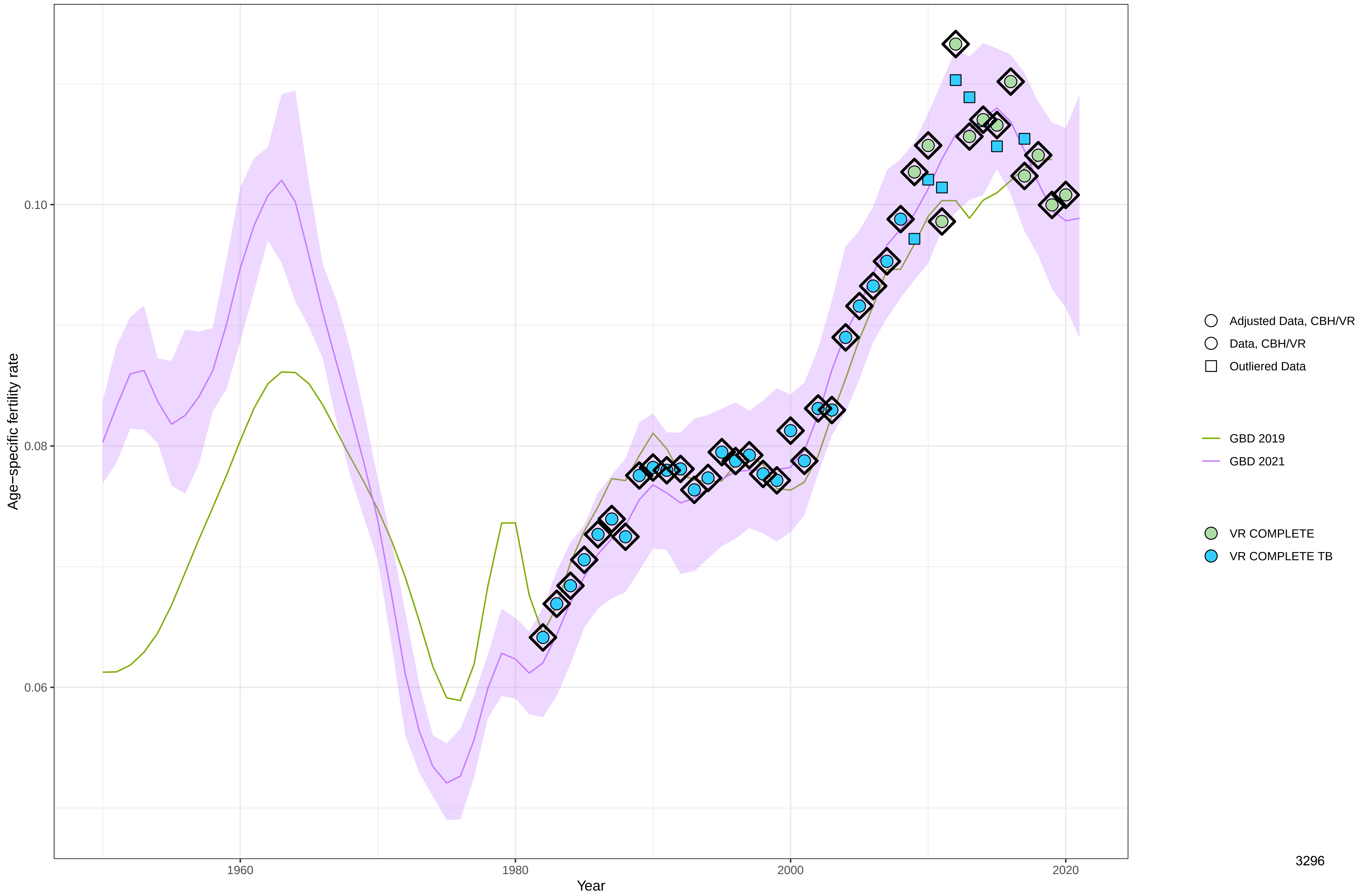

Age−specific fertility fitted trend lines, 1950−2021: Dudley, Ages: 35−39
Data points enclosed with a diamond are reference sources

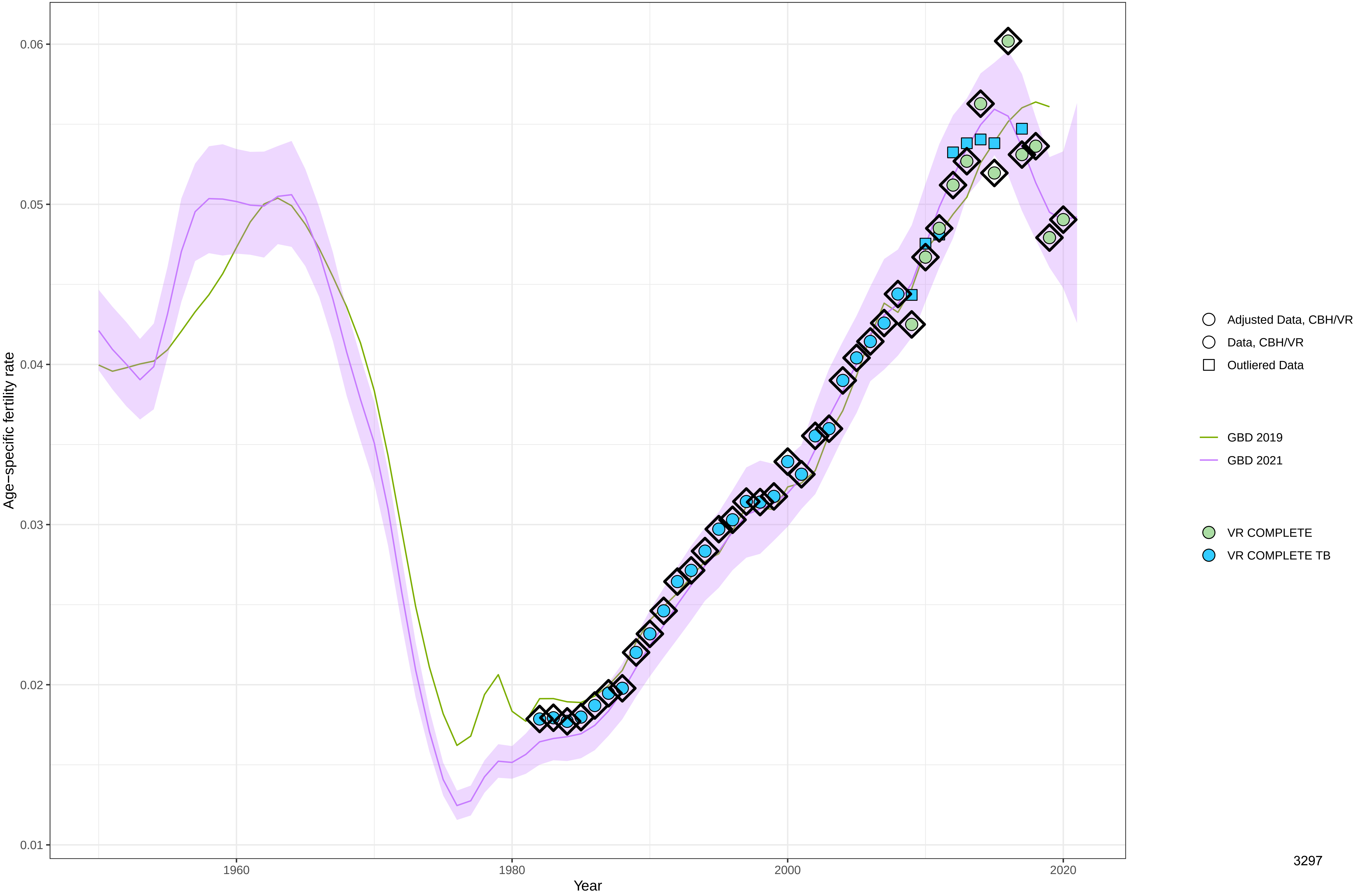

Age−specific fertility fitted trend lines, 1950−2021: Dudley, Ages: 40−44
Data points enclosed with a diamond are reference sources

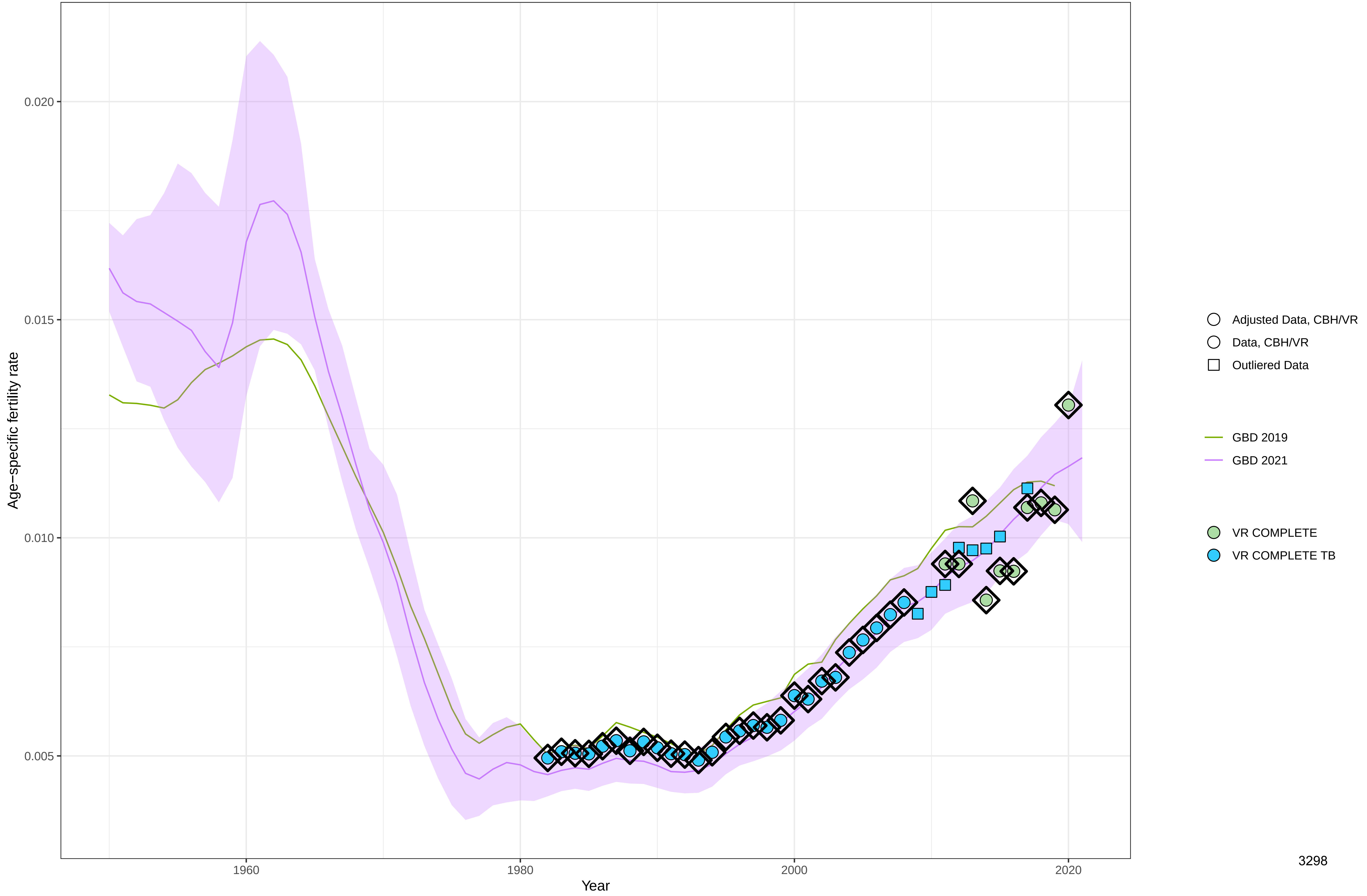

Age−specific fertility fitted trend lines, 1950−2021: Dudley, Ages: 45−49
Data points enclosed with a diamond are reference sources

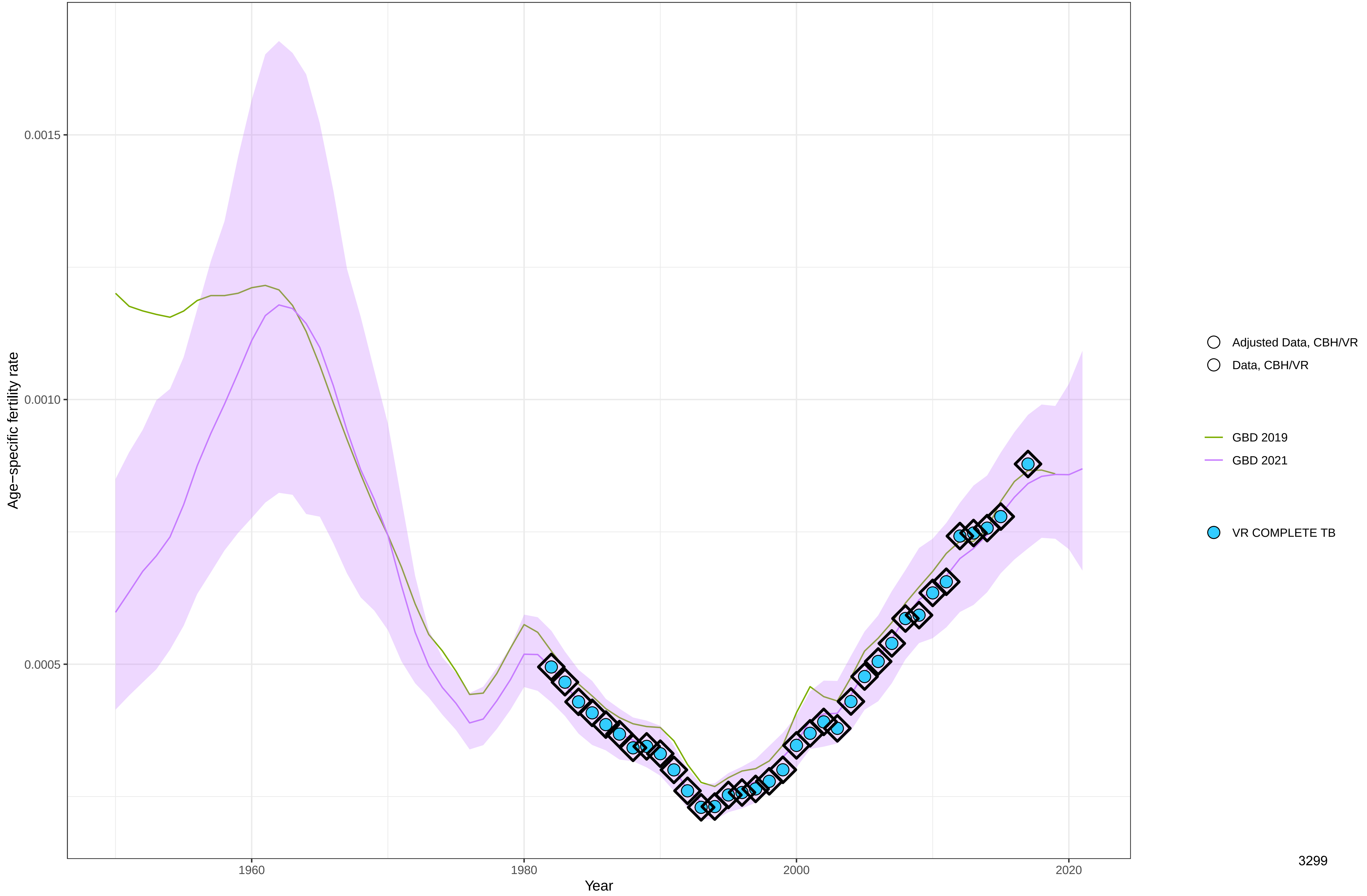

Age−specific fertility fitted trend lines, 1950−2021: Dudley, Ages: 50−54
Data points enclosed with a diamond are reference sources

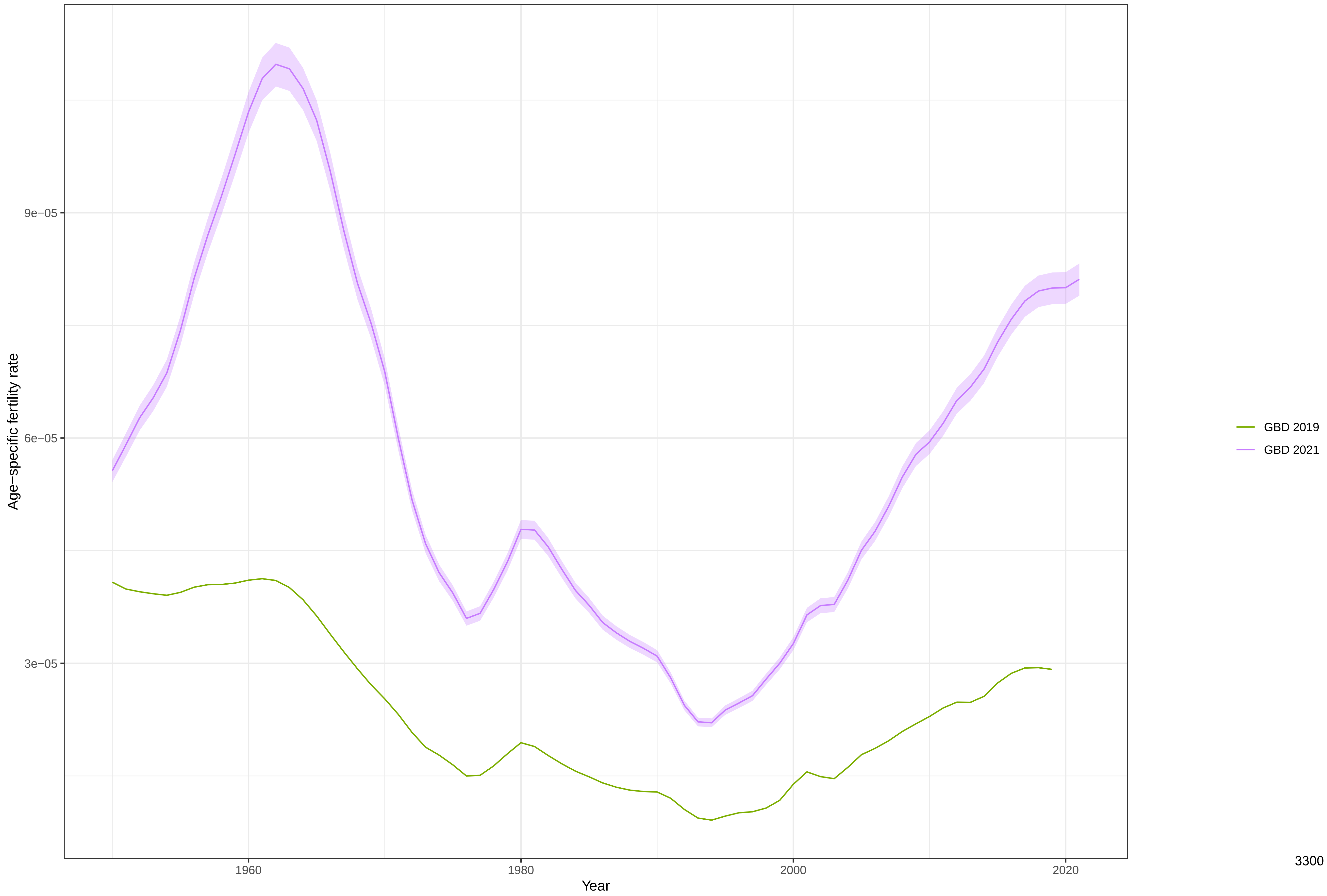

Total fertility rate fitted trend lines, 1950−2021: Dudley TFR
Data points enclosed with a diamond are reference sources

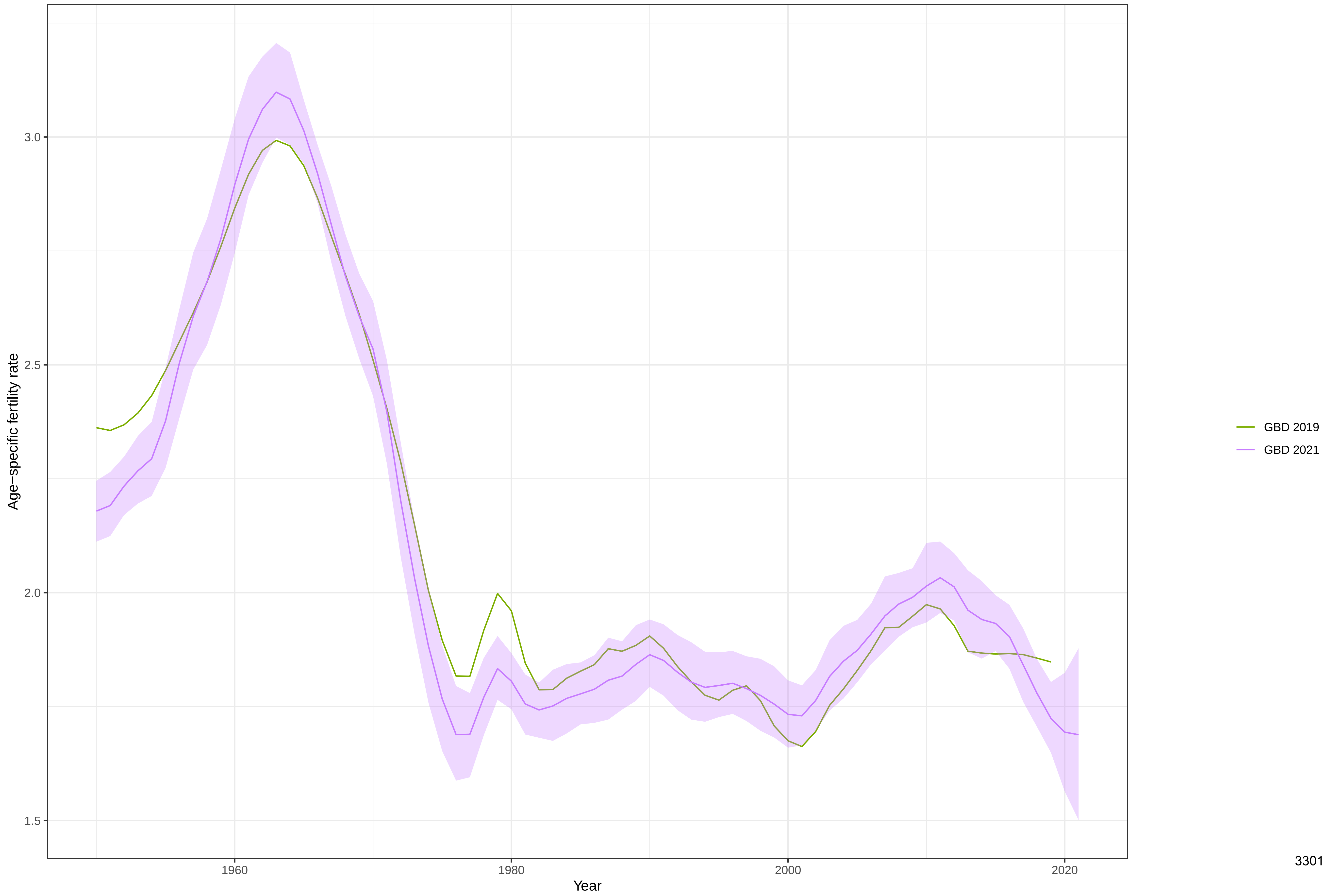

Age−specific fertility fitted trend lines, 1950−2021: Herefordshire, County of, Ages: 10−14
Data points enclosed with a diamond are reference sources

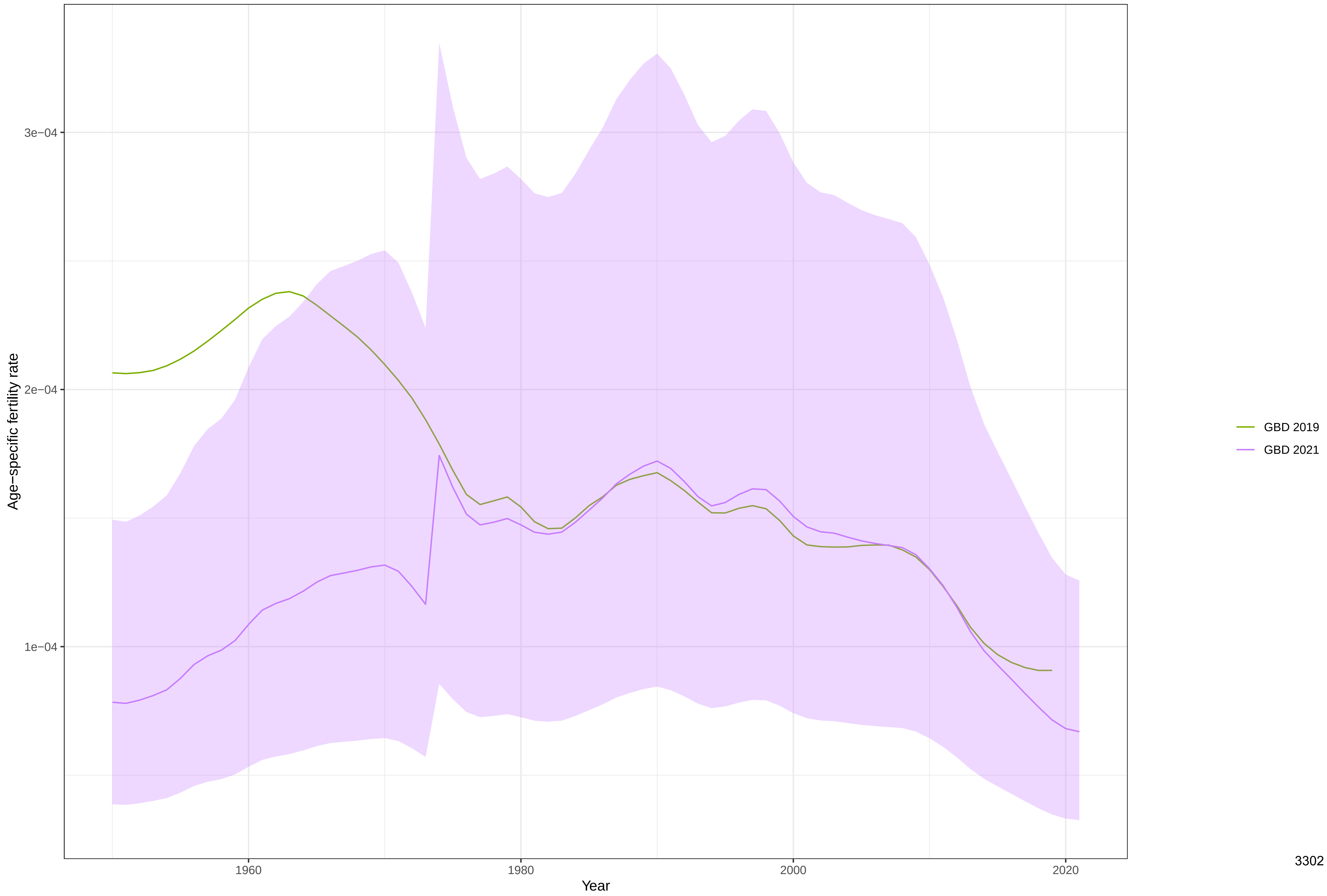

Age−specific fertility fitted trend lines, 1950−2021: Herefordshire, County of, Ages: 15−19
Data points enclosed with a diamond are reference sources

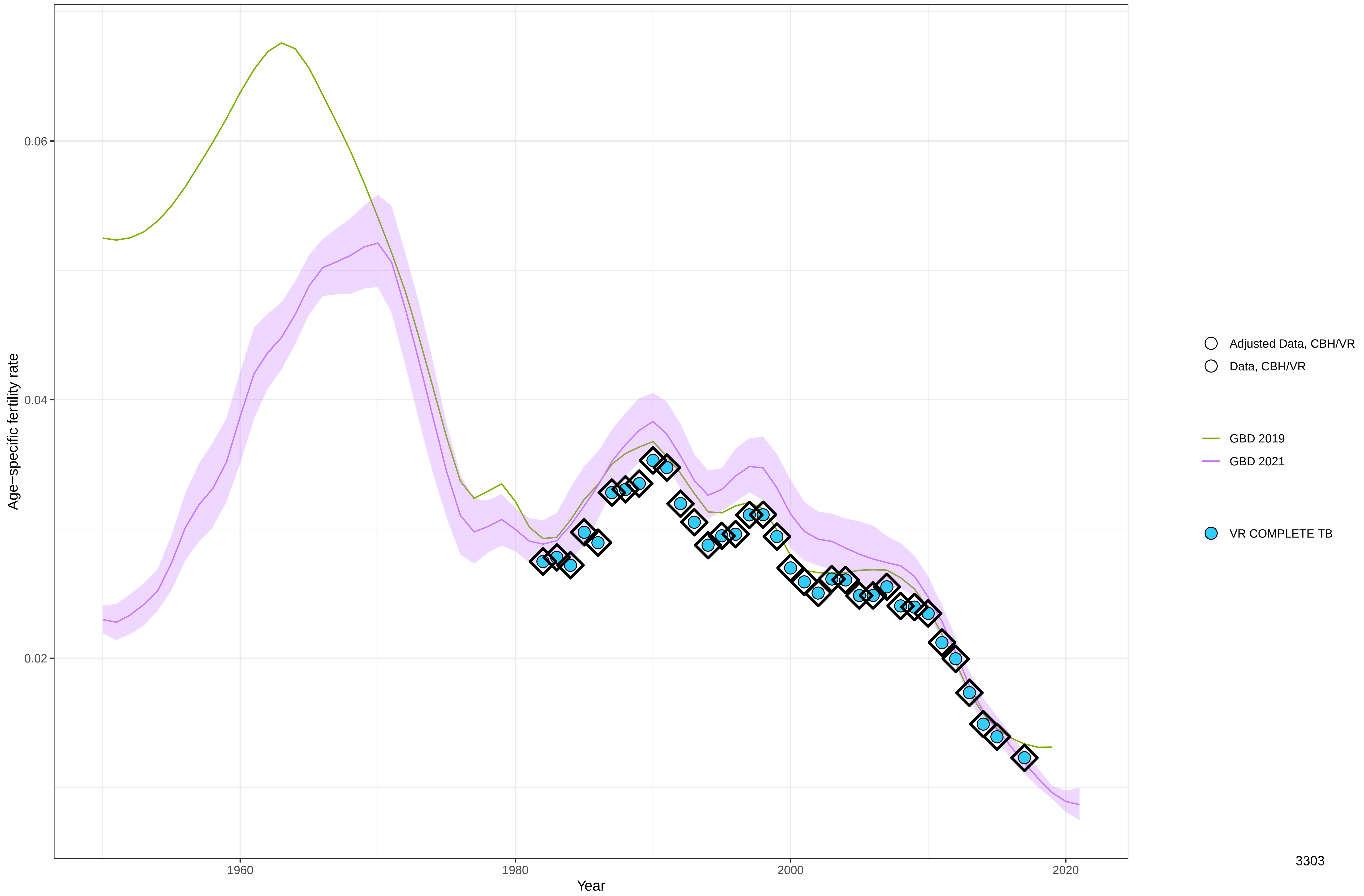

Age−specific fertility fitted trend lines, 1950−2021: Herefordshire, County of, Ages: 20−24
Data points enclosed with a diamond are reference sources

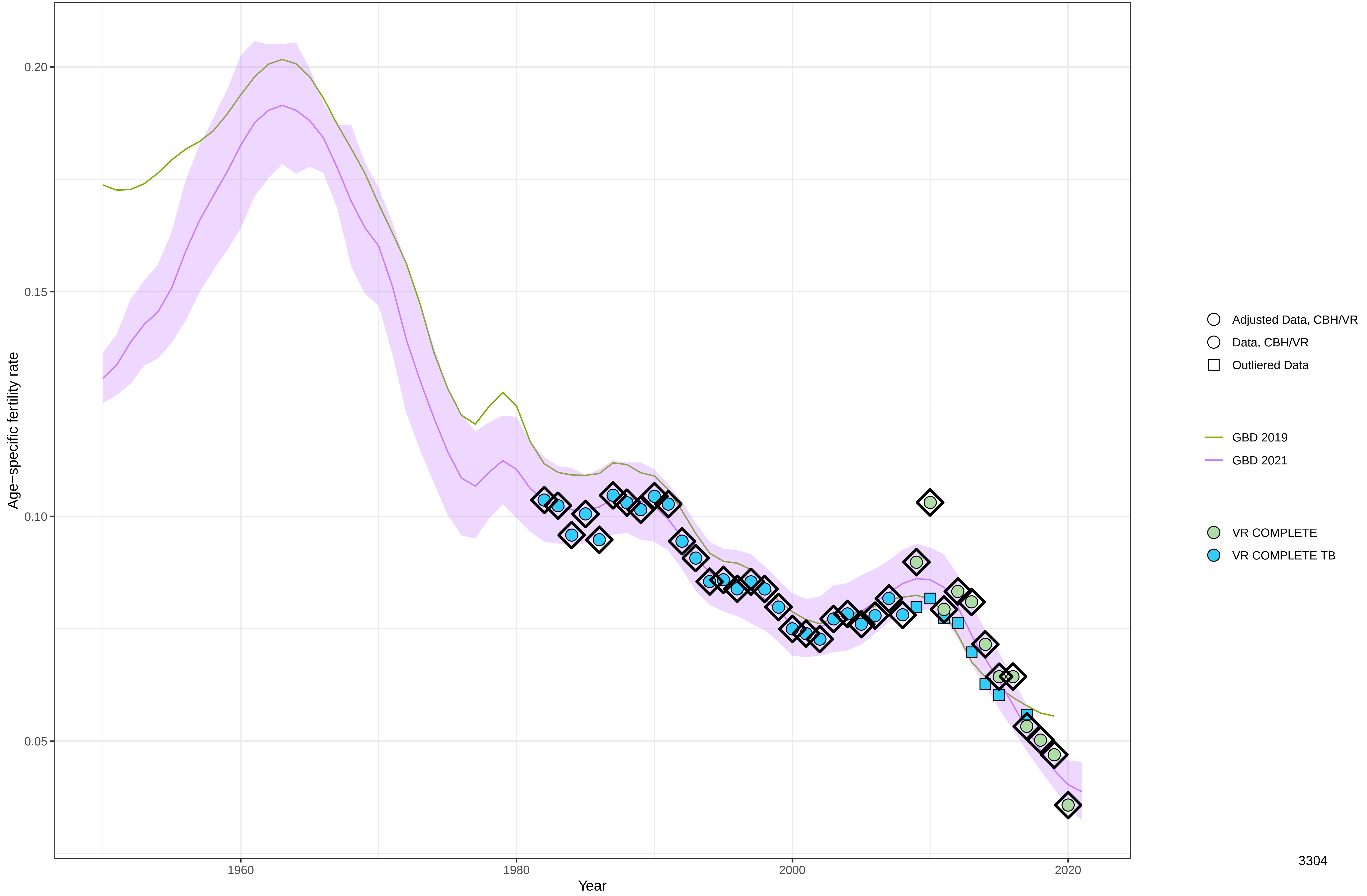

Age−specific fertility fitted trend lines, 1950−2021: Herefordshire, County of, Ages: 25−29
Data points enclosed with a diamond are reference sources

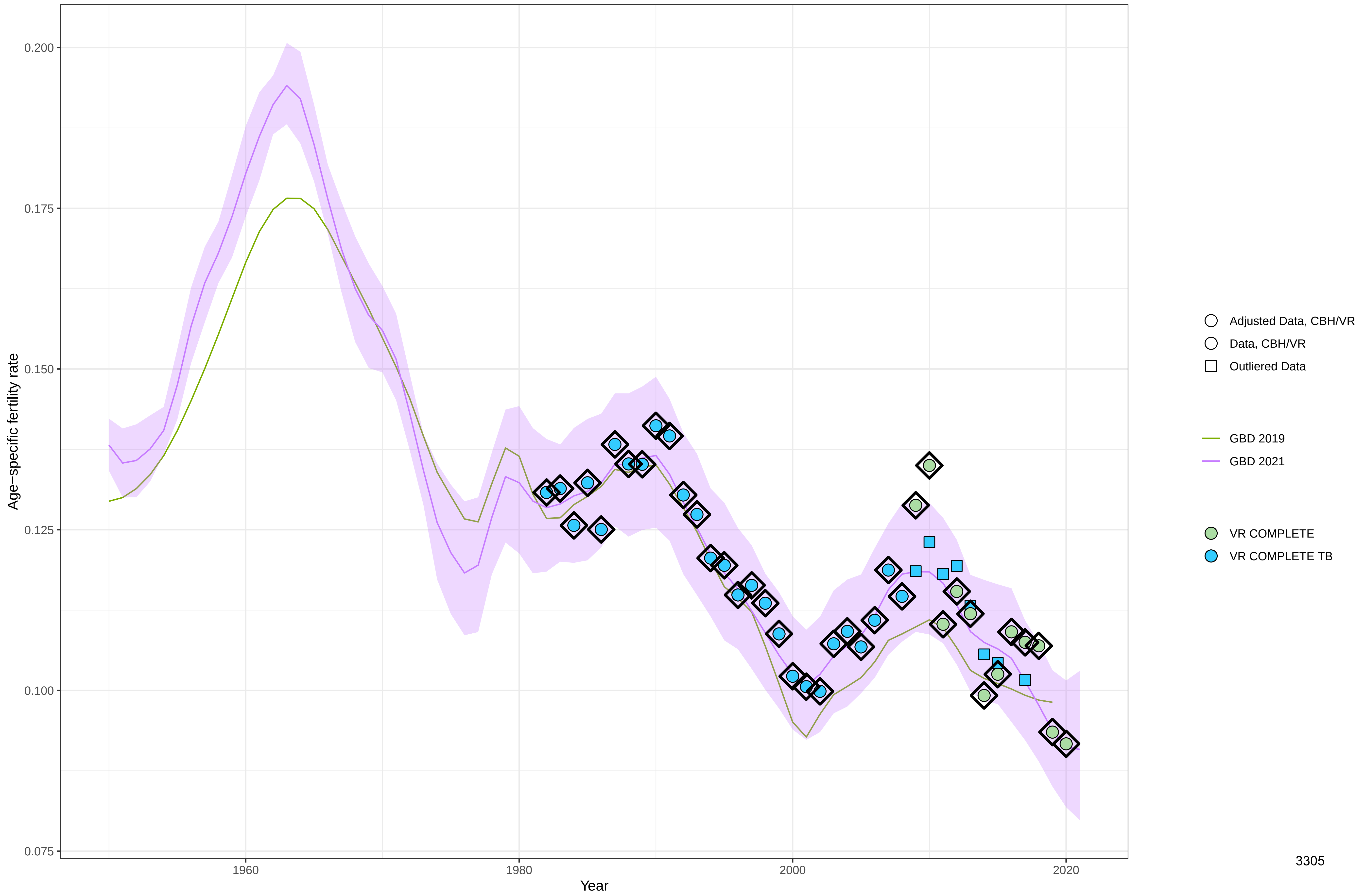

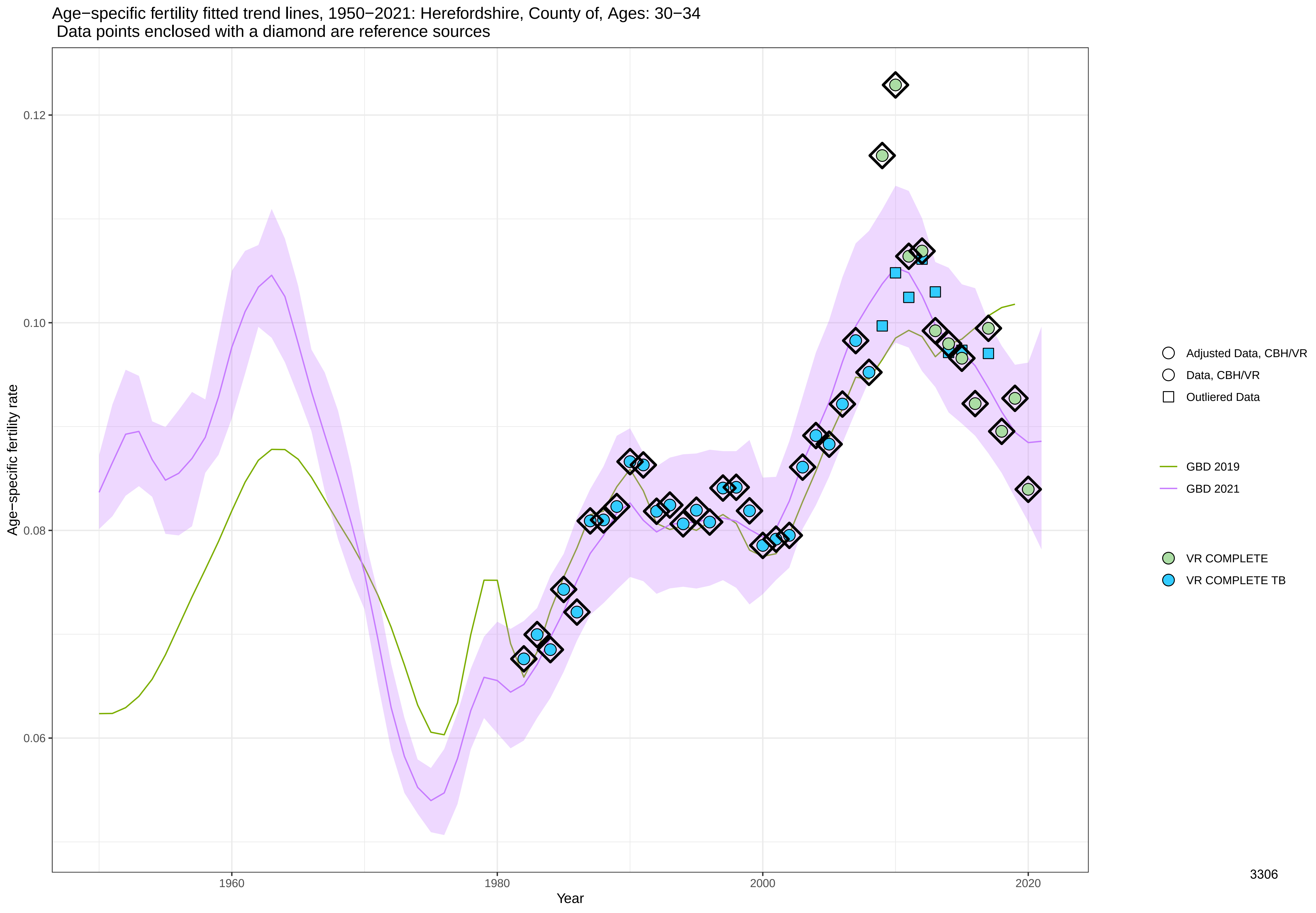

Age−specific fertility fitted trend lines, 1950−2021: Herefordshire, County of, Ages: 30−34
Data points enclosed with a diamond are reference sources
Age−specific fertility rate
Year
0.06
0.08
0.10
0.12
1960
1980
2000
2020
Adjusted Data, CBH/VR
Data, CBH/VR
Outliered Data
GBD 2019
GBD 2021
VR COMPLETE
VR COMPLETE TB

Age−specific fertility fitted trend lines, 1950−2021: Herefordshire, County of, Ages: 35−39
Data points enclosed with a diamond are reference sources

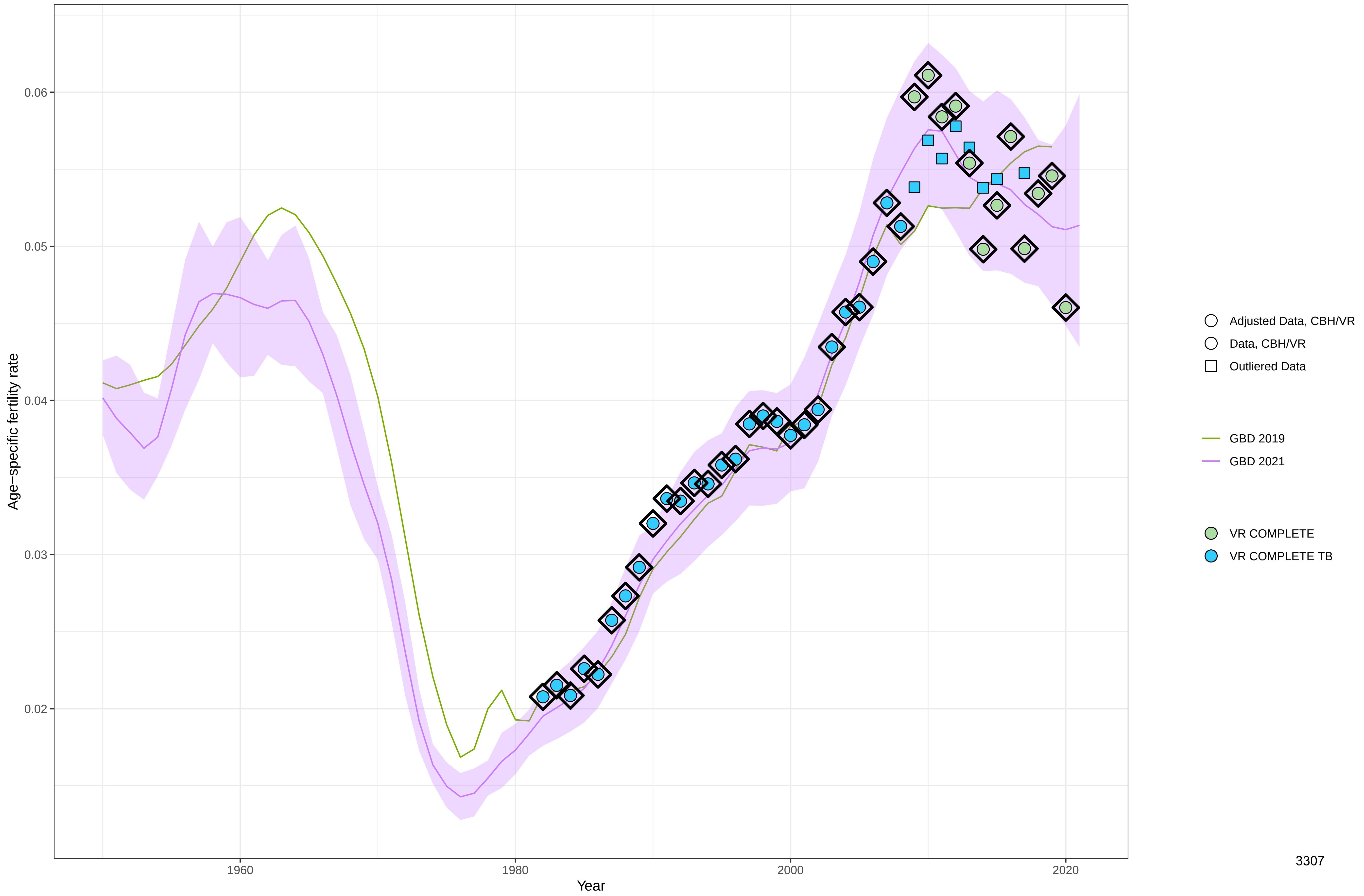

Age−specific fertility fitted trend lines, 1950−2021: Herefordshire, County of, Ages: 40−44
Data points enclosed with a diamond are reference sources

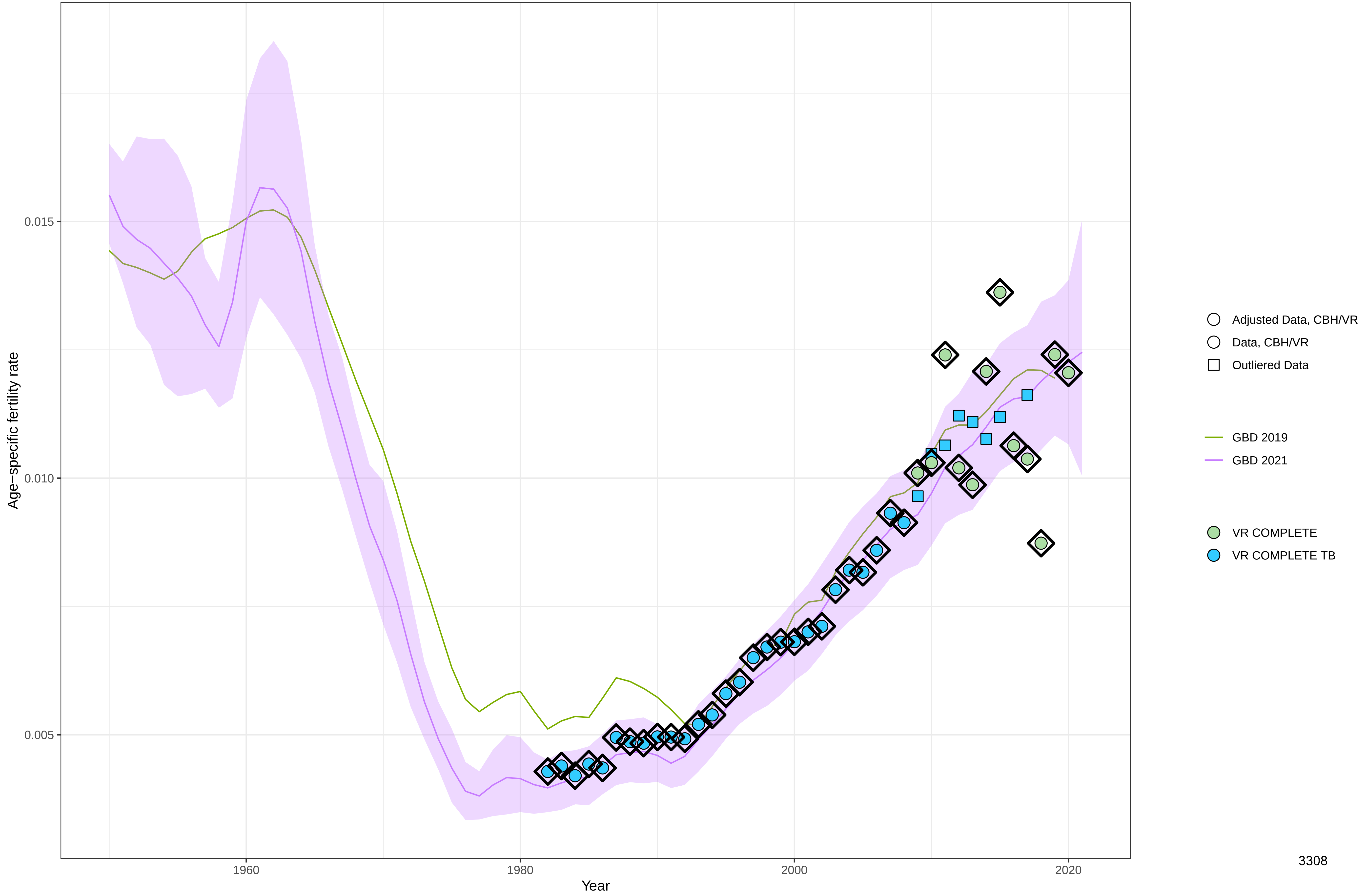

Age−specific fertility fitted trend lines, 1950−2021: Herefordshire, County of, Ages: 45−49
Data points enclosed with a diamond are reference sources

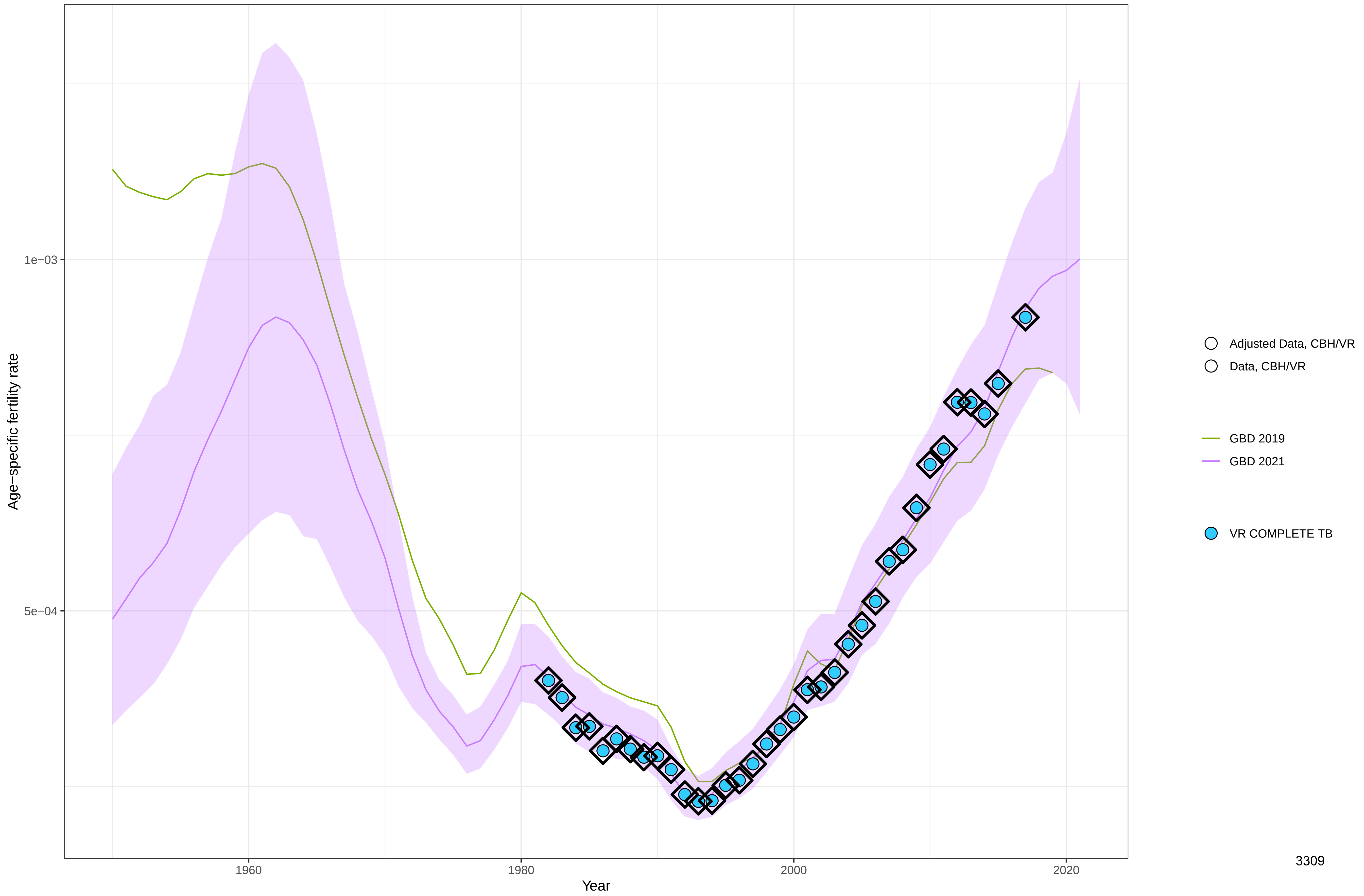

Age−specific fertility fitted trend lines, 1950−2021: Herefordshire, County of, Ages: 50−54
Data points enclosed with a diamond are reference sources

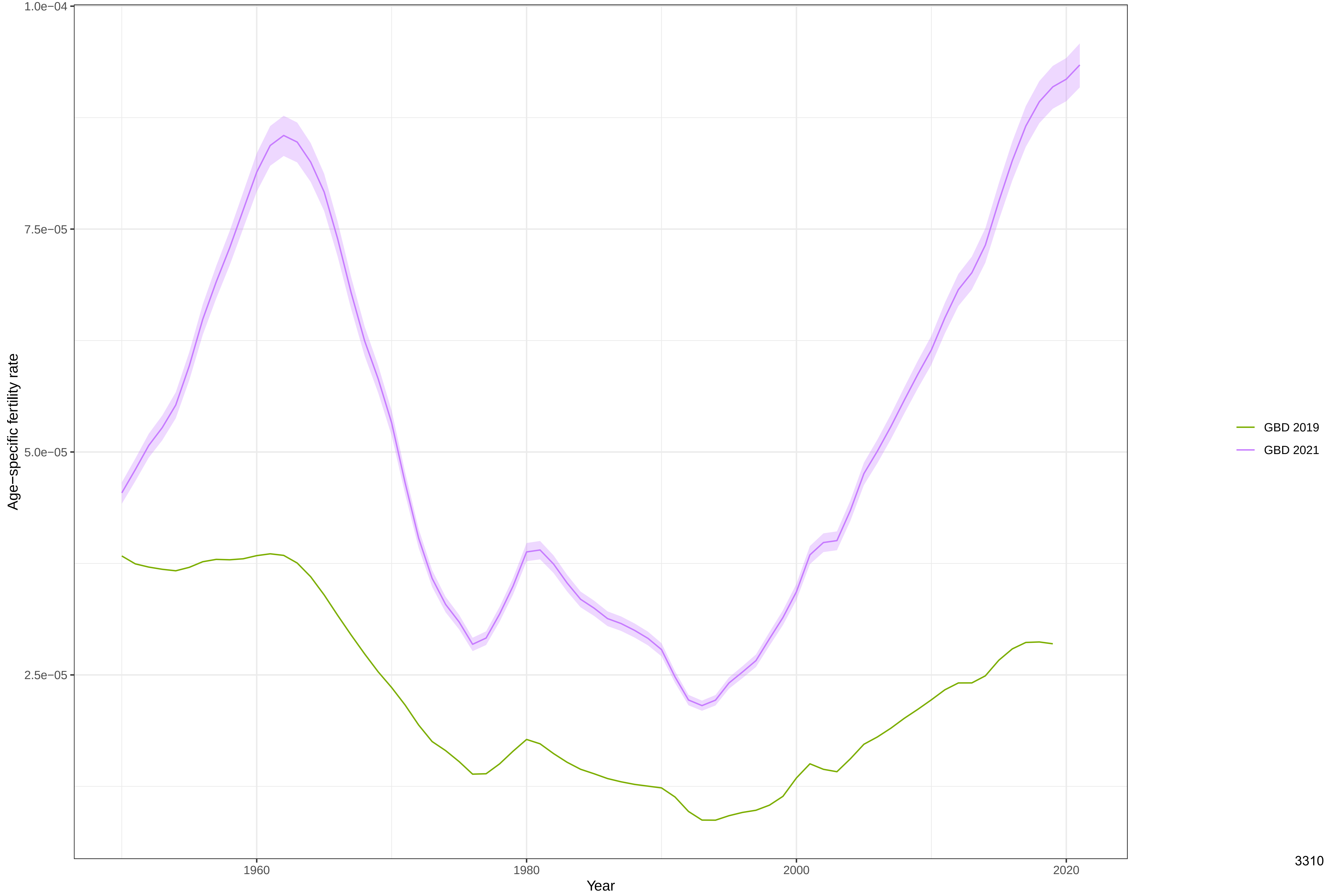

Total fertility rate fitted trend lines, 1950−2021: Herefordshire, County of TFR
Data points enclosed with a diamond are reference sources

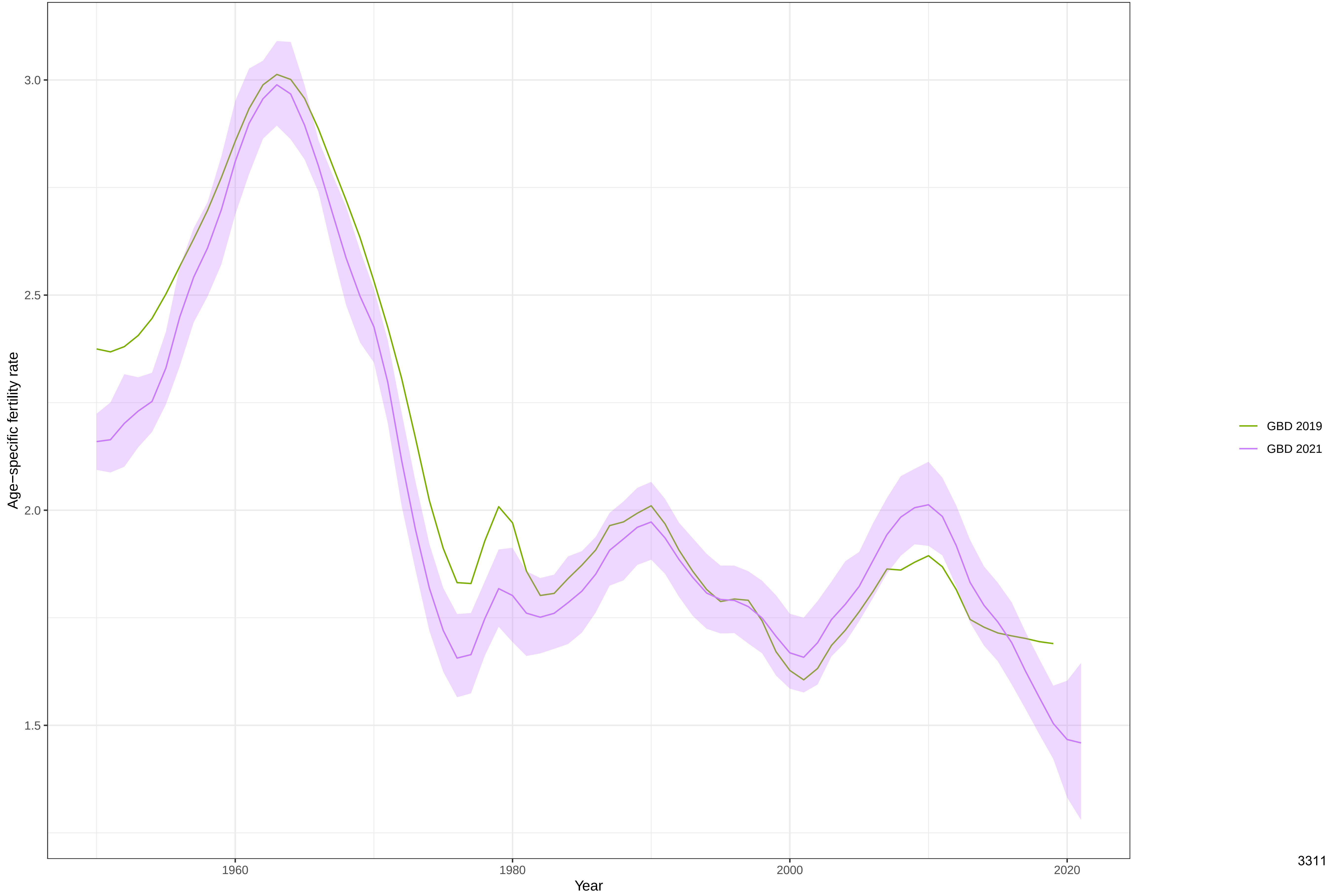

Age−specific fertility fitted trend lines, 1950−2021: Sandwell, Ages: 10−14
Data points enclosed with a diamond are reference sources

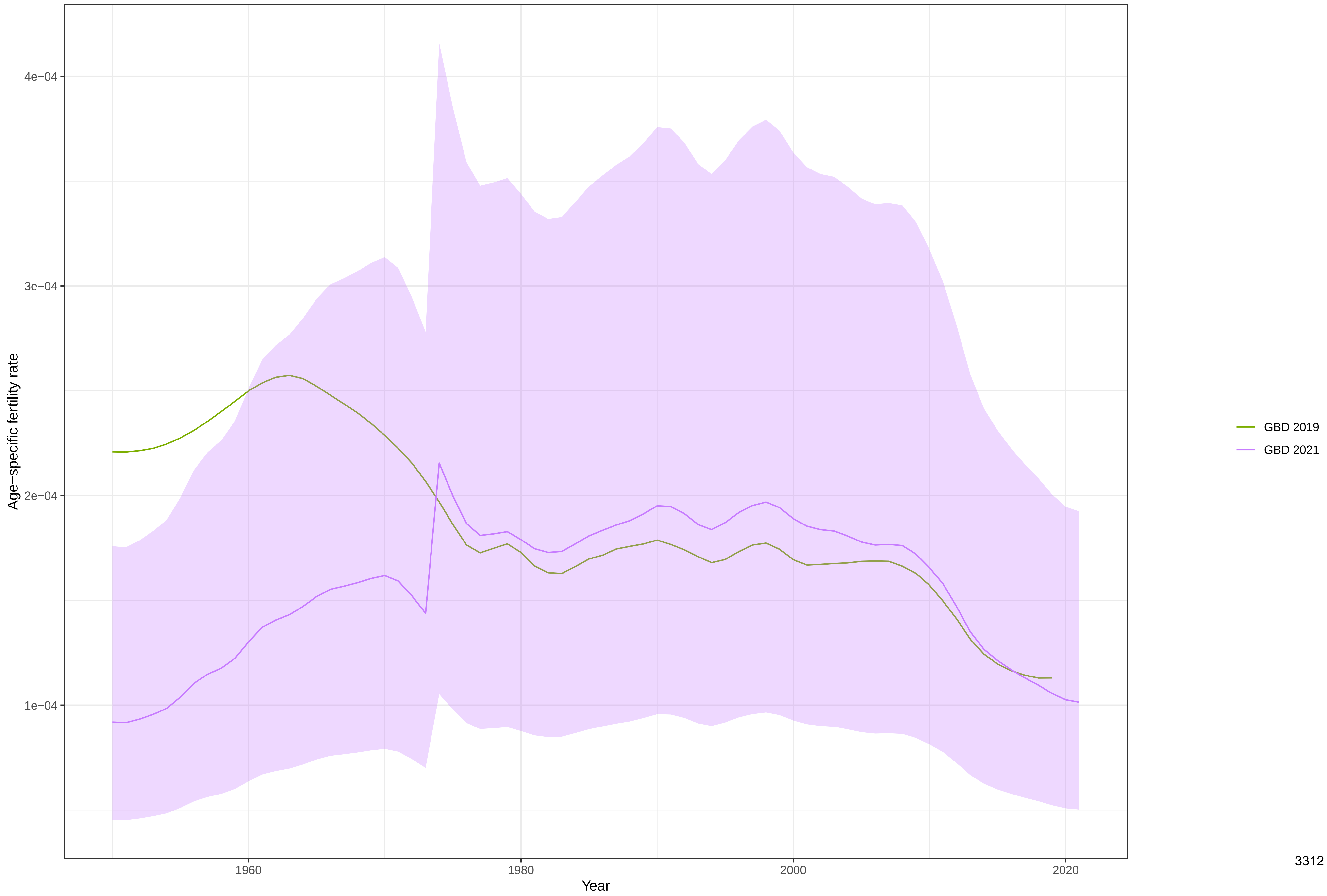

Age−specific fertility fitted trend lines, 1950−2021: Sandwell, Ages: 15−19
Data points enclosed with a diamond are reference sources

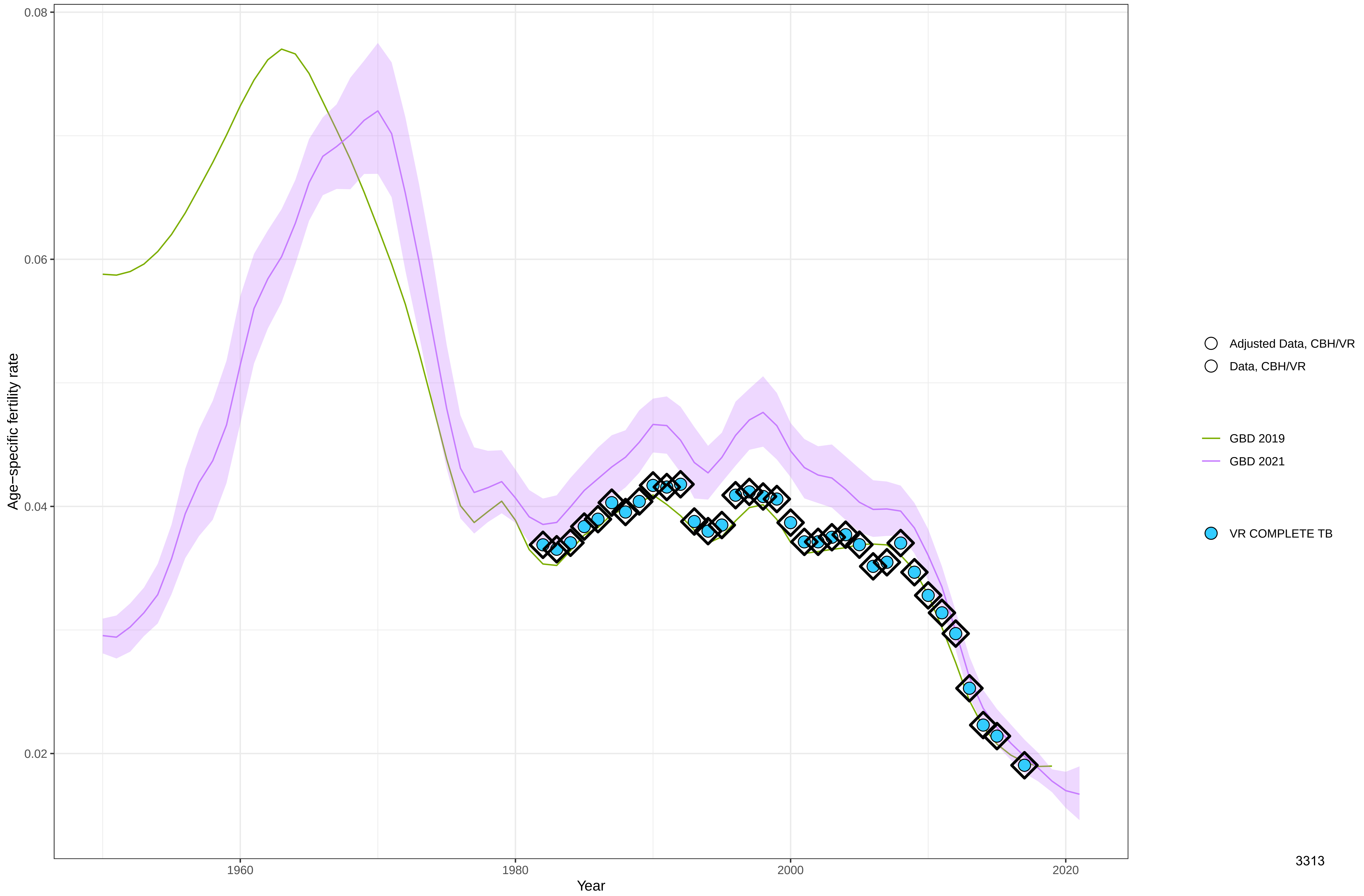

Age−specific fertility fitted trend lines, 1950−2021: Sandwell, Ages: 20−24
Data points enclosed with a diamond are reference sources

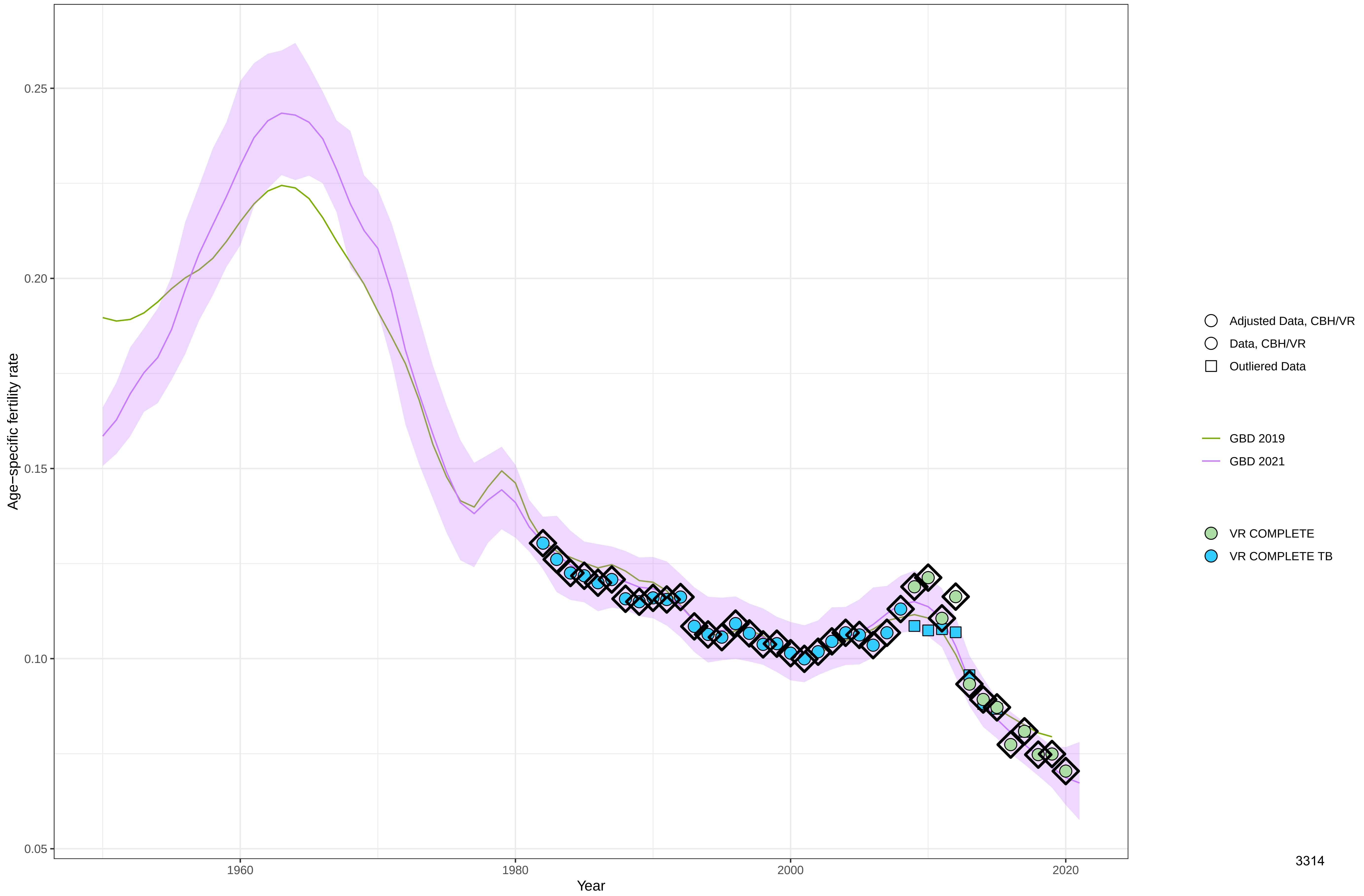

Age−specific fertility fitted trend lines, 1950−2021: Sandwell, Ages: 25−29
Data points enclosed with a diamond are reference sources

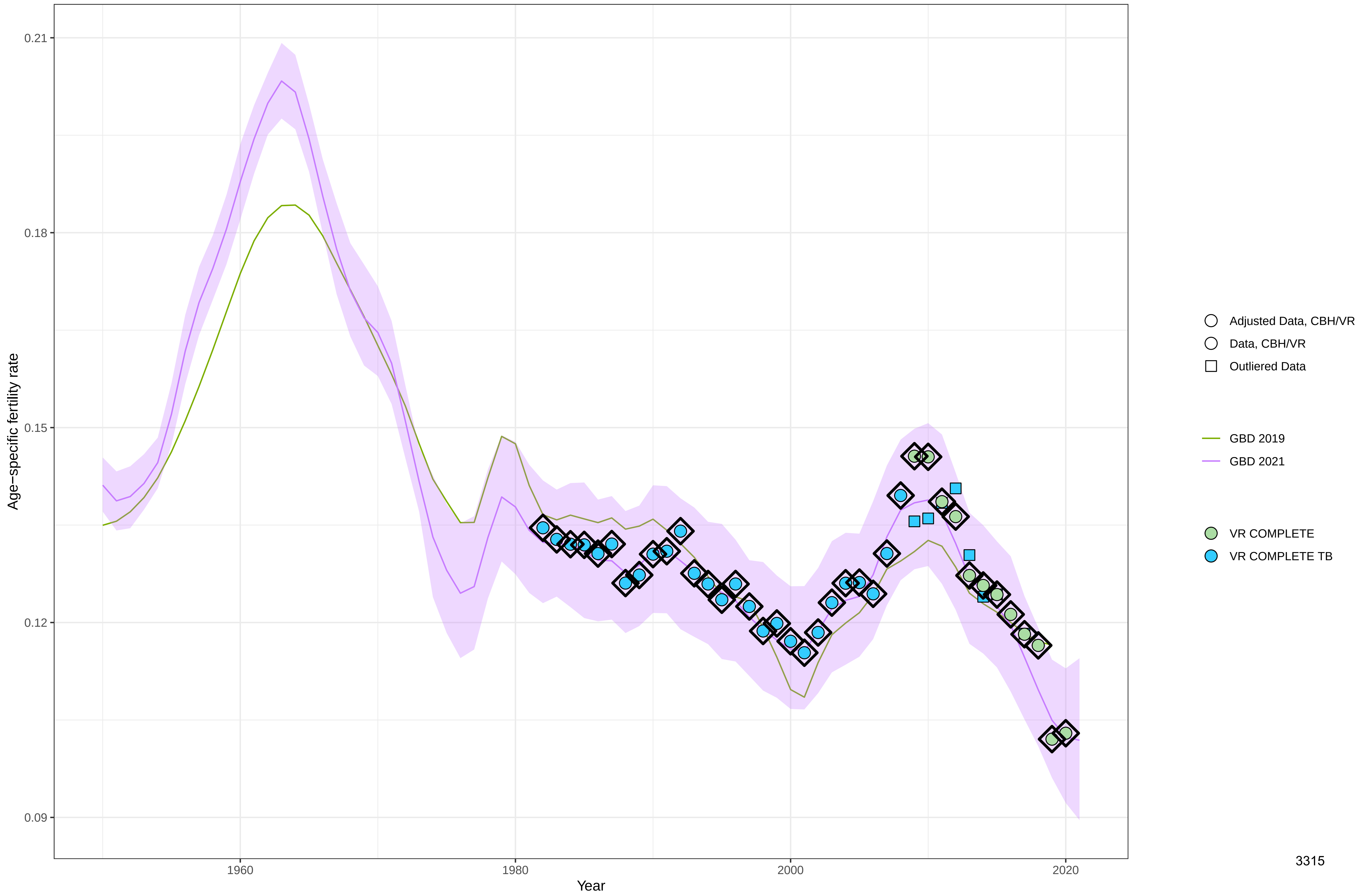

Age−specific fertility fitted trend lines, 1950−2021: Sandwell, Ages: 30−34
Data points enclosed with a diamond are reference sources

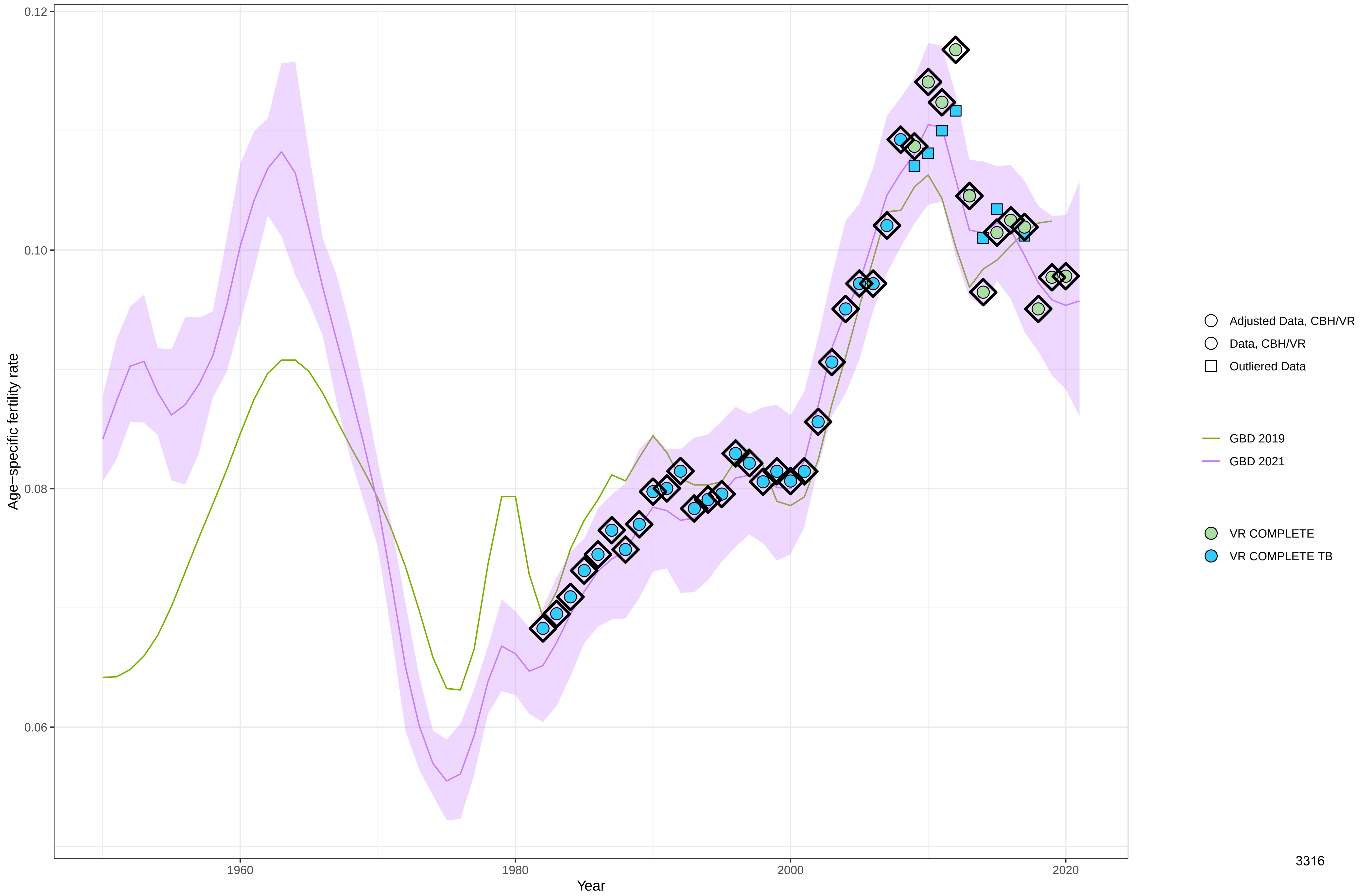

Age−specific fertility fitted trend lines, 1950−2021: Sandwell, Ages: 35−39
Data points enclosed with a diamond are reference sources

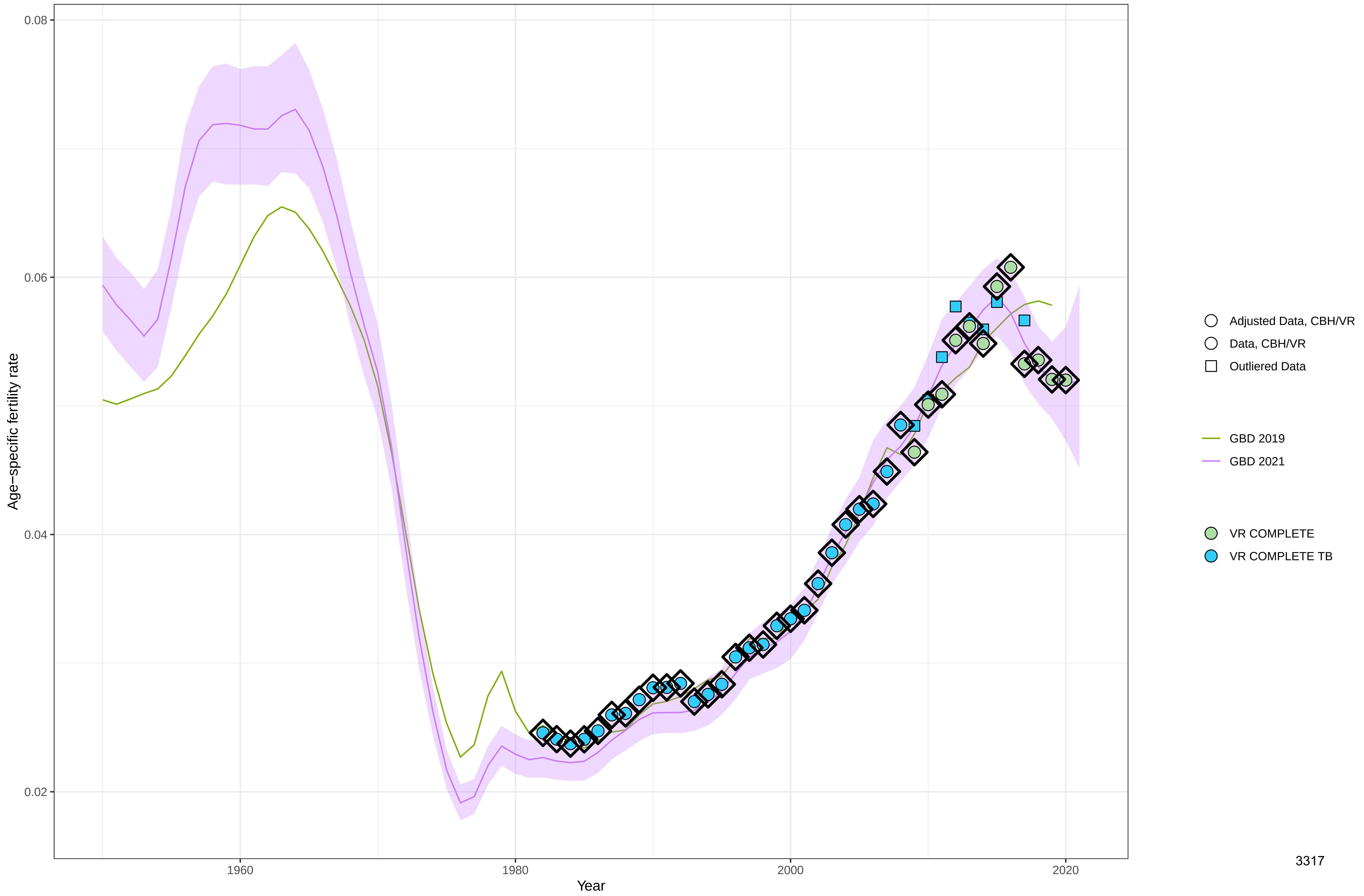

Age−specific fertility fitted trend lines, 1950−2021: Sandwell, Ages: 40−44
Data points enclosed with a diamond are reference sources

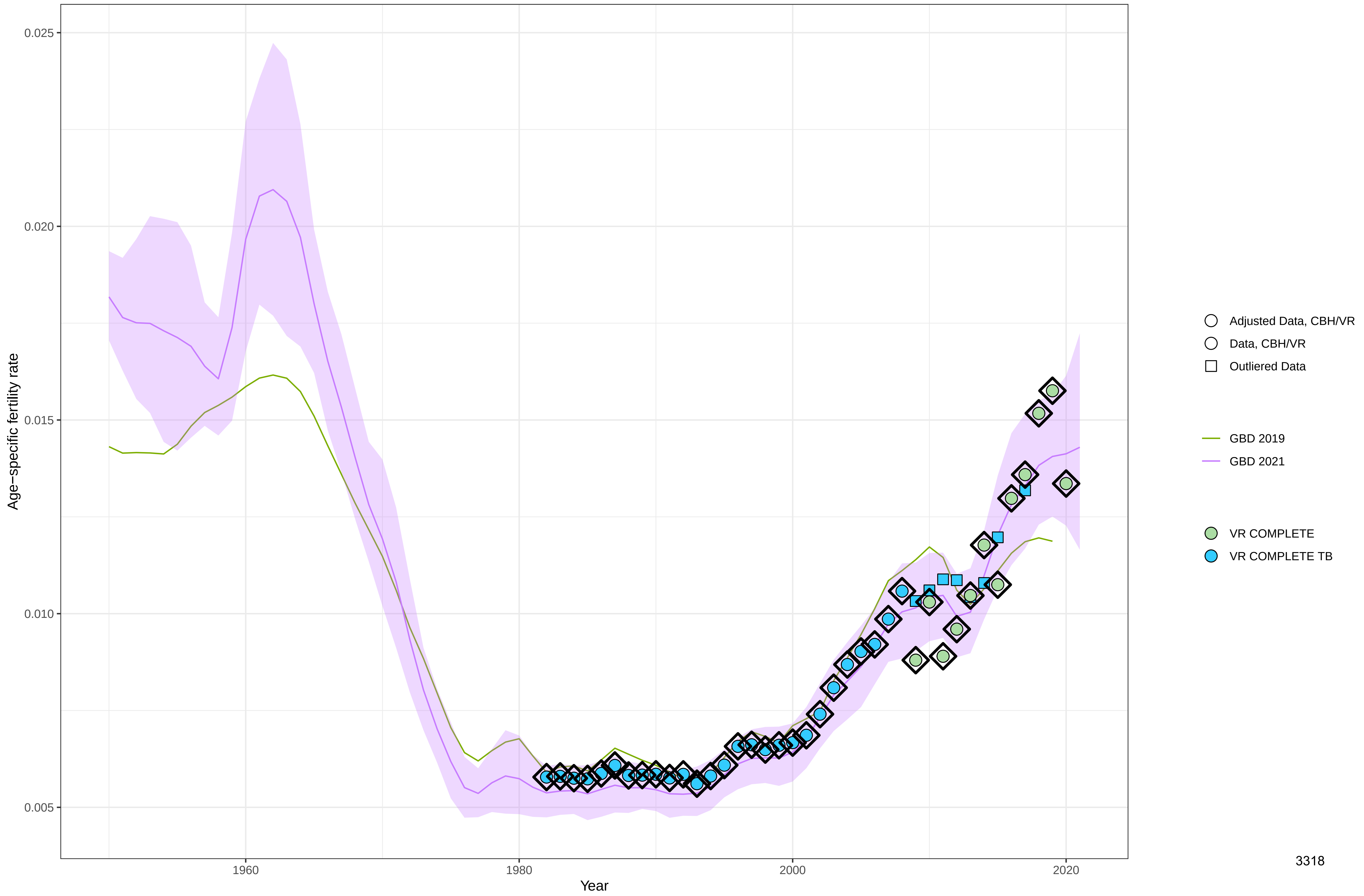

Age−specific fertility fitted trend lines, 1950−2021: Sandwell, Ages: 45−49
Data points enclosed with a diamond are reference sources

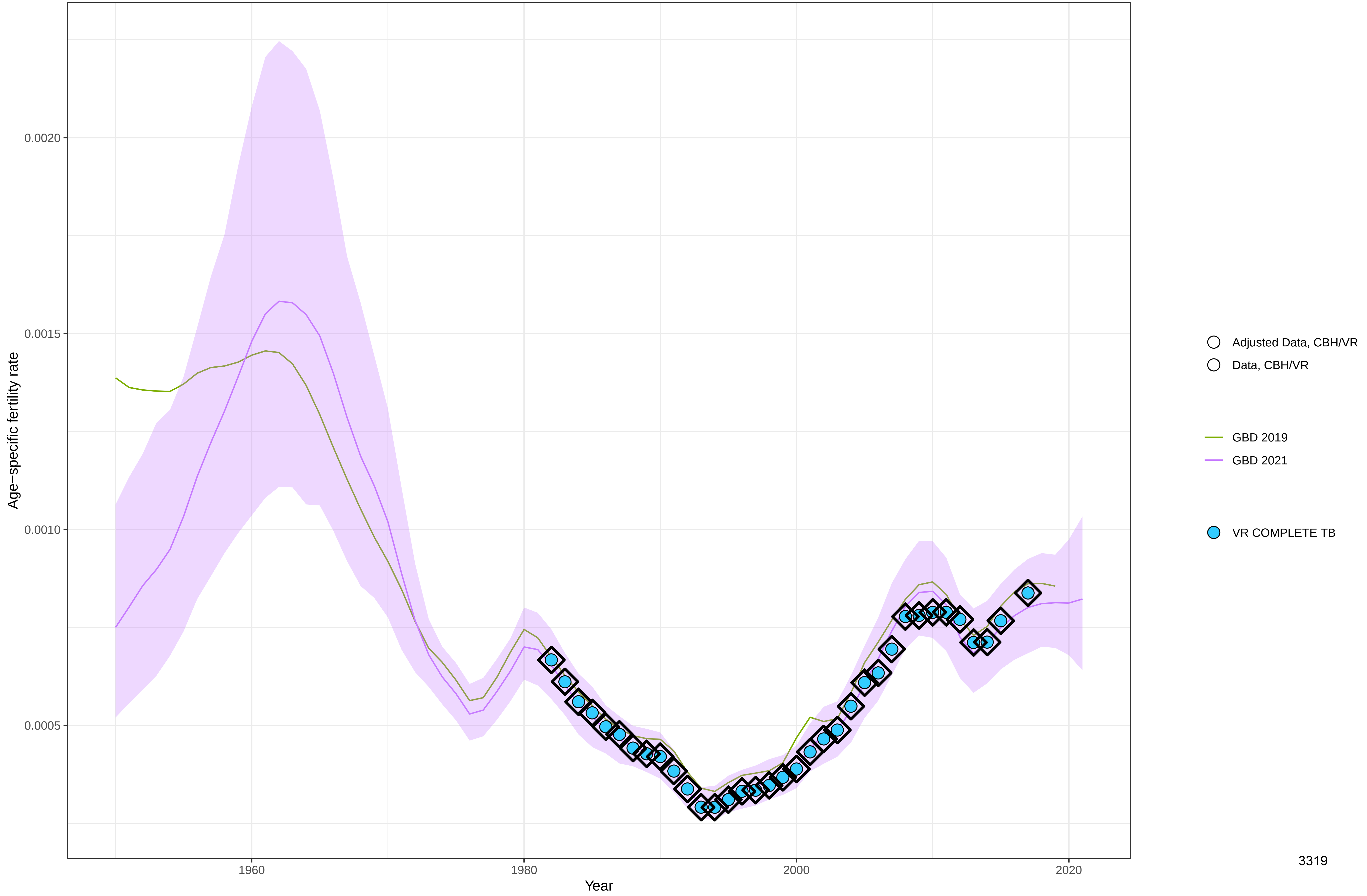

Age−specific fertility fitted trend lines, 1950−2021: Sandwell, Ages: 50−54
Data points enclosed with a diamond are reference sources

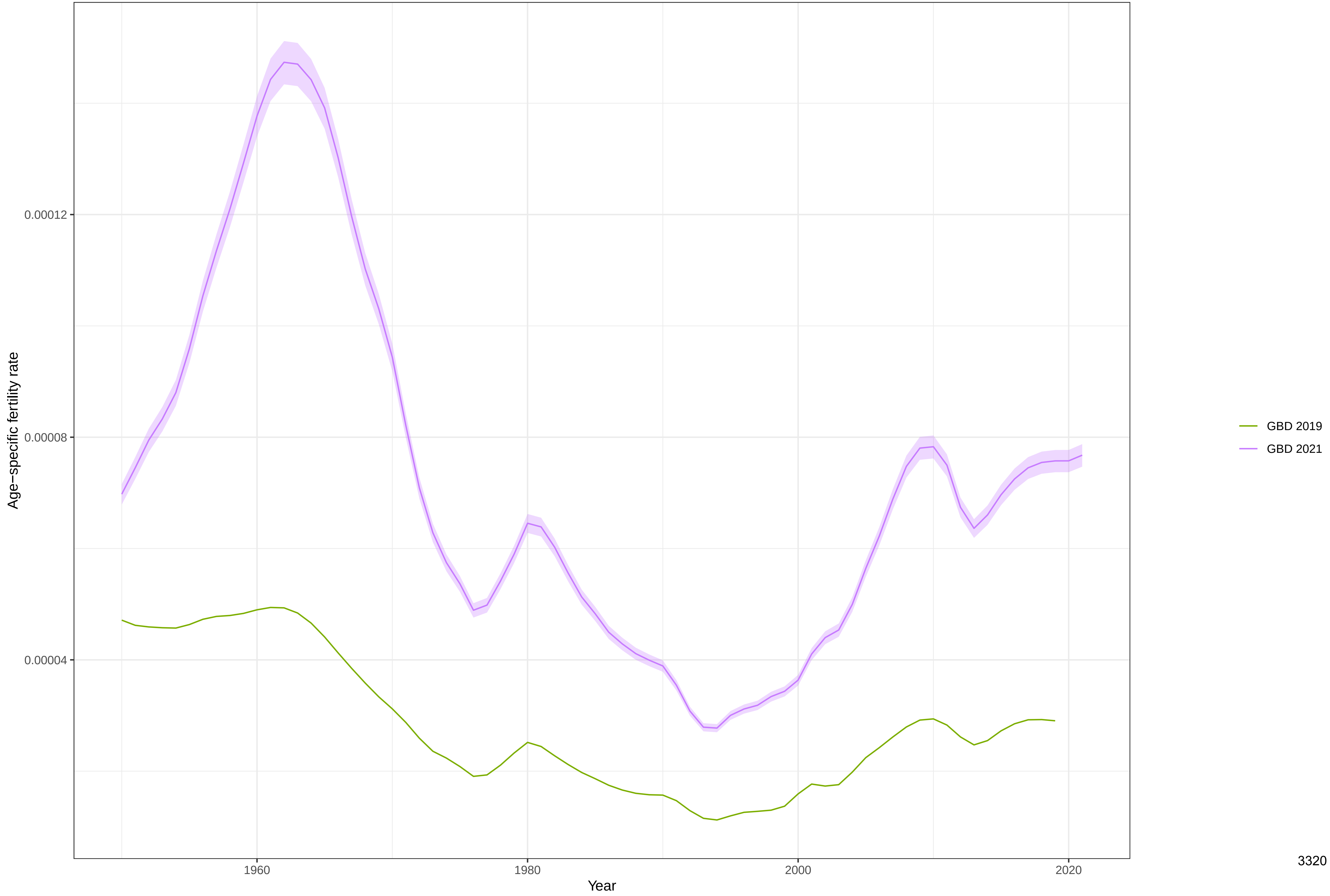

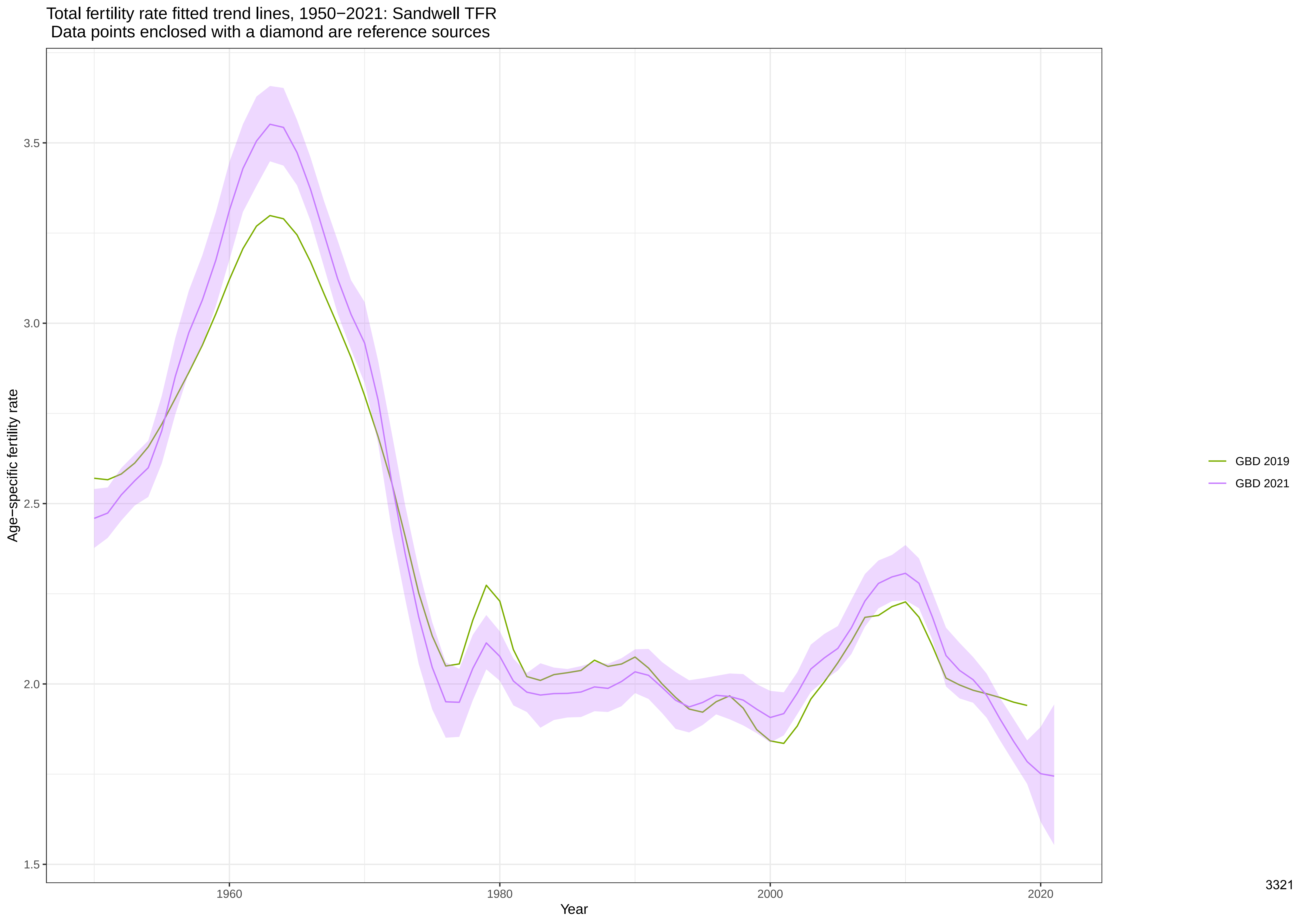

Total fertility rate fitted trend lines, 1950−2021: Sandwell TFR
Data points enclosed with a diamond are reference sources
Age−specific fertility rate
3.5
3.0
2.5
2.0
1.5
1960
1980
2000
2020
Year
GBD 2019
GBD 2021

Age−specific fertility fitted trend lines, 1950−2021: Shropshire, Ages: 10−14
Data points enclosed with a diamond are reference sources

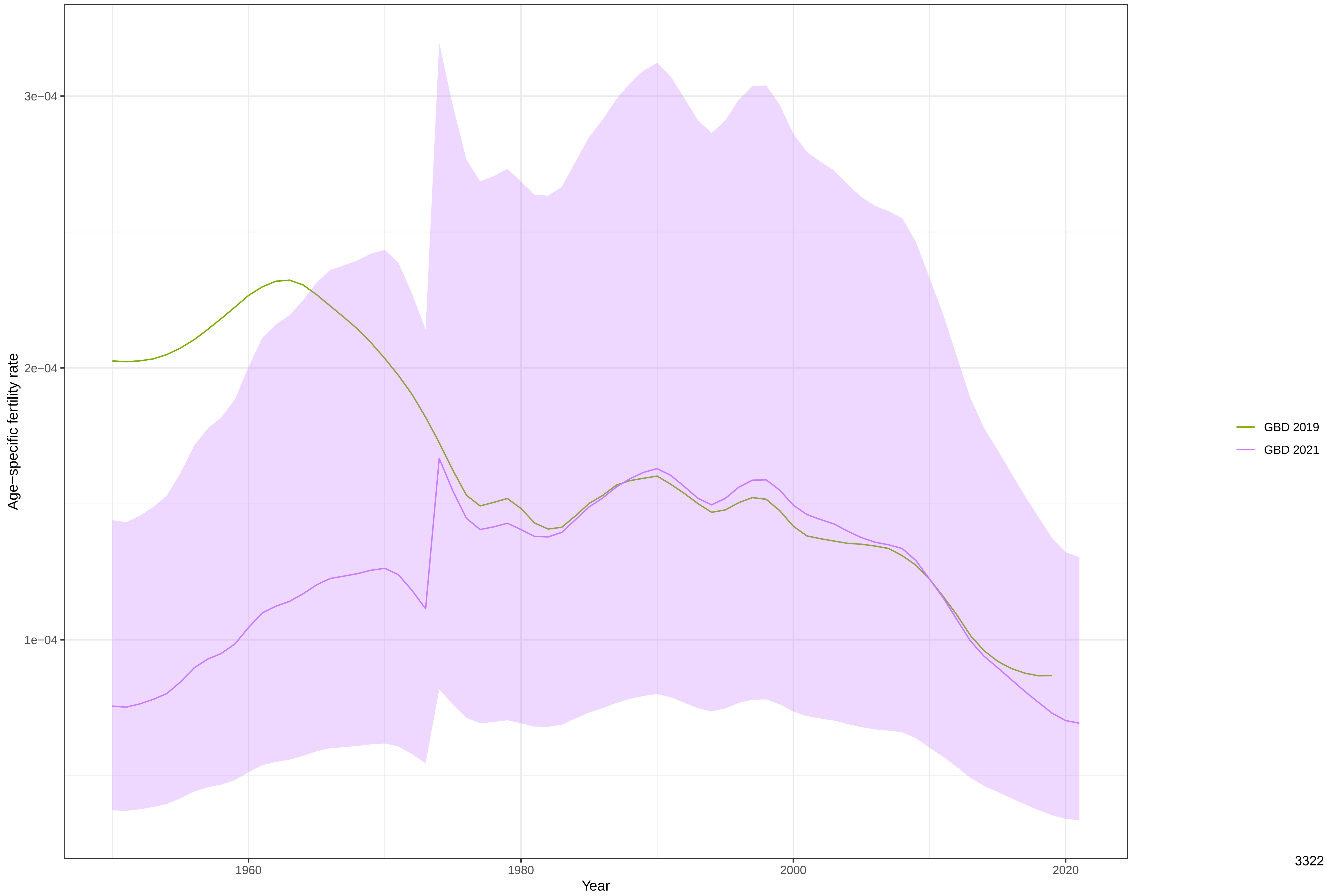

Age−specific fertility fitted trend lines, 1950−2021: Shropshire, Ages: 15−19
Data points enclosed with a diamond are reference sources

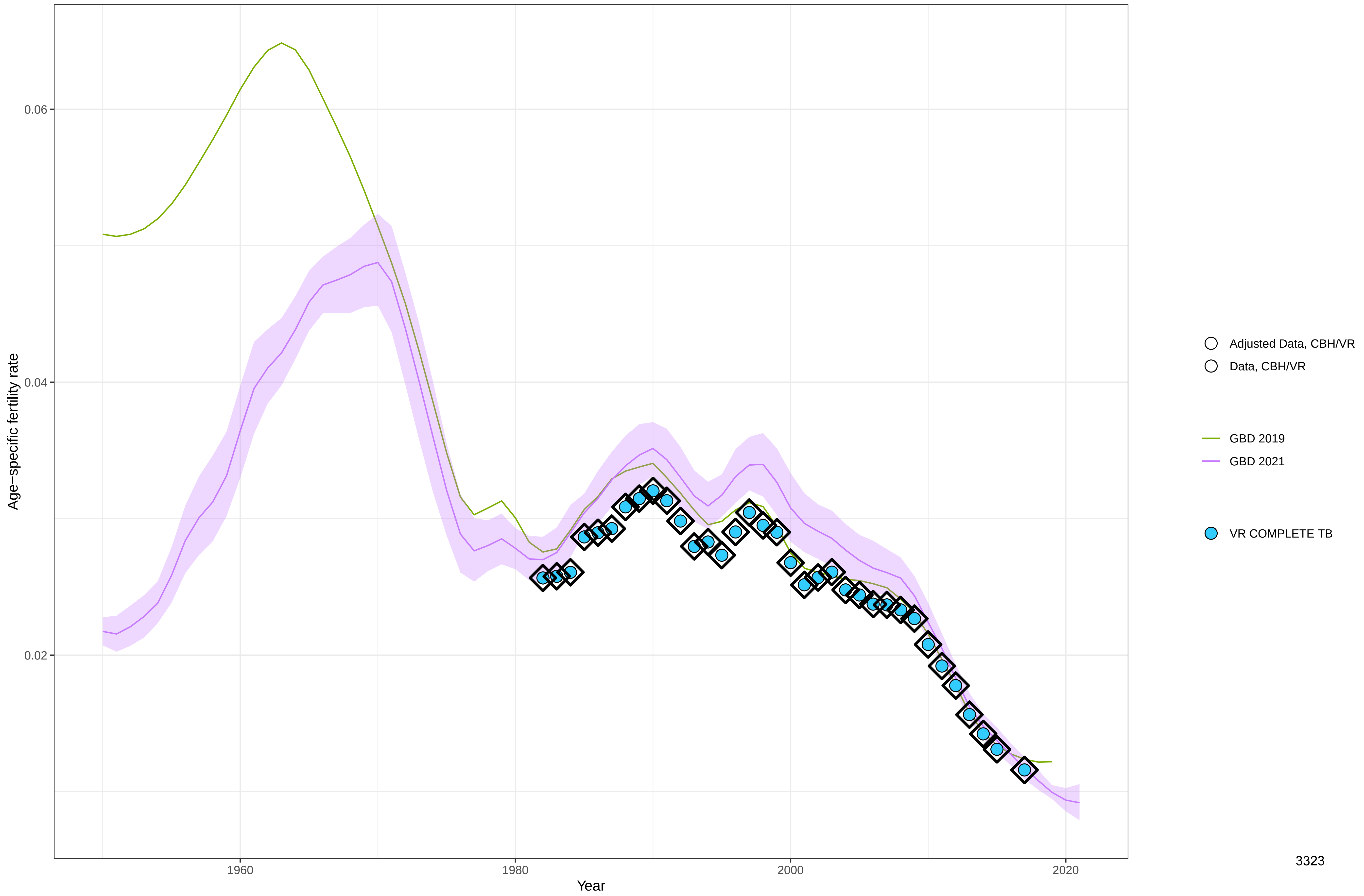

Age−specific fertility fitted trend lines, 1950−2021: Shropshire, Ages: 20−24
Data points enclosed with a diamond are reference sources

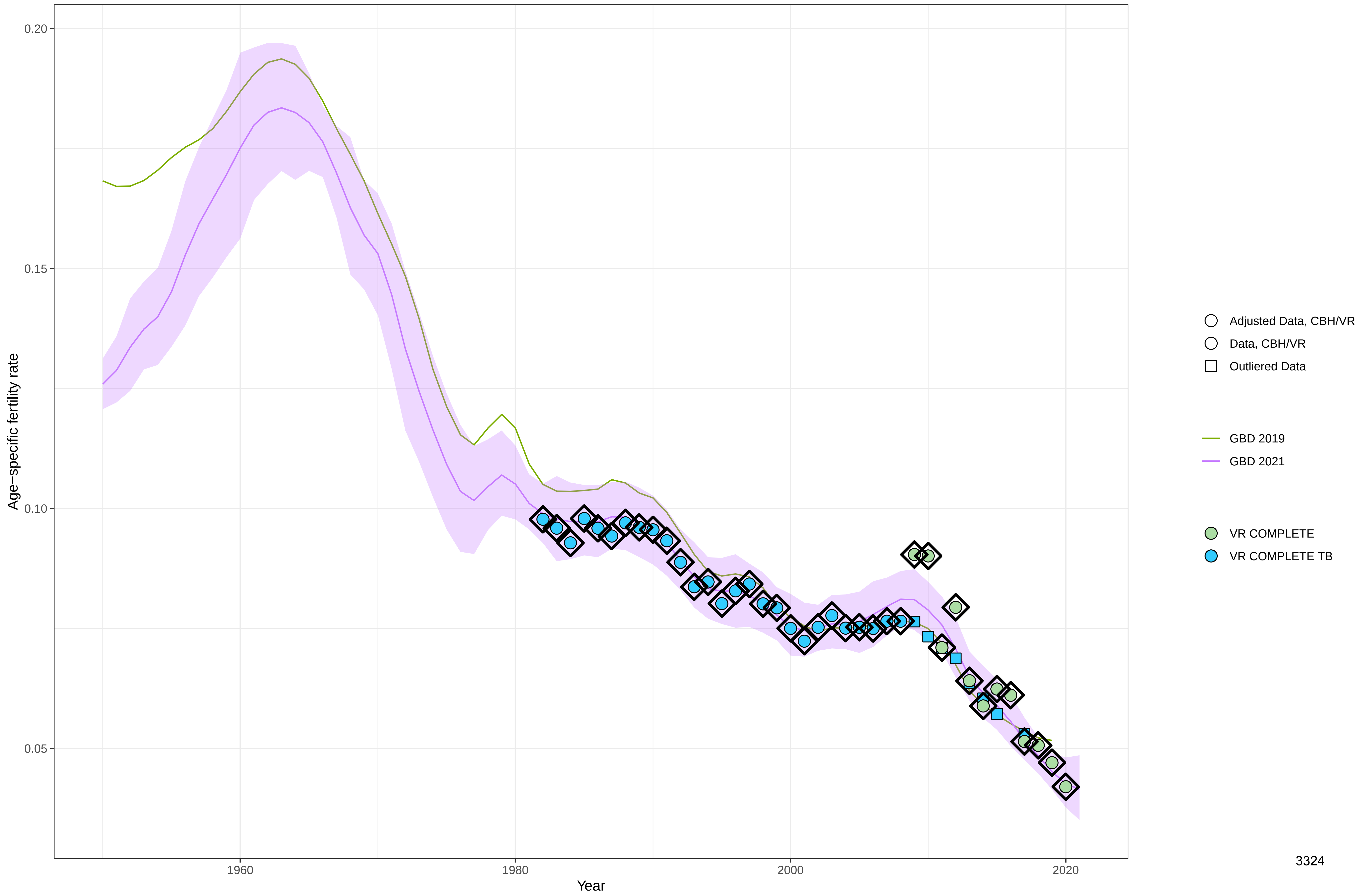

Age−specific fertility fitted trend lines, 1950−2021: Shropshire, Ages: 25−29
Data points enclosed with a diamond are reference sources

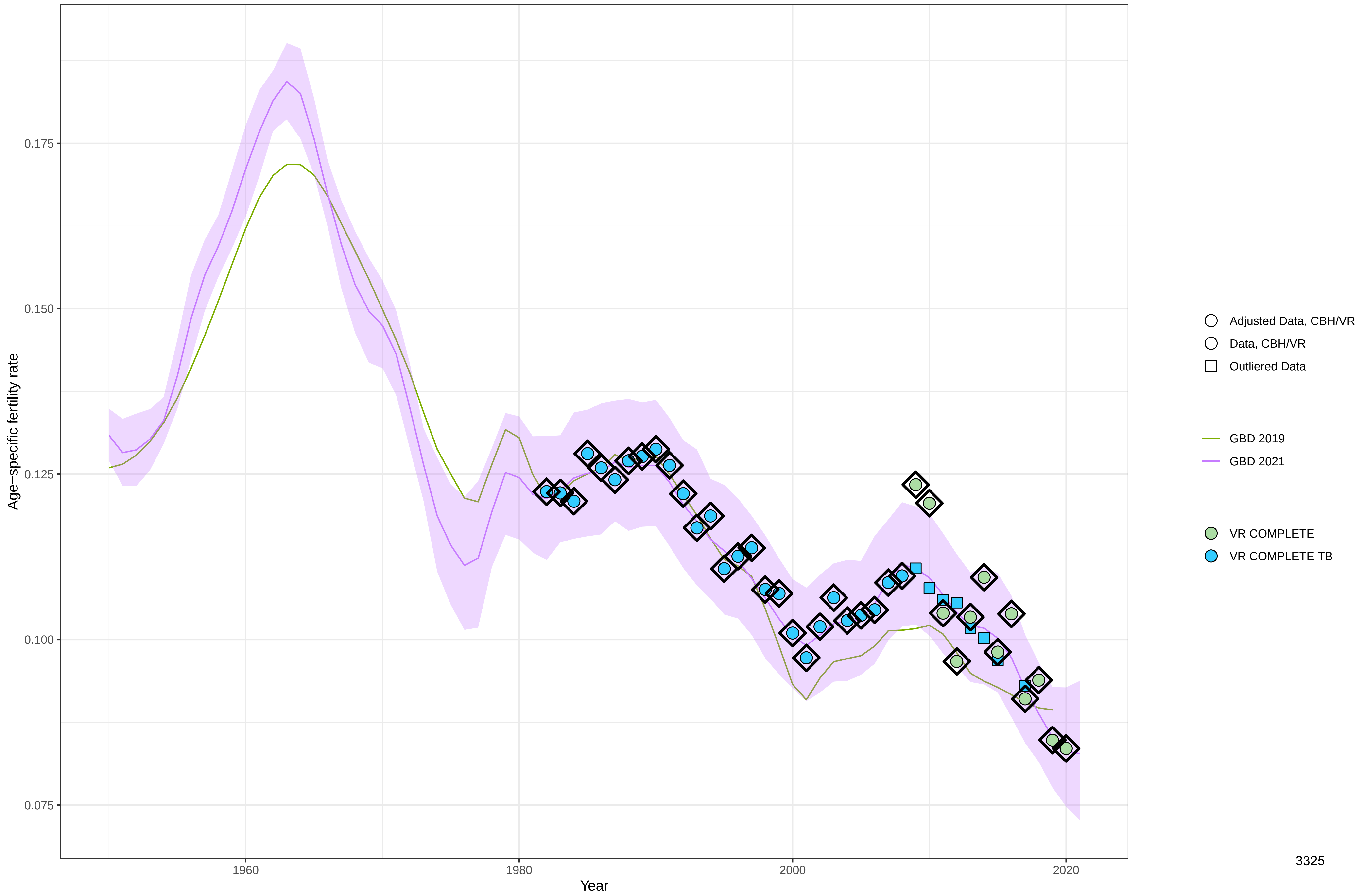

Age−specific fertility fitted trend lines, 1950−2021: Shropshire, Ages: 30−34
Data points enclosed with a diamond are reference sources

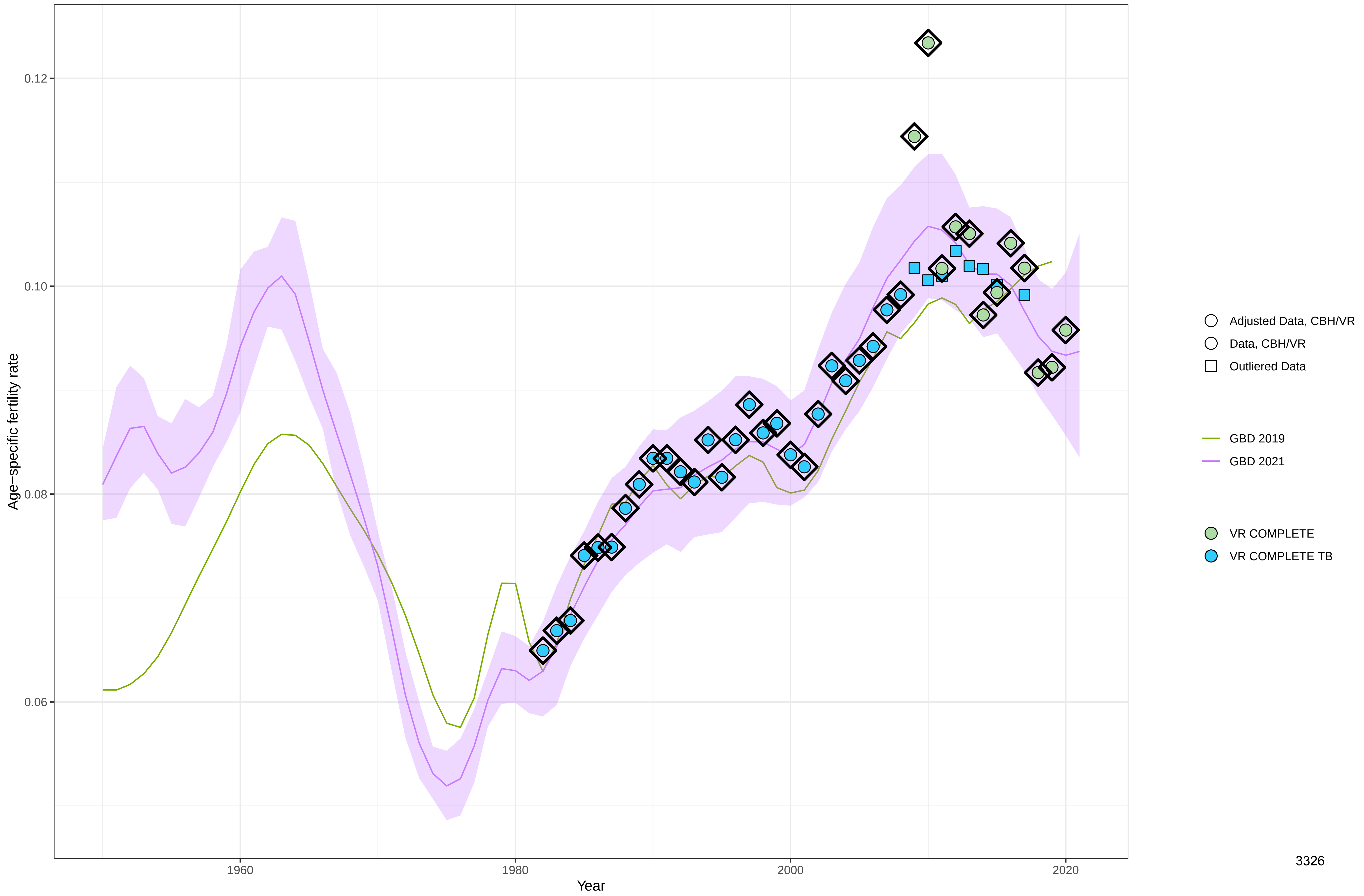

Age−specific fertility fitted trend lines, 1950−2021: Shropshire, Ages: 35−39
Data points enclosed with a diamond are reference sources

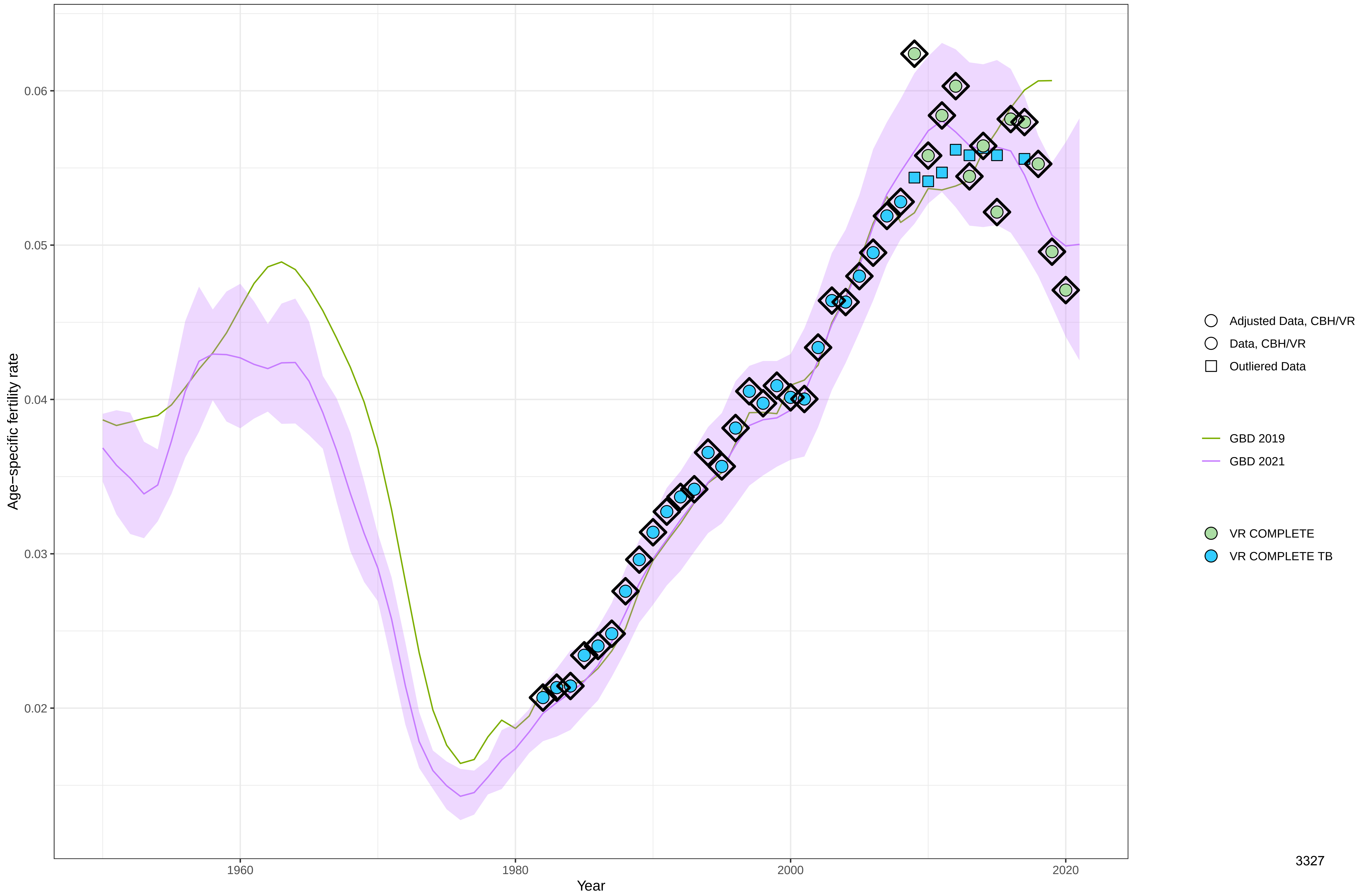

Age−specific fertility fitted trend lines, 1950−2021: Shropshire, Ages: 40−44
Data points enclosed with a diamond are reference sources

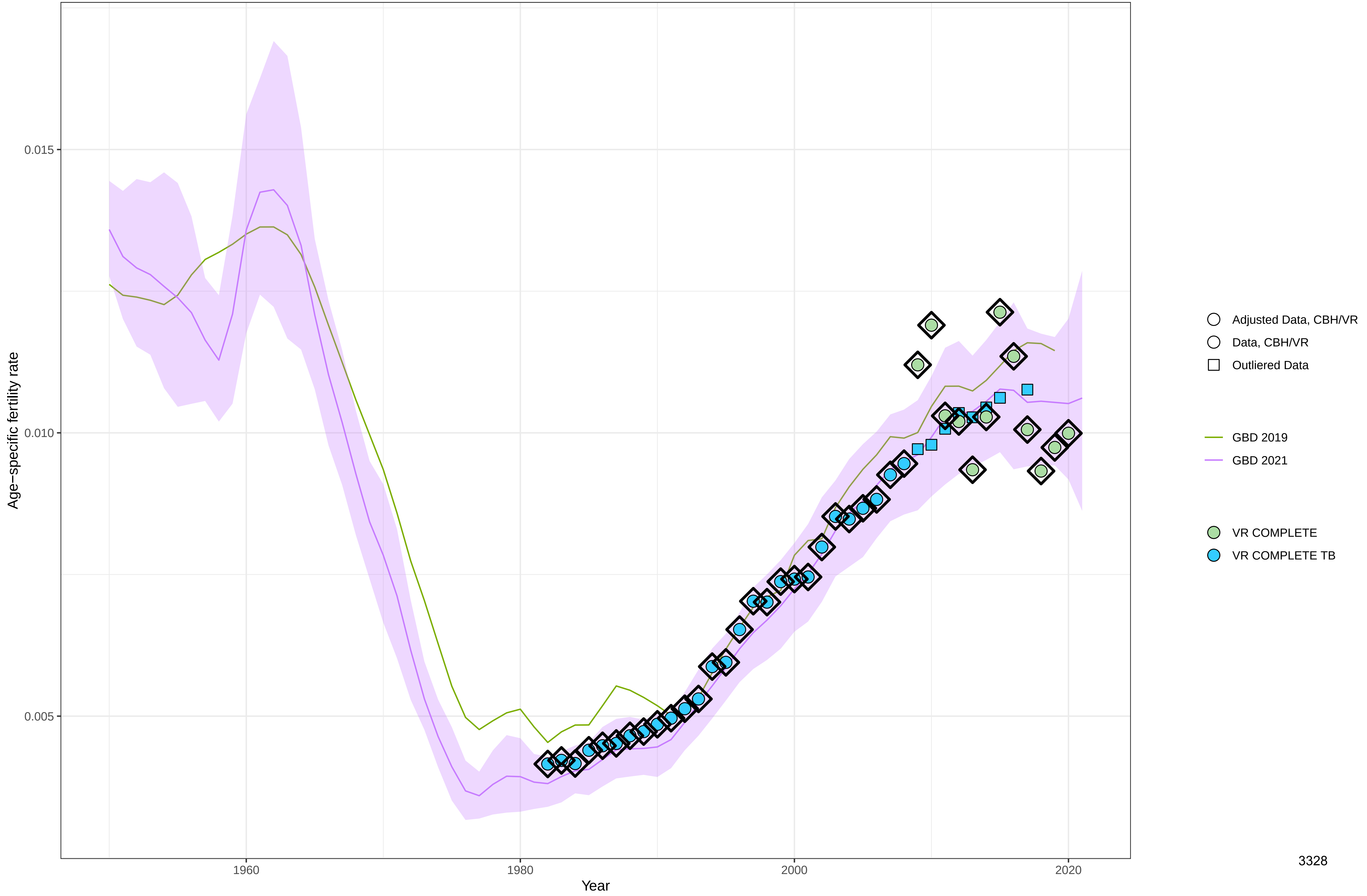

Age−specific fertility fitted trend lines, 1950−2021: Shropshire, Ages: 45−49
Data points enclosed with a diamond are reference sources

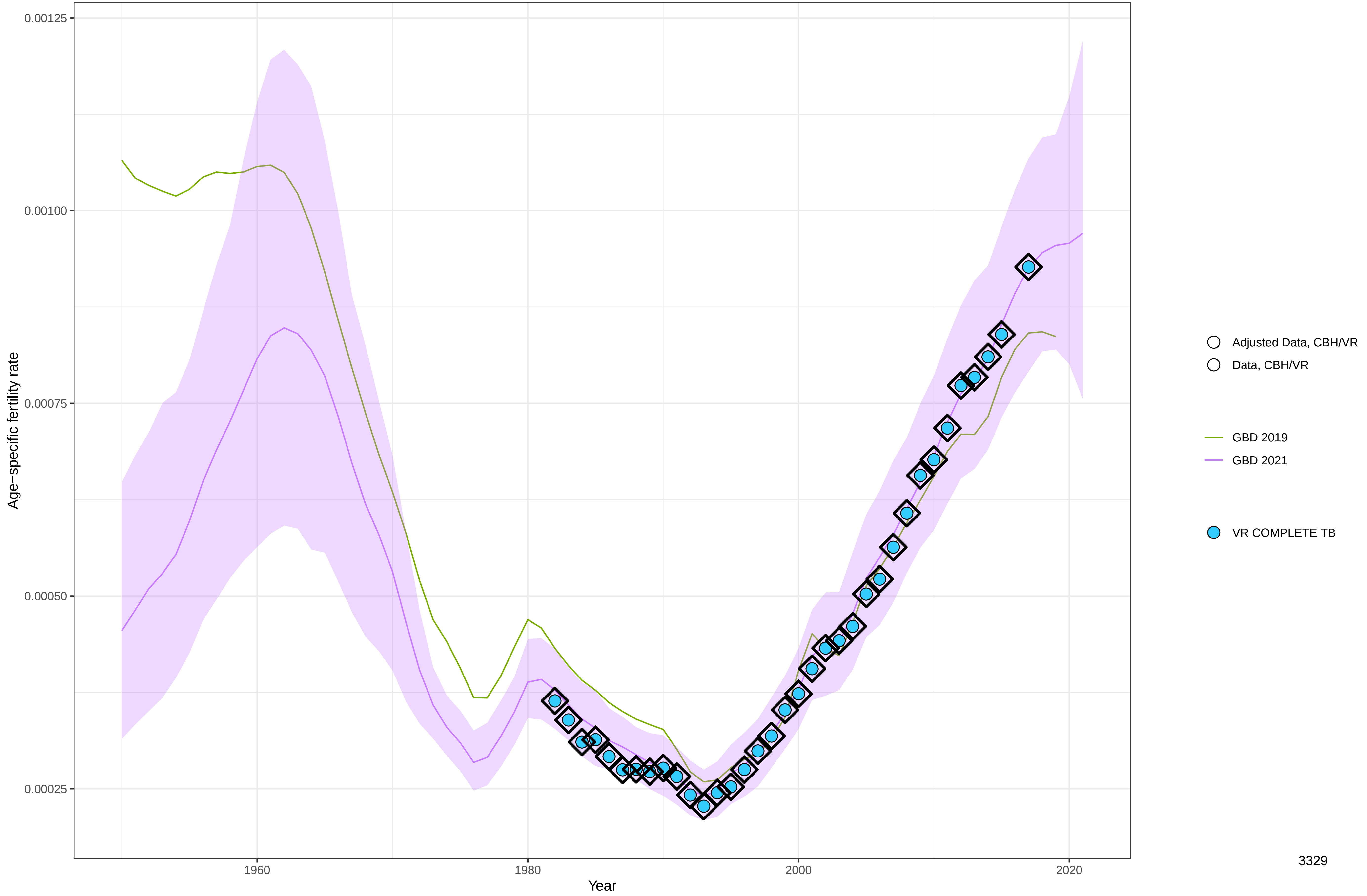

Age−specific fertility fitted trend lines, 1950−2021: Shropshire, Ages: 50−54
Data points enclosed with a diamond are reference sources

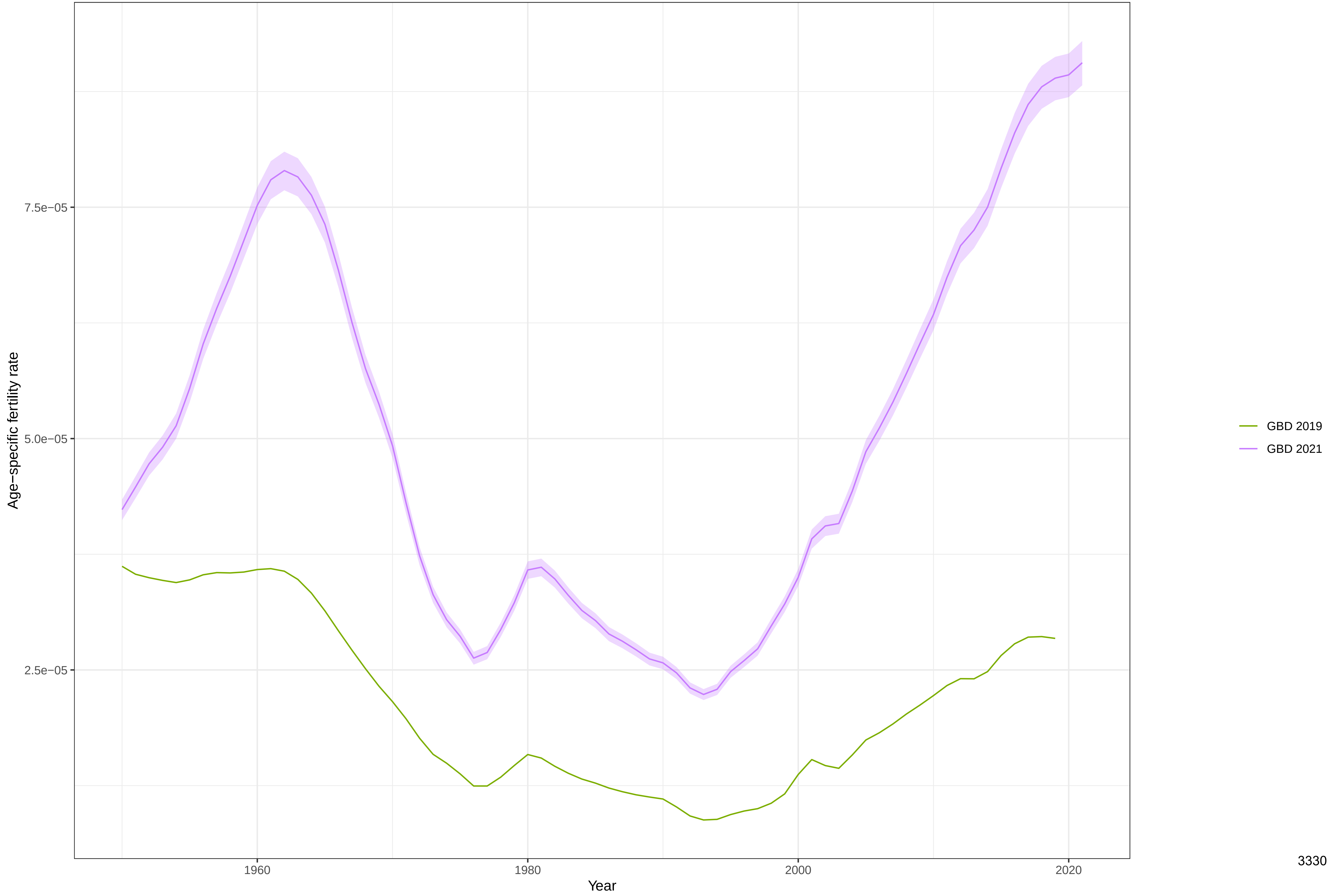

Total fertility rate fitted trend lines, 1950−2021: Shropshire TFR
Data points enclosed with a diamond are reference sources

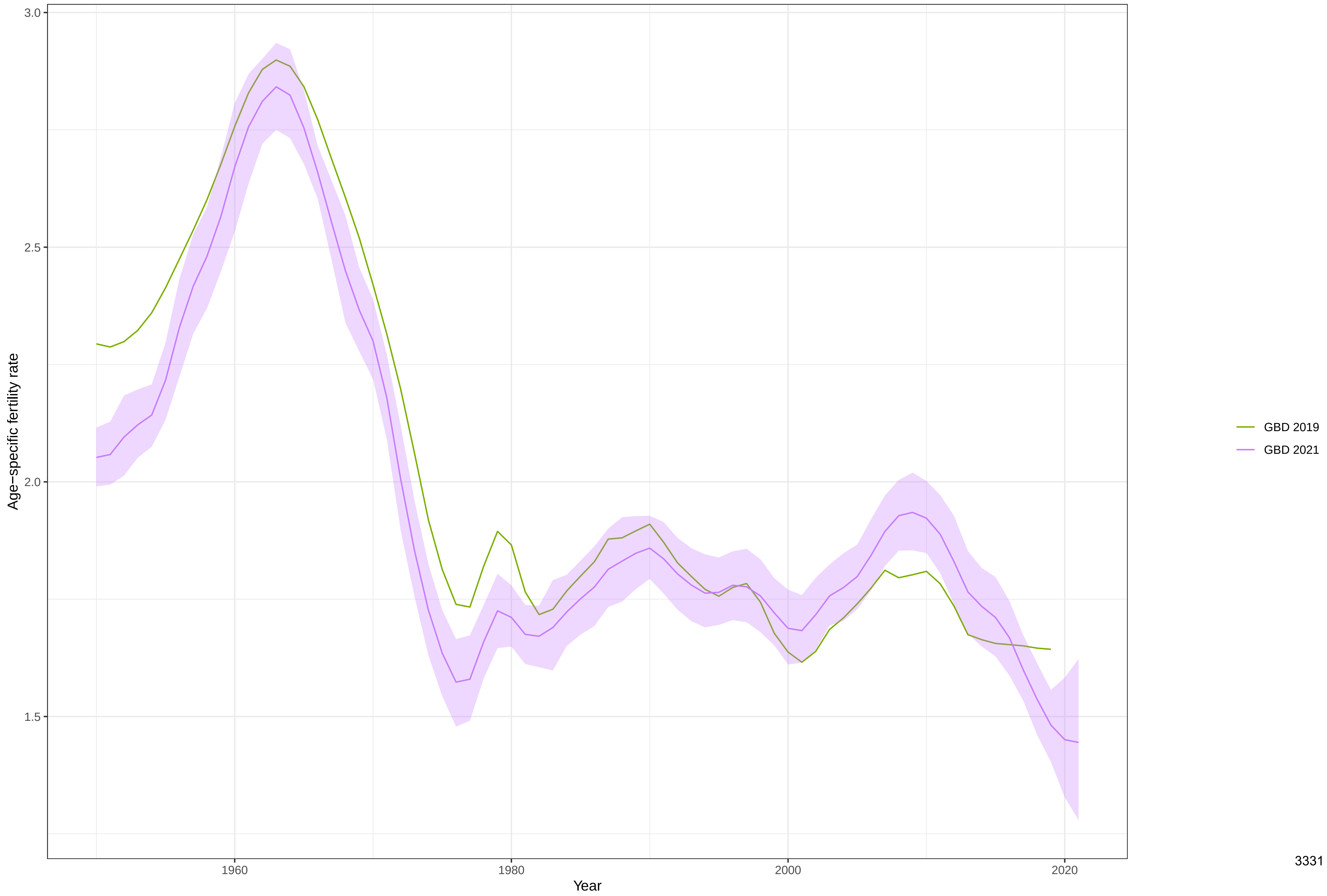

Age−specific fertility fitted trend lines, 1950−2021: Solihull, Ages: 10−14
Data points enclosed with a diamond are reference sources

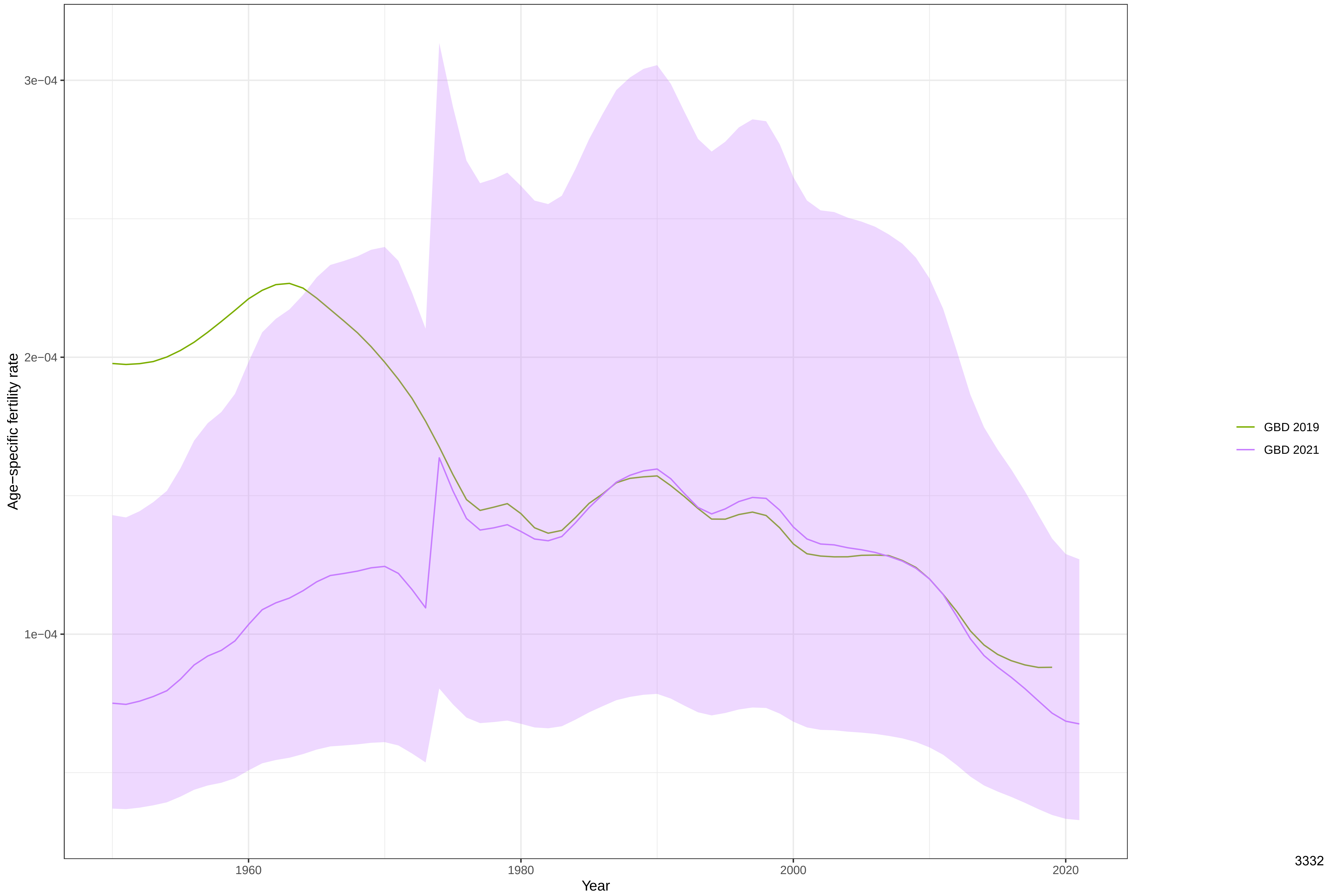

3332

Age−specific fertility fitted trend lines, 1950−2021: Solihull, Ages: 15−19
Data points enclosed with a diamond are reference sources

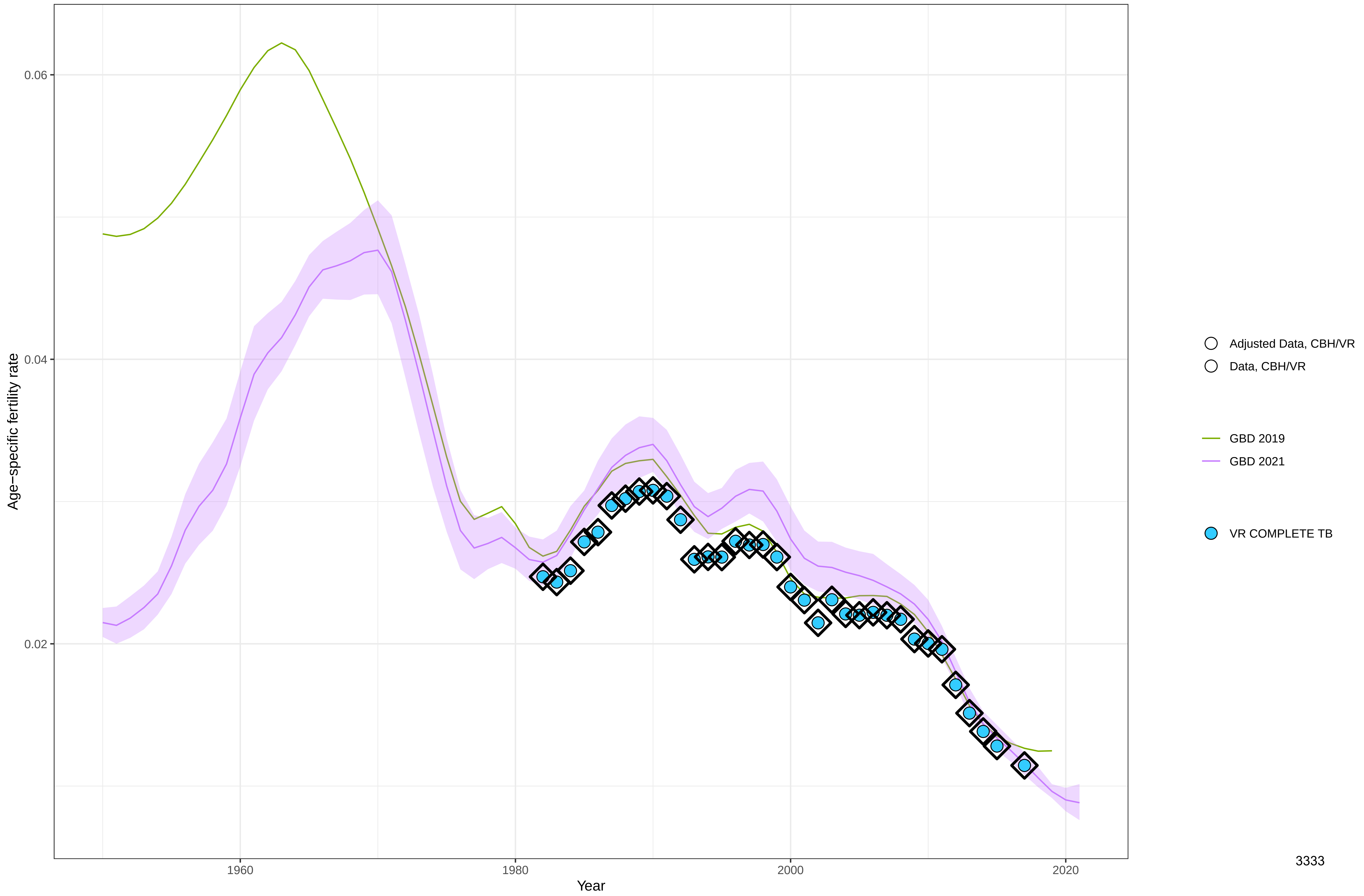

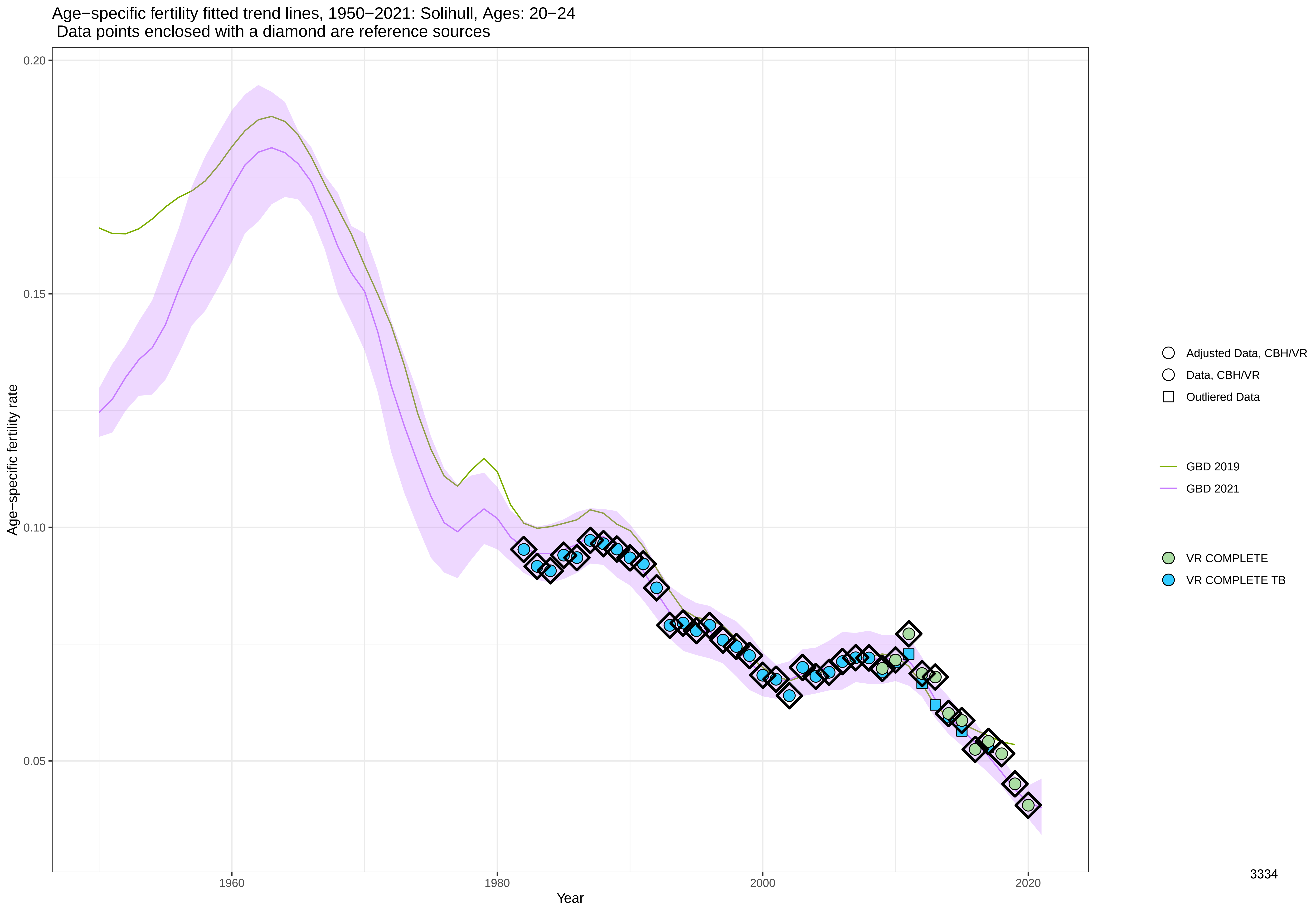
Age−specific fertility fitted trend lines, 1950−2021: Solihull, Ages: 20−24
Data points enclosed with a diamond are reference sources
Age−specific fertility rate
0.20
0.15
0.10
0.05
1960
1980
2000
2020
Year
Adjusted Data, CBH/VR
Data, CBH/VR
Outliered Data
GBD 2019
GBD 2021
VR COMPLETE
VR COMPLETE TB

Age−specific fertility fitted trend lines, 1950−2021: Solihull, Ages: 25−29
Data points enclosed with a diamond are reference sources

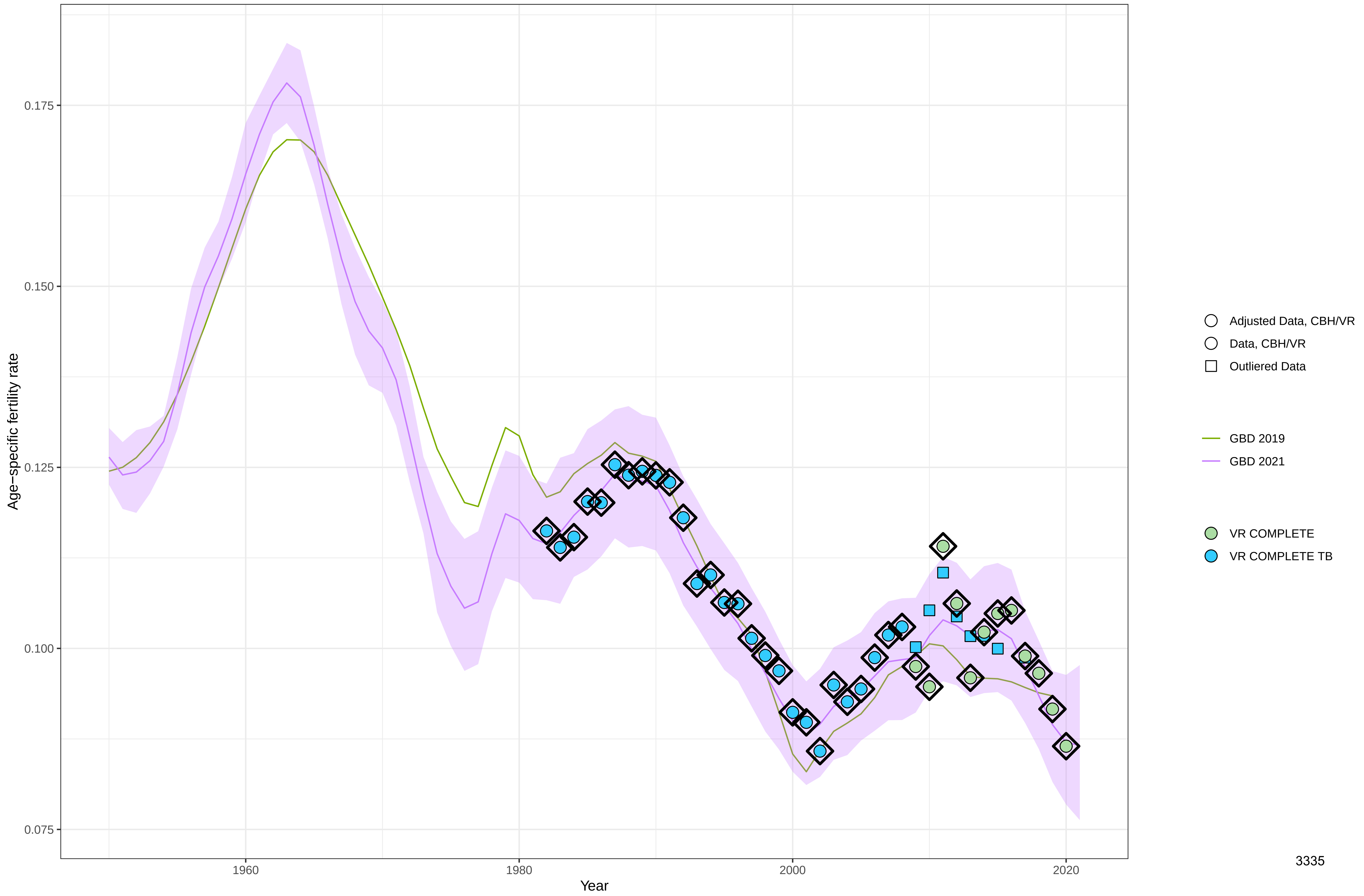

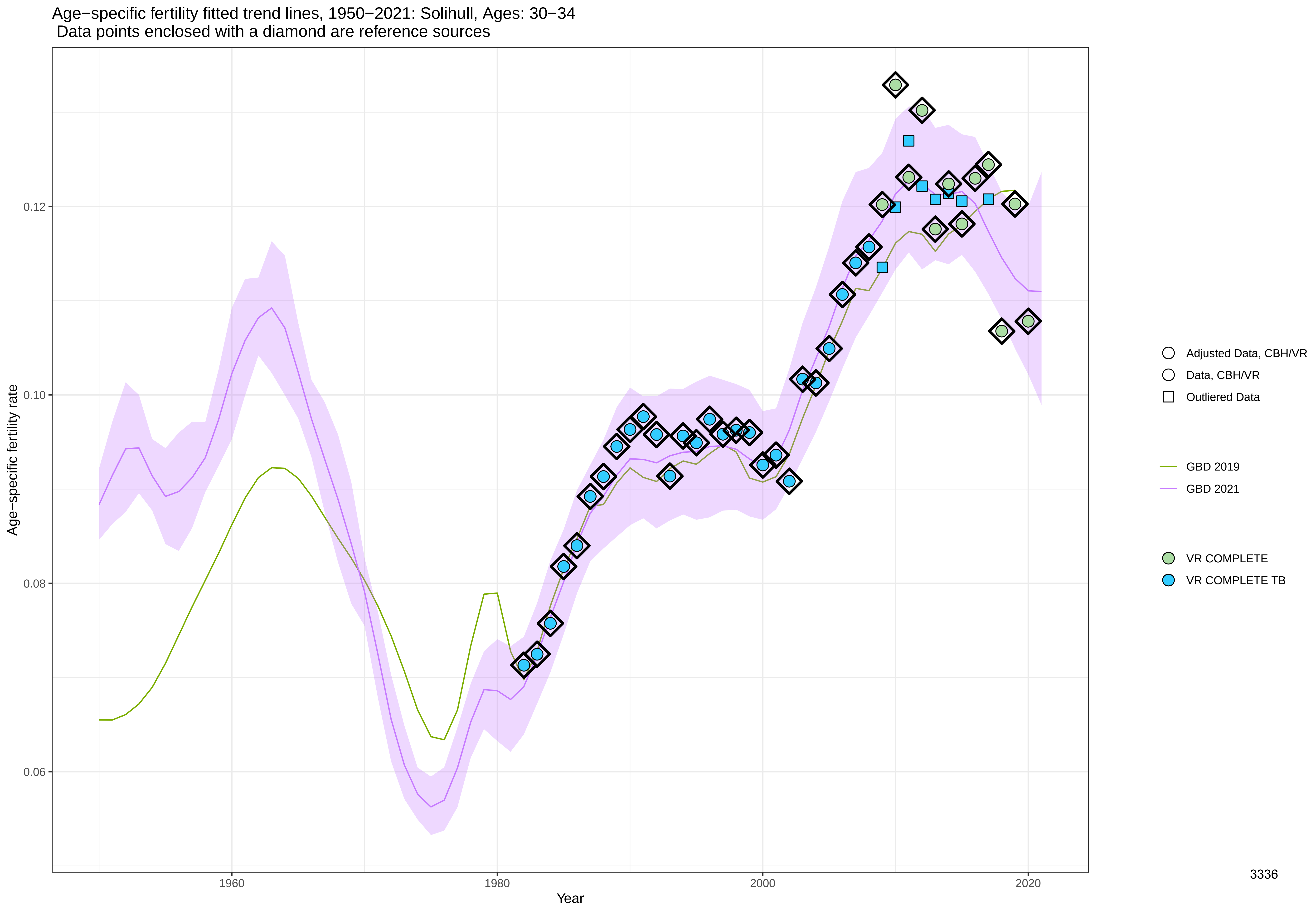

Age−specific fertility fitted trend lines, 1950−2021: Solihull, Ages: 30−34
Data points enclosed with a diamond are reference sources
Age−specific fertility rate
0.06
0.08
0.10
0.12
1960
1980
2000
2020
Year
Adjusted Data, CBH/VR
Data, CBH/VR
Outliered Data
GBD 2019
GBD 2021
VR COMPLETE
VR COMPLETE TB

3336

Age−specific fertility fitted trend lines, 1950−2021: Solihull, Ages: 35−39
Data points enclosed with a diamond are reference sources

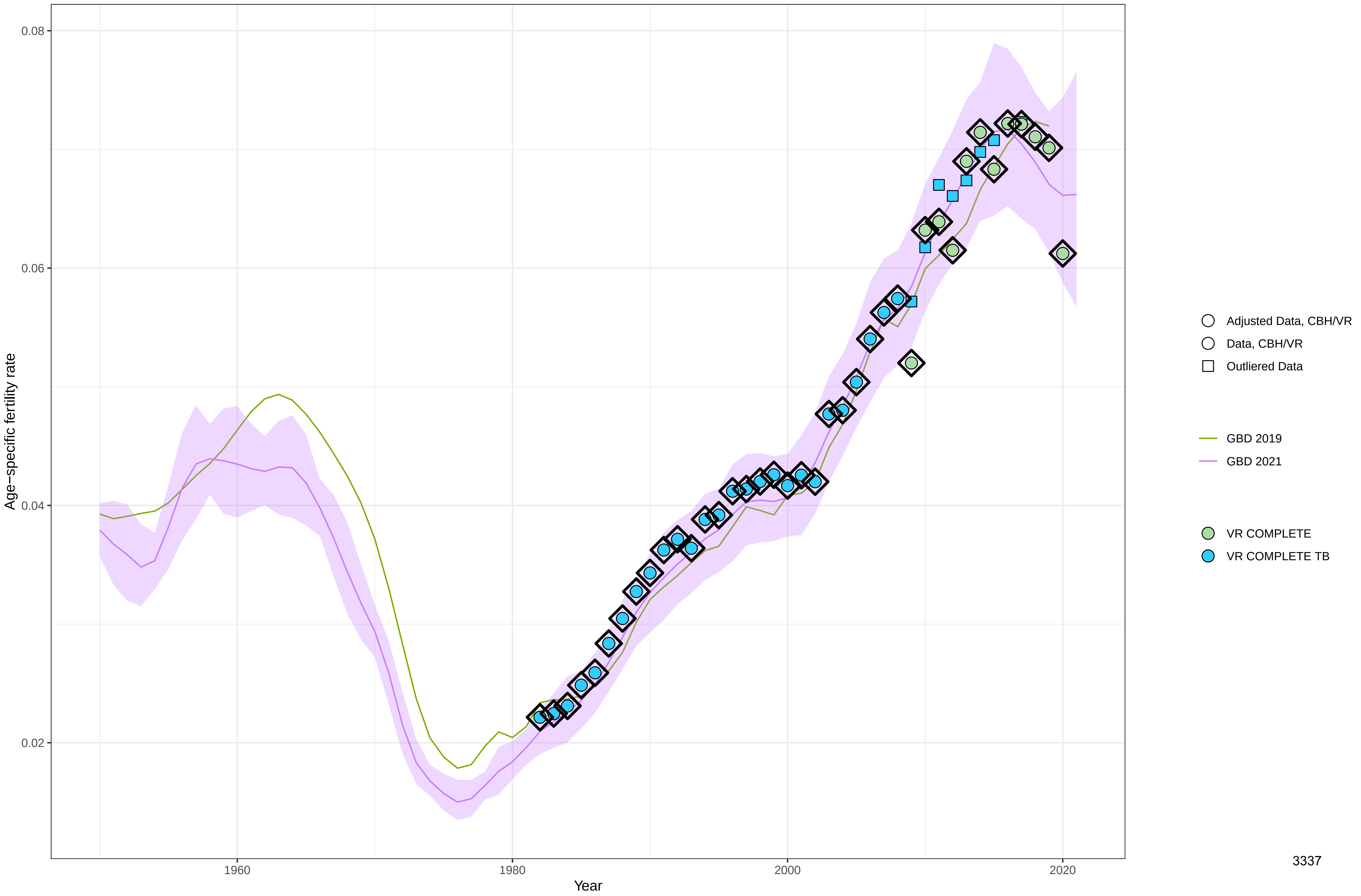

Age−specific fertility fitted trend lines, 1950−2021: Solihull, Ages: 40−44
Data points enclosed with a diamond are reference sources

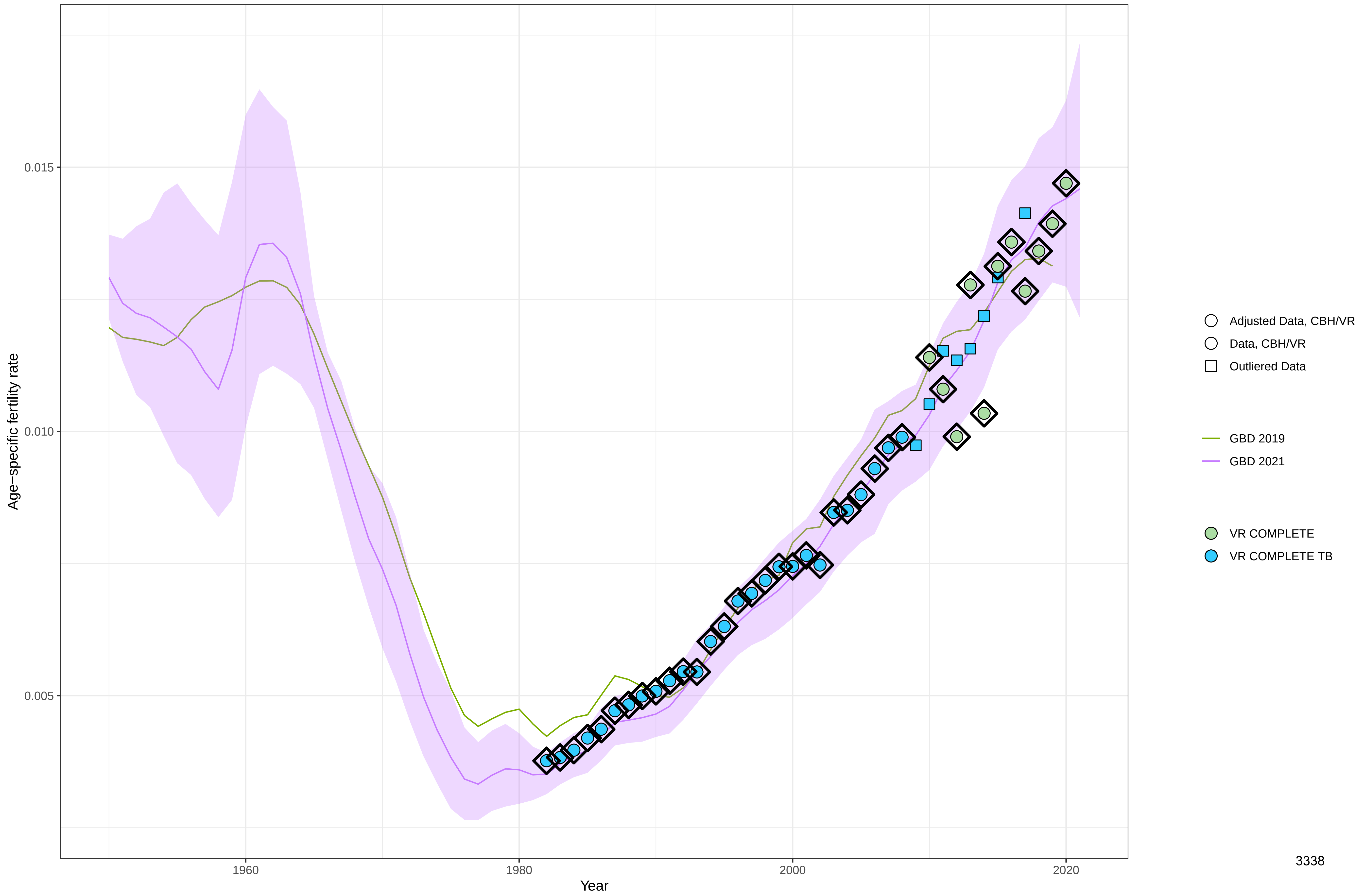

Age−specific fertility fitted trend lines, 1950−2021: Solihull, Ages: 45−49
Data points enclosed with a diamond are reference sources

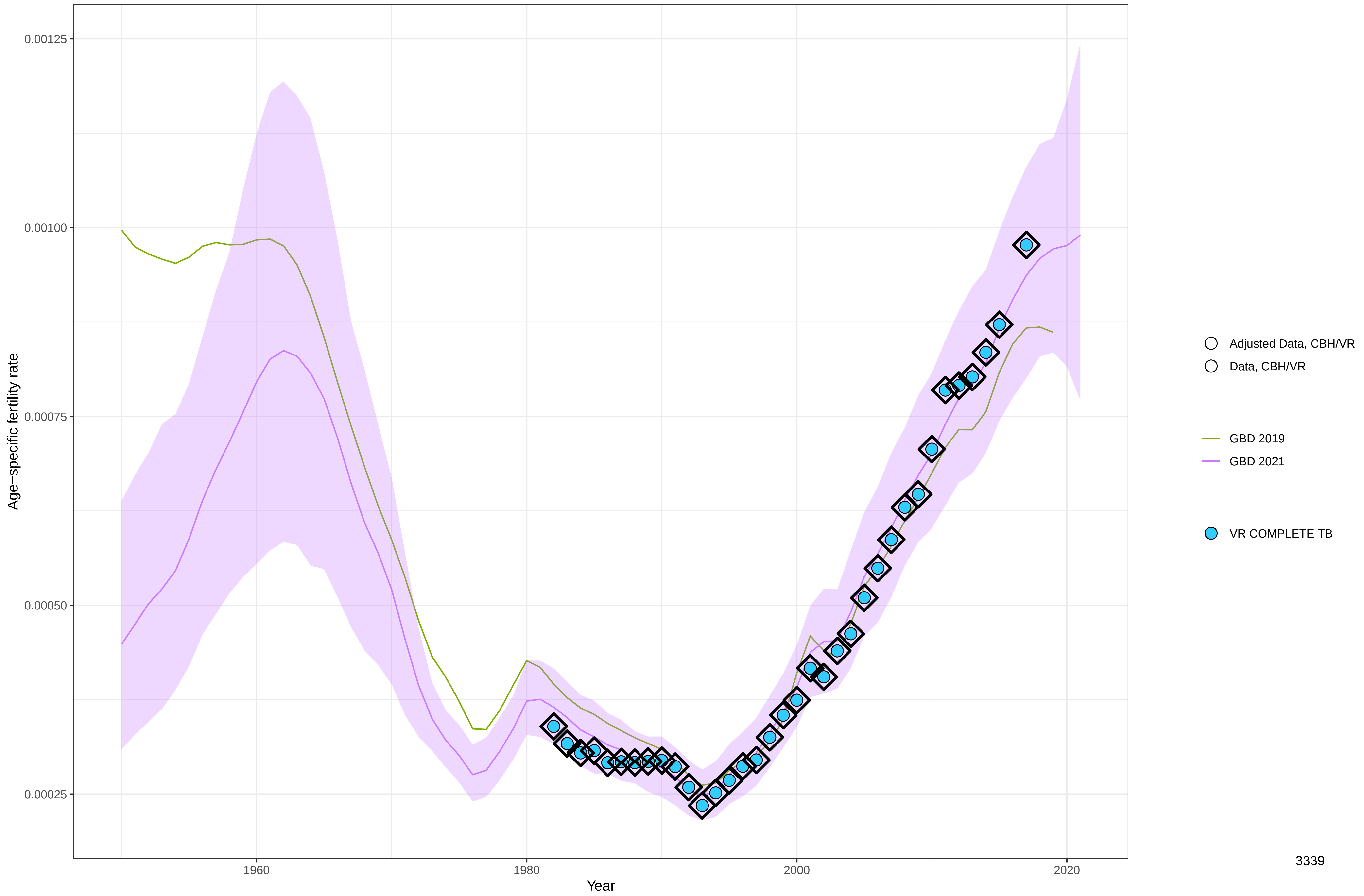

Age−specific fertility fitted trend lines, 1950−2021: Solihull, Ages: 50−54
Data points enclosed with a diamond are reference sources

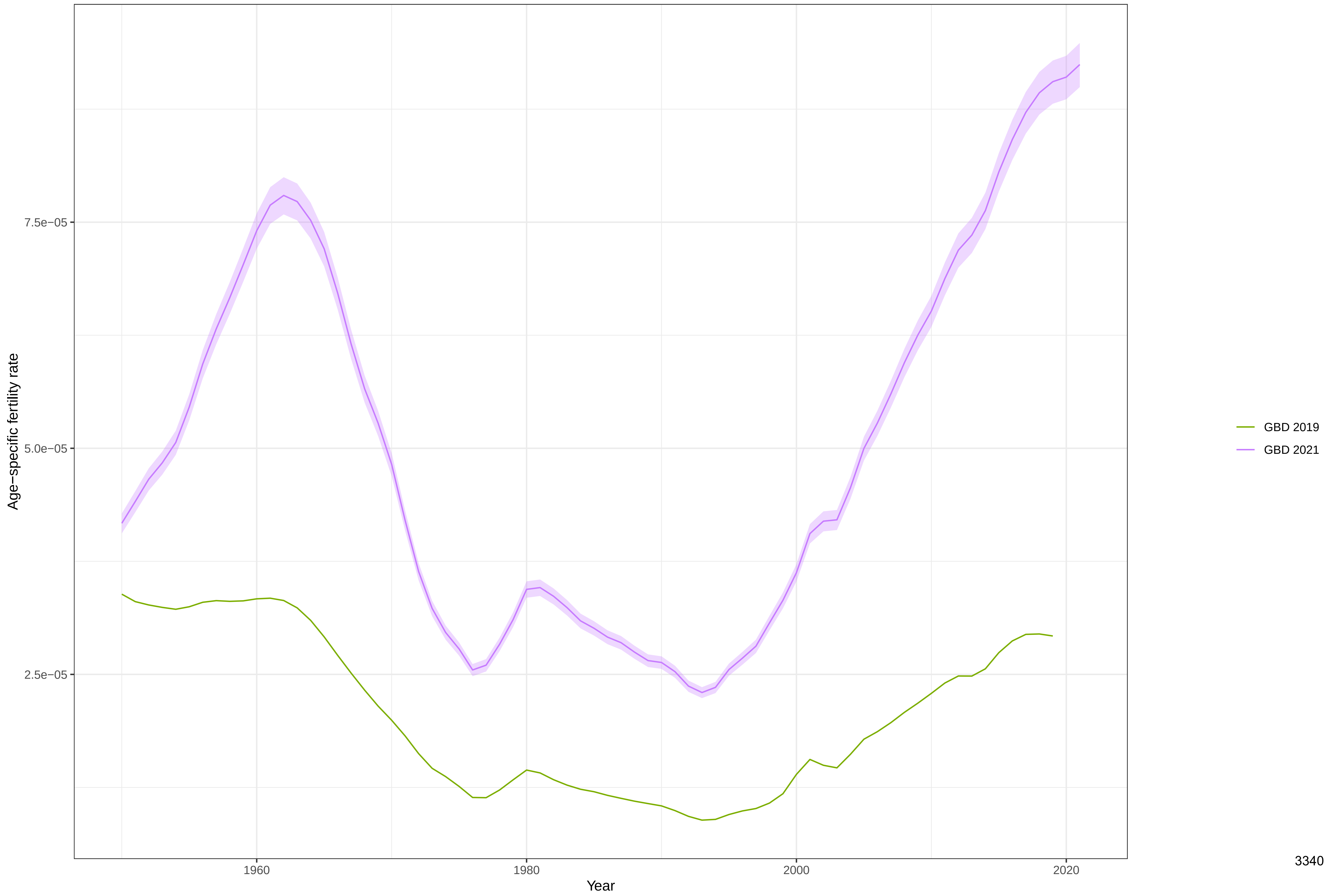

Total fertility rate fitted trend lines, 1950−2021: Solihull TFR
Data points enclosed with a diamond are reference sources

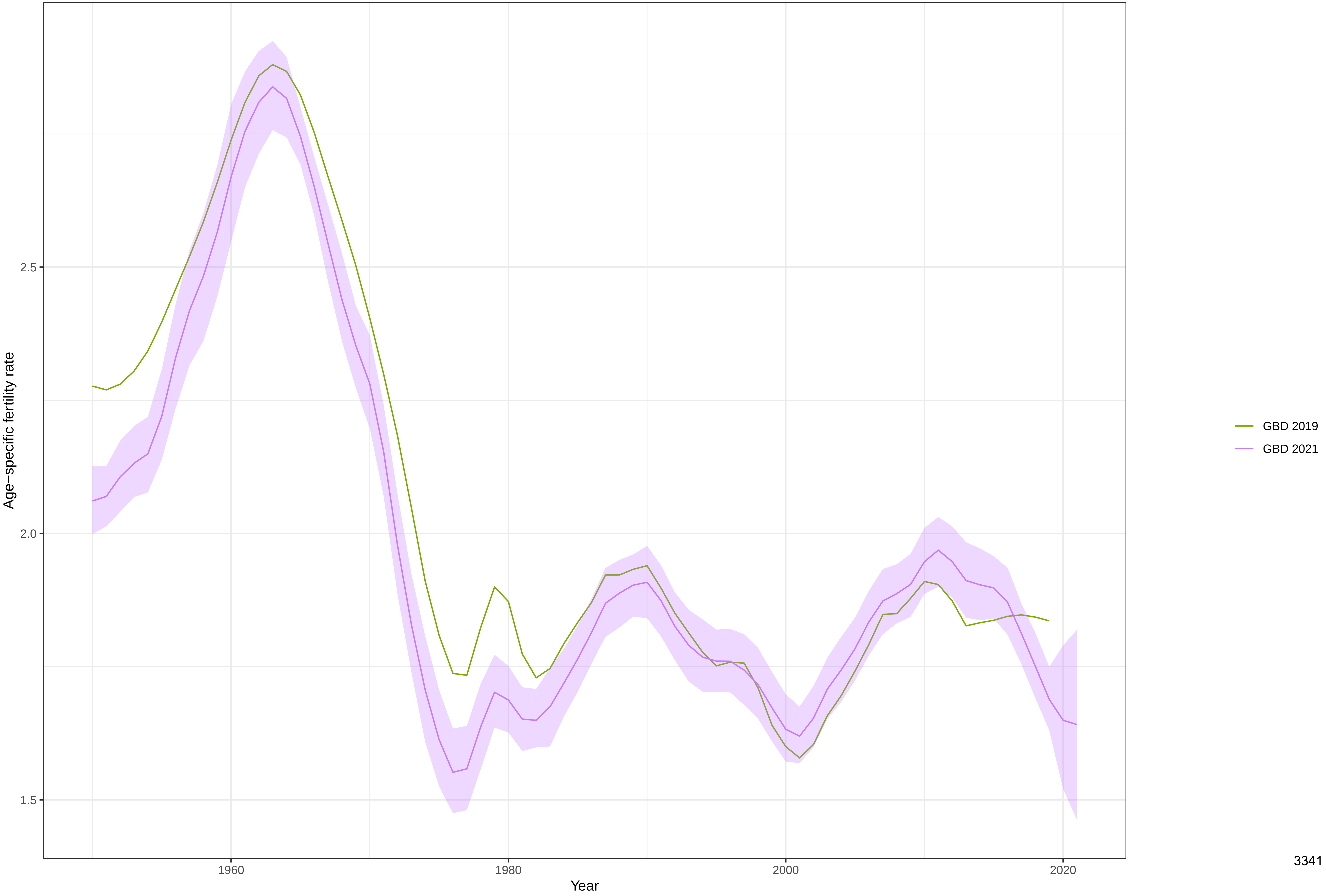

Age−specific fertility fitted trend lines, 1950−2021: Staffordshire, Ages: 10−14
Data points enclosed with a diamond are reference sources

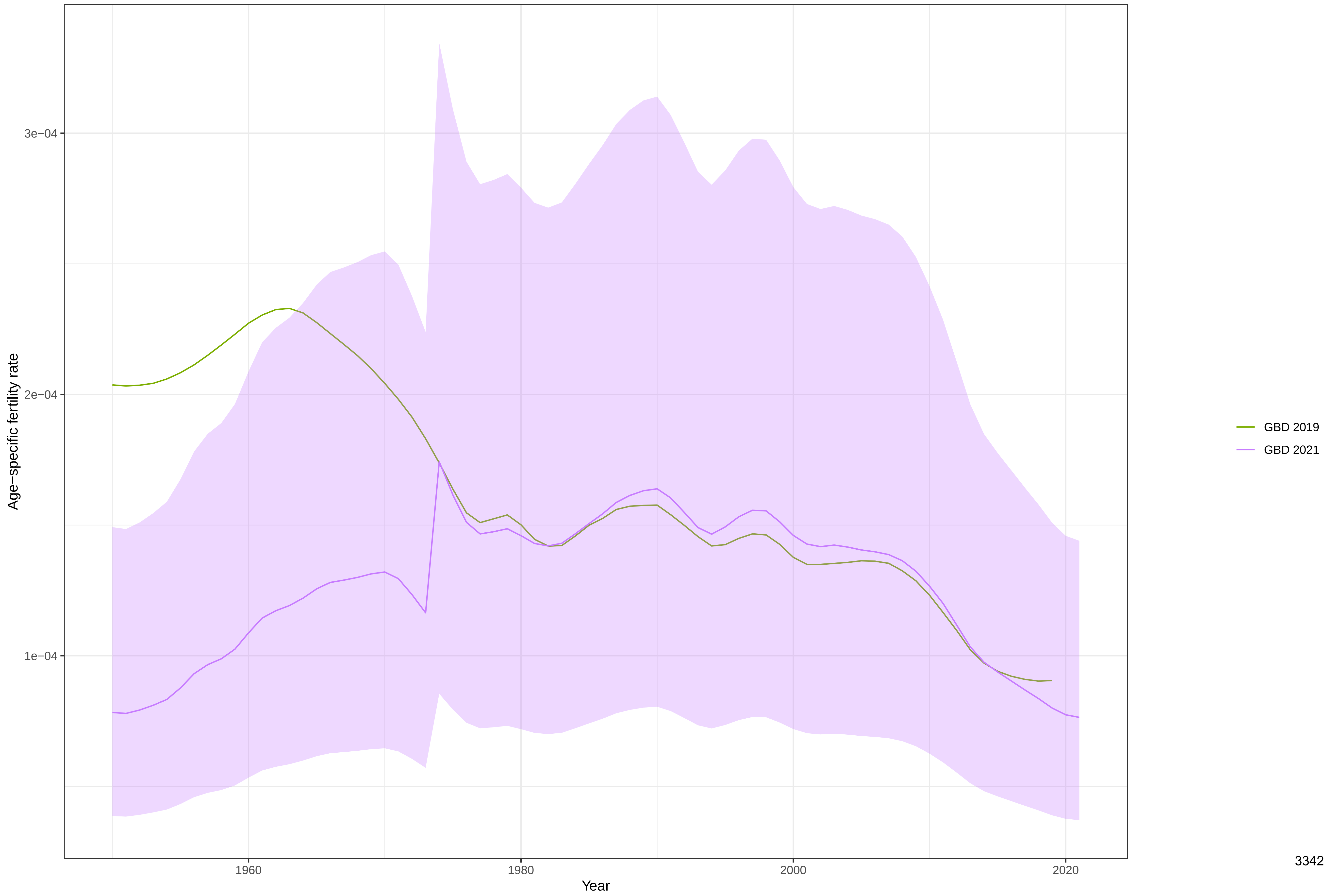

3342

Age−specific fertility fitted trend lines, 1950−2021: Staffordshire, Ages: 15−19
Data points enclosed with a diamond are reference sources

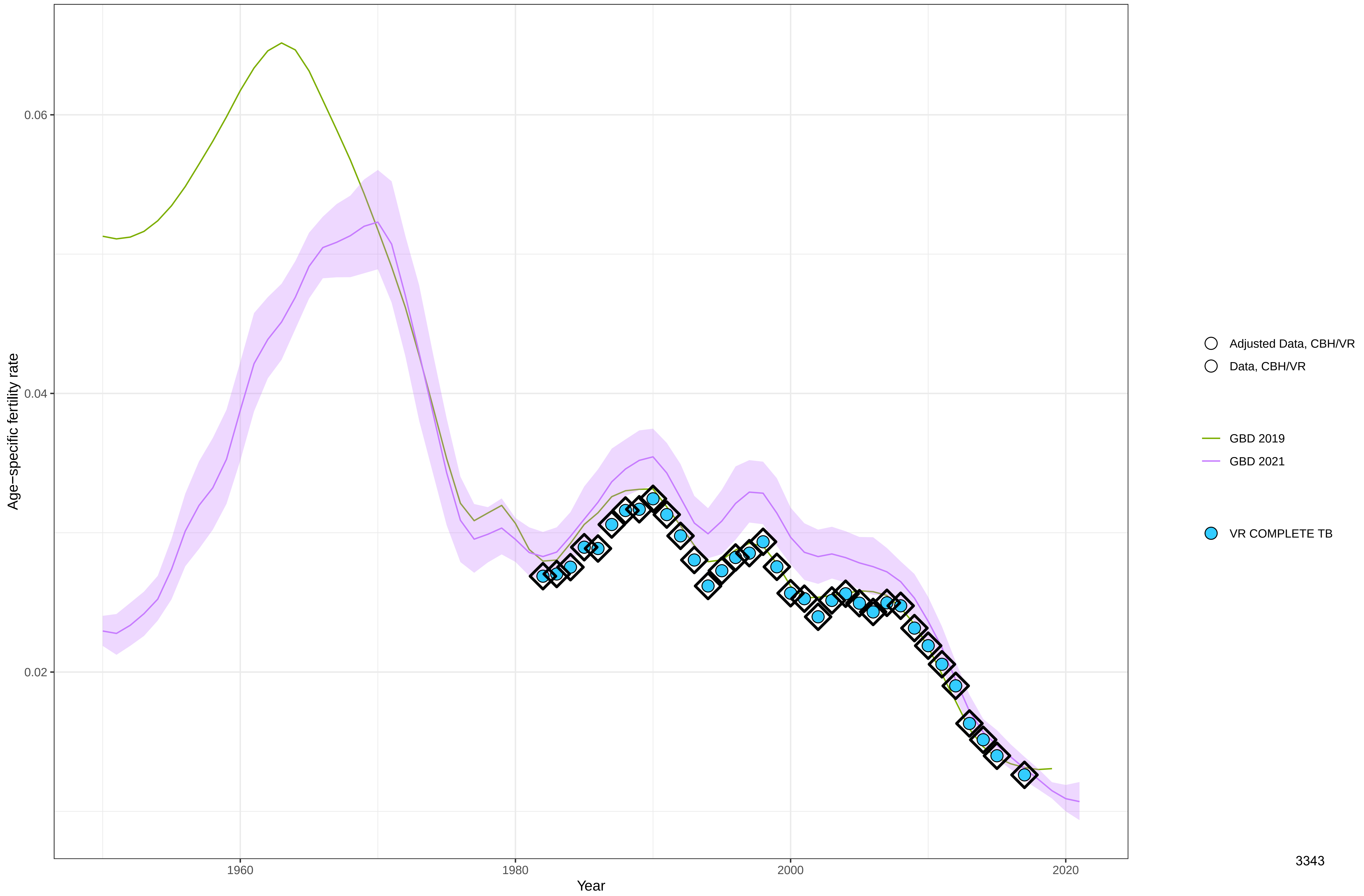

Age−specific fertility fitted trend lines, 1950−2021: Staffordshire, Ages: 20−24
Data points enclosed with a diamond are reference sources

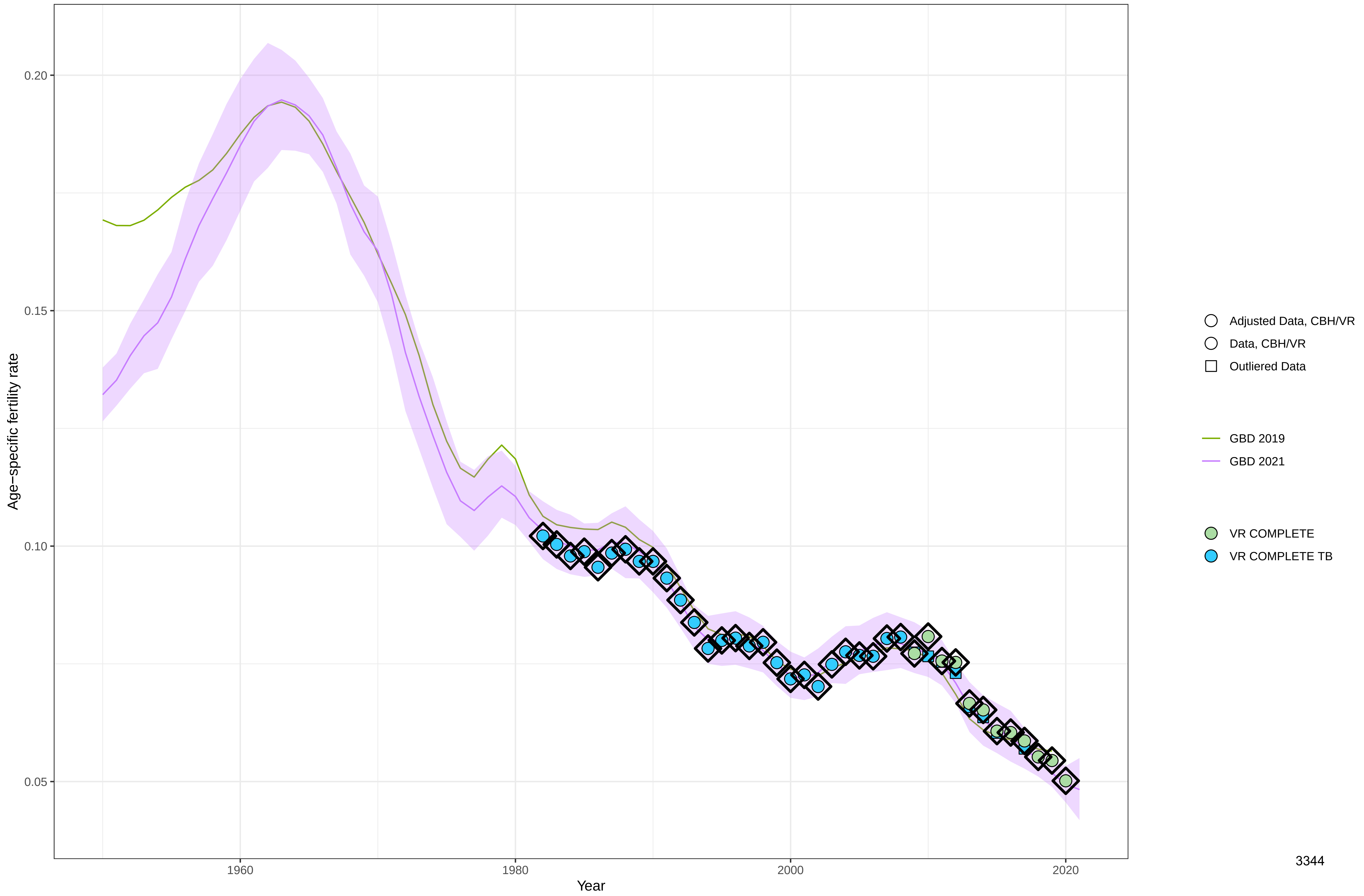

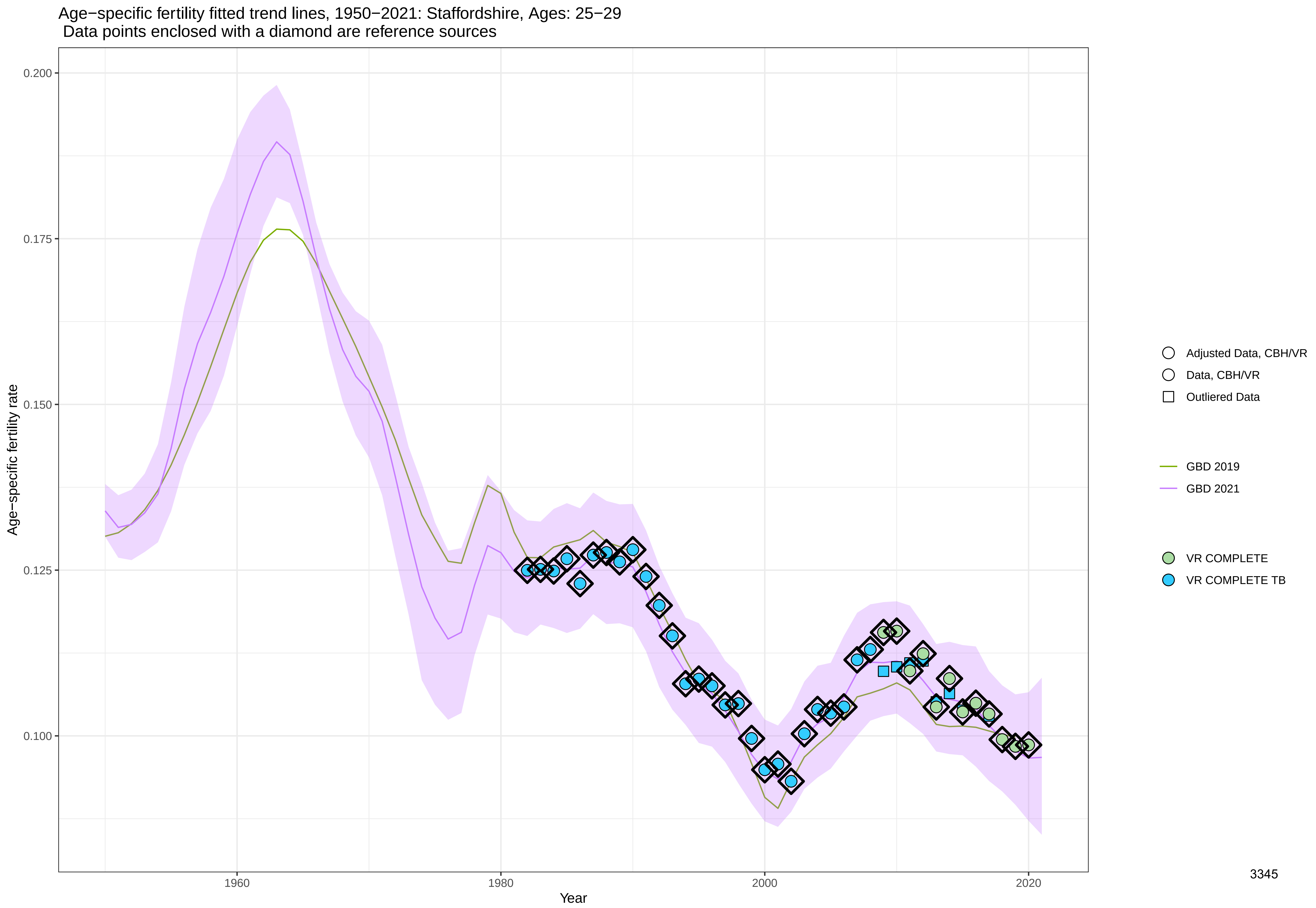

Age−specific fertility fitted trend lines, 1950−2021: Staffordshire, Ages: 25−29
Data points enclosed with a diamond are reference sources
0.200
0.175
0.150
0.125
0.100
Age−specific fertility rate
1960
1980
2000
2020
Year
Adjusted Data, CBH/VR
Data, CBH/VR
Outliered Data
GBD 2019
GBD 2021
VR COMPLETE
VR COMPLETE TB

Age−specific fertility fitted trend lines, 1950−2021: Staffordshire, Ages: 30−34
Data points enclosed with a diamond are reference sources

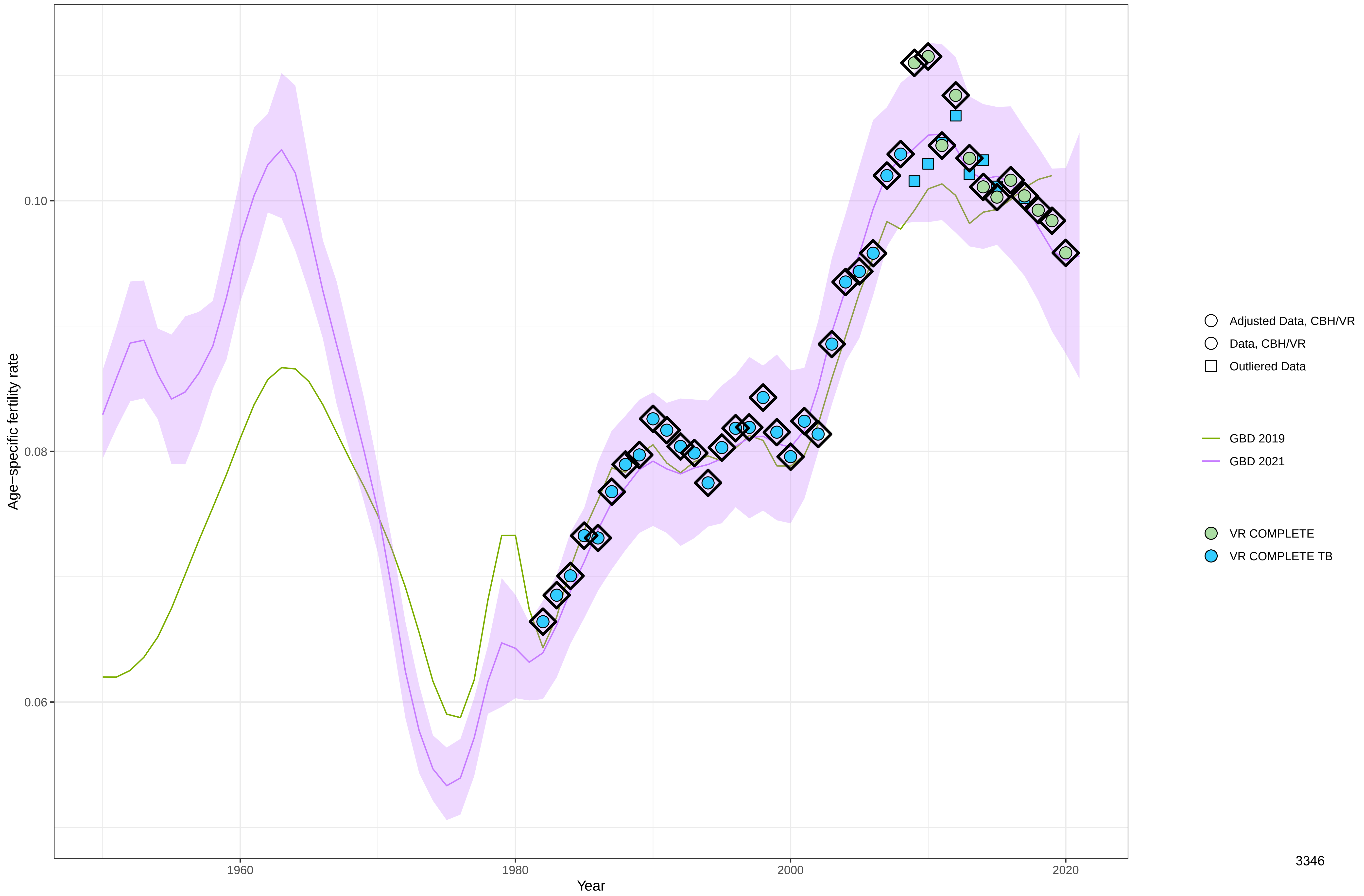

Age−specific fertility fitted trend lines, 1950−2021: Staffordshire, Ages: 35−39
Data points enclosed with a diamond are reference sources

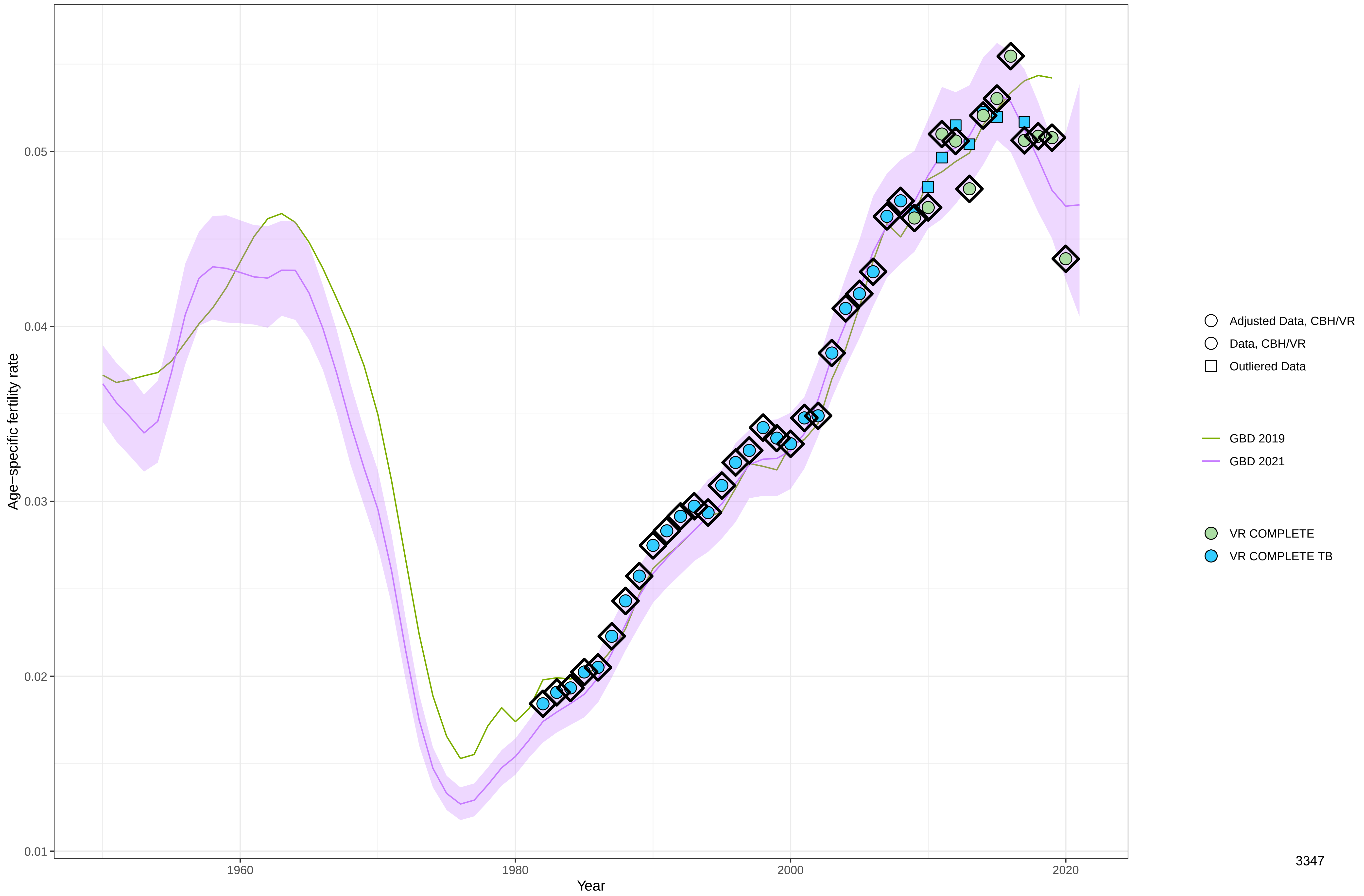

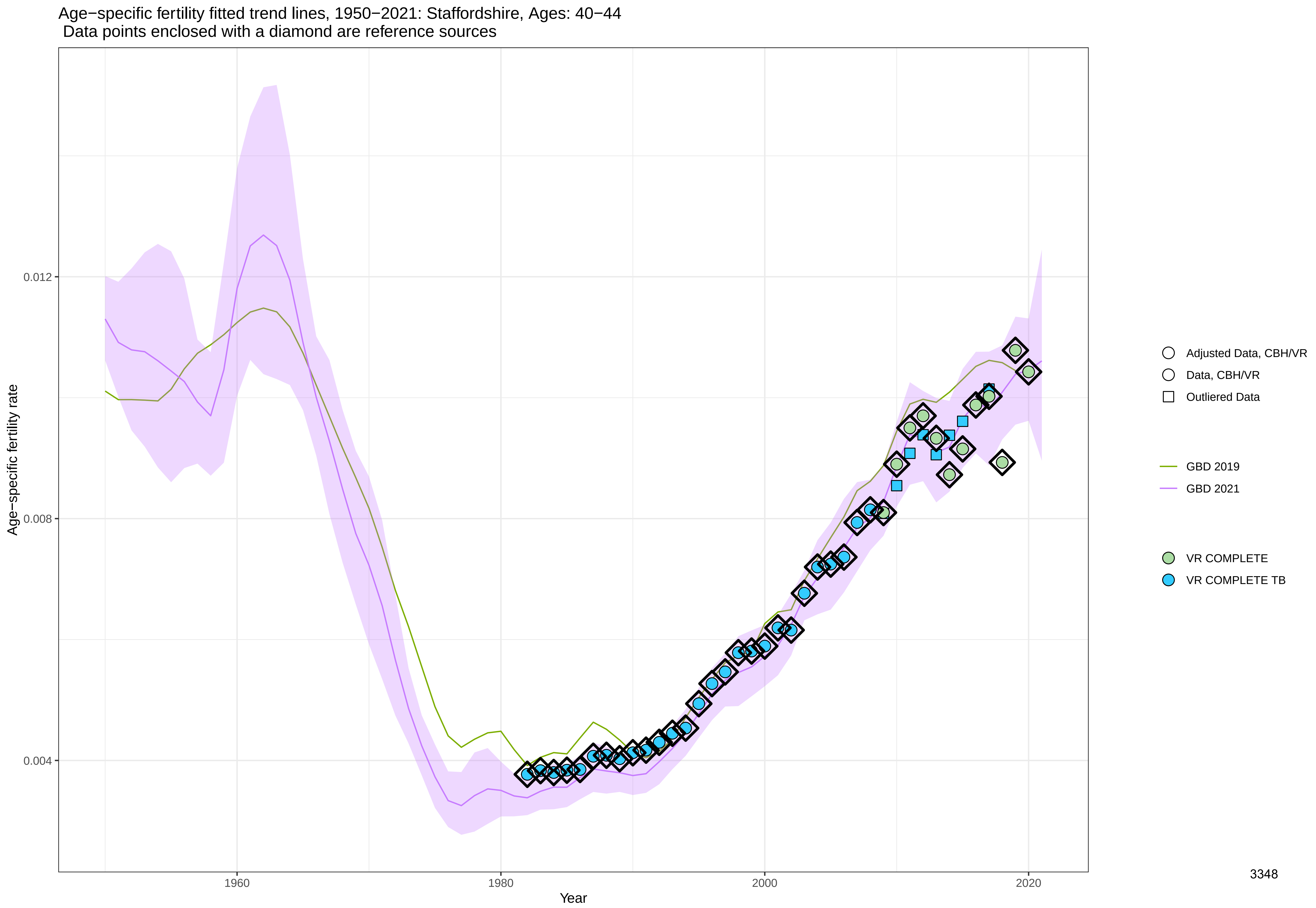

Age−specific fertility fitted trend lines, 1950−2021: Staffordshire, Ages: 40−44
Data points enclosed with a diamond are reference sources
Age−specific fertility rate
0.012
0.008
0.004
1960
1980
2000
2020
Year
Adjusted Data, CBH/VR
Data, CBH/VR
Outliered Data
GBD 2019
GBD 2021
VR COMPLETE
VR COMPLETE TB

Age−specific fertility fitted trend lines, 1950−2021: Staffordshire, Ages: 45−49
Data points enclosed with a diamond are reference sources

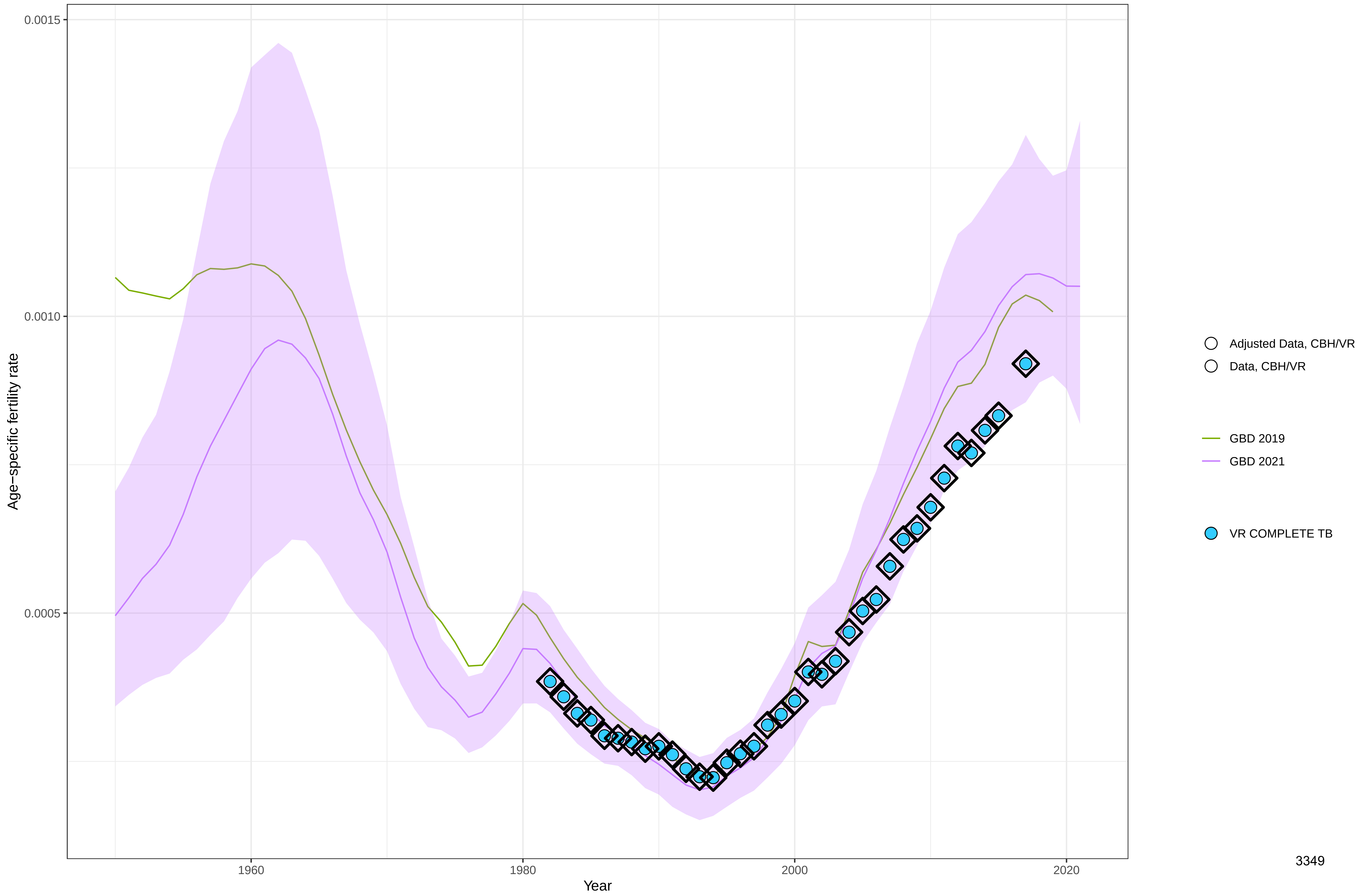

Age−specific fertility fitted trend lines, 1950−2021: Staffordshire, Ages: 50−54
Data points enclosed with a diamond are reference sources

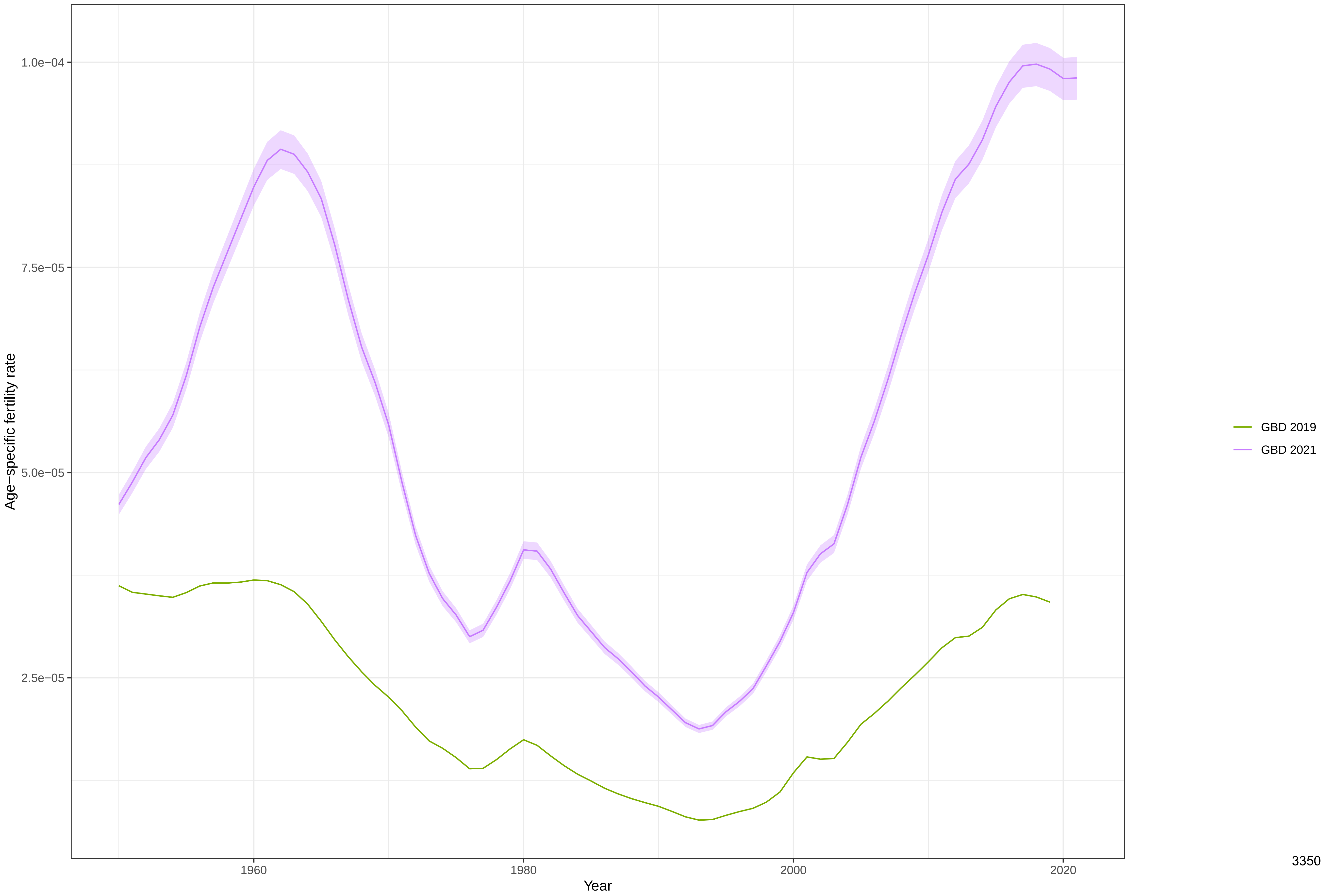

Total fertility rate fitted trend lines, 1950−2021: Staffordshire TFR
Data points enclosed with a diamond are reference sources

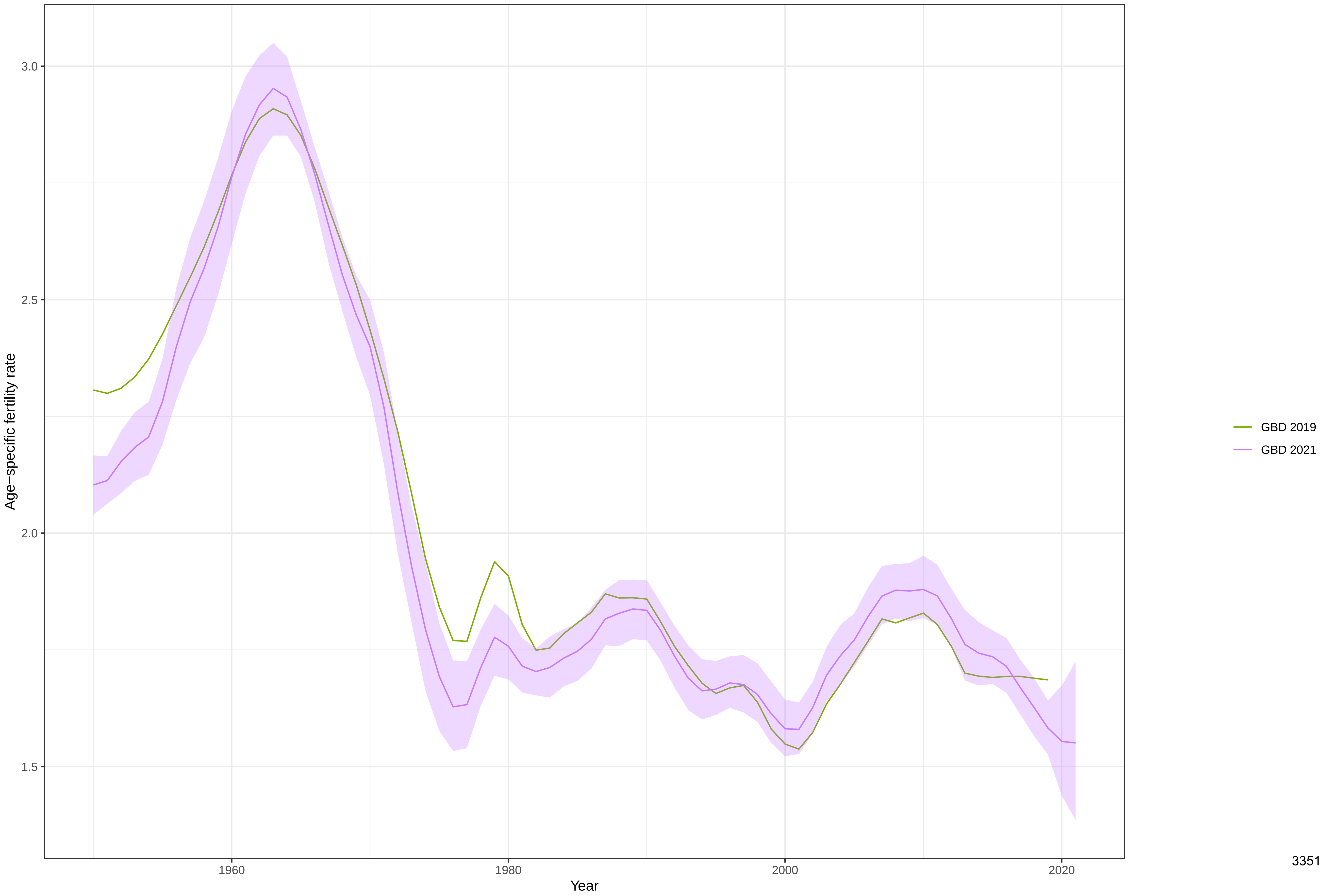

Age−specific fertility fitted trend lines, 1950−2021: Stoke−on−Trent, Ages: 10−14
Data points enclosed with a diamond are reference sources

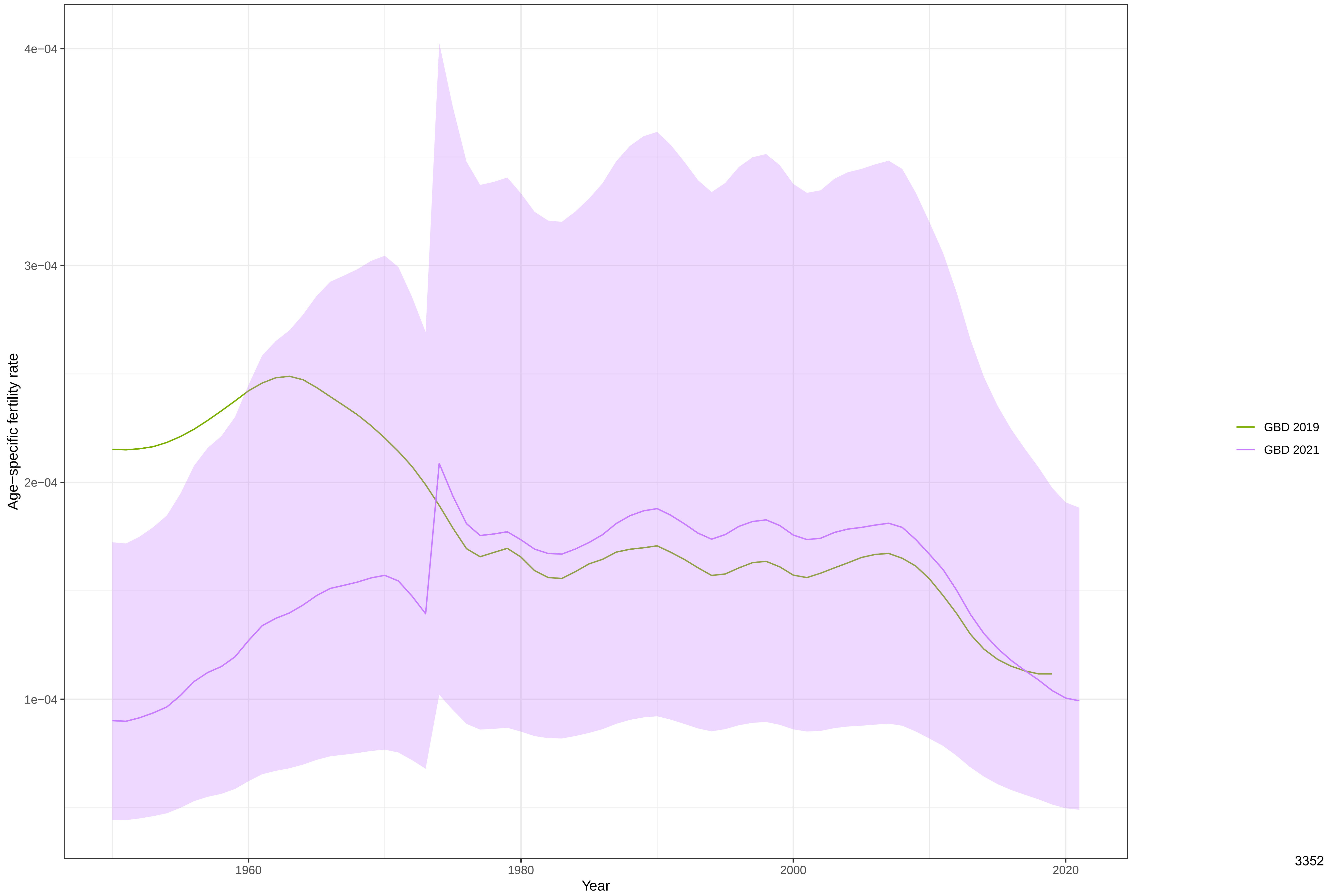

Age−specific fertility fitted trend lines, 1950−2021: Stoke−on−Trent, Ages: 15−19
Data points enclosed with a diamond are reference sources

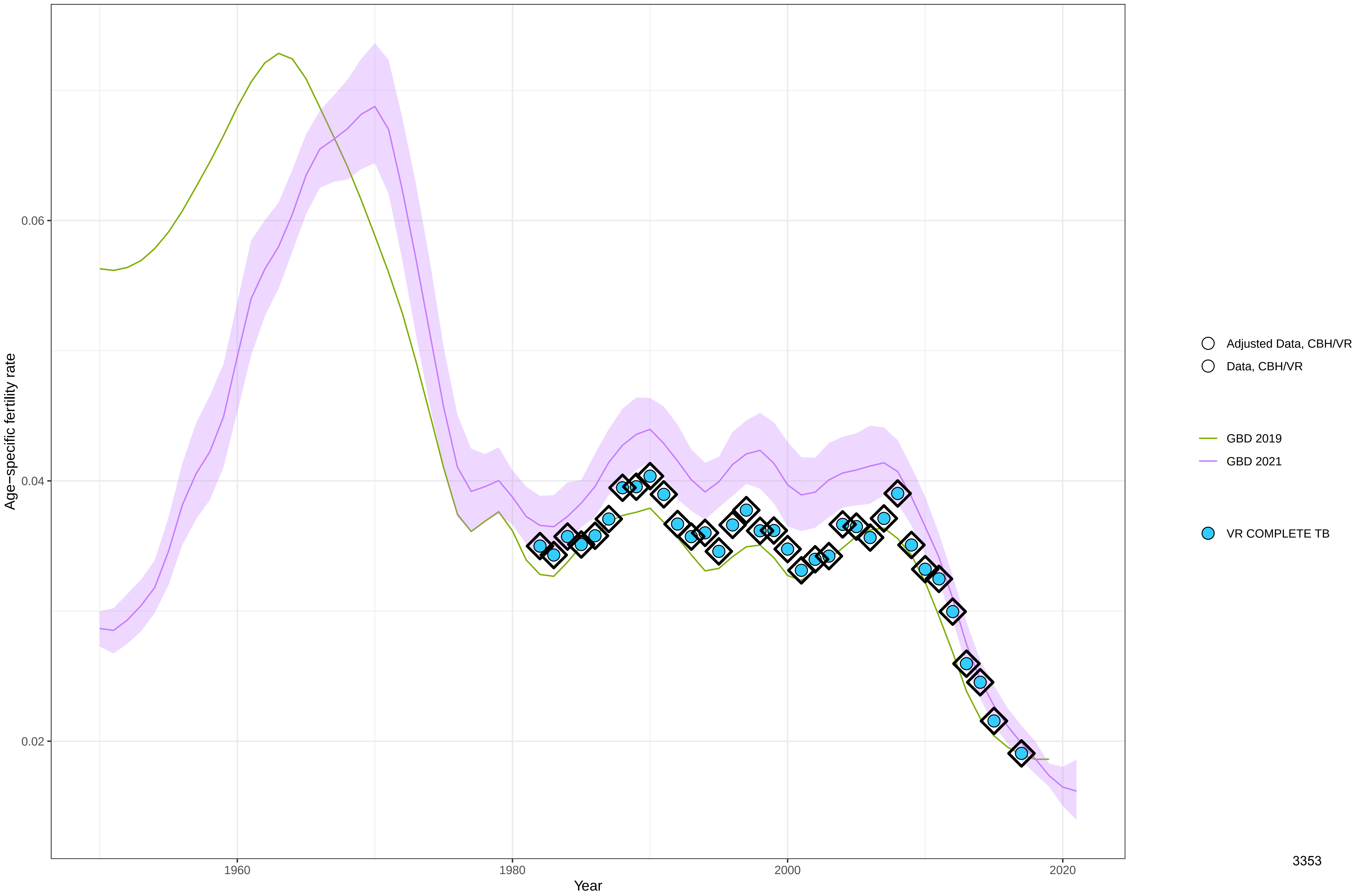

Age−specific fertility fitted trend lines, 1950−2021: Stoke−on−Trent, Ages: 20−24
Data points enclosed with a diamond are reference sources

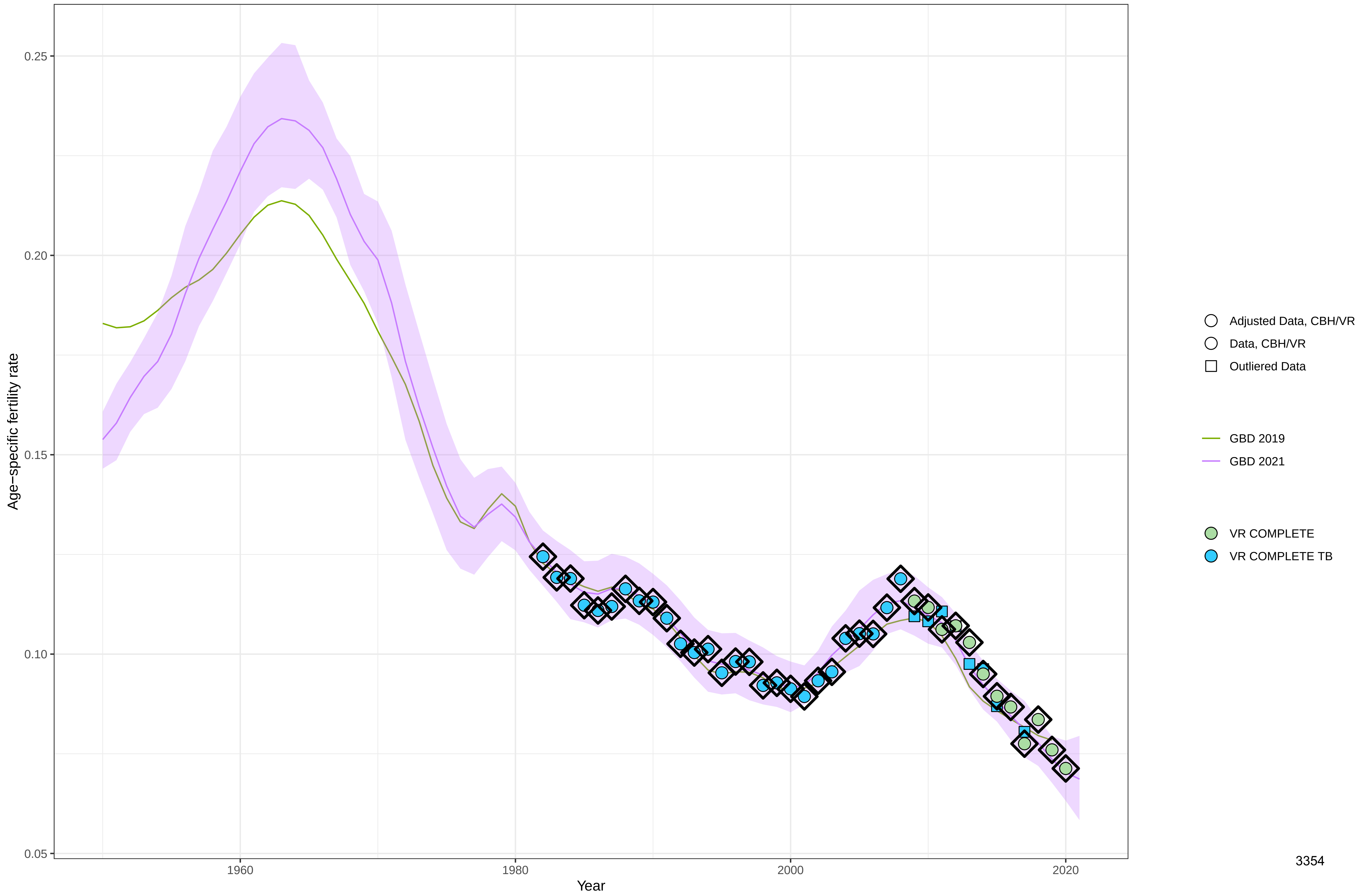

Age−specific fertility fitted trend lines, 1950−2021: Stoke−on−Trent, Ages: 25−29
Data points enclosed with a diamond are reference sources

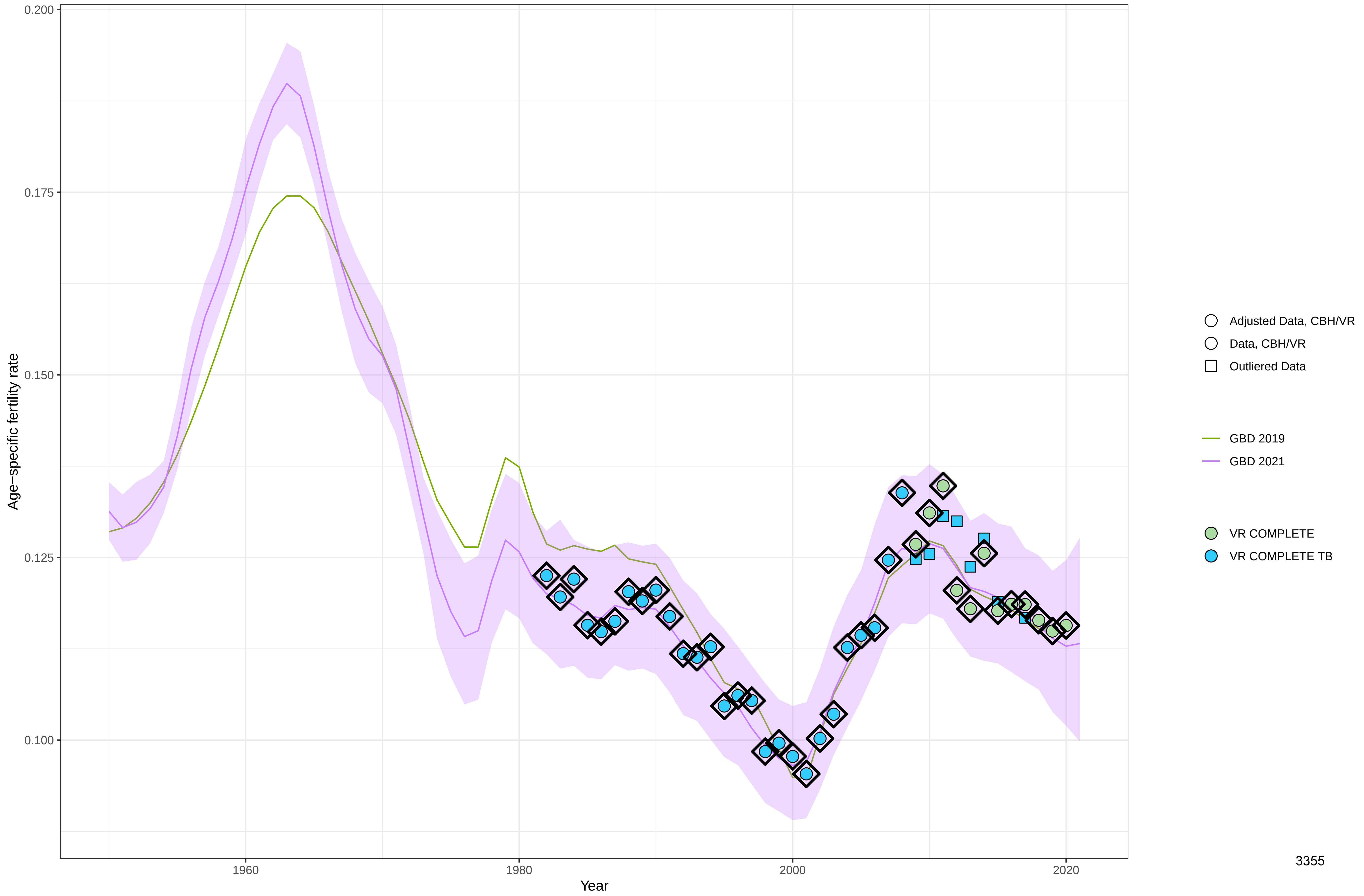

Age−specific fertility fitted trend lines, 1950−2021: Stoke−on−Trent, Ages: 30−34
Data points enclosed with a diamond are reference sources

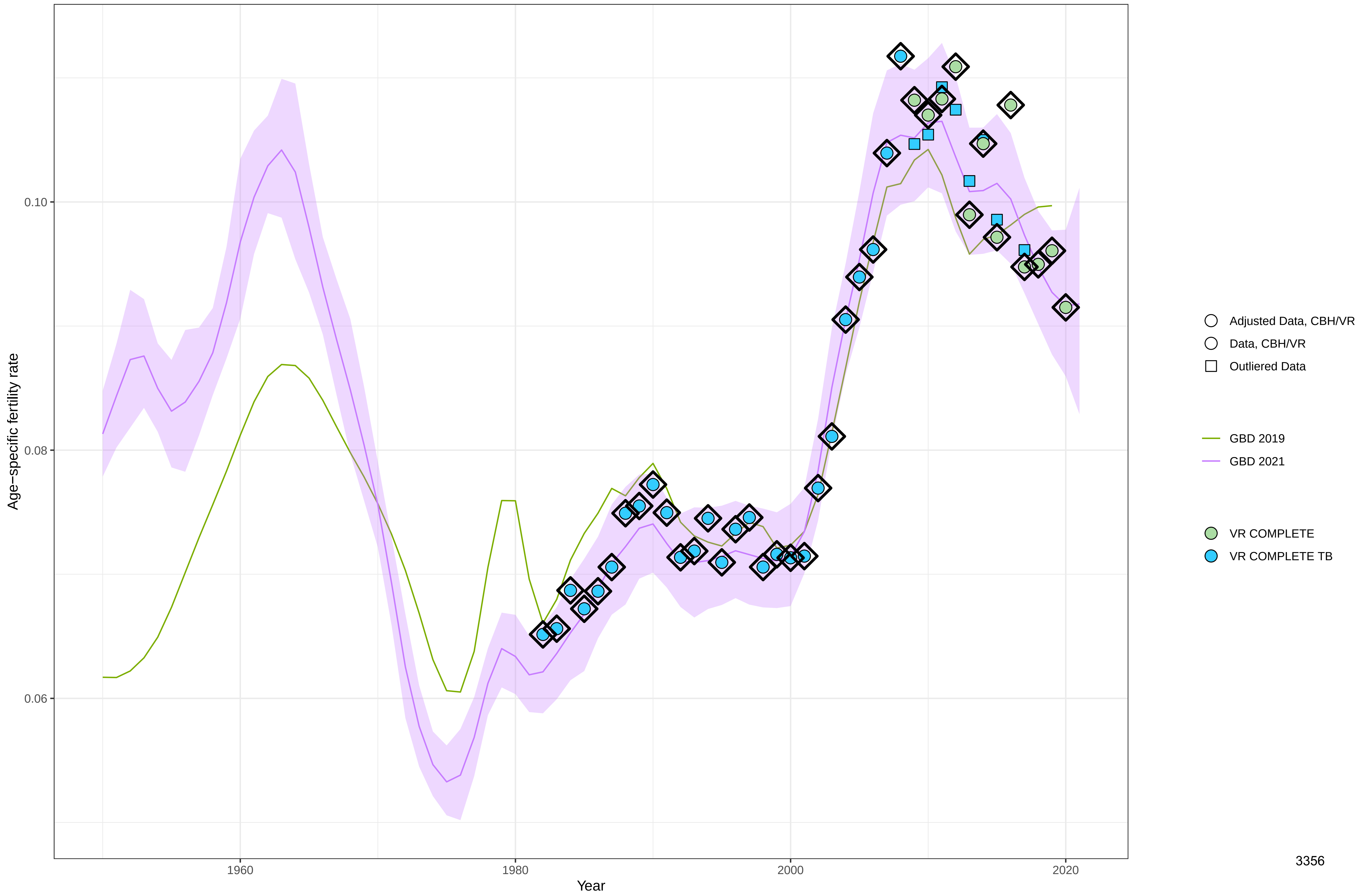

Age−specific fertility fitted trend lines, 1950−2021: Stoke−on−Trent, Ages: 35−39
Data points enclosed with a diamond are reference sources

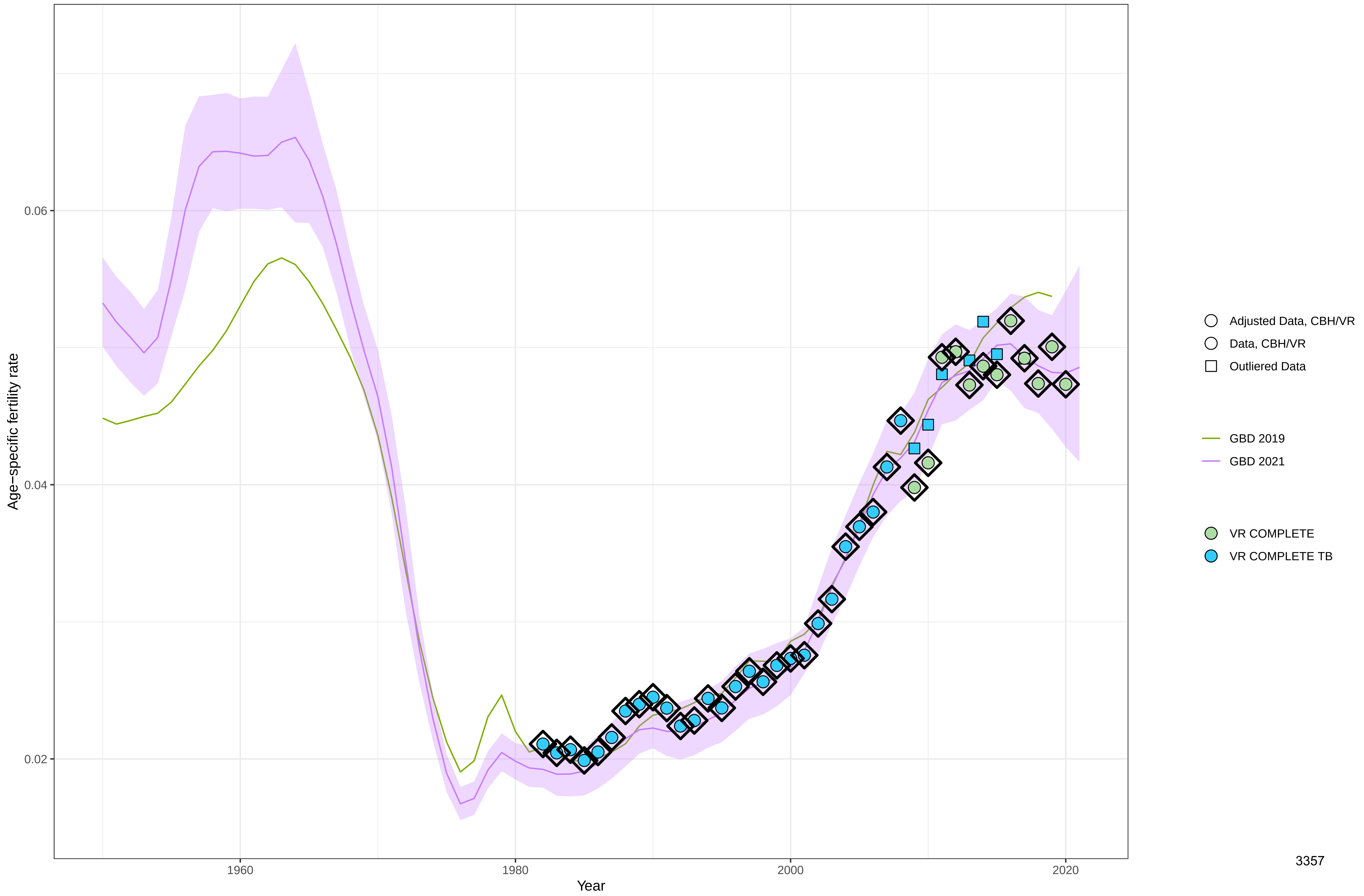

Age−specific fertility fitted trend lines, 1950−2021: Stoke−on−Trent, Ages: 40−44
Data points enclosed with a diamond are reference sources

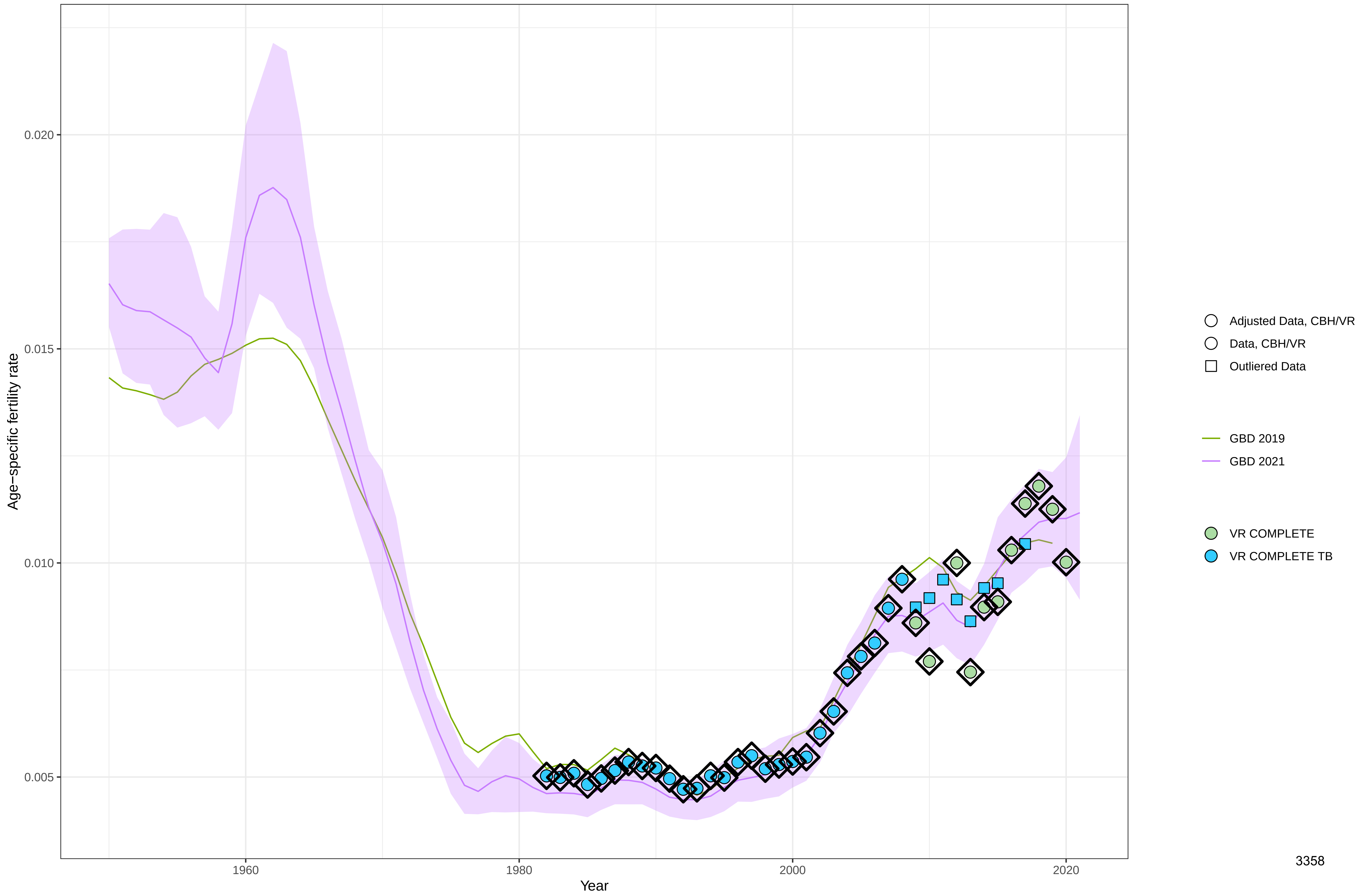

Age−specific fertility fitted trend lines, 1950−2021: Stoke−on−Trent, Ages: 45−49
Data points enclosed with a diamond are reference sources

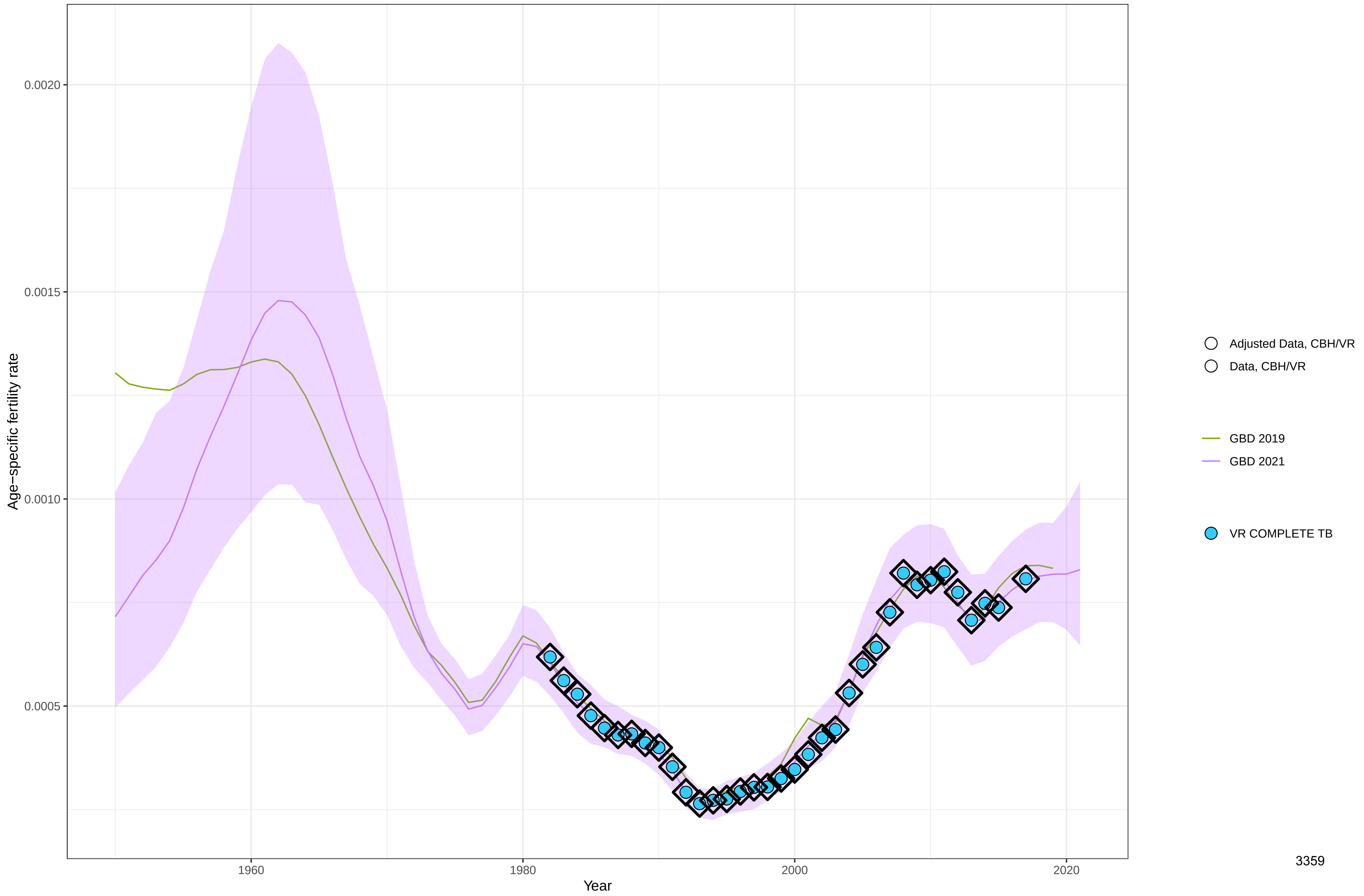

Age−specific fertility fitted trend lines, 1950−2021: Stoke−on−Trent, Ages: 50−54
Data points enclosed with a diamond are reference sources

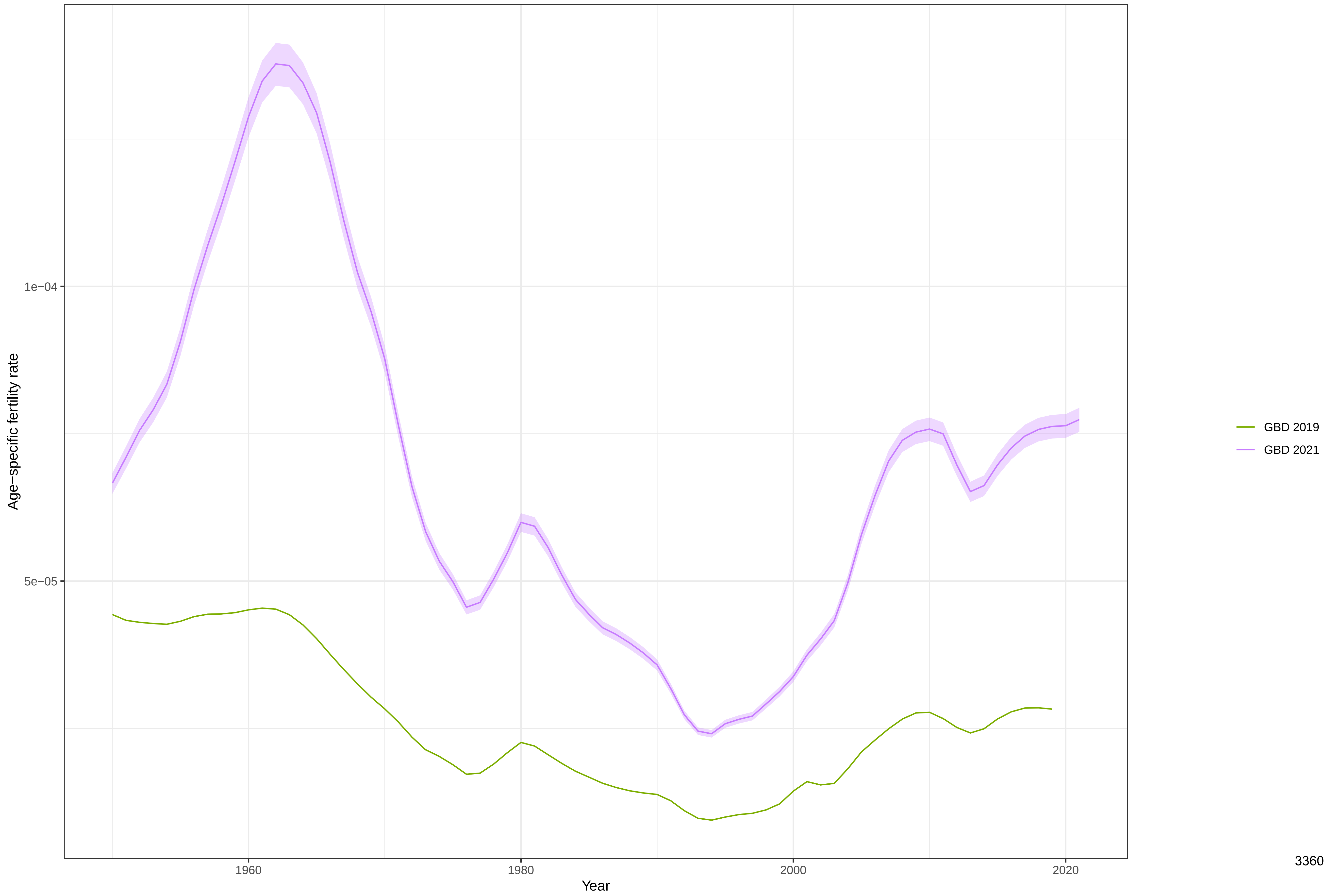

Total fertility rate fitted trend lines, 1950−2021: Stoke−on−Trent TFR
Data points enclosed with a diamond are reference sources

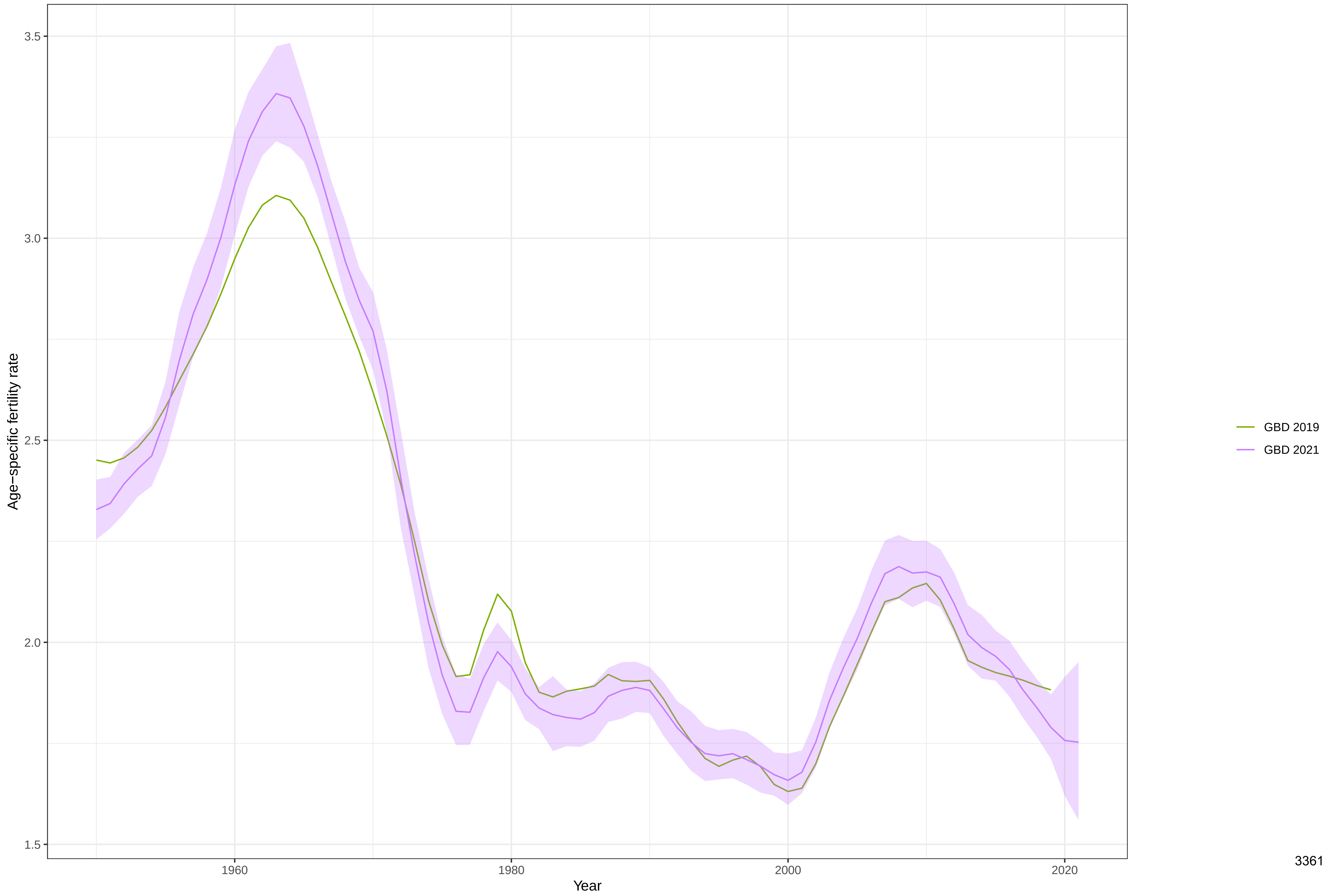

Age−specific fertility fitted trend lines, 1950−2021: Telford and Wrekin, Ages: 10−14
Data points enclosed with a diamond are reference sources

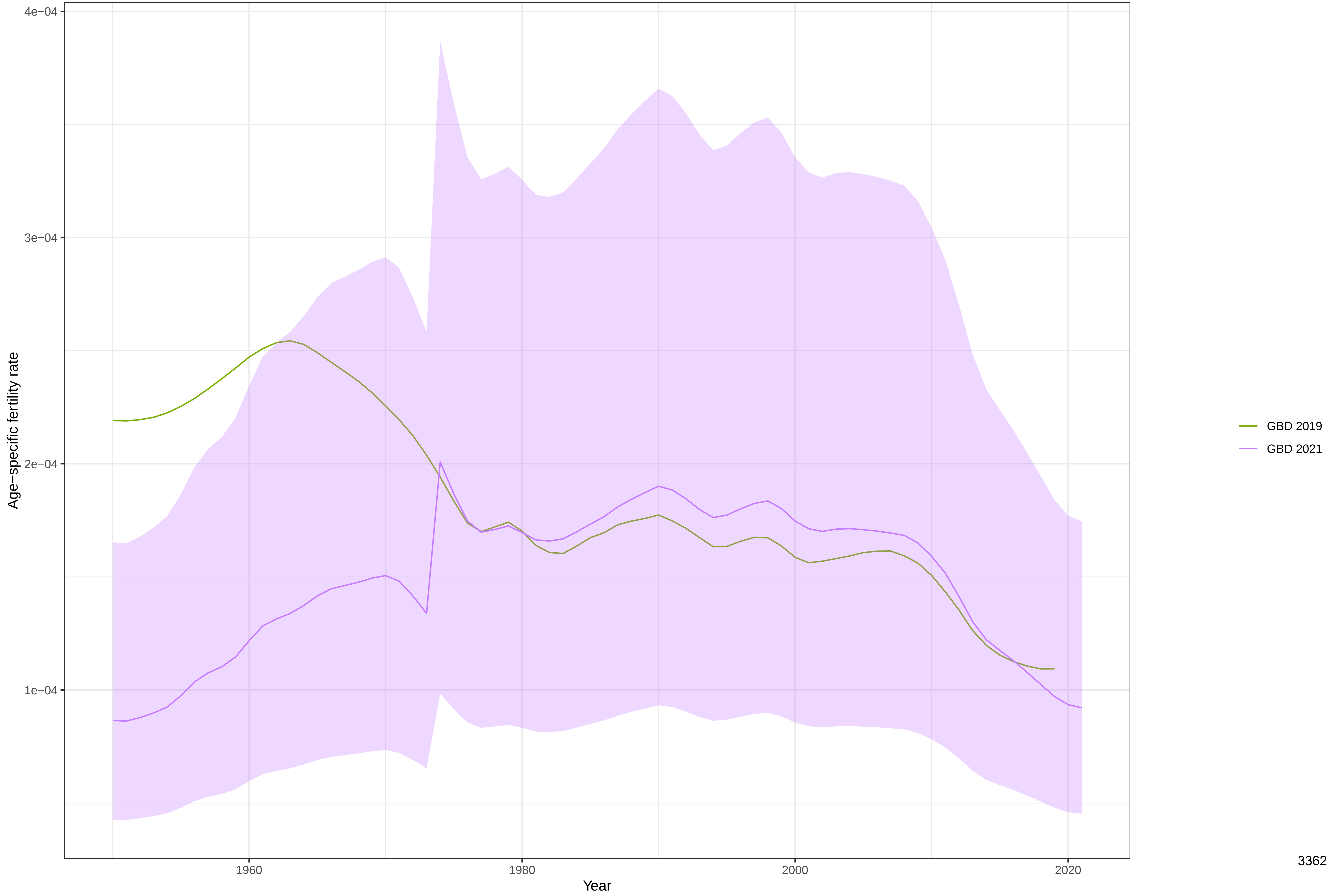

Age−specific fertility fitted trend lines, 1950−2021: Telford and Wrekin, Ages: 15−19
Data points enclosed with a diamond are reference sources

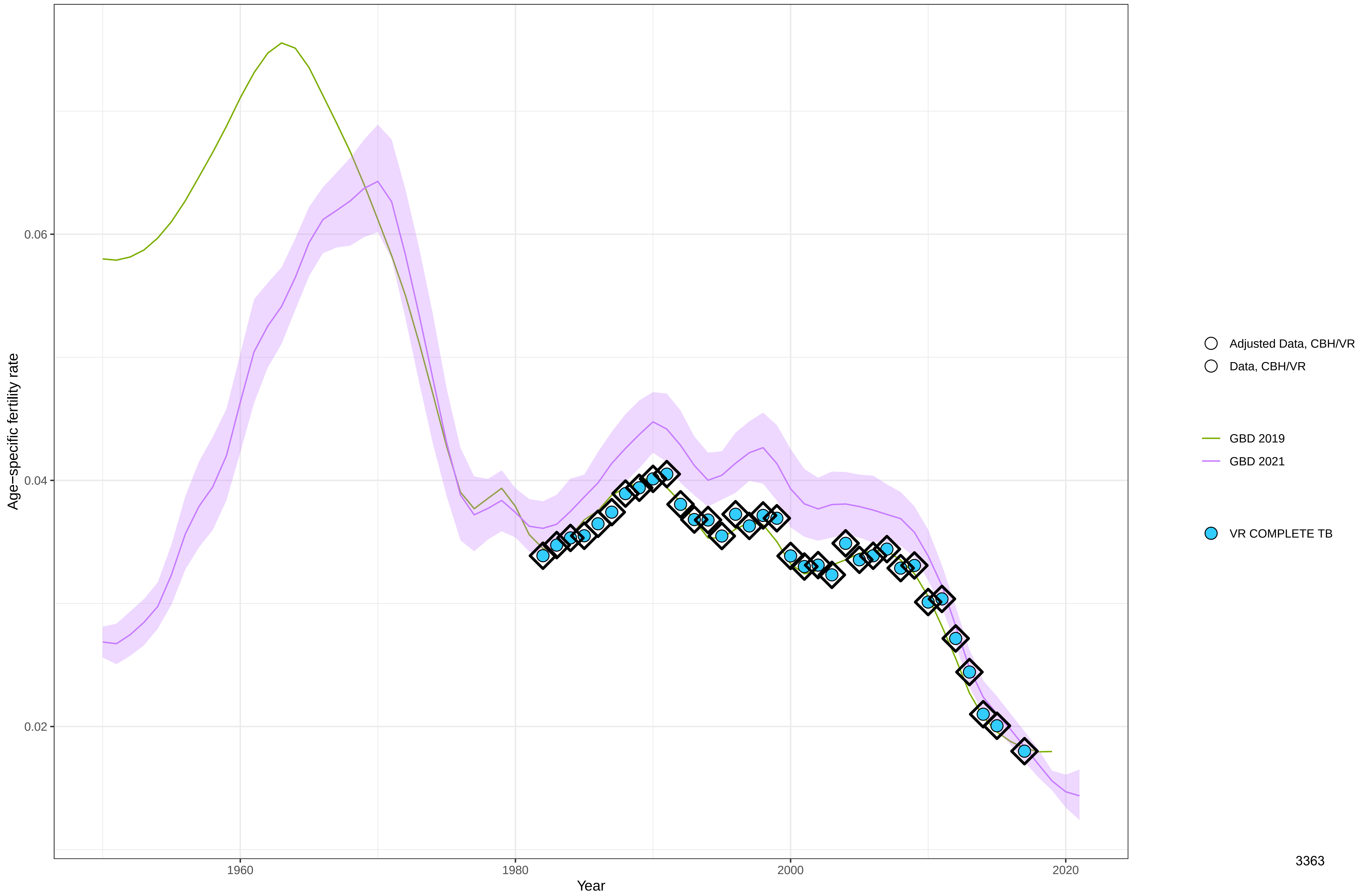

Age−specific fertility fitted trend lines, 1950−2021: Telford and Wrekin, Ages: 20−24
Data points enclosed with a diamond are reference sources

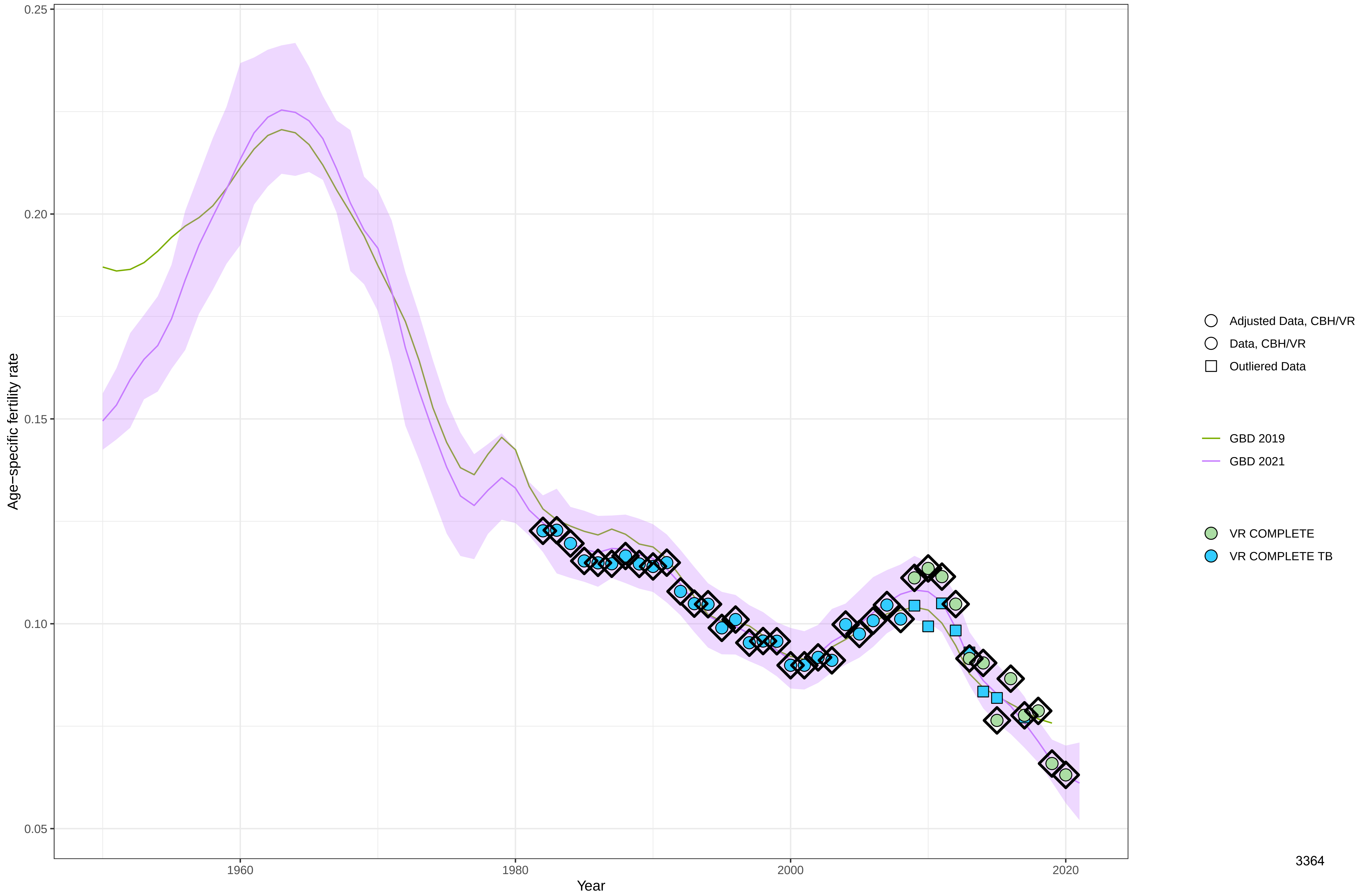

Age−specific fertility fitted trend lines, 1950−2021: Telford and Wrekin, Ages: 25−29
Data points enclosed with a diamond are reference sources

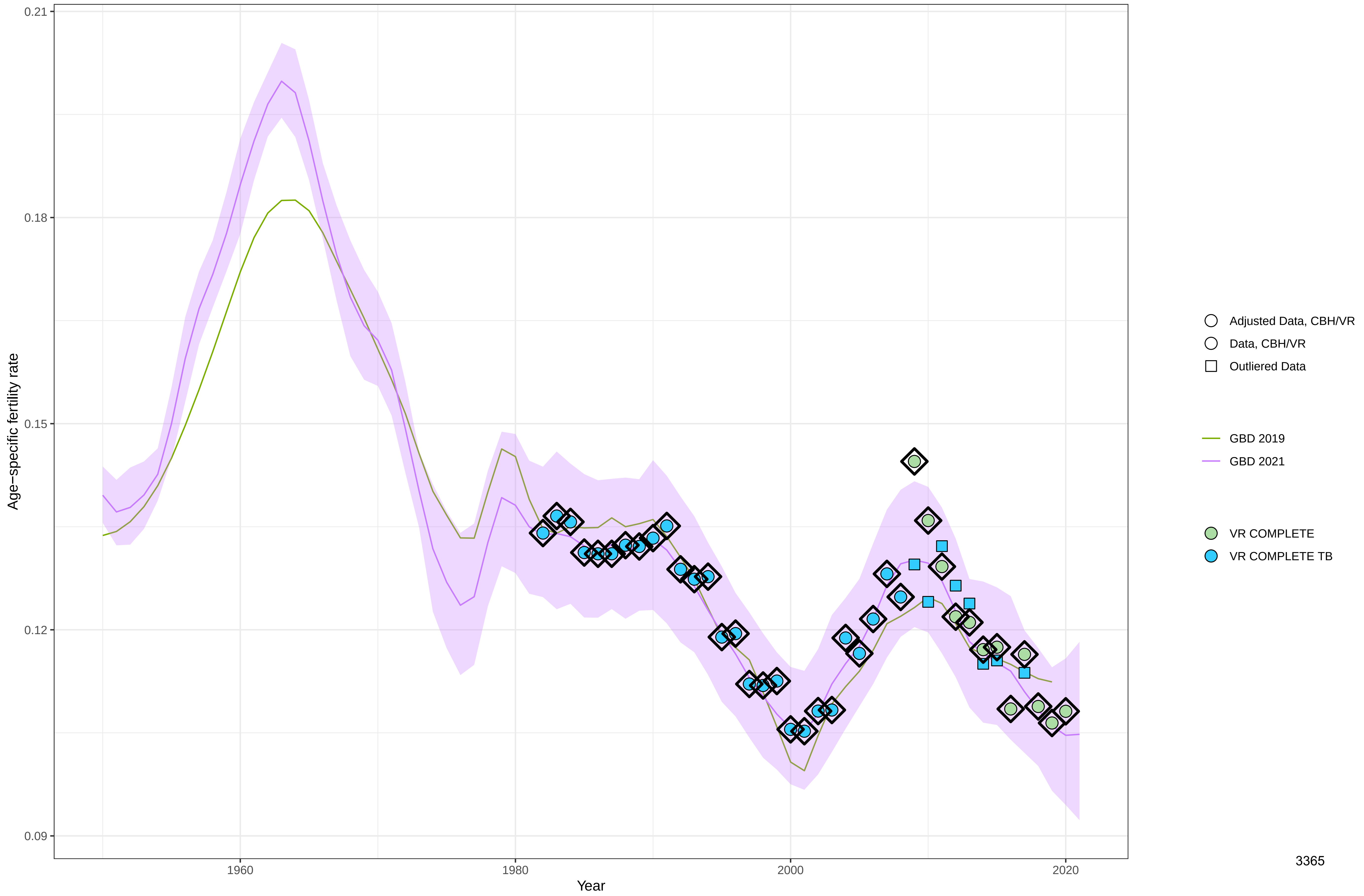

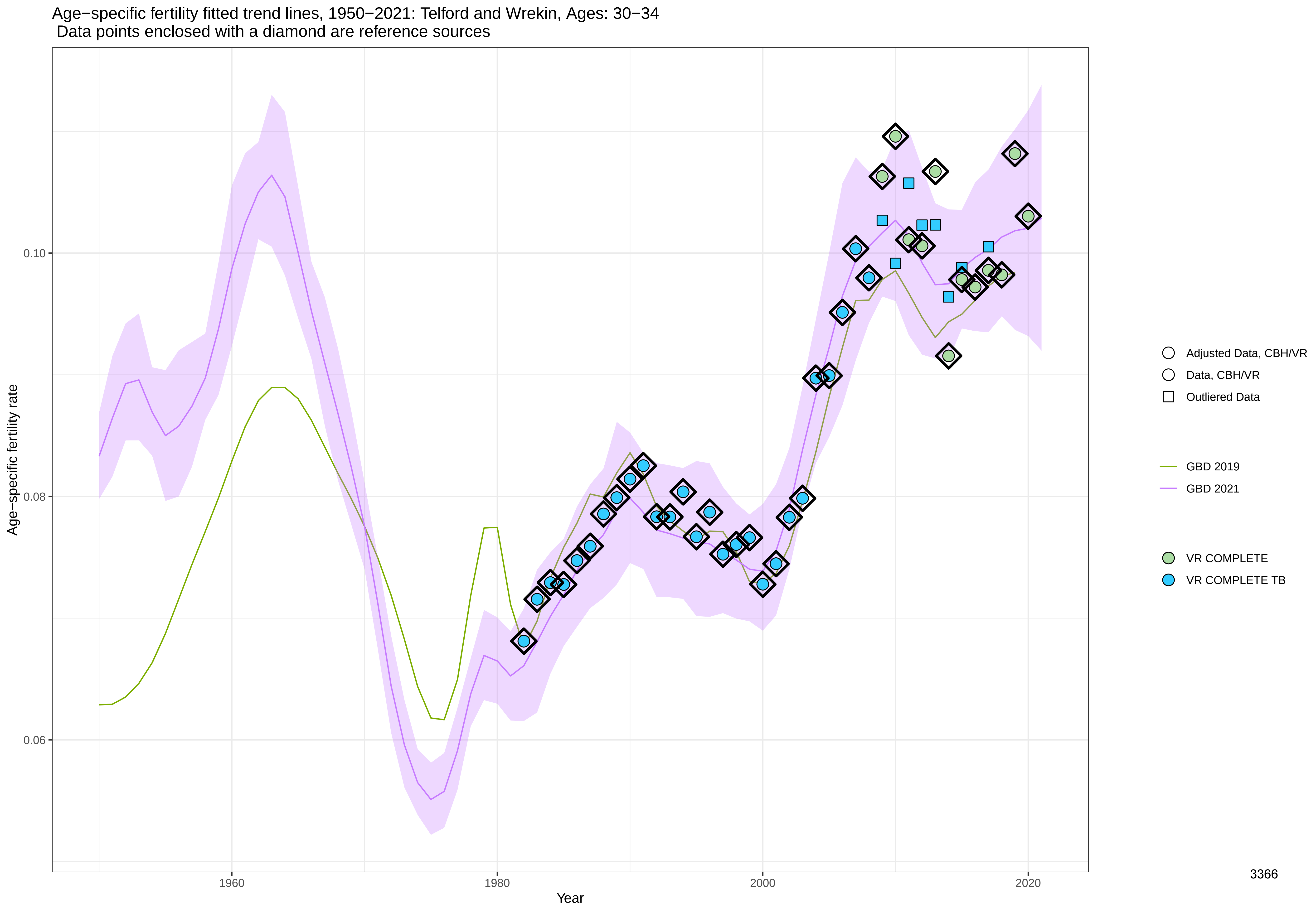
Age−specific fertility fitted trend lines, 1950−2021: Telford and Wrekin, Ages: 30−34
Data points enclosed with a diamond are reference sources
Age−specific fertility rate
0.10
0.08
0.06
1960
1980
2000
2020
Year
Adjusted Data, CBH/VR
Data, CBH/VR
Outliered Data
GBD 2019
GBD 2021
VR COMPLETE
VR COMPLETE TB

Age−specific fertility fitted trend lines, 1950−2021: Telford and Wrekin, Ages: 35−39
Data points enclosed with a diamond are reference sources

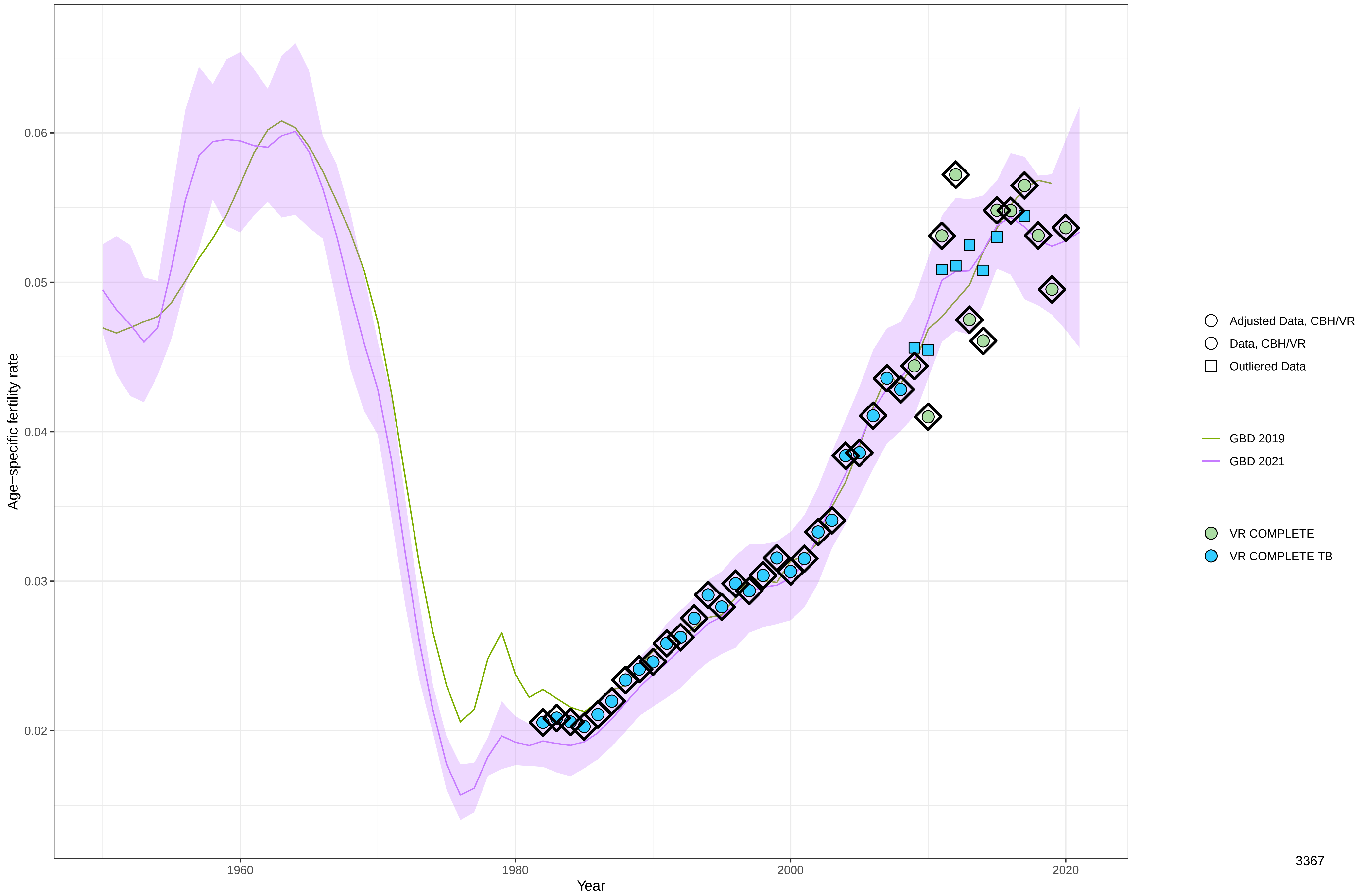

Age−specific fertility fitted trend lines, 1950−2021: Telford and Wrekin, Ages: 40−44
Data points enclosed with a diamond are reference sources

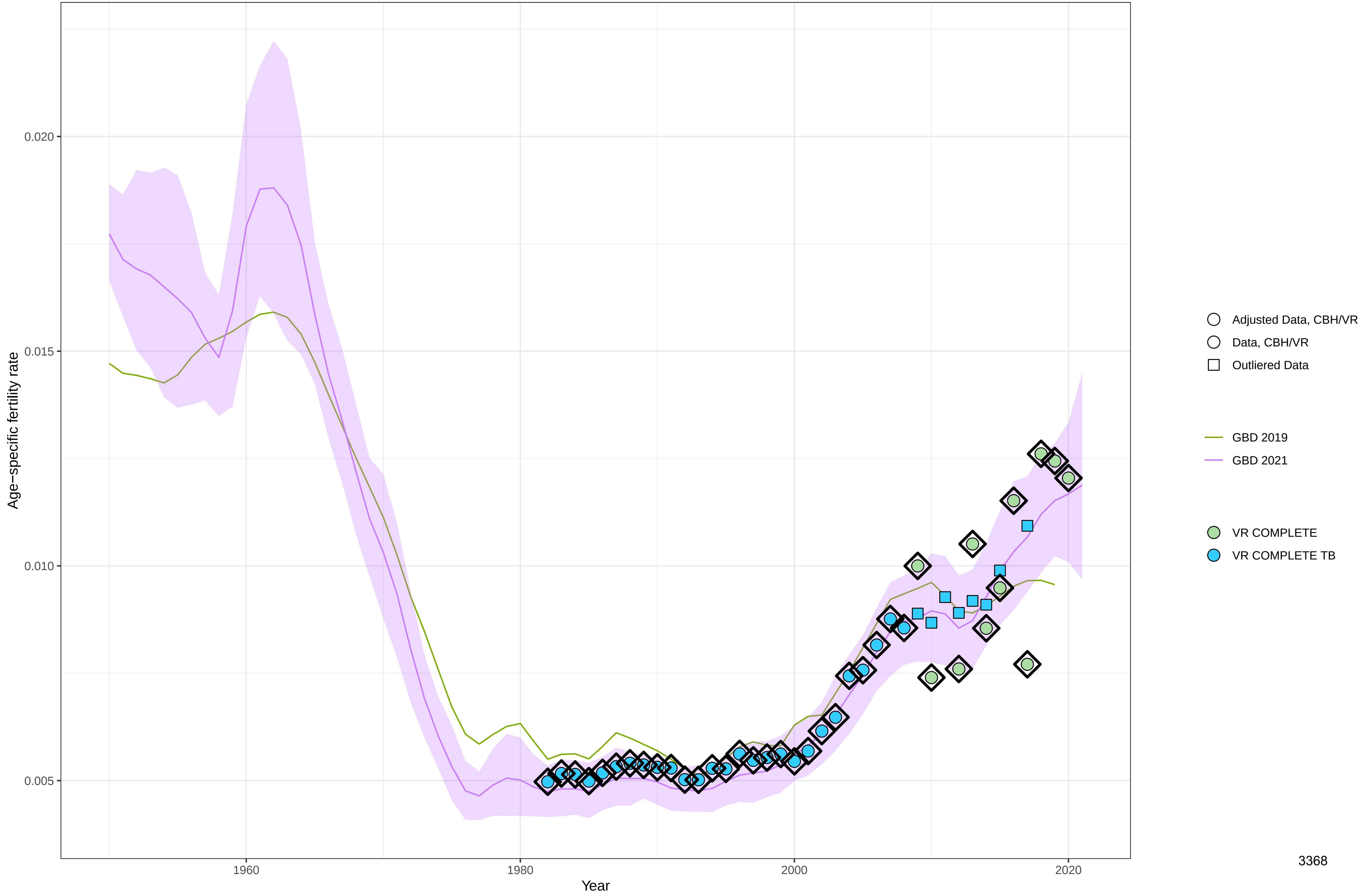

Age−specific fertility fitted trend lines, 1950−2021: Telford and Wrekin, Ages: 45−49
Data points enclosed with a diamond are reference sources

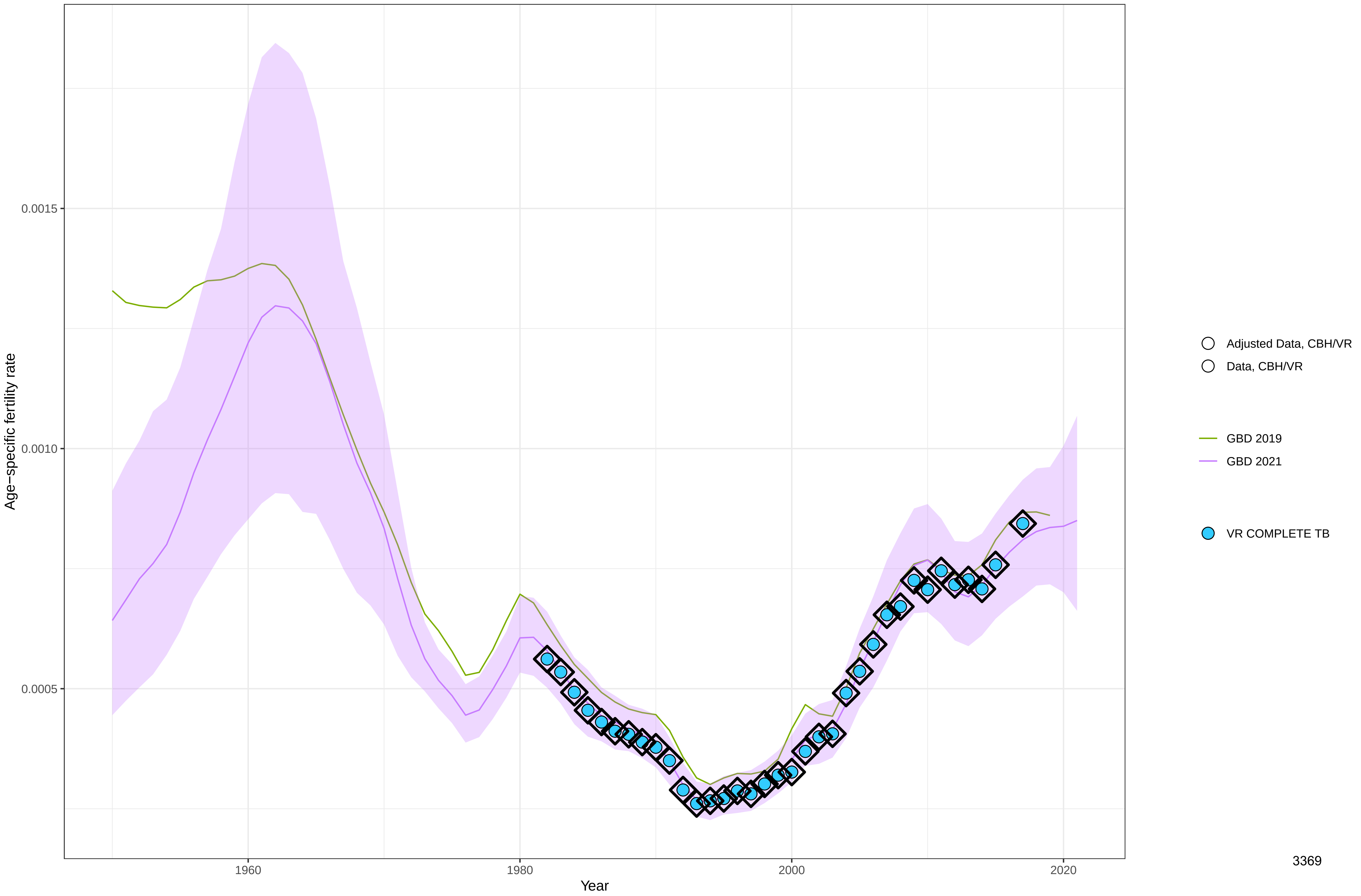

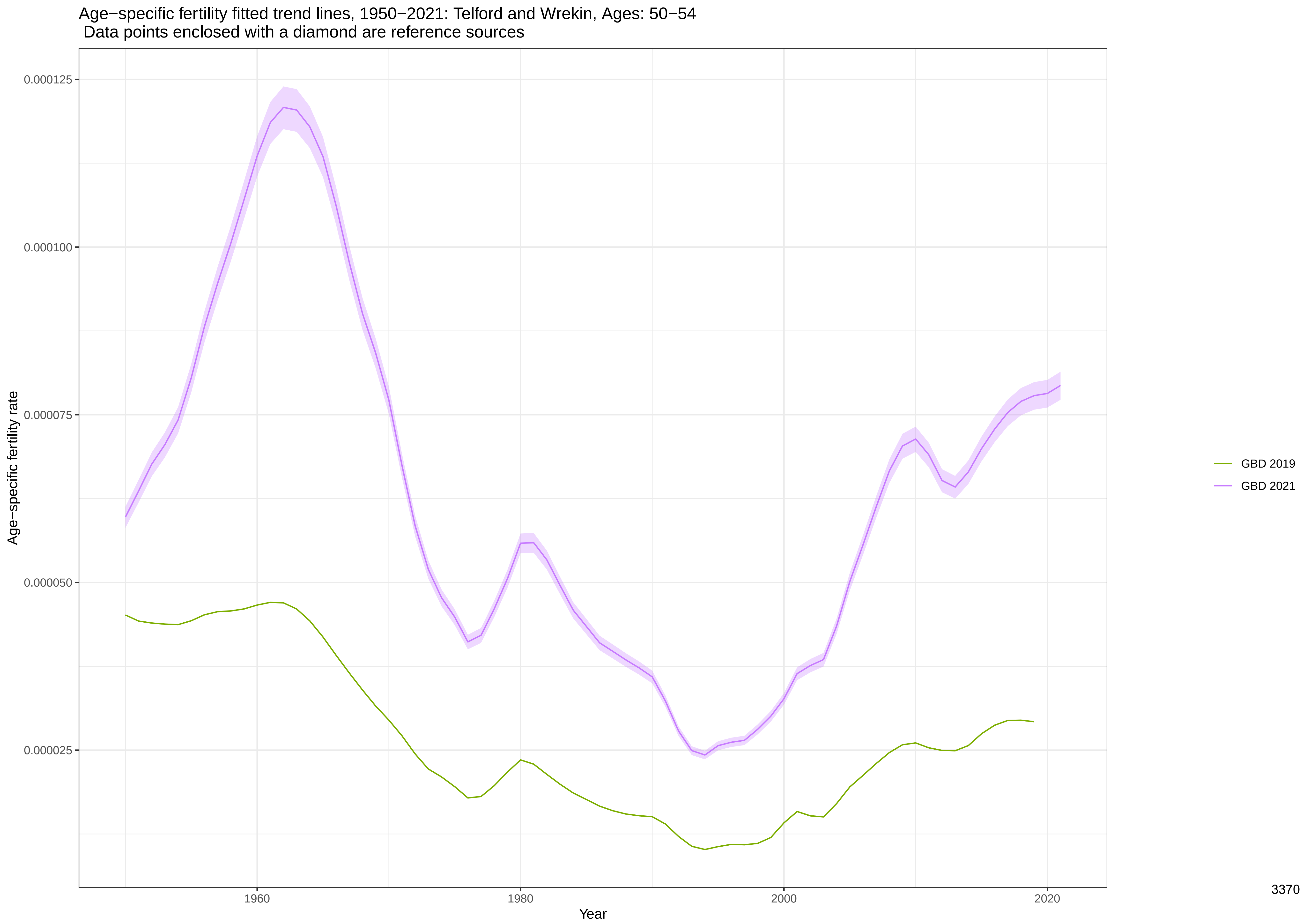
Age−specific fertility fitted trend lines, 1950−2021: Telford and Wrekin, Ages: 50−54
Data points enclosed with a diamond are reference sources
Age−specific fertility rate
0.000125
0.000100
0.000075
0.000050
0.000025
Year
1960
1980
2000
2020
GBD 2019
GBD 2021

Total fertility rate fitted trend lines, 1950−2021: Telford and Wrekin TFR
Data points enclosed with a diamond are reference sources

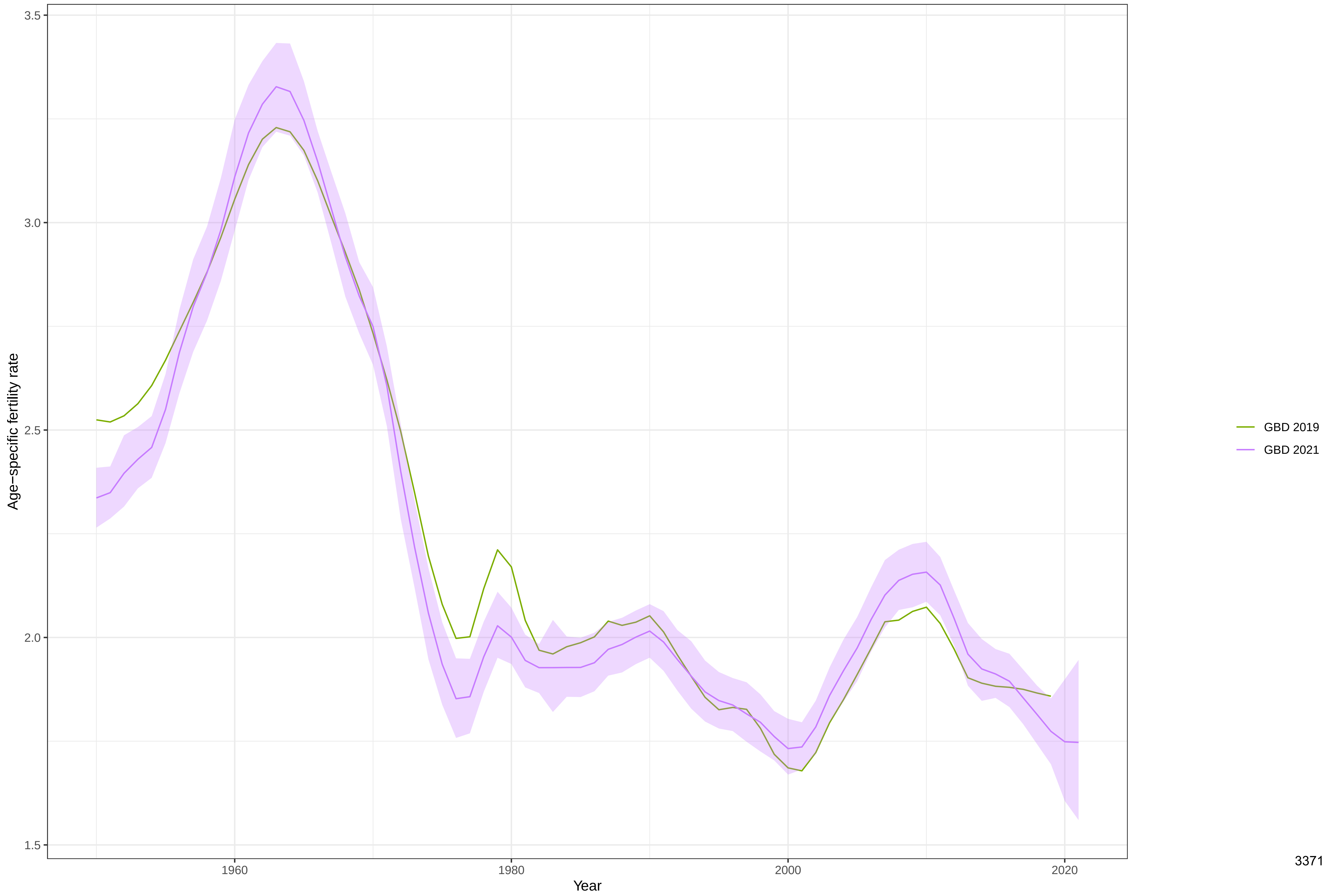

Age−specific fertility fitted trend lines, 1950−2021: Walsall, Ages: 10−14
Data points enclosed with a diamond are reference sources

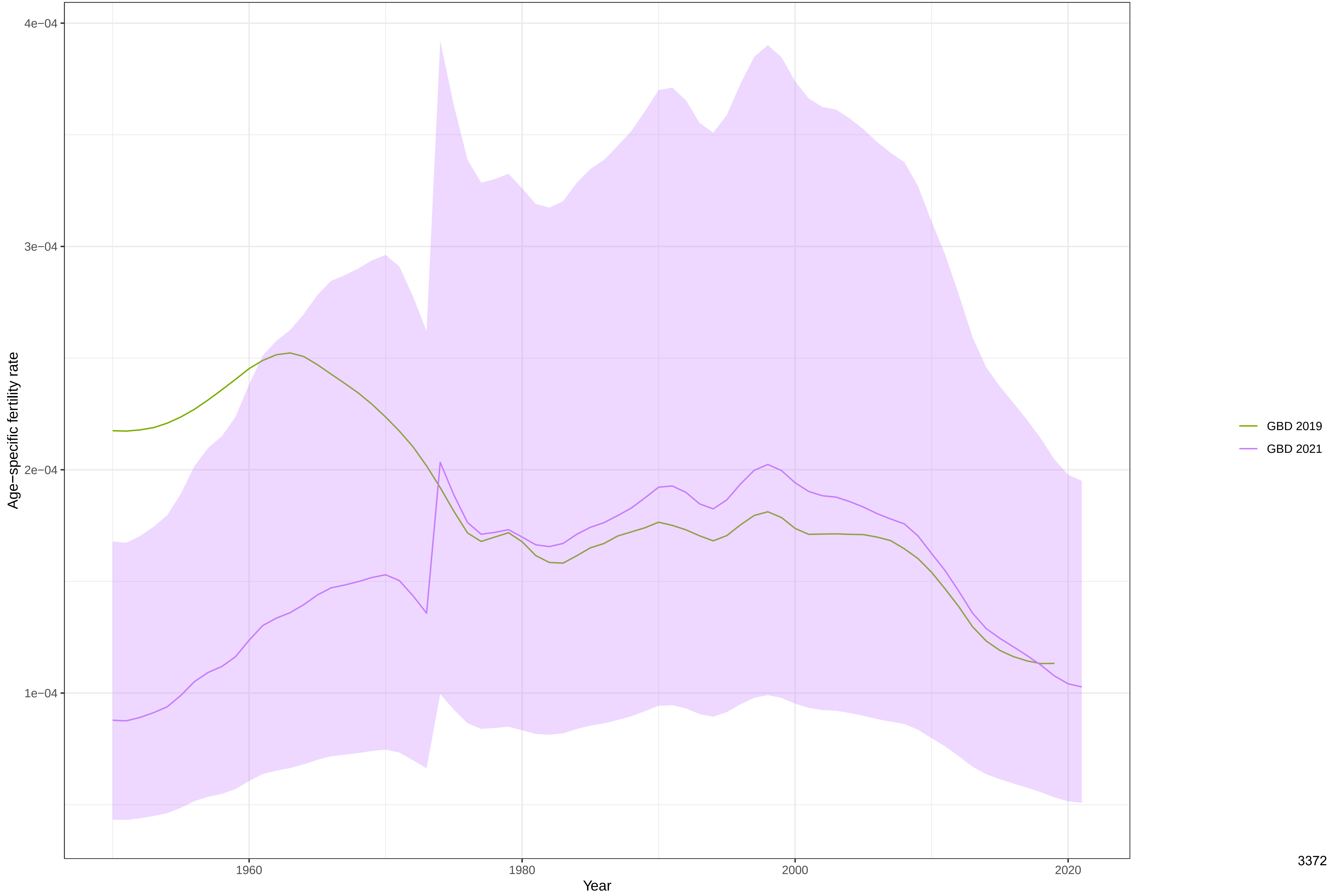

Age−specific fertility fitted trend lines, 1950−2021: Walsall, Ages: 15−19
Data points enclosed with a diamond are reference sources

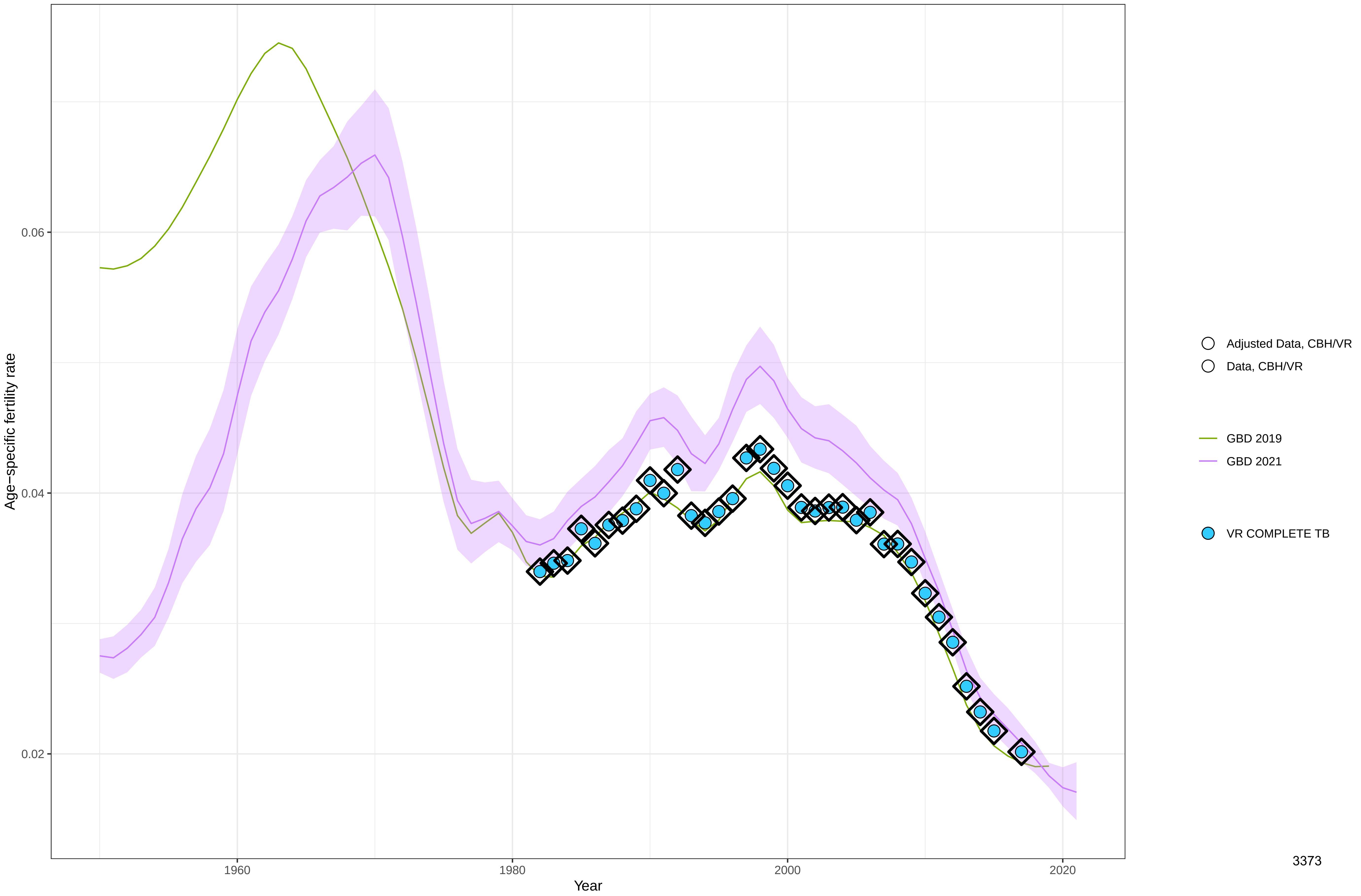

Age−specific fertility fitted trend lines, 1950−2021: Walsall, Ages: 20−24
Data points enclosed with a diamond are reference sources

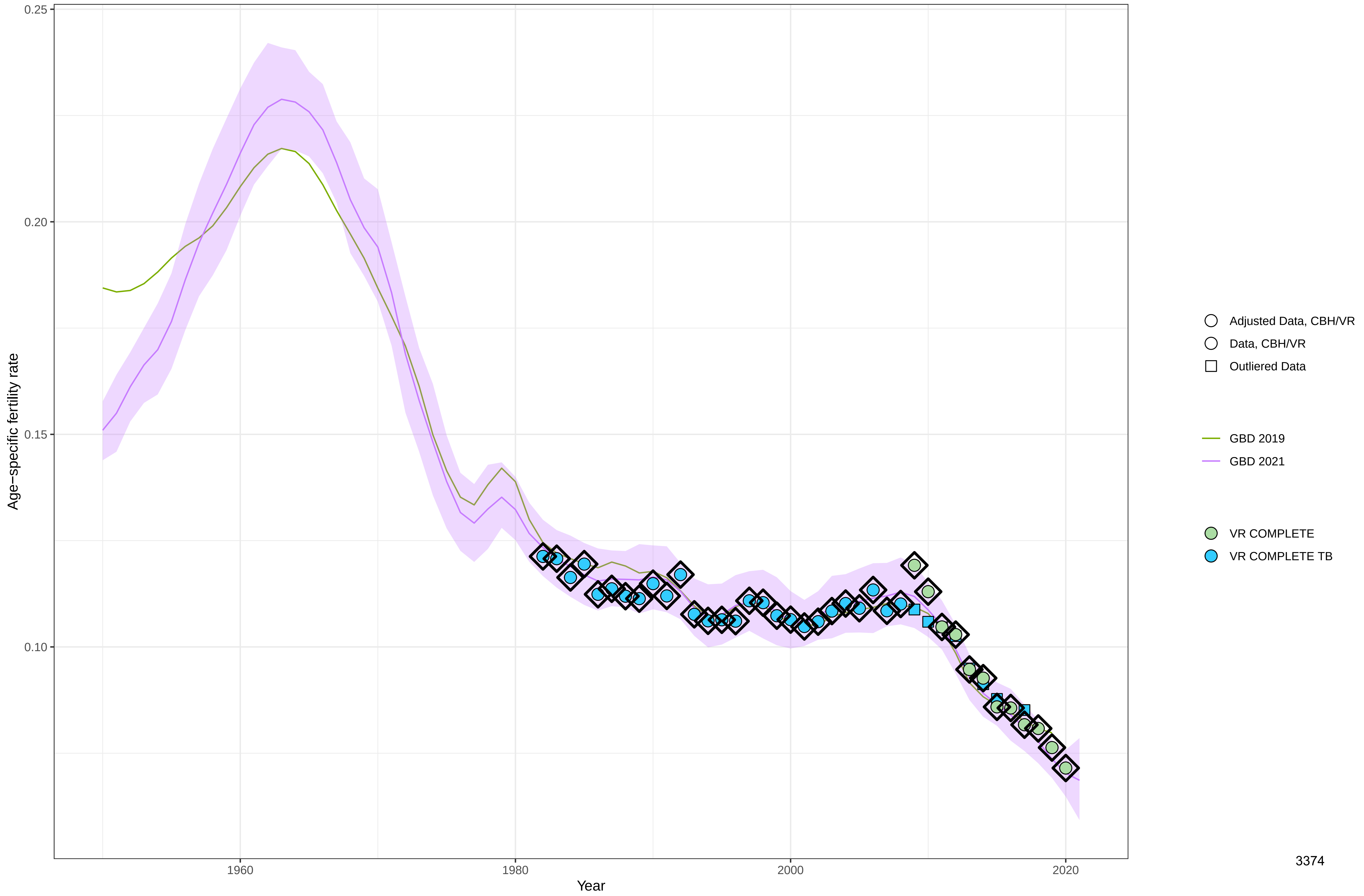

Age−specific fertility fitted trend lines, 1950−2021: Walsall, Ages: 25−29
Data points enclosed with a diamond are reference sources

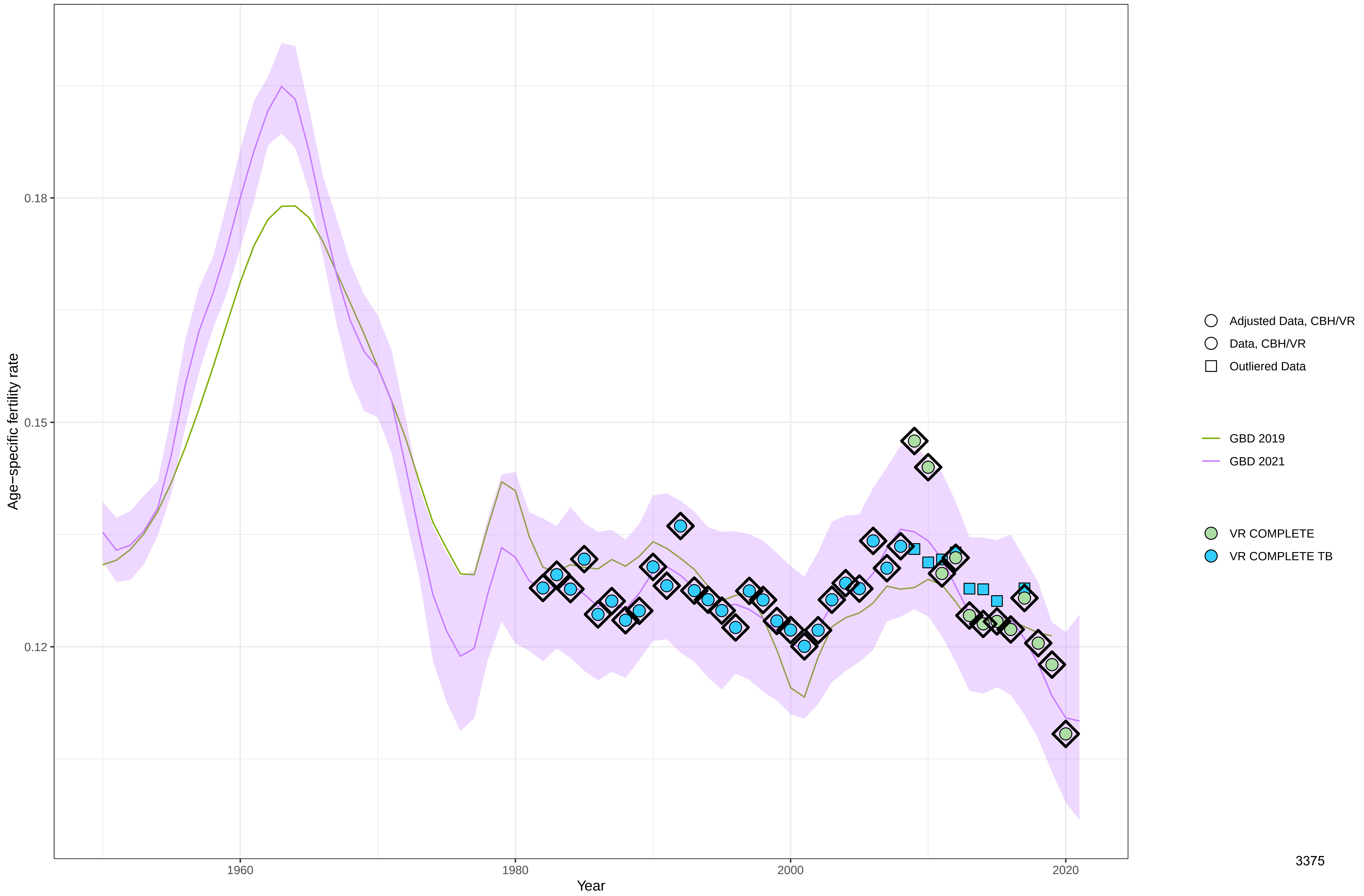

Age−specific fertility fitted trend lines, 1950−2021: Walsall, Ages: 30−34
Data points enclosed with a diamond are reference sources

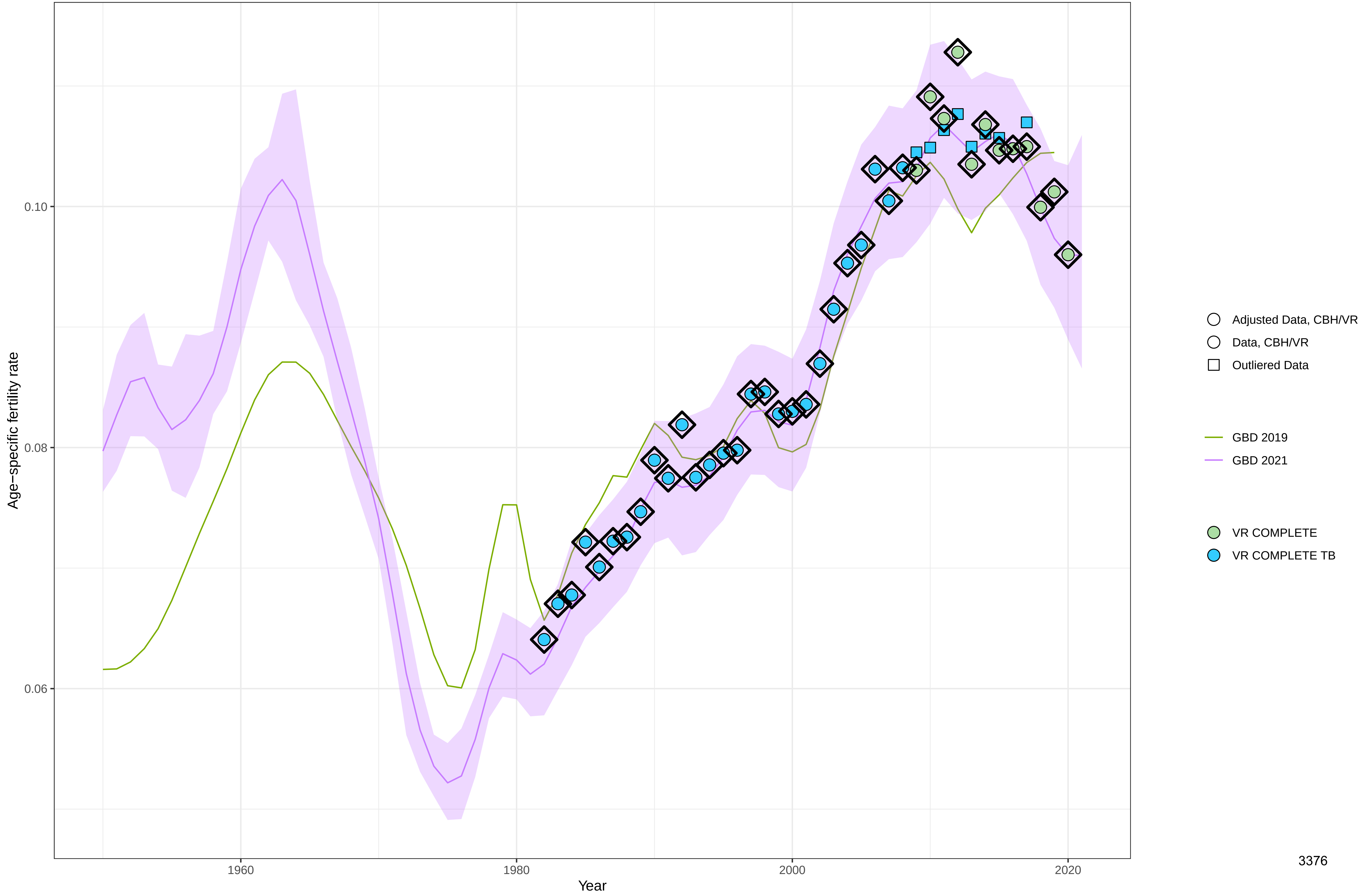

Age−specific fertility fitted trend lines, 1950−2021: Walsall, Ages: 35−39
Data points enclosed with a diamond are reference sources

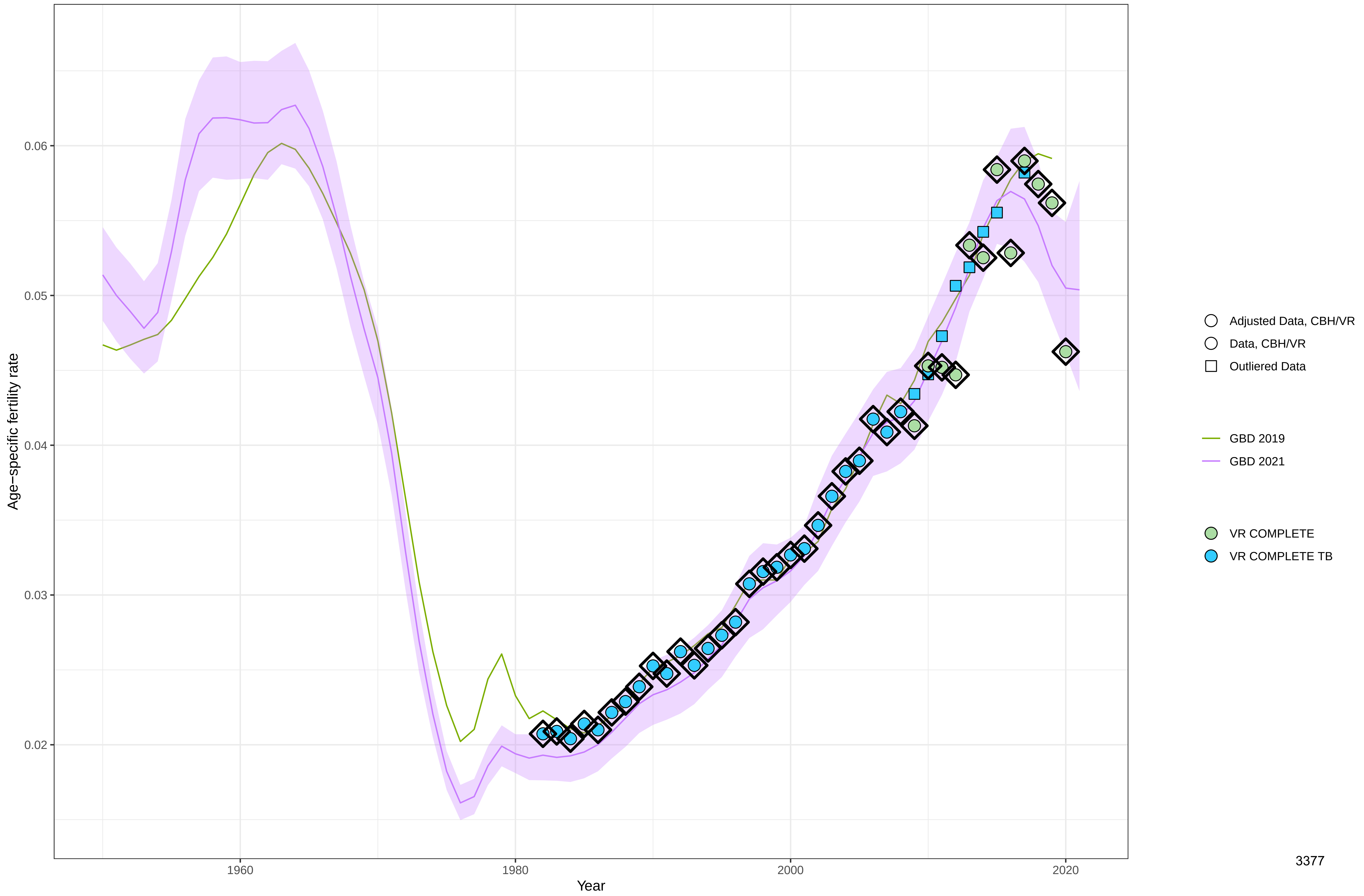

Age−specific fertility fitted trend lines, 1950−2021: Walsall, Ages: 40−44
Data points enclosed with a diamond are reference sources

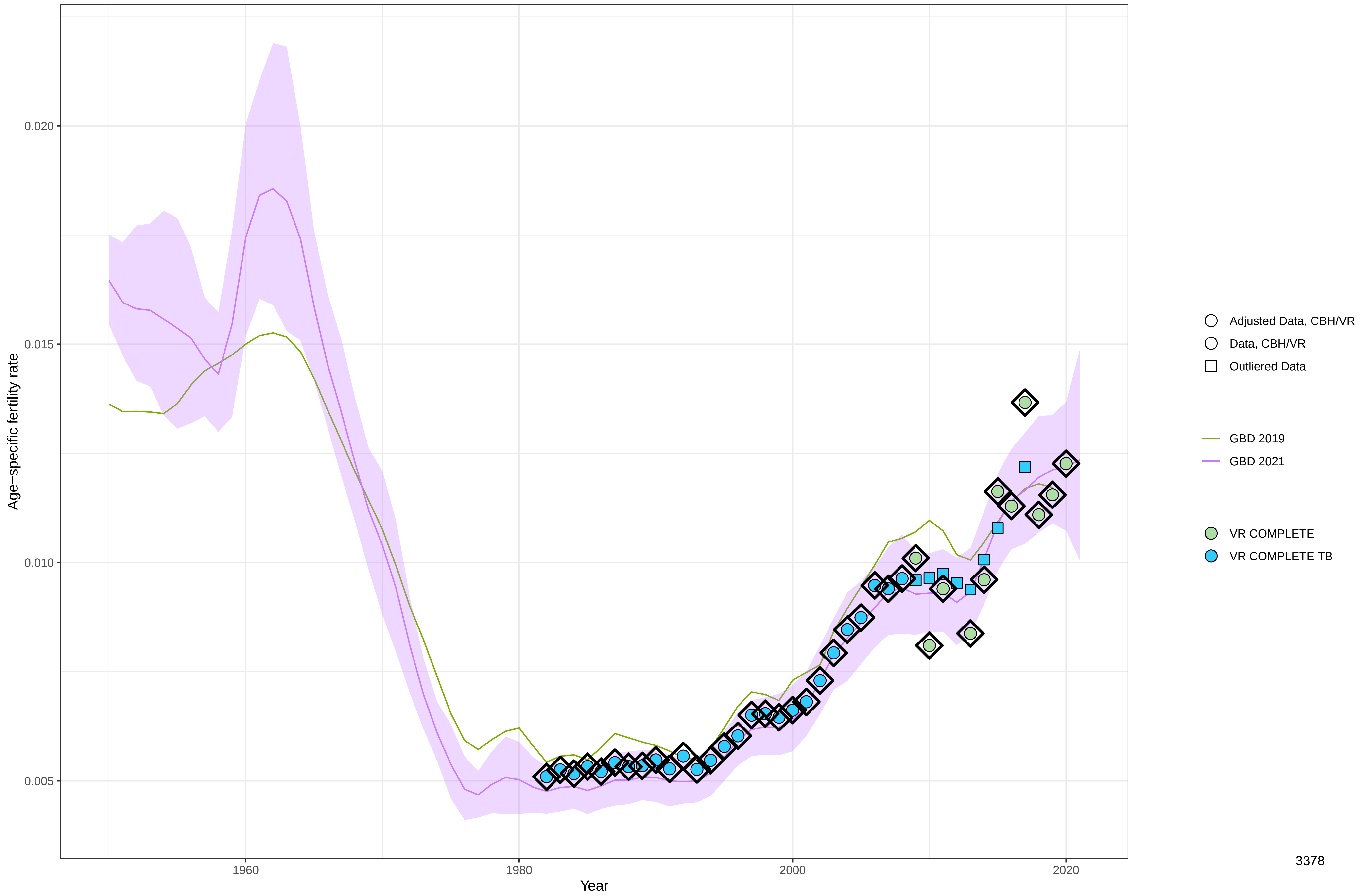

Age−specific fertility fitted trend lines, 1950−2021: Walsall, Ages: 45−49
Data points enclosed with a diamond are reference sources

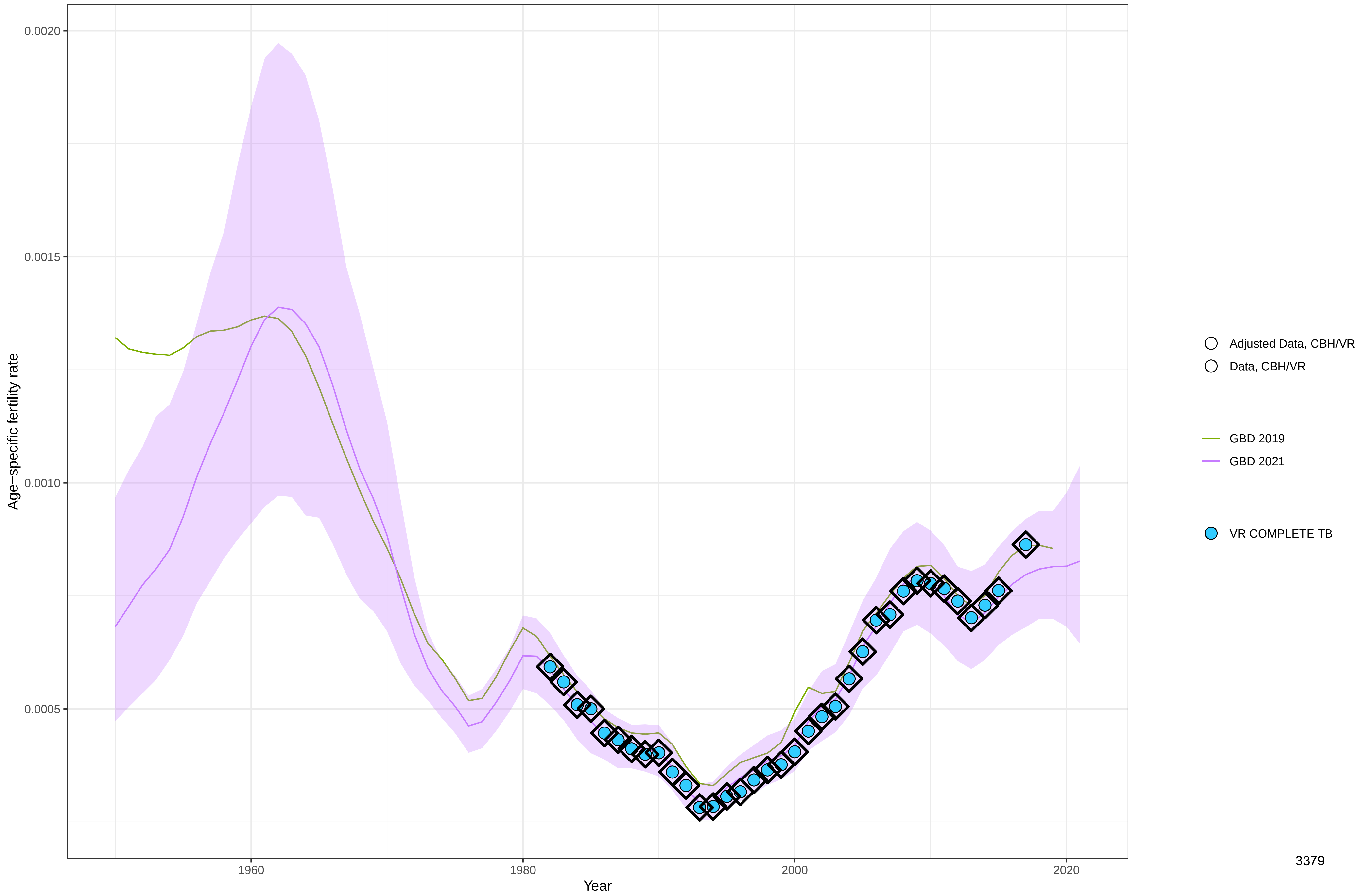

3379

Age−specific fertility fitted trend lines, 1950−2021: Walsall, Ages: 50−54
Data points enclosed with a diamond are reference sources

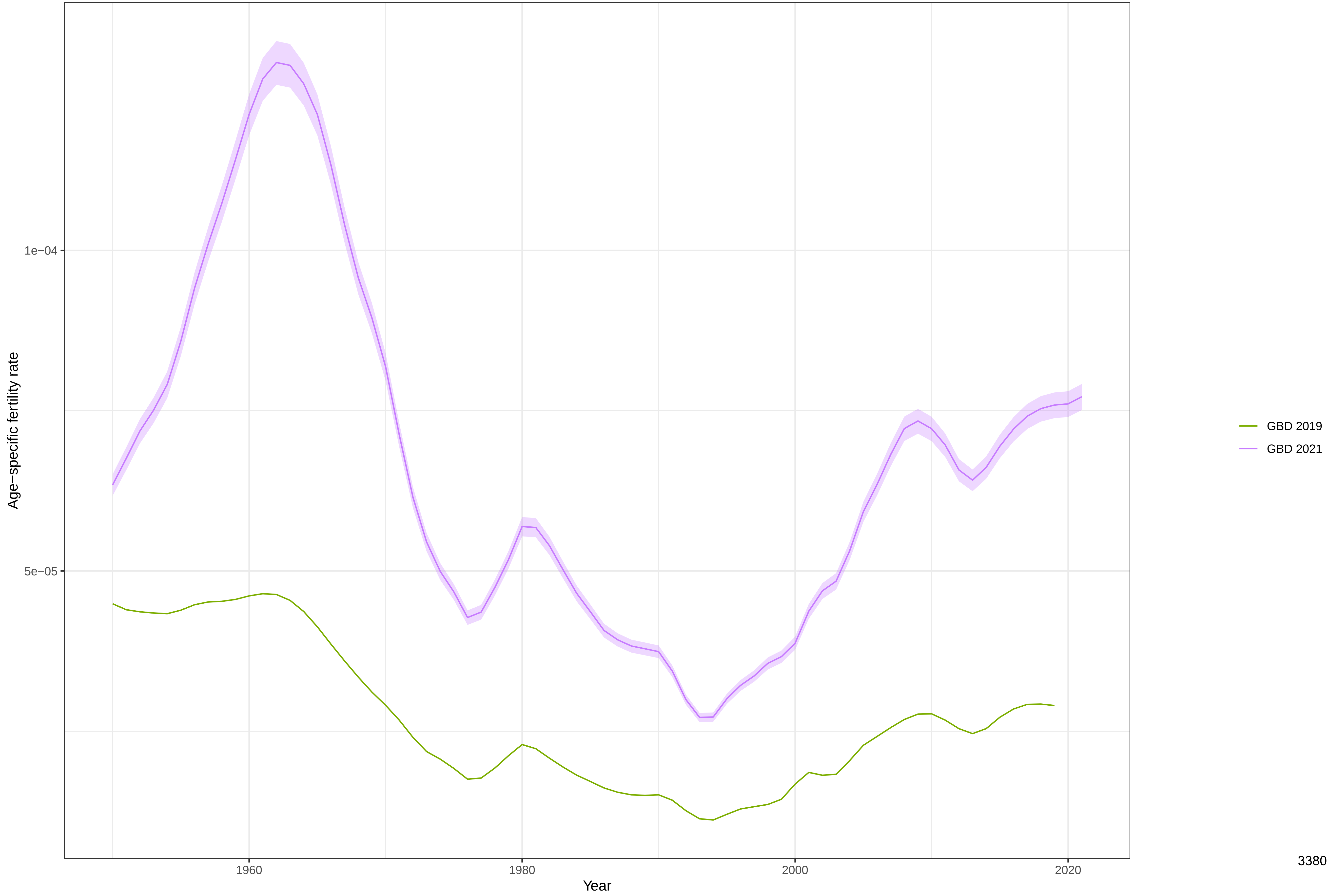

Total fertility rate fitted trend lines, 1950−2021: Walsall TFR
Data points enclosed with a diamond are reference sources

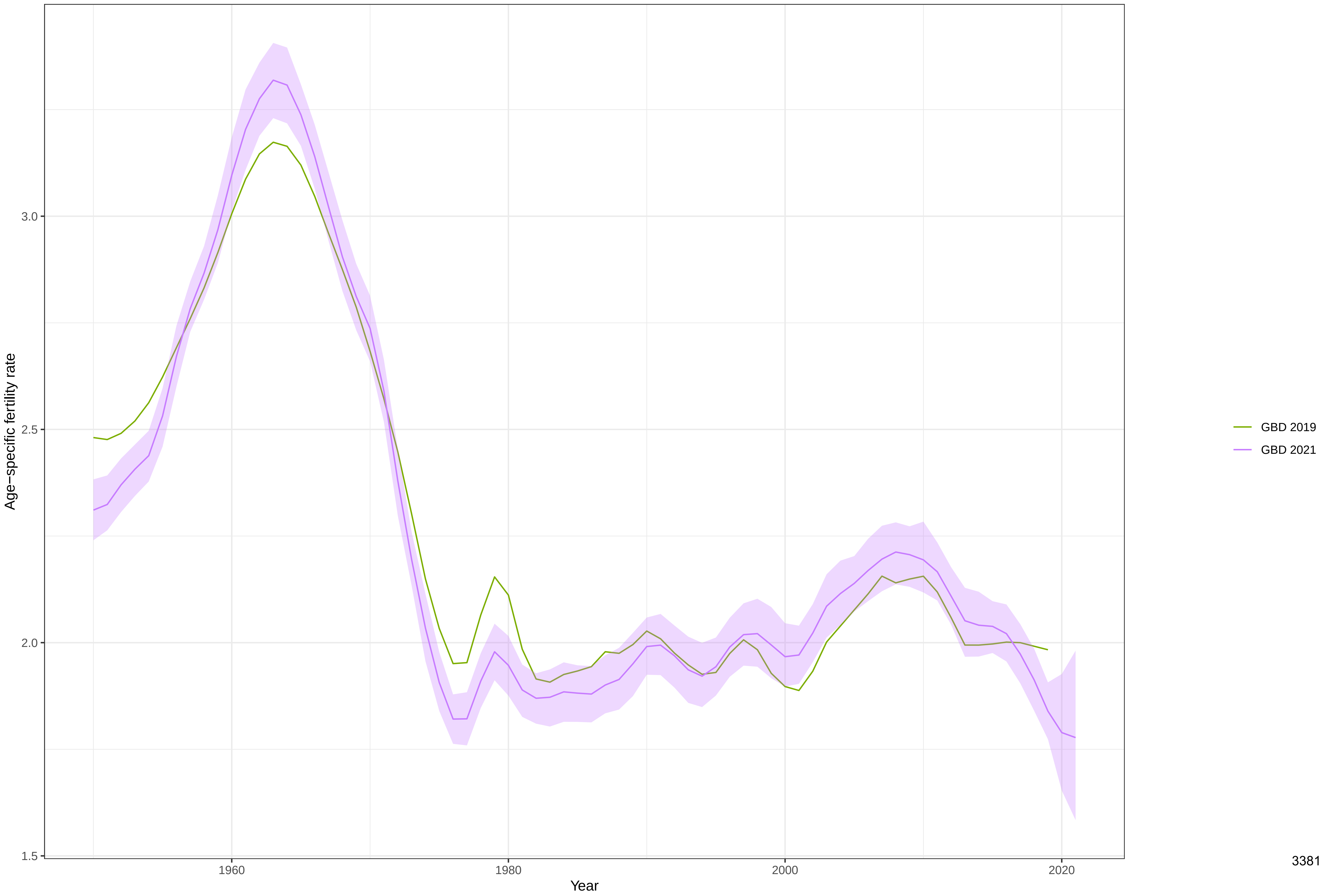

Age−specific fertility fitted trend lines, 1950−2021: Warwickshire, Ages: 10−14
Data points enclosed with a diamond are reference sources

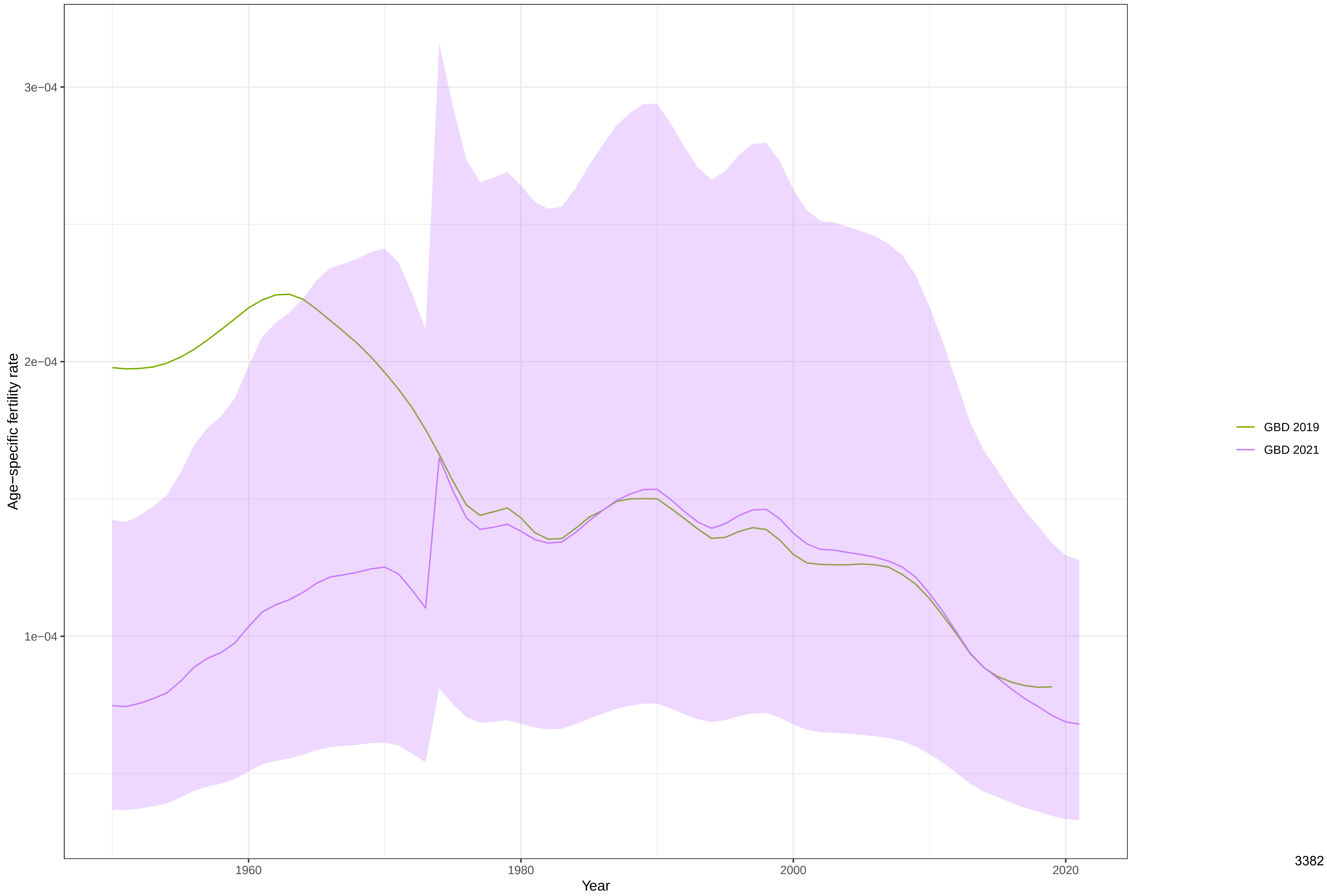

Age−specific fertility fitted trend lines, 1950−2021: Warwickshire, Ages: 15−19
Data points enclosed with a diamond are reference sources

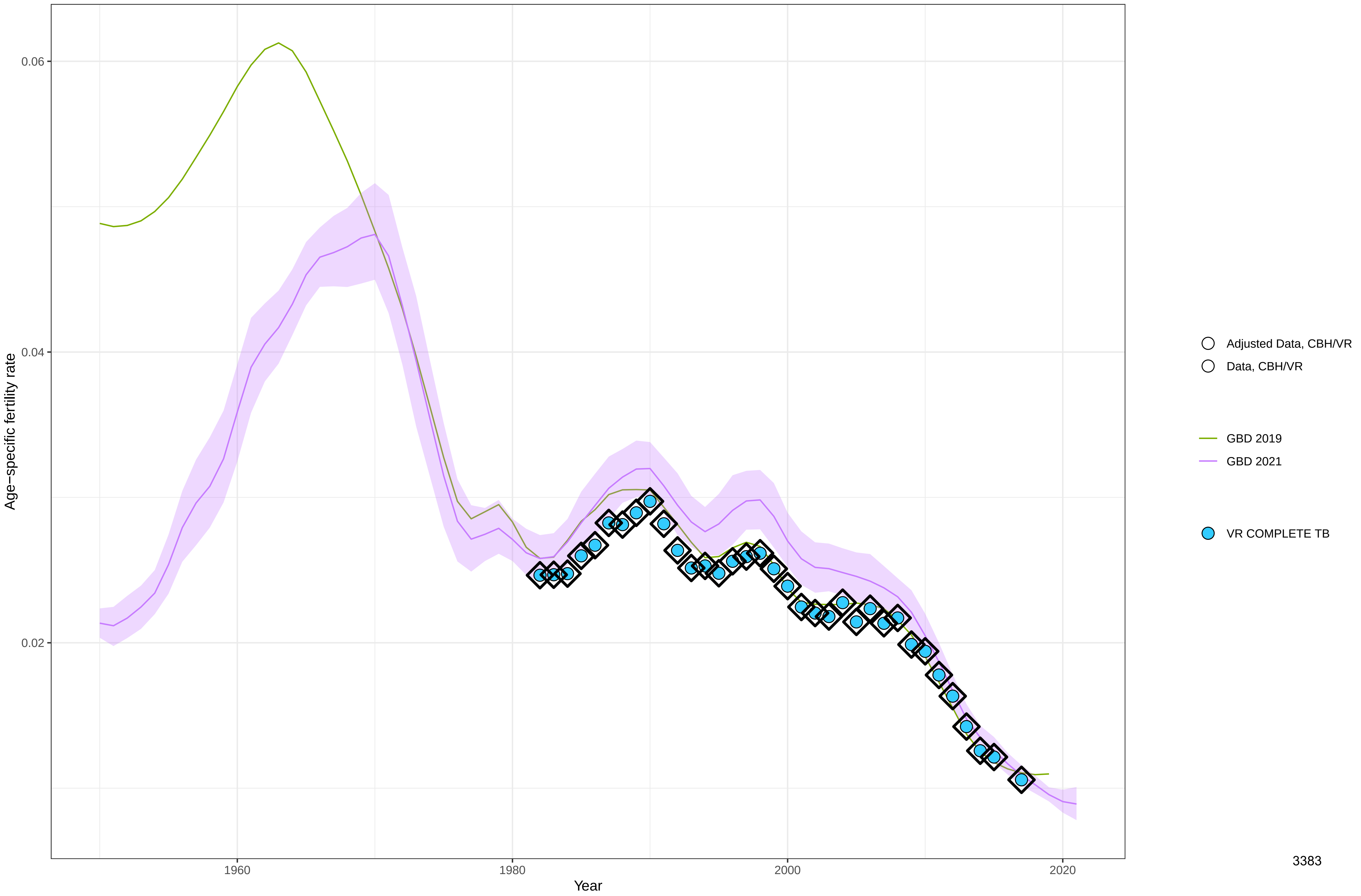

Age−specific fertility fitted trend lines, 1950−2021: Warwickshire, Ages: 20−24
Data points enclosed with a diamond are reference sources

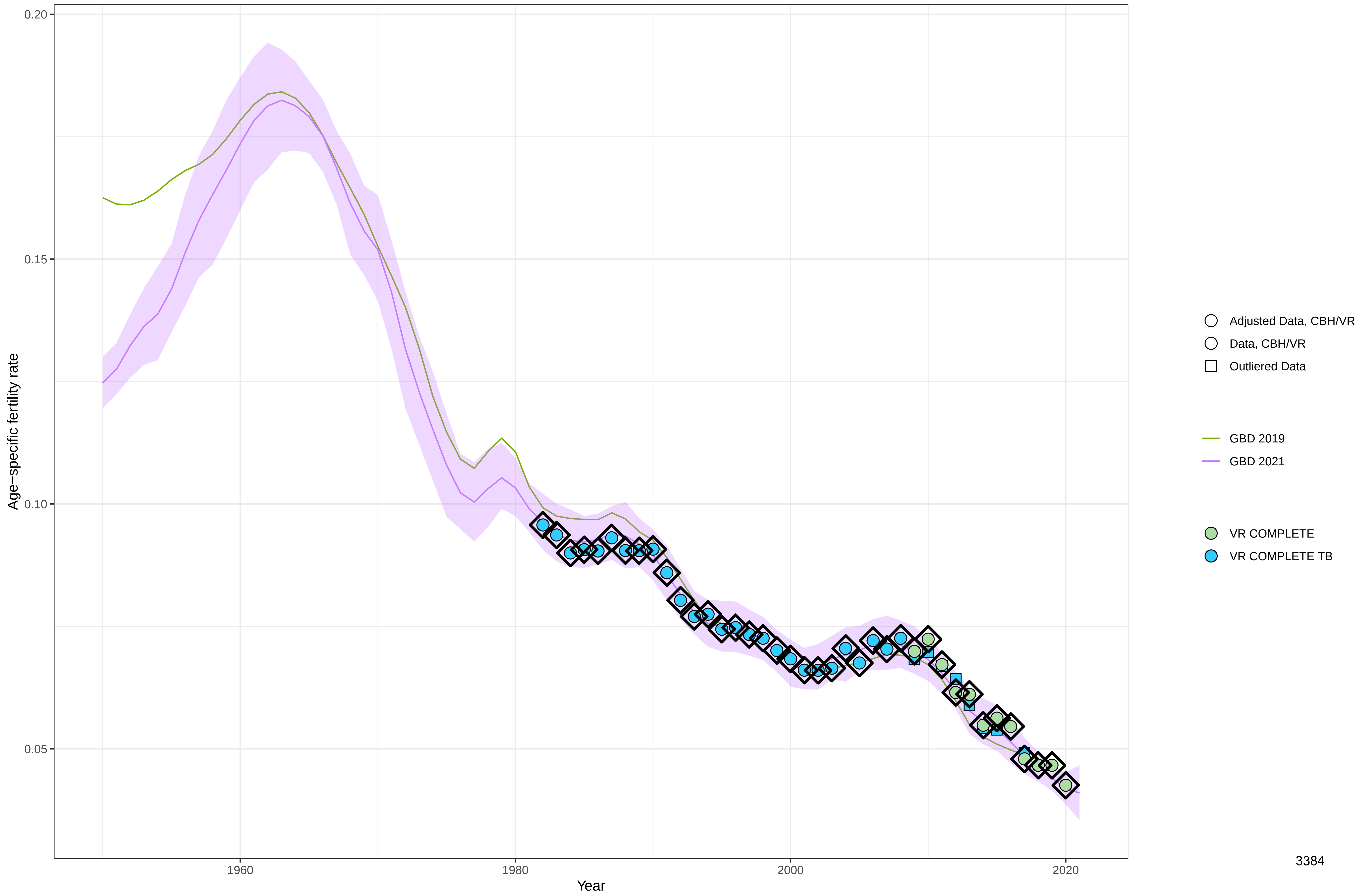

Age−specific fertility fitted trend lines, 1950−2021: Warwickshire, Ages: 25−29
Data points enclosed with a diamond are reference sources

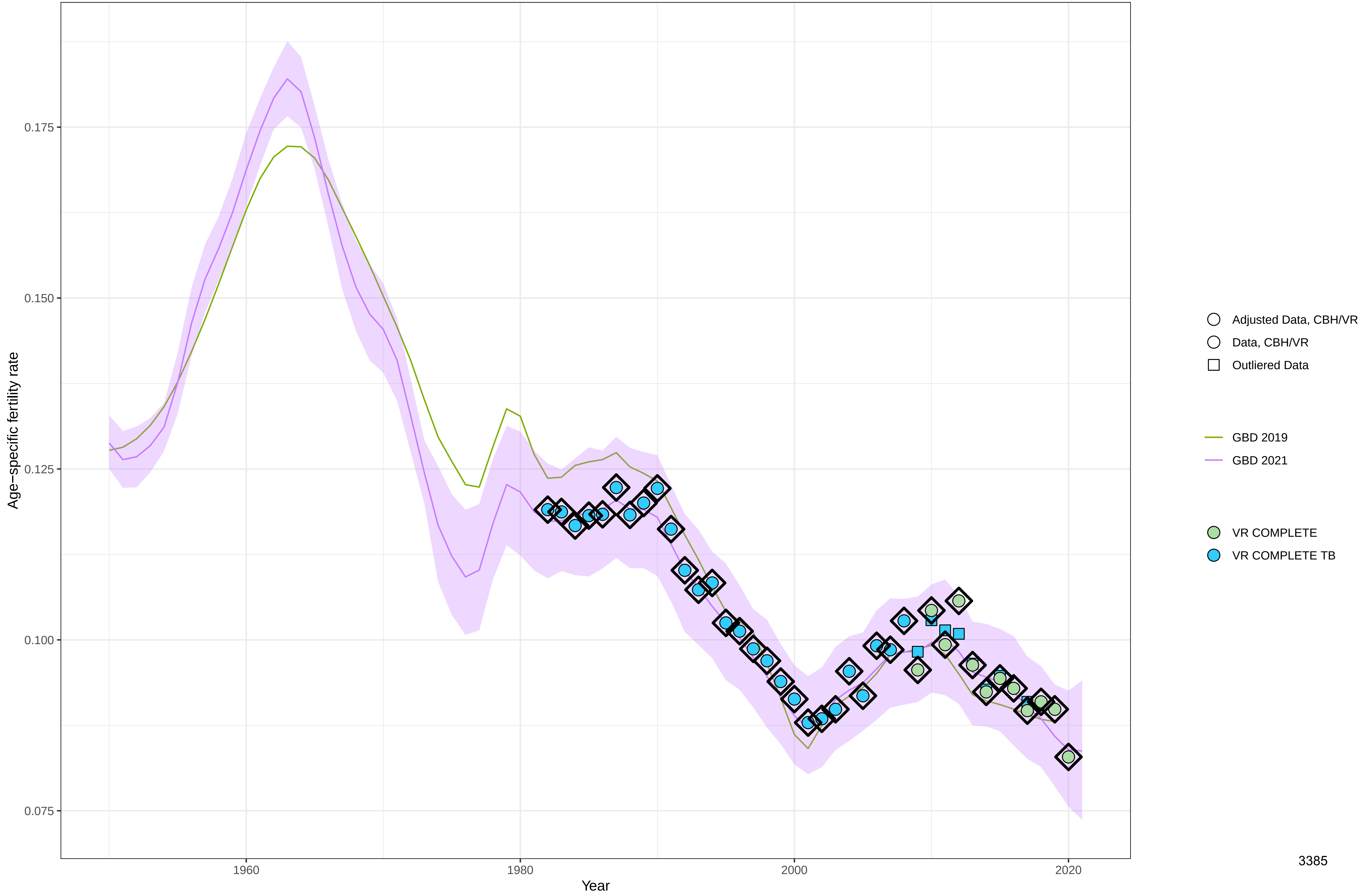

Age−specific fertility fitted trend lines, 1950−2021: Warwickshire, Ages: 30−34
Data points enclosed with a diamond are reference sources

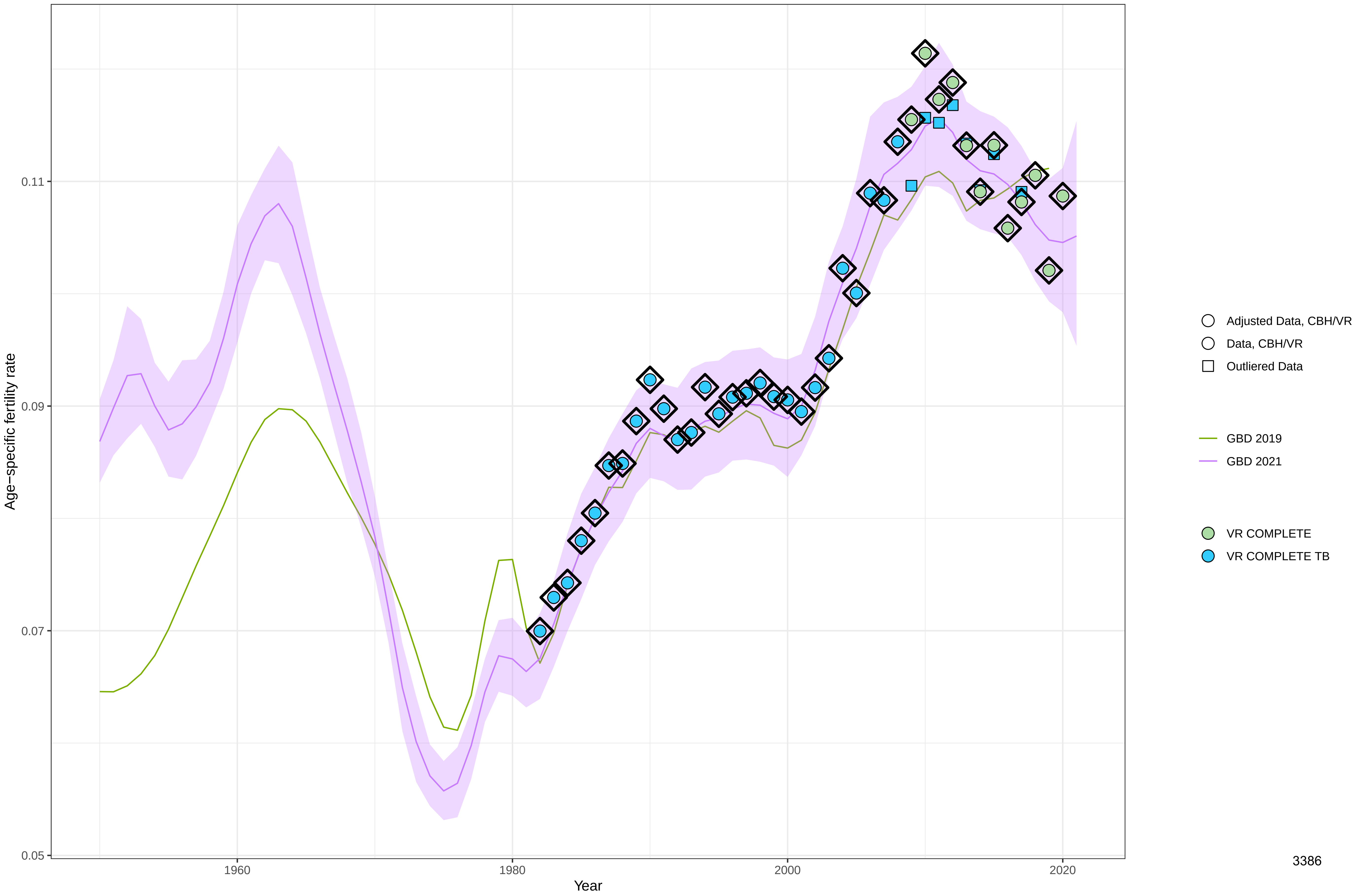

Age−specific fertility fitted trend lines, 1950−2021: Warwickshire, Ages: 35−39
Data points enclosed with a diamond are reference sources

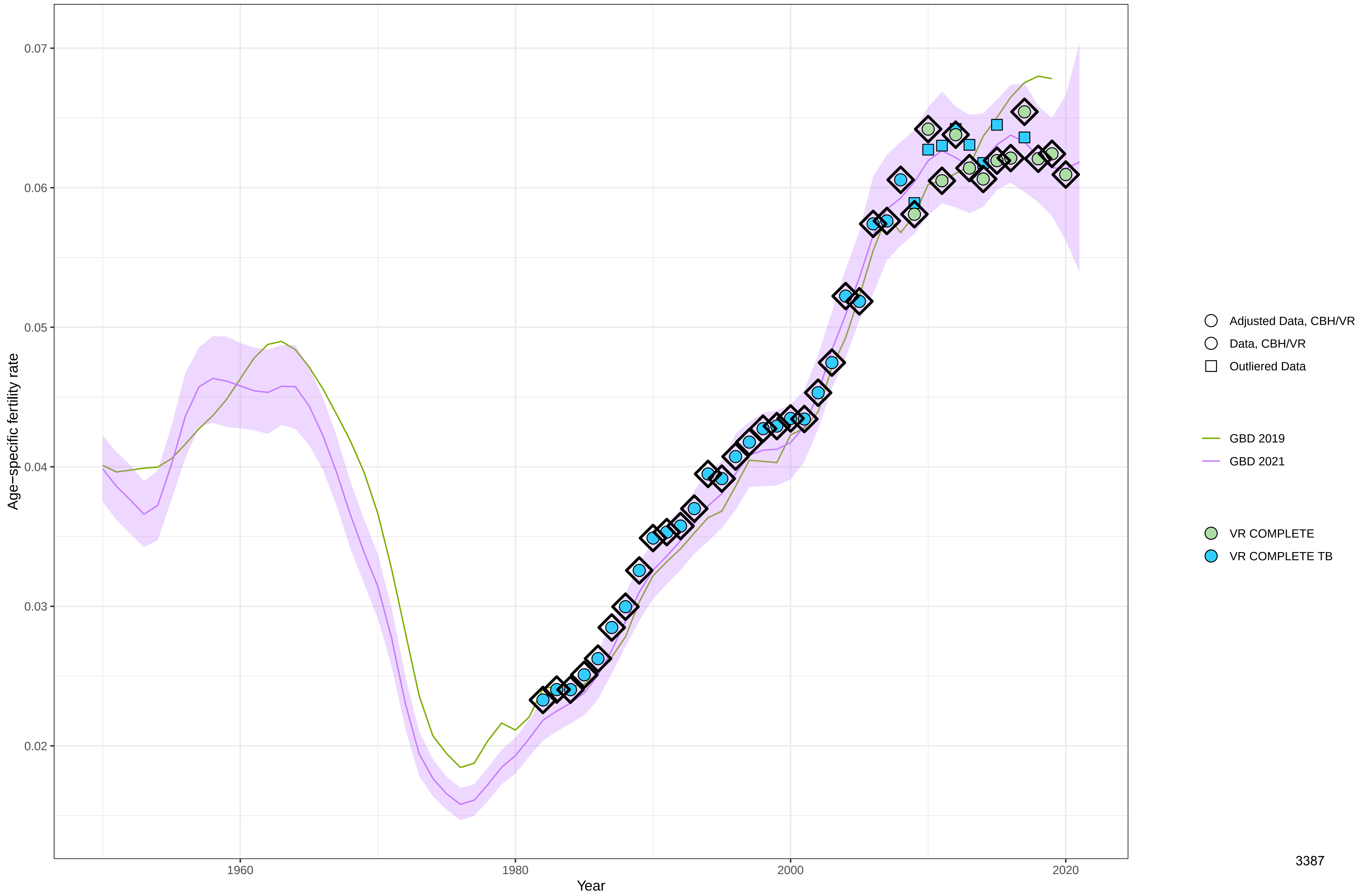

Age−specific fertility fitted trend lines, 1950−2021: Warwickshire, Ages: 40−44
Data points enclosed with a diamond are reference sources

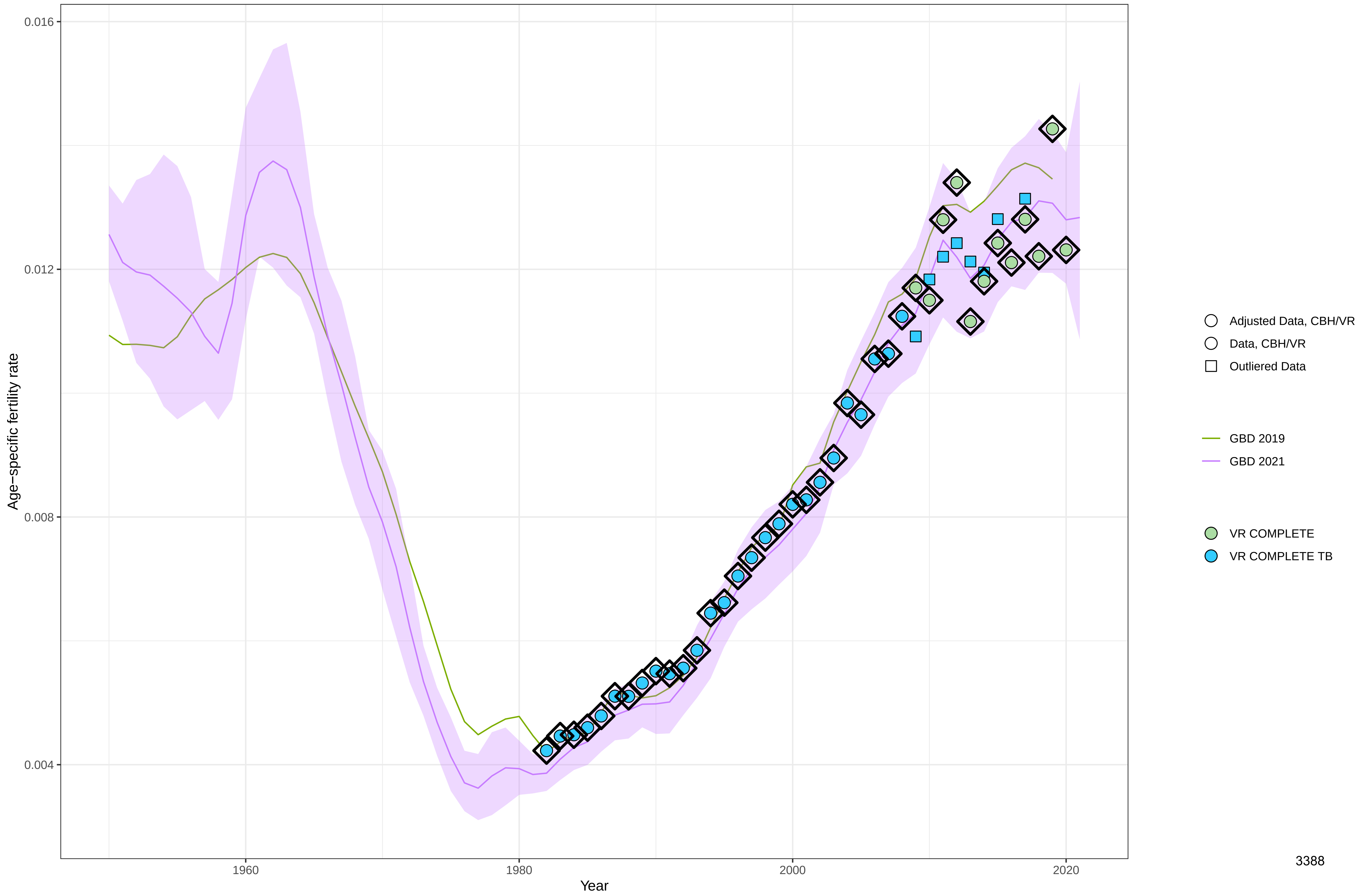

Age−specific fertility fitted trend lines, 1950−2021: Warwickshire, Ages: 45−49
Data points enclosed with a diamond are reference sources

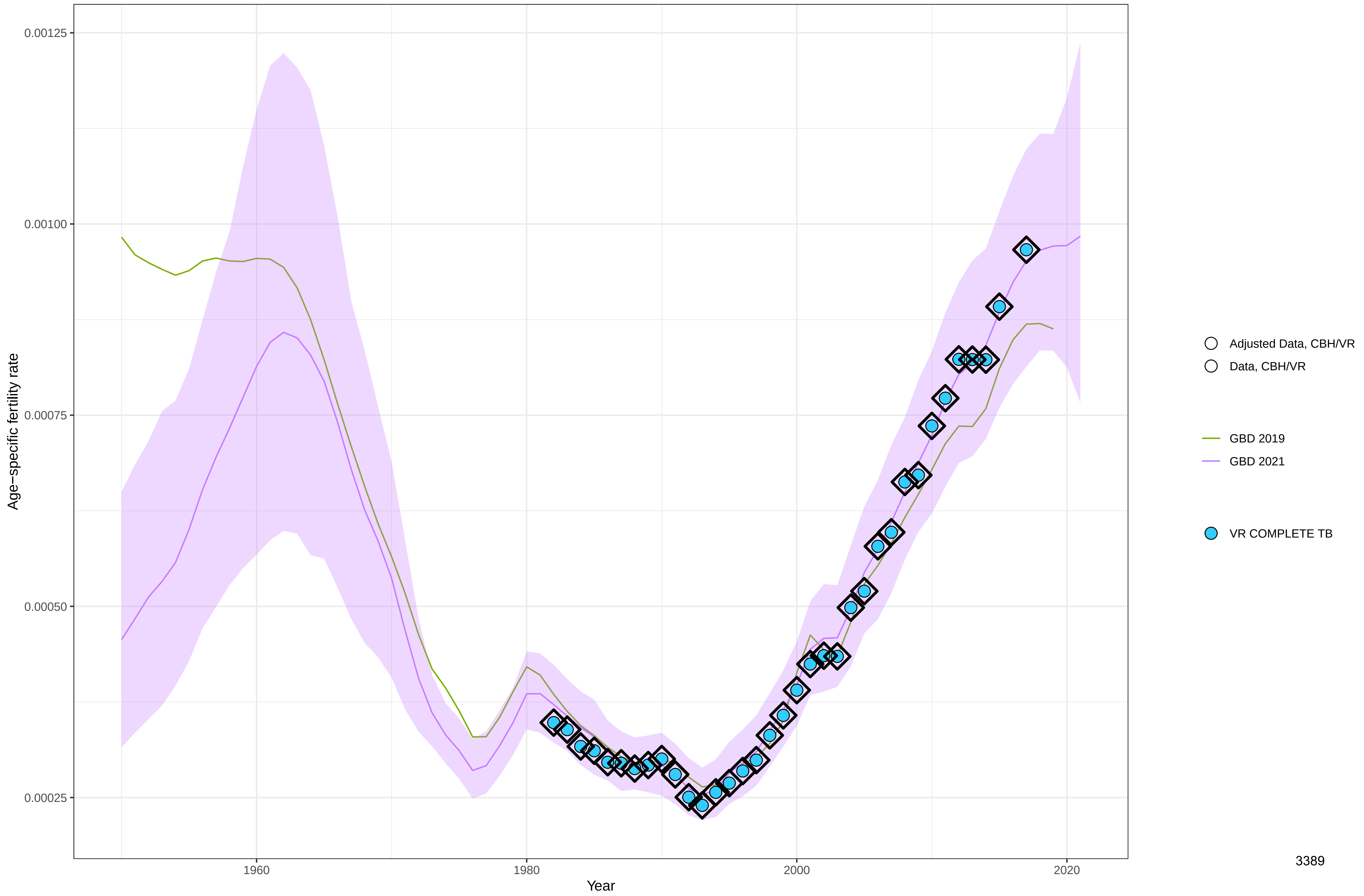

Age−specific fertility fitted trend lines, 1950−2021: Warwickshire, Ages: 50−54
Data points enclosed with a diamond are reference sources

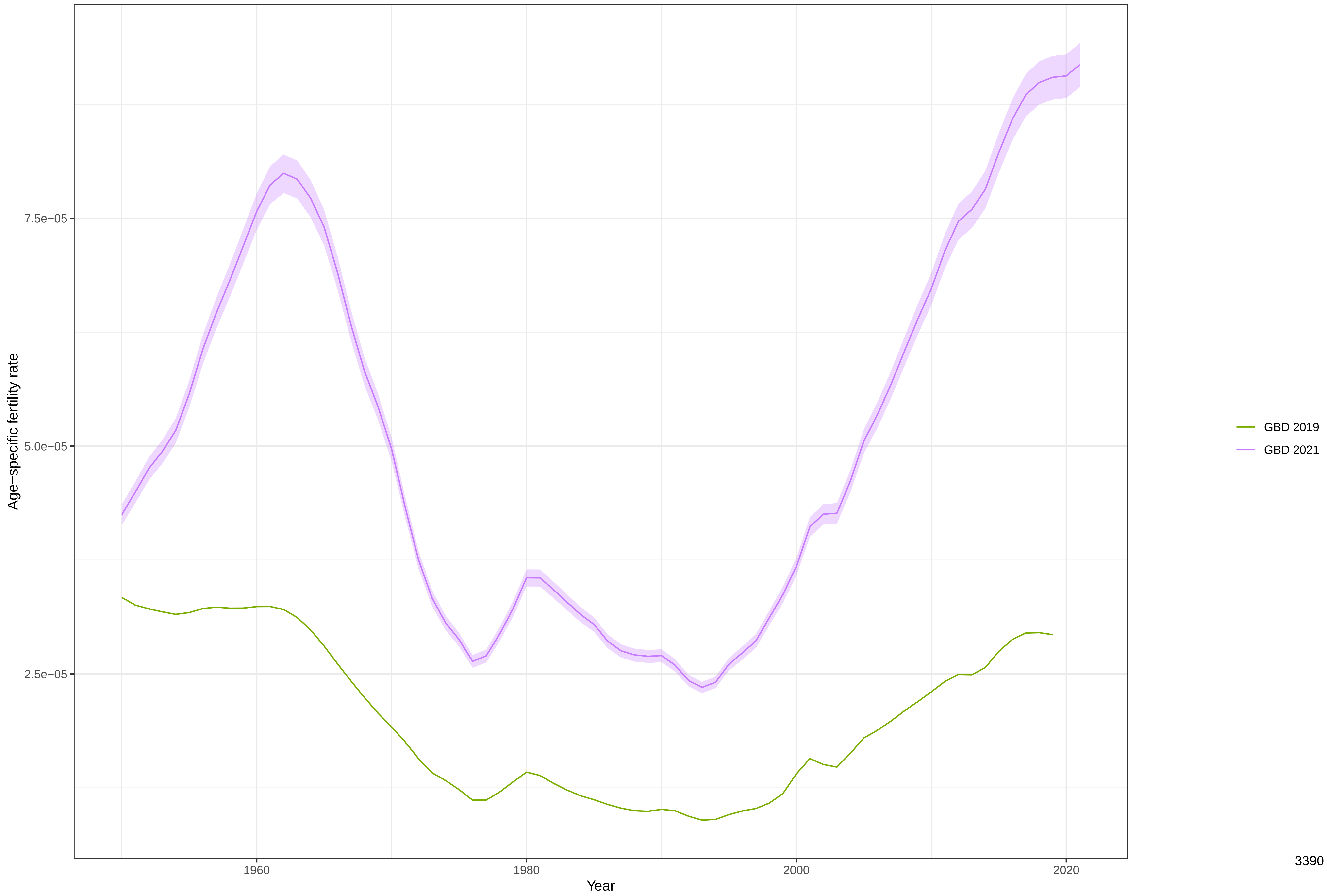

Total fertility rate fitted trend lines, 1950−2021: Warwickshire TFR
Data points enclosed with a diamond are reference sources

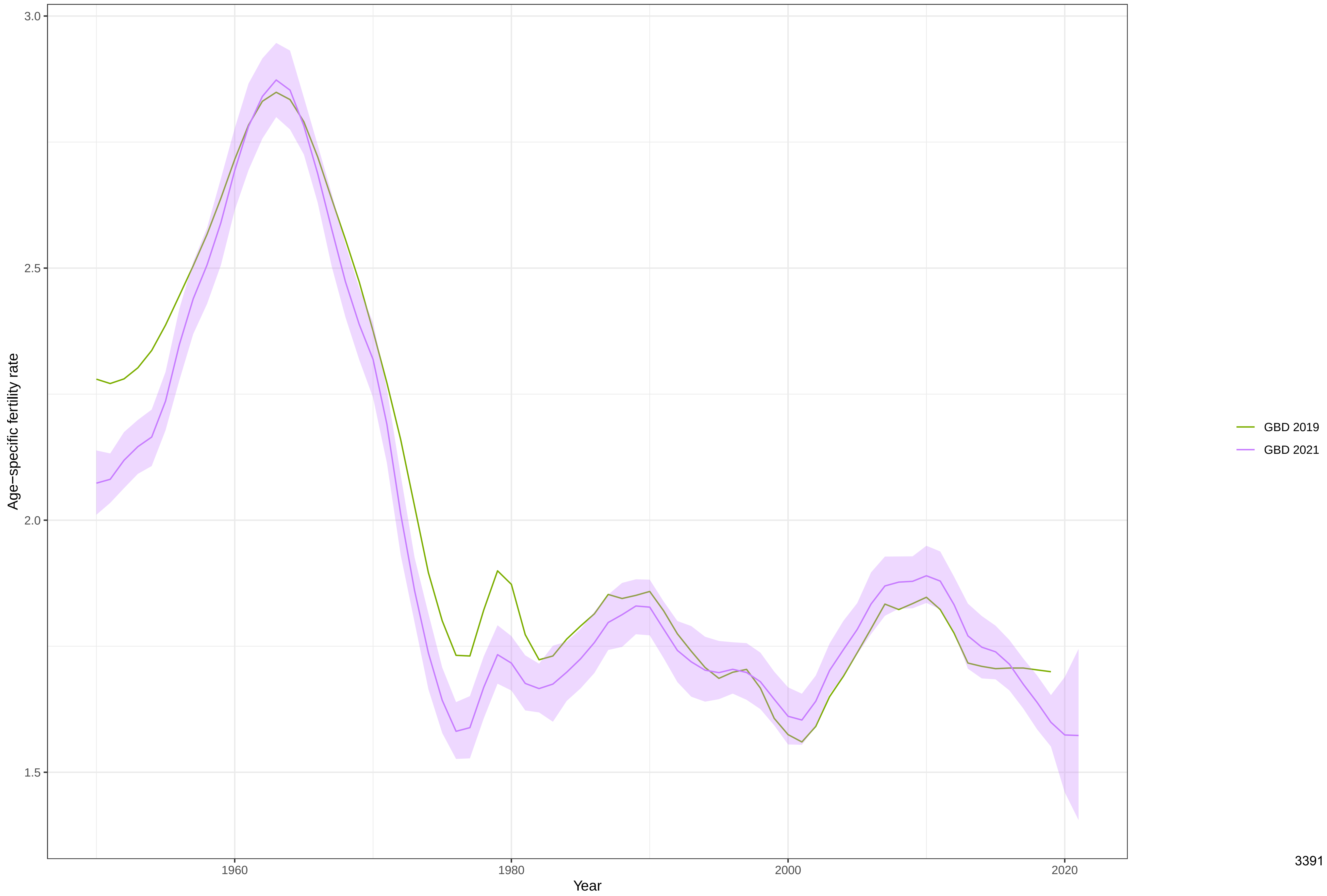

Age−specific fertility fitted trend lines, 1950−2021: Wolverhampton, Ages: 10−14
Data points enclosed with a diamond are reference sources

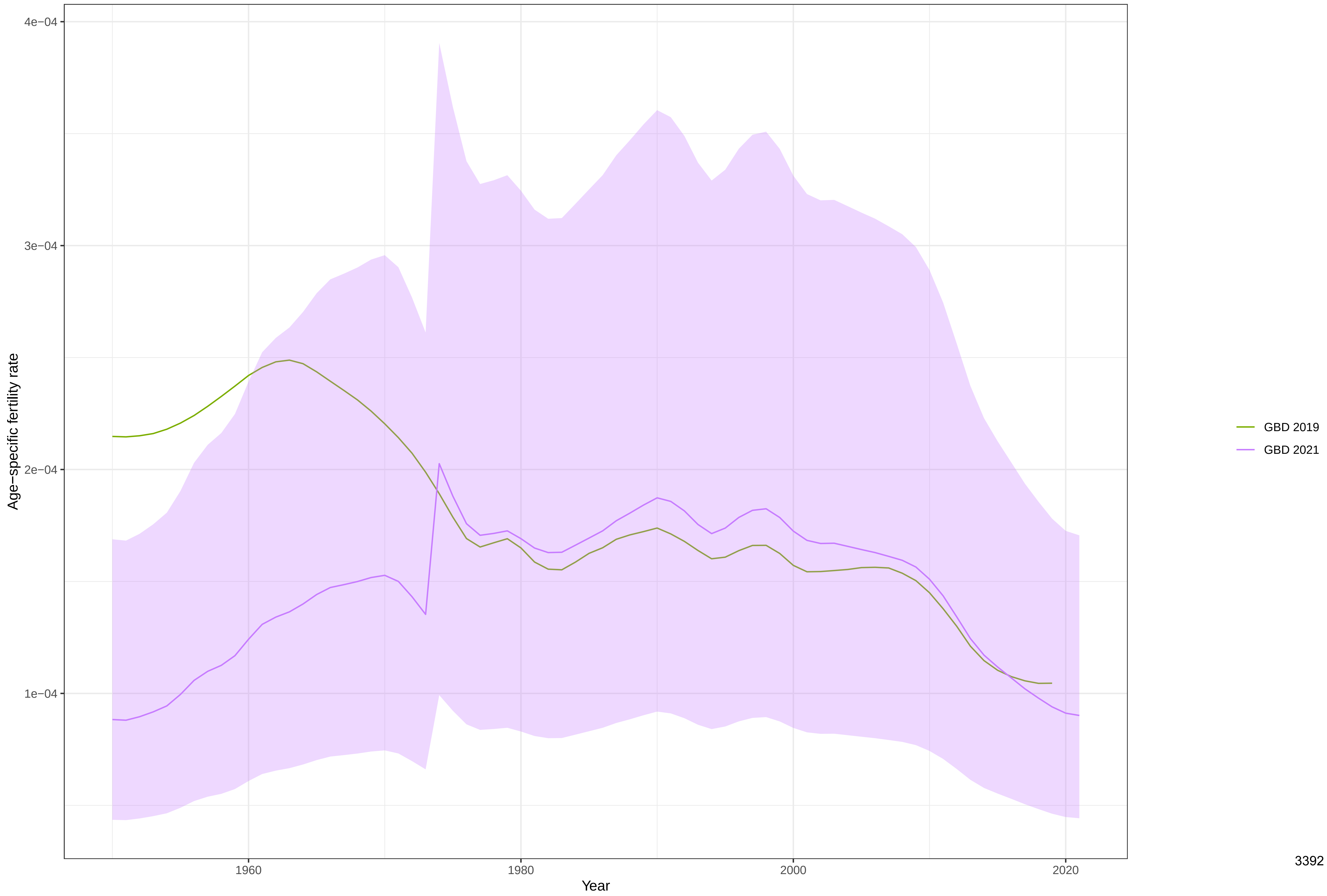

Age−specific fertility fitted trend lines, 1950−2021: Wolverhampton, Ages: 15−19
Data points enclosed with a diamond are reference sources

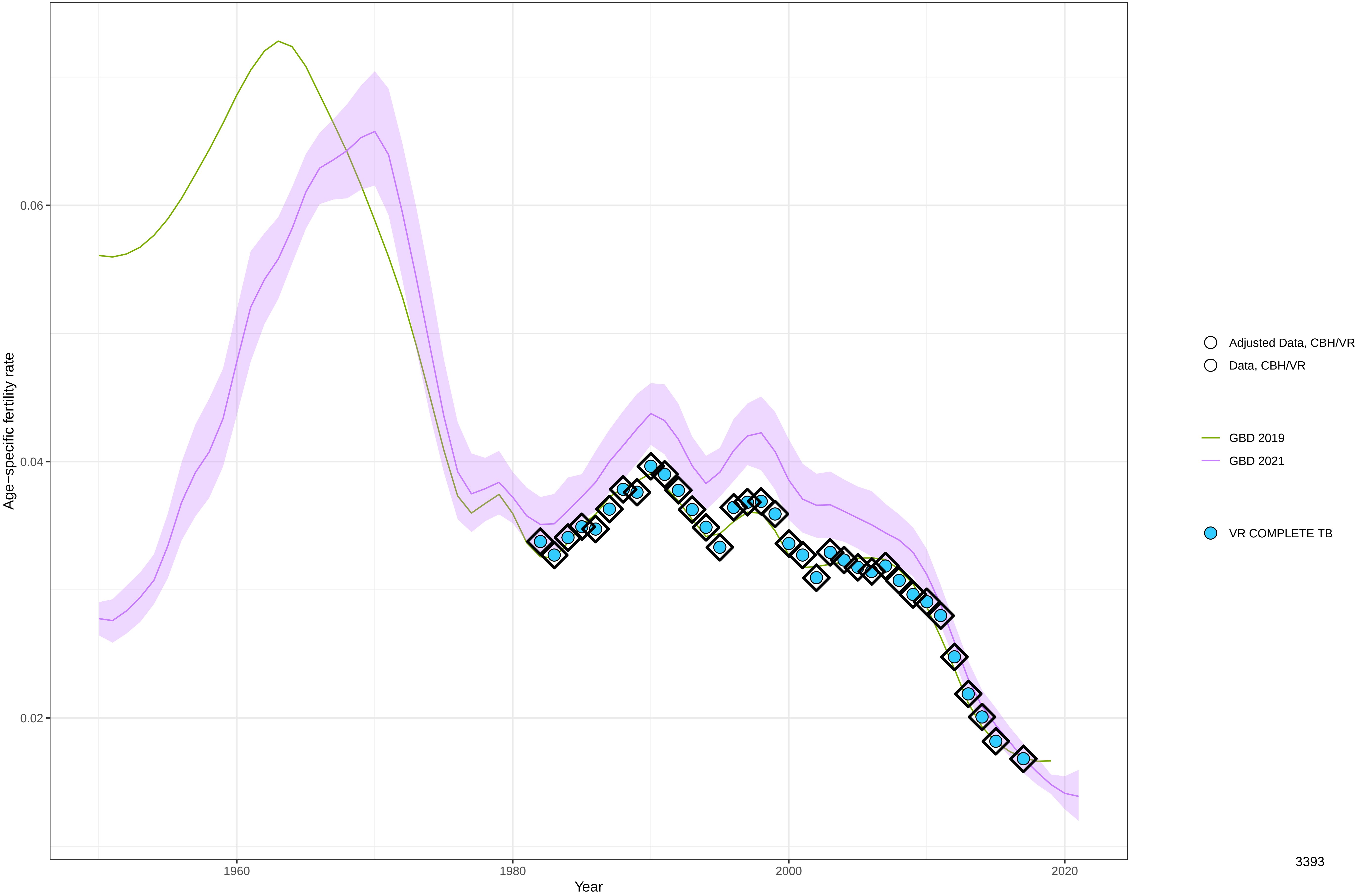

Age−specific fertility fitted trend lines, 1950−2021: Wolverhampton, Ages: 20−24
Data points enclosed with a diamond are reference sources

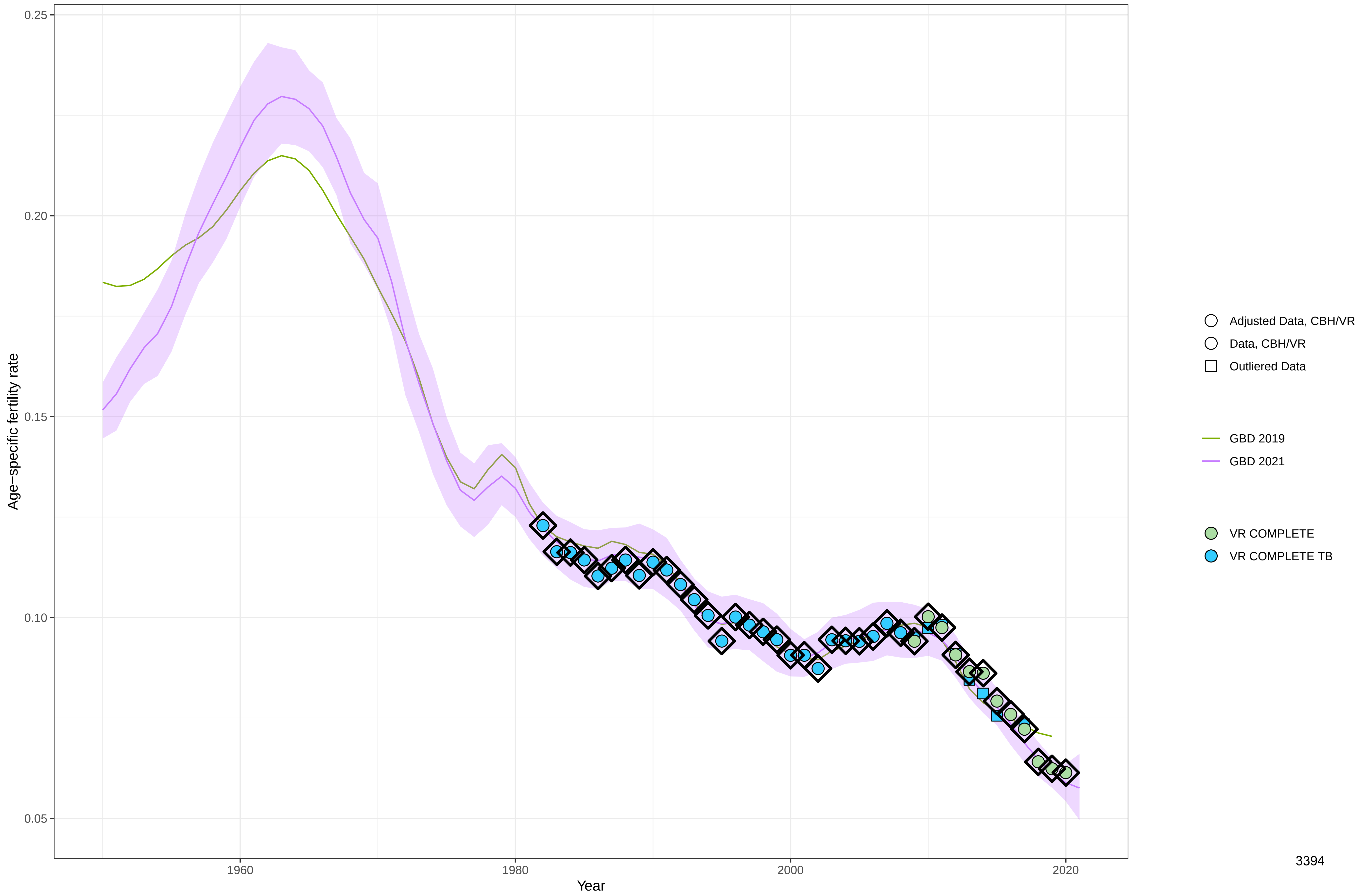

Age−specific fertility fitted trend lines, 1950−2021: Wolverhampton, Ages: 25−29
Data points enclosed with a diamond are reference sources

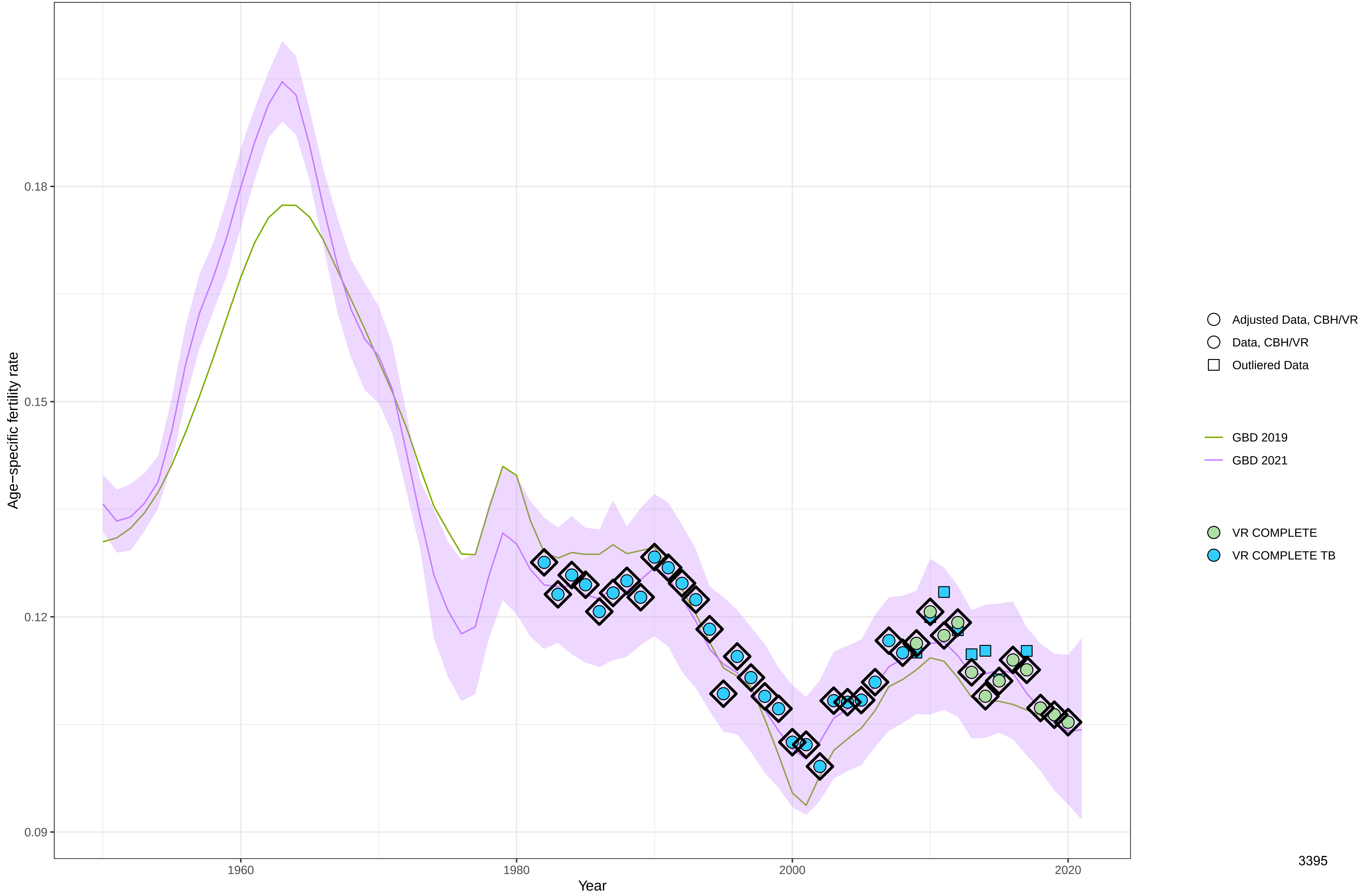

Age−specific fertility fitted trend lines, 1950−2021: Wolverhampton, Ages: 30−34
Data points enclosed with a diamond are reference sources

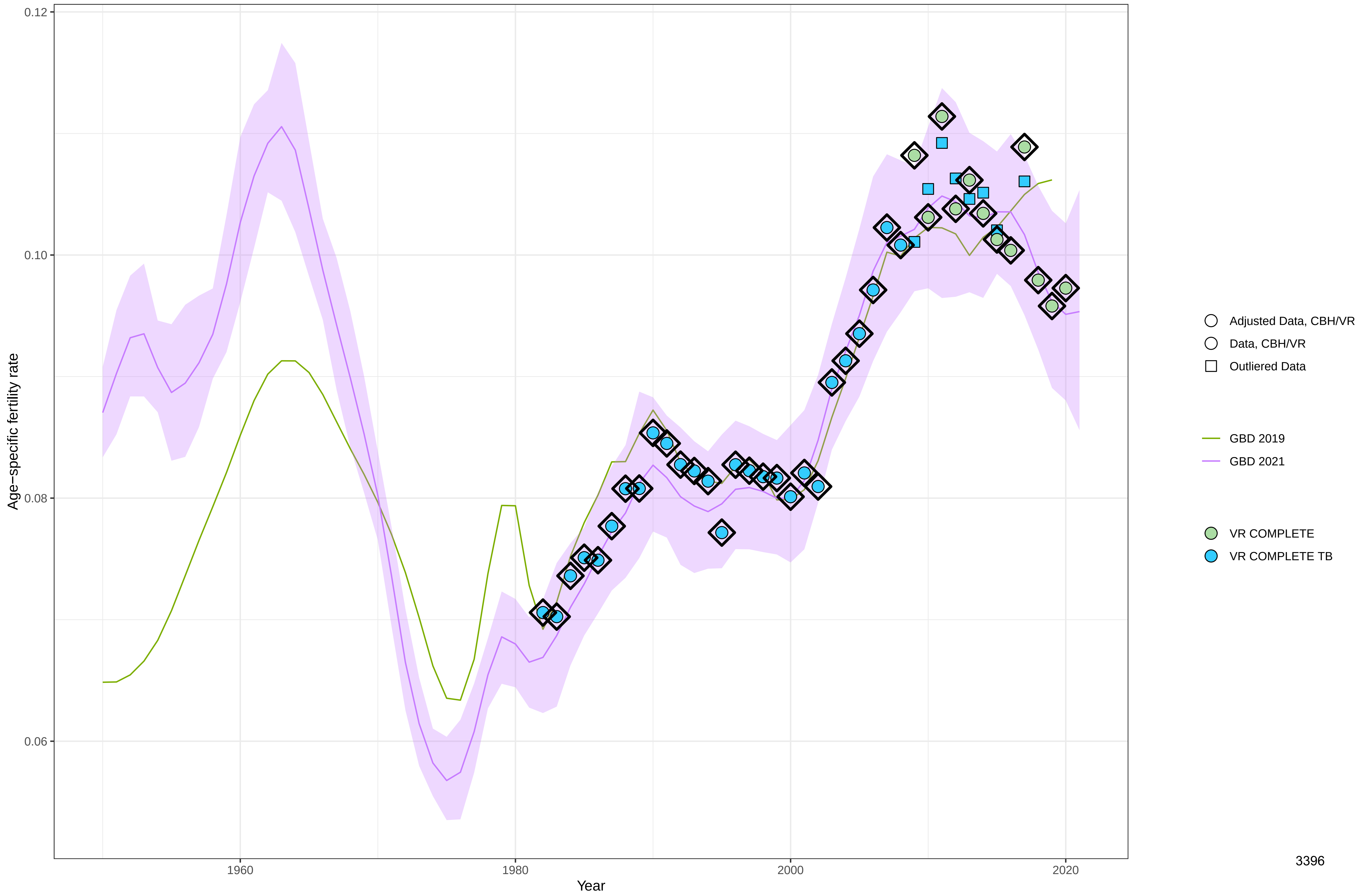

Age−specific fertility fitted trend lines, 1950−2021: Wolverhampton, Ages: 35−39
Data points enclosed with a diamond are reference sources

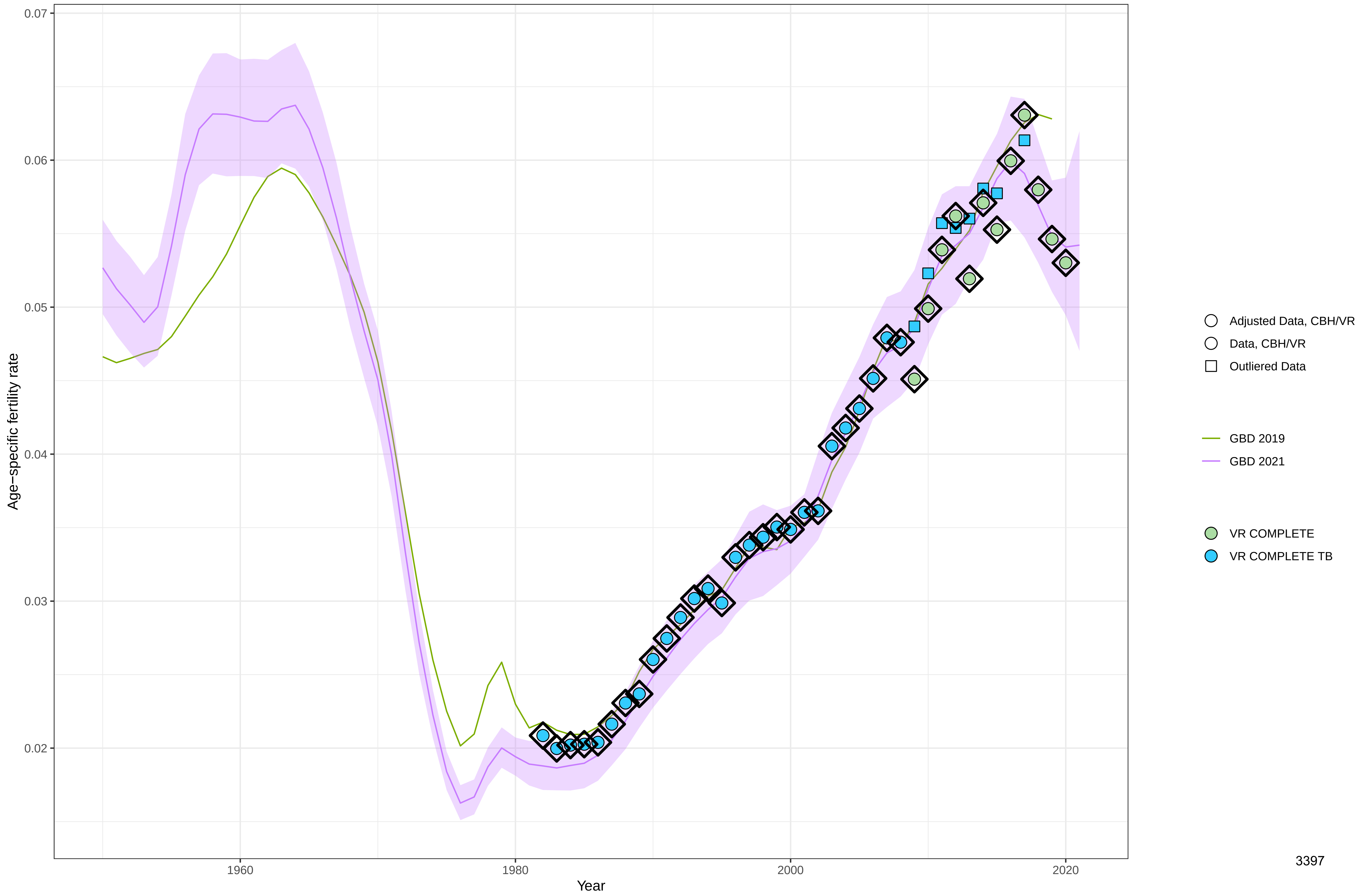

Age−specific fertility fitted trend lines, 1950−2021: Wolverhampton, Ages: 40−44
Data points enclosed with a diamond are reference sources

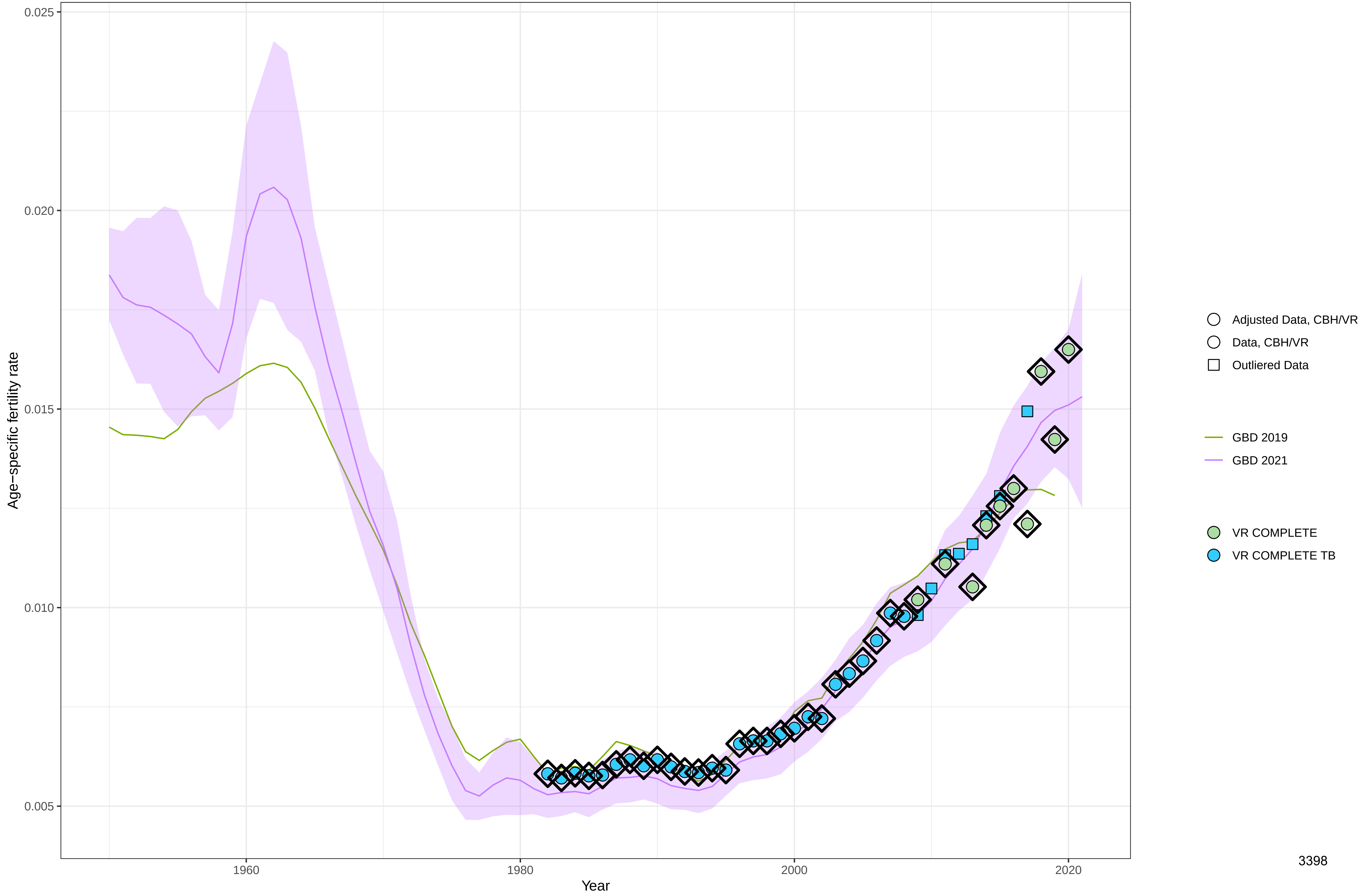

Age−specific fertility fitted trend lines, 1950−2021: Wolverhampton, Ages: 45−49
Data points enclosed with a diamond are reference sources

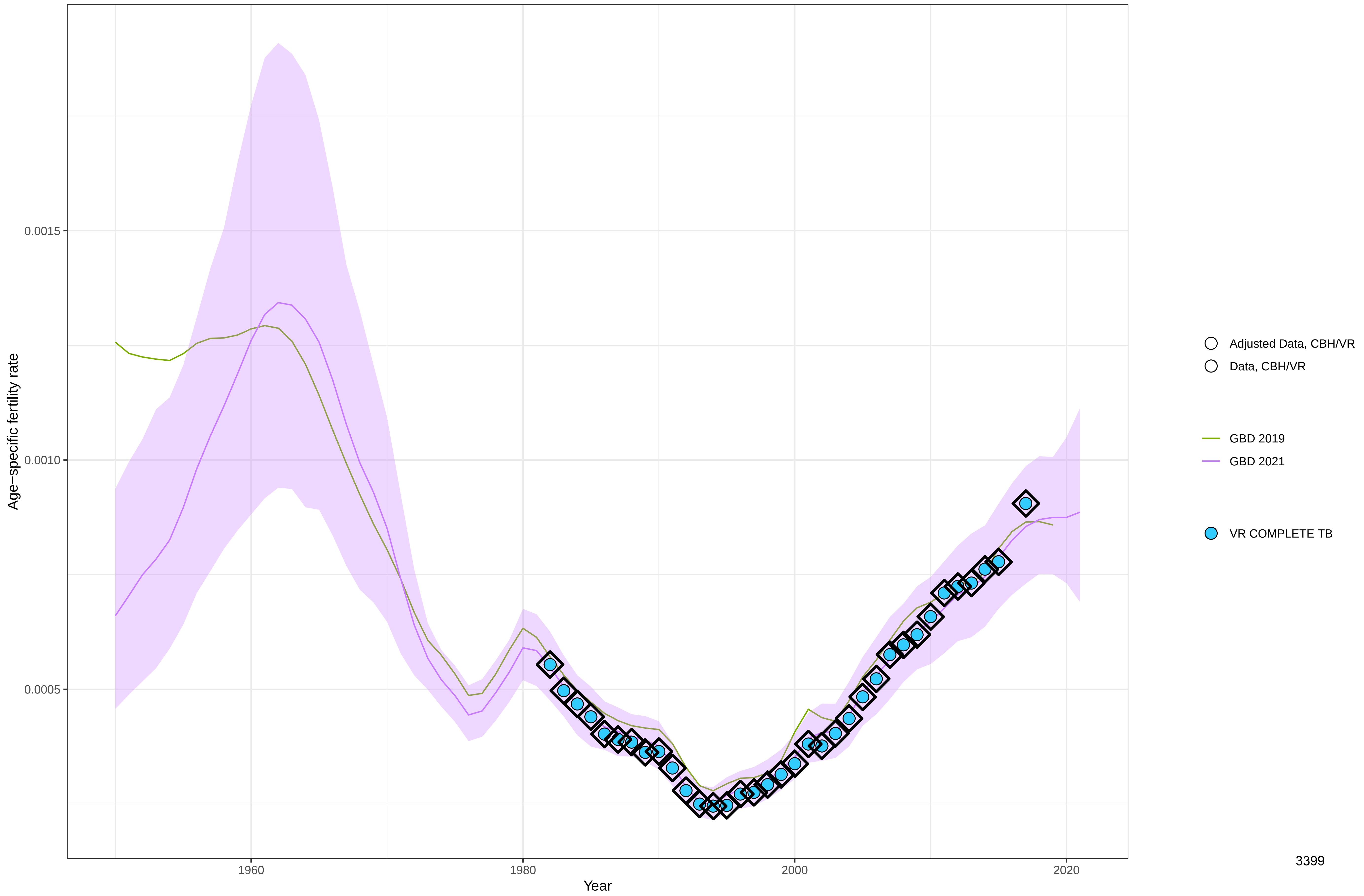

Age−specific fertility fitted trend lines, 1950−2021: Wolverhampton, Ages: 50−54
Data points enclosed with a diamond are reference sources

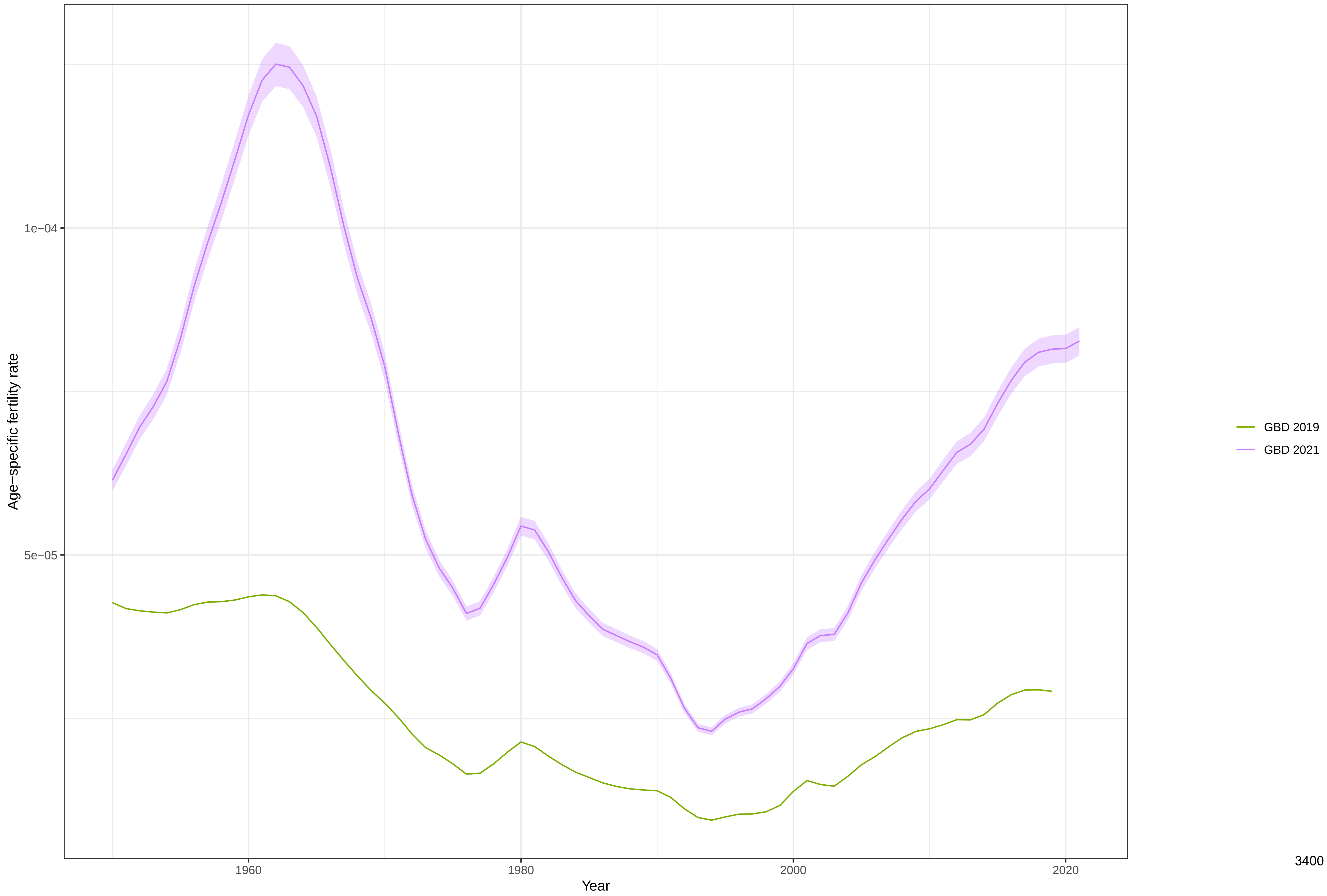

Total fertility rate fitted trend lines, 1950−2021: Wolverhampton TFR
Data points enclosed with a diamond are reference sources

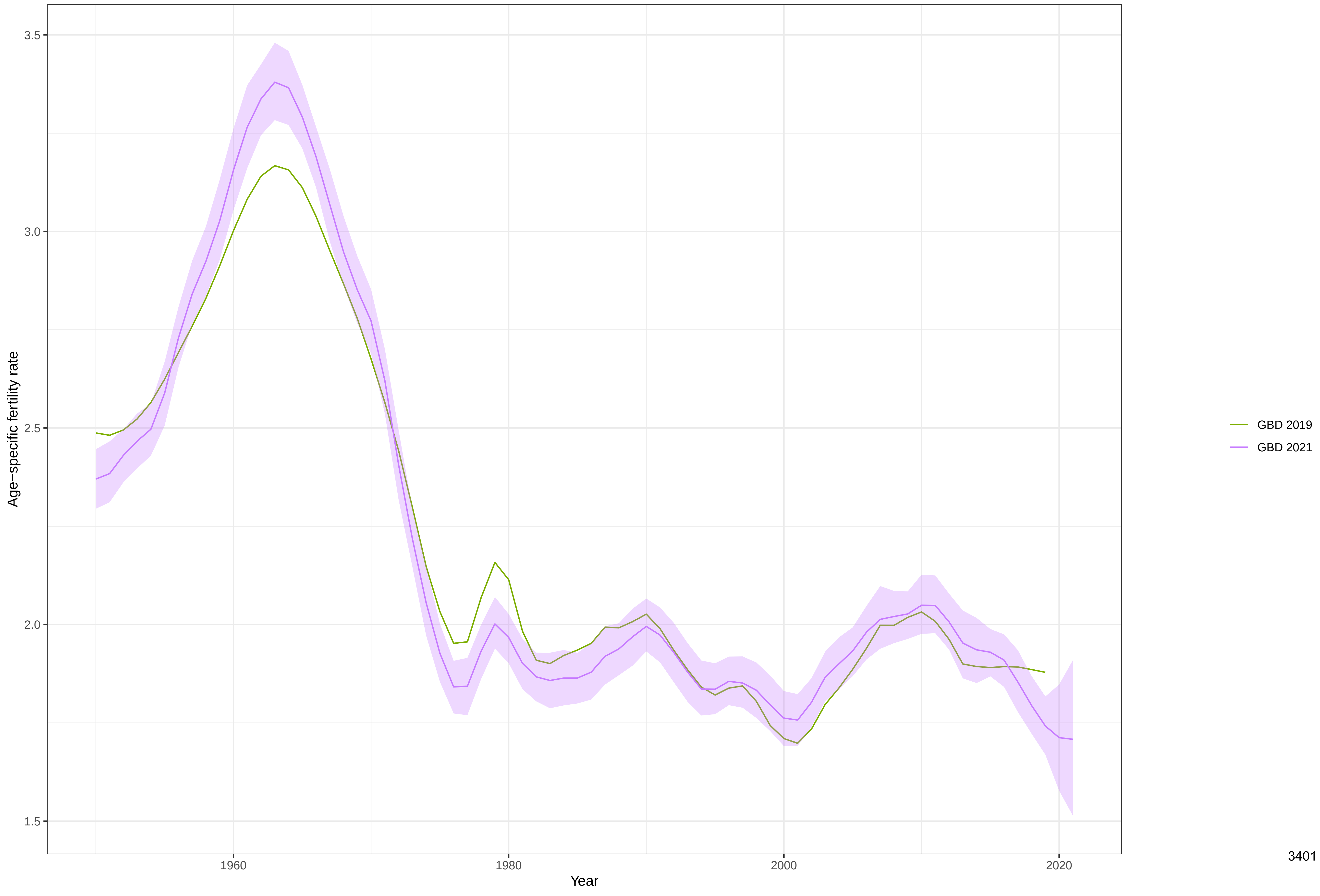

3401

Age−specific fertility fitted trend lines, 1950−2021: Worcestershire, Ages: 10−14
Data points enclosed with a diamond are reference sources

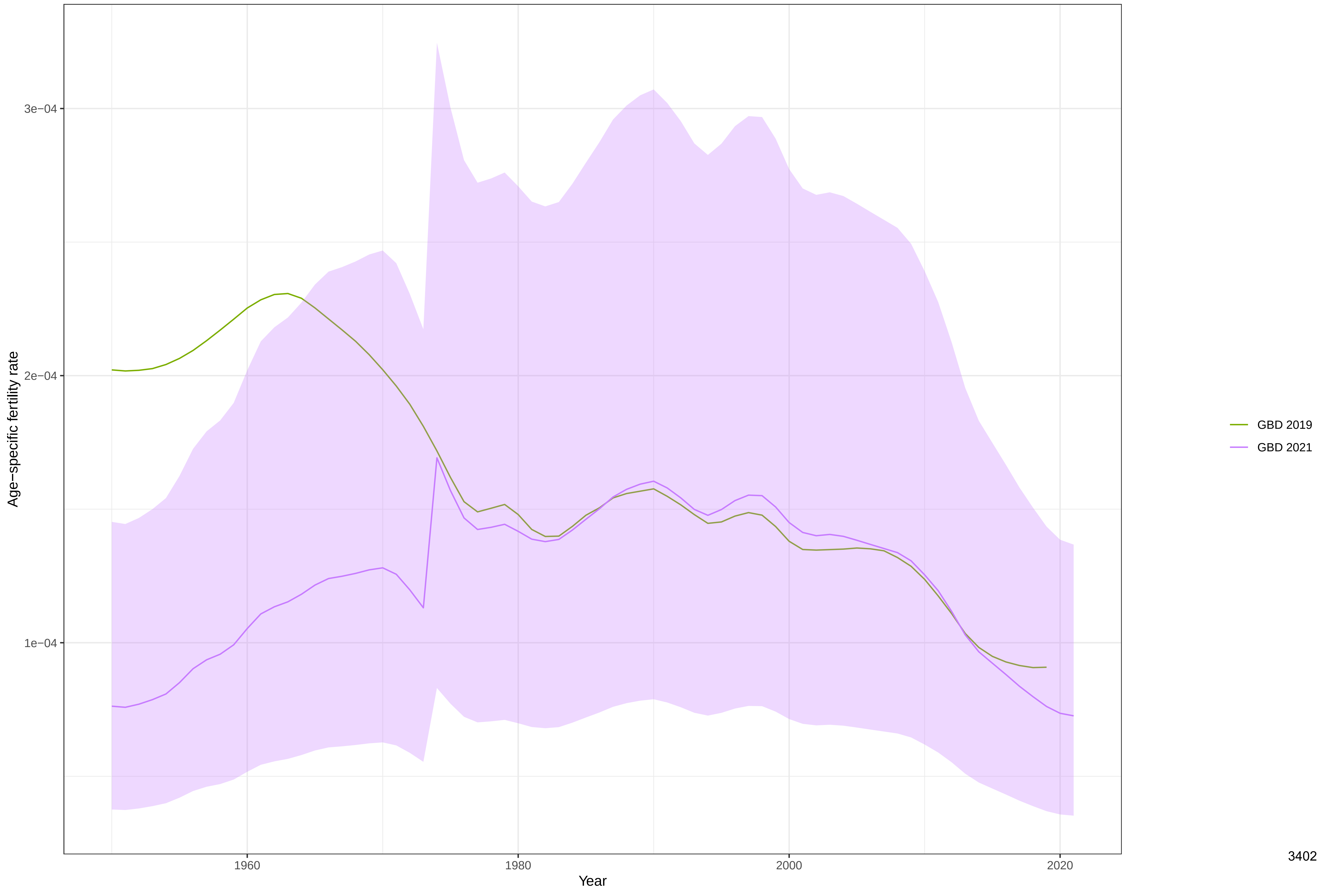

Age−specific fertility fitted trend lines, 1950−2021: Worcestershire, Ages: 15−19
Data points enclosed with a diamond are reference sources

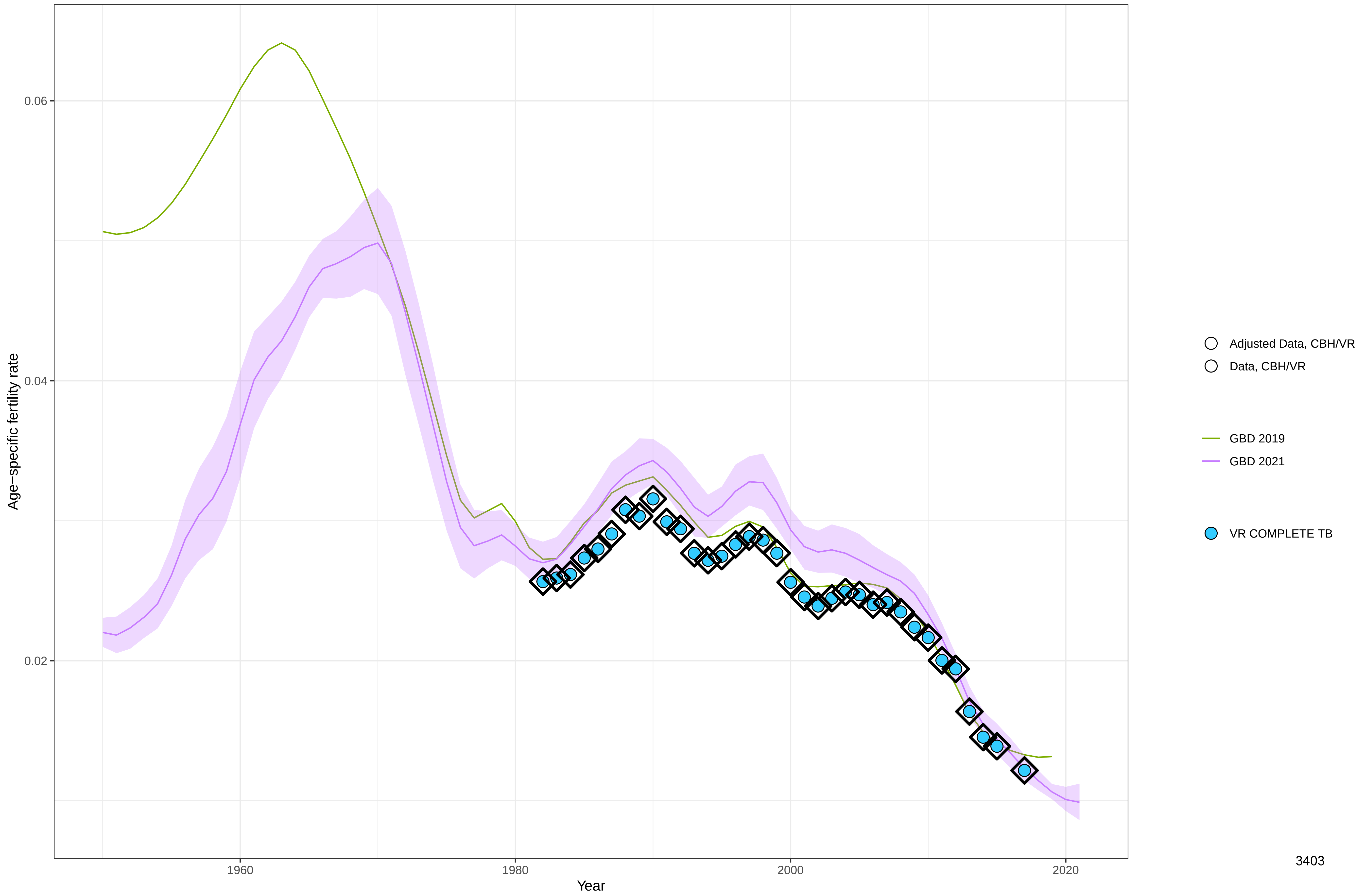

Age−specific fertility fitted trend lines, 1950−2021: Worcestershire, Ages: 20−24
Data points enclosed with a diamond are reference sources

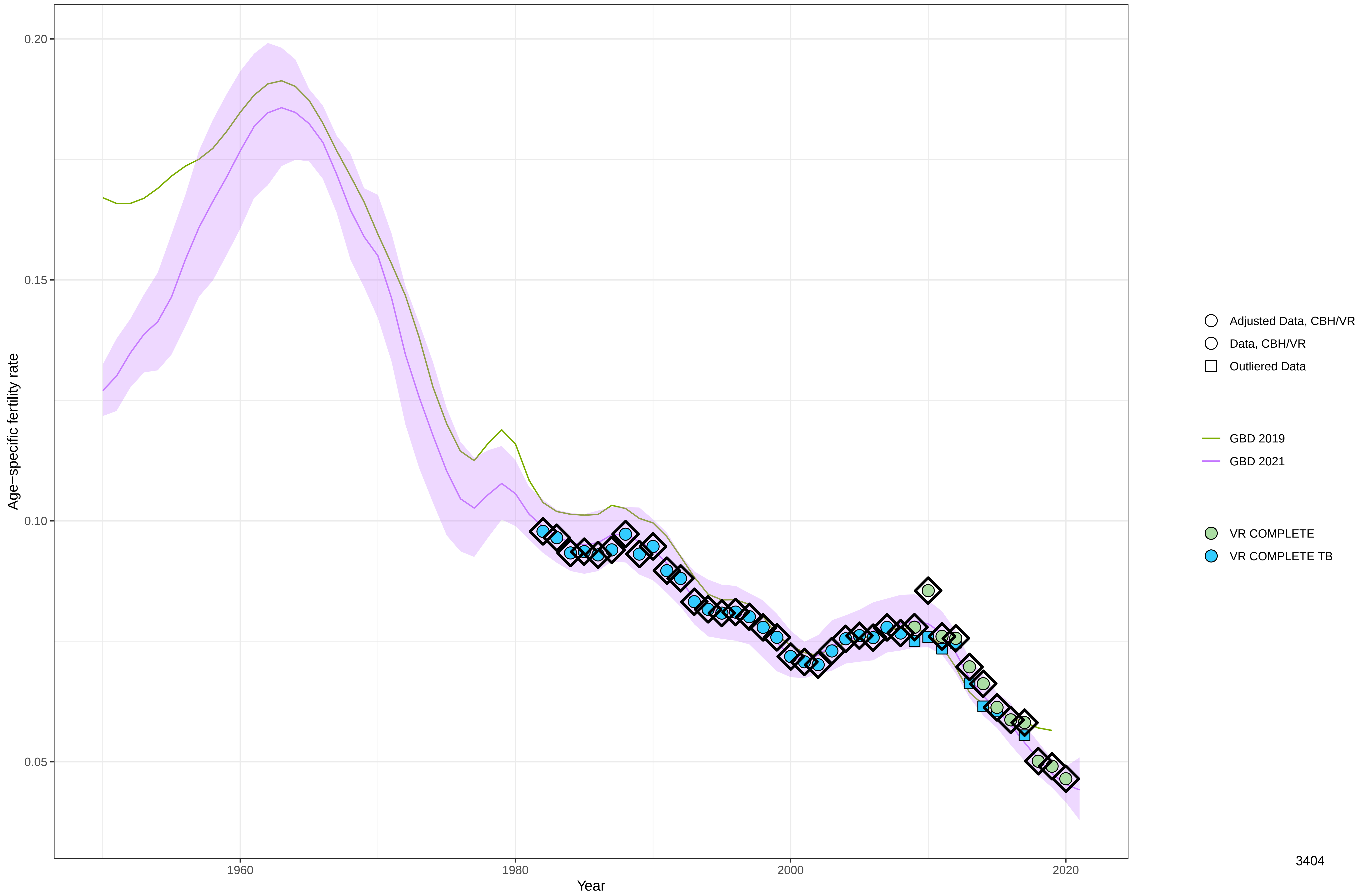

Age−specific fertility fitted trend lines, 1950−2021: Worcestershire, Ages: 25−29
Data points enclosed with a diamond are reference sources

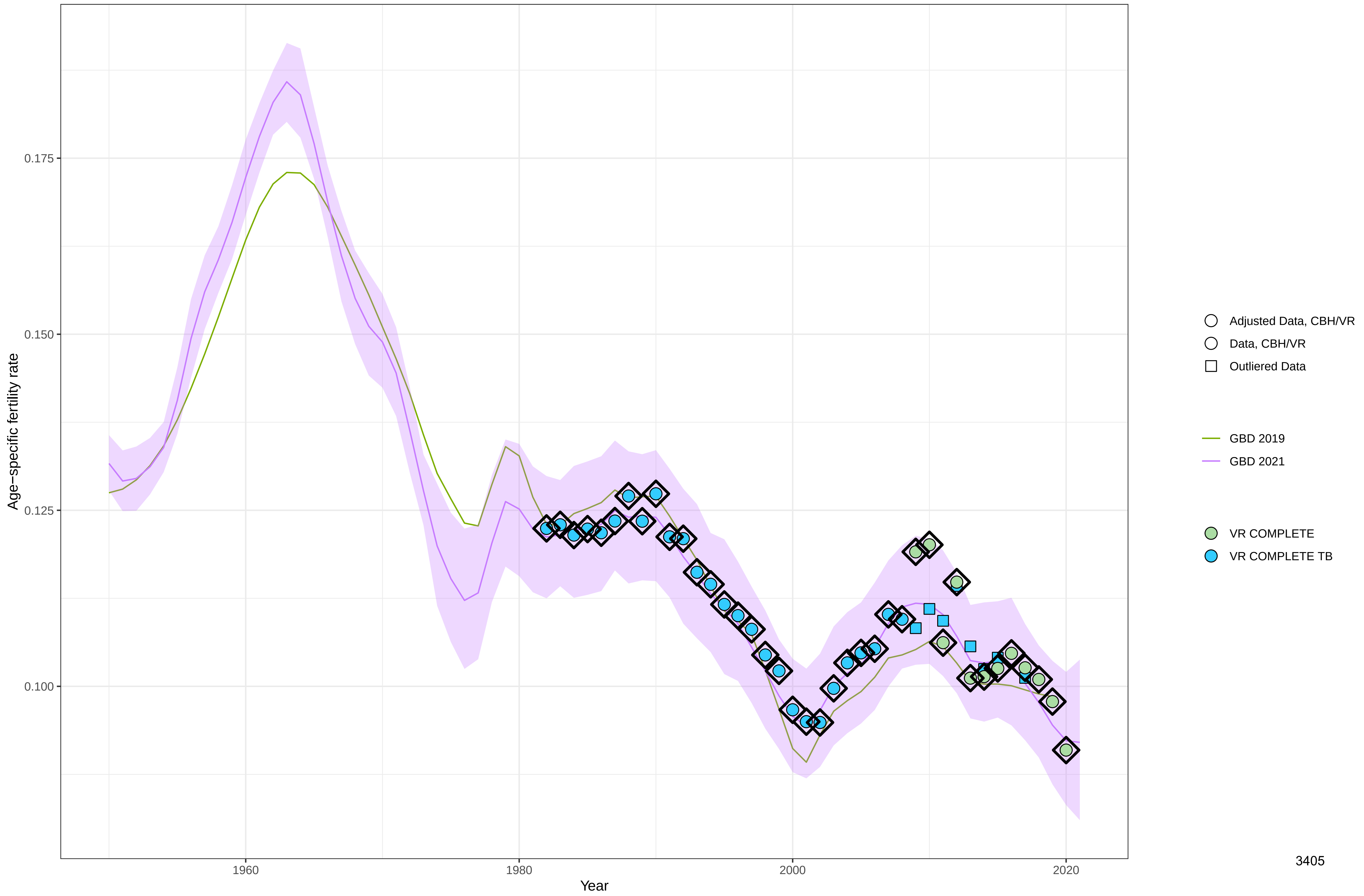

Age−specific fertility fitted trend lines, 1950−2021: Worcestershire, Ages: 30−34
Data points enclosed with a diamond are reference sources

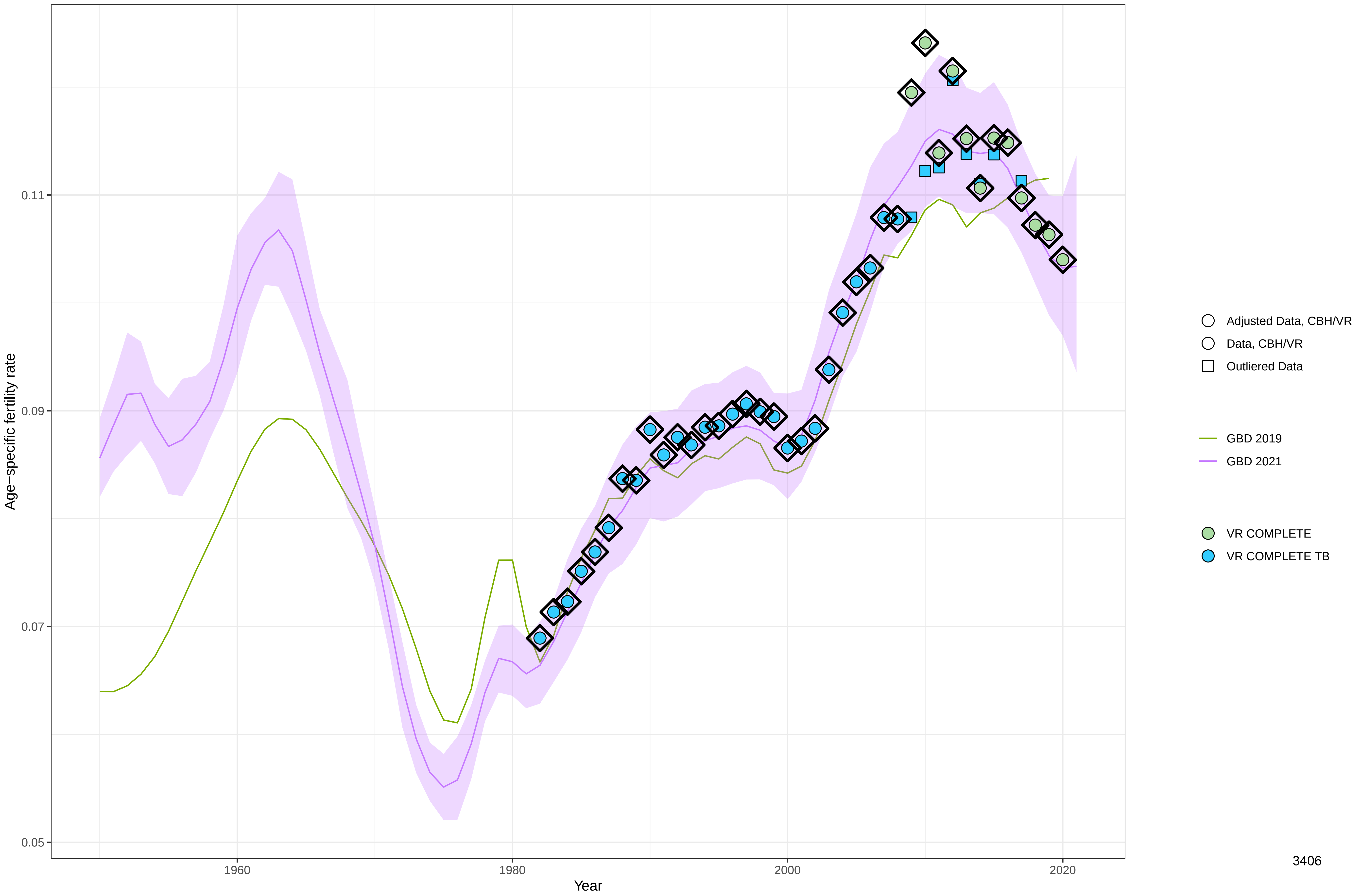

Age−specific fertility fitted trend lines, 1950−2021: Worcestershire, Ages: 35−39
Data points enclosed with a diamond are reference sources

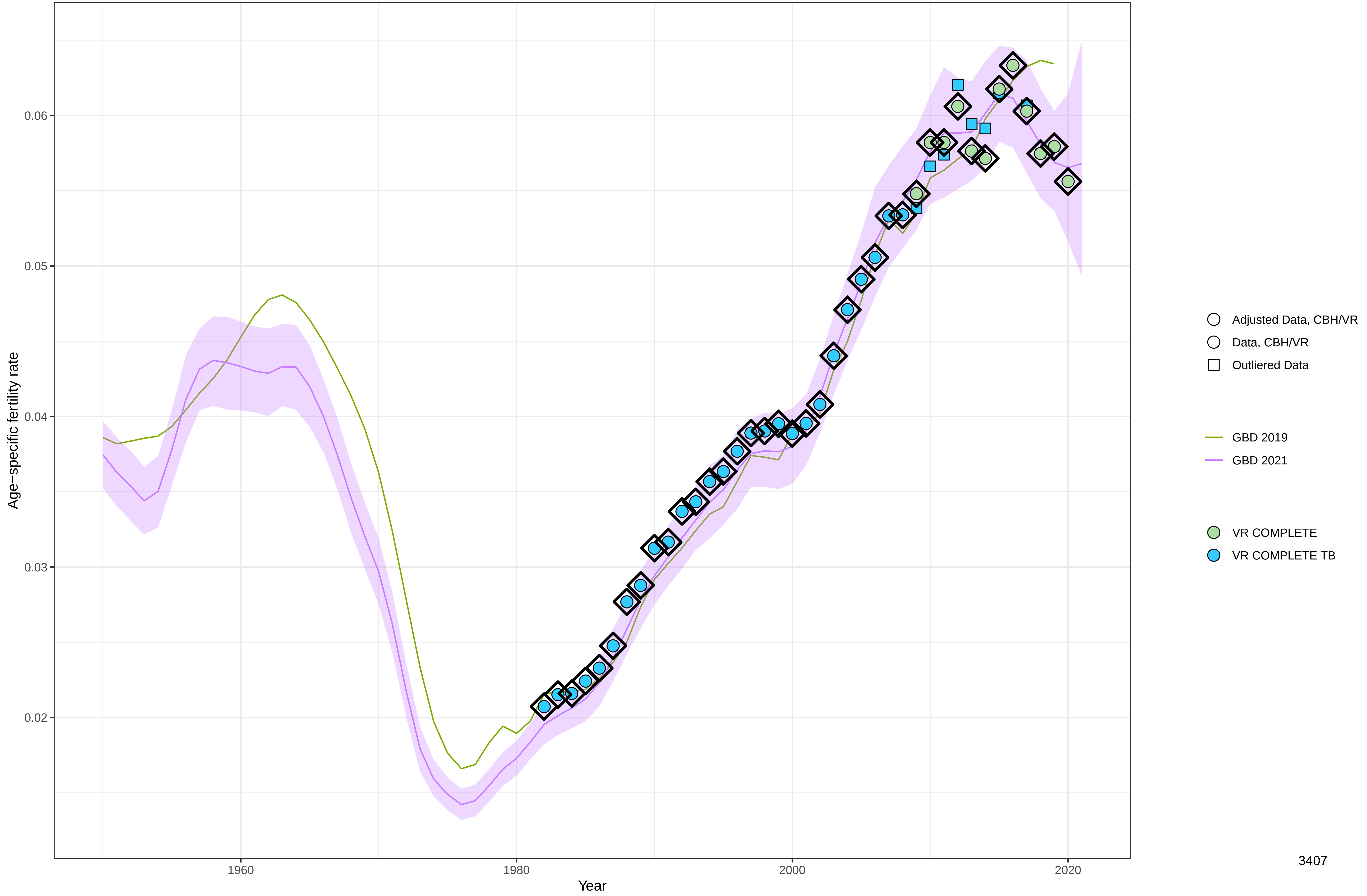

Age−specific fertility fitted trend lines, 1950−2021: Worcestershire, Ages: 40−44
Data points enclosed with a diamond are reference sources

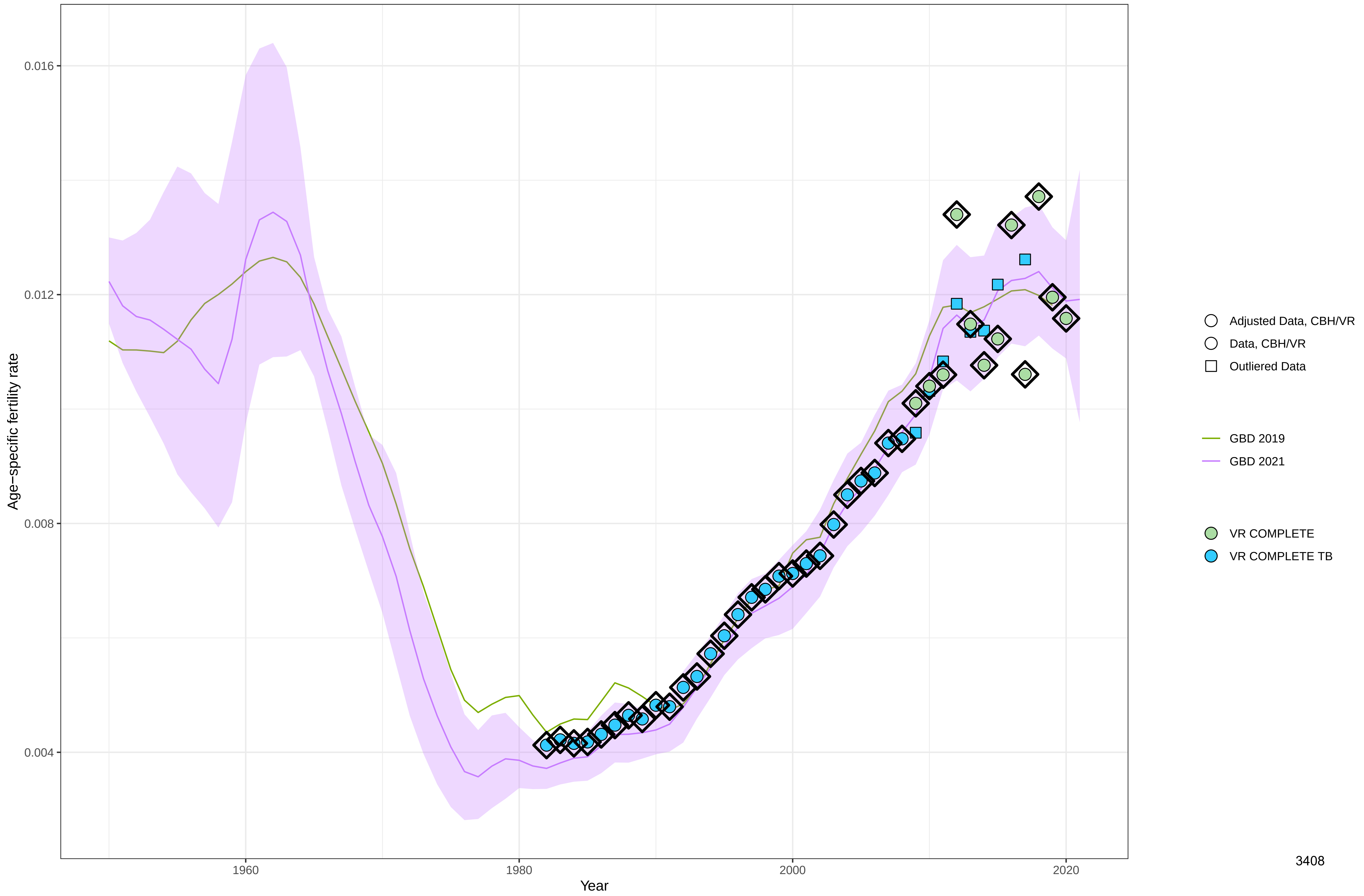

Age−specific fertility fitted trend lines, 1950−2021: Worcestershire, Ages: 45−49
Data points enclosed with a diamond are reference sources

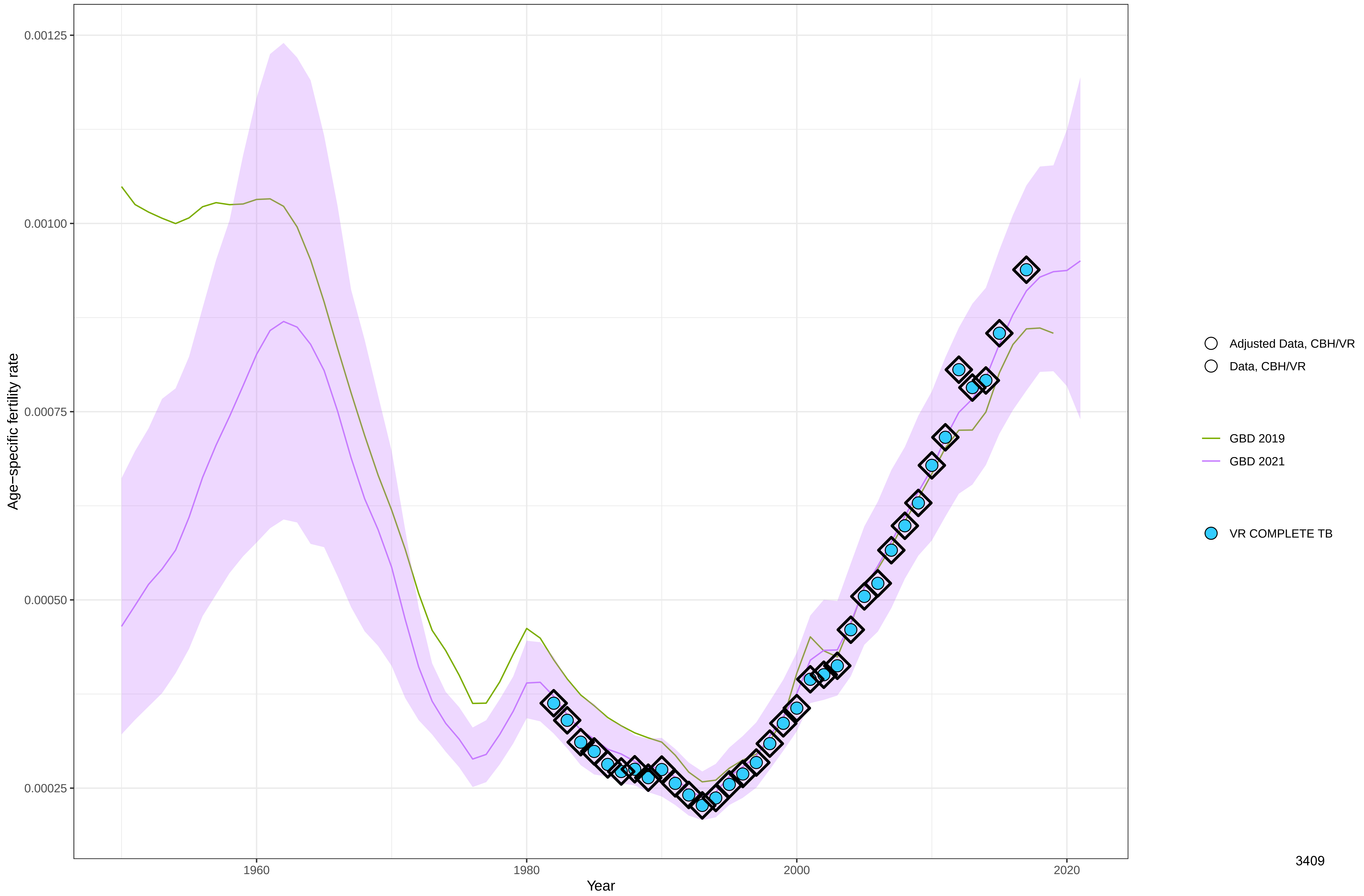

Age−specific fertility fitted trend lines, 1950−2021: Worcestershire, Ages: 50−54
Data points enclosed with a diamond are reference sources

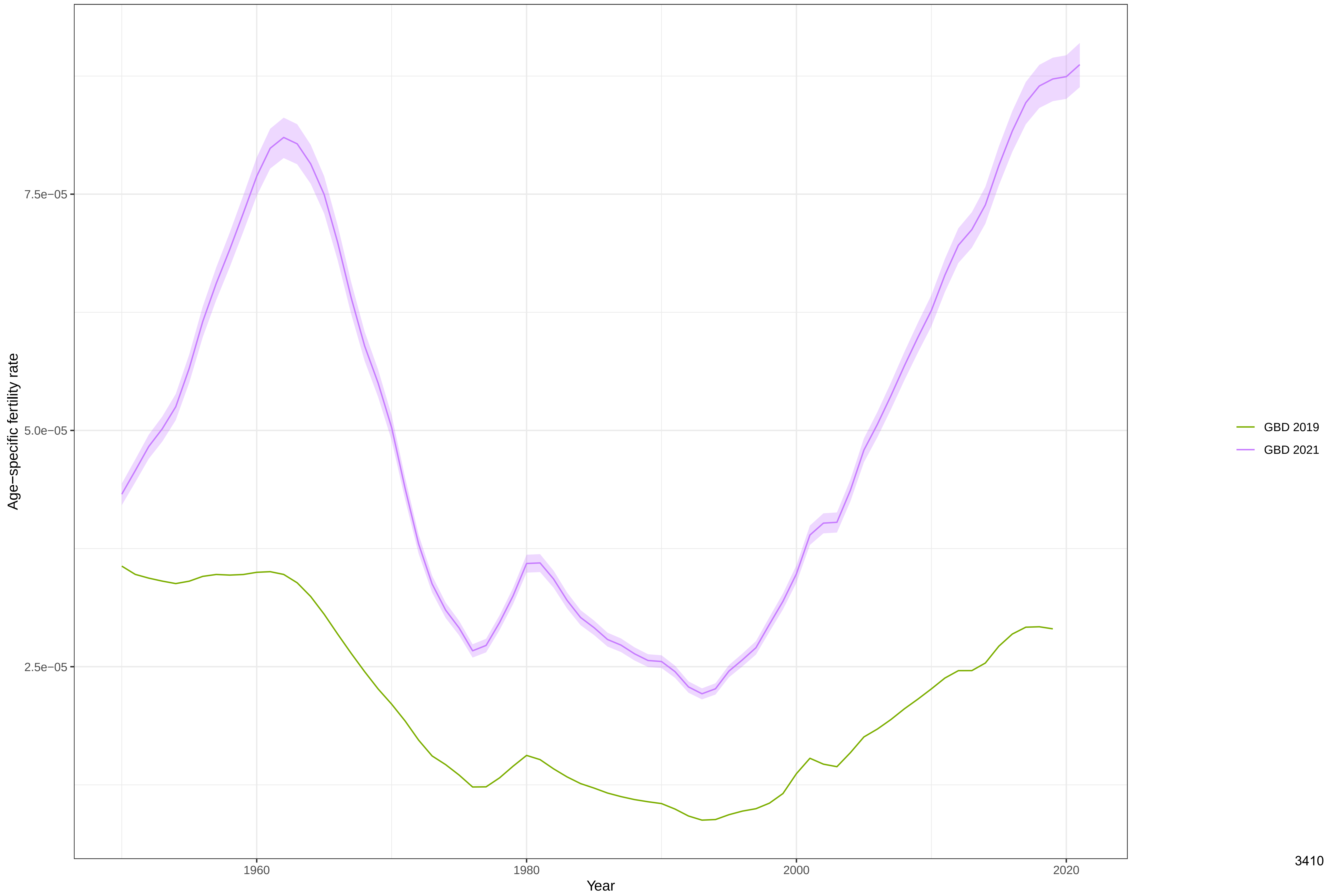

Total fertility rate fitted trend lines, 1950−2021: Worcestershire TFR
Data points enclosed with a diamond are reference sources

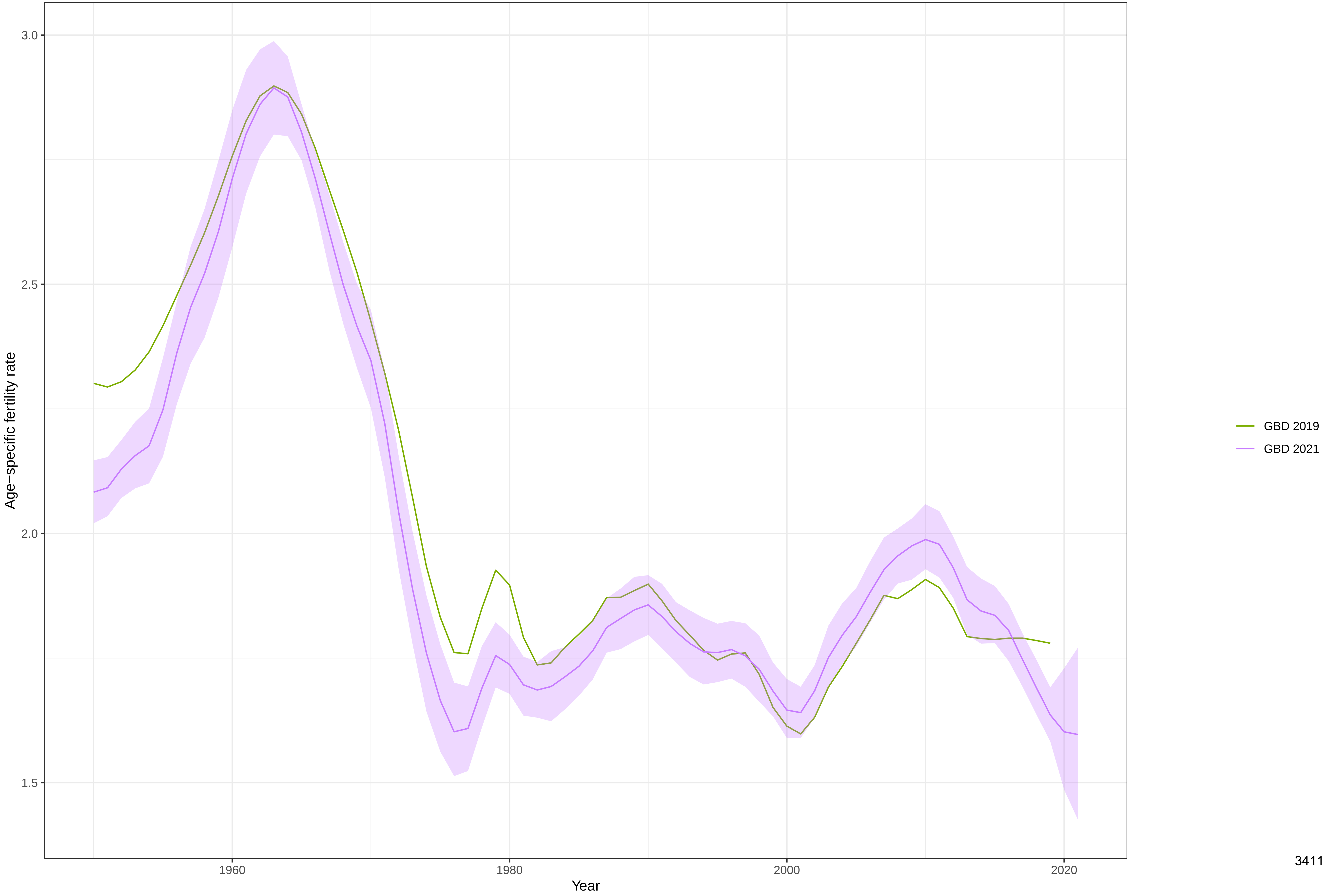

Age−specific fertility fitted trend lines, 1950−2021: Yorkshire and the Humber, Ages: 10−14
Data points enclosed with a diamond are reference sources

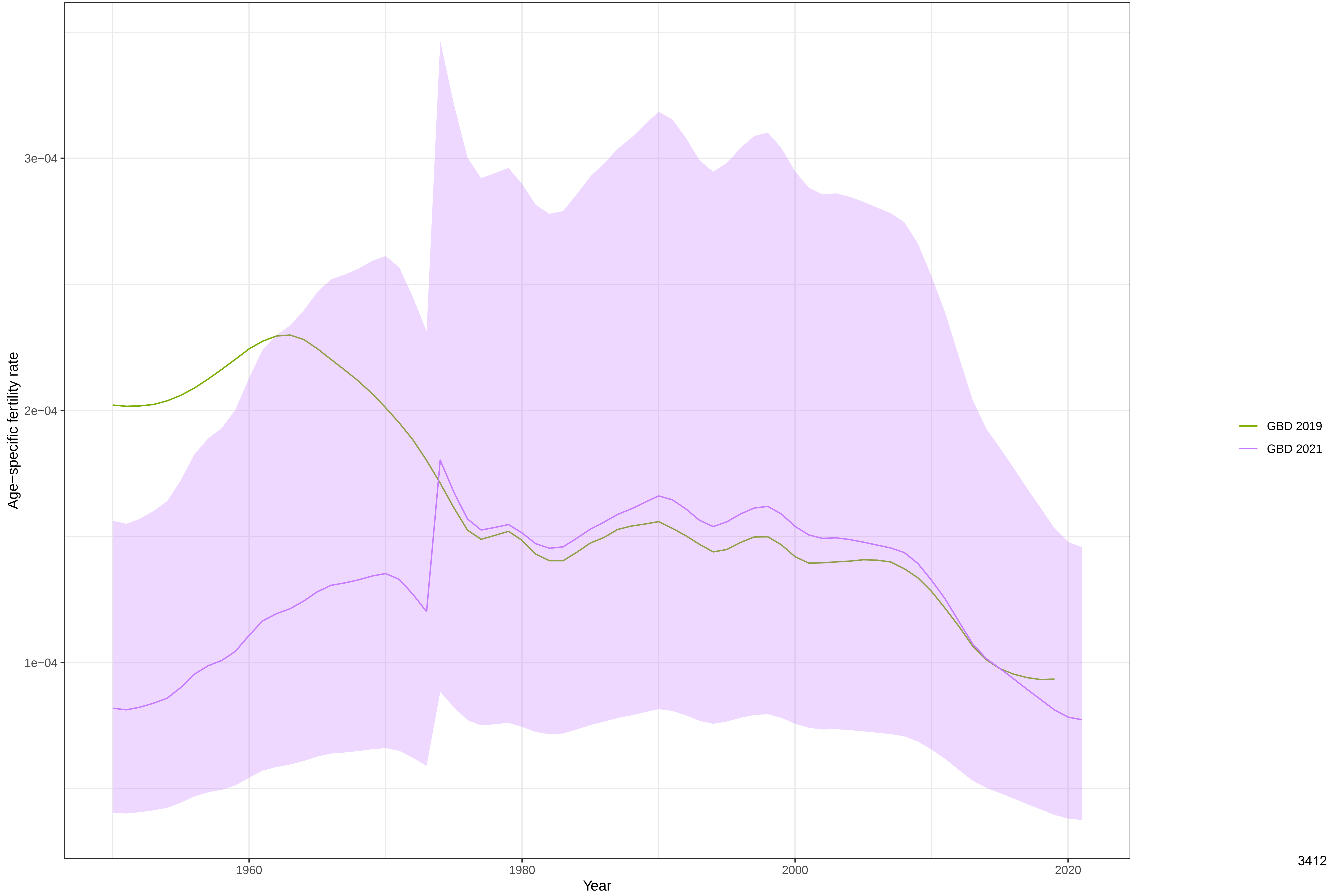

Age−specific fertility fitted trend lines, 1950−2021: Yorkshire and the Humber, Ages: 15−19
Data points enclosed with a diamond are reference sources

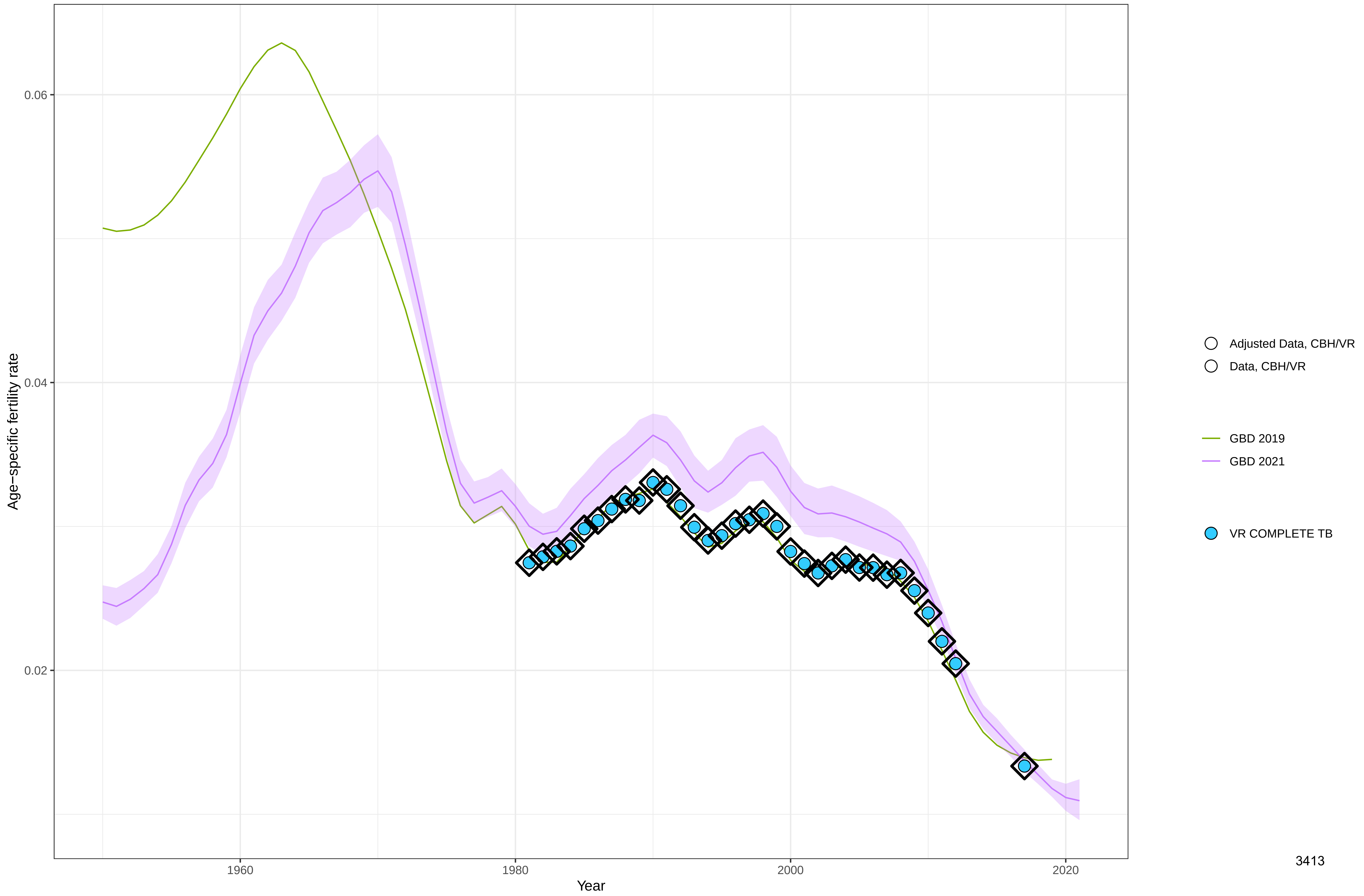

Age−specific fertility fitted trend lines, 1950−2021: Yorkshire and the Humber, Ages: 20−24
Data points enclosed with a diamond are reference sources

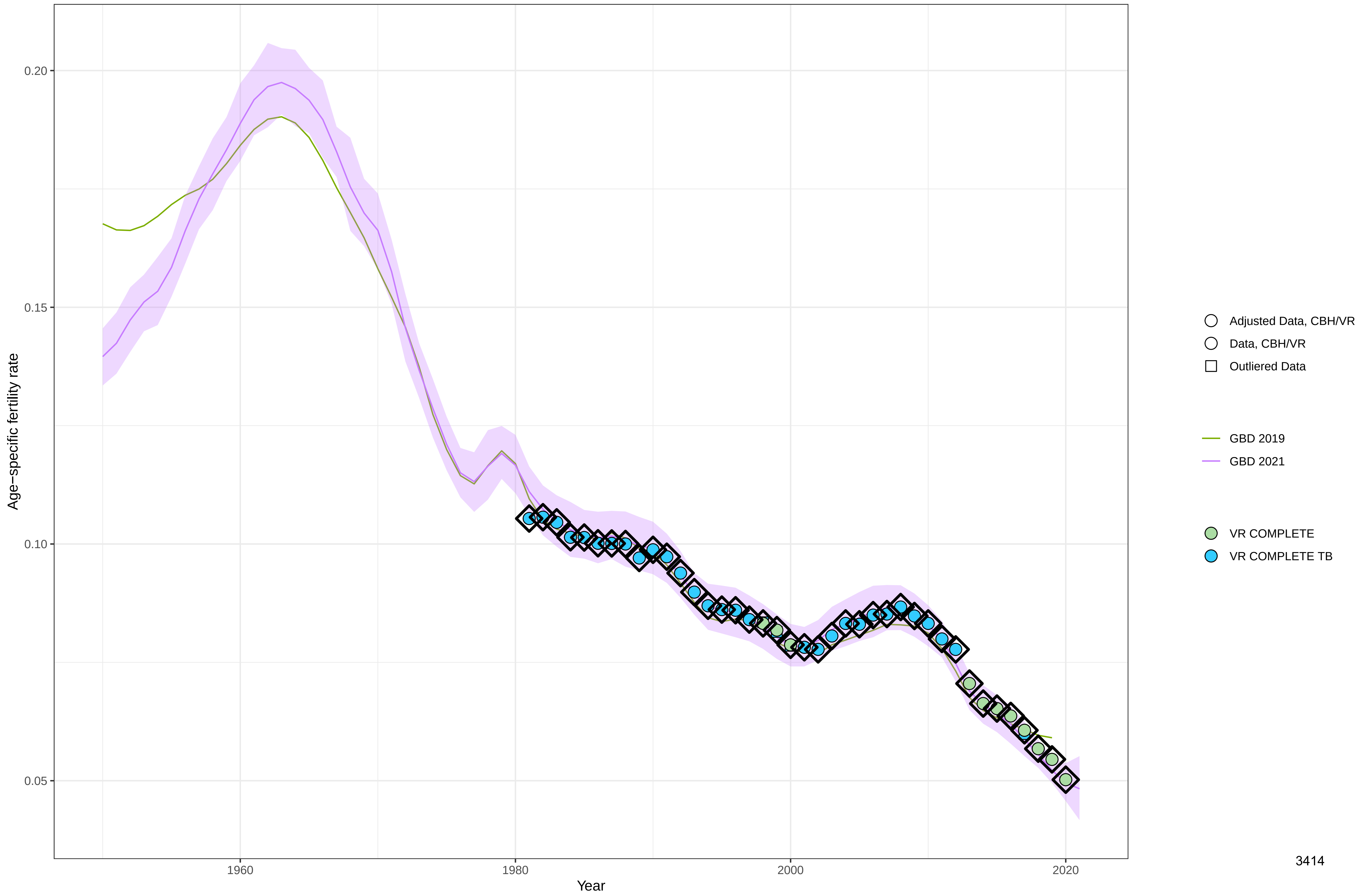

Age−specific fertility fitted trend lines, 1950−2021: Yorkshire and the Humber, Ages: 25−29
Data points enclosed with a diamond are reference sources

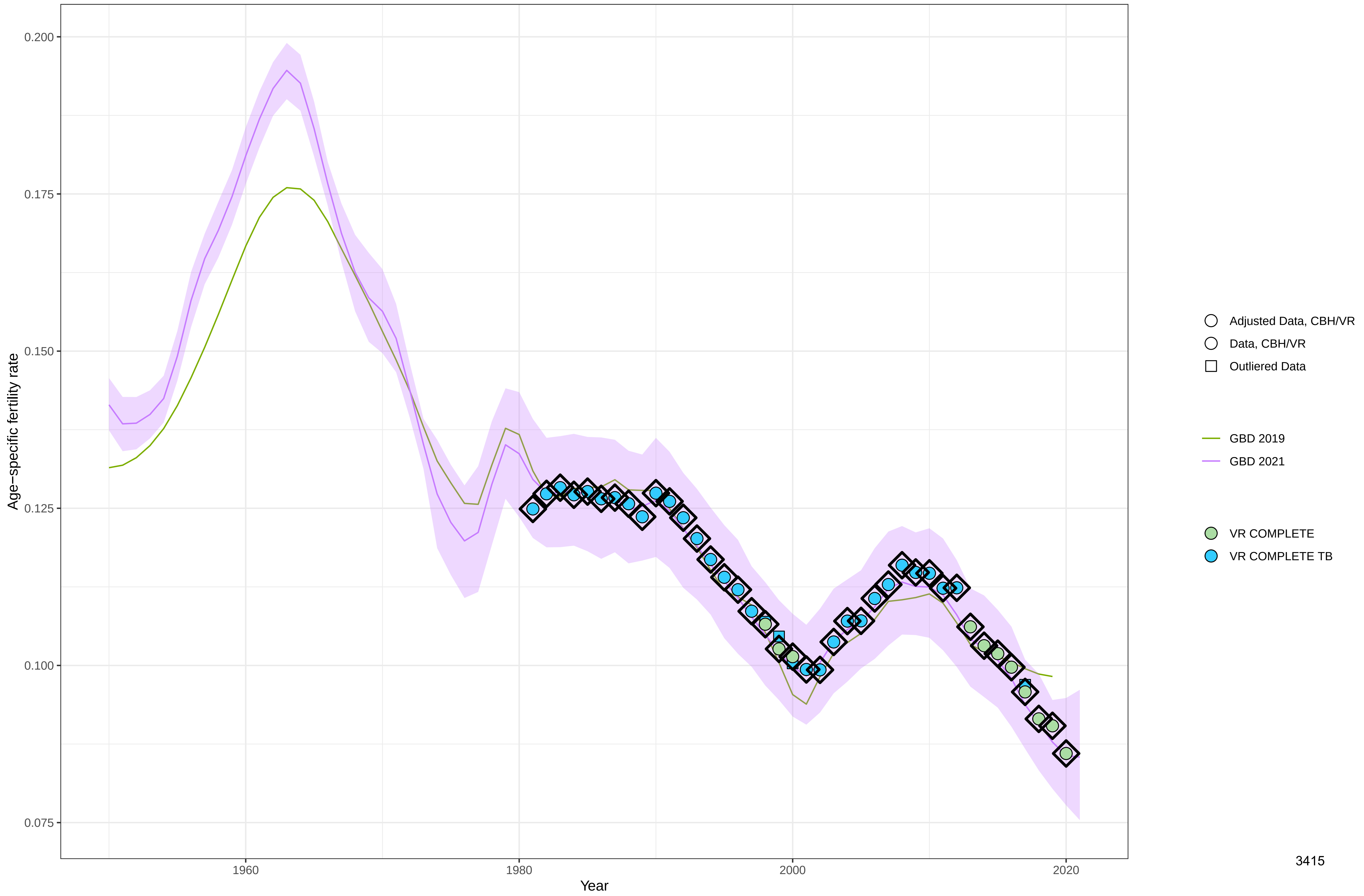

Age−specific fertility fitted trend lines, 1950−2021: Yorkshire and the Humber, Ages: 30−34
Data points enclosed with a diamond are reference sources

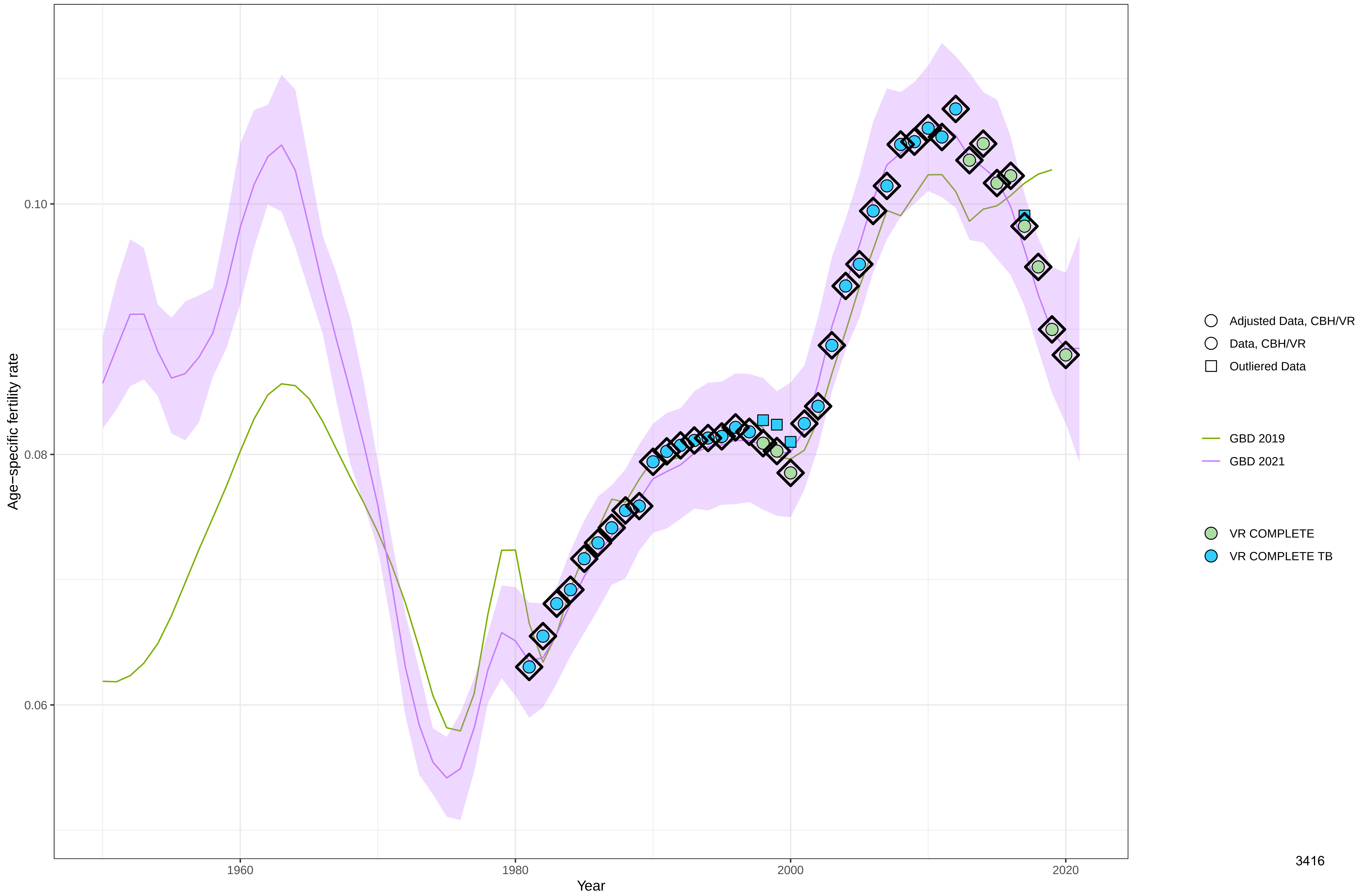

Age−specific fertility fitted trend lines, 1950−2021: Yorkshire and the Humber, Ages: 35−39
Data points enclosed with a diamond are reference sources

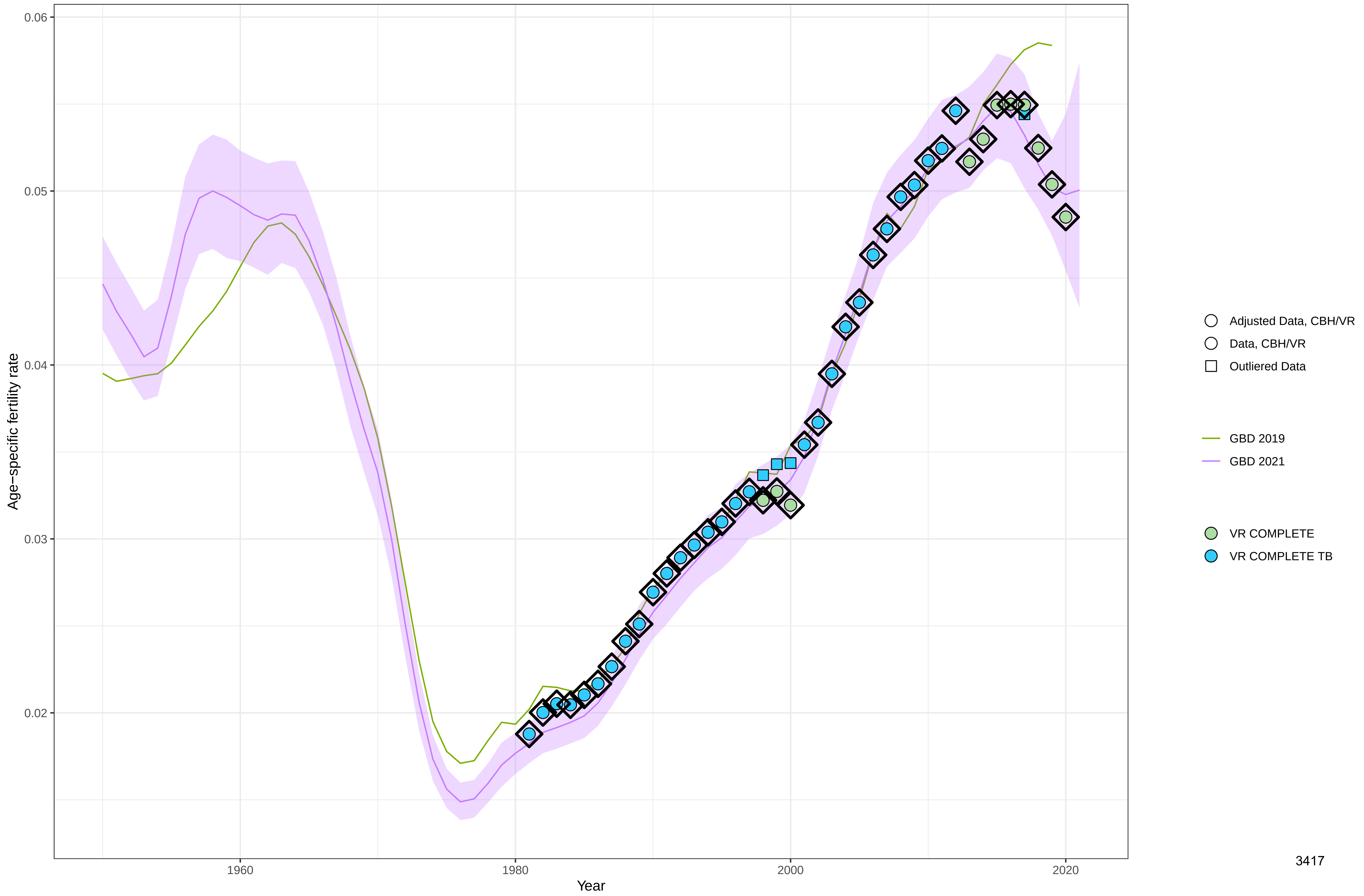

Age−specific fertility fitted trend lines, 1950−2021: Yorkshire and the Humber, Ages: 40−44
Data points enclosed with a diamond are reference sources

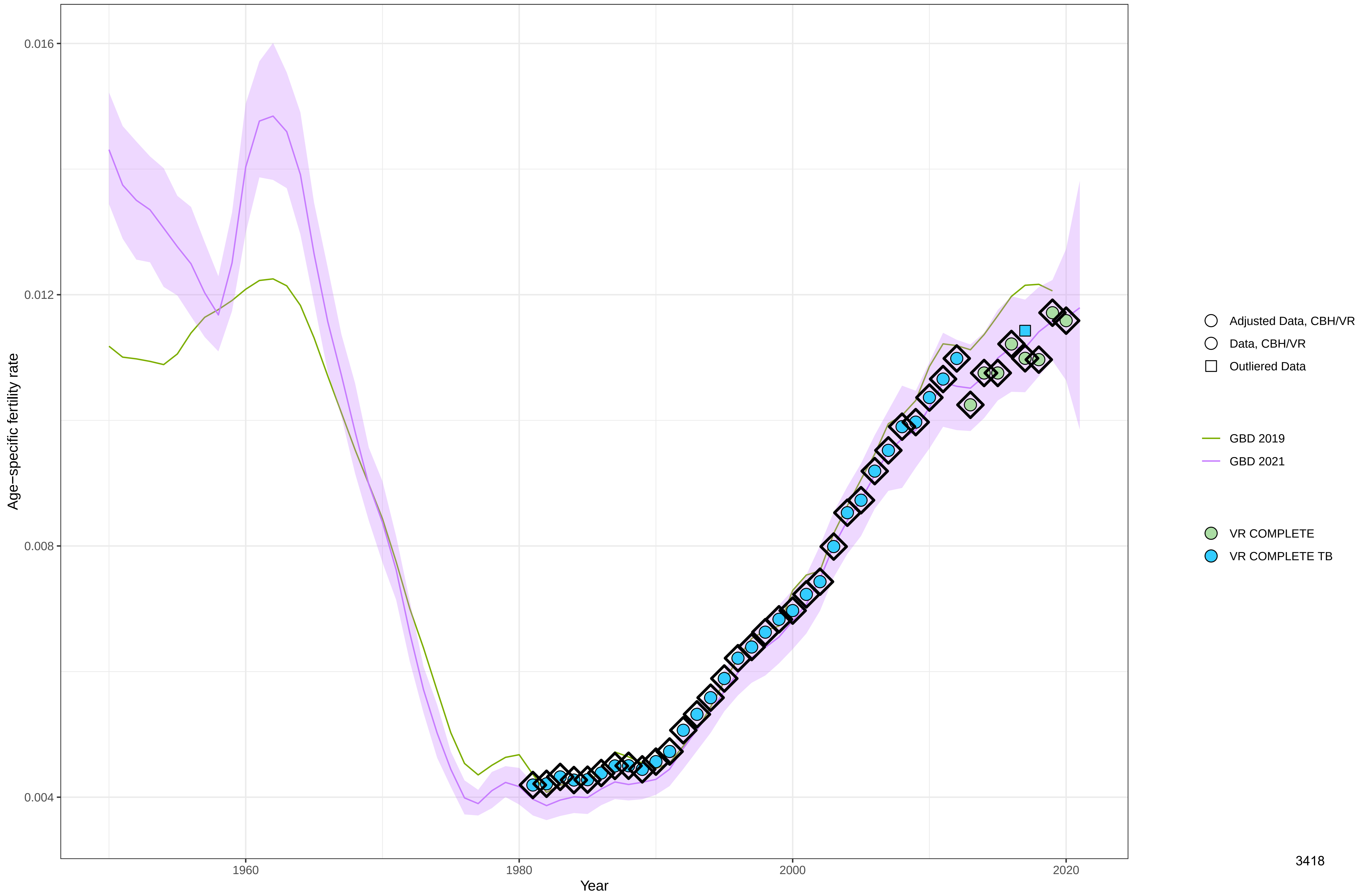

Age−specific fertility fitted trend lines, 1950−2021: Yorkshire and the Humber, Ages: 45−49
Data points enclosed with a diamond are reference sources

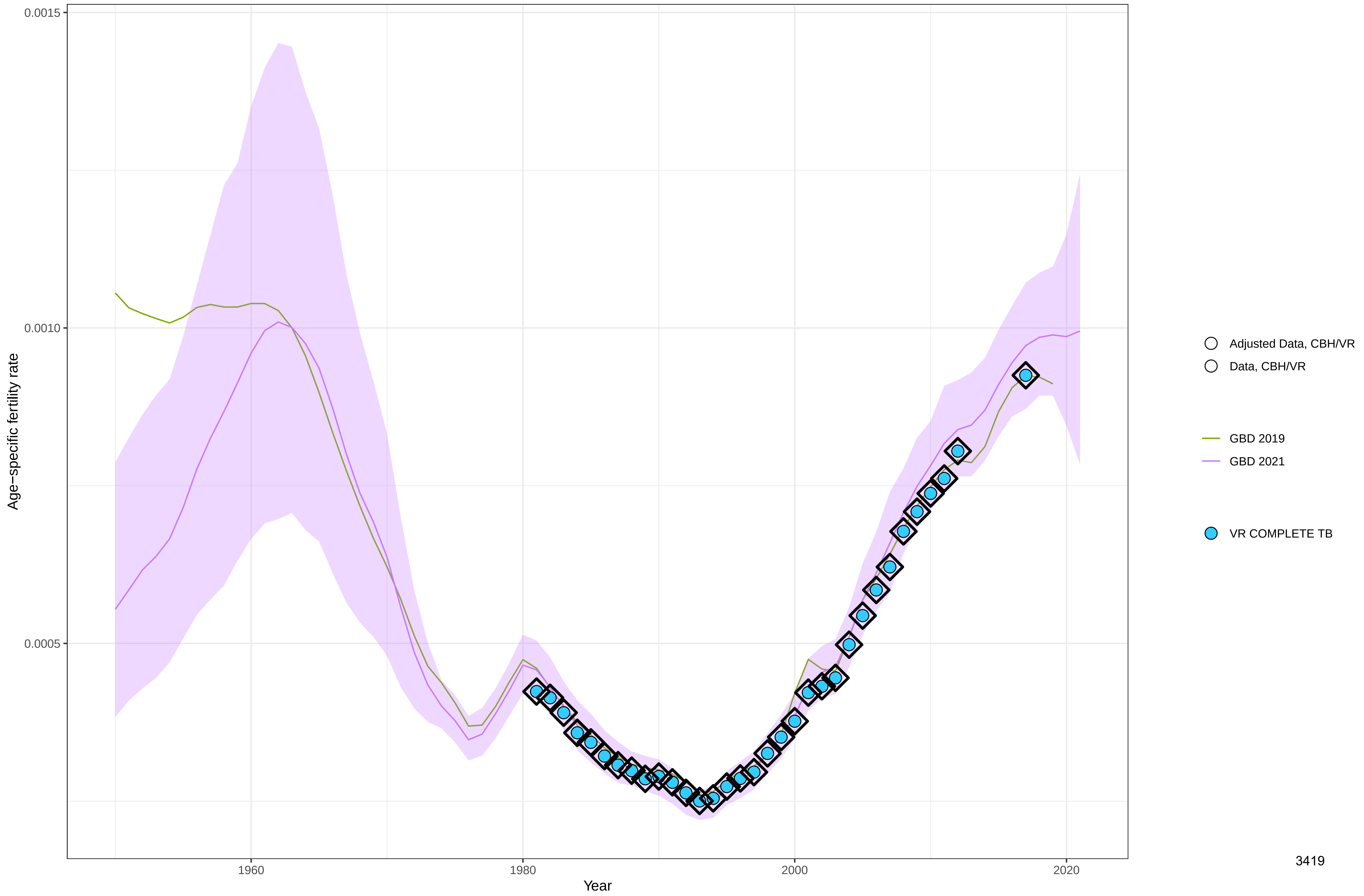

Age−specific fertility fitted trend lines, 1950−2021: Yorkshire and the Humber, Ages: 50−54
Data points enclosed with a diamond are reference sources

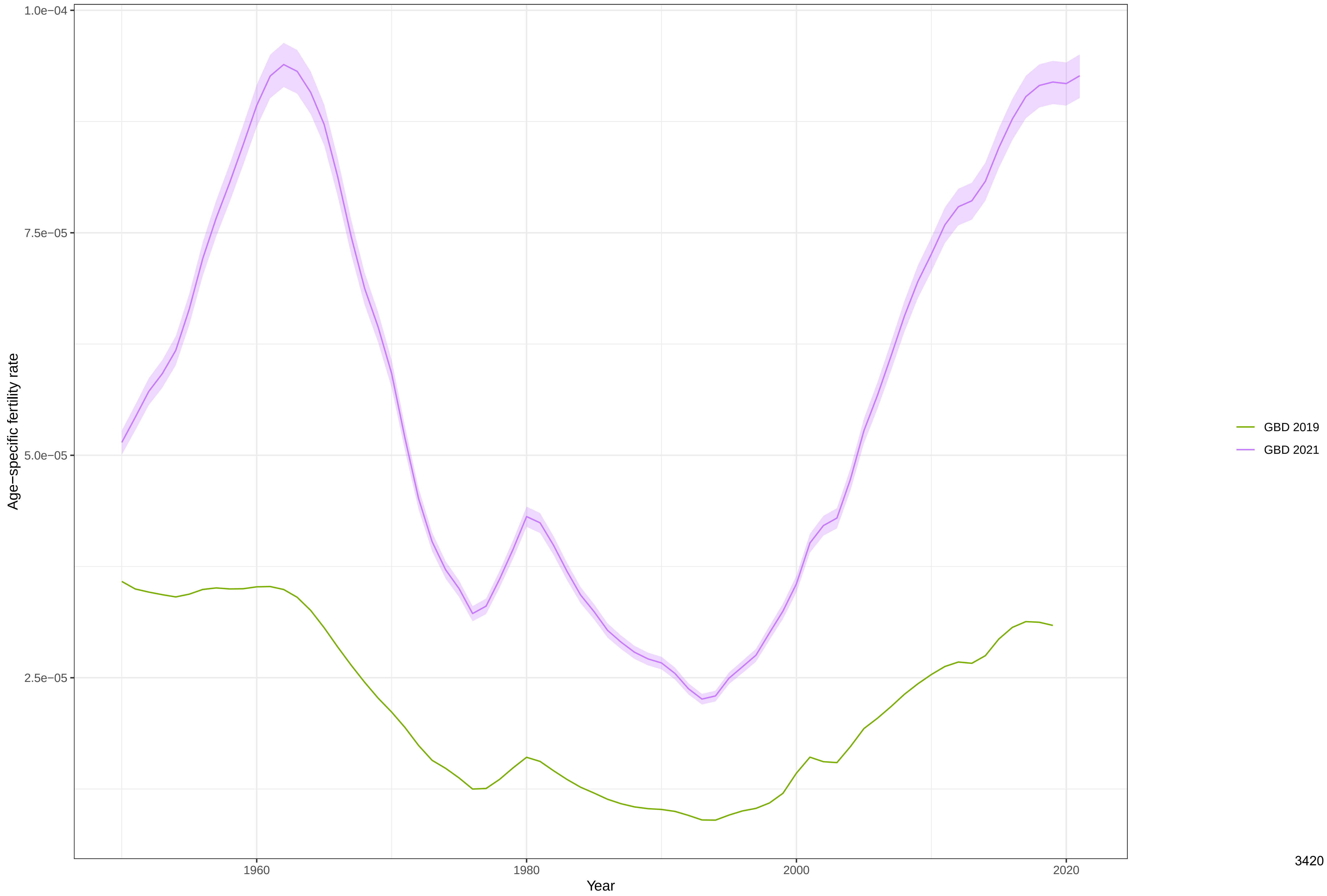

Total fertility rate fitted trend lines, 1950−2021: Yorkshire and the Humber TFR
Data points enclosed with a diamond are reference sources

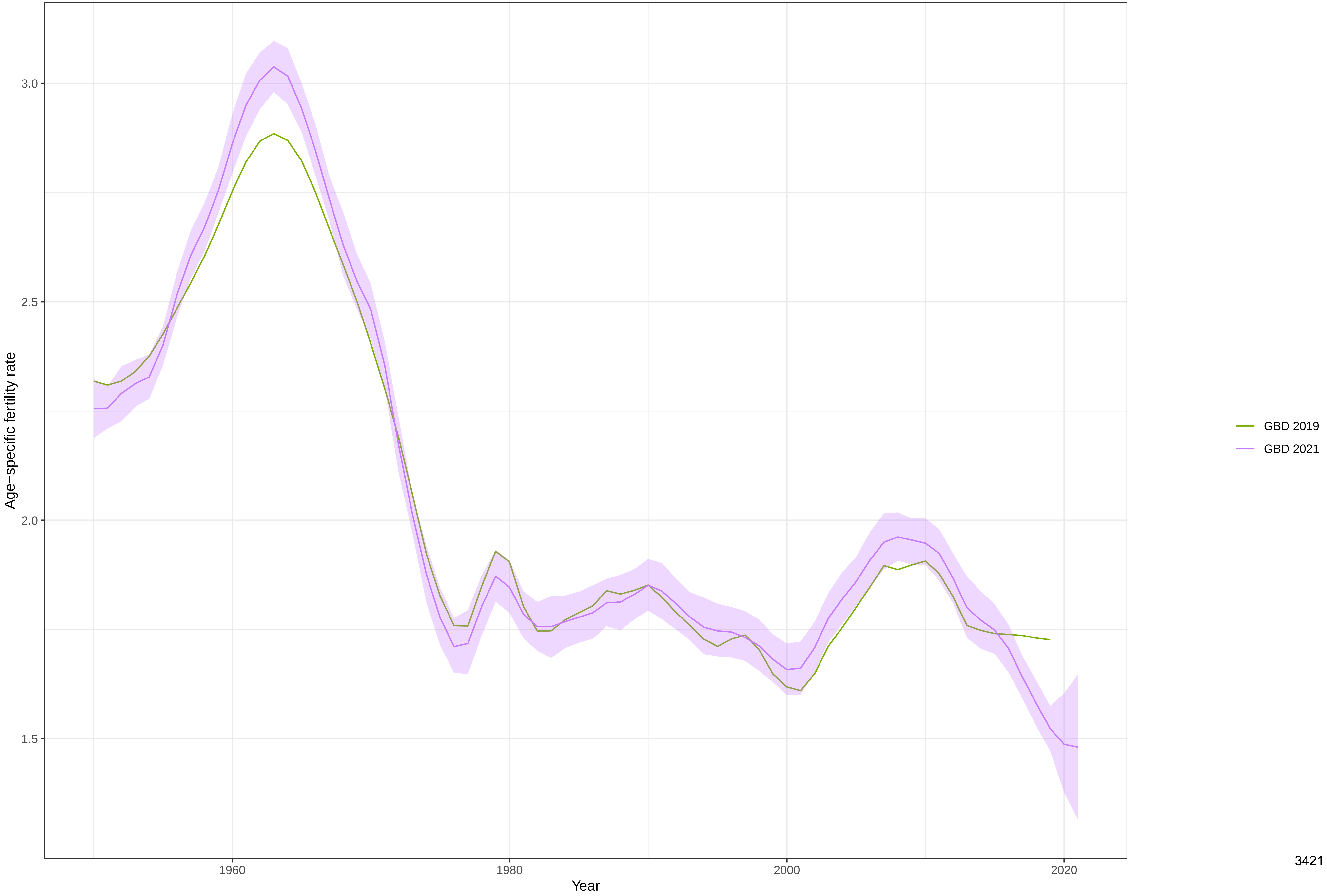

Age−specific fertility fitted trend lines, 1950−2021: Barnsley, Ages: 10−14
Data points enclosed with a diamond are reference sources

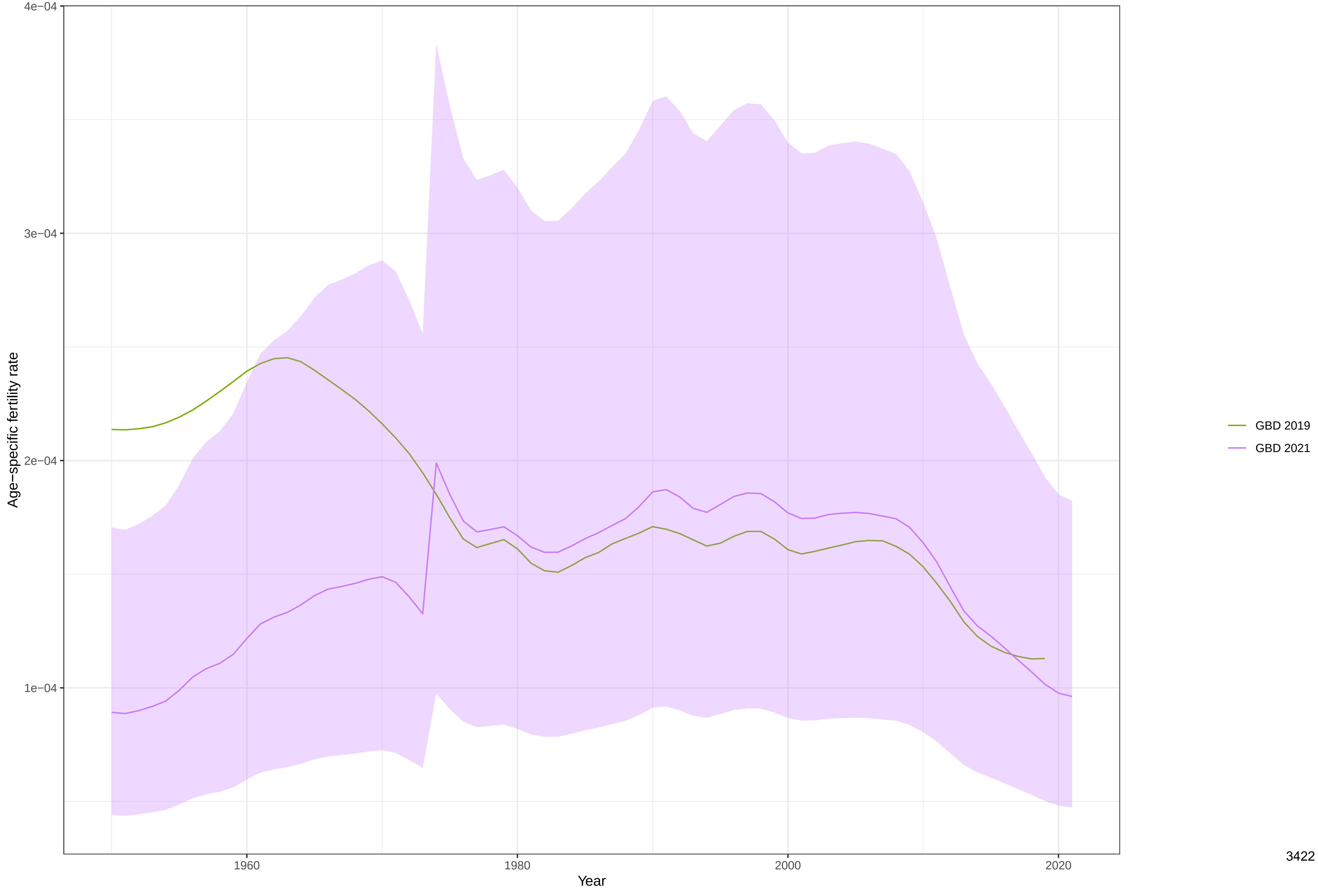

3422

Age−specific fertility fitted trend lines, 1950−2021: Barnsley, Ages: 15−19
Data points enclosed with a diamond are reference sources

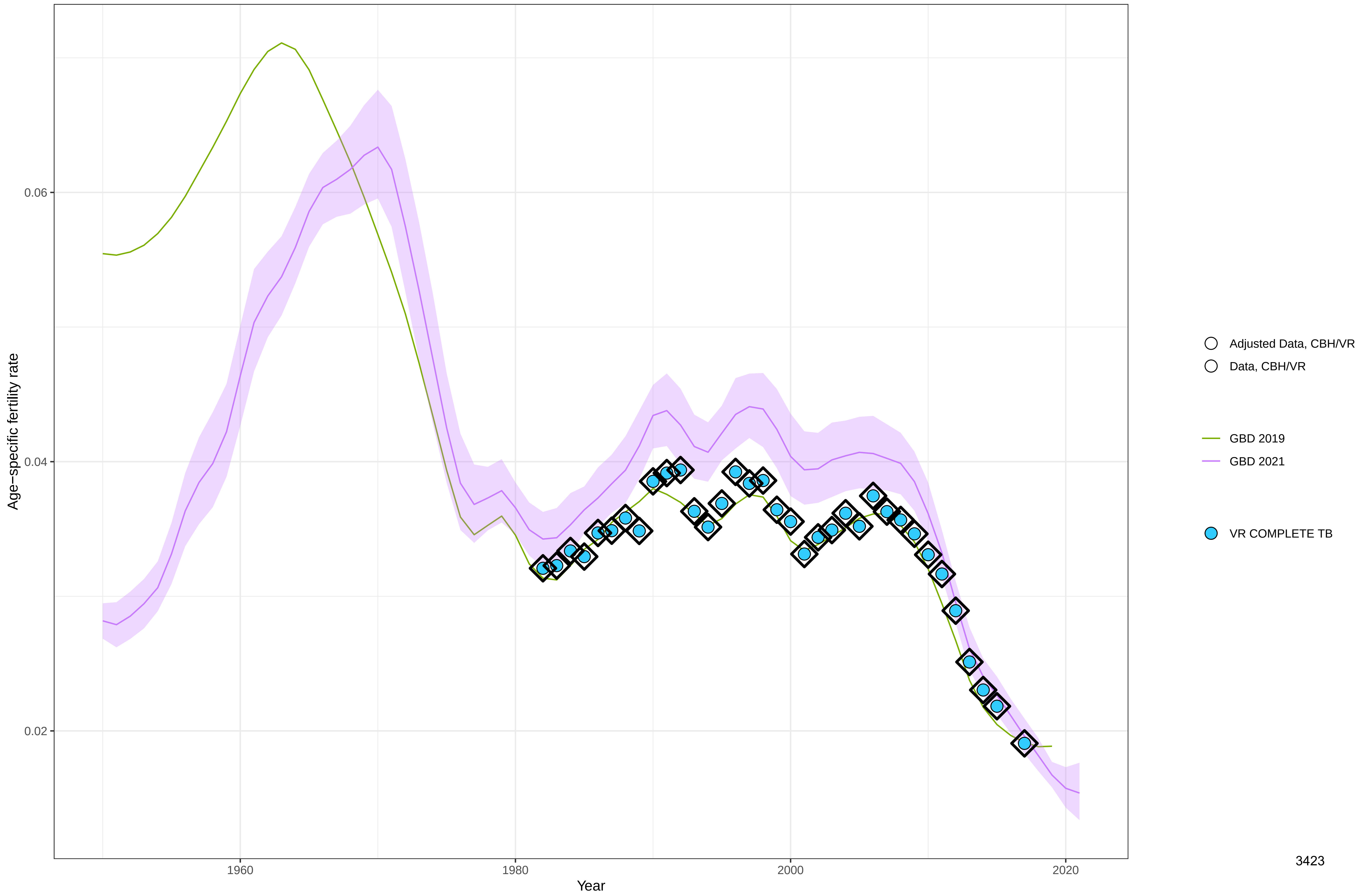

Age−specific fertility fitted trend lines, 1950−2021: Barnsley, Ages: 20−24
Data points enclosed with a diamond are reference sources

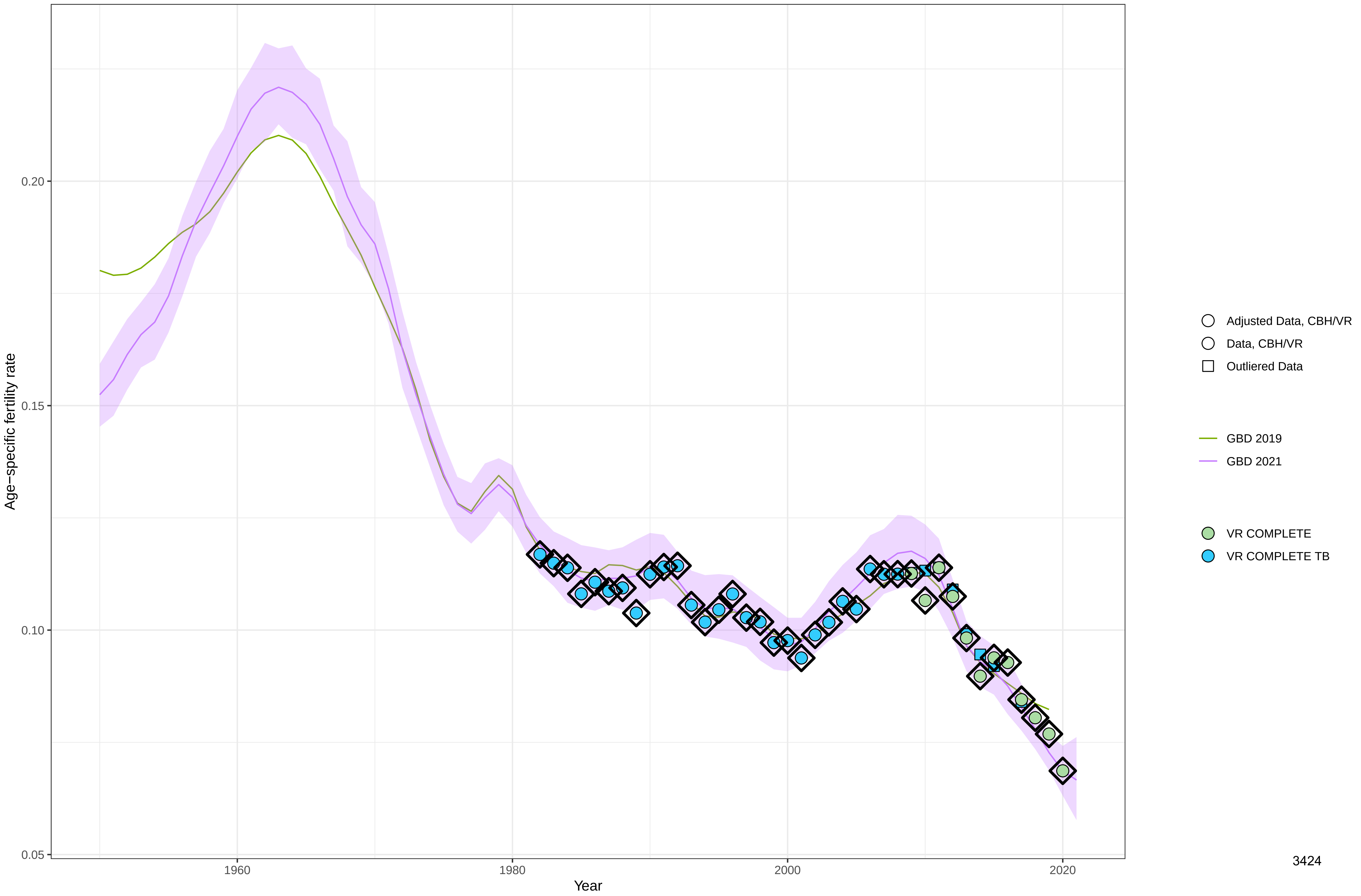

Age−specific fertility fitted trend lines, 1950−2021: Barnsley, Ages: 25−29
Data points enclosed with a diamond are reference sources

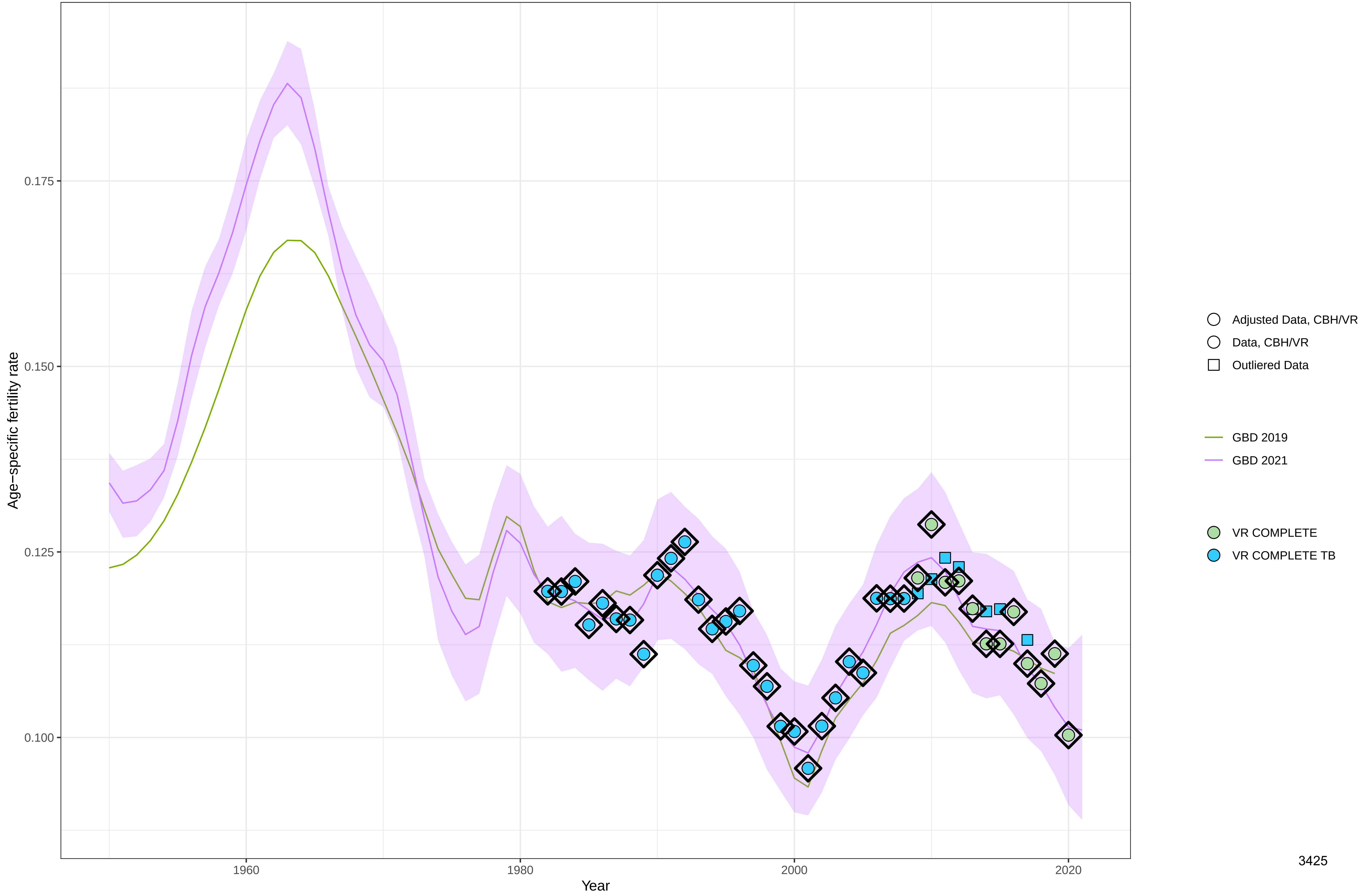

Age−specific fertility fitted trend lines, 1950−2021: Barnsley, Ages: 30−34
Data points enclosed with a diamond are reference sources

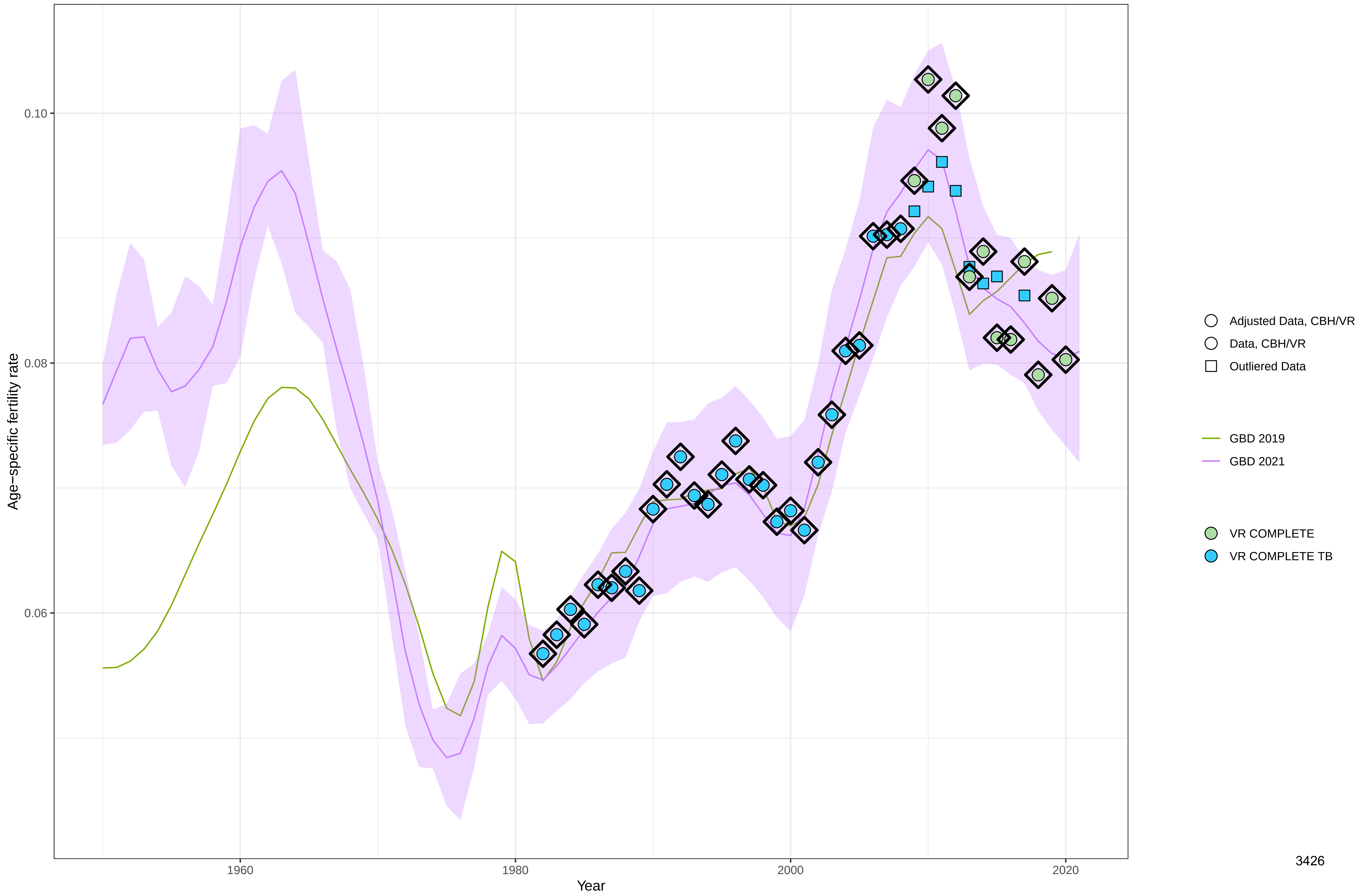

Age−specific fertility fitted trend lines, 1950−2021: Barnsley, Ages: 35−39
Data points enclosed with a diamond are reference sources

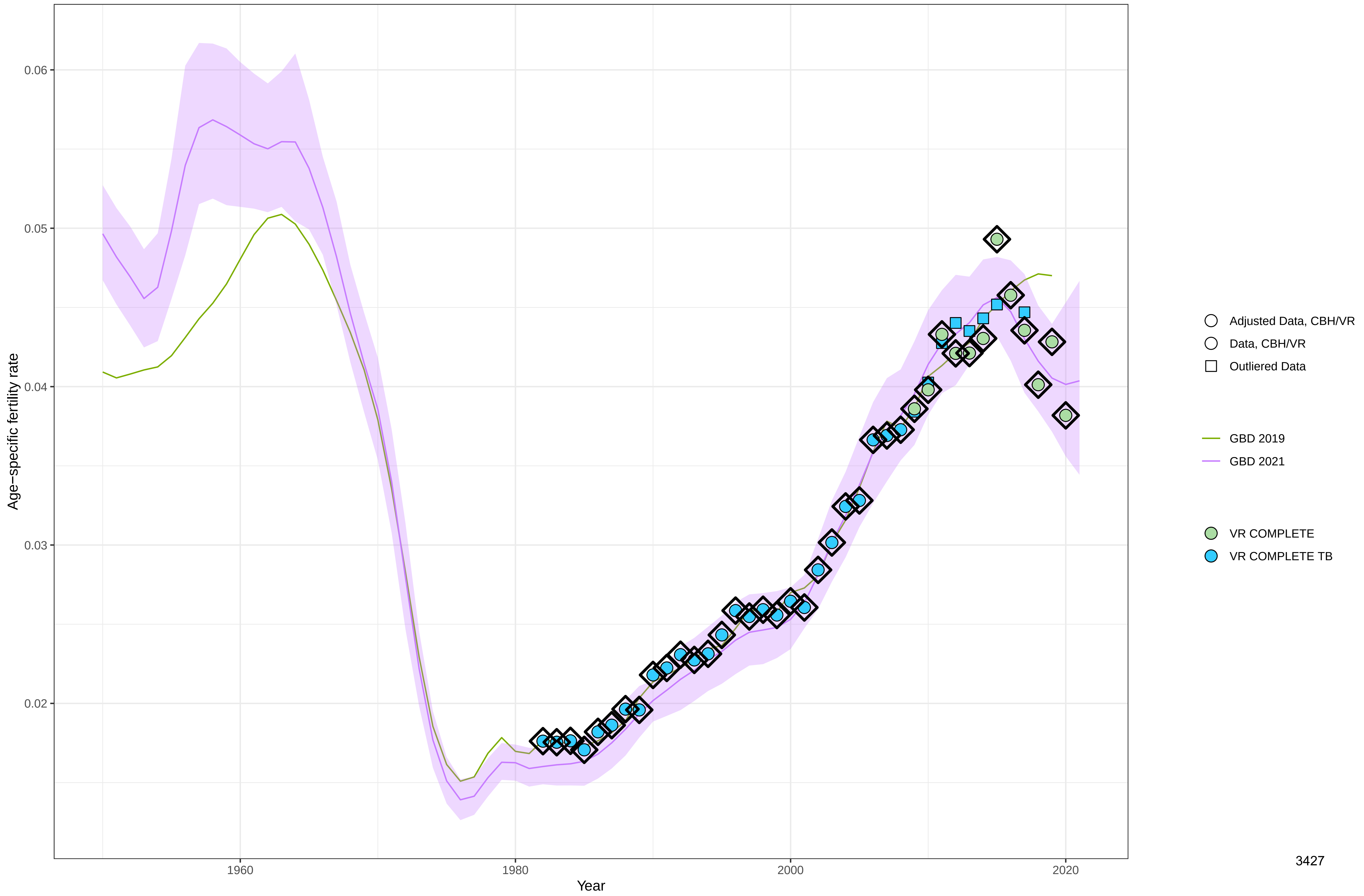

Age−specific fertility fitted trend lines, 1950−2021: Barnsley, Ages: 40−44
Data points enclosed with a diamond are reference sources

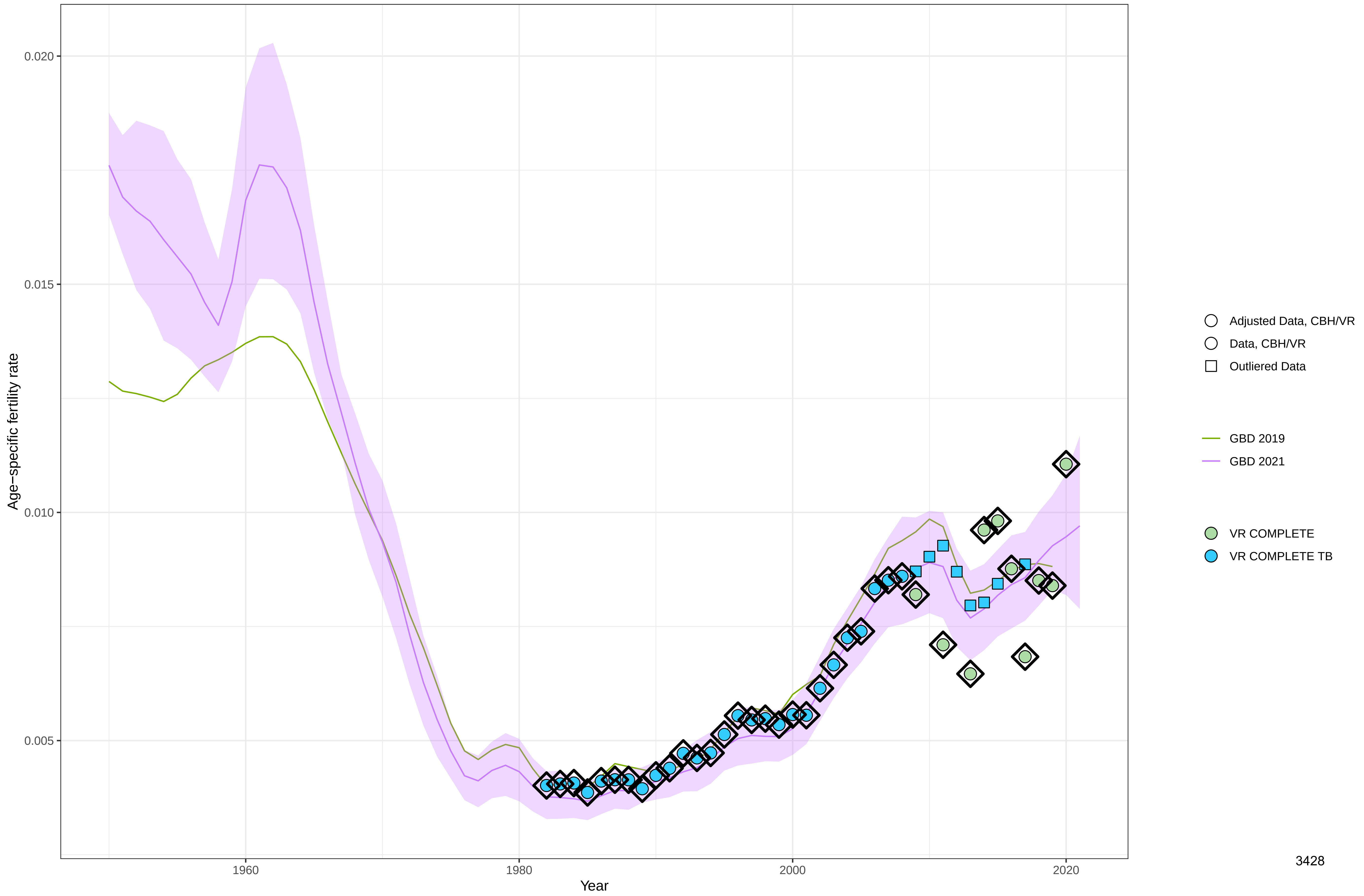

Age−specific fertility fitted trend lines, 1950−2021: Barnsley, Ages: 45−49
Data points enclosed with a diamond are reference sources

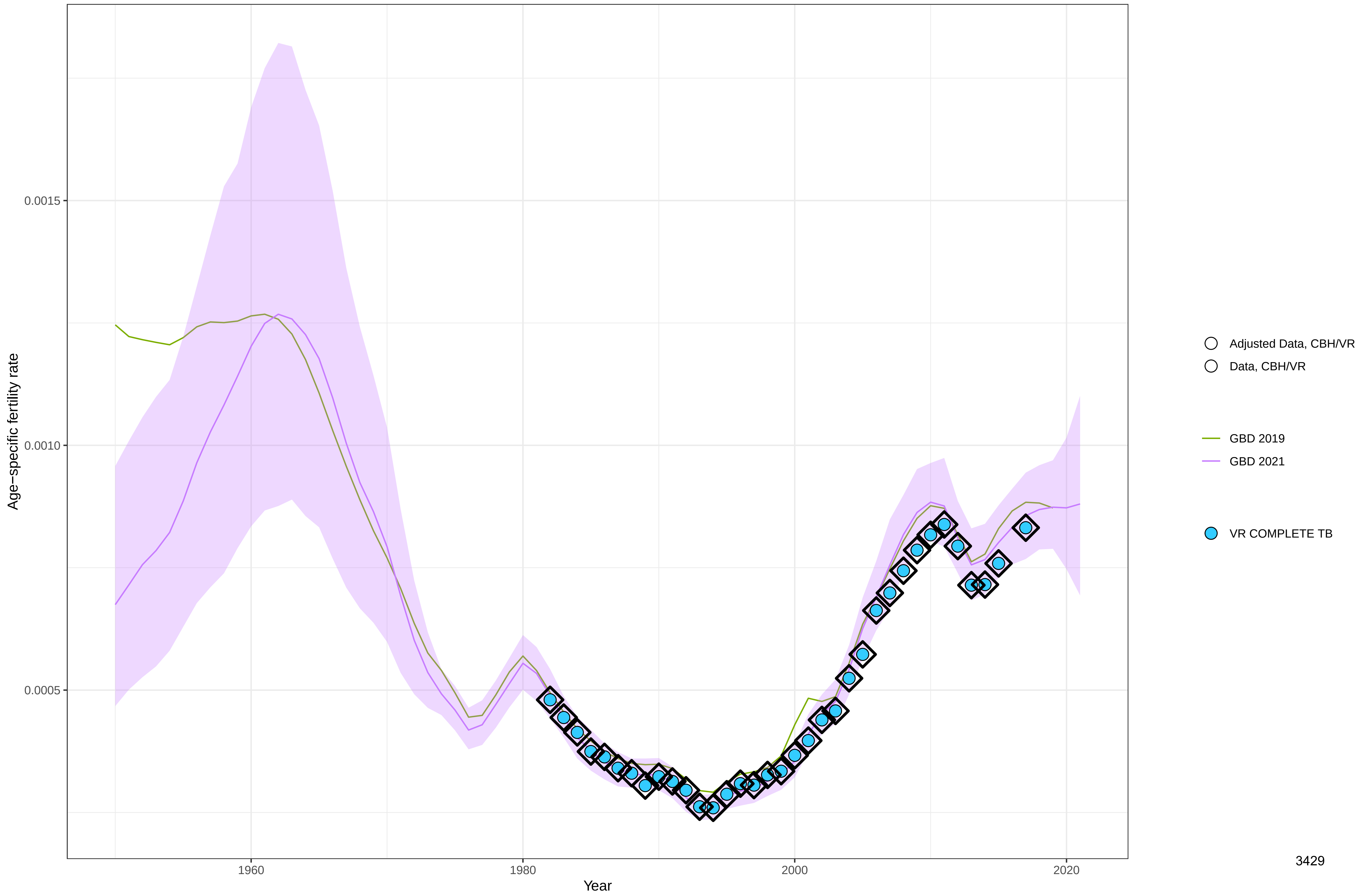

Age−specific fertility fitted trend lines, 1950−2021: Barnsley, Ages: 50−54
Data points enclosed with a diamond are reference sources

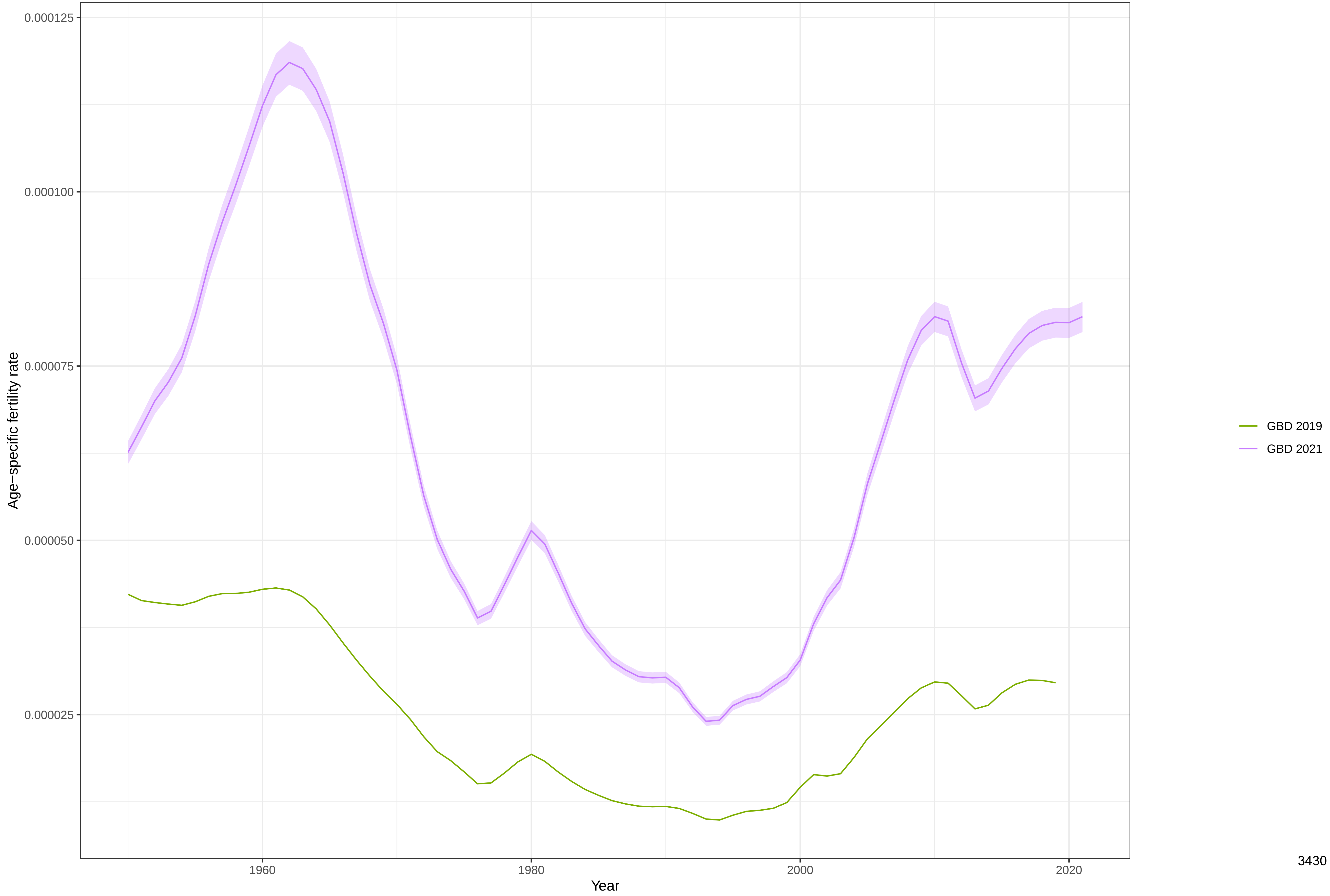

Total fertility rate fitted trend lines, 1950−2021: Barnsley TFR
Data points enclosed with a diamond are reference sources

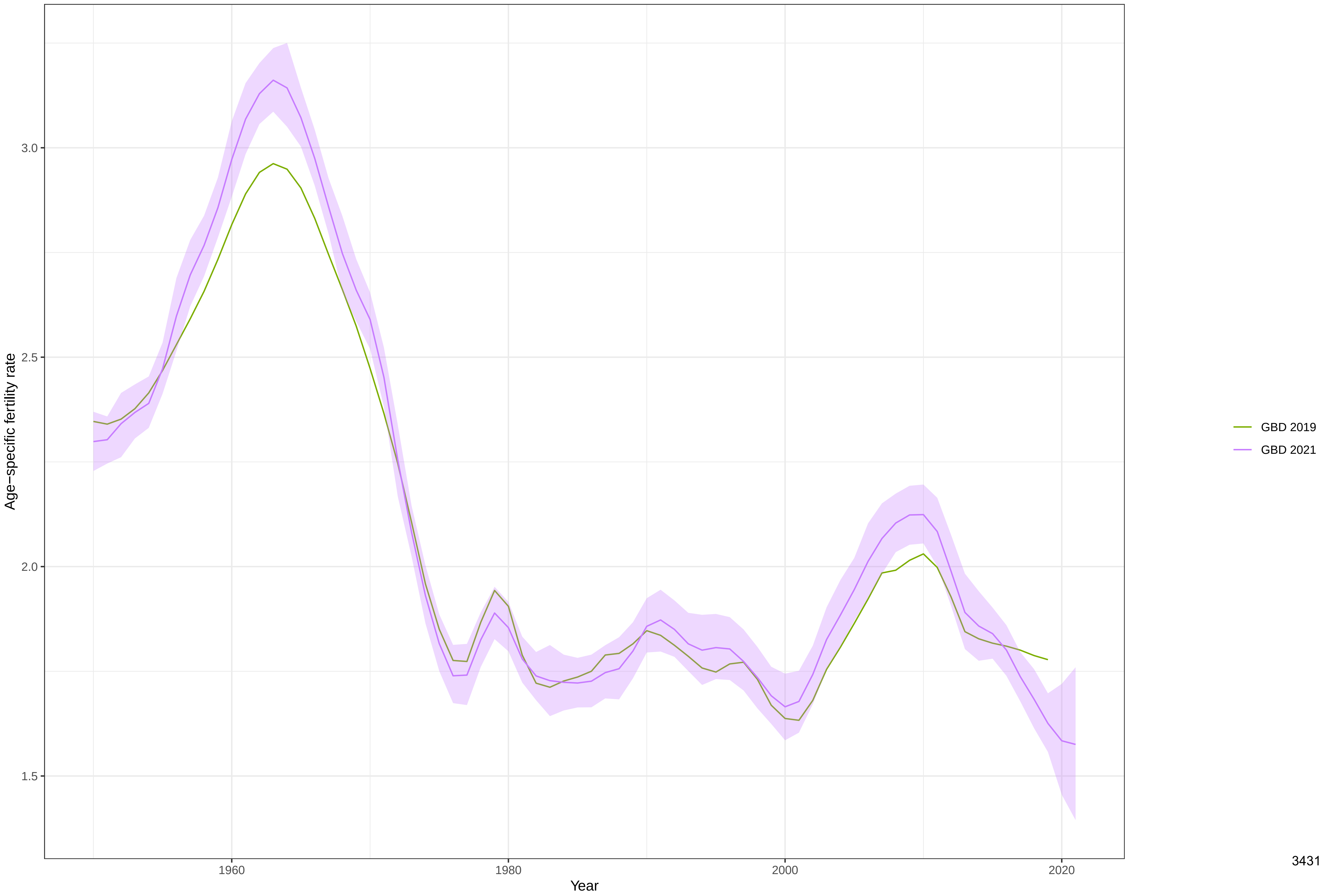

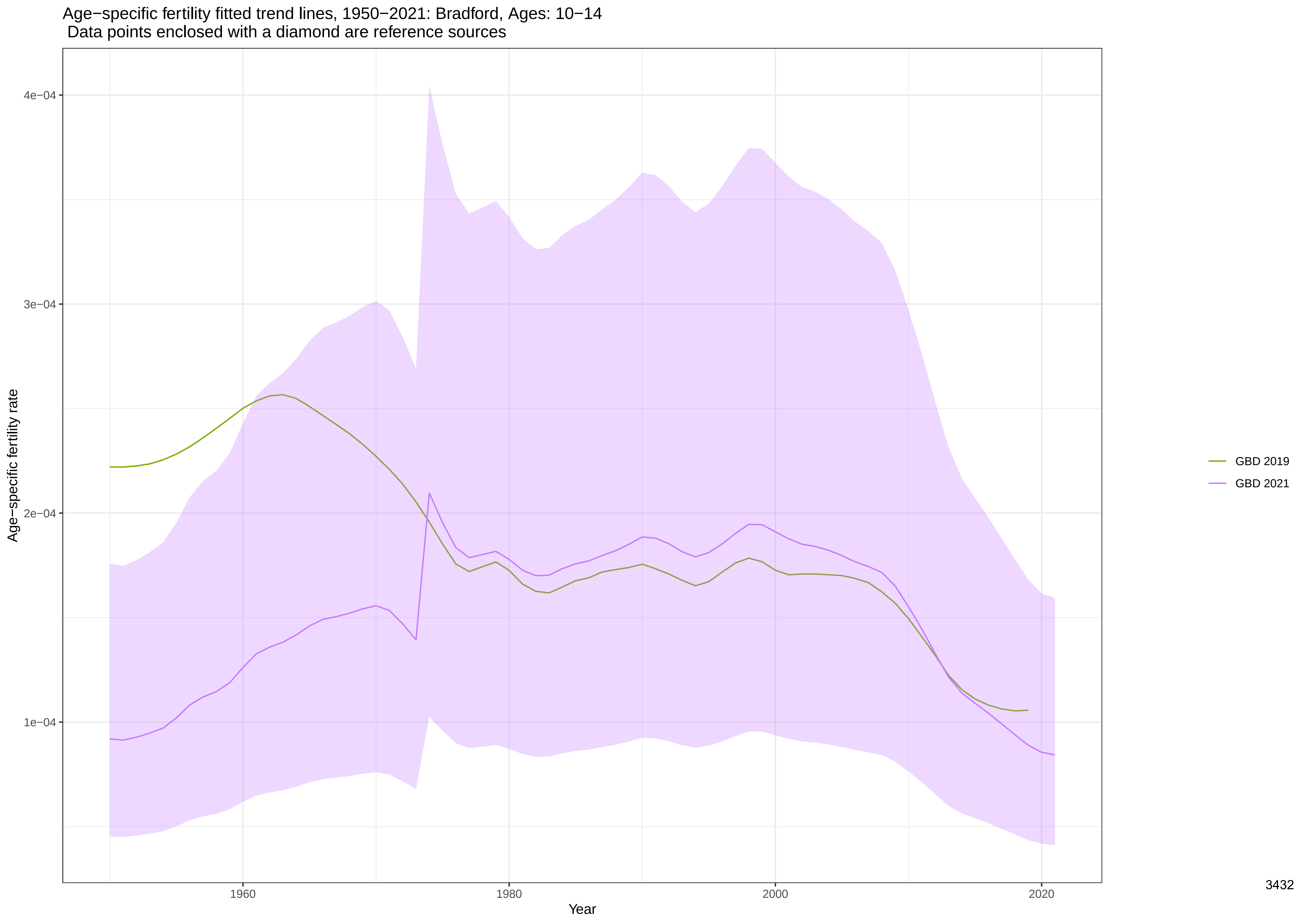

Age−specific fertility fitted trend lines, 1950−2021: Bradford, Ages: 10−14
Data points enclosed with a diamond are reference sources
4e−04
3e−04
2e−04
1e−04
1960
1980
2000
2020
Year
Age−specific fertility rate
GBD 2019
GBD 2021

Age−specific fertility fitted trend lines, 1950−2021: Bradford, Ages: 15−19
Data points enclosed with a diamond are reference sources

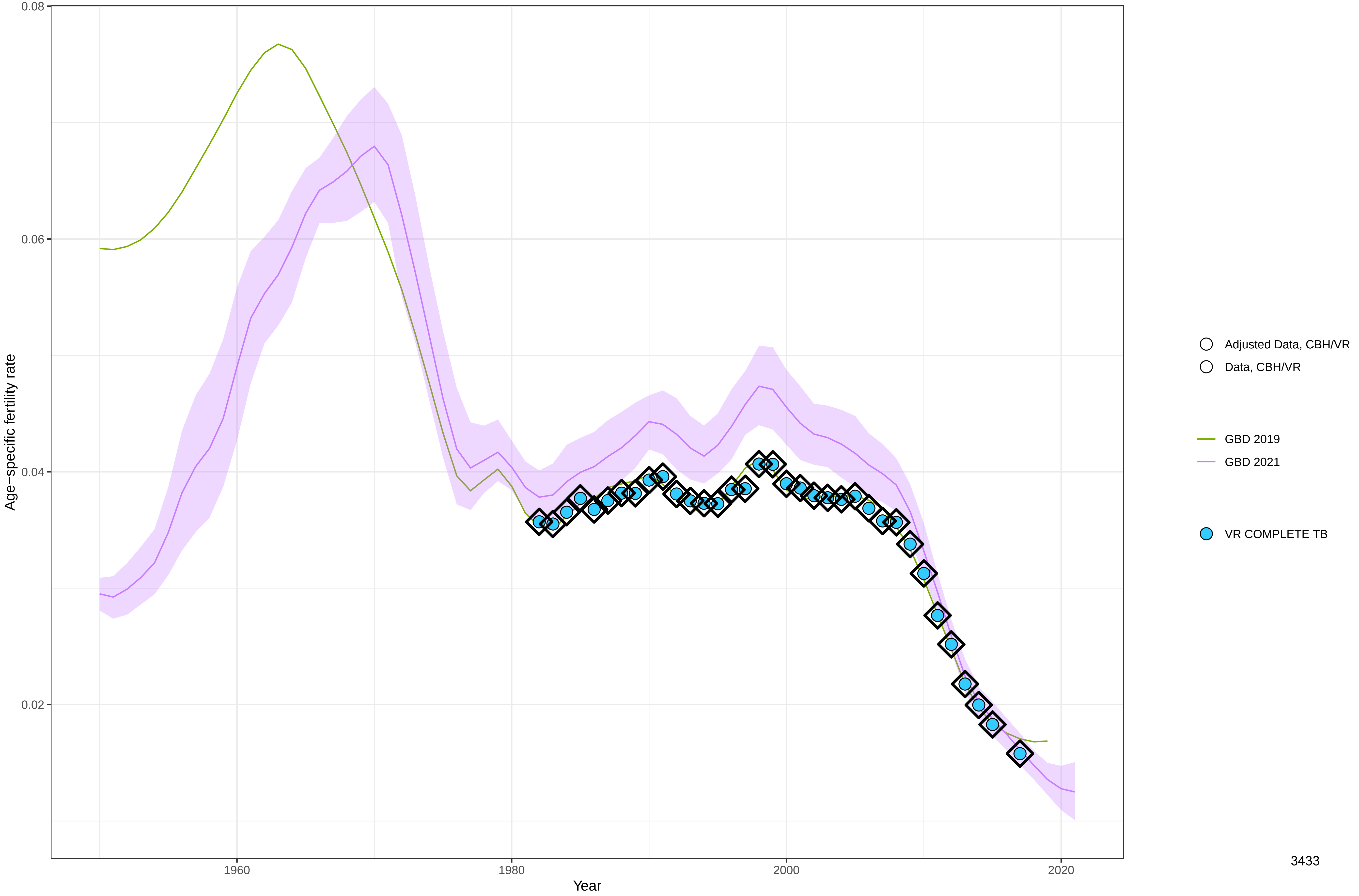

Age−specific fertility fitted trend lines, 1950−2021: Bradford, Ages: 20−24
Data points enclosed with a diamond are reference sources

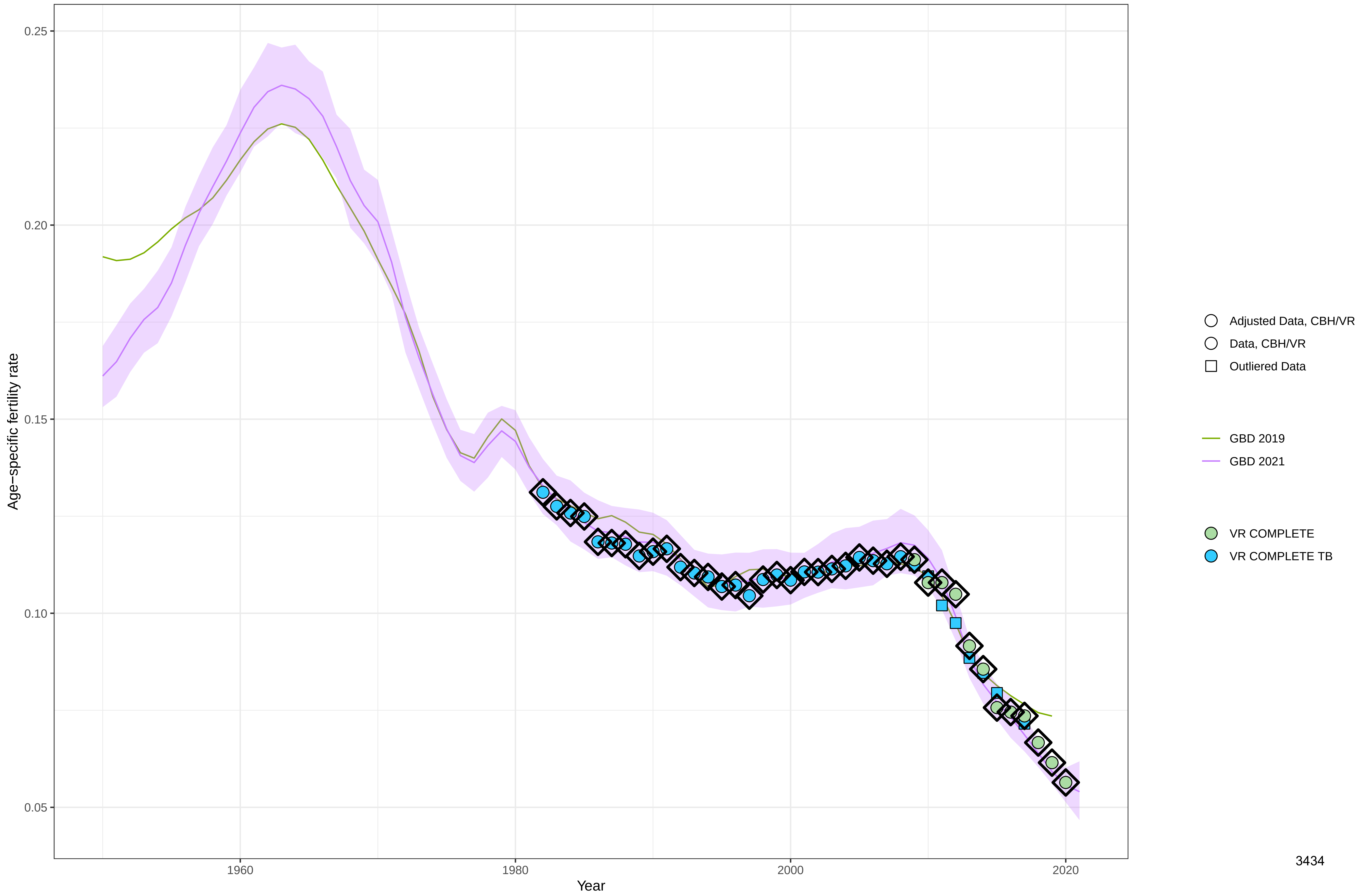

Age−specific fertility fitted trend lines, 1950−2021: Bradford, Ages: 25−29
Data points enclosed with a diamond are reference sources

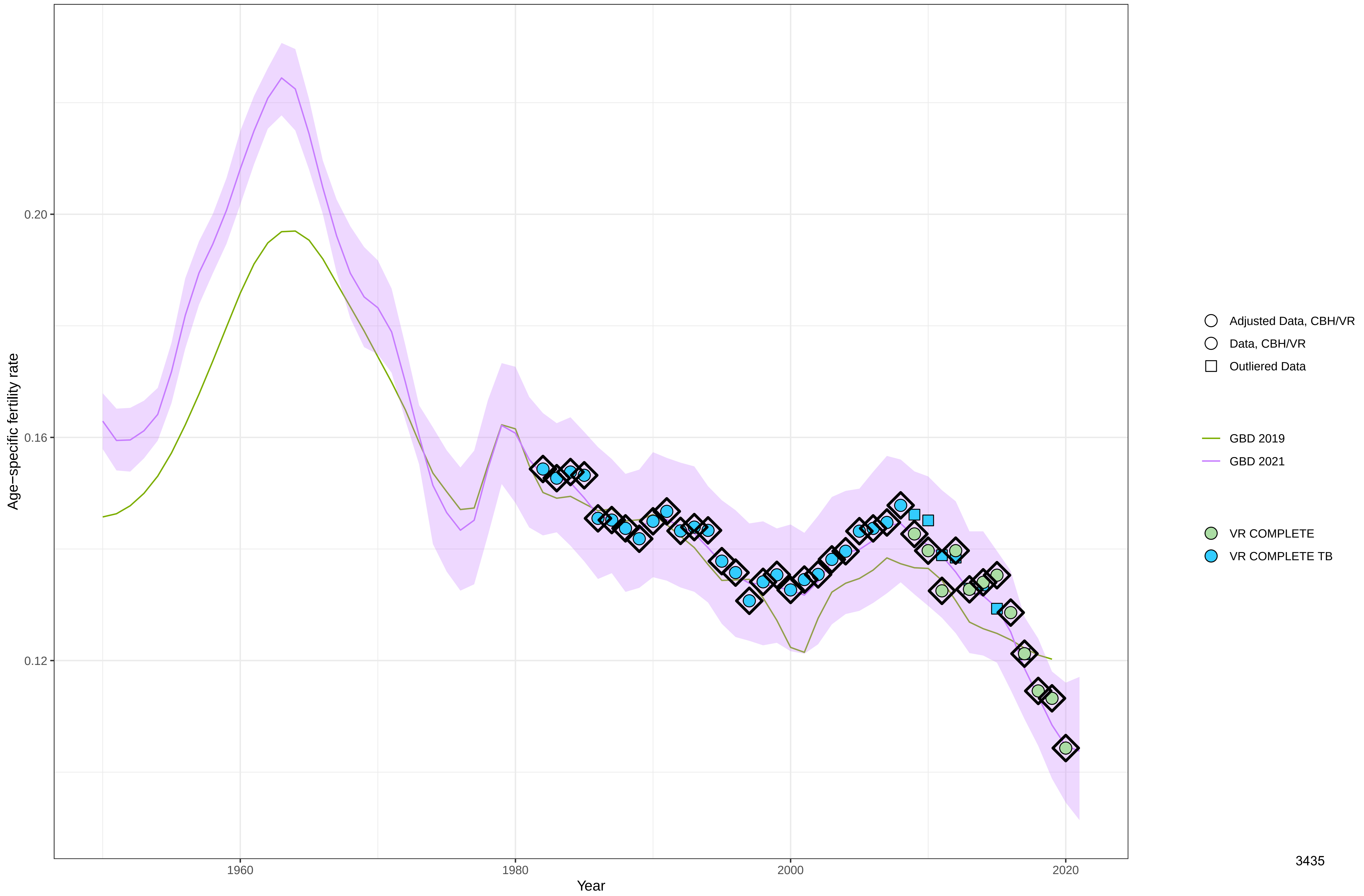

Age−specific fertility fitted trend lines, 1950−2021: Bradford, Ages: 30−34
Data points enclosed with a diamond are reference sources

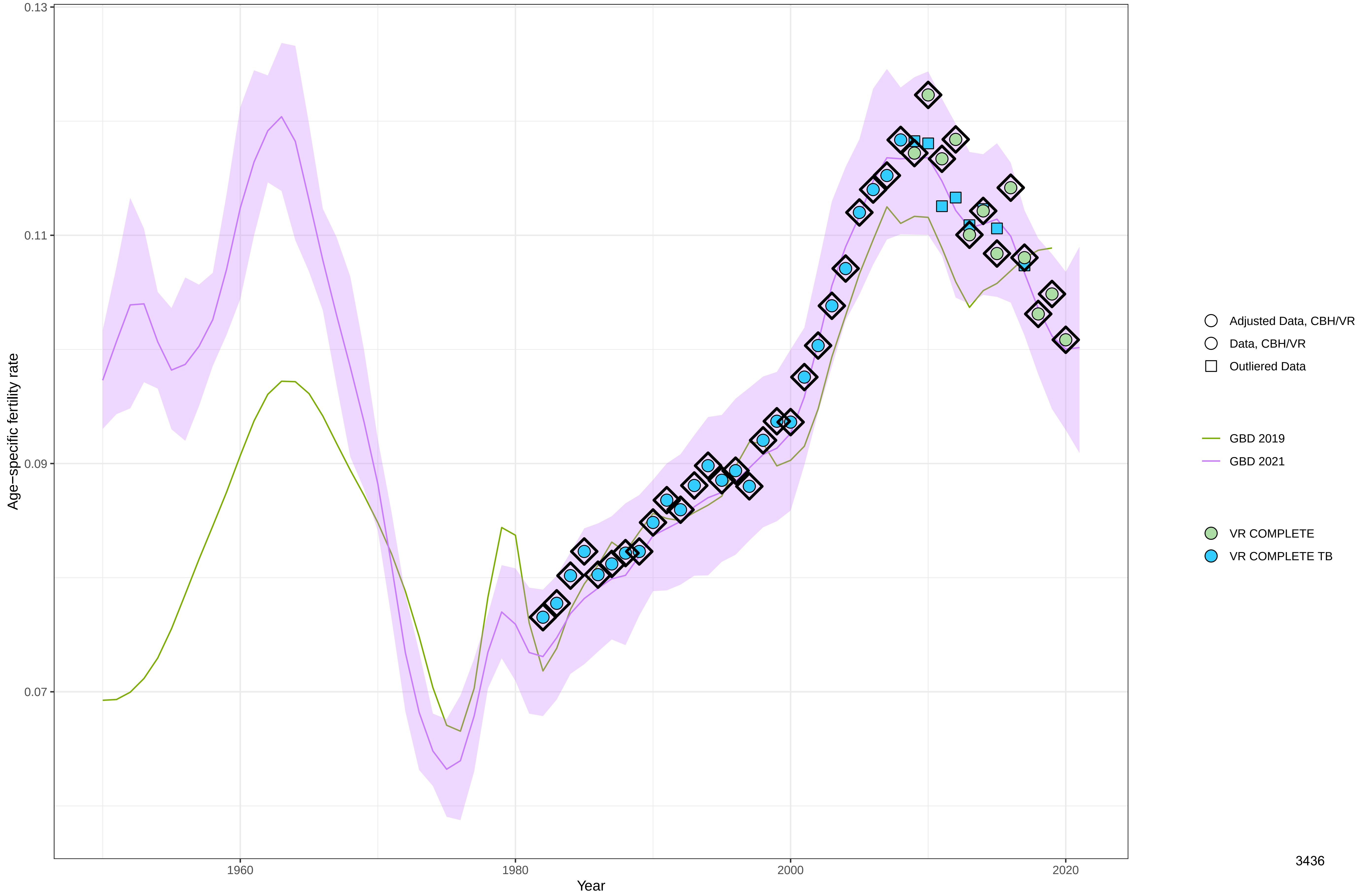

Age−specific fertility fitted trend lines, 1950−2021: Bradford, Ages: 35−39
Data points enclosed with a diamond are reference sources

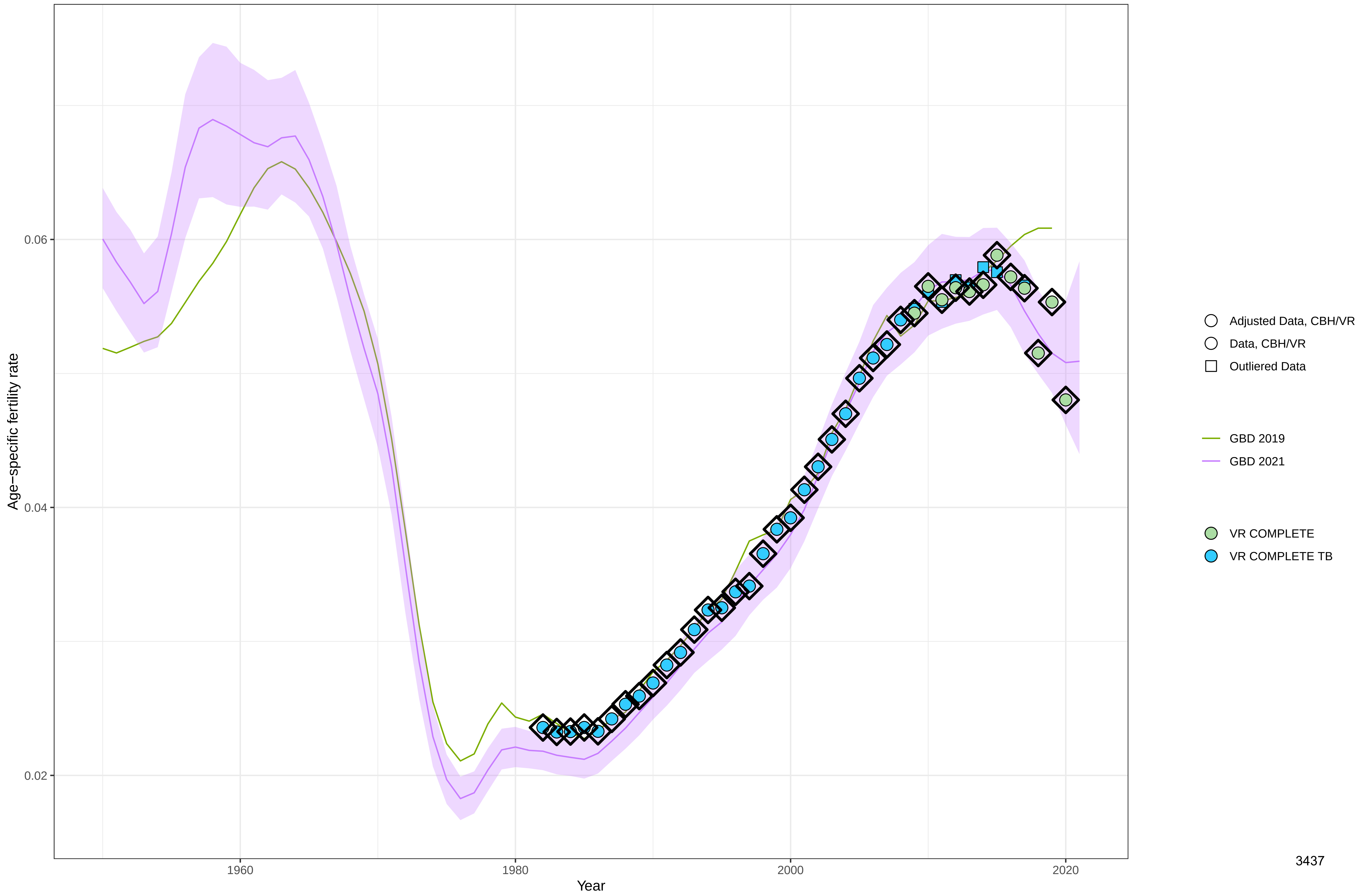

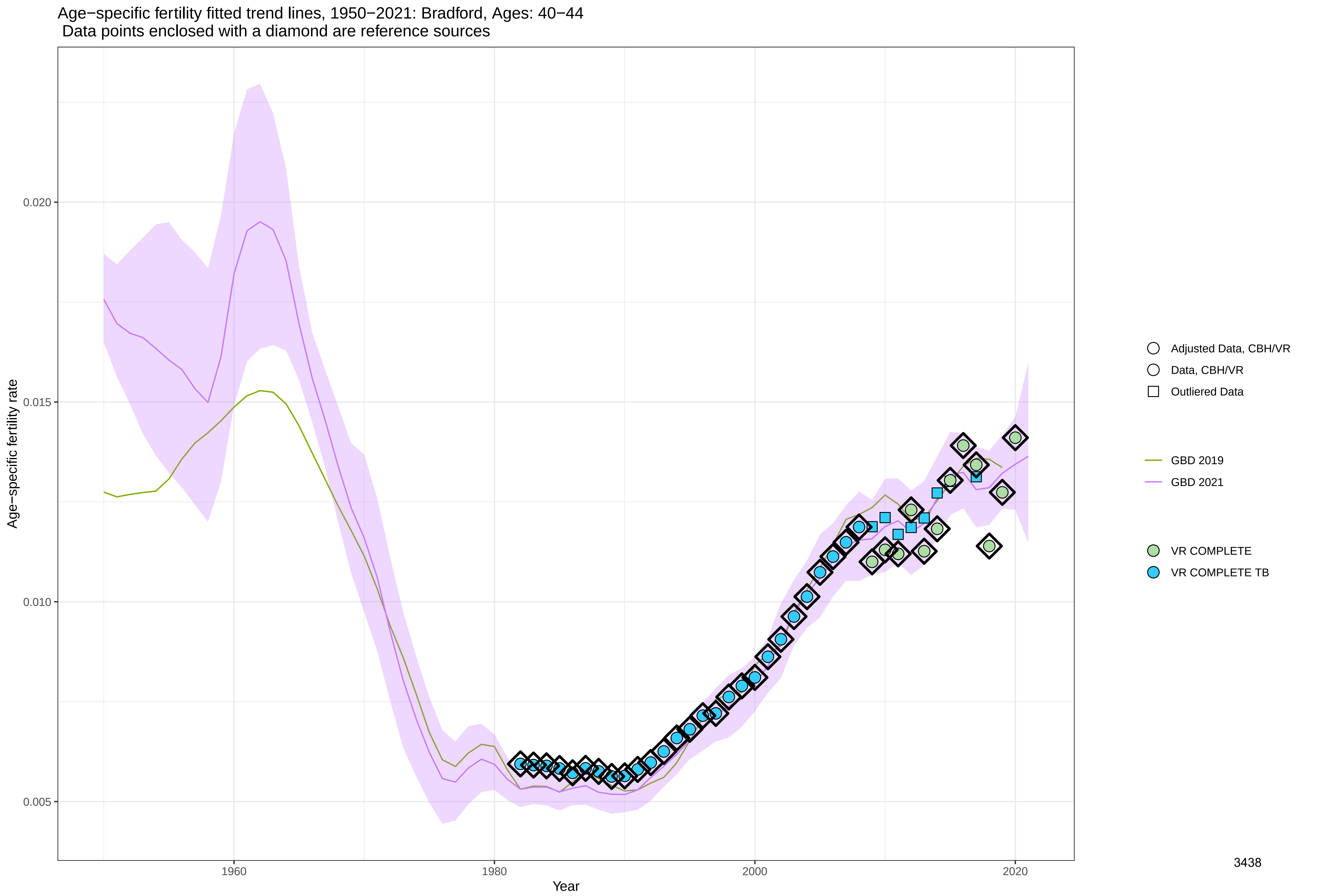

Age−specific fertility fitted trend lines, 1950−2021: Bradford, Ages: 40−44
Data points enclosed with a diamond are reference sources
Age−specific fertility rate
0.020
0.015
0.010
0.005
1960
1980
2000
2020
Year
Adjusted Data, CBH/VR
Data, CBH/VR
Outliered Data
GBD 2019
GBD 2021
VR COMPLETE
VR COMPLETE TB

Age−specific fertility fitted trend lines, 1950−2021: Bradford, Ages: 45−49
Data points enclosed with a diamond are reference sources

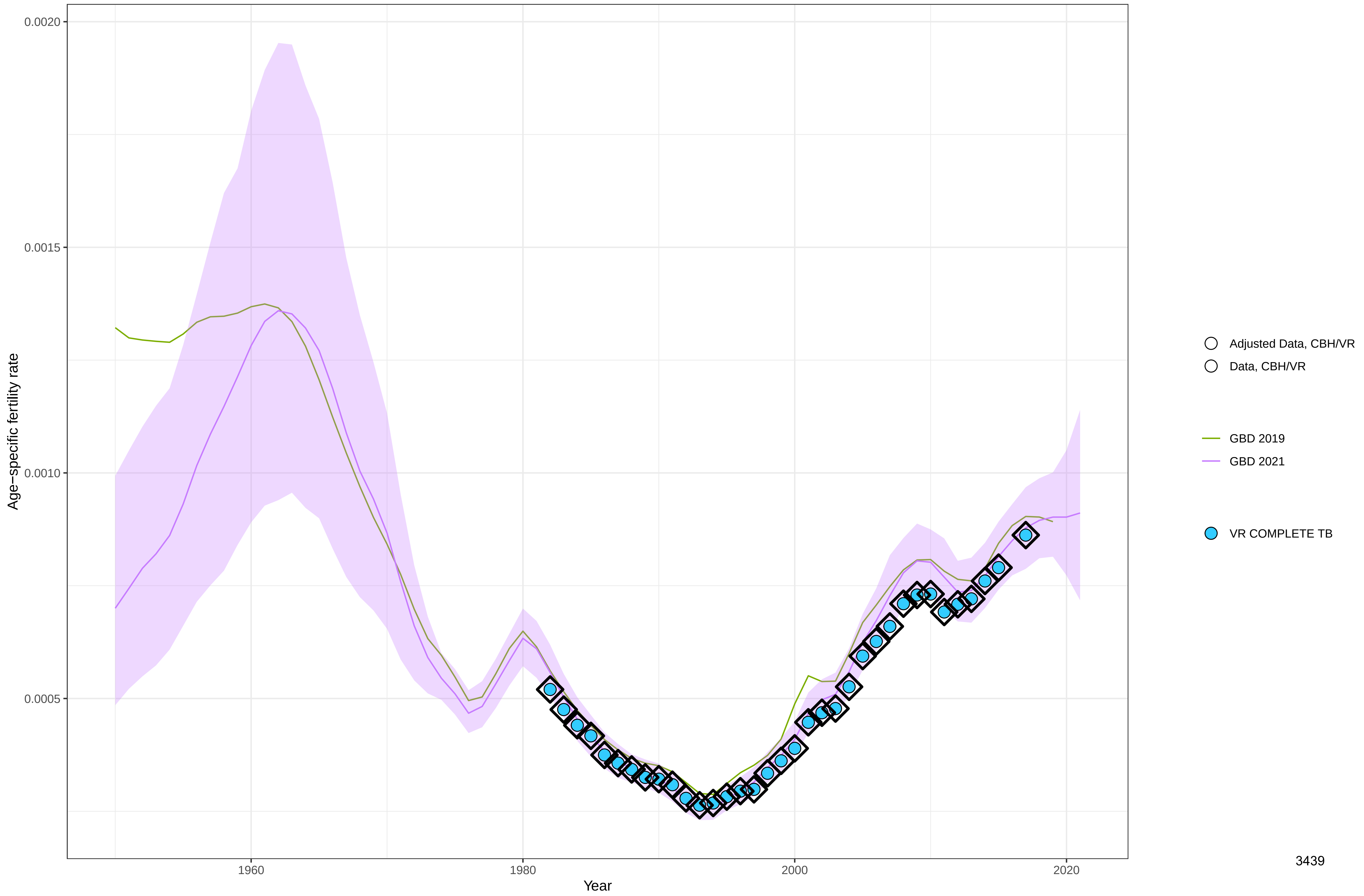

Age−specific fertility fitted trend lines, 1950−2021: Bradford, Ages: 50−54
Data points enclosed with a diamond are reference sources

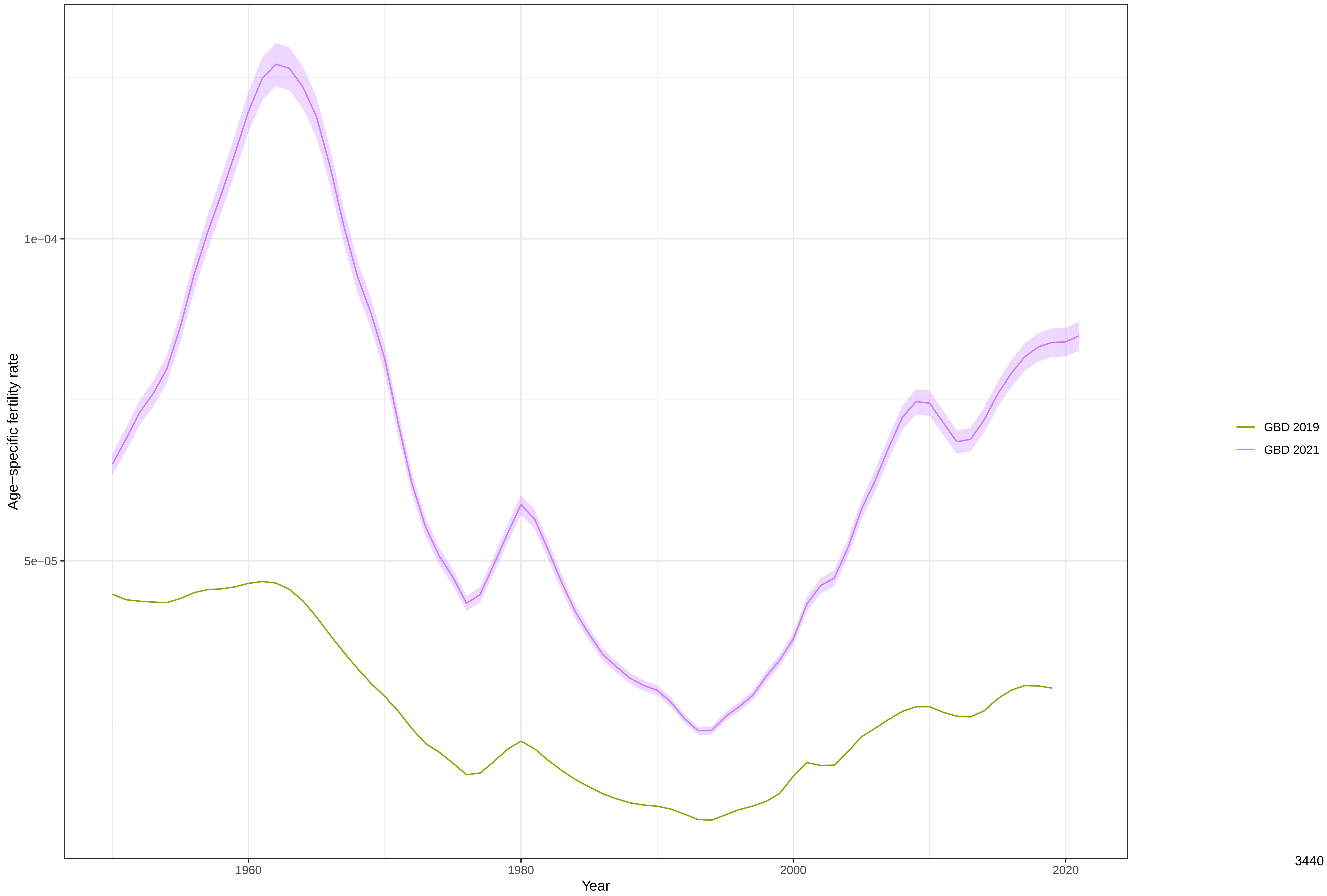

3440

Total fertility rate fitted trend lines, 1950−2021: Bradford TFR
Data points enclosed with a diamond are reference sources

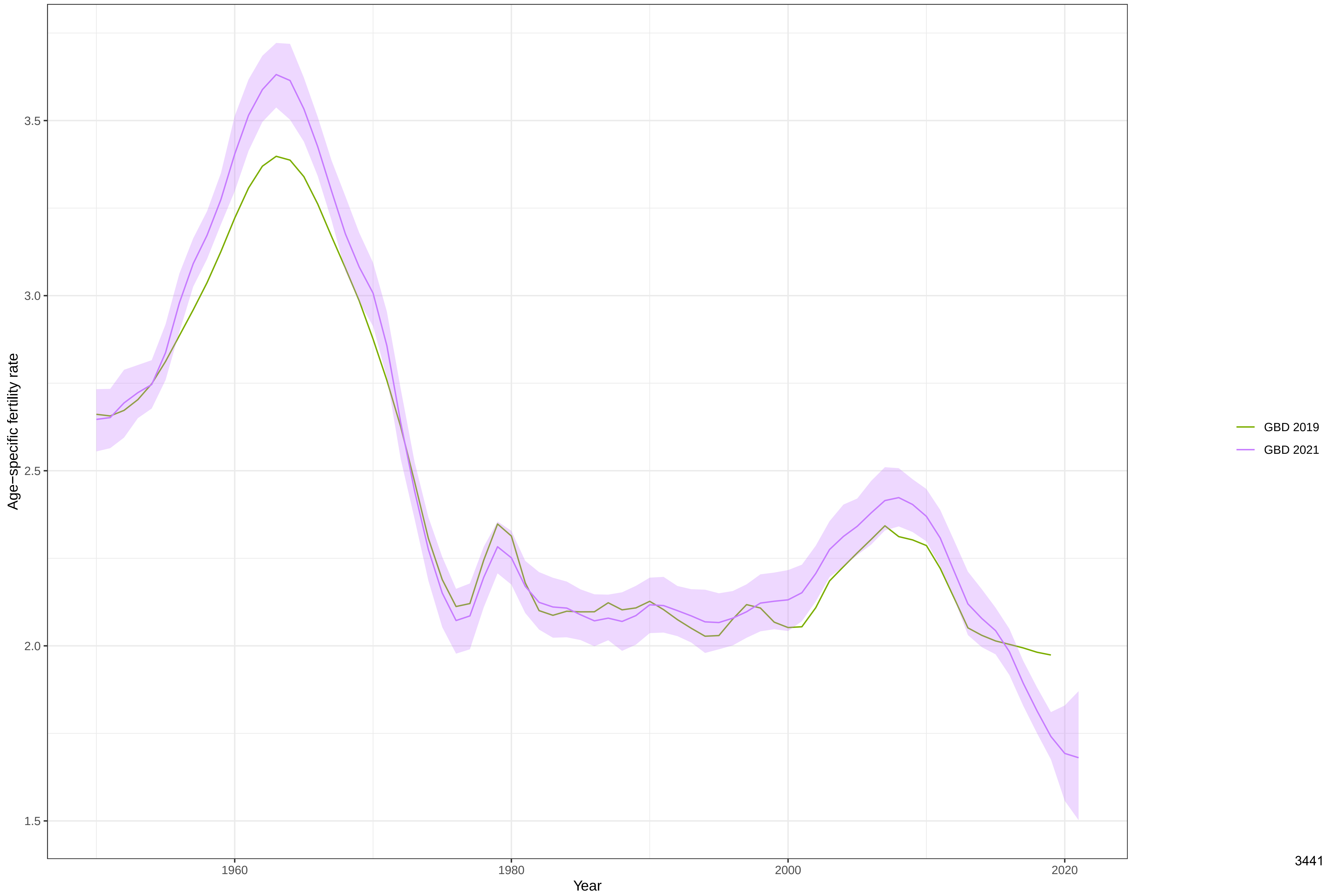

Age−specific fertility fitted trend lines, 1950−2021: Calderdale, Ages: 10−14
Data points enclosed with a diamond are reference sources

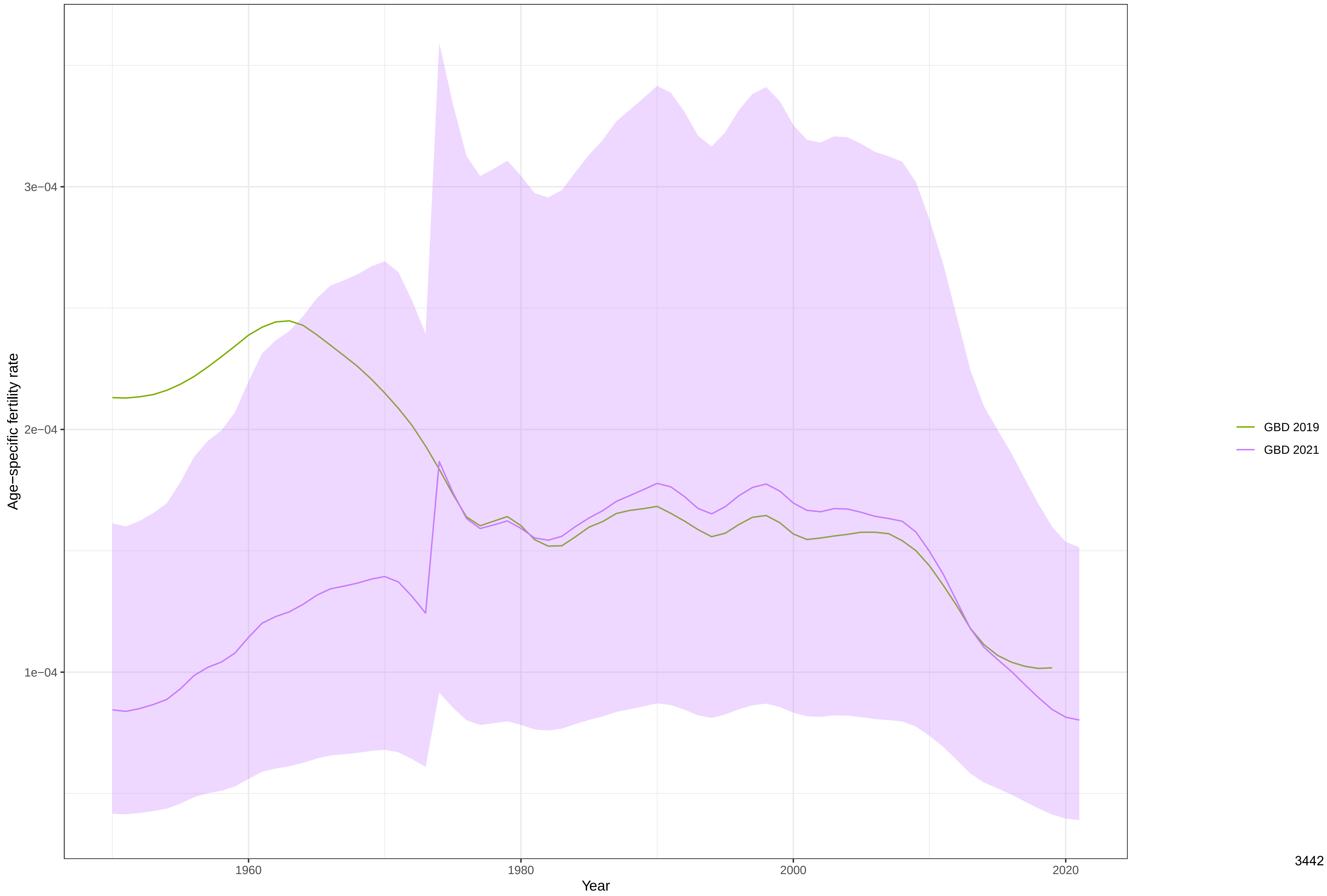

Age−specific fertility fitted trend lines, 1950−2021: Calderdale, Ages: 15−19
Data points enclosed with a diamond are reference sources

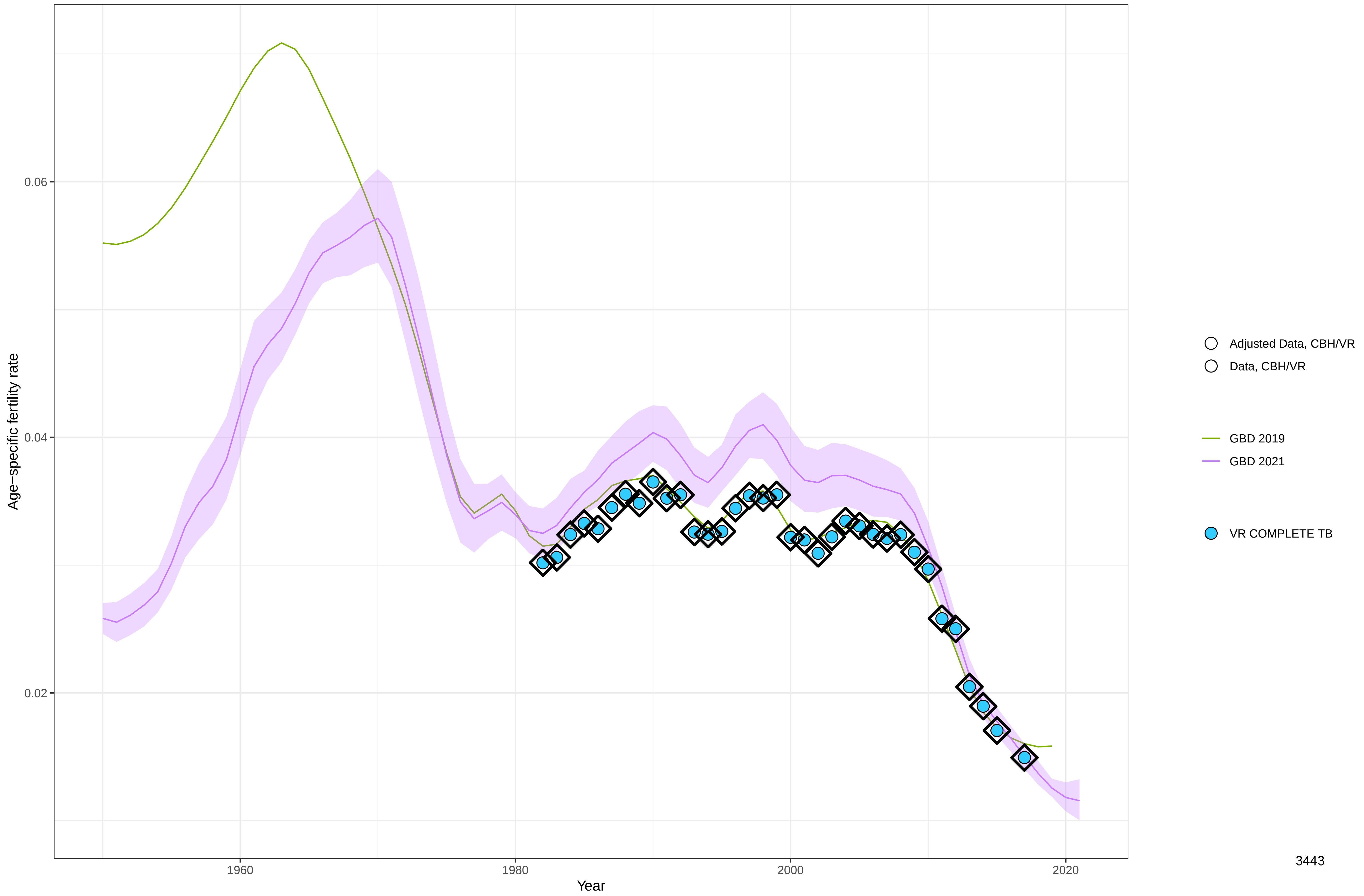

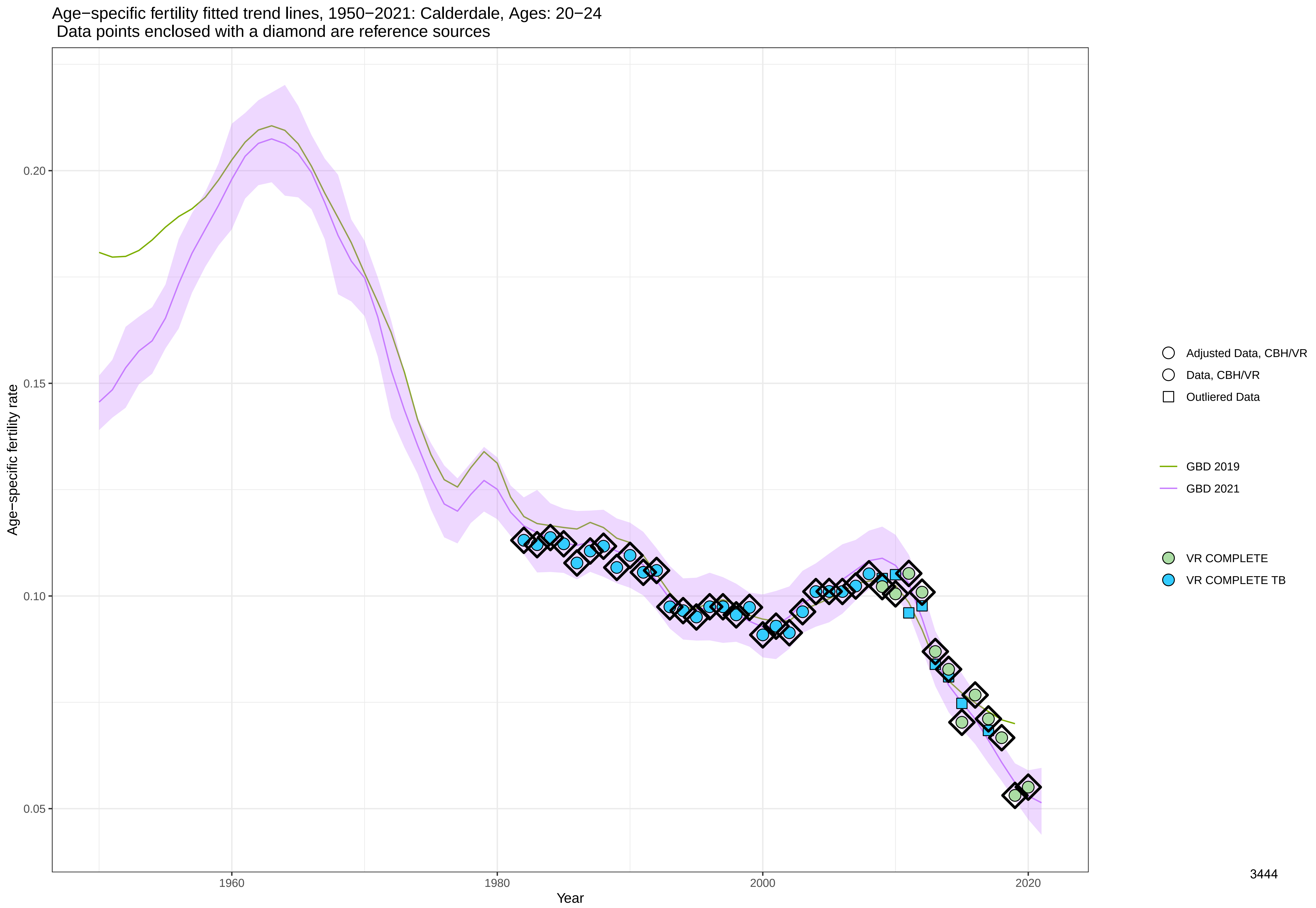

Age−specific fertility fitted trend lines, 1950−2021: Calderdale, Ages: 20−24
Data points enclosed with a diamond are reference sources
Age−specific fertility rate
0.05
0.10
0.15
0.20
1960
1980
2000
2020
Year
Adjusted Data, CBH/VR
Data, CBH/VR
Outliered Data
GBD 2019
GBD 2021
VR COMPLETE
VR COMPLETE TB

Age−specific fertility fitted trend lines, 1950−2021: Calderdale, Ages: 25−29
Data points enclosed with a diamond are reference sources

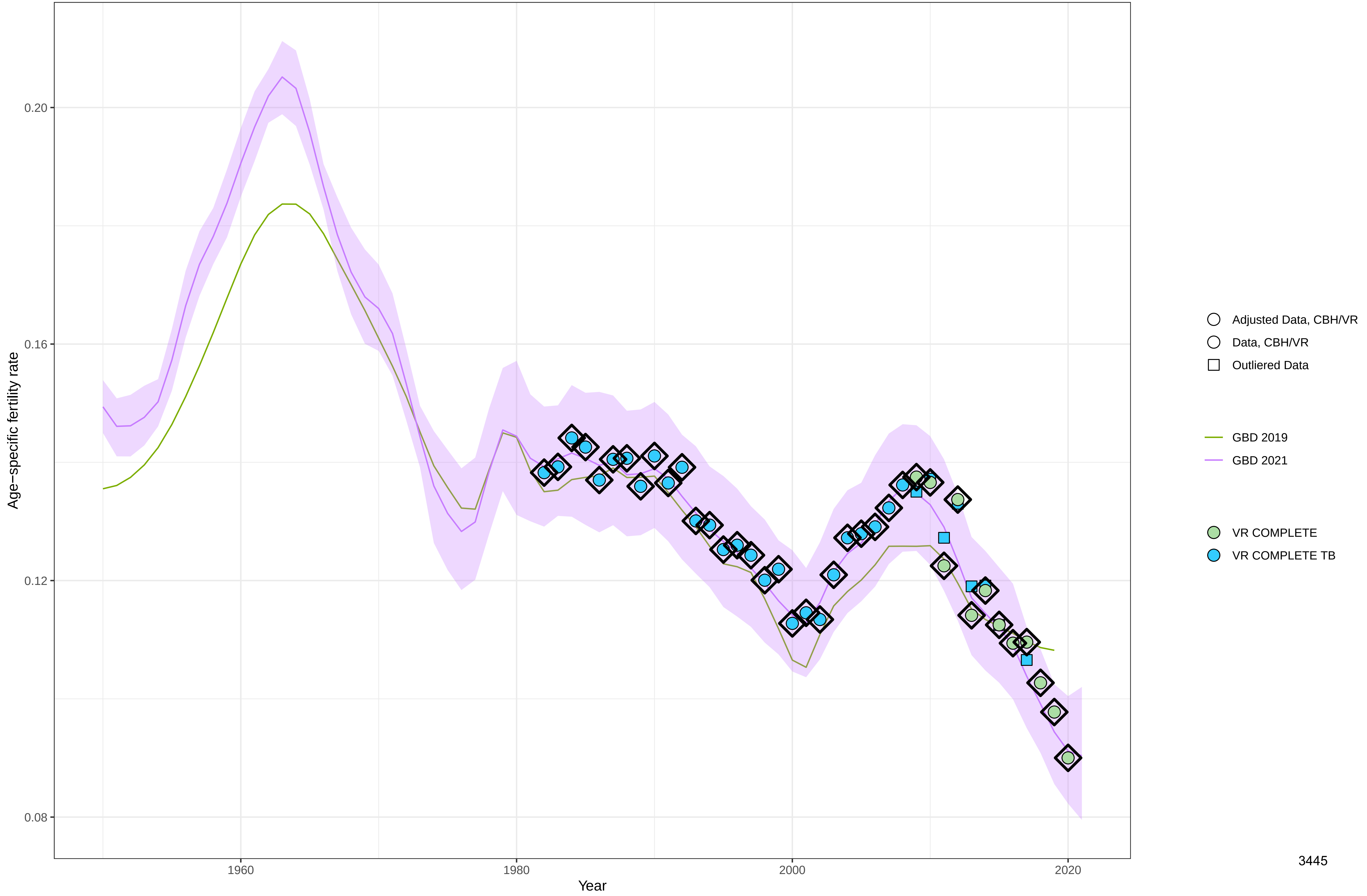

Age−specific fertility fitted trend lines, 1950−2021: Calderdale, Ages: 30−34
Data points enclosed with a diamond are reference sources

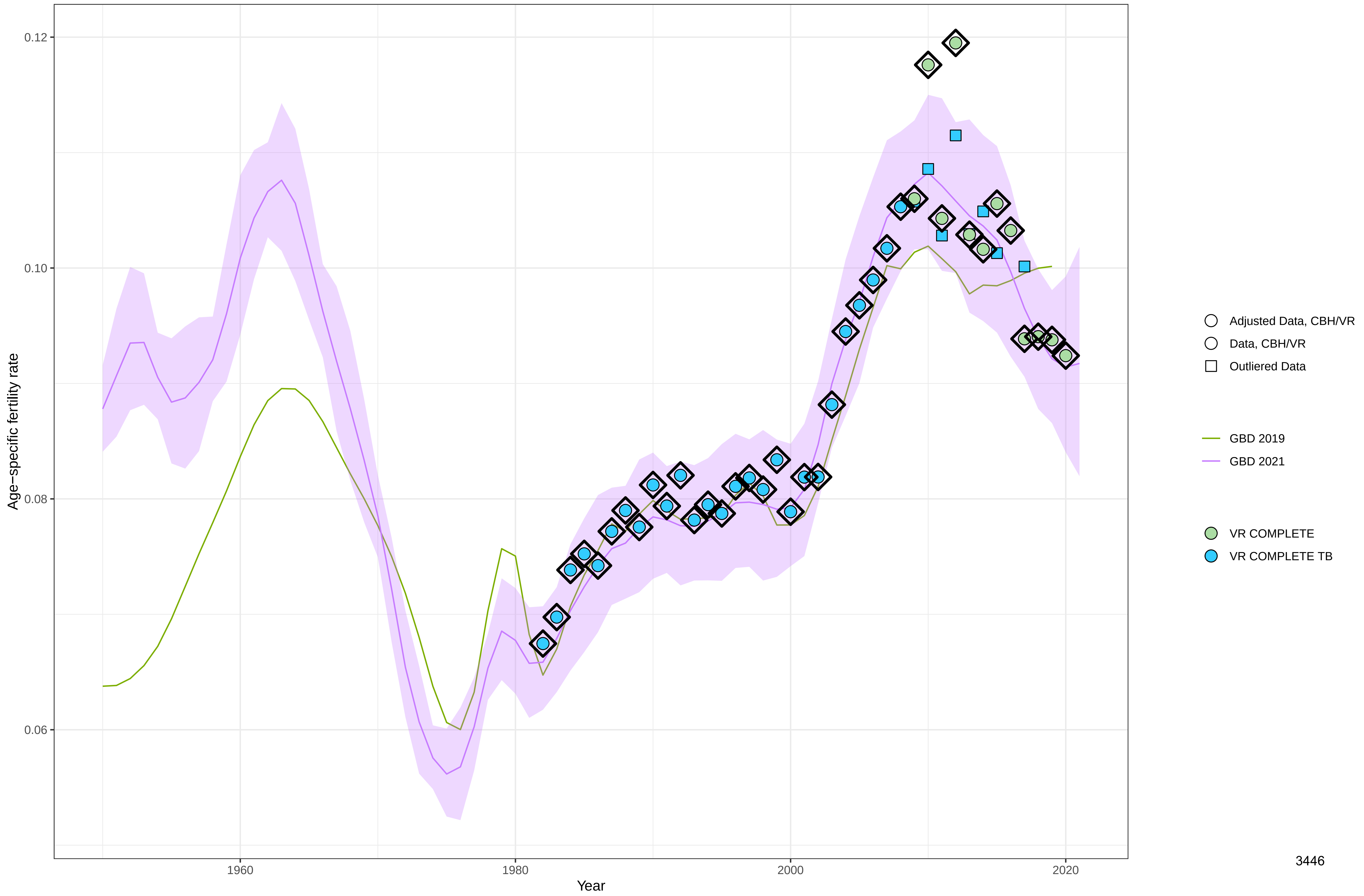

Age−specific fertility fitted trend lines, 1950−2021: Calderdale, Ages: 35−39
Data points enclosed with a diamond are reference sources

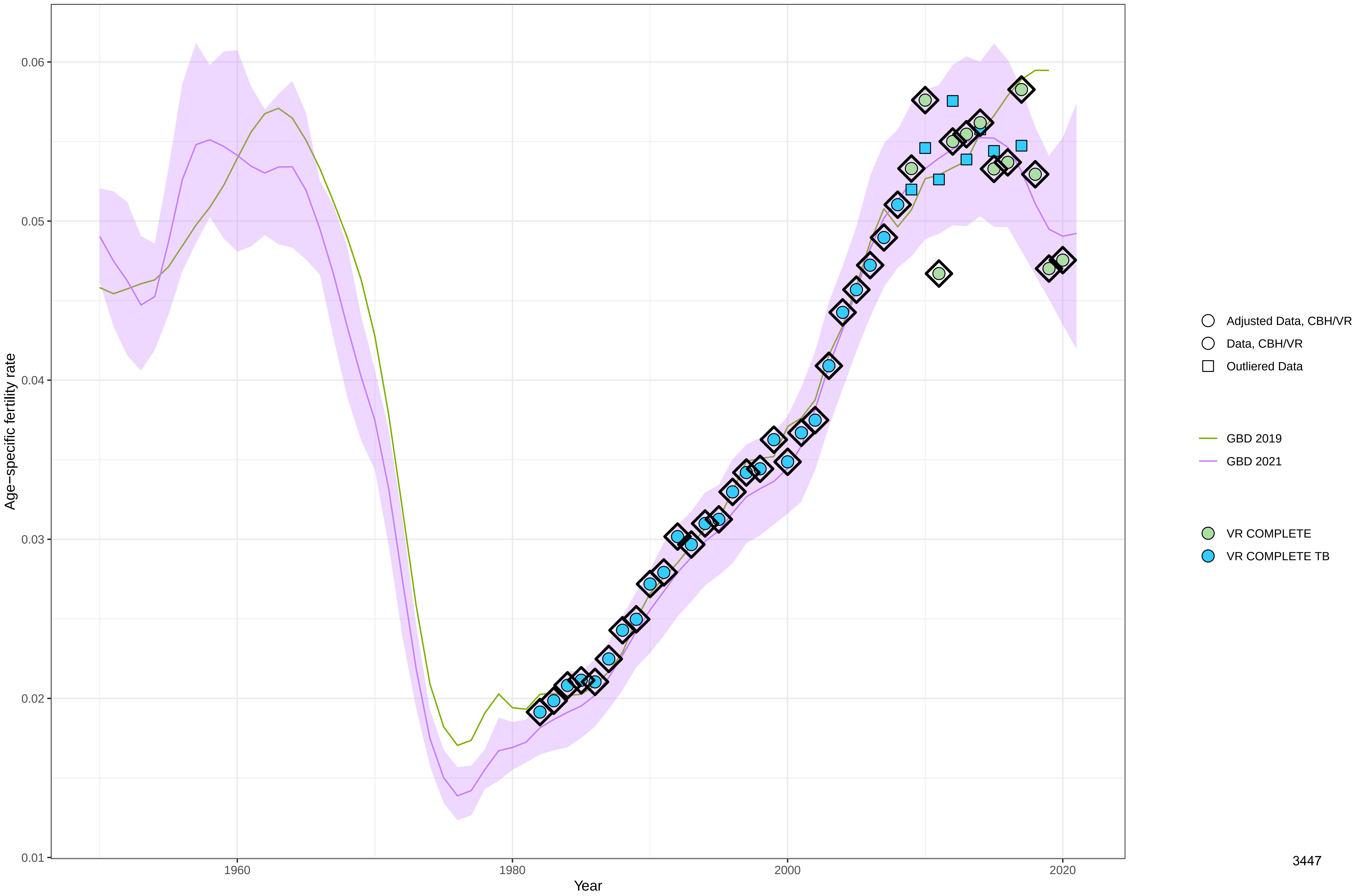

Age−specific fertility fitted trend lines, 1950−2021: Calderdale, Ages: 40−44
Data points enclosed with a diamond are reference sources

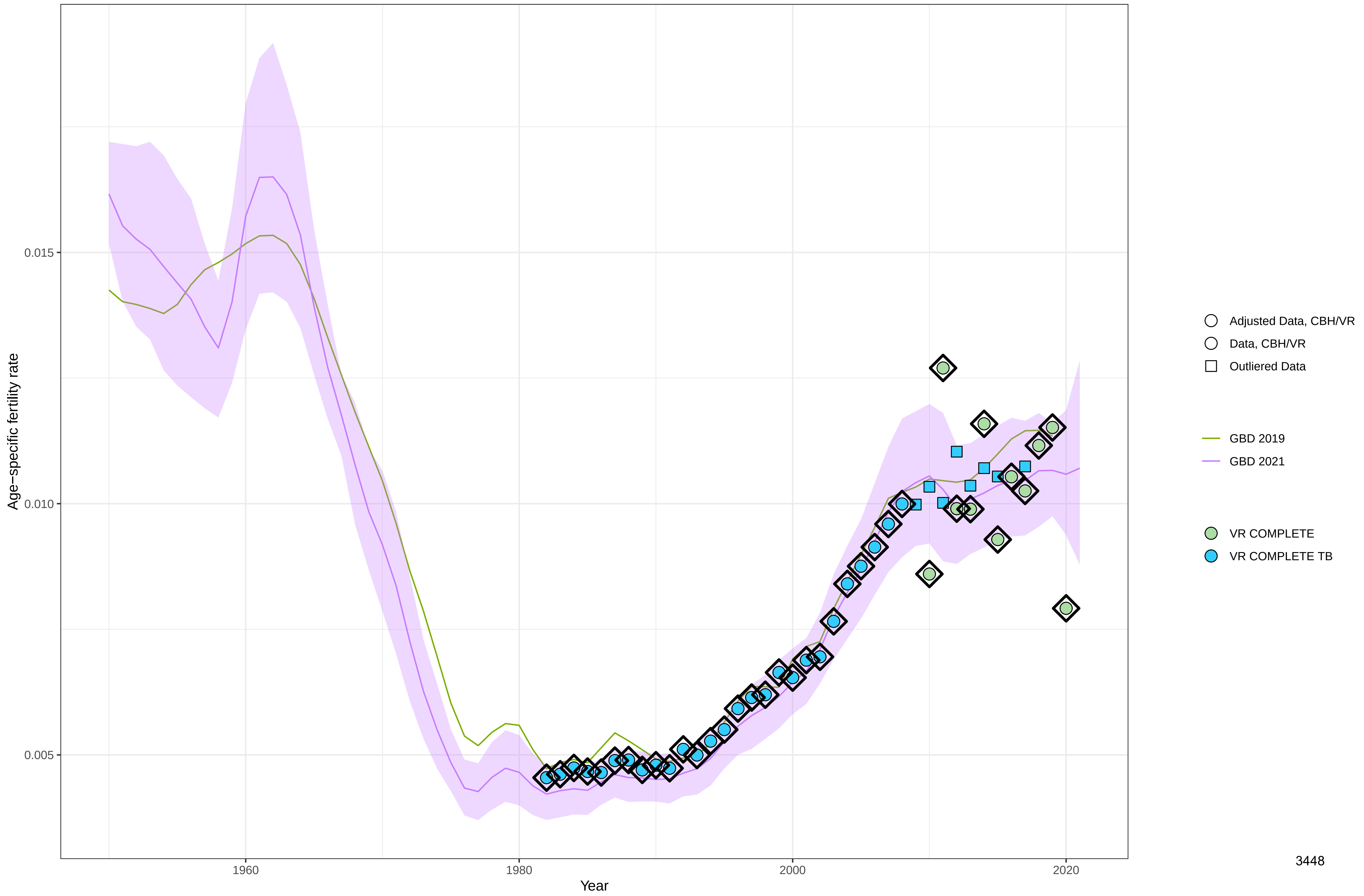

Age−specific fertility fitted trend lines, 1950−2021: Calderdale, Ages: 45−49
Data points enclosed with a diamond are reference sources

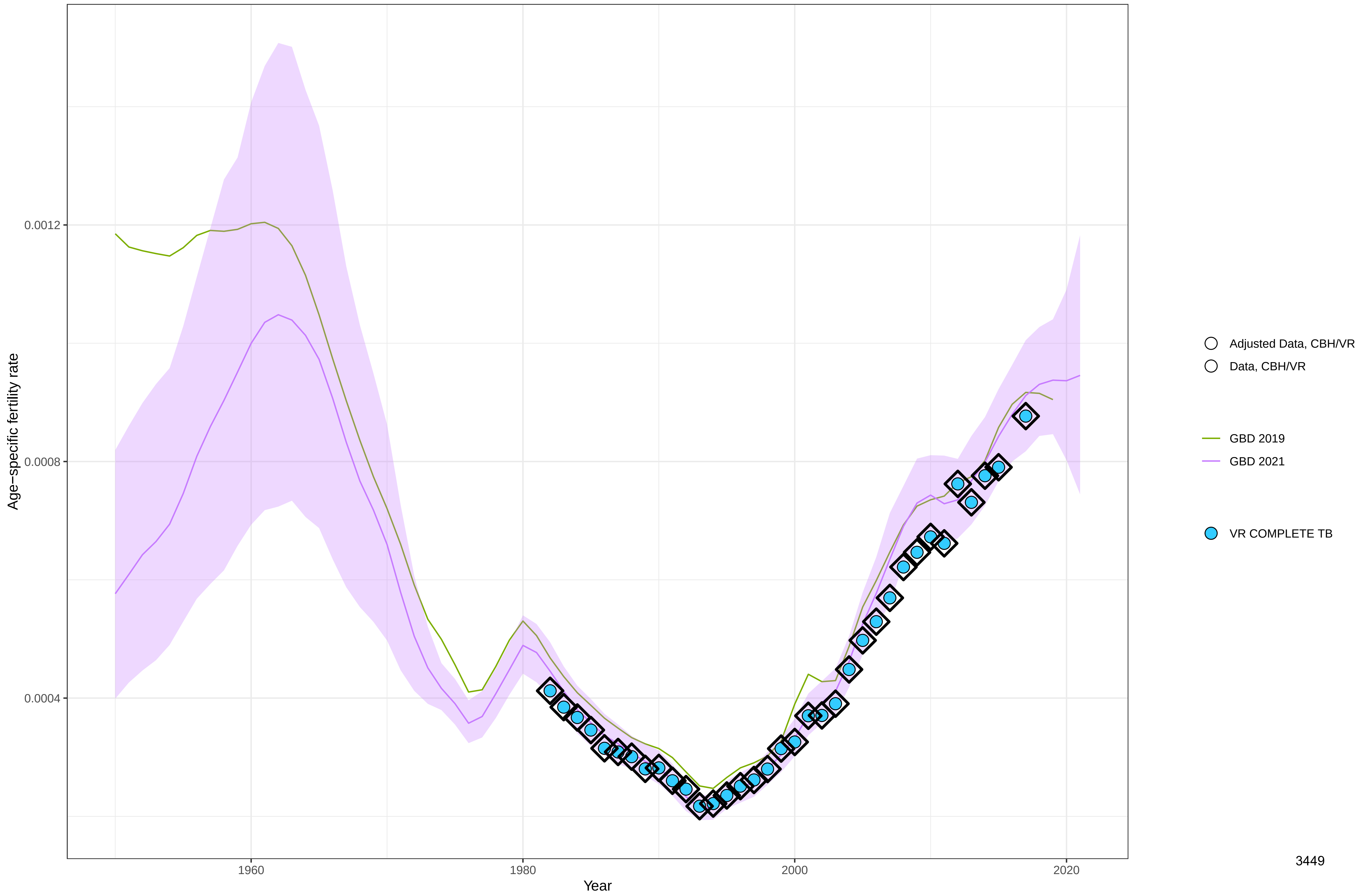

Age−specific fertility fitted trend lines, 1950−2021: Calderdale, Ages: 50−54
Data points enclosed with a diamond are reference sources

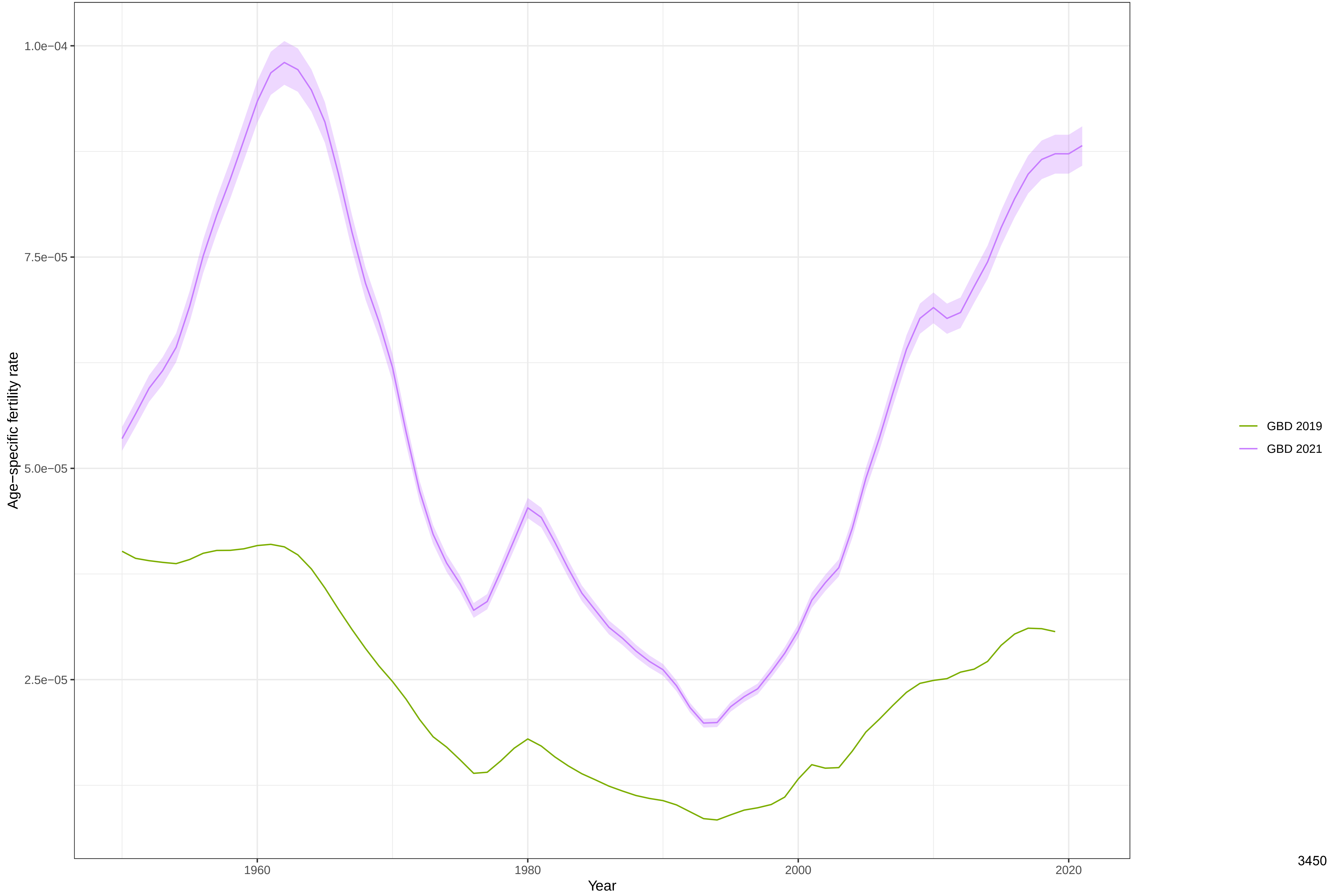

Total fertility rate fitted trend lines, 1950−2021: Calderdale TFR
Data points enclosed with a diamond are reference sources

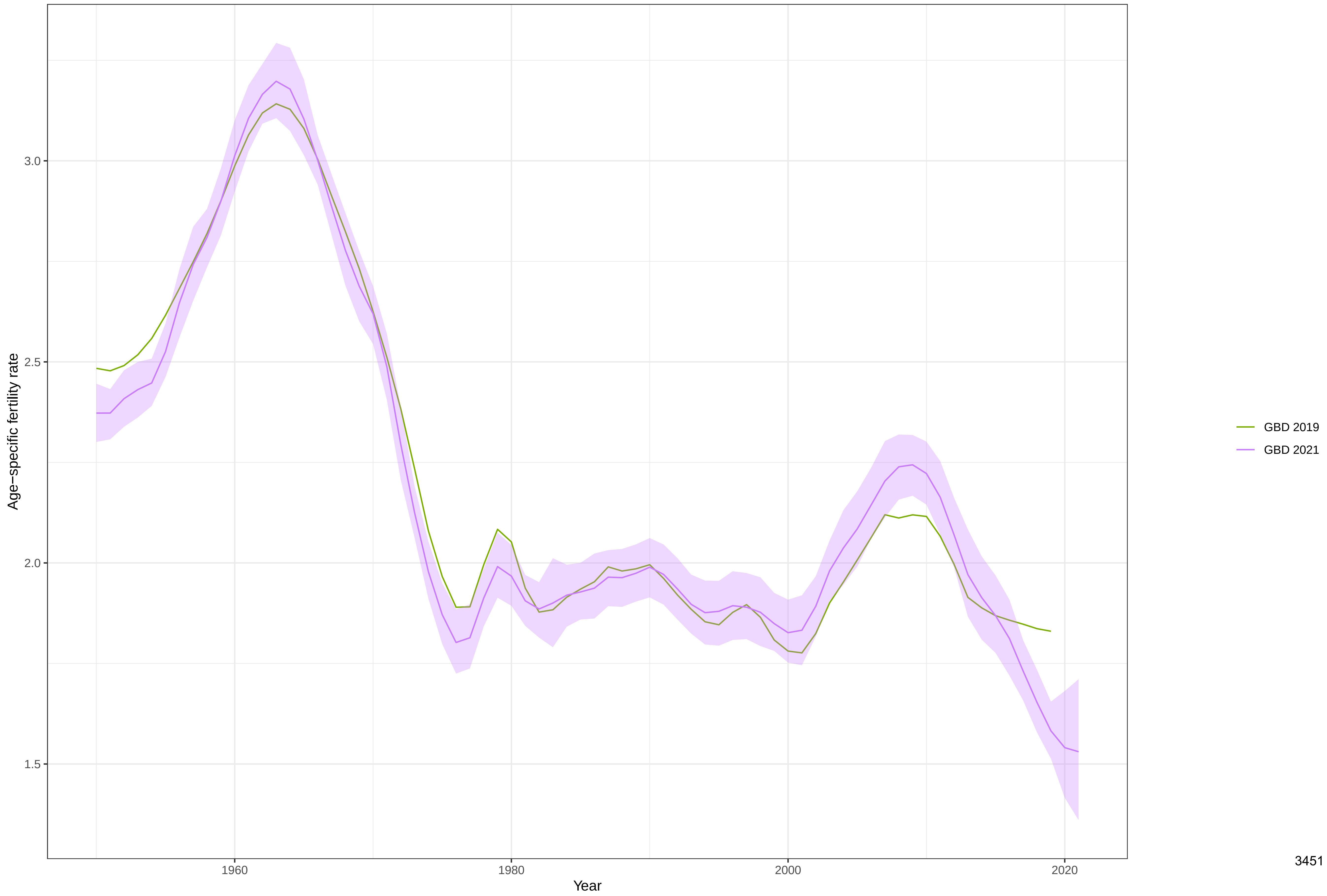

Age−specific fertility fitted trend lines, 1950−2021: Doncaster, Ages: 10−14
Data points enclosed with a diamond are reference sources

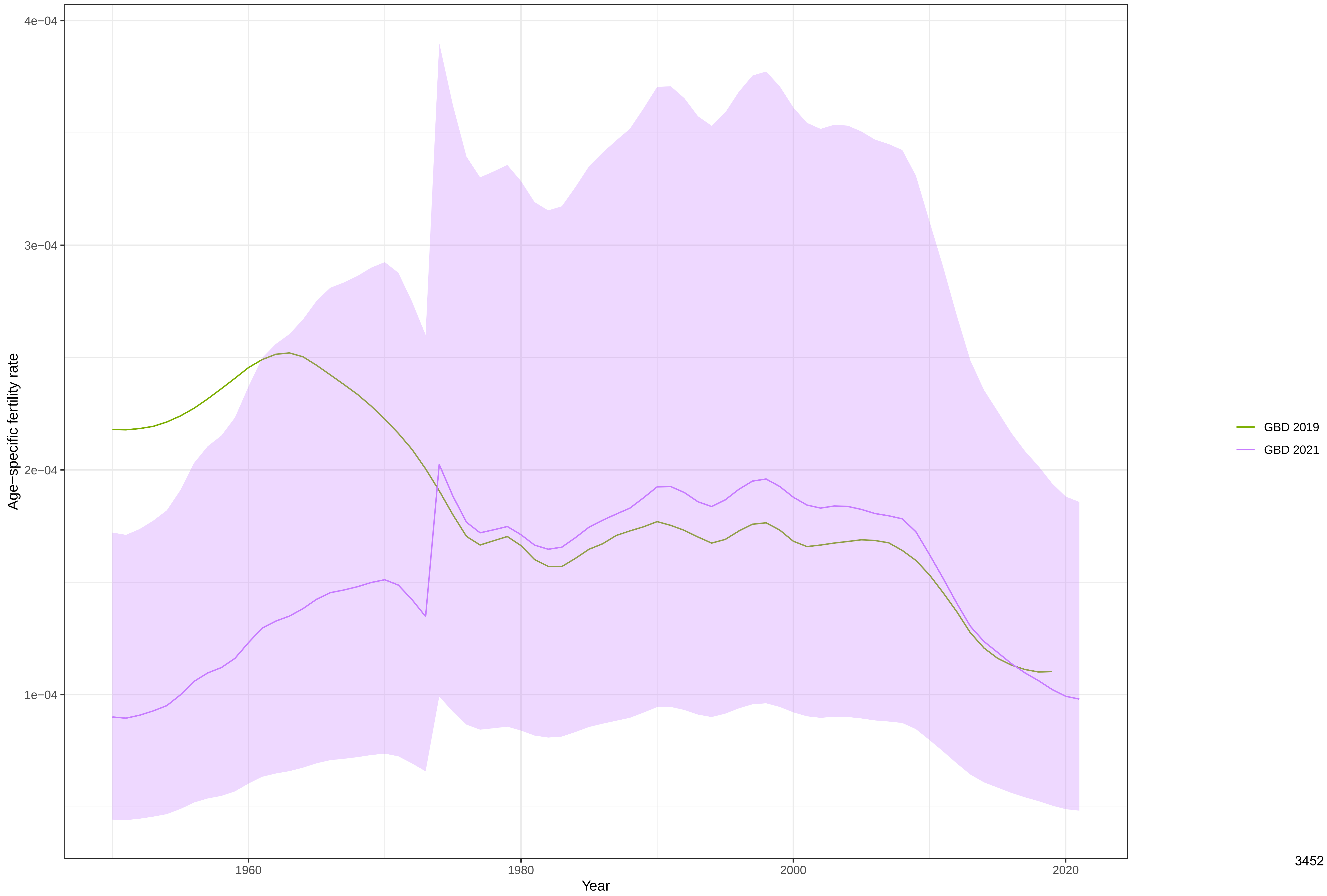

Age−specific fertility fitted trend lines, 1950−2021: Doncaster, Ages: 15−19
Data points enclosed with a diamond are reference sources

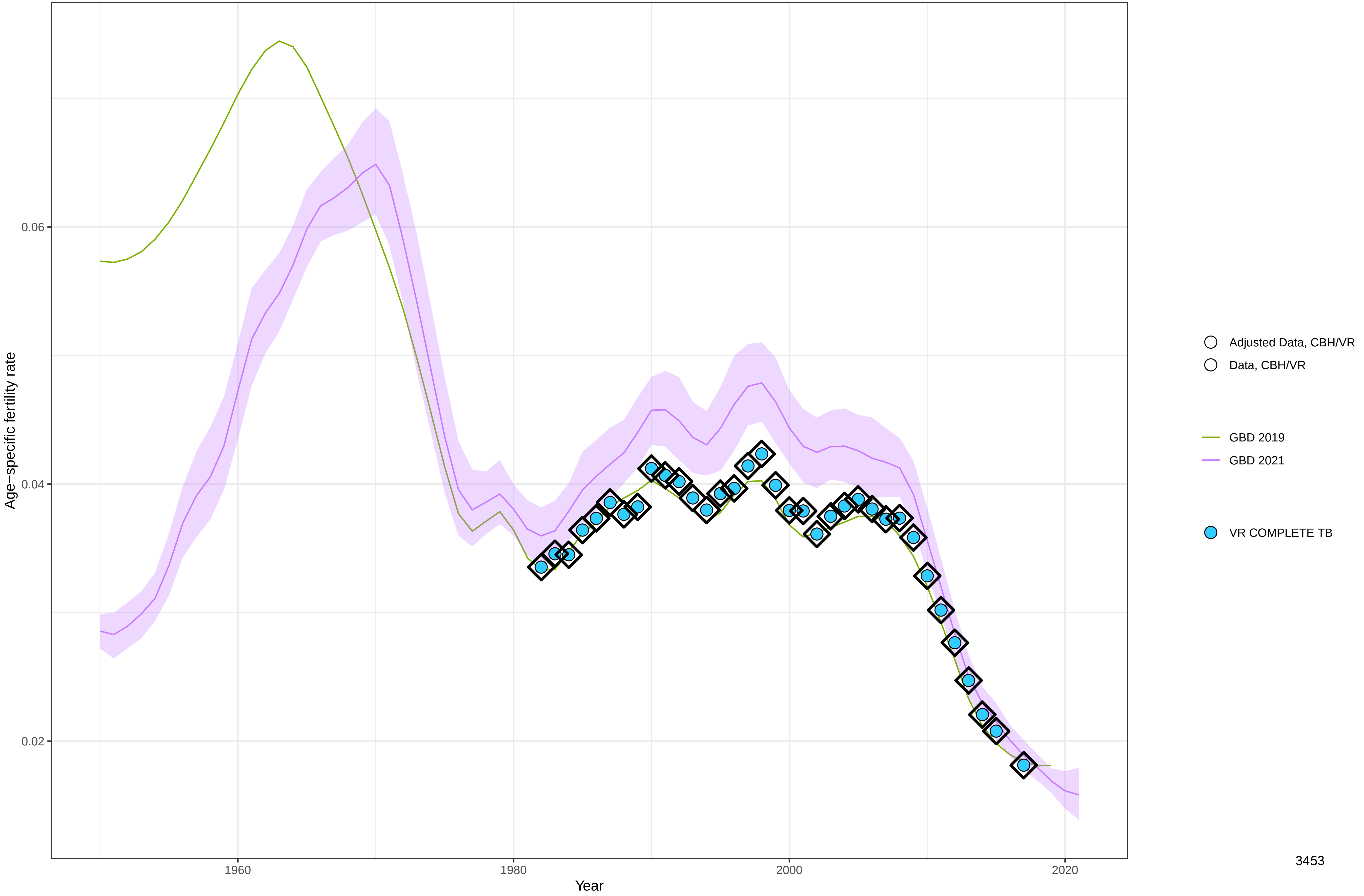

Age−specific fertility fitted trend lines, 1950−2021: Doncaster, Ages: 20−24
Data points enclosed with a diamond are reference sources

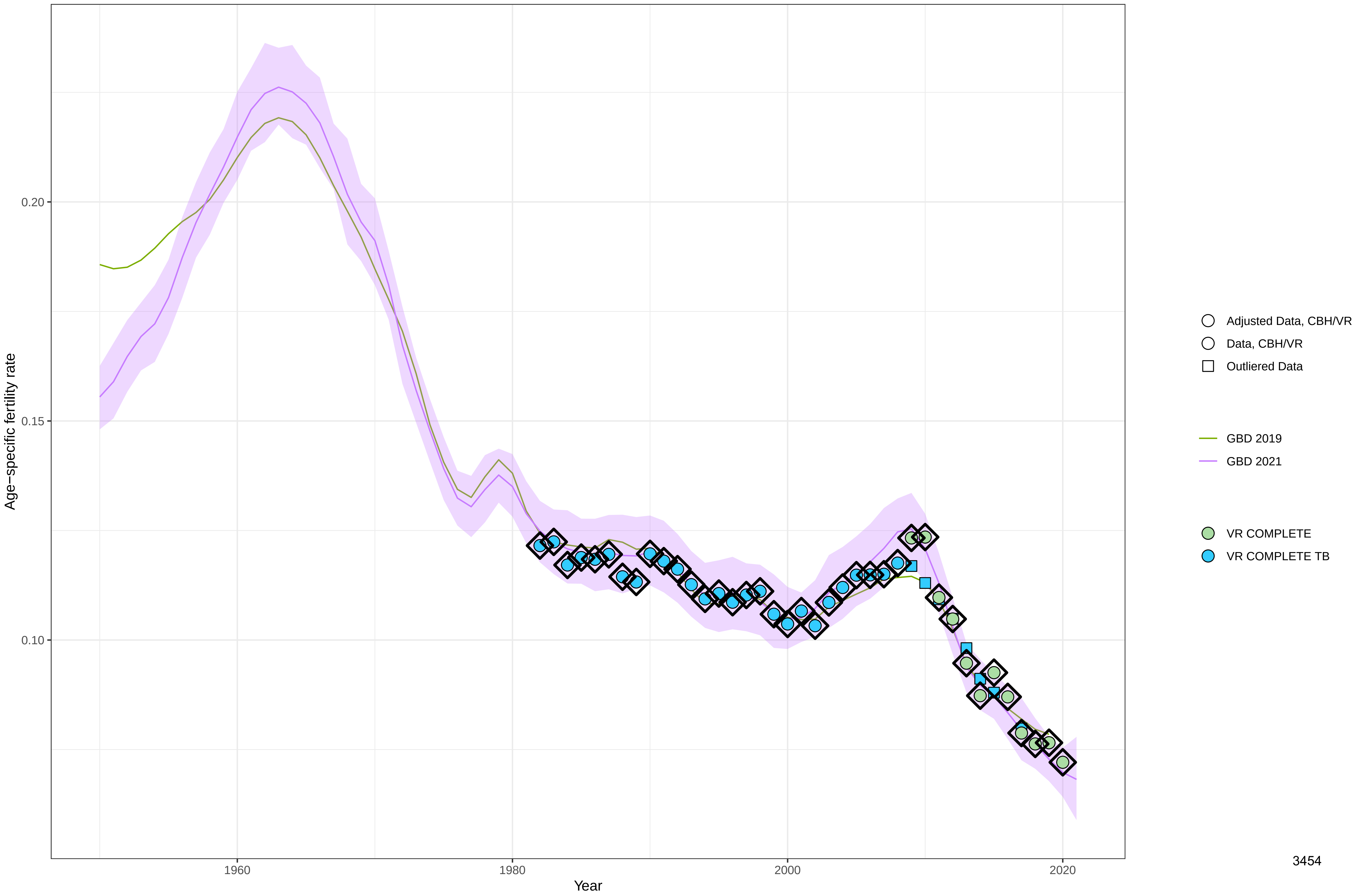

Age−specific fertility fitted trend lines, 1950−2021: Doncaster, Ages: 25−29
Data points enclosed with a diamond are reference sources

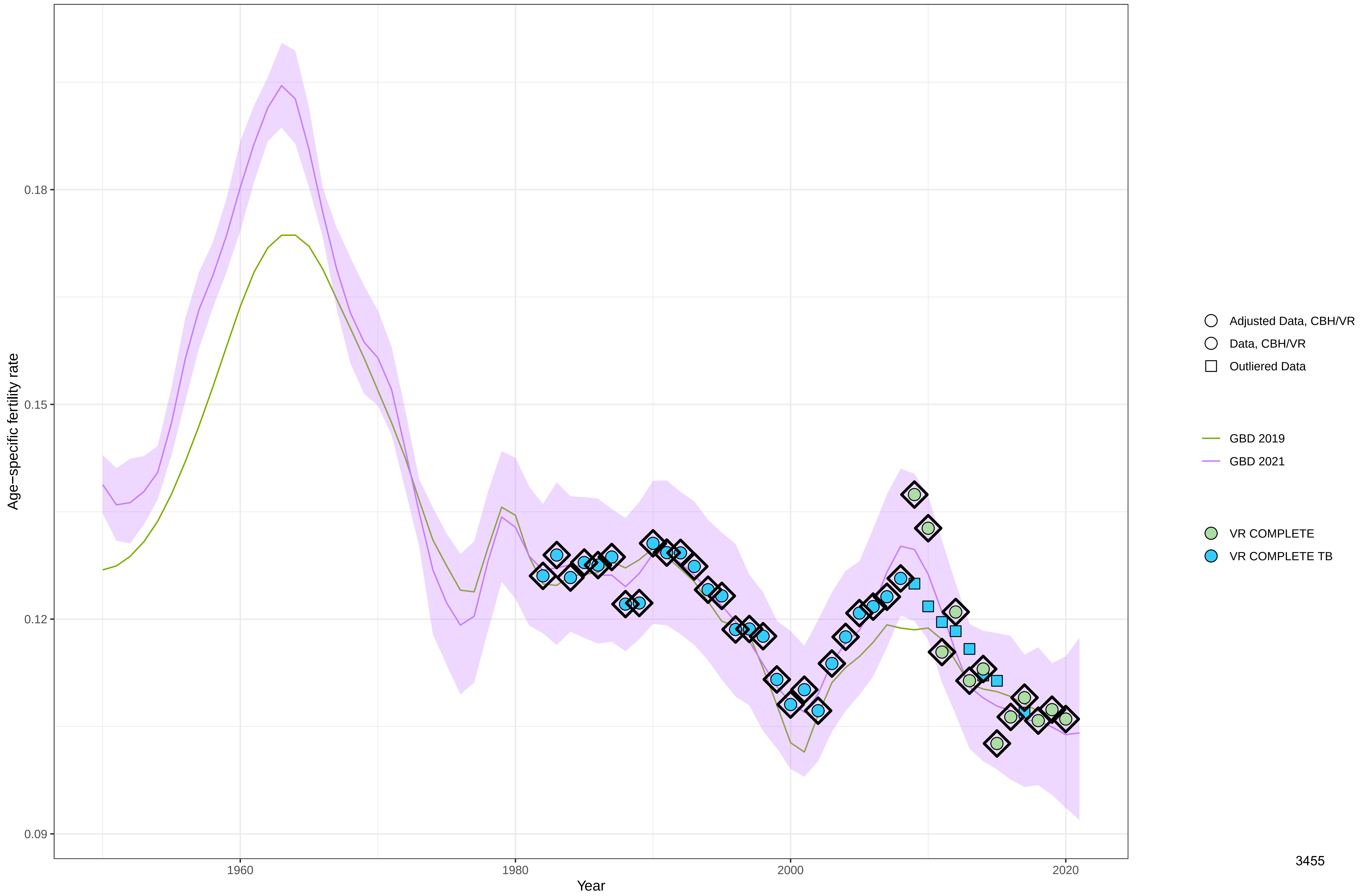

Age−specific fertility fitted trend lines, 1950−2021: Doncaster, Ages: 30−34
Data points enclosed with a diamond are reference sources

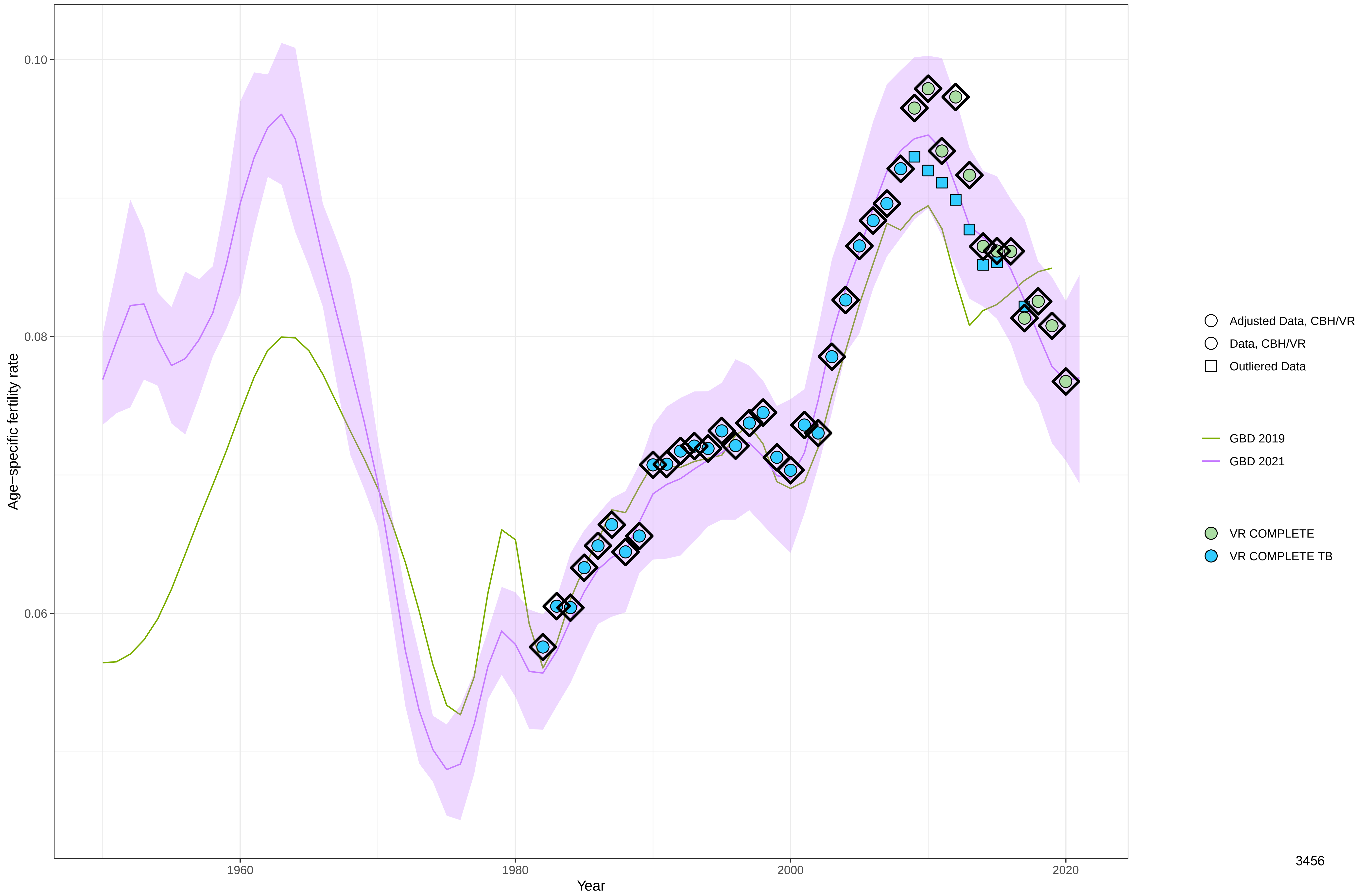

Age−specific fertility fitted trend lines, 1950−2021: Doncaster, Ages: 35−39
Data points enclosed with a diamond are reference sources

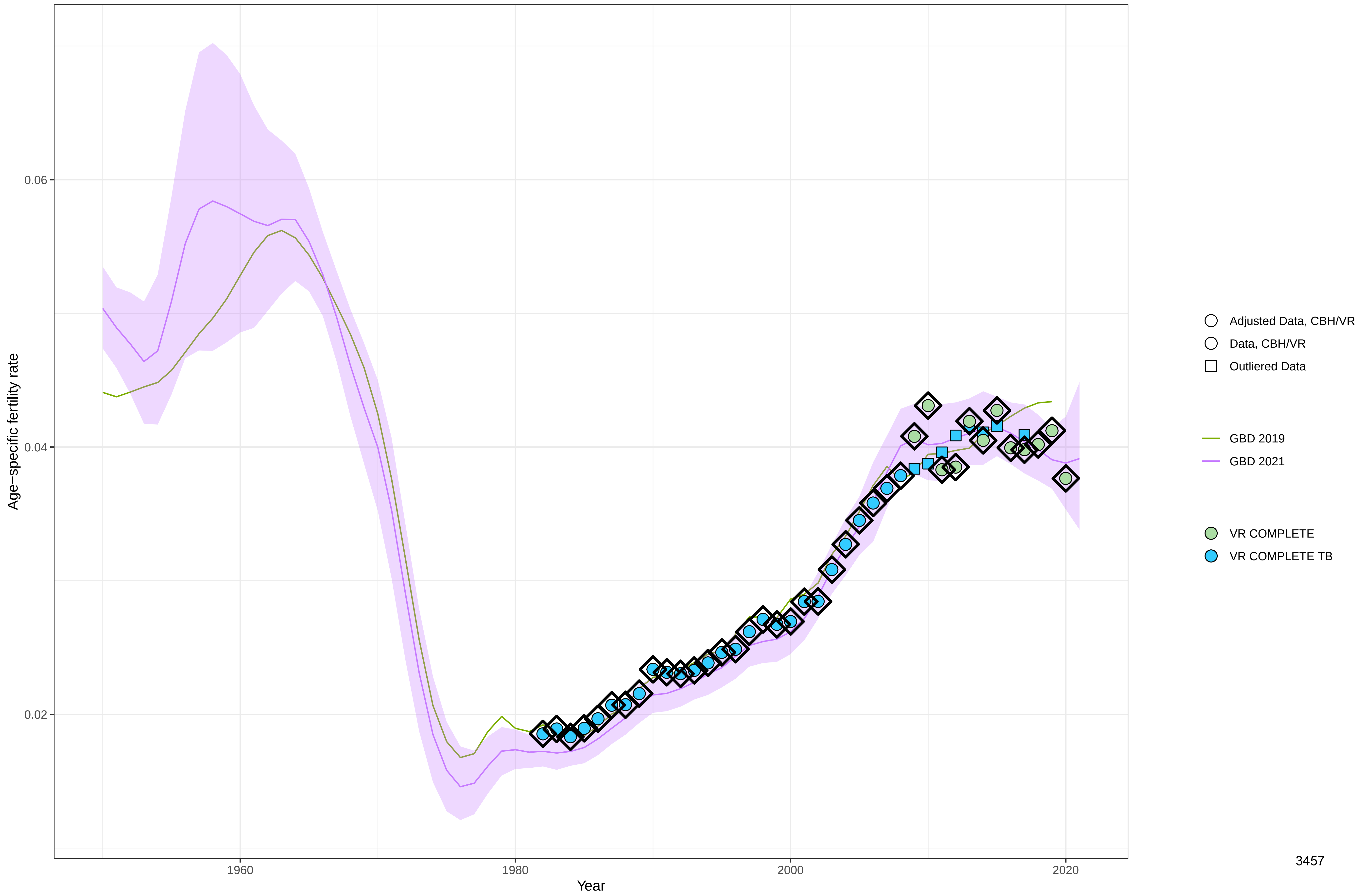

Age−specific fertility fitted trend lines, 1950−2021: Doncaster, Ages: 40−44
Data points enclosed with a diamond are reference sources

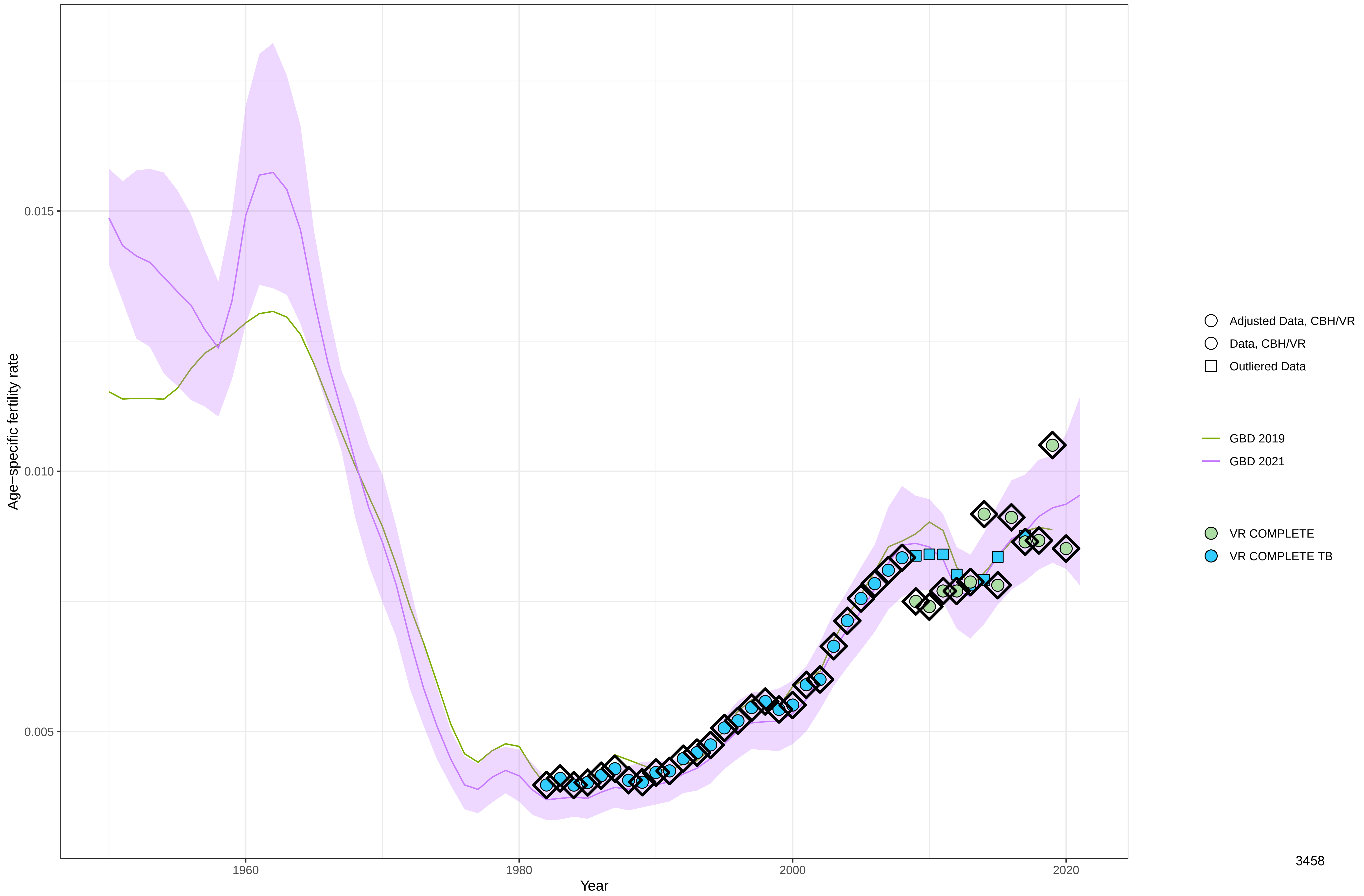

Age−specific fertility fitted trend lines, 1950−2021: Doncaster, Ages: 45−49
Data points enclosed with a diamond are reference sources

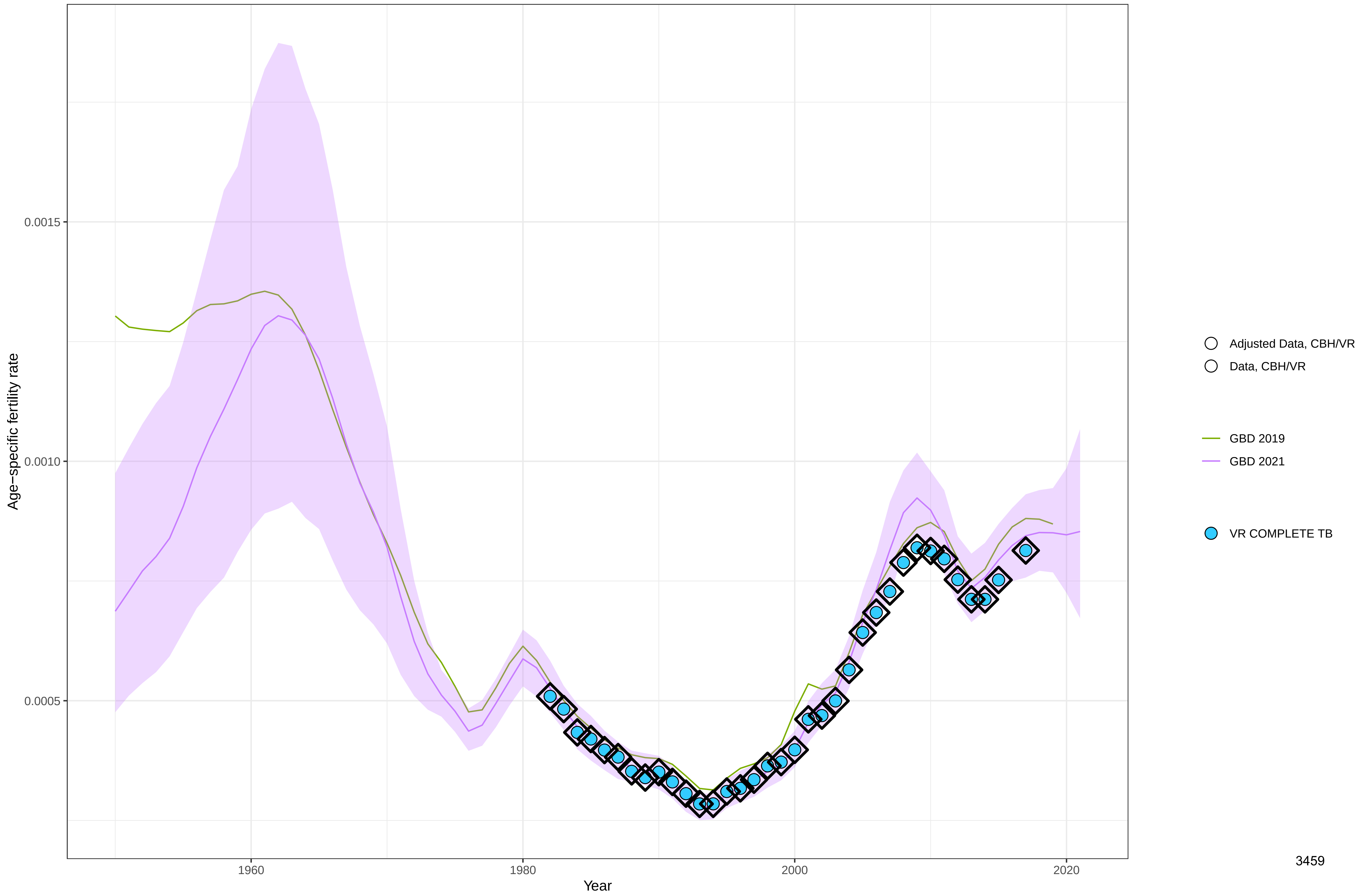

Age−specific fertility fitted trend lines, 1950−2021: Doncaster, Ages: 50−54
Data points enclosed with a diamond are reference sources

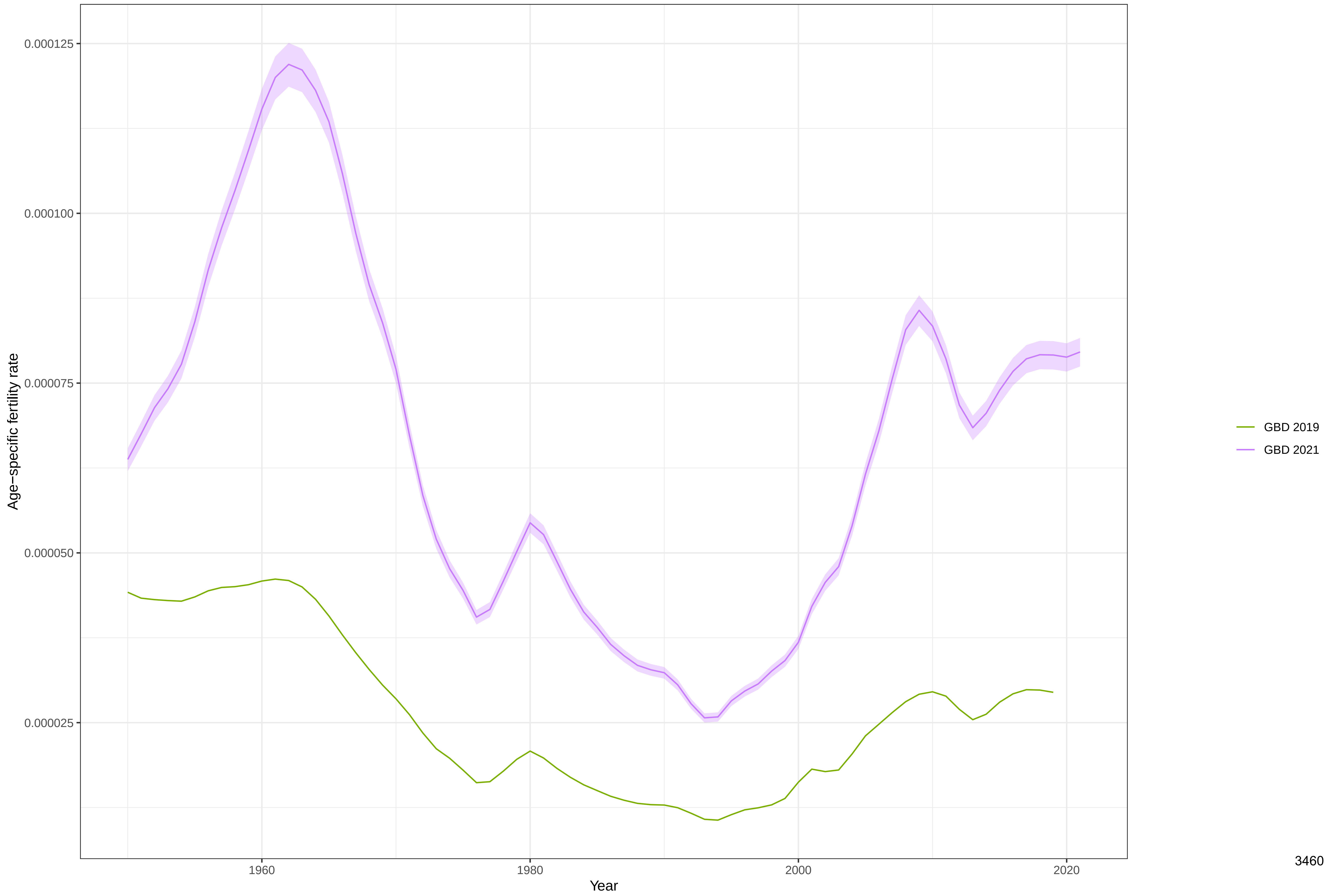

Total fertility rate fitted trend lines, 1950−2021: Doncaster TFR
Data points enclosed with a diamond are reference sources

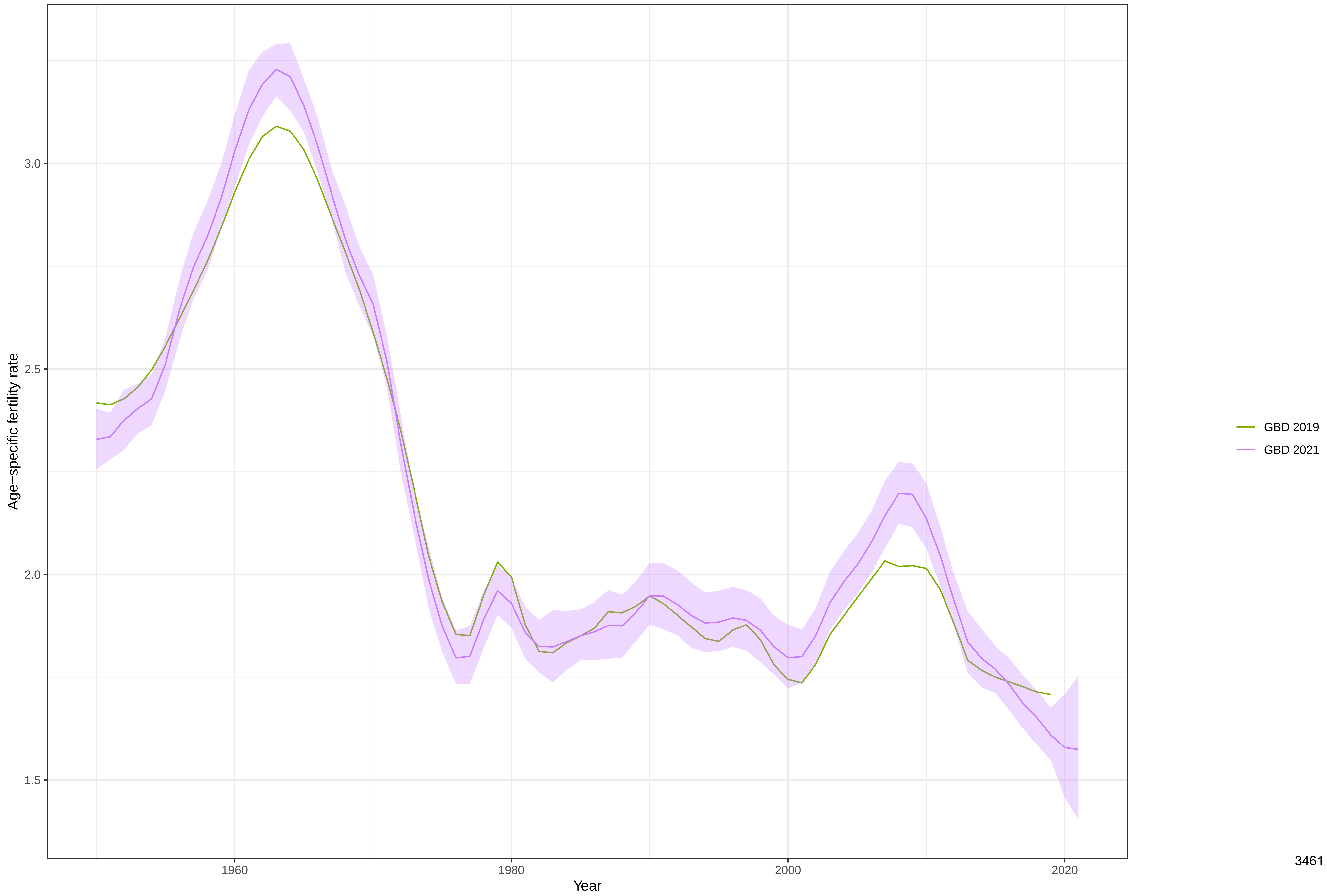

Age−specific fertility fitted trend lines, 1950−2021: East Riding of Yorkshire, Ages: 10−14
Data points enclosed with a diamond are reference sources

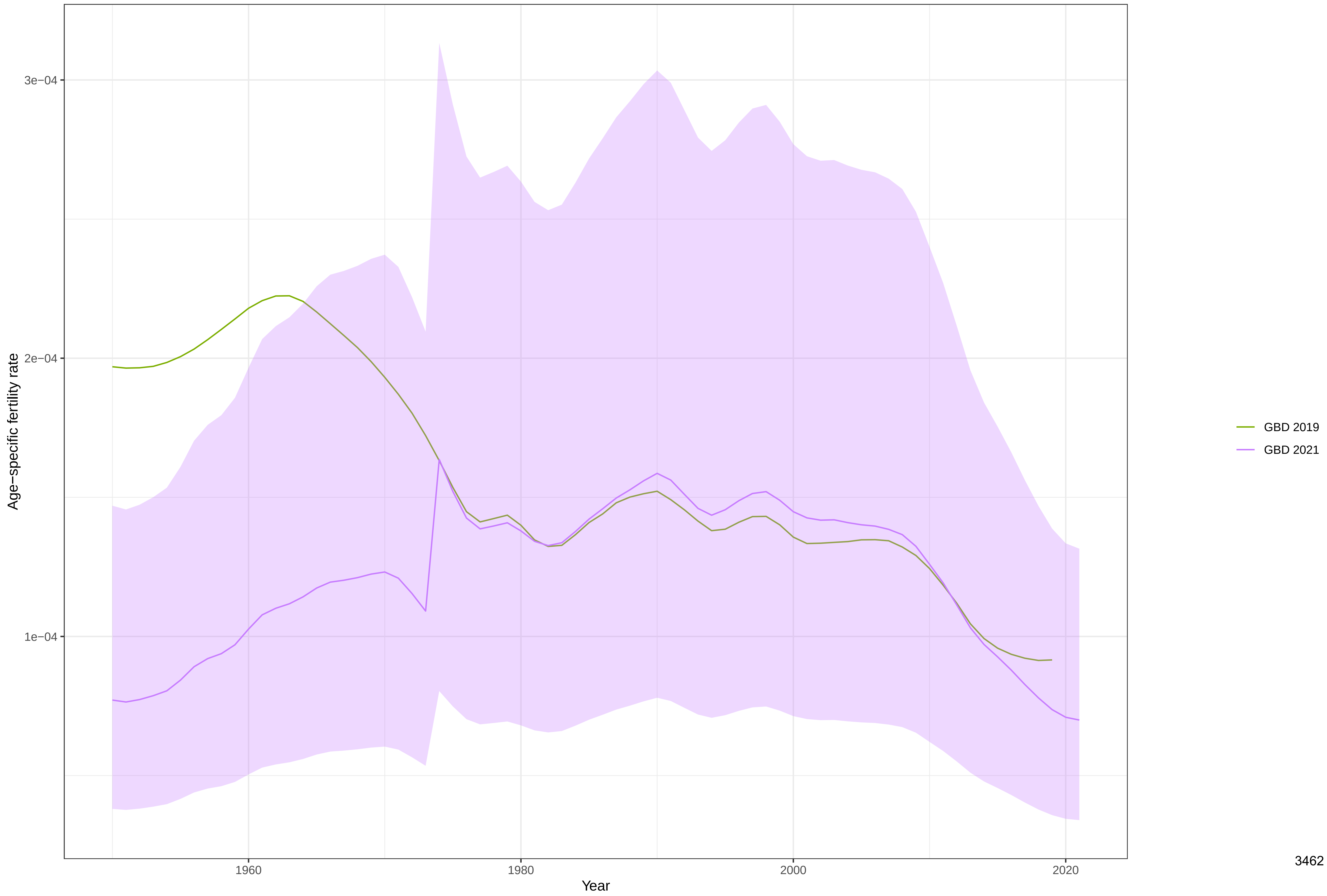

Age−specific fertility fitted trend lines, 1950−2021: East Riding of Yorkshire, Ages: 15−19
Data points enclosed with a diamond are reference sources

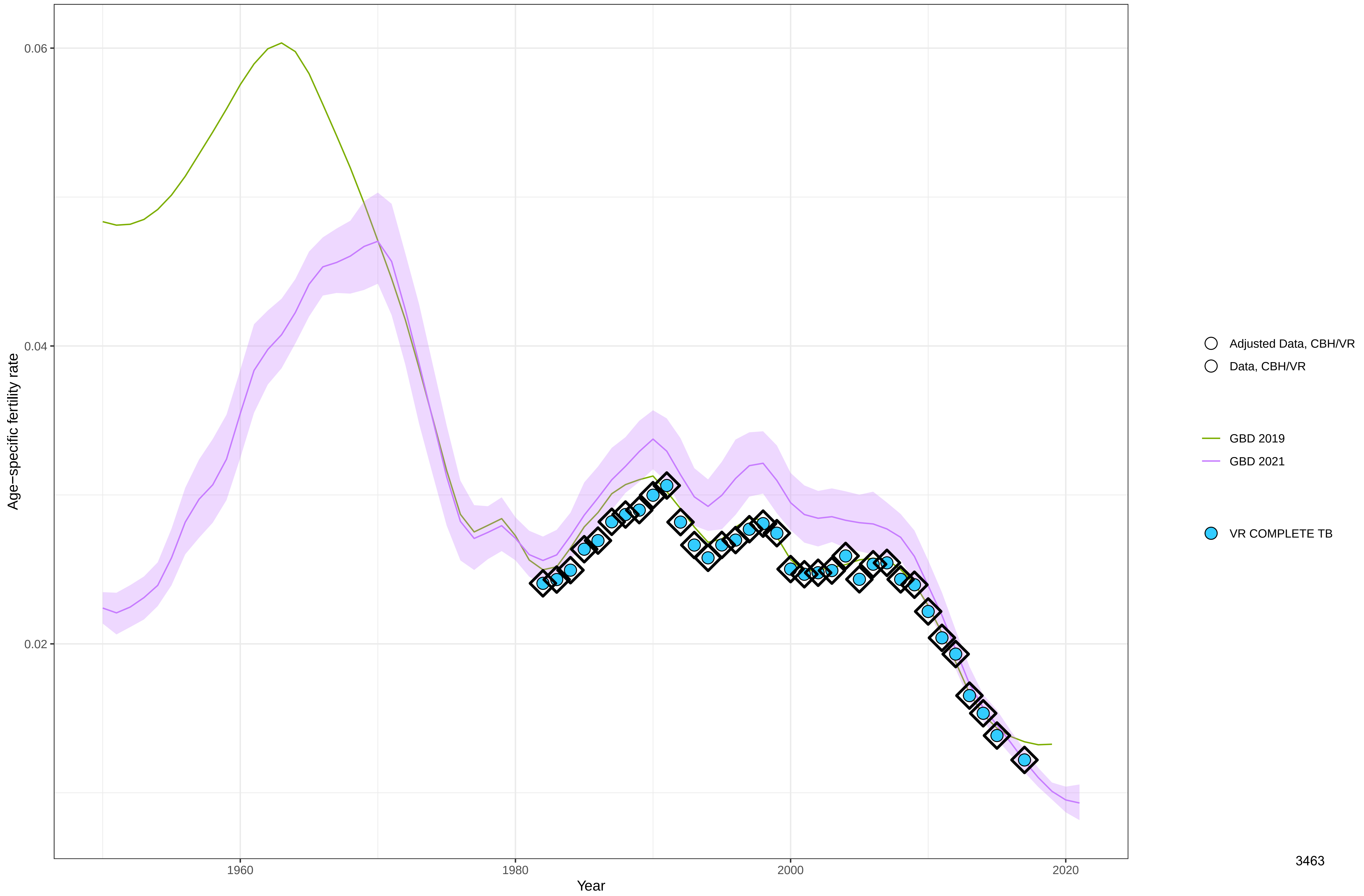

Age−specific fertility fitted trend lines, 1950−2021: East Riding of Yorkshire, Ages: 20−24
Data points enclosed with a diamond are reference sources

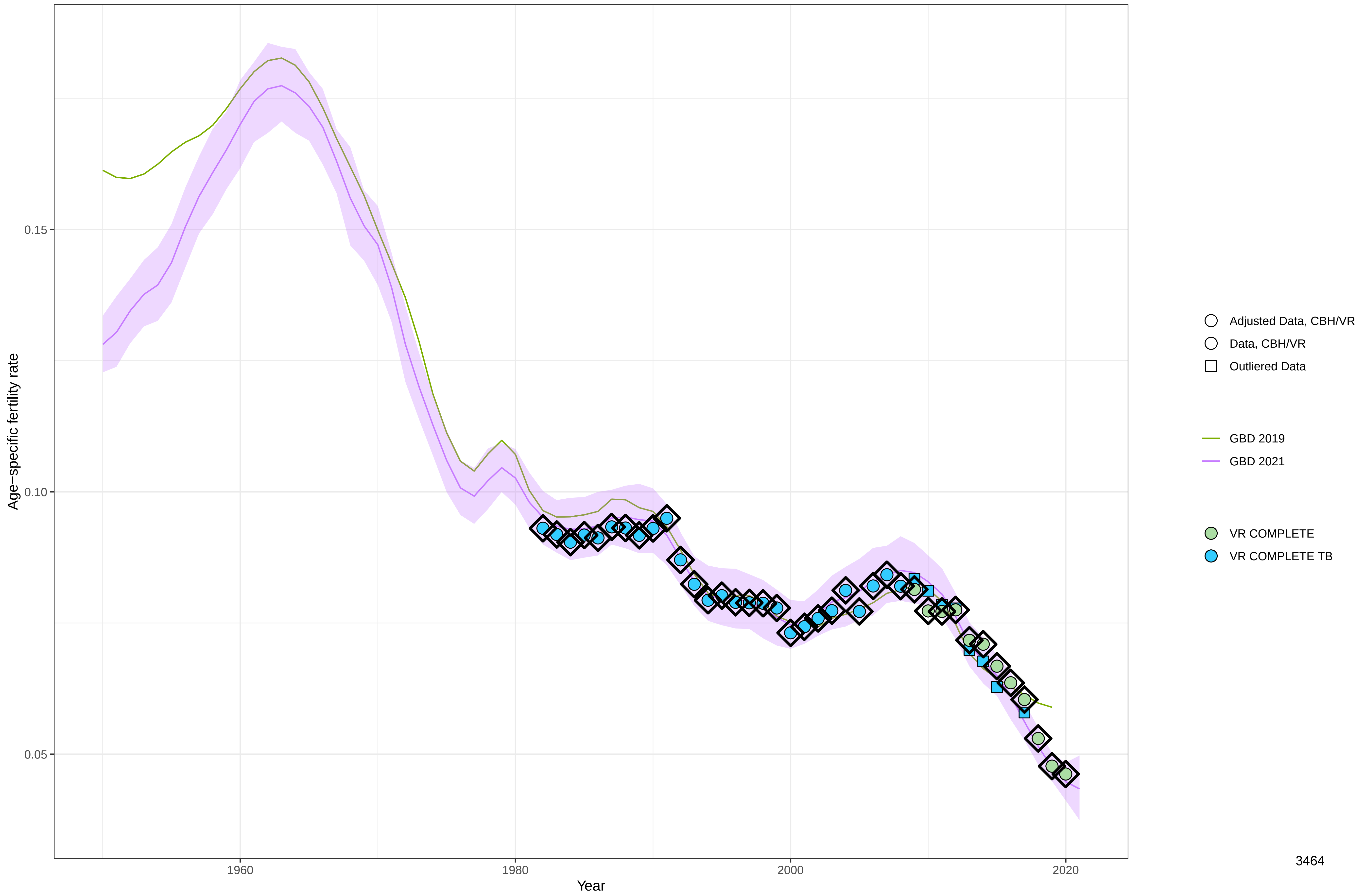

Age−specific fertility fitted trend lines, 1950−2021: East Riding of Yorkshire, Ages: 25−29
Data points enclosed with a diamond are reference sources

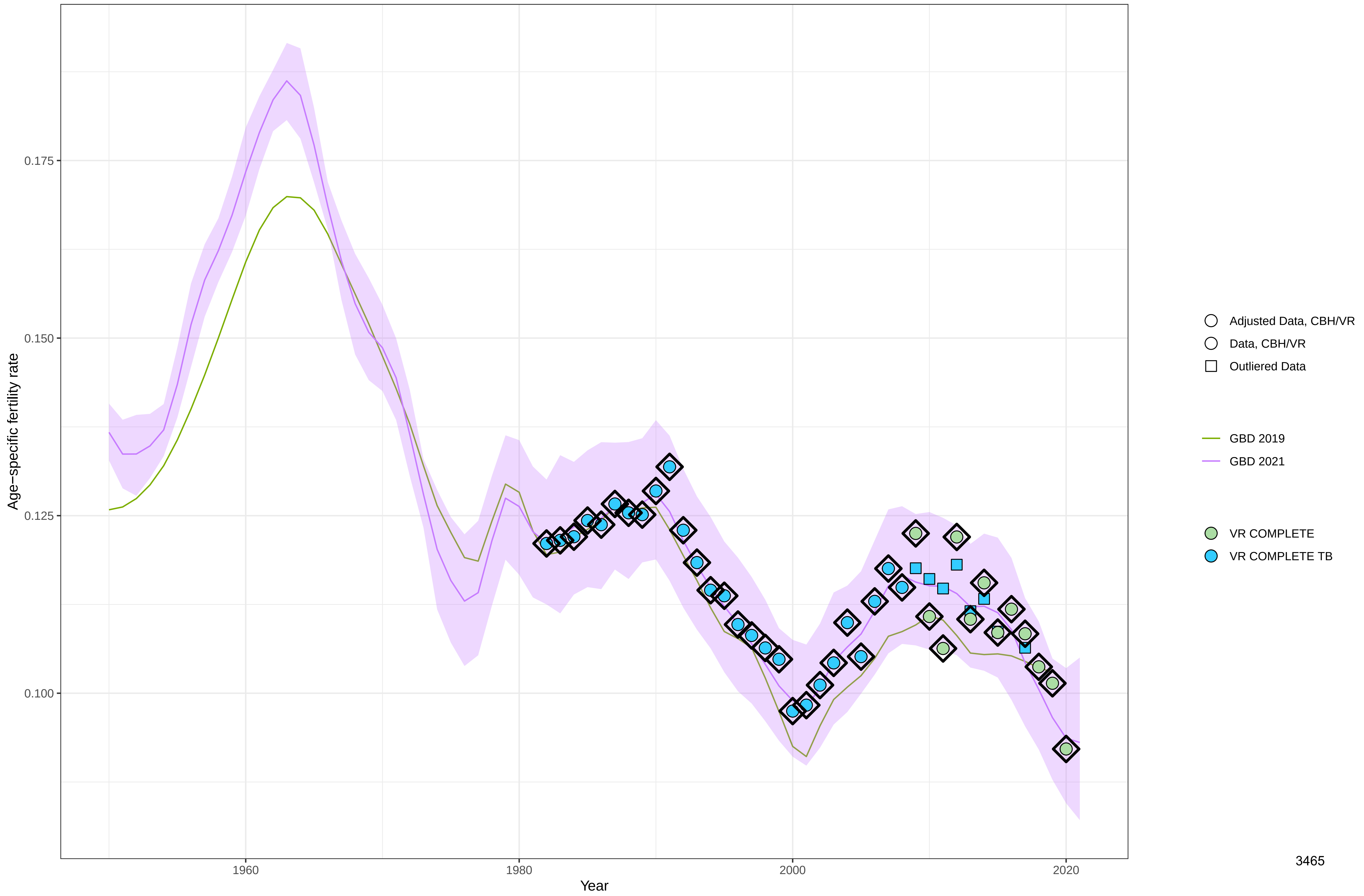

Age−specific fertility fitted trend lines, 1950−2021: East Riding of Yorkshire, Ages: 30−34
Data points enclosed with a diamond are reference sources

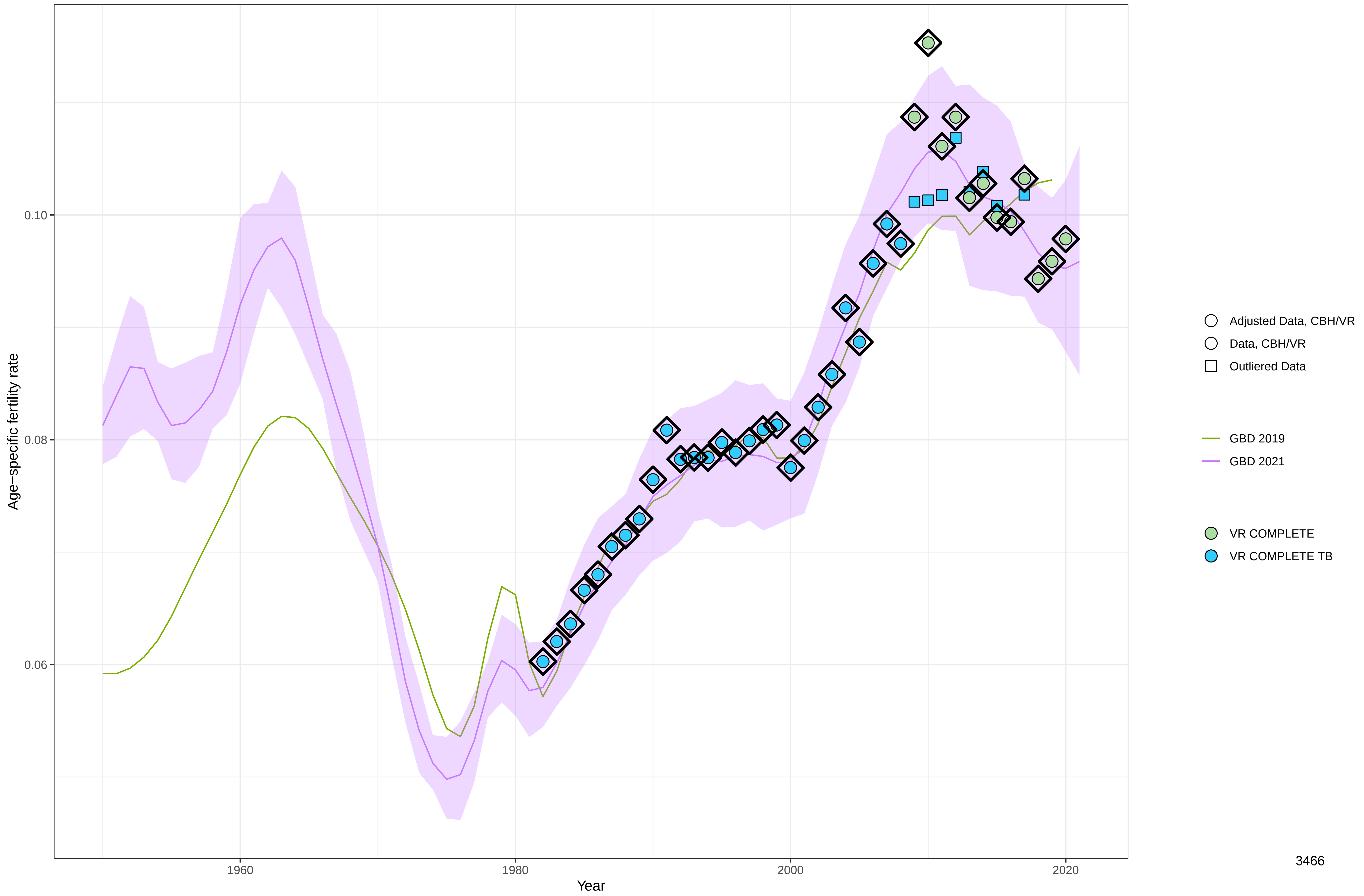

Age−specific fertility fitted trend lines, 1950−2021: East Riding of Yorkshire, Ages: 35−39
Data points enclosed with a diamond are reference sources

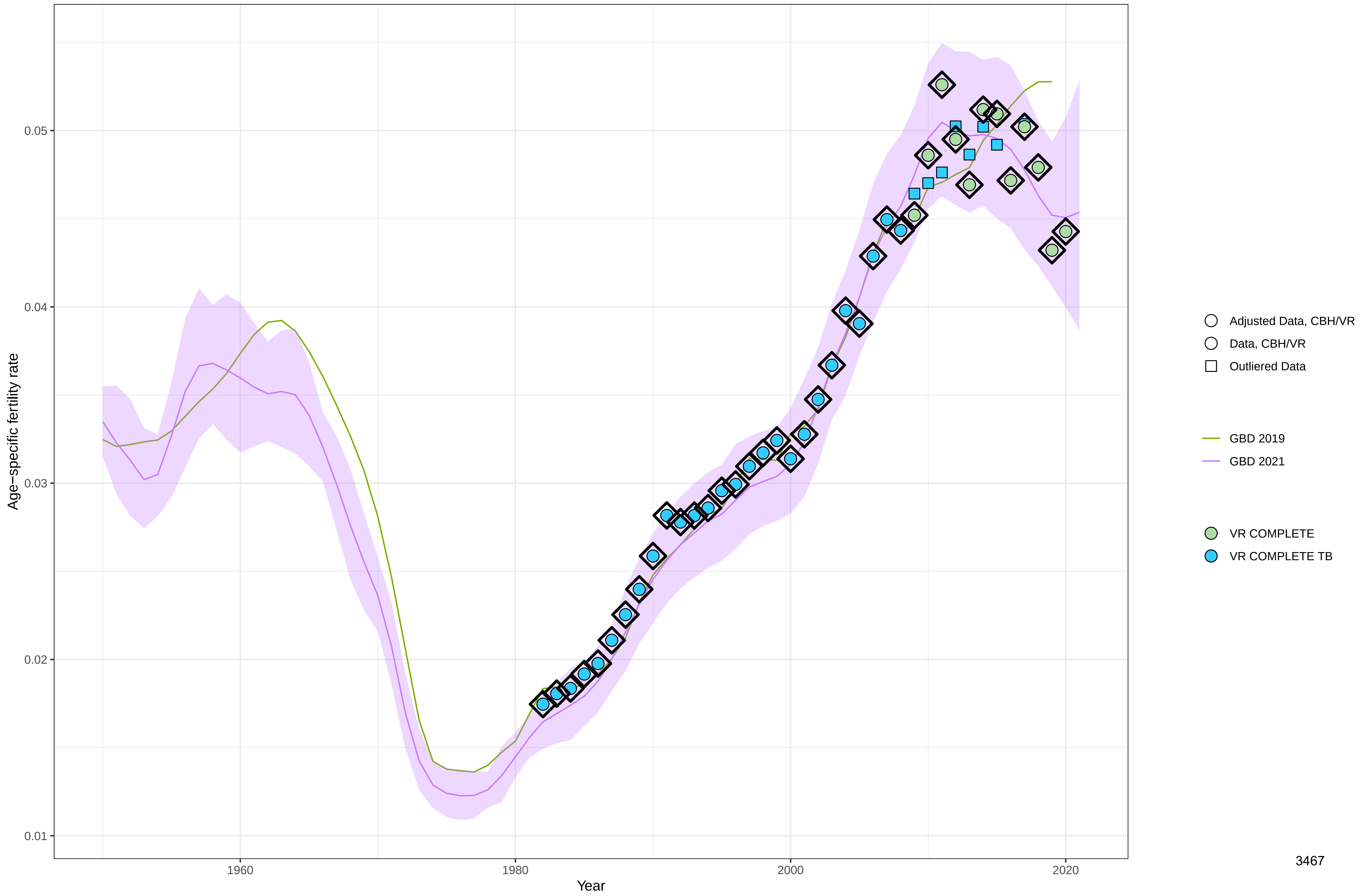

Age−specific fertility fitted trend lines, 1950−2021: East Riding of Yorkshire, Ages: 40−44
Data points enclosed with a diamond are reference sources

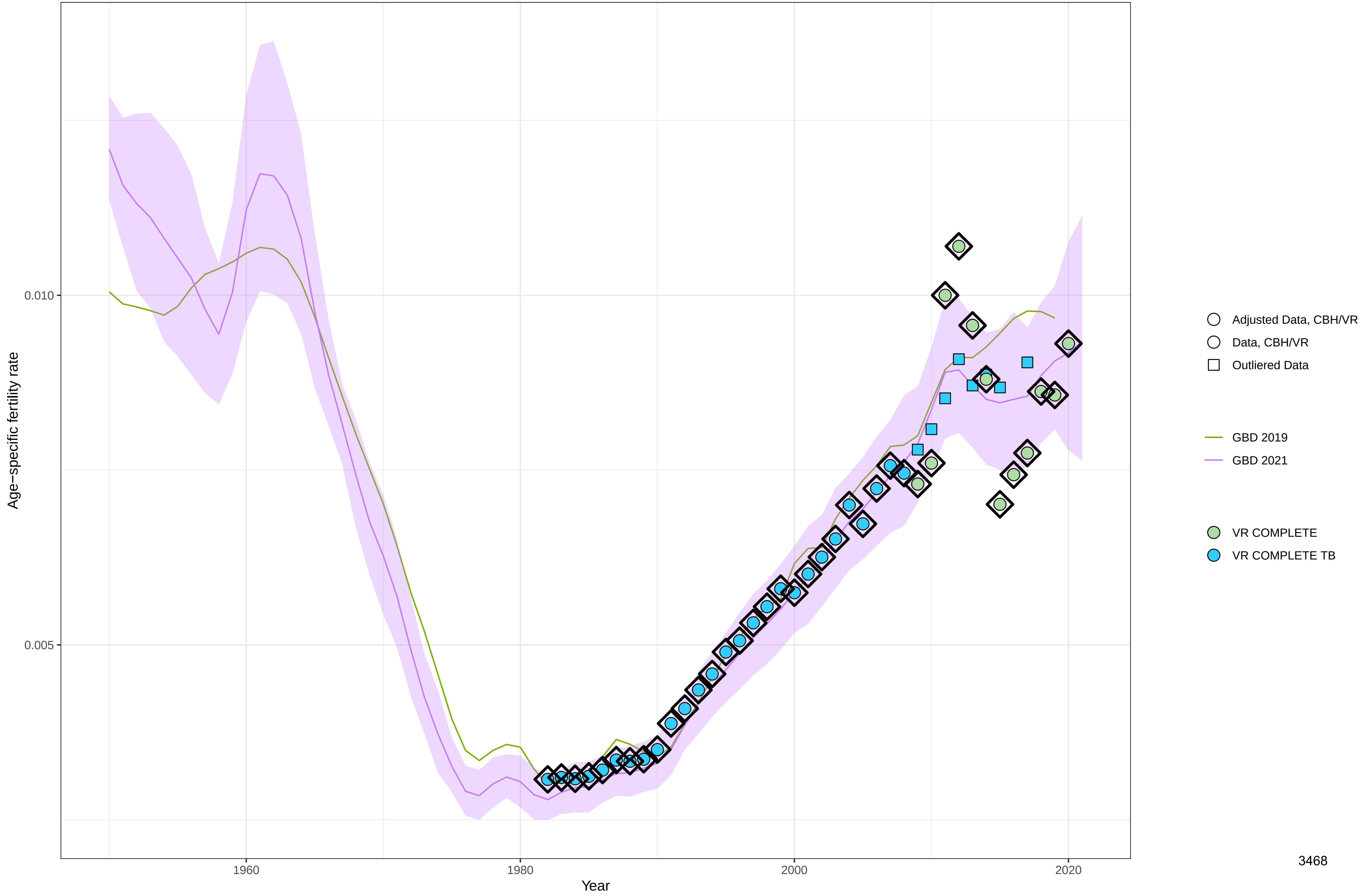

Age−specific fertility fitted trend lines, 1950−2021: East Riding of Yorkshire, Ages: 45−49
Data points enclosed with a diamond are reference sources

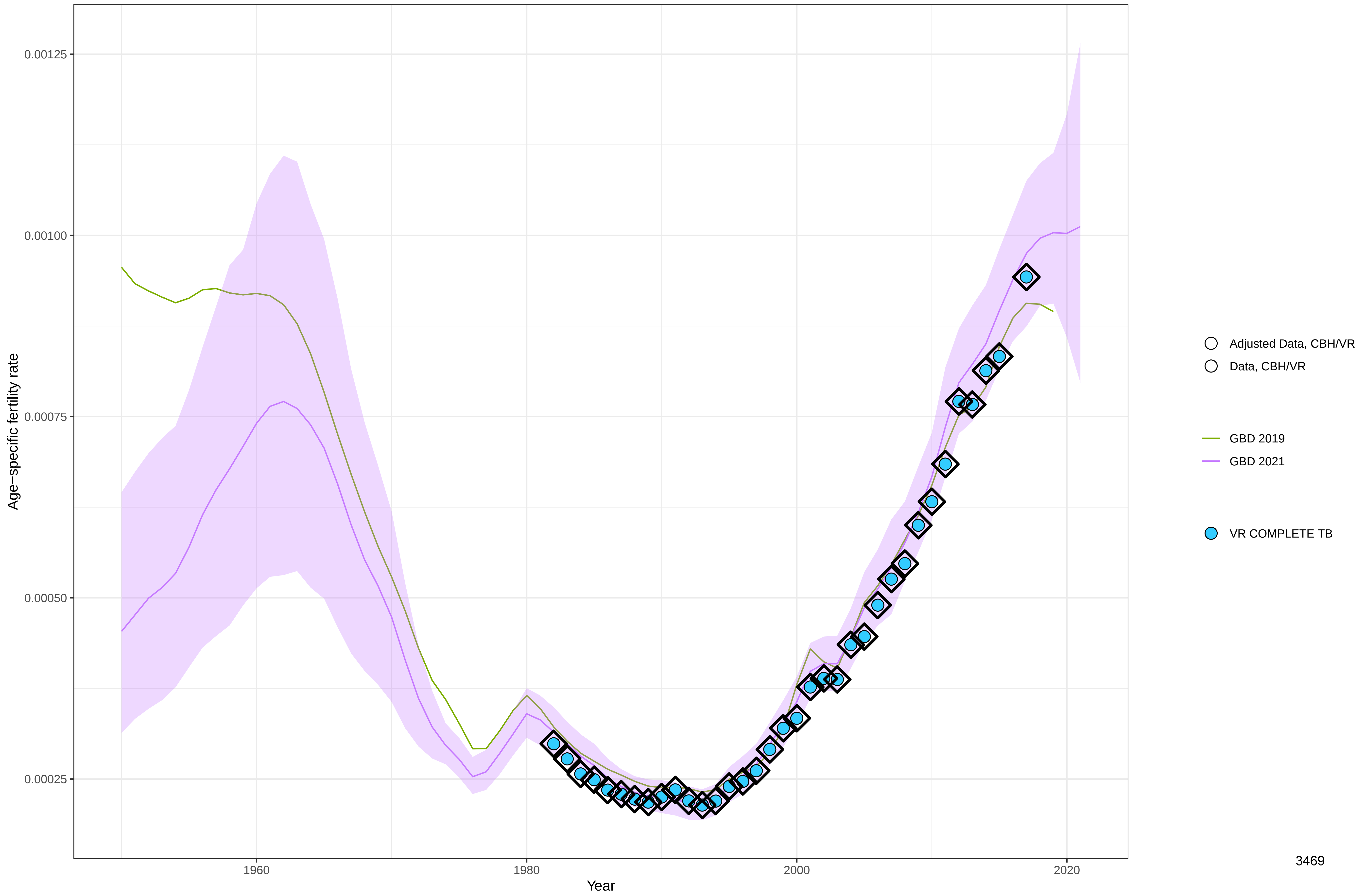

Age−specific fertility fitted trend lines, 1950−2021: East Riding of Yorkshire, Ages: 50−54
Data points enclosed with a diamond are reference sources

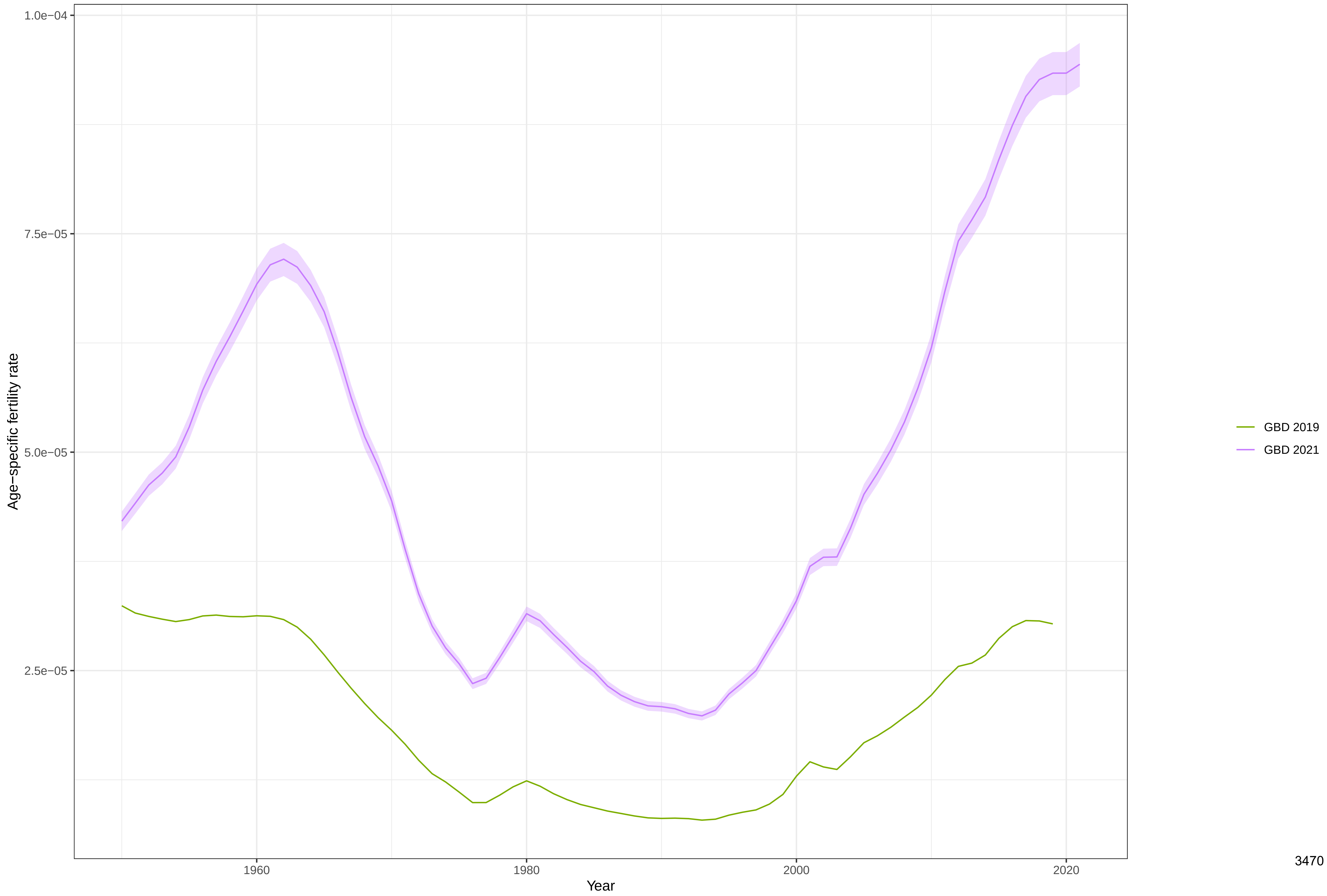

Total fertility rate fitted trend lines, 1950−2021: East Riding of Yorkshire TFR
Data points enclosed with a diamond are reference sources

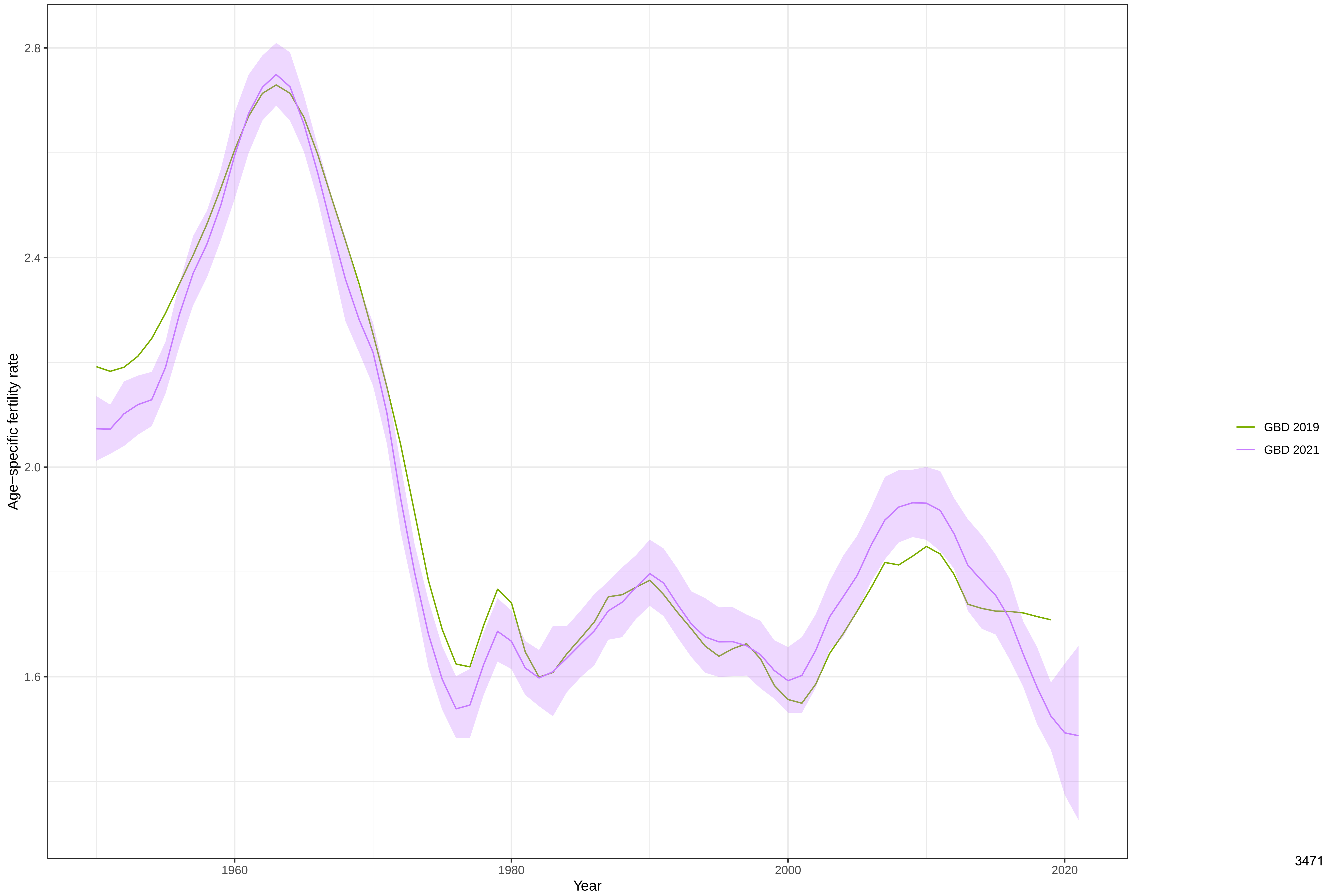

Age−specific fertility fitted trend lines, 1950−2021: Kingston upon Hull, City of, Ages: 10−14
Data points enclosed with a diamond are reference sources

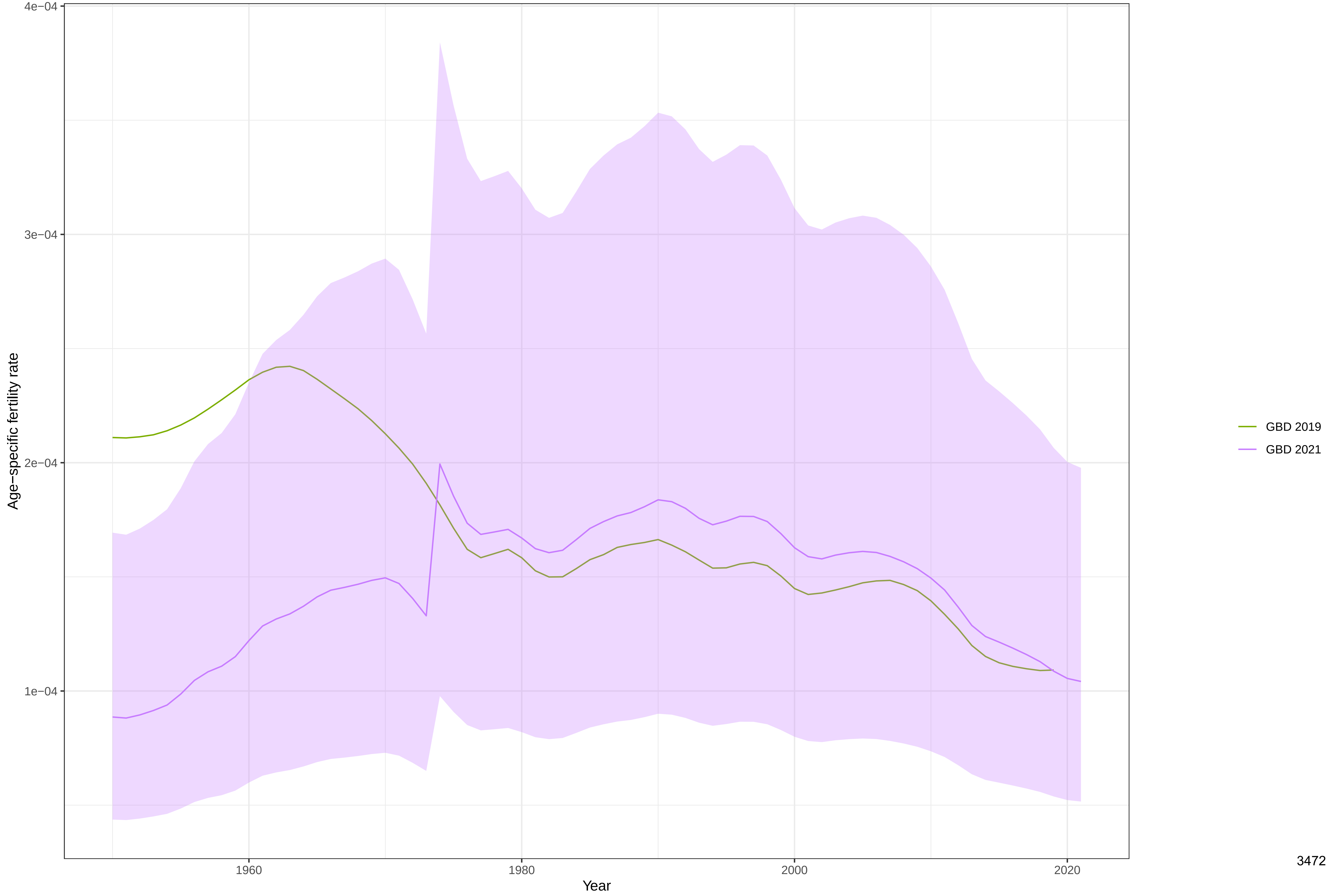

Age−specific fertility fitted trend lines, 1950−2021: Kingston upon Hull, City of, Ages: 15−19
Data points enclosed with a diamond are reference sources

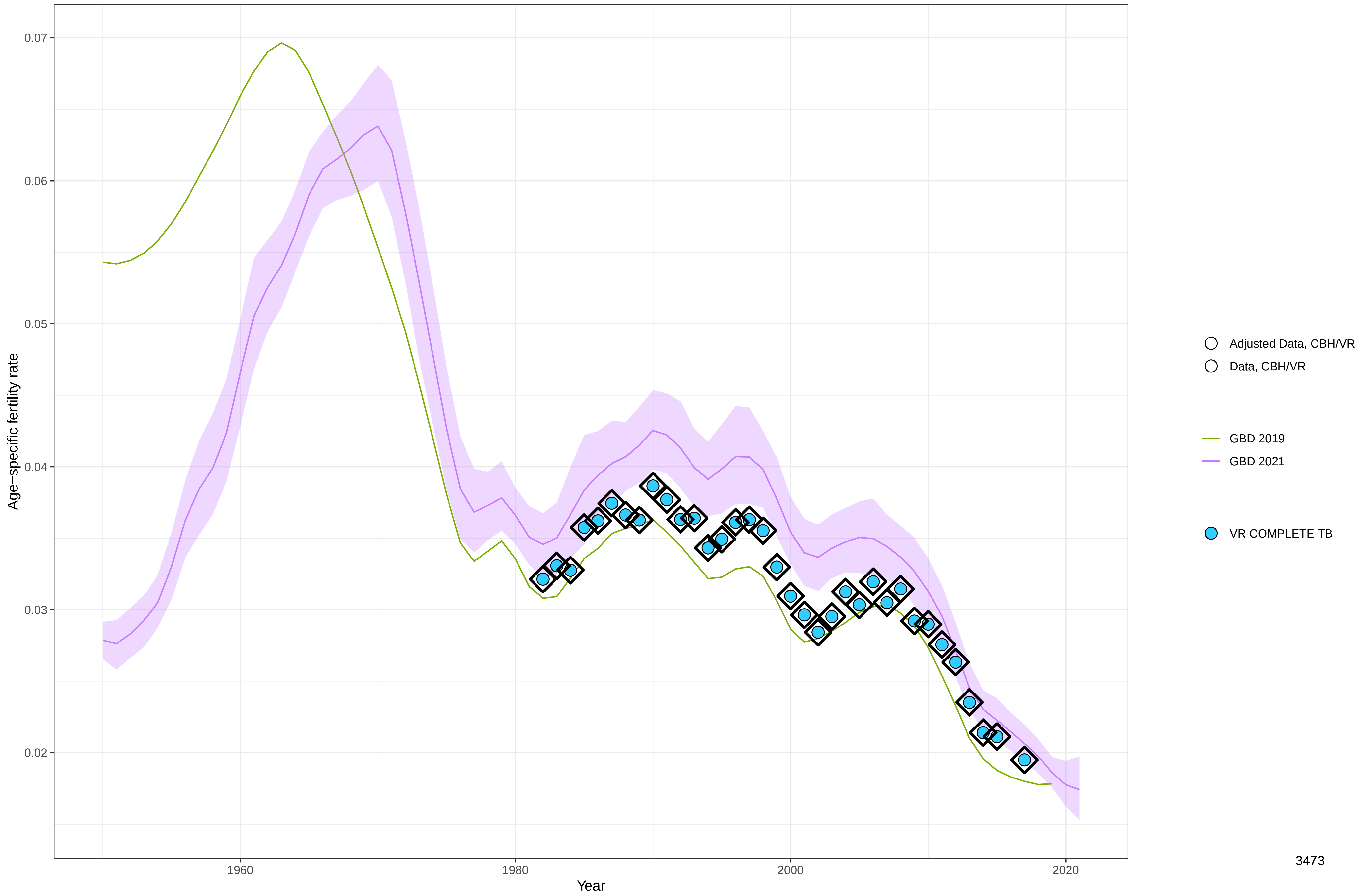

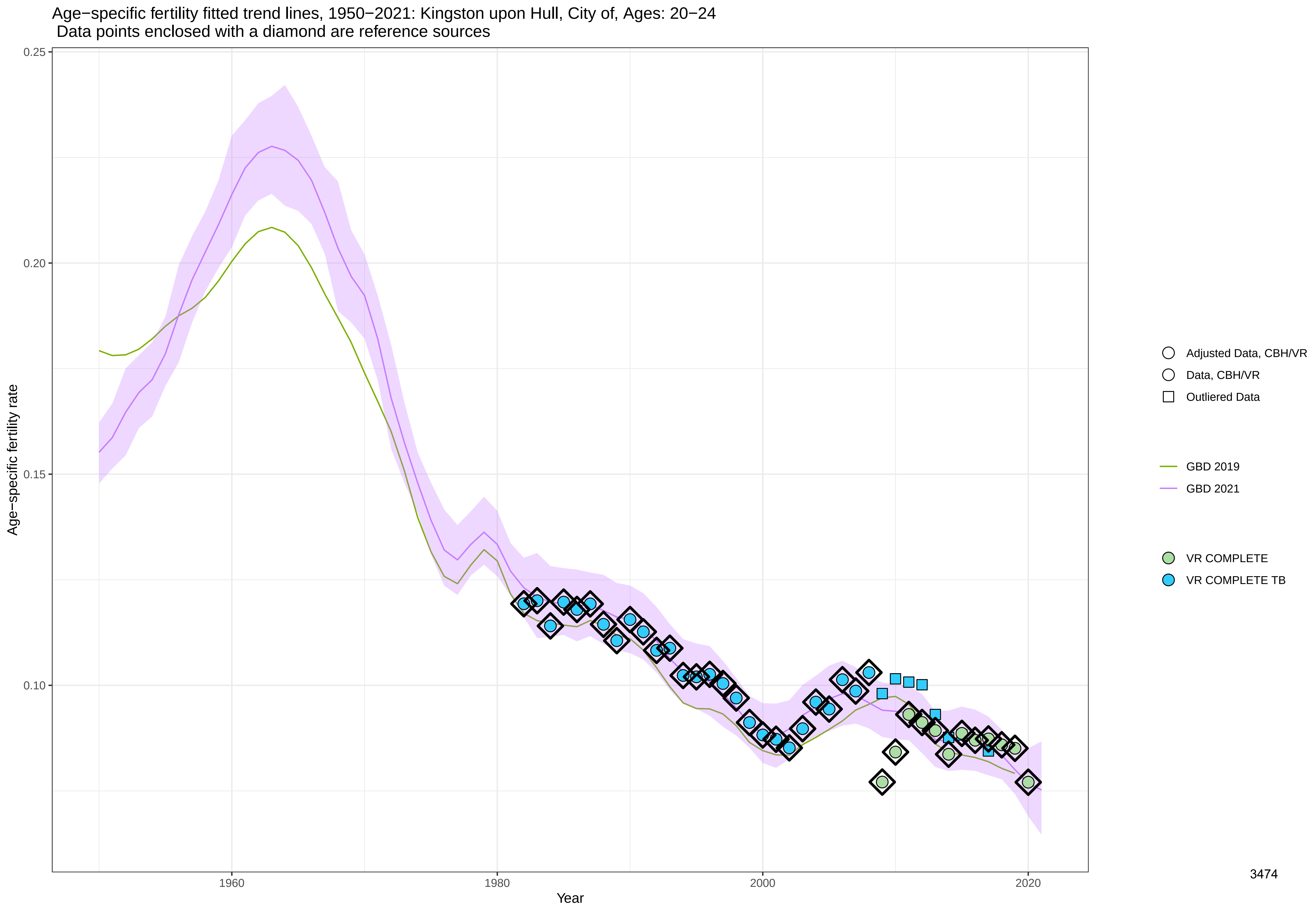

Age−specific fertility fitted trend lines, 1950−2021: Kingston upon Hull, City of, Ages: 20−24
Data points enclosed with a diamond are reference sources
Age−specific fertility rate
0.25
0.20
0.15
0.10
1960
1980
2000
2020
Year
Adjusted Data, CBH/VR
Data, CBH/VR
Outliered Data
GBD 2019
GBD 2021
VR COMPLETE
VR COMPLETE TB

3474

Age−specific fertility fitted trend lines, 1950−2021: Kingston upon Hull, City of, Ages: 25−29
Data points enclosed with a diamond are reference sources

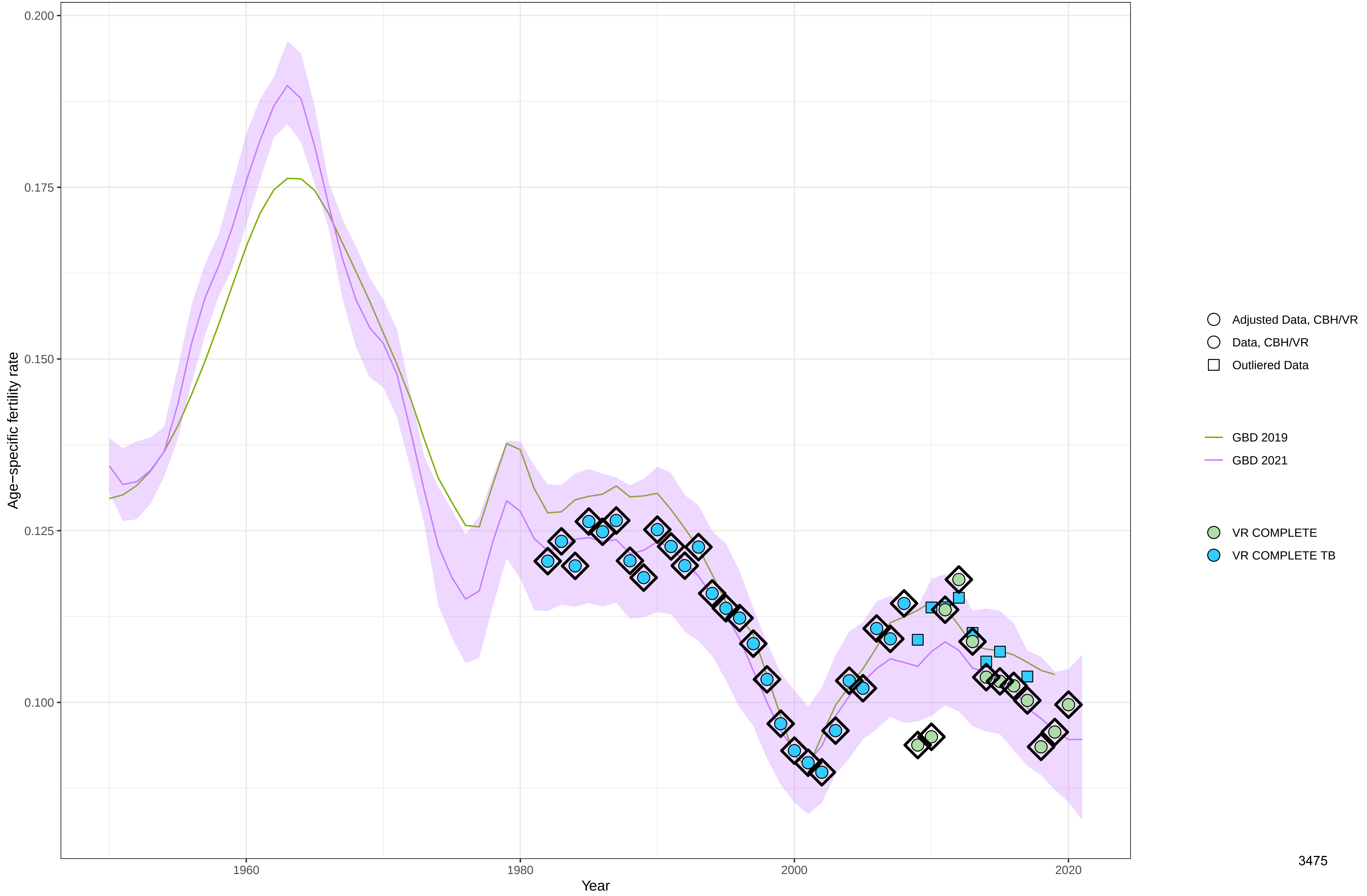

Age−specific fertility fitted trend lines, 1950−2021: Kingston upon Hull, City of, Ages: 30−34
Data points enclosed with a diamond are reference sources

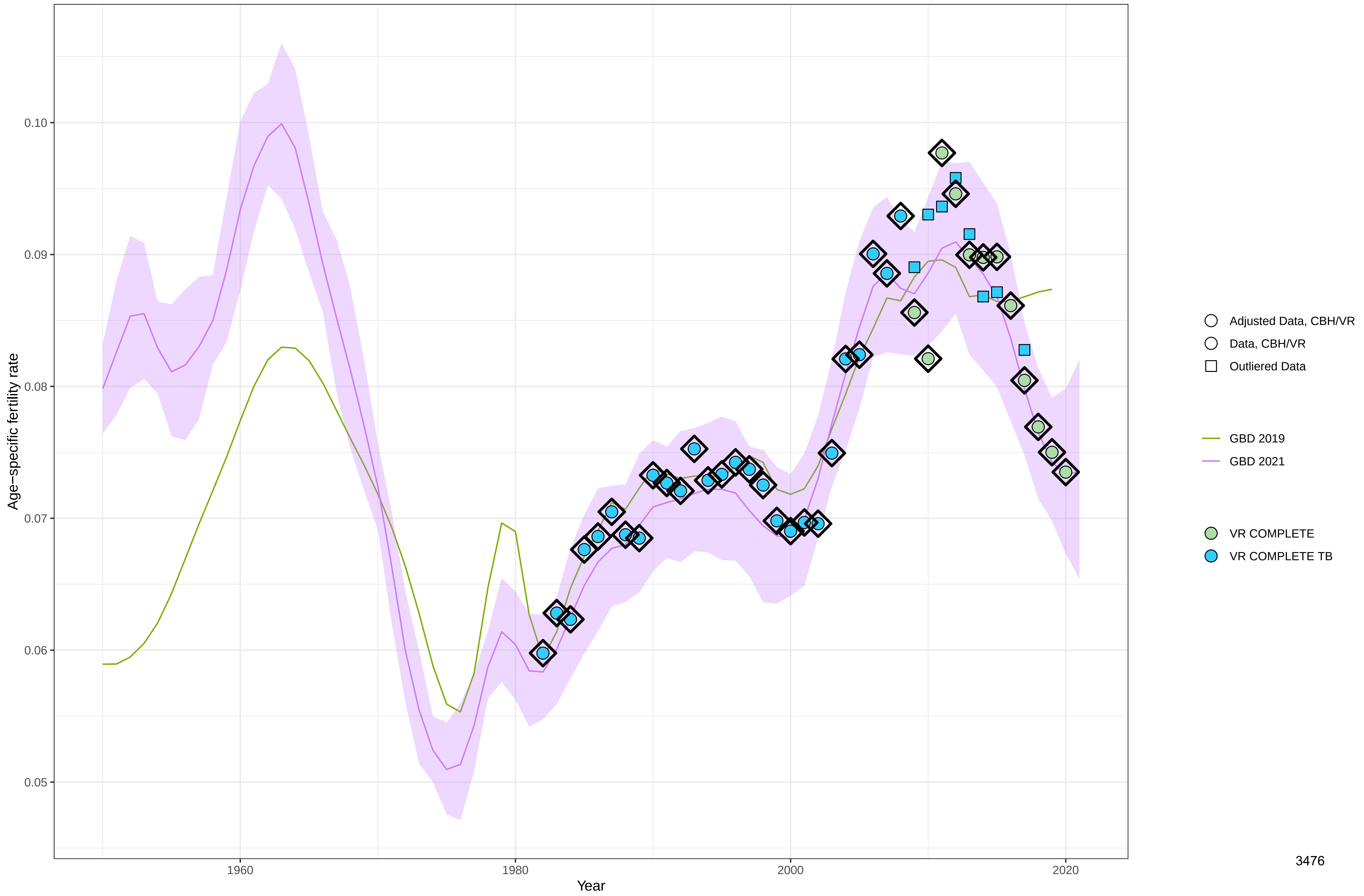

Age−specific fertility fitted trend lines, 1950−2021: Kingston upon Hull, City of, Ages: 35−39
Data points enclosed with a diamond are reference sources

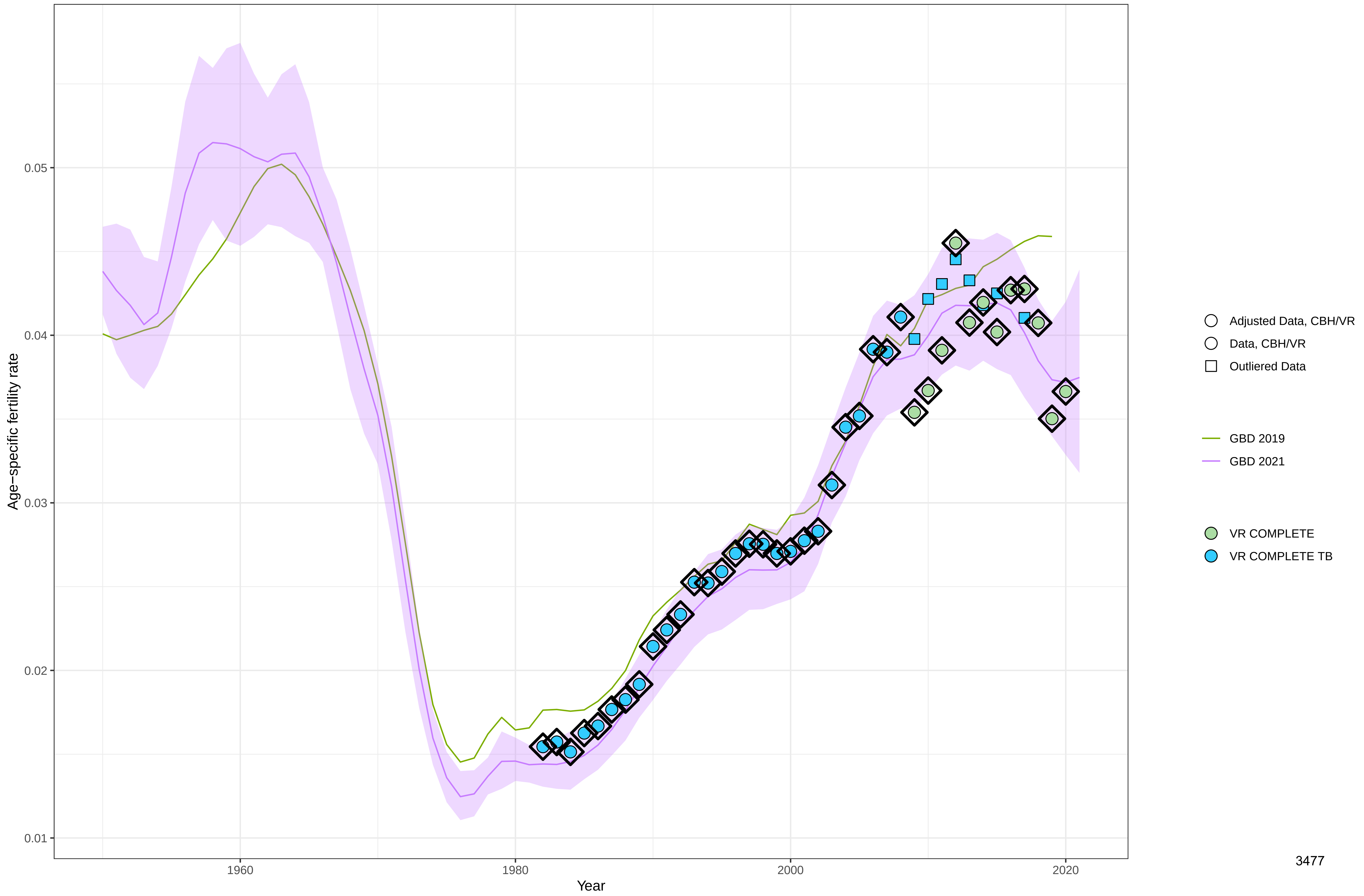

Age−specific fertility fitted trend lines, 1950−2021: Kingston upon Hull, City of, Ages: 40−44
Data points enclosed with a diamond are reference sources

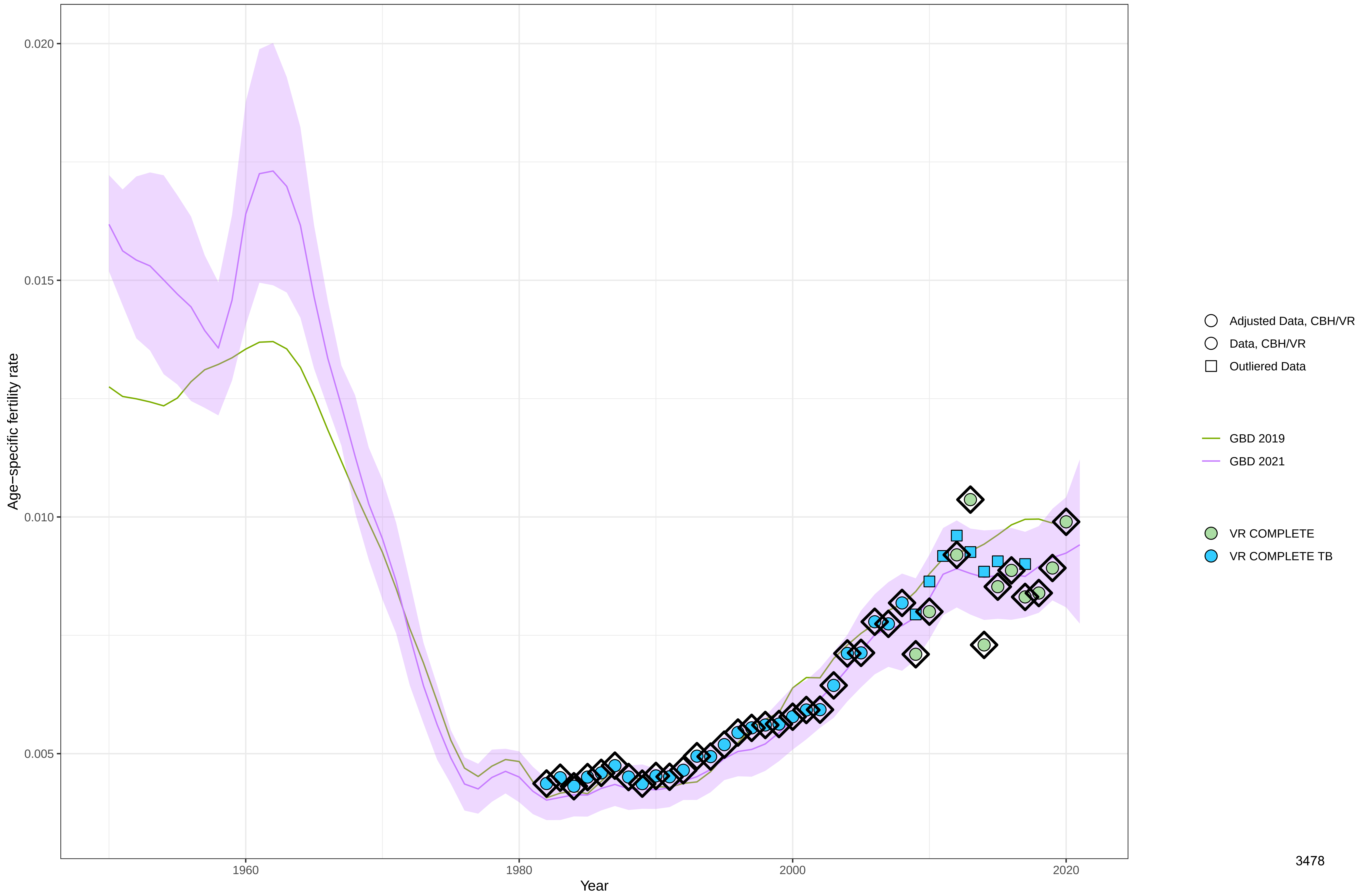

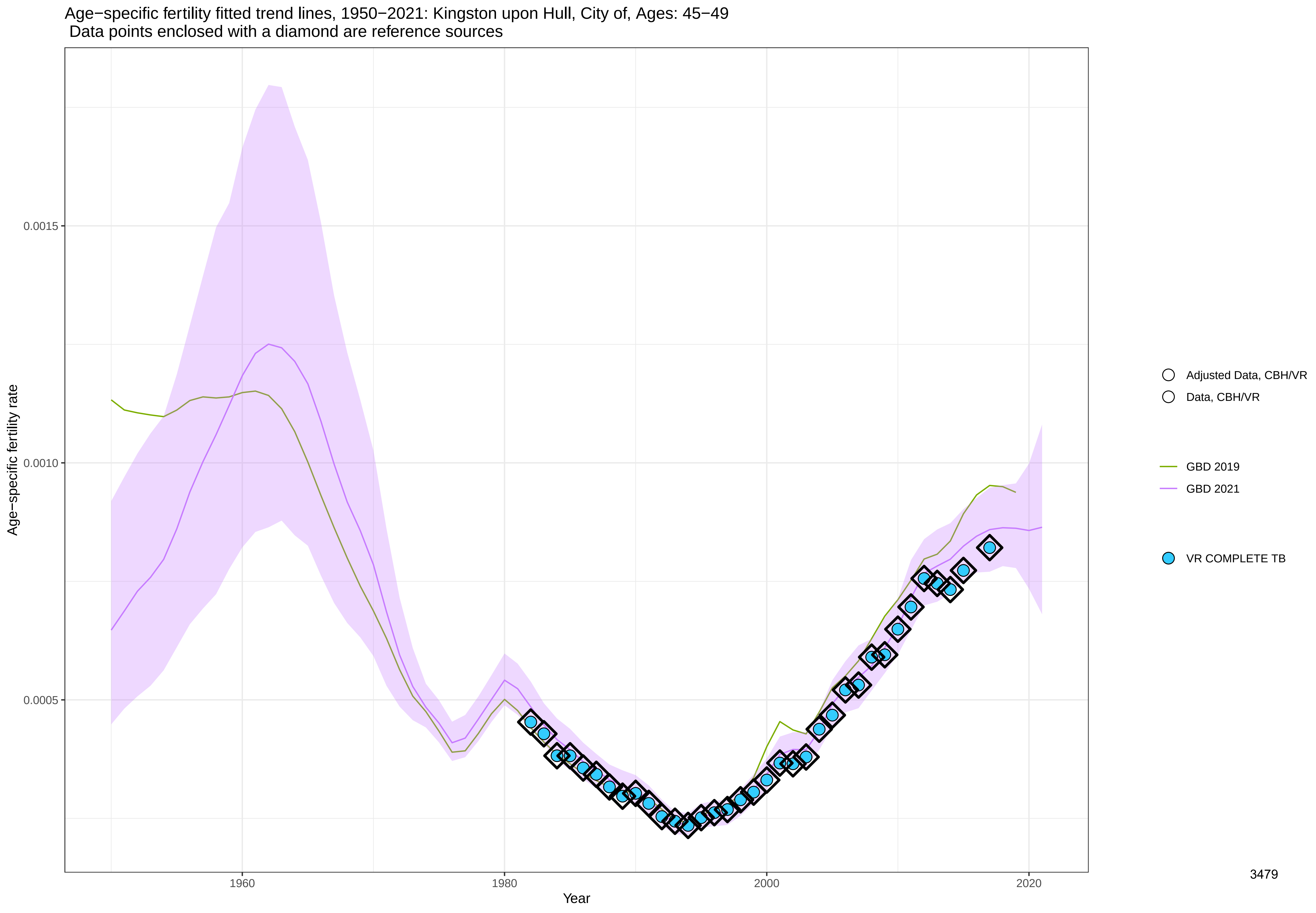
Age−specific fertility fitted trend lines, 1950−2021: Kingston upon Hull, City of, Ages: 45−49
Data points enclosed with a diamond are reference sources
Age−specific fertility rate
0.0015
0.0010
0.0005
1960
1980
2000
2020
Year
Adjusted Data, CBH/VR
Data, CBH/VR
GBD 2019
GBD 2021
VR COMPLETE TB

3479

Age−specific fertility fitted trend lines, 1950−2021: Kingston upon Hull, City of, Ages: 50−54
Data points enclosed with a diamond are reference sources

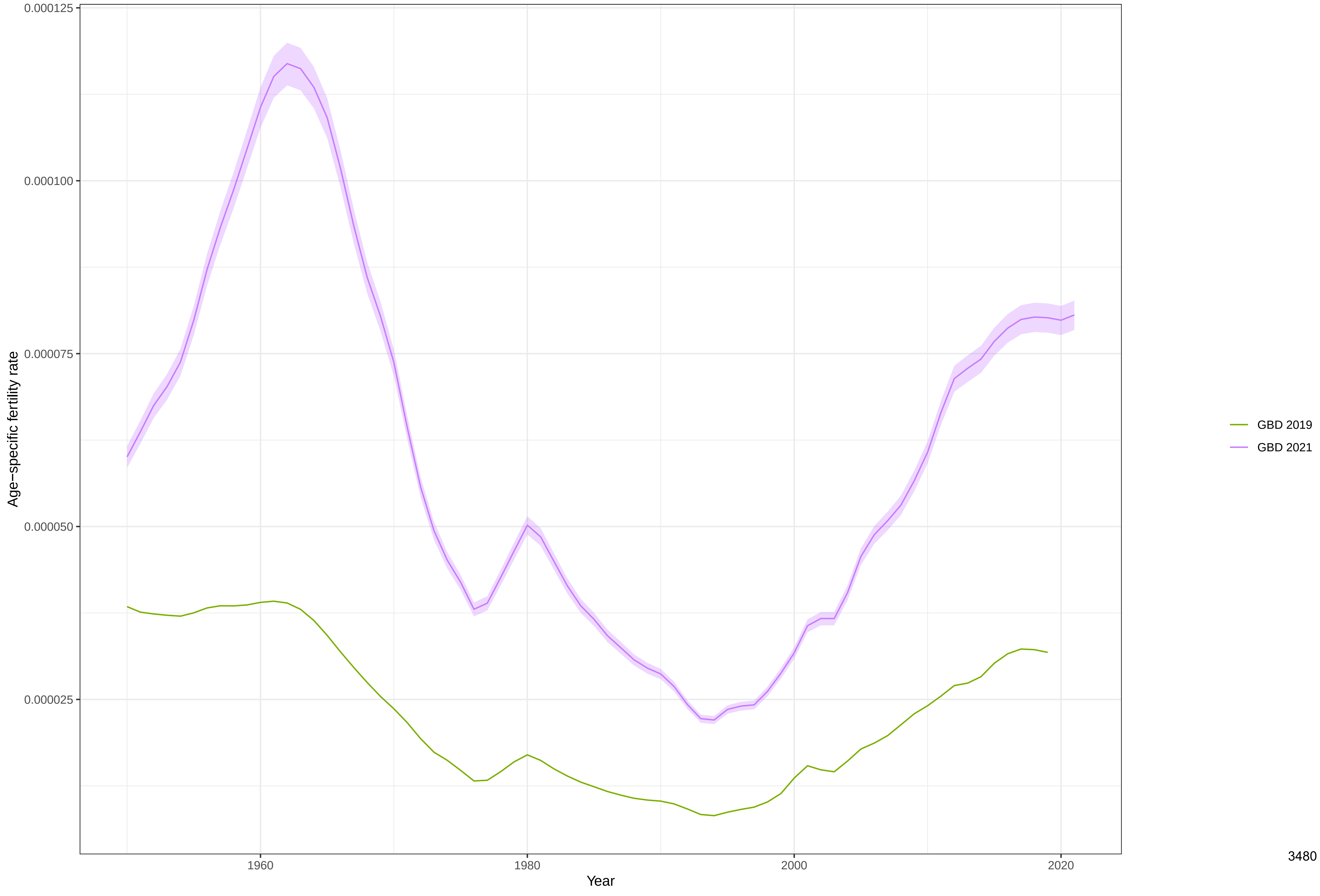

Total fertility rate fitted trend lines, 1950−2021: Kingston upon Hull, City of TFR
Data points enclosed with a diamond are reference sources

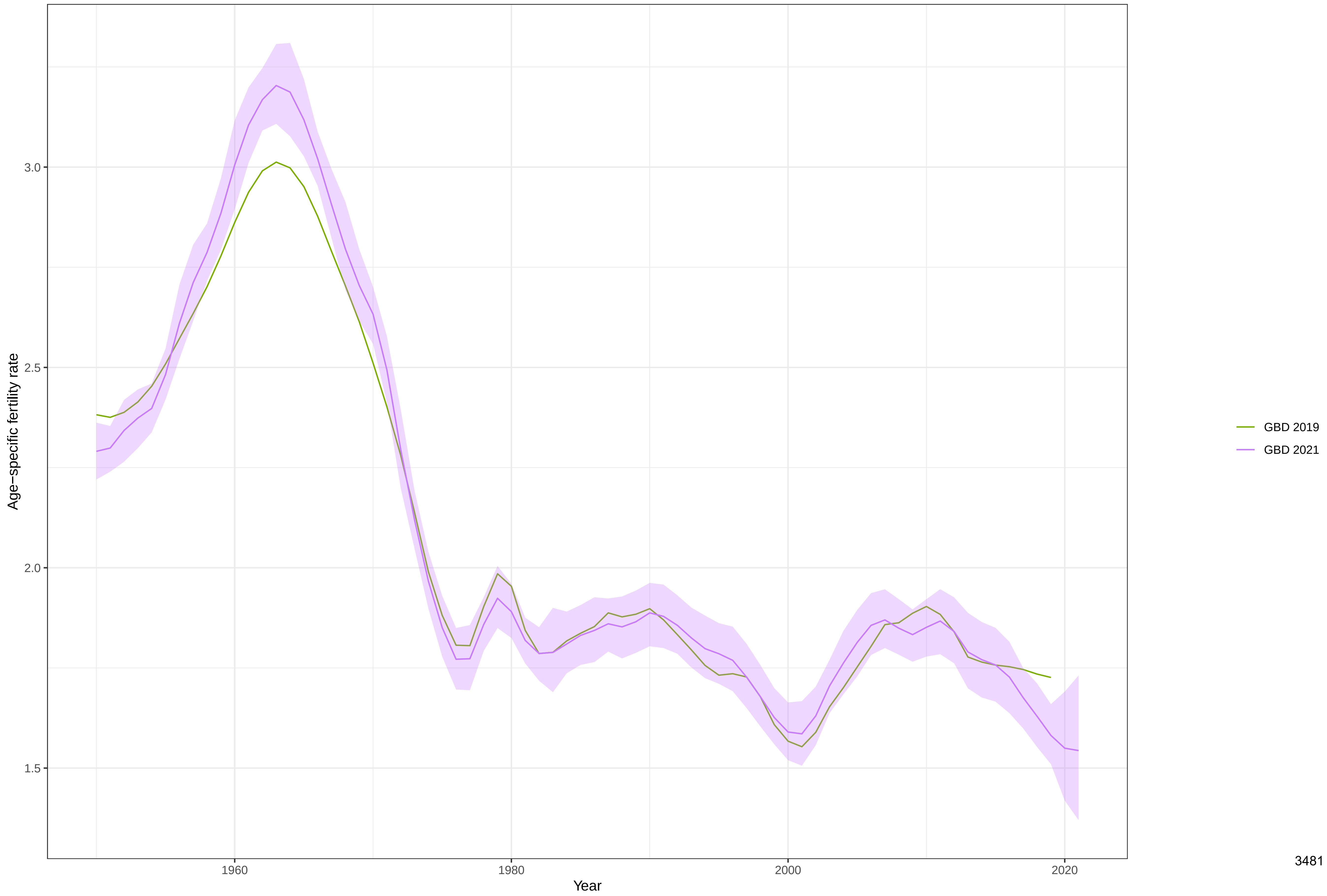

Age−specific fertility fitted trend lines, 1950−2021: Kirklees, Ages: 10−14
Data points enclosed with a diamond are reference sources

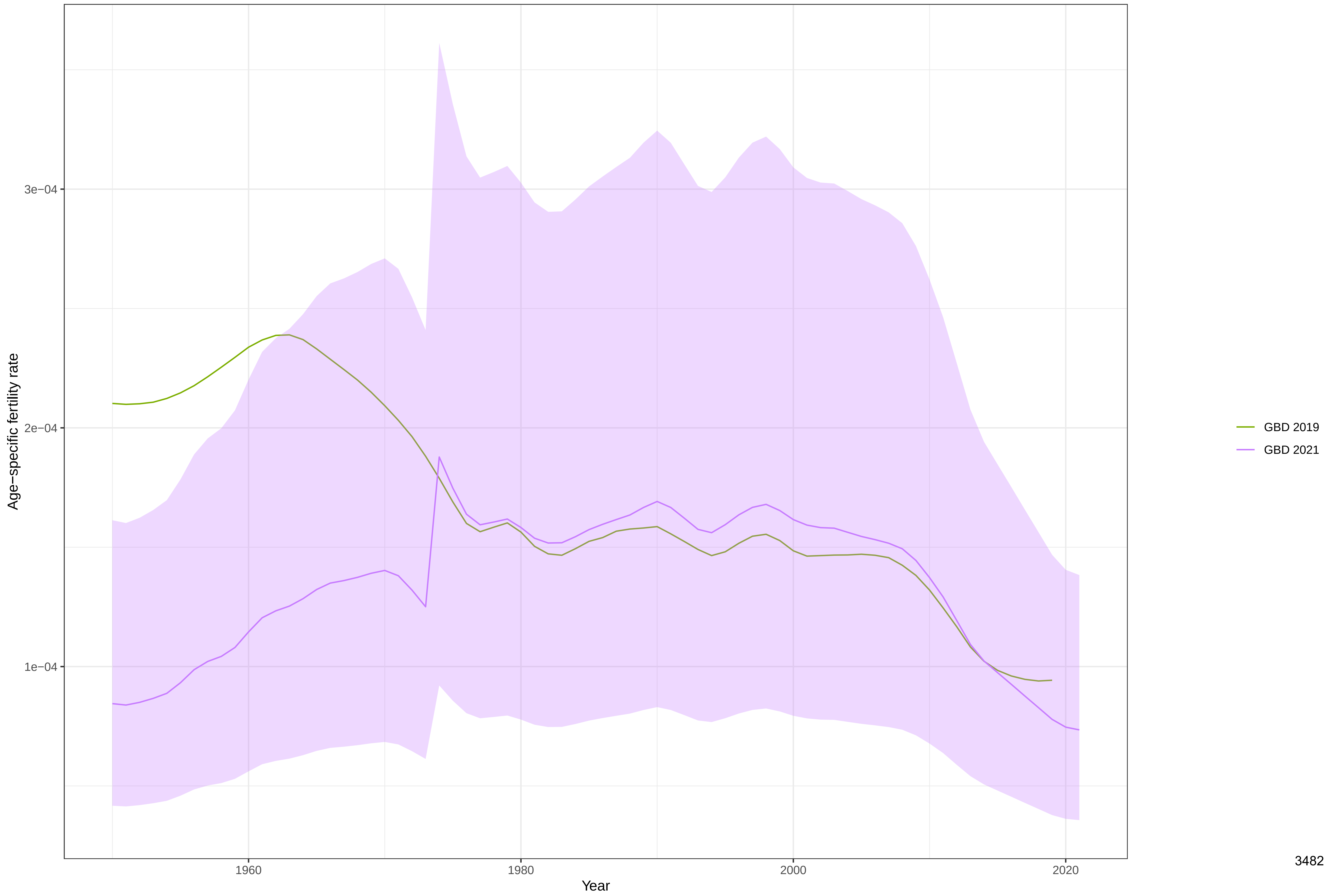

Age−specific fertility fitted trend lines, 1950−2021: Kirklees, Ages: 15−19
Data points enclosed with a diamond are reference sources

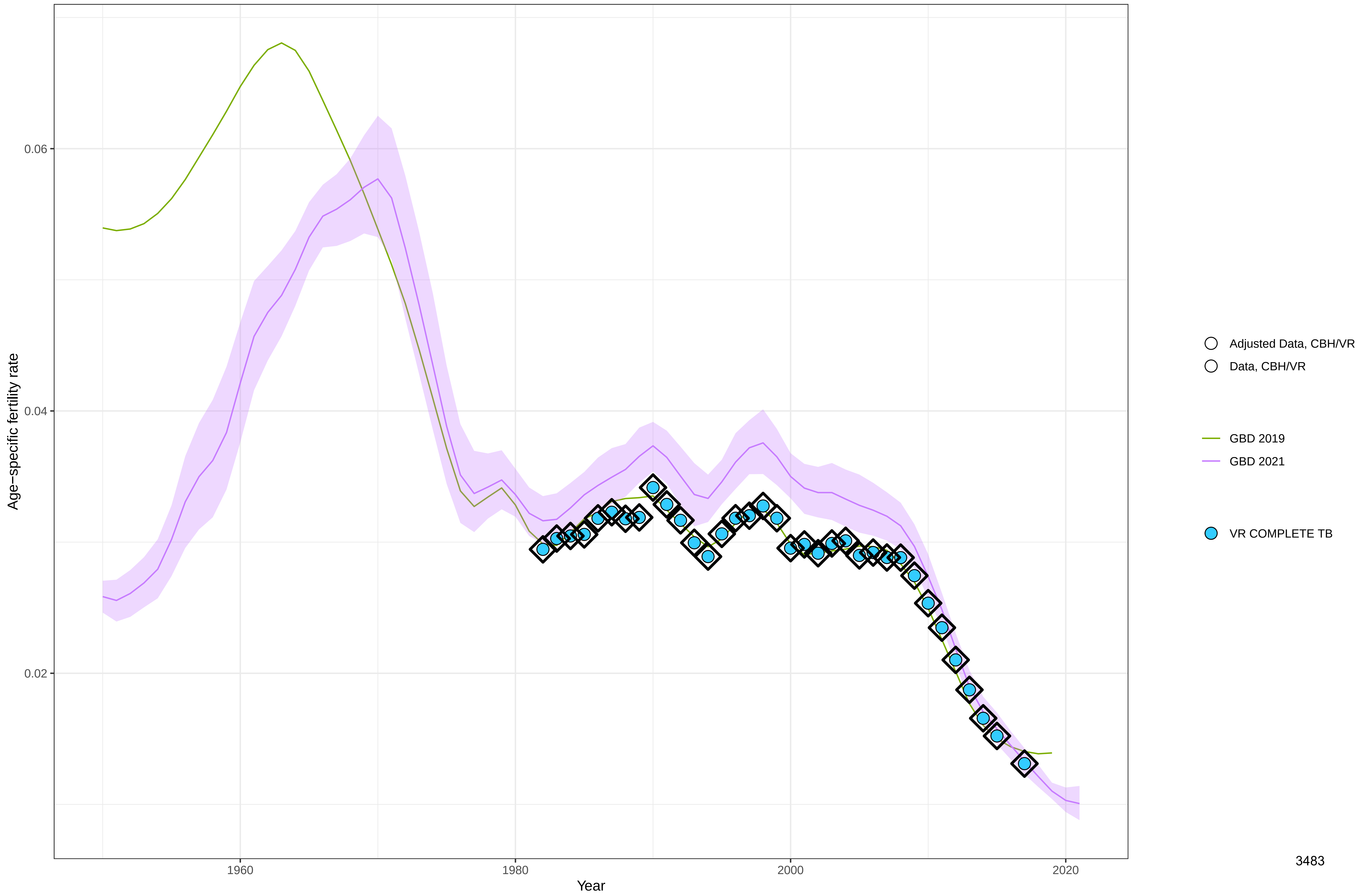

Age−specific fertility fitted trend lines, 1950−2021: Kirklees, Ages: 20−24
Data points enclosed with a diamond are reference sources

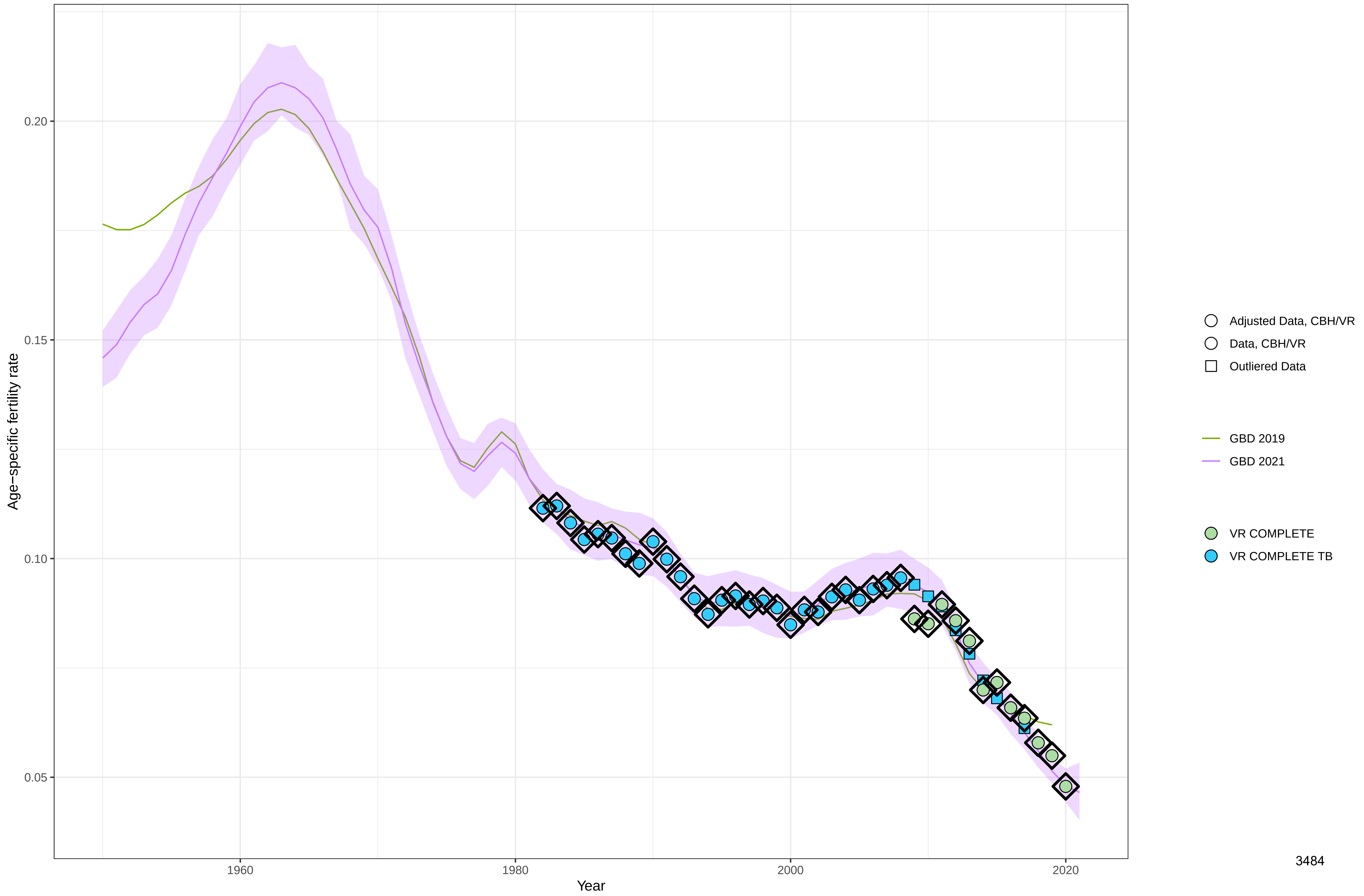

Age−specific fertility fitted trend lines, 1950−2021: Kirklees, Ages: 25−29
Data points enclosed with a diamond are reference sources

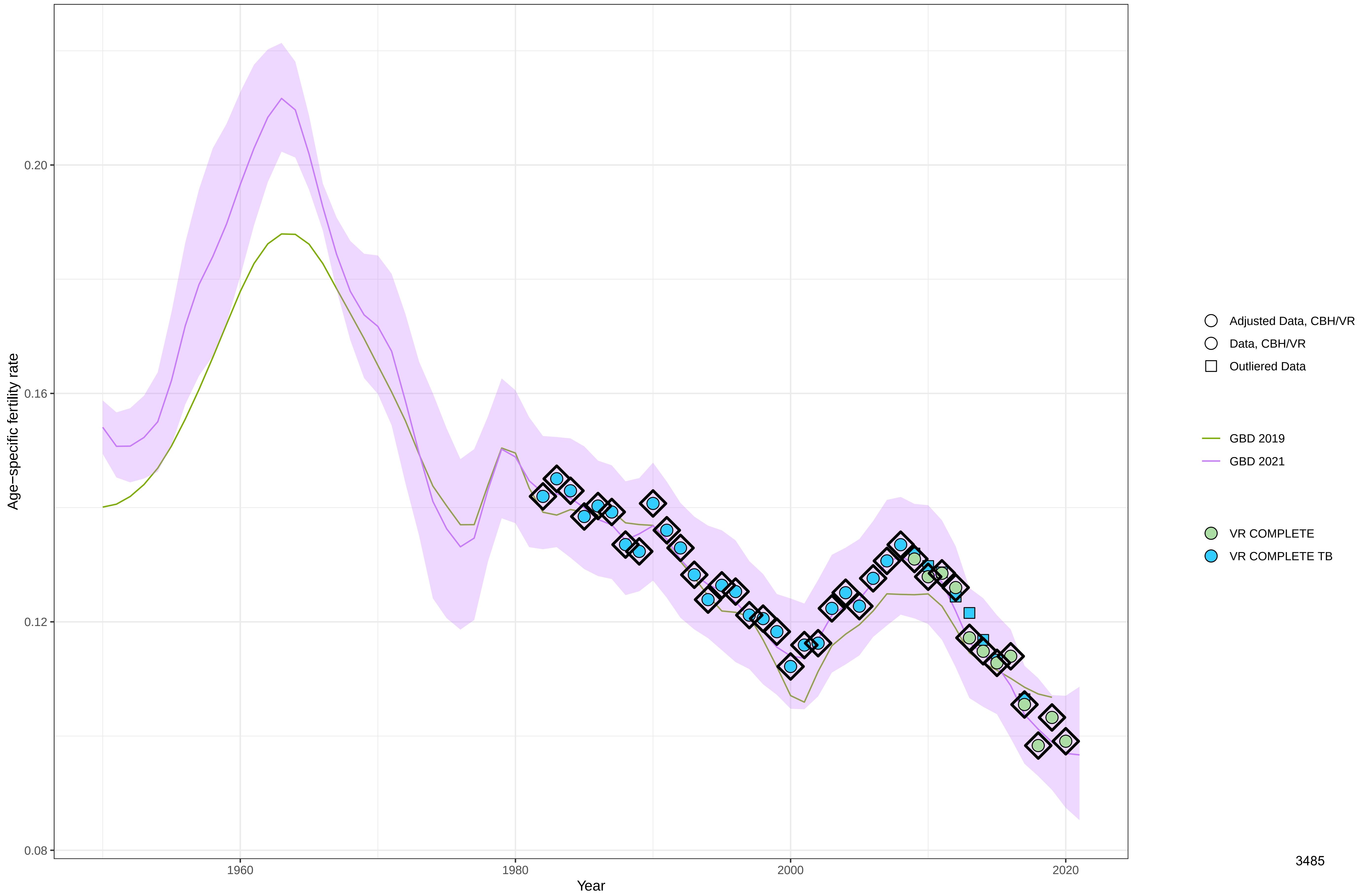

Age−specific fertility fitted trend lines, 1950−2021: Kirklees, Ages: 30−34
Data points enclosed with a diamond are reference sources

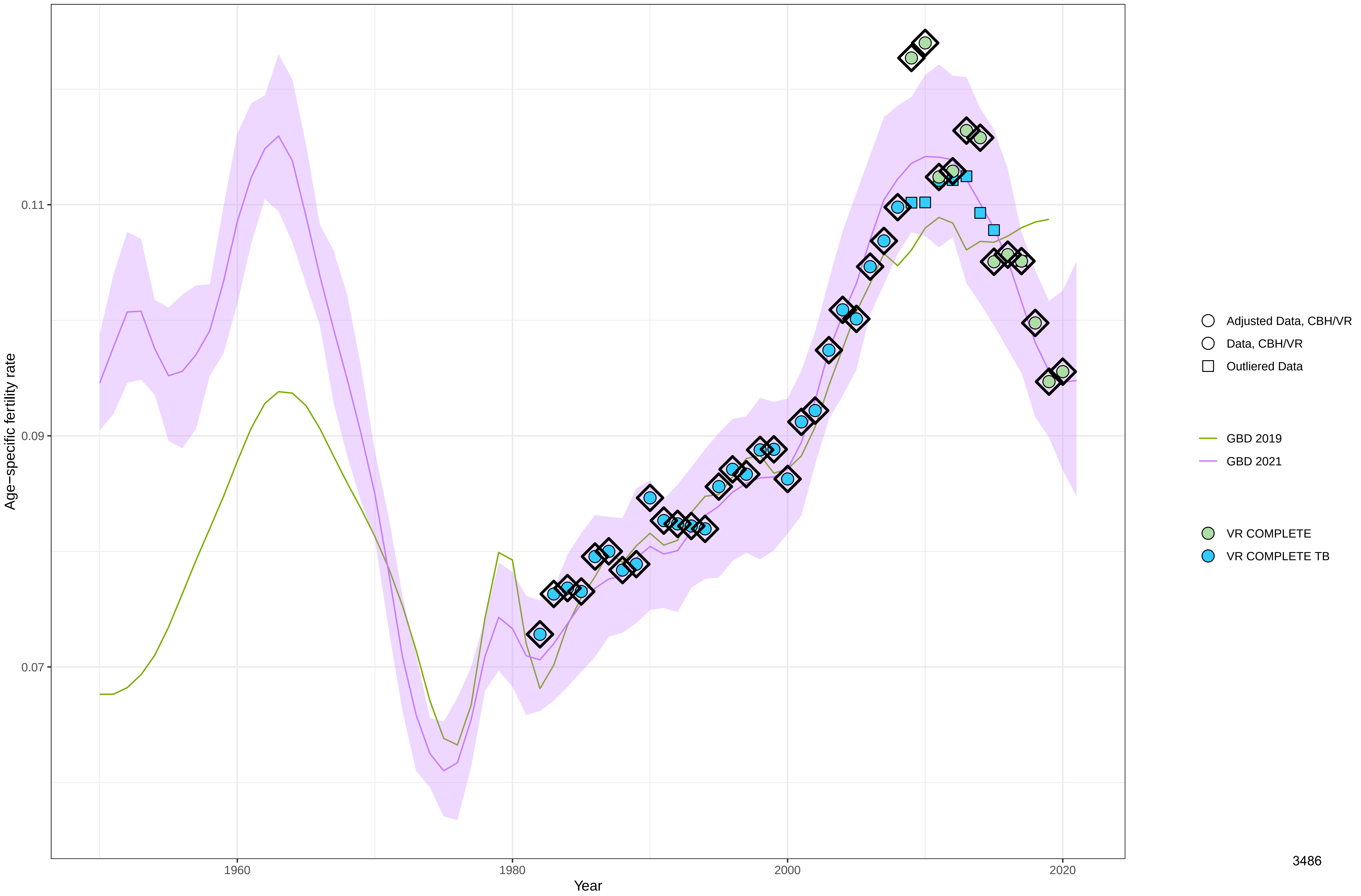

Age−specific fertility fitted trend lines, 1950−2021: Kirklees, Ages: 35−39
Data points enclosed with a diamond are reference sources

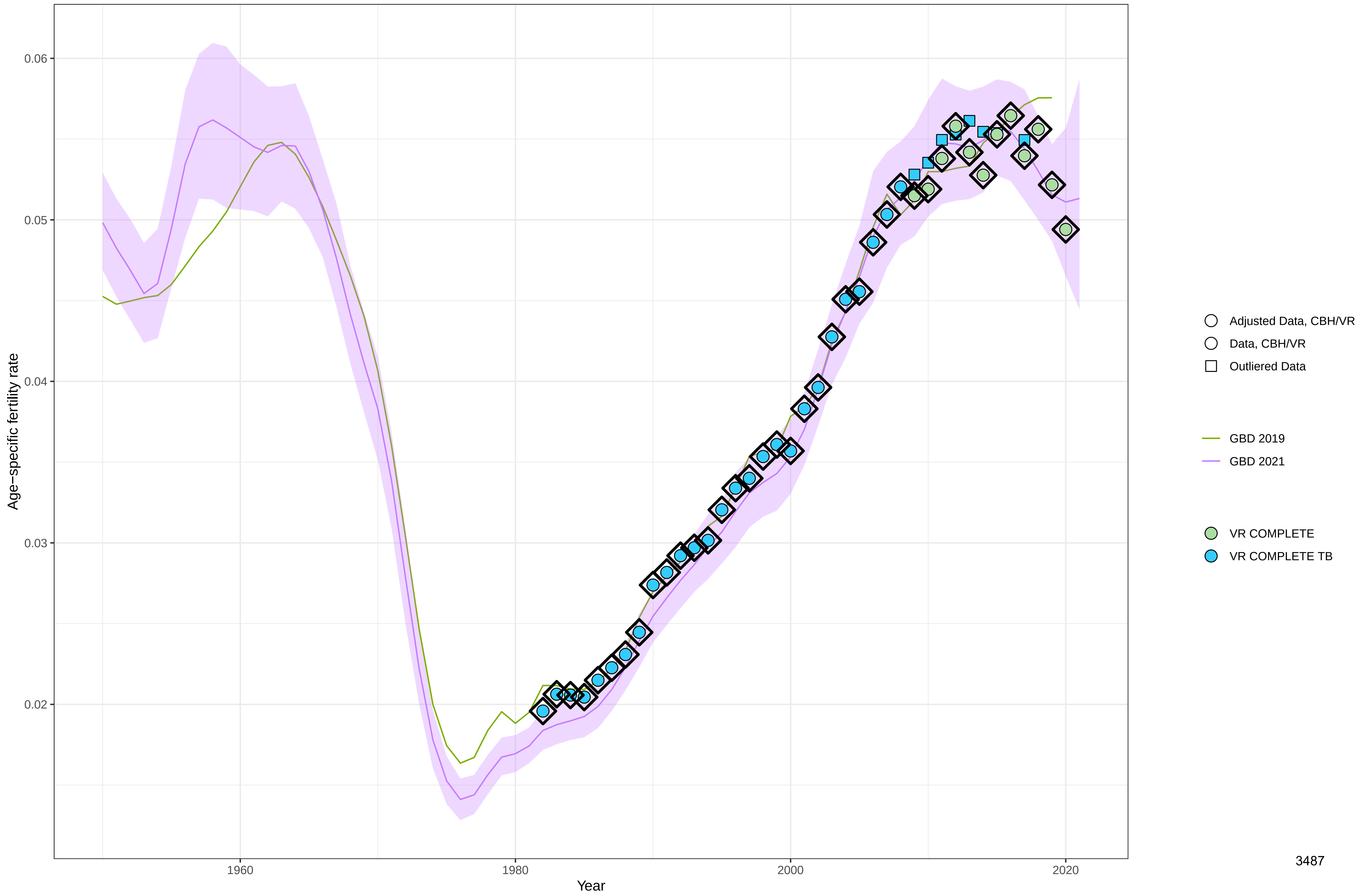

Age−specific fertility fitted trend lines, 1950−2021: Kirklees, Ages: 40−44
Data points enclosed with a diamond are reference sources

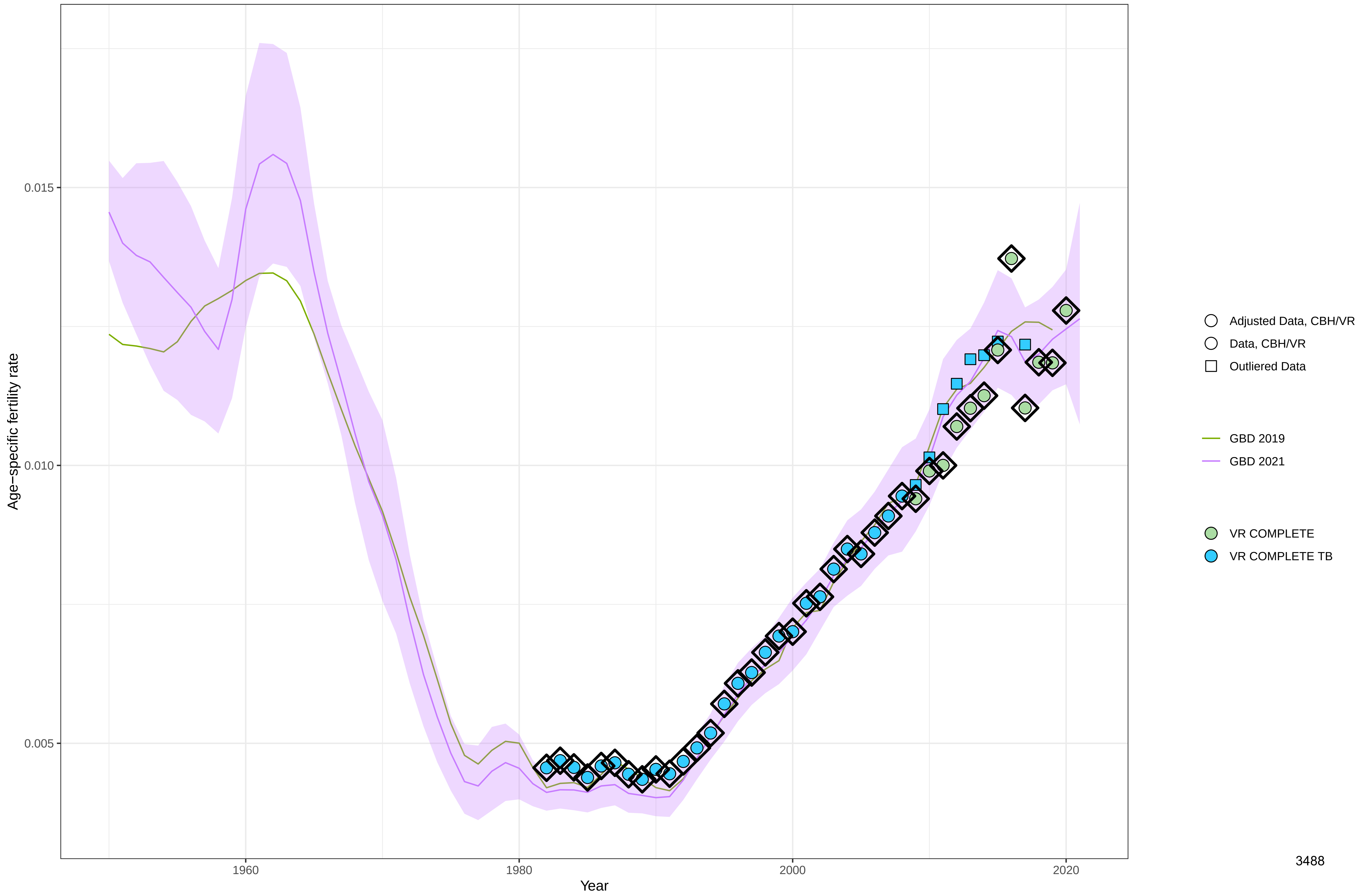

Age−specific fertility fitted trend lines, 1950−2021: Kirklees, Ages: 45−49
Data points enclosed with a diamond are reference sources

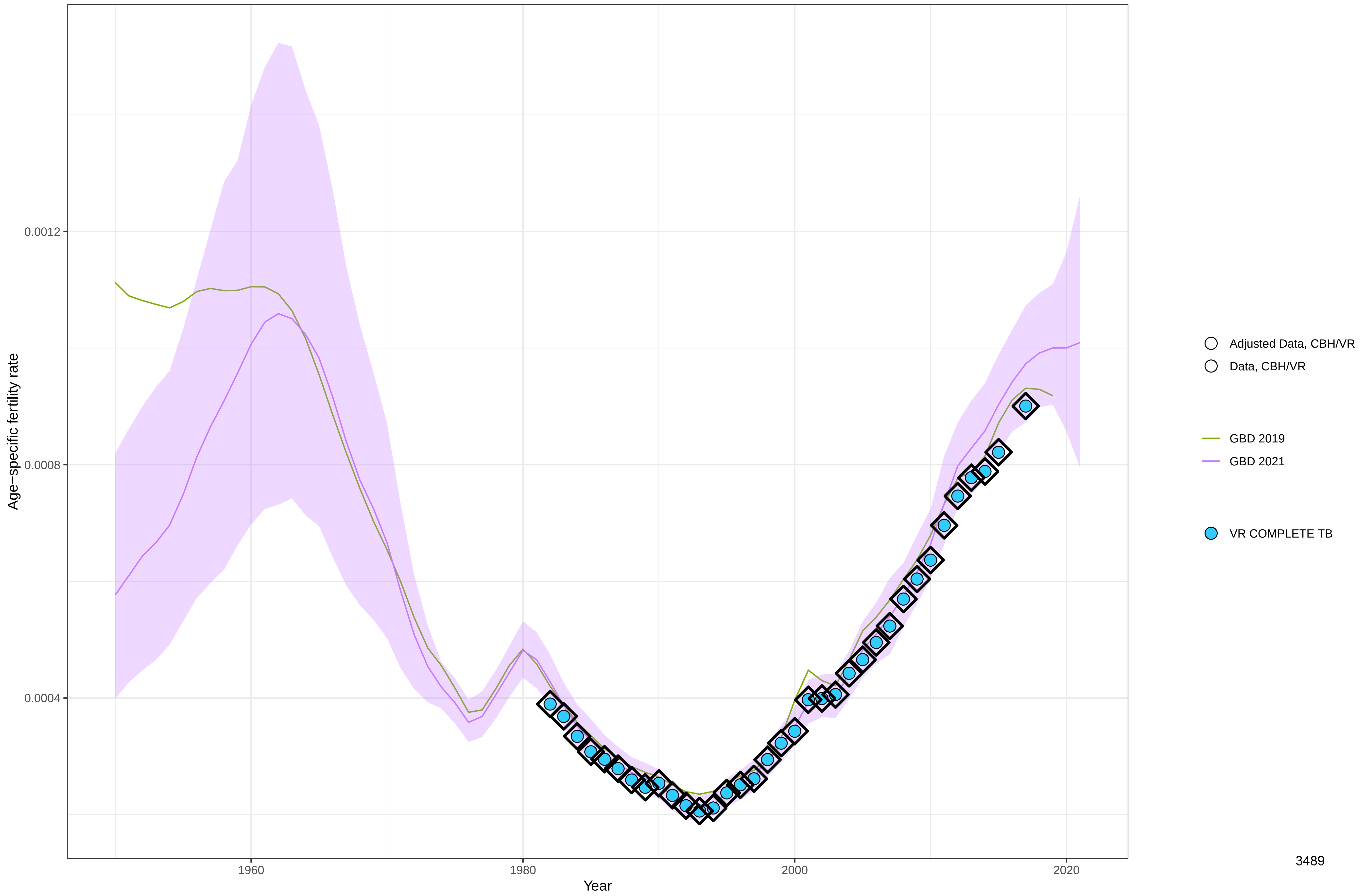

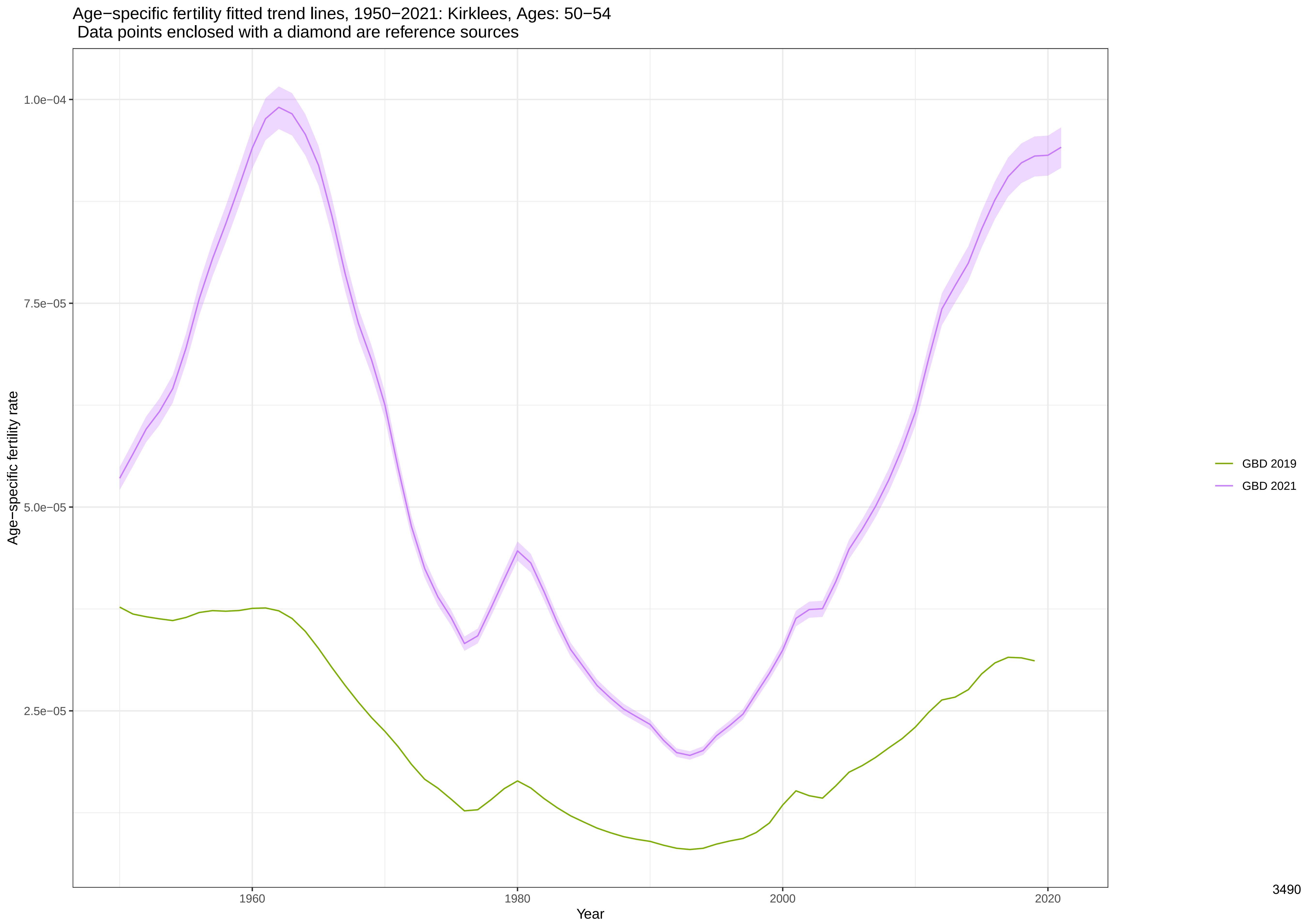

Age−specific fertility fitted trend lines, 1950−2021: Kirklees, Ages: 50−54
Data points enclosed with a diamond are reference sources
1.0e−04
7.5e−05
5.0e−05
2.5e−05
Age−specific fertility rate
1960
1980
2000
2020
Year
GBD 2019
GBD 2021

Total fertility rate fitted trend lines, 1950−2021: Kirklees TFR
Data points enclosed with a diamond are reference sources

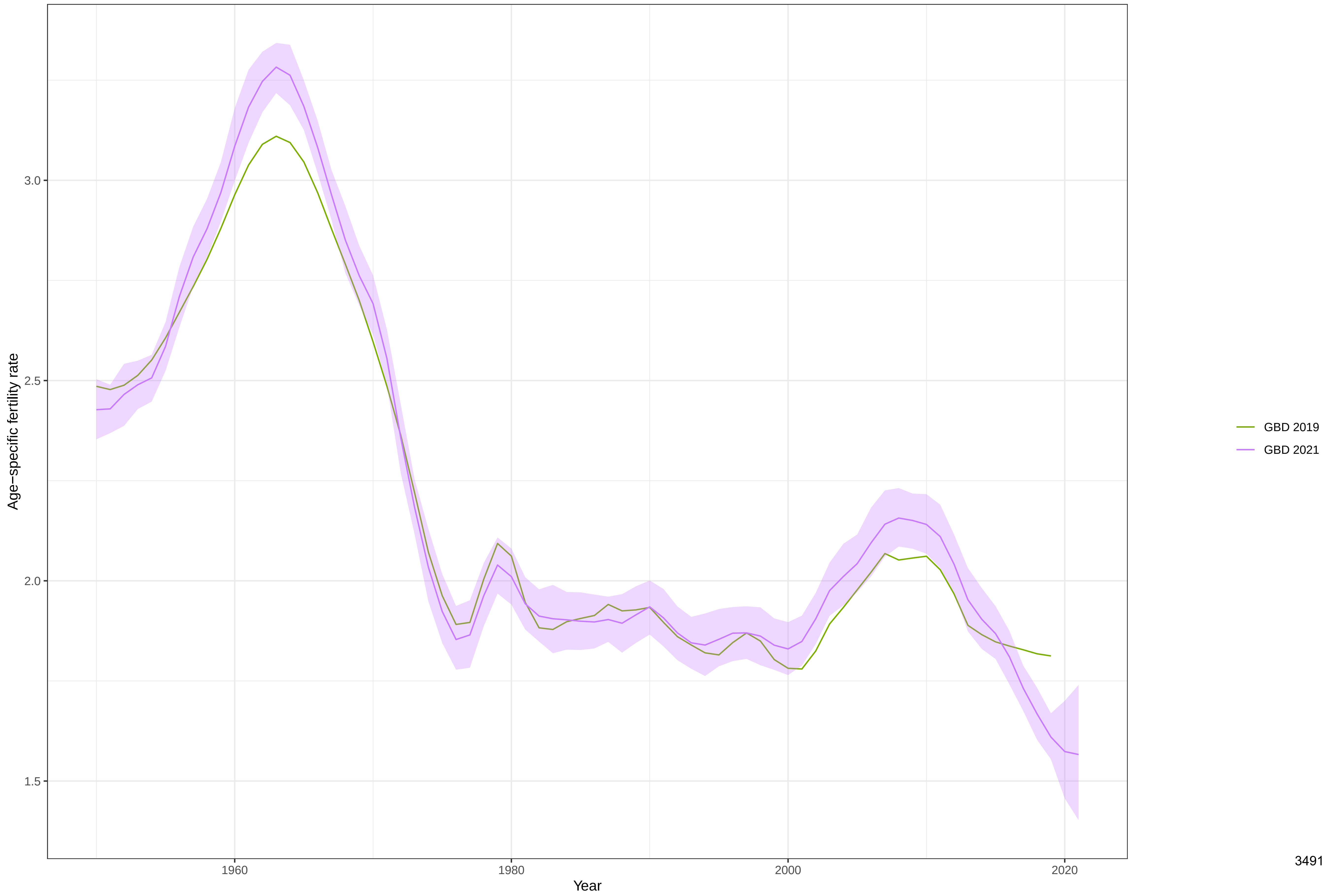

Age−specific fertility fitted trend lines, 1950−2021: Leeds, Ages: 10−14
Data points enclosed with a diamond are reference sources

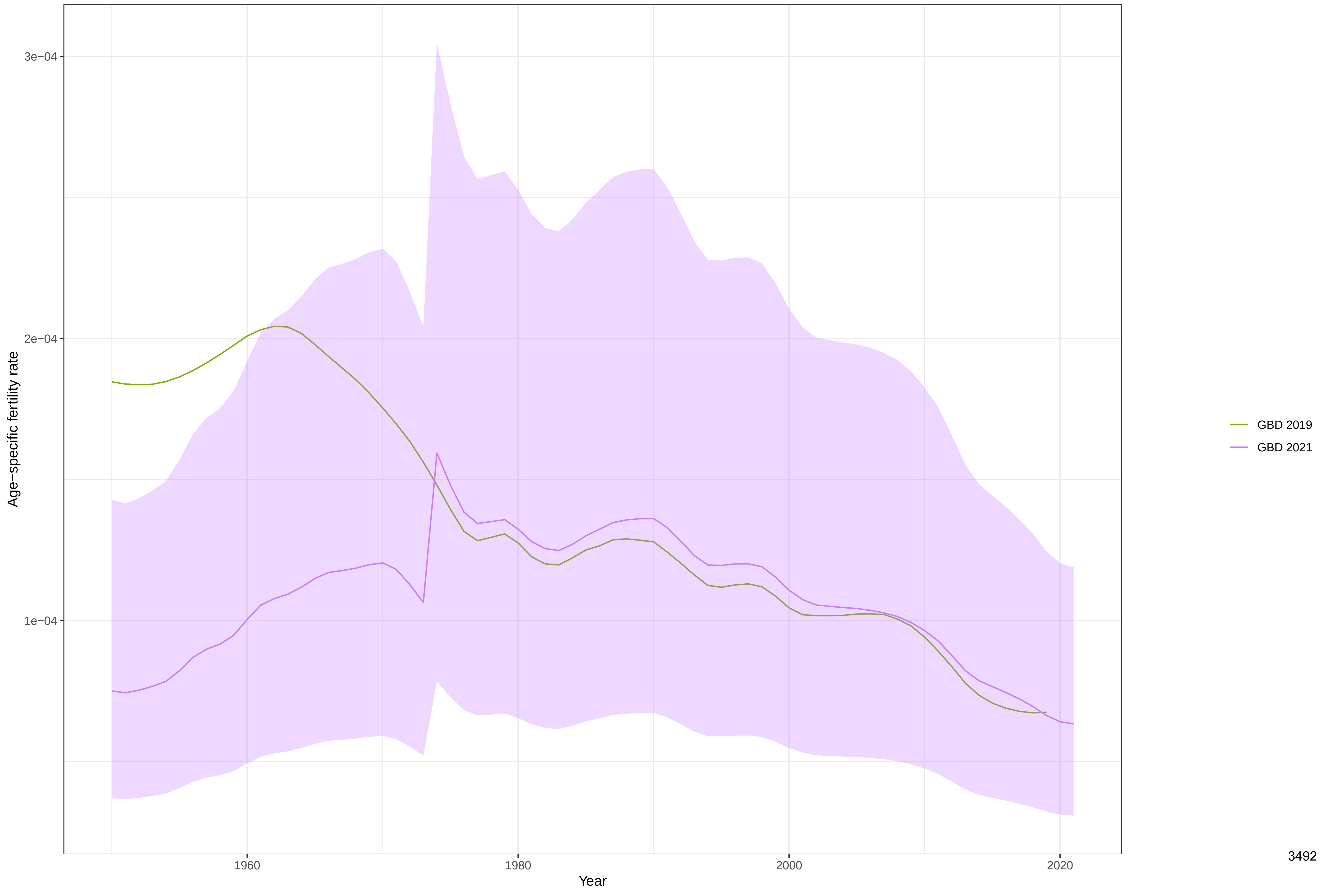

3492

Age−specific fertility fitted trend lines, 1950−2021: Leeds, Ages: 15−19
Data points enclosed with a diamond are reference sources

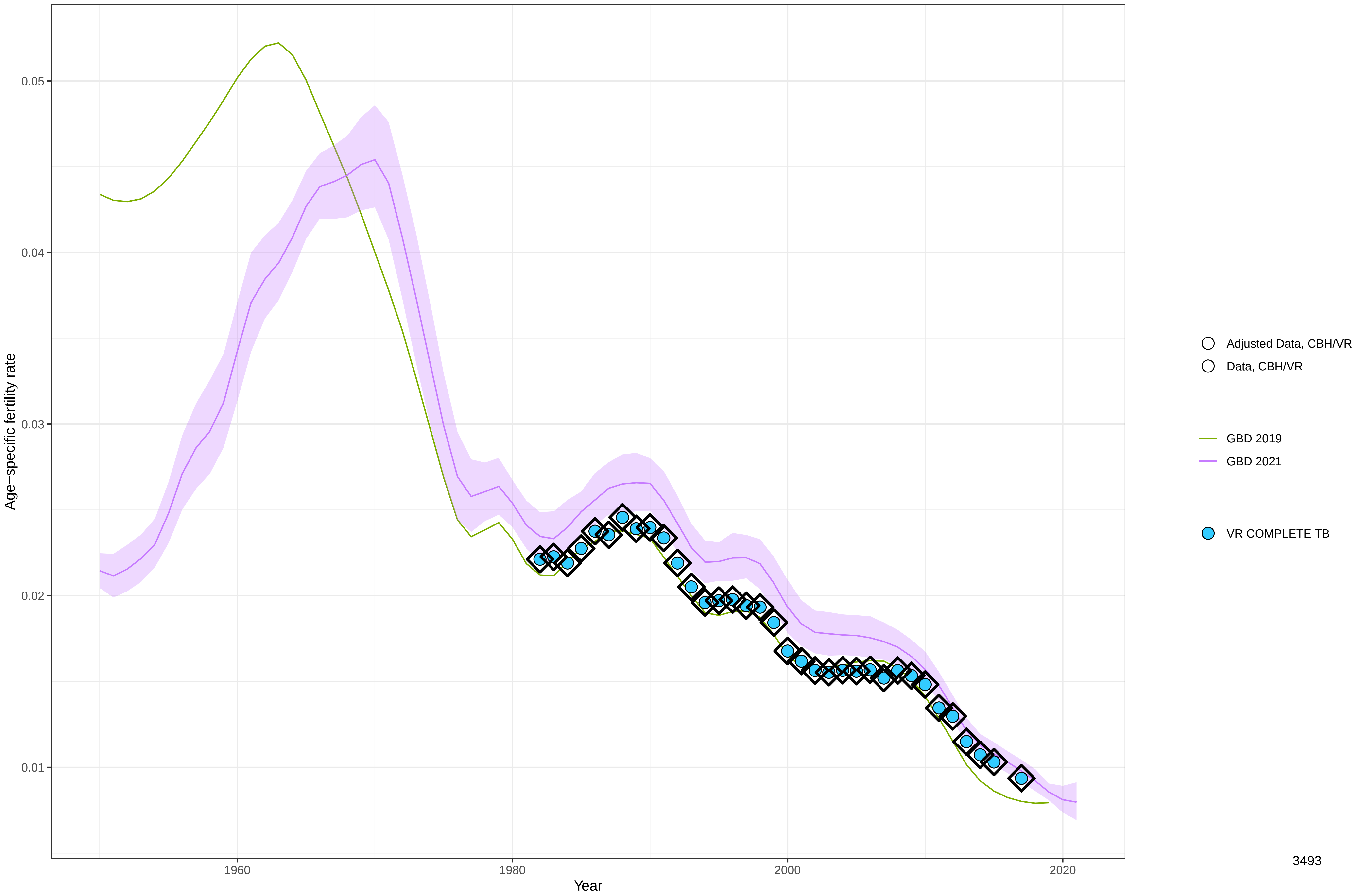

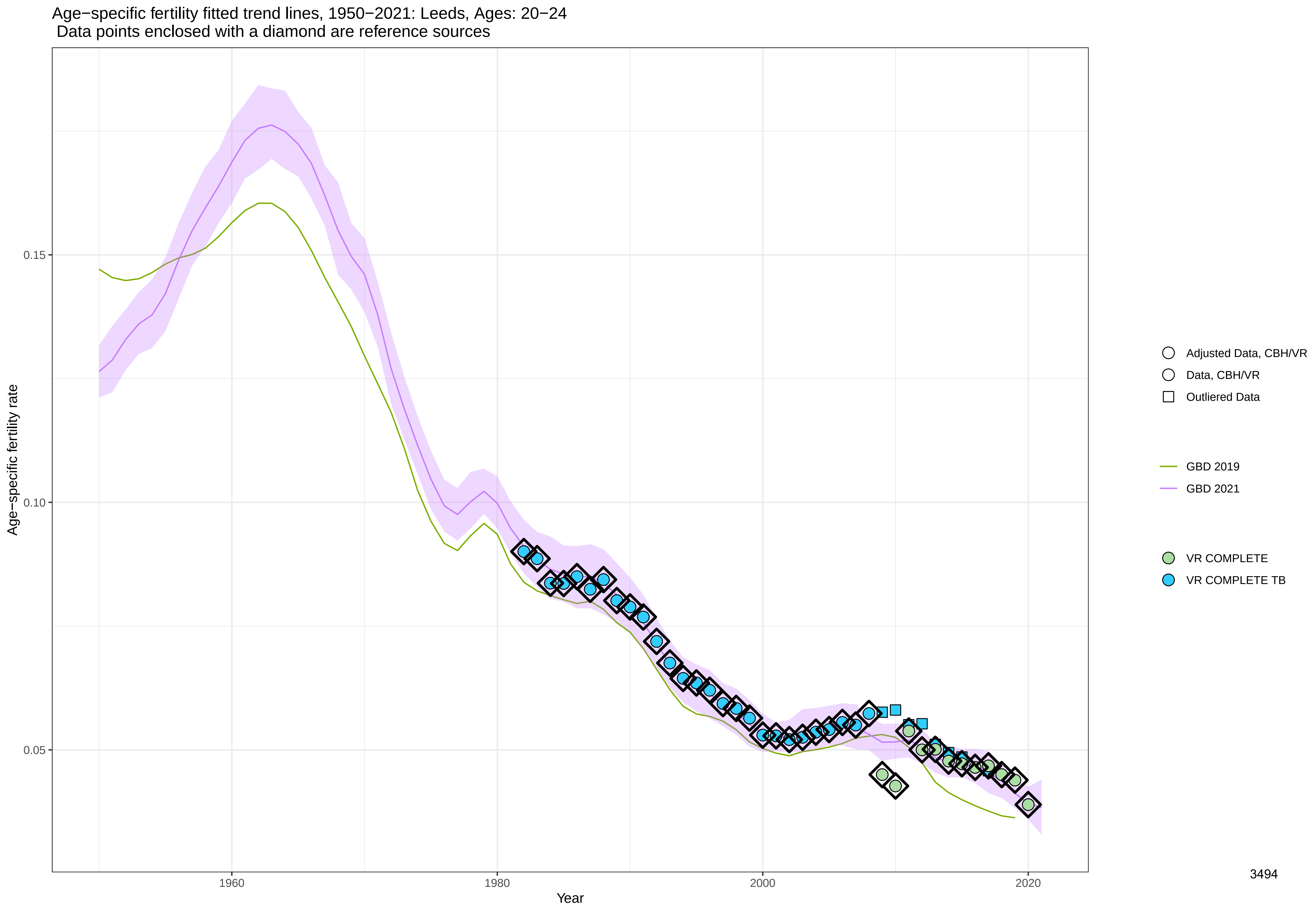

Age−specific fertility fitted trend lines, 1950−2021: Leeds, Ages: 20−24
Data points enclosed with a diamond are reference sources
Age−specific fertility rate
0.15
0.10
0.05
1960
1980
2000
2020
Year
Adjusted Data, CBH/VR
Data, CBH/VR
Outliered Data
GBD 2019
GBD 2021
VR COMPLETE
VR COMPLETE TB

Age−specific fertility fitted trend lines, 1950−2021: Leeds, Ages: 25−29
Data points enclosed with a diamond are reference sources

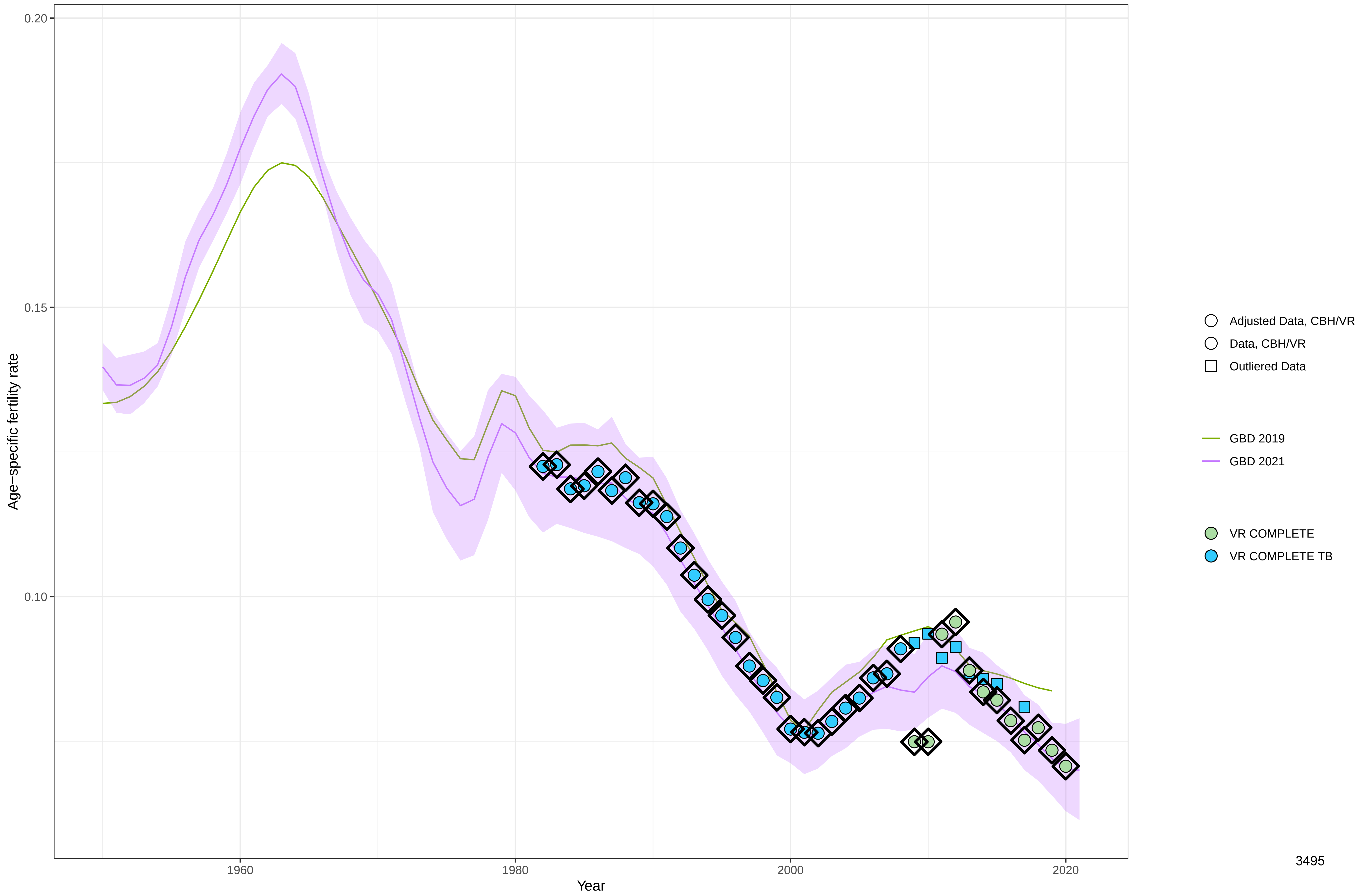

Age−specific fertility fitted trend lines, 1950−2021: Leeds, Ages: 30−34
Data points enclosed with a diamond are reference sources

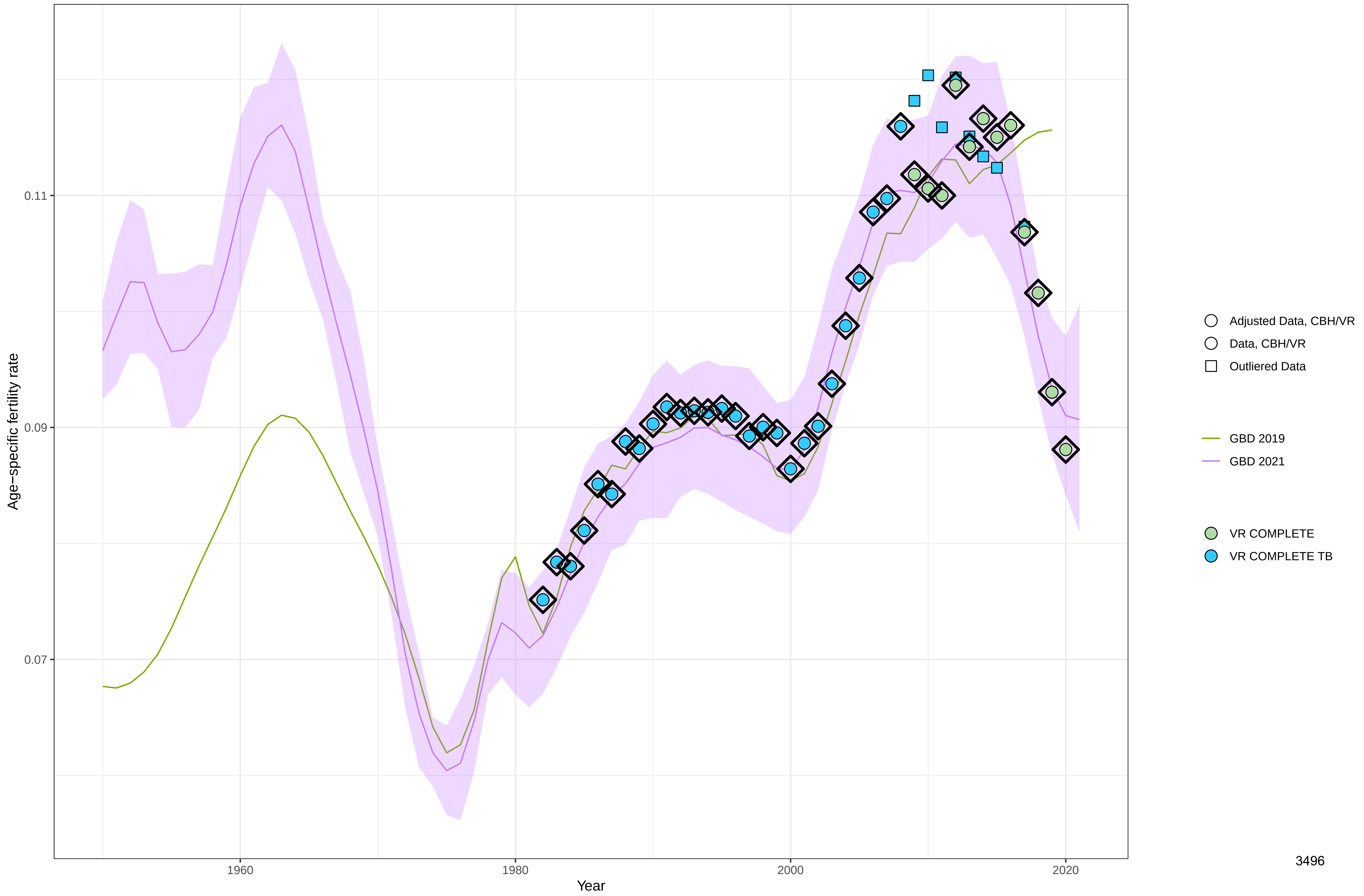

Age−specific fertility fitted trend lines, 1950−2021: Leeds, Ages: 35−39
Data points enclosed with a diamond are reference sources

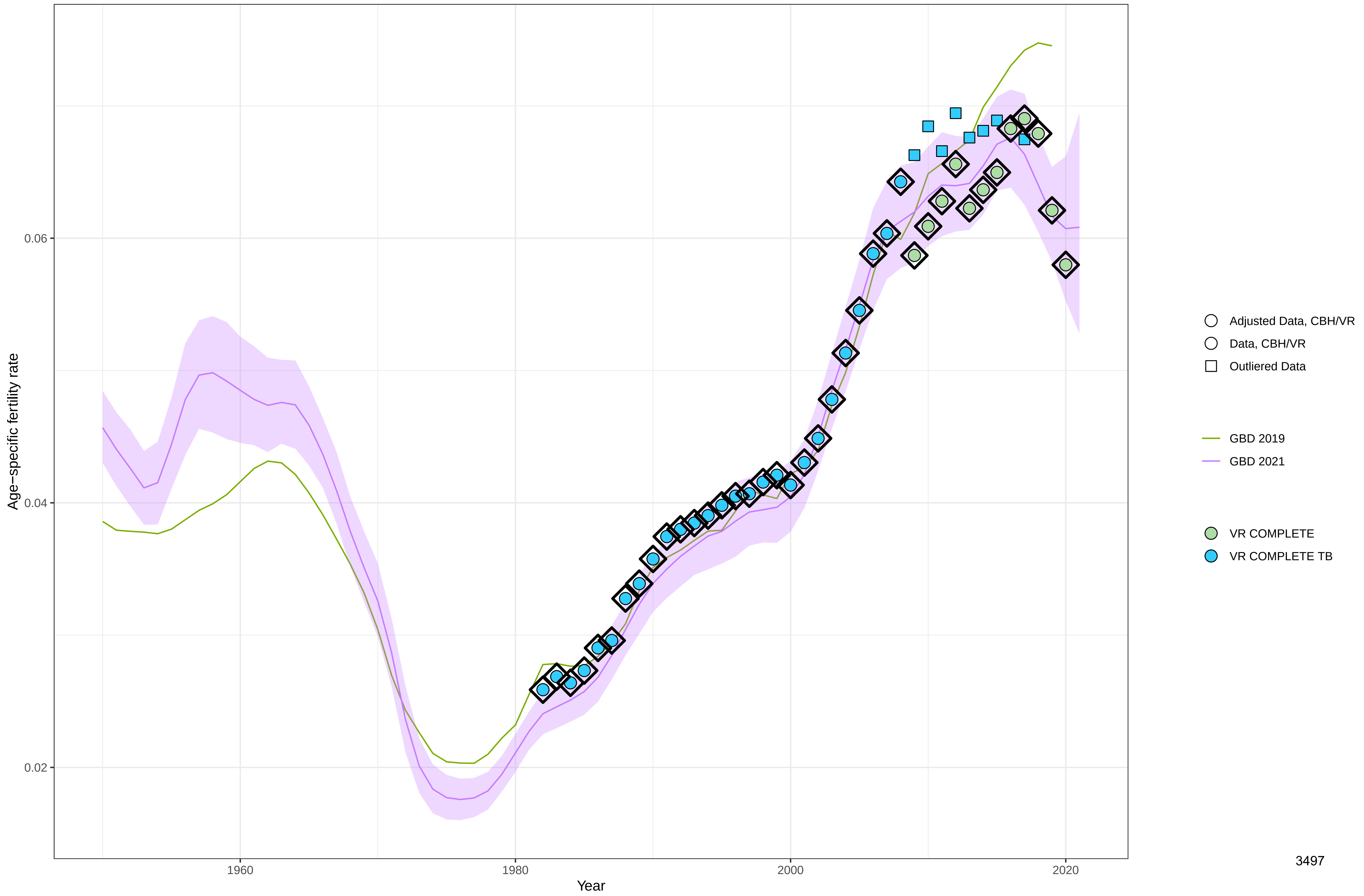

Age−specific fertility fitted trend lines, 1950−2021: Leeds, Ages: 40−44
Data points enclosed with a diamond are reference sources

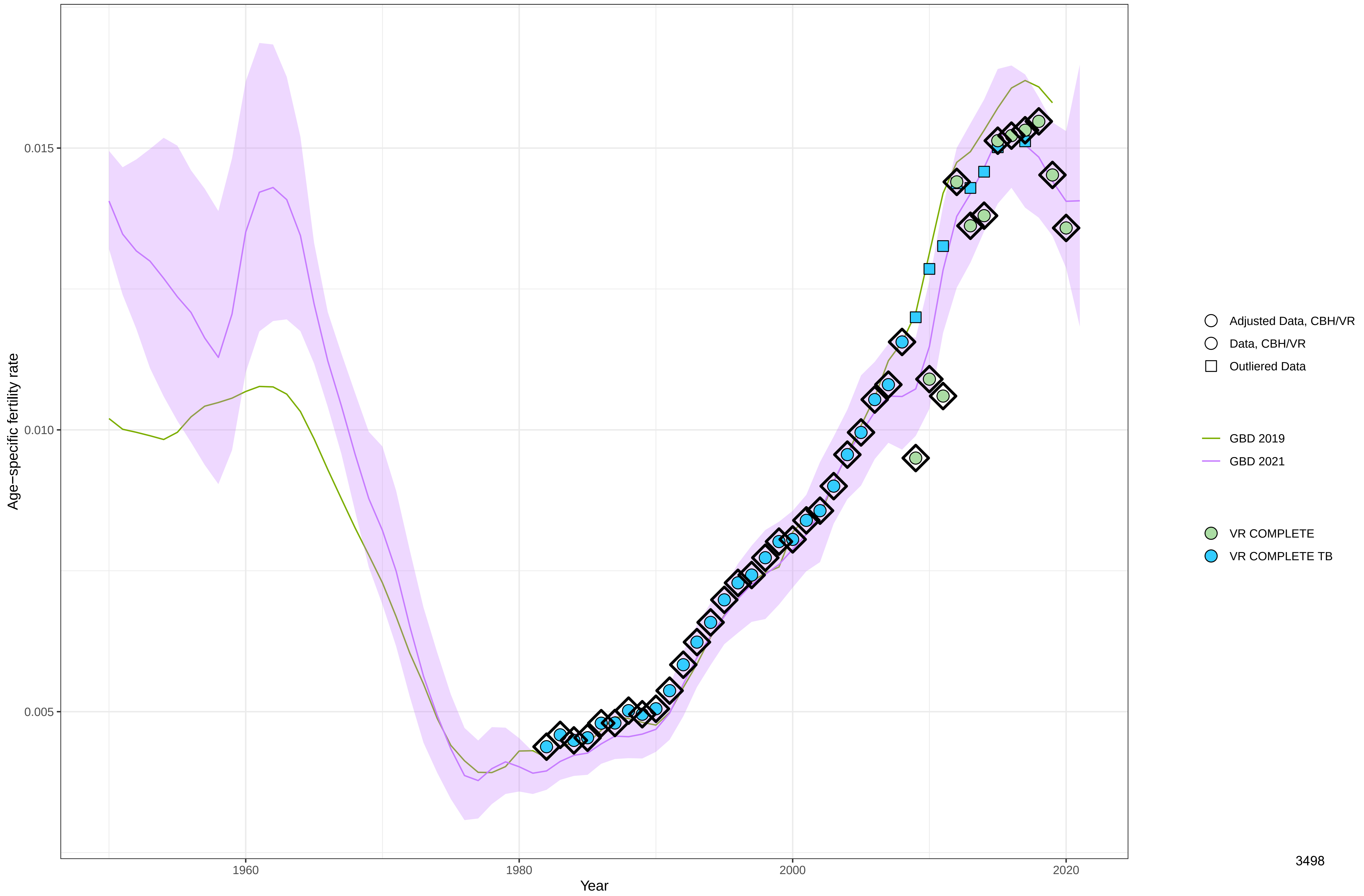

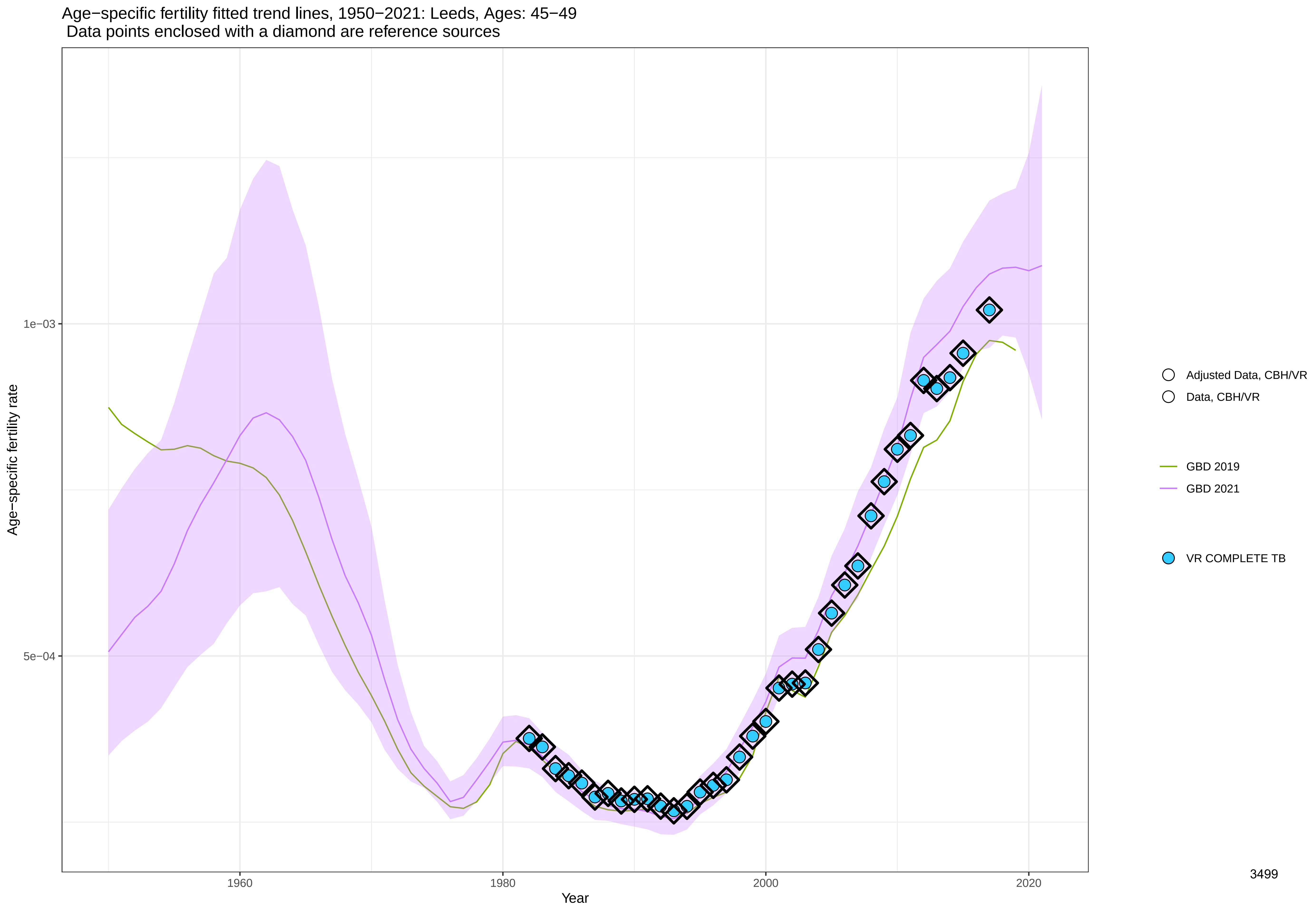
Age−specific fertility fitted trend lines, 1950−2021: Leeds, Ages: 45−49
Data points enclosed with a diamond are reference sources
Age−specific fertility rate
1e−03
5e−04
1960
1980
2000
2020
Year
Adjusted Data, CBH/VR
Data, CBH/VR
GBD 2019
GBD 2021
VR COMPLETE TB

Age−specific fertility fitted trend lines, 1950−2021: Leeds, Ages: 50−54
Data points enclosed with a diamond are reference sources

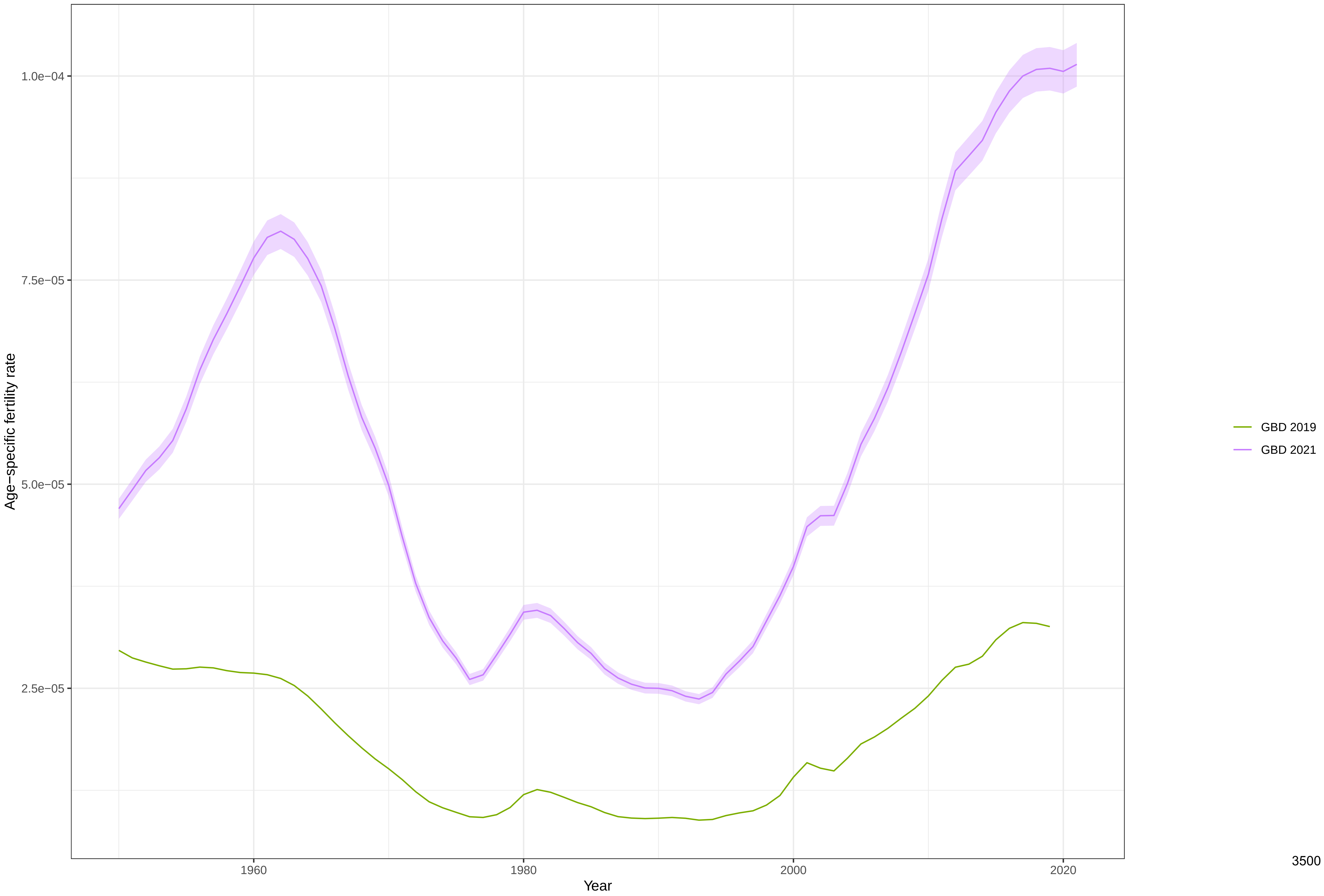

Total fertility rate fitted trend lines, 1950−2021: Leeds TFR
Data points enclosed with a diamond are reference sources

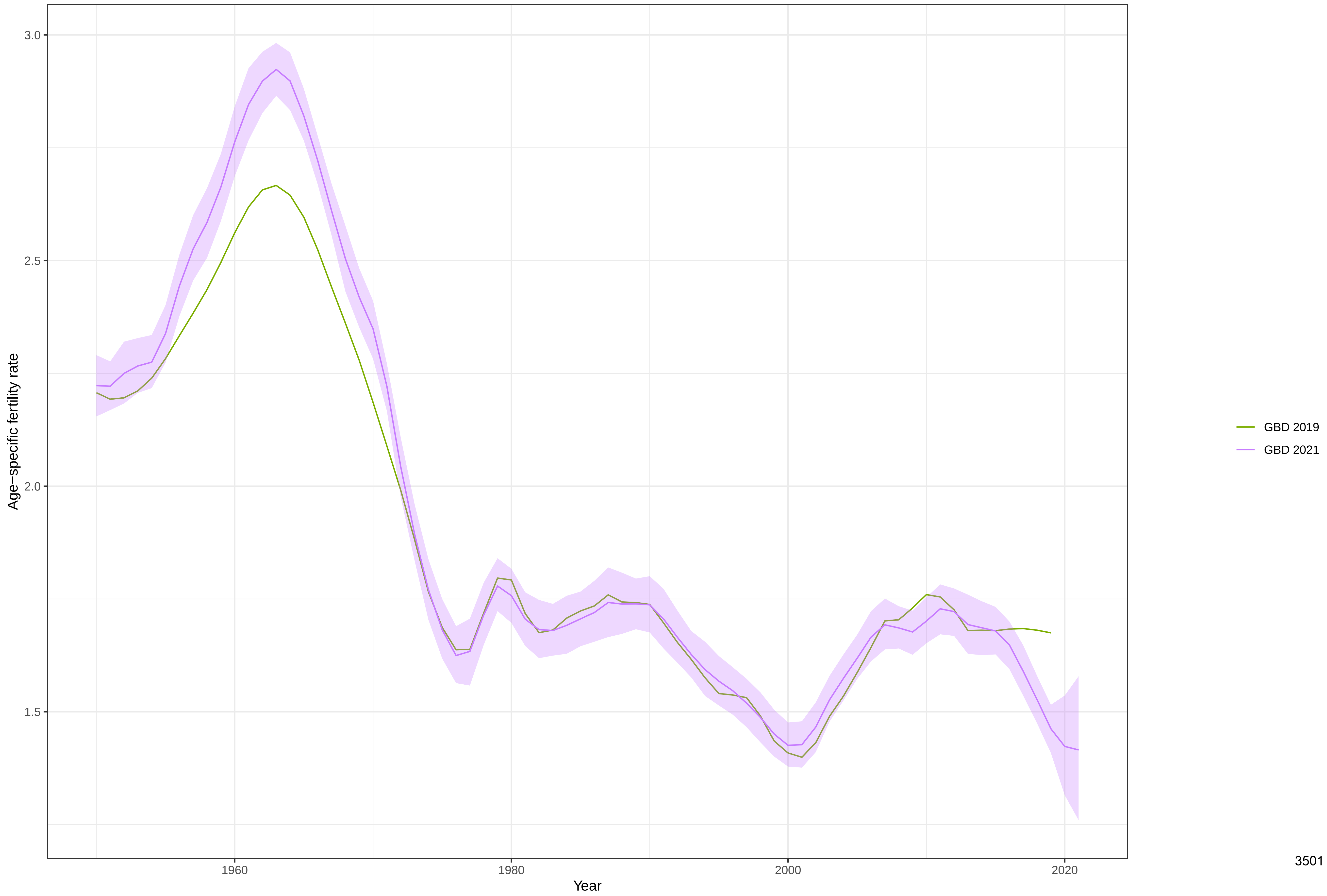

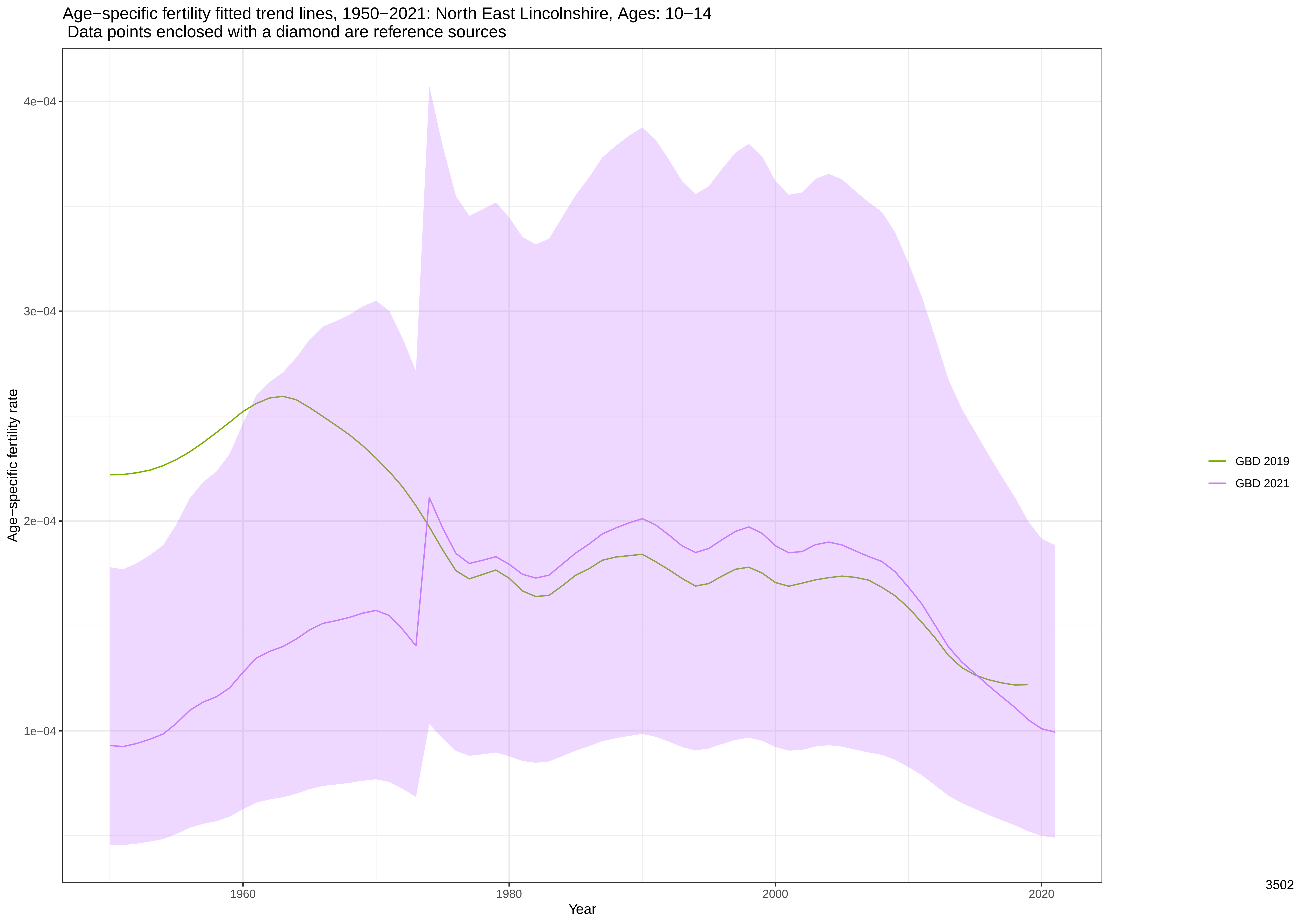

Age−specific fertility fitted trend lines, 1950−2021: North East Lincolnshire, Ages: 10−14
Data points enclosed with a diamond are reference sources
4e−04
3e−04
2e−04
1e−04
Age−specific fertility rate
1960
1980
2000
2020
Year
GBD 2019
GBD 2021

Age−specific fertility fitted trend lines, 1950−2021: North East Lincolnshire, Ages: 15−19
Data points enclosed with a diamond are reference sources

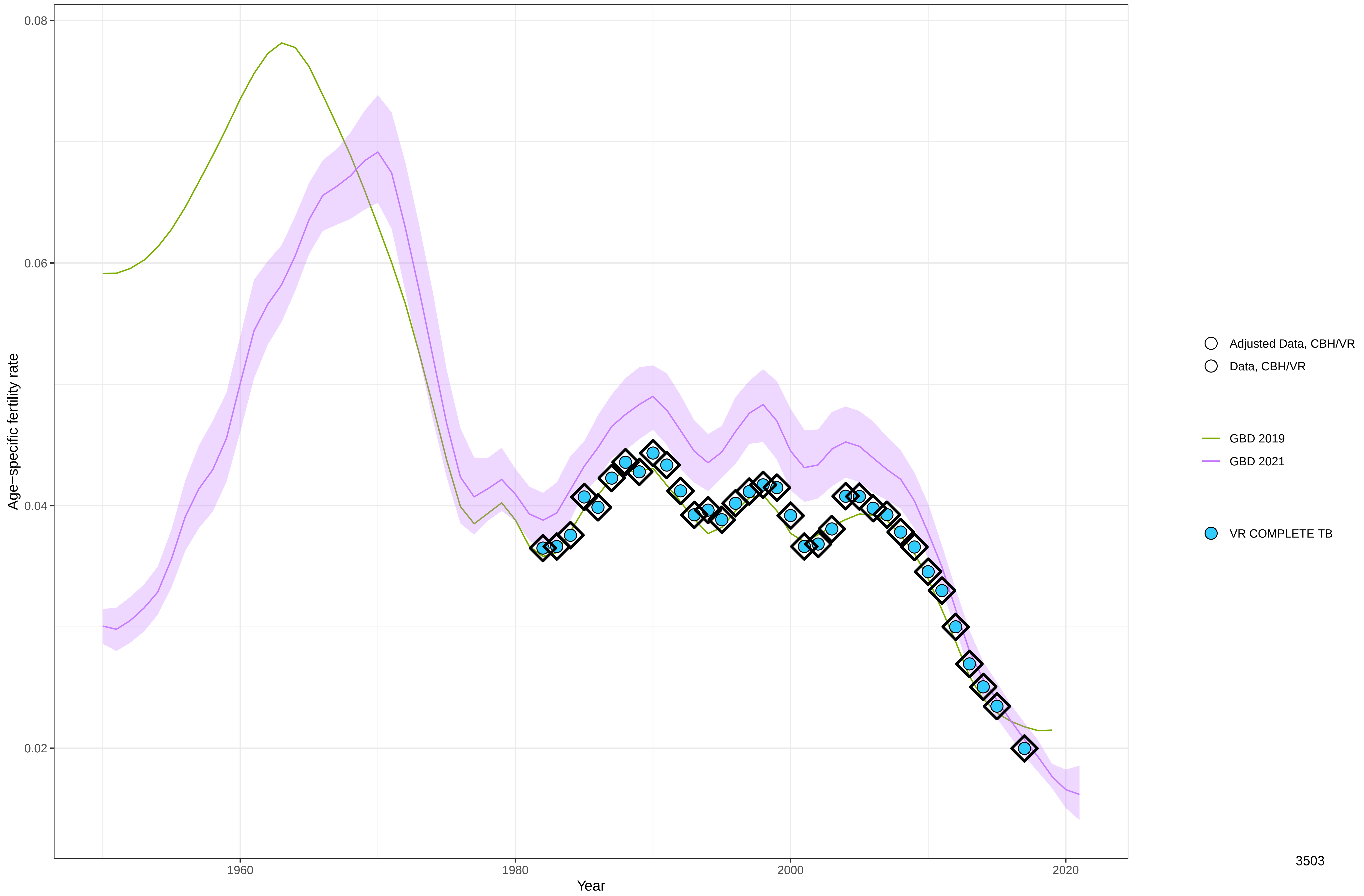

Age−specific fertility fitted trend lines, 1950−2021: North East Lincolnshire, Ages: 20−24
Data points enclosed with a diamond are reference sources

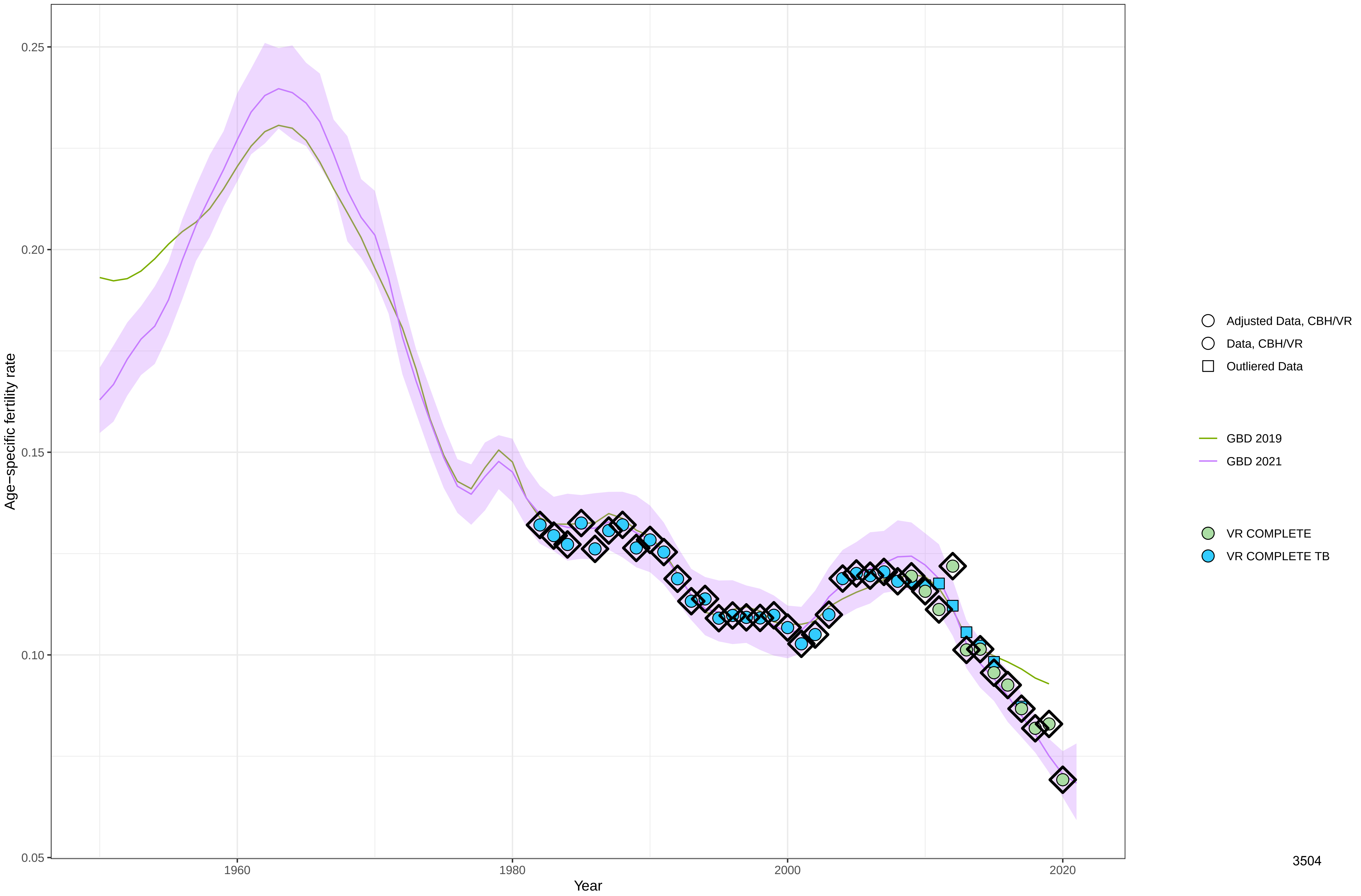

Age−specific fertility fitted trend lines, 1950−2021: North East Lincolnshire, Ages: 25−29
Data points enclosed with a diamond are reference sources

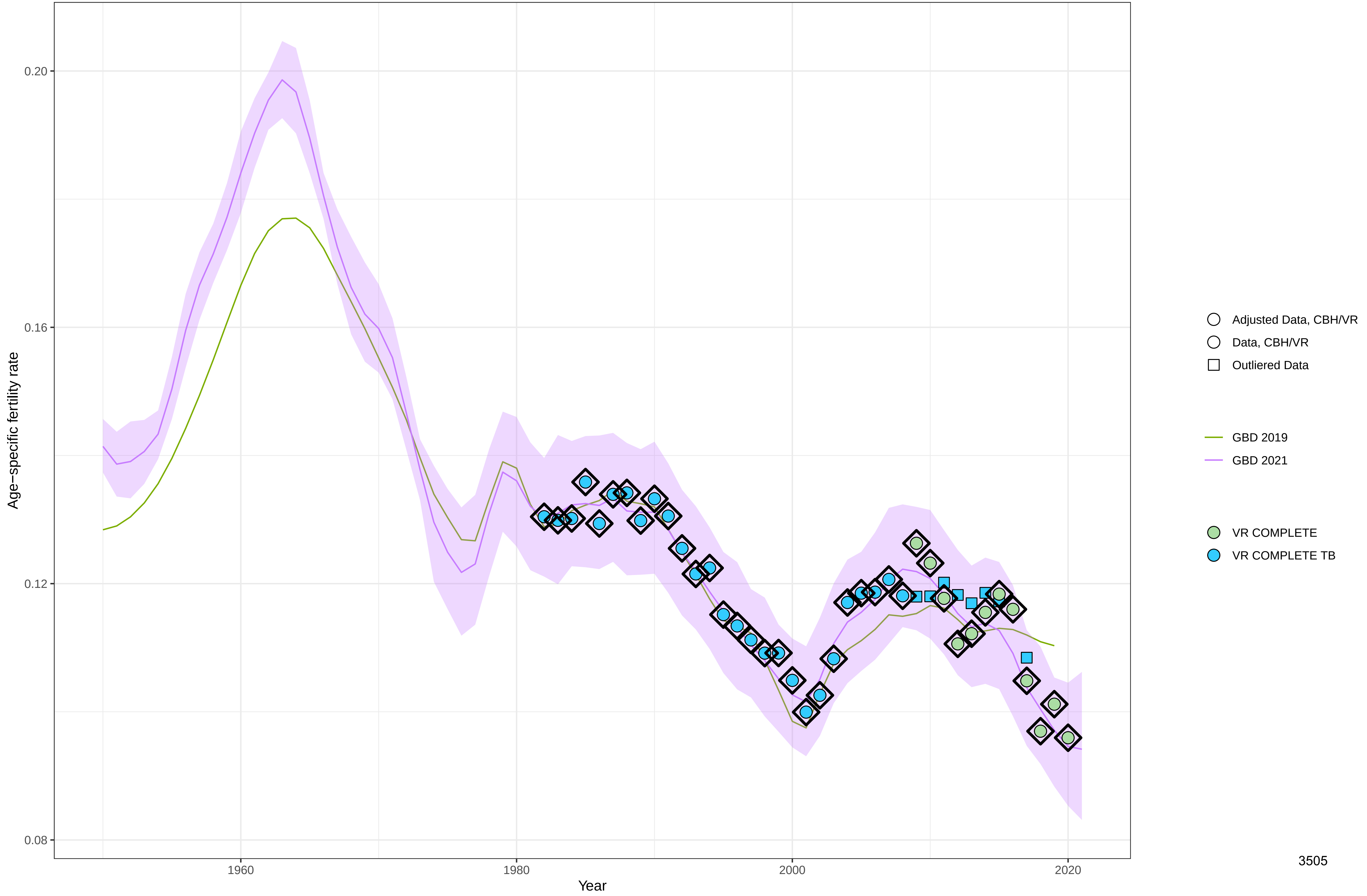

Age−specific fertility fitted trend lines, 1950−2021: North East Lincolnshire, Ages: 30−34
Data points enclosed with a diamond are reference sources

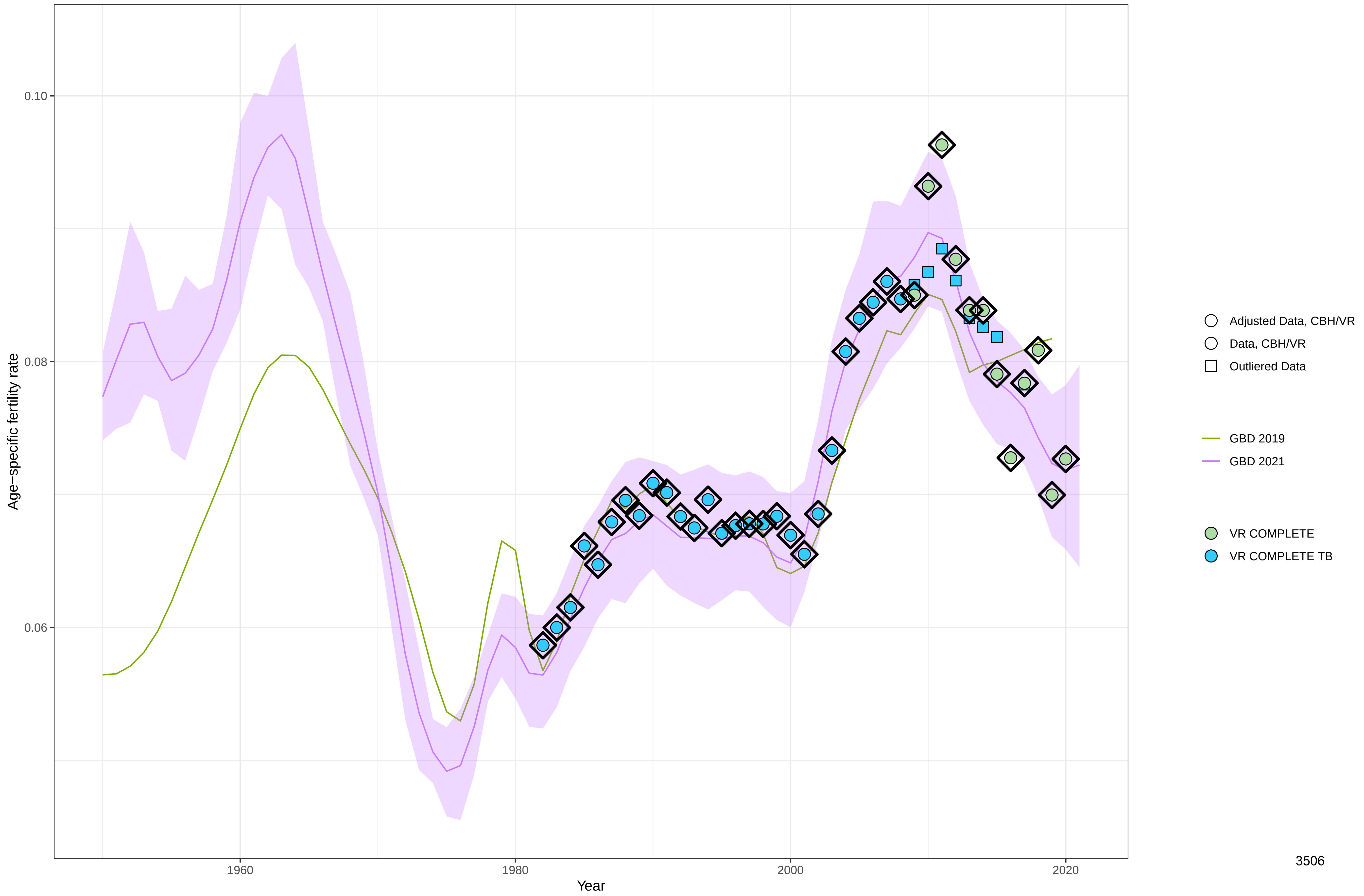

Age−specific fertility fitted trend lines, 1950−2021: North East Lincolnshire, Ages: 35−39
Data points enclosed with a diamond are reference sources

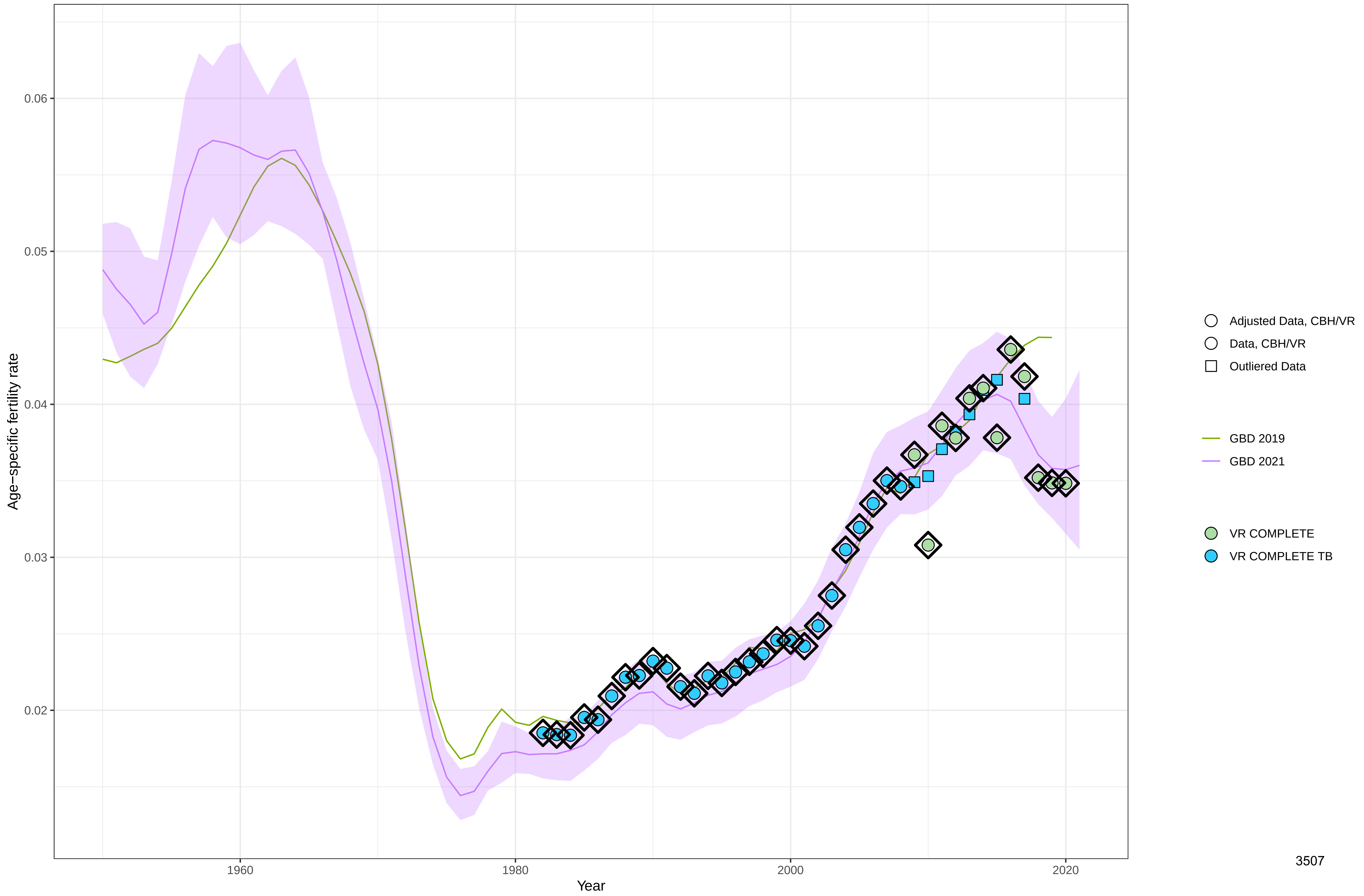

Age−specific fertility fitted trend lines, 1950−2021: North East Lincolnshire, Ages: 40−44
Data points enclosed with a diamond are reference sources

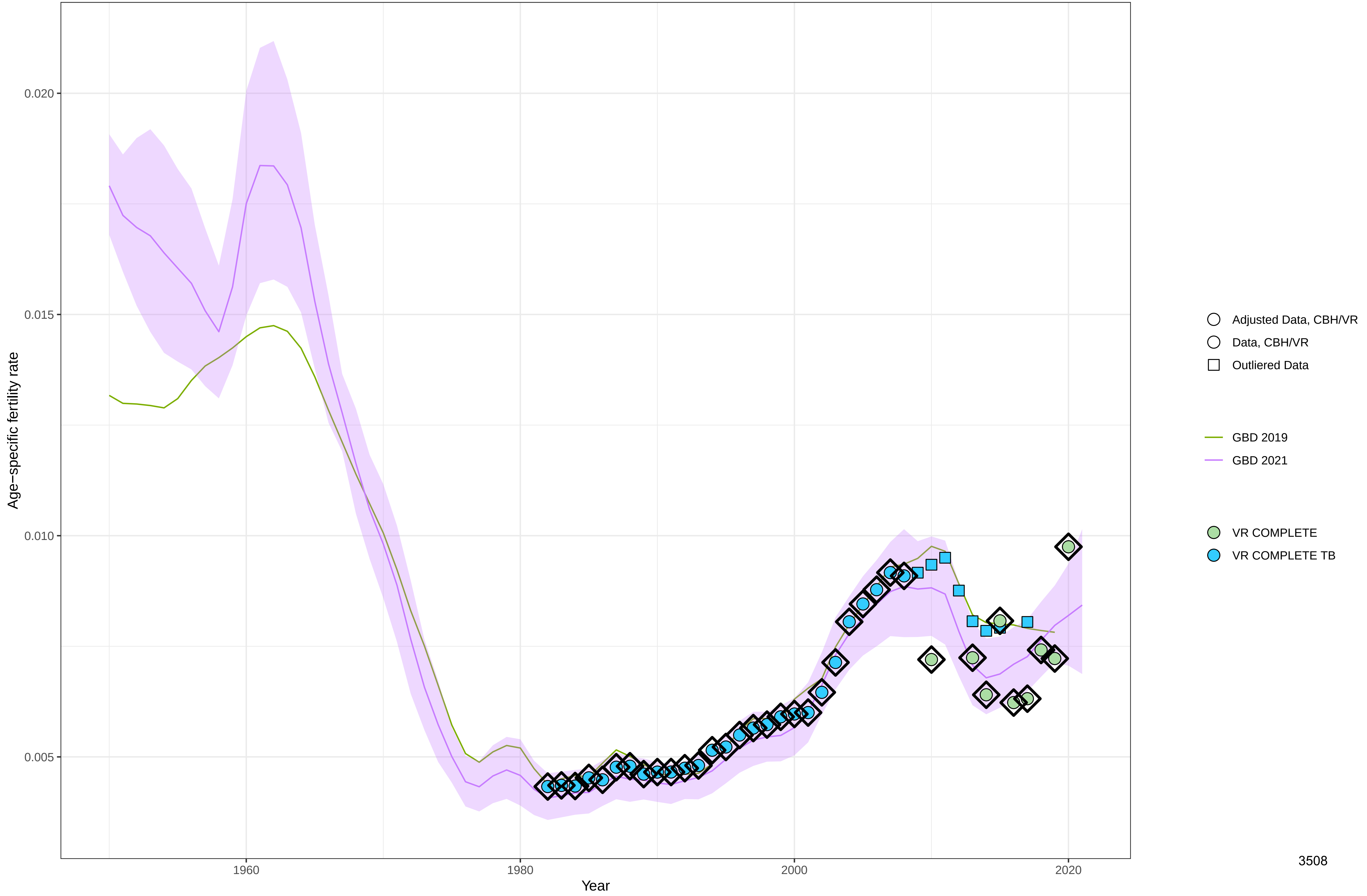

Age−specific fertility fitted trend lines, 1950−2021: North East Lincolnshire, Ages: 45−49
Data points enclosed with a diamond are reference sources

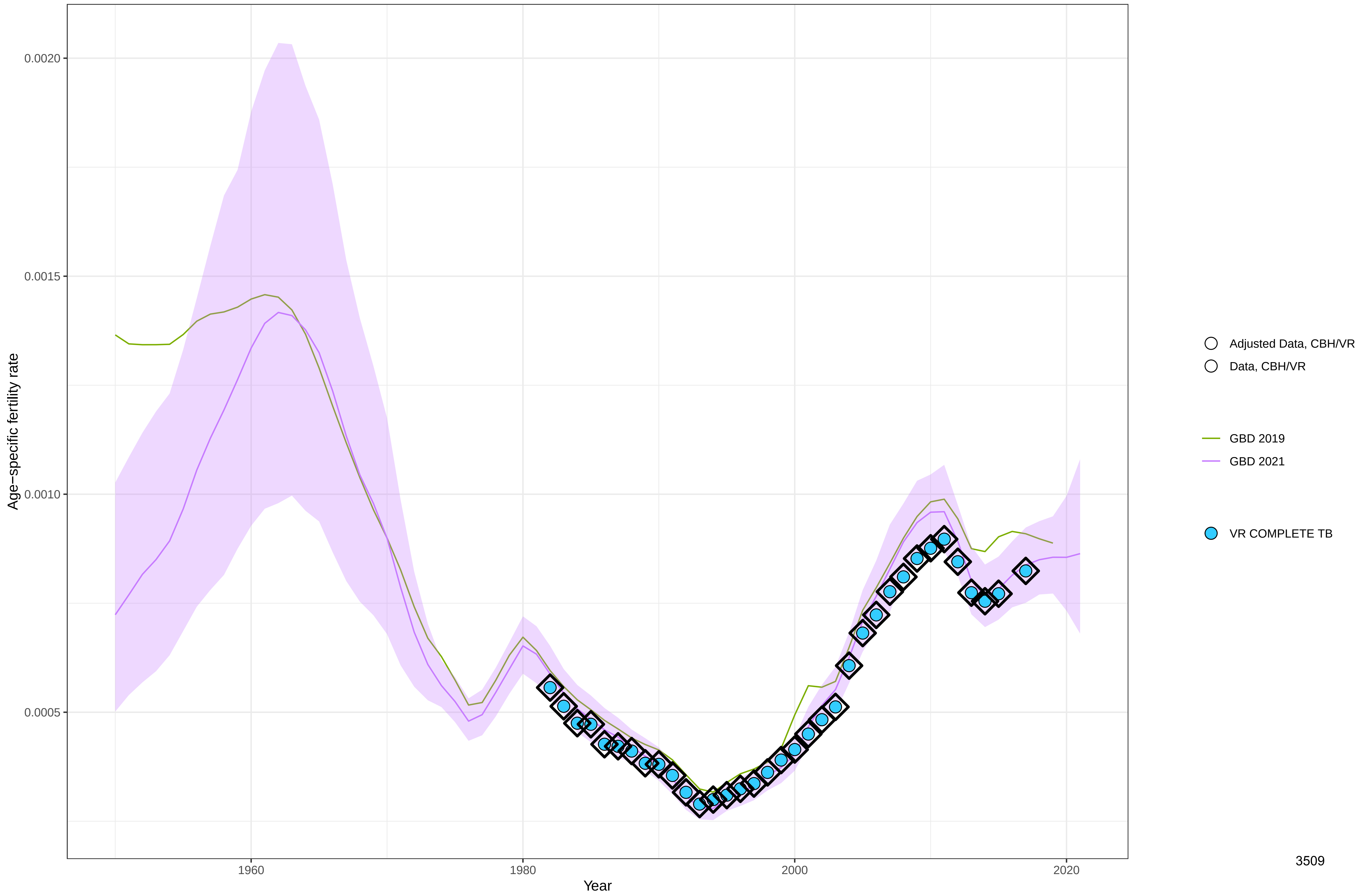

Age−specific fertility fitted trend lines, 1950−2021: North East Lincolnshire, Ages: 50−54
Data points enclosed with a diamond are reference sources

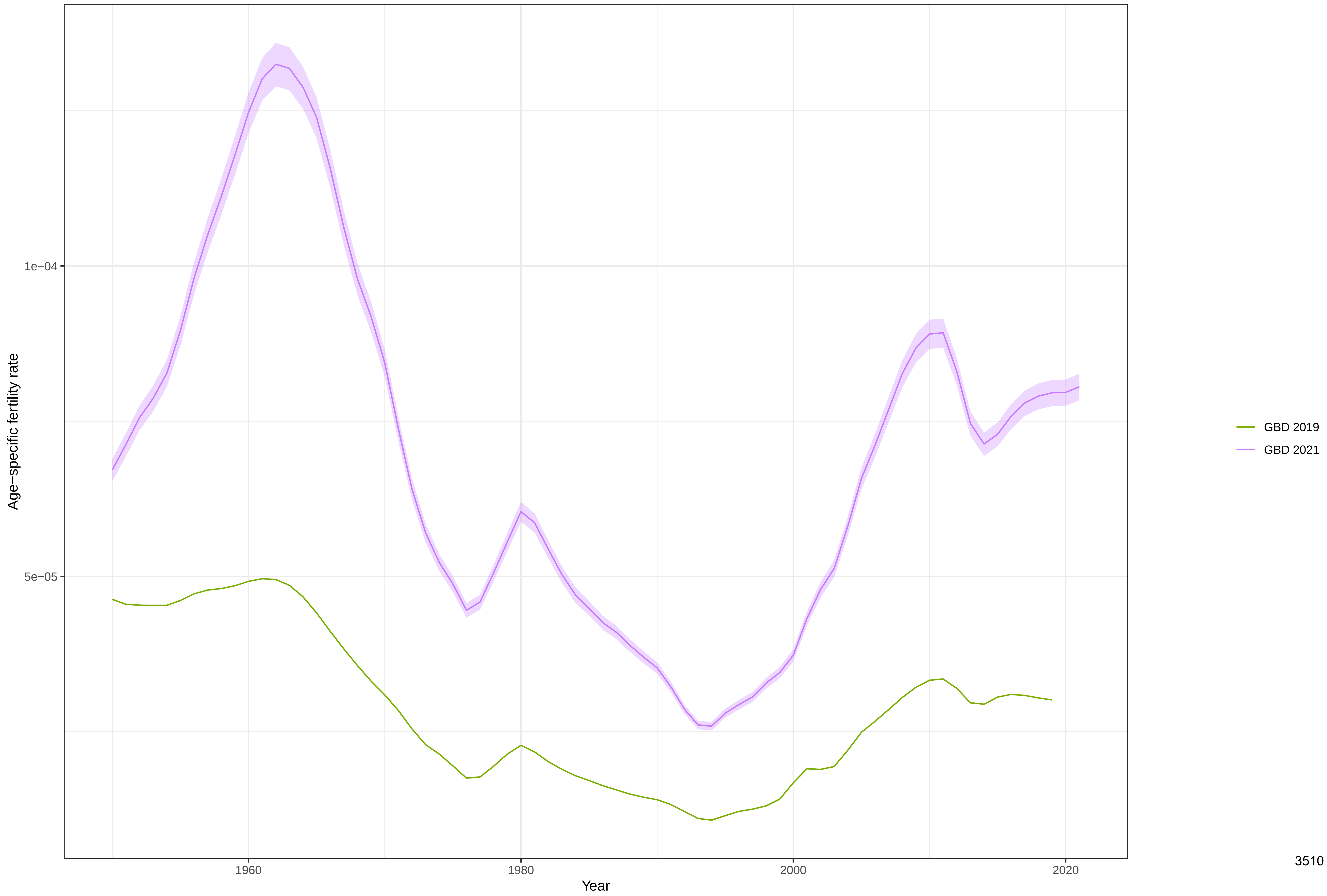

Total fertility rate fitted trend lines, 1950−2021: North East Lincolnshire TFR
Data points enclosed with a diamond are reference sources

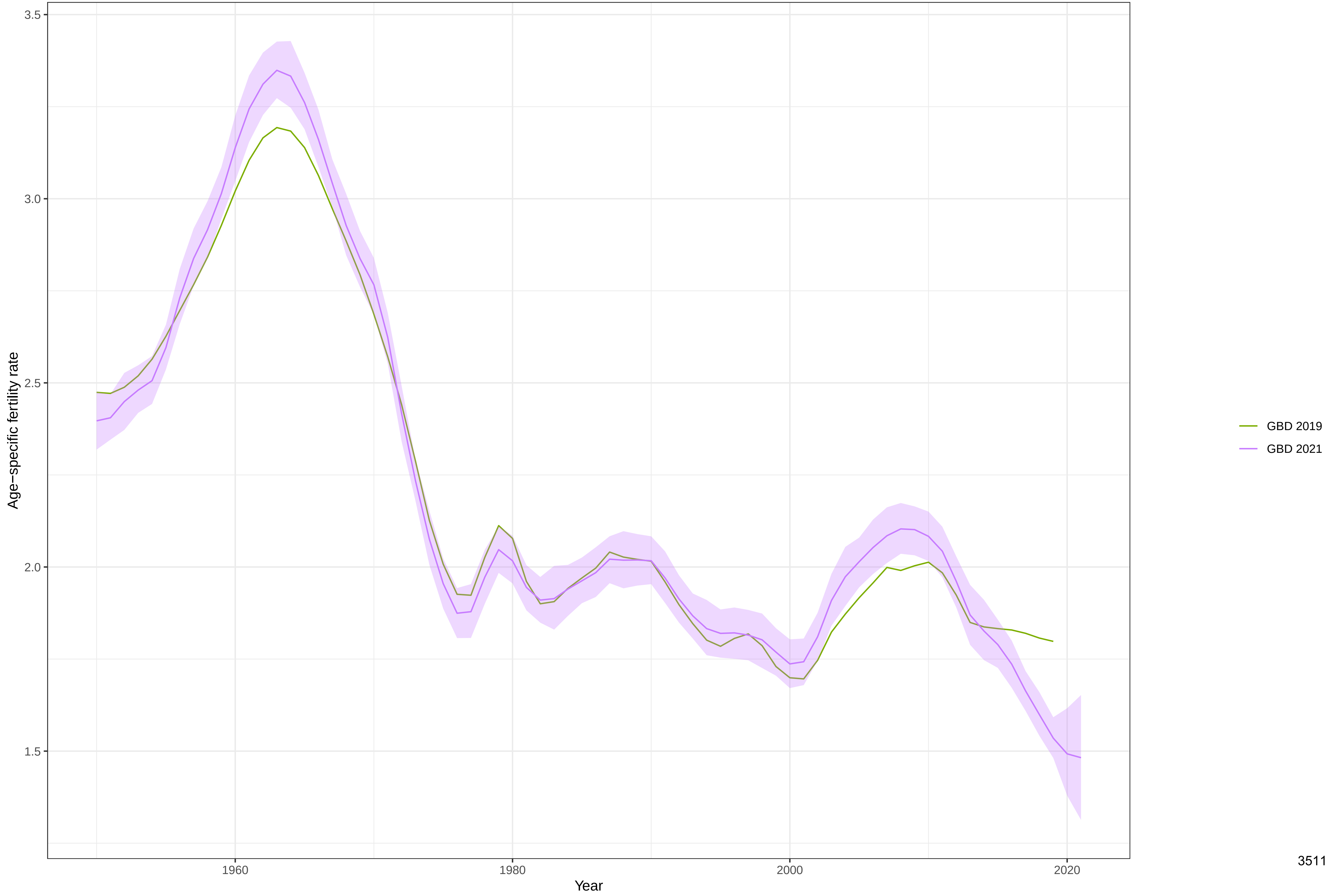

Age−specific fertility fitted trend lines, 1950−2021: North Lincolnshire, Ages: 10−14
Data points enclosed with a diamond are reference sources

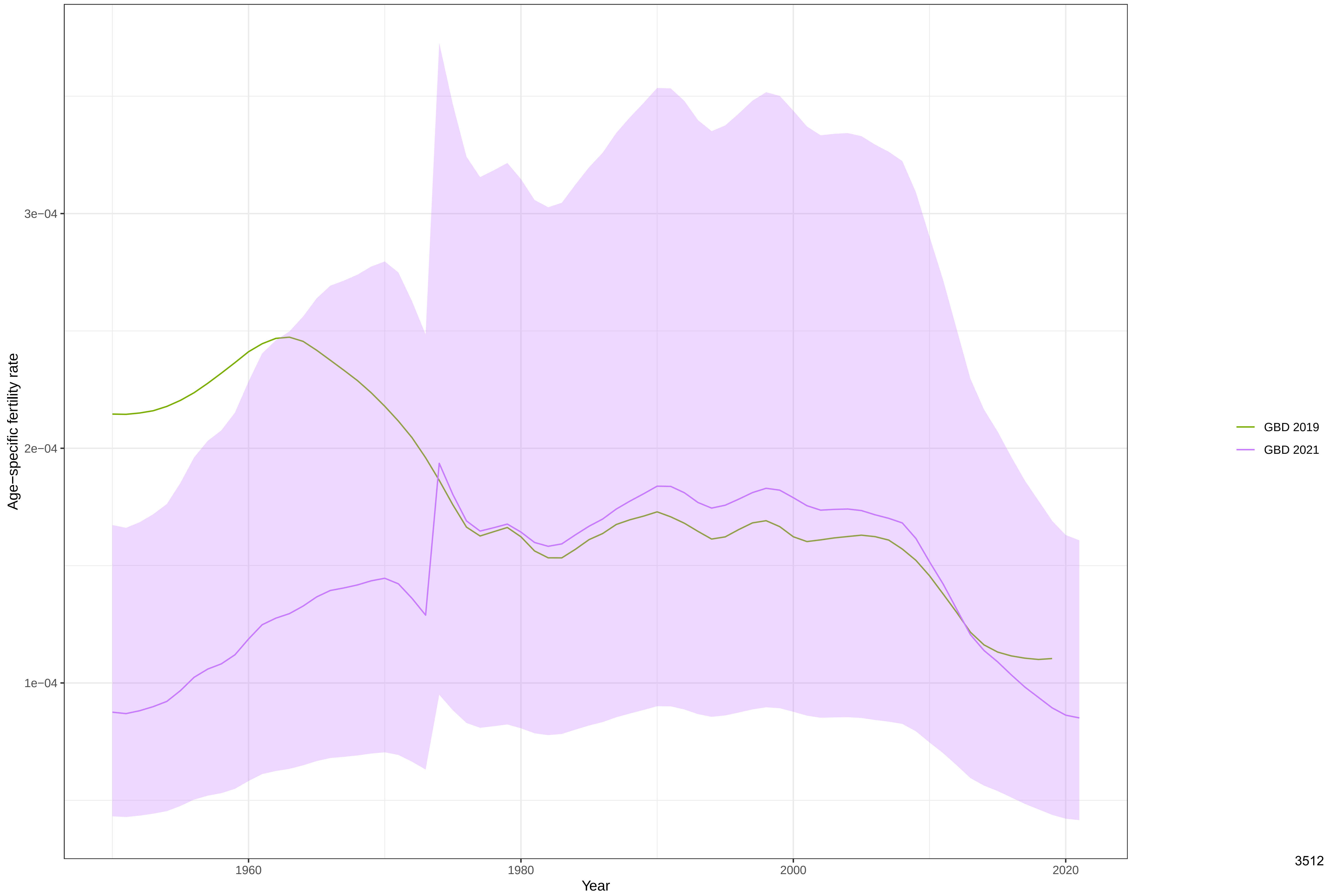

Age−specific fertility fitted trend lines, 1950−2021: North Lincolnshire, Ages: 15−19
Data points enclosed with a diamond are reference sources

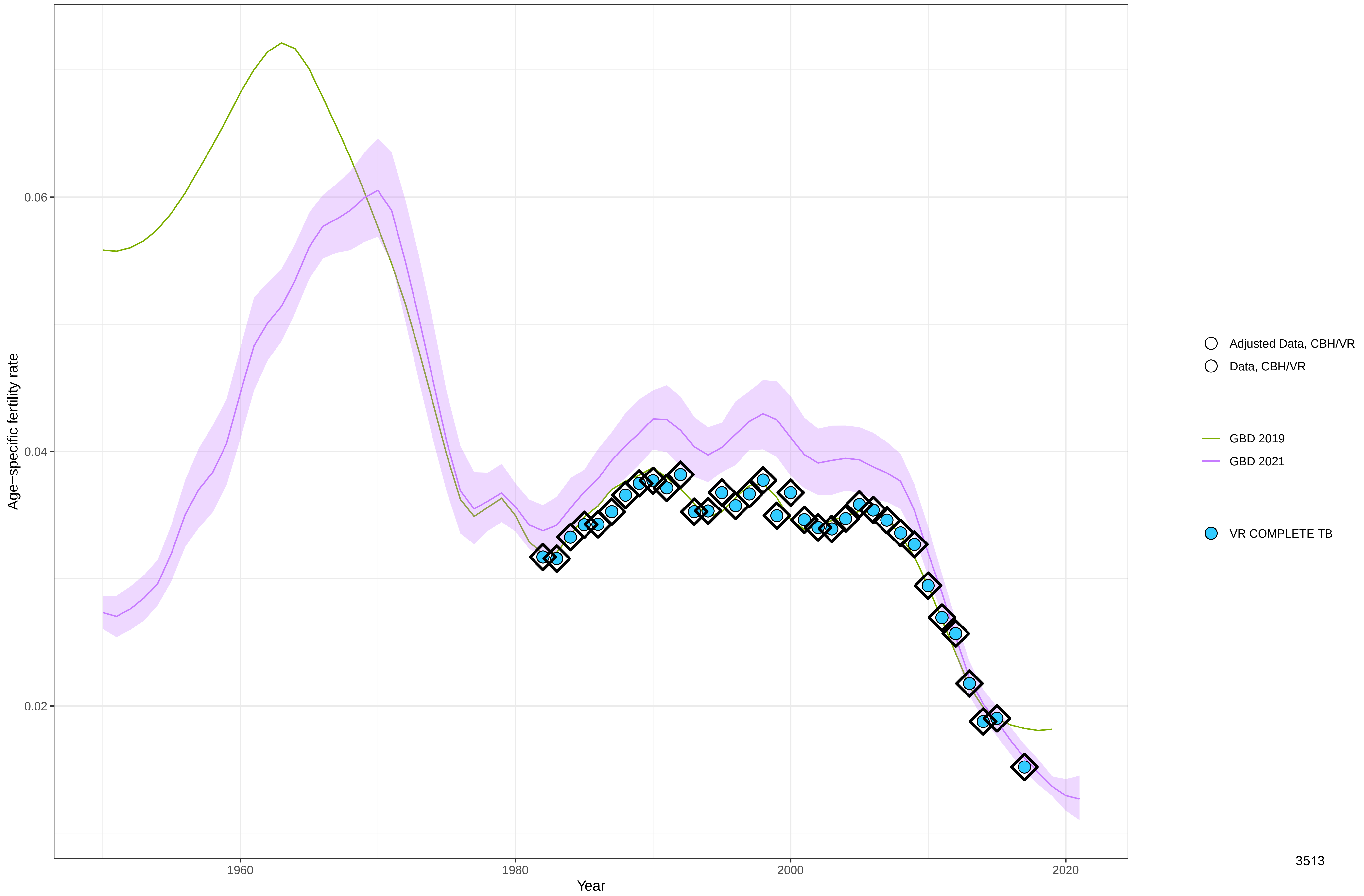

Age−specific fertility fitted trend lines, 1950−2021: North Lincolnshire, Ages: 20−24
Data points enclosed with a diamond are reference sources

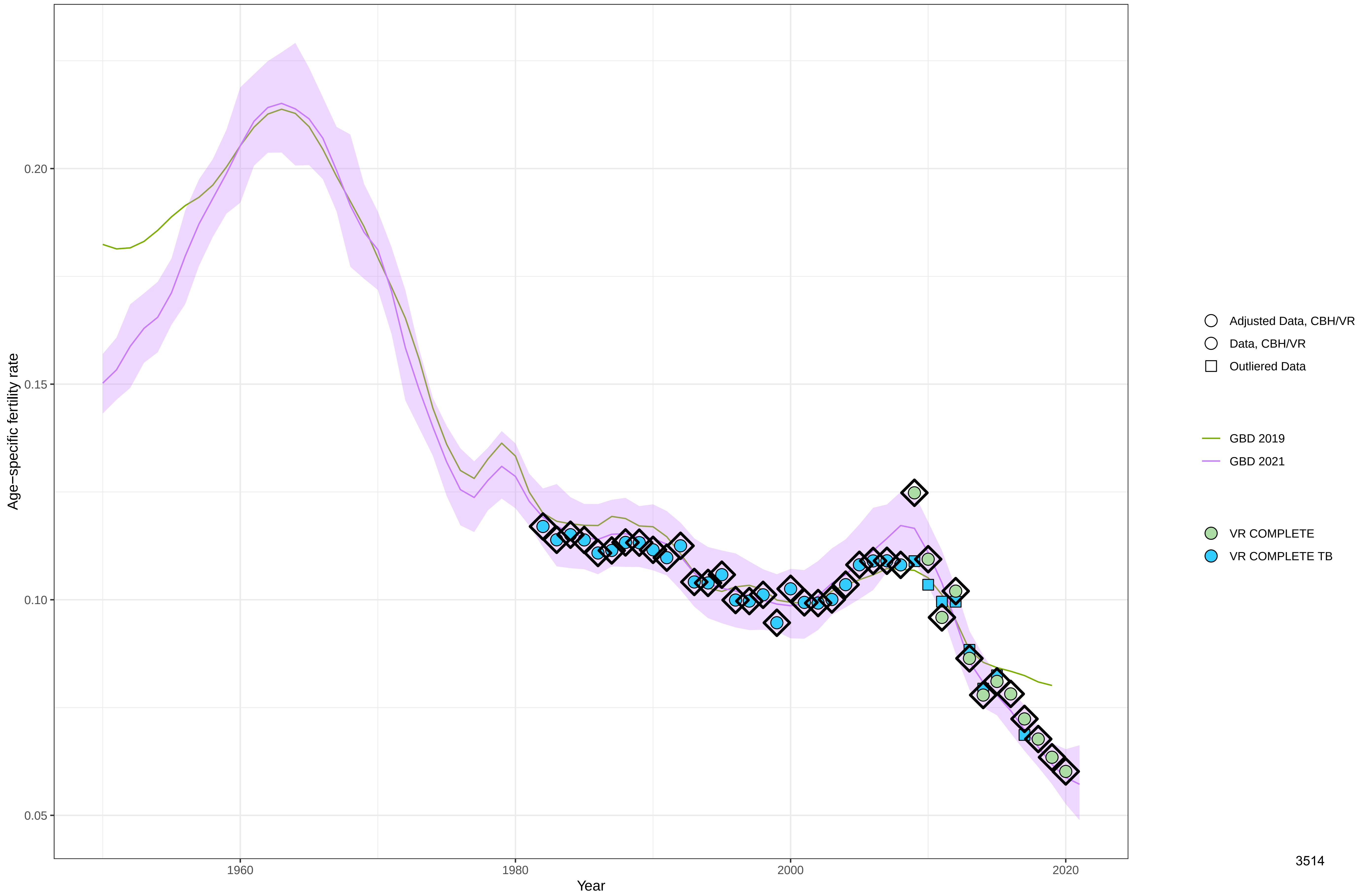

Age−specific fertility fitted trend lines, 1950−2021: North Lincolnshire, Ages: 25−29
Data points enclosed with a diamond are reference sources

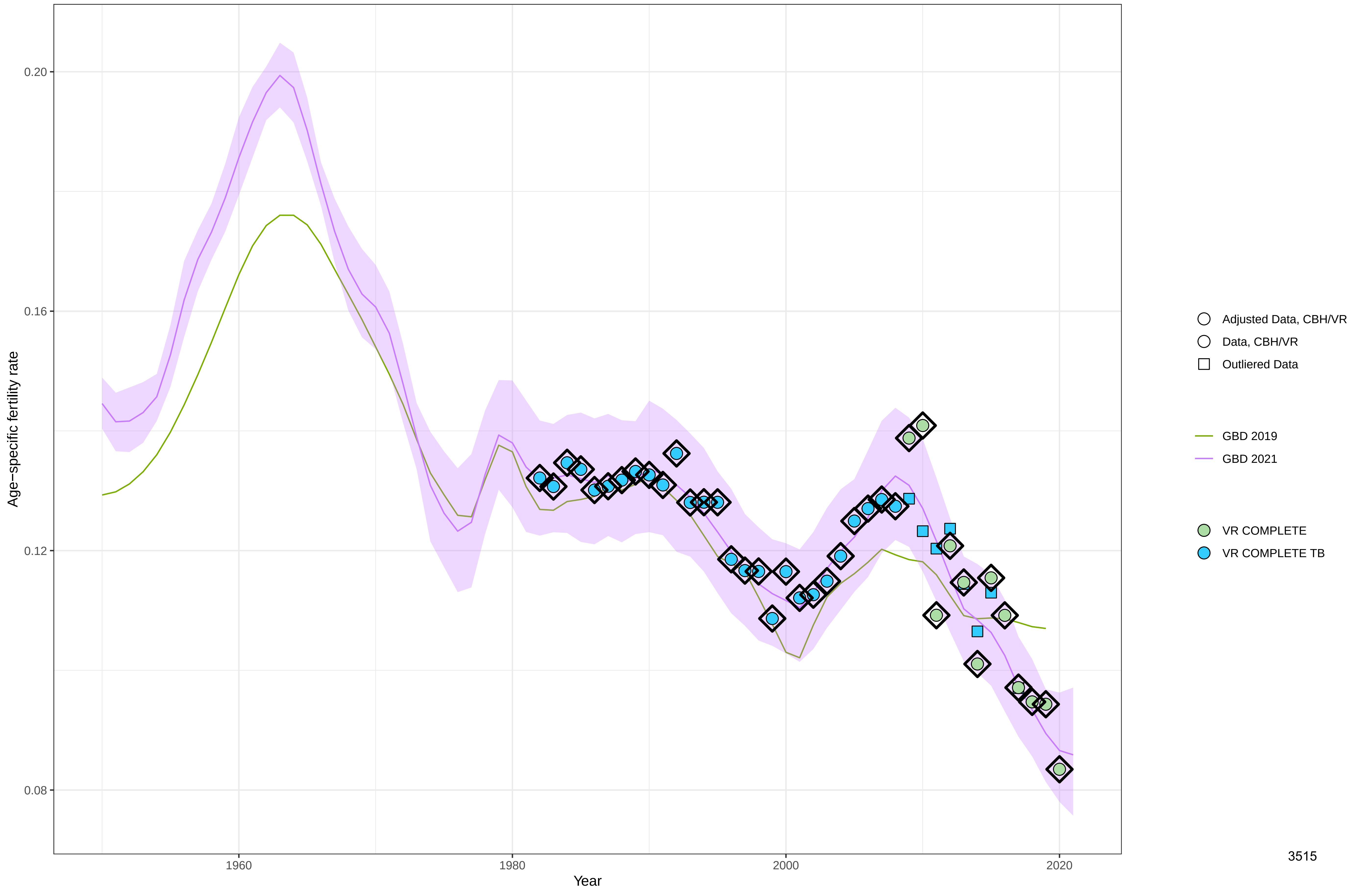

Age−specific fertility fitted trend lines, 1950−2021: North Lincolnshire, Ages: 30−34
Data points enclosed with a diamond are reference sources

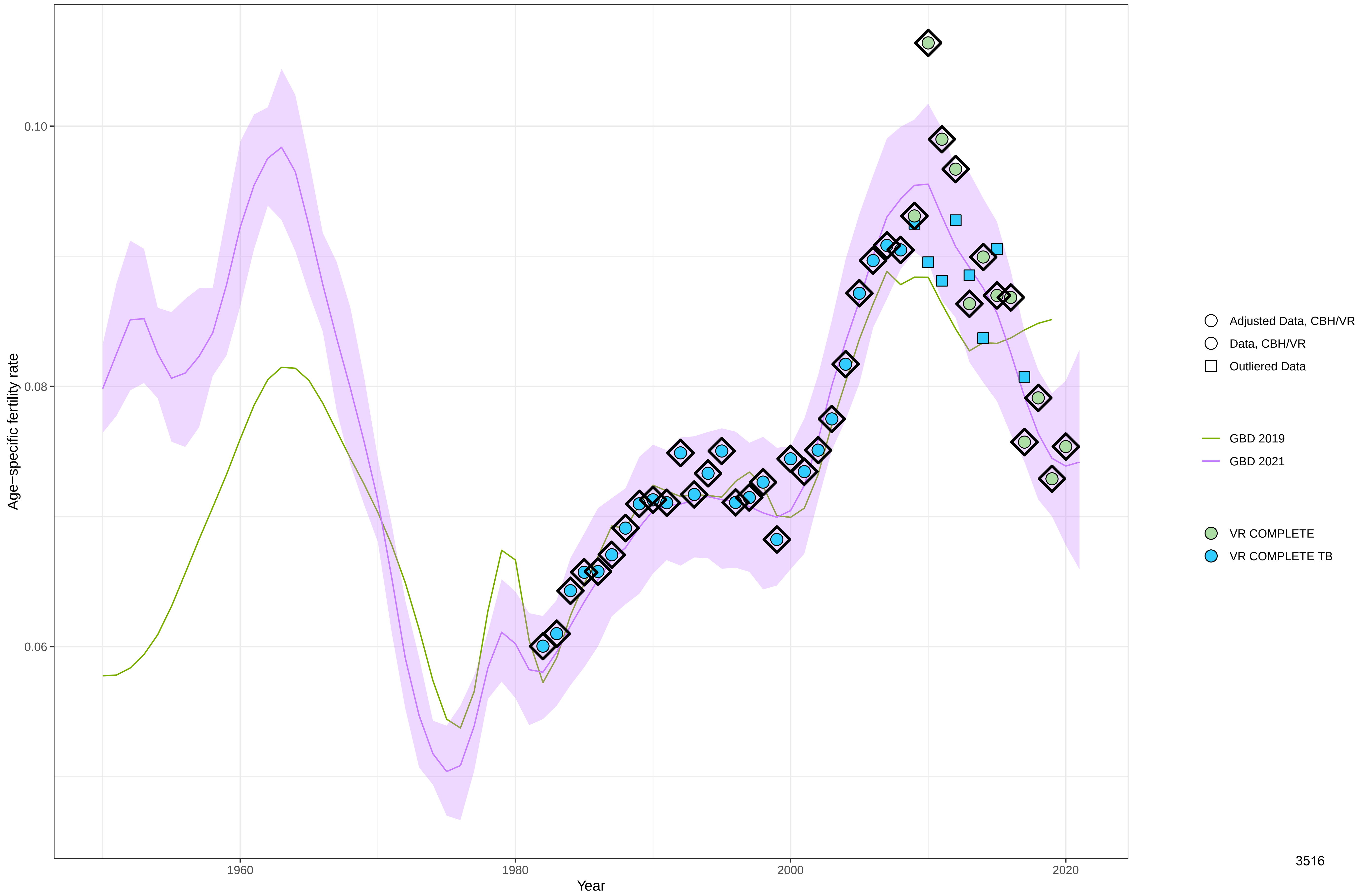

Age−specific fertility fitted trend lines, 1950−2021: North Lincolnshire, Ages: 35−39
Data points enclosed with a diamond are reference sources

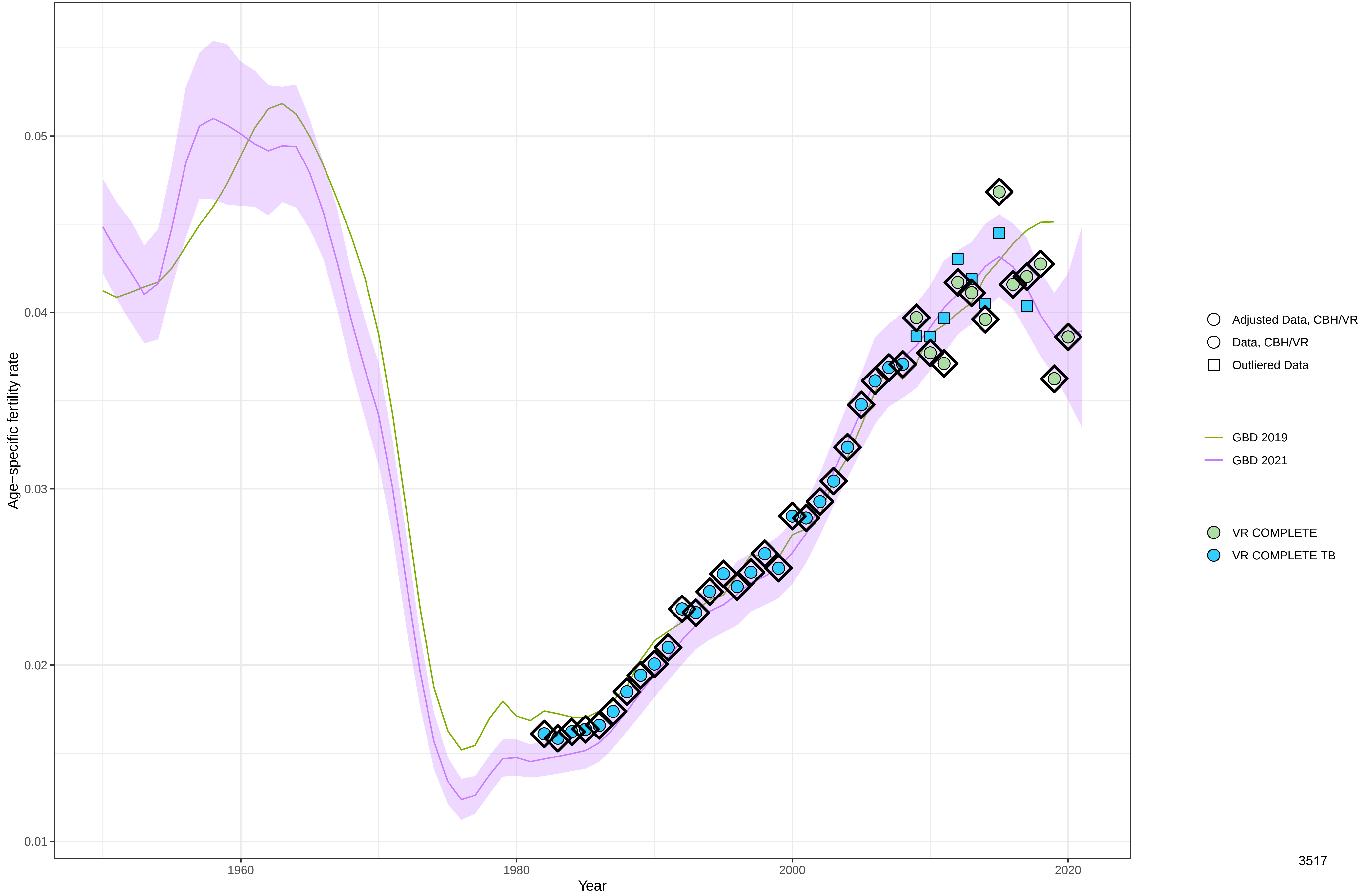

Age−specific fertility fitted trend lines, 1950−2021: North Lincolnshire, Ages: 40−44
Data points enclosed with a diamond are reference sources

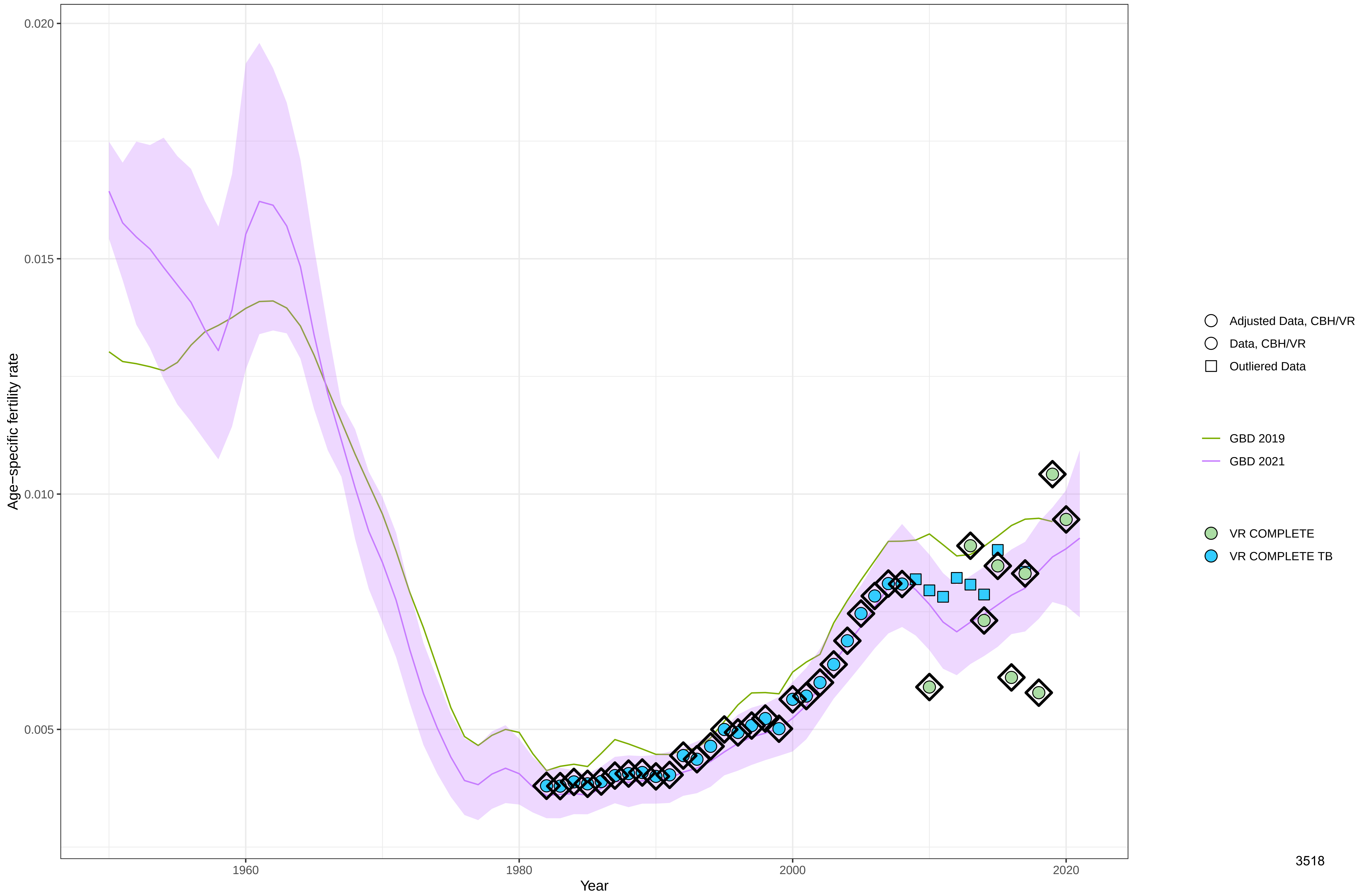

Age−specific fertility fitted trend lines, 1950−2021: North Lincolnshire, Ages: 45−49
Data points enclosed with a diamond are reference sources

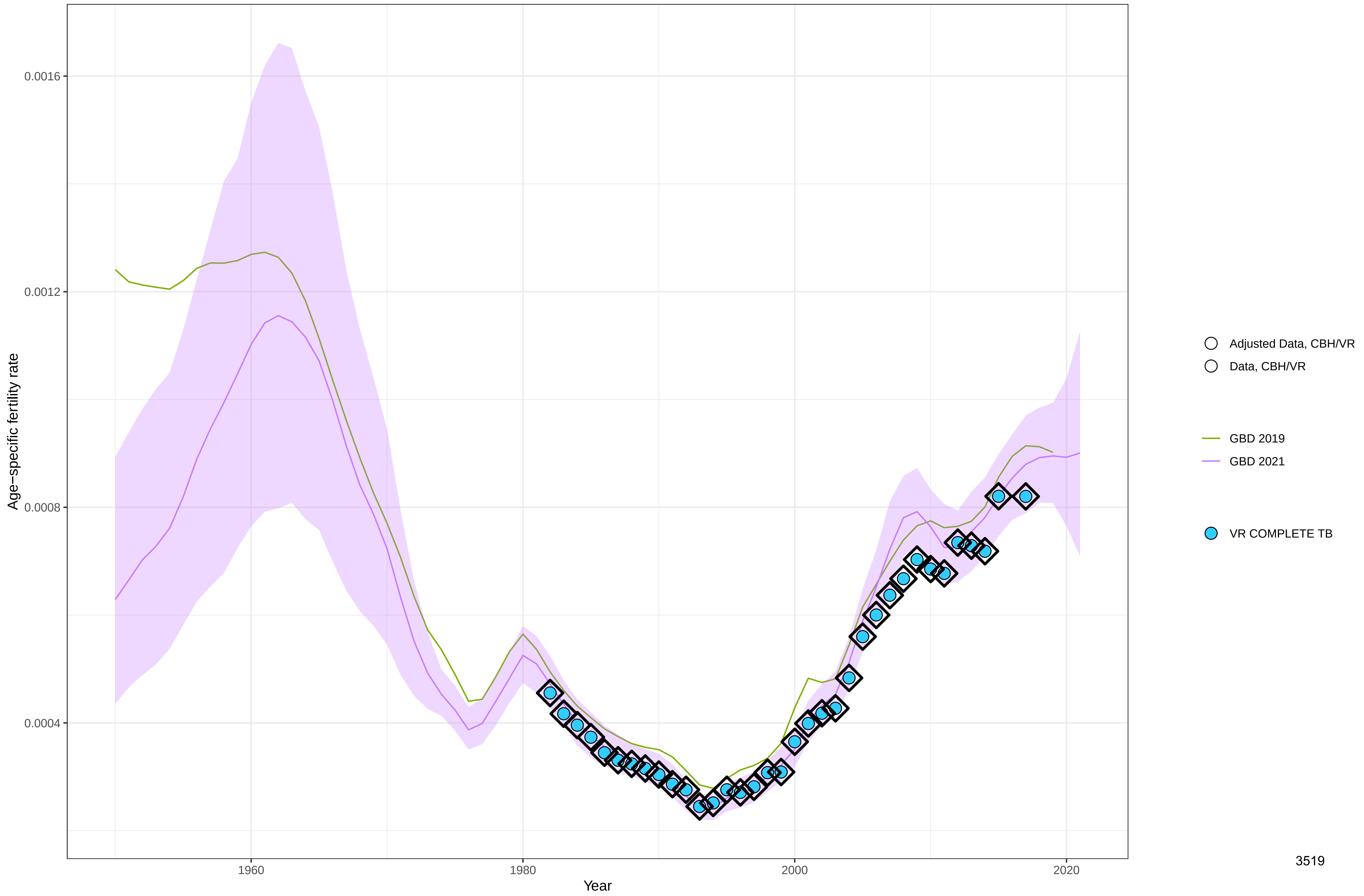

Age−specific fertility fitted trend lines, 1950−2021: North Lincolnshire, Ages: 50−54
Data points enclosed with a diamond are reference sources

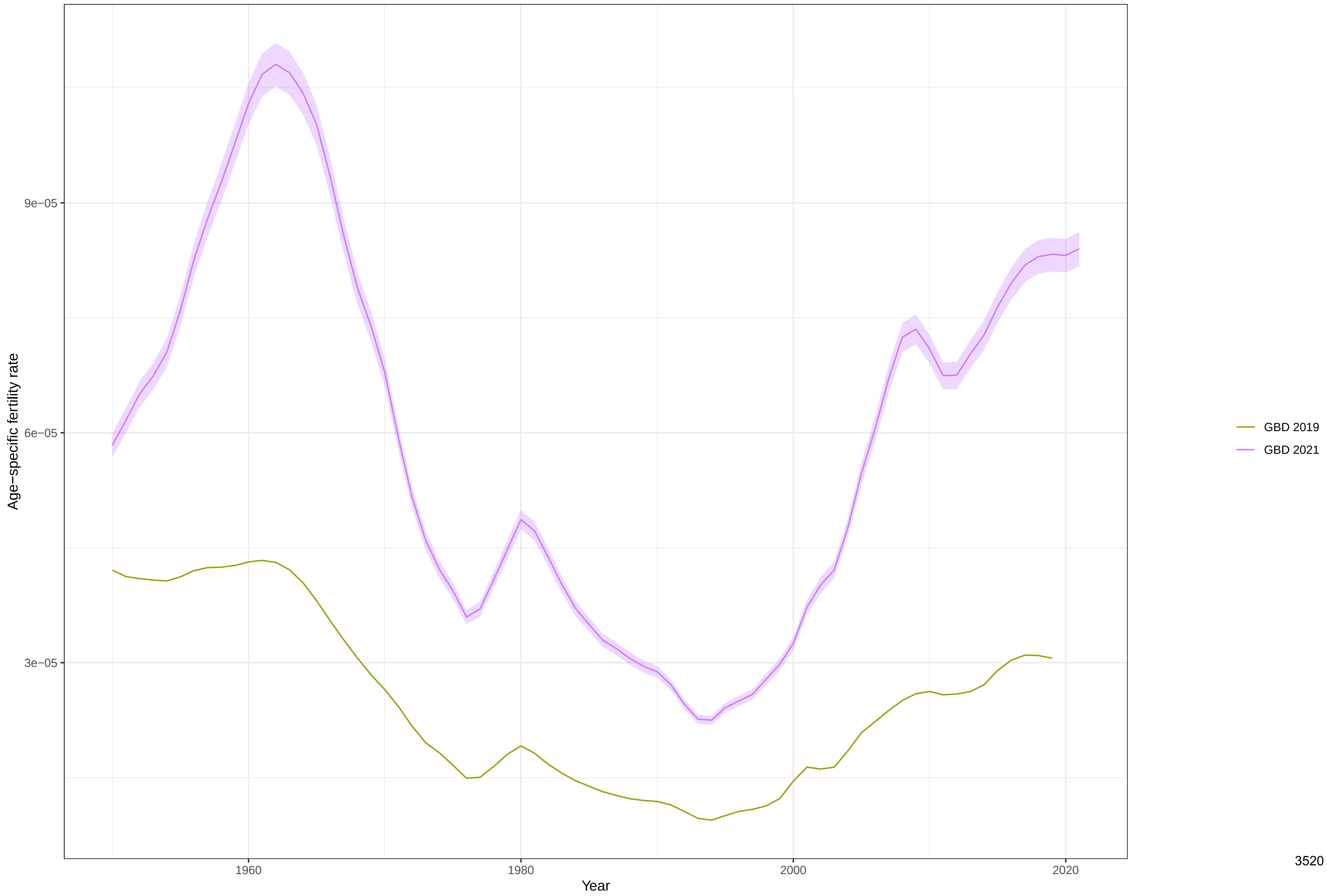

Total fertility rate fitted trend lines, 1950−2021: North Lincolnshire TFR
Data points enclosed with a diamond are reference sources

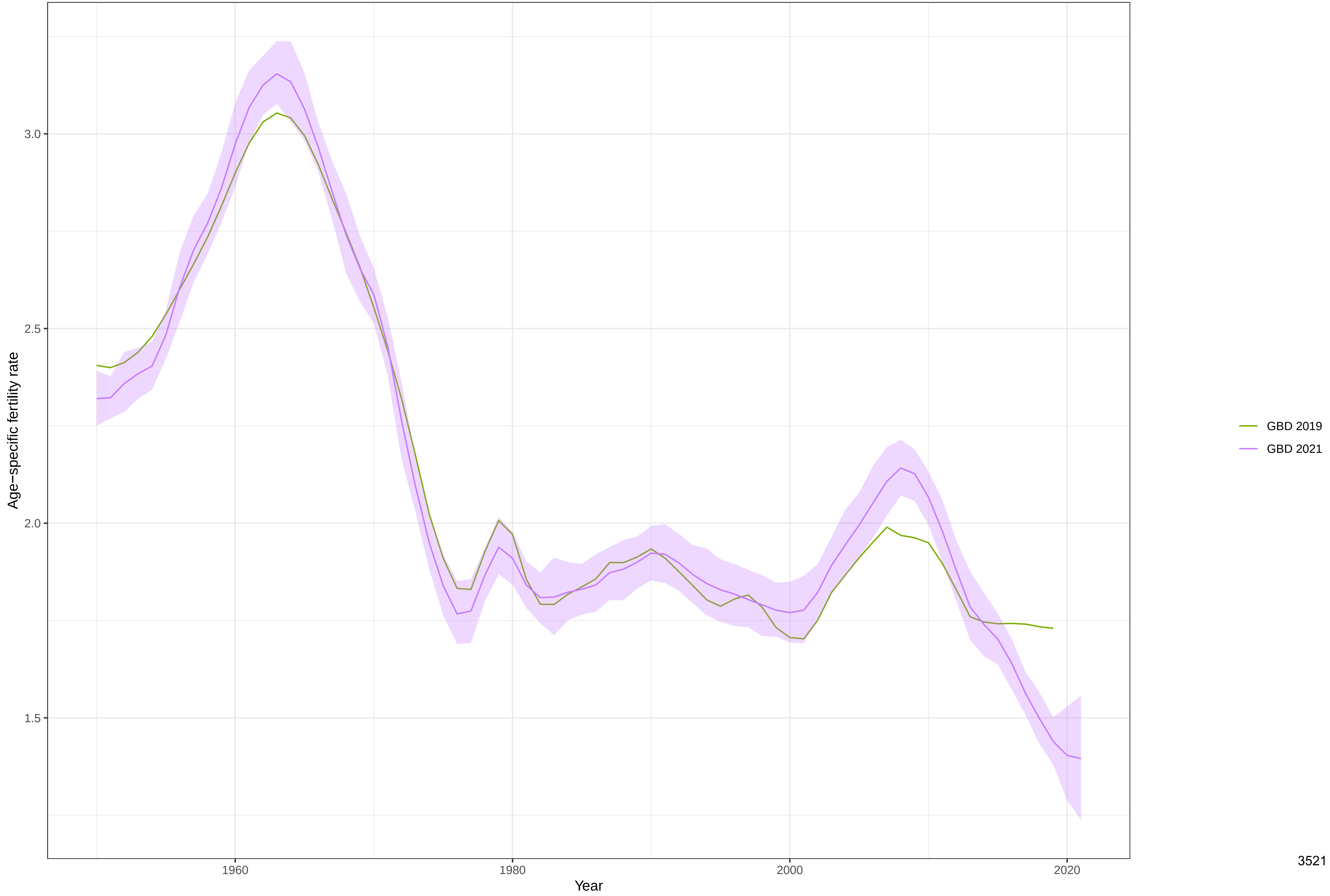

Age−specific fertility fitted trend lines, 1950−2021: North Yorkshire, Ages: 10−14
Data points enclosed with a diamond are reference sources

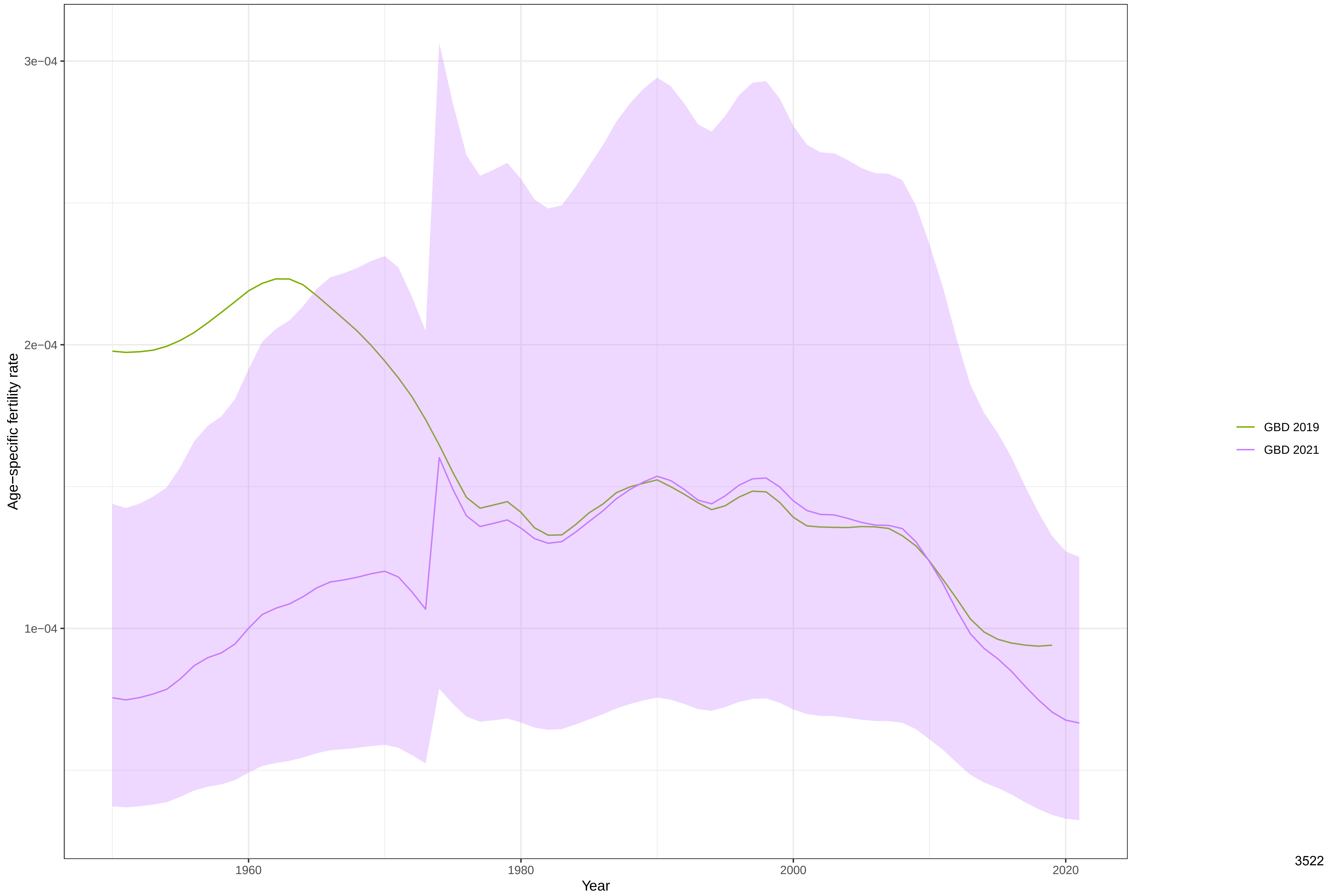

Age−specific fertility fitted trend lines, 1950−2021: North Yorkshire, Ages: 15−19
Data points enclosed with a diamond are reference sources

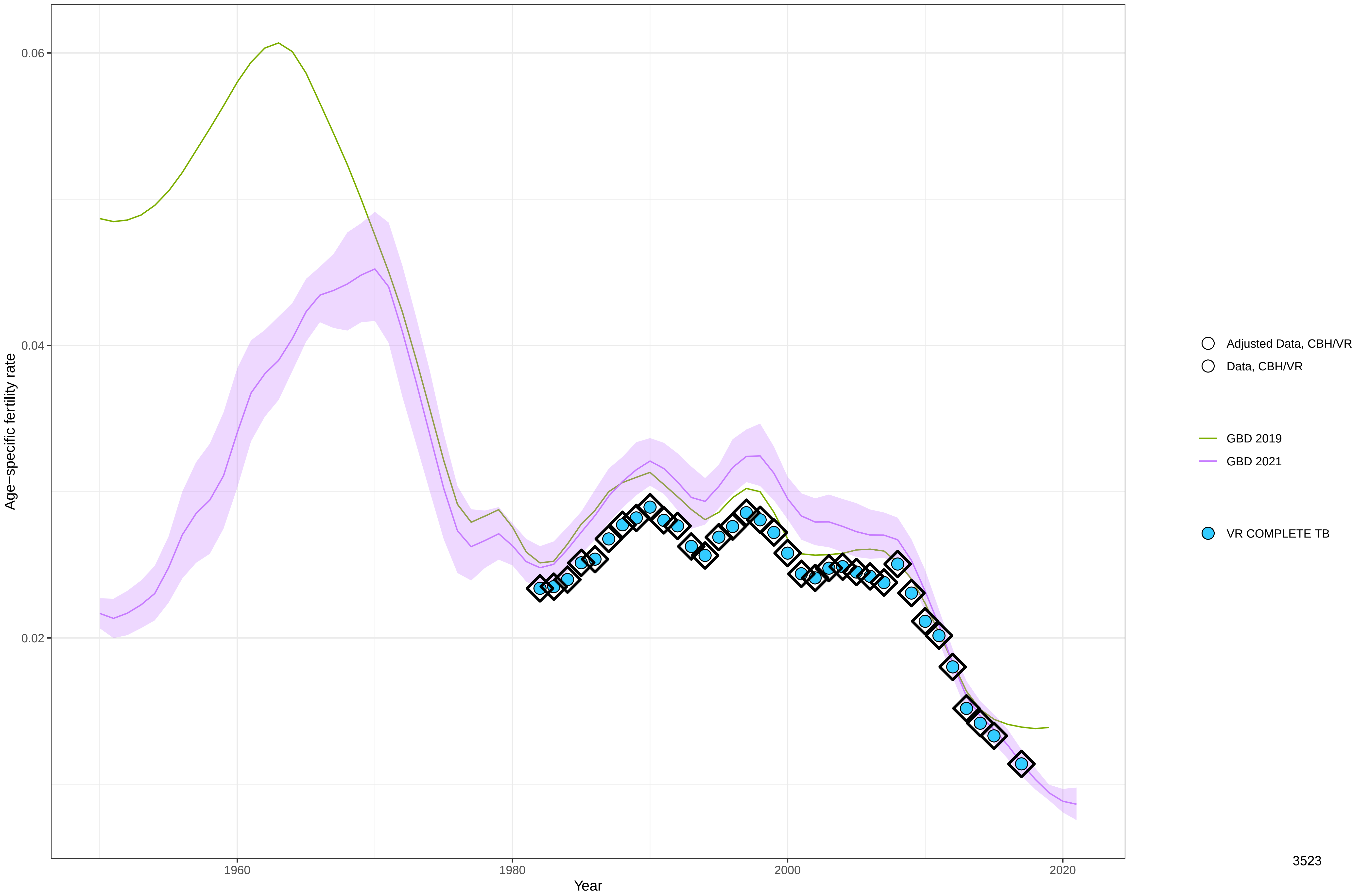

Age−specific fertility fitted trend lines, 1950−2021: North Yorkshire, Ages: 20−24
Data points enclosed with a diamond are reference sources

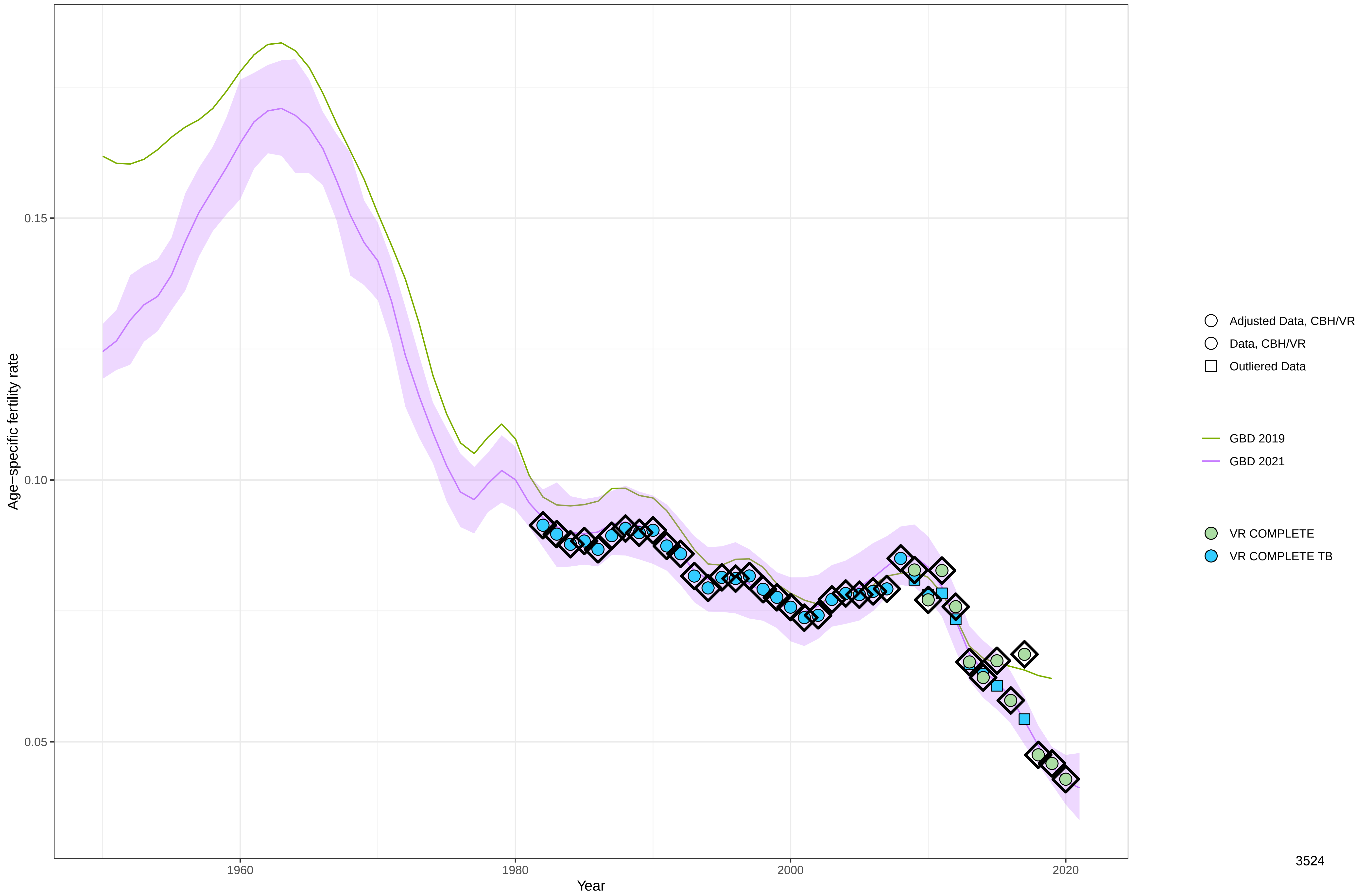

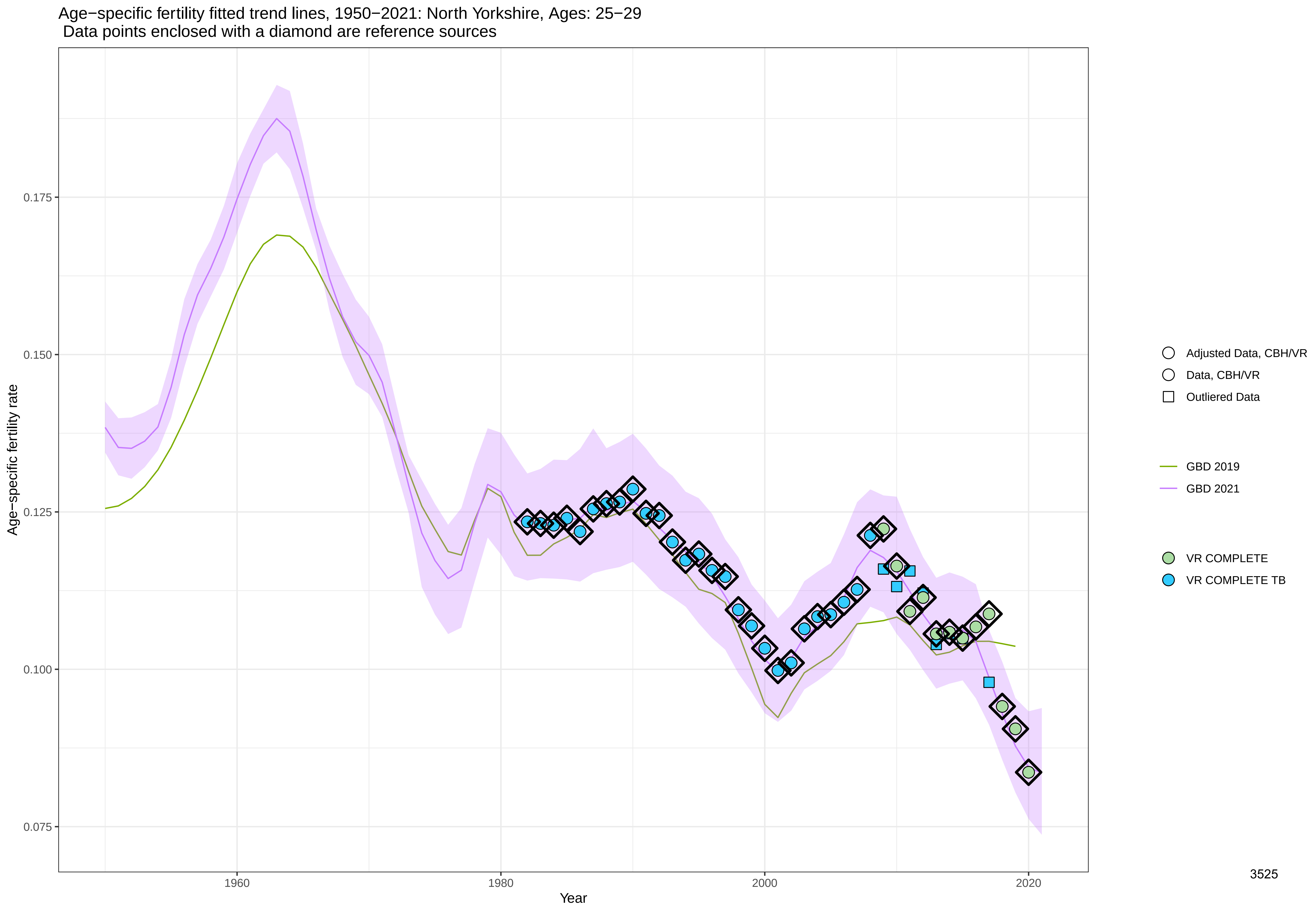

Age−specific fertility fitted trend lines, 1950−2021: North Yorkshire, Ages: 25−29
Data points enclosed with a diamond are reference sources
Age−specific fertility rate
0.175
0.150
0.125
0.100
0.075
Year
1960
1980
2000
2020
Adjusted Data, CBH/VR
Data, CBH/VR
Outliered Data
GBD 2019
GBD 2021
VR COMPLETE
VR COMPLETE TB

Age−specific fertility fitted trend lines, 1950−2021: North Yorkshire, Ages: 30−34
Data points enclosed with a diamond are reference sources

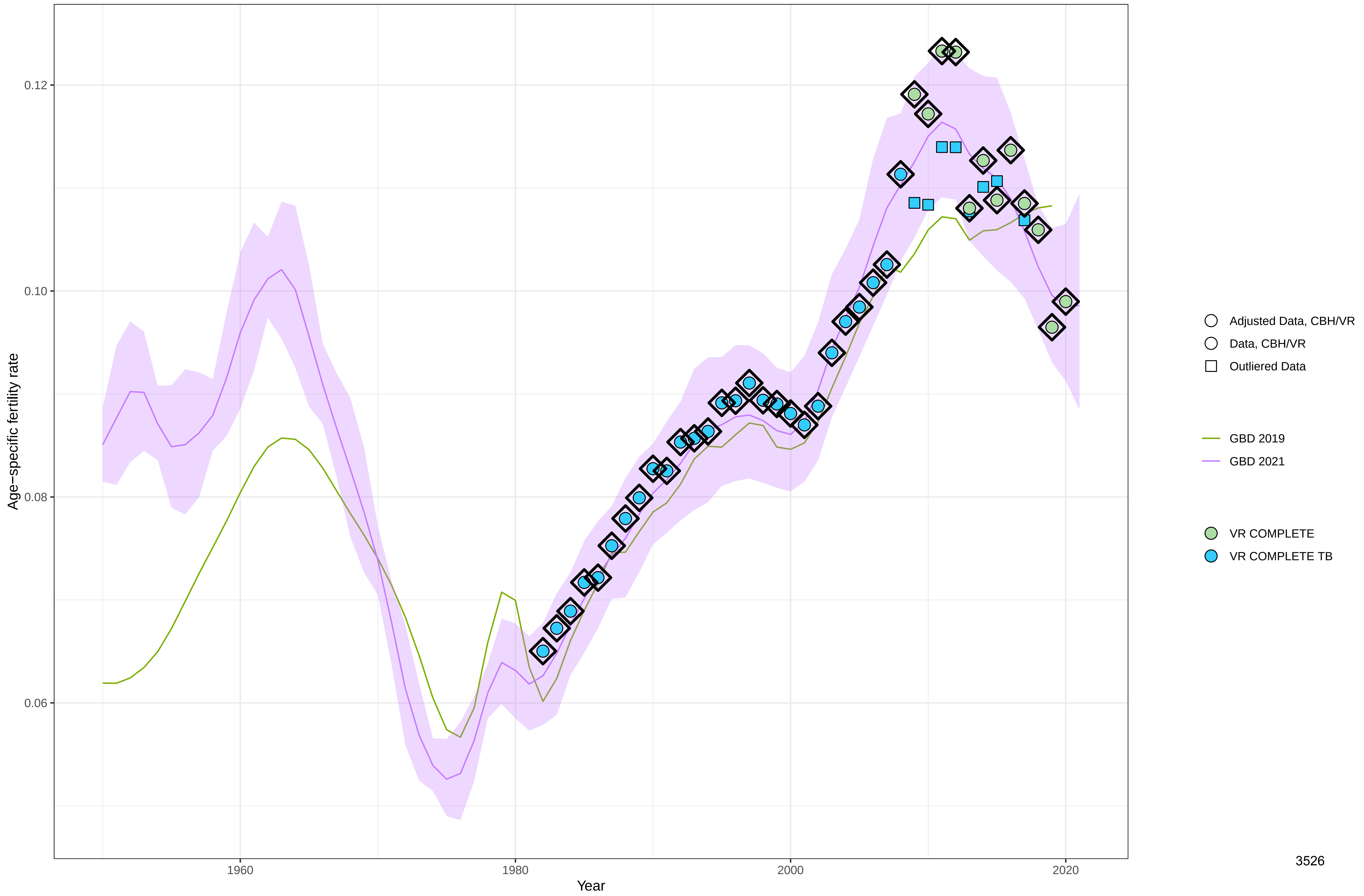

Age−specific fertility fitted trend lines, 1950−2021: North Yorkshire, Ages: 35−39
Data points enclosed with a diamond are reference sources

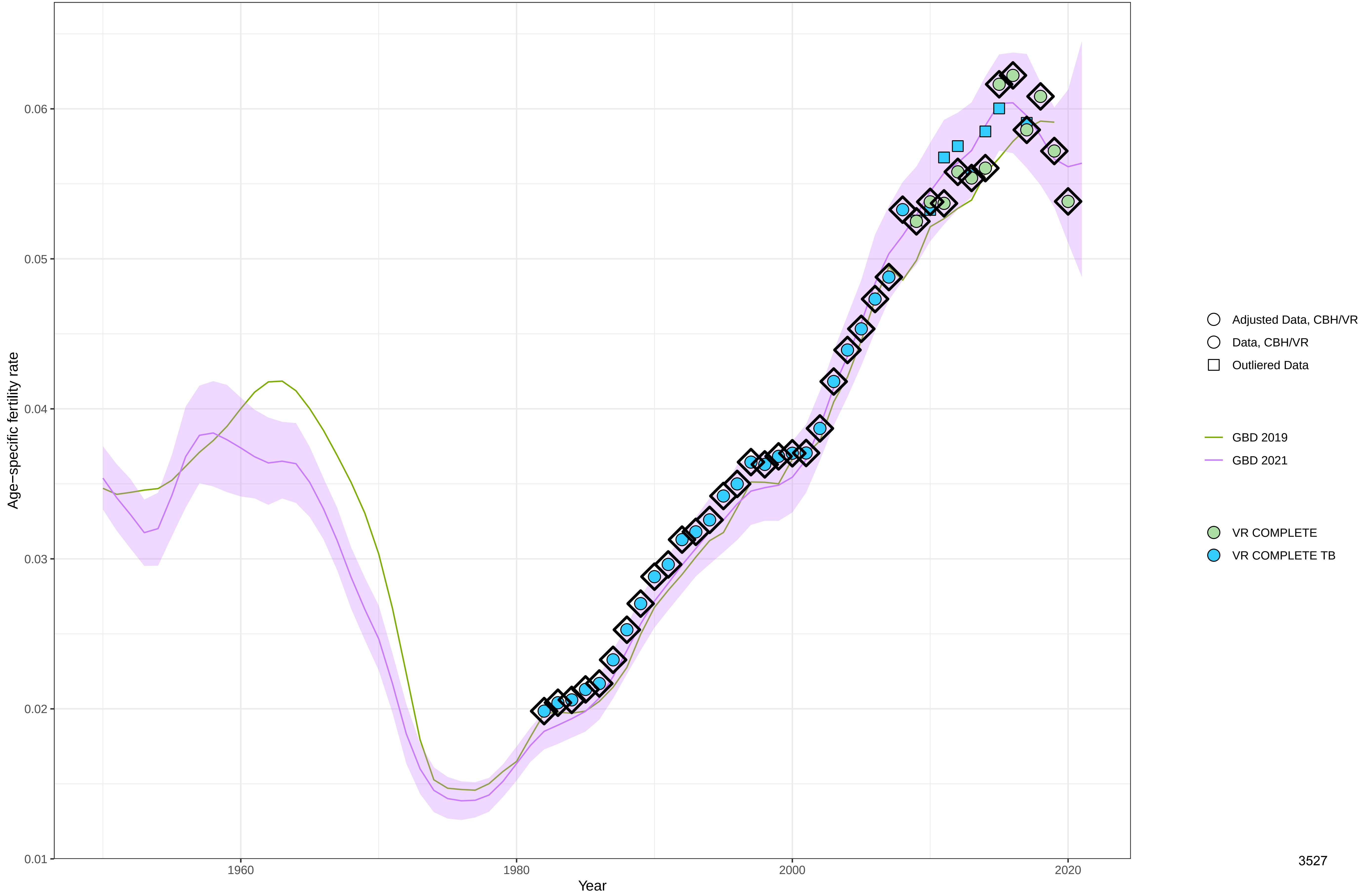

Age−specific fertility fitted trend lines, 1950−2021: North Yorkshire, Ages: 40−44
Data points enclosed with a diamond are reference sources

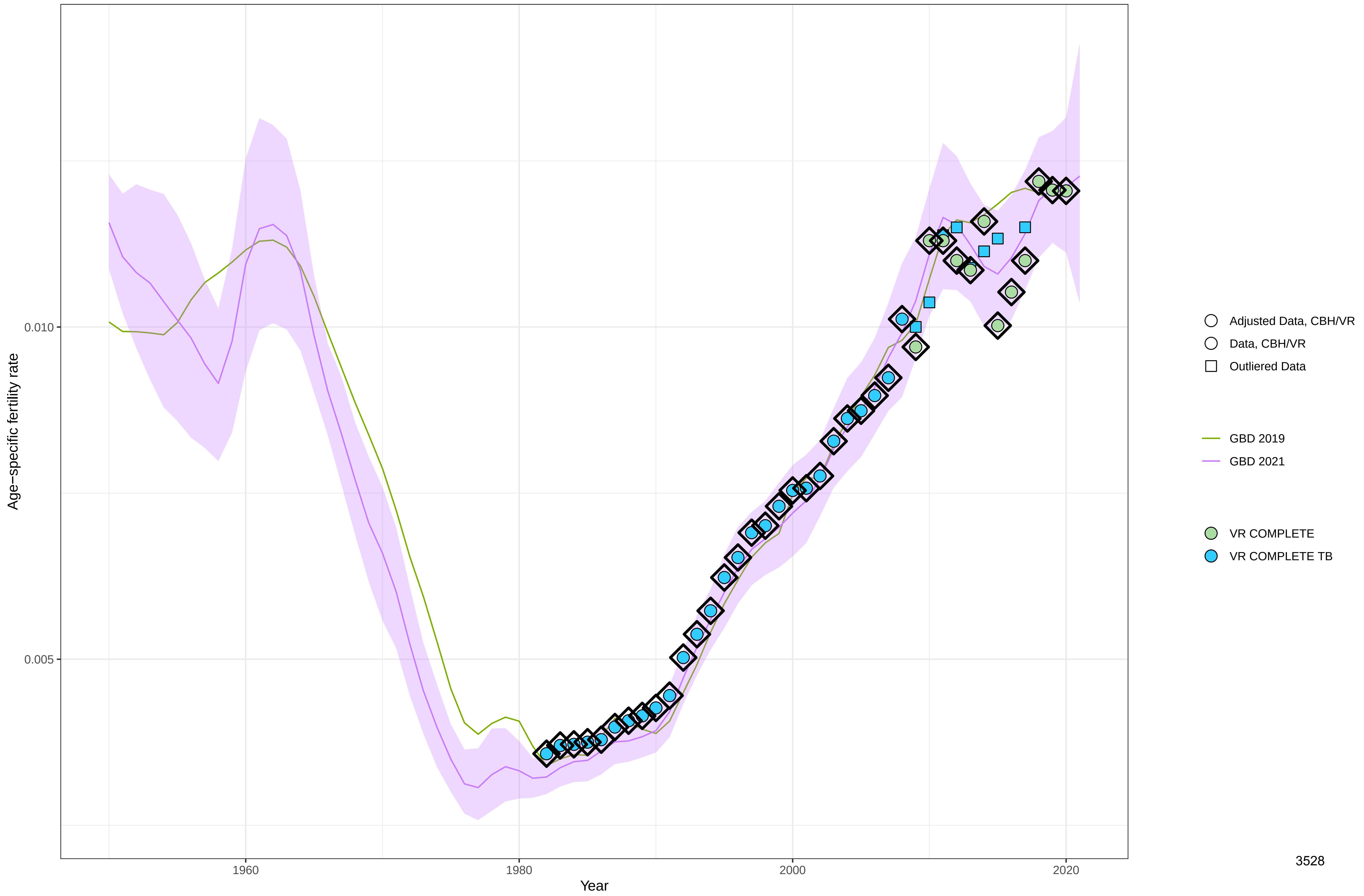

Age−specific fertility fitted trend lines, 1950−2021: North Yorkshire, Ages: 45−49
Data points enclosed with a diamond are reference sources

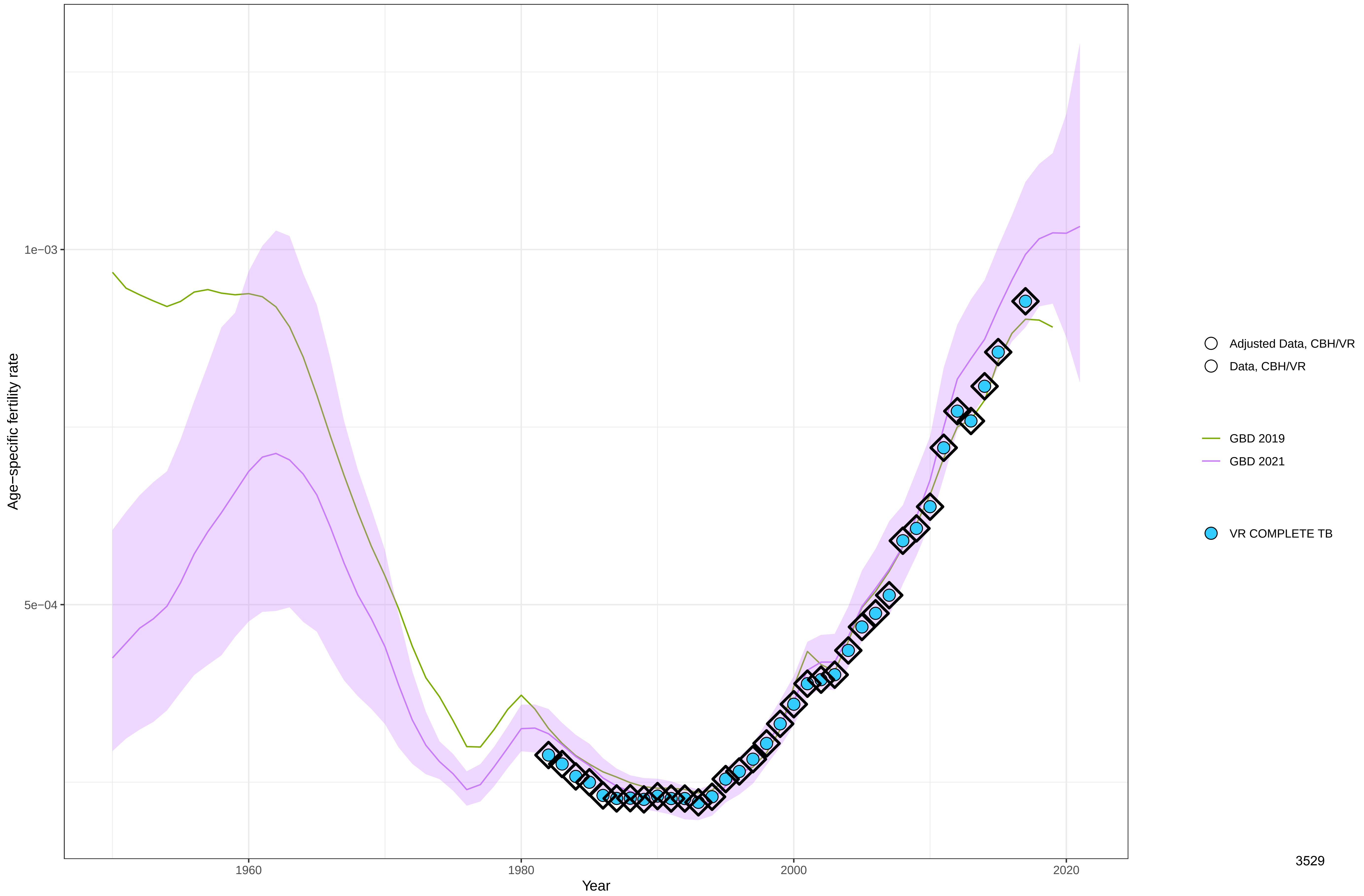

Age−specific fertility fitted trend lines, 1950−2021: North Yorkshire, Ages: 50−54
Data points enclosed with a diamond are reference sources

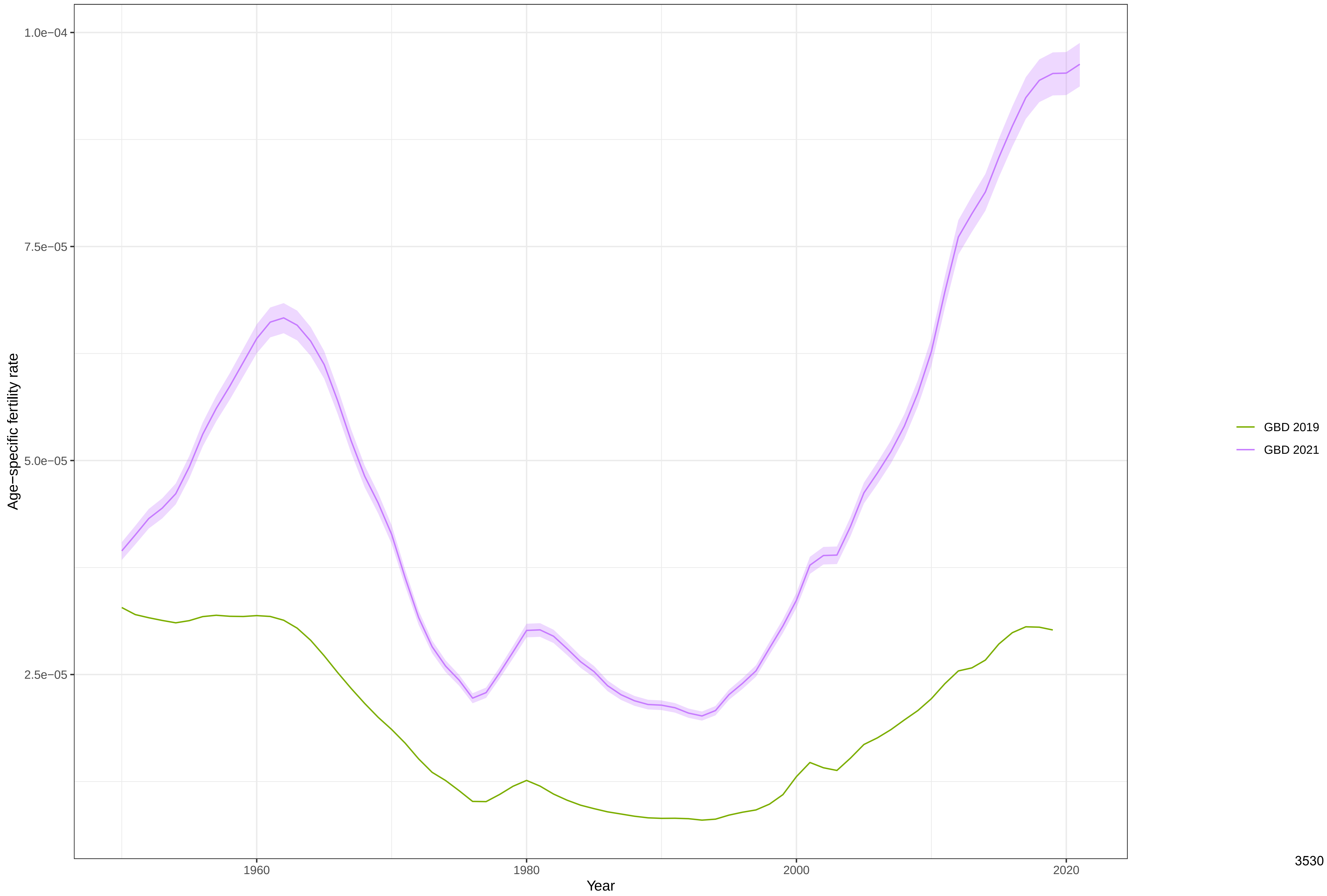

Total fertility rate fitted trend lines, 1950−2021: North Yorkshire TFR
Data points enclosed with a diamond are reference sources

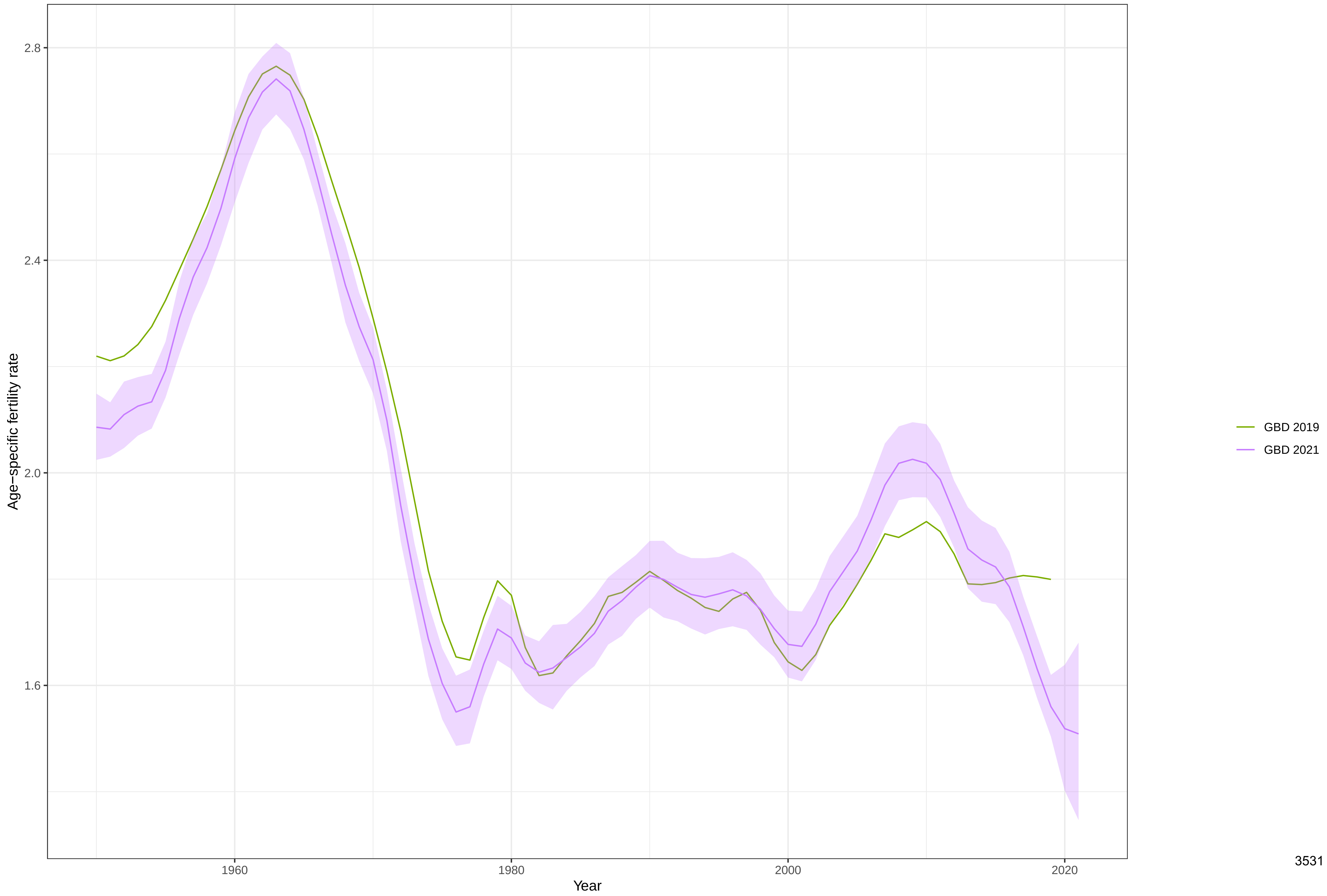

Age−specific fertility fitted trend lines, 1950−2021: Rotherham, Ages: 10−14
Data points enclosed with a diamond are reference sources

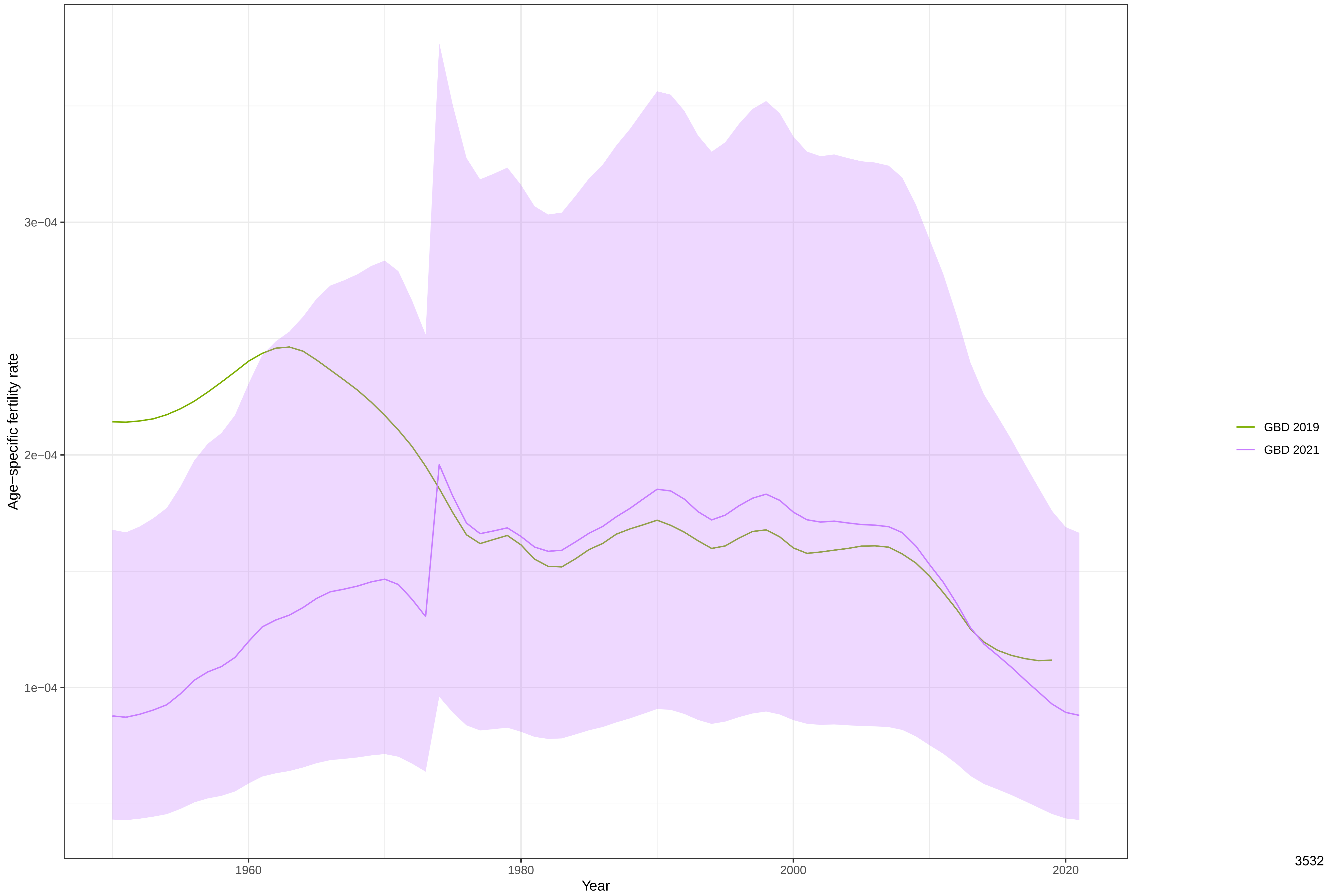

3532

Age−specific fertility fitted trend lines, 1950−2021: Rotherham, Ages: 15−19
Data points enclosed with a diamond are reference sources

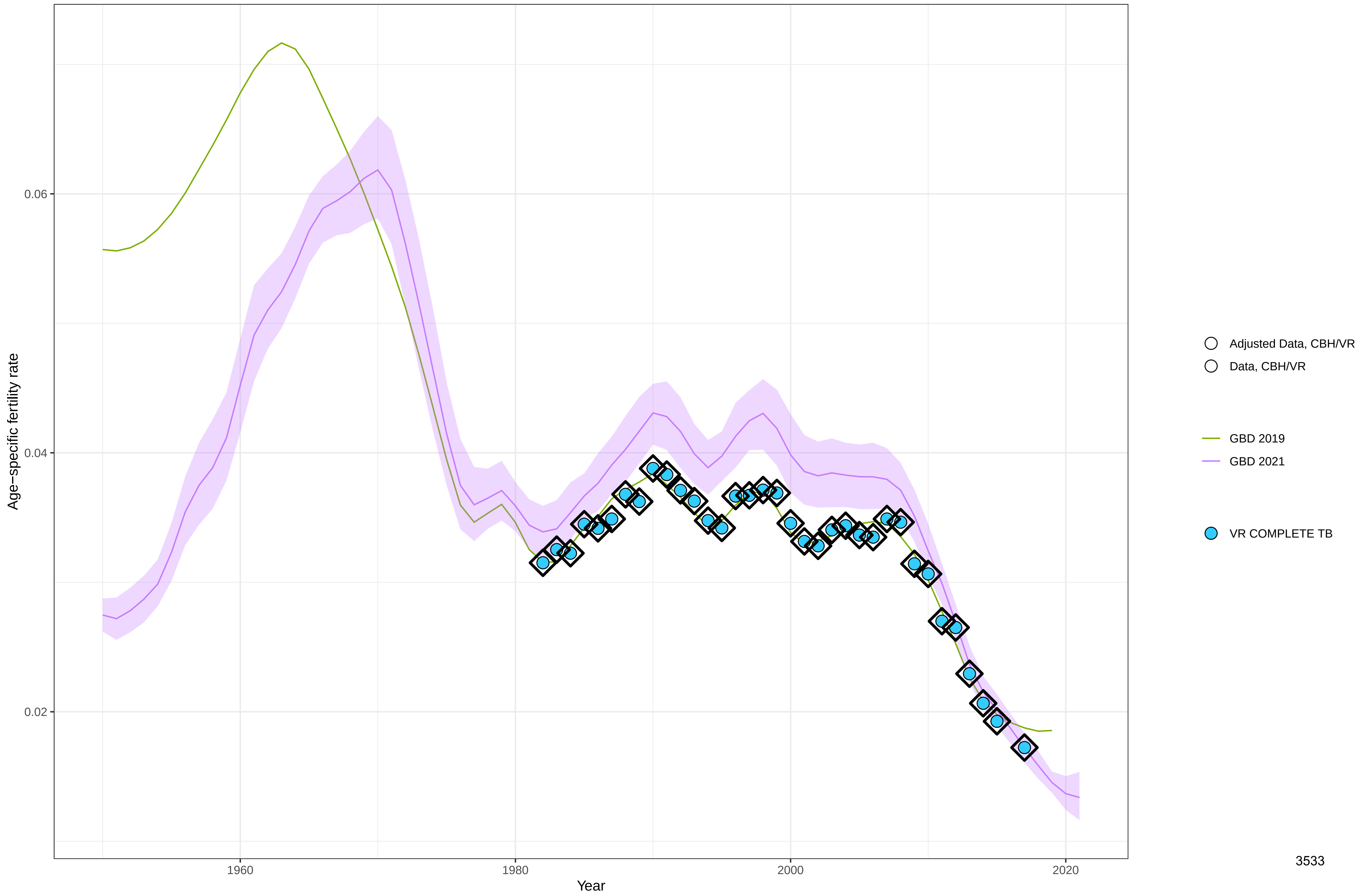

Age−specific fertility fitted trend lines, 1950−2021: Rotherham, Ages: 20−24
Data points enclosed with a diamond are reference sources

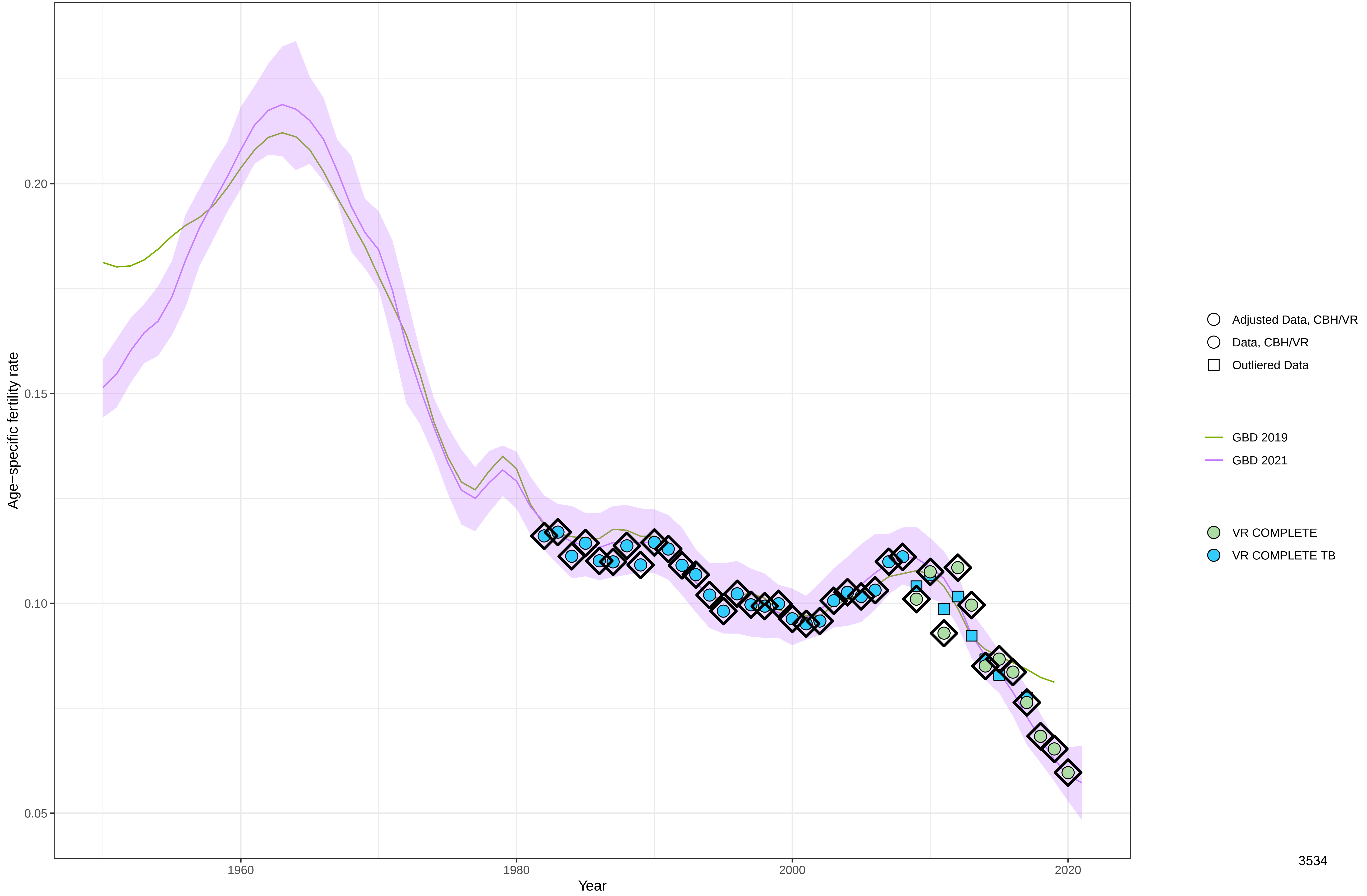

Age−specific fertility fitted trend lines, 1950−2021: Rotherham, Ages: 25−29
Data points enclosed with a diamond are reference sources

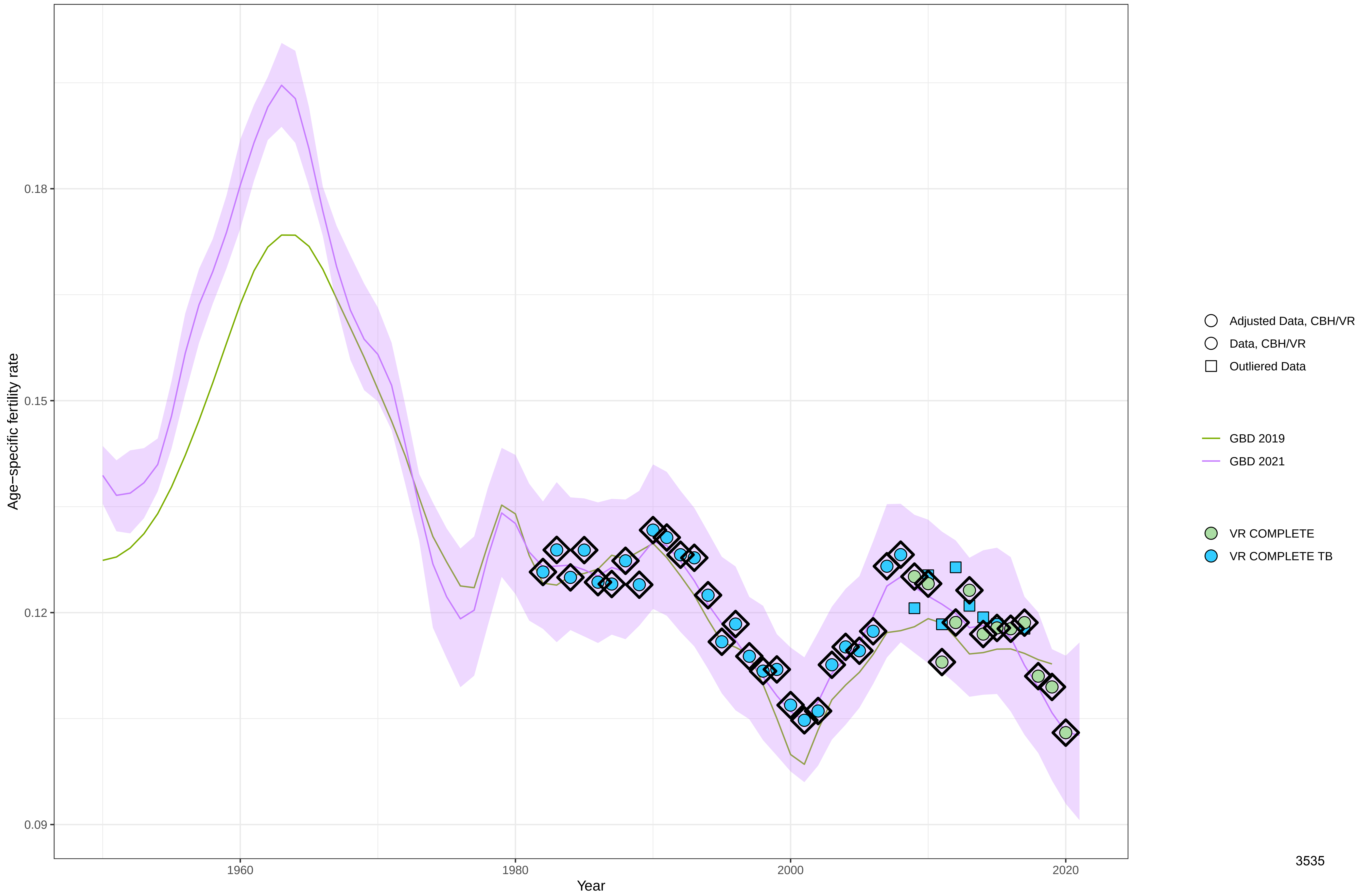

Age−specific fertility fitted trend lines, 1950−2021: Rotherham, Ages: 30−34
Data points enclosed with a diamond are reference sources

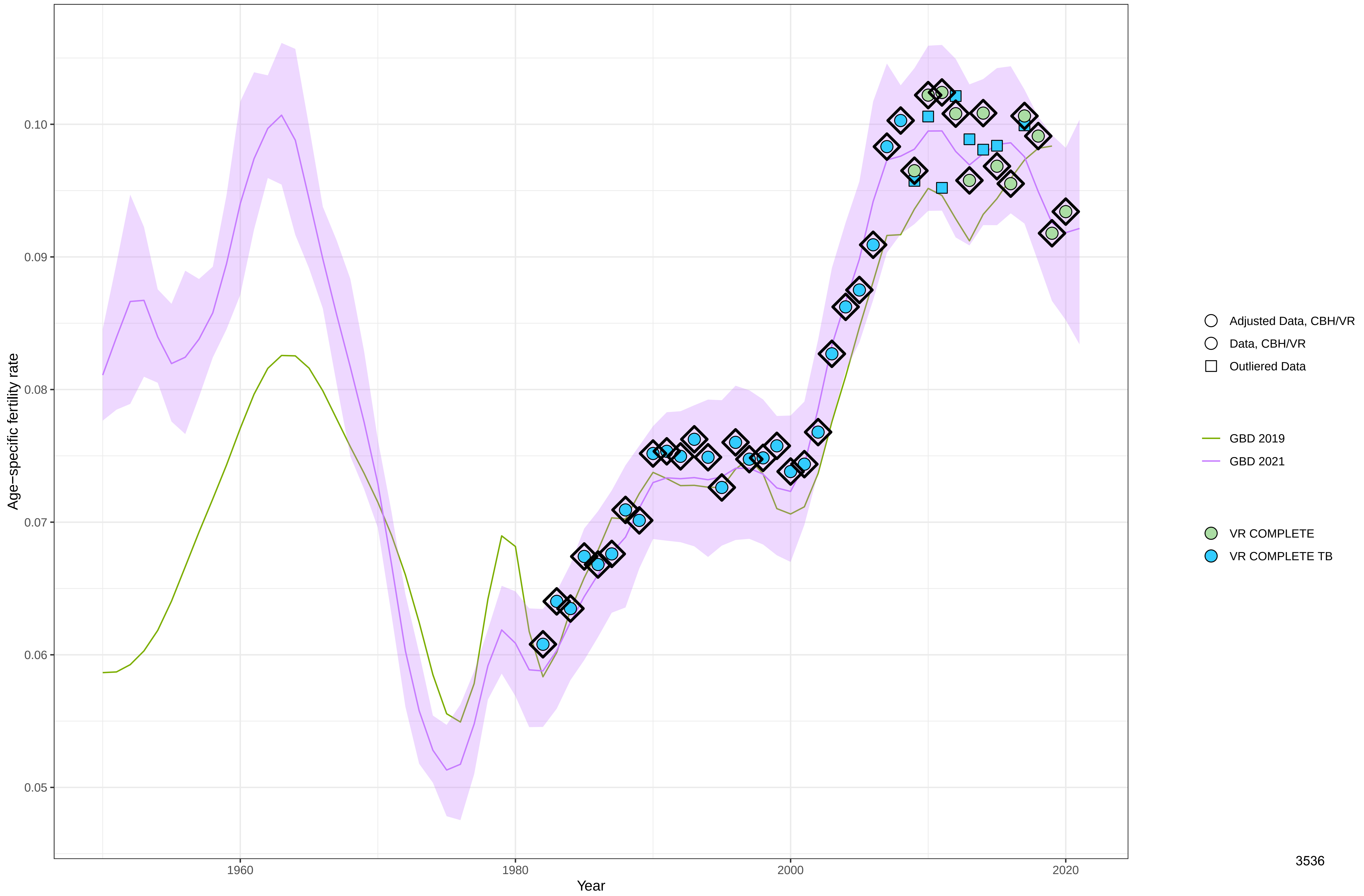

Age−specific fertility fitted trend lines, 1950−2021: Rotherham, Ages: 35−39
Data points enclosed with a diamond are reference sources

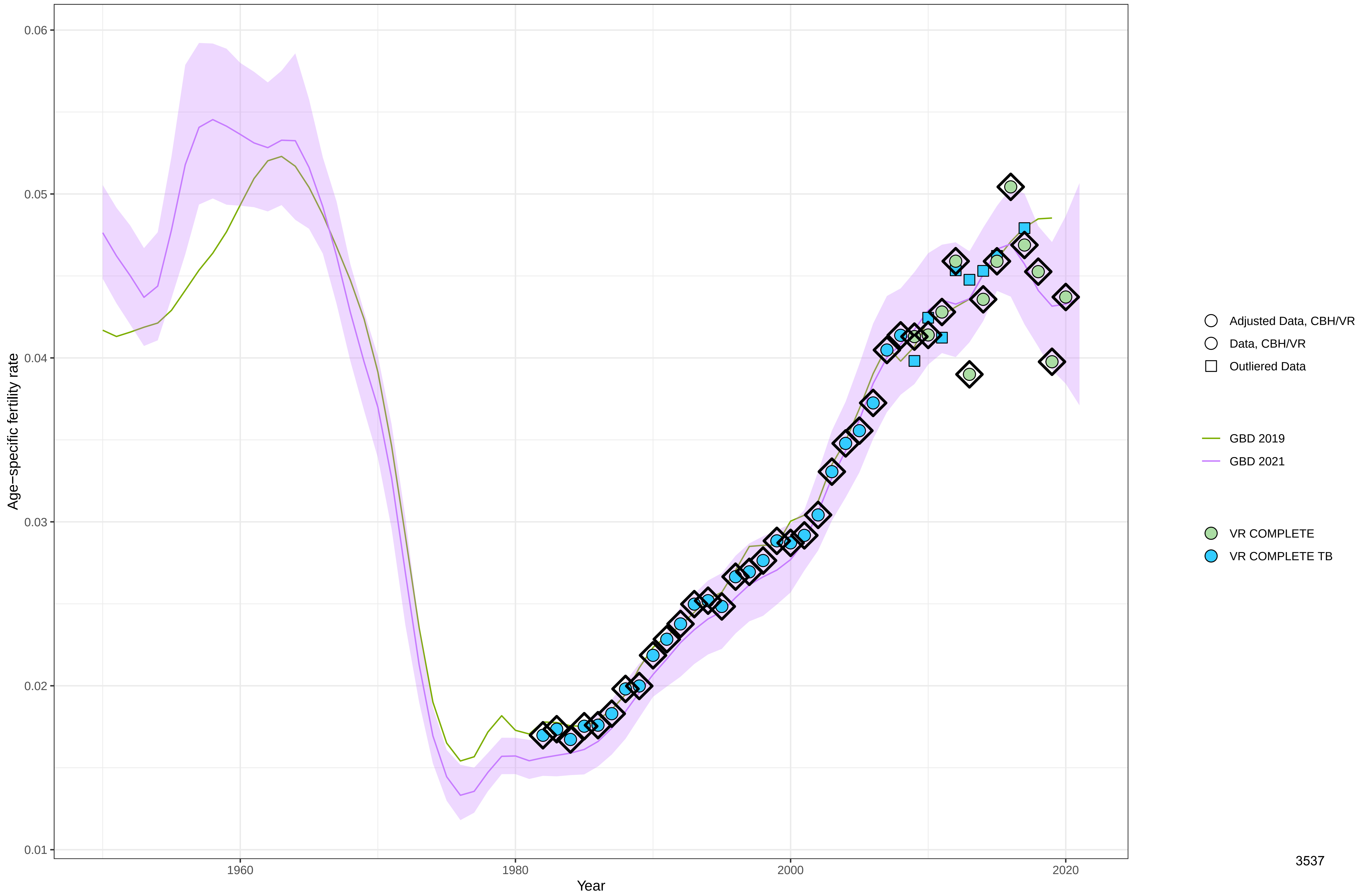

Age−specific fertility fitted trend lines, 1950−2021: Rotherham, Ages: 40−44
Data points enclosed with a diamond are reference sources

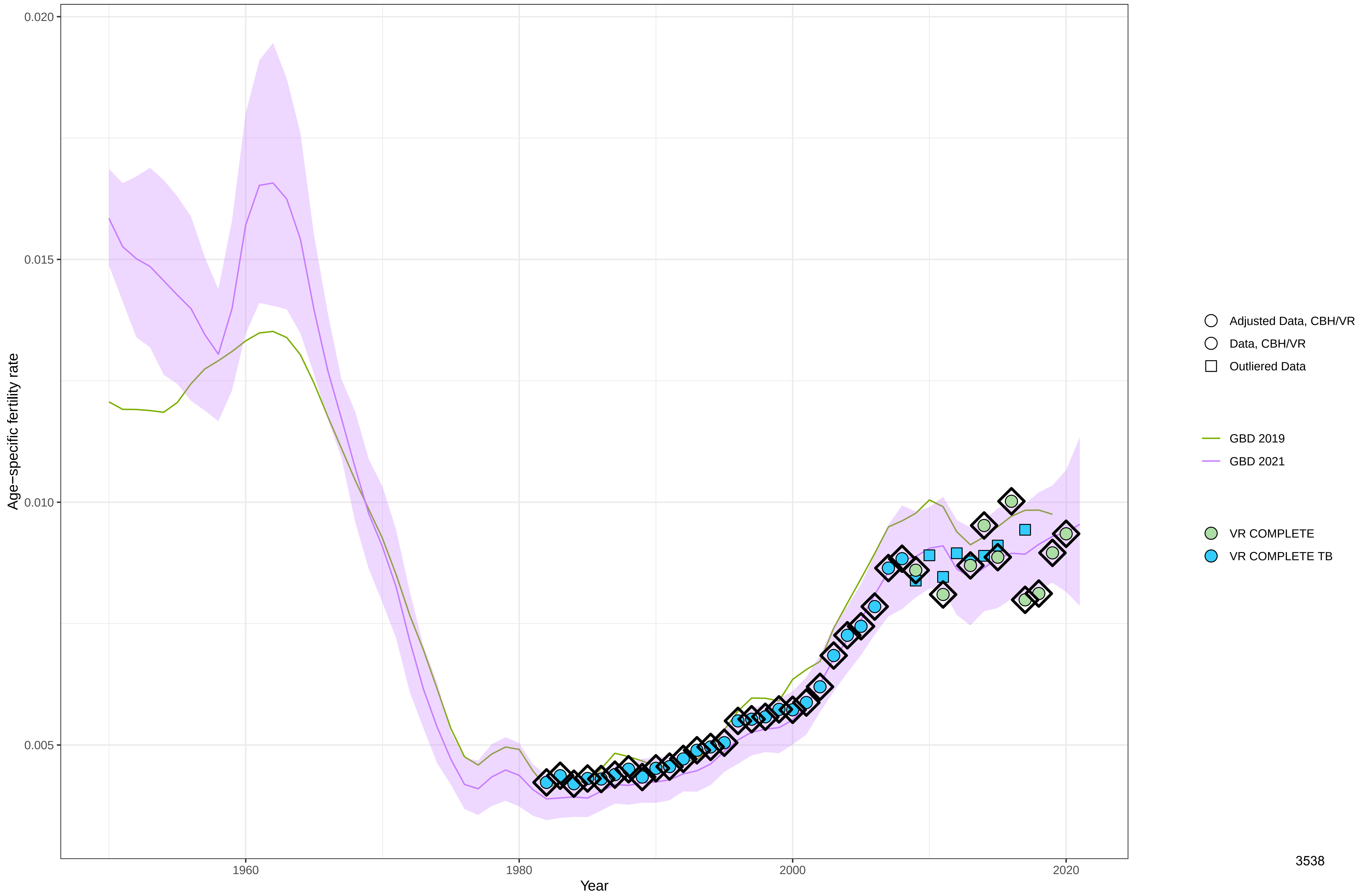

Age−specific fertility fitted trend lines, 1950−2021: Rotherham, Ages: 45−49
Data points enclosed with a diamond are reference sources

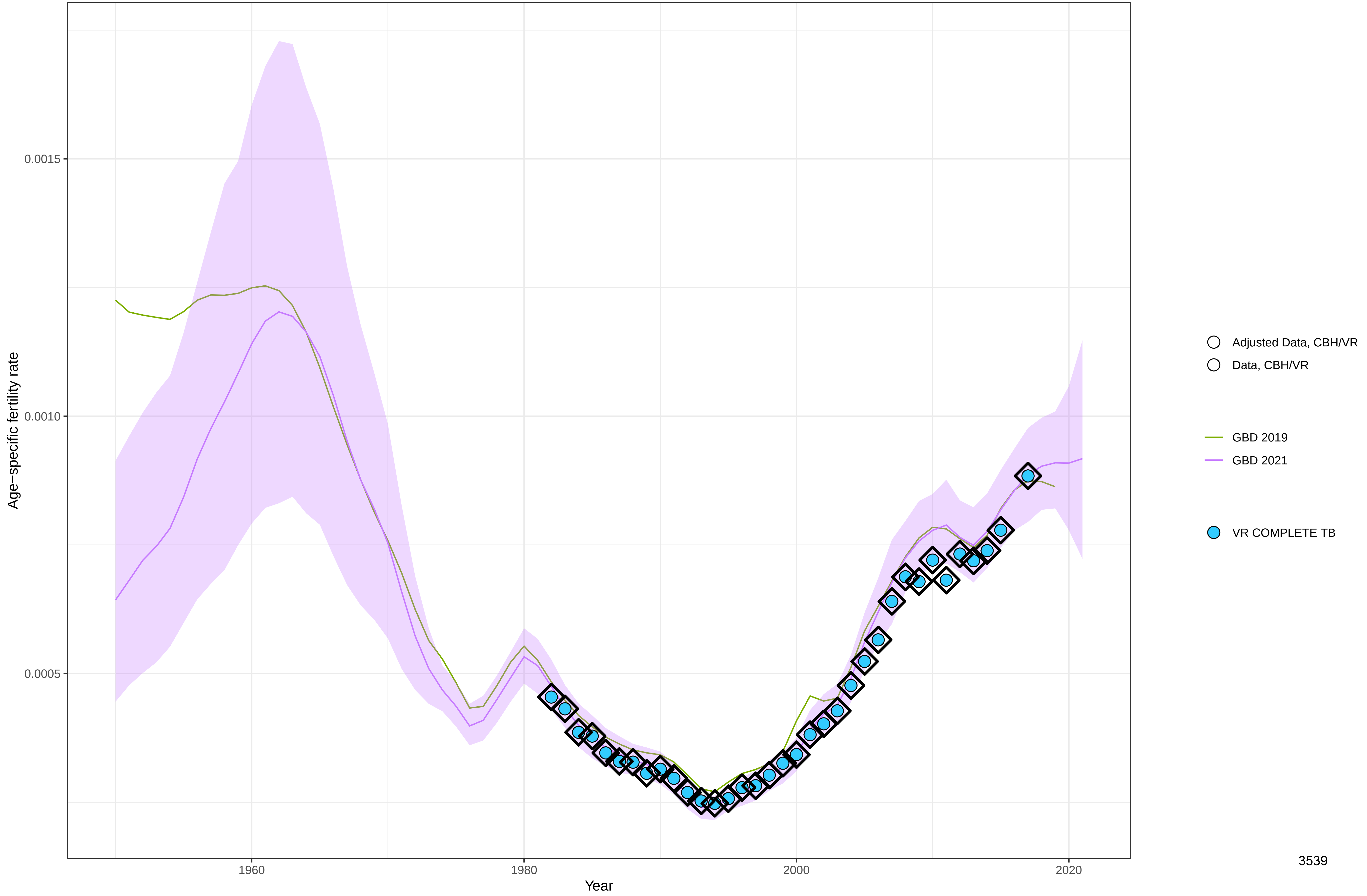

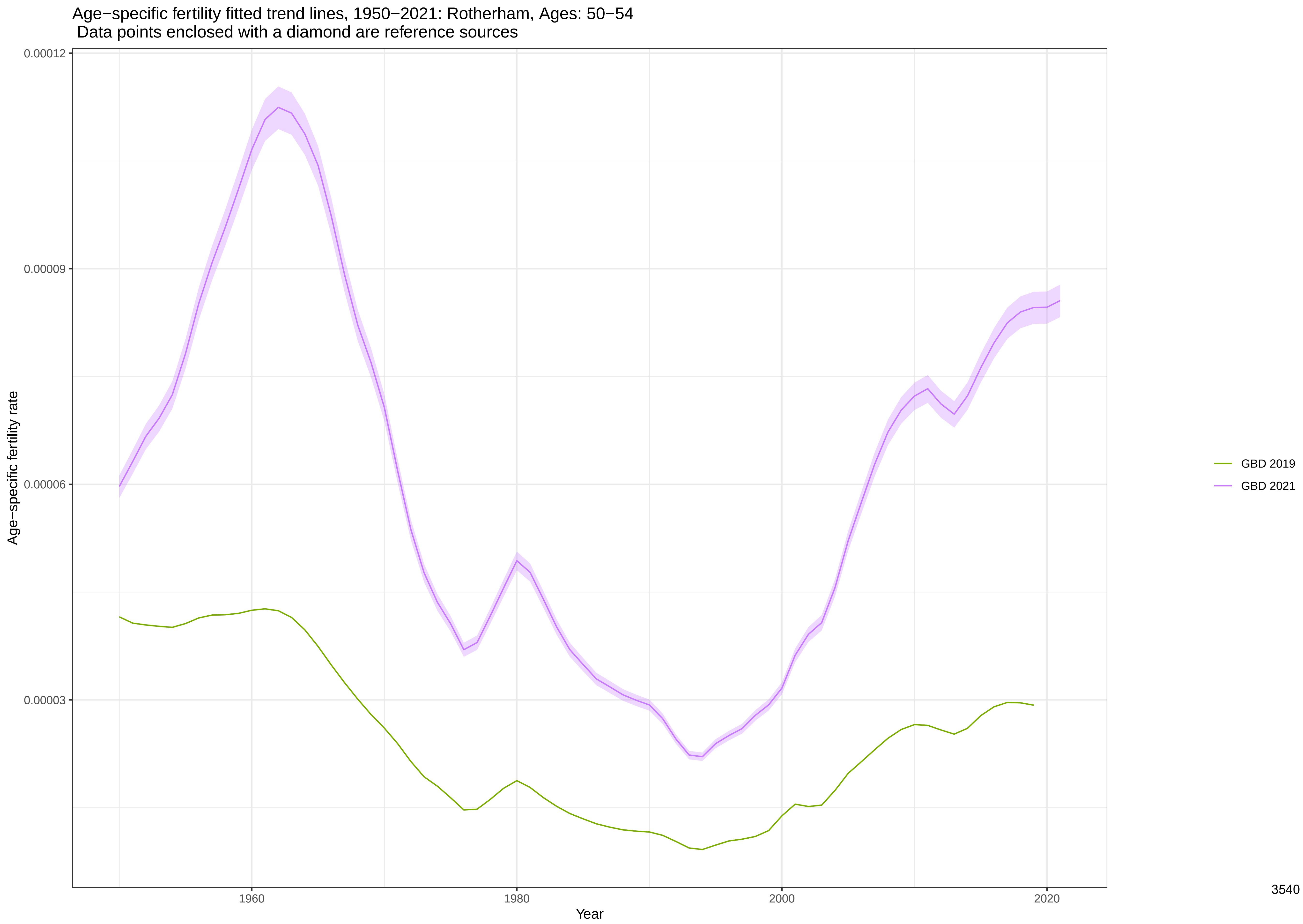

Age−specific fertility fitted trend lines, 1950−2021: Rotherham, Ages: 50−54
Data points enclosed with a diamond are reference sources
0.00012
0.00009
0.00006
0.00003
Age−specific fertility rate
1960
1980
2000
2020
Year
GBD 2019
GBD 2021

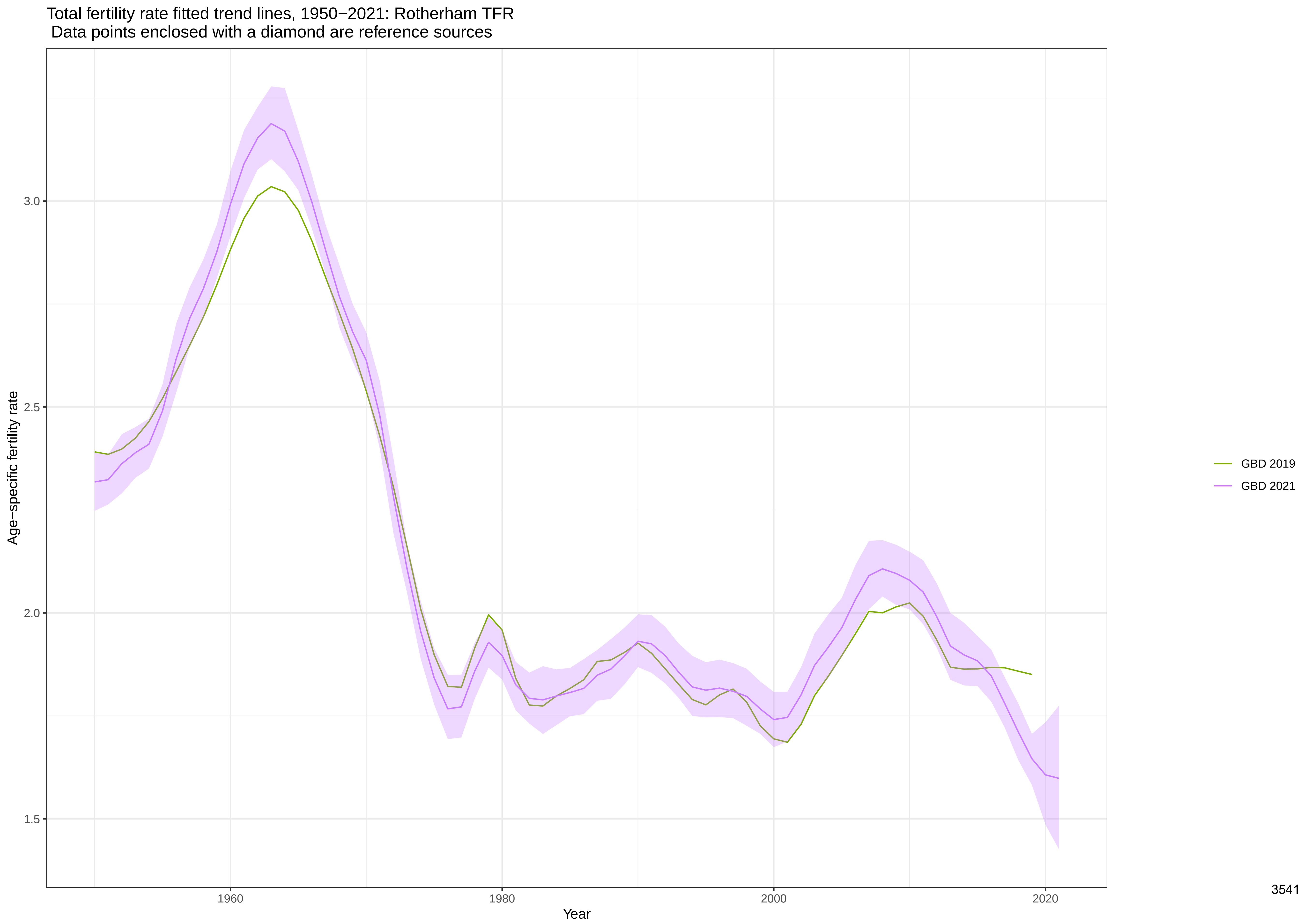
Total fertility rate fitted trend lines, 1950−2021: Rotherham TFR
Data points enclosed with a diamond are reference sources
Age−specific fertility rate
3.0
2.5
2.0
1.5
1960
1980
2000
2020
Year
GBD 2019
GBD 2021

Age−specific fertility fitted trend lines, 1950−2021: Sheffield, Ages: 10−14
Data points enclosed with a diamond are reference sources

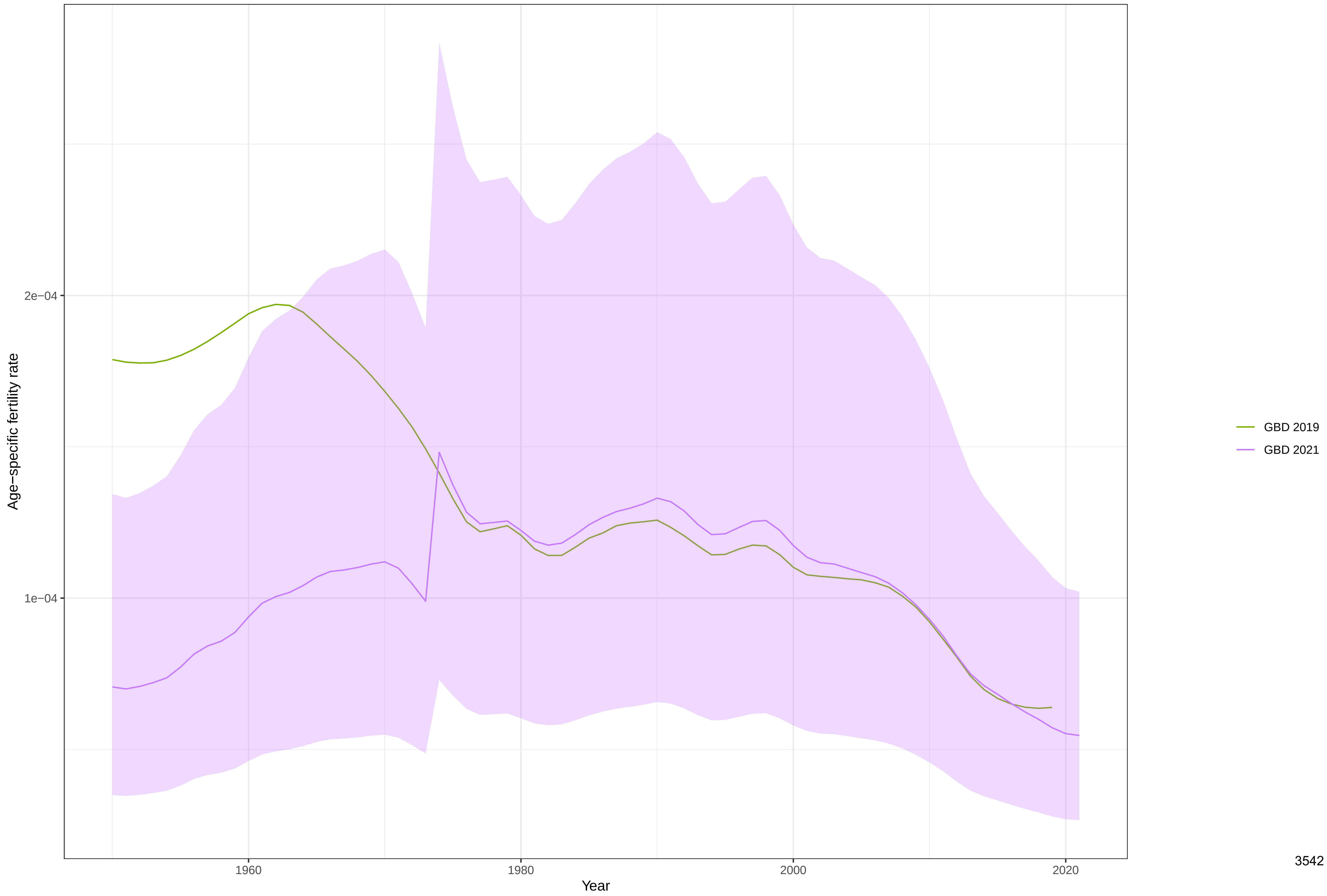

Age−specific fertility fitted trend lines, 1950−2021: Sheffield, Ages: 15−19
Data points enclosed with a diamond are reference sources

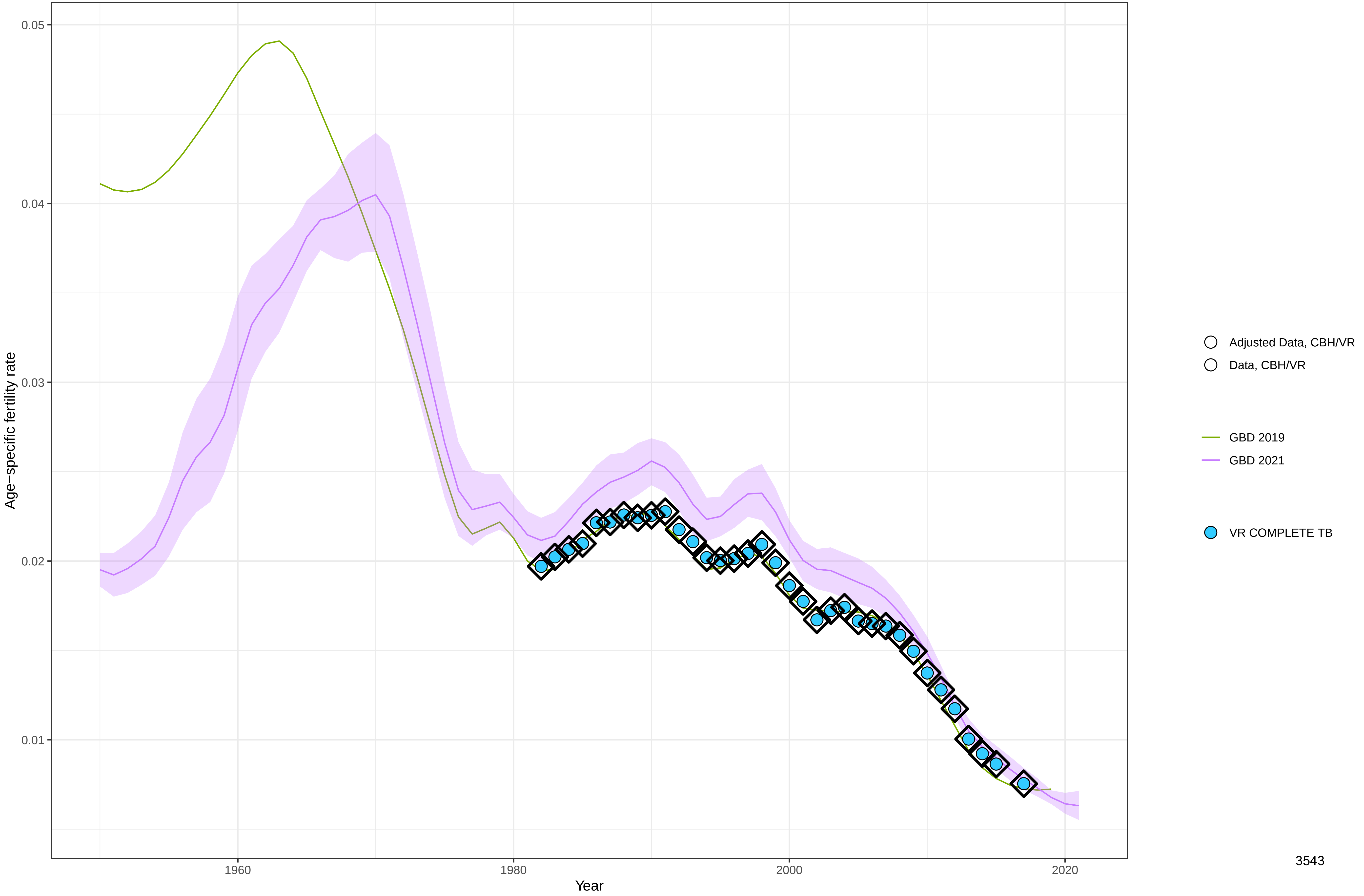

Age−specific fertility fitted trend lines, 1950−2021: Sheffield, Ages: 20−24
Data points enclosed with a diamond are reference sources

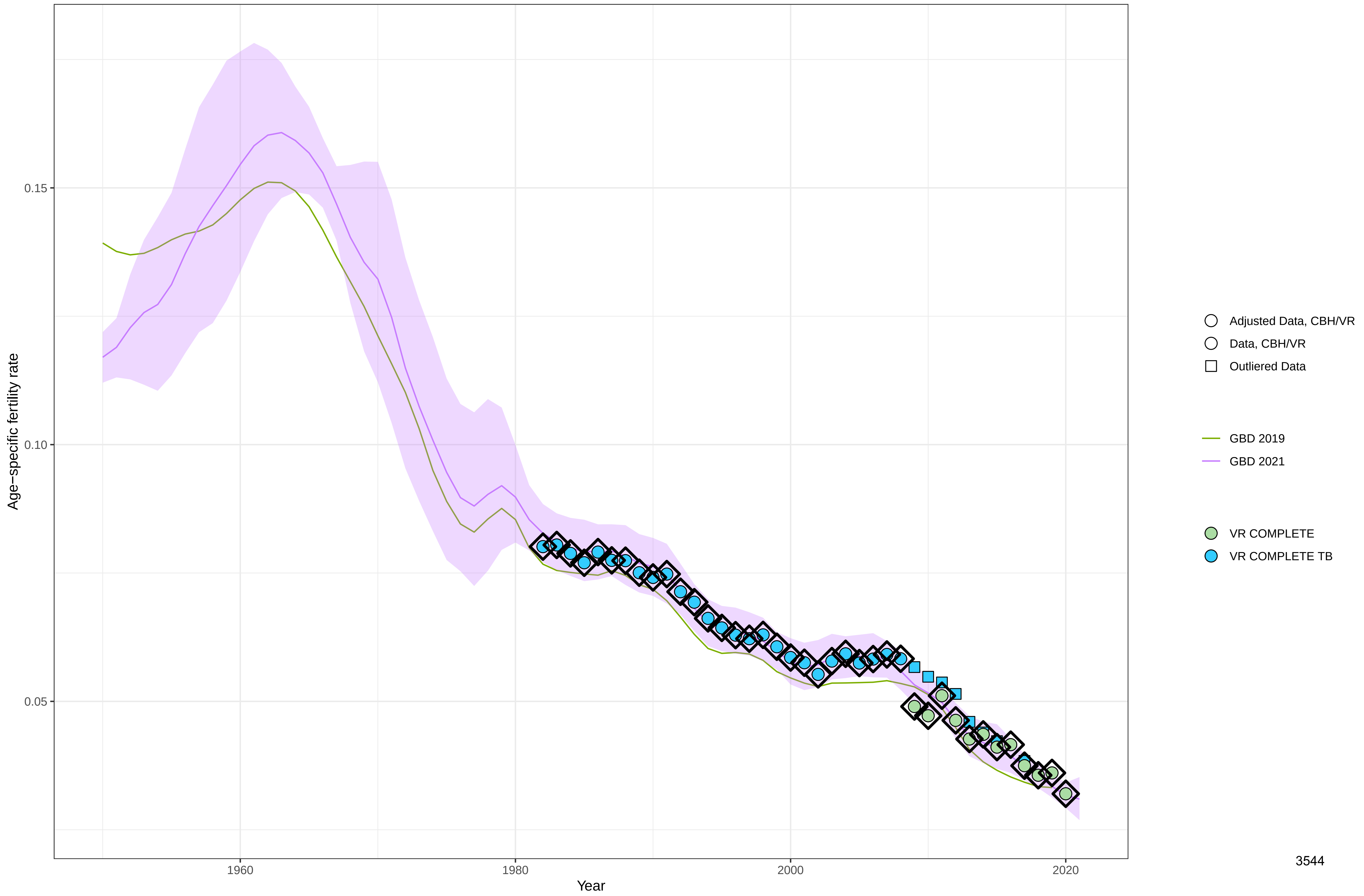

Age−specific fertility fitted trend lines, 1950−2021: Sheffield, Ages: 25−29
Data points enclosed with a diamond are reference sources

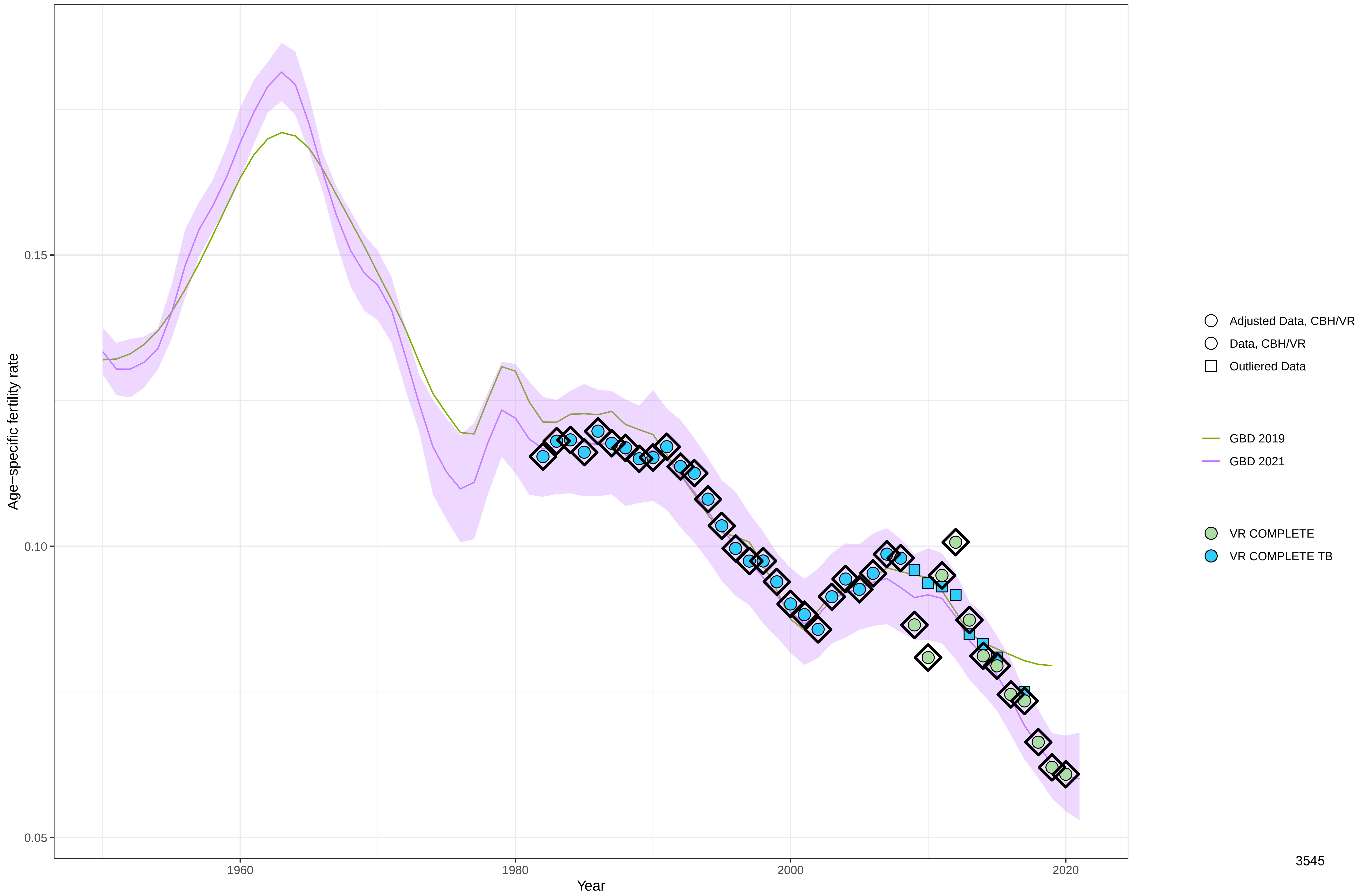

Age−specific fertility fitted trend lines, 1950−2021: Sheffield, Ages: 30−34
Data points enclosed with a diamond are reference sources

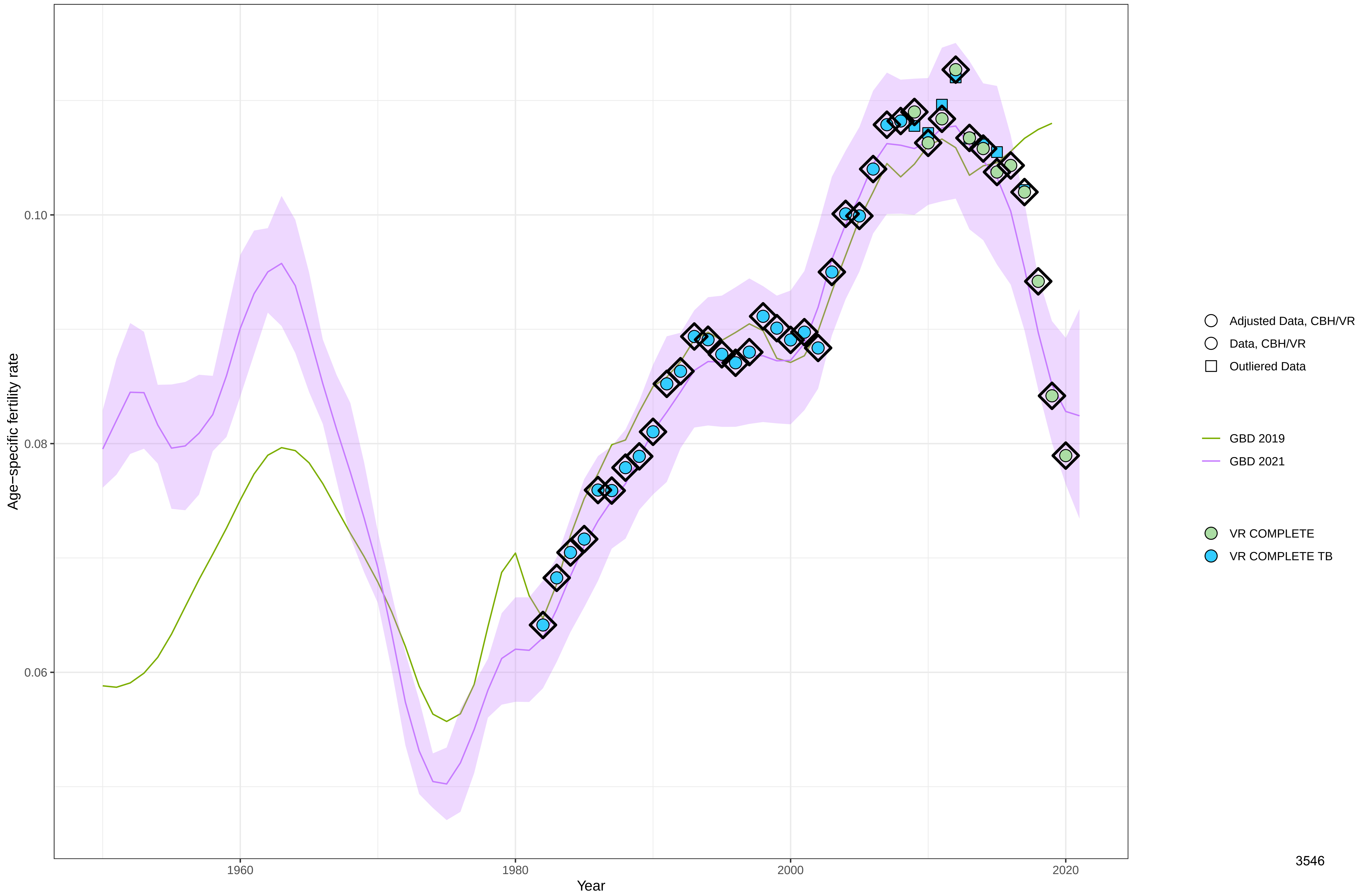

Age−specific fertility fitted trend lines, 1950−2021: Sheffield, Ages: 35−39
Data points enclosed with a diamond are reference sources

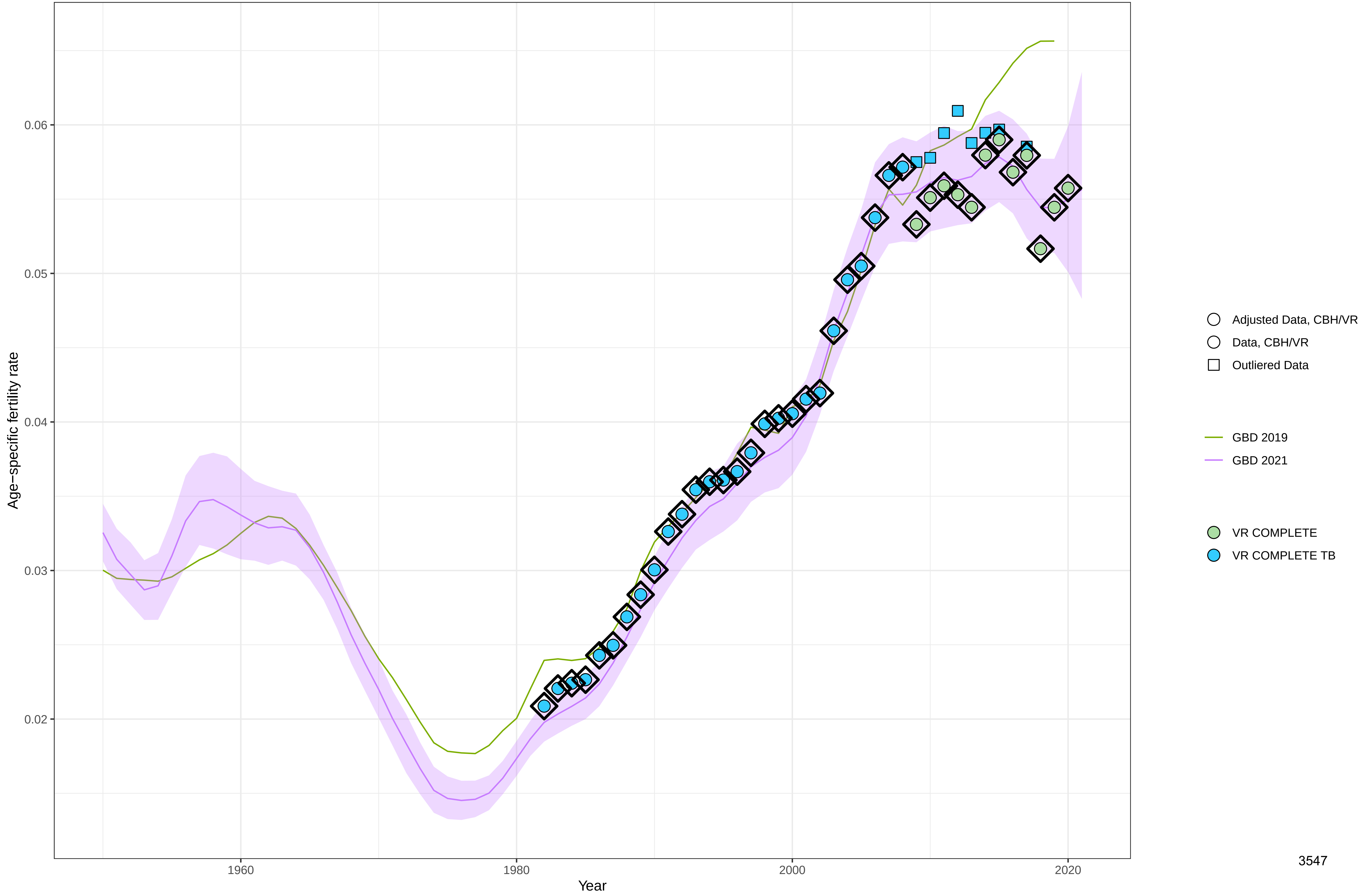

Age−specific fertility fitted trend lines, 1950−2021: Sheffield, Ages: 40−44
Data points enclosed with a diamond are reference sources

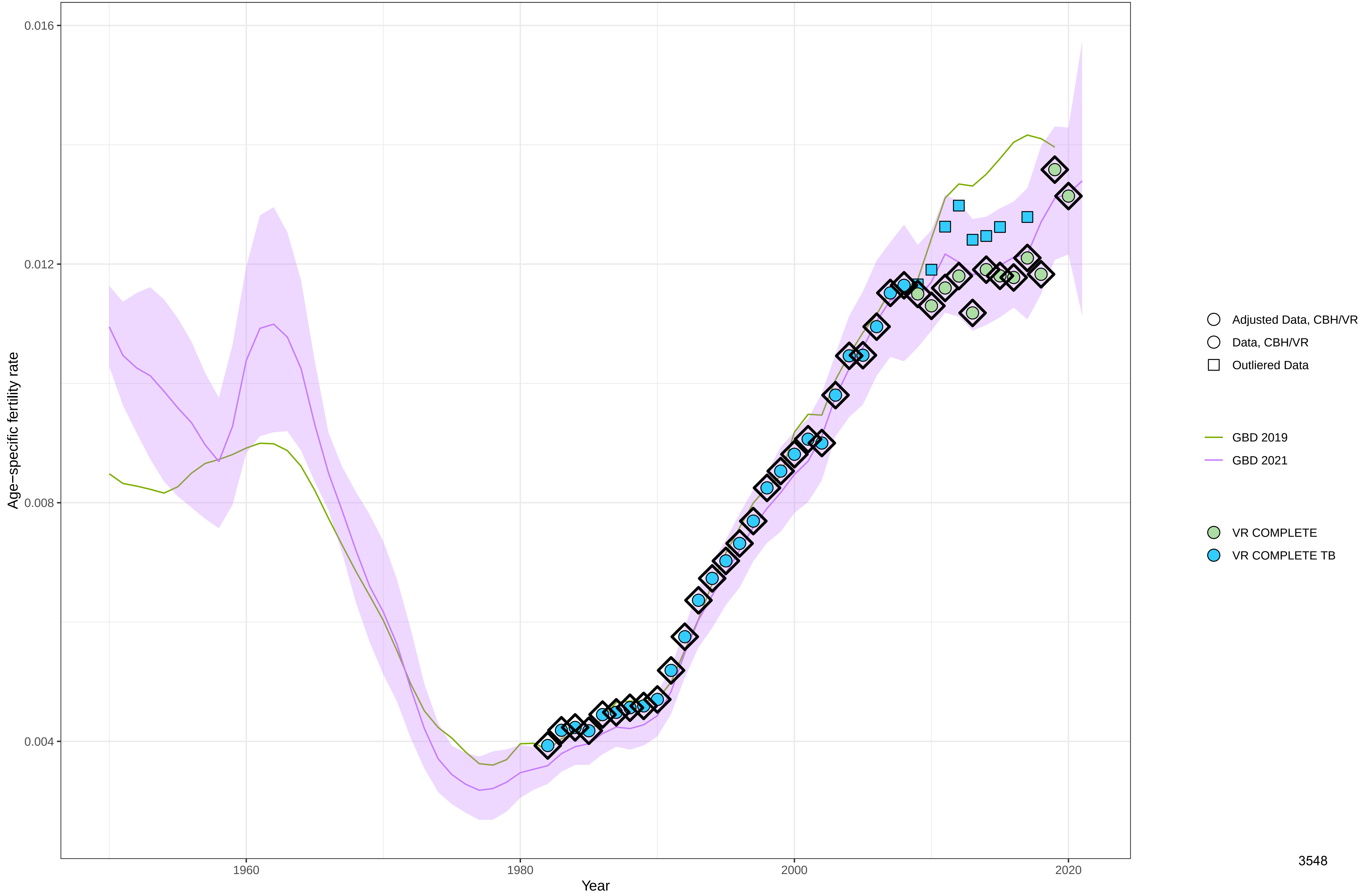

Age−specific fertility fitted trend lines, 1950−2021: Sheffield, Ages: 45−49
Data points enclosed with a diamond are reference sources

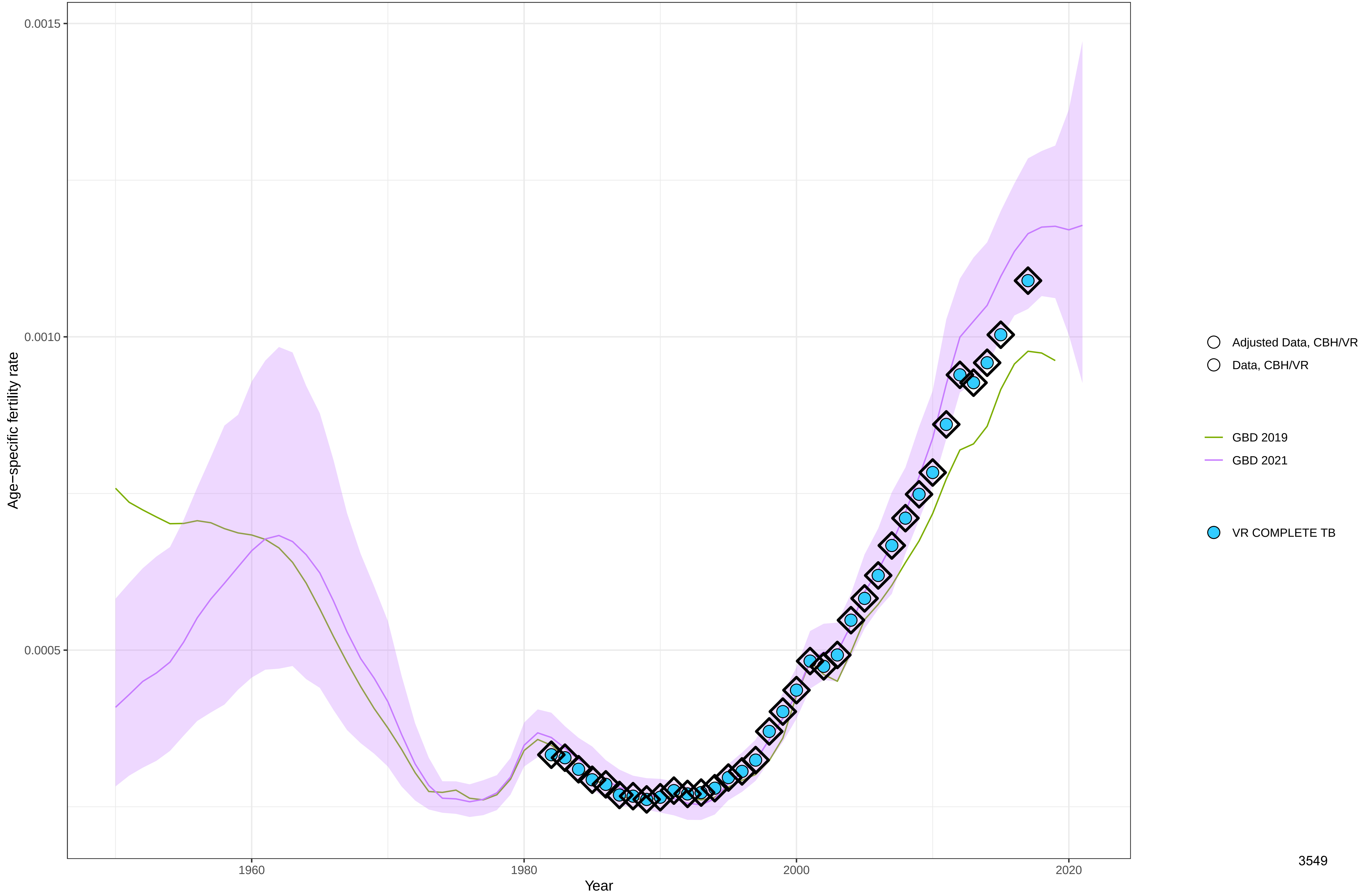

Age−specific fertility fitted trend lines, 1950−2021: Sheffield, Ages: 50−54
Data points enclosed with a diamond are reference sources

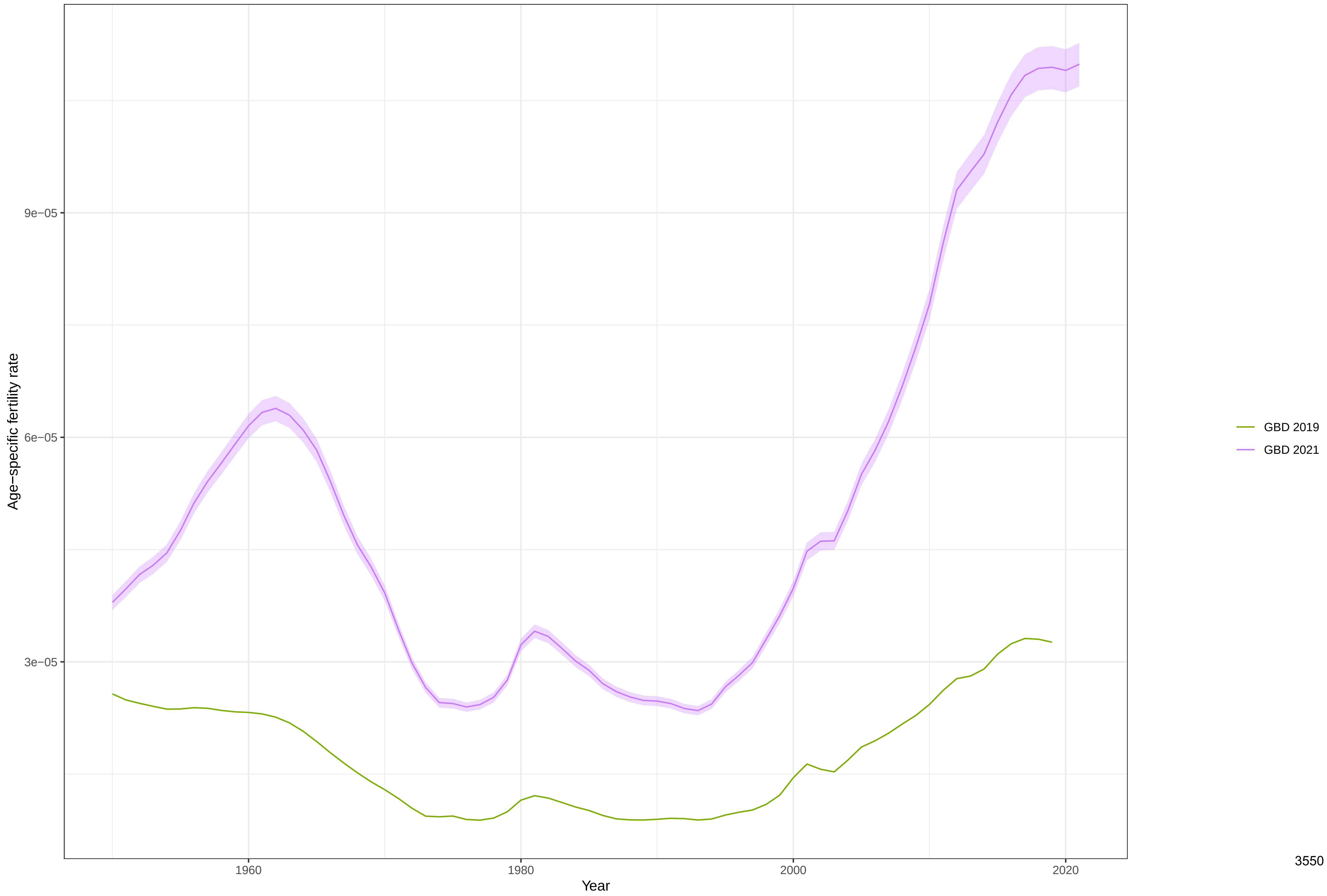

Total fertility rate fitted trend lines, 1950−2021: Sheffield TFR
Data points enclosed with a diamond are reference sources

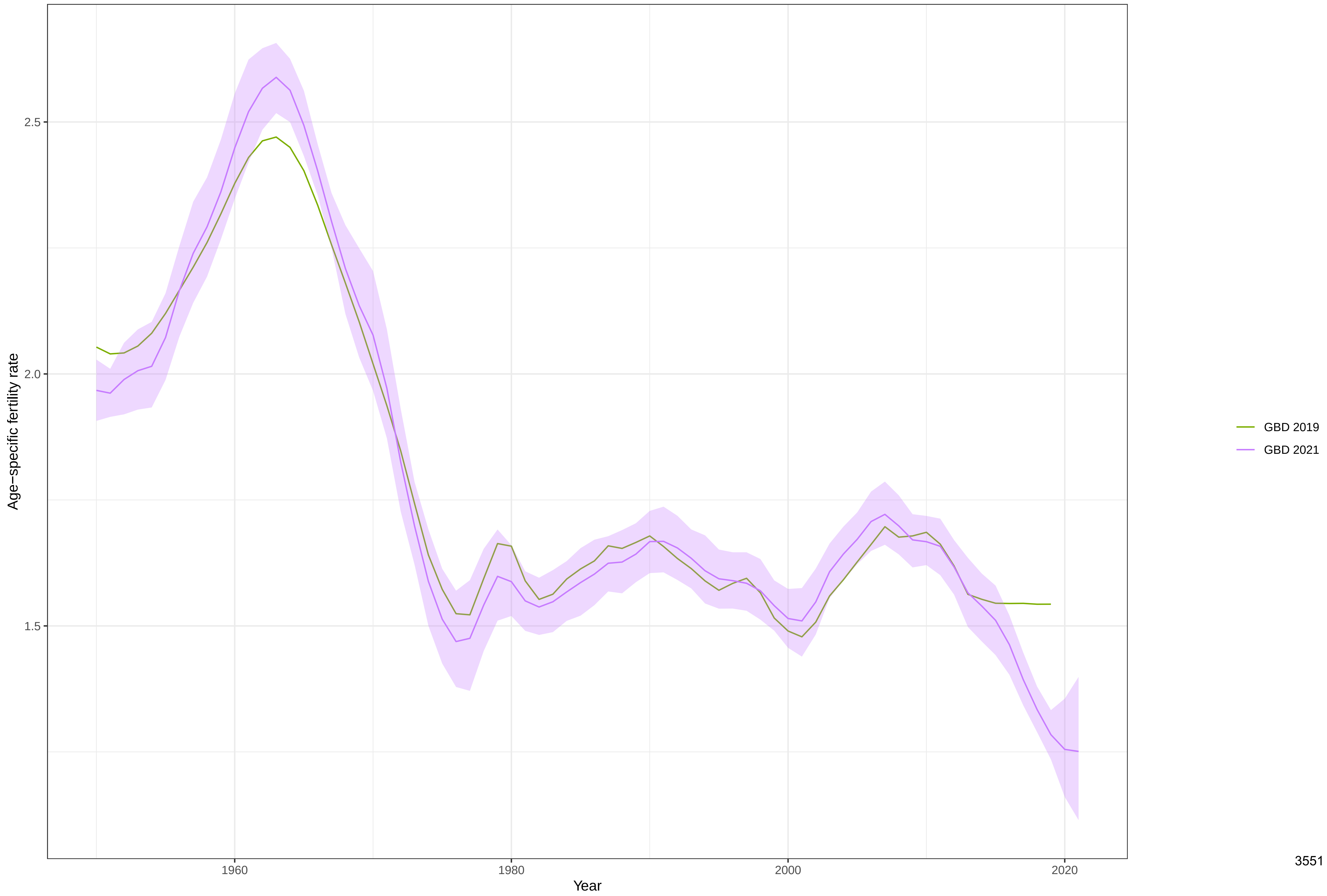

Age−specific fertility fitted trend lines, 1950−2021: Wakefield, Ages: 10−14
Data points enclosed with a diamond are reference sources

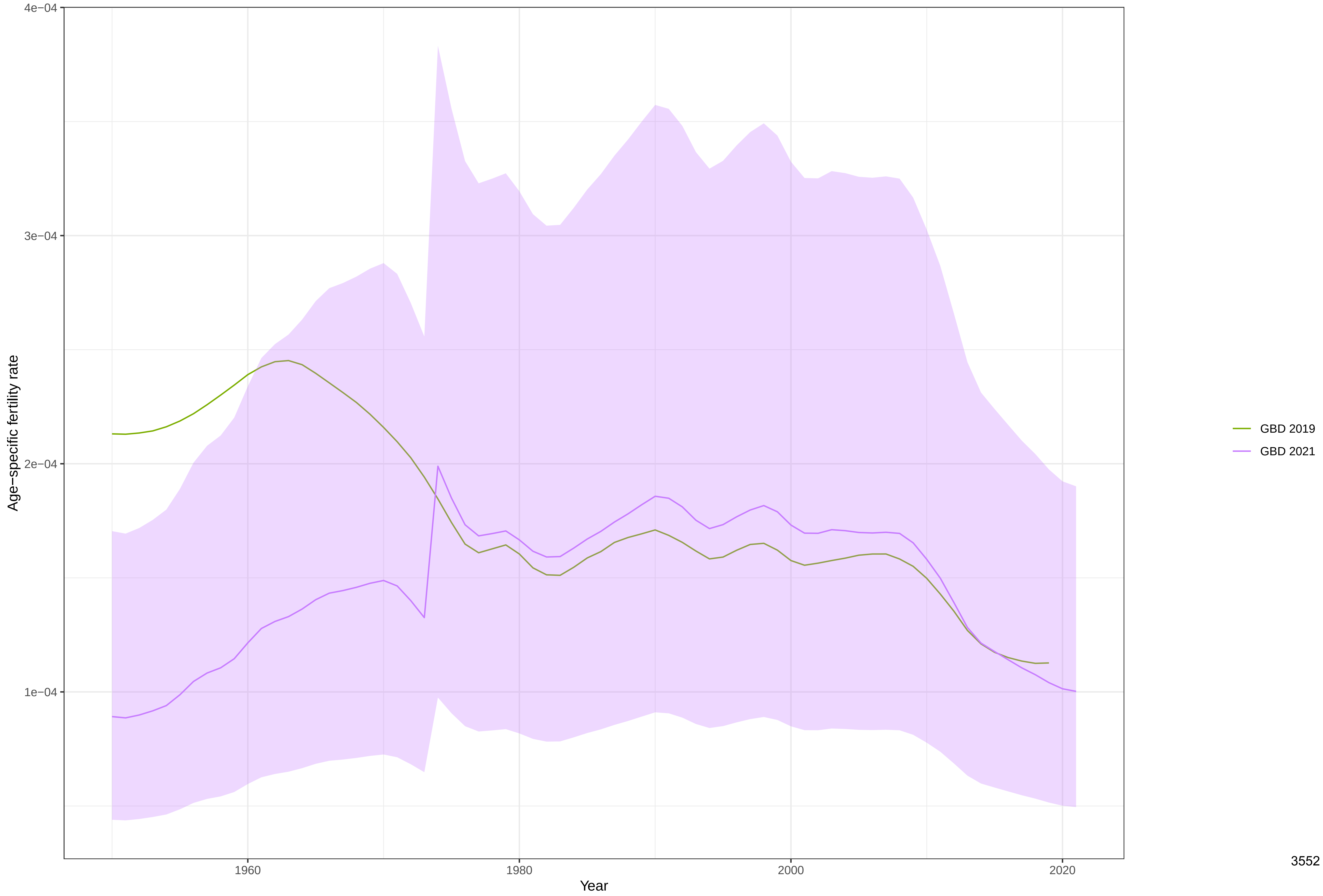

Age−specific fertility fitted trend lines, 1950−2021: Wakefield, Ages: 15−19
Data points enclosed with a diamond are reference sources

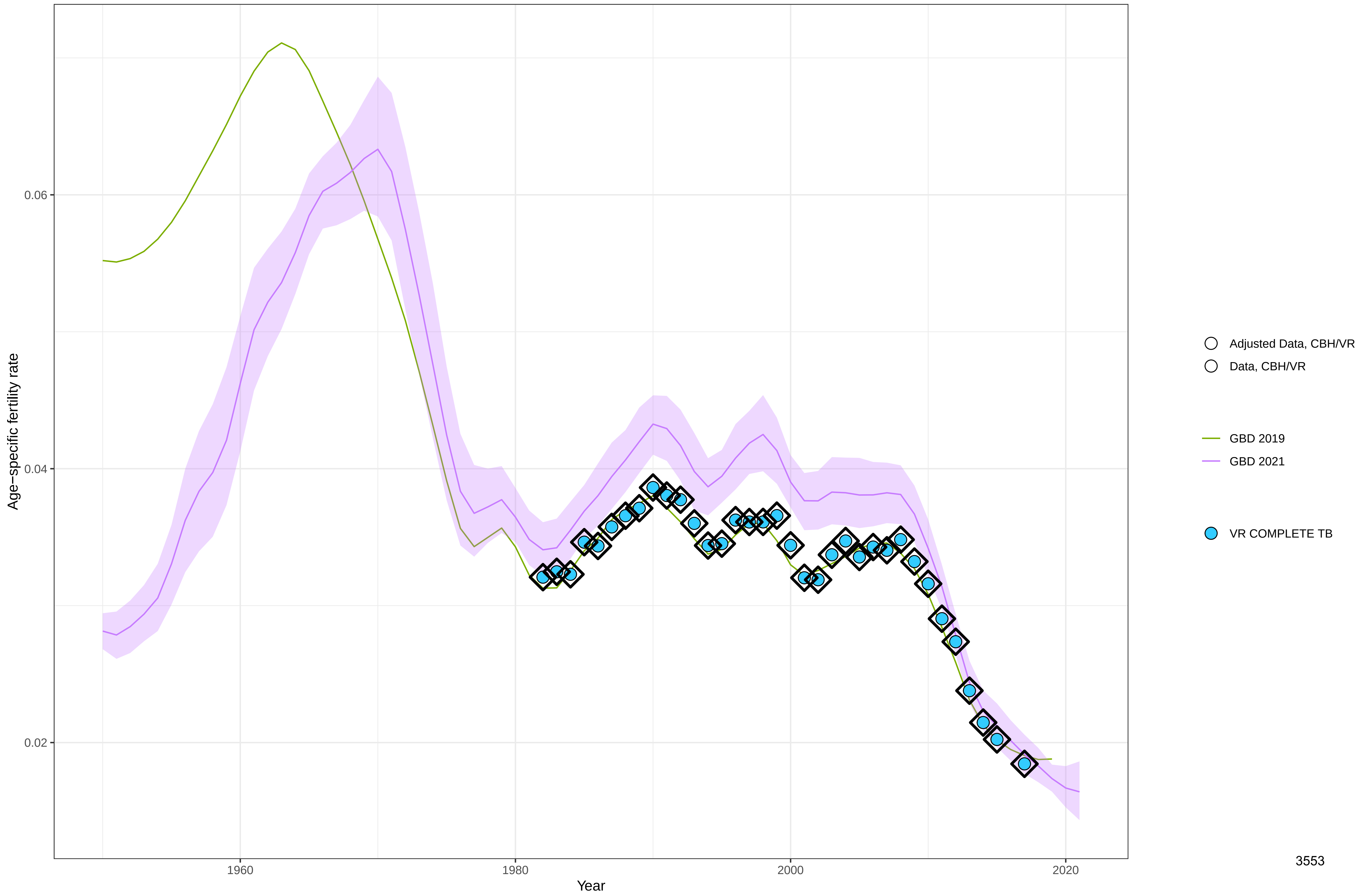

Age−specific fertility fitted trend lines, 1950−2021: Wakefield, Ages: 20−24
Data points enclosed with a diamond are reference sources

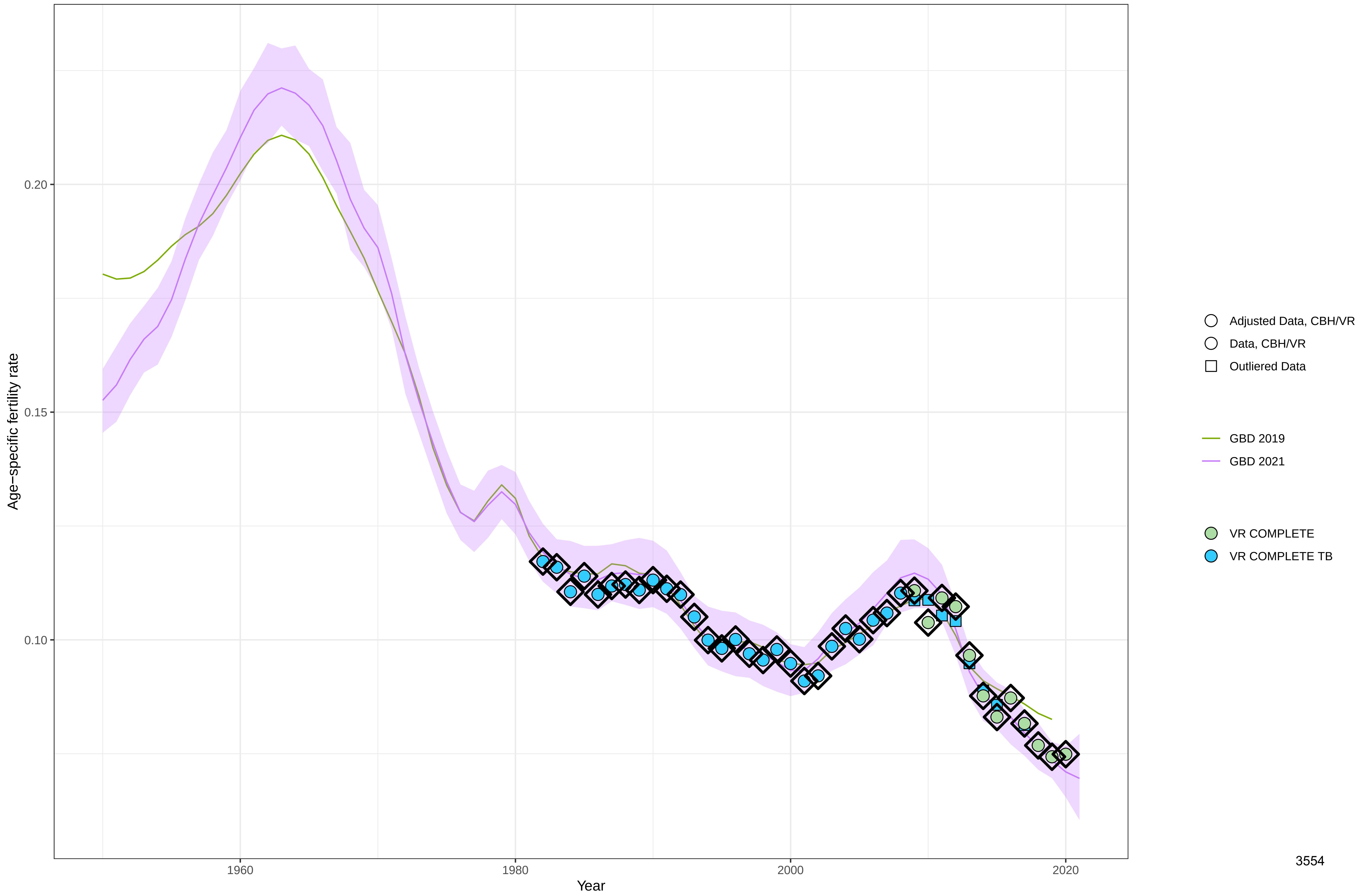

Age−specific fertility fitted trend lines, 1950−2021: Wakefield, Ages: 25−29
Data points enclosed with a diamond are reference sources

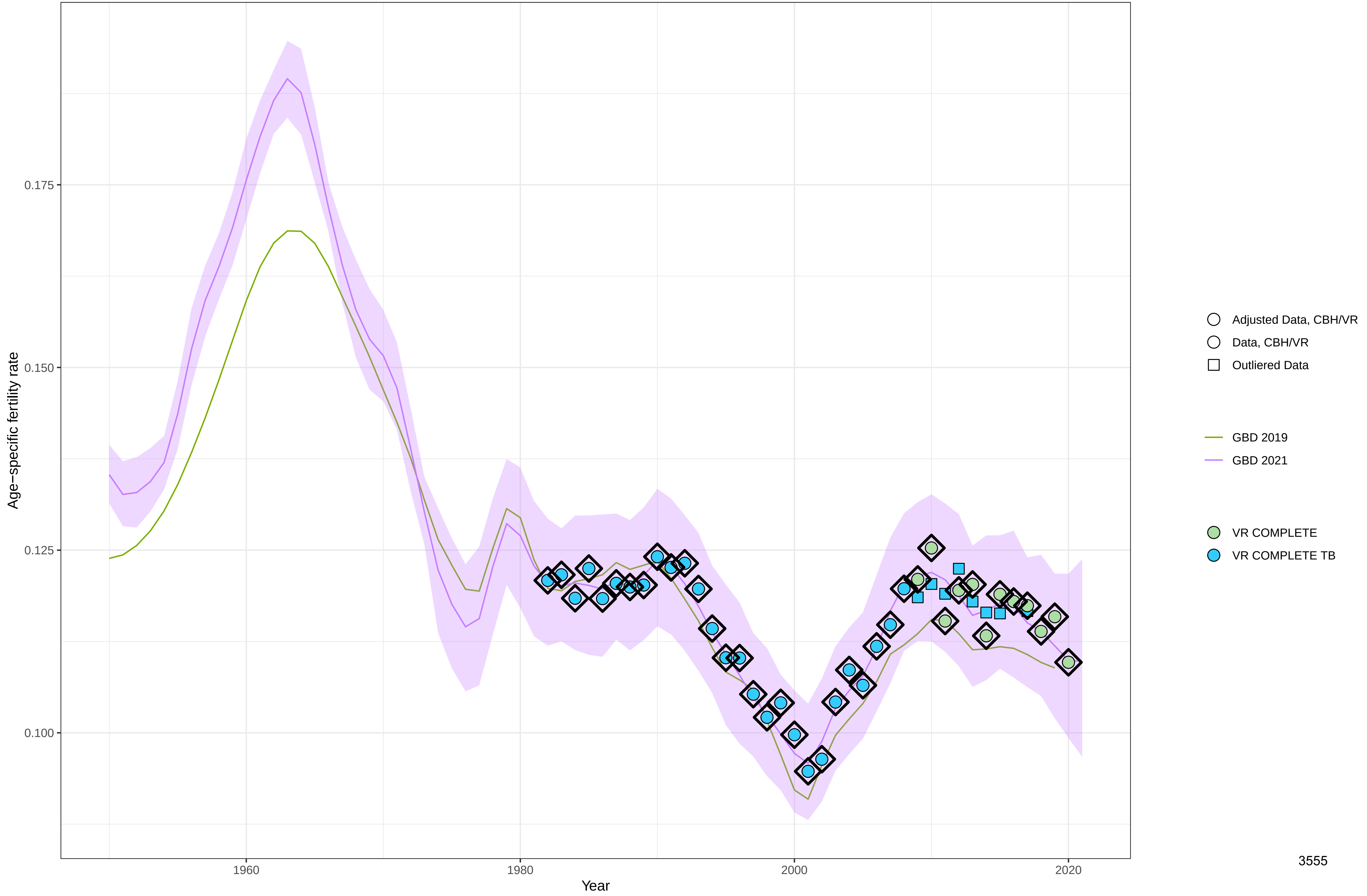

Age−specific fertility fitted trend lines, 1950−2021: Wakefield, Ages: 30−34
Data points enclosed with a diamond are reference sources

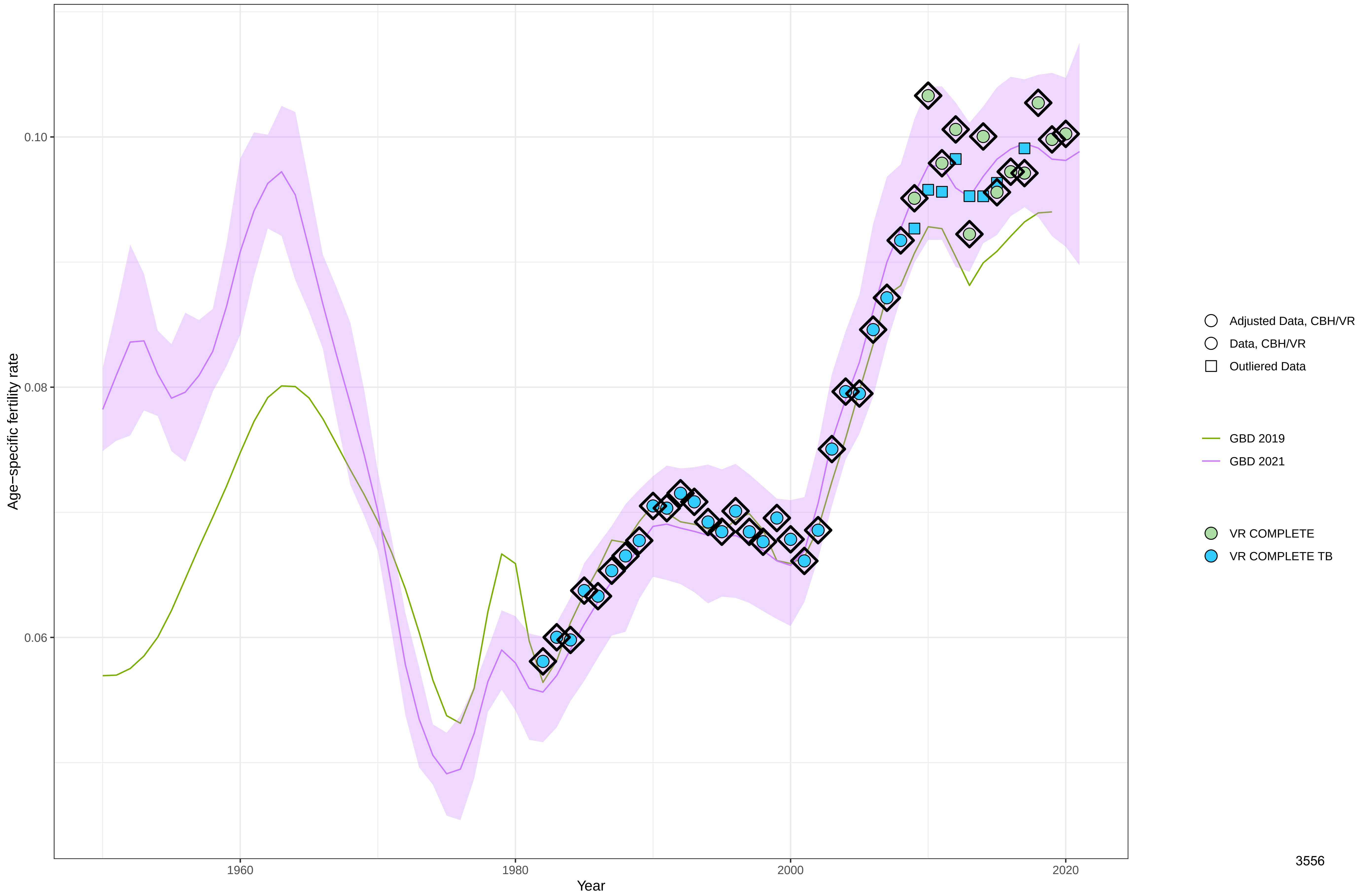

Age−specific fertility fitted trend lines, 1950−2021: Wakefield, Ages: 35−39
Data points enclosed with a diamond are reference sources

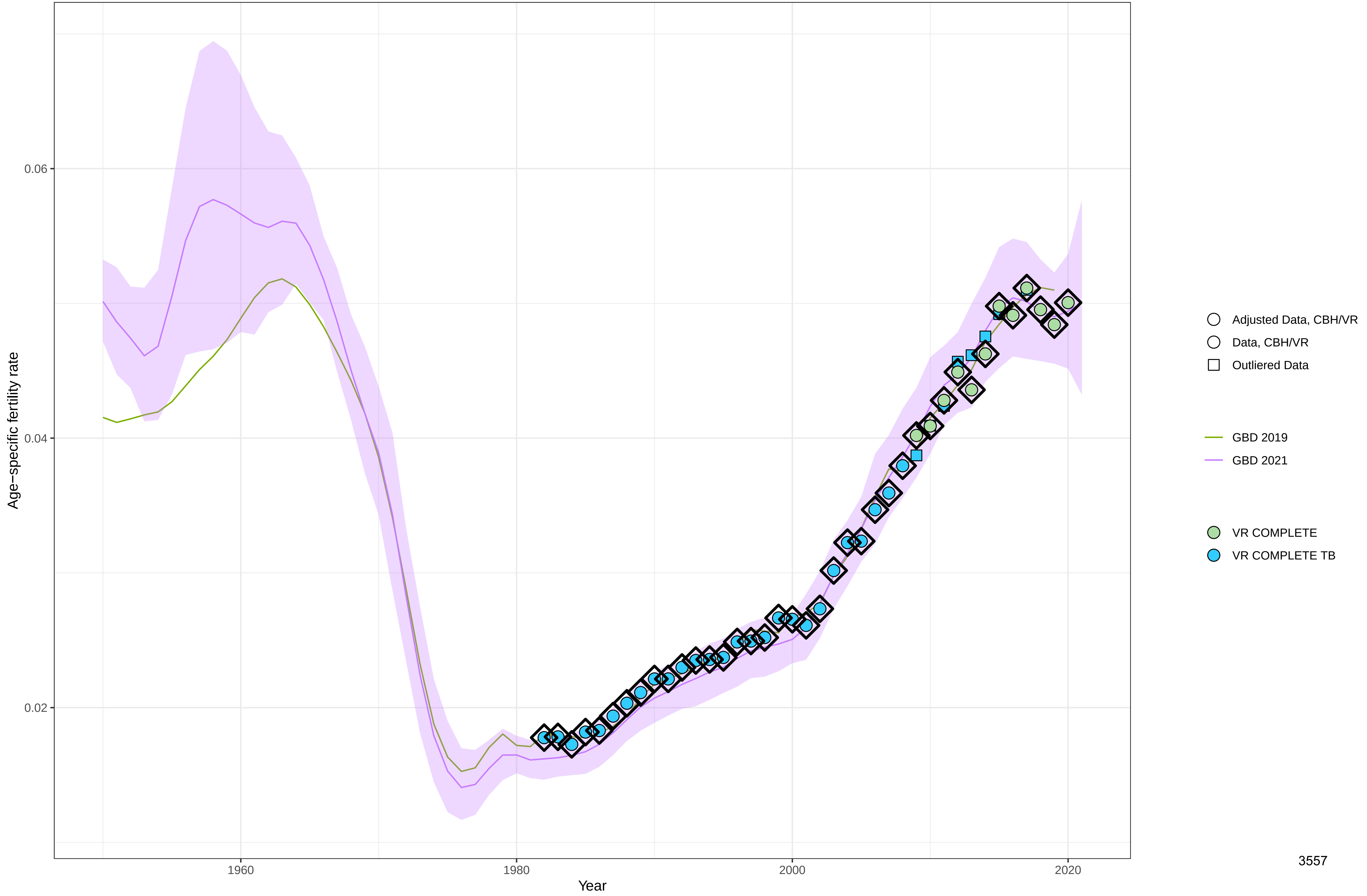

Age−specific fertility fitted trend lines, 1950−2021: Wakefield, Ages: 40−44
Data points enclosed with a diamond are reference sources

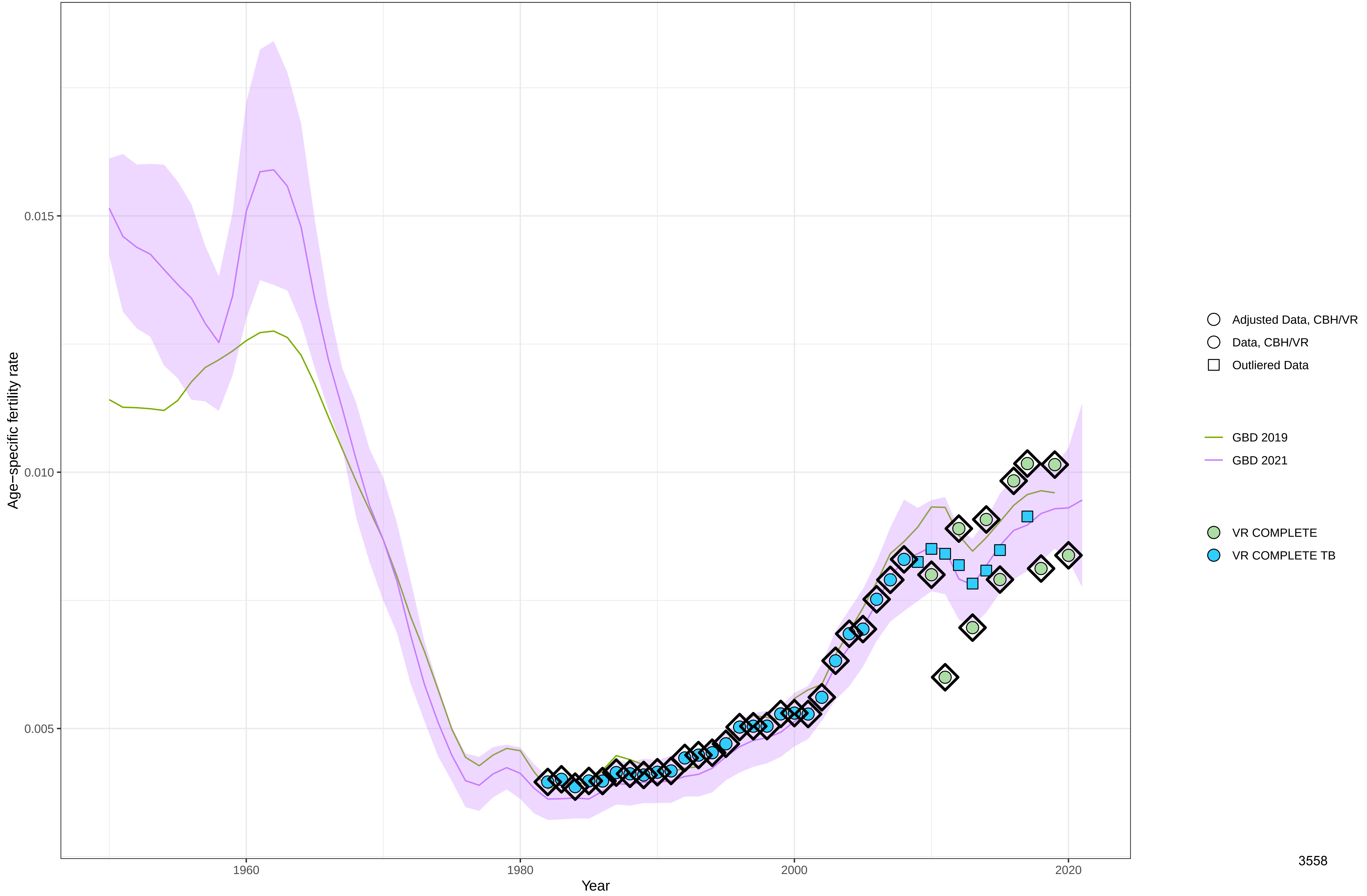

Age−specific fertility fitted trend lines, 1950−2021: Wakefield, Ages: 45−49
Data points enclosed with a diamond are reference sources

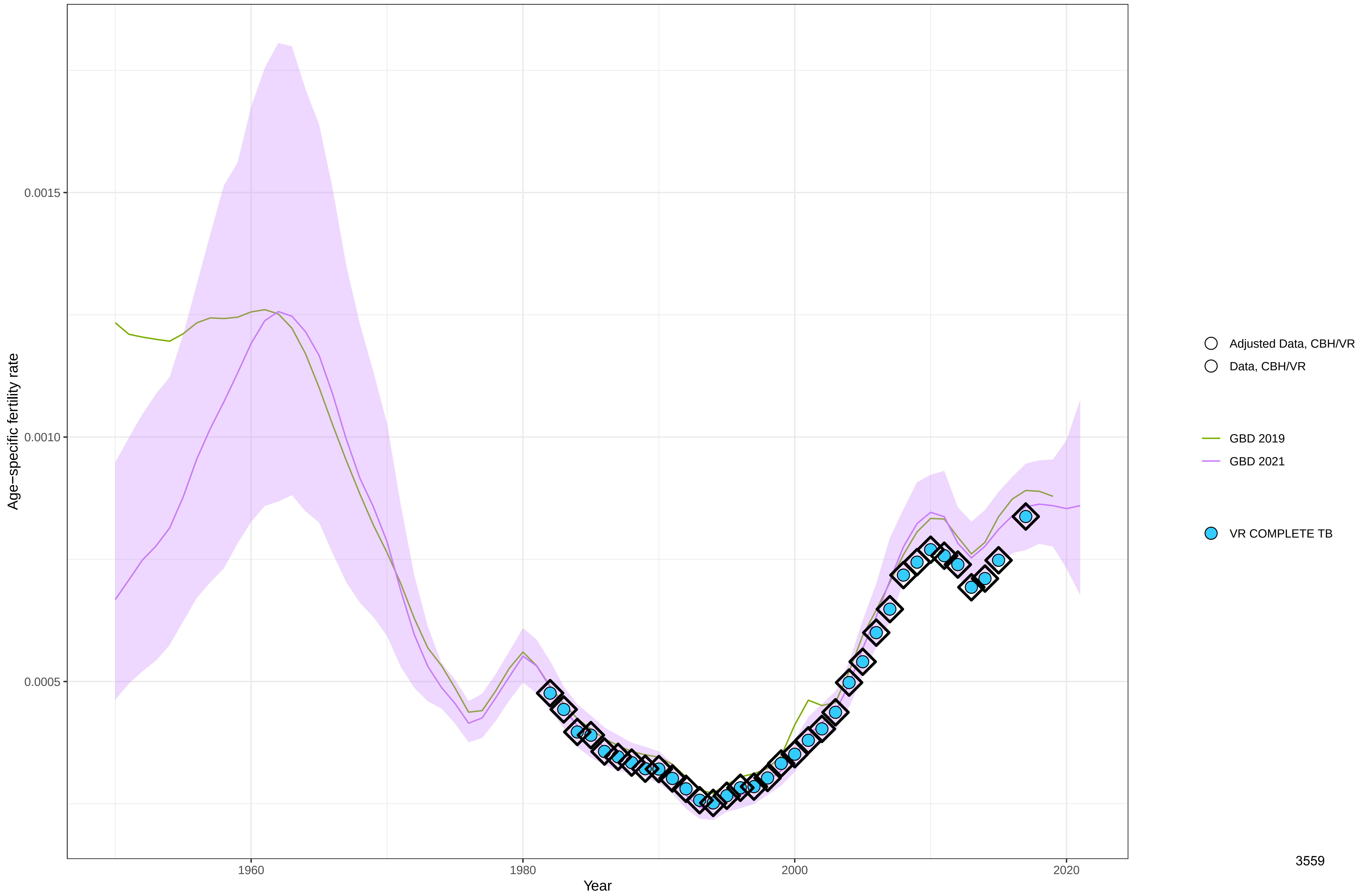

Age−specific fertility fitted trend lines, 1950−2021: Wakefield, Ages: 50−54
Data points enclosed with a diamond are reference sources

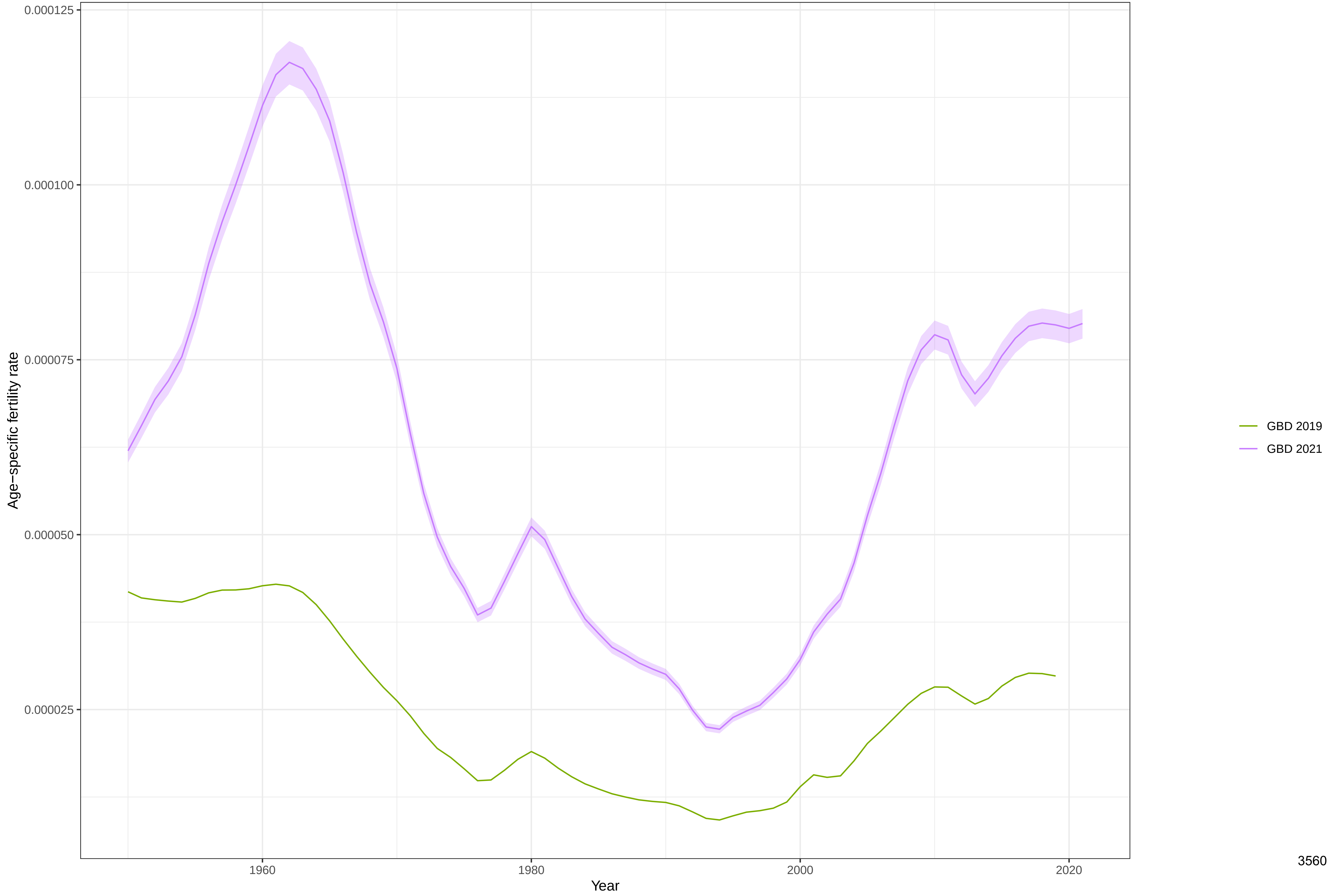

Total fertility rate fitted trend lines, 1950−2021: Wakefield TFR
Data points enclosed with a diamond are reference sources

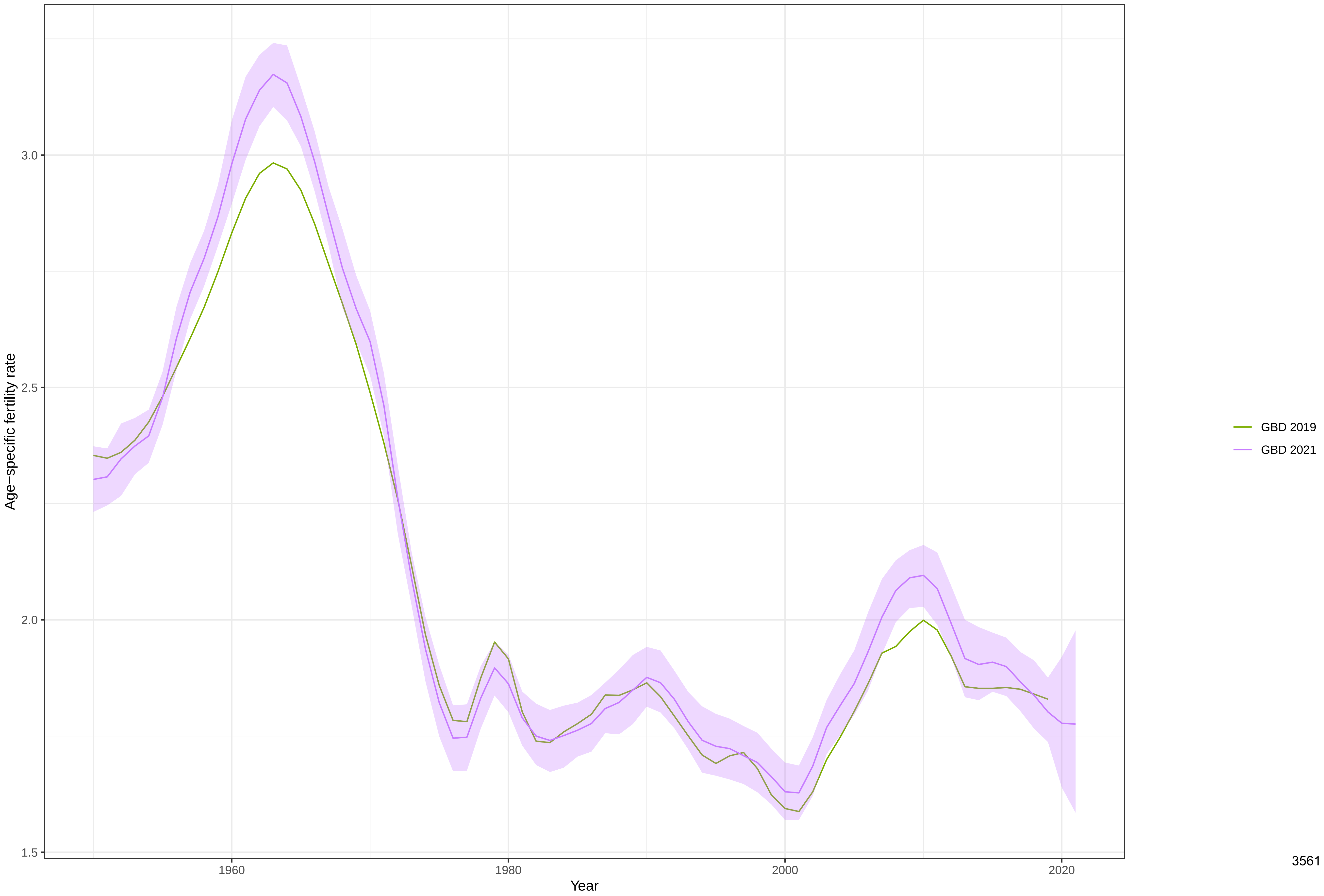

Age−specific fertility fitted trend lines, 1950−2021: York, Ages: 10−14
Data points enclosed with a diamond are reference sources

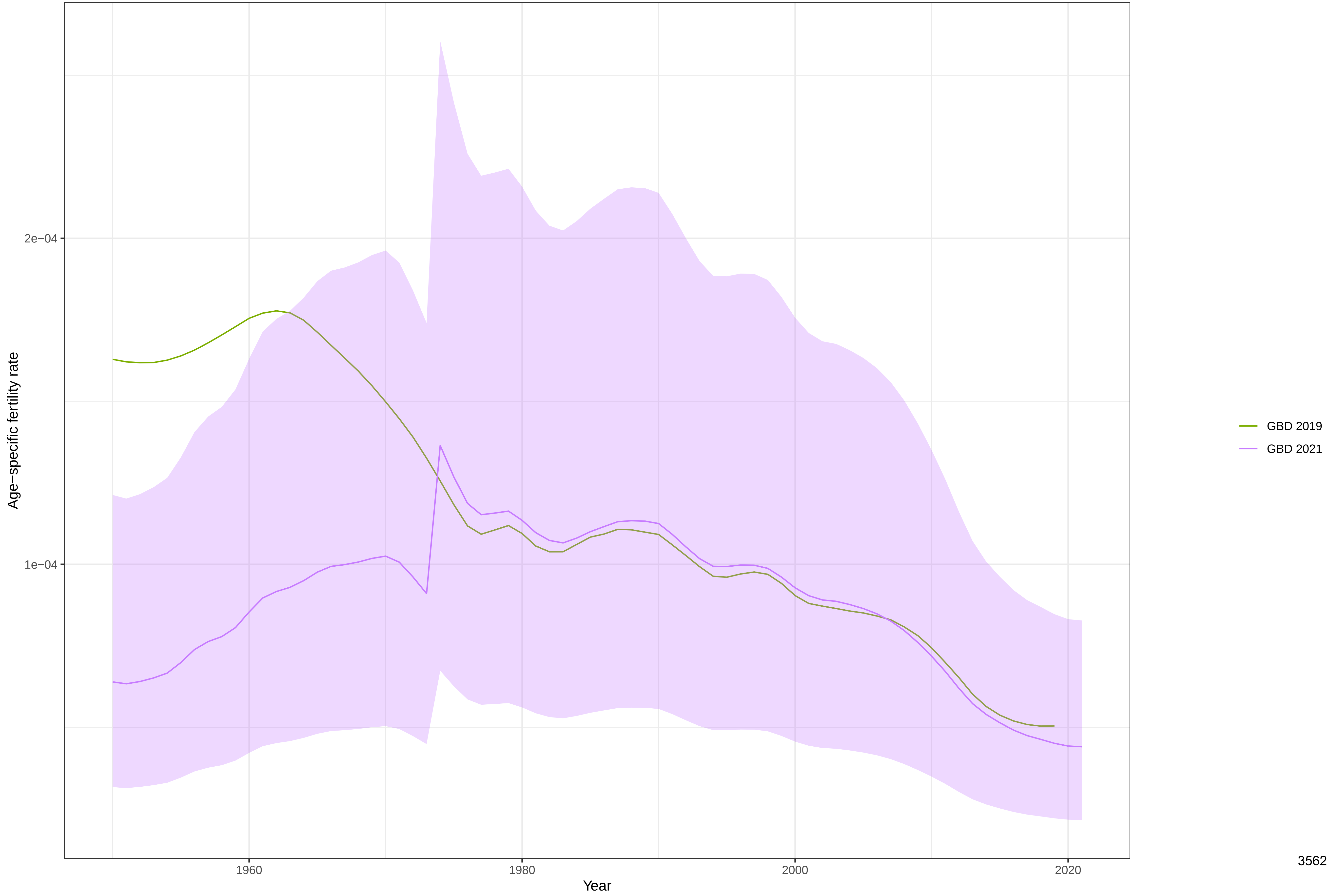

Age−specific fertility fitted trend lines, 1950−2021: York, Ages: 15−19
Data points enclosed with a diamond are reference sources

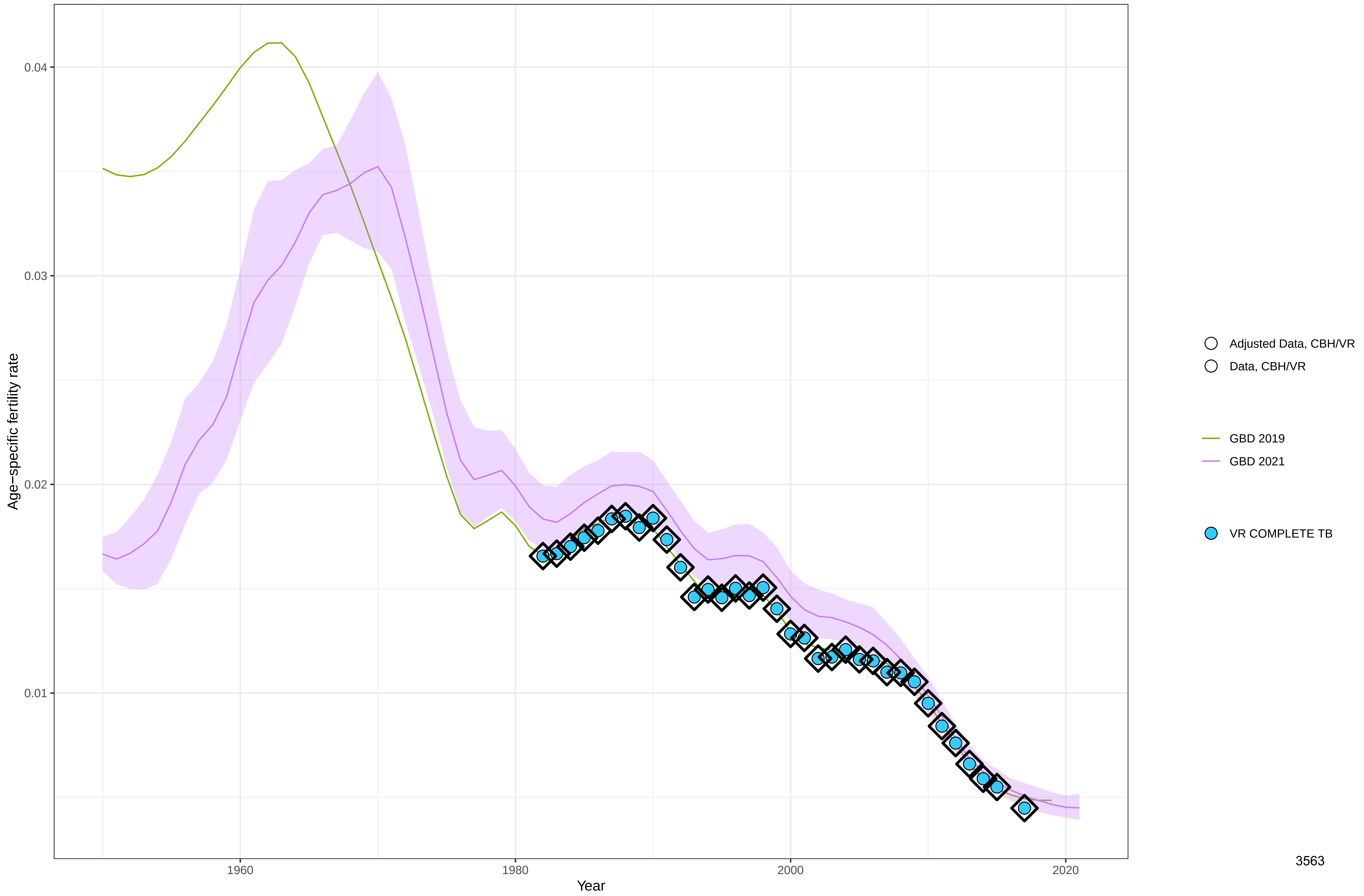

Age−specific fertility fitted trend lines, 1950−2021: York, Ages: 20−24
Data points enclosed with a diamond are reference sources

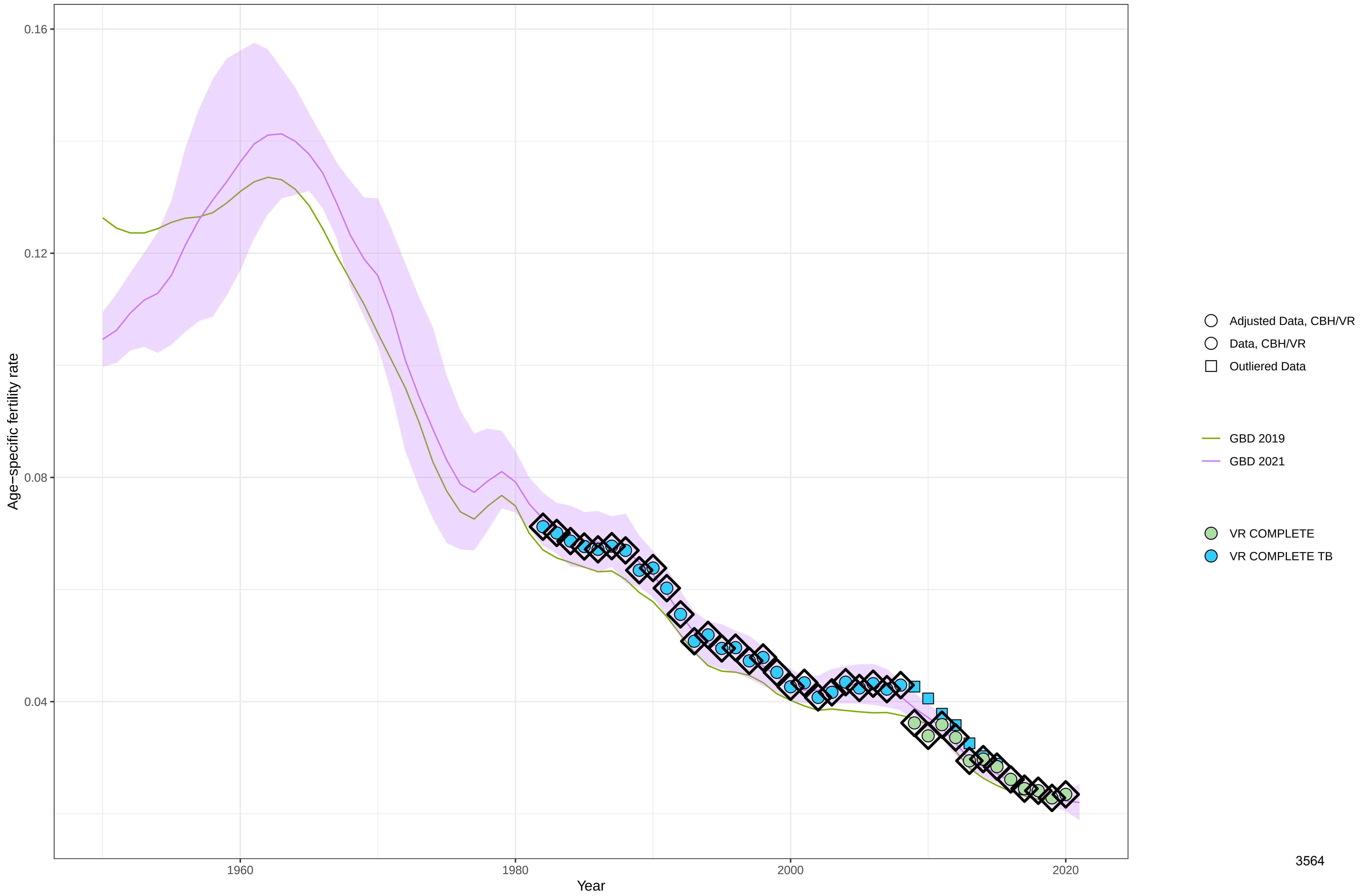

Age−specific fertility fitted trend lines, 1950−2021: York, Ages: 25−29
Data points enclosed with a diamond are reference sources

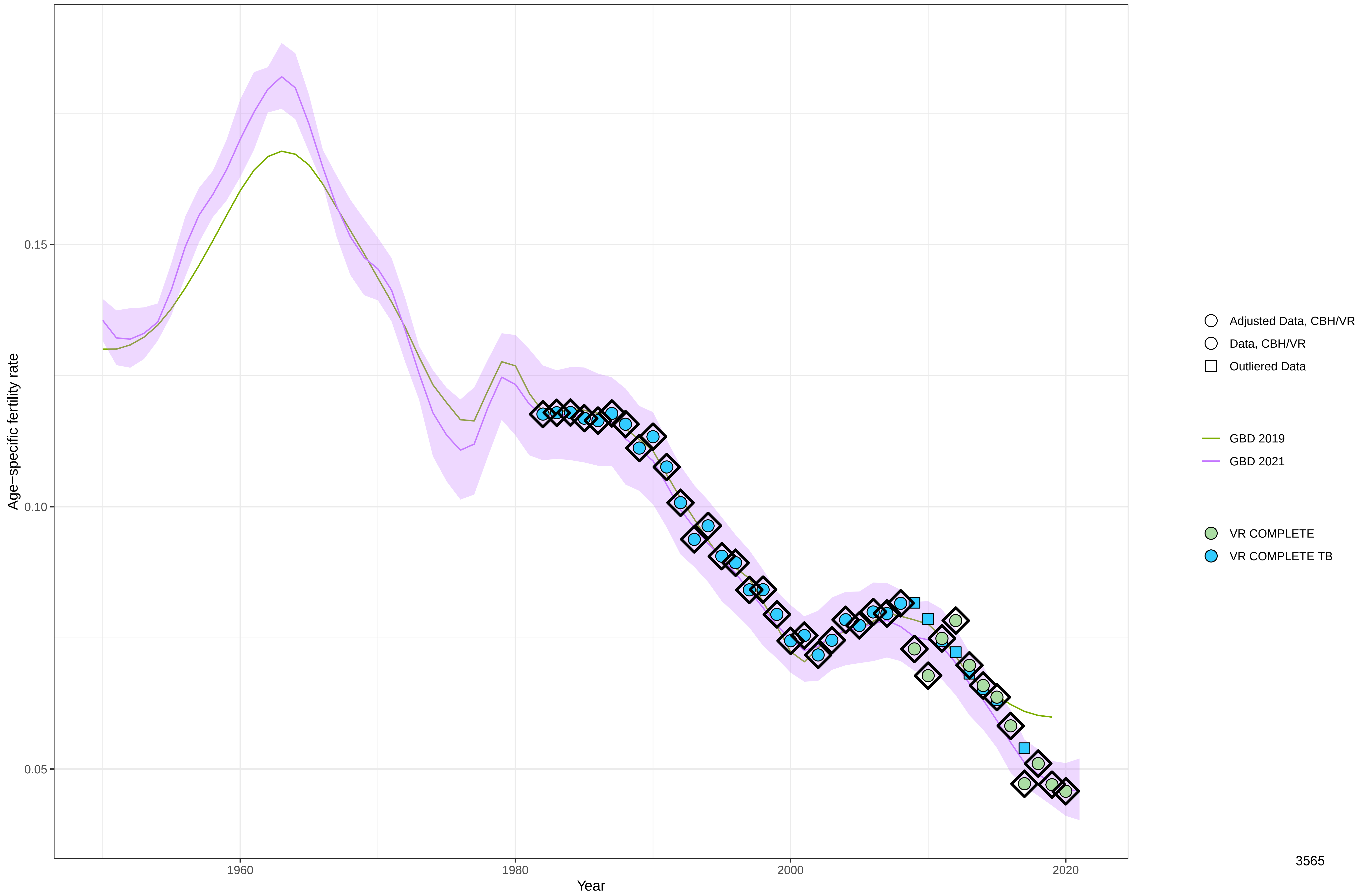

Age−specific fertility fitted trend lines, 1950−2021: York, Ages: 30−34
Data points enclosed with a diamond are reference sources

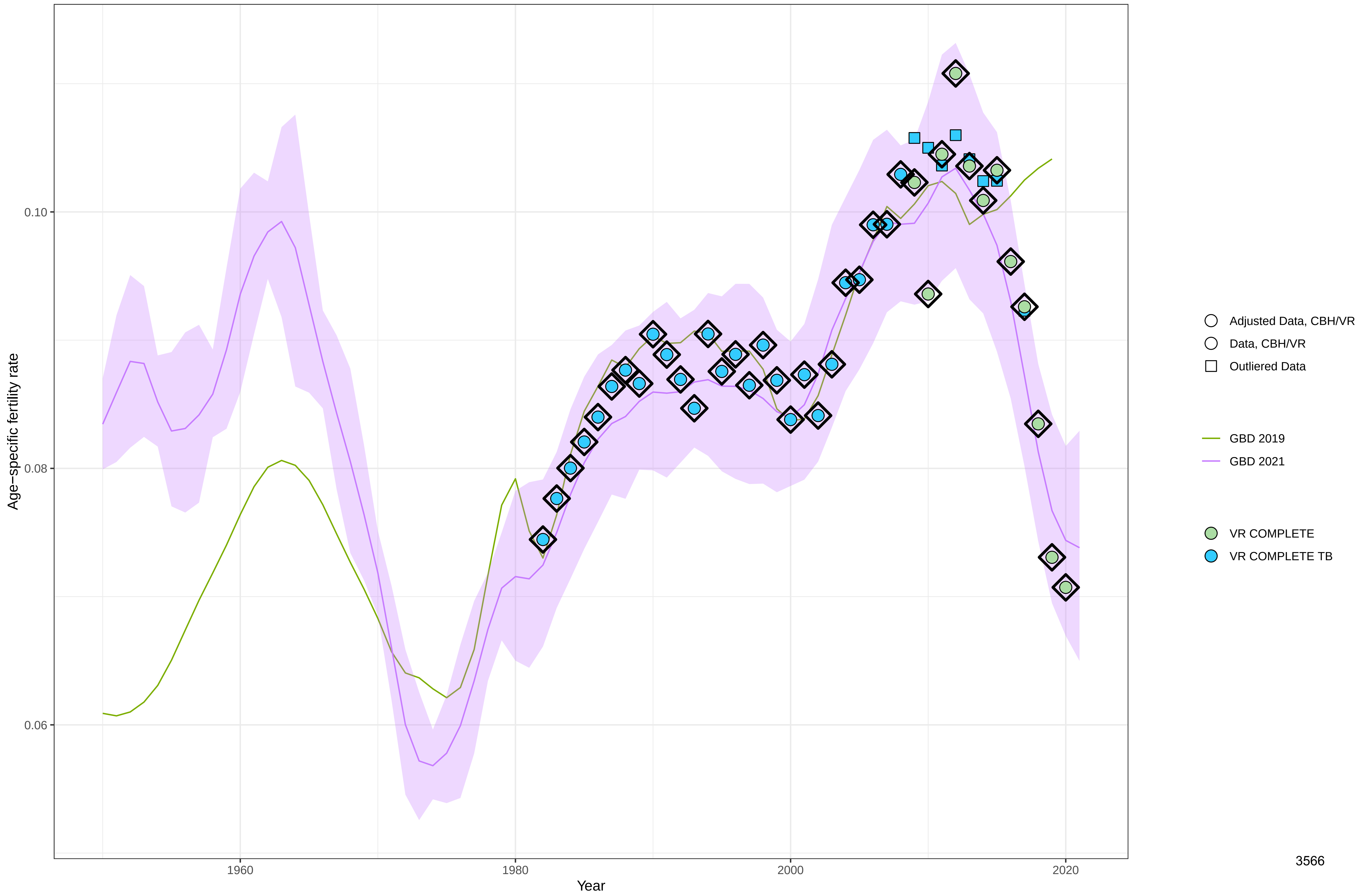

Age−specific fertility fitted trend lines, 1950−2021: York, Ages: 35−39
Data points enclosed with a diamond are reference sources

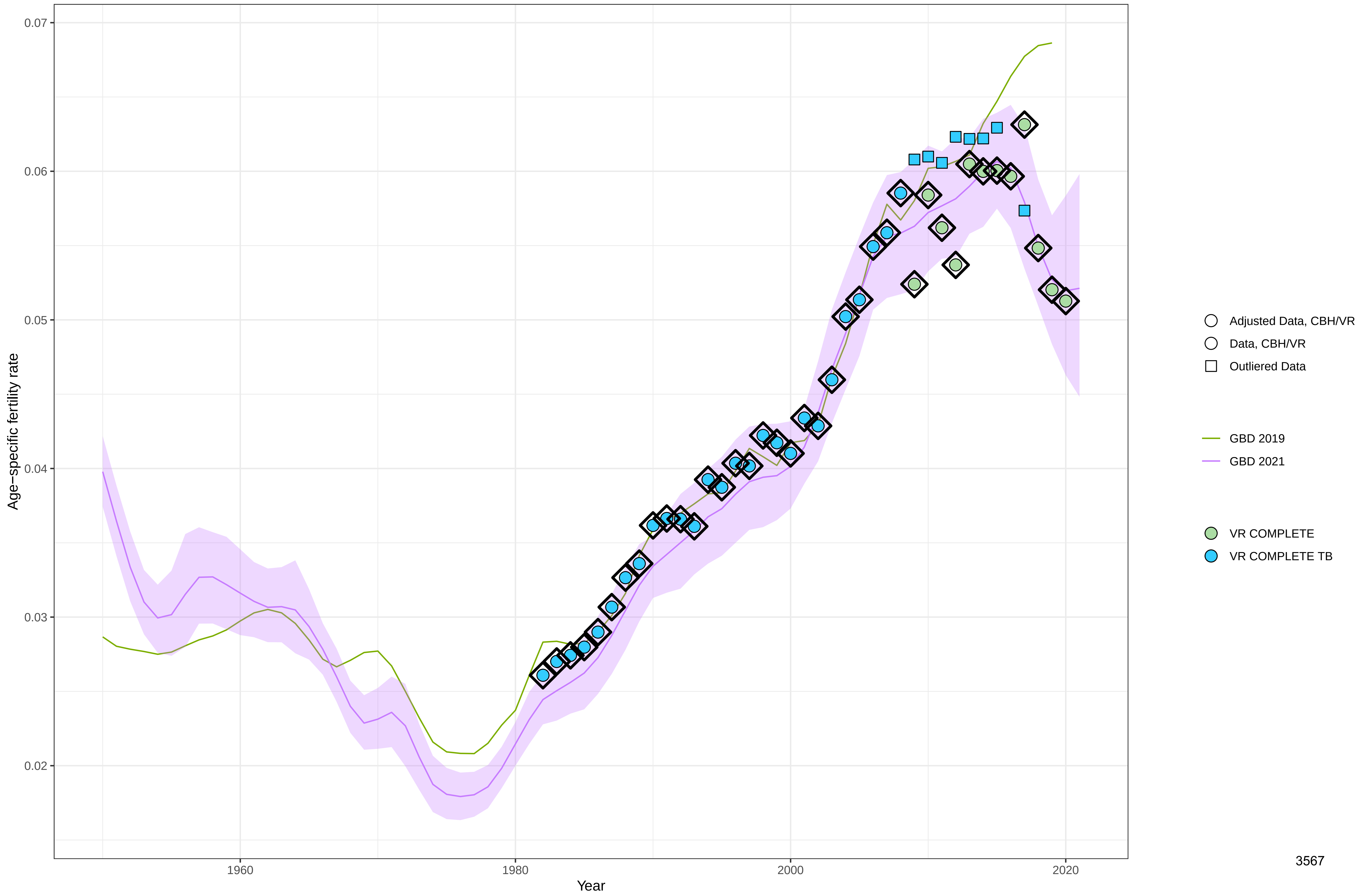

Age−specific fertility fitted trend lines, 1950−2021: York, Ages: 40−44
Data points enclosed with a diamond are reference sources

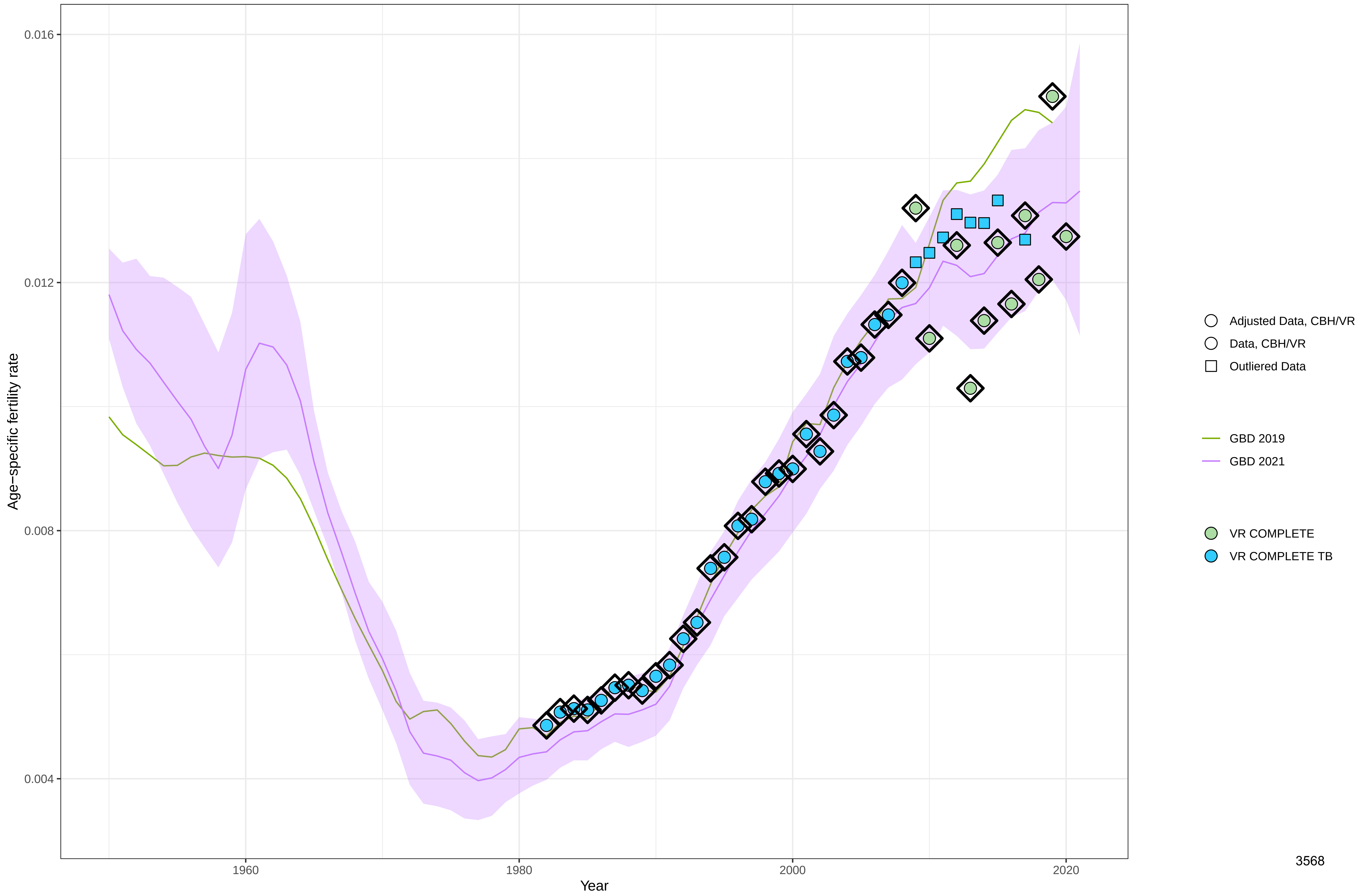

Age−specific fertility fitted trend lines, 1950−2021: York, Ages: 45−49
Data points enclosed with a diamond are reference sources

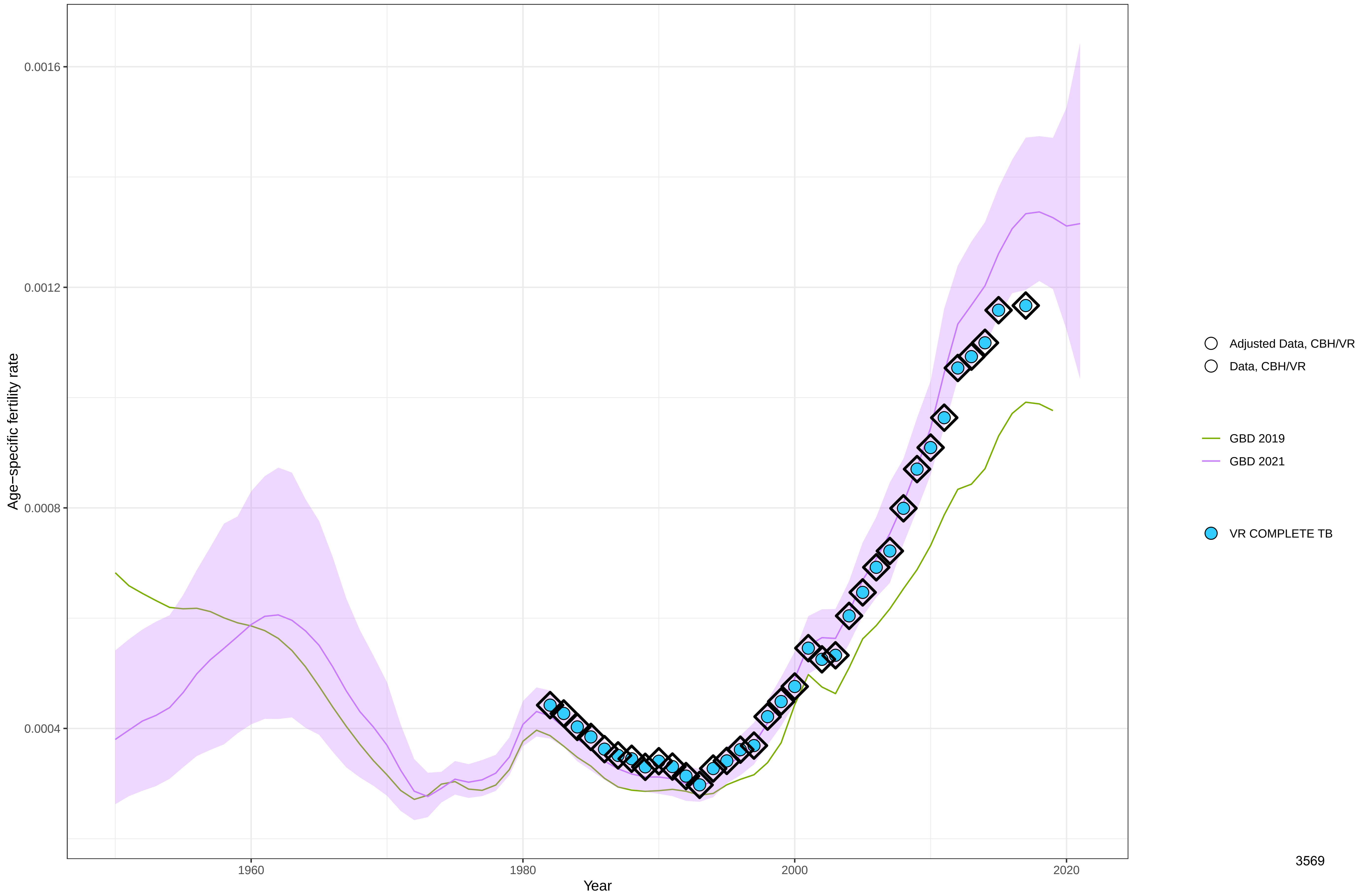

Age−specific fertility fitted trend lines, 1950−2021: York, Ages: 50−54
 Data points enclosed with a diamond are reference sources

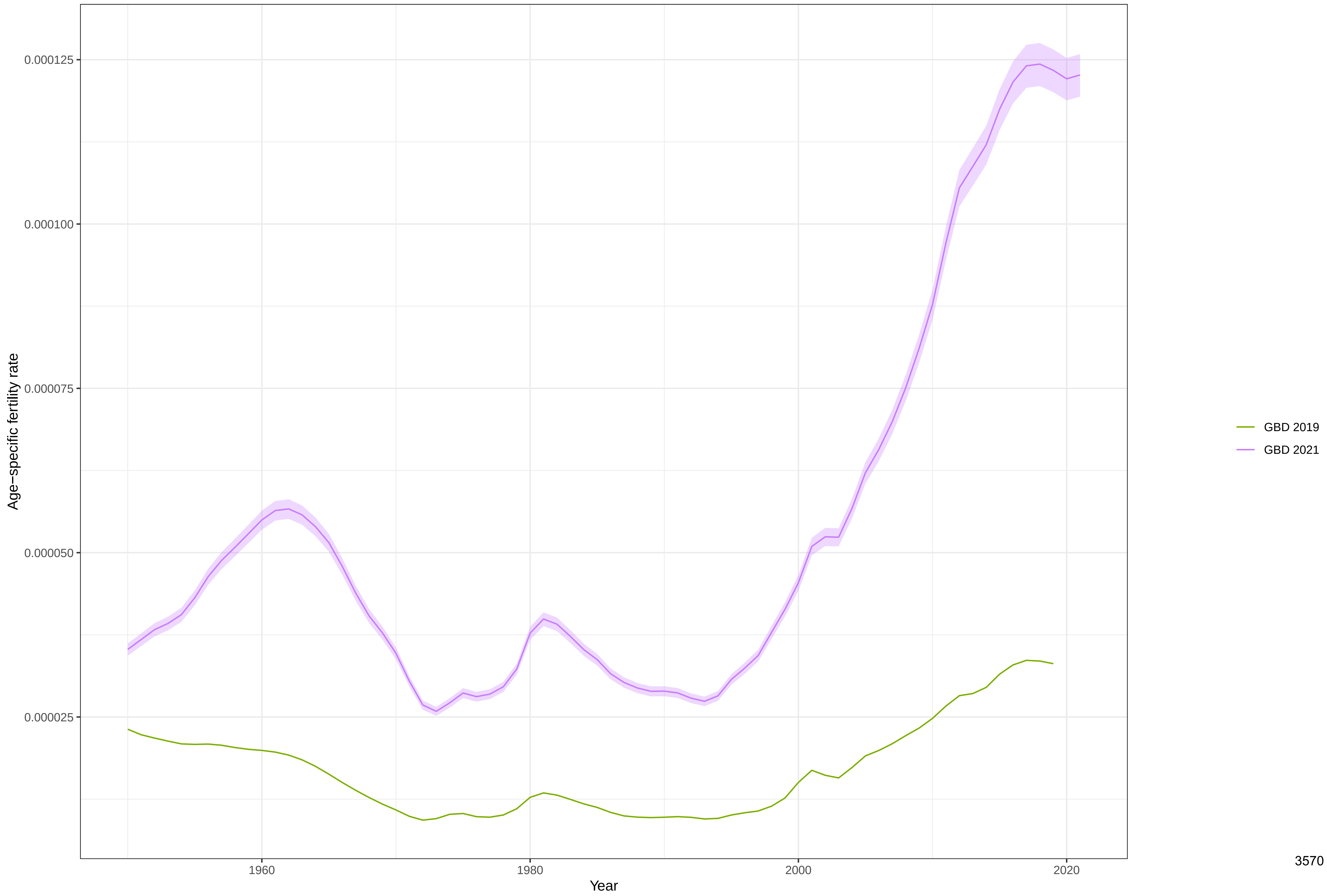

Total fertility rate fitted trend lines, 1950−2021: York TFR
Data points enclosed with a diamond are reference sources

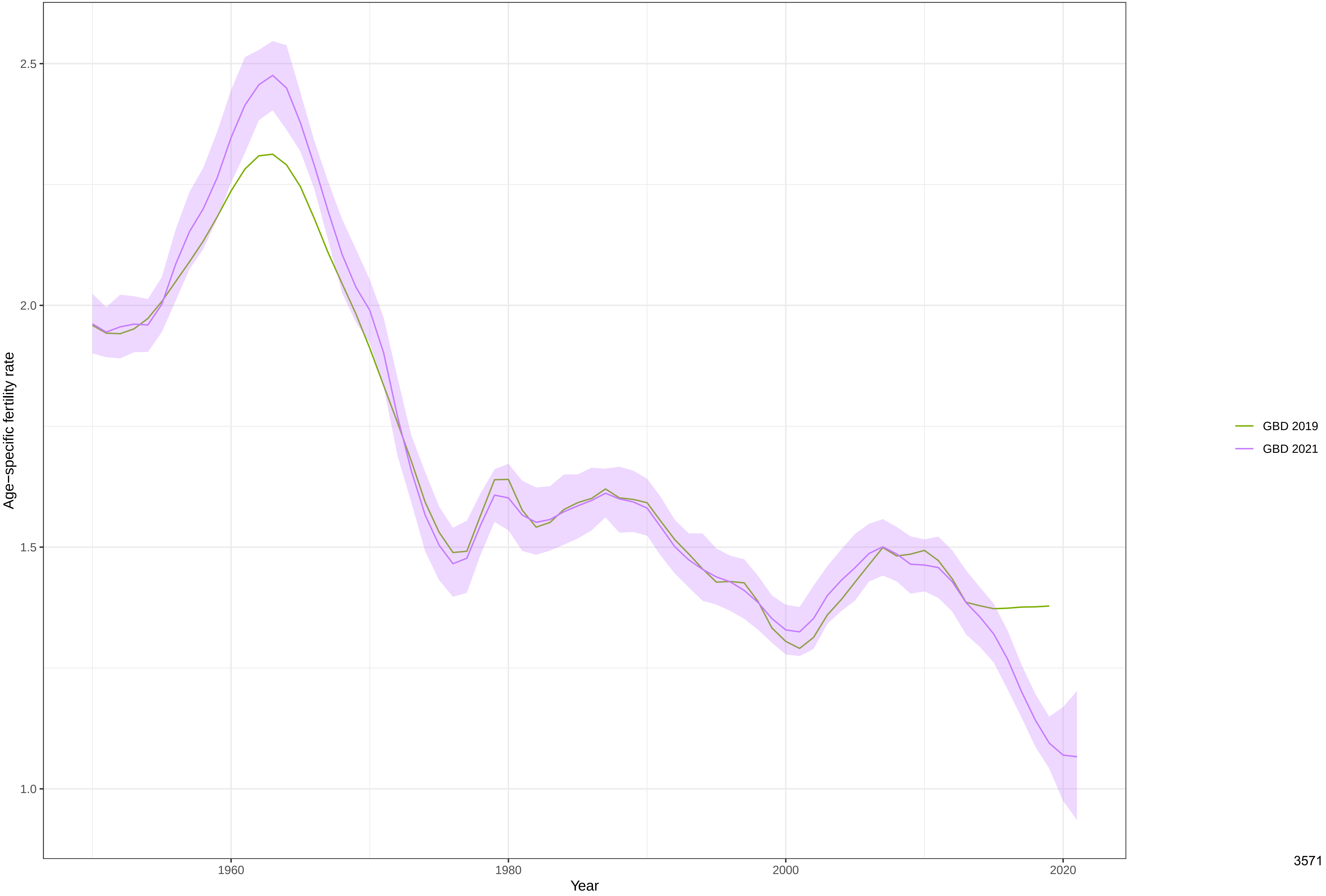

Age−specific fertility fitted trend lines, 1950−2021: Northern Ireland, Ages: 10−14
Data points enclosed with a diamond are reference sources

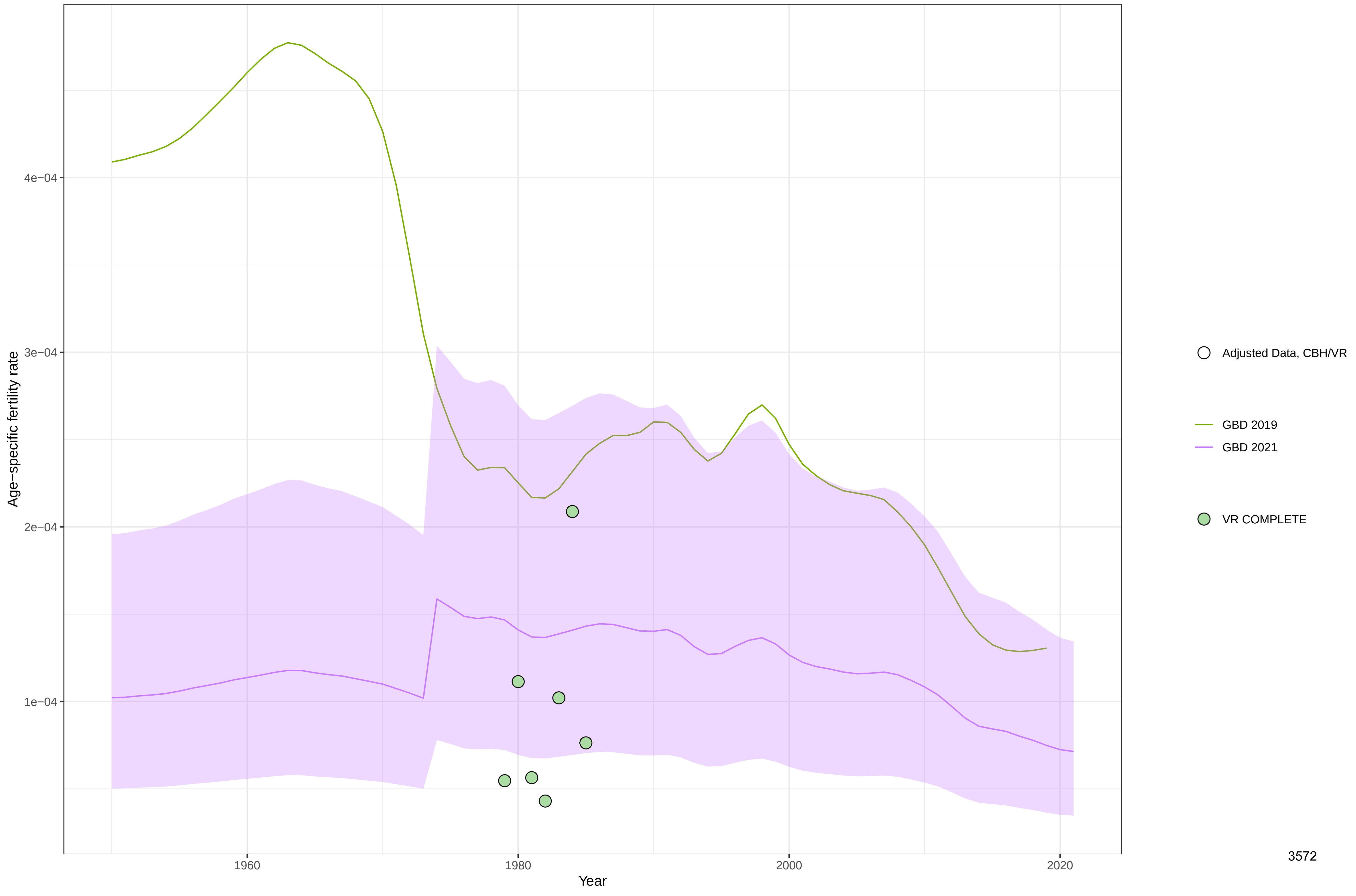

Age−specific fertility fitted trend lines, 1950−2021: Northern Ireland, Ages: 15−19
Data points enclosed with a diamond are reference sources

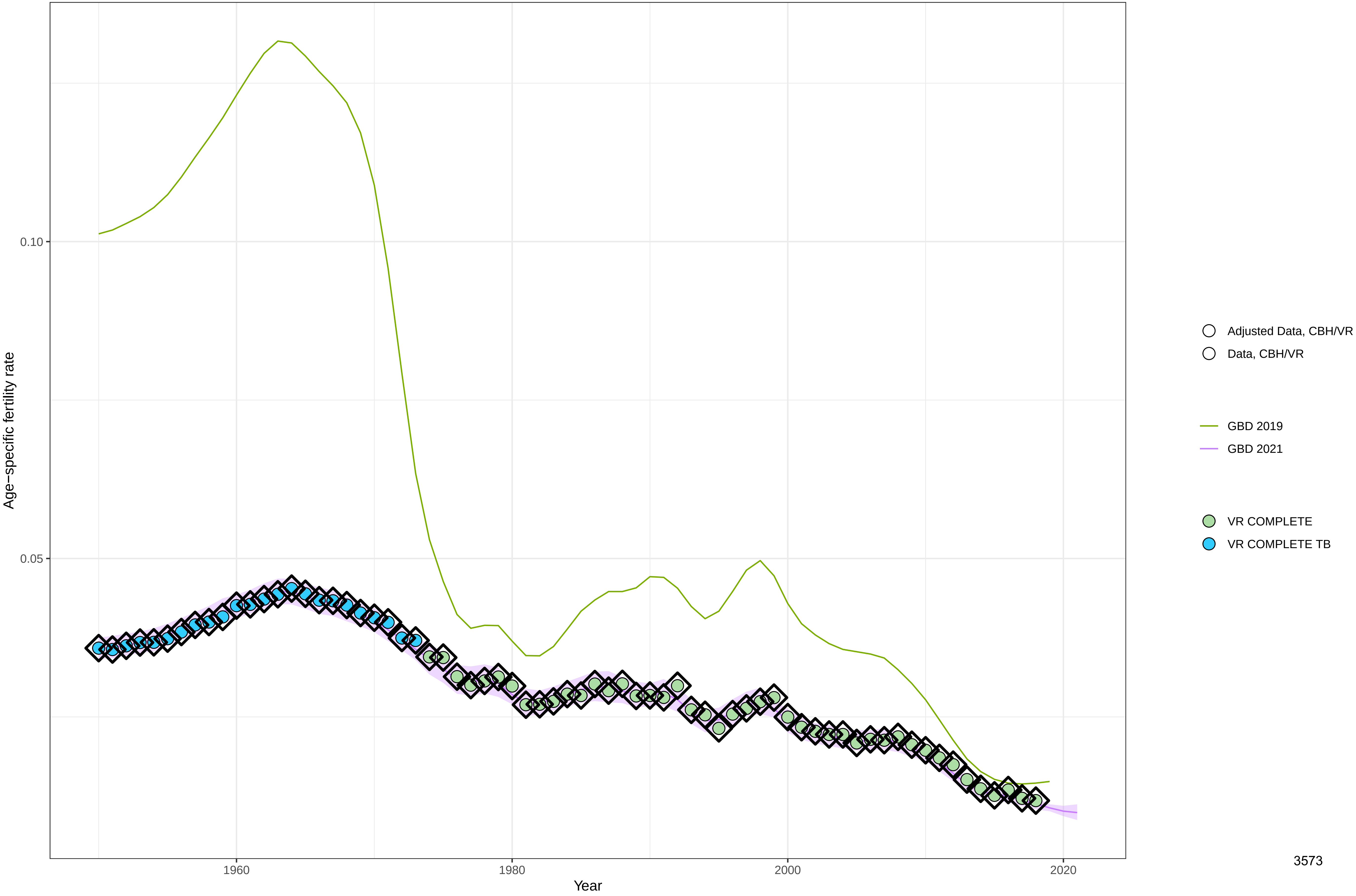

Age−specific fertility fitted trend lines, 1950−2021: Northern Ireland, Ages: 20−24
Data points enclosed with a diamond are reference sources

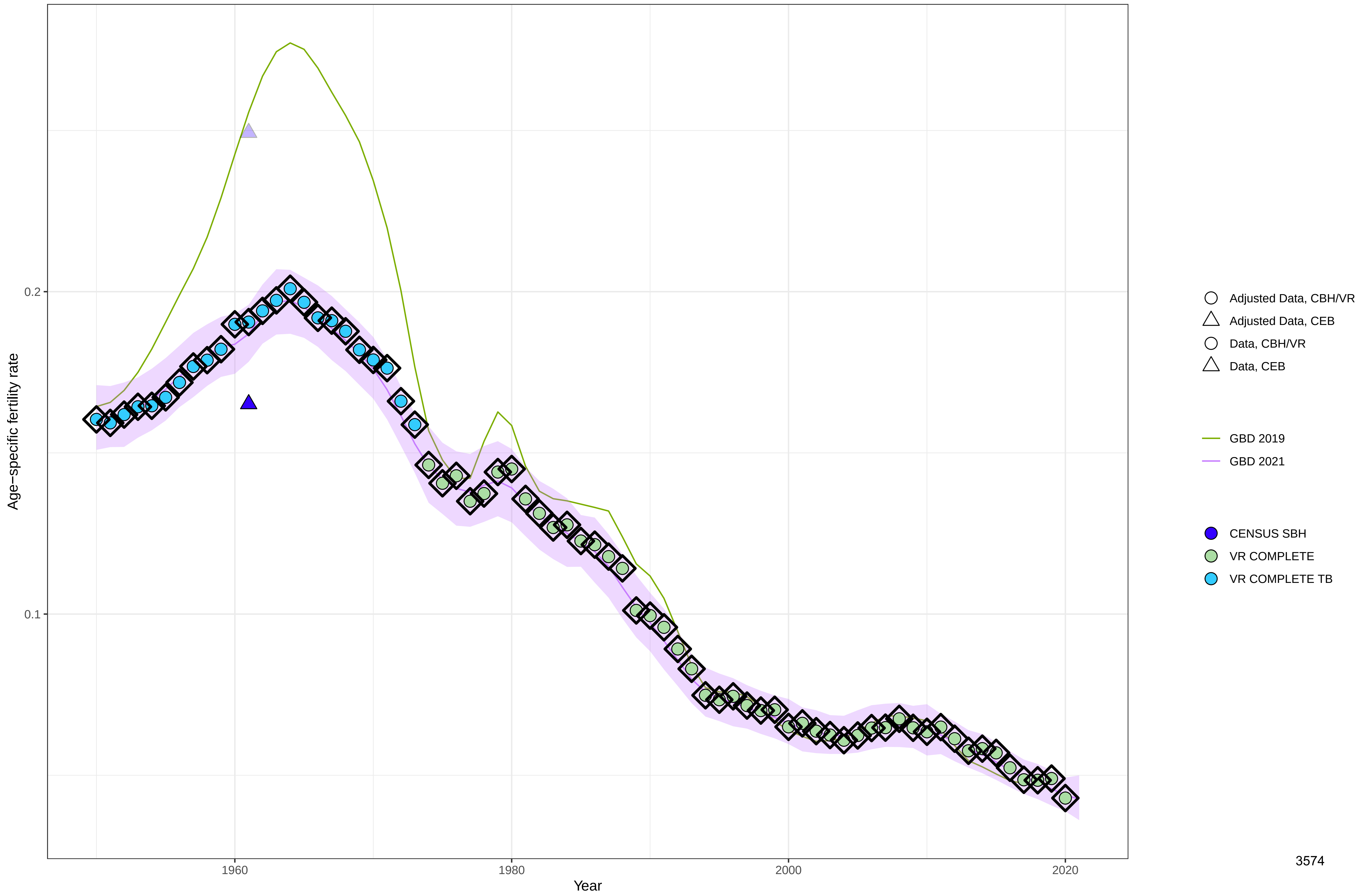

Age−specific fertility fitted trend lines, 1950−2021: Northern Ireland, Ages: 25−29
Data points enclosed with a diamond are reference sources

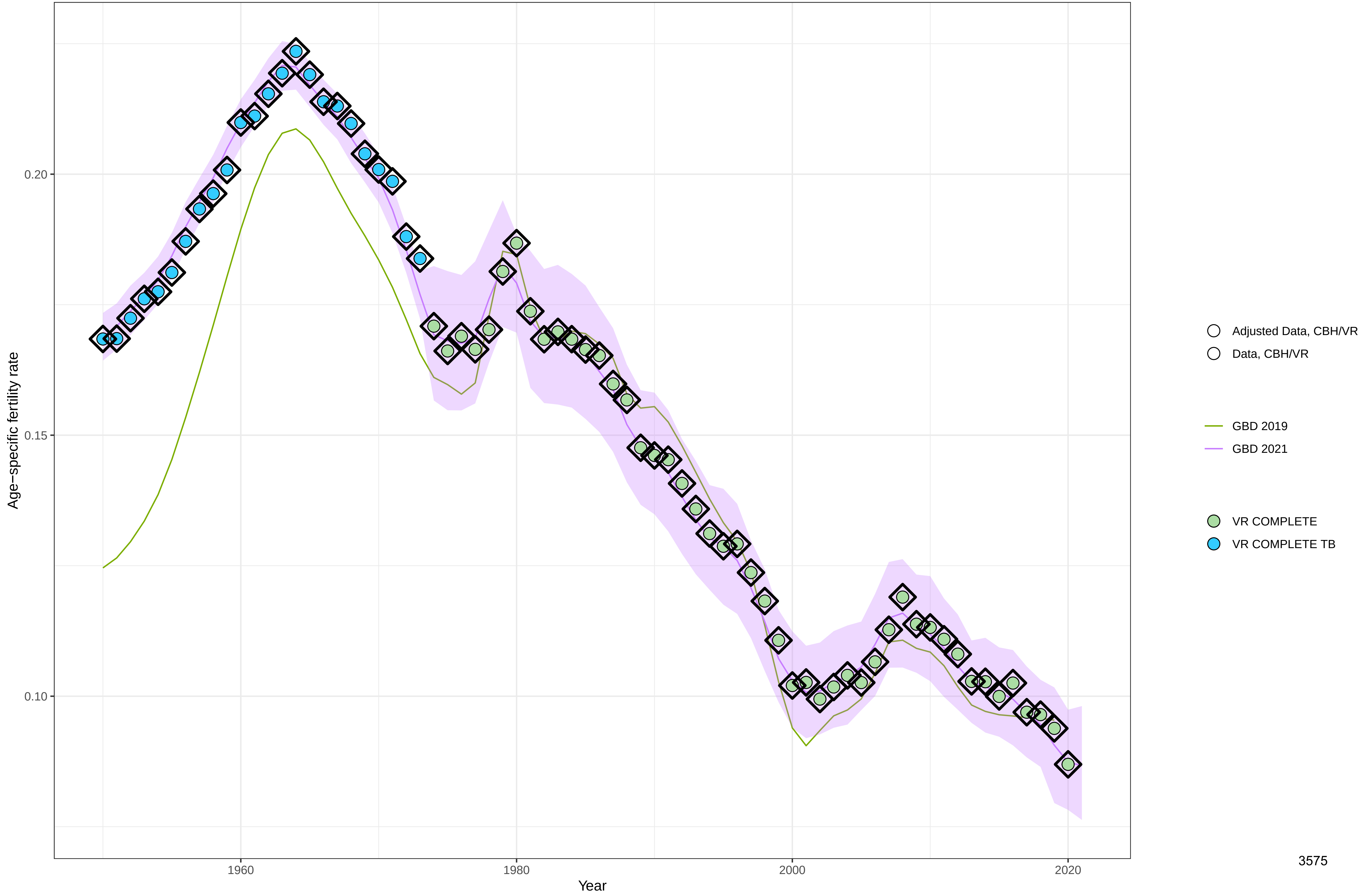

Age−specific fertility fitted trend lines, 1950−2021: Northern Ireland, Ages: 30−34
Data points enclosed with a diamond are reference sources

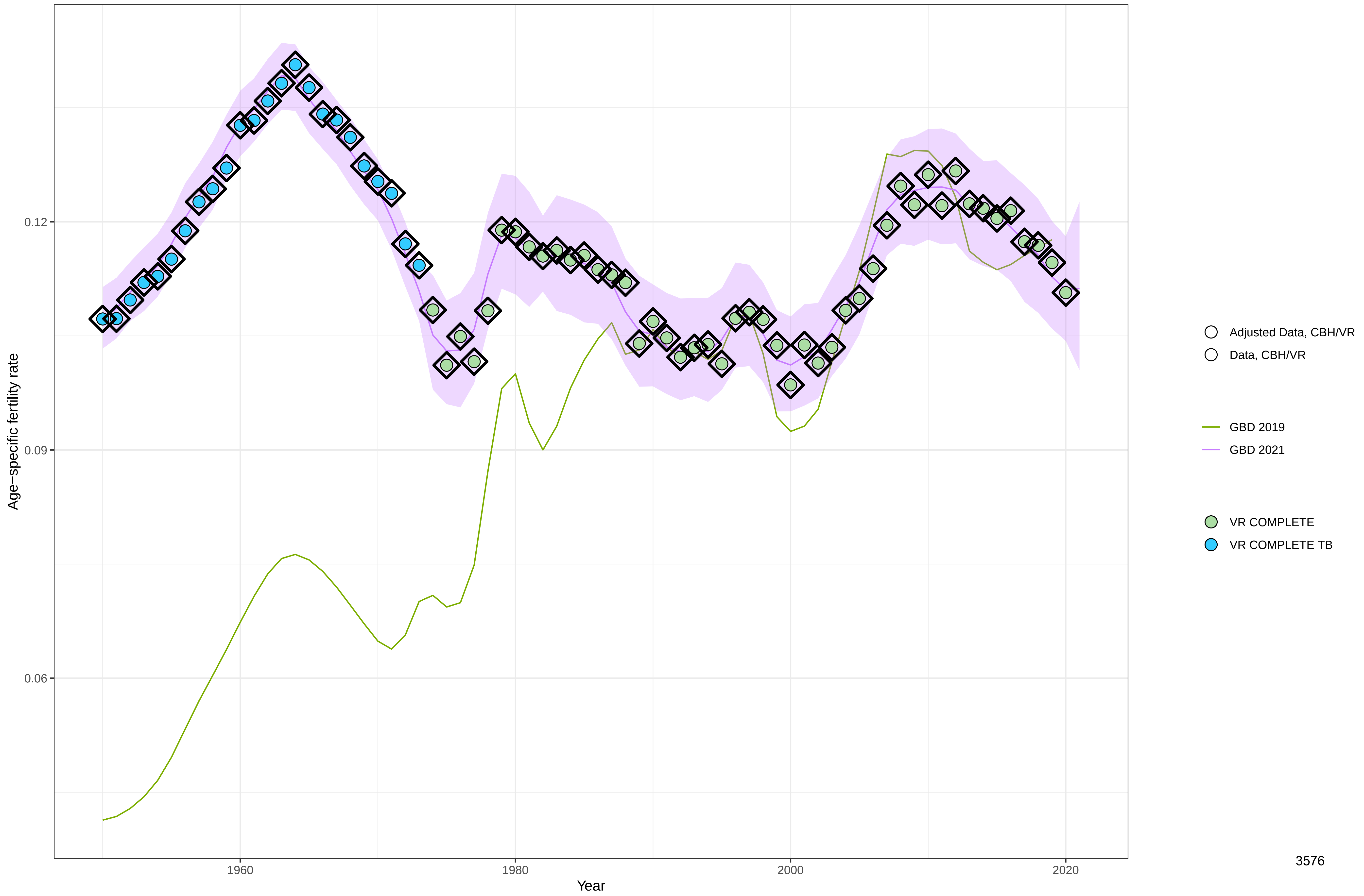

Age−specific fertility fitted trend lines, 1950−2021: Northern Ireland, Ages: 35−39
Data points enclosed with a diamond are reference sources

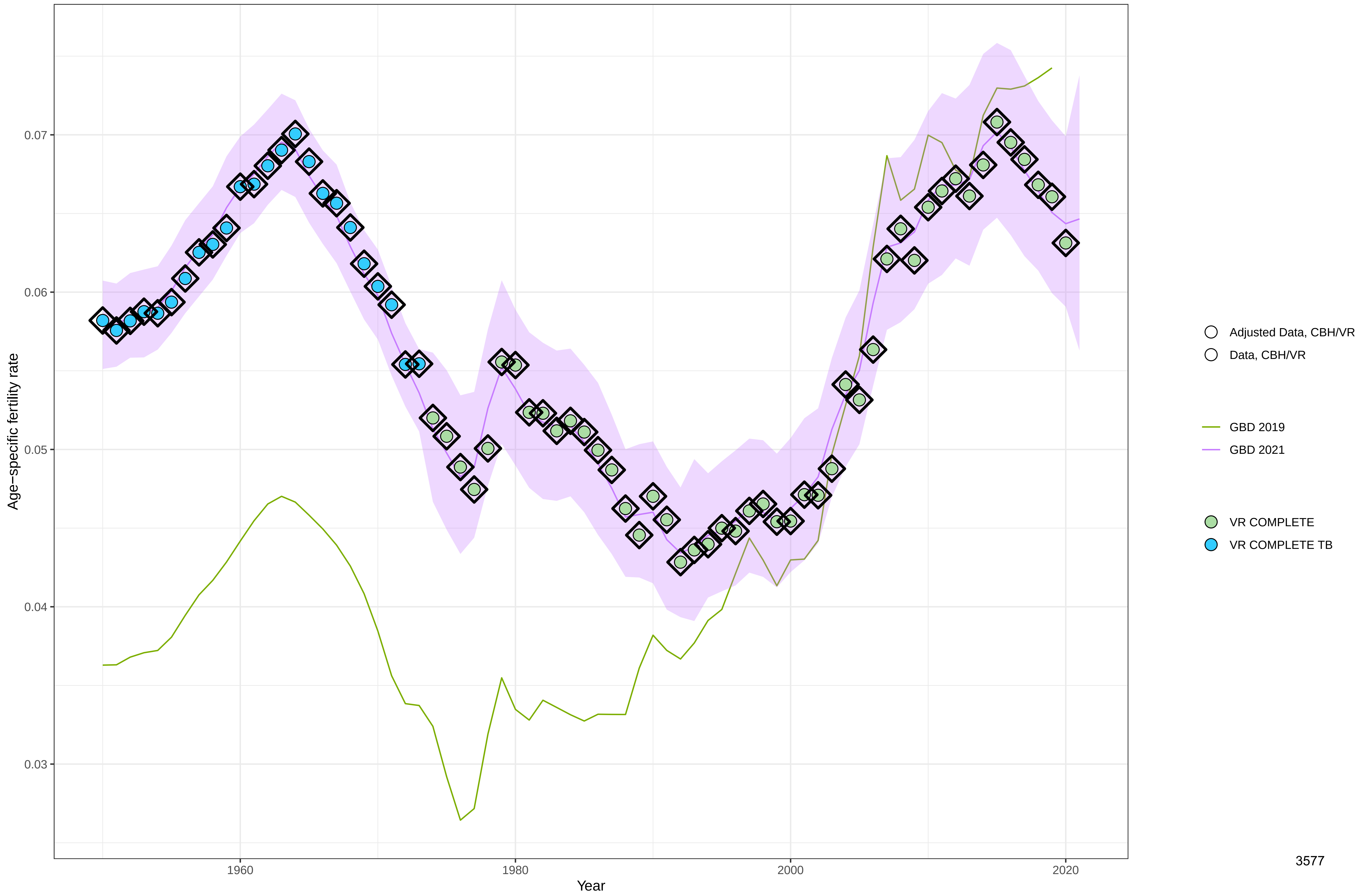

Age−specific fertility fitted trend lines, 1950−2021: Northern Ireland, Ages: 40−44
Data points enclosed with a diamond are reference sources

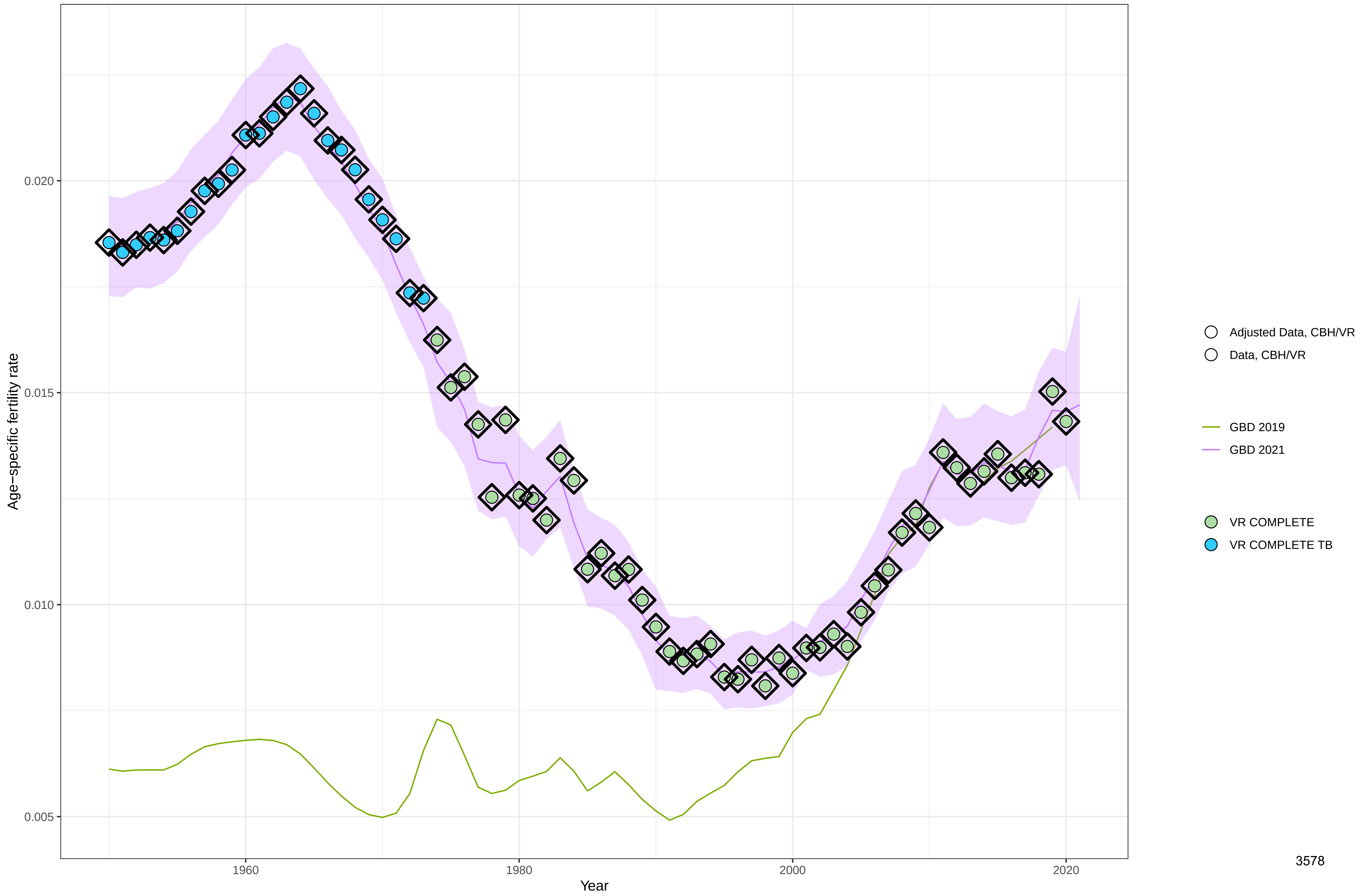

Age−specific fertility fitted trend lines, 1950−2021: Northern Ireland, Ages: 45−49
Data points enclosed with a diamond are reference sources

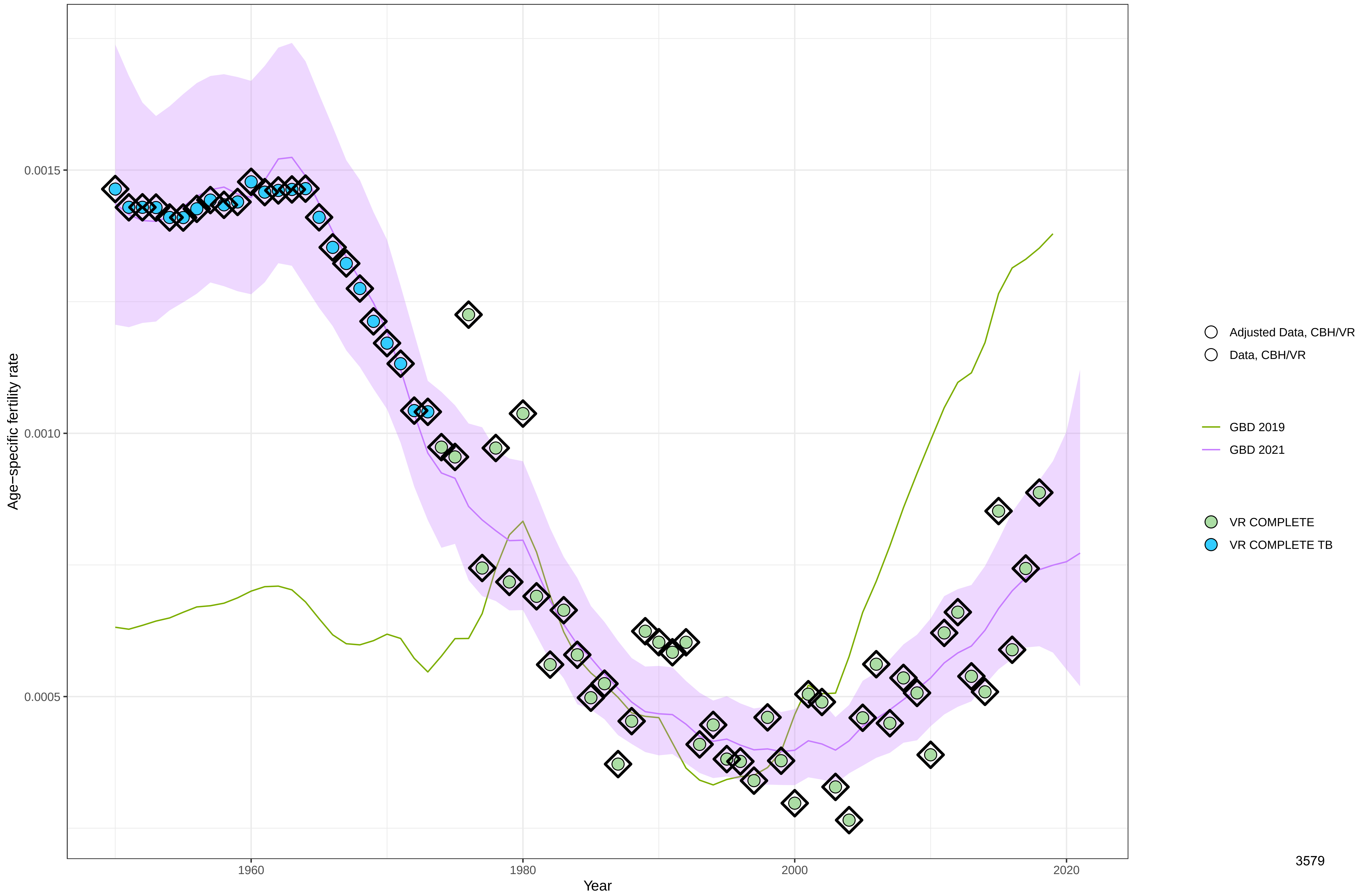

Age−specific fertility fitted trend lines, 1950−2021: Northern Ireland, Ages: 50−54
Data points enclosed with a diamond are reference sources

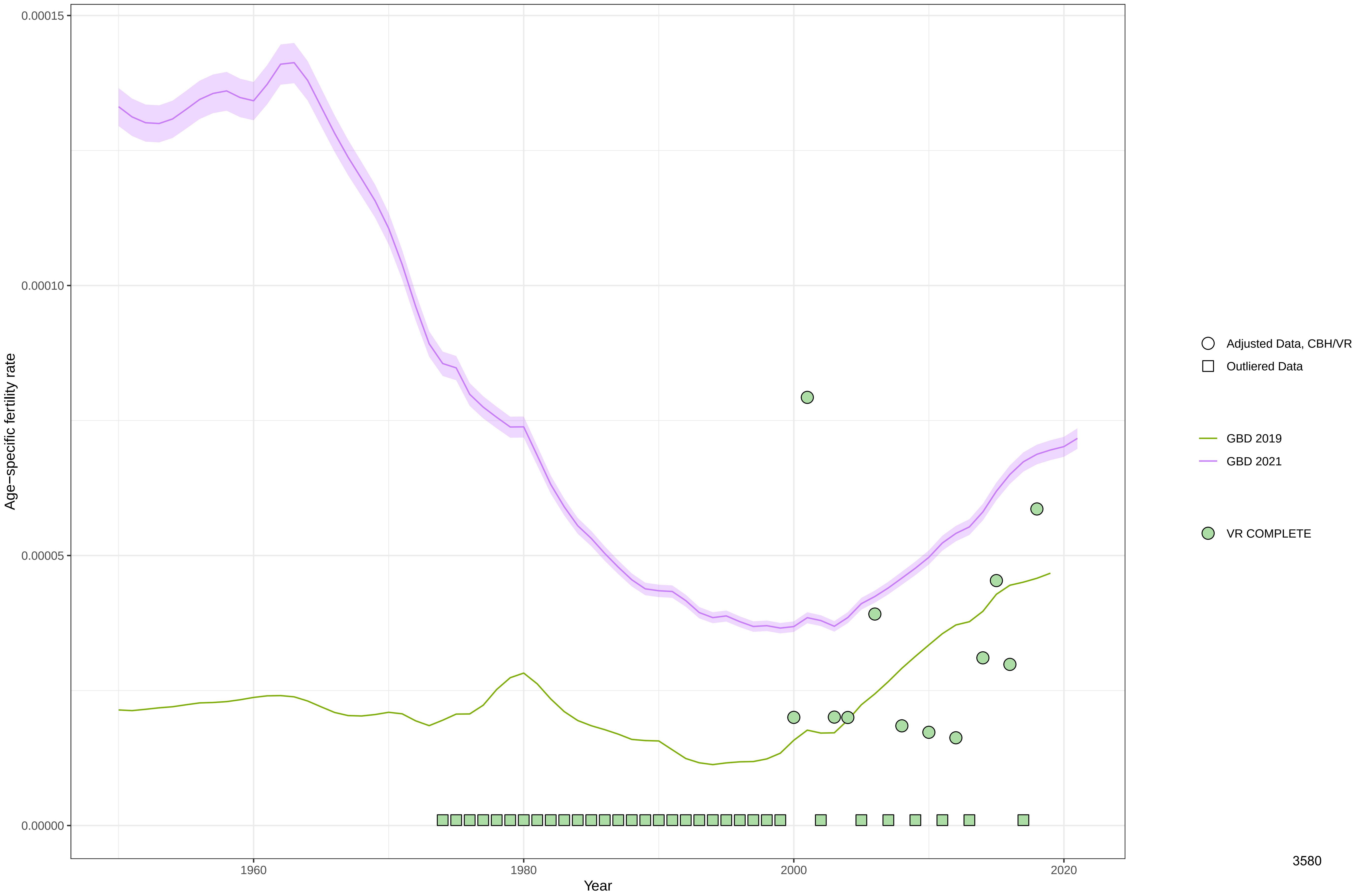

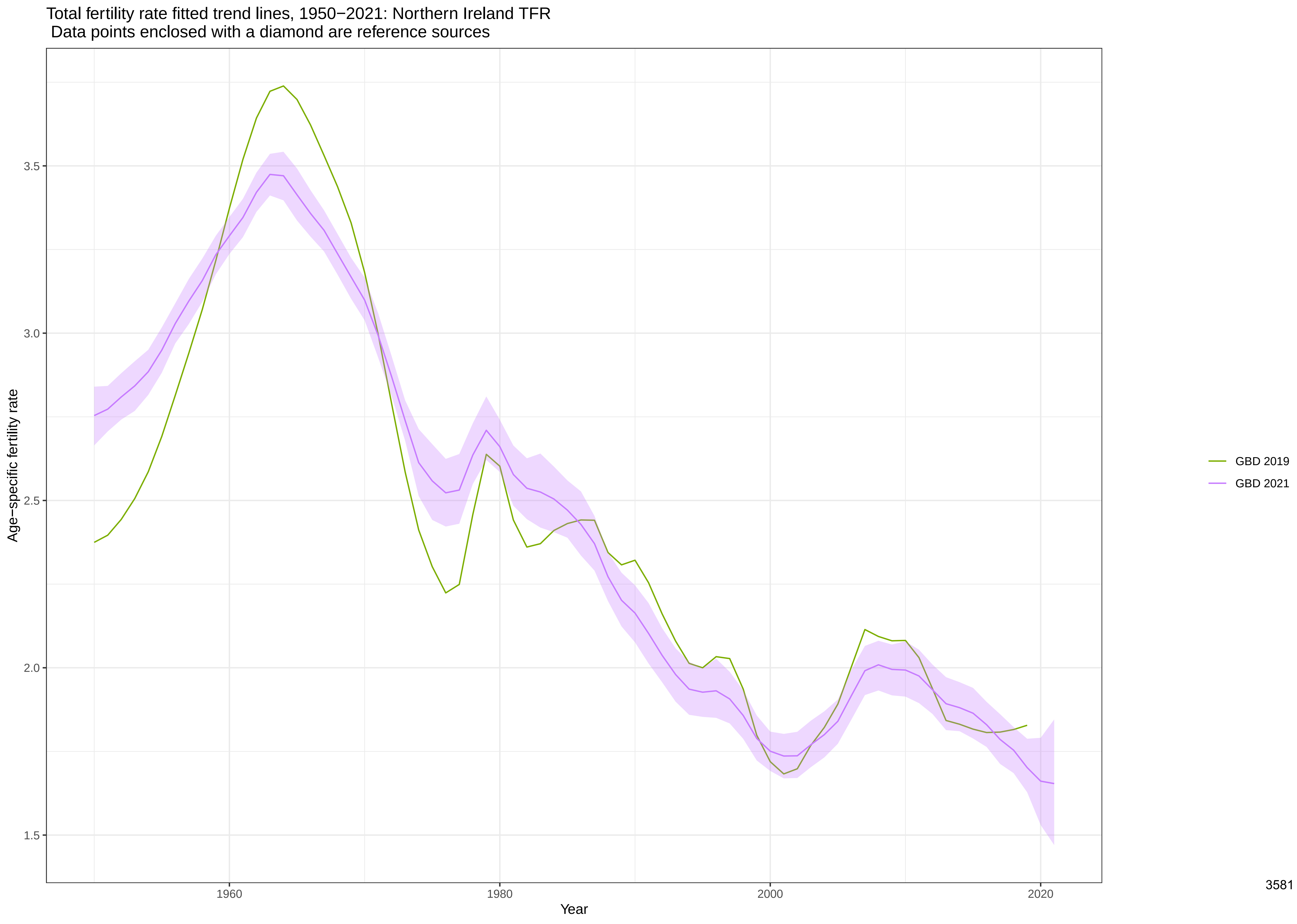
Total fertility rate fitted trend lines, 1950−2021: Northern Ireland TFR
Data points enclosed with a diamond are reference sources
Age−specific fertility rate
3.5
3.0
2.5
2.0
1.5
1960
1980
2000
2020
Year
GBD 2019
GBD 2021

Age−specific fertility fitted trend lines, 1950−2021: Scotland, Ages: 10−14
Data points enclosed with a diamond are reference sources

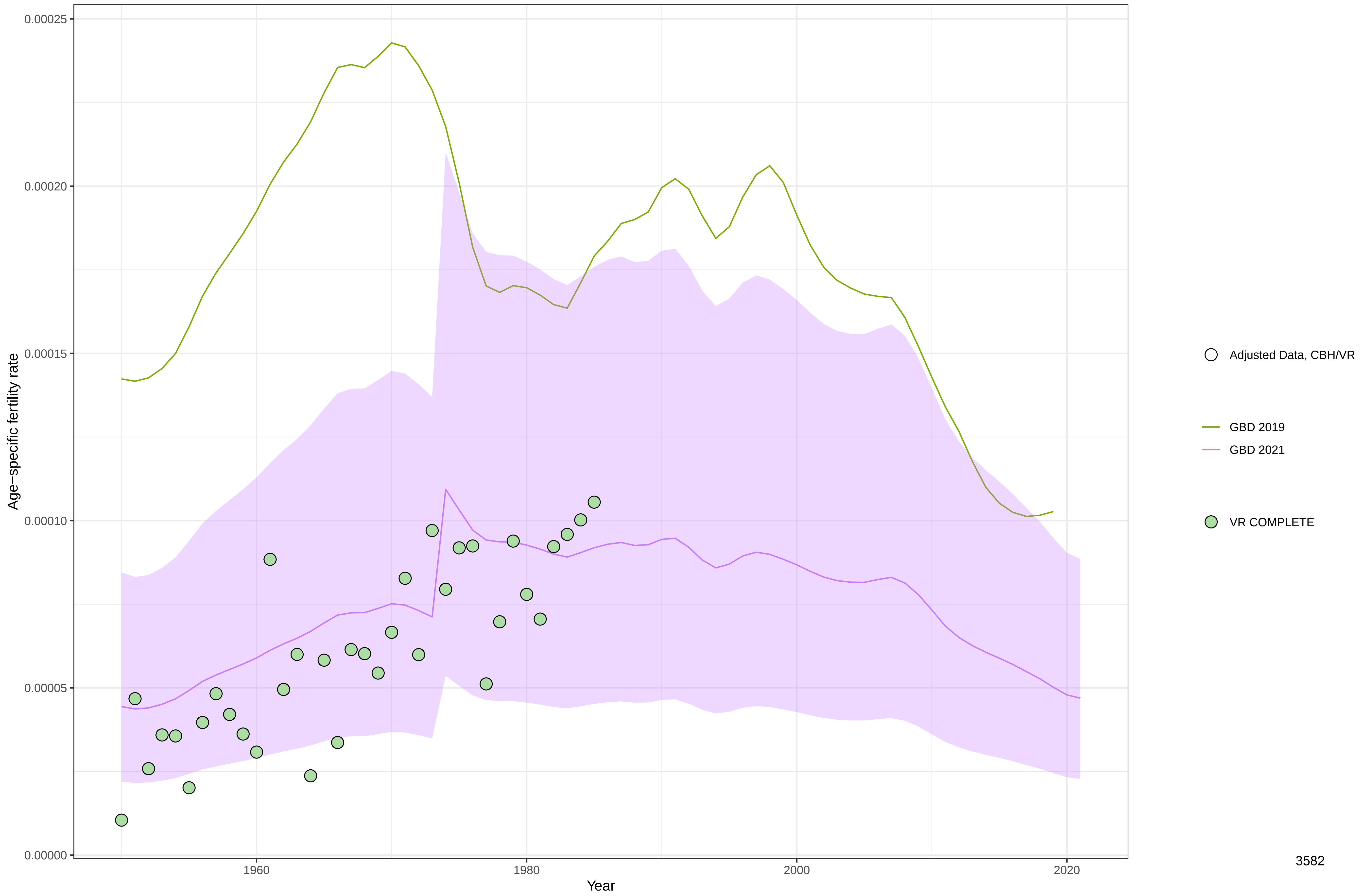

Age−specific fertility fitted trend lines, 1950−2021: Scotland, Ages: 15−19
Data points enclosed with a diamond are reference sources

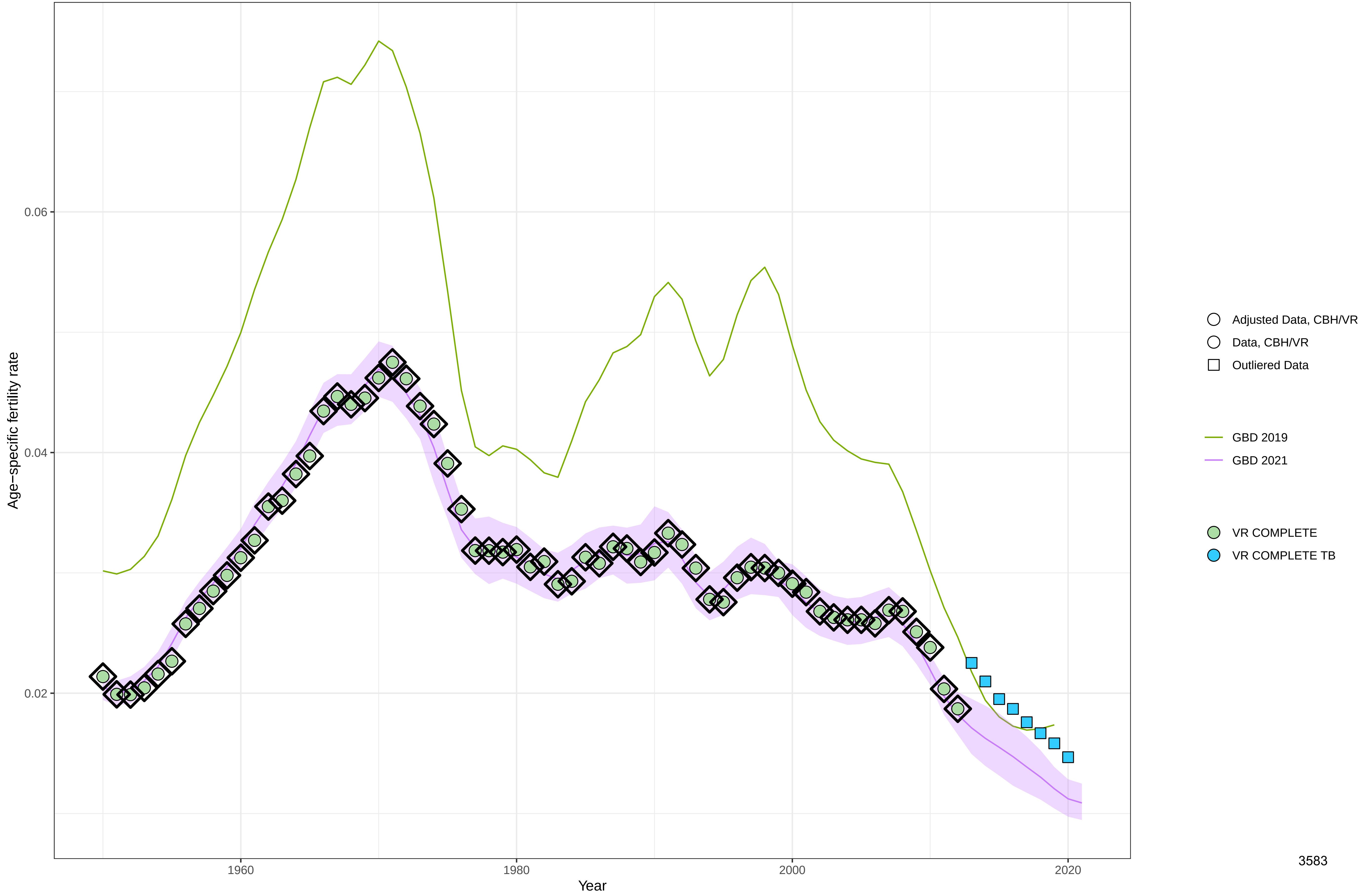

Age−specific fertility fitted trend lines, 1950−2021: Scotland, Ages: 20−24
Data points enclosed with a diamond are reference sources

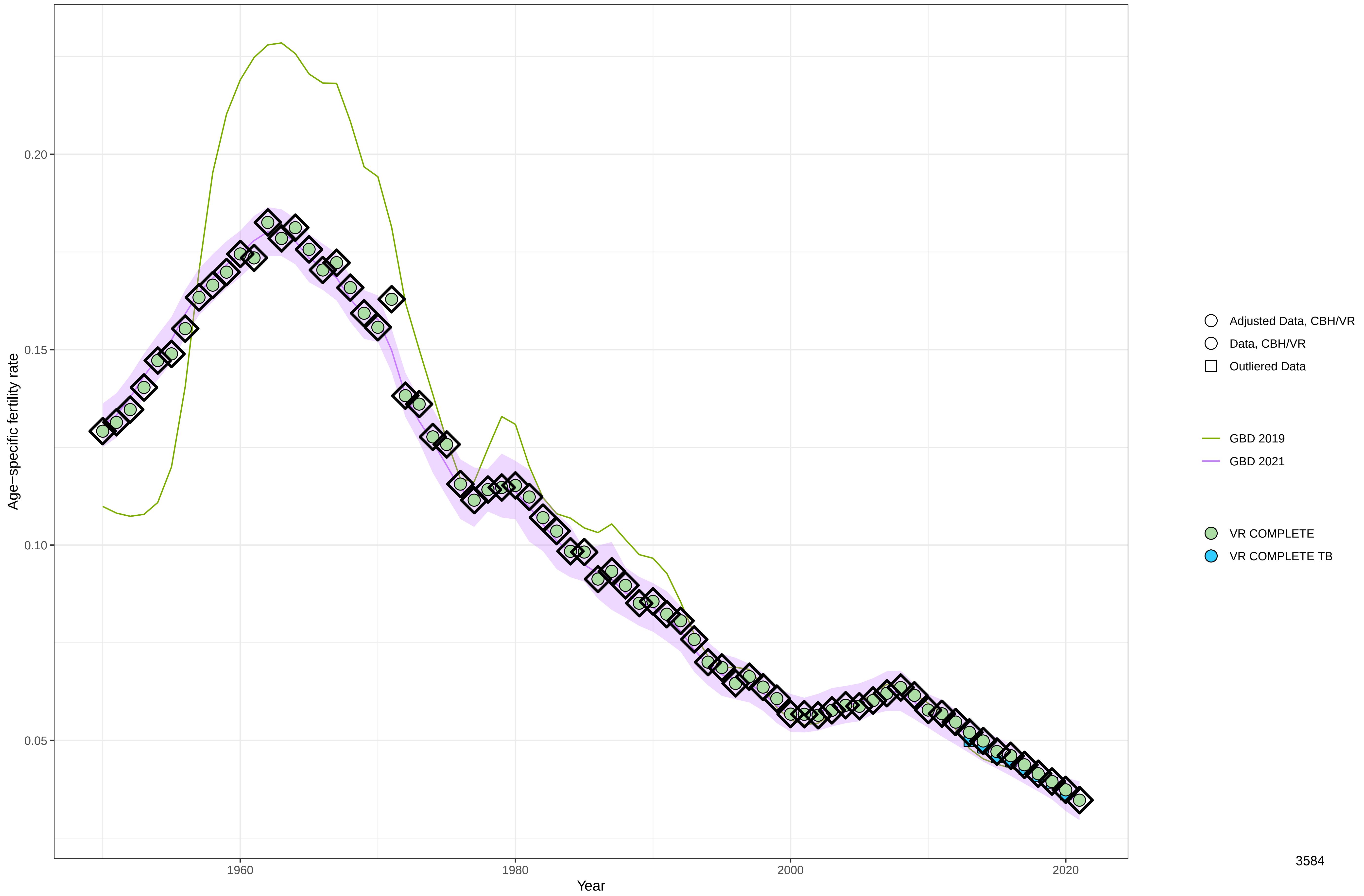

Age−specific fertility fitted trend lines, 1950−2021: Scotland, Ages: 25−29
Data points enclosed with a diamond are reference sources

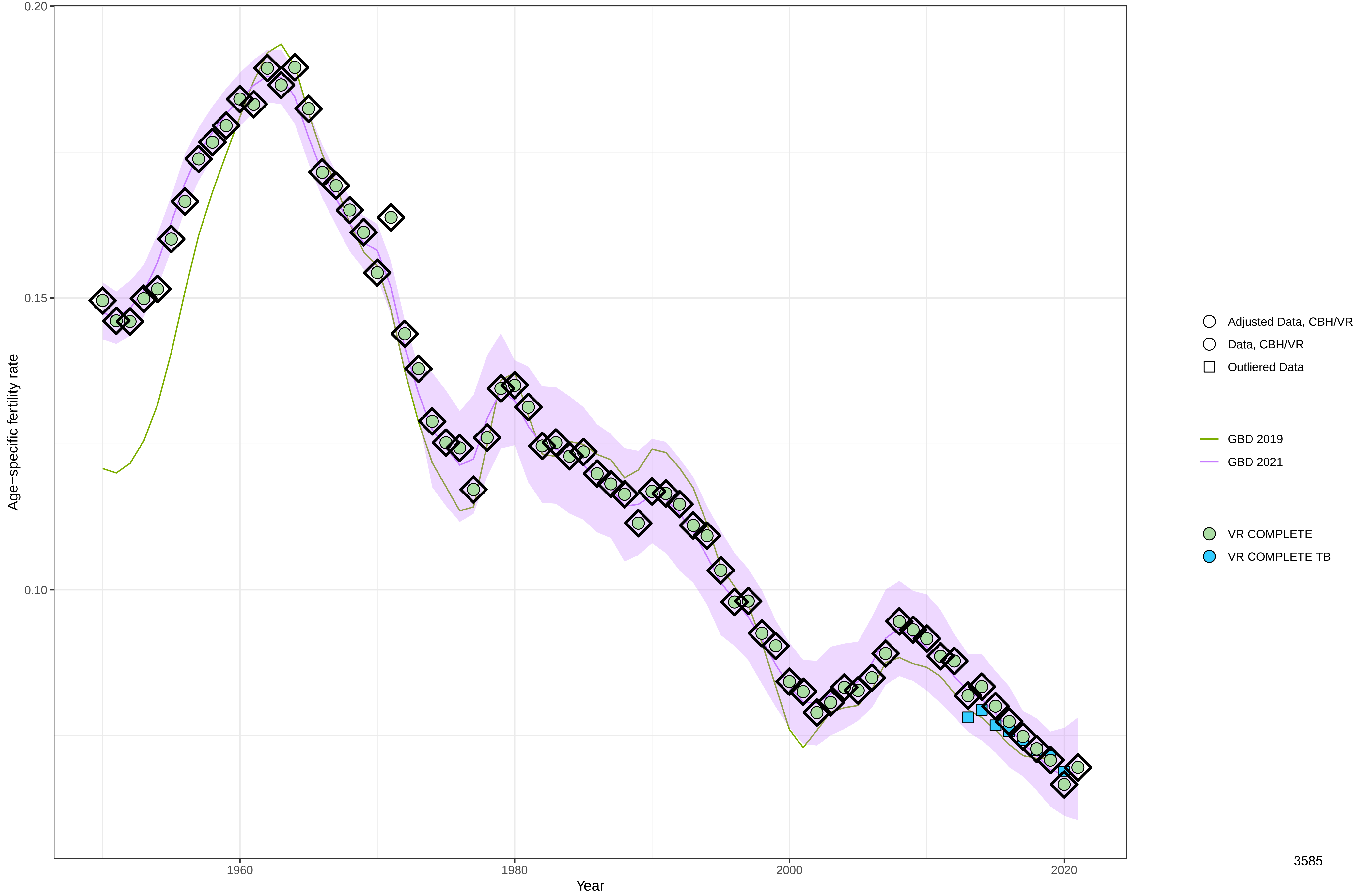

Age−specific fertility fitted trend lines, 1950−2021: Scotland, Ages: 30−34
Data points enclosed with a diamond are reference sources

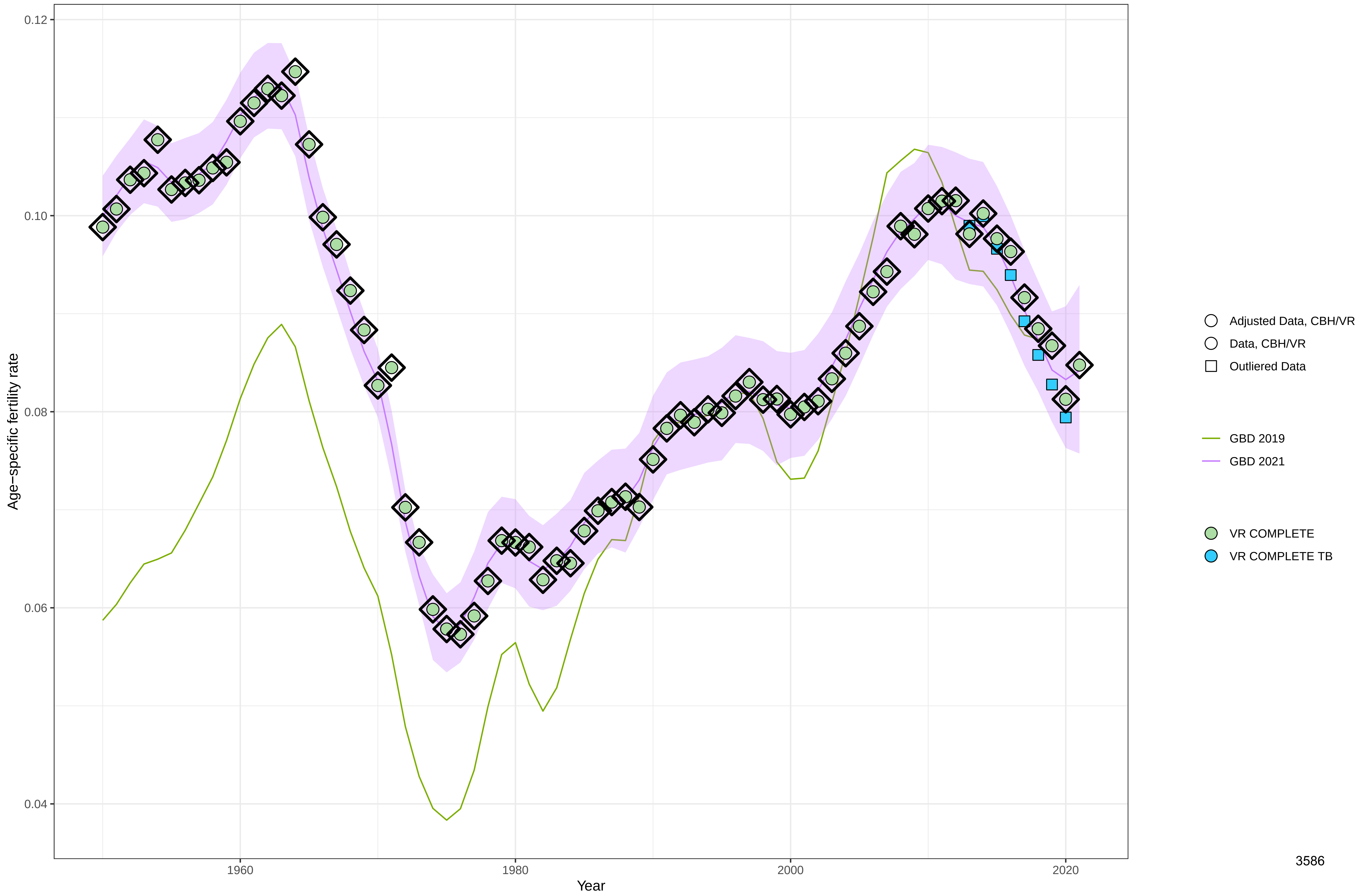

Age−specific fertility fitted trend lines, 1950−2021: Scotland, Ages: 35−39
Data points enclosed with a diamond are reference sources

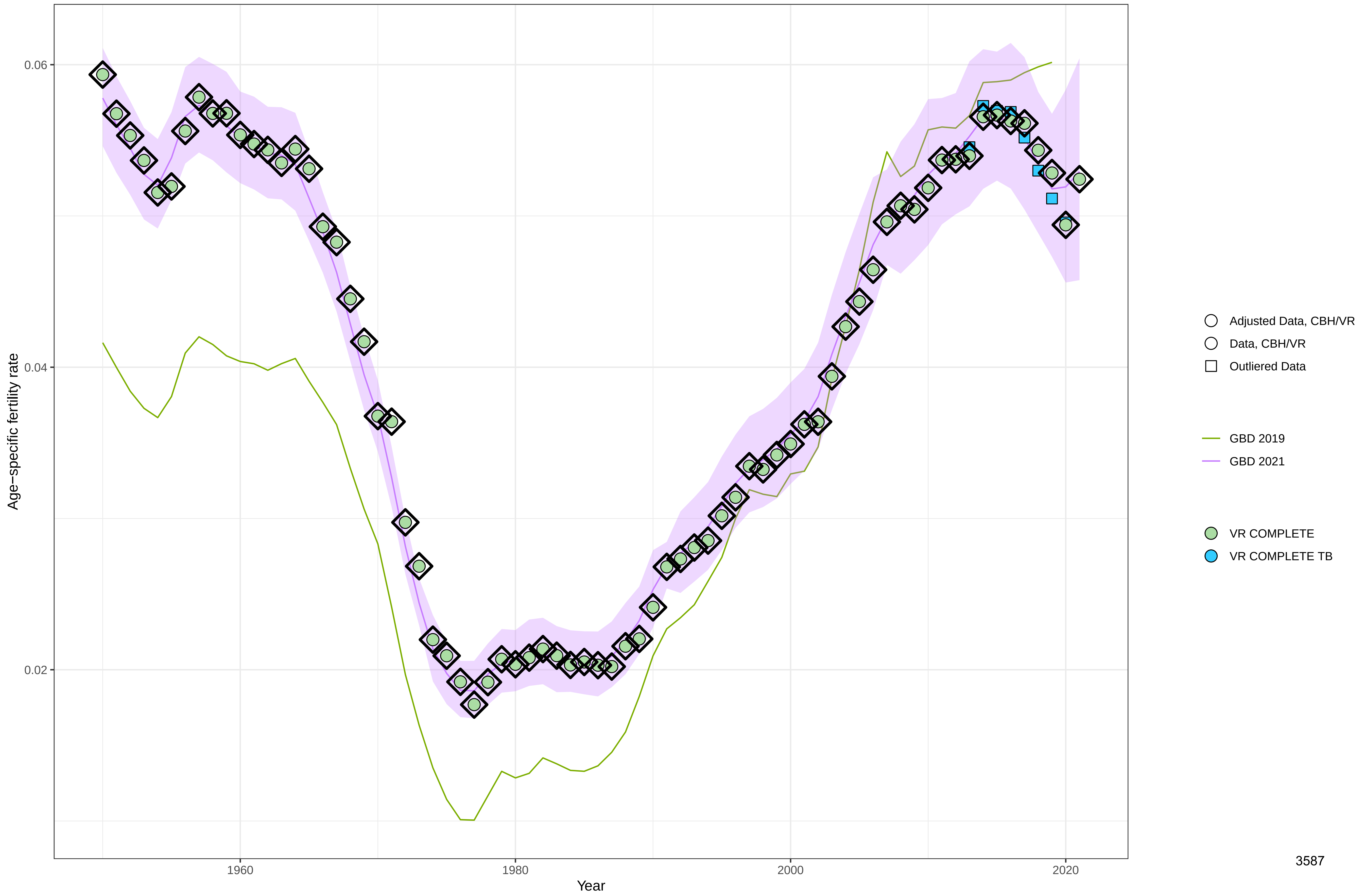

Age−specific fertility fitted trend lines, 1950−2021: Scotland, Ages: 40−44
Data points enclosed with a diamond are reference sources

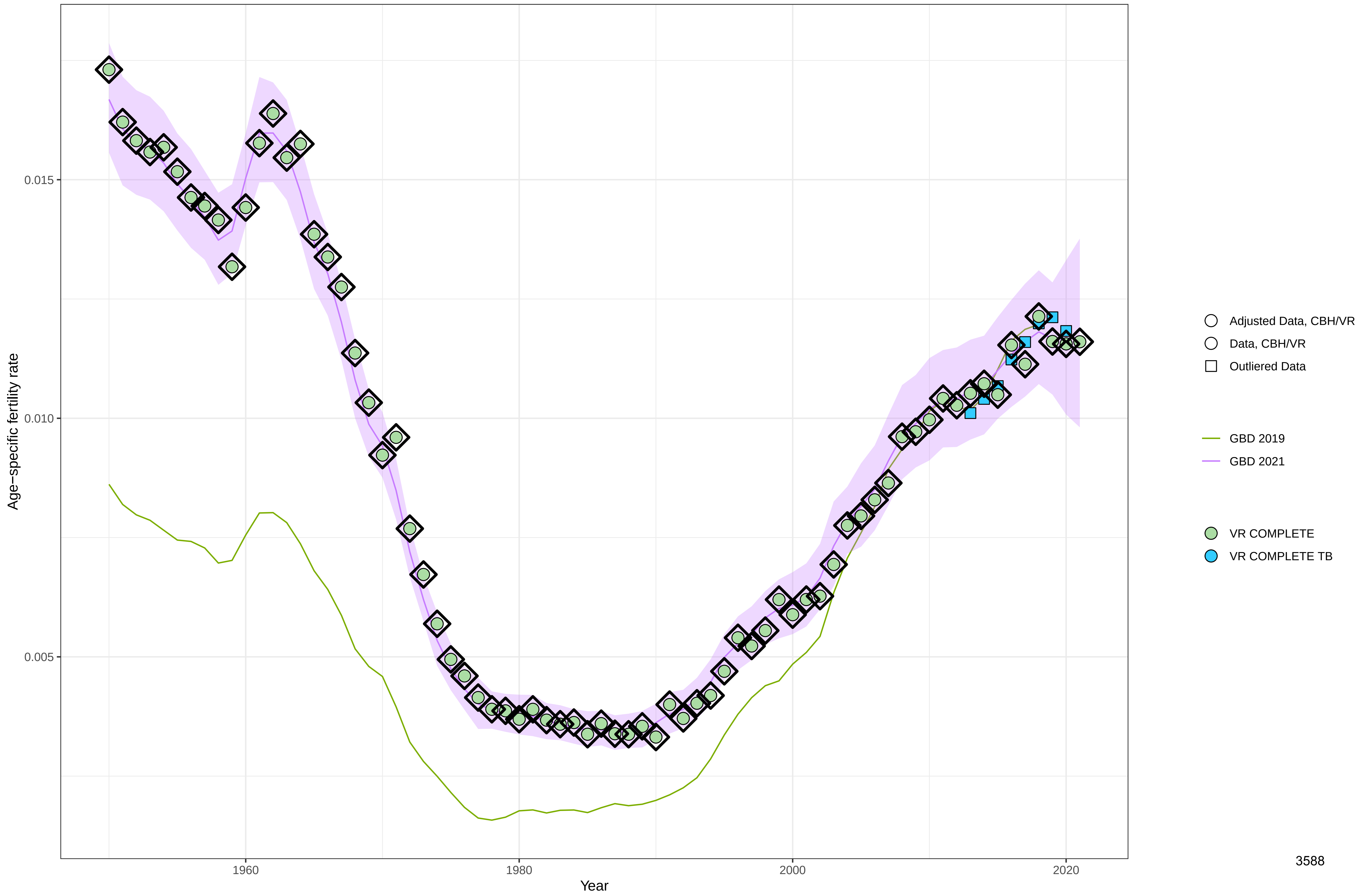

Age−specific fertility fitted trend lines, 1950−2021: Scotland, Ages: 45−49
Data points enclosed with a diamond are reference sources

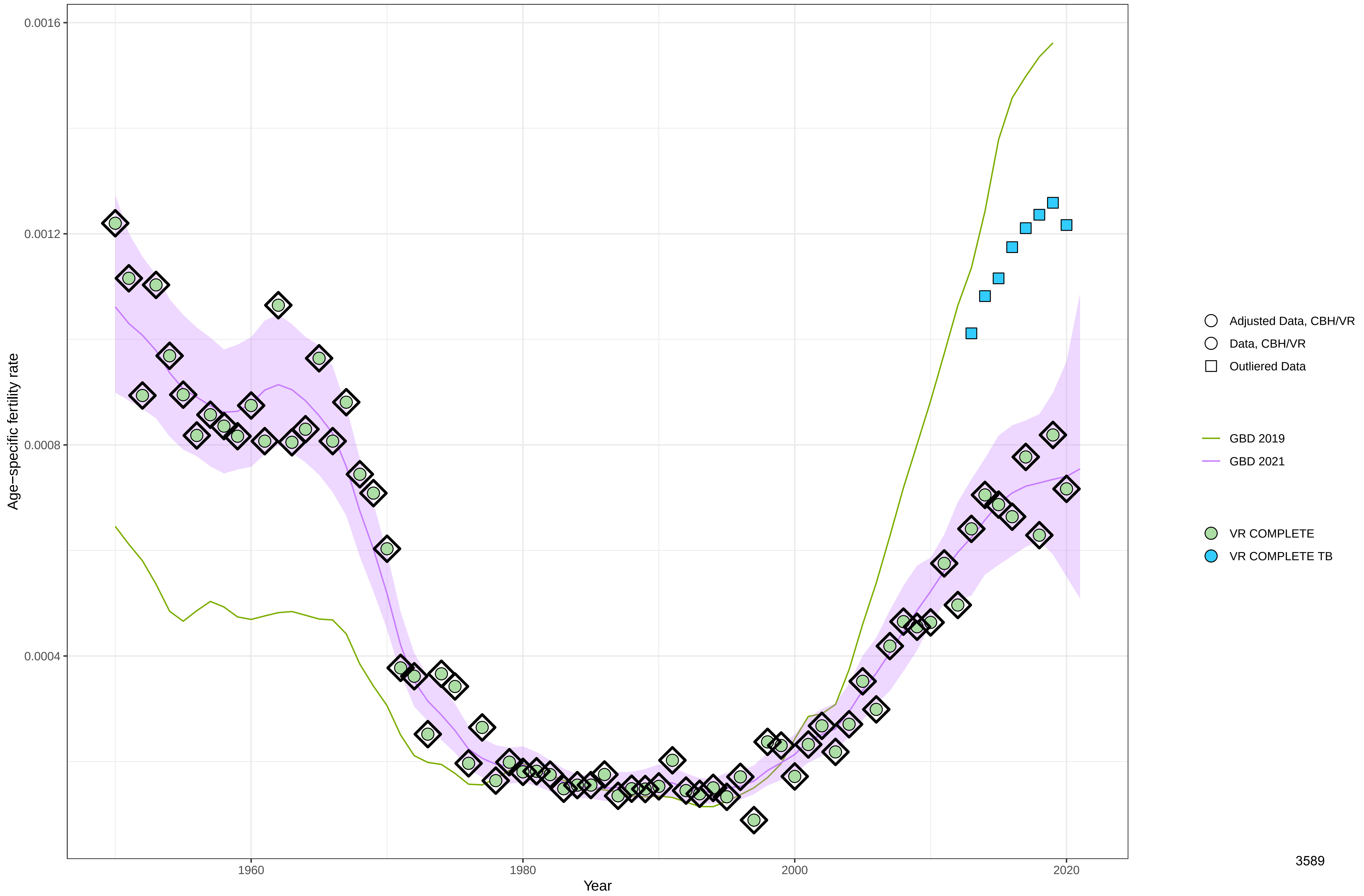

Age−specific fertility fitted trend lines, 1950−2021: Scotland, Ages: 50−54
Data points enclosed with a diamond are reference sources

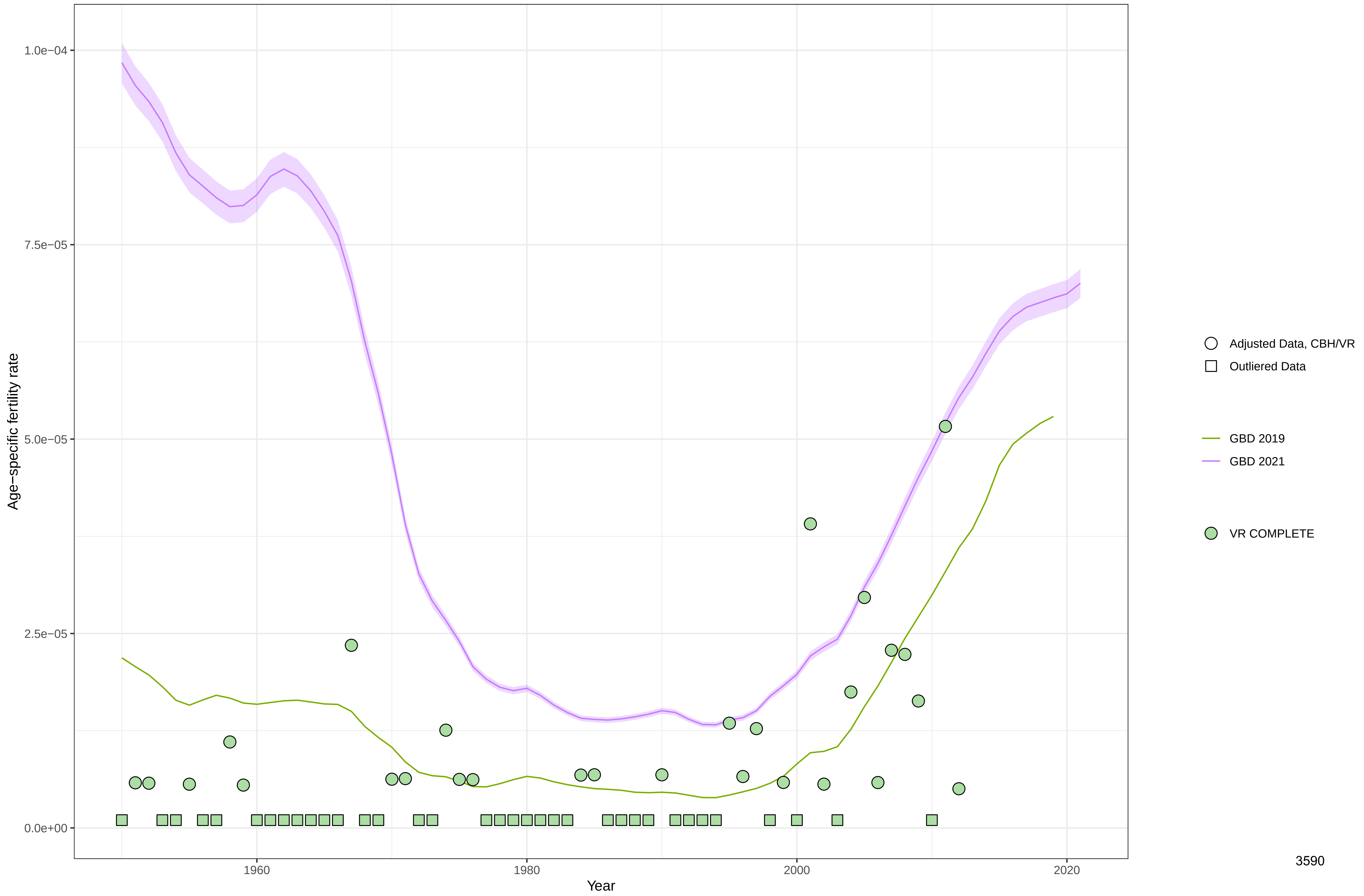

Total fertility rate fitted trend lines, 1950−2021: Scotland TFR
Data points enclosed with a diamond are reference sources

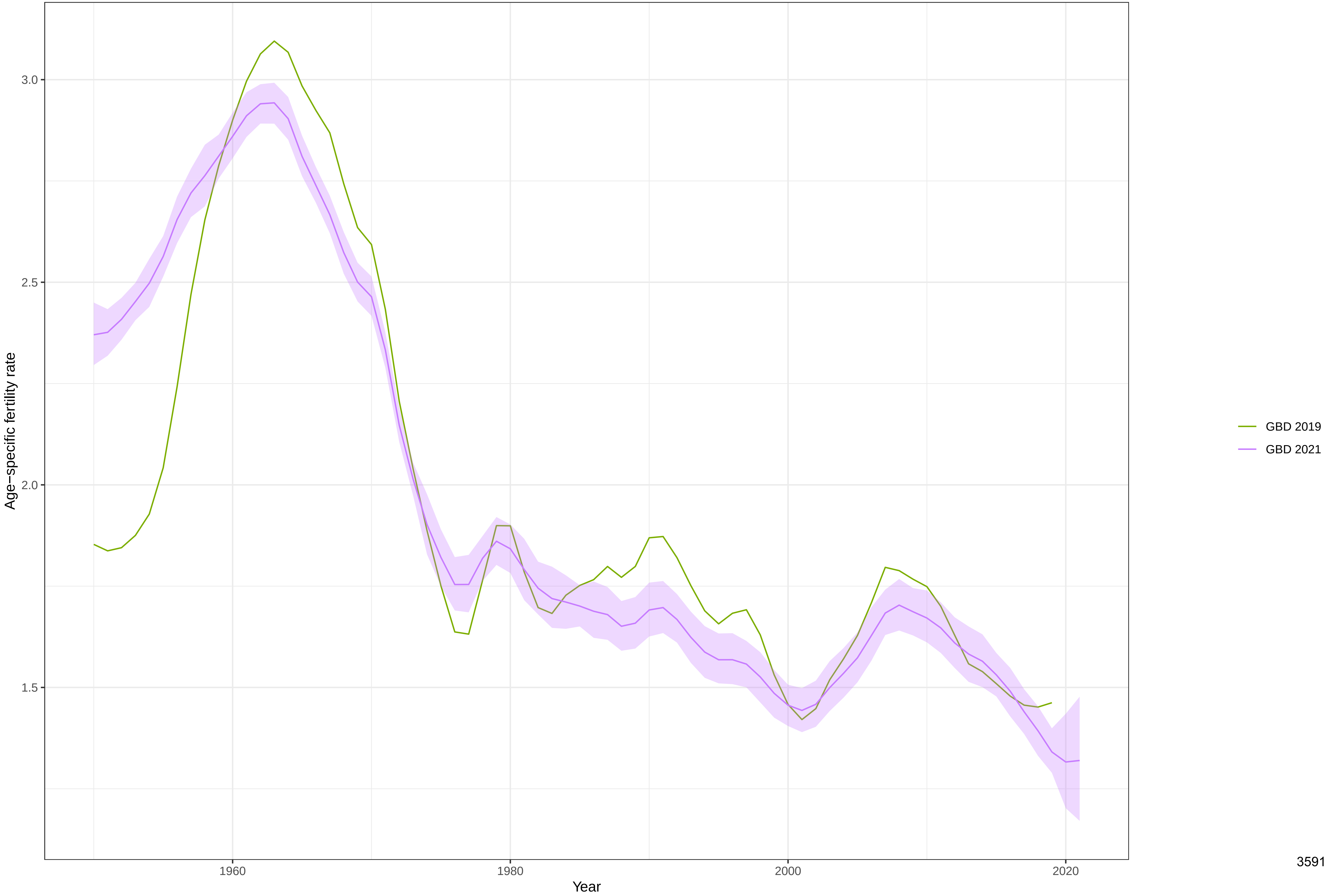

Age−specific fertility fitted trend lines, 1950−2021: Wales, Ages: 10−14
 Data points enclosed with a diamond are reference sources

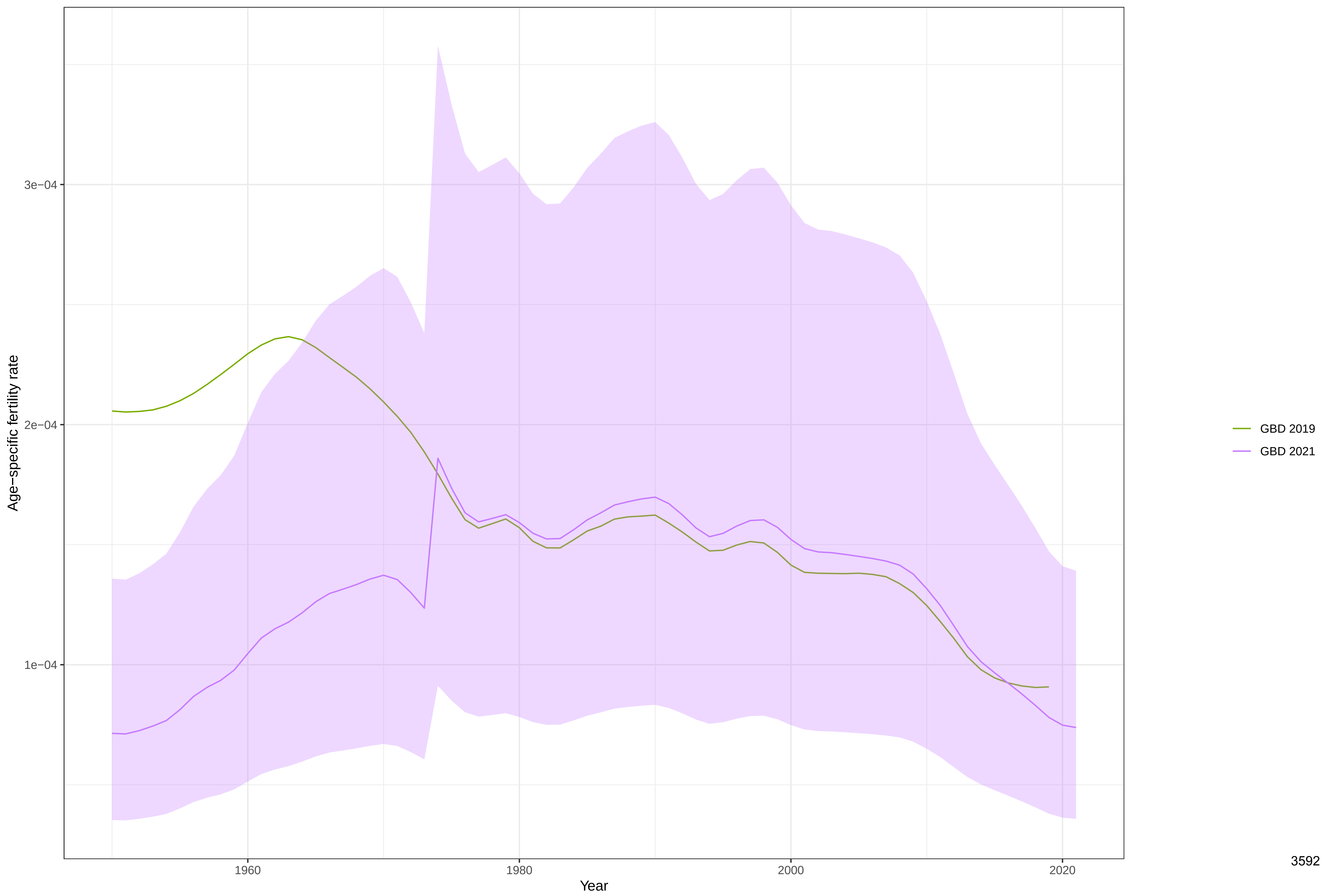

3592

Age−specific fertility fitted trend lines, 1950−2021: Wales, Ages: 15−19
Data points enclosed with a diamond are reference sources

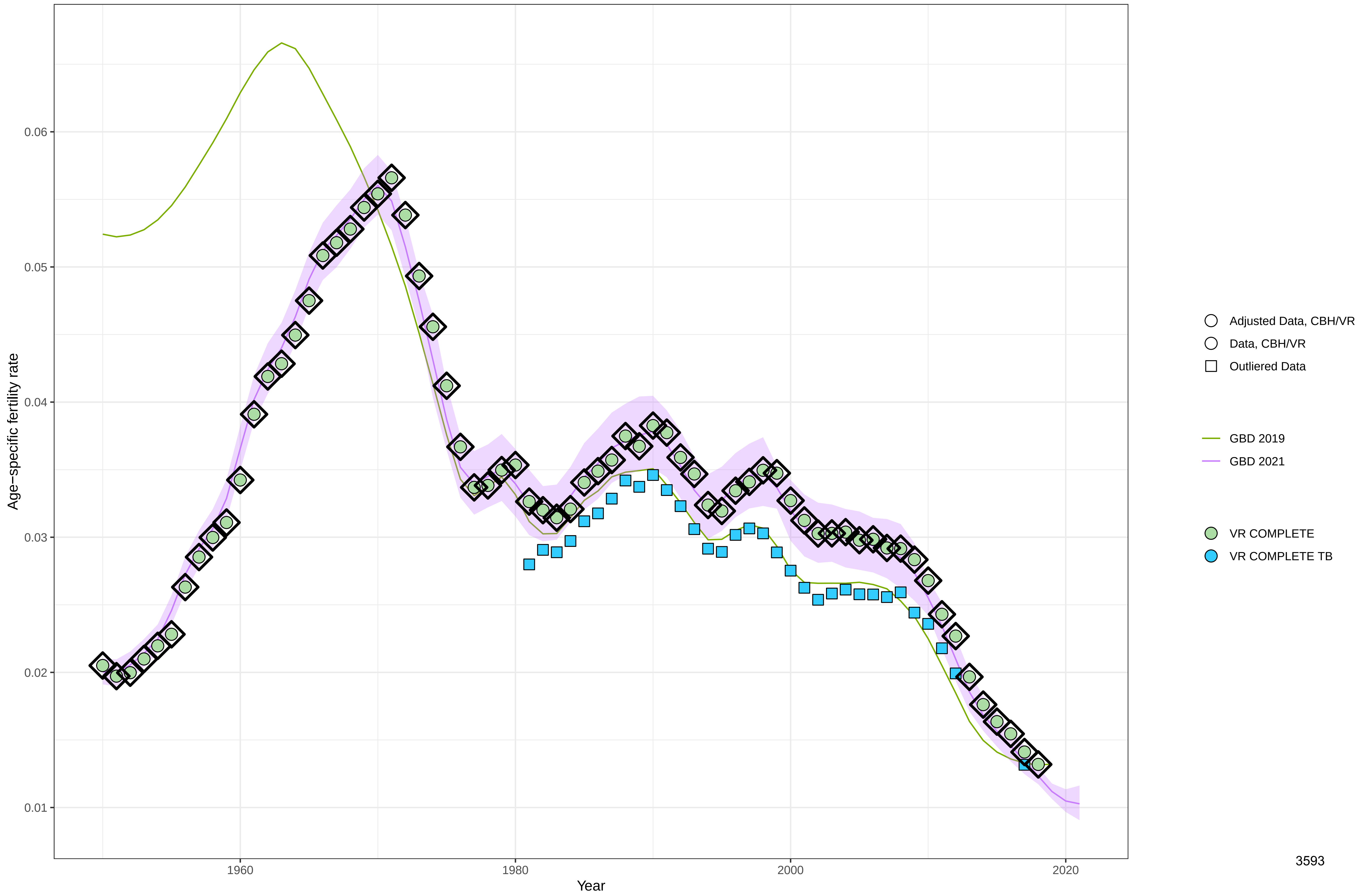

Age−specific fertility fitted trend lines, 1950−2021: Wales, Ages: 20−24
Data points enclosed with a diamond are reference sources

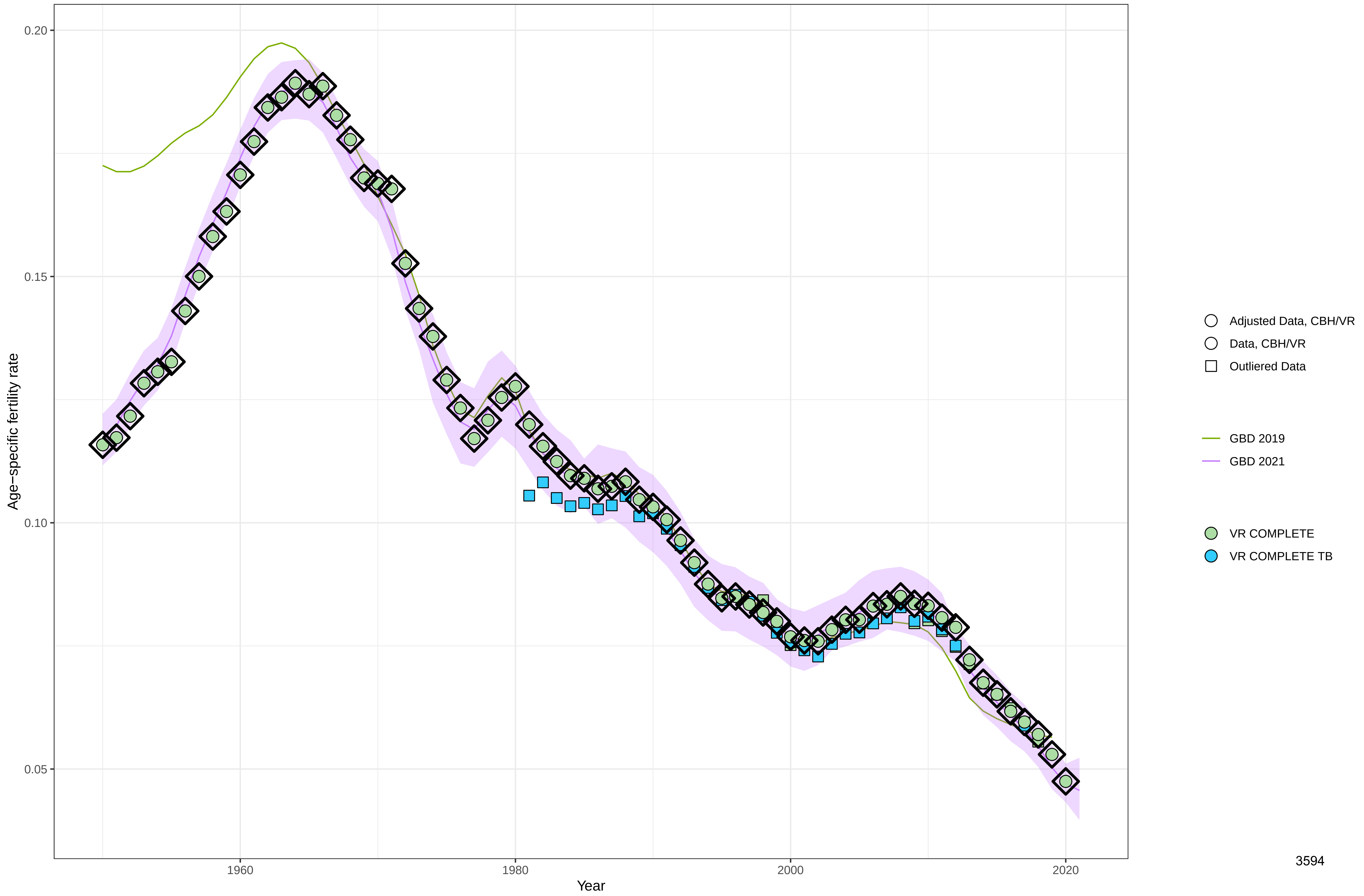

Age−specific fertility fitted trend lines, 1950−2021: Wales, Ages: 25−29
Data points enclosed with a diamond are reference sources

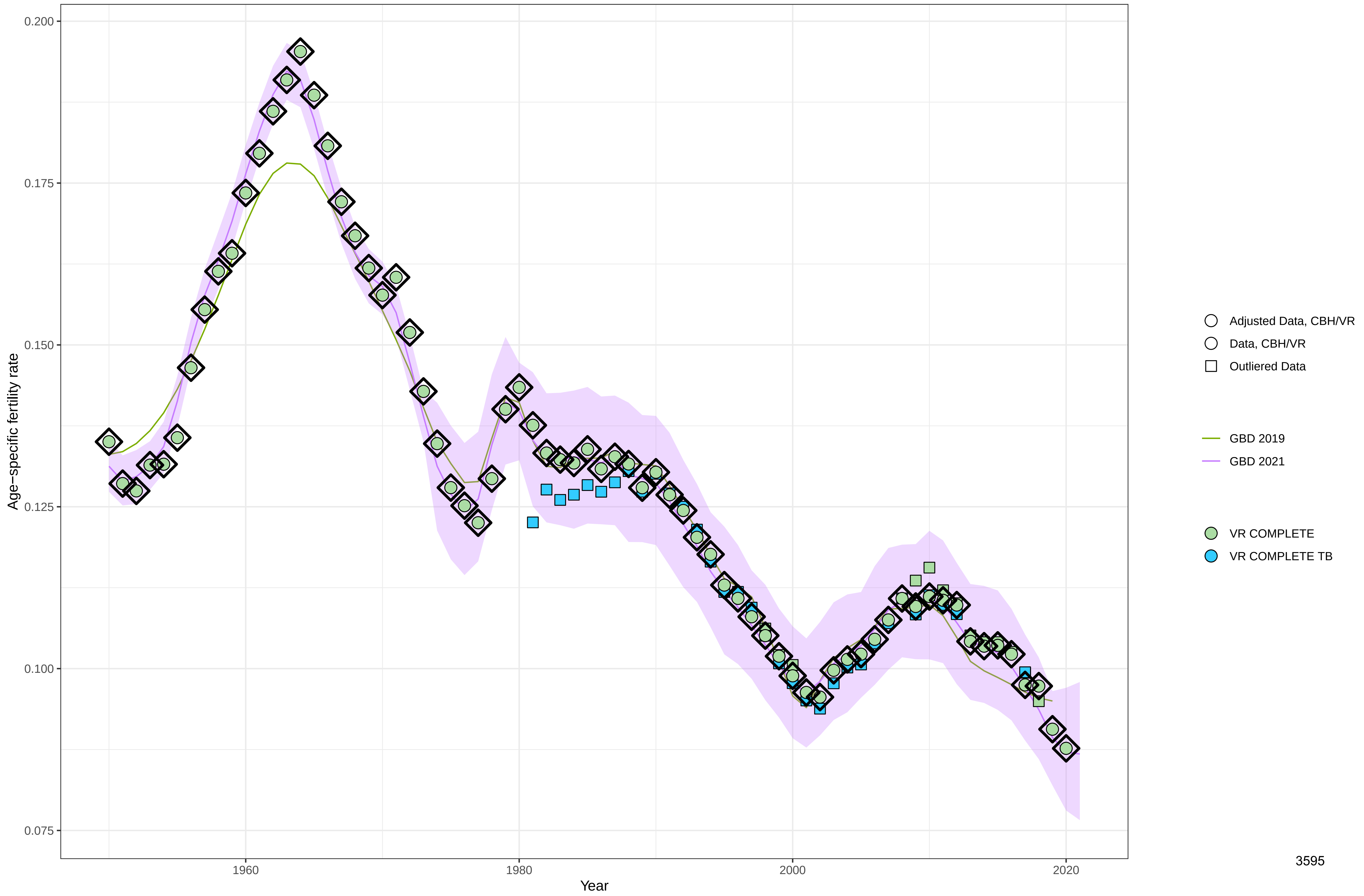

Age−specific fertility fitted trend lines, 1950−2021: Wales, Ages: 30−34
Data points enclosed with a diamond are reference sources

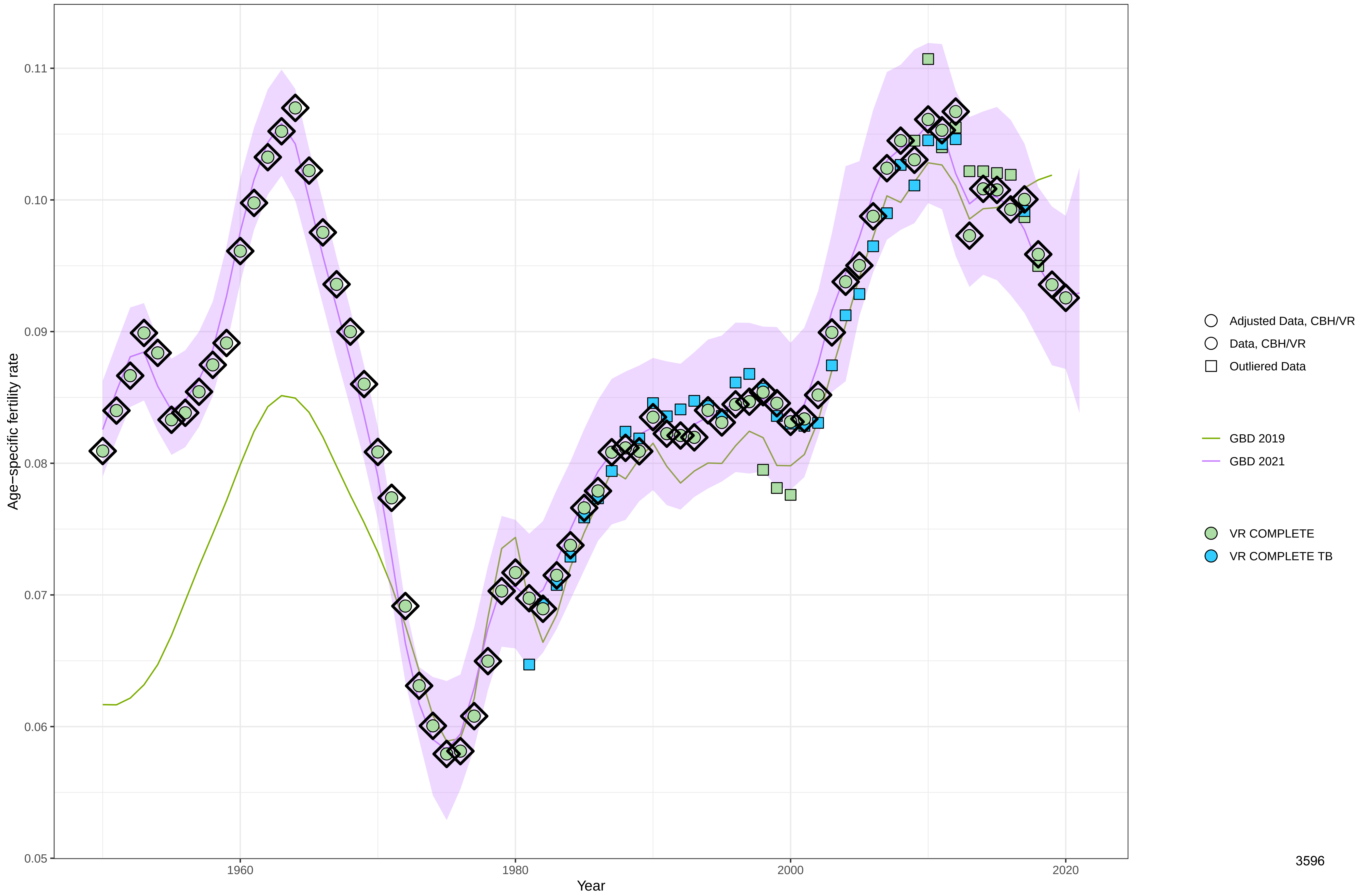

Age−specific fertility fitted trend lines, 1950−2021: Wales, Ages: 35−39
Data points enclosed with a diamond are reference sources

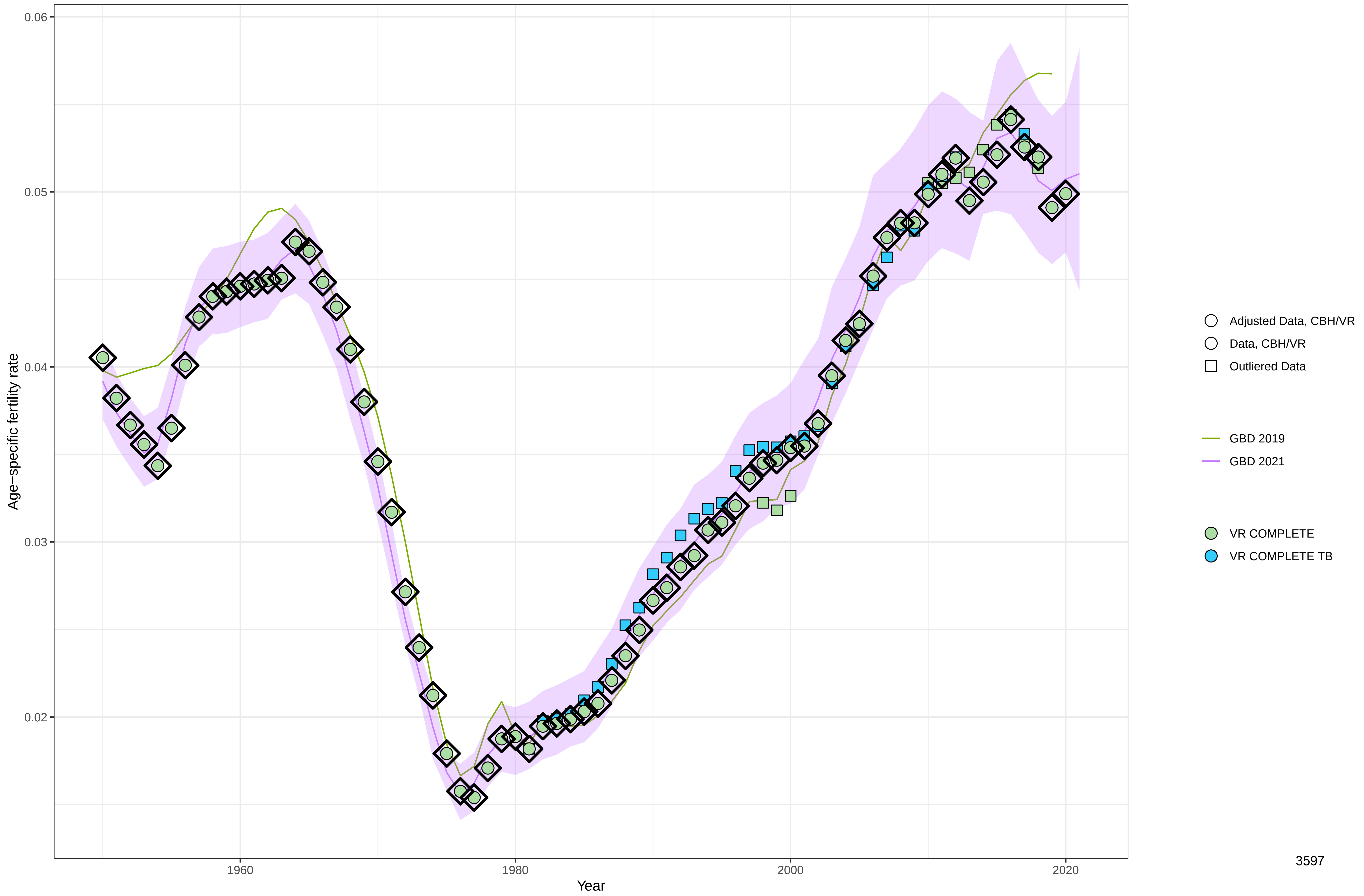

Age−specific fertility fitted trend lines, 1950−2021: Wales, Ages: 40−44
Data points enclosed with a diamond are reference sources

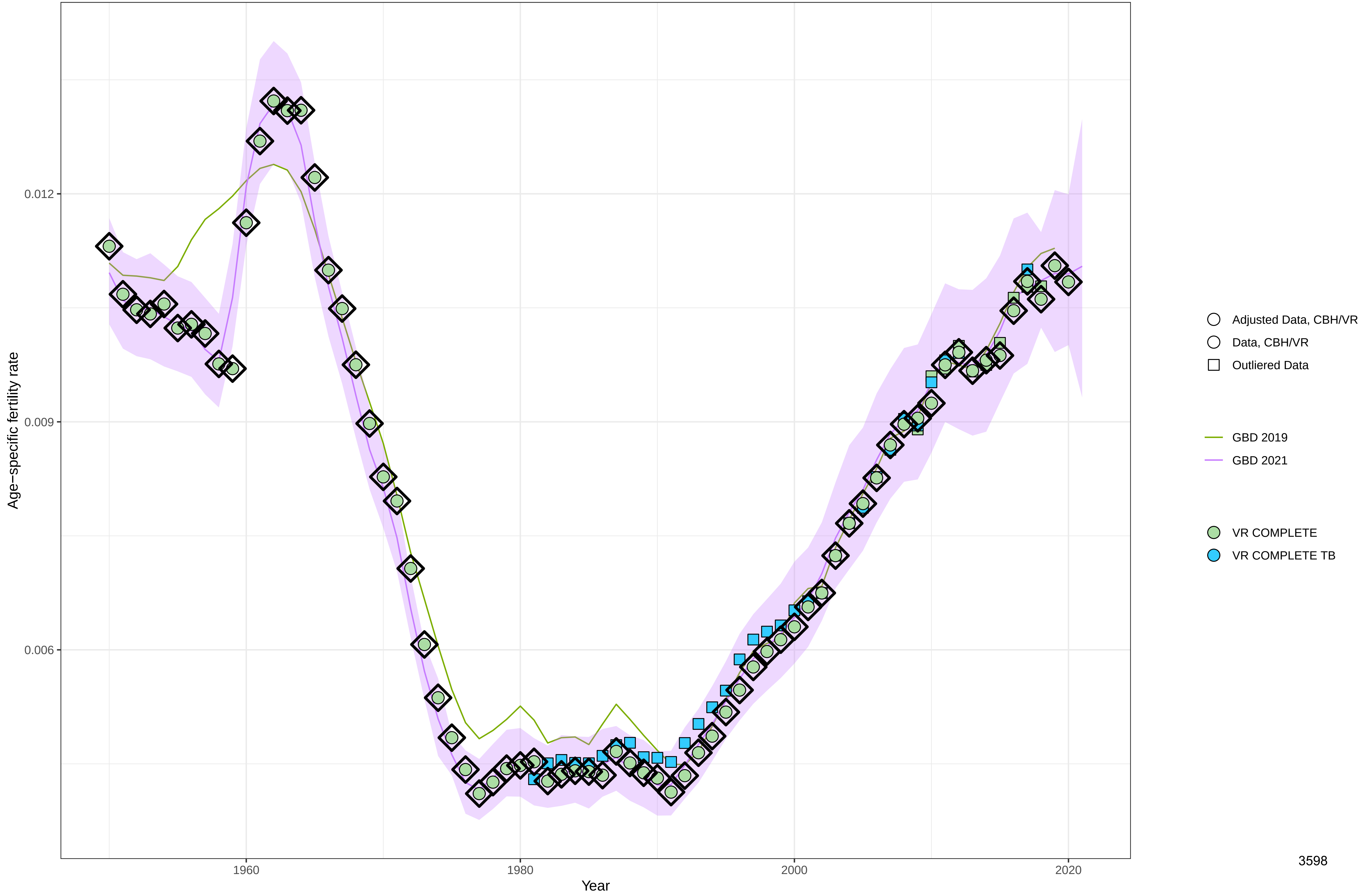

Age−specific fertility fitted trend lines, 1950−2021: Wales, Ages: 45−49
Data points enclosed with a diamond are reference sources

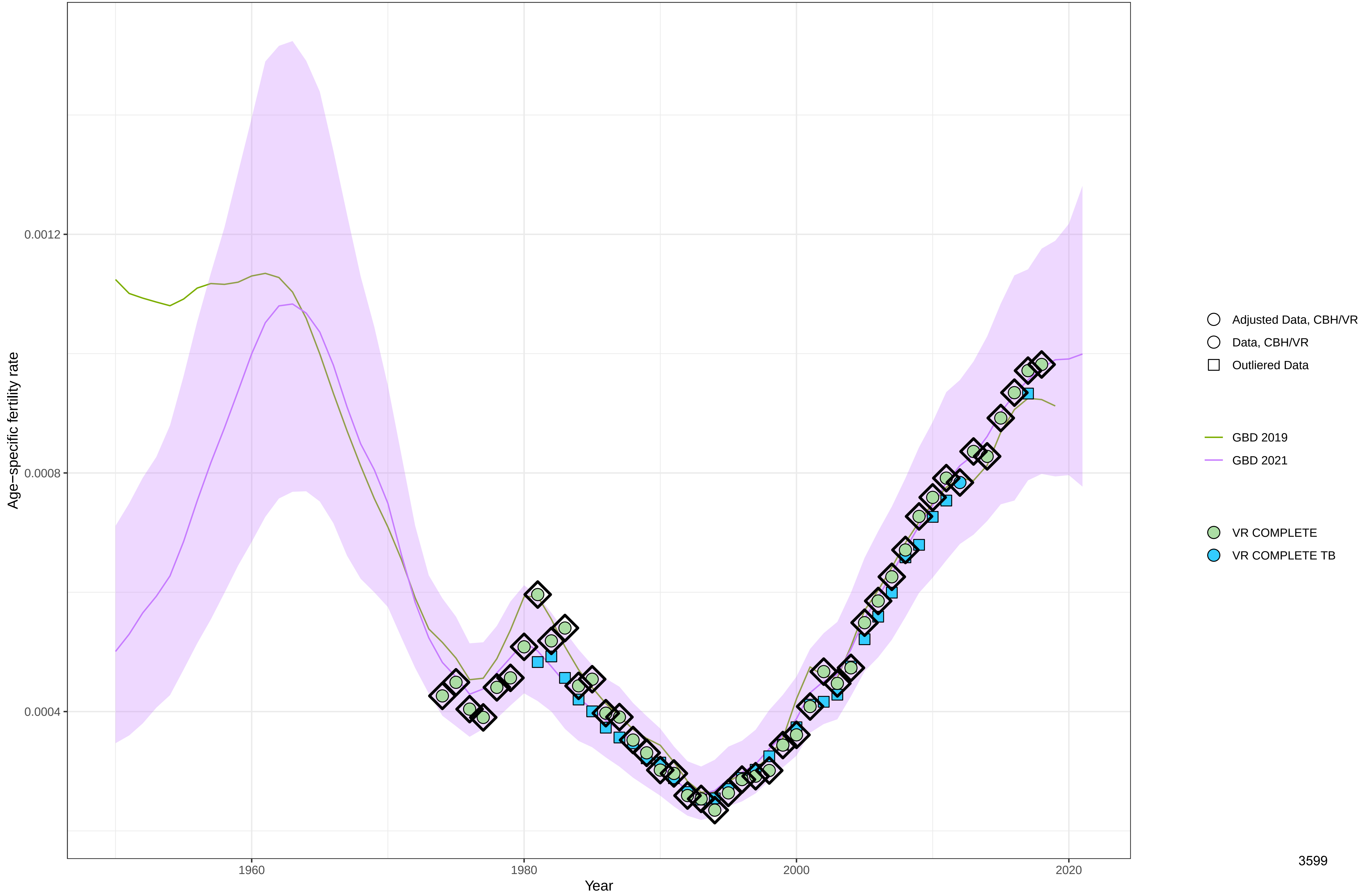

Age−specific fertility fitted trend lines, 1950−2021: Wales, Ages: 50−54
Data points enclosed with a diamond are reference sources

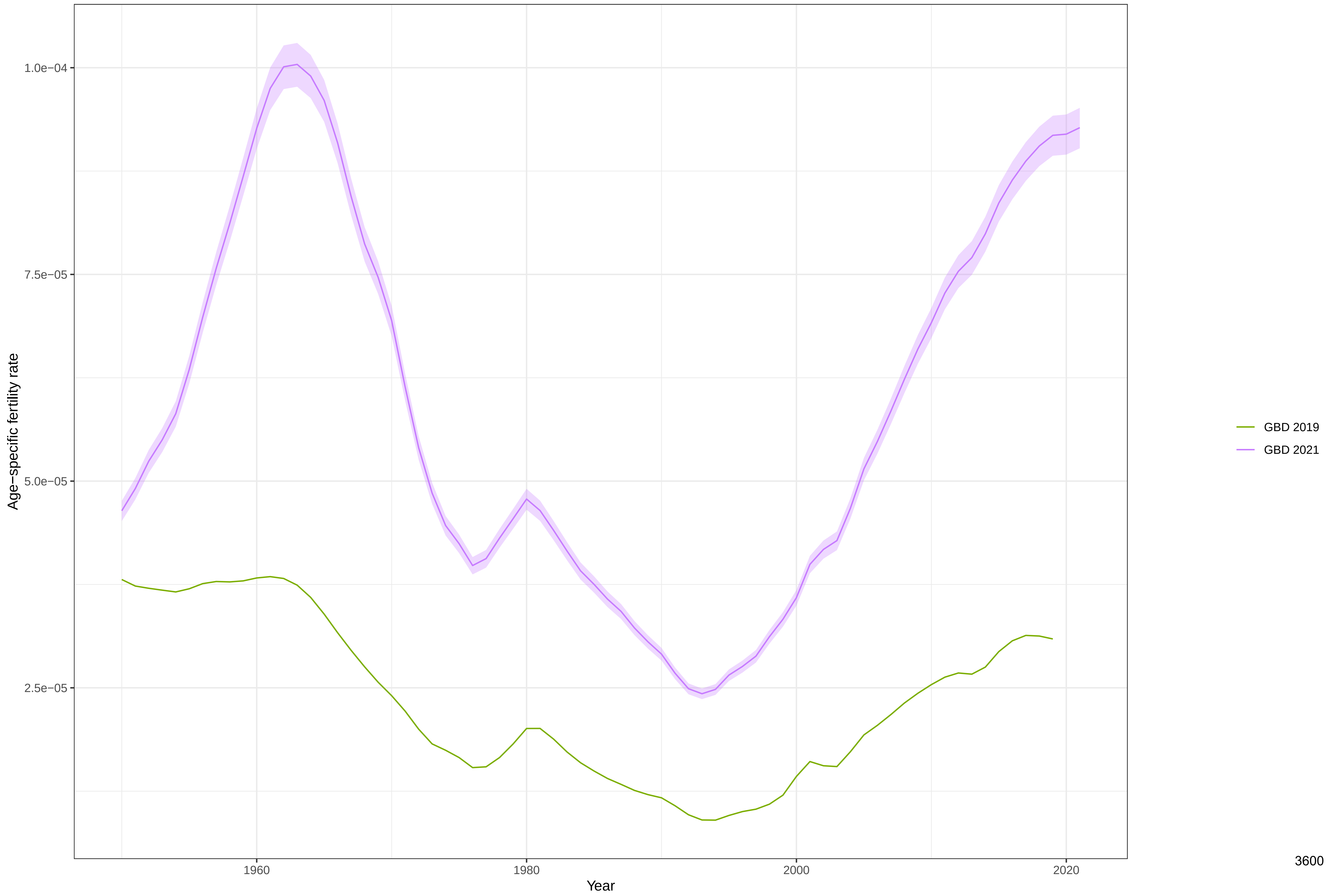

Total fertility rate fitted trend lines, 1950−2021: Wales TFR
Data points enclosed with a diamond are reference sources

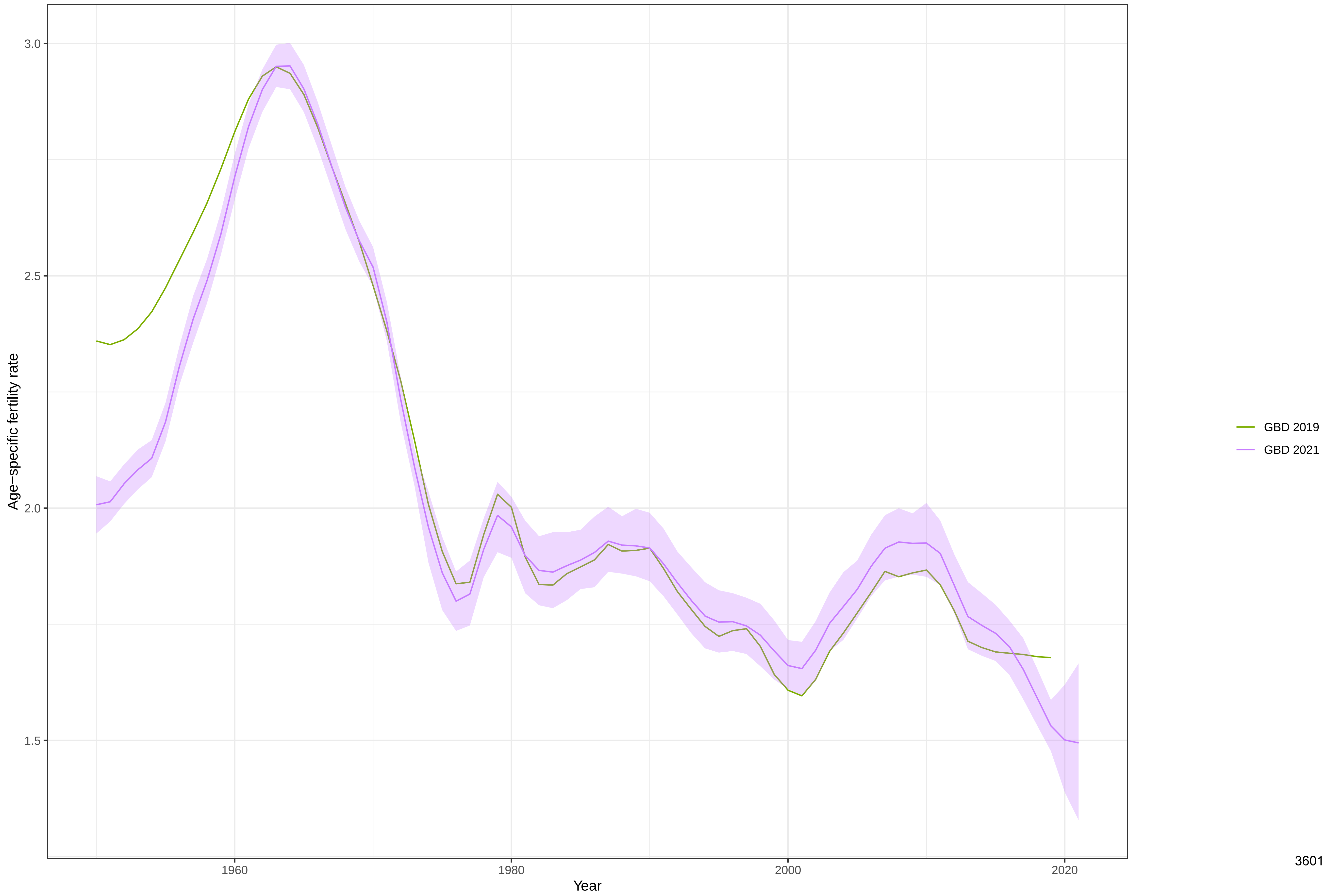

Age−specific fertility fitted trend lines, 1950−2021: Bolivia, Ages: 10−14
Data points enclosed with a diamond are reference sources

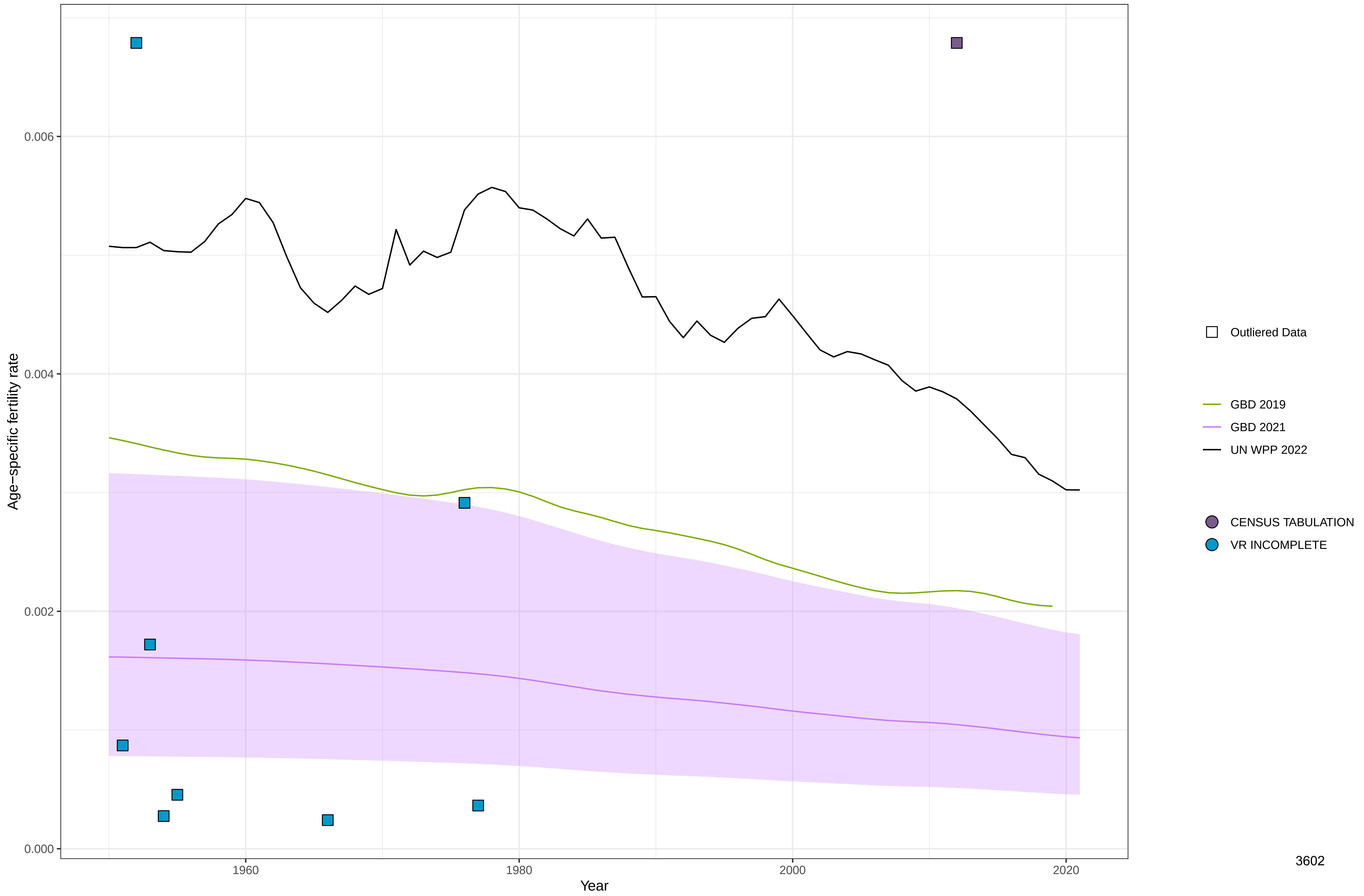

Age−specific fertility fitted trend lines, 1950−2021: Bolivia, Ages: 15−19
Data points enclosed with a diamond are reference sources

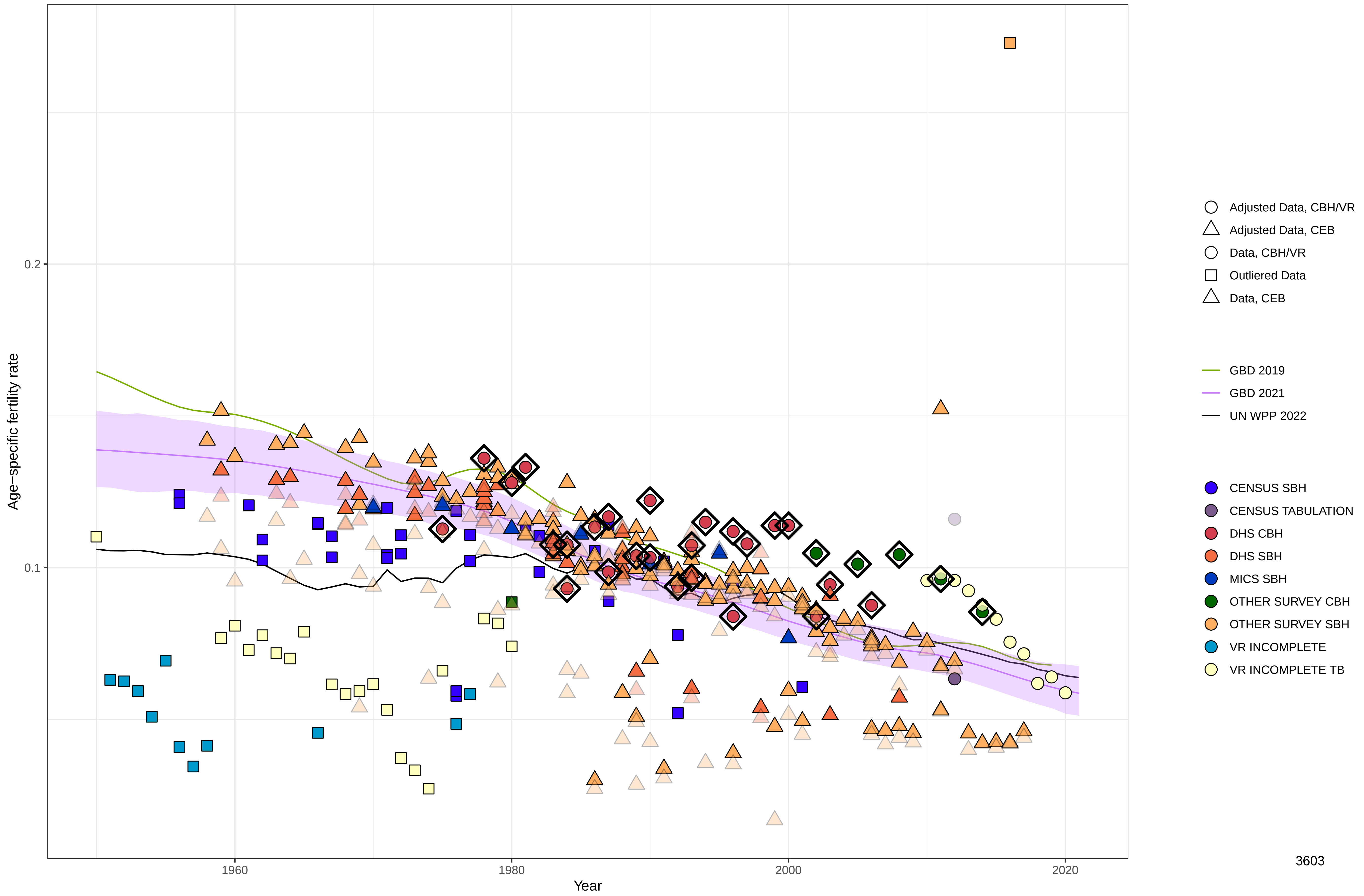

Age−specific fertility fitted trend lines, 1950−2021: Bolivia, Ages: 20−24
Data points enclosed with a diamond are reference sources

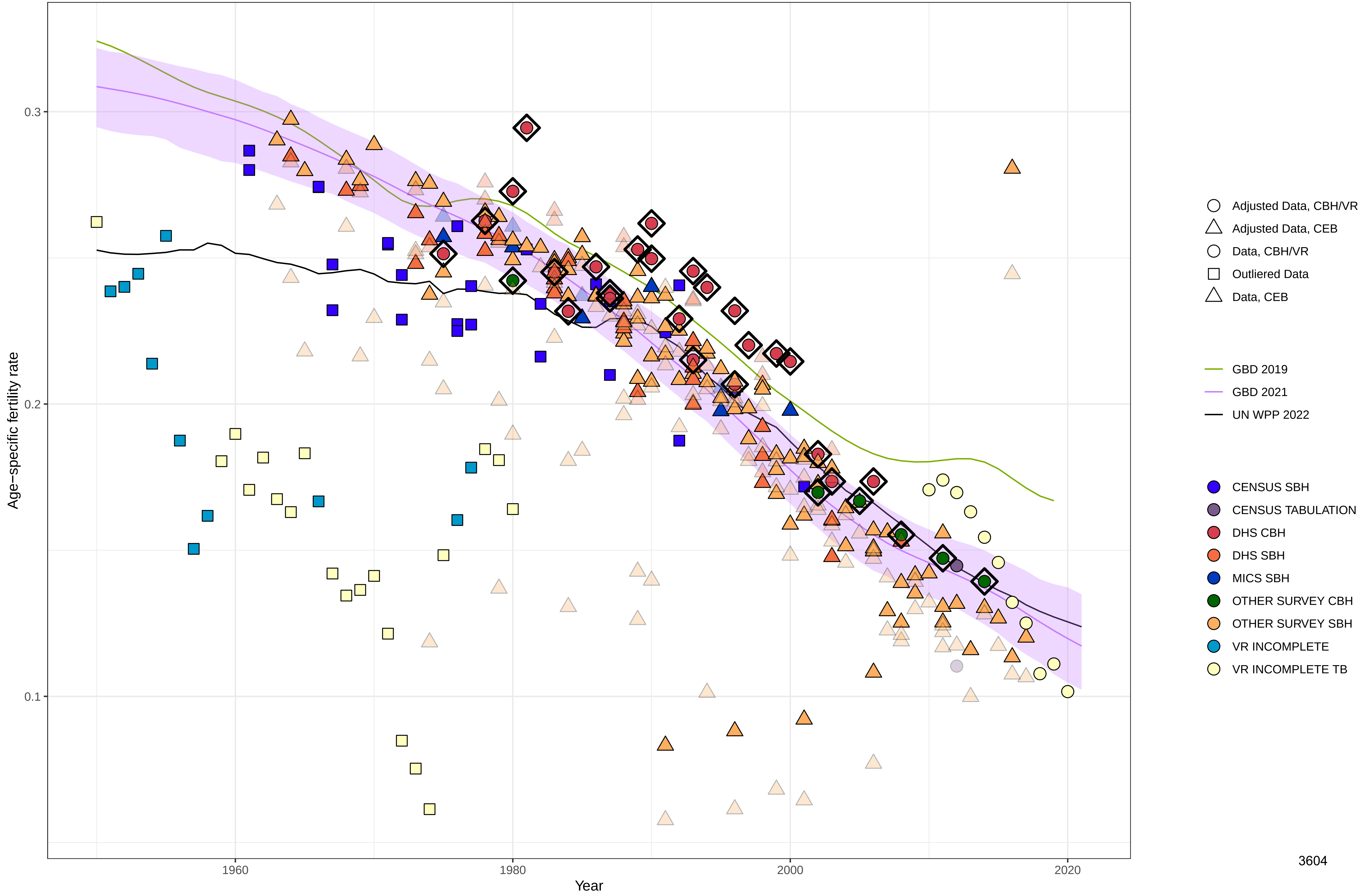

Age−specific fertility fitted trend lines, 1950−2021: Bolivia, Ages: 25−29
Data points enclosed with a diamond are reference sources

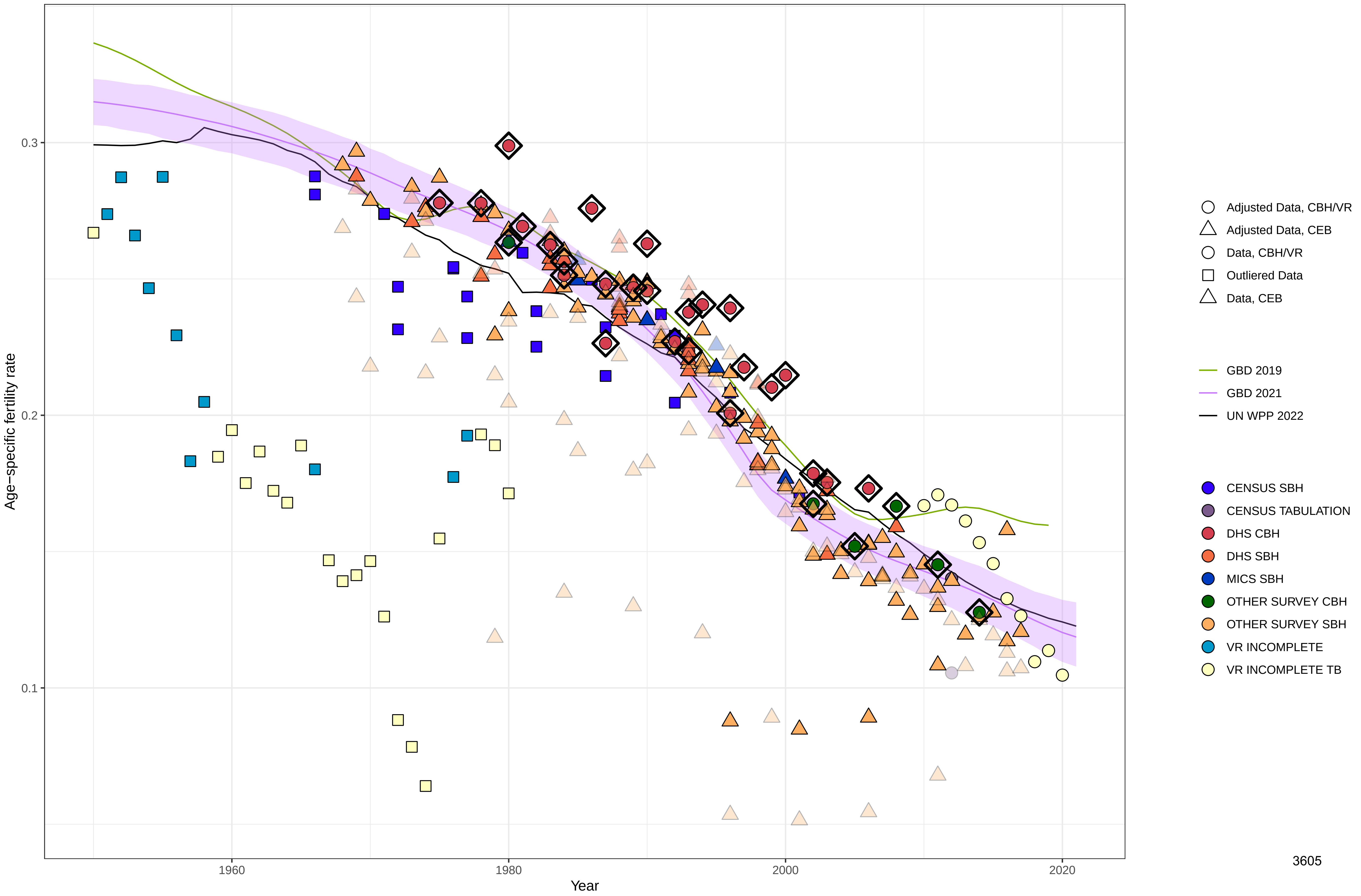

Age−specific fertility fitted trend lines, 1950−2021: Bolivia, Ages: 30−34
Data points enclosed with a diamond are reference sources

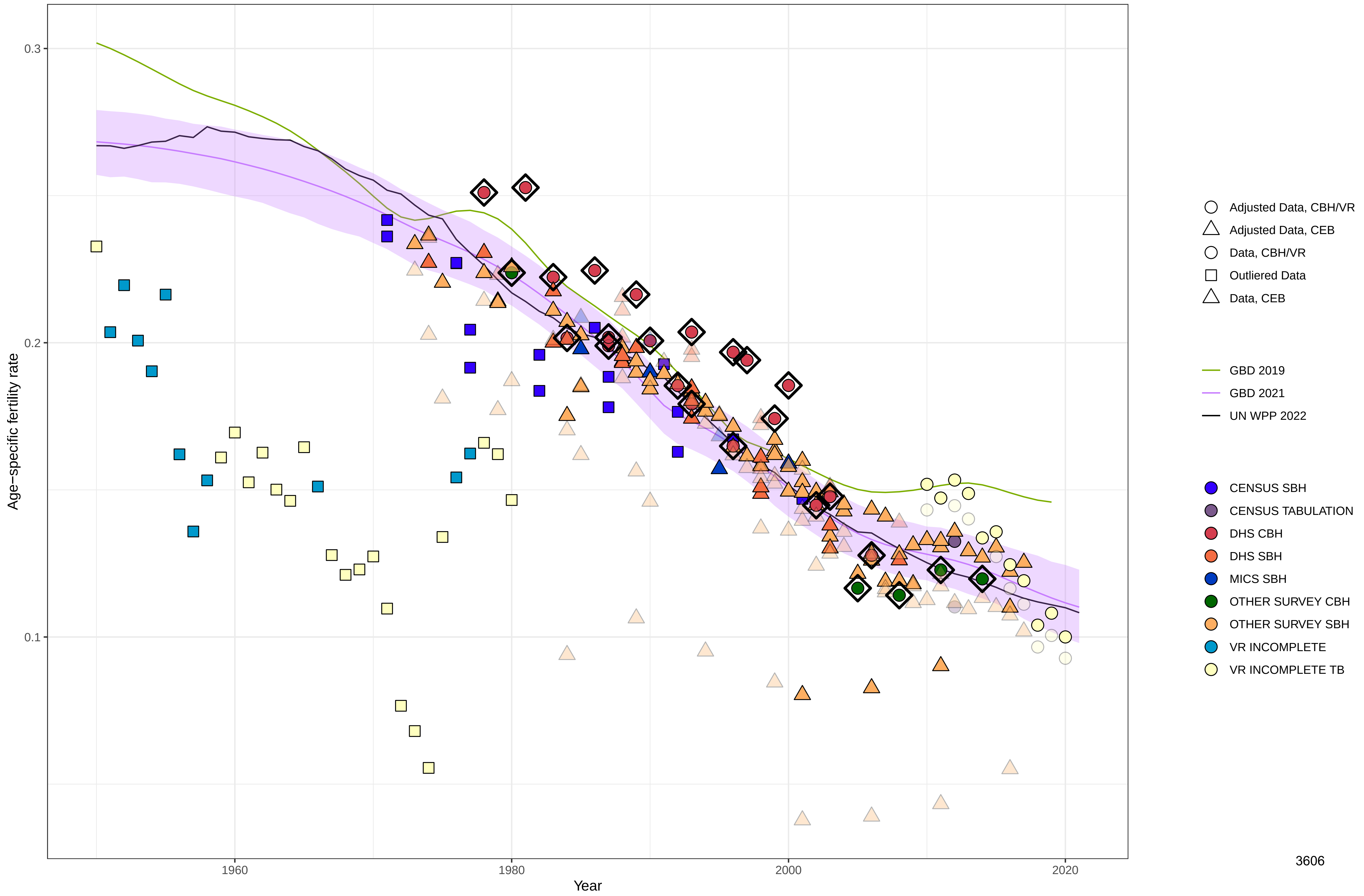

Age−specific fertility fitted trend lines, 1950−2021: Bolivia, Ages: 35−39
Data points enclosed with a diamond are reference sources

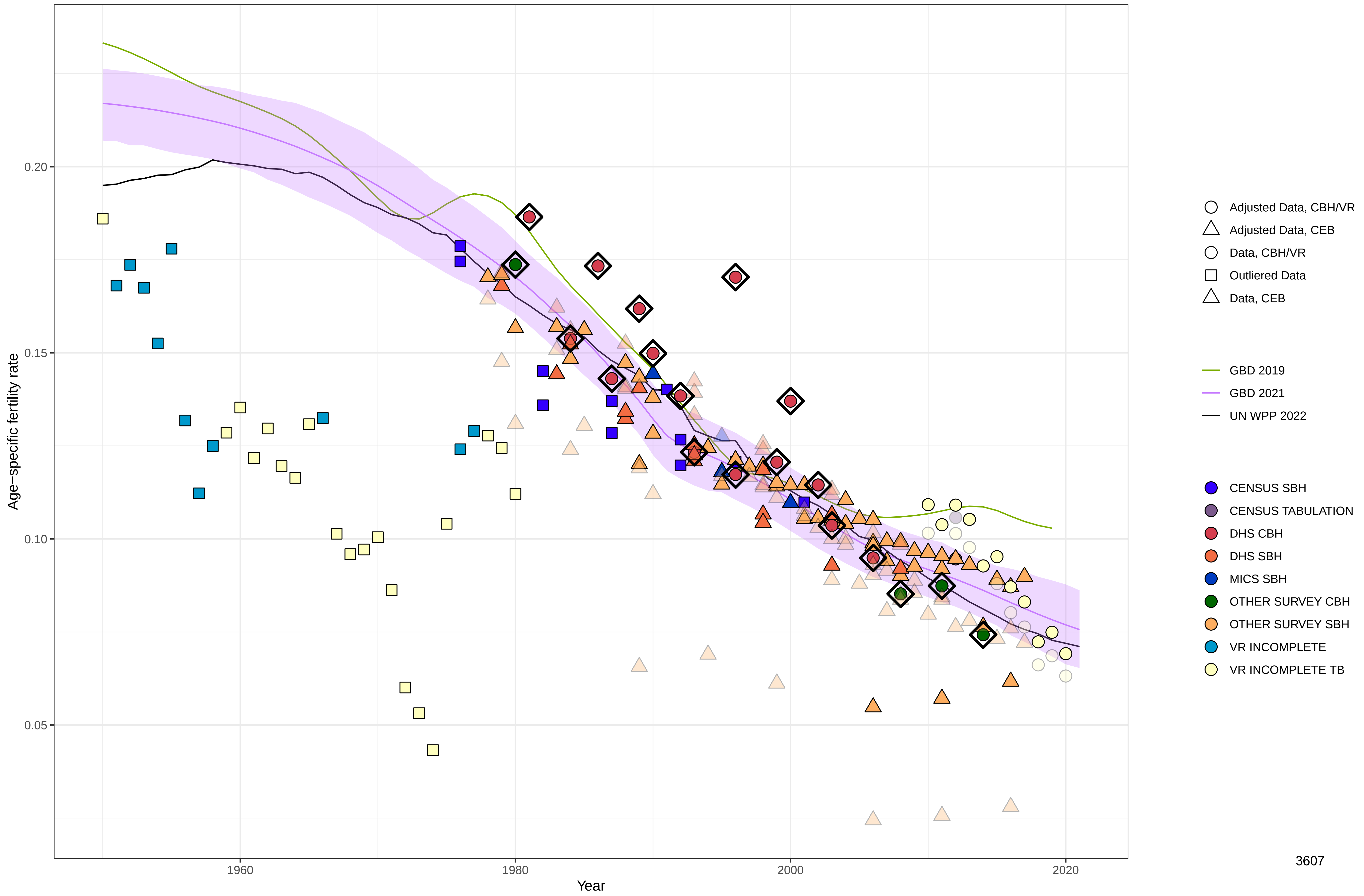

Age−specific fertility fitted trend lines, 1950−2021: Bolivia, Ages: 40−44
Data points enclosed with a diamond are reference sources

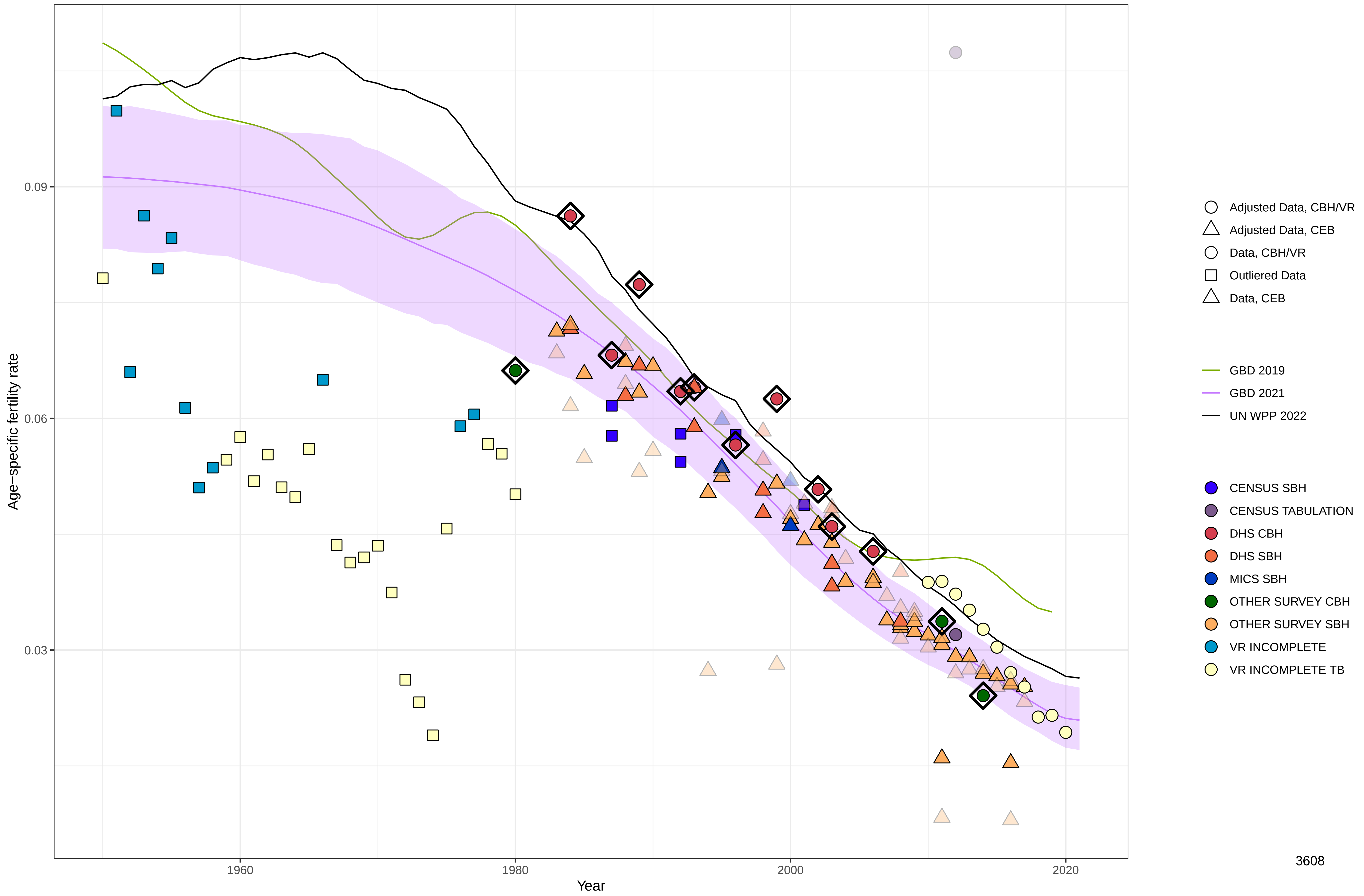

Age−specific fertility fitted trend lines, 1950−2021: Bolivia, Ages: 45−49
Data points enclosed with a diamond are reference sources

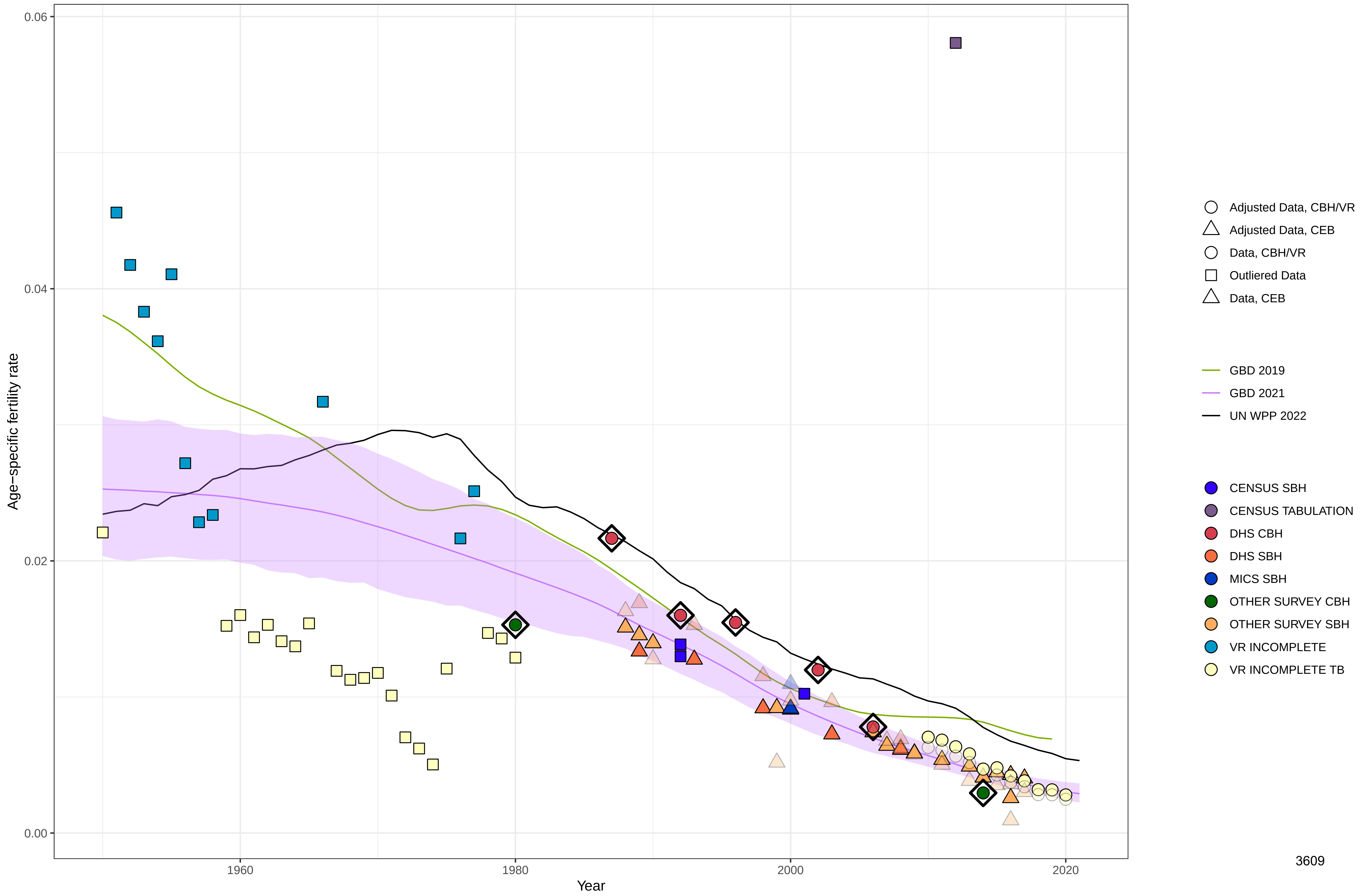

Age−specific fertility fitted trend lines, 1950−2021: Bolivia, Ages: 50−54
Data points enclosed with a diamond are reference sources

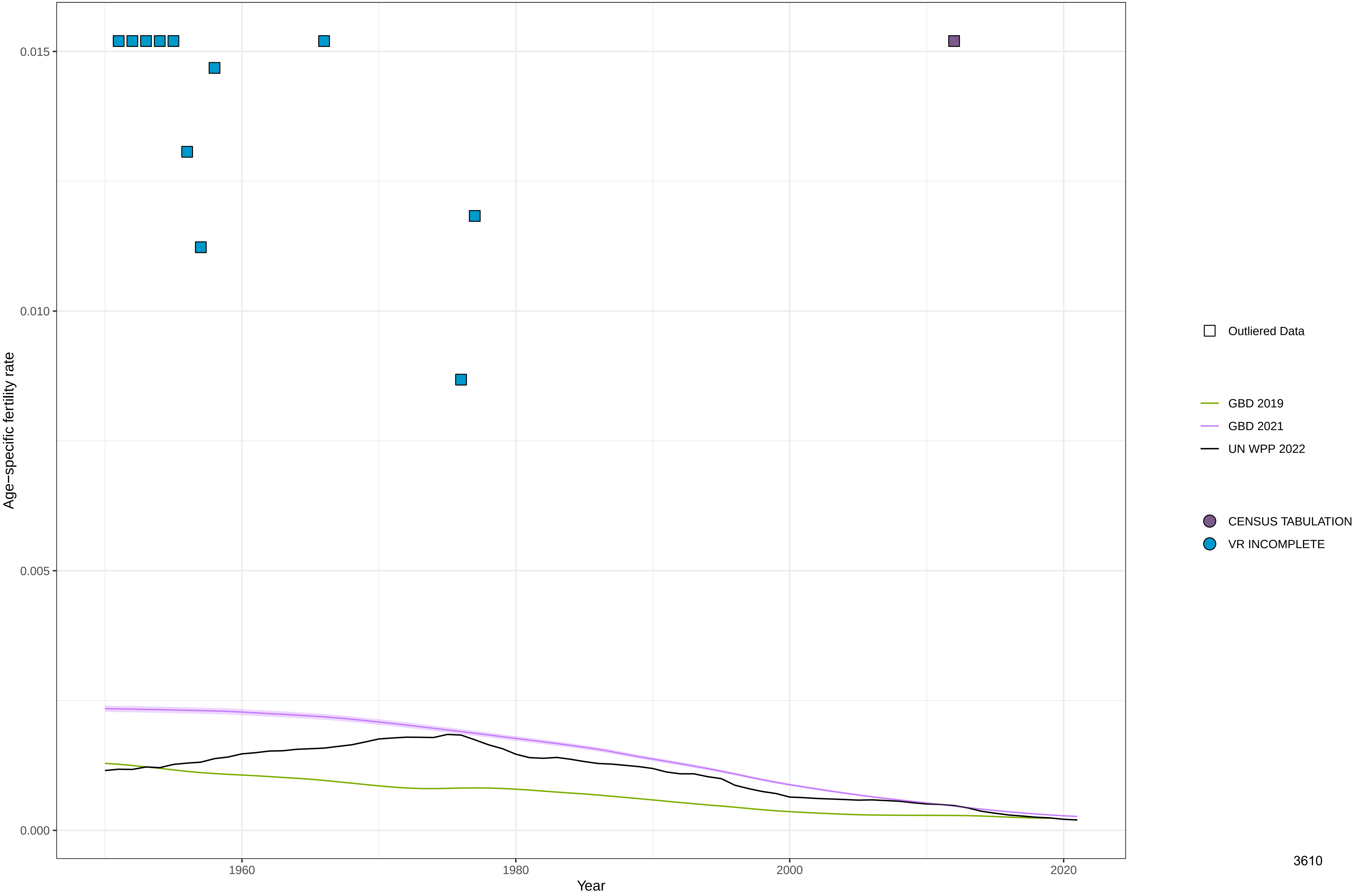

Total fertility rate fitted trend lines, 1950−2021: Bolivia TFR
Data points enclosed with a diamond are reference sources

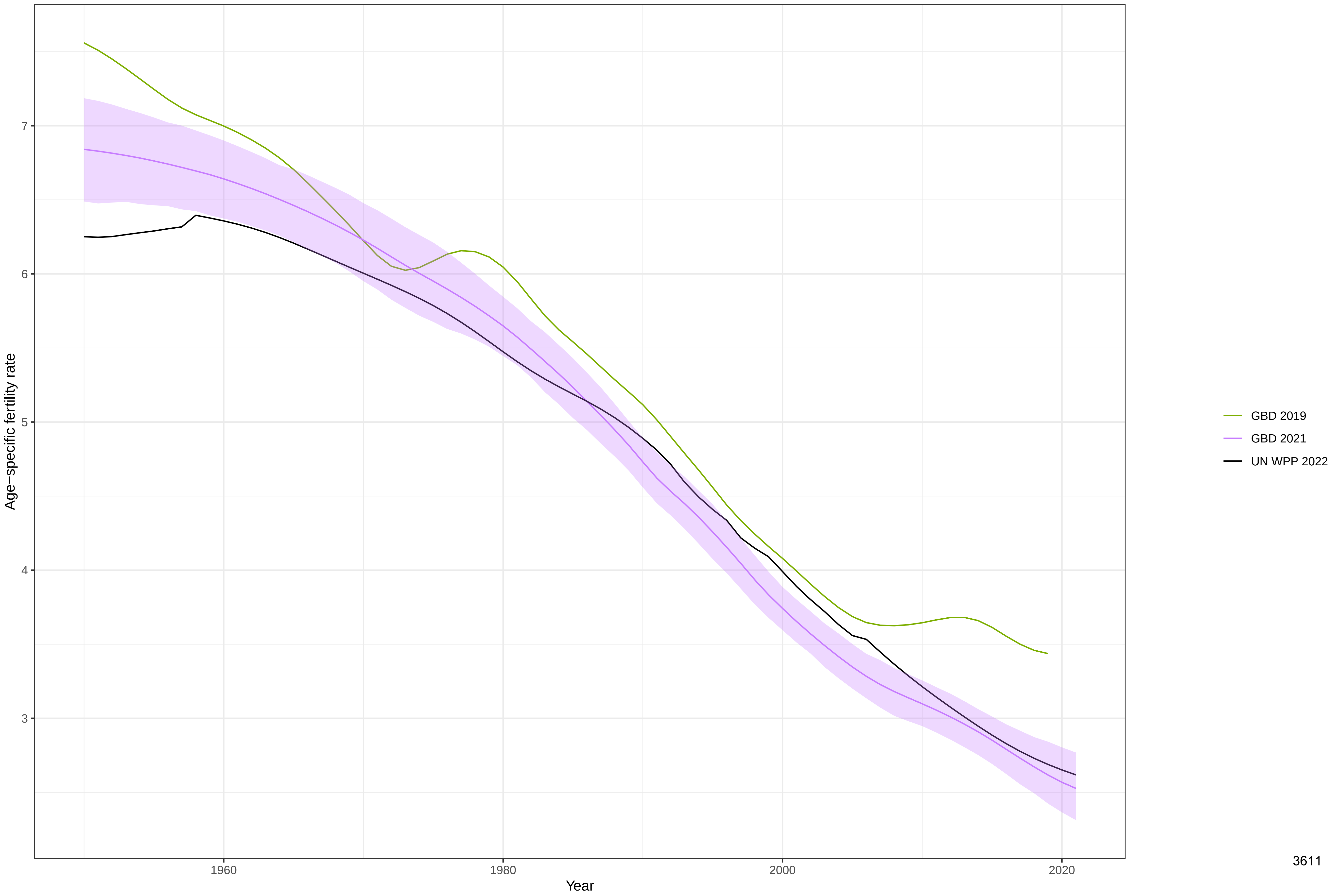

Age−specific fertility fitted trend lines, 1950−2021: Ecuador, Ages: 10−14
Data points enclosed with a diamond are reference sources

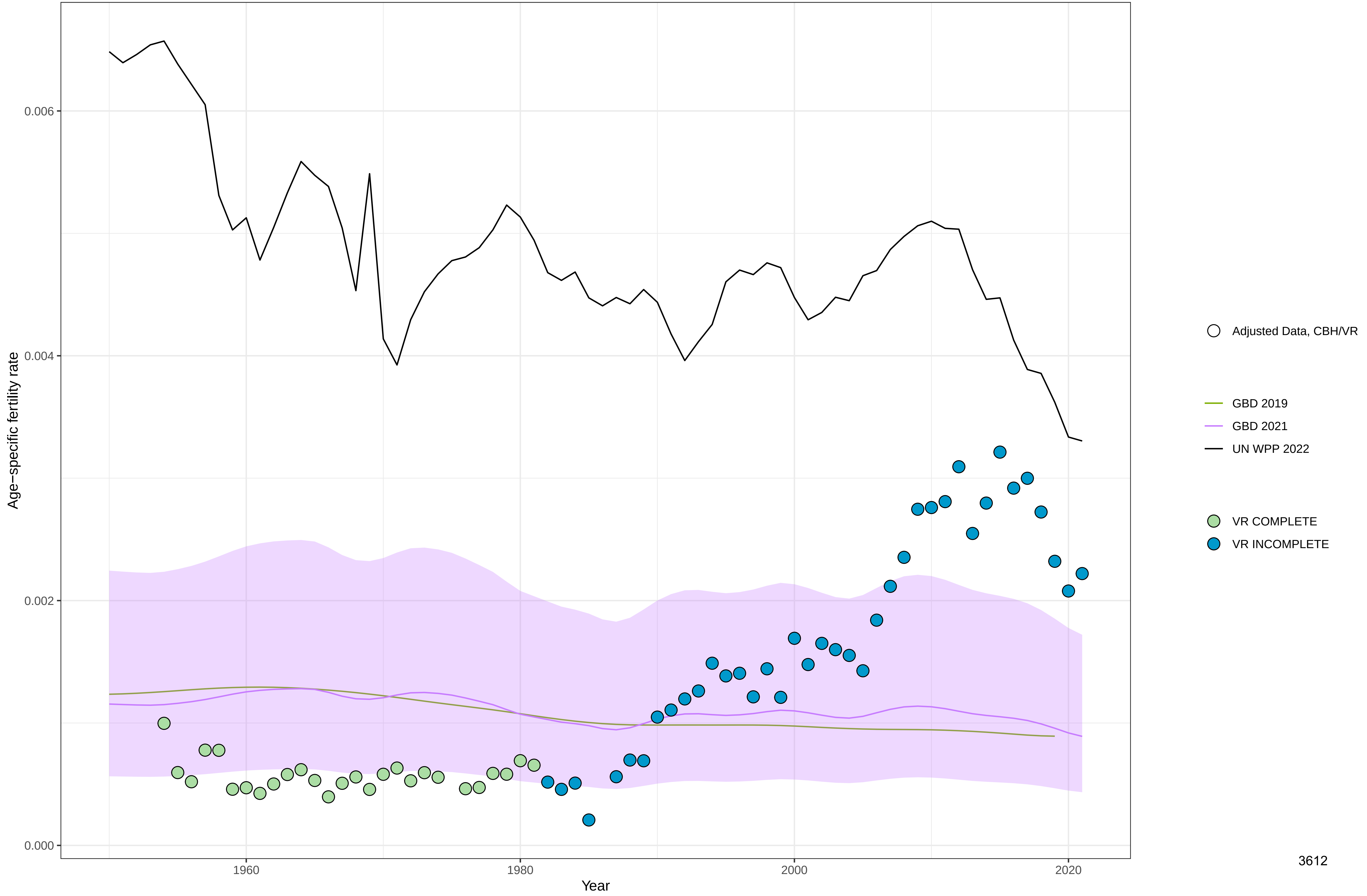

Age−specific fertility fitted trend lines, 1950−2021: Ecuador, Ages: 15−19
Data points enclosed with a diamond are reference sources

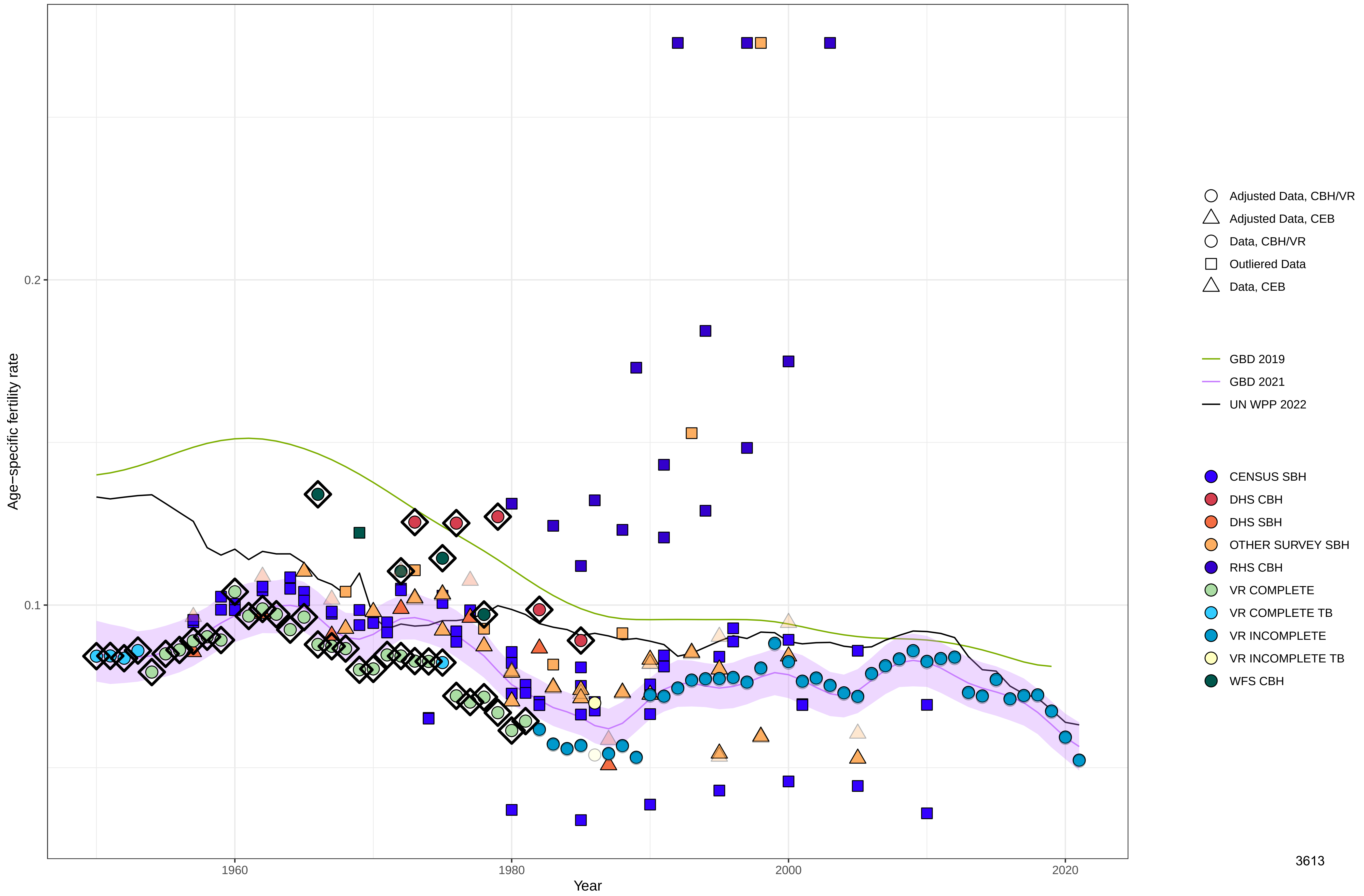

Age−specific fertility fitted trend lines, 1950−2021: Ecuador, Ages: 20−24
Data points enclosed with a diamond are reference sources

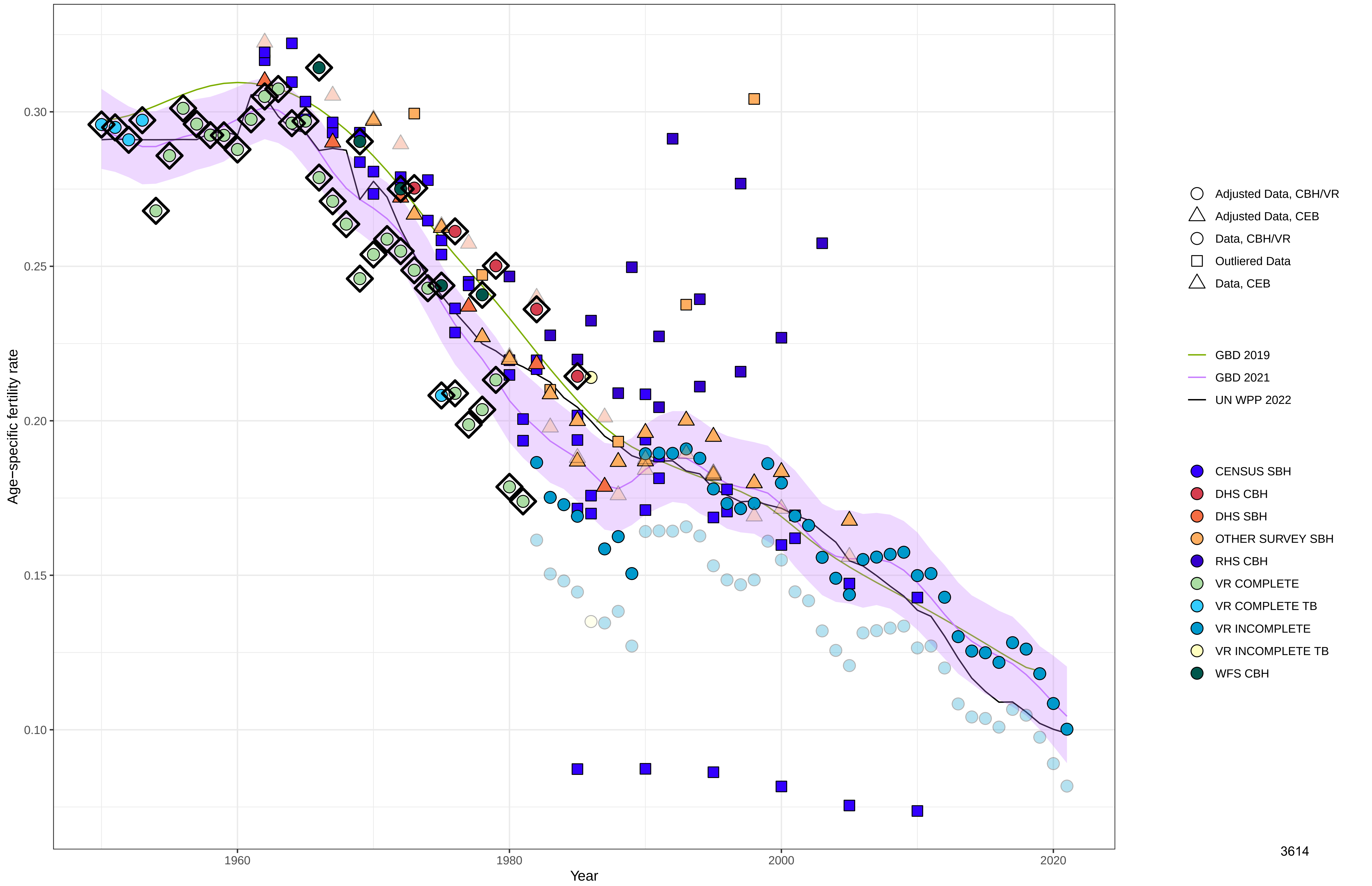

Age−specific fertility fitted trend lines, 1950−2021: Ecuador, Ages: 25−29
Data points enclosed with a diamond are reference sources

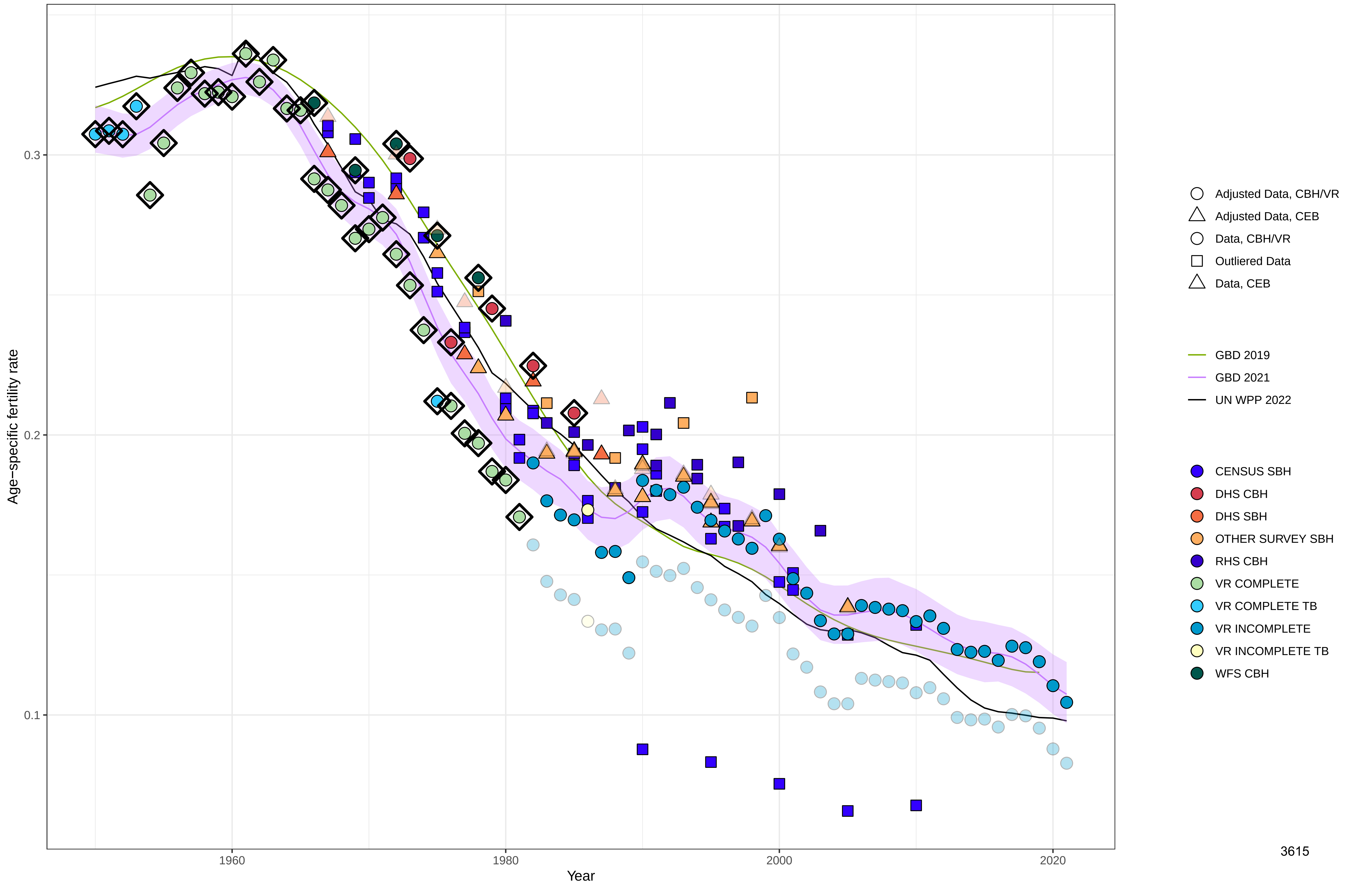

Age−specific fertility fitted trend lines, 1950−2021: Ecuador, Ages: 30−34
Data points enclosed with a diamond are reference sources

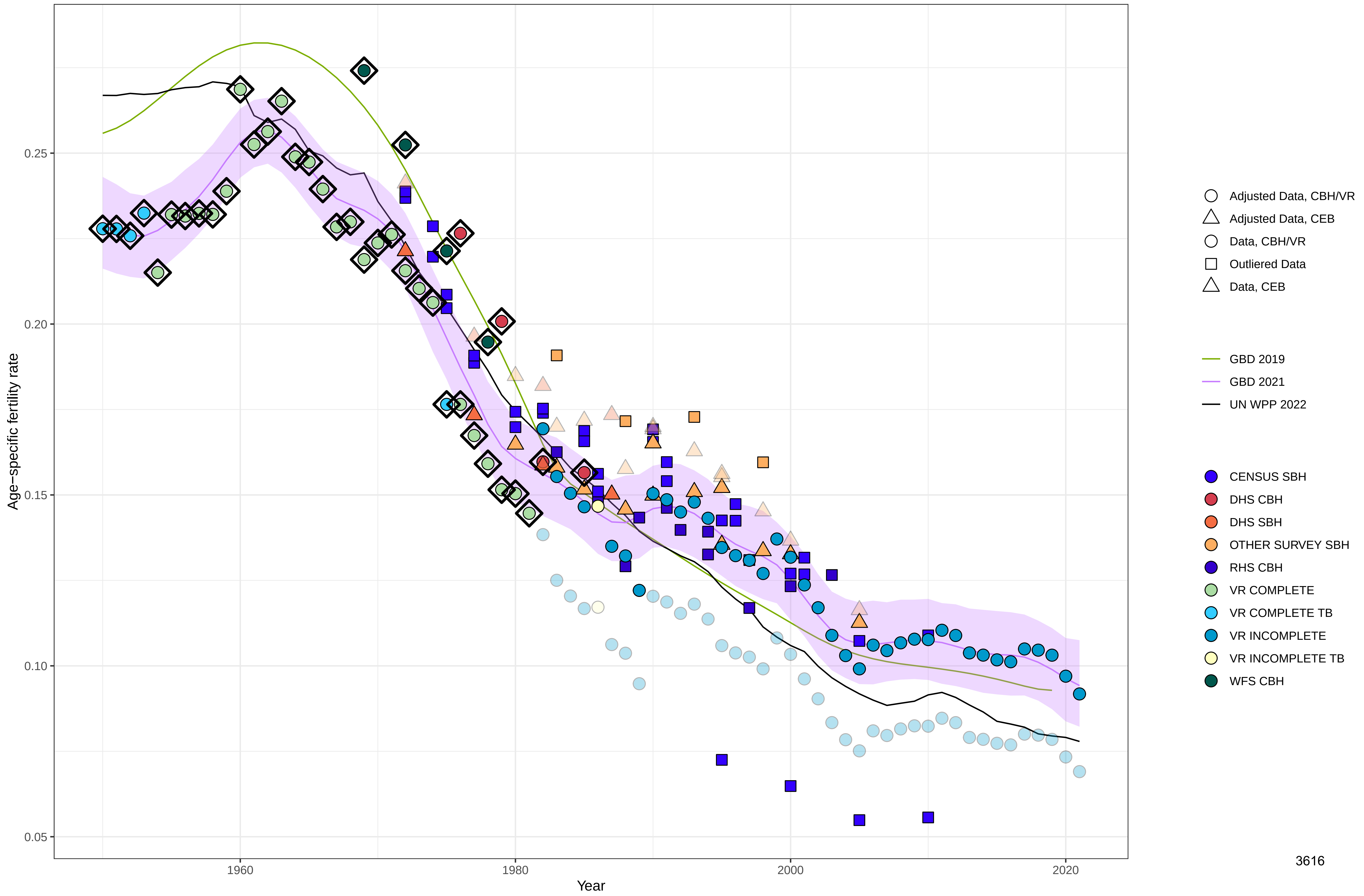

Age−specific fertility fitted trend lines, 1950−2021: Ecuador, Ages: 35−39
Data points enclosed with a diamond are reference sources

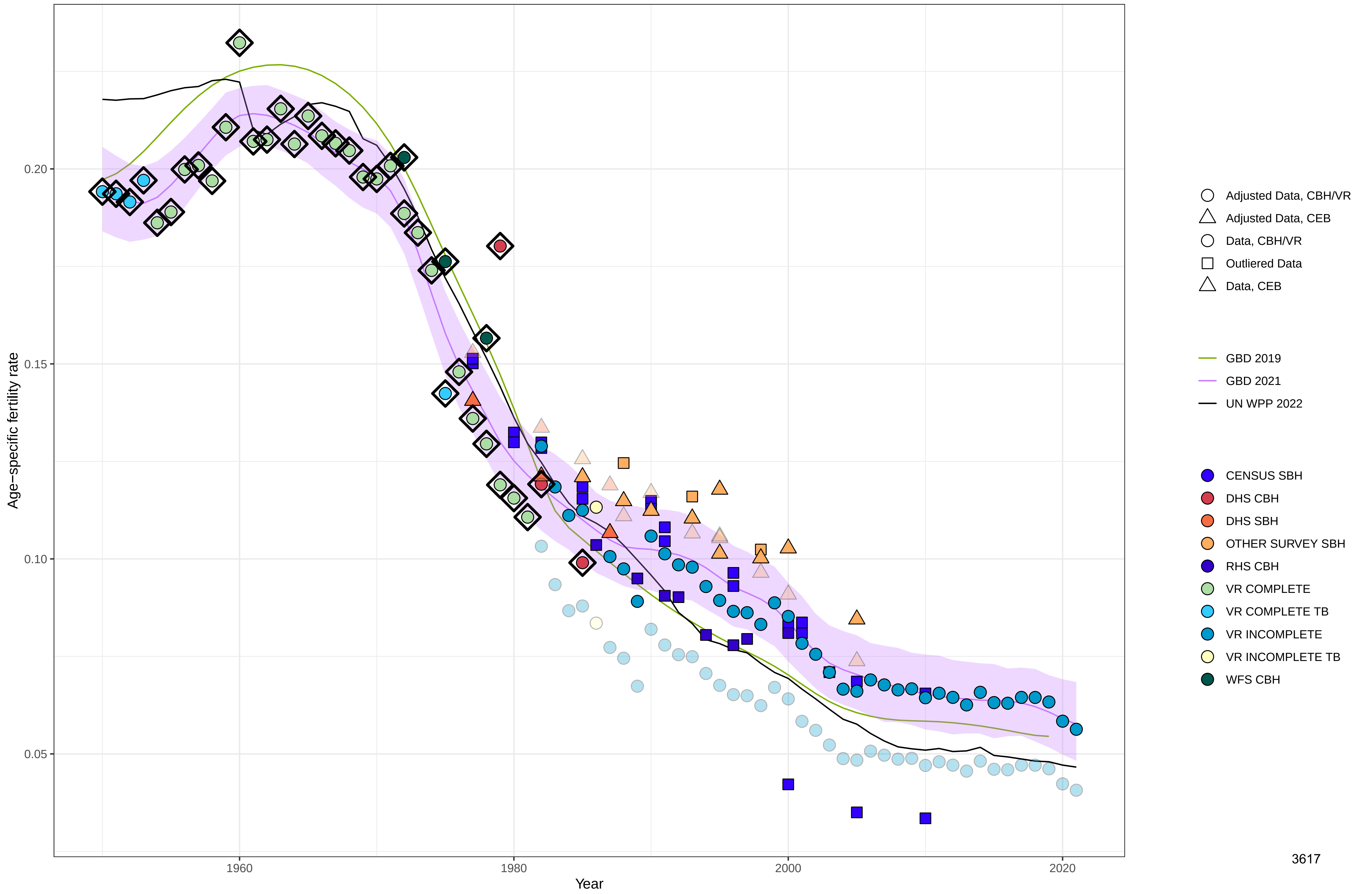

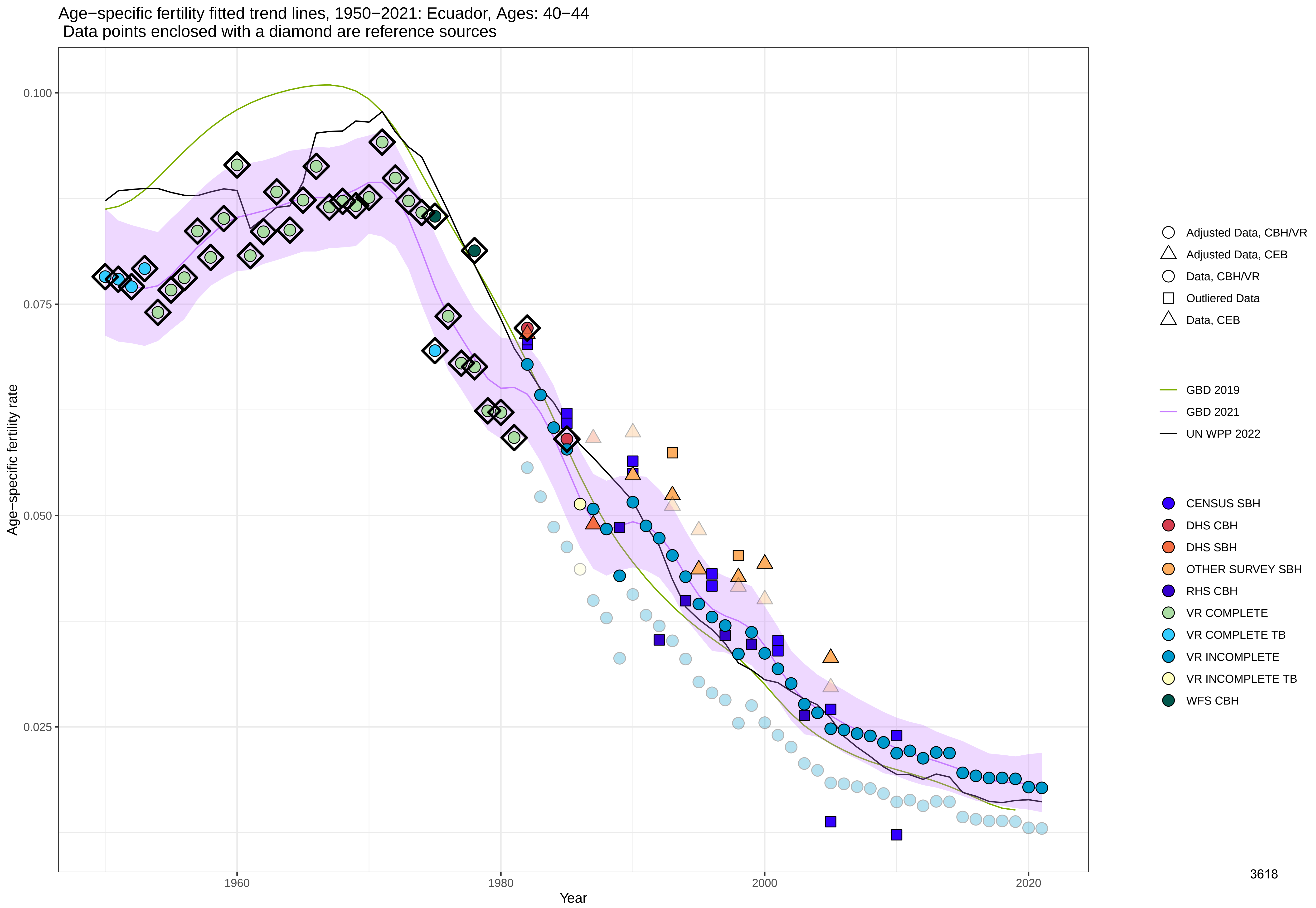

Age−specific fertility fitted trend lines, 1950−2021: Ecuador, Ages: 40−44
Data points enclosed with a diamond are reference sources
0.100
0.075
0.050
0.025
Age−specific fertility rate
1960
1980
2000
2020
Year
Adjusted Data, CBH/VR
Adjusted Data, CEB
Data, CBH/VR
Outliered Data
Data, CEB
GBD 2019
GBD 2021
UN WPP 2022
CENSUS SBH
DHS CBH
DHS SBH
OTHER SURVEY SBH
RHS CBH
VR COMPLETE
VR COMPLETE TB
VR INCOMPLETE
VR INCOMPLETE TB
WFS CBH

Age−specific fertility fitted trend lines, 1950−2021: Ecuador, Ages: 45−49
Data points enclosed with a diamond are reference sources

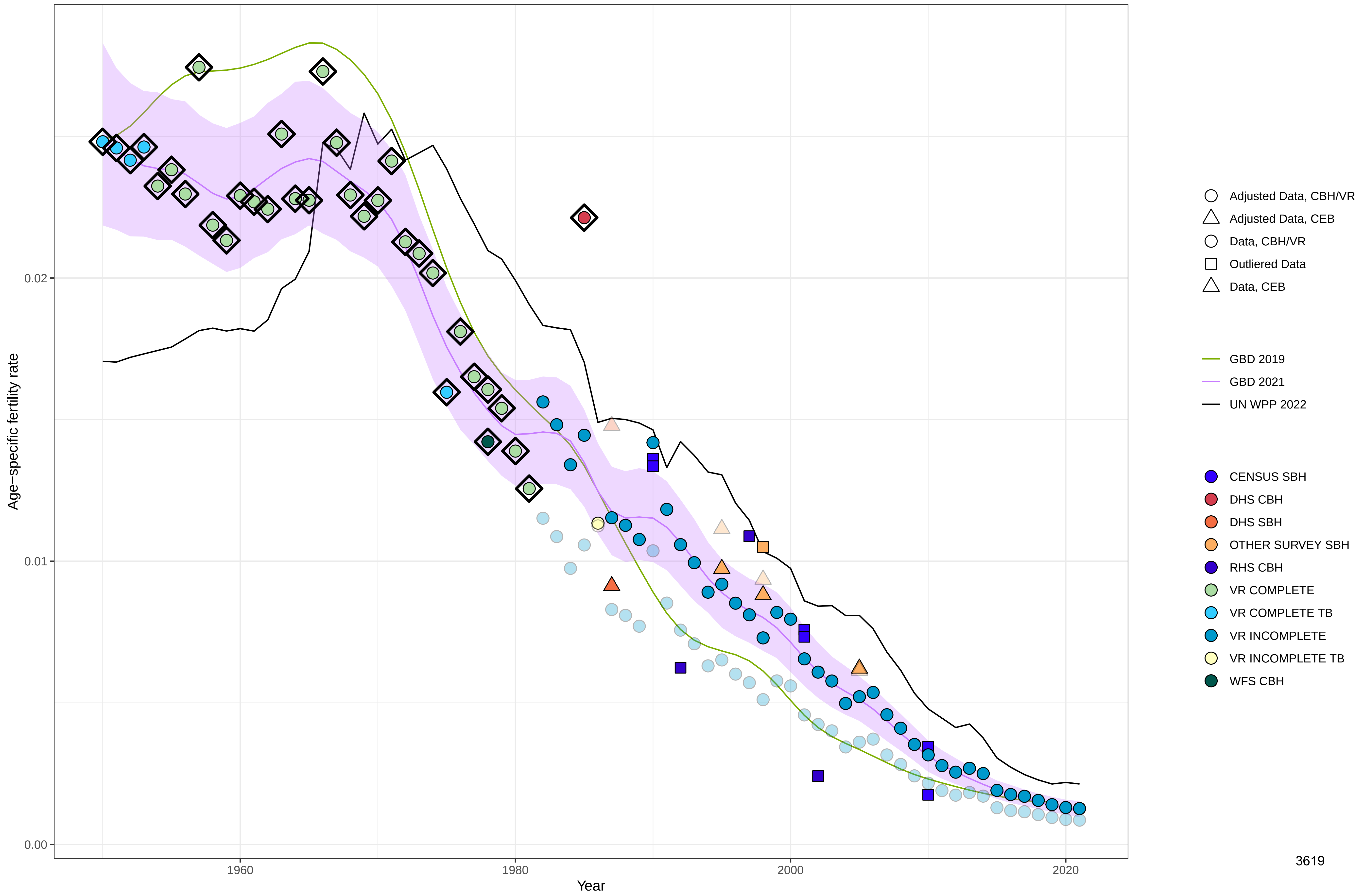

Age−specific fertility fitted trend lines, 1950−2021: Ecuador, Ages: 50−54
Data points enclosed with a diamond are reference sources

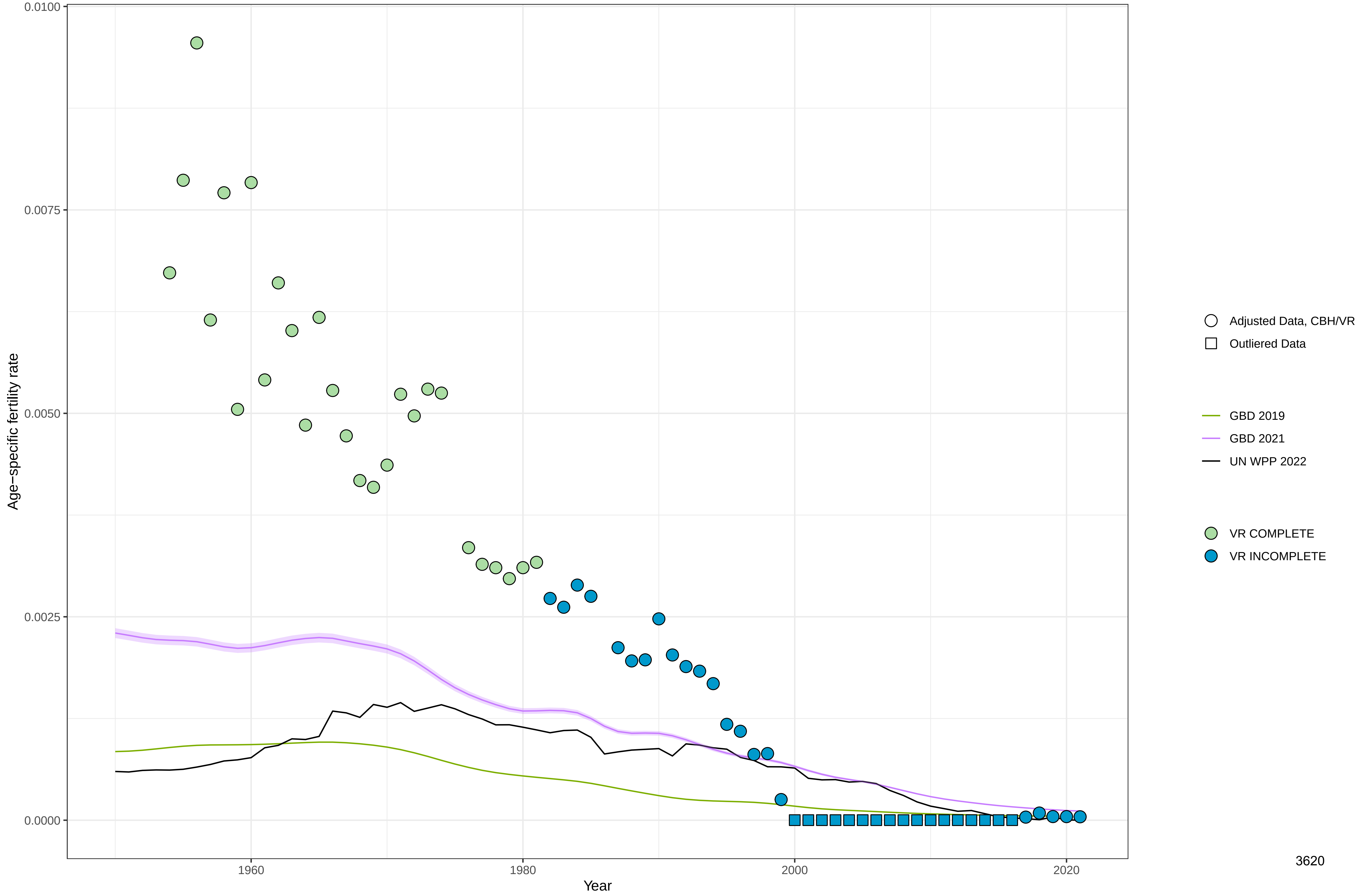

Total fertility rate fitted trend lines, 1950−2021: Ecuador TFR
Data points enclosed with a diamond are reference sources

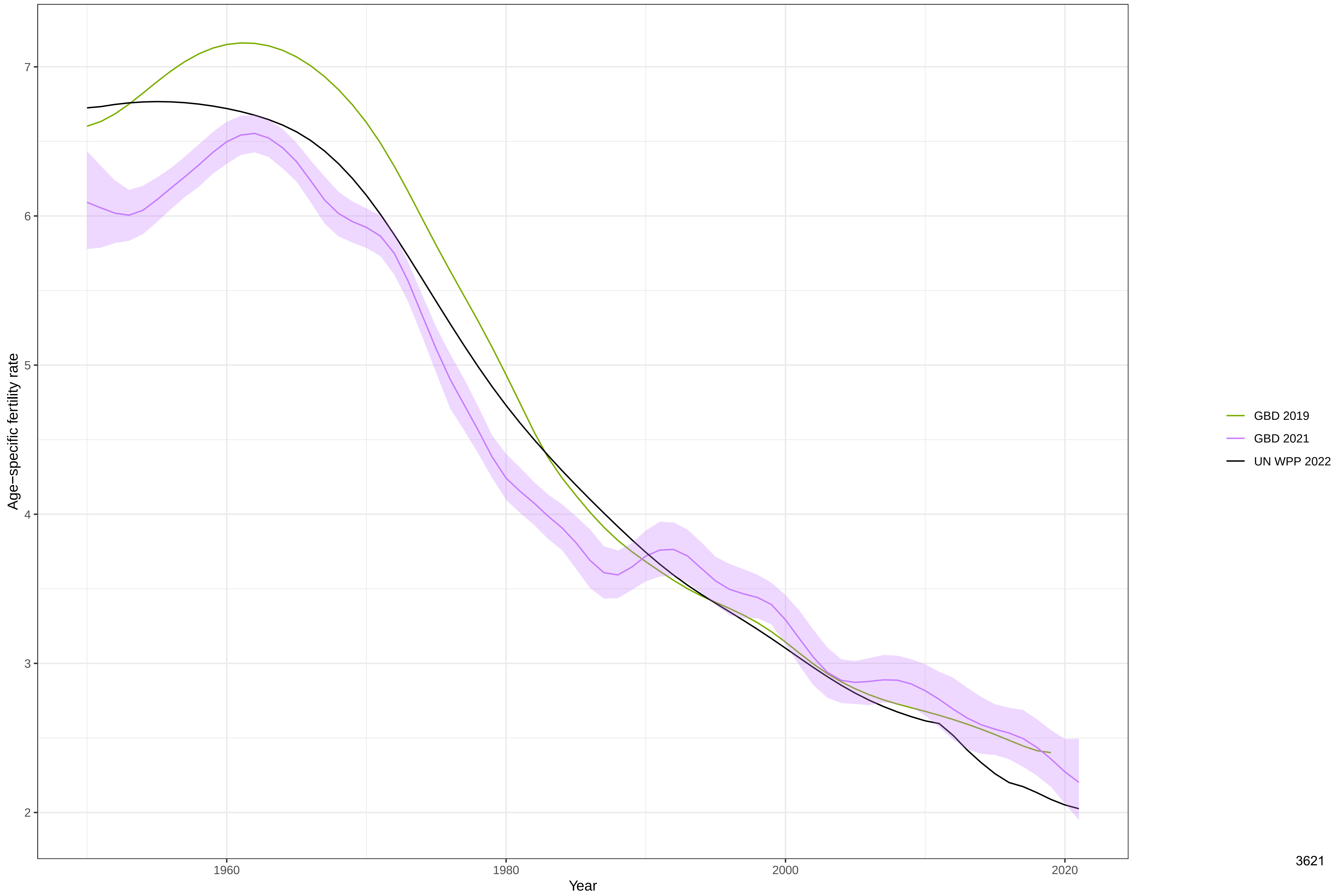

Age−specific fertility fitted trend lines, 1950−2021: Peru, Ages: 10−14
Data points enclosed with a diamond are reference sources

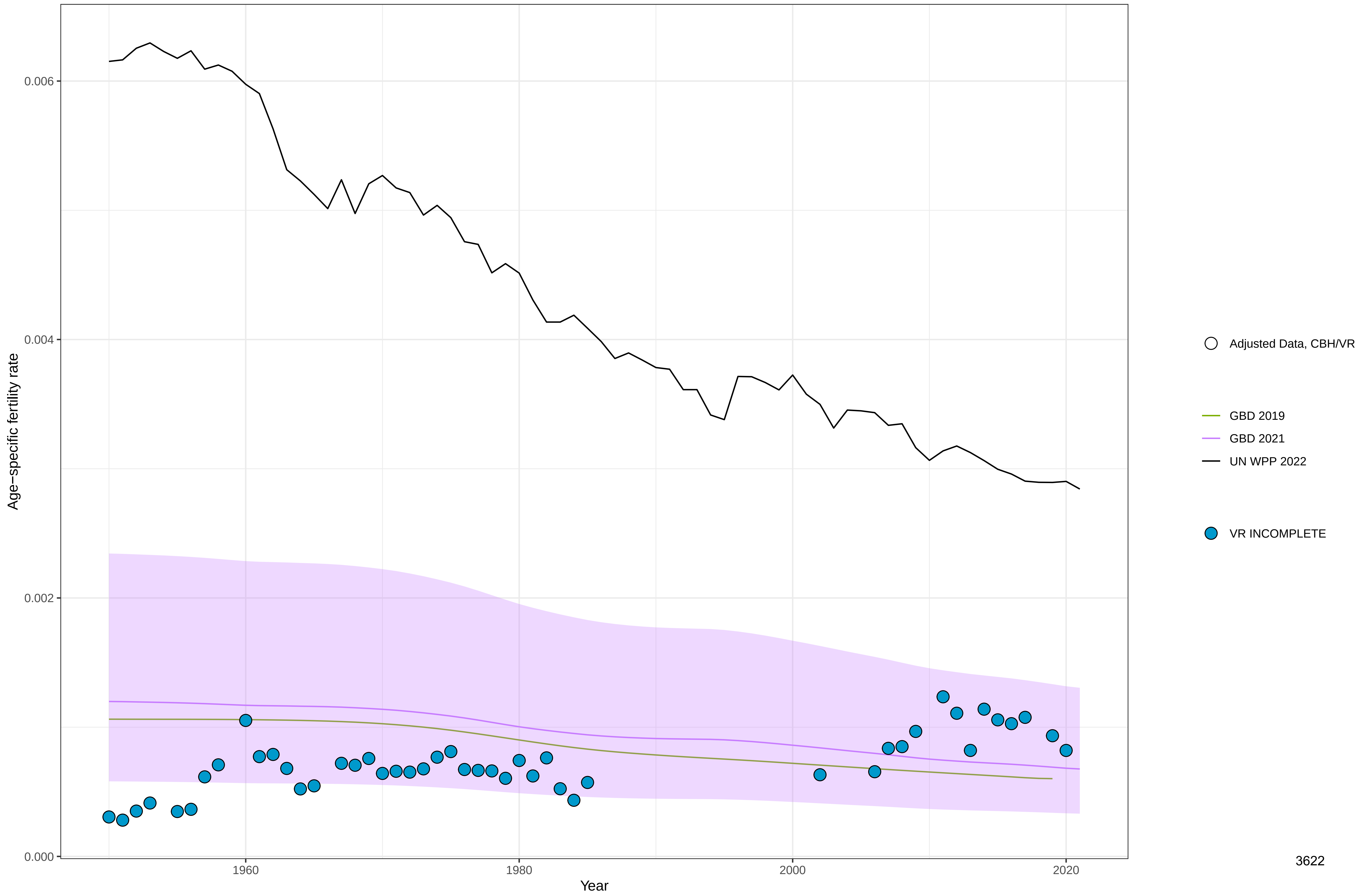

Age−specific fertility fitted trend lines, 1950−2021: Peru, Ages: 15−19
Data points enclosed with a diamond are reference sources

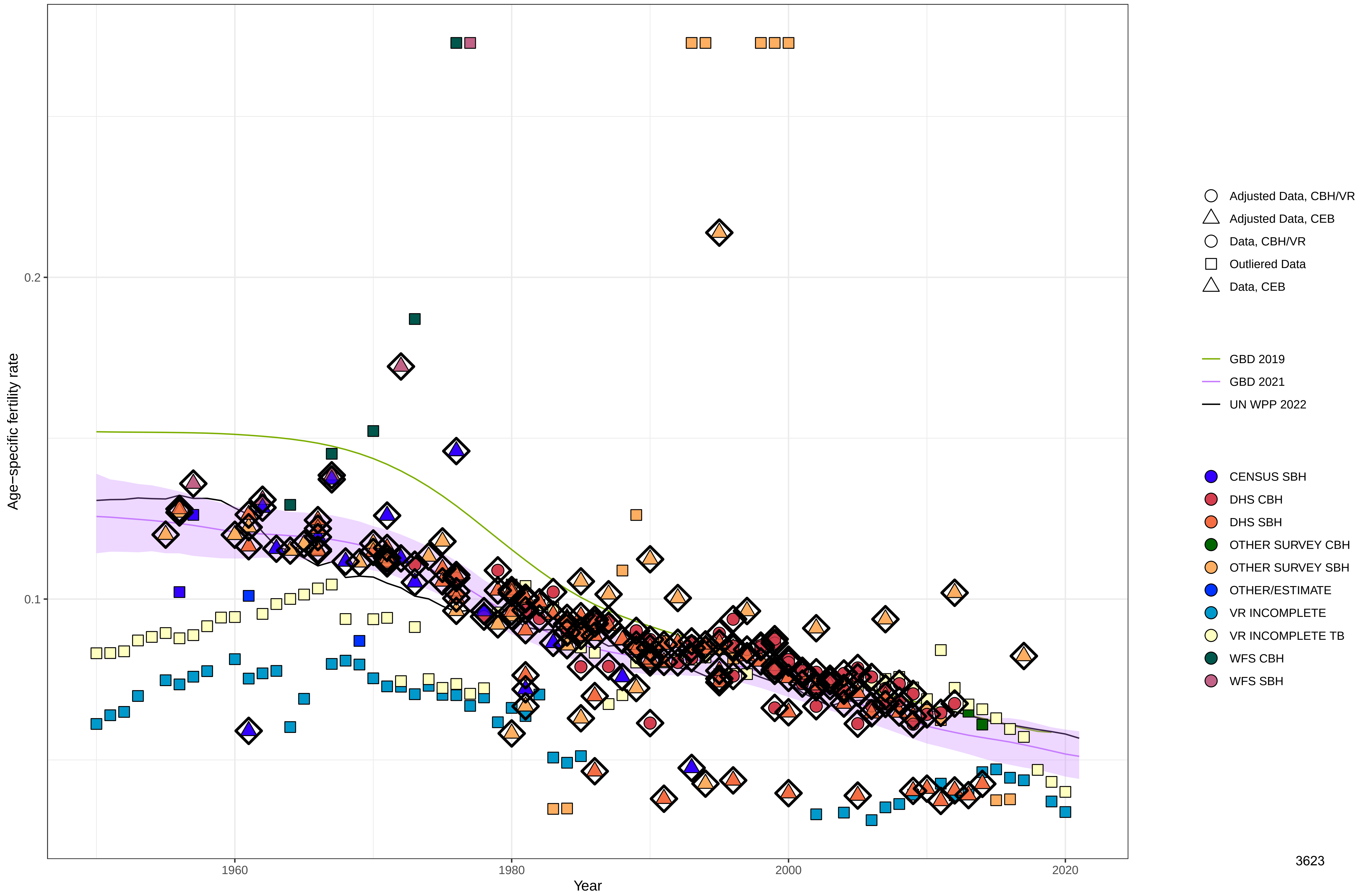

Age−specific fertility fitted trend lines, 1950−2021: Peru, Ages: 20−24
Data points enclosed with a diamond are reference sources

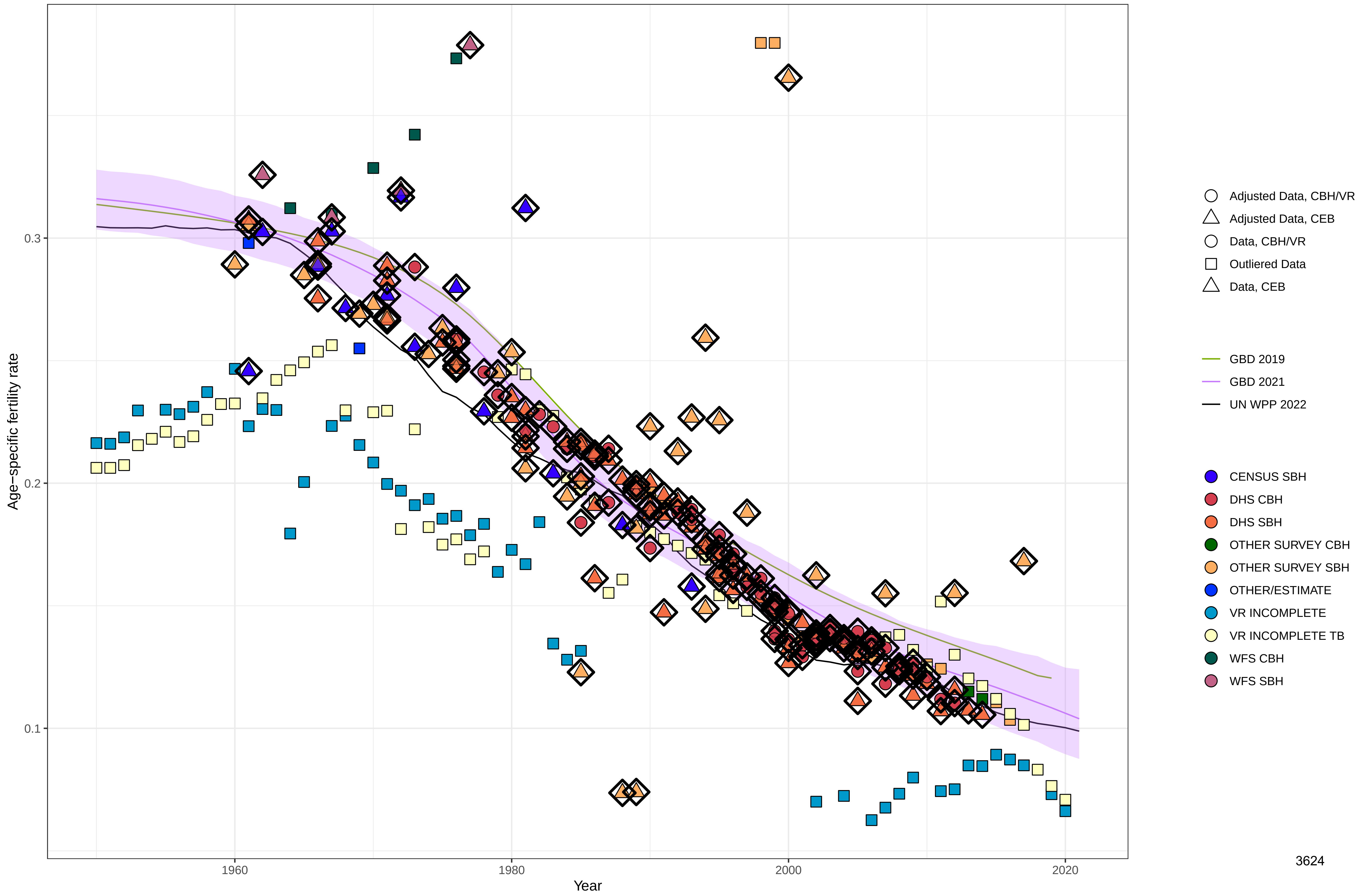

Age−specific fertility fitted trend lines, 1950−2021: Peru, Ages: 25−29
Data points enclosed with a diamond are reference sources

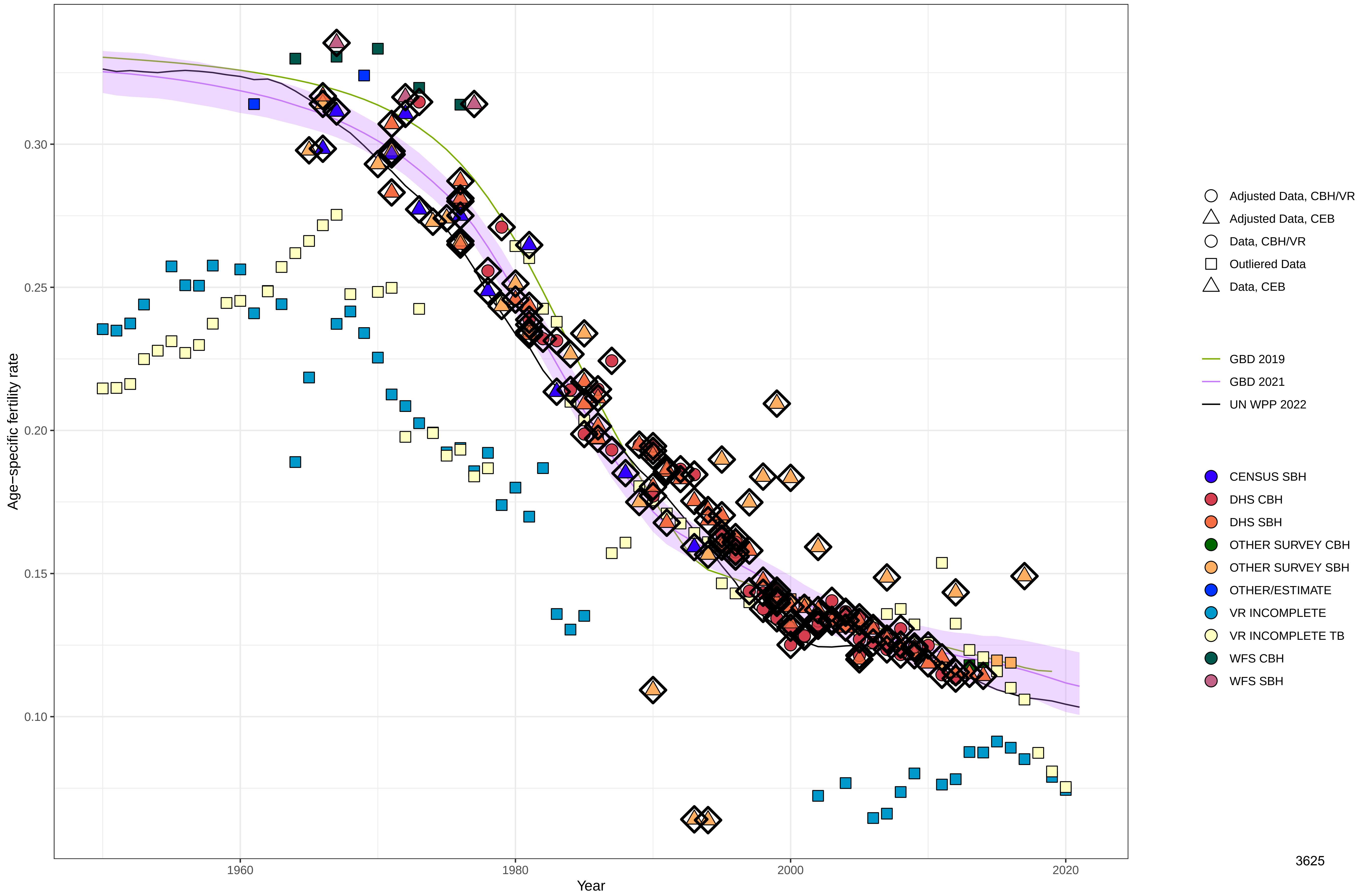

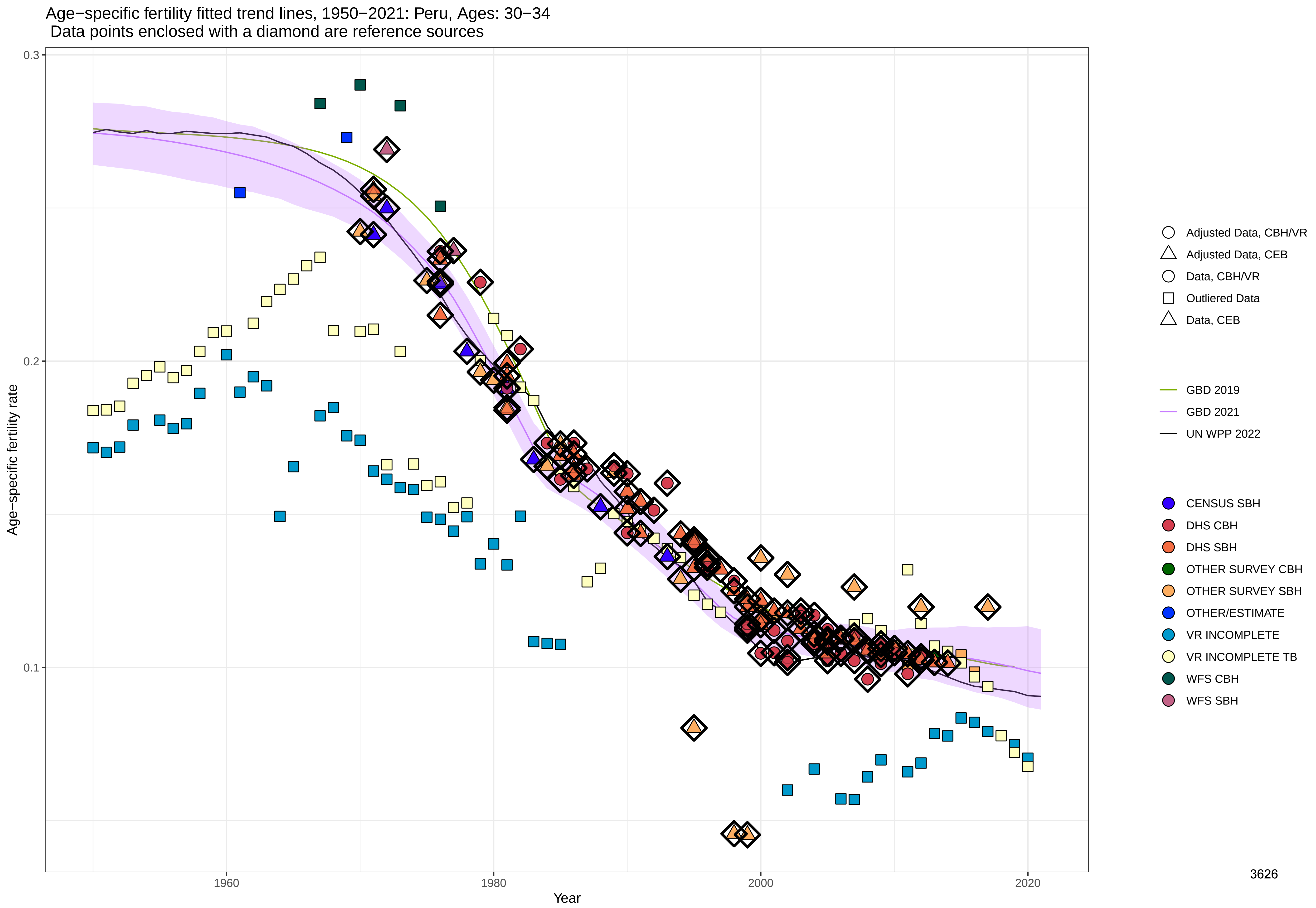
Age−specific fertility fitted trend lines, 1950−2021: Peru, Ages: 30−34
Data points enclosed with a diamond are reference sources
Age−specific fertility rate
Year
0.3
0.2
0.1
1960
1980
2000
2020
Adjusted Data, CBH/VR
Adjusted Data, CEB
Data, CBH/VR
Outliered Data
Data, CEB
GBD 2019
GBD 2021
UN WPP 2022
CENSUS SBH
DHS CBH
DHS SBH
OTHER SURVEY CBH
OTHER SURVEY SBH
OTHER/ESTIMATE
VR INCOMPLETE
VR INCOMPLETE TB
WFS CBH
WFS SBH

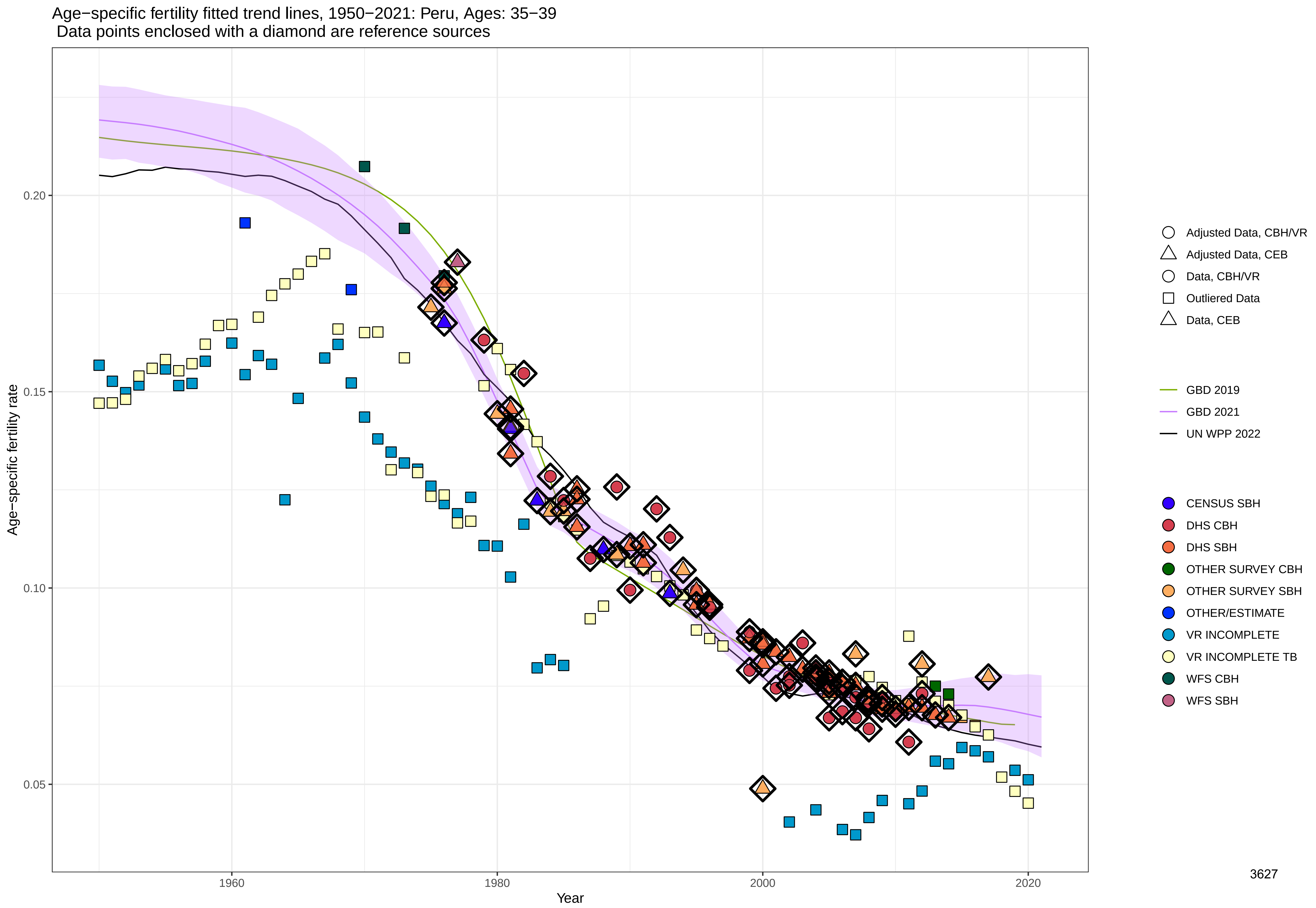
Age−specific fertility fitted trend lines, 1950−2021: Peru, Ages: 35−39
Data points enclosed with a diamond are reference sources
Age−specific fertility rate
Year
0.05
0.10
0.15
0.20
1960
1980
2000
2020
Adjusted Data, CBH/VR
Adjusted Data, CEB
Data, CBH/VR
Outliered Data
Data, CEB
GBD 2019
GBD 2021
UN WPP 2022
CENSUS SBH
DHS CBH
DHS SBH
OTHER SURVEY CBH
OTHER SURVEY SBH
OTHER/ESTIMATE
VR INCOMPLETE
VR INCOMPLETE TB
WFS CBH
WFS SBH

Age−specific fertility fitted trend lines, 1950−2021: Peru, Ages: 40−44
Data points enclosed with a diamond are reference sources

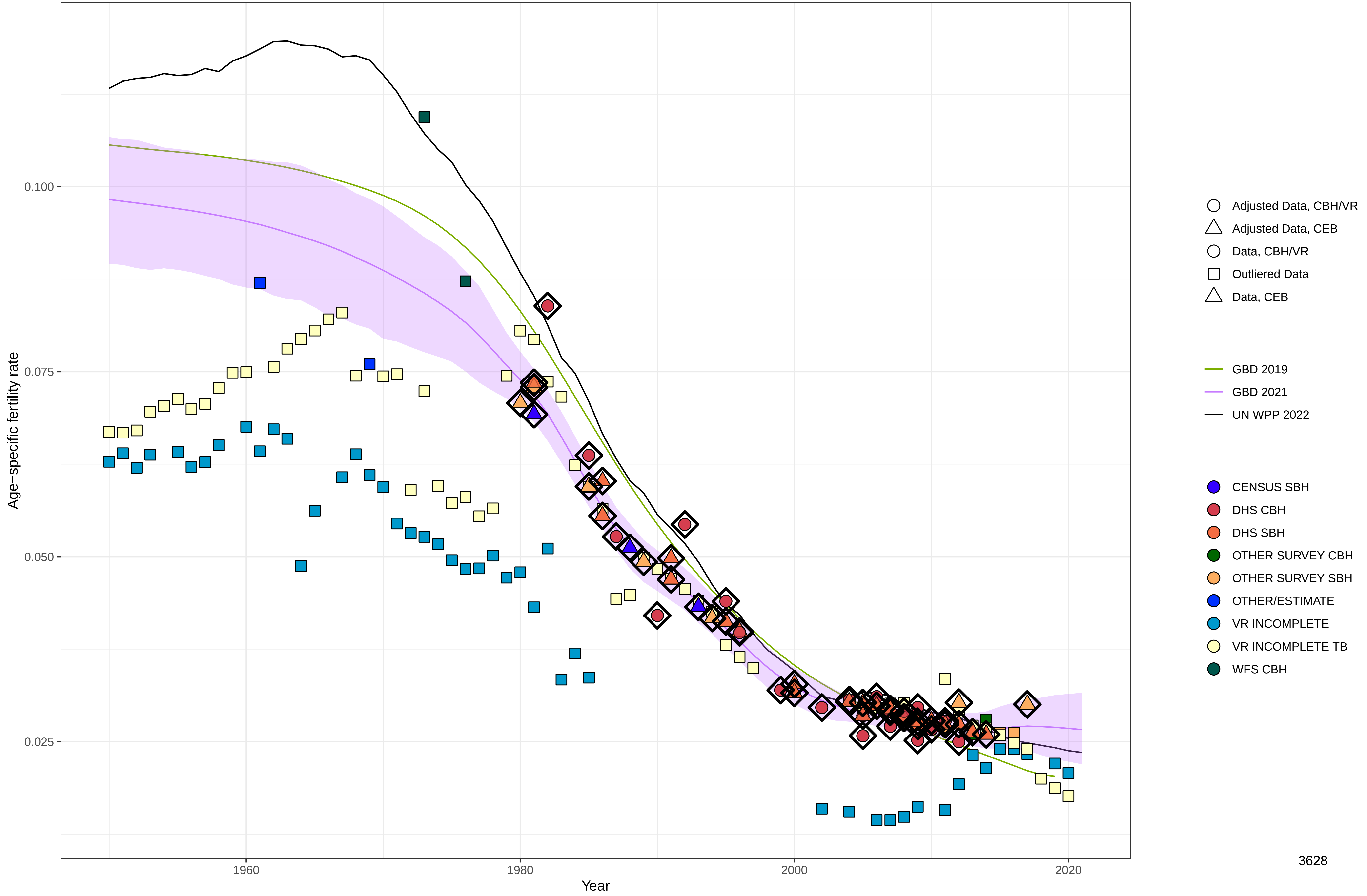

Age−specific fertility fitted trend lines, 1950−2021: Peru, Ages: 45−49
Data points enclosed with a diamond are reference sources

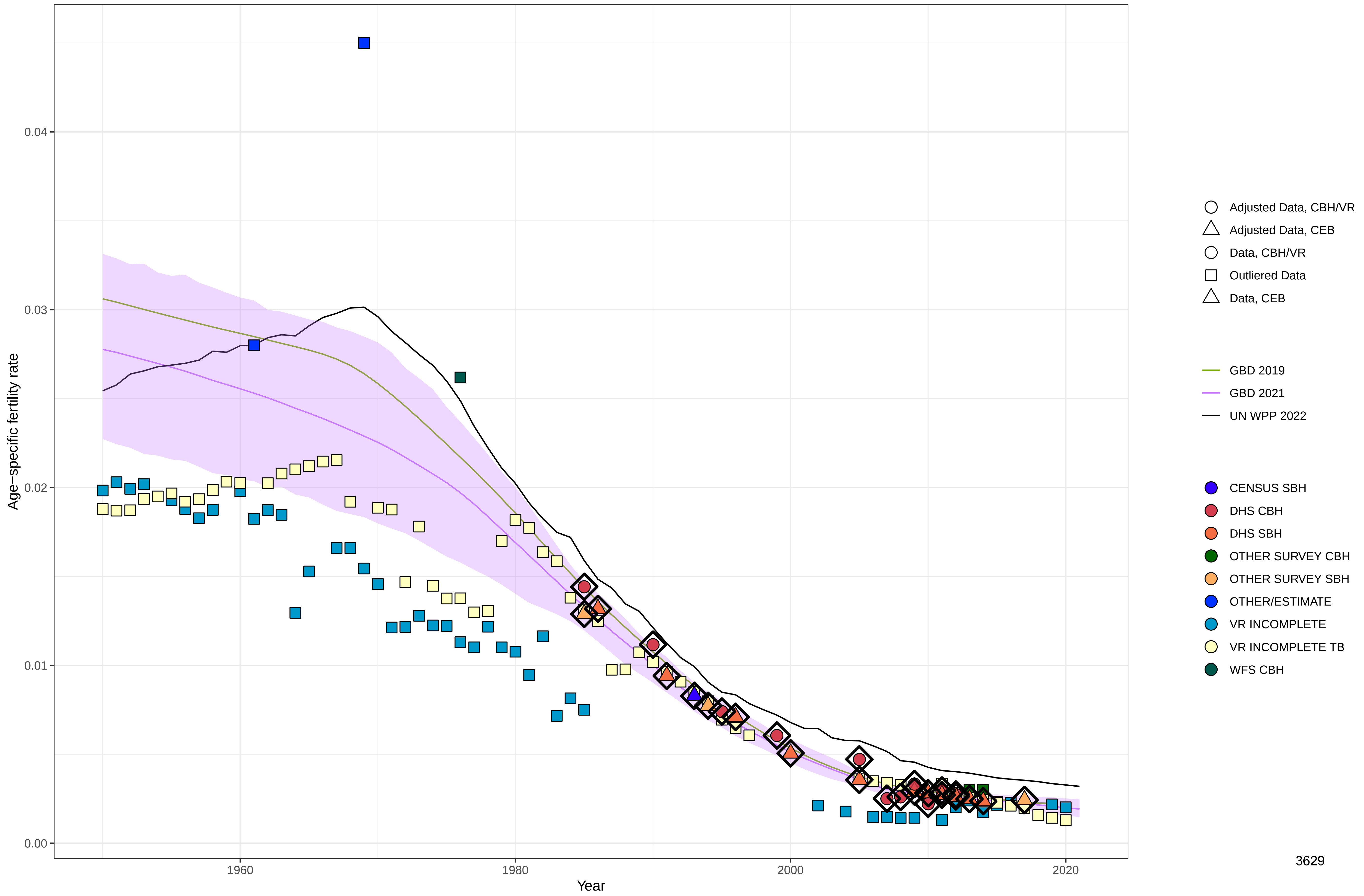

Age−specific fertility fitted trend lines, 1950−2021: Peru, Ages: 50−54
Data points enclosed with a diamond are reference sources

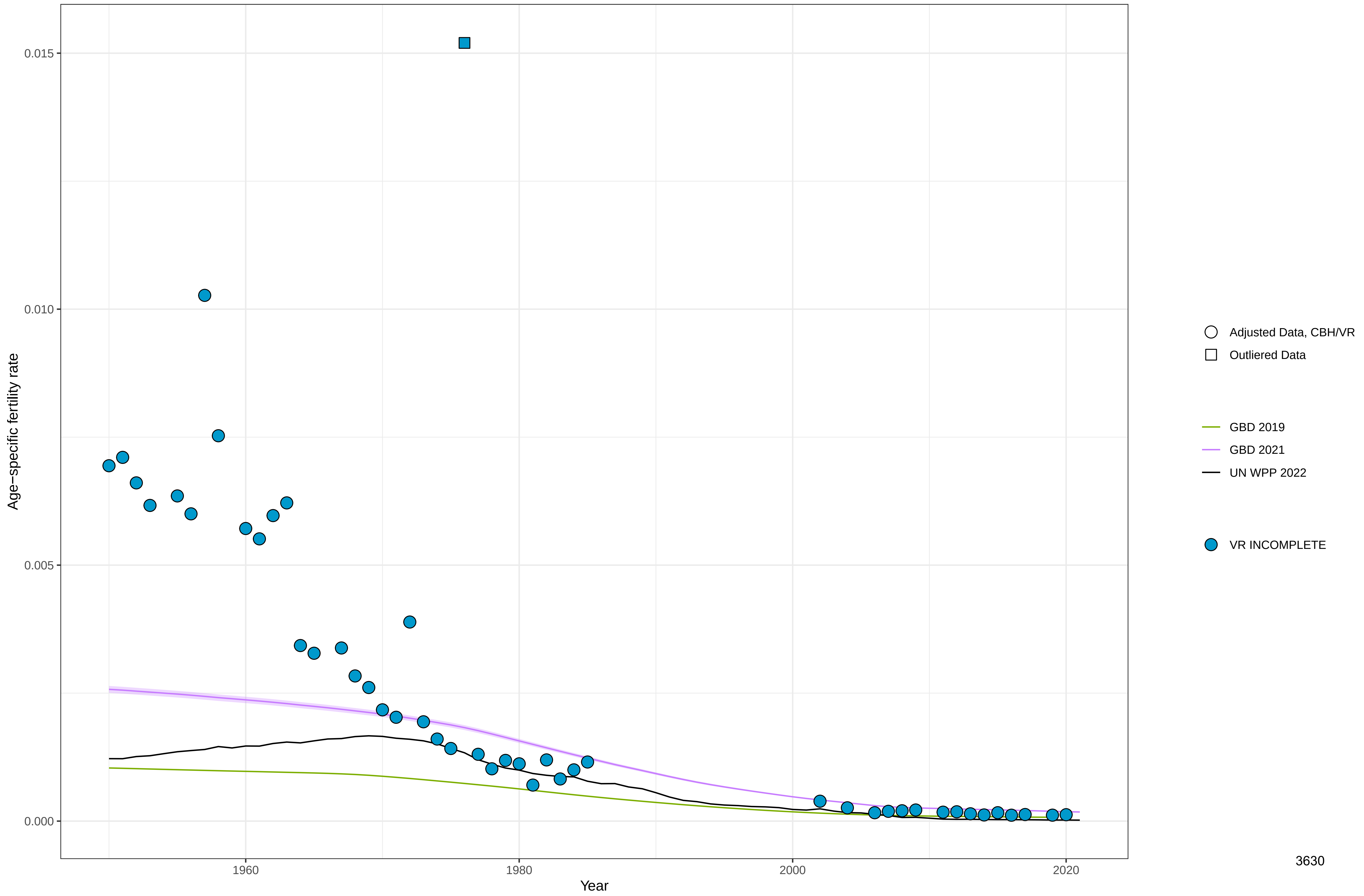

Total fertility rate fitted trend lines, 1950−2021: Peru TFR
Data points enclosed with a diamond are reference sources

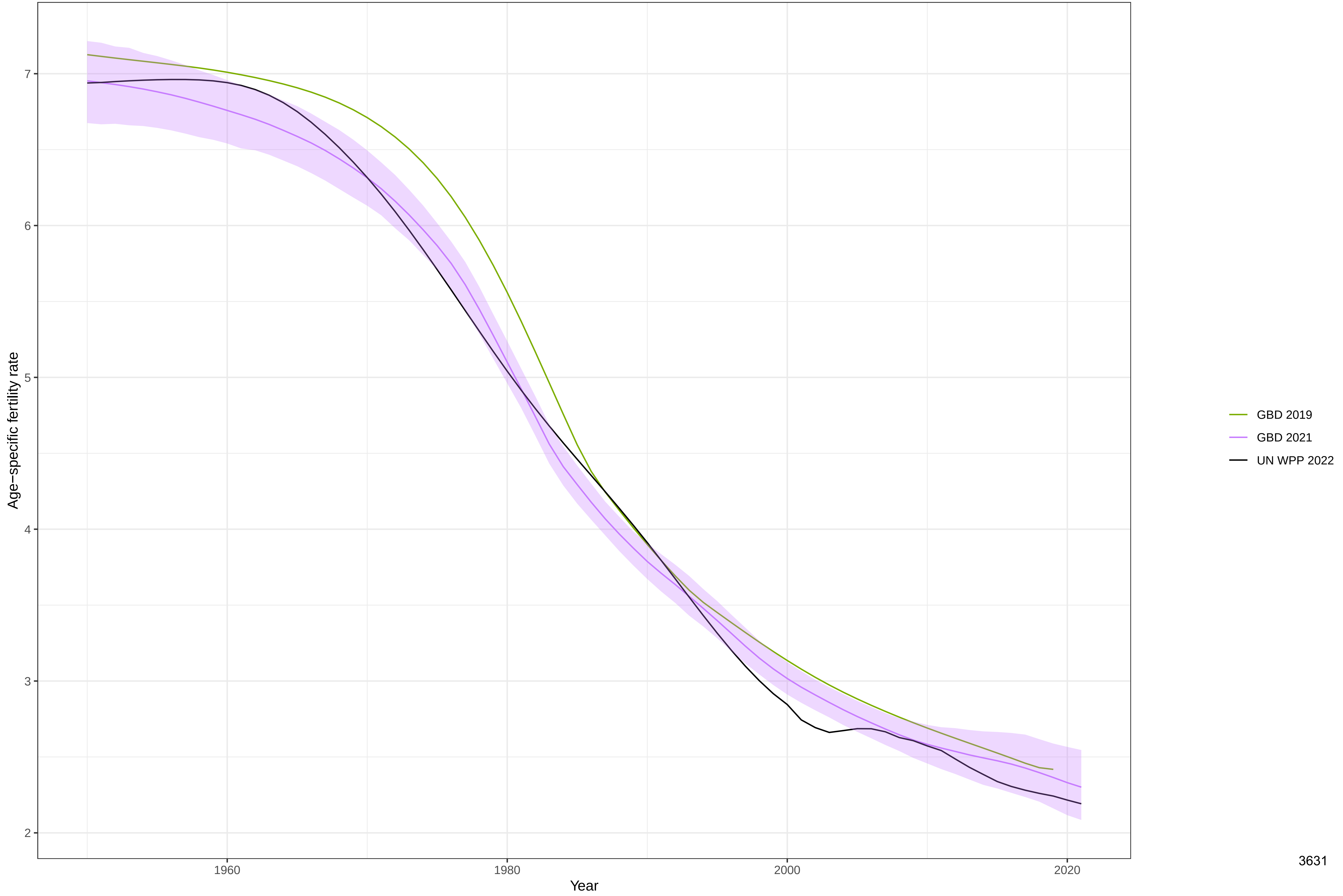

Age−specific fertility fitted trend lines, 1950−2021: Antigua and Barbuda, Ages: 10−14
Data points enclosed with a diamond are reference sources

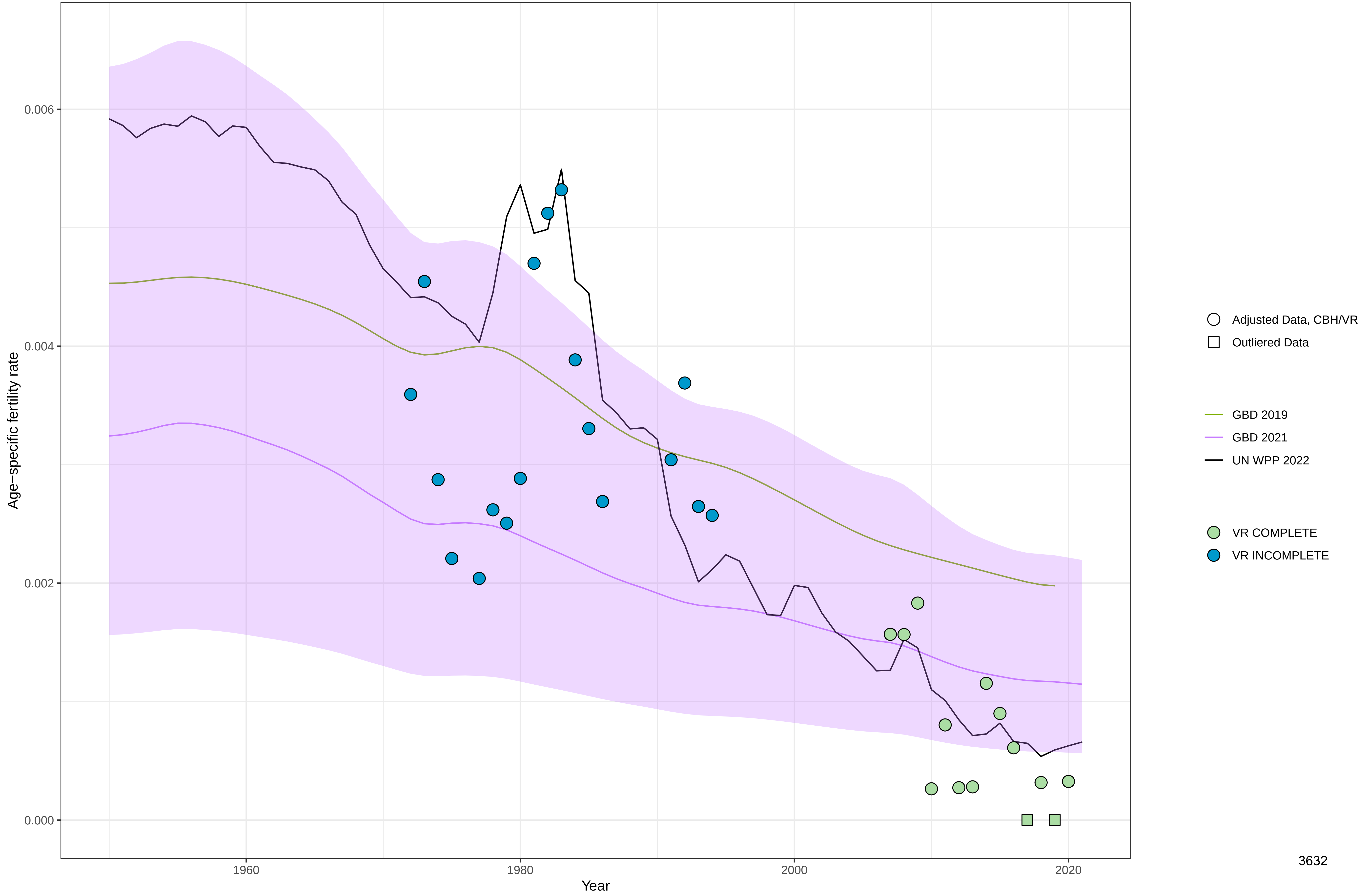

Age−specific fertility fitted trend lines, 1950−2021: Antigua and Barbuda, Ages: 15−19
Data points enclosed with a diamond are reference sources

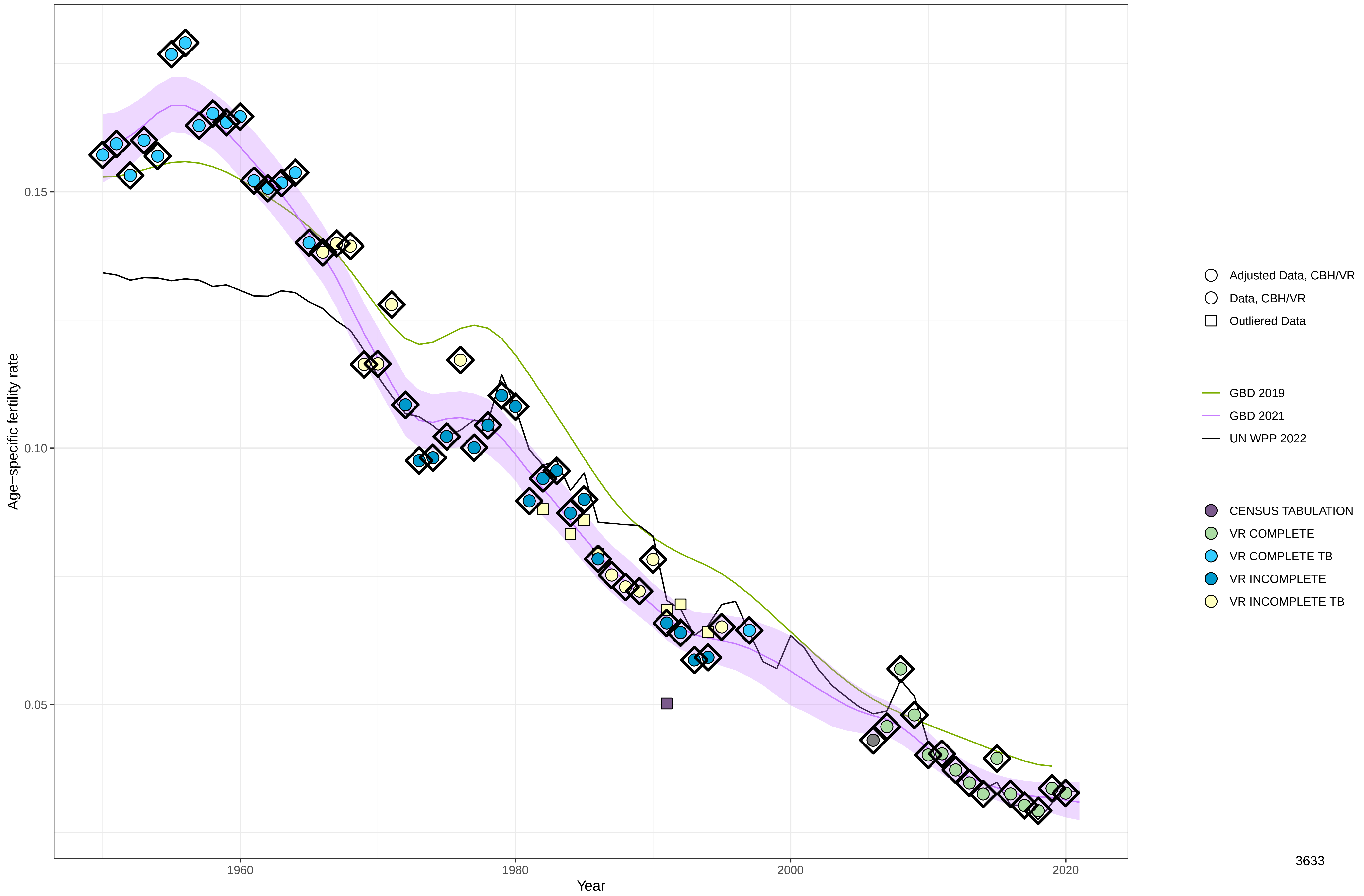

Age−specific fertility fitted trend lines, 1950−2021: Antigua and Barbuda, Ages: 20−24
Data points enclosed with a diamond are reference sources

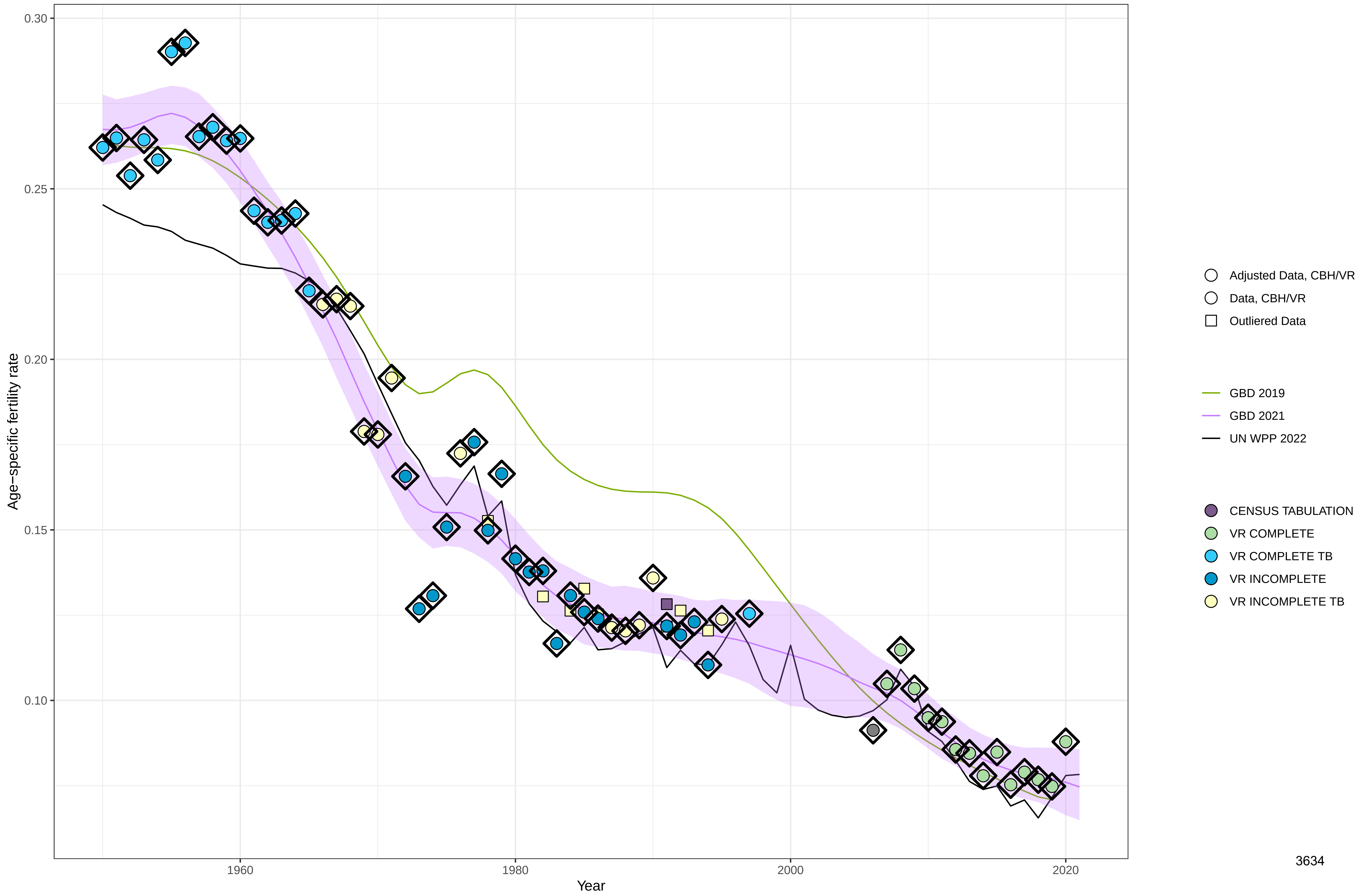

Age−specific fertility fitted trend lines, 1950−2021: Antigua and Barbuda, Ages: 25−29
Data points enclosed with a diamond are reference sources

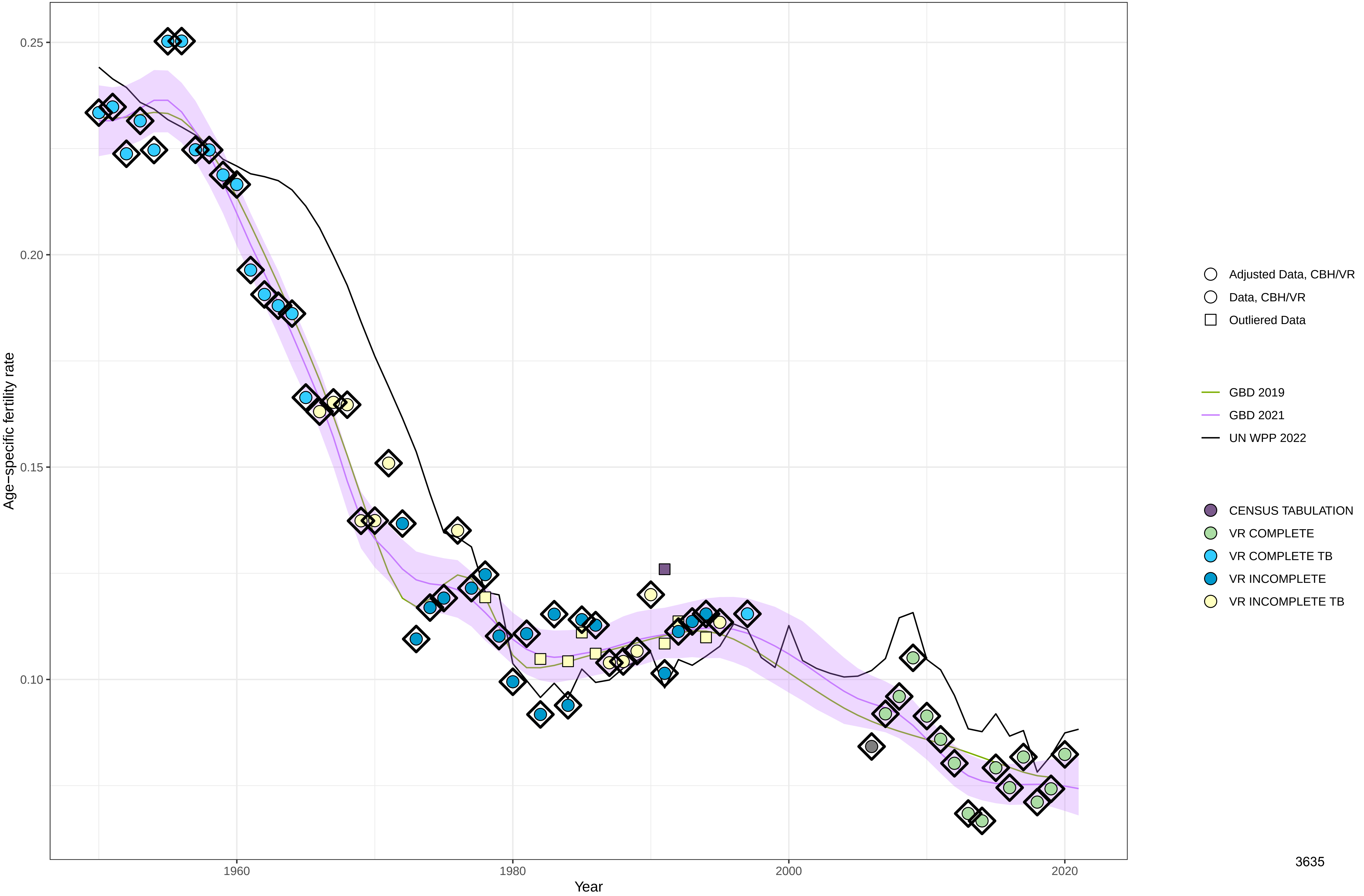

Age−specific fertility fitted trend lines, 1950−2021: Antigua and Barbuda, Ages: 30−34
Data points enclosed with a diamond are reference sources

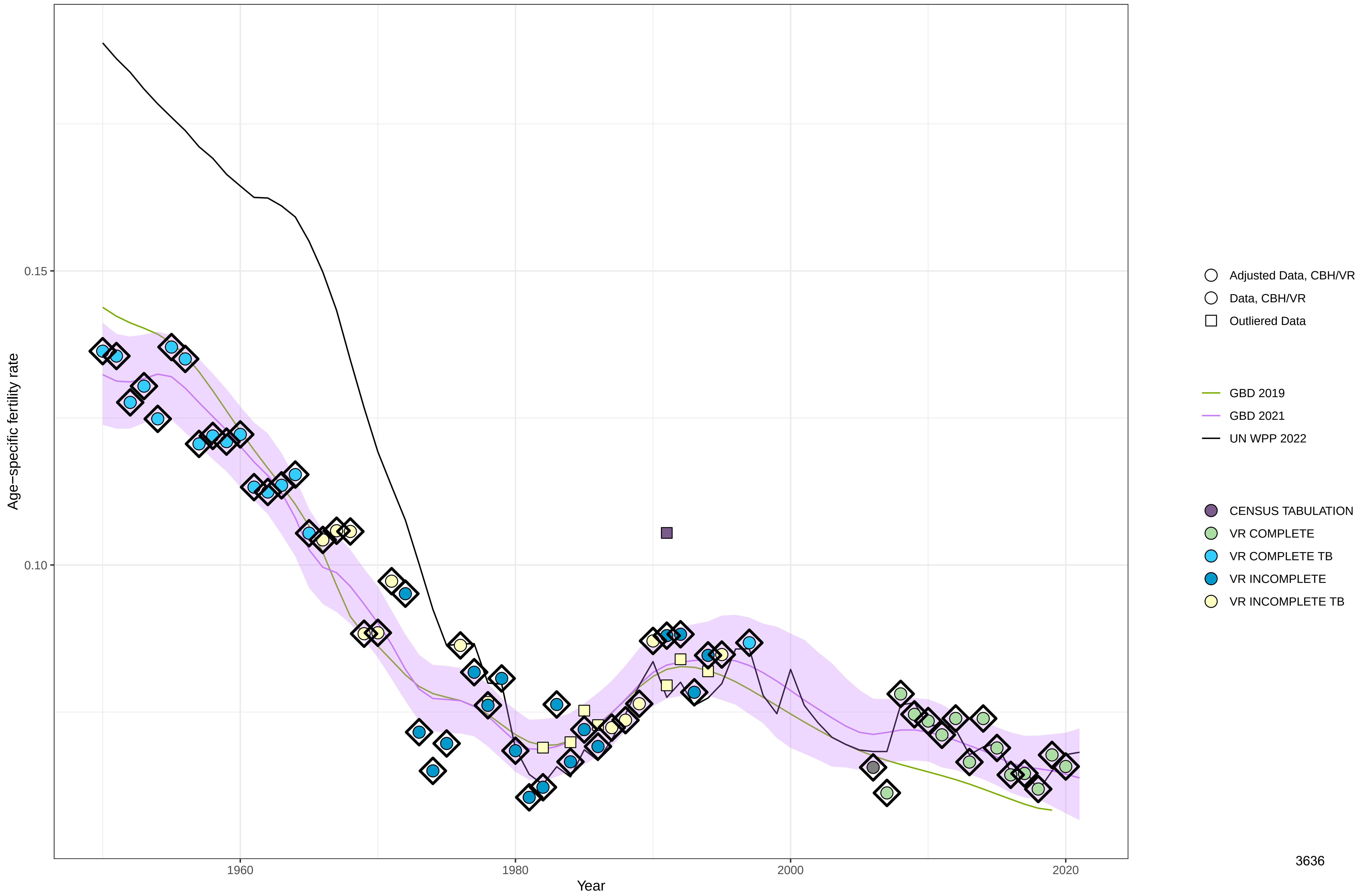

Age−specific fertility fitted trend lines, 1950−2021: Antigua and Barbuda, Ages: 35−39
Data points enclosed with a diamond are reference sources

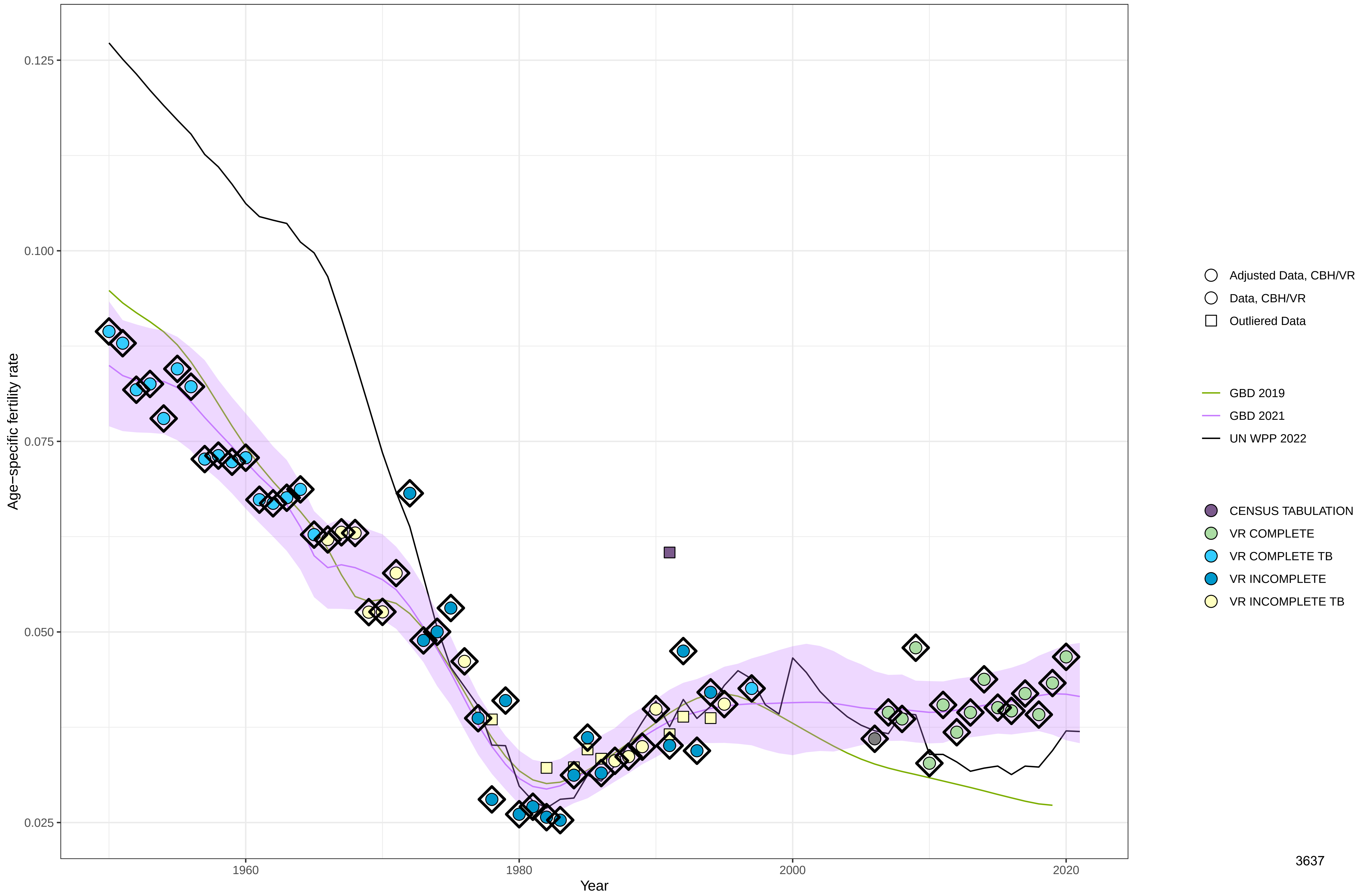

Age−specific fertility fitted trend lines, 1950−2021: Antigua and Barbuda, Ages: 40−44
Data points enclosed with a diamond are reference sources

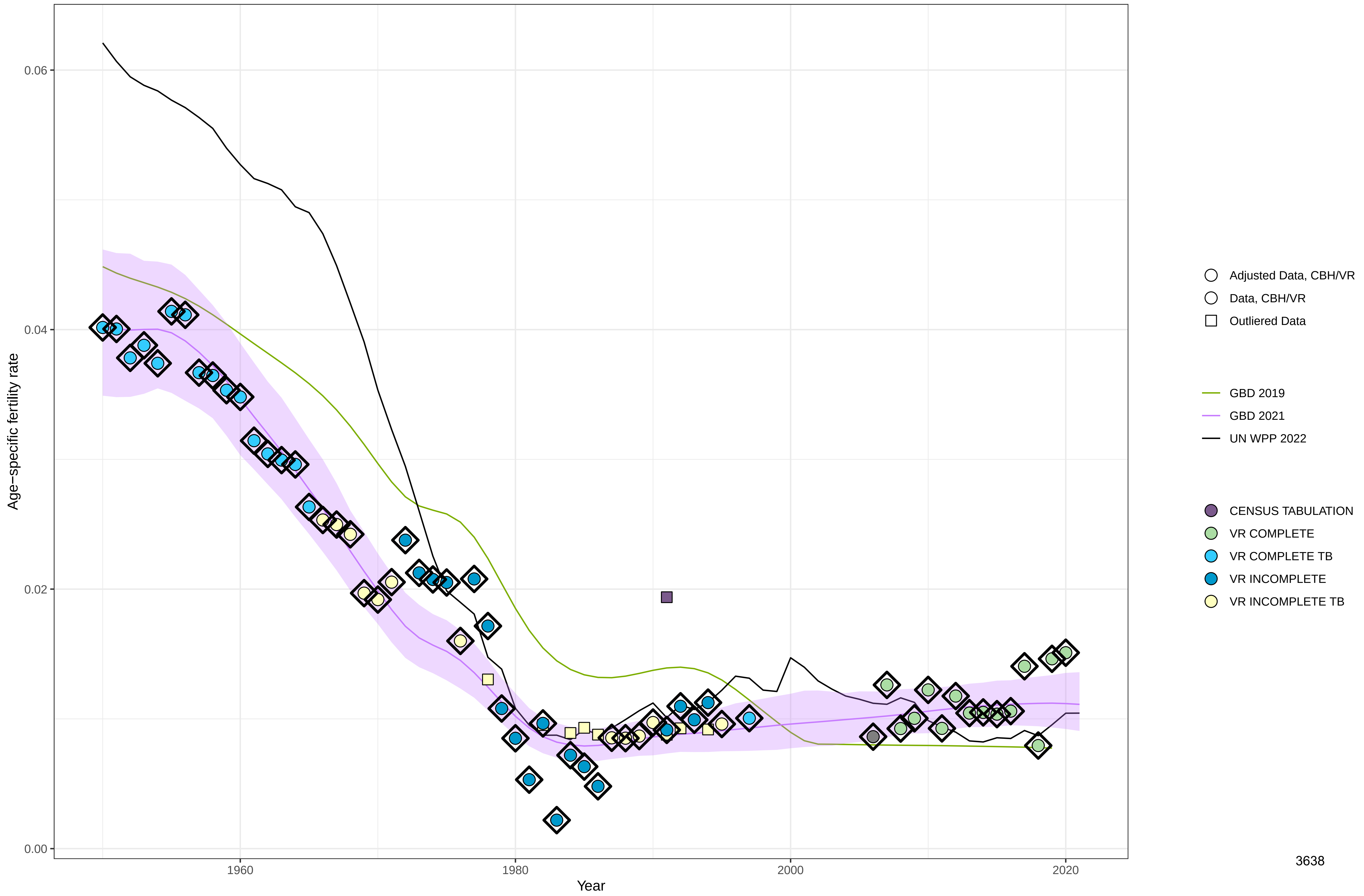

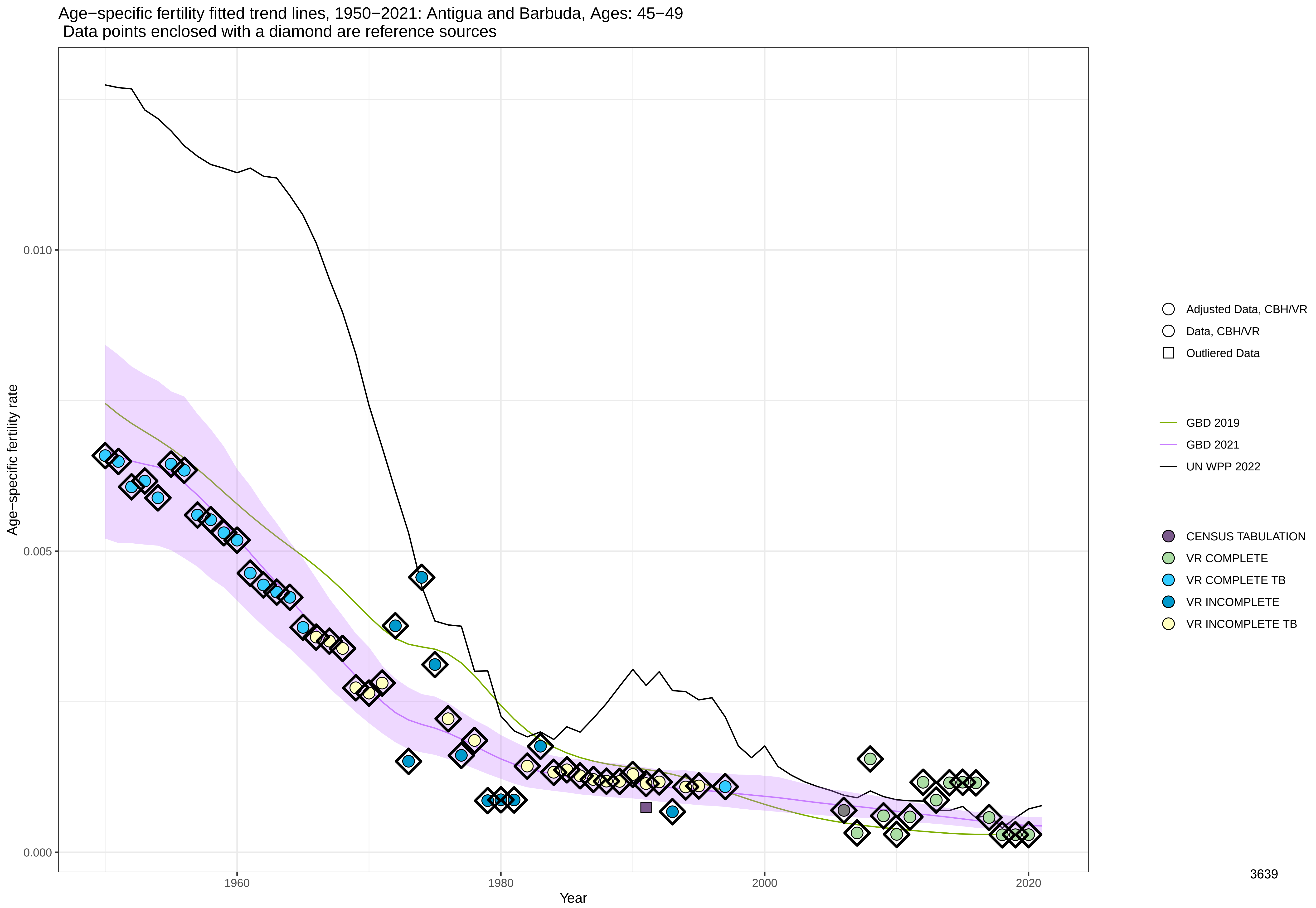

Age−specific fertility fitted trend lines, 1950−2021: Antigua and Barbuda, Ages: 45−49
Data points enclosed with a diamond are reference sources
Age−specific fertility rate
0.000
0.005
0.010
Year
1960
1980
2000
2020
Adjusted Data, CBH/VR
Data, CBH/VR
Outliered Data
GBD 2019
GBD 2021
UN WPP 2022
CENSUS TABULATION
VR COMPLETE
VR COMPLETE TB
VR INCOMPLETE
VR INCOMPLETE TB

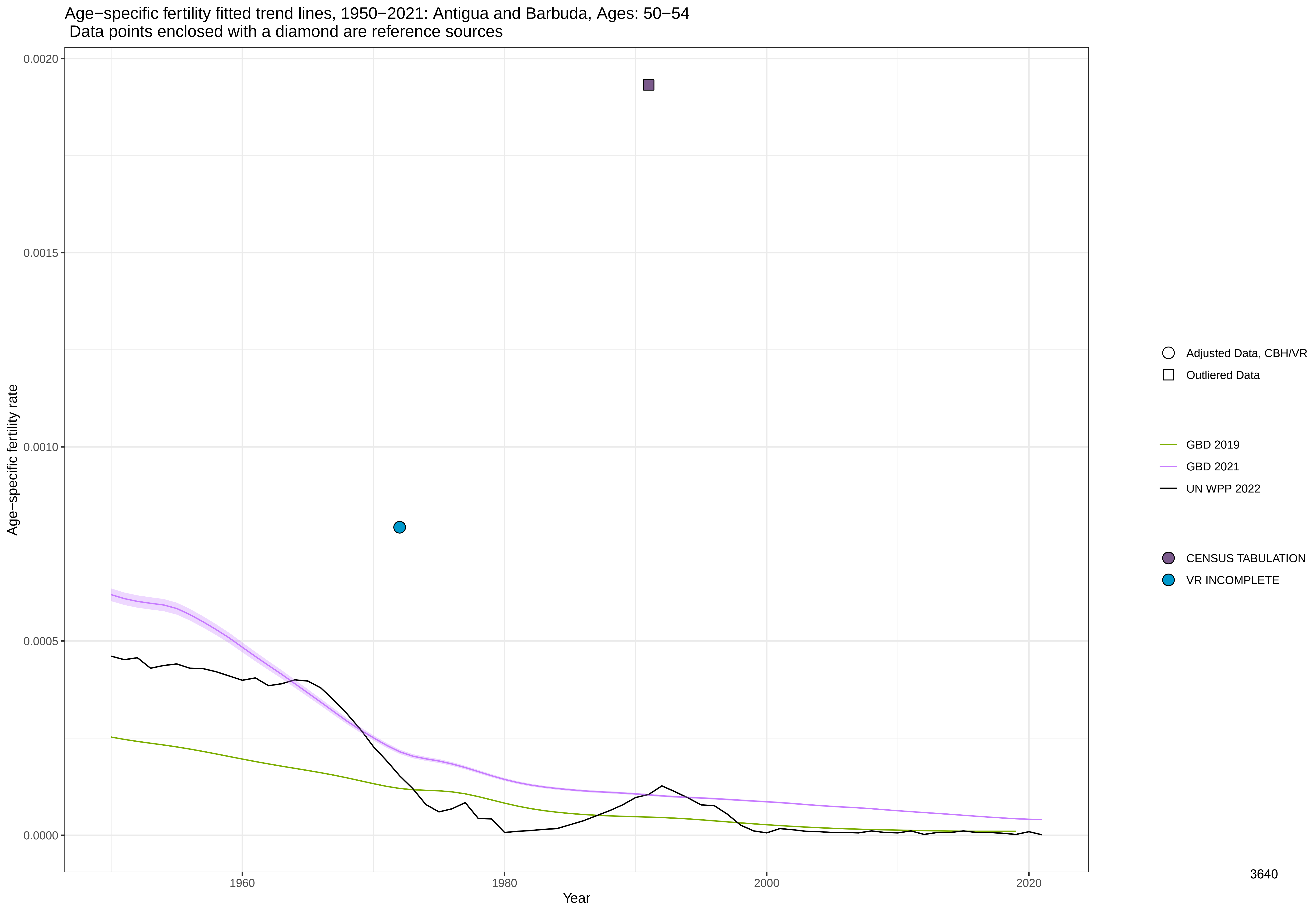

Age−specific fertility fitted trend lines, 1950−2021: Antigua and Barbuda, Ages: 50−54
Data points enclosed with a diamond are reference sources
0.0020
0.0015
0.0010
0.0005
0.0000
Age−specific fertility rate
1960
1980
2000
2020
Year
Adjusted Data, CBH/VR
Outliered Data
GBD 2019
GBD 2021
UN WPP 2022
CENSUS TABULATION
VR INCOMPLETE

Total fertility rate fitted trend lines, 1950−2021: Antigua and Barbuda TFR
Data points enclosed with a diamond are reference sources

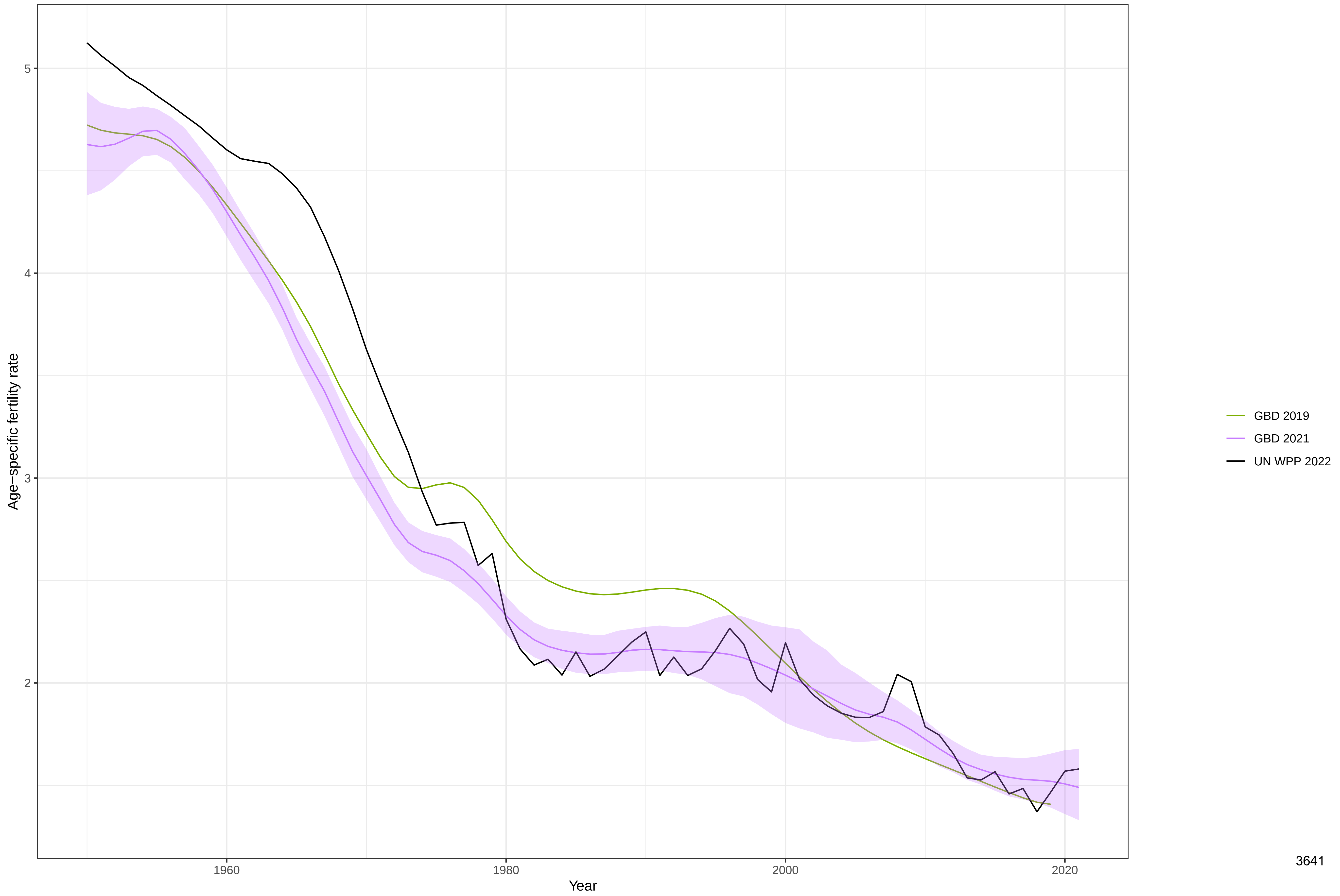

Age−specific fertility fitted trend lines, 1950−2021: The Bahamas, Ages: 10−14
Data points enclosed with a diamond are reference sources

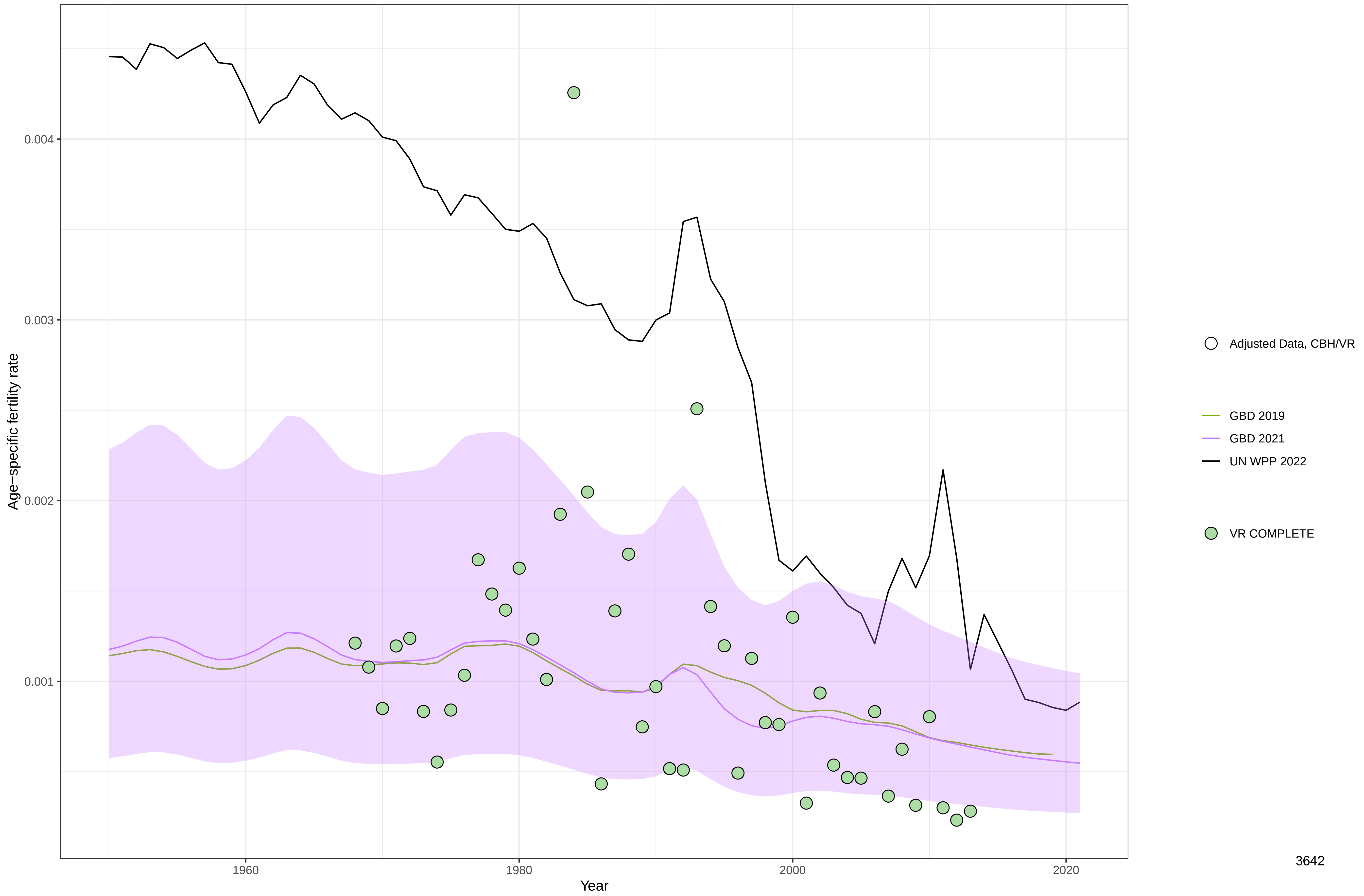

Age−specific fertility fitted trend lines, 1950−2021: The Bahamas, Ages: 15−19
Data points enclosed with a diamond are reference sources

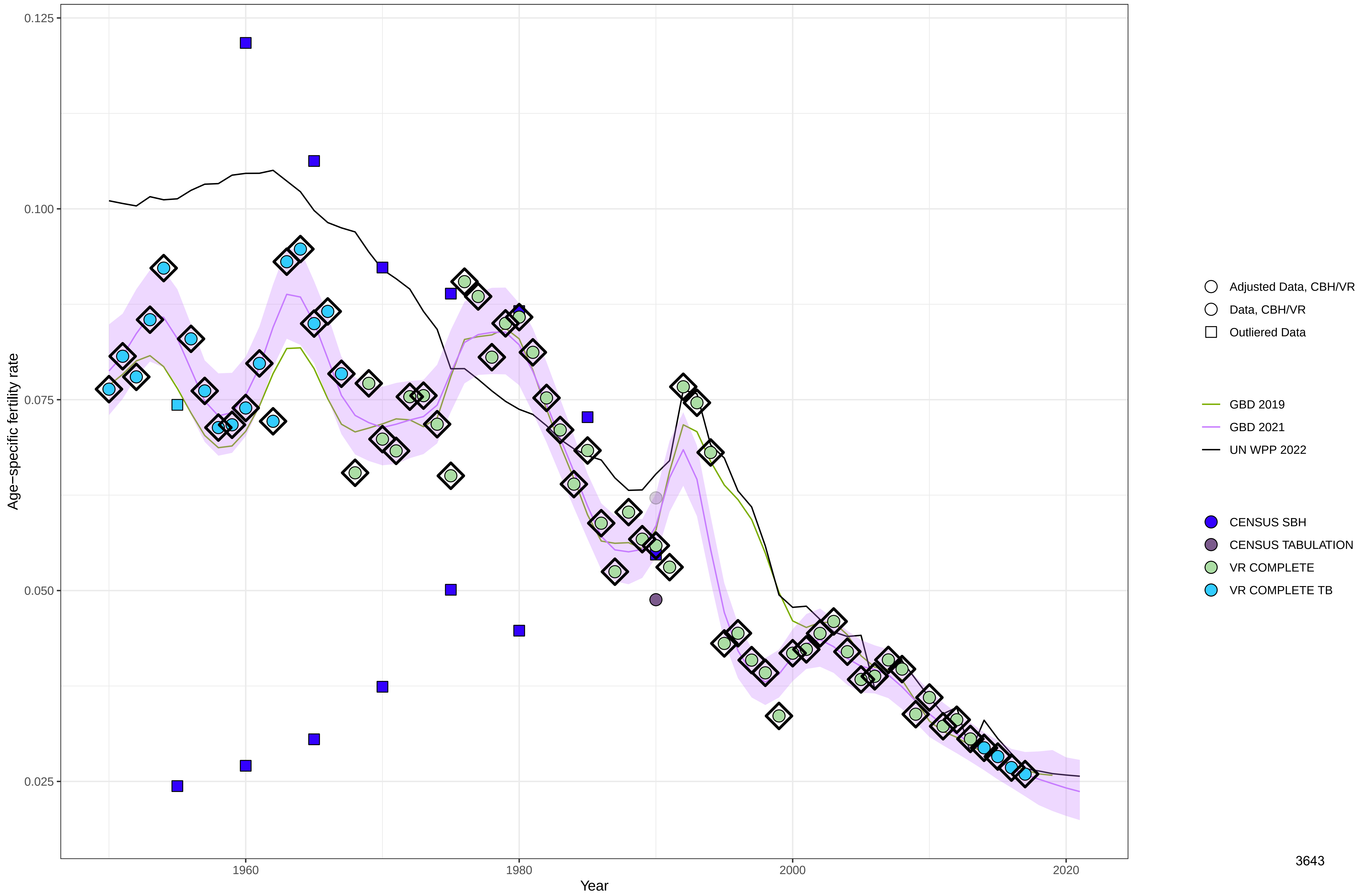

Age−specific fertility fitted trend lines, 1950−2021: The Bahamas, Ages: 20−24
Data points enclosed with a diamond are reference sources

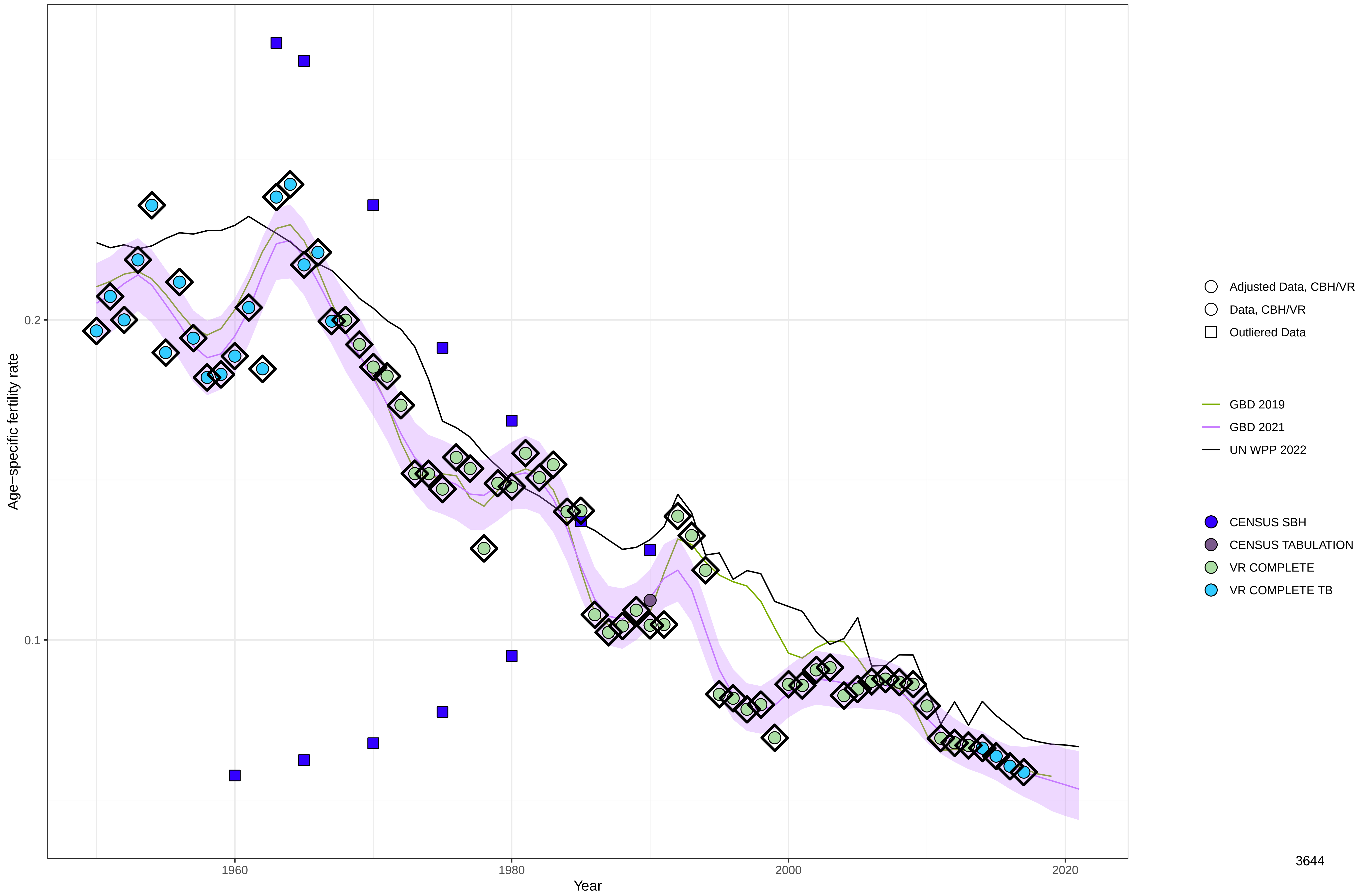

Age−specific fertility fitted trend lines, 1950−2021: The Bahamas, Ages: 25−29
Data points enclosed with a diamond are reference sources

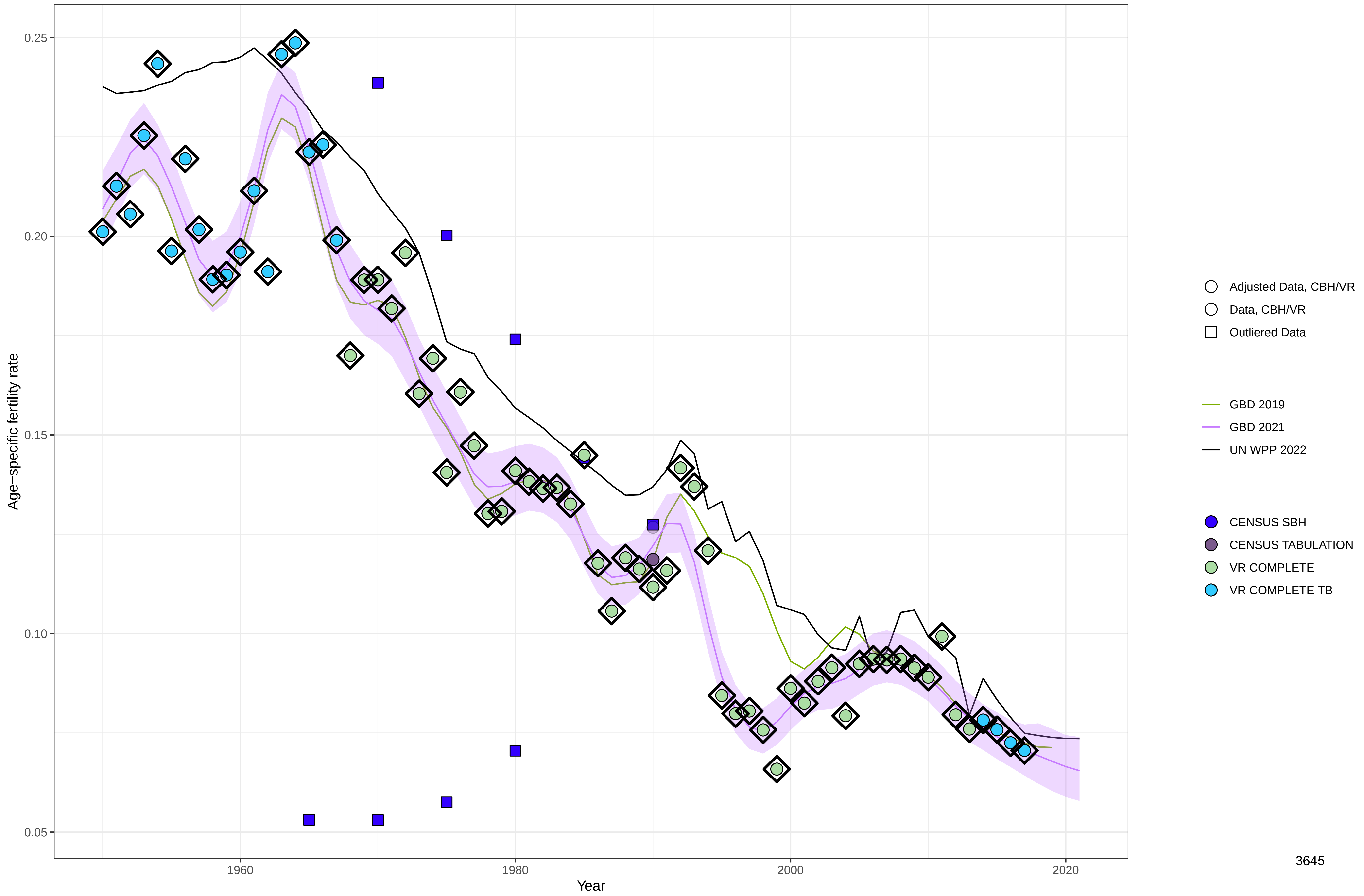

Age−specific fertility fitted trend lines, 1950−2021: The Bahamas, Ages: 30−34
Data points enclosed with a diamond are reference sources

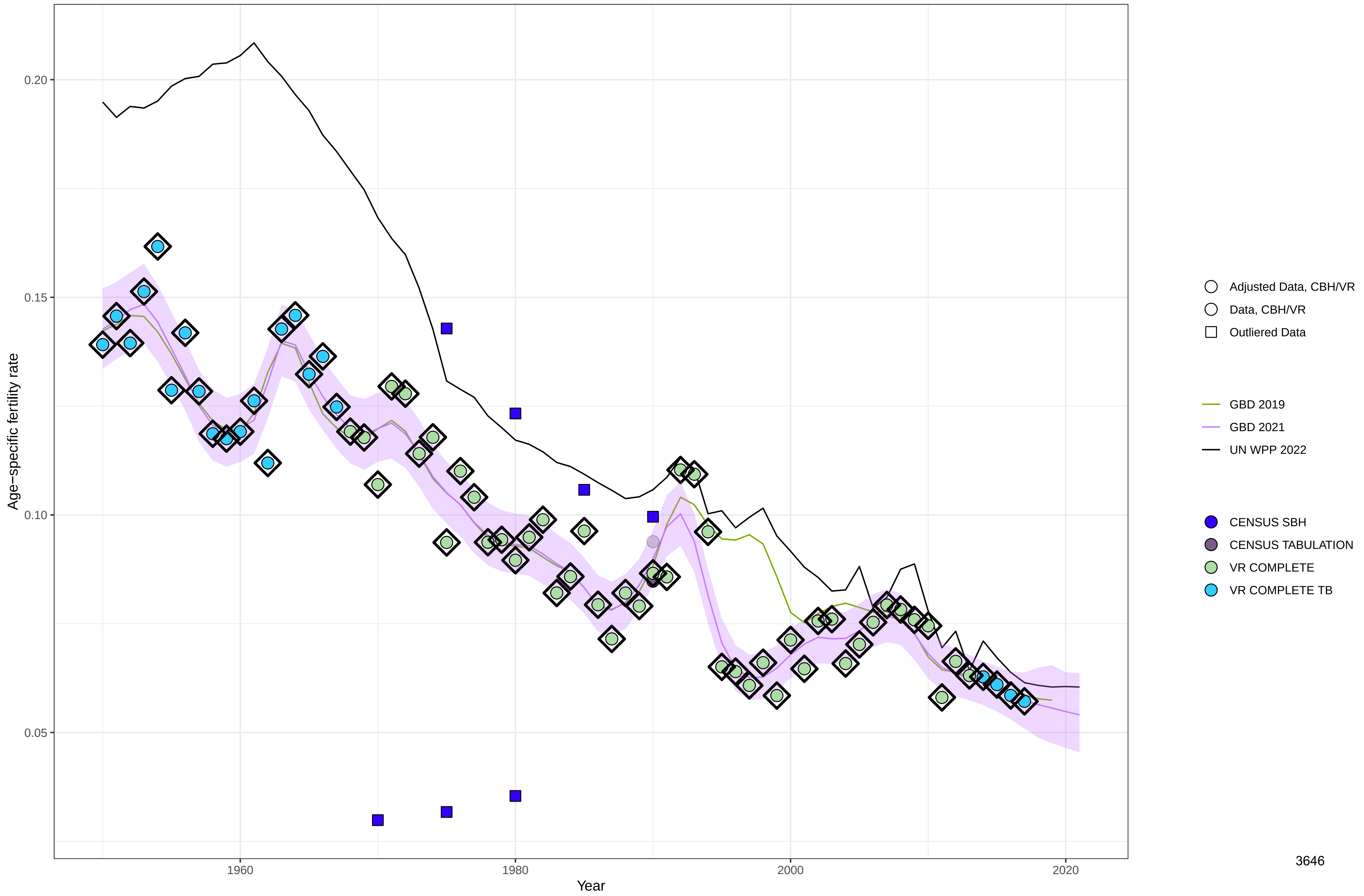

Age−specific fertility fitted trend lines, 1950−2021: The Bahamas, Ages: 35−39
Data points enclosed with a diamond are reference sources

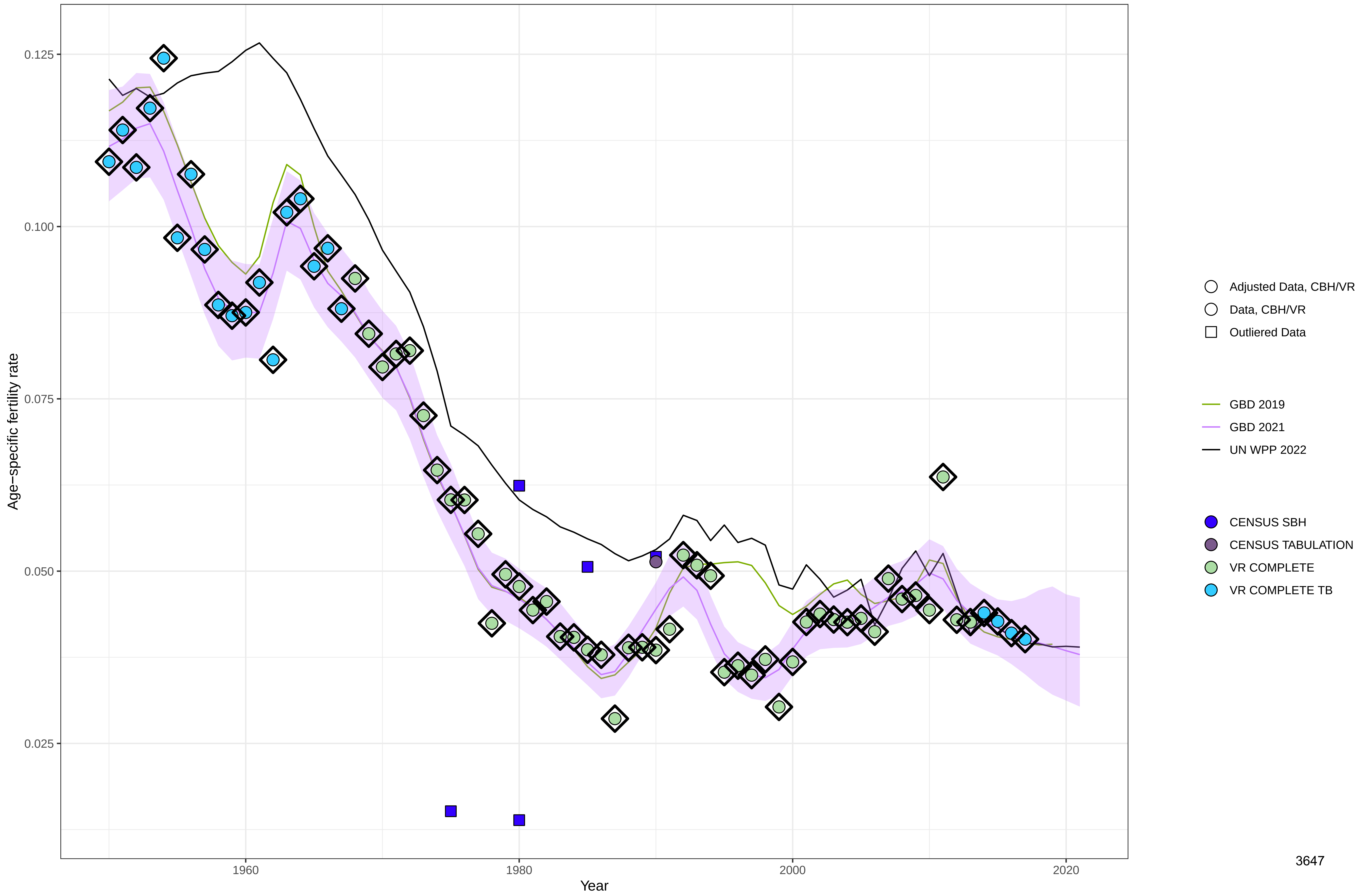

Age−specific fertility fitted trend lines, 1950−2021: The Bahamas, Ages: 40−44
Data points enclosed with a diamond are reference sources

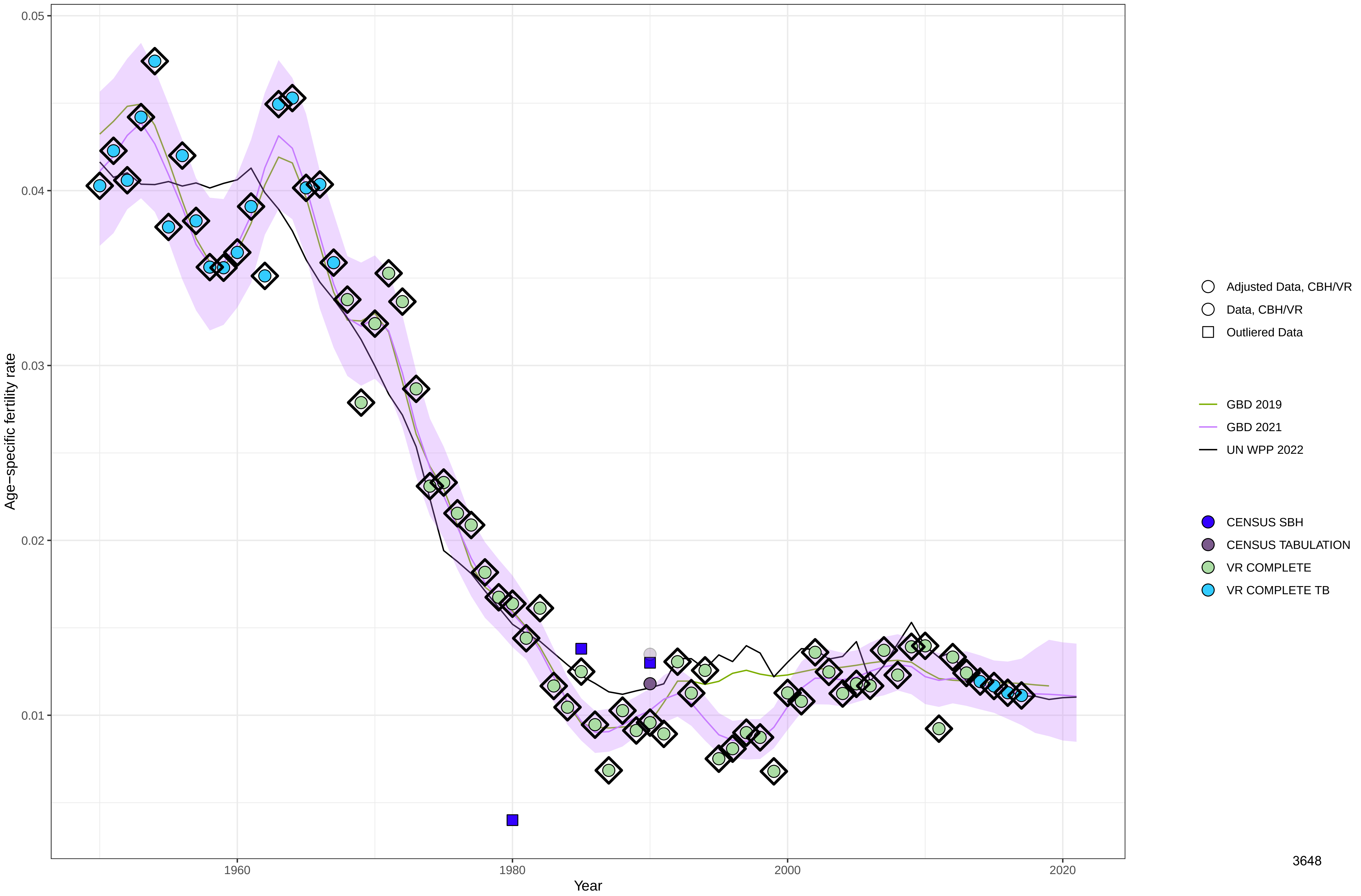

Age−specific fertility fitted trend lines, 1950−2021: The Bahamas, Ages: 45−49
Data points enclosed with a diamond are reference sources

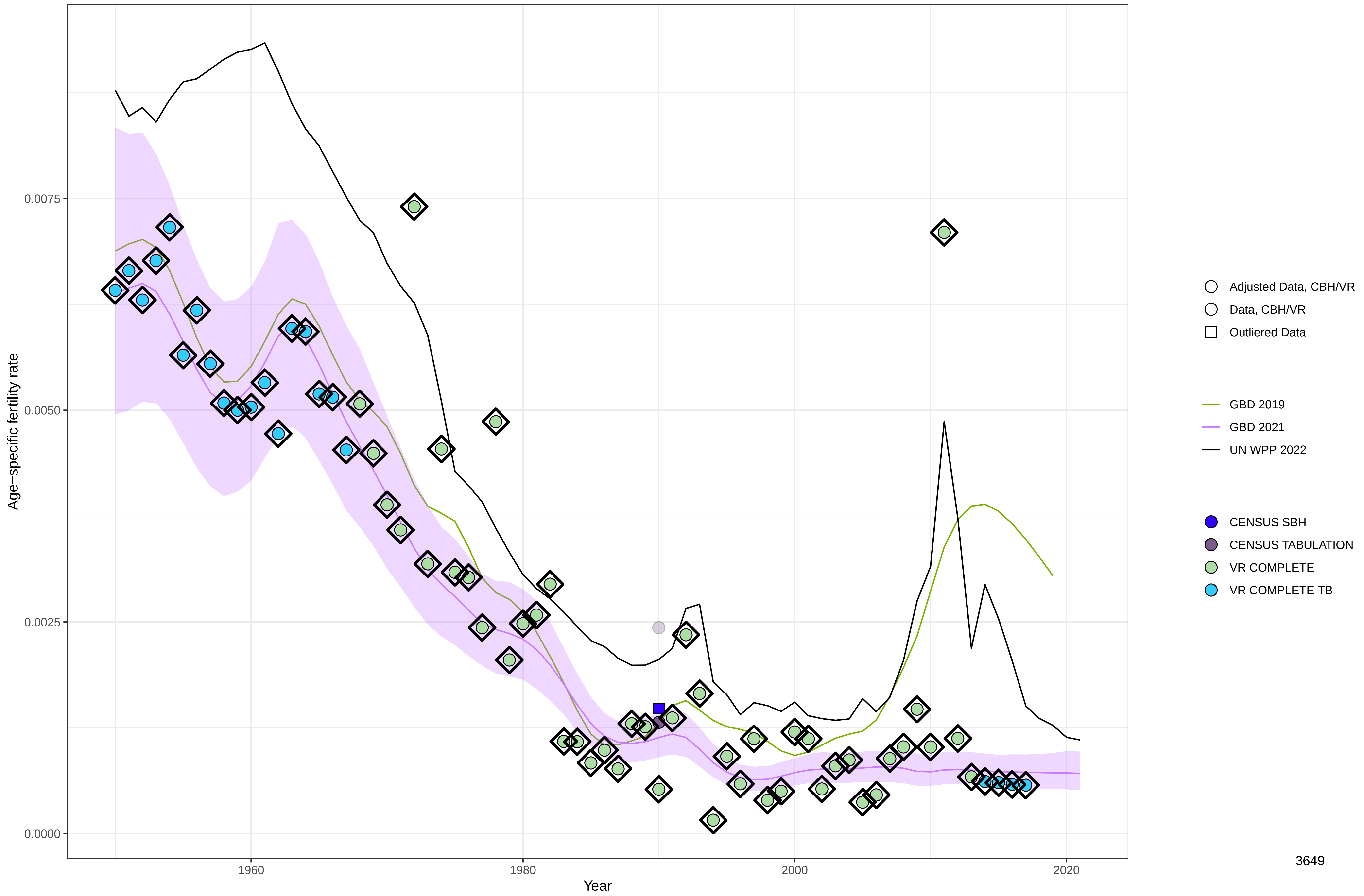

Age−specific fertility fitted trend lines, 1950−2021: The Bahamas, Ages: 50−54
Data points enclosed with a diamond are reference sources

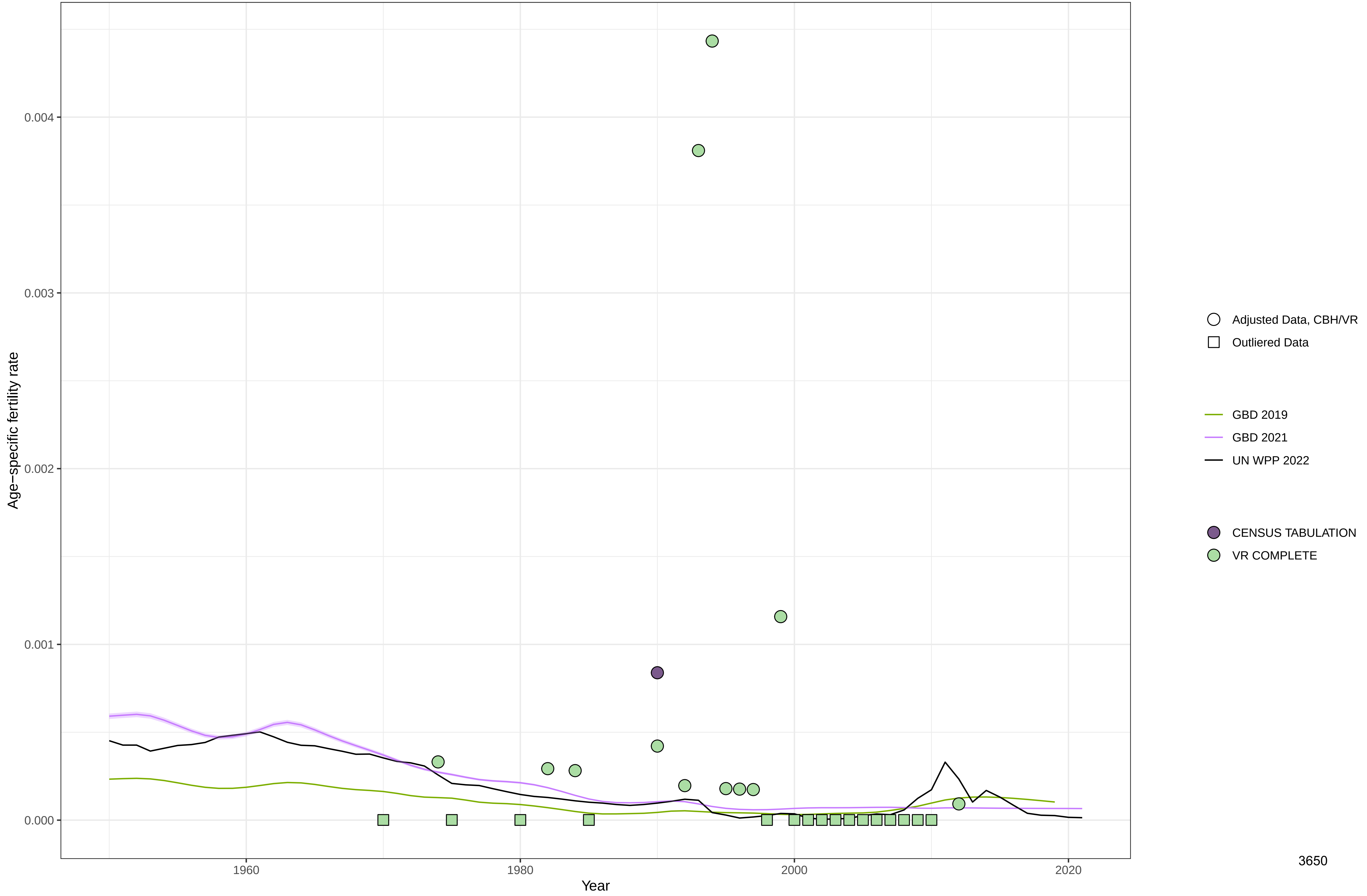

Total fertility rate fitted trend lines, 1950−2021: The Bahamas TFR
Data points enclosed with a diamond are reference sources

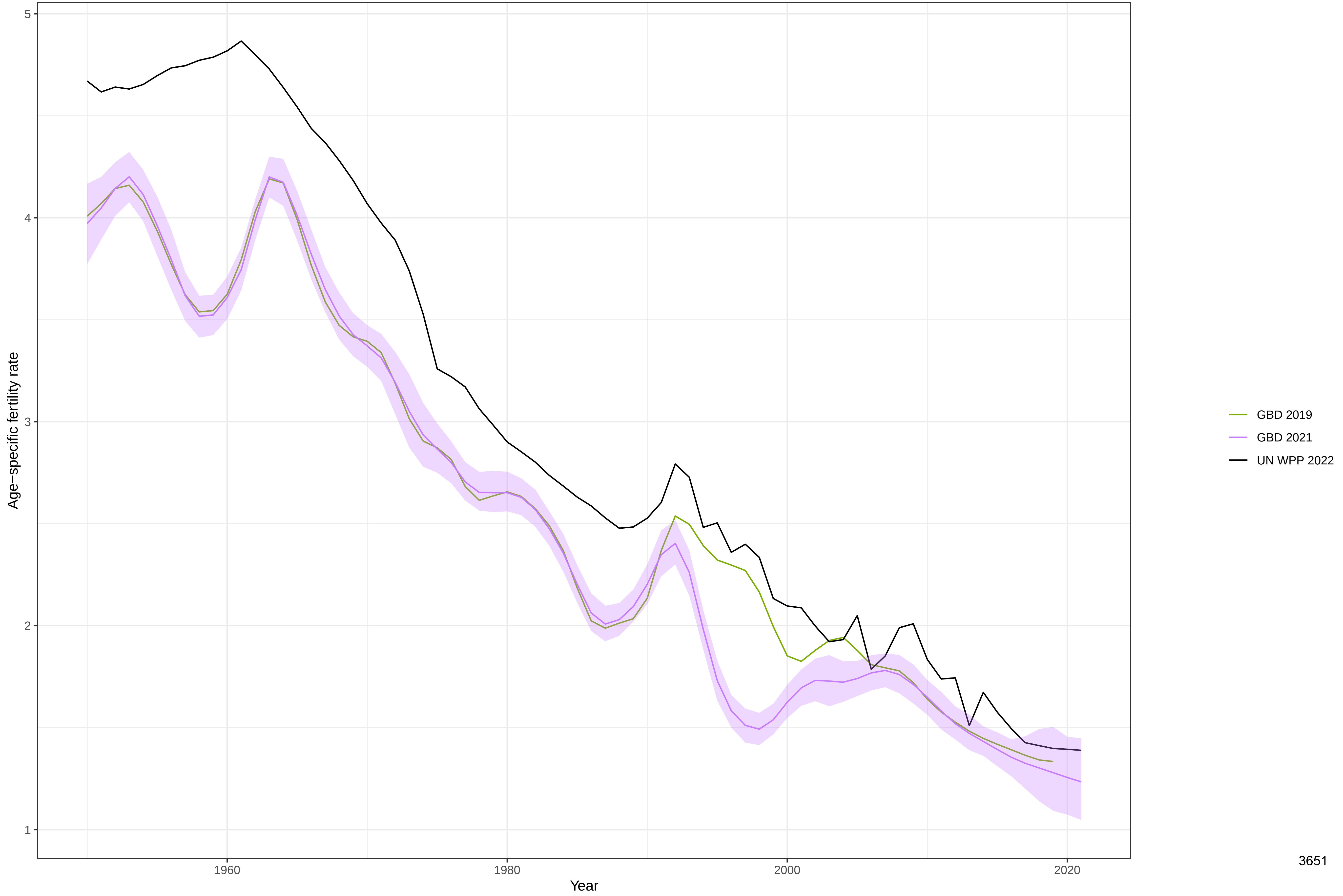

Age−specific fertility fitted trend lines, 1950−2021: Barbados, Ages: 10−14
Data points enclosed with a diamond are reference sources

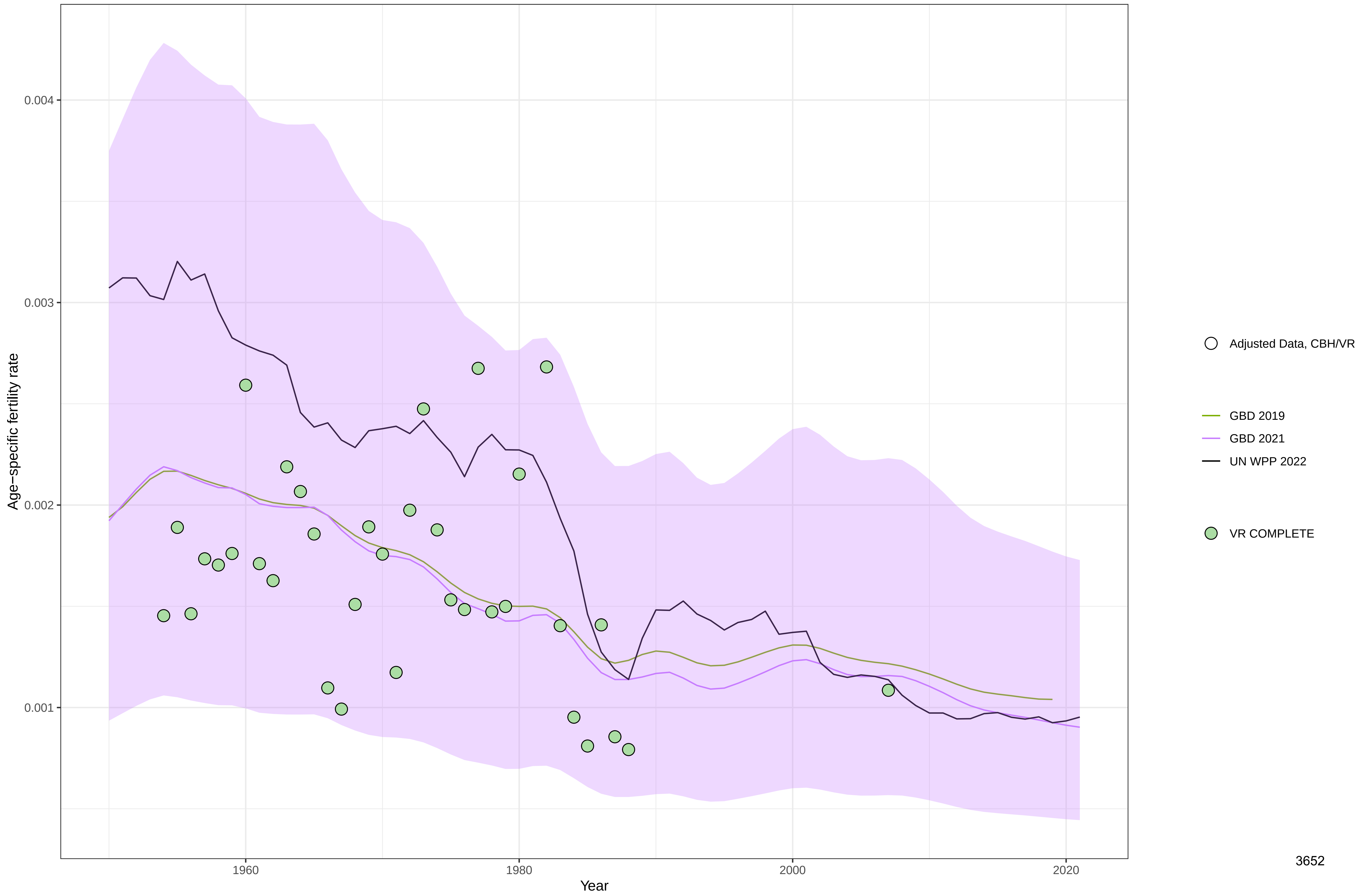

Age−specific fertility fitted trend lines, 1950−2021: Barbados, Ages: 15−19
Data points enclosed with a diamond are reference sources

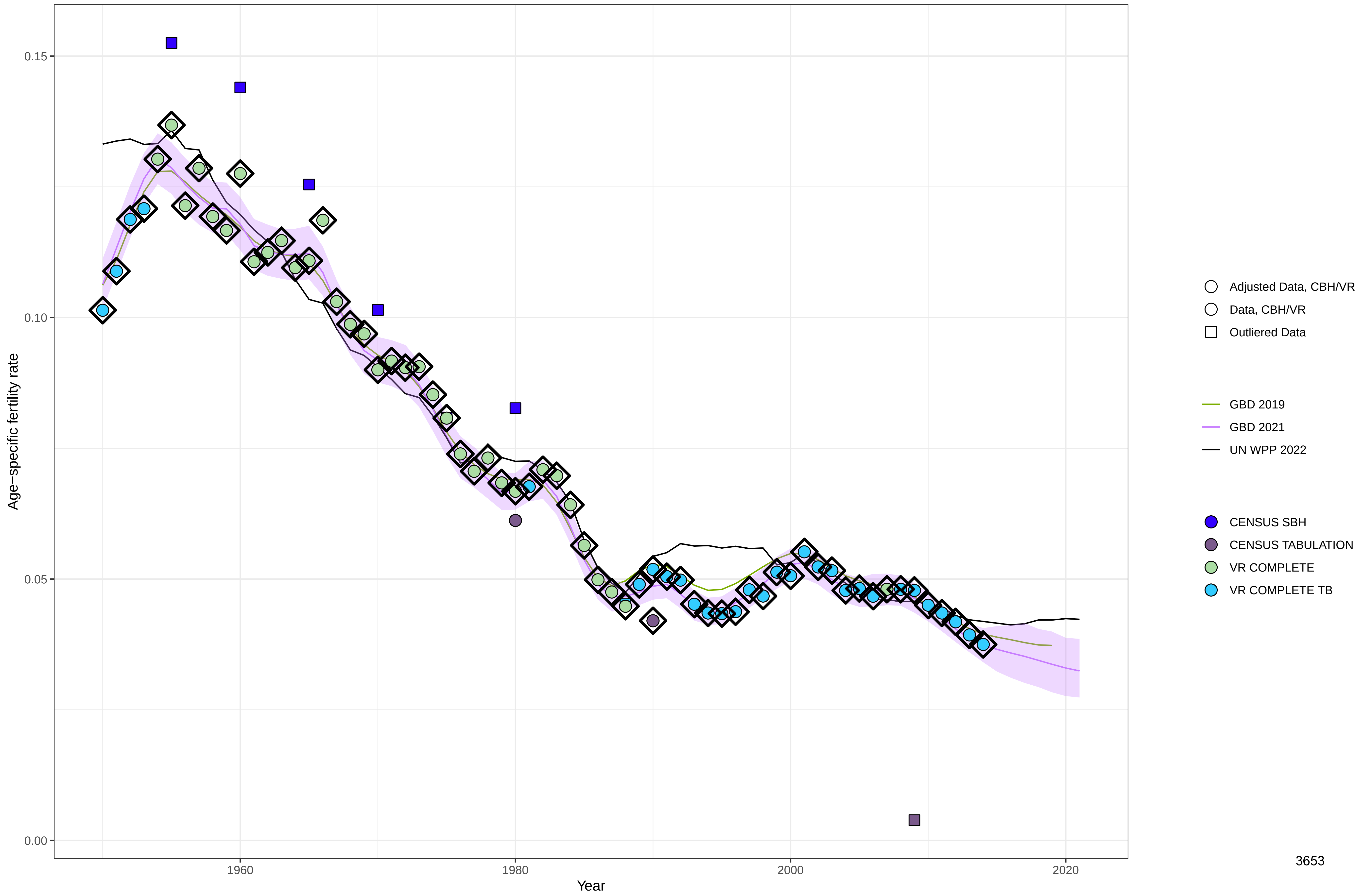

Age−specific fertility fitted trend lines, 1950−2021: Barbados, Ages: 20−24
Data points enclosed with a diamond are reference sources

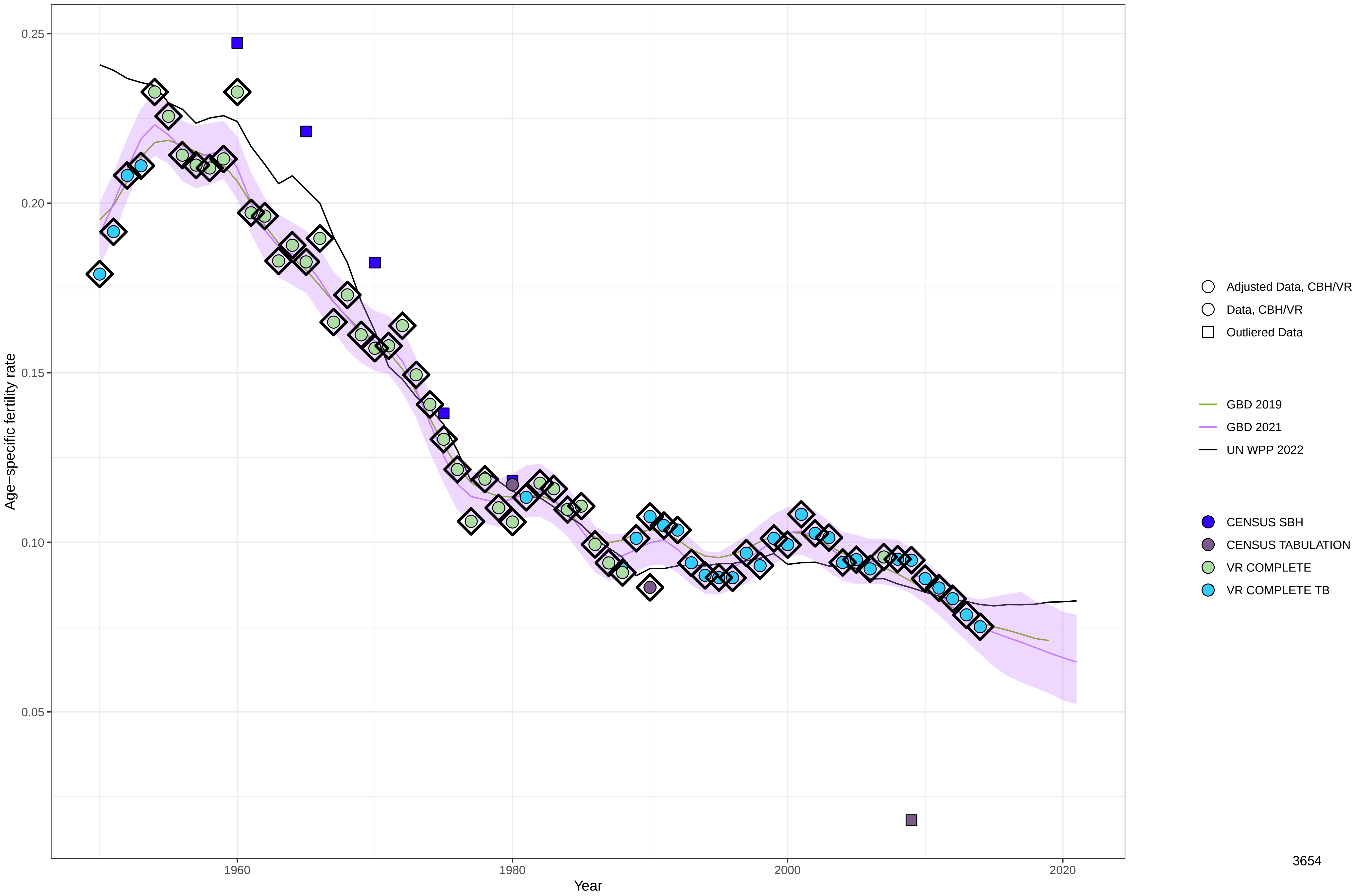

Age−specific fertility fitted trend lines, 1950−2021: Barbados, Ages: 25−29
Data points enclosed with a diamond are reference sources

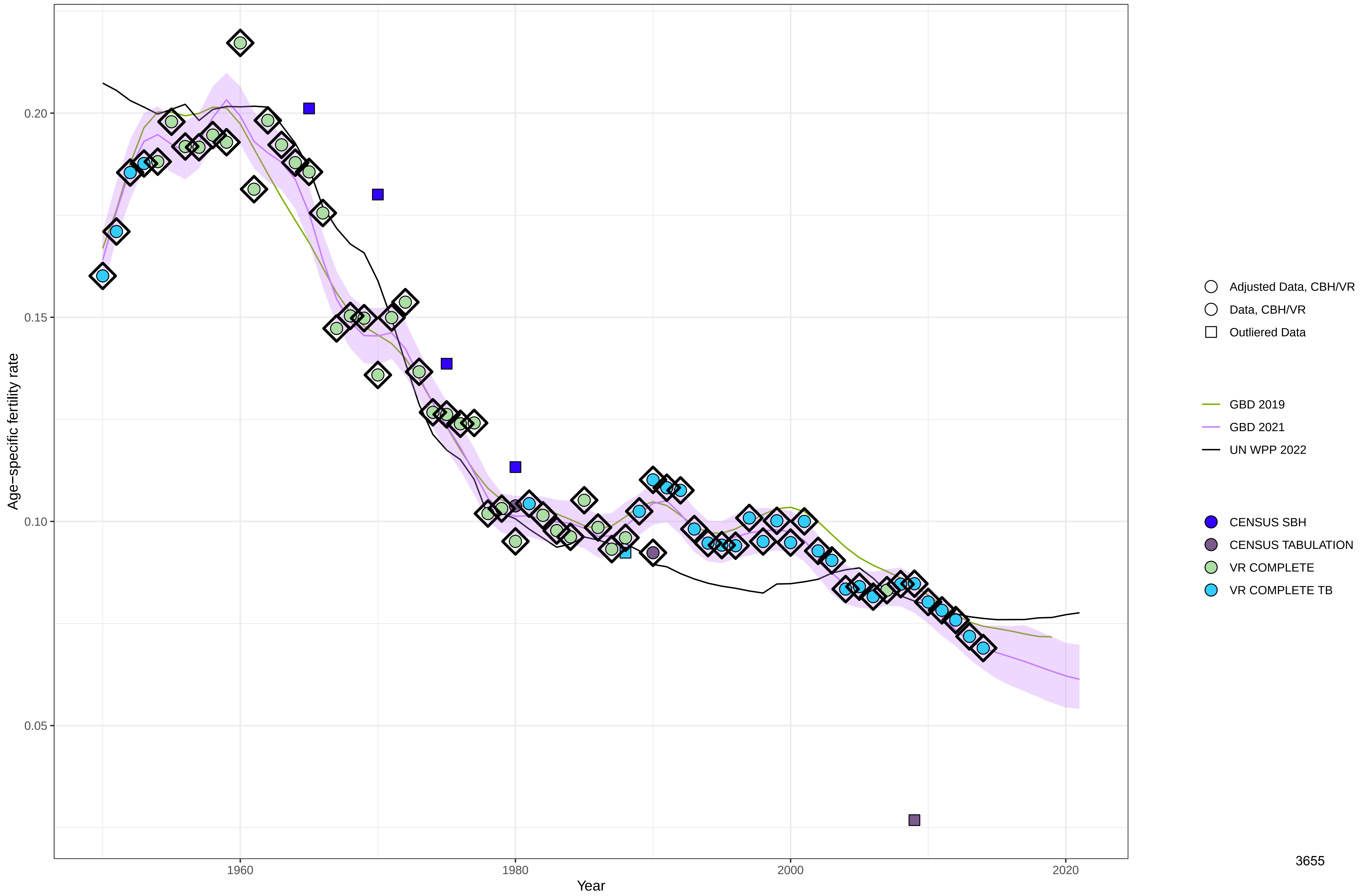

Age−specific fertility fitted trend lines, 1950−2021: Barbados, Ages: 30−34
Data points enclosed with a diamond are reference sources

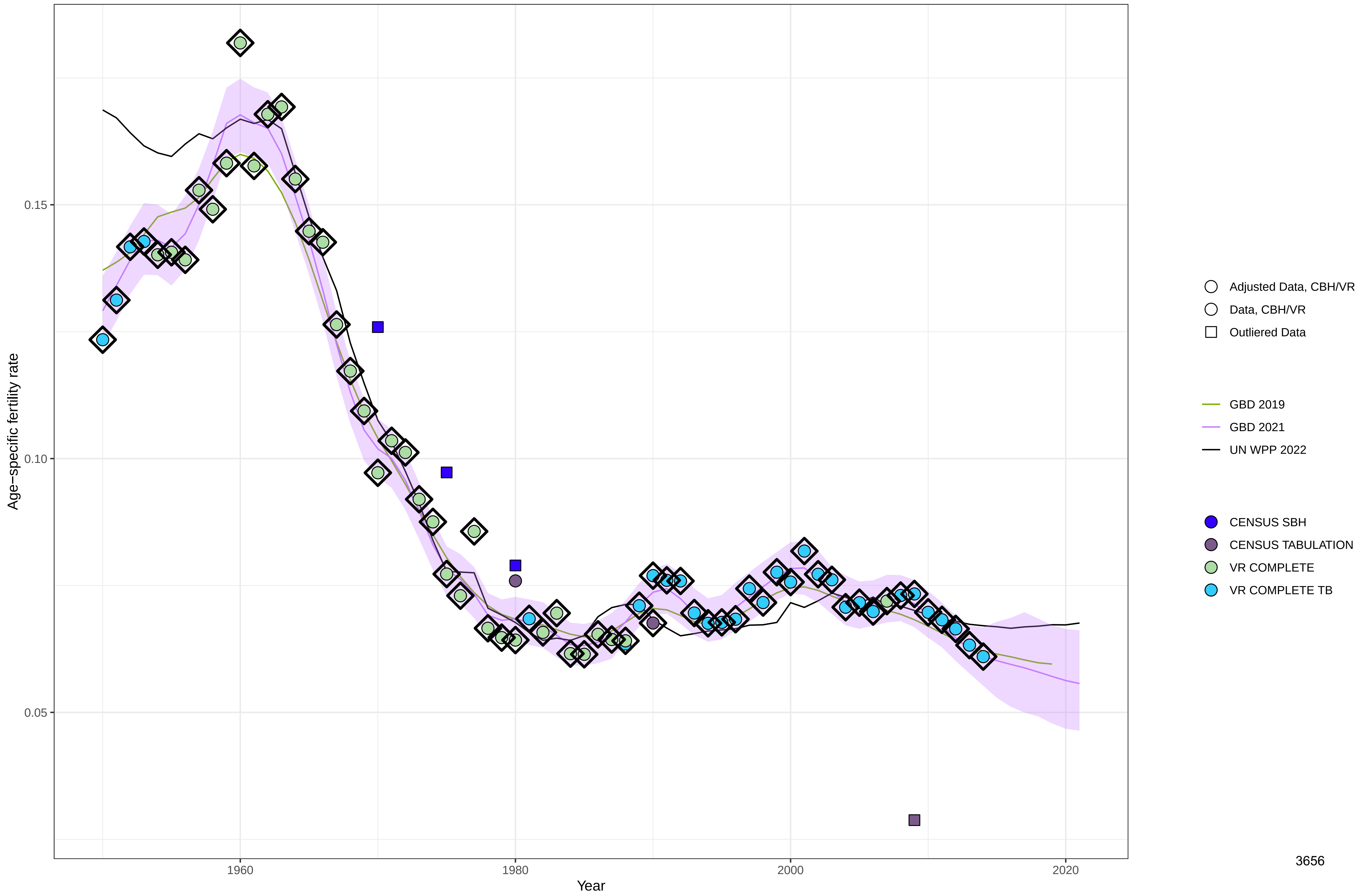

Age−specific fertility fitted trend lines, 1950−2021: Barbados, Ages: 35−39
Data points enclosed with a diamond are reference sources

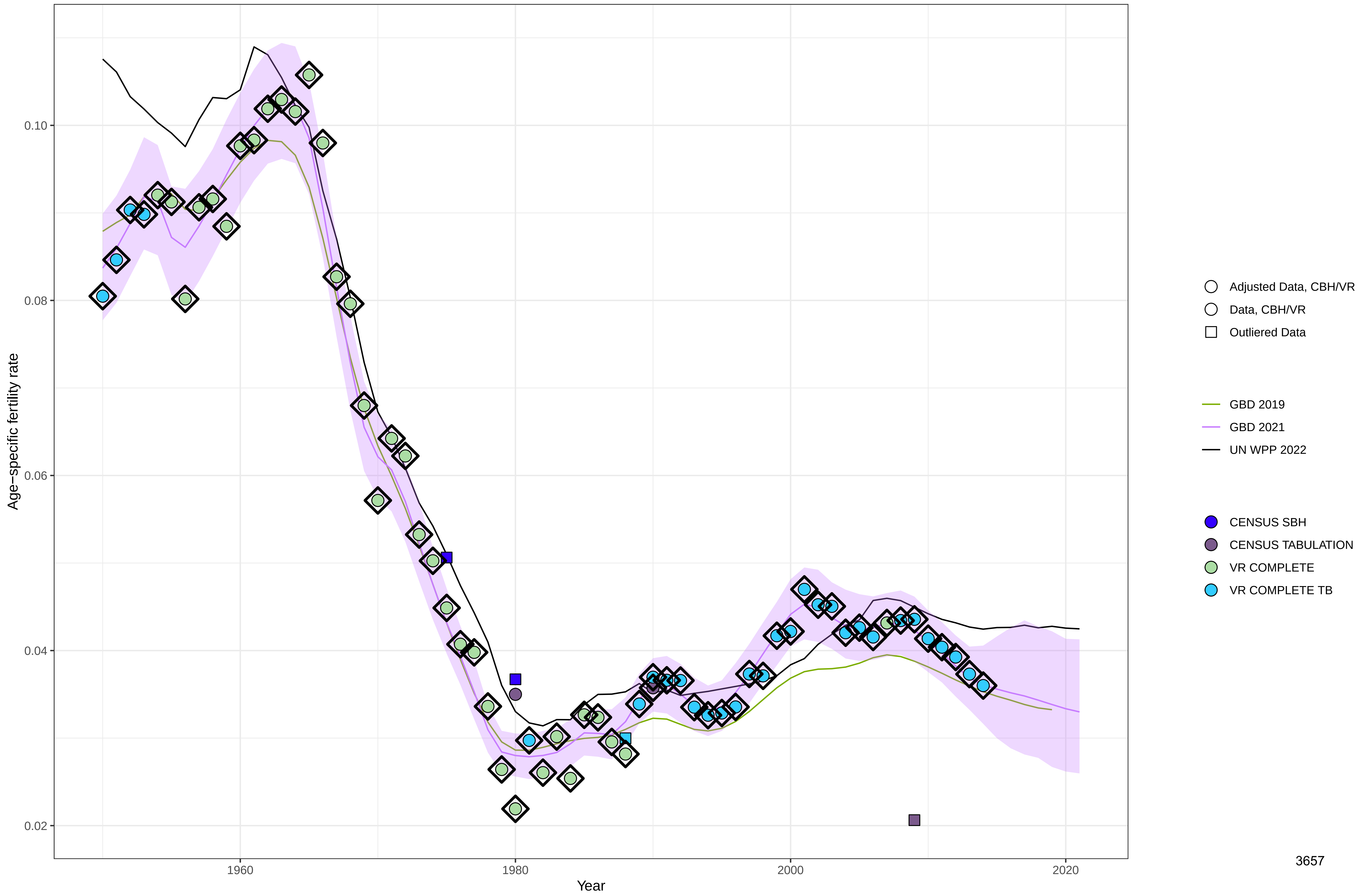

Age−specific fertility fitted trend lines, 1950−2021: Barbados, Ages: 40−44
Data points enclosed with a diamond are reference sources

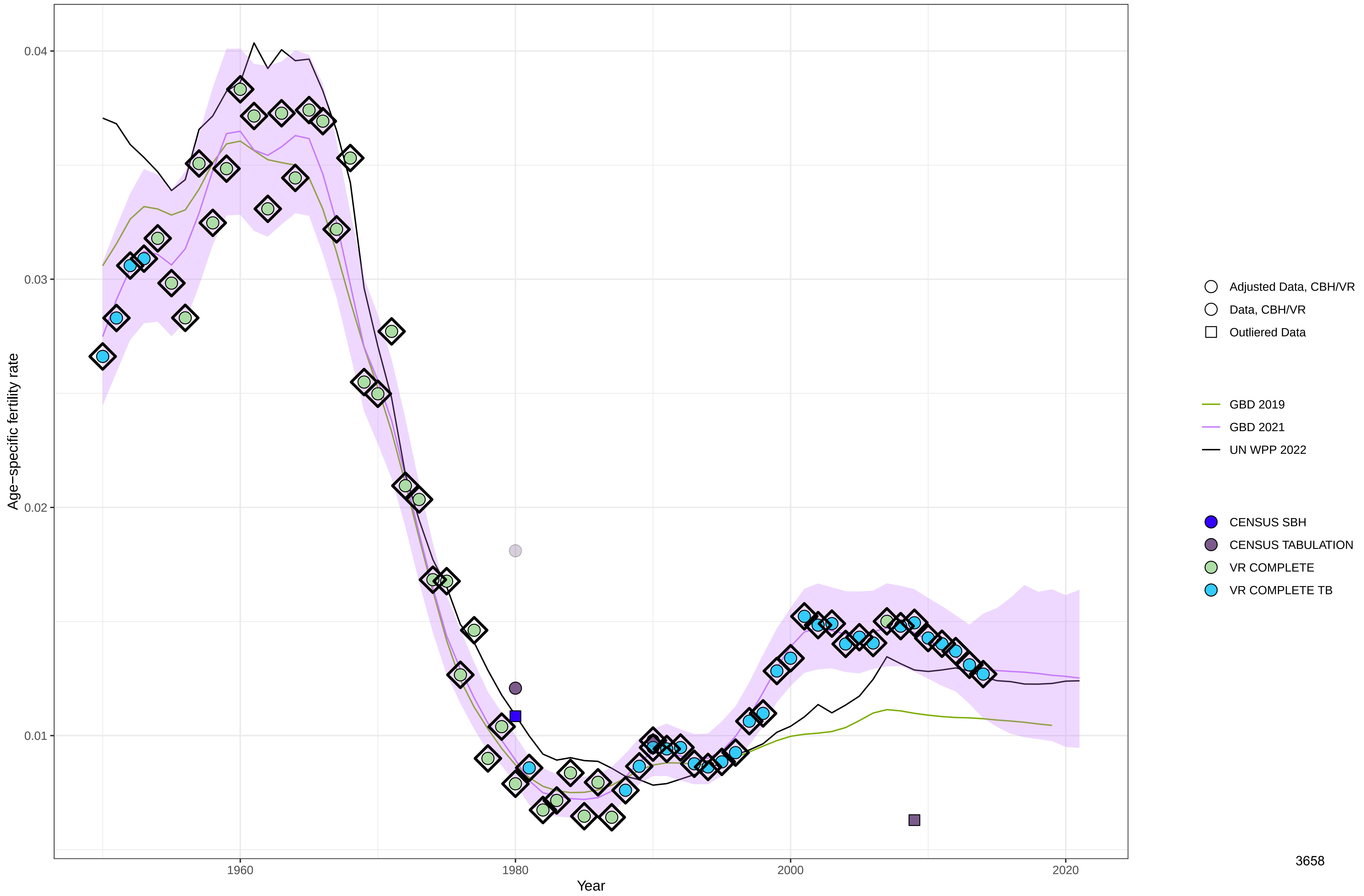

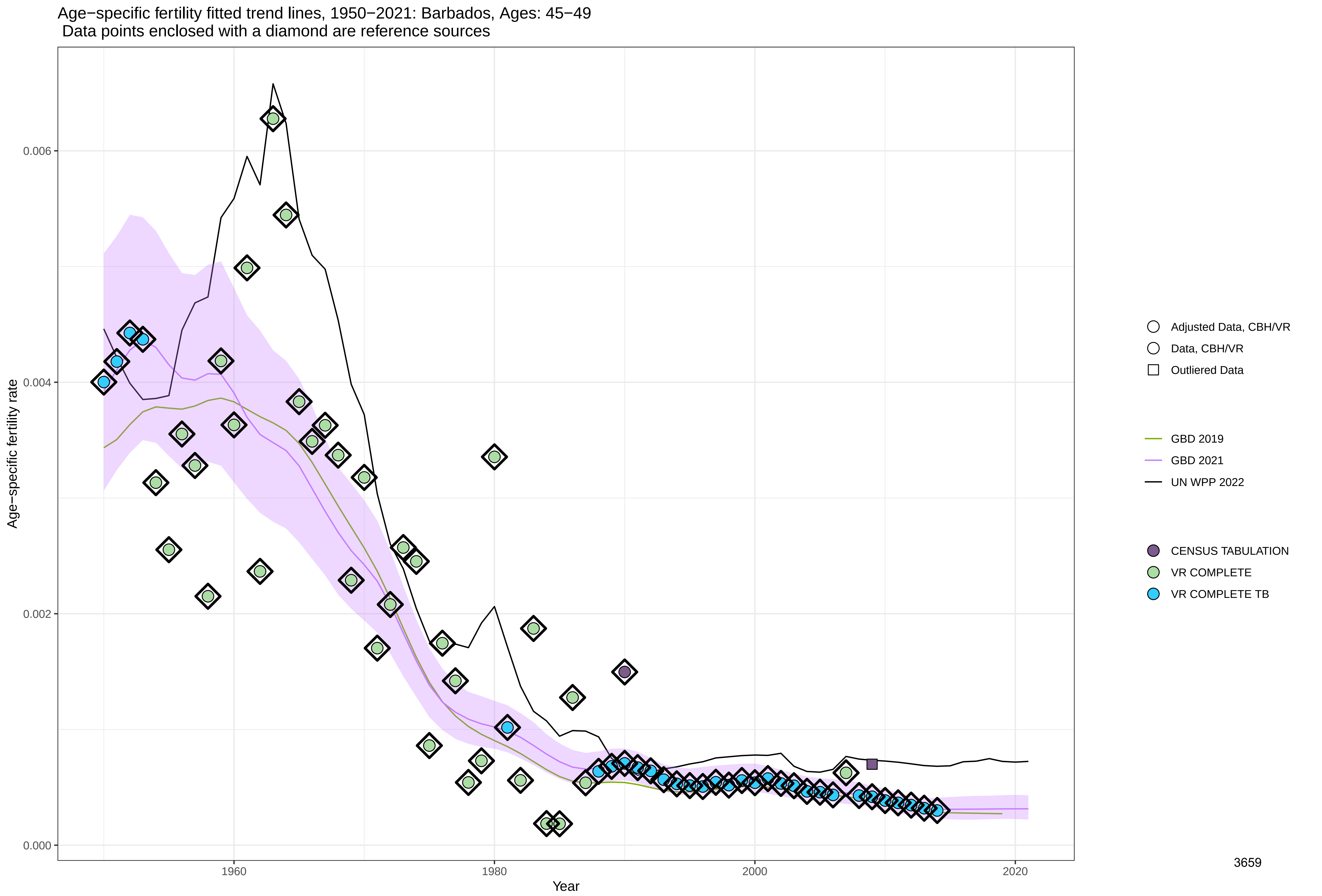

Age−specific fertility fitted trend lines, 1950−2021: Barbados, Ages: 45−49
Data points enclosed with a diamond are reference sources
Age−specific fertility rate
0.000
0.002
0.004
0.006
1960
1980
2000
2020
Year
Adjusted Data, CBH/VR
Data, CBH/VR
Outliered Data
GBD 2019
GBD 2021
UN WPP 2022
CENSUS TABULATION
VR COMPLETE
VR COMPLETE TB

Age−specific fertility fitted trend lines, 1950−2021: Barbados, Ages: 50−54
Data points enclosed with a diamond are reference sources

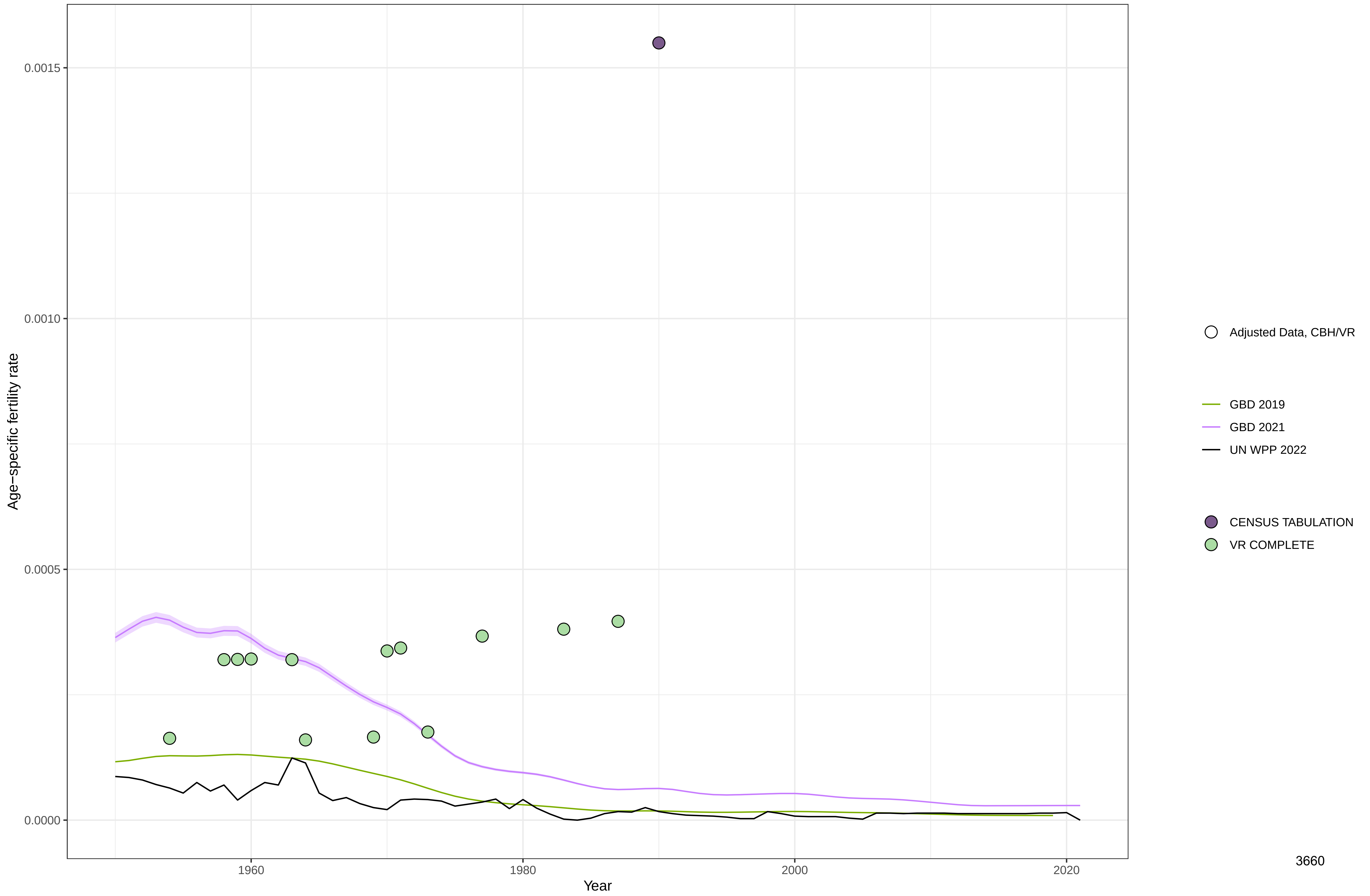

Total fertility rate fitted trend lines, 1950−2021: Barbados TFR
Data points enclosed with a diamond are reference sources

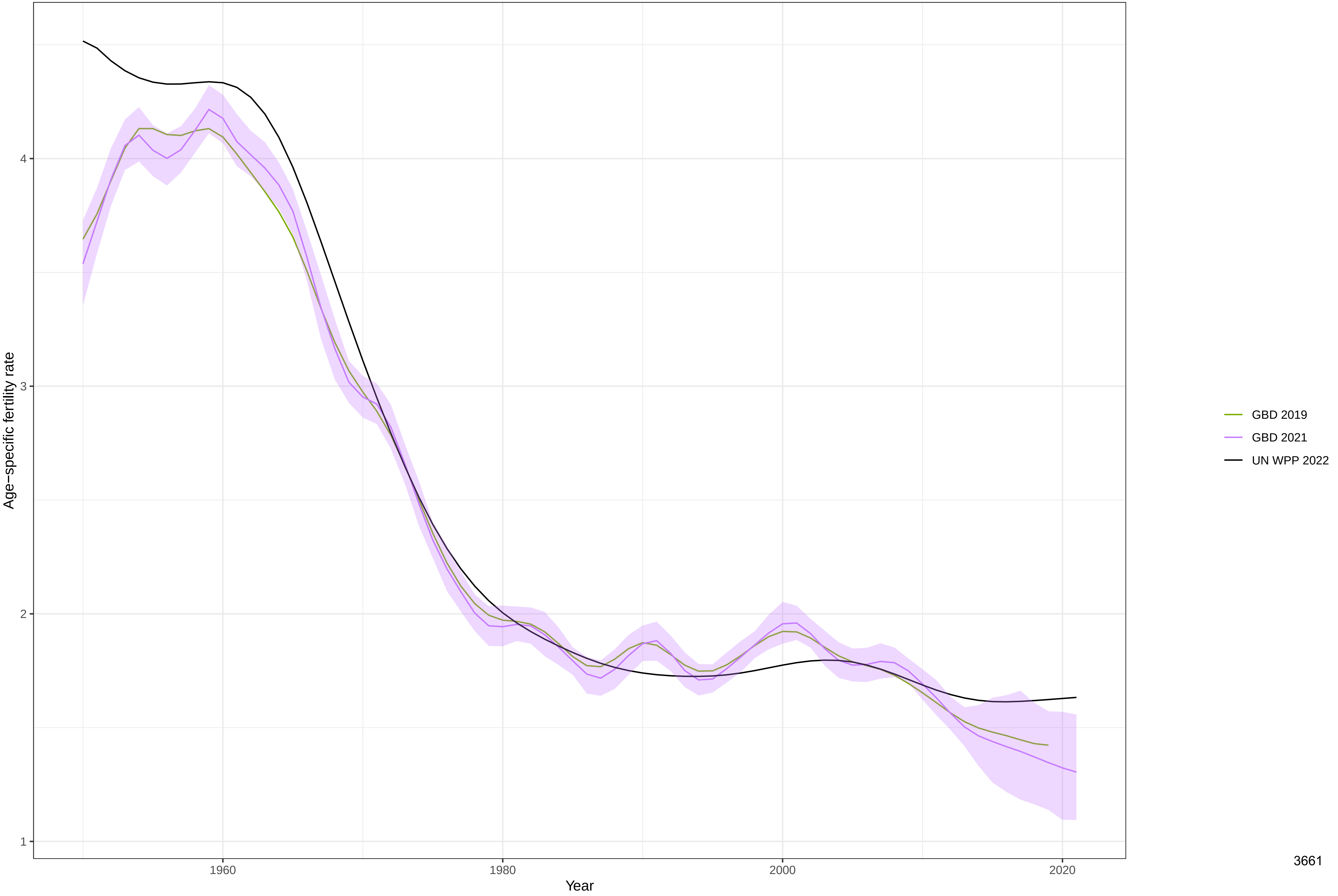

Age−specific fertility fitted trend lines, 1950−2021: Belize, Ages: 10−14
Data points enclosed with a diamond are reference sources

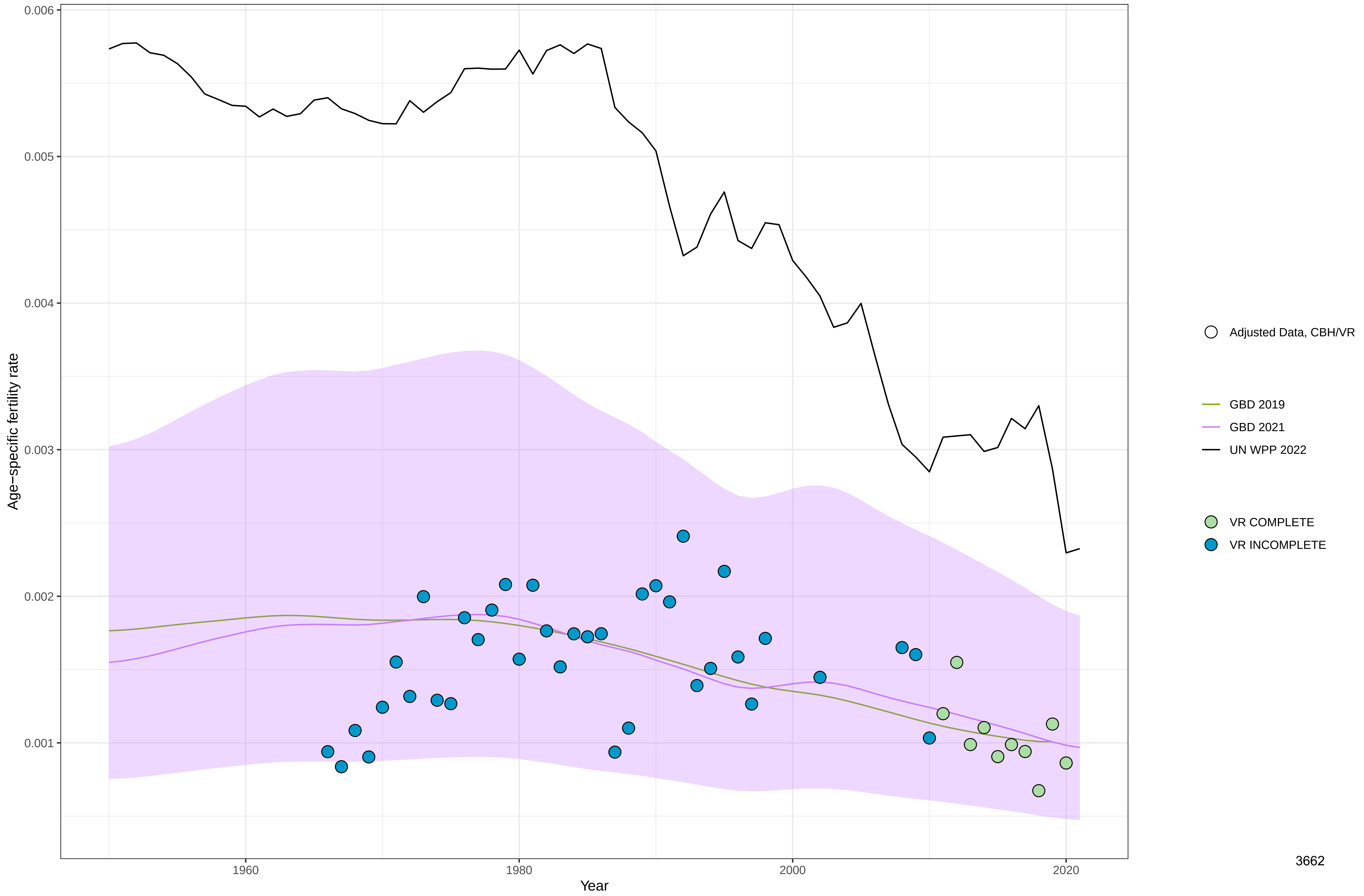

Age−specific fertility fitted trend lines, 1950−2021: Belize, Ages: 15−19
Data points enclosed with a diamond are reference sources

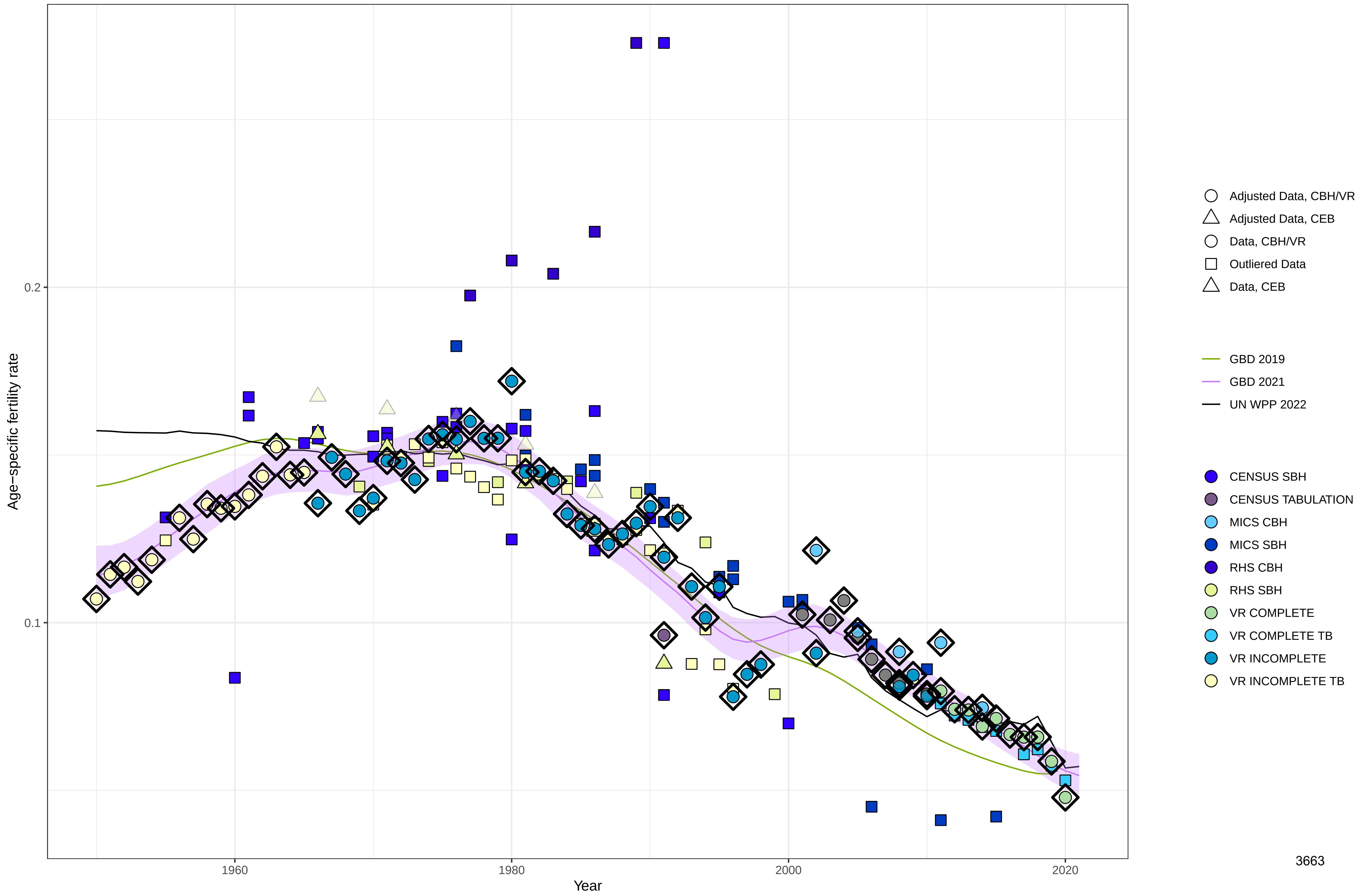

Age−specific fertility fitted trend lines, 1950−2021: Belize, Ages: 20−24
Data points enclosed with a diamond are reference sources

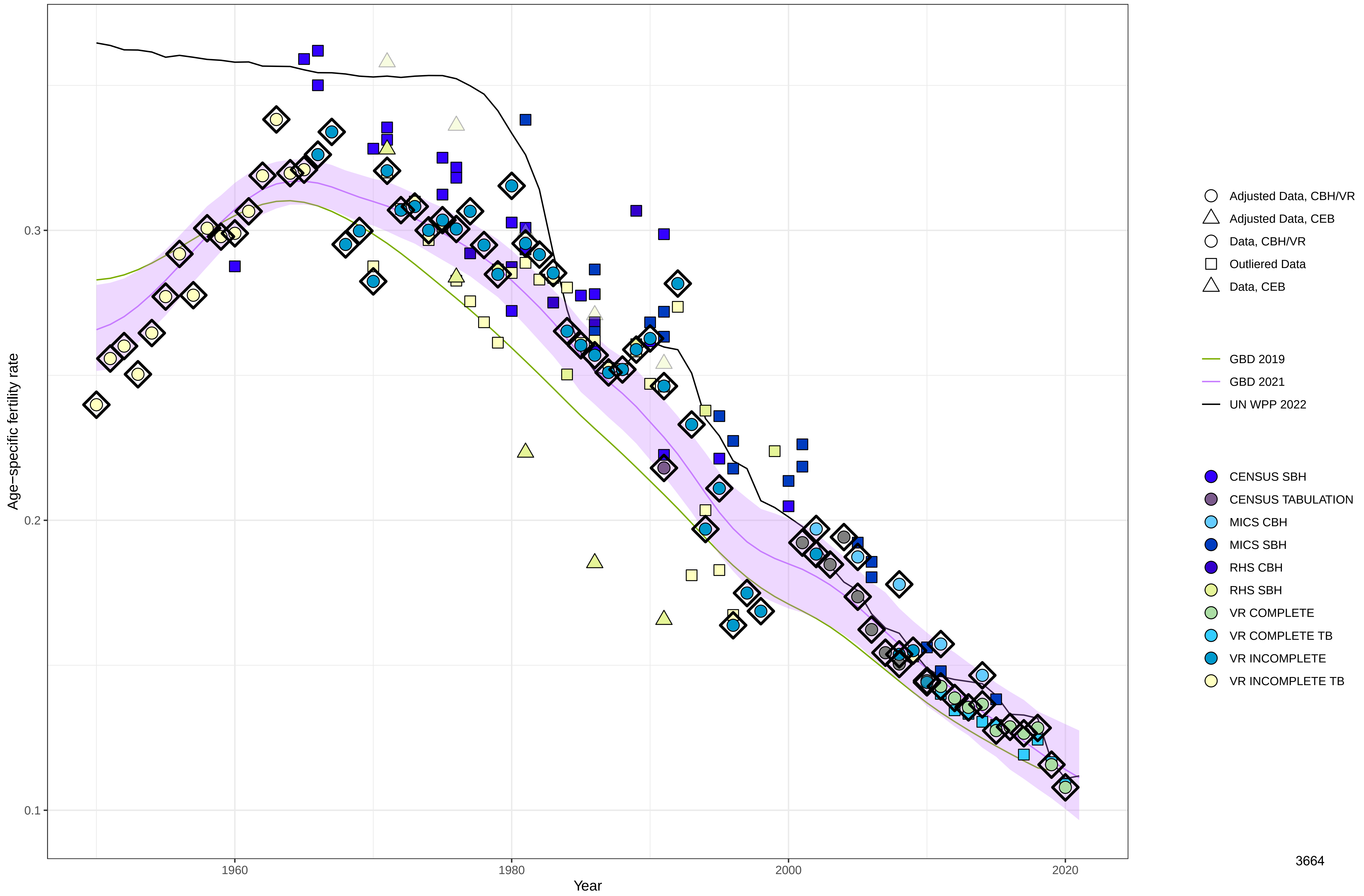

Age−specific fertility fitted trend lines, 1950−2021: Belize, Ages: 25−29
Data points enclosed with a diamond are reference sources

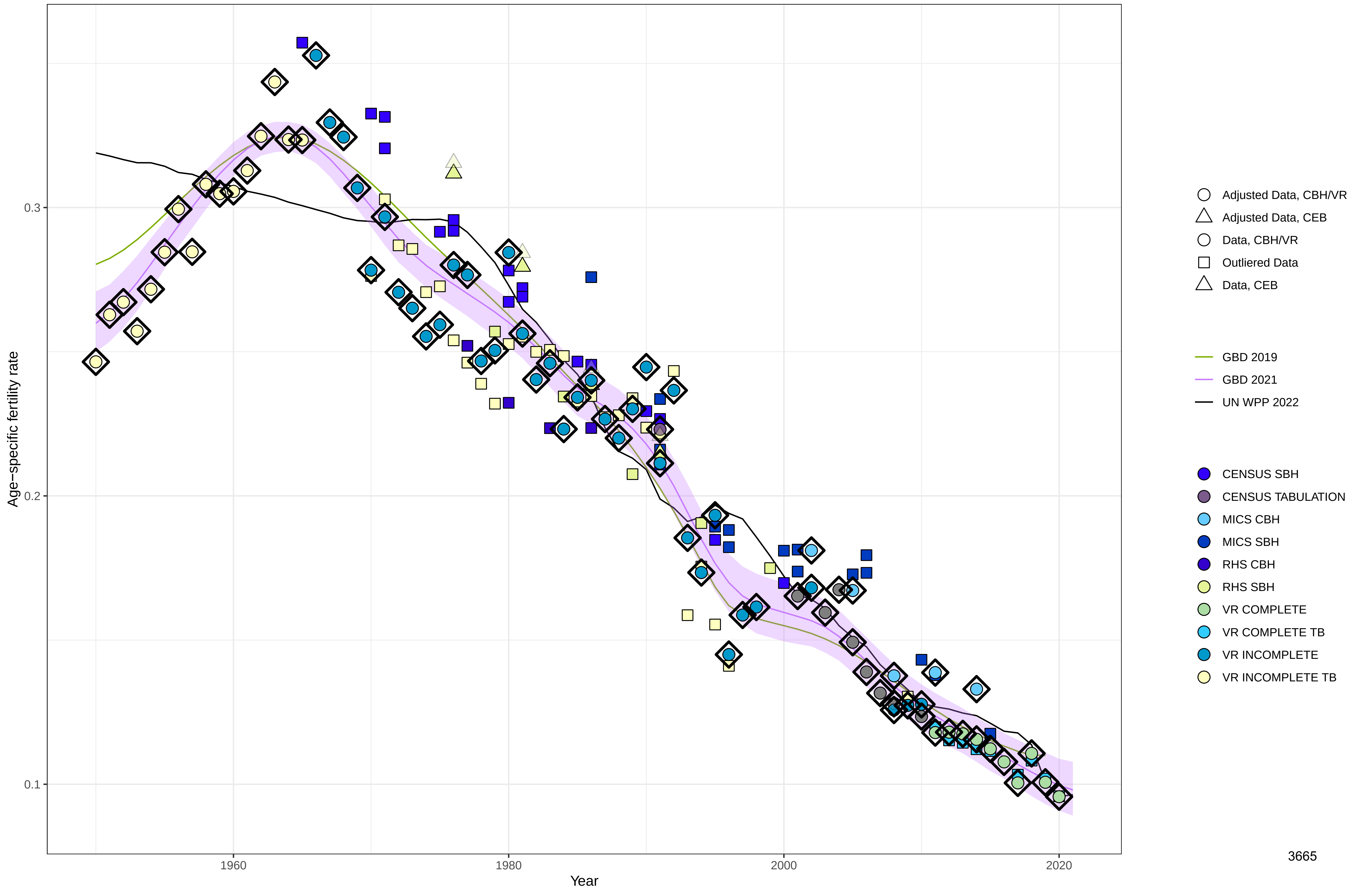

Age−specific fertility fitted trend lines, 1950−2021: Belize, Ages: 30−34
Data points enclosed with a diamond are reference sources

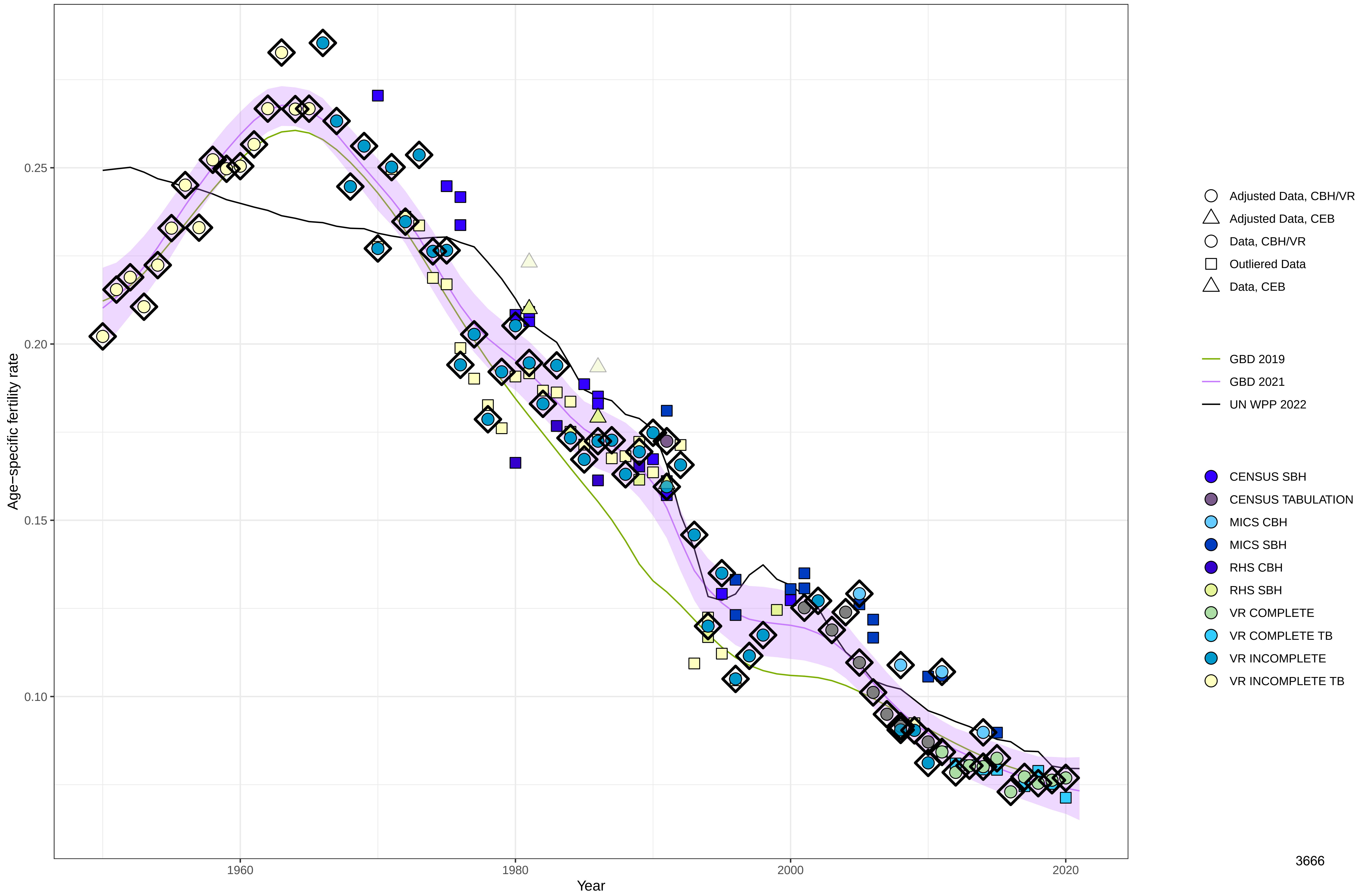

Age−specific fertility fitted trend lines, 1950−2021: Belize, Ages: 35−39
Data points enclosed with a diamond are reference sources

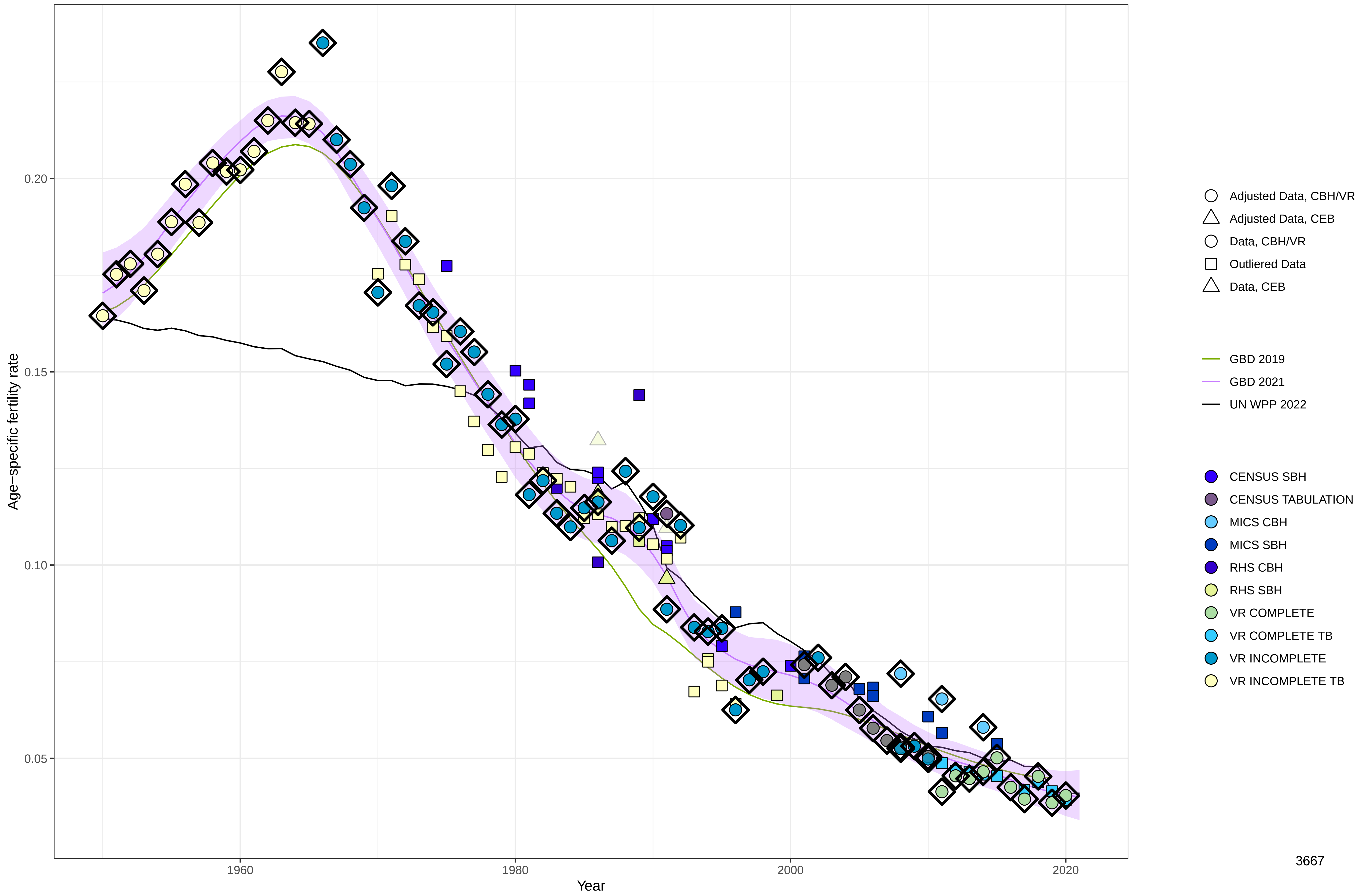

Age−specific fertility fitted trend lines, 1950−2021: Belize, Ages: 40−44
Data points enclosed with a diamond are reference sources

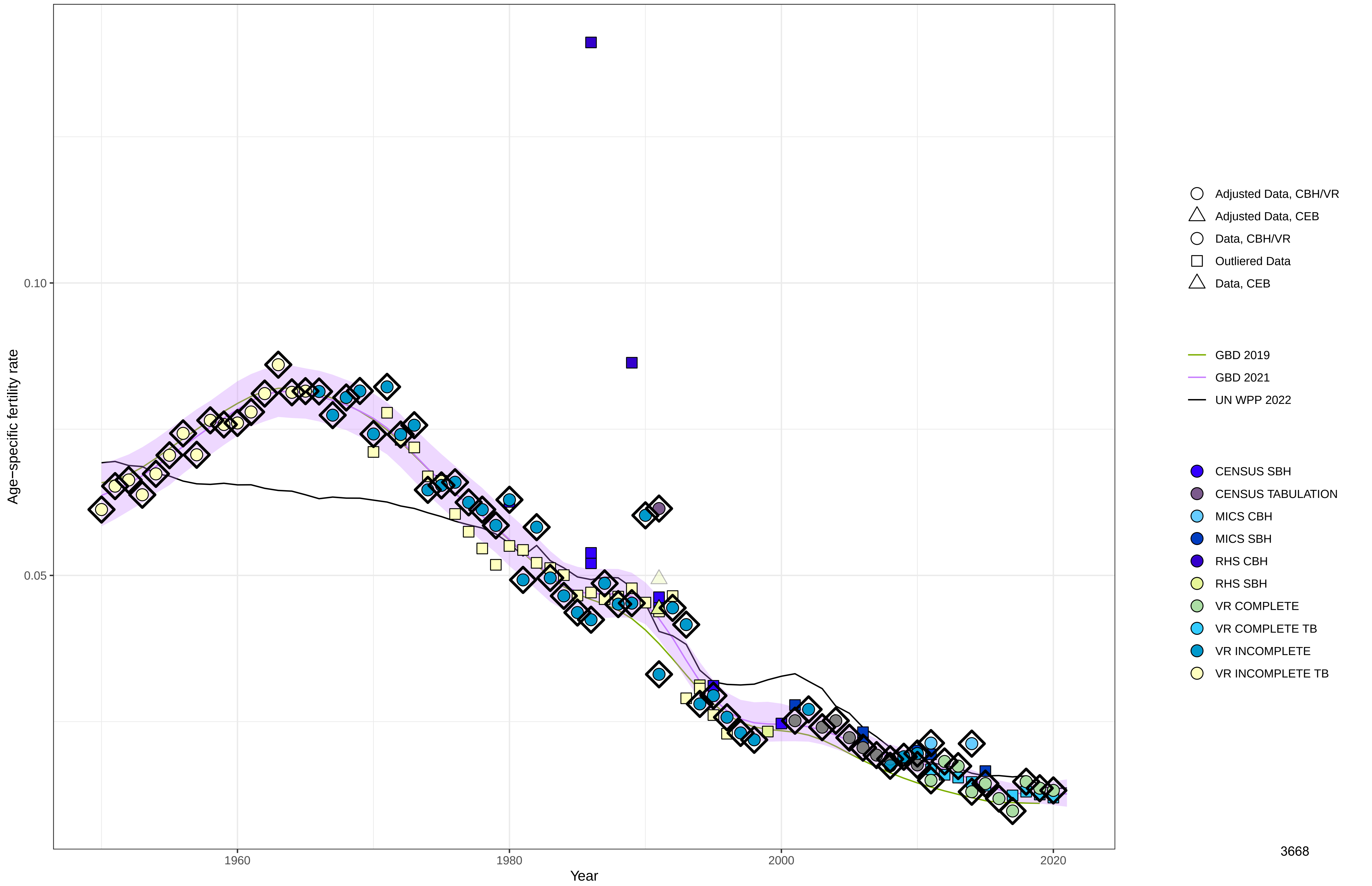

Age−specific fertility fitted trend lines, 1950−2021: Belize, Ages: 45−49
Data points enclosed with a diamond are reference sources

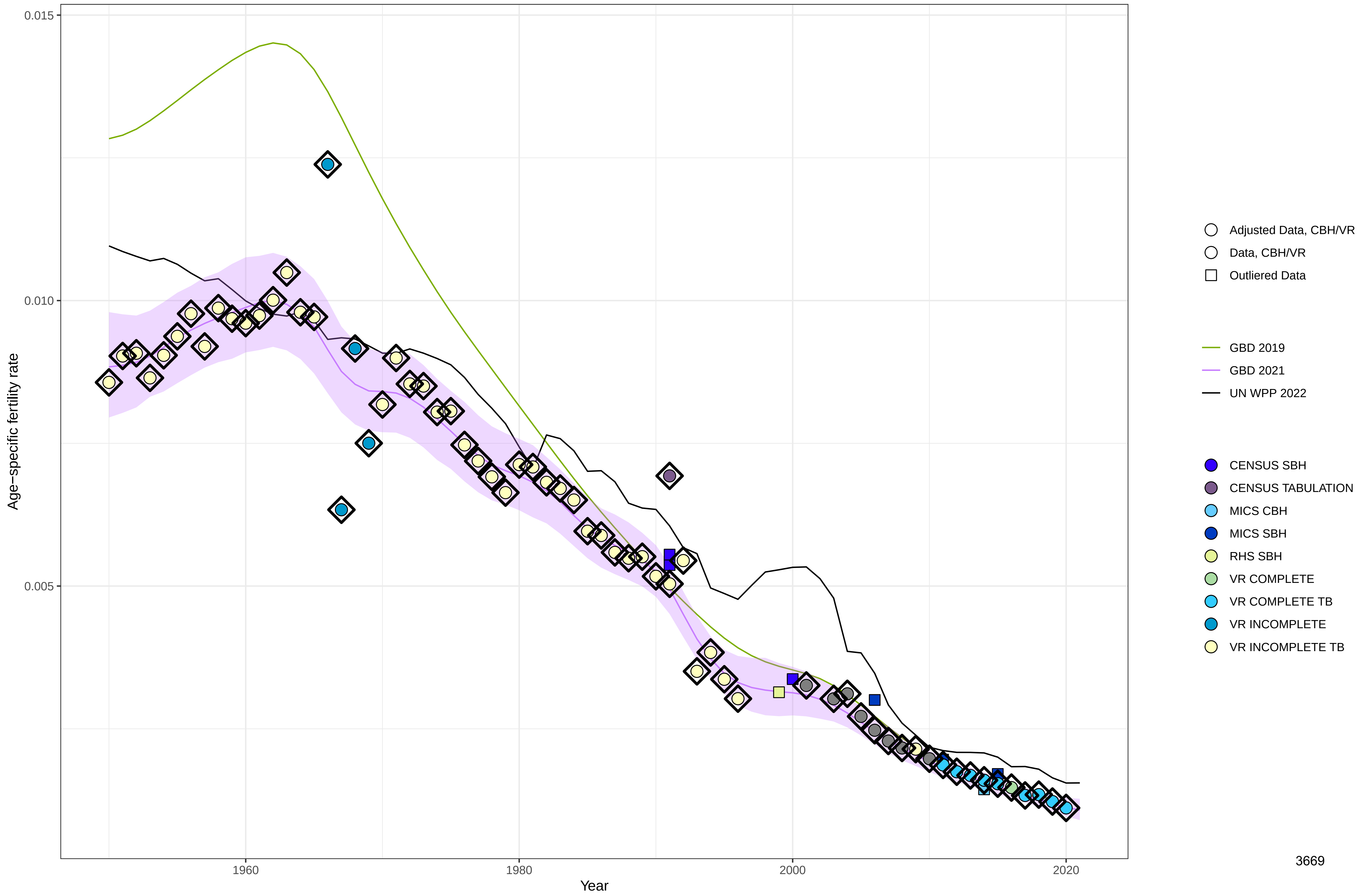

Age−specific fertility fitted trend lines, 1950−2021: Belize, Ages: 50−54
Data points enclosed with a diamond are reference sources

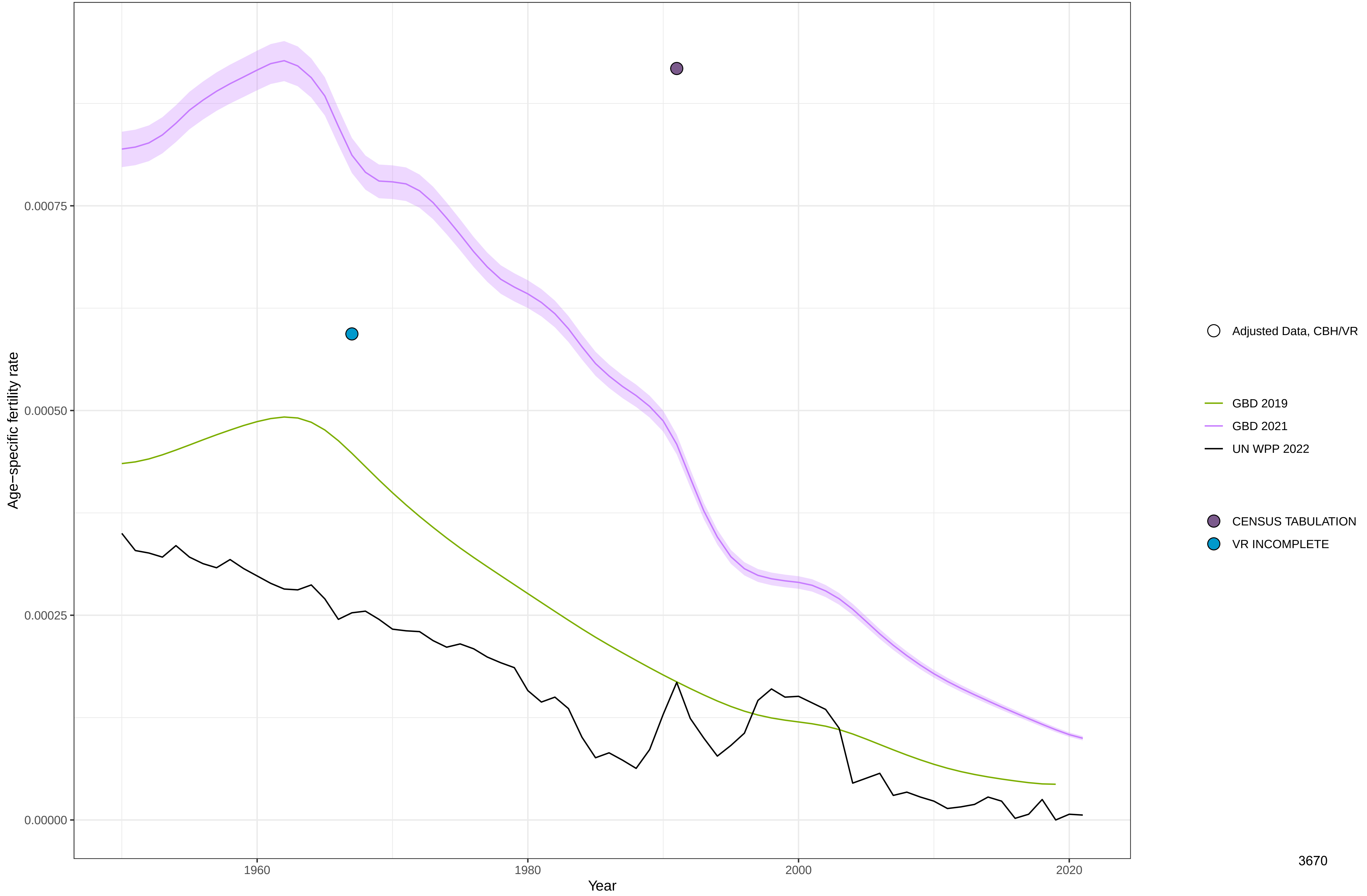

Total fertility rate fitted trend lines, 1950−2021: Belize TFR
Data points enclosed with a diamond are reference sources

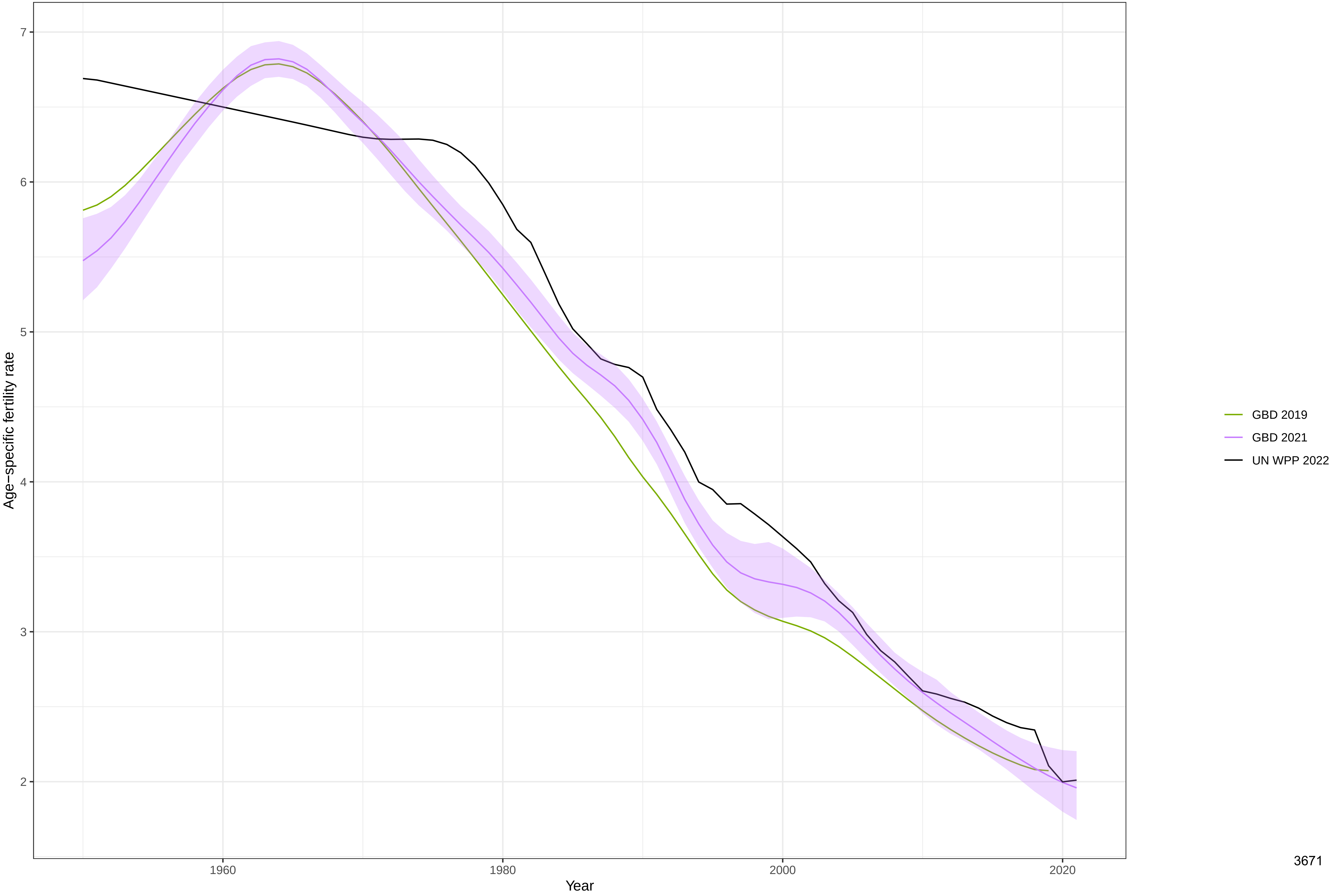

Age−specific fertility fitted trend lines, 1950−2021: Bermuda, Ages: 10−14
Data points enclosed with a diamond are reference sources

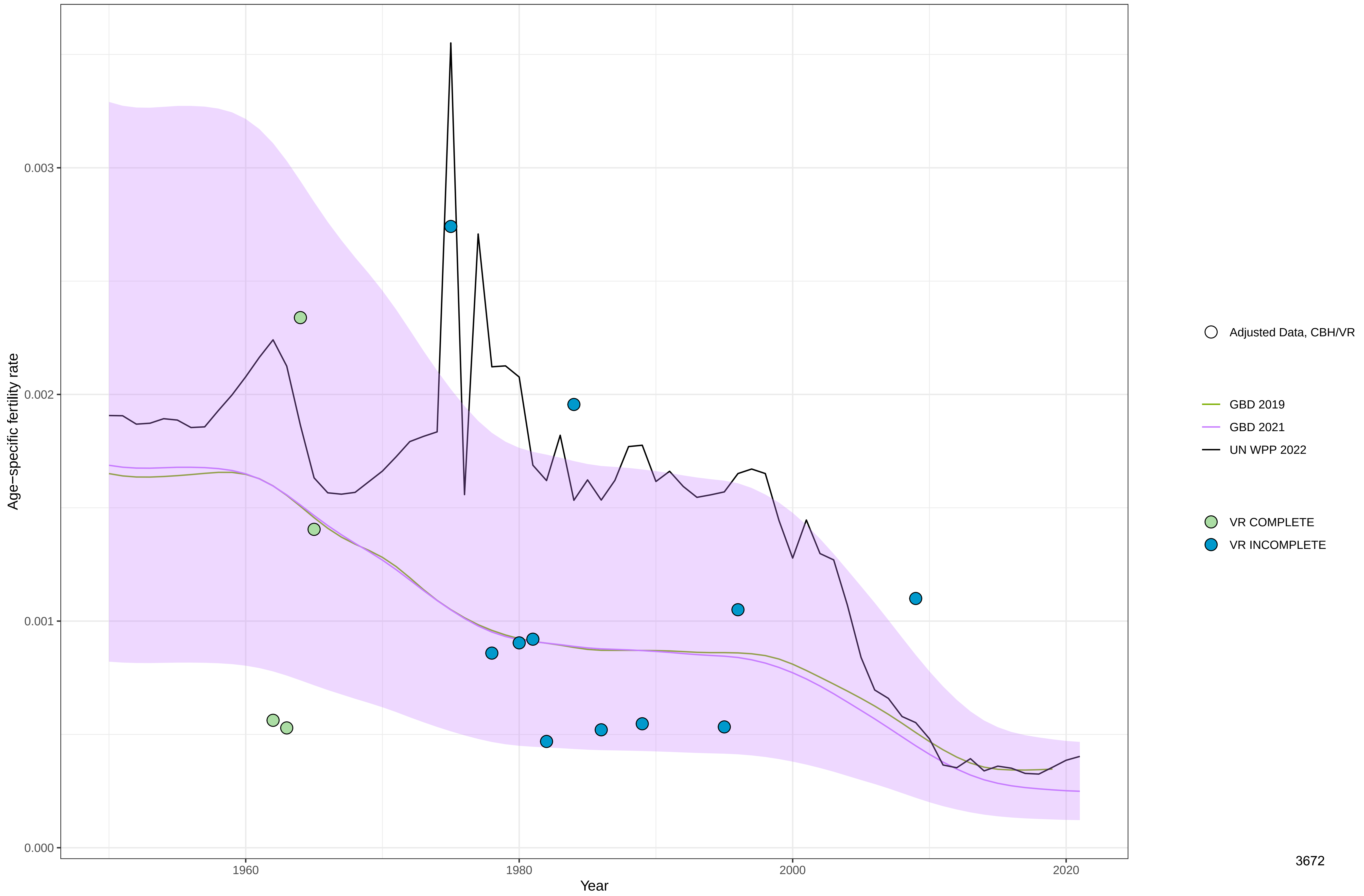

Age−specific fertility fitted trend lines, 1950−2021: Bermuda, Ages: 15−19
Data points enclosed with a diamond are reference sources

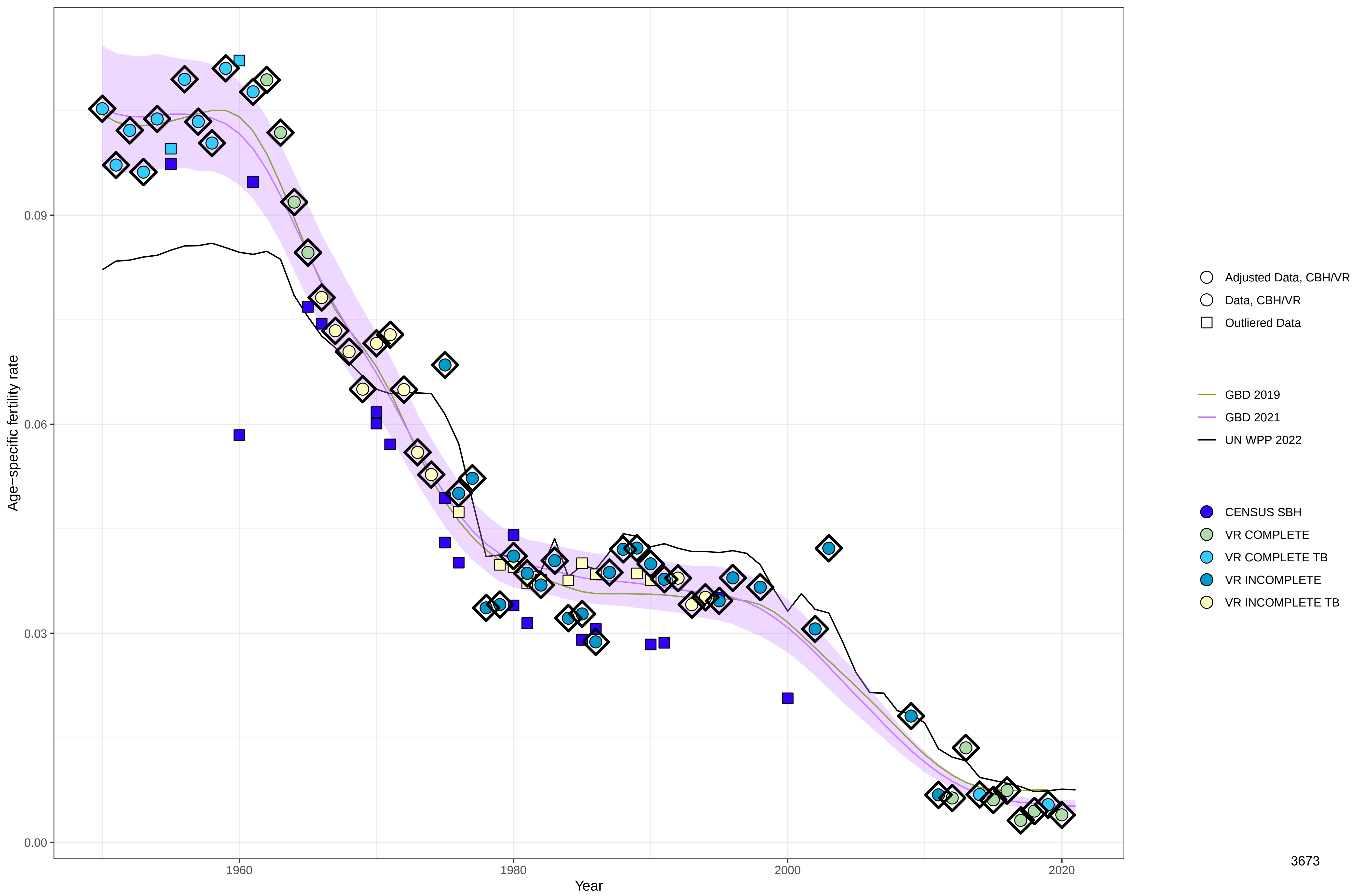

Age−specific fertility fitted trend lines, 1950−2021: Bermuda, Ages: 20−24
Data points enclosed with a diamond are reference sources

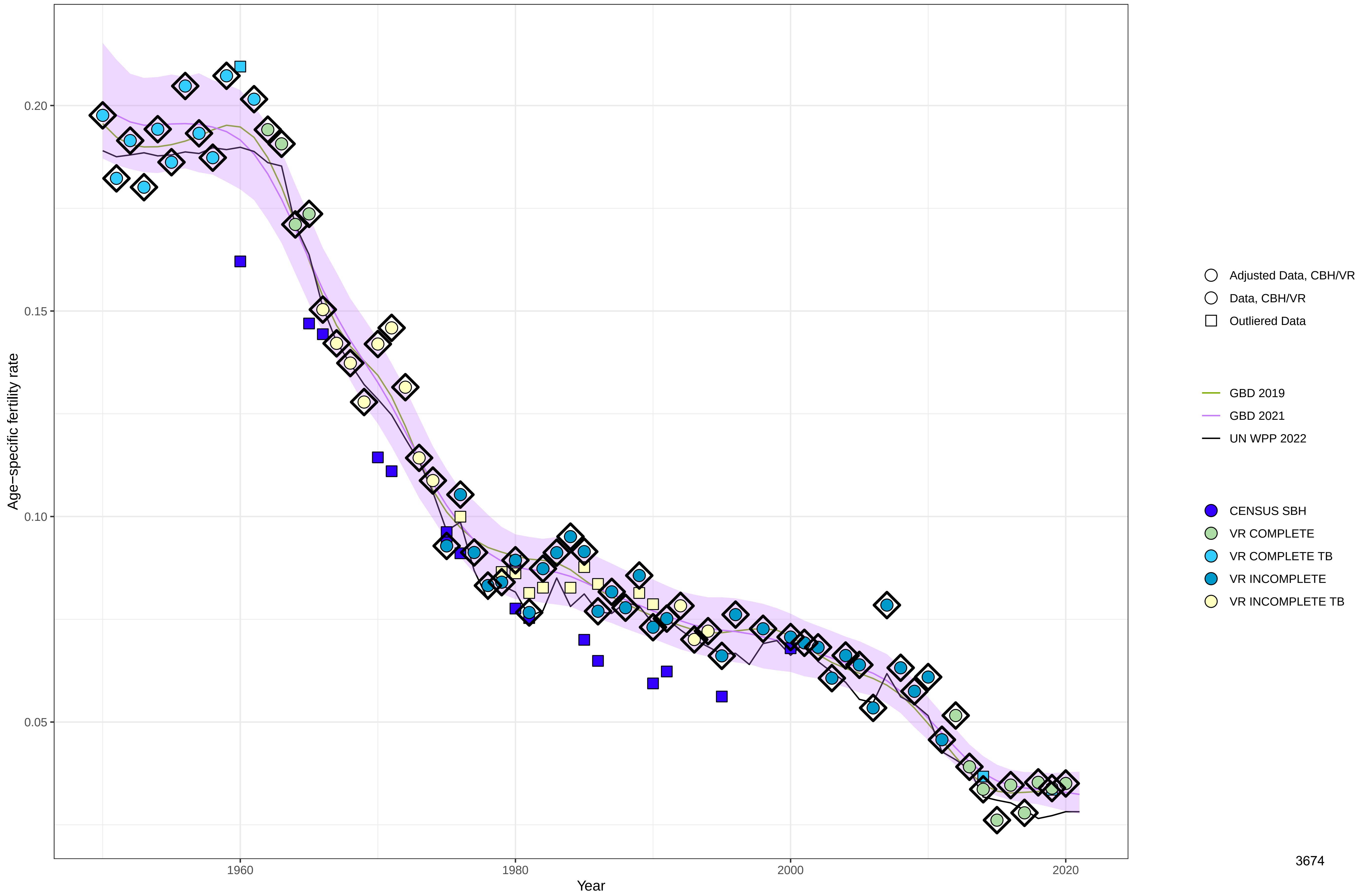

Age−specific fertility fitted trend lines, 1950−2021: Bermuda, Ages: 25−29
Data points enclosed with a diamond are reference sources

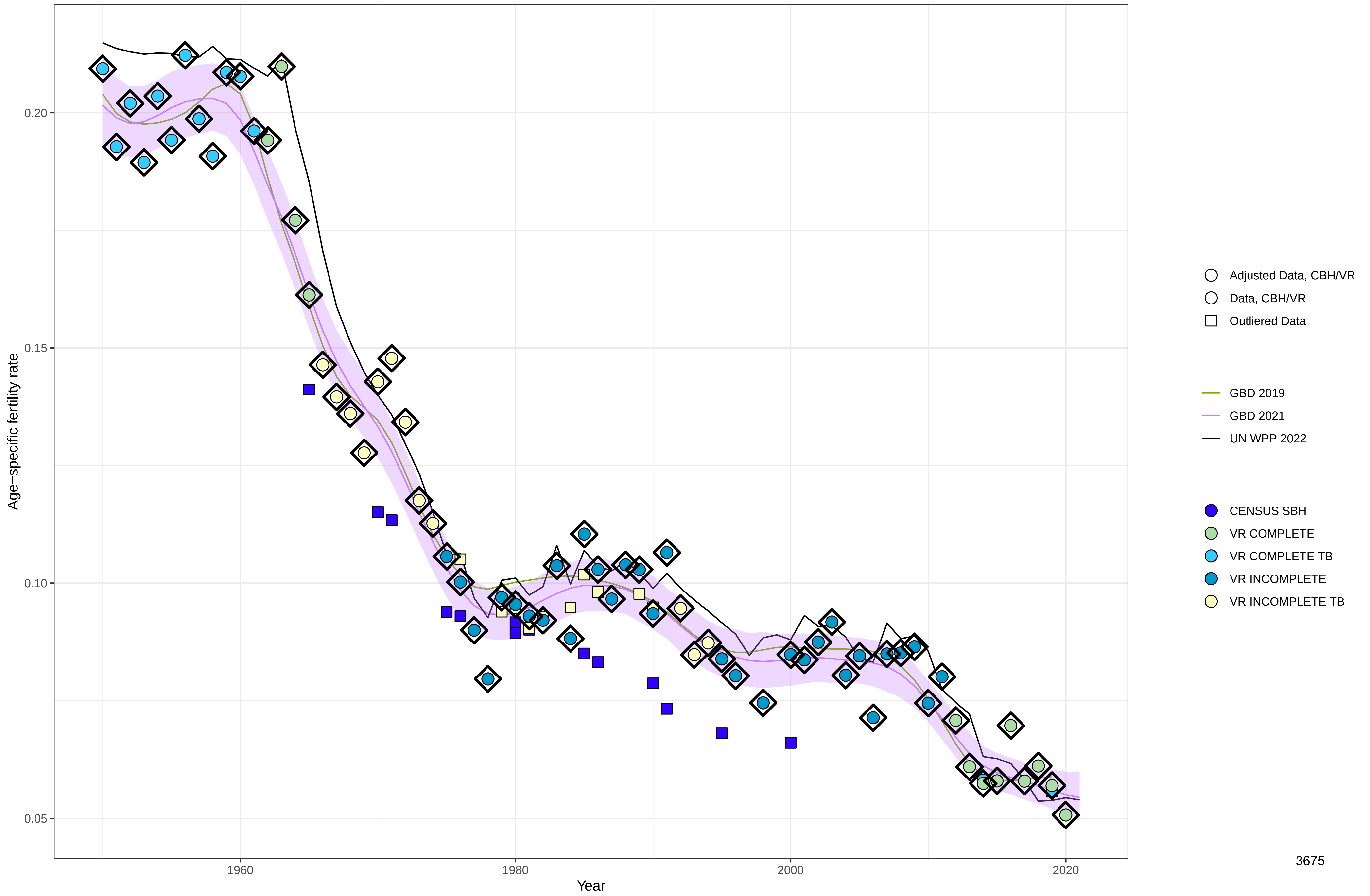

Age−specific fertility fitted trend lines, 1950−2021: Bermuda, Ages: 30−34
Data points enclosed with a diamond are reference sources

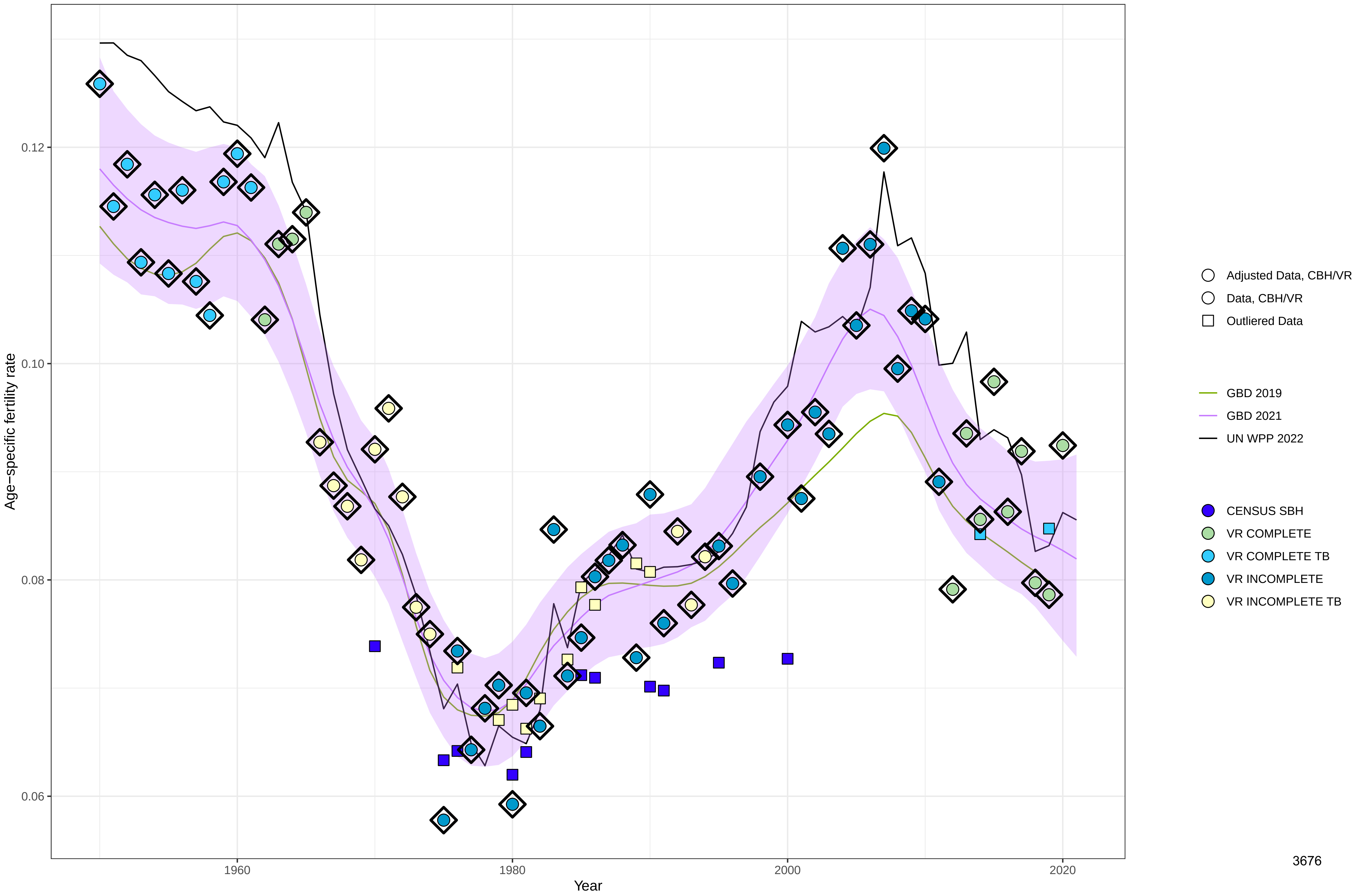

Age−specific fertility fitted trend lines, 1950−2021: Bermuda, Ages: 35−39
Data points enclosed with a diamond are reference sources

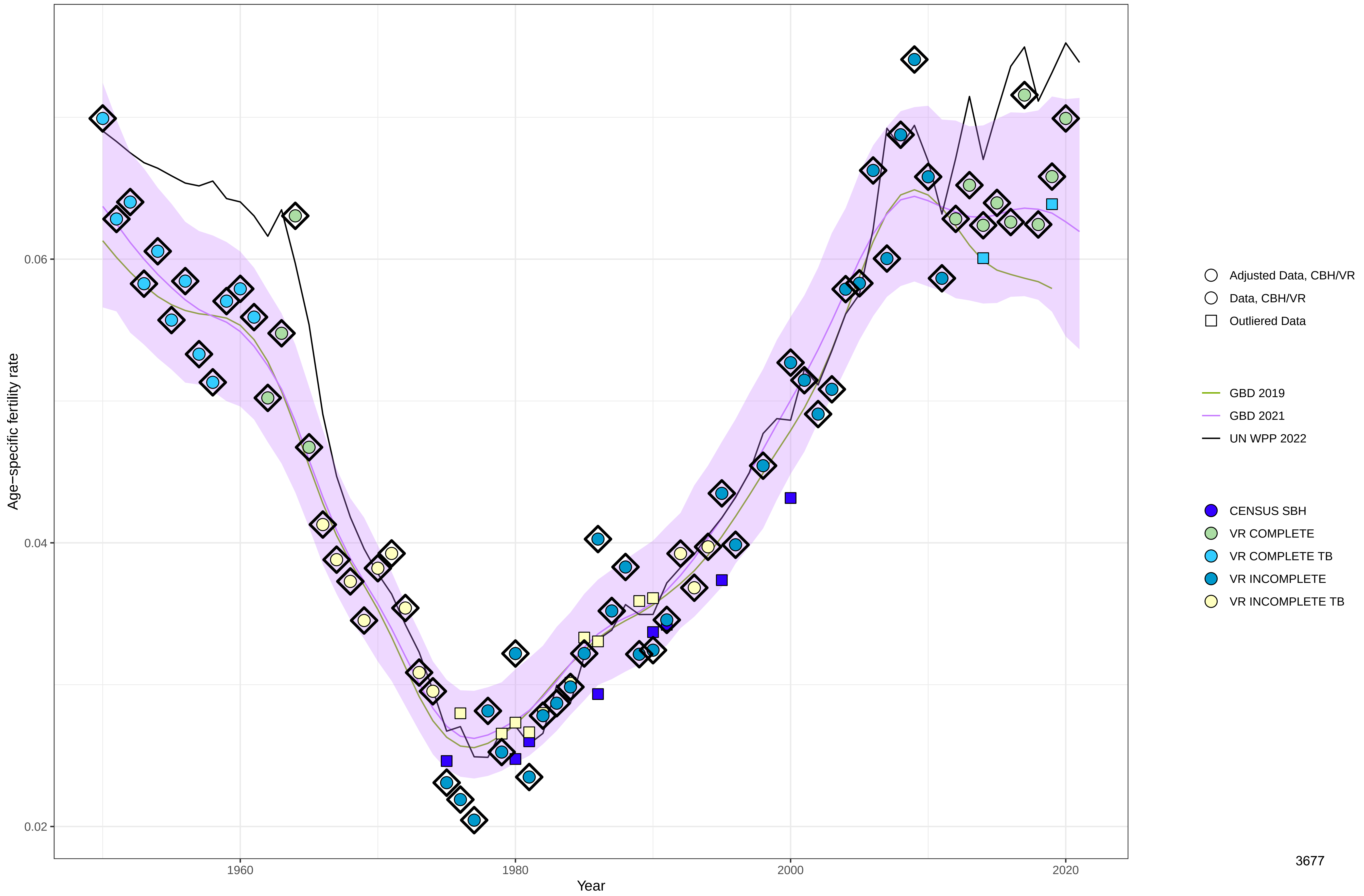

Age−specific fertility fitted trend lines, 1950−2021: Bermuda, Ages: 40−44
Data points enclosed with a diamond are reference sources

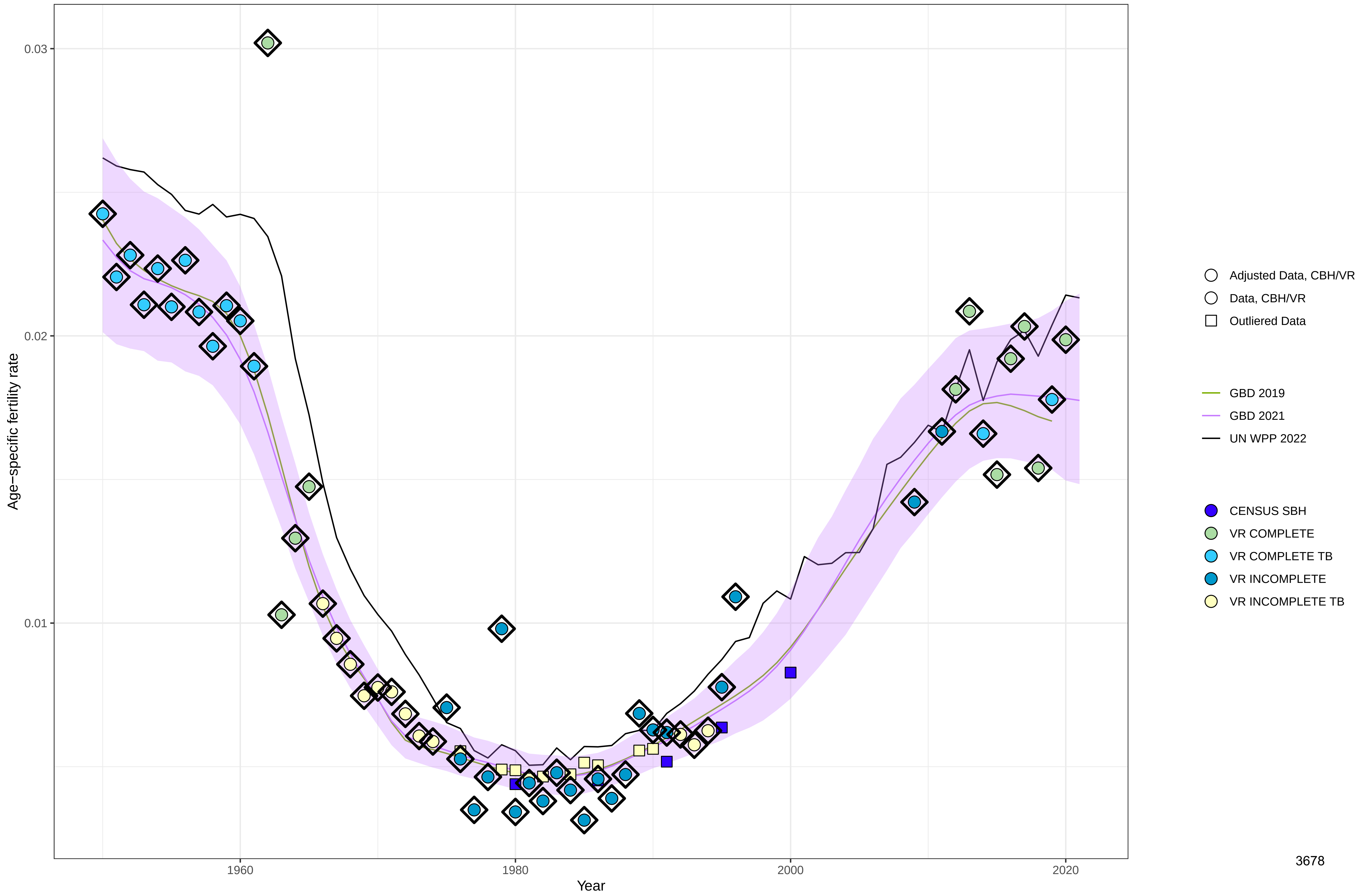

Age−specific fertility fitted trend lines, 1950−2021: Bermuda, Ages: 45−49
Data points enclosed with a diamond are reference sources

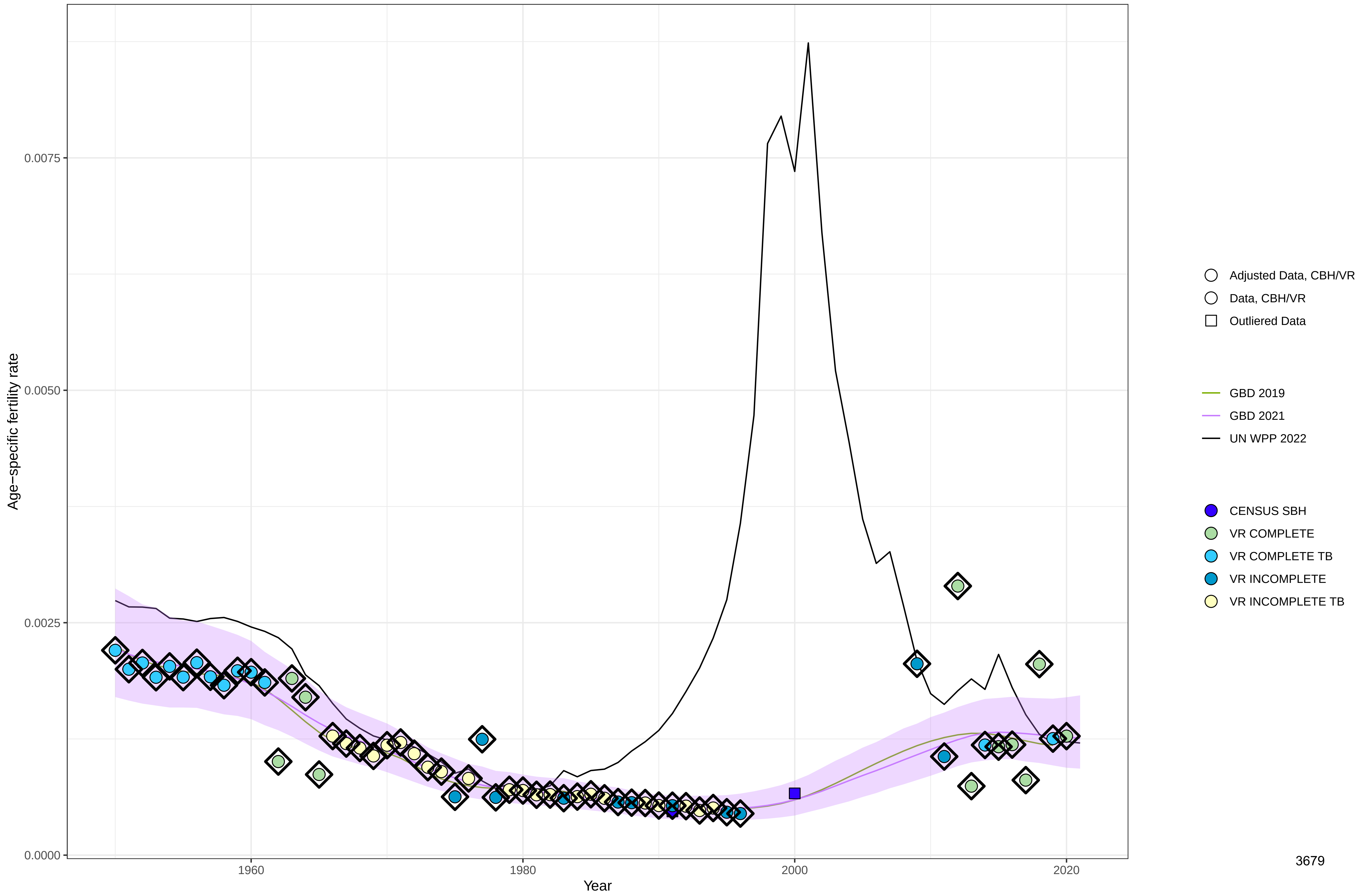

Age−specific fertility fitted trend lines, 1950−2021: Bermuda, Ages: 50−54
Data points enclosed with a diamond are reference sources

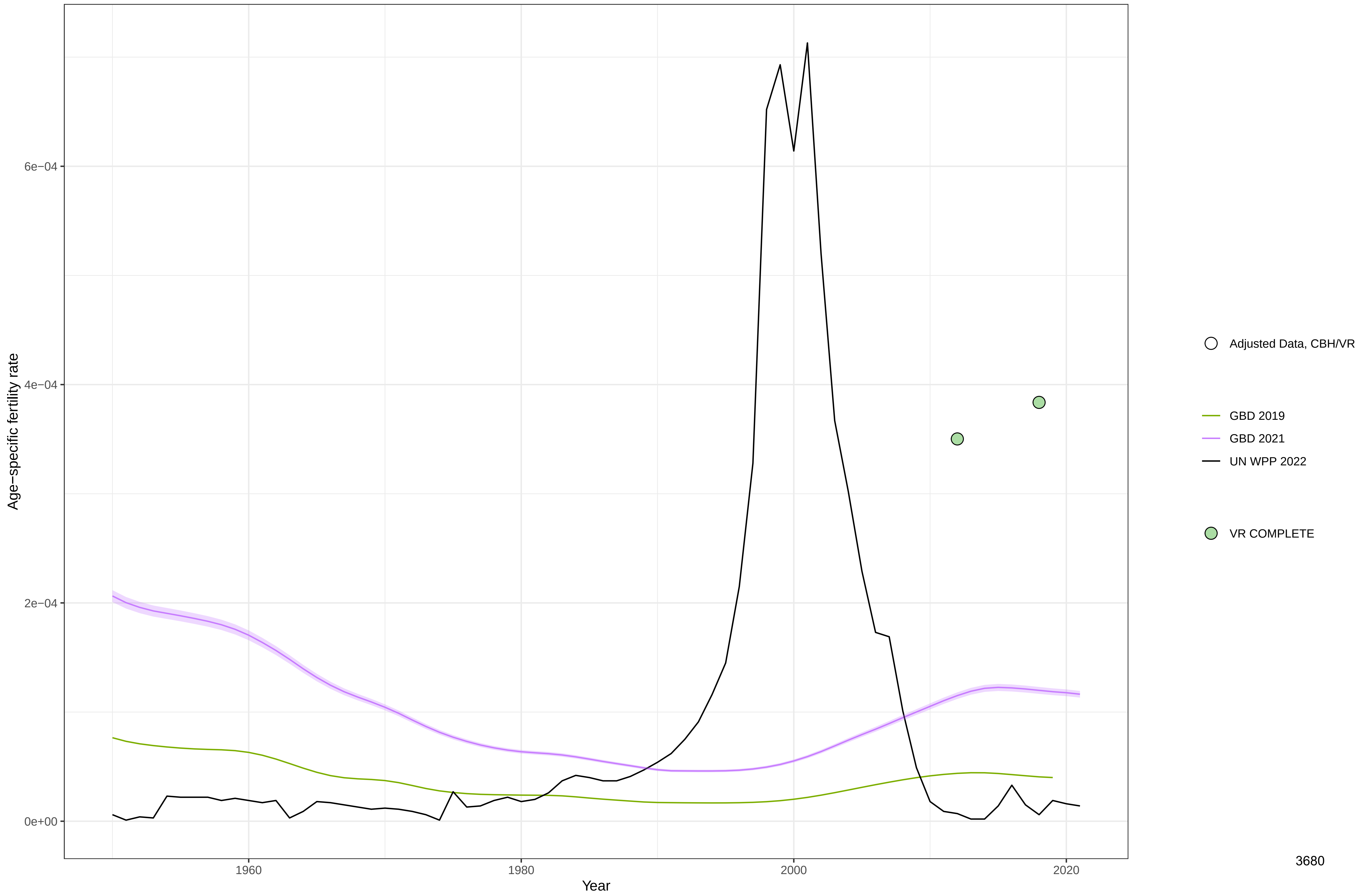

Total fertility rate fitted trend lines, 1950−2021: Bermuda TFR
Data points enclosed with a diamond are reference sources

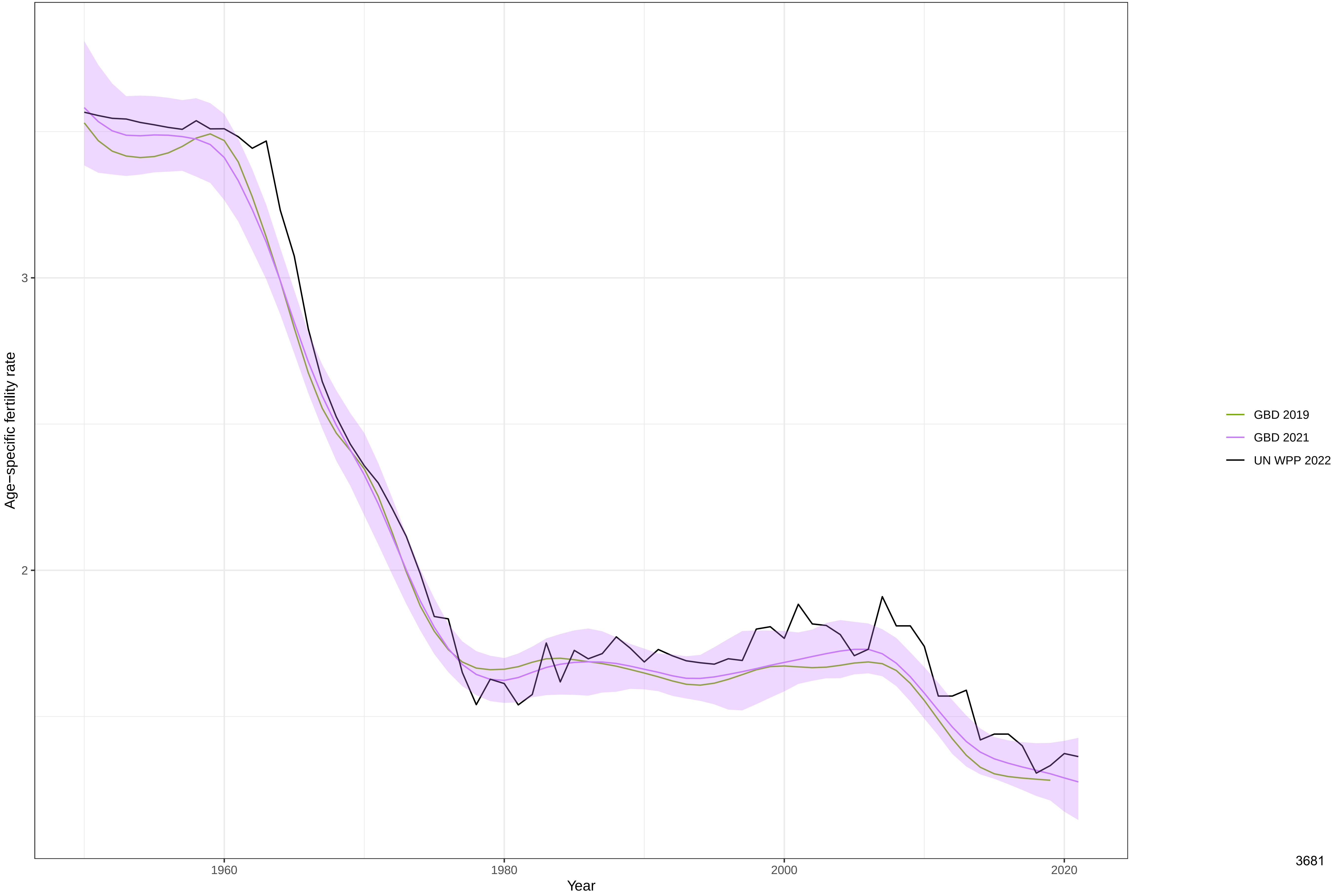

Age−specific fertility fitted trend lines, 1950−2021: Cuba, Ages: 10−14
Data points enclosed with a diamond are reference sources

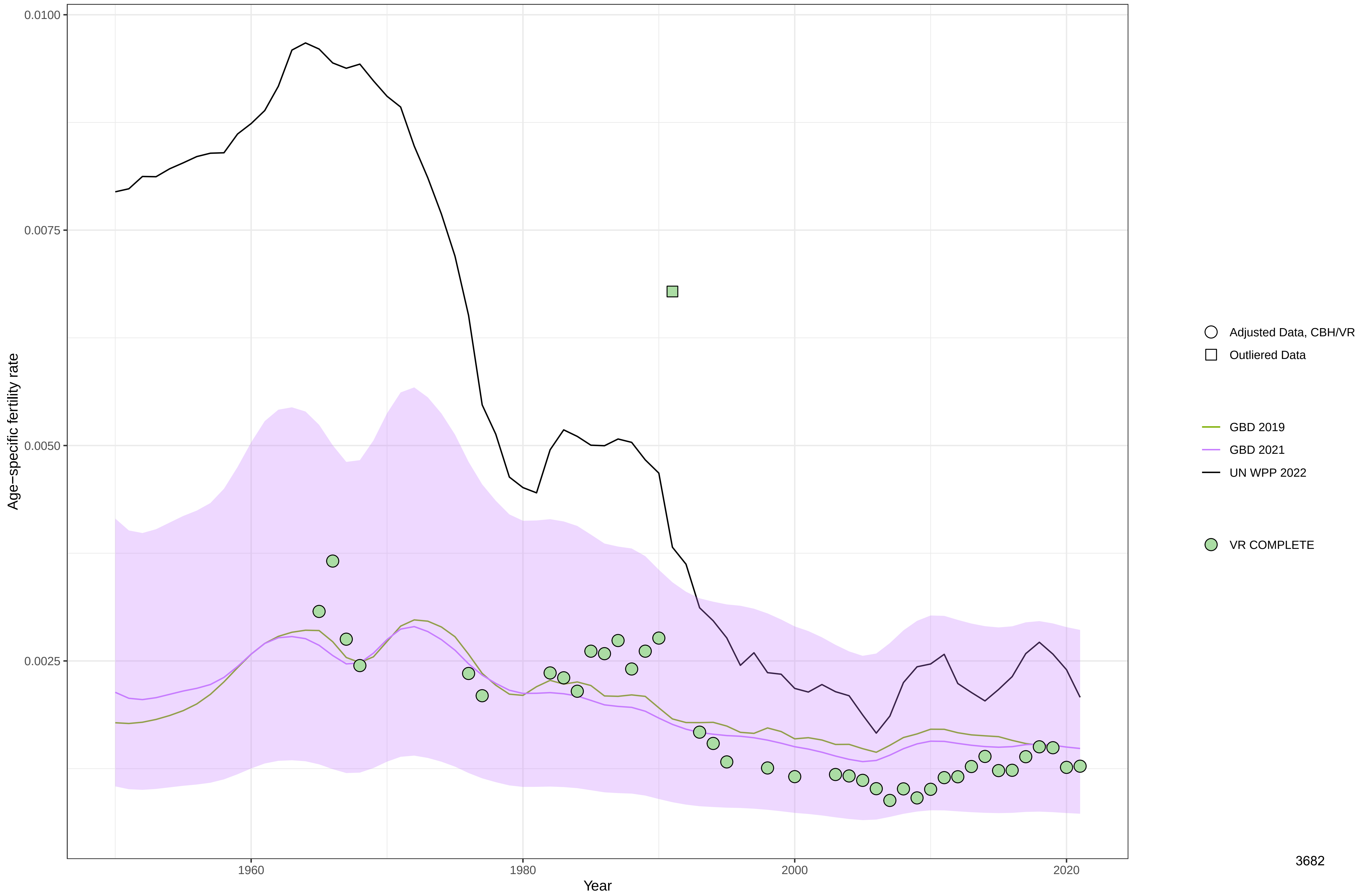

Age−specific fertility fitted trend lines, 1950−2021: Cuba, Ages: 15−19
Data points enclosed with a diamond are reference sources

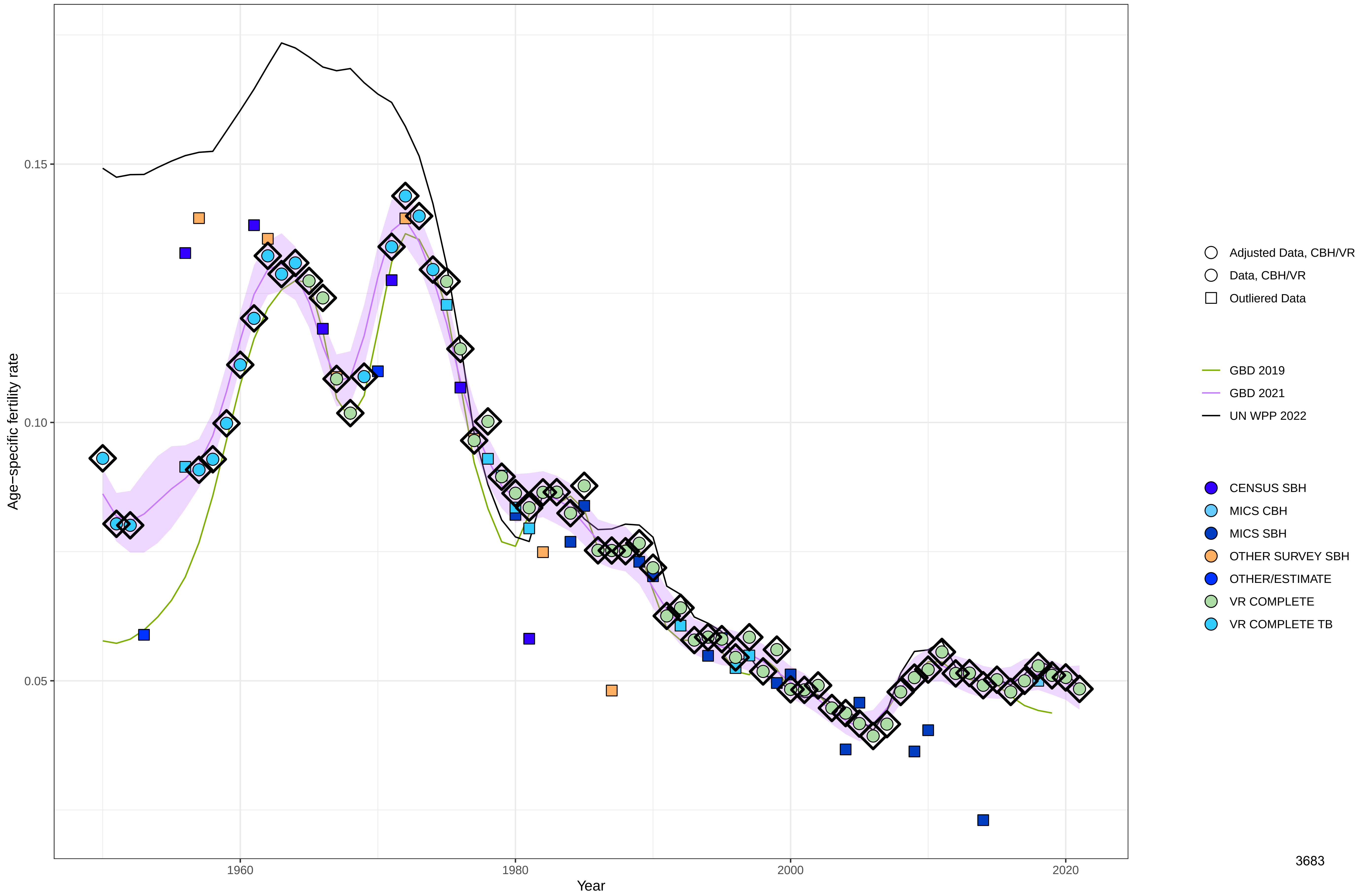

Age−specific fertility fitted trend lines, 1950−2021: Cuba, Ages: 20−24
Data points enclosed with a diamond are reference sources

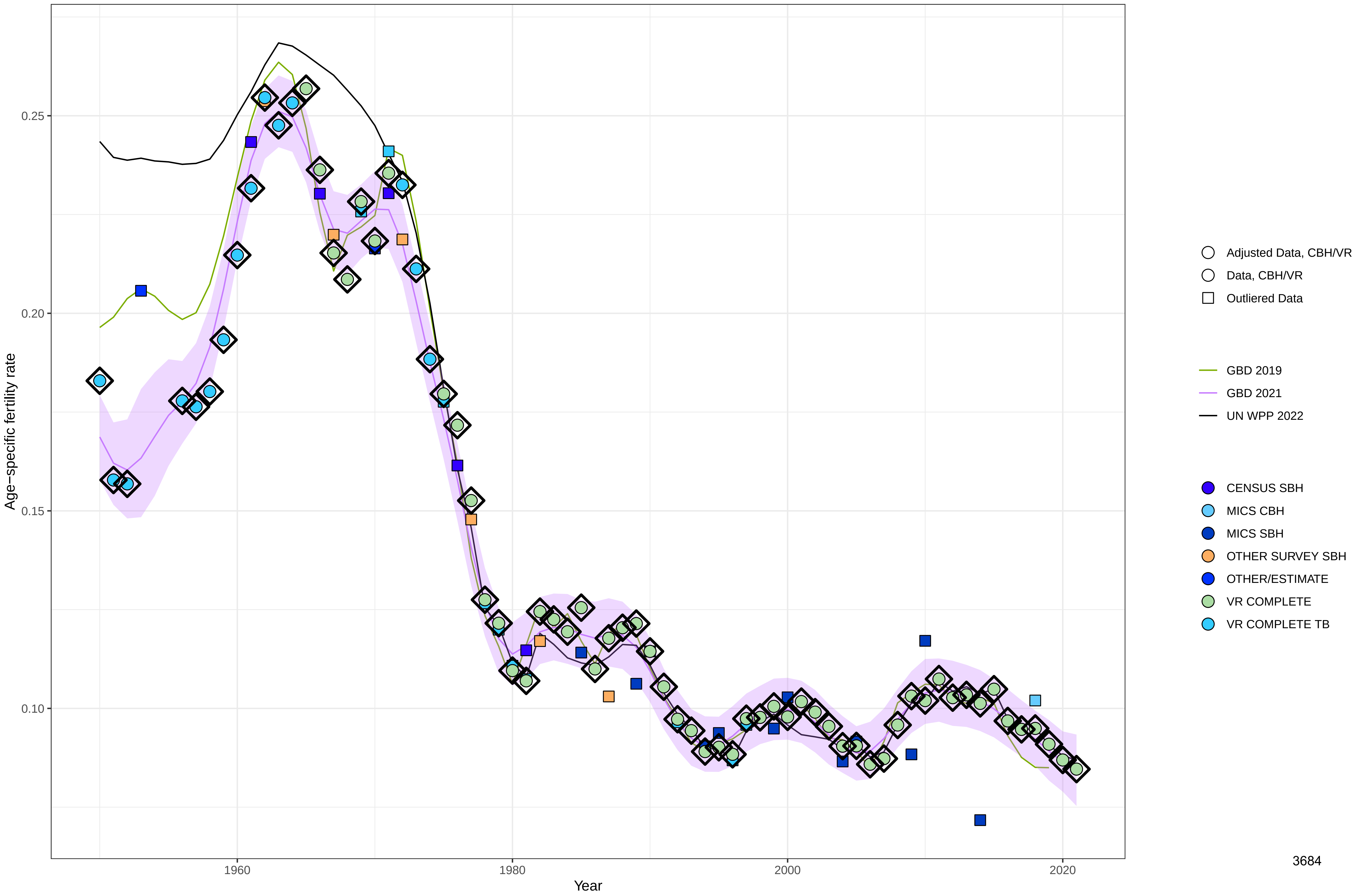

Age−specific fertility fitted trend lines, 1950−2021: Cuba, Ages: 25−29
Data points enclosed with a diamond are reference sources

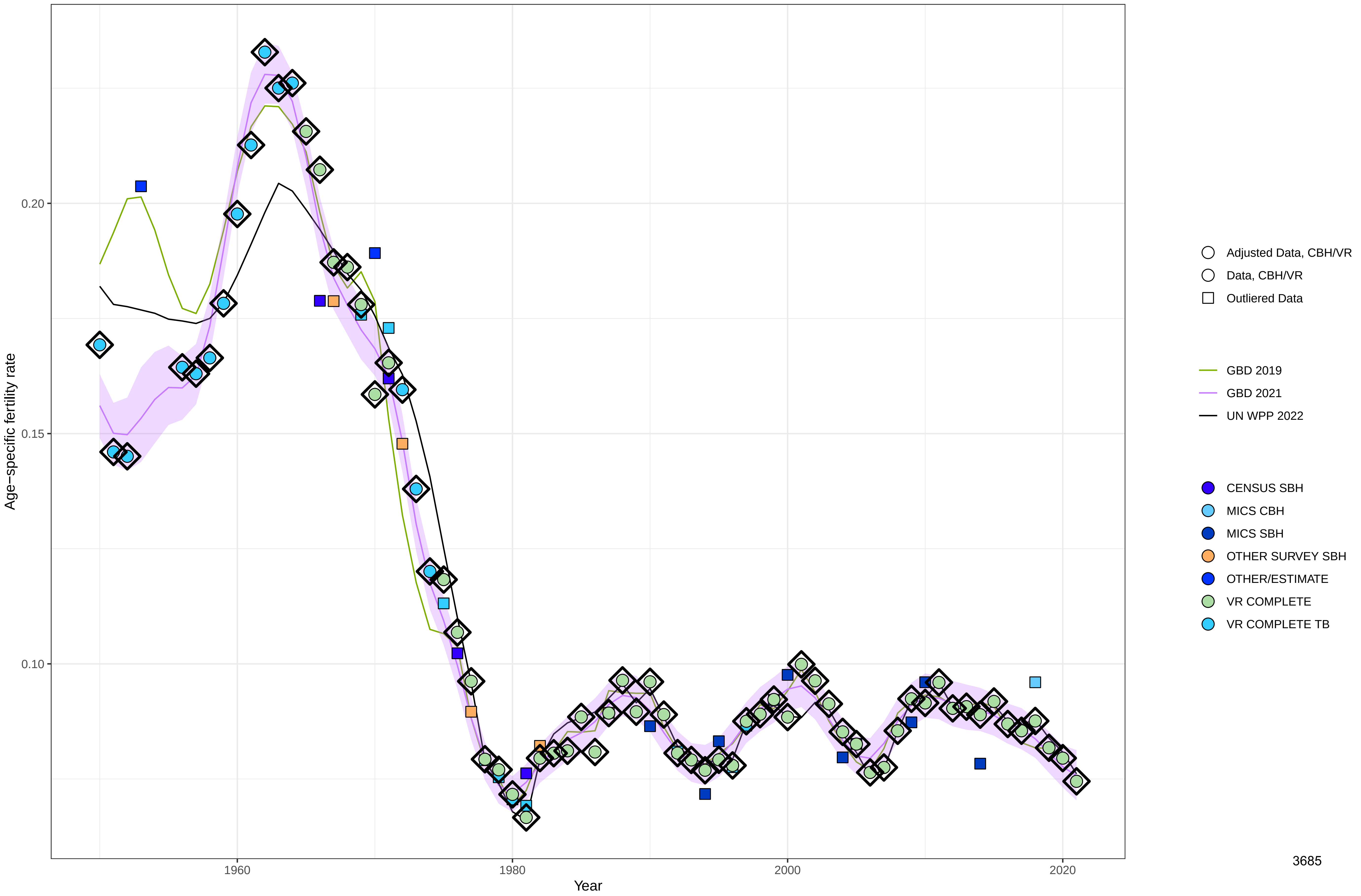

Age−specific fertility fitted trend lines, 1950−2021: Cuba, Ages: 30−34
Data points enclosed with a diamond are reference sources

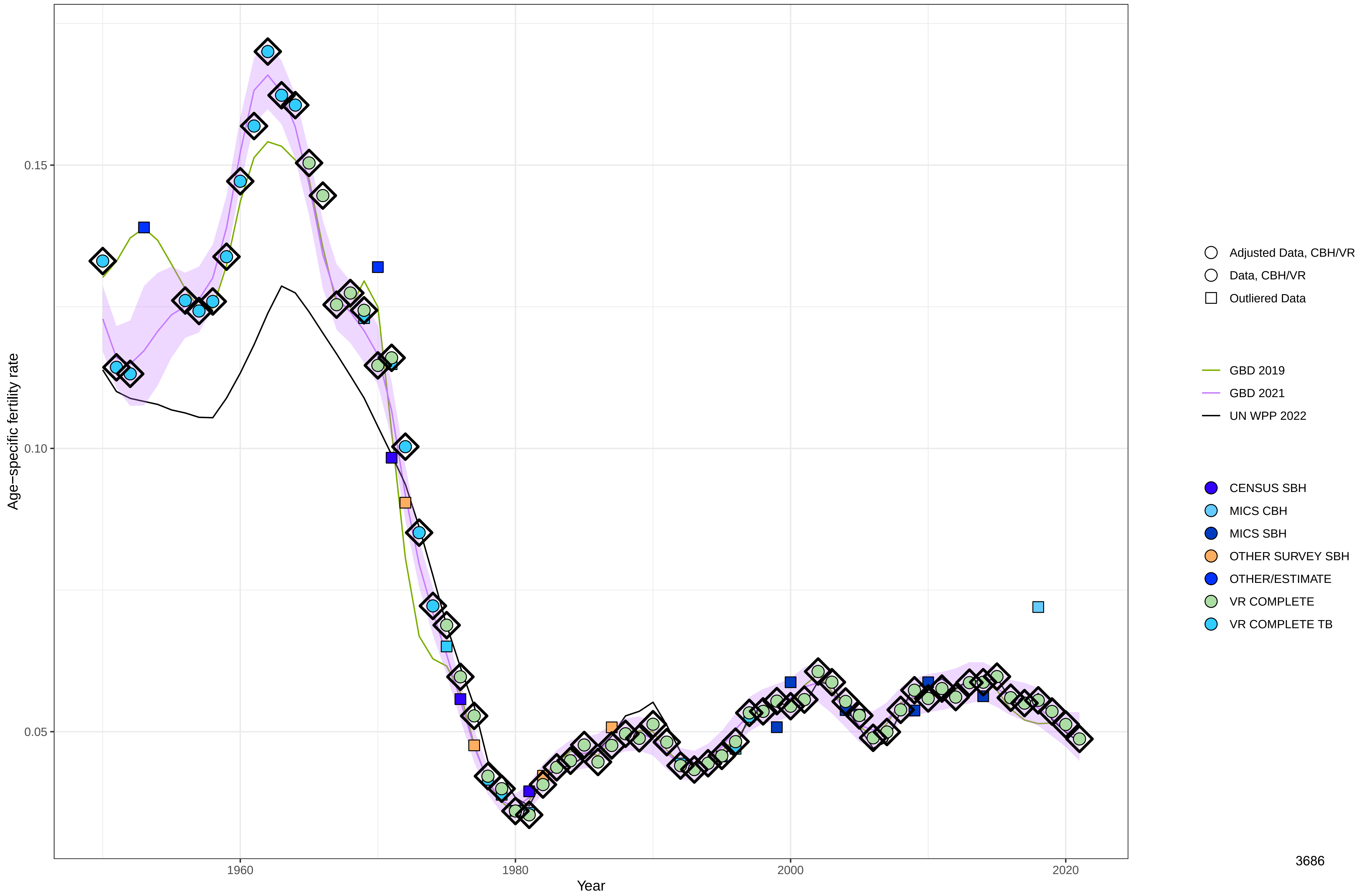

Age−specific fertility fitted trend lines, 1950−2021: Cuba, Ages: 35−39
Data points enclosed with a diamond are reference sources

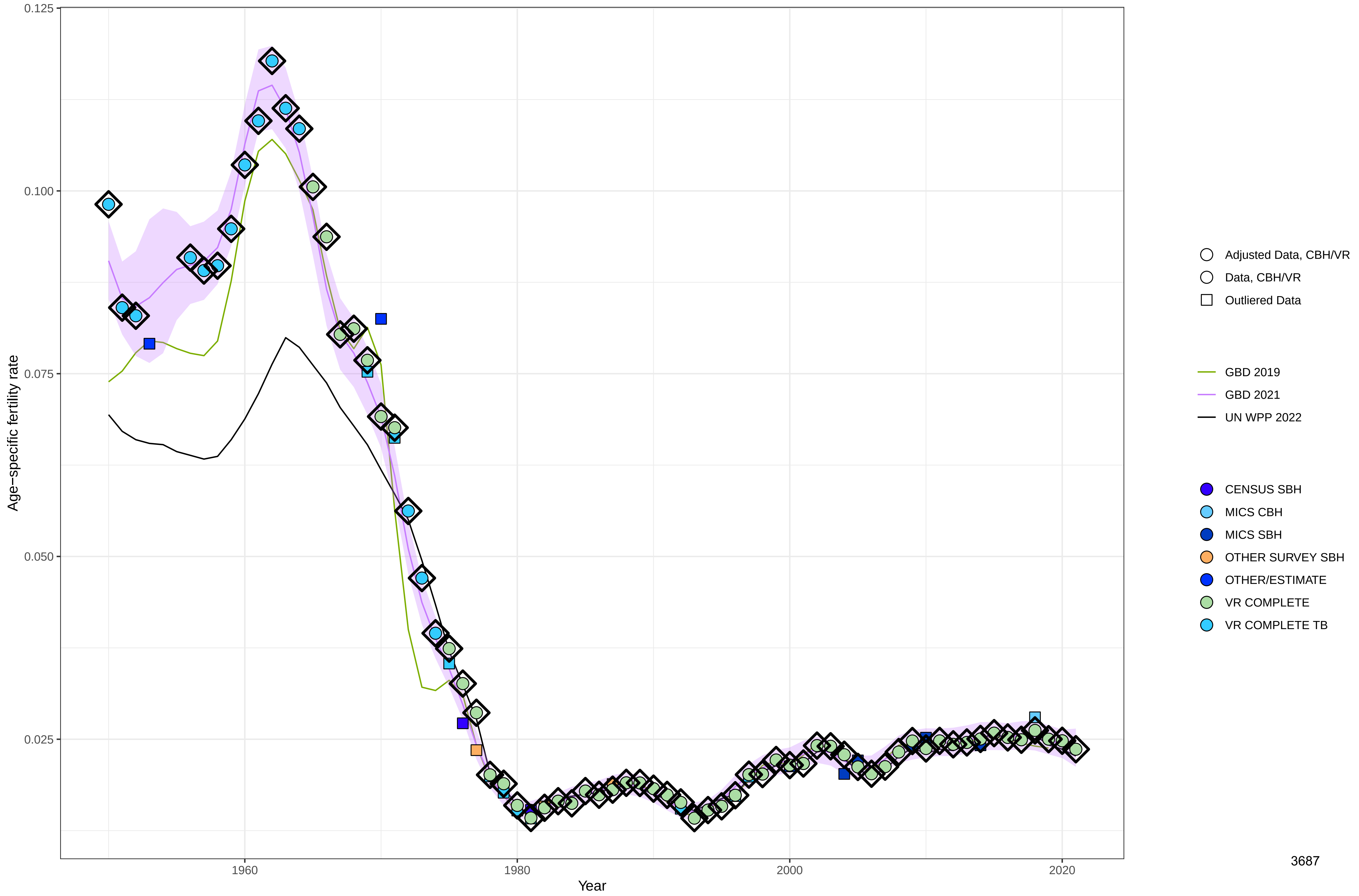

Age−specific fertility fitted trend lines, 1950−2021: Cuba, Ages: 40−44
Data points enclosed with a diamond are reference sources

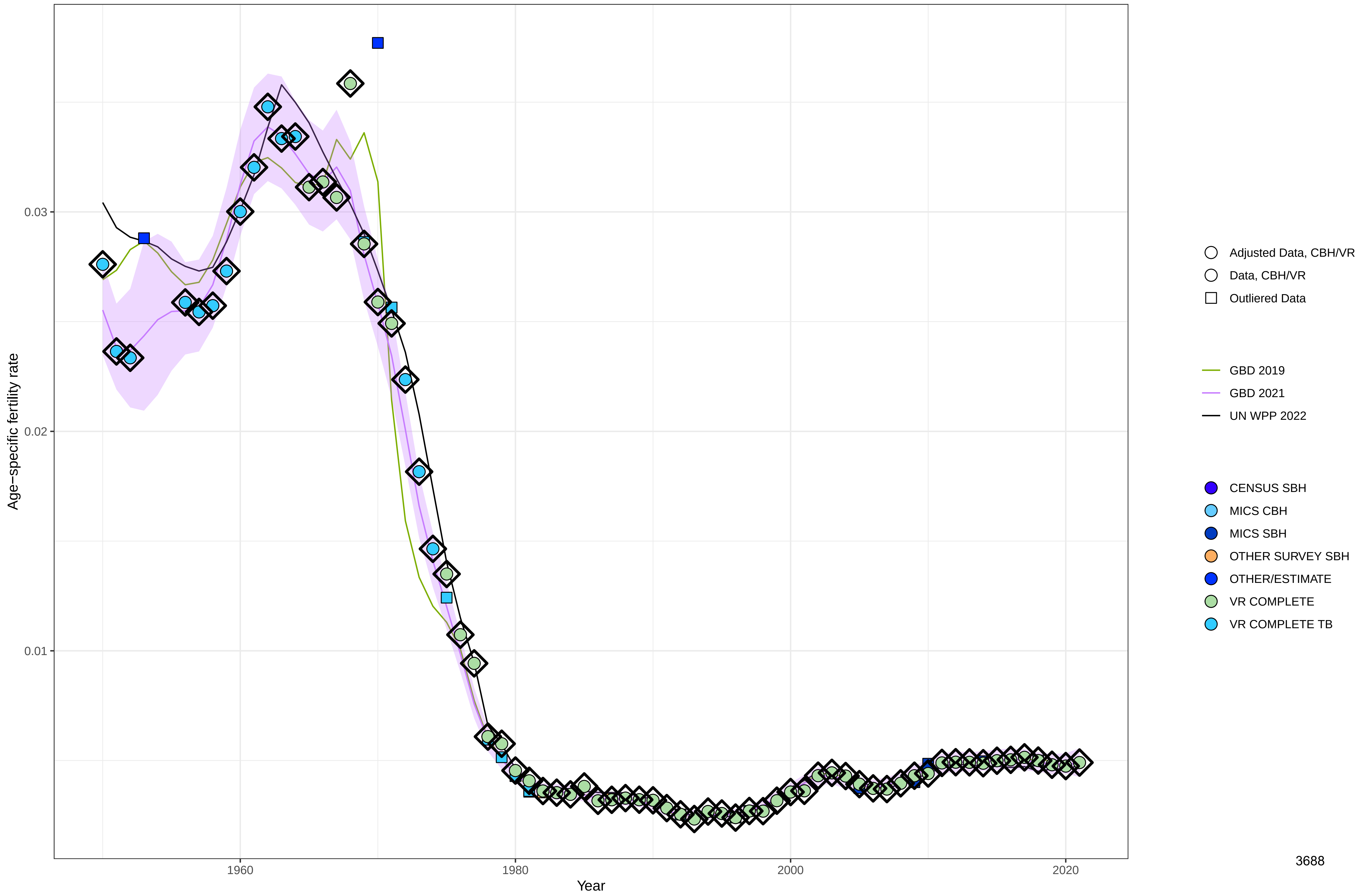

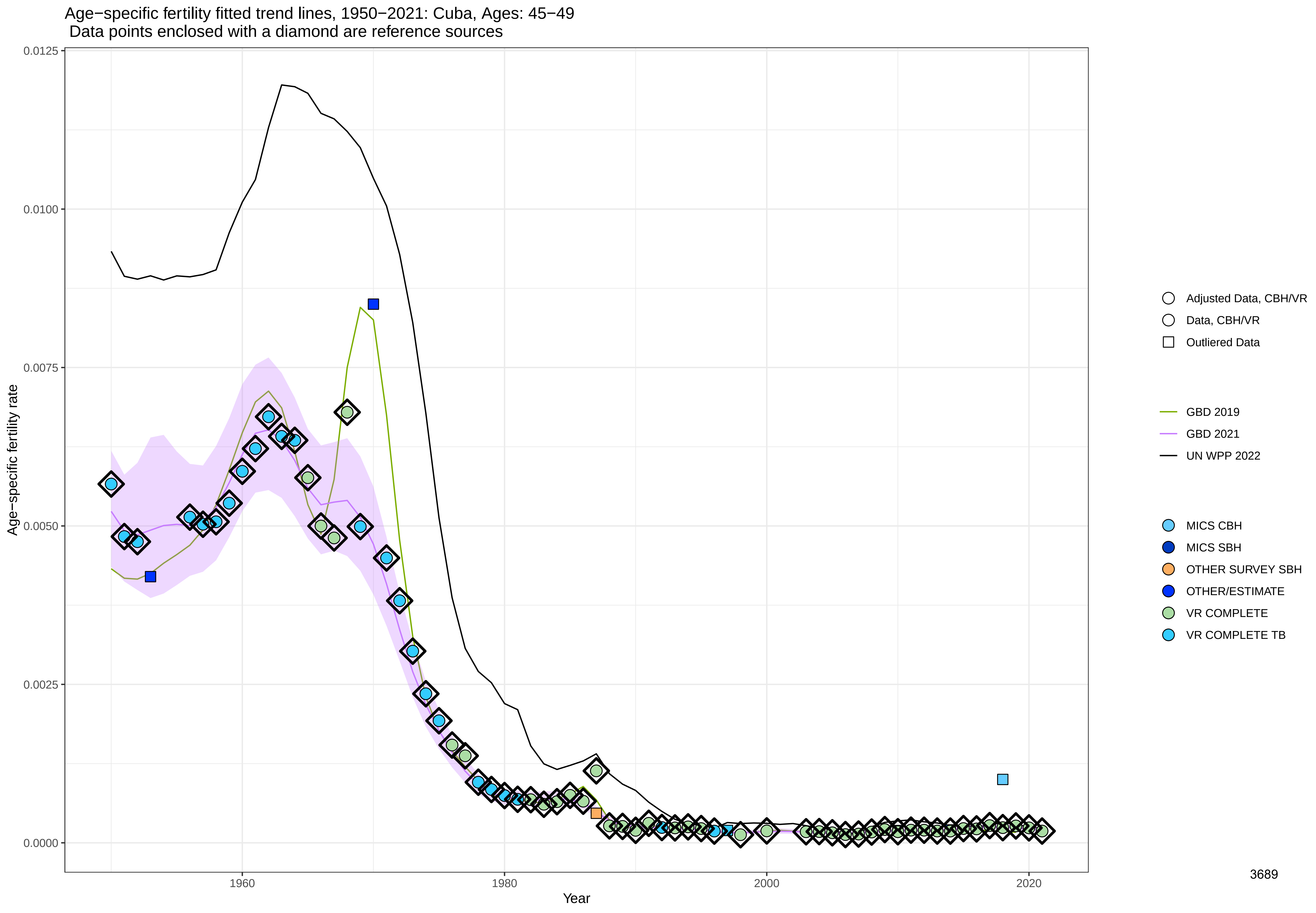

Age−specific fertility fitted trend lines, 1950−2021: Cuba, Ages: 45−49
Data points enclosed with a diamond are reference sources
Age−specific fertility rate
Year
0.0000
0.0025
0.0050
0.0075
0.0100
0.0125
1960
1980
2000
2020
Adjusted Data, CBH/VR
Data, CBH/VR
Outliered Data
GBD 2019
GBD 2021
UN WPP 2022
MICS CBH
MICS SBH
OTHER SURVEY SBH
OTHER/ESTIMATE
VR COMPLETE
VR COMPLETE TB

Age−specific fertility fitted trend lines, 1950−2021: Cuba, Ages: 50−54
Data points enclosed with a diamond are reference sources

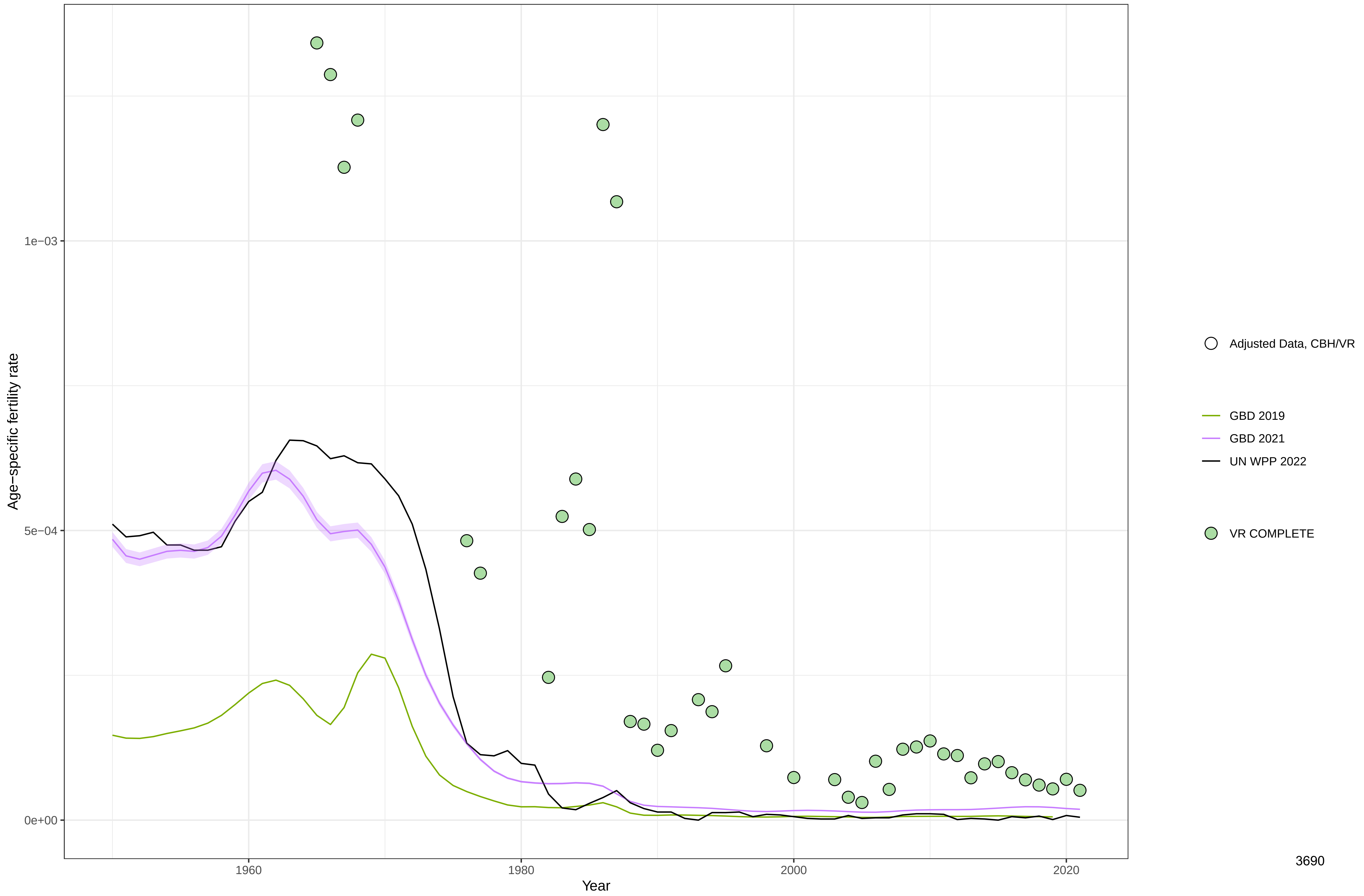

Total fertility rate fitted trend lines, 1950−2021: Cuba TFR
Data points enclosed with a diamond are reference sources

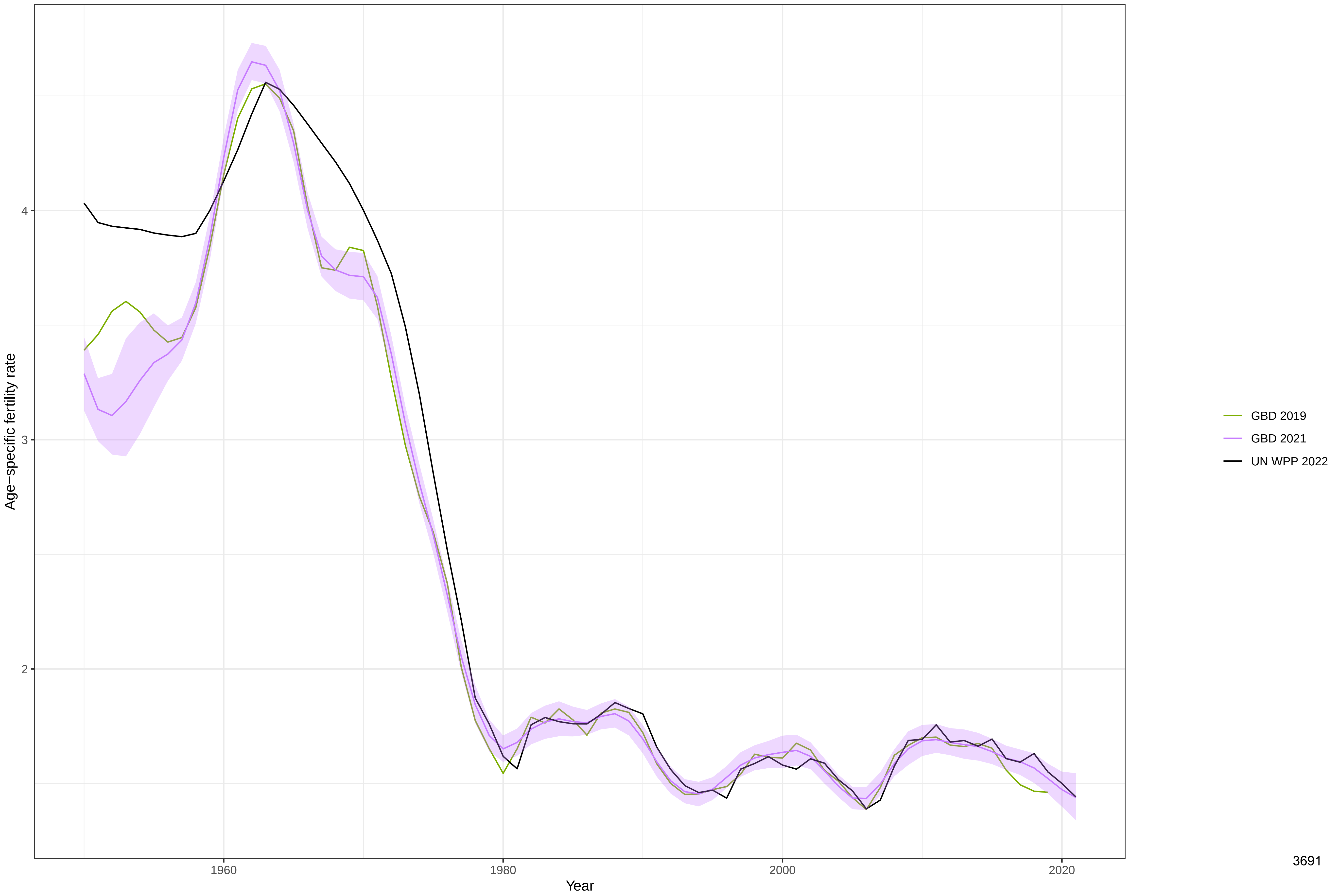

Age−specific fertility fitted trend lines, 1950−2021: Dominica, Ages: 10−14
Data points enclosed with a diamond are reference sources

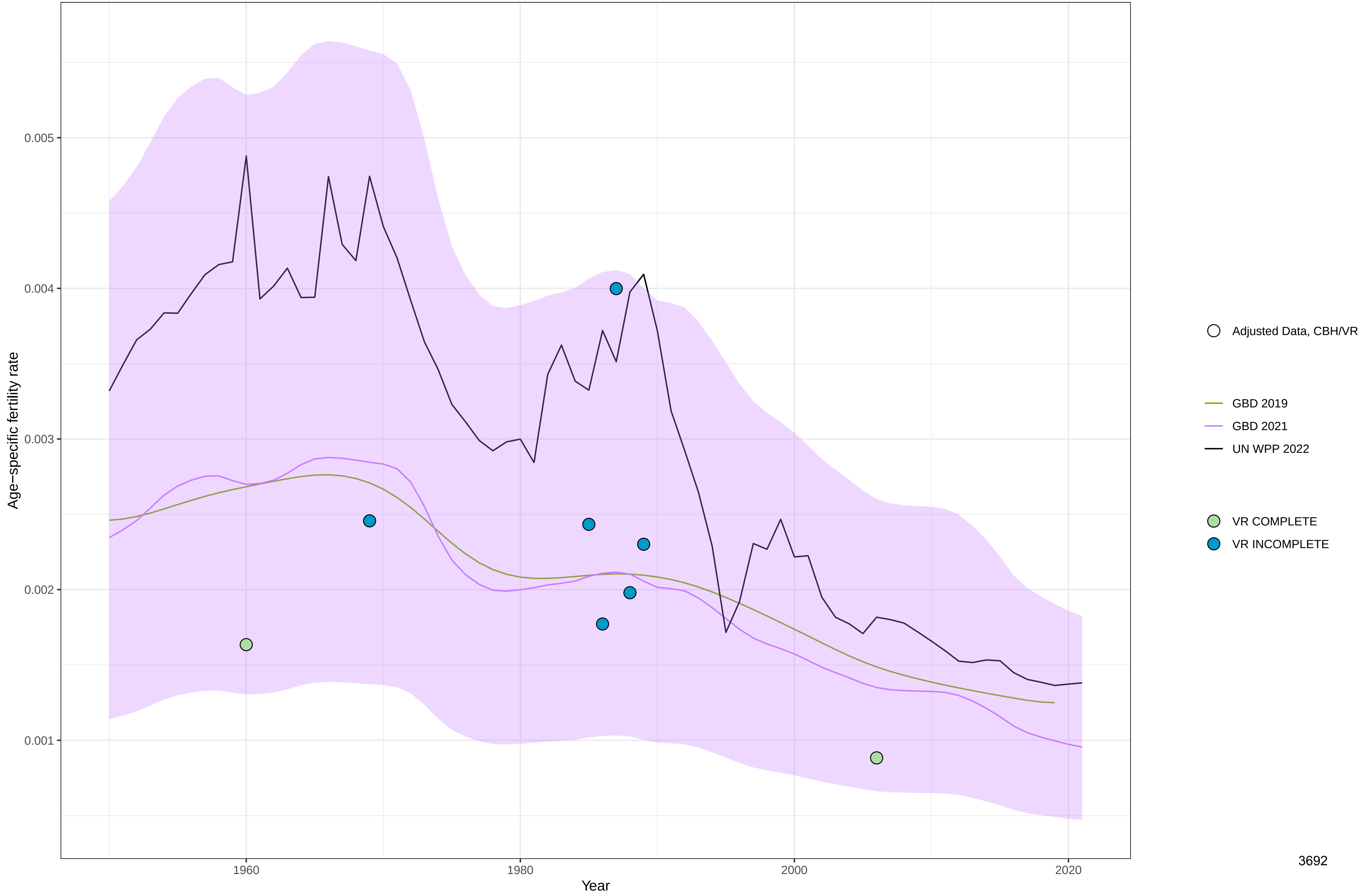

Age−specific fertility fitted trend lines, 1950−2021: Dominica, Ages: 15−19
Data points enclosed with a diamond are reference sources

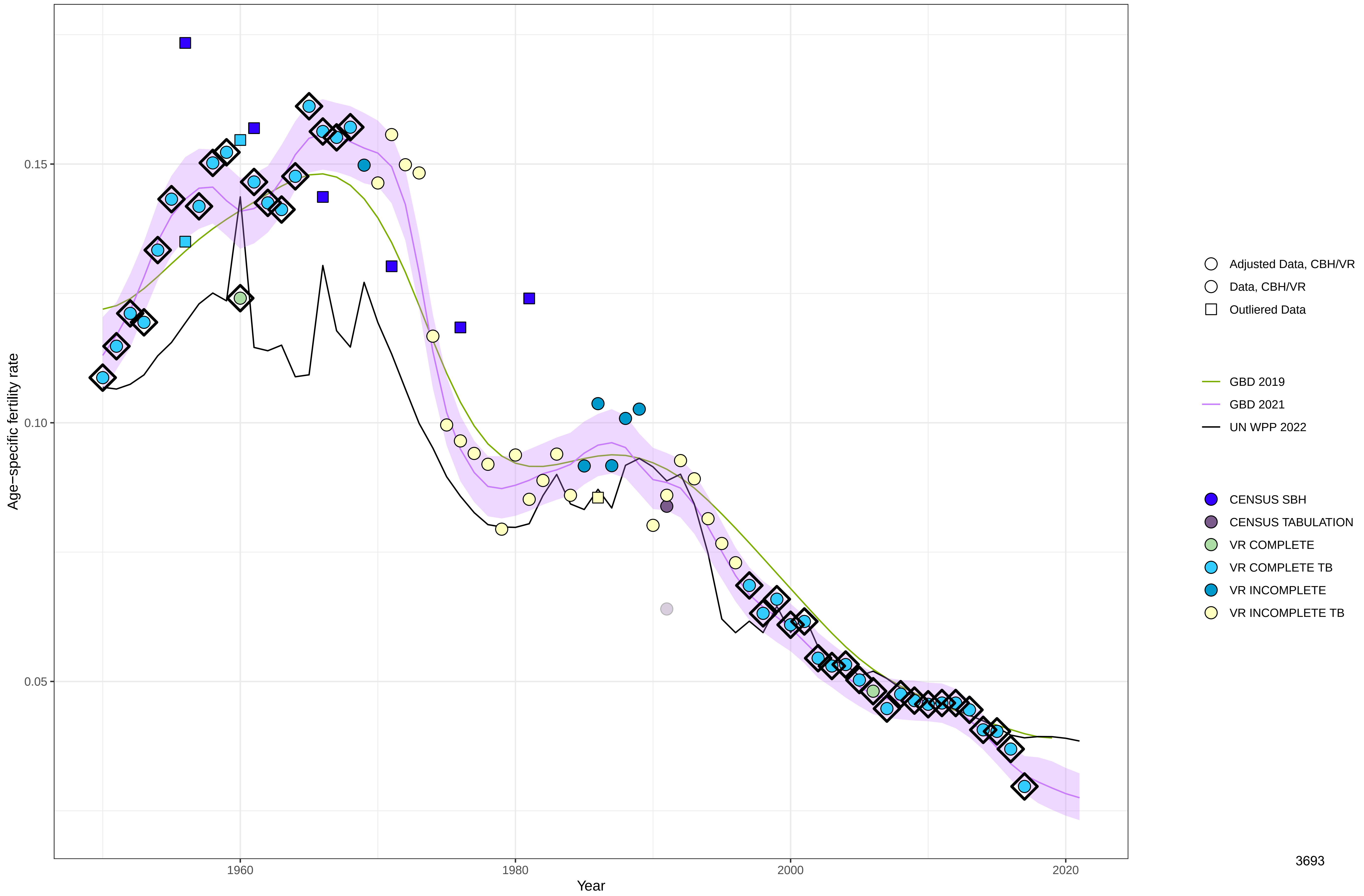

Age−specific fertility fitted trend lines, 1950−2021: Dominica, Ages: 20−24
Data points enclosed with a diamond are reference sources

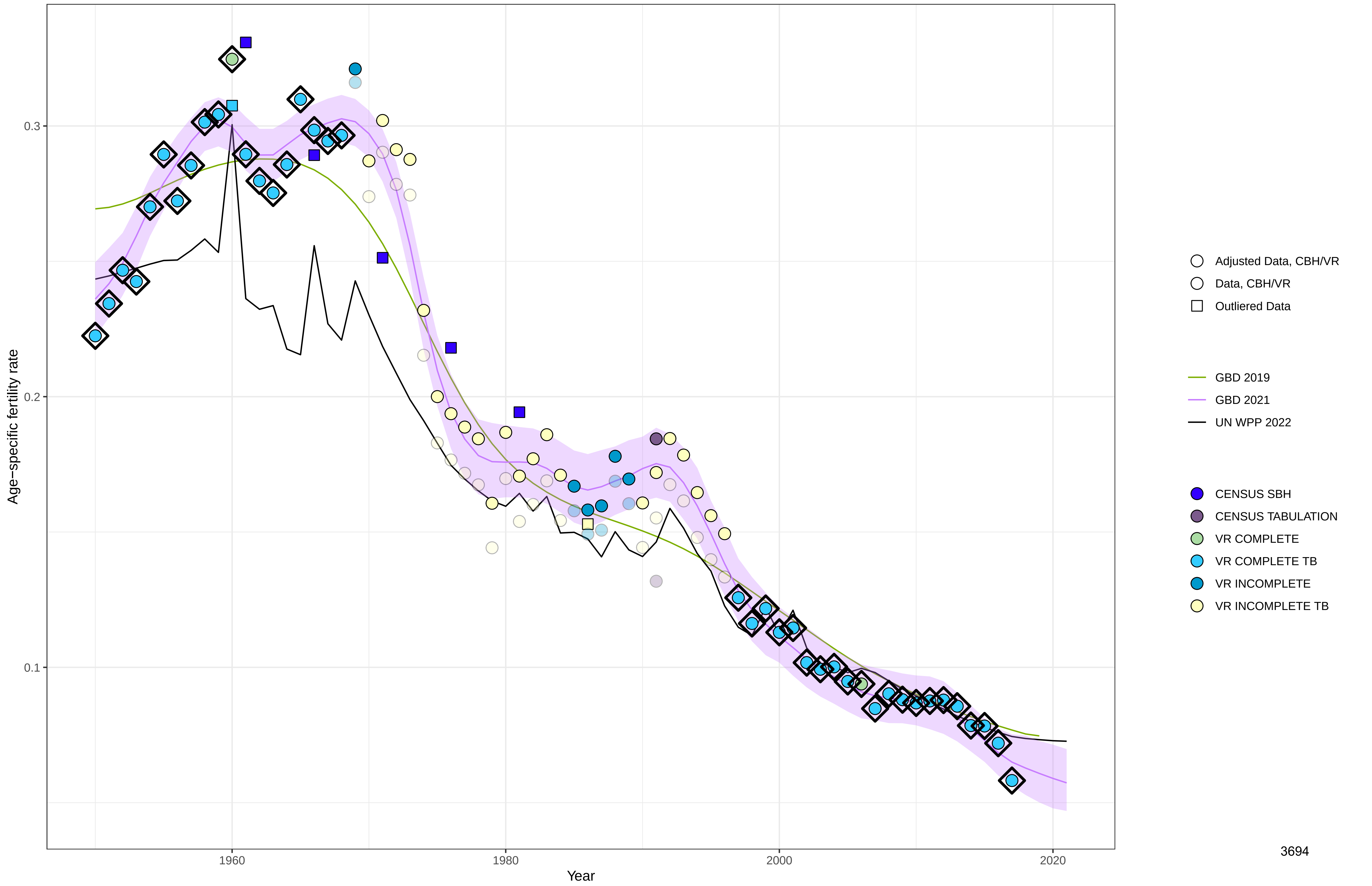

Age−specific fertility fitted trend lines, 1950−2021: Dominica, Ages: 25−29
Data points enclosed with a diamond are reference sources

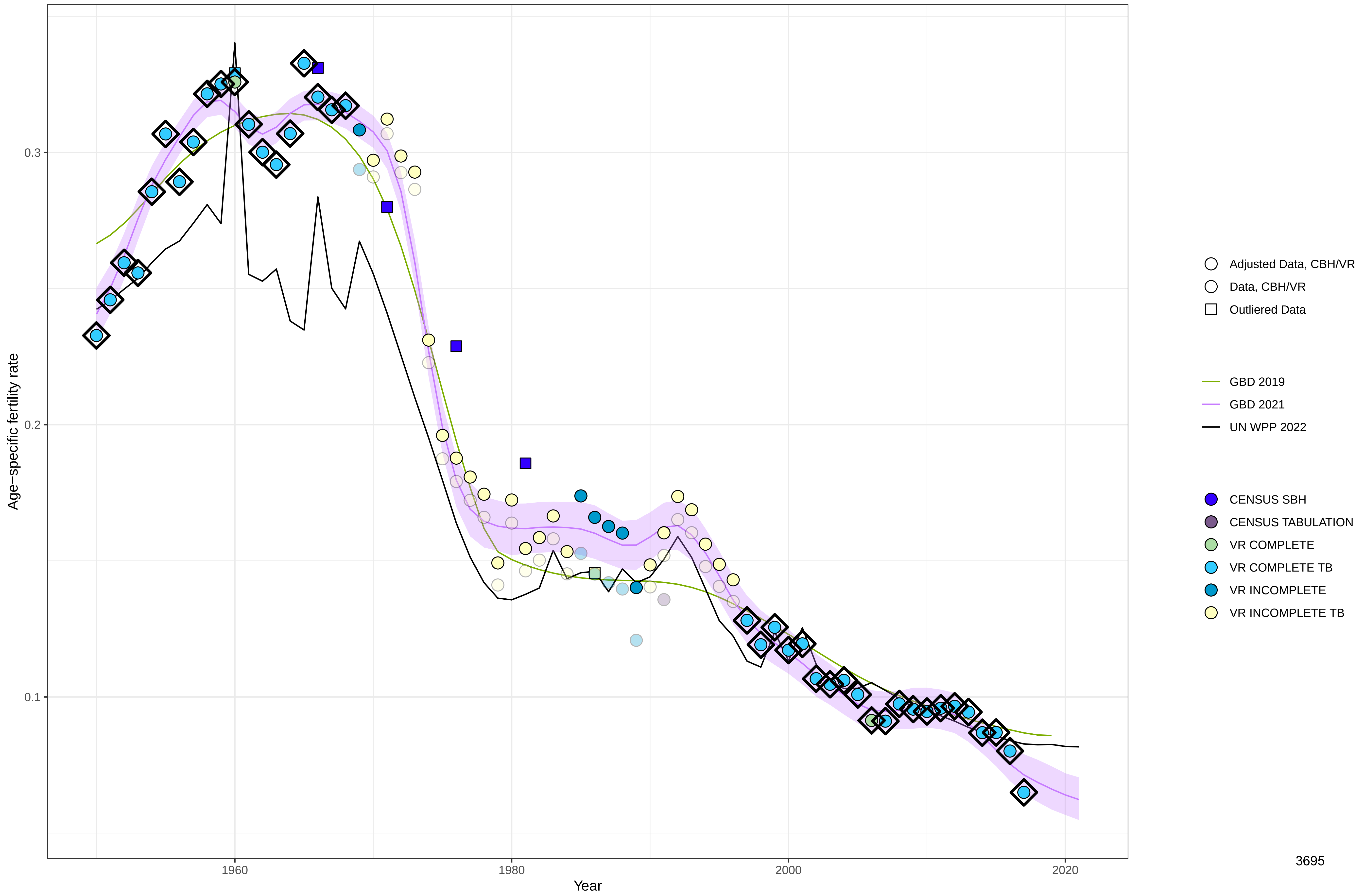

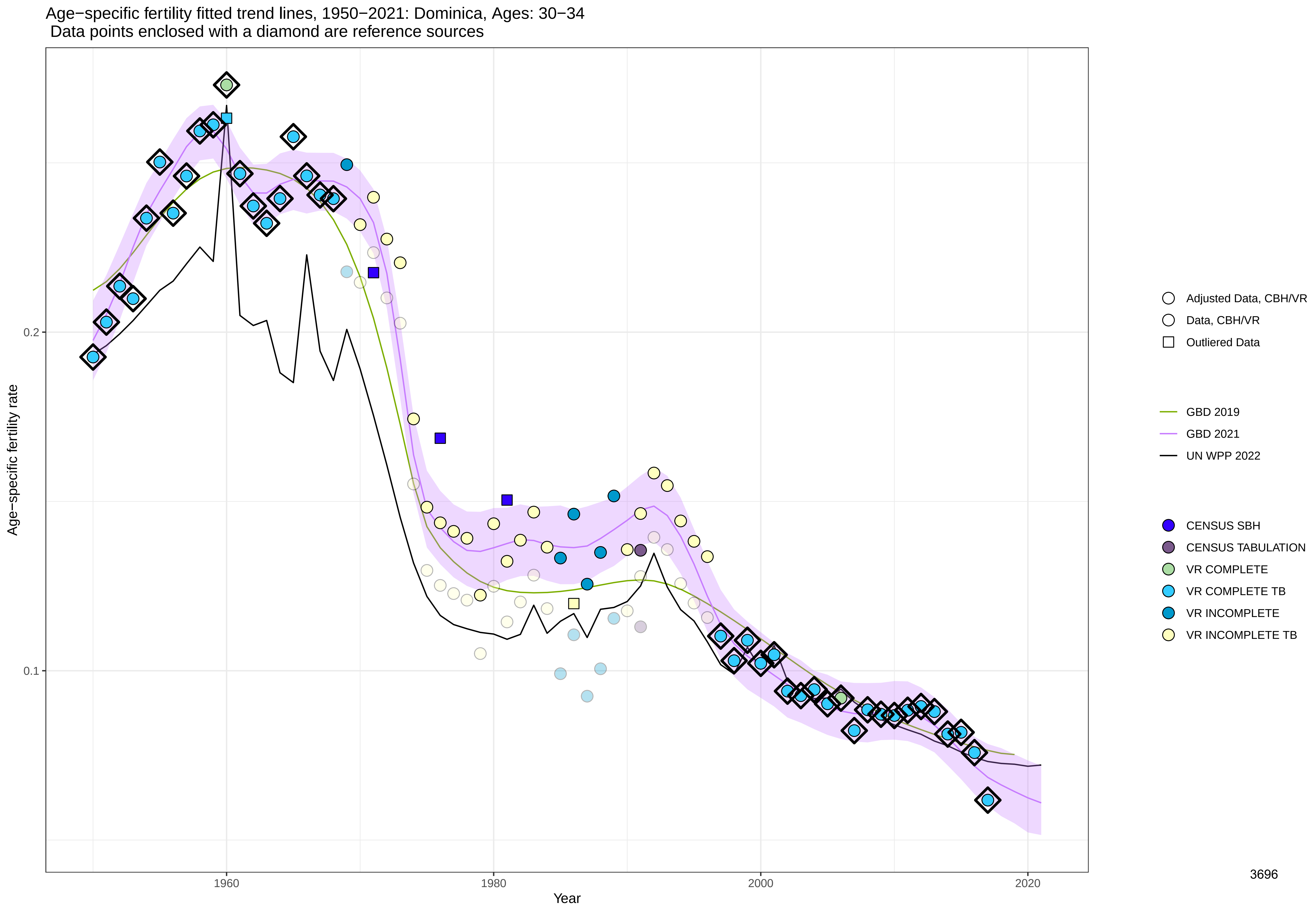
Age−specific fertility fitted trend lines, 1950−2021: Dominica, Ages: 30−34
Data points enclosed with a diamond are reference sources
Age−specific fertility rate
0.2
0.1
1960
1980
2000
2020
Year
Adjusted Data, CBH/VR
Data, CBH/VR
Outliered Data
GBD 2019
GBD 2021
UN WPP 2022
CENSUS SBH
CENSUS TABULATION
VR COMPLETE
VR COMPLETE TB
VR INCOMPLETE
VR INCOMPLETE TB

Age−specific fertility fitted trend lines, 1950−2021: Dominica, Ages: 35−39
Data points enclosed with a diamond are reference sources

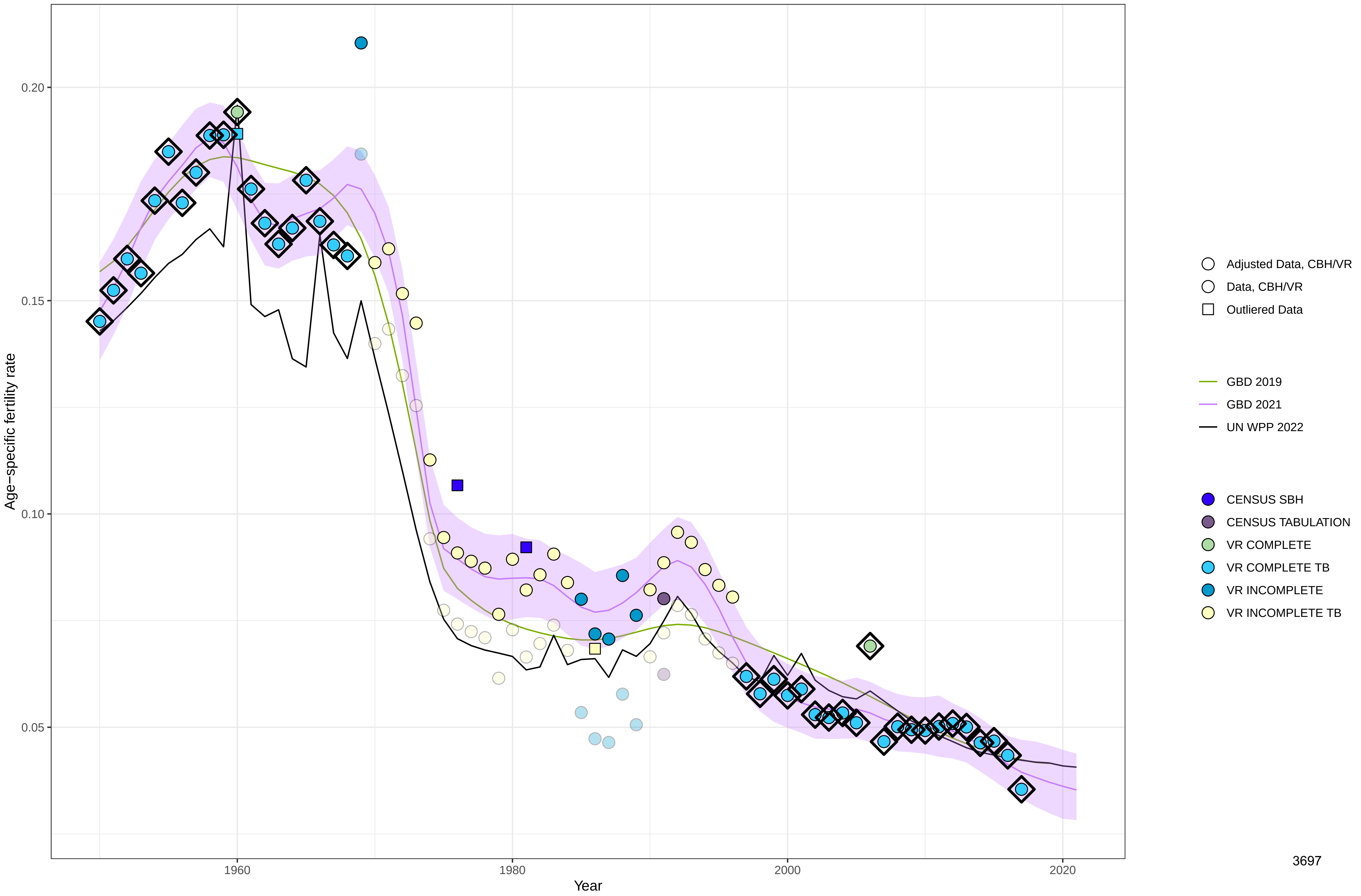

Age−specific fertility fitted trend lines, 1950−2021: Dominica, Ages: 40−44
Data points enclosed with a diamond are reference sources

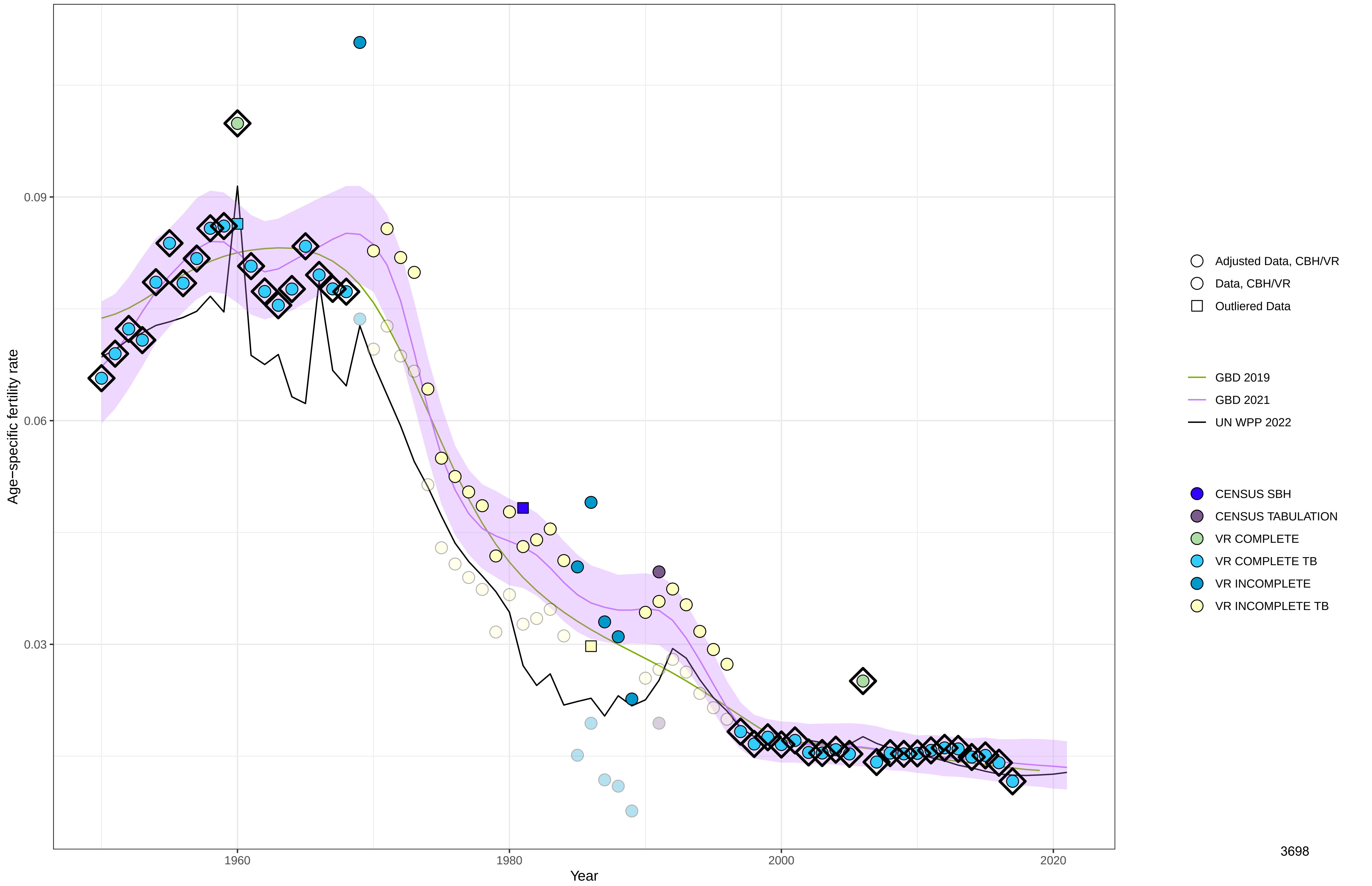

Age−specific fertility fitted trend lines, 1950−2021: Dominica, Ages: 45−49
Data points enclosed with a diamond are reference sources

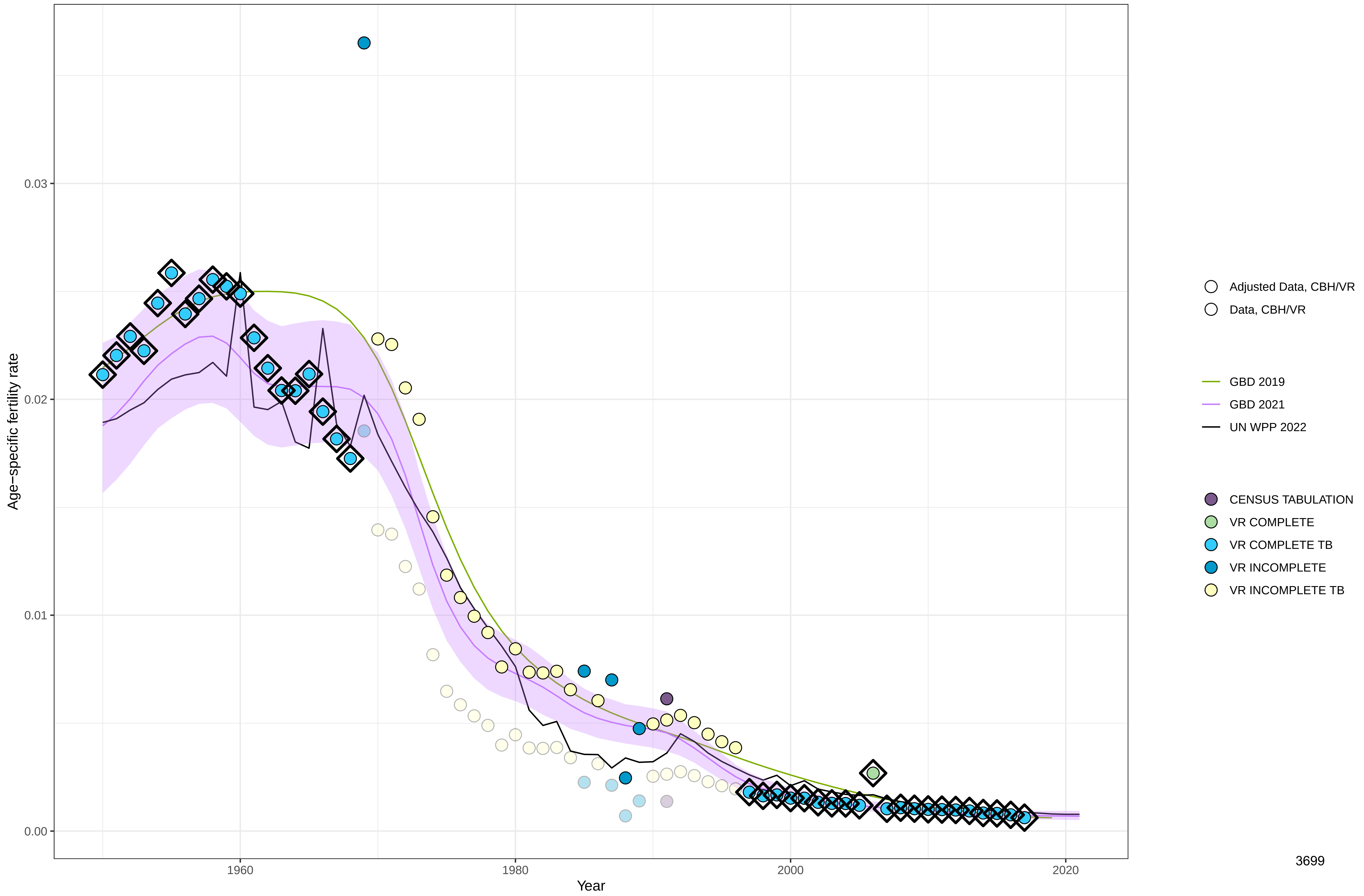

Age−specific fertility fitted trend lines, 1950−2021: Dominica, Ages: 50−54
Data points enclosed with a diamond are reference sources

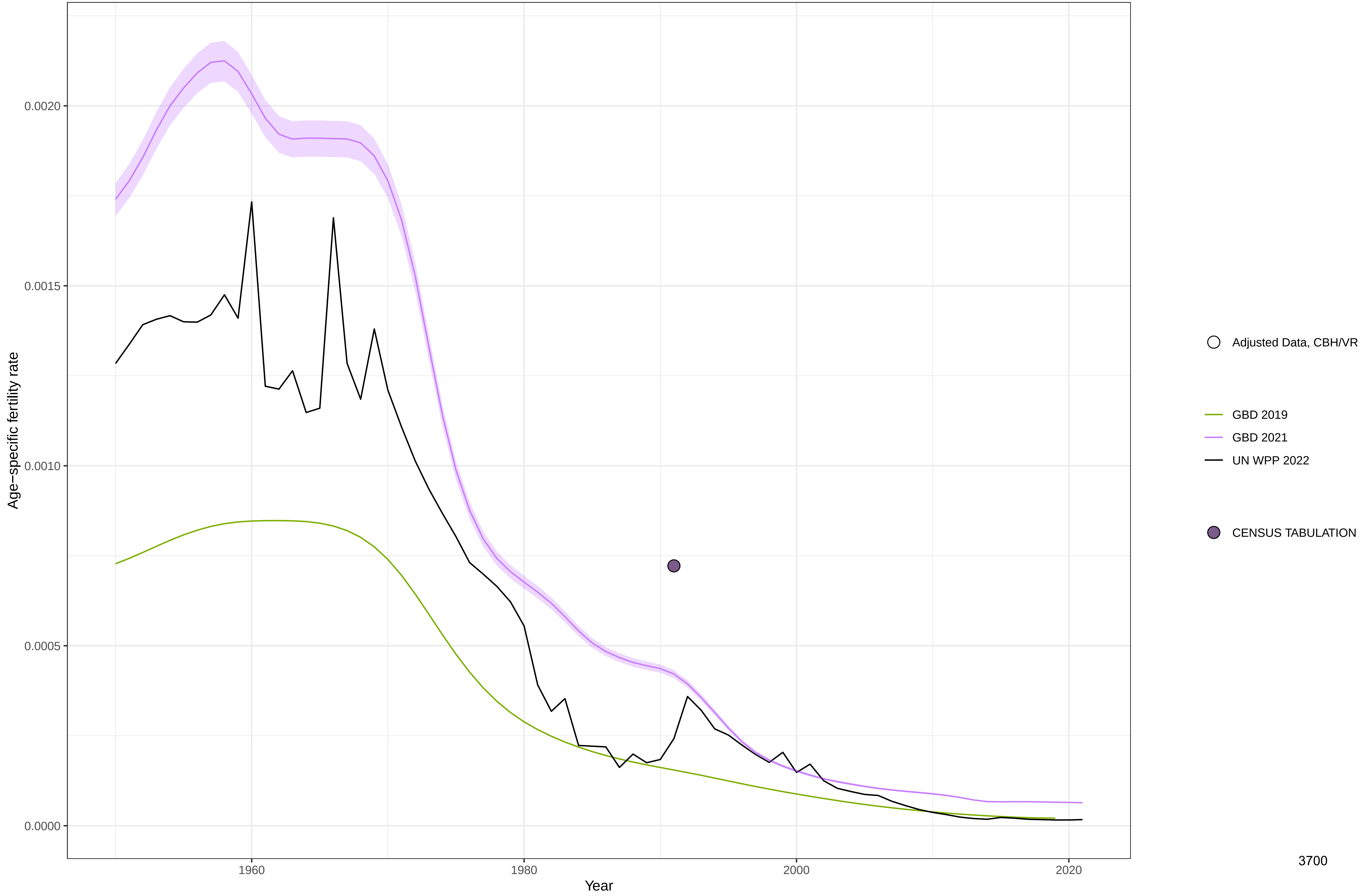

Total fertility rate fitted trend lines, 1950−2021: Dominica TFR
Data points enclosed with a diamond are reference sources

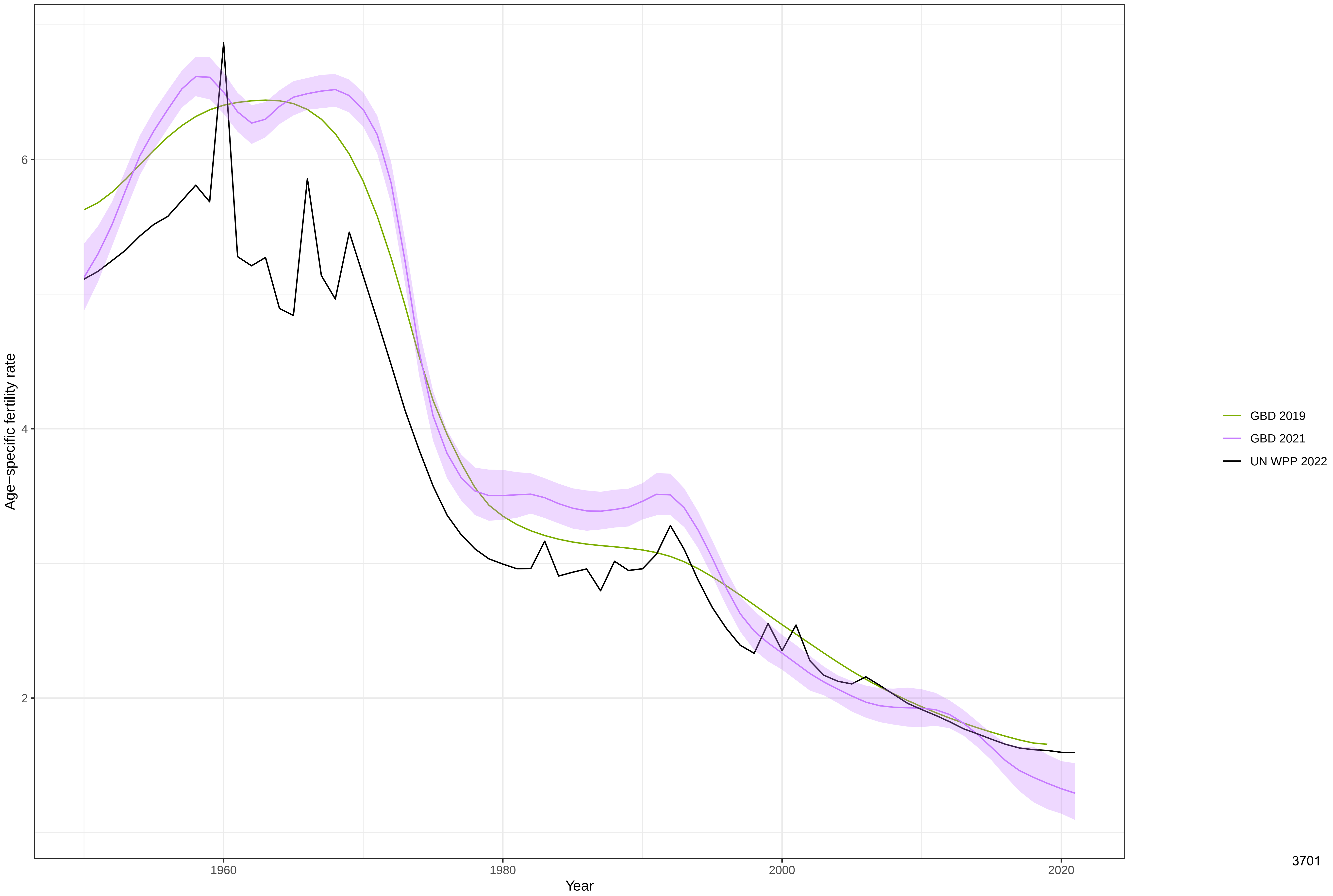

Age−specific fertility fitted trend lines, 1950−2021: Dominican Republic, Ages: 10−14
Data points enclosed with a diamond are reference sources

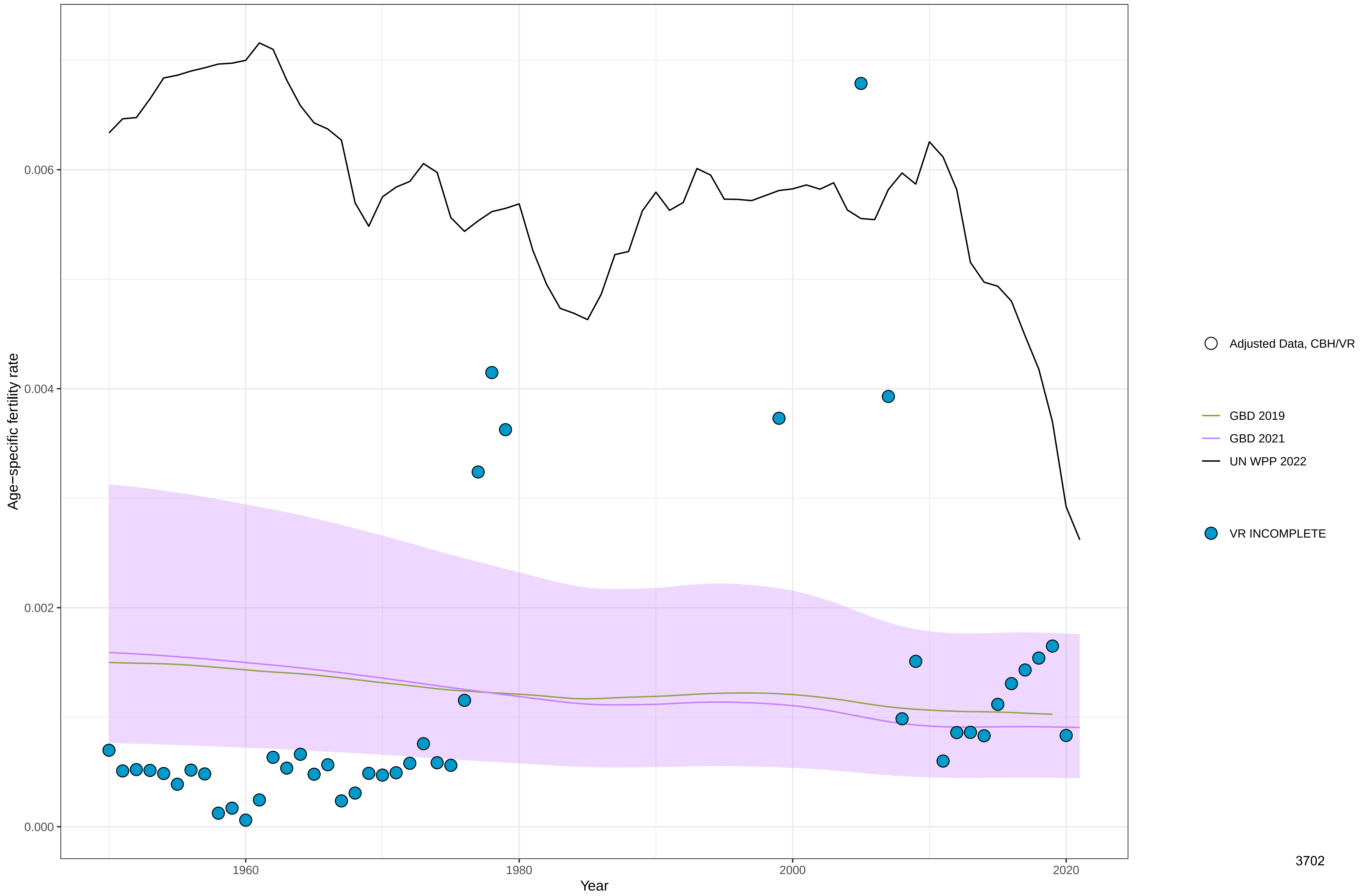

Age−specific fertility fitted trend lines, 1950−2021: Dominican Republic, Ages: 15−19
Data points enclosed with a diamond are reference sources

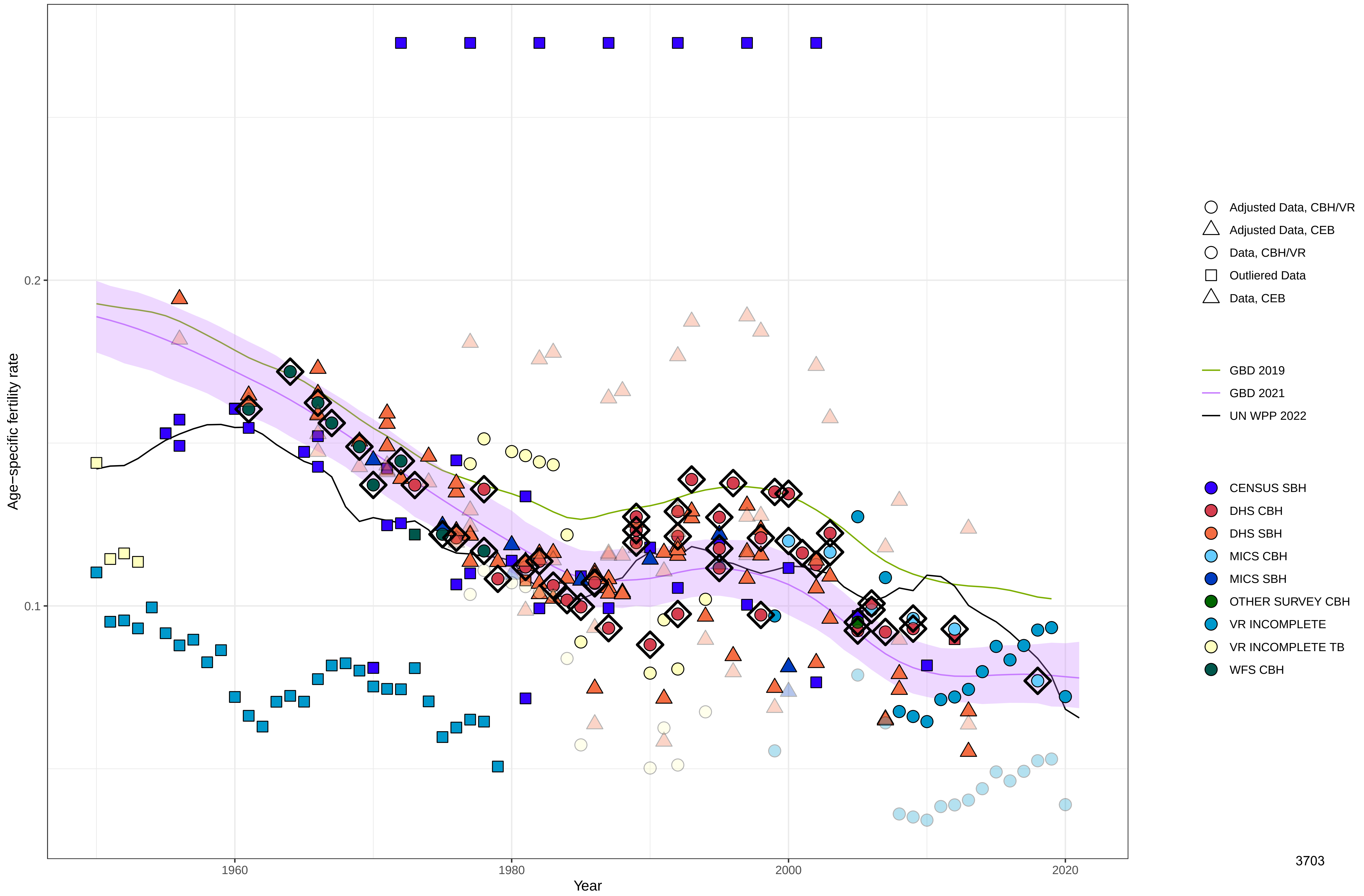

Age−specific fertility fitted trend lines, 1950−2021: Dominican Republic, Ages: 20−24
Data points enclosed with a diamond are reference sources

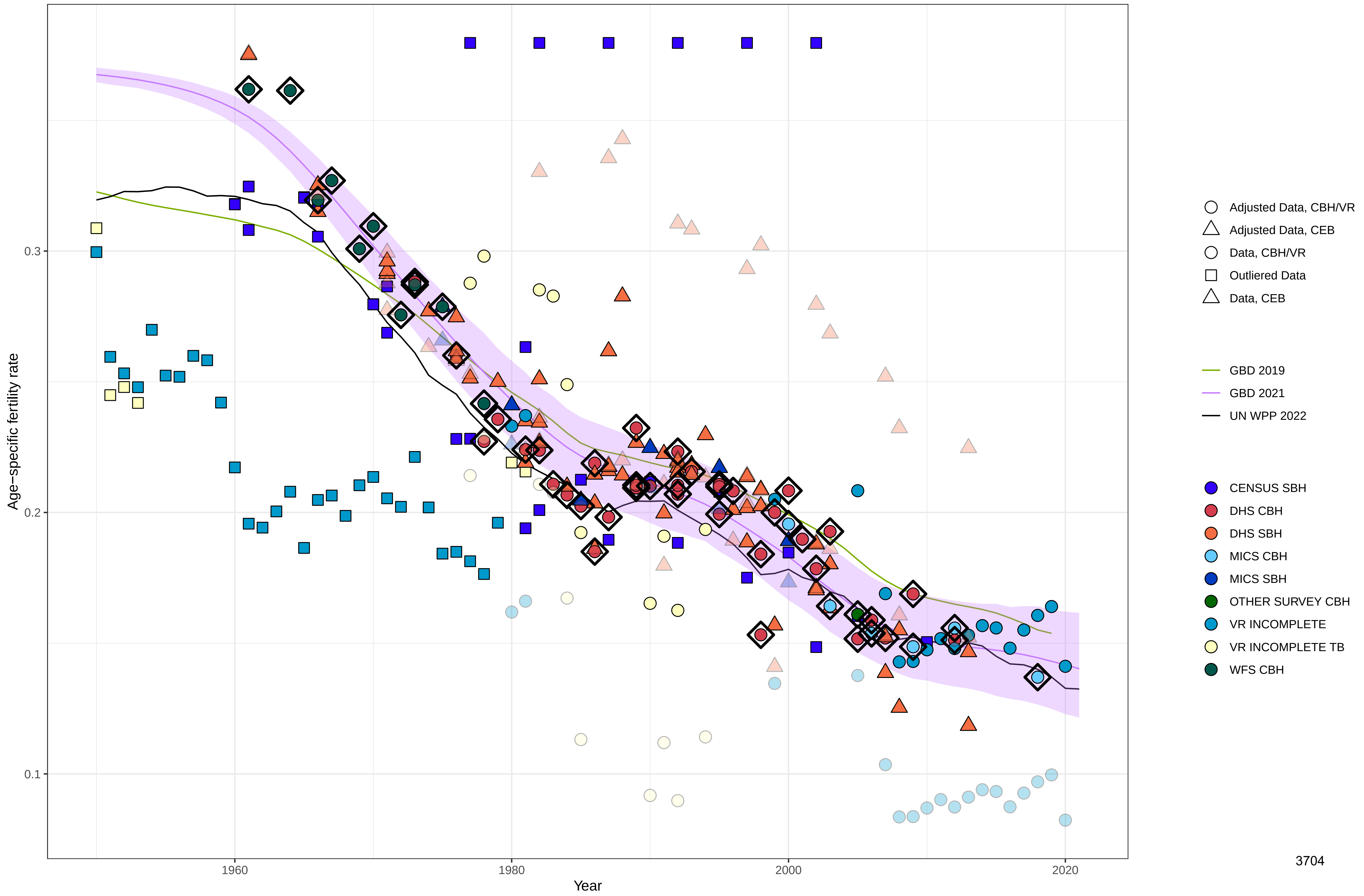

Age−specific fertility fitted trend lines, 1950−2021: Dominican Republic, Ages: 25−29
Data points enclosed with a diamond are reference sources

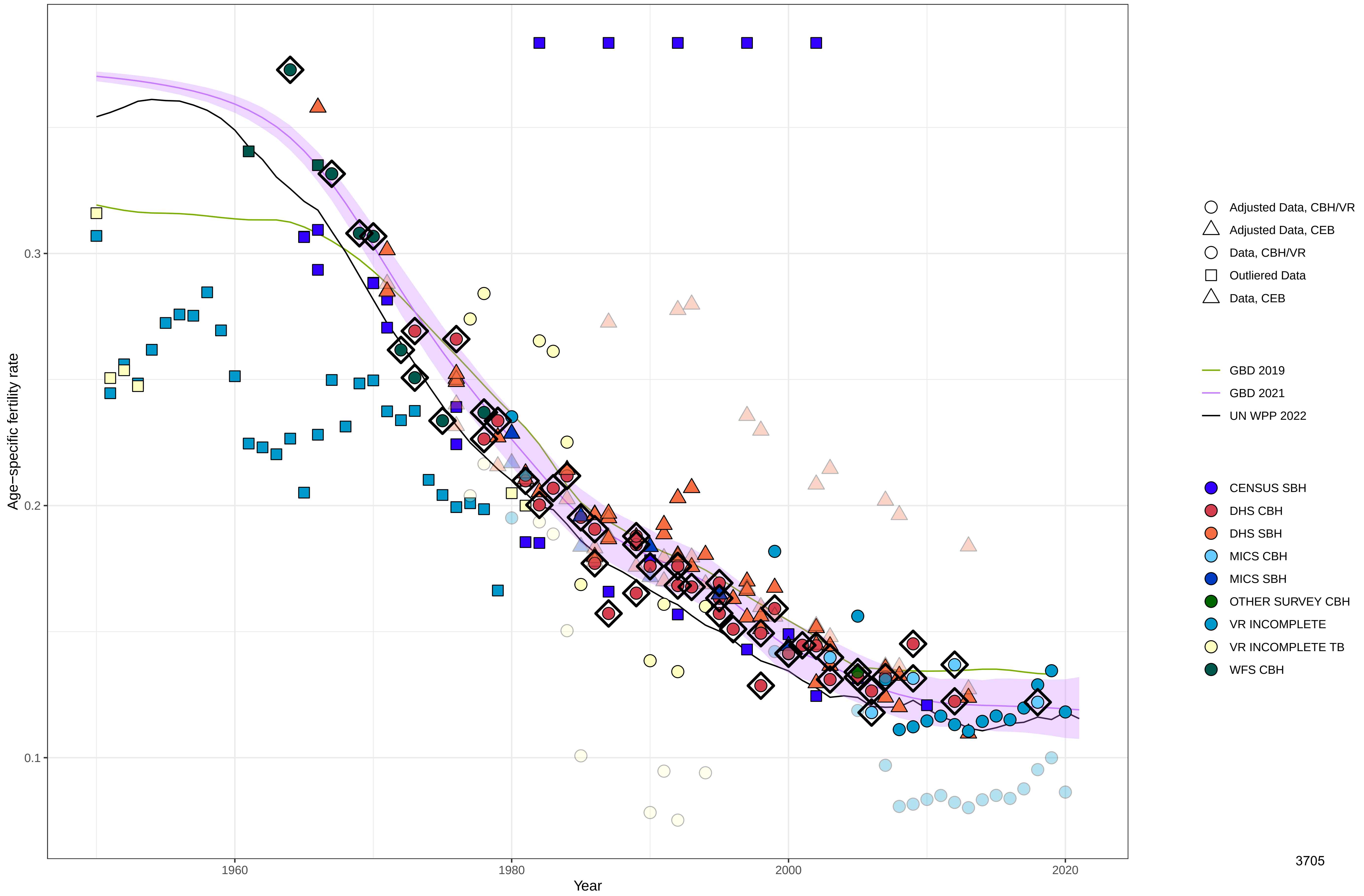

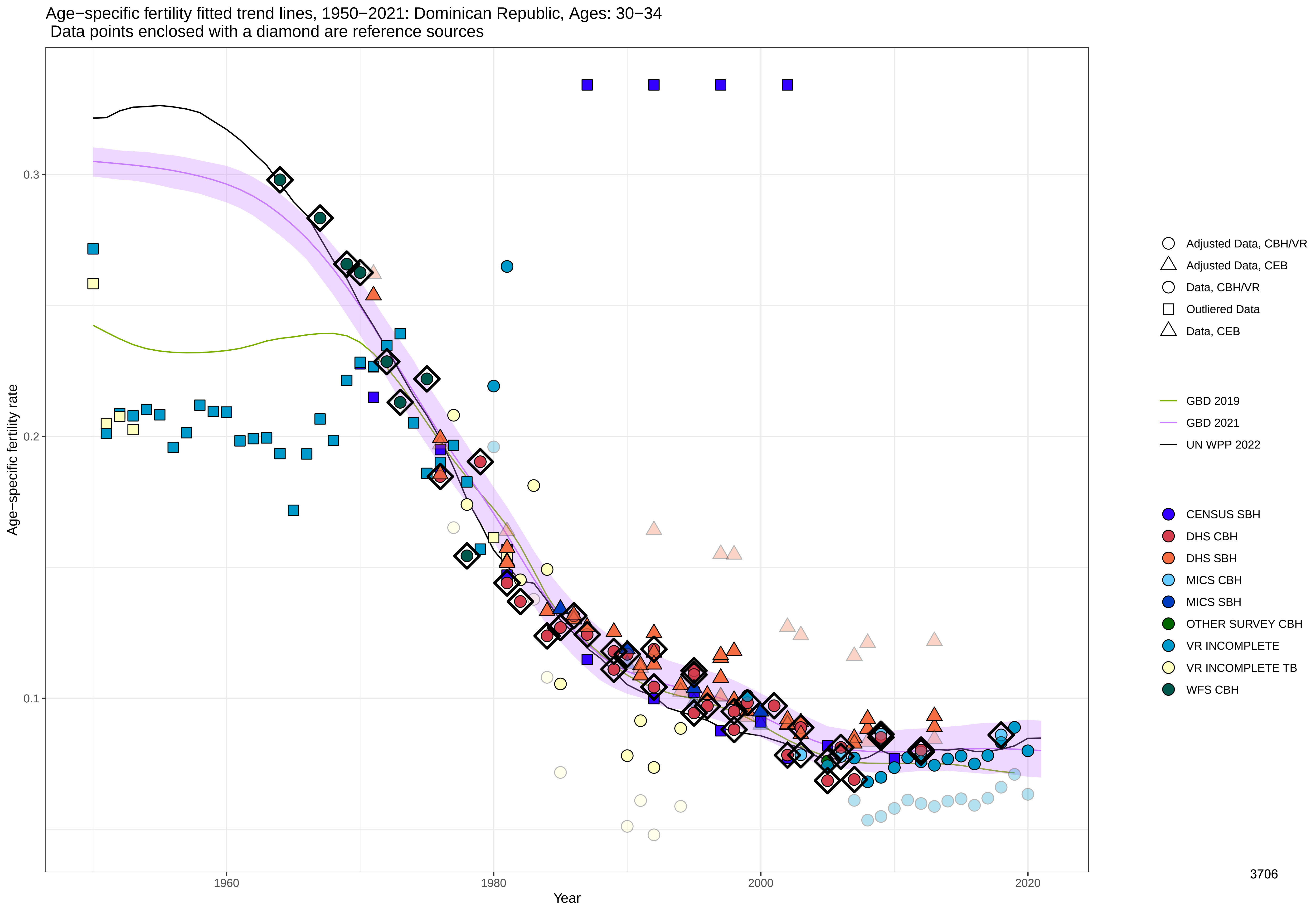

Age−specific fertility fitted trend lines, 1950−2021: Dominican Republic, Ages: 30−34
Data points enclosed with a diamond are reference sources
Age−specific fertility rate
Year
0.1
0.2
0.3
1960
1980
2000
2020
Adjusted Data, CBH/VR
Adjusted Data, CEB
Data, CBH/VR
Outliered Data
Data, CEB
GBD 2019
GBD 2021
UN WPP 2022
CENSUS SBH
DHS CBH
DHS SBH
MICS CBH
MICS SBH
OTHER SURVEY CBH
VR INCOMPLETE
VR INCOMPLETE TB
WFS CBH

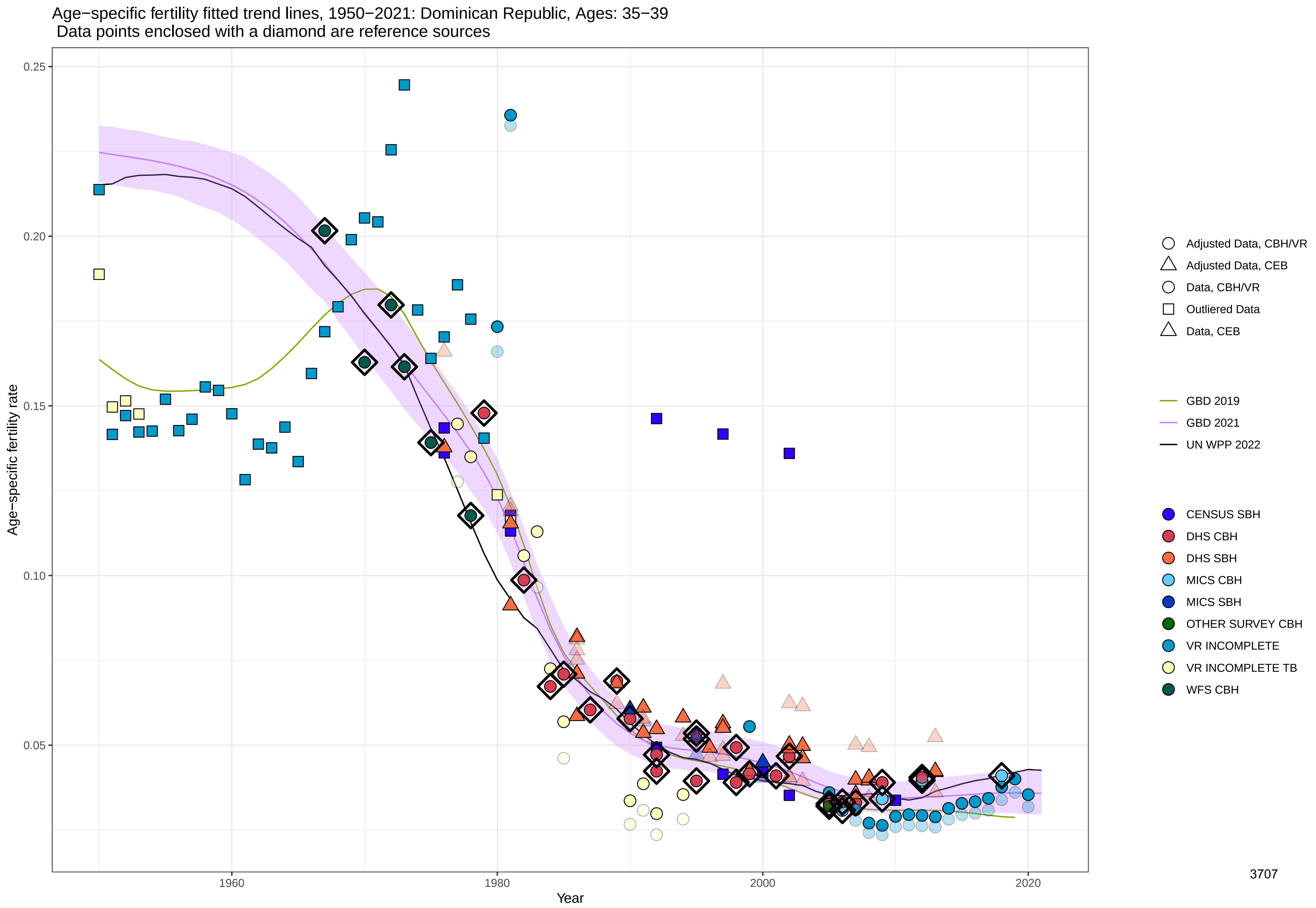

Age−specific fertility fitted trend lines, 1950−2021: Dominican Republic, Ages: 35−39
Data points enclosed with a diamond are reference sources
Age−specific fertility rate
0.25
0.20
0.15
0.10
0.05
1960
1980
2000
2020
Year
Adjusted Data, CBH/VR
Adjusted Data, CEB
Data, CBH/VR
Outliered Data
Data, CEB
GBD 2019
GBD 2021
UN WPP 2022
CENSUS SBH
DHS CBH
DHS SBH
MICS CBH
MICS SBH
OTHER SURVEY CBH
VR INCOMPLETE
VR INCOMPLETE TB
WFS CBH

Age−specific fertility fitted trend lines, 1950−2021: Dominican Republic, Ages: 40−44
Data points enclosed with a diamond are reference sources

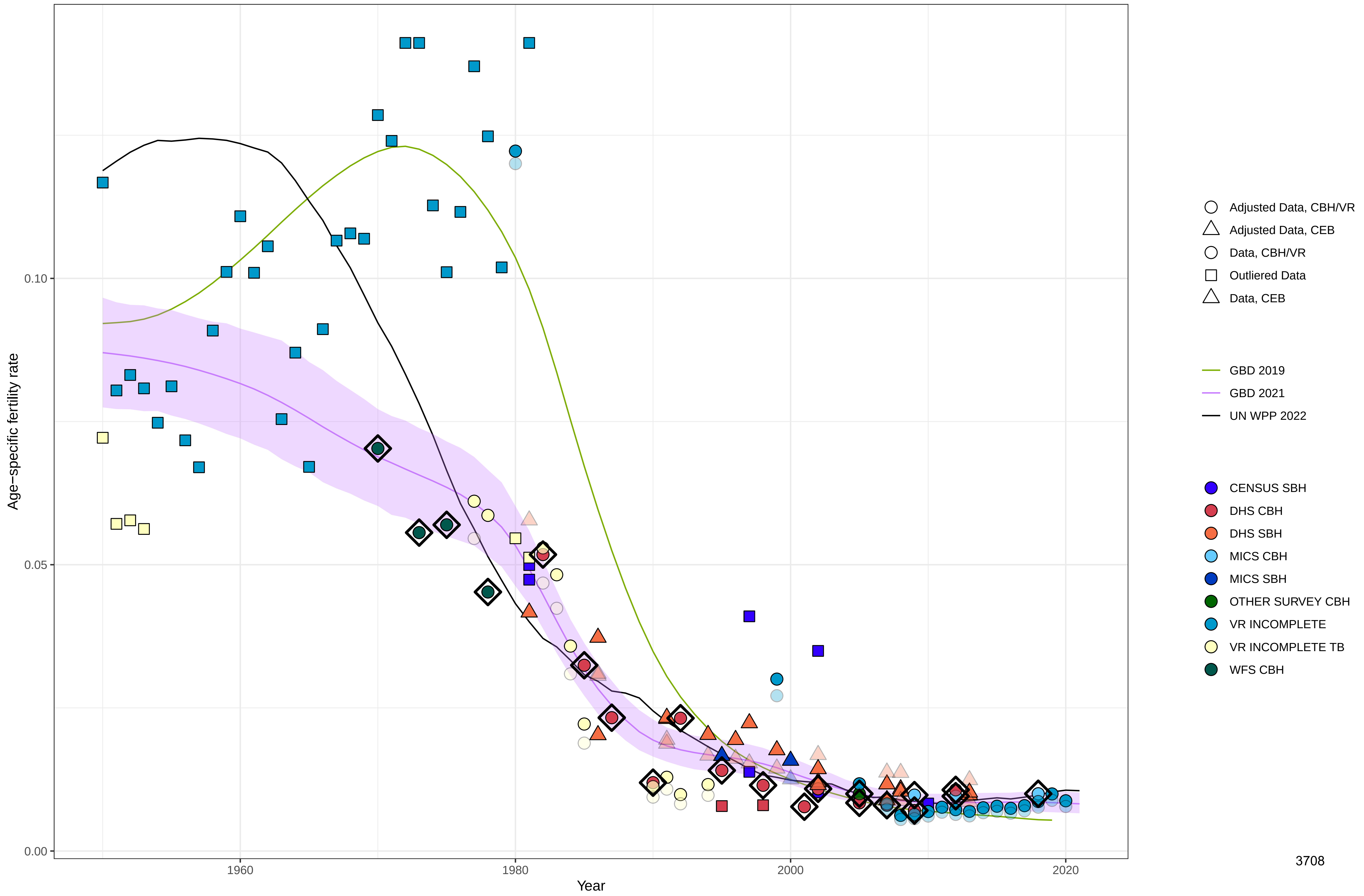

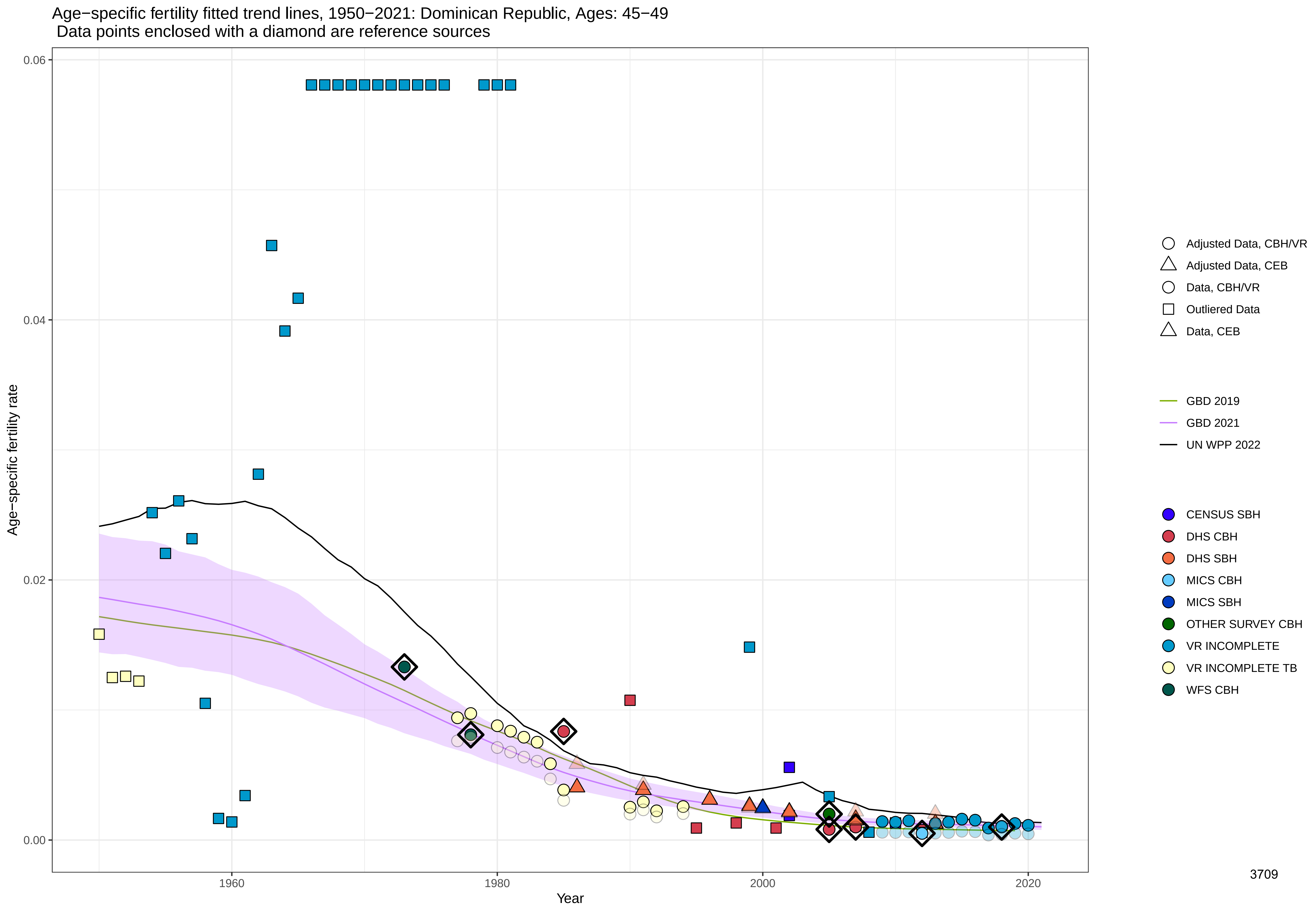

Age−specific fertility fitted trend lines, 1950−2021: Dominican Republic, Ages: 45−49
Data points enclosed with a diamond are reference sources
Age−specific fertility rate
0.06
0.04
0.02
0.00
1960
1980
2000
2020
Year
Adjusted Data, CBH/VR
Adjusted Data, CEB
Data, CBH/VR
Outliered Data
Data, CEB
GBD 2019
GBD 2021
UN WPP 2022
CENSUS SBH
DHS CBH
DHS SBH
MICS CBH
MICS SBH
OTHER SURVEY CBH
VR INCOMPLETE
VR INCOMPLETE TB
WFS CBH

Age−specific fertility fitted trend lines, 1950−2021: Dominican Republic, Ages: 50−54
Data points enclosed with a diamond are reference sources

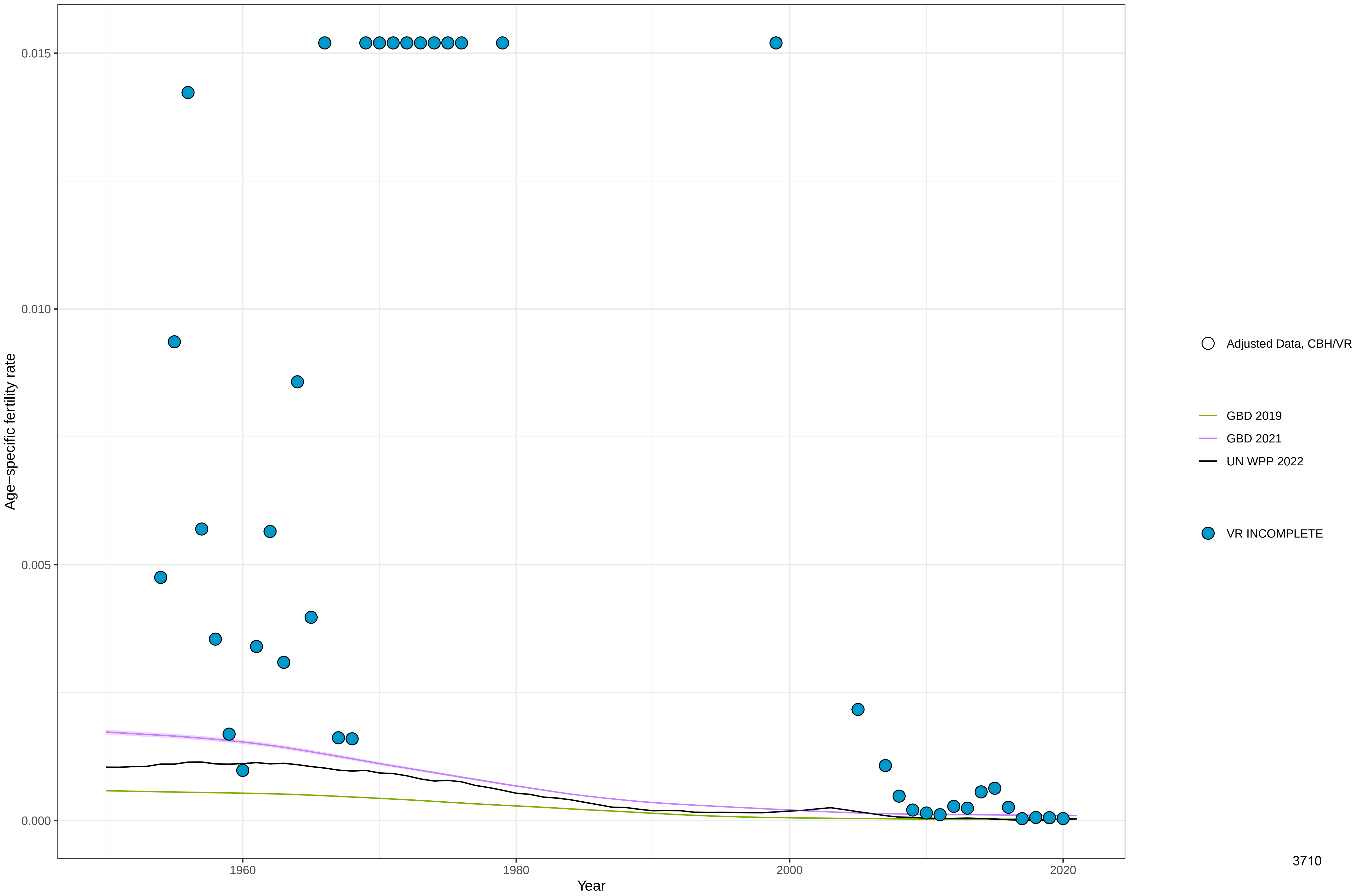

Total fertility rate fitted trend lines, 1950−2021: Dominican Republic TFR
Data points enclosed with a diamond are reference sources

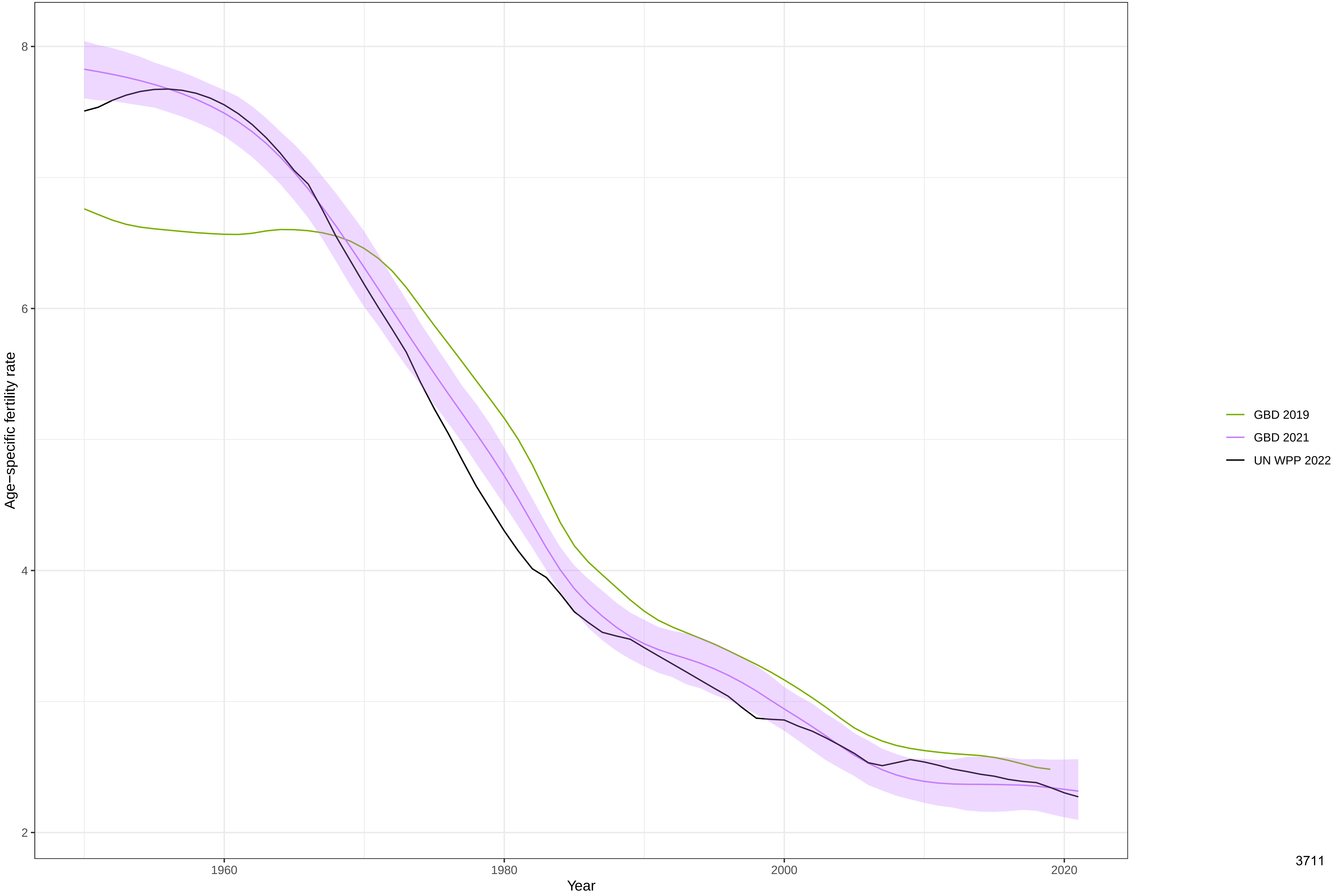

Age−specific fertility fitted trend lines, 1950−2021: Grenada, Ages: 10−14
Data points enclosed with a diamond are reference sources

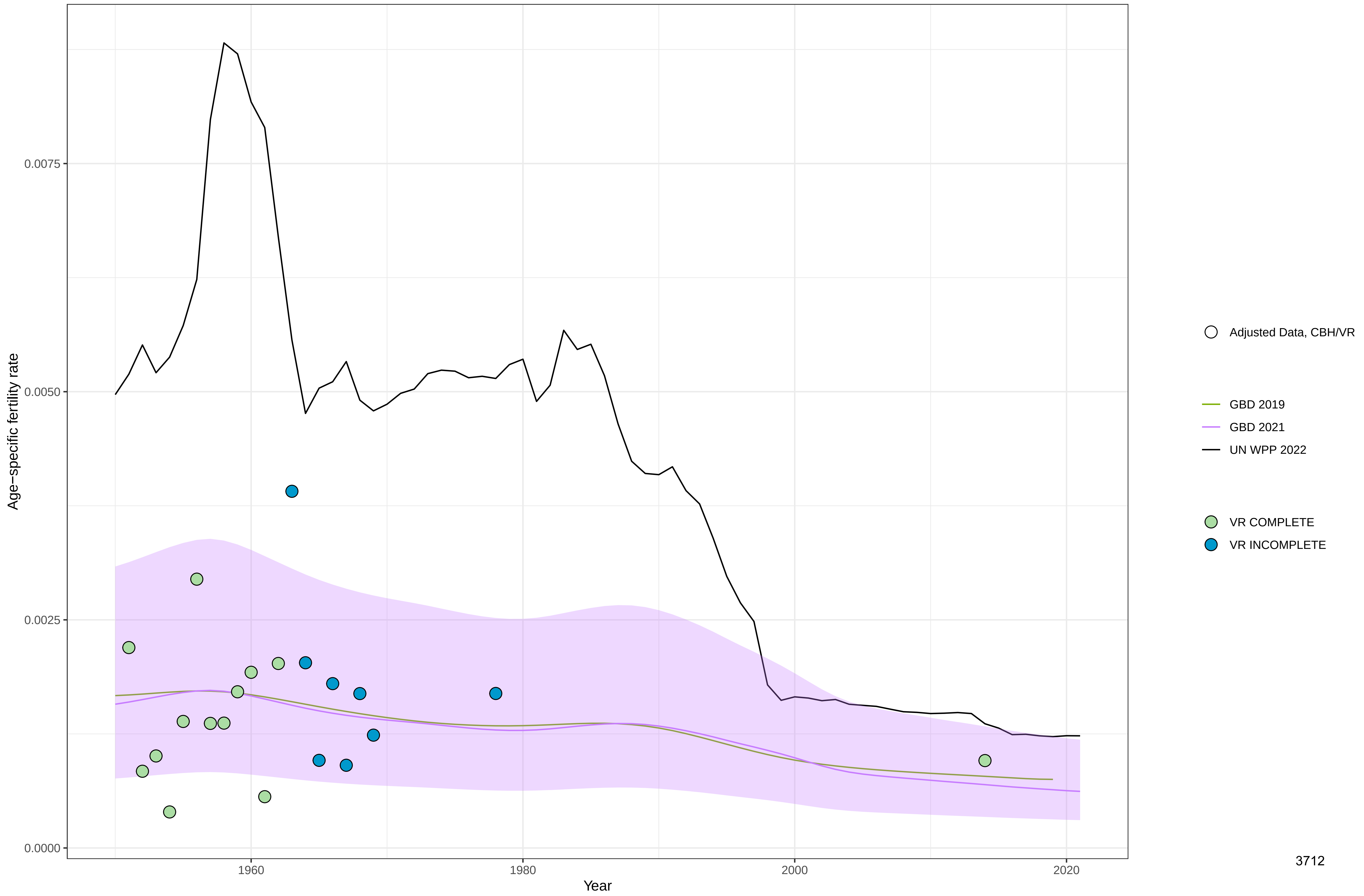

Age−specific fertility fitted trend lines, 1950−2021: Grenada, Ages: 15−19
Data points enclosed with a diamond are reference sources

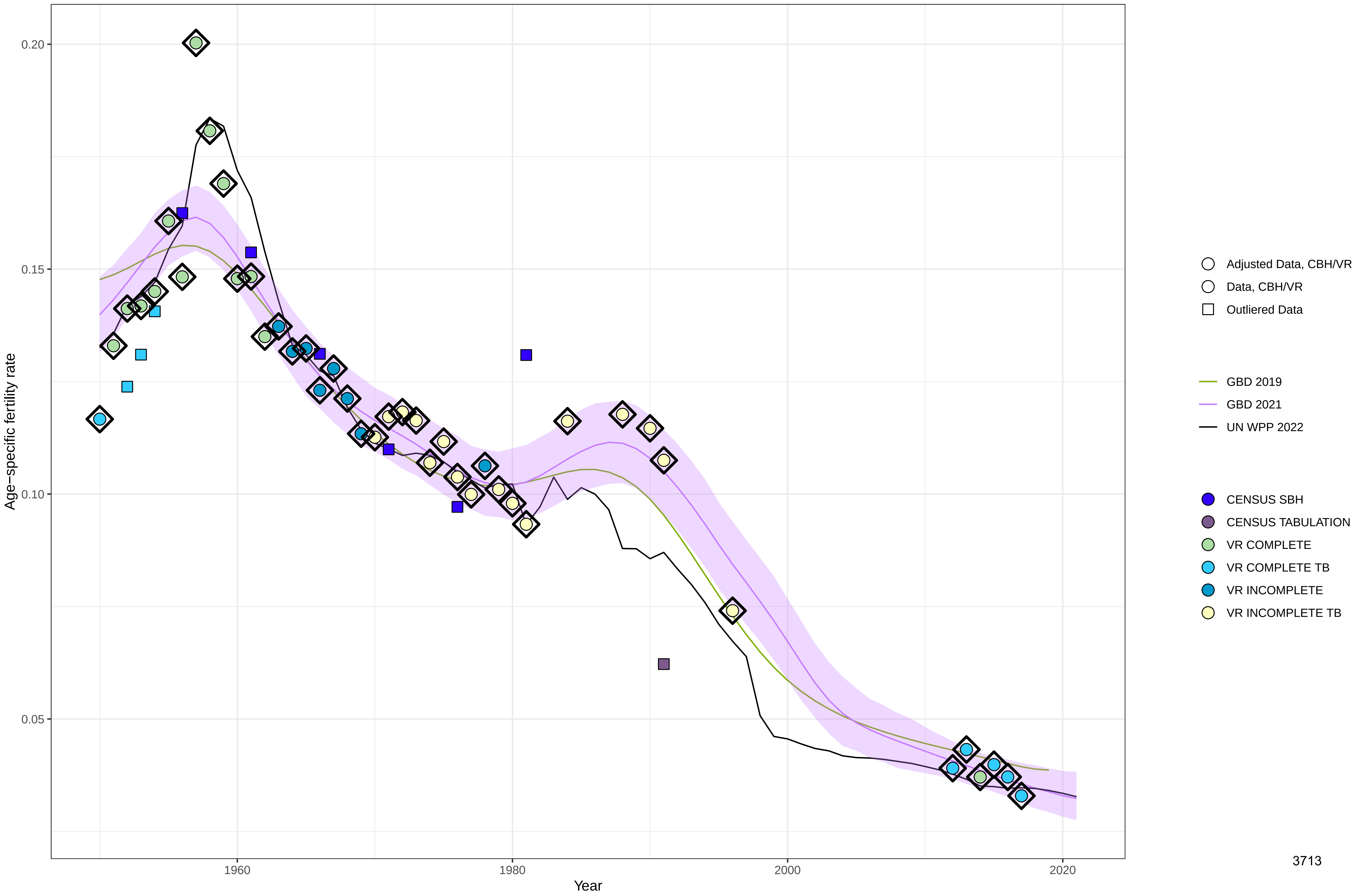

Age−specific fertility fitted trend lines, 1950−2021: Grenada, Ages: 20−24
Data points enclosed with a diamond are reference sources

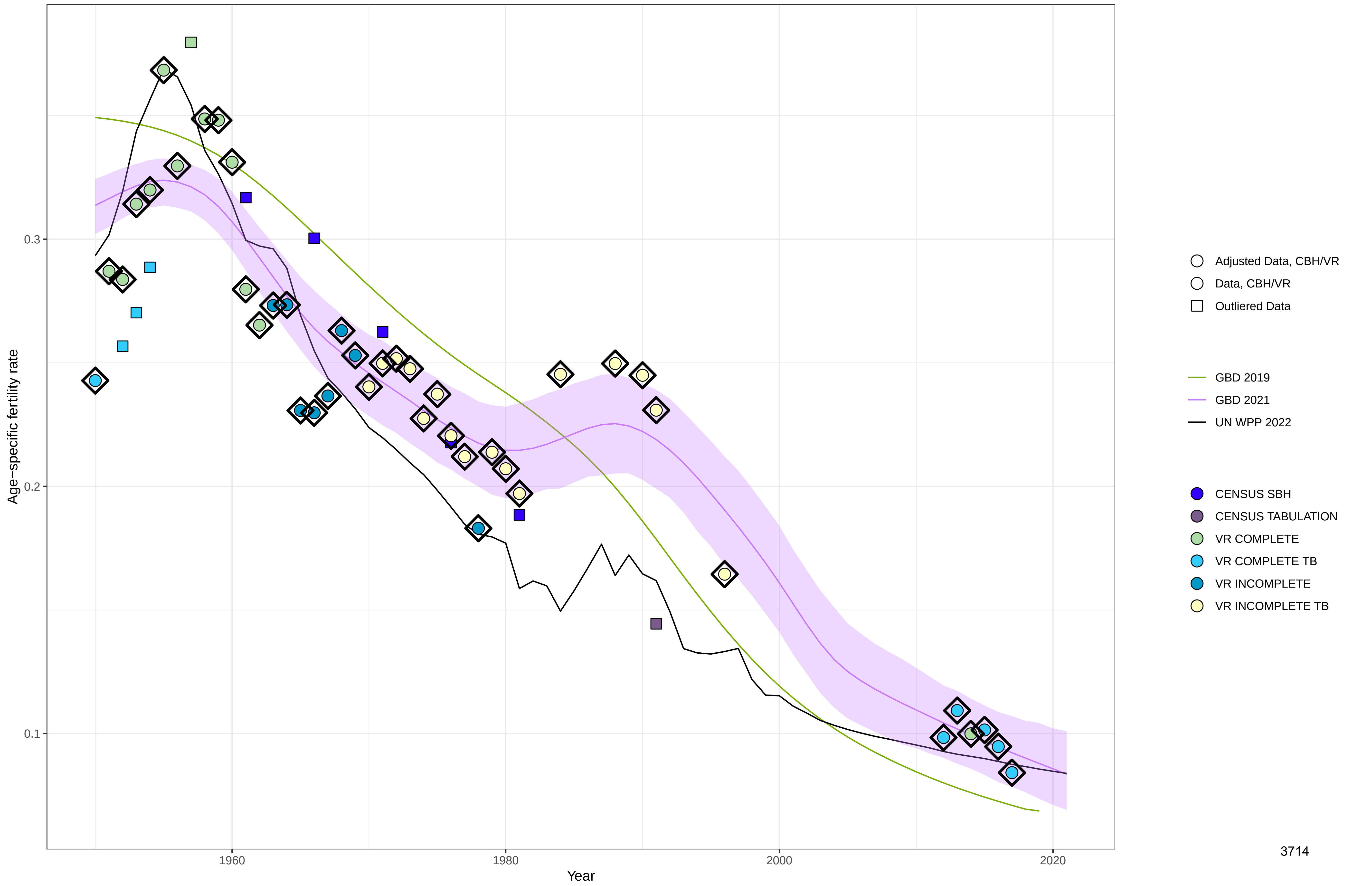

Age−specific fertility fitted trend lines, 1950−2021: Grenada, Ages: 25−29
Data points enclosed with a diamond are reference sources

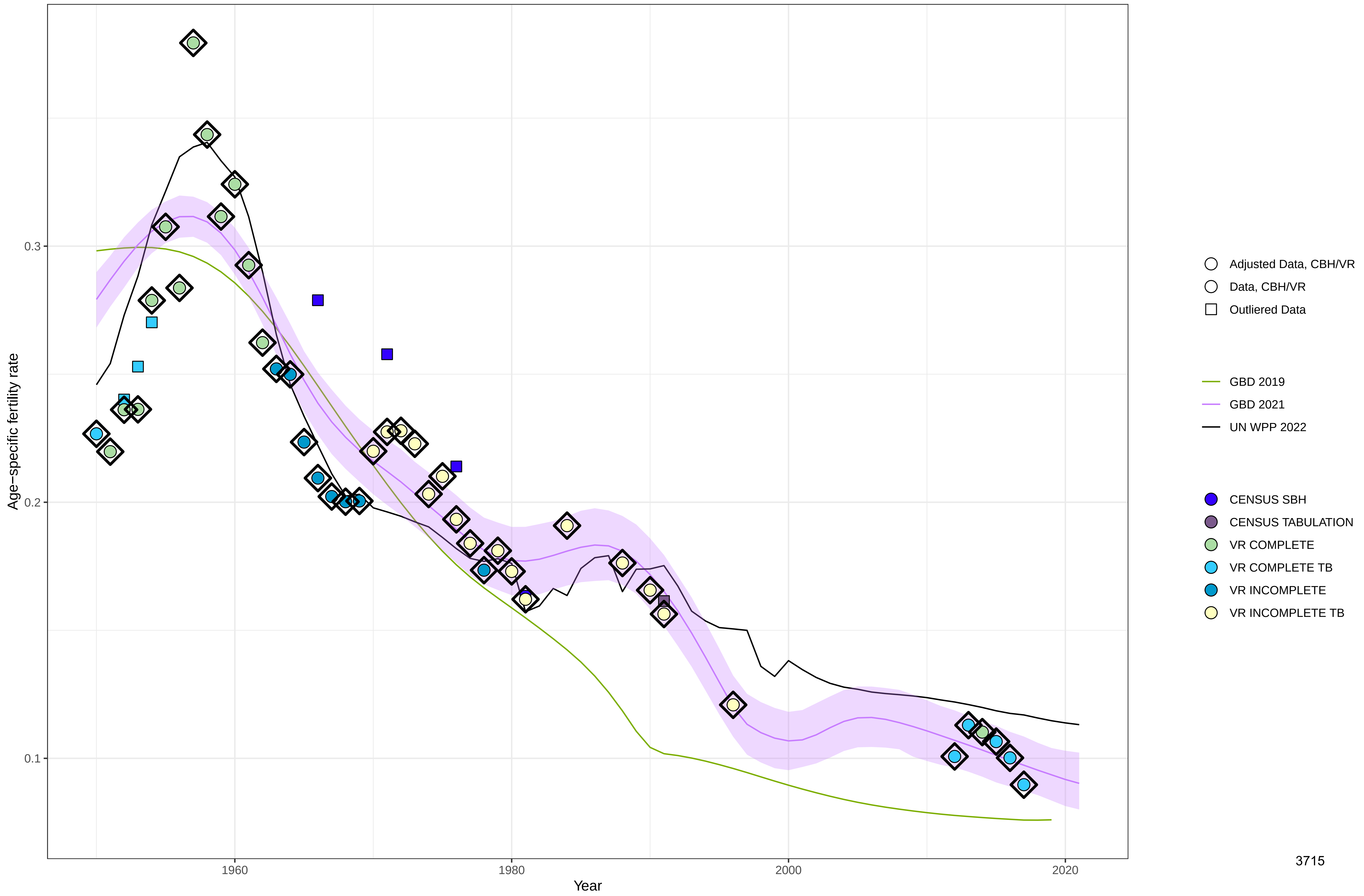

Age−specific fertility fitted trend lines, 1950−2021: Grenada, Ages: 30−34
Data points enclosed with a diamond are reference sources

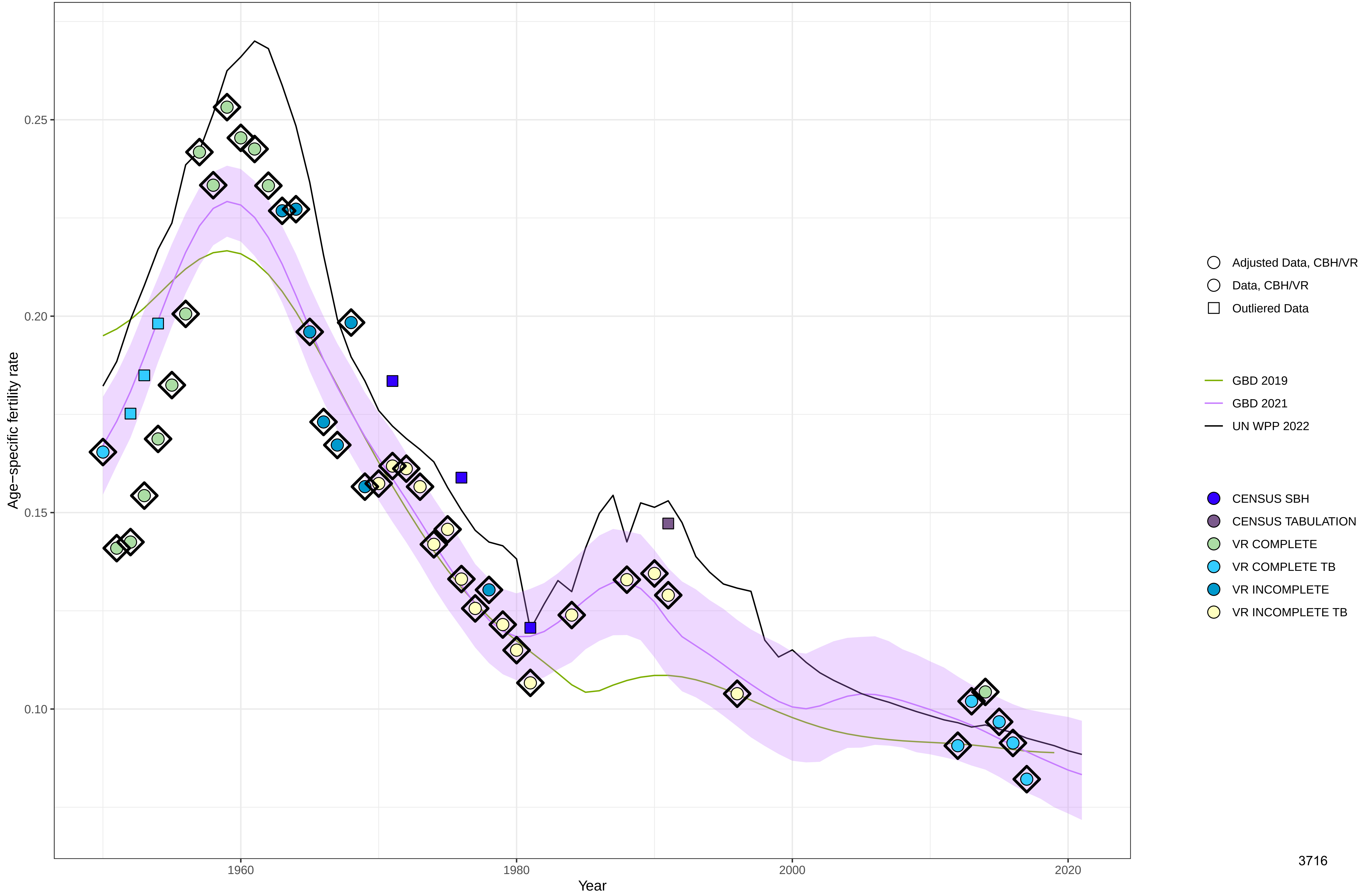

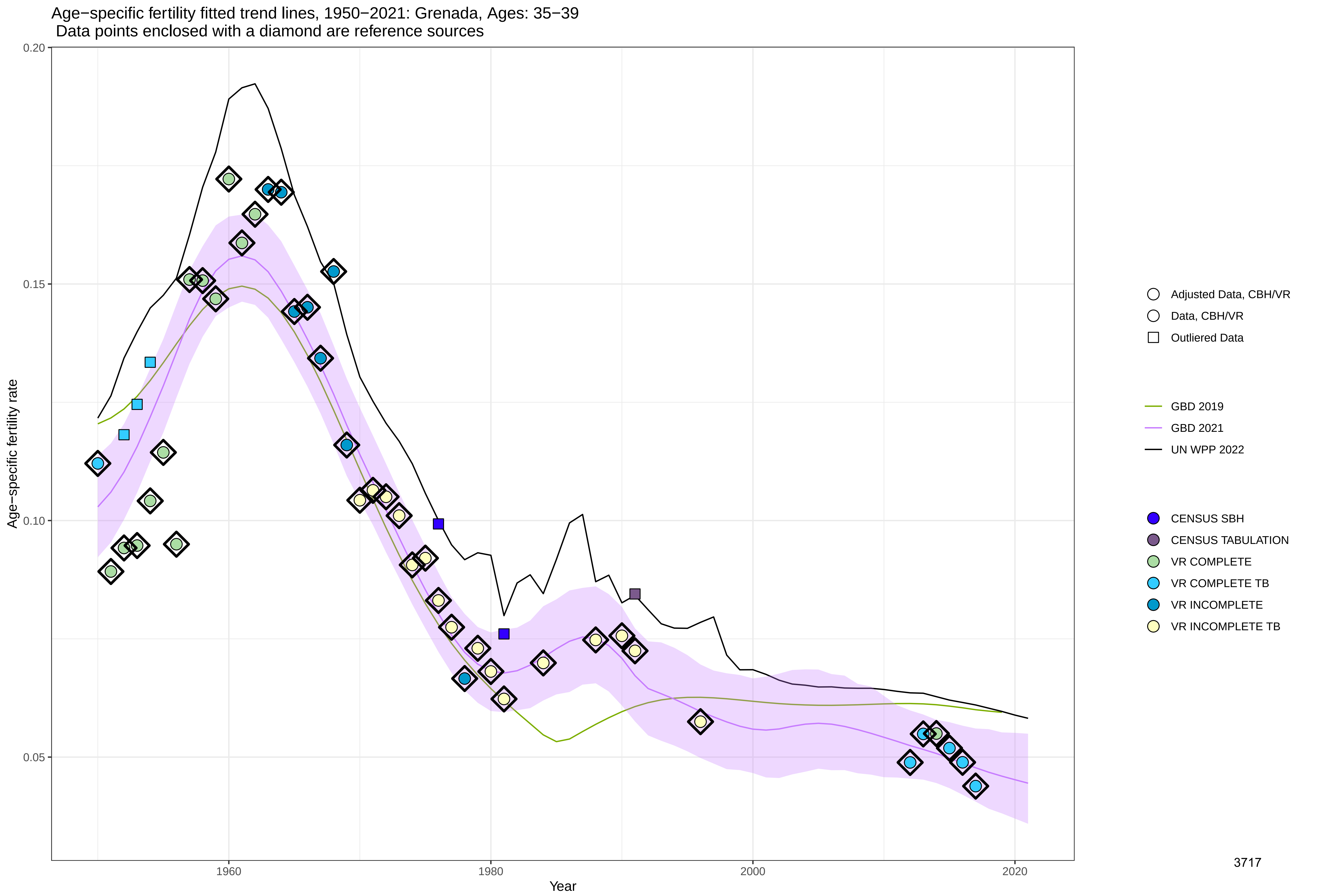

Age−specific fertility fitted trend lines, 1950−2021: Grenada, Ages: 35−39
Data points enclosed with a diamond are reference sources
Age−specific fertility rate
0.20
0.15
0.10
0.05
1960
1980
2000
2020
Year
Adjusted Data, CBH/VR
Data, CBH/VR
Outliered Data
GBD 2019
GBD 2021
UN WPP 2022
CENSUS SBH
CENSUS TABULATION
VR COMPLETE
VR COMPLETE TB
VR INCOMPLETE
VR INCOMPLETE TB

3717

Age−specific fertility fitted trend lines, 1950−2021: Grenada, Ages: 40−44
Data points enclosed with a diamond are reference sources

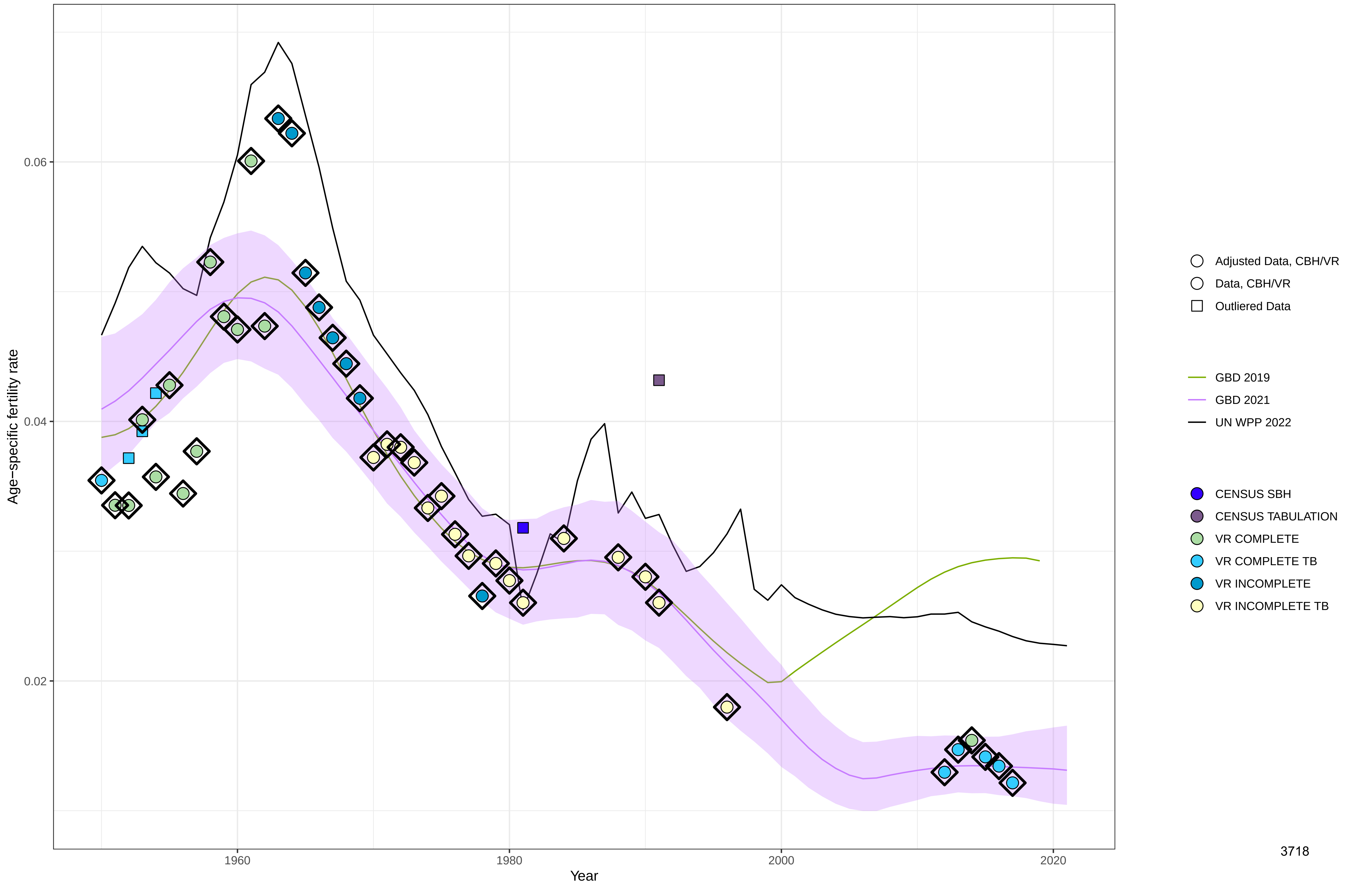

Age−specific fertility fitted trend lines, 1950−2021: Grenada, Ages: 45−49
Data points enclosed with a diamond are reference sources

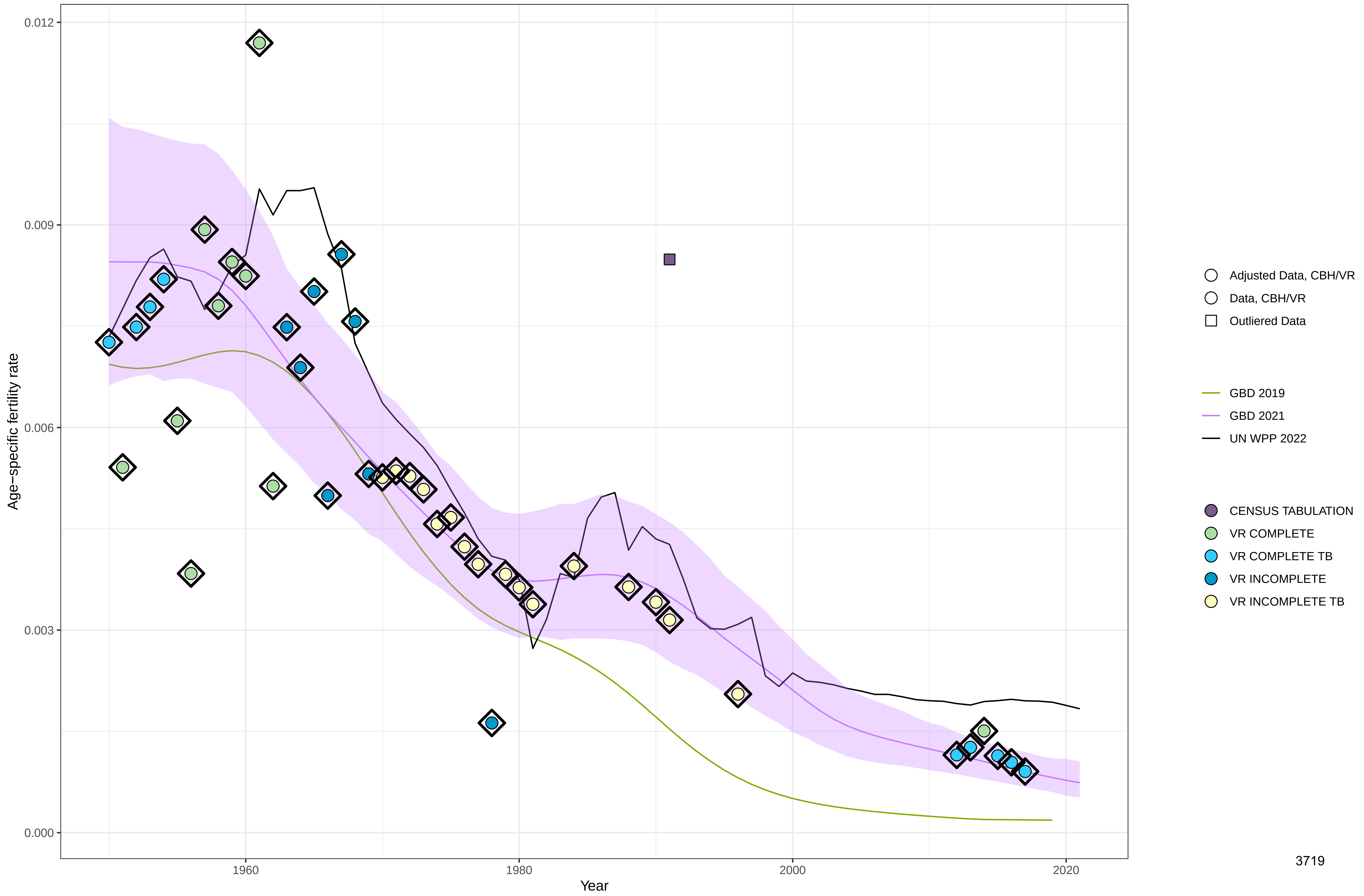

Age−specific fertility fitted trend lines, 1950−2021: Grenada, Ages: 50−54
Data points enclosed with a diamond are reference sources

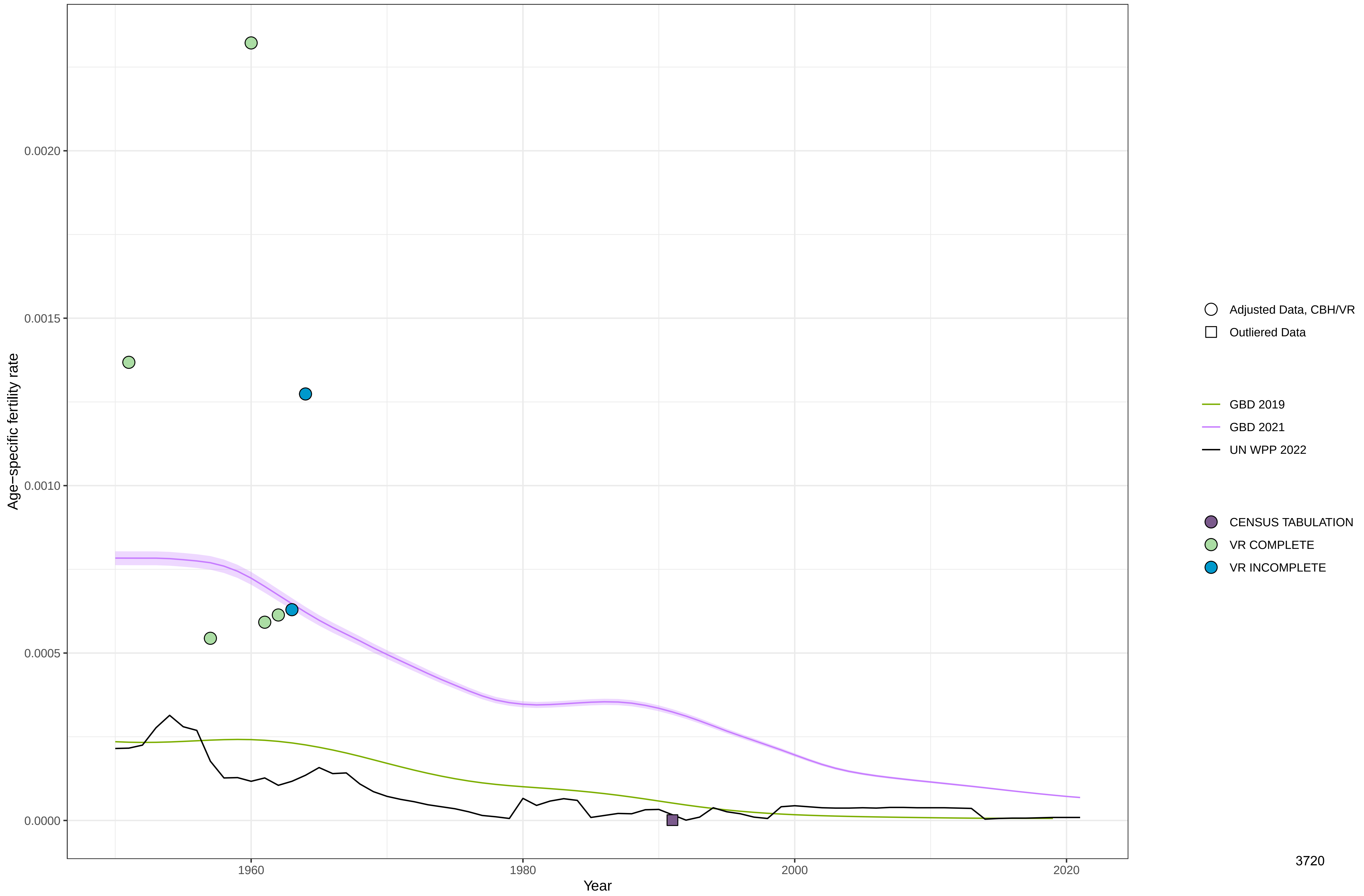

Total fertility rate fitted trend lines, 1950−2021: Grenada TFR
Data points enclosed with a diamond are reference sources

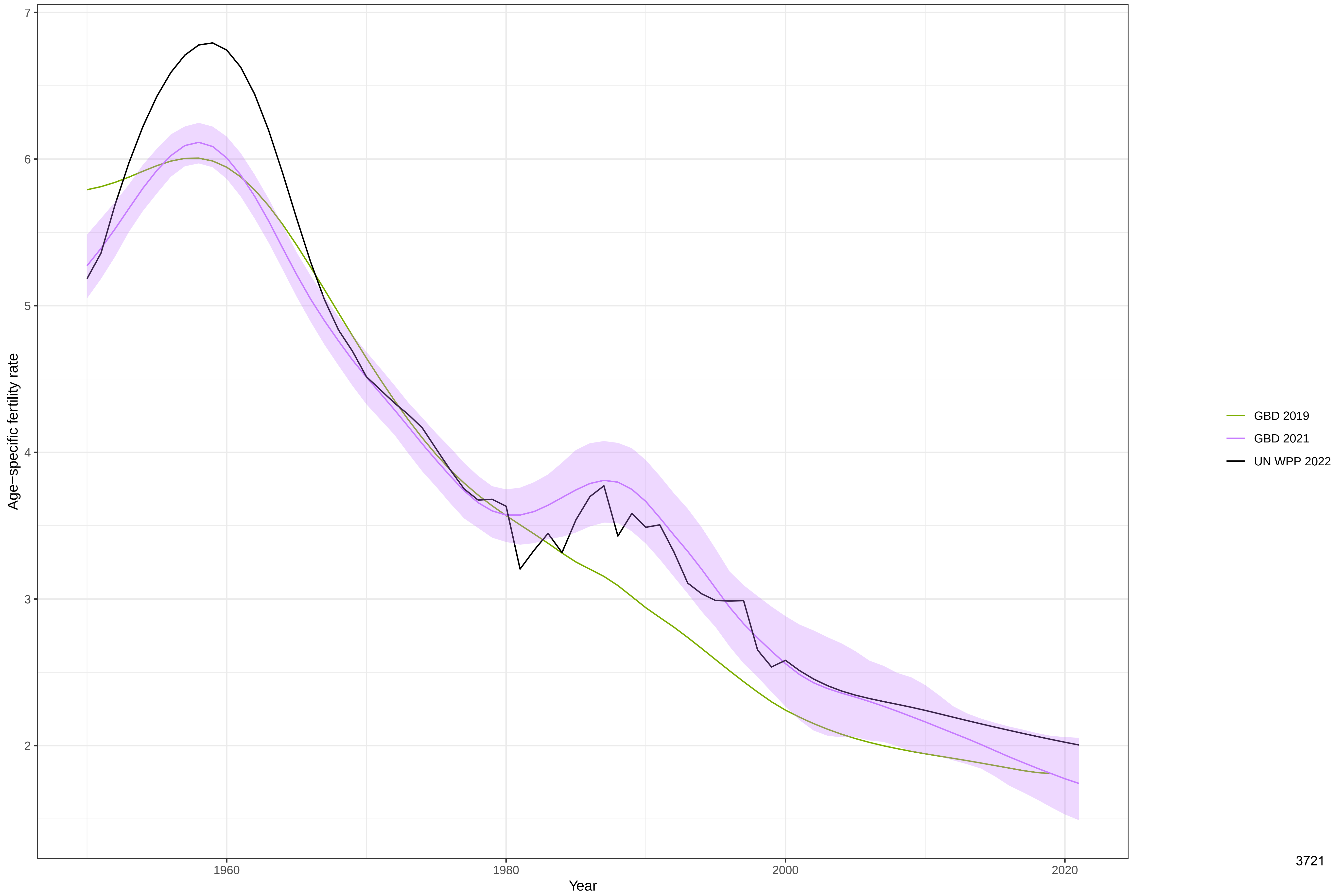

Age−specific fertility fitted trend lines, 1950−2021: Guyana, Ages: 10−14
Data points enclosed with a diamond are reference sources

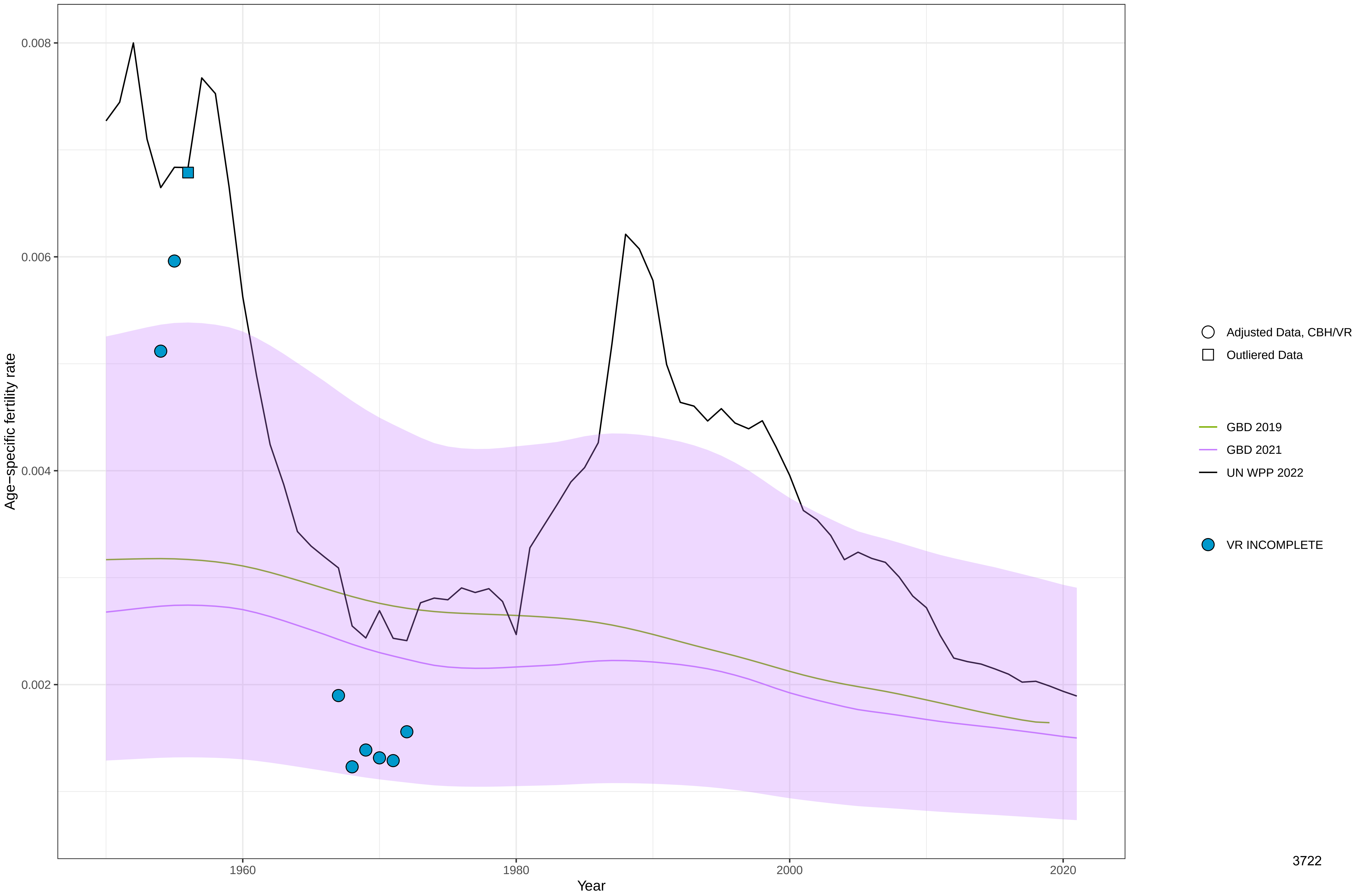

Age−specific fertility fitted trend lines, 1950−2021: Guyana, Ages: 15−19
Data points enclosed with a diamond are reference sources

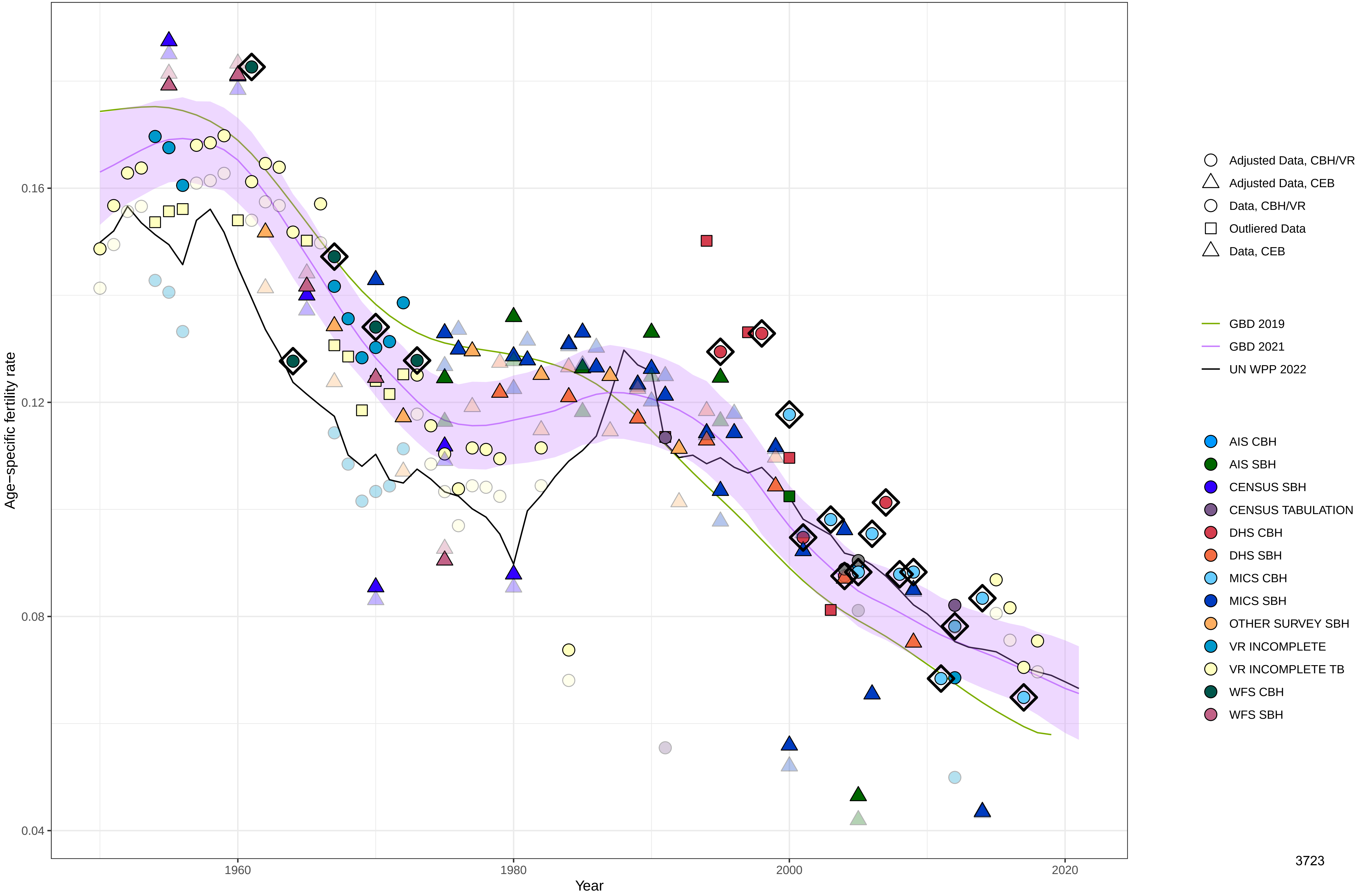

Age−specific fertility fitted trend lines, 1950−2021: Guyana, Ages: 20−24
Data points enclosed with a diamond are reference sources

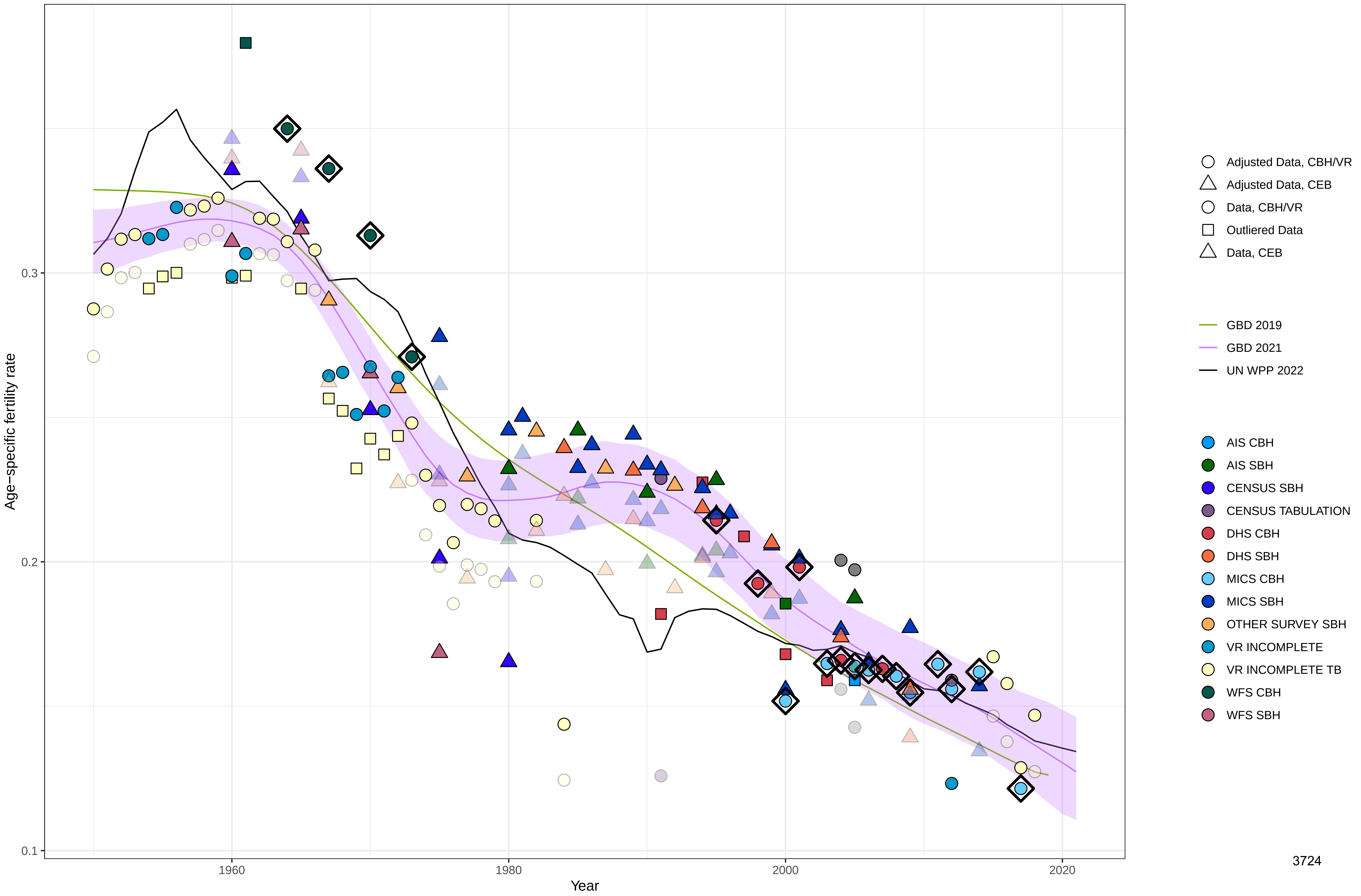

Age−specific fertility fitted trend lines, 1950−2021: Guyana, Ages: 25−29
Data points enclosed with a diamond are reference sources

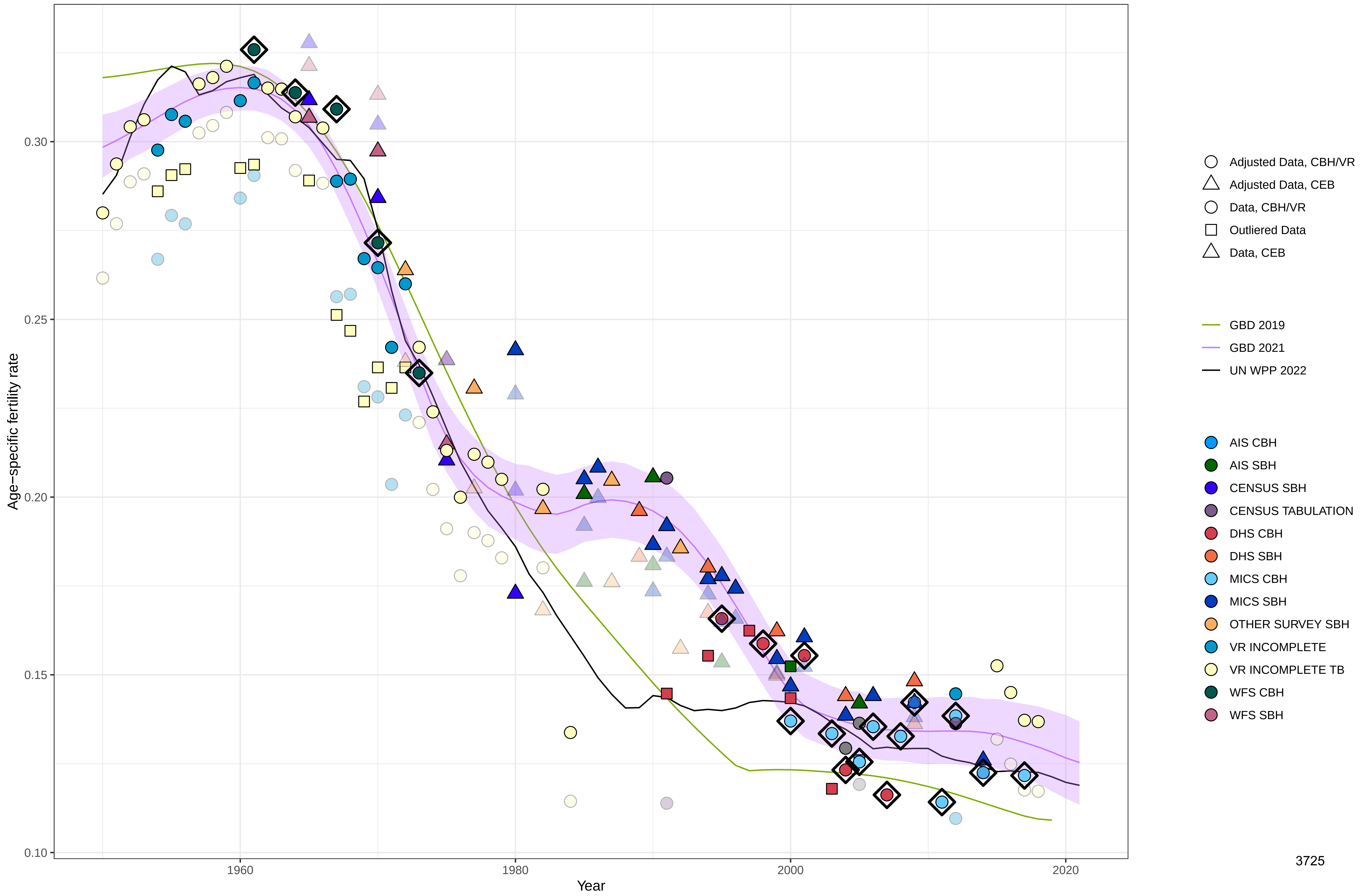

Age−specific fertility fitted trend lines, 1950−2021: Guyana, Ages: 30−34
Data points enclosed with a diamond are reference sources

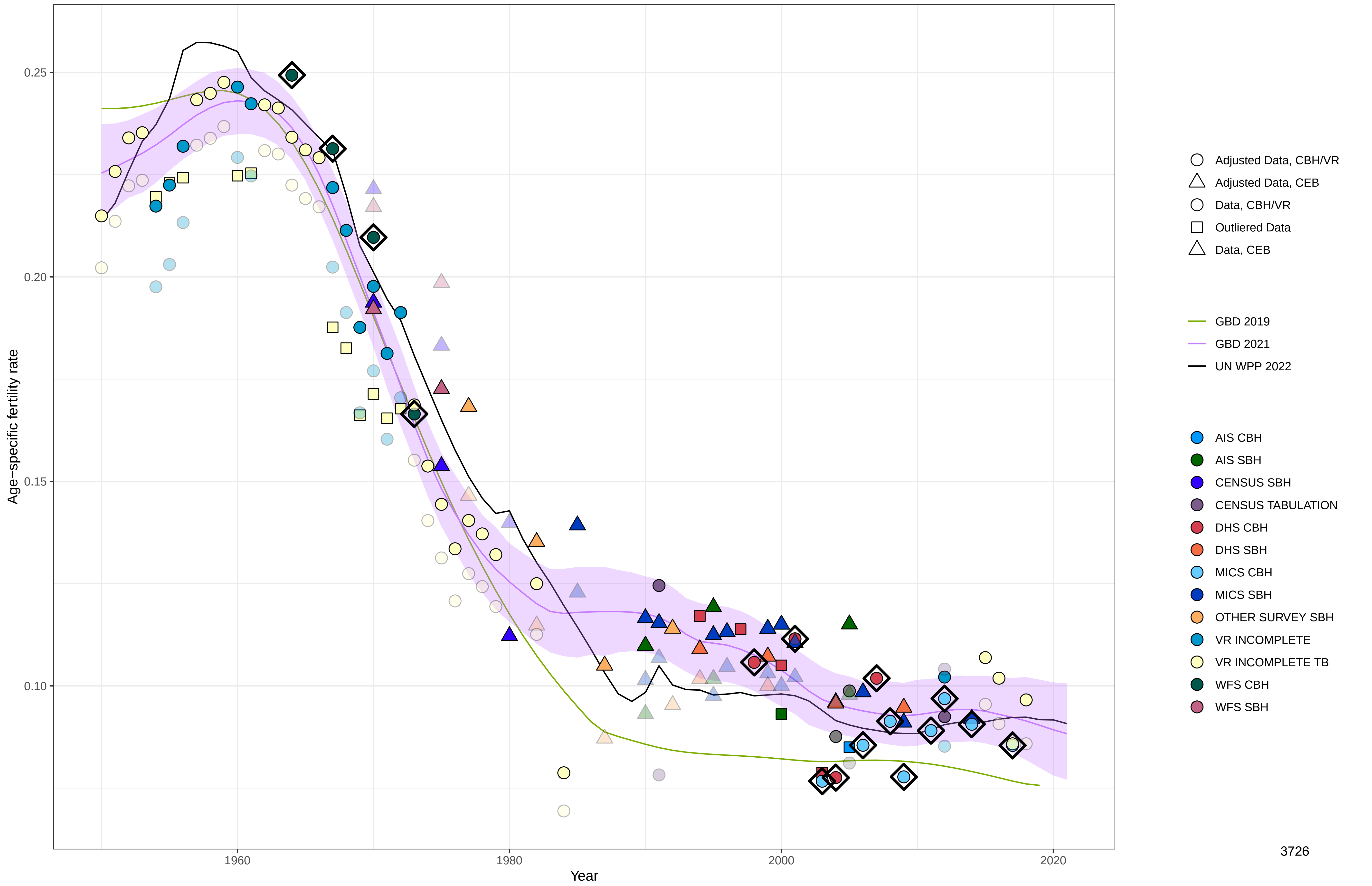

Age−specific fertility fitted trend lines, 1950−2021: Guyana, Ages: 35−39
Data points enclosed with a diamond are reference sources

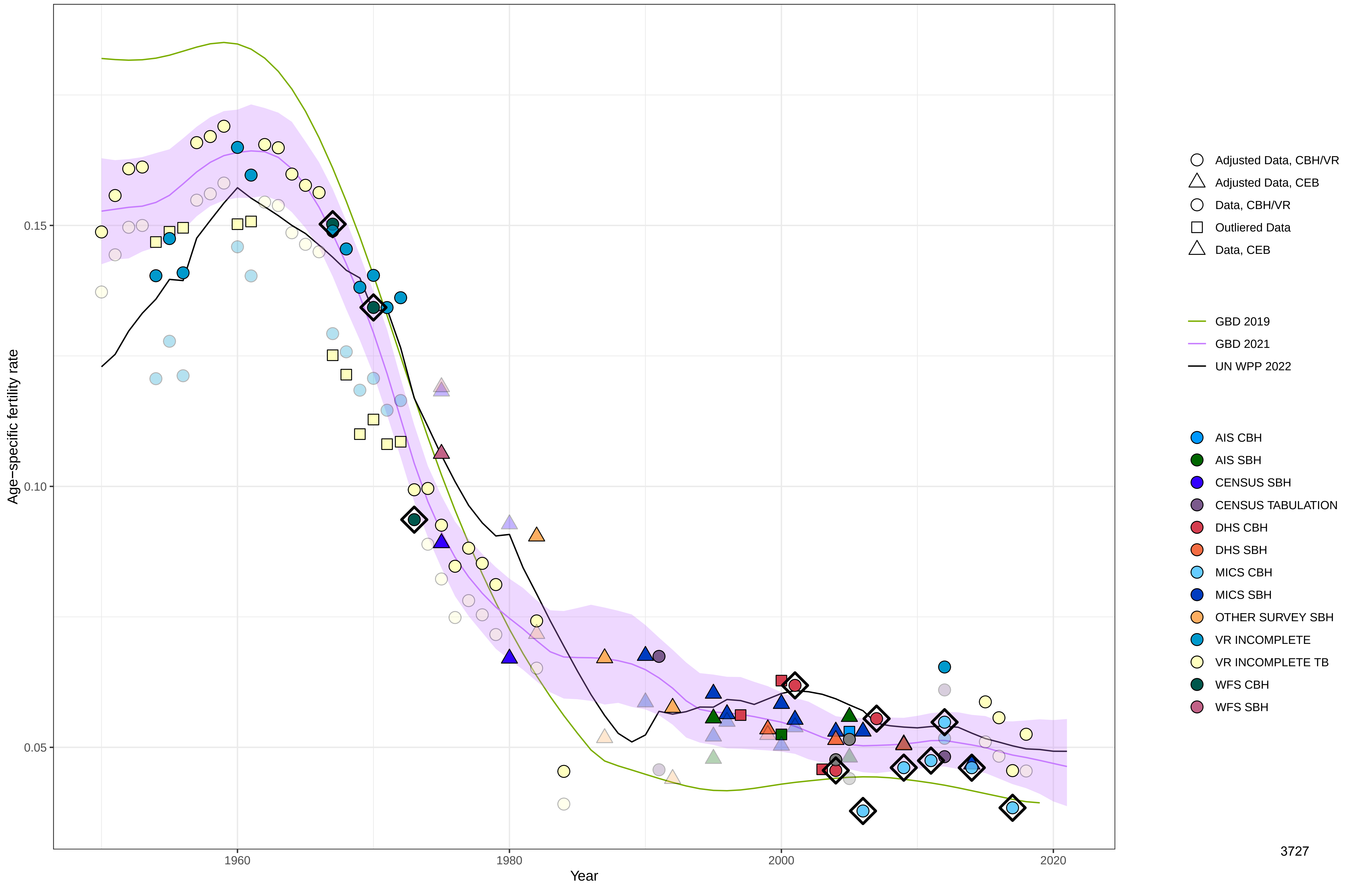

Age−specific fertility fitted trend lines, 1950−2021: Guyana, Ages: 40−44
Data points enclosed with a diamond are reference sources

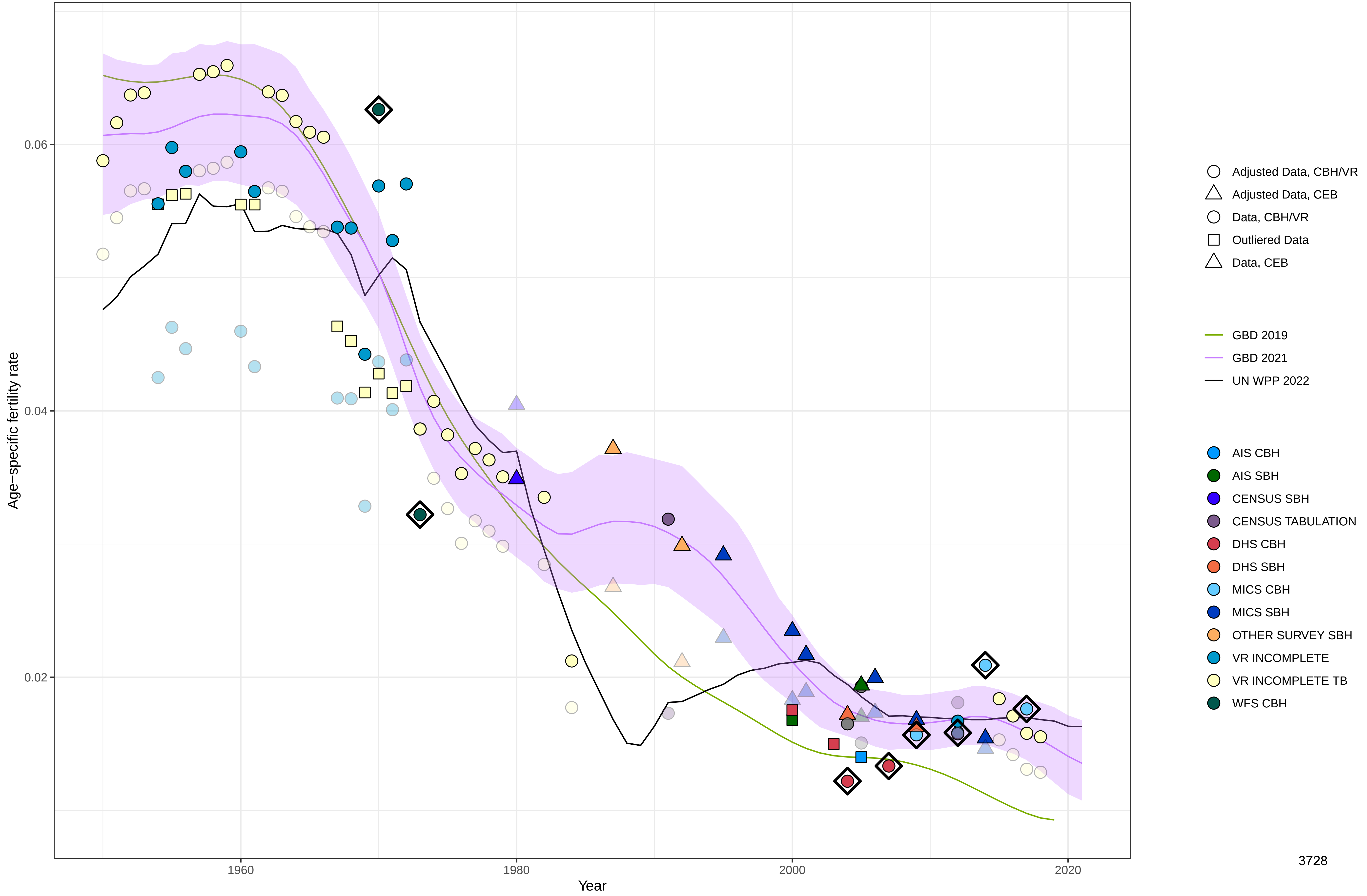

Age−specific fertility fitted trend lines, 1950−2021: Guyana, Ages: 45−49
Data points enclosed with a diamond are reference sources

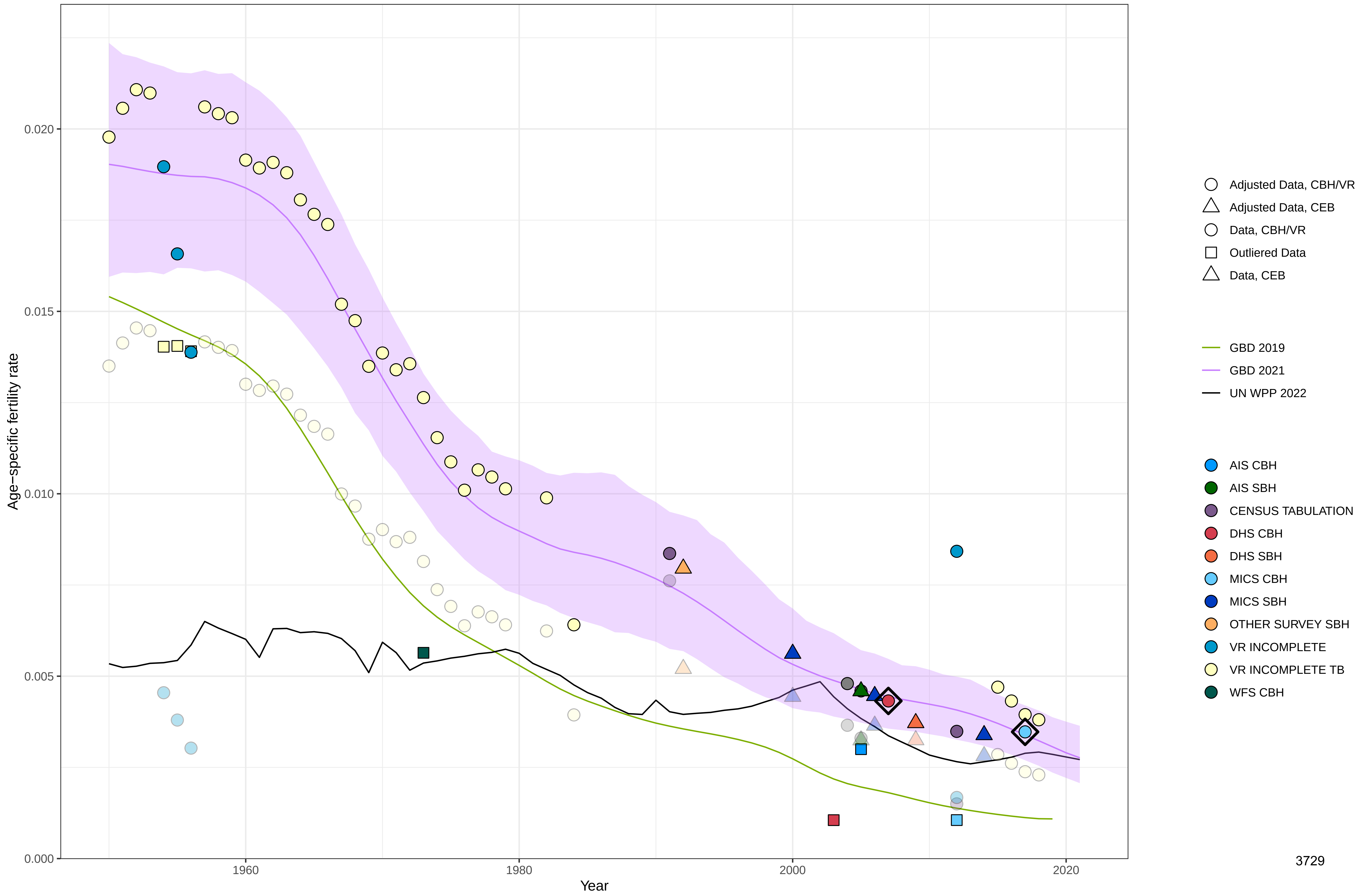

Age−specific fertility fitted trend lines, 1950−2021: Guyana, Ages: 50−54
Data points enclosed with a diamond are reference sources

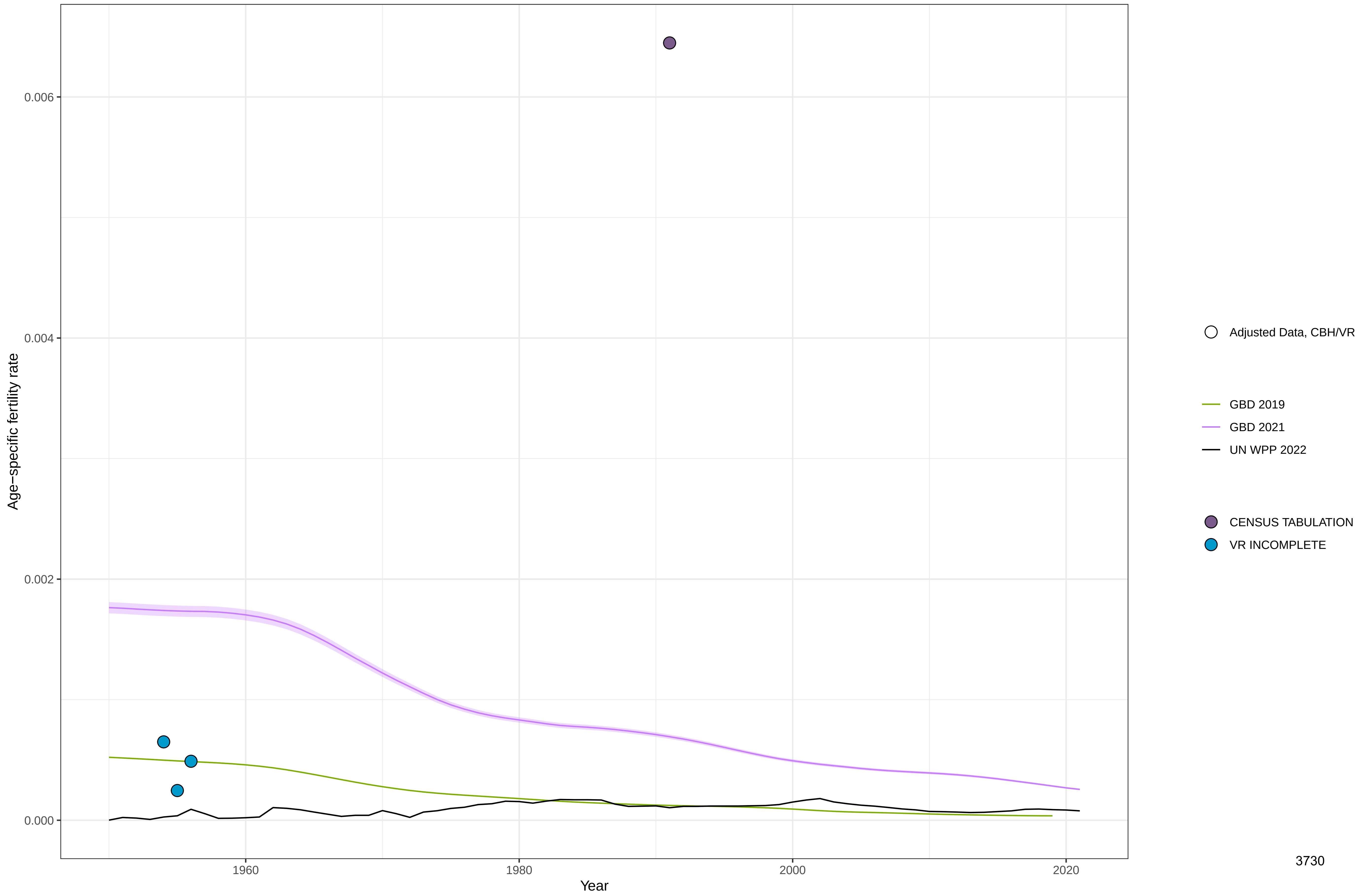

Total fertility rate fitted trend lines, 1950−2021: Guyana TFR
Data points enclosed with a diamond are reference sources

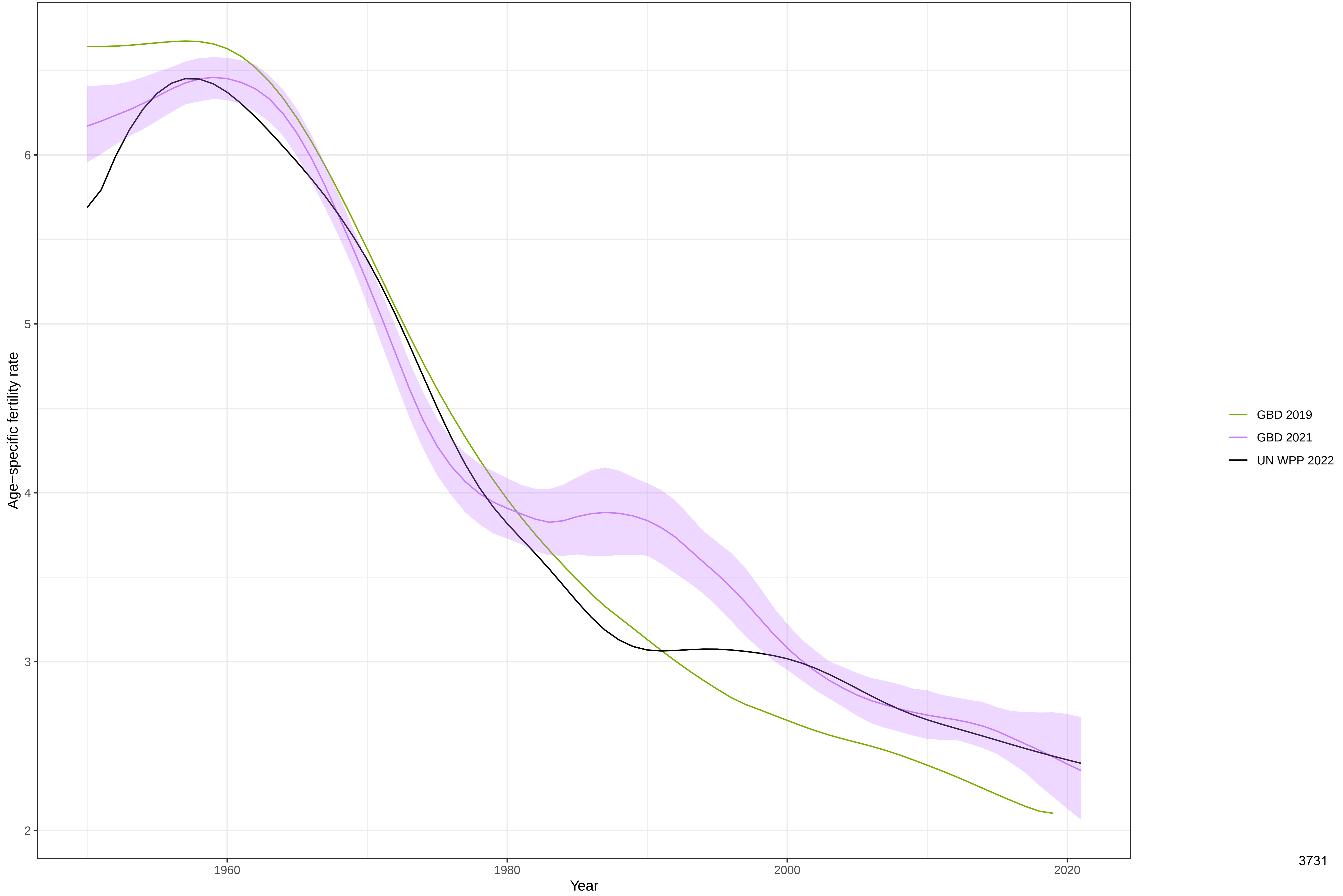

Age−specific fertility fitted trend lines, 1950−2021: Haiti, Ages: 10−14
Data points enclosed with a diamond are reference sources

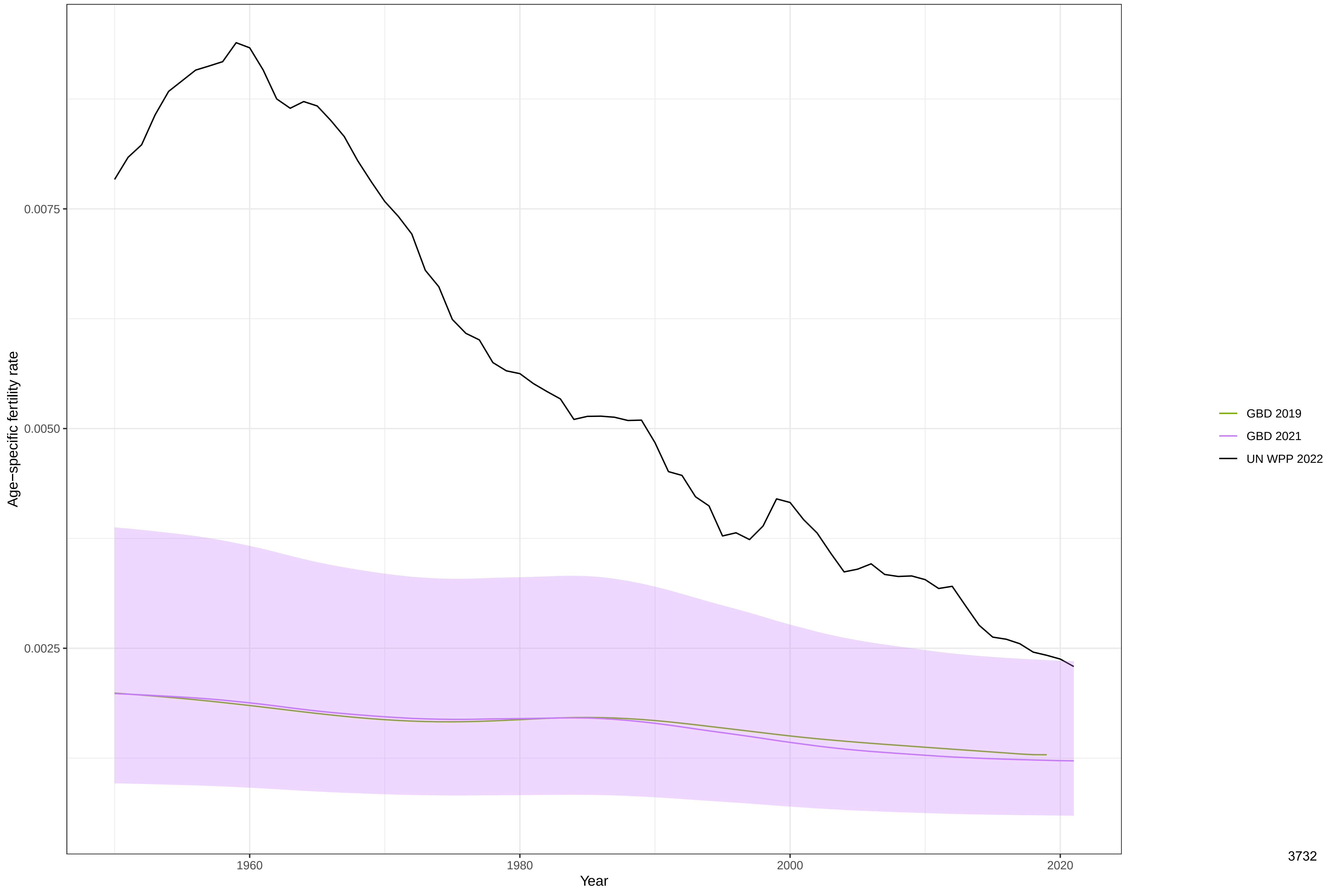

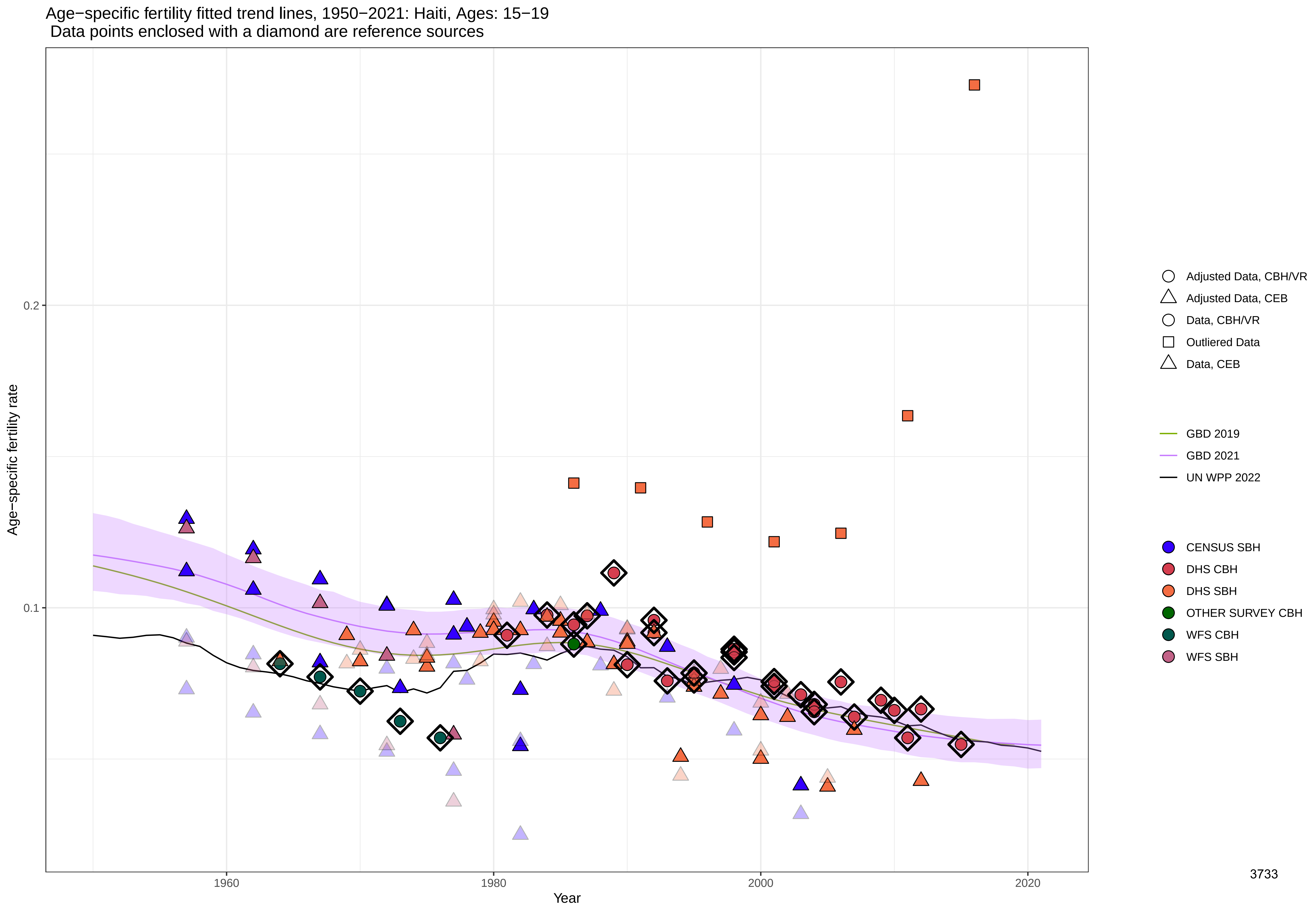

Age−specific fertility fitted trend lines, 1950−2021: Haiti, Ages: 15−19
Data points enclosed with a diamond are reference sources
Age−specific fertility rate
0.2
0.1
1960
1980
2000
2020
Year
Adjusted Data, CBH/VR
Adjusted Data, CEB
Data, CBH/VR
Outliered Data
Data, CEB
GBD 2019
GBD 2021
UN WPP 2022
CENSUS SBH
DHS CBH
DHS SBH
OTHER SURVEY CBH
WFS CBH
WFS SBH

Age−specific fertility fitted trend lines, 1950−2021: Haiti, Ages: 20−24
Data points enclosed with a diamond are reference sources

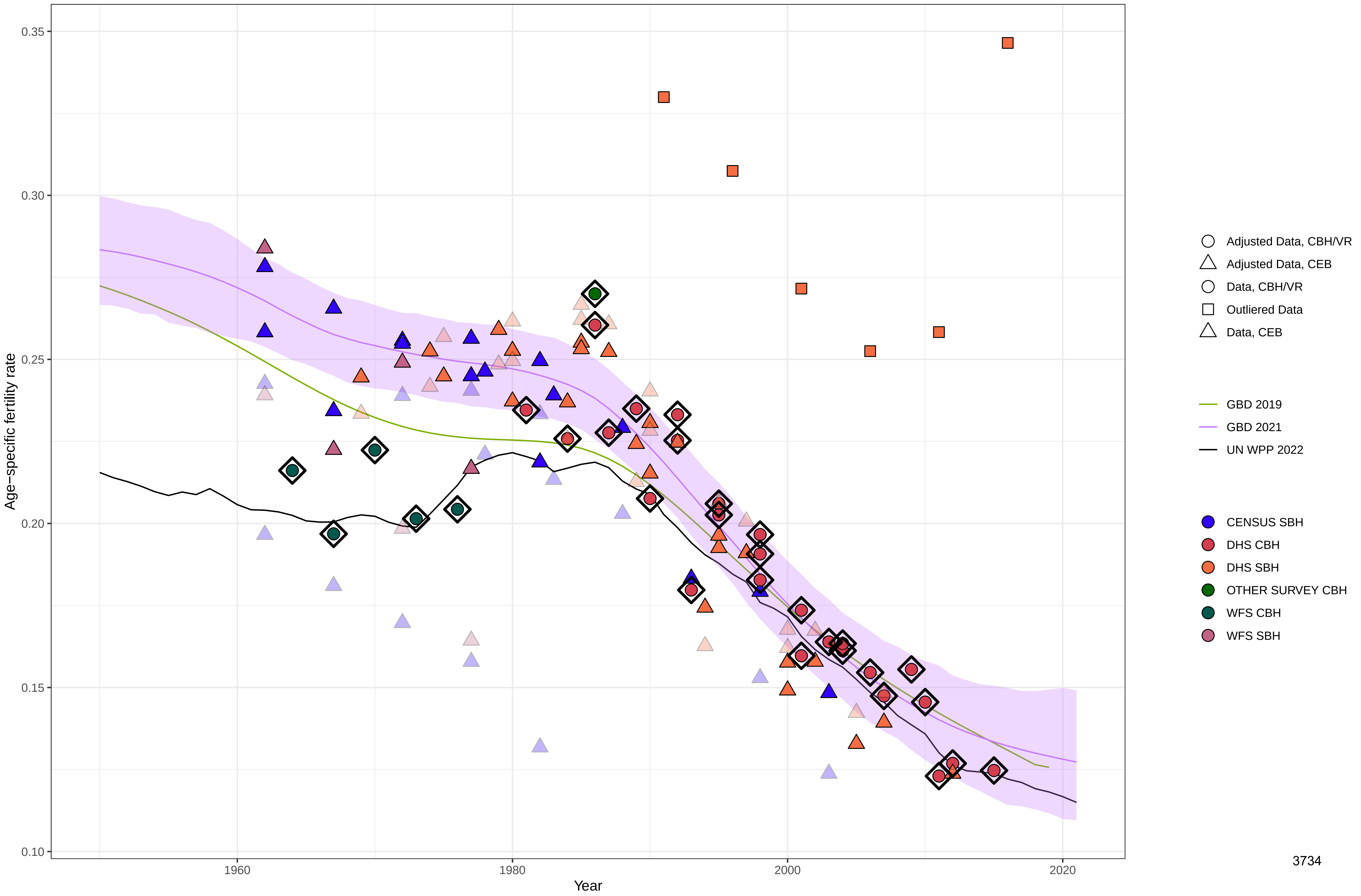

Age−specific fertility fitted trend lines, 1950−2021: Haiti, Ages: 25−29
Data points enclosed with a diamond are reference sources

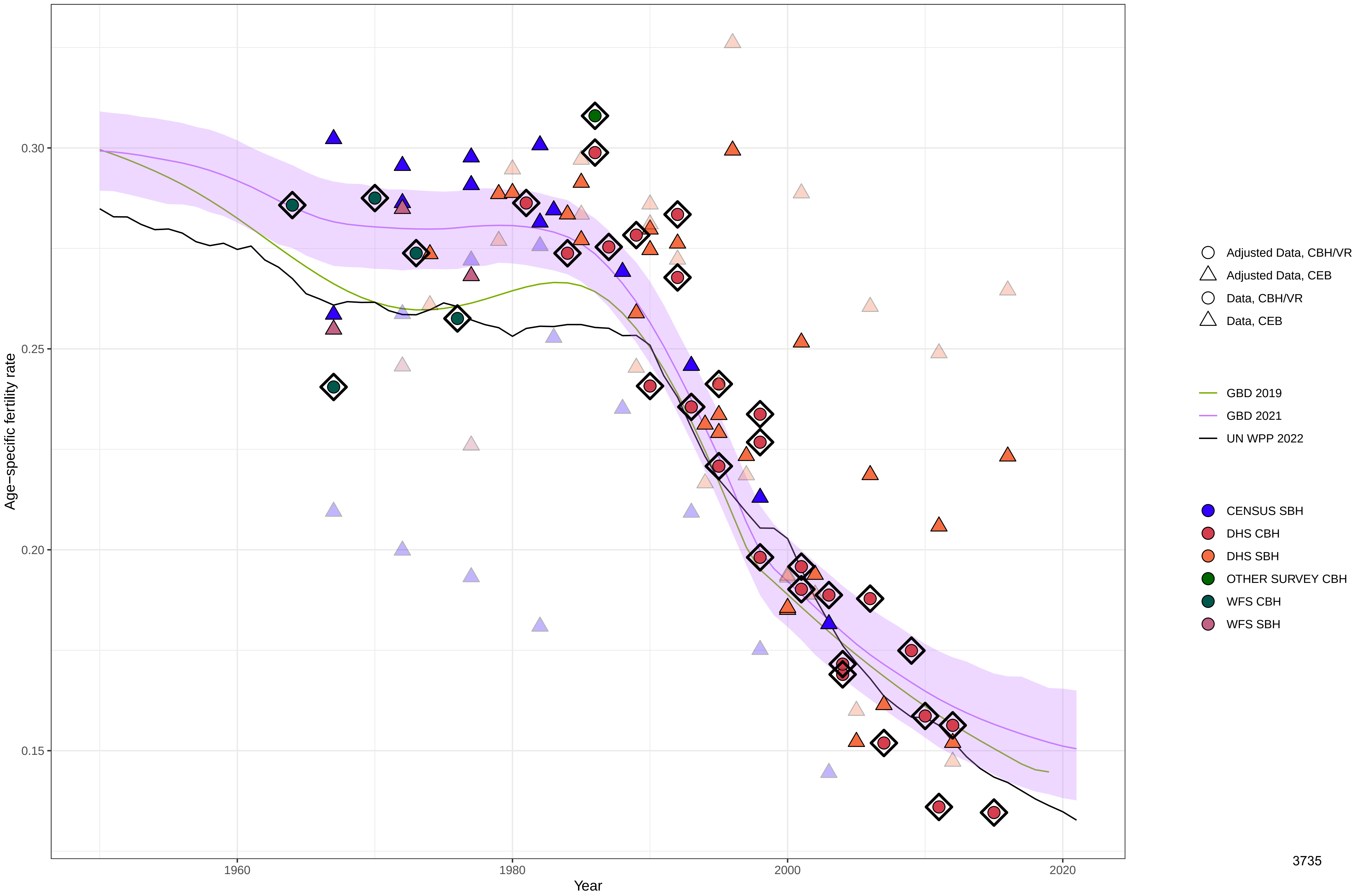

Age−specific fertility fitted trend lines, 1950−2021: Haiti, Ages: 30−34
Data points enclosed with a diamond are reference sources

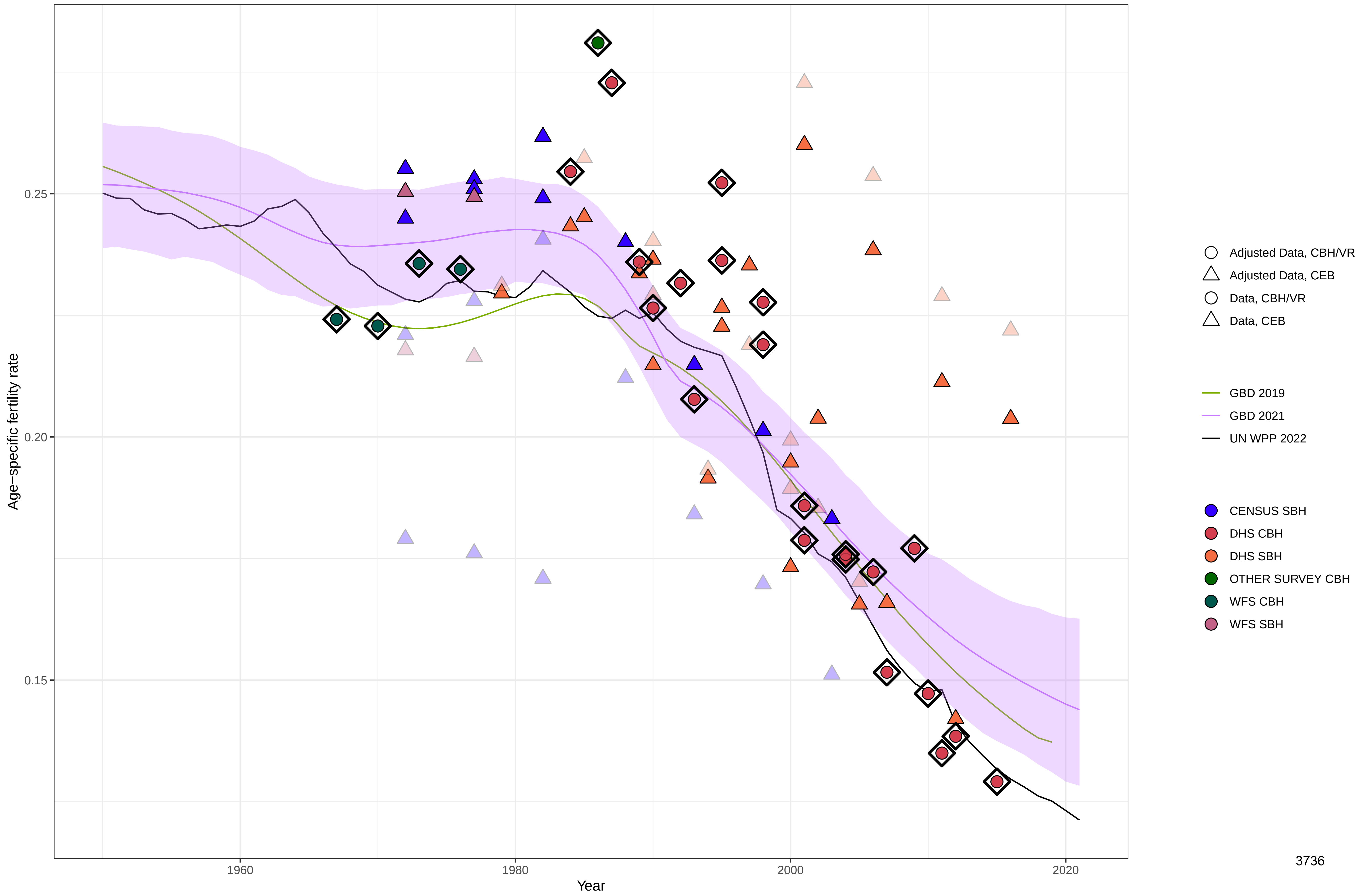

Age−specific fertility fitted trend lines, 1950−2021: Haiti, Ages: 35−39
Data points enclosed with a diamond are reference sources

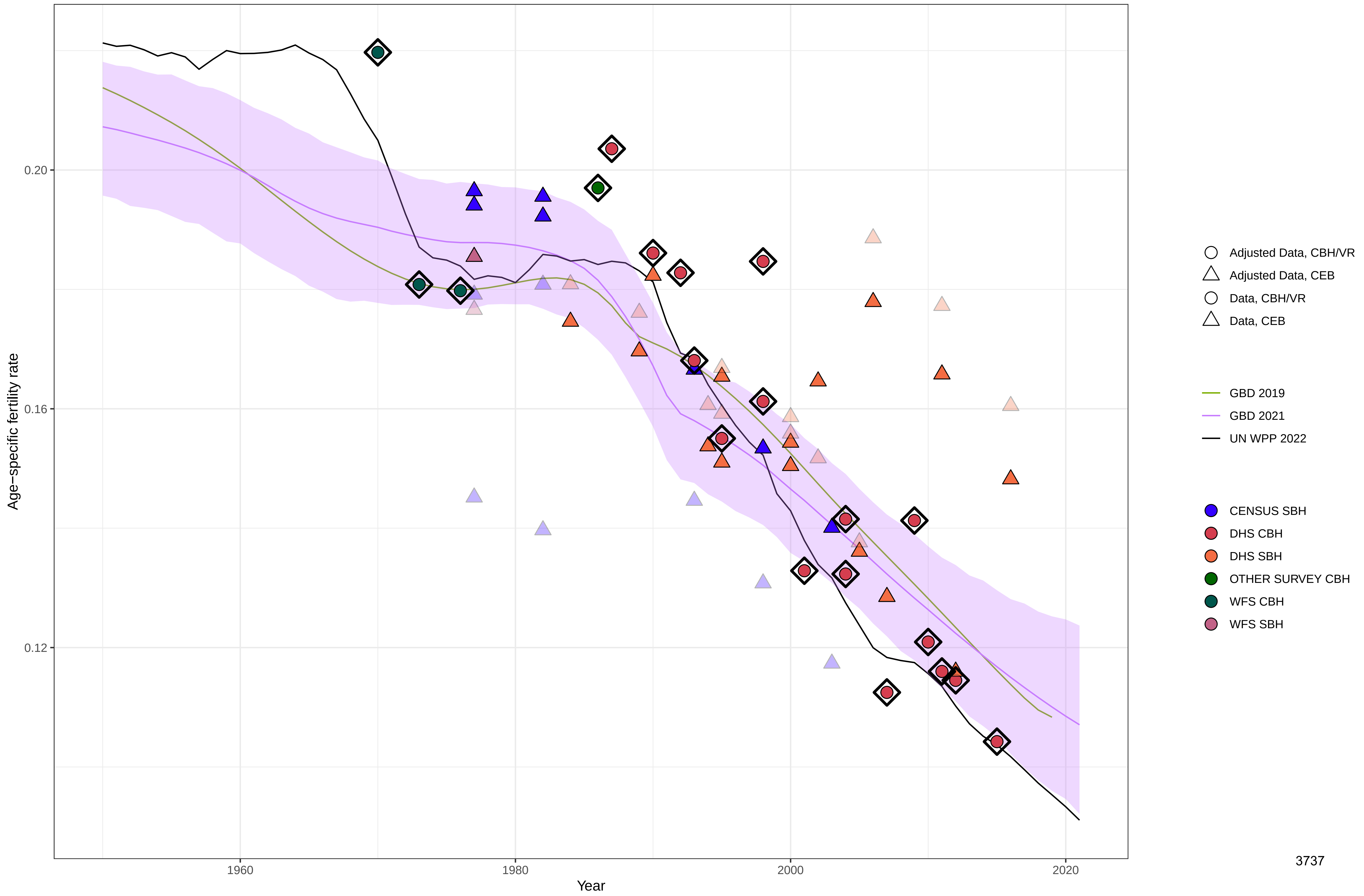

Age−specific fertility fitted trend lines, 1950−2021: Haiti, Ages: 40−44
Data points enclosed with a diamond are reference sources

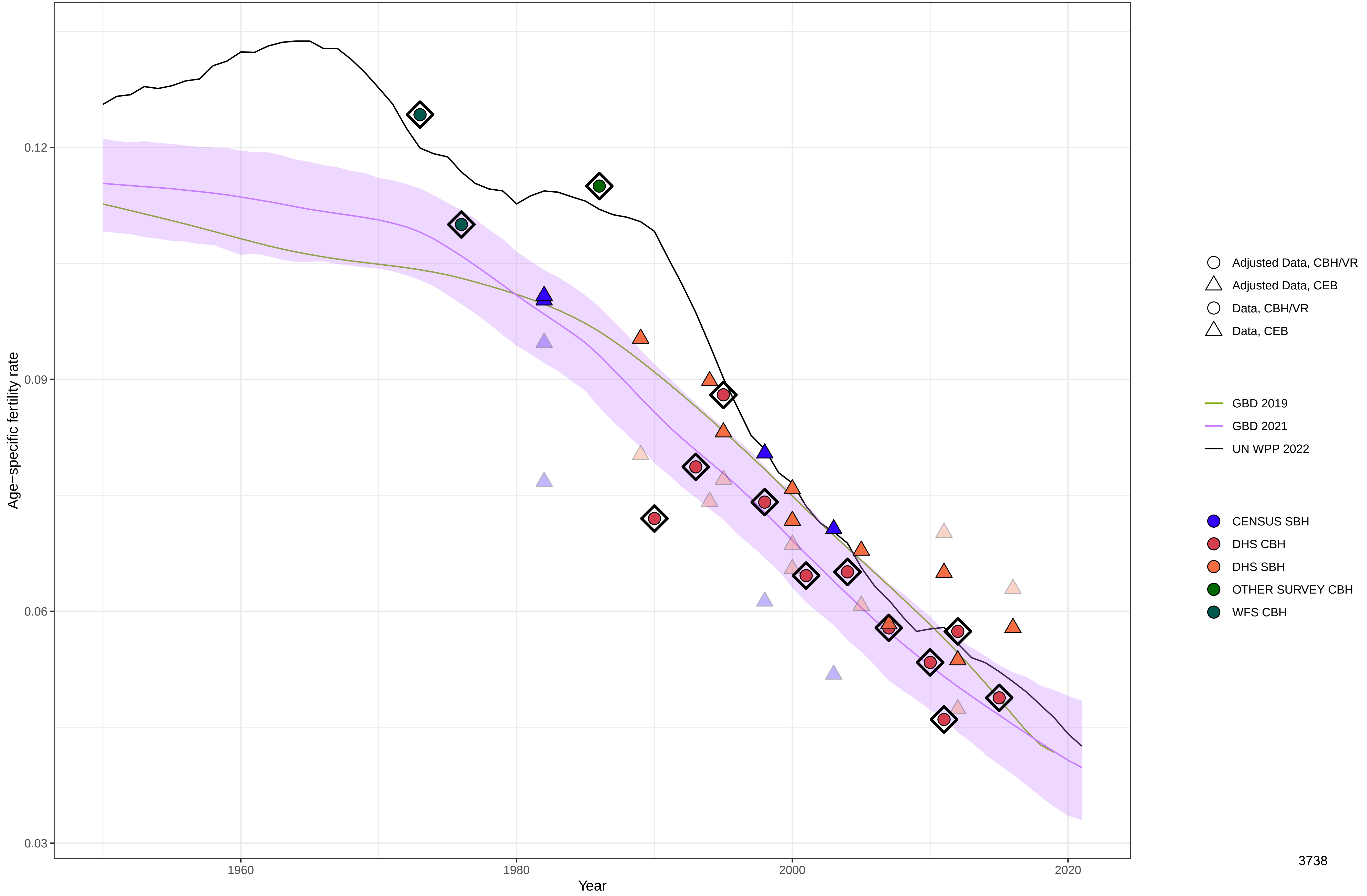

Age−specific fertility fitted trend lines, 1950−2021: Haiti, Ages: 45−49
Data points enclosed with a diamond are reference sources

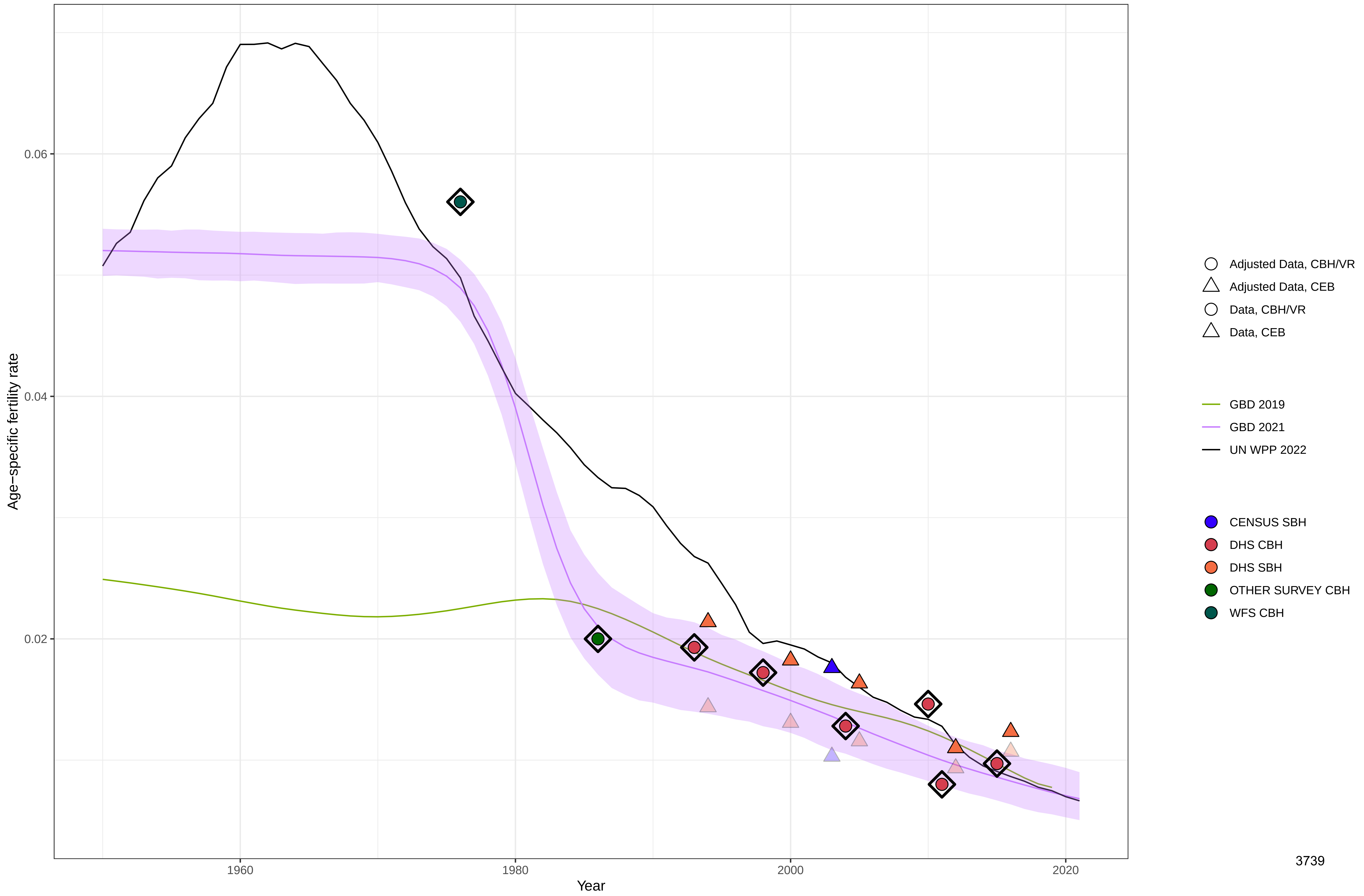

Age−specific fertility fitted trend lines, 1950−2021: Haiti, Ages: 50−54
Data points enclosed with a diamond are reference sources

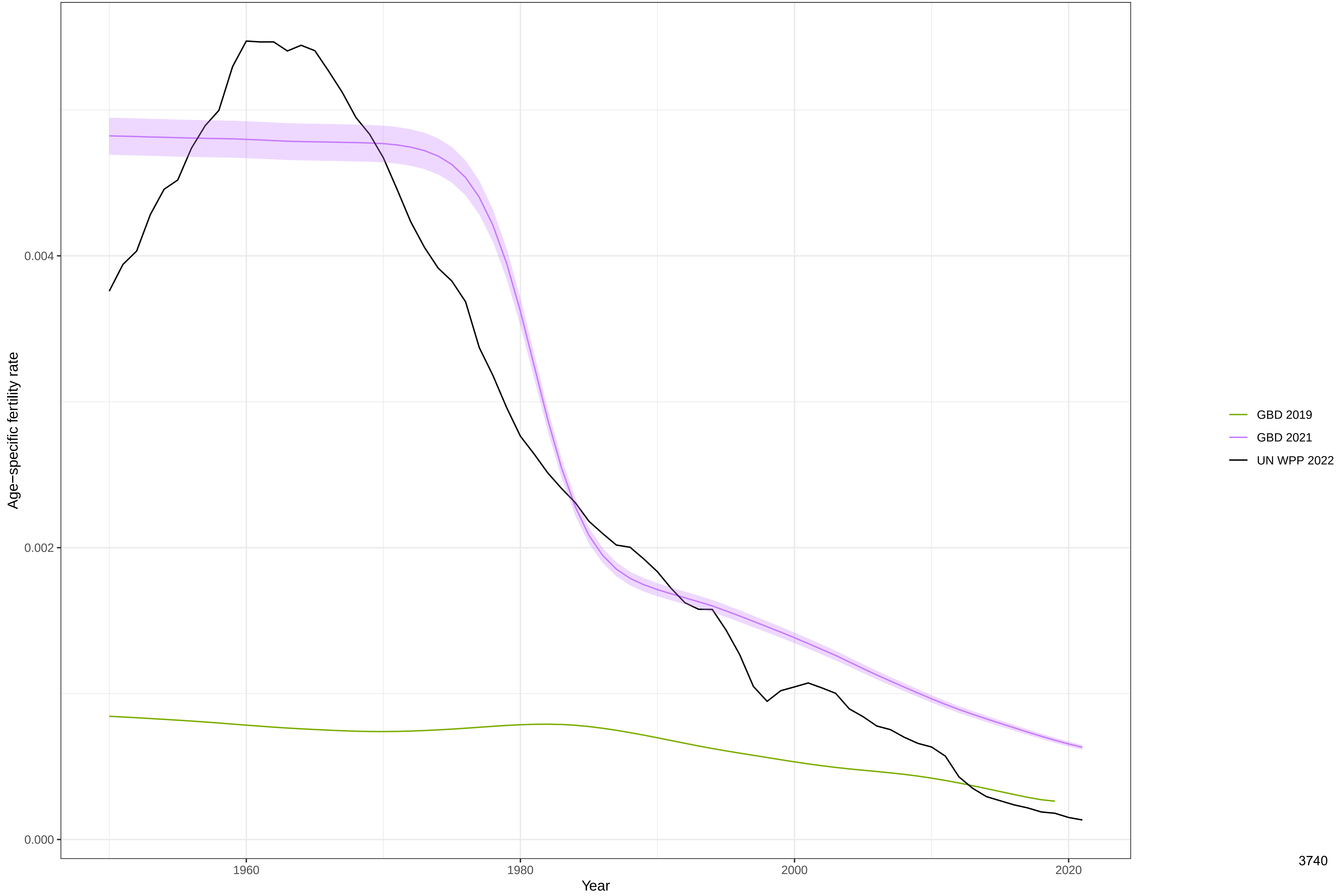

Total fertility rate fitted trend lines, 1950−2021: Haiti TFR
Data points enclosed with a diamond are reference sources

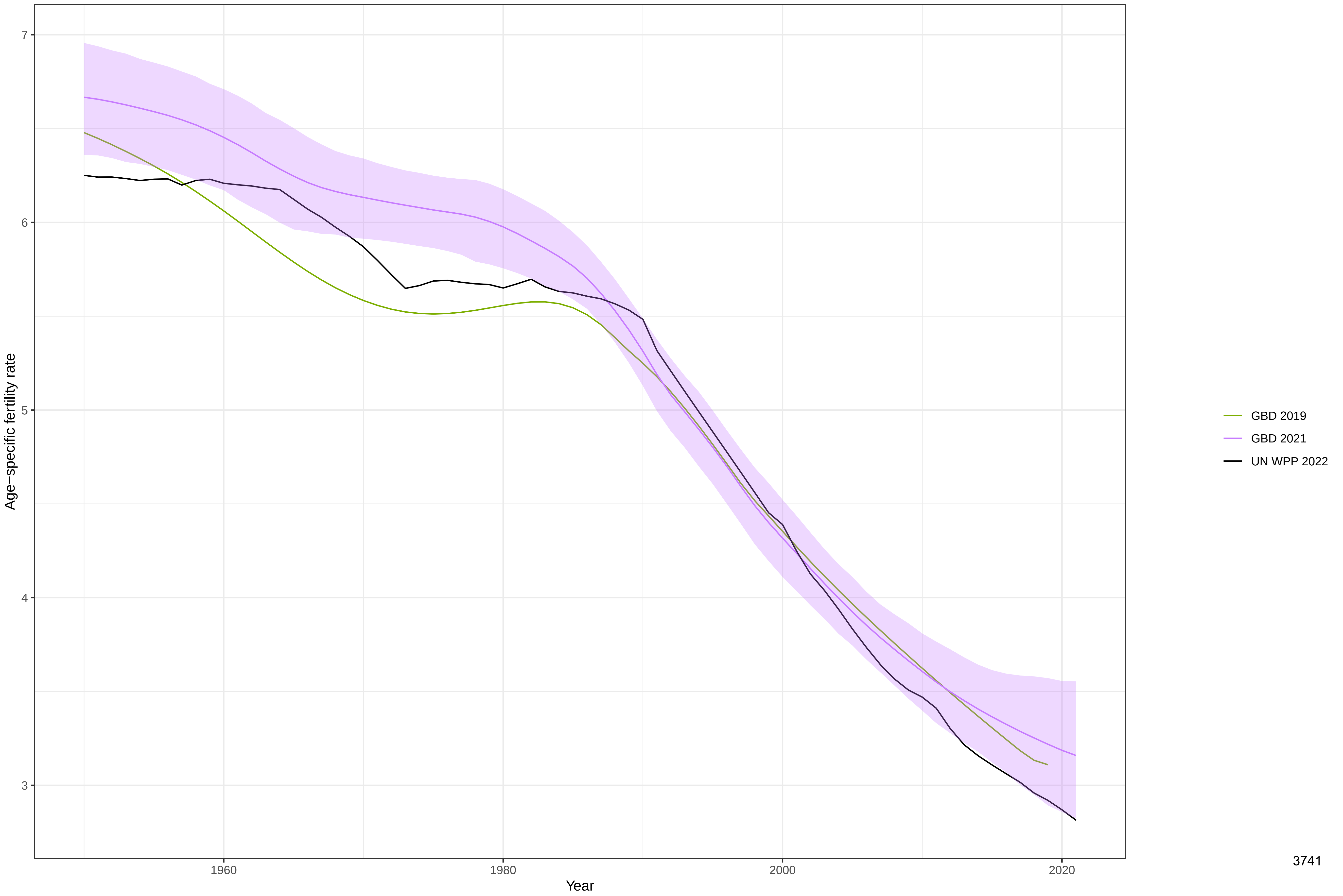

Age−specific fertility fitted trend lines, 1950−2021: Jamaica, Ages: 10−14
Data points enclosed with a diamond are reference sources

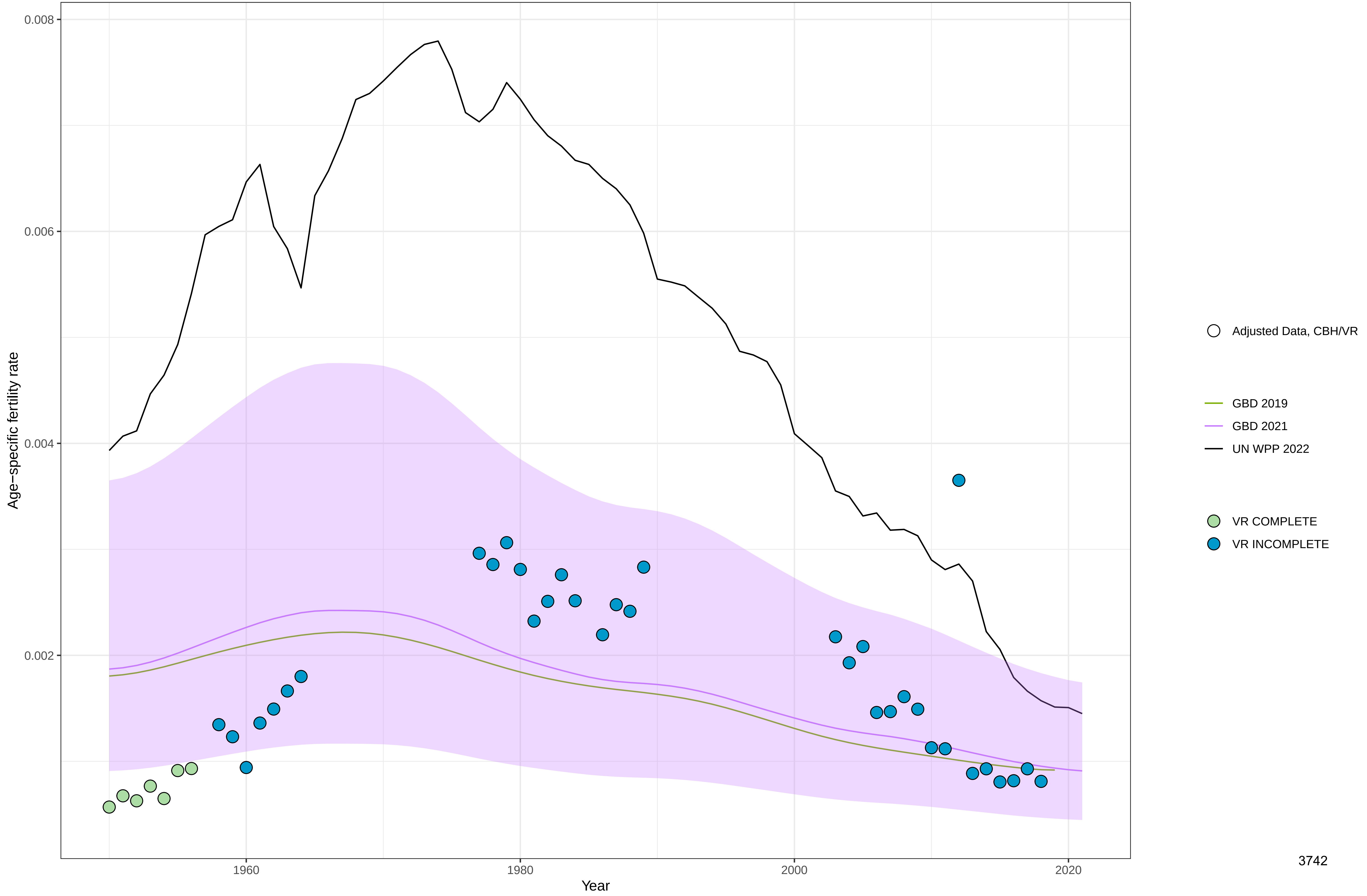

Age−specific fertility fitted trend lines, 1950−2021: Jamaica, Ages: 15−19
Data points enclosed with a diamond are reference sources

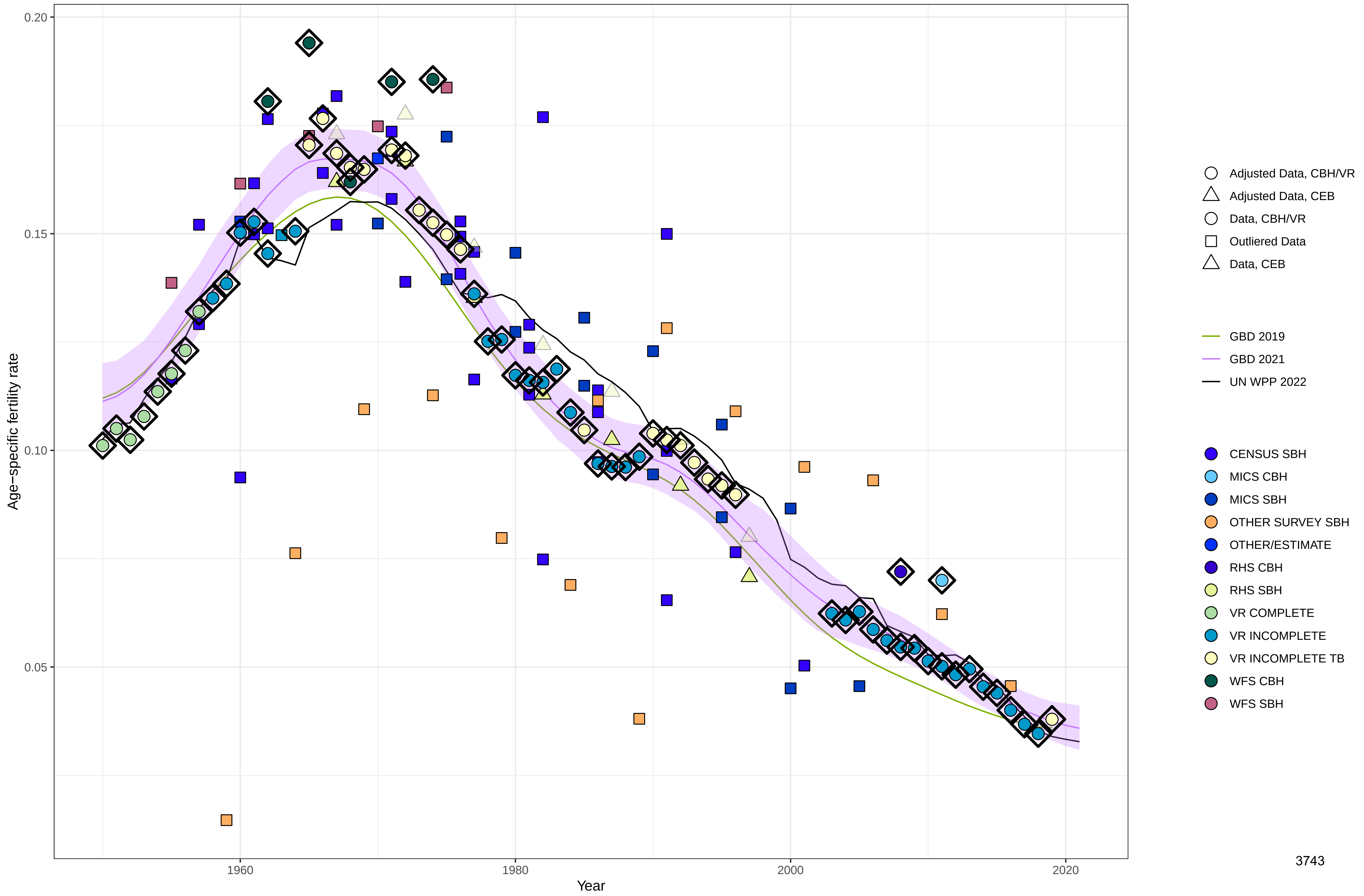

Age−specific fertility fitted trend lines, 1950−2021: Jamaica, Ages: 20−24
Data points enclosed with a diamond are reference sources

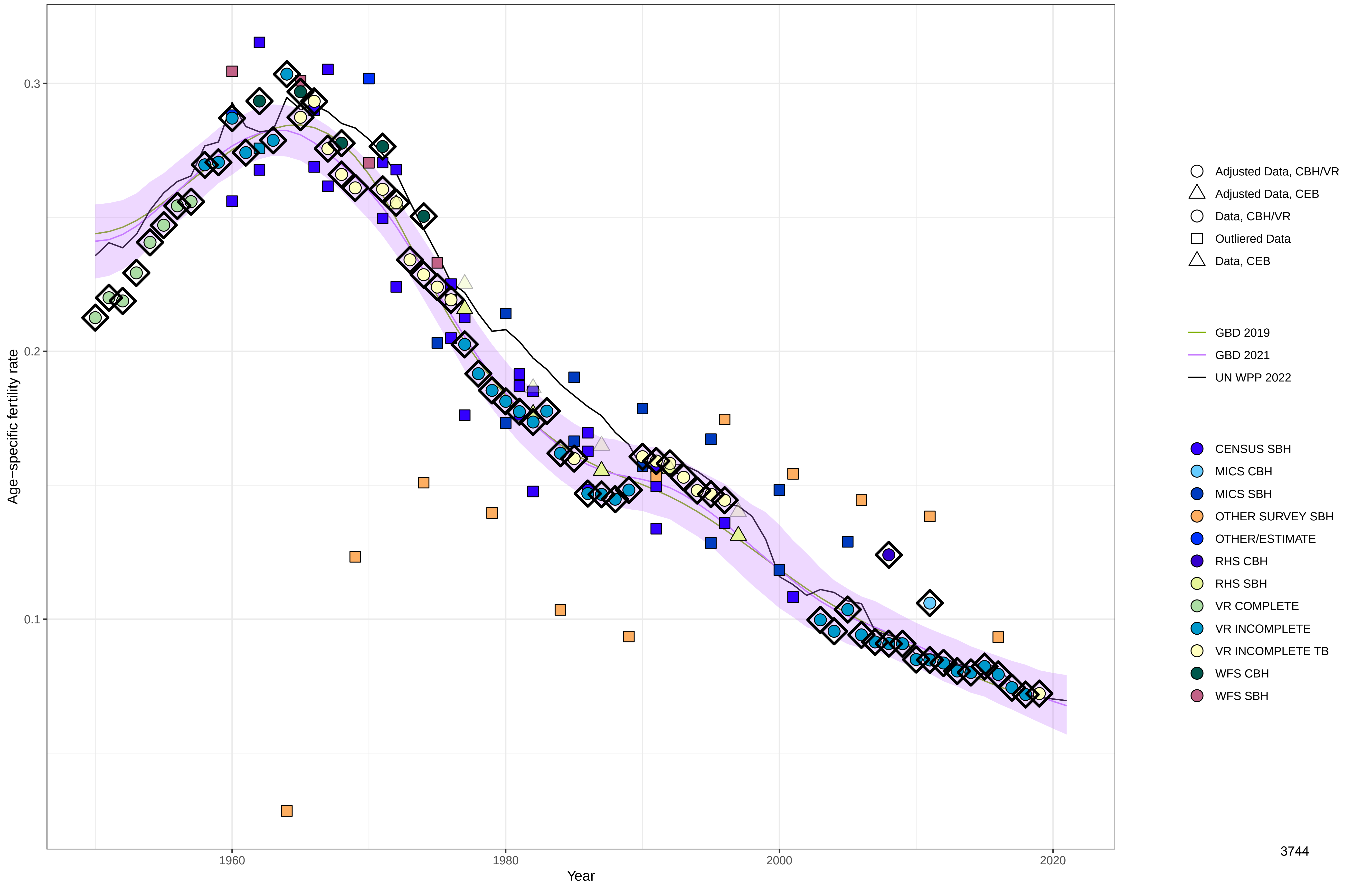

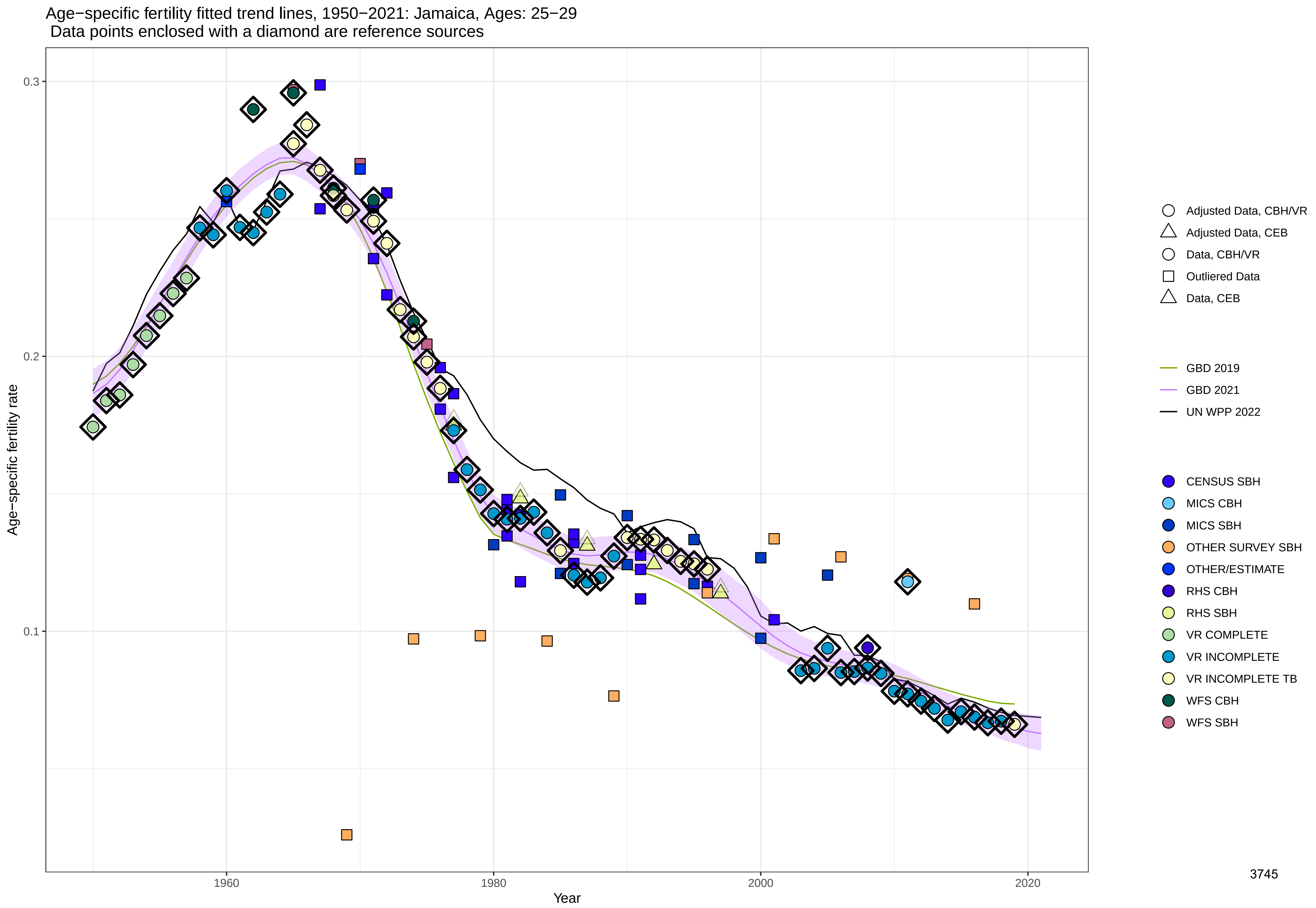
Age−specific fertility fitted trend lines, 1950−2021: Jamaica, Ages: 25−29
Data points enclosed with a diamond are reference sources
Age−specific fertility rate
0.3
0.2
0.1
1960
1980
2000
2020
Year
Adjusted Data, CBH/VR
Adjusted Data, CEB
Data, CBH/VR
Outliered Data
Data, CEB
GBD 2019
GBD 2021
UN WPP 2022
CENSUS SBH
MICS CBH
MICS SBH
OTHER SURVEY SBH
OTHER/ESTIMATE
RHS CBH
RHS SBH
VR COMPLETE
VR INCOMPLETE
VR INCOMPLETE TB
WFS CBH
WFS SBH

Age−specific fertility fitted trend lines, 1950−2021: Jamaica, Ages: 30−34
Data points enclosed with a diamond are reference sources

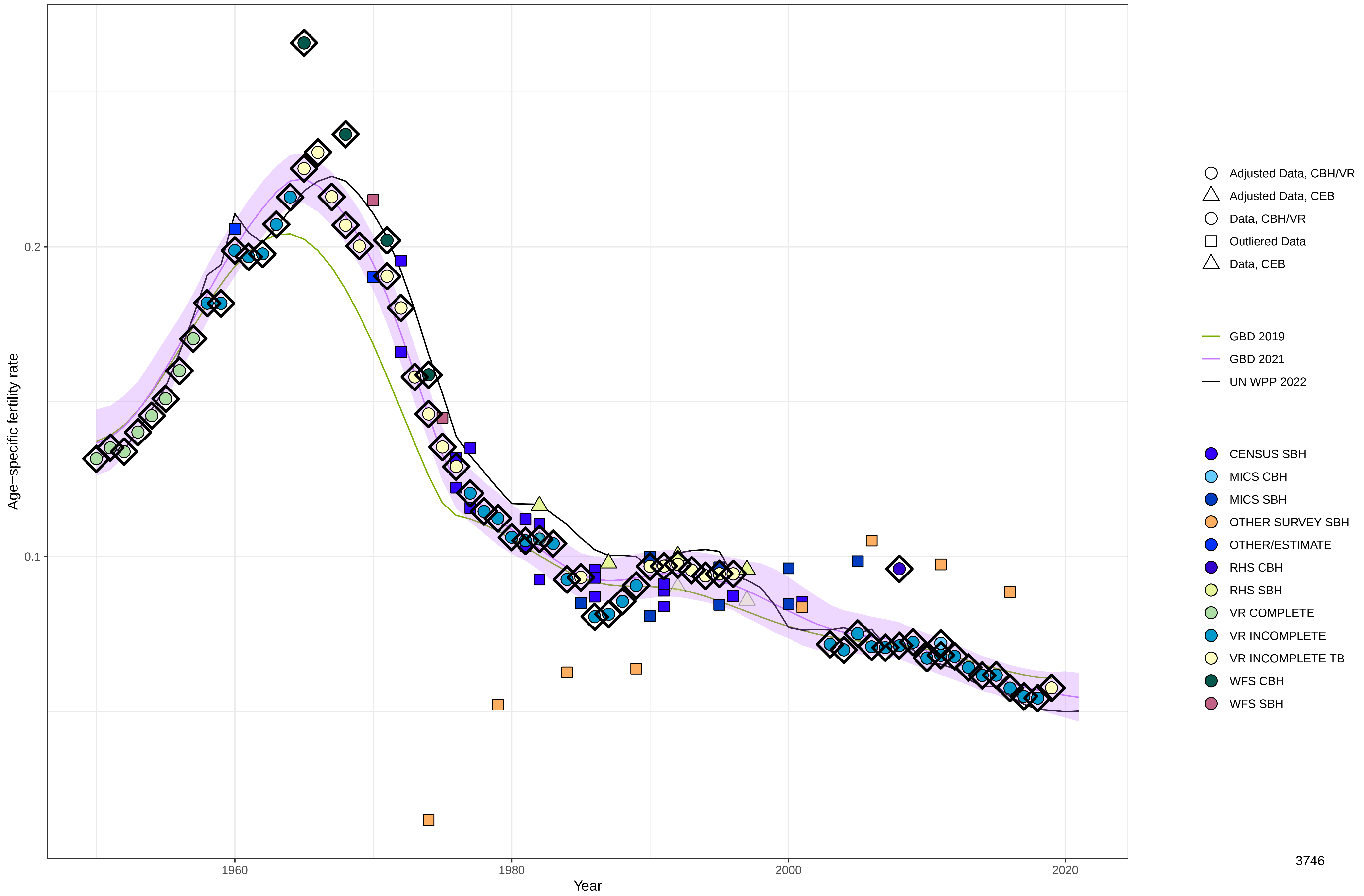

Age−specific fertility fitted trend lines, 1950−2021: Jamaica, Ages: 35−39
Data points enclosed with a diamond are reference sources

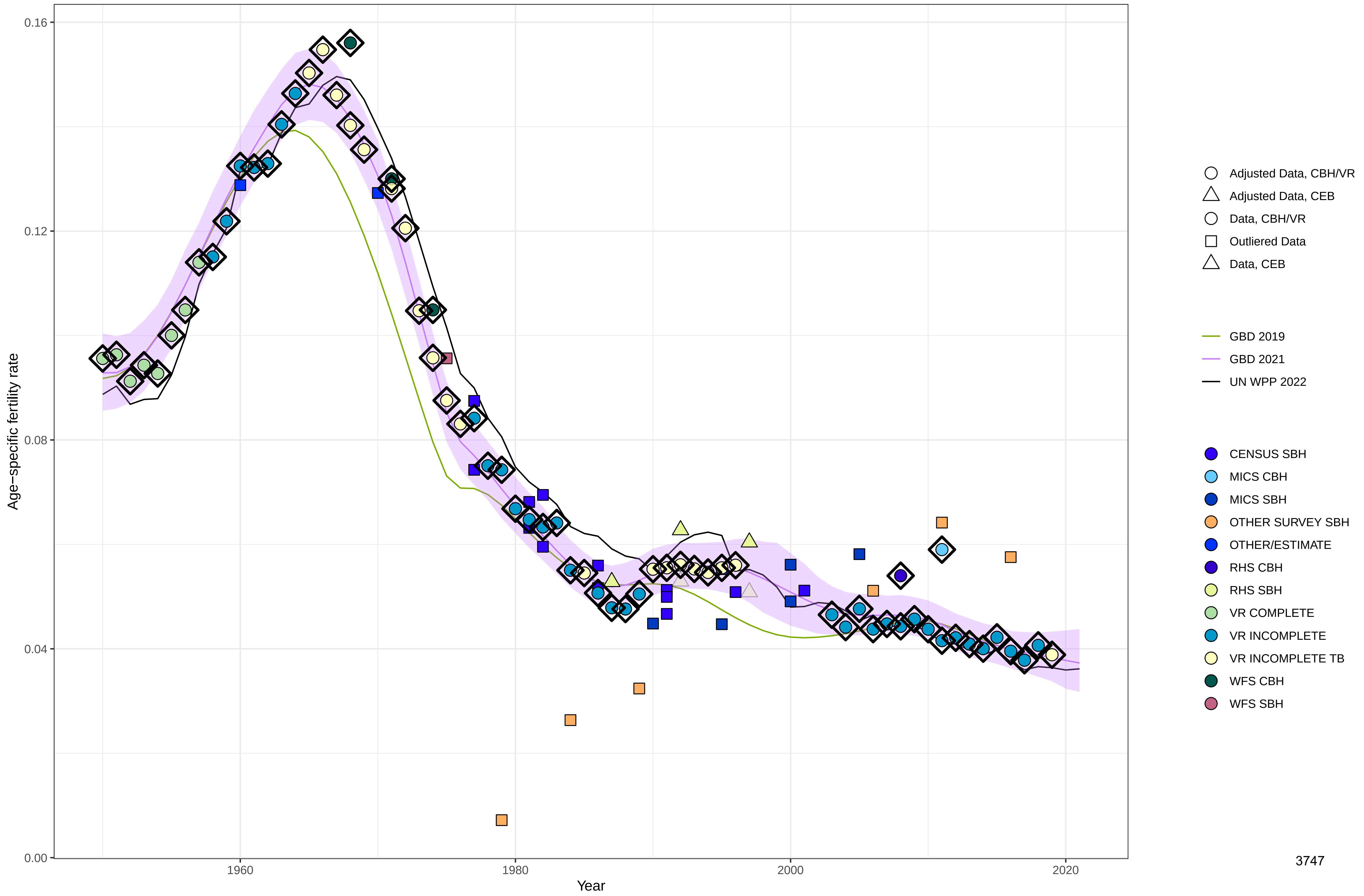

Age−specific fertility fitted trend lines, 1950−2021: Jamaica, Ages: 40−44
Data points enclosed with a diamond are reference sources

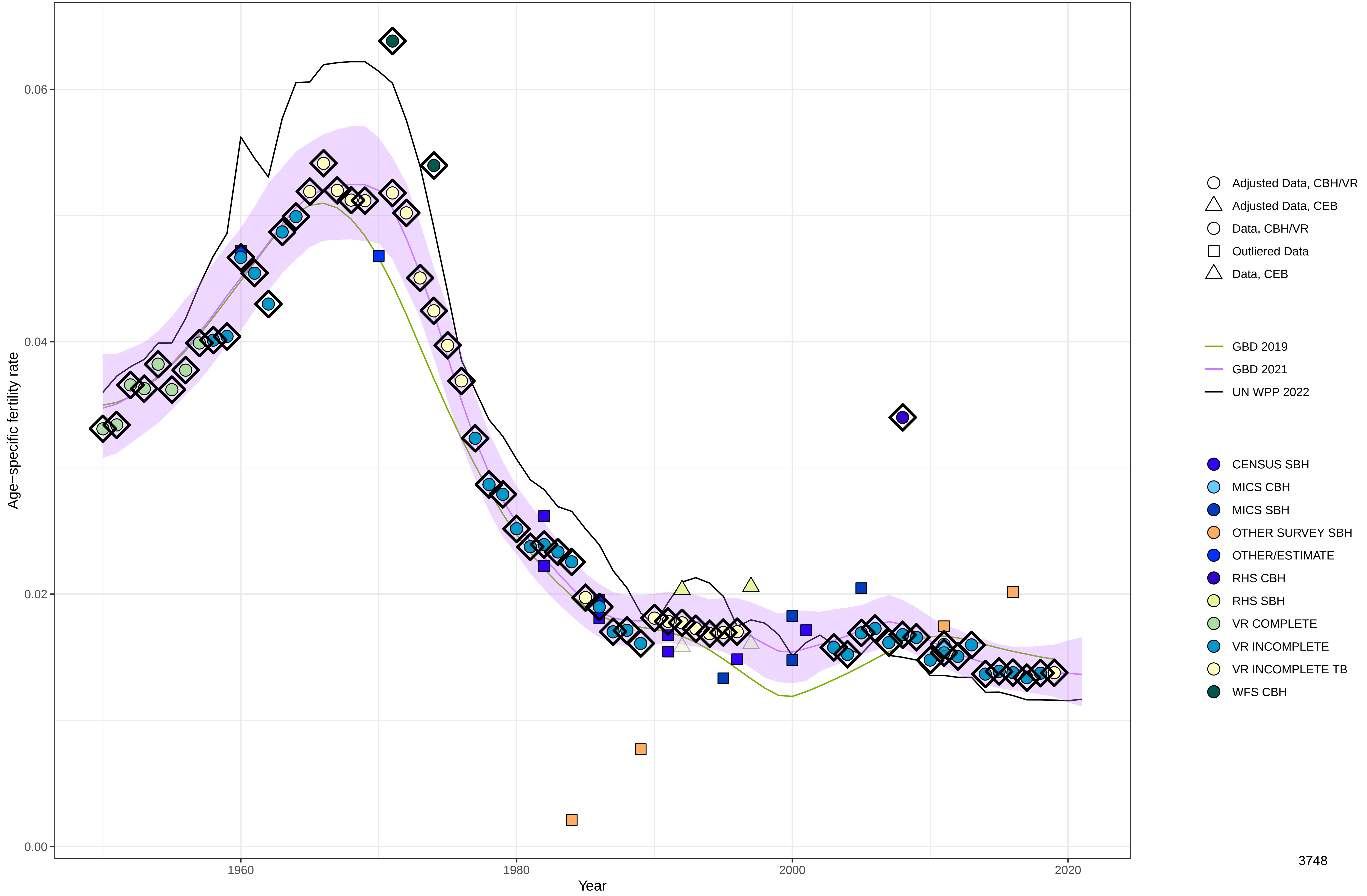

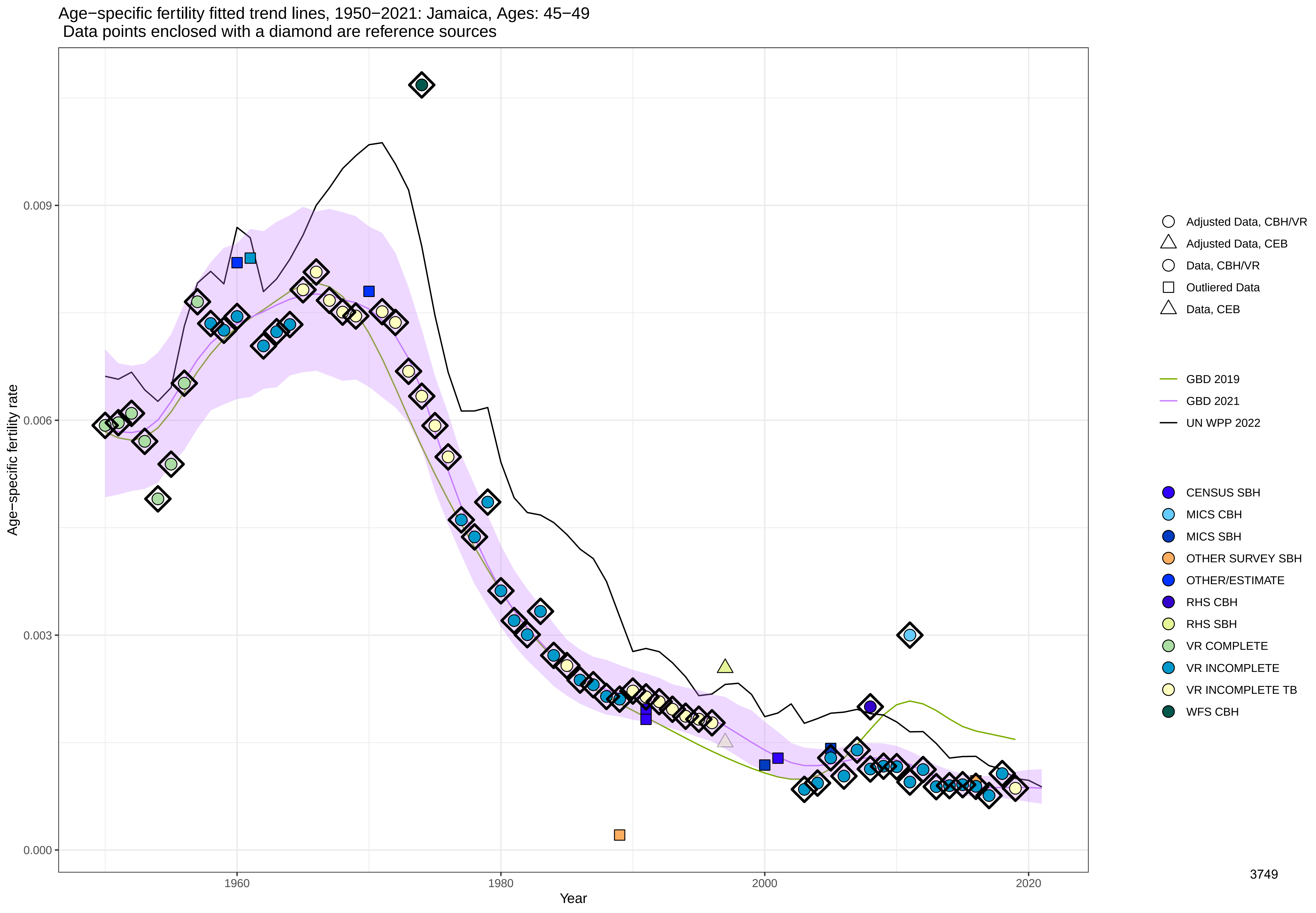

Age−specific fertility fitted trend lines, 1950−2021: Jamaica, Ages: 45−49
Data points enclosed with a diamond are reference sources
Age−specific fertility rate
0.009
0.006
0.003
0.000
1960
1980
2000
2020
Year
Adjusted Data, CBH/VR
Adjusted Data, CEB
Data, CBH/VR
Outliered Data
Data, CEB
GBD 2019
GBD 2021
UN WPP 2022
CENSUS SBH
MICS CBH
MICS SBH
OTHER SURVEY SBH
OTHER/ESTIMATE
RHS CBH
RHS SBH
VR COMPLETE
VR INCOMPLETE
VR INCOMPLETE TB
WFS CBH

Age−specific fertility fitted trend lines, 1950−2021: Jamaica, Ages: 50−54
Data points enclosed with a diamond are reference sources

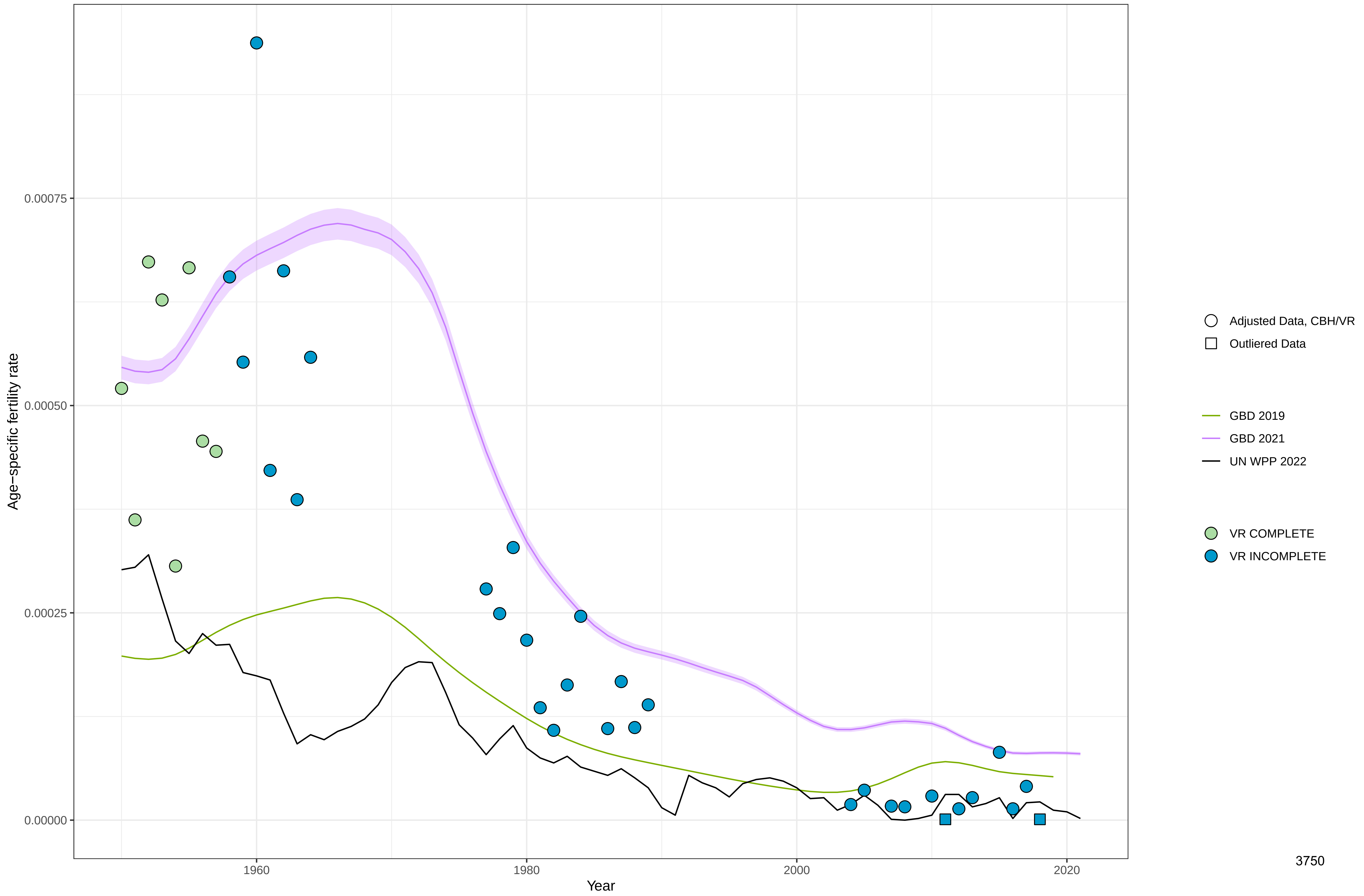

Total fertility rate fitted trend lines, 1950−2021: Jamaica TFR
Data points enclosed with a diamond are reference sources

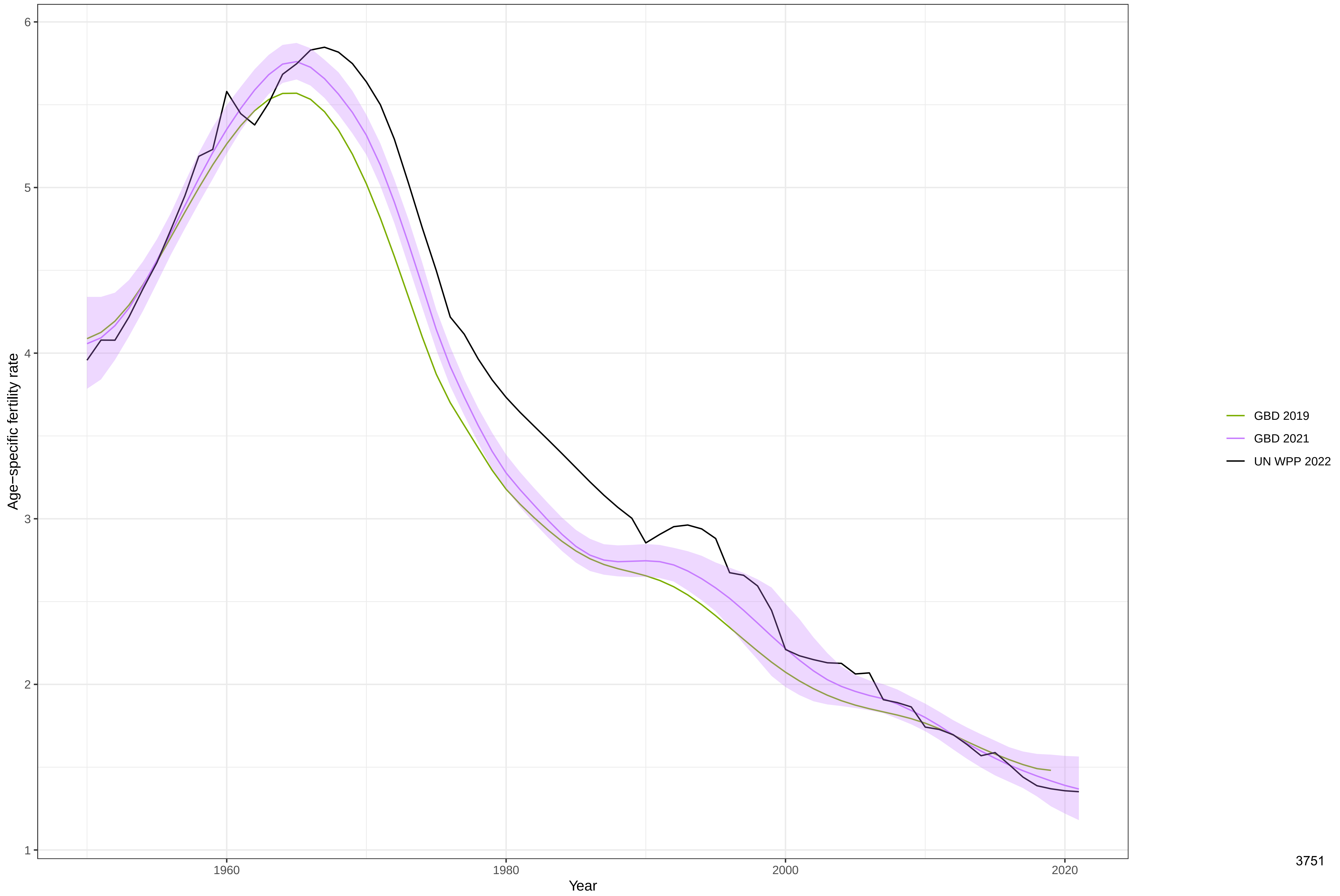

Age−specific fertility fitted trend lines, 1950−2021: Puerto Rico, Ages: 10−14
Data points enclosed with a diamond are reference sources

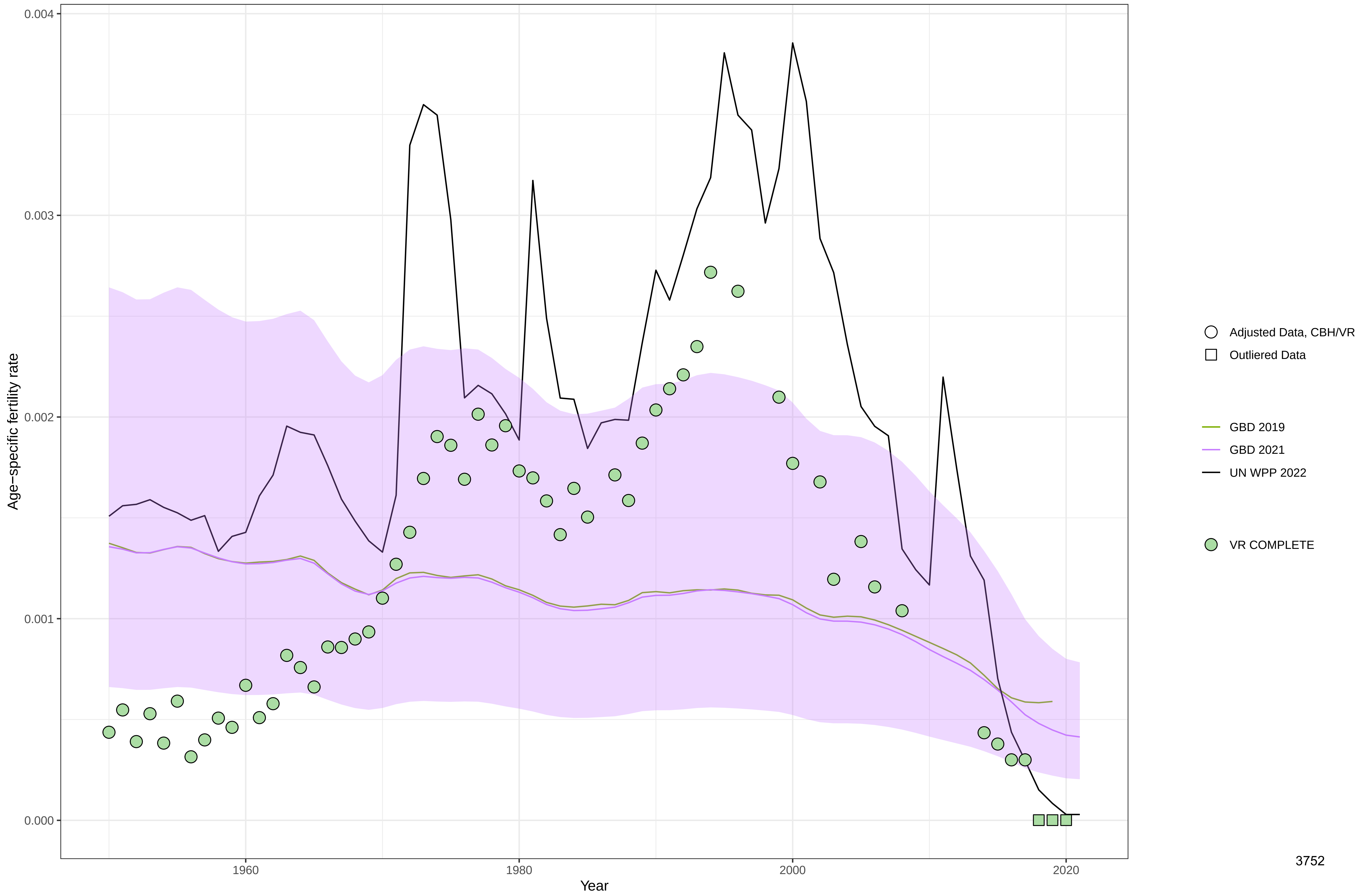

Age−specific fertility fitted trend lines, 1950−2021: Puerto Rico, Ages: 15−19
Data points enclosed with a diamond are reference sources

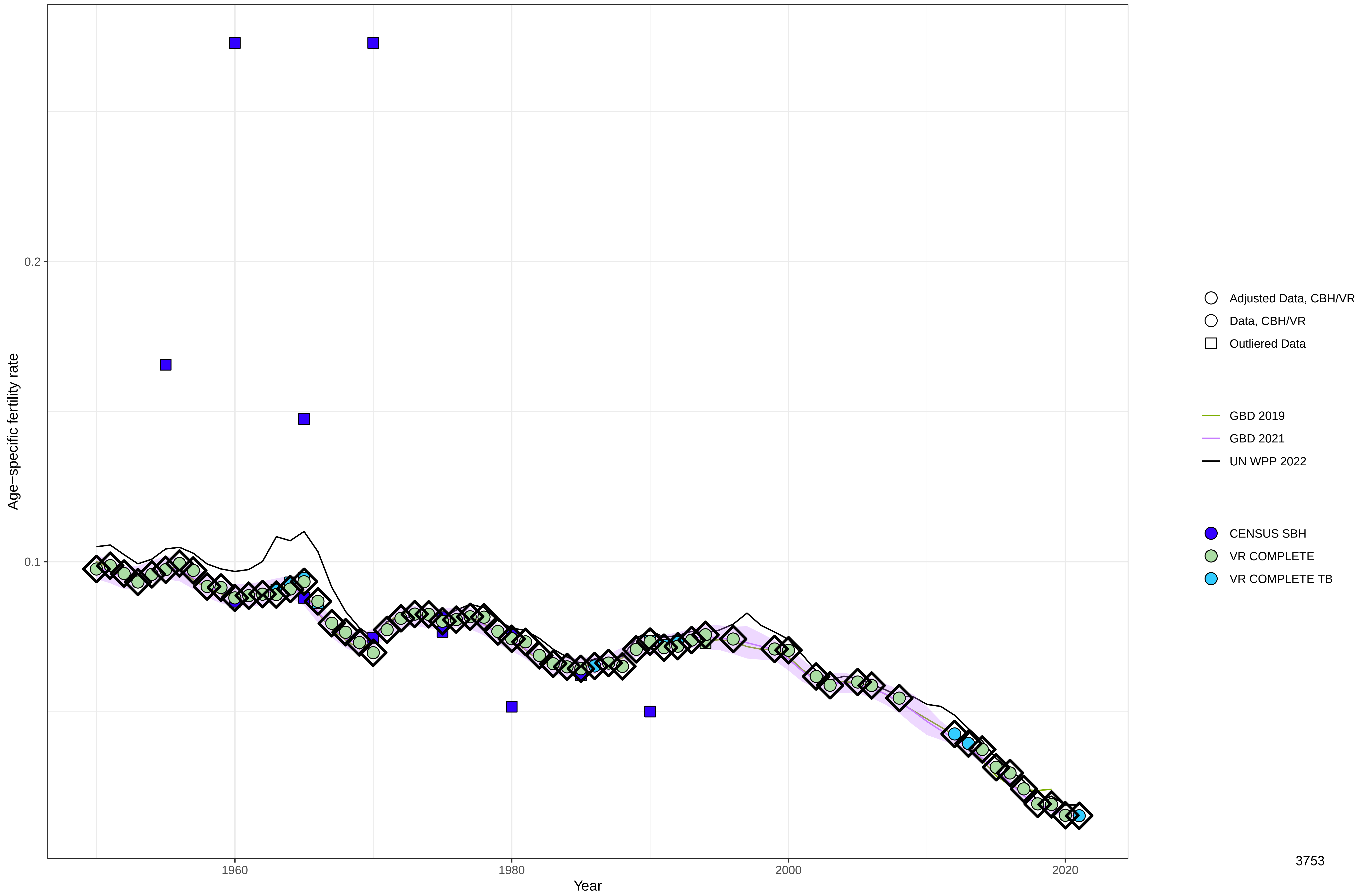

Age−specific fertility fitted trend lines, 1950−2021: Puerto Rico, Ages: 20−24
Data points enclosed with a diamond are reference sources

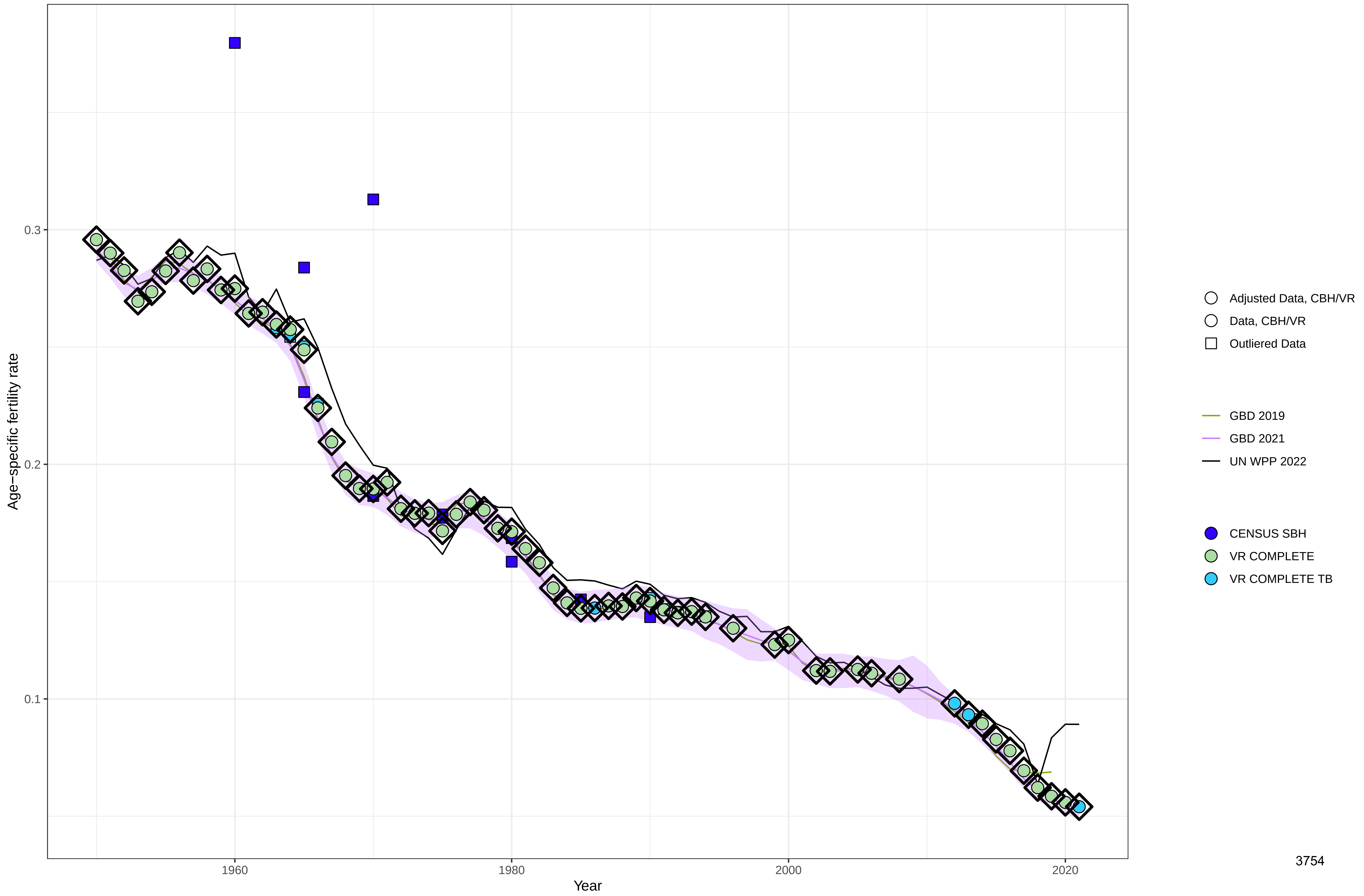

Age−specific fertility fitted trend lines, 1950−2021: Puerto Rico, Ages: 25−29
Data points enclosed with a diamond are reference sources

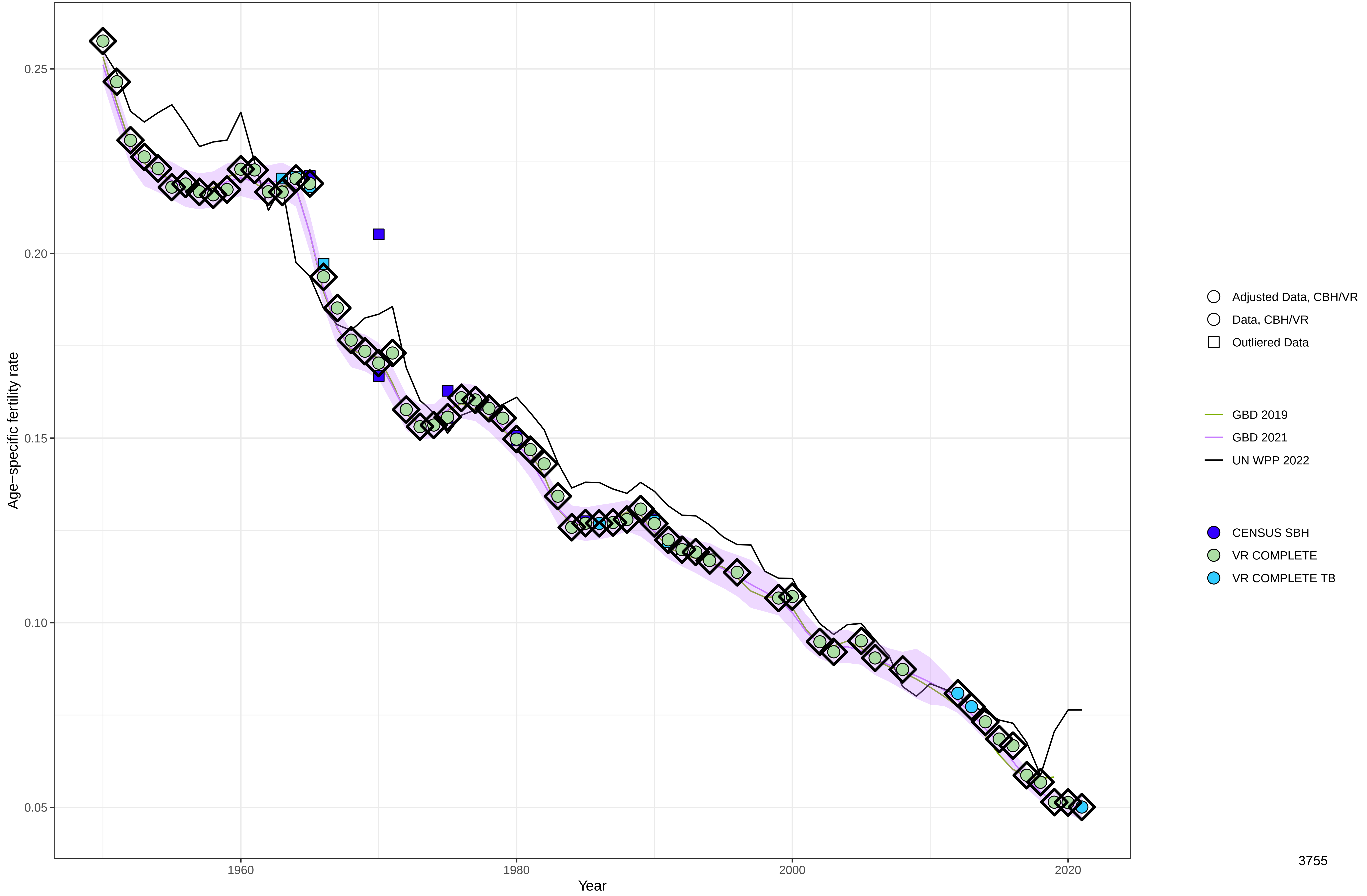

Age−specific fertility fitted trend lines, 1950−2021: Puerto Rico, Ages: 30−34
Data points enclosed with a diamond are reference sources

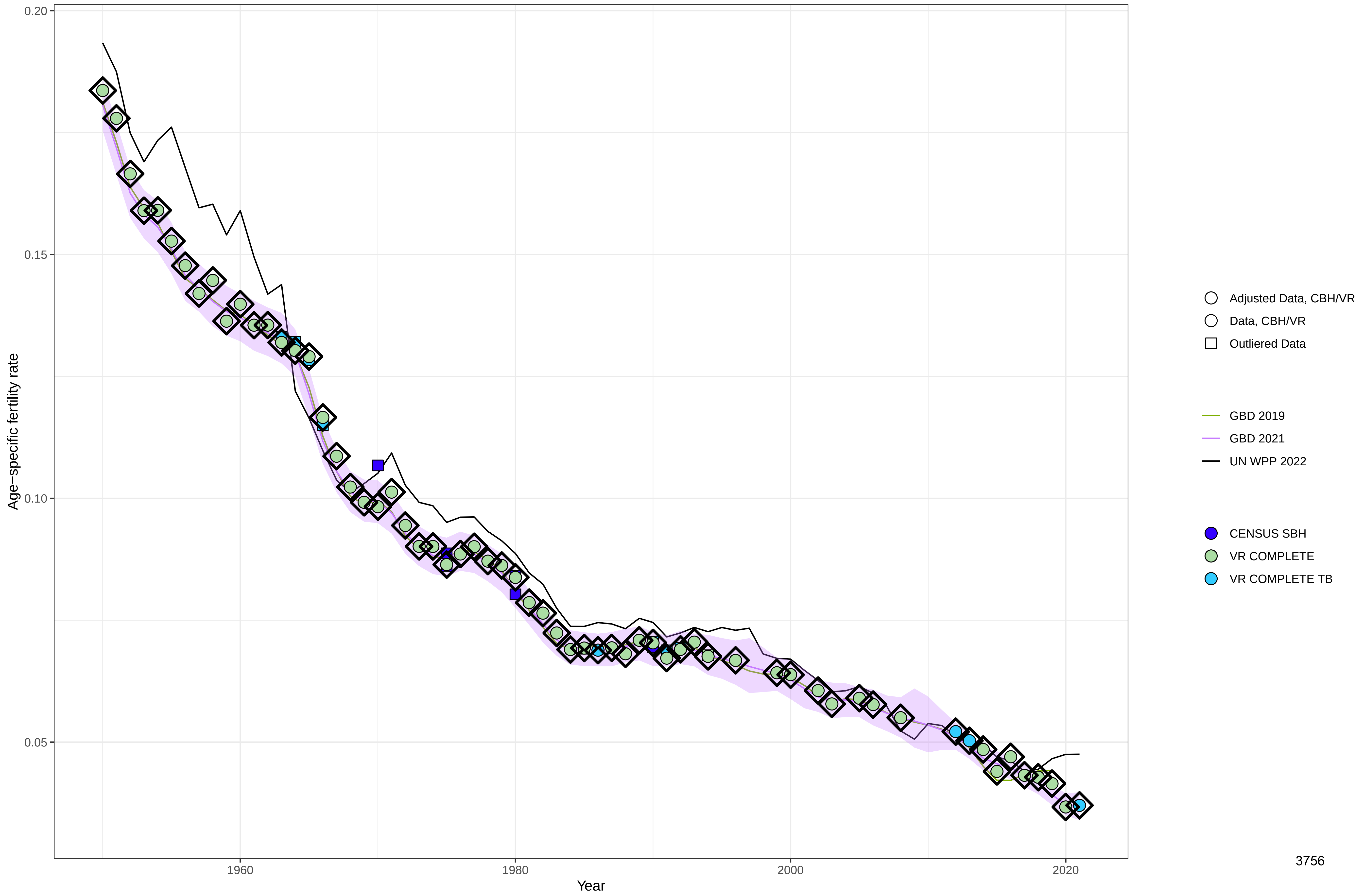

Age−specific fertility fitted trend lines, 1950−2021: Puerto Rico, Ages: 35−39
Data points enclosed with a diamond are reference sources

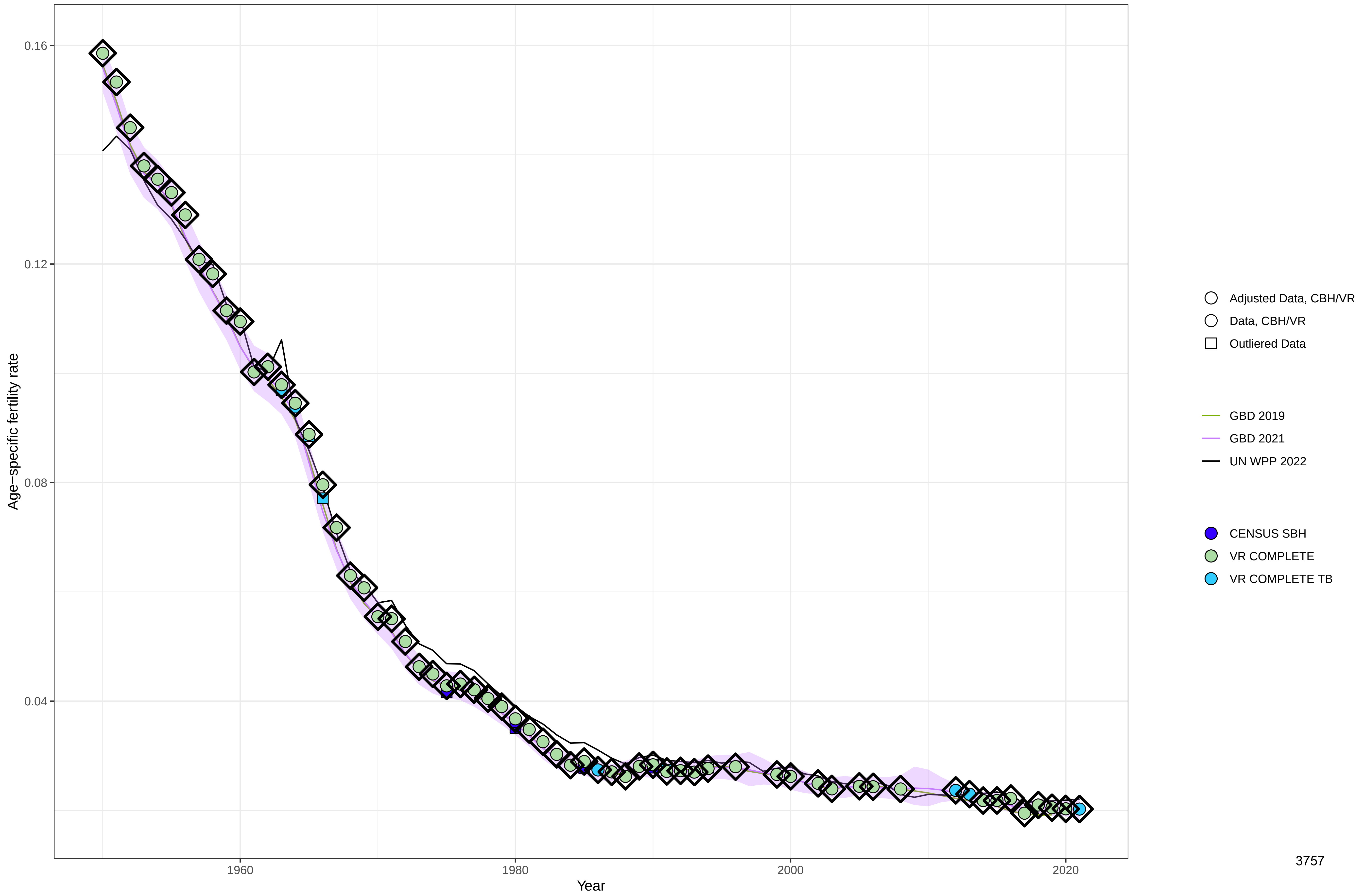

Age−specific fertility fitted trend lines, 1950−2021: Puerto Rico, Ages: 40−44
Data points enclosed with a diamond are reference sources

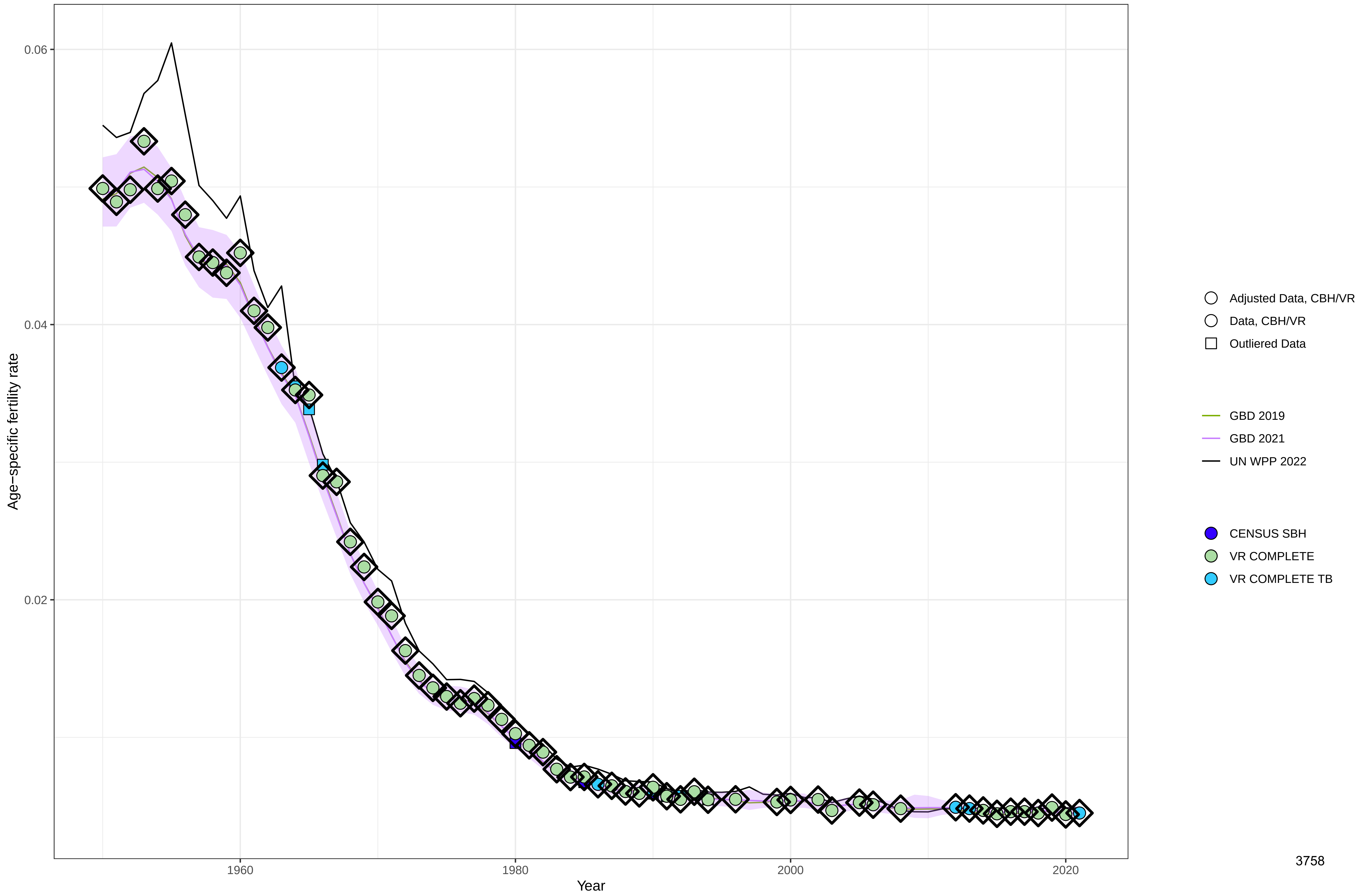

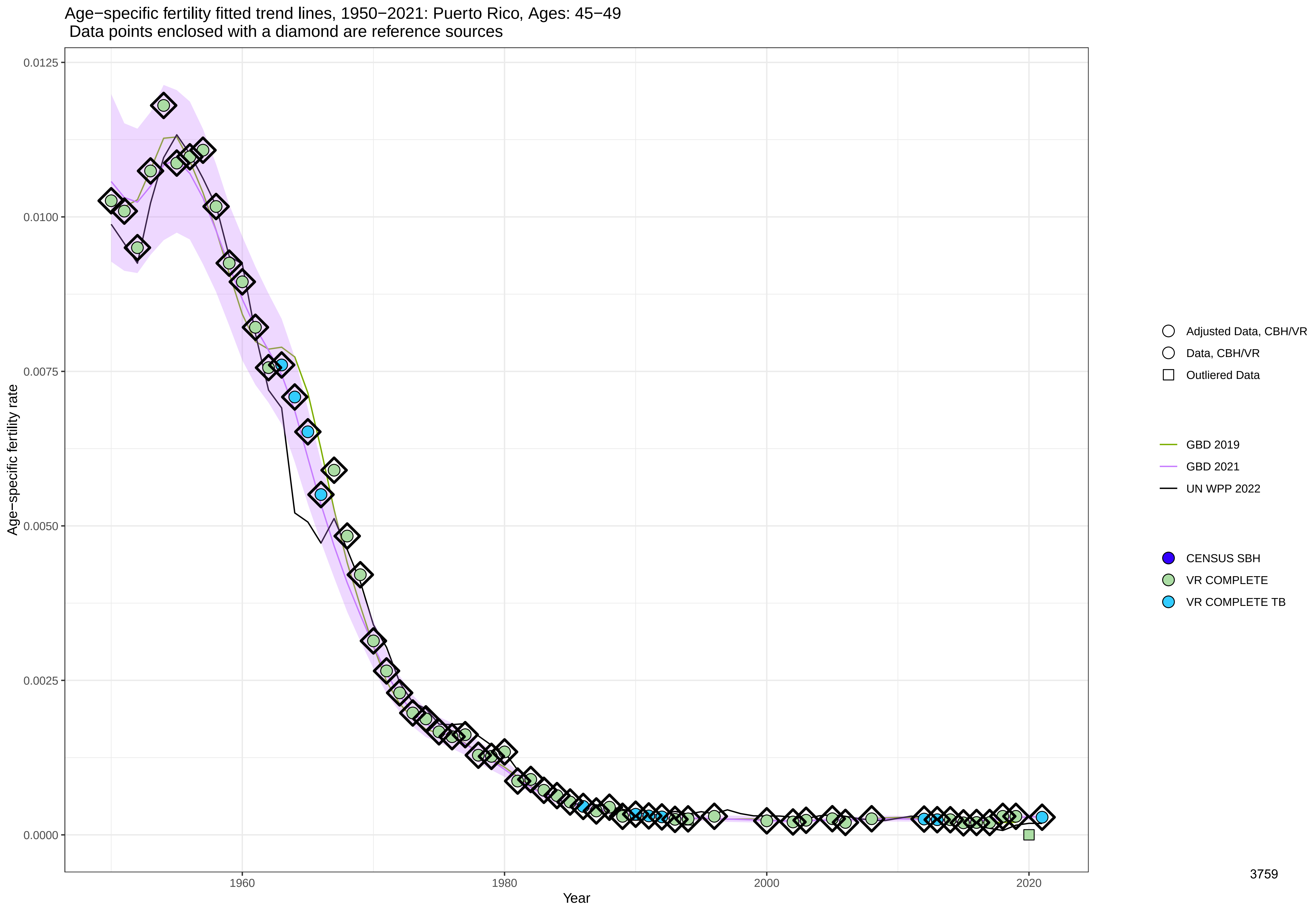

Age−specific fertility fitted trend lines, 1950−2021: Puerto Rico, Ages: 45−49
Data points enclosed with a diamond are reference sources
Age−specific fertility rate
0.0125
0.0100
0.0075
0.0050
0.0025
0.0000
1960
1980
2000
2020
Year
Adjusted Data, CBH/VR
Data, CBH/VR
Outliered Data
GBD 2019
GBD 2021
UN WPP 2022
CENSUS SBH
VR COMPLETE
VR COMPLETE TB

Age−specific fertility fitted trend lines, 1950−2021: Puerto Rico, Ages: 50−54
Data points enclosed with a diamond are reference sources

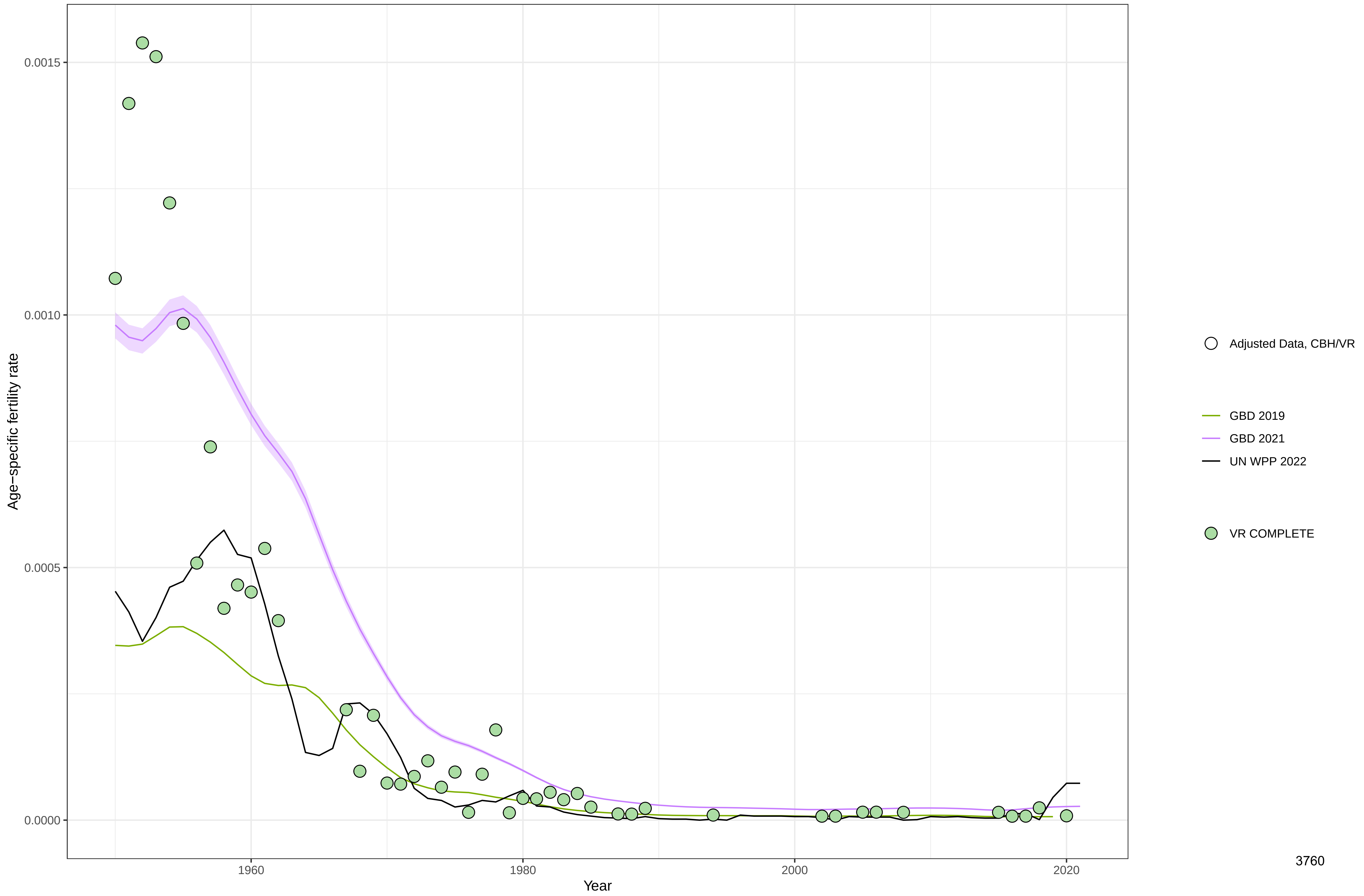

Total fertility rate fitted trend lines, 1950−2021: Puerto Rico TFR
Data points enclosed with a diamond are reference sources

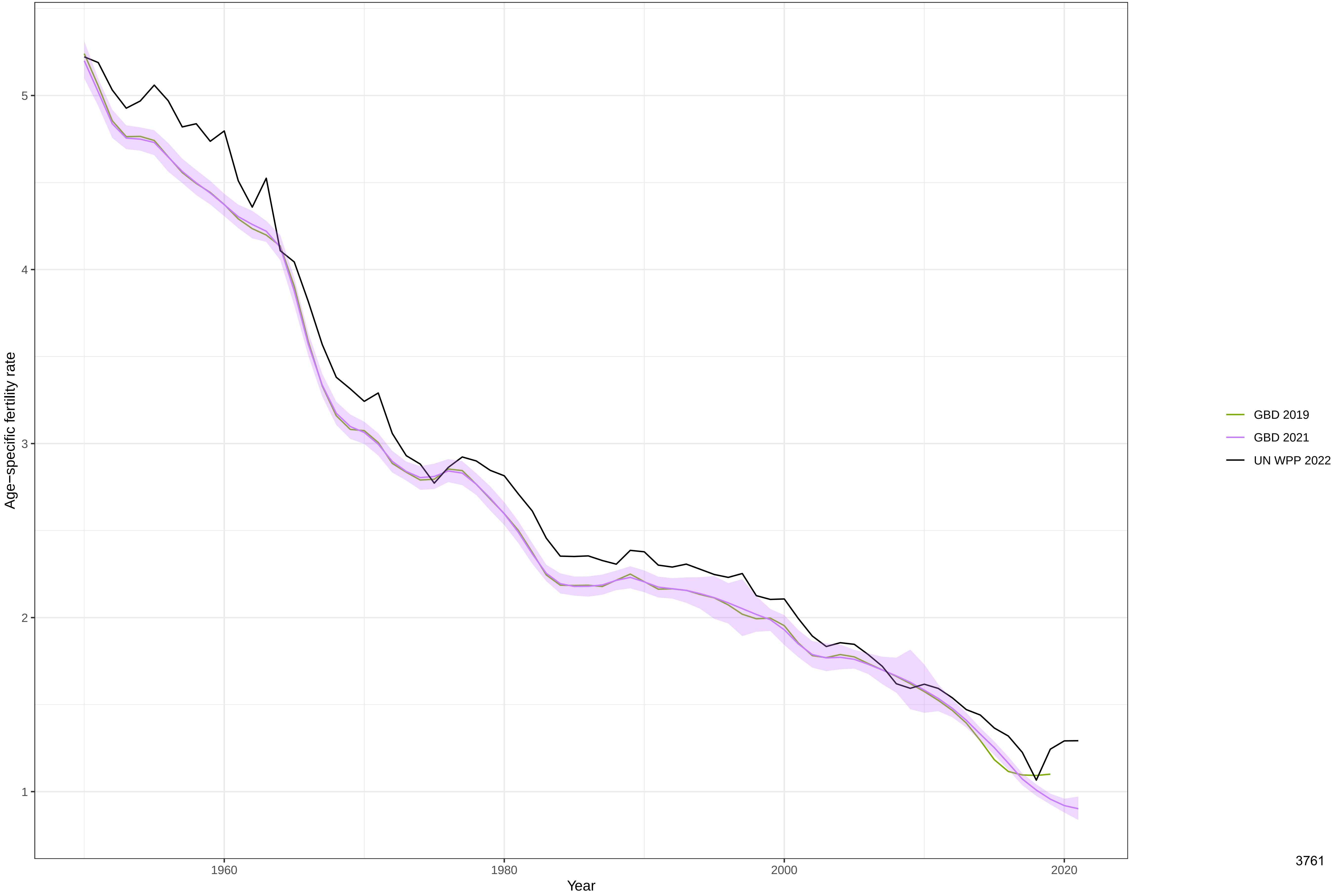

Age−specific fertility fitted trend lines, 1950−2021: Saint Kitts and Nevis, Ages: 10−14
Data points enclosed with a diamond are reference sources

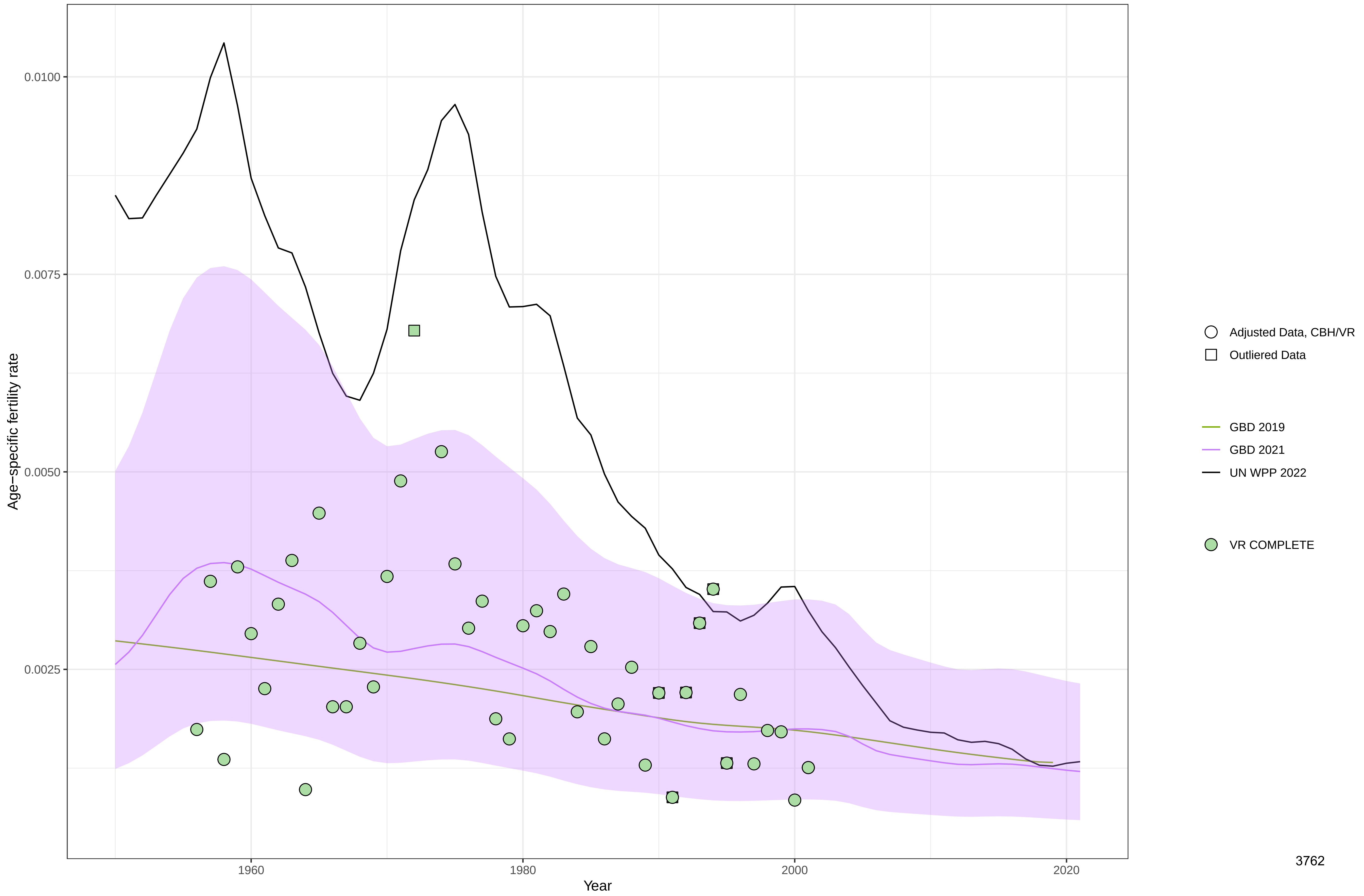

Age−specific fertility fitted trend lines, 1950−2021: Saint Kitts and Nevis, Ages: 15−19
Data points enclosed with a diamond are reference sources

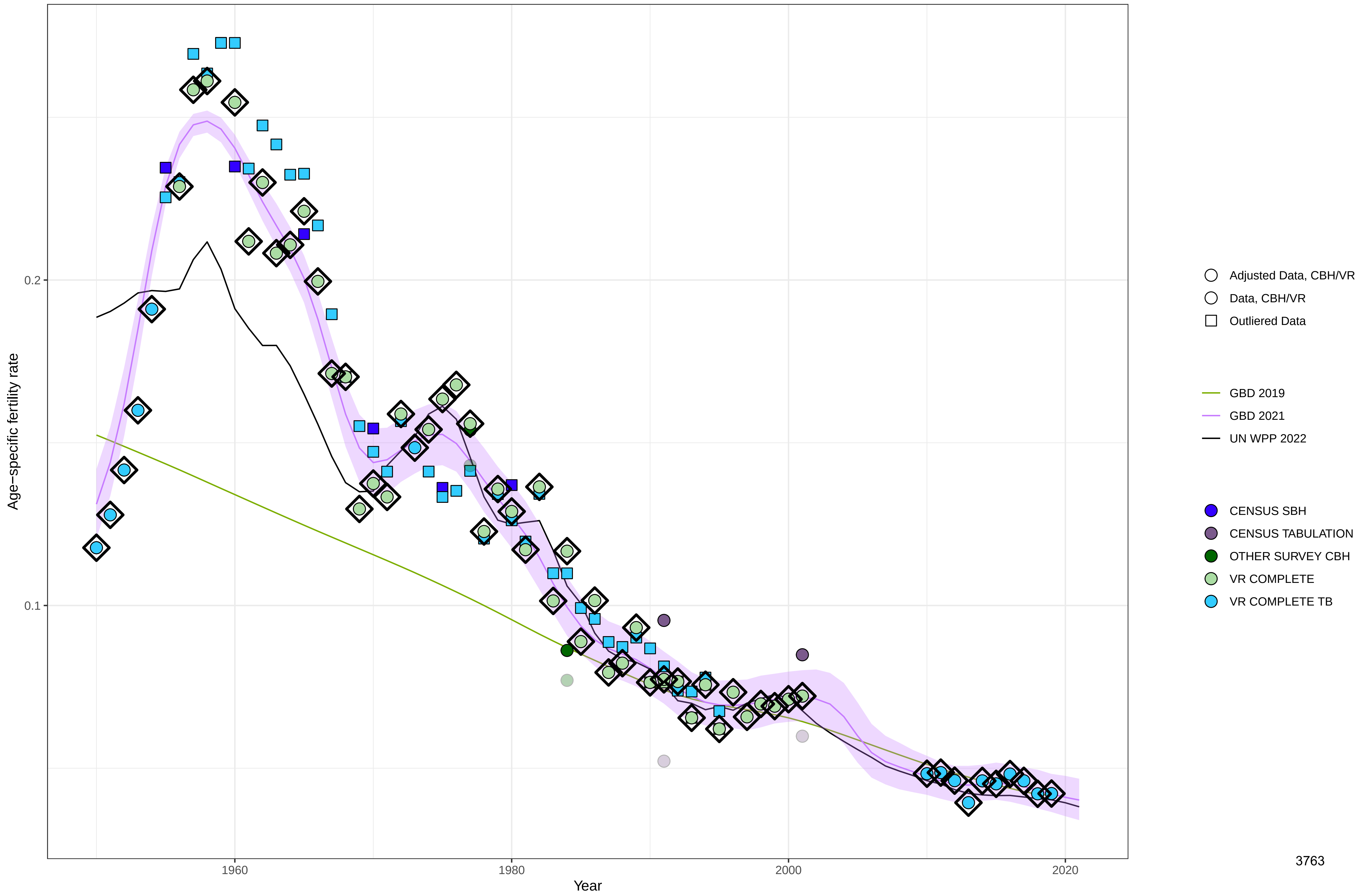

Age−specific fertility fitted trend lines, 1950−2021: Saint Kitts and Nevis, Ages: 20−24
Data points enclosed with a diamond are reference sources

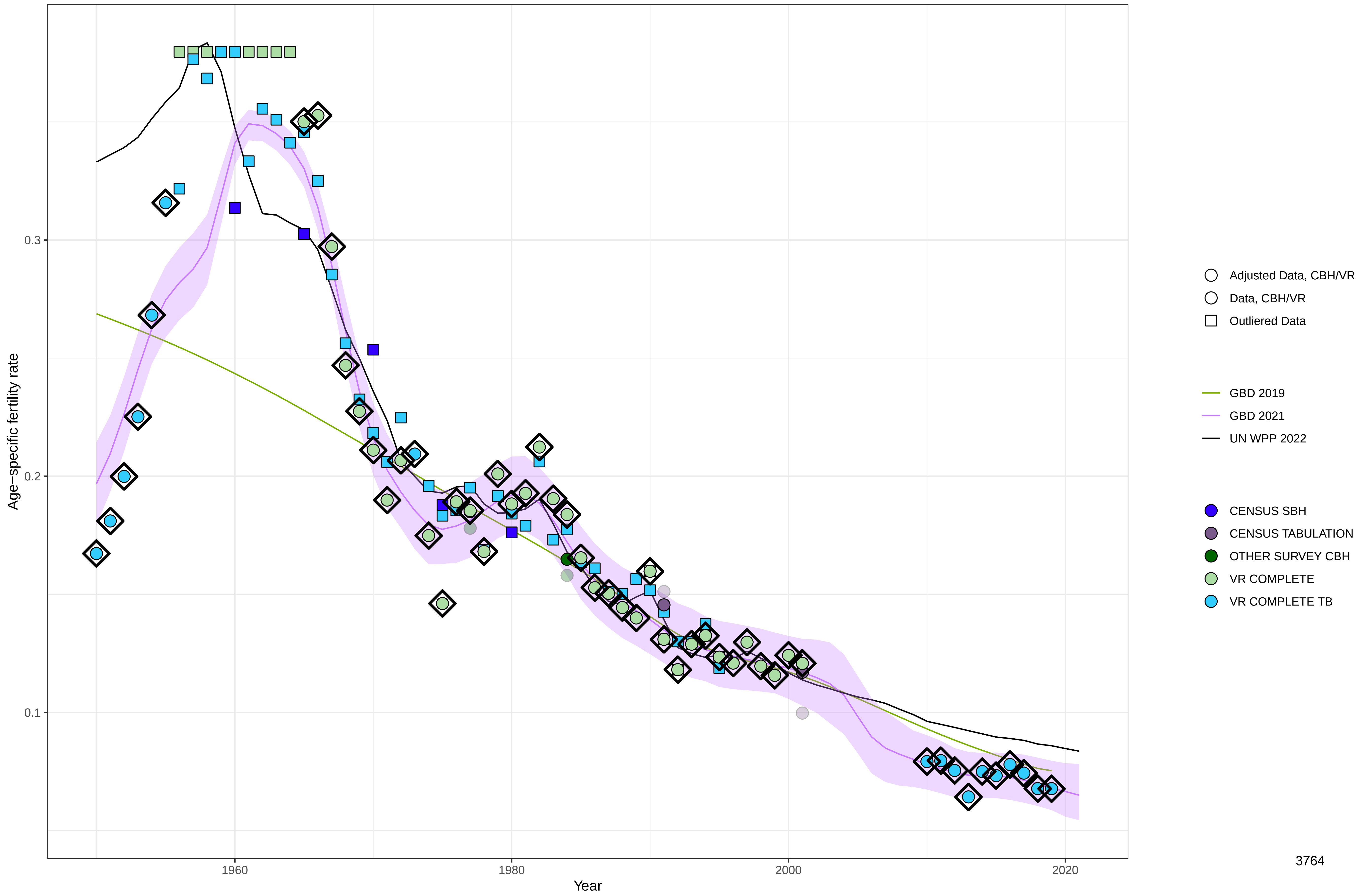

Age−specific fertility fitted trend lines, 1950−2021: Saint Kitts and Nevis, Ages: 25−29
Data points enclosed with a diamond are reference sources

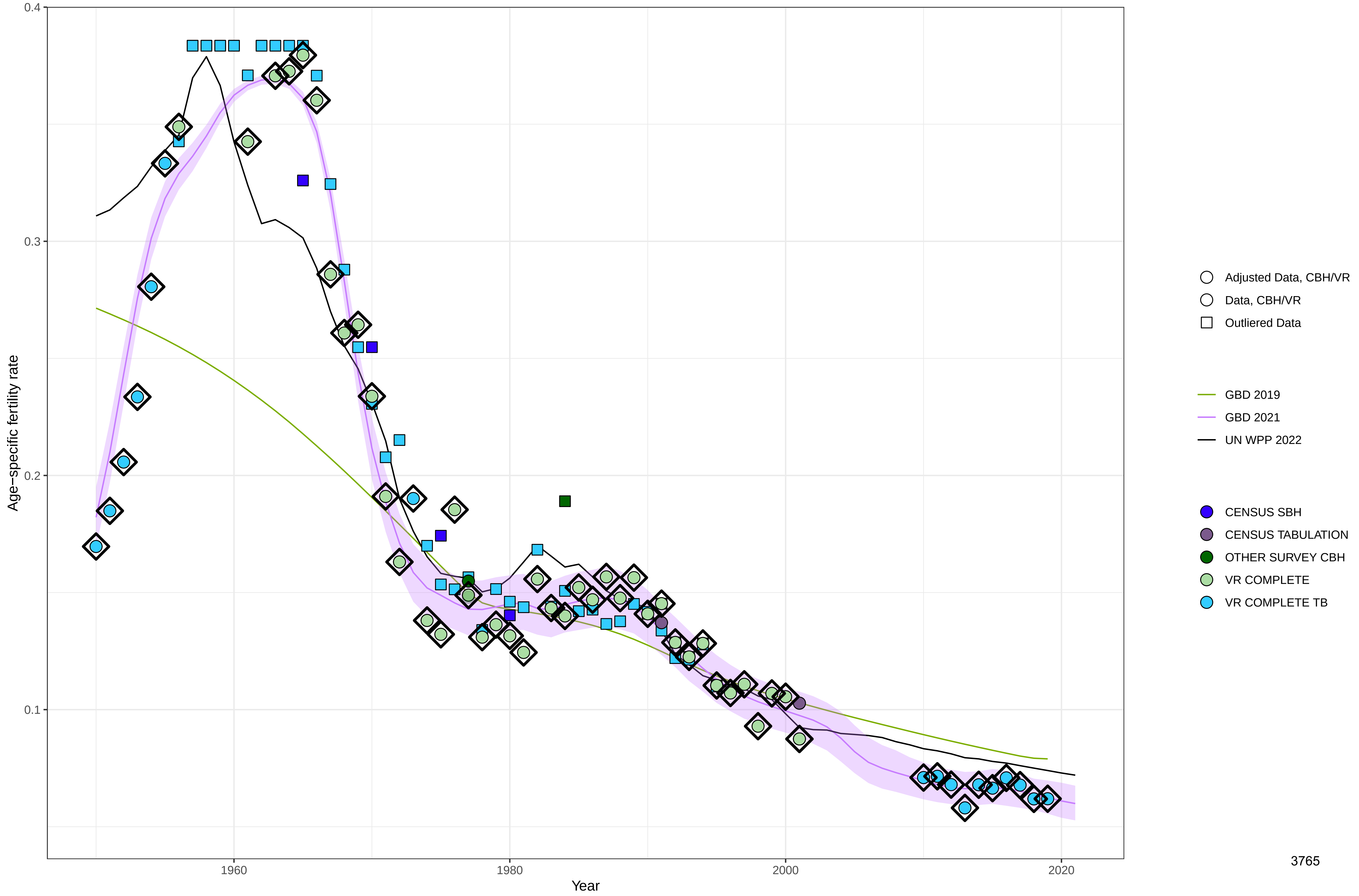

Age−specific fertility fitted trend lines, 1950−2021: Saint Kitts and Nevis, Ages: 30−34
Data points enclosed with a diamond are reference sources

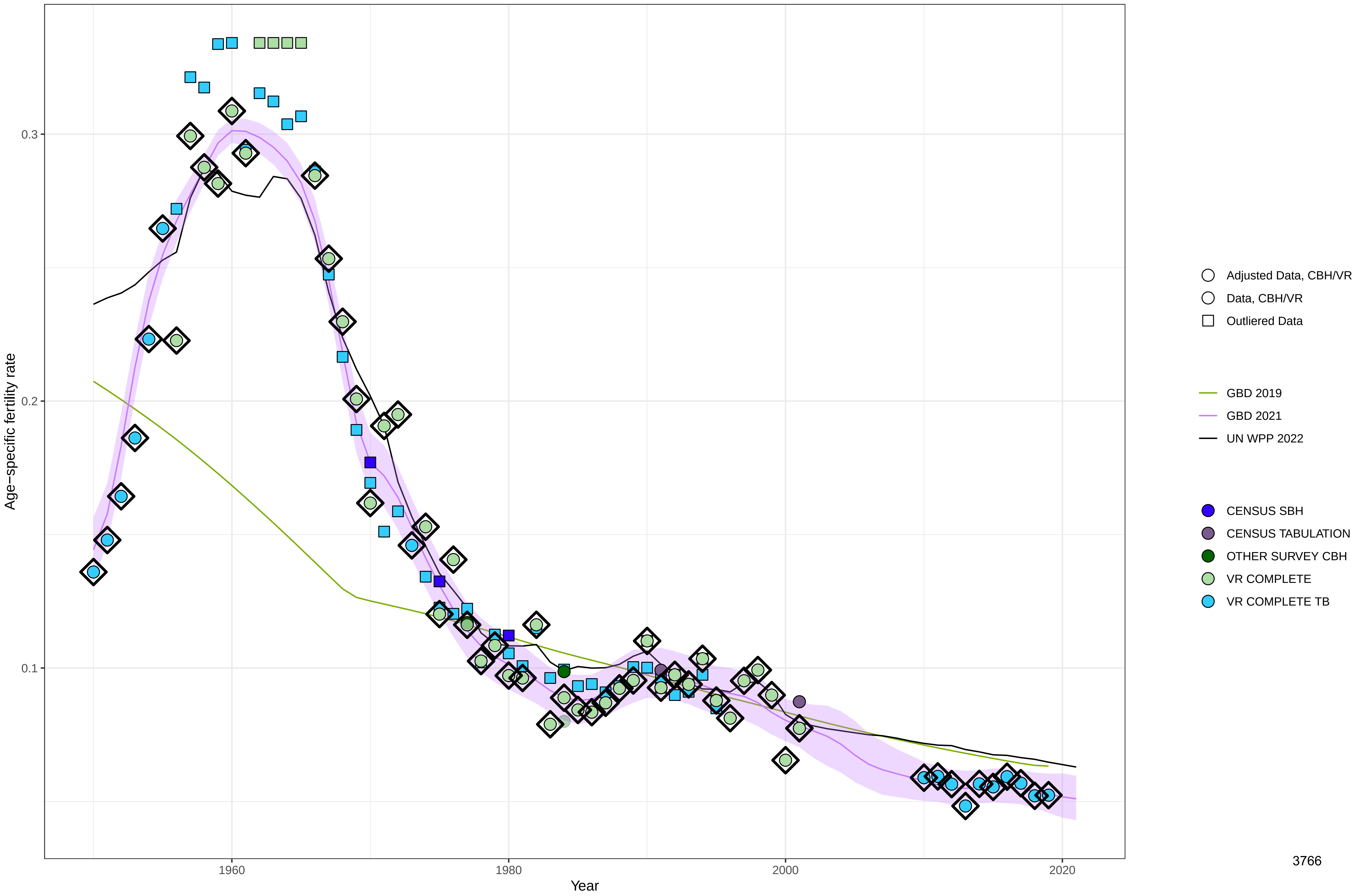

Age−specific fertility fitted trend lines, 1950−2021: Saint Kitts and Nevis, Ages: 35−39
Data points enclosed with a diamond are reference sources

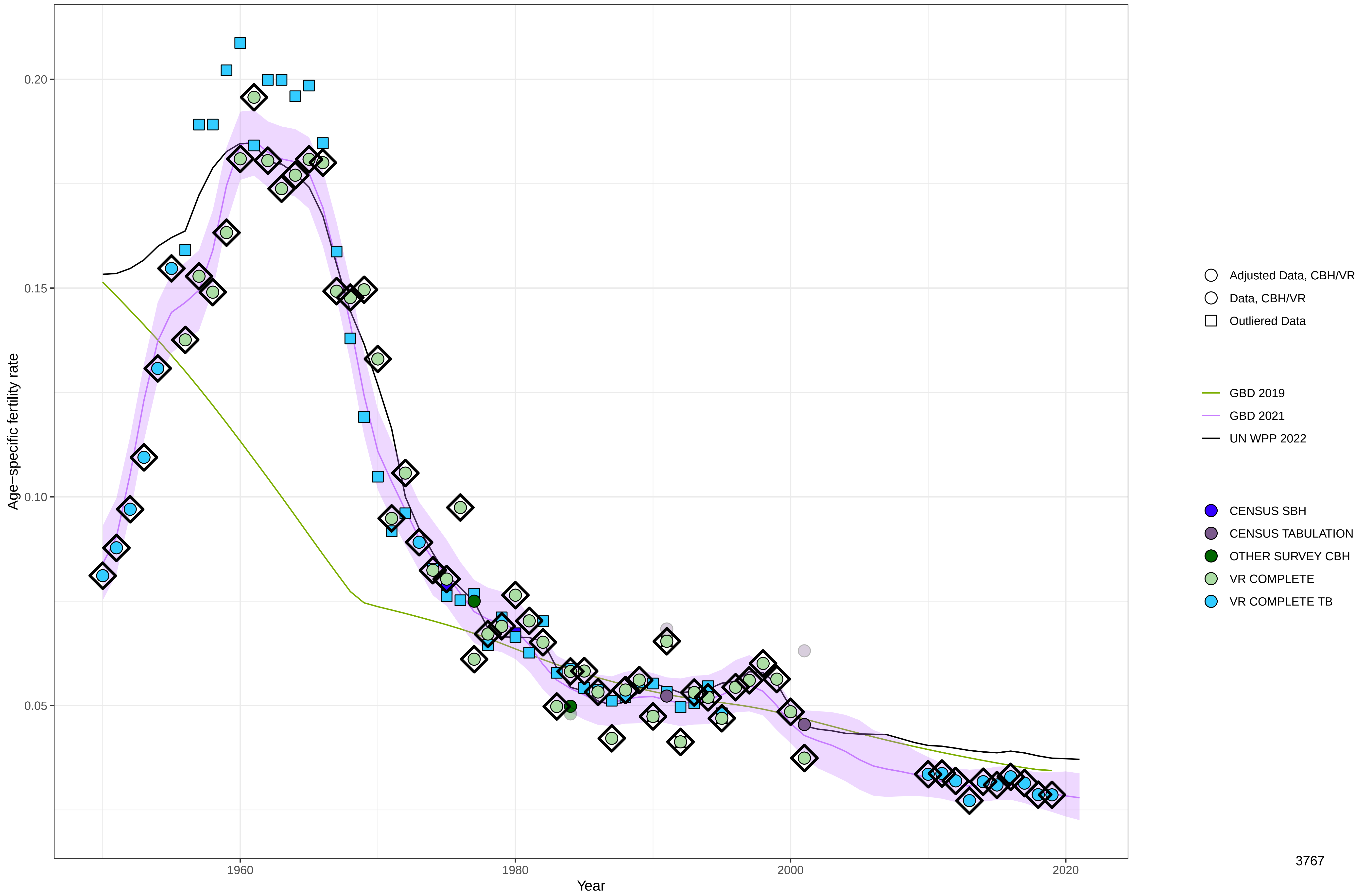

Age−specific fertility fitted trend lines, 1950−2021: Saint Kitts and Nevis, Ages: 40−44
Data points enclosed with a diamond are reference sources

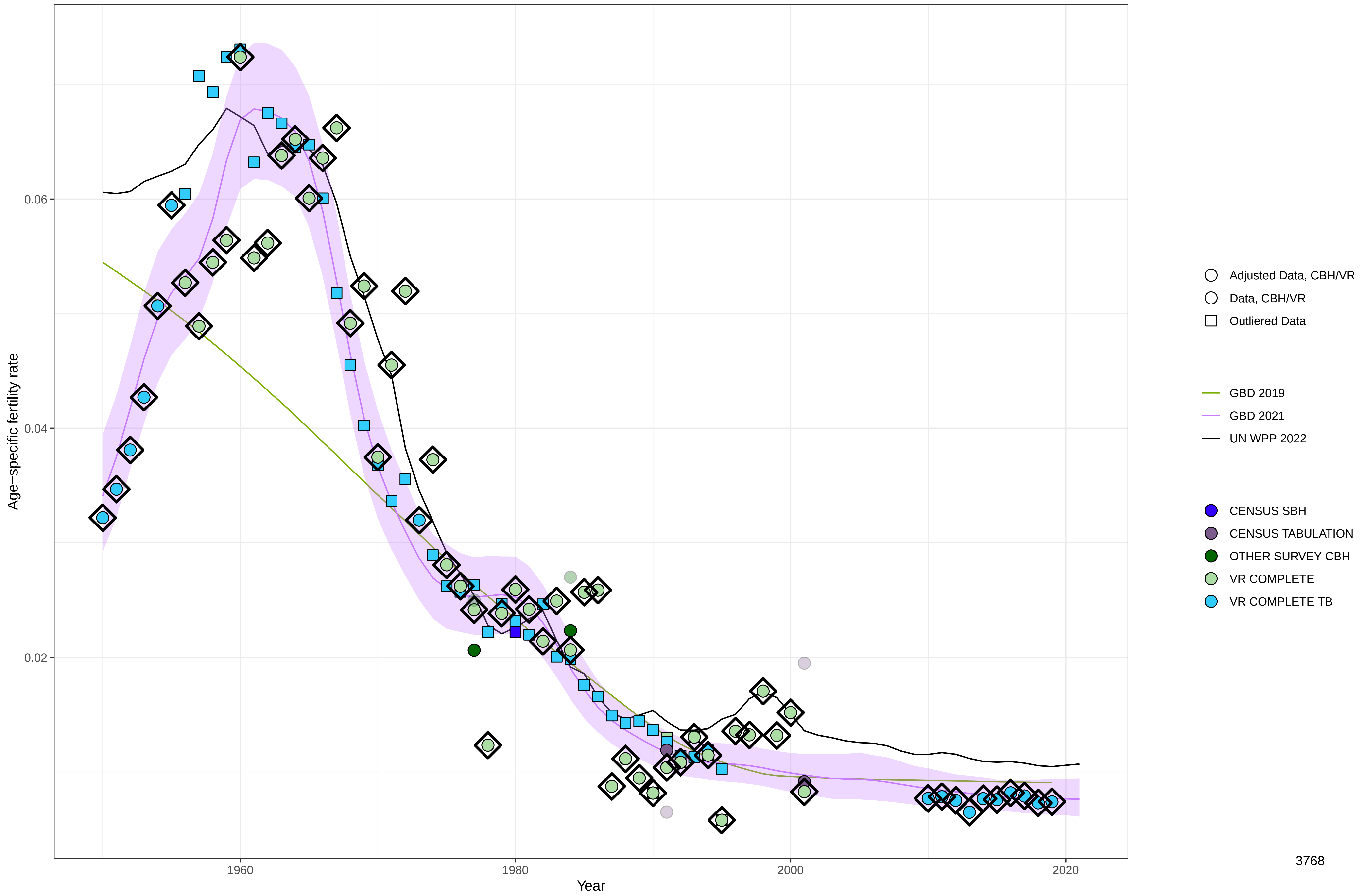

Age−specific fertility fitted trend lines, 1950−2021: Saint Kitts and Nevis, Ages: 45−49
Data points enclosed with a diamond are reference sources

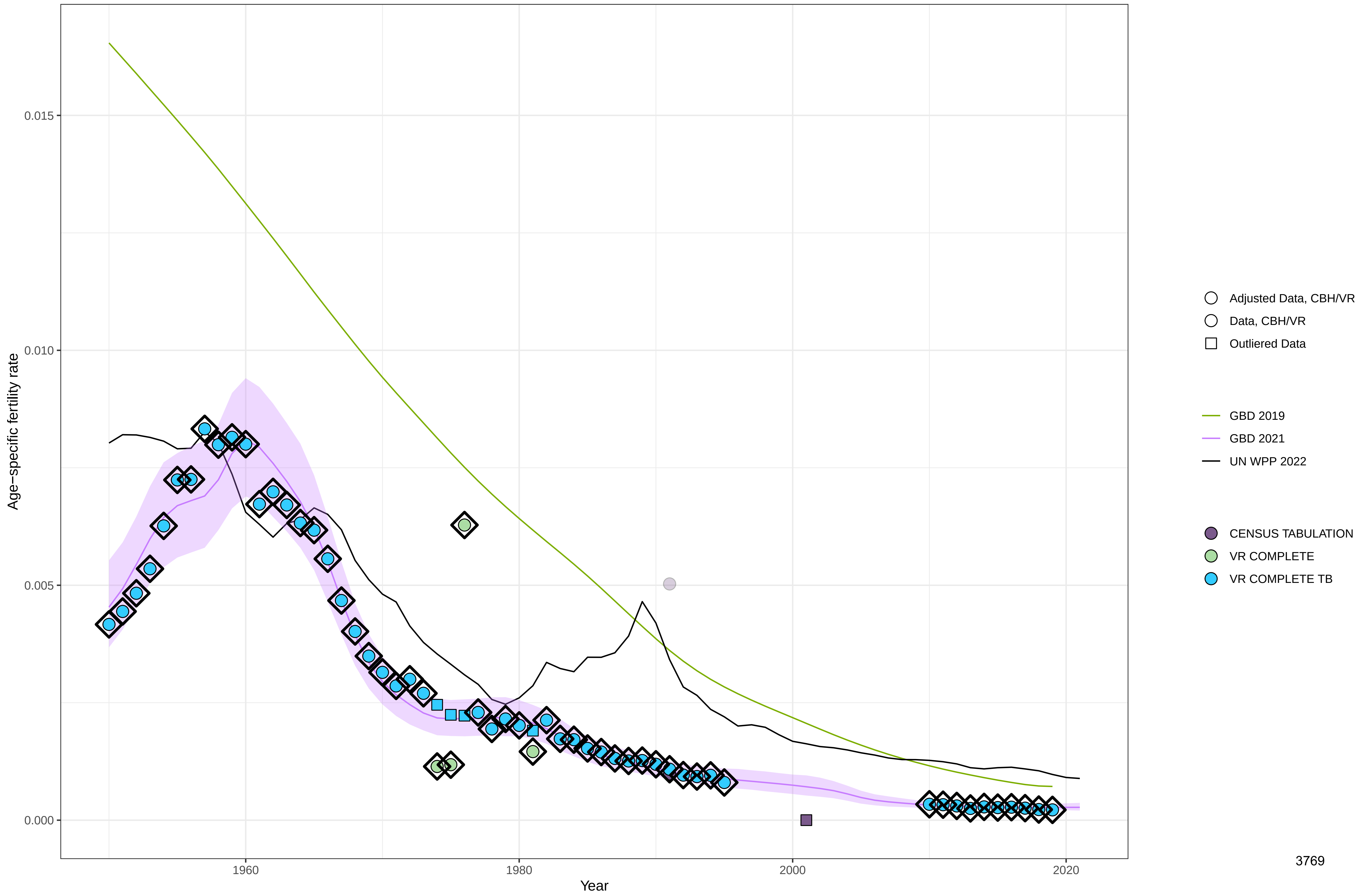

Age−specific fertility fitted trend lines, 1950−2021: Saint Kitts and Nevis, Ages: 50−54
Data points enclosed with a diamond are reference sources

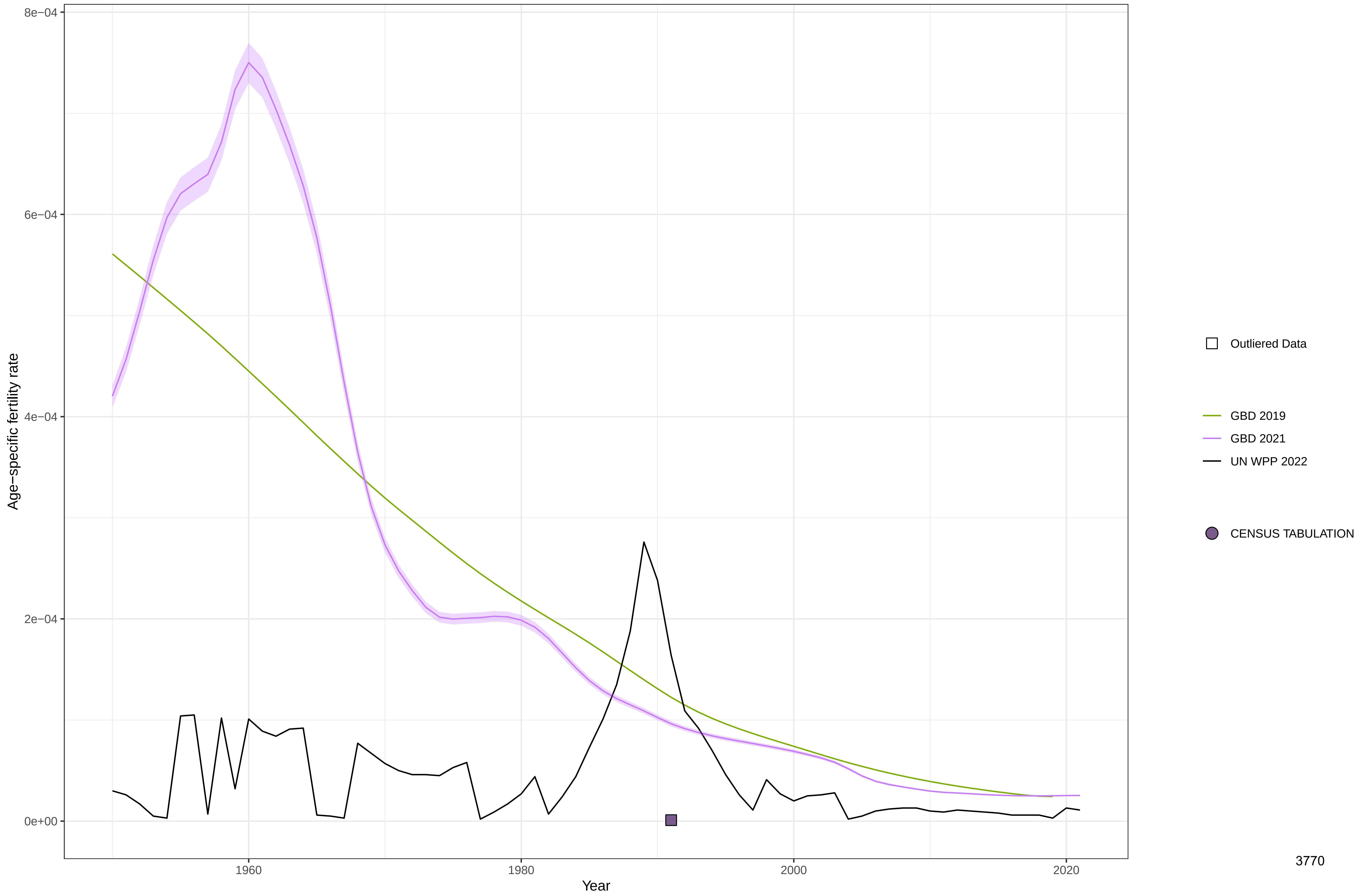

Total fertility rate fitted trend lines, 1950−2021: Saint Kitts and Nevis TFR
Data points enclosed with a diamond are reference sources

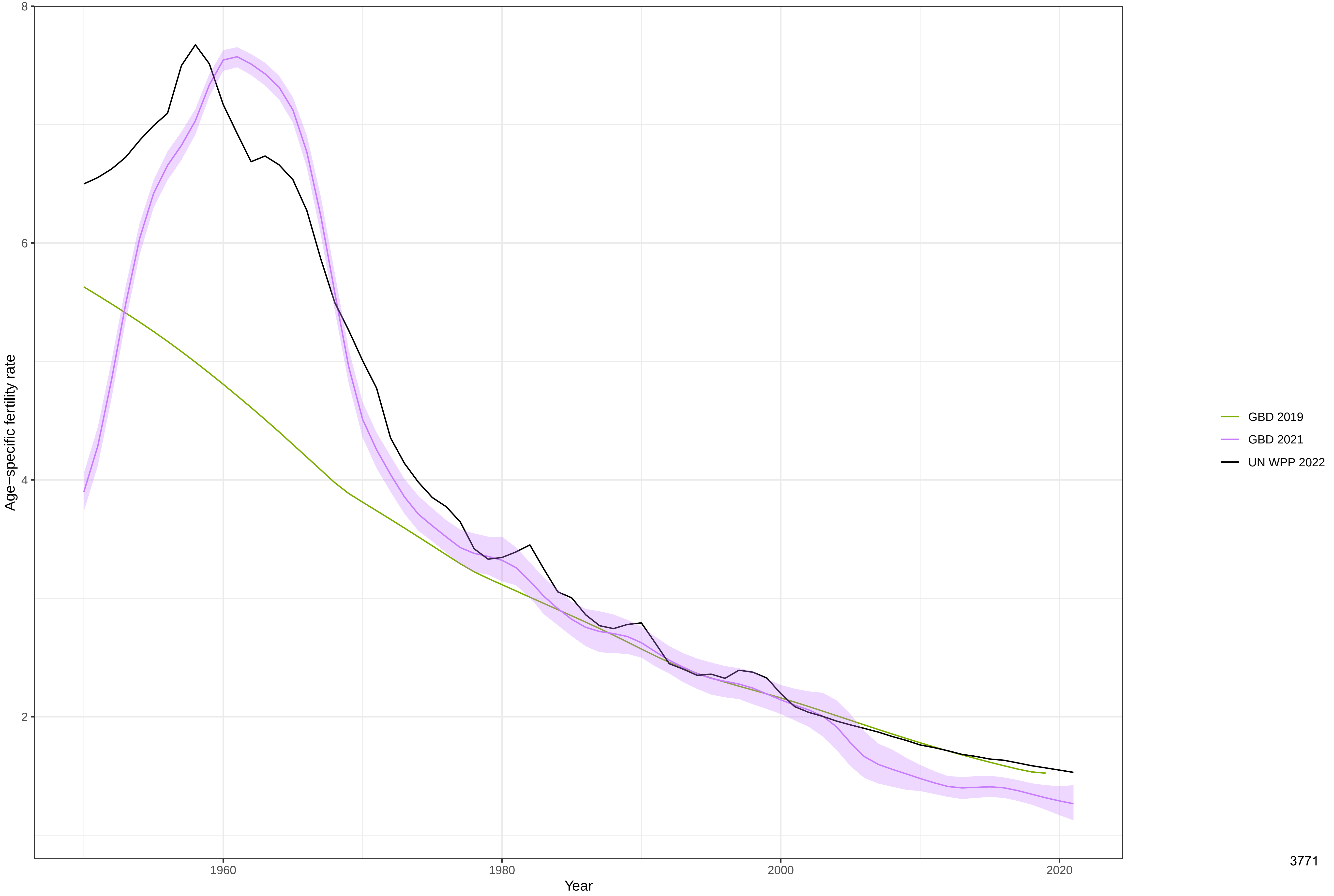

Age−specific fertility fitted trend lines, 1950−2021: Saint Lucia, Ages: 10−14
Data points enclosed with a diamond are reference sources

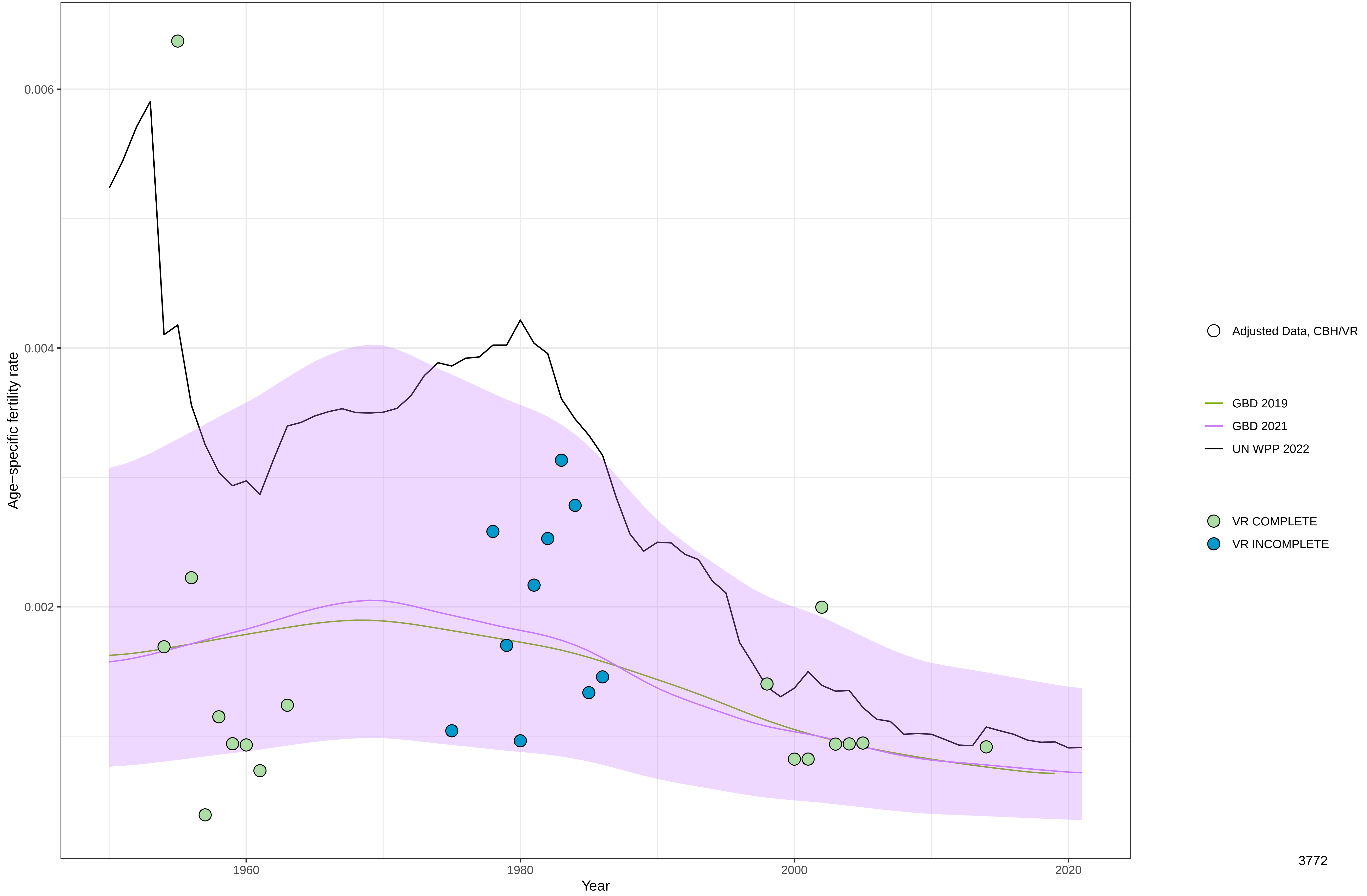

Age−specific fertility fitted trend lines, 1950−2021: Saint Lucia, Ages: 15−19
Data points enclosed with a diamond are reference sources

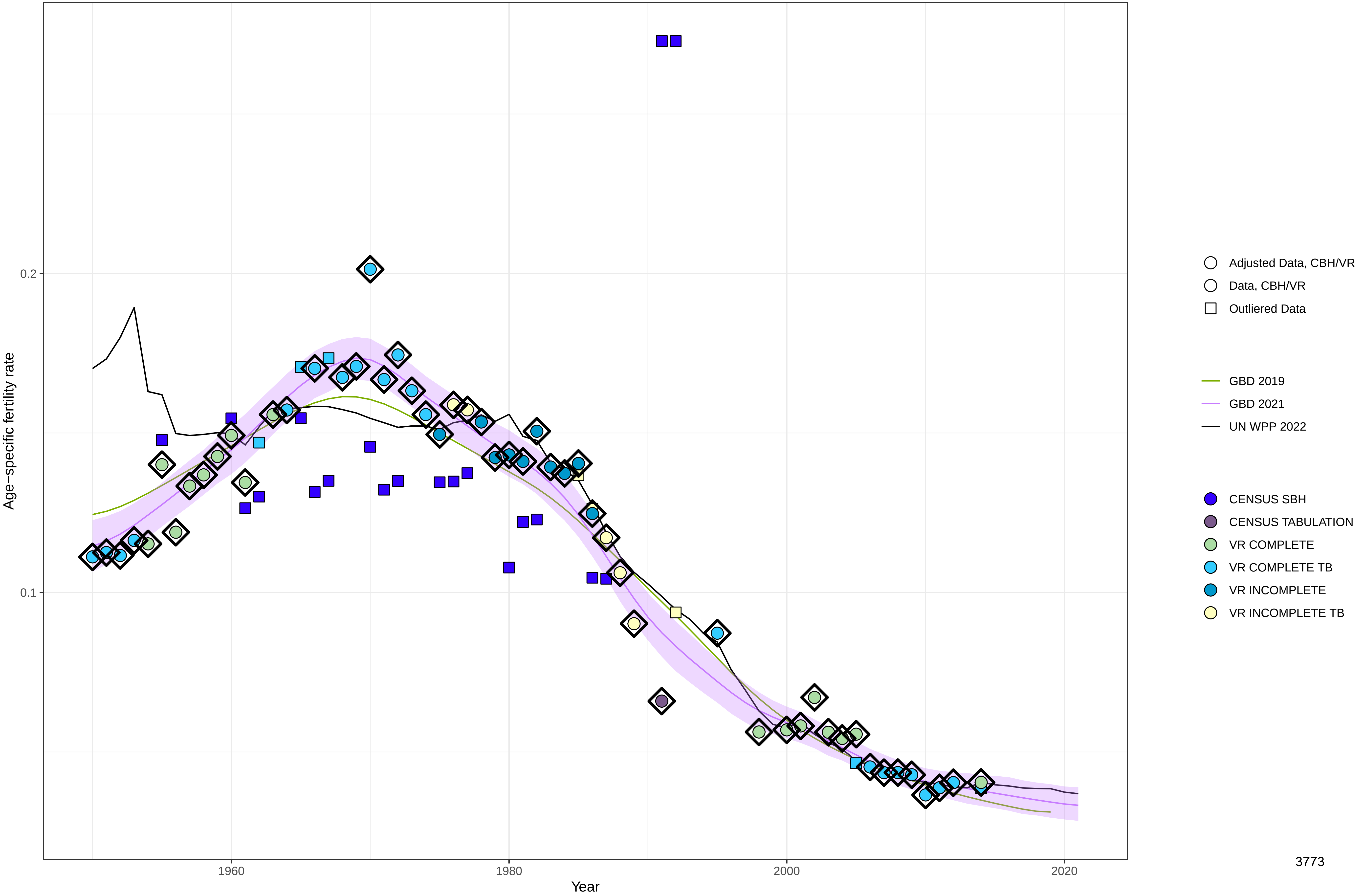

Age−specific fertility fitted trend lines, 1950−2021: Saint Lucia, Ages: 20−24
Data points enclosed with a diamond are reference sources

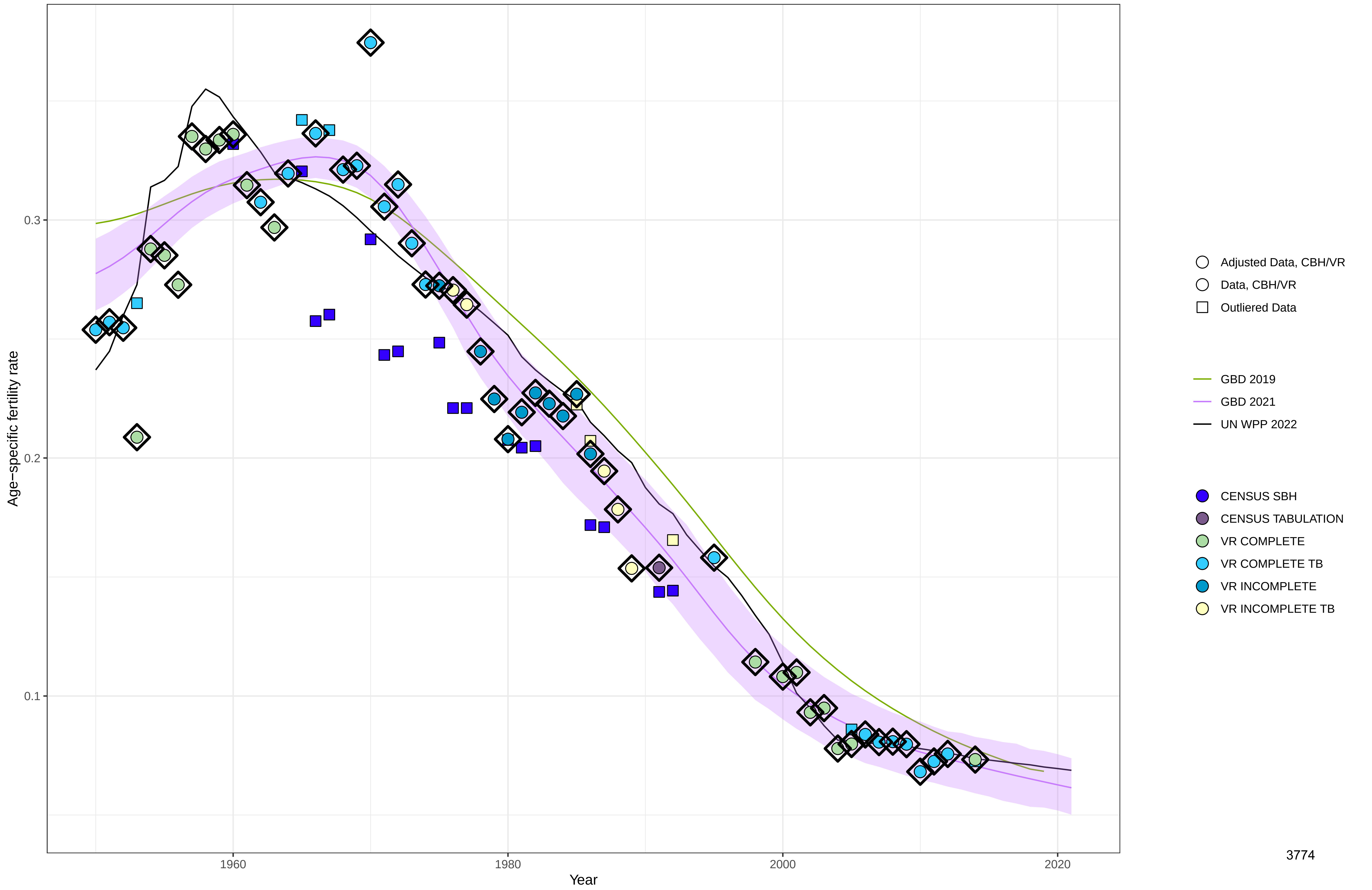

Age−specific fertility fitted trend lines, 1950−2021: Saint Lucia, Ages: 25−29
Data points enclosed with a diamond are reference sources

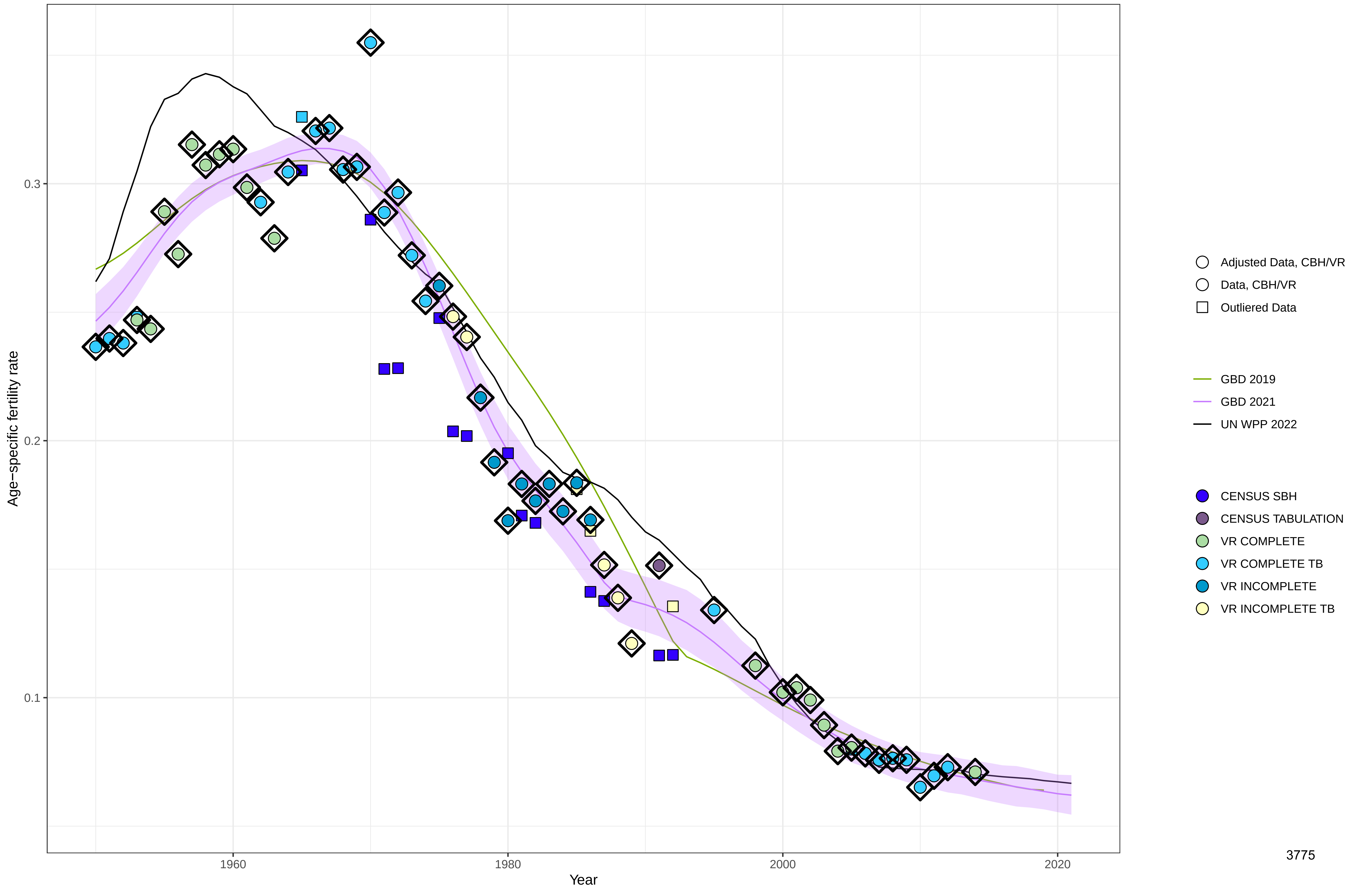

Age−specific fertility fitted trend lines, 1950−2021: Saint Lucia, Ages: 30−34
Data points enclosed with a diamond are reference sources

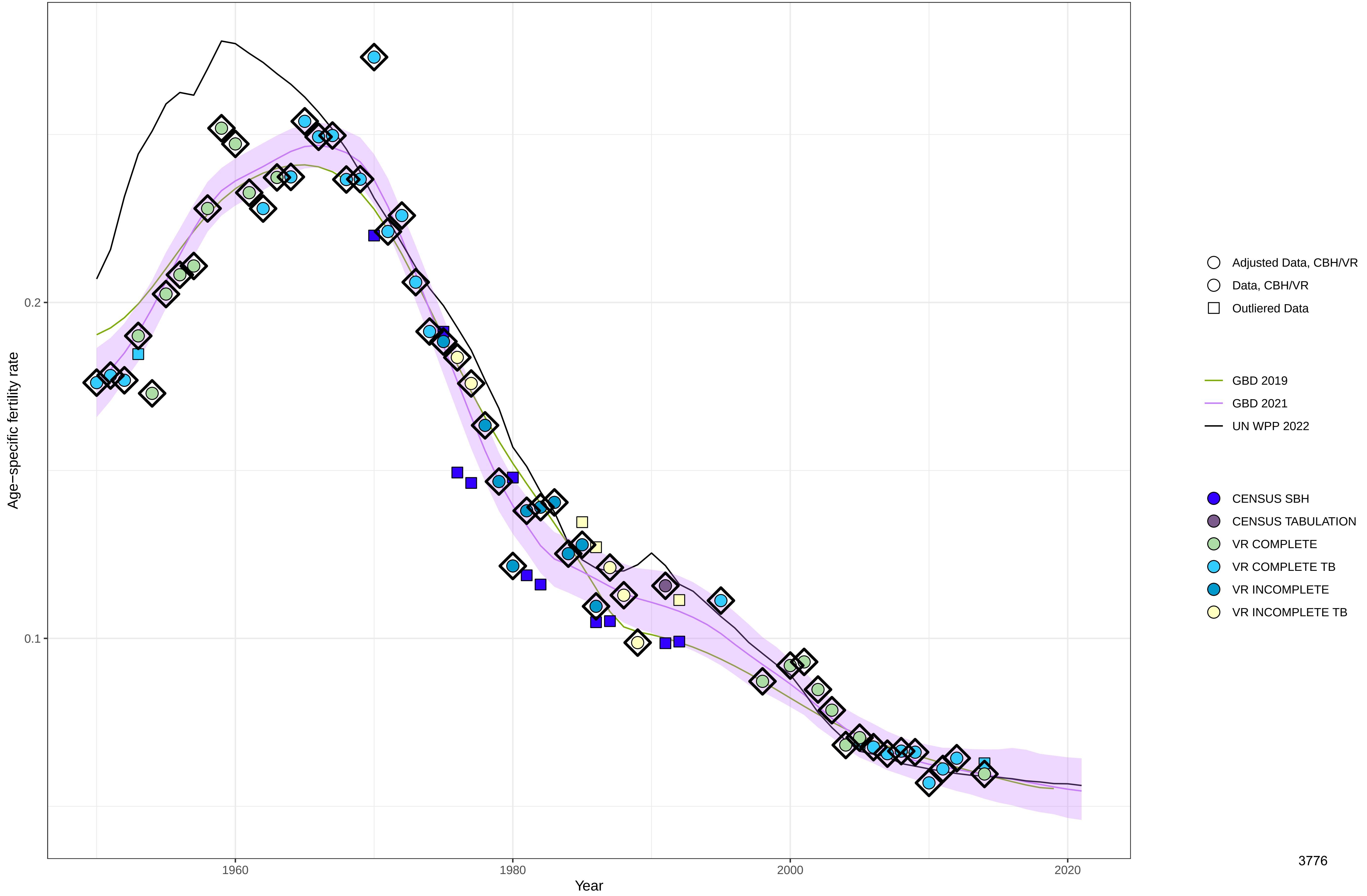

Age−specific fertility fitted trend lines, 1950−2021: Saint Lucia, Ages: 35−39
Data points enclosed with a diamond are reference sources

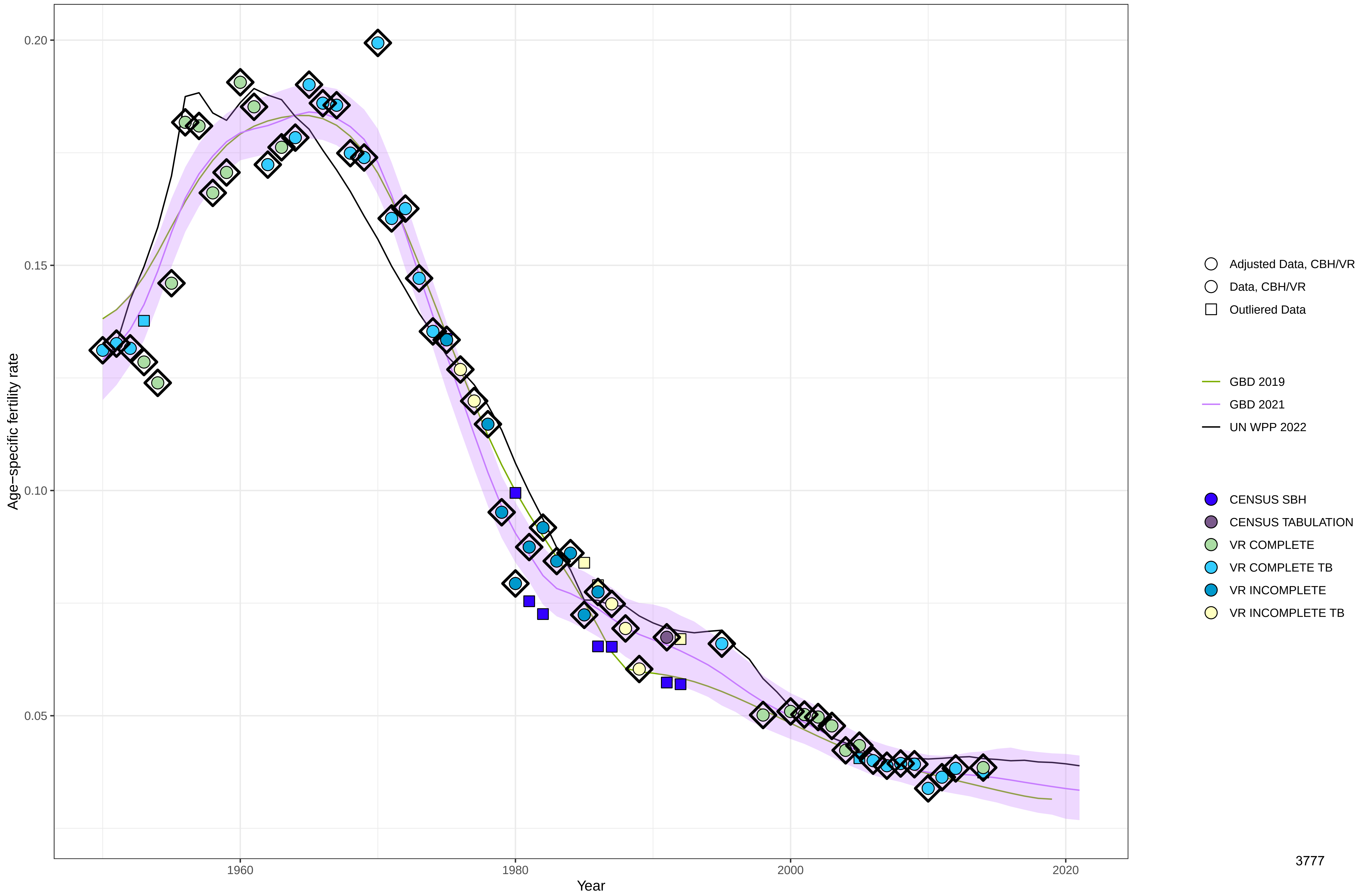

Age−specific fertility fitted trend lines, 1950−2021: Saint Lucia, Ages: 40−44
Data points enclosed with a diamond are reference sources

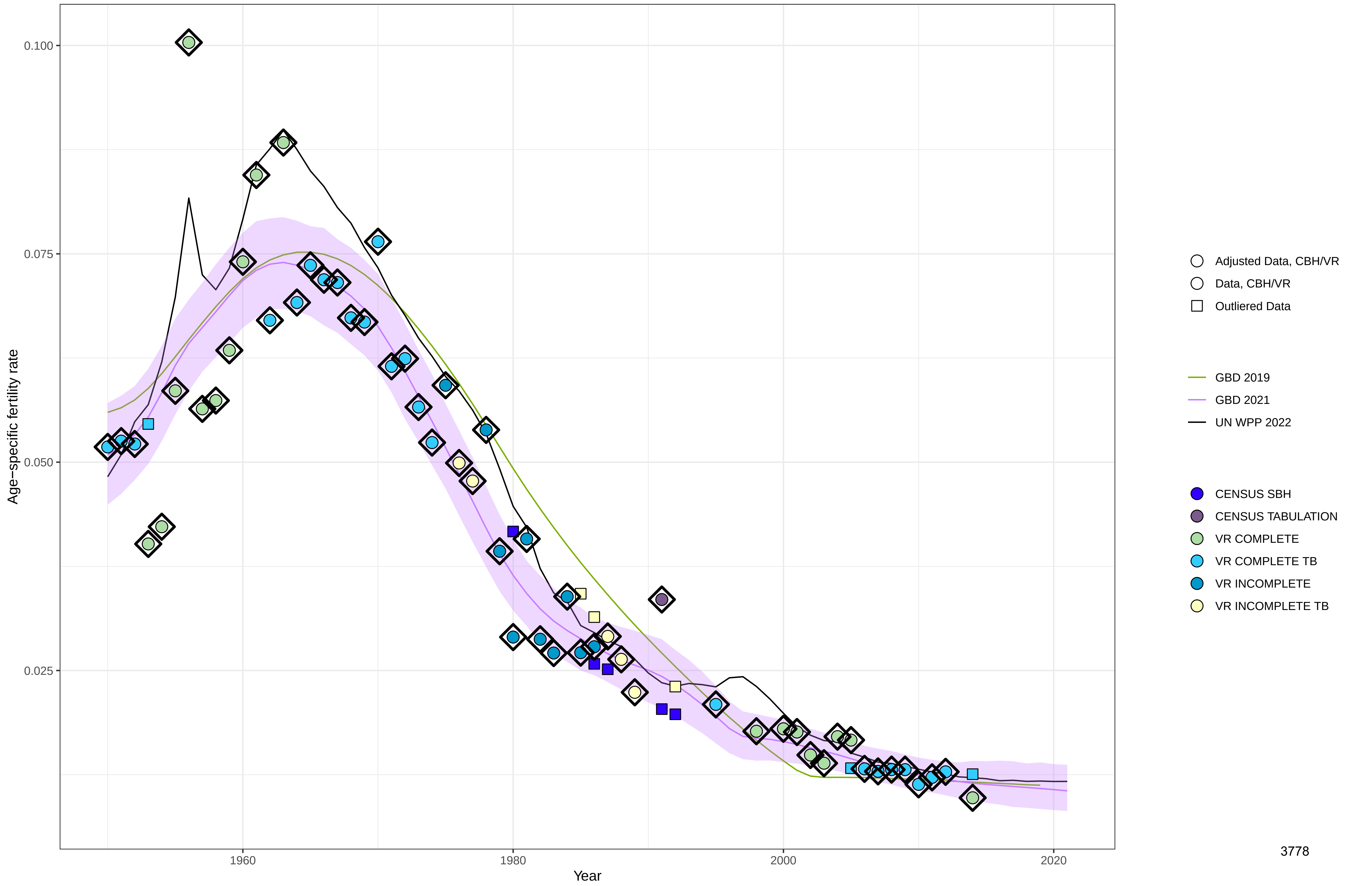

Age−specific fertility fitted trend lines, 1950−2021: Saint Lucia, Ages: 45−49
Data points enclosed with a diamond are reference sources

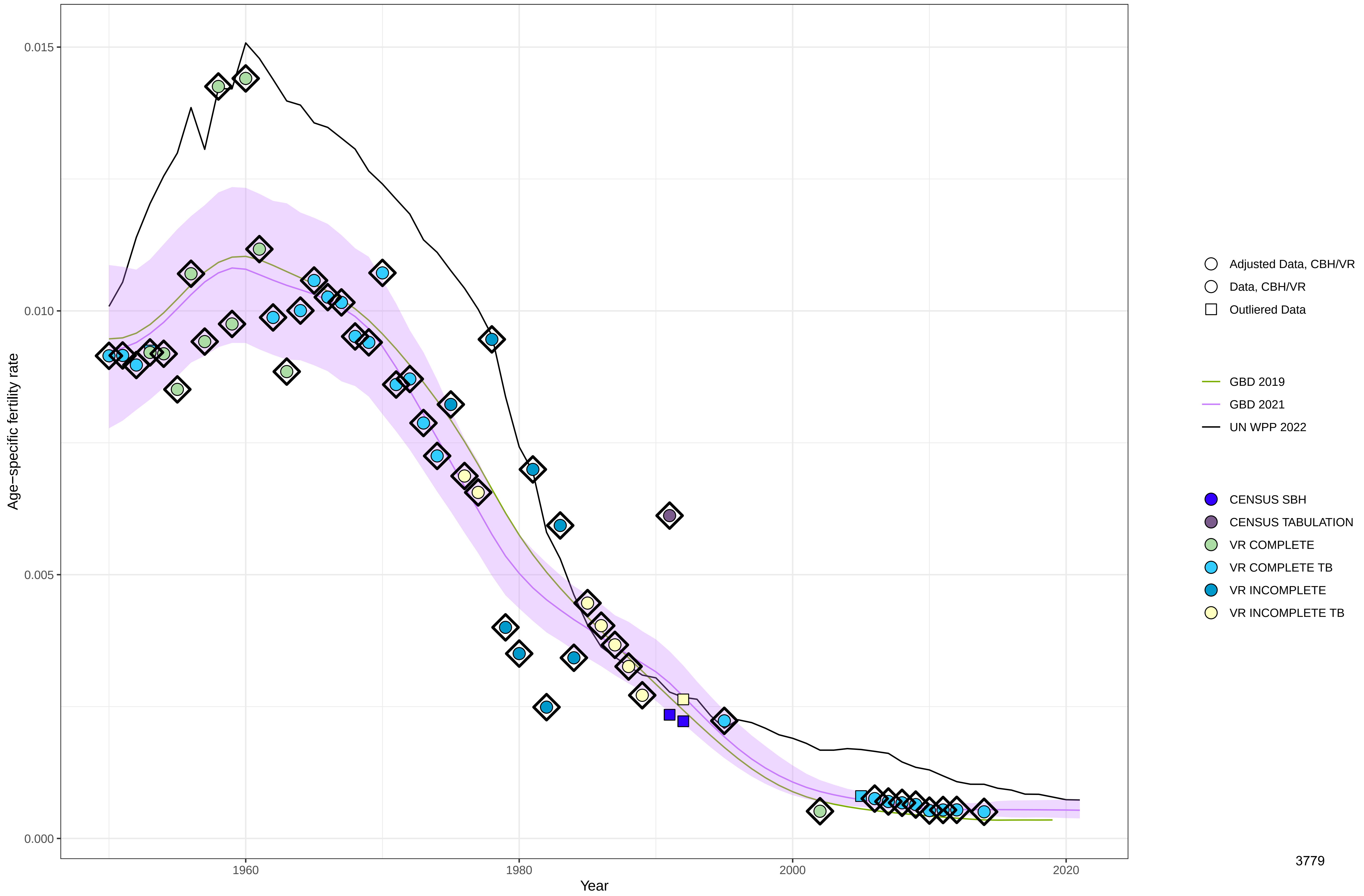

Age−specific fertility fitted trend lines, 1950−2021: Saint Lucia, Ages: 50−54
Data points enclosed with a diamond are reference sources

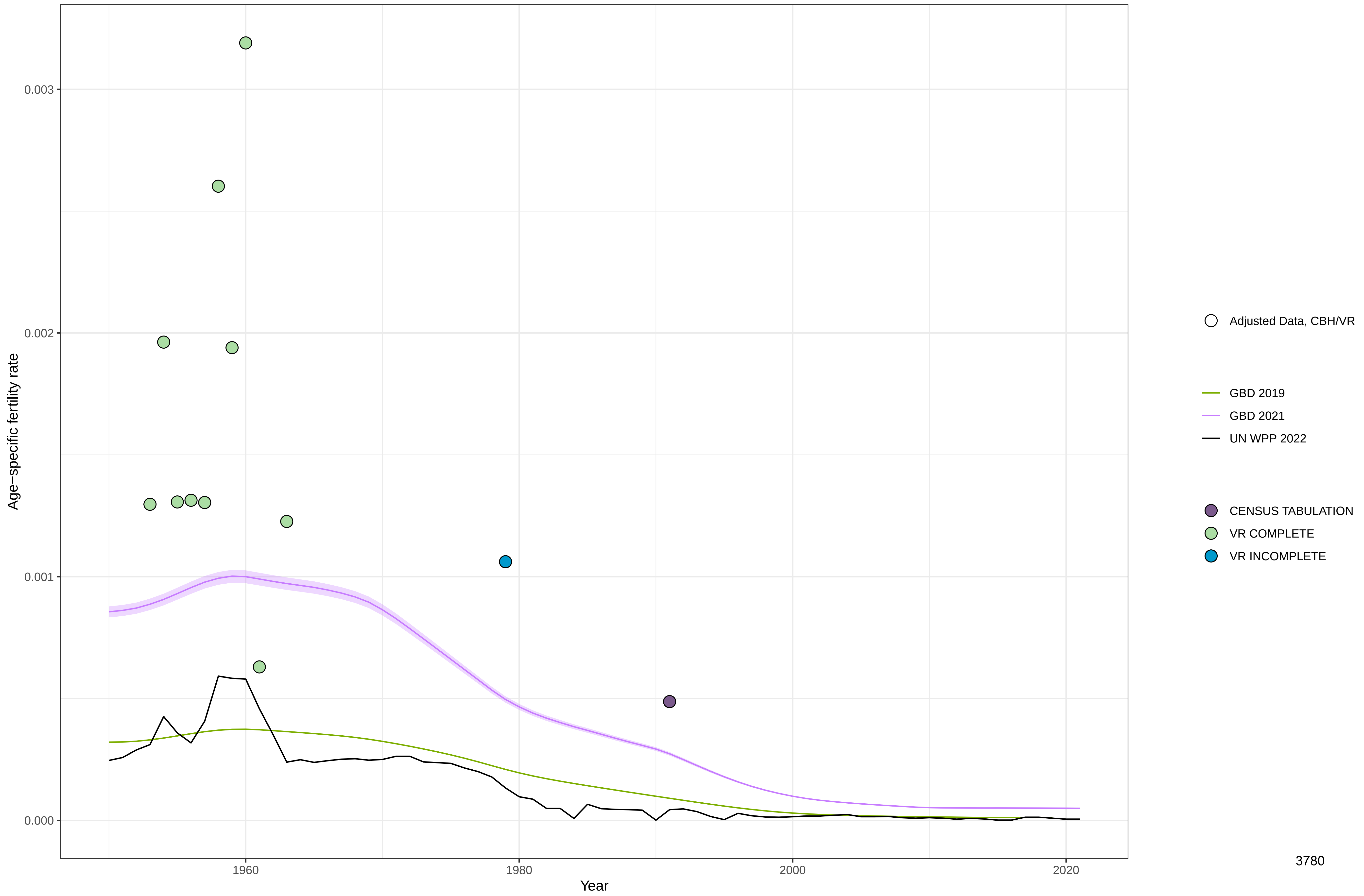

Total fertility rate fitted trend lines, 1950−2021: Saint Lucia TFR
Data points enclosed with a diamond are reference sources

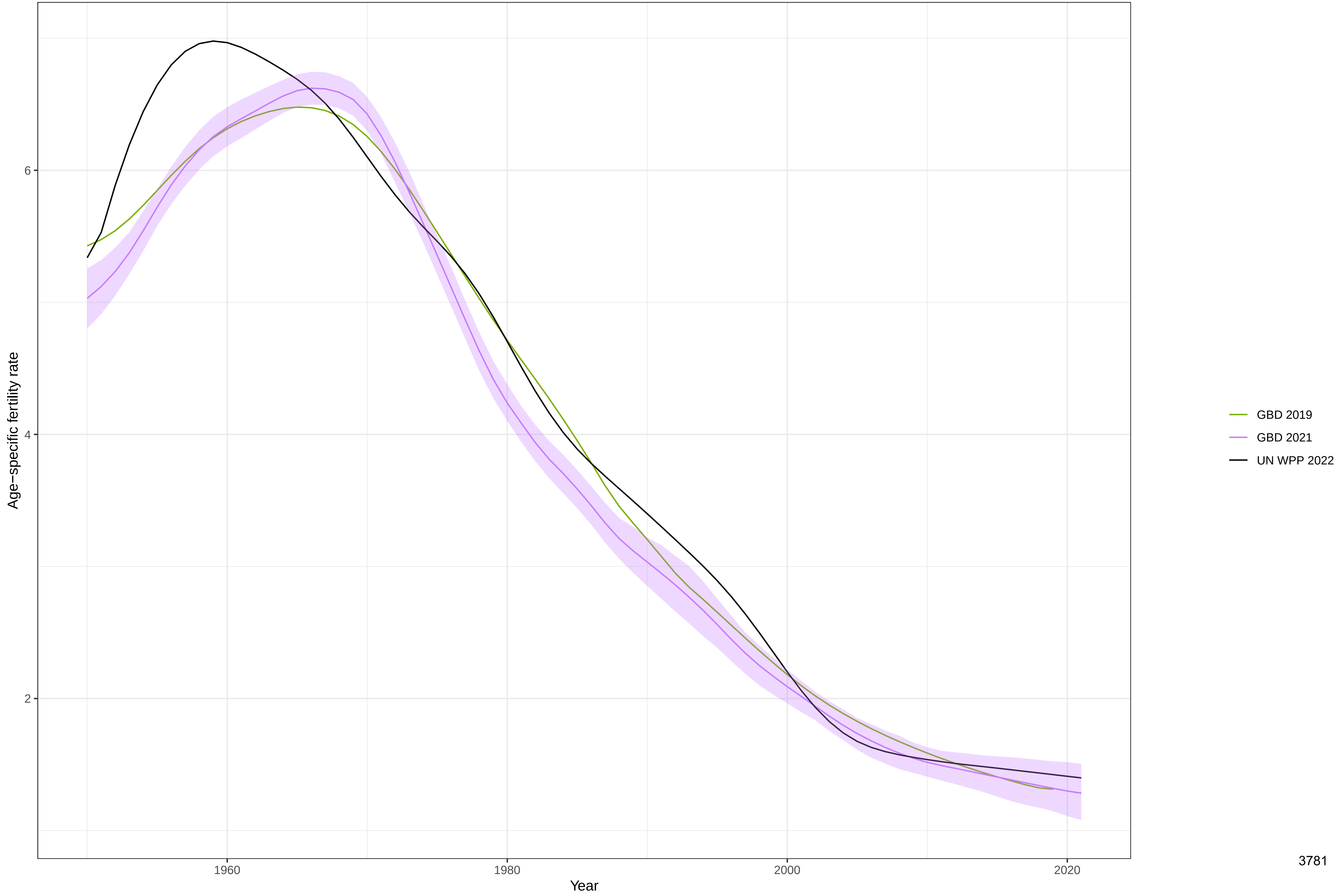

Age−specific fertility fitted trend lines, 1950−2021: Saint Vincent and the Grenadines, Ages: 10−14
Data points enclosed with a diamond are reference sources

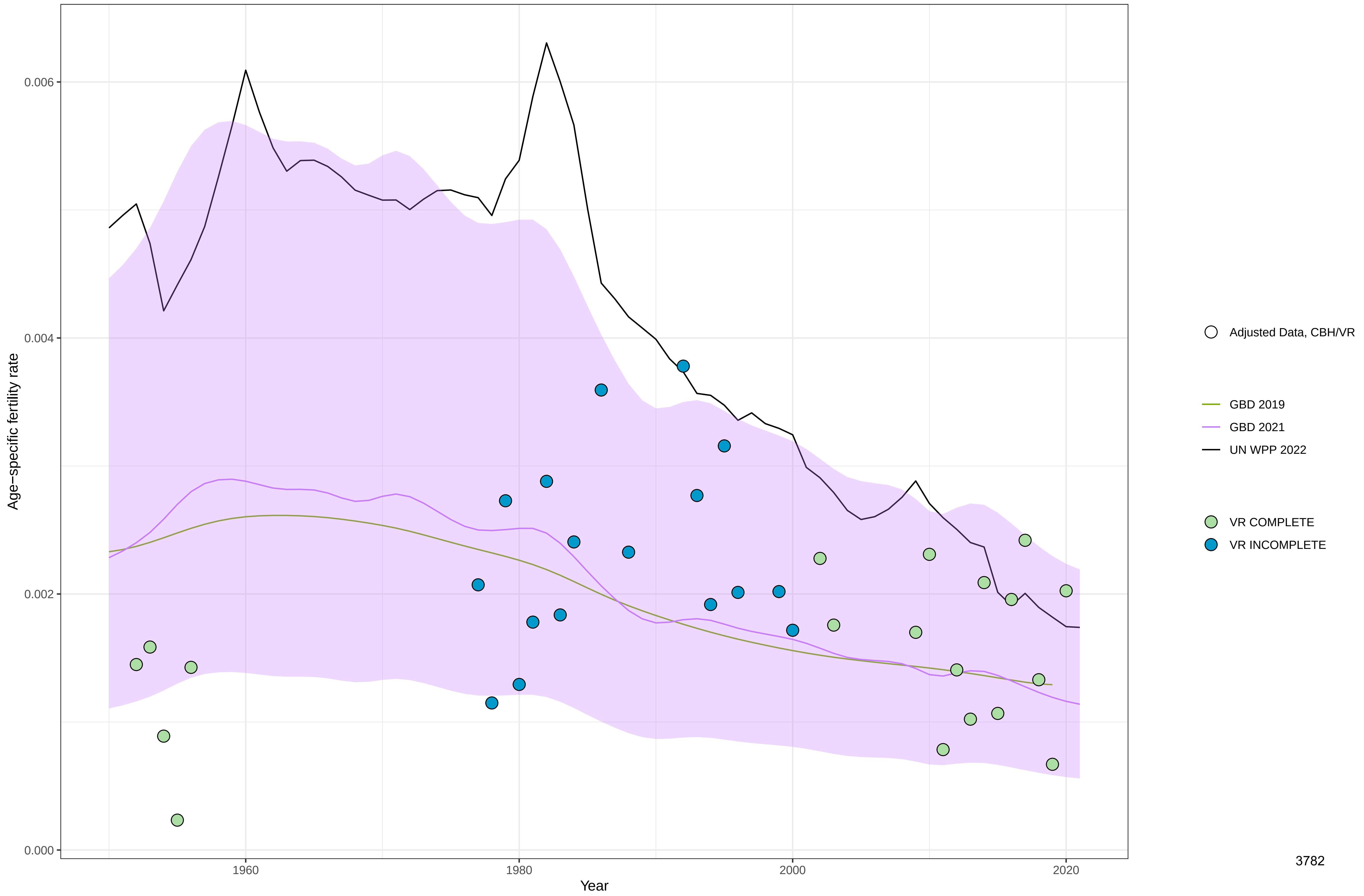

Age−specific fertility fitted trend lines, 1950−2021: Saint Vincent and the Grenadines, Ages: 15−19
Data points enclosed with a diamond are reference sources

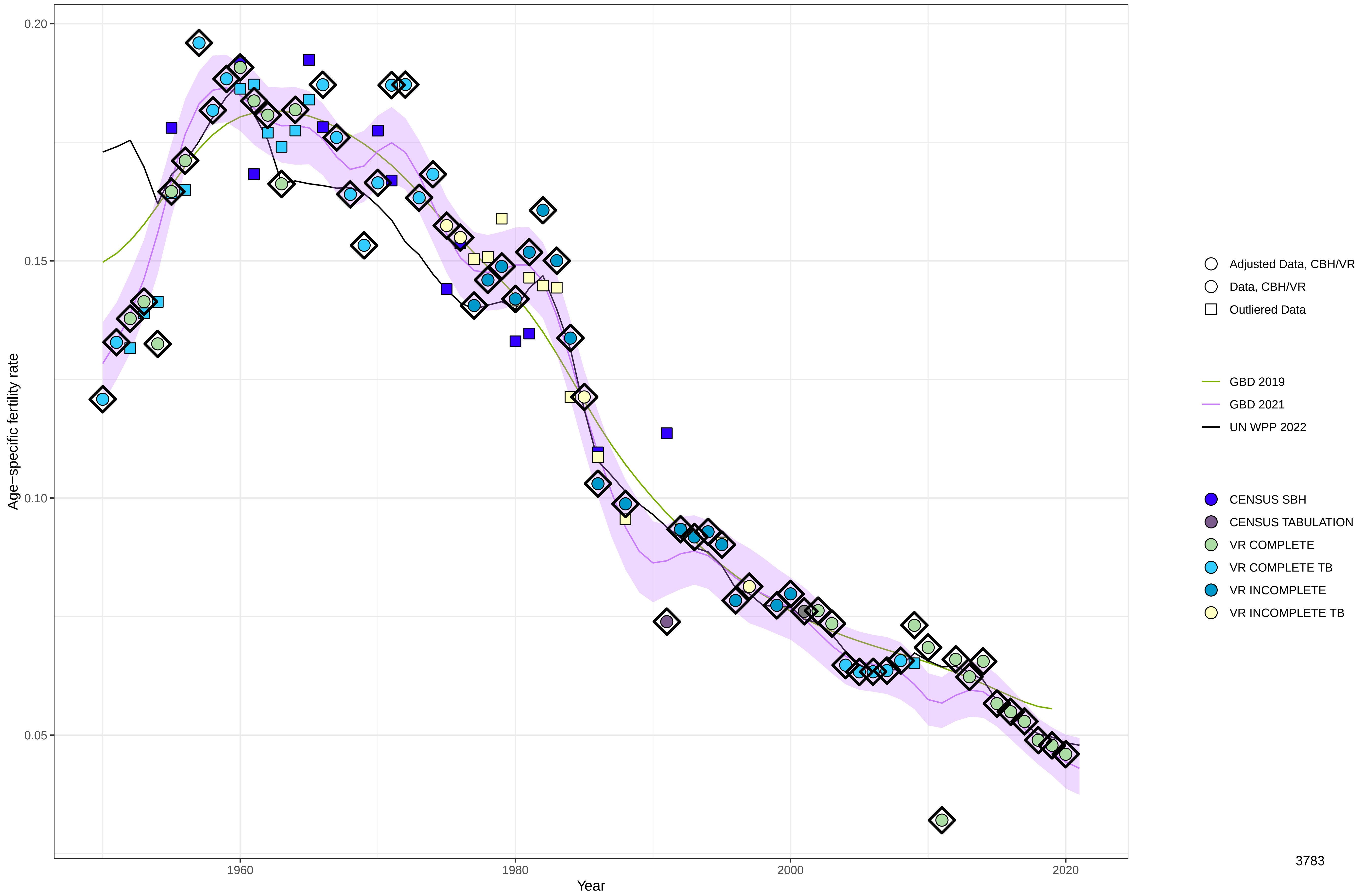

Age−specific fertility fitted trend lines, 1950−2021: Saint Vincent and the Grenadines, Ages: 20−24
Data points enclosed with a diamond are reference sources

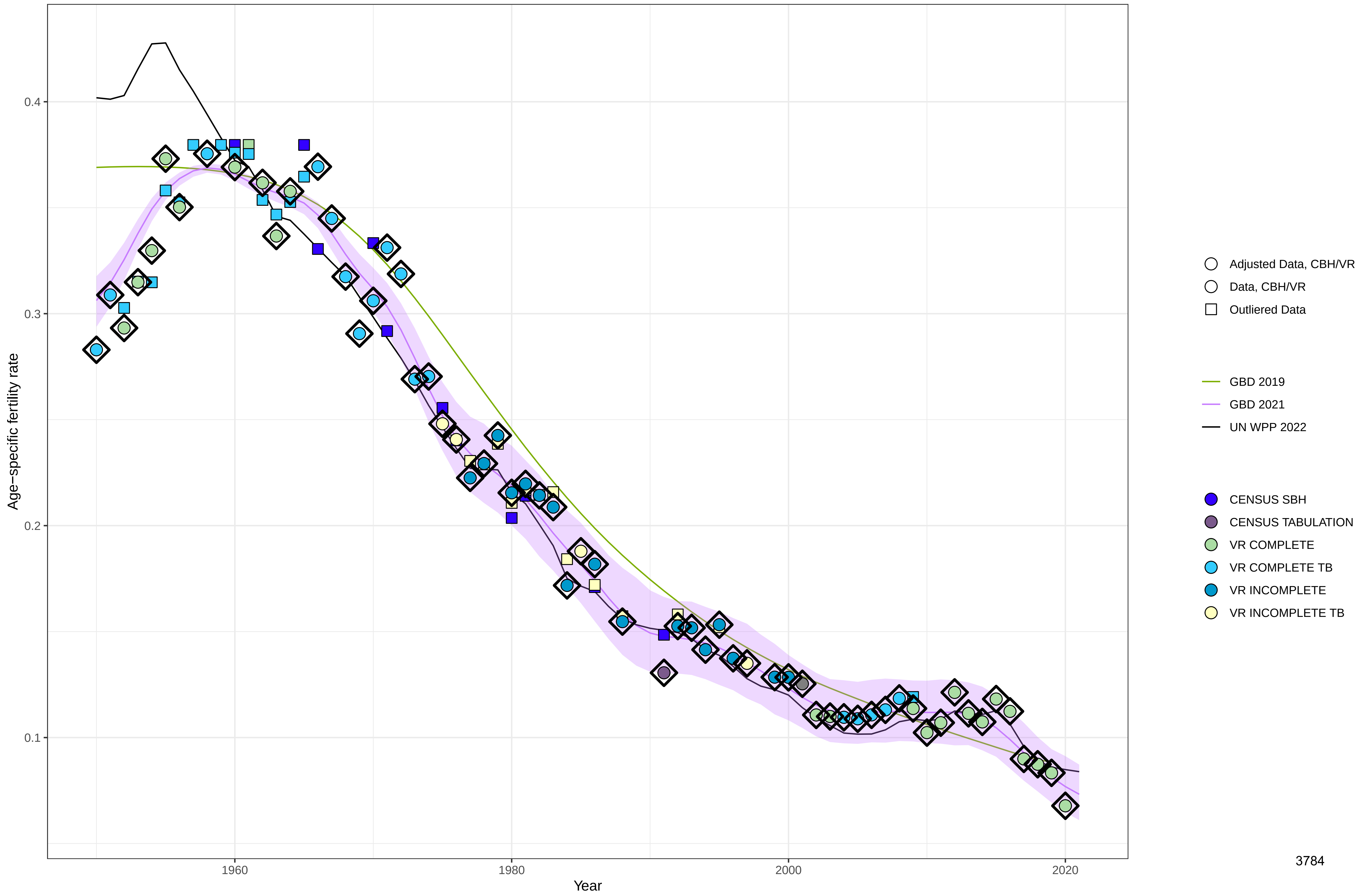

Age−specific fertility fitted trend lines, 1950−2021: Saint Vincent and the Grenadines, Ages: 25−29
Data points enclosed with a diamond are reference sources

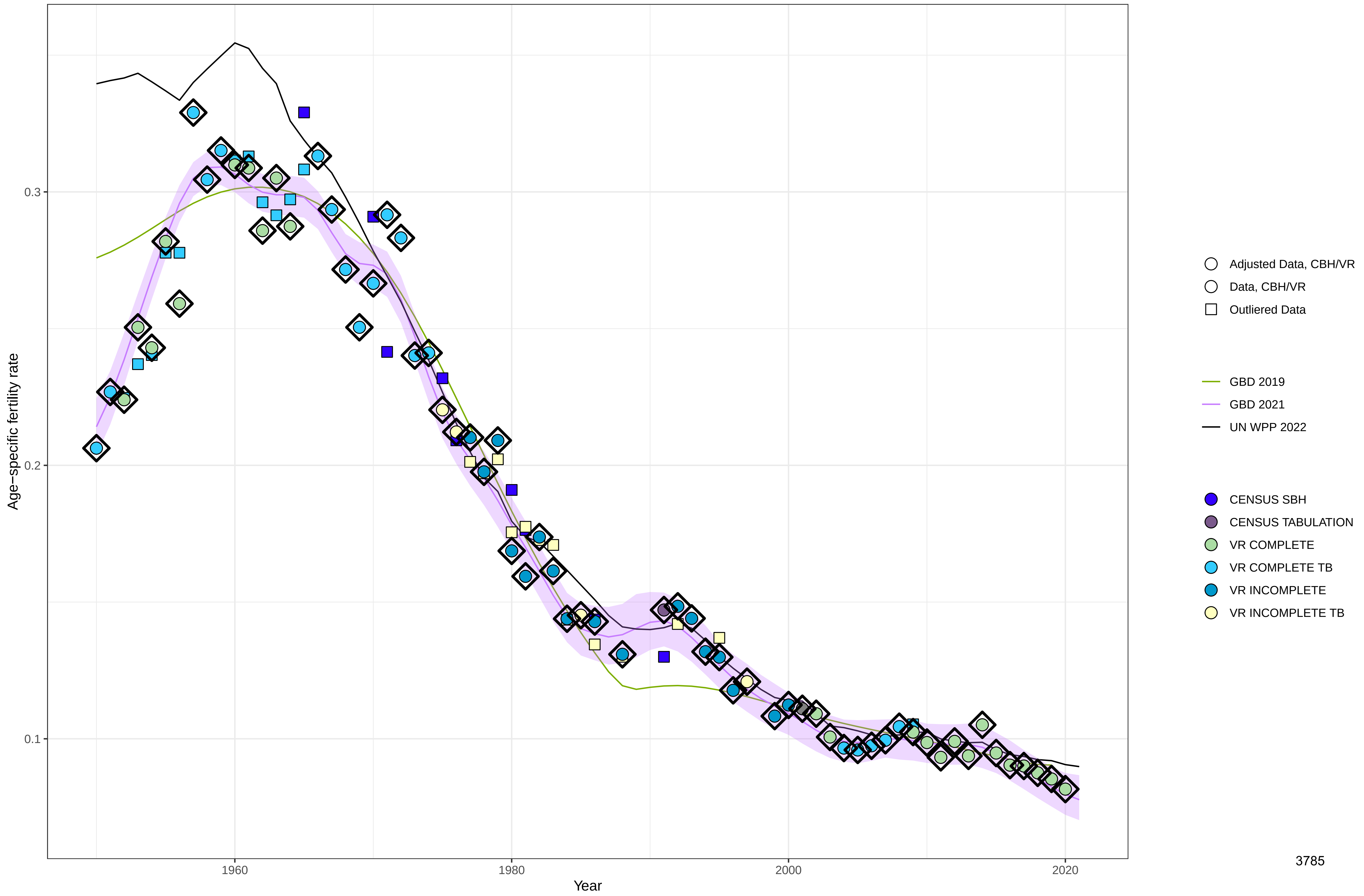

Age−specific fertility fitted trend lines, 1950−2021: Saint Vincent and the Grenadines, Ages: 30−34
Data points enclosed with a diamond are reference sources

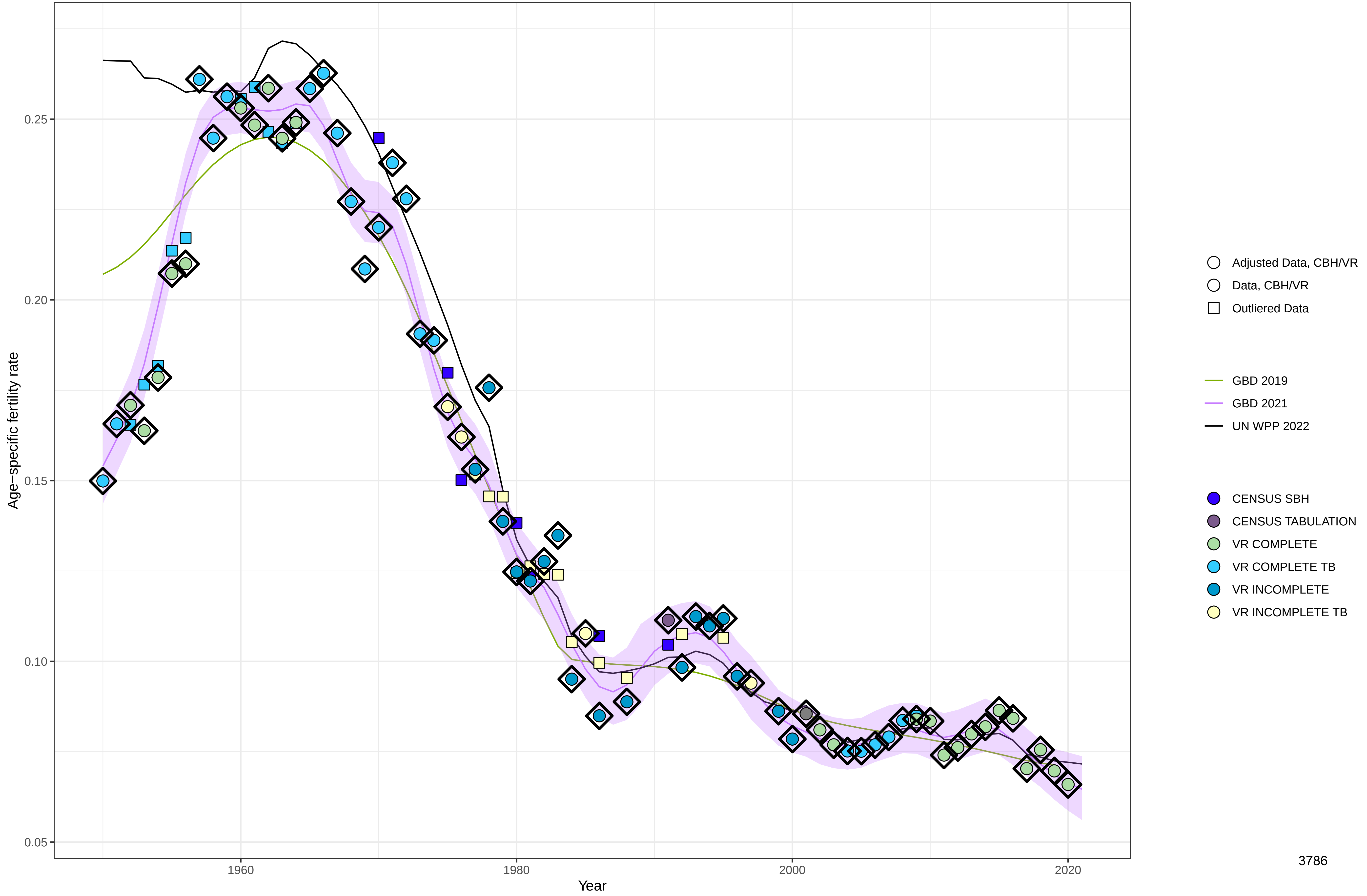

Age−specific fertility fitted trend lines, 1950−2021: Saint Vincent and the Grenadines, Ages: 35−39
Data points enclosed with a diamond are reference sources

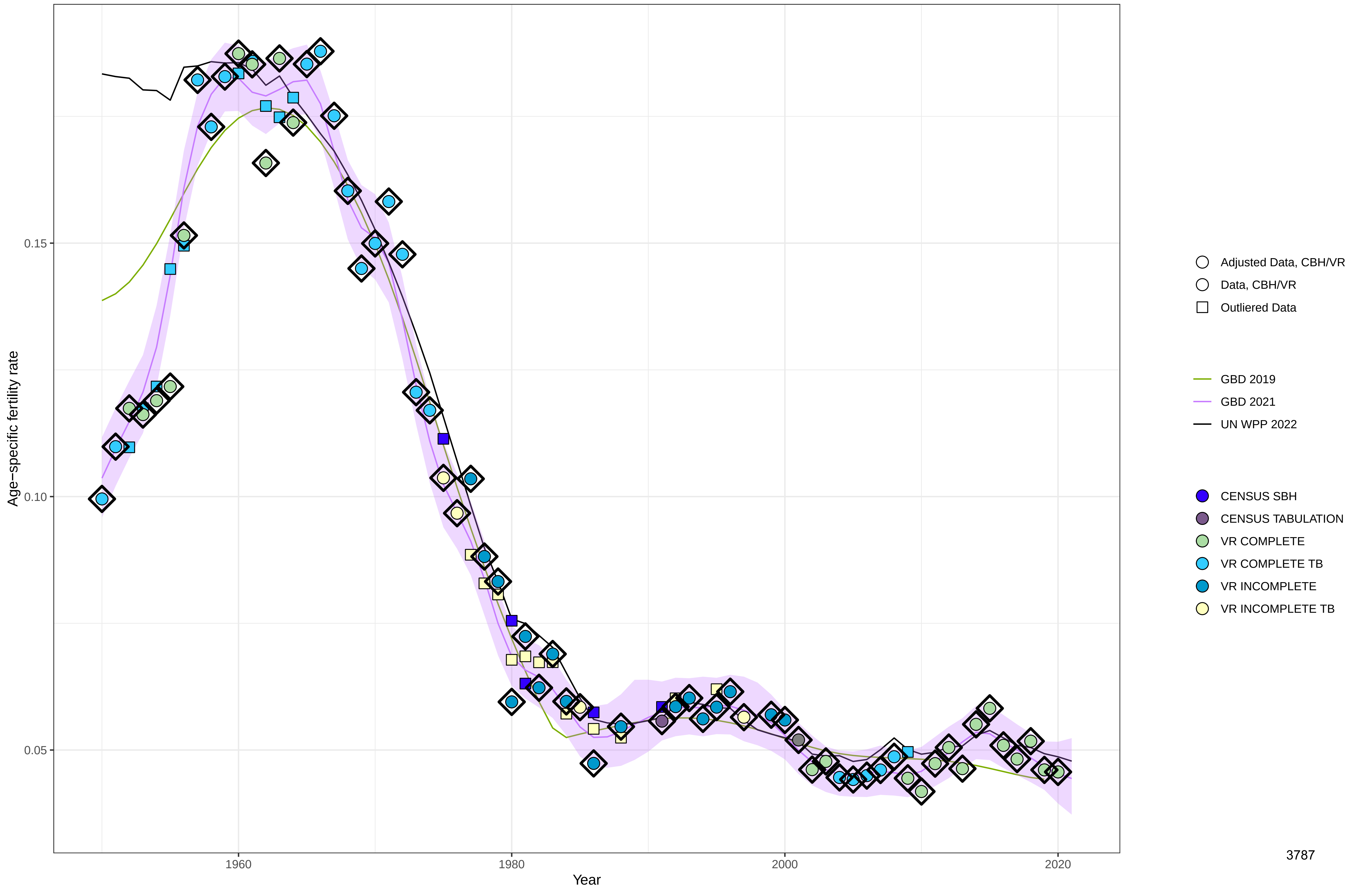

Age−specific fertility fitted trend lines, 1950−2021: Saint Vincent and the Grenadines, Ages: 40−44
Data points enclosed with a diamond are reference sources

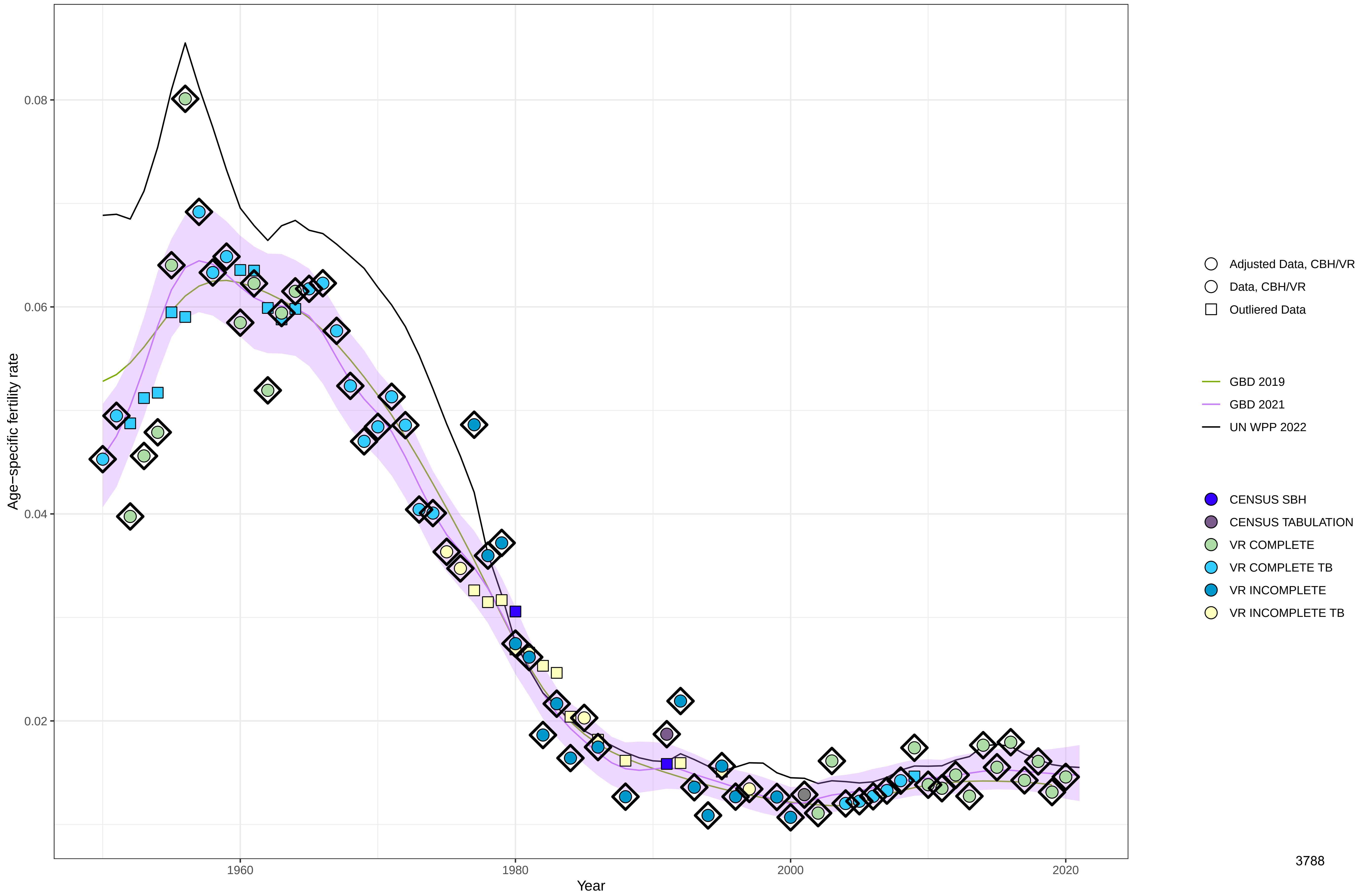

Age−specific fertility fitted trend lines, 1950−2021: Saint Vincent and the Grenadines, Ages: 45−49
Data points enclosed with a diamond are reference sources

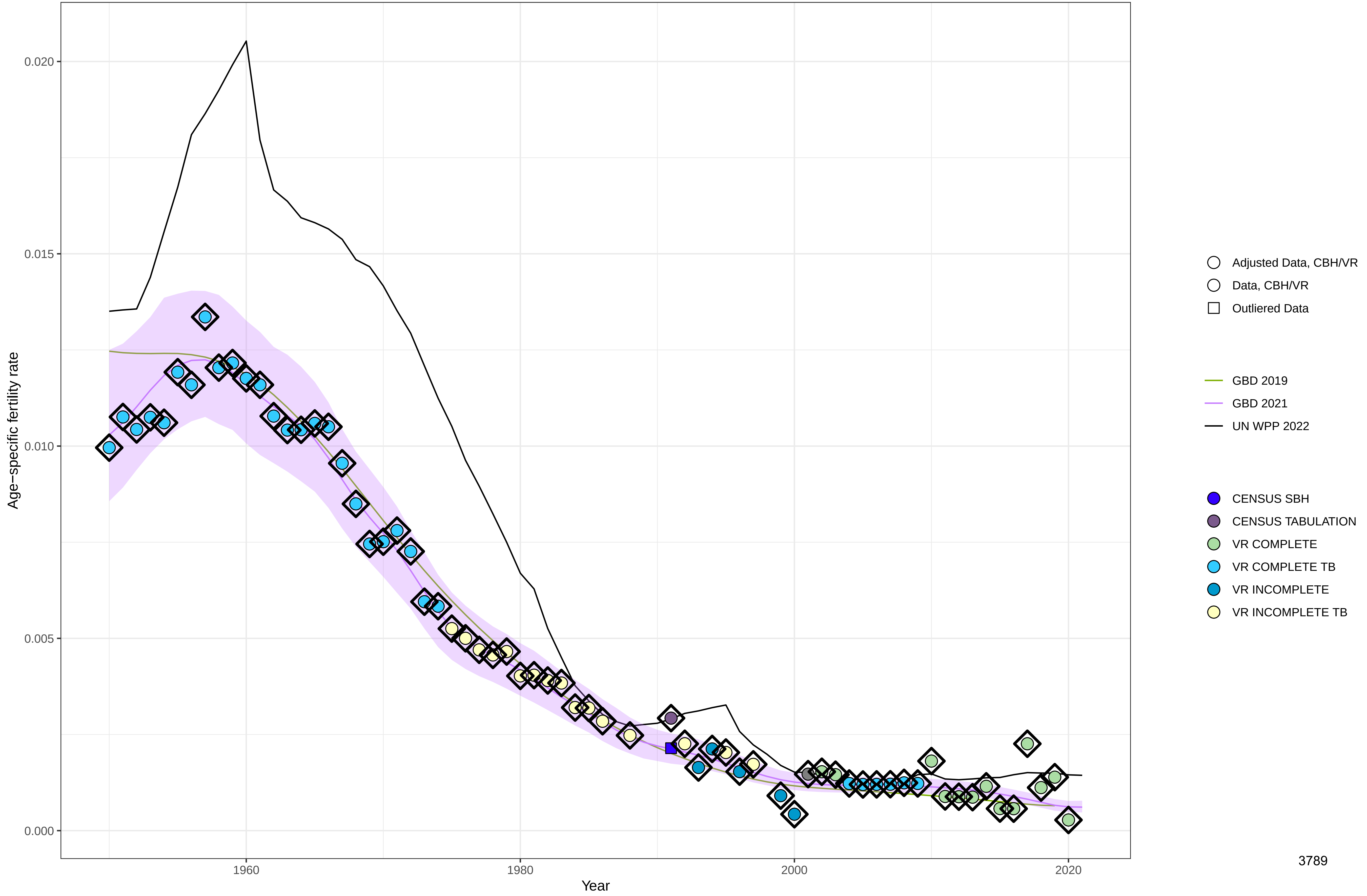

Age−specific fertility fitted trend lines, 1950−2021: Saint Vincent and the Grenadines, Ages: 50−54
Data points enclosed with a diamond are reference sources

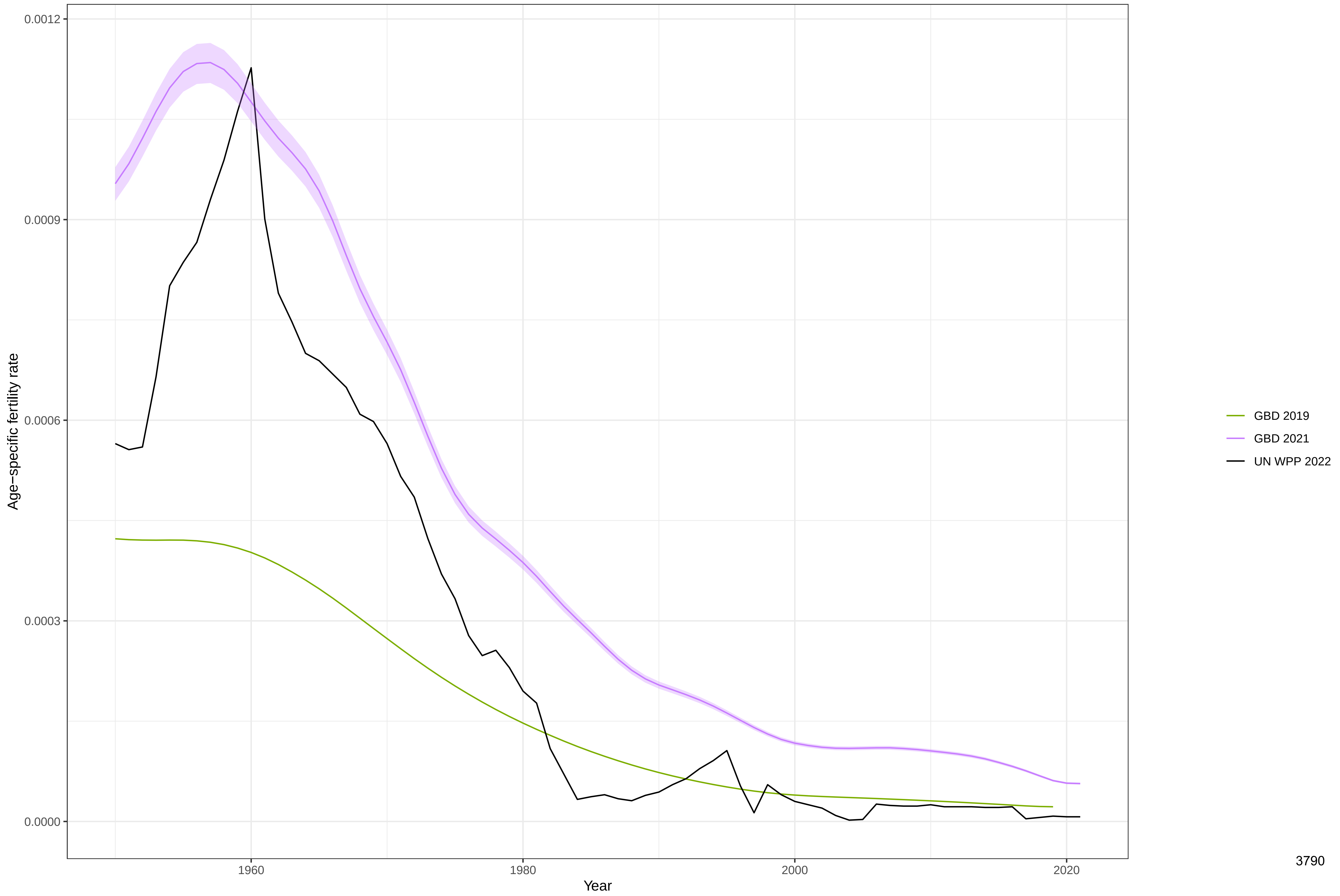

Total fertility rate fitted trend lines, 1950−2021: Saint Vincent and the Grenadines TFR
Data points enclosed with a diamond are reference sources

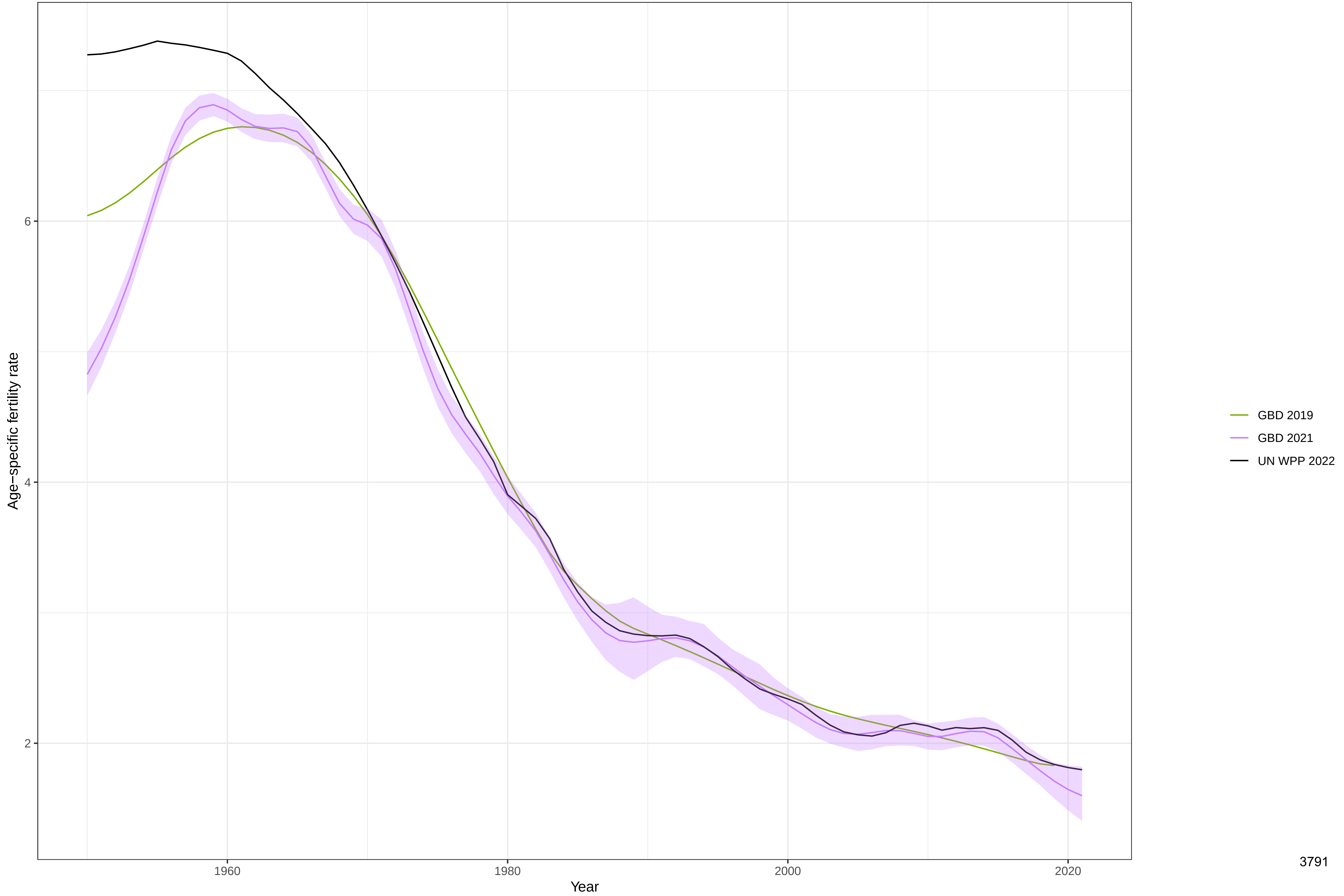

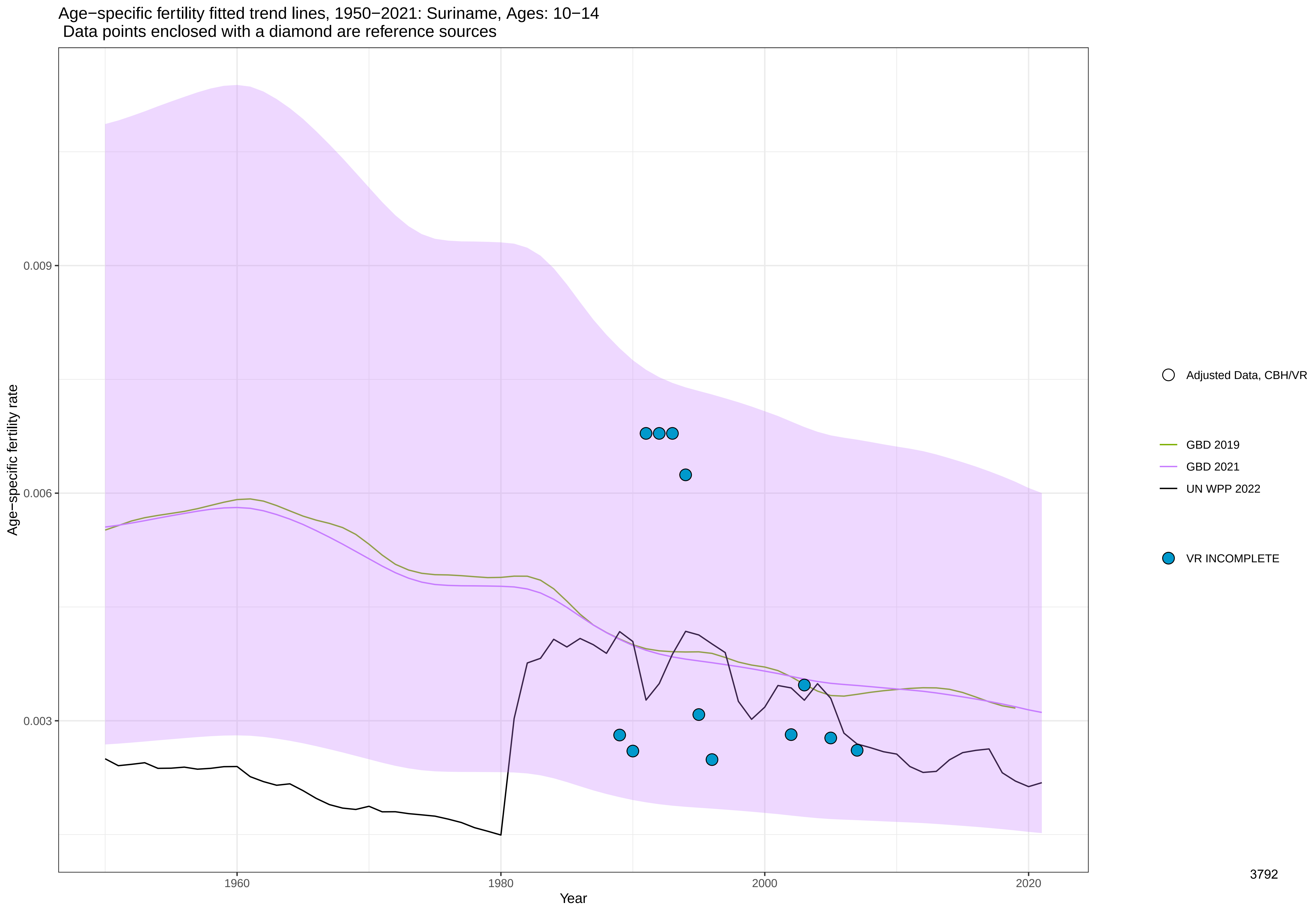

Age−specific fertility fitted trend lines, 1950−2021: Suriname, Ages: 10−14
Data points enclosed with a diamond are reference sources
Age−specific fertility rate
0.009
0.006
0.003
1960
1980
2000
2020
Year
Adjusted Data, CBH/VR
GBD 2019
GBD 2021
UN WPP 2022
VR INCOMPLETE

Age−specific fertility fitted trend lines, 1950−2021: Suriname, Ages: 15−19
Data points enclosed with a diamond are reference sources

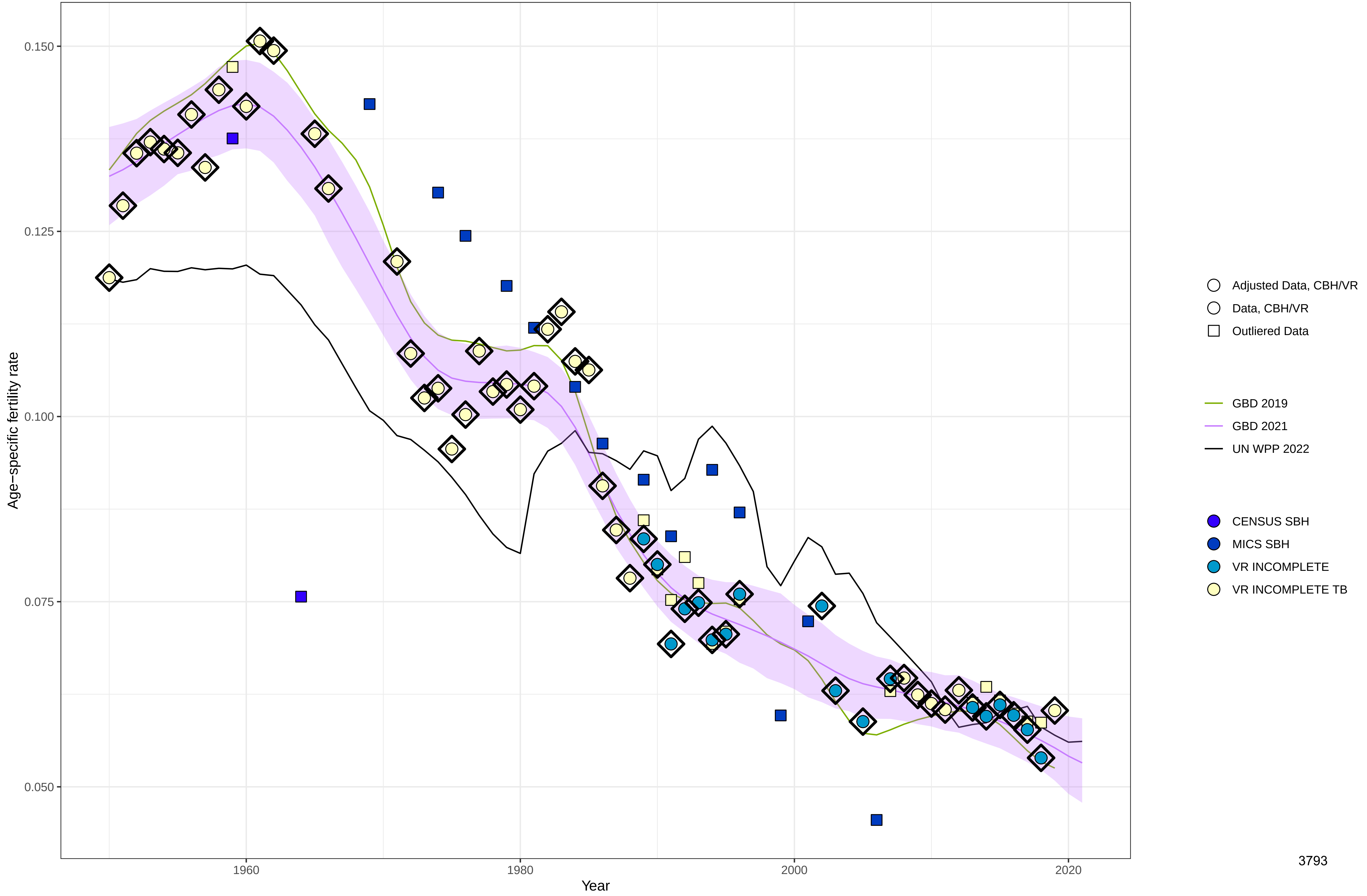

Age−specific fertility fitted trend lines, 1950−2021: Suriname, Ages: 20−24
Data points enclosed with a diamond are reference sources

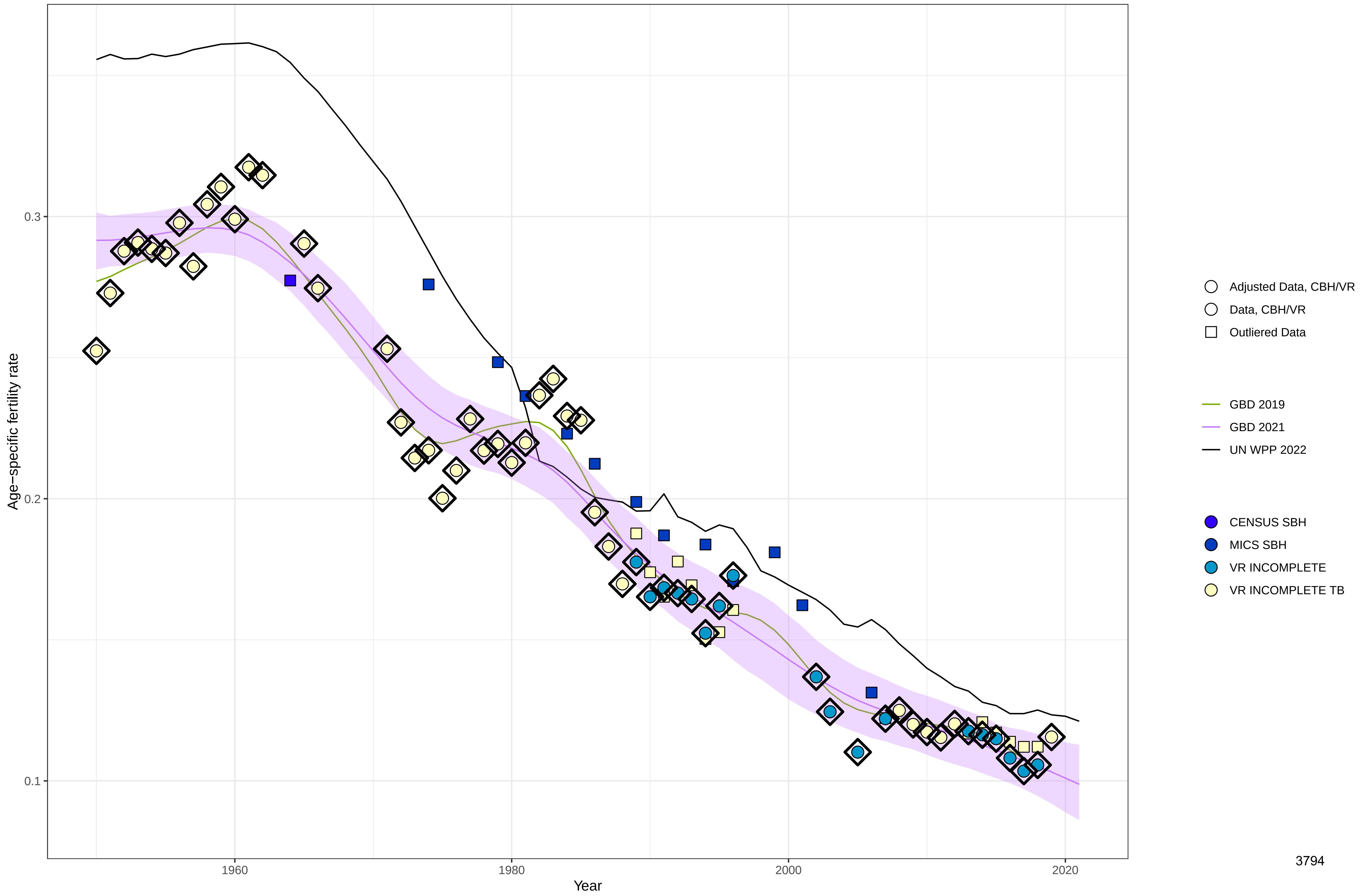

Age−specific fertility fitted trend lines, 1950−2021: Suriname, Ages: 25−29
Data points enclosed with a diamond are reference sources

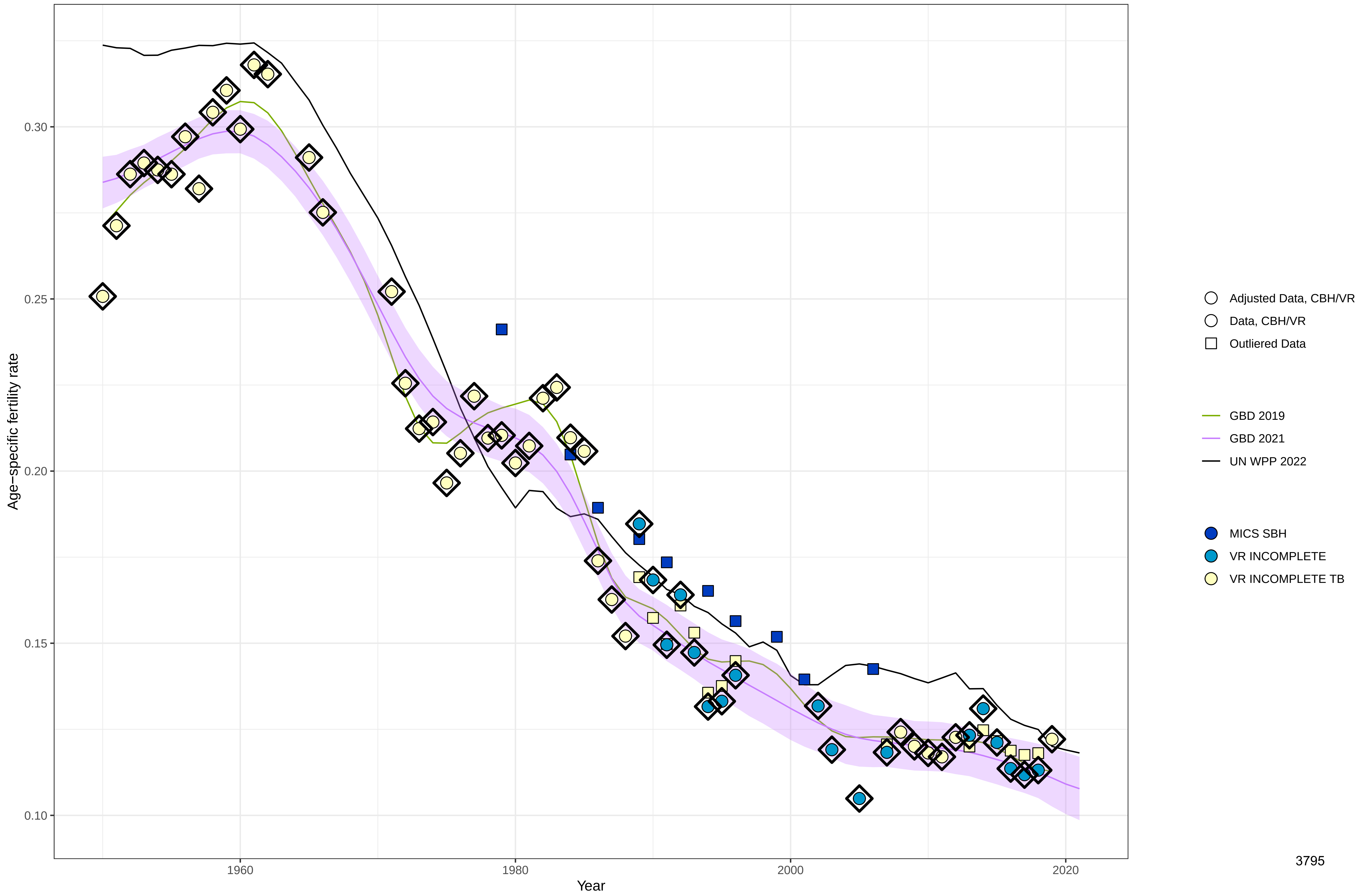

Age−specific fertility fitted trend lines, 1950−2021: Suriname, Ages: 30−34
Data points enclosed with a diamond are reference sources

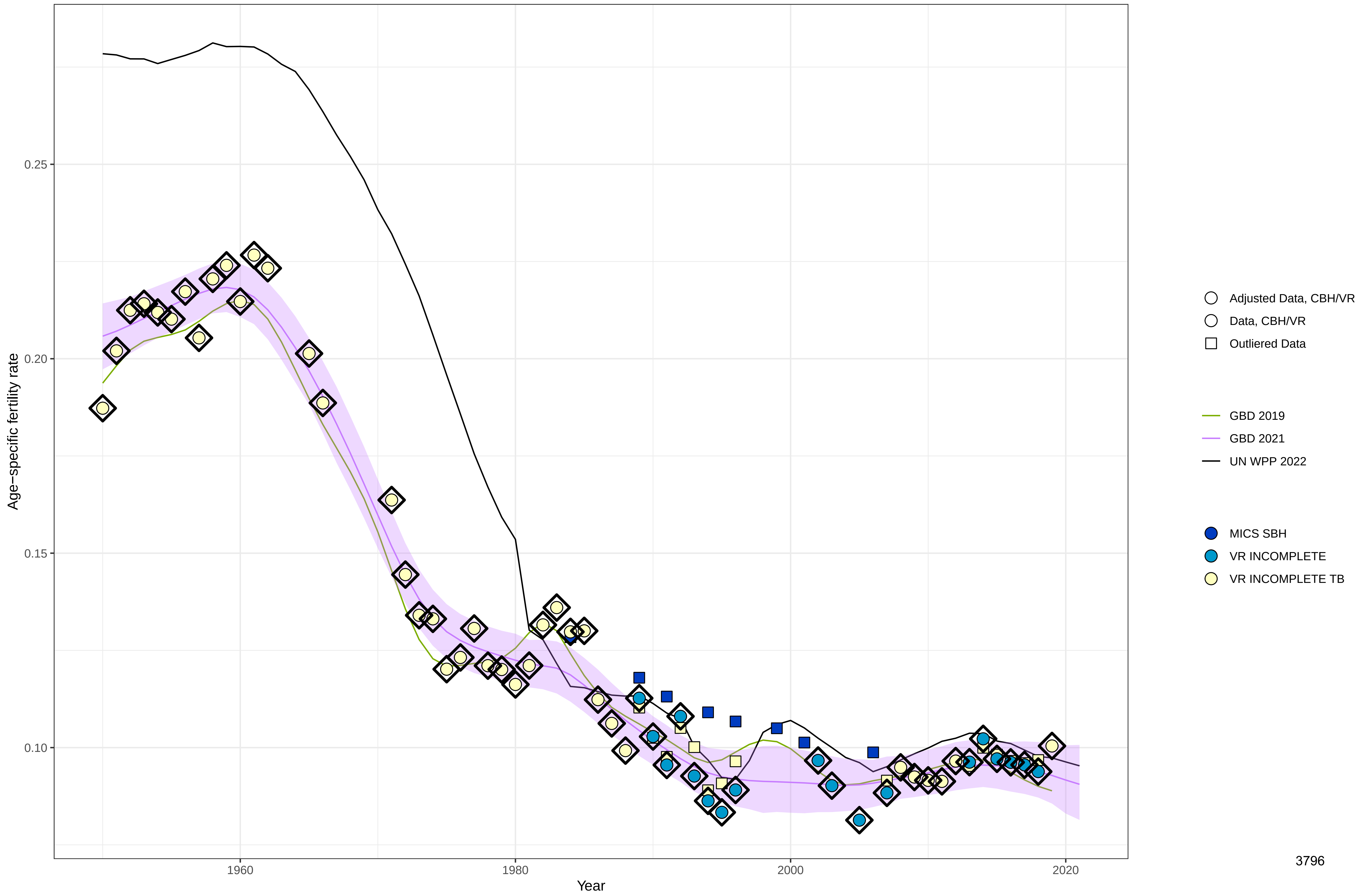

Age−specific fertility fitted trend lines, 1950−2021: Suriname, Ages: 35−39
Data points enclosed with a diamond are reference sources

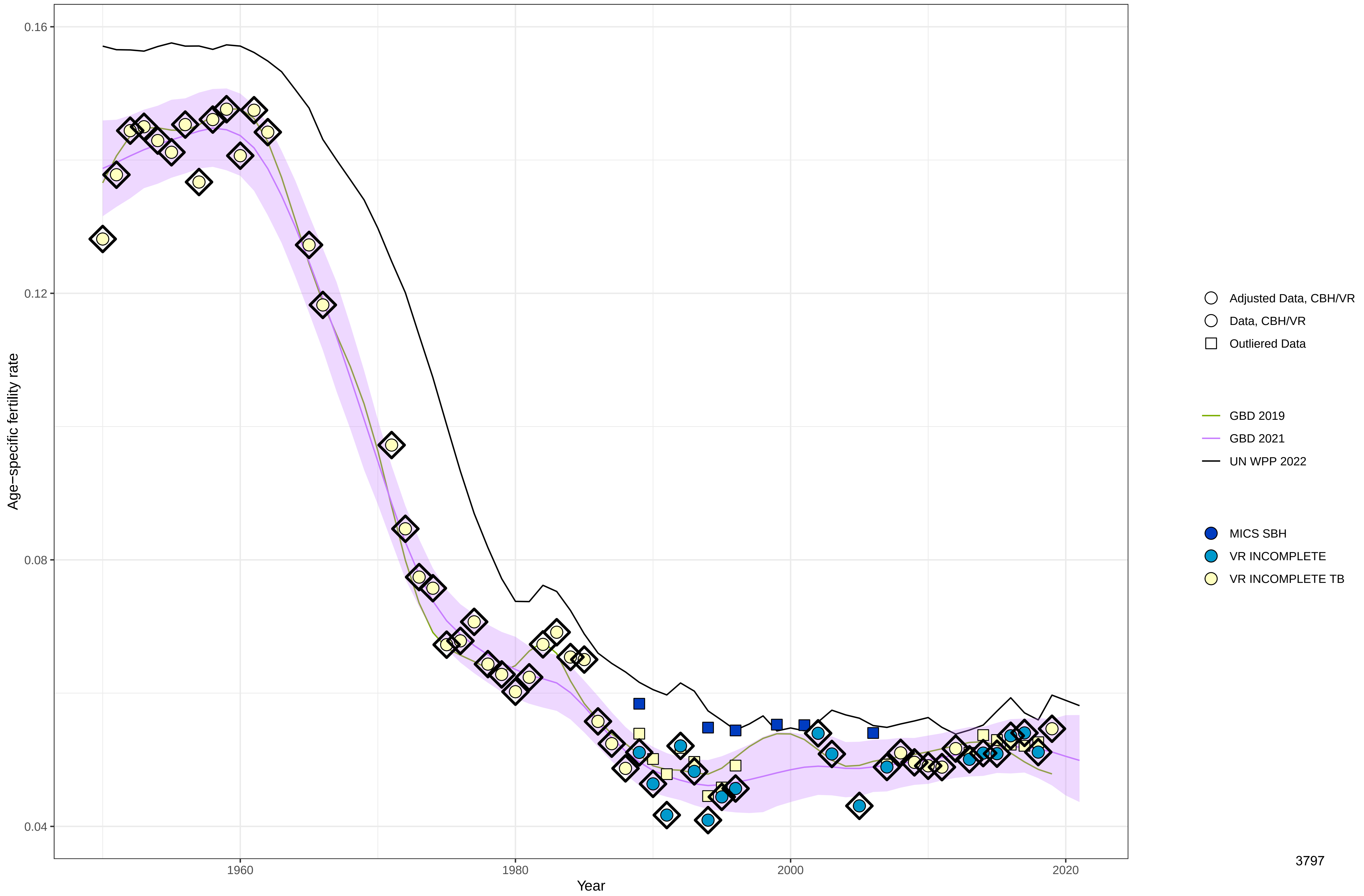

Age−specific fertility fitted trend lines, 1950−2021: Suriname, Ages: 40−44
Data points enclosed with a diamond are reference sources

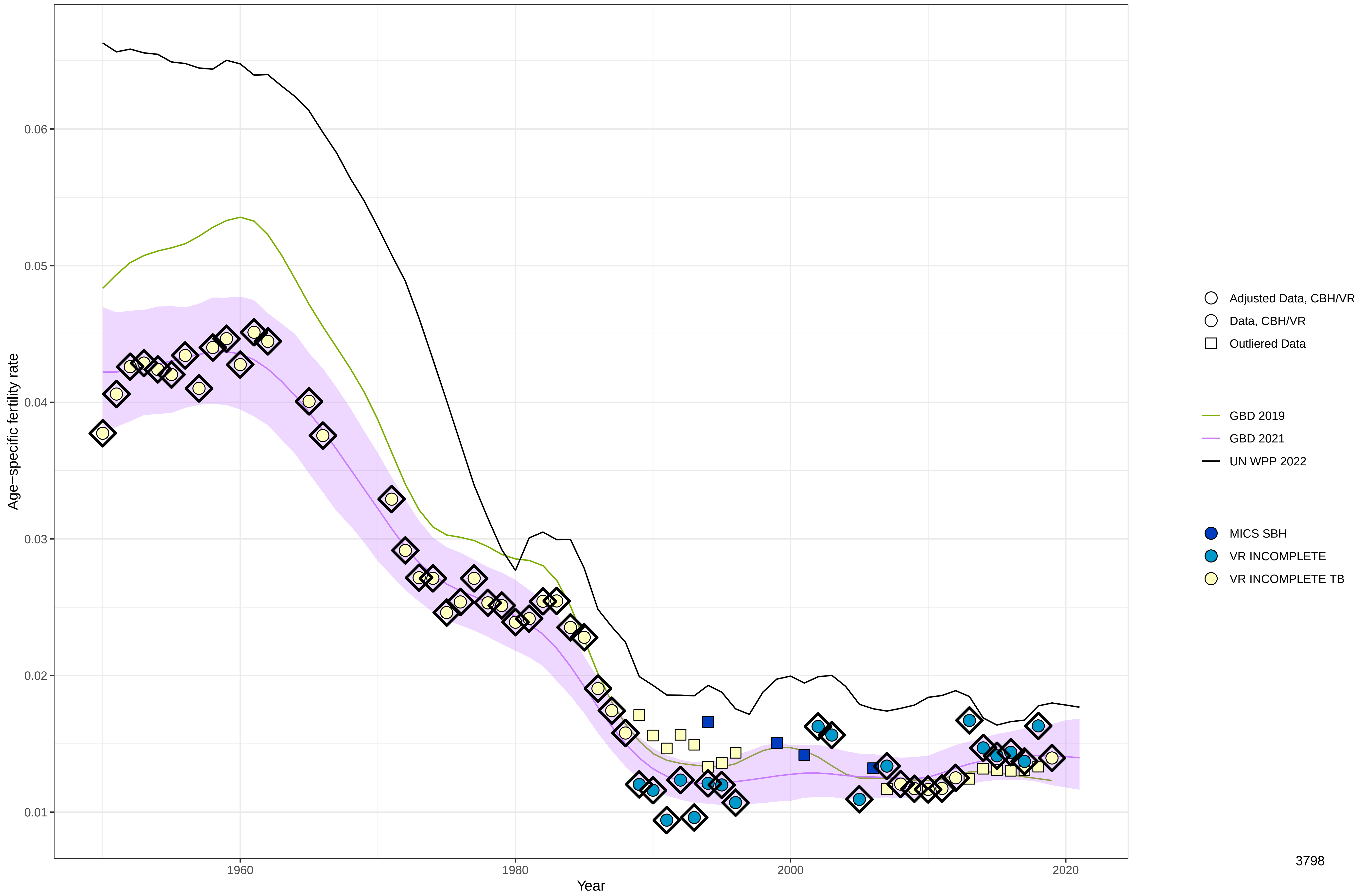

Age−specific fertility fitted trend lines, 1950−2021: Suriname, Ages: 45−49
Data points enclosed with a diamond are reference sources

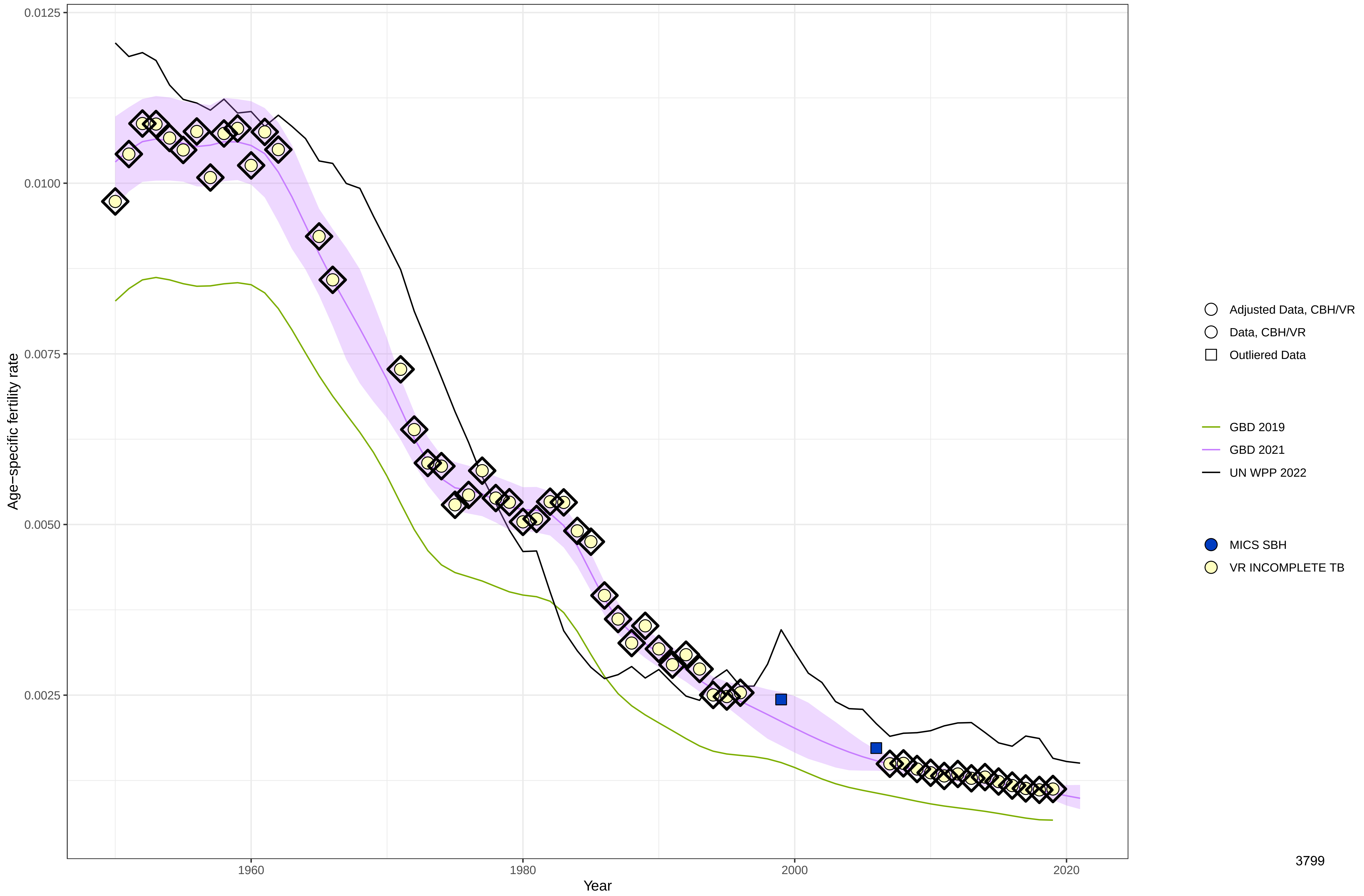

Age−specific fertility fitted trend lines, 1950−2021: Suriname, Ages: 50−54
Data points enclosed with a diamond are reference sources

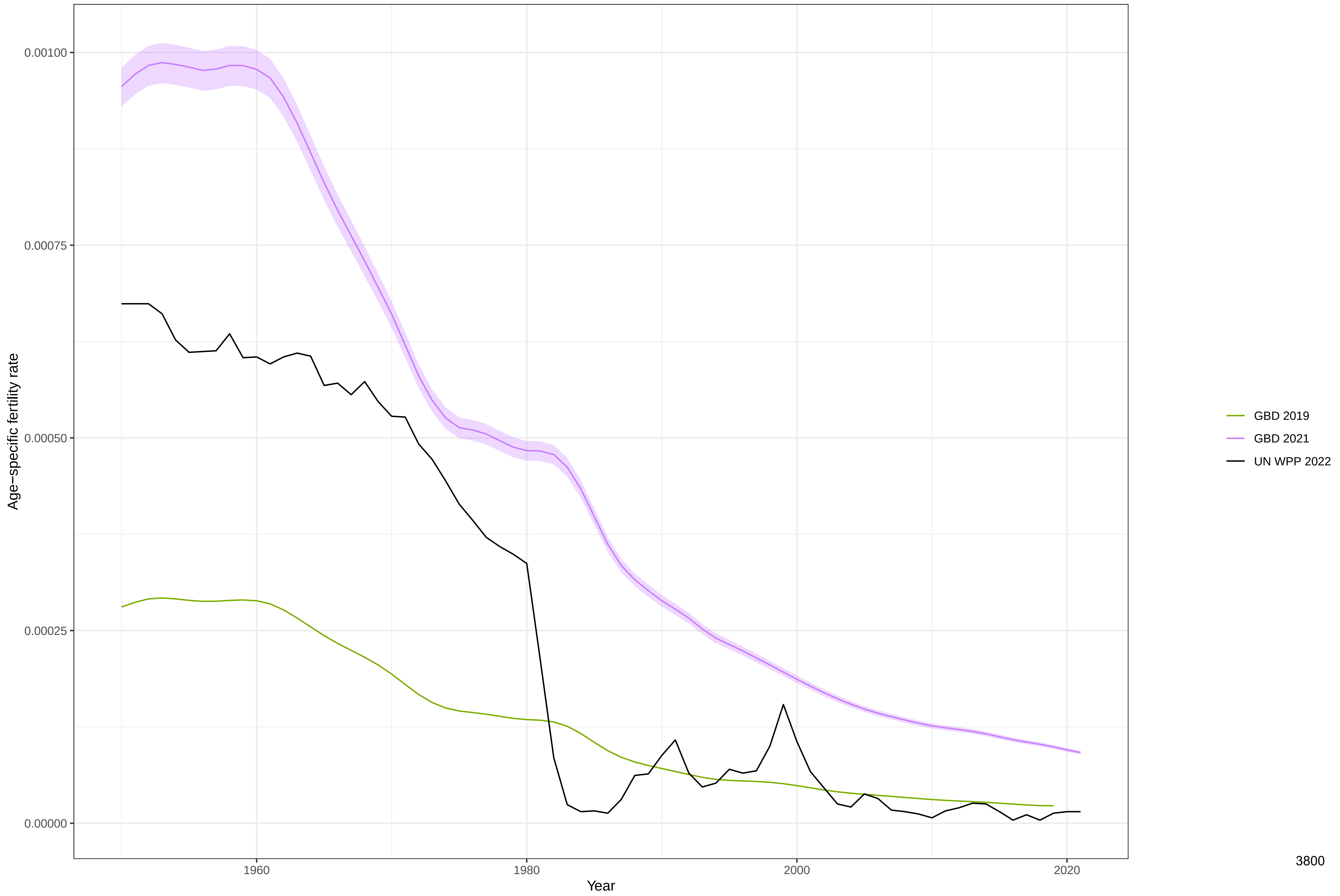

Total fertility rate fitted trend lines, 1950−2021: Suriname TFR
Data points enclosed with a diamond are reference sources

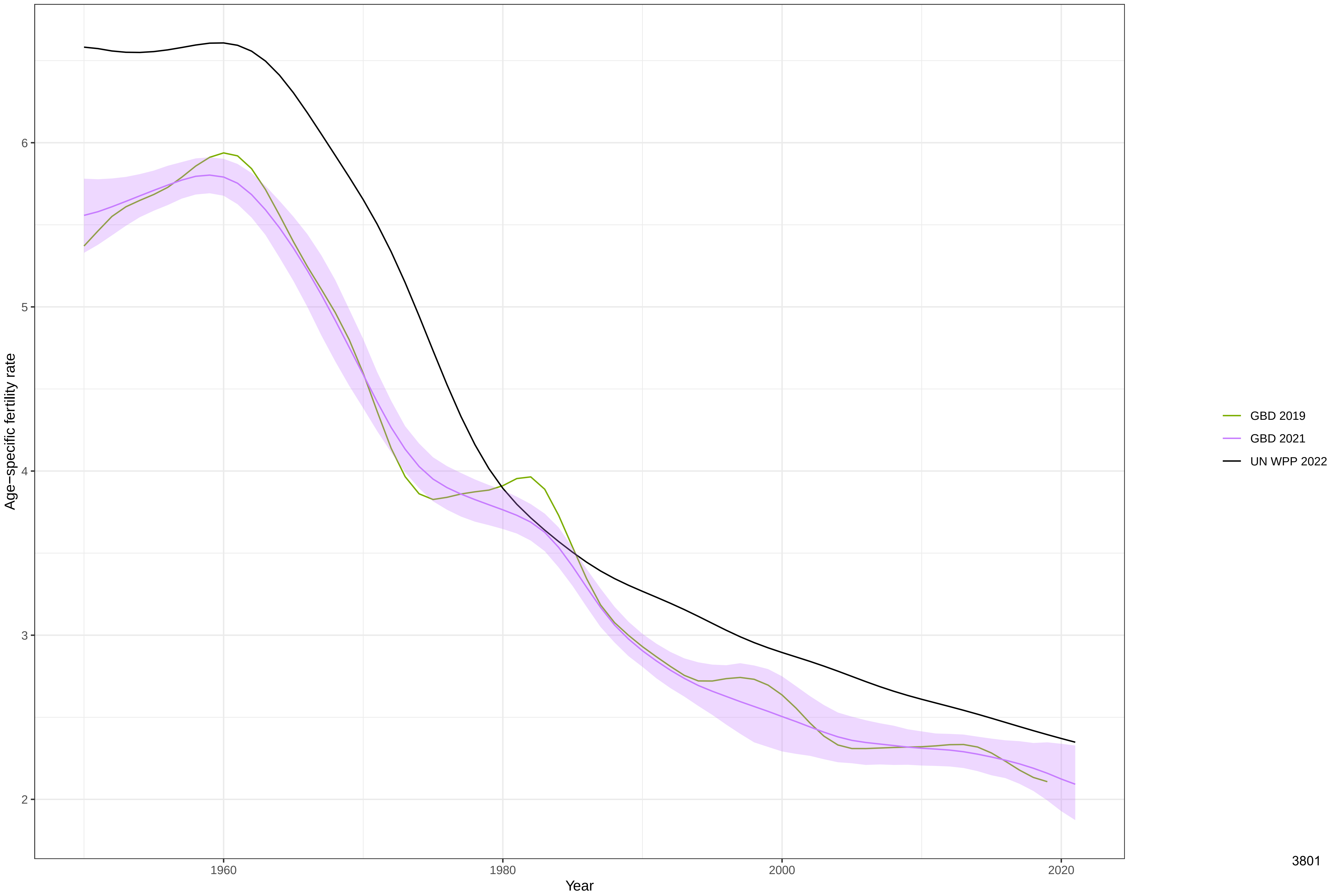

Age−specific fertility fitted trend lines, 1950−2021: Trinidad and Tobago, Ages: 10−14
Data points enclosed with a diamond are reference sources

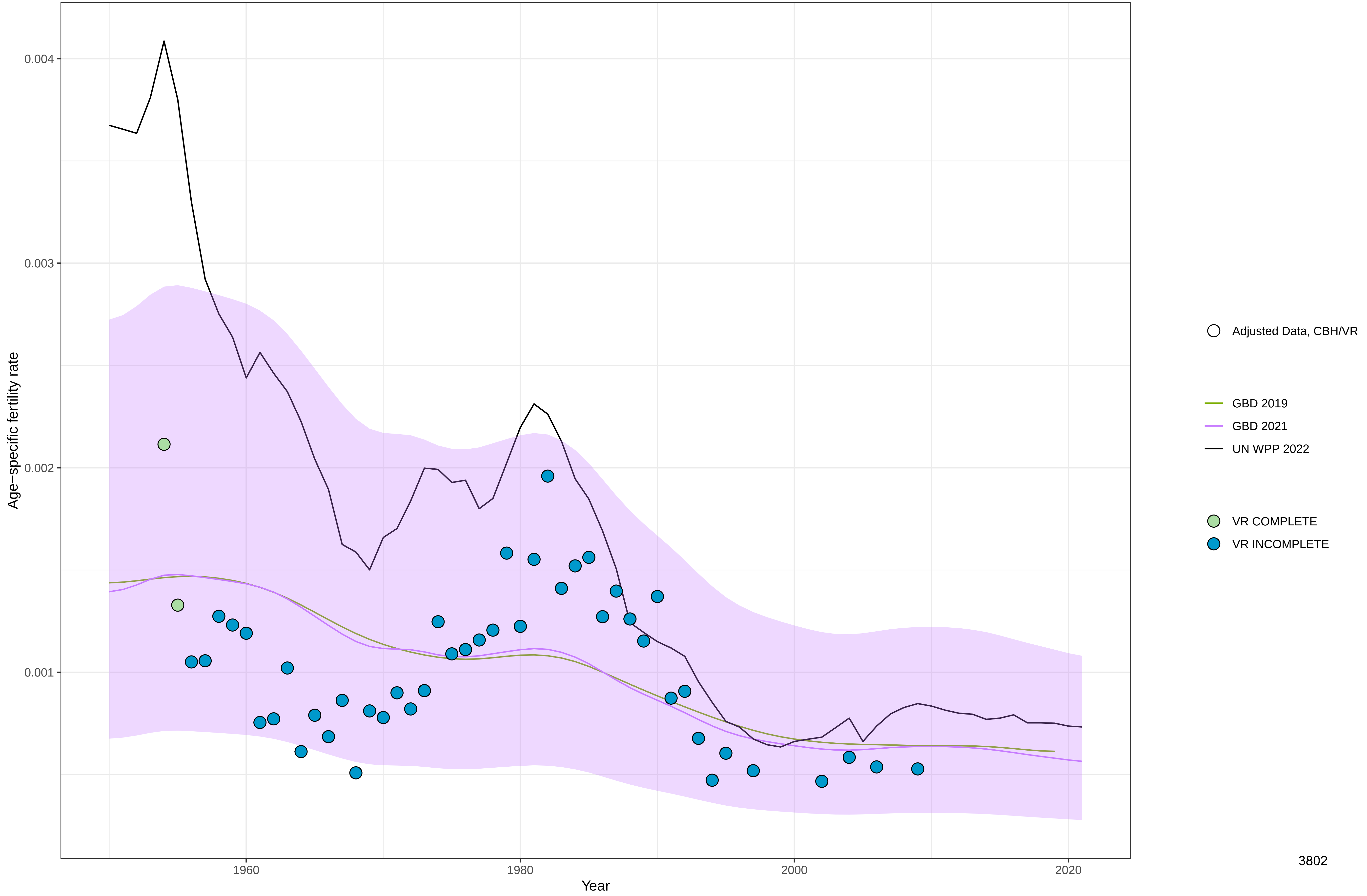

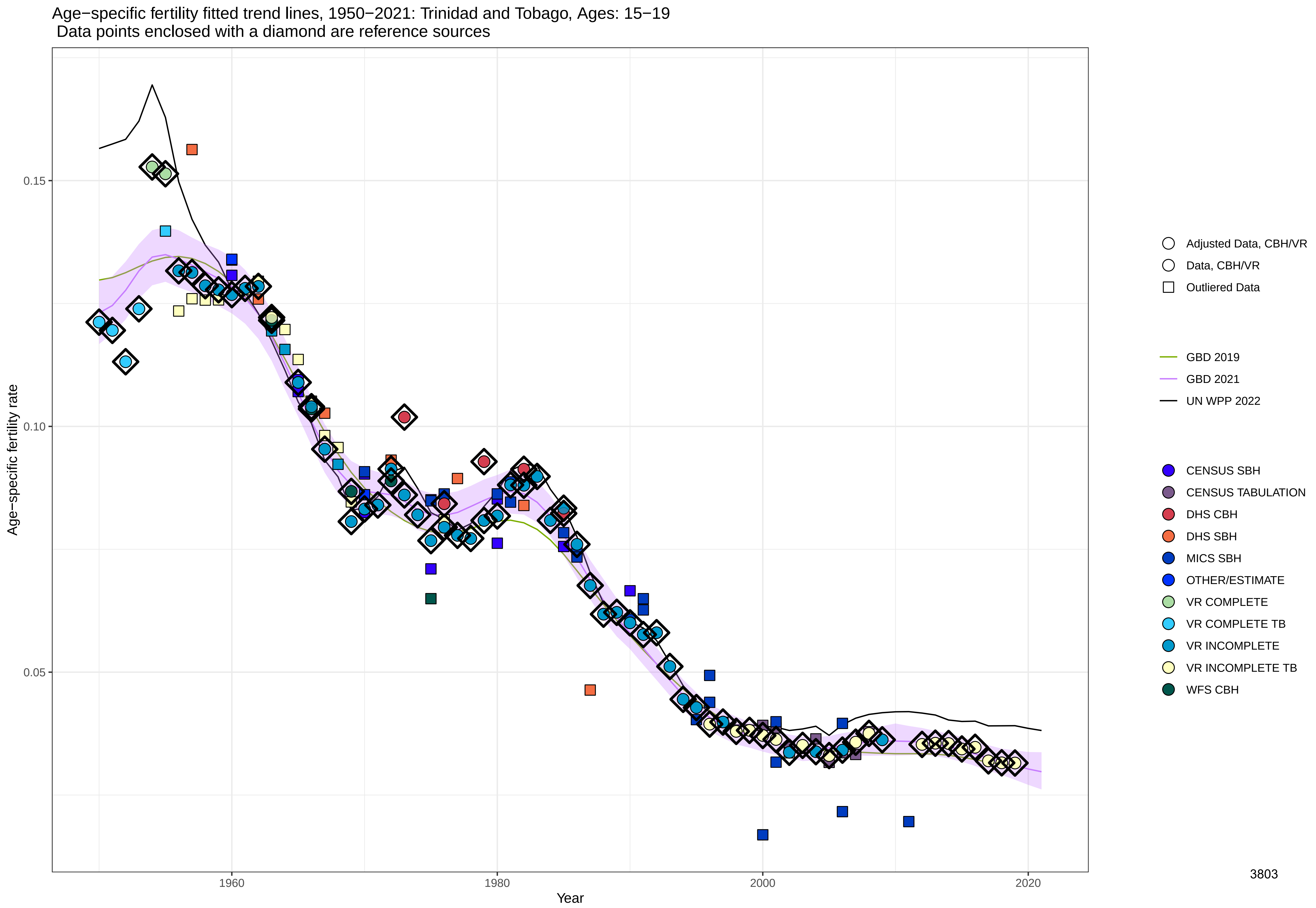

Age−specific fertility fitted trend lines, 1950−2021: Trinidad and Tobago, Ages: 15−19
Data points enclosed with a diamond are reference sources
Age−specific fertility rate
0.15
0.10
0.05
1960
1980
2000
2020
Year
Adjusted Data, CBH/VR
Data, CBH/VR
Outliered Data
GBD 2019
GBD 2021
UN WPP 2022
CENSUS SBH
CENSUS TABULATION
DHS CBH
DHS SBH
MICS SBH
OTHER/ESTIMATE
VR COMPLETE
VR COMPLETE TB
VR INCOMPLETE
VR INCOMPLETE TB
WFS CBH

Age−specific fertility fitted trend lines, 1950−2021: Trinidad and Tobago, Ages: 20−24
Data points enclosed with a diamond are reference sources

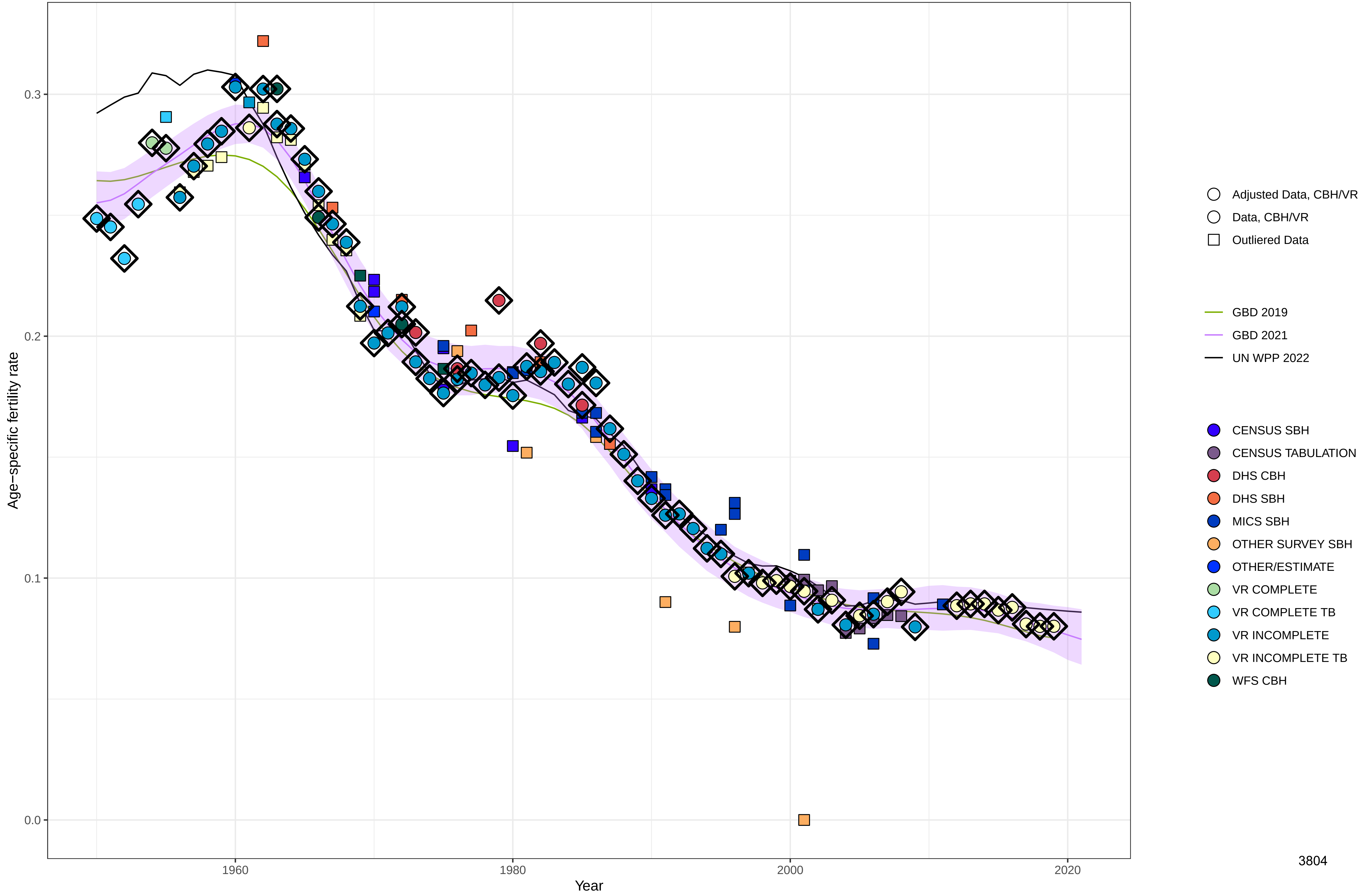

Age−specific fertility fitted trend lines, 1950−2021: Trinidad and Tobago, Ages: 25−29
Data points enclosed with a diamond are reference sources

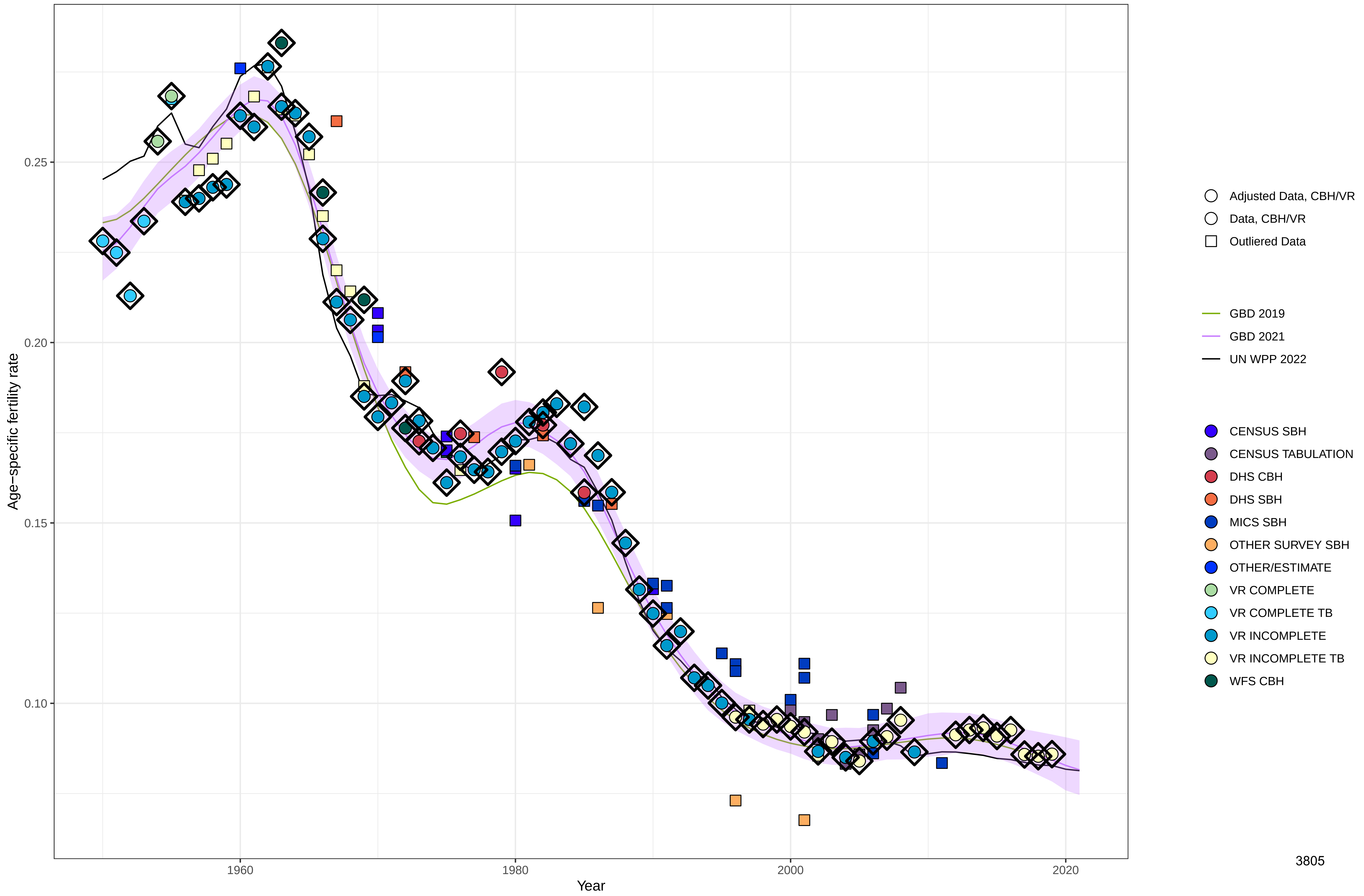

Age−specific fertility fitted trend lines, 1950−2021: Trinidad and Tobago, Ages: 30−34
Data points enclosed with a diamond are reference sources

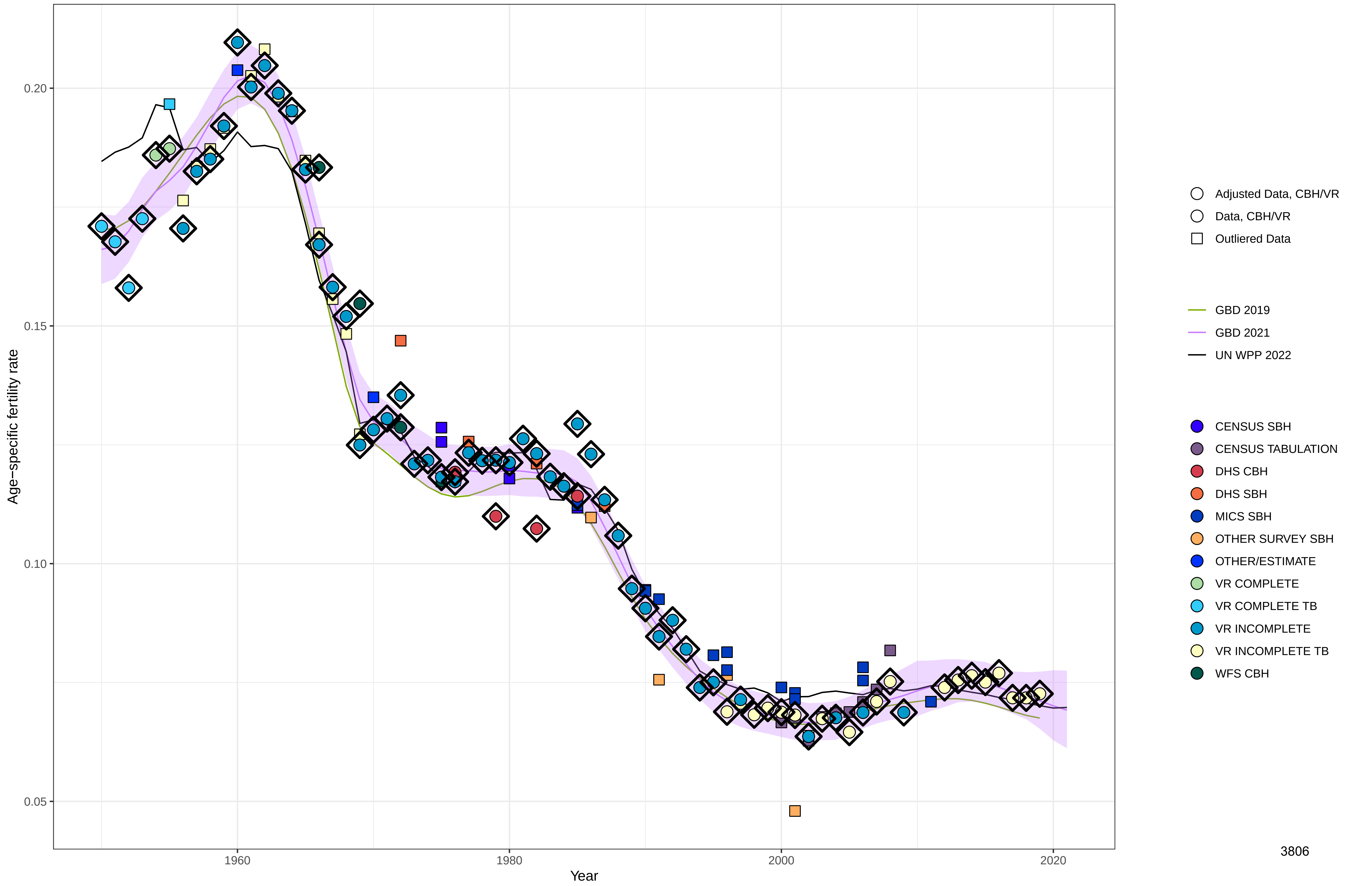

Age−specific fertility fitted trend lines, 1950−2021: Trinidad and Tobago, Ages: 35−39
Data points enclosed with a diamond are reference sources

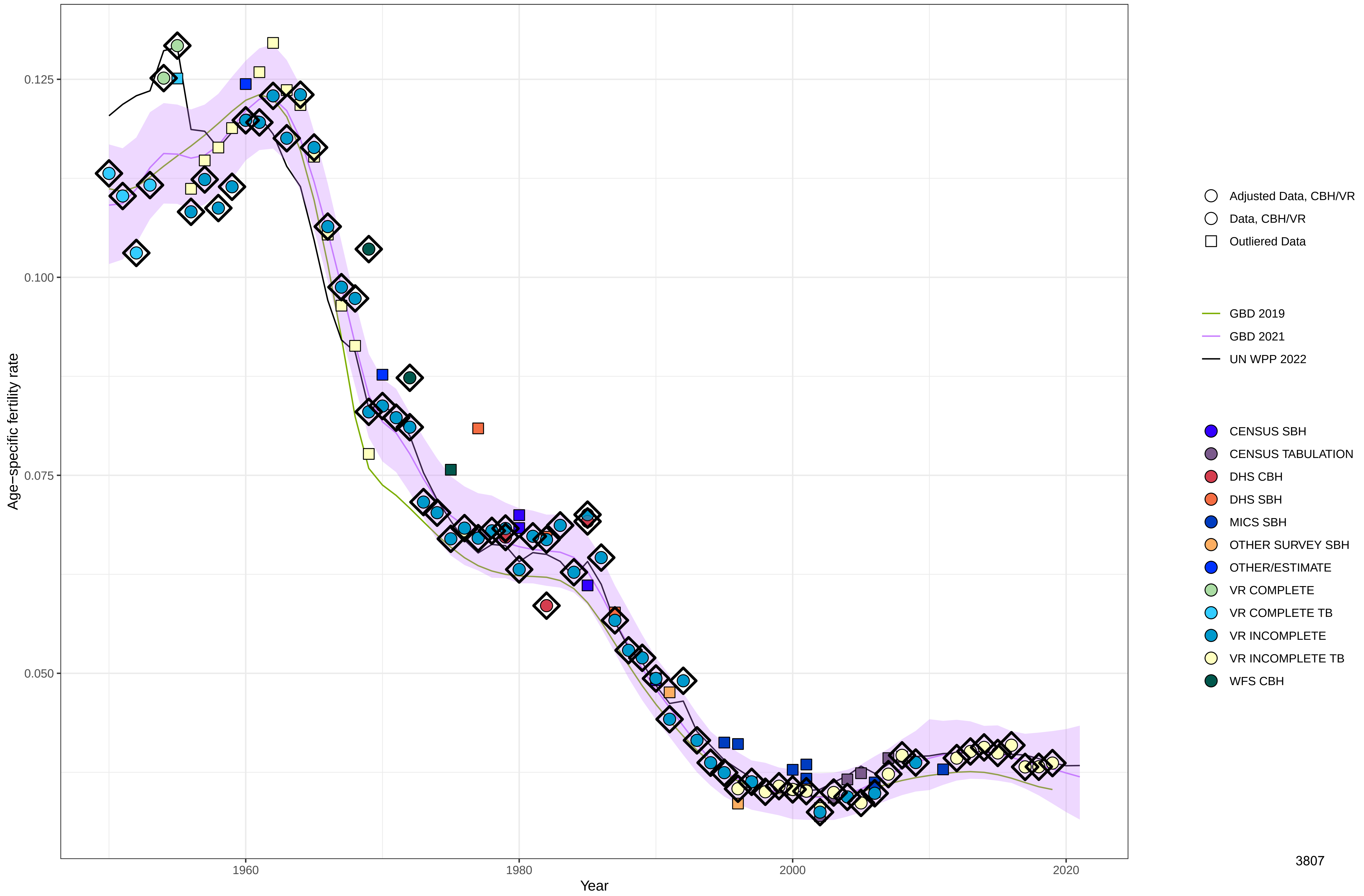

Age−specific fertility fitted trend lines, 1950−2021: Trinidad and Tobago, Ages: 40−44
Data points enclosed with a diamond are reference sources

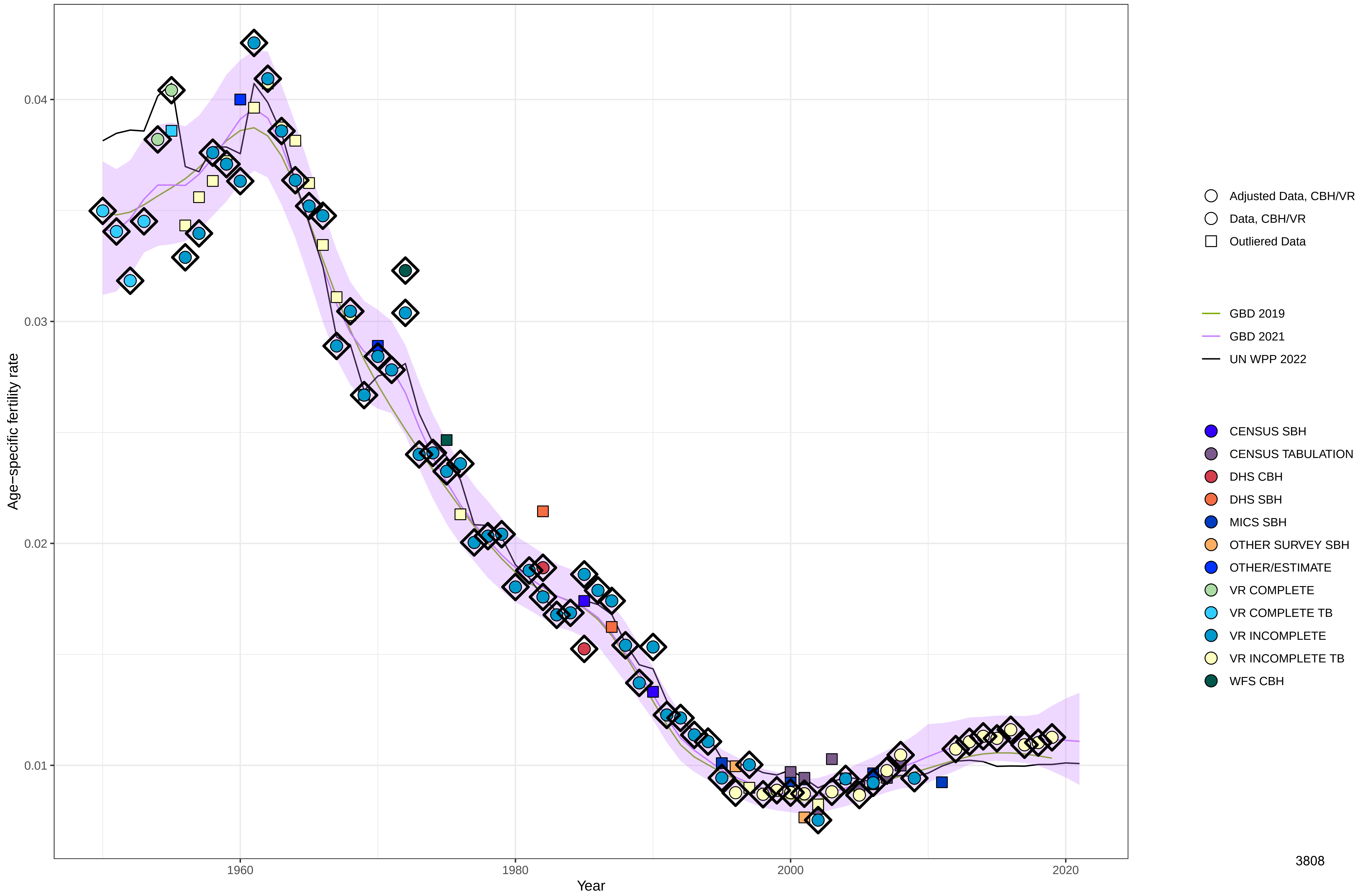

Age−specific fertility fitted trend lines, 1950−2021: Trinidad and Tobago, Ages: 45−49
Data points enclosed with a diamond are reference sources

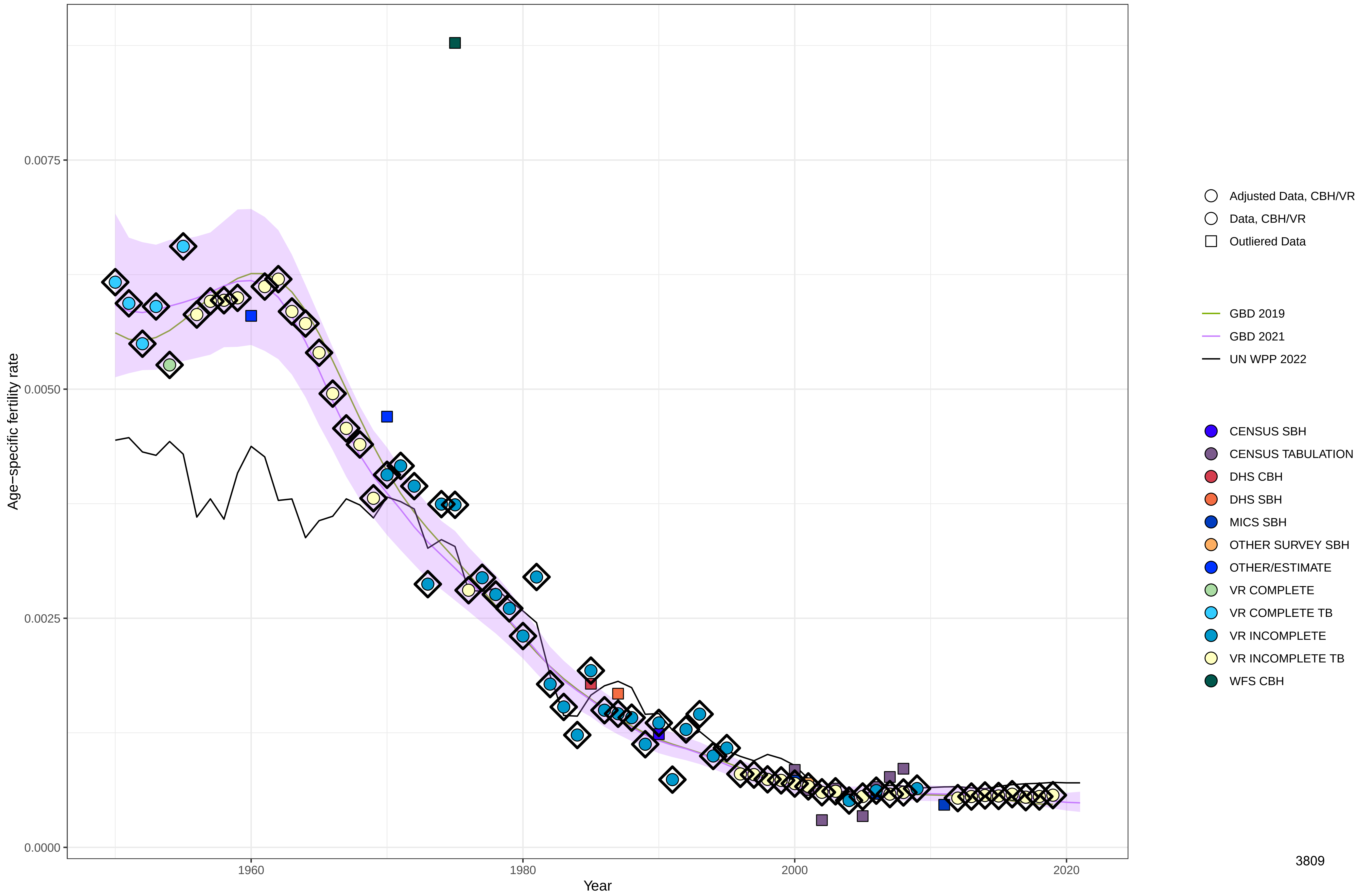

Age−specific fertility fitted trend lines, 1950−2021: Trinidad and Tobago, Ages: 50−54
Data points enclosed with a diamond are reference sources

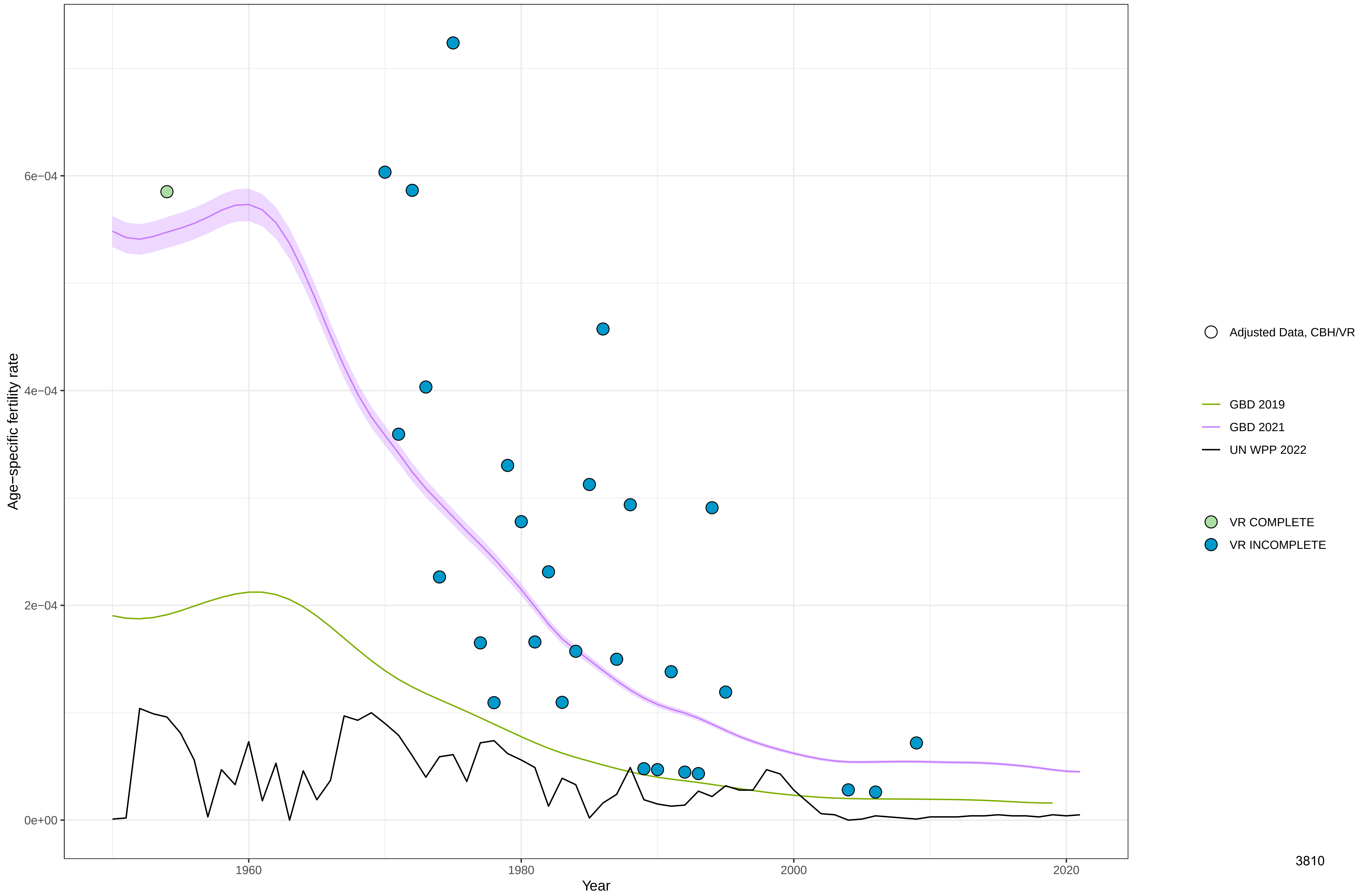

Total fertility rate fitted trend lines, 1950−2021: Trinidad and Tobago TFR
Data points enclosed with a diamond are reference sources

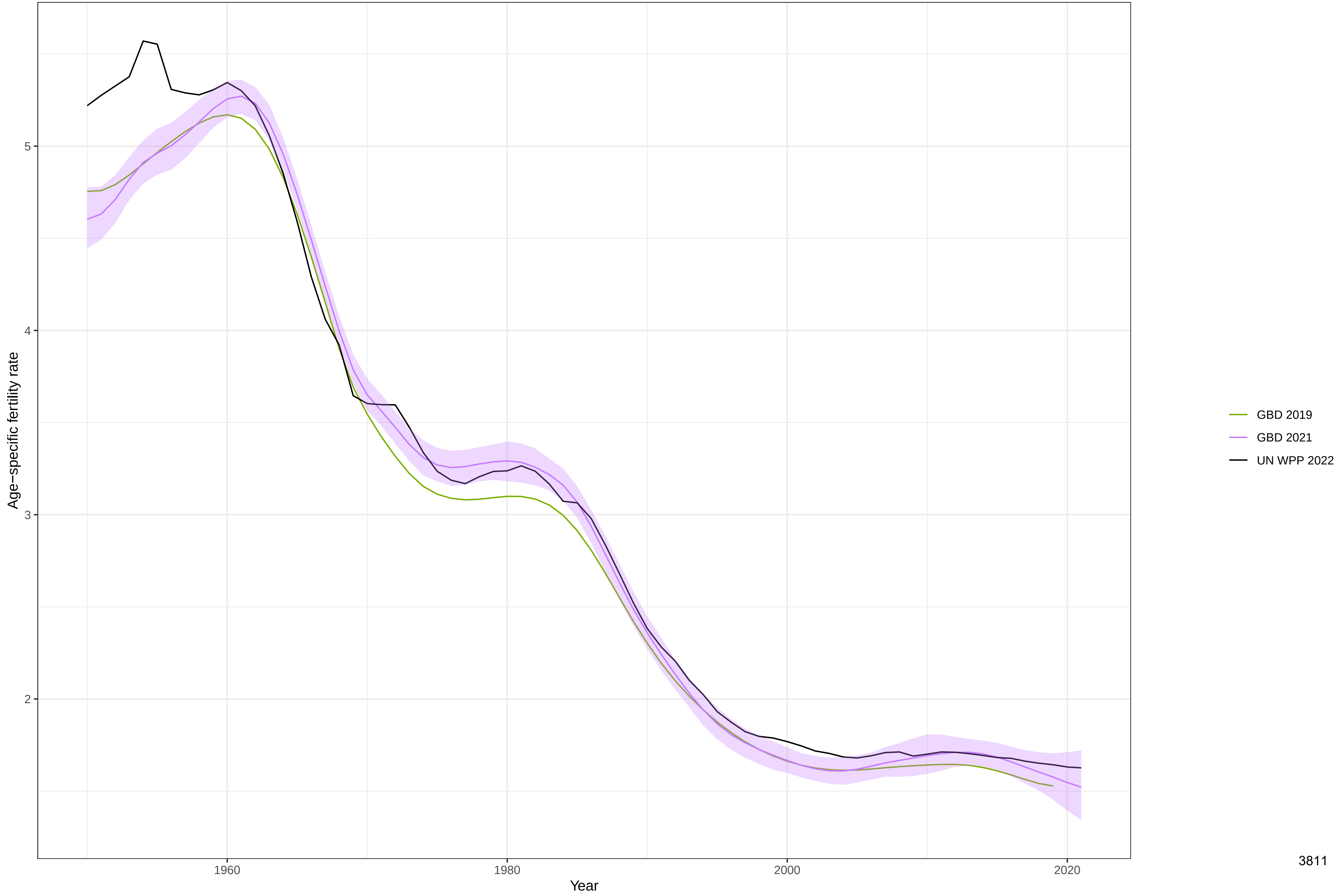

Age−specific fertility fitted trend lines, 1950−2021: Virgin Islands, Ages: 10−14
Data points enclosed with a diamond are reference sources

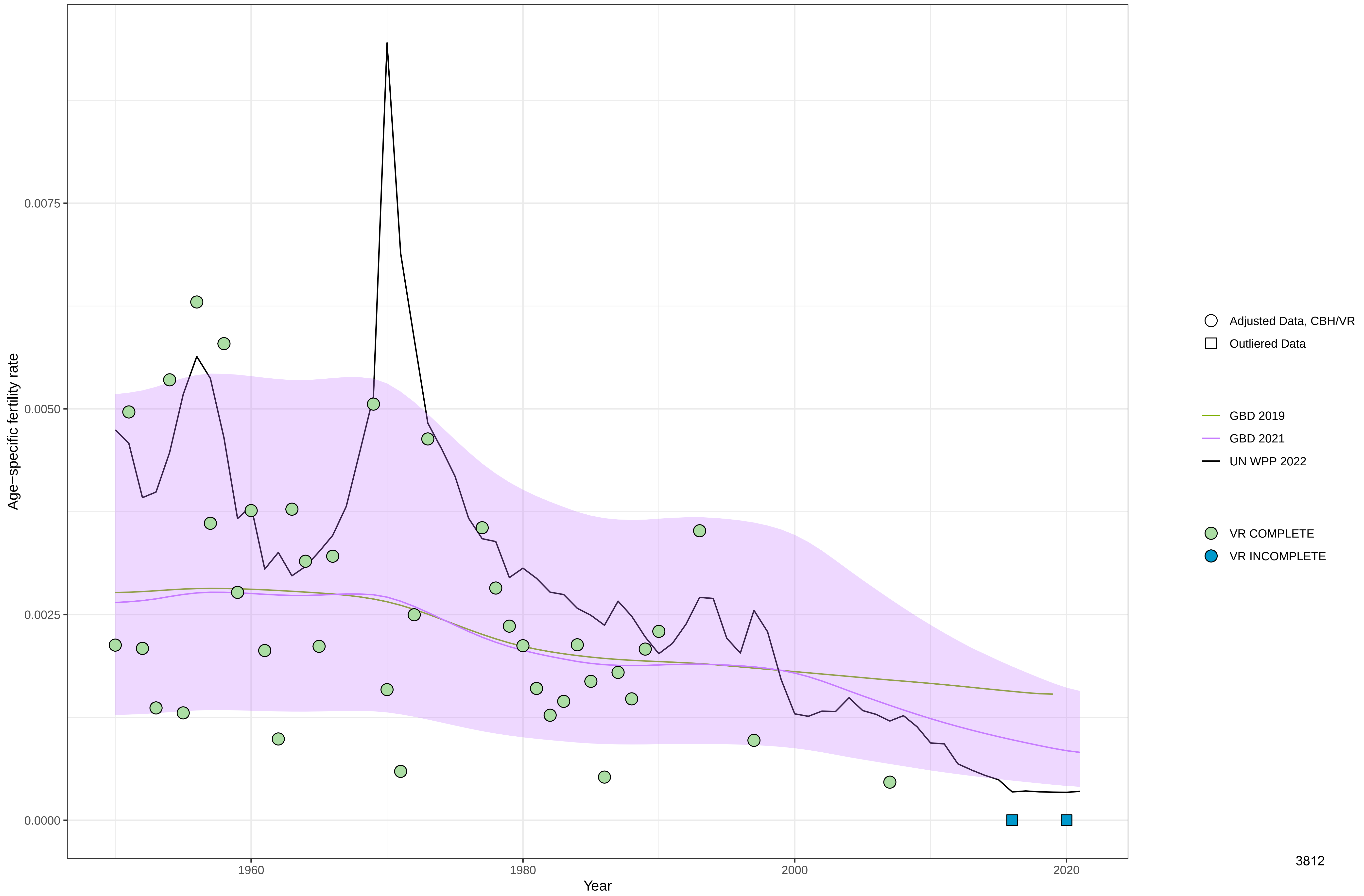

Age−specific fertility fitted trend lines, 1950−2021: Virgin Islands, Ages: 15−19
Data points enclosed with a diamond are reference sources

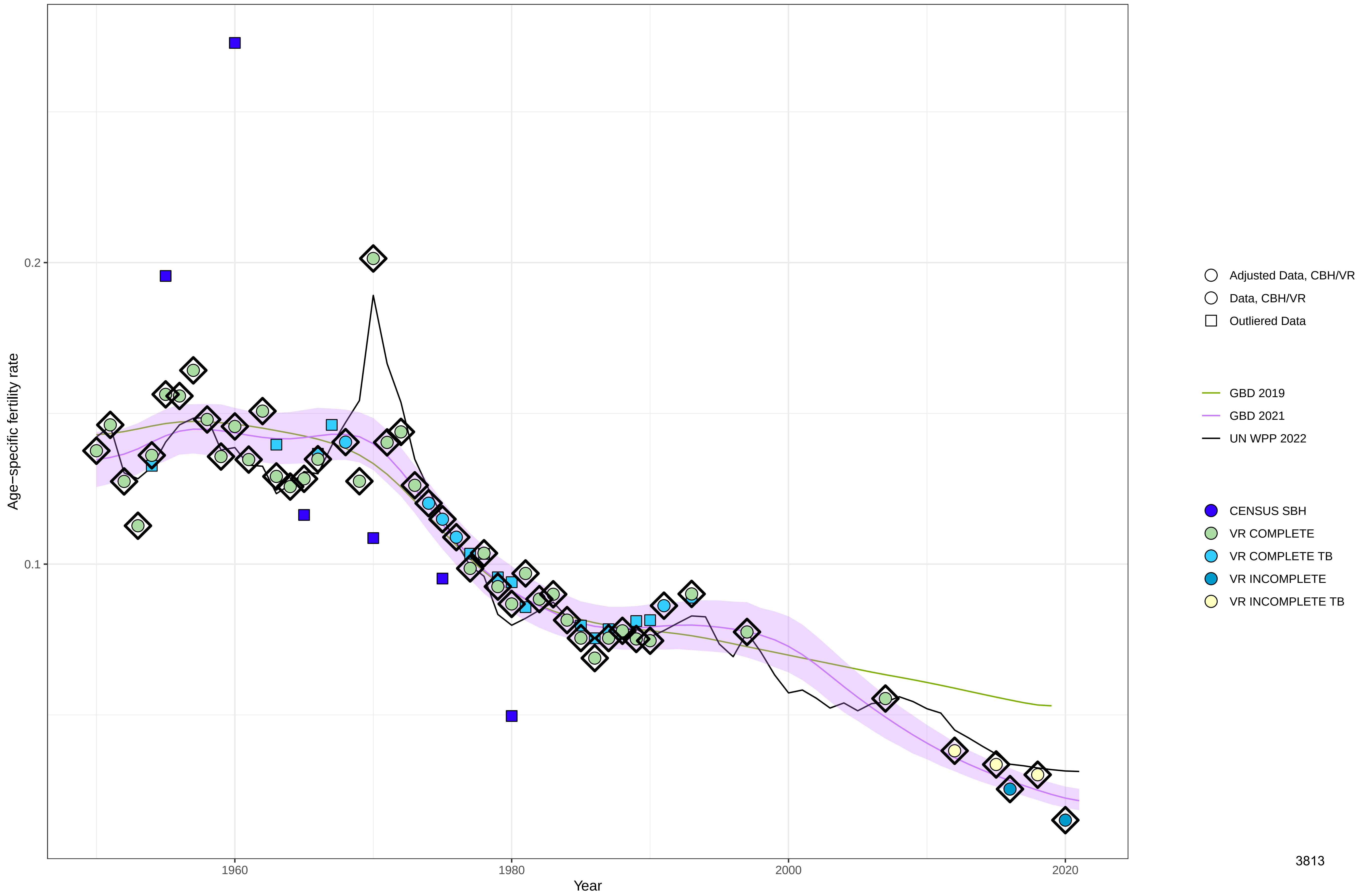

Age−specific fertility fitted trend lines, 1950−2021: Virgin Islands, Ages: 20−24
Data points enclosed with a diamond are reference sources

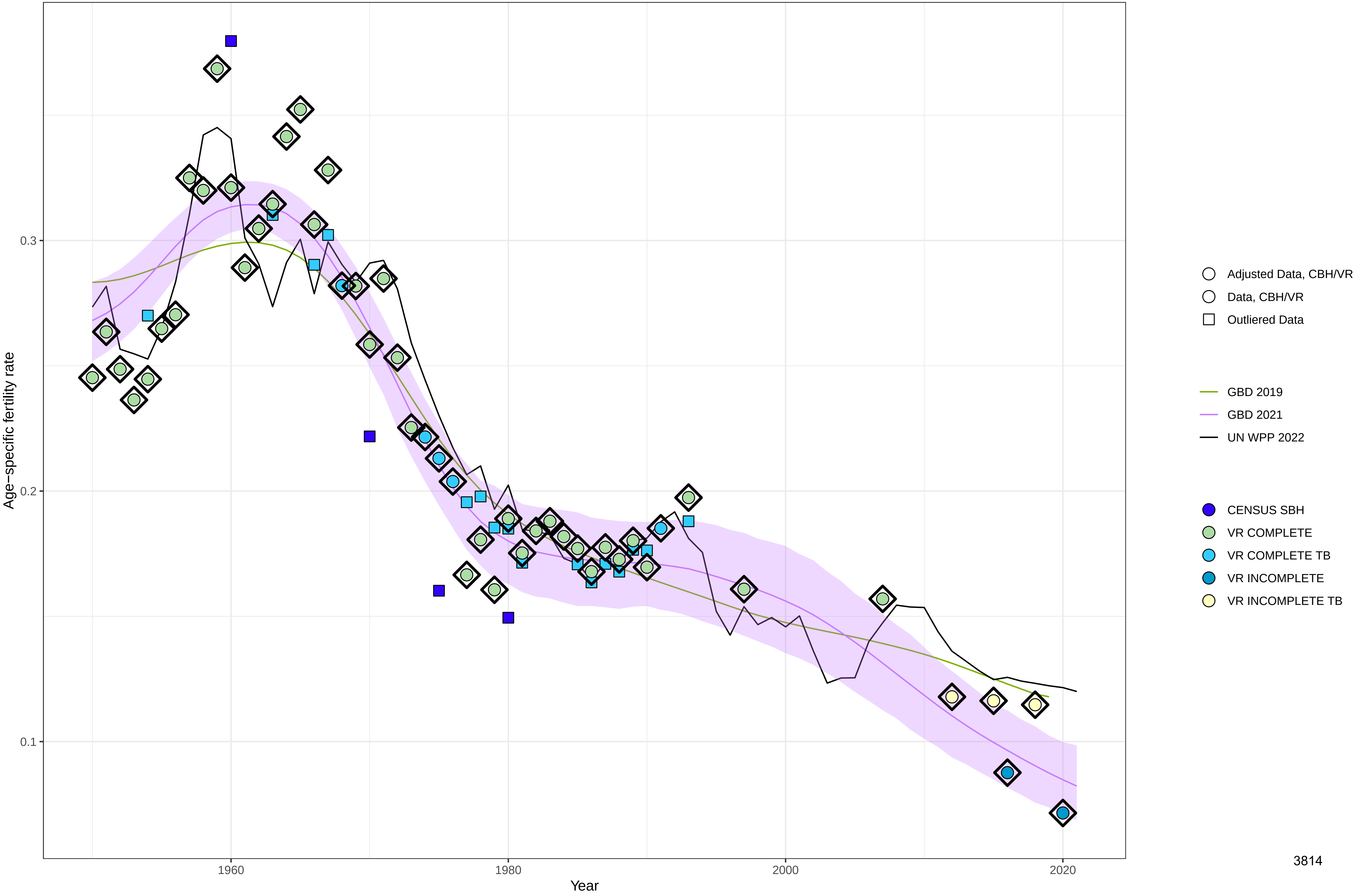

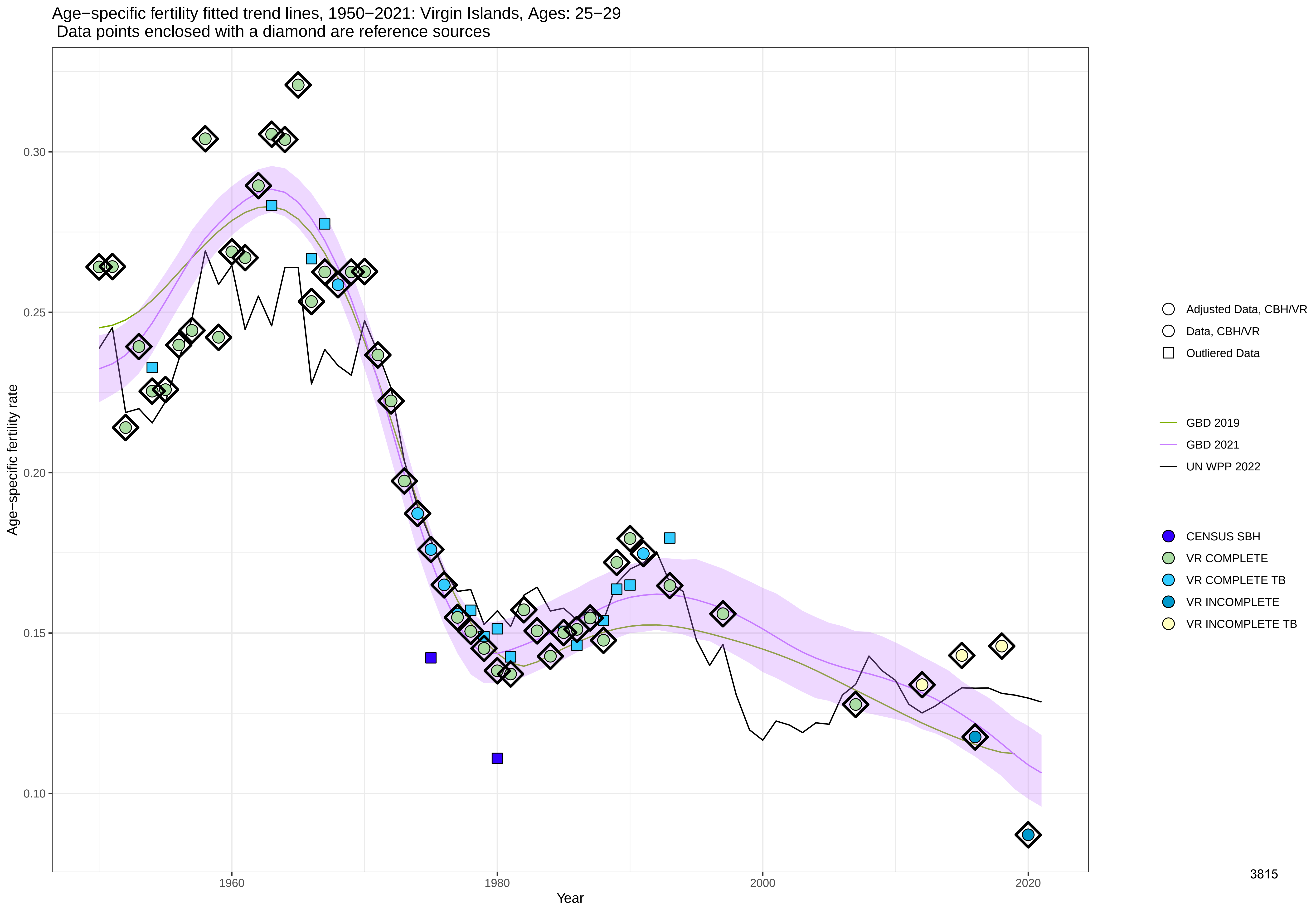
Age−specific fertility fitted trend lines, 1950−2021: Virgin Islands, Ages: 25−29
Data points enclosed with a diamond are reference sources
Age−specific fertility rate
0.10
0.15
0.20
0.25
0.30
Year
1960
1980
2000
2020
Adjusted Data, CBH/VR
Data, CBH/VR
Outliered Data
GBD 2019
GBD 2021
UN WPP 2022
CENSUS SBH
VR COMPLETE
VR COMPLETE TB
VR INCOMPLETE
VR INCOMPLETE TB

Age−specific fertility fitted trend lines, 1950−2021: Virgin Islands, Ages: 30−34
Data points enclosed with a diamond are reference sources

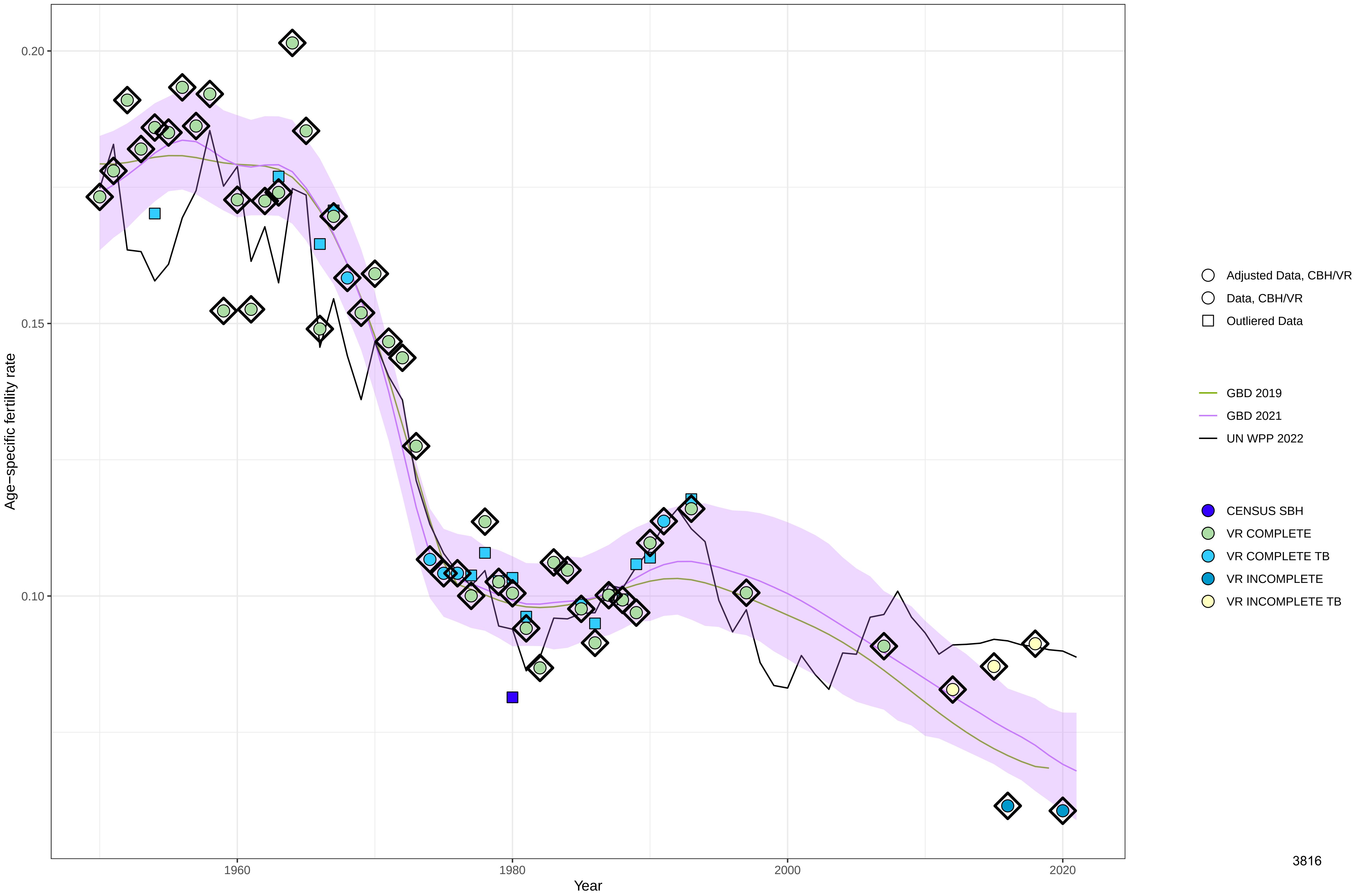

Age−specific fertility fitted trend lines, 1950−2021: Virgin Islands, Ages: 35−39
Data points enclosed with a diamond are reference sources

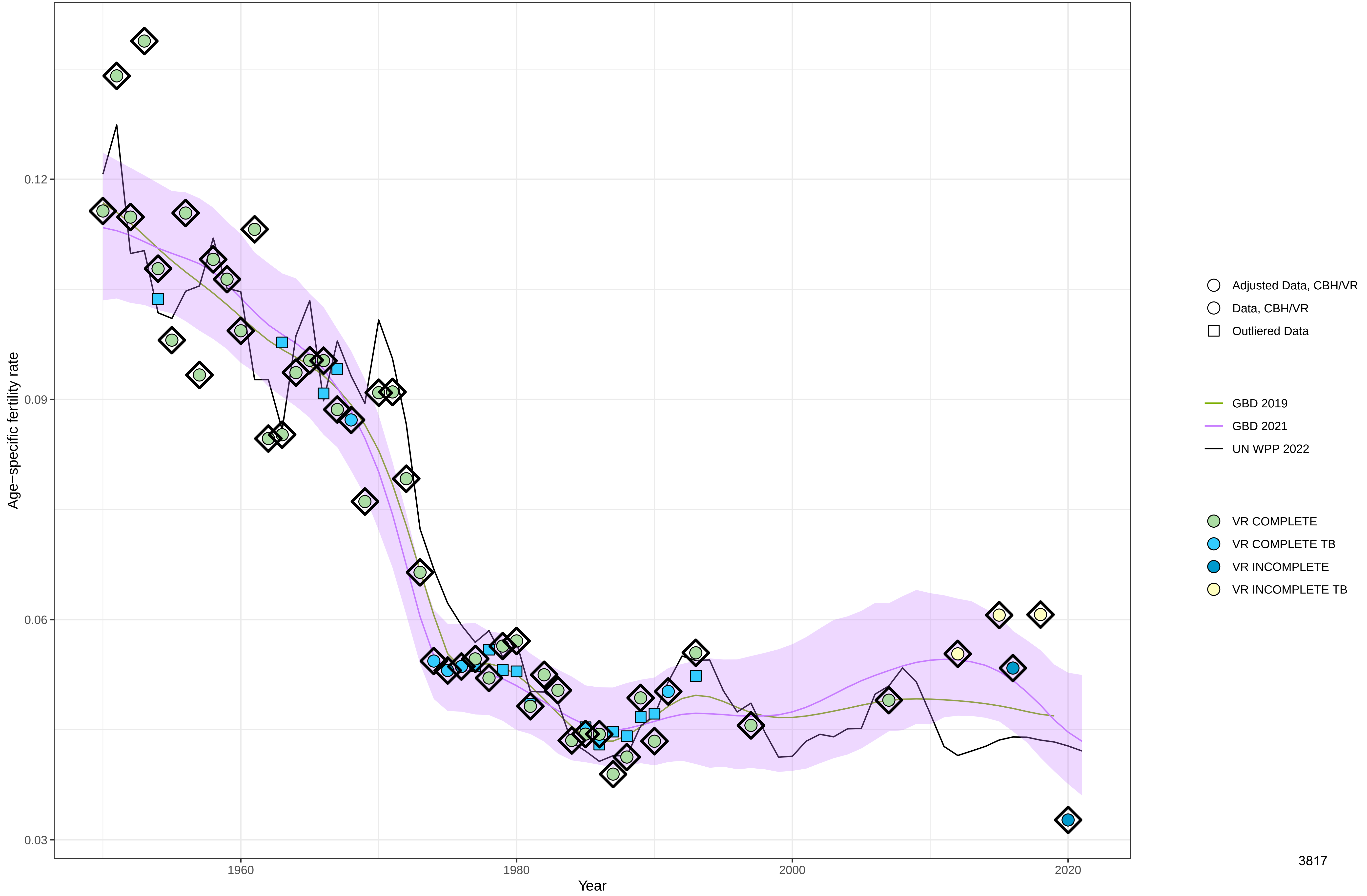

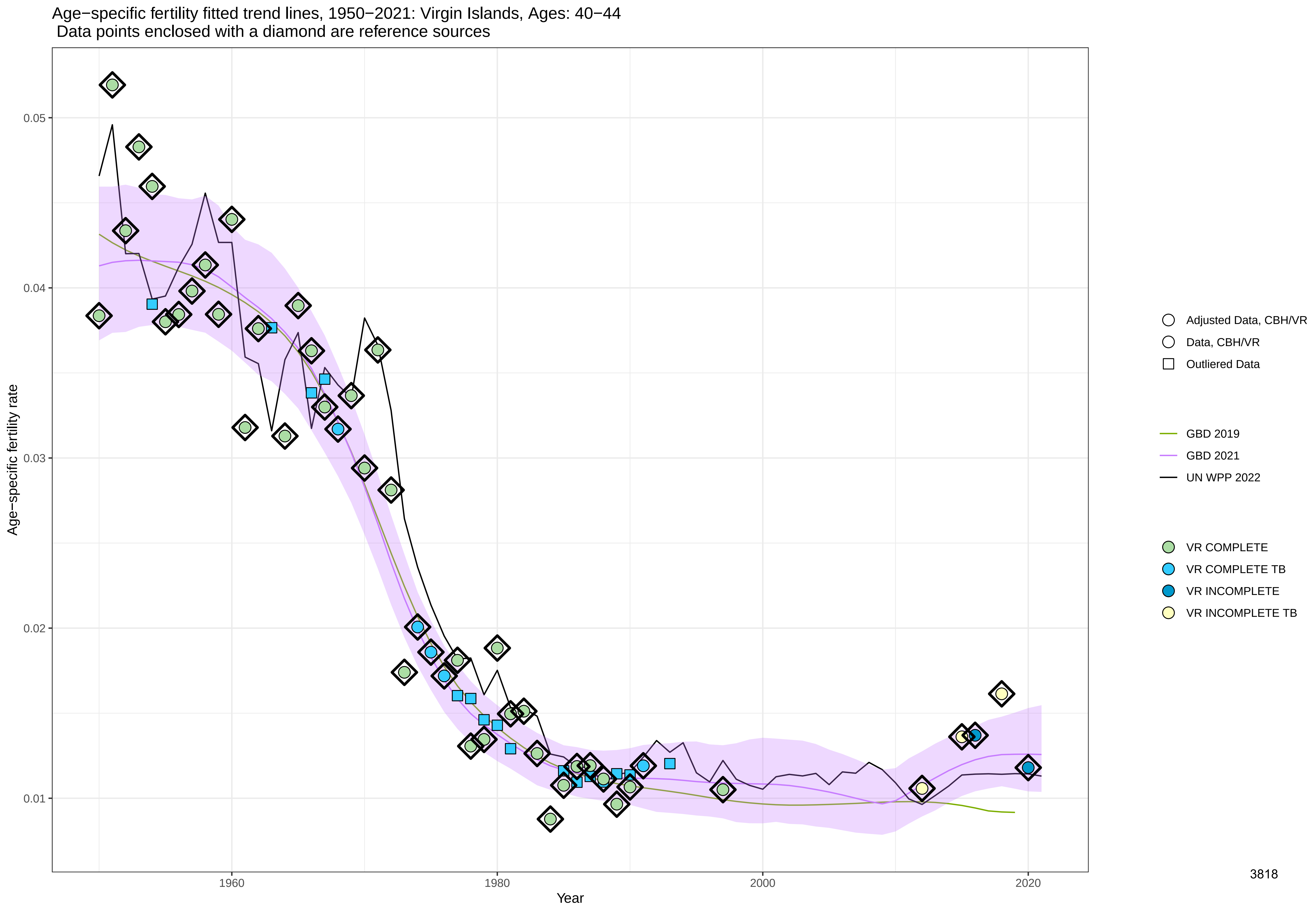

Age−specific fertility fitted trend lines, 1950−2021: Virgin Islands, Ages: 40−44
Data points enclosed with a diamond are reference sources
Age−specific fertility rate
Year
0.01
0.02
0.03
0.04
0.05
1960
1980
2000
2020
Adjusted Data, CBH/VR
Data, CBH/VR
Outliered Data
GBD 2019
GBD 2021
UN WPP 2022
VR COMPLETE
VR COMPLETE TB
VR INCOMPLETE
VR INCOMPLETE TB

Age−specific fertility fitted trend lines, 1950−2021: Virgin Islands, Ages: 45−49
Data points enclosed with a diamond are reference sources

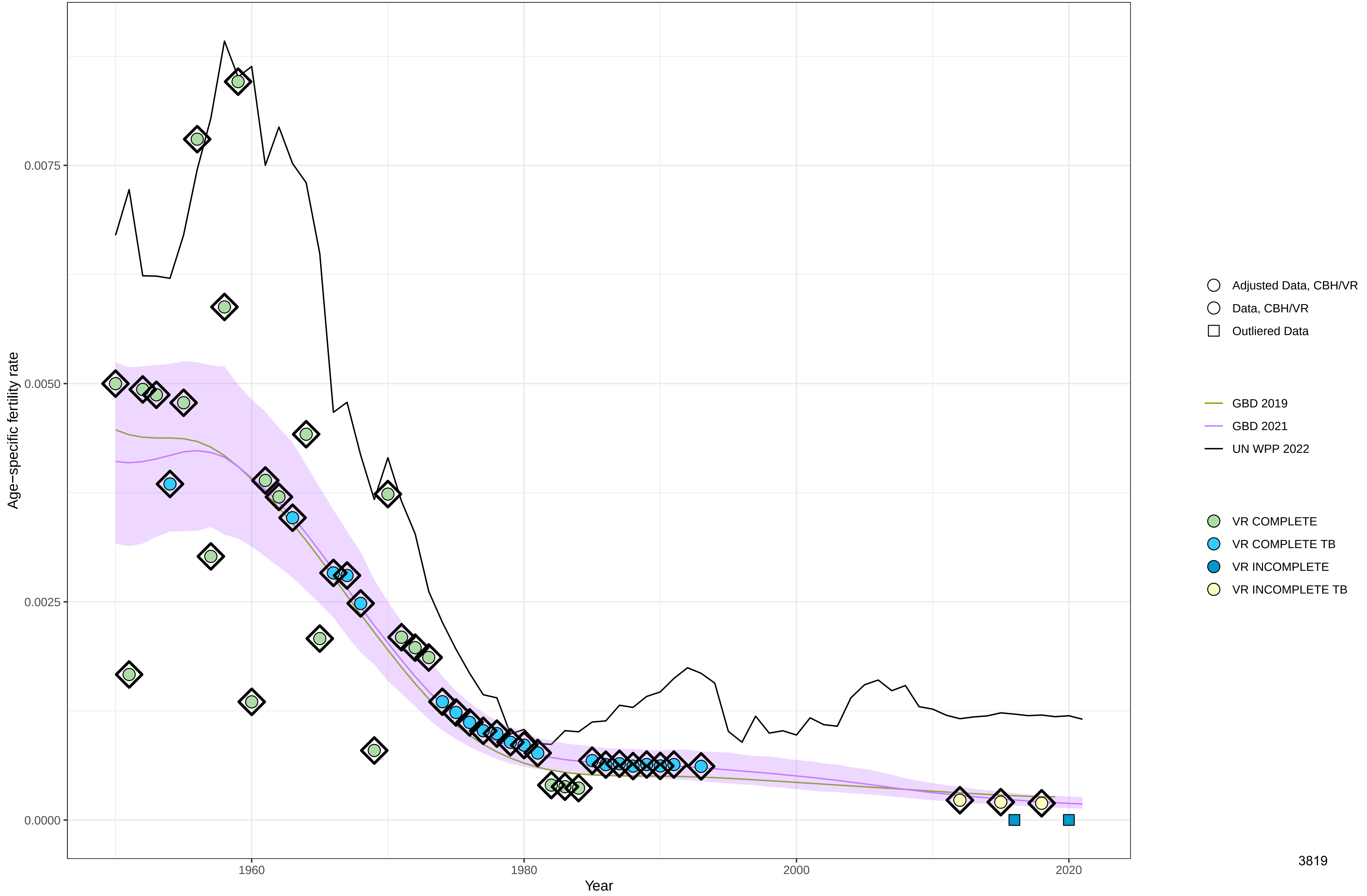

Age−specific fertility fitted trend lines, 1950−2021: Virgin Islands, Ages: 50−54
Data points enclosed with a diamond are reference sources

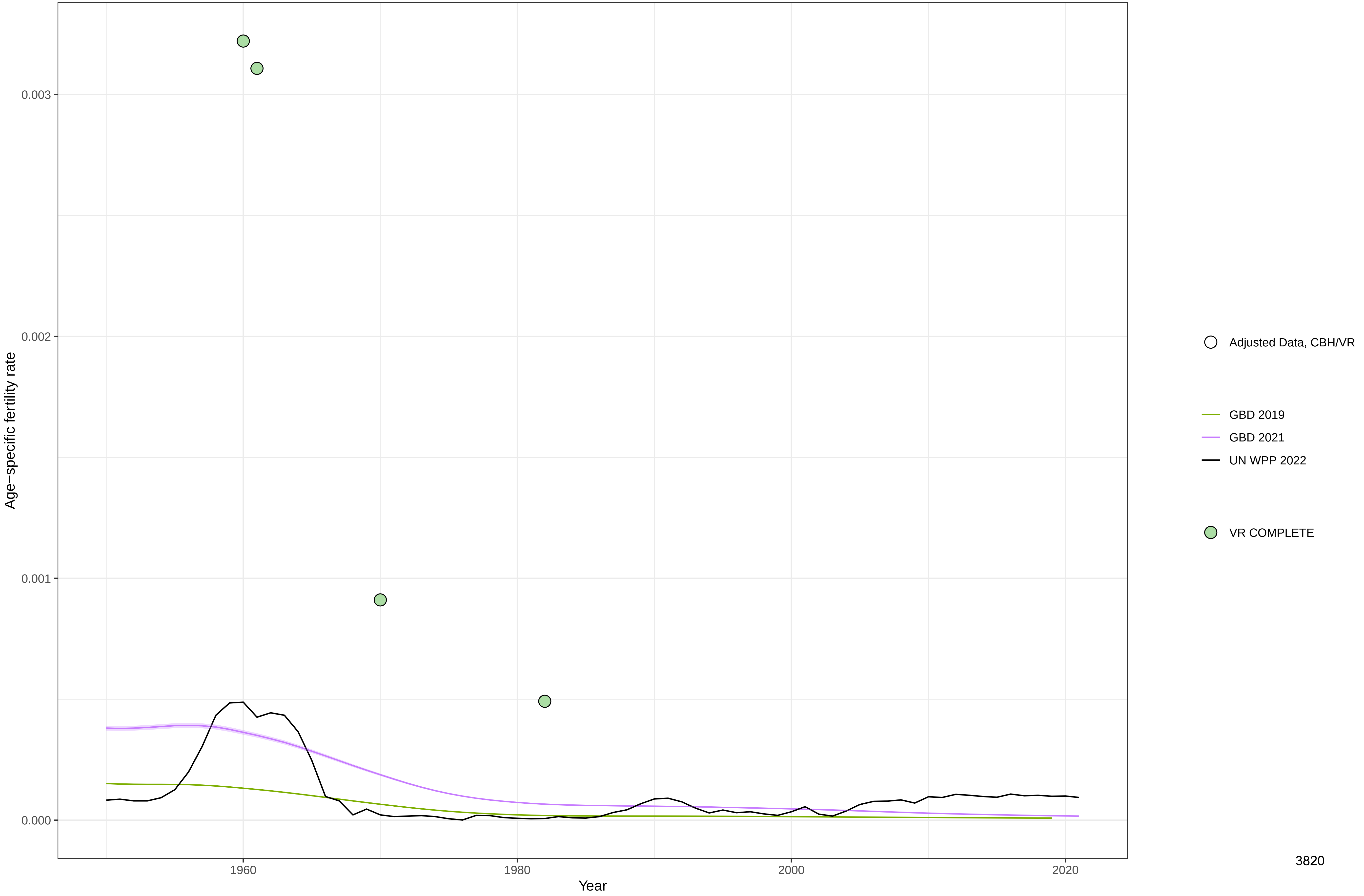

Total fertility rate fitted trend lines, 1950−2021: Virgin Islands TFR
Data points enclosed with a diamond are reference sources

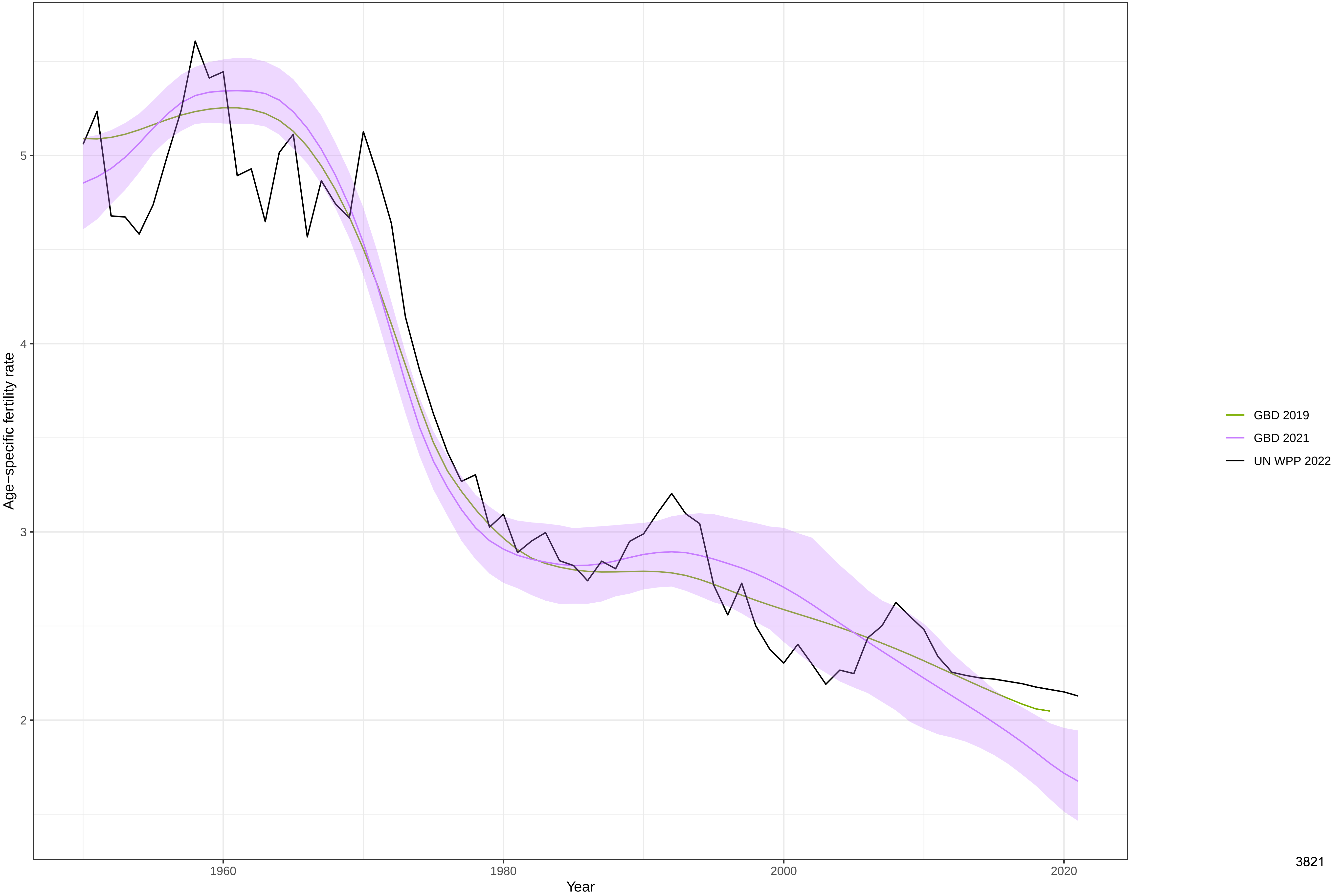

Age−specific fertility fitted trend lines, 1950−2021: Colombia, Ages: 10−14
Data points enclosed with a diamond are reference sources

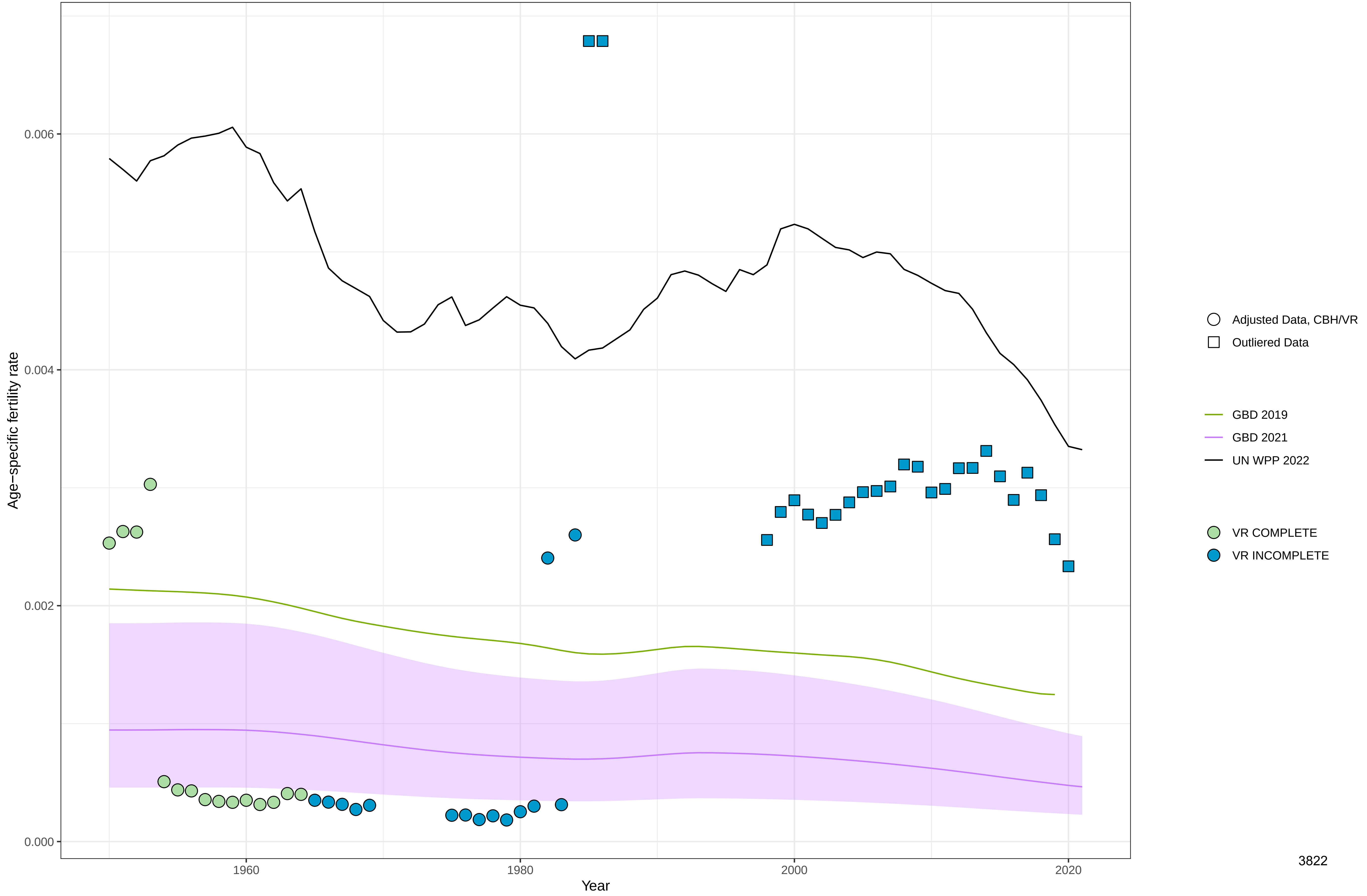

Age−specific fertility fitted trend lines, 1950−2021: Colombia, Ages: 15−19
Data points enclosed with a diamond are reference sources

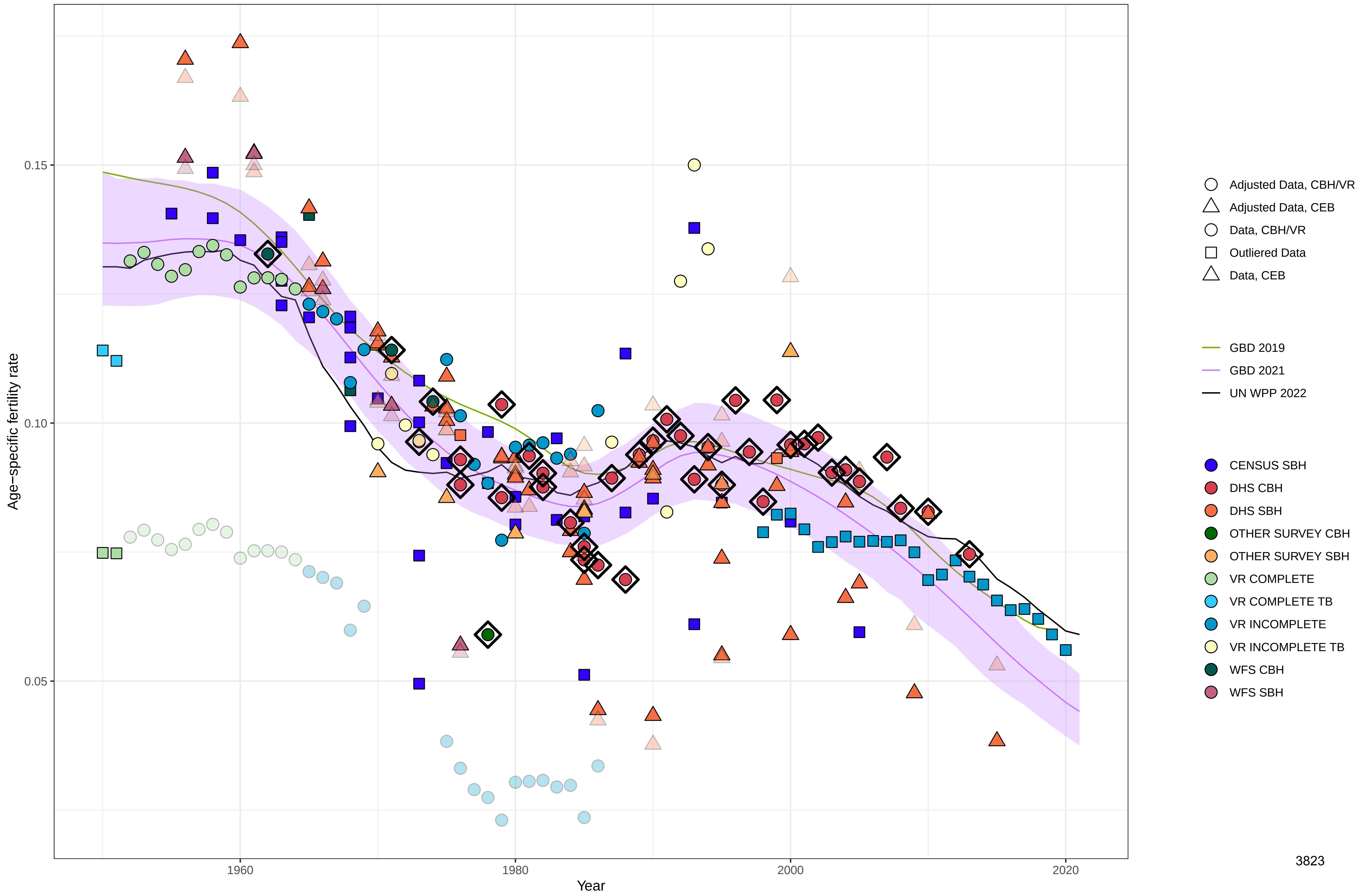

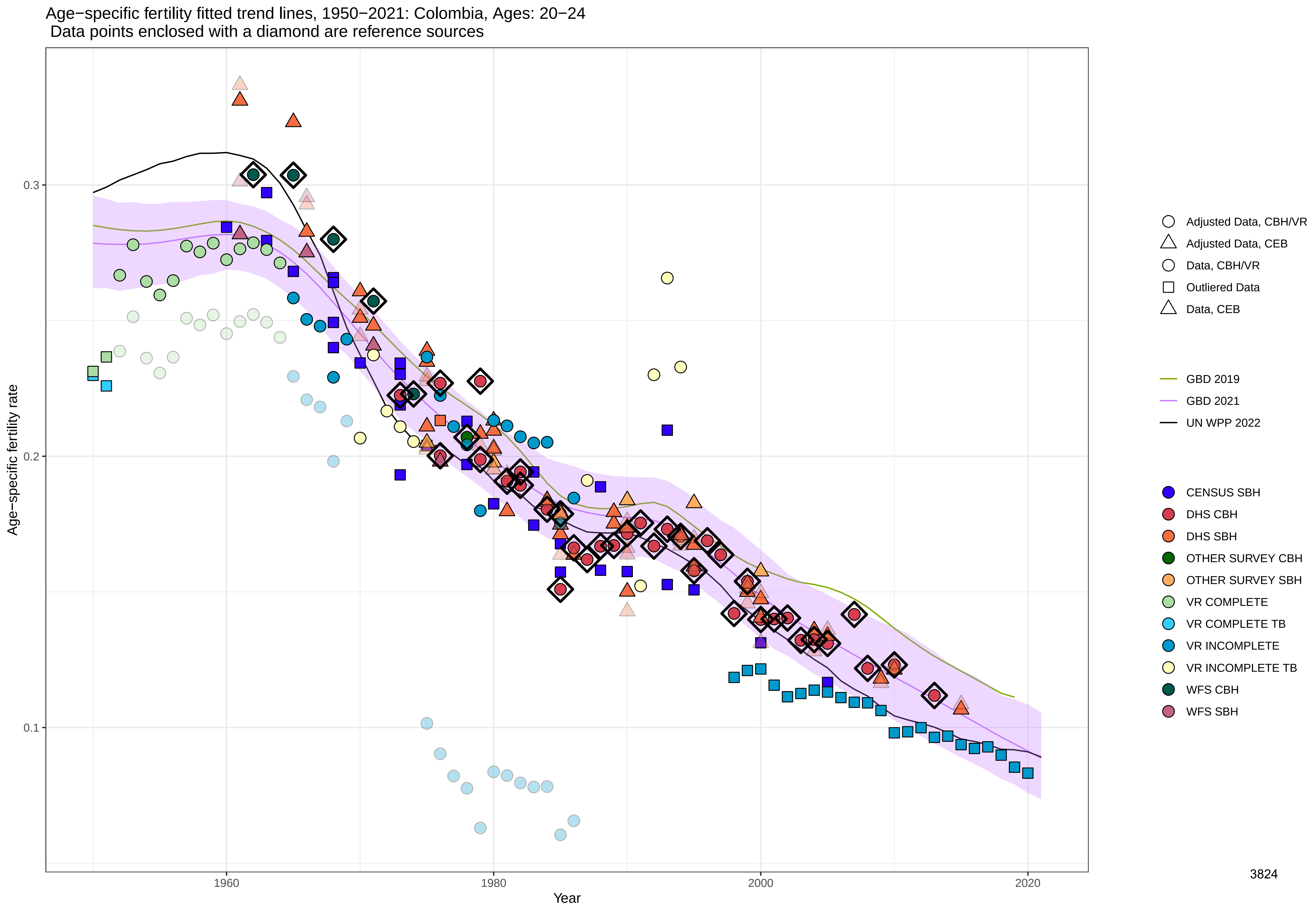

Age−specific fertility fitted trend lines, 1950−2021: Colombia, Ages: 20−24
Data points enclosed with a diamond are reference sources
Age−specific fertility rate
Year
0.1
0.2
0.3
1960
1980
2000
2020
Adjusted Data, CBH/VR
Adjusted Data, CEB
Data, CBH/VR
Outliered Data
Data, CEB
GBD 2019
GBD 2021
UN WPP 2022
CENSUS SBH
DHS CBH
DHS SBH
OTHER SURVEY CBH
OTHER SURVEY SBH
VR COMPLETE
VR COMPLETE TB
VR INCOMPLETE
VR INCOMPLETE TB
WFS CBH
WFS SBH

Age−specific fertility fitted trend lines, 1950−2021: Colombia, Ages: 25−29
Data points enclosed with a diamond are reference sources

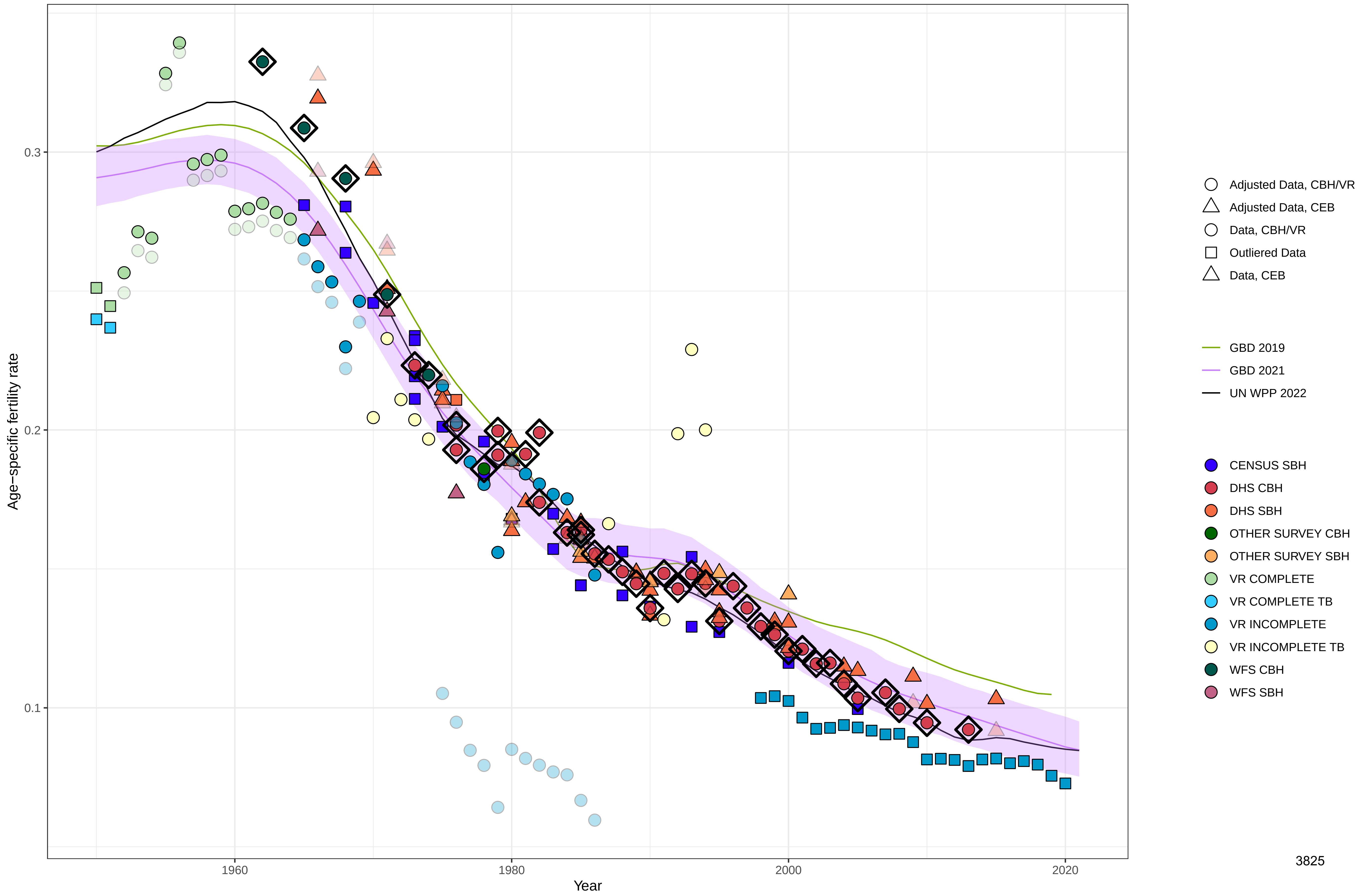

Age−specific fertility fitted trend lines, 1950−2021: Colombia, Ages: 30−34
Data points enclosed with a diamond are reference sources

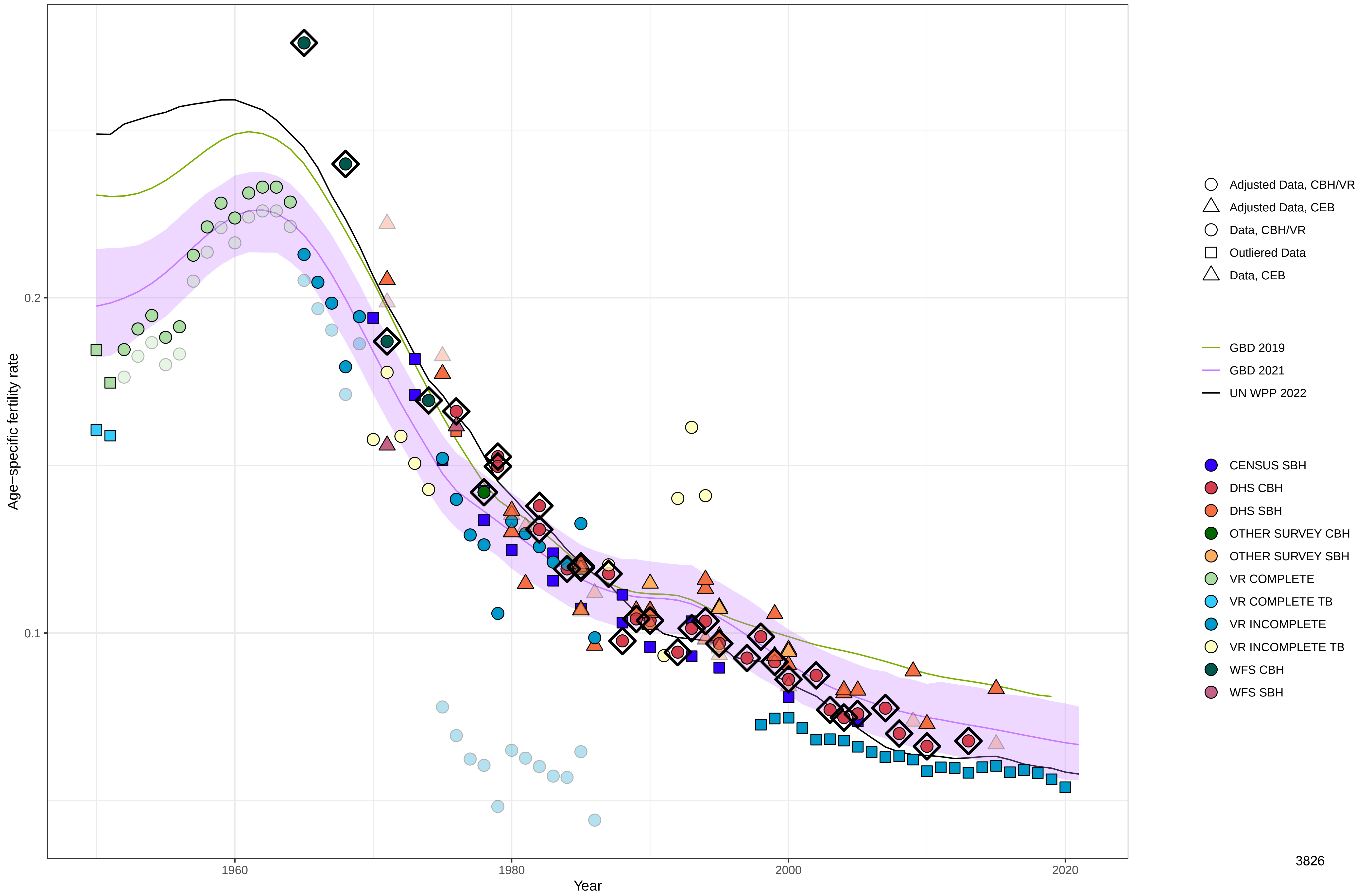

Age−specific fertility fitted trend lines, 1950−2021: Colombia, Ages: 35−39
Data points enclosed with a diamond are reference sources

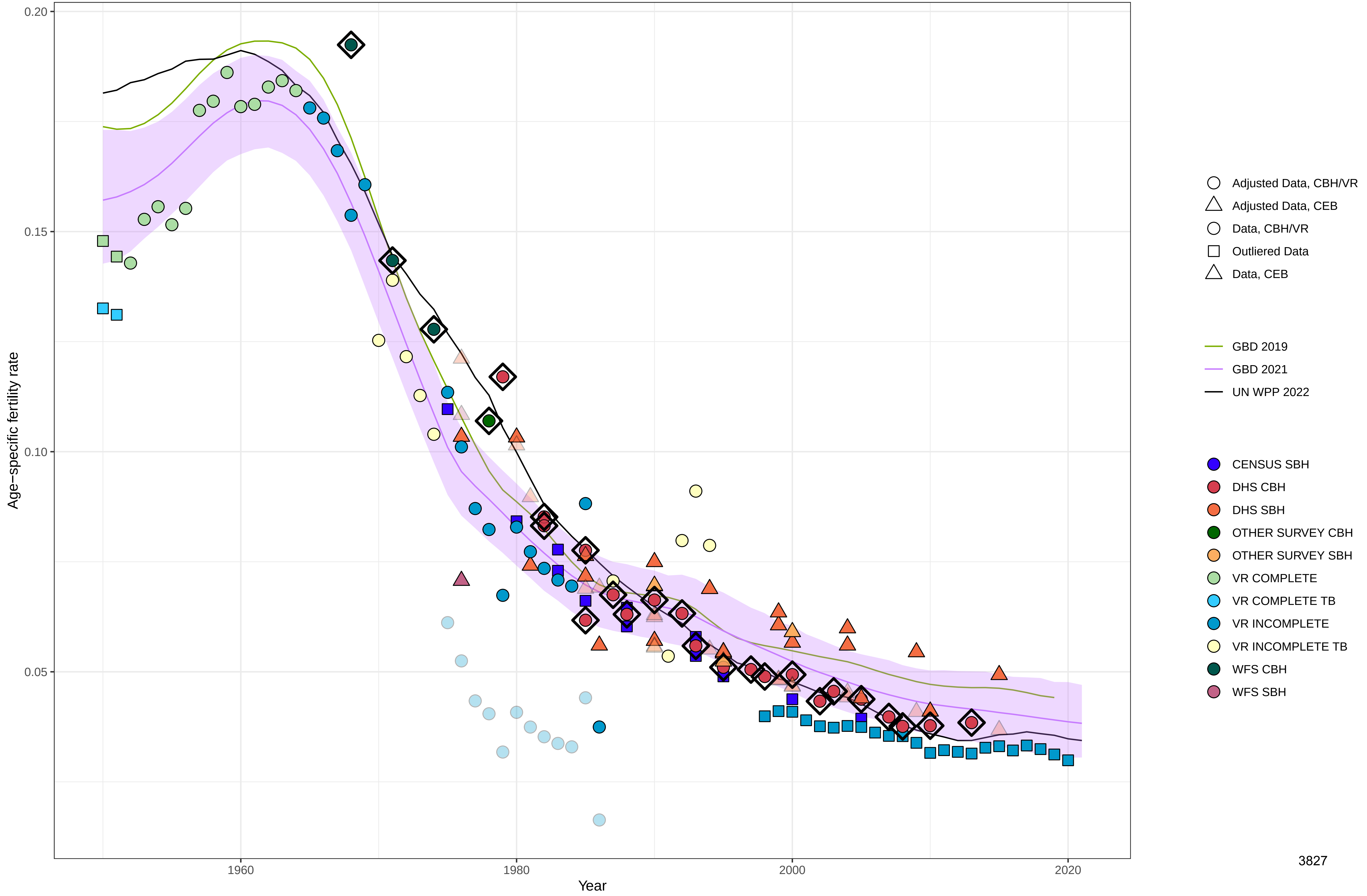

Age−specific fertility fitted trend lines, 1950−2021: Colombia, Ages: 40−44
Data points enclosed with a diamond are reference sources

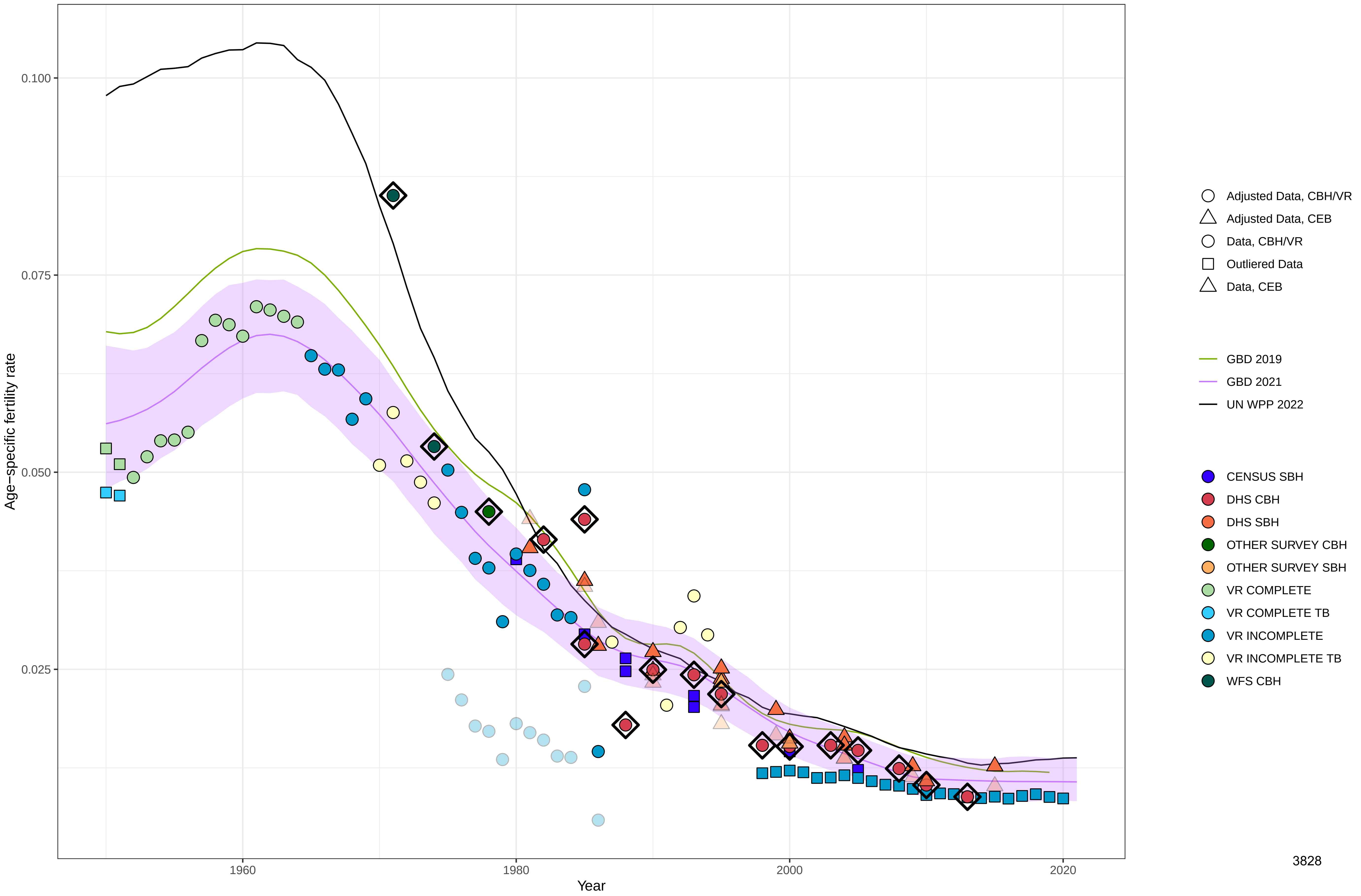

Age−specific fertility fitted trend lines, 1950−2021: Colombia, Ages: 45−49
Data points enclosed with a diamond are reference sources

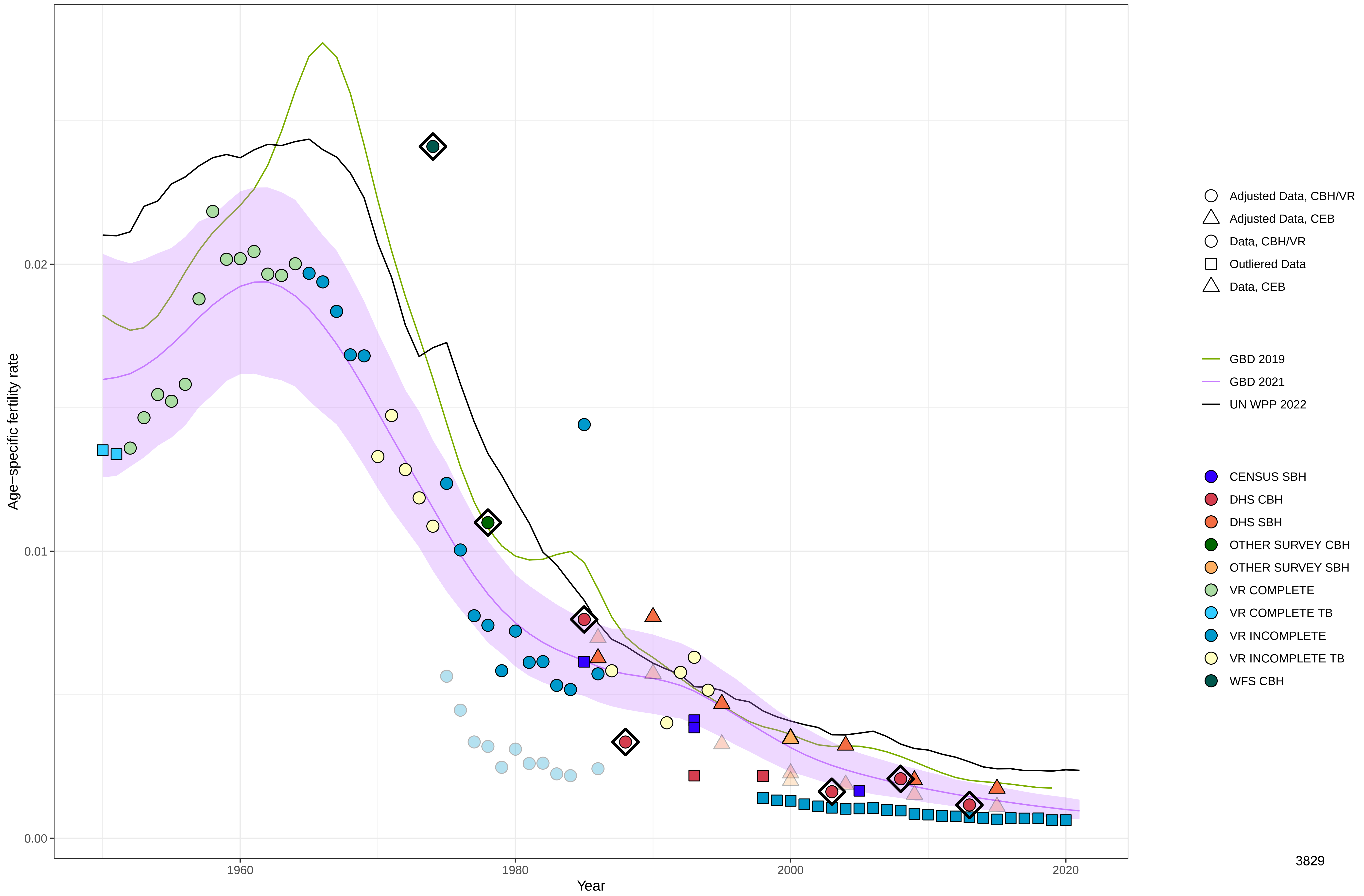

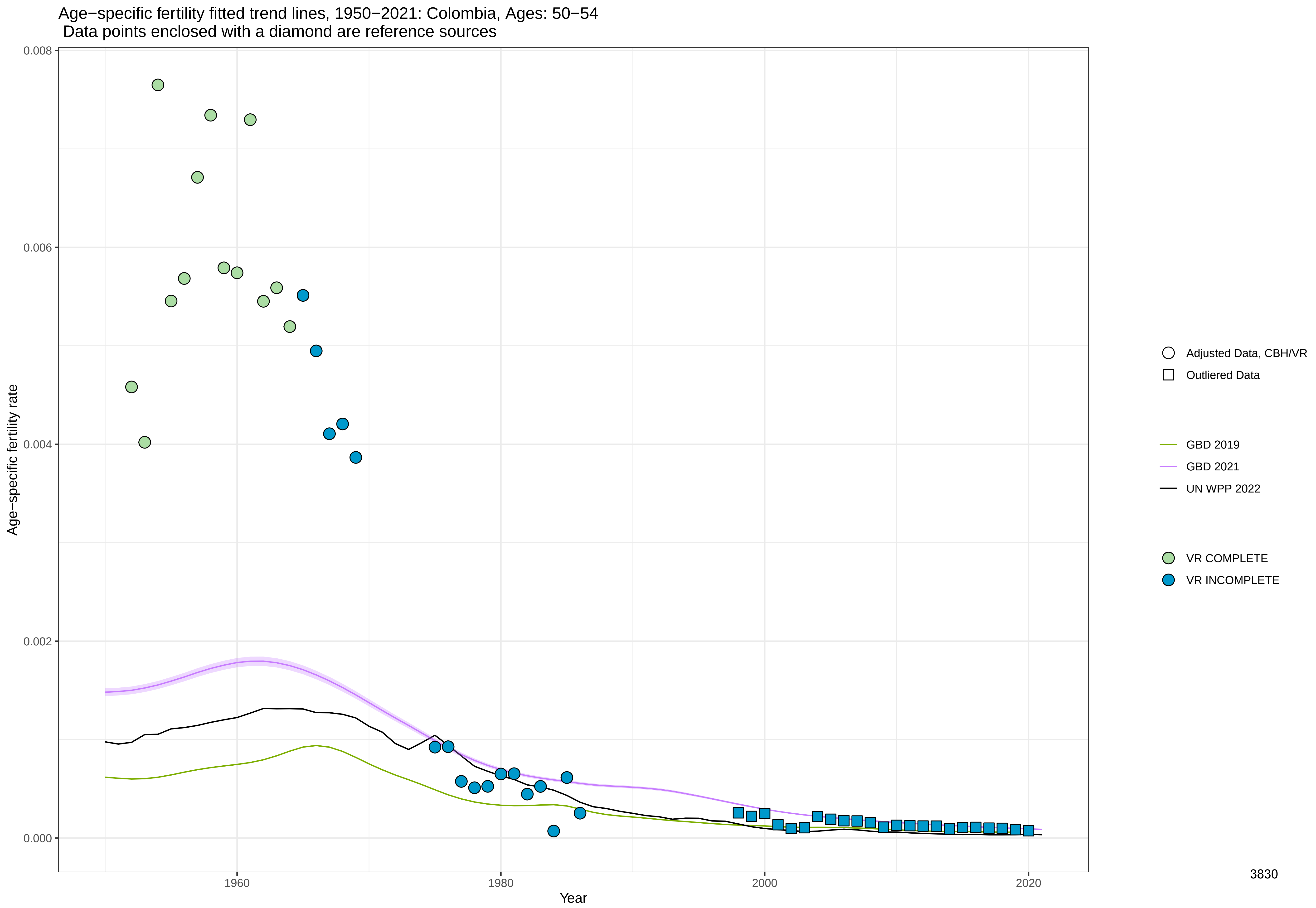

Age−specific fertility fitted trend lines, 1950−2021: Colombia, Ages: 50−54
Data points enclosed with a diamond are reference sources
0.008
0.006
0.004
0.002
0.000
Age−specific fertility rate
1960
1980
2000
2020
Year
Adjusted Data, CBH/VR
Outliered Data
GBD 2019
GBD 2021
UN WPP 2022
VR COMPLETE
VR INCOMPLETE

Total fertility rate fitted trend lines, 1950−2021: Colombia TFR
Data points enclosed with a diamond are reference sources

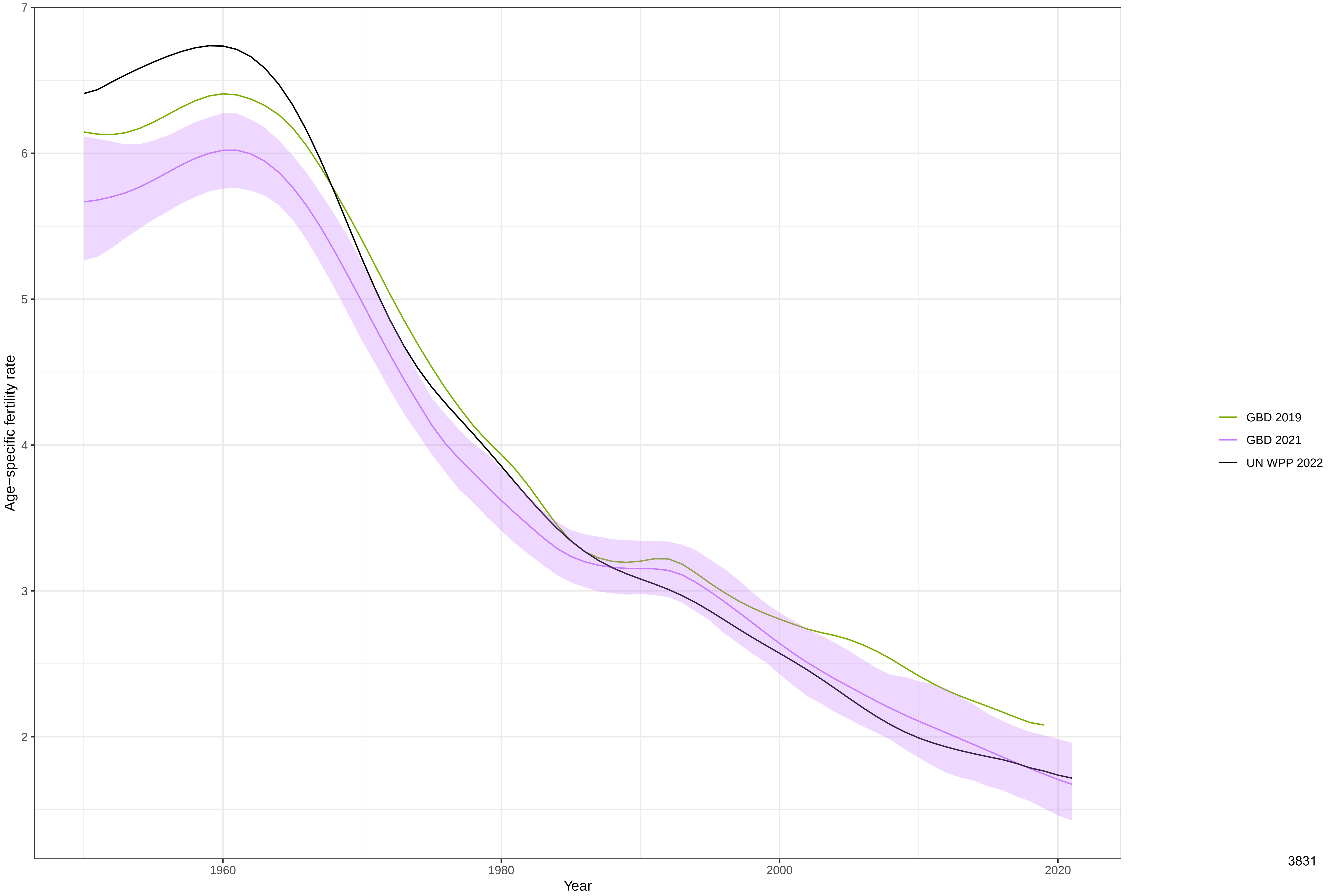

Age−specific fertility fitted trend lines, 1950−2021: Costa Rica, Ages: 10−14
Data points enclosed with a diamond are reference sources

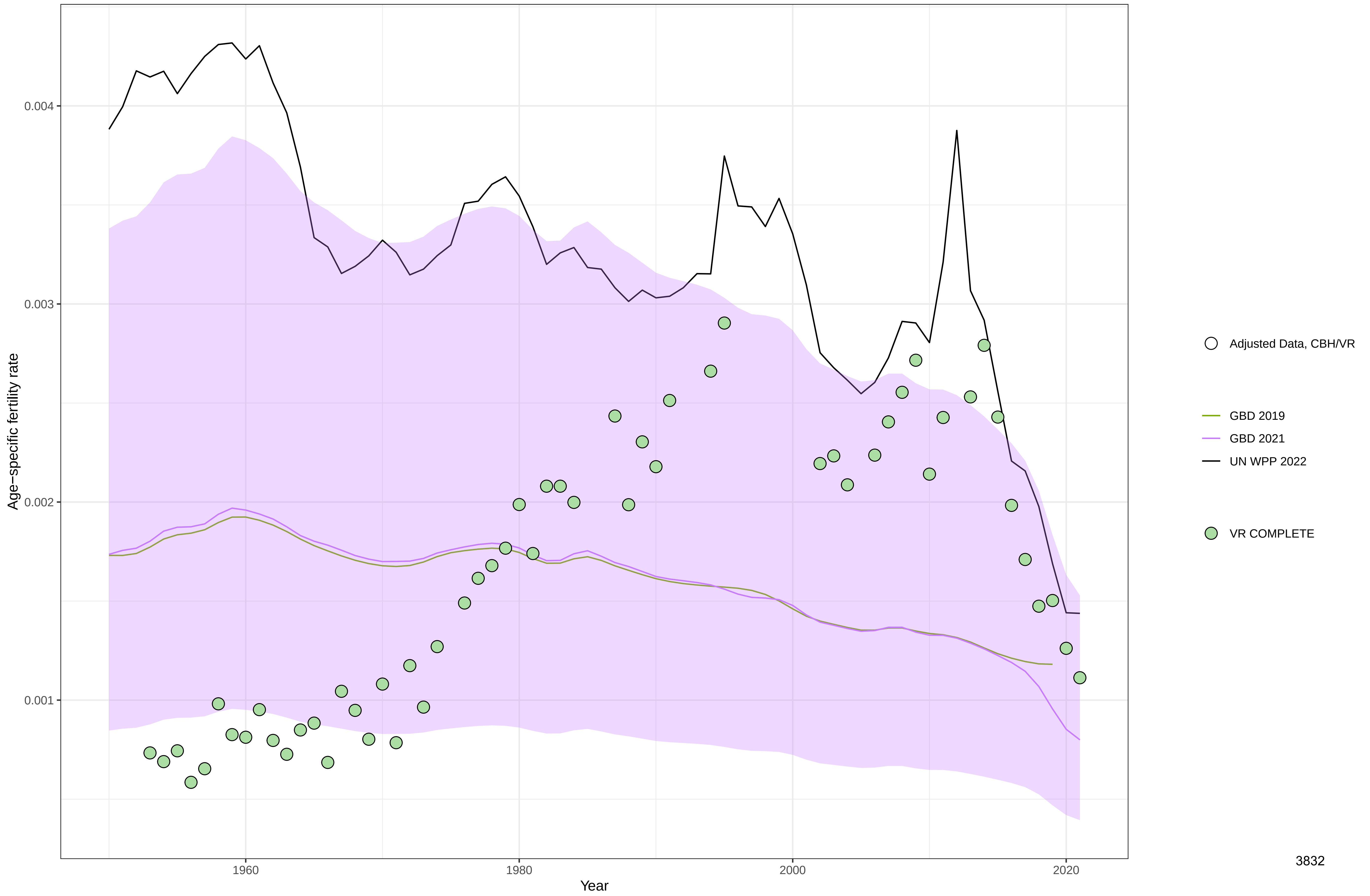

Age−specific fertility fitted trend lines, 1950−2021: Costa Rica, Ages: 15−19
Data points enclosed with a diamond are reference sources

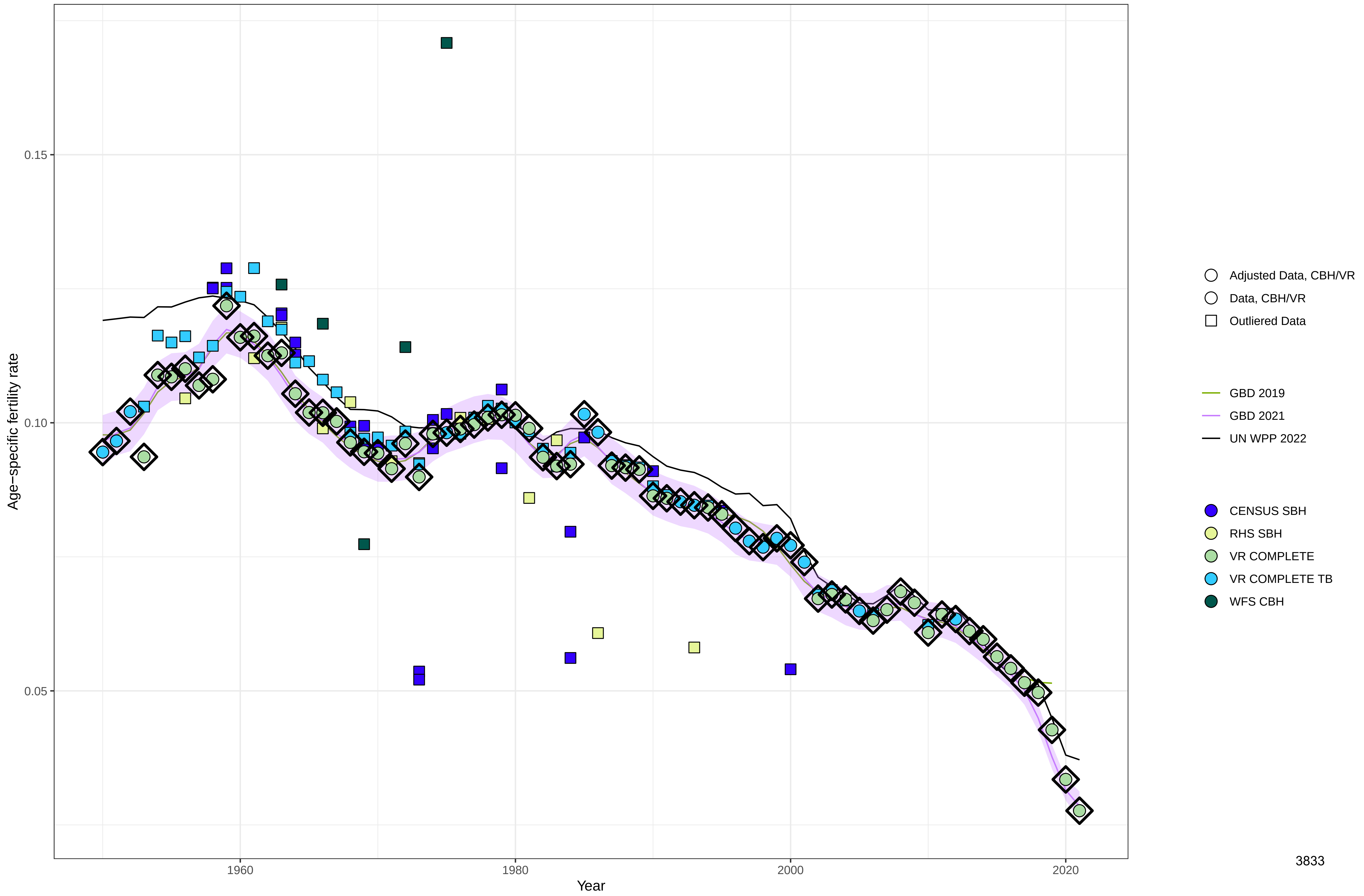

Age−specific fertility fitted trend lines, 1950−2021: Costa Rica, Ages: 20−24
Data points enclosed with a diamond are reference sources

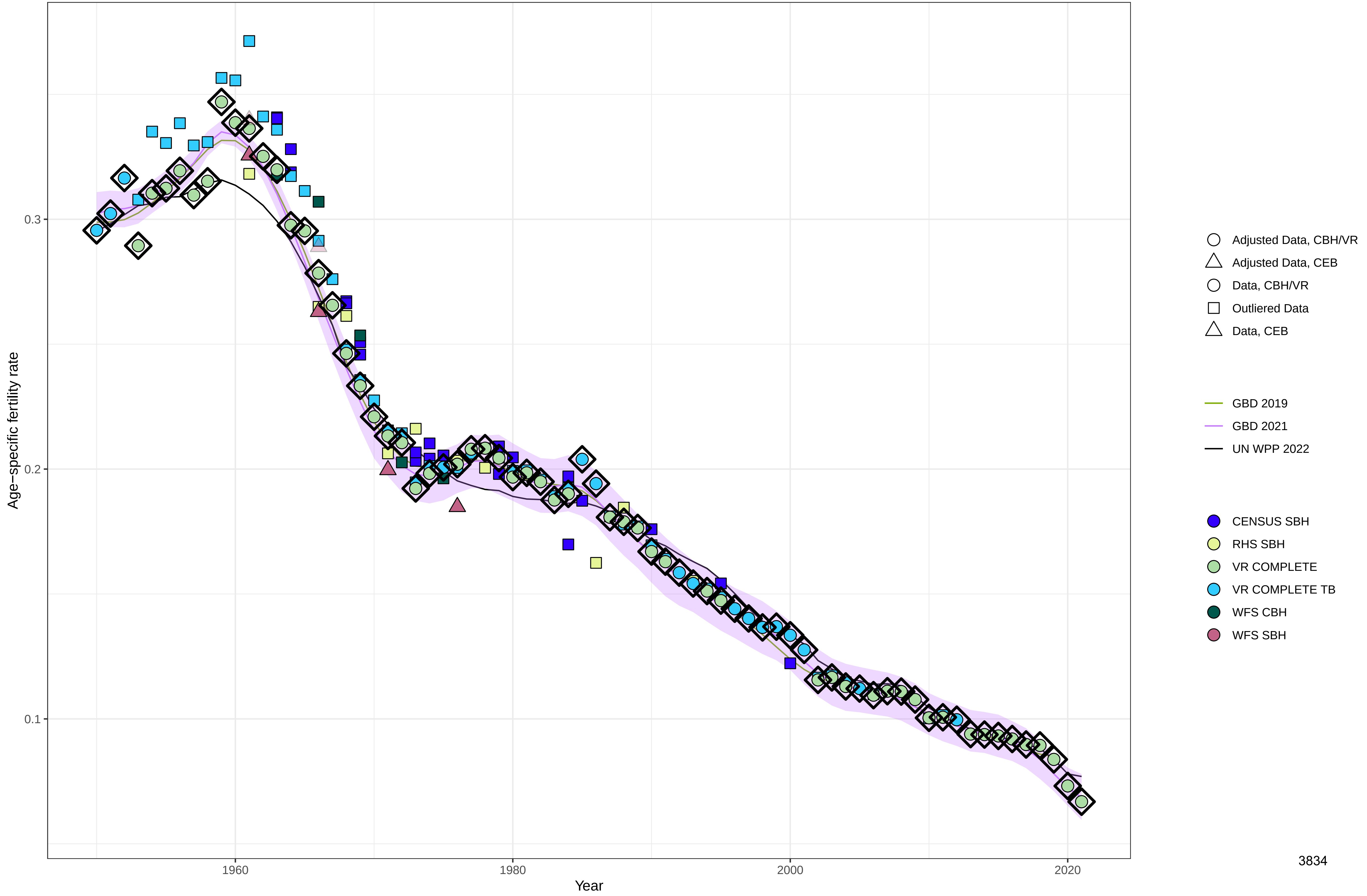

Age−specific fertility fitted trend lines, 1950−2021: Costa Rica, Ages: 25−29
Data points enclosed with a diamond are reference sources

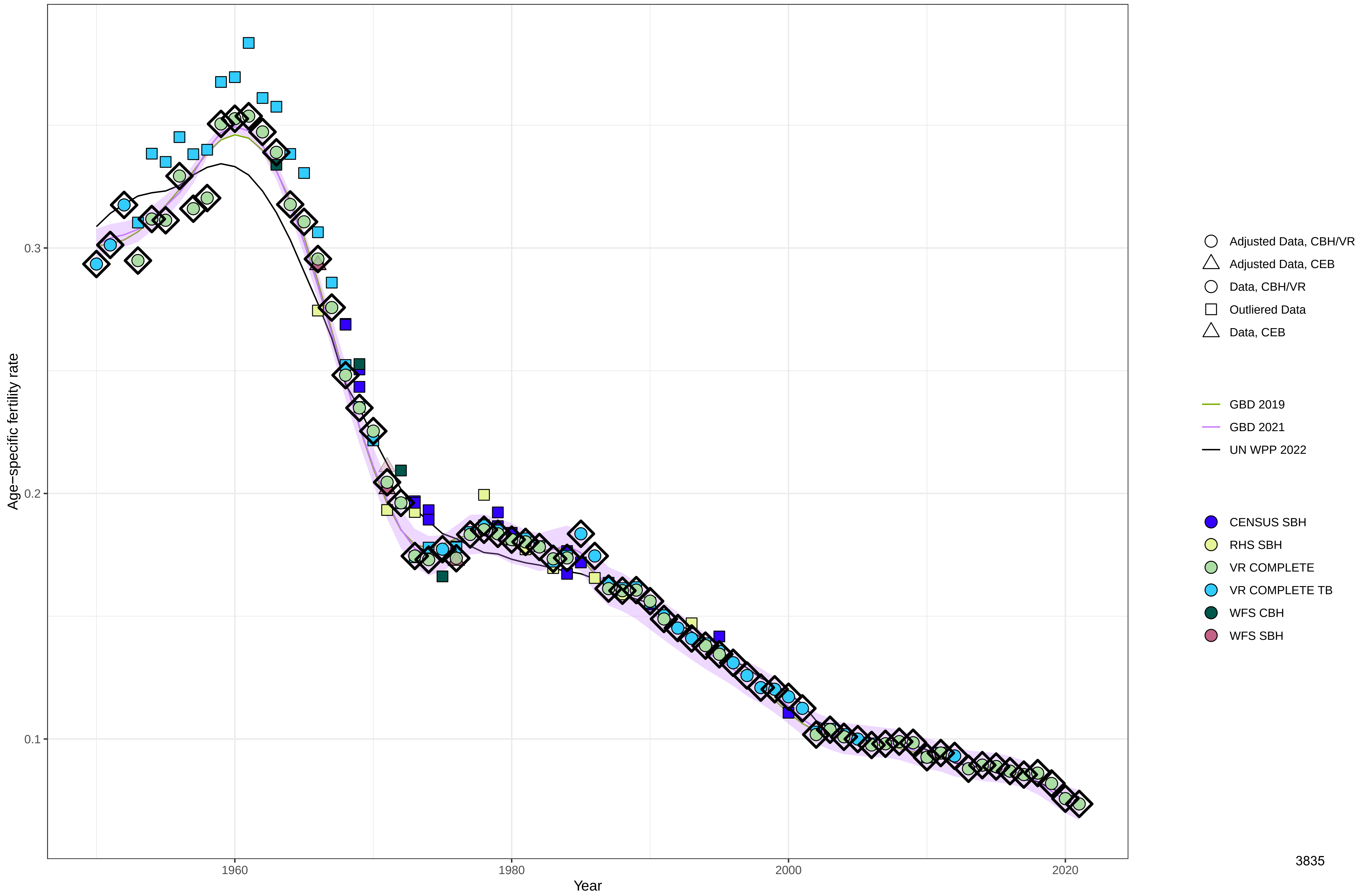

Age−specific fertility fitted trend lines, 1950−2021: Costa Rica, Ages: 30−34
Data points enclosed with a diamond are reference sources

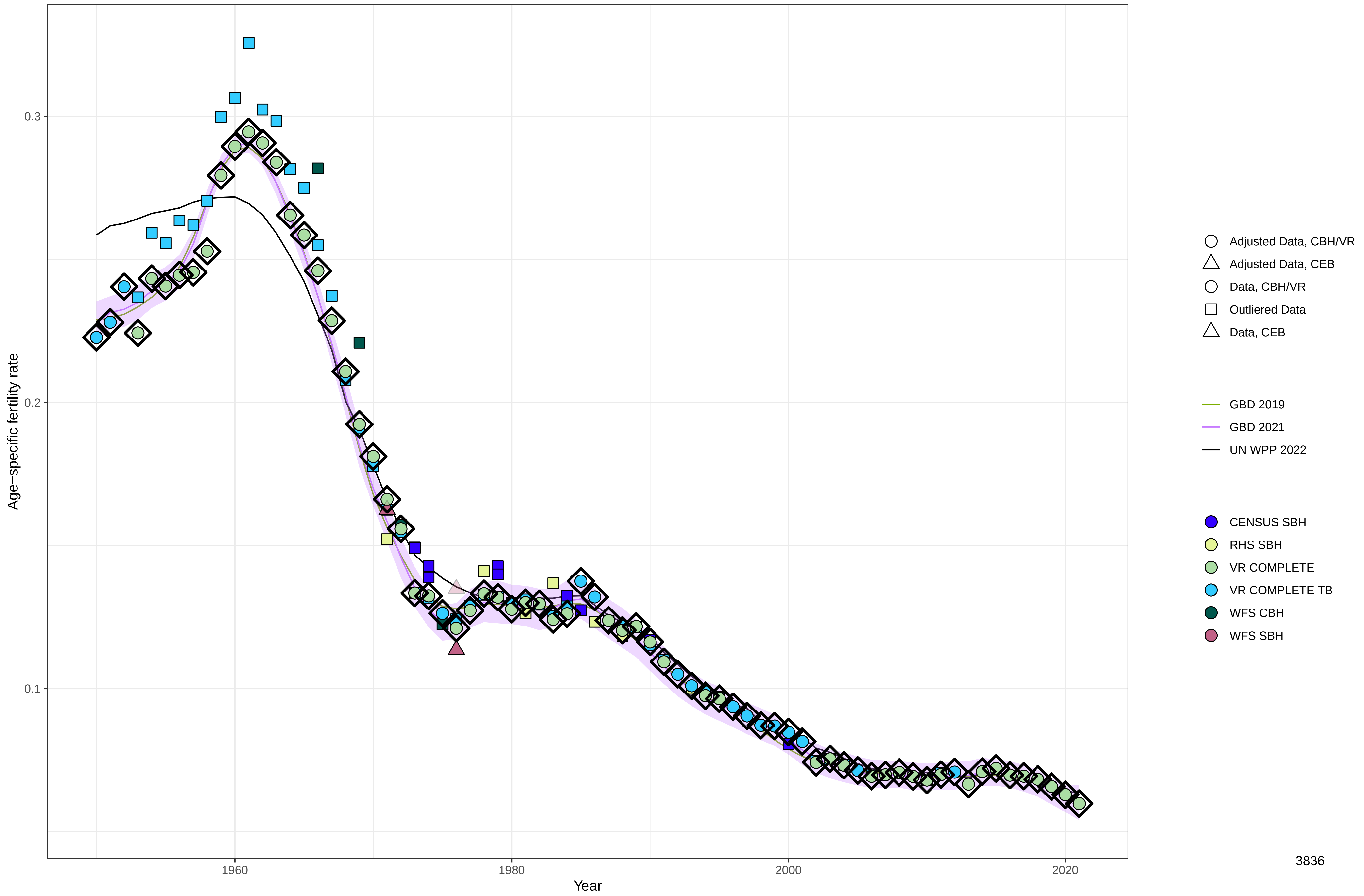

Age−specific fertility fitted trend lines, 1950−2021: Costa Rica, Ages: 35−39
Data points enclosed with a diamond are reference sources

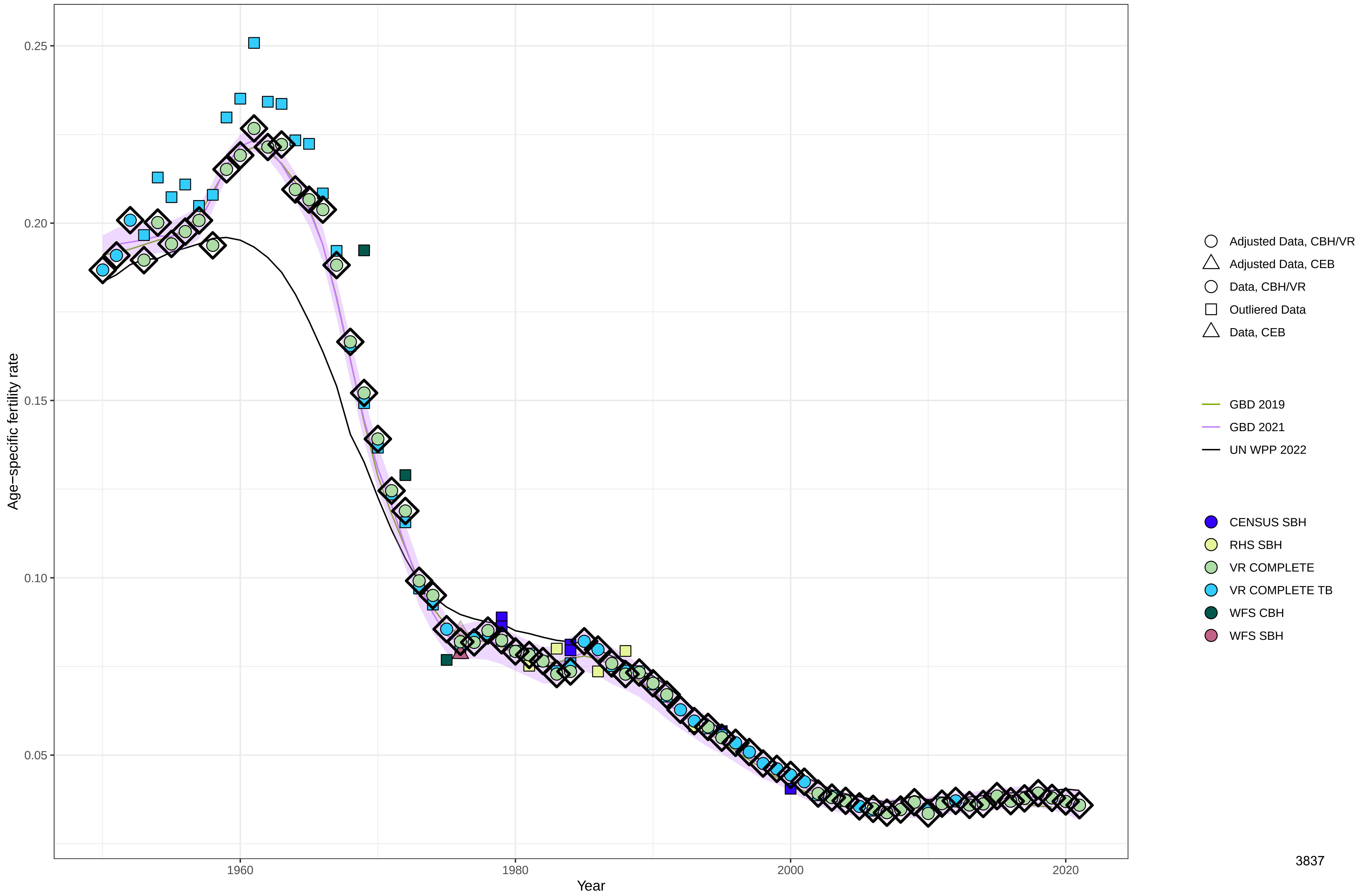

Age−specific fertility fitted trend lines, 1950−2021: Costa Rica, Ages: 40−44
Data points enclosed with a diamond are reference sources

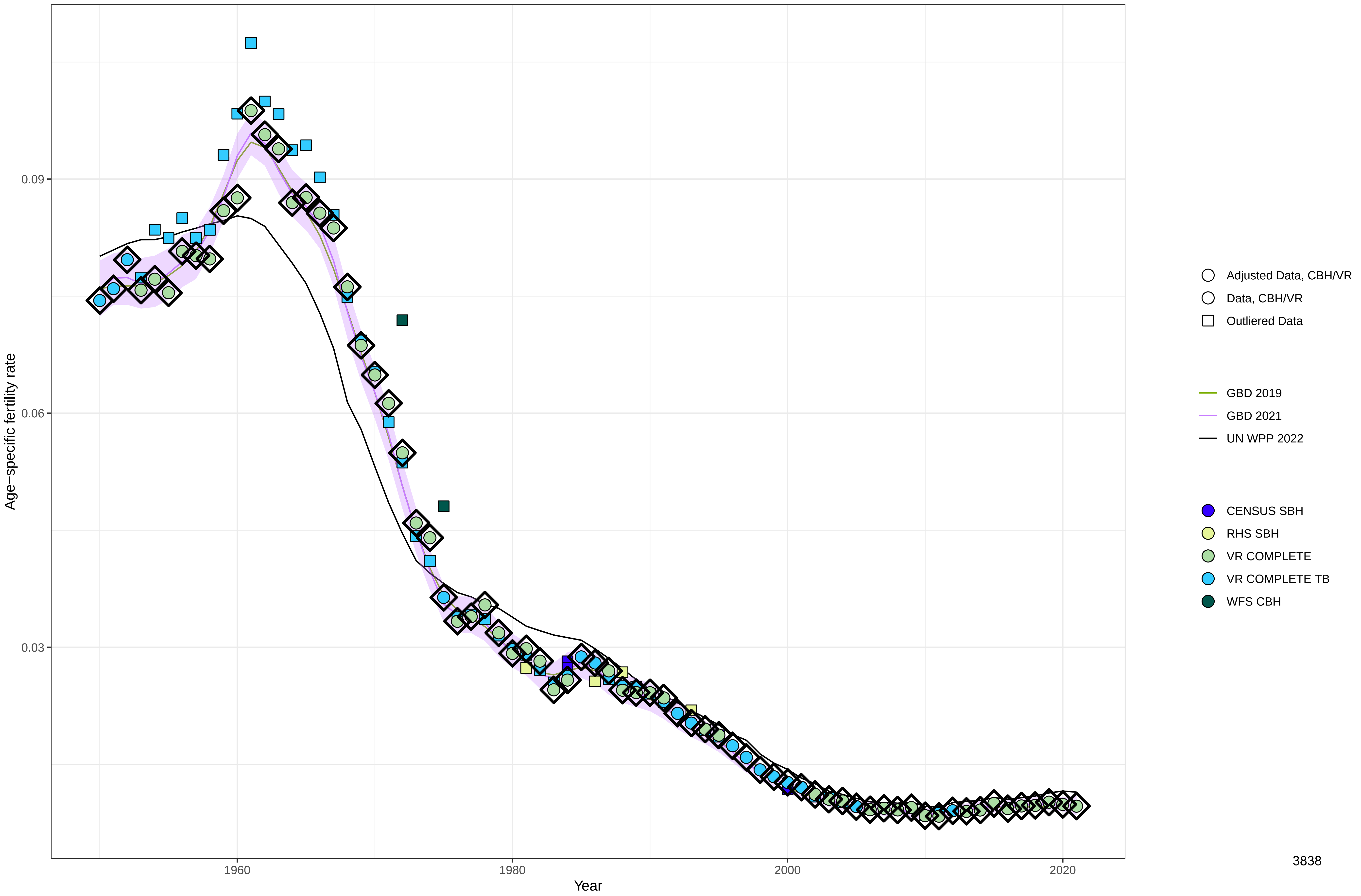

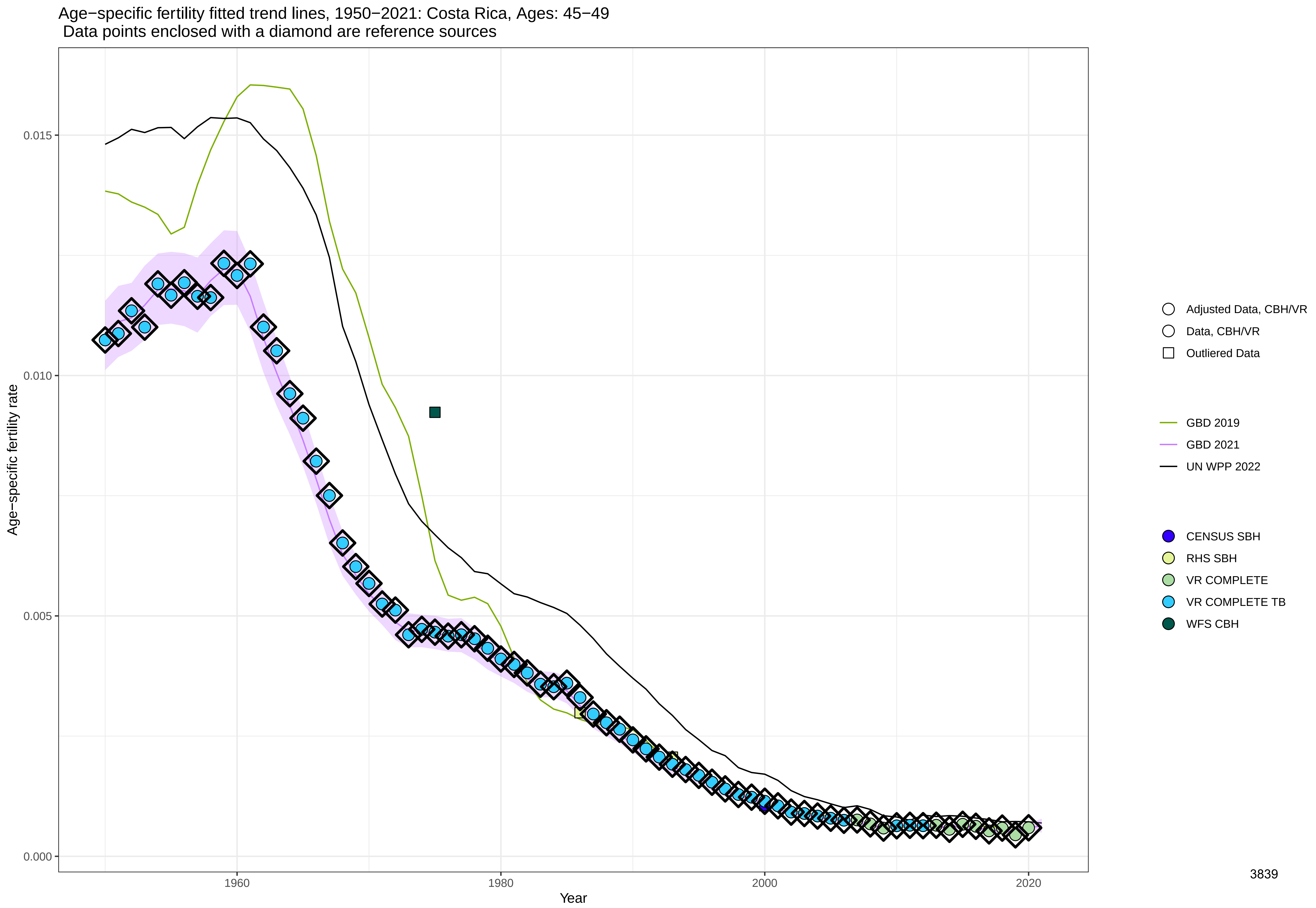

Age−specific fertility fitted trend lines, 1950−2021: Costa Rica, Ages: 45−49
Data points enclosed with a diamond are reference sources
Age−specific fertility rate
0.015
0.010
0.005
0.000
1960
1980
2000
2020
Year
Adjusted Data, CBH/VR
Data, CBH/VR
Outliered Data
GBD 2019
GBD 2021
UN WPP 2022
CENSUS SBH
RHS SBH
VR COMPLETE
VR COMPLETE TB
WFS CBH

Age−specific fertility fitted trend lines, 1950−2021: Costa Rica, Ages: 50−54
 Data points enclosed with a diamond are reference sources

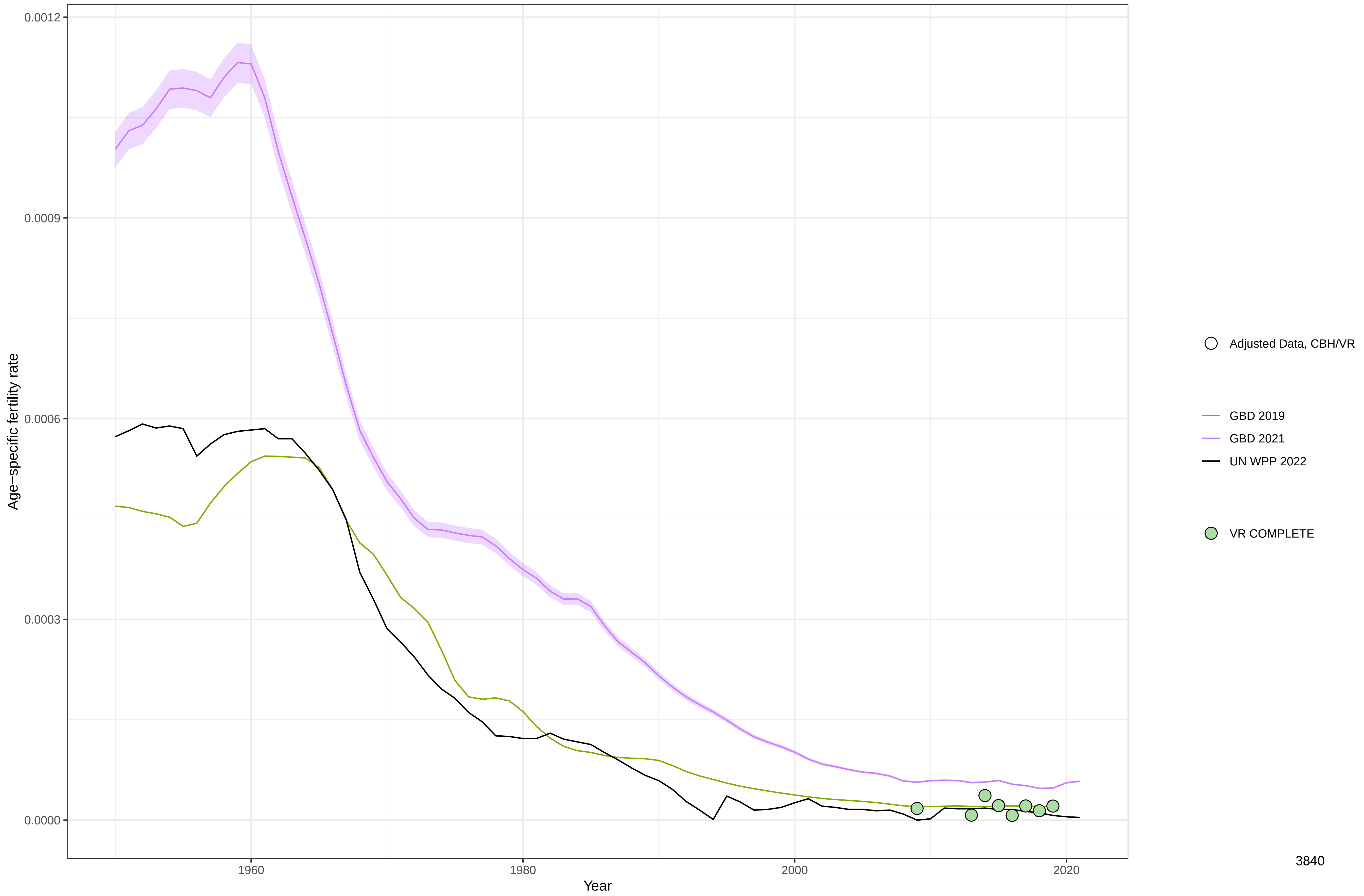

Total fertility rate fitted trend lines, 1950−2021: Costa Rica TFR
Data points enclosed with a diamond are reference sources

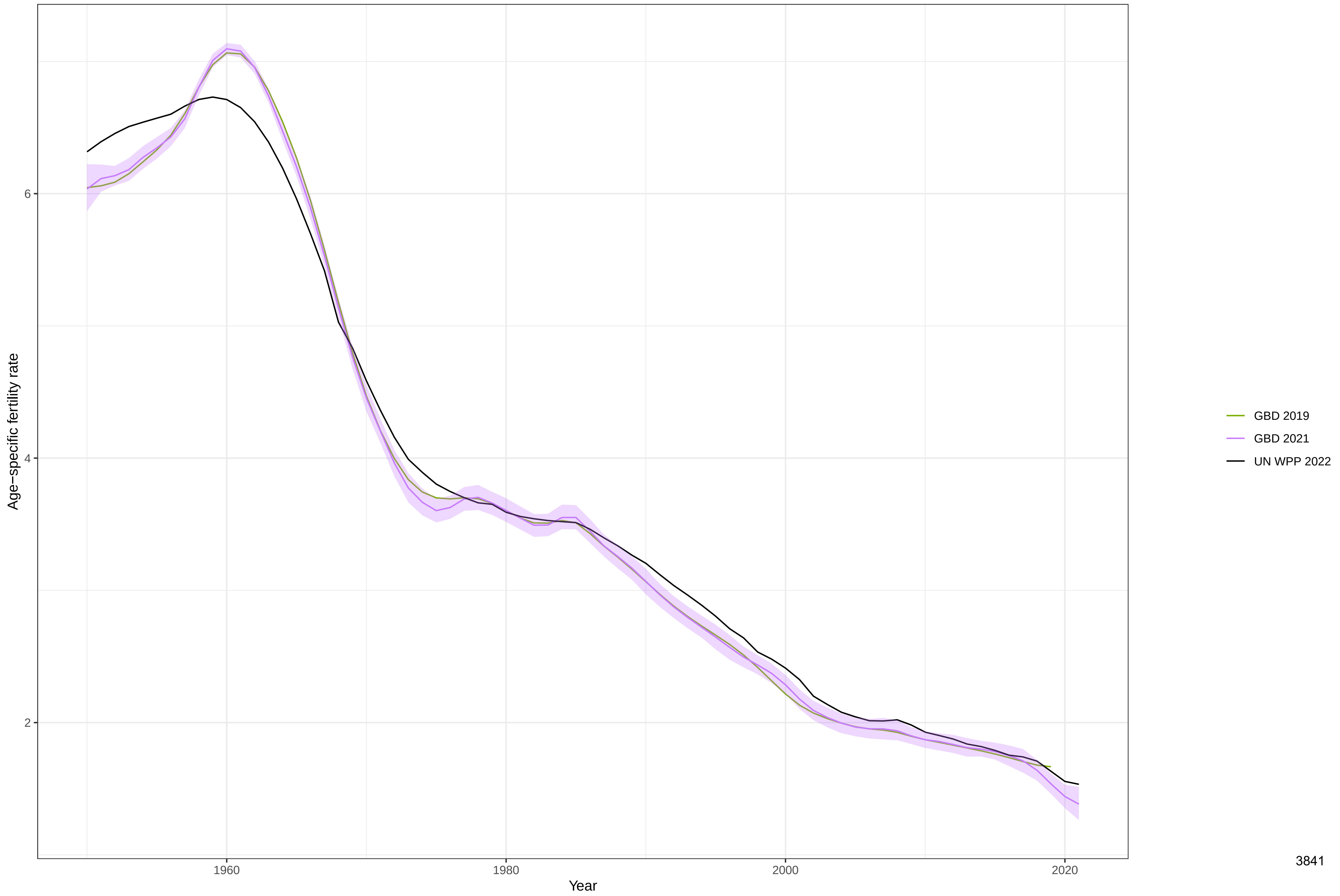

Age−specific fertility fitted trend lines, 1950−2021: El Salvador, Ages: 10−14
Data points enclosed with a diamond are reference sources

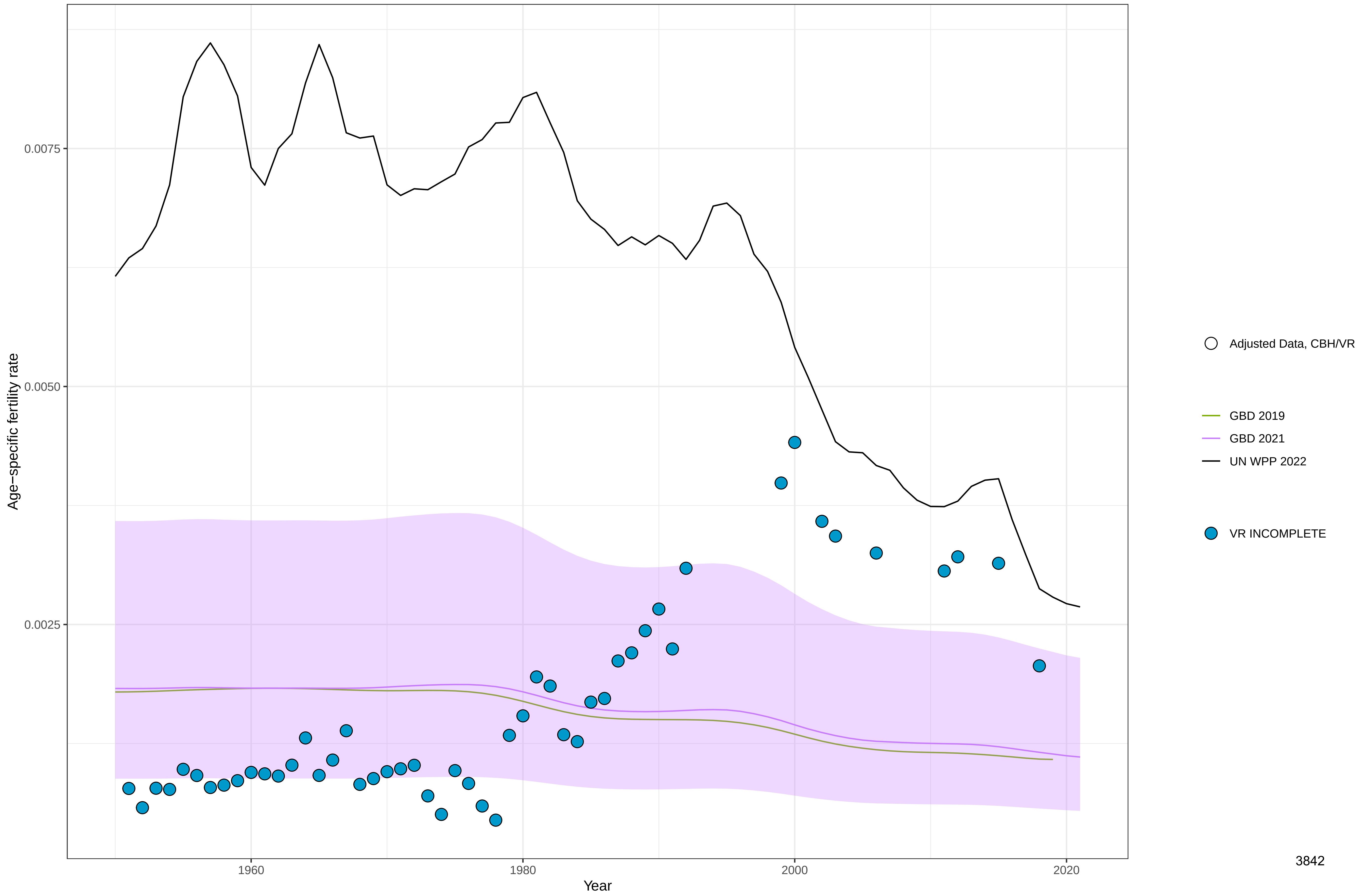

Age−specific fertility fitted trend lines, 1950−2021: El Salvador, Ages: 15−19
Data points enclosed with a diamond are reference sources

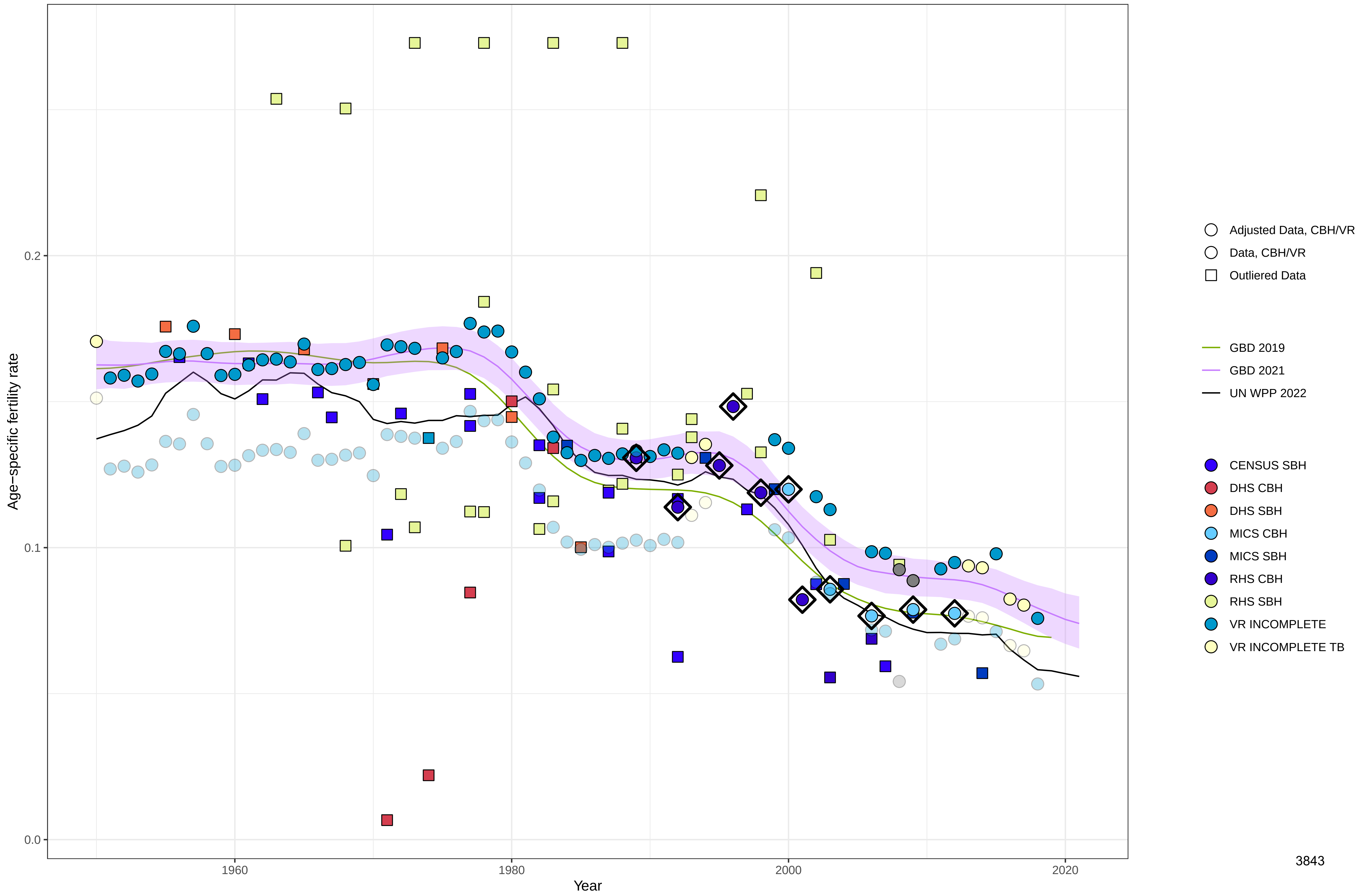

Age−specific fertility fitted trend lines, 1950−2021: El Salvador, Ages: 20−24
Data points enclosed with a diamond are reference sources

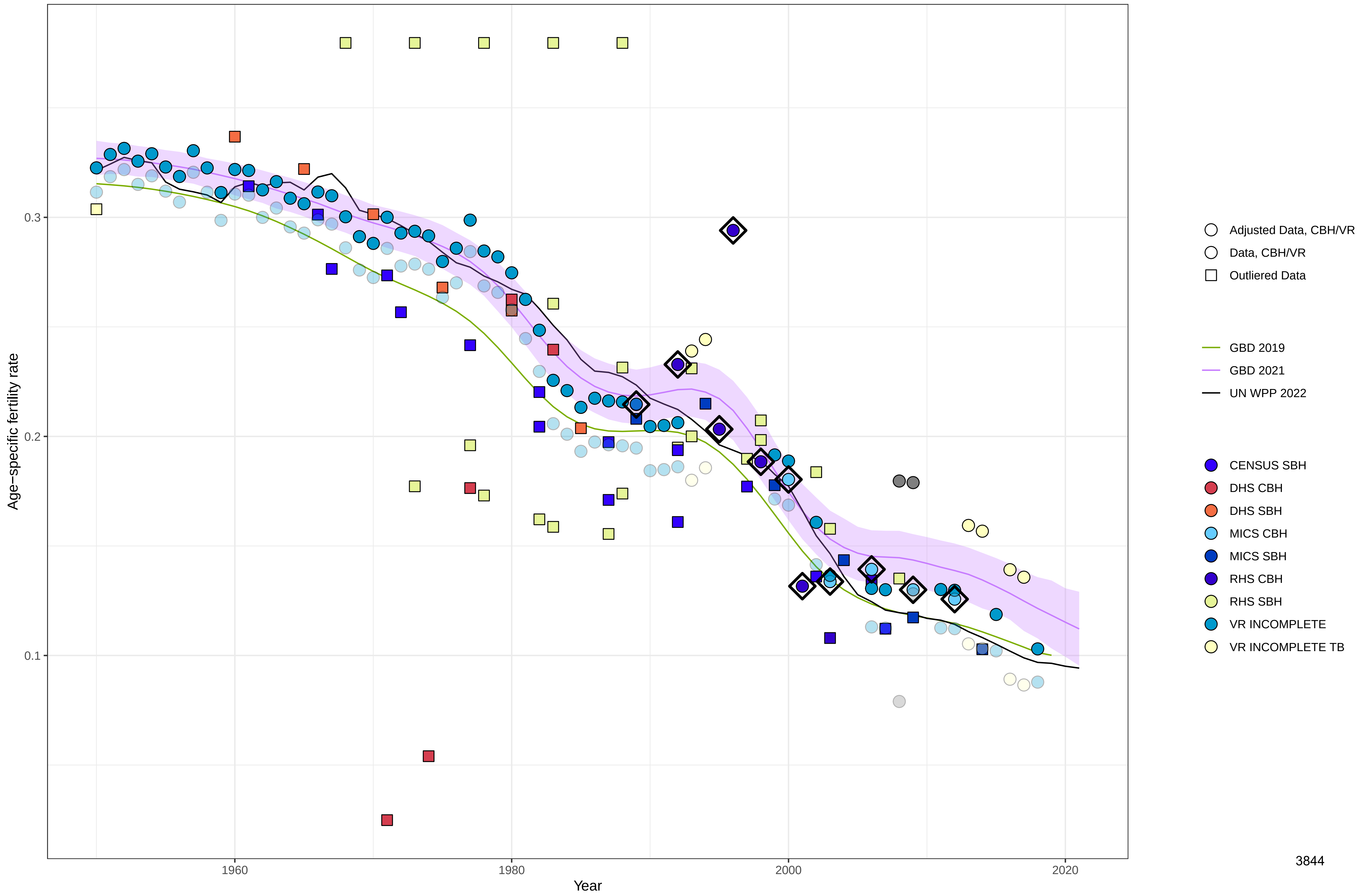

Age−specific fertility fitted trend lines, 1950−2021: El Salvador, Ages: 25−29
Data points enclosed with a diamond are reference sources

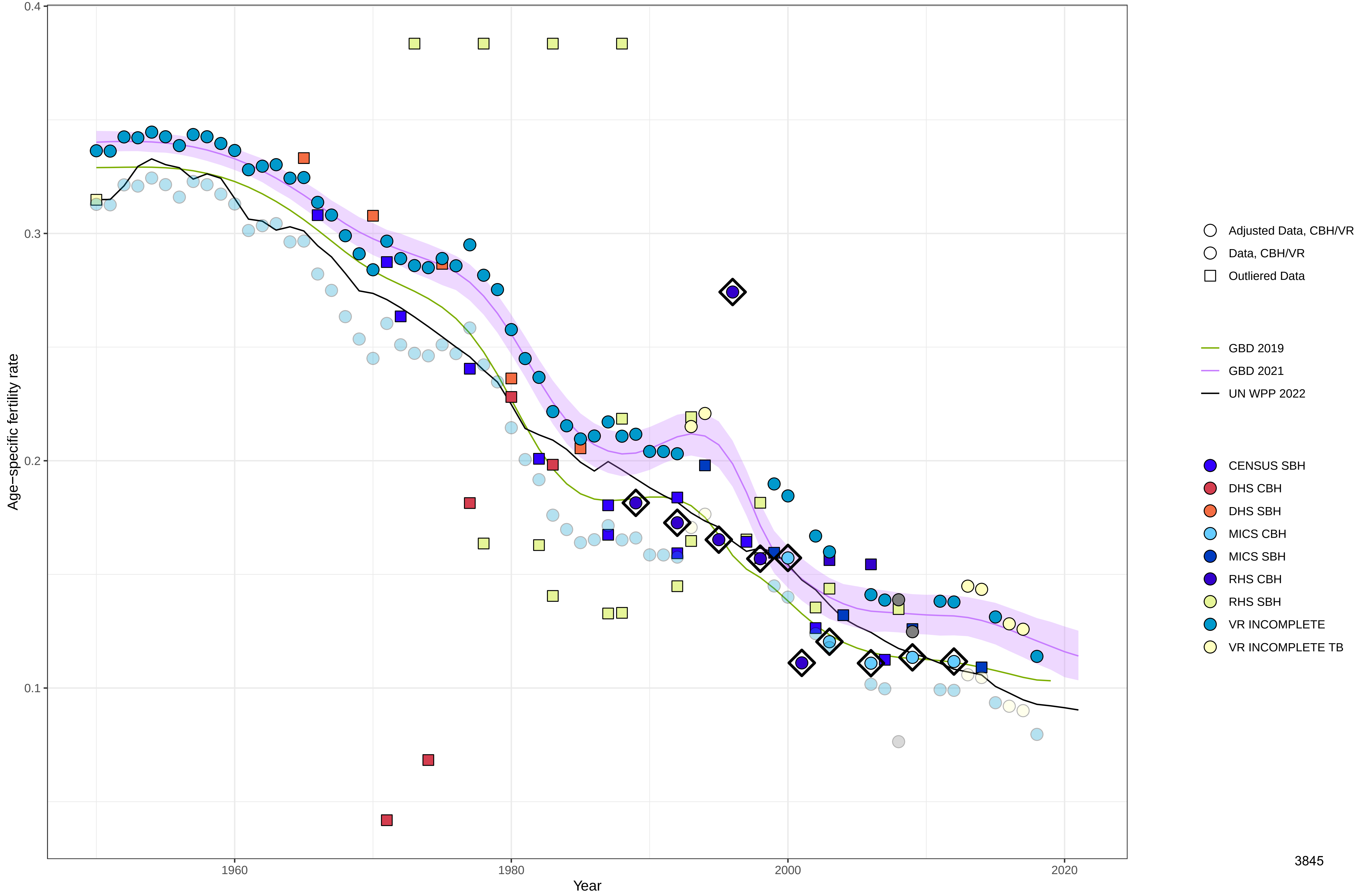

Age−specific fertility fitted trend lines, 1950−2021: El Salvador, Ages: 30−34
Data points enclosed with a diamond are reference sources

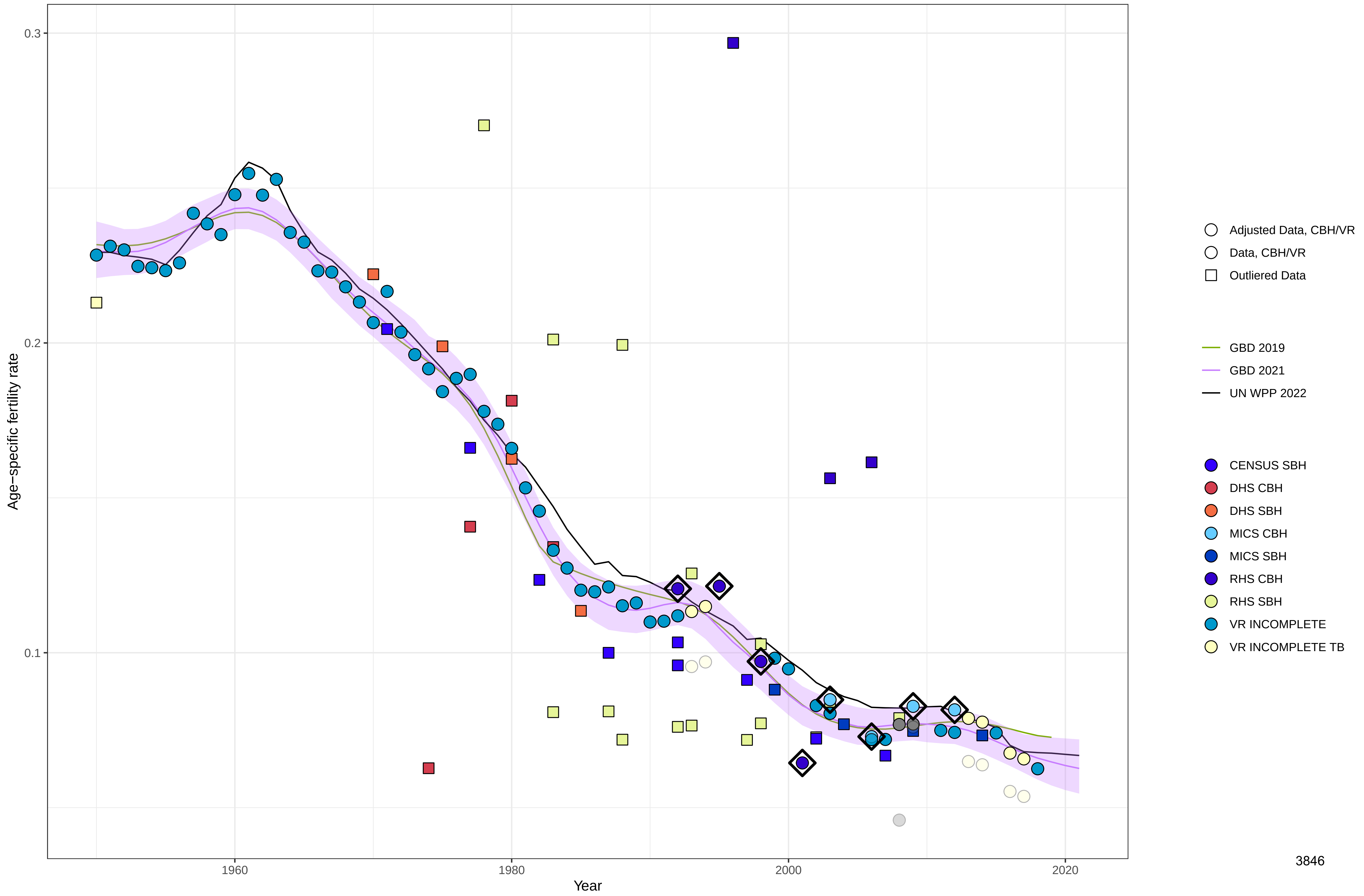

3846

Age−specific fertility fitted trend lines, 1950−2021: El Salvador, Ages: 35−39
Data points enclosed with a diamond are reference sources

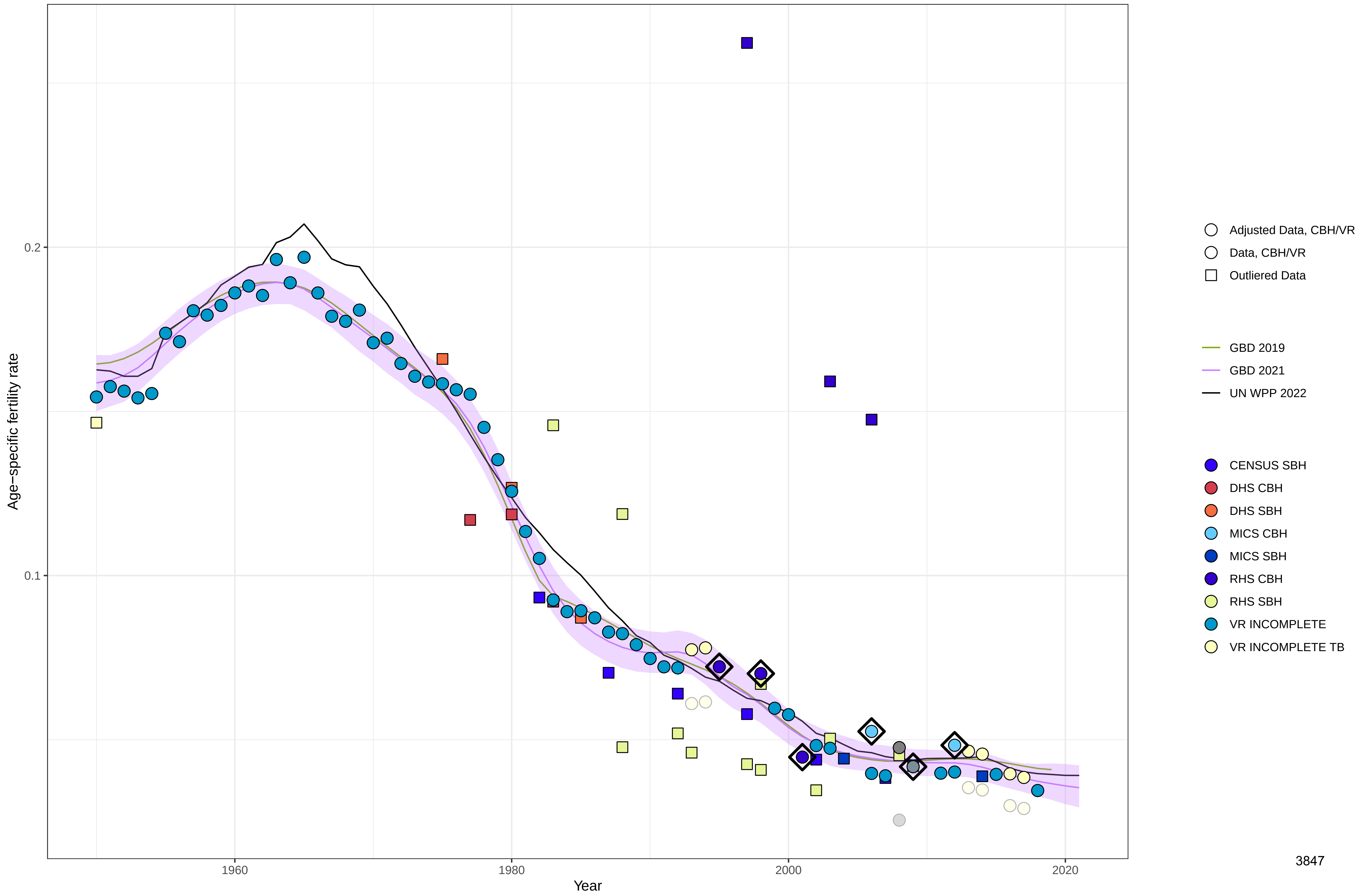

Age−specific fertility fitted trend lines, 1950−2021: El Salvador, Ages: 40−44
Data points enclosed with a diamond are reference sources

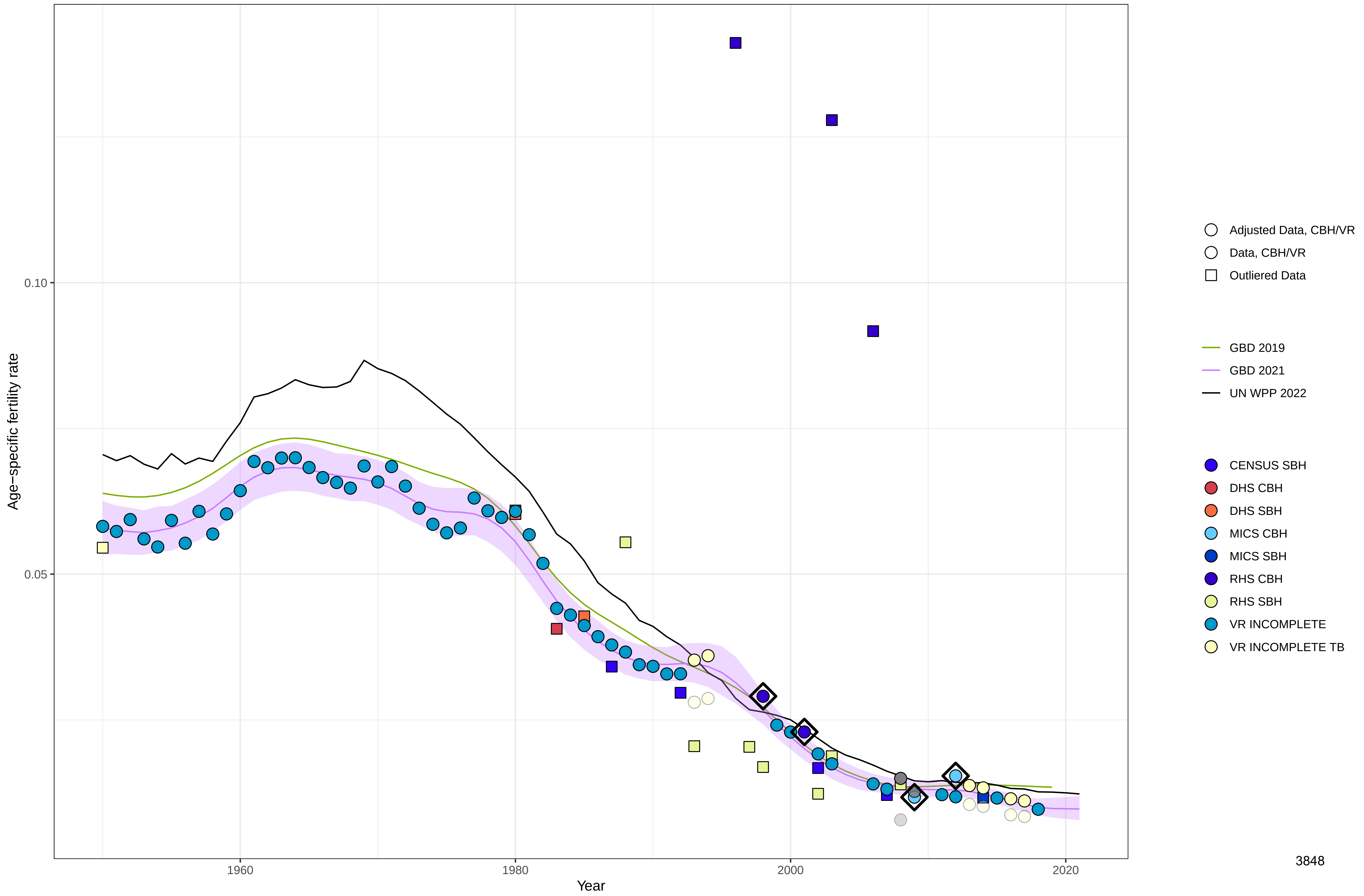

Age−specific fertility fitted trend lines, 1950−2021: El Salvador, Ages: 45−49
Data points enclosed with a diamond are reference sources

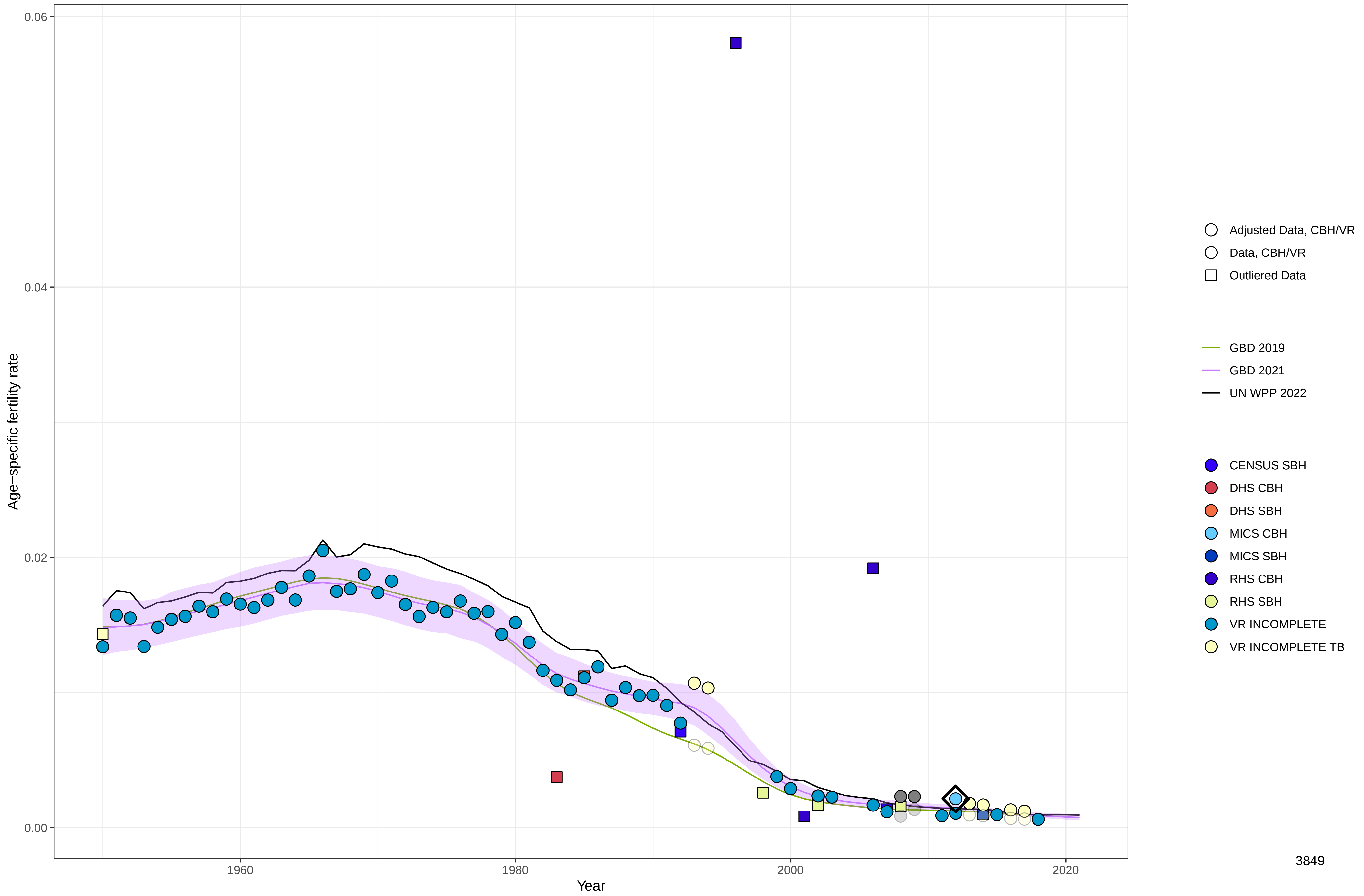

Age−specific fertility fitted trend lines, 1950−2021: El Salvador, Ages: 50−54
Data points enclosed with a diamond are reference sources

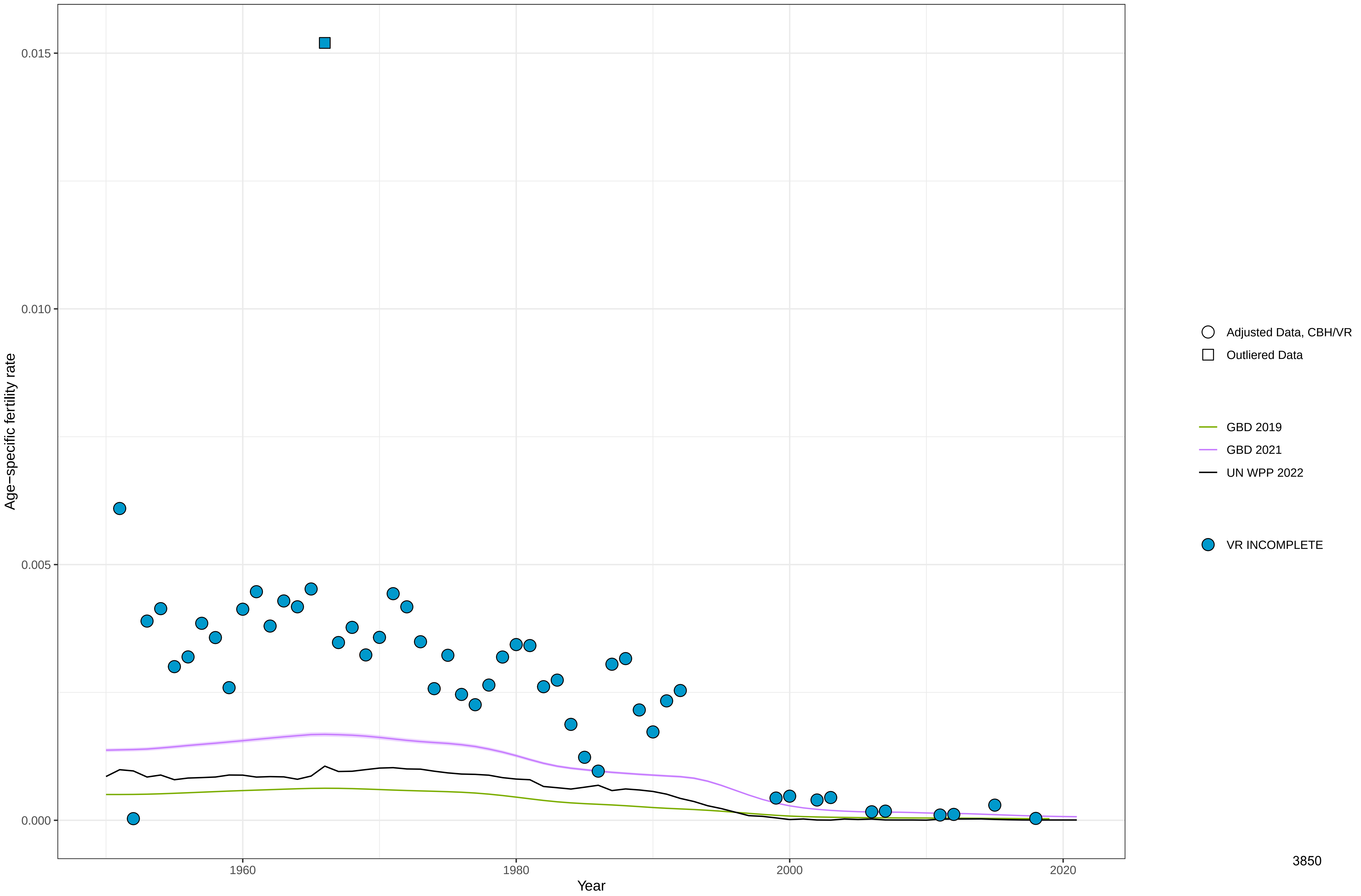

Total fertility rate fitted trend lines, 1950−2021: El Salvador TFR
Data points enclosed with a diamond are reference sources

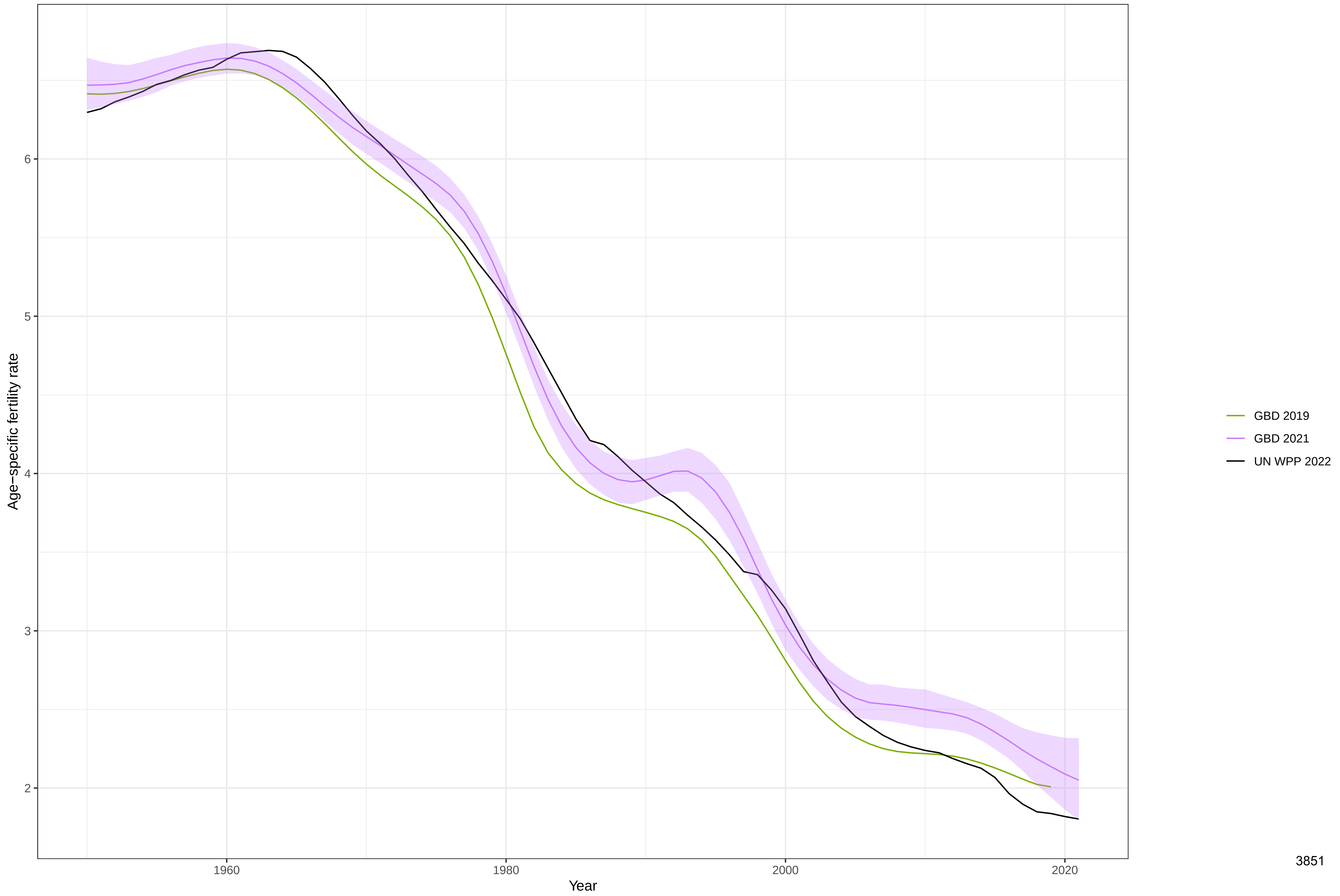

Age−specific fertility fitted trend lines, 1950−2021: Guatemala, Ages: 10−14
Data points enclosed with a diamond are reference sources

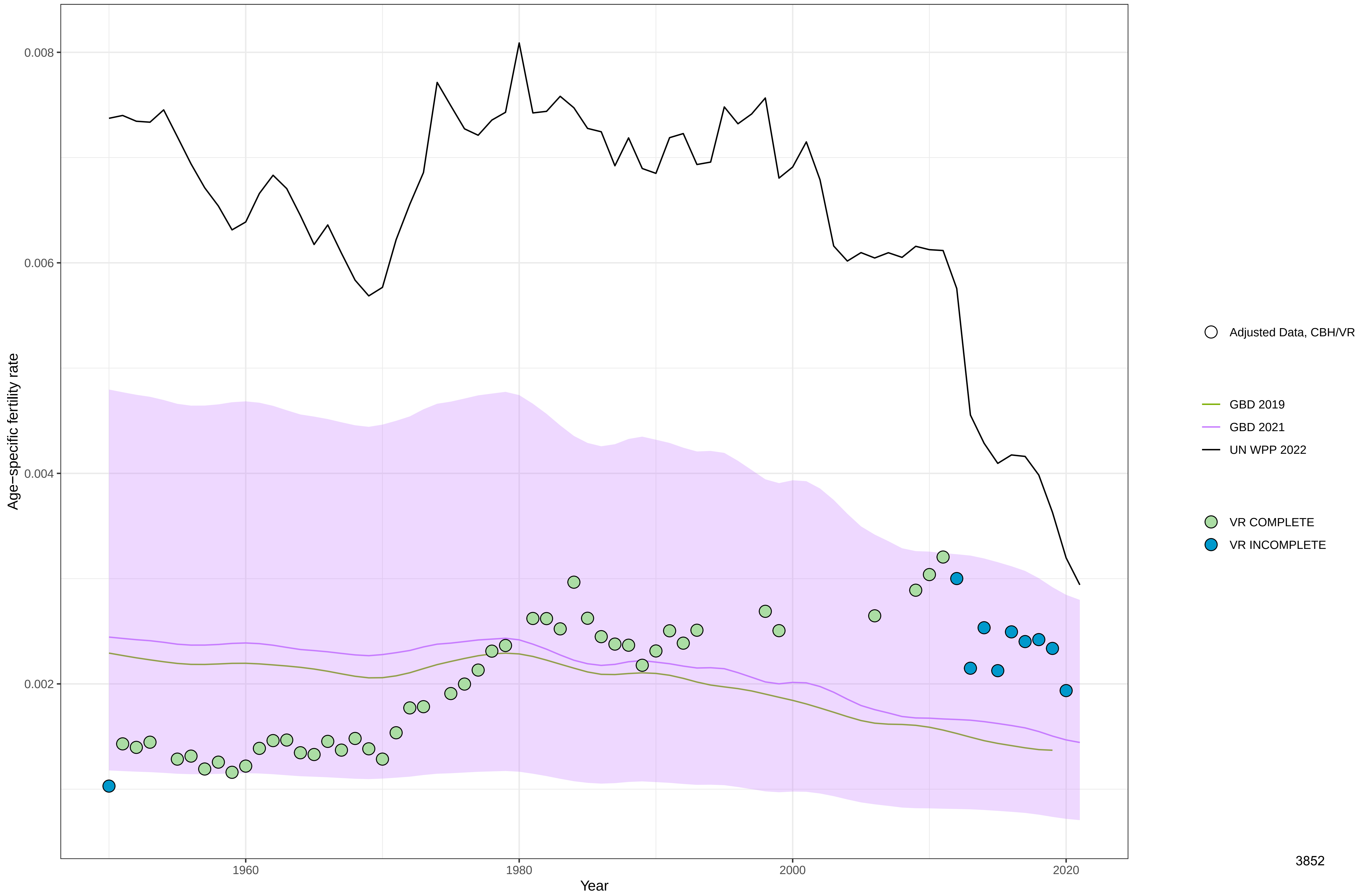

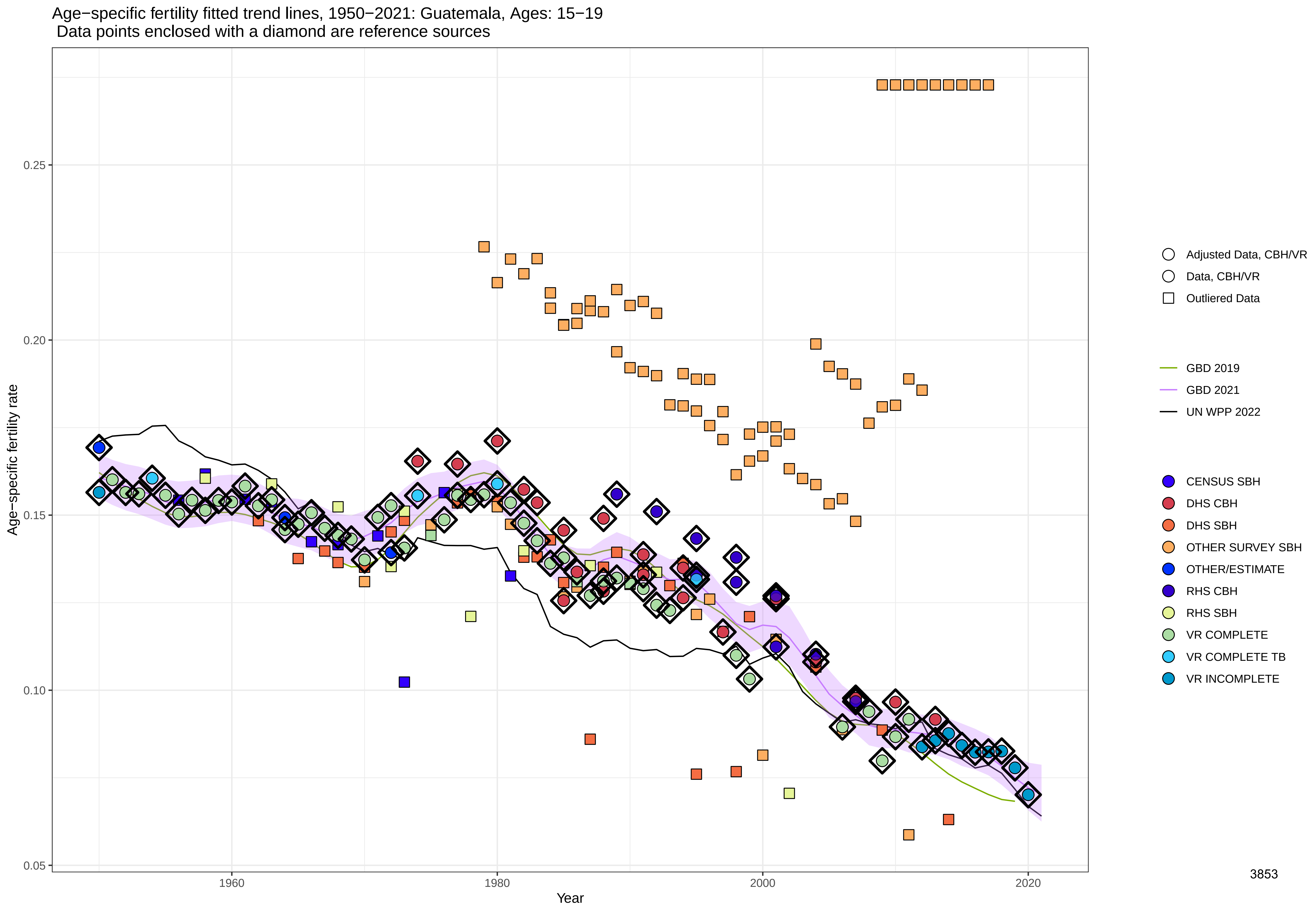

Age−specific fertility fitted trend lines, 1950−2021: Guatemala, Ages: 15−19
Data points enclosed with a diamond are reference sources
Age−specific fertility rate
Year
0.05
0.10
0.15
0.20
0.25
1960
1980
2000
2020
Adjusted Data, CBH/VR
Data, CBH/VR
Outliered Data
GBD 2019
GBD 2021
UN WPP 2022
CENSUS SBH
DHS CBH
DHS SBH
OTHER SURVEY SBH
OTHER/ESTIMATE
RHS CBH
RHS SBH
VR COMPLETE
VR COMPLETE TB
VR INCOMPLETE

Age−specific fertility fitted trend lines, 1950−2021: Guatemala, Ages: 20−24
Data points enclosed with a diamond are reference sources

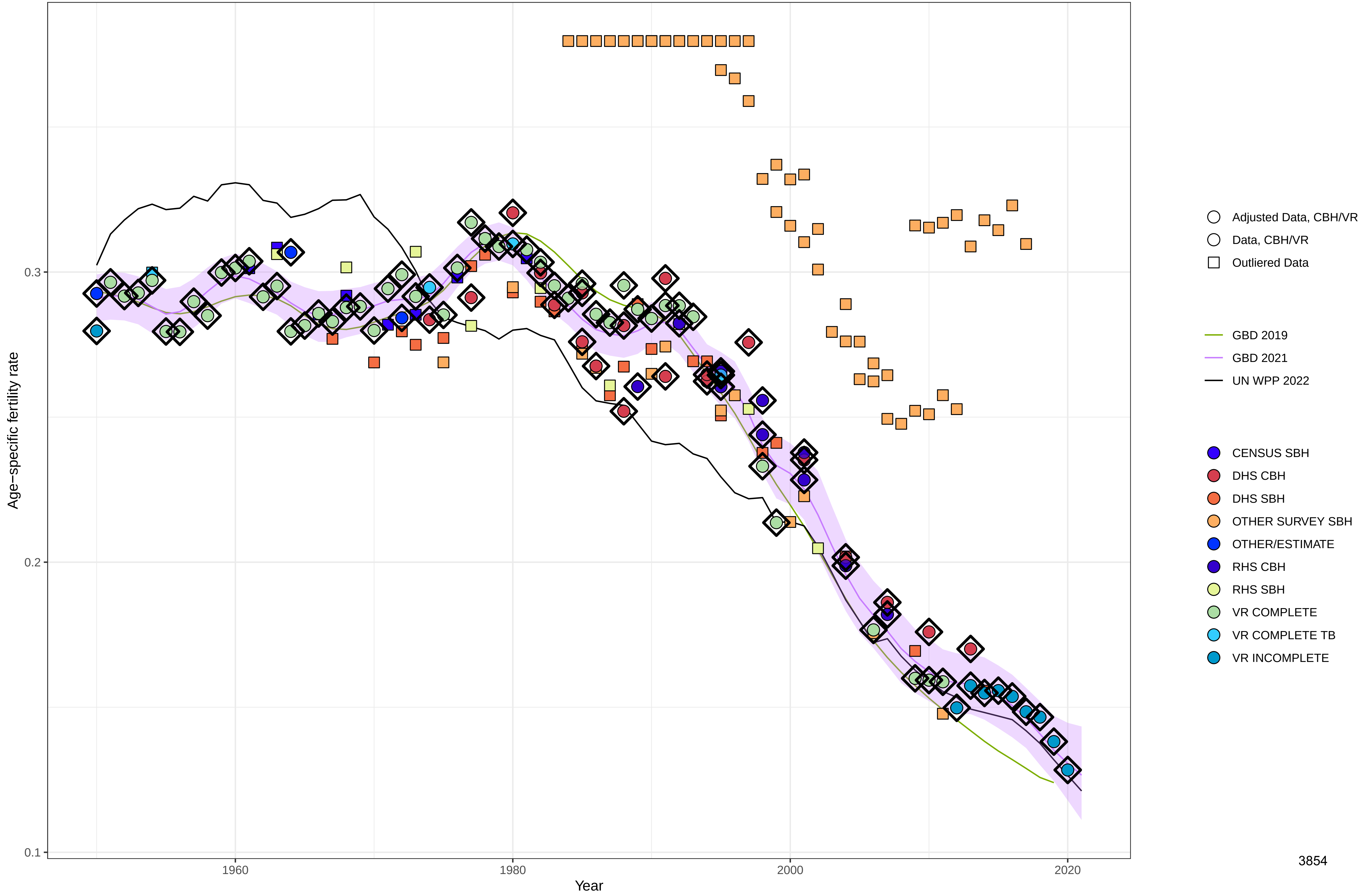

Age−specific fertility fitted trend lines, 1950−2021: Guatemala, Ages: 25−29
Data points enclosed with a diamond are reference sources

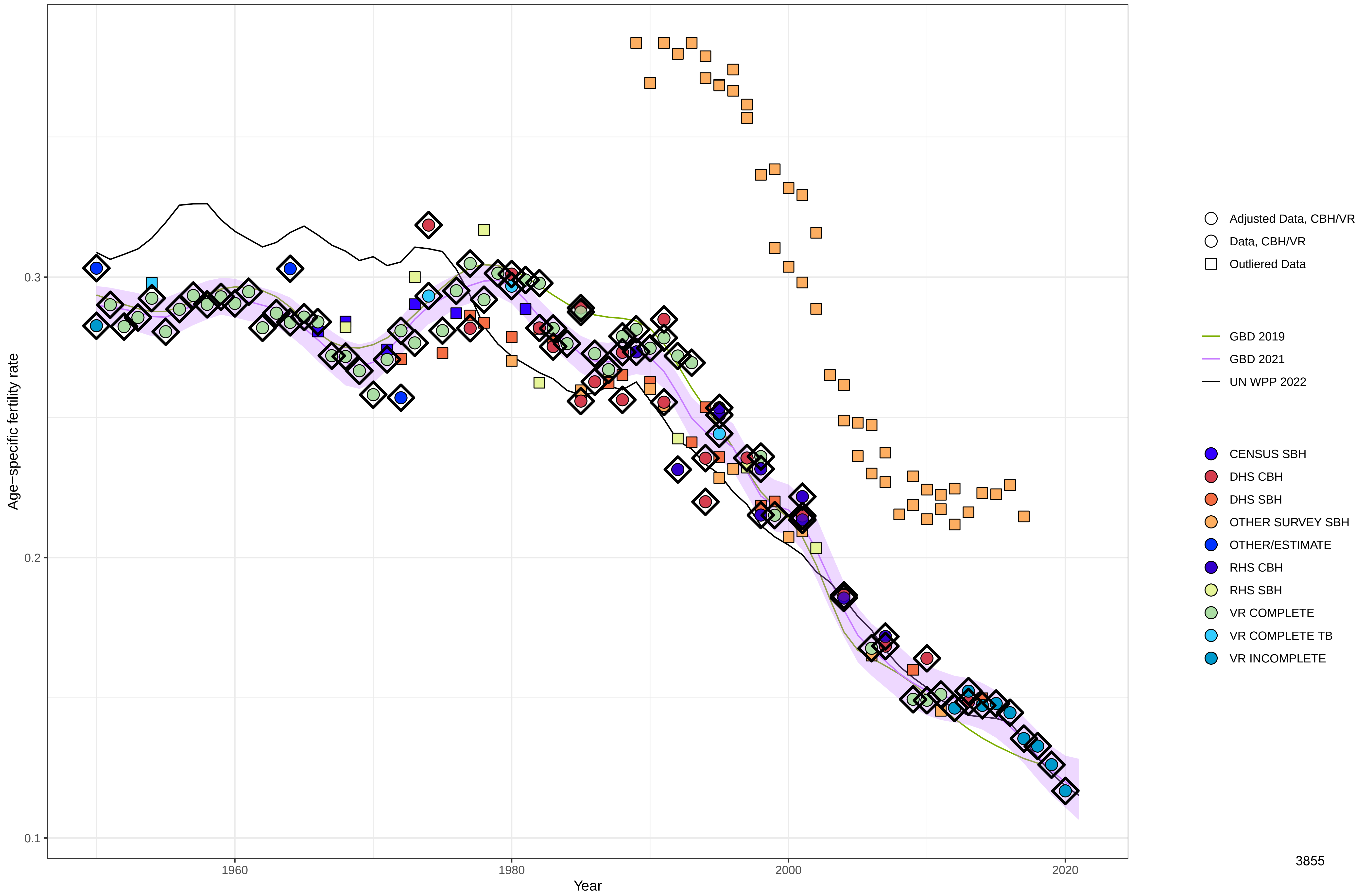

Age−specific fertility fitted trend lines, 1950−2021: Guatemala, Ages: 30−34
Data points enclosed with a diamond are reference sources

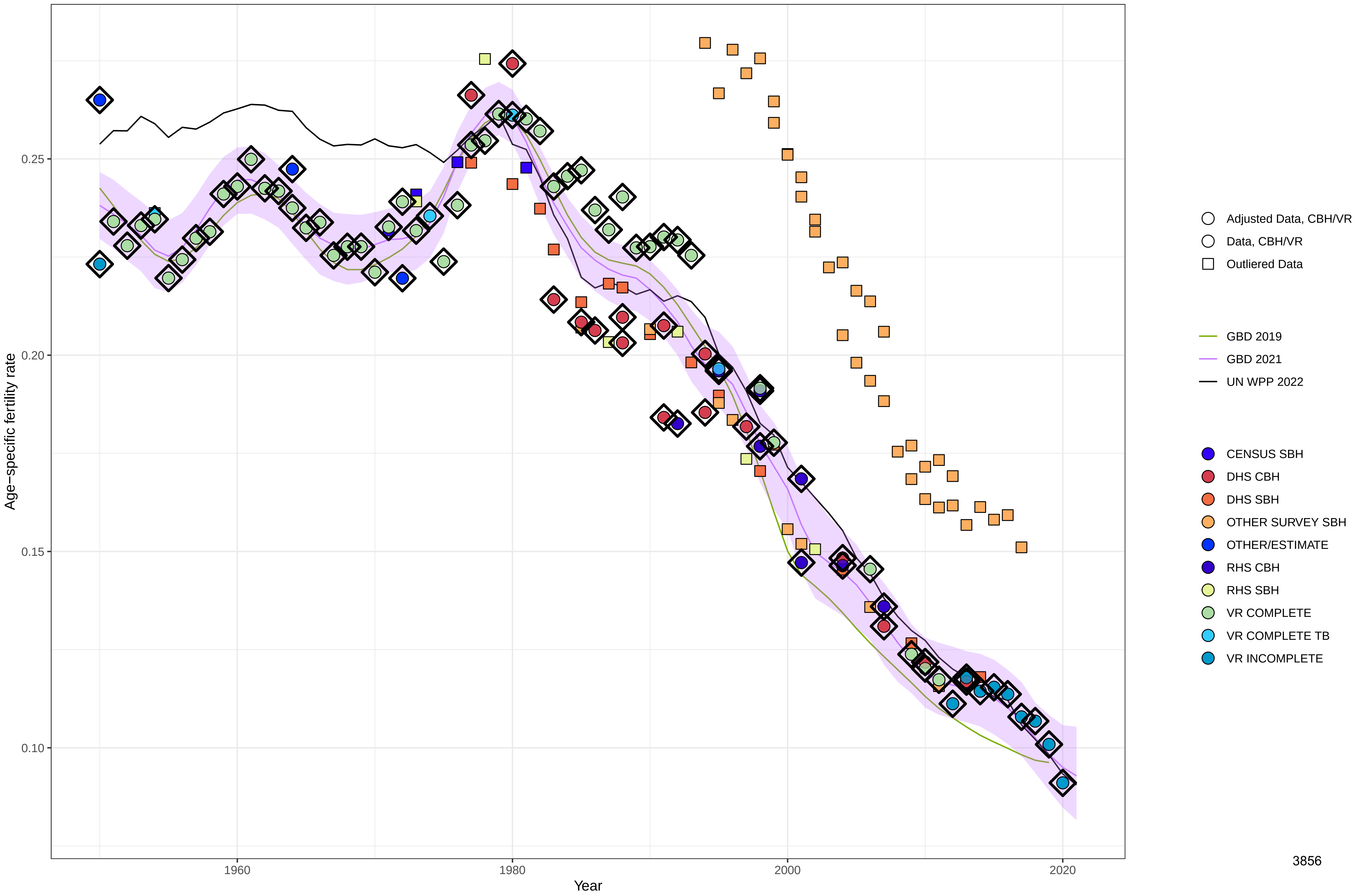

Age−specific fertility fitted trend lines, 1950−2021: Guatemala, Ages: 35−39
Data points enclosed with a diamond are reference sources

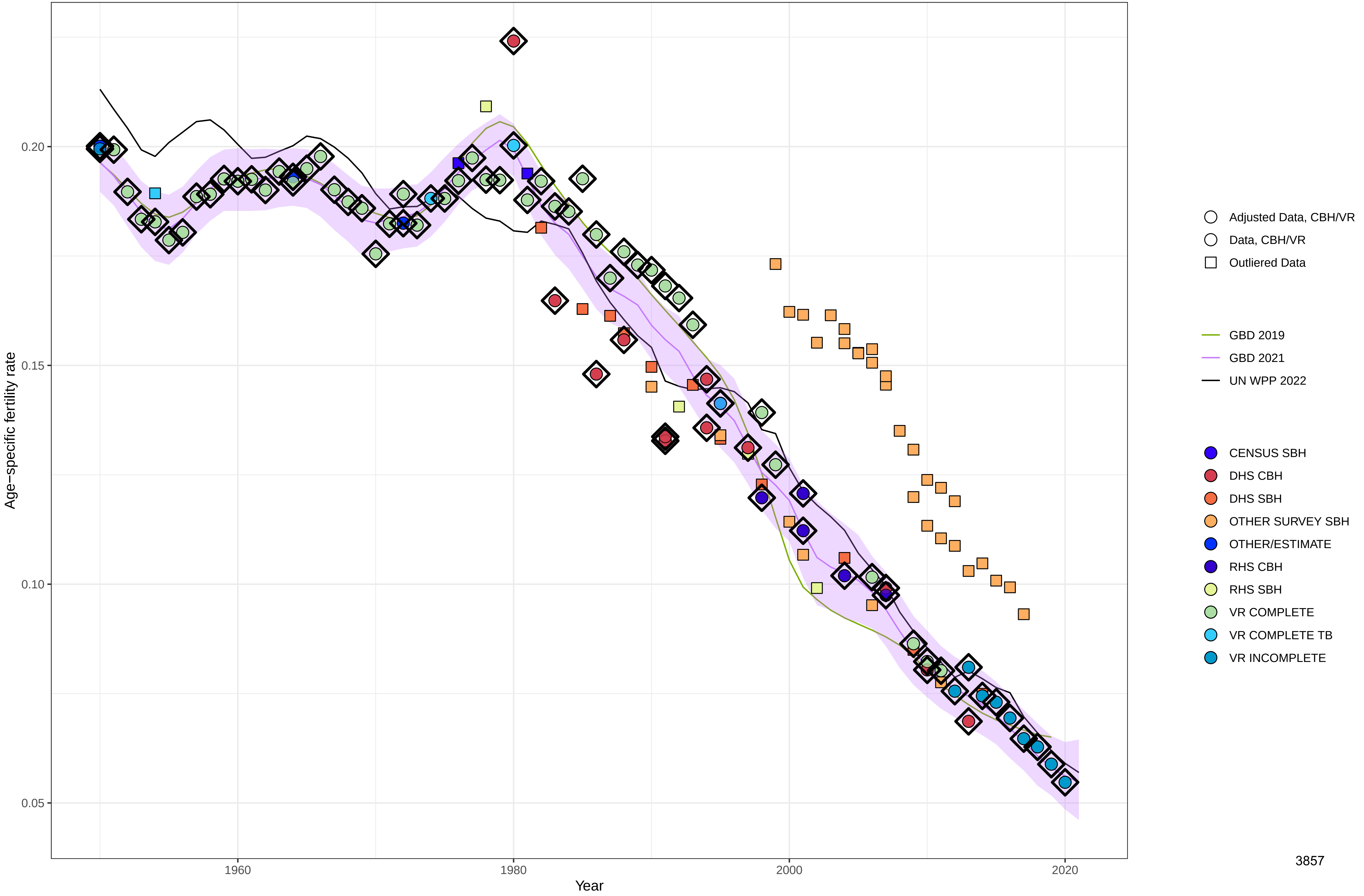

Age−specific fertility fitted trend lines, 1950−2021: Guatemala, Ages: 40−44
Data points enclosed with a diamond are reference sources

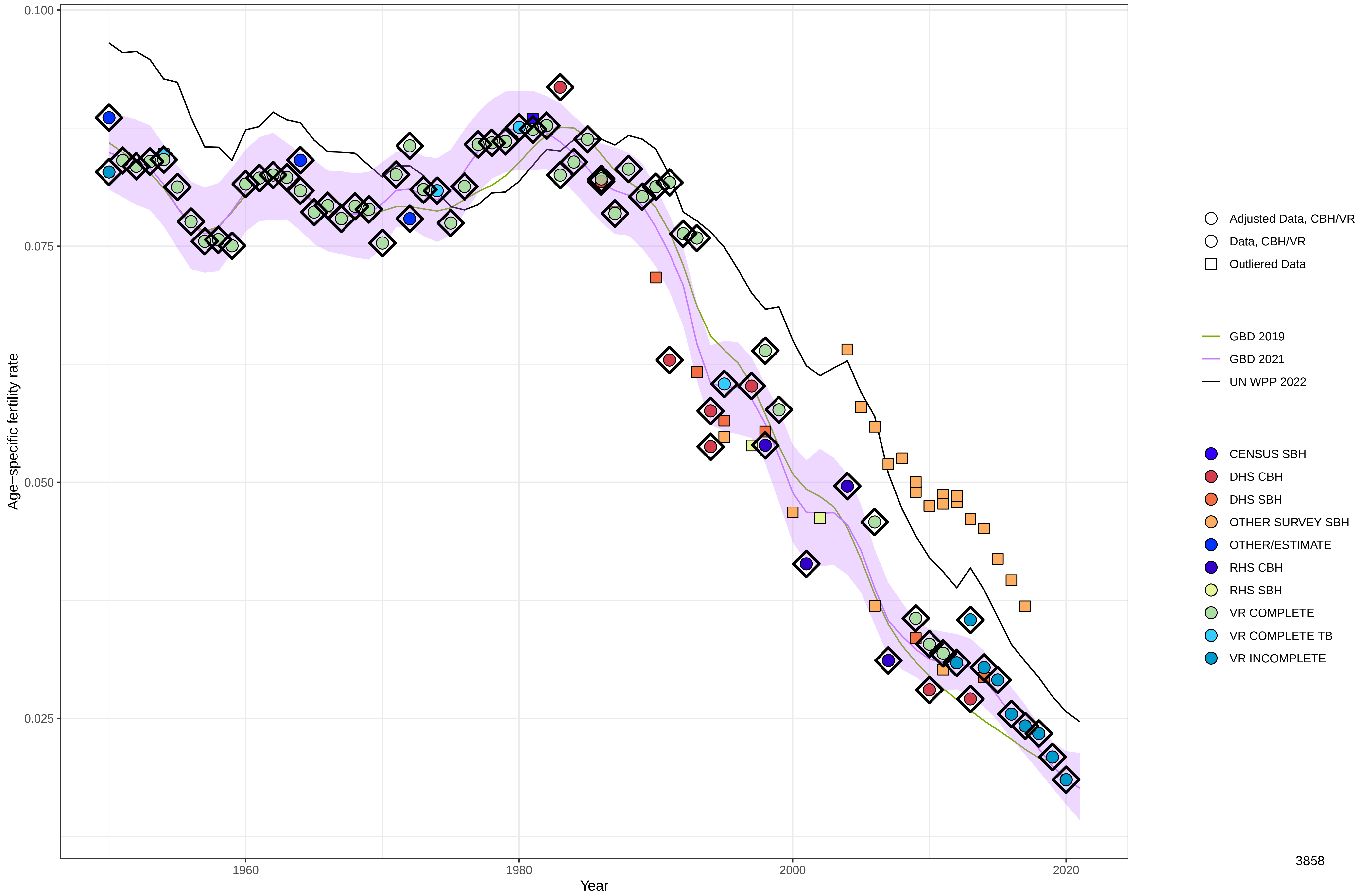

Age−specific fertility fitted trend lines, 1950−2021: Guatemala, Ages: 45−49
Data points enclosed with a diamond are reference sources

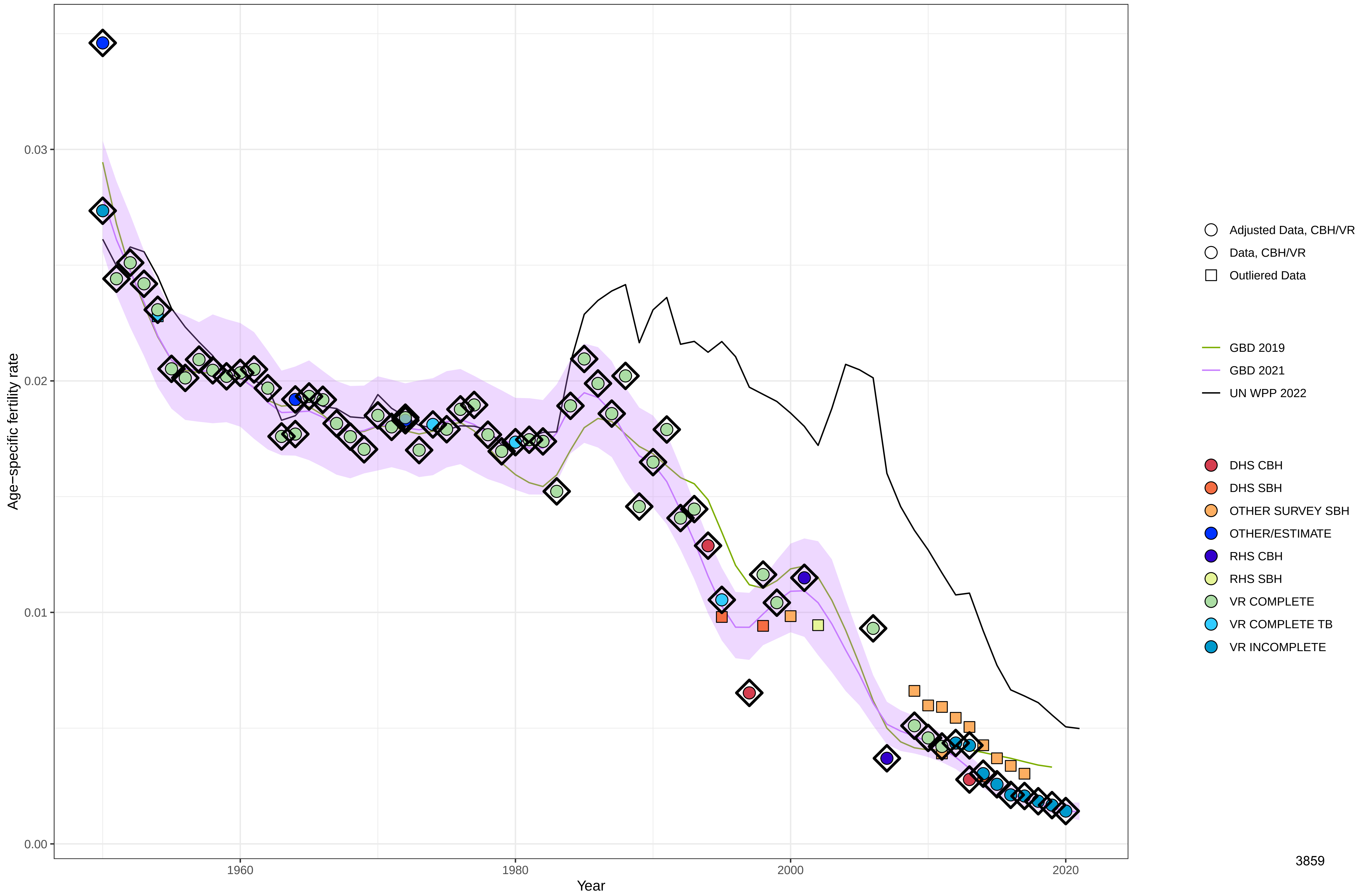

Age−specific fertility fitted trend lines, 1950−2021: Guatemala, Ages: 50−54
Data points enclosed with a diamond are reference sources

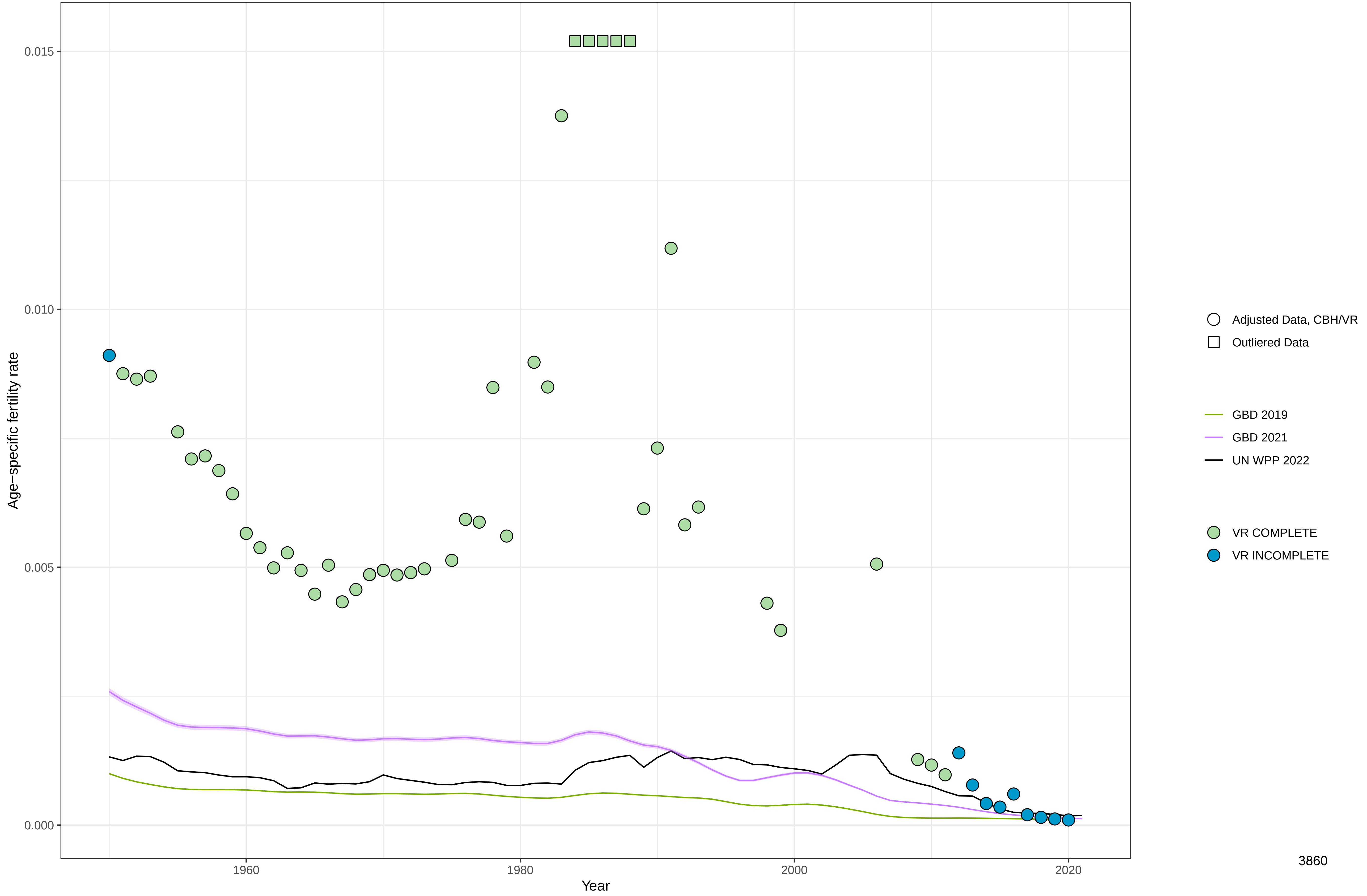

Total fertility rate fitted trend lines, 1950−2021: Guatemala TFR
Data points enclosed with a diamond are reference sources

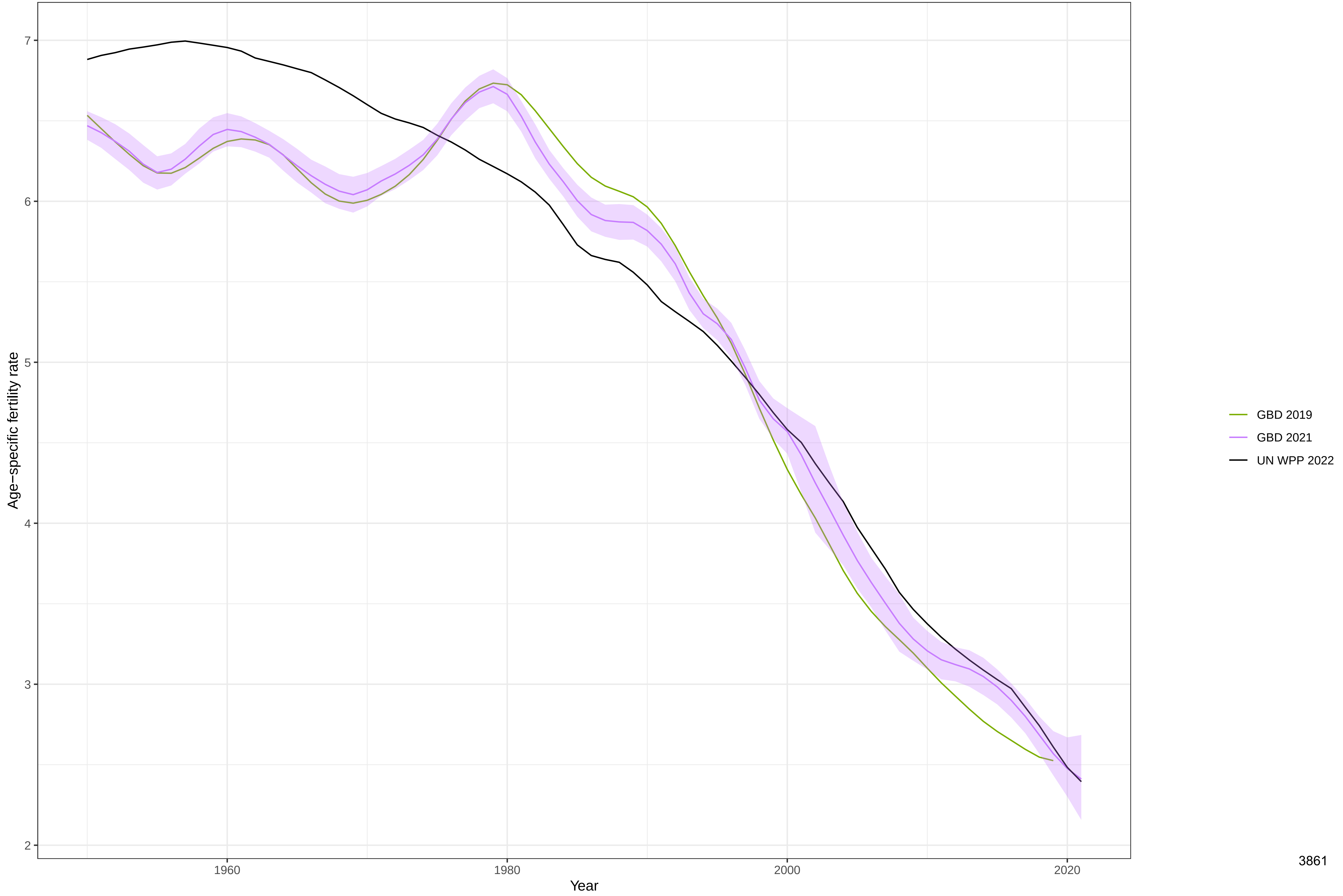

Age−specific fertility fitted trend lines, 1950−2021: Honduras, Ages: 10−14
Data points enclosed with a diamond are reference sources

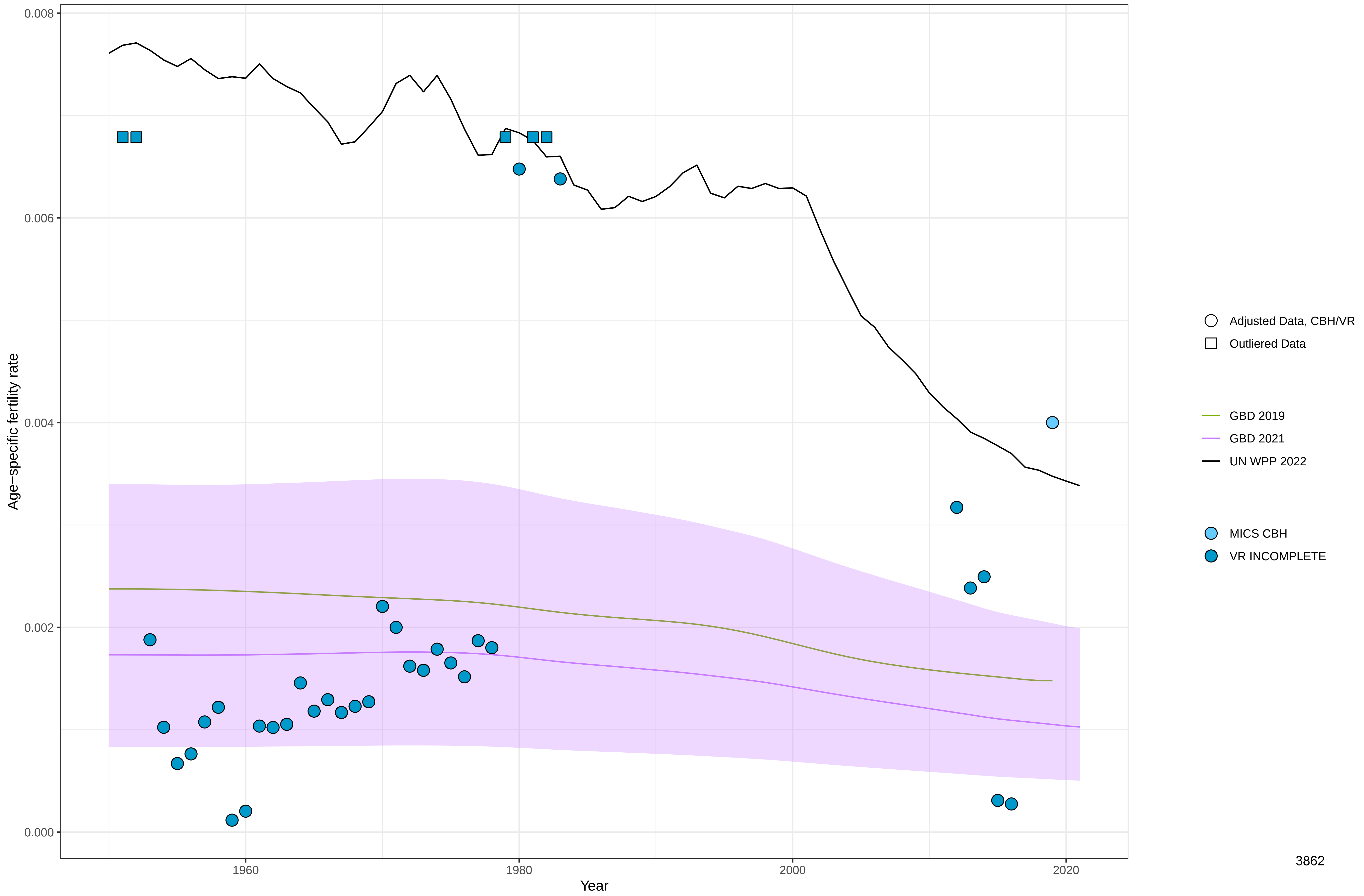

Age−specific fertility fitted trend lines, 1950−2021: Honduras, Ages: 15−19
Data points enclosed with a diamond are reference sources

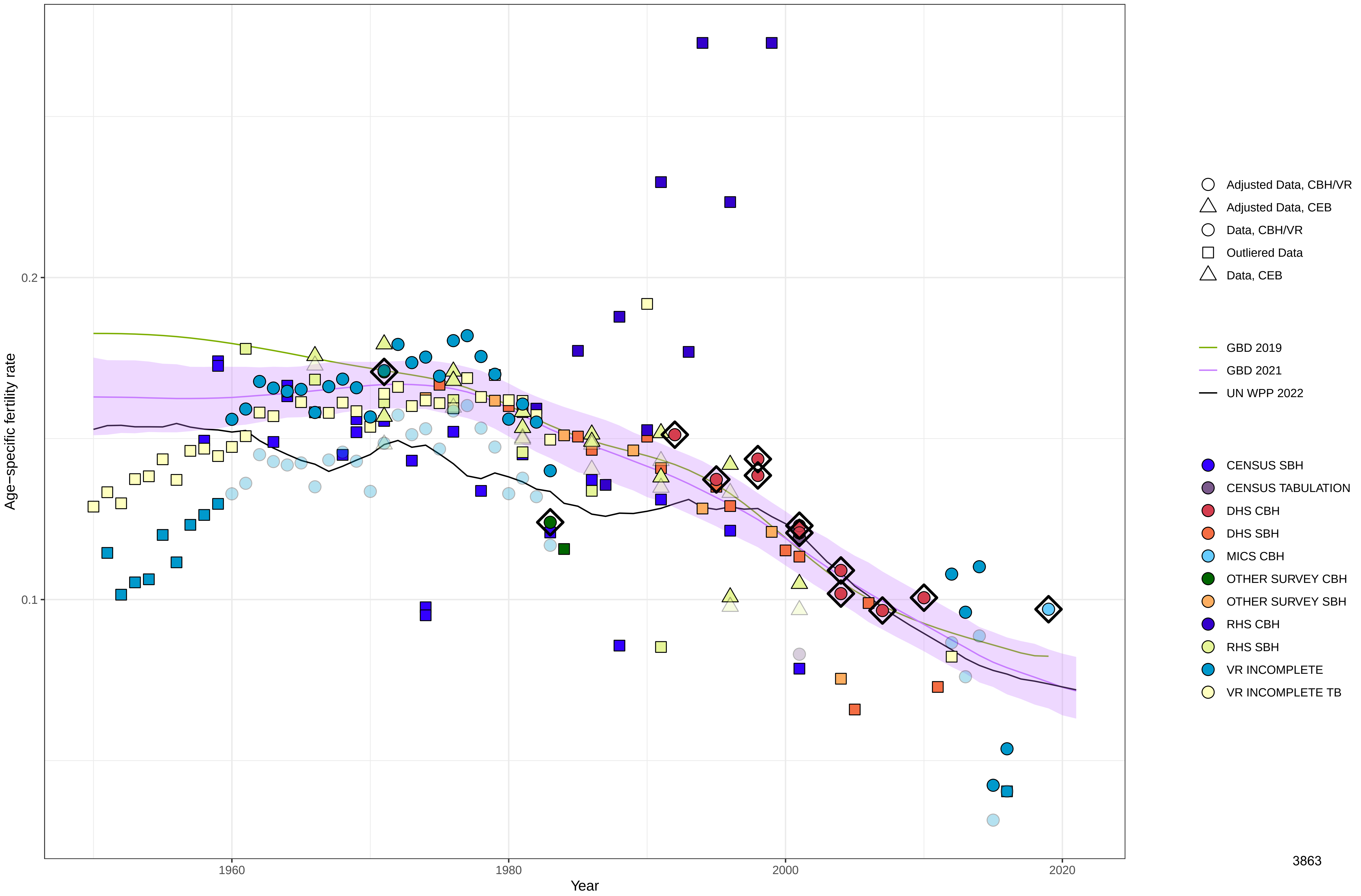

Age−specific fertility fitted trend lines, 1950−2021: Honduras, Ages: 20−24
Data points enclosed with a diamond are reference sources

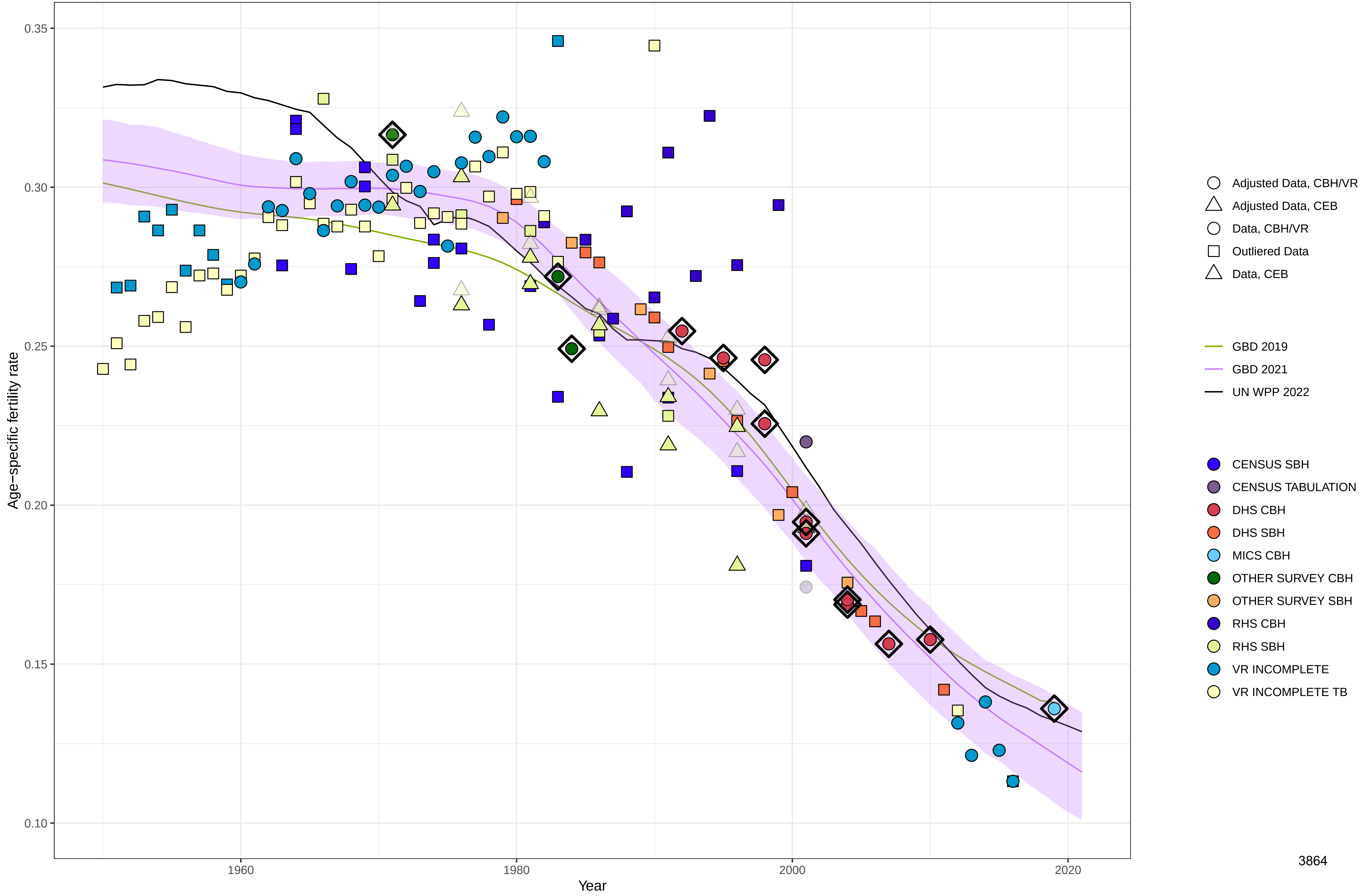

Age−specific fertility fitted trend lines, 1950−2021: Honduras, Ages: 25−29
Data points enclosed with a diamond are reference sources

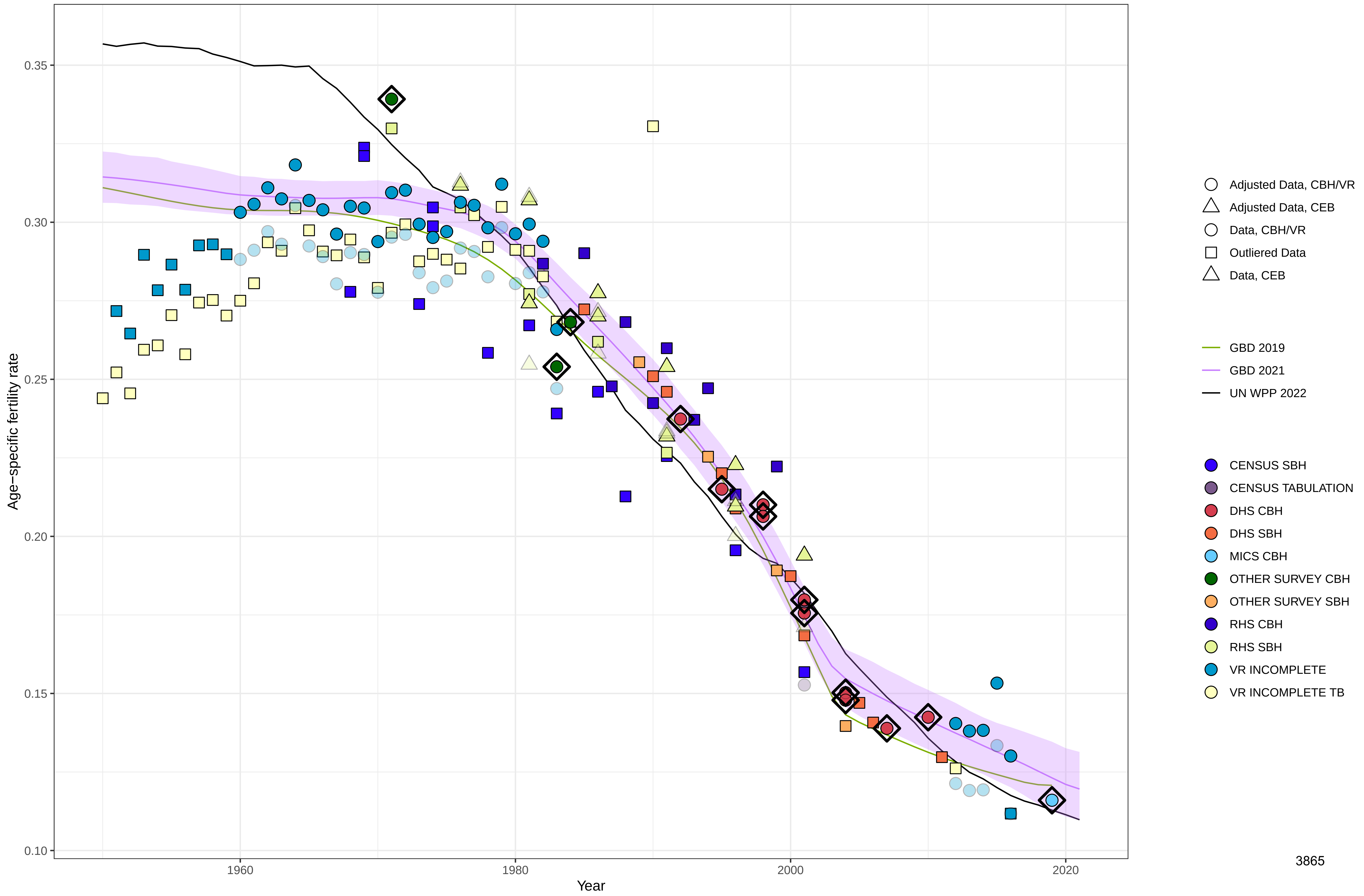

Age−specific fertility fitted trend lines, 1950−2021: Honduras, Ages: 30−34
Data points enclosed with a diamond are reference sources

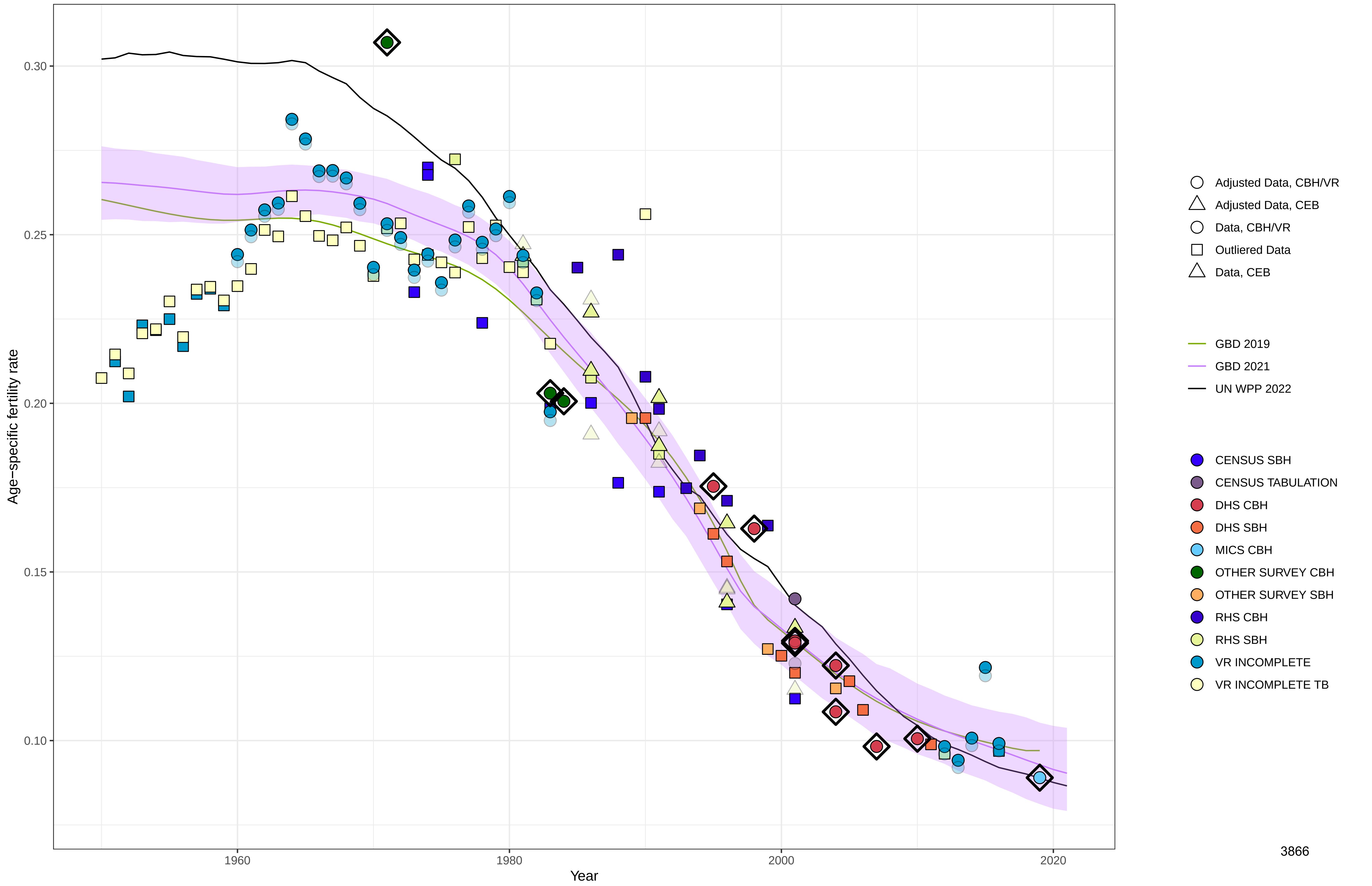

Age−specific fertility fitted trend lines, 1950−2021: Honduras, Ages: 35−39
Data points enclosed with a diamond are reference sources

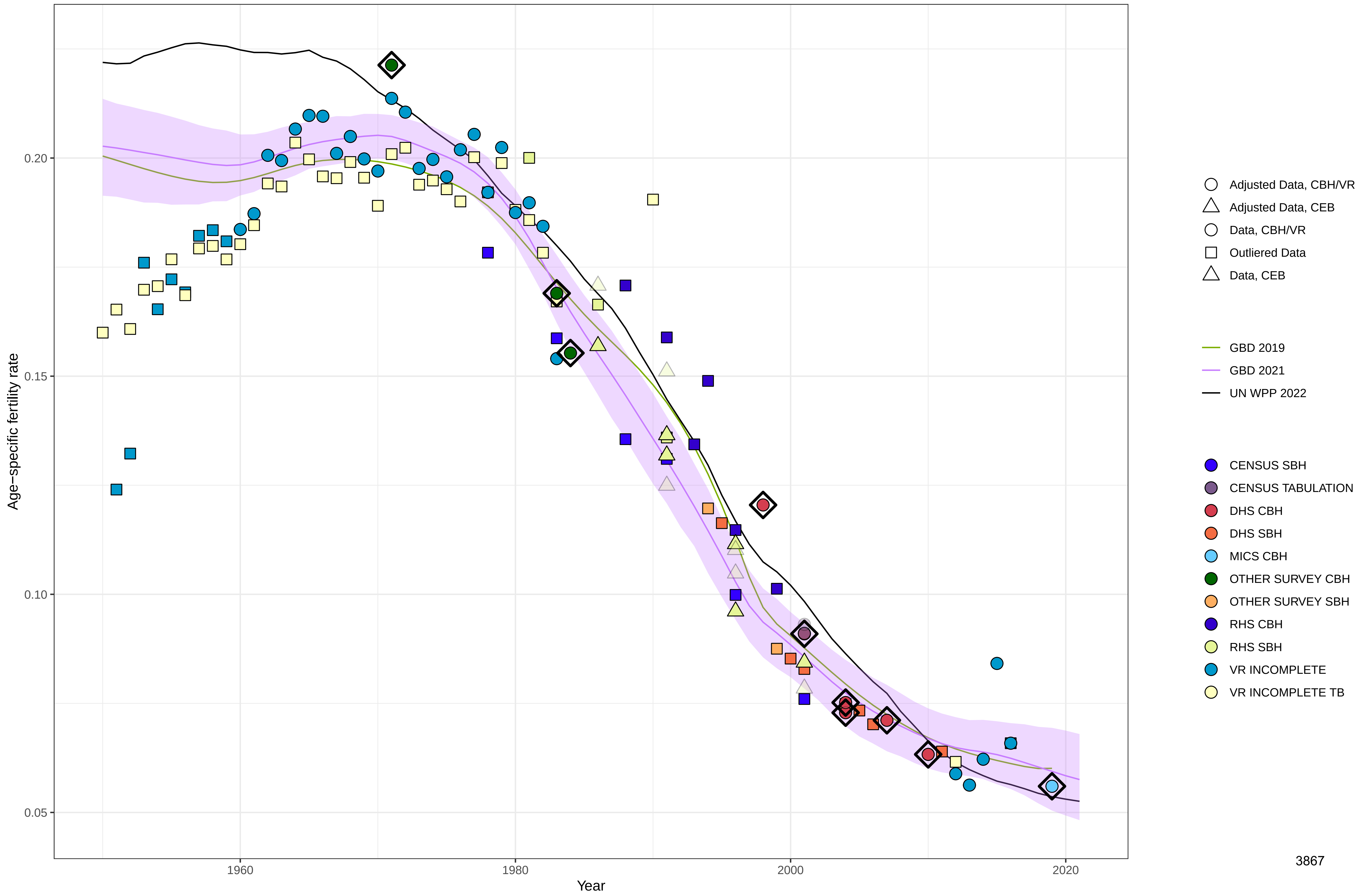

Age−specific fertility fitted trend lines, 1950−2021: Honduras, Ages: 40−44
Data points enclosed with a diamond are reference sources

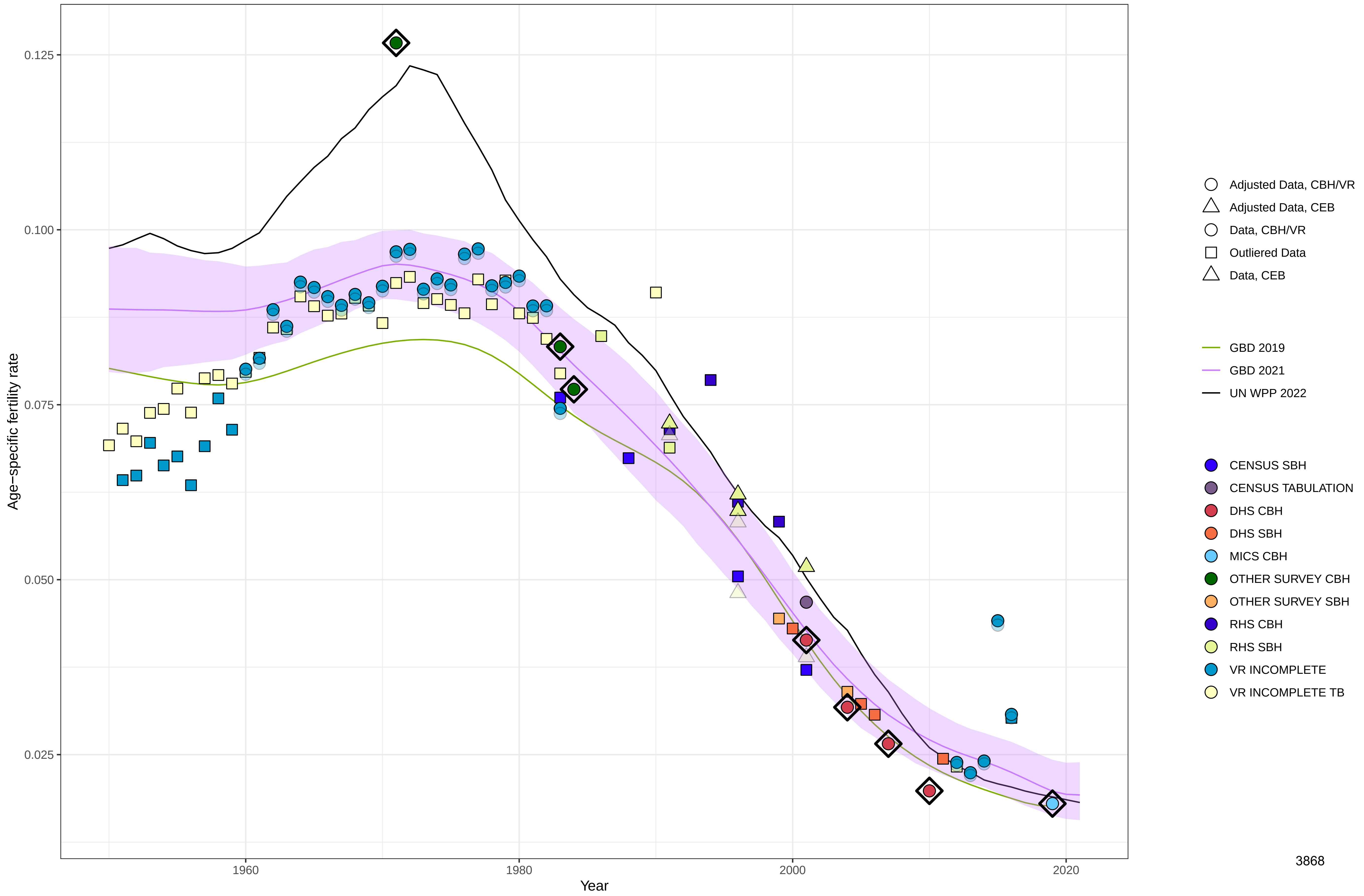

Age−specific fertility fitted trend lines, 1950−2021: Honduras, Ages: 45−49
Data points enclosed with a diamond are reference sources

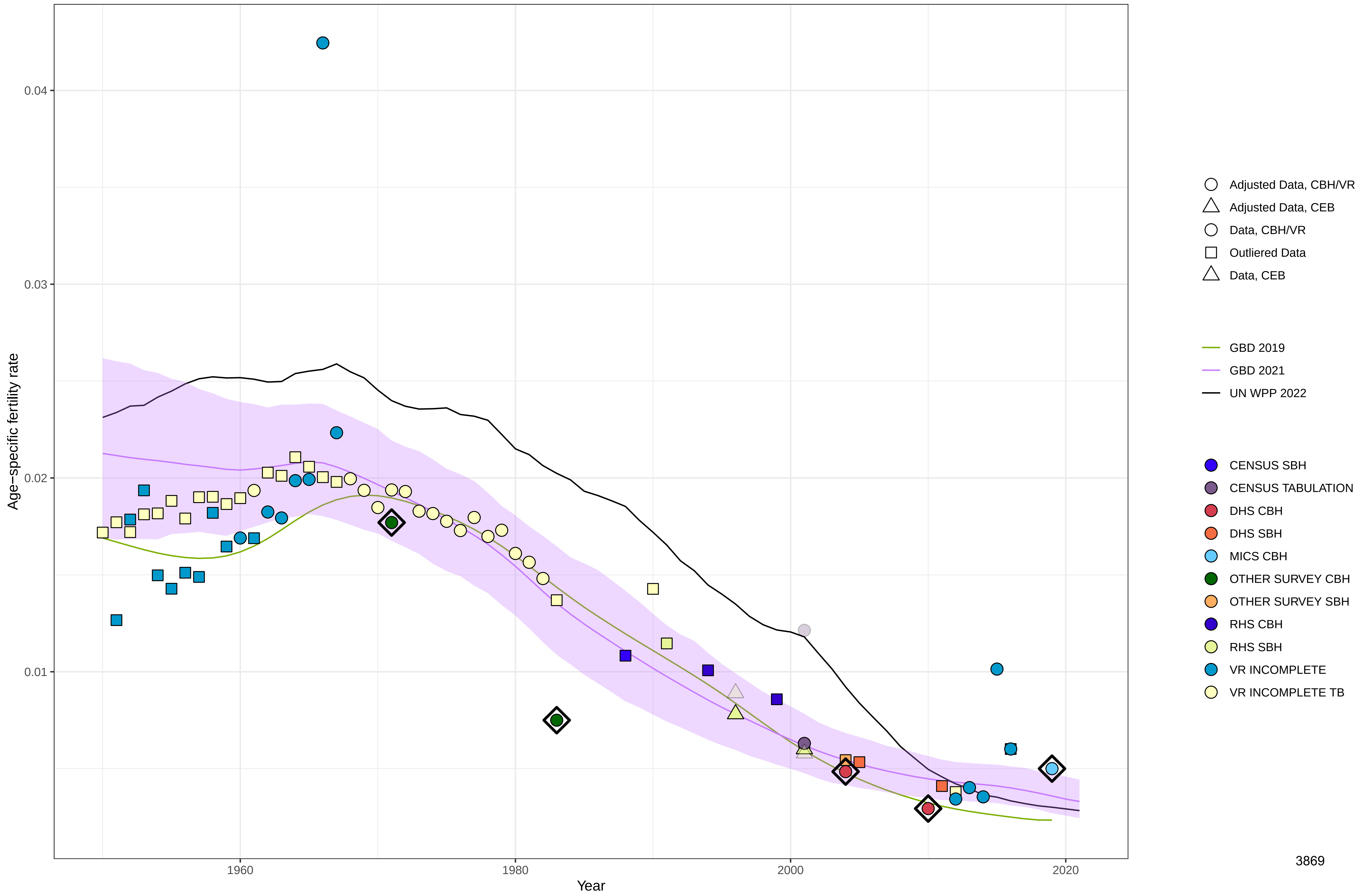

Age−specific fertility fitted trend lines, 1950−2021: Honduras, Ages: 50−54
Data points enclosed with a diamond are reference sources

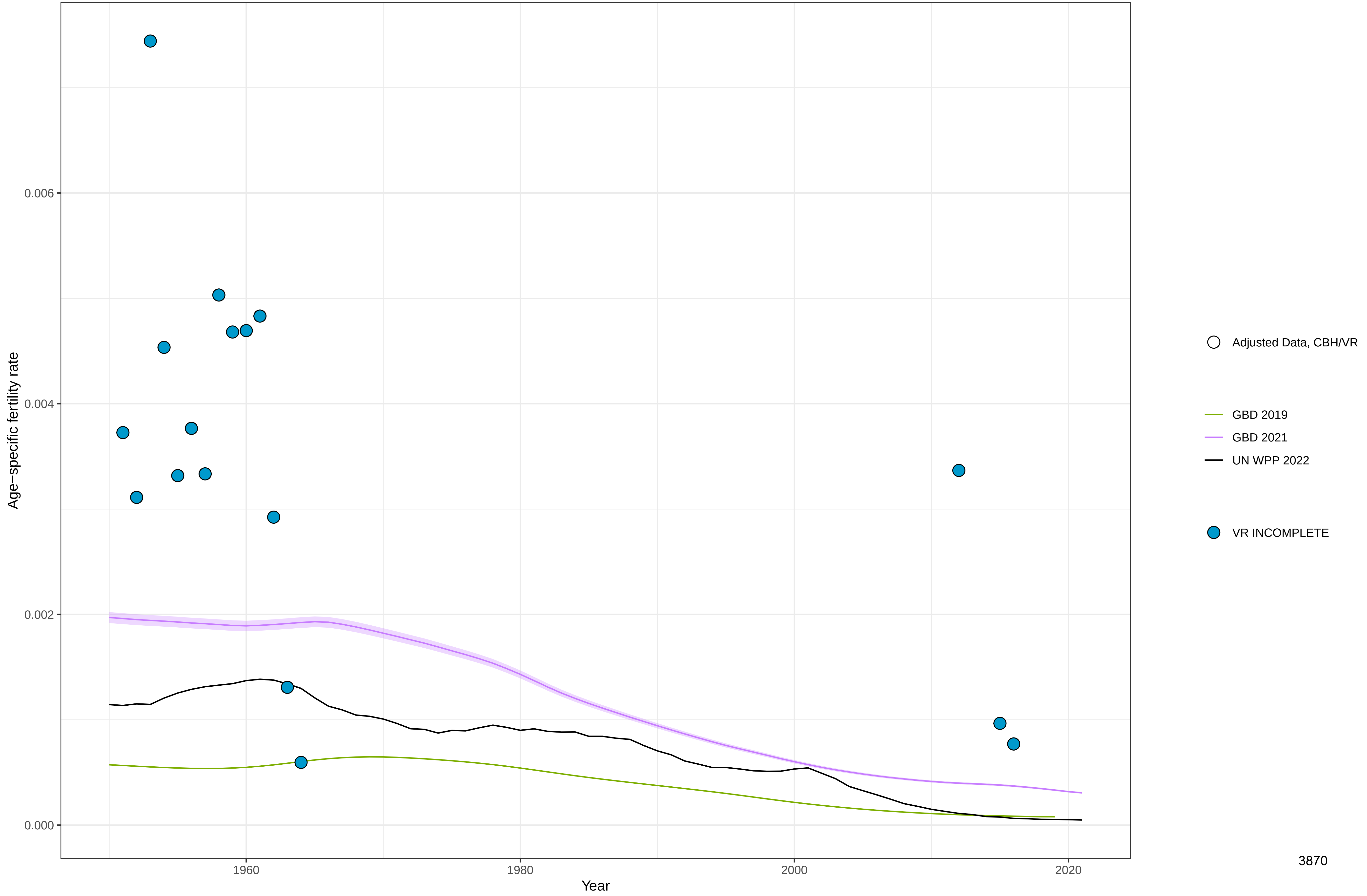

Total fertility rate fitted trend lines, 1950−2021: Honduras TFR
Data points enclosed with a diamond are reference sources

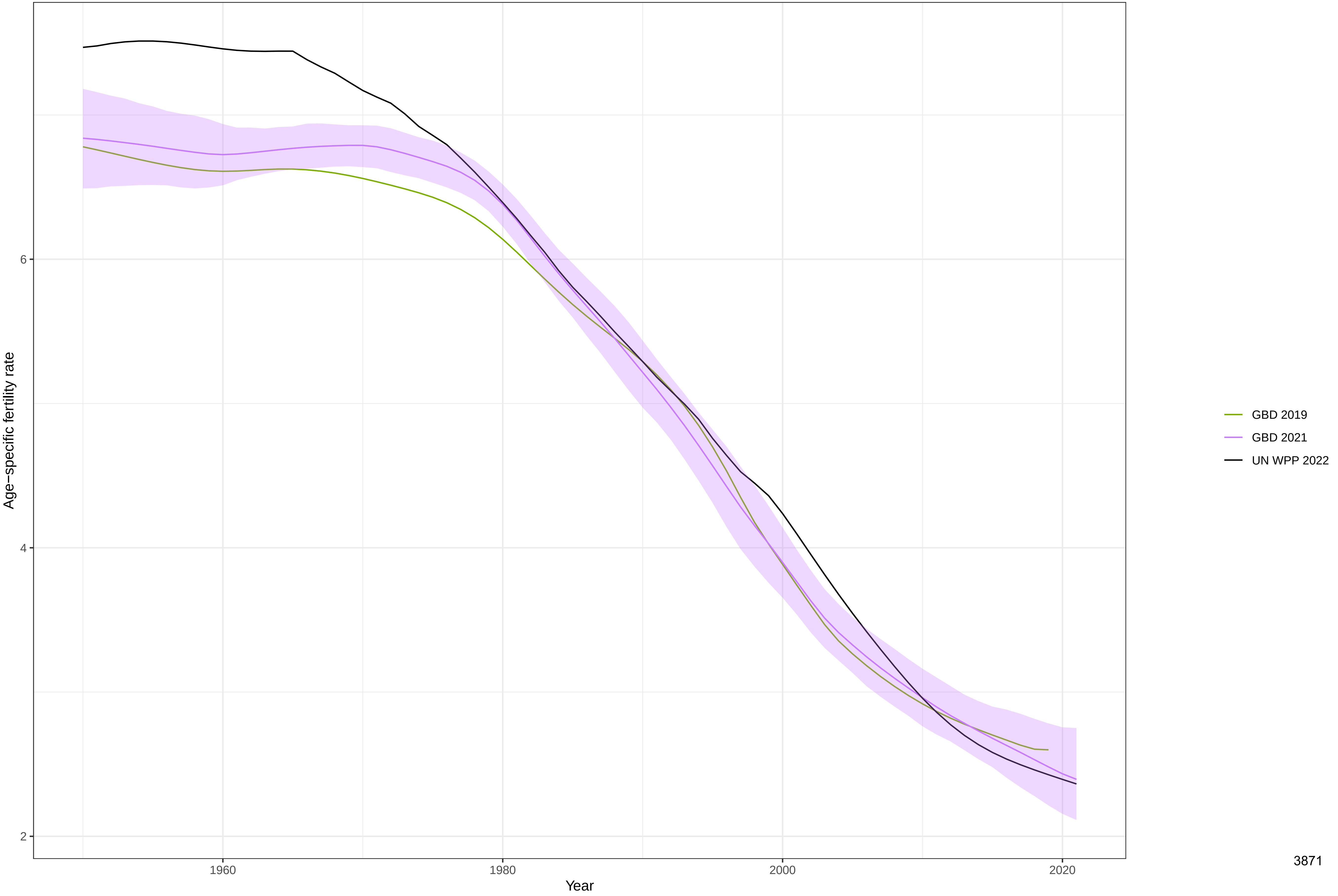

Age−specific fertility fitted trend lines, 1950−2021: Mexico, Ages: 10−14
Data points enclosed with a diamond are reference sources

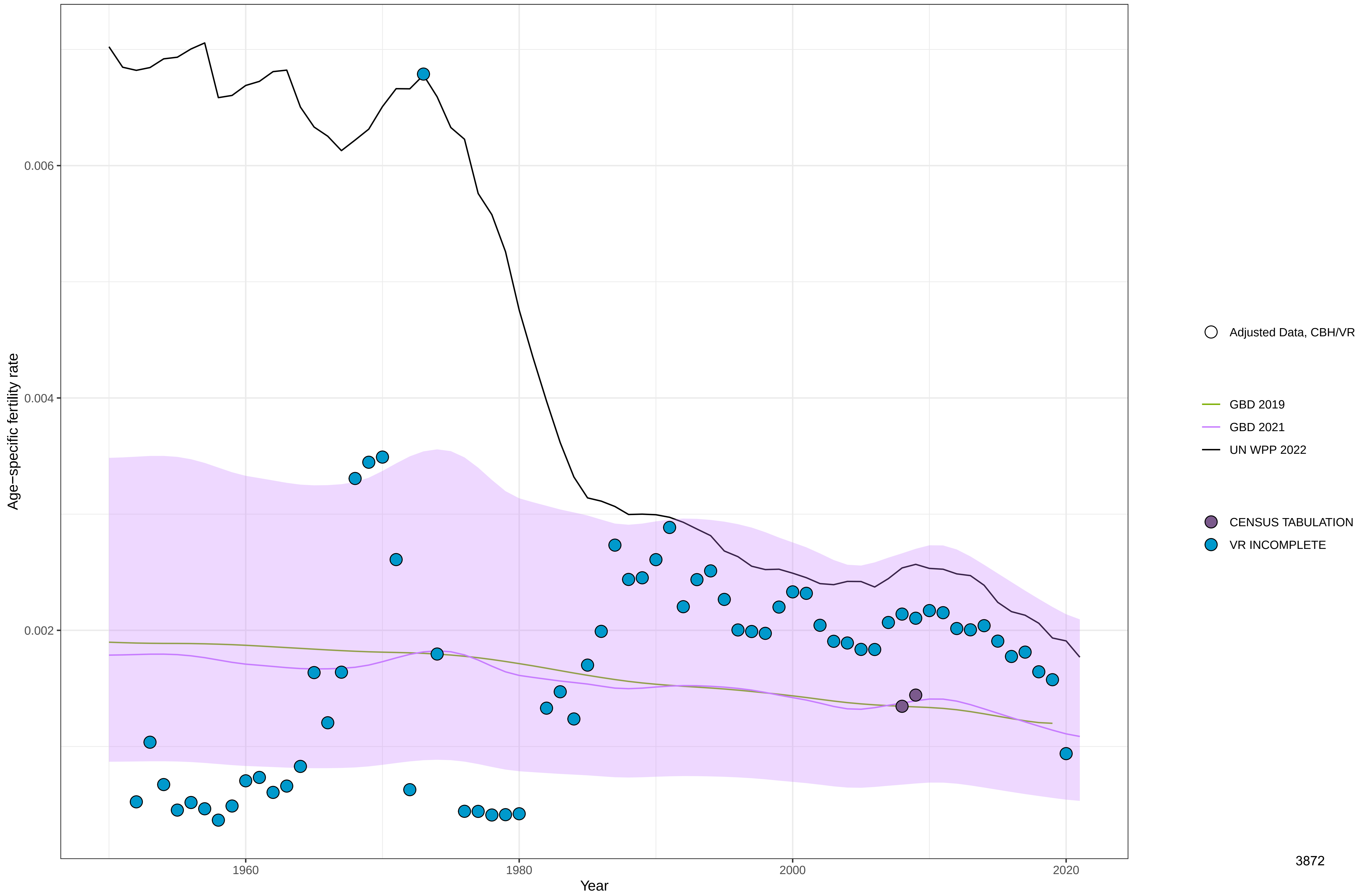

Age−specific fertility fitted trend lines, 1950−2021: Mexico, Ages: 15−19
Data points enclosed with a diamond are reference sources

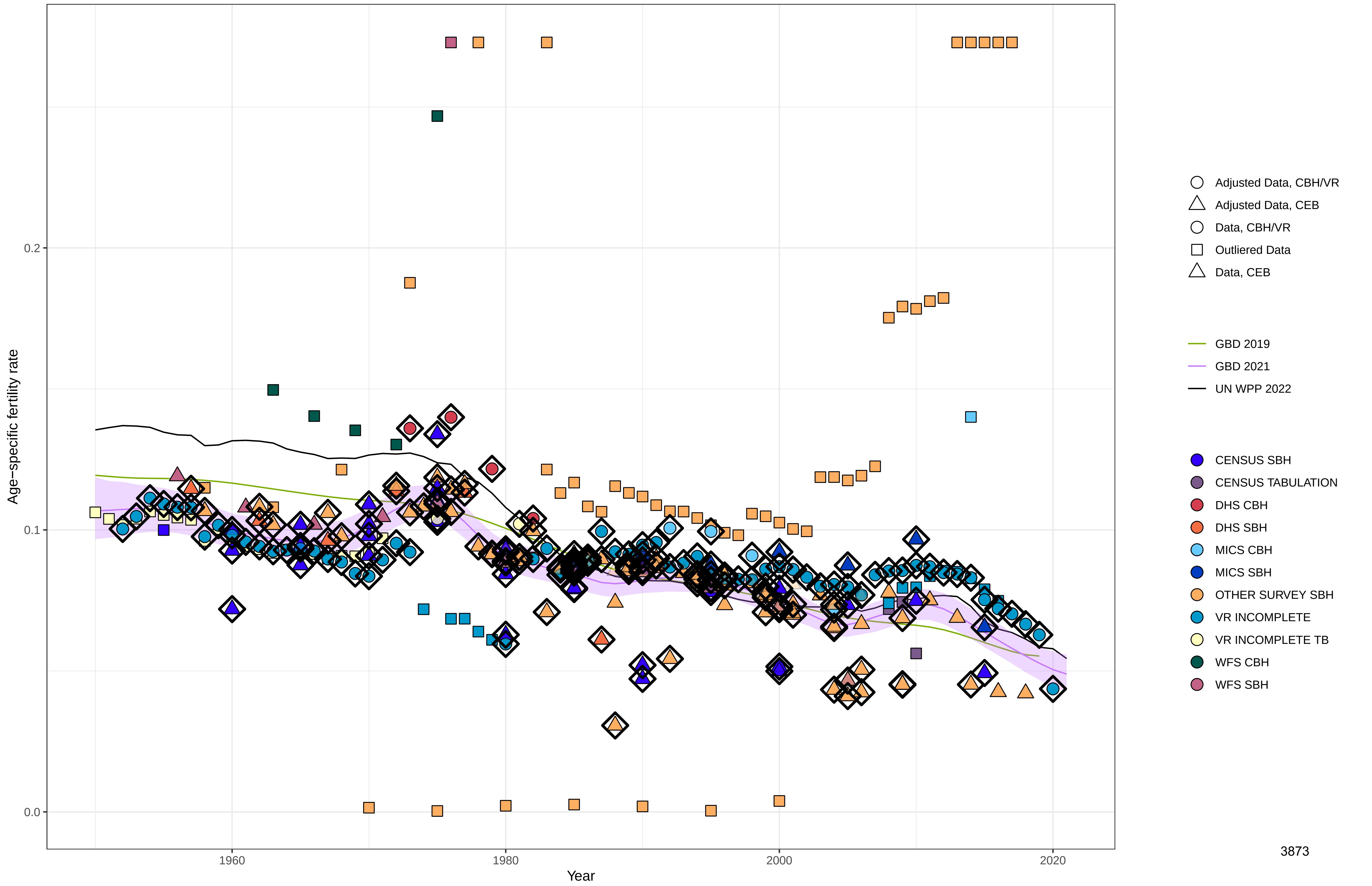

Age−specific fertility fitted trend lines, 1950−2021: Mexico, Ages: 20−24
Data points enclosed with a diamond are reference sources

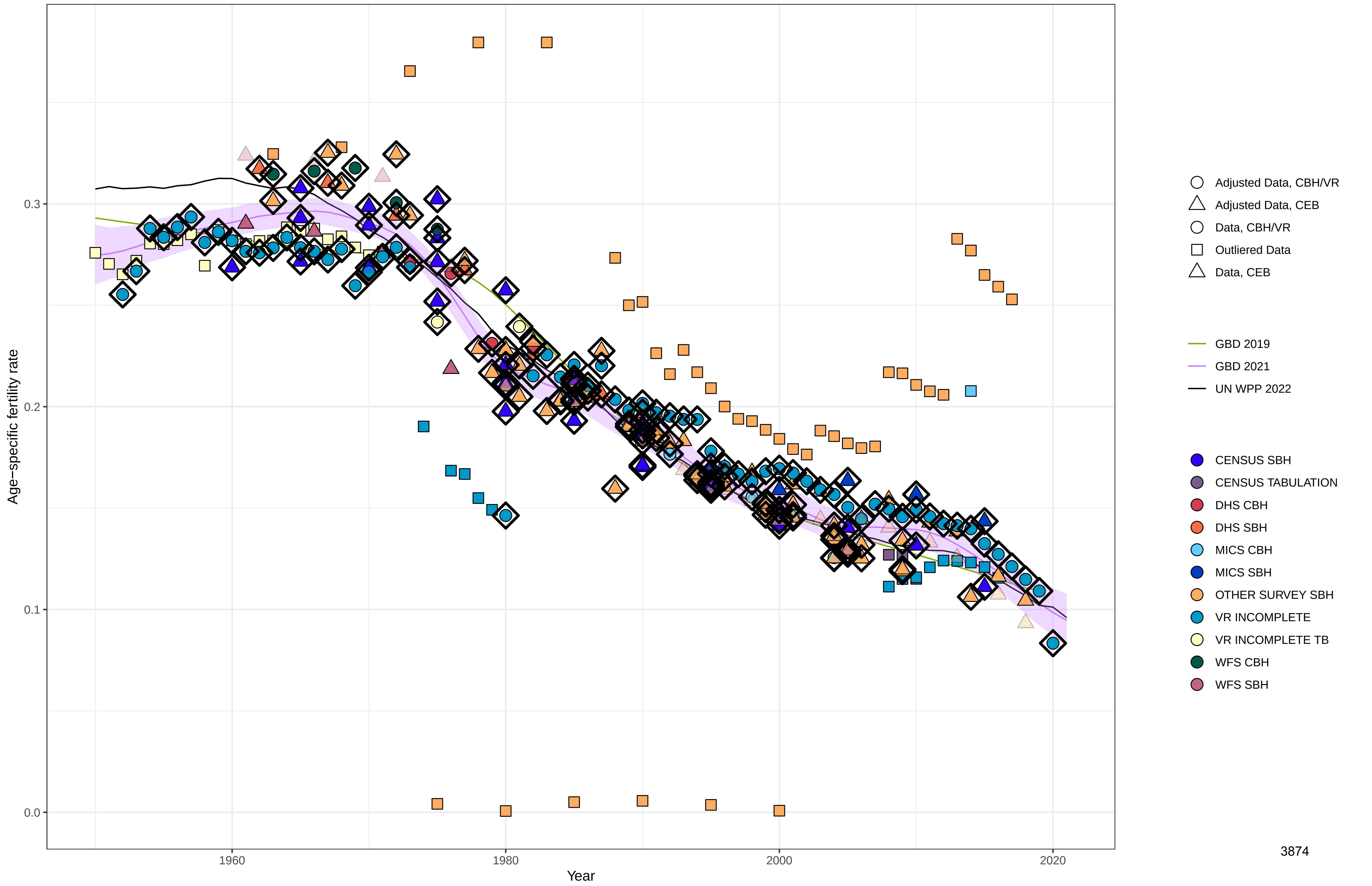

Age−specific fertility fitted trend lines, 1950−2021: Mexico, Ages: 25−29
Data points enclosed with a diamond are reference sources

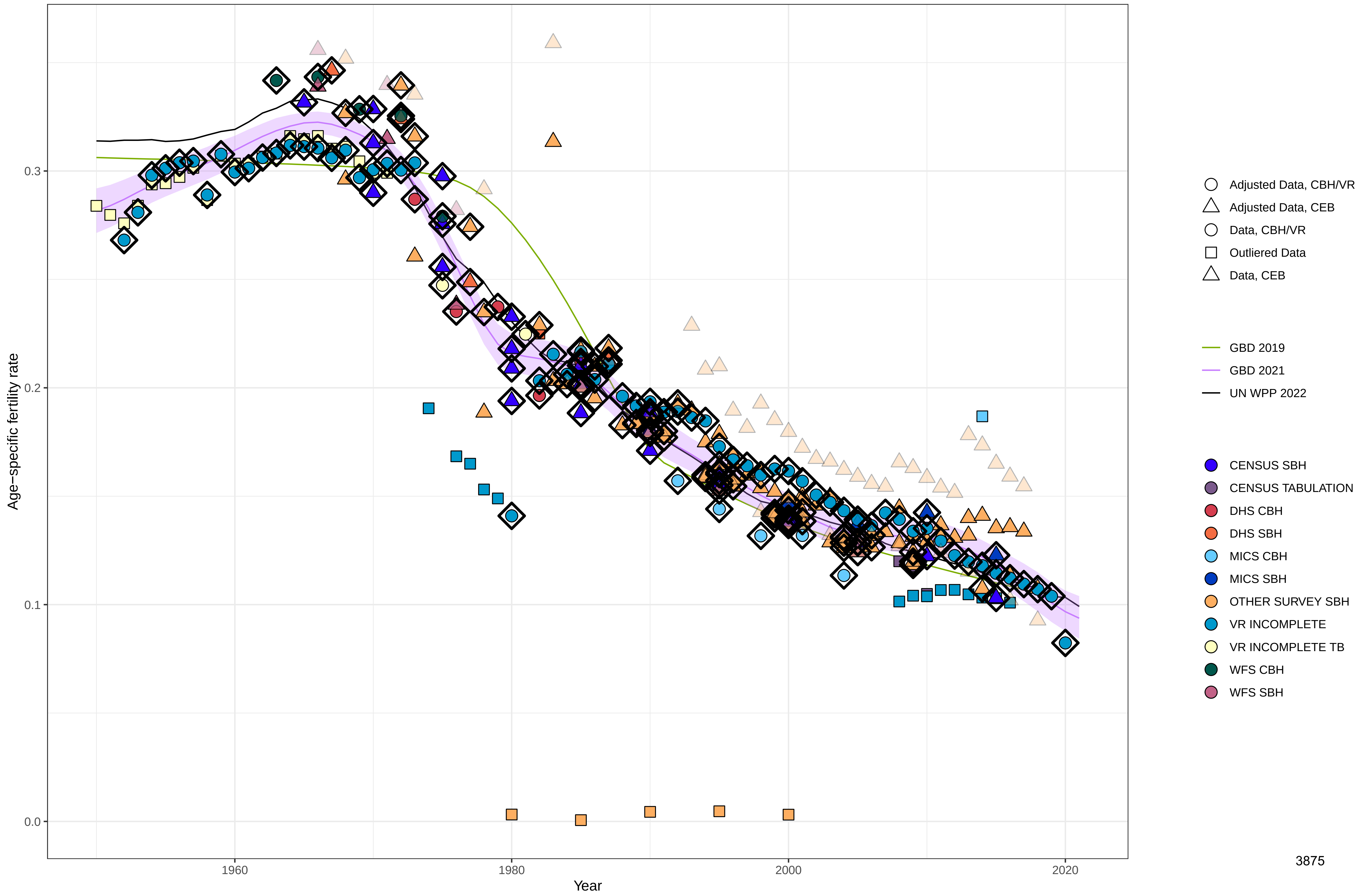

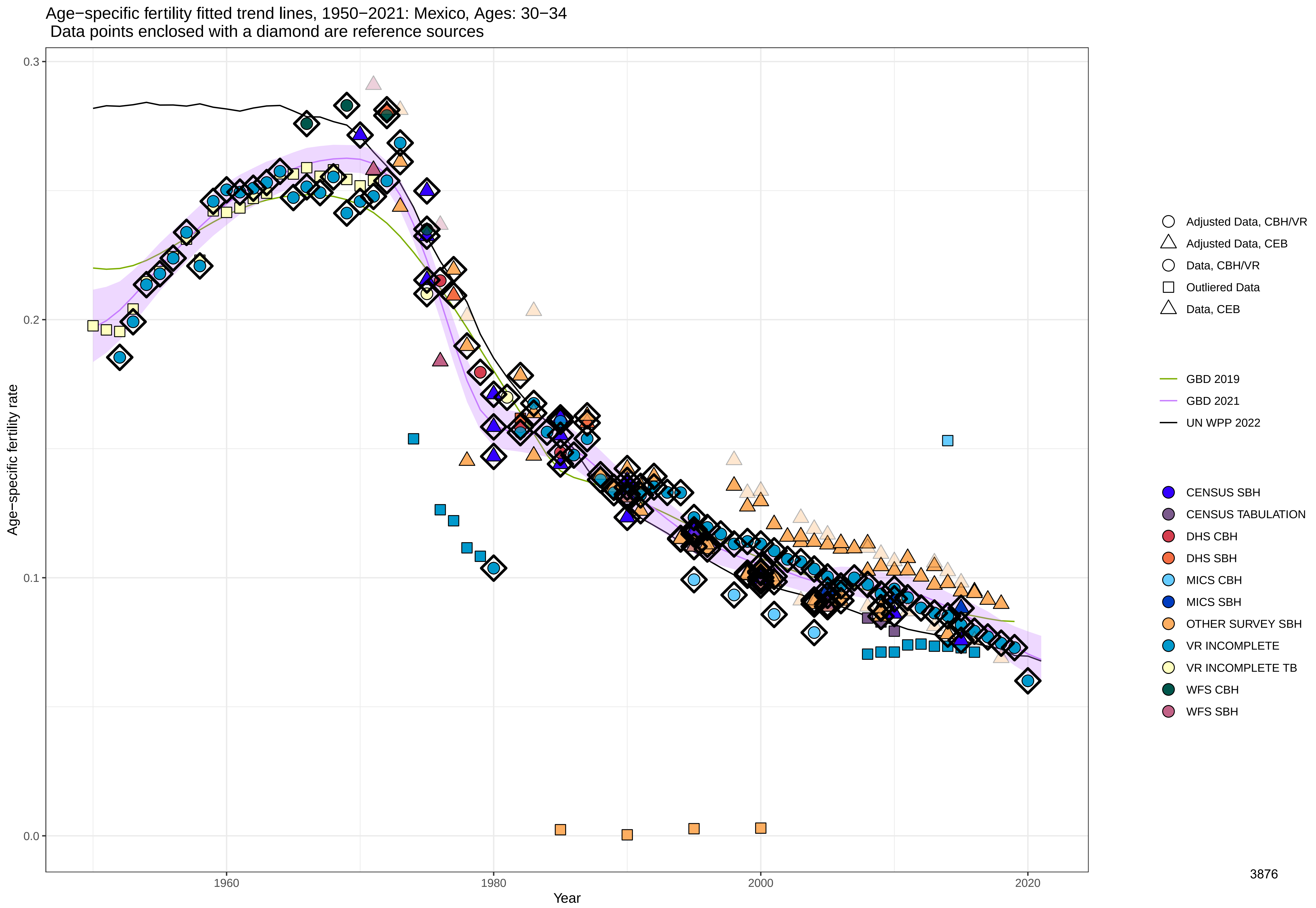
Age−specific fertility fitted trend lines, 1950−2021: Mexico, Ages: 30−34
Data points enclosed with a diamond are reference sources
Age−specific fertility rate
Year
0.0
0.1
0.2
0.3
1960
1980
2000
2020
Adjusted Data, CBH/VR
Adjusted Data, CEB
Data, CBH/VR
Outliered Data
Data, CEB
GBD 2019
GBD 2021
UN WPP 2022
CENSUS SBH
CENSUS TABULATION
DHS CBH
DHS SBH
MICS CBH
MICS SBH
OTHER SURVEY SBH
VR INCOMPLETE
VR INCOMPLETE TB
WFS CBH
WFS SBH

Age−specific fertility fitted trend lines, 1950−2021: Mexico, Ages: 35−39
Data points enclosed with a diamond are reference sources

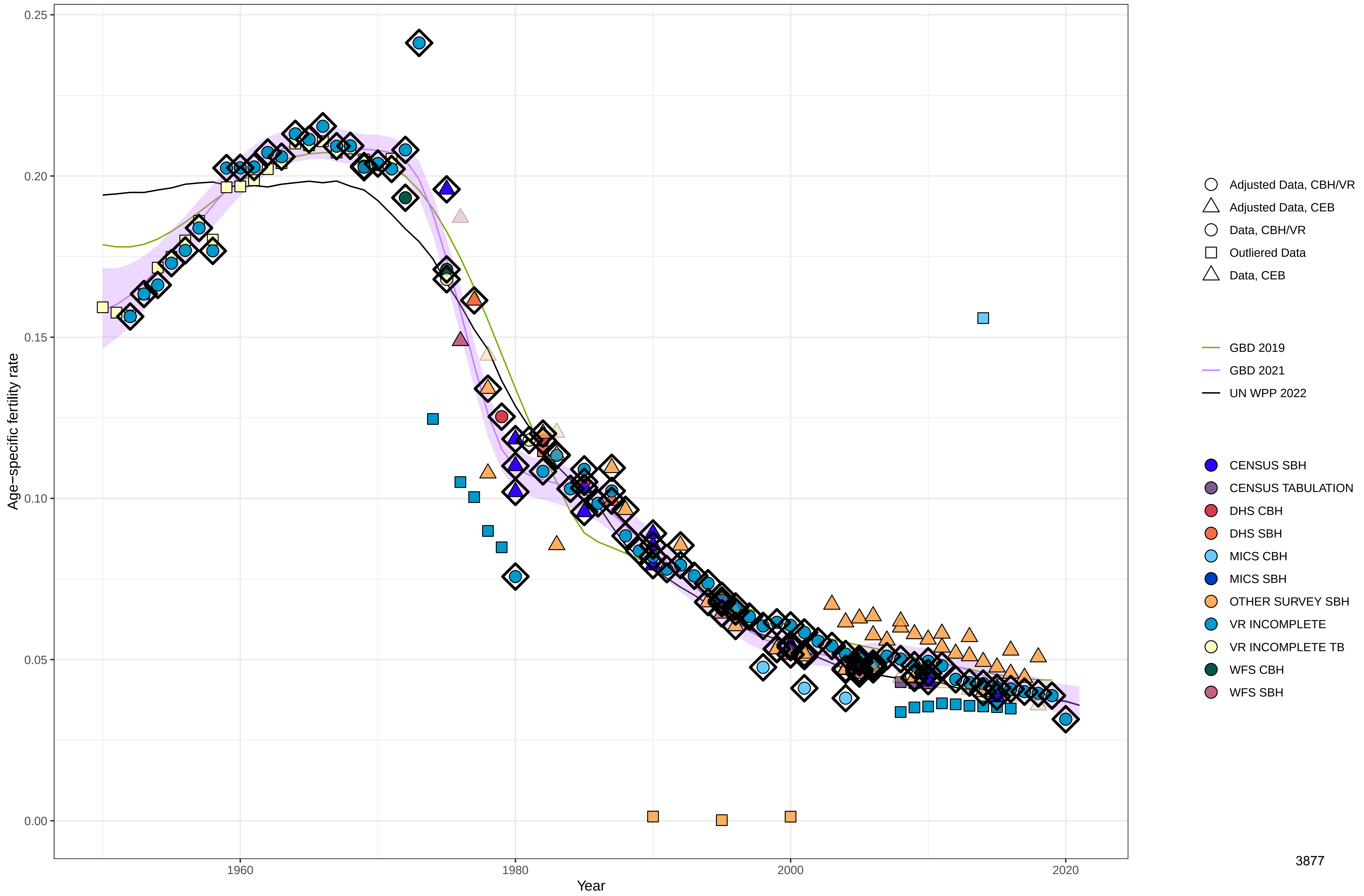

Age−specific fertility fitted trend lines, 1950−2021: Mexico, Ages: 40−44
Data points enclosed with a diamond are reference sources

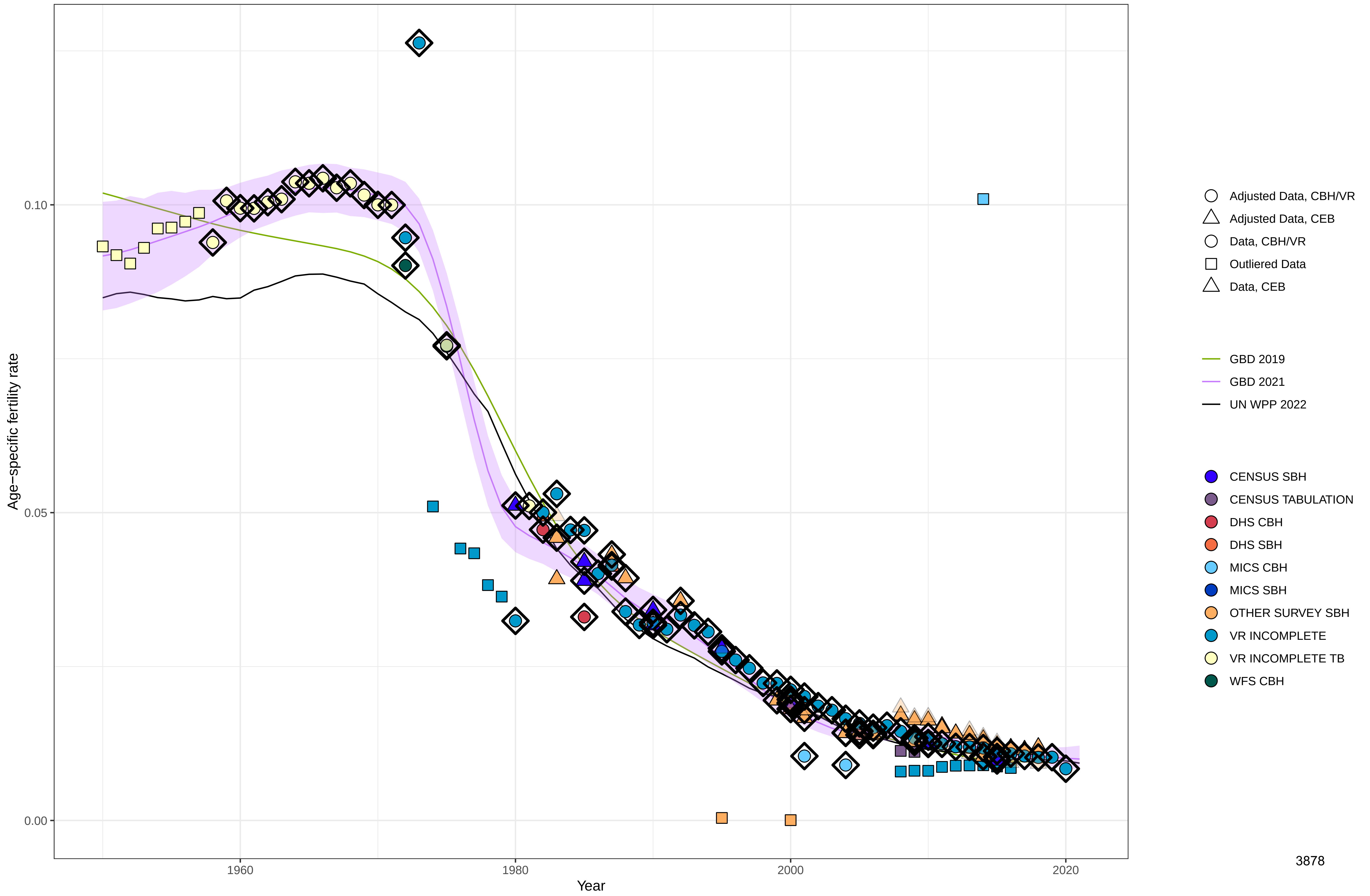

Age−specific fertility fitted trend lines, 1950−2021: Mexico, Ages: 45−49
Data points enclosed with a diamond are reference sources

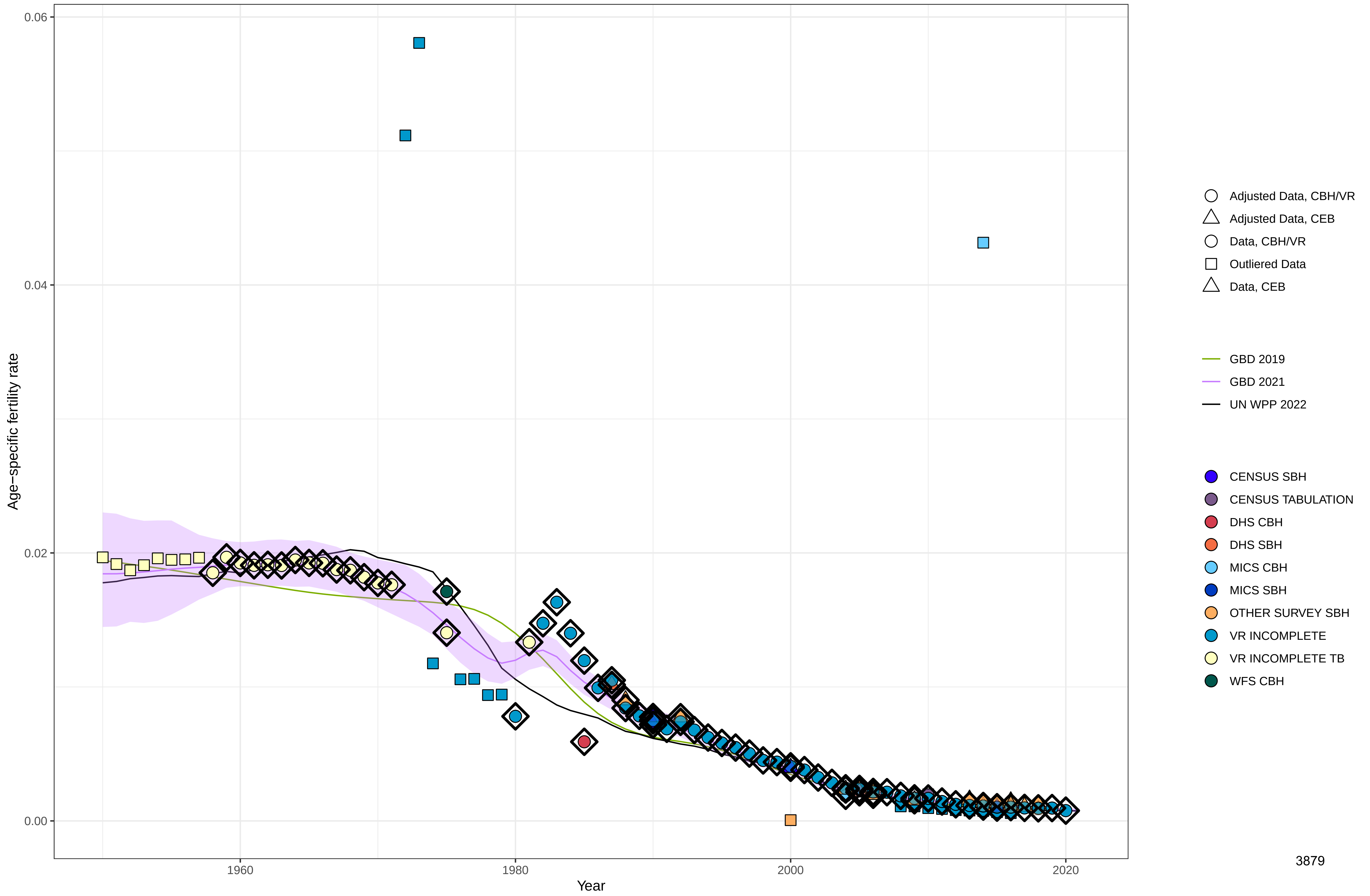

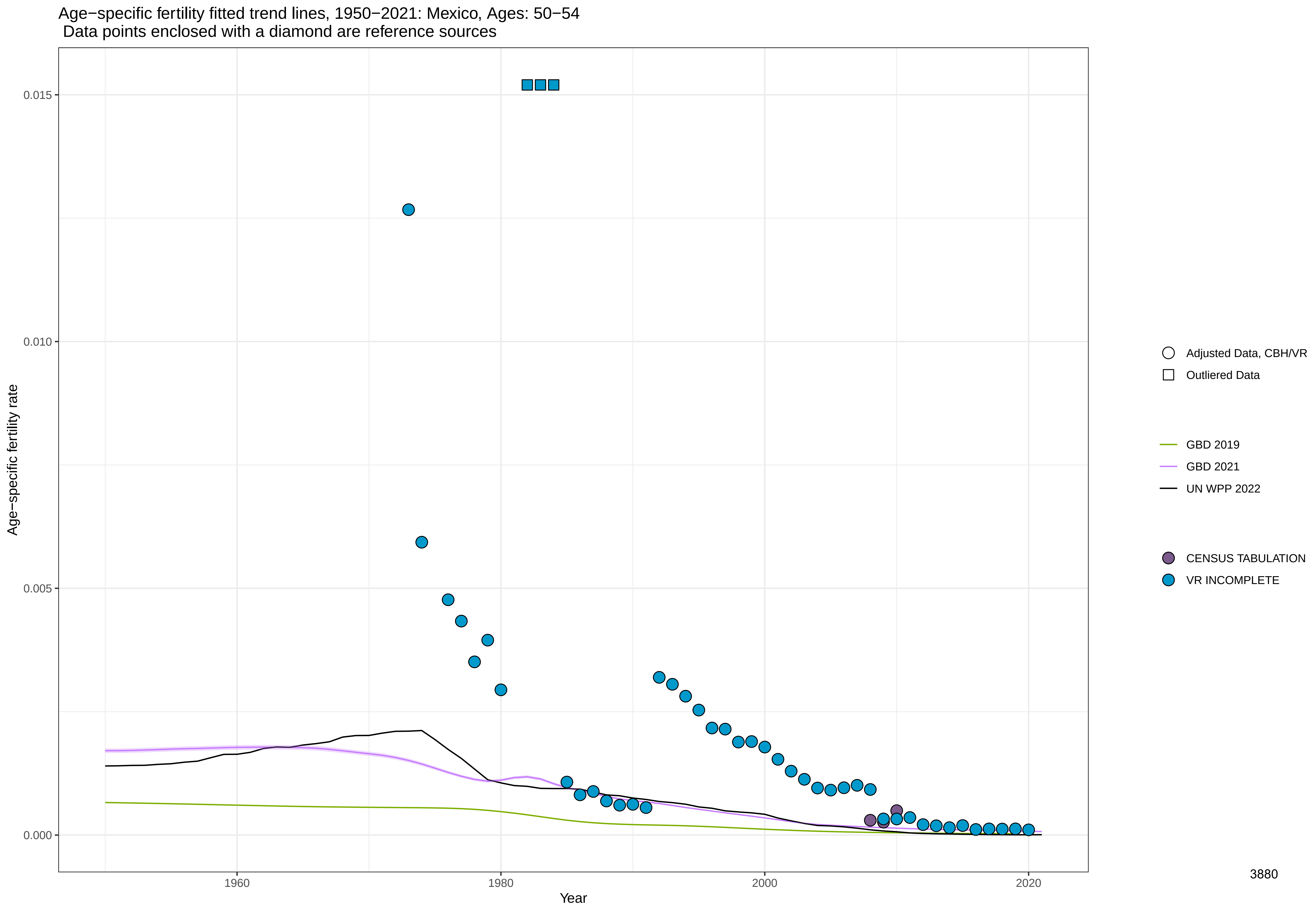

Age−specific fertility fitted trend lines, 1950−2021: Mexico, Ages: 50−54
Data points enclosed with a diamond are reference sources
Age−specific fertility rate
0.015
0.010
0.005
0.000
1960
1980
2000
2020
Year
Adjusted Data, CBH/VR
Outliered Data
GBD 2019
GBD 2021
UN WPP 2022
CENSUS TABULATION
VR INCOMPLETE

Total fertility rate fitted trend lines, 1950−2021: Mexico TFR
Data points enclosed with a diamond are reference sources

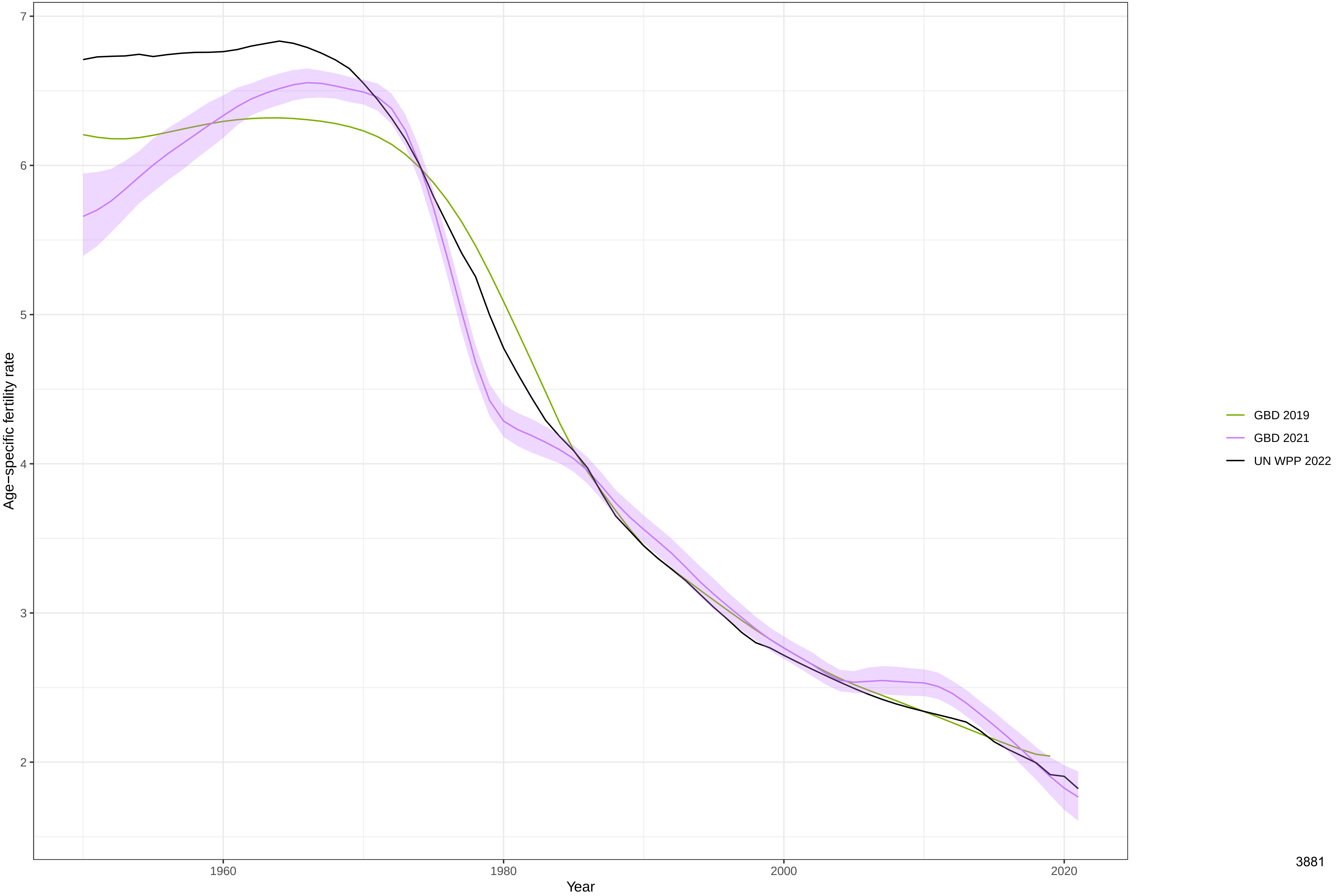

Age−specific fertility fitted trend lines, 1950−2021: Aguascalientes, Ages: 10−14
Data points enclosed with a diamond are reference sources

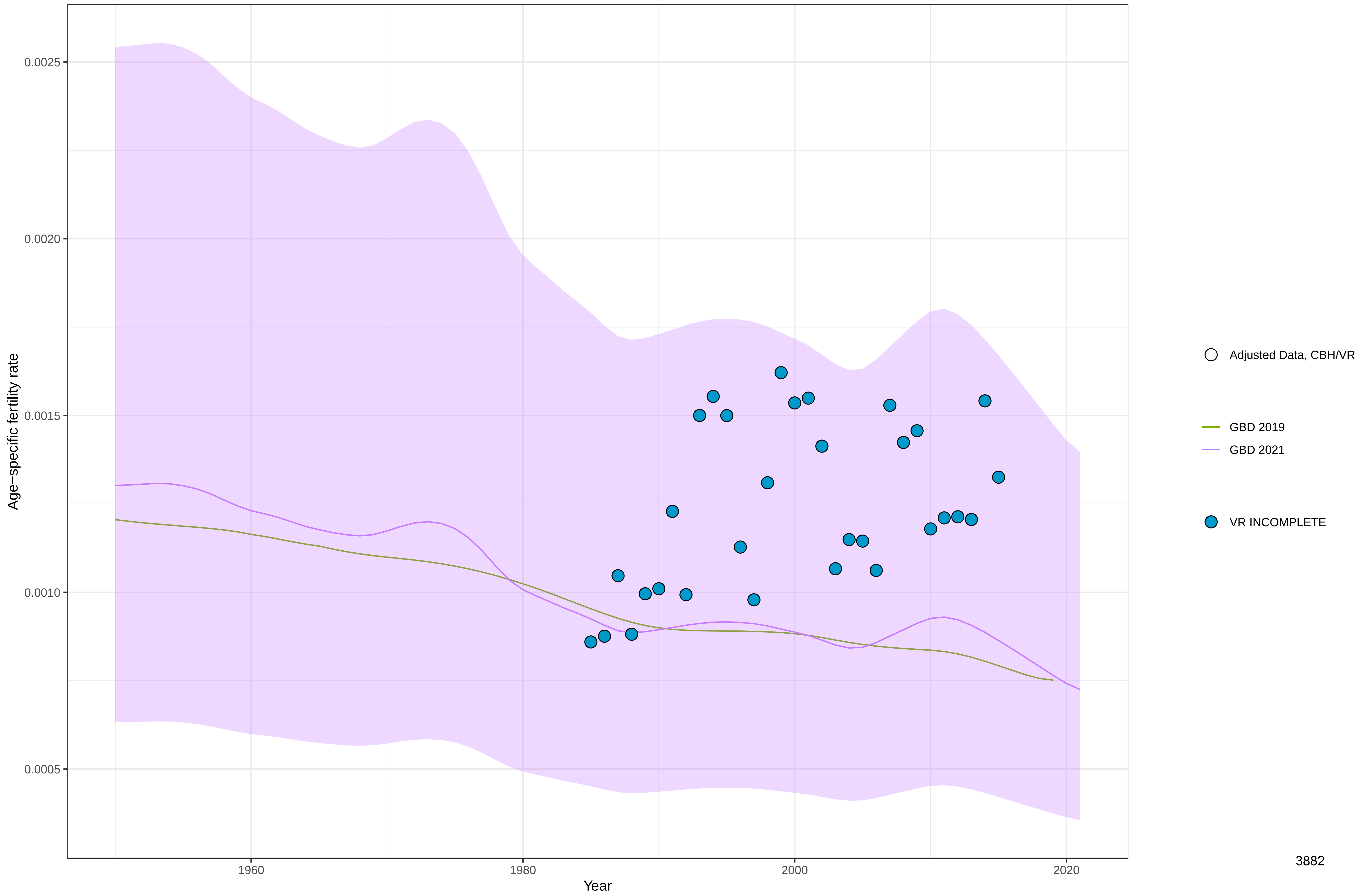

Age−specific fertility fitted trend lines, 1950−2021: Aguascalientes, Ages: 15−19
Data points enclosed with a diamond are reference sources

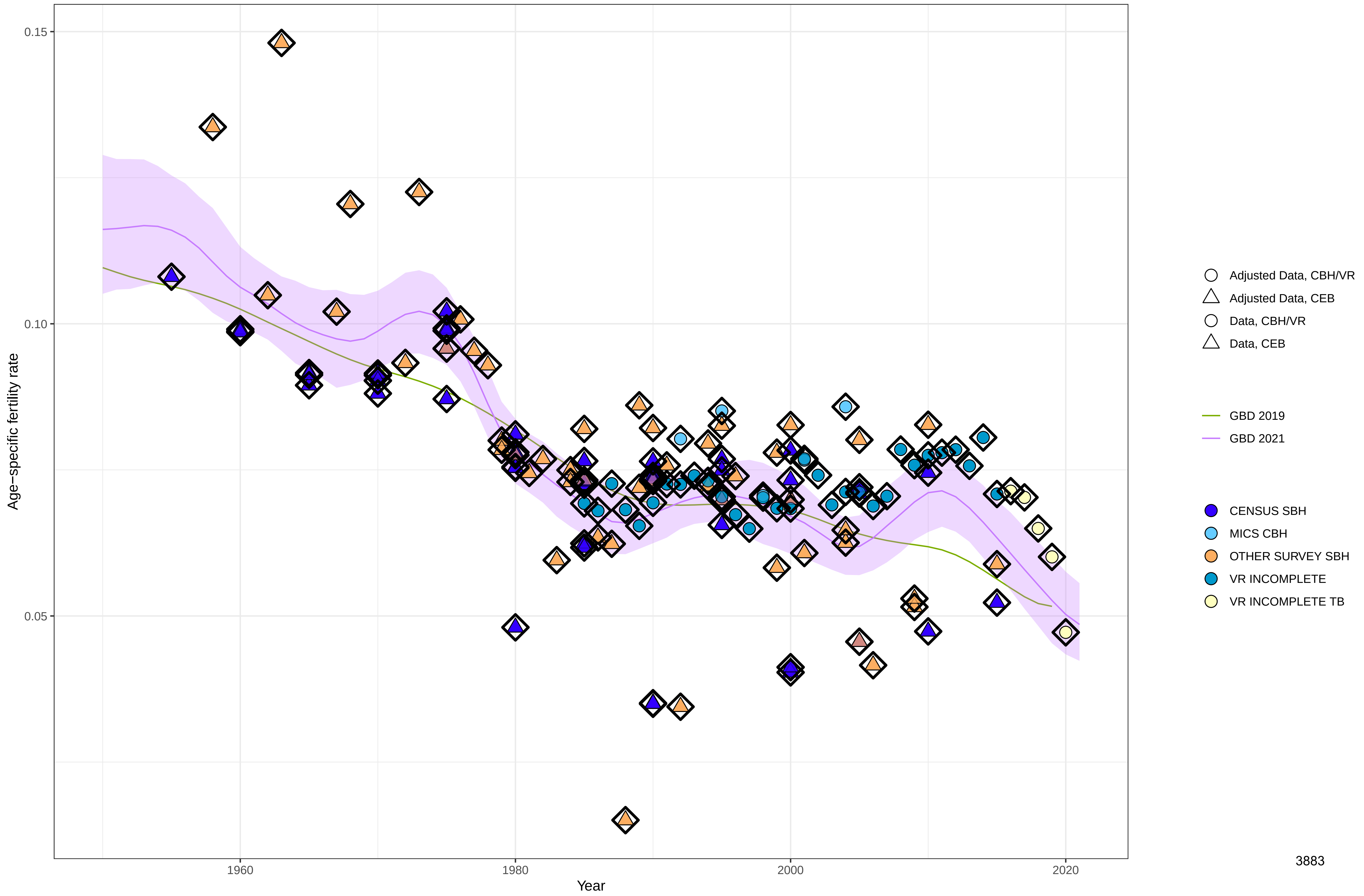

Age−specific fertility fitted trend lines, 1950−2021: Aguascalientes, Ages: 20−24
Data points enclosed with a diamond are reference sources

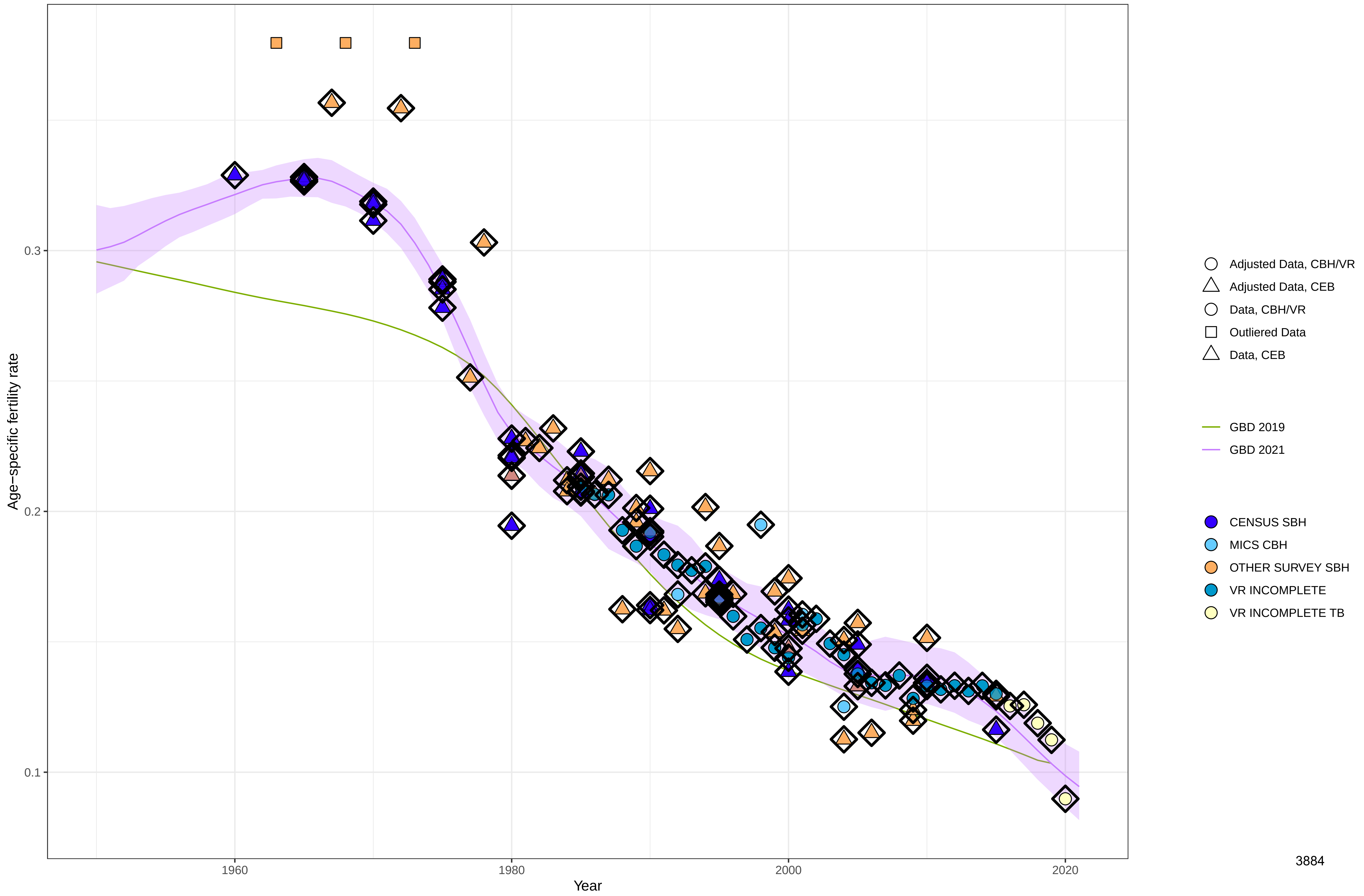

Age−specific fertility fitted trend lines, 1950−2021: Aguascalientes, Ages: 25−29
Data points enclosed with a diamond are reference sources

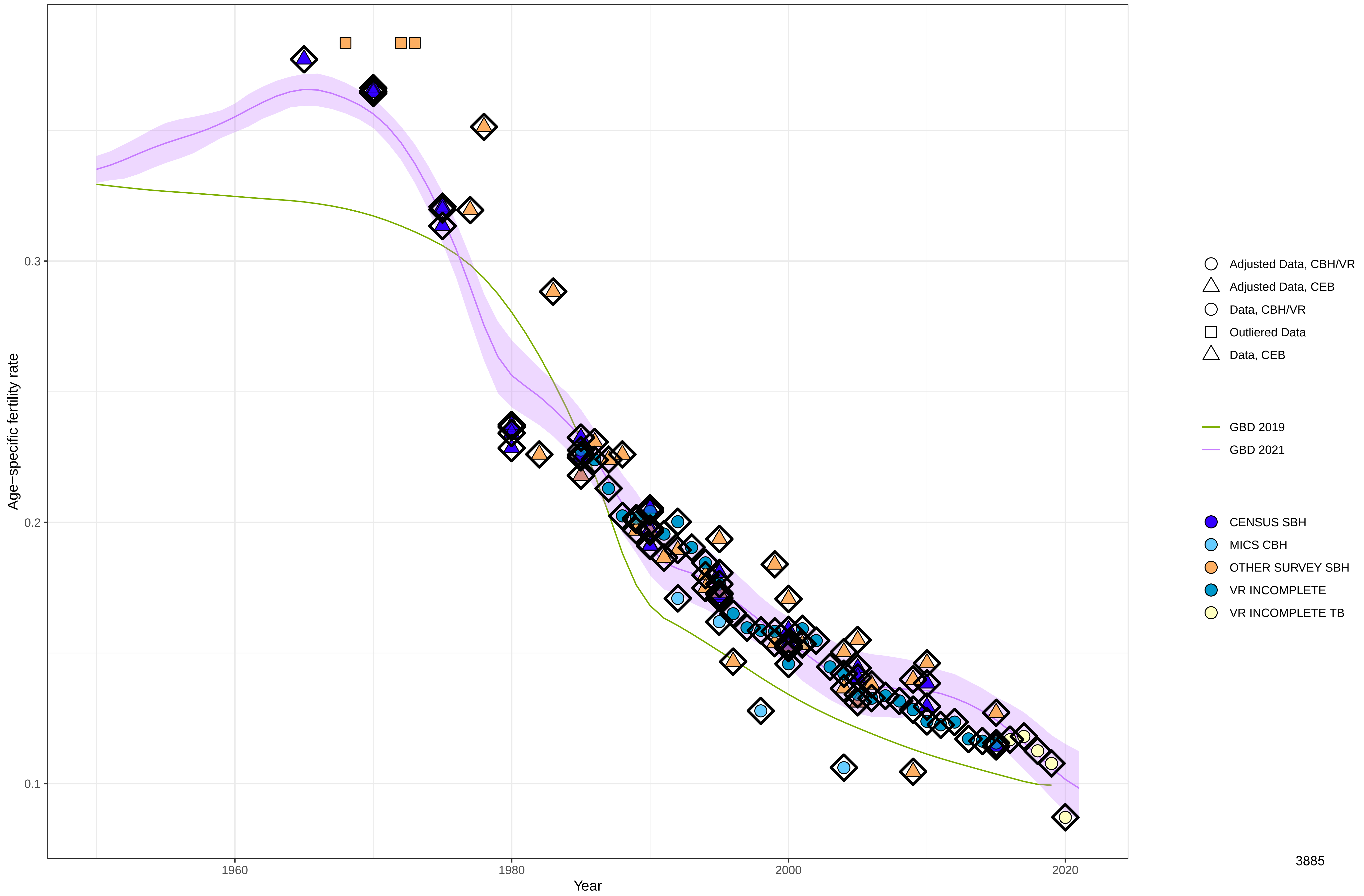

Age−specific fertility fitted trend lines, 1950−2021: Aguascalientes, Ages: 30−34
Data points enclosed with a diamond are reference sources

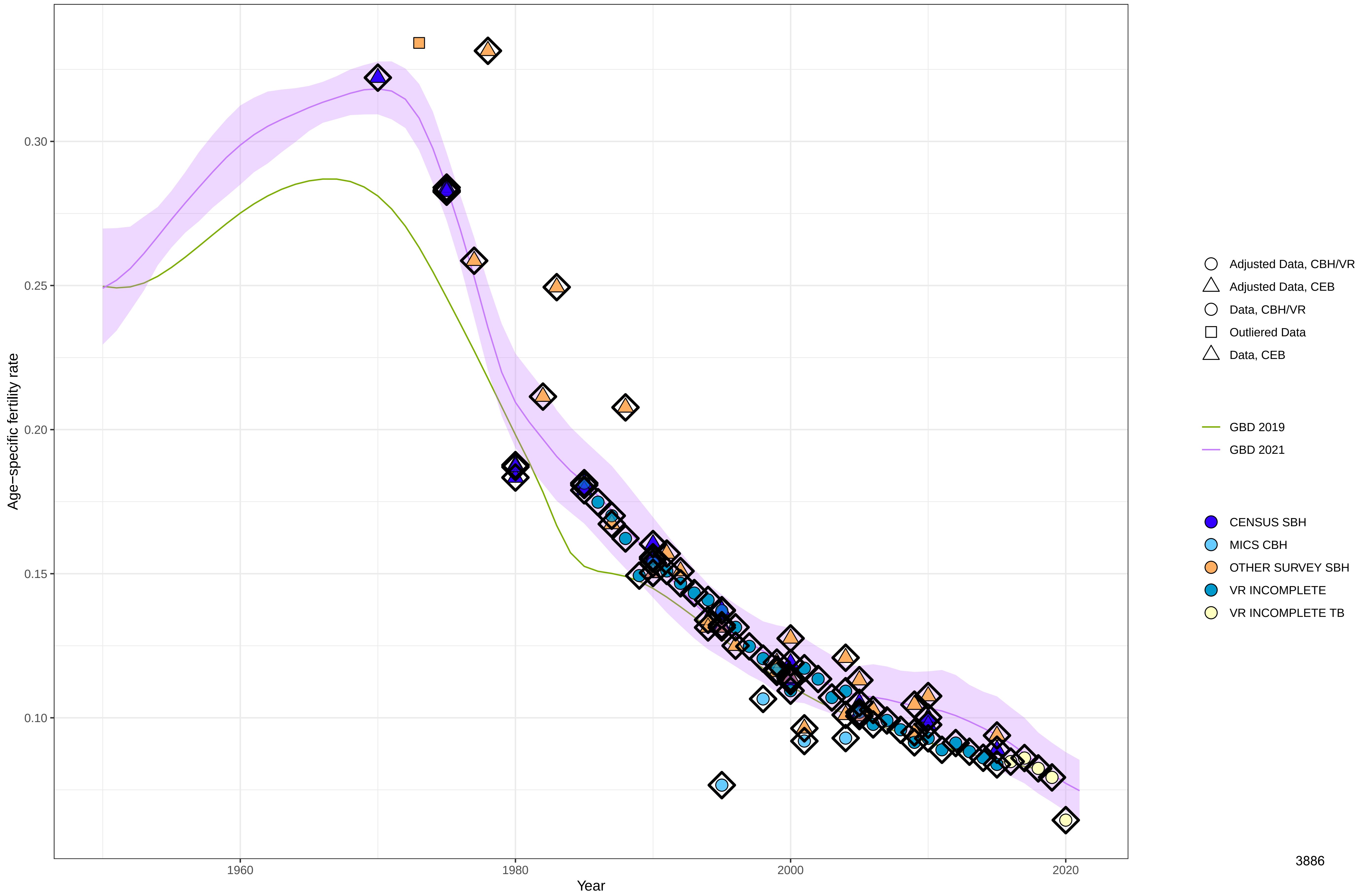

Age−specific fertility fitted trend lines, 1950−2021: Aguascalientes, Ages: 35−39
Data points enclosed with a diamond are reference sources

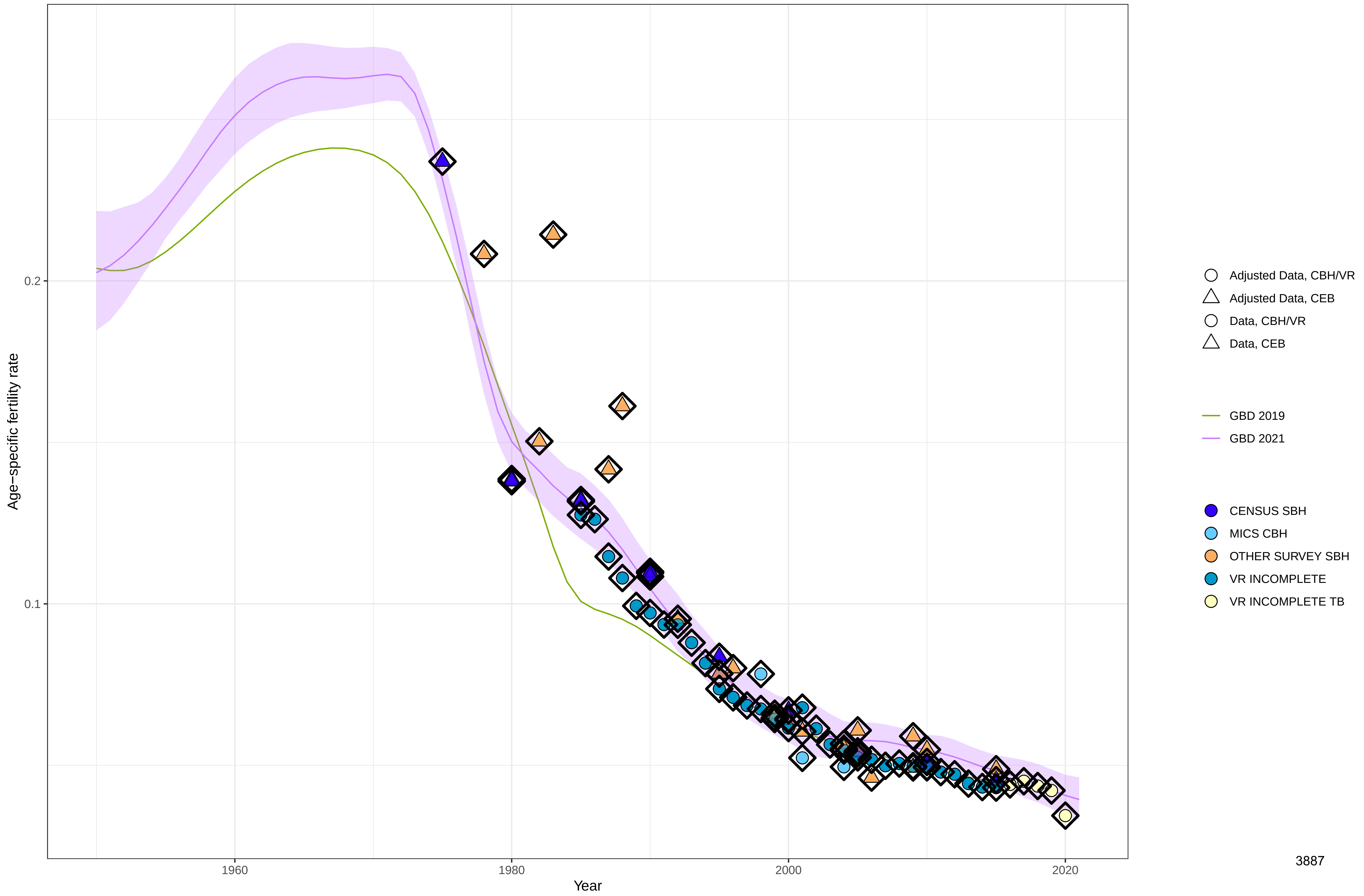

Age−specific fertility fitted trend lines, 1950−2021: Aguascalientes, Ages: 40−44
Data points enclosed with a diamond are reference sources

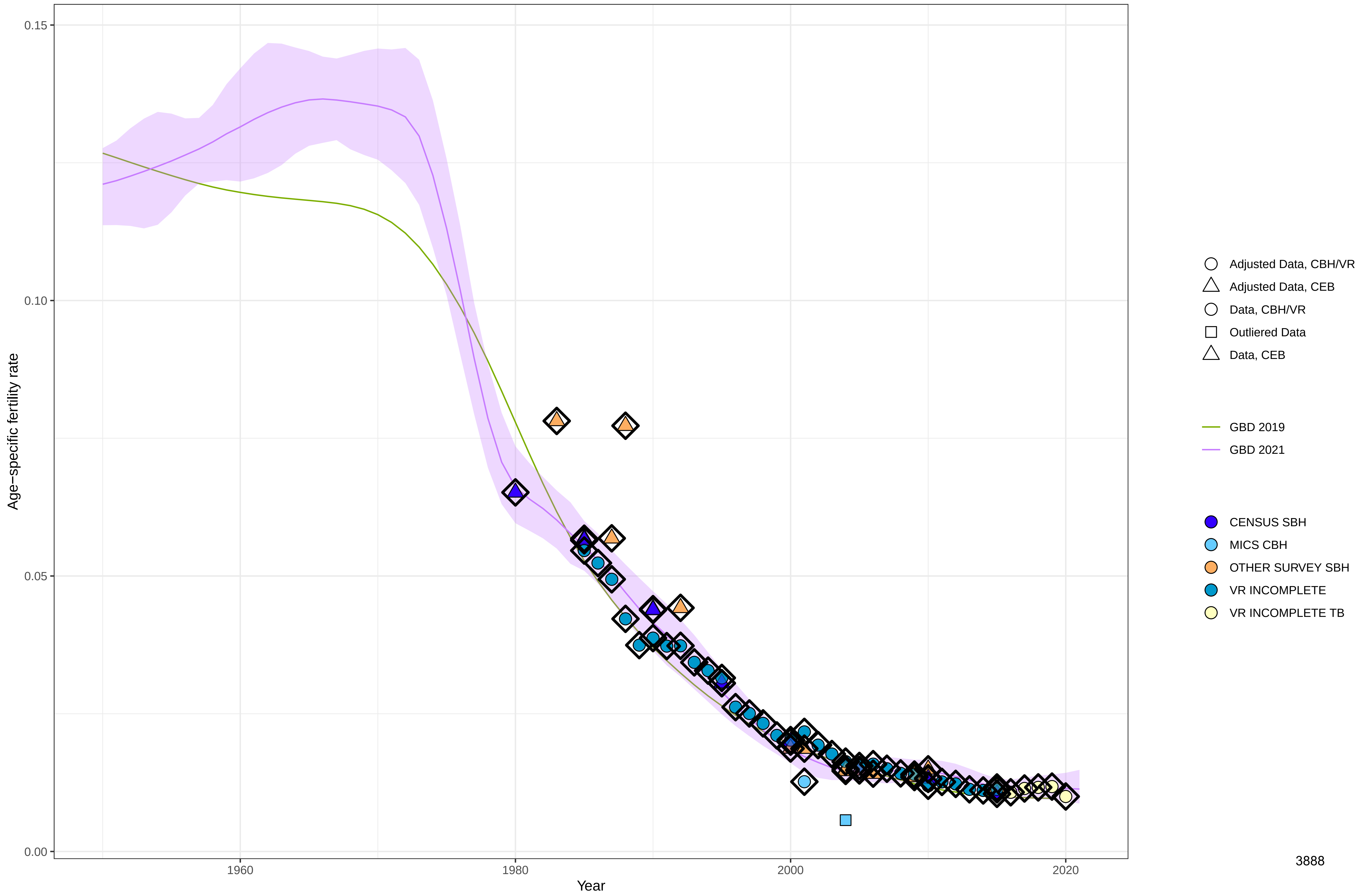

Age−specific fertility fitted trend lines, 1950−2021: Aguascalientes, Ages: 45−49
Data points enclosed with a diamond are reference sources

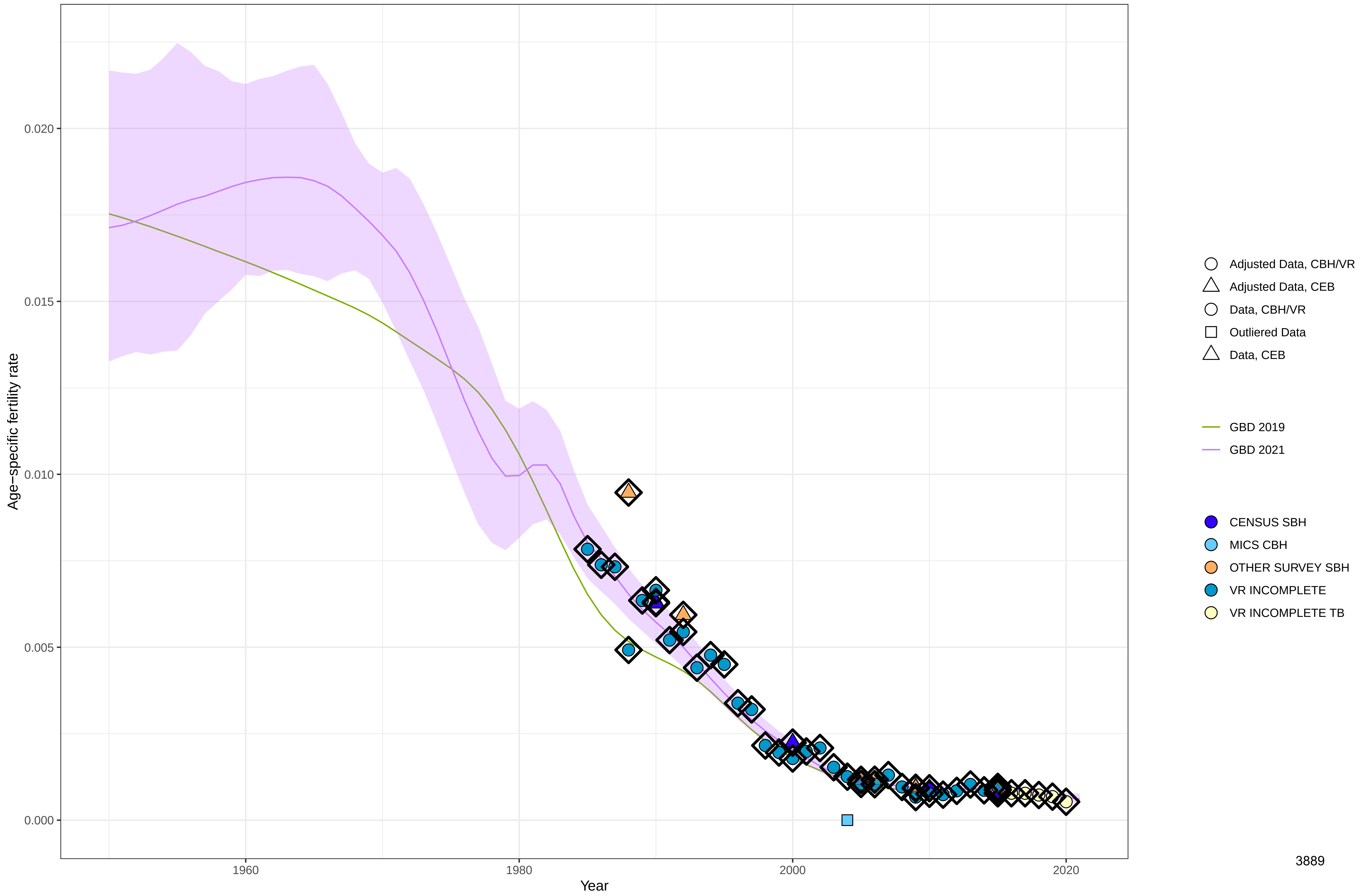

Age−specific fertility fitted trend lines, 1950−2021: Aguascalientes, Ages: 50−54
Data points enclosed with a diamond are reference sources

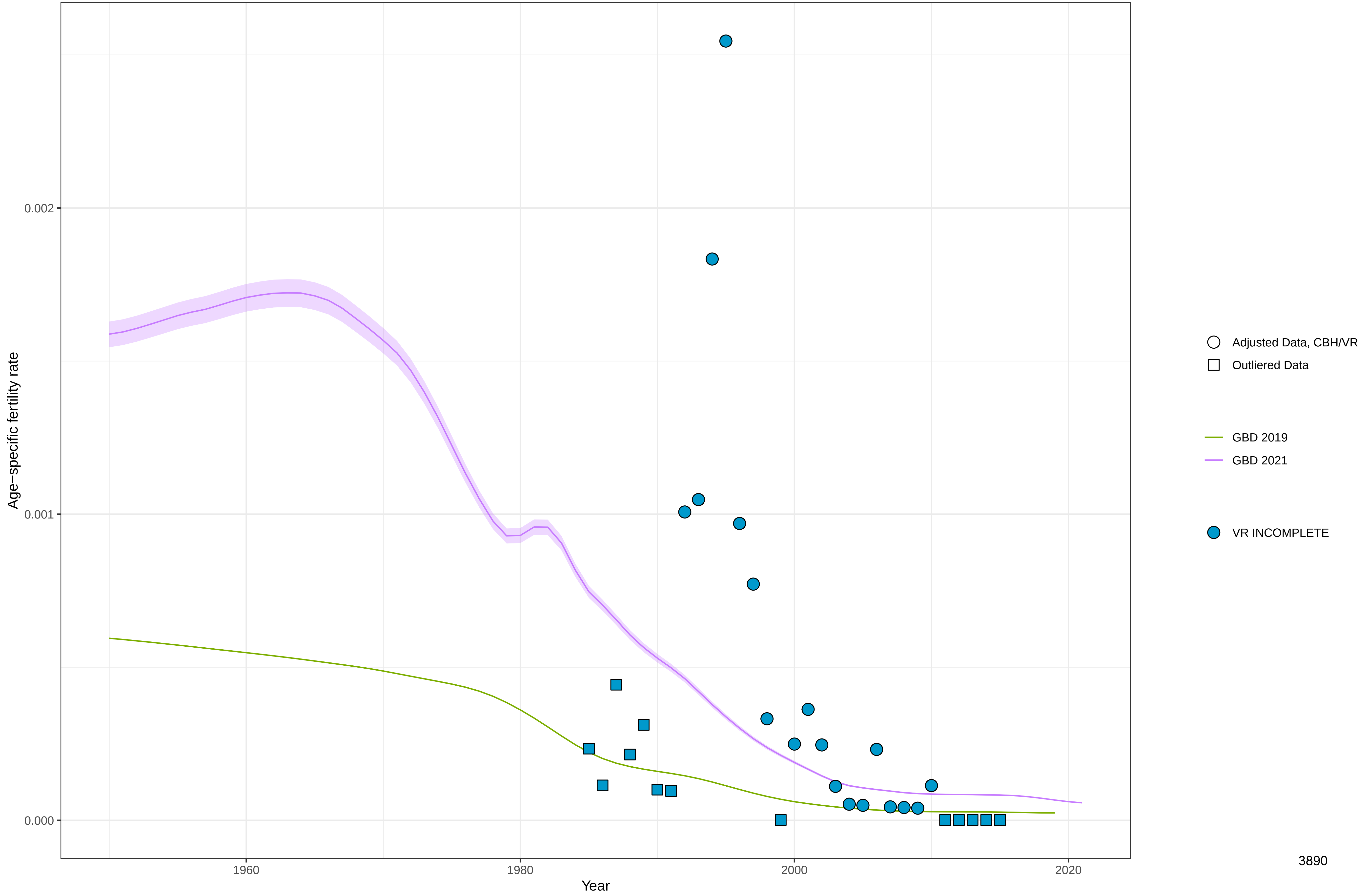

3890

Total fertility rate fitted trend lines, 1950−2021: Aguascalientes TFR
Data points enclosed with a diamond are reference sources

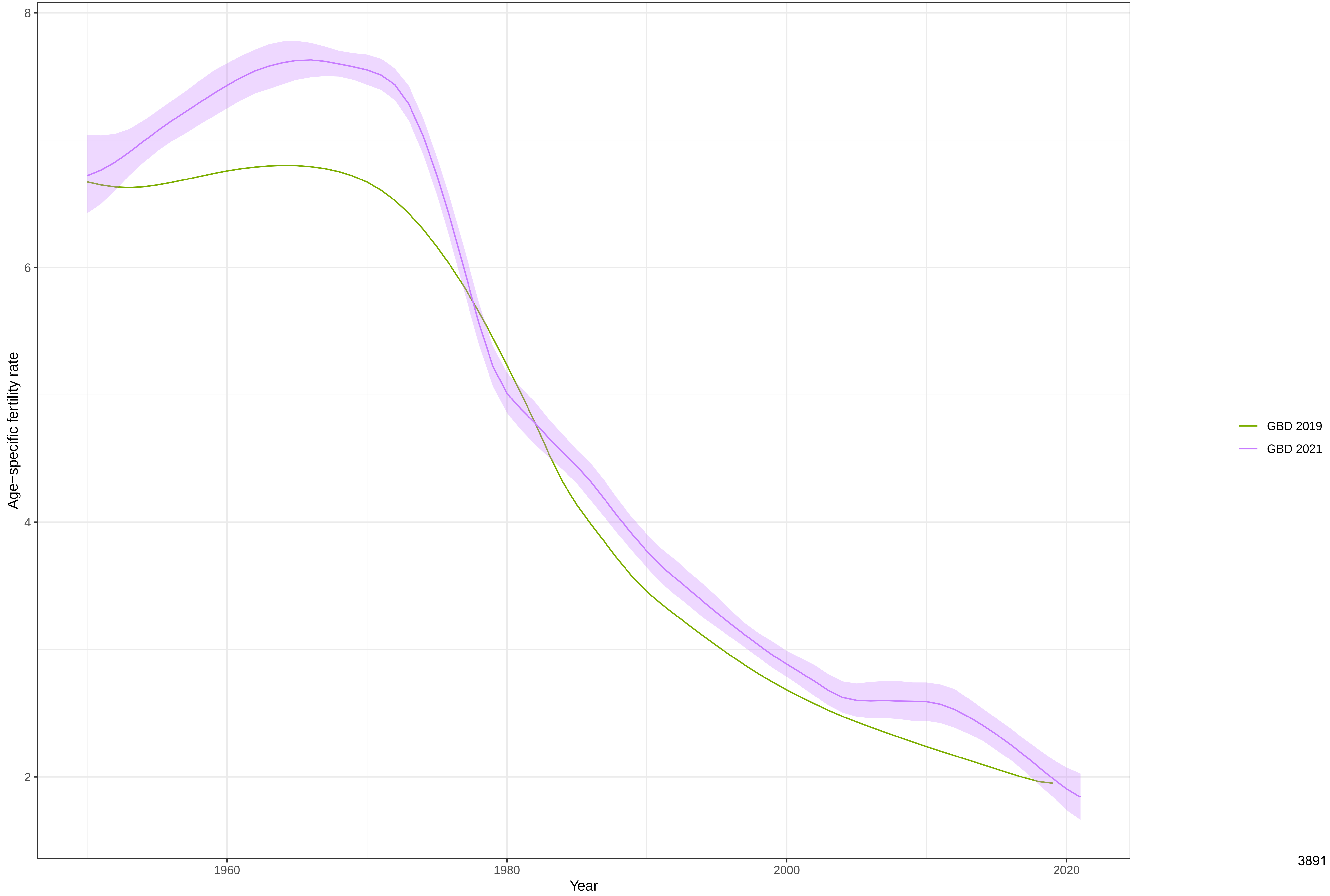

Age−specific fertility fitted trend lines, 1950−2021: Baja California, Ages: 10−14
Data points enclosed with a diamond are reference sources

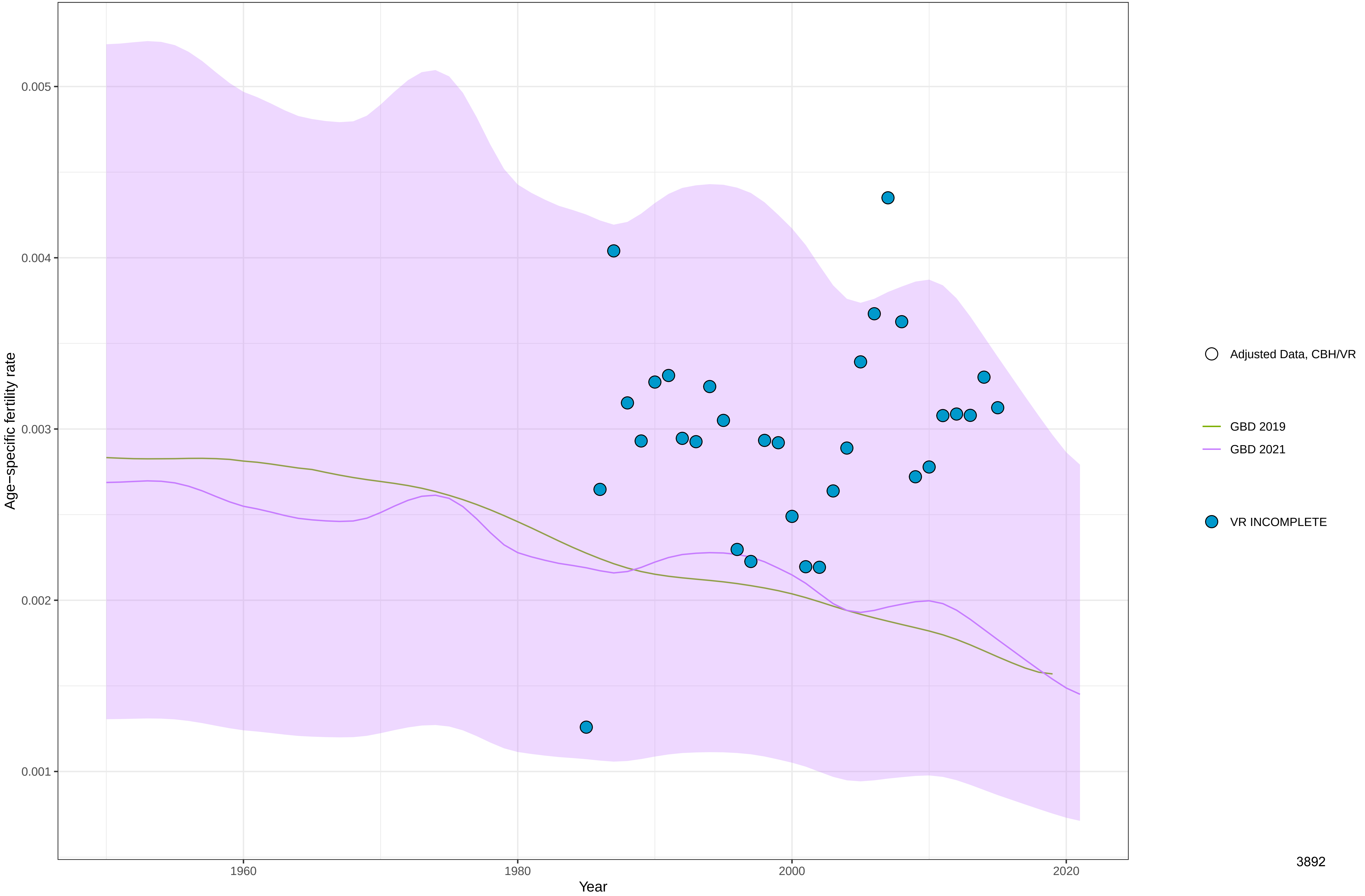

Age−specific fertility fitted trend lines, 1950−2021: Baja California, Ages: 15−19
Data points enclosed with a diamond are reference sources

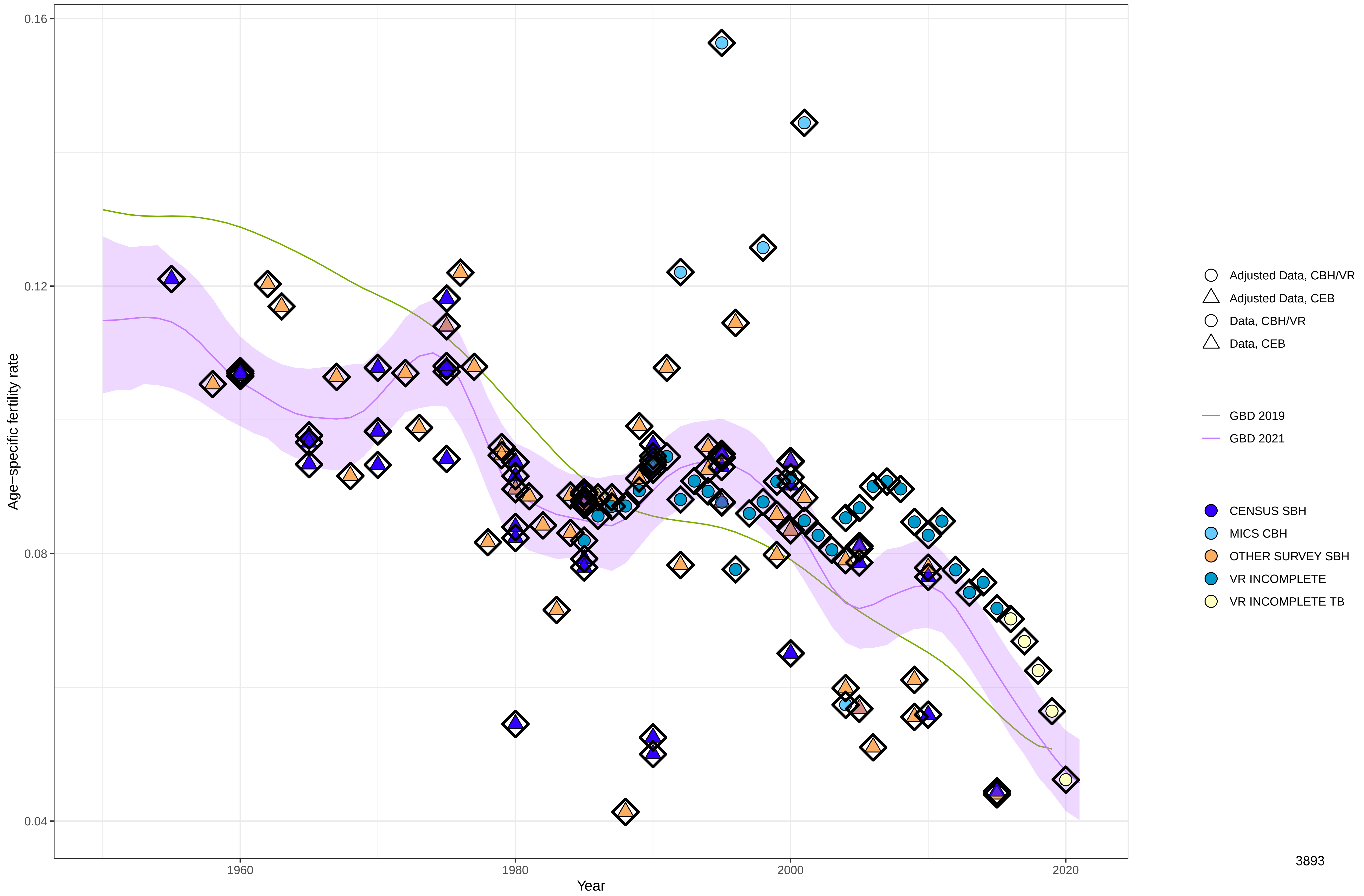

Age−specific fertility fitted trend lines, 1950−2021: Baja California, Ages: 20−24
Data points enclosed with a diamond are reference sources

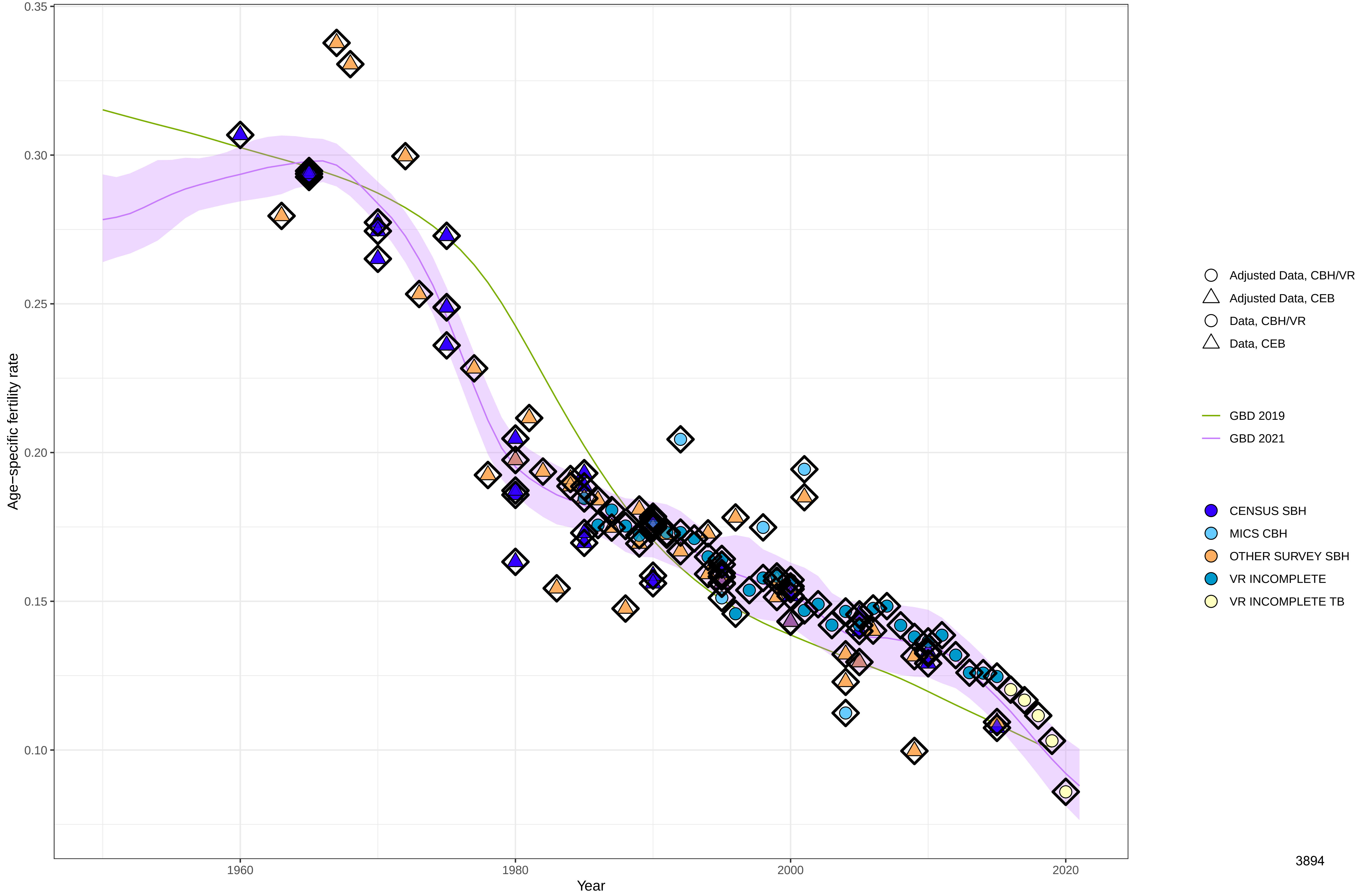

Age−specific fertility fitted trend lines, 1950−2021: Baja California, Ages: 25−29
Data points enclosed with a diamond are reference sources

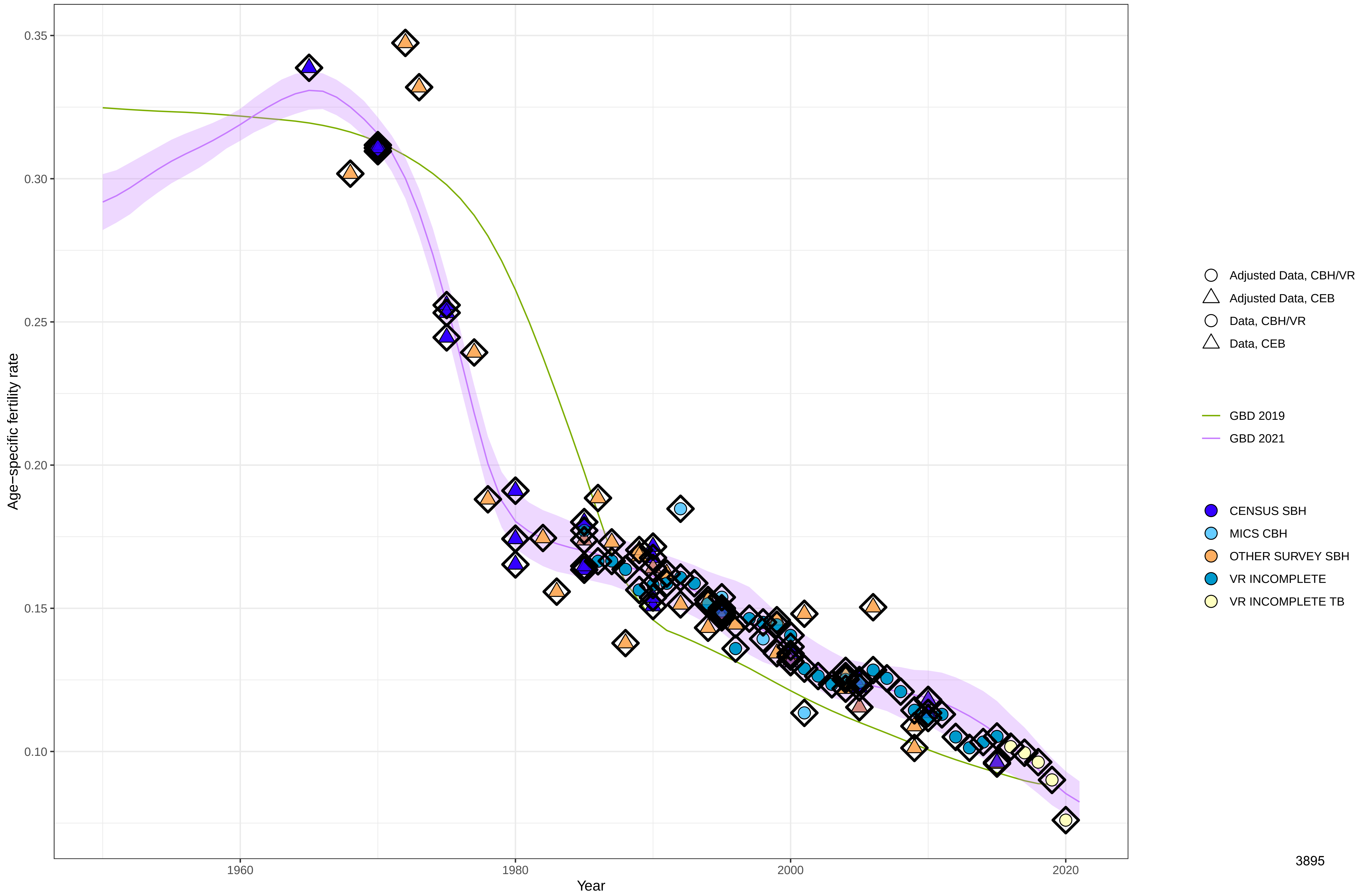

Age−specific fertility fitted trend lines, 1950−2021: Baja California, Ages: 30−34
Data points enclosed with a diamond are reference sources

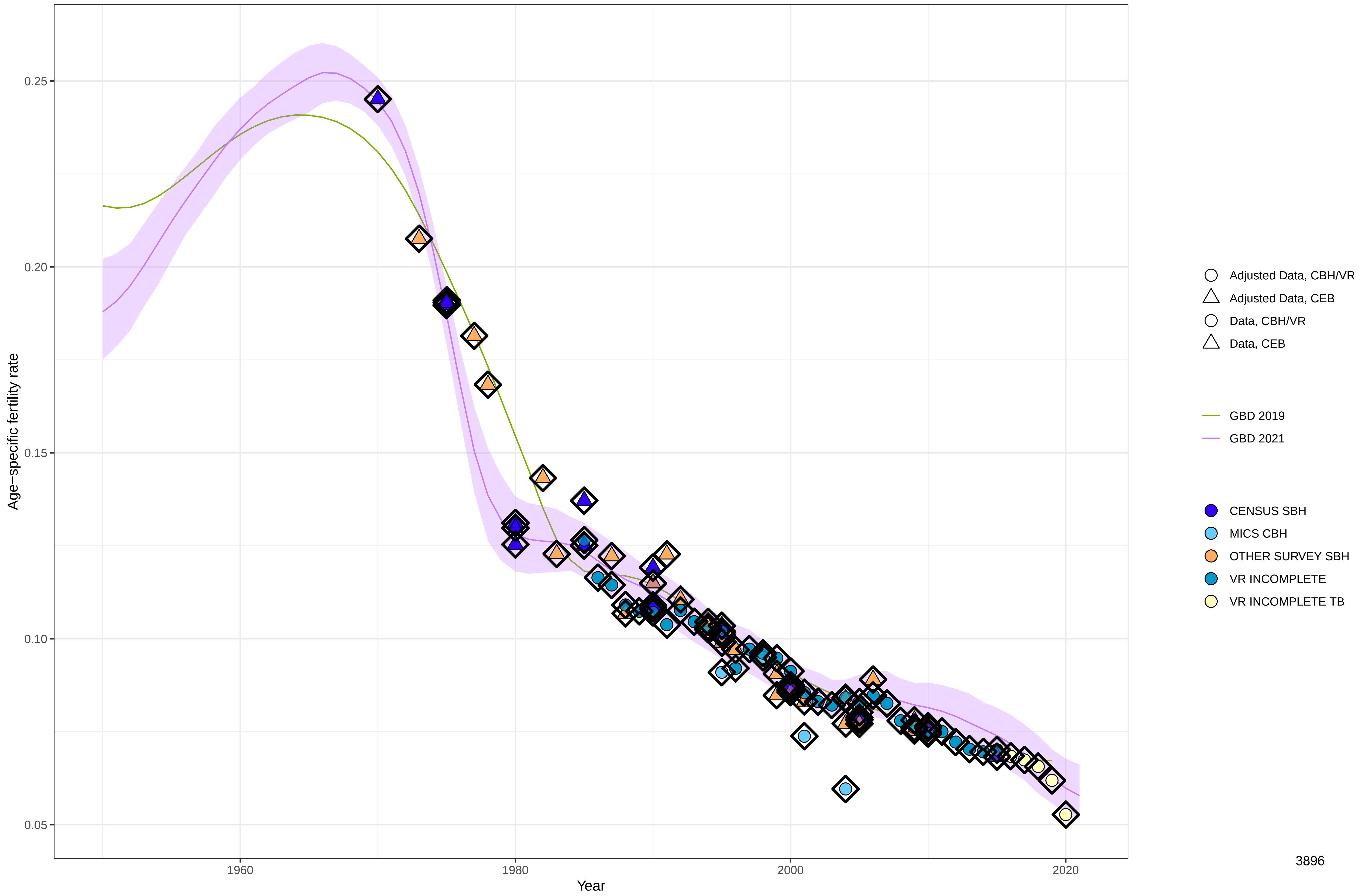

Age−specific fertility fitted trend lines, 1950−2021: Baja California, Ages: 35−39
Data points enclosed with a diamond are reference sources

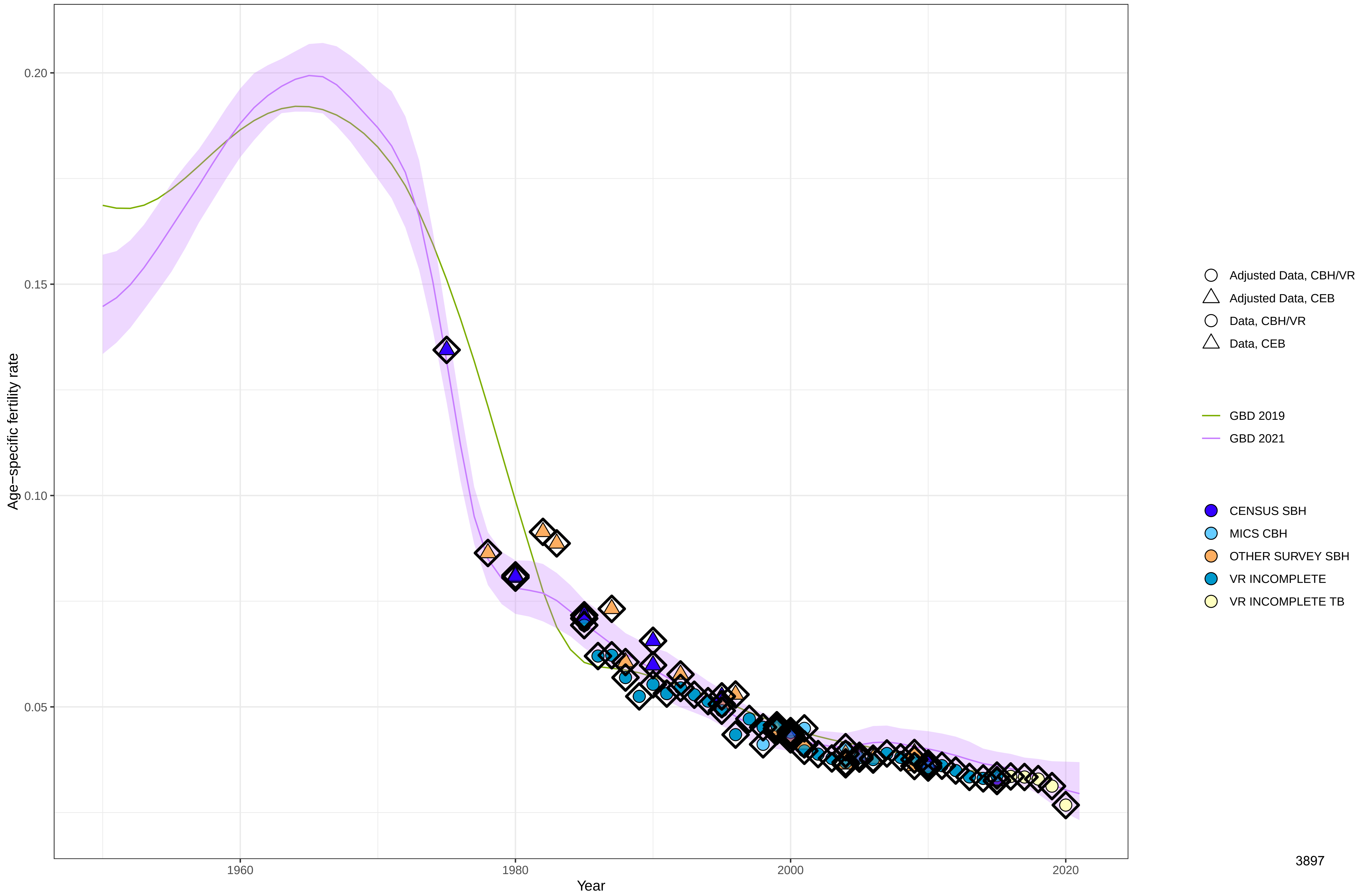

Age−specific fertility fitted trend lines, 1950−2021: Baja California, Ages: 40−44
Data points enclosed with a diamond are reference sources

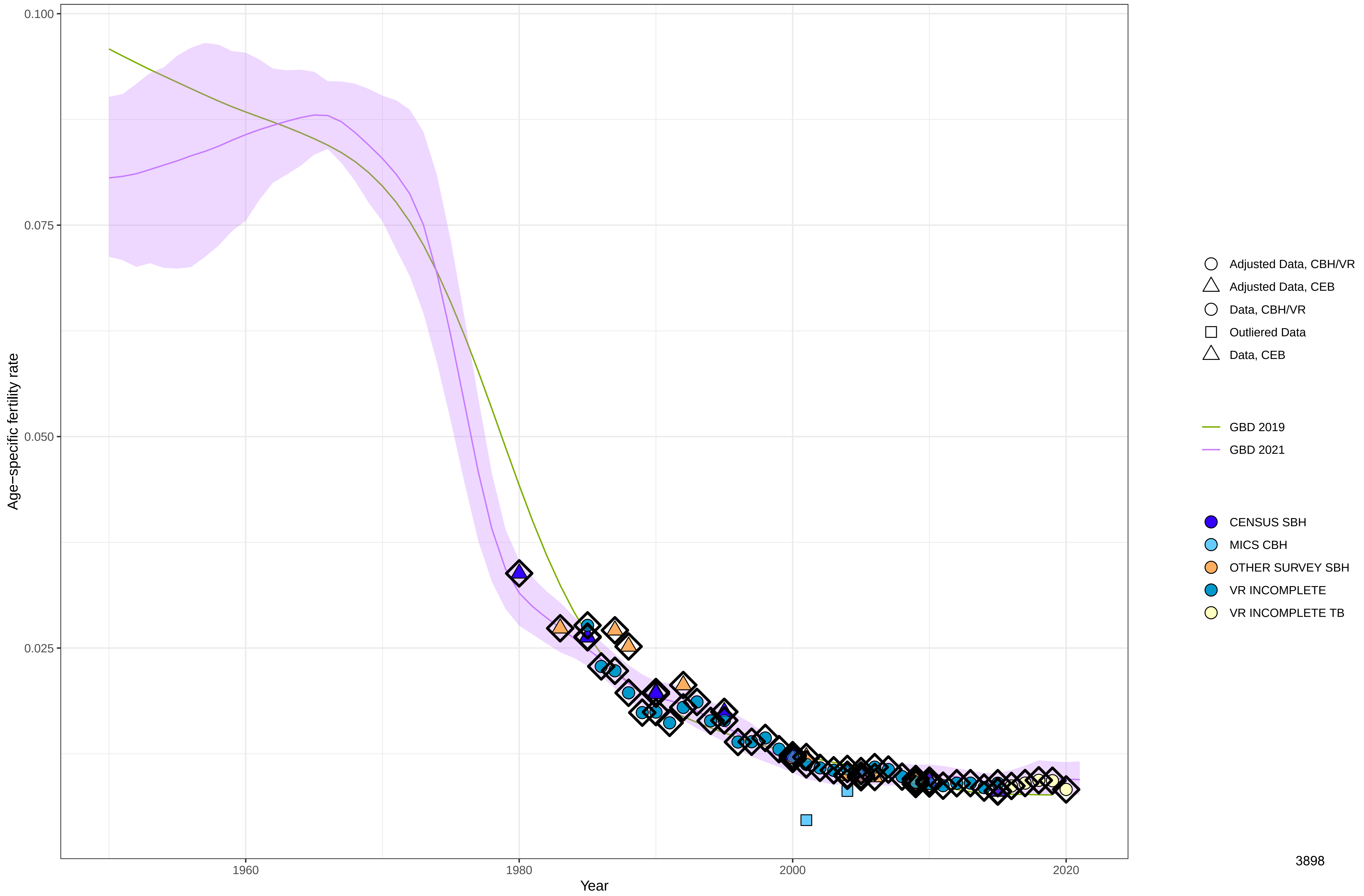

Age−specific fertility fitted trend lines, 1950−2021: Baja California, Ages: 45−49
Data points enclosed with a diamond are reference sources

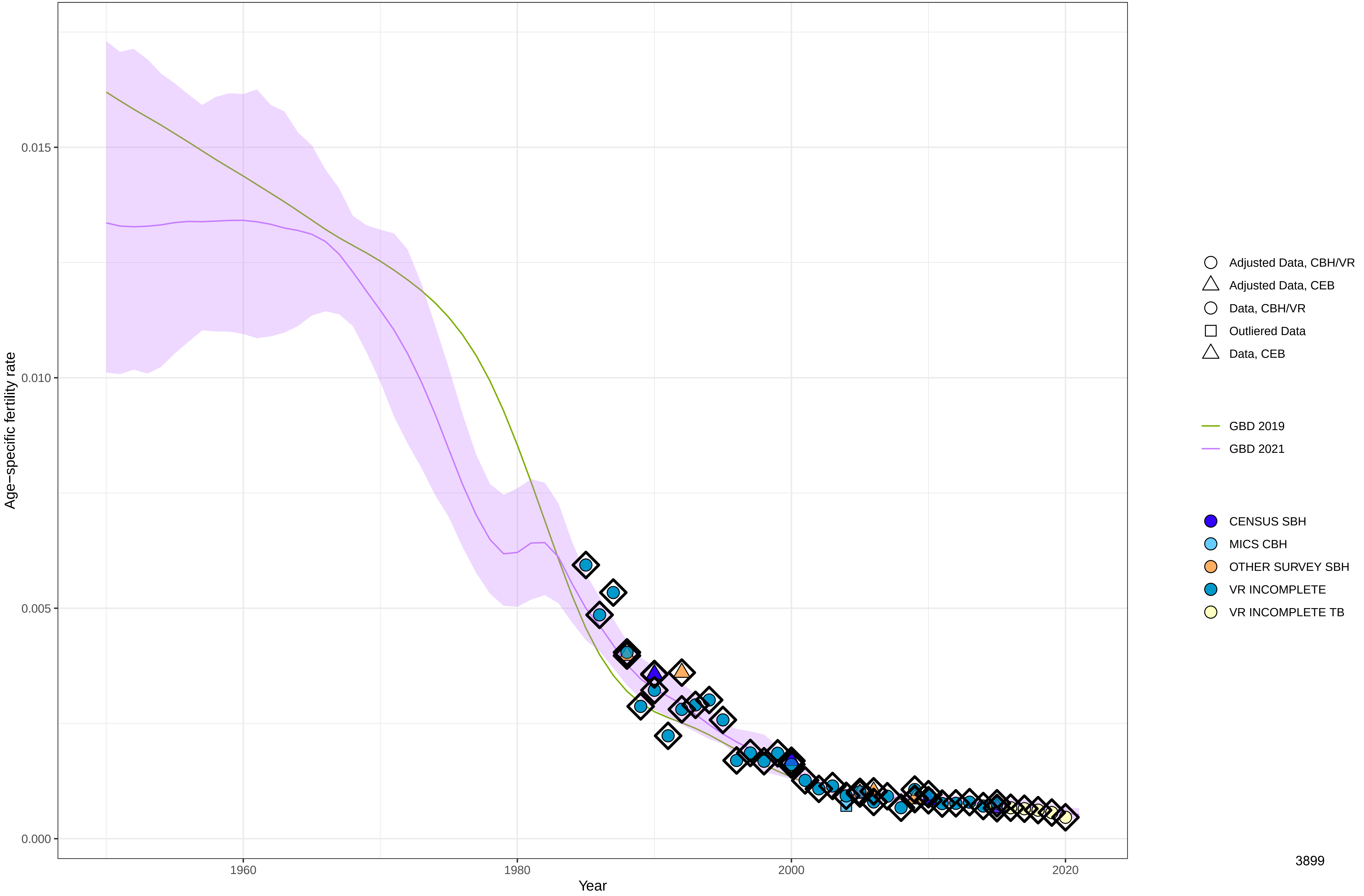

Age−specific fertility fitted trend lines, 1950−2021: Baja California, Ages: 50−54
Data points enclosed with a diamond are reference sources

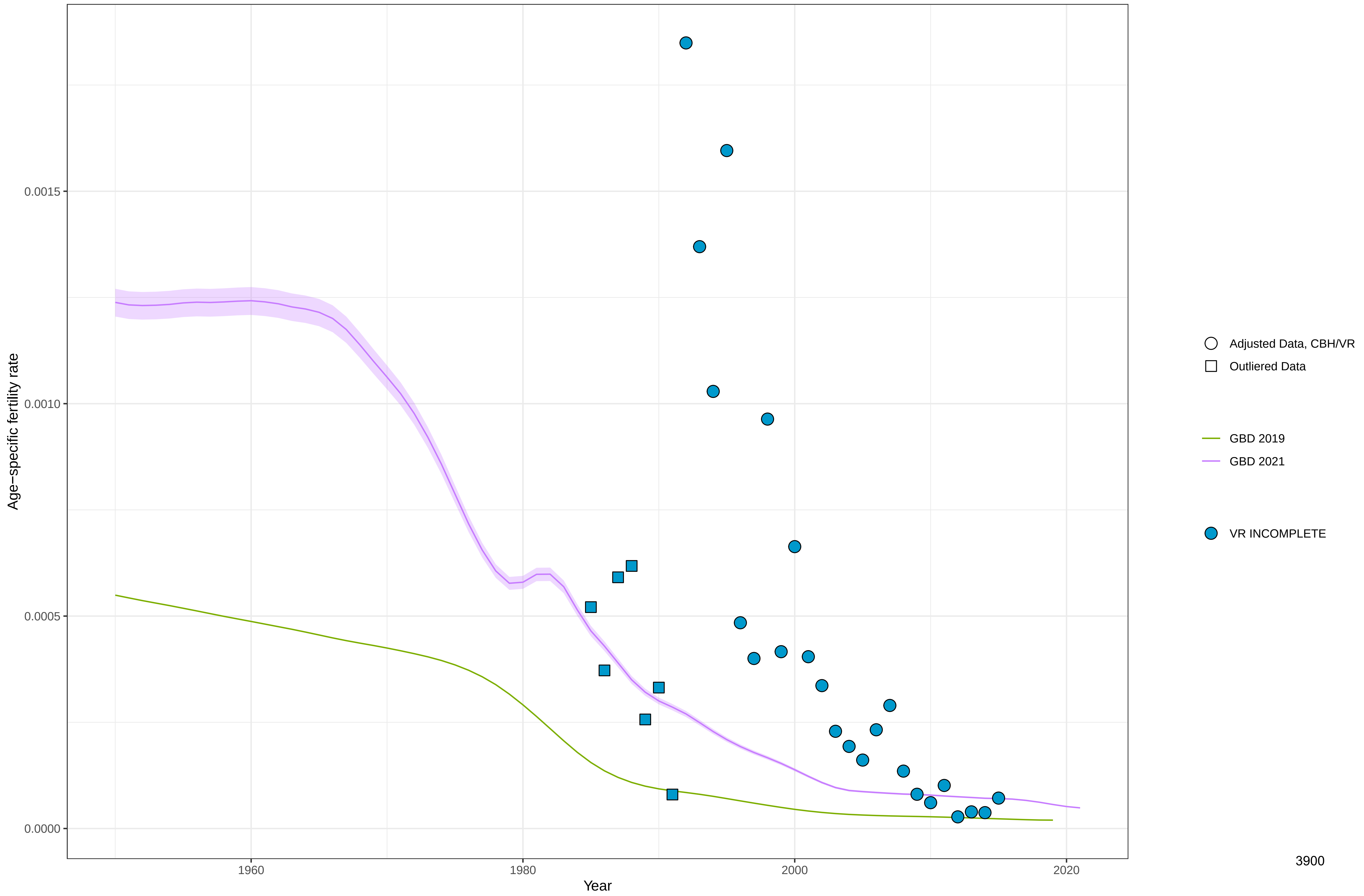

Total fertility rate fitted trend lines, 1950−2021: Baja California TFR
Data points enclosed with a diamond are reference sources

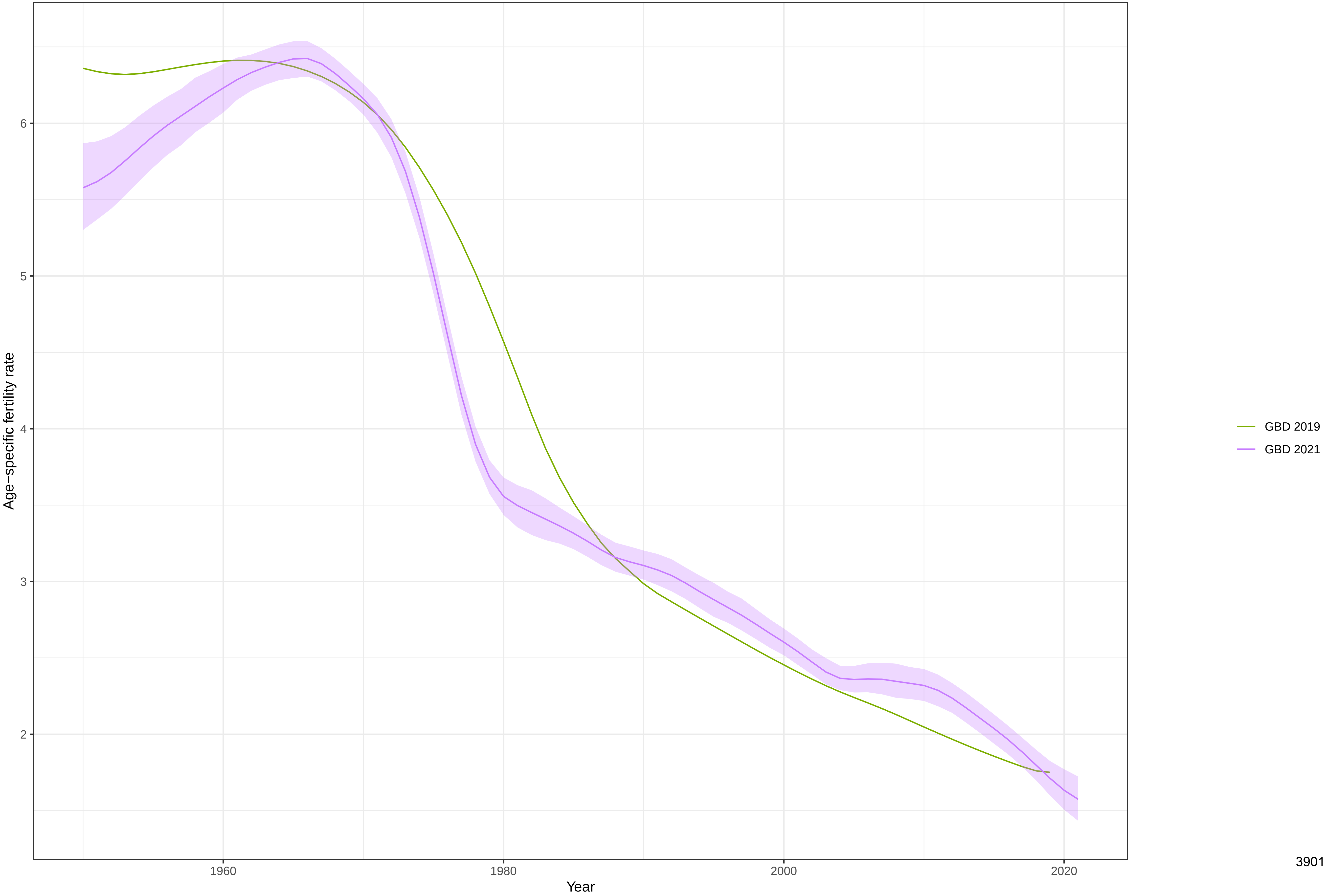

Age−specific fertility fitted trend lines, 1950−2021: Baja California Sur, Ages: 10−14
Data points enclosed with a diamond are reference sources

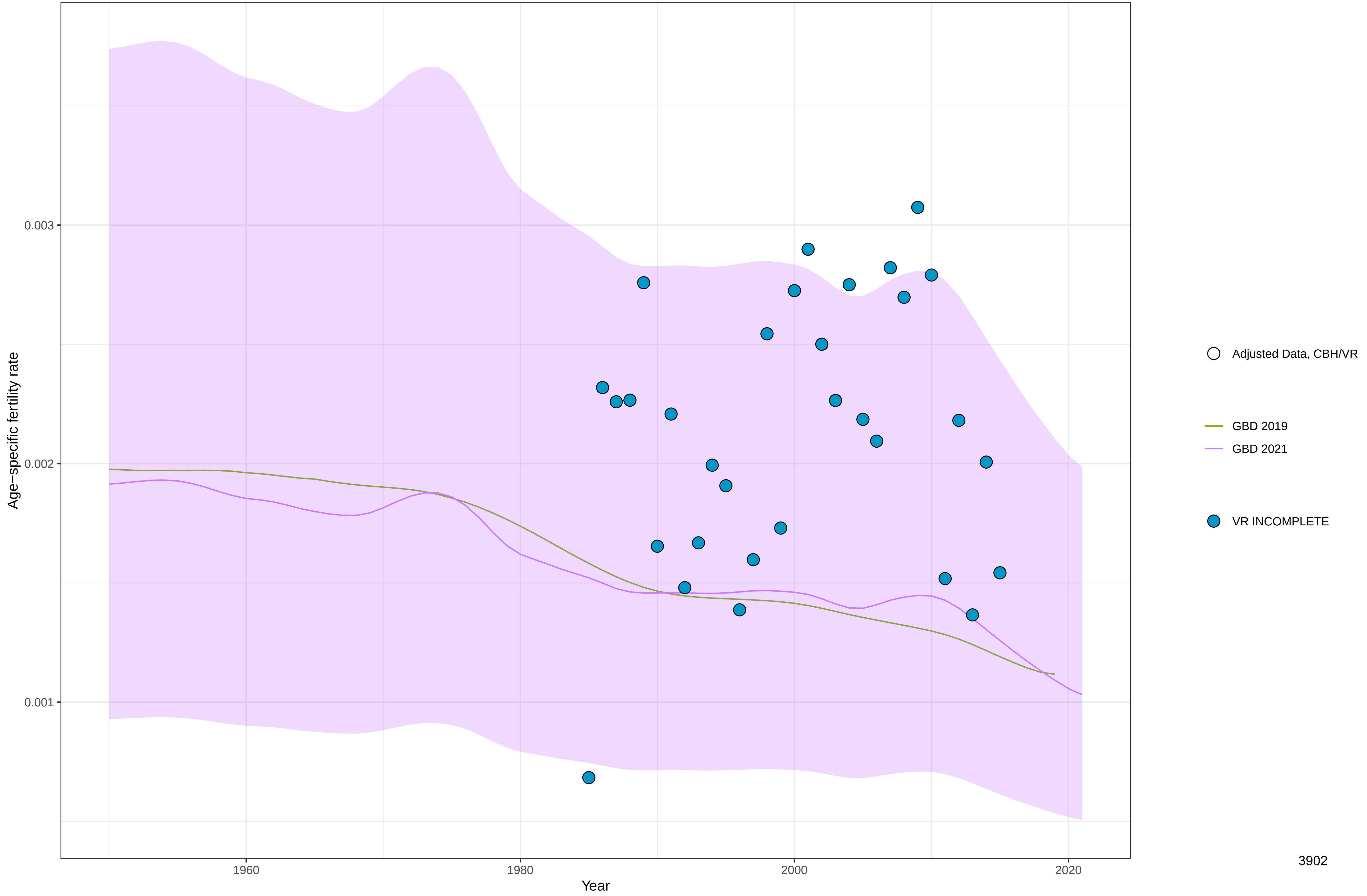

Age−specific fertility fitted trend lines, 1950−2021: Baja California Sur, Ages: 15−19
Data points enclosed with a diamond are reference sources

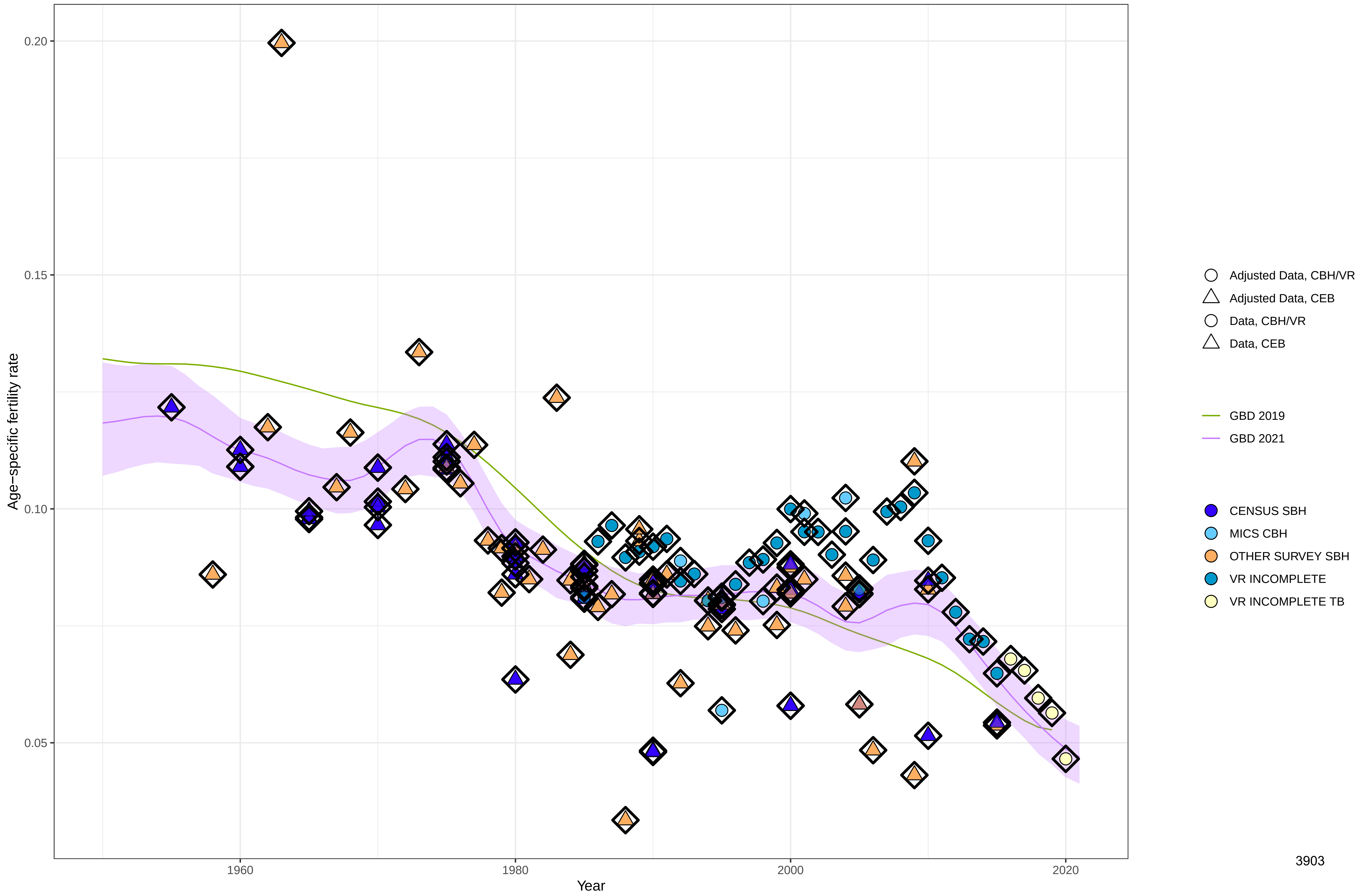

Age−specific fertility fitted trend lines, 1950−2021: Baja California Sur, Ages: 20−24
Data points enclosed with a diamond are reference sources

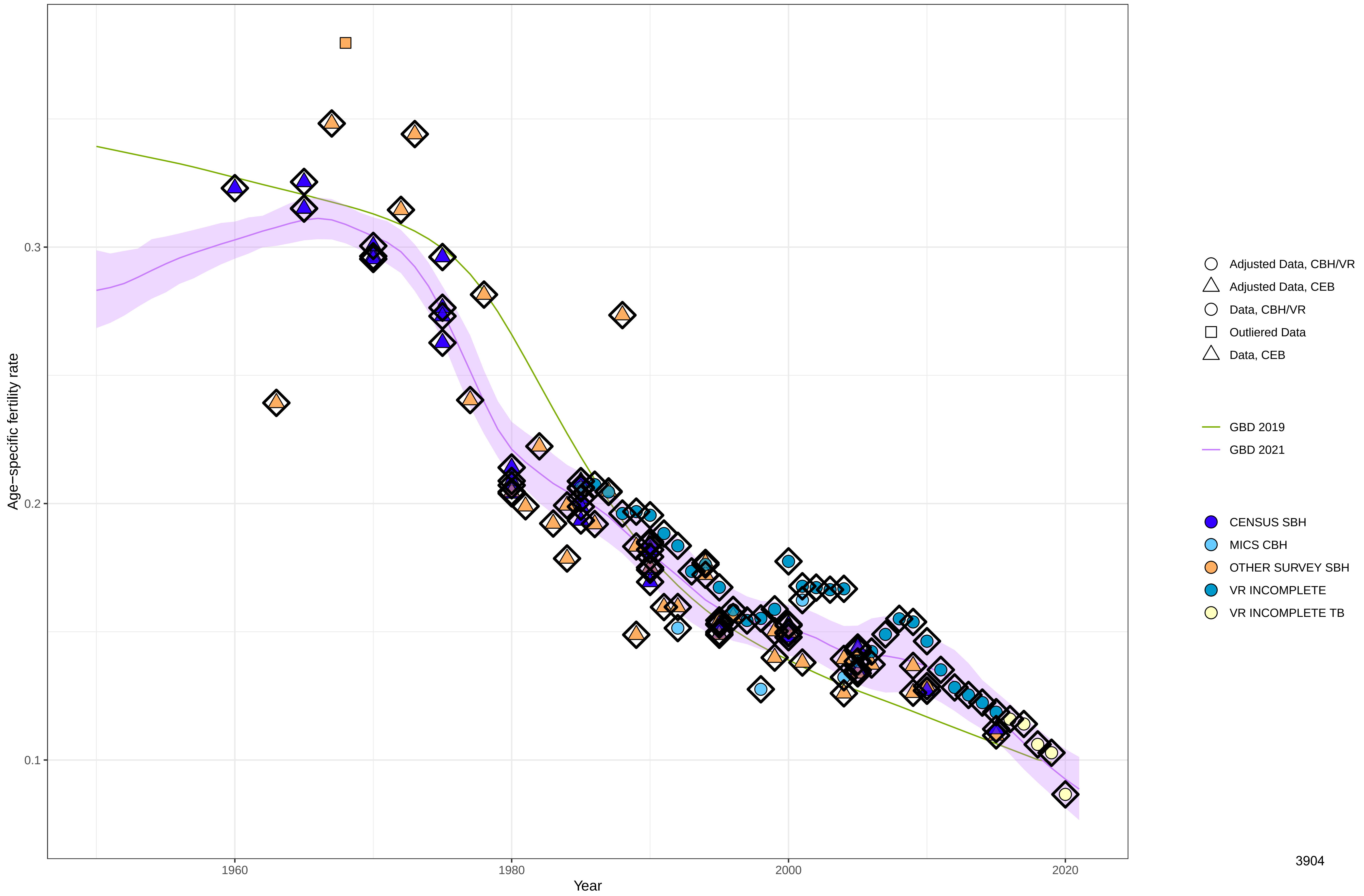

Age−specific fertility fitted trend lines, 1950−2021: Baja California Sur, Ages: 25−29
Data points enclosed with a diamond are reference sources

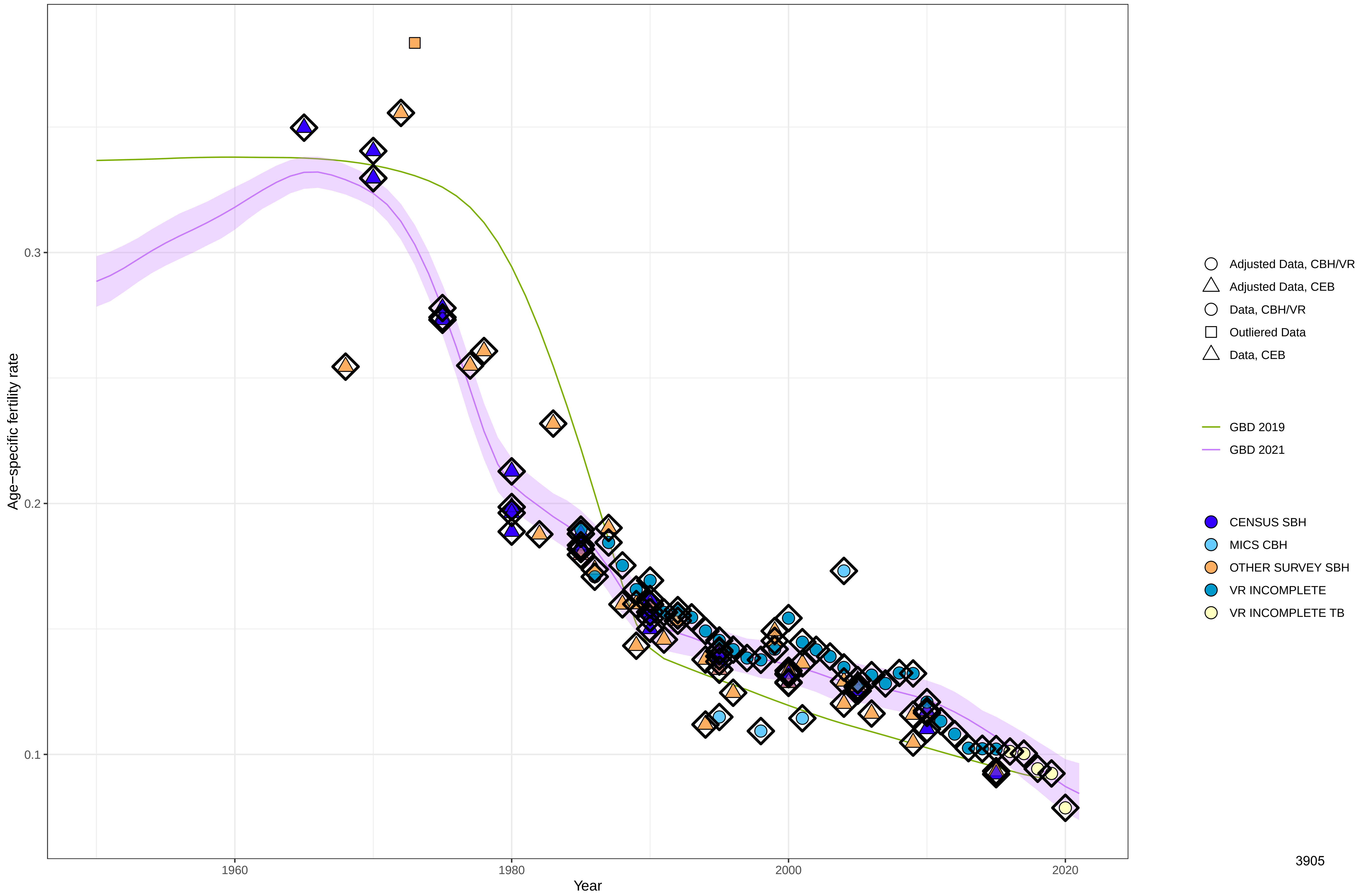

Age−specific fertility fitted trend lines, 1950−2021: Baja California Sur, Ages: 30−34
Data points enclosed with a diamond are reference sources

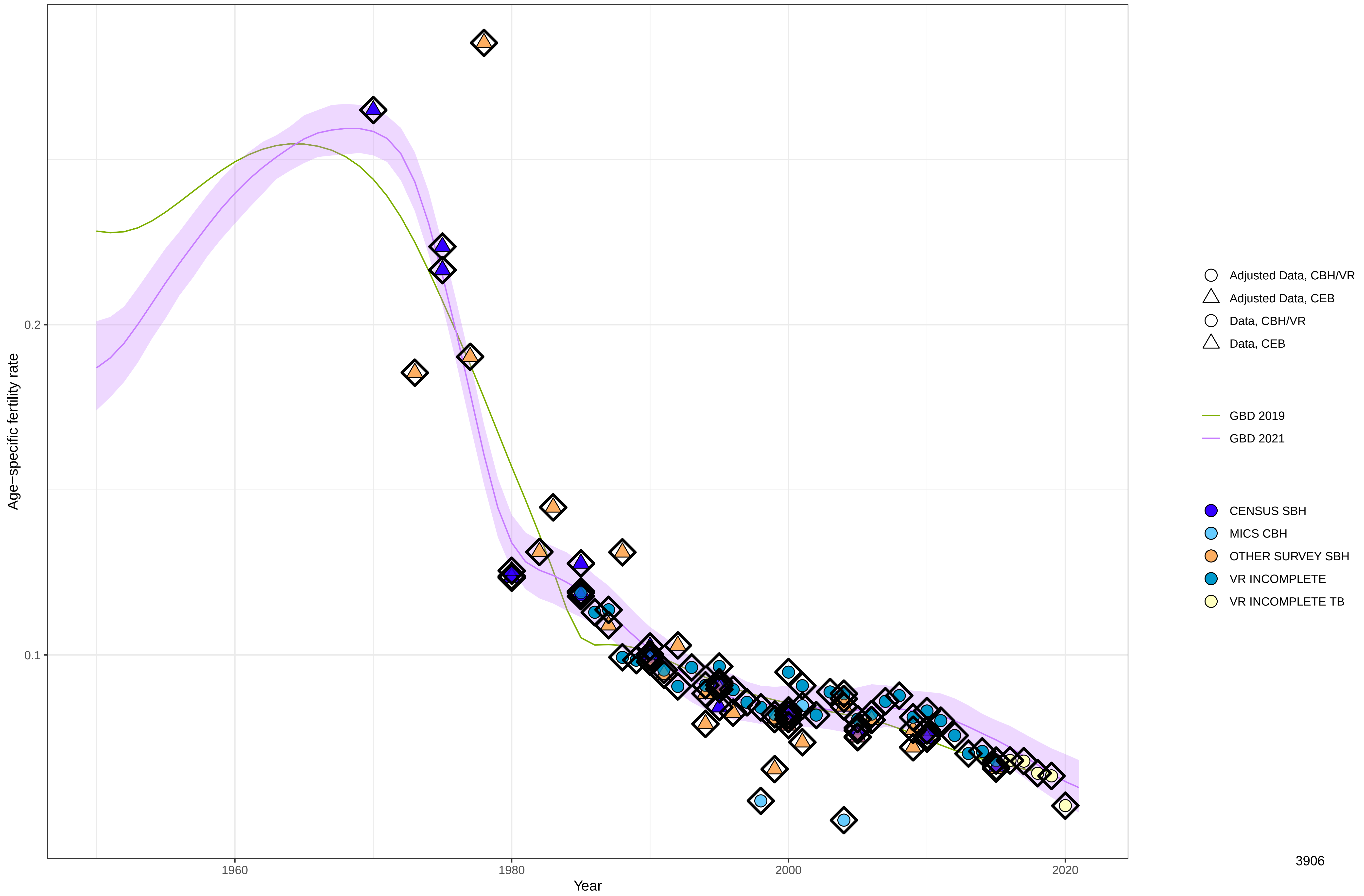

Age−specific fertility fitted trend lines, 1950−2021: Baja California Sur, Ages: 35−39
Data points enclosed with a diamond are reference sources

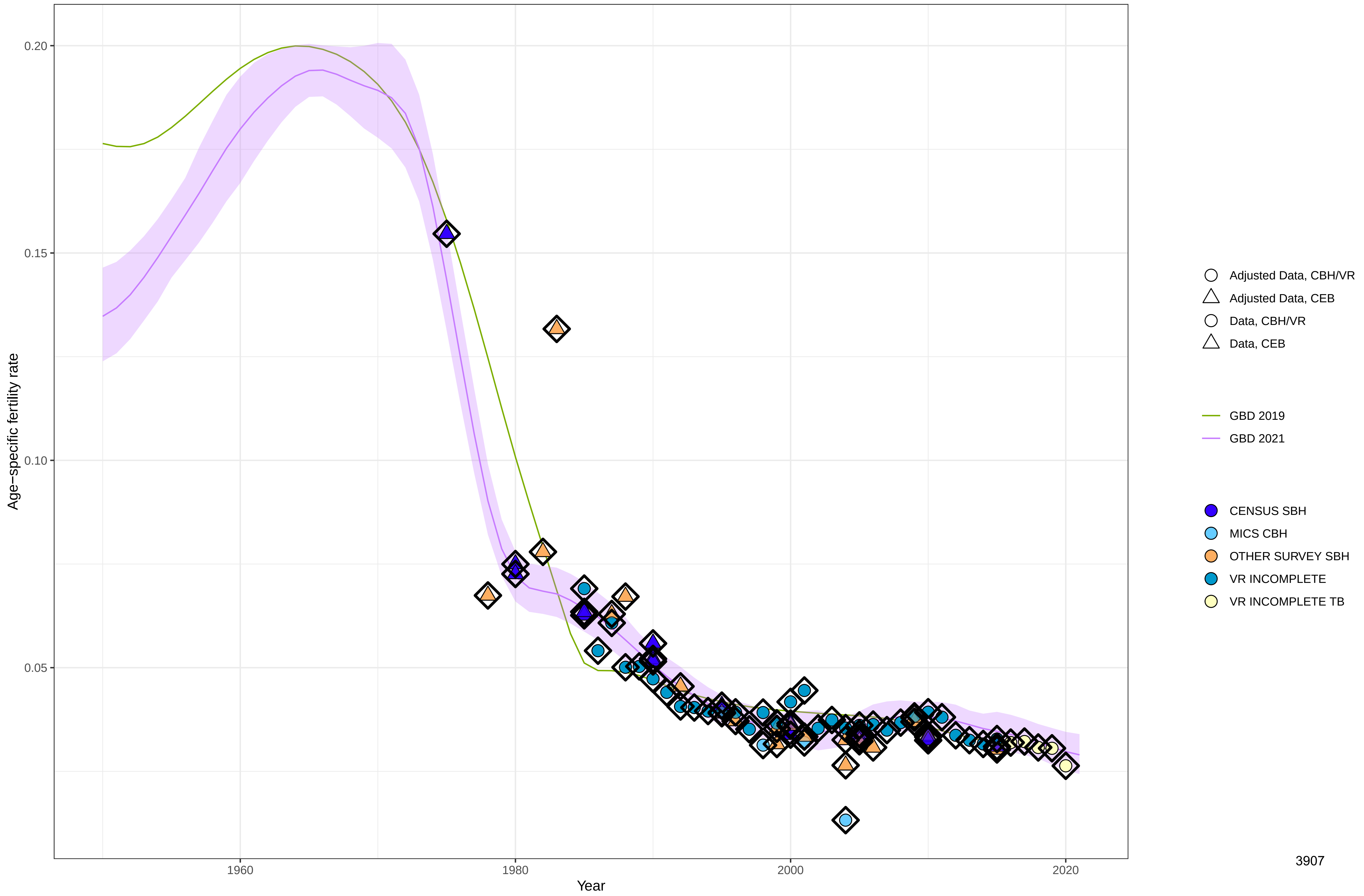

Age−specific fertility fitted trend lines, 1950−2021: Baja California Sur, Ages: 40−44
Data points enclosed with a diamond are reference sources

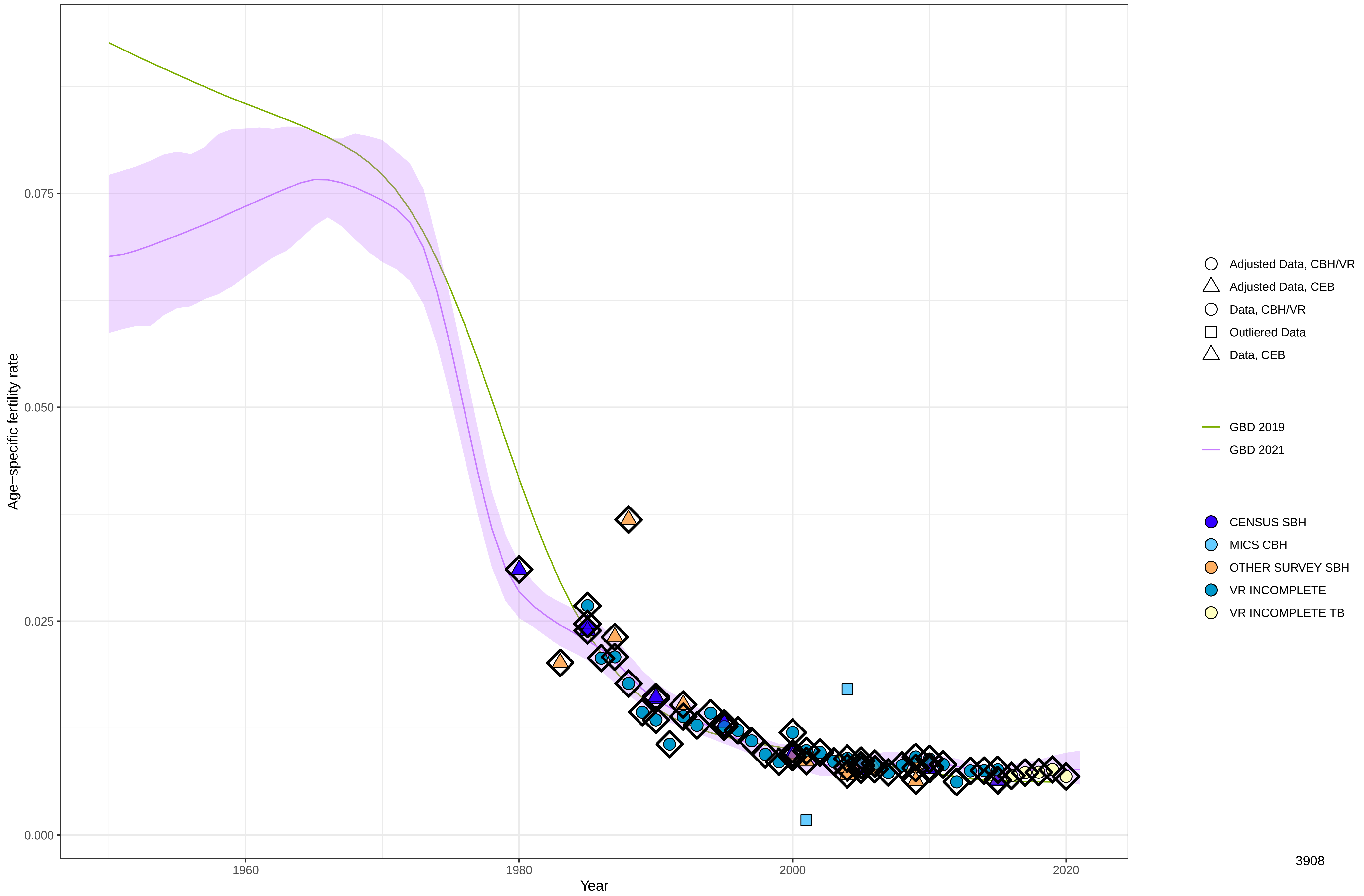

Age−specific fertility fitted trend lines, 1950−2021: Baja California Sur, Ages: 45−49
Data points enclosed with a diamond are reference sources

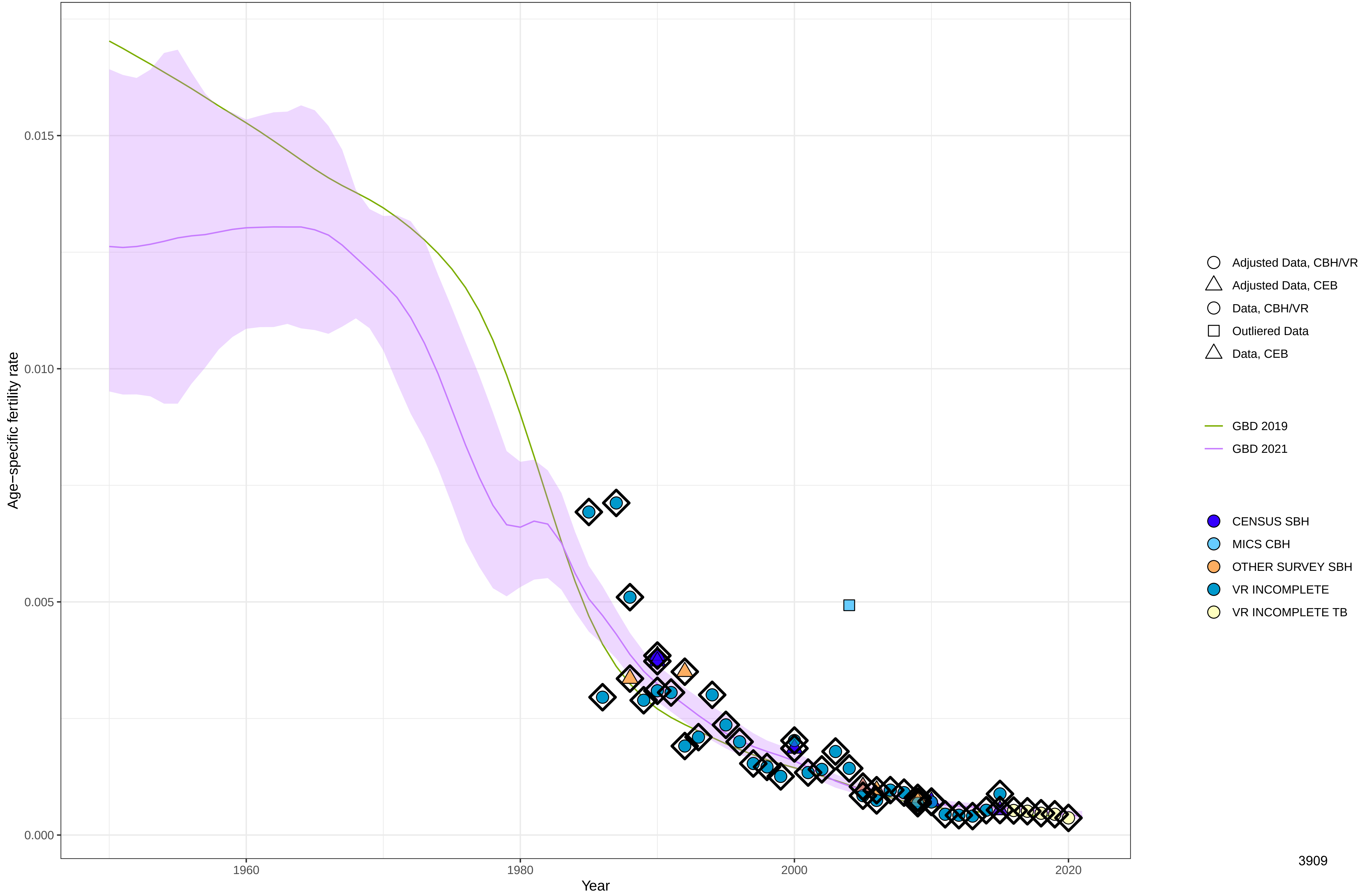

Age−specific fertility fitted trend lines, 1950−2021: Baja California Sur, Ages: 50−54
Data points enclosed with a diamond are reference sources

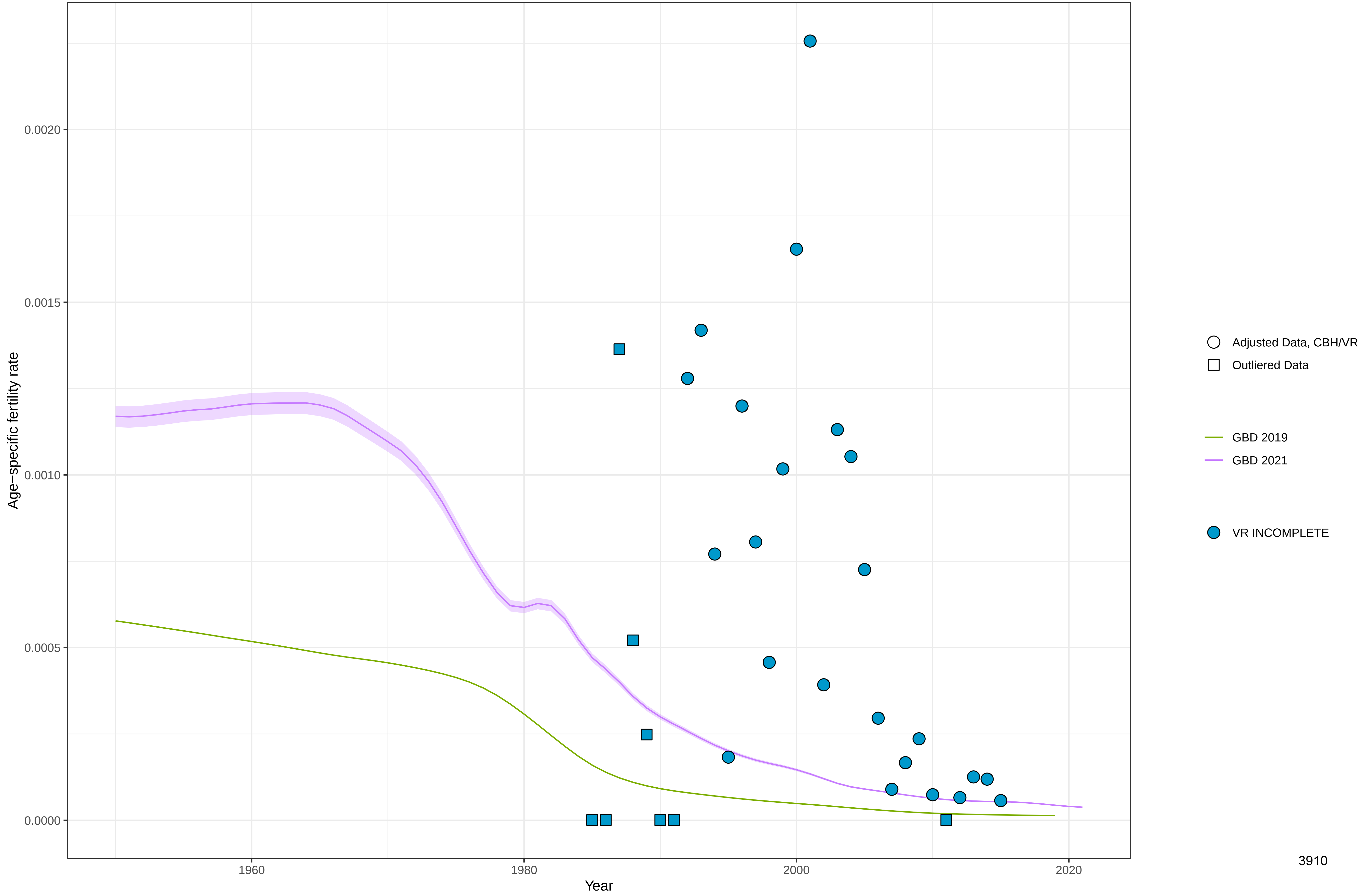

3910

Total fertility rate fitted trend lines, 1950−2021: Baja California Sur TFR
Data points enclosed with a diamond are reference sources

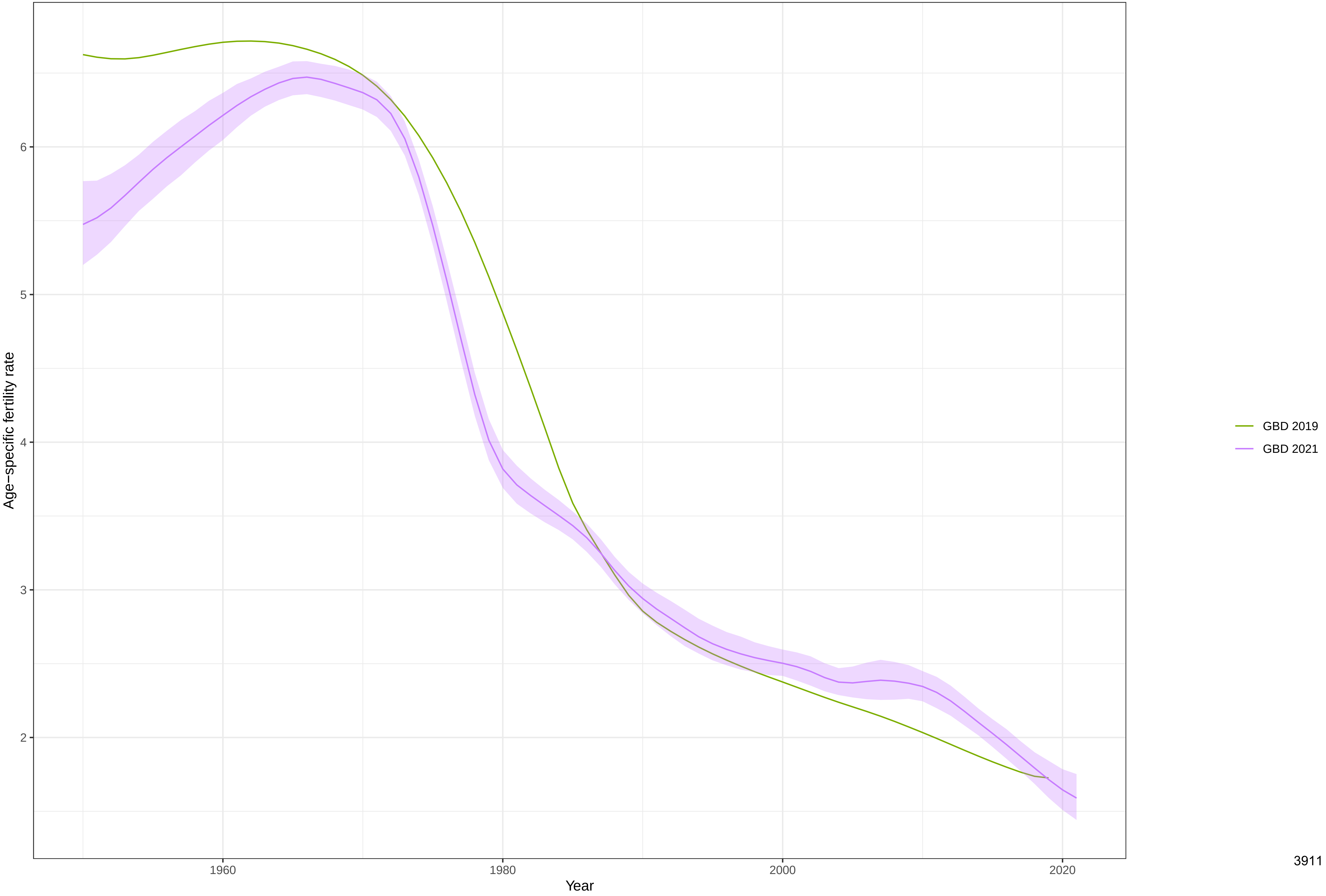

Age−specific fertility fitted trend lines, 1950−2021: Campeche, Ages: 10−14
 Data points enclosed with a diamond are reference sources

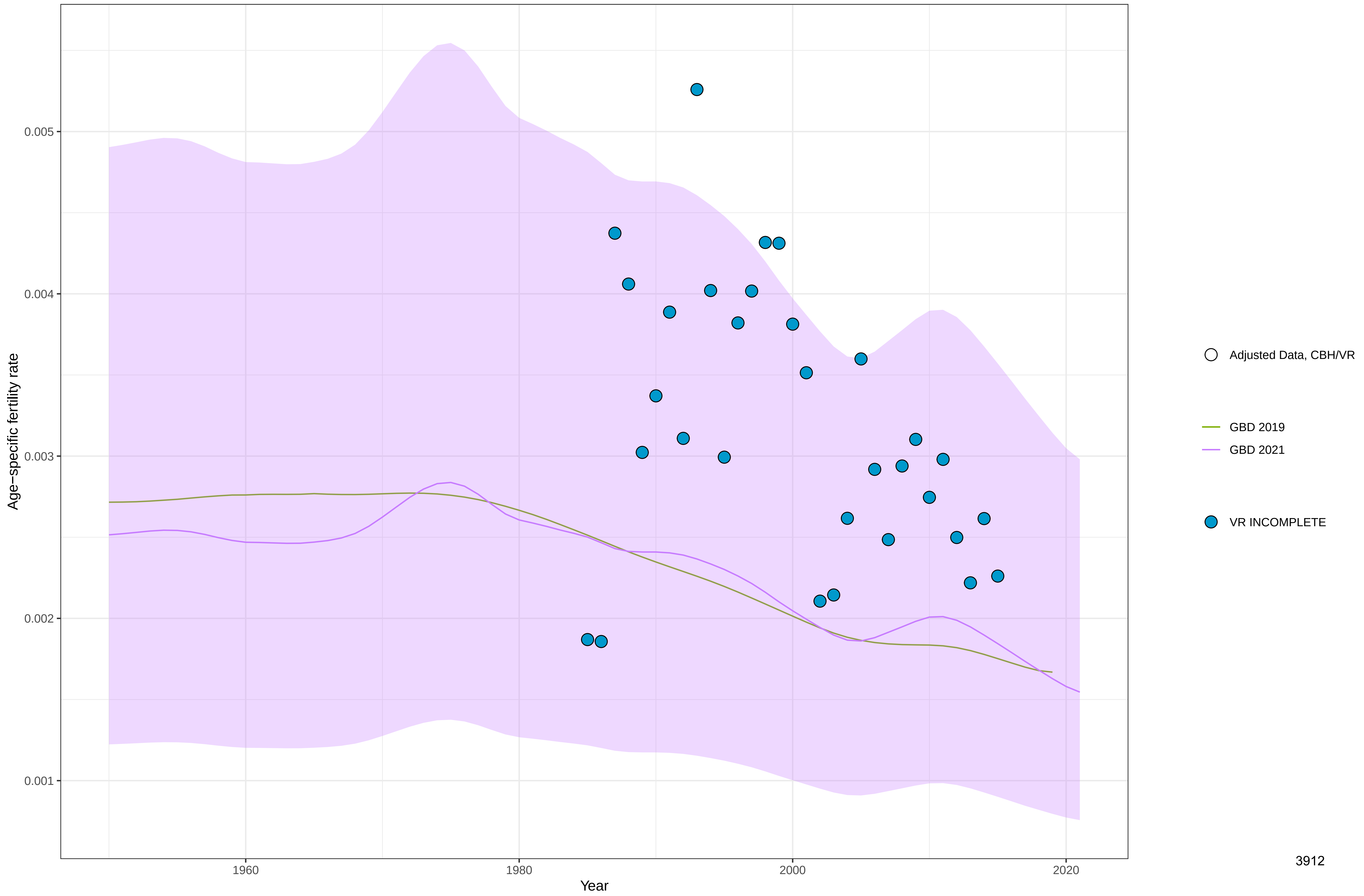

Age−specific fertility fitted trend lines, 1950−2021: Campeche, Ages: 15−19
Data points enclosed with a diamond are reference sources

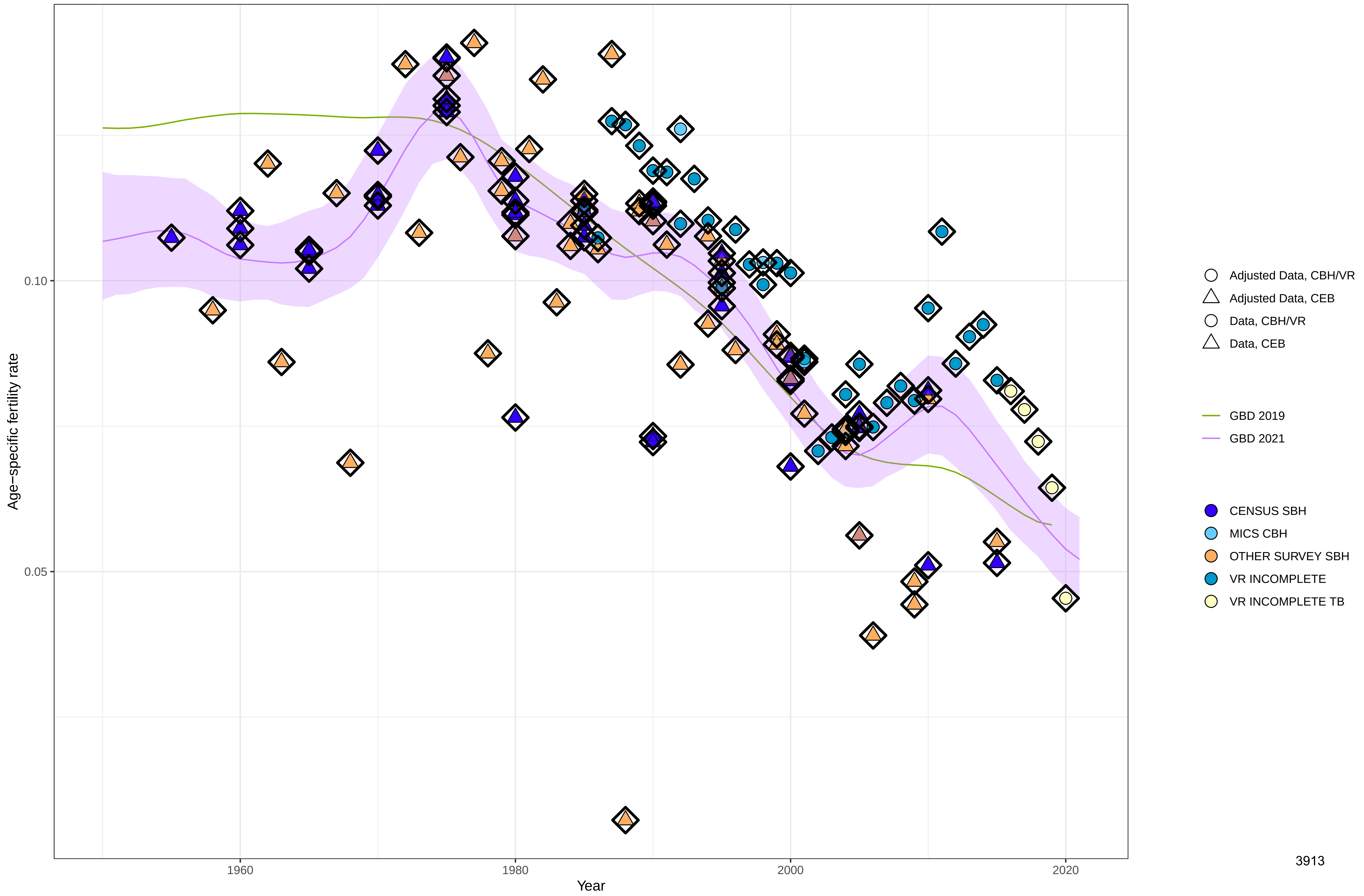

Age−specific fertility fitted trend lines, 1950−2021: Campeche, Ages: 20−24
Data points enclosed with a diamond are reference sources

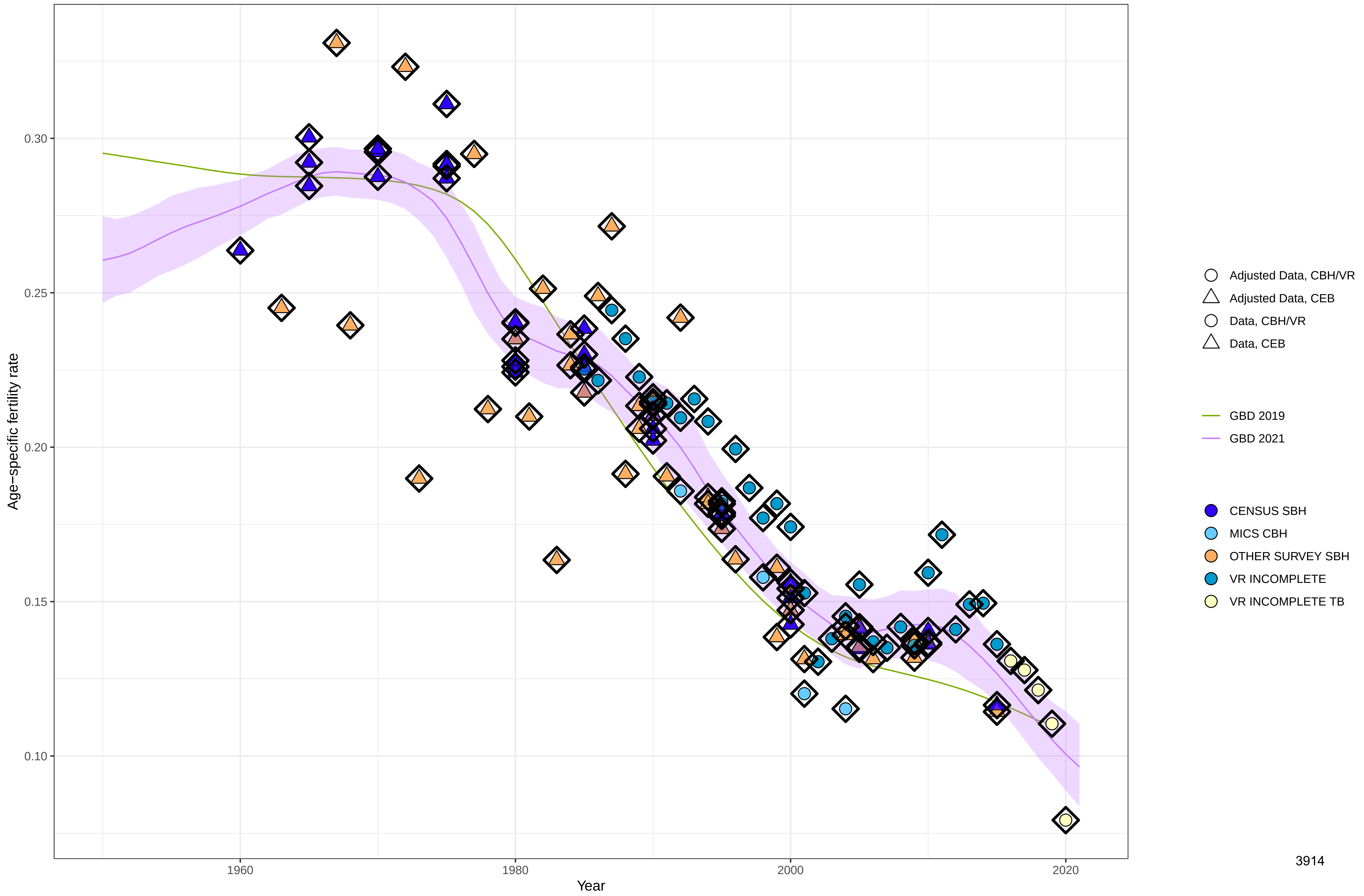

Age−specific fertility fitted trend lines, 1950−2021: Campeche, Ages: 25−29
Data points enclosed with a diamond are reference sources

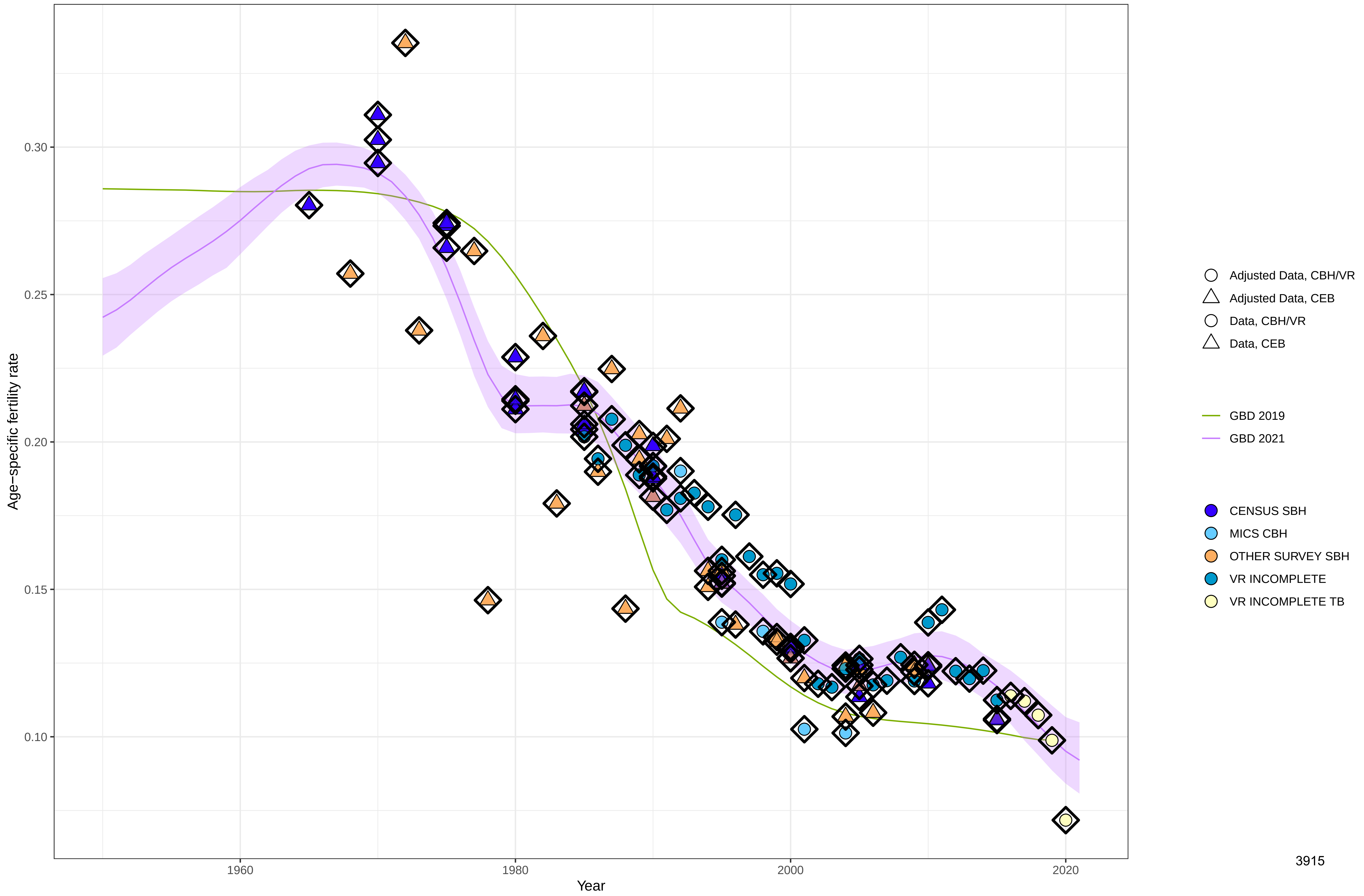

Age−specific fertility fitted trend lines, 1950−2021: Campeche, Ages: 30−34
Data points enclosed with a diamond are reference sources

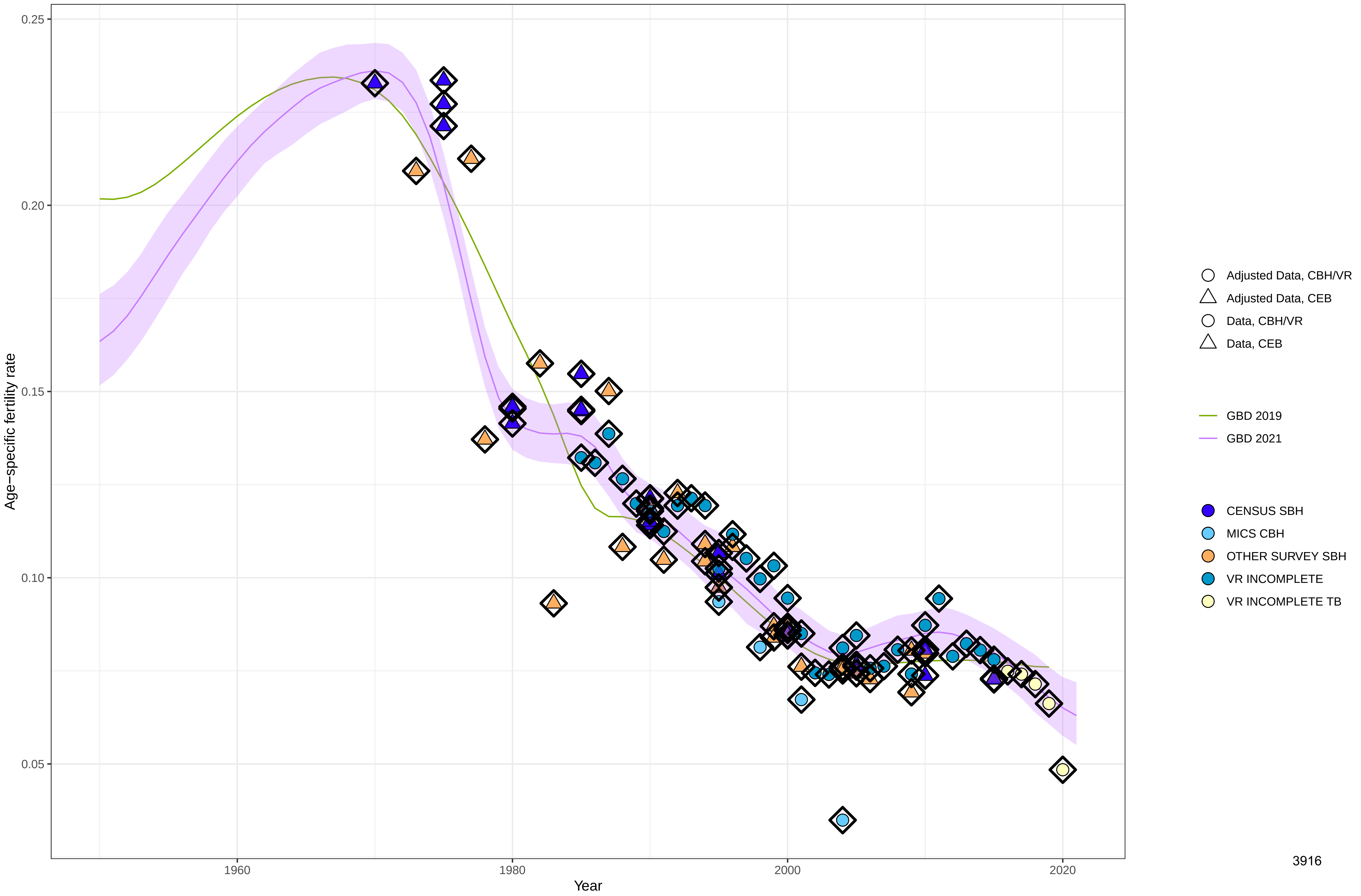

Age−specific fertility fitted trend lines, 1950−2021: Campeche, Ages: 35−39
Data points enclosed with a diamond are reference sources

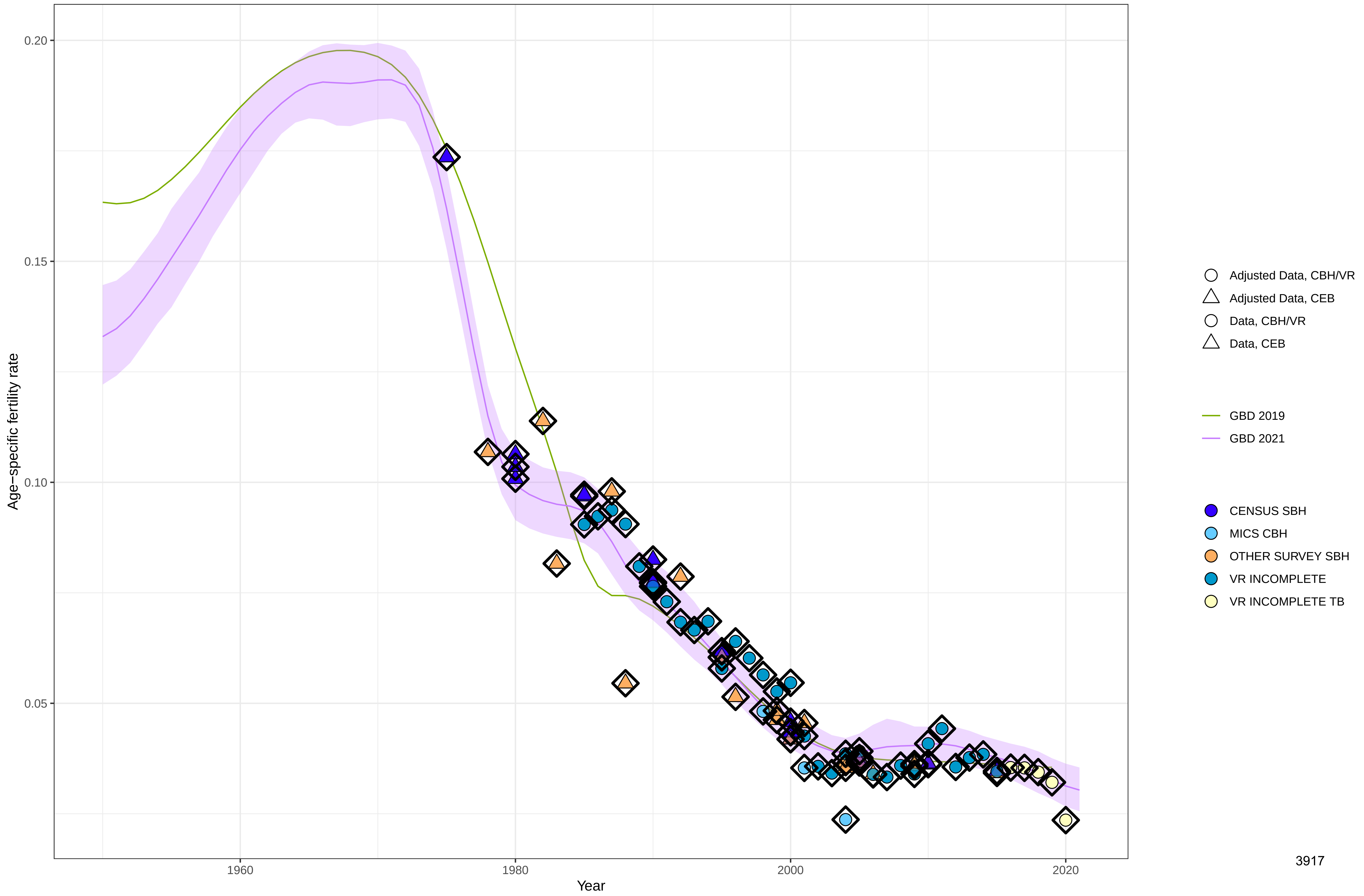

Age−specific fertility fitted trend lines, 1950−2021: Campeche, Ages: 40−44
Data points enclosed with a diamond are reference sources

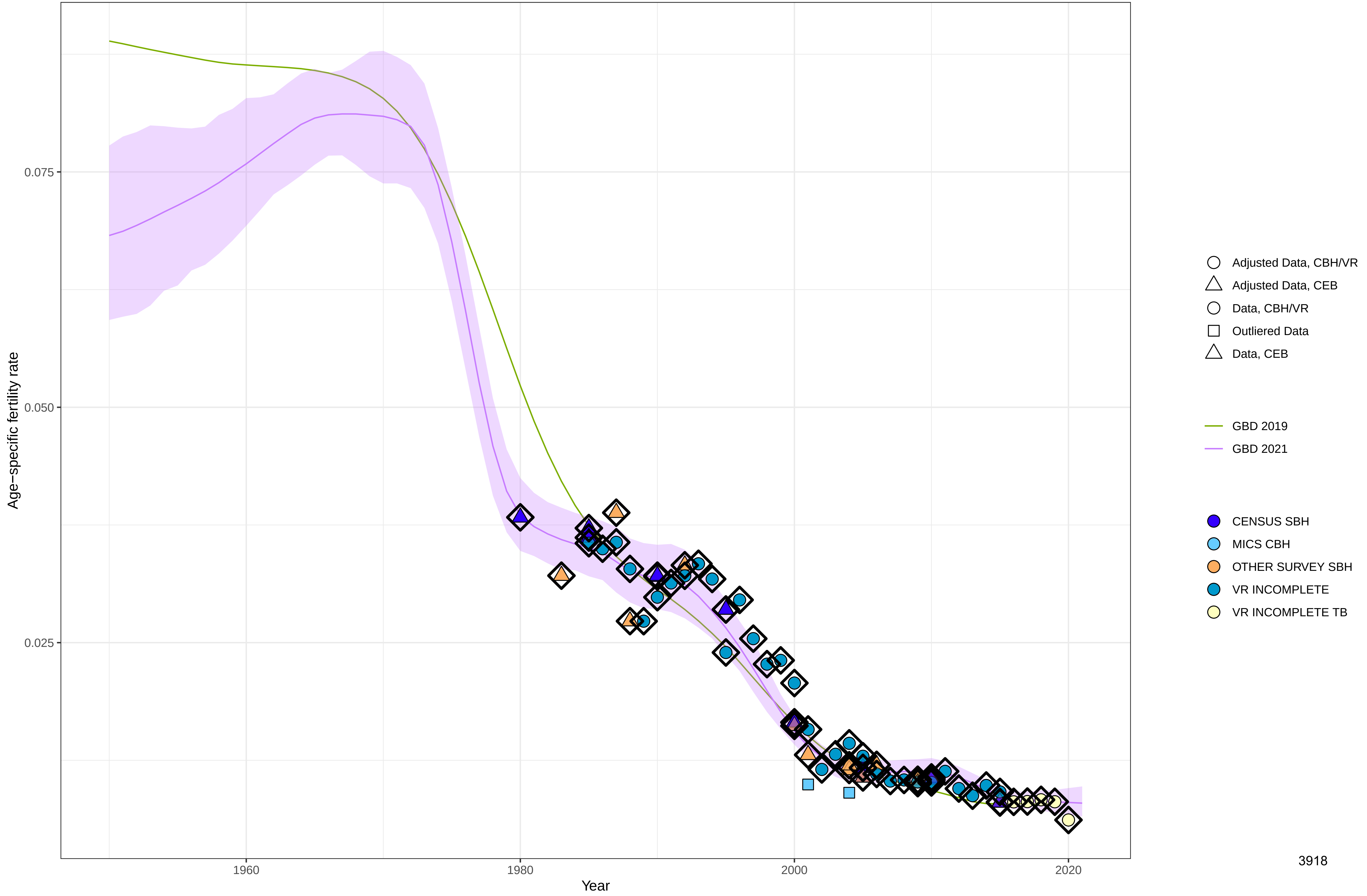

Age−specific fertility fitted trend lines, 1950−2021: Campeche, Ages: 45−49
Data points enclosed with a diamond are reference sources

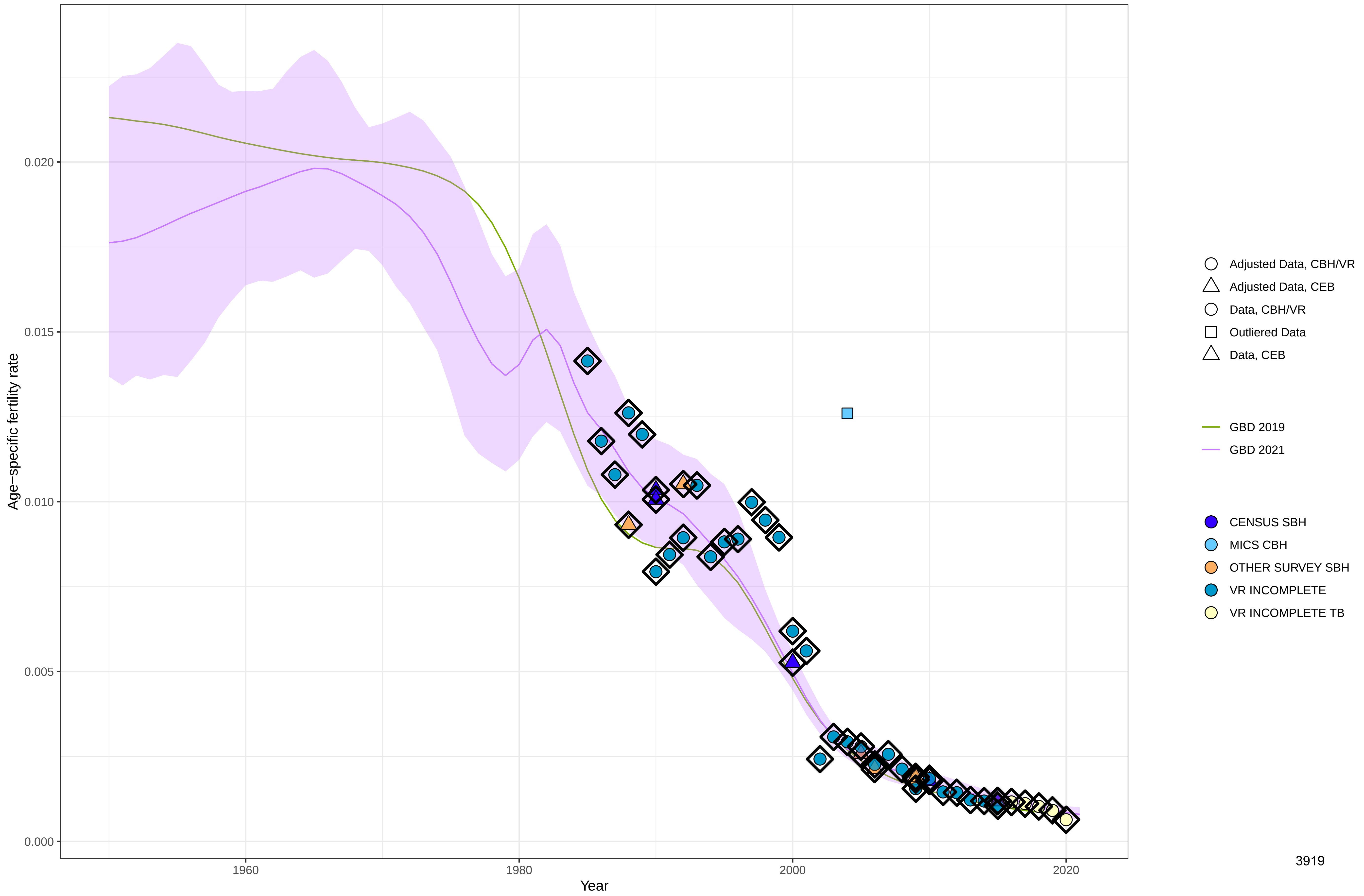

Age−specific fertility fitted trend lines, 1950−2021: Campeche, Ages: 50−54
Data points enclosed with a diamond are reference sources

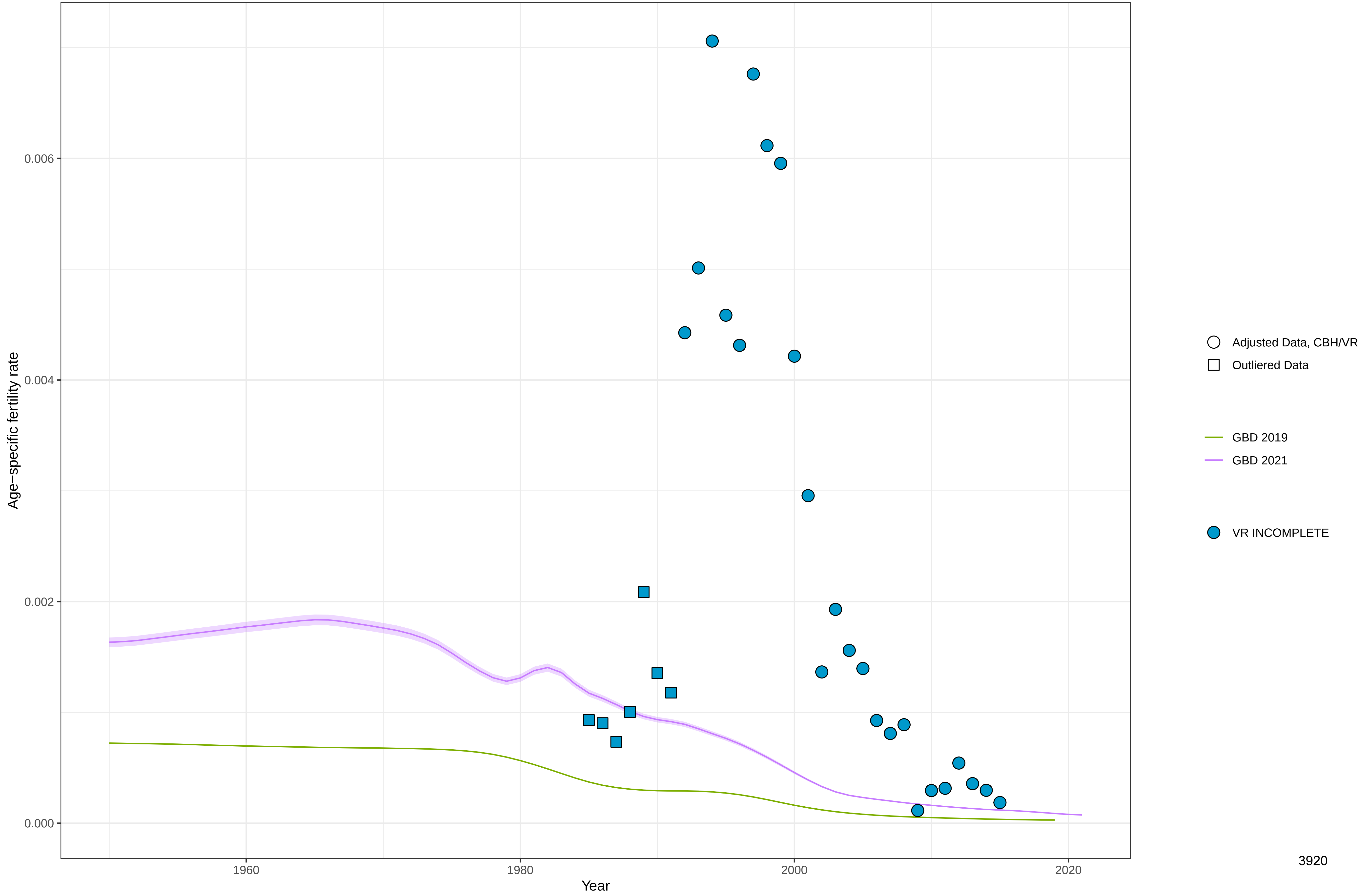

3920

Total fertility rate fitted trend lines, 1950−2021: Campeche TFR
Data points enclosed with a diamond are reference sources

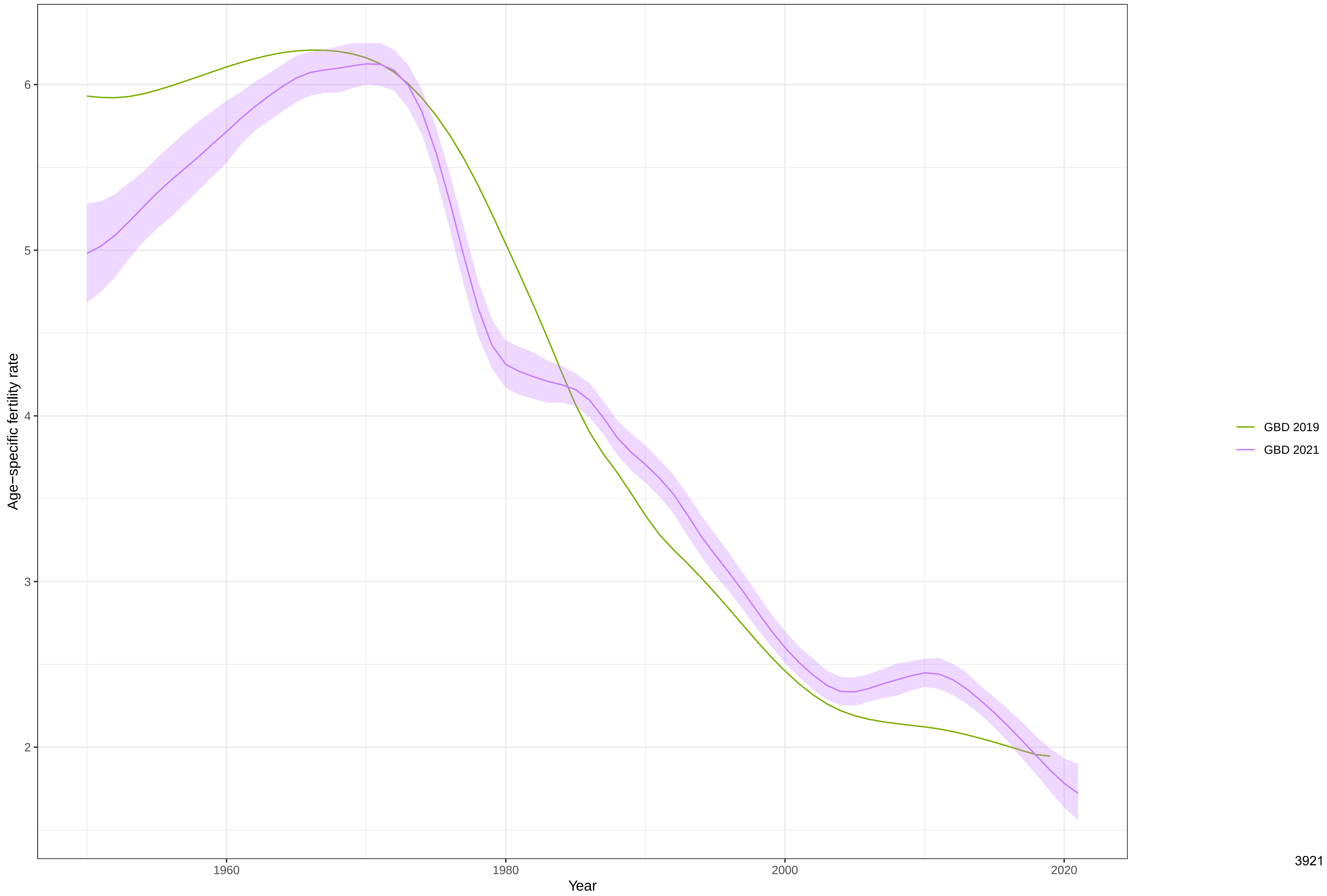

Age−specific fertility fitted trend lines, 1950−2021: Chiapas, Ages: 10−14
Data points enclosed with a diamond are reference sources

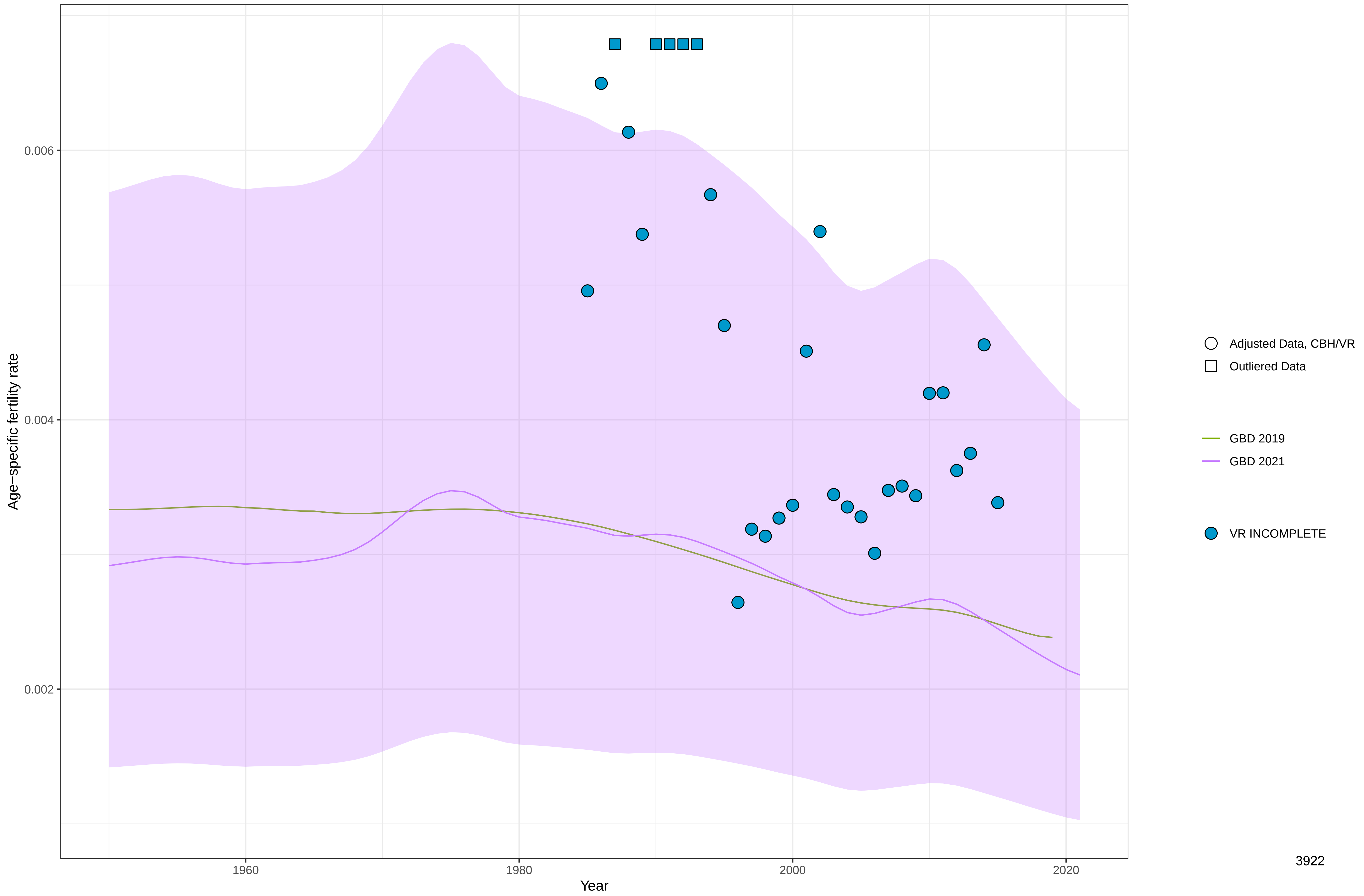

Age−specific fertility fitted trend lines, 1950−2021: Chiapas, Ages: 15−19
Data points enclosed with a diamond are reference sources

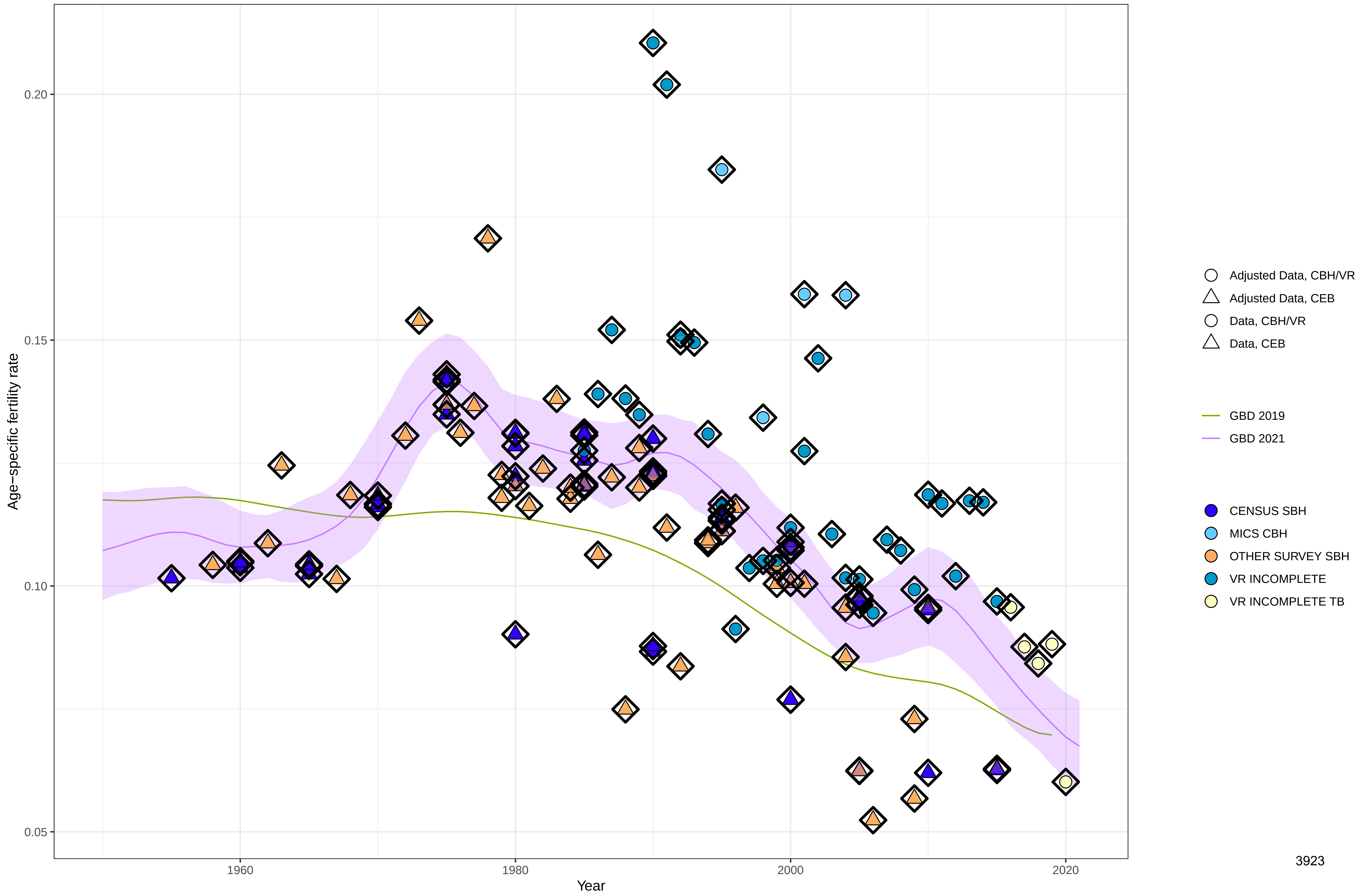

Age−specific fertility fitted trend lines, 1950−2021: Chiapas, Ages: 20−24
Data points enclosed with a diamond are reference sources

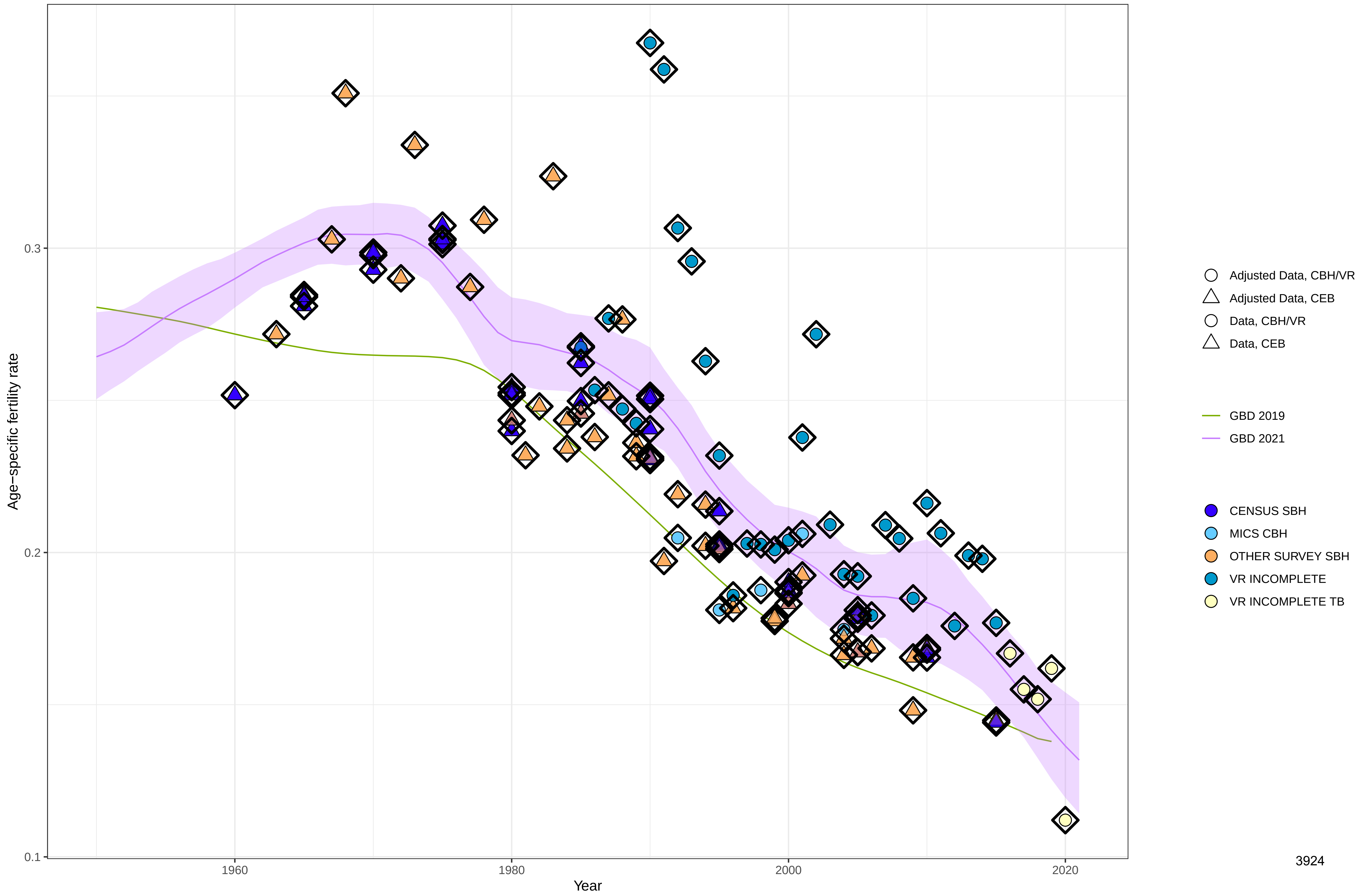

Age−specific fertility fitted trend lines, 1950−2021: Chiapas, Ages: 25−29
Data points enclosed with a diamond are reference sources

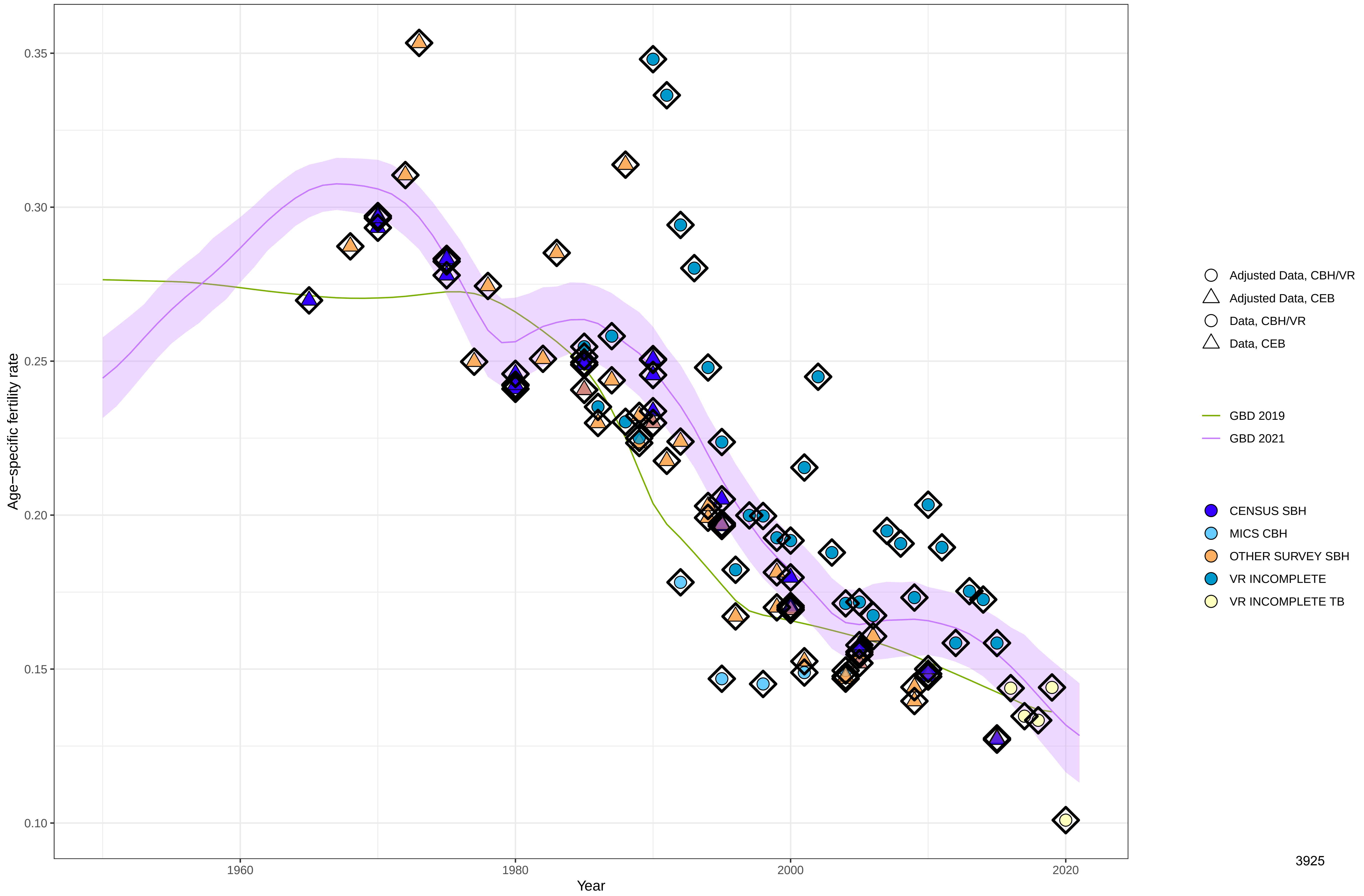

Age−specific fertility fitted trend lines, 1950−2021: Chiapas, Ages: 30−34
Data points enclosed with a diamond are reference sources

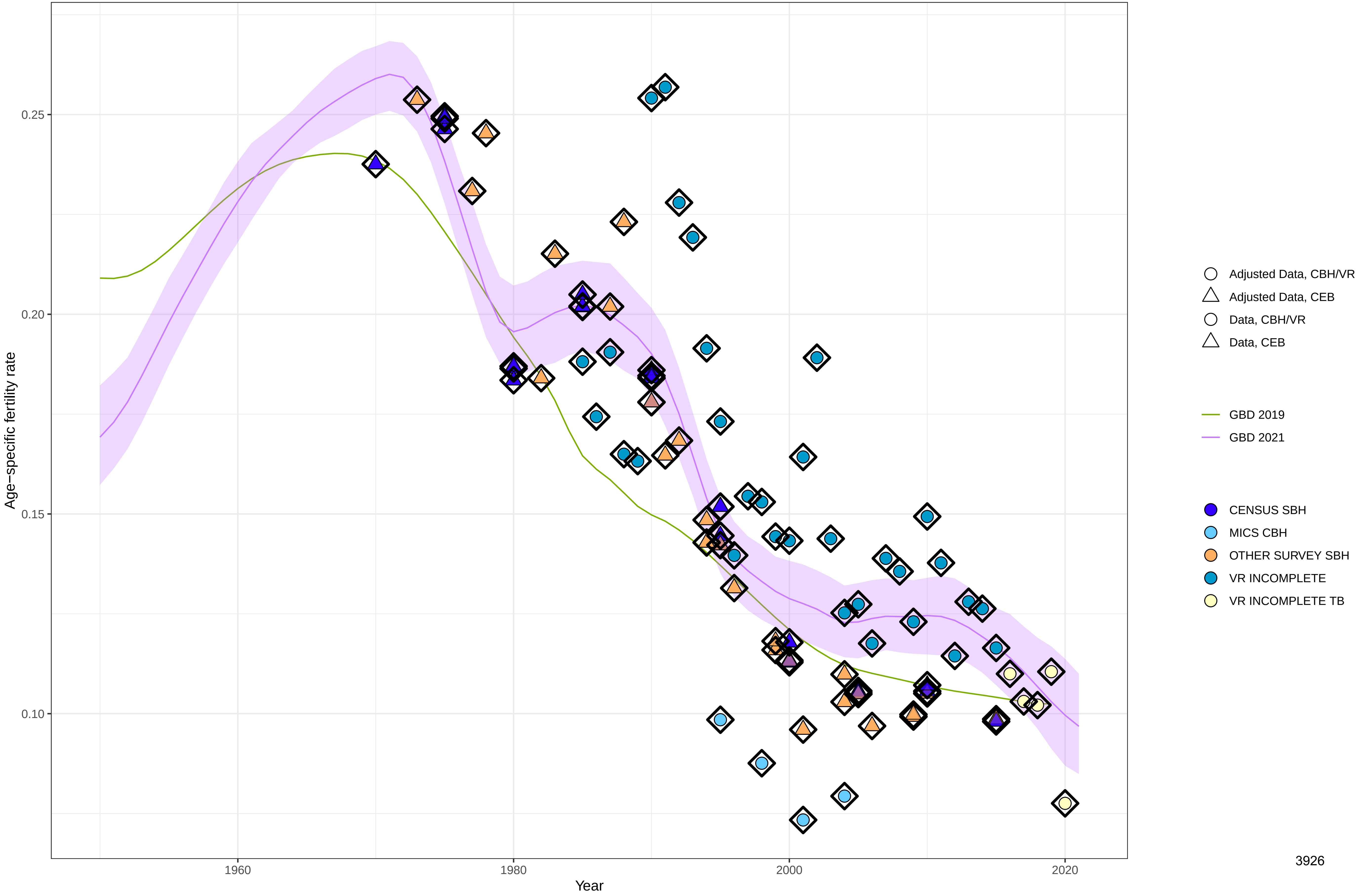

Age−specific fertility fitted trend lines, 1950−2021: Chiapas, Ages: 35−39
Data points enclosed with a diamond are reference sources

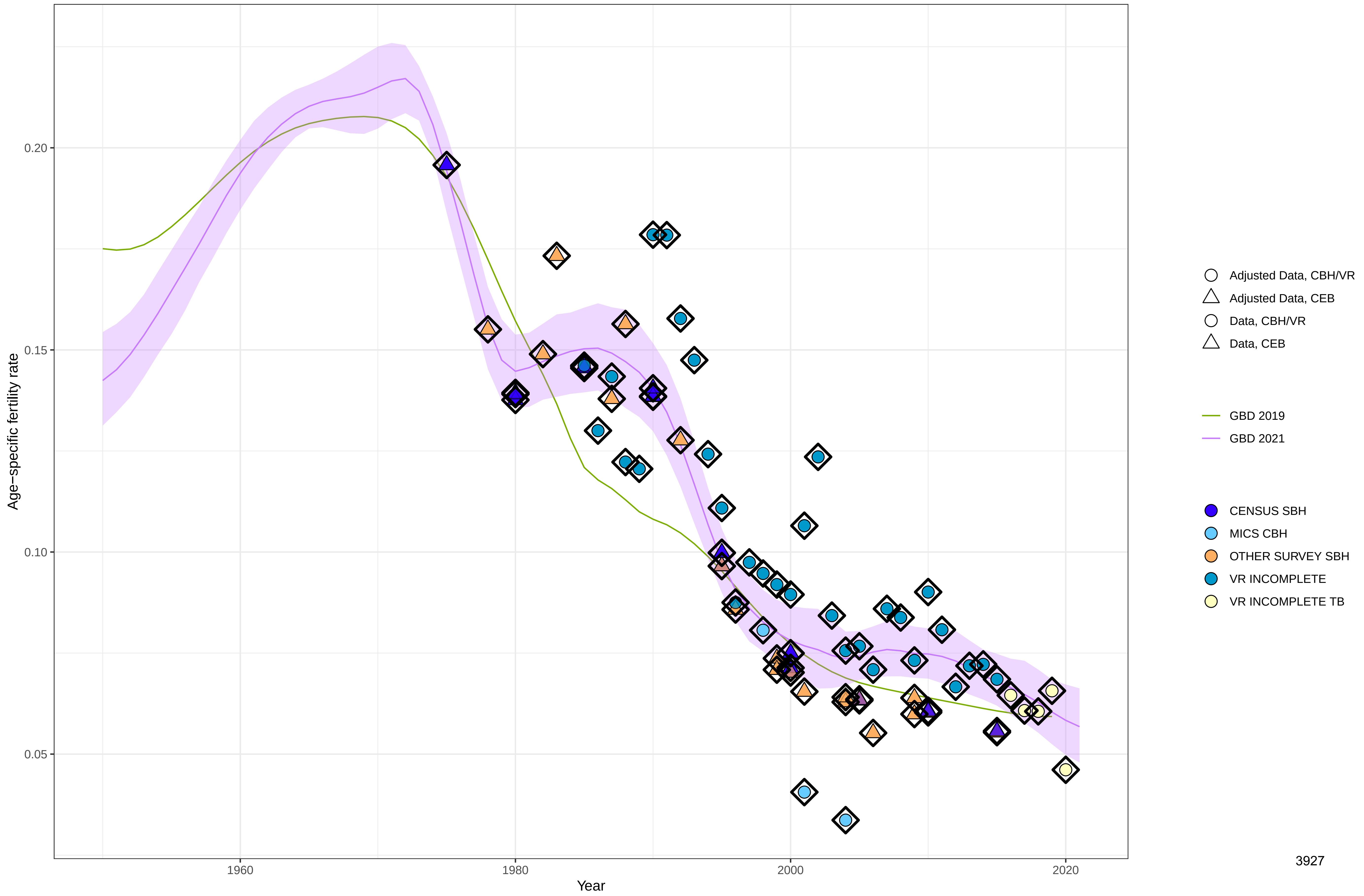

Age−specific fertility fitted trend lines, 1950−2021: Chiapas, Ages: 40−44
Data points enclosed with a diamond are reference sources

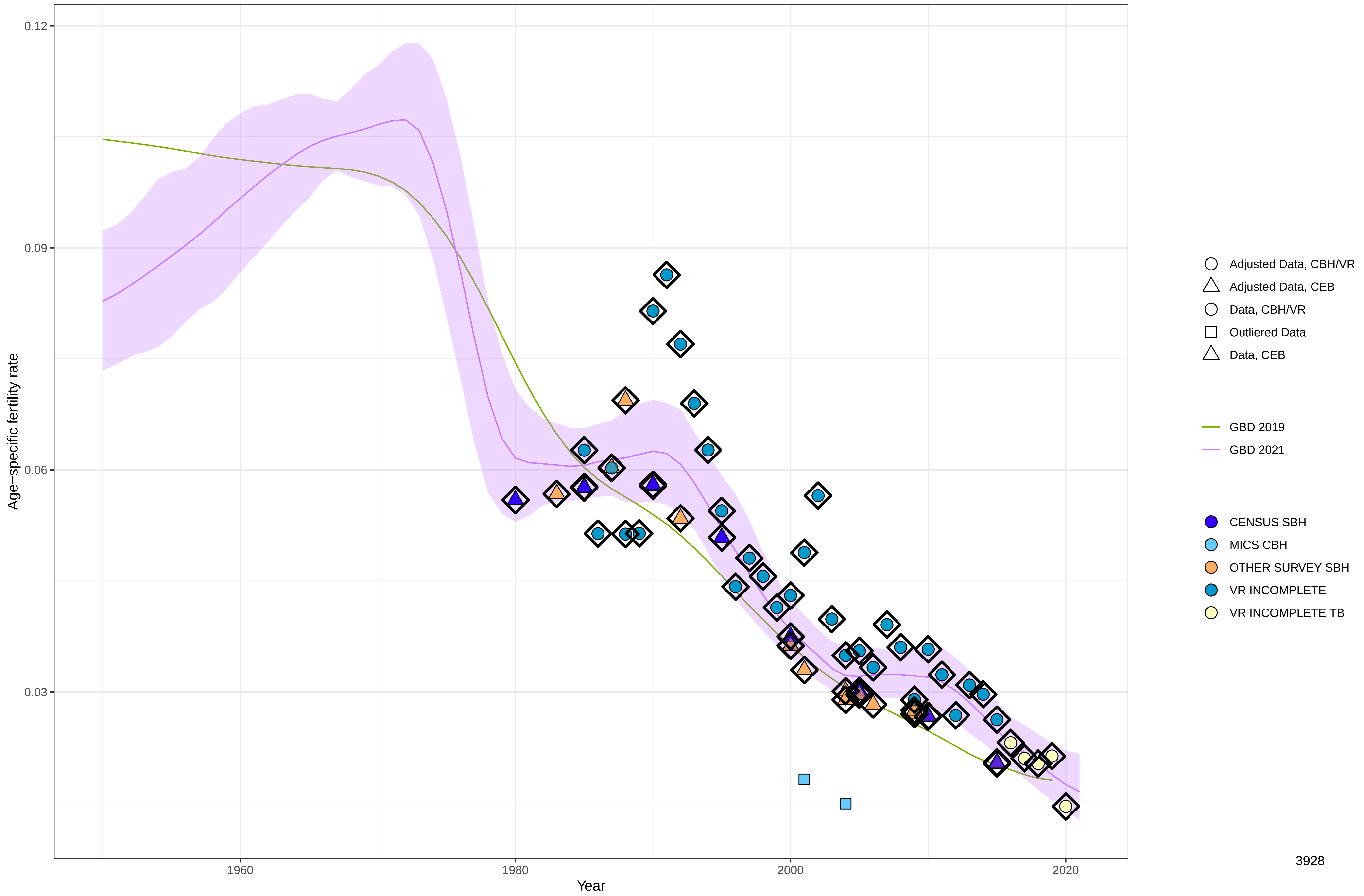

Age−specific fertility fitted trend lines, 1950−2021: Chiapas, Ages: 45−49
Data points enclosed with a diamond are reference sources

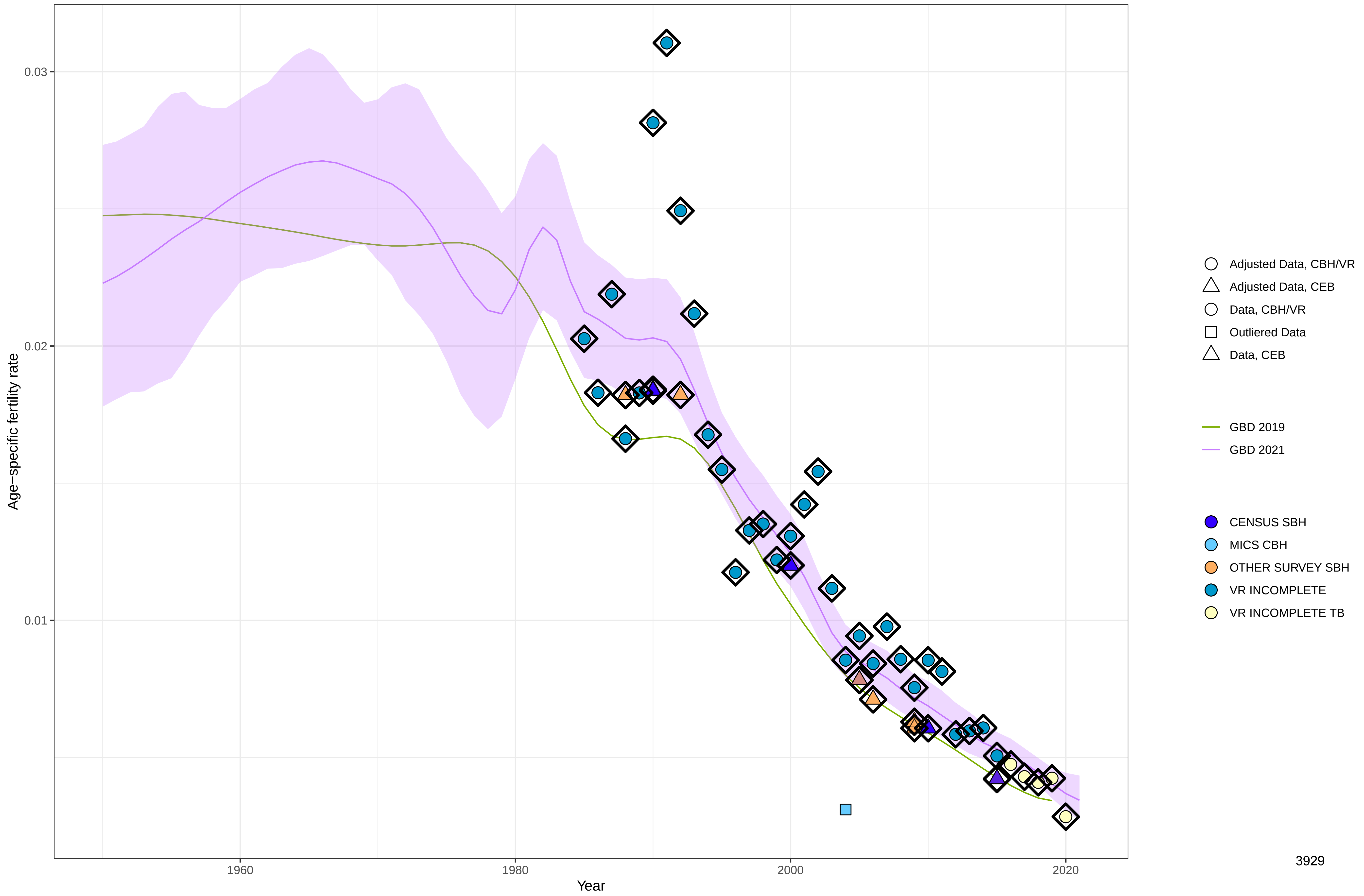

Age−specific fertility fitted trend lines, 1950−2021: Chiapas, Ages: 50−54
Data points enclosed with a diamond are reference sources

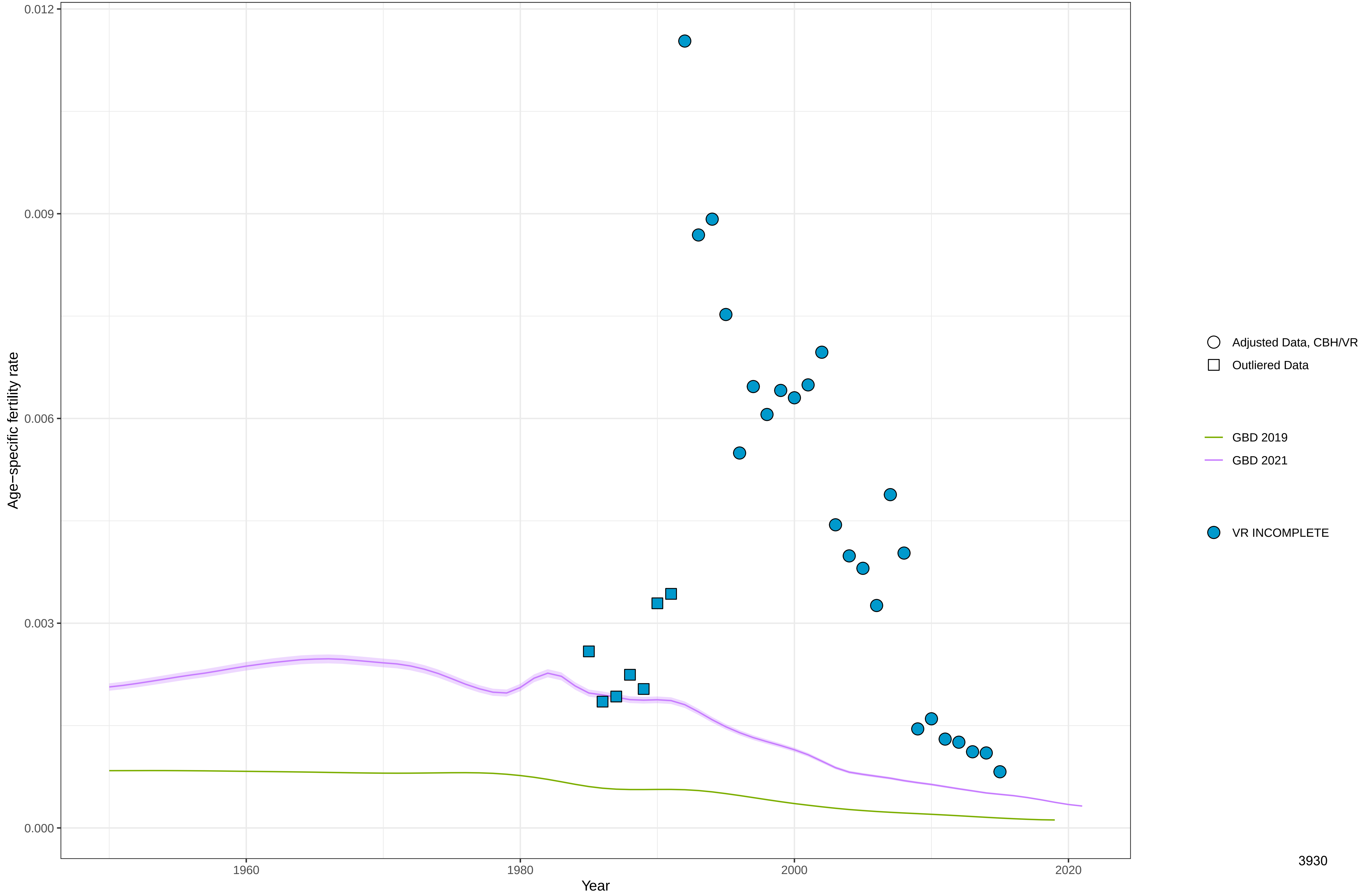

Total fertility rate fitted trend lines, 1950−2021: Chiapas TFR
Data points enclosed with a diamond are reference sources

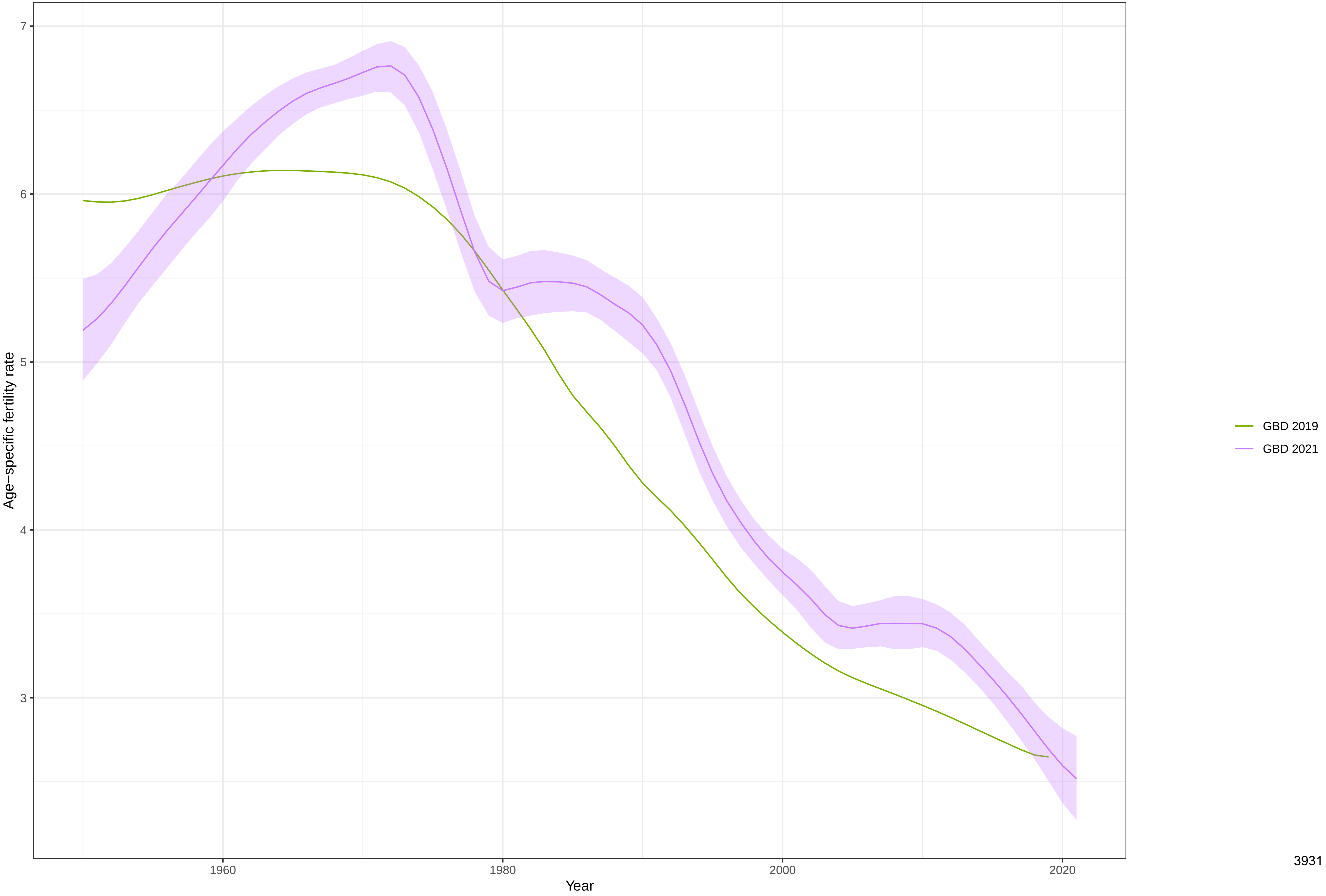

Age−specific fertility fitted trend lines, 1950−2021: Chihuahua, Ages: 10−14
Data points enclosed with a diamond are reference sources

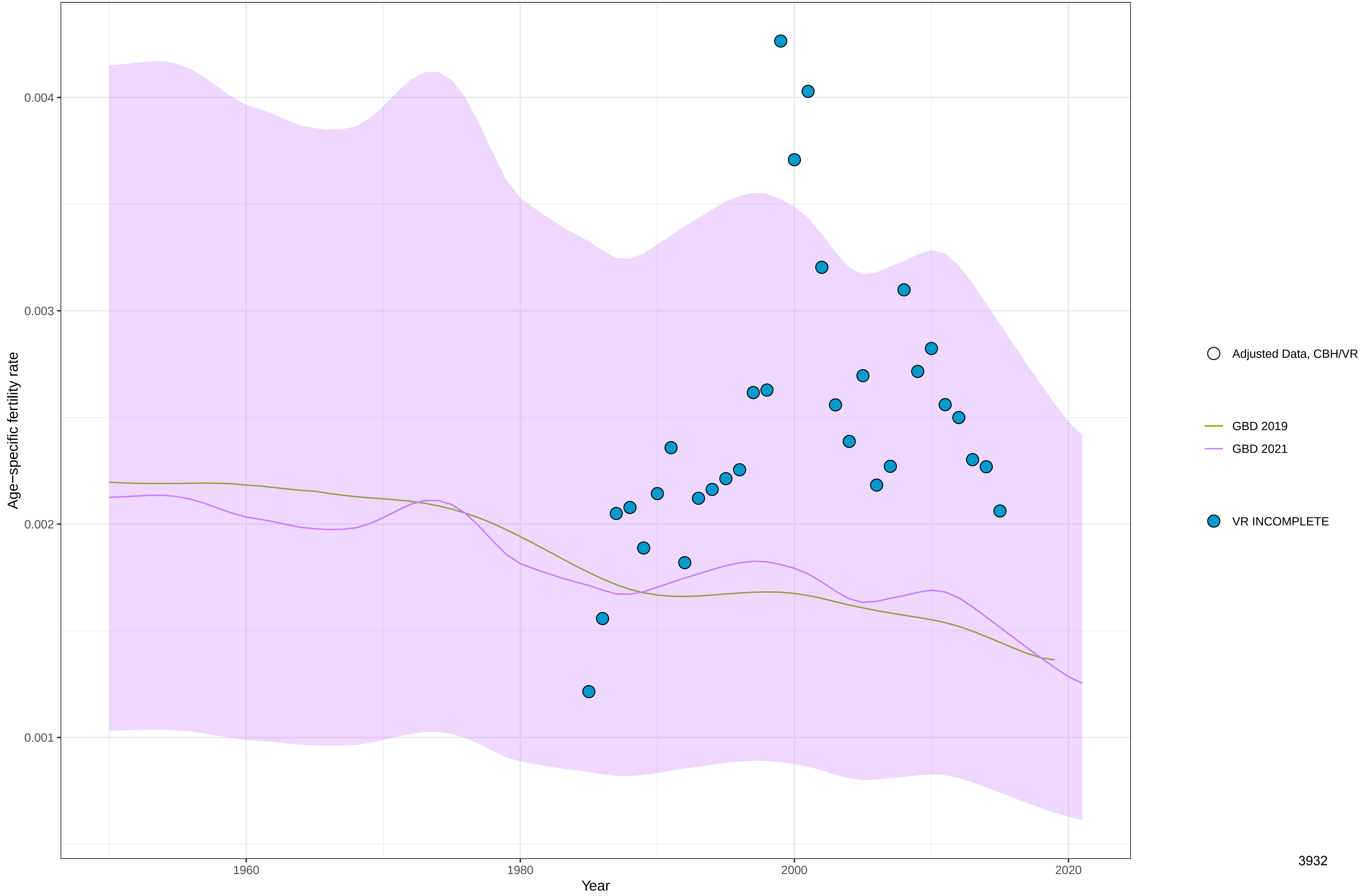

Age−specific fertility fitted trend lines, 1950−2021: Chihuahua, Ages: 15−19
Data points enclosed with a diamond are reference sources

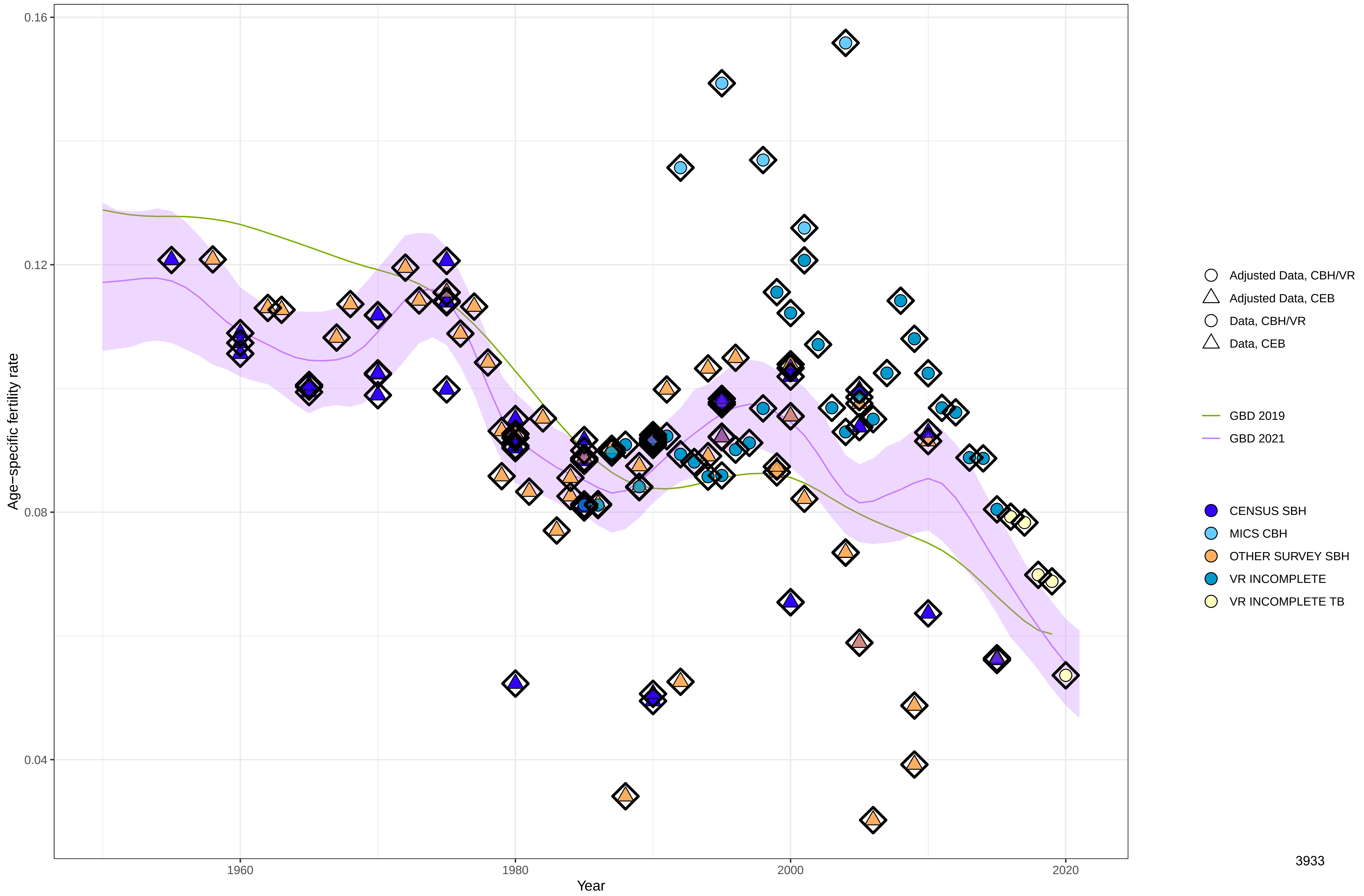

Age−specific fertility fitted trend lines, 1950−2021: Chihuahua, Ages: 20−24
Data points enclosed with a diamond are reference sources

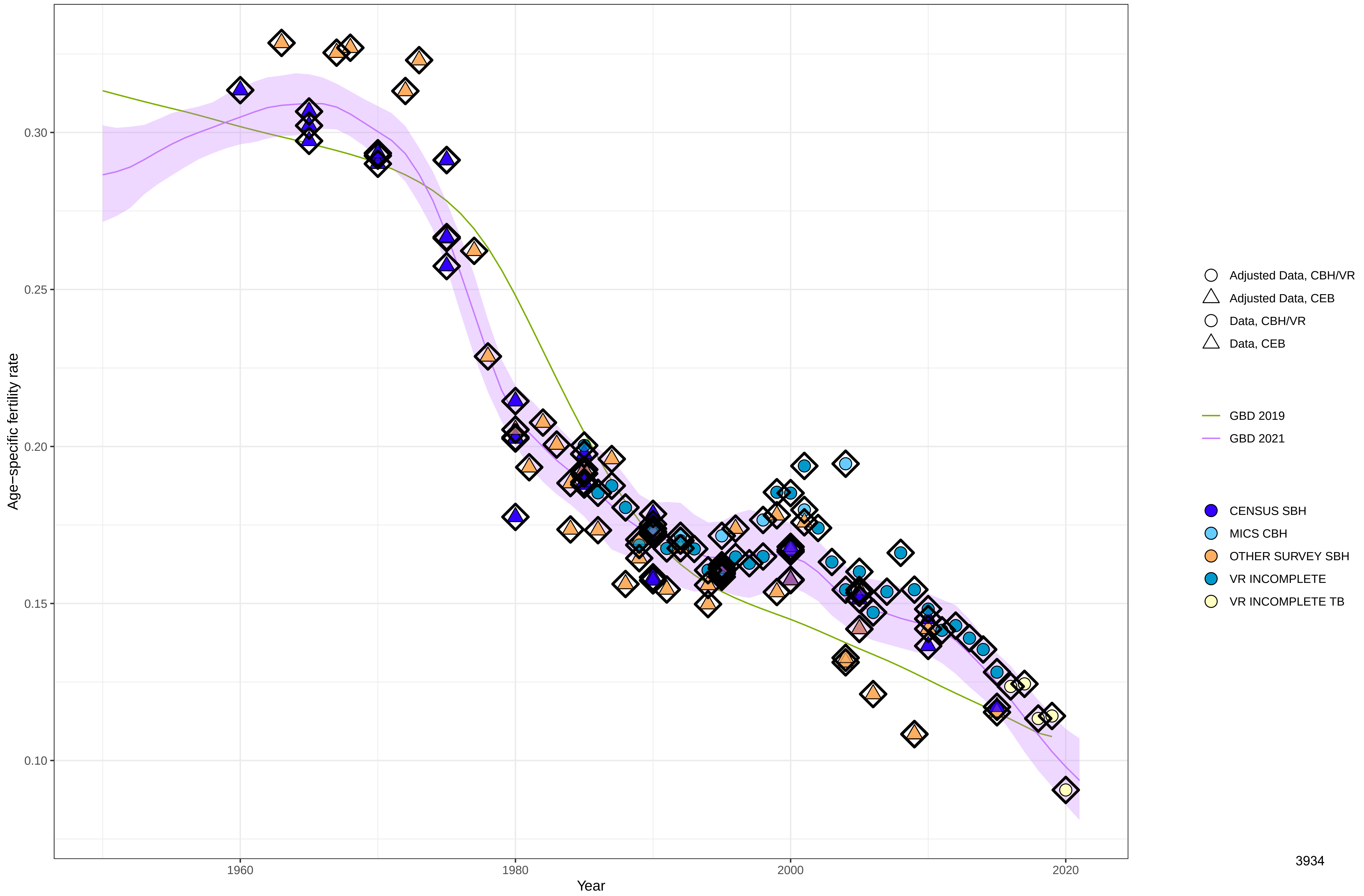

Age−specific fertility fitted trend lines, 1950−2021: Chihuahua, Ages: 25−29
Data points enclosed with a diamond are reference sources

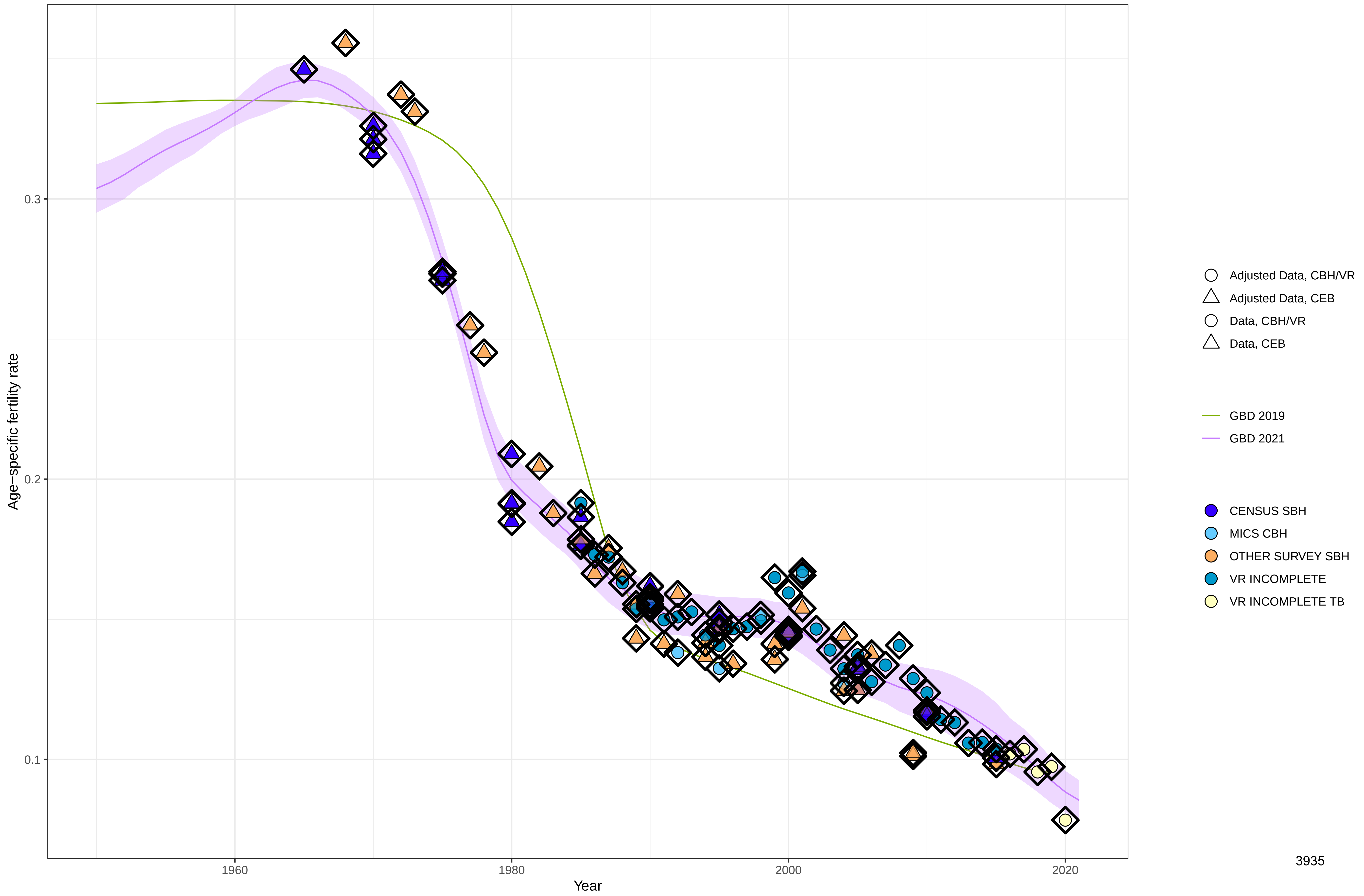

Age−specific fertility fitted trend lines, 1950−2021: Chihuahua, Ages: 30−34
Data points enclosed with a diamond are reference sources

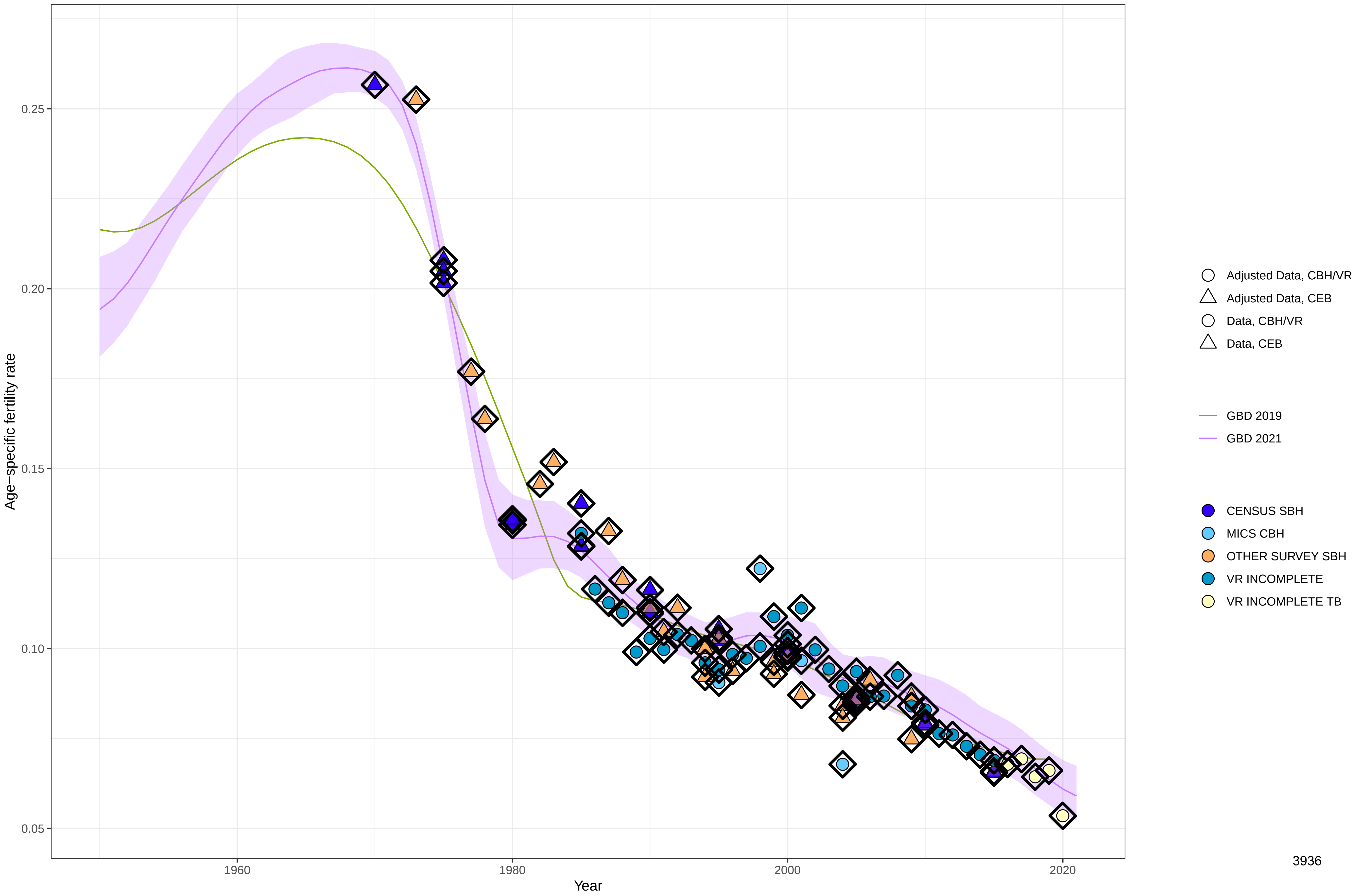

Age−specific fertility fitted trend lines, 1950−2021: Chihuahua, Ages: 35−39
Data points enclosed with a diamond are reference sources

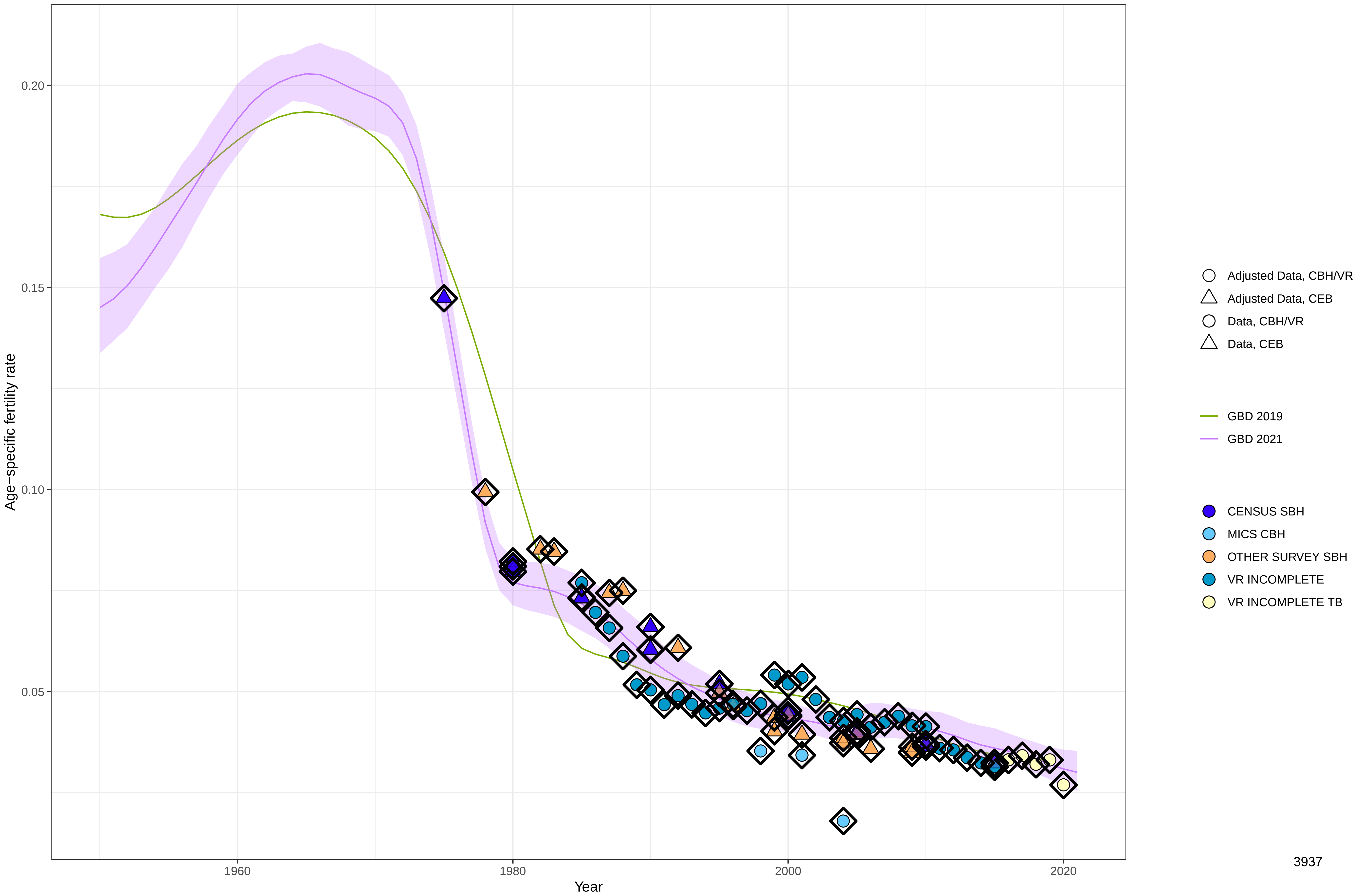

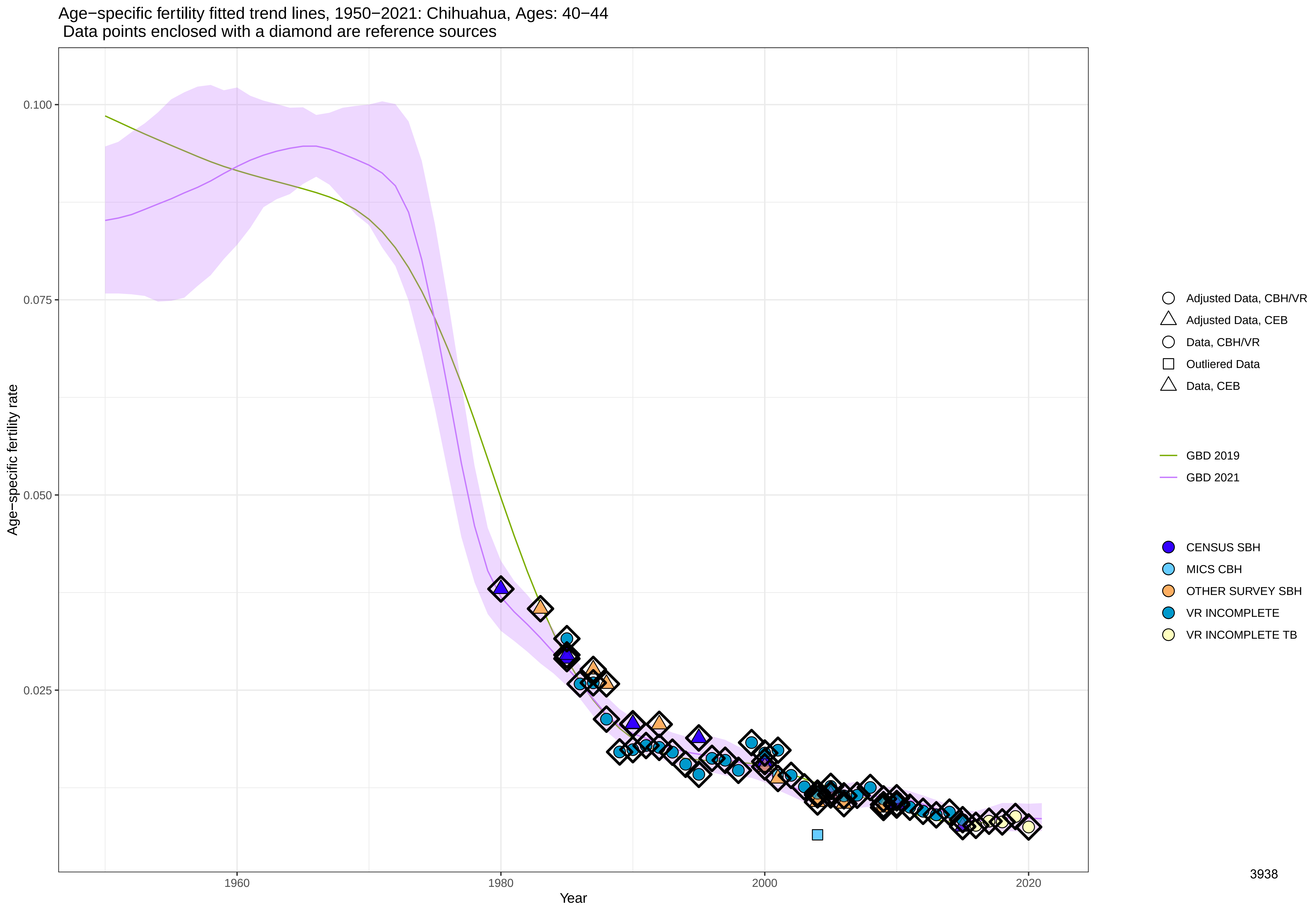
Age−specific fertility fitted trend lines, 1950−2021: Chihuahua, Ages: 40−44
Data points enclosed with a diamond are reference sources
Age−specific fertility rate
0.100
0.075
0.050
0.025
1960
1980
2000
2020
Year
Adjusted Data, CBH/VR
Adjusted Data, CEB
Data, CBH/VR
Outliered Data
Data, CEB
GBD 2019
GBD 2021
CENSUS SBH
MICS CBH
OTHER SURVEY SBH
VR INCOMPLETE
VR INCOMPLETE TB

Age−specific fertility fitted trend lines, 1950−2021: Chihuahua, Ages: 45−49
Data points enclosed with a diamond are reference sources

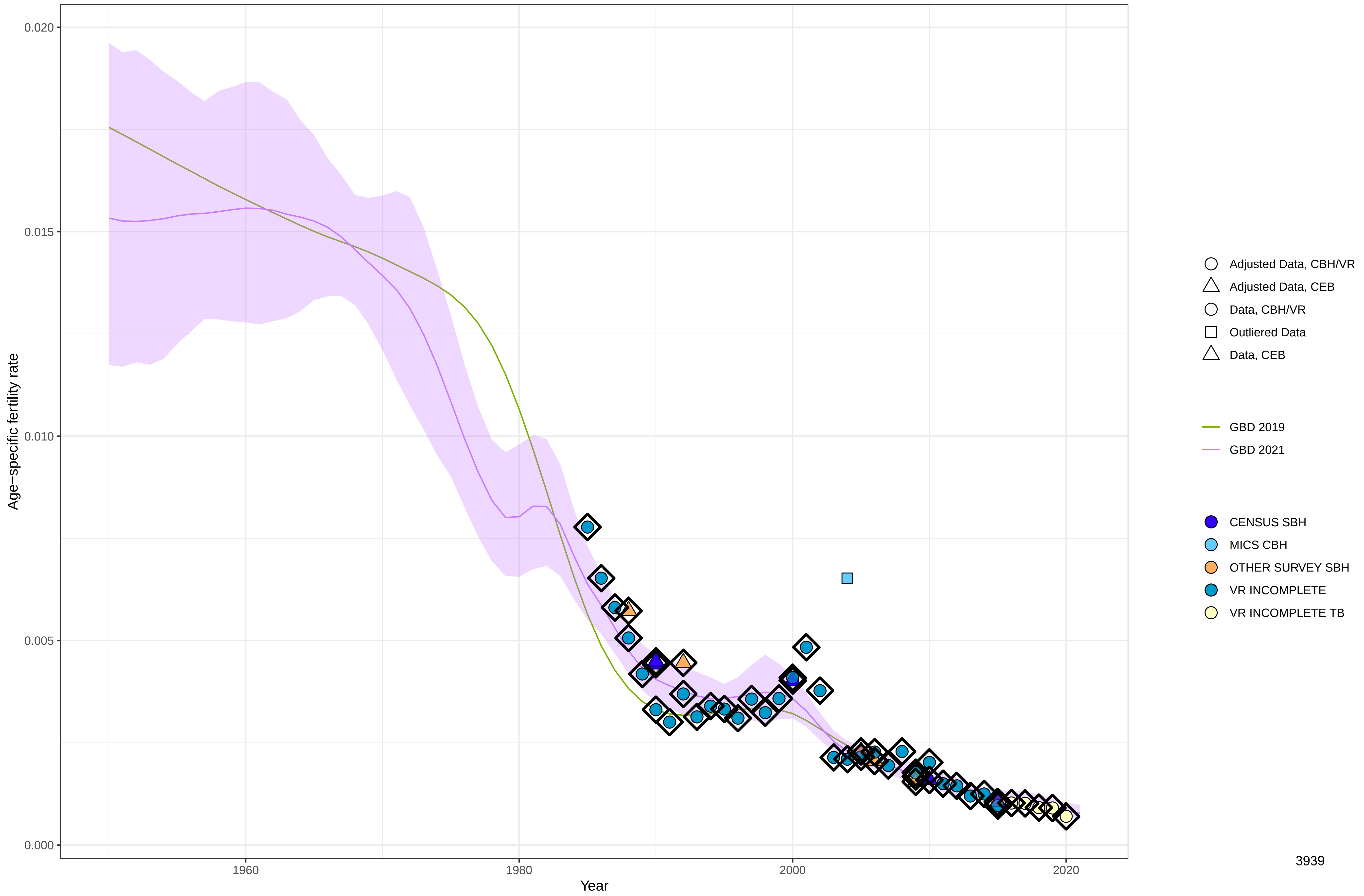

Age−specific fertility fitted trend lines, 1950−2021: Chihuahua, Ages: 50−54
Data points enclosed with a diamond are reference sources

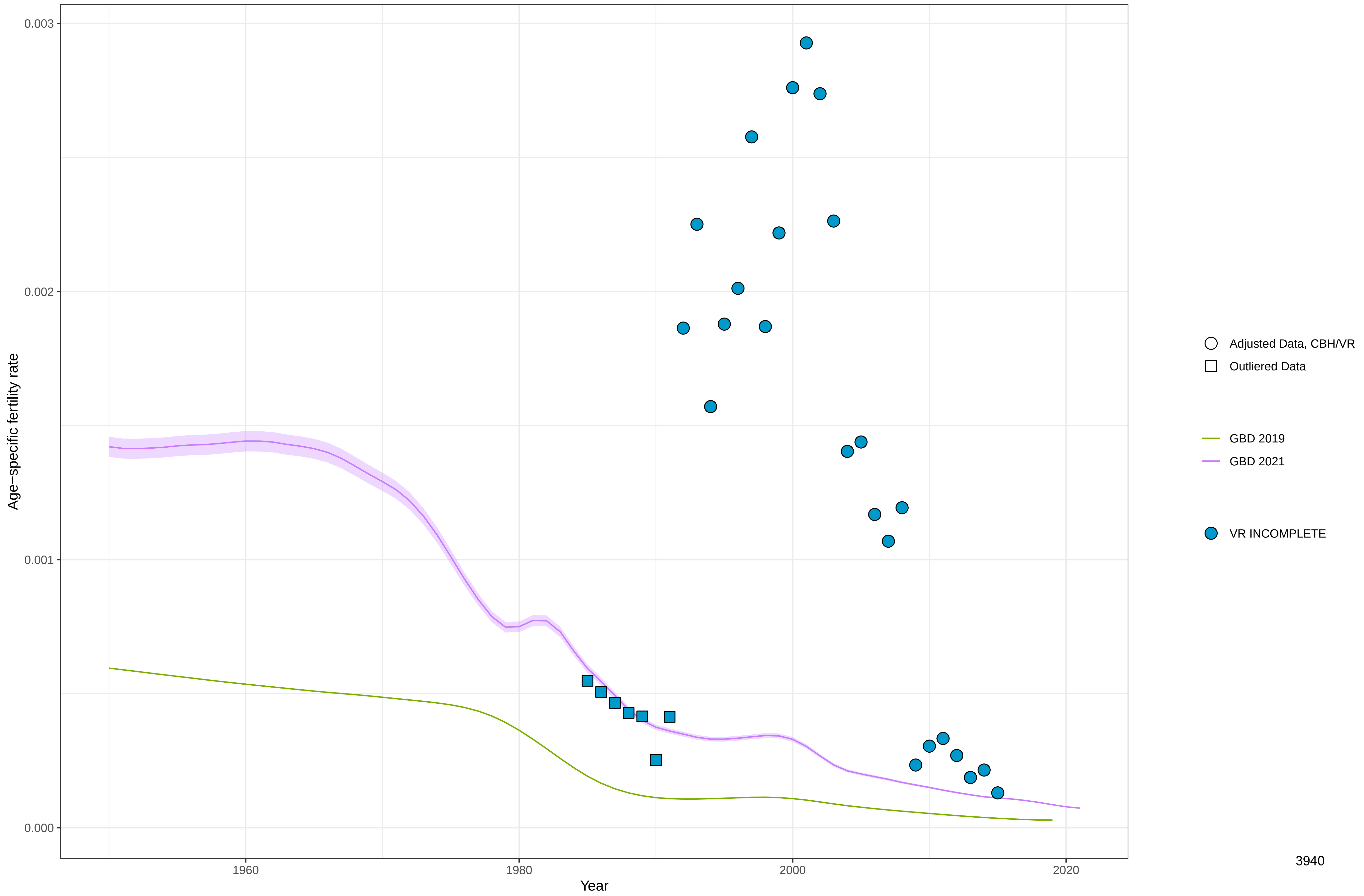

Total fertility rate fitted trend lines, 1950−2021: Chihuahua TFR
Data points enclosed with a diamond are reference sources

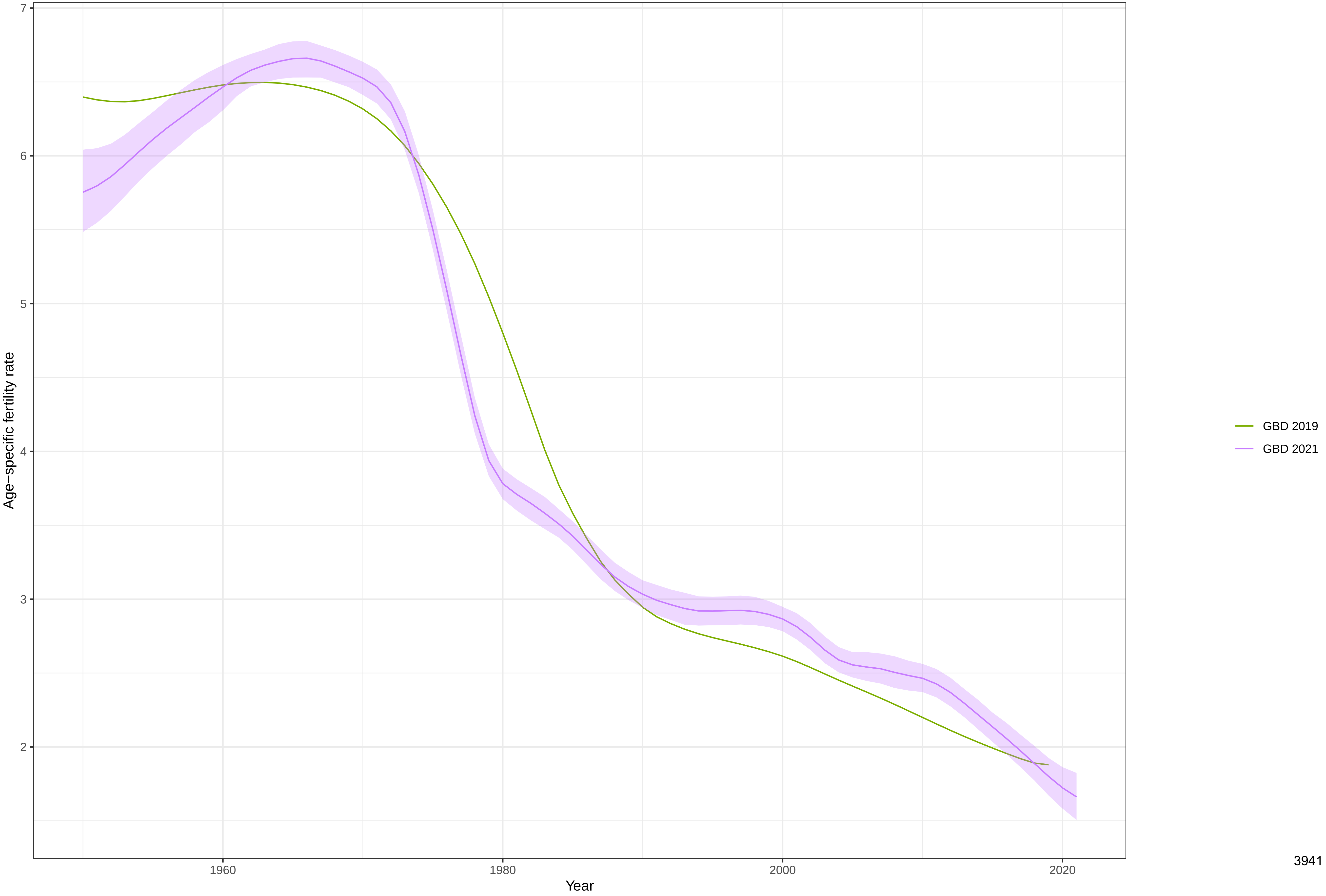

Age−specific fertility fitted trend lines, 1950−2021: Coahuila, Ages: 10−14
Data points enclosed with a diamond are reference sources

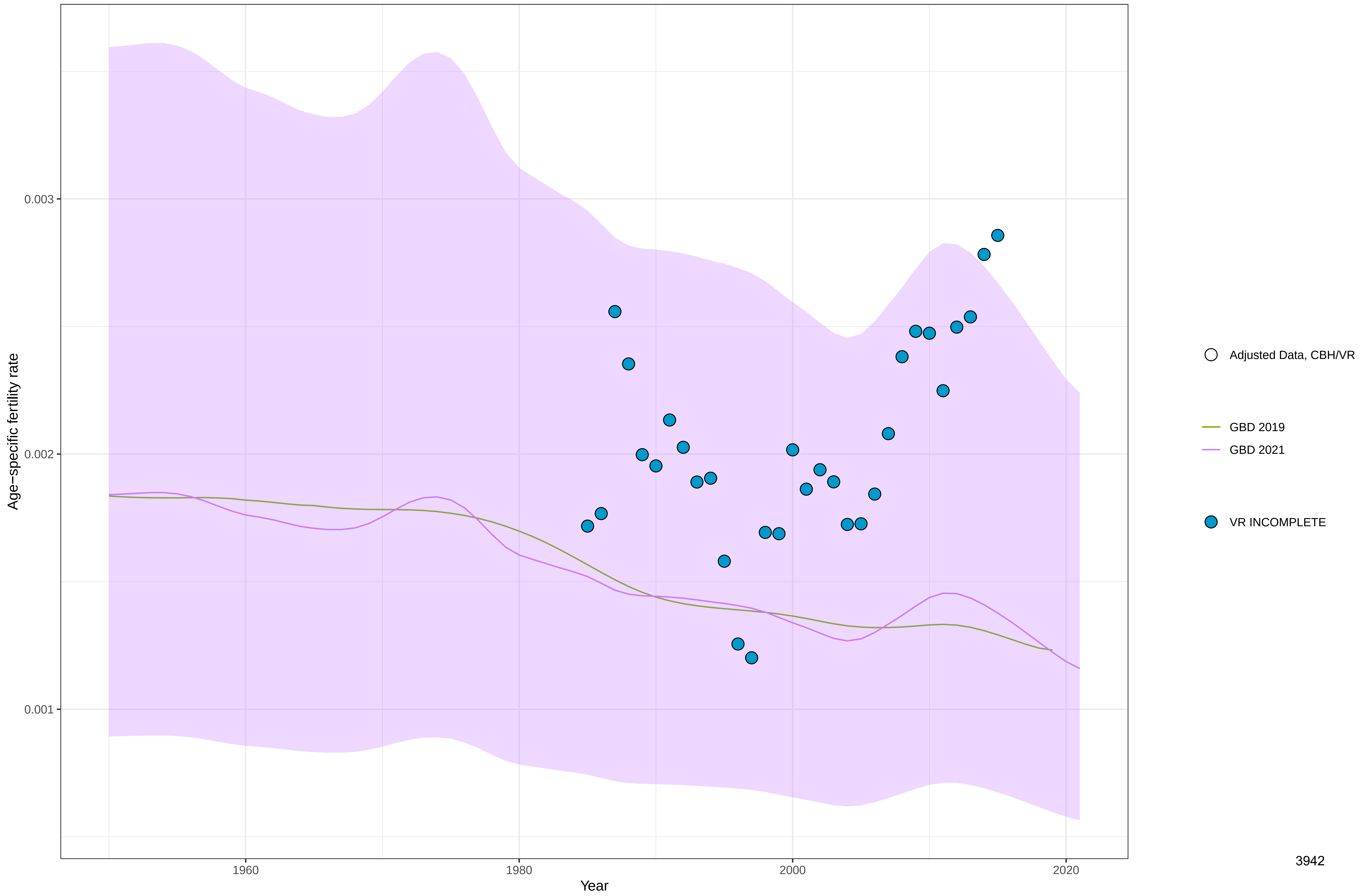

Age−specific fertility fitted trend lines, 1950−2021: Coahuila, Ages: 15−19
Data points enclosed with a diamond are reference sources

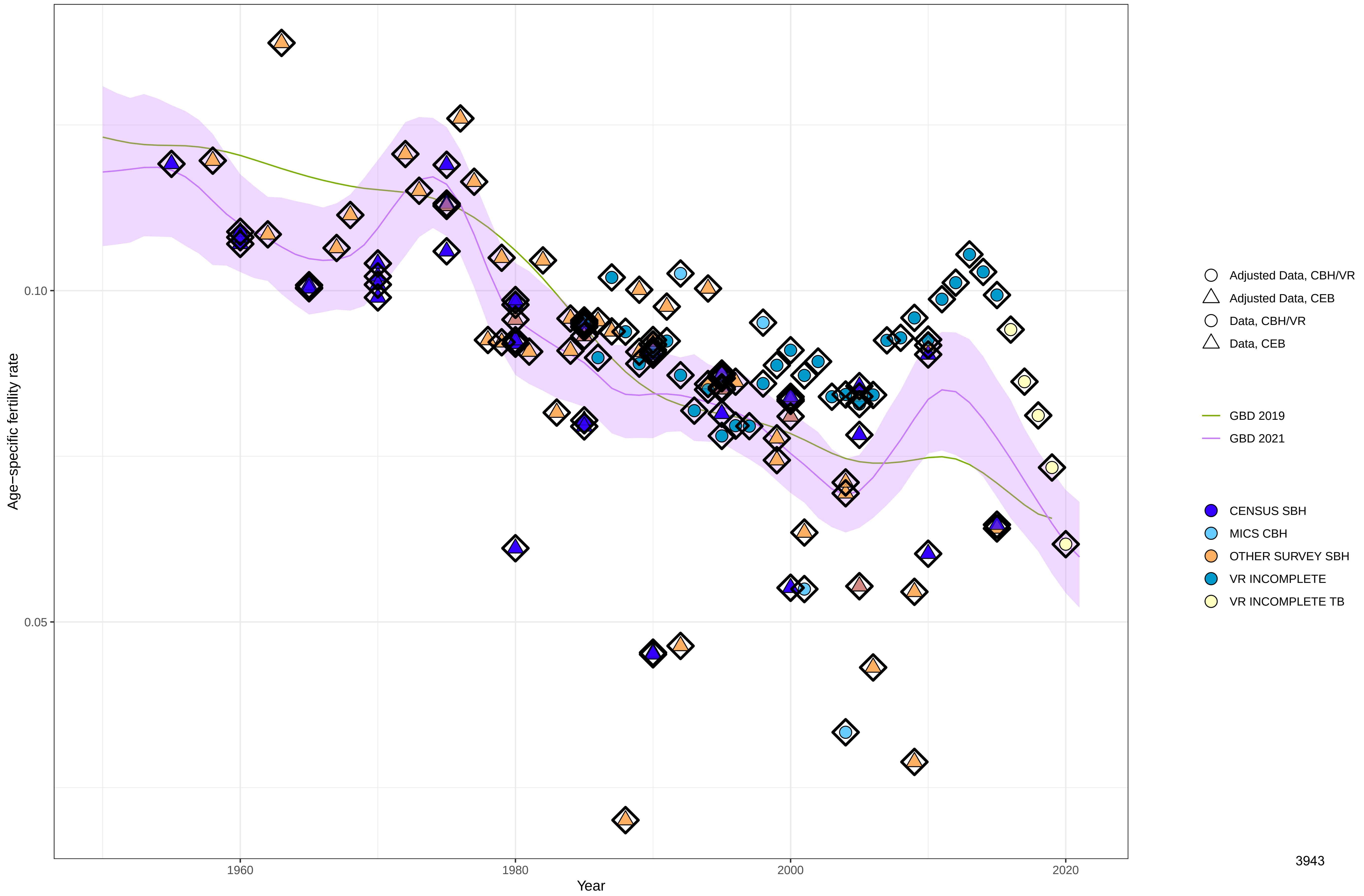

Age−specific fertility fitted trend lines, 1950−2021: Coahuila, Ages: 20−24
Data points enclosed with a diamond are reference sources

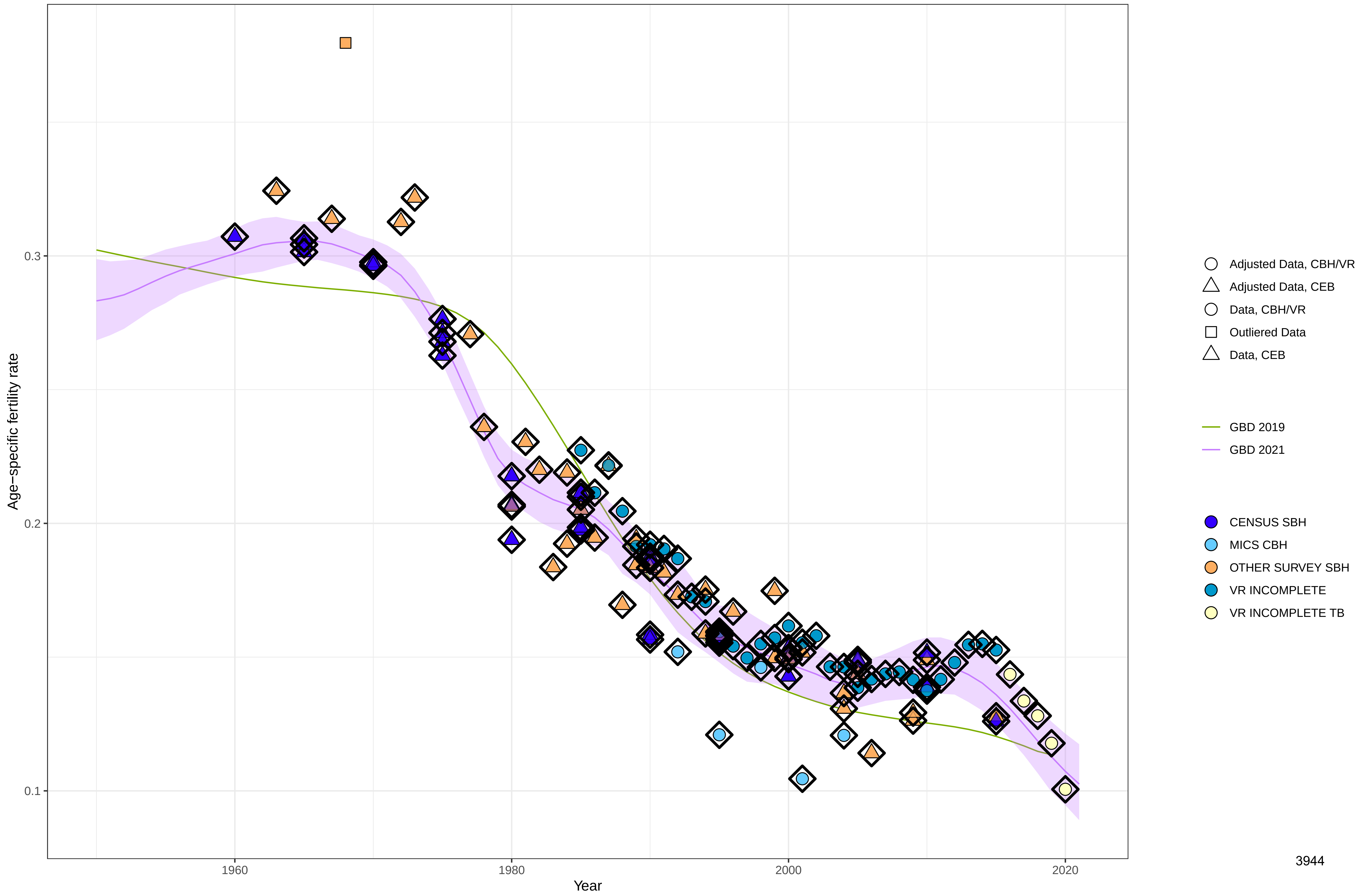

3944

Age−specific fertility fitted trend lines, 1950−2021: Coahuila, Ages: 25−29
Data points enclosed with a diamond are reference sources

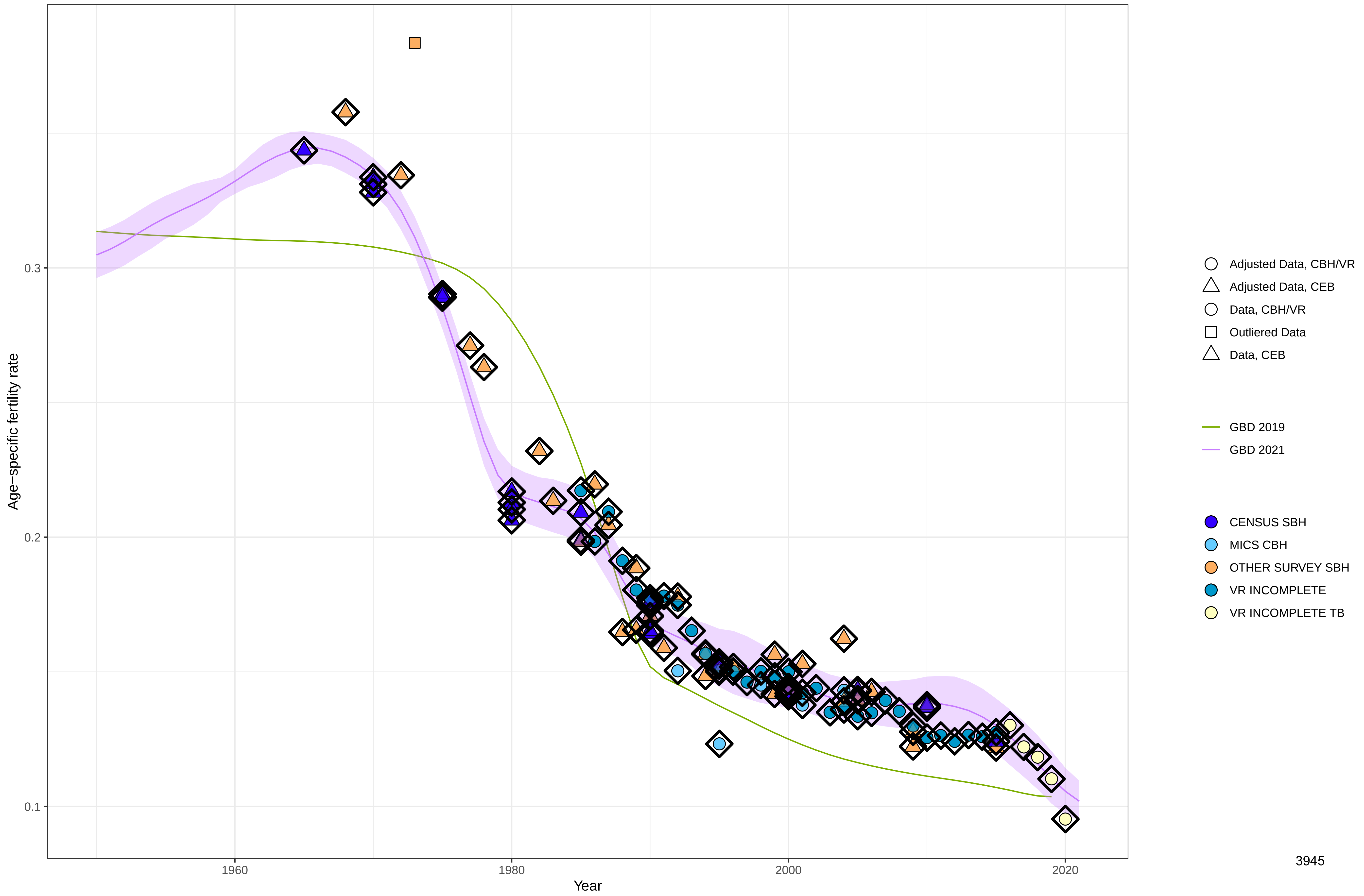

Age−specific fertility fitted trend lines, 1950−2021: Coahuila, Ages: 30−34
Data points enclosed with a diamond are reference sources

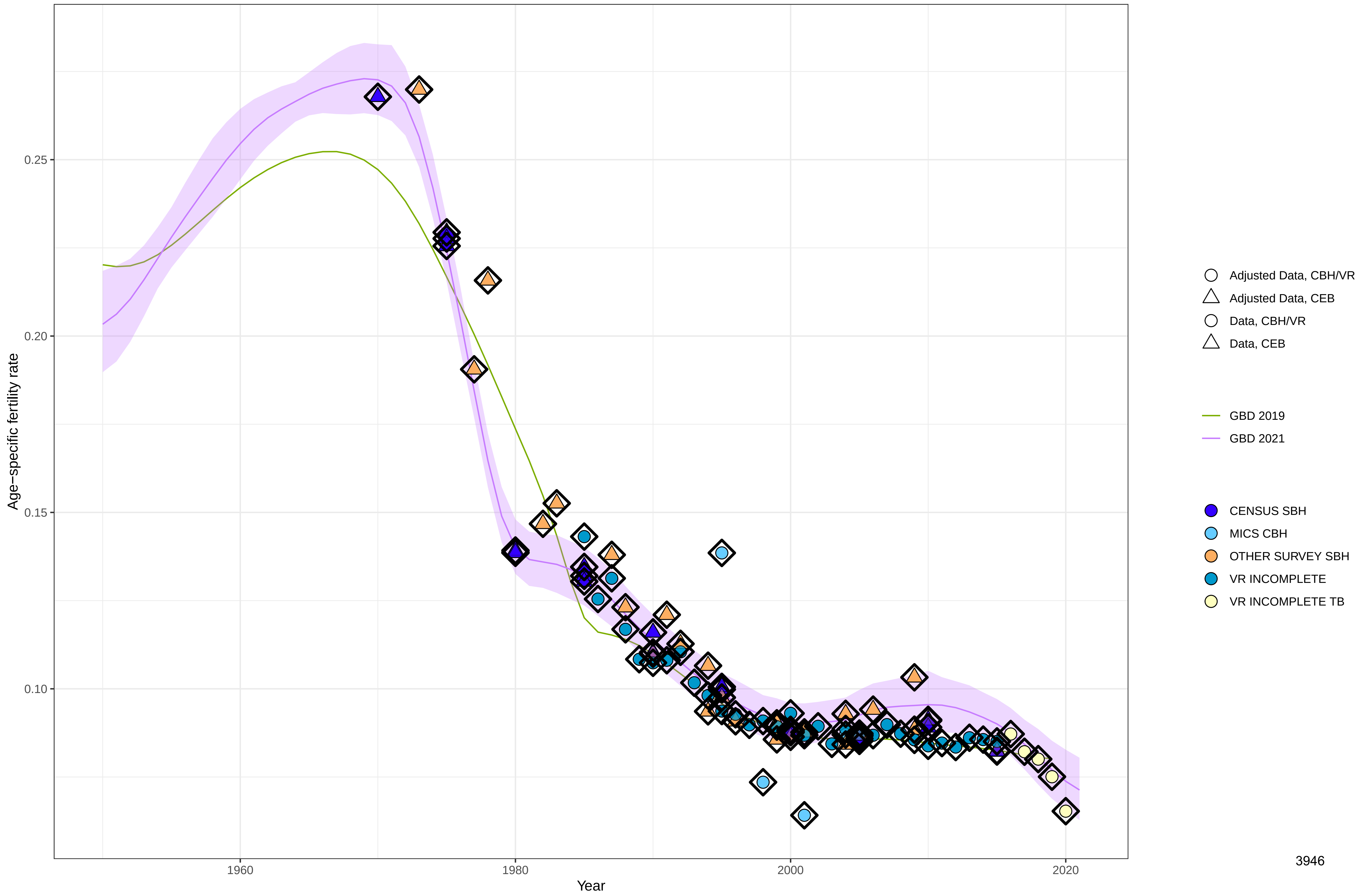

Age−specific fertility fitted trend lines, 1950−2021: Coahuila, Ages: 35−39
Data points enclosed with a diamond are reference sources

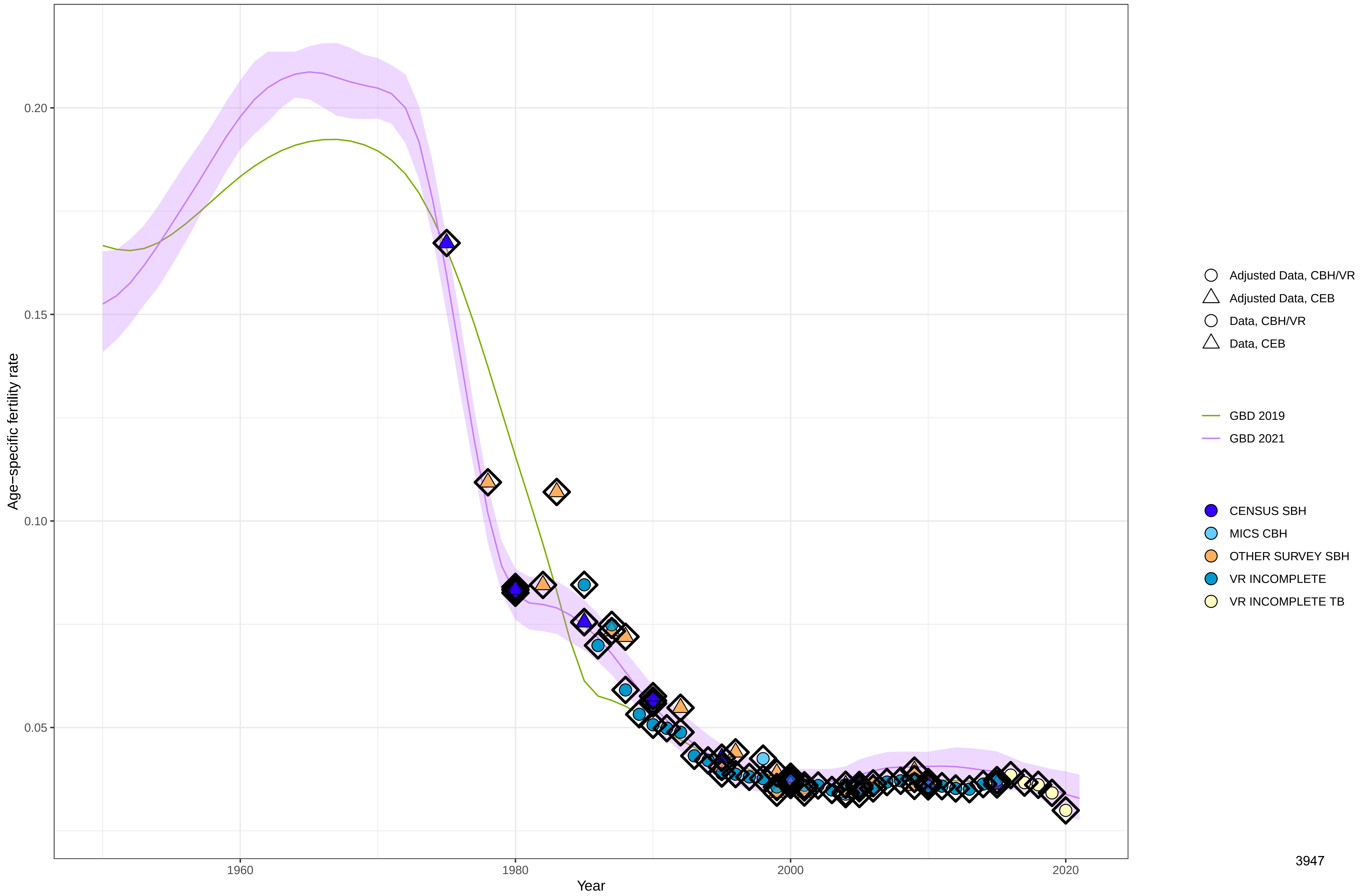

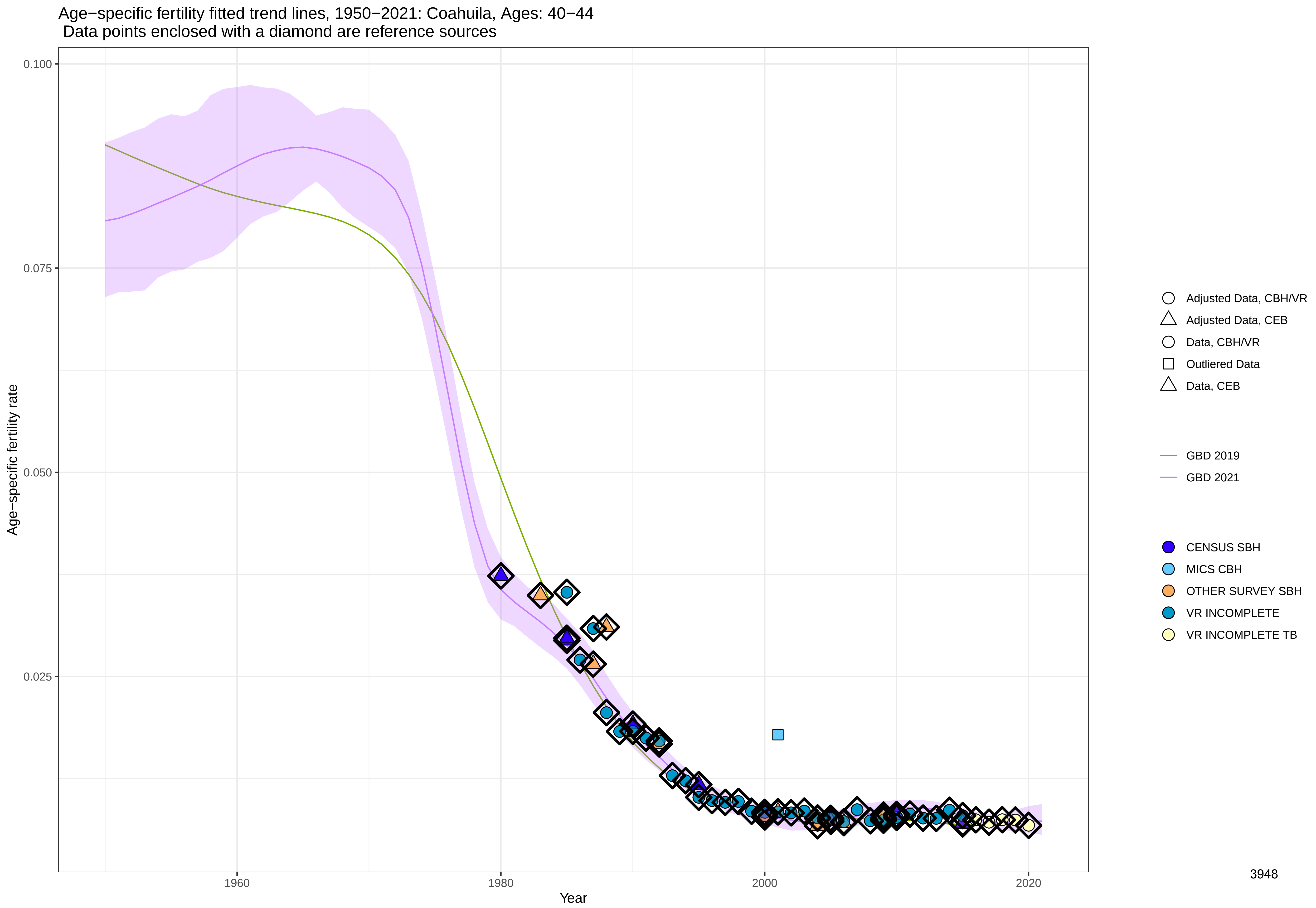

Age−specific fertility fitted trend lines, 1950−2021: Coahuila, Ages: 40−44
Data points enclosed with a diamond are reference sources
Age−specific fertility rate
Year
0.100
0.075
0.050
0.025
1960
1980
2000
2020
Adjusted Data, CBH/VR
Adjusted Data, CEB
Data, CBH/VR
Outliered Data
Data, CEB
GBD 2019
GBD 2021
CENSUS SBH
MICS CBH
OTHER SURVEY SBH
VR INCOMPLETE
VR INCOMPLETE TB

Age−specific fertility fitted trend lines, 1950−2021: Coahuila, Ages: 45−49
Data points enclosed with a diamond are reference sources

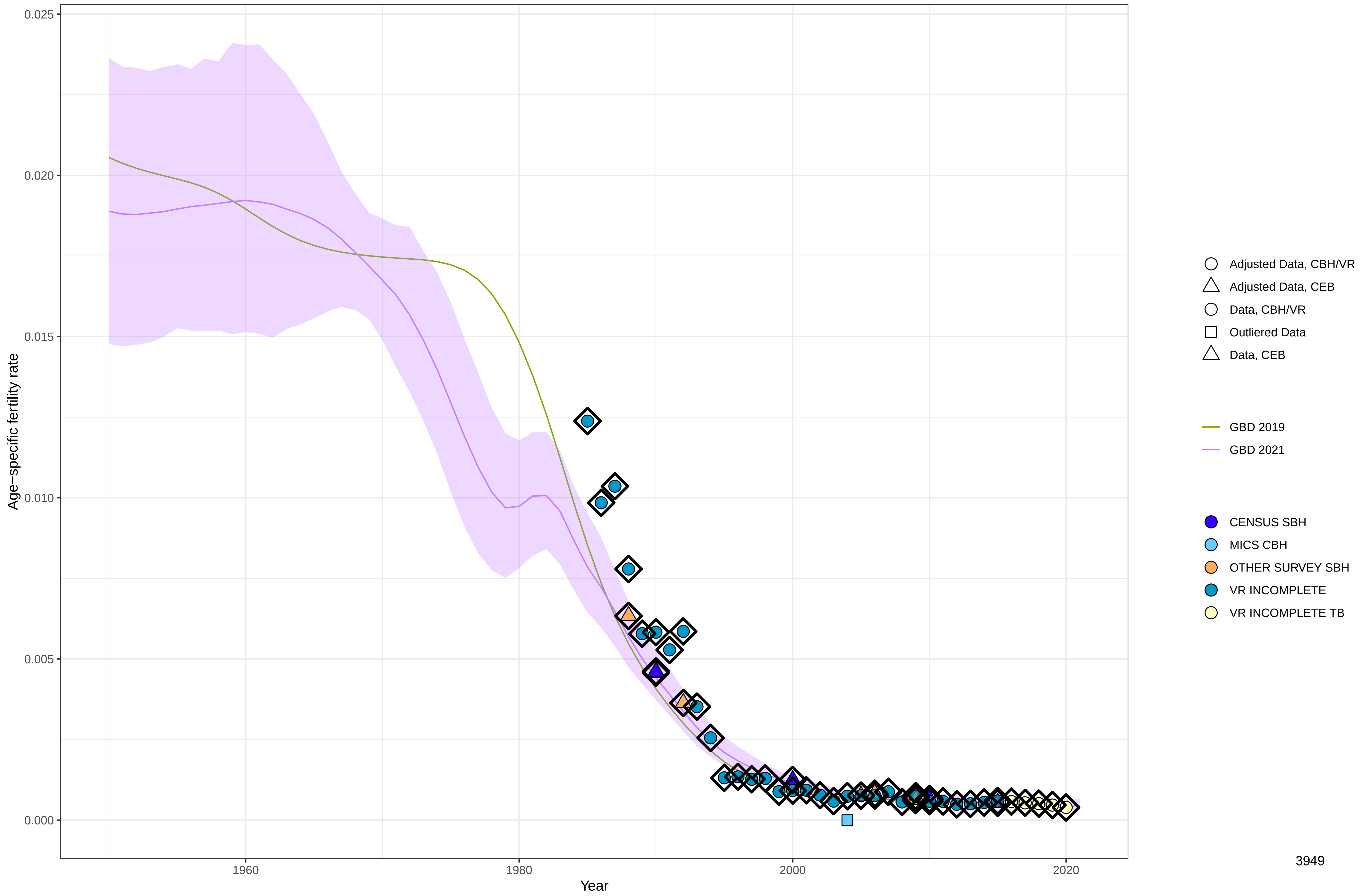

Age−specific fertility fitted trend lines, 1950−2021: Coahuila, Ages: 50−54
Data points enclosed with a diamond are reference sources

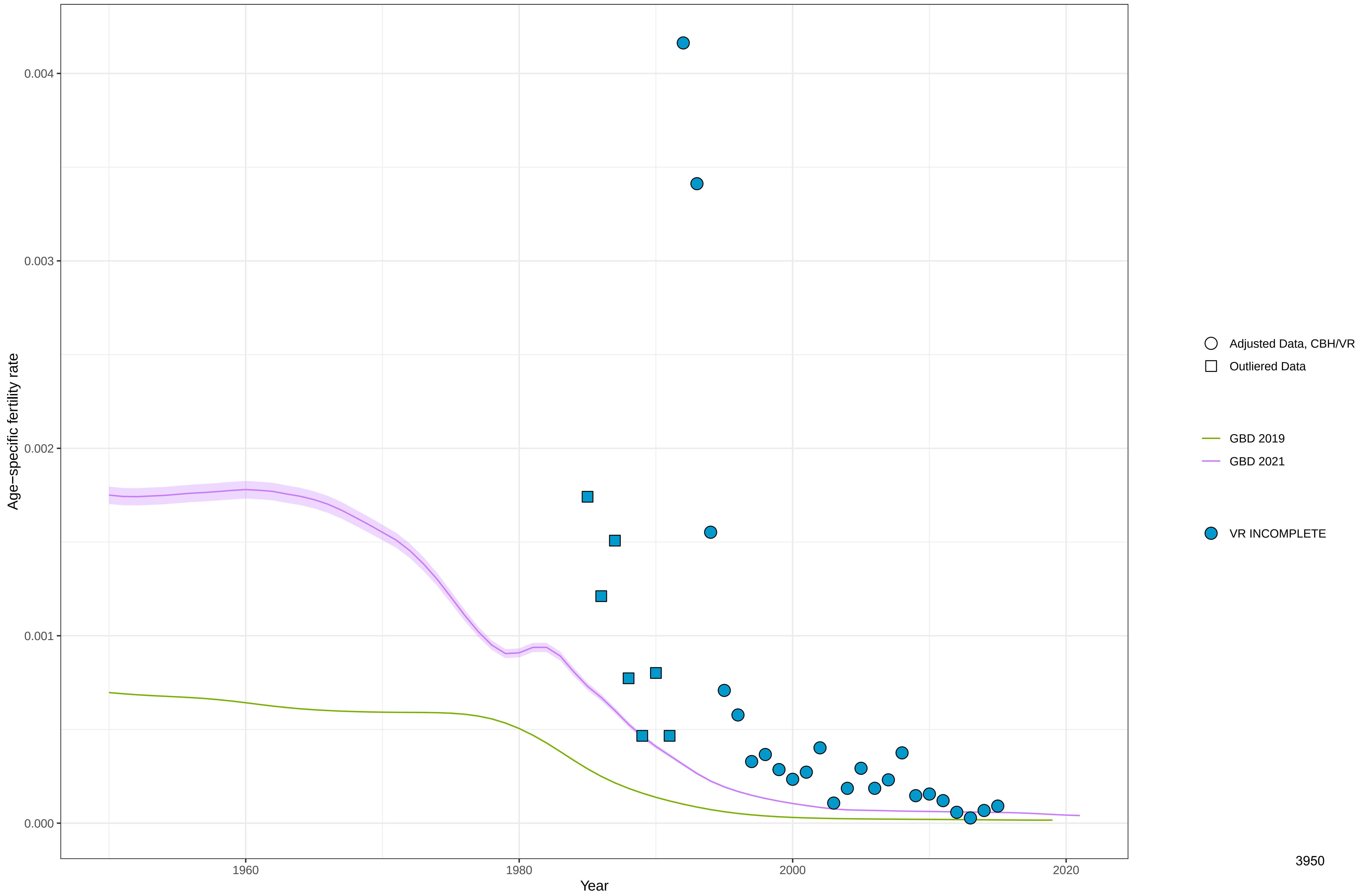

Total fertility rate fitted trend lines, 1950−2021: Coahuila TFR
Data points enclosed with a diamond are reference sources

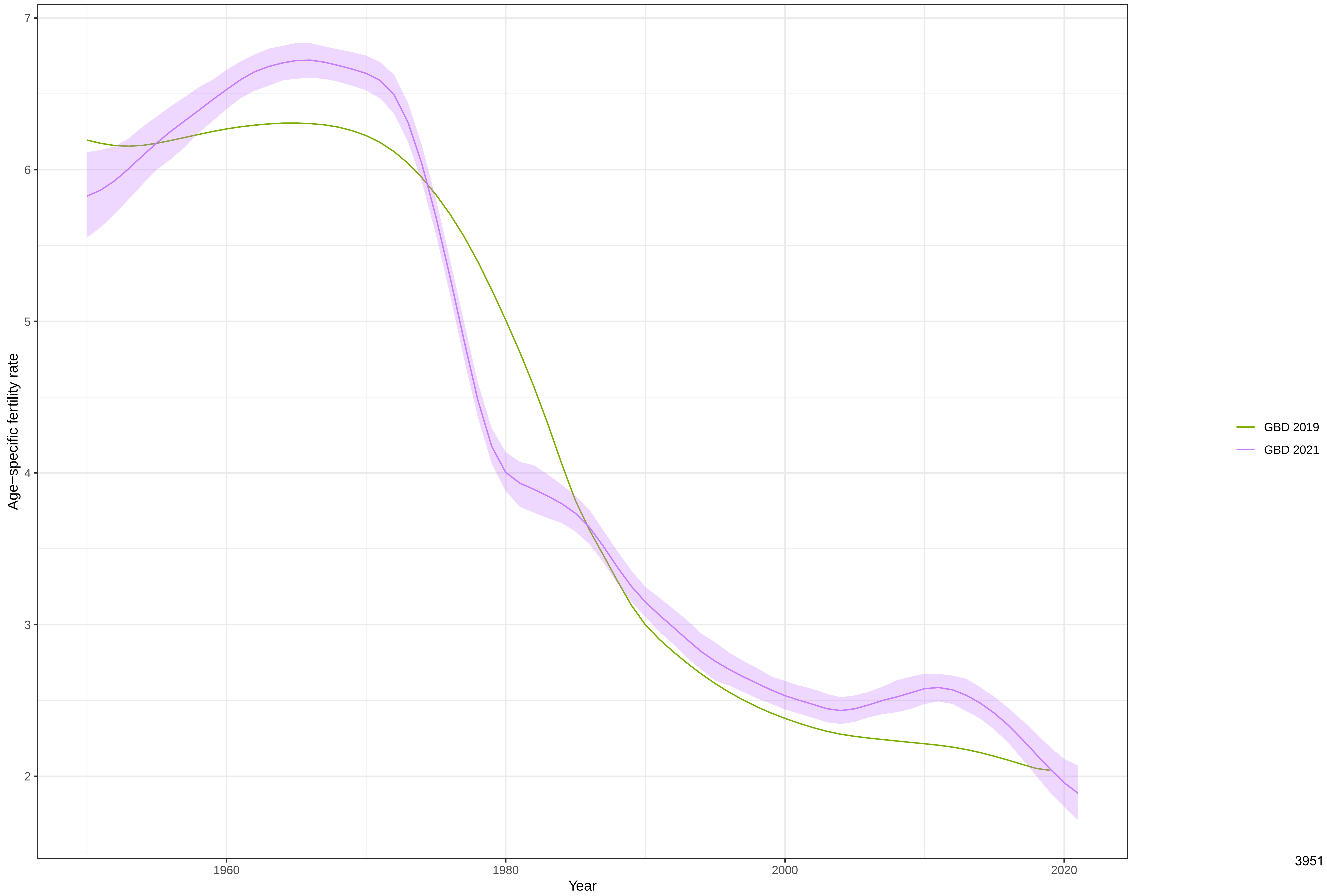

Age−specific fertility fitted trend lines, 1950−2021: Colima, Ages: 10−14
Data points enclosed with a diamond are reference sources

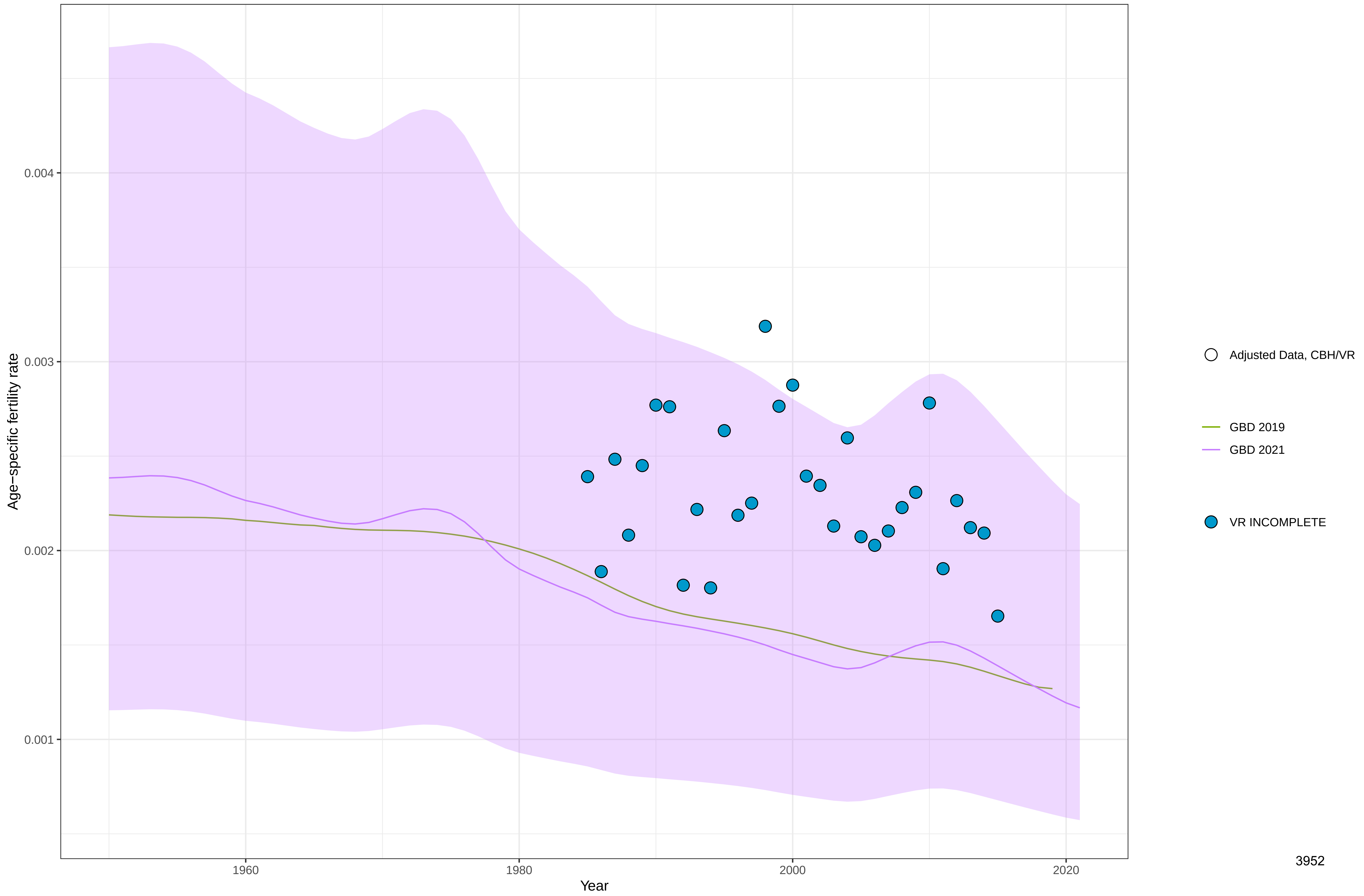

3952

Age−specific fertility fitted trend lines, 1950−2021: Colima, Ages: 15−19
Data points enclosed with a diamond are reference sources

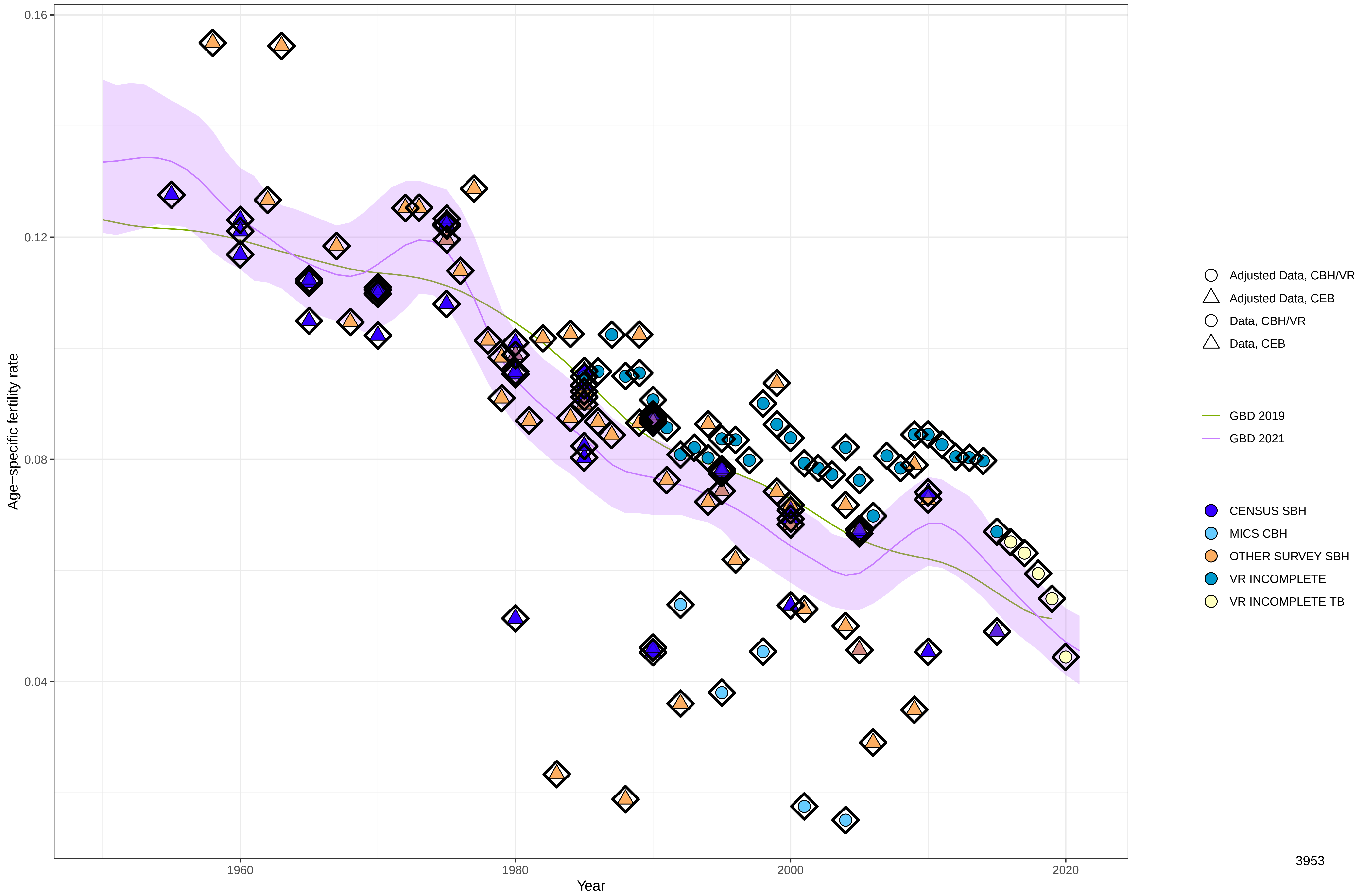

Age−specific fertility fitted trend lines, 1950−2021: Colima, Ages: 20−24
Data points enclosed with a diamond are reference sources

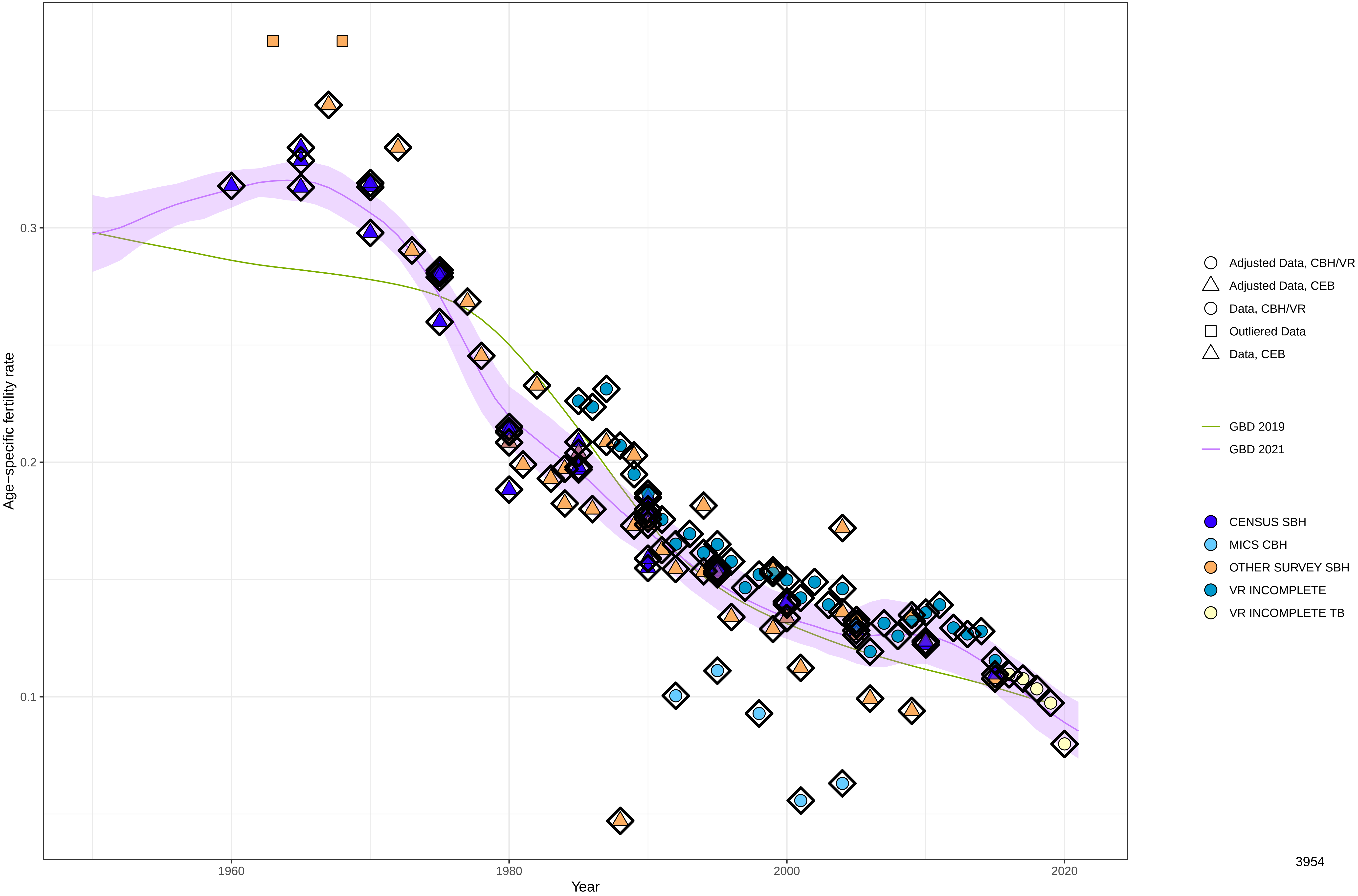

Age−specific fertility fitted trend lines, 1950−2021: Colima, Ages: 25−29
Data points enclosed with a diamond are reference sources

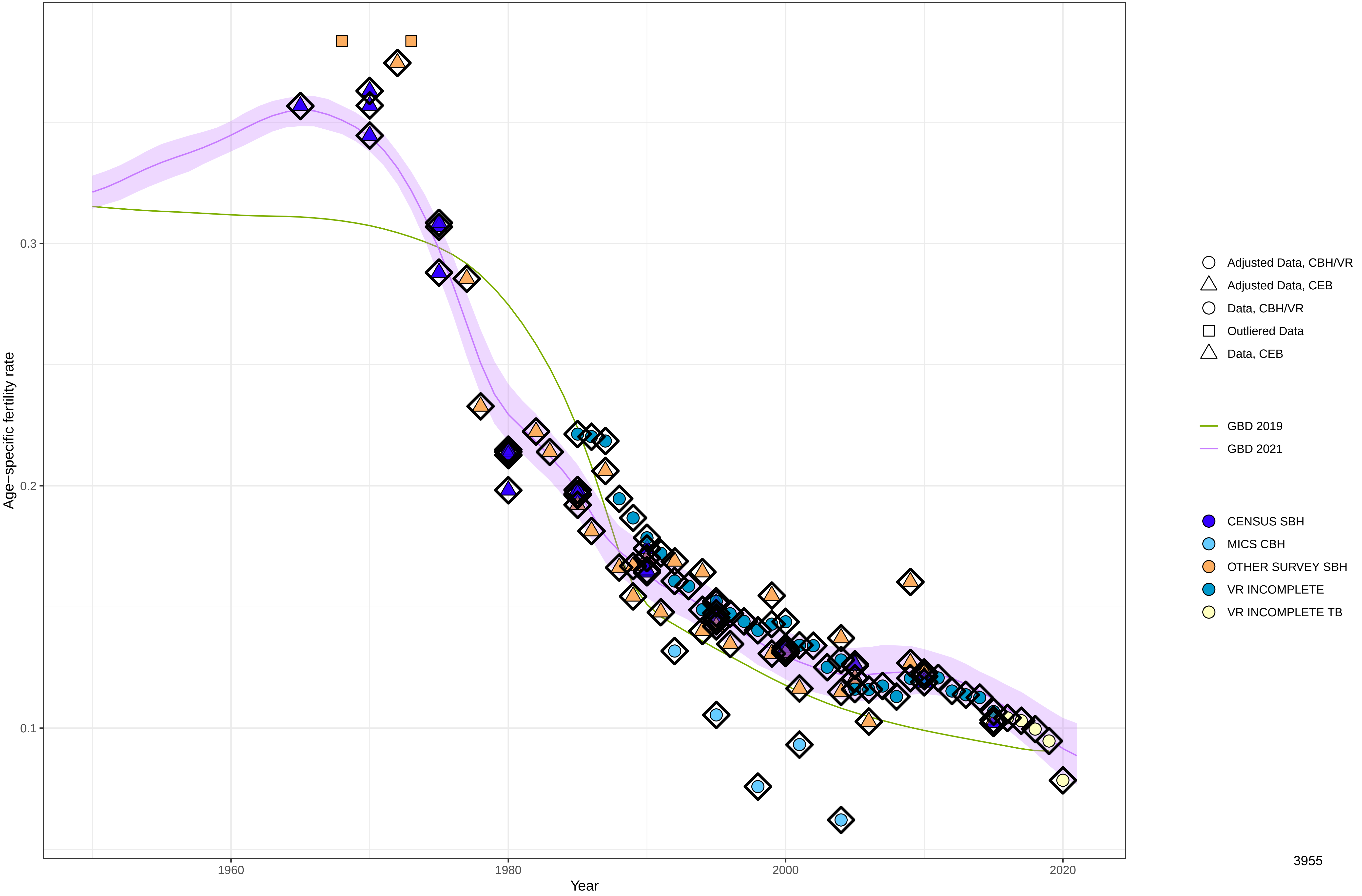

Age−specific fertility fitted trend lines, 1950−2021: Colima, Ages: 30−34
Data points enclosed with a diamond are reference sources

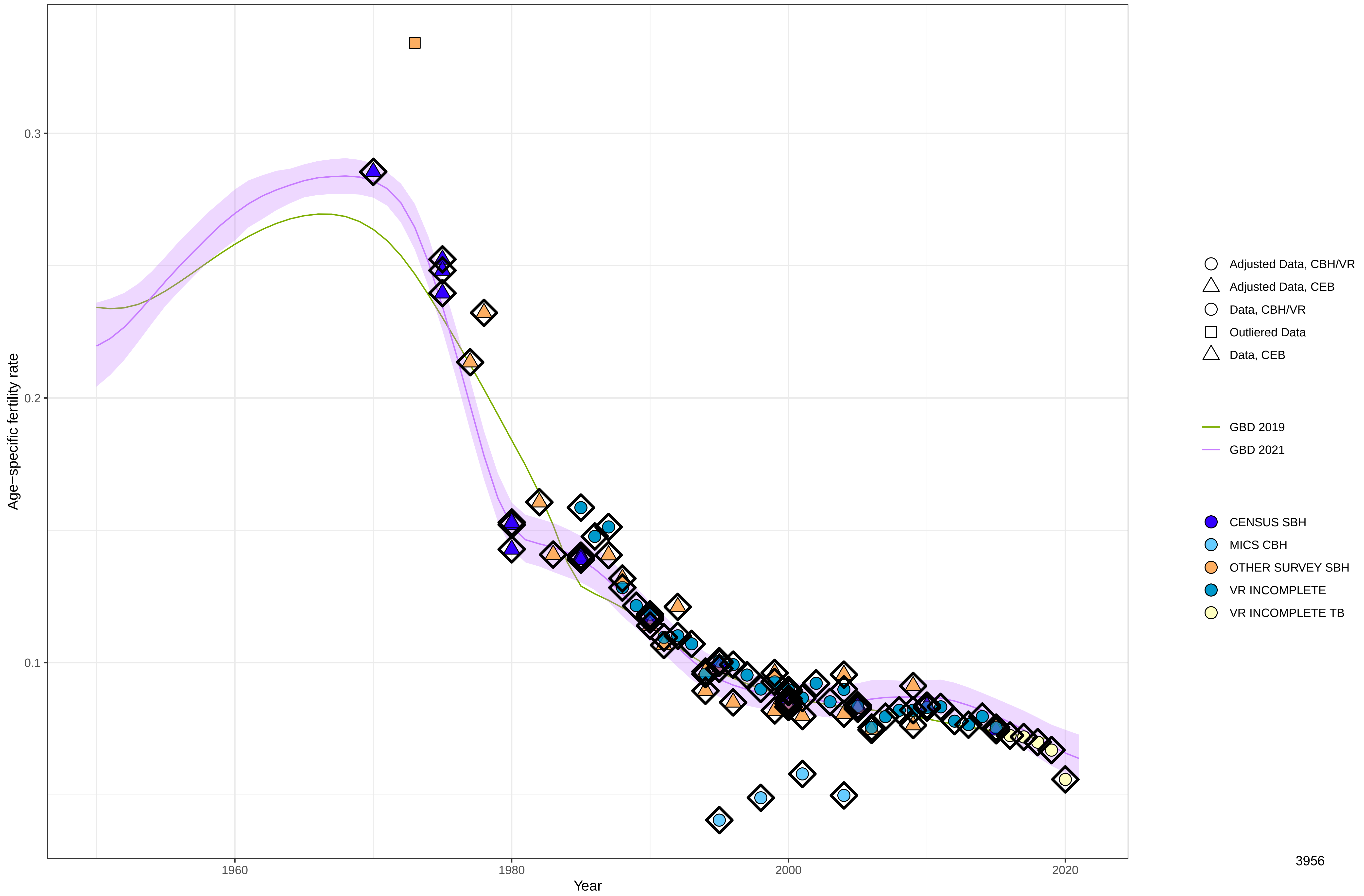

Age−specific fertility fitted trend lines, 1950−2021: Colima, Ages: 35−39
Data points enclosed with a diamond are reference sources

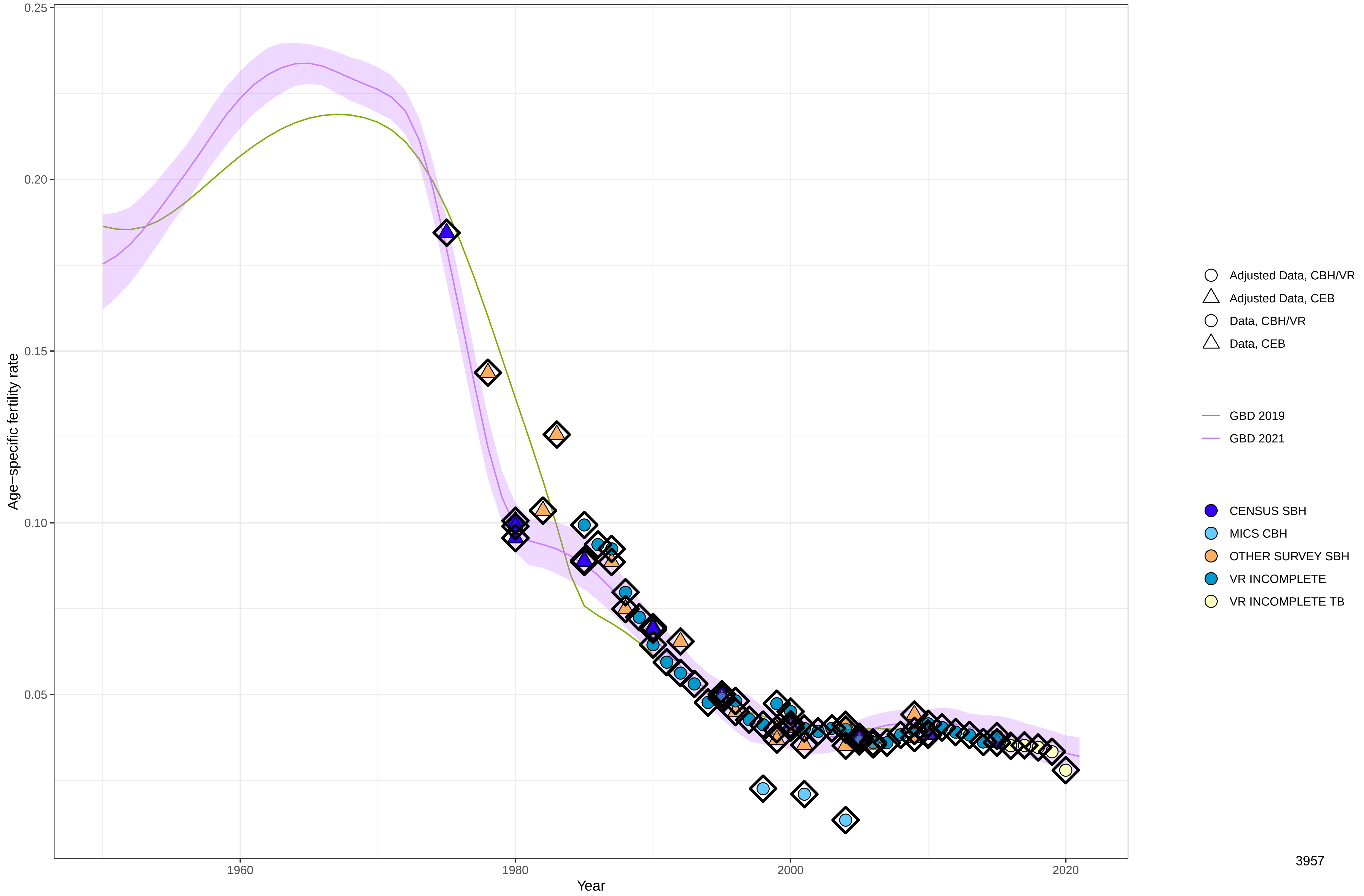

Age−specific fertility fitted trend lines, 1950−2021: Colima, Ages: 40−44
Data points enclosed with a diamond are reference sources

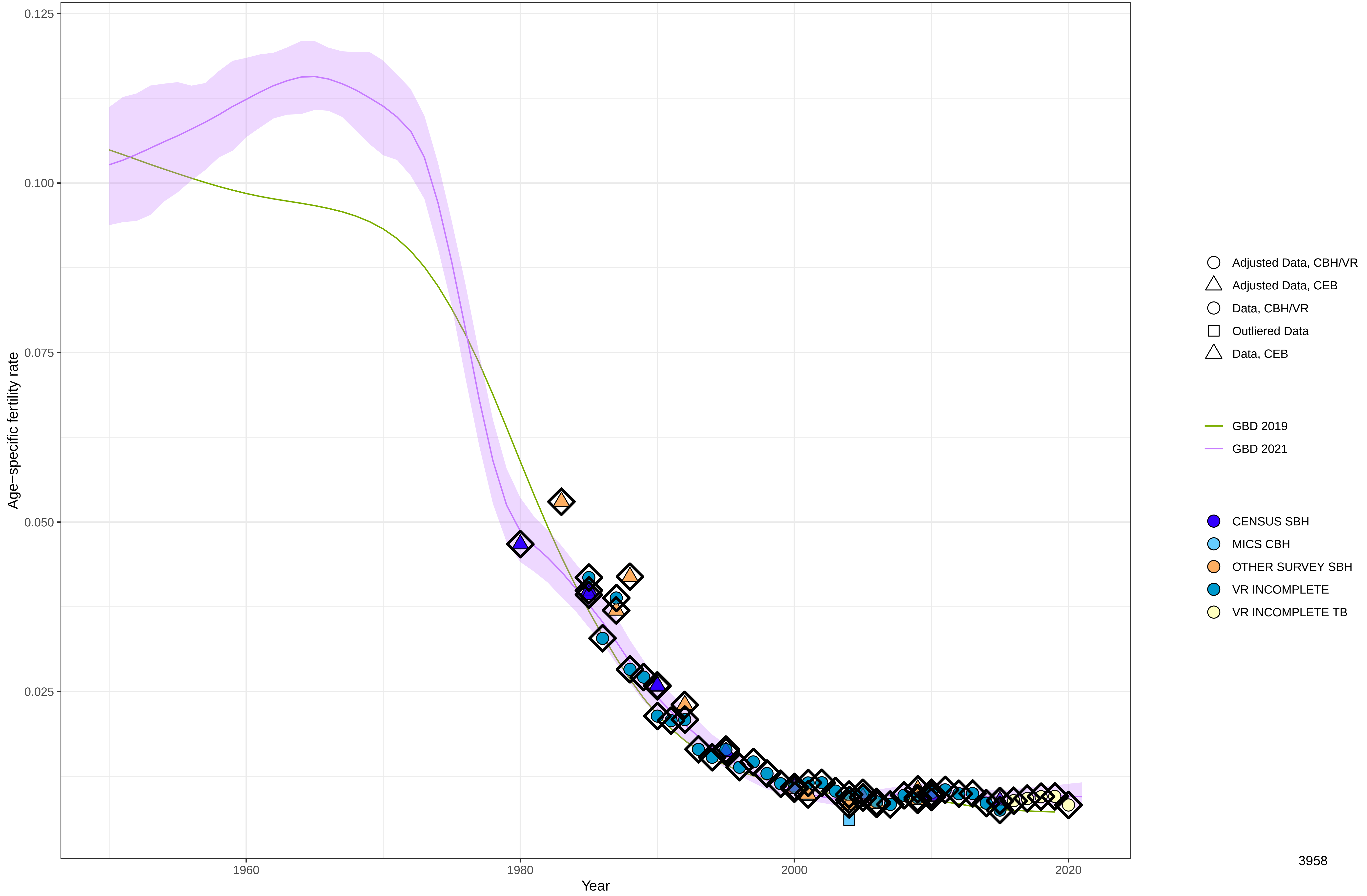

3958

Age−specific fertility fitted trend lines, 1950−2021: Colima, Ages: 45−49
Data points enclosed with a diamond are reference sources

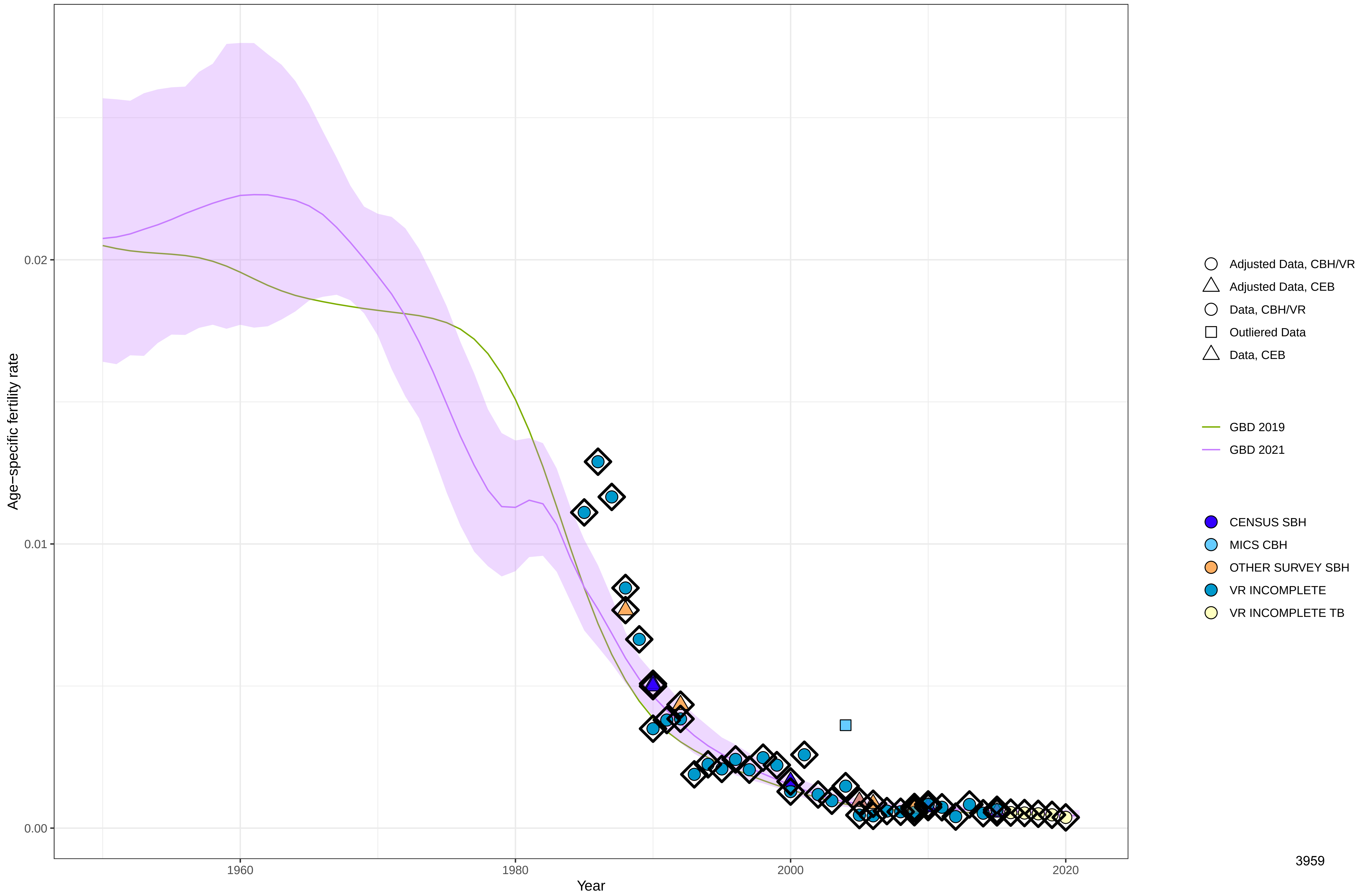

Age−specific fertility fitted trend lines, 1950−2021: Colima, Ages: 50−54
Data points enclosed with a diamond are reference sources

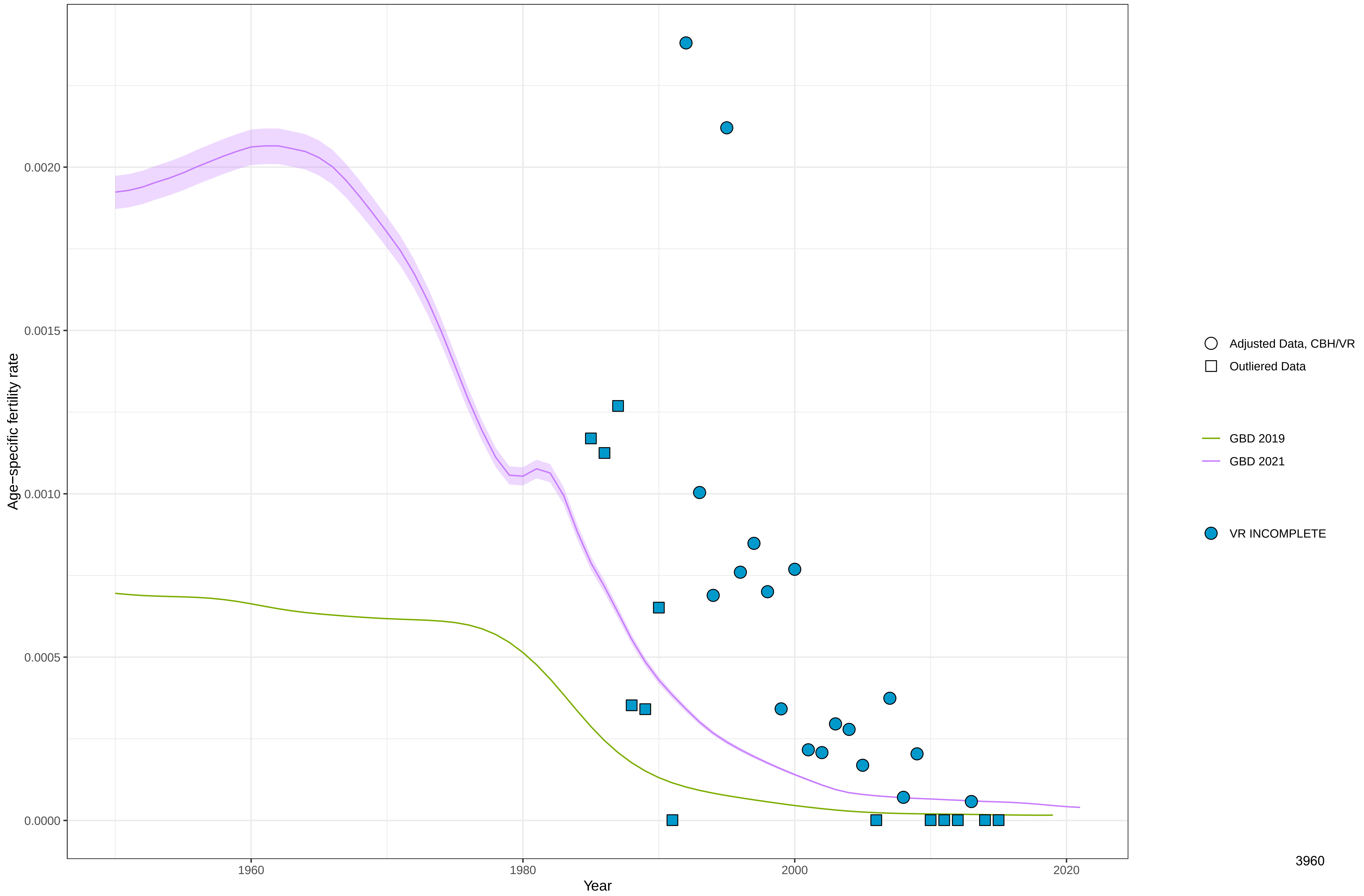

Total fertility rate fitted trend lines, 1950−2021: Colima TFR
Data points enclosed with a diamond are reference sources

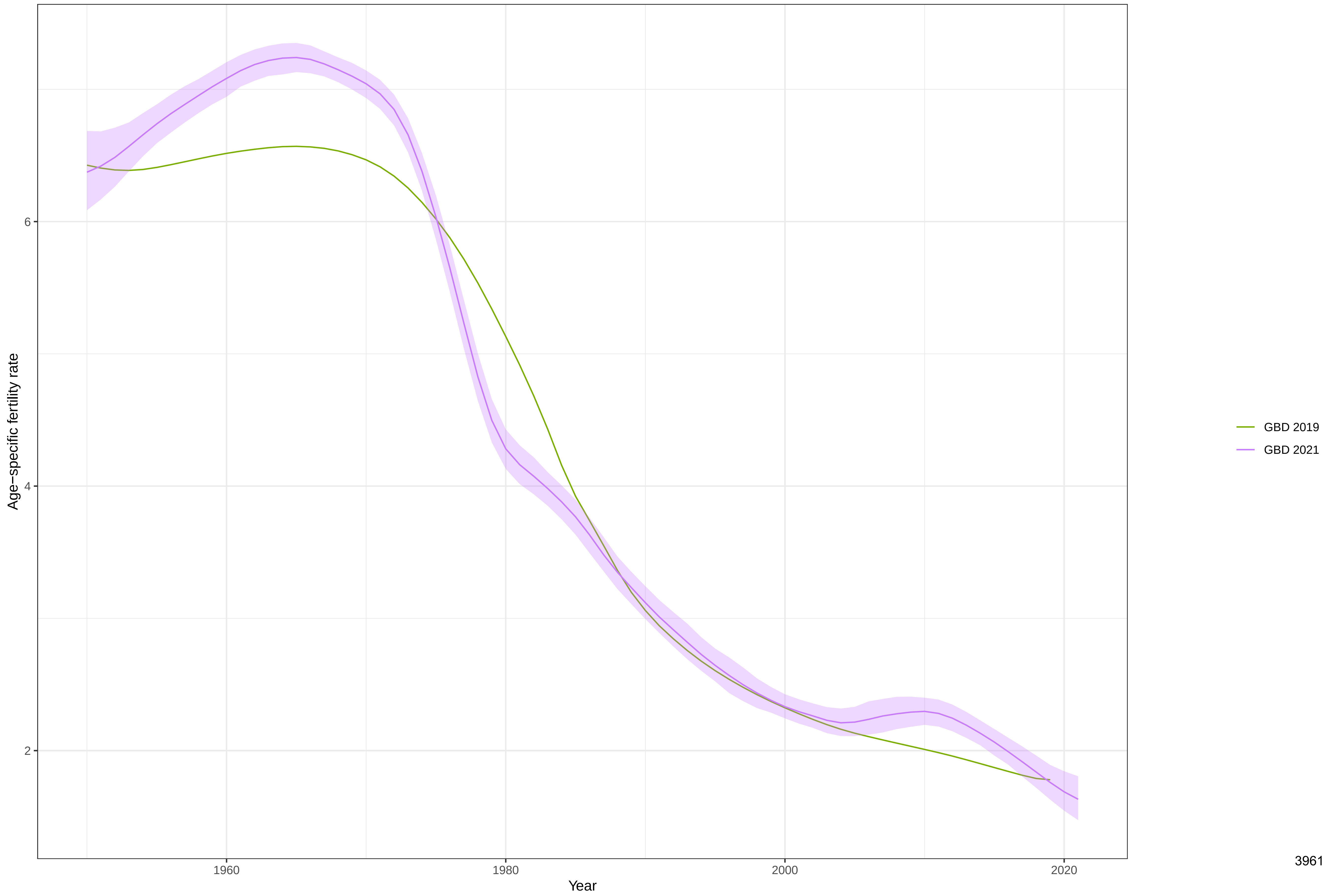

Age−specific fertility fitted trend lines, 1950−2021: Durango, Ages: 10−14
Data points enclosed with a diamond are reference sources

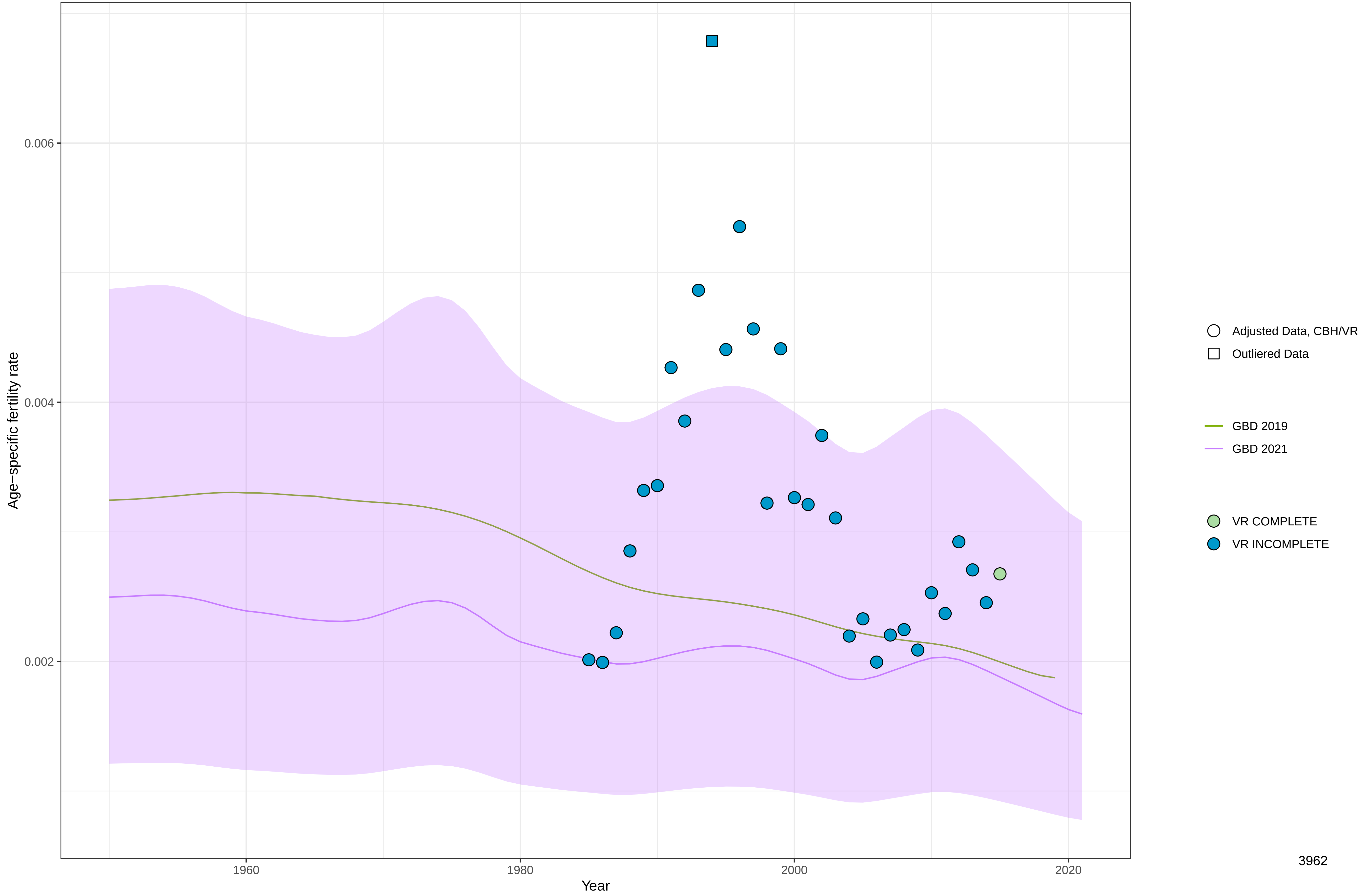

Age−specific fertility fitted trend lines, 1950−2021: Durango, Ages: 15−19
Data points enclosed with a diamond are reference sources

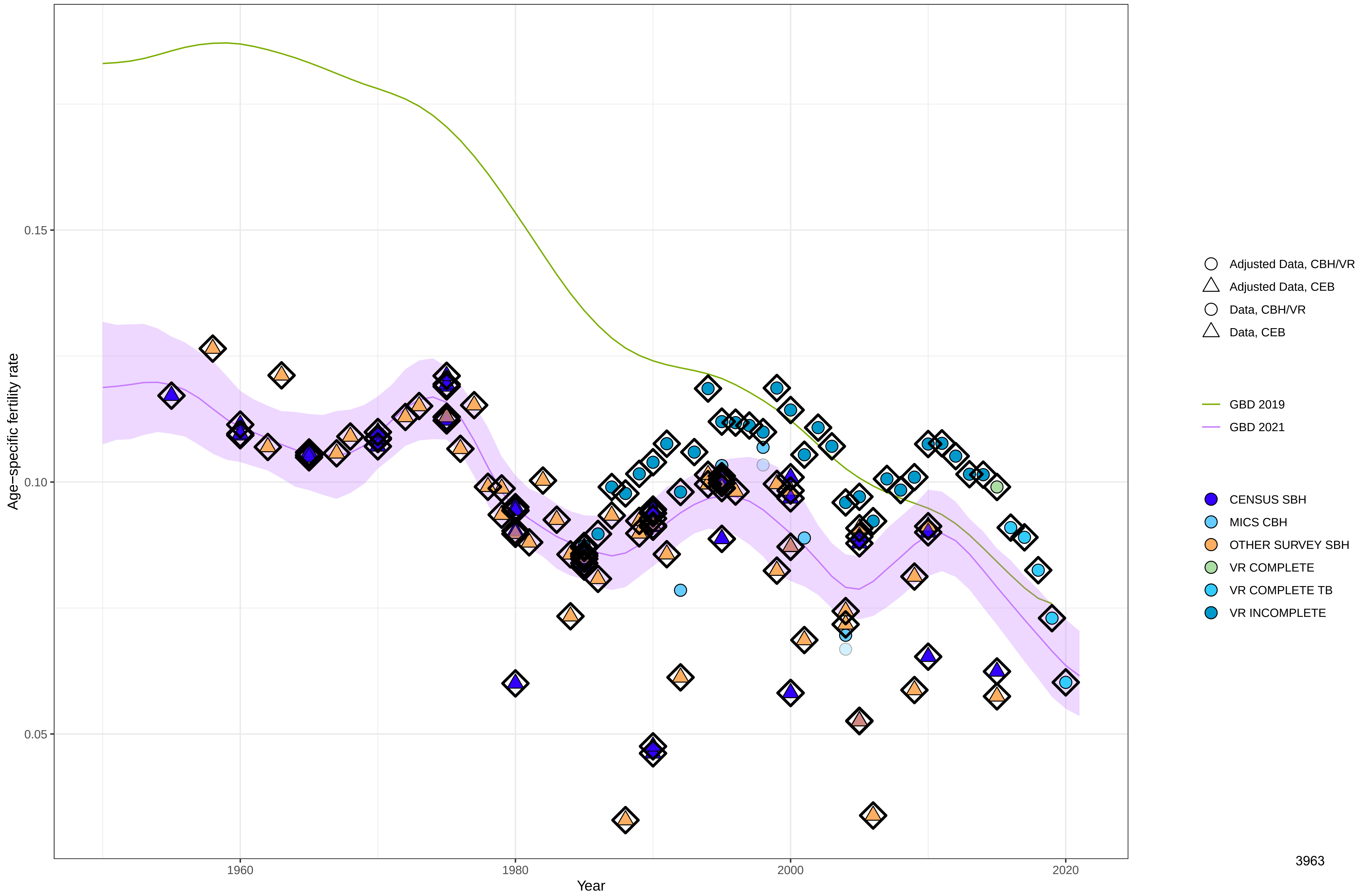

Age−specific fertility fitted trend lines, 1950−2021: Durango, Ages: 20−24
Data points enclosed with a diamond are reference sources

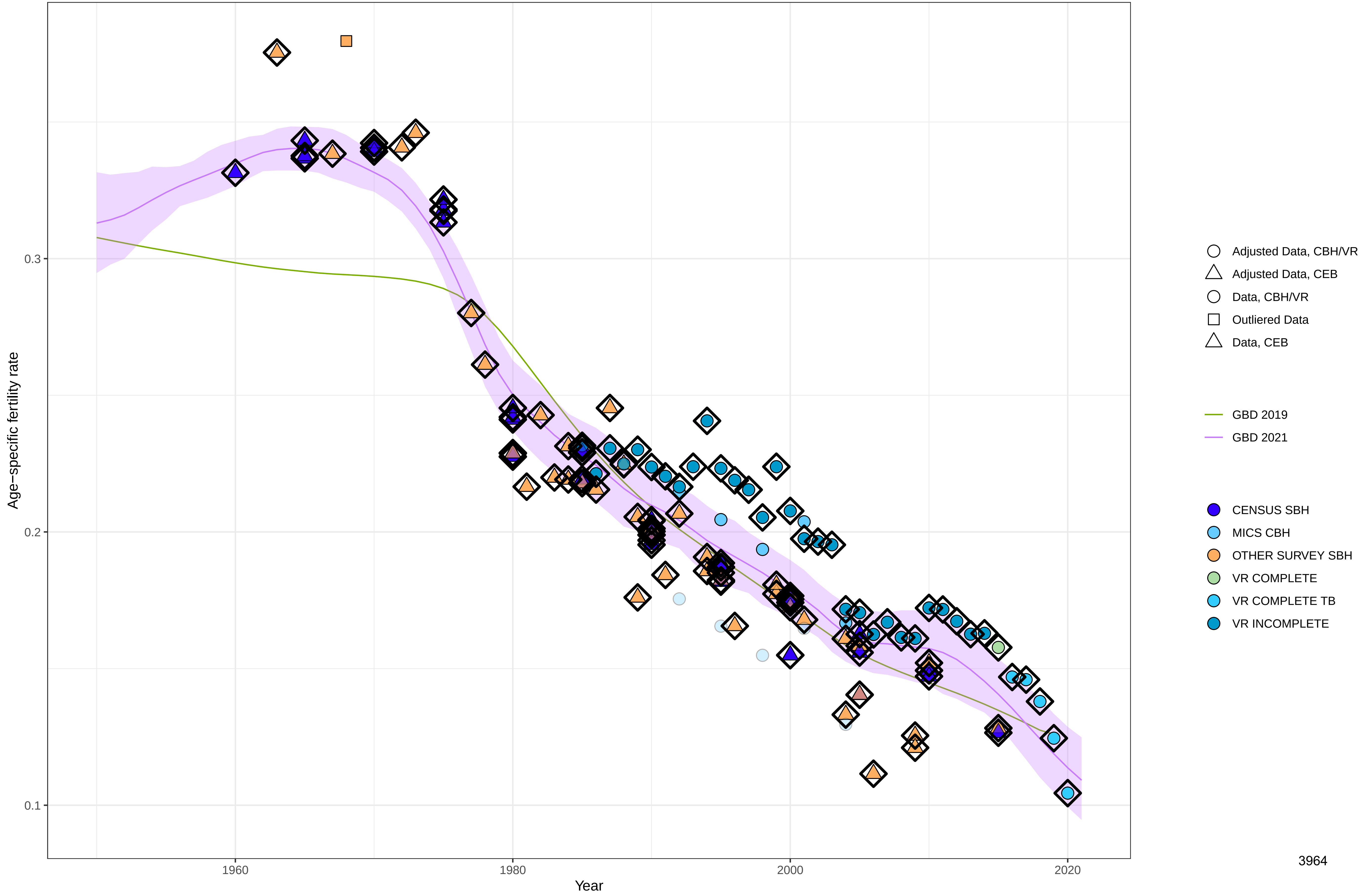

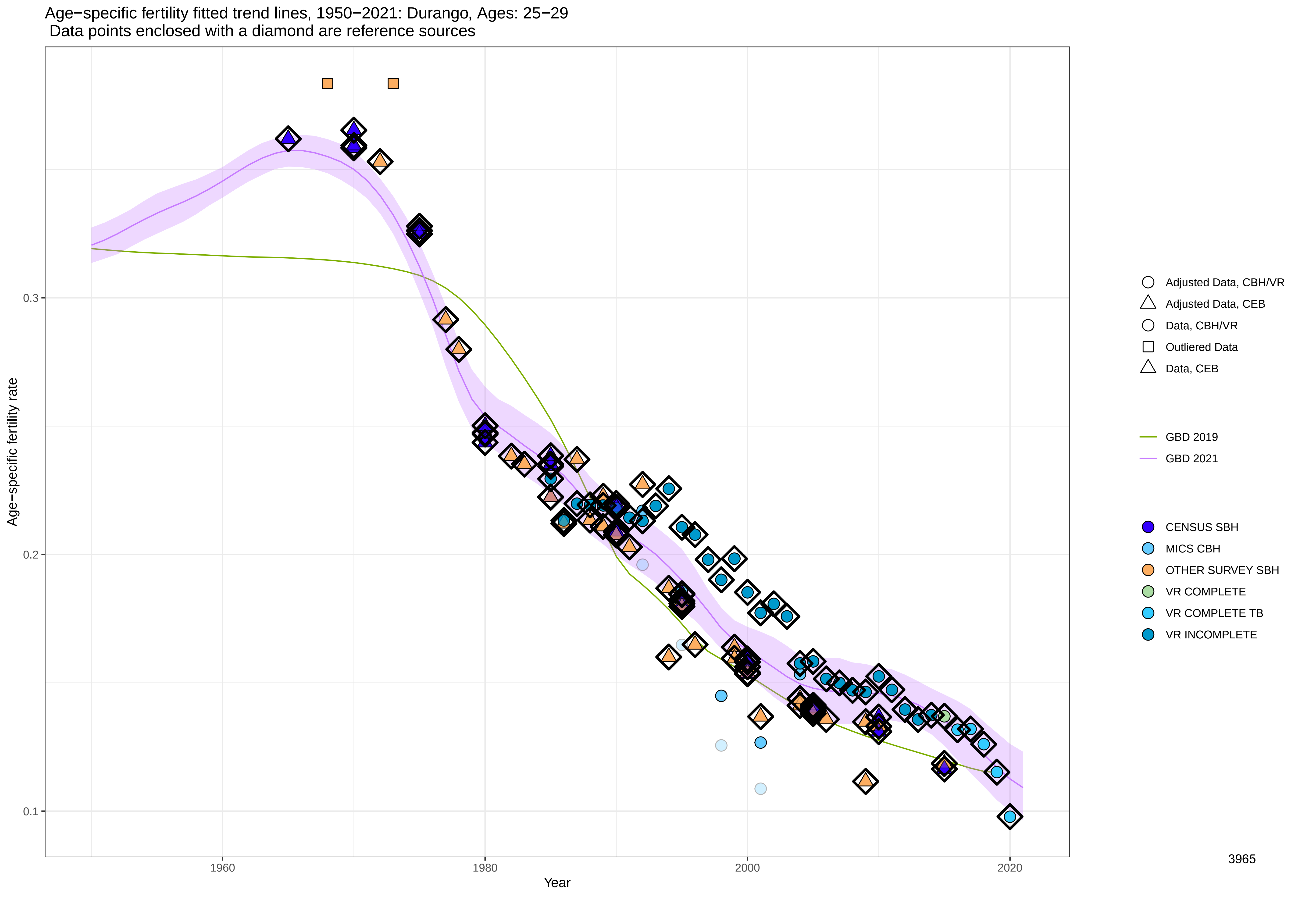

Age−specific fertility fitted trend lines, 1950−2021: Durango, Ages: 25−29
Data points enclosed with a diamond are reference sources
Age−specific fertility rate
0.1
0.2
0.3
Year
1960
1980
2000
2020
Adjusted Data, CBH/VR
Adjusted Data, CEB
Data, CBH/VR
Outliered Data
Data, CEB
GBD 2019
GBD 2021
CENSUS SBH
MICS CBH
OTHER SURVEY SBH
VR COMPLETE
VR COMPLETE TB
VR INCOMPLETE

3965

Age−specific fertility fitted trend lines, 1950−2021: Durango, Ages: 30−34
Data points enclosed with a diamond are reference sources

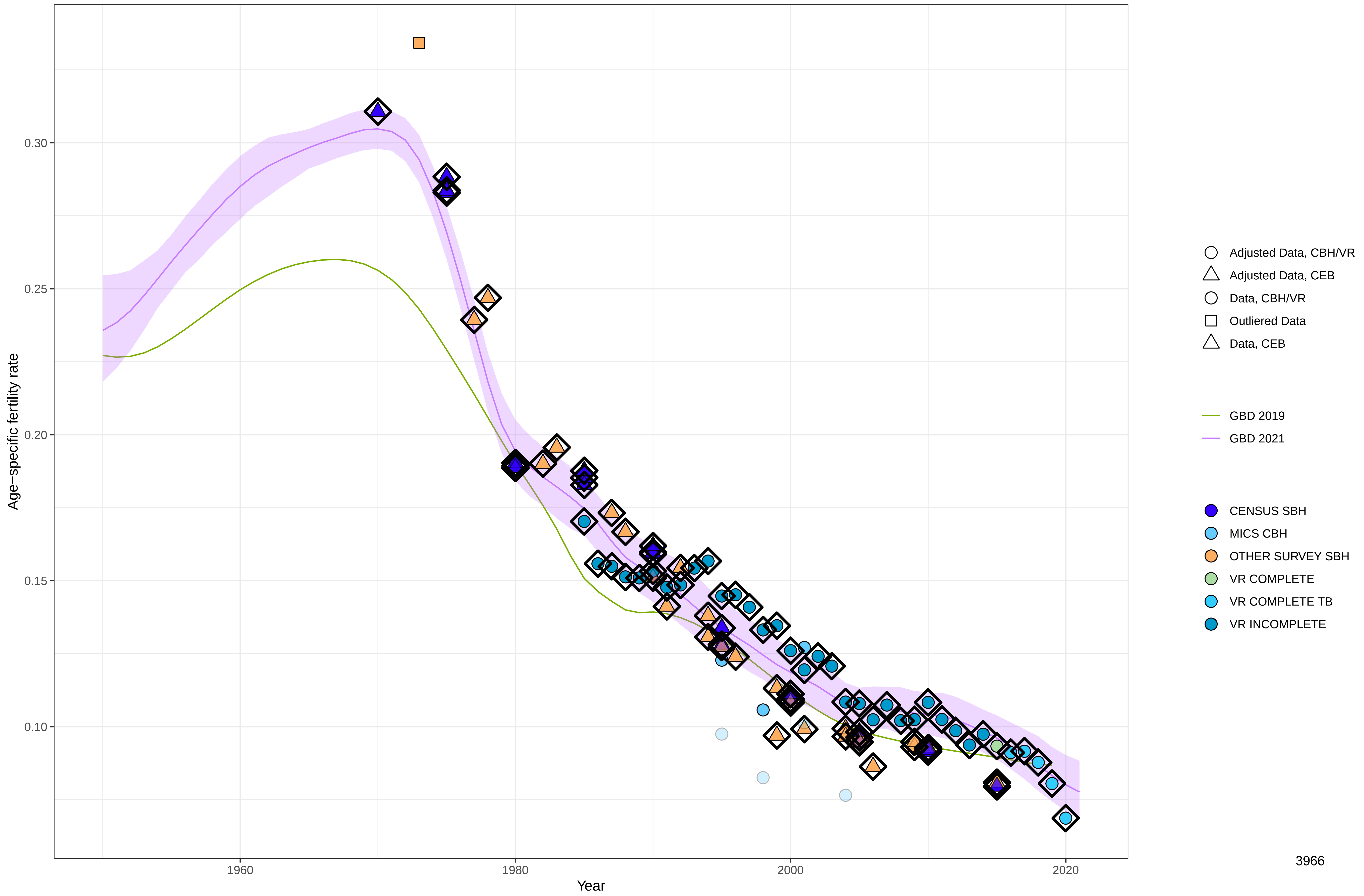

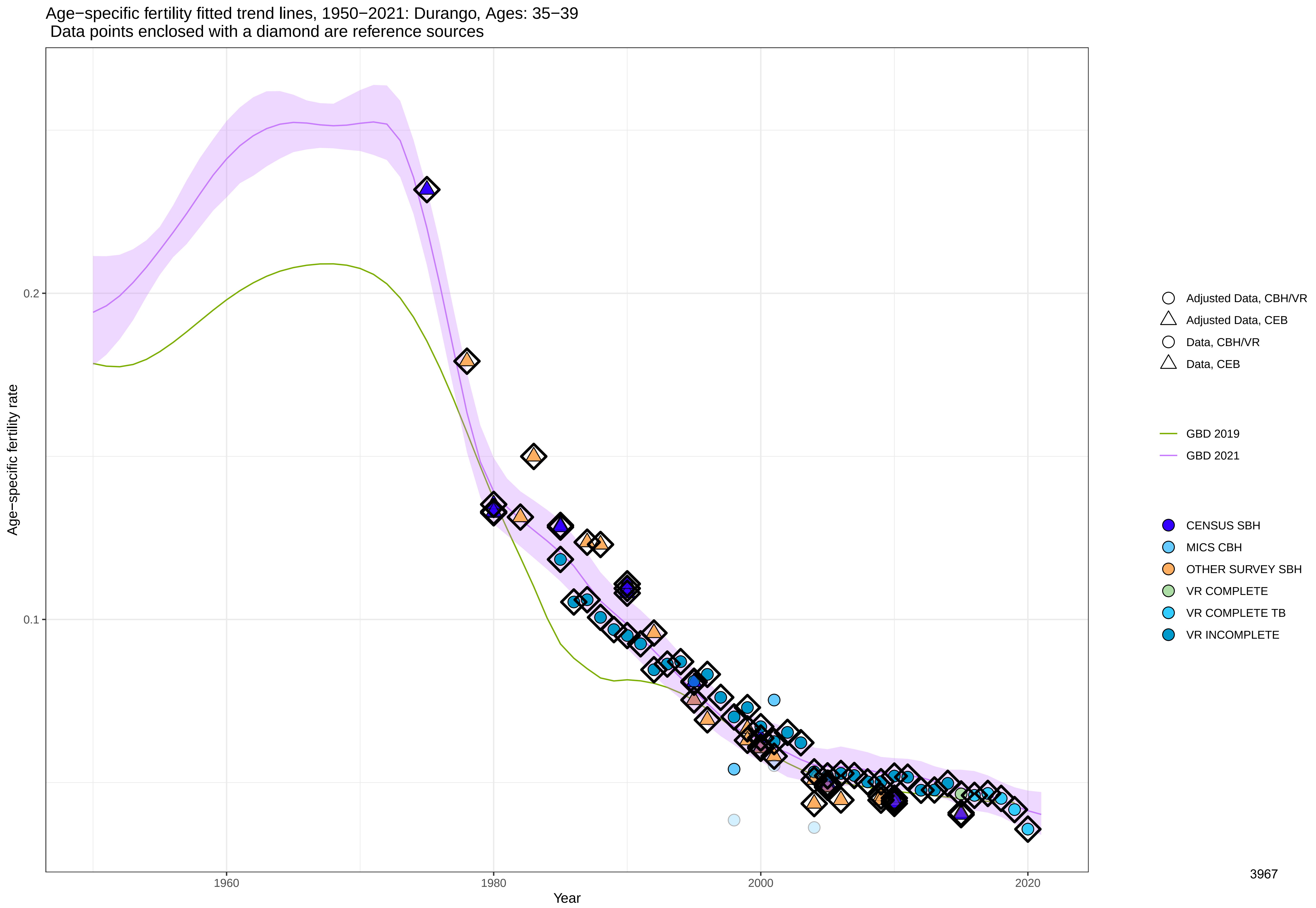

Age−specific fertility fitted trend lines, 1950−2021: Durango, Ages: 35−39
Data points enclosed with a diamond are reference sources
Age−specific fertility rate
Year
0.1
0.2
1960
1980
2000
2020
Adjusted Data, CBH/VR
Adjusted Data, CEB
Data, CBH/VR
Data, CEB
GBD 2019
GBD 2021
CENSUS SBH
MICS CBH
OTHER SURVEY SBH
VR COMPLETE
VR COMPLETE TB
VR INCOMPLETE

Age−specific fertility fitted trend lines, 1950−2021: Durango, Ages: 40−44
Data points enclosed with a diamond are reference sources

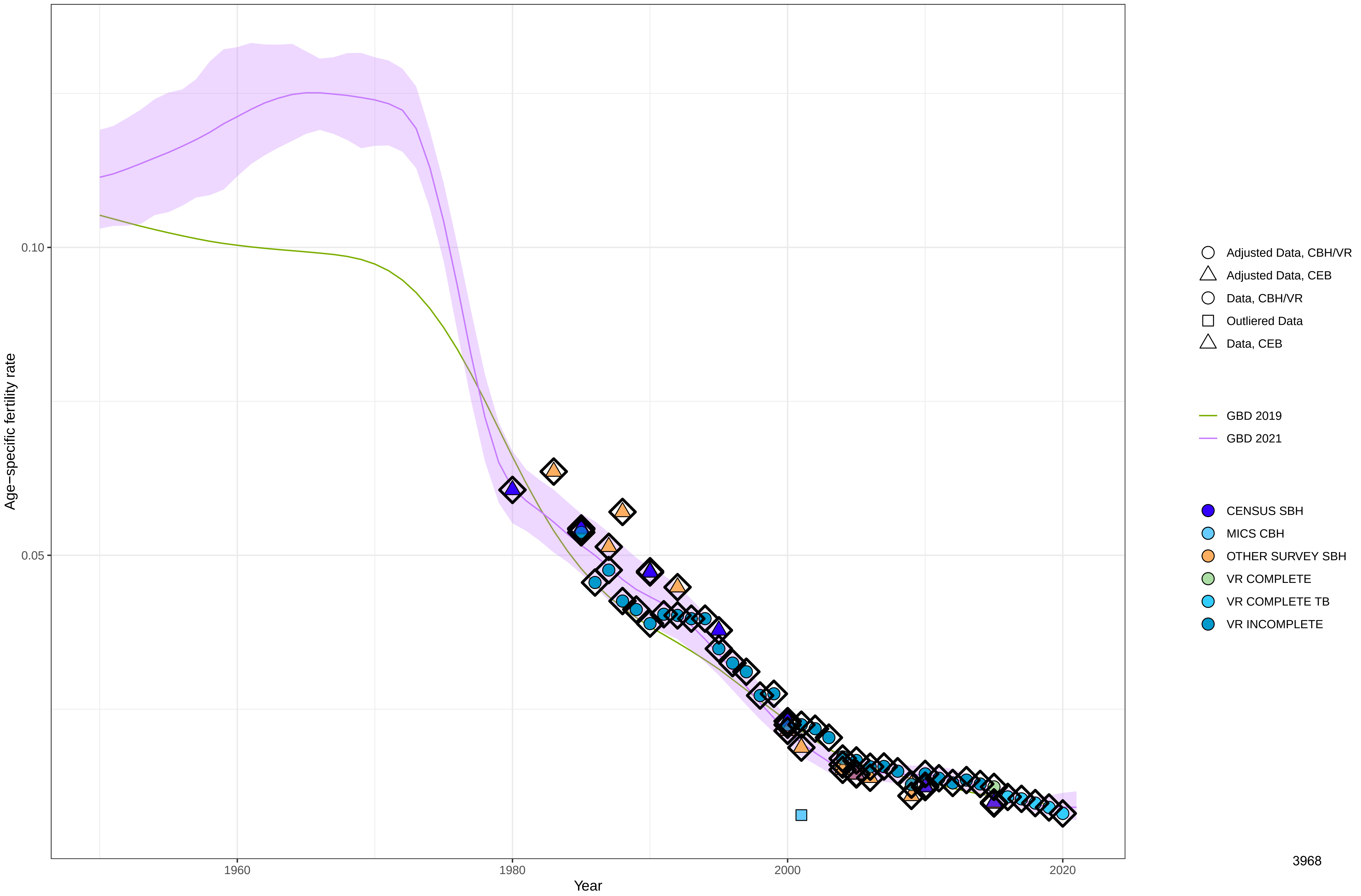

Age−specific fertility fitted trend lines, 1950−2021: Durango, Ages: 45−49
Data points enclosed with a diamond are reference sources

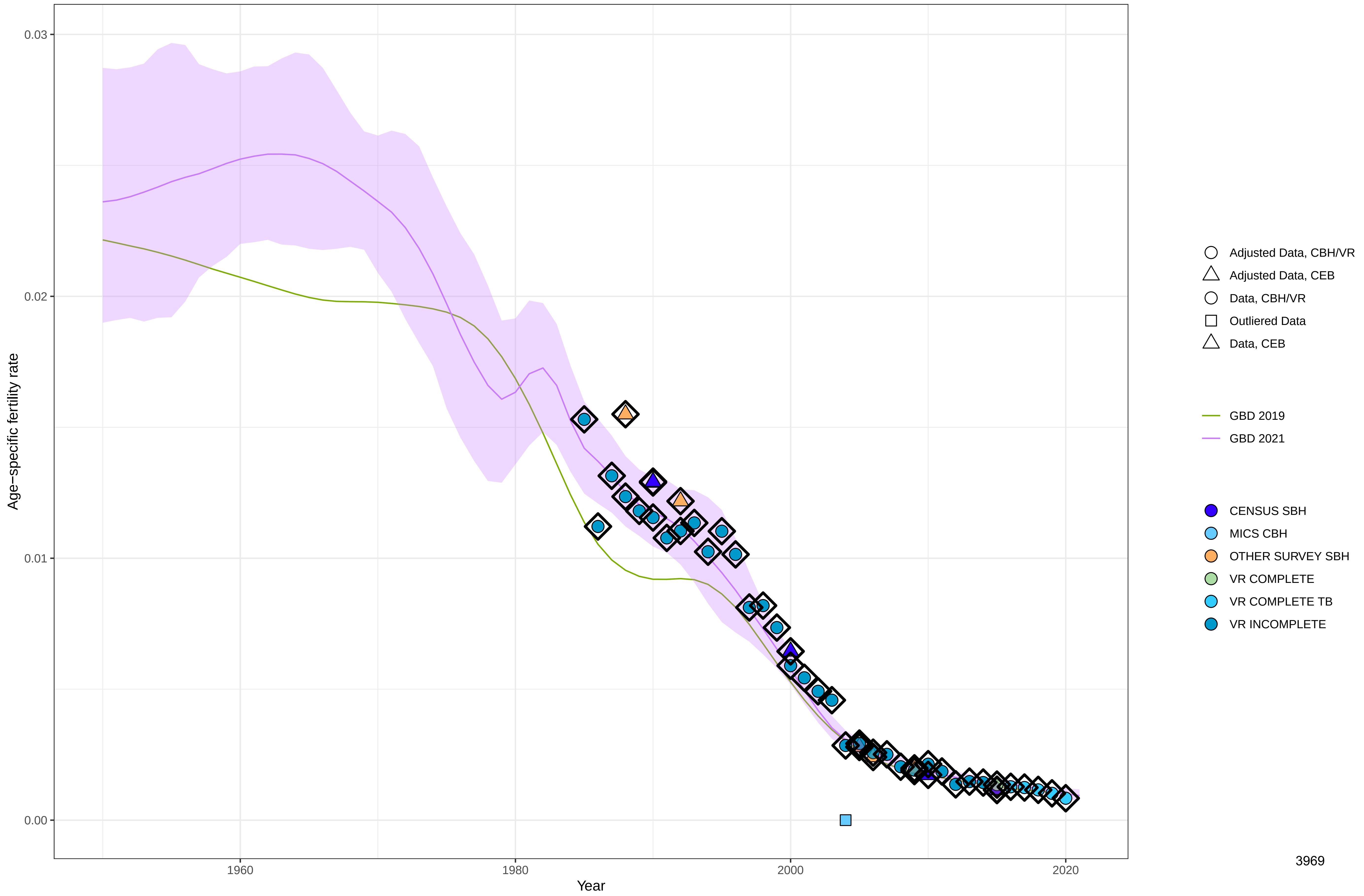

Age−specific fertility fitted trend lines, 1950−2021: Durango, Ages: 50−54
Data points enclosed with a diamond are reference sources

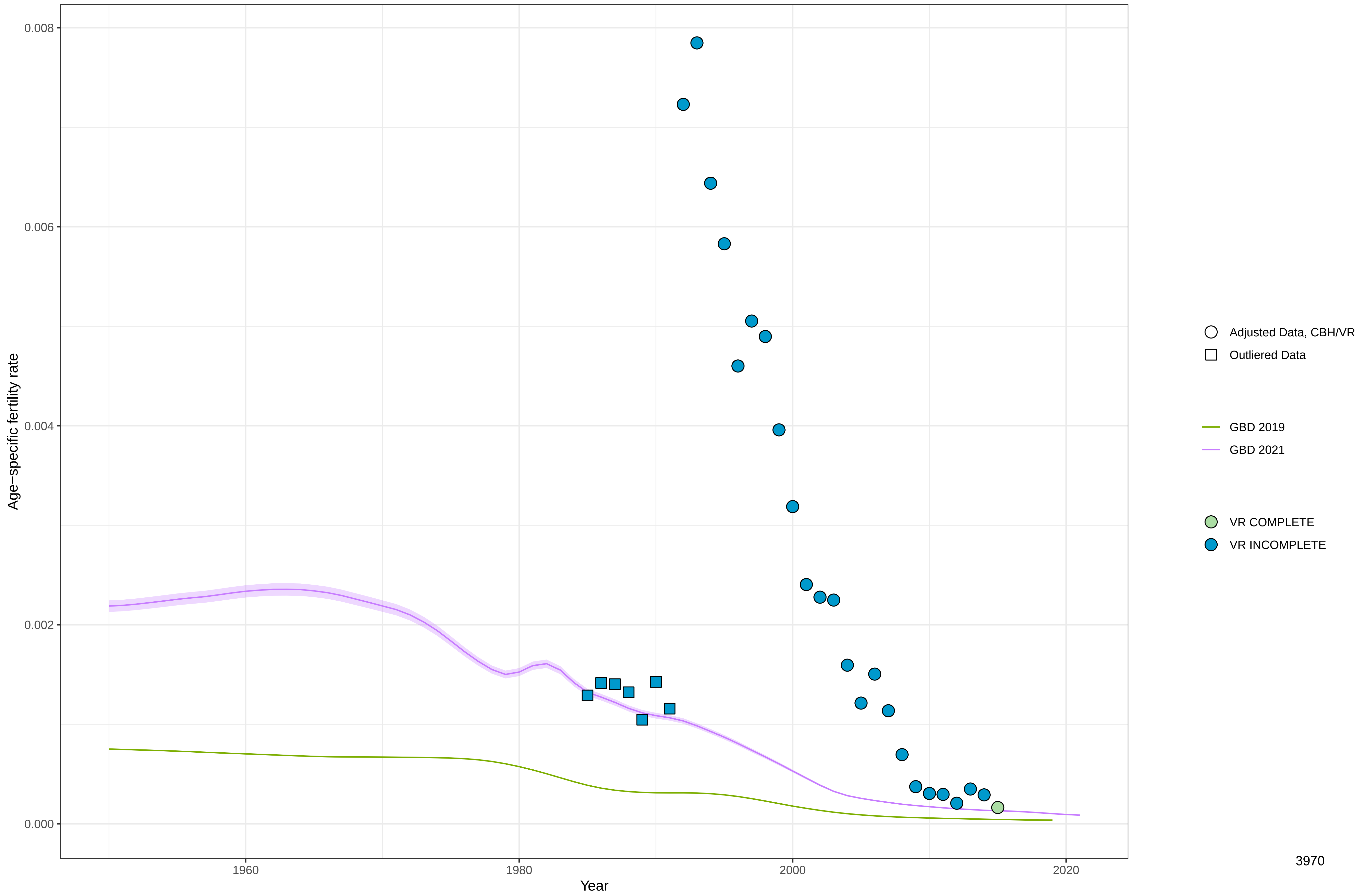

Total fertility rate fitted trend lines, 1950−2021: Durango TFR
Data points enclosed with a diamond are reference sources

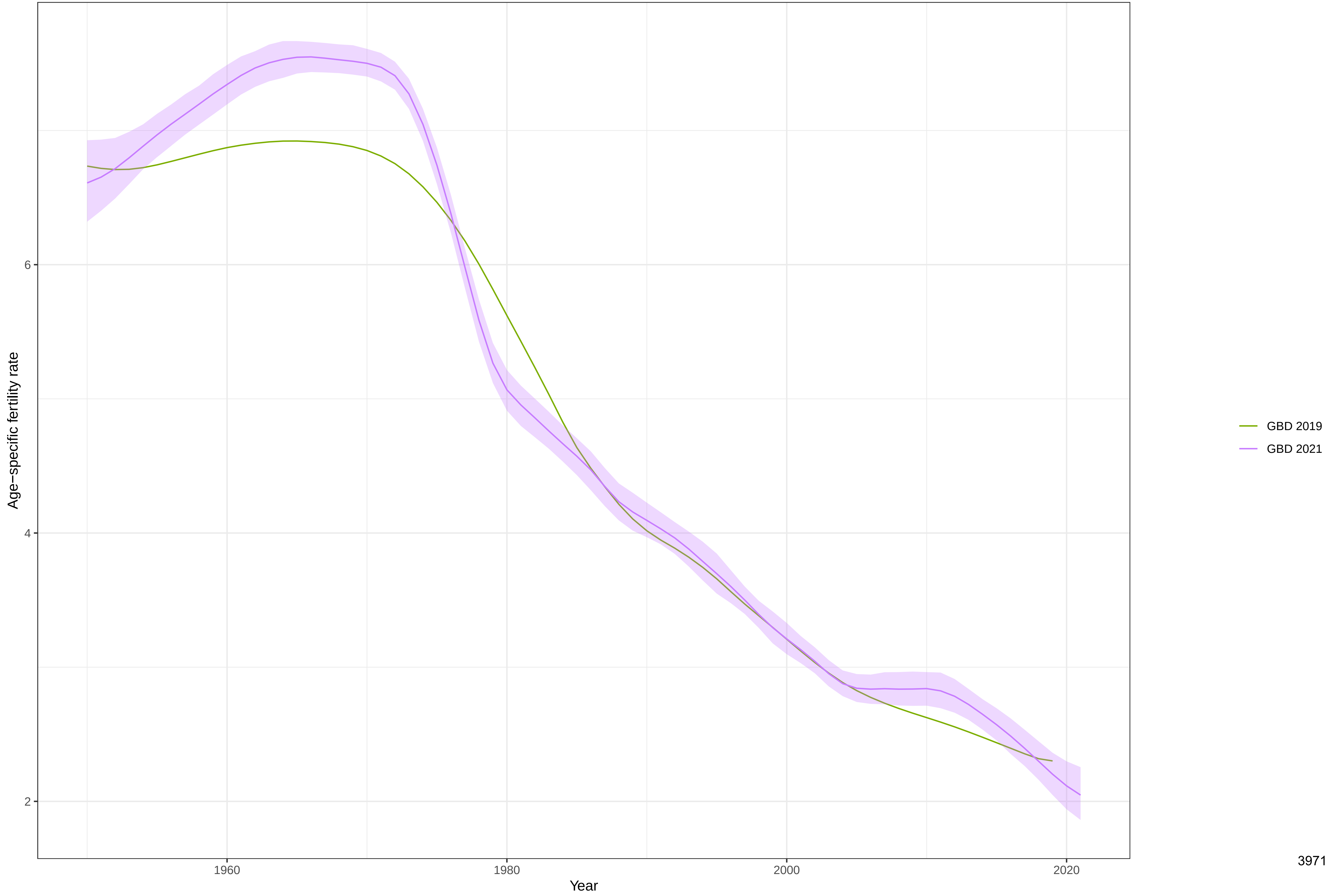

Age−specific fertility fitted trend lines, 1950−2021: Guanajuato, Ages: 10−14
Data points enclosed with a diamond are reference sources

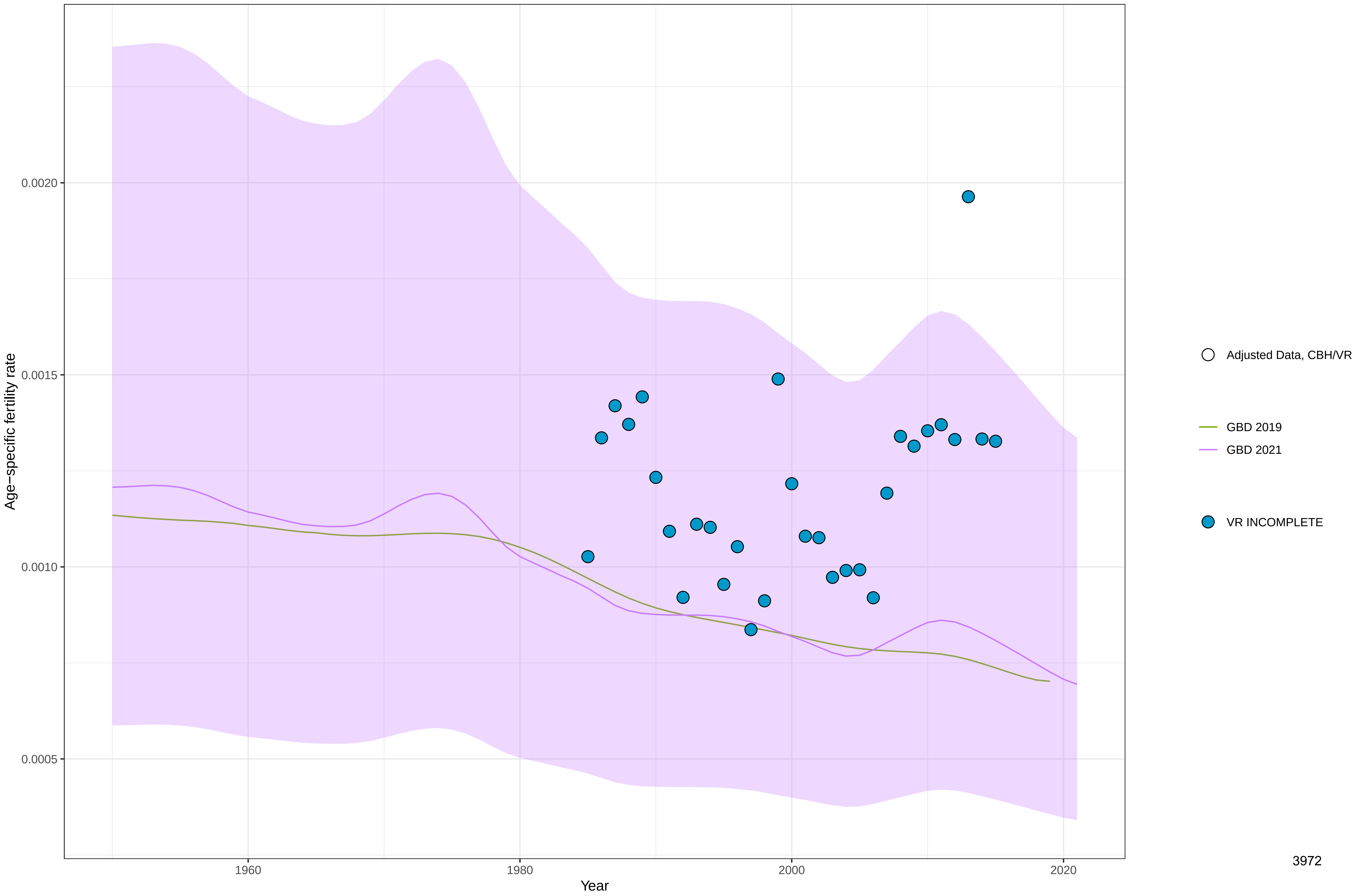

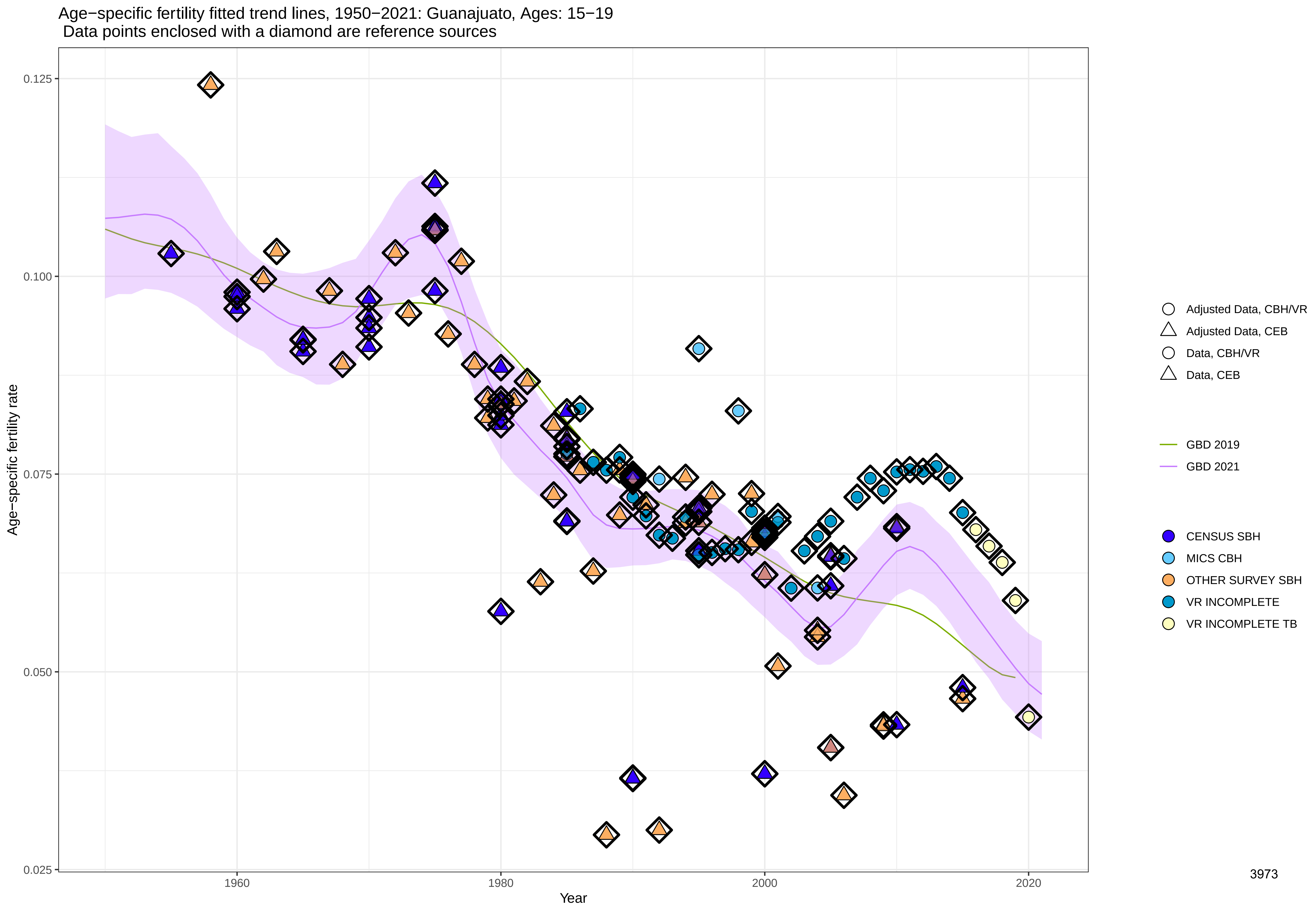

Age−specific fertility fitted trend lines, 1950−2021: Guanajuato, Ages: 15−19
Data points enclosed with a diamond are reference sources
Age−specific fertility rate
Year
0.125
0.100
0.075
0.050
0.025
1960
1980
2000
2020
Adjusted Data, CBH/VR
Adjusted Data, CEB
Data, CBH/VR
Data, CEB
GBD 2019
GBD 2021
CENSUS SBH
MICS CBH
OTHER SURVEY SBH
VR INCOMPLETE
VR INCOMPLETE TB

Age−specific fertility fitted trend lines, 1950−2021: Guanajuato, Ages: 20−24
Data points enclosed with a diamond are reference sources

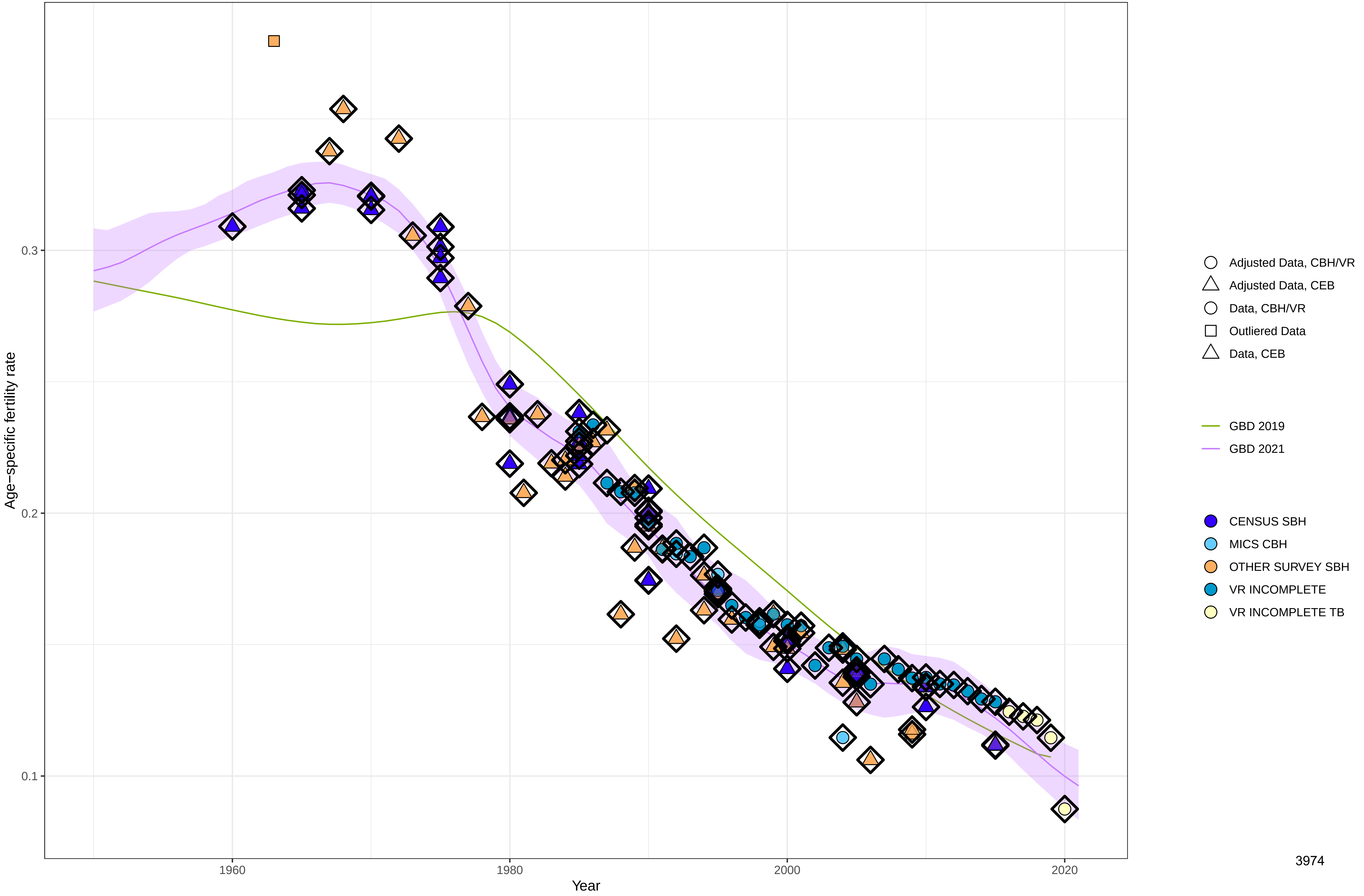

Age−specific fertility fitted trend lines, 1950−2021: Guanajuato, Ages: 25−29
Data points enclosed with a diamond are reference sources

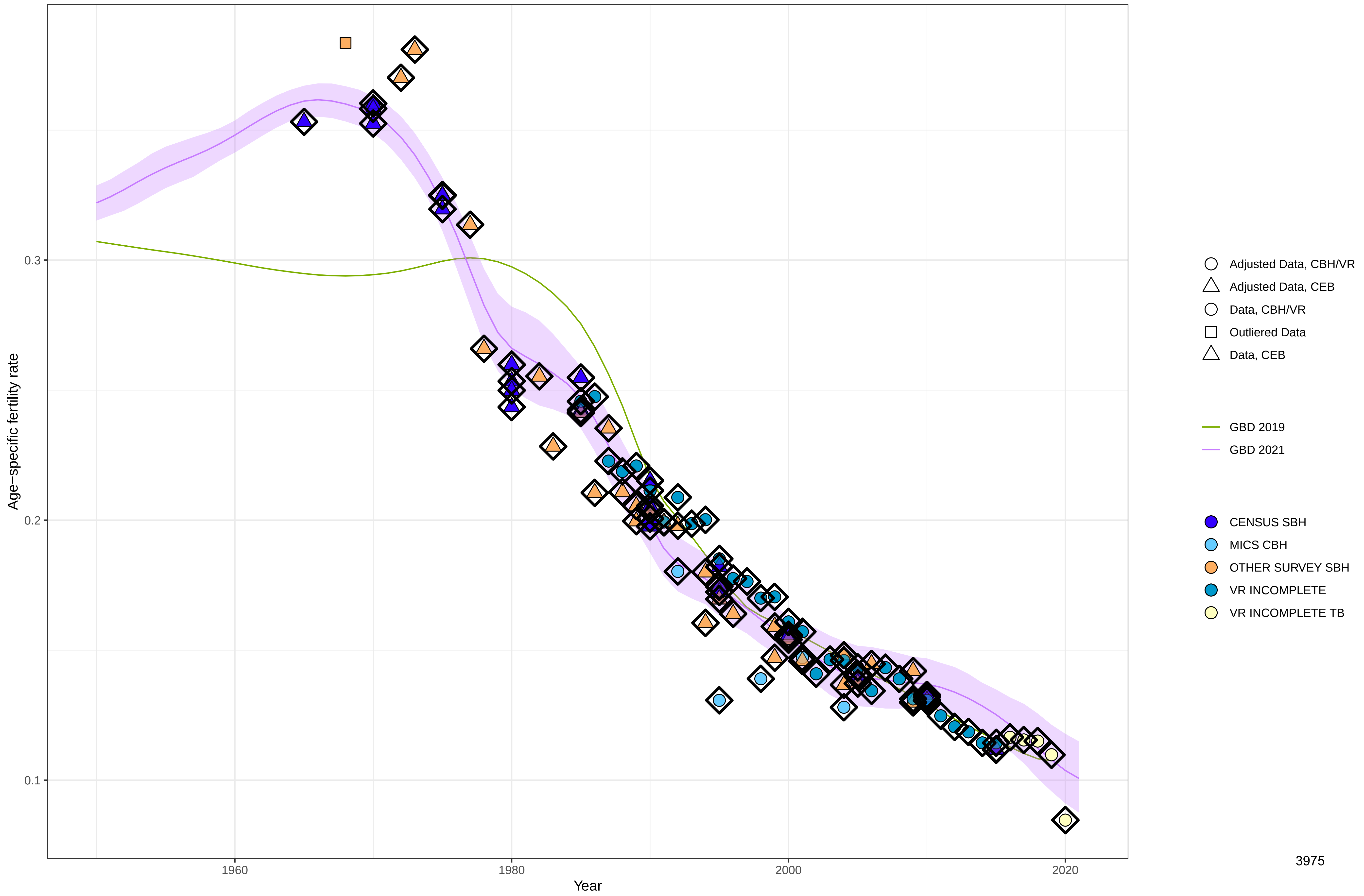

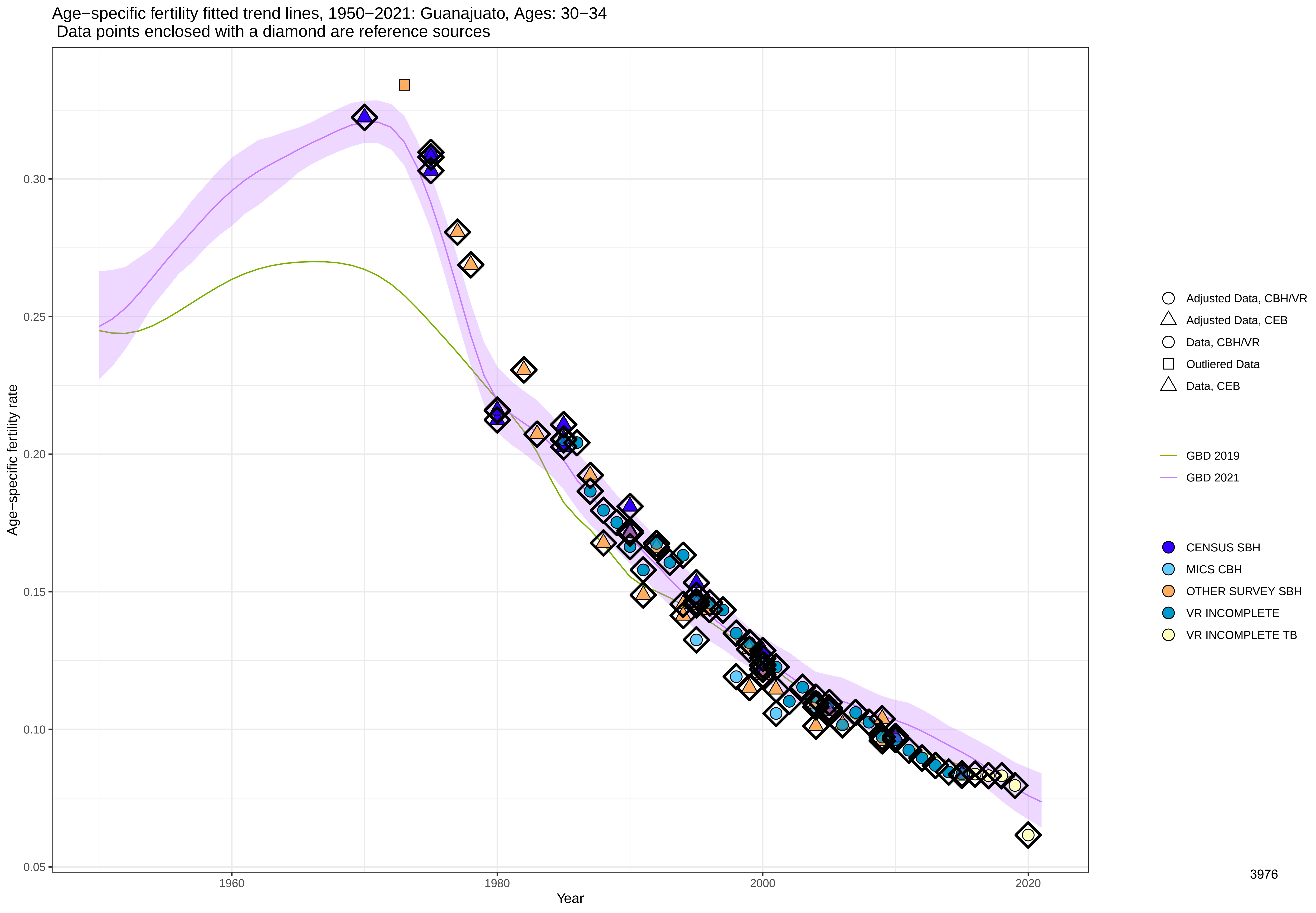

Age−specific fertility fitted trend lines, 1950−2021: Guanajuato, Ages: 30−34
Data points enclosed with a diamond are reference sources
Age−specific fertility rate
Year
0.05
0.10
0.15
0.20
0.25
0.30
1960
1980
2000
2020
Adjusted Data, CBH/VR
Adjusted Data, CEB
Data, CBH/VR
Outliered Data
Data, CEB
GBD 2019
GBD 2021
CENSUS SBH
MICS CBH
OTHER SURVEY SBH
VR INCOMPLETE
VR INCOMPLETE TB

3976

Age−specific fertility fitted trend lines, 1950−2021: Guanajuato, Ages: 35−39
Data points enclosed with a diamond are reference sources

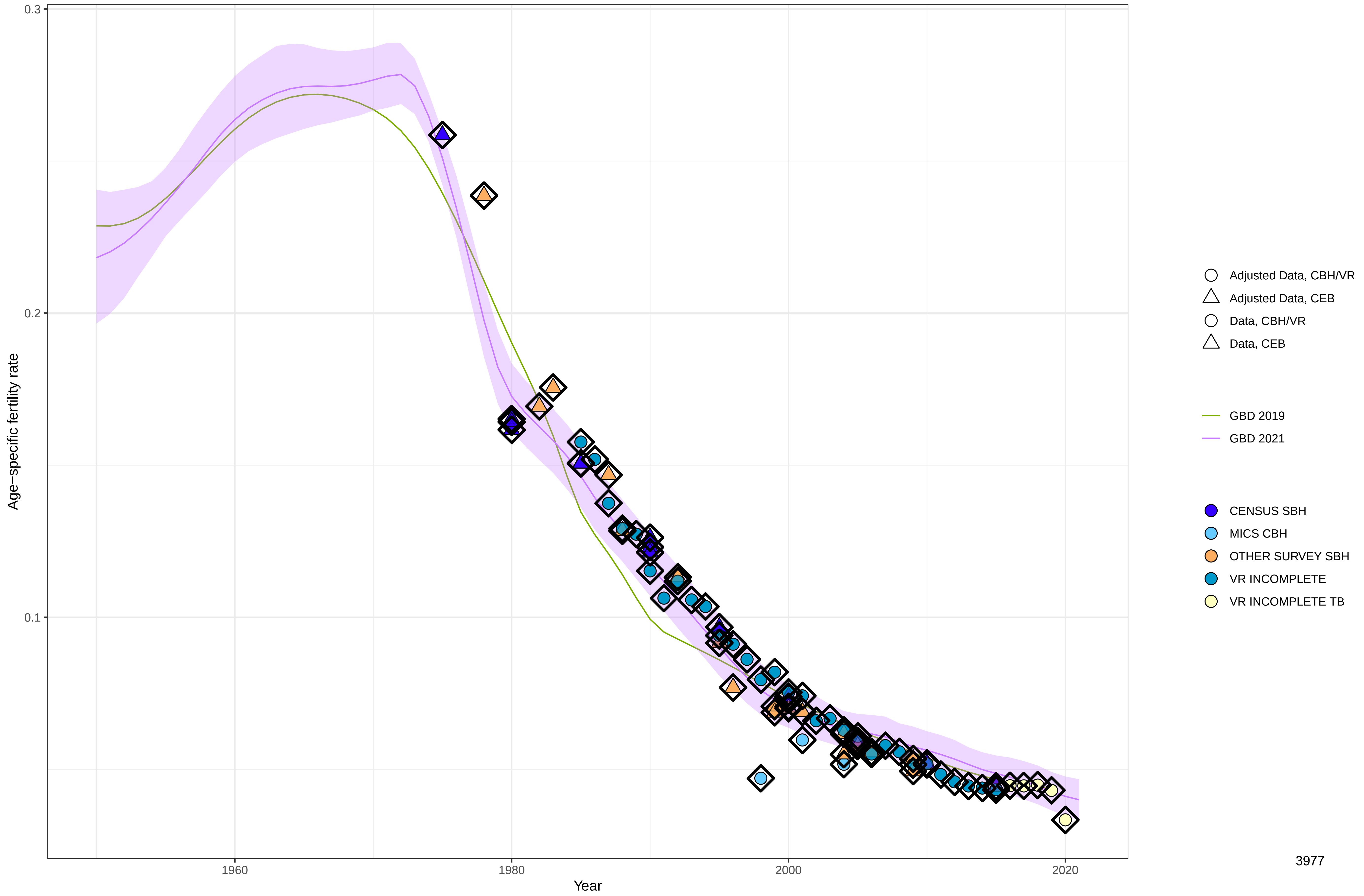

Age−specific fertility fitted trend lines, 1950−2021: Guanajuato, Ages: 40−44
Data points enclosed with a diamond are reference sources

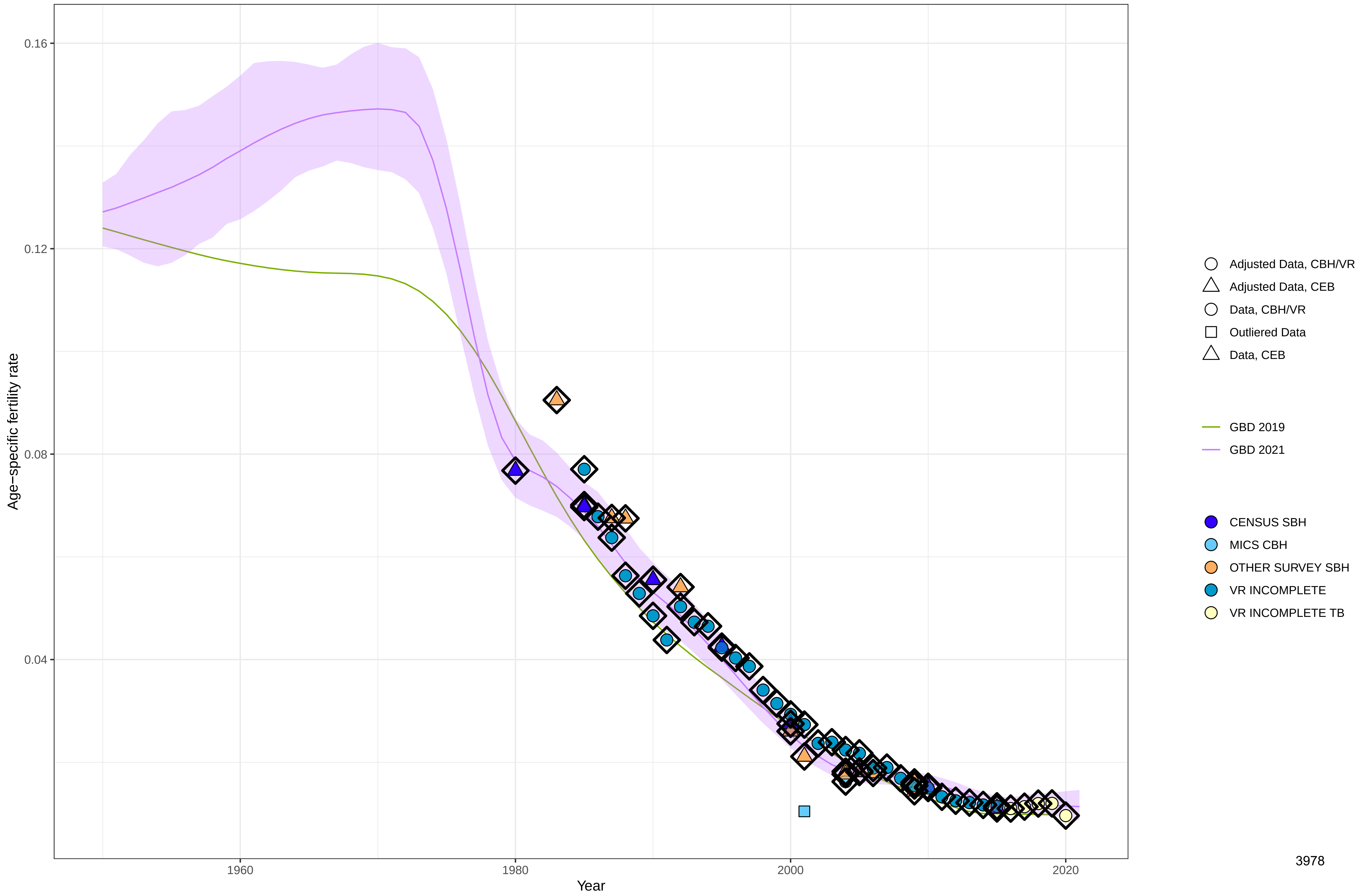

Age−specific fertility fitted trend lines, 1950−2021: Guanajuato, Ages: 45−49
Data points enclosed with a diamond are reference sources

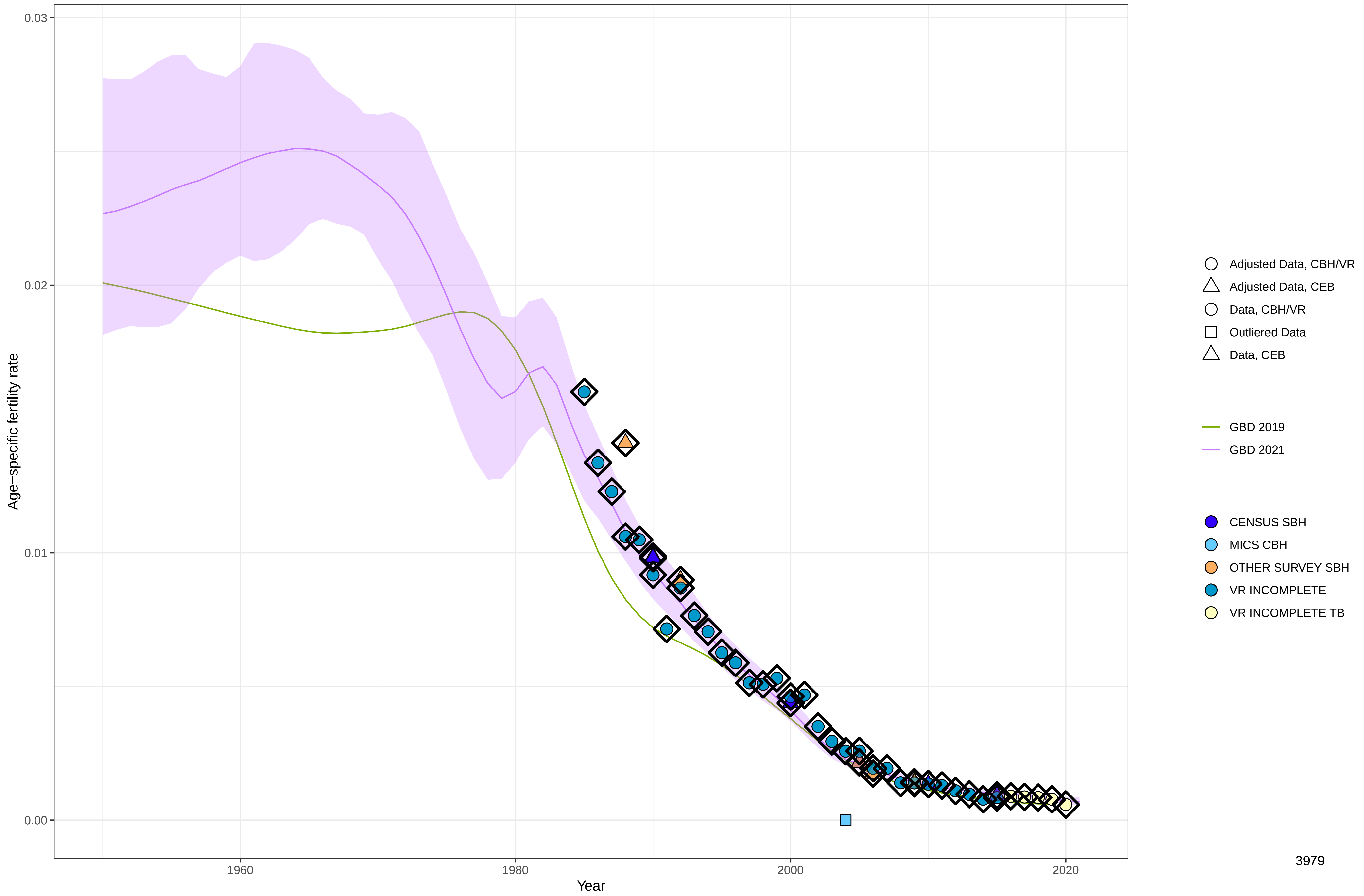

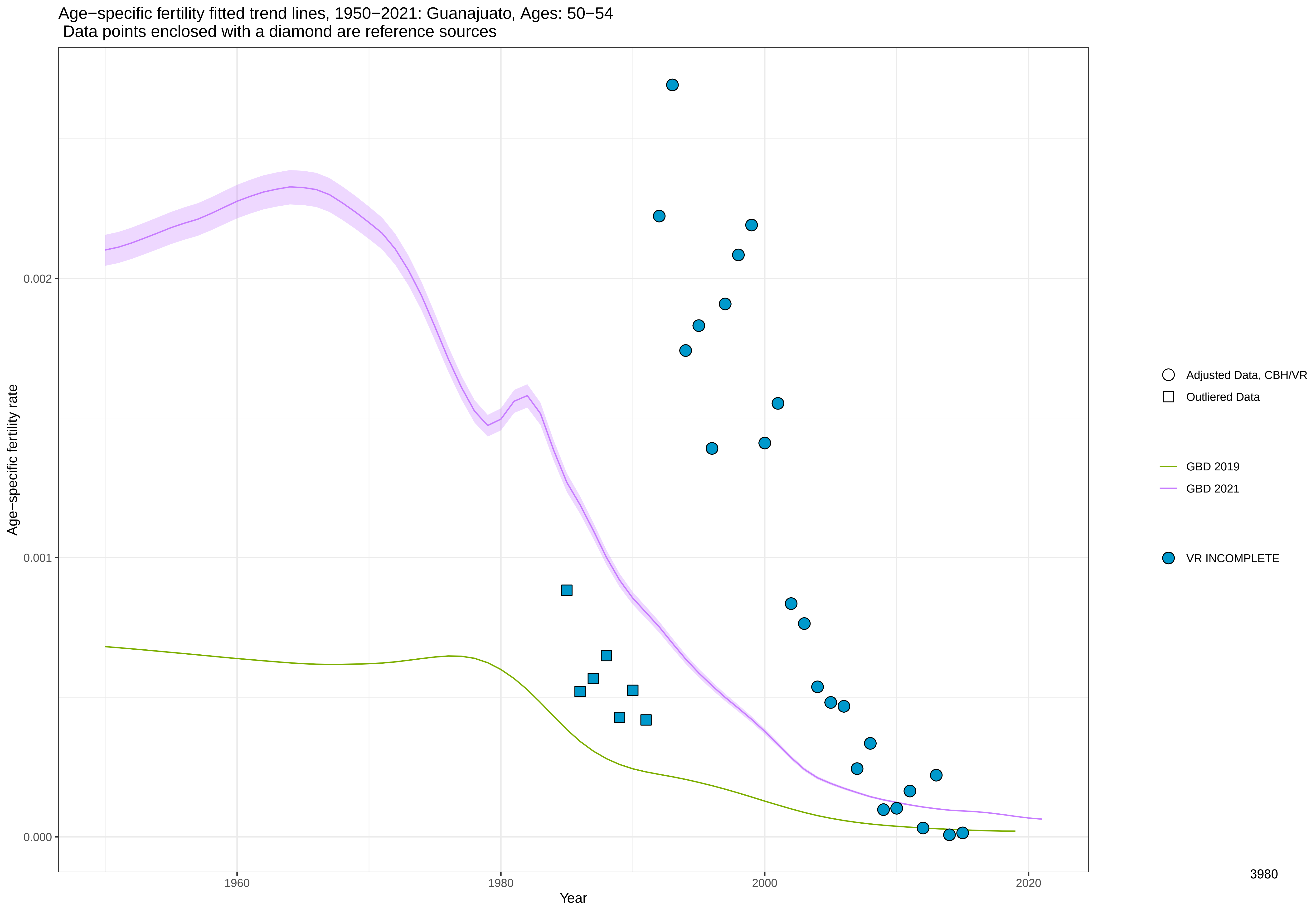

Age−specific fertility fitted trend lines, 1950−2021: Guanajuato, Ages: 50−54
Data points enclosed with a diamond are reference sources
Age−specific fertility rate
0.000
0.001
0.002
1960
1980
2000
2020
Year
Adjusted Data, CBH/VR
Outliered Data
GBD 2019
GBD 2021
VR INCOMPLETE

Total fertility rate fitted trend lines, 1950−2021: Guanajuato TFR
Data points enclosed with a diamond are reference sources

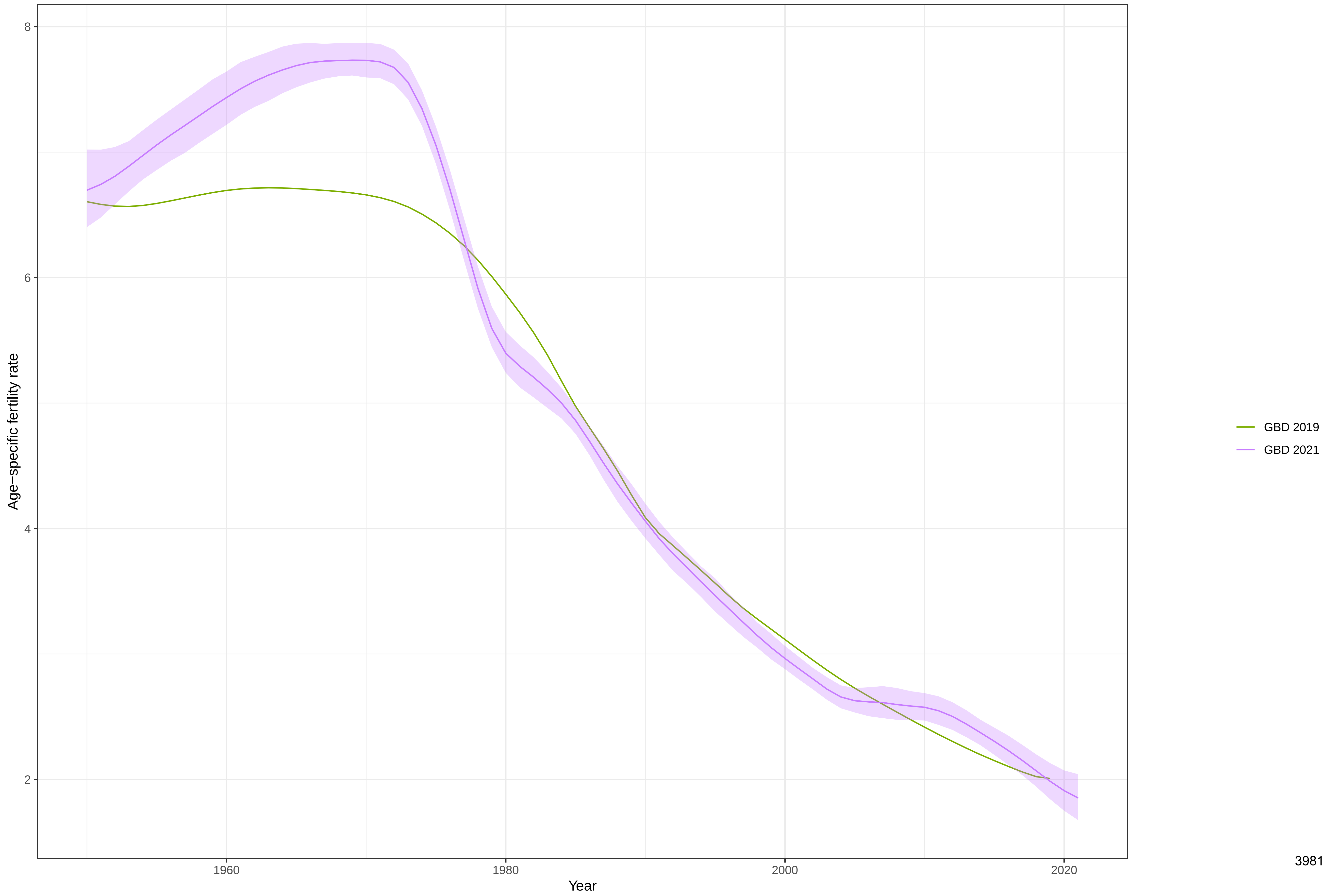

Age−specific fertility fitted trend lines, 1950−2021: Guerrero, Ages: 10−14
Data points enclosed with a diamond are reference sources

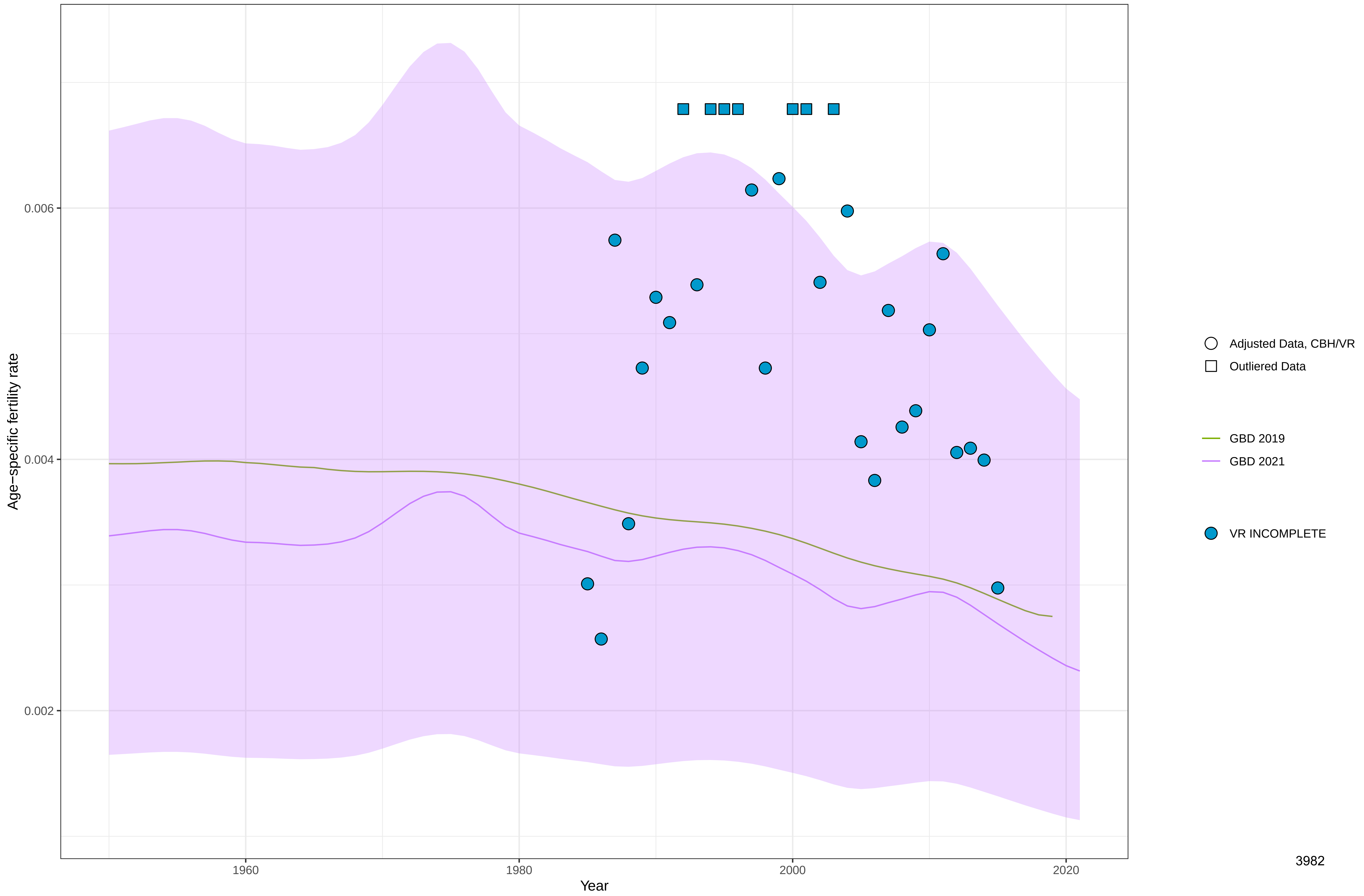

Age−specific fertility fitted trend lines, 1950−2021: Guerrero, Ages: 15−19
Data points enclosed with a diamond are reference sources

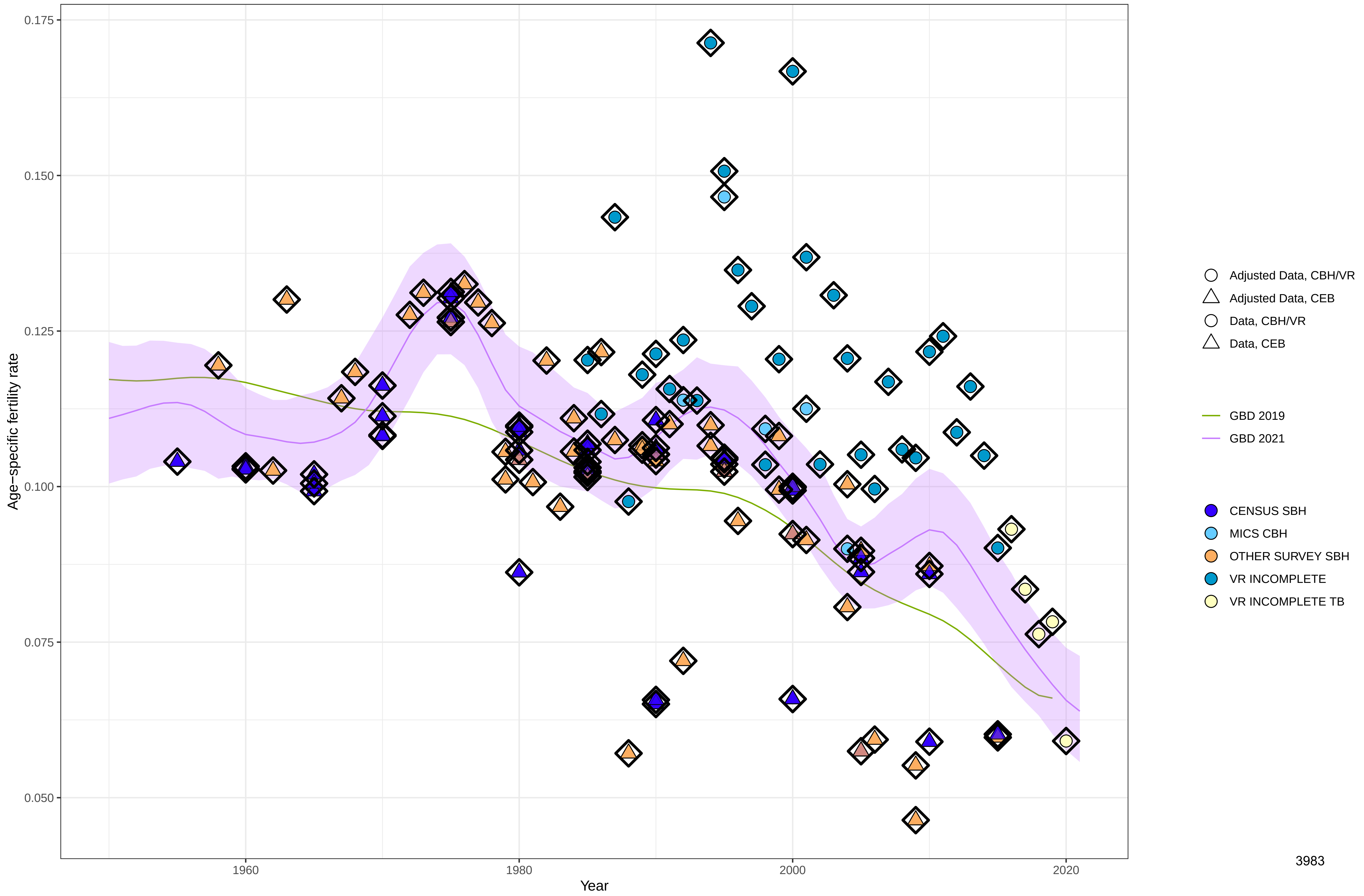

Age−specific fertility fitted trend lines, 1950−2021: Guerrero, Ages: 20−24
Data points enclosed with a diamond are reference sources

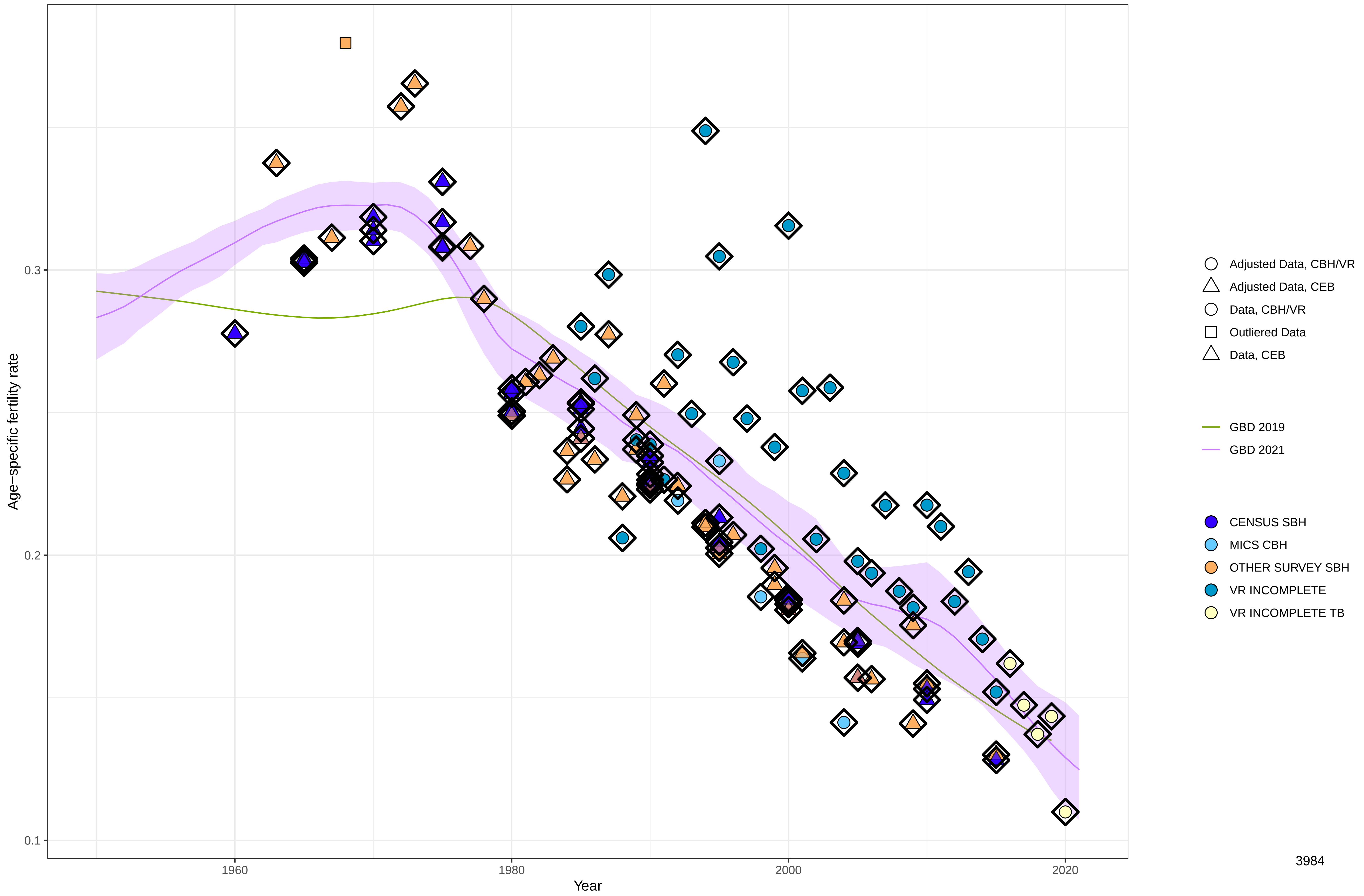

Age−specific fertility fitted trend lines, 1950−2021: Guerrero, Ages: 25−29
Data points enclosed with a diamond are reference sources

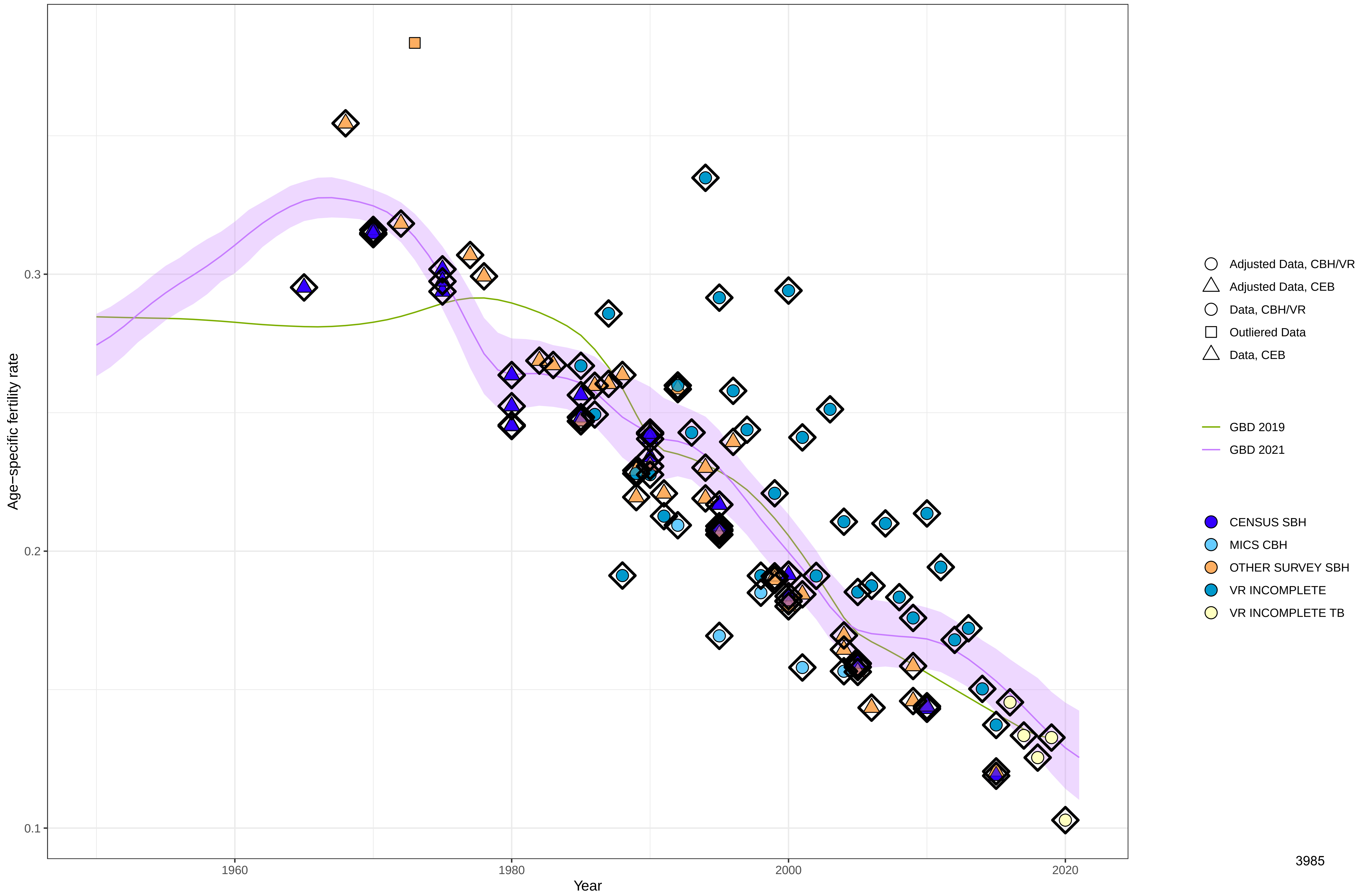

Age−specific fertility fitted trend lines, 1950−2021: Guerrero, Ages: 30−34
Data points enclosed with a diamond are reference sources

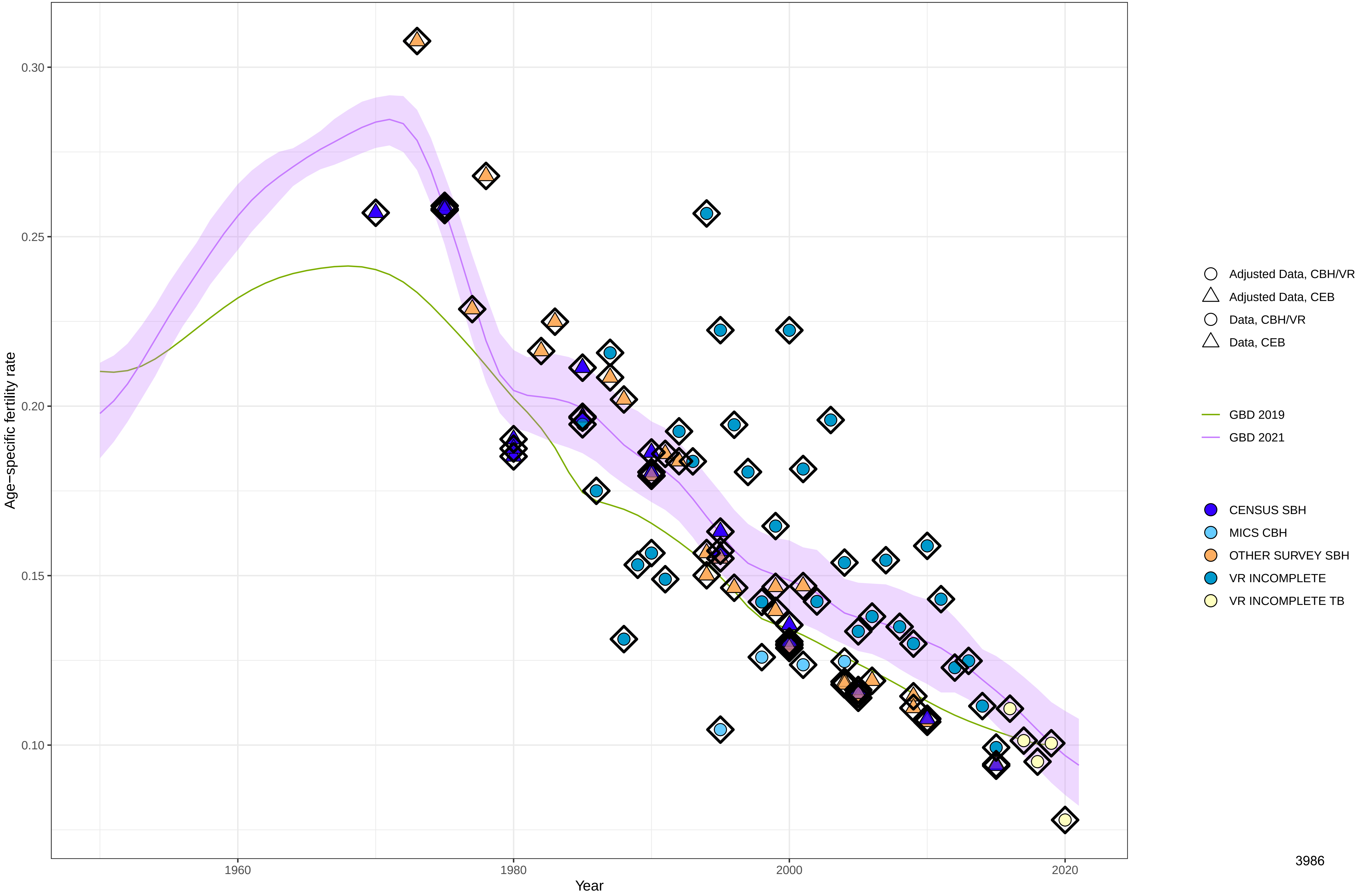

Age−specific fertility fitted trend lines, 1950−2021: Guerrero, Ages: 35−39
Data points enclosed with a diamond are reference sources

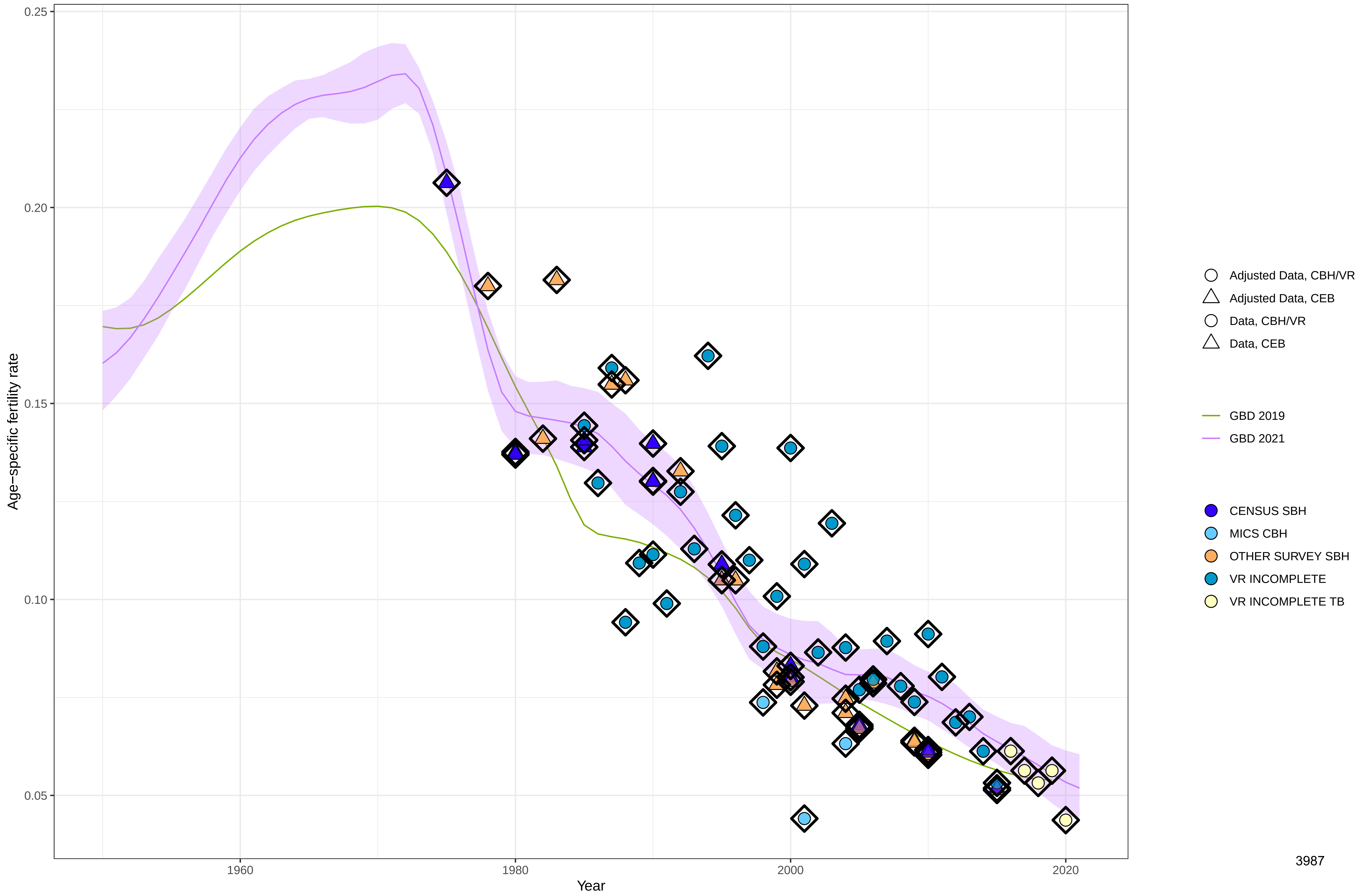

Age−specific fertility fitted trend lines, 1950−2021: Guerrero, Ages: 40−44
Data points enclosed with a diamond are reference sources

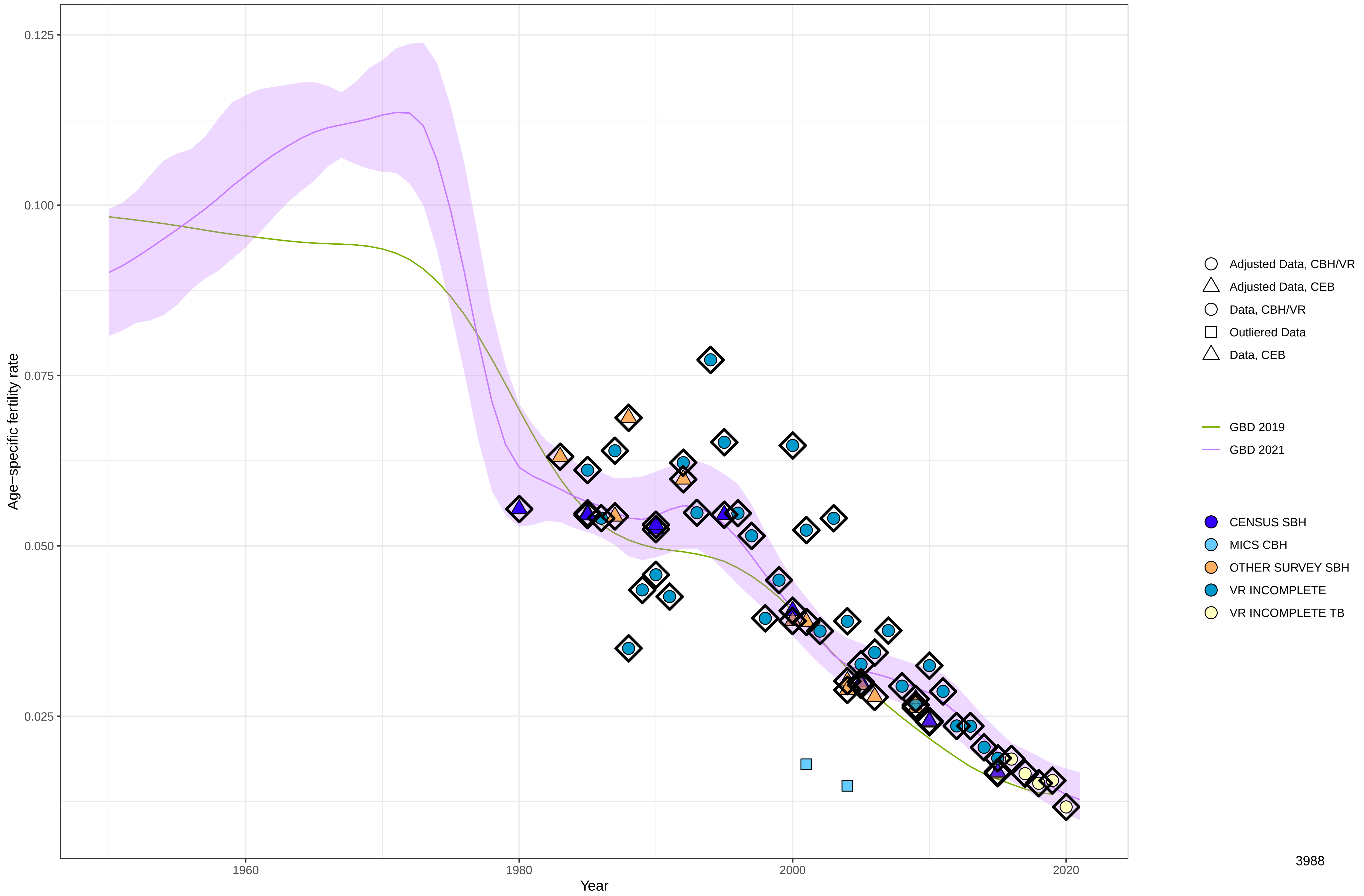

Age−specific fertility fitted trend lines, 1950−2021: Guerrero, Ages: 45−49
Data points enclosed with a diamond are reference sources

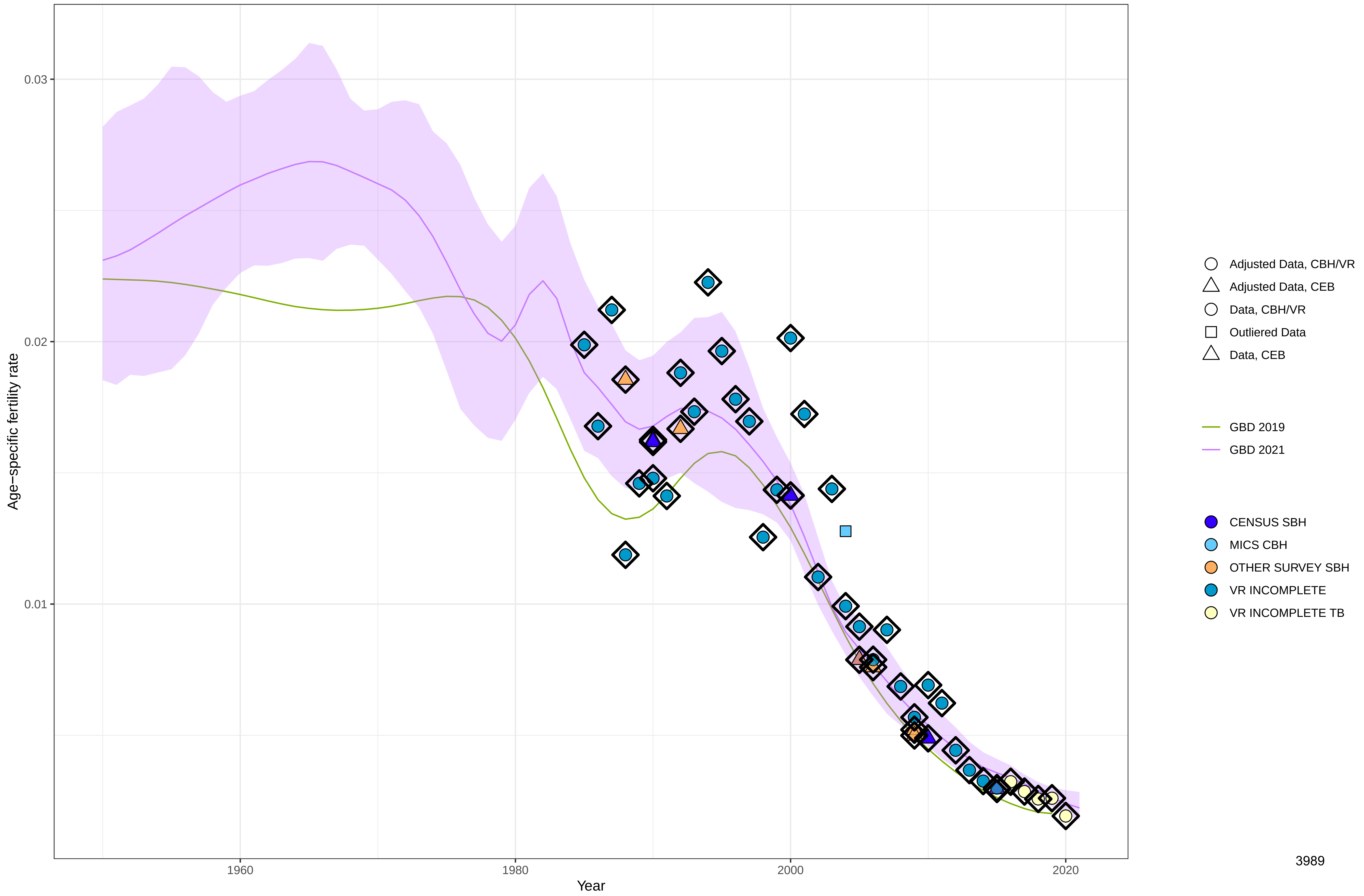

Age−specific fertility fitted trend lines, 1950−2021: Guerrero, Ages: 50−54
Data points enclosed with a diamond are reference sources

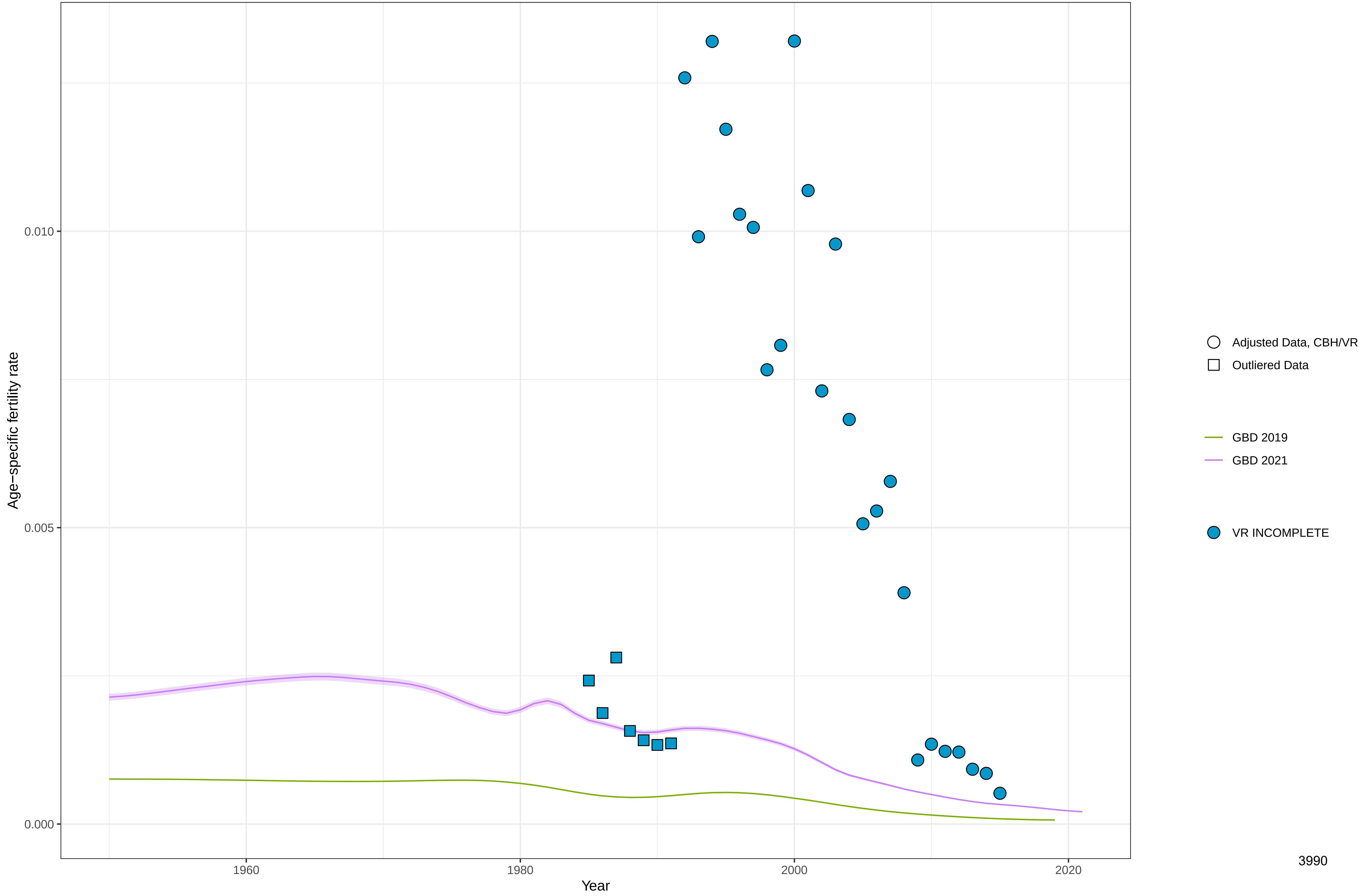

Total fertility rate fitted trend lines, 1950−2021: Guerrero TFR
Data points enclosed with a diamond are reference sources

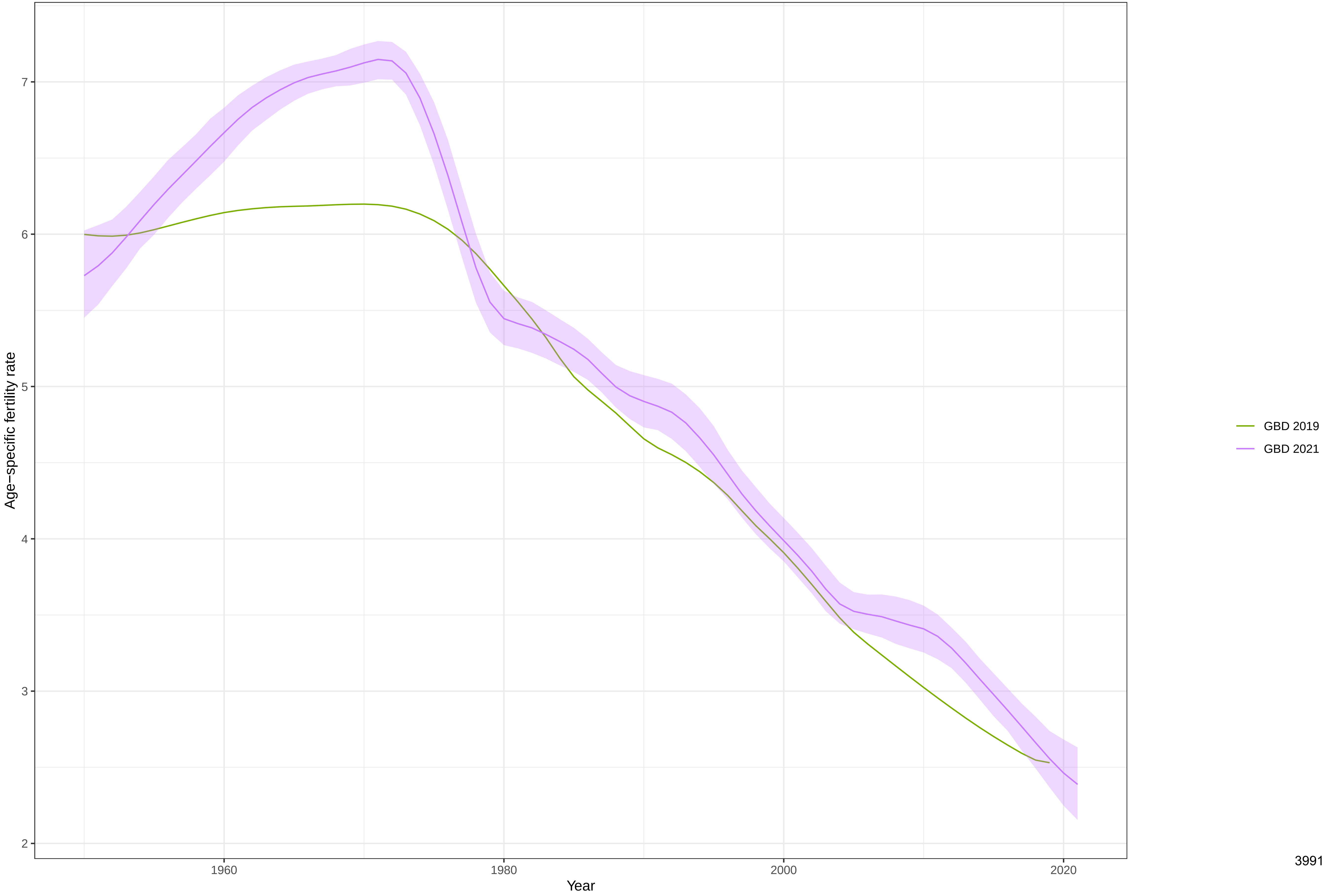

Age−specific fertility fitted trend lines, 1950−2021: Hidalgo, Ages: 10−14
Data points enclosed with a diamond are reference sources

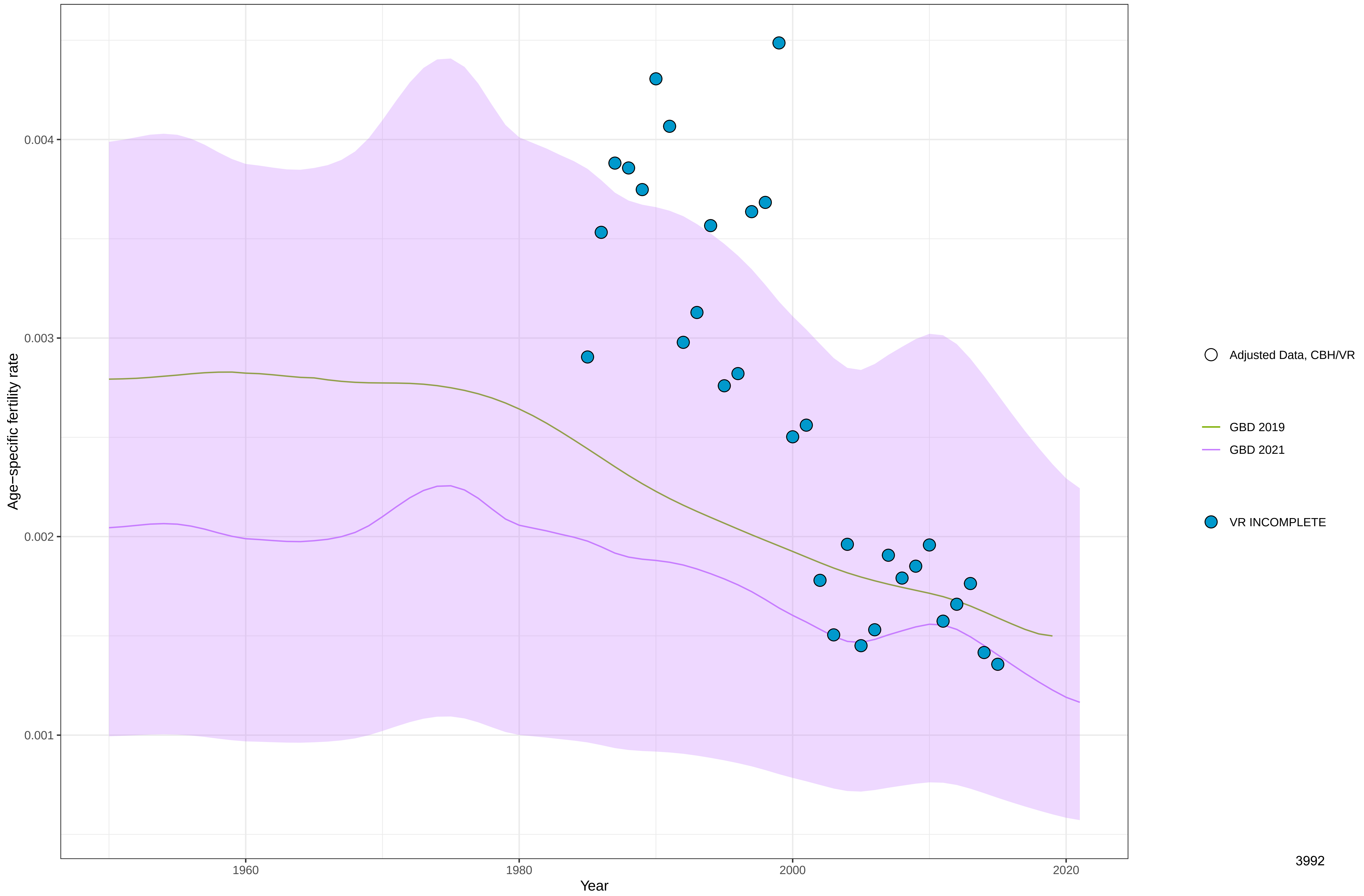

Age−specific fertility fitted trend lines, 1950−2021: Hidalgo, Ages: 15−19
Data points enclosed with a diamond are reference sources

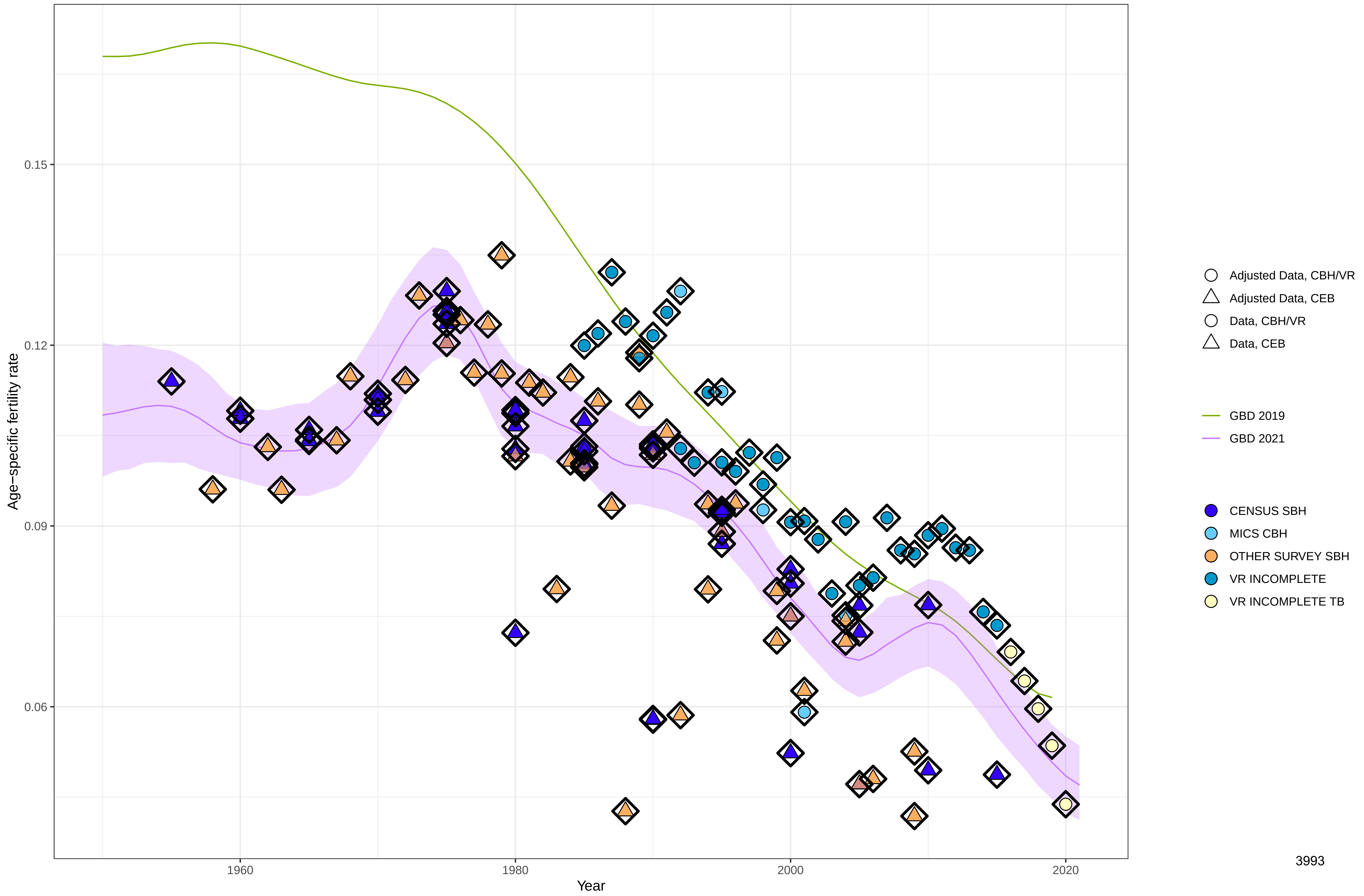

Age−specific fertility fitted trend lines, 1950−2021: Hidalgo, Ages: 20−24
Data points enclosed with a diamond are reference sources

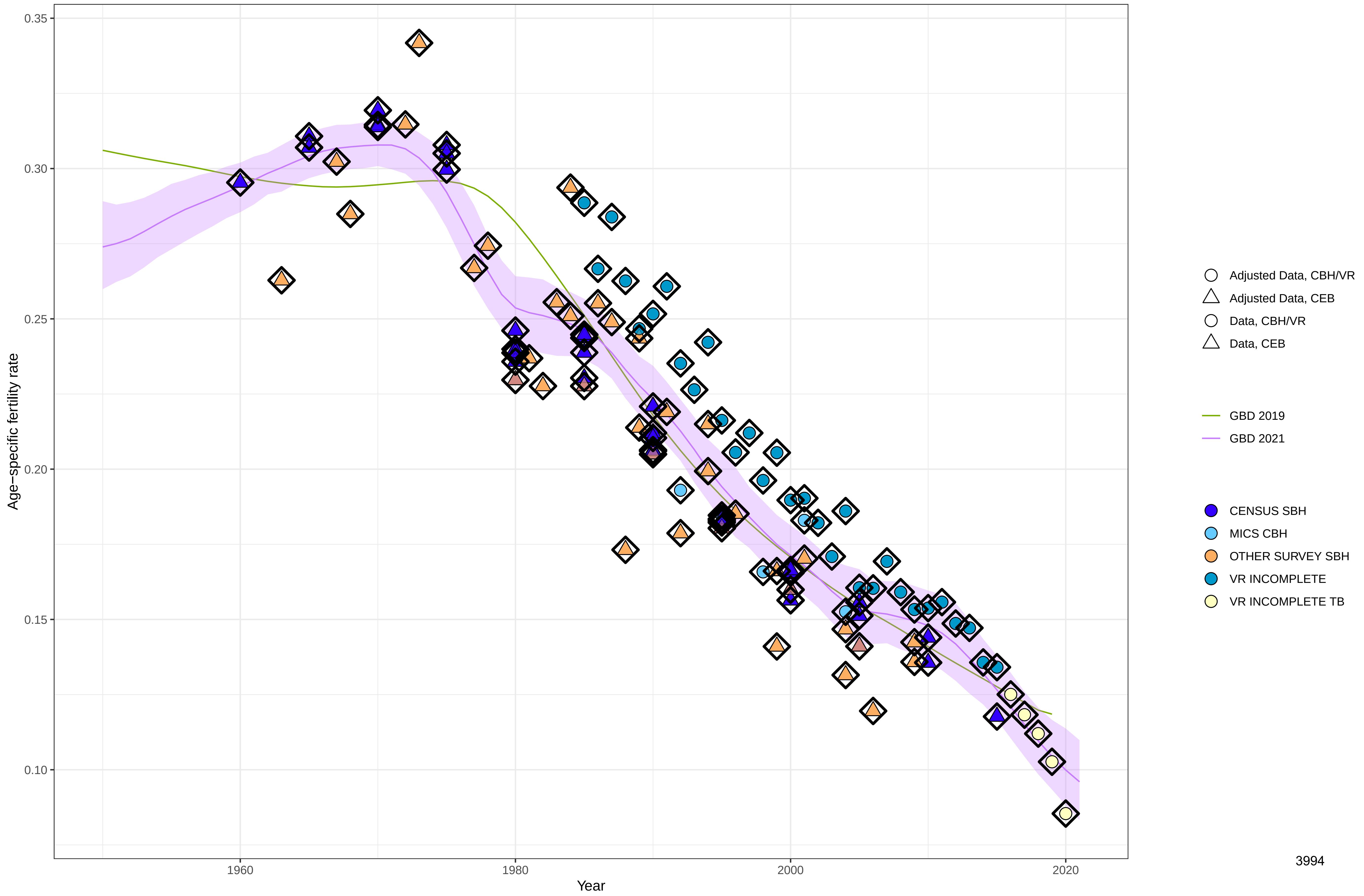

Age−specific fertility fitted trend lines, 1950−2021: Hidalgo, Ages: 25−29
Data points enclosed with a diamond are reference sources

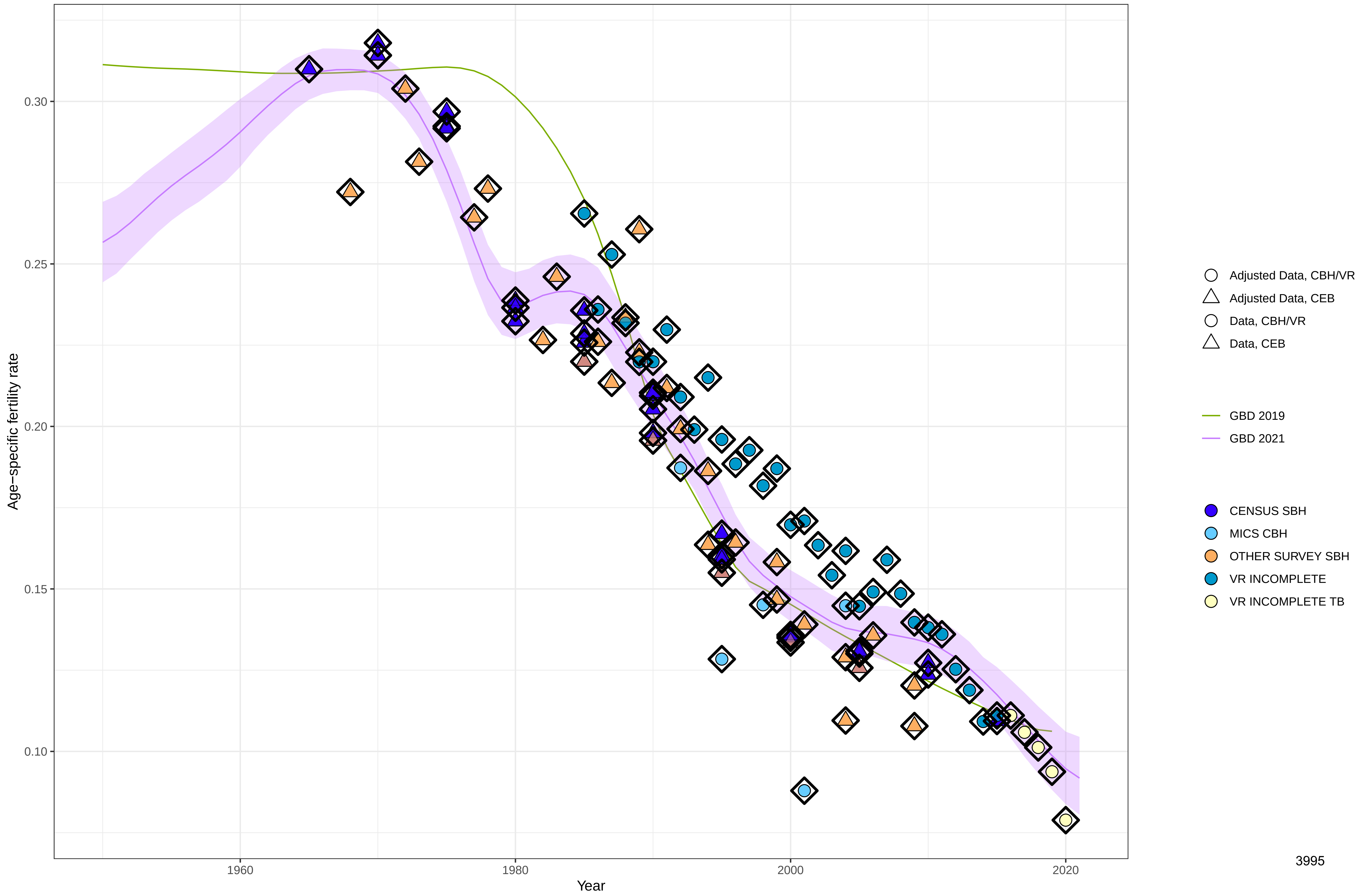

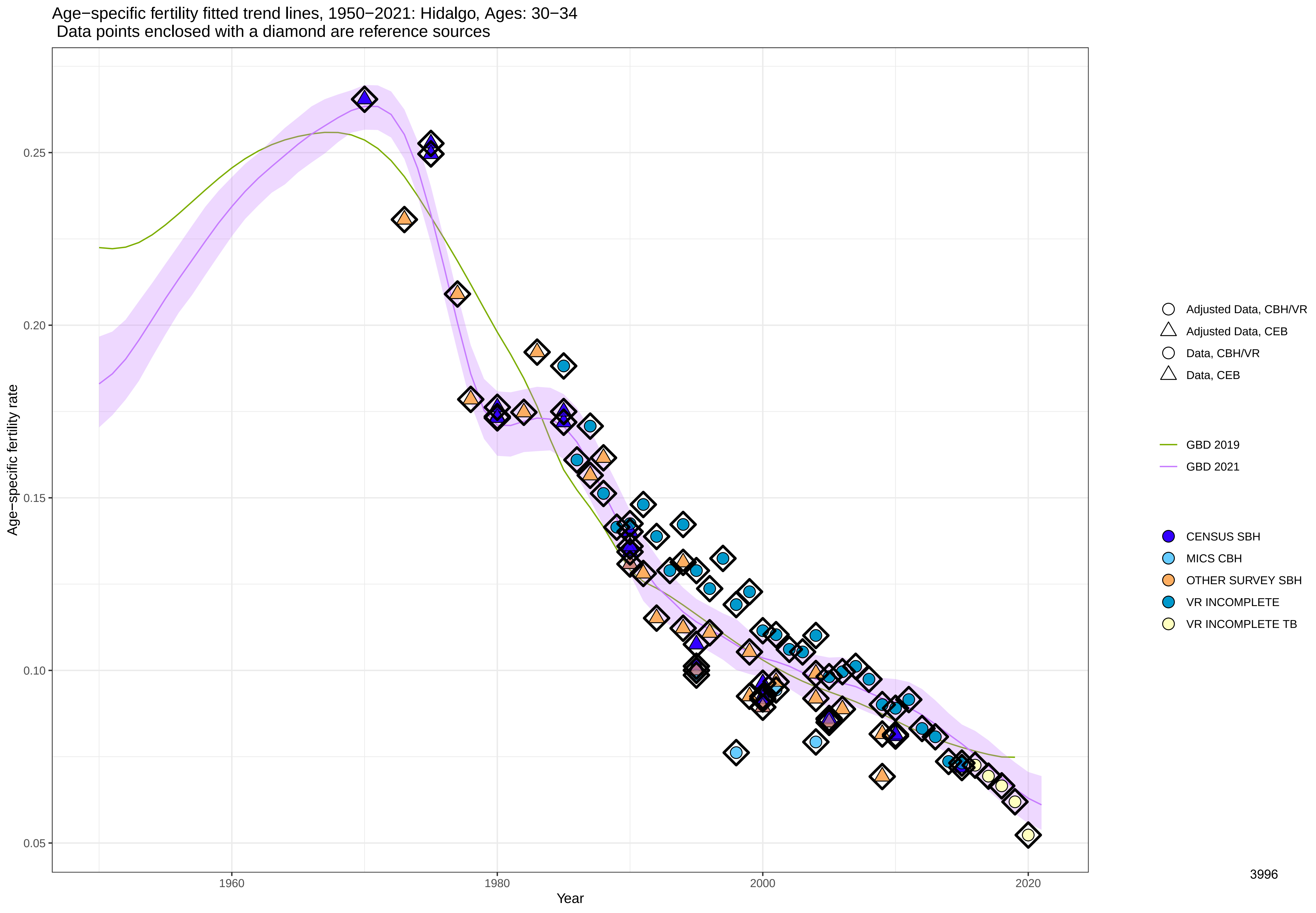

Age−specific fertility fitted trend lines, 1950−2021: Hidalgo, Ages: 30−34
Data points enclosed with a diamond are reference sources
Age−specific fertility rate
0.05
0.10
0.15
0.20
0.25
1960
1980
2000
2020
Year
Adjusted Data, CBH/VR
Adjusted Data, CEB
Data, CBH/VR
Data, CEB
GBD 2019
GBD 2021
CENSUS SBH
MICS CBH
OTHER SURVEY SBH
VR INCOMPLETE
VR INCOMPLETE TB

Age−specific fertility fitted trend lines, 1950−2021: Hidalgo, Ages: 35−39
Data points enclosed with a diamond are reference sources

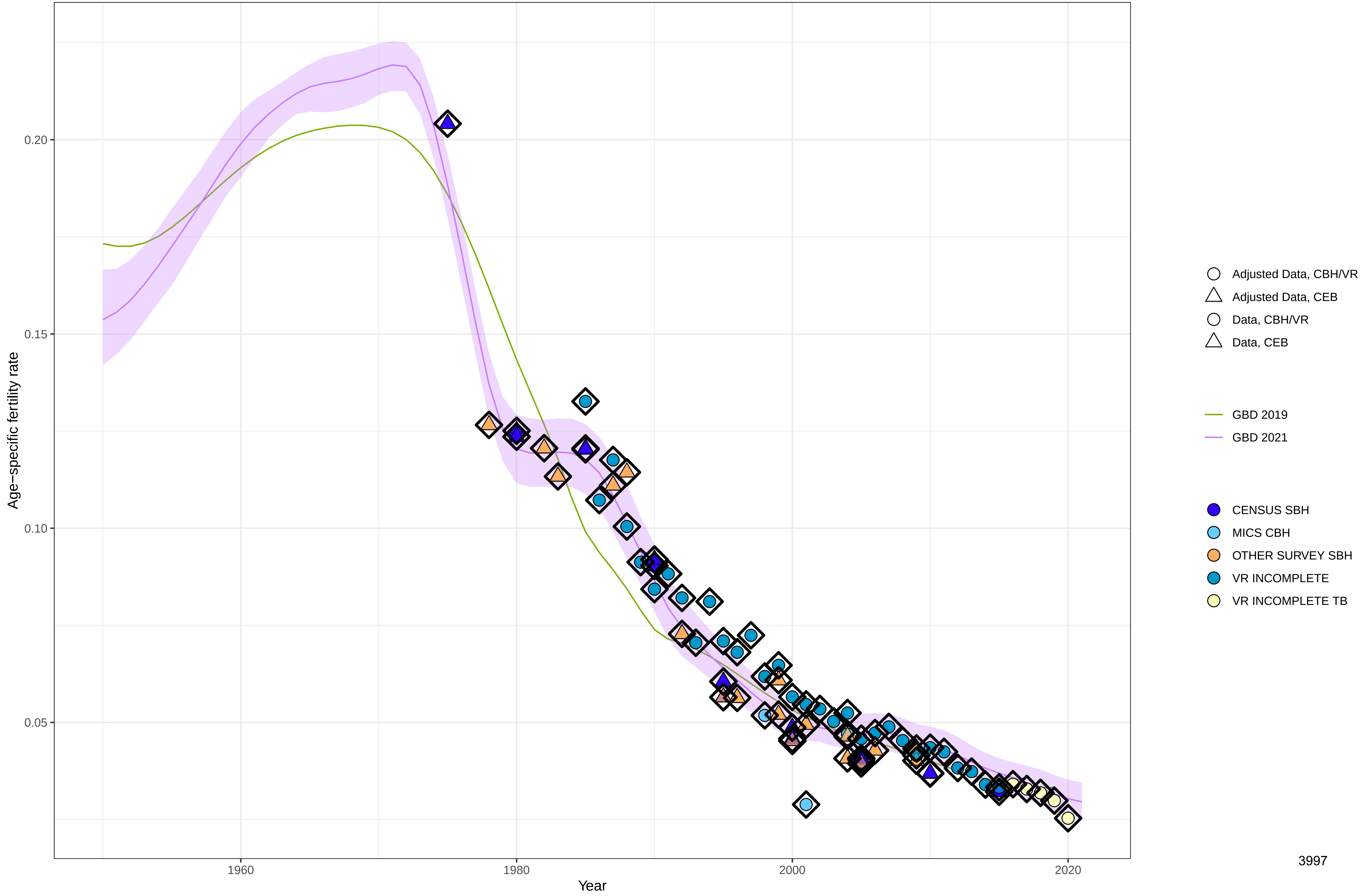

Age−specific fertility fitted trend lines, 1950−2021: Hidalgo, Ages: 40−44
Data points enclosed with a diamond are reference sources

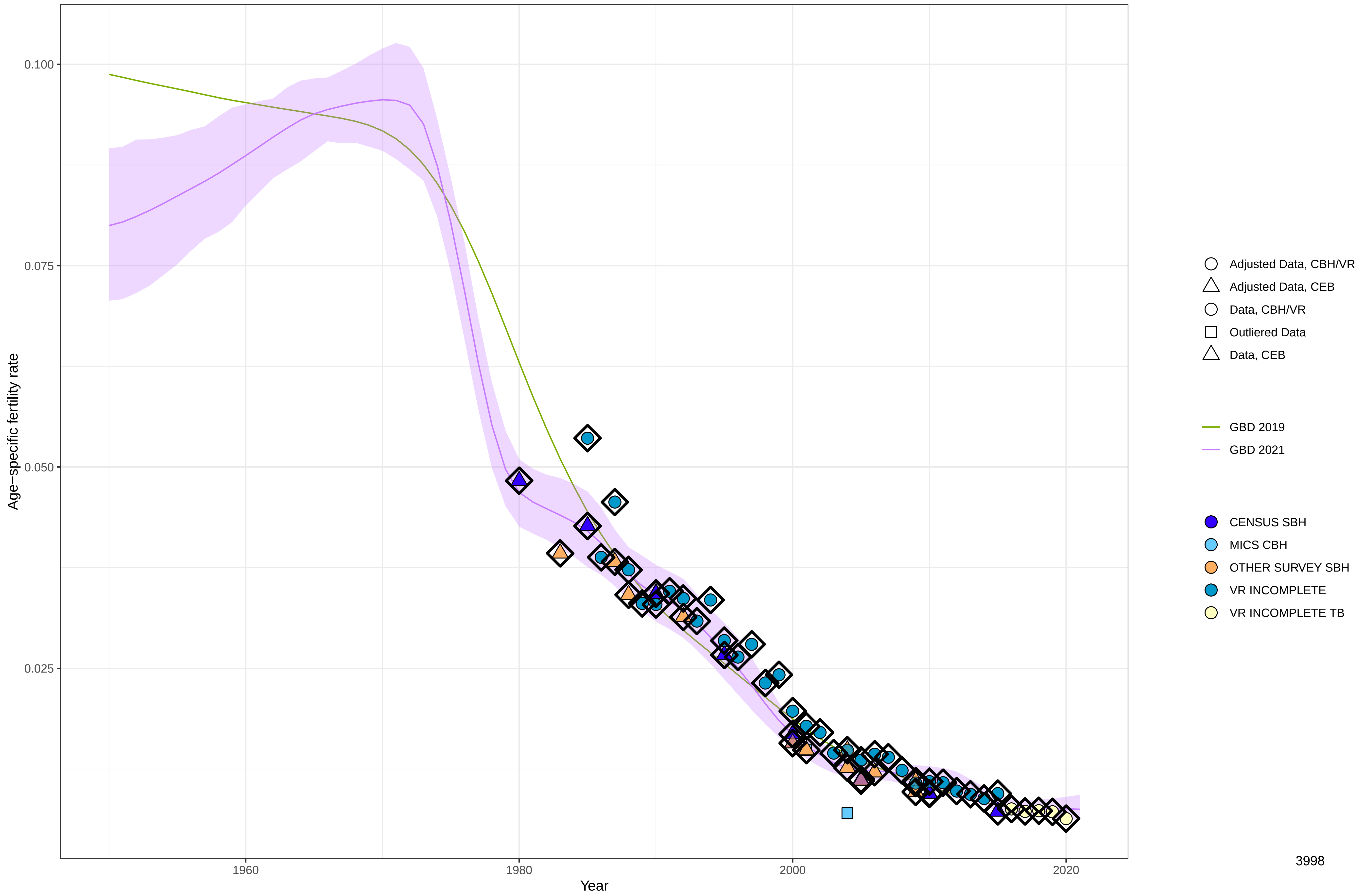

Age−specific fertility fitted trend lines, 1950−2021: Hidalgo, Ages: 45−49
Data points enclosed with a diamond are reference sources

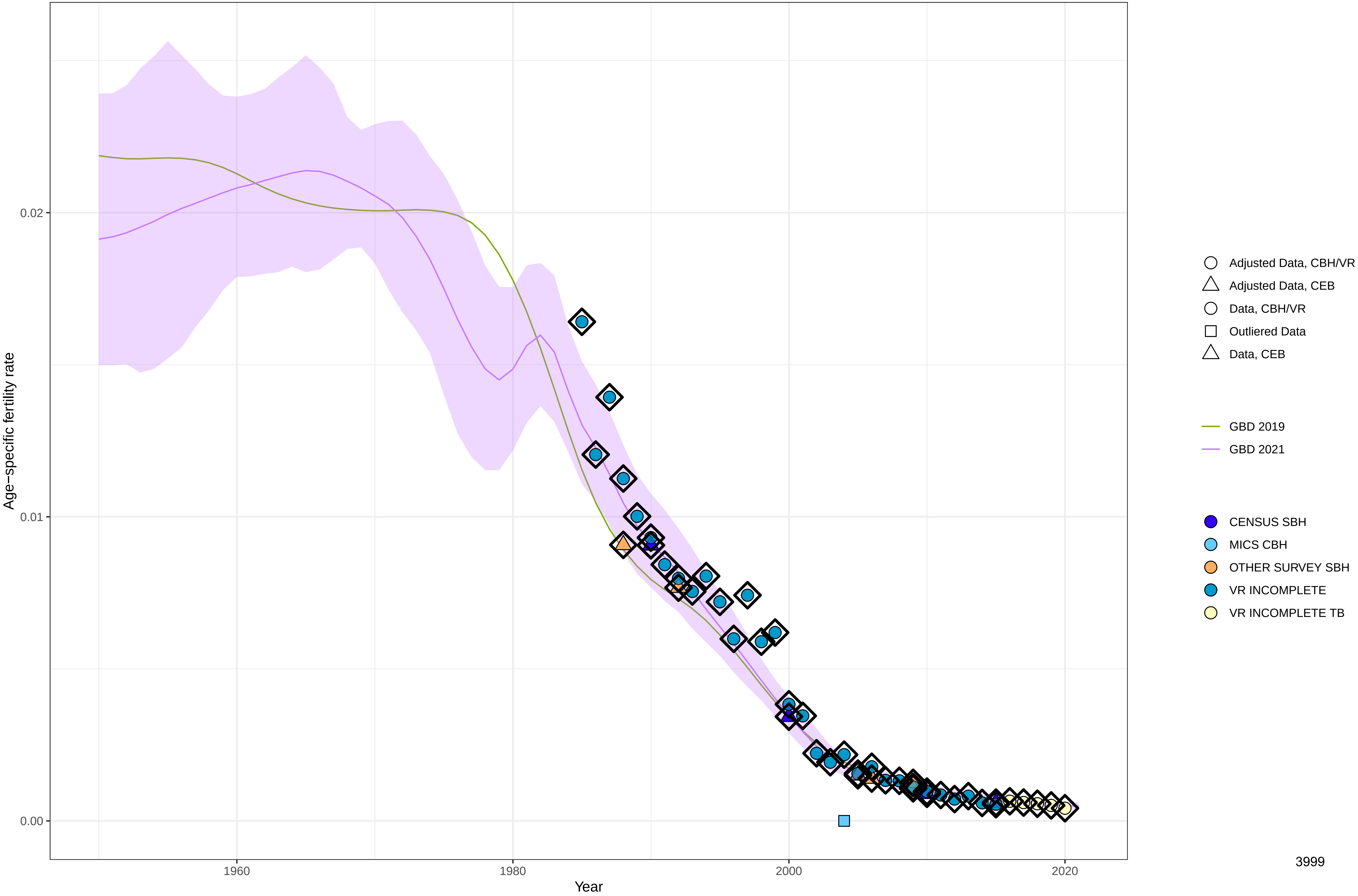

Age−specific fertility fitted trend lines, 1950−2021: Hidalgo, Ages: 50−54
Data points enclosed with a diamond are reference sources

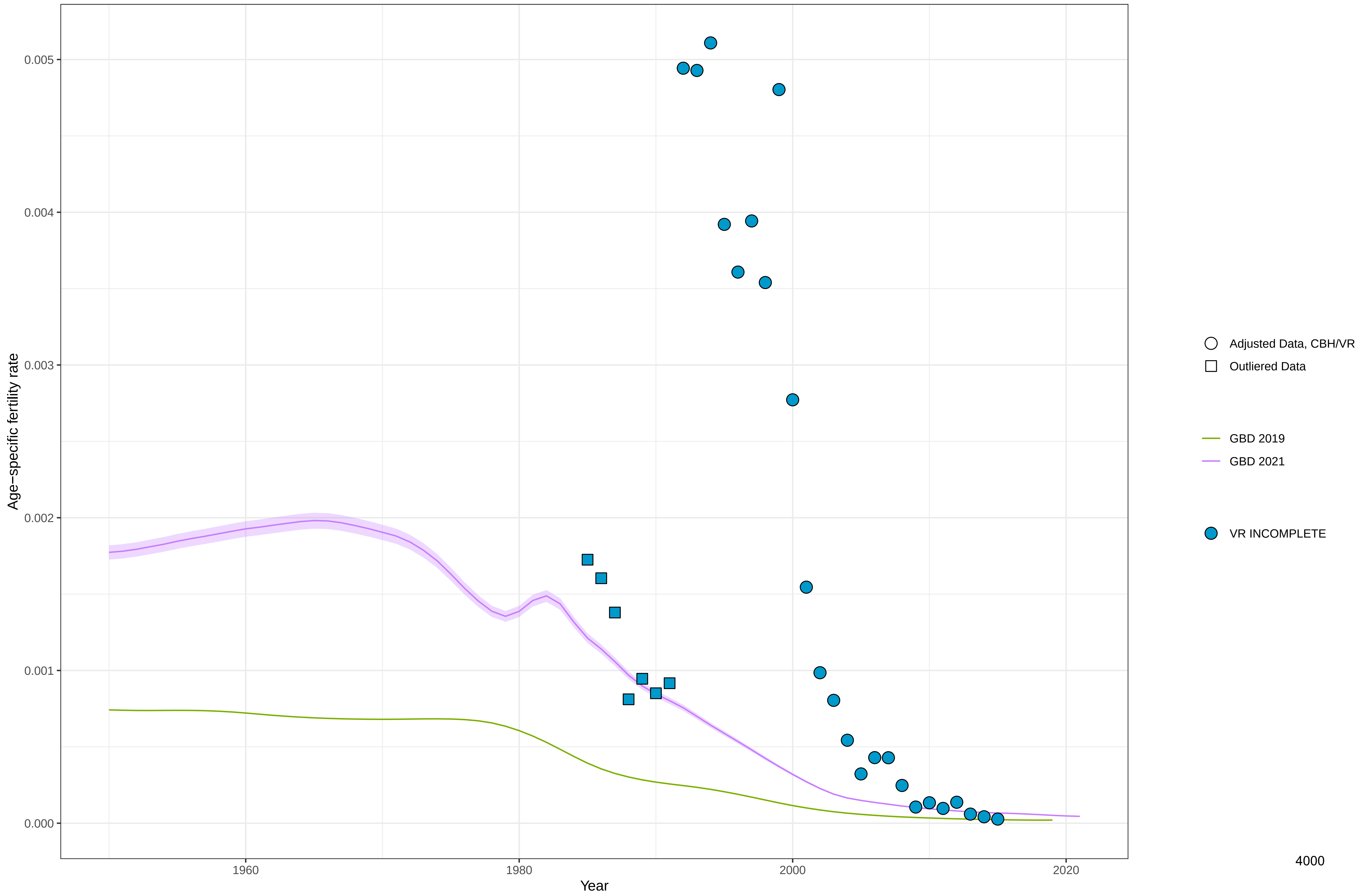

Total fertility rate fitted trend lines, 1950−2021: Hidalgo TFR
Data points enclosed with a diamond are reference sources

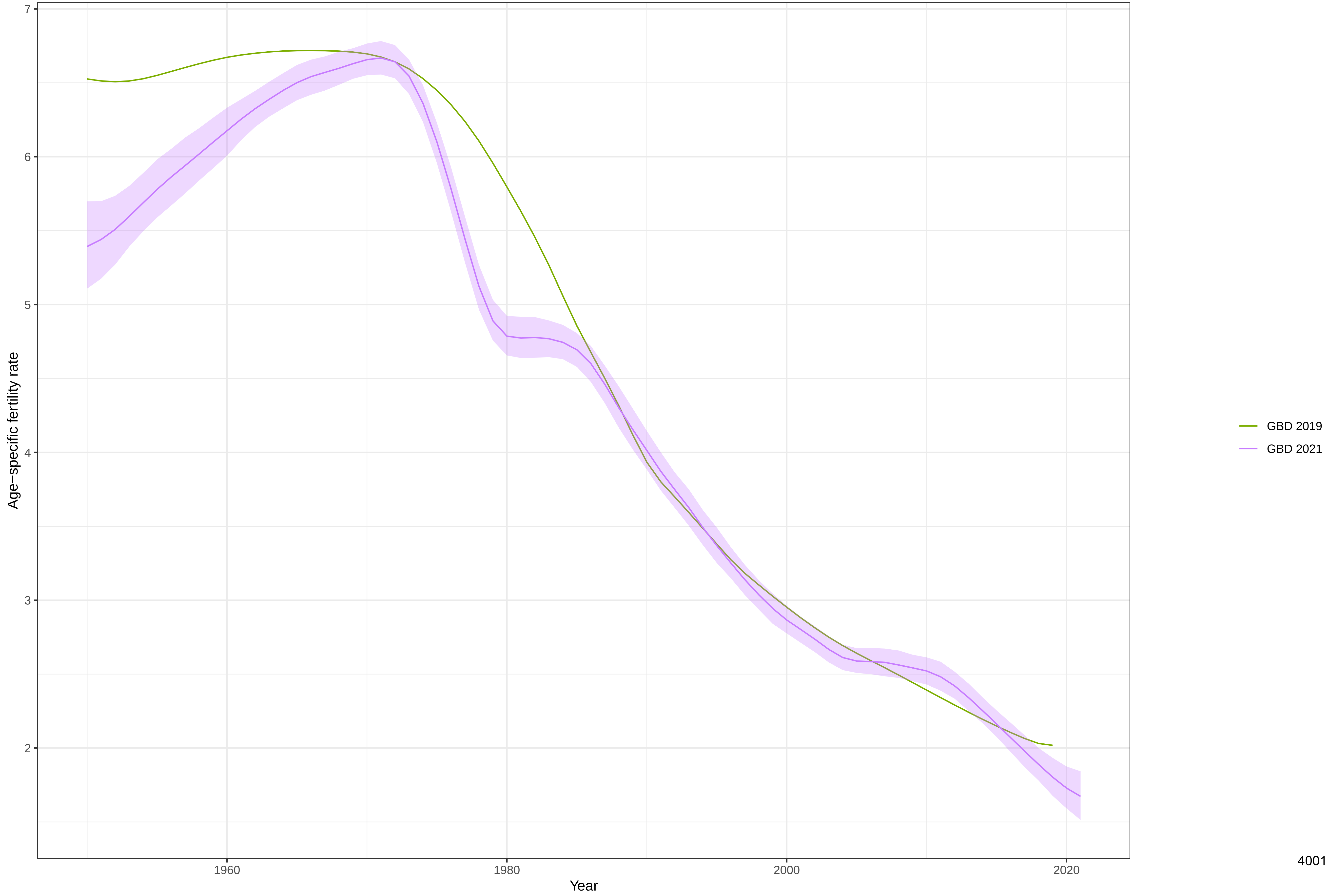

Age−specific fertility fitted trend lines, 1950−2021: Jalisco, Ages: 10−14
Data points enclosed with a diamond are reference sources

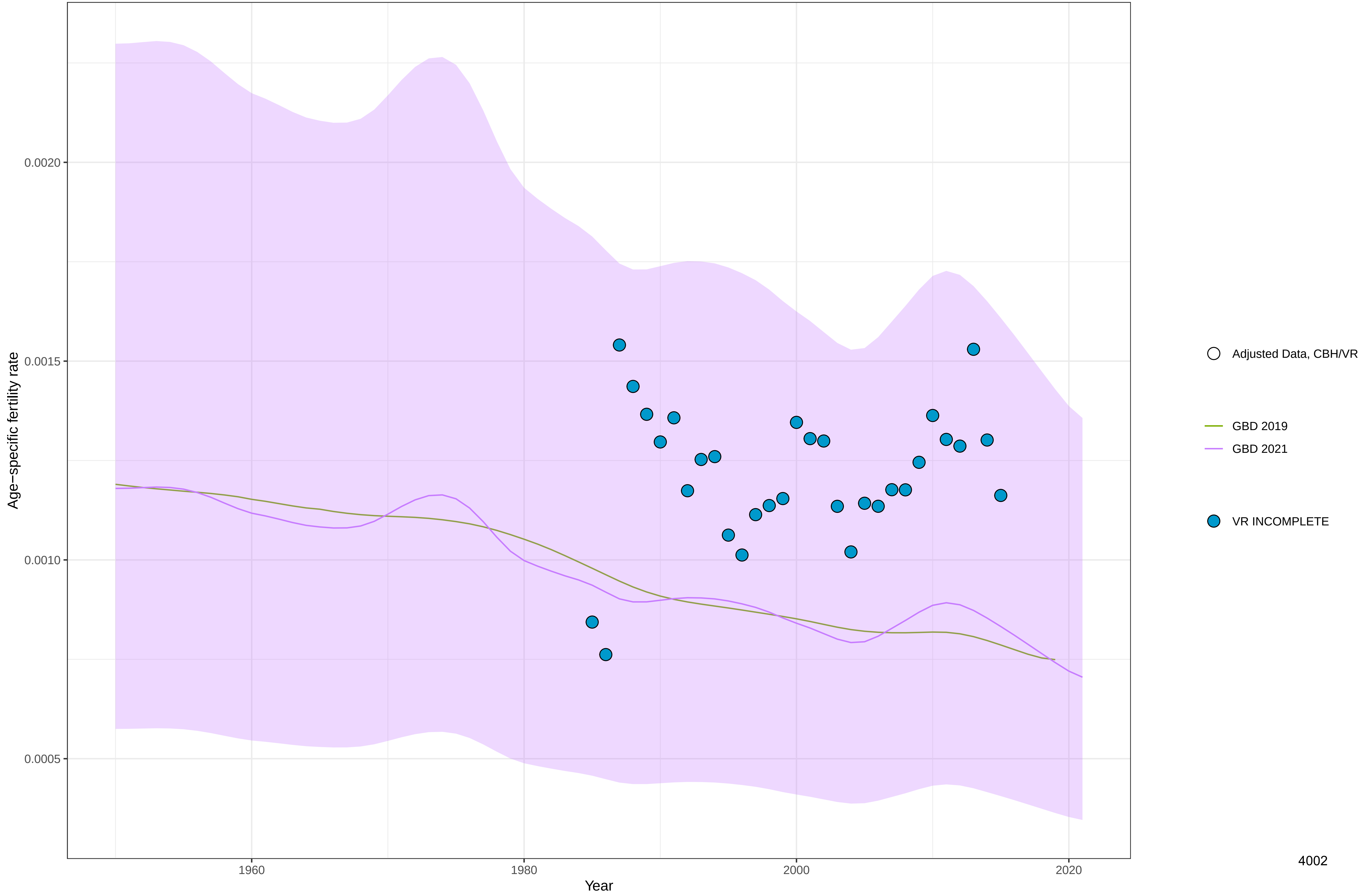

Age−specific fertility fitted trend lines, 1950−2021: Jalisco, Ages: 15−19
Data points enclosed with a diamond are reference sources

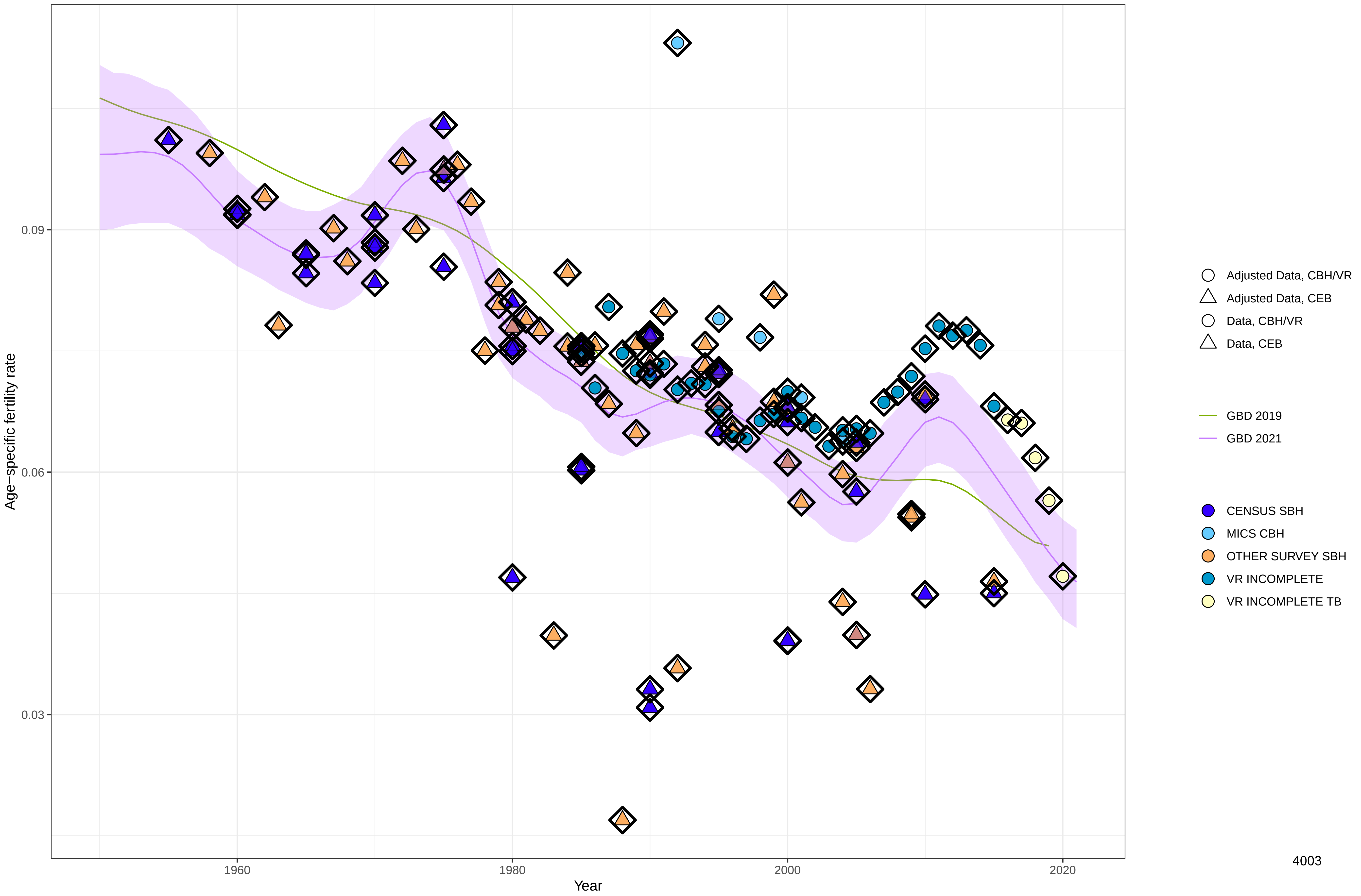

Age−specific fertility fitted trend lines, 1950−2021: Jalisco, Ages: 20−24
Data points enclosed with a diamond are reference sources

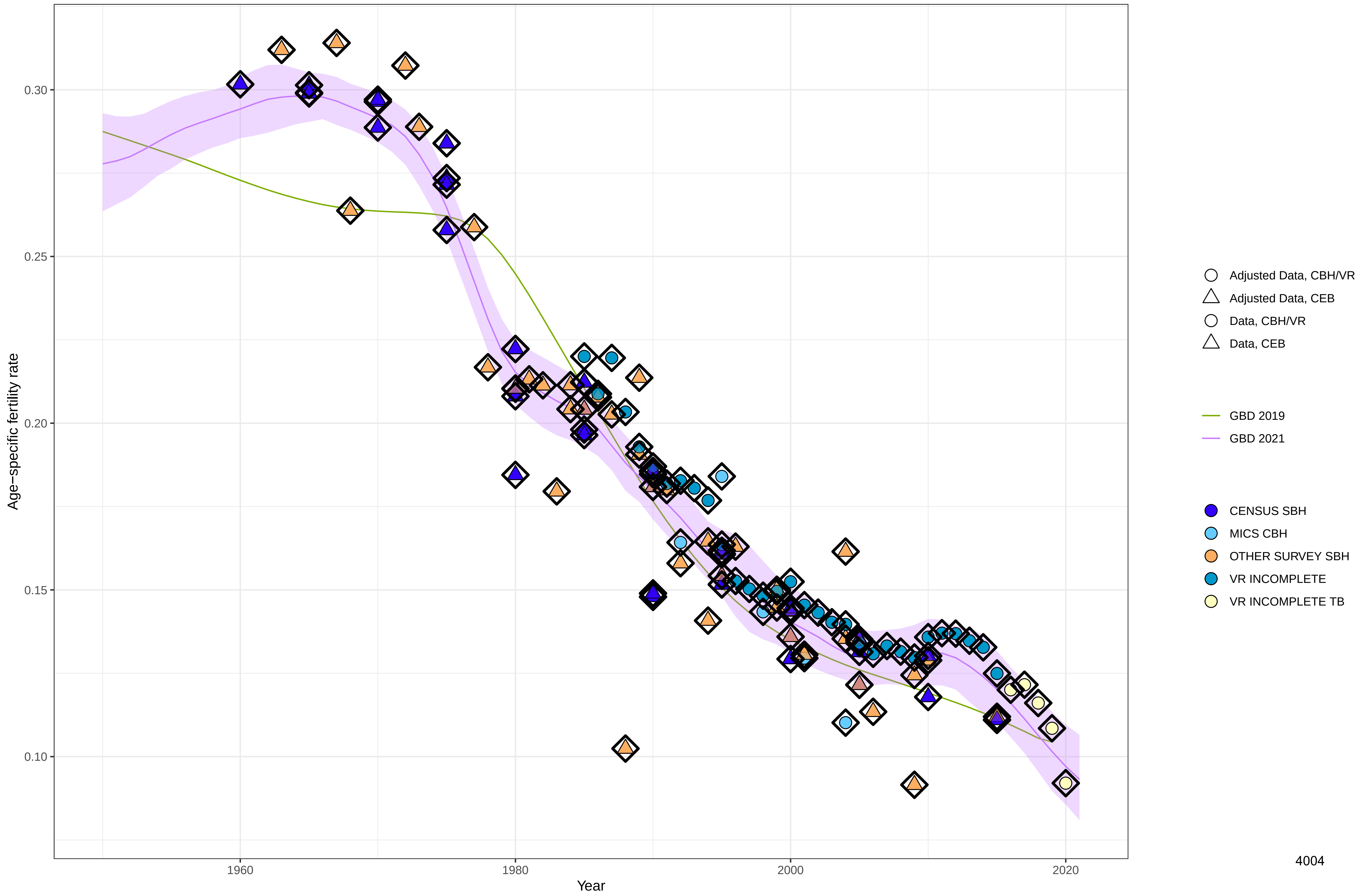

Age−specific fertility fitted trend lines, 1950−2021: Jalisco, Ages: 25−29
Data points enclosed with a diamond are reference sources

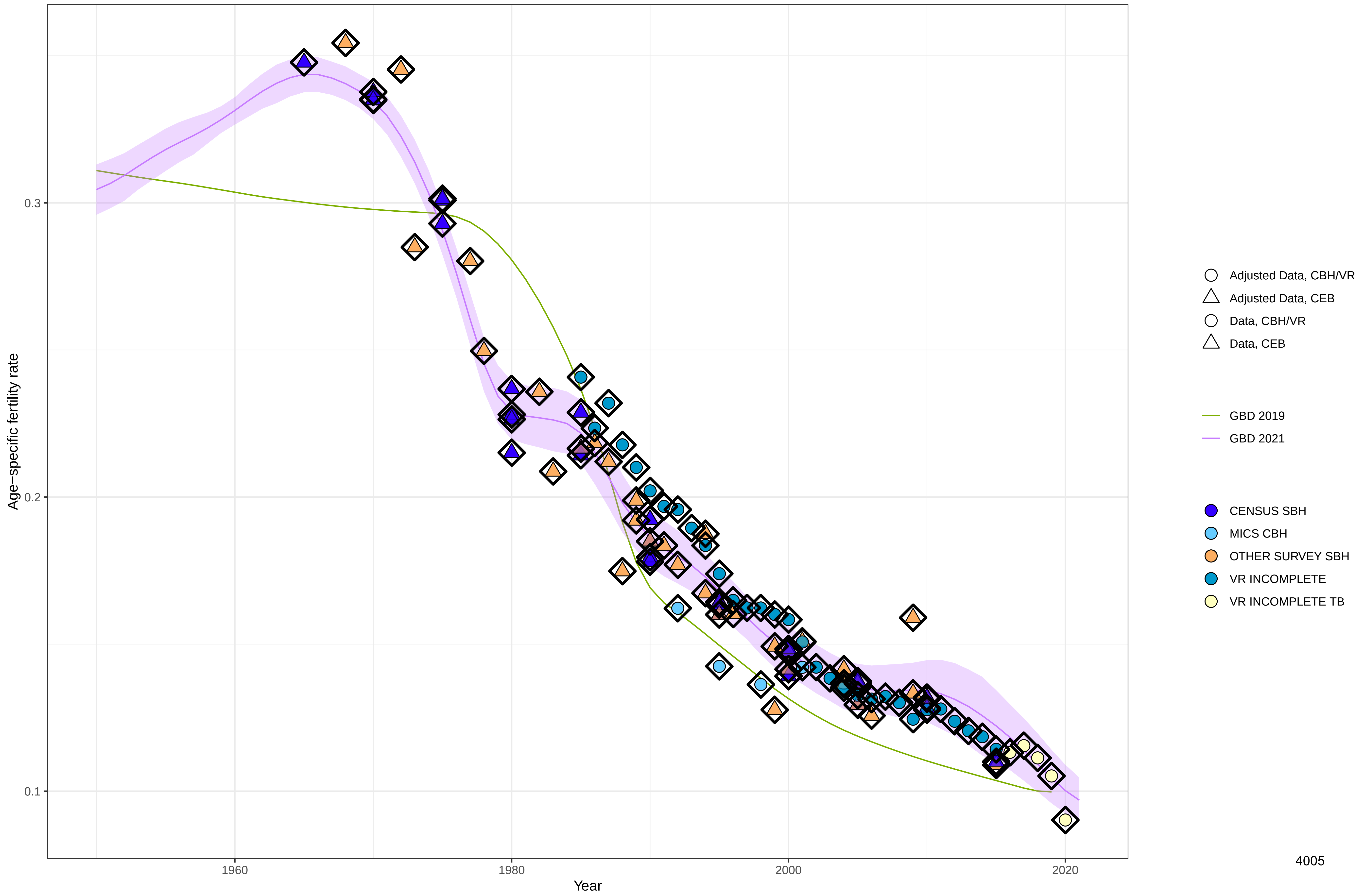

Age−specific fertility fitted trend lines, 1950−2021: Jalisco, Ages: 30−34
Data points enclosed with a diamond are reference sources

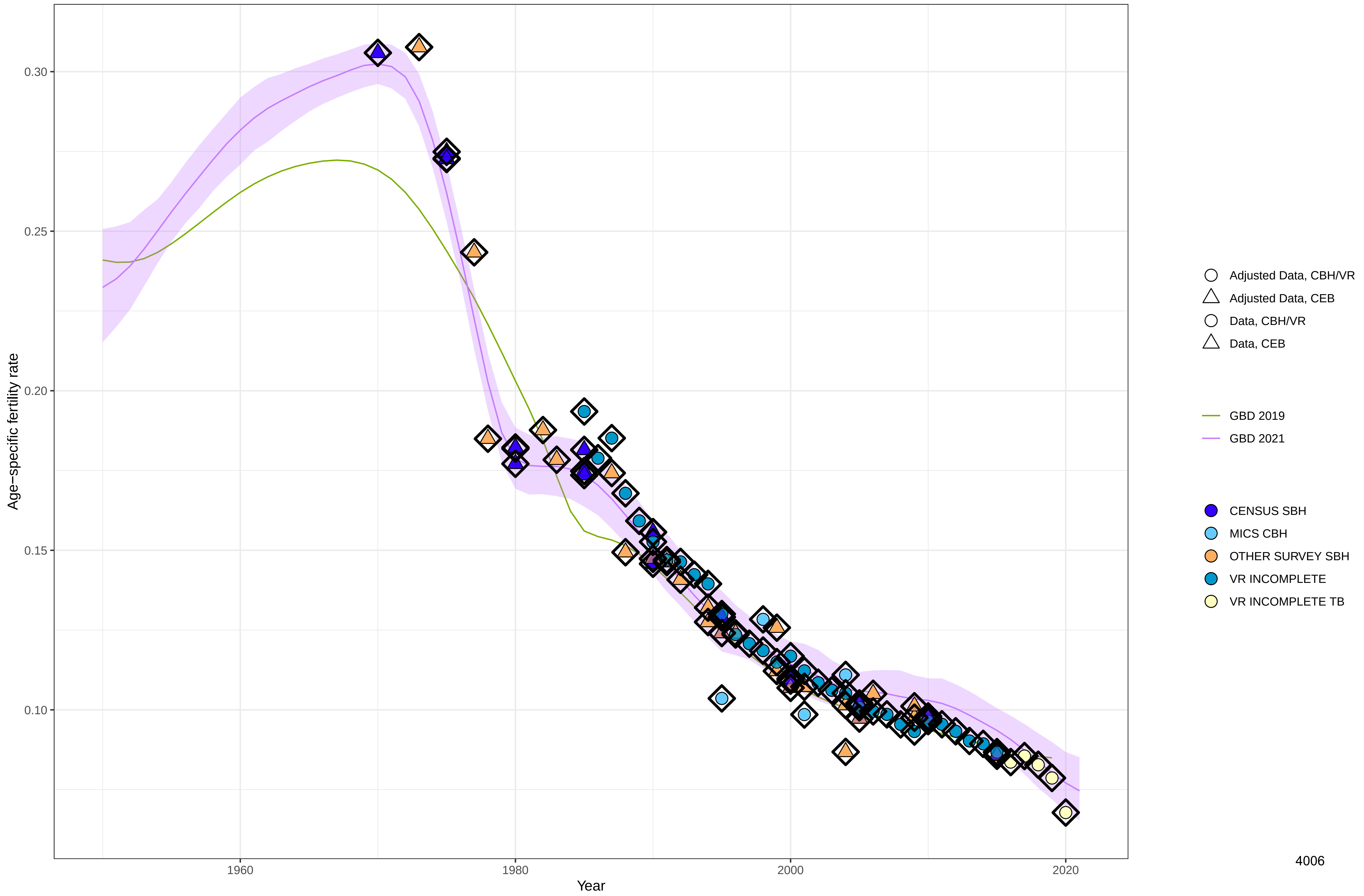

Age−specific fertility fitted trend lines, 1950−2021: Jalisco, Ages: 35−39
Data points enclosed with a diamond are reference sources

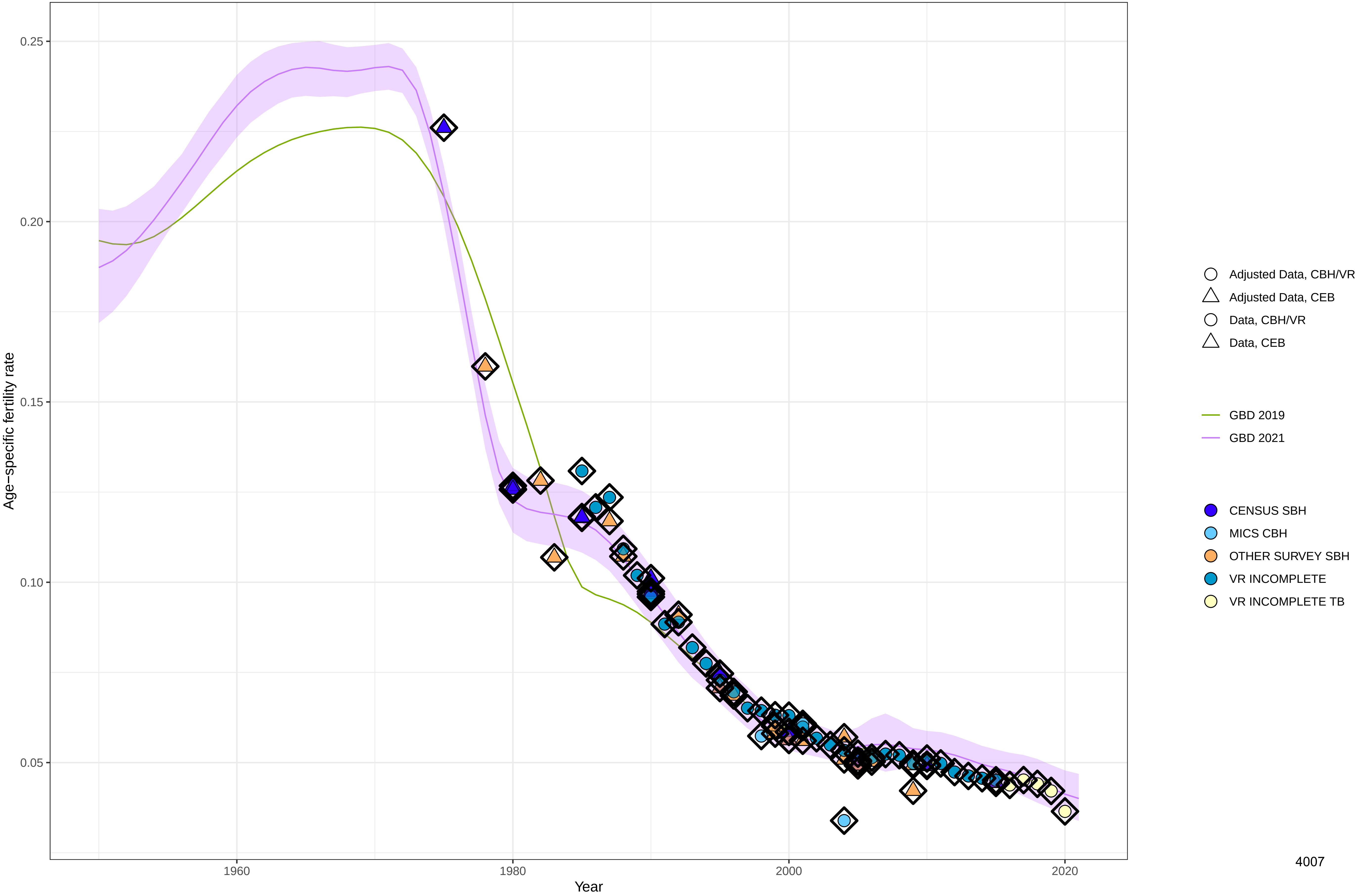

Age−specific fertility fitted trend lines, 1950−2021: Jalisco, Ages: 40−44
Data points enclosed with a diamond are reference sources

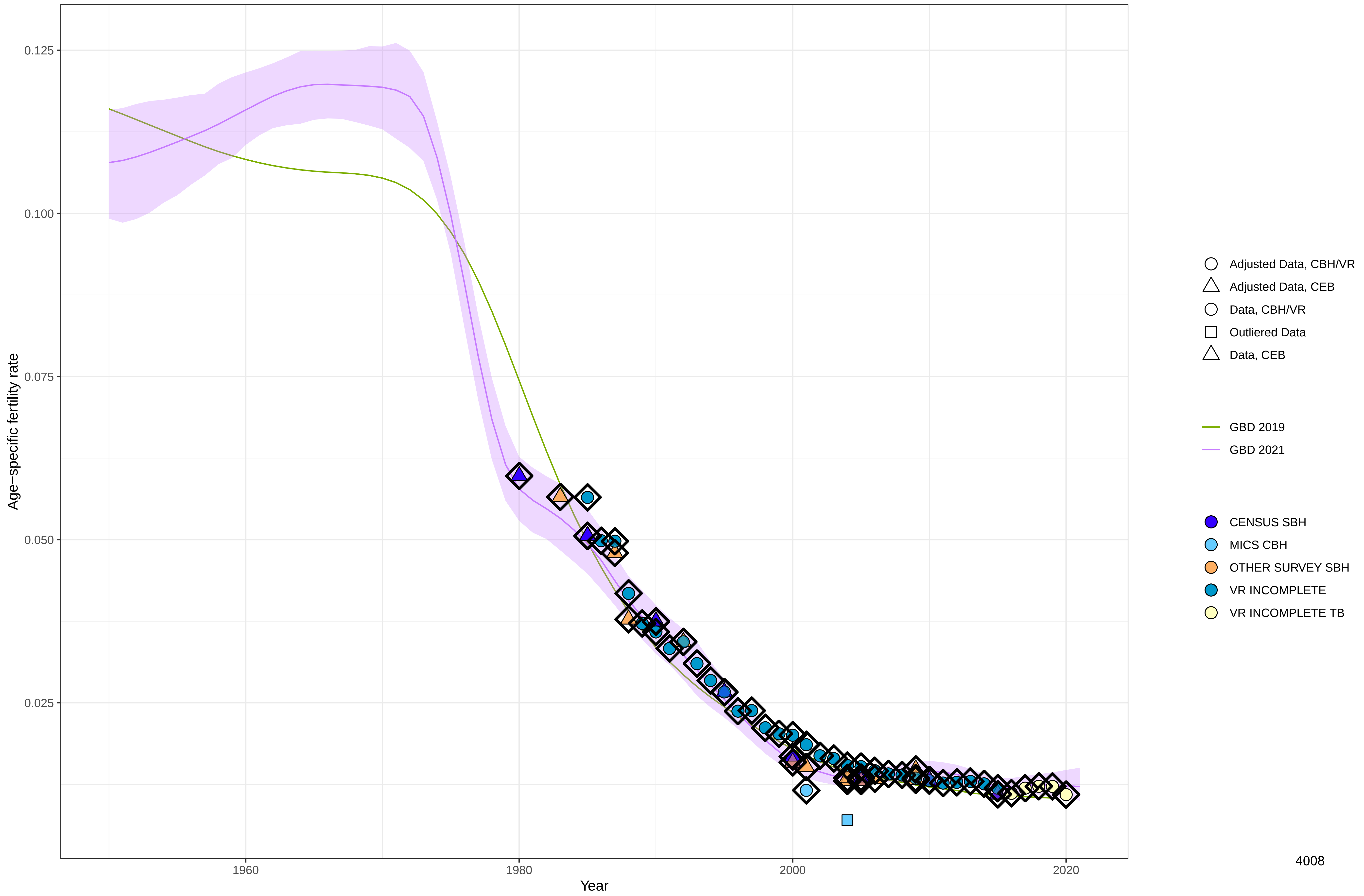

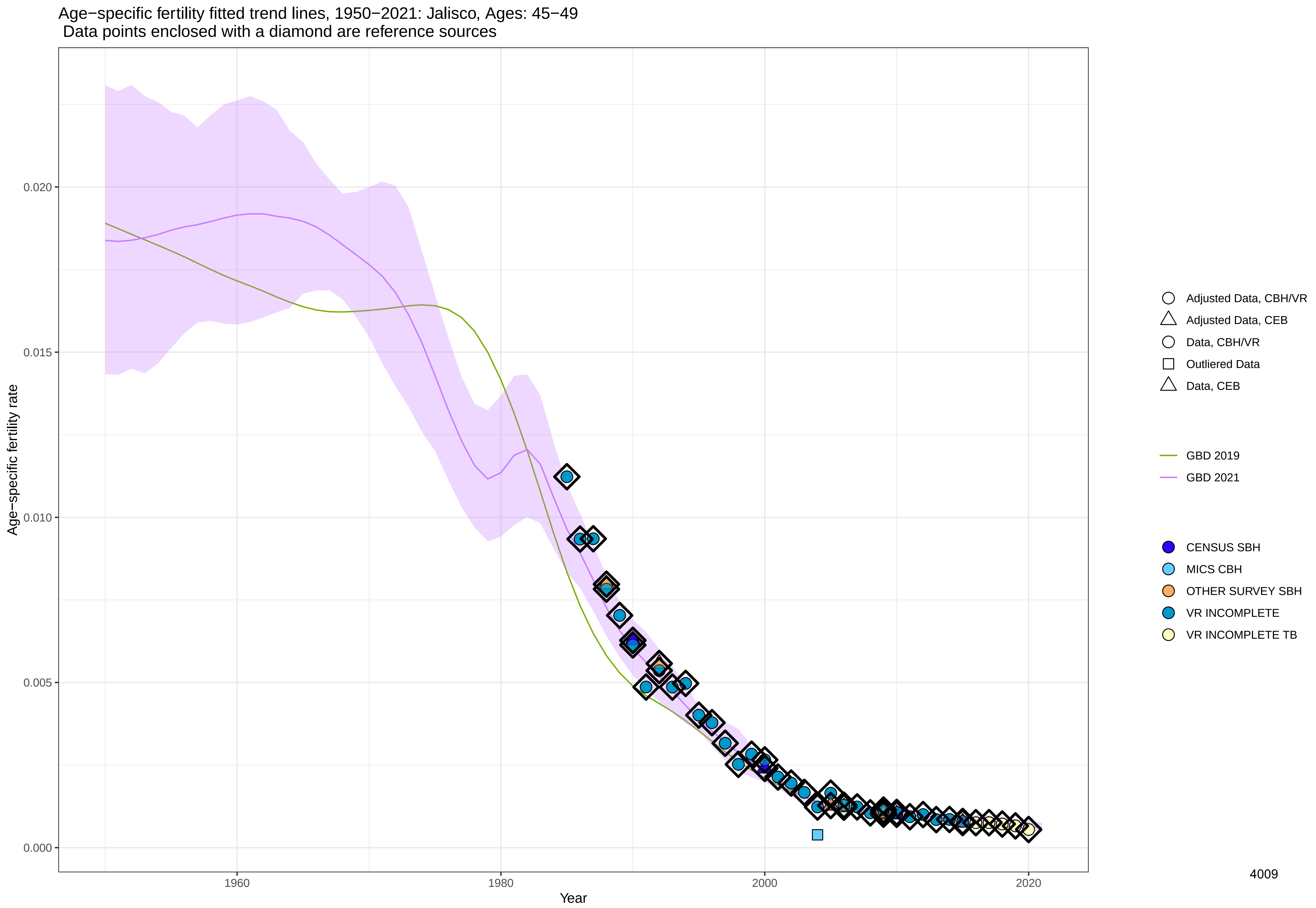

Age−specific fertility fitted trend lines, 1950−2021: Jalisco, Ages: 45−49
Data points enclosed with a diamond are reference sources
Age−specific fertility rate
0.000
0.005
0.010
0.015
0.020
Year
1960
1980
2000
2020
Adjusted Data, CBH/VR
Adjusted Data, CEB
Data, CBH/VR
Outliered Data
Data, CEB
GBD 2019
GBD 2021
CENSUS SBH
MICS CBH
OTHER SURVEY SBH
VR INCOMPLETE
VR INCOMPLETE TB

4009

Age−specific fertility fitted trend lines, 1950−2021: Jalisco, Ages: 50−54
Data points enclosed with a diamond are reference sources

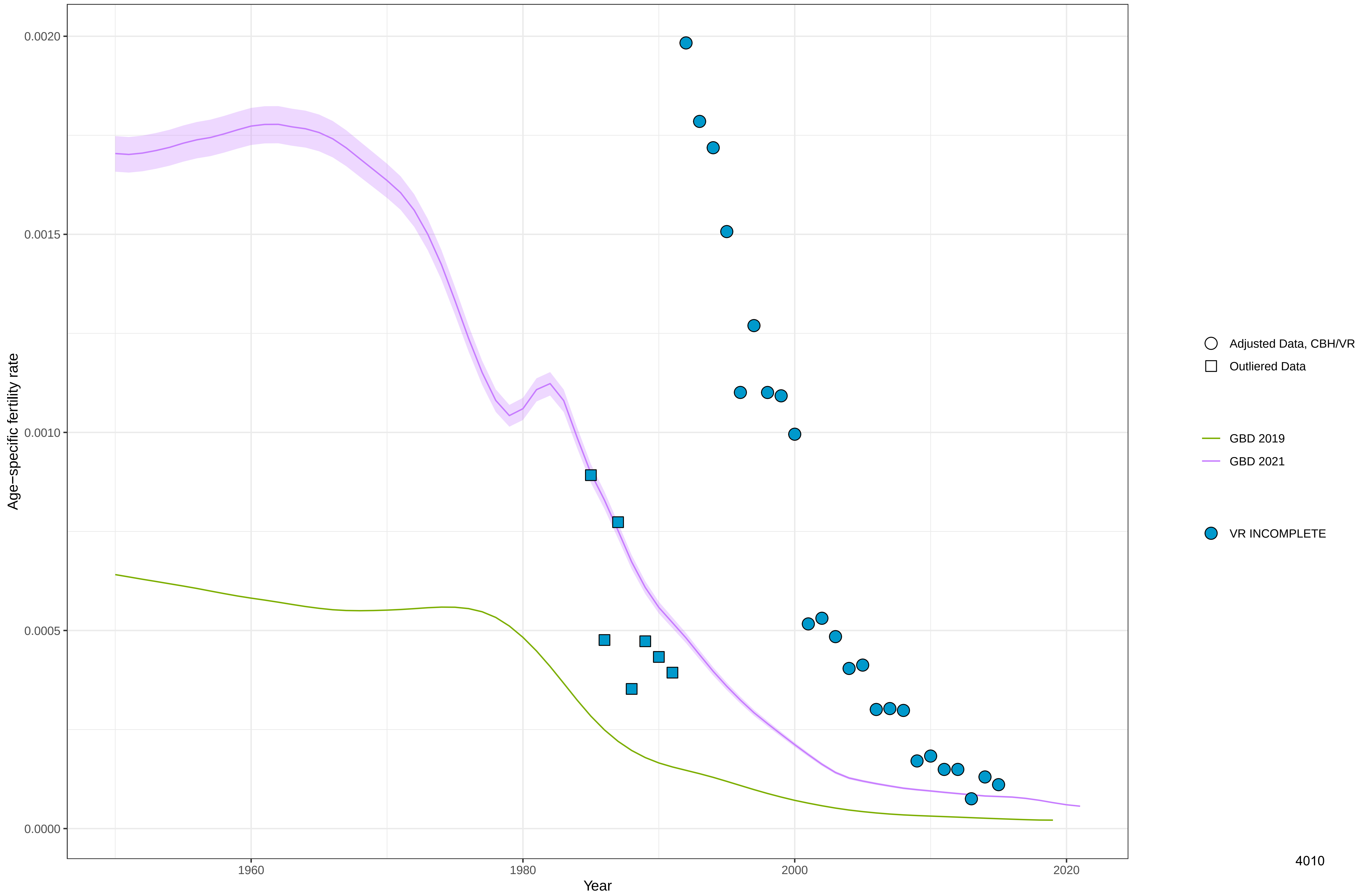

Total fertility rate fitted trend lines, 1950−2021: Jalisco TFR
Data points enclosed with a diamond are reference sources

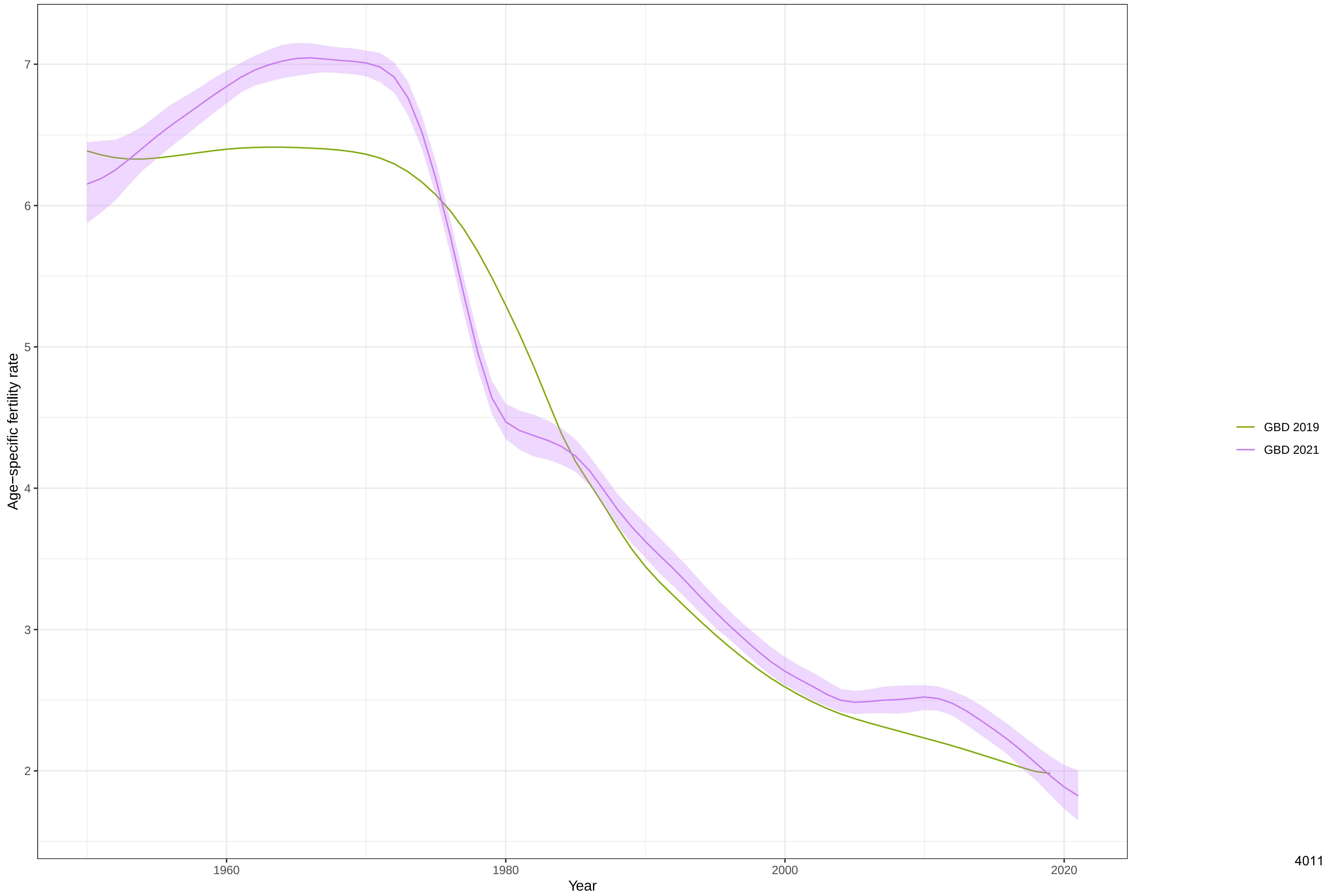

Age−specific fertility fitted trend lines, 1950−2021: México, Ages: 10−14
Data points enclosed with a diamond are reference sources

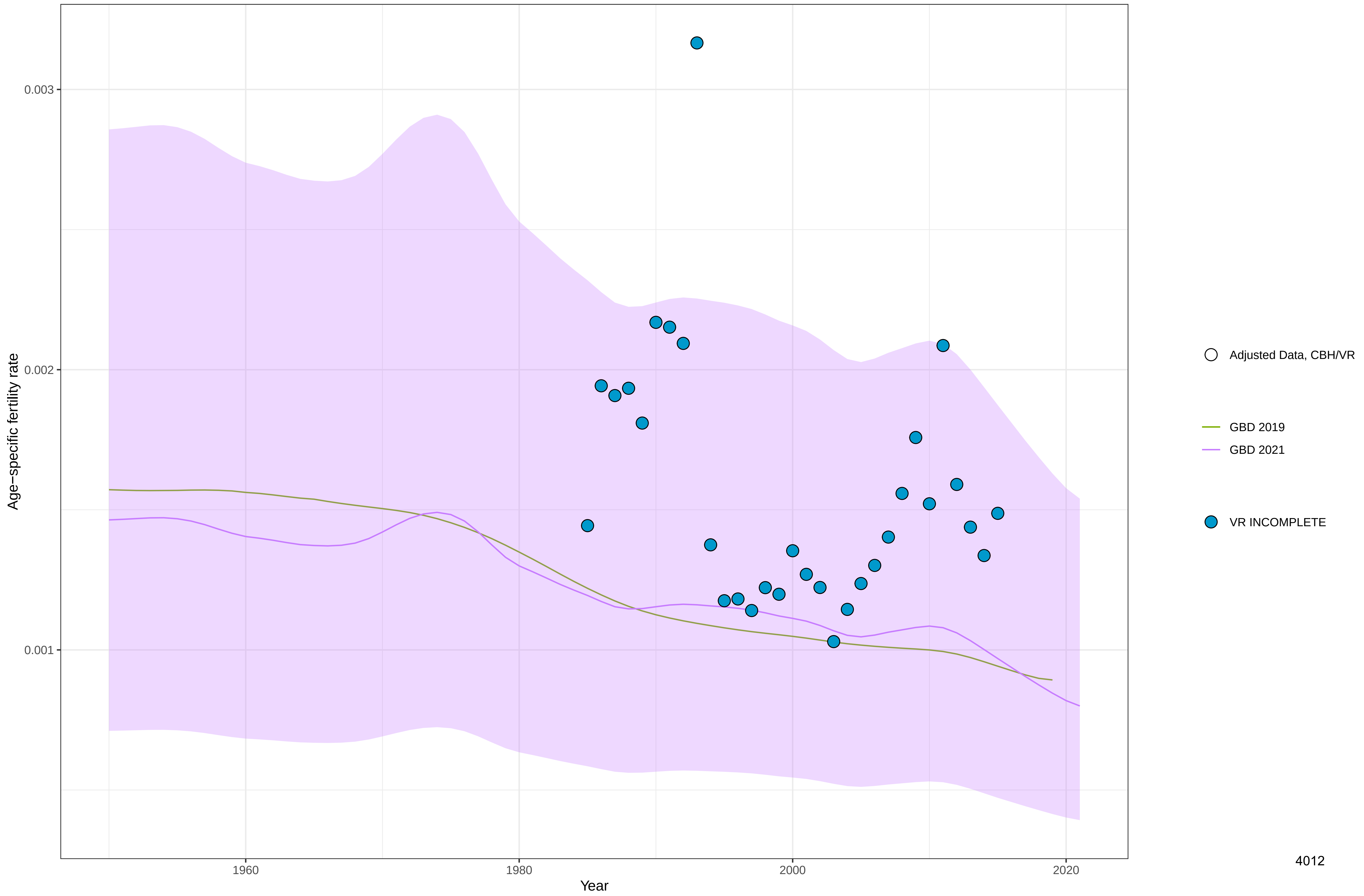

Age−specific fertility fitted trend lines, 1950−2021: México, Ages: 15−19
Data points enclosed with a diamond are reference sources

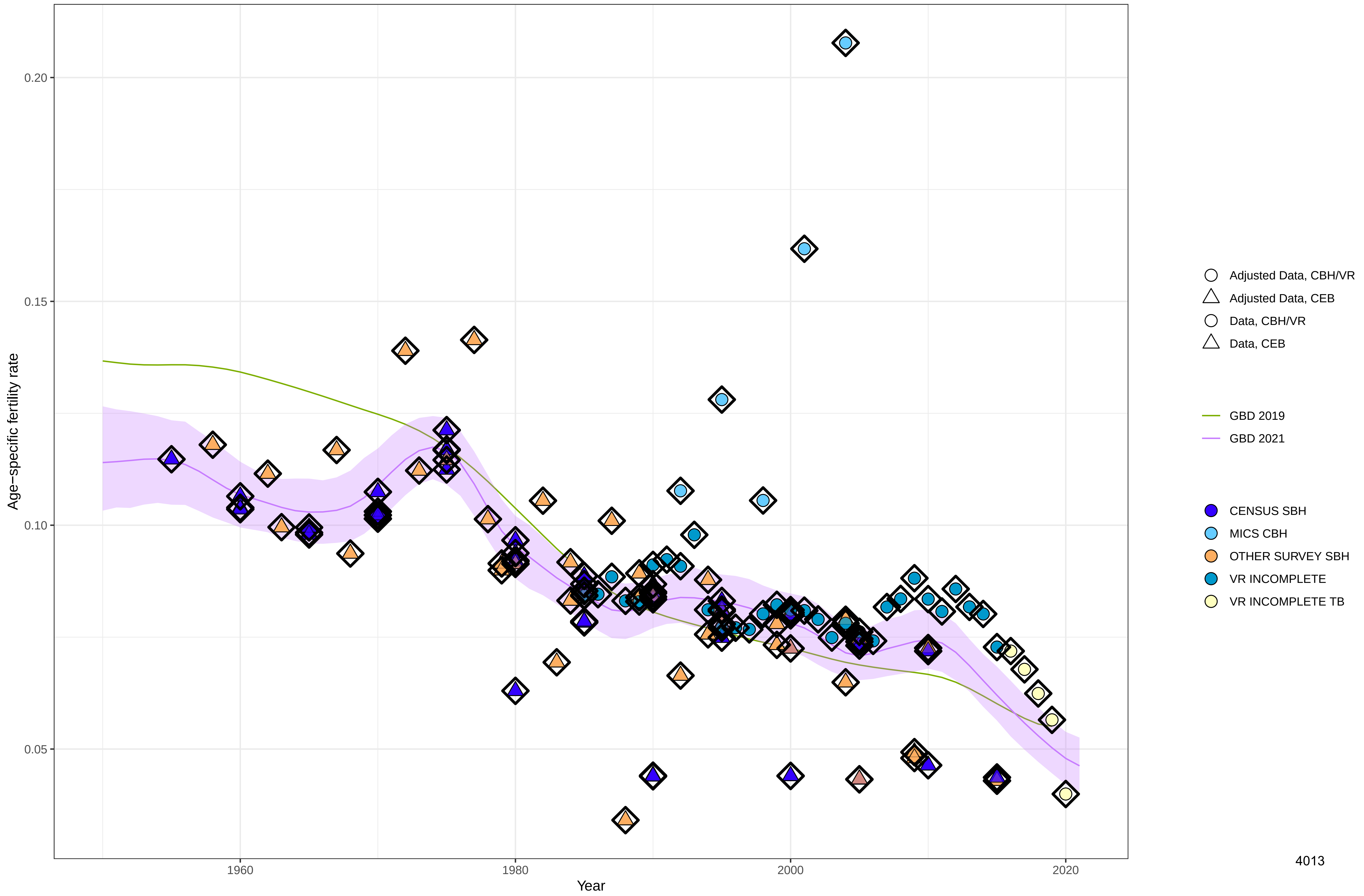

Age−specific fertility fitted trend lines, 1950−2021: México, Ages: 20−24
Data points enclosed with a diamond are reference sources

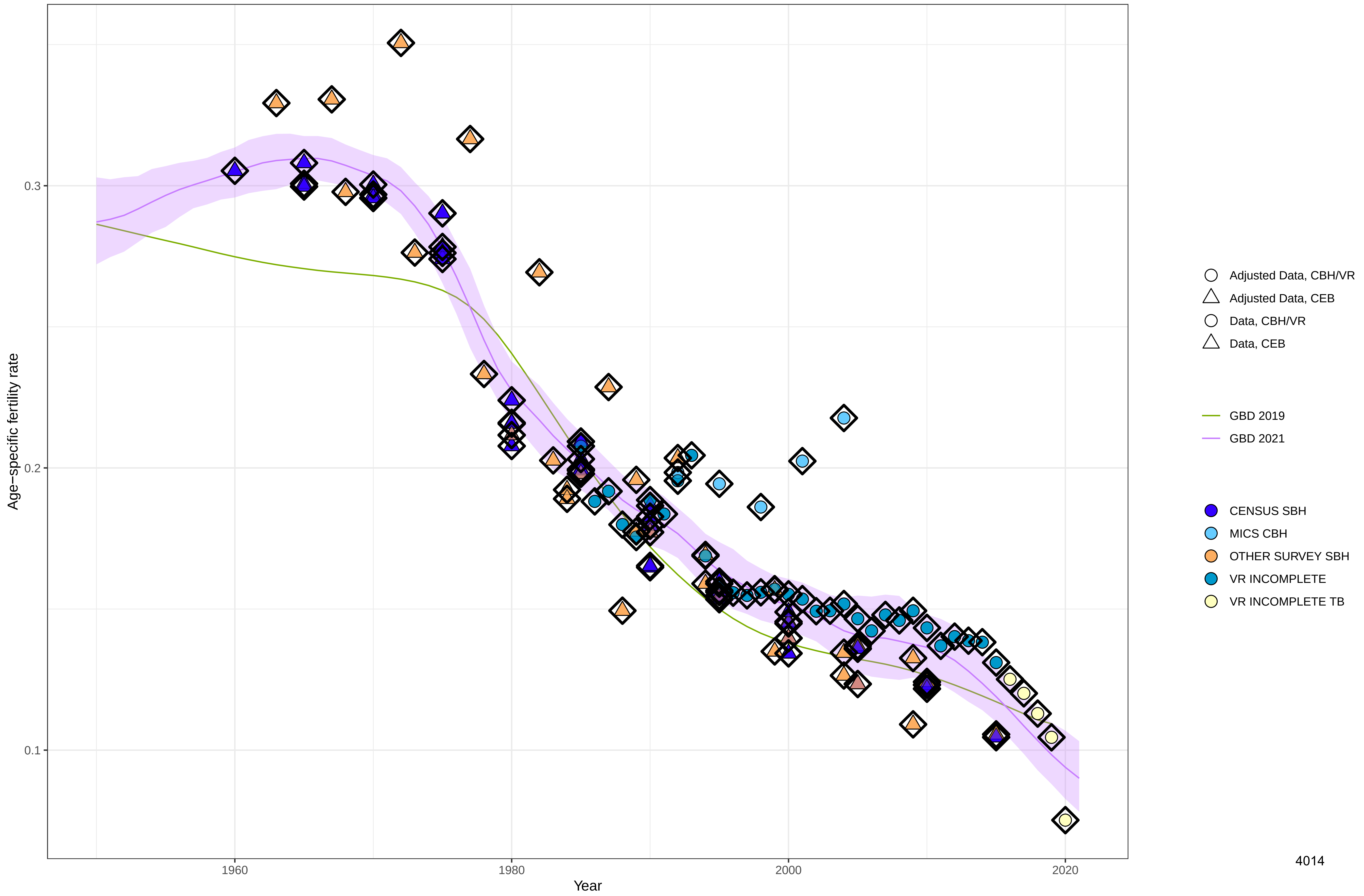

Age−specific fertility fitted trend lines, 1950−2021: México, Ages: 25−29
Data points enclosed with a diamond are reference sources

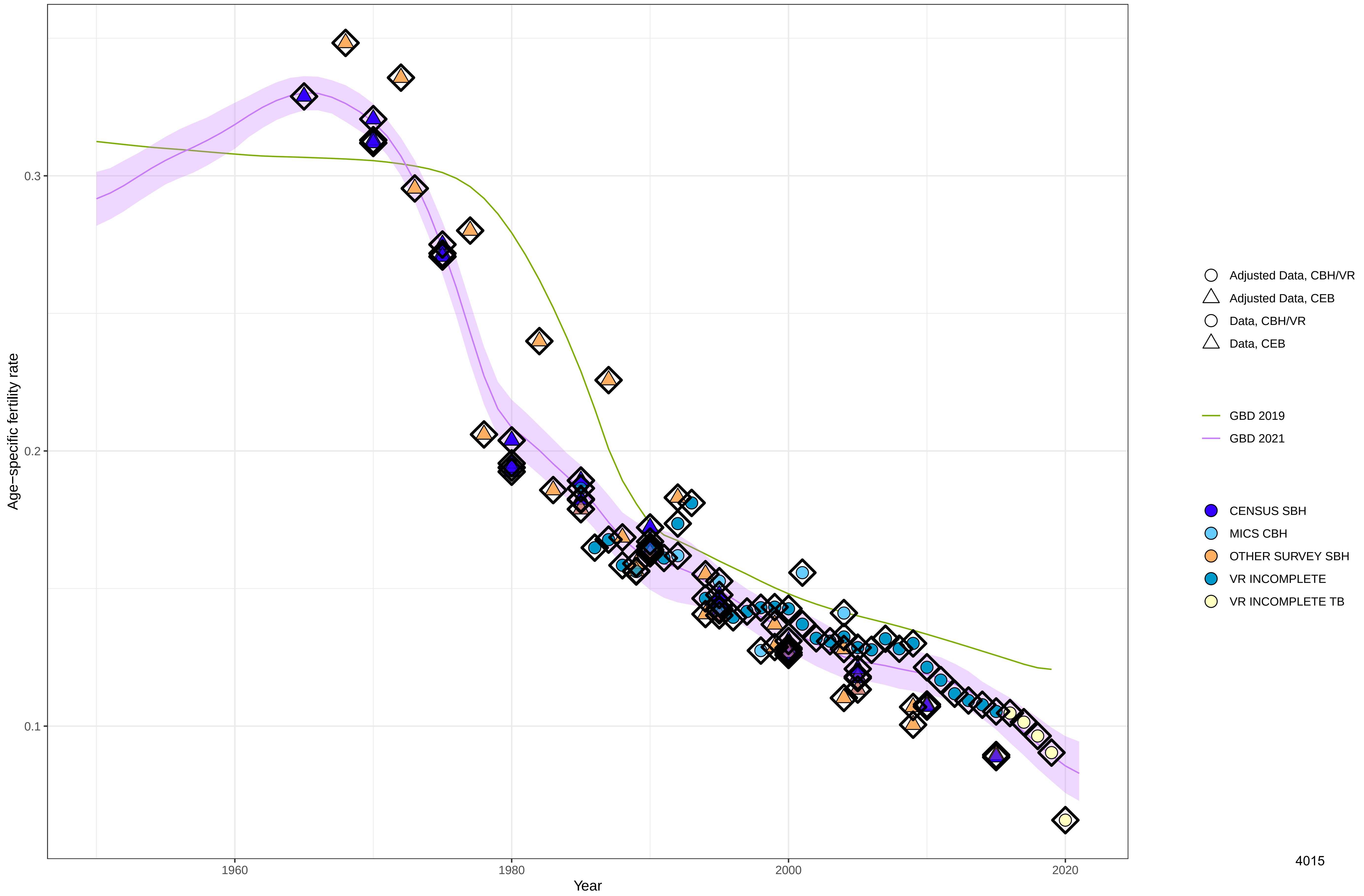

Age−specific fertility fitted trend lines, 1950−2021: México, Ages: 30−34
Data points enclosed with a diamond are reference sources

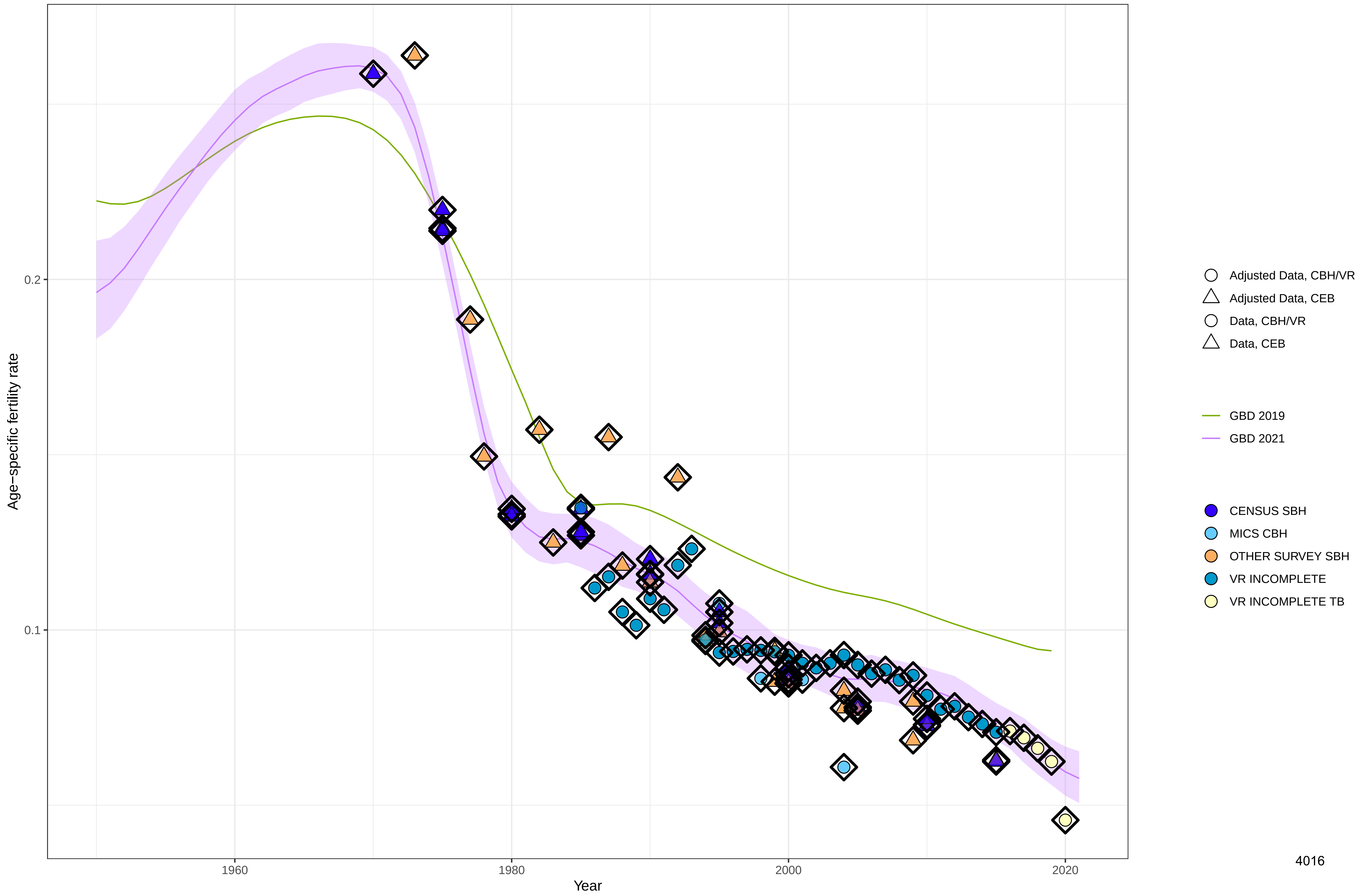

Age−specific fertility fitted trend lines, 1950−2021: México, Ages: 35−39
Data points enclosed with a diamond are reference sources

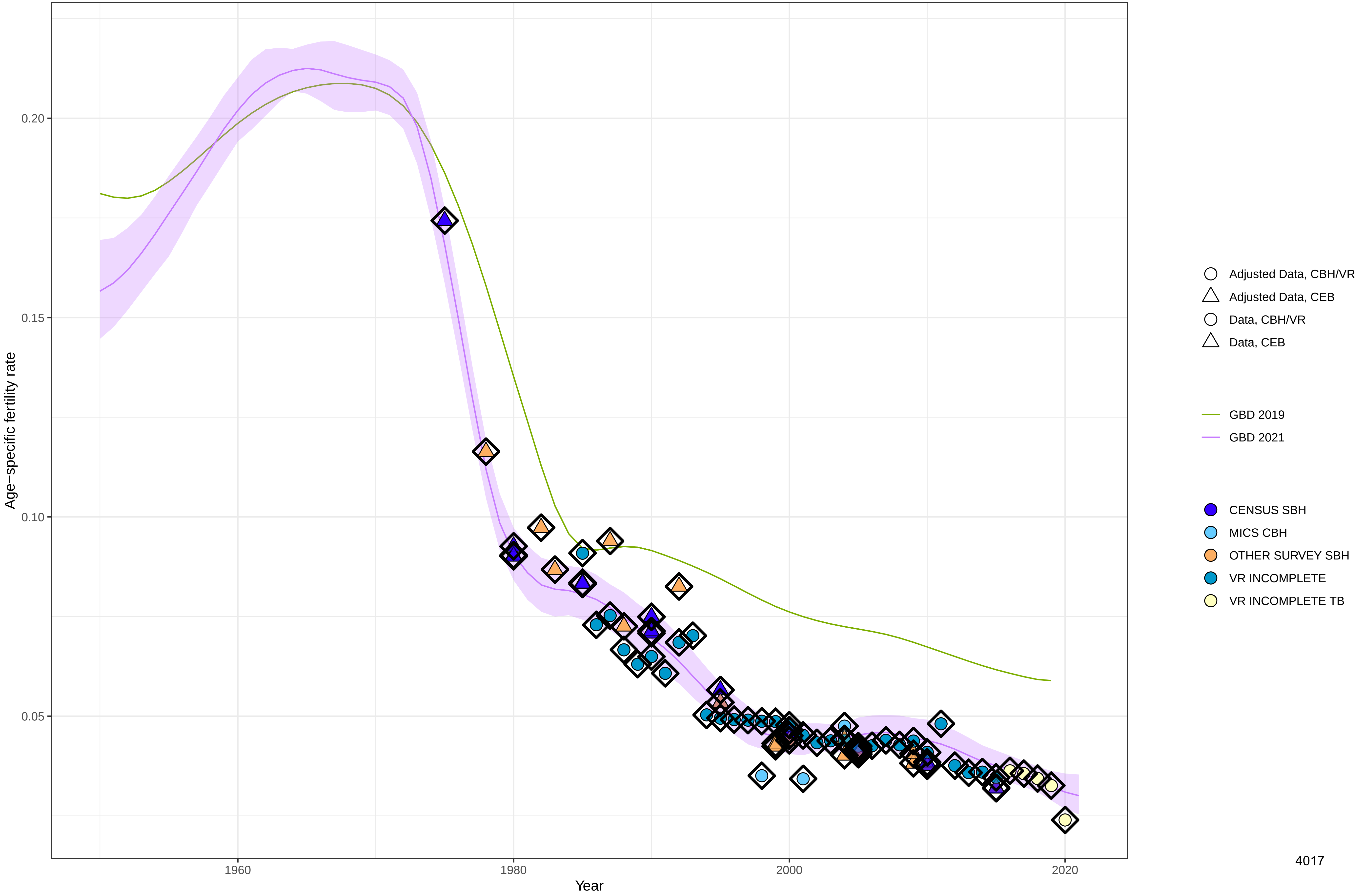

Age−specific fertility fitted trend lines, 1950−2021: México, Ages: 40−44
Data points enclosed with a diamond are reference sources

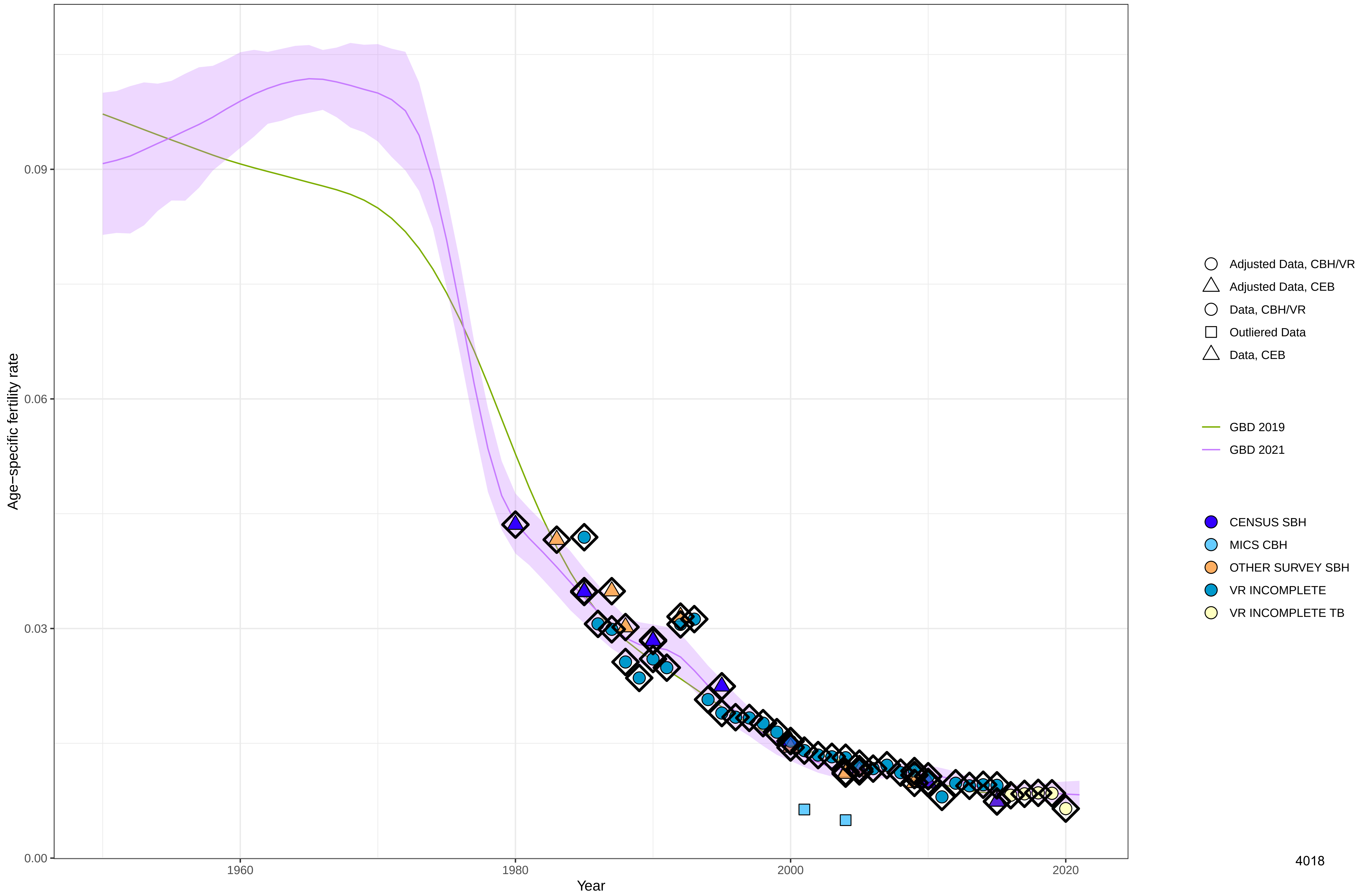

Age−specific fertility fitted trend lines, 1950−2021: México, Ages: 45−49
Data points enclosed with a diamond are reference sources

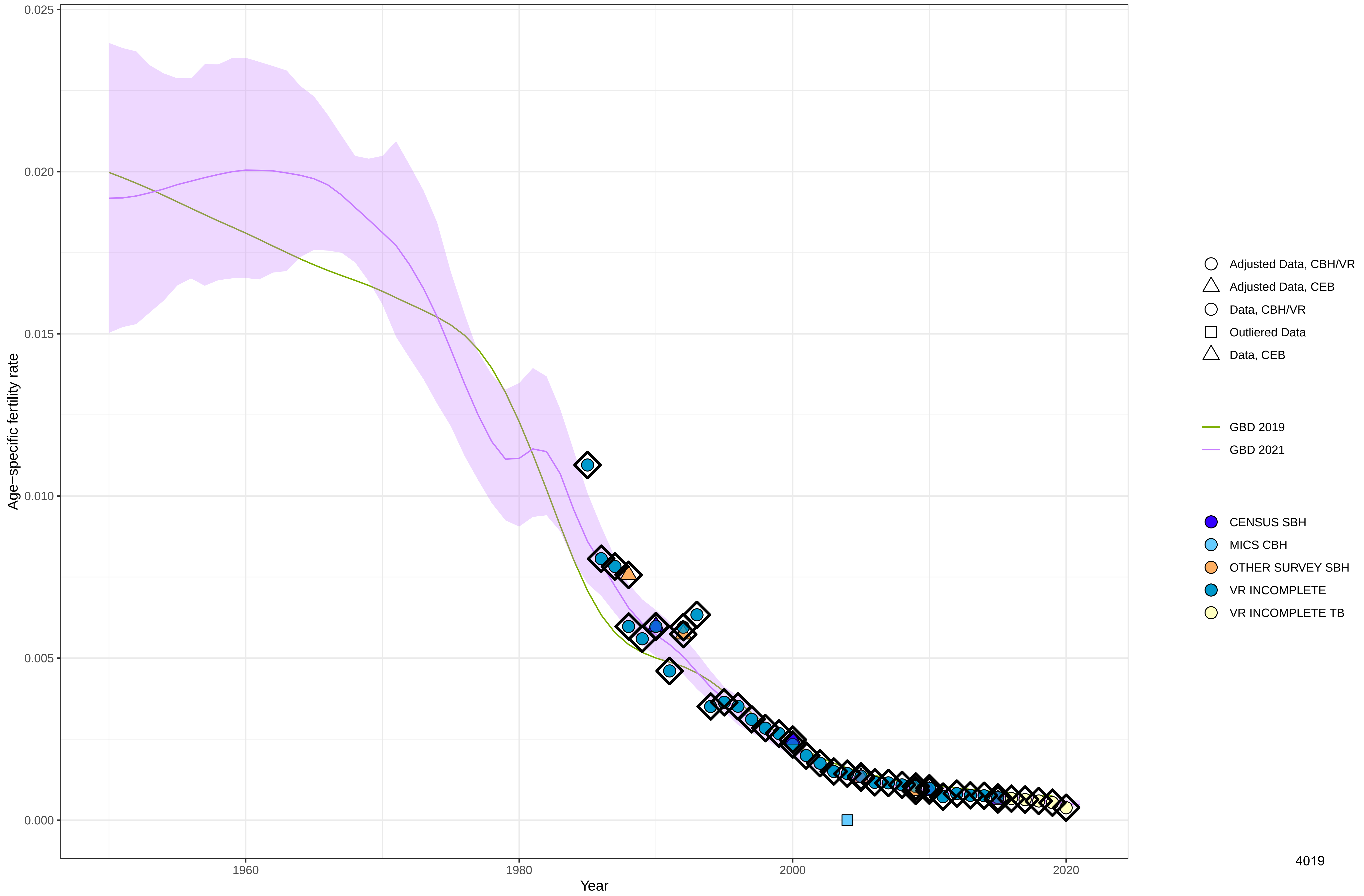

Age−specific fertility fitted trend lines, 1950−2021: México, Ages: 50−54
Data points enclosed with a diamond are reference sources

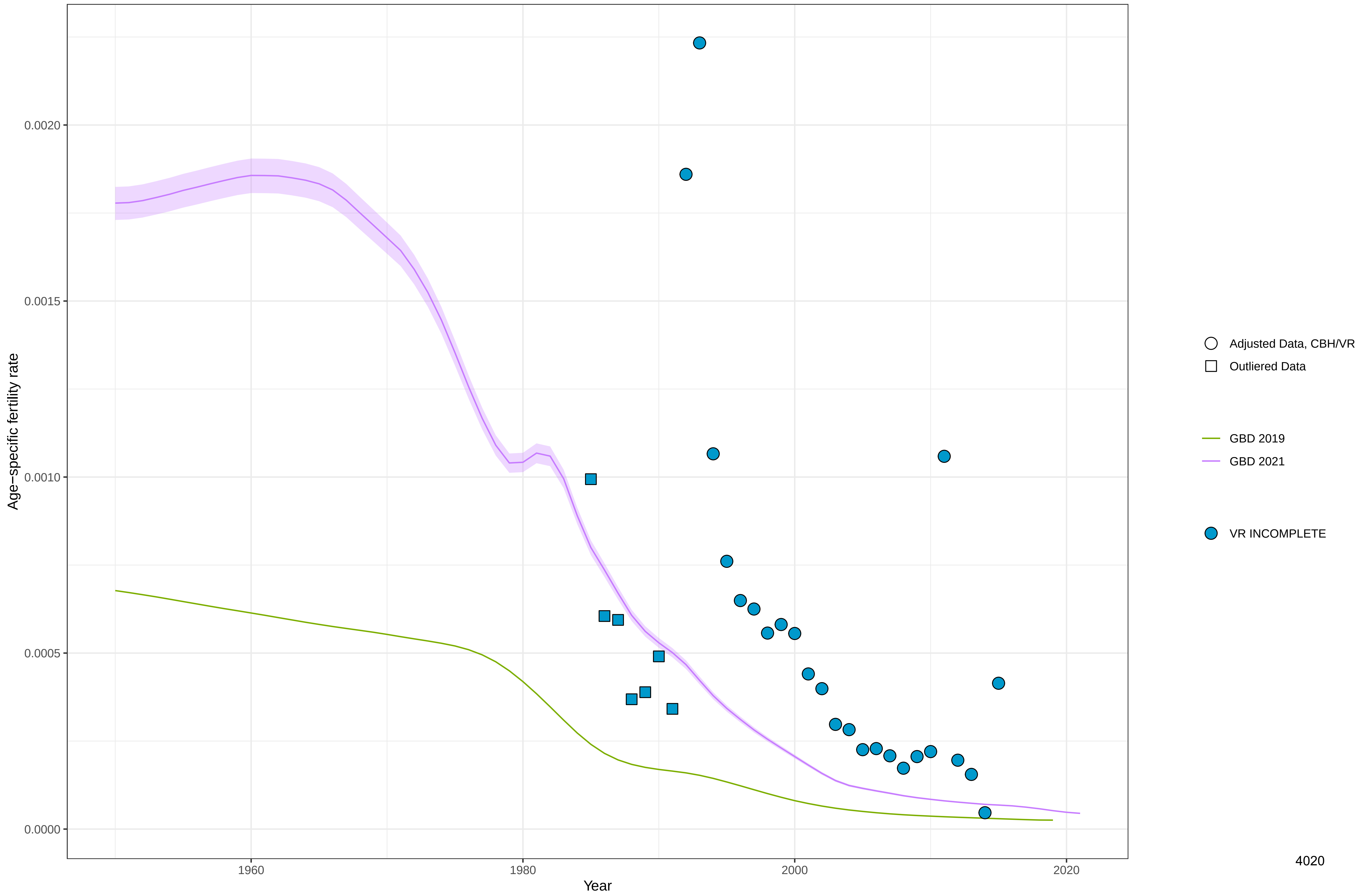

Total fertility rate fitted trend lines, 1950−2021: México TFR
Data points enclosed with a diamond are reference sources

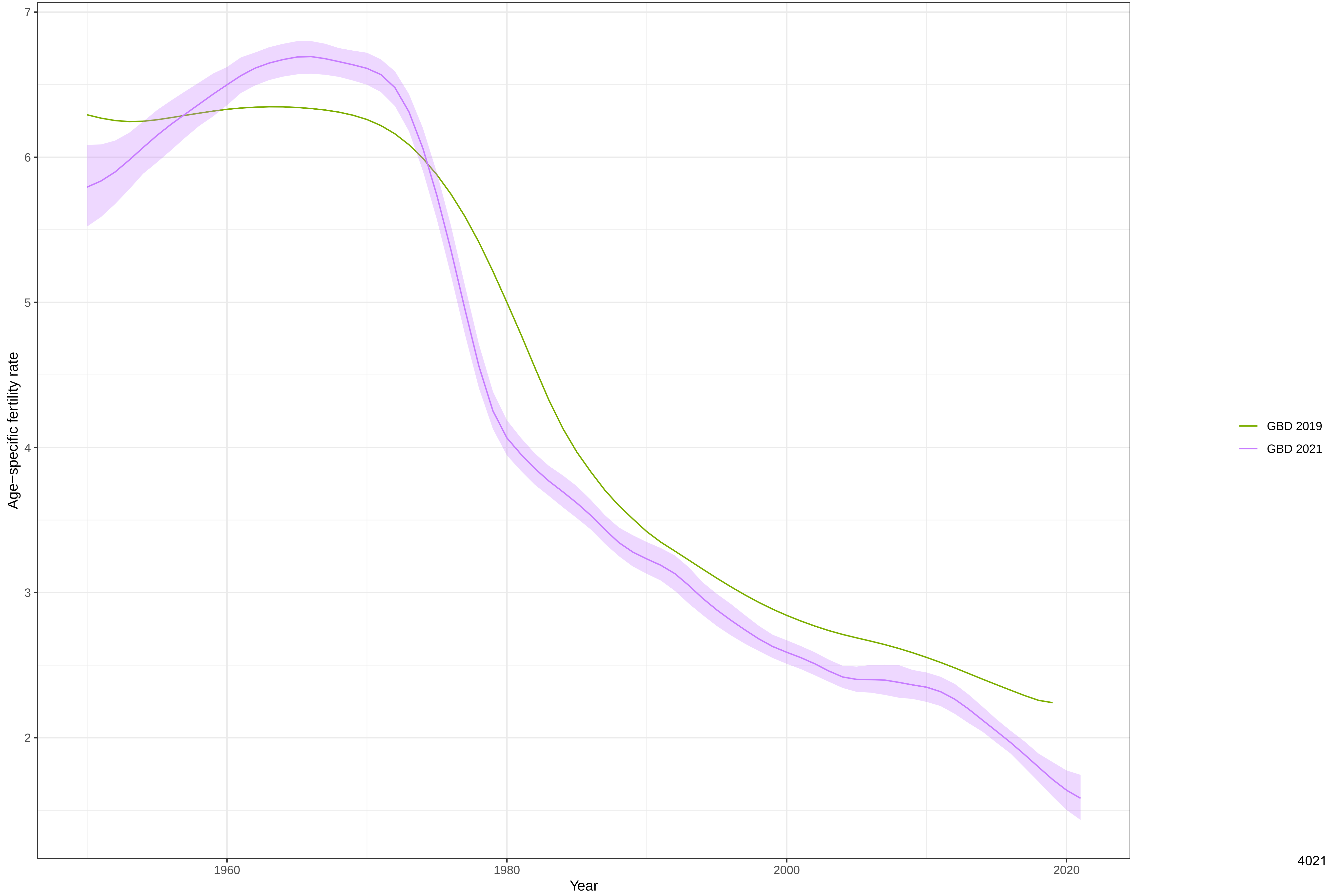

Age−specific fertility fitted trend lines, 1950−2021: Mexico City, Ages: 10−14
Data points enclosed with a diamond are reference sources

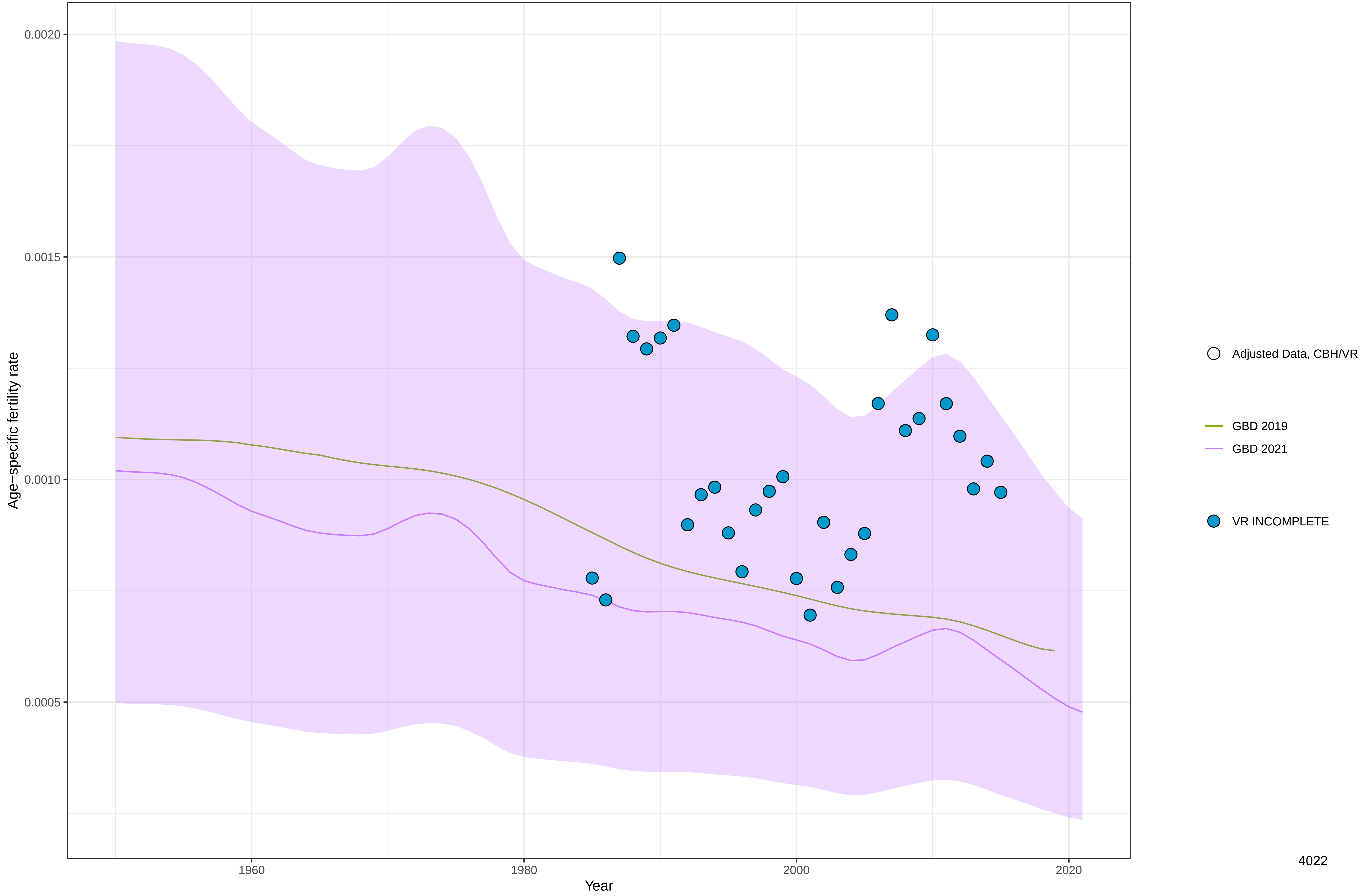

Age−specific fertility fitted trend lines, 1950−2021: Mexico City, Ages: 15−19
Data points enclosed with a diamond are reference sources

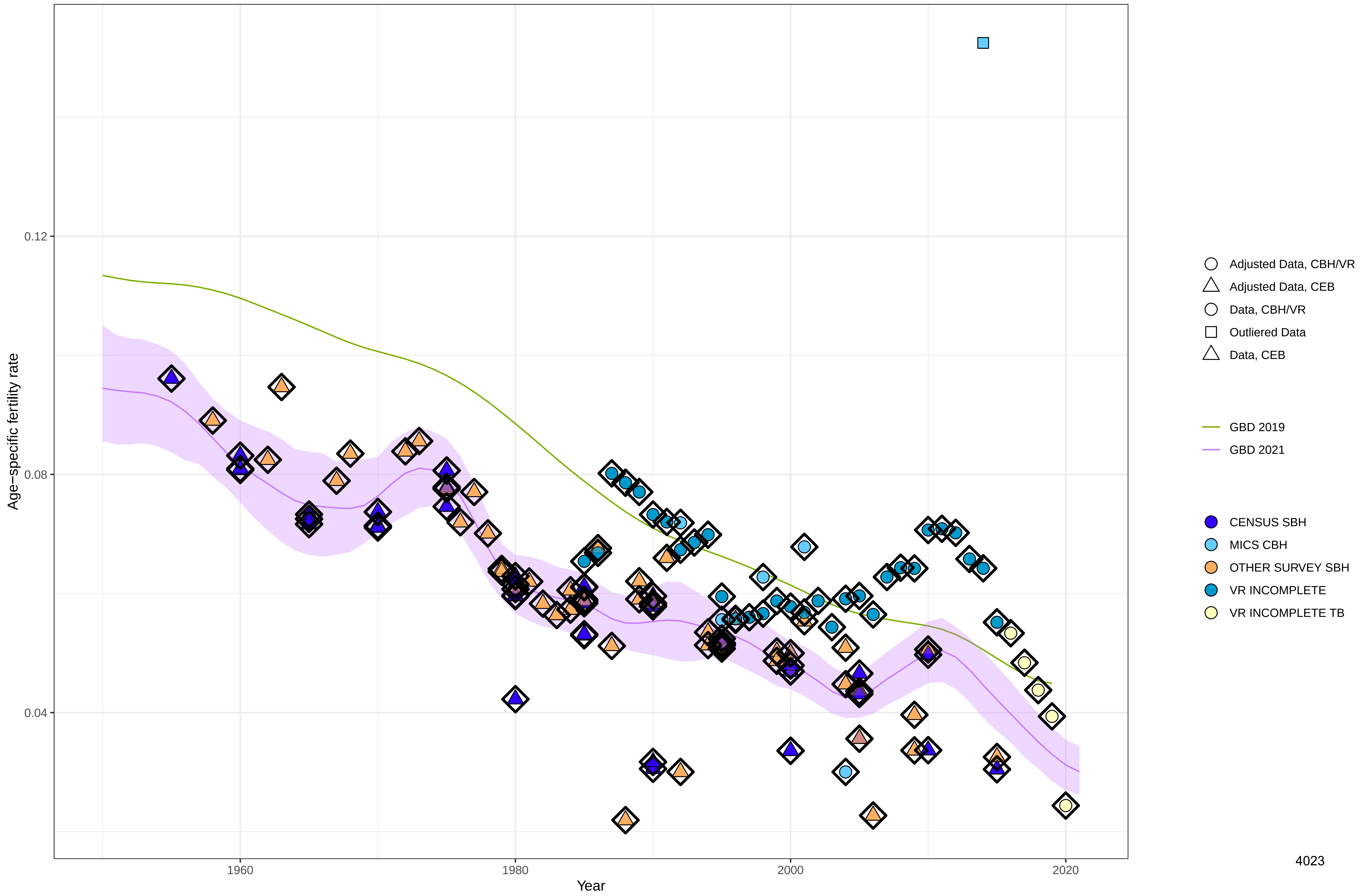

Age−specific fertility fitted trend lines, 1950−2021: Mexico City, Ages: 20−24
Data points enclosed with a diamond are reference sources

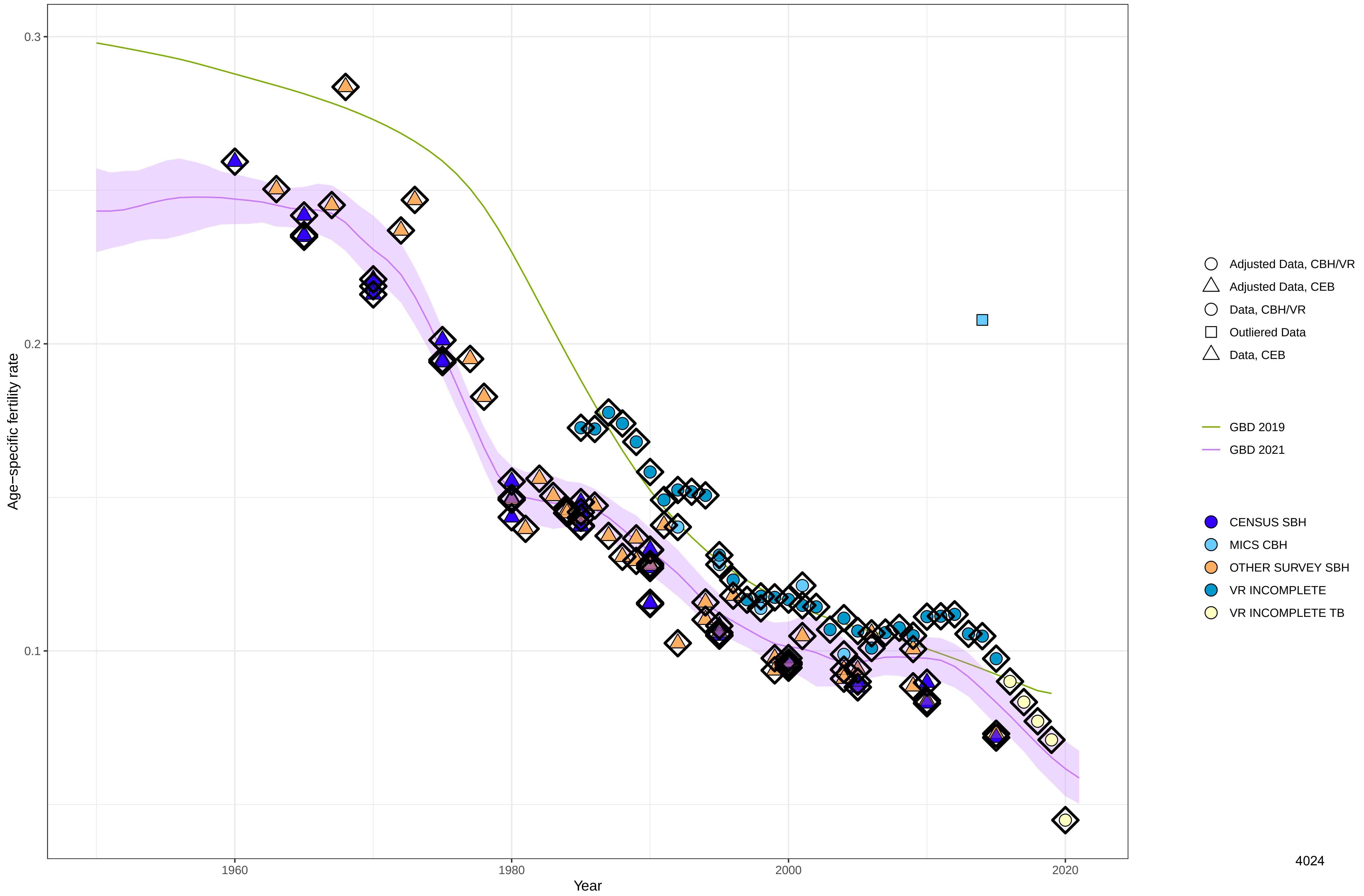

Age−specific fertility fitted trend lines, 1950−2021: Mexico City, Ages: 25−29
Data points enclosed with a diamond are reference sources

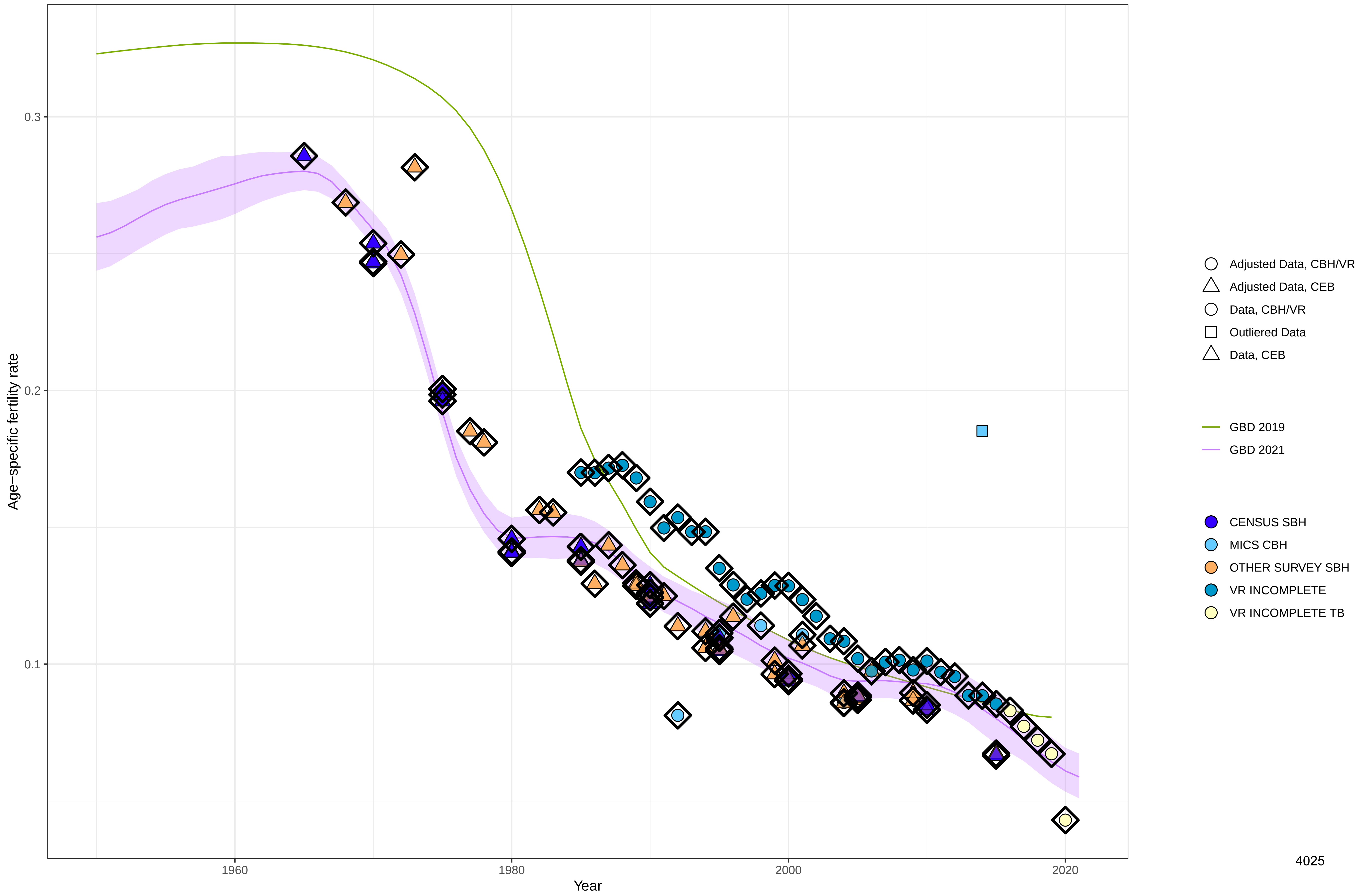

Age−specific fertility fitted trend lines, 1950−2021: Mexico City, Ages: 30−34
Data points enclosed with a diamond are reference sources

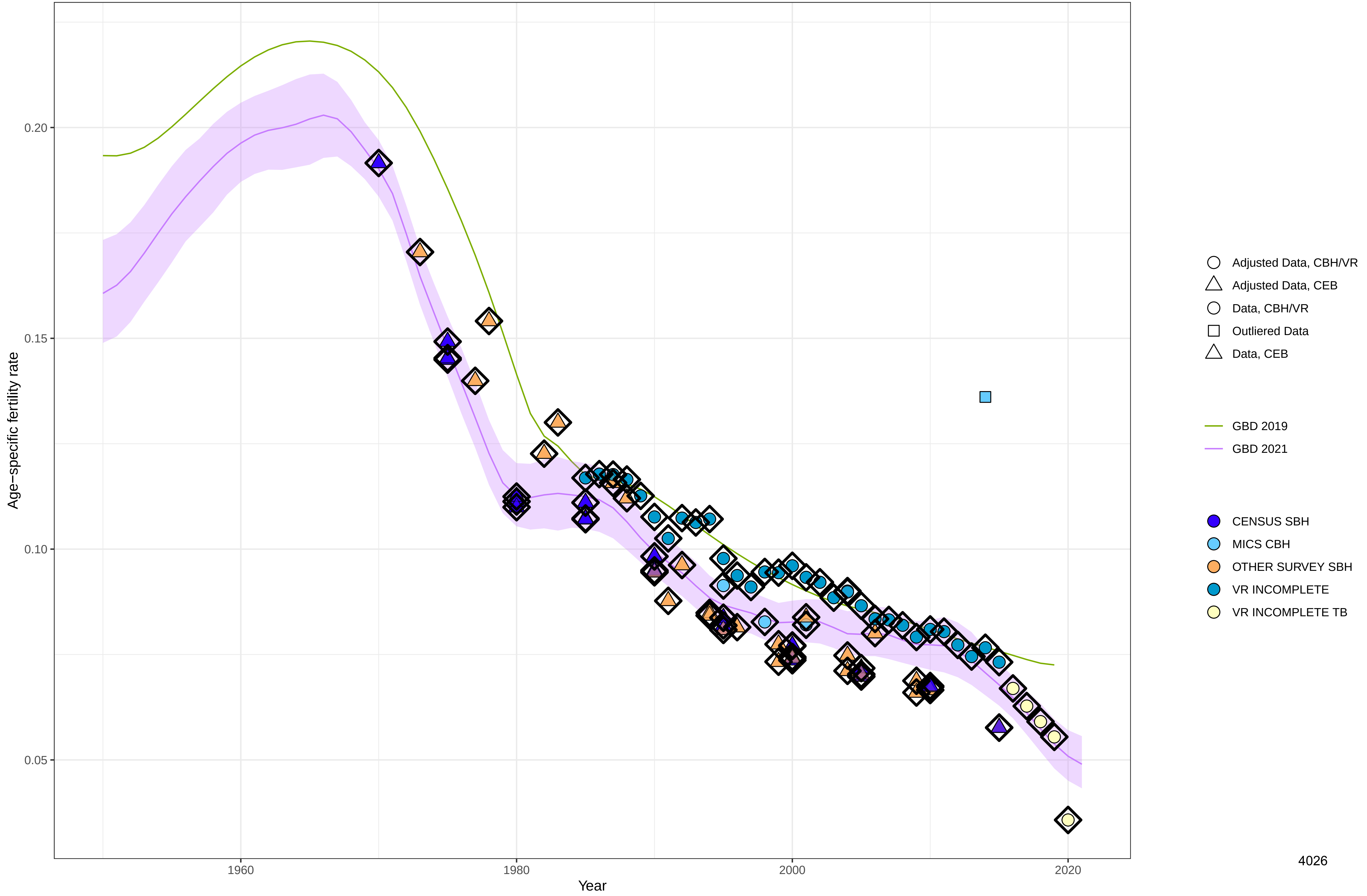

4026

Age−specific fertility fitted trend lines, 1950−2021: Mexico City, Ages: 35−39
Data points enclosed with a diamond are reference sources

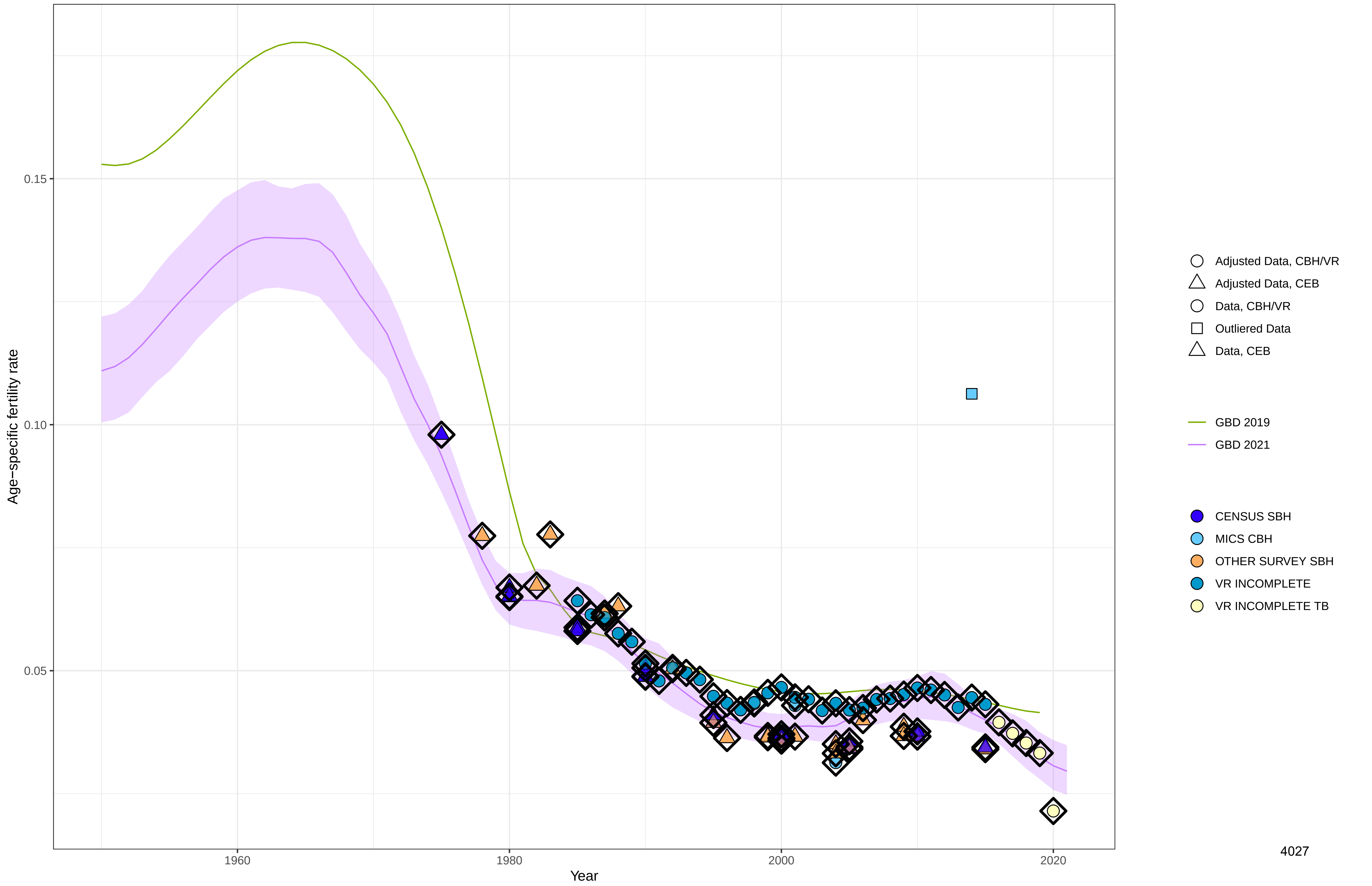

Age−specific fertility fitted trend lines, 1950−2021: Mexico City, Ages: 40−44
Data points enclosed with a diamond are reference sources

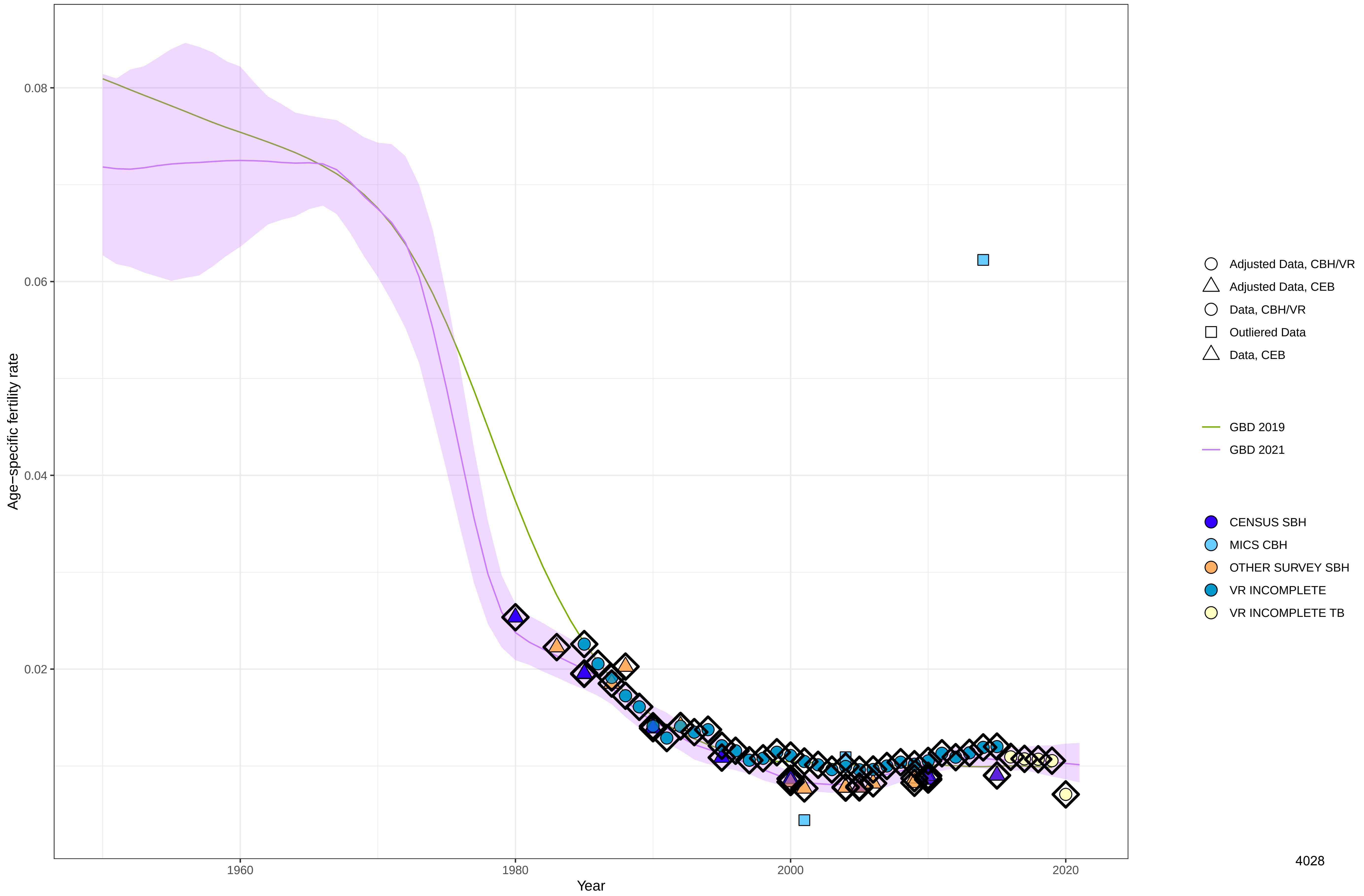

Age−specific fertility fitted trend lines, 1950−2021: Mexico City, Ages: 45−49
Data points enclosed with a diamond are reference sources

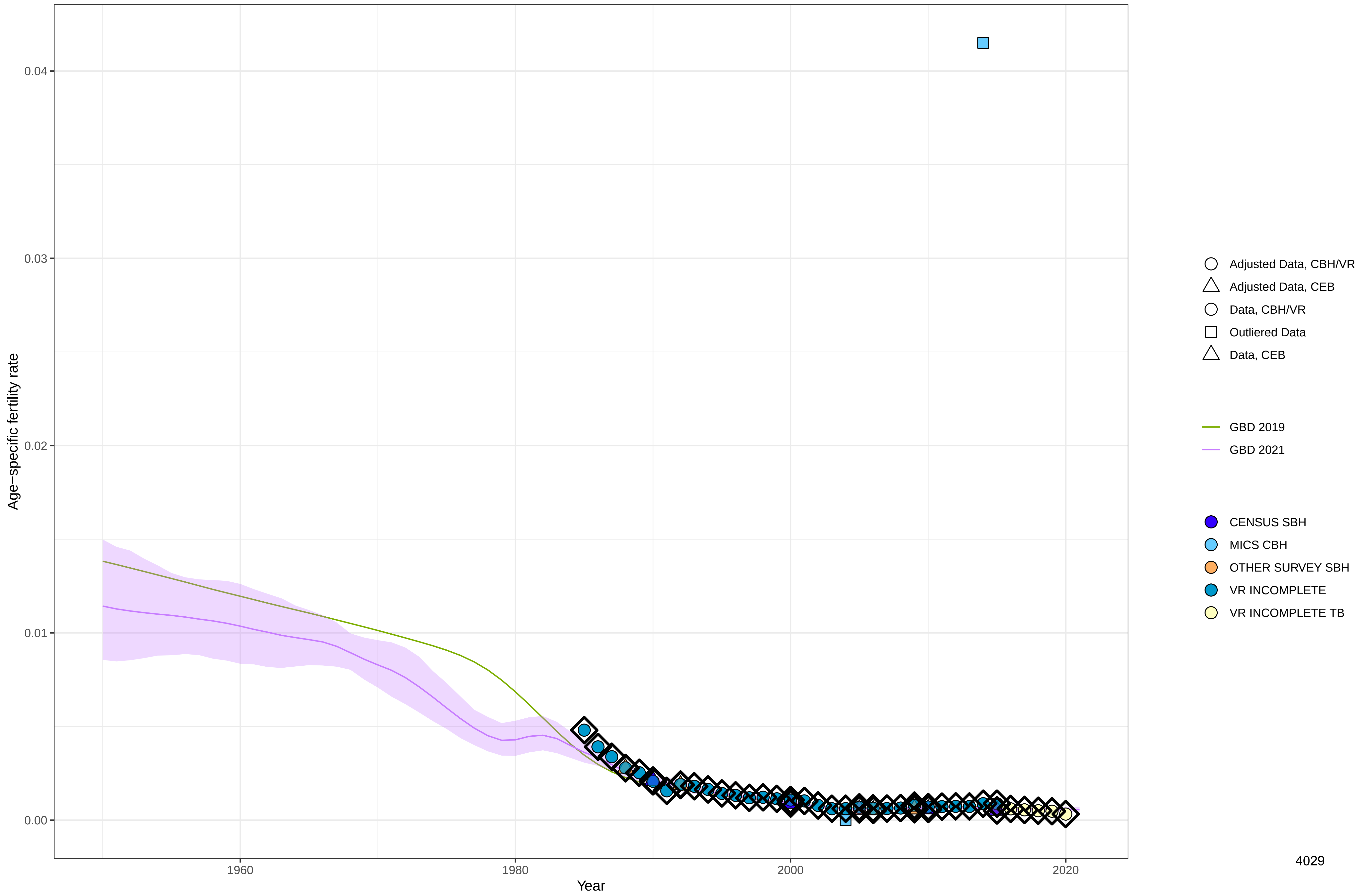

Age−specific fertility fitted trend lines, 1950−2021: Mexico City, Ages: 50−54
Data points enclosed with a diamond are reference sources

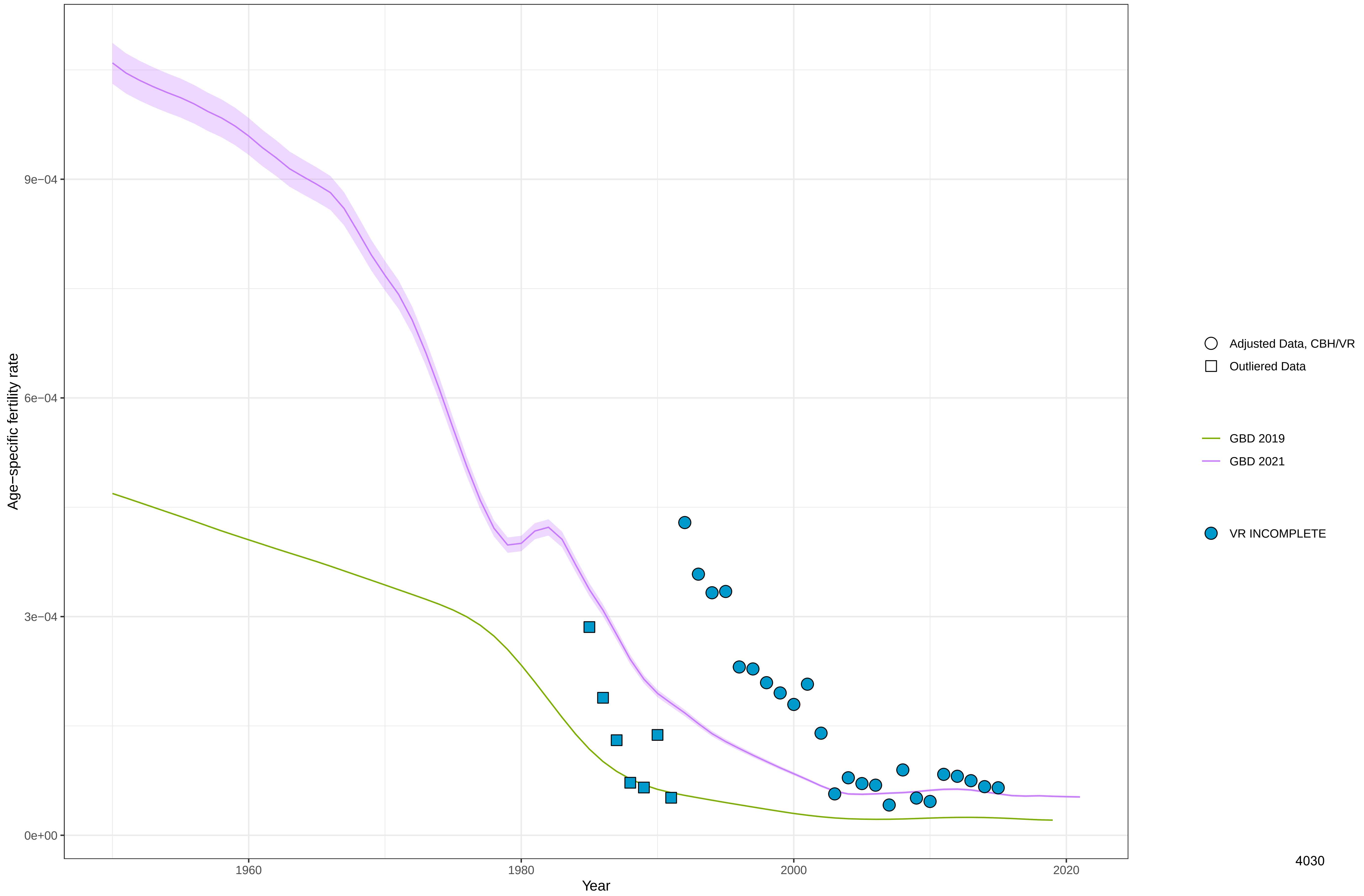

Total fertility rate fitted trend lines, 1950−2021: Mexico City TFR
Data points enclosed with a diamond are reference sources

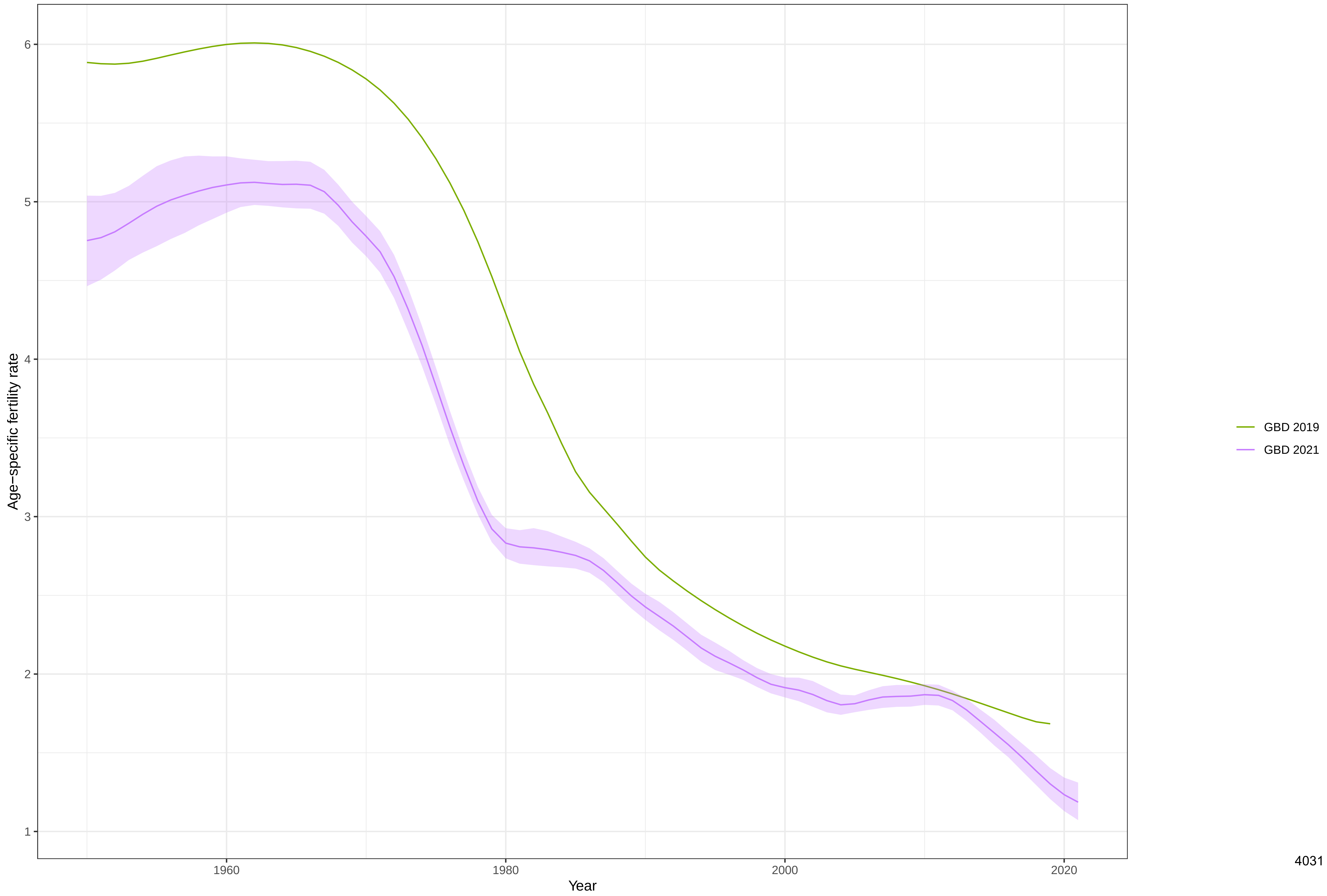

Age−specific fertility fitted trend lines, 1950−2021: Michoacán de Ocampo, Ages: 10−14
Data points enclosed with a diamond are reference sources

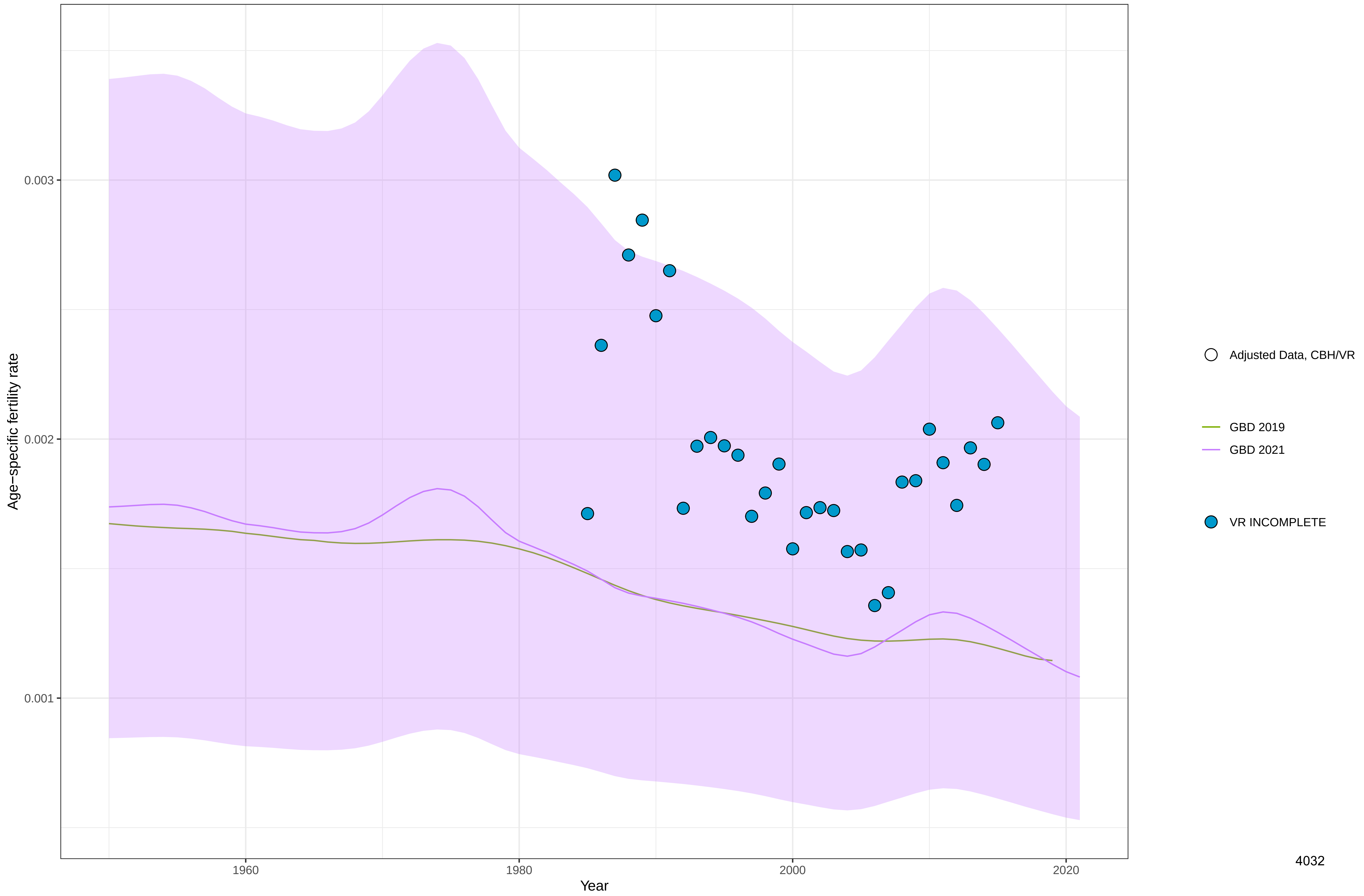

Age−specific fertility fitted trend lines, 1950−2021: Michoacán de Ocampo, Ages: 15−19
Data points enclosed with a diamond are reference sources

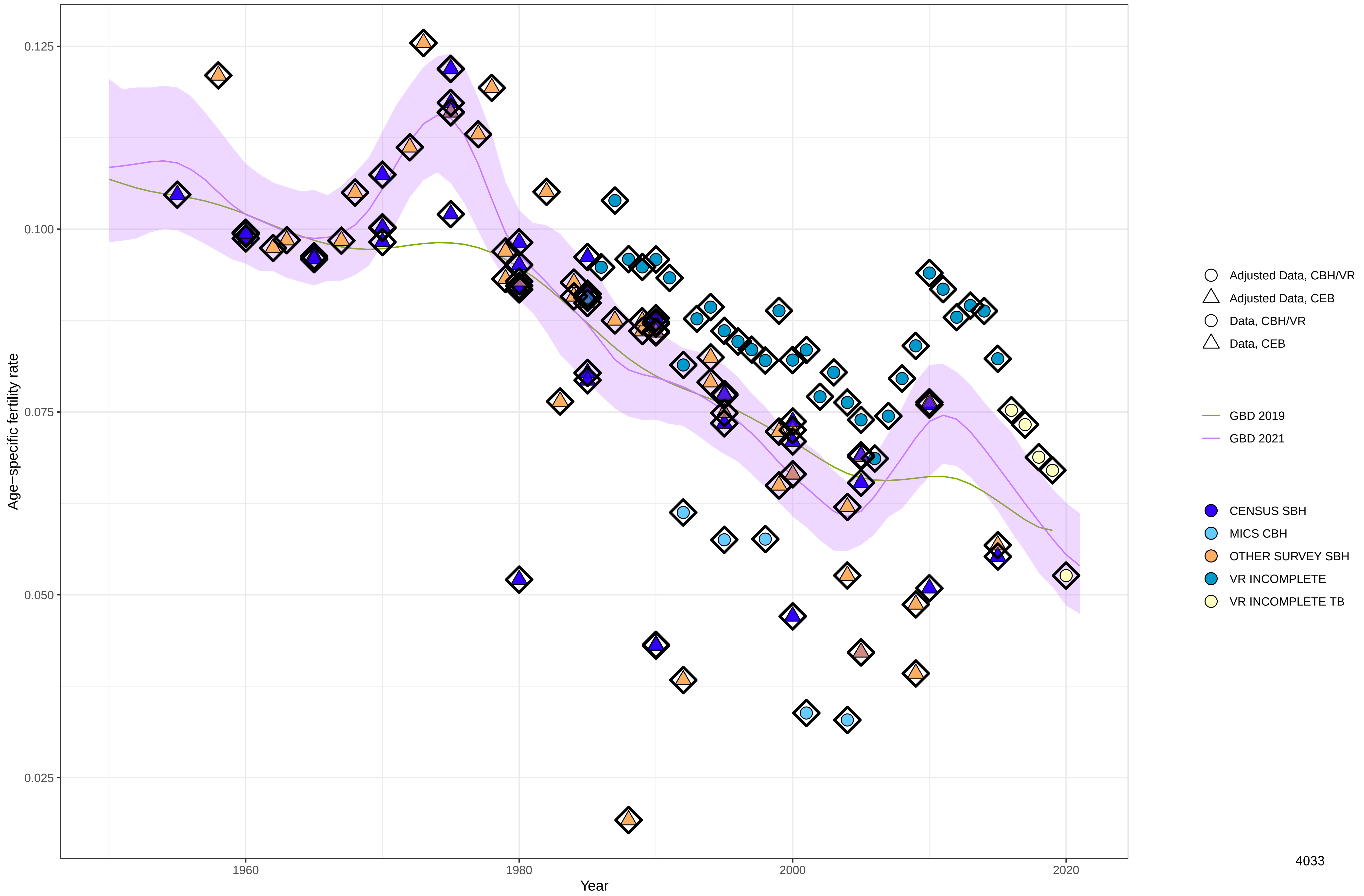

Age−specific fertility fitted trend lines, 1950−2021: Michoacán de Ocampo, Ages: 20−24
Data points enclosed with a diamond are reference sources

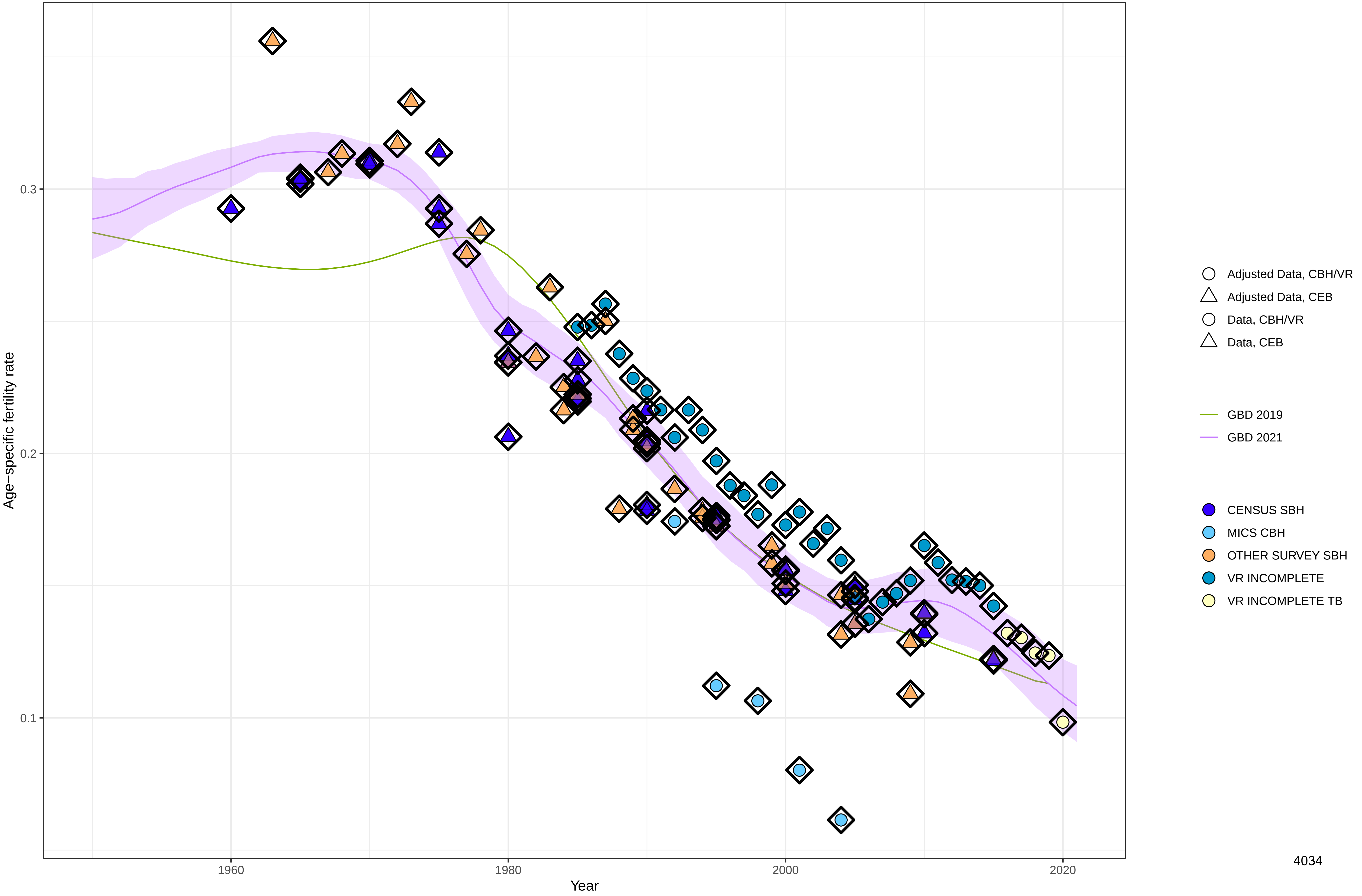

Age−specific fertility fitted trend lines, 1950−2021: Michoacán de Ocampo, Ages: 25−29
Data points enclosed with a diamond are reference sources

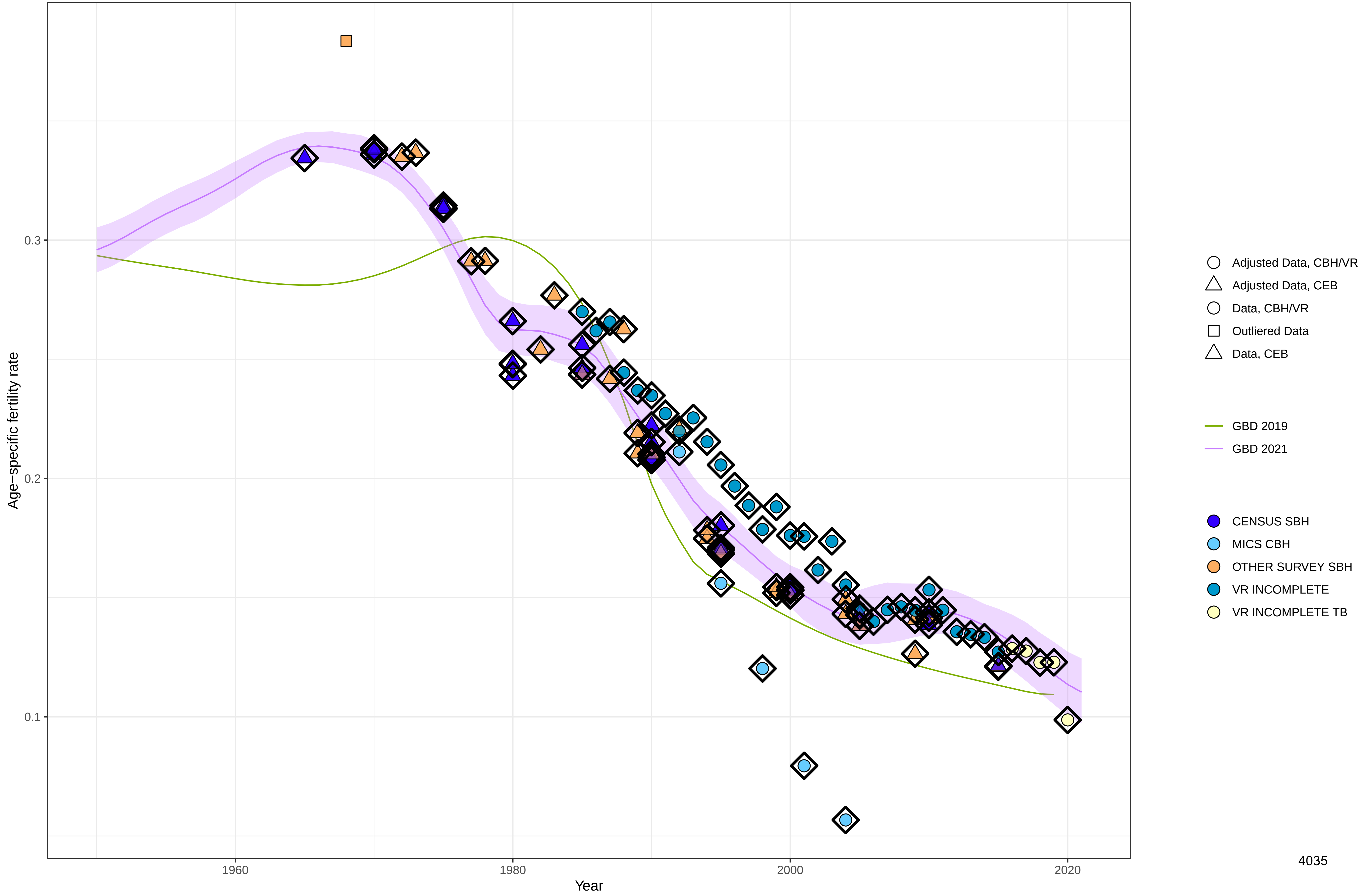

Age−specific fertility fitted trend lines, 1950−2021: Michoacán de Ocampo, Ages: 30−34
Data points enclosed with a diamond are reference sources

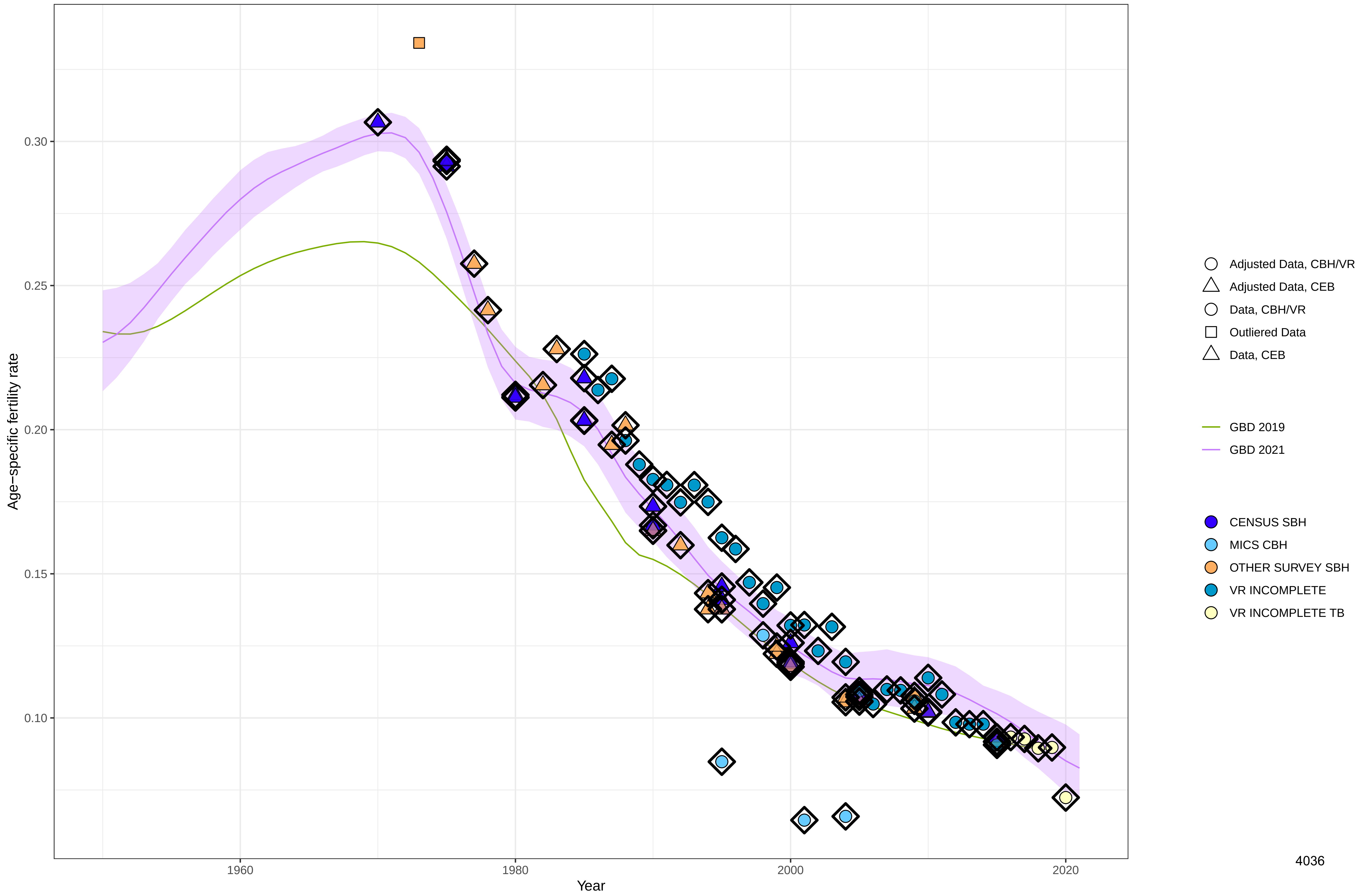

Age−specific fertility fitted trend lines, 1950−2021: Michoacán de Ocampo, Ages: 35−39
Data points enclosed with a diamond are reference sources

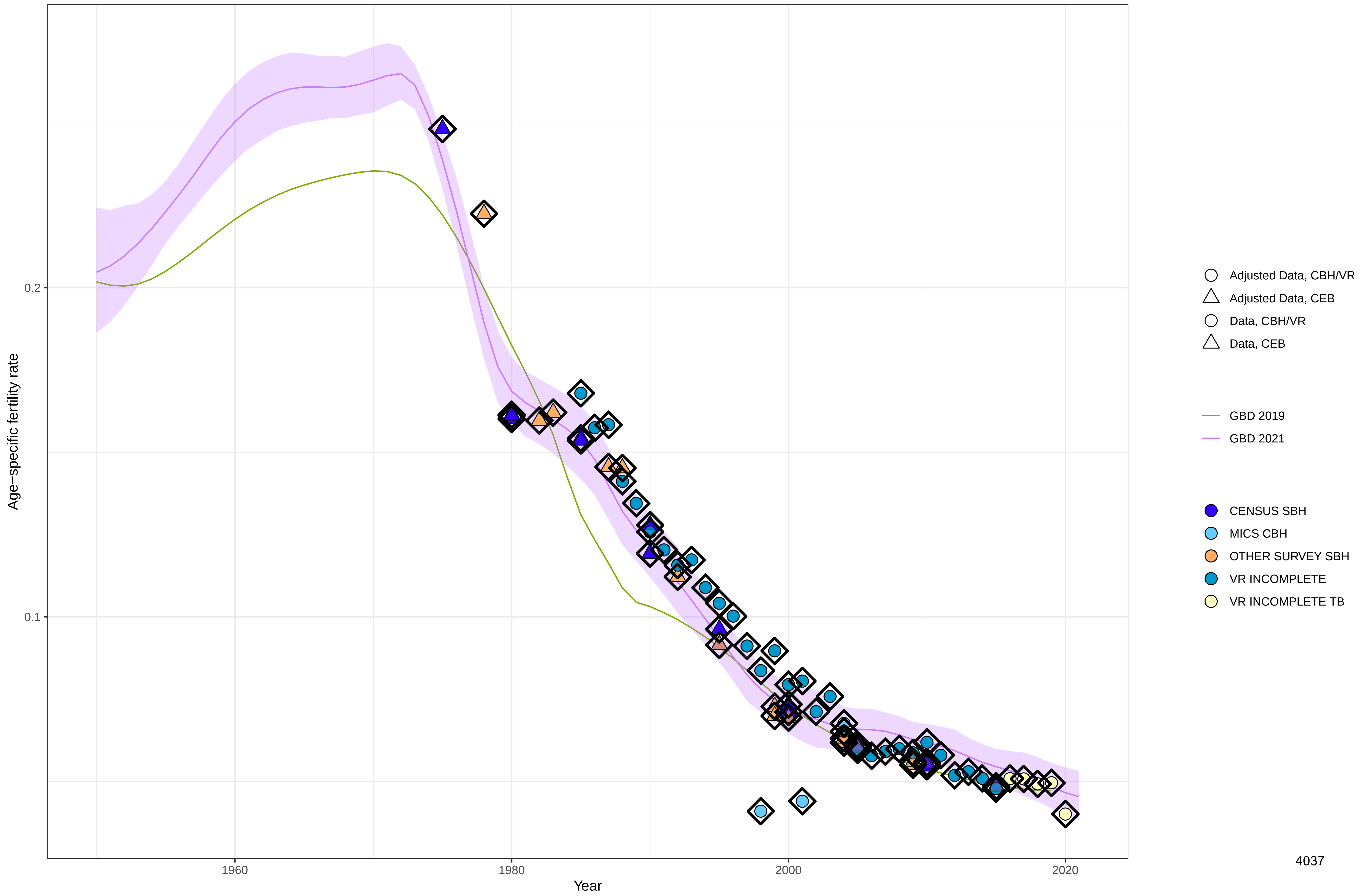

Age−specific fertility fitted trend lines, 1950−2021: Michoacán de Ocampo, Ages: 40−44
Data points enclosed with a diamond are reference sources

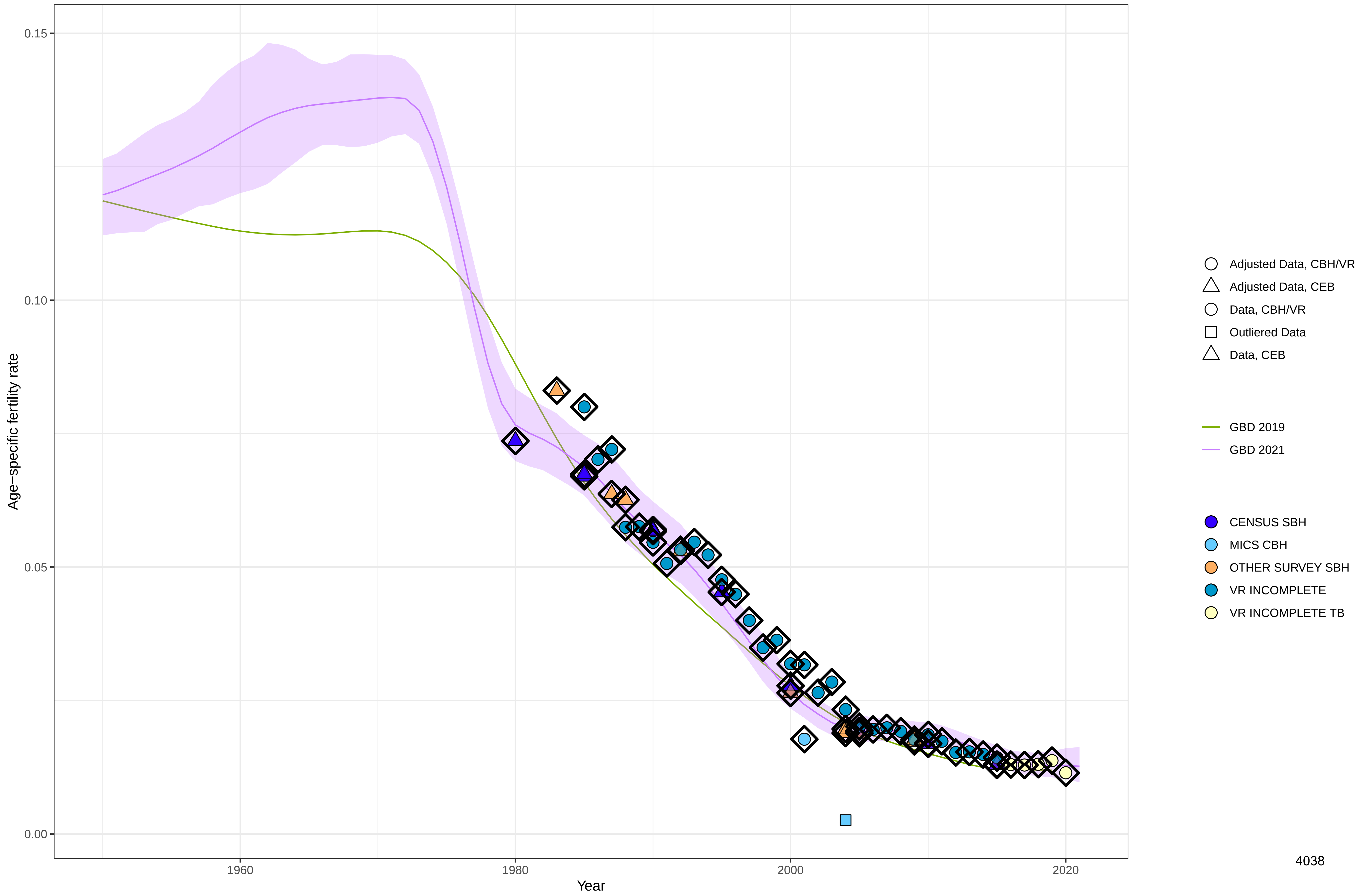

Age−specific fertility fitted trend lines, 1950−2021: Michoacán de Ocampo, Ages: 45−49
Data points enclosed with a diamond are reference sources

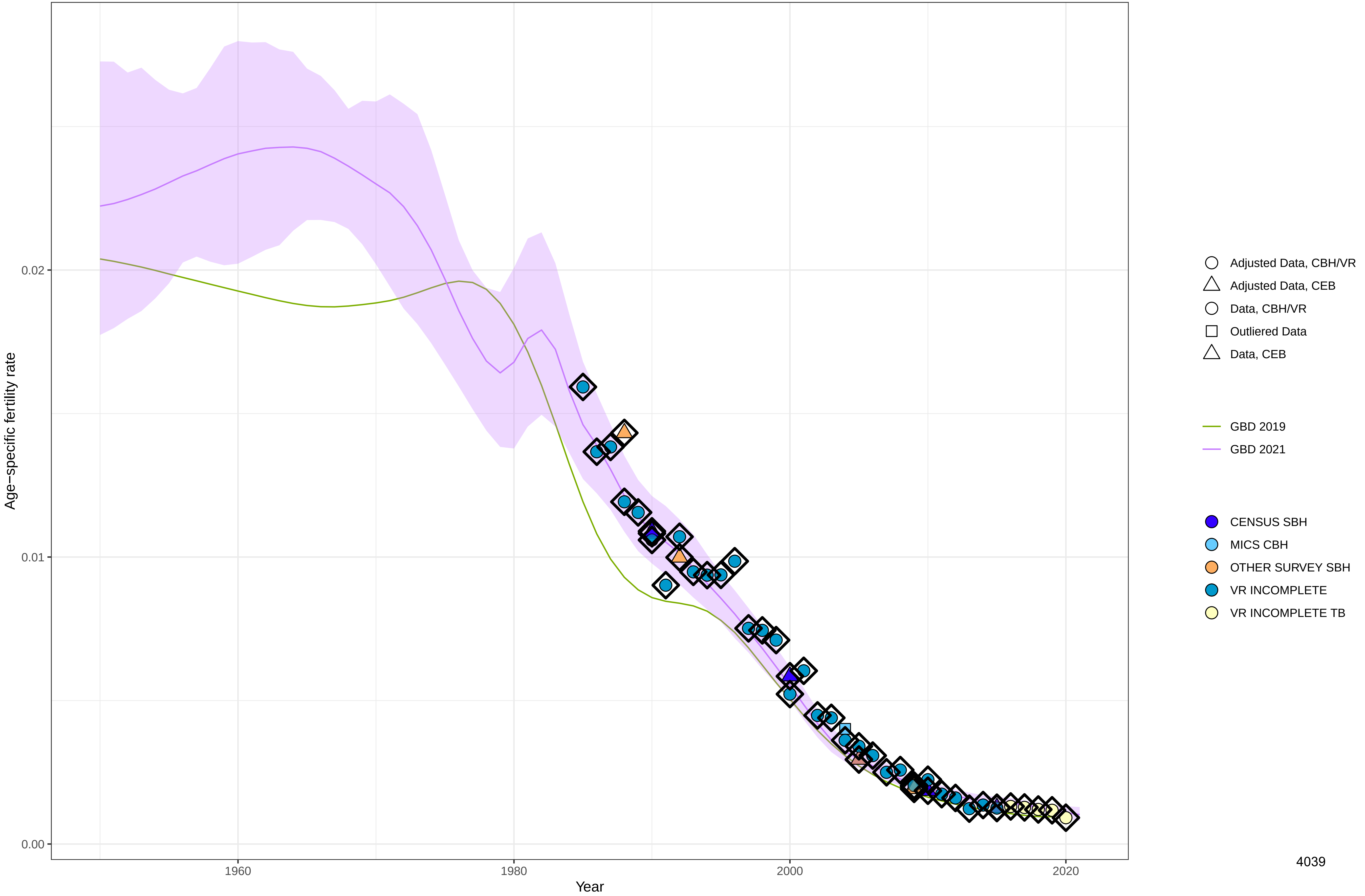

Age−specific fertility fitted trend lines, 1950−2021: Michoacán de Ocampo, Ages: 50−54
Data points enclosed with a diamond are reference sources

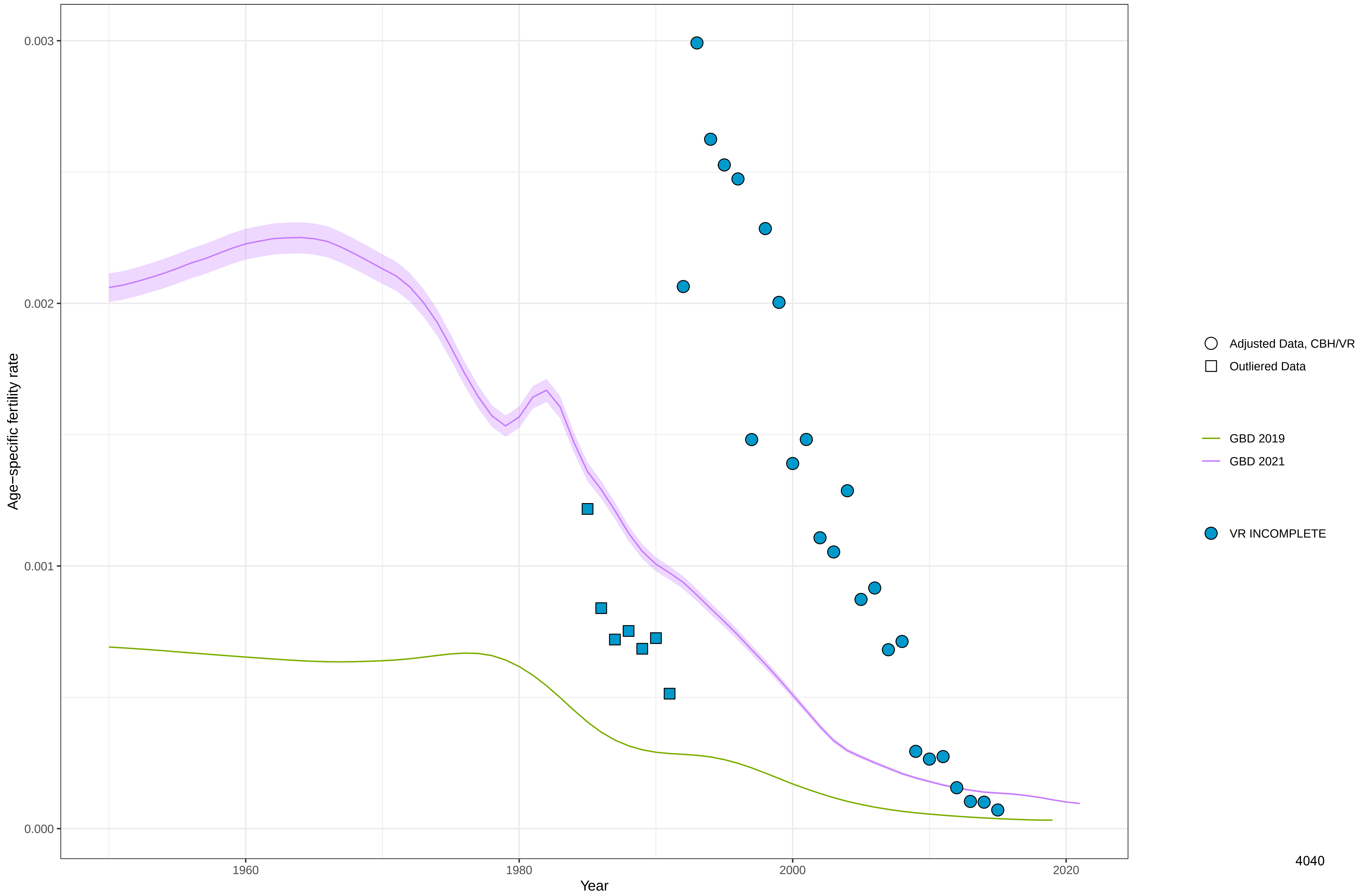

Total fertility rate fitted trend lines, 1950−2021: Michoacán de Ocampo TFR
Data points enclosed with a diamond are reference sources

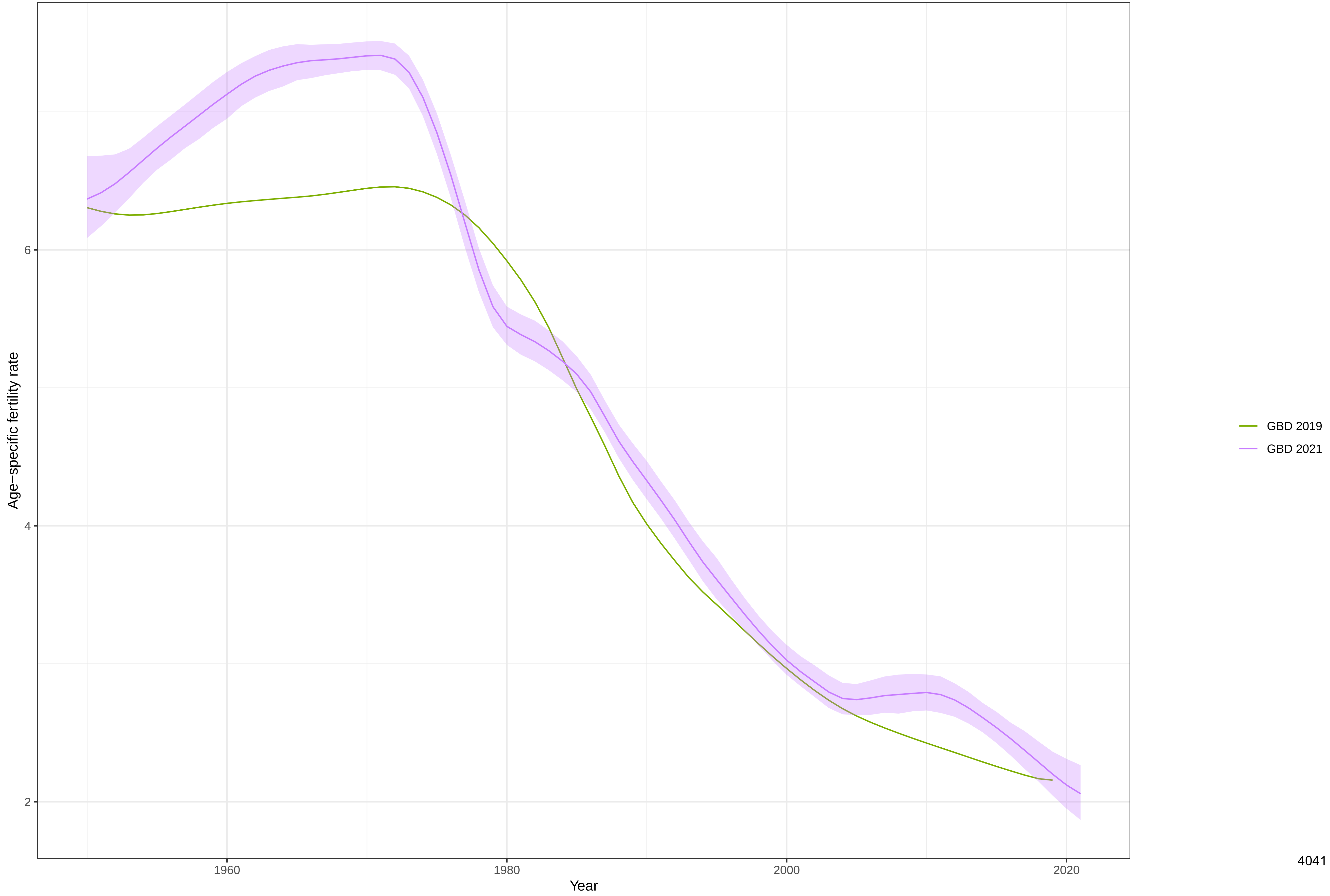

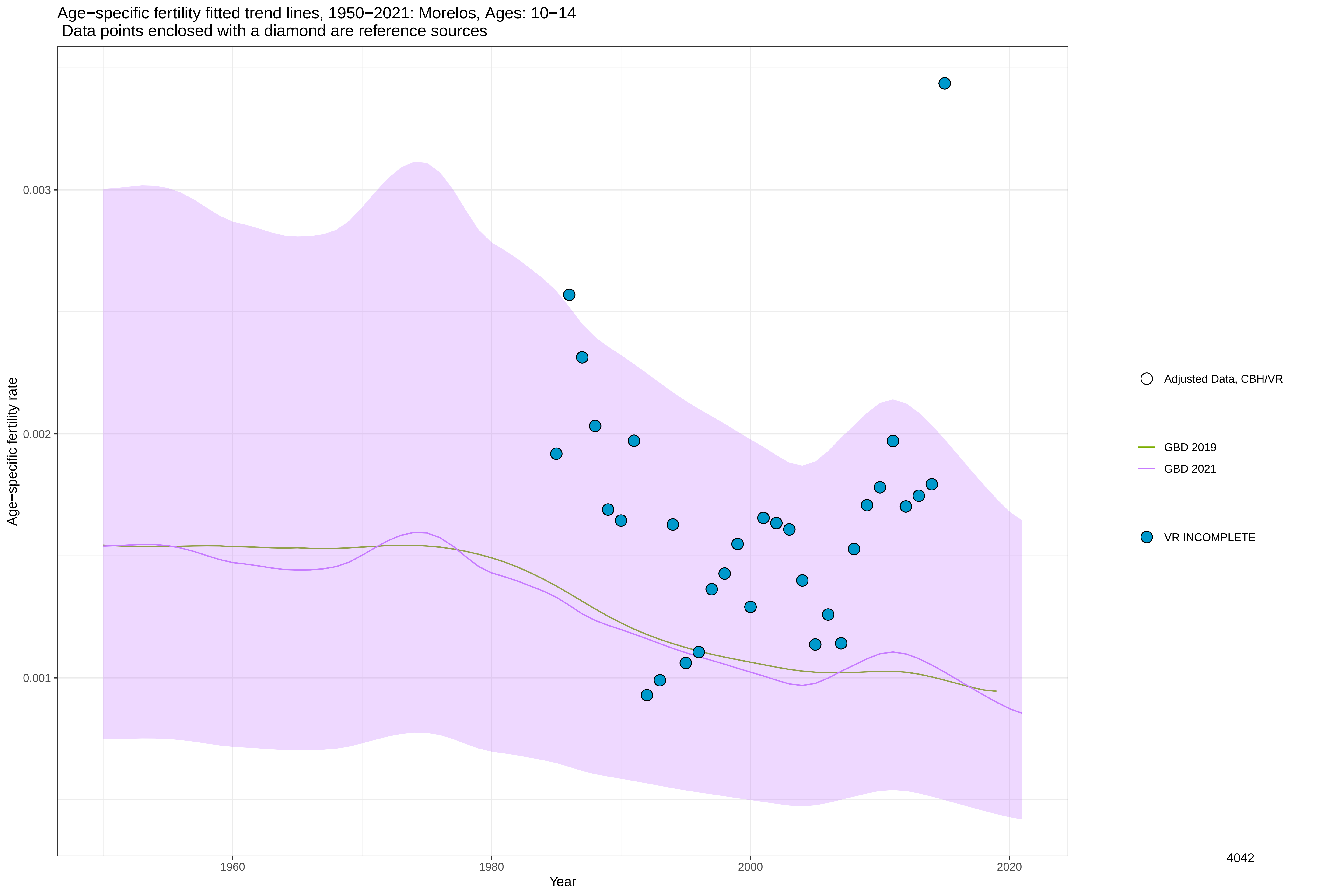

Age−specific fertility fitted trend lines, 1950−2021: Morelos, Ages: 10−14
Data points enclosed with a diamond are reference sources
Age−specific fertility rate
Year
0.003
0.002
0.001
1960
1980
2000
2020
Adjusted Data, CBH/VR
GBD 2019
GBD 2021
VR INCOMPLETE

4042

Age−specific fertility fitted trend lines, 1950−2021: Morelos, Ages: 15−19
Data points enclosed with a diamond are reference sources

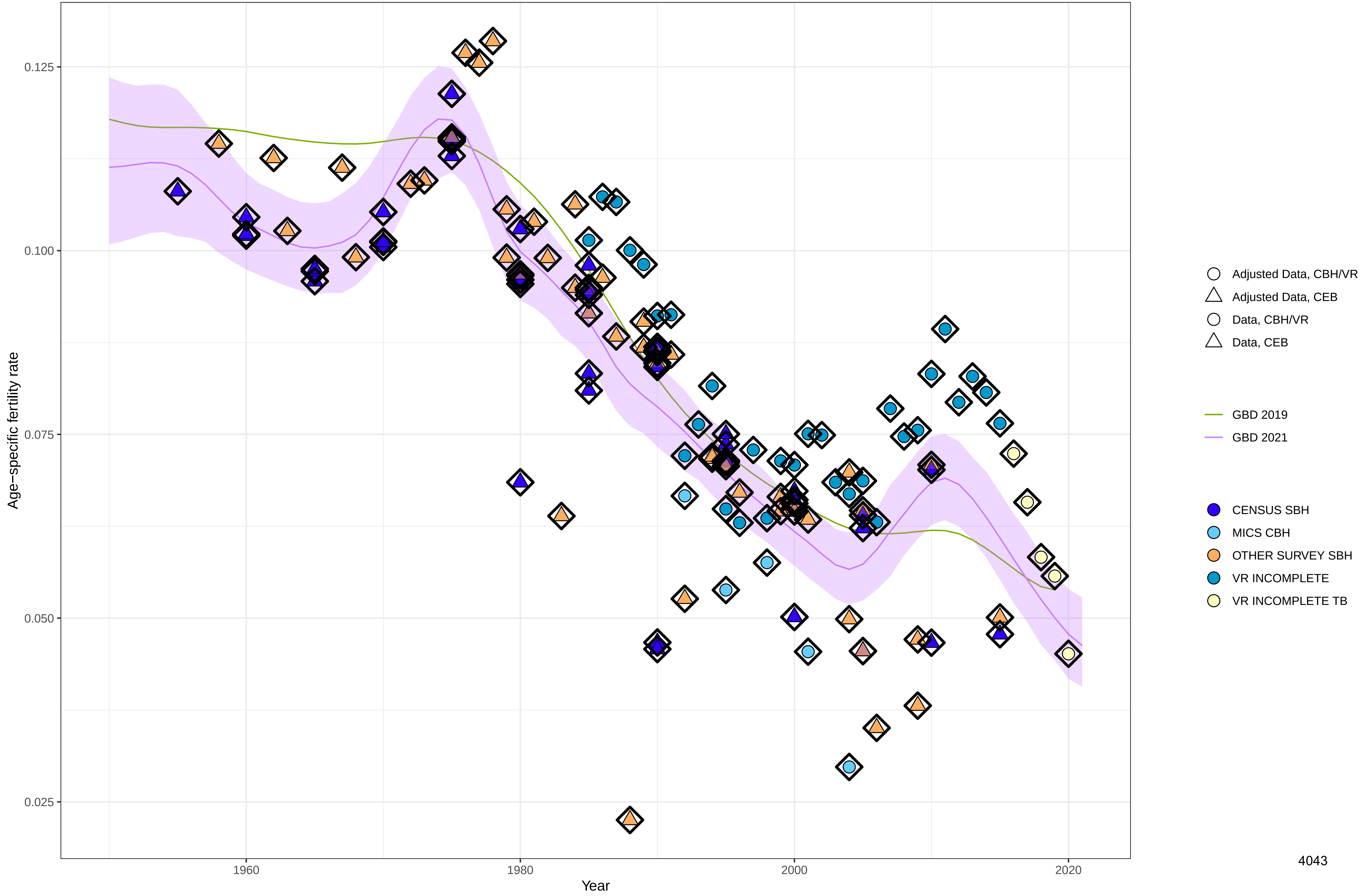

Age−specific fertility fitted trend lines, 1950−2021: Morelos, Ages: 20−24
Data points enclosed with a diamond are reference sources

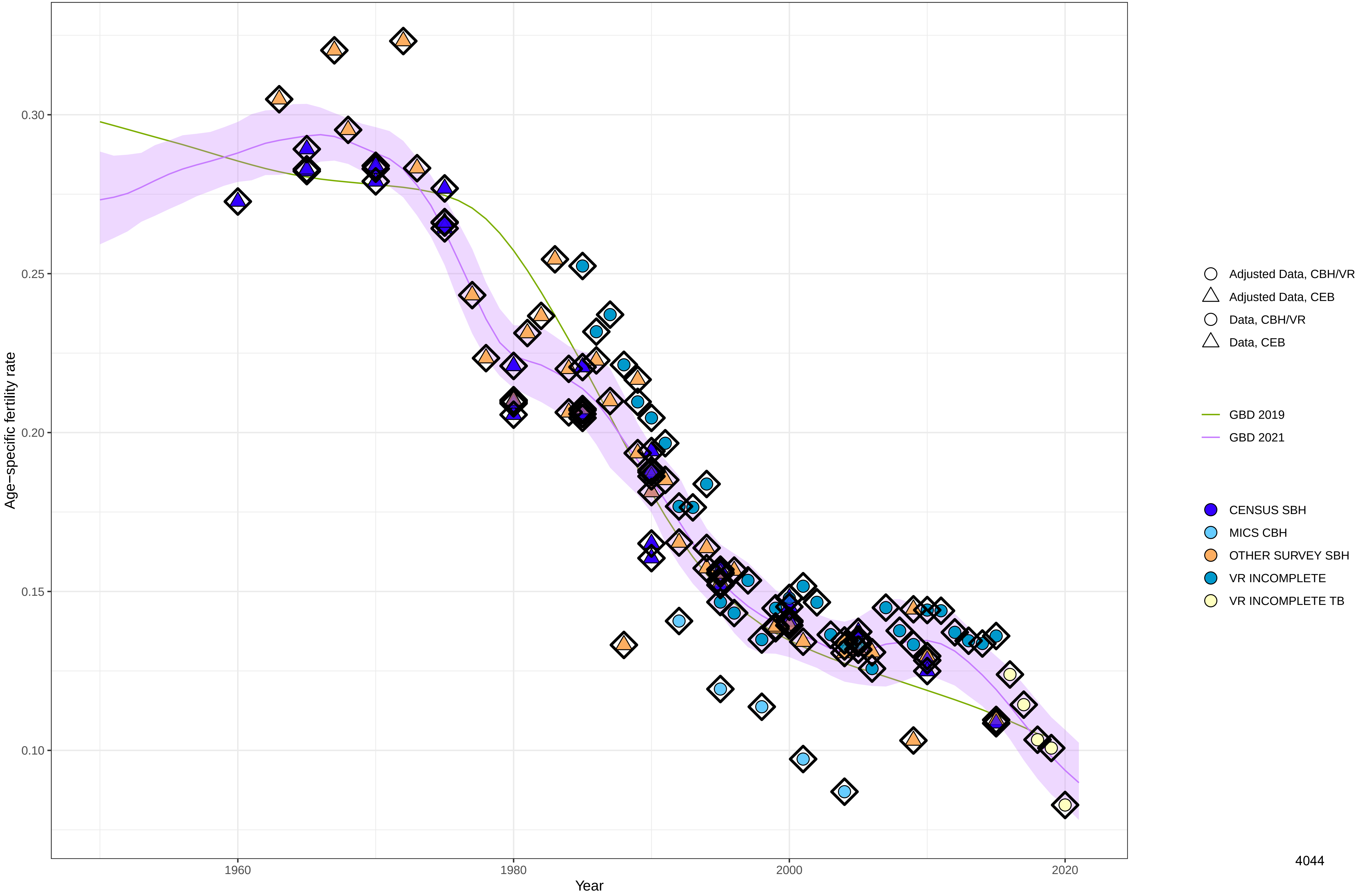

Age−specific fertility fitted trend lines, 1950−2021: Morelos, Ages: 25−29
Data points enclosed with a diamond are reference sources

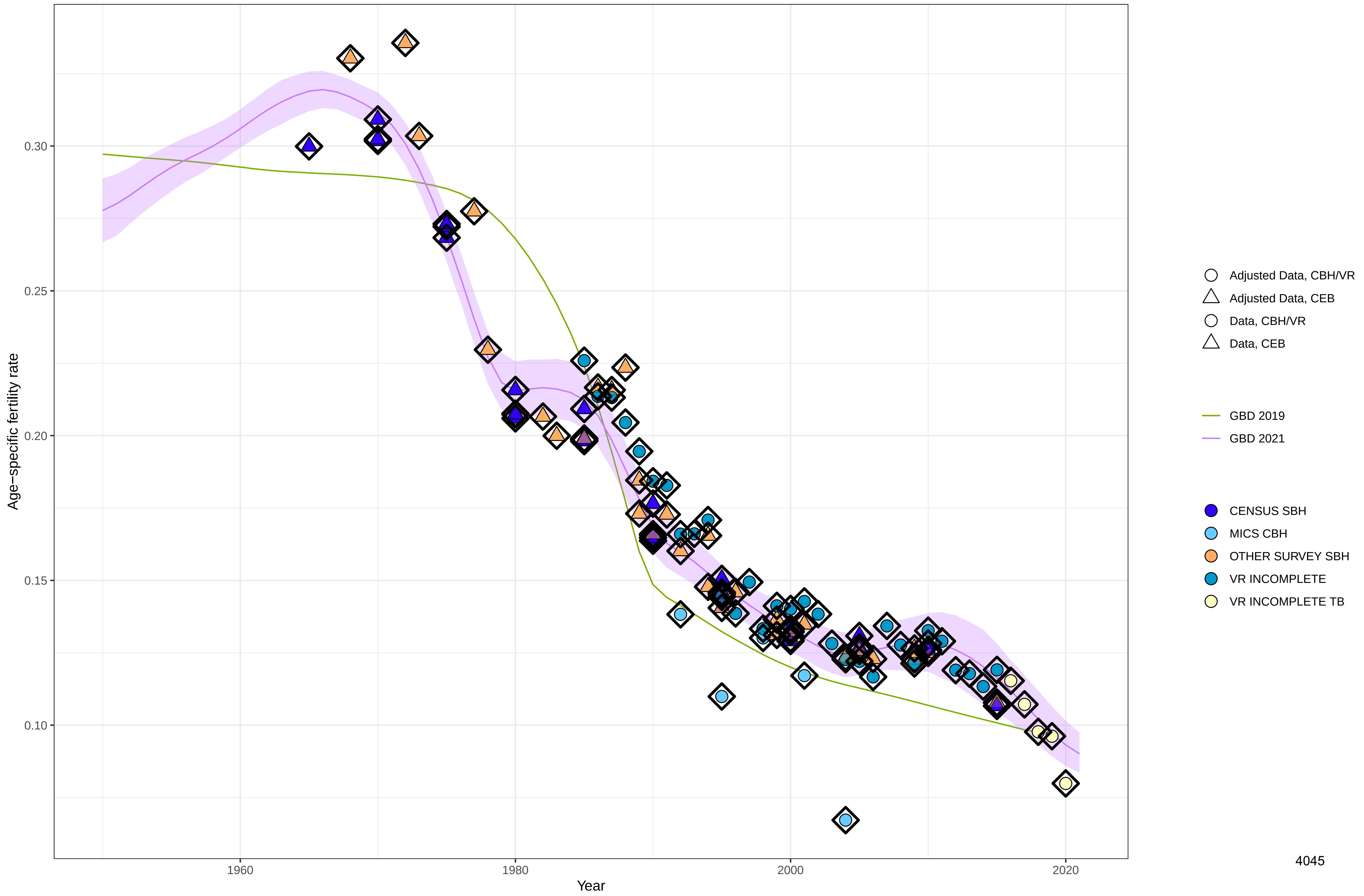

Age−specific fertility fitted trend lines, 1950−2021: Morelos, Ages: 30−34
Data points enclosed with a diamond are reference sources

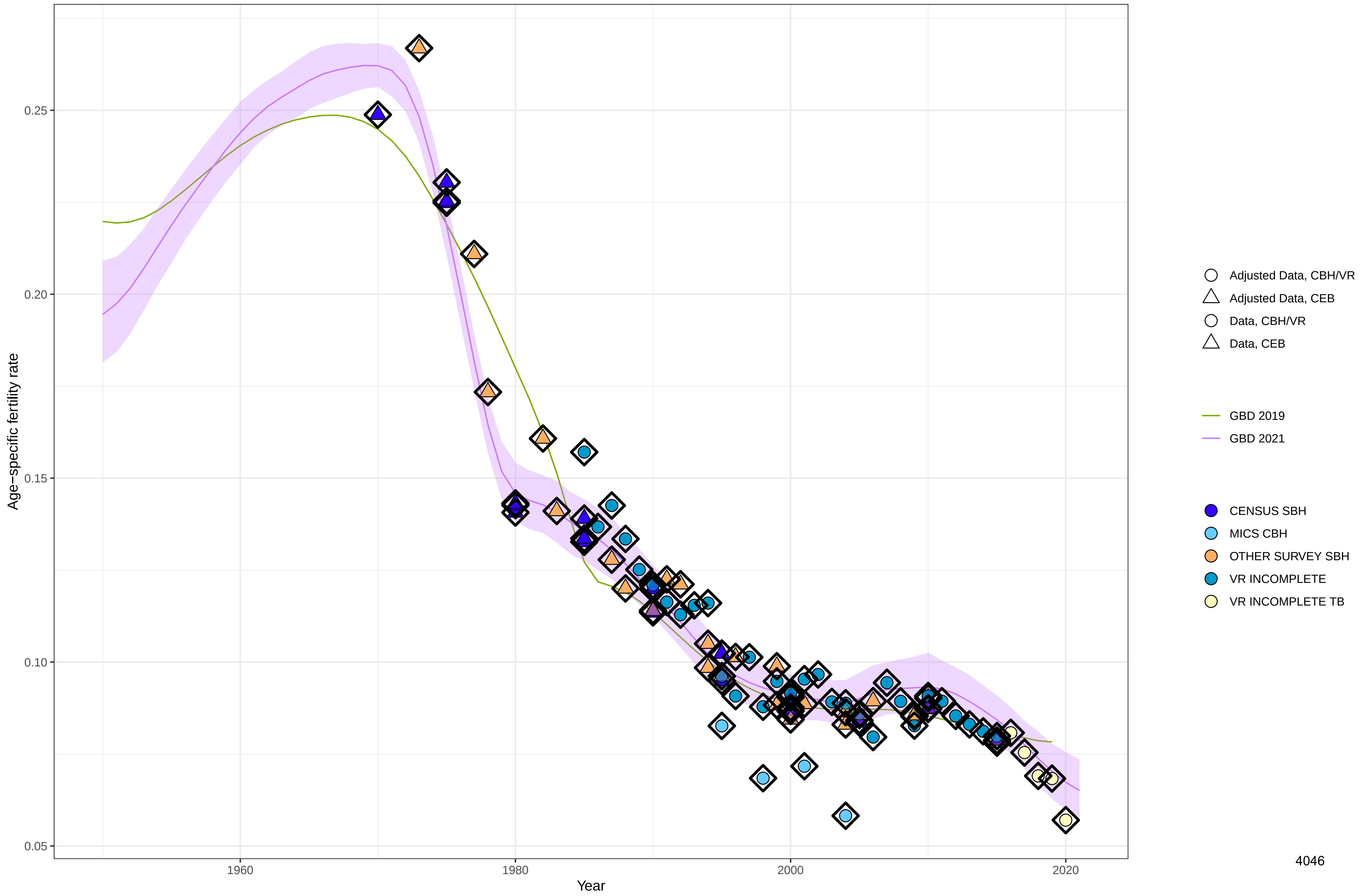

Age−specific fertility fitted trend lines, 1950−2021: Morelos, Ages: 35−39
Data points enclosed with a diamond are reference sources

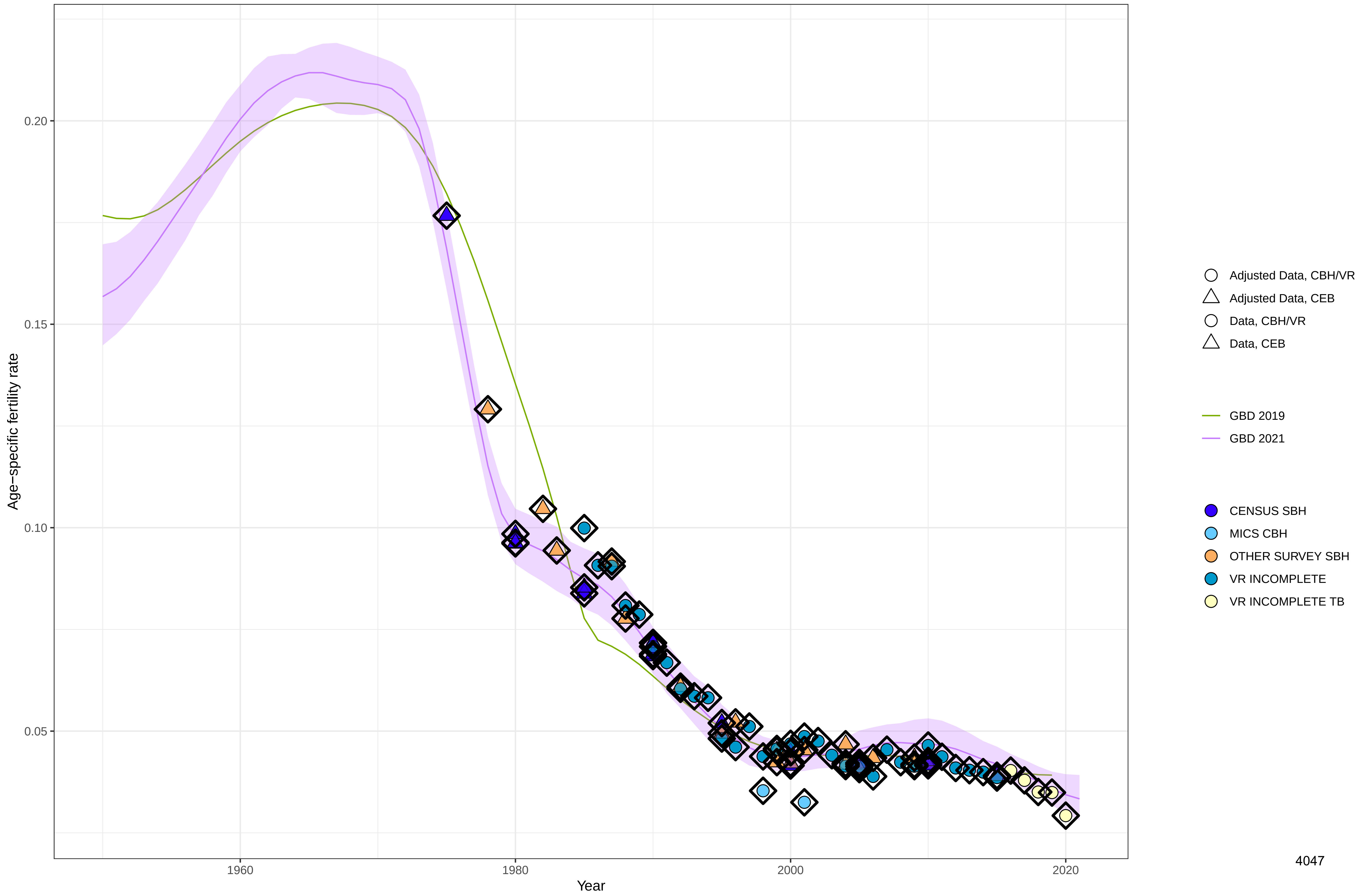

Age−specific fertility fitted trend lines, 1950−2021: Morelos, Ages: 40−44
Data points enclosed with a diamond are reference sources

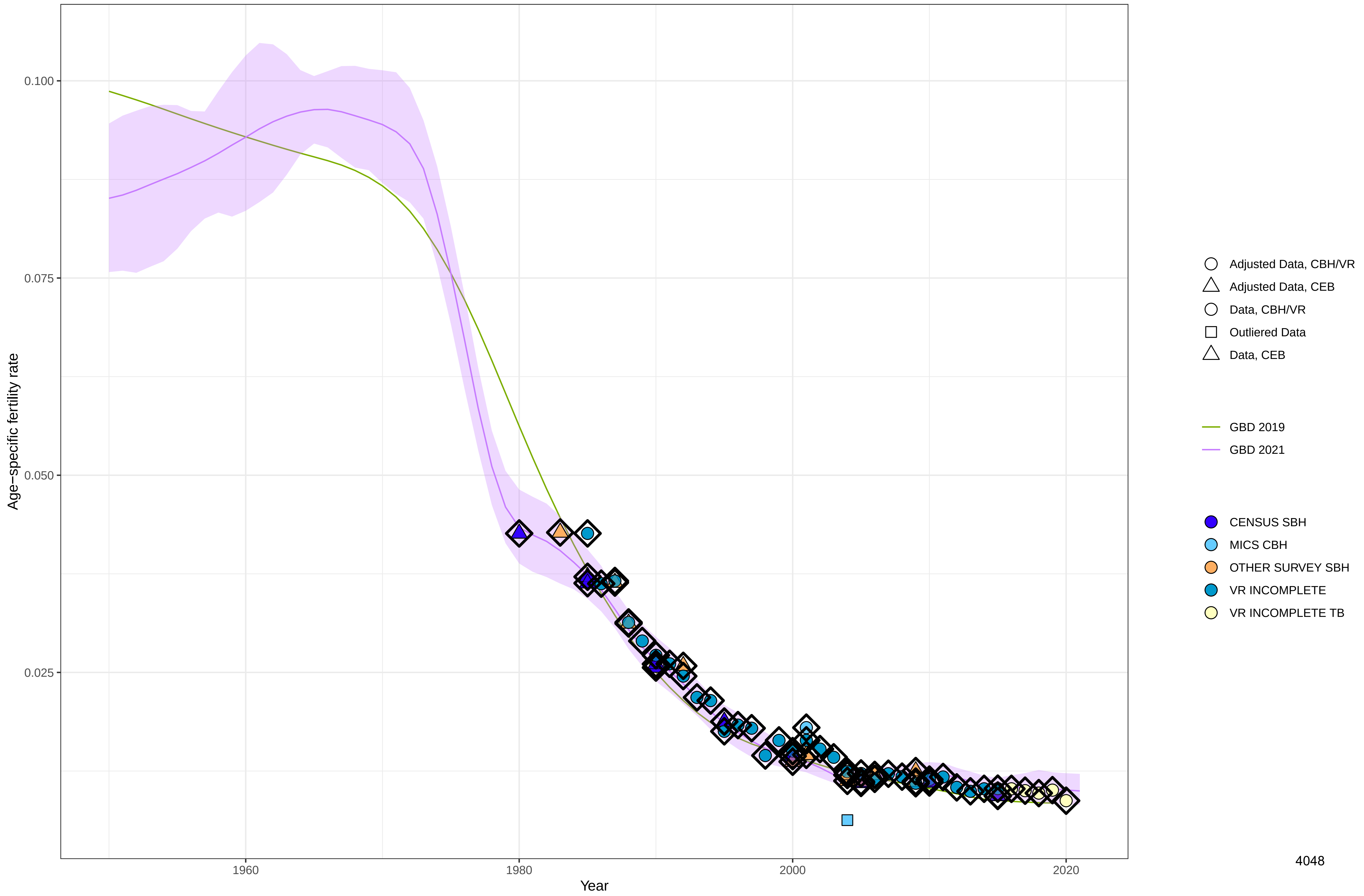

Age−specific fertility fitted trend lines, 1950−2021: Morelos, Ages: 45−49
Data points enclosed with a diamond are reference sources

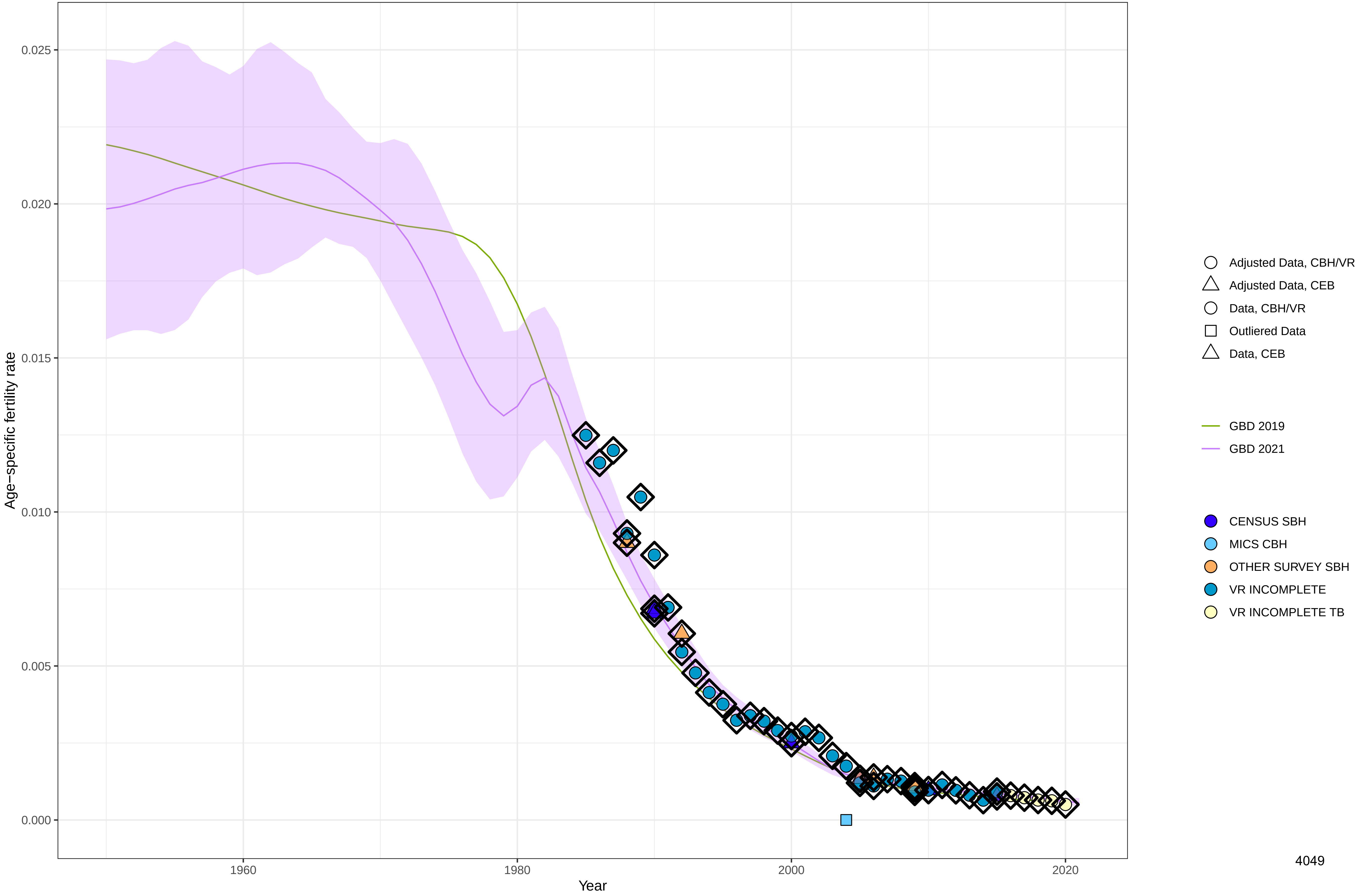

Age−specific fertility fitted trend lines, 1950−2021: Morelos, Ages: 50−54
Data points enclosed with a diamond are reference sources

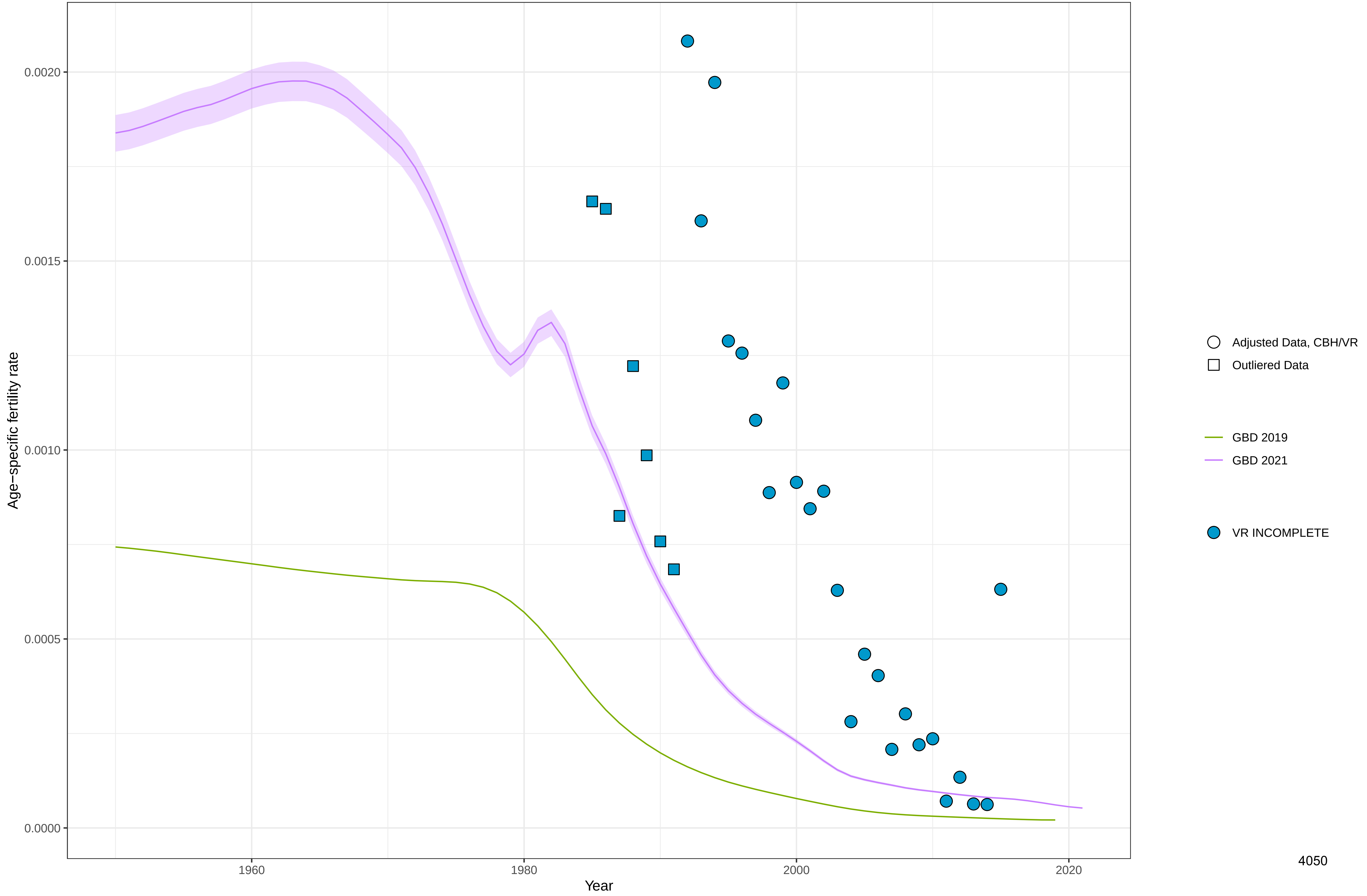

Total fertility rate fitted trend lines, 1950−2021: Morelos TFR
Data points enclosed with a diamond are reference sources

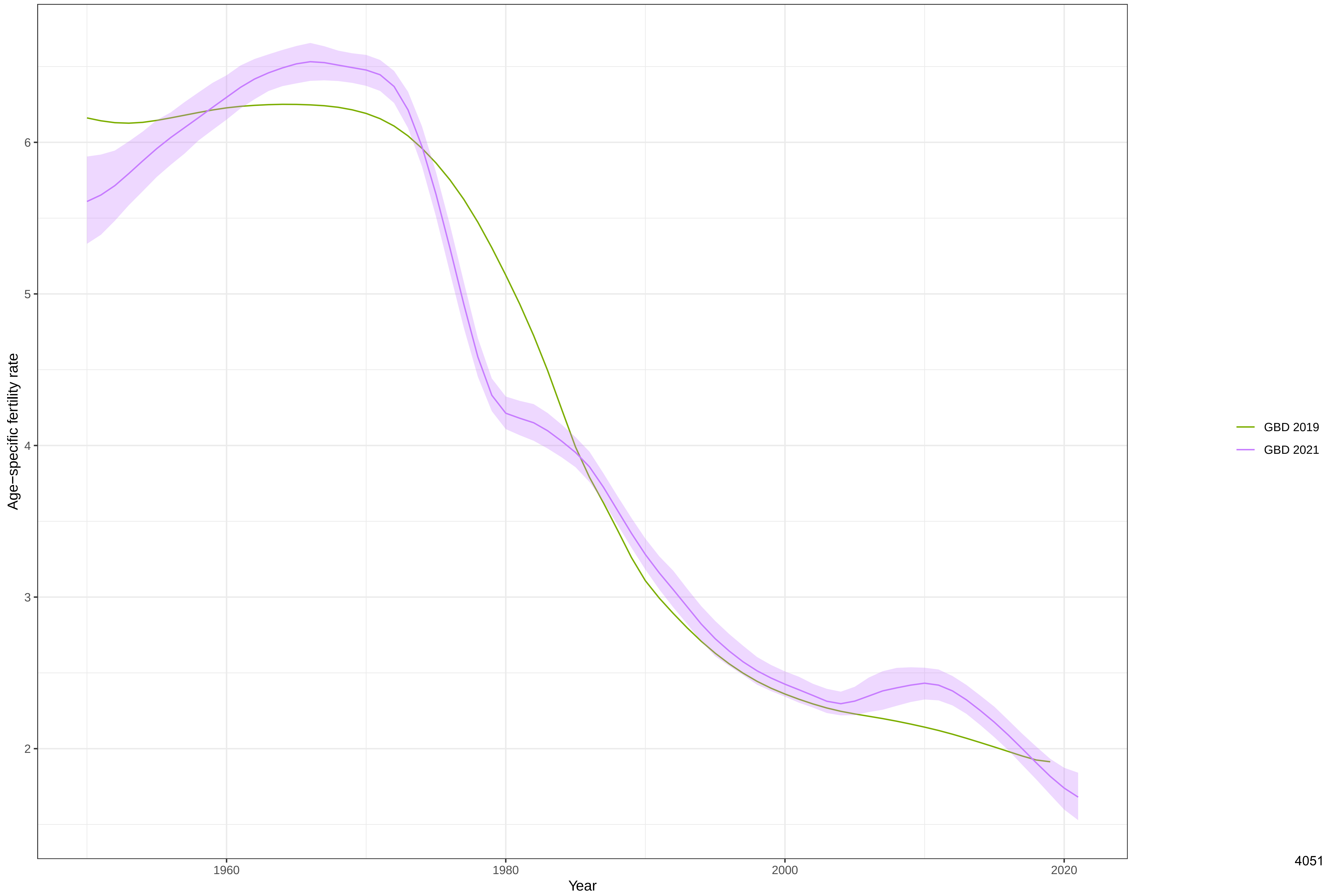

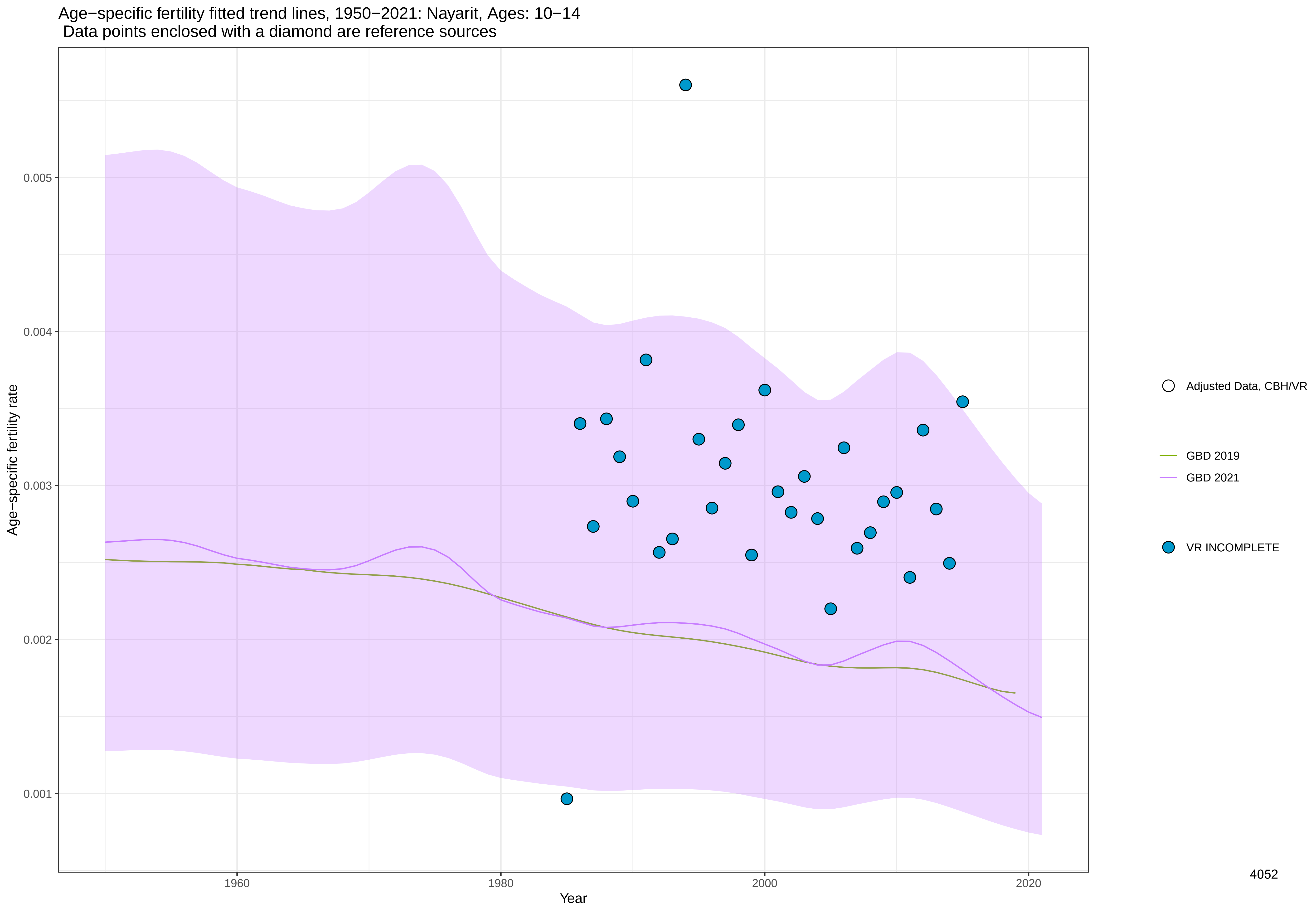
Age−specific fertility fitted trend lines, 1950−2021: Nayarit, Ages: 10−14
Data points enclosed with a diamond are reference sources
Age−specific fertility rate
0.001
0.002
0.003
0.004
0.005
Year
1960
1980
2000
2020
Adjusted Data, CBH/VR
GBD 2019
GBD 2021
VR INCOMPLETE

Age−specific fertility fitted trend lines, 1950−2021: Nayarit, Ages: 15−19
Data points enclosed with a diamond are reference sources

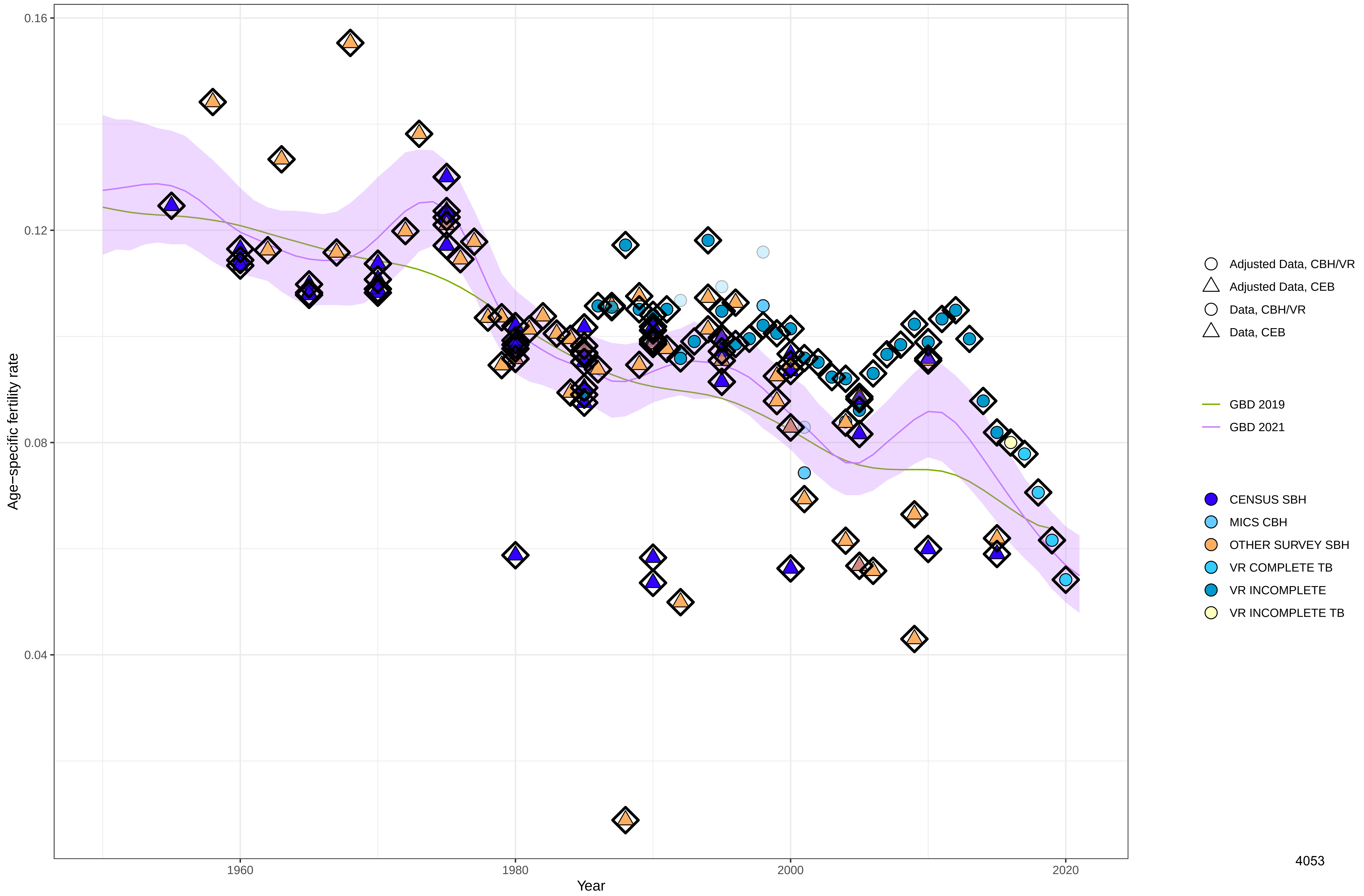

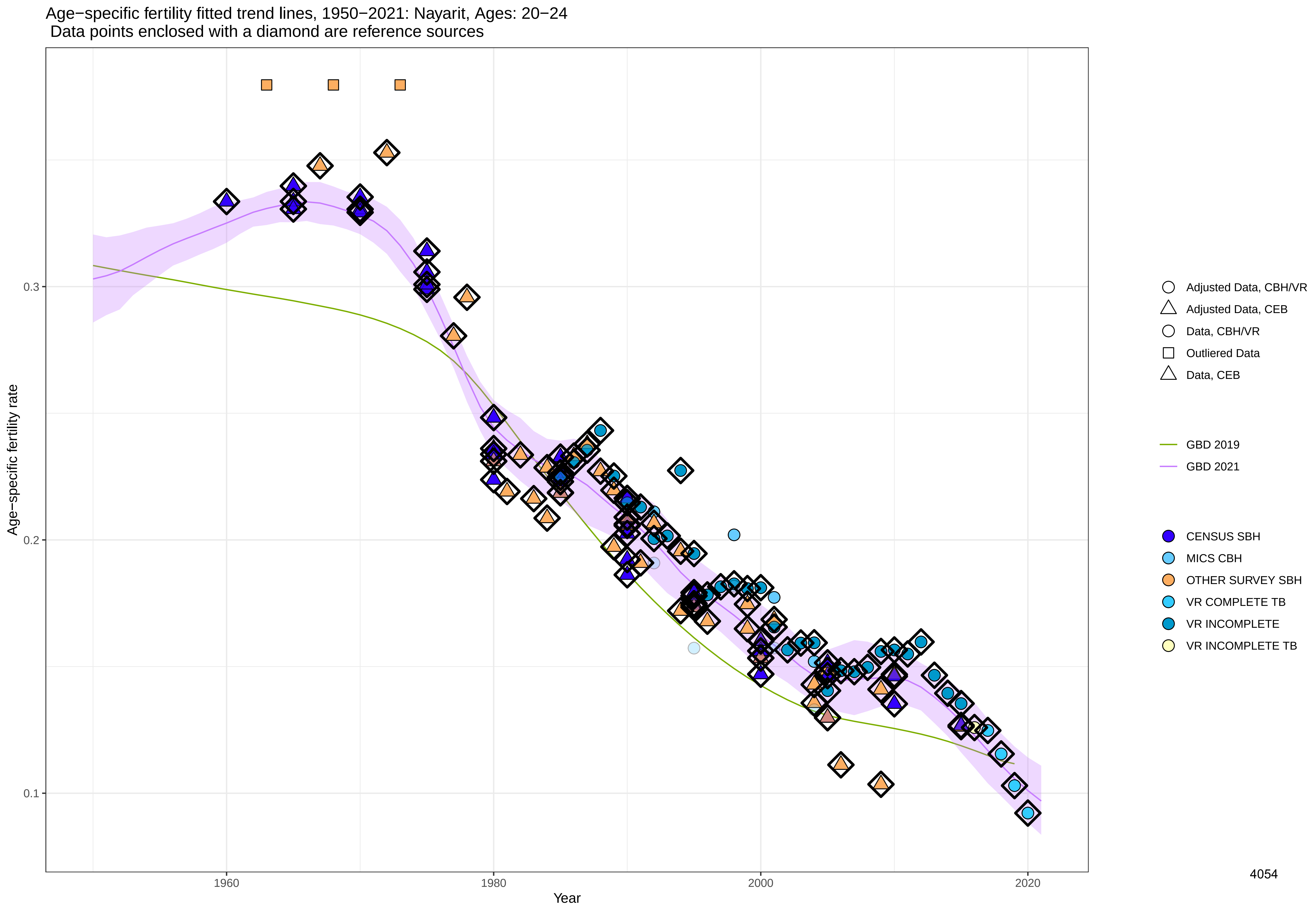

Age−specific fertility fitted trend lines, 1950−2021: Nayarit, Ages: 20−24
Data points enclosed with a diamond are reference sources
Age−specific fertility rate
0.3
0.2
0.1
1960
1980
2000
2020
Year
Adjusted Data, CBH/VR
Adjusted Data, CEB
Data, CBH/VR
Outliered Data
Data, CEB
GBD 2019
GBD 2021
CENSUS SBH
MICS CBH
OTHER SURVEY SBH
VR COMPLETE TB
VR INCOMPLETE
VR INCOMPLETE TB

Age−specific fertility fitted trend lines, 1950−2021: Nayarit, Ages: 25−29
Data points enclosed with a diamond are reference sources

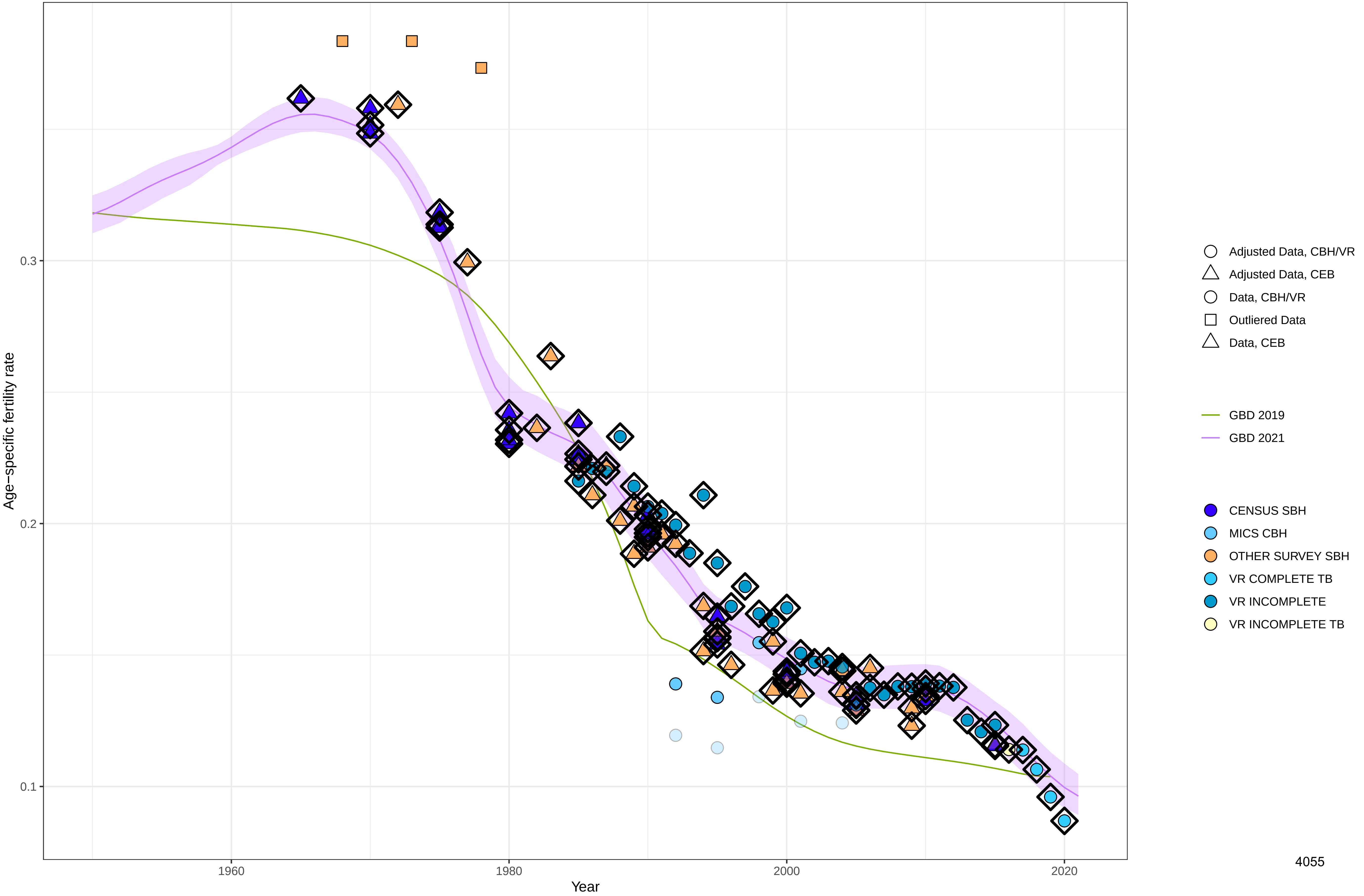

Age−specific fertility fitted trend lines, 1950−2021: Nayarit, Ages: 30−34
Data points enclosed with a diamond are reference sources

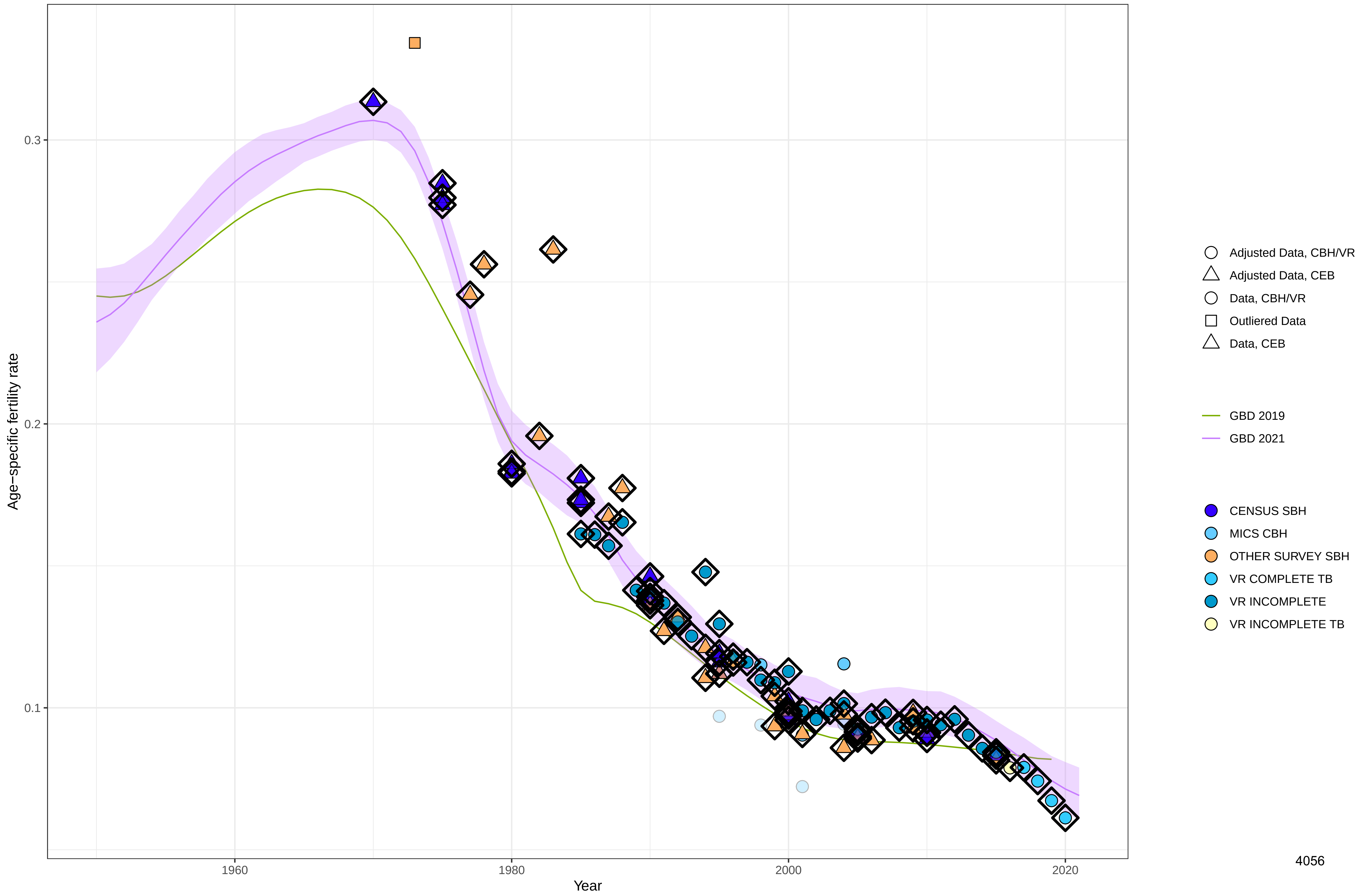

Age−specific fertility fitted trend lines, 1950−2021: Nayarit, Ages: 35−39
Data points enclosed with a diamond are reference sources

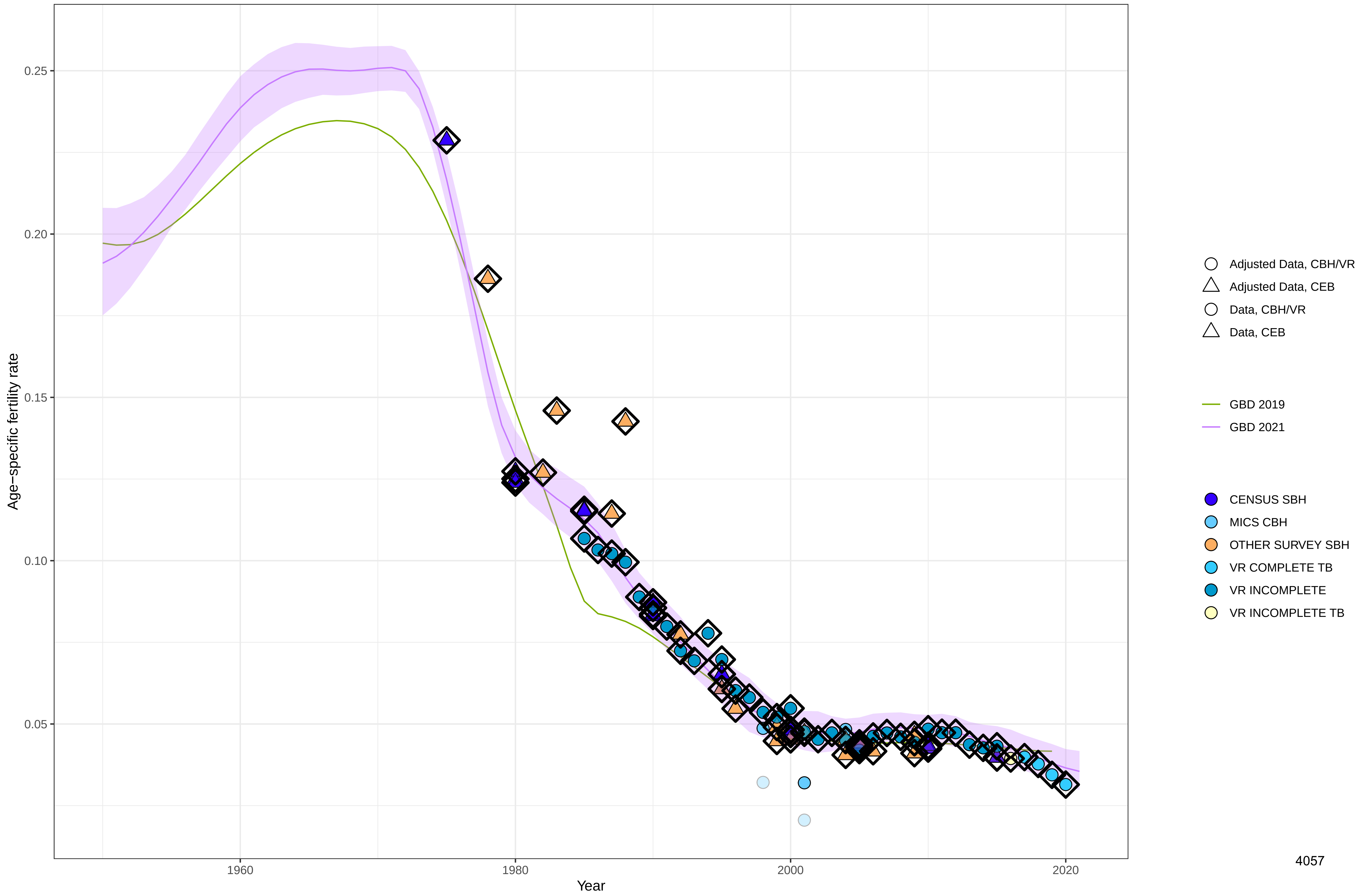

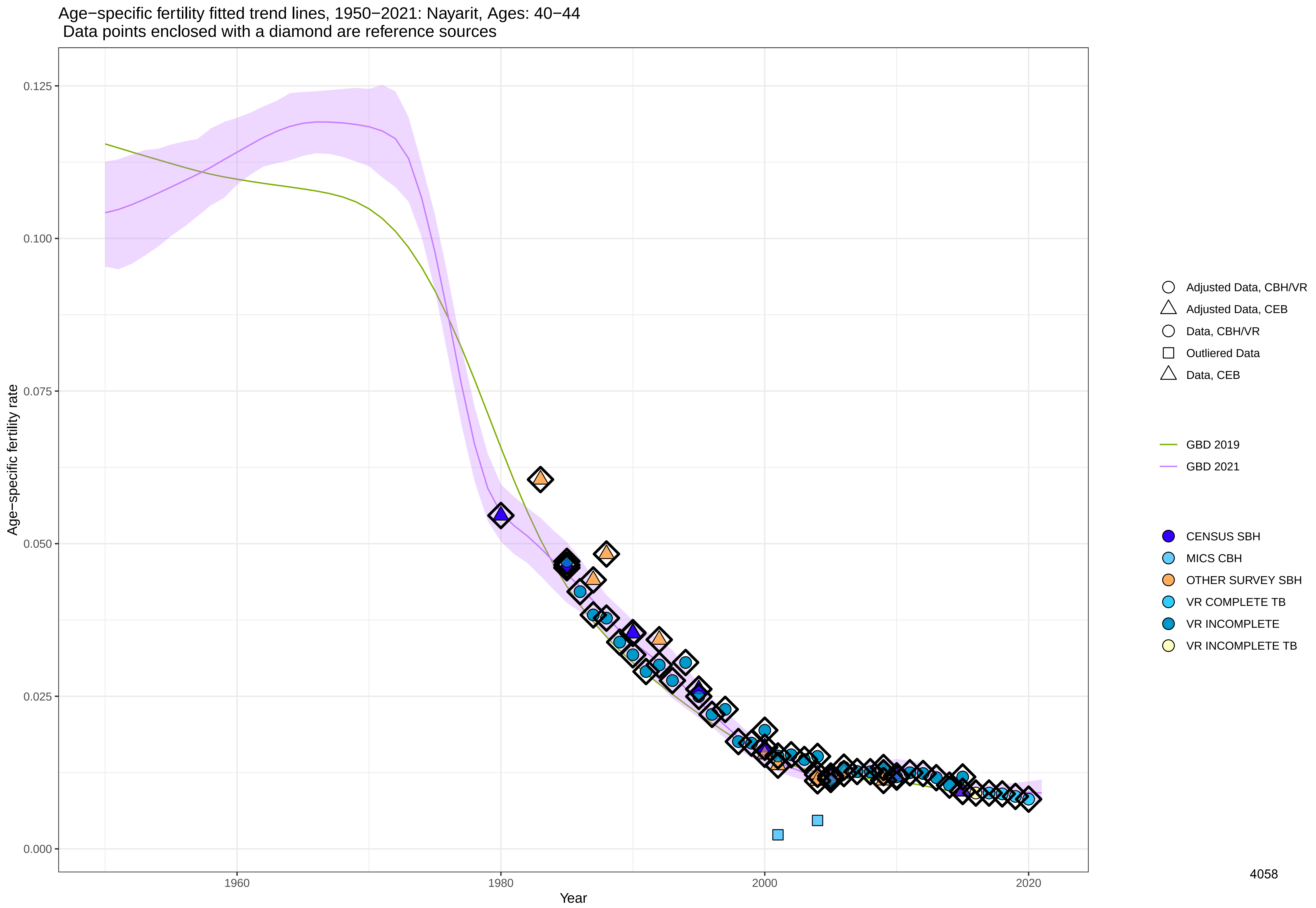

Age−specific fertility fitted trend lines, 1950−2021: Nayarit, Ages: 40−44
Data points enclosed with a diamond are reference sources
Age−specific fertility rate
Year
0.000
0.025
0.050
0.075
0.100
0.125
1960
1980
2000
2020
Adjusted Data, CBH/VR
Adjusted Data, CEB
Data, CBH/VR
Outliered Data
Data, CEB
GBD 2019
GBD 2021
CENSUS SBH
MICS CBH
OTHER SURVEY SBH
VR COMPLETE TB
VR INCOMPLETE
VR INCOMPLETE TB

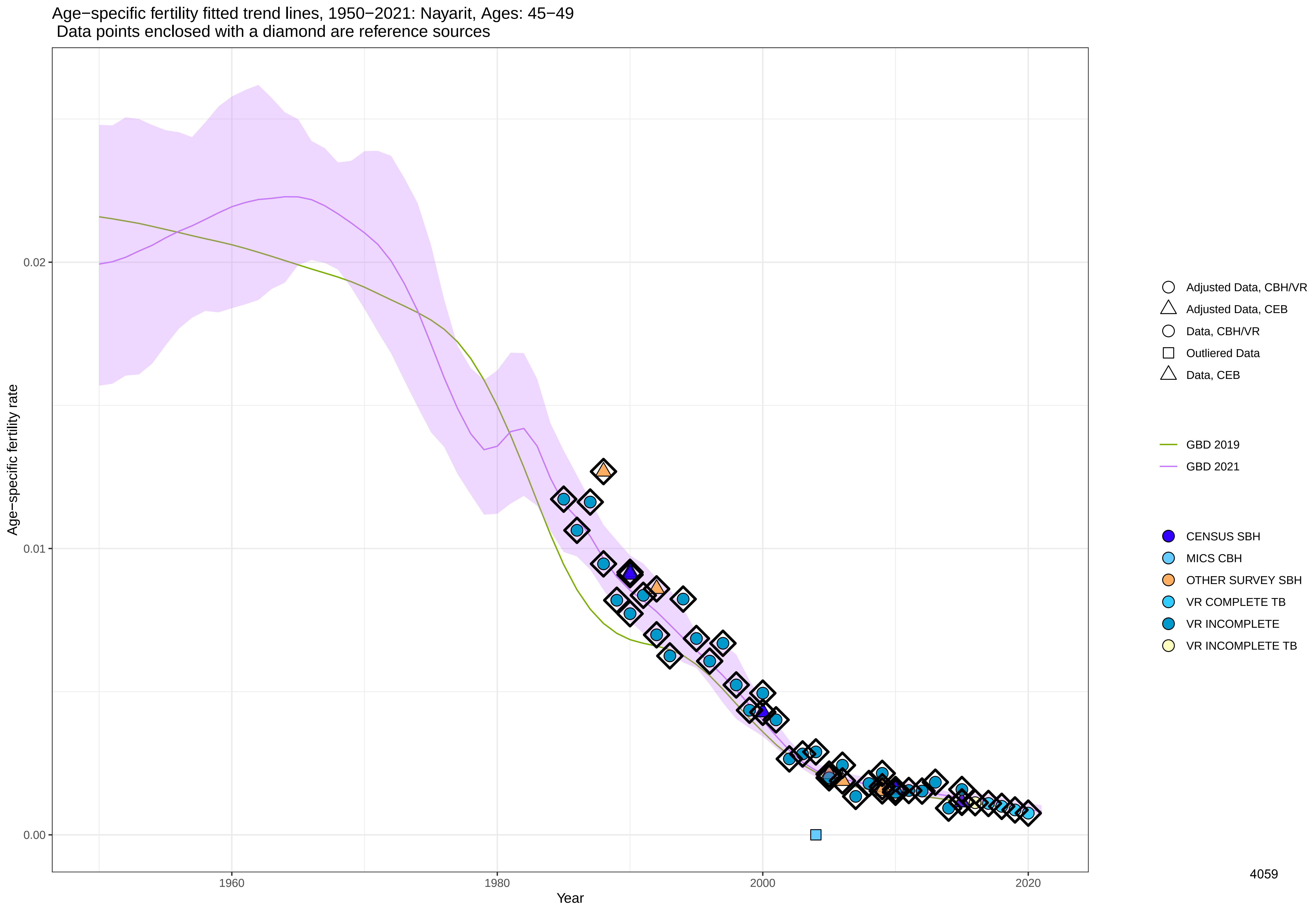

Age−specific fertility fitted trend lines, 1950−2021: Nayarit, Ages: 45−49
Data points enclosed with a diamond are reference sources
Age−specific fertility rate
0.00
0.01
0.02
1960
1980
2000
2020
Year
Adjusted Data, CBH/VR
Adjusted Data, CEB
Data, CBH/VR
Outliered Data
Data, CEB
GBD 2019
GBD 2021
CENSUS SBH
MICS CBH
OTHER SURVEY SBH
VR COMPLETE TB
VR INCOMPLETE
VR INCOMPLETE TB

Age−specific fertility fitted trend lines, 1950−2021: Nayarit, Ages: 50−54
Data points enclosed with a diamond are reference sources

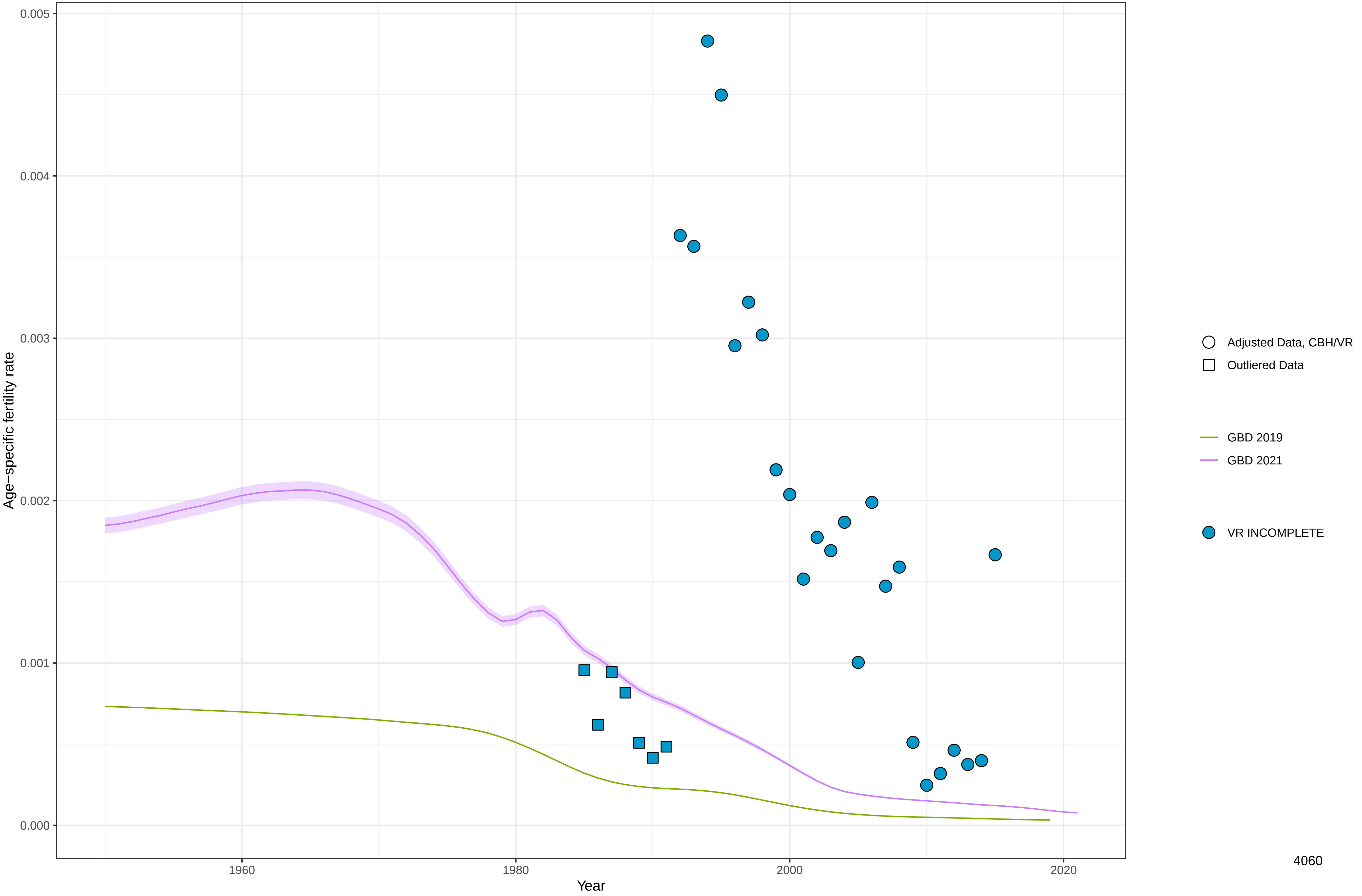

Total fertility rate fitted trend lines, 1950−2021: Nayarit TFR
Data points enclosed with a diamond are reference sources

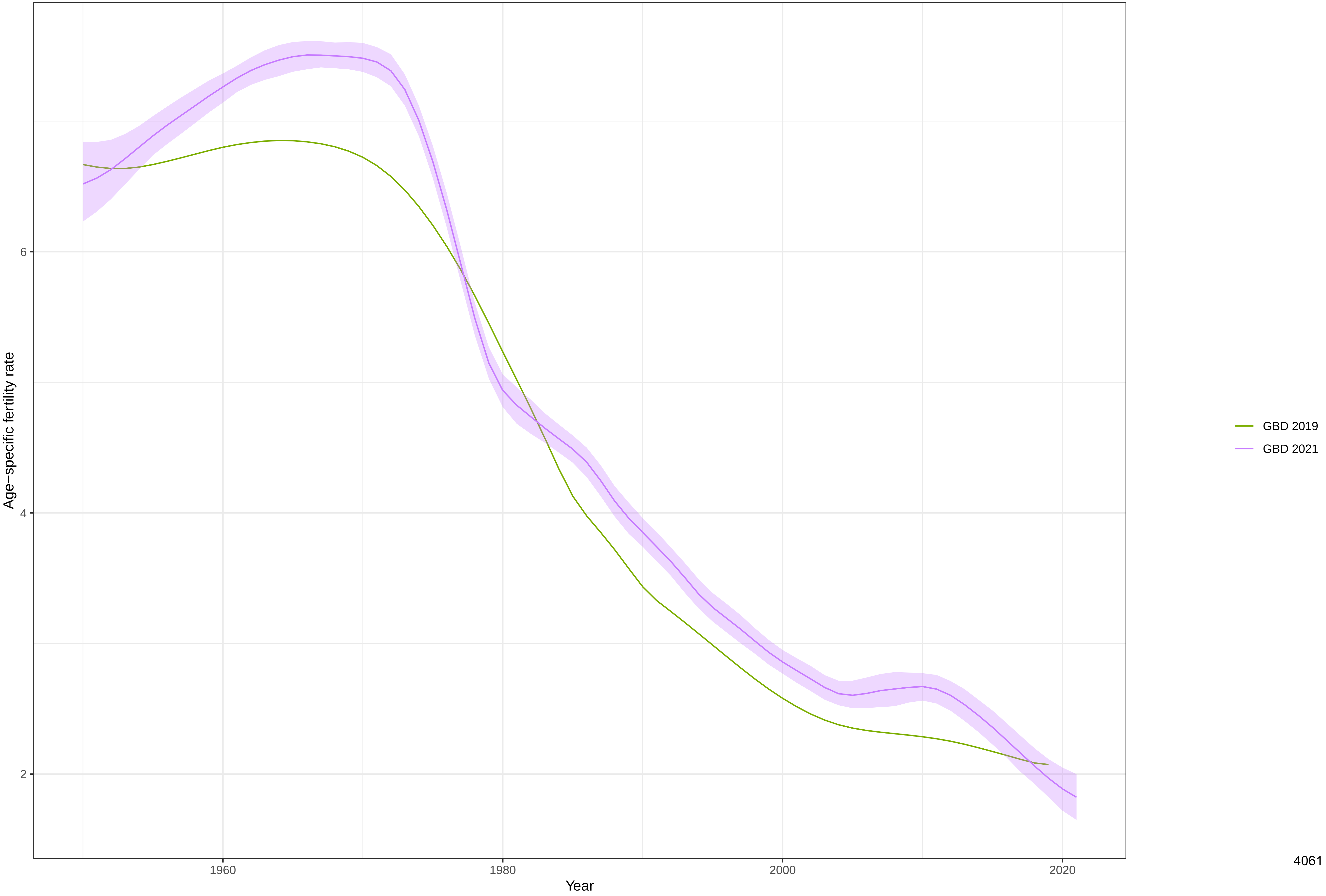

4061

Age−specific fertility fitted trend lines, 1950−2021: Nuevo León, Ages: 10−14
Data points enclosed with a diamond are reference sources

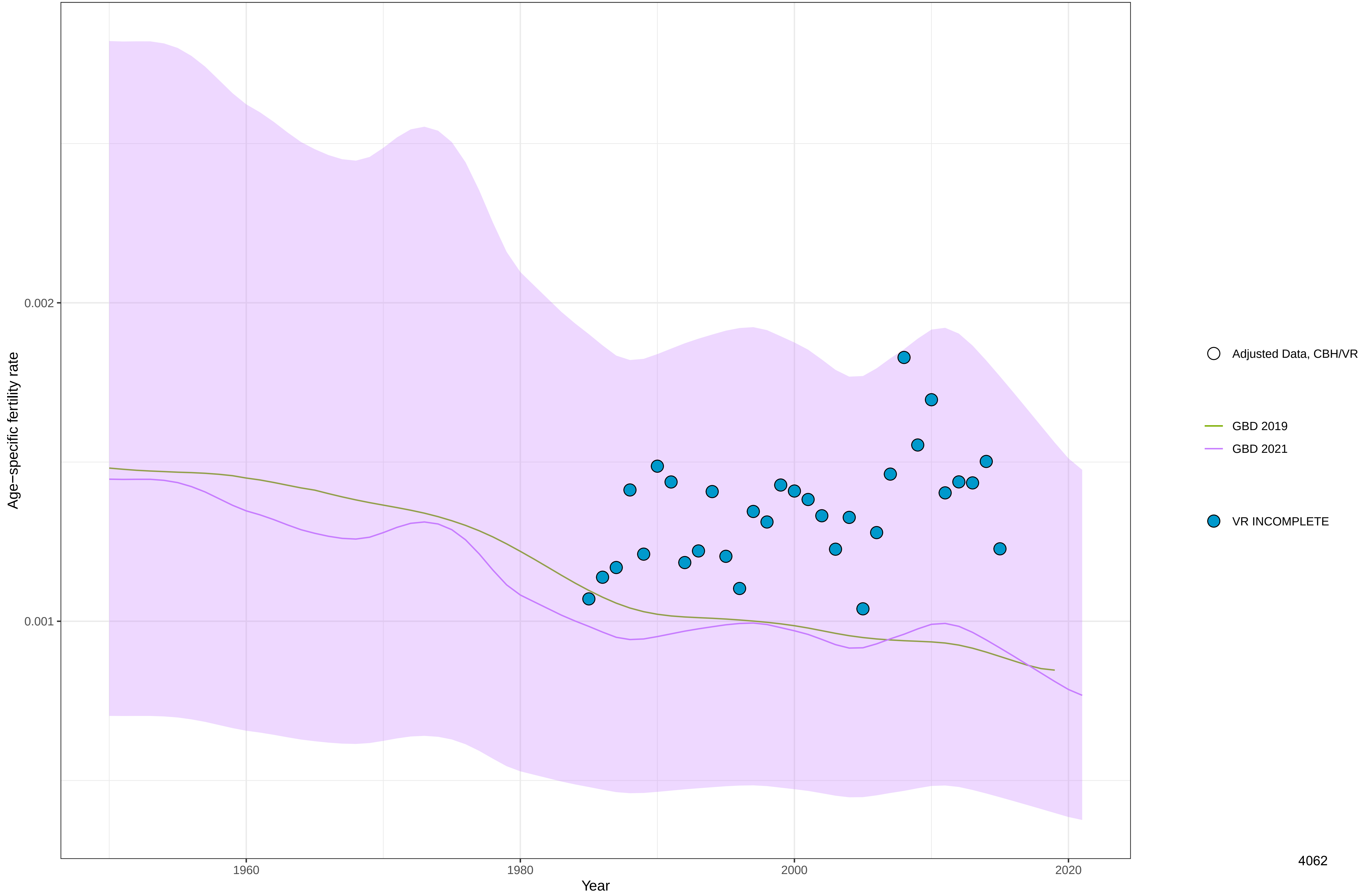

Age−specific fertility fitted trend lines, 1950−2021: Nuevo León, Ages: 15−19
Data points enclosed with a diamond are reference sources

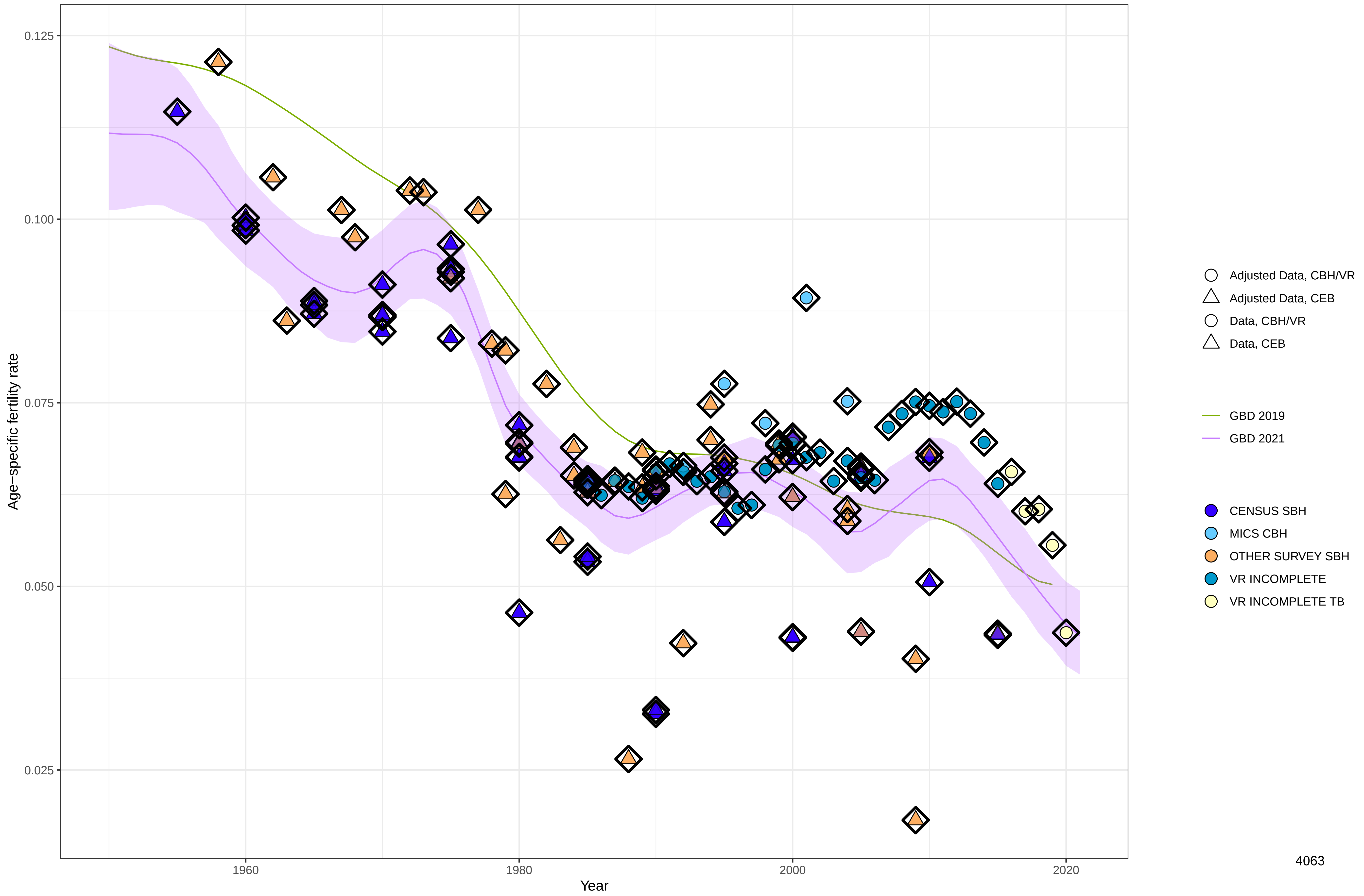

Age−specific fertility fitted trend lines, 1950−2021: Nuevo León, Ages: 20−24
Data points enclosed with a diamond are reference sources

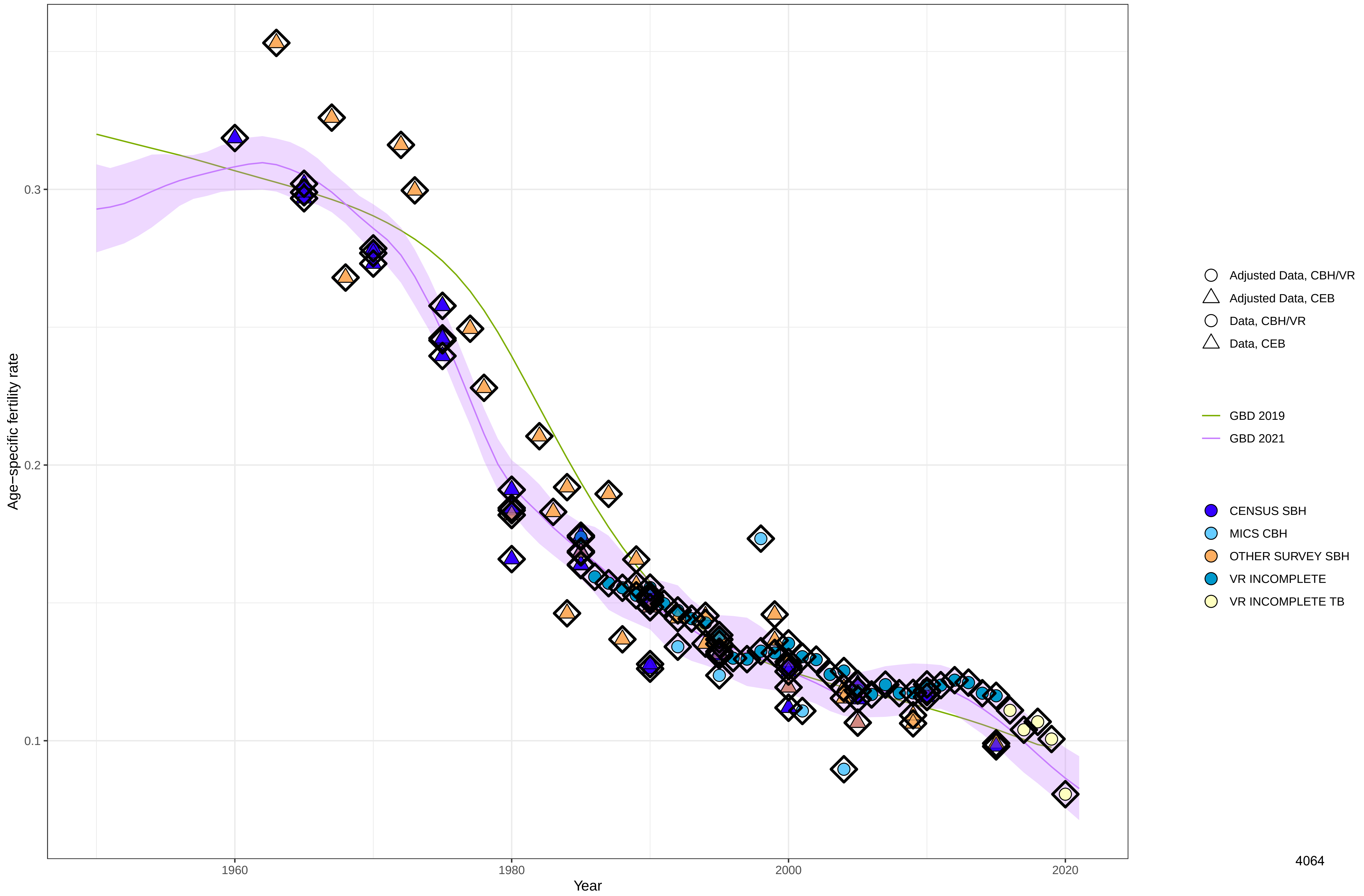

Age−specific fertility fitted trend lines, 1950−2021: Nuevo León, Ages: 25−29
Data points enclosed with a diamond are reference sources

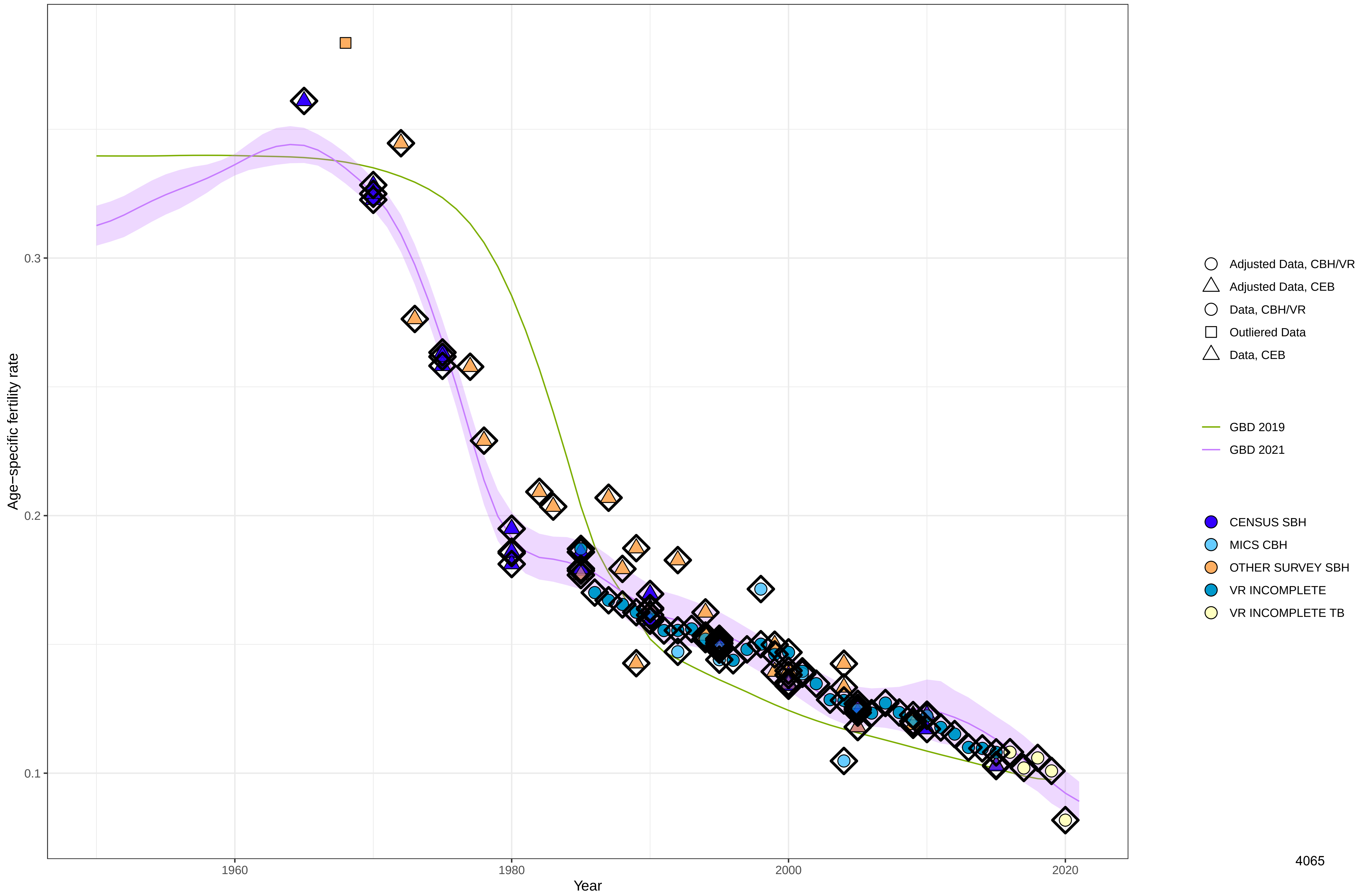

Age−specific fertility fitted trend lines, 1950−2021: Nuevo León, Ages: 30−34
Data points enclosed with a diamond are reference sources

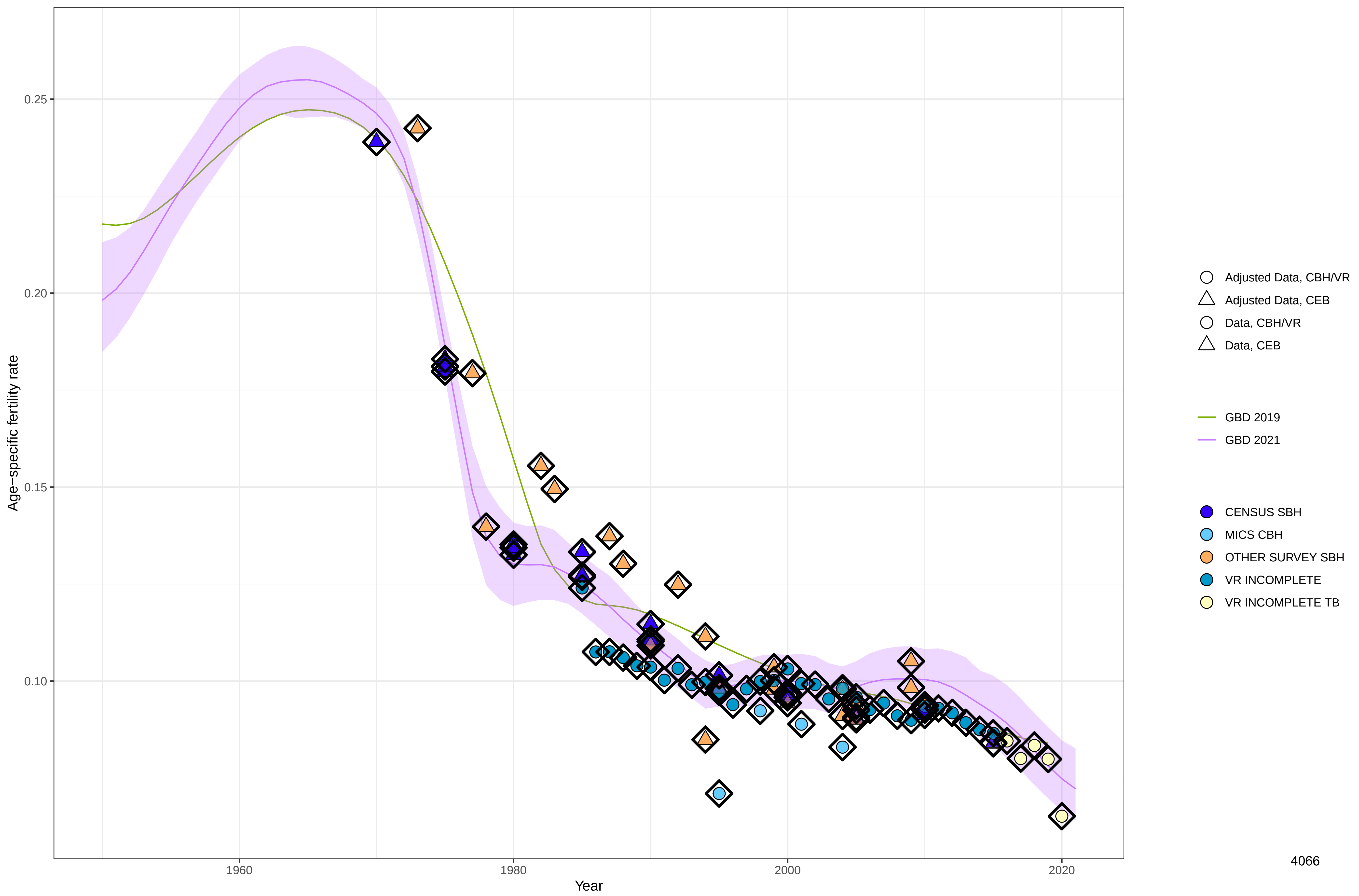

Age−specific fertility fitted trend lines, 1950−2021: Nuevo León, Ages: 35−39
Data points enclosed with a diamond are reference sources

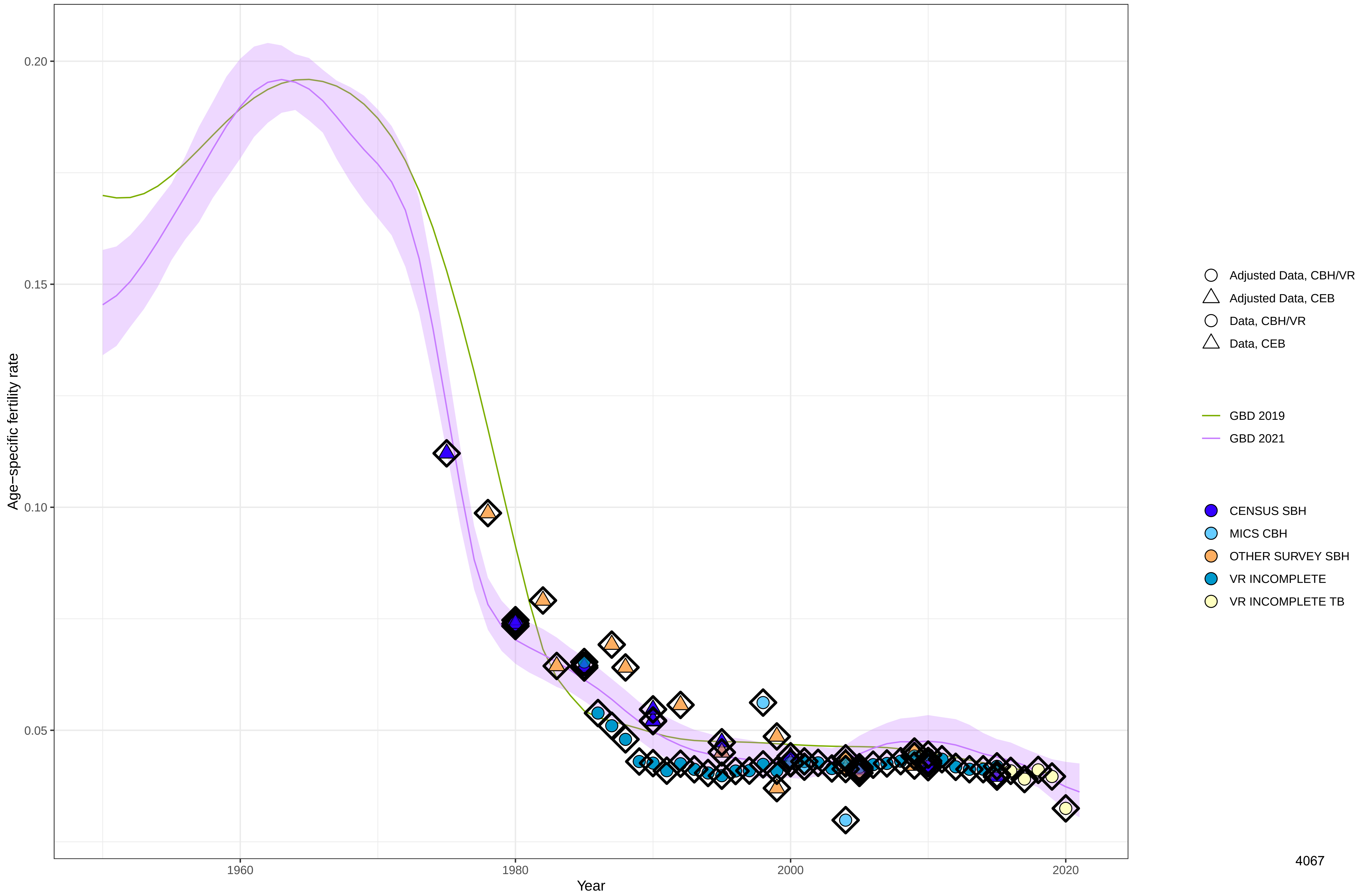

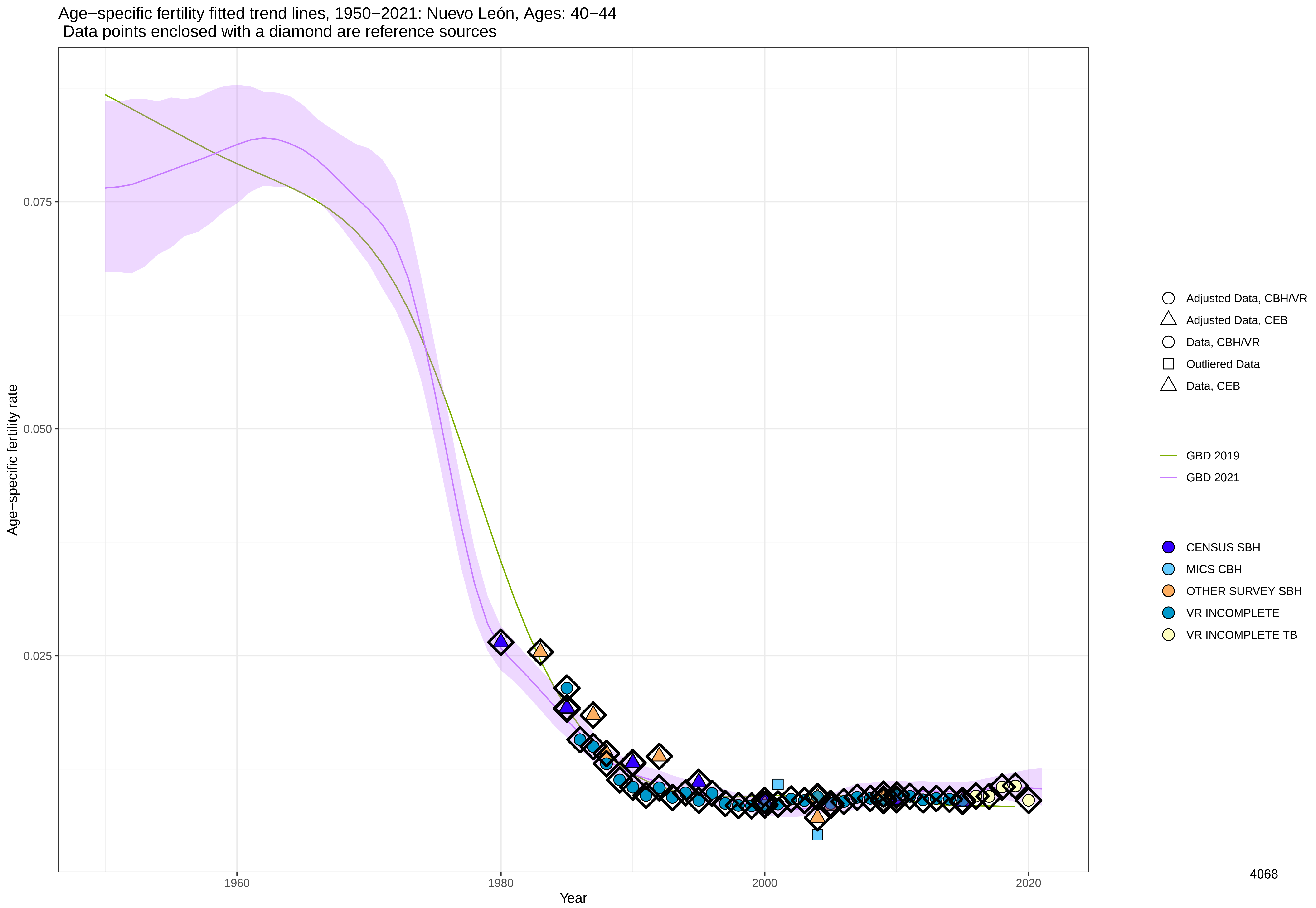

Age−specific fertility fitted trend lines, 1950−2021: Nuevo León, Ages: 40−44
Data points enclosed with a diamond are reference sources
Age−specific fertility rate
0.075
0.050
0.025
Year
1960
1980
2000
2020
Adjusted Data, CBH/VR
Adjusted Data, CEB
Data, CBH/VR
Outliered Data
Data, CEB
GBD 2019
GBD 2021
CENSUS SBH
MICS CBH
OTHER SURVEY SBH
VR INCOMPLETE
VR INCOMPLETE TB

Age−specific fertility fitted trend lines, 1950−2021: Nuevo León, Ages: 45−49
Data points enclosed with a diamond are reference sources

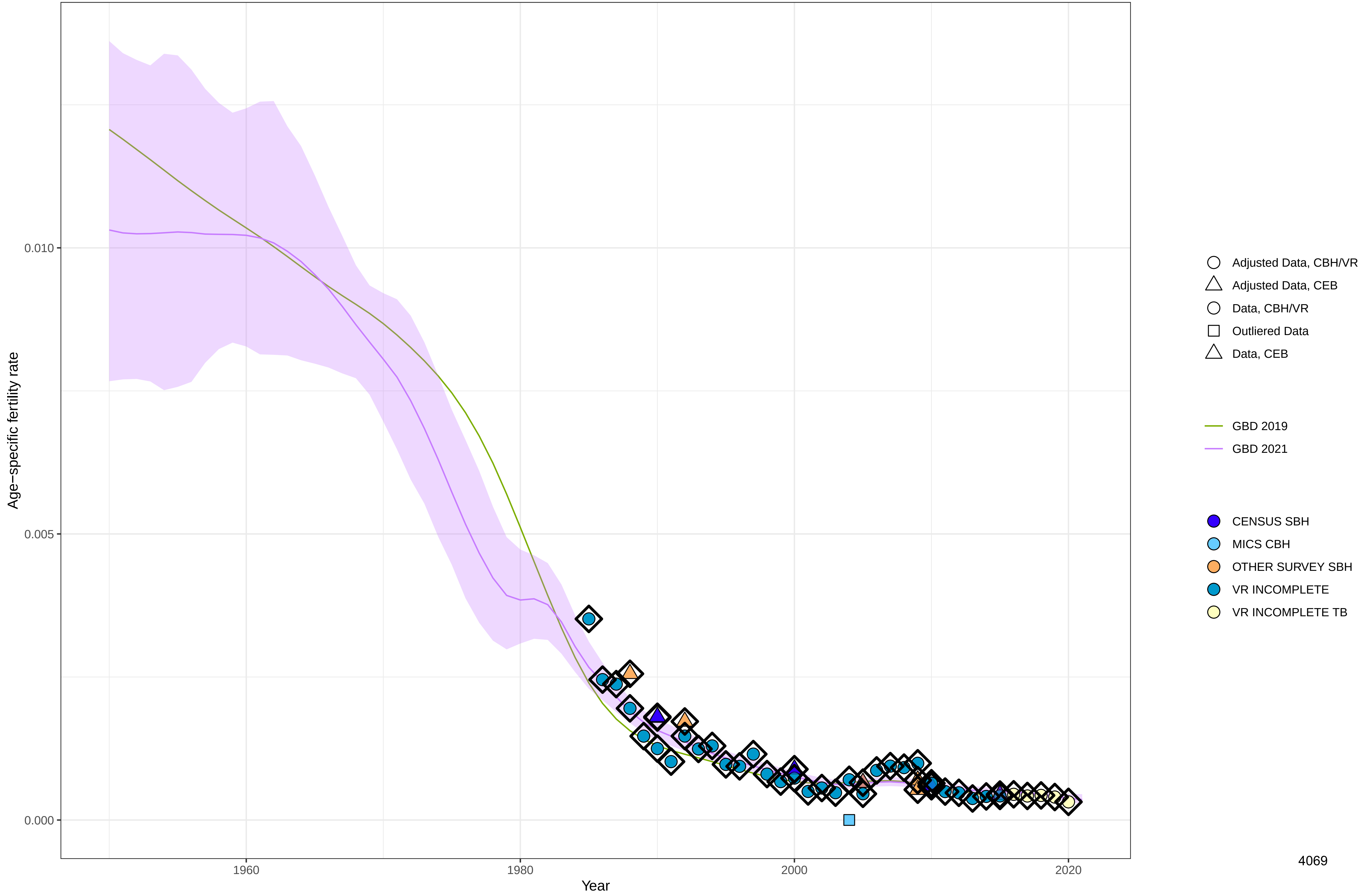

Age−specific fertility fitted trend lines, 1950−2021: Nuevo León, Ages: 50−54
Data points enclosed with a diamond are reference sources

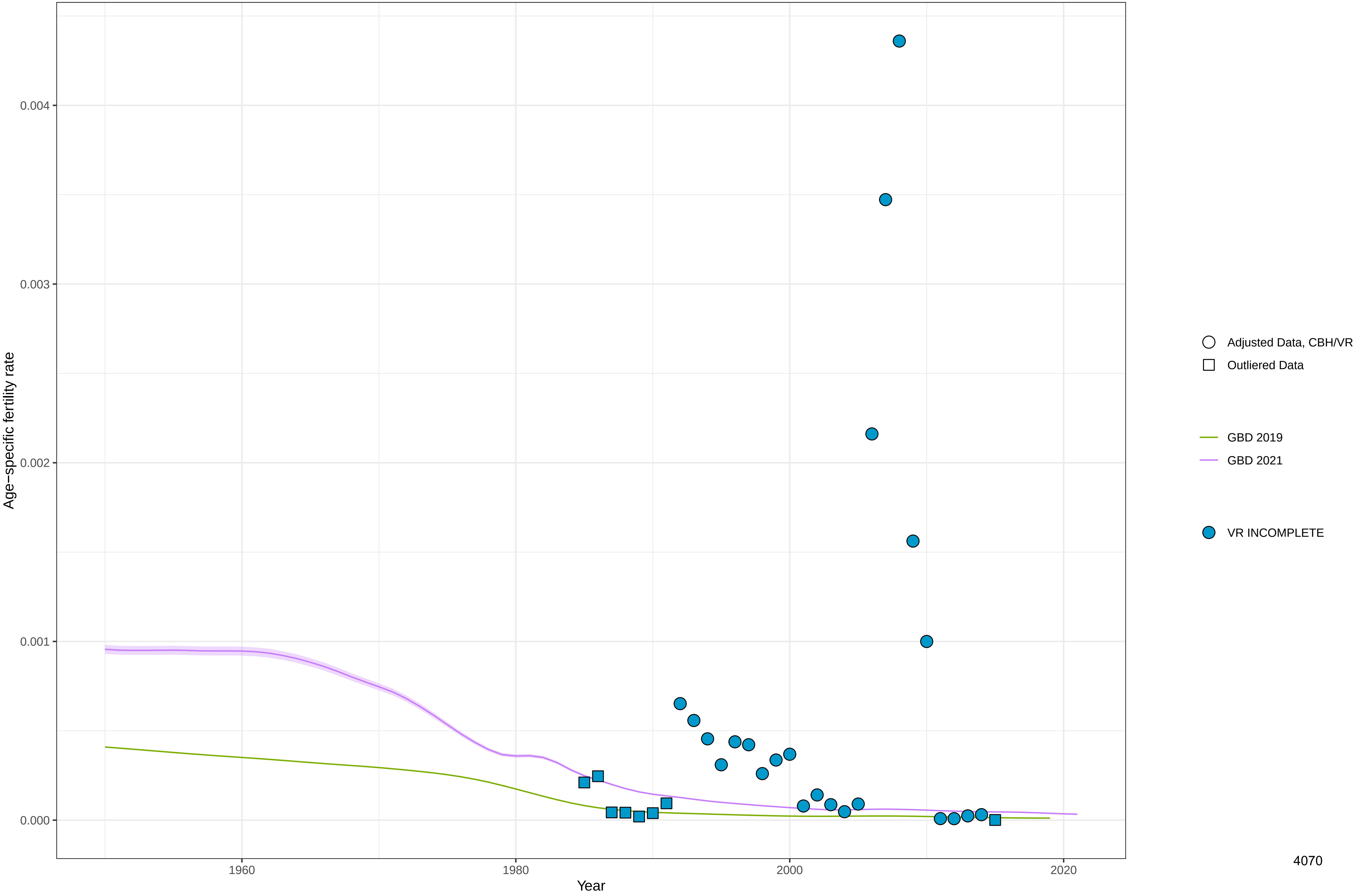

Total fertility rate fitted trend lines, 1950−2021: Nuevo León TFR
Data points enclosed with a diamond are reference sources

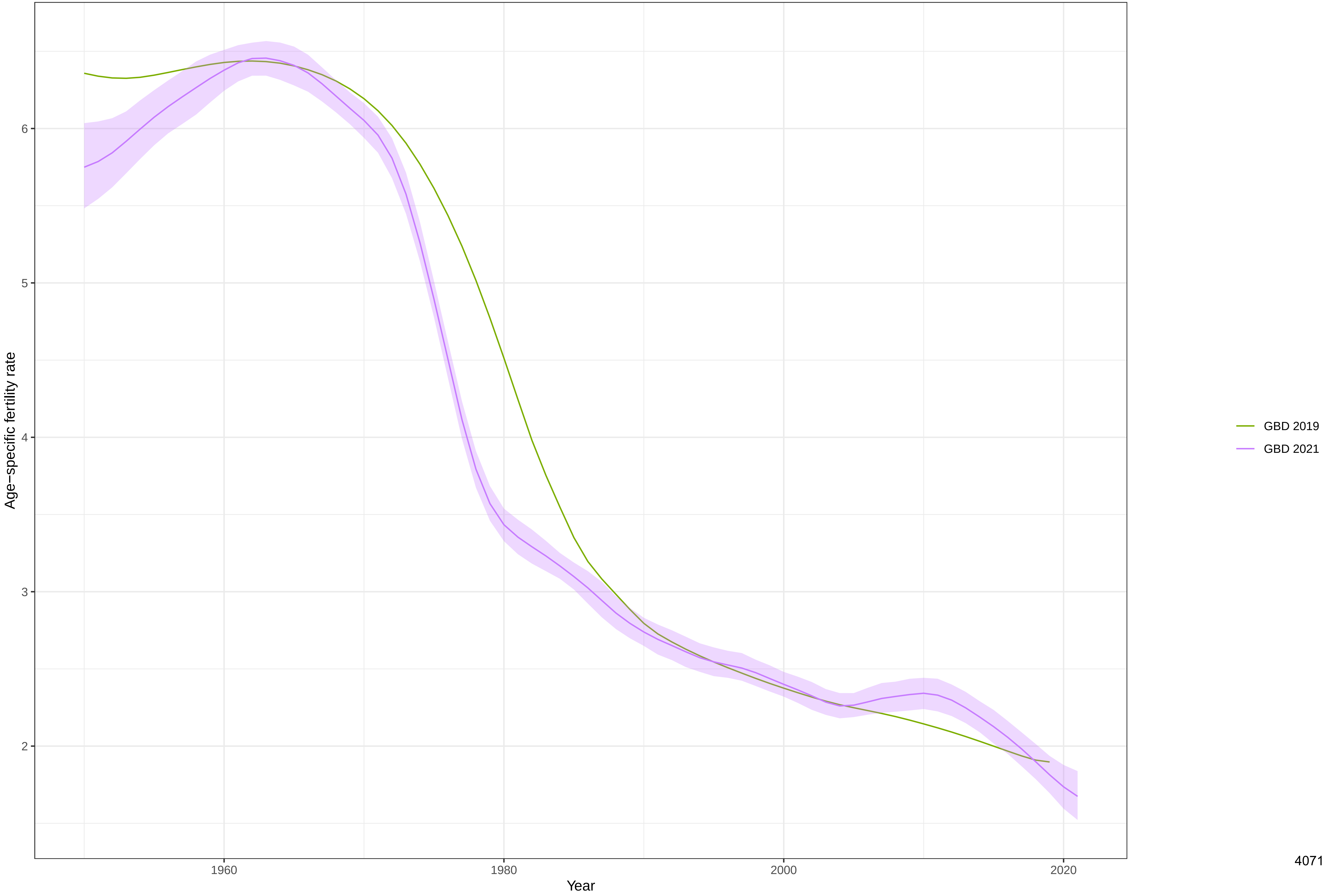

Age−specific fertility fitted trend lines, 1950−2021: Oaxaca, Ages: 10−14
Data points enclosed with a diamond are reference sources

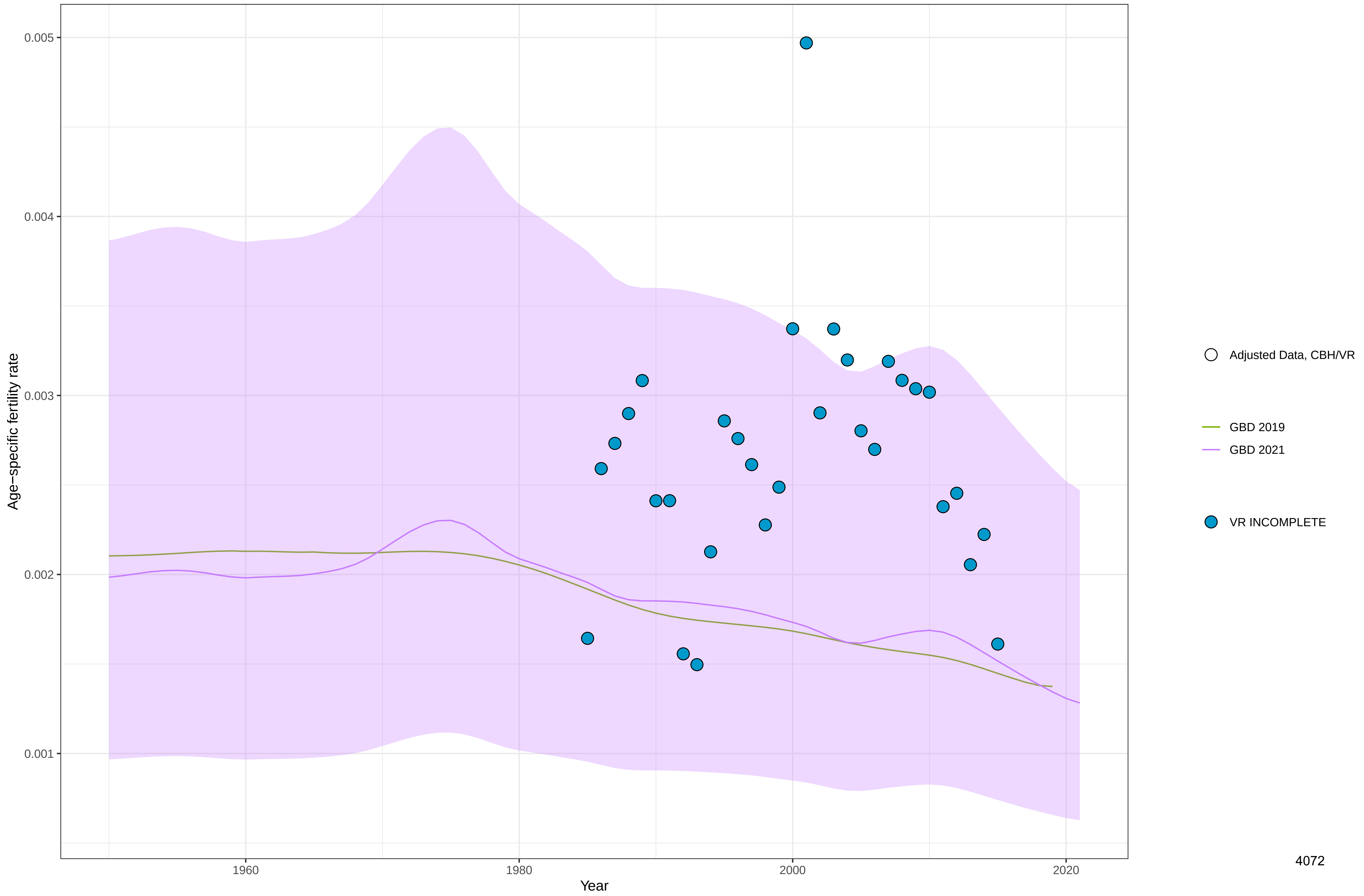

Age−specific fertility fitted trend lines, 1950−2021: Oaxaca, Ages: 15−19
Data points enclosed with a diamond are reference sources

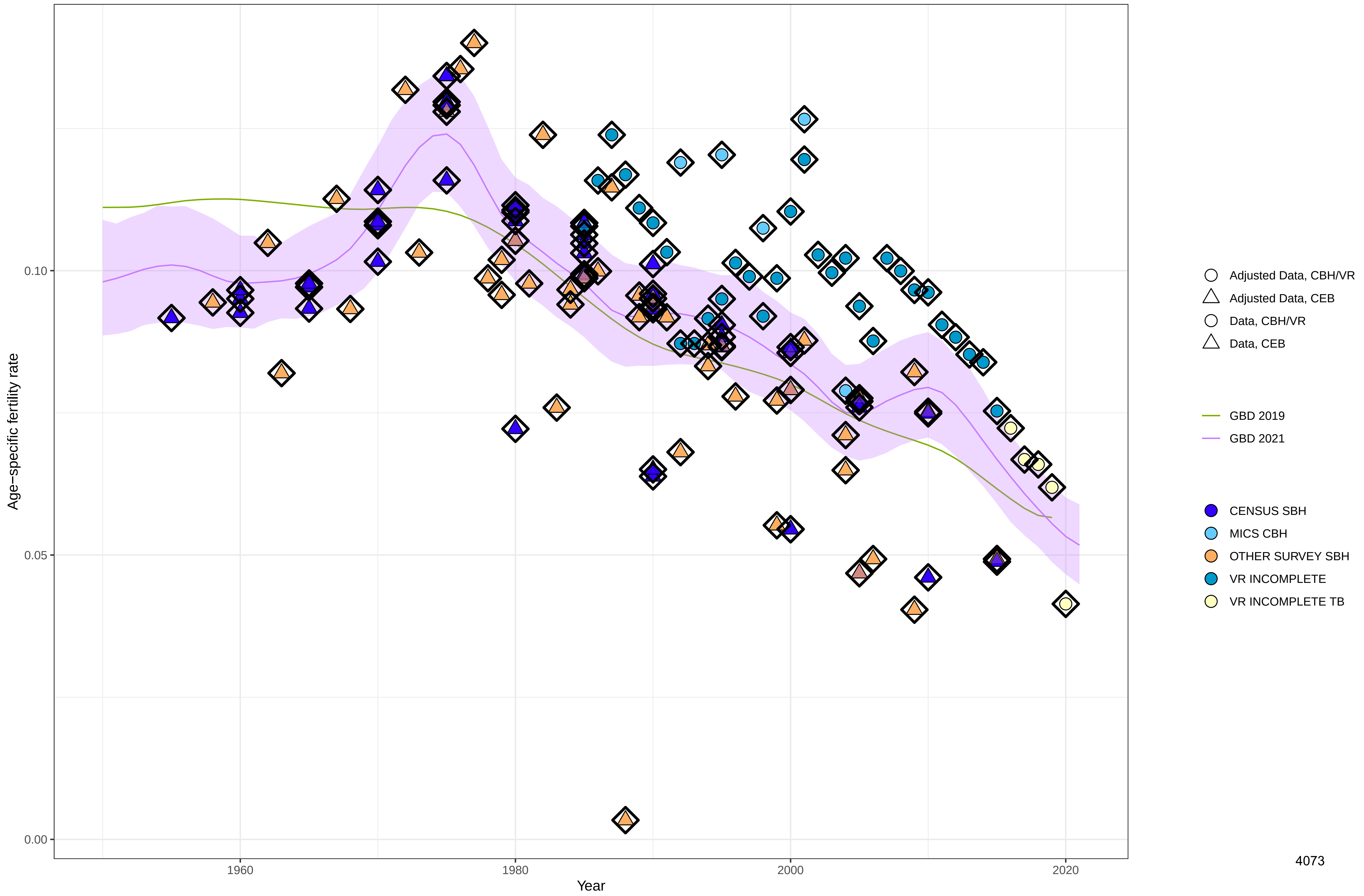

Age−specific fertility fitted trend lines, 1950−2021: Oaxaca, Ages: 20−24
Data points enclosed with a diamond are reference sources

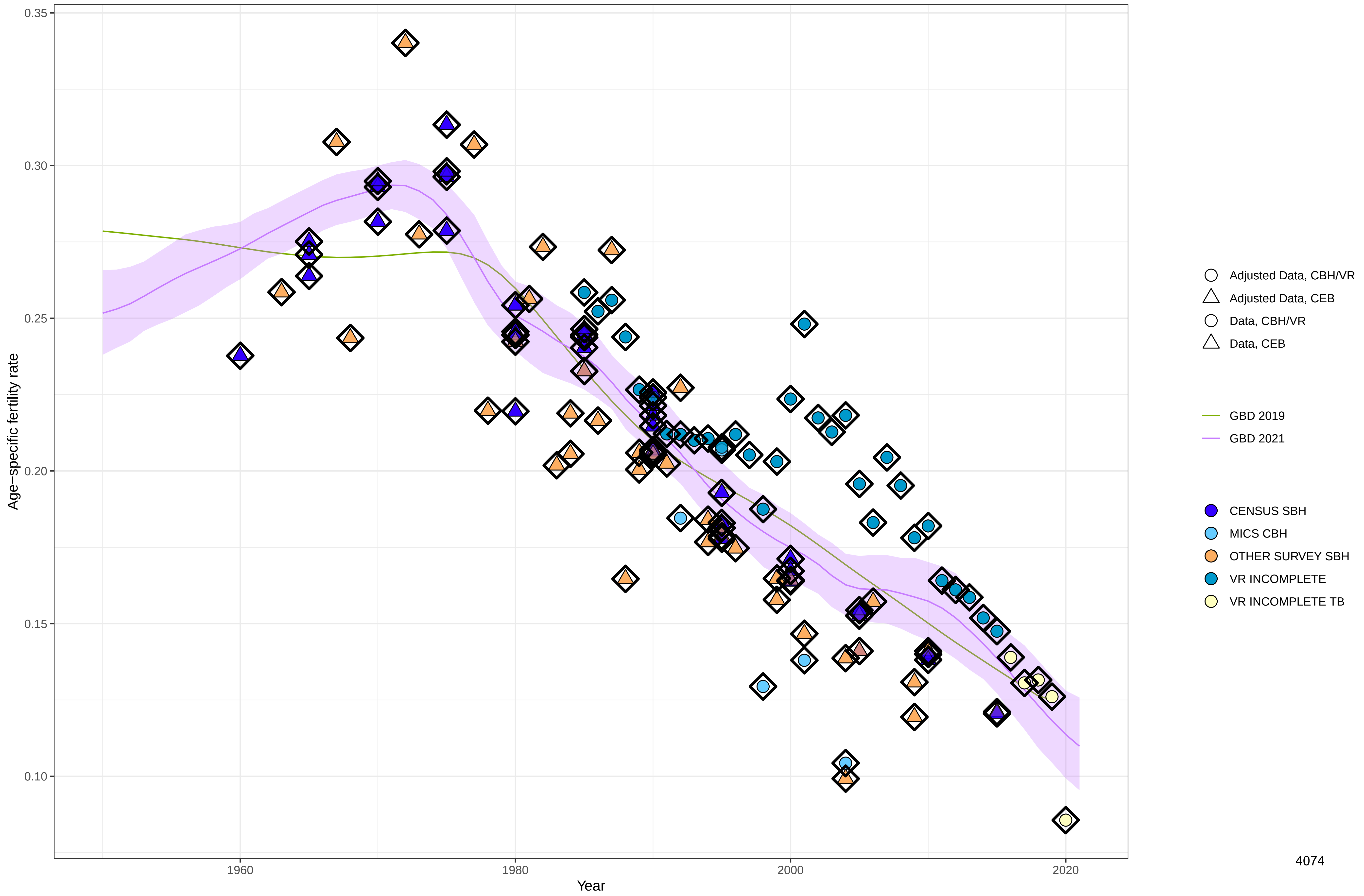

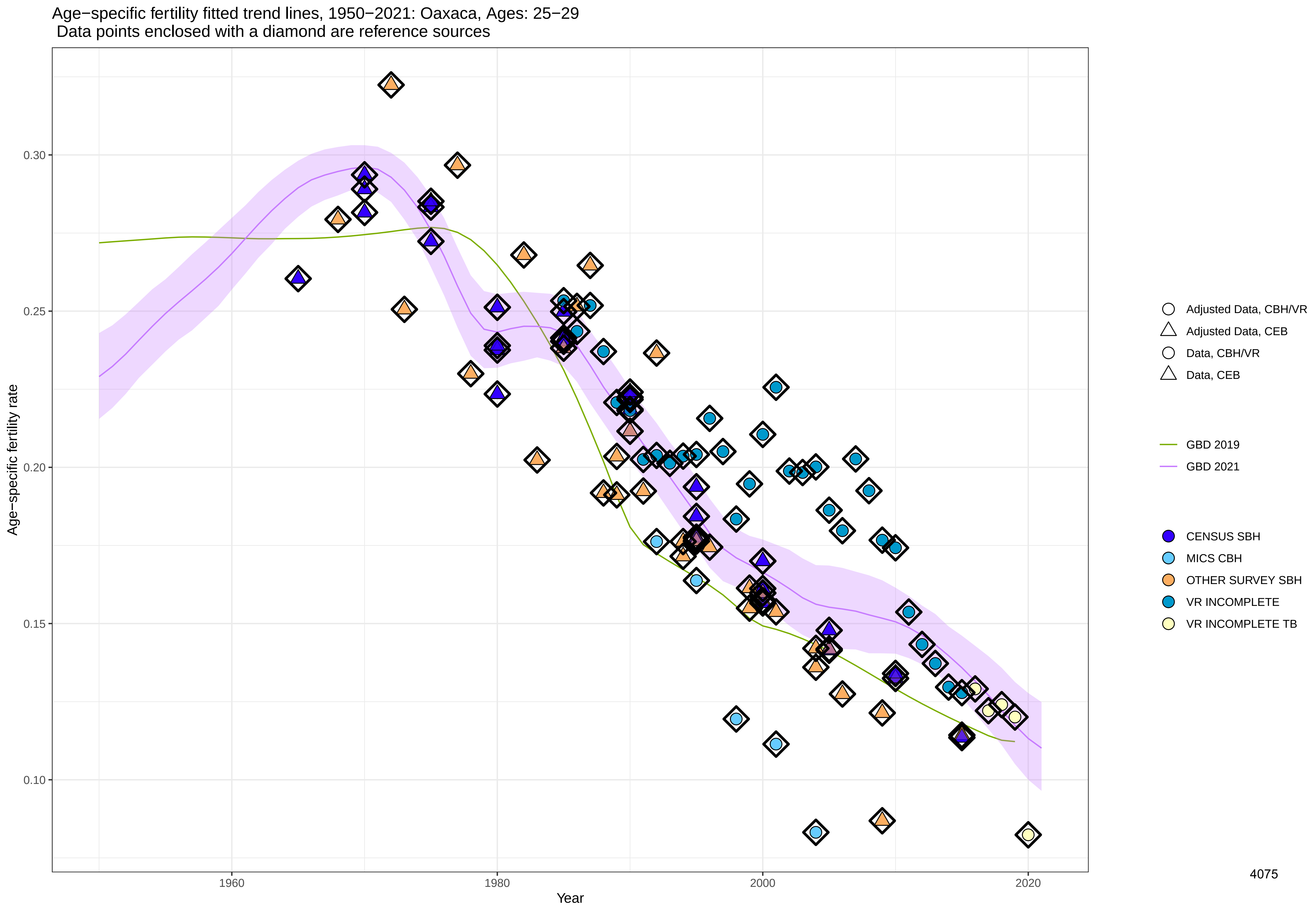

Age−specific fertility fitted trend lines, 1950−2021: Oaxaca, Ages: 25−29
Data points enclosed with a diamond are reference sources
0.30
0.25
0.20
0.15
0.10
Age−specific fertility rate
1960
1980
2000
2020
Year
Adjusted Data, CBH/VR
Adjusted Data, CEB
Data, CBH/VR
Data, CEB
GBD 2019
GBD 2021
CENSUS SBH
MICS CBH
OTHER SURVEY SBH
VR INCOMPLETE
VR INCOMPLETE TB

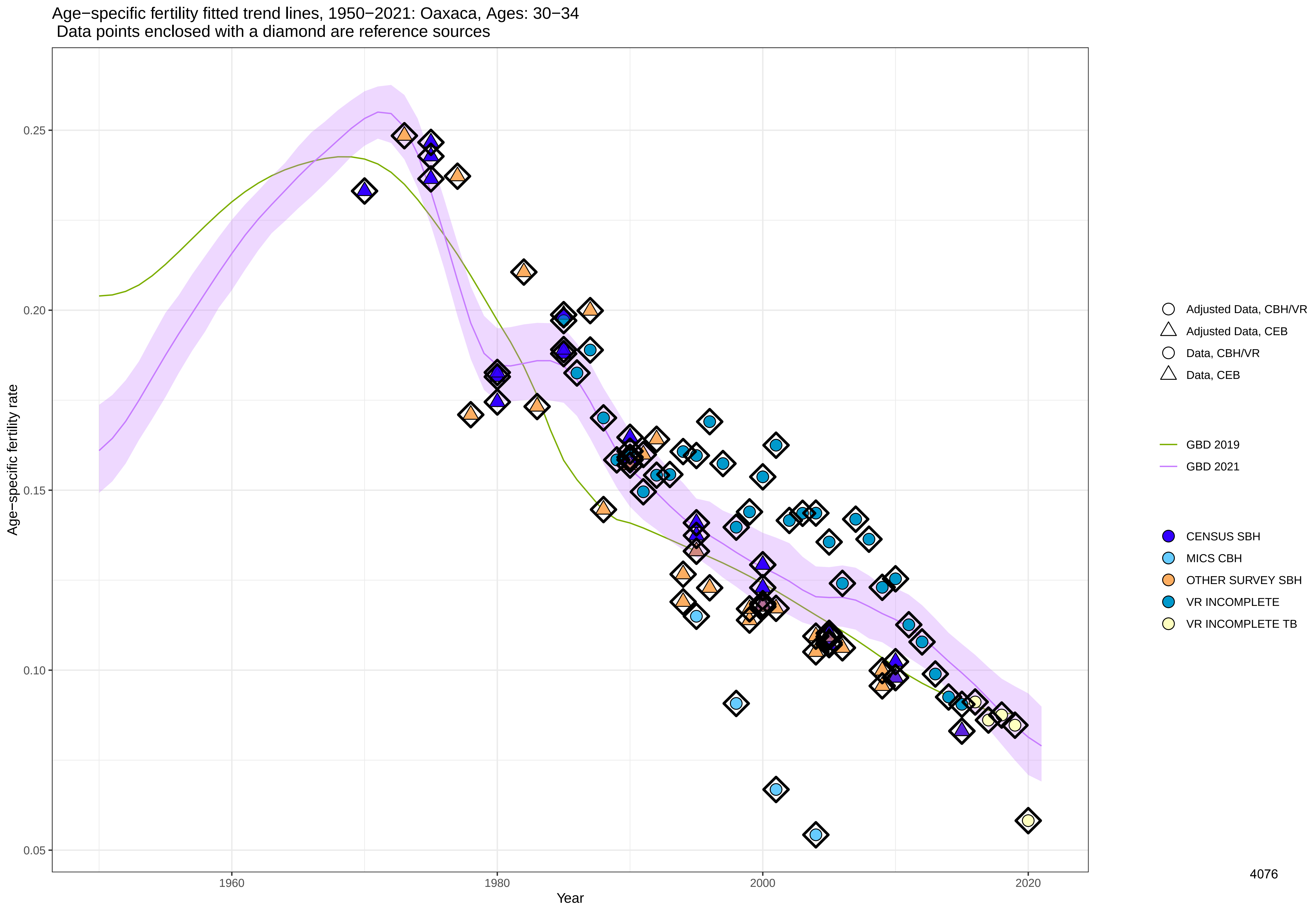

Age−specific fertility fitted trend lines, 1950−2021: Oaxaca, Ages: 30−34
Data points enclosed with a diamond are reference sources
Age−specific fertility rate
0.25
0.20
0.15
0.10
0.05
1960
1980
2000
2020
Year
Adjusted Data, CBH/VR
Adjusted Data, CEB
Data, CBH/VR
Data, CEB
GBD 2019
GBD 2021
CENSUS SBH
MICS CBH
OTHER SURVEY SBH
VR INCOMPLETE
VR INCOMPLETE TB

Age−specific fertility fitted trend lines, 1950−2021: Oaxaca, Ages: 35−39
Data points enclosed with a diamond are reference sources

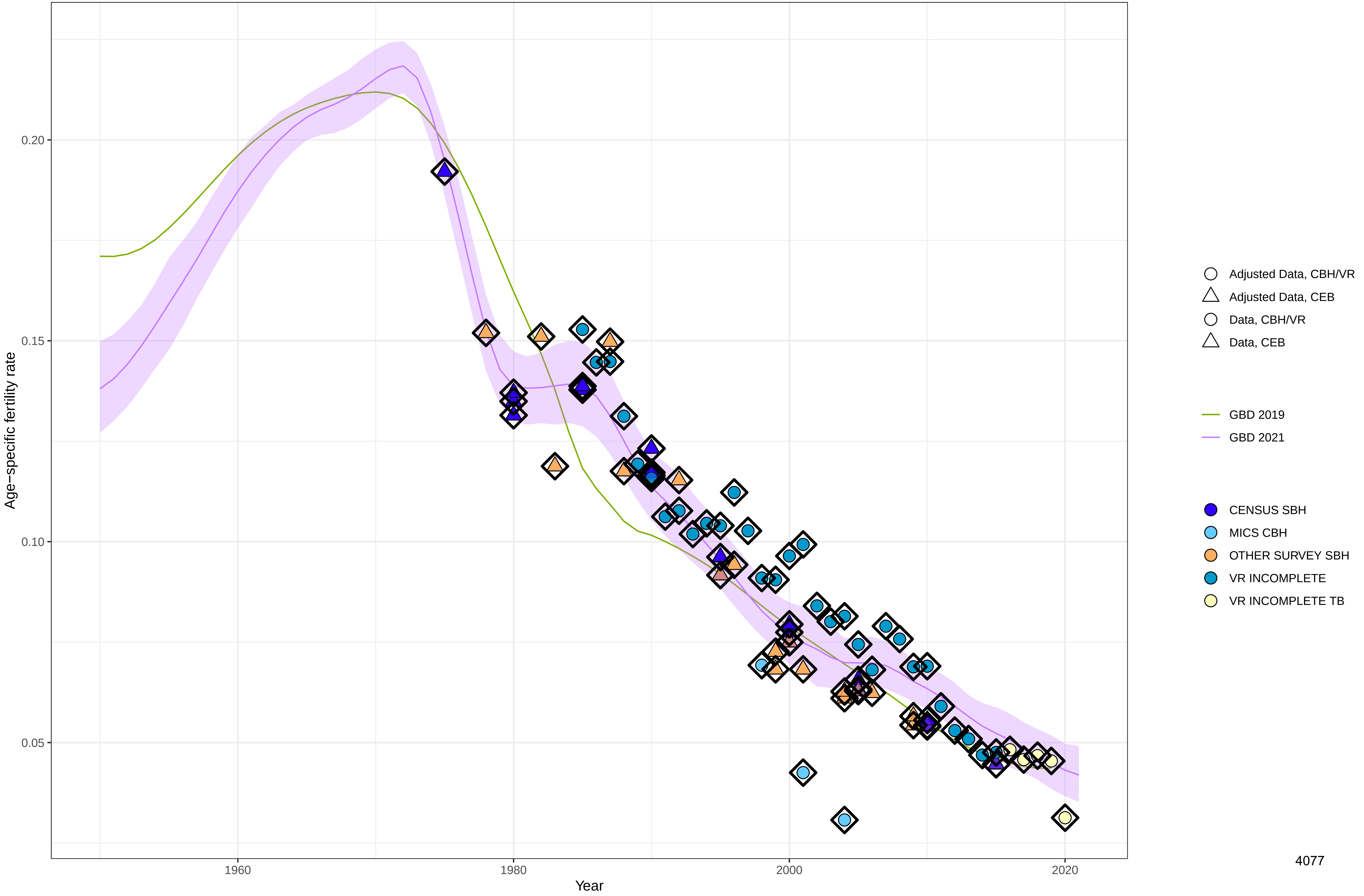

Age−specific fertility fitted trend lines, 1950−2021: Oaxaca, Ages: 40−44
Data points enclosed with a diamond are reference sources

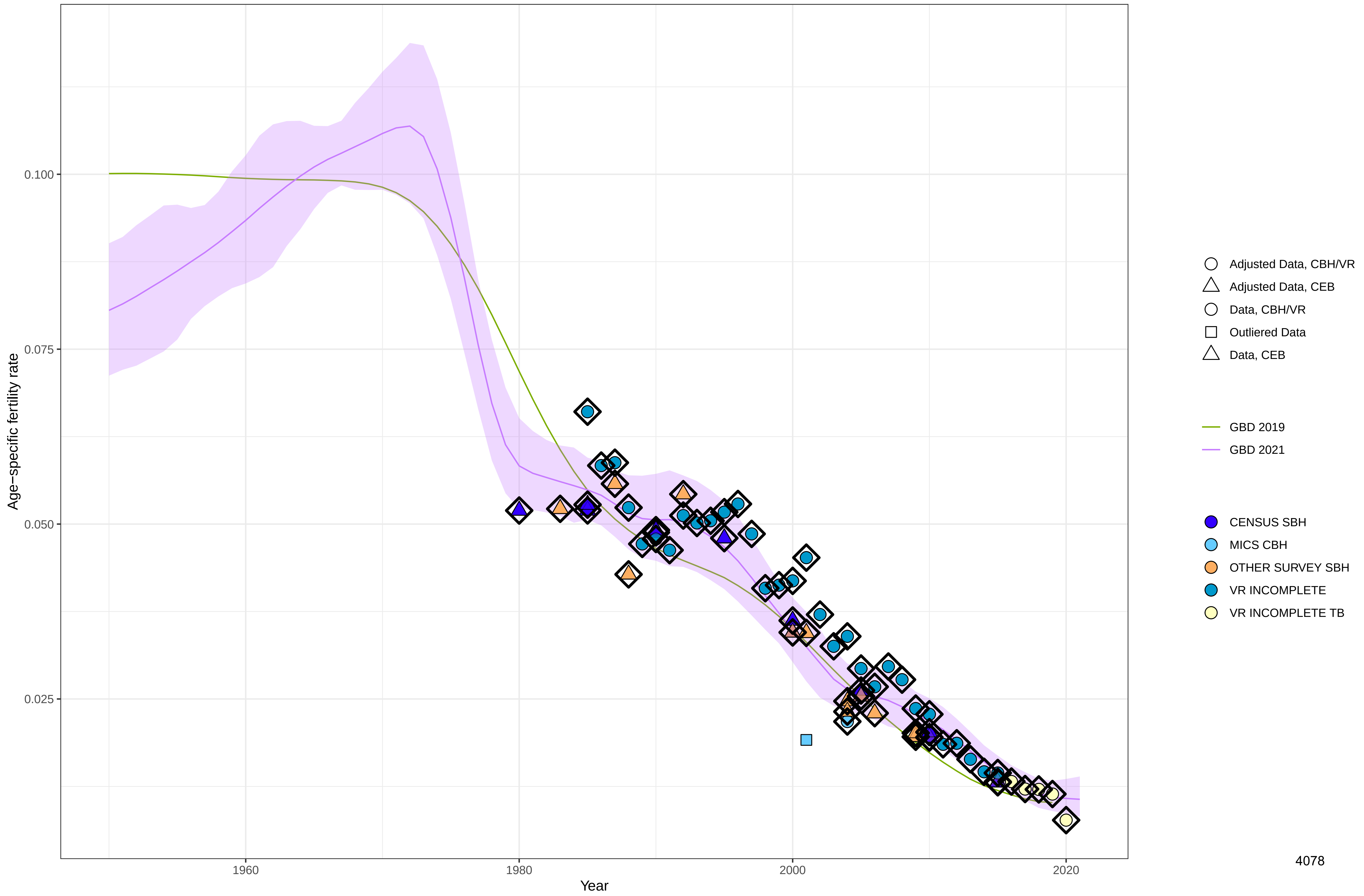

Age−specific fertility fitted trend lines, 1950−2021: Oaxaca, Ages: 45−49
Data points enclosed with a diamond are reference sources

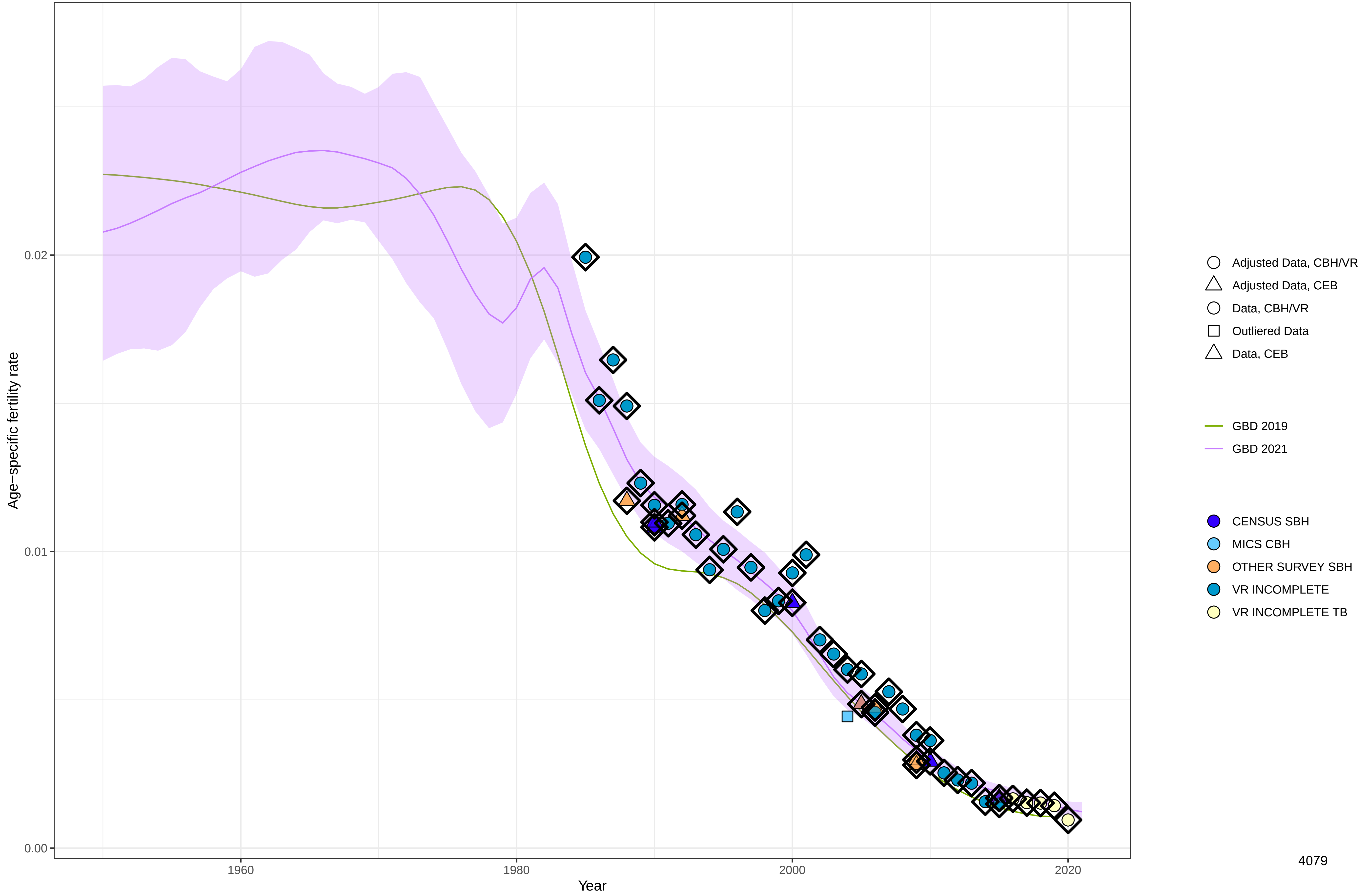

Age−specific fertility fitted trend lines, 1950−2021: Oaxaca, Ages: 50−54
Data points enclosed with a diamond are reference sources

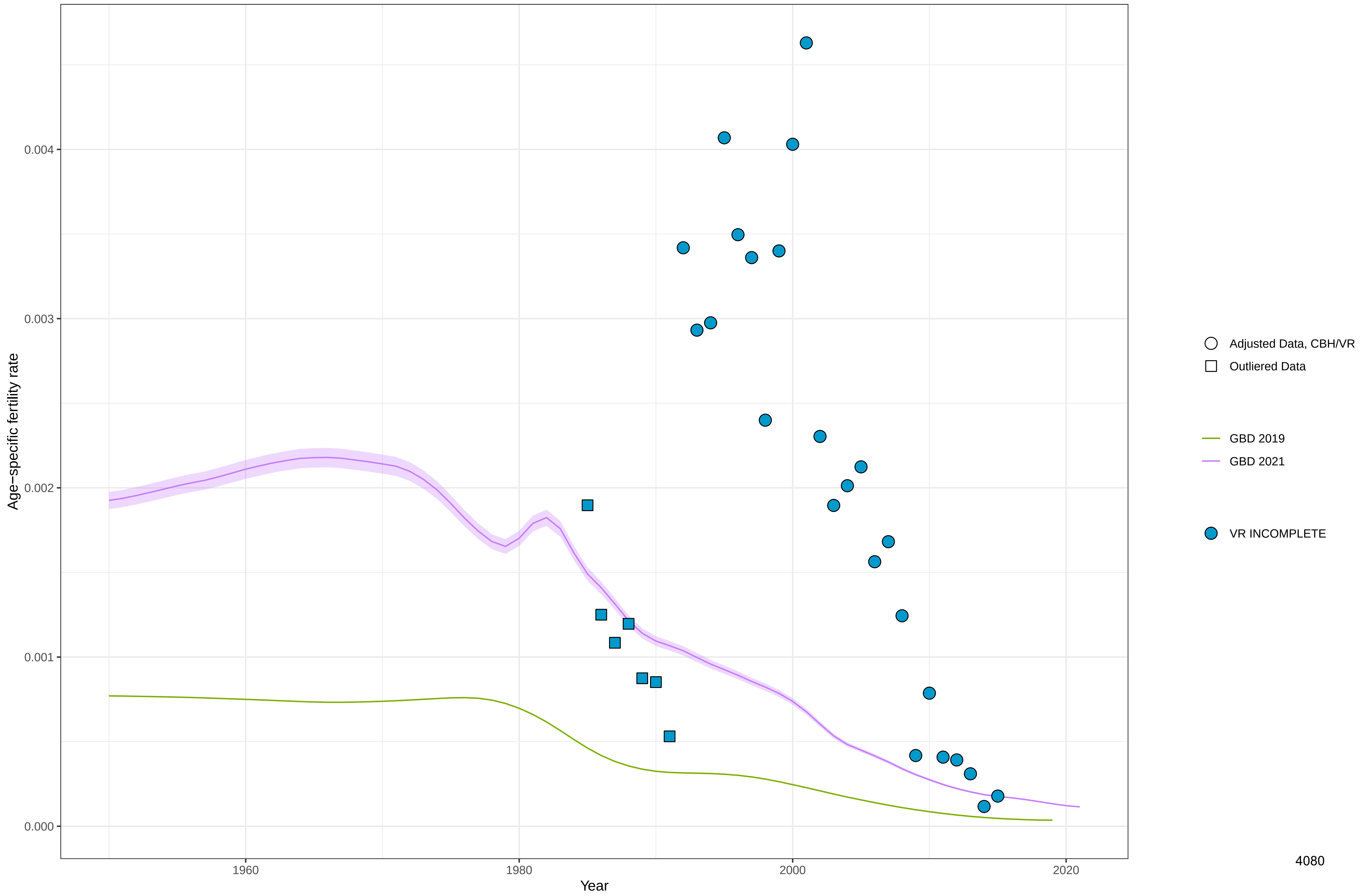

Total fertility rate fitted trend lines, 1950−2021: Oaxaca TFR
Data points enclosed with a diamond are reference sources

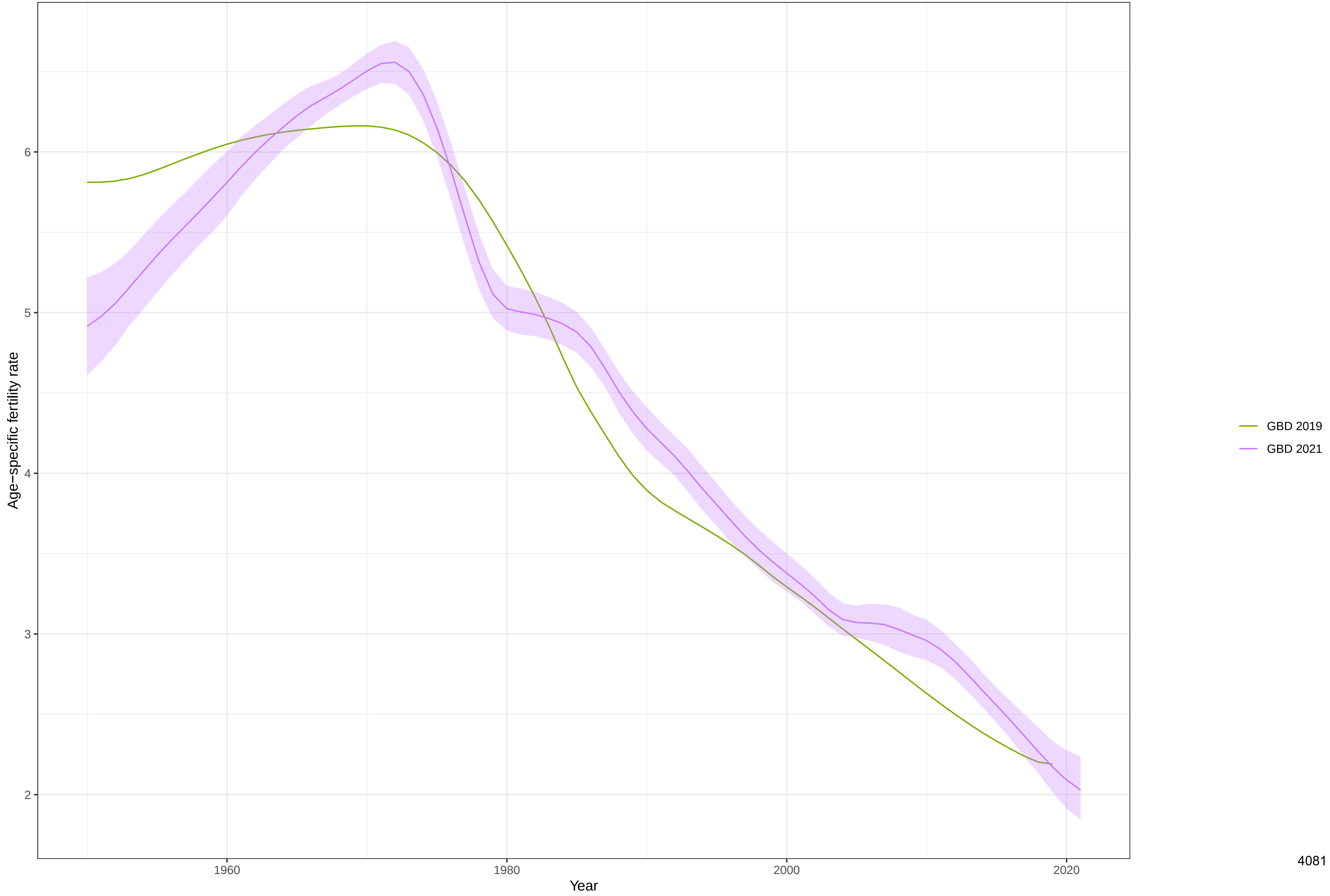

Age−specific fertility fitted trend lines, 1950−2021: Puebla, Ages: 10−14
Data points enclosed with a diamond are reference sources

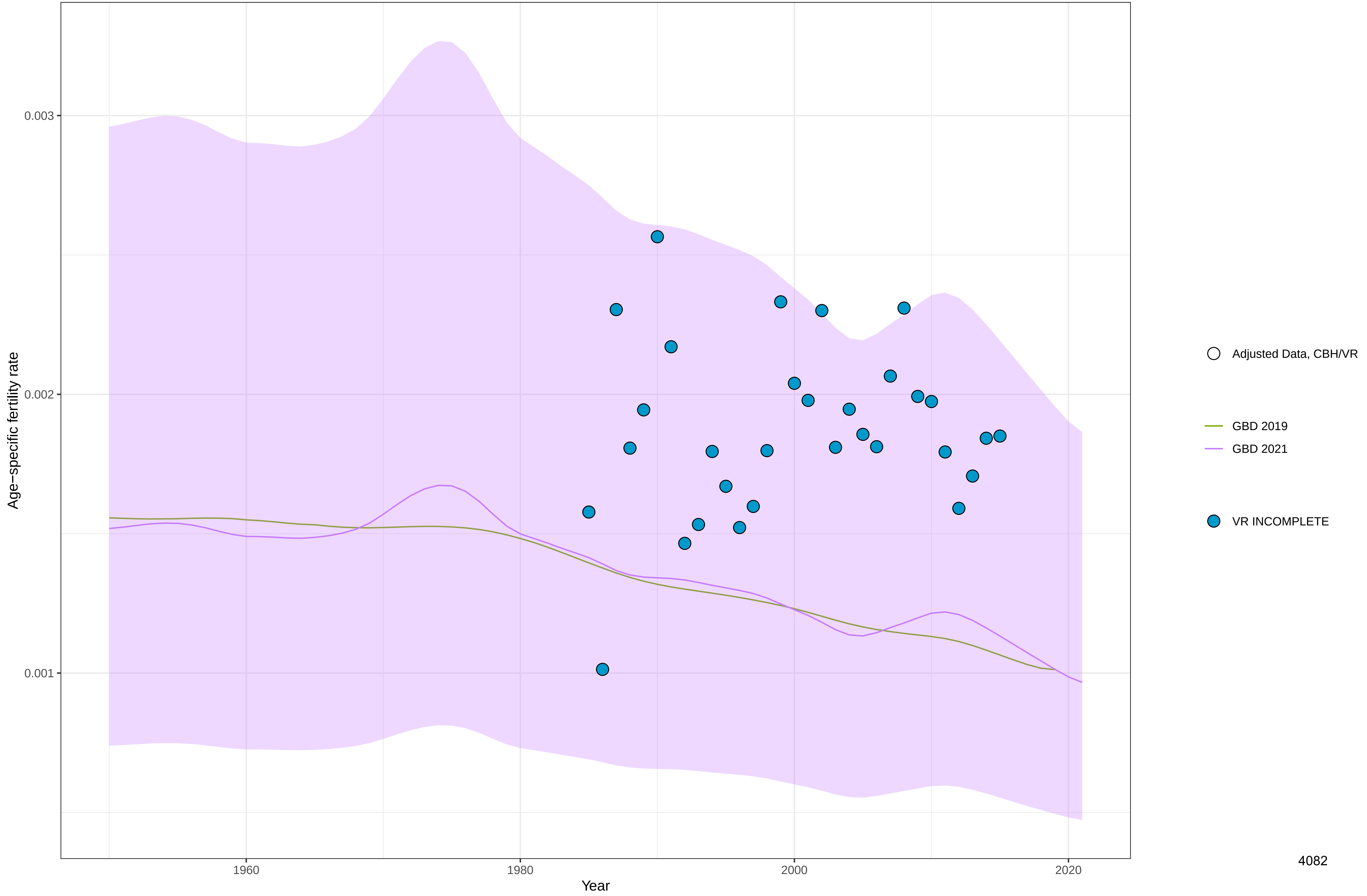

Age−specific fertility fitted trend lines, 1950−2021: Puebla, Ages: 15−19
Data points enclosed with a diamond are reference sources

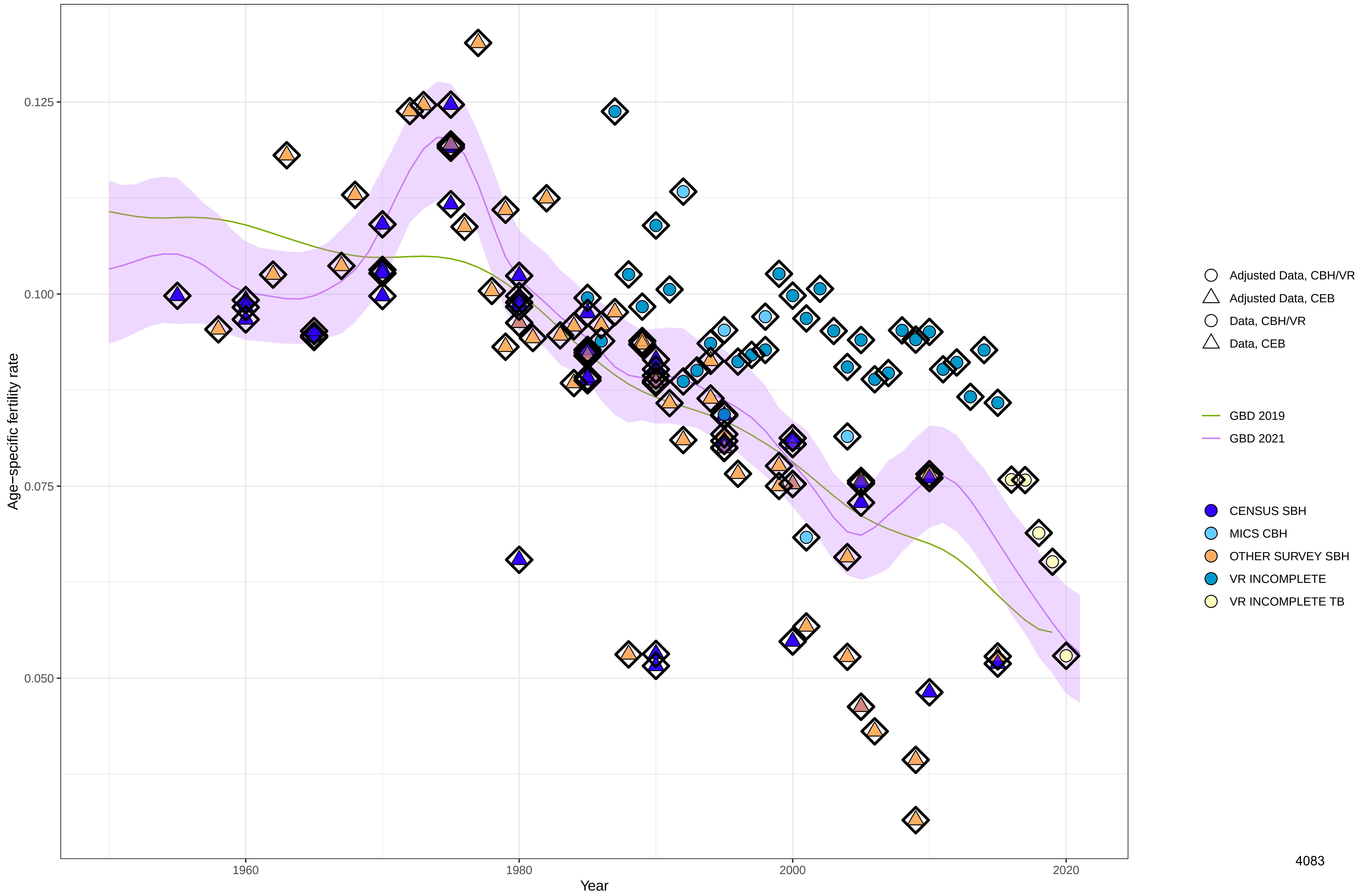

Age−specific fertility fitted trend lines, 1950−2021: Puebla, Ages: 20−24
Data points enclosed with a diamond are reference sources

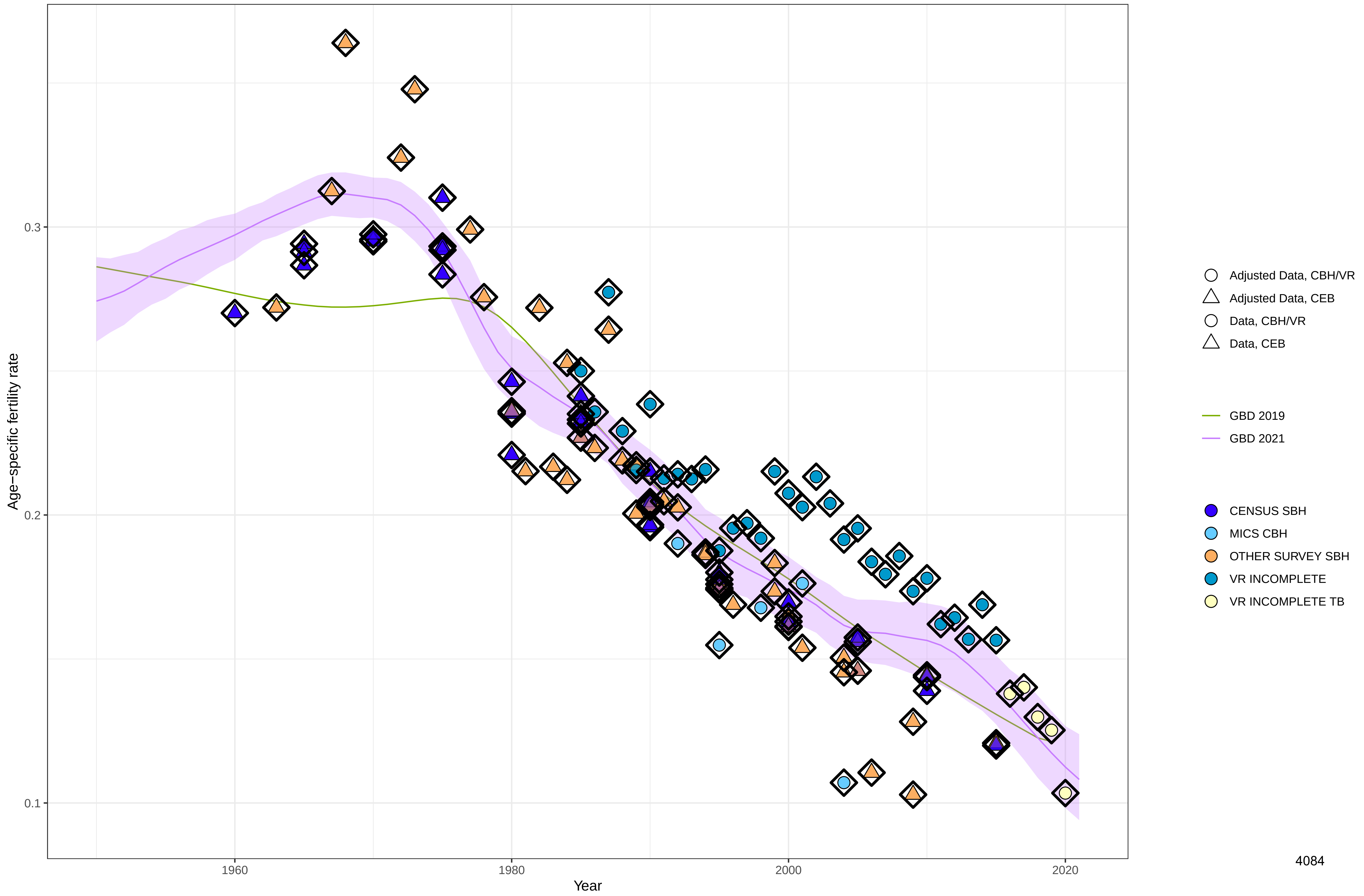

Age−specific fertility fitted trend lines, 1950−2021: Puebla, Ages: 25−29
Data points enclosed with a diamond are reference sources

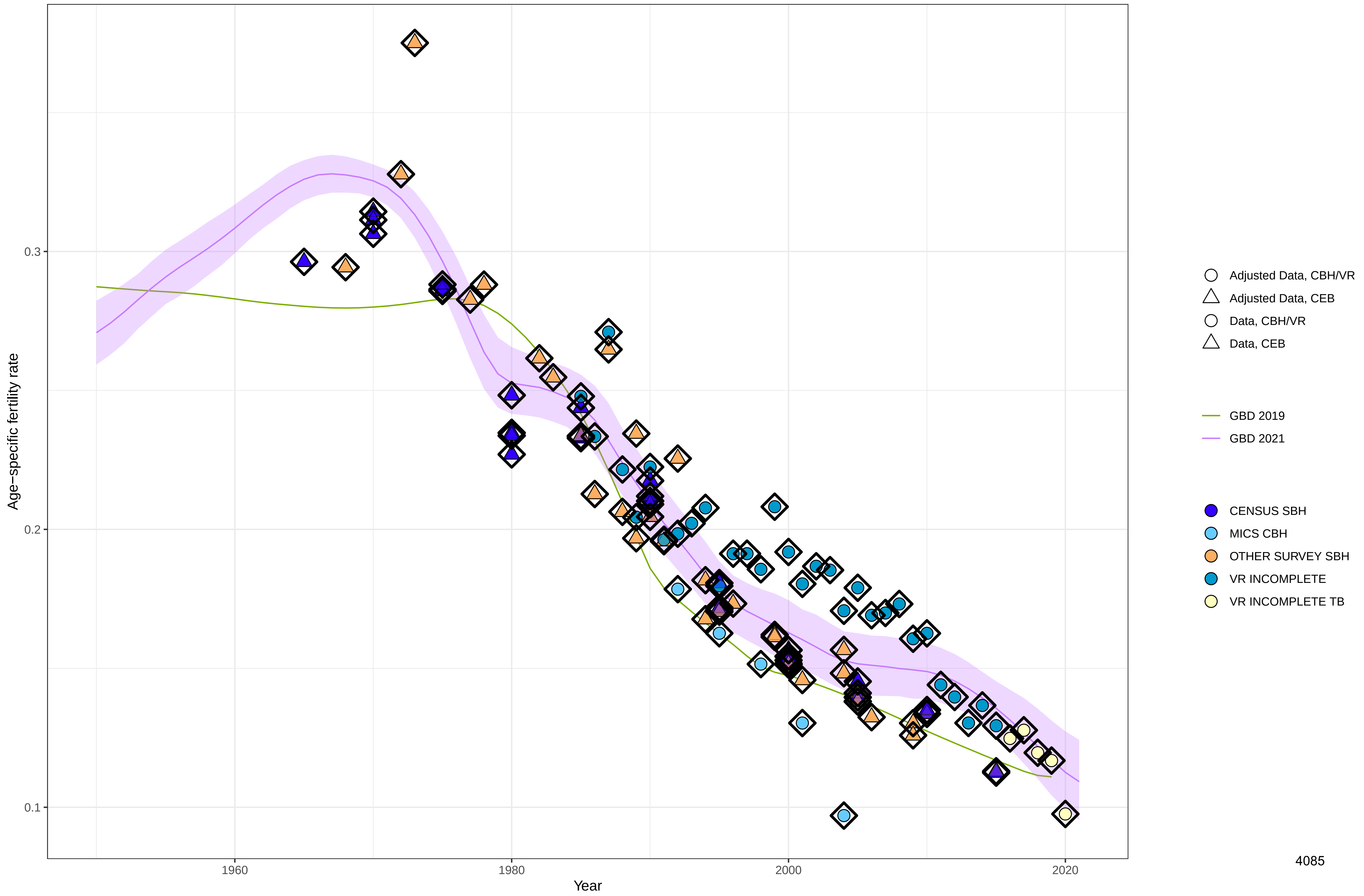

Age−specific fertility fitted trend lines, 1950−2021: Puebla, Ages: 30−34
Data points enclosed with a diamond are reference sources

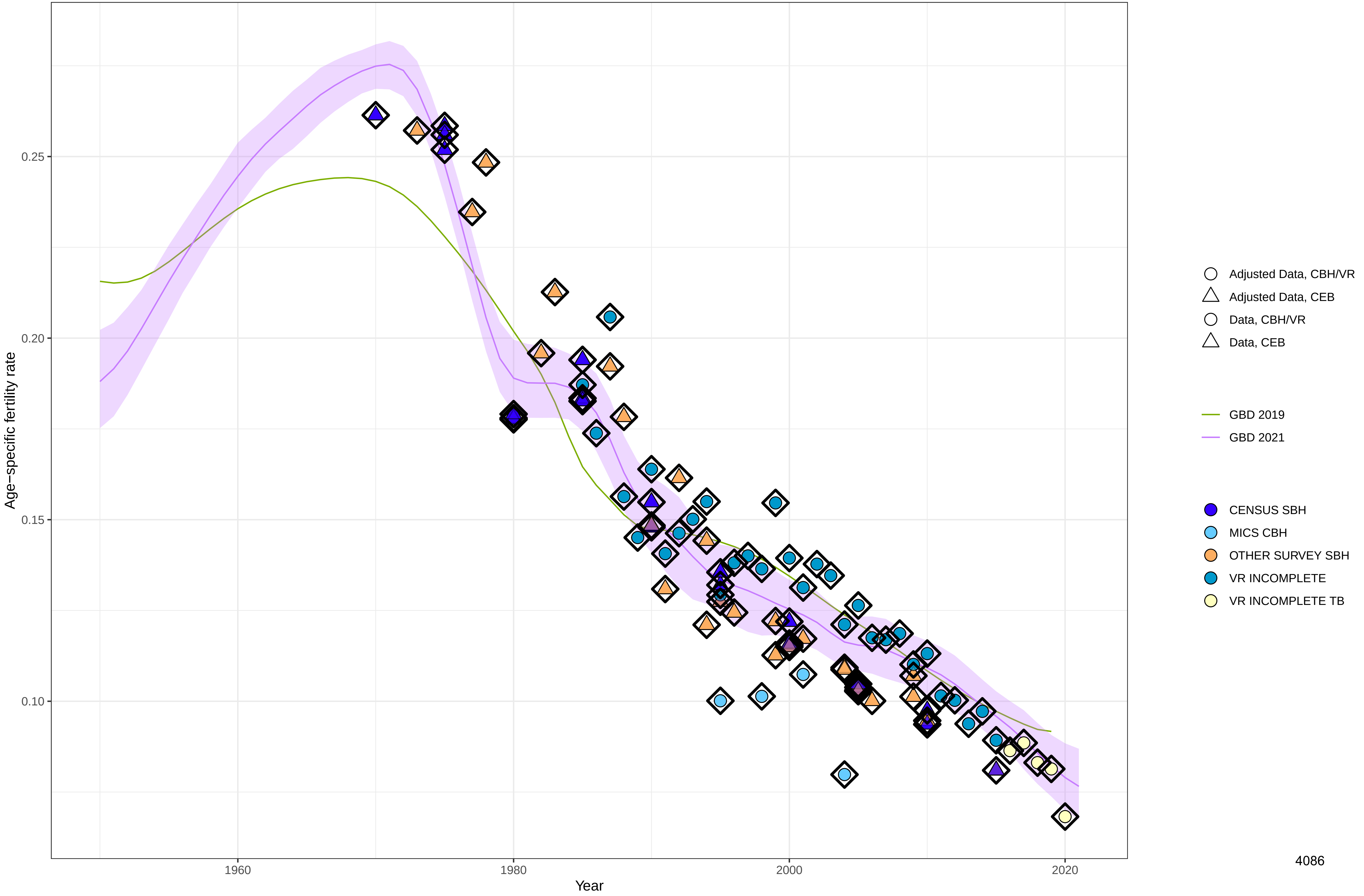

Age−specific fertility fitted trend lines, 1950−2021: Puebla, Ages: 35−39
Data points enclosed with a diamond are reference sources

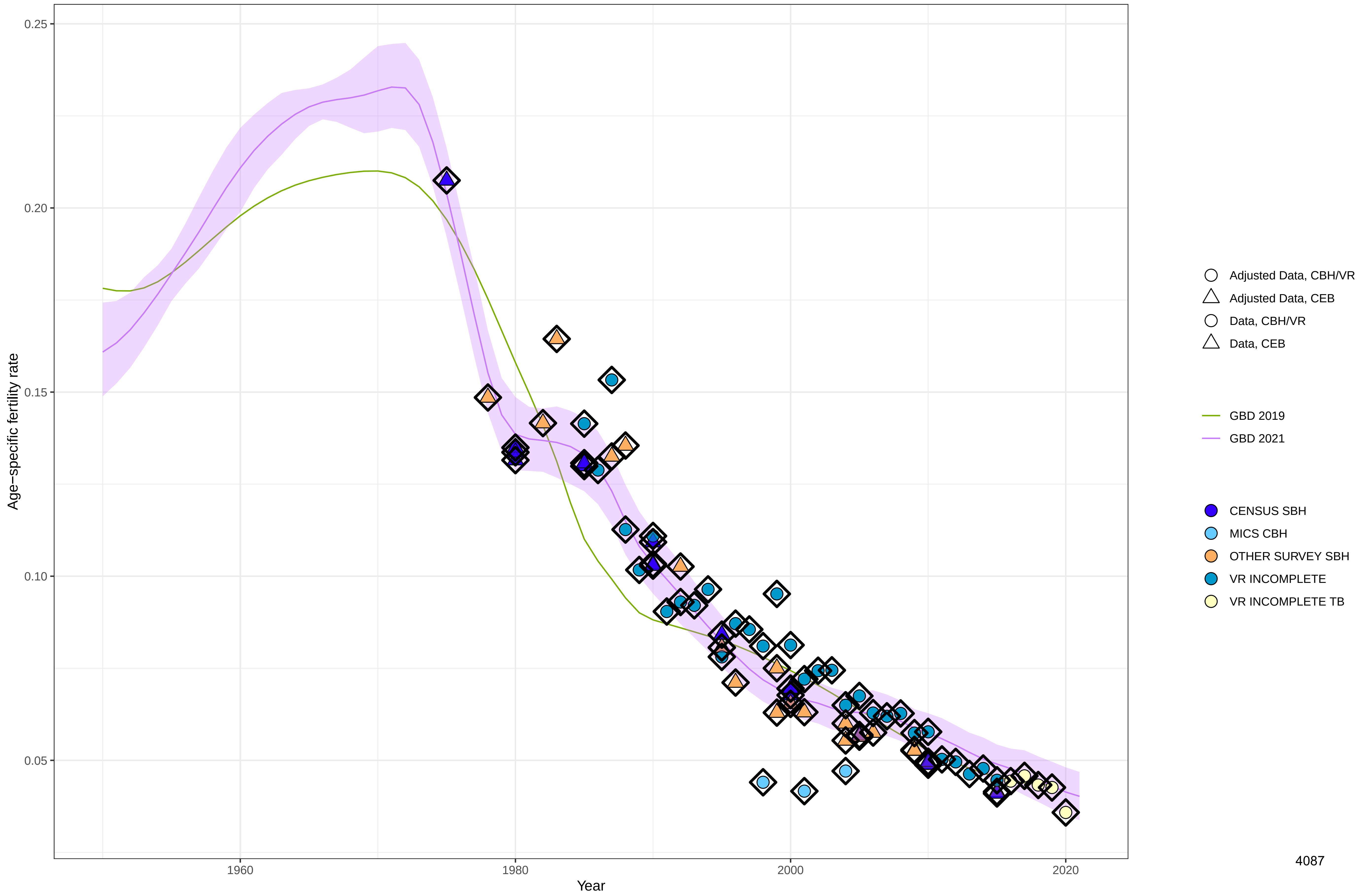

Age−specific fertility fitted trend lines, 1950−2021: Puebla, Ages: 40−44
Data points enclosed with a diamond are reference sources

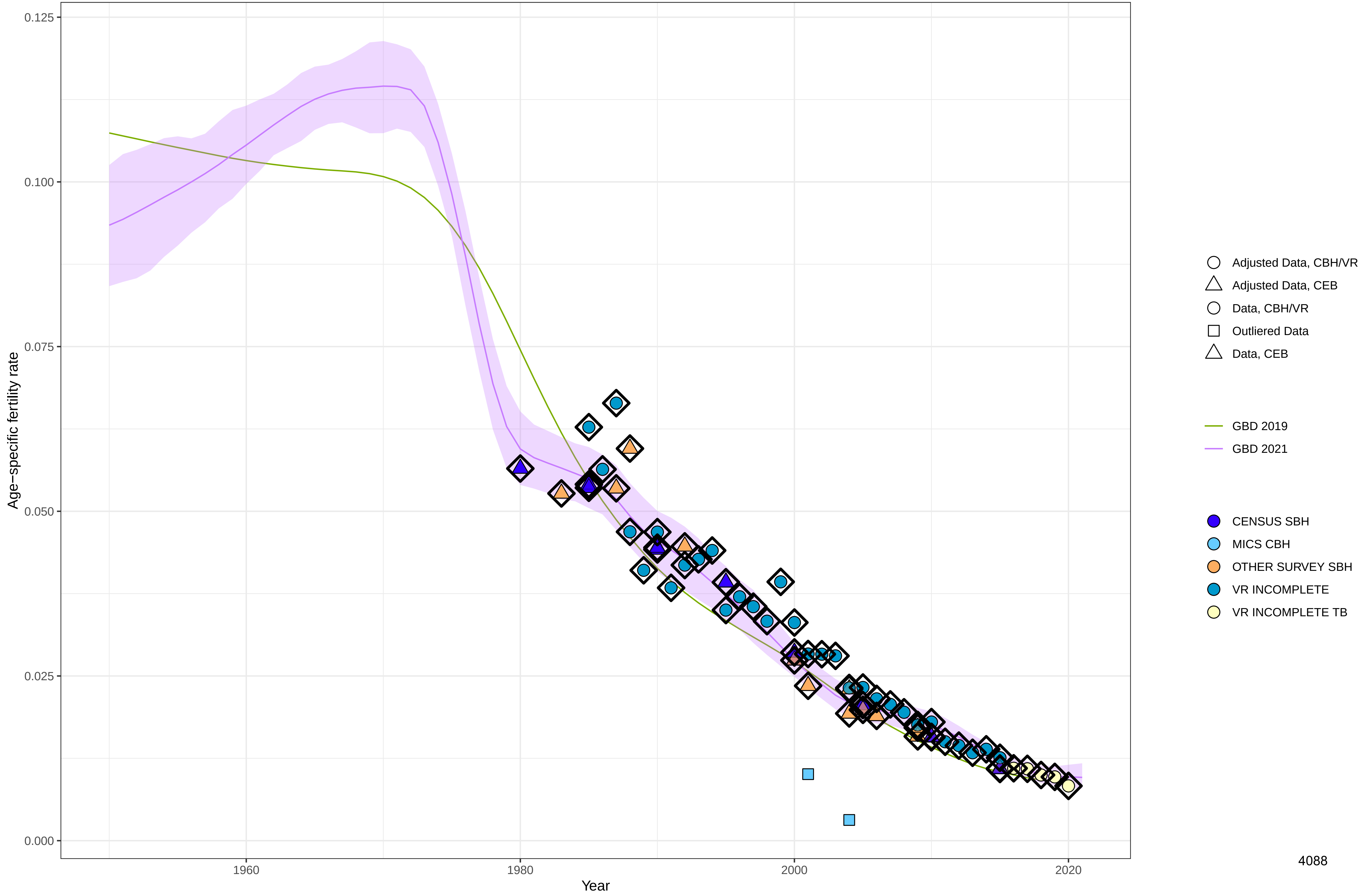

Age−specific fertility fitted trend lines, 1950−2021: Puebla, Ages: 45−49
Data points enclosed with a diamond are reference sources

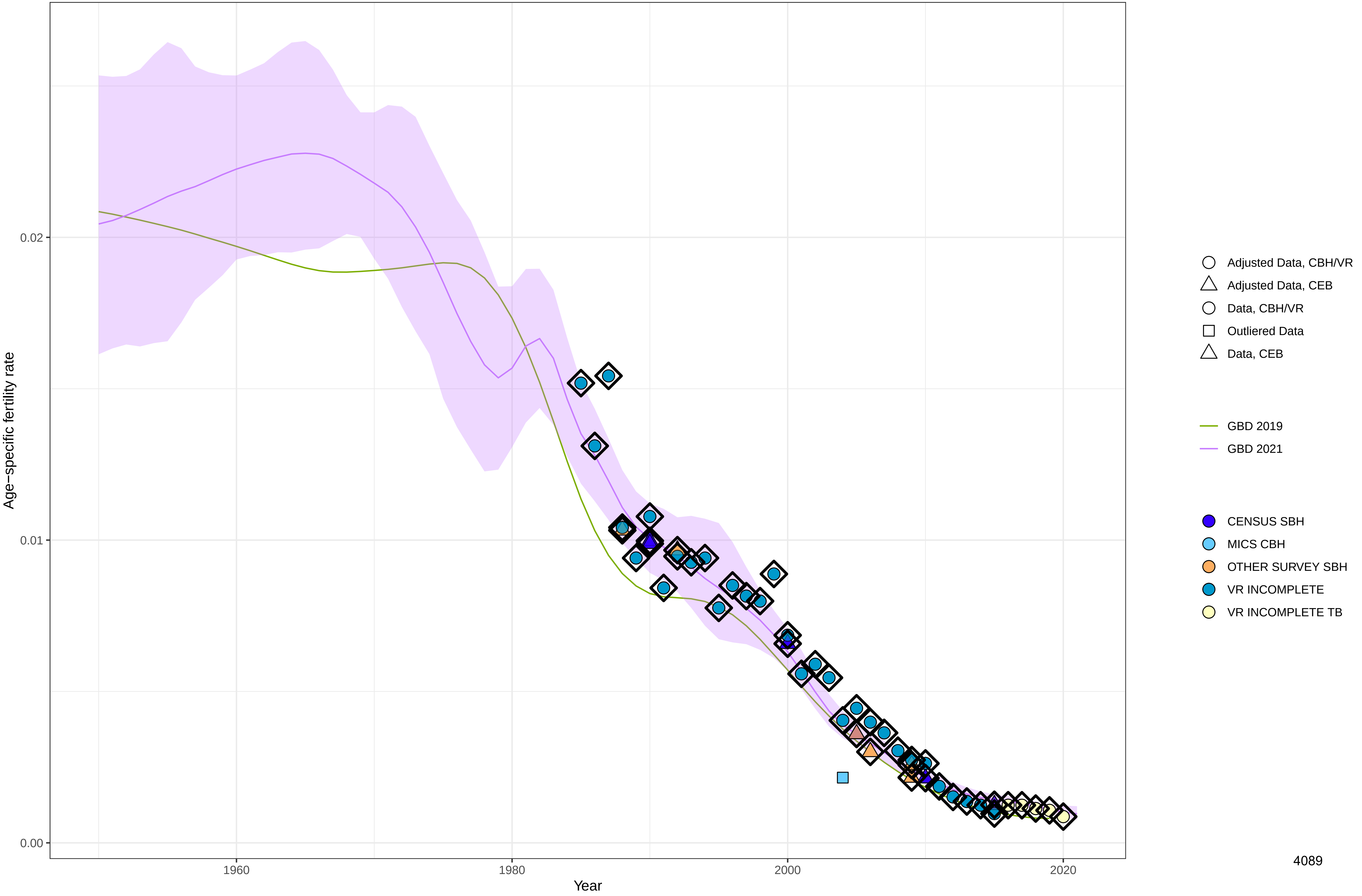

Age−specific fertility fitted trend lines, 1950−2021: Puebla, Ages: 50−54
Data points enclosed with a diamond are reference sources

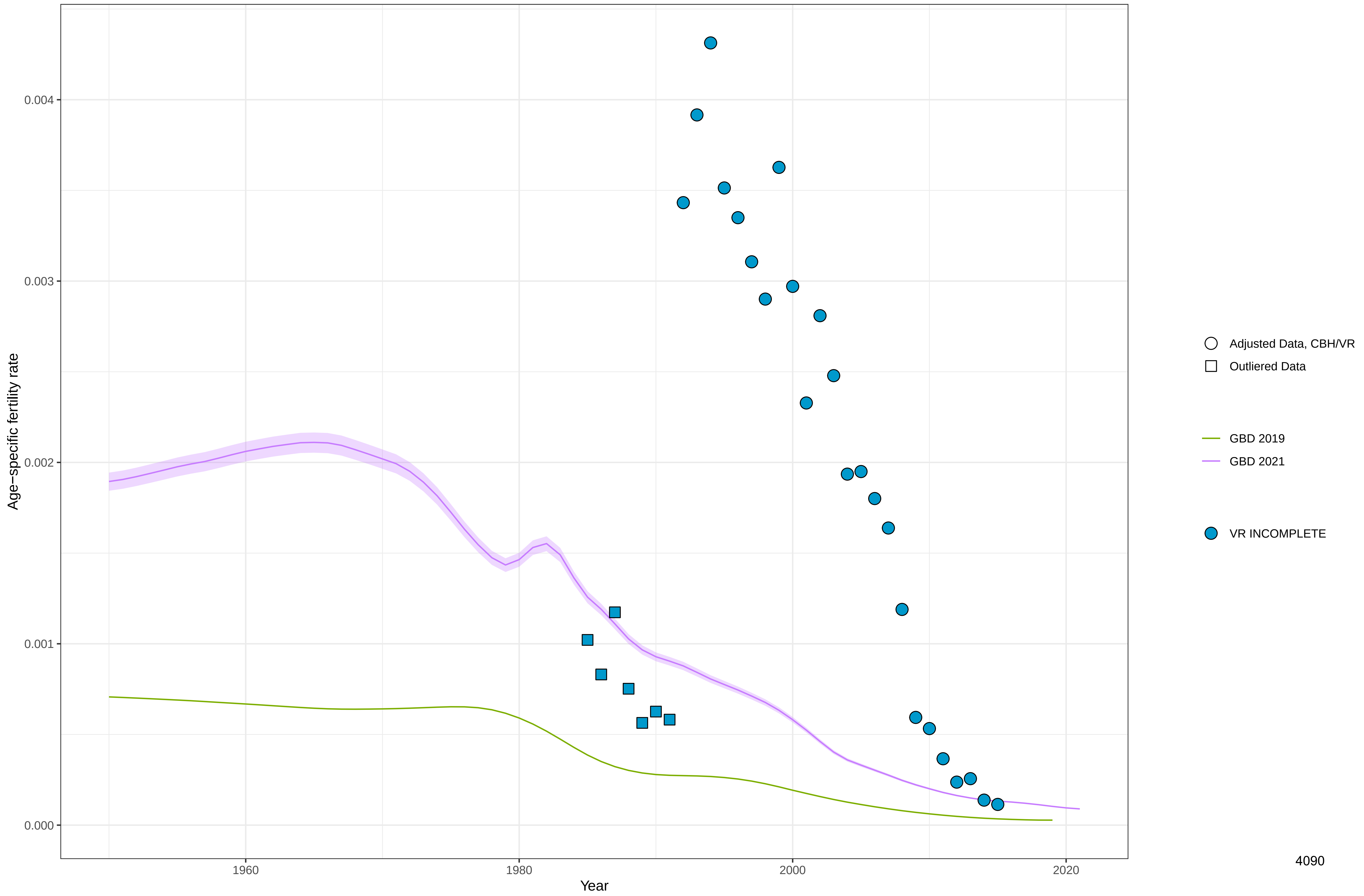

Total fertility rate fitted trend lines, 1950−2021: Puebla TFR
Data points enclosed with a diamond are reference sources

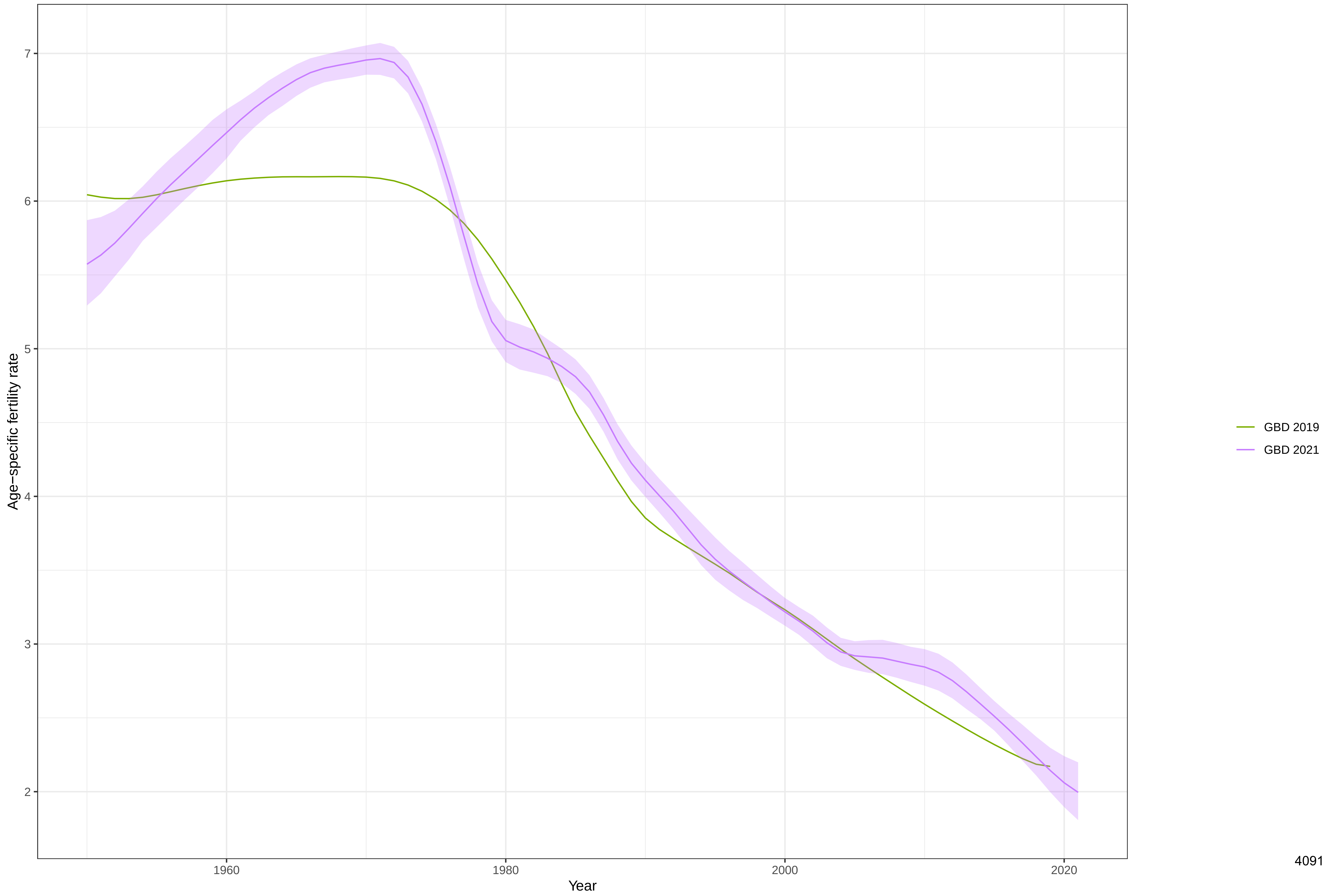

Age−specific fertility fitted trend lines, 1950−2021: Querétaro, Ages: 10−14
Data points enclosed with a diamond are reference sources

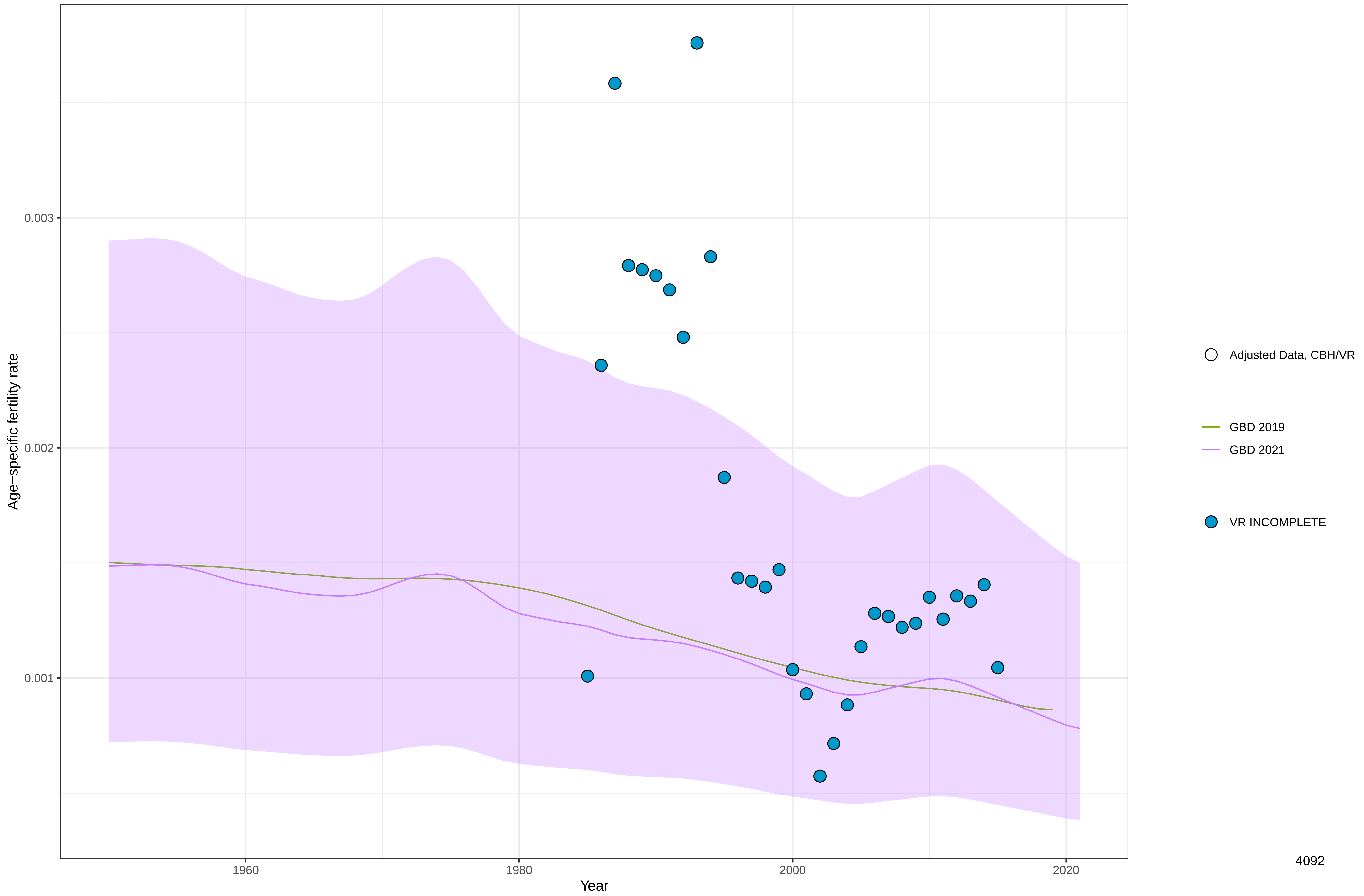

Age−specific fertility fitted trend lines, 1950−2021: Querétaro, Ages: 15−19
Data points enclosed with a diamond are reference sources

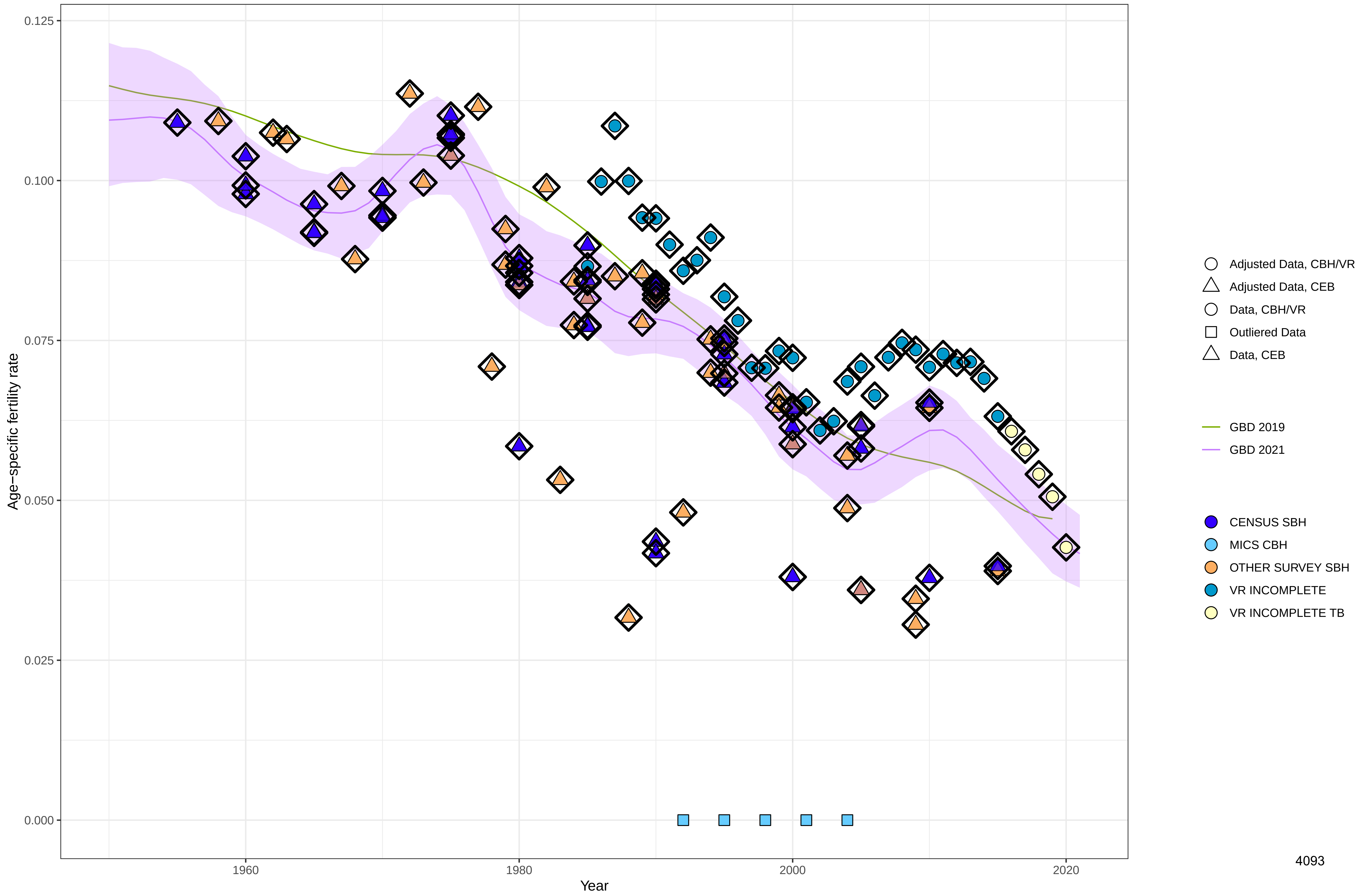

Age−specific fertility fitted trend lines, 1950−2021: Querétaro, Ages: 20−24
Data points enclosed with a diamond are reference sources

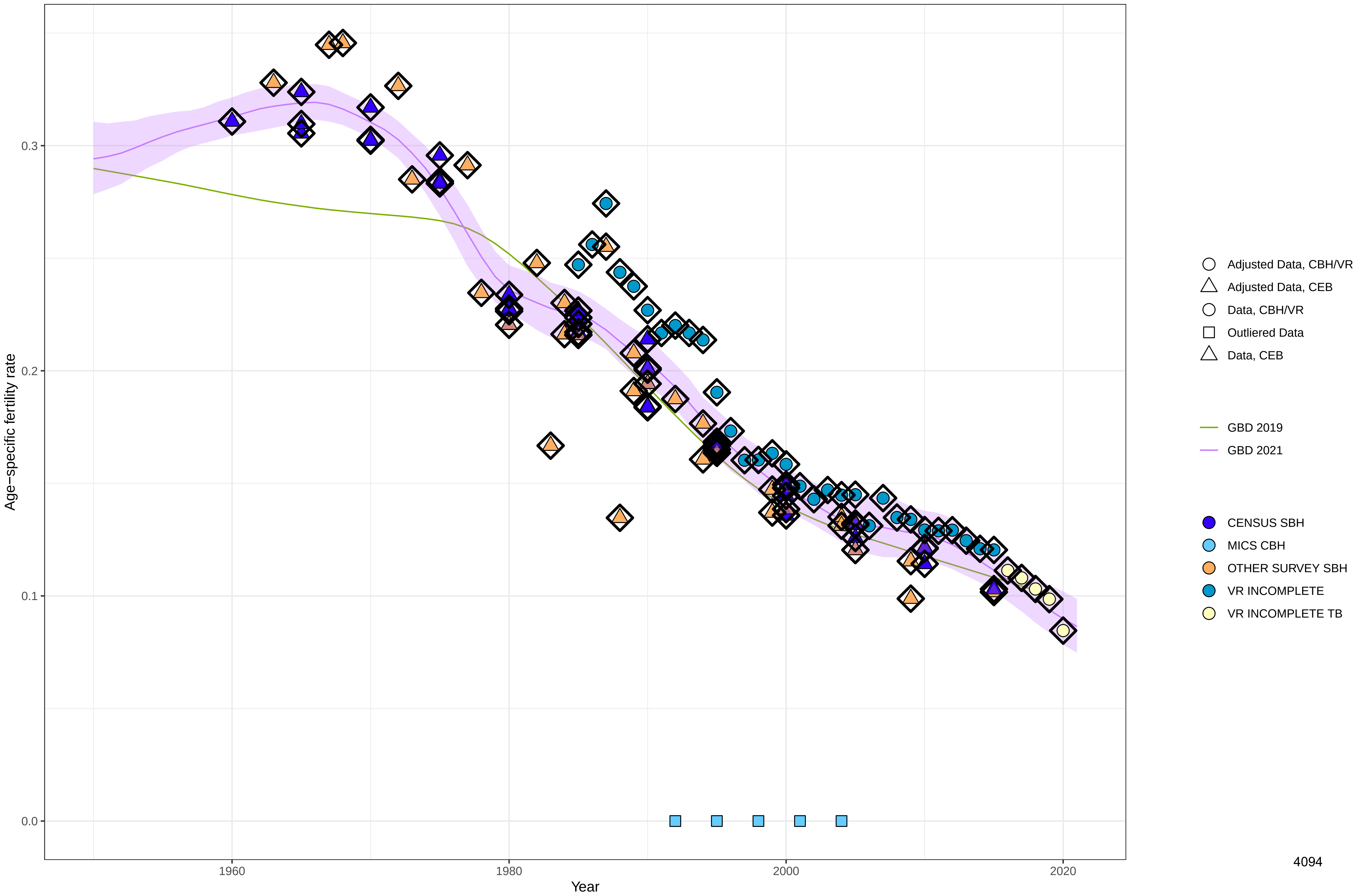

Age−specific fertility fitted trend lines, 1950−2021: Querétaro, Ages: 25−29
Data points enclosed with a diamond are reference sources

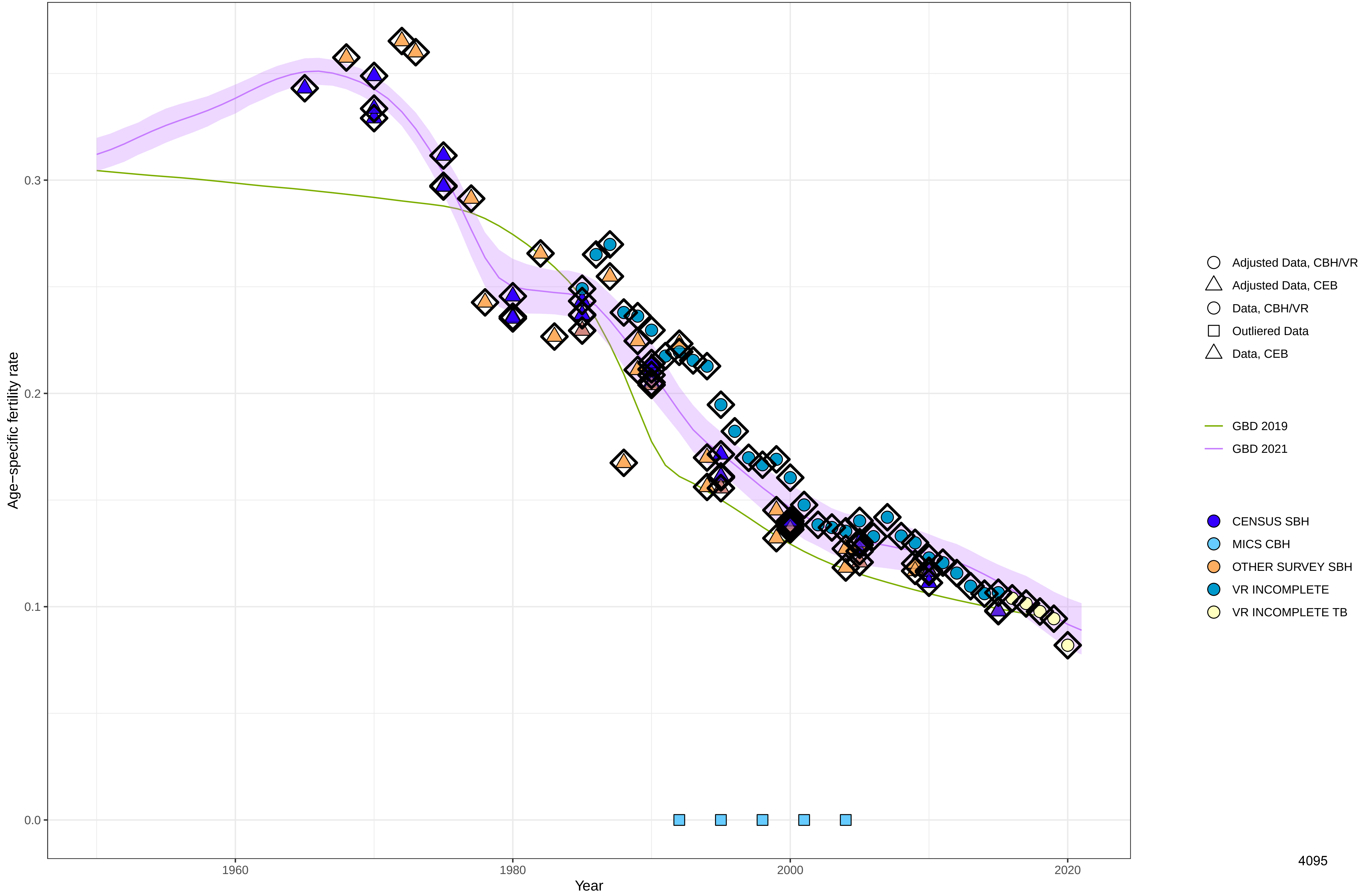

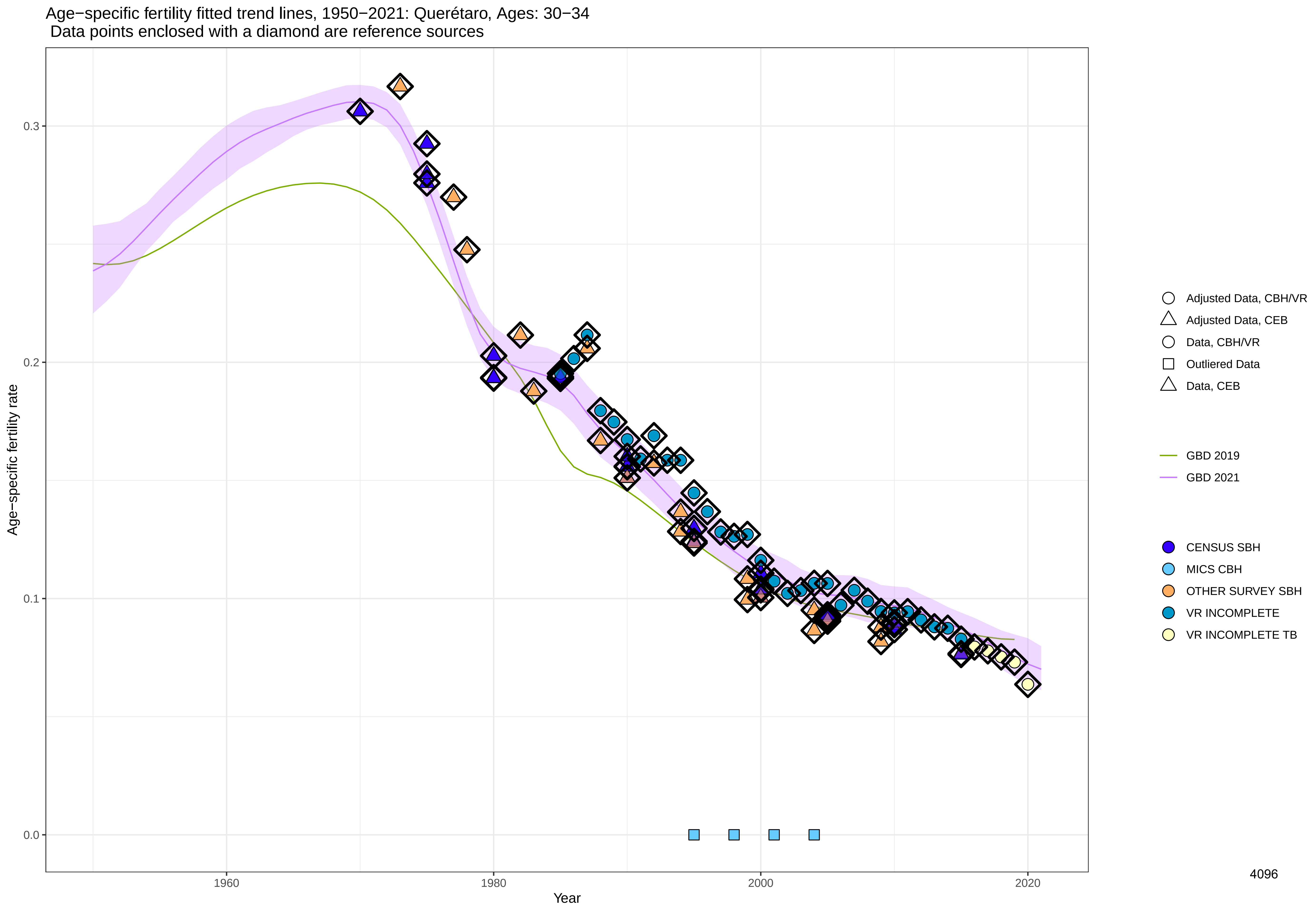

Age−specific fertility fitted trend lines, 1950−2021: Querétaro, Ages: 30−34
Data points enclosed with a diamond are reference sources
Age−specific fertility rate
Year
0.0
0.1
0.2
0.3
1960
1980
2000
2020
Adjusted Data, CBH/VR
Adjusted Data, CEB
Data, CBH/VR
Outliered Data
Data, CEB
GBD 2019
GBD 2021
CENSUS SBH
MICS CBH
OTHER SURVEY SBH
VR INCOMPLETE
VR INCOMPLETE TB

Age−specific fertility fitted trend lines, 1950−2021: Querétaro, Ages: 35−39
Data points enclosed with a diamond are reference sources

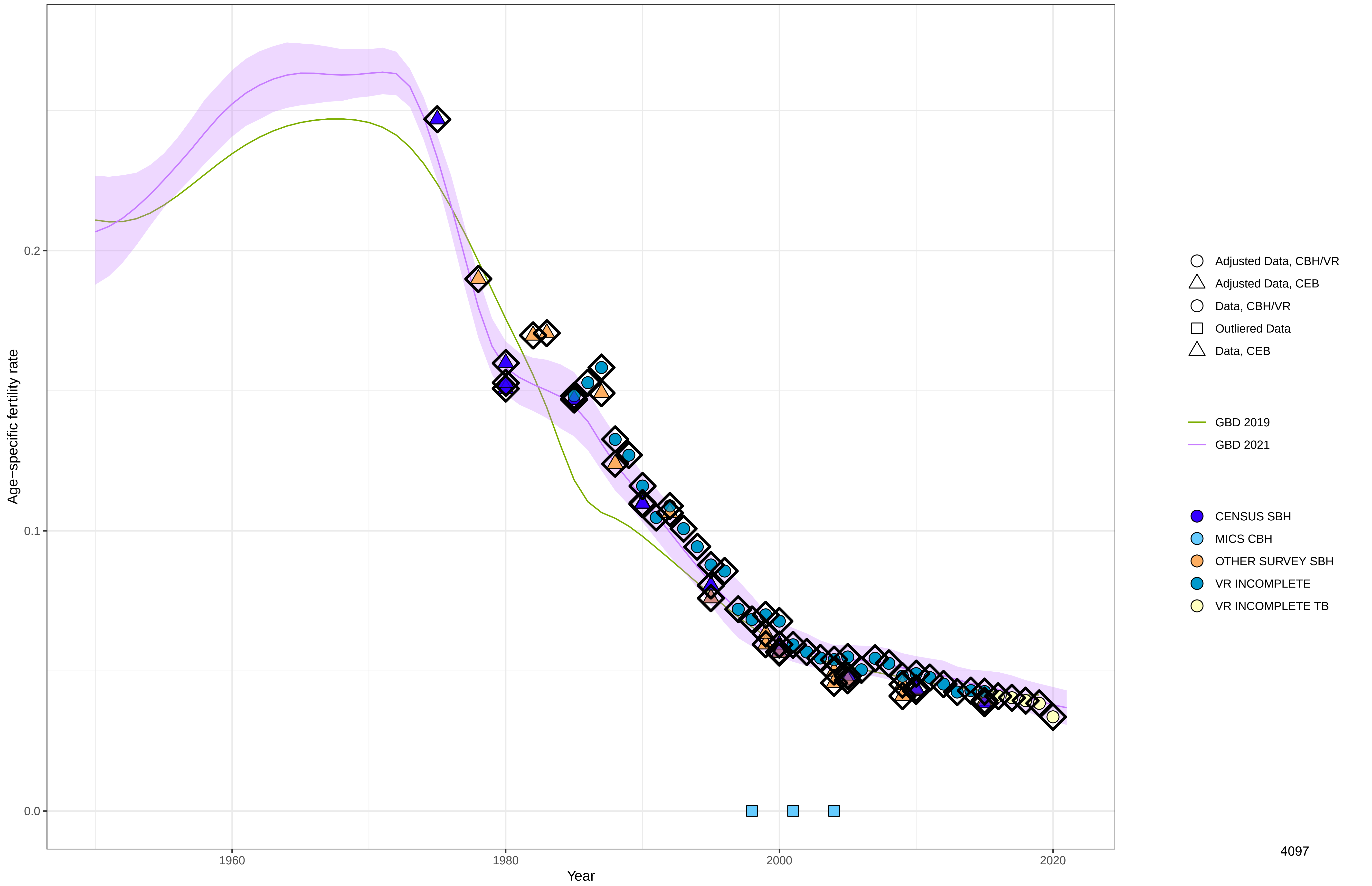

4097

Age−specific fertility fitted trend lines, 1950−2021: Querétaro, Ages: 40−44
Data points enclosed with a diamond are reference sources

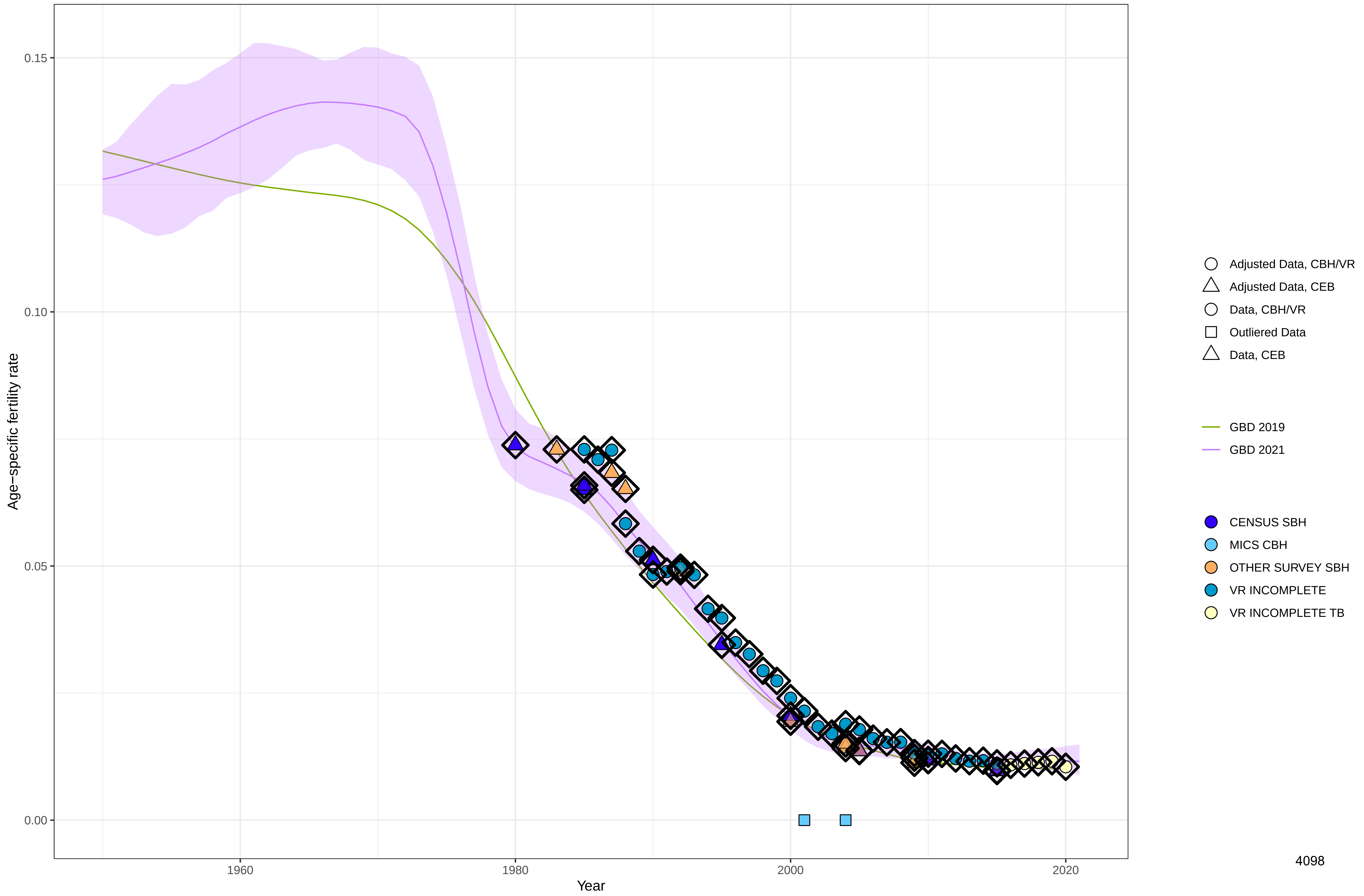

Age−specific fertility fitted trend lines, 1950−2021: Querétaro, Ages: 45−49
Data points enclosed with a diamond are reference sources

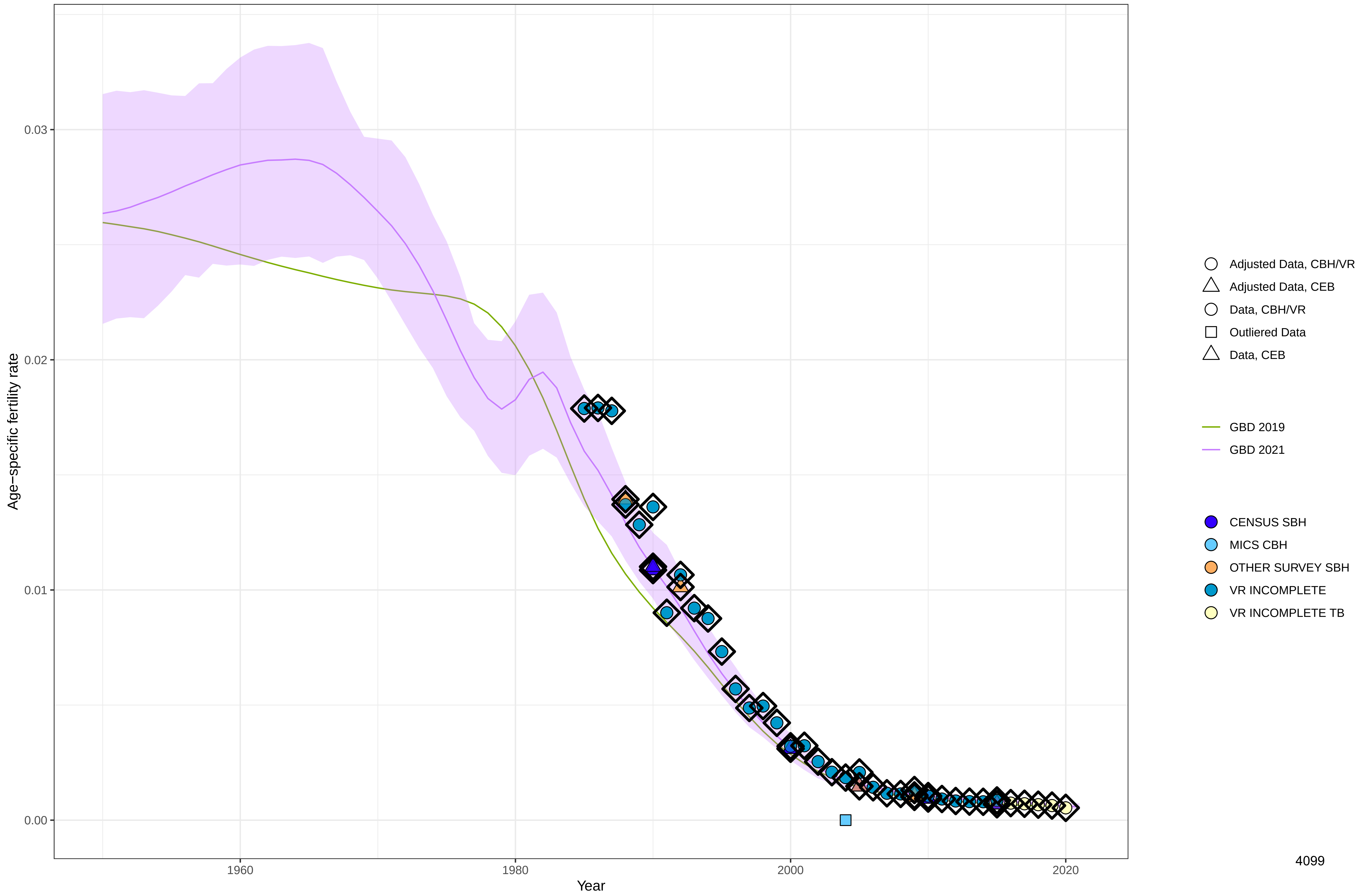

Age−specific fertility fitted trend lines, 1950−2021: Querétaro, Ages: 50−54
Data points enclosed with a diamond are reference sources

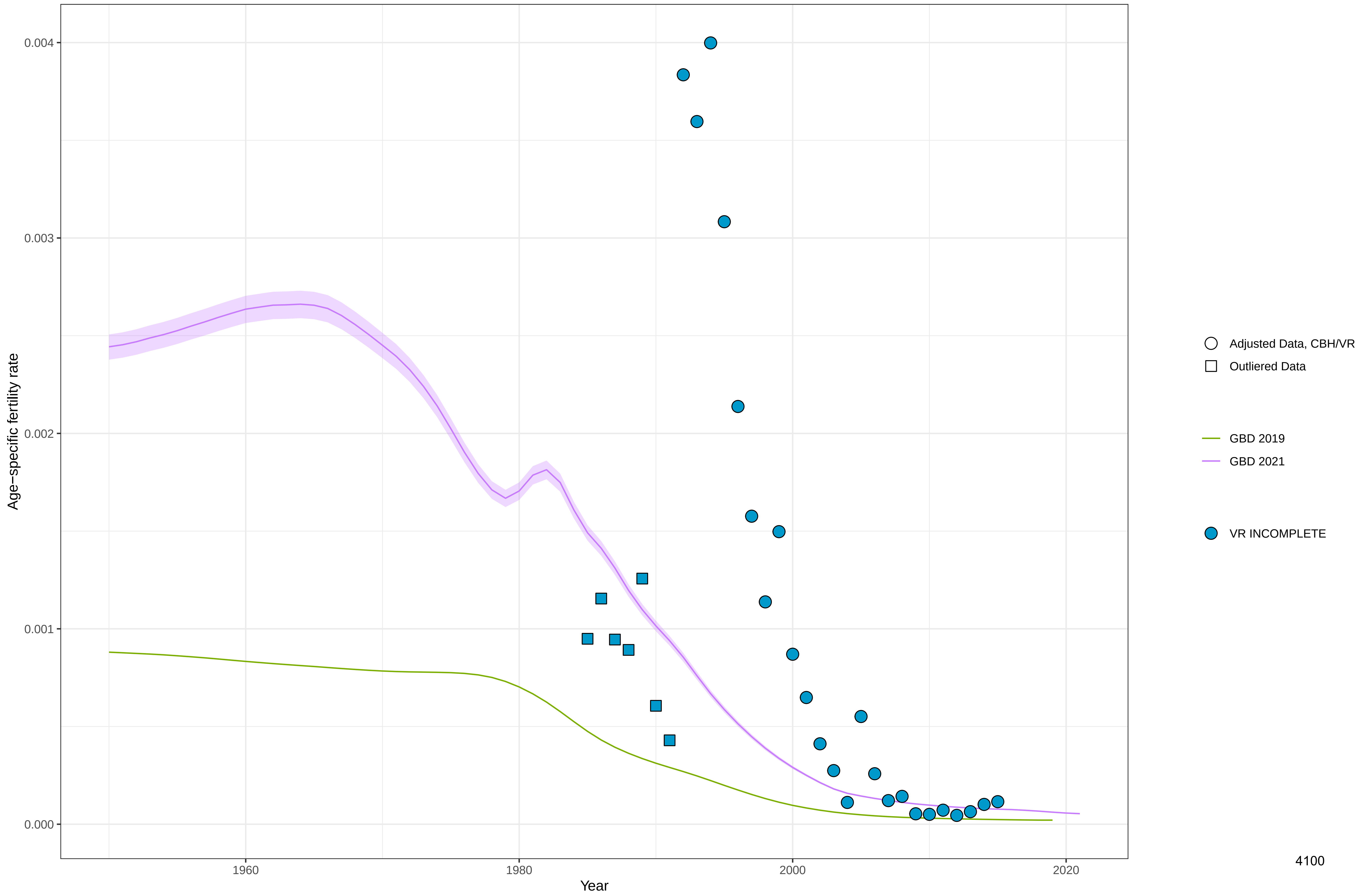

Total fertility rate fitted trend lines, 1950−2021: Querétaro TFR
Data points enclosed with a diamond are reference sources

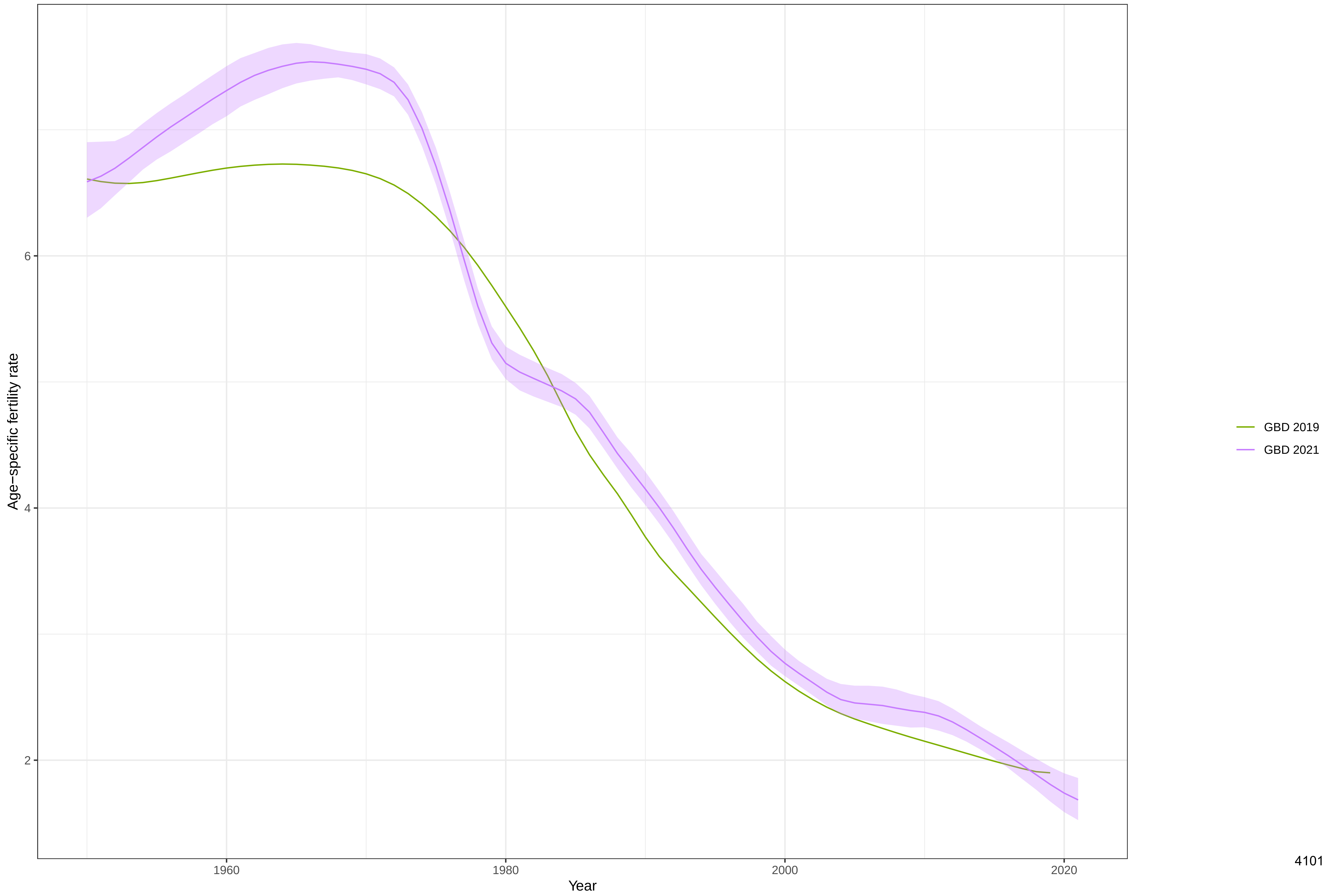

Age−specific fertility fitted trend lines, 1950−2021: Quintana Roo, Ages: 10−14
Data points enclosed with a diamond are reference sources

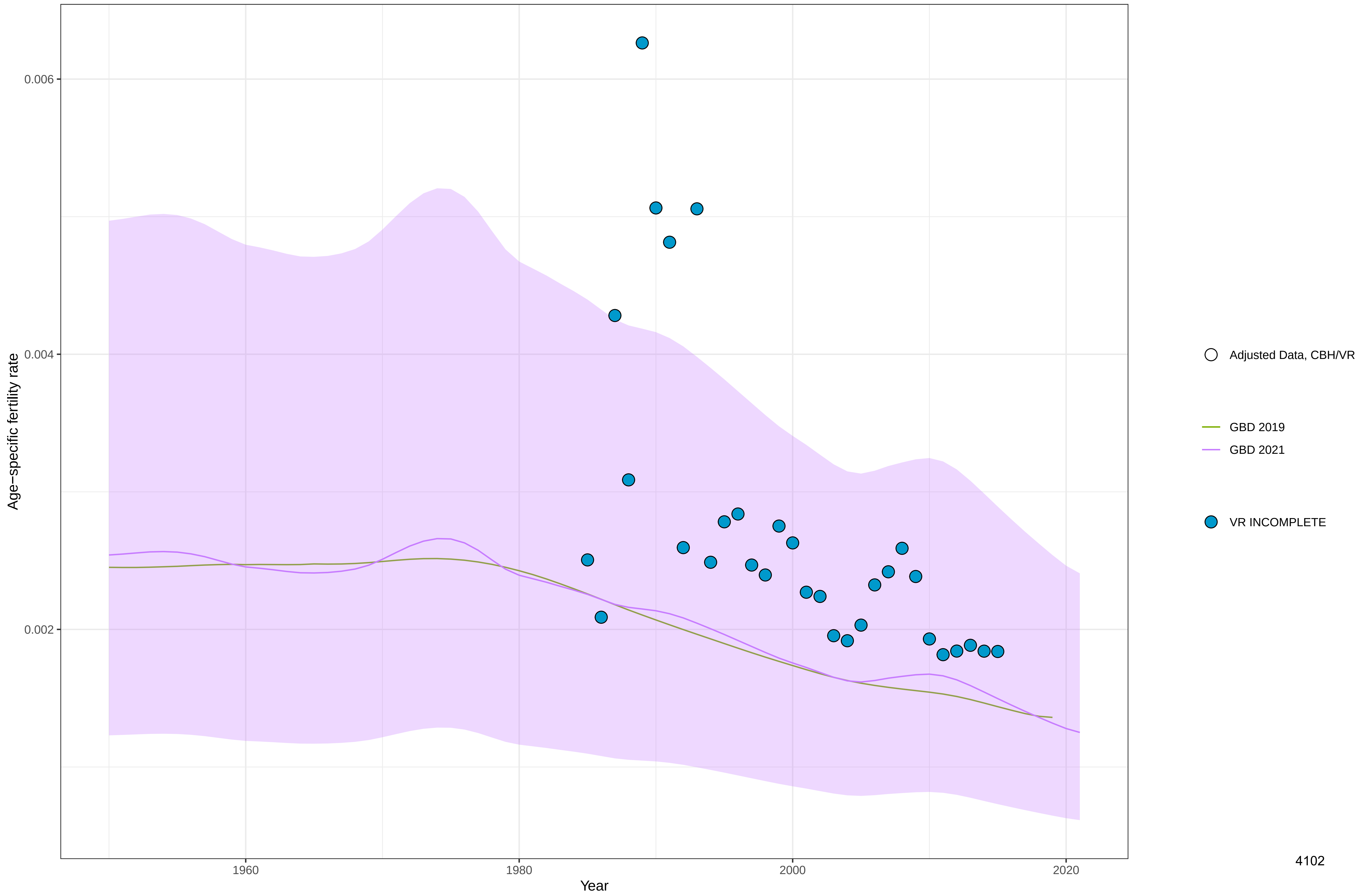

Age−specific fertility fitted trend lines, 1950−2021: Quintana Roo, Ages: 15−19
Data points enclosed with a diamond are reference sources

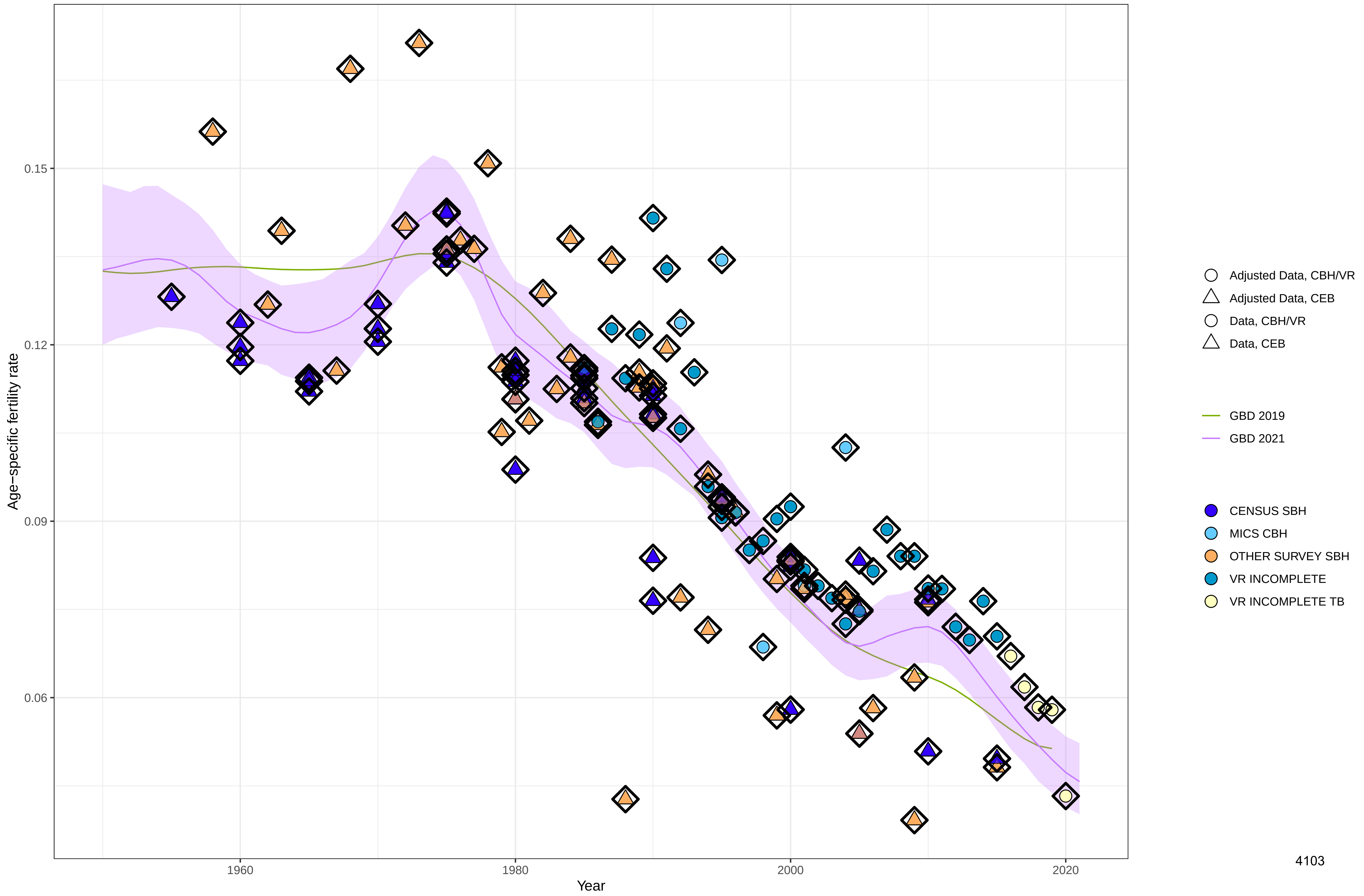

Age−specific fertility fitted trend lines, 1950−2021: Quintana Roo, Ages: 20−24
Data points enclosed with a diamond are reference sources

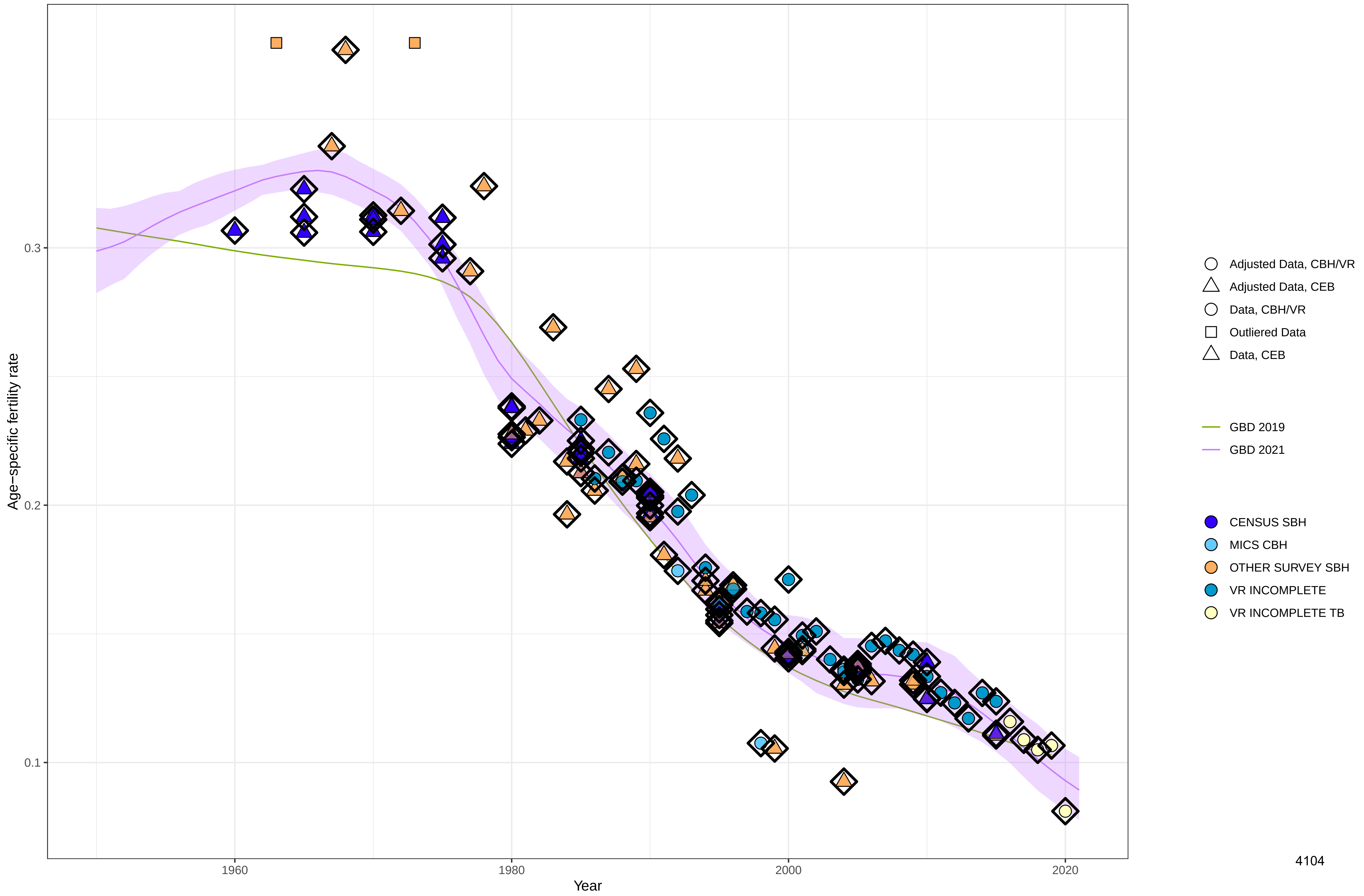

Age−specific fertility fitted trend lines, 1950−2021: Quintana Roo, Ages: 25−29
Data points enclosed with a diamond are reference sources

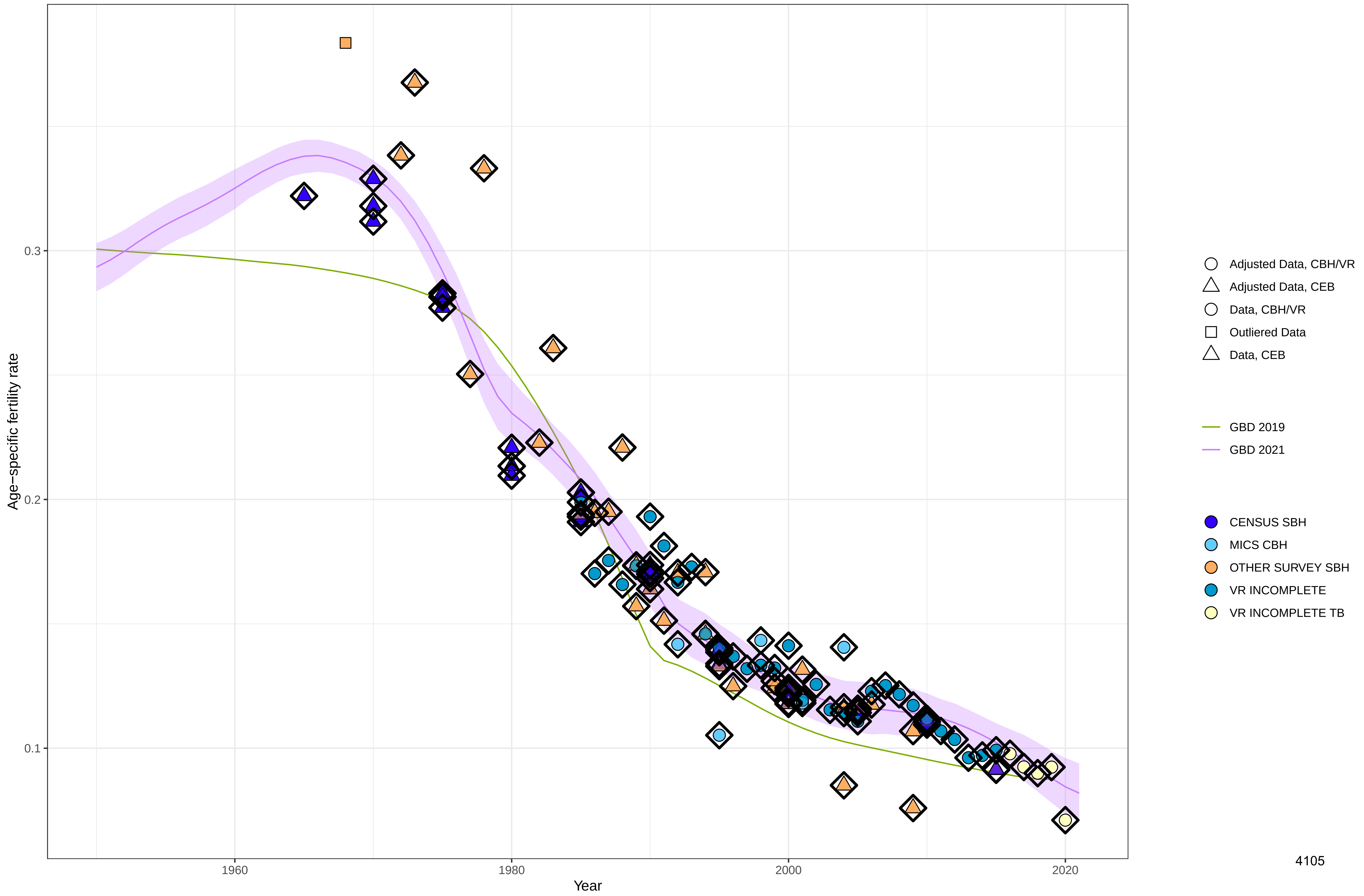

Age−specific fertility fitted trend lines, 1950−2021: Quintana Roo, Ages: 30−34
Data points enclosed with a diamond are reference sources

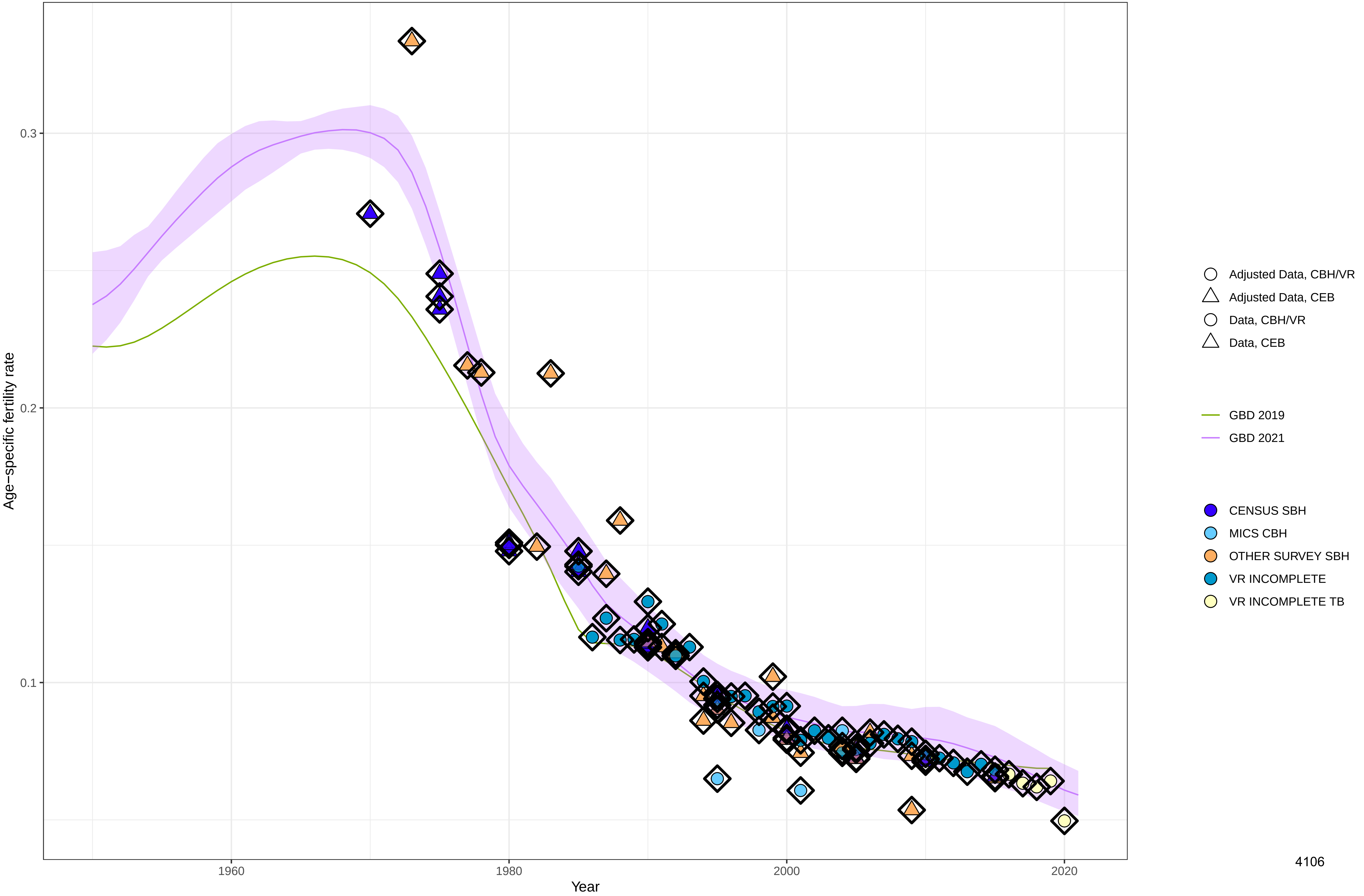

Age−specific fertility fitted trend lines, 1950−2021: Quintana Roo, Ages: 35−39
Data points enclosed with a diamond are reference sources

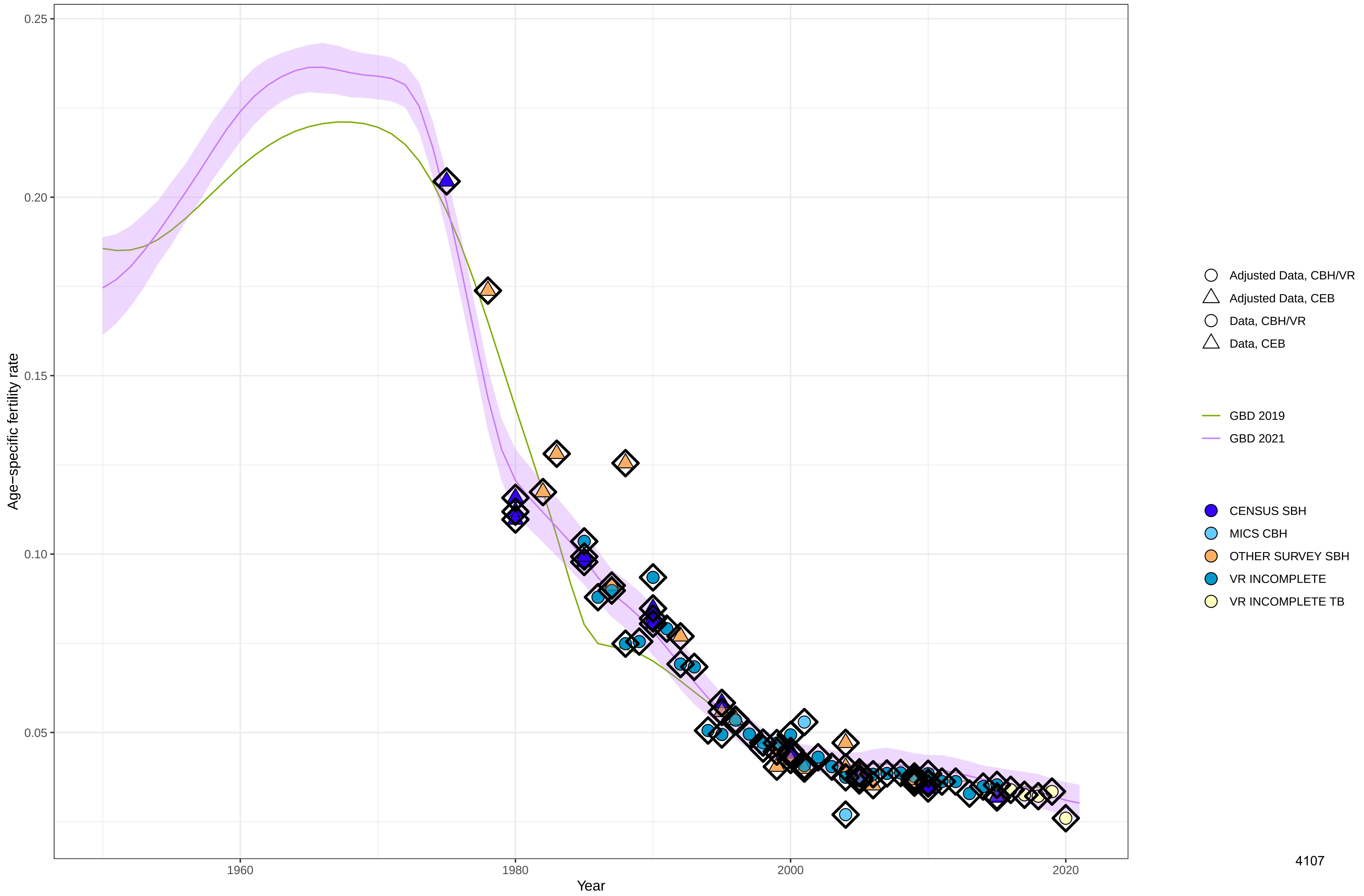

Age−specific fertility fitted trend lines, 1950−2021: Quintana Roo, Ages: 40−44
Data points enclosed with a diamond are reference sources

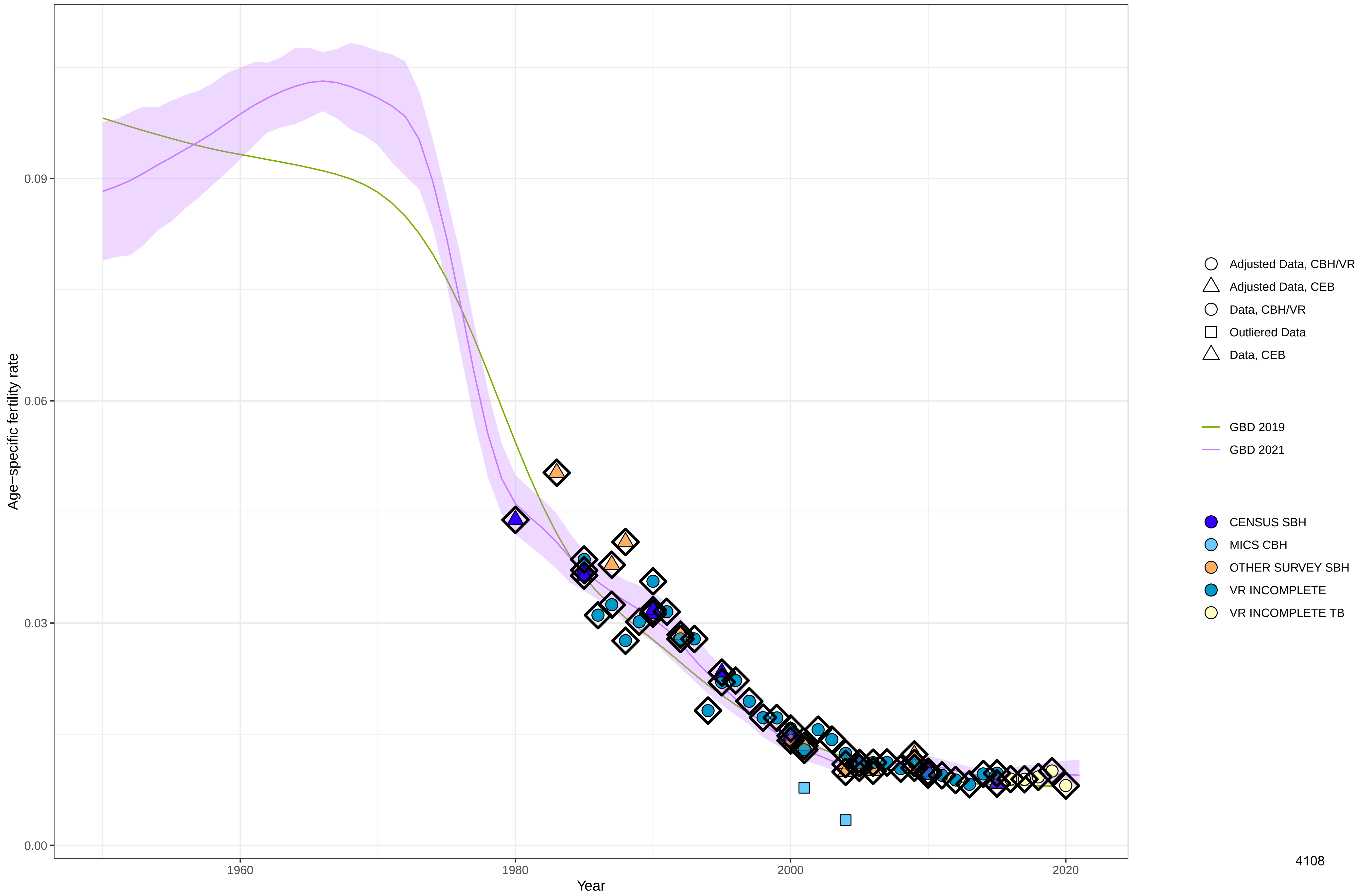

Age−specific fertility fitted trend lines, 1950−2021: Quintana Roo, Ages: 45−49
Data points enclosed with a diamond are reference sources

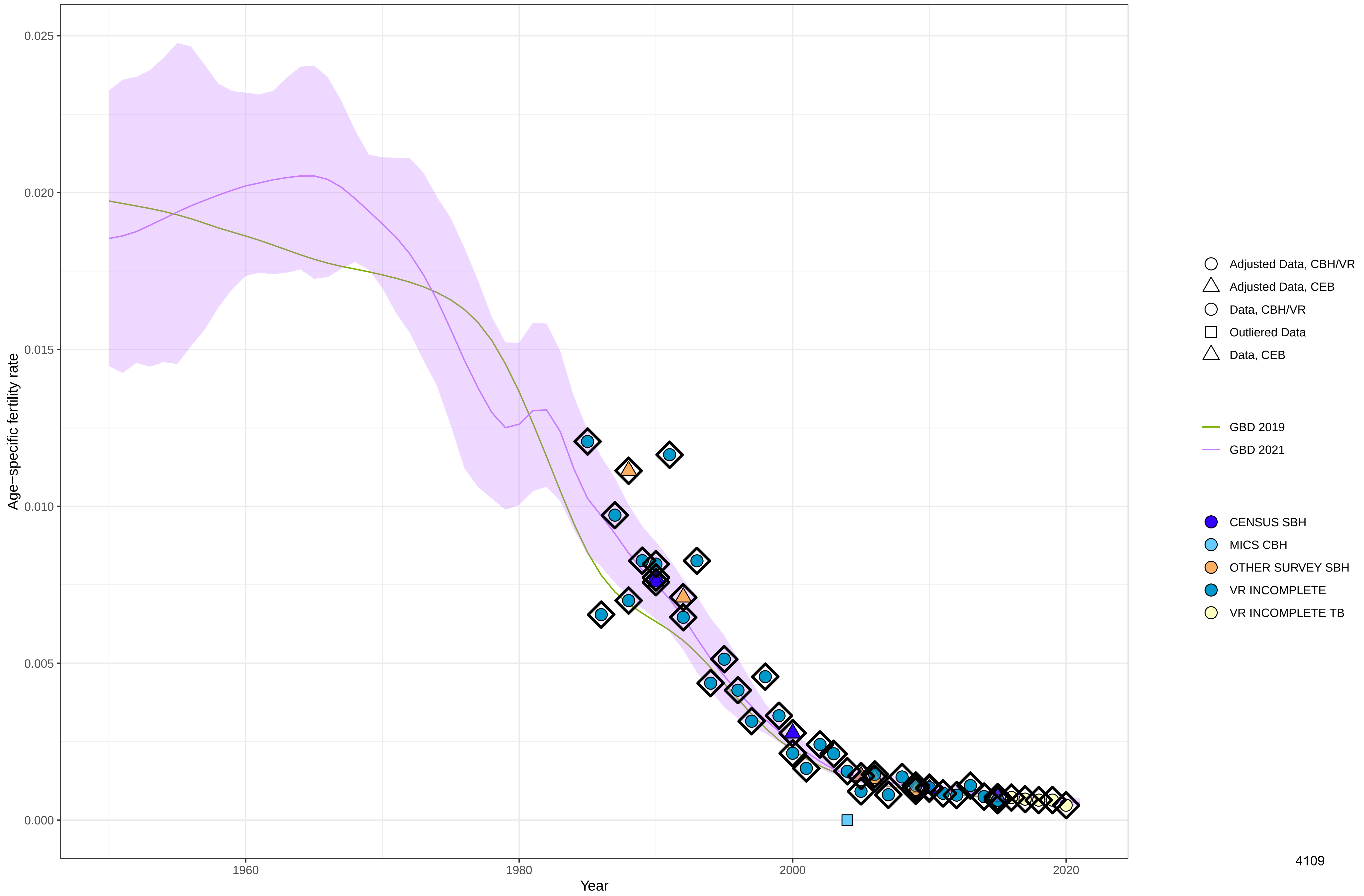

Age−specific fertility fitted trend lines, 1950−2021: Quintana Roo, Ages: 50−54
Data points enclosed with a diamond are reference sources

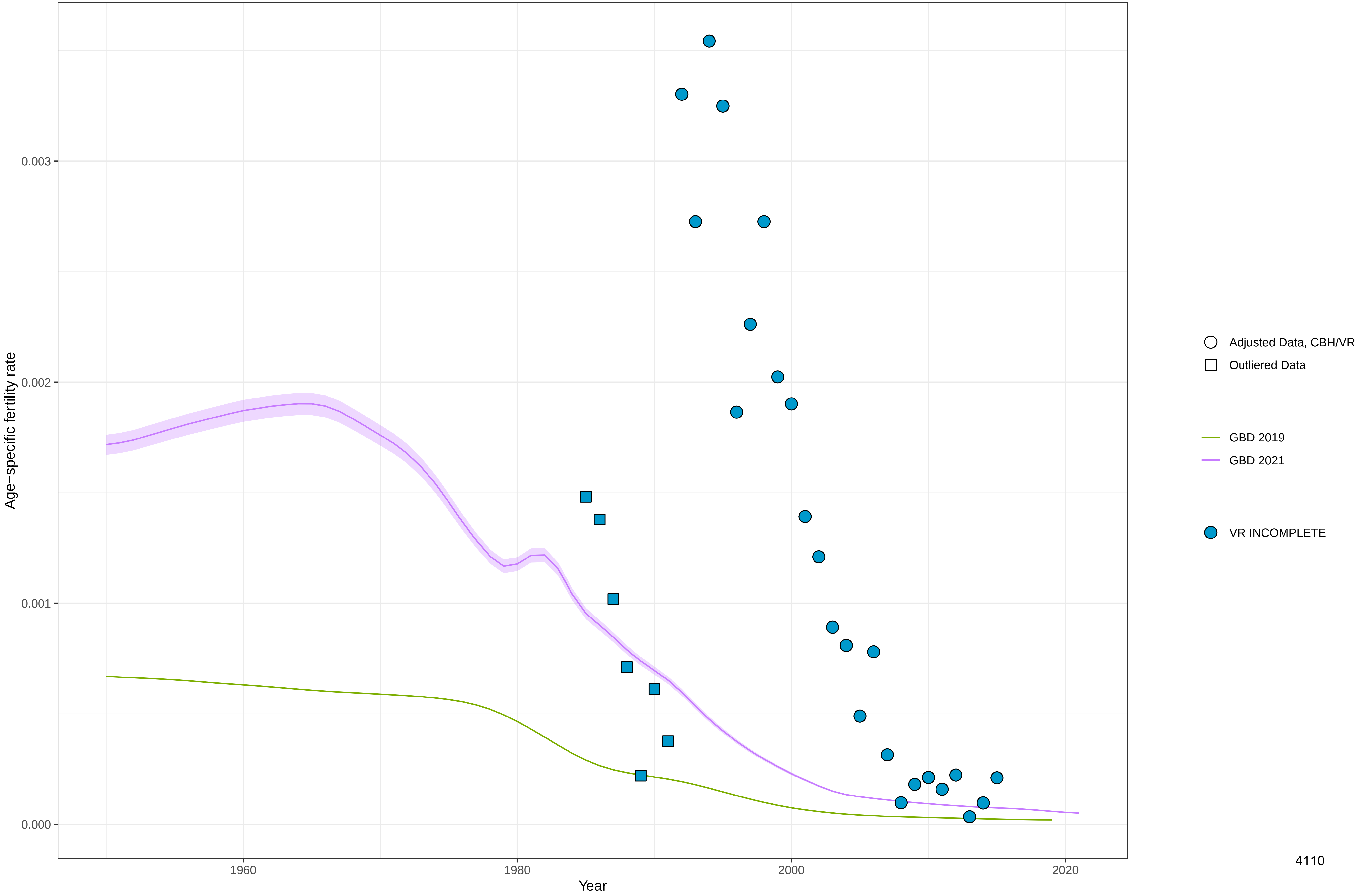

Total fertility rate fitted trend lines, 1950−2021: Quintana Roo TFR
Data points enclosed with a diamond are reference sources

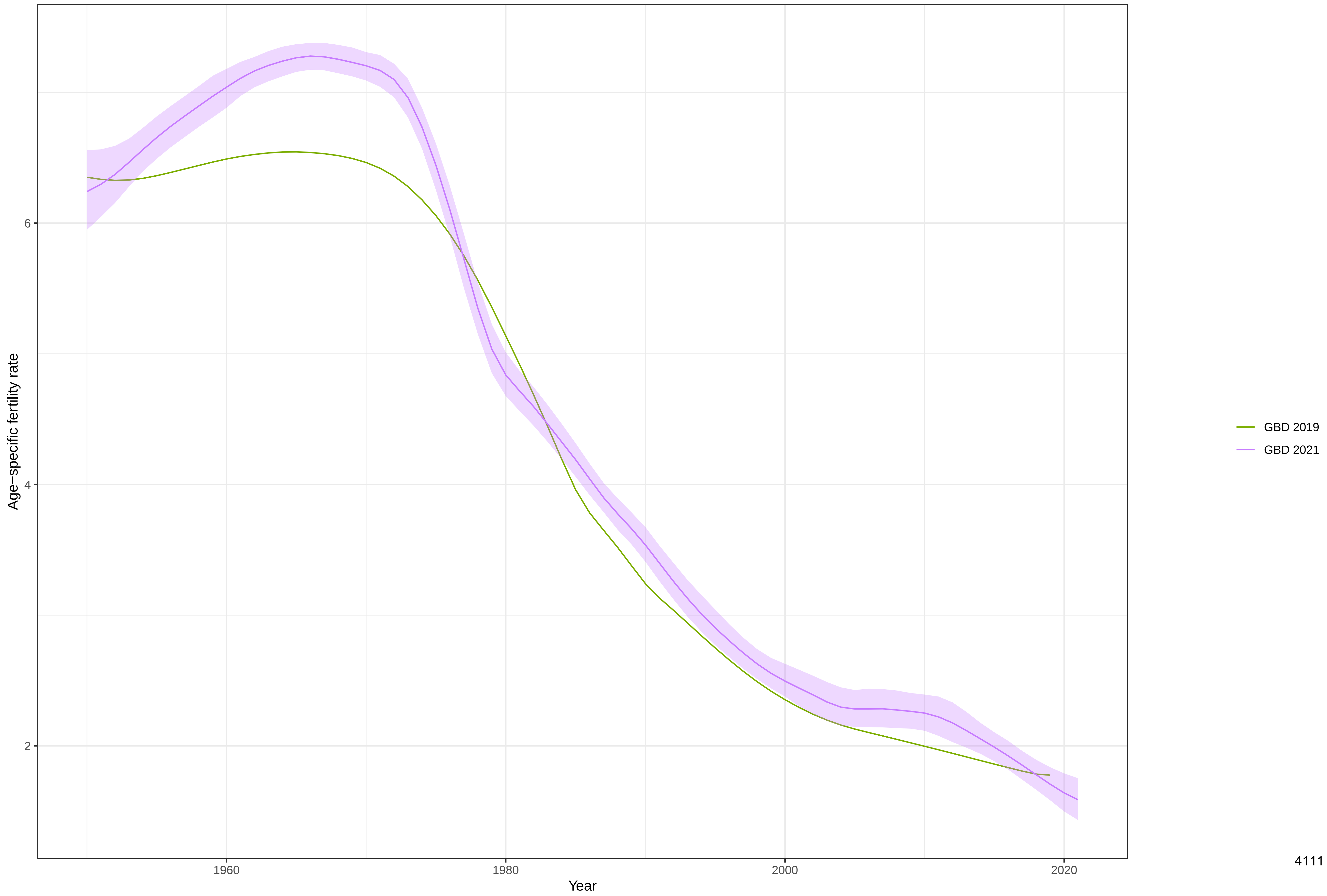

Age−specific fertility fitted trend lines, 1950−2021: San Luis Potosí, Ages: 10−14
Data points enclosed with a diamond are reference sources

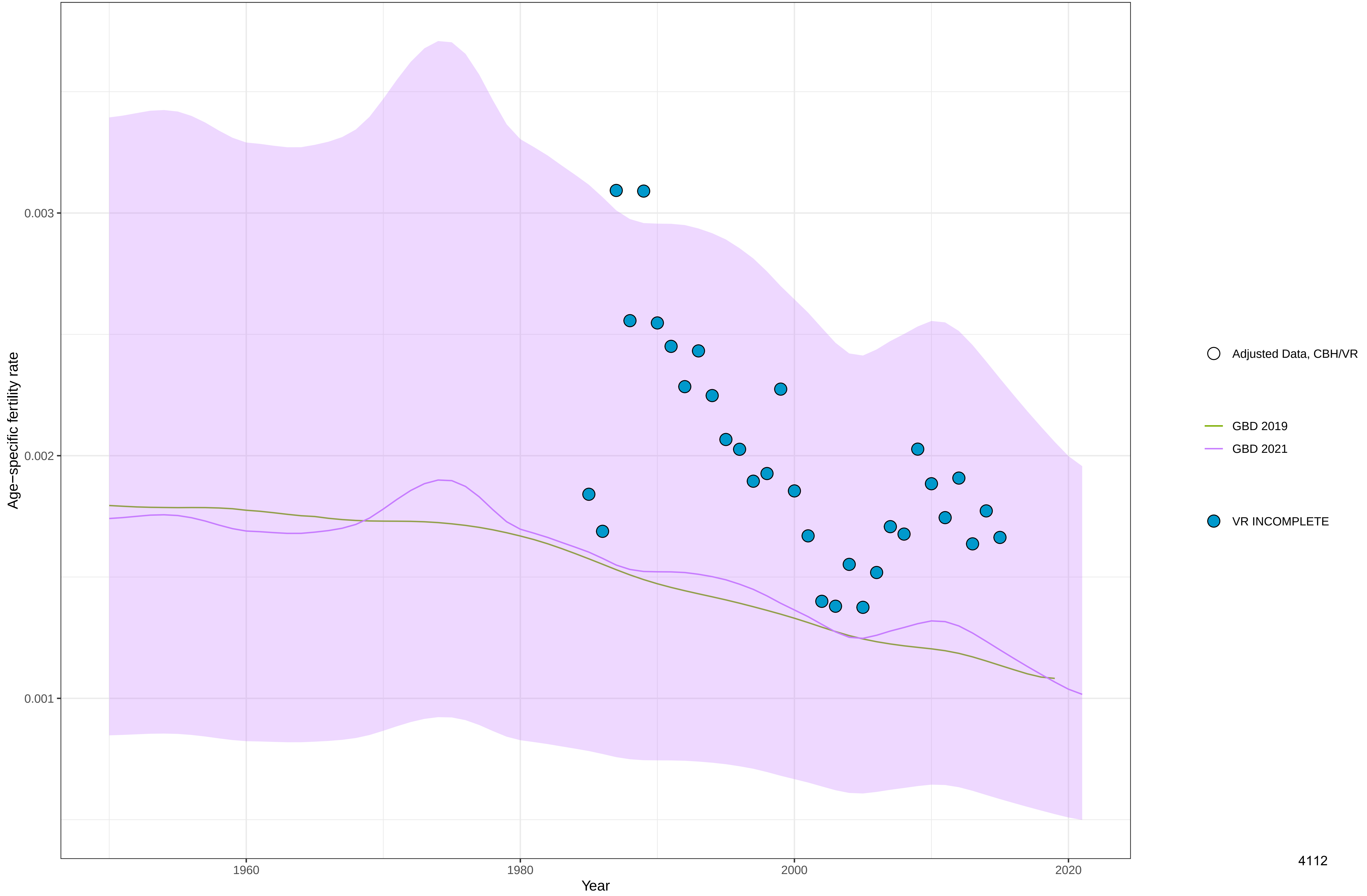

Age−specific fertility fitted trend lines, 1950−2021: San Luis Potosí, Ages: 15−19
Data points enclosed with a diamond are reference sources

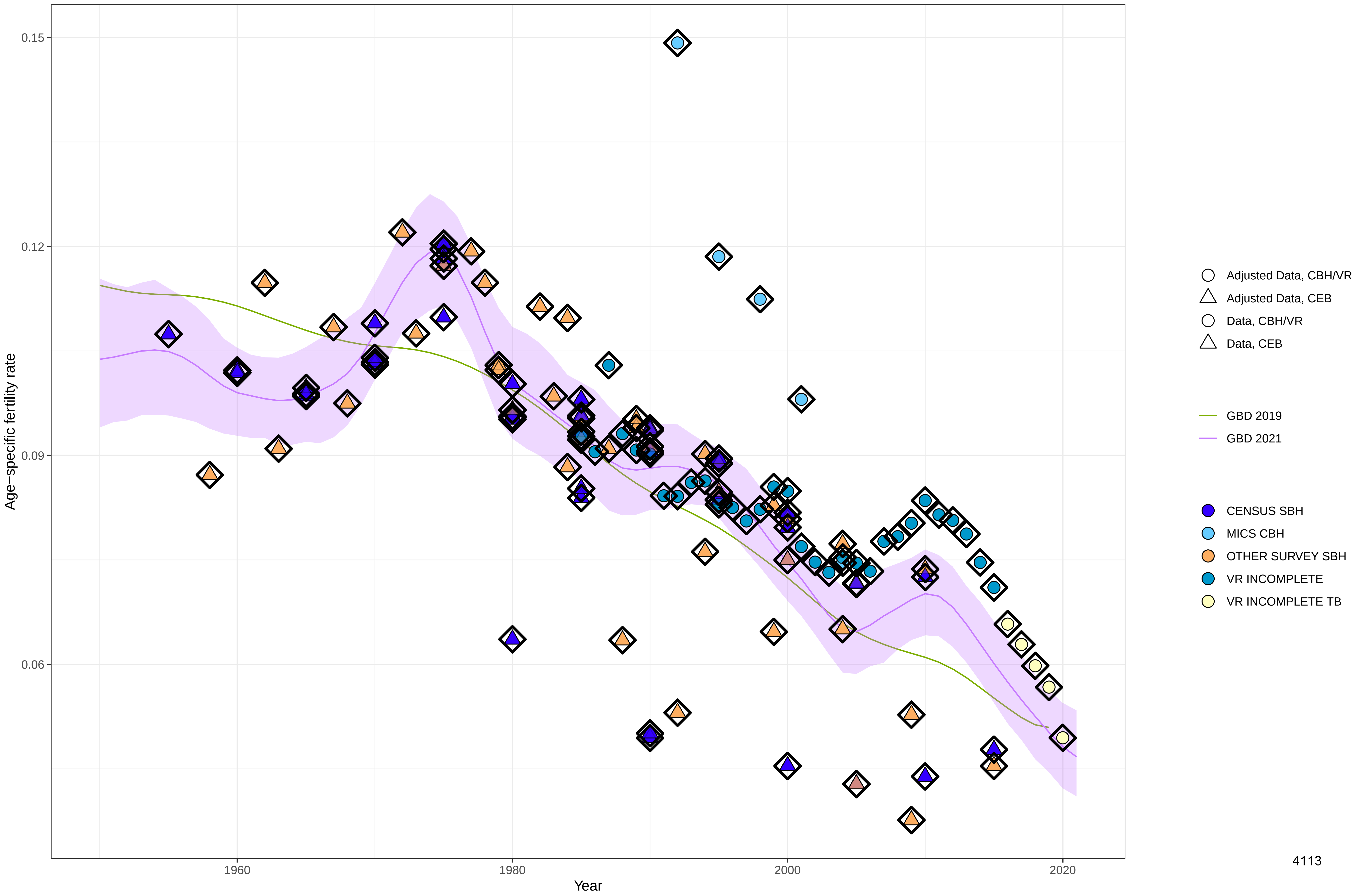

Age−specific fertility fitted trend lines, 1950−2021: San Luis Potosí, Ages: 20−24
Data points enclosed with a diamond are reference sources

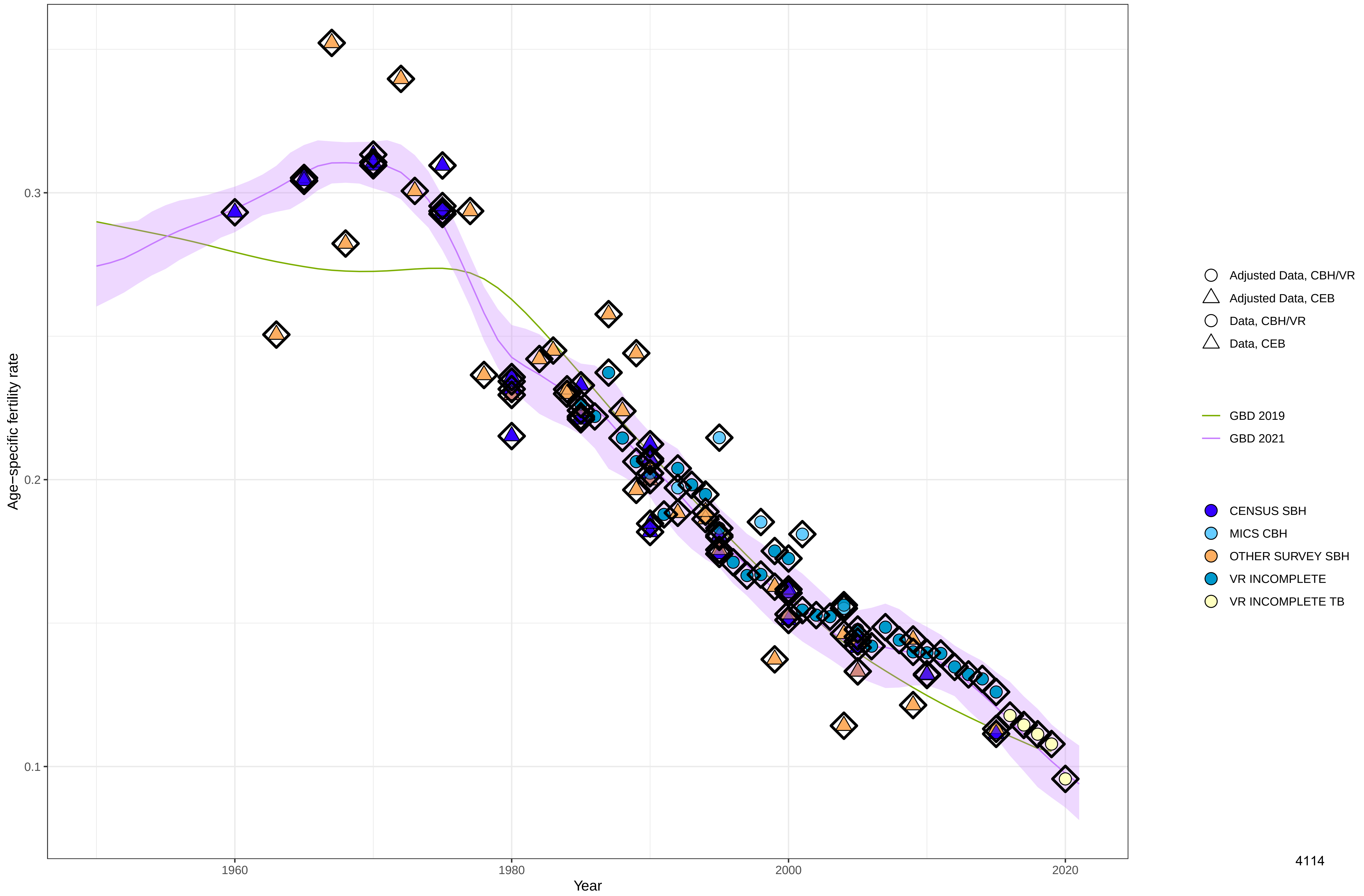

Age−specific fertility fitted trend lines, 1950−2021: San Luis Potosí, Ages: 25−29
Data points enclosed with a diamond are reference sources

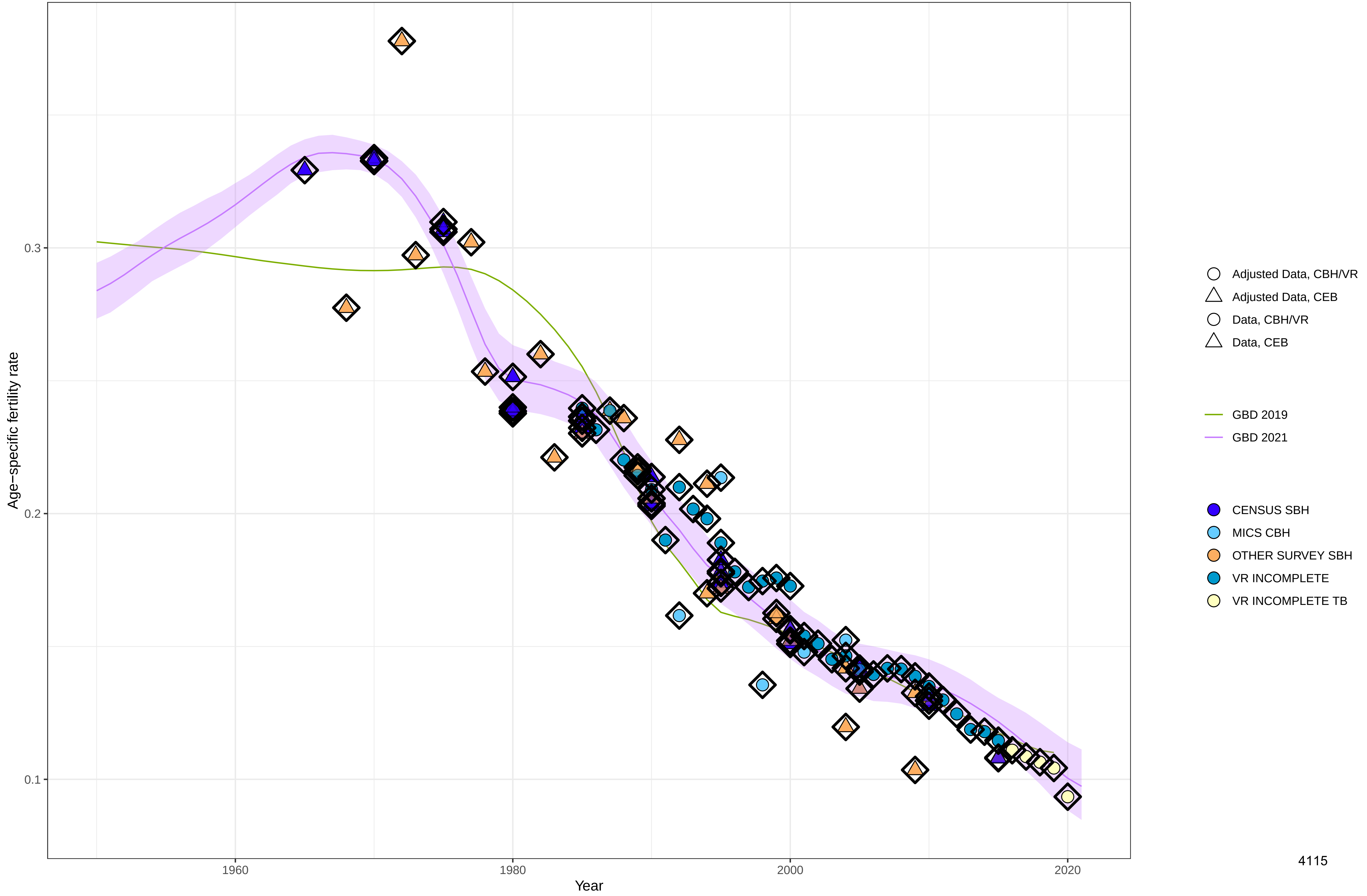

Age−specific fertility fitted trend lines, 1950−2021: San Luis Potosí, Ages: 30−34
Data points enclosed with a diamond are reference sources

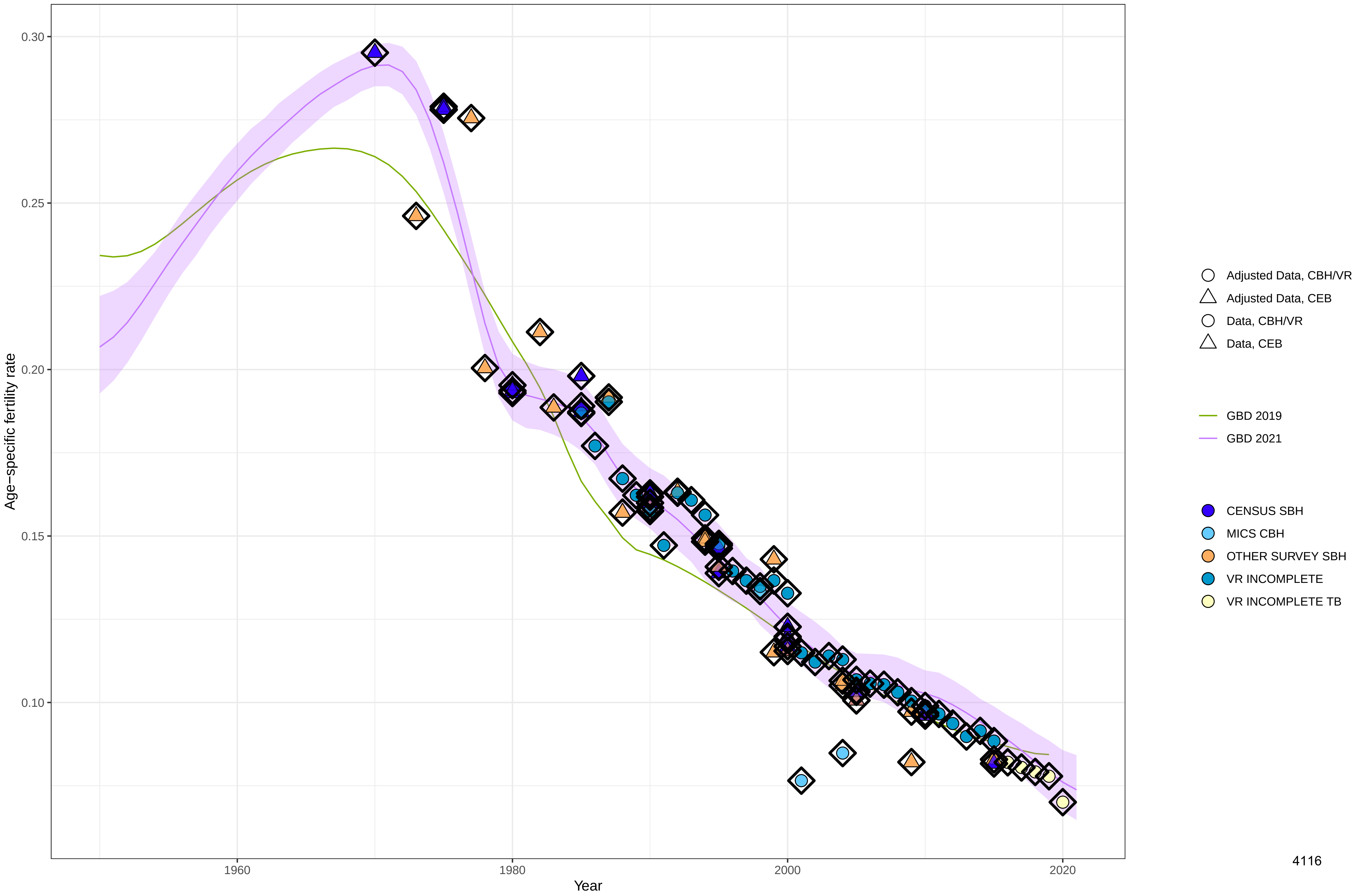

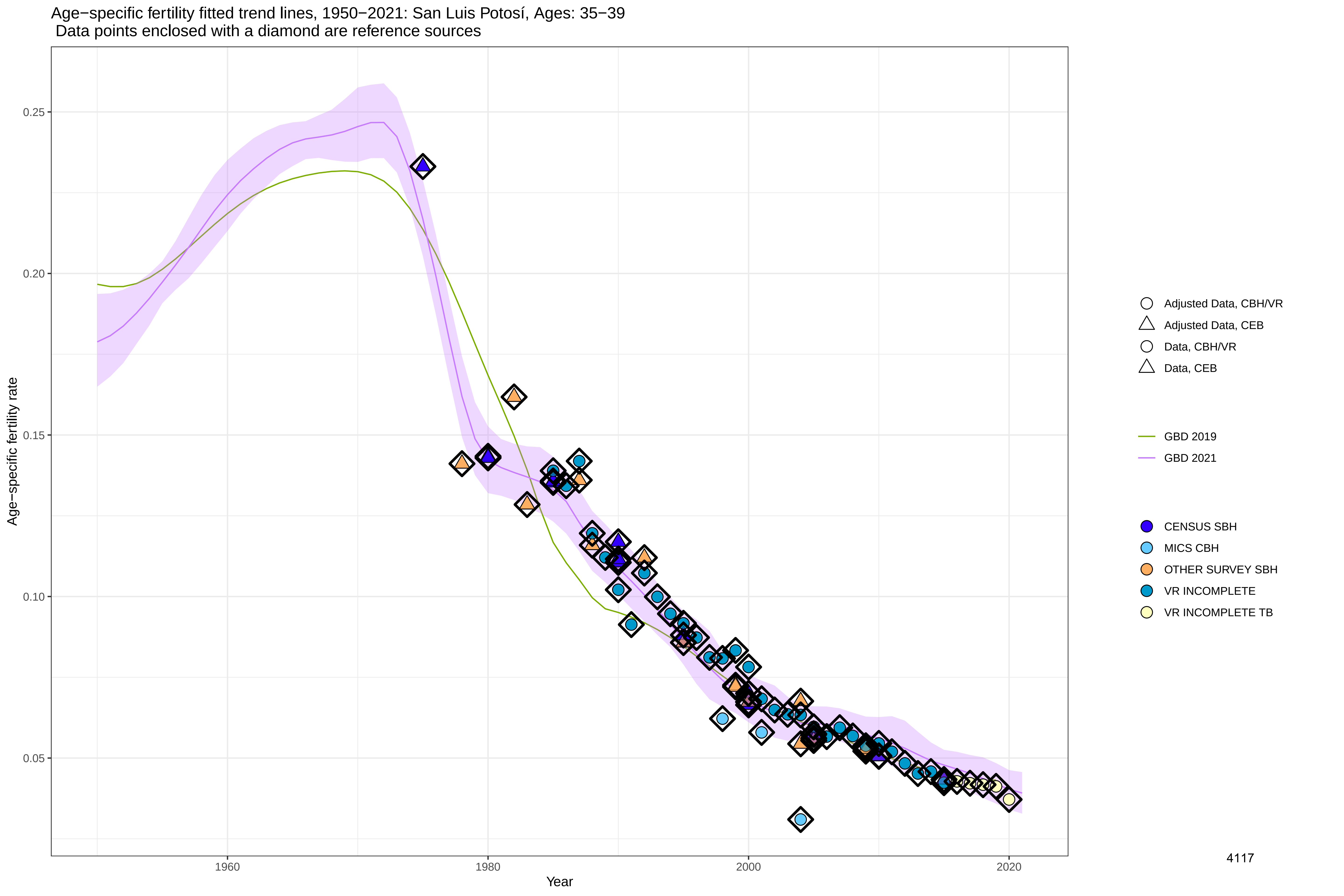

Age−specific fertility fitted trend lines, 1950−2021: San Luis Potosí, Ages: 35−39
Data points enclosed with a diamond are reference sources
Age−specific fertility rate
Year
0.05
0.10
0.15
0.20
0.25
1960
1980
2000
2020
Adjusted Data, CBH/VR
Adjusted Data, CEB
Data, CBH/VR
Data, CEB
GBD 2019
GBD 2021
CENSUS SBH
MICS CBH
OTHER SURVEY SBH
VR INCOMPLETE
VR INCOMPLETE TB

Age−specific fertility fitted trend lines, 1950−2021: San Luis Potosí, Ages: 40−44
Data points enclosed with a diamond are reference sources

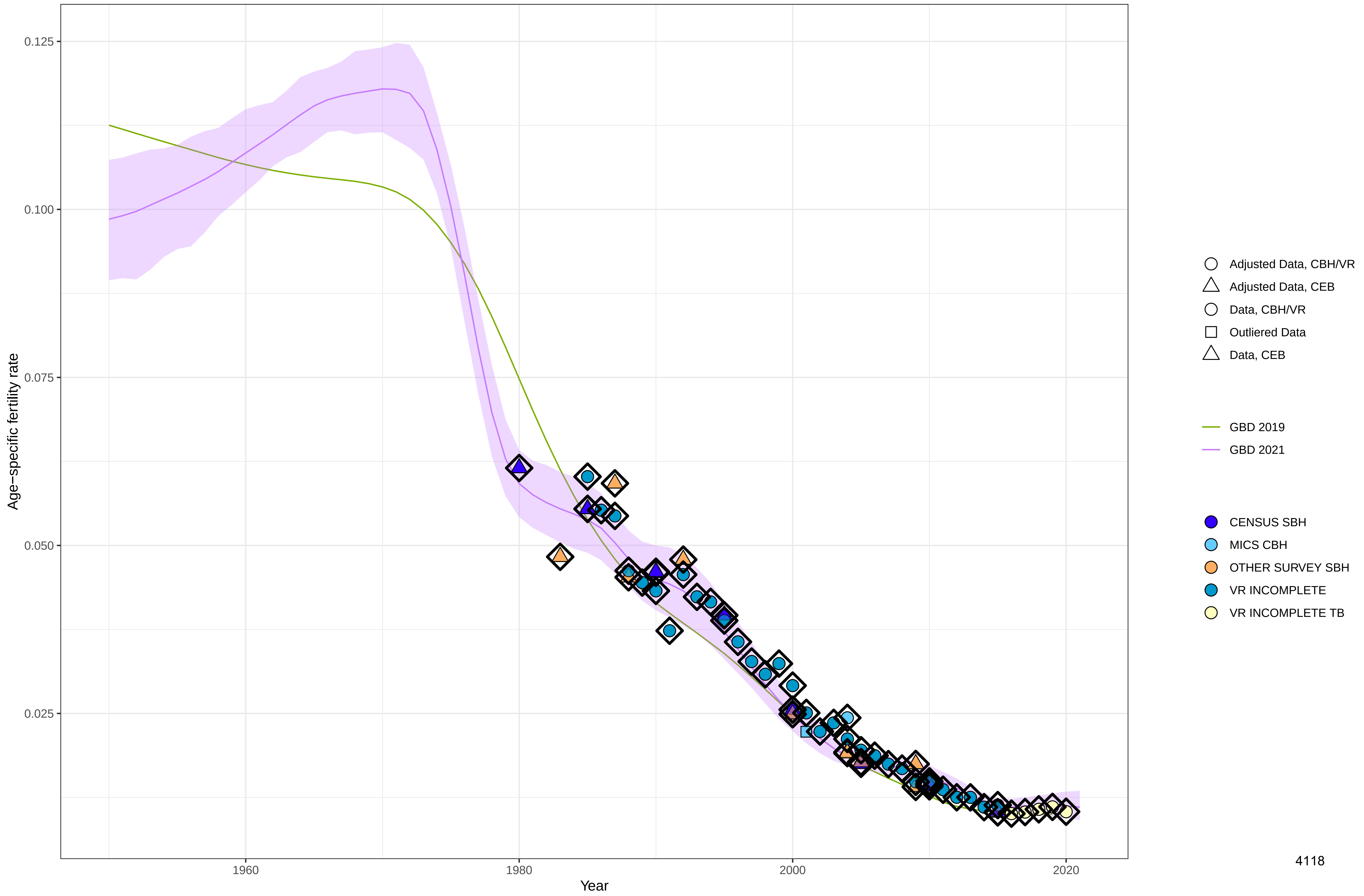

Age−specific fertility fitted trend lines, 1950−2021: San Luis Potosí, Ages: 45−49
Data points enclosed with a diamond are reference sources

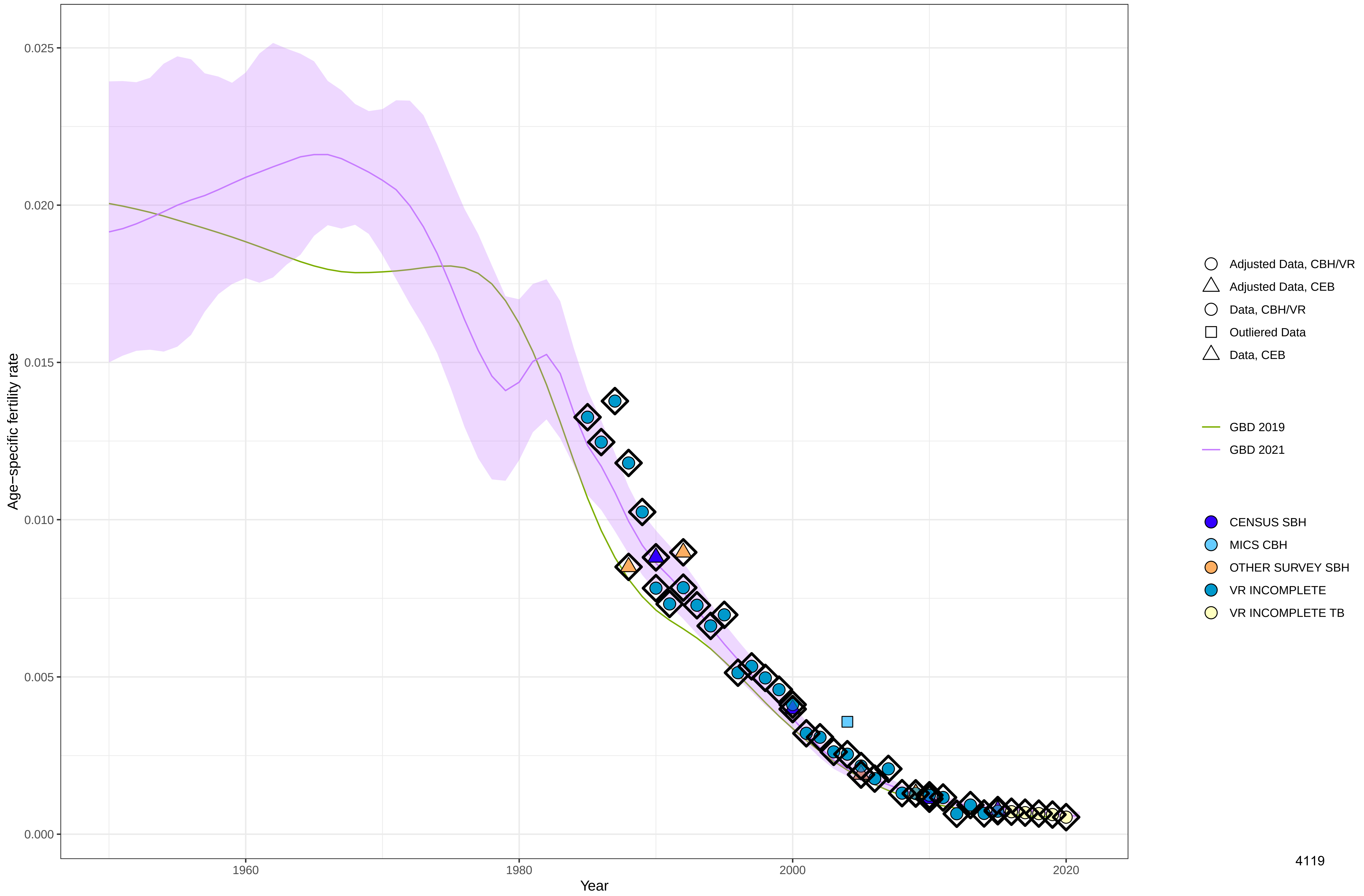

Age−specific fertility fitted trend lines, 1950−2021: San Luis Potosí, Ages: 50−54
Data points enclosed with a diamond are reference sources

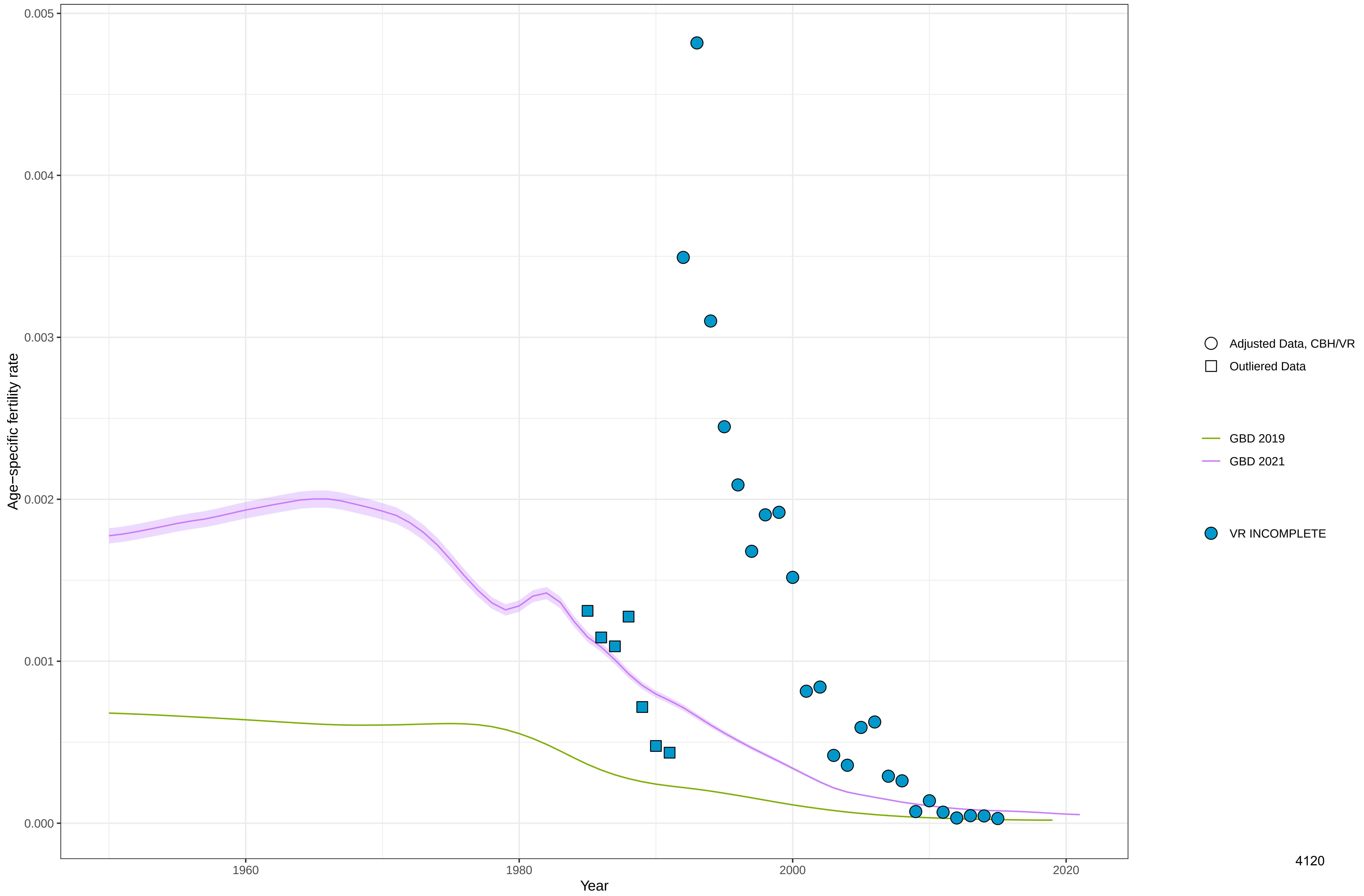

Total fertility rate fitted trend lines, 1950−2021: San Luis Potosí TFR
Data points enclosed with a diamond are reference sources

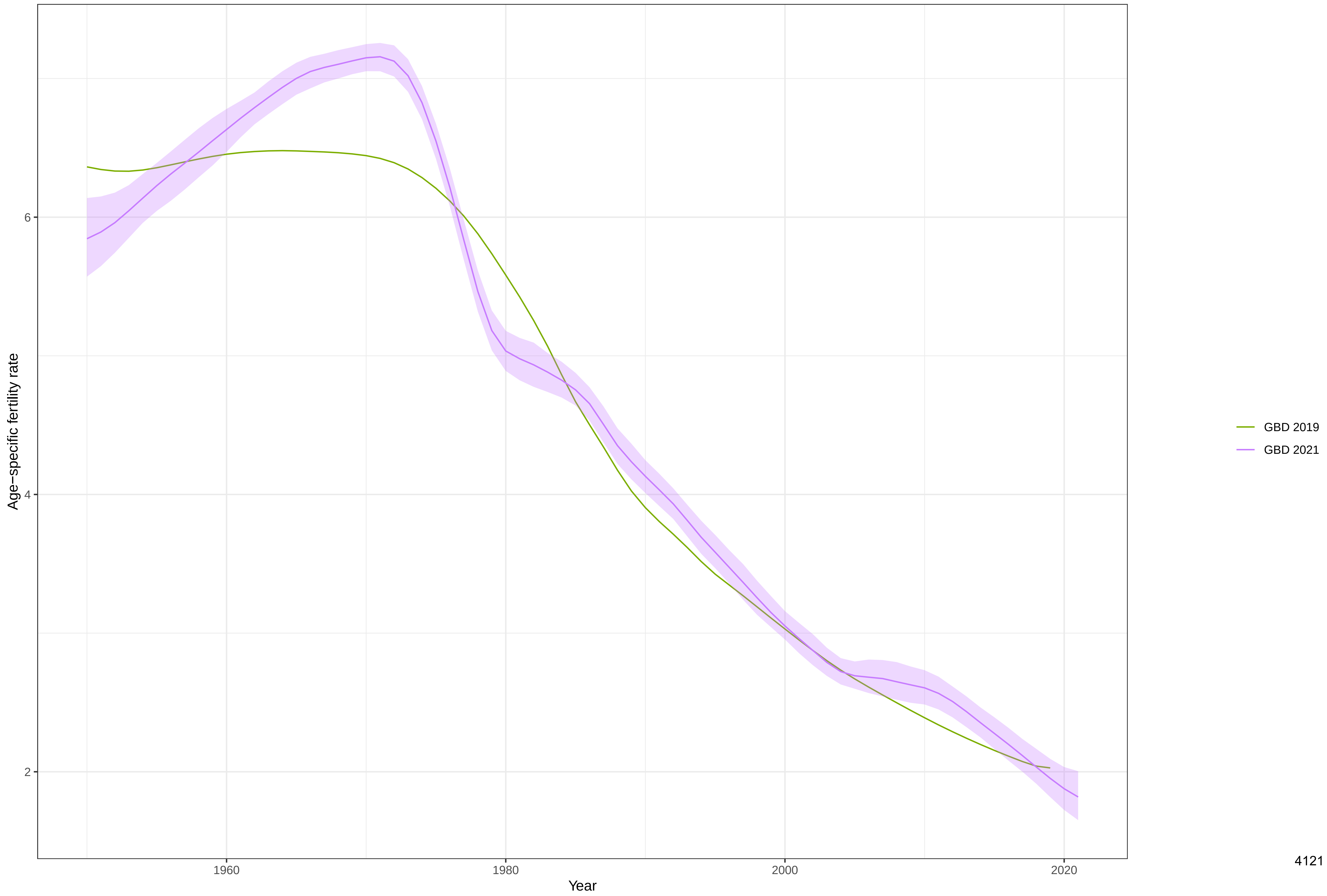

Age−specific fertility fitted trend lines, 1950−2021: Sinaloa, Ages: 10−14
Data points enclosed with a diamond are reference sources

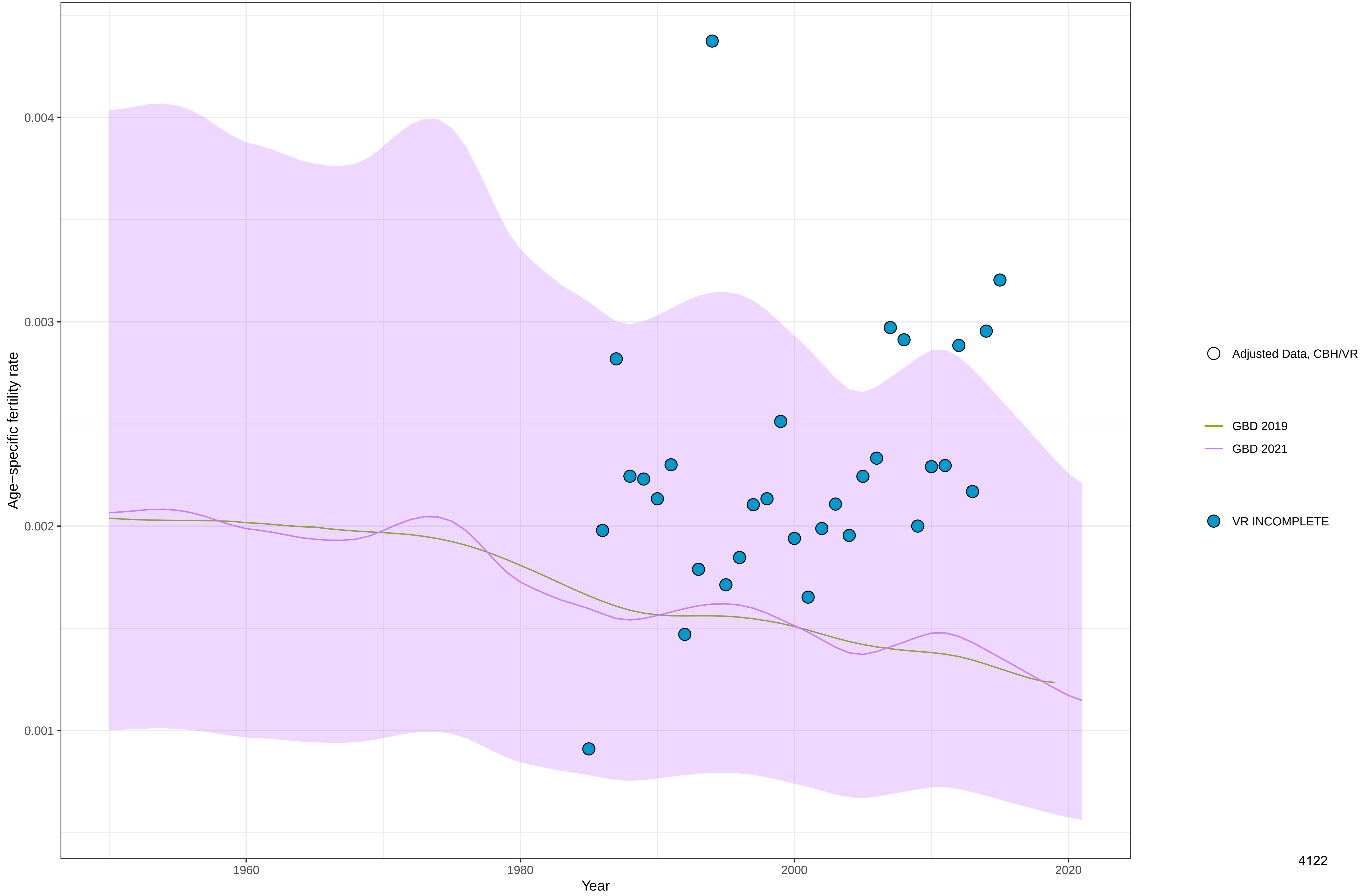

Age−specific fertility fitted trend lines, 1950−2021: Sinaloa, Ages: 15−19
Data points enclosed with a diamond are reference sources

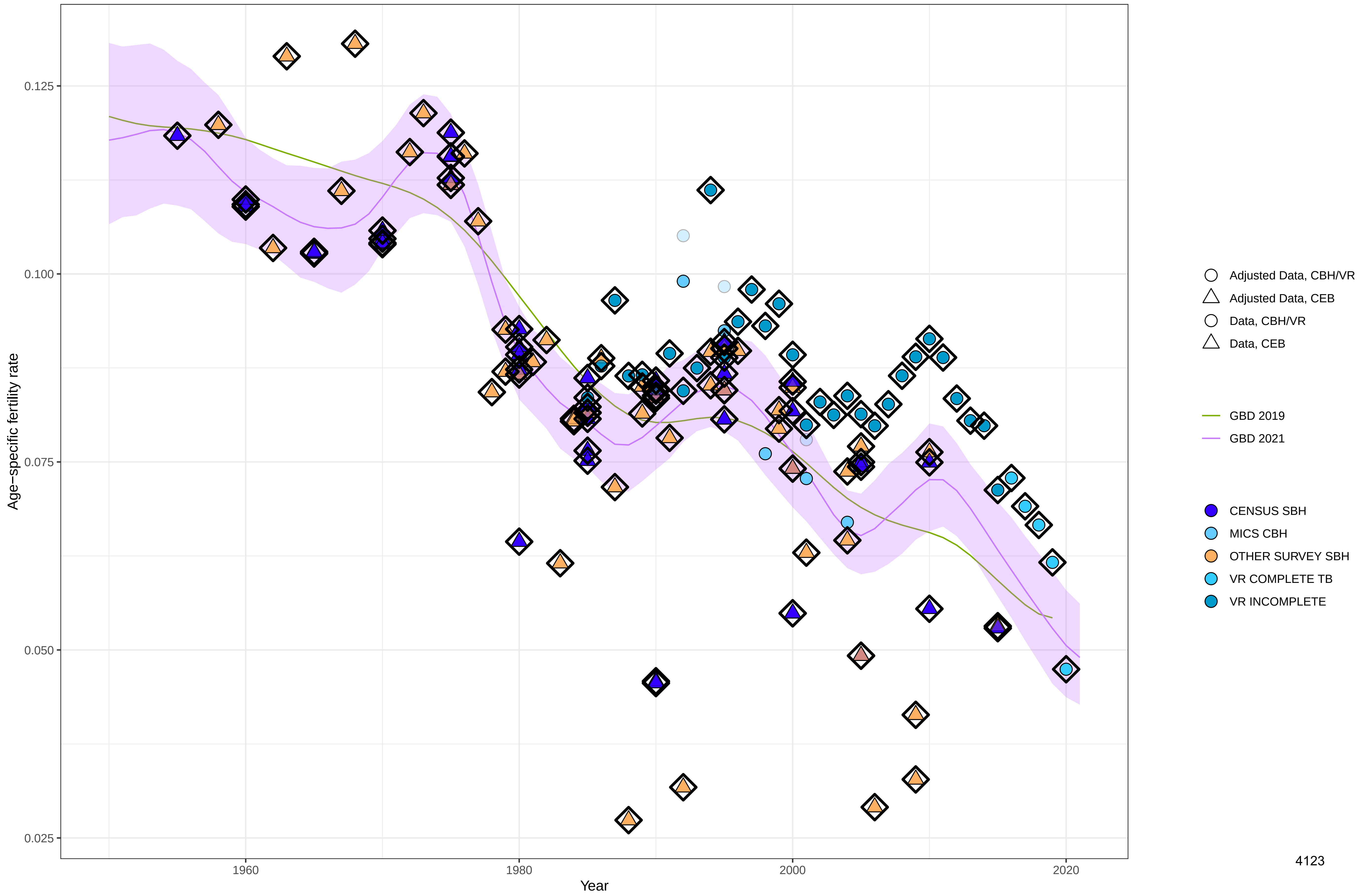

Age−specific fertility fitted trend lines, 1950−2021: Sinaloa, Ages: 20−24
Data points enclosed with a diamond are reference sources

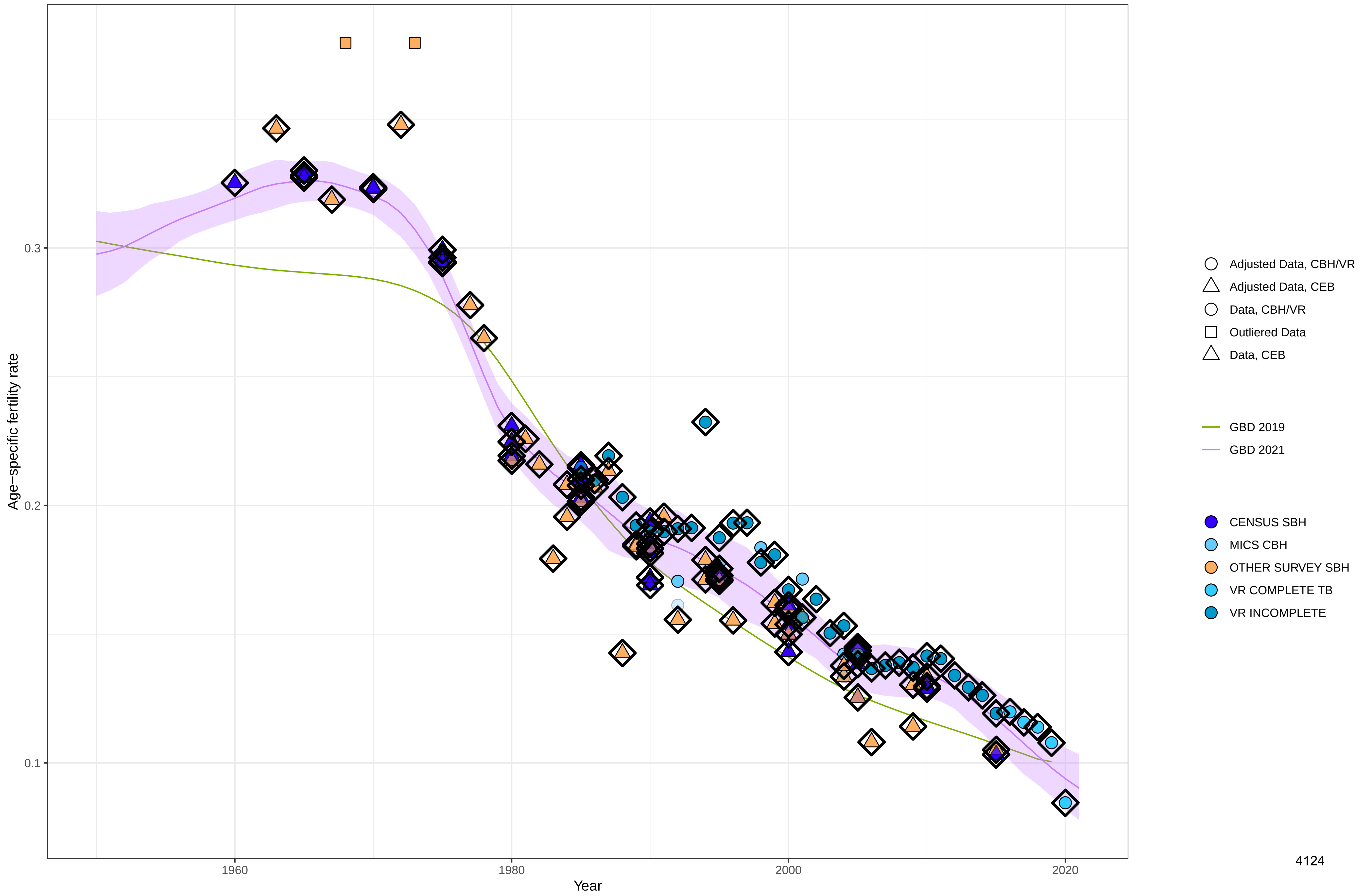

Age−specific fertility fitted trend lines, 1950−2021: Sinaloa, Ages: 25−29
Data points enclosed with a diamond are reference sources

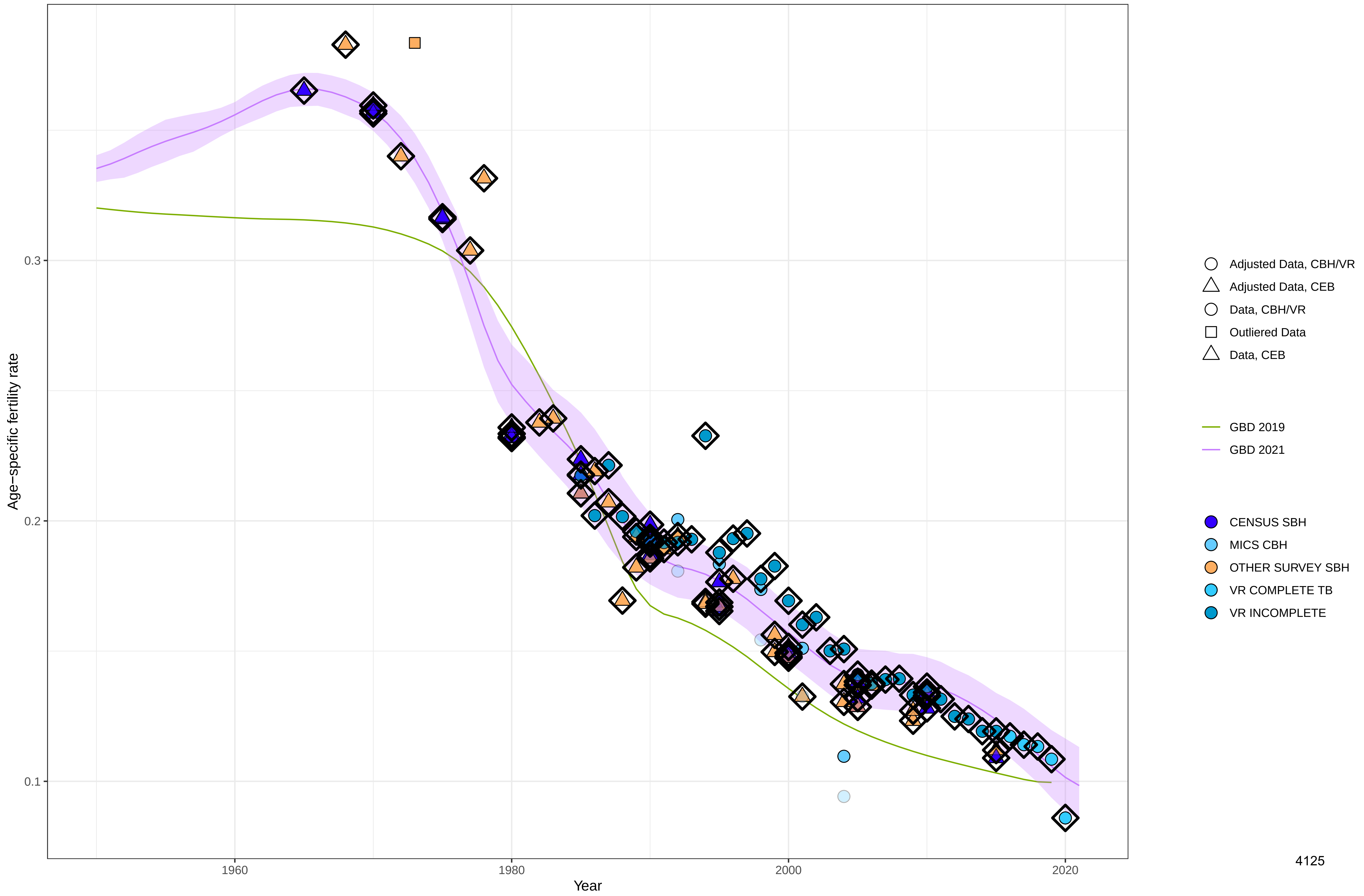

Age−specific fertility fitted trend lines, 1950−2021: Sinaloa, Ages: 30−34
Data points enclosed with a diamond are reference sources

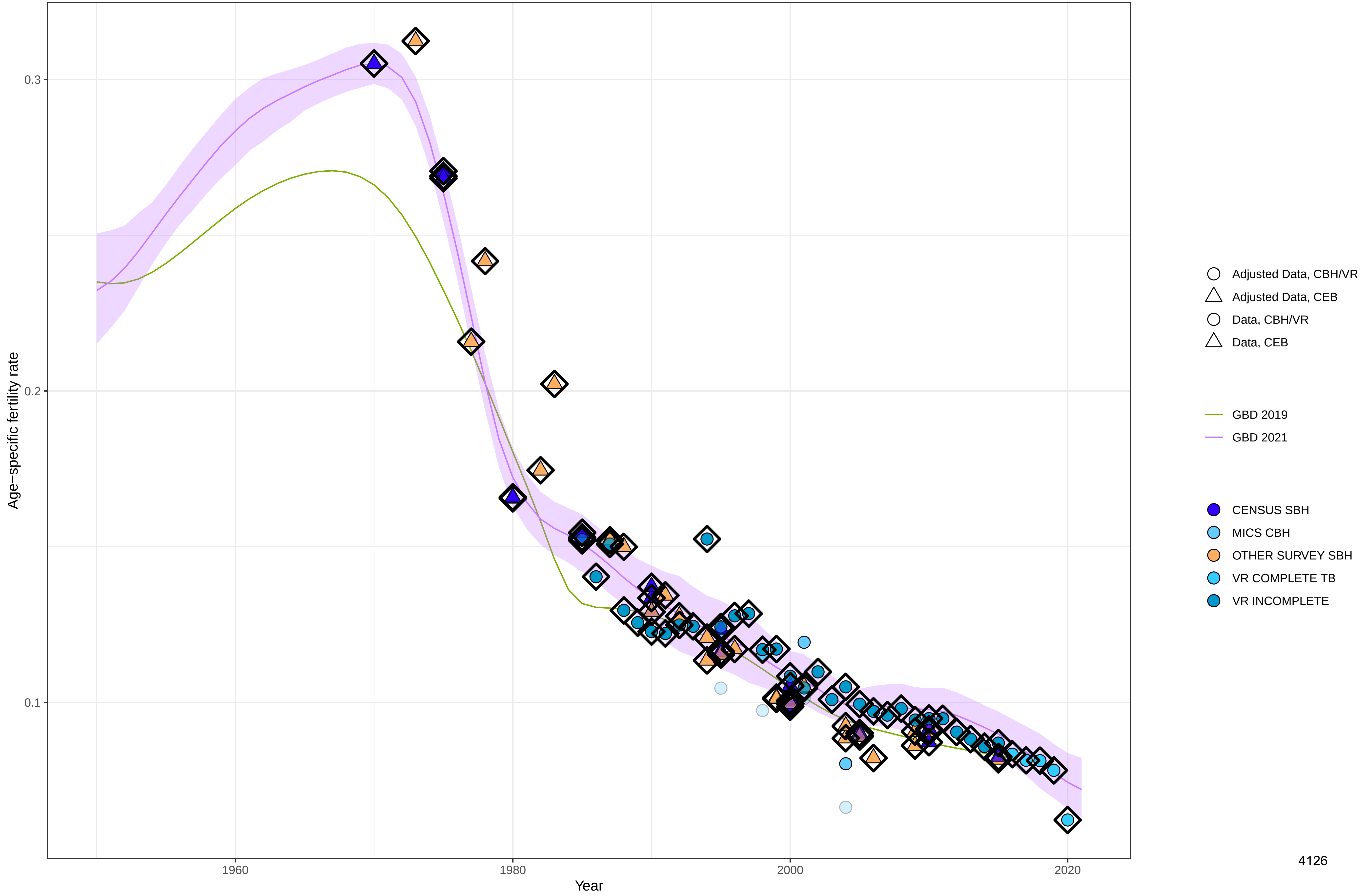

Age−specific fertility fitted trend lines, 1950−2021: Sinaloa, Ages: 35−39
Data points enclosed with a diamond are reference sources

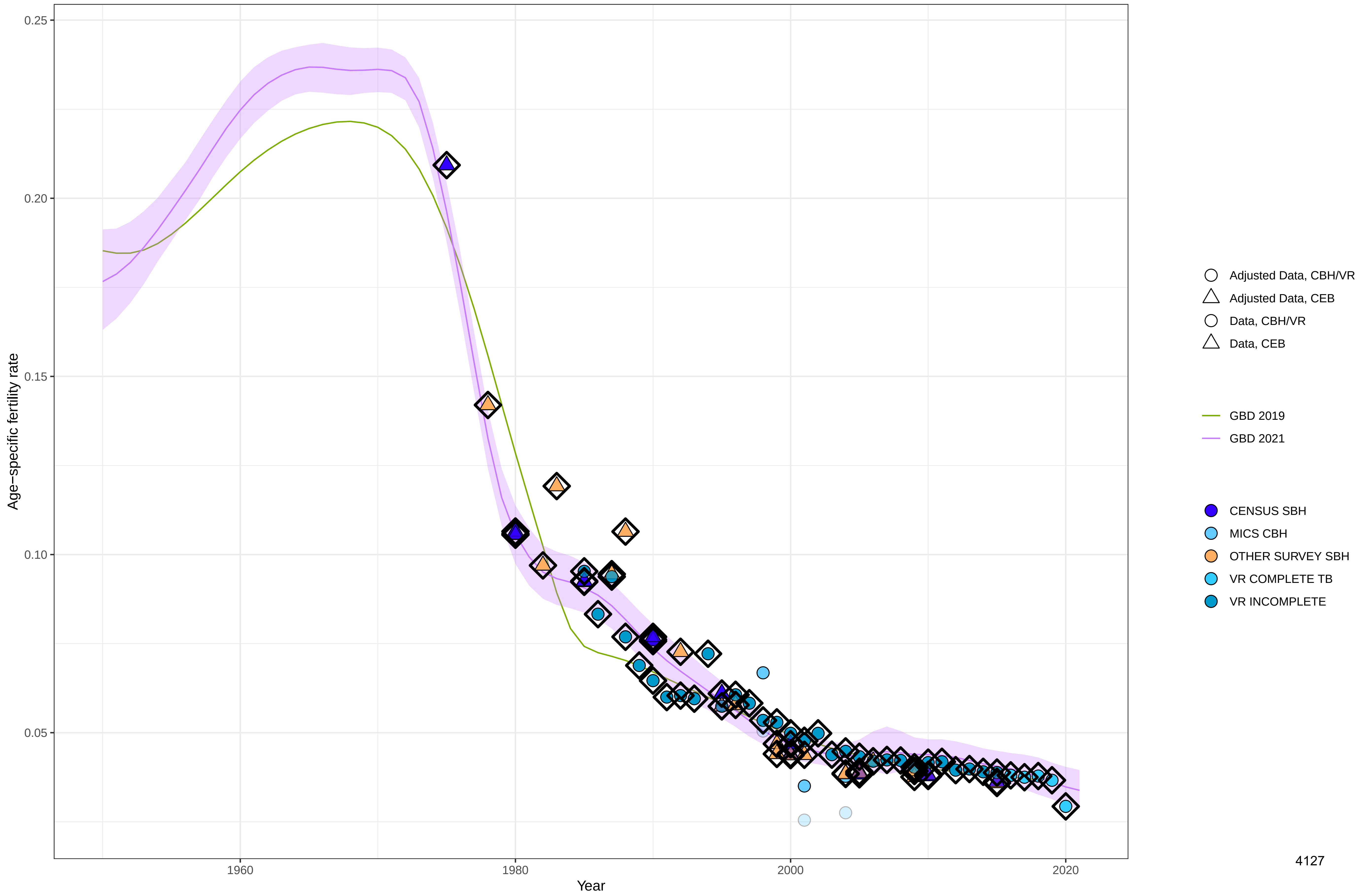

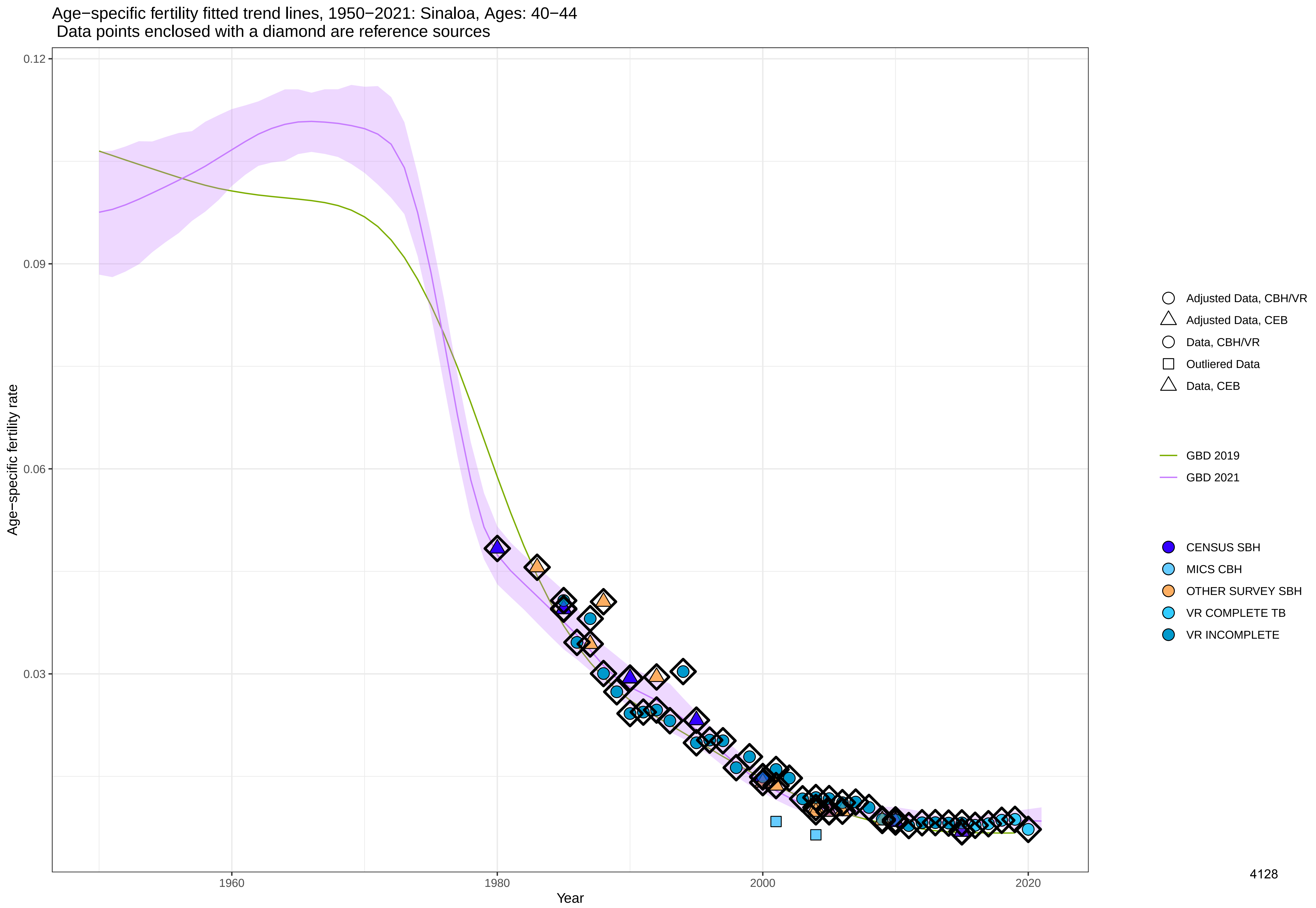

Age−specific fertility fitted trend lines, 1950−2021: Sinaloa, Ages: 40−44
Data points enclosed with a diamond are reference sources
0.12
0.09
0.06
0.03
Age−specific fertility rate
1960
1980
2000
2020
Year
Adjusted Data, CBH/VR
Adjusted Data, CEB
Data, CBH/VR
Outliered Data
Data, CEB
GBD 2019
GBD 2021
CENSUS SBH
MICS CBH
OTHER SURVEY SBH
VR COMPLETE TB
VR INCOMPLETE

Age−specific fertility fitted trend lines, 1950−2021: Sinaloa, Ages: 45−49
Data points enclosed with a diamond are reference sources

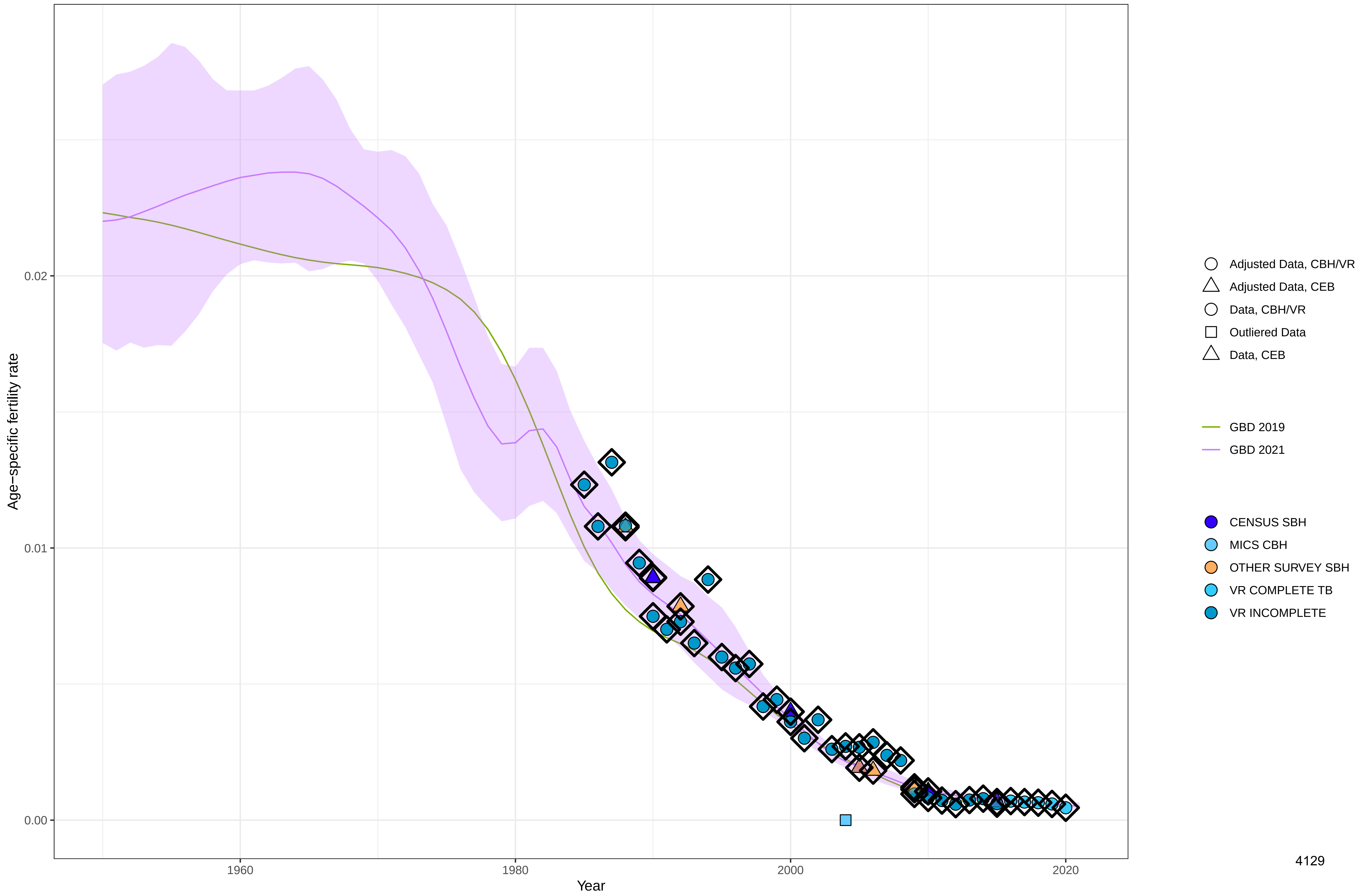

Age−specific fertility fitted trend lines, 1950−2021: Sinaloa, Ages: 50−54
Data points enclosed with a diamond are reference sources

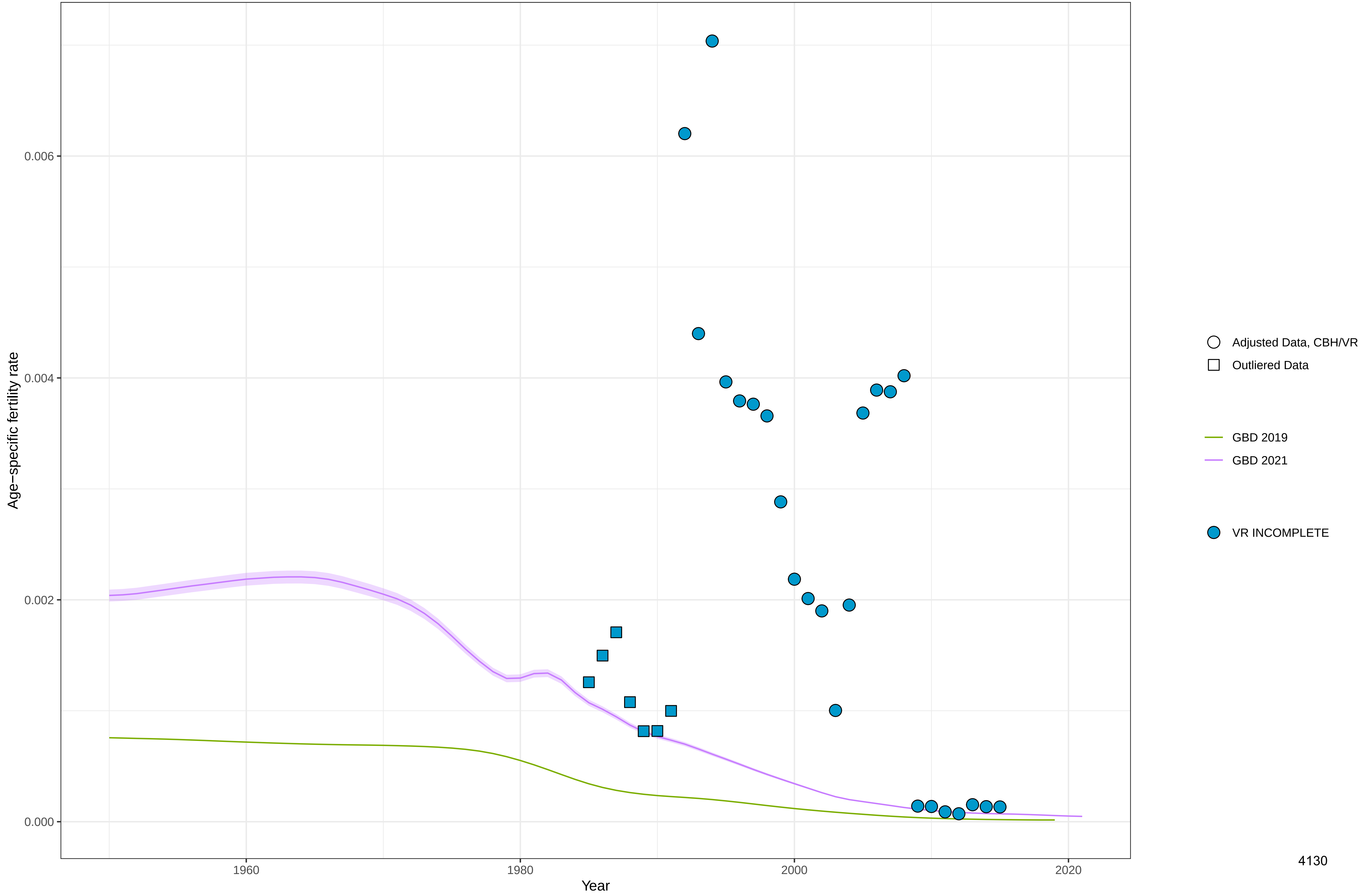

Total fertility rate fitted trend lines, 1950−2021: Sinaloa TFR
Data points enclosed with a diamond are reference sources

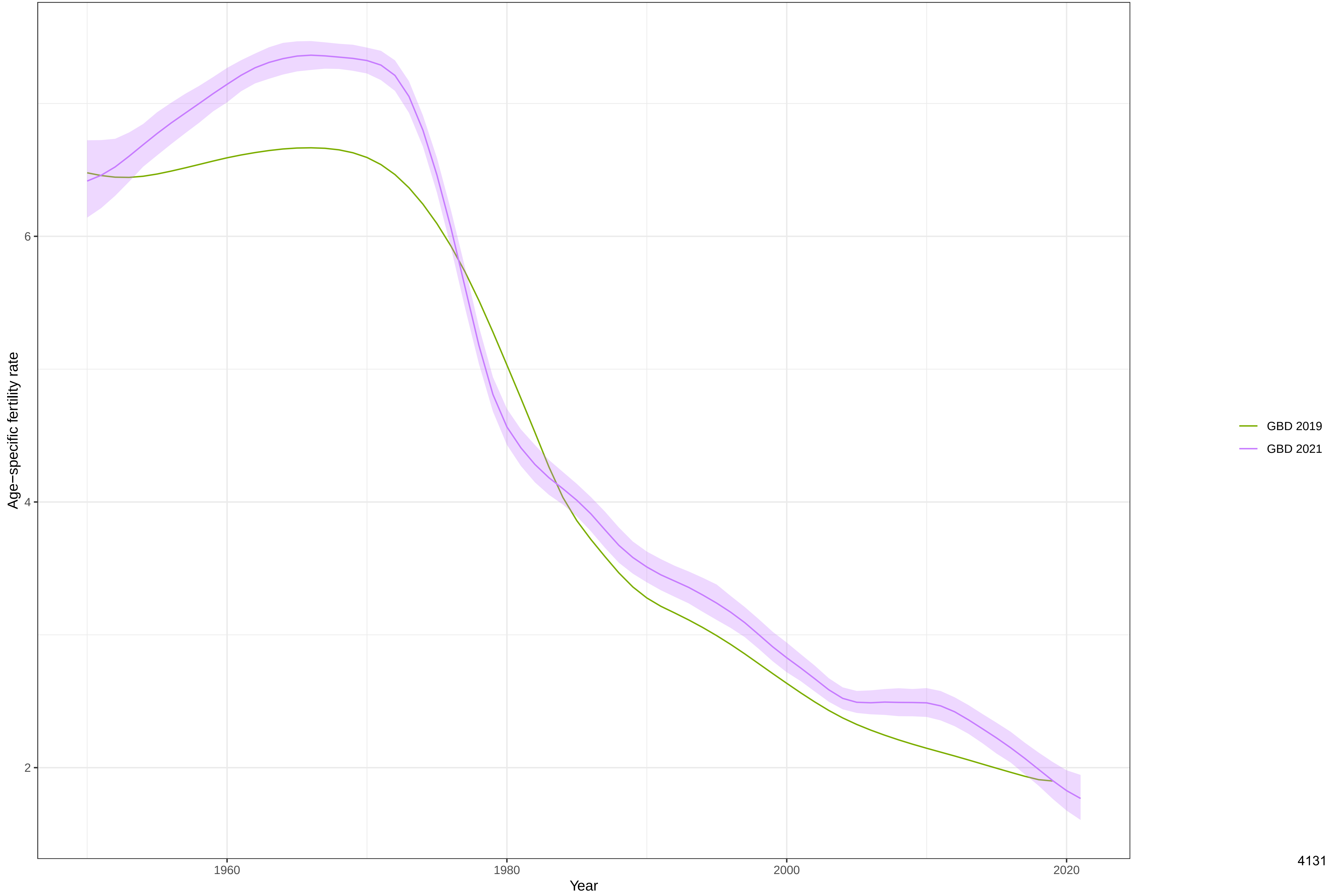

Age−specific fertility fitted trend lines, 1950−2021: Sonora, Ages: 10−14
Data points enclosed with a diamond are reference sources

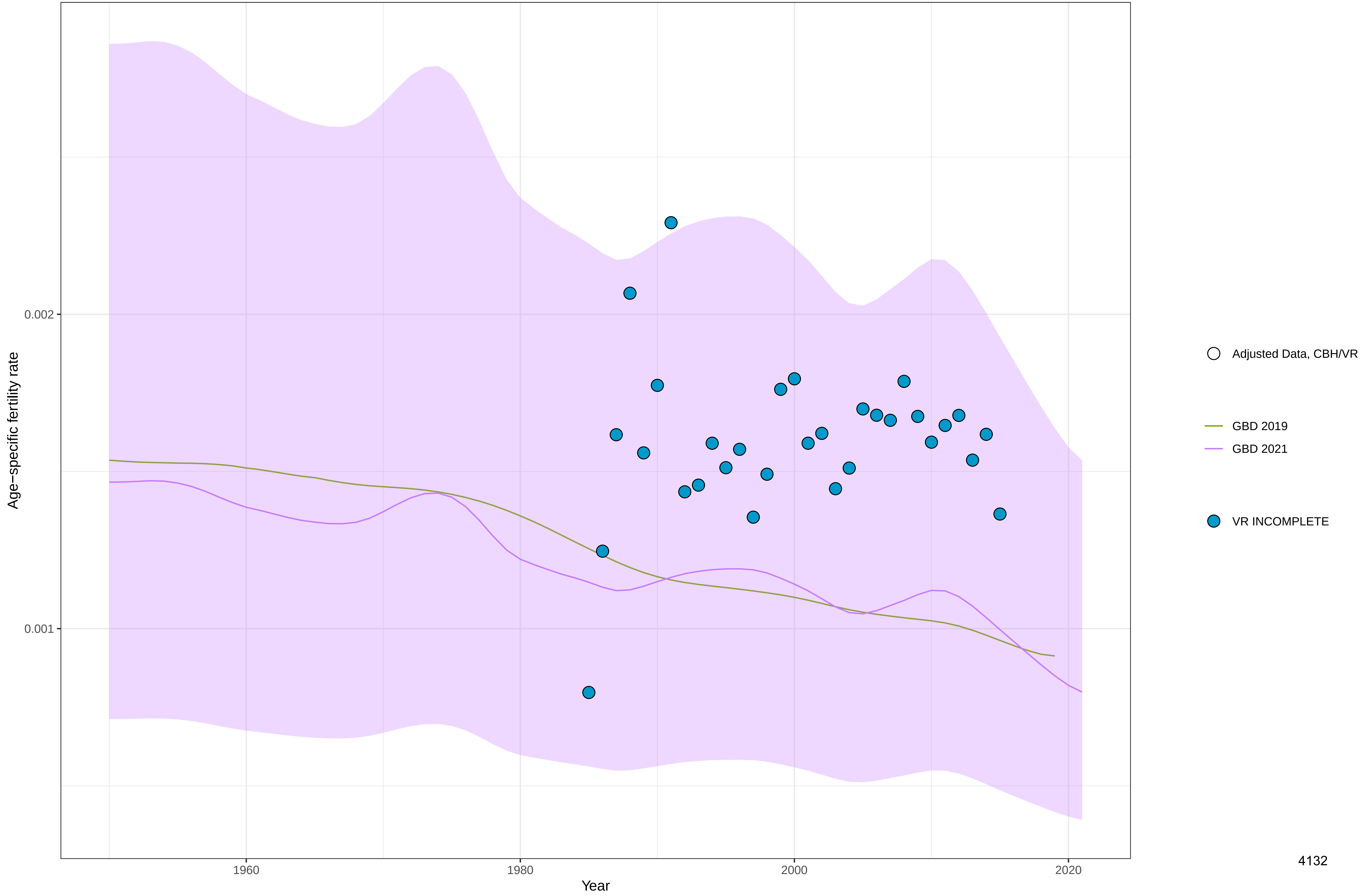

Age−specific fertility fitted trend lines, 1950−2021: Sonora, Ages: 15−19
Data points enclosed with a diamond are reference sources

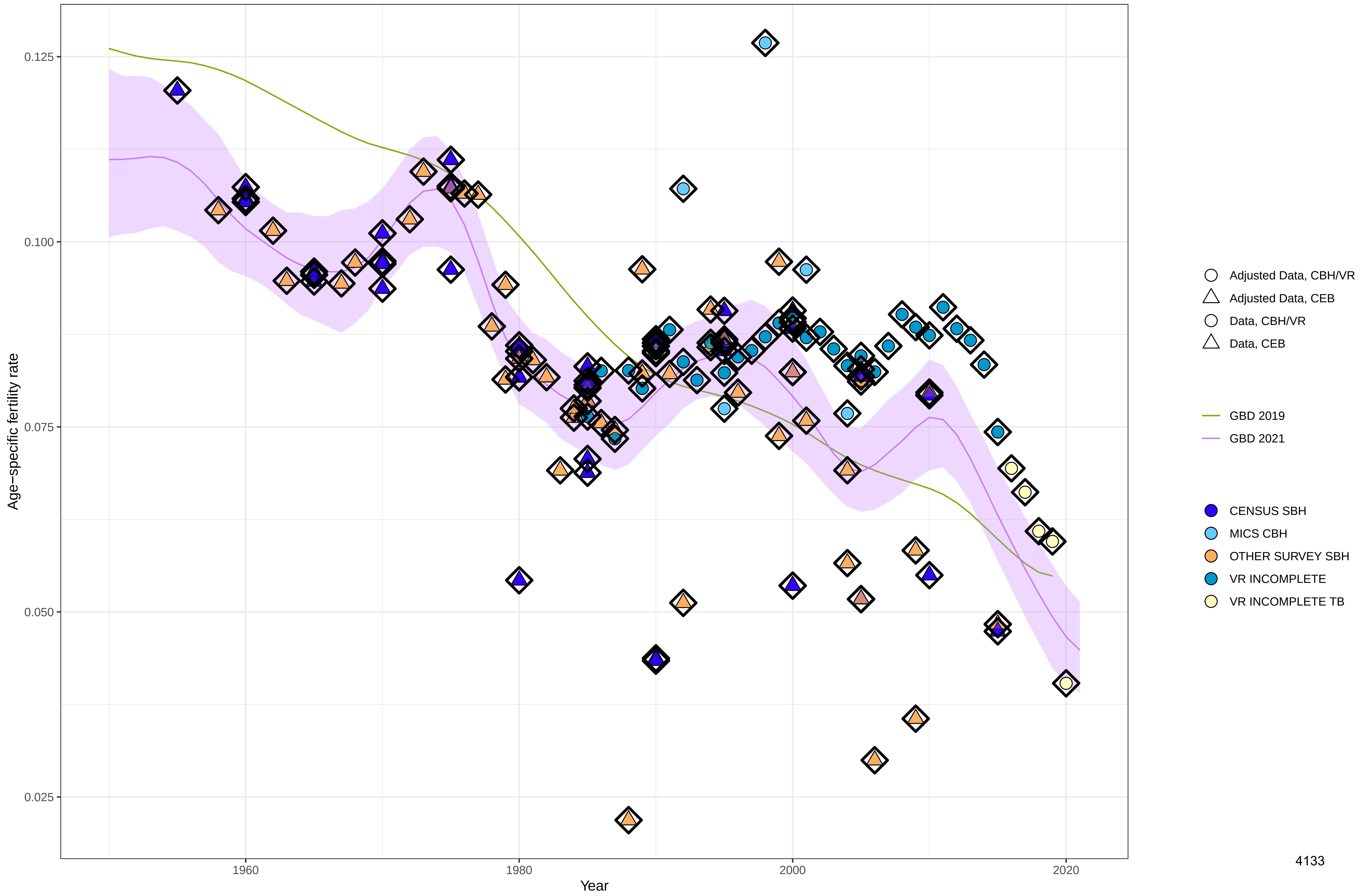

Age−specific fertility fitted trend lines, 1950−2021: Sonora, Ages: 20−24
Data points enclosed with a diamond are reference sources

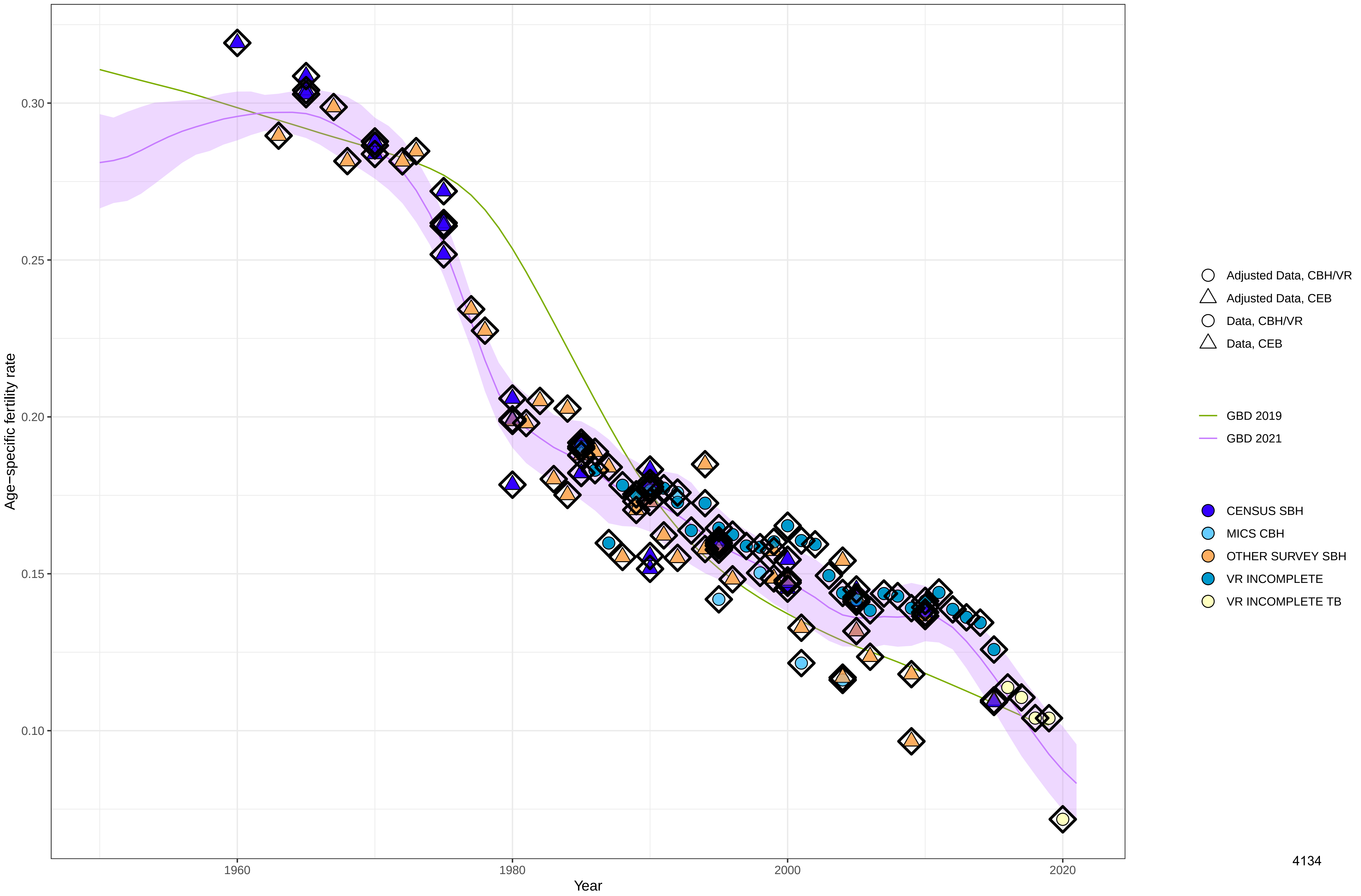

Age−specific fertility fitted trend lines, 1950−2021: Sonora, Ages: 25−29
Data points enclosed with a diamond are reference sources

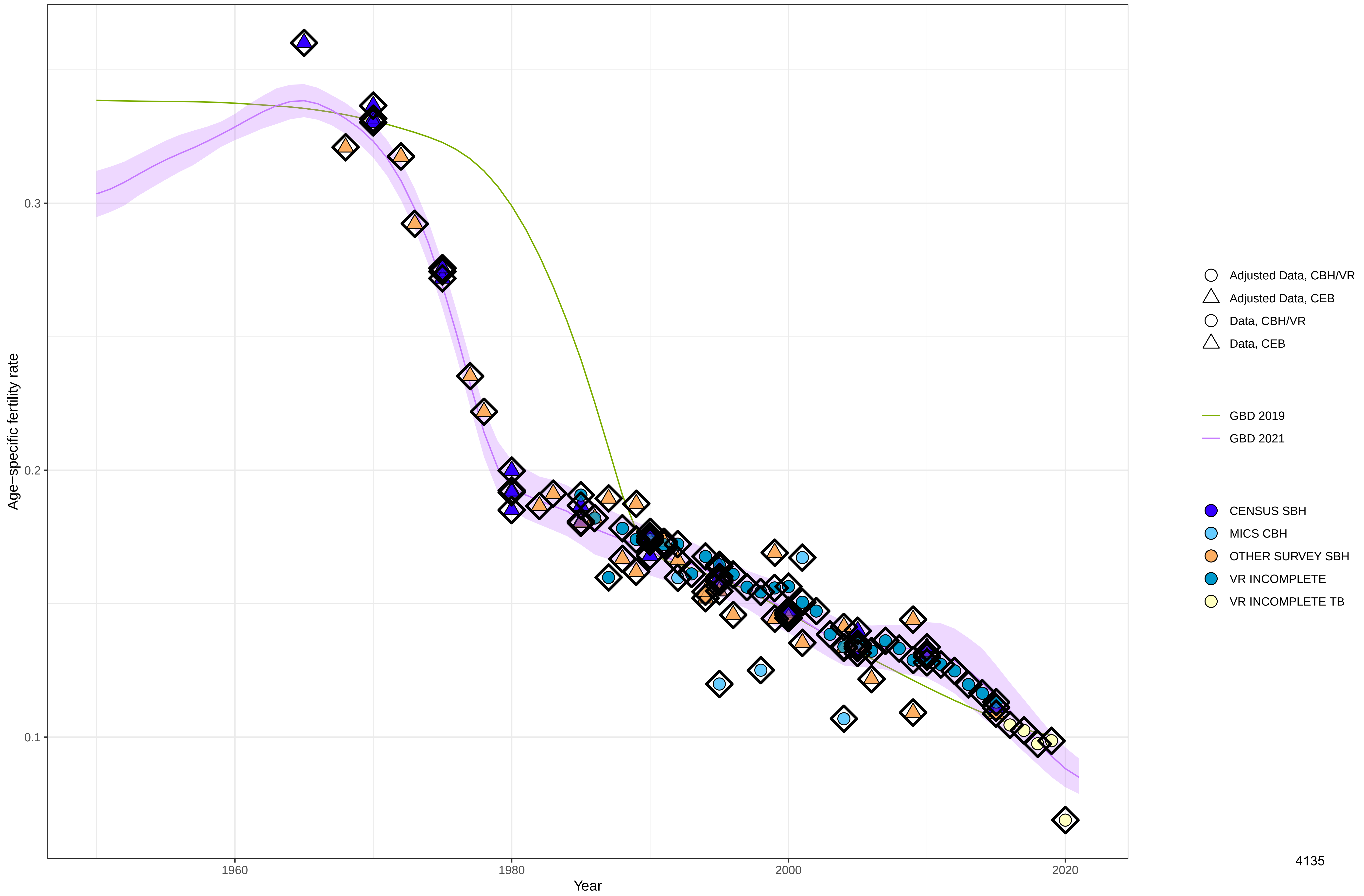

Age−specific fertility fitted trend lines, 1950−2021: Sonora, Ages: 30−34
Data points enclosed with a diamond are reference sources

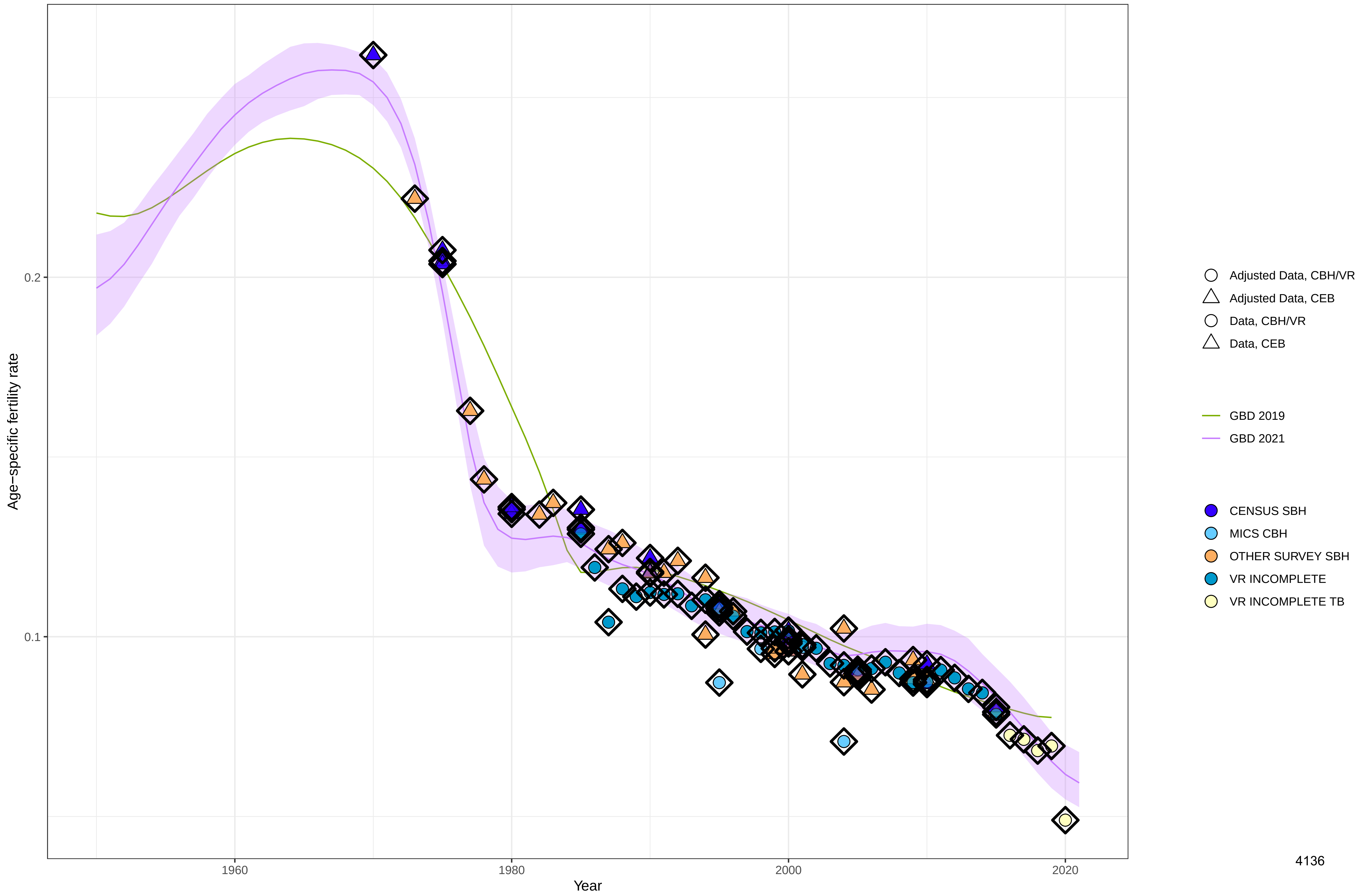

Age−specific fertility fitted trend lines, 1950−2021: Sonora, Ages: 35−39
Data points enclosed with a diamond are reference sources

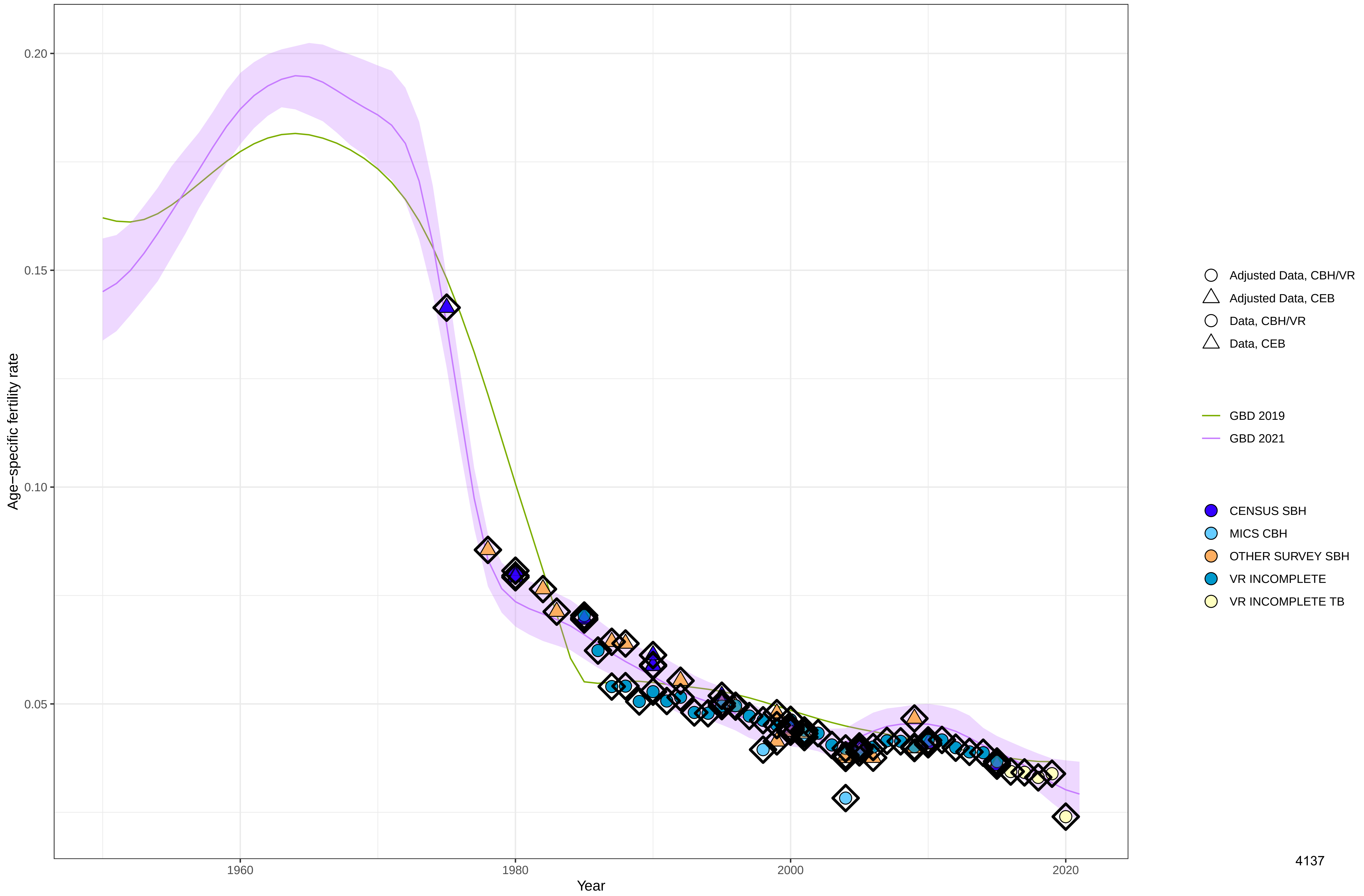

Age−specific fertility fitted trend lines, 1950−2021: Sonora, Ages: 40−44
Data points enclosed with a diamond are reference sources

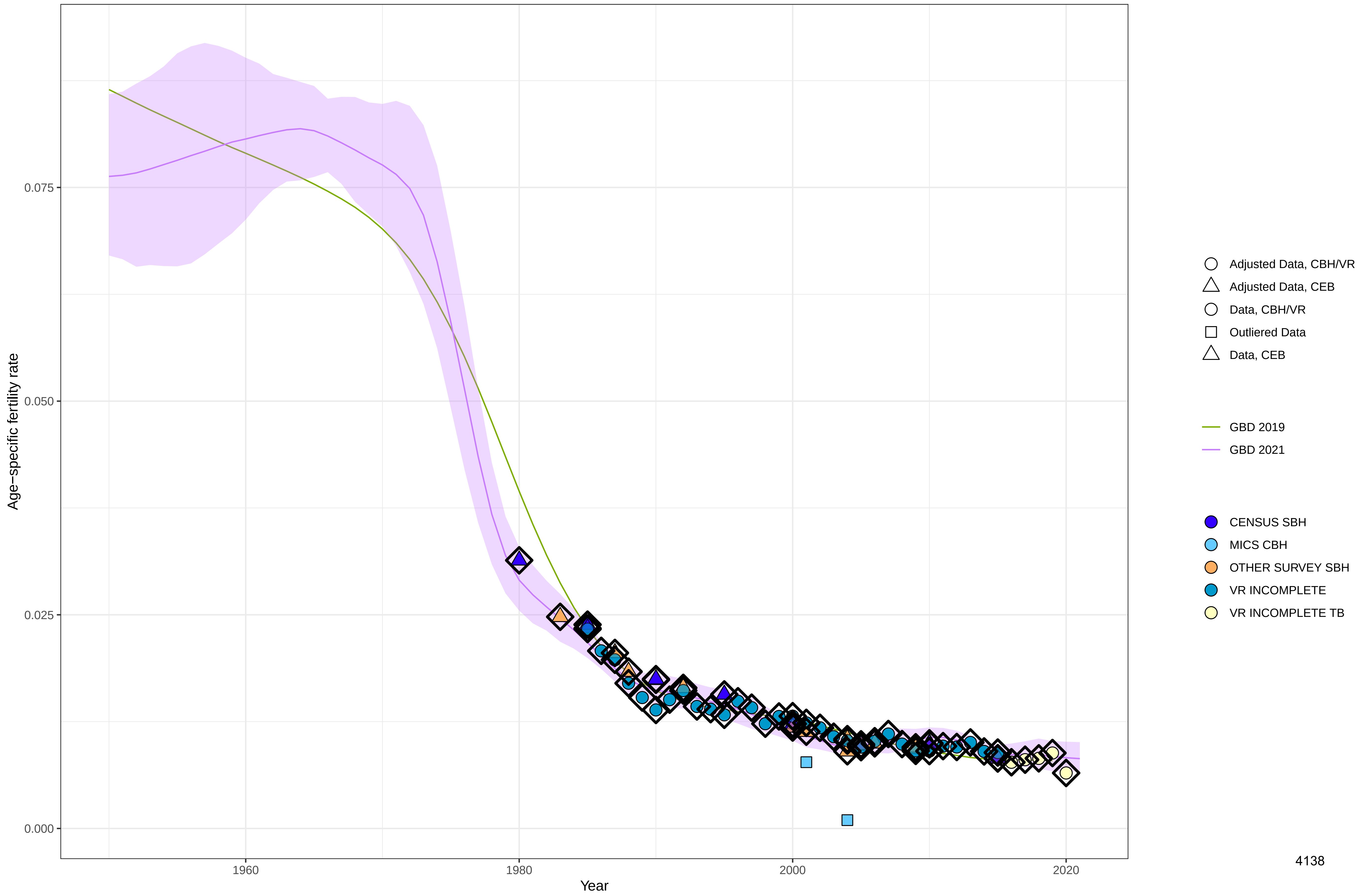

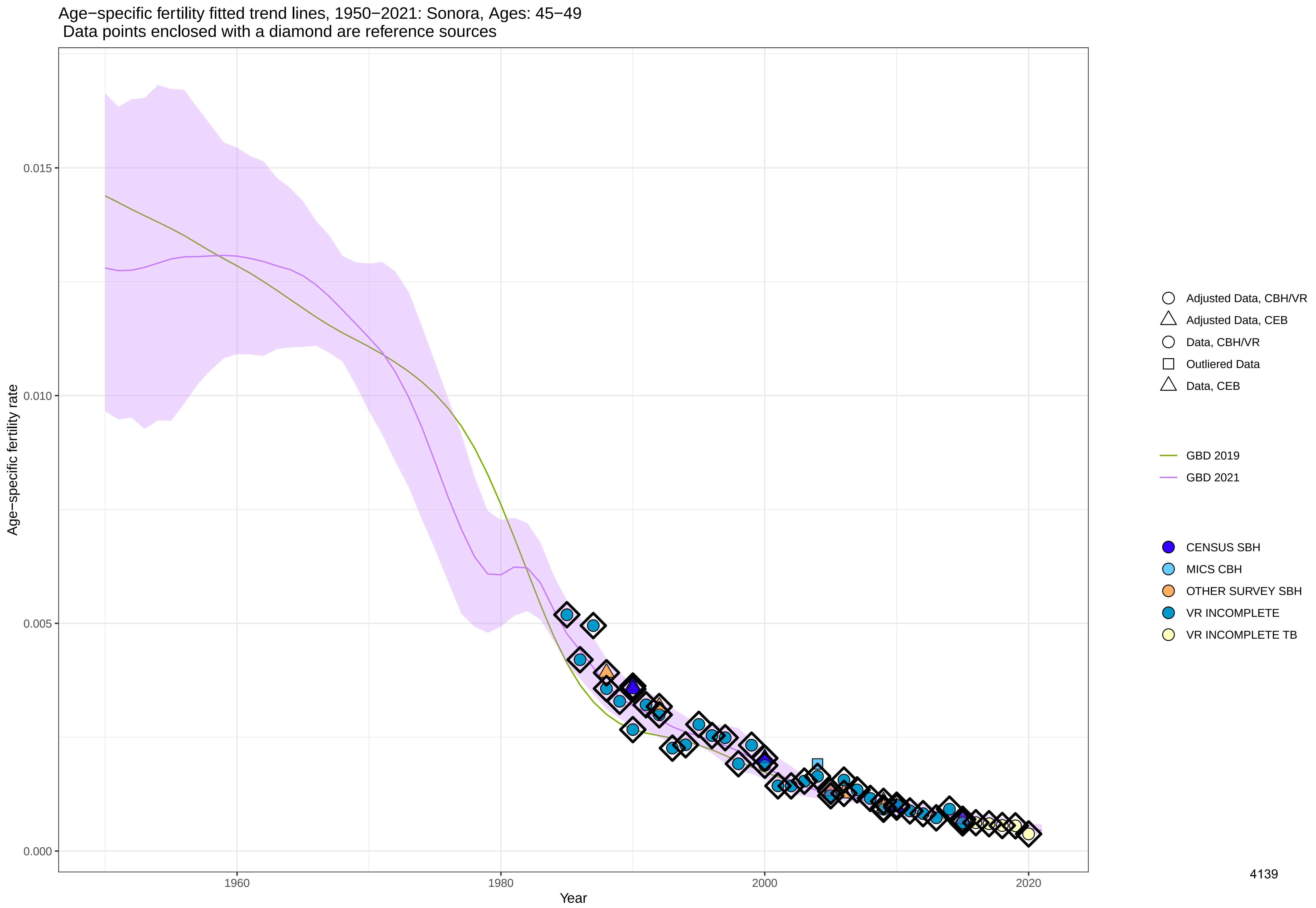

Age−specific fertility fitted trend lines, 1950−2021: Sonora, Ages: 45−49
Data points enclosed with a diamond are reference sources
Age−specific fertility rate
0.000
0.005
0.010
0.015
Year
1960
1980
2000
2020
Adjusted Data, CBH/VR
Adjusted Data, CEB
Data, CBH/VR
Outliered Data
Data, CEB
GBD 2019
GBD 2021
CENSUS SBH
MICS CBH
OTHER SURVEY SBH
VR INCOMPLETE
VR INCOMPLETE TB

Age−specific fertility fitted trend lines, 1950−2021: Sonora, Ages: 50−54
Data points enclosed with a diamond are reference sources

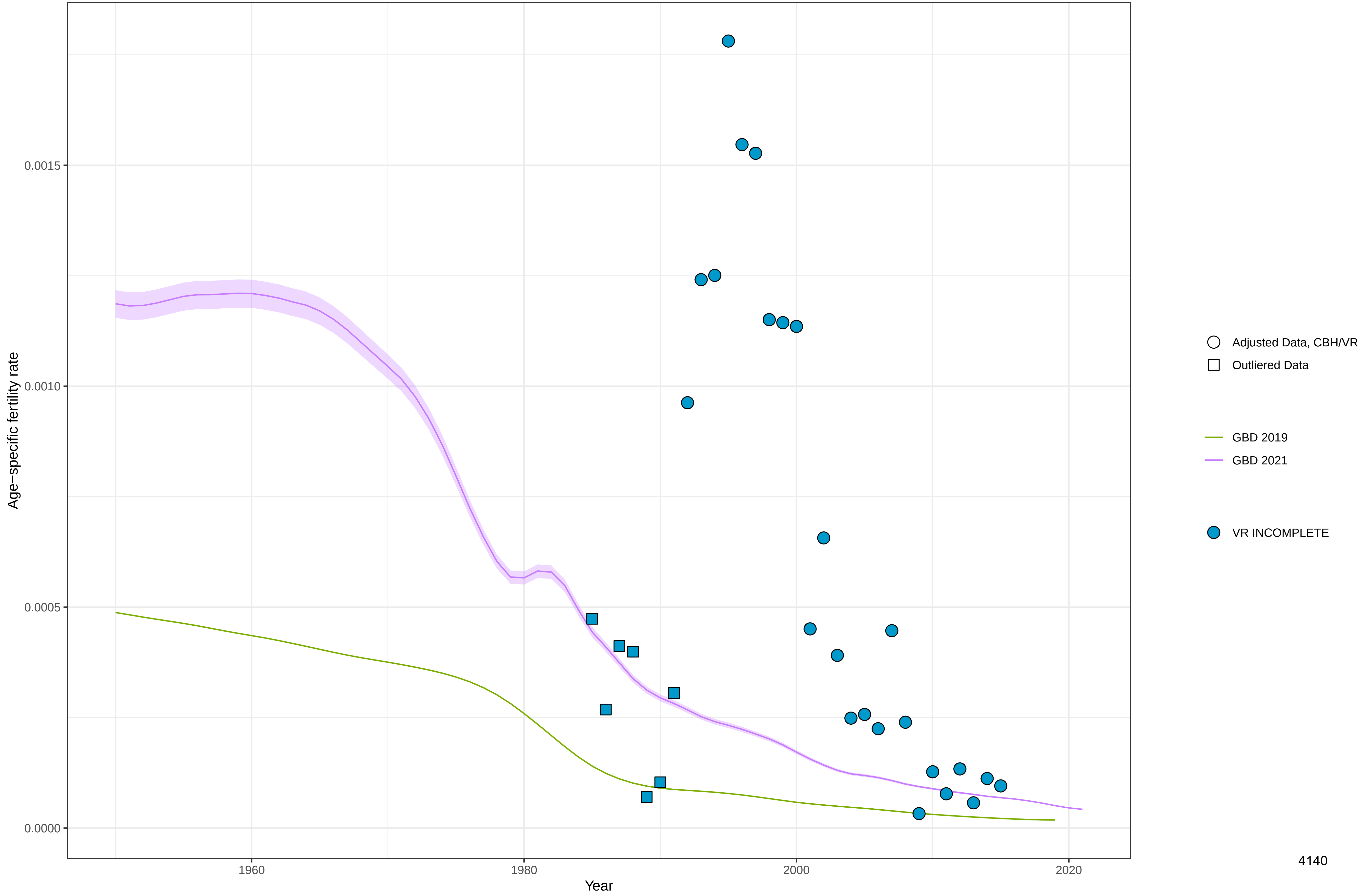

Total fertility rate fitted trend lines, 1950−2021: Sonora TFR
Data points enclosed with a diamond are reference sources

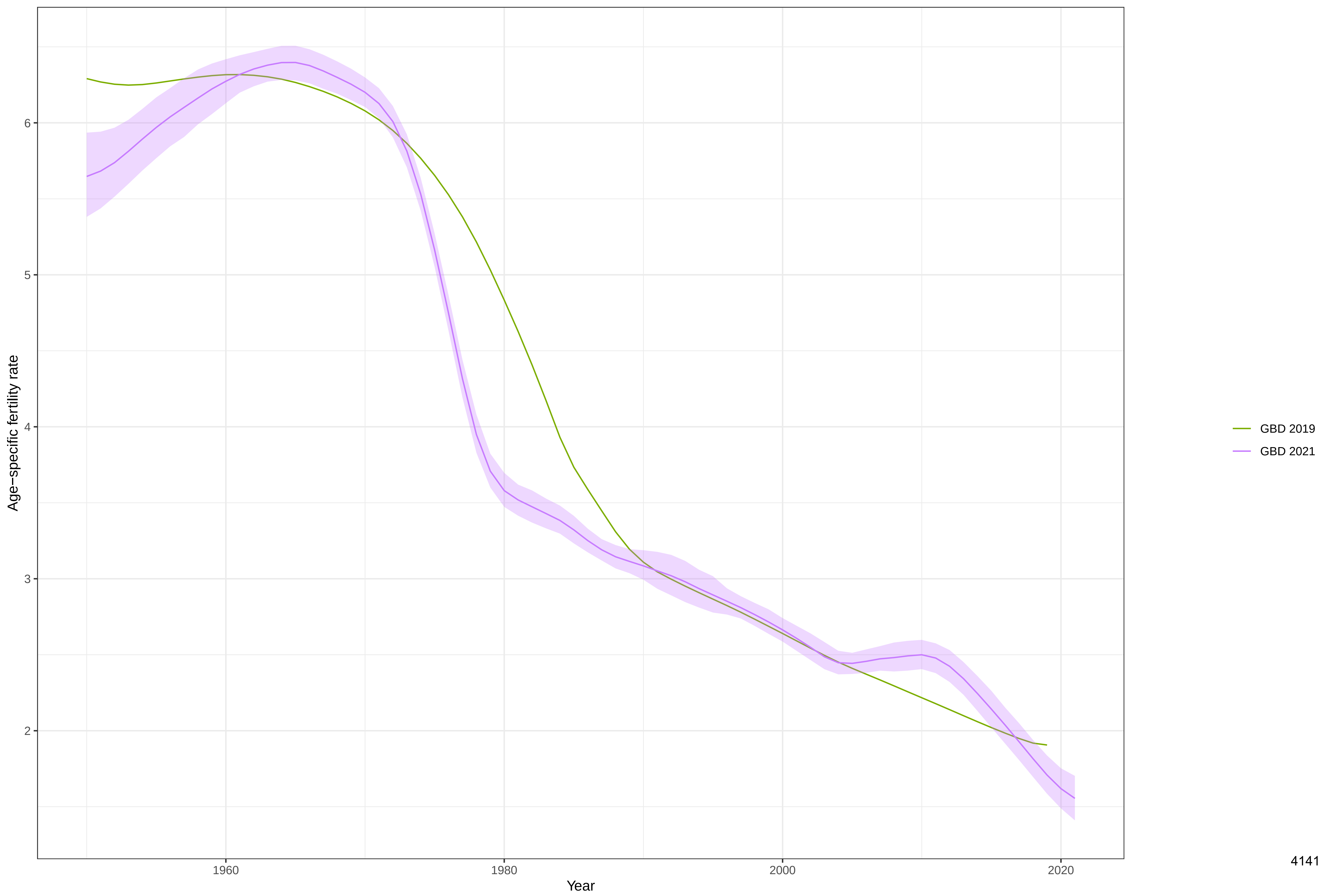

4141

Age−specific fertility fitted trend lines, 1950−2021: Tabasco, Ages: 10−14
Data points enclosed with a diamond are reference sources

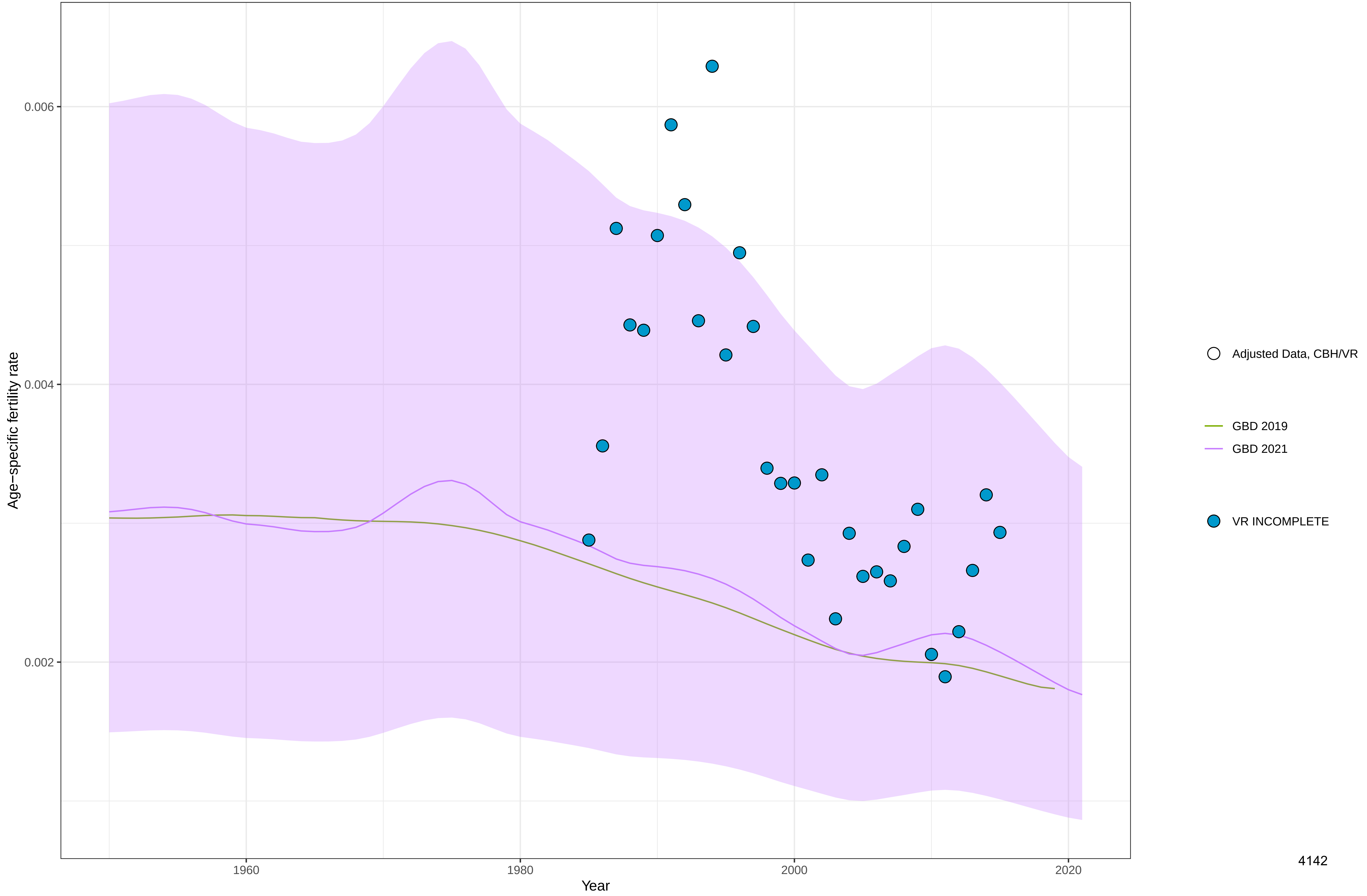

Age−specific fertility fitted trend lines, 1950−2021: Tabasco, Ages: 15−19
Data points enclosed with a diamond are reference sources

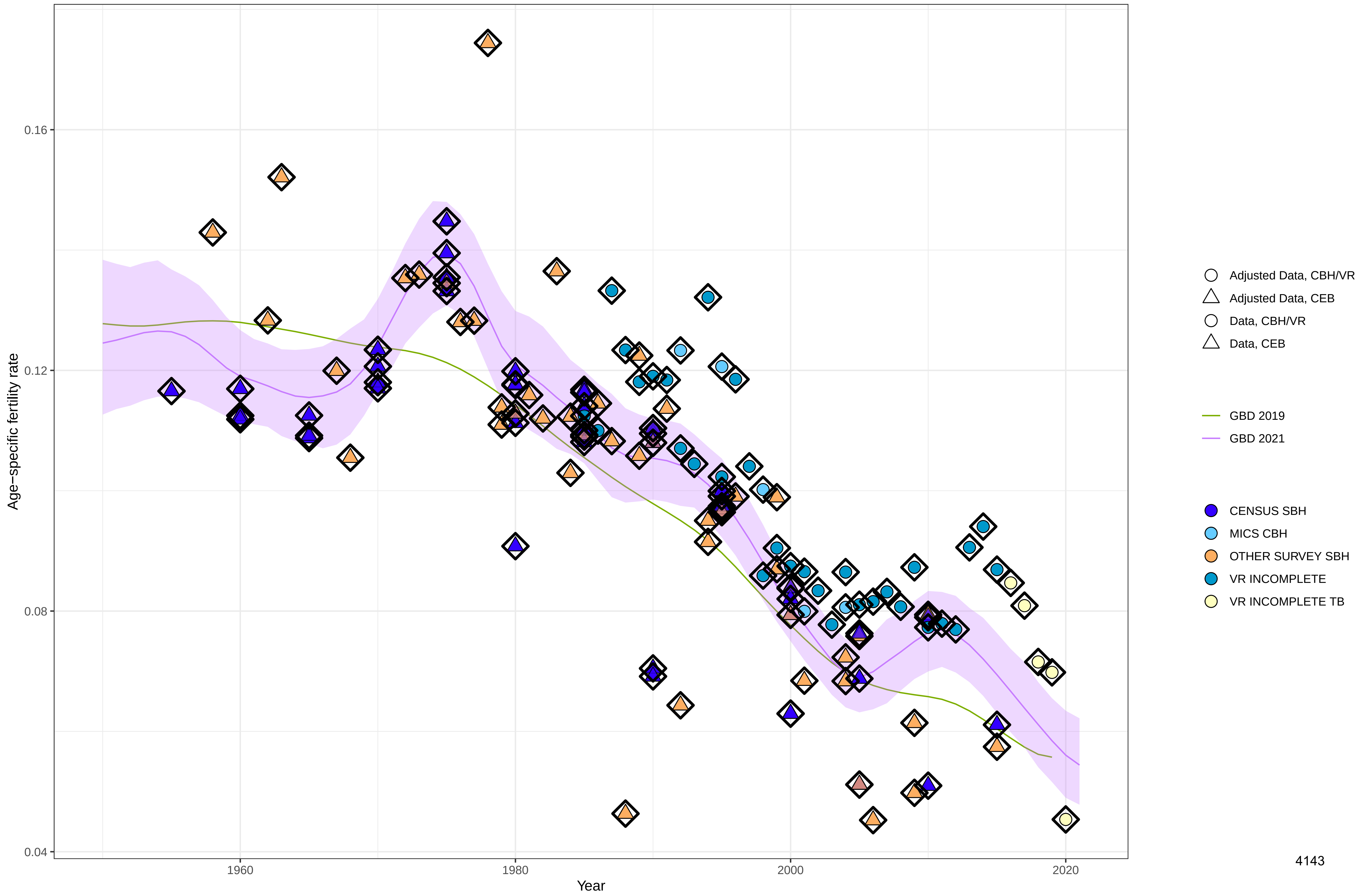

Age−specific fertility fitted trend lines, 1950−2021: Tabasco, Ages: 20−24
Data points enclosed with a diamond are reference sources

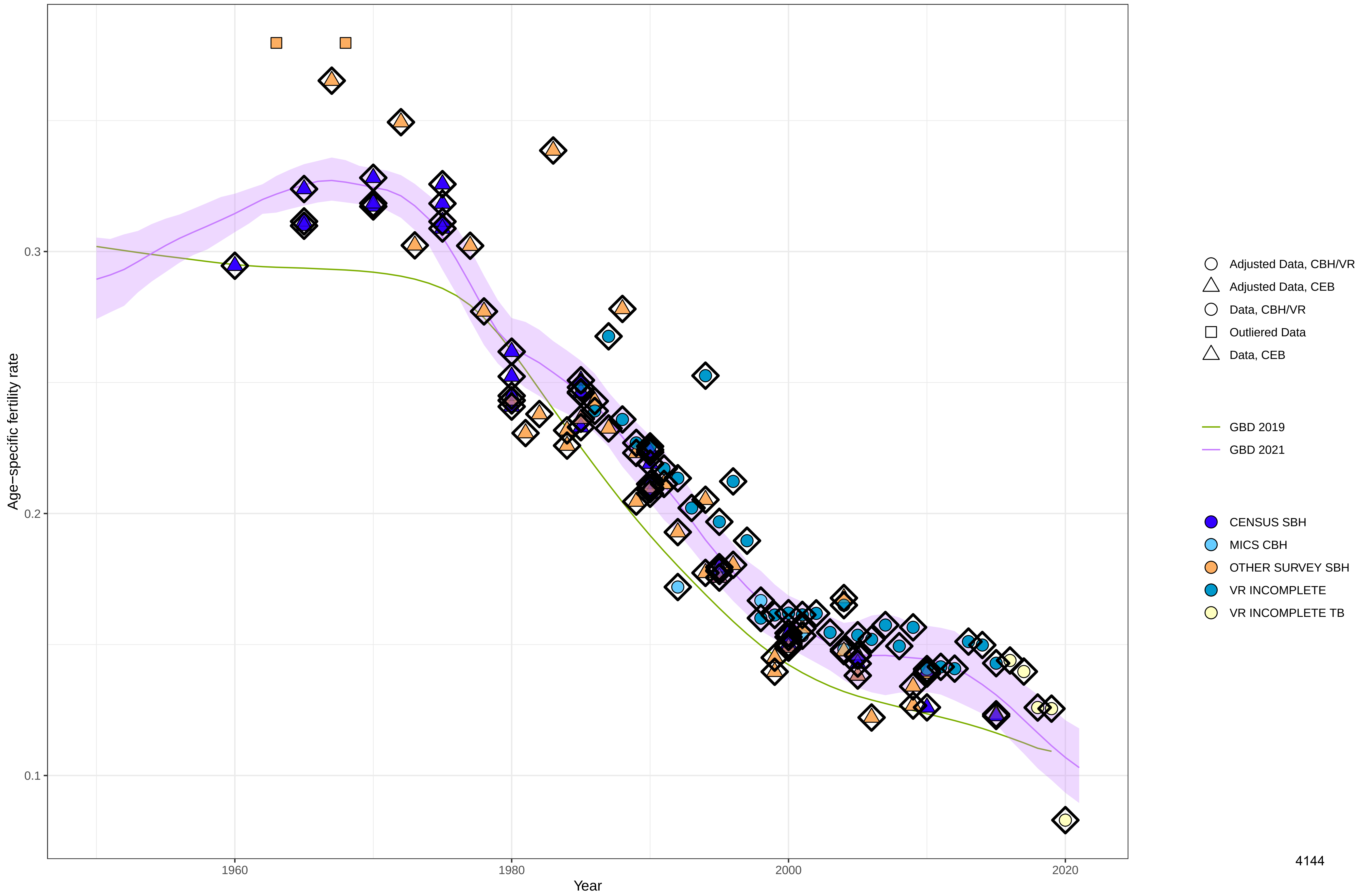

Age−specific fertility fitted trend lines, 1950−2021: Tabasco, Ages: 25−29
Data points enclosed with a diamond are reference sources

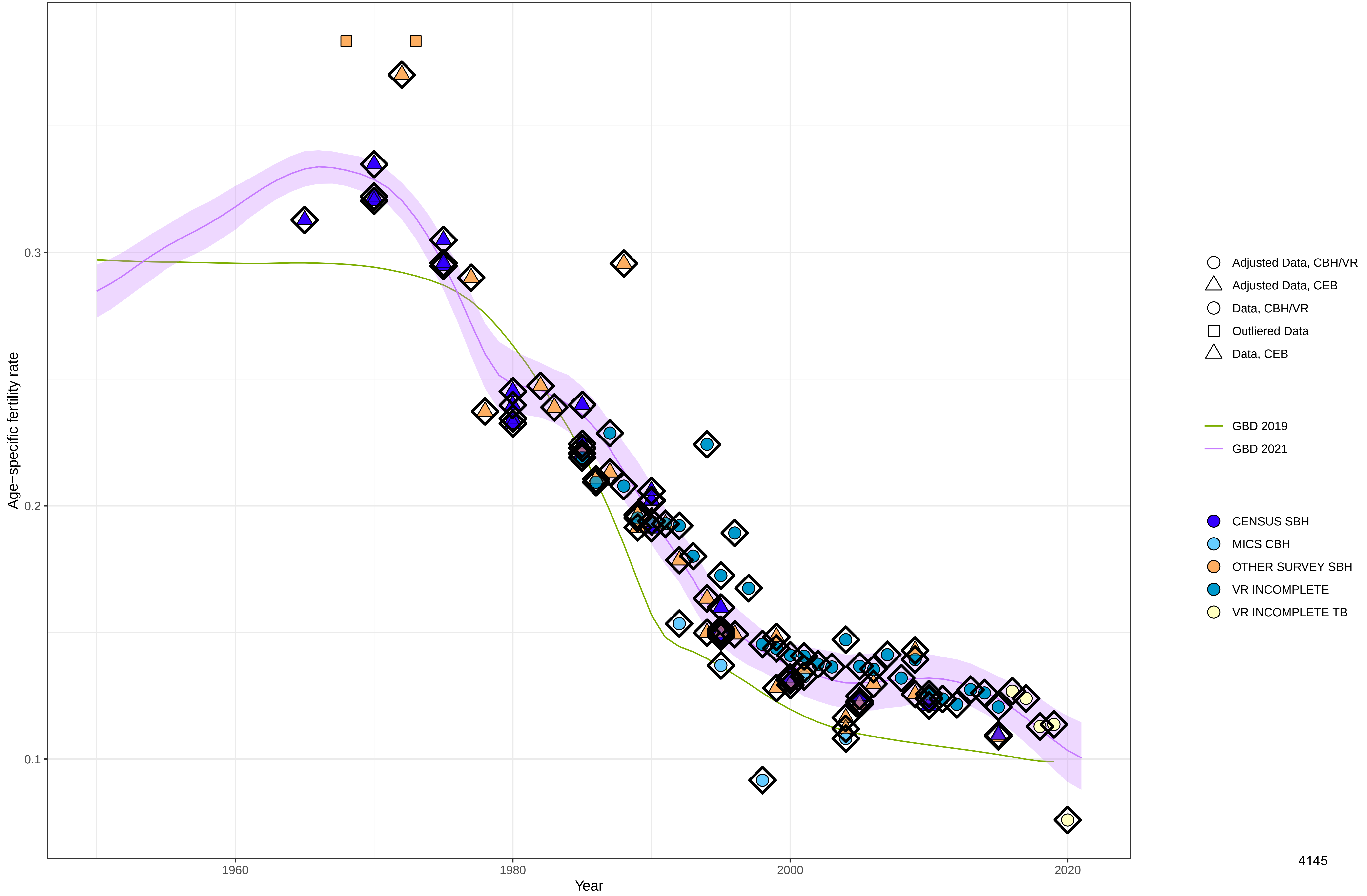

4145

Age−specific fertility fitted trend lines, 1950−2021: Tabasco, Ages: 30−34
Data points enclosed with a diamond are reference sources

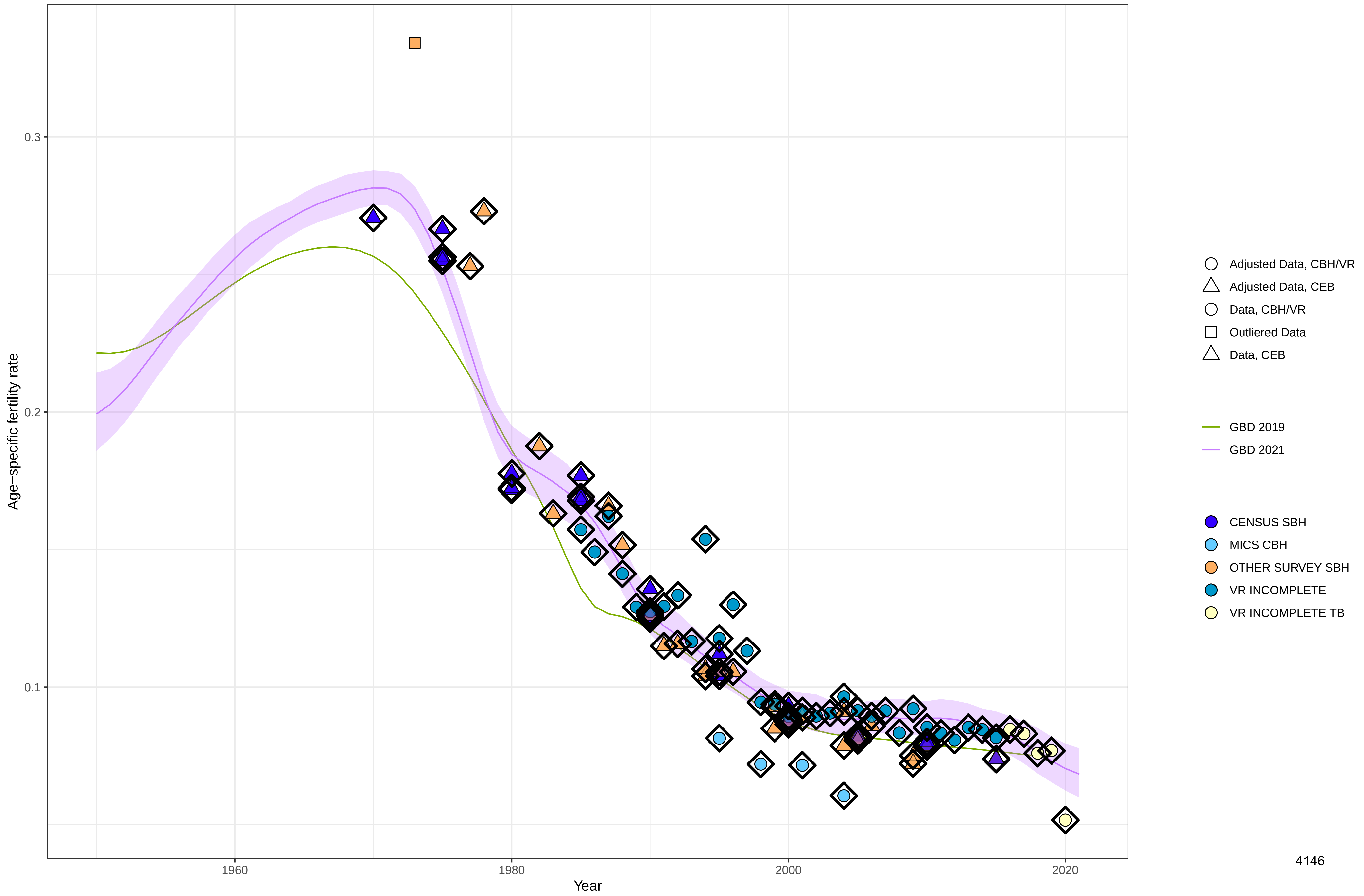

Age−specific fertility fitted trend lines, 1950−2021: Tabasco, Ages: 35−39
Data points enclosed with a diamond are reference sources

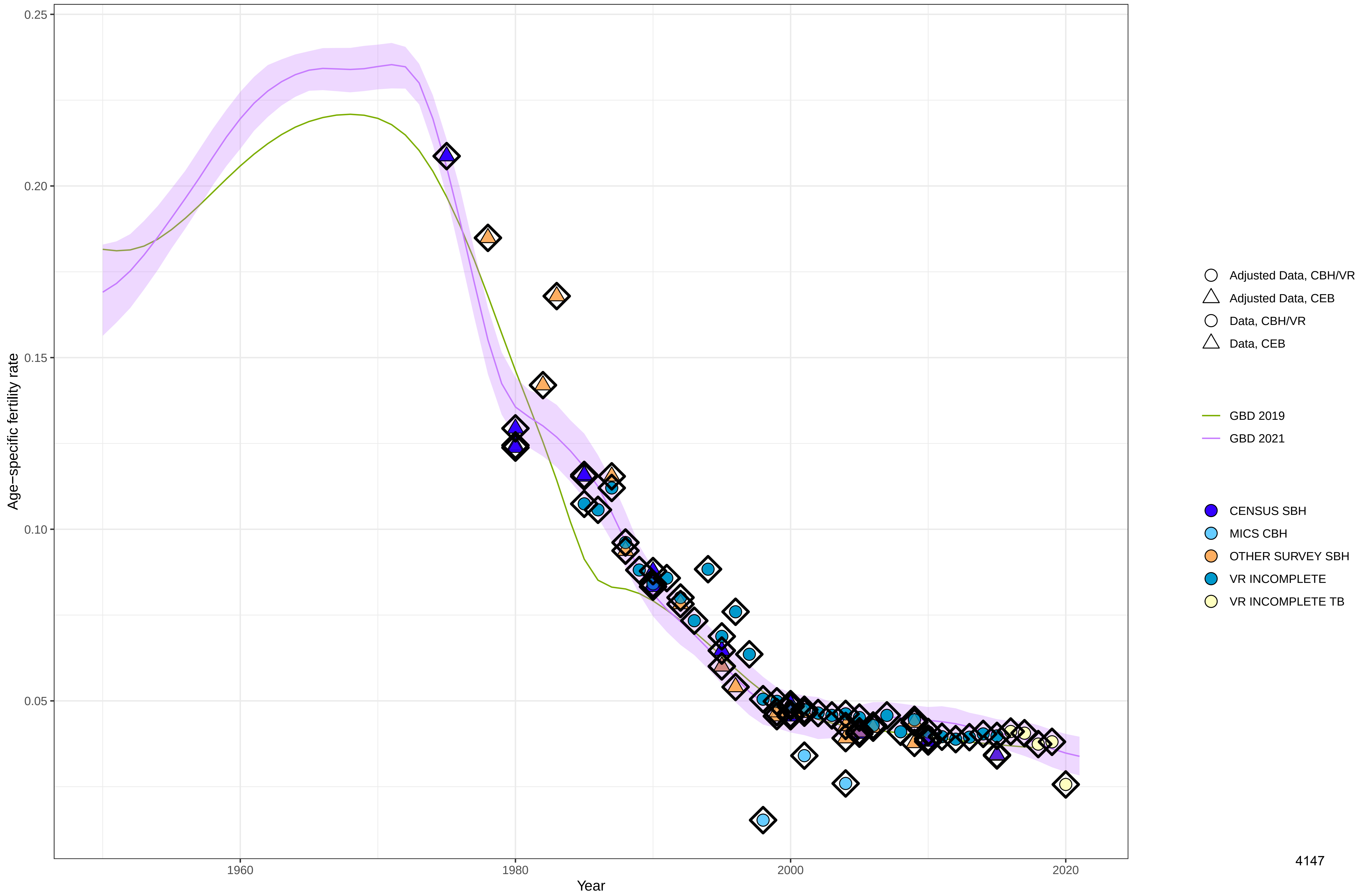

Age−specific fertility fitted trend lines, 1950−2021: Tabasco, Ages: 40−44
Data points enclosed with a diamond are reference sources

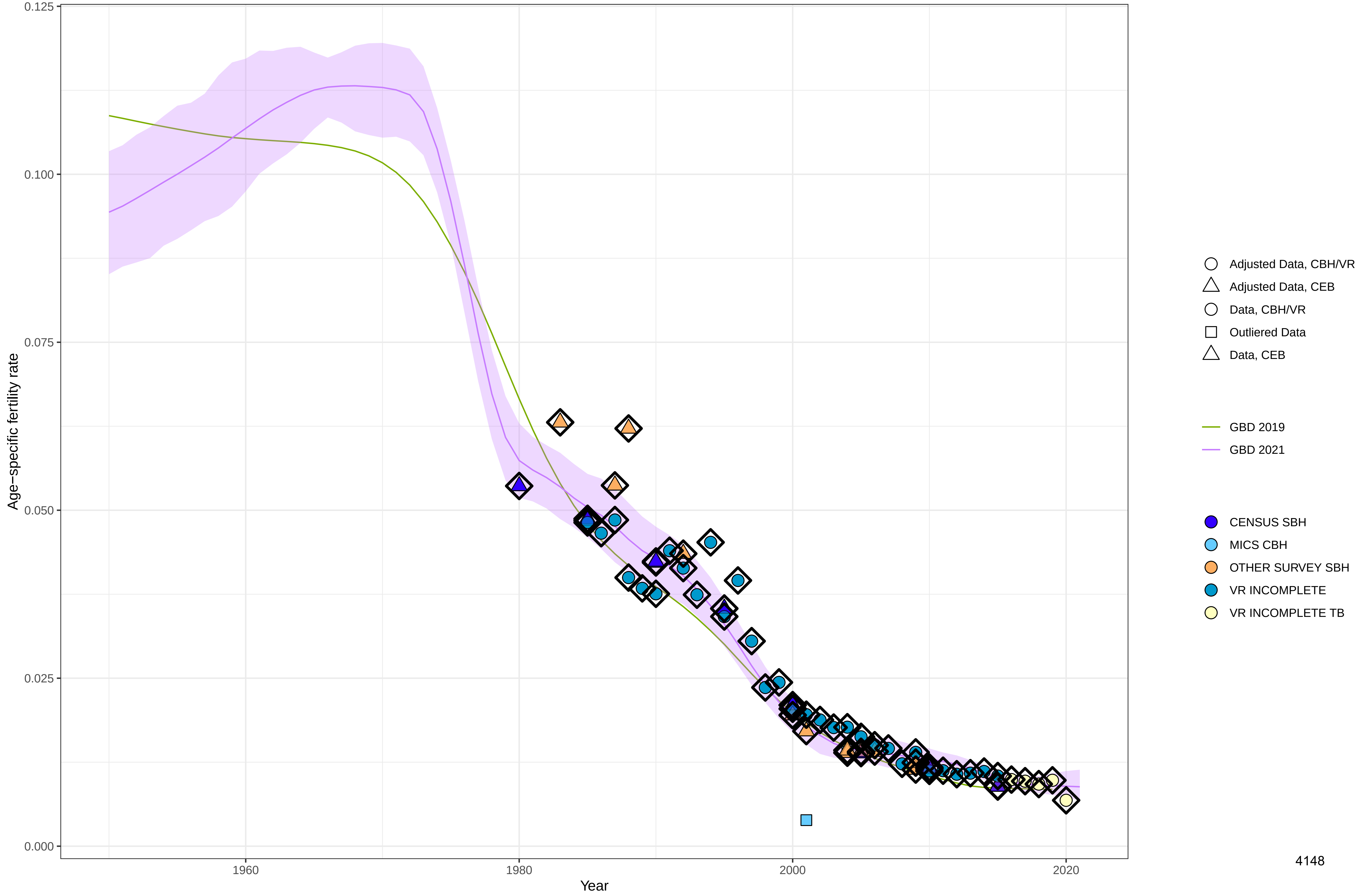

Age−specific fertility fitted trend lines, 1950−2021: Tabasco, Ages: 45−49
Data points enclosed with a diamond are reference sources

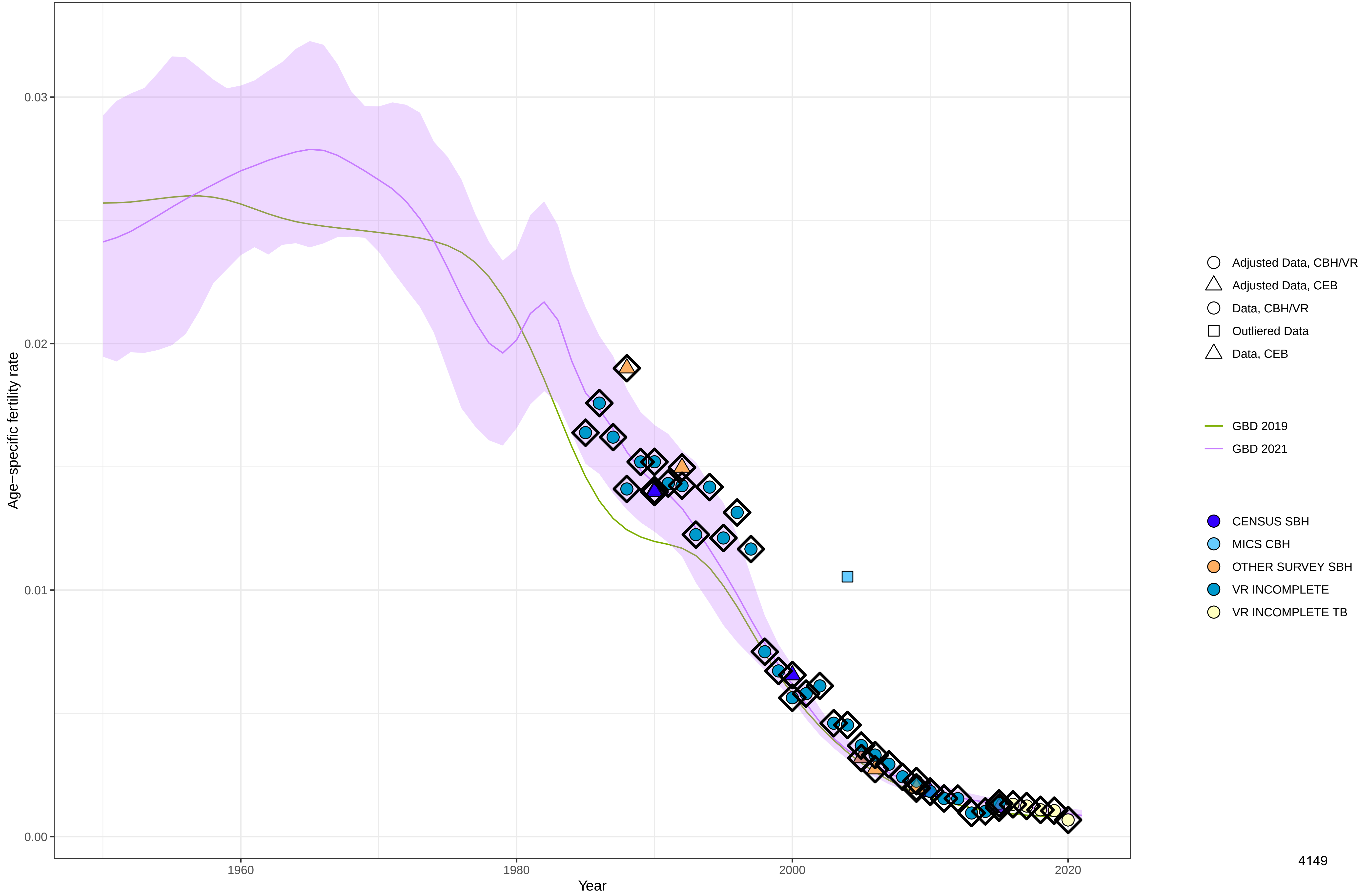

Age−specific fertility fitted trend lines, 1950−2021: Tabasco, Ages: 50−54
Data points enclosed with a diamond are reference sources

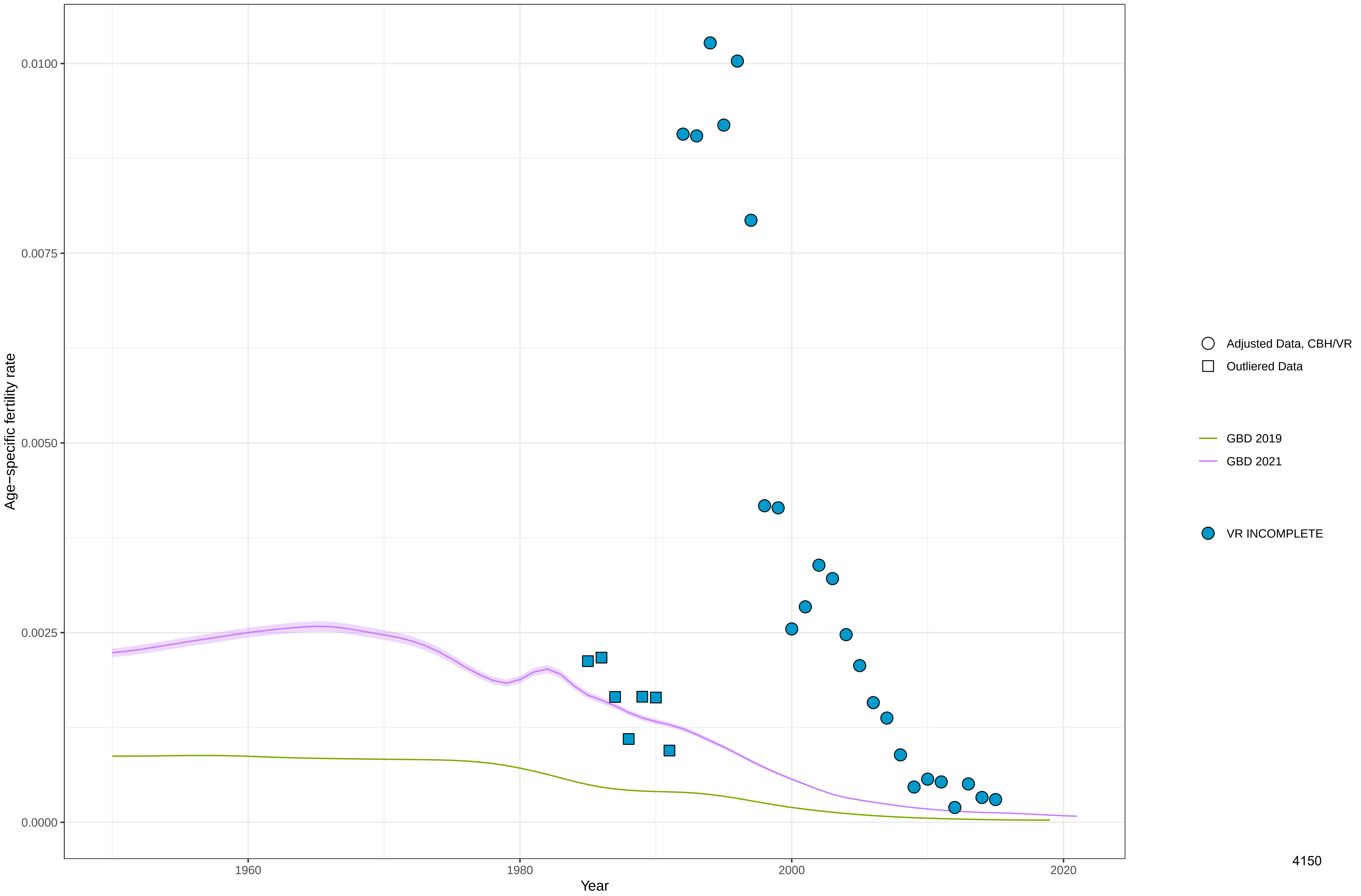

Total fertility rate fitted trend lines, 1950−2021: Tabasco TFR
Data points enclosed with a diamond are reference sources

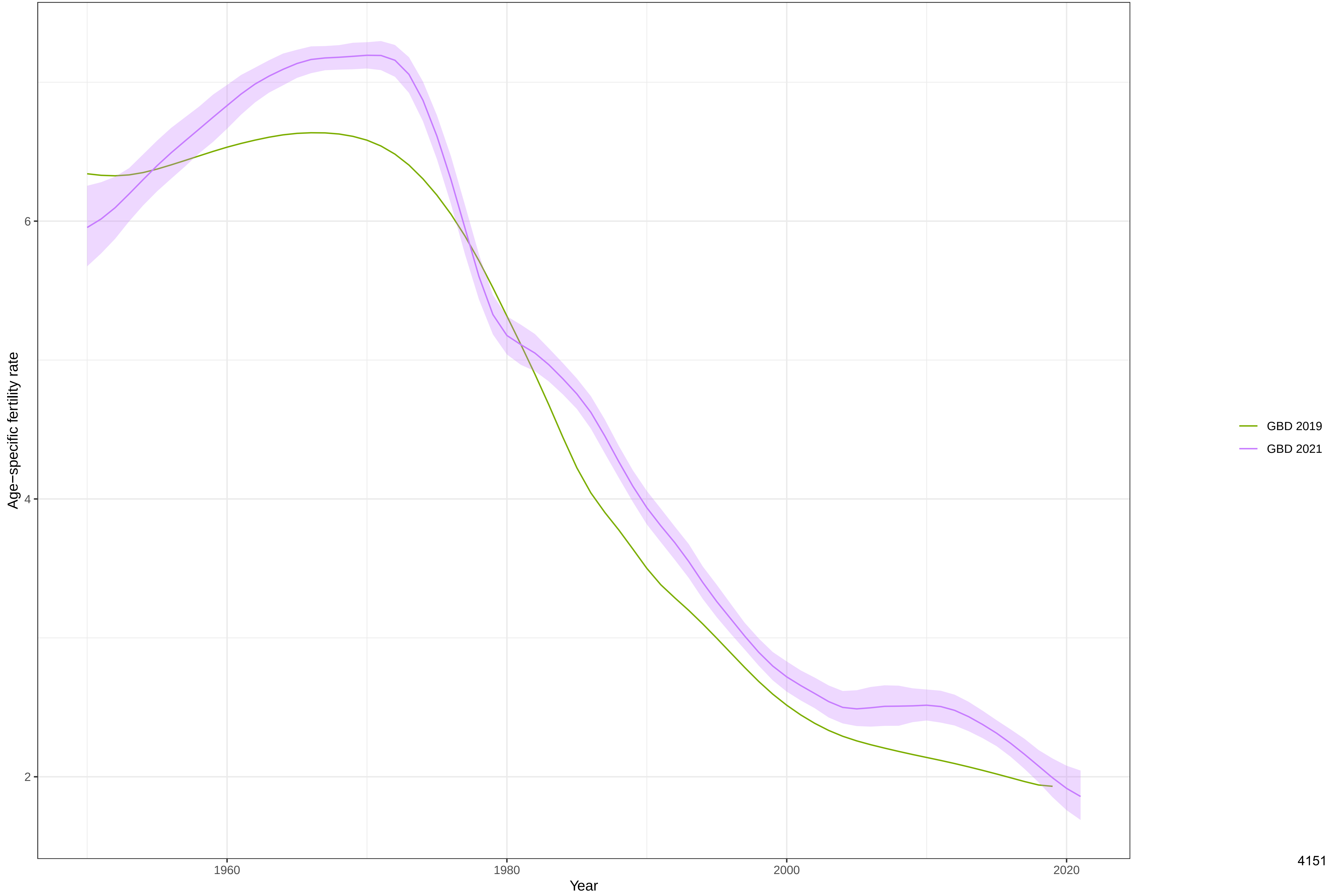

Age−specific fertility fitted trend lines, 1950−2021: Tamaulipas, Ages: 10−14
Data points enclosed with a diamond are reference sources

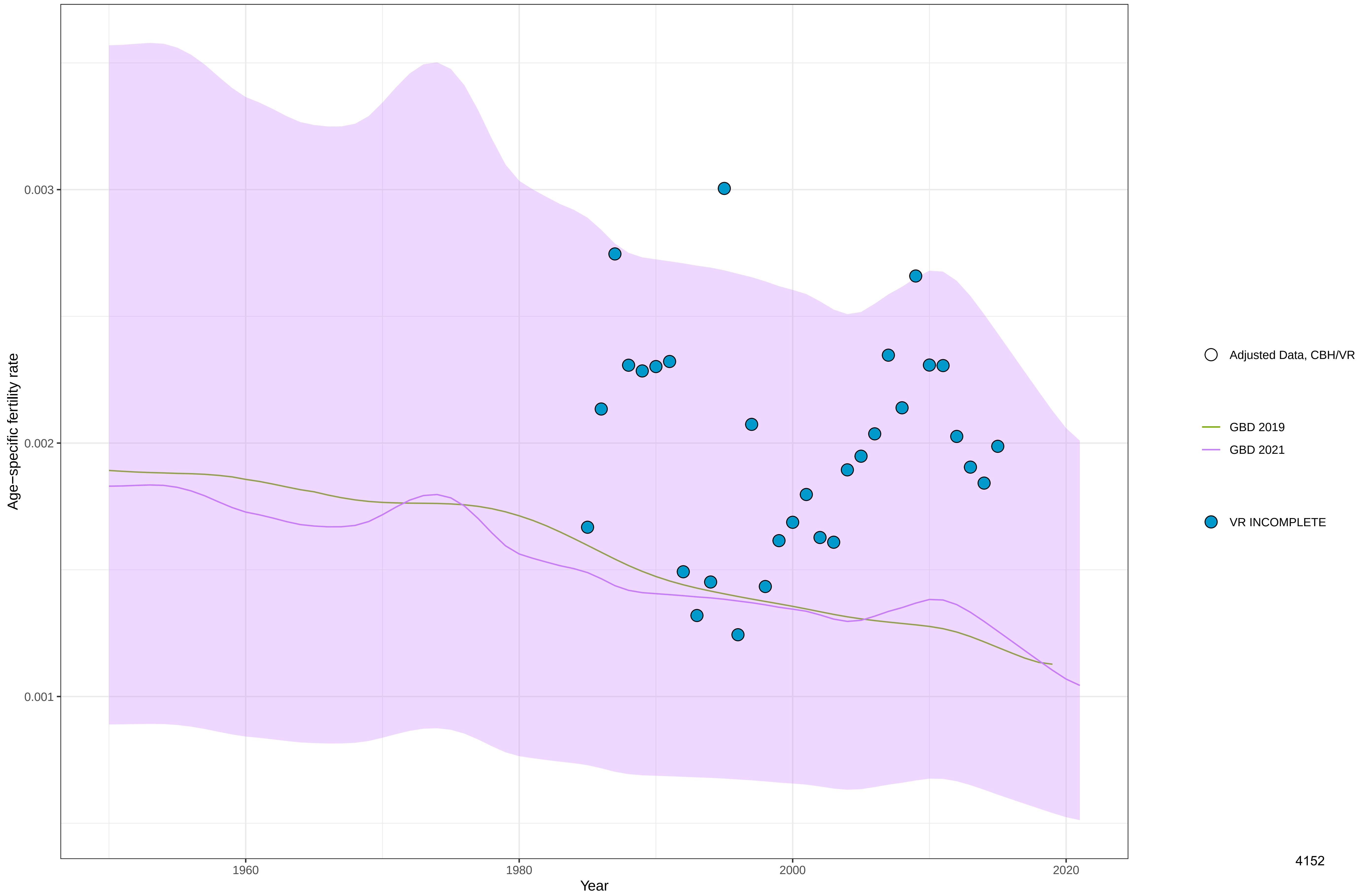

Age−specific fertility fitted trend lines, 1950−2021: Tamaulipas, Ages: 15−19
Data points enclosed with a diamond are reference sources

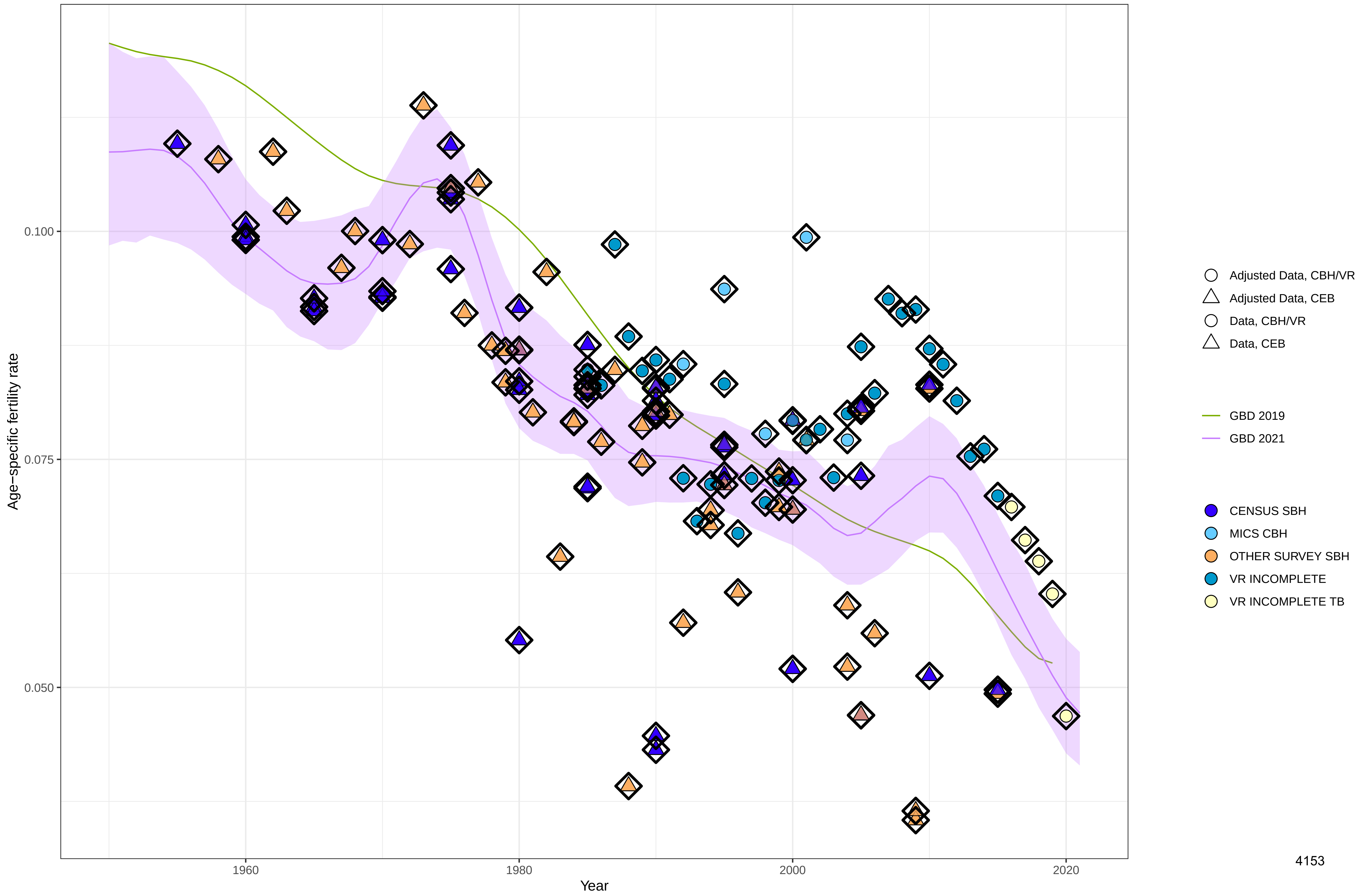

Age−specific fertility fitted trend lines, 1950−2021: Tamaulipas, Ages: 20−24
Data points enclosed with a diamond are reference sources

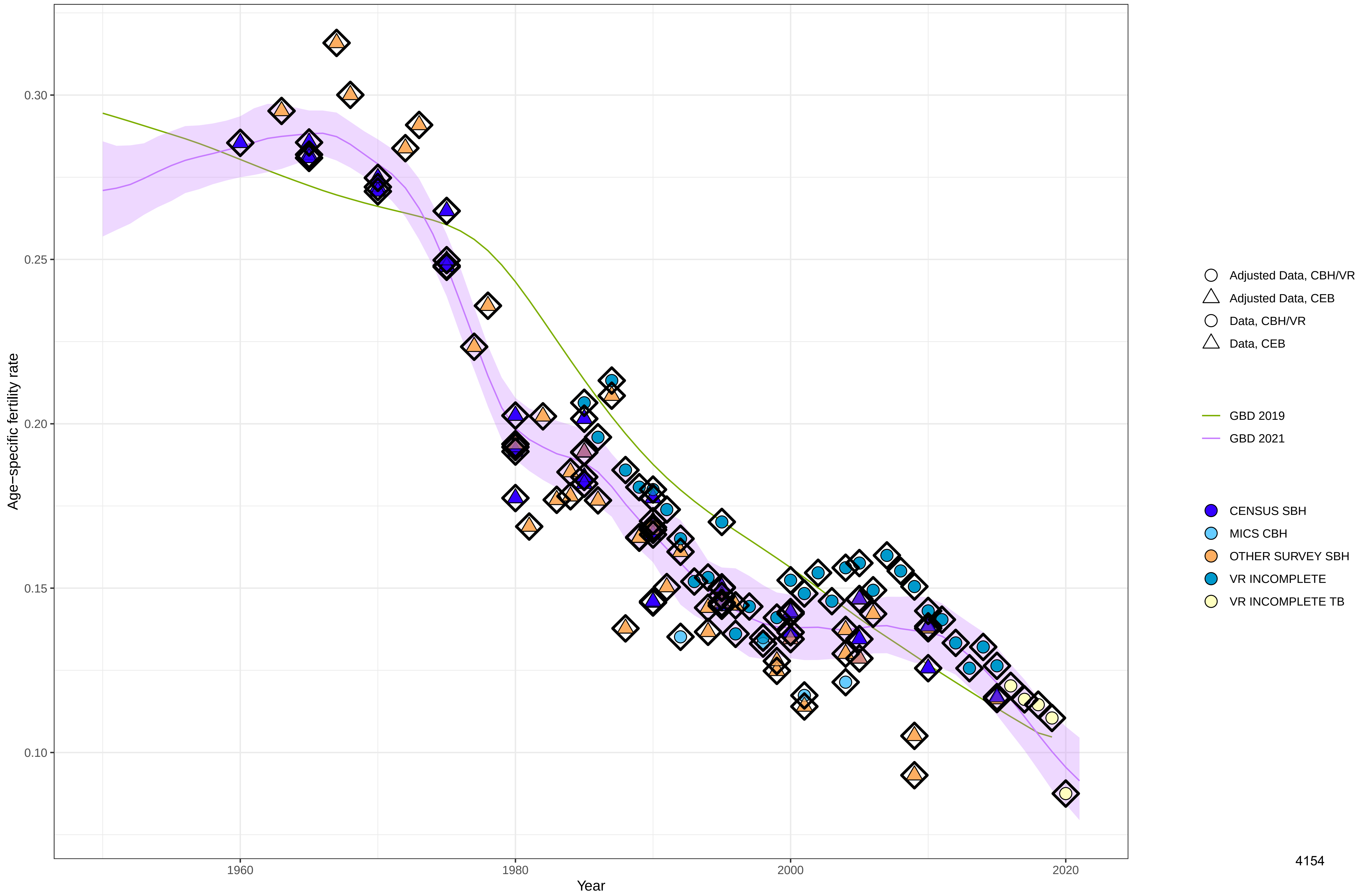

Age−specific fertility fitted trend lines, 1950−2021: Tamaulipas, Ages: 25−29
Data points enclosed with a diamond are reference sources

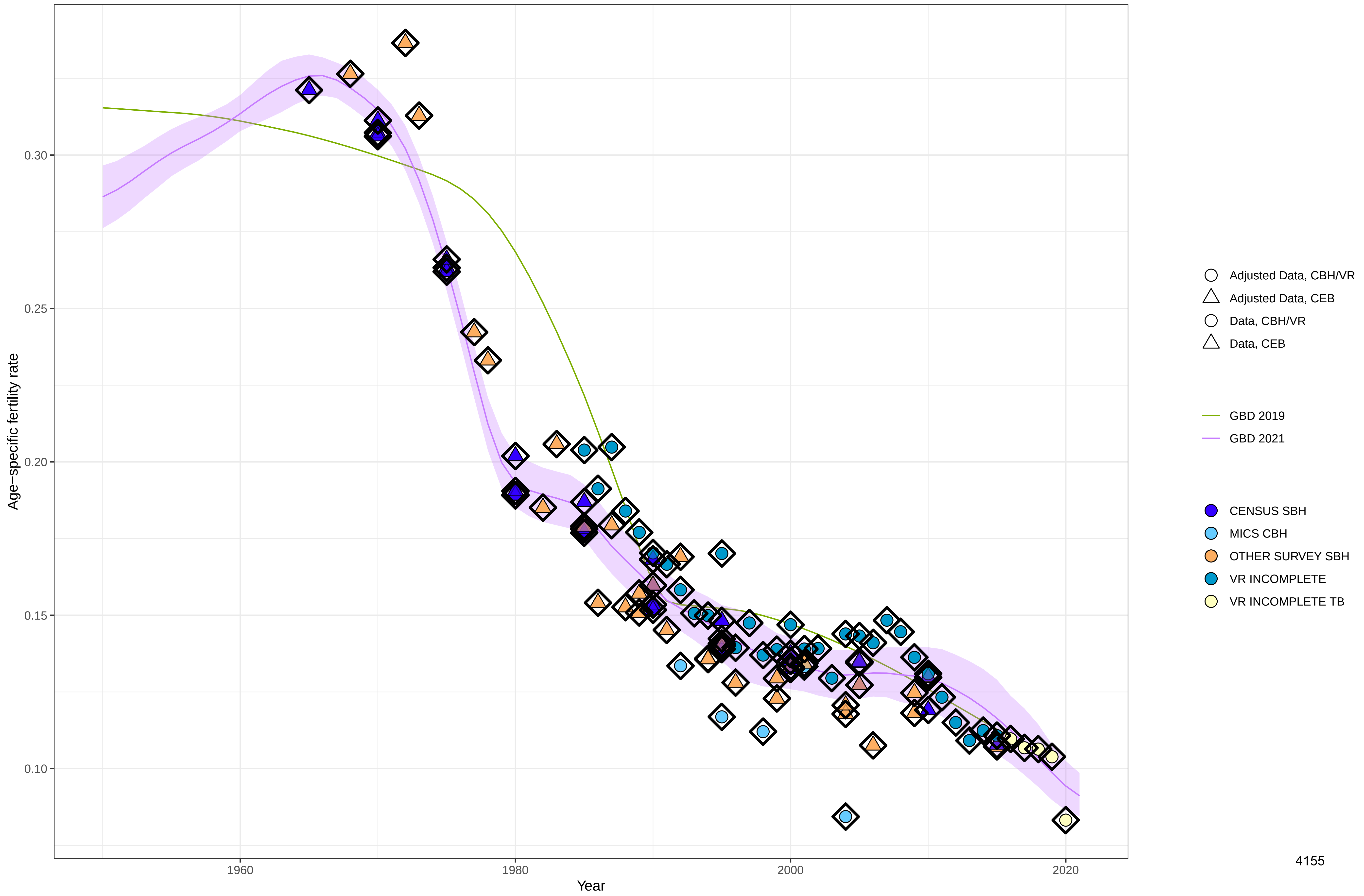

Age−specific fertility fitted trend lines, 1950−2021: Tamaulipas, Ages: 30−34
Data points enclosed with a diamond are reference sources

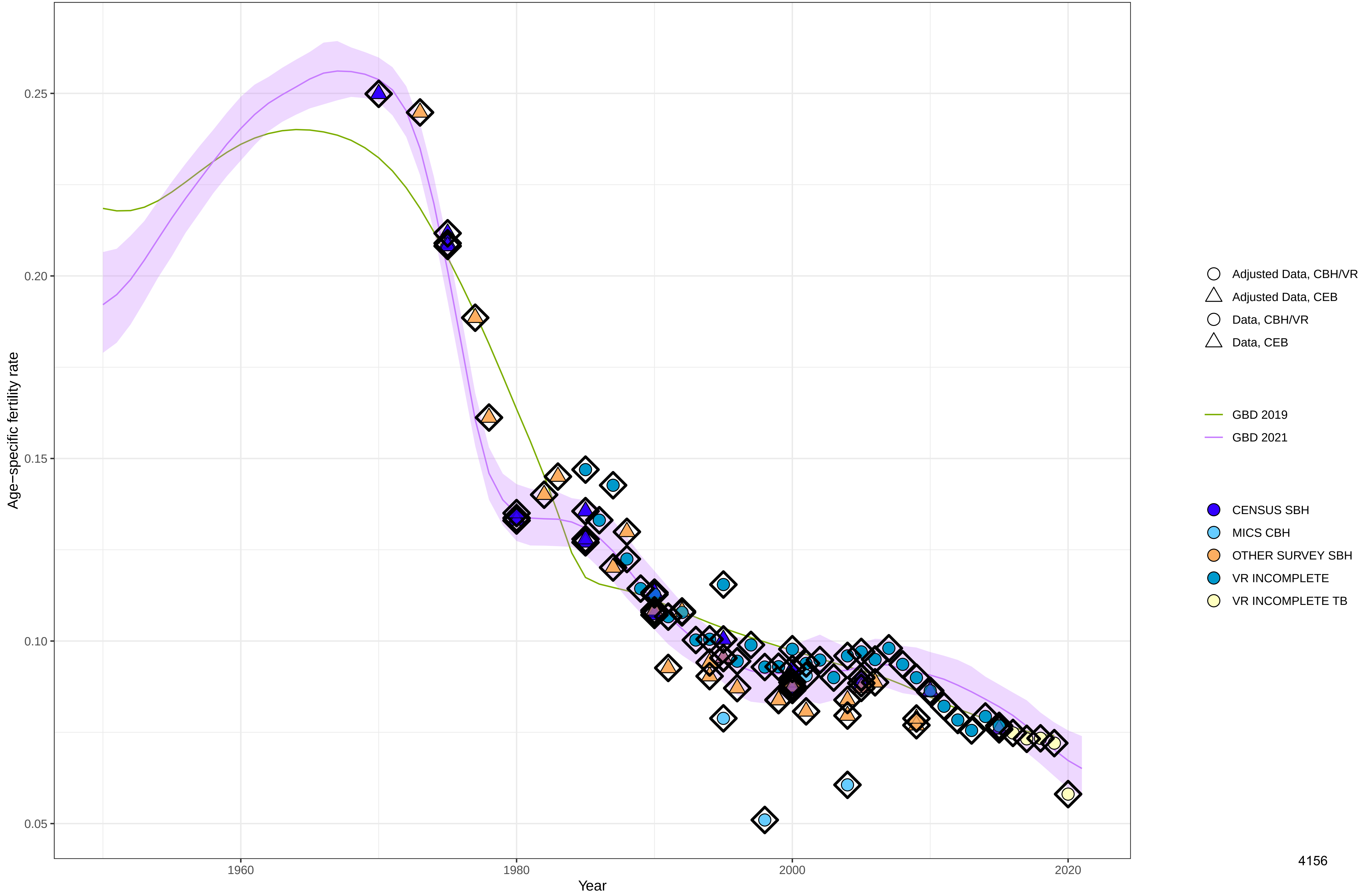

Age−specific fertility fitted trend lines, 1950−2021: Tamaulipas, Ages: 35−39
Data points enclosed with a diamond are reference sources

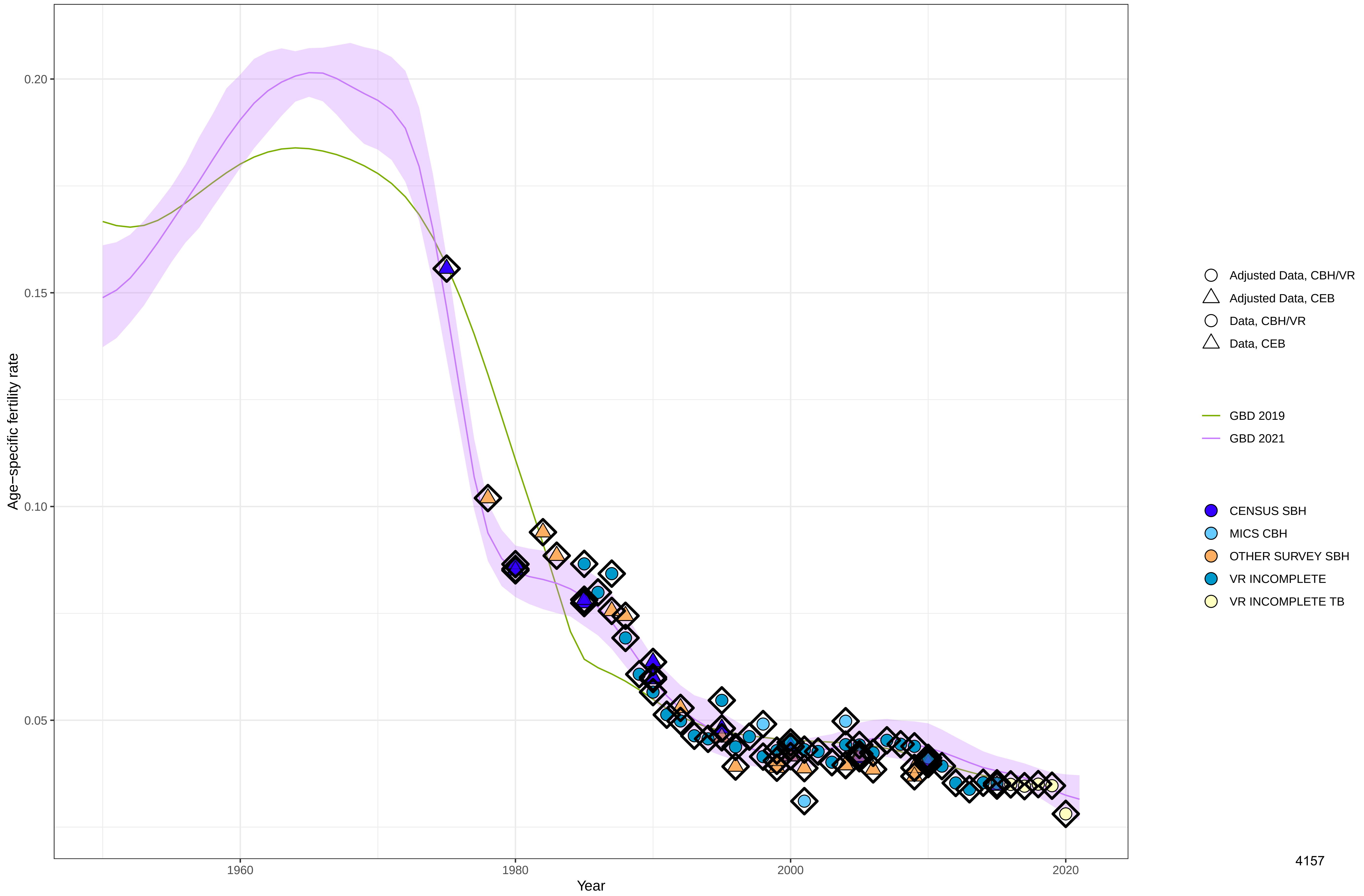

Age−specific fertility fitted trend lines, 1950−2021: Tamaulipas, Ages: 40−44
Data points enclosed with a diamond are reference sources

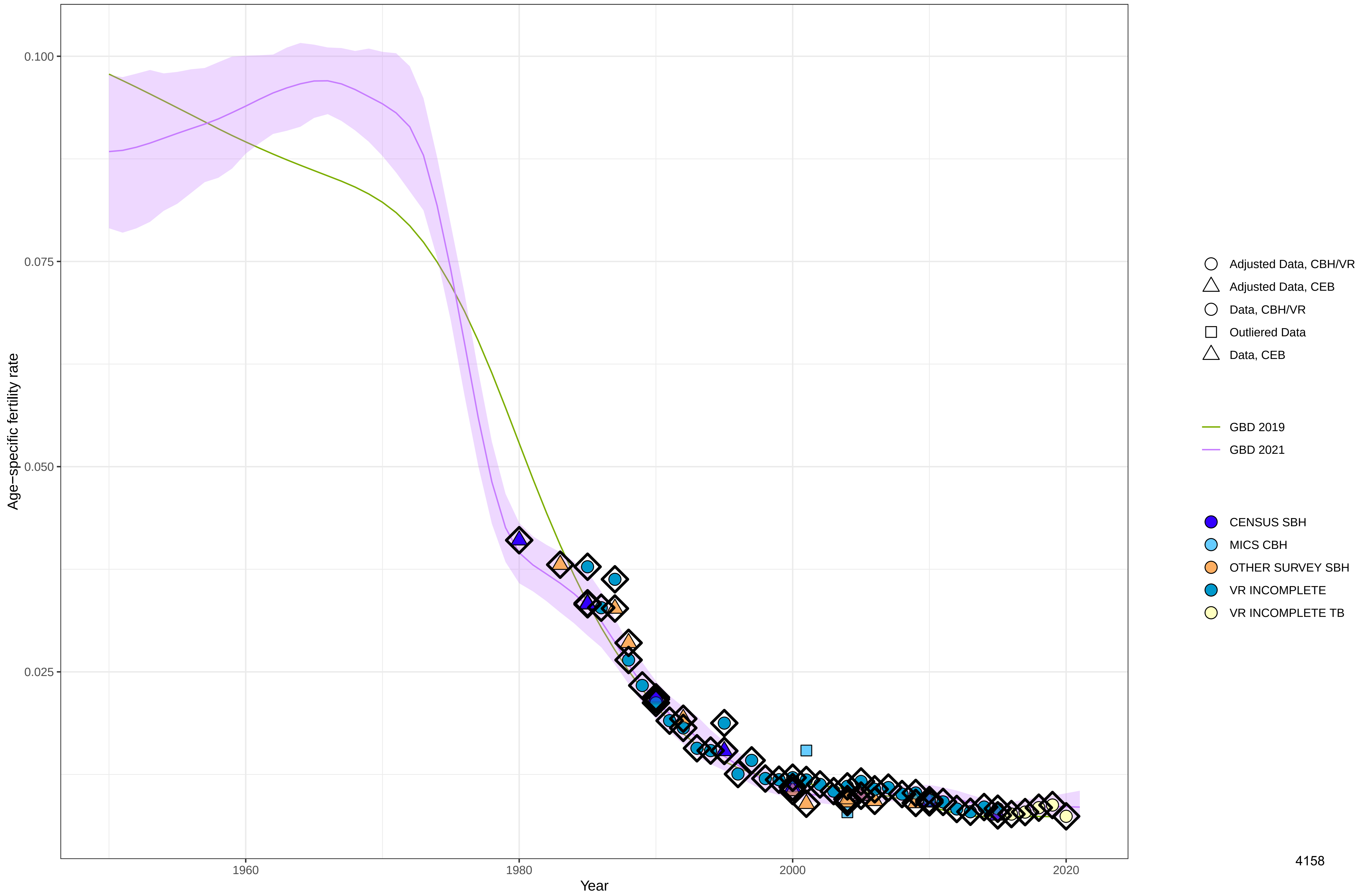

Age−specific fertility fitted trend lines, 1950−2021: Tamaulipas, Ages: 45−49
Data points enclosed with a diamond are reference sources

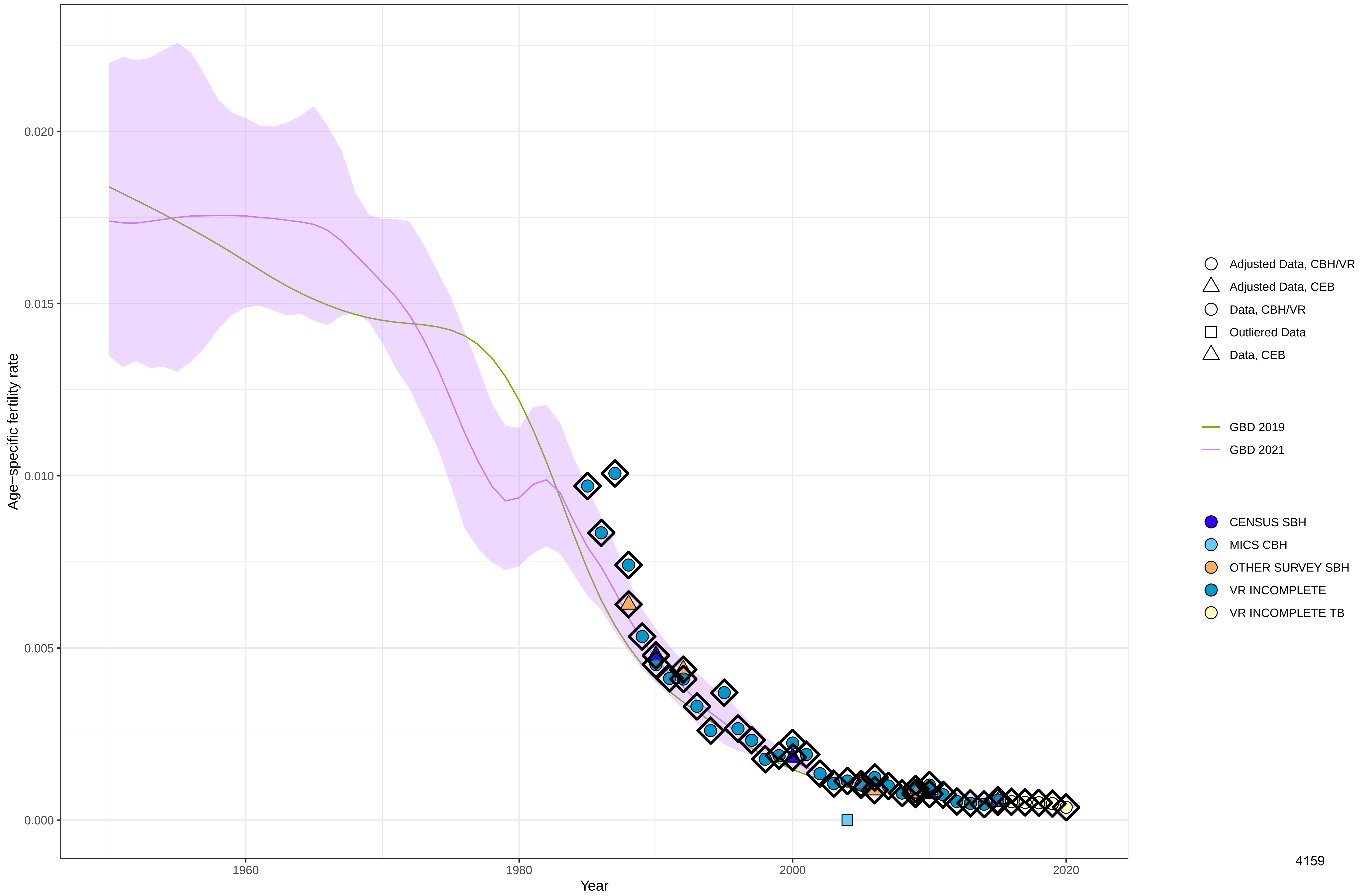

Age−specific fertility fitted trend lines, 1950−2021: Tamaulipas, Ages: 50−54
Data points enclosed with a diamond are reference sources

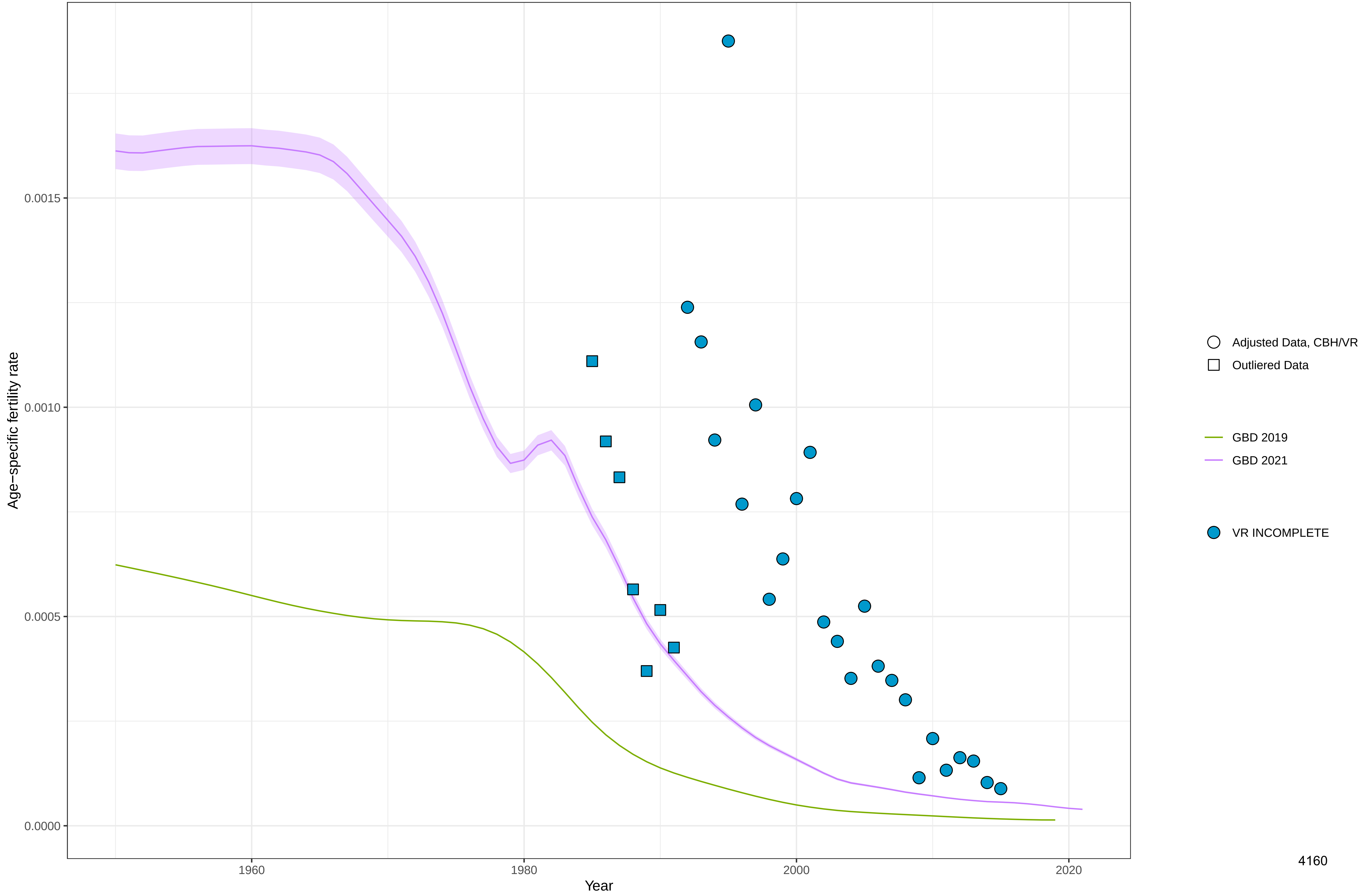

Total fertility rate fitted trend lines, 1950−2021: Tamaulipas TFR
Data points enclosed with a diamond are reference sources

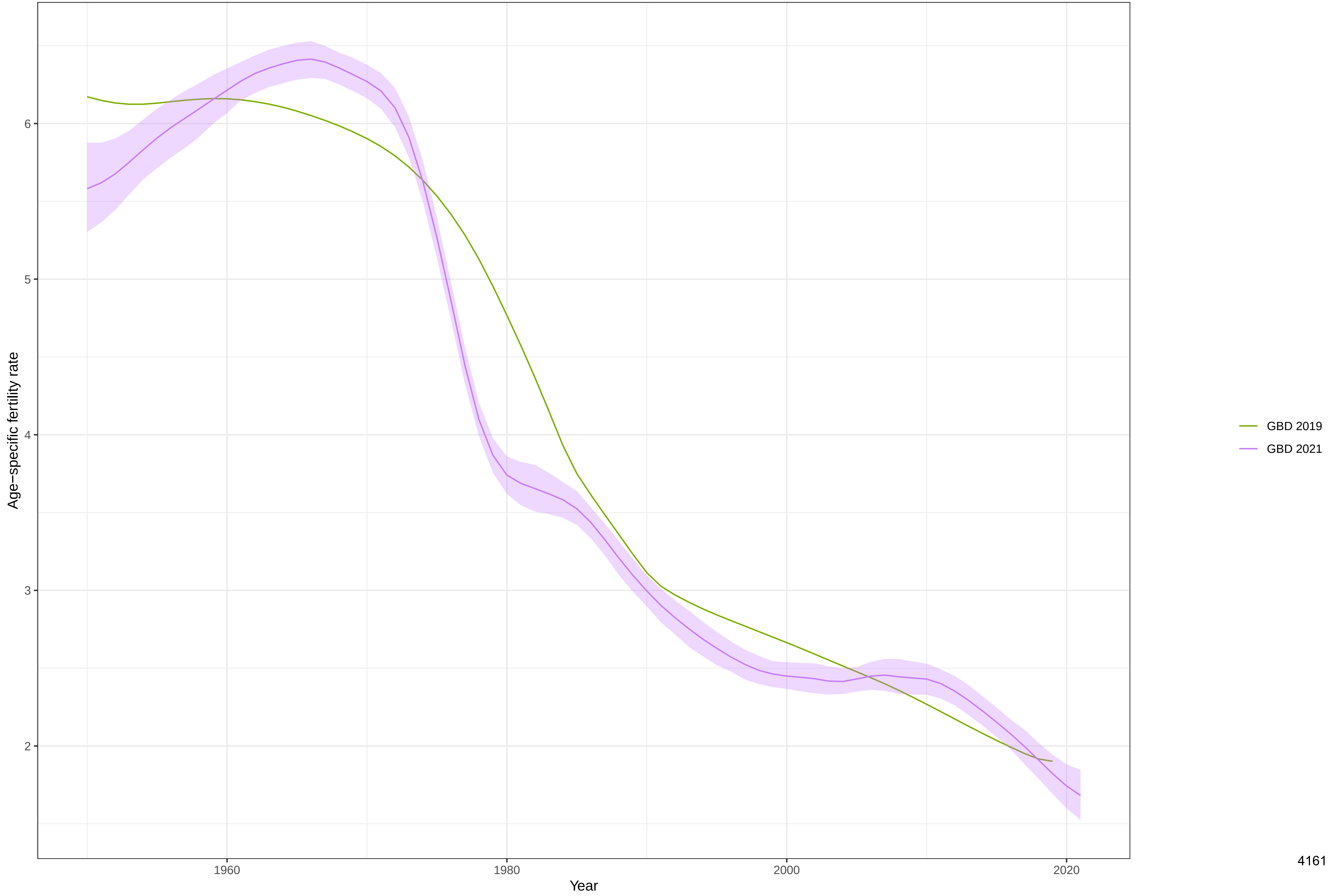

Age−specific fertility fitted trend lines, 1950−2021: Tlaxcala, Ages: 10−14
Data points enclosed with a diamond are reference sources

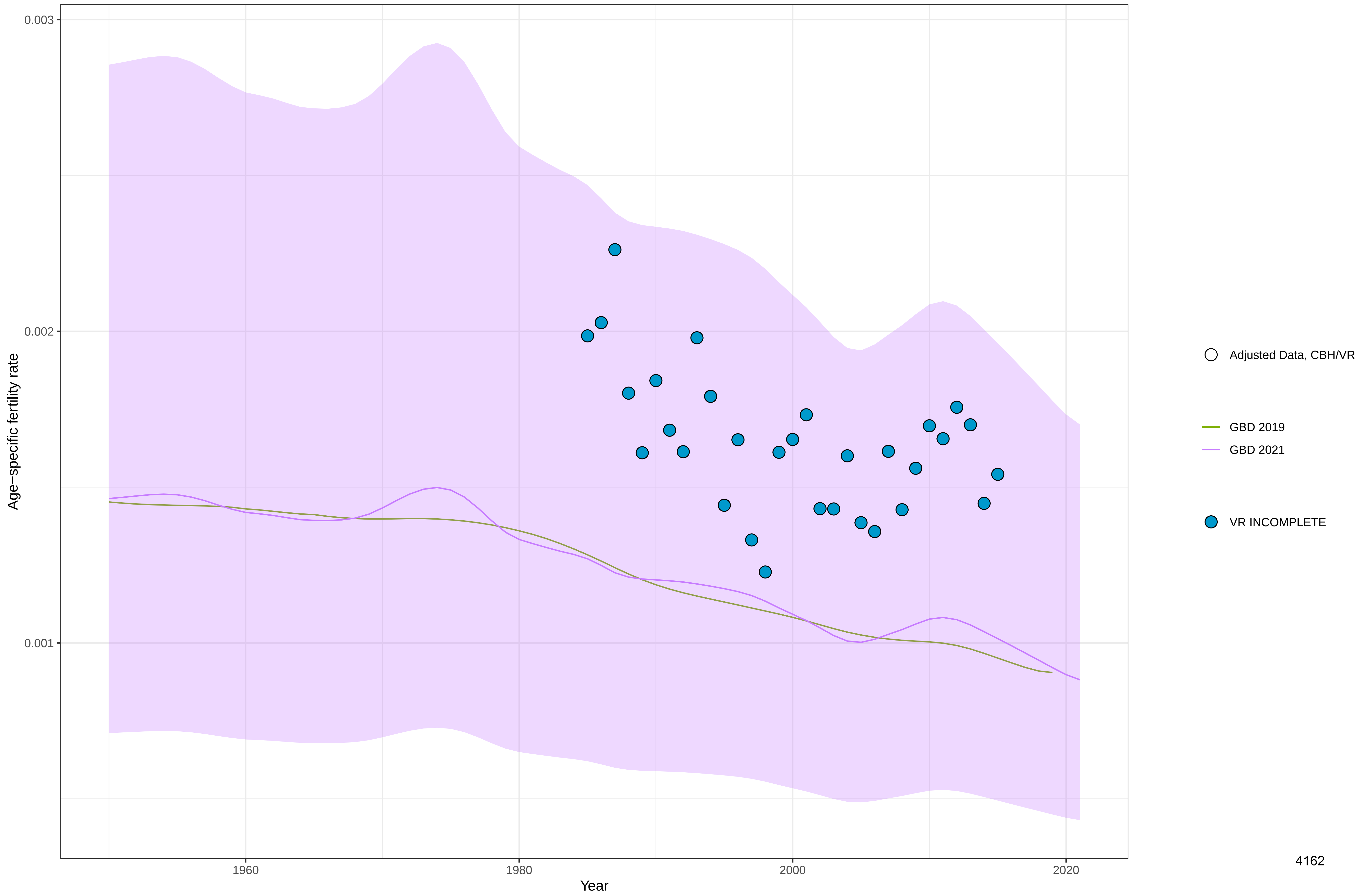

Age−specific fertility fitted trend lines, 1950−2021: Tlaxcala, Ages: 15−19
Data points enclosed with a diamond are reference sources

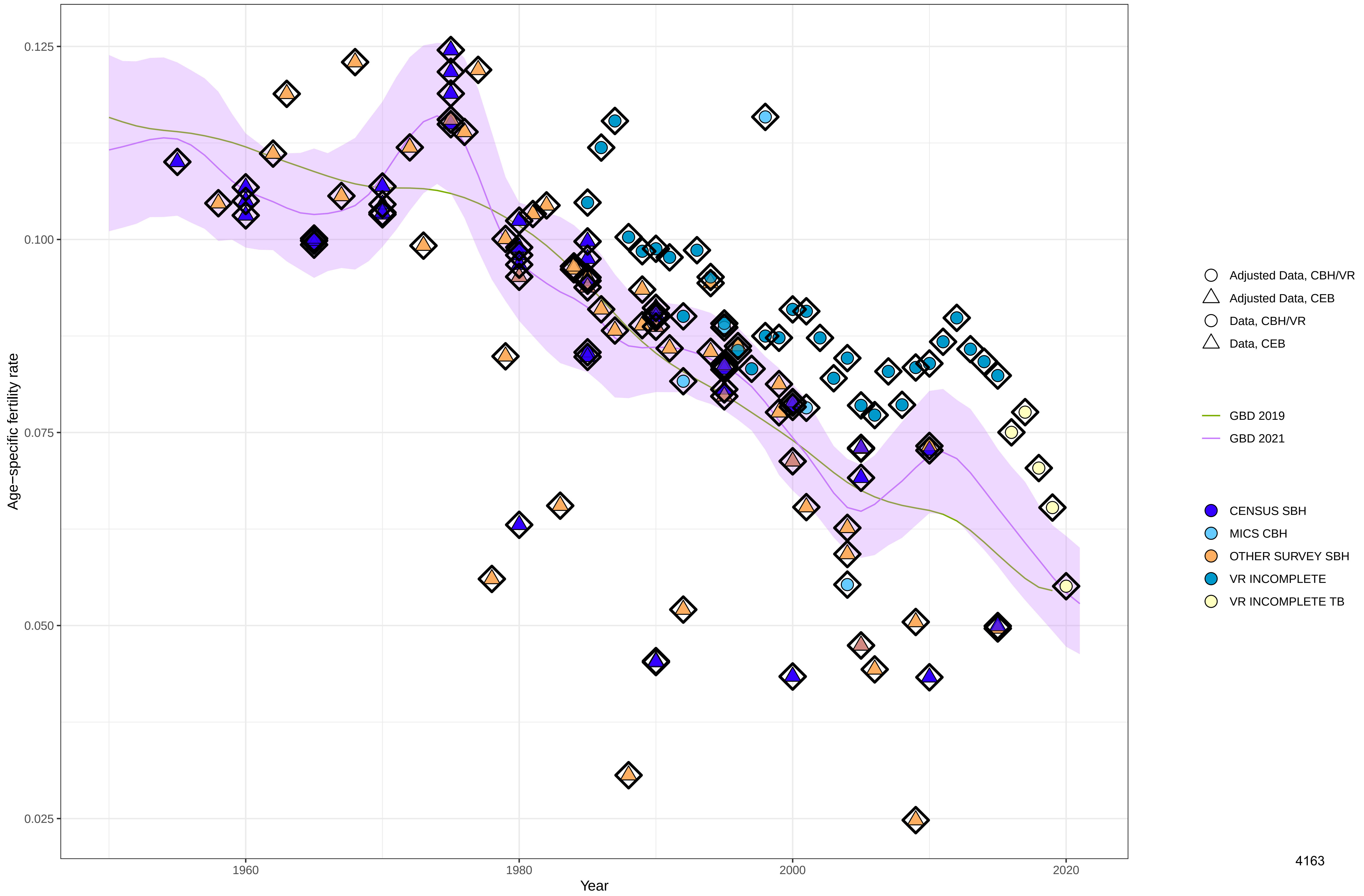

Age−specific fertility fitted trend lines, 1950−2021: Tlaxcala, Ages: 20−24
Data points enclosed with a diamond are reference sources

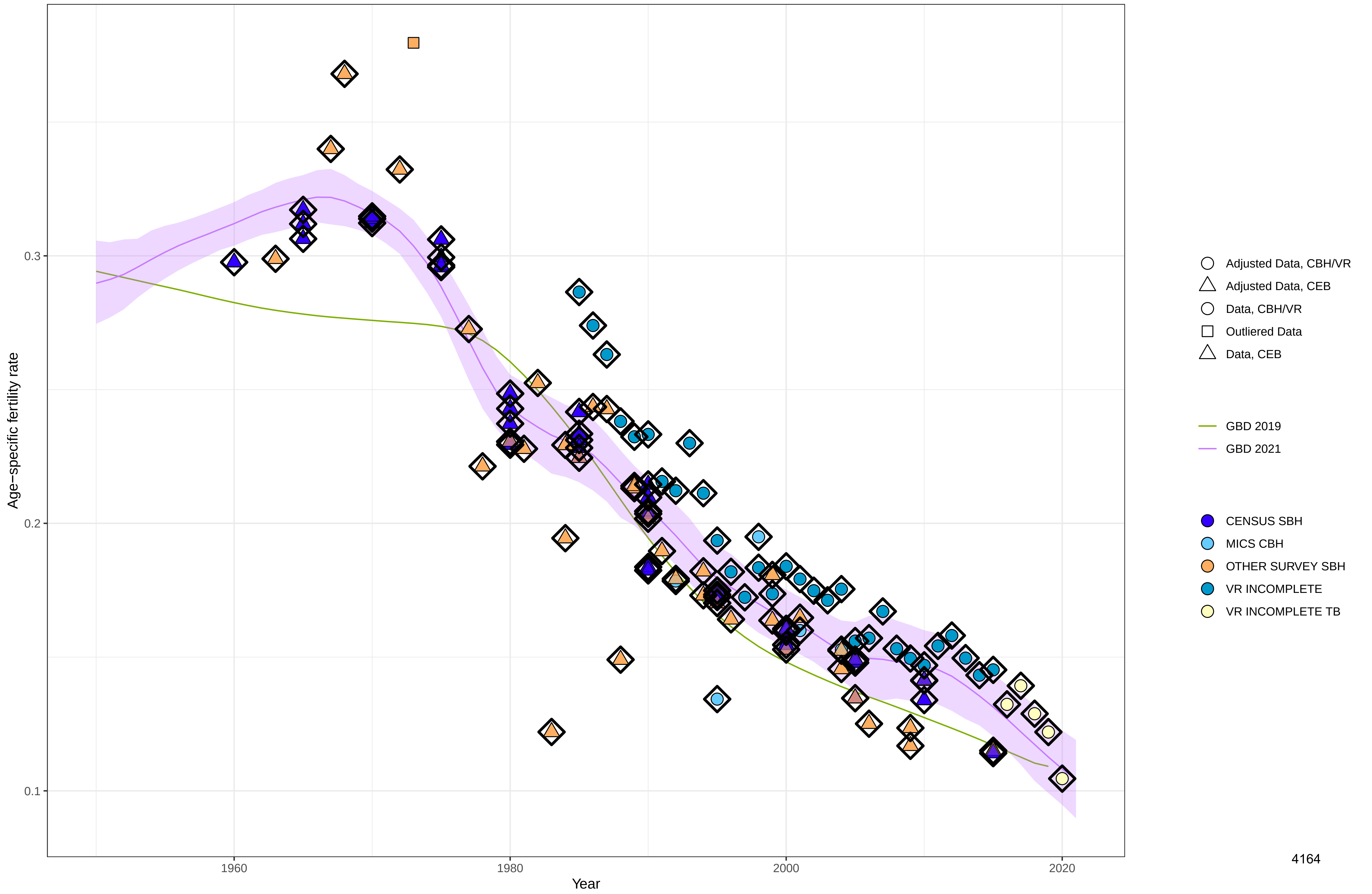

Age−specific fertility fitted trend lines, 1950−2021: Tlaxcala, Ages: 25−29
Data points enclosed with a diamond are reference sources

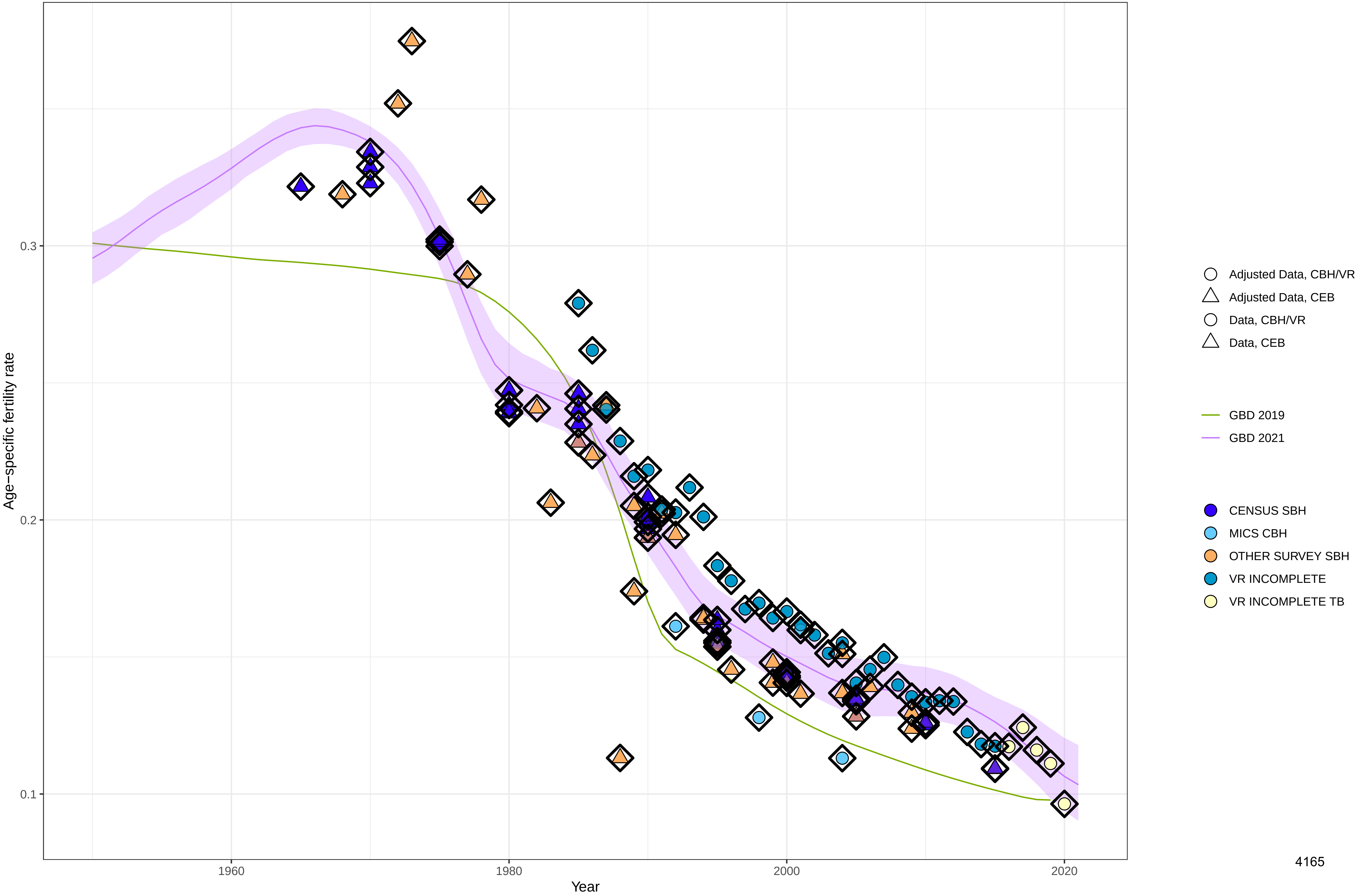

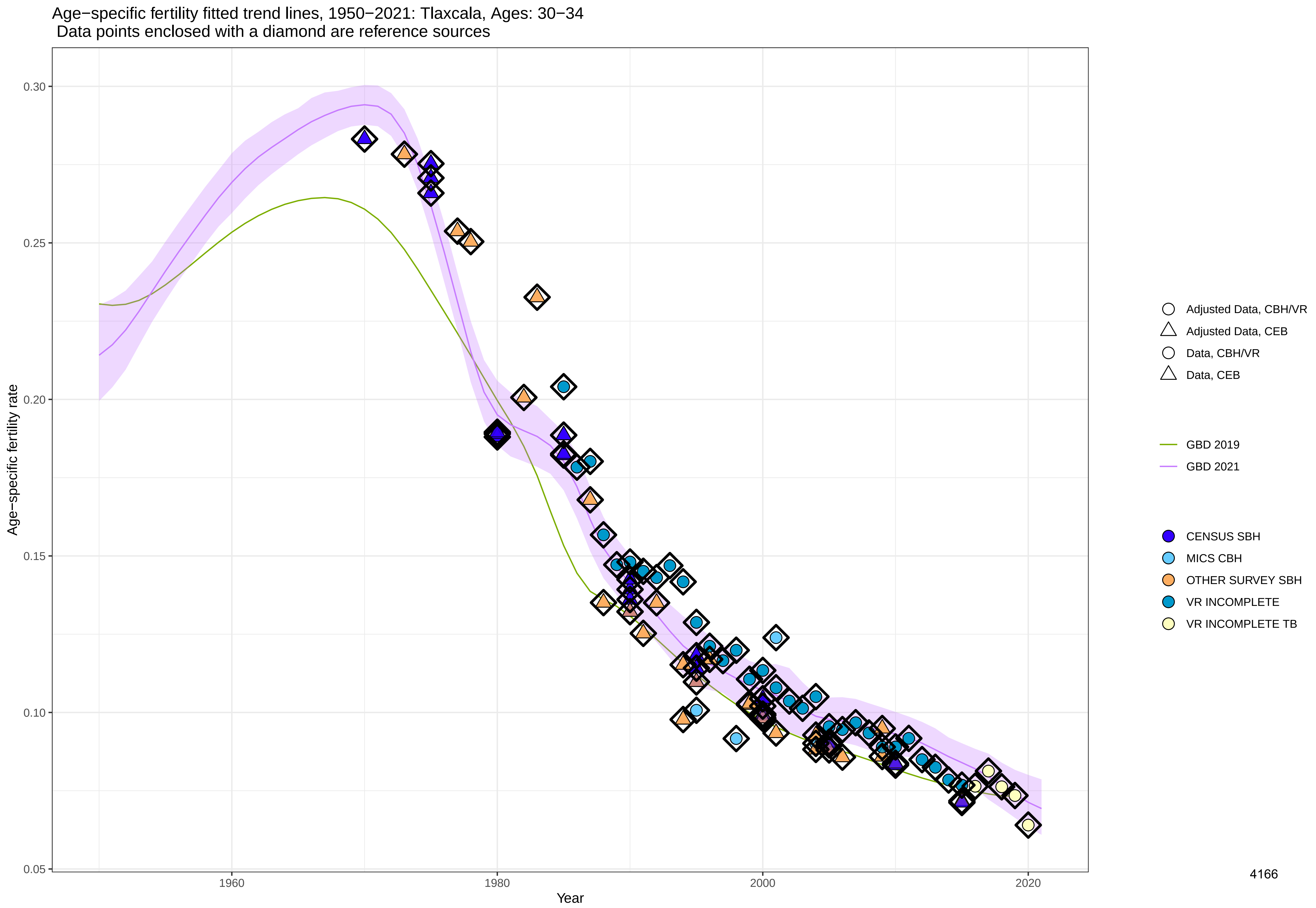

Age−specific fertility fitted trend lines, 1950−2021: Tlaxcala, Ages: 30−34
Data points enclosed with a diamond are reference sources
Age−specific fertility rate
Year
0.05
0.10
0.15
0.20
0.25
0.30
1960
1980
2000
2020
Adjusted Data, CBH/VR
Adjusted Data, CEB
Data, CBH/VR
Data, CEB
GBD 2019
GBD 2021
CENSUS SBH
MICS CBH
OTHER SURVEY SBH
VR INCOMPLETE
VR INCOMPLETE TB

Age−specific fertility fitted trend lines, 1950−2021: Tlaxcala, Ages: 35−39
Data points enclosed with a diamond are reference sources

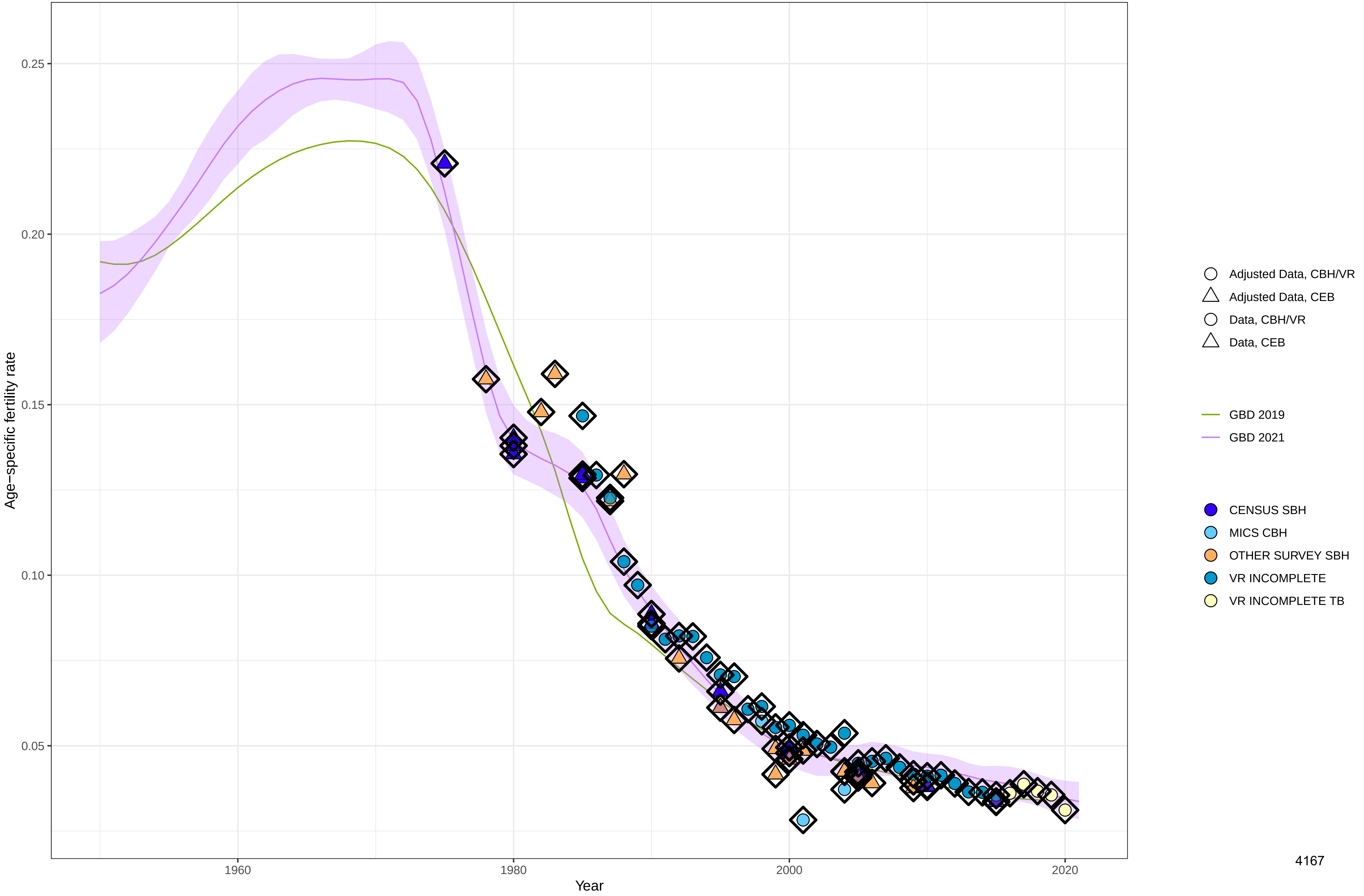

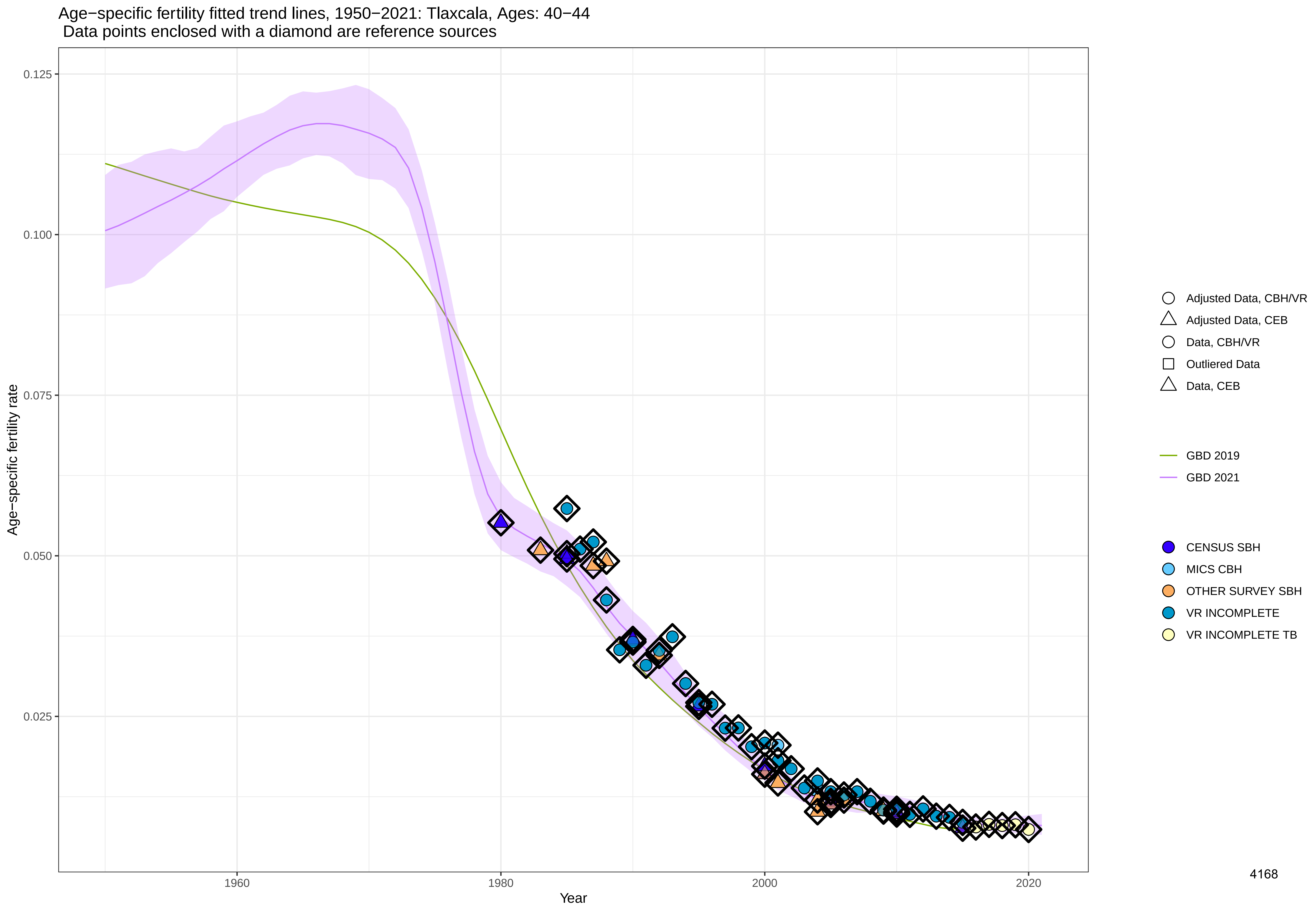

Age−specific fertility fitted trend lines, 1950−2021: Tlaxcala, Ages: 40−44
Data points enclosed with a diamond are reference sources
Age−specific fertility rate
0.125
0.100
0.075
0.050
0.025
1960
1980
2000
2020
Year
Adjusted Data, CBH/VR
Adjusted Data, CEB
Data, CBH/VR
Outliered Data
Data, CEB
GBD 2019
GBD 2021
CENSUS SBH
MICS CBH
OTHER SURVEY SBH
VR INCOMPLETE
VR INCOMPLETE TB

4168

Age−specific fertility fitted trend lines, 1950−2021: Tlaxcala, Ages: 45−49
Data points enclosed with a diamond are reference sources

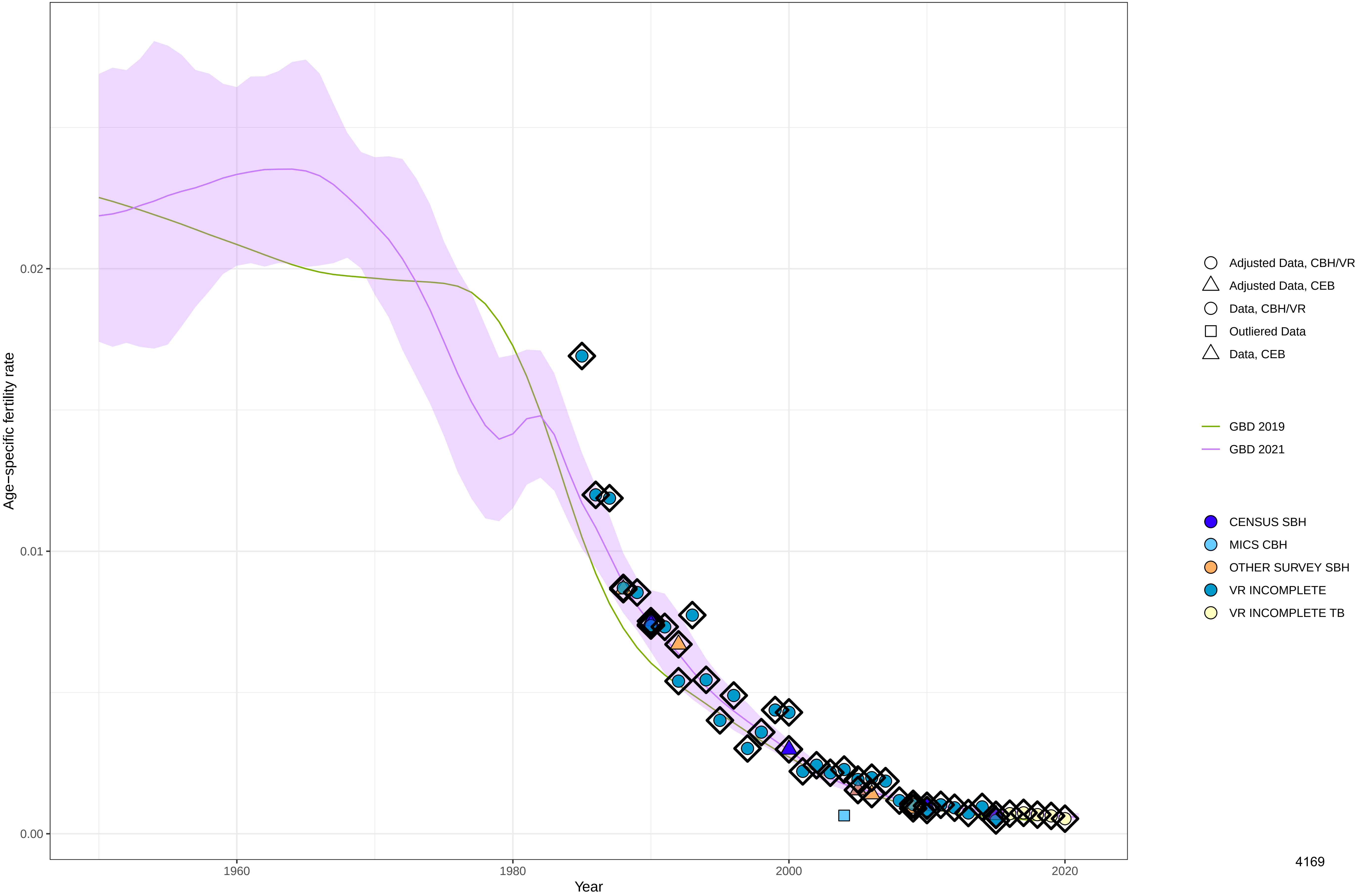

Age−specific fertility fitted trend lines, 1950−2021: Tlaxcala, Ages: 50−54
Data points enclosed with a diamond are reference sources

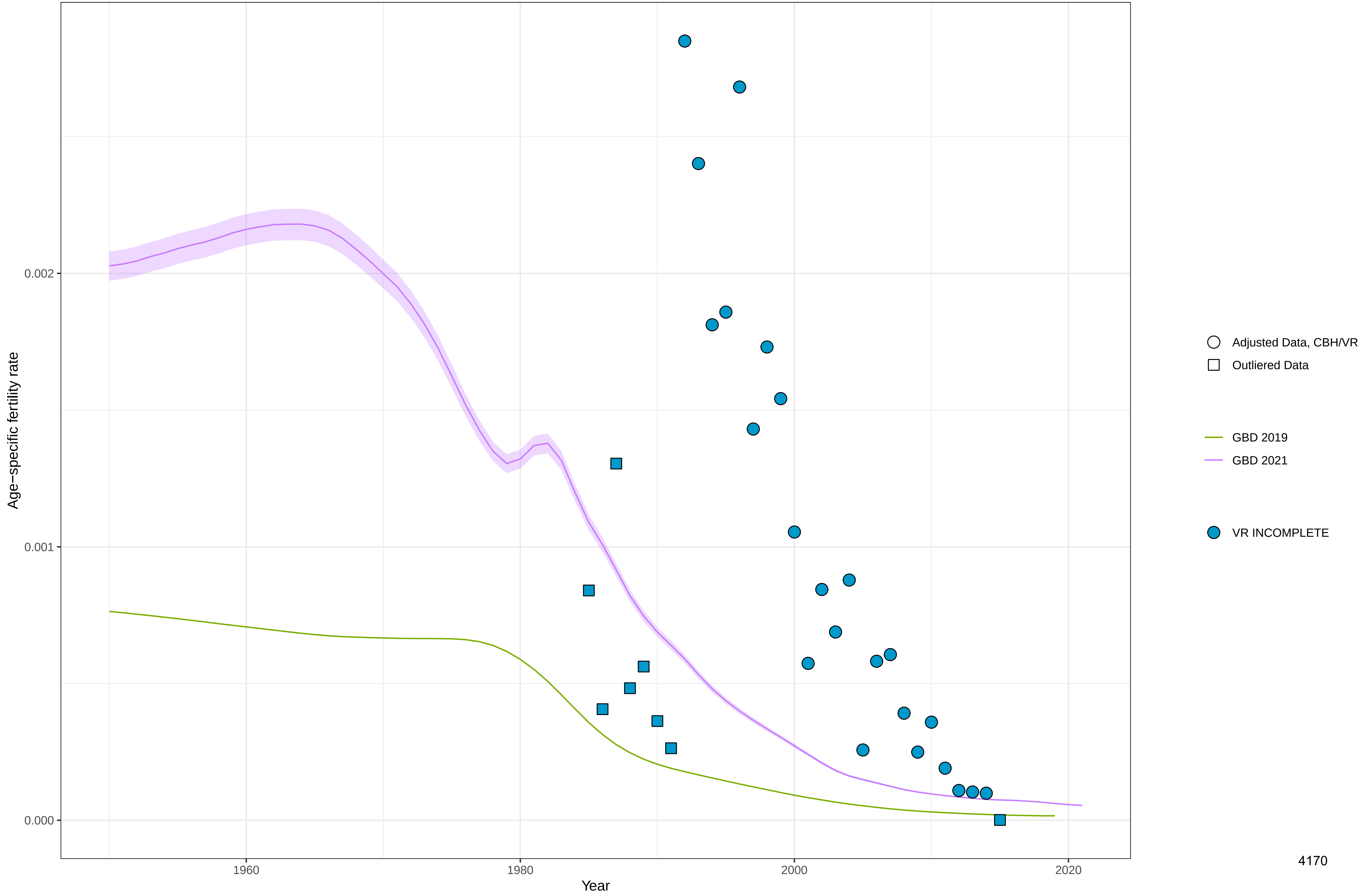

Total fertility rate fitted trend lines, 1950−2021: Tlaxcala TFR
Data points enclosed with a diamond are reference sources

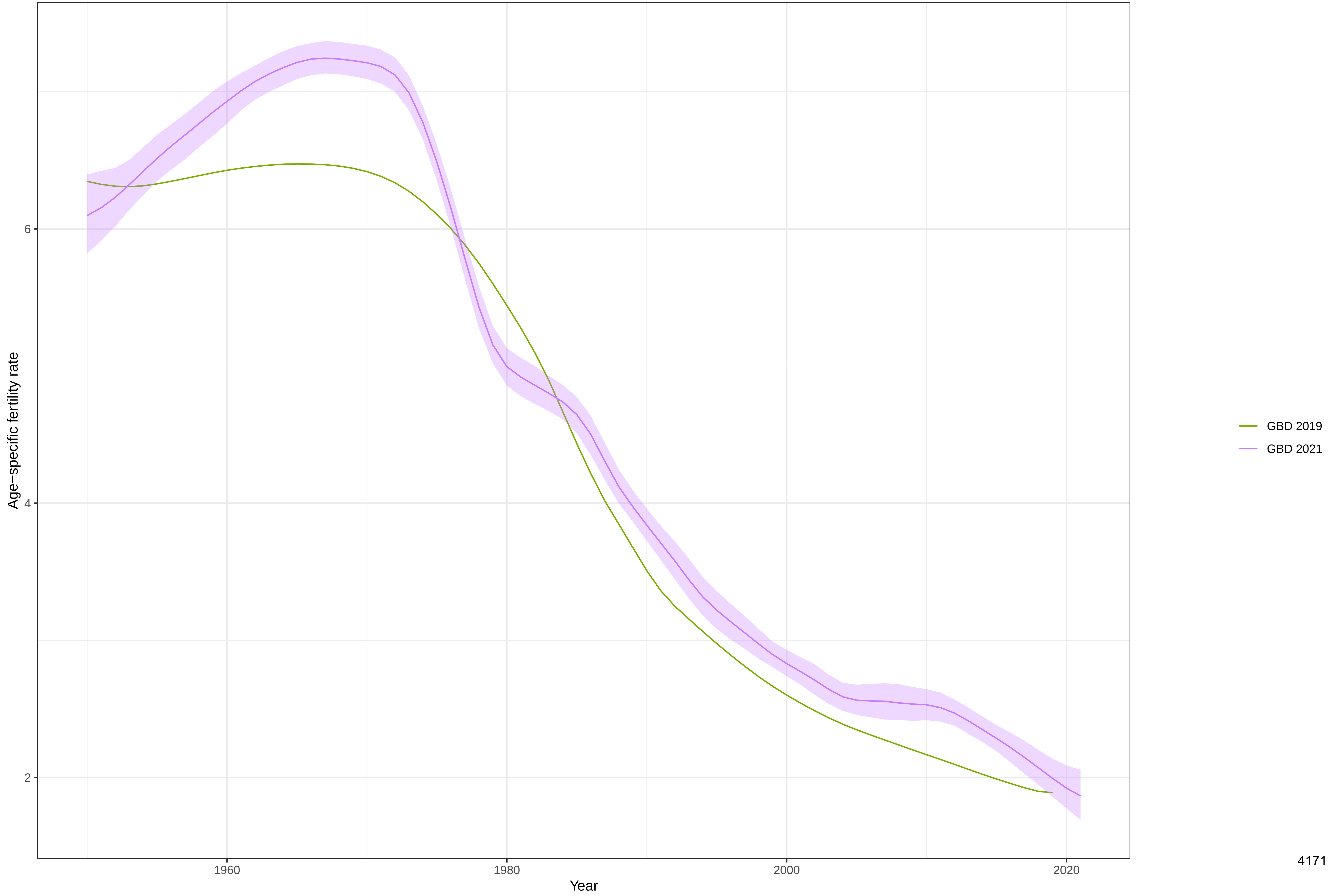

Age−specific fertility fitted trend lines, 1950−2021: Veracruz de Ignacio de la Llave, Ages: 10−14
Data points enclosed with a diamond are reference sources

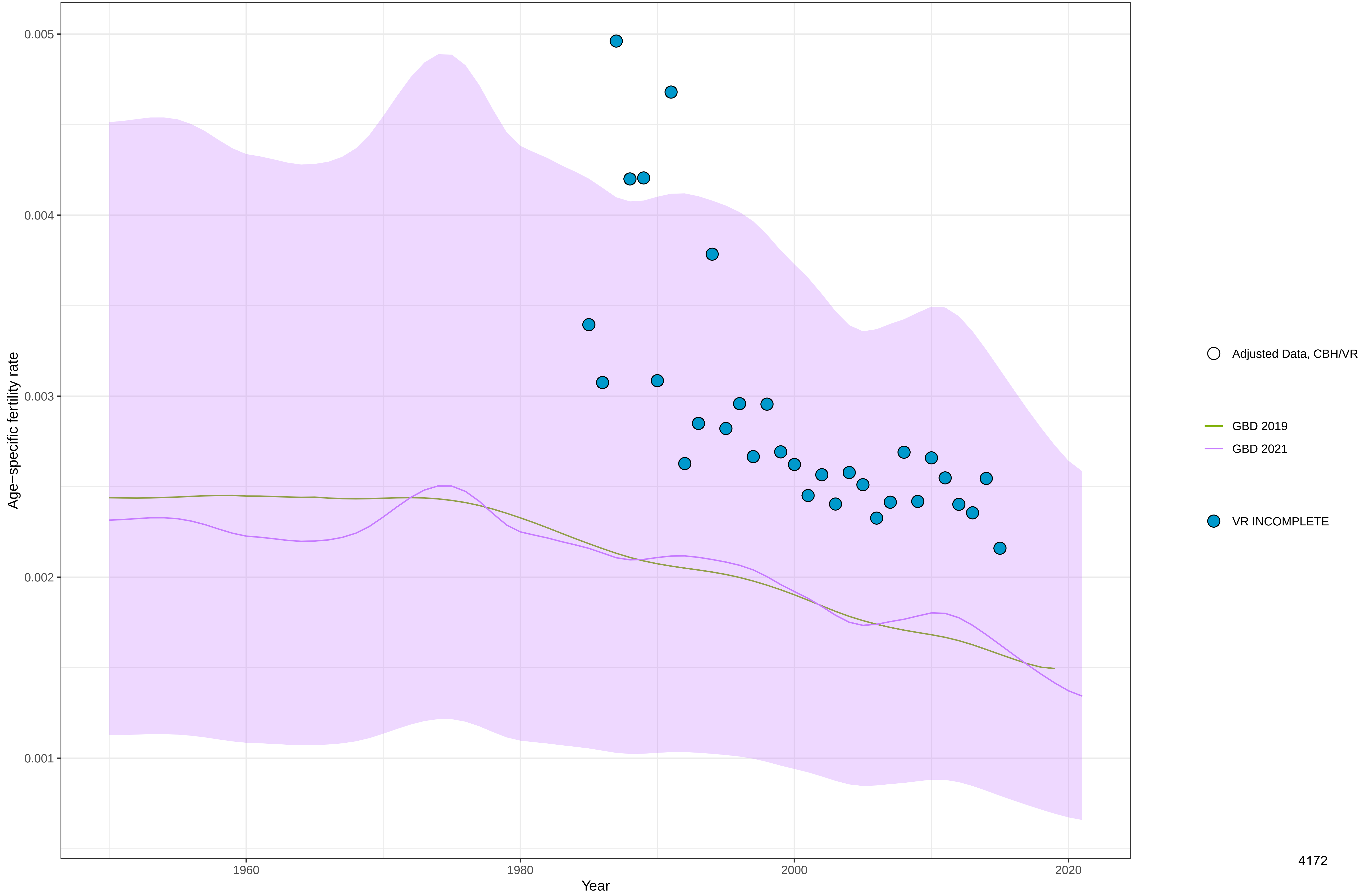

Age−specific fertility fitted trend lines, 1950−2021: Veracruz de Ignacio de la Llave, Ages: 15−19
Data points enclosed with a diamond are reference sources

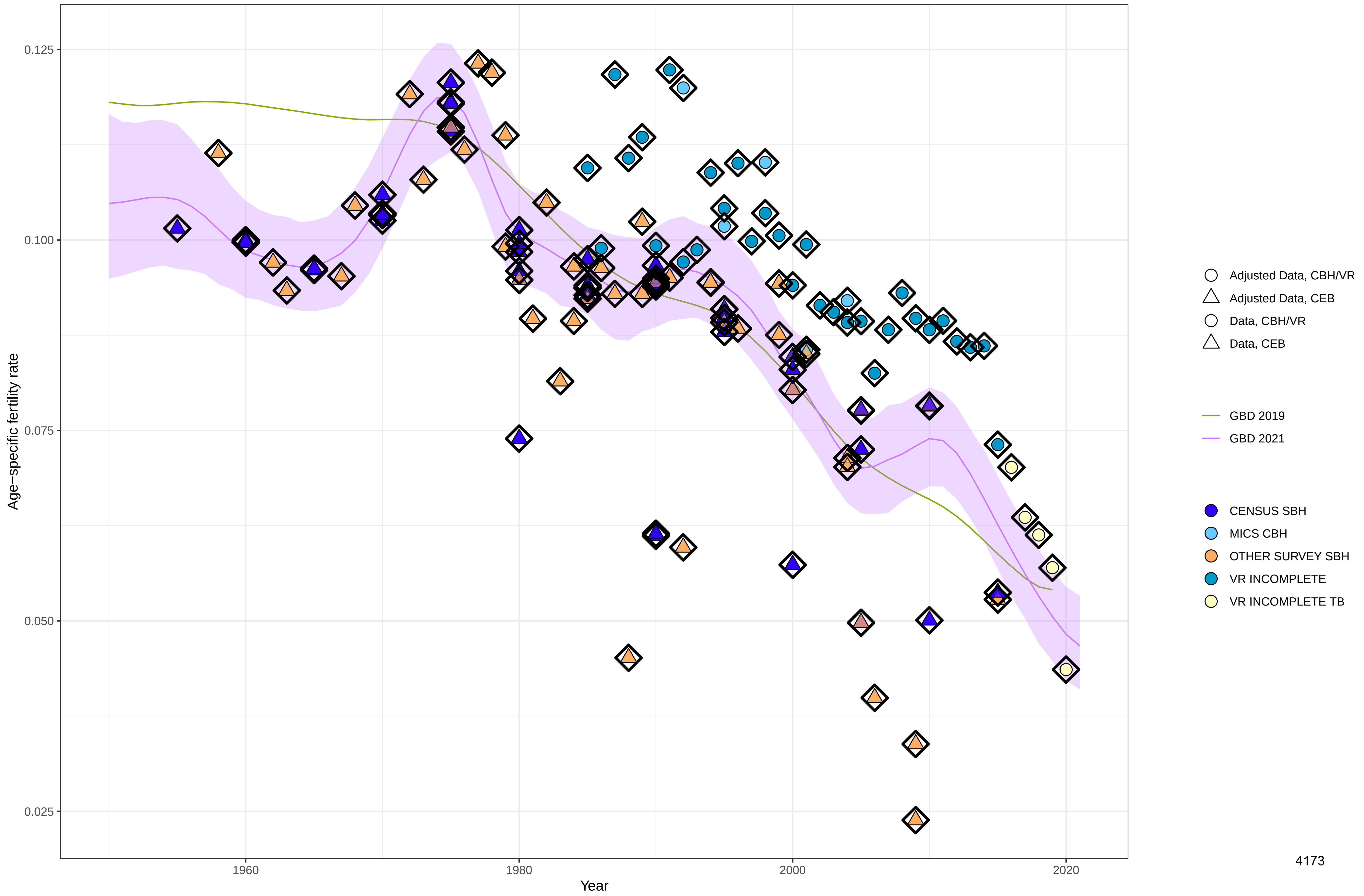

Age−specific fertility fitted trend lines, 1950−2021: Veracruz de Ignacio de la Llave, Ages: 20−24
Data points enclosed with a diamond are reference sources

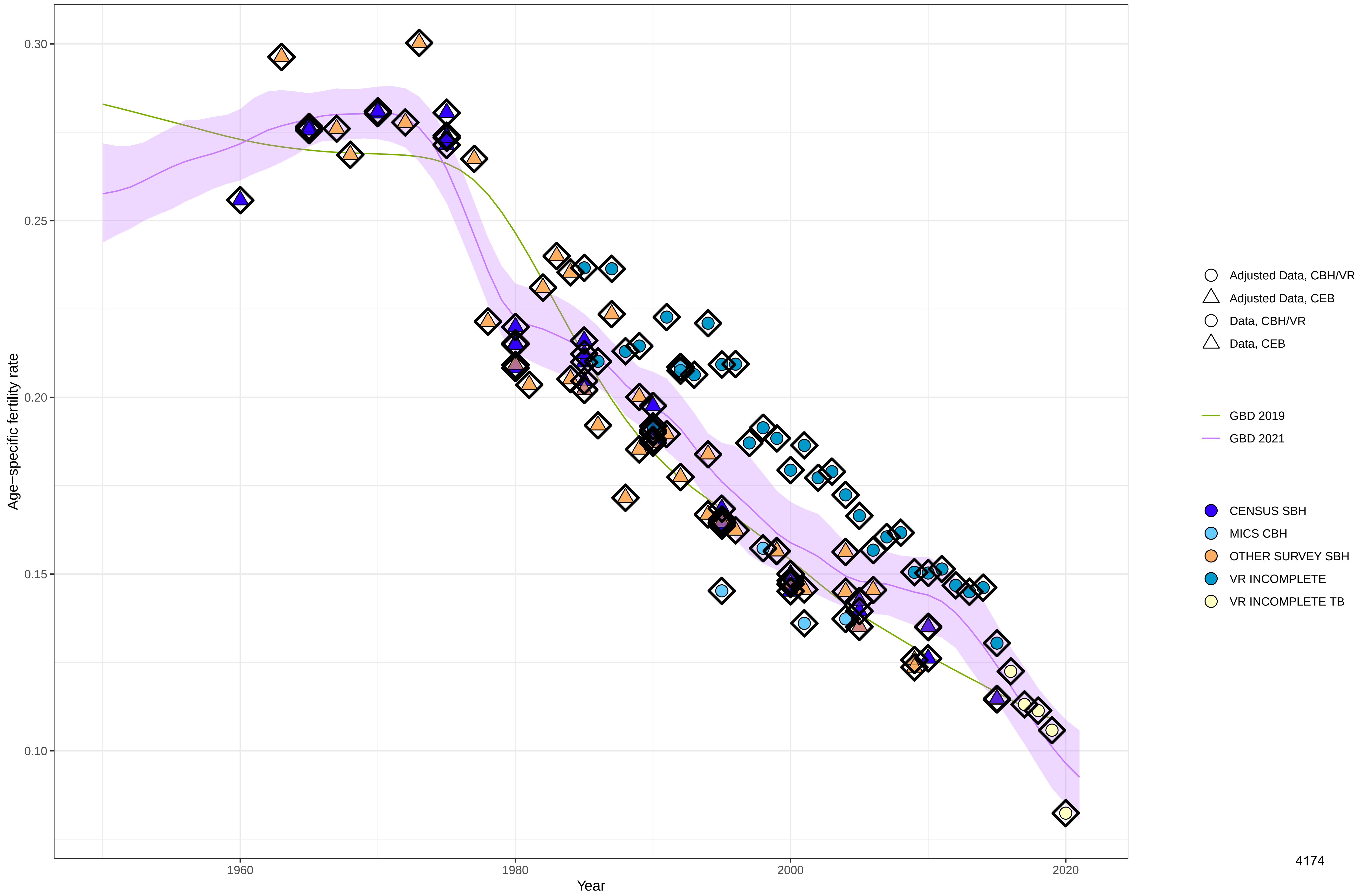

Age−specific fertility fitted trend lines, 1950−2021: Veracruz de Ignacio de la Llave, Ages: 25−29
Data points enclosed with a diamond are reference sources

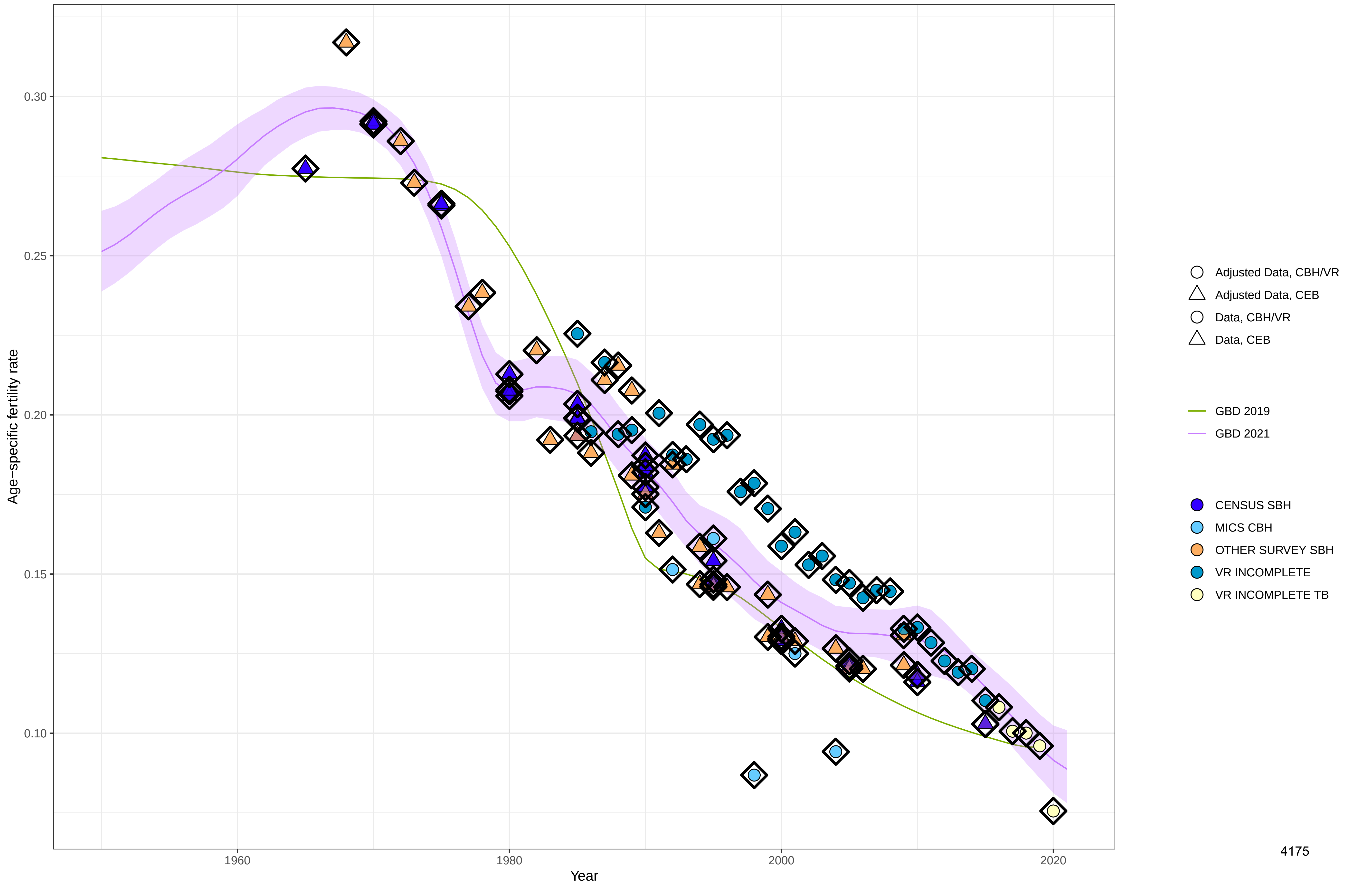

Age−specific fertility fitted trend lines, 1950−2021: Veracruz de Ignacio de la Llave, Ages: 30−34
Data points enclosed with a diamond are reference sources

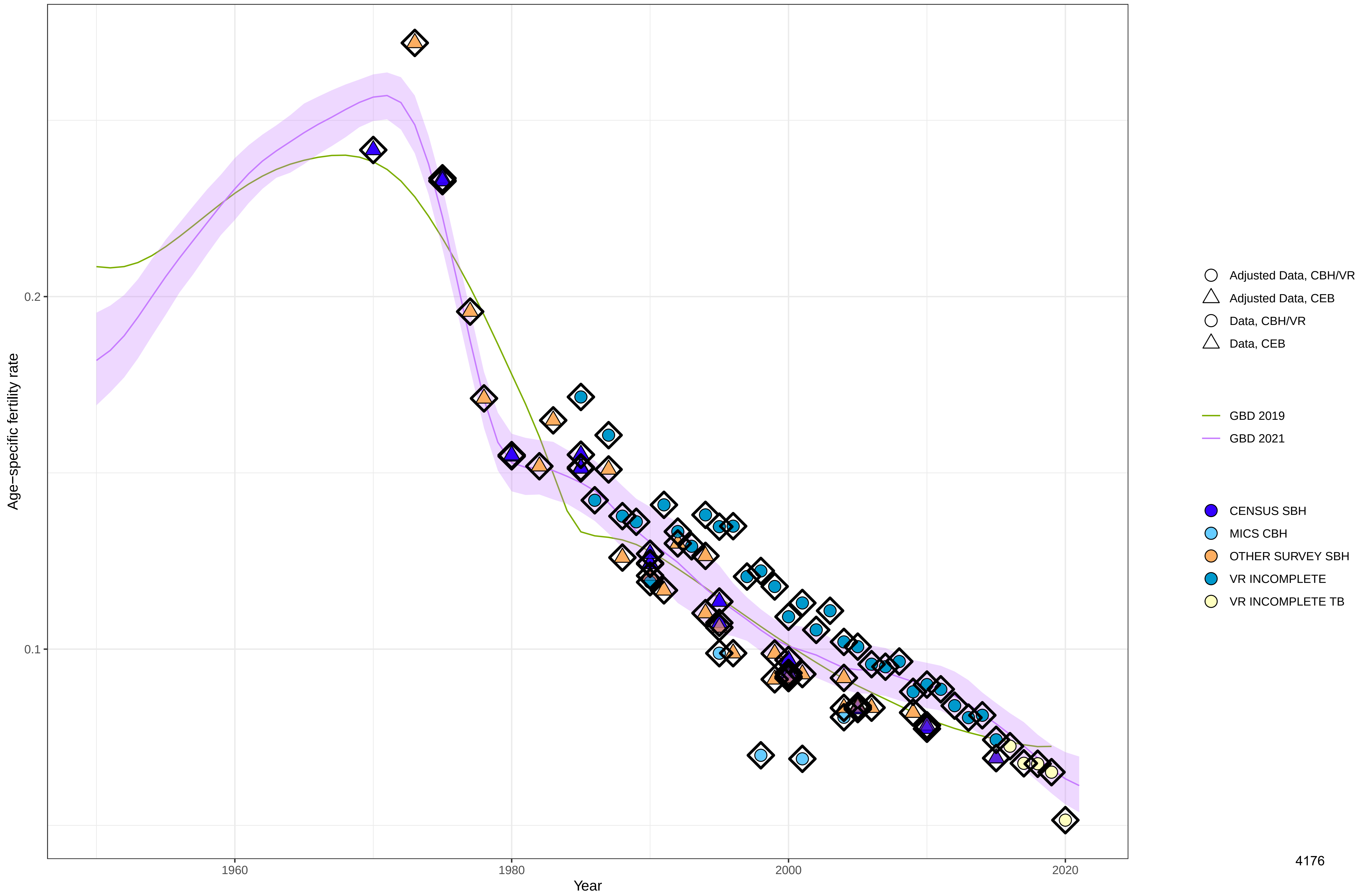

Age−specific fertility fitted trend lines, 1950−2021: Veracruz de Ignacio de la Llave, Ages: 35−39
Data points enclosed with a diamond are reference sources

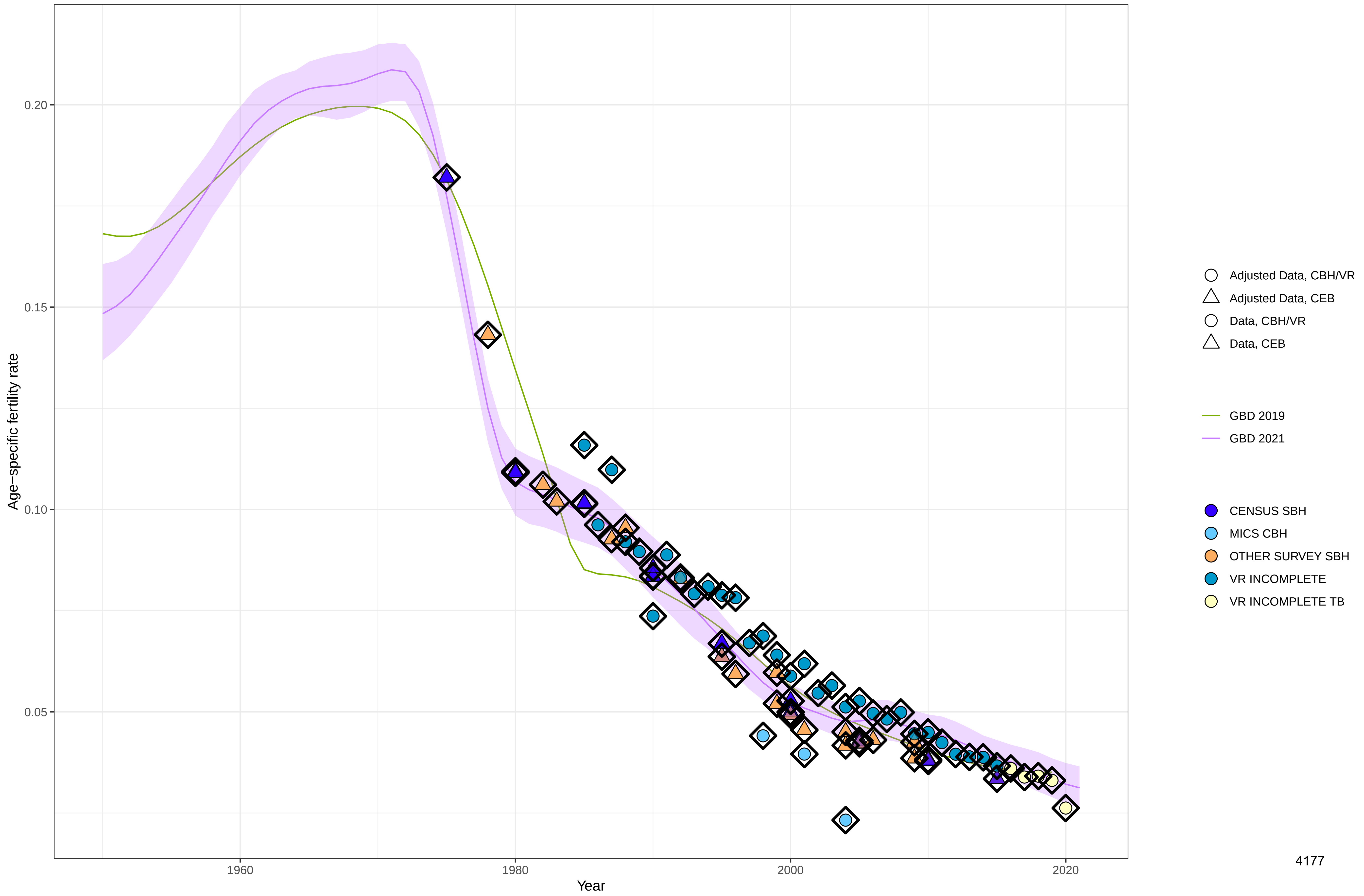

Age−specific fertility fitted trend lines, 1950−2021: Veracruz de Ignacio de la Llave, Ages: 40−44
Data points enclosed with a diamond are reference sources

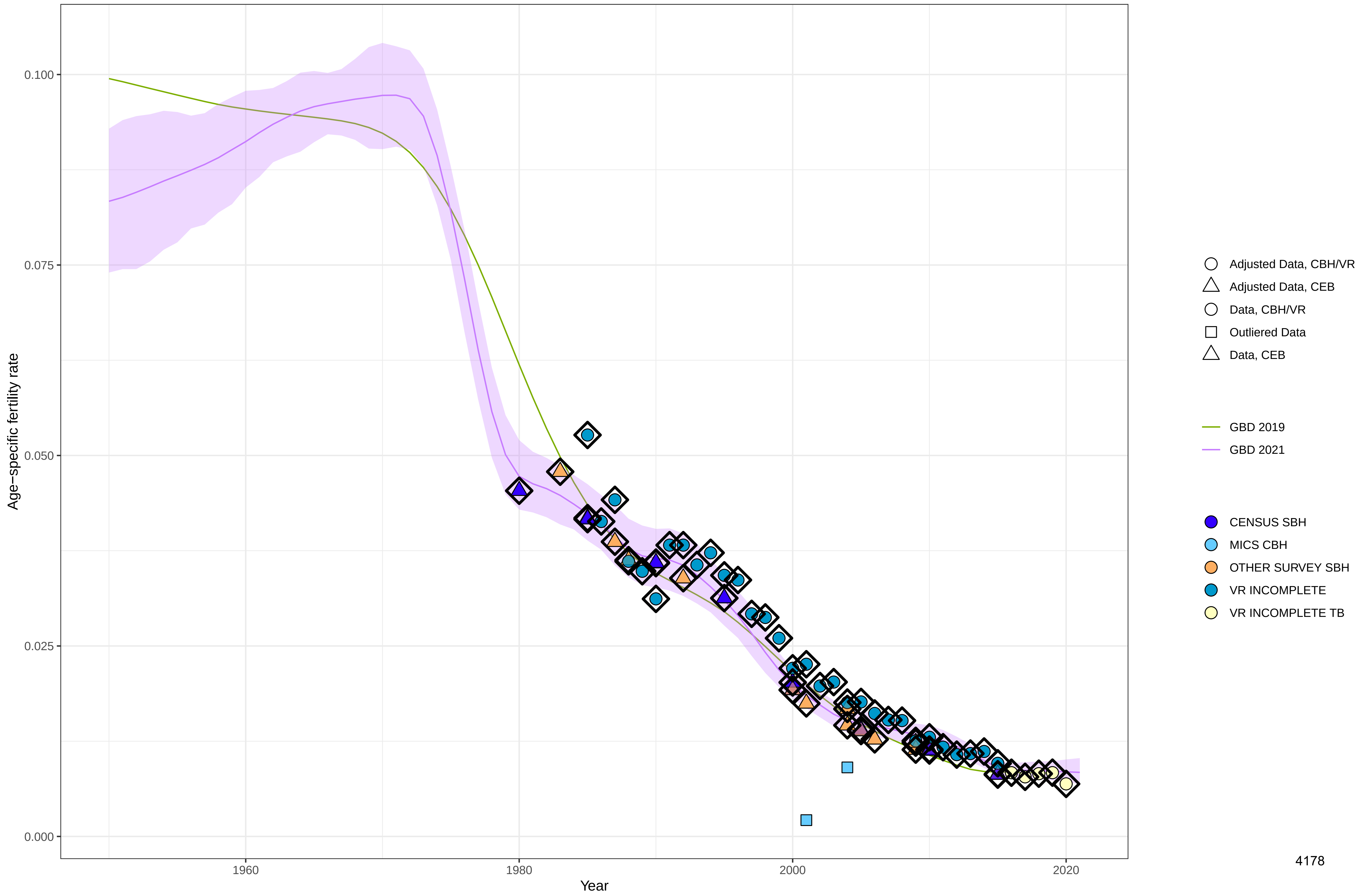

Age−specific fertility fitted trend lines, 1950−2021: Veracruz de Ignacio de la Llave, Ages: 45−49
Data points enclosed with a diamond are reference sources

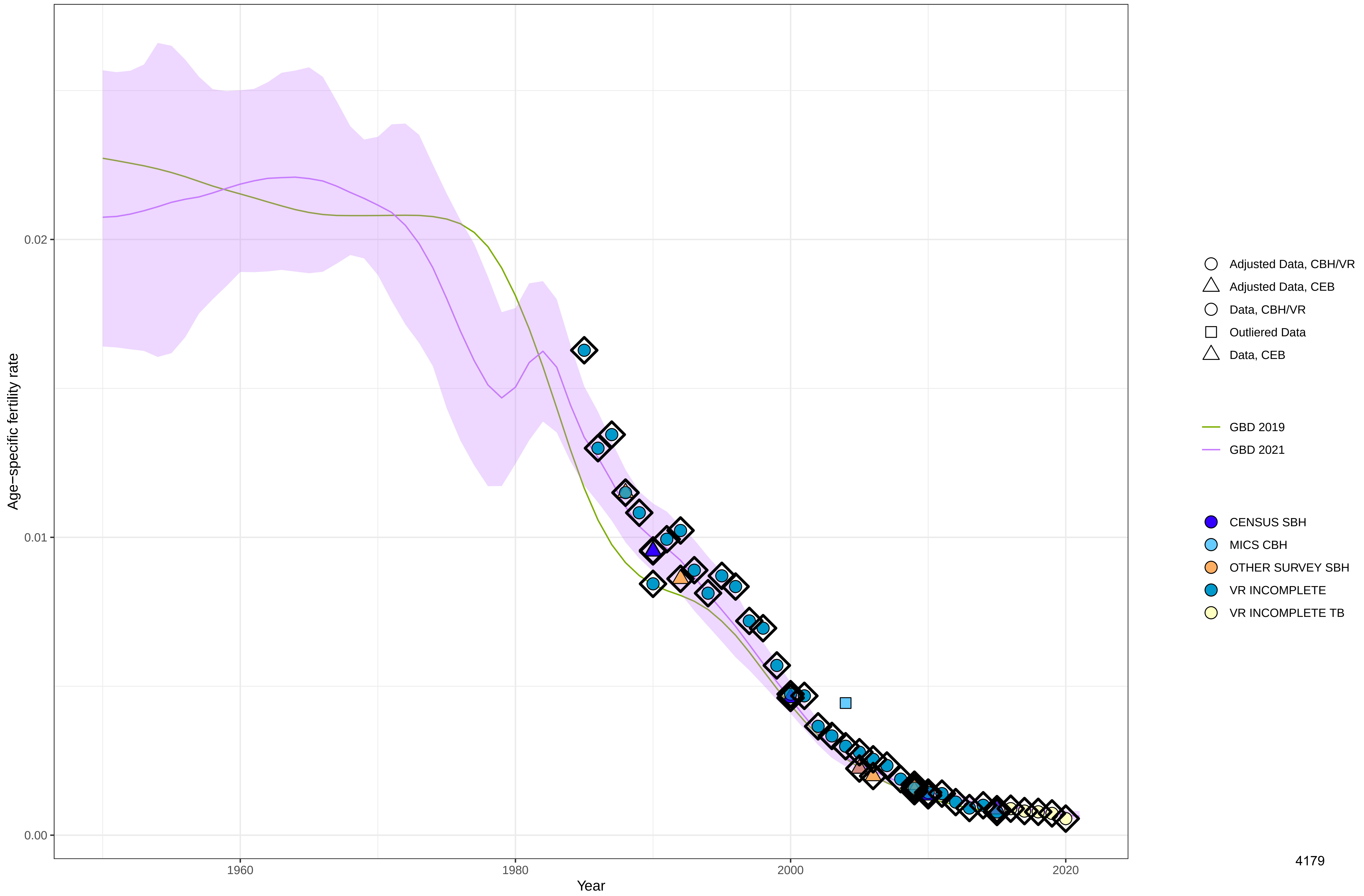

Age−specific fertility fitted trend lines, 1950−2021: Veracruz de Ignacio de la Llave, Ages: 50−54
Data points enclosed with a diamond are reference sources

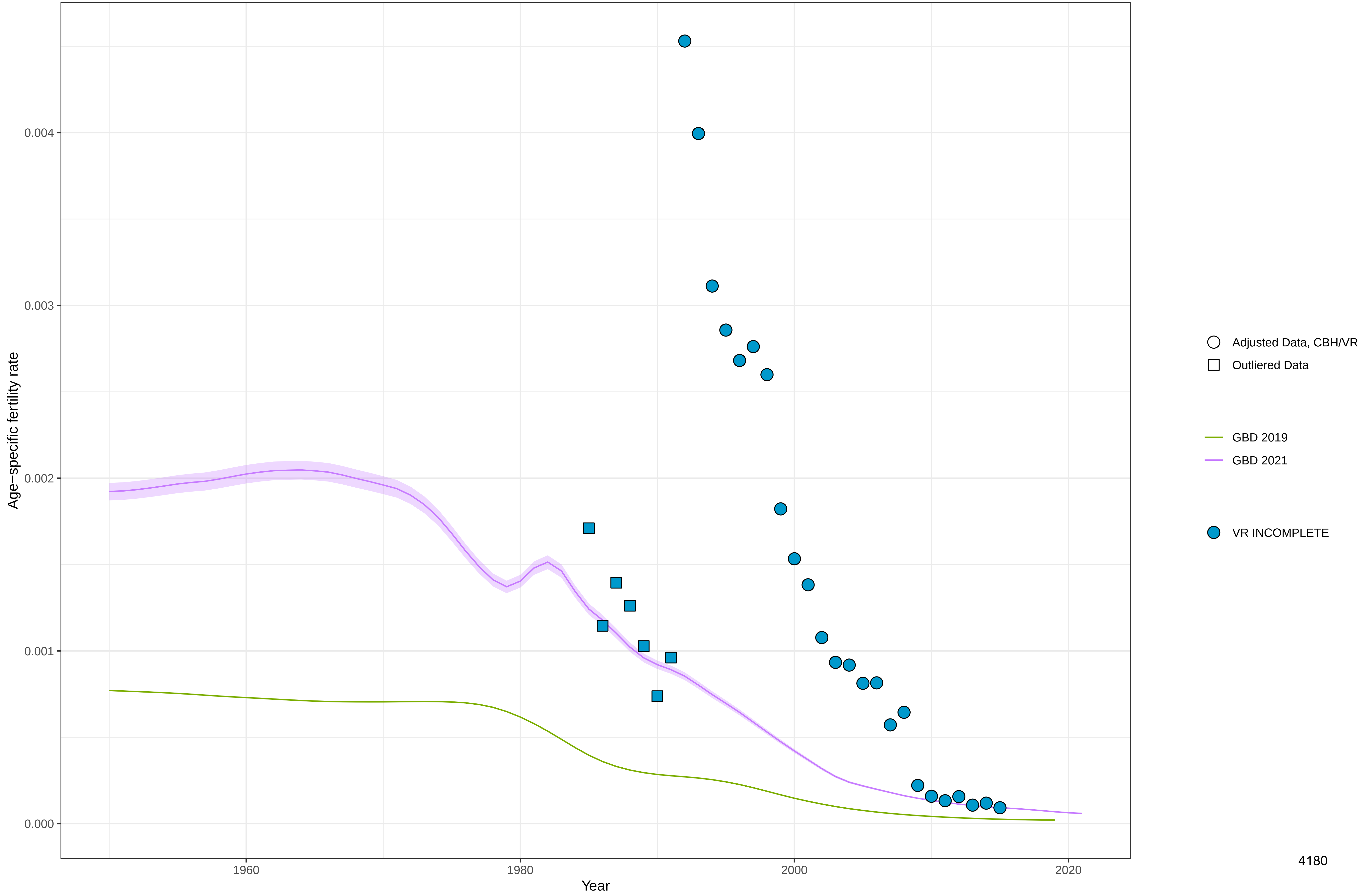

Total fertility rate fitted trend lines, 1950−2021: Veracruz de Ignacio de la Llave TFR
Data points enclosed with a diamond are reference sources

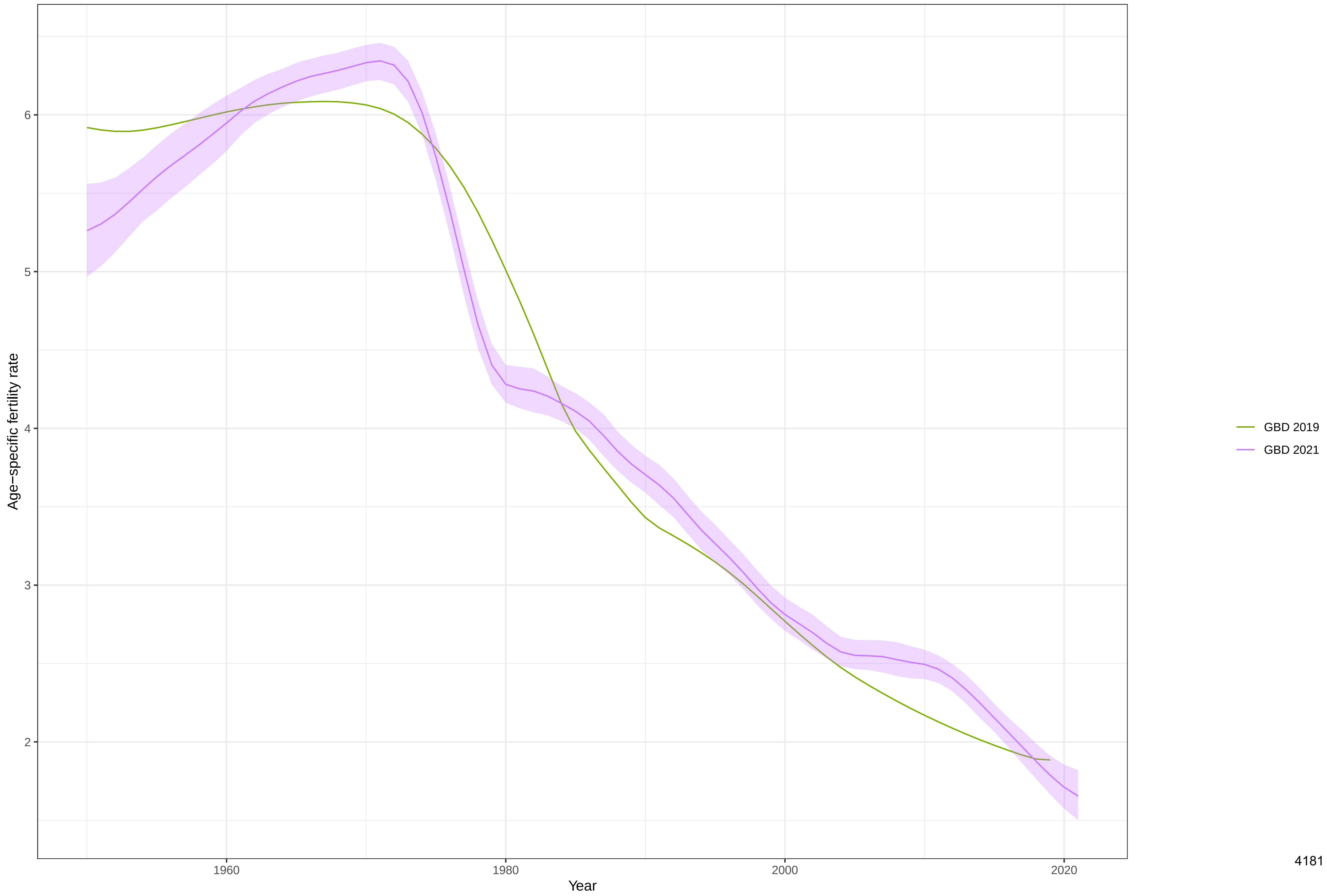

Age−specific fertility fitted trend lines, 1950−2021: Yucatán, Ages: 10−14
Data points enclosed with a diamond are reference sources

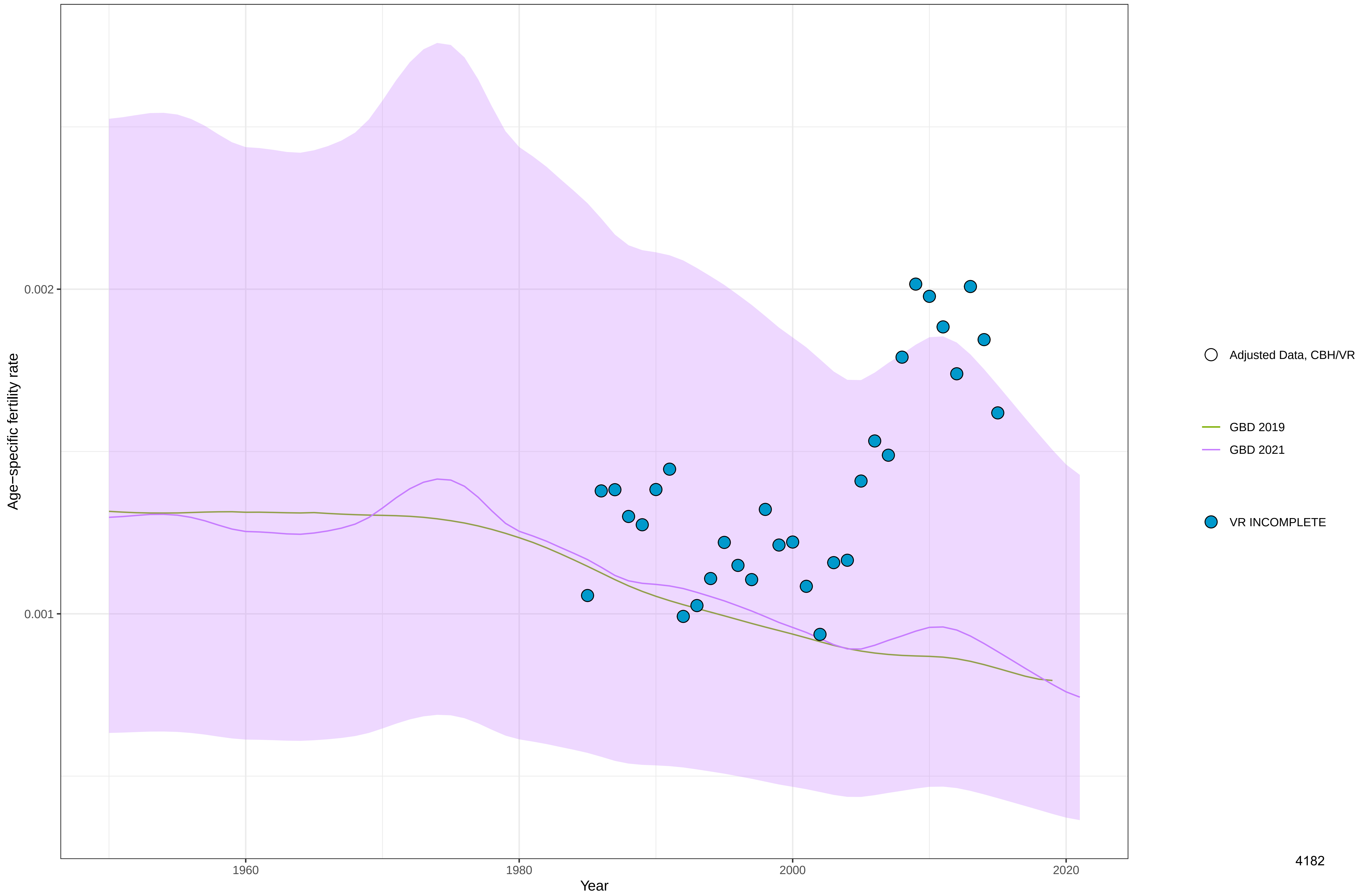

Age−specific fertility fitted trend lines, 1950−2021: Yucatán, Ages: 15−19
Data points enclosed with a diamond are reference sources

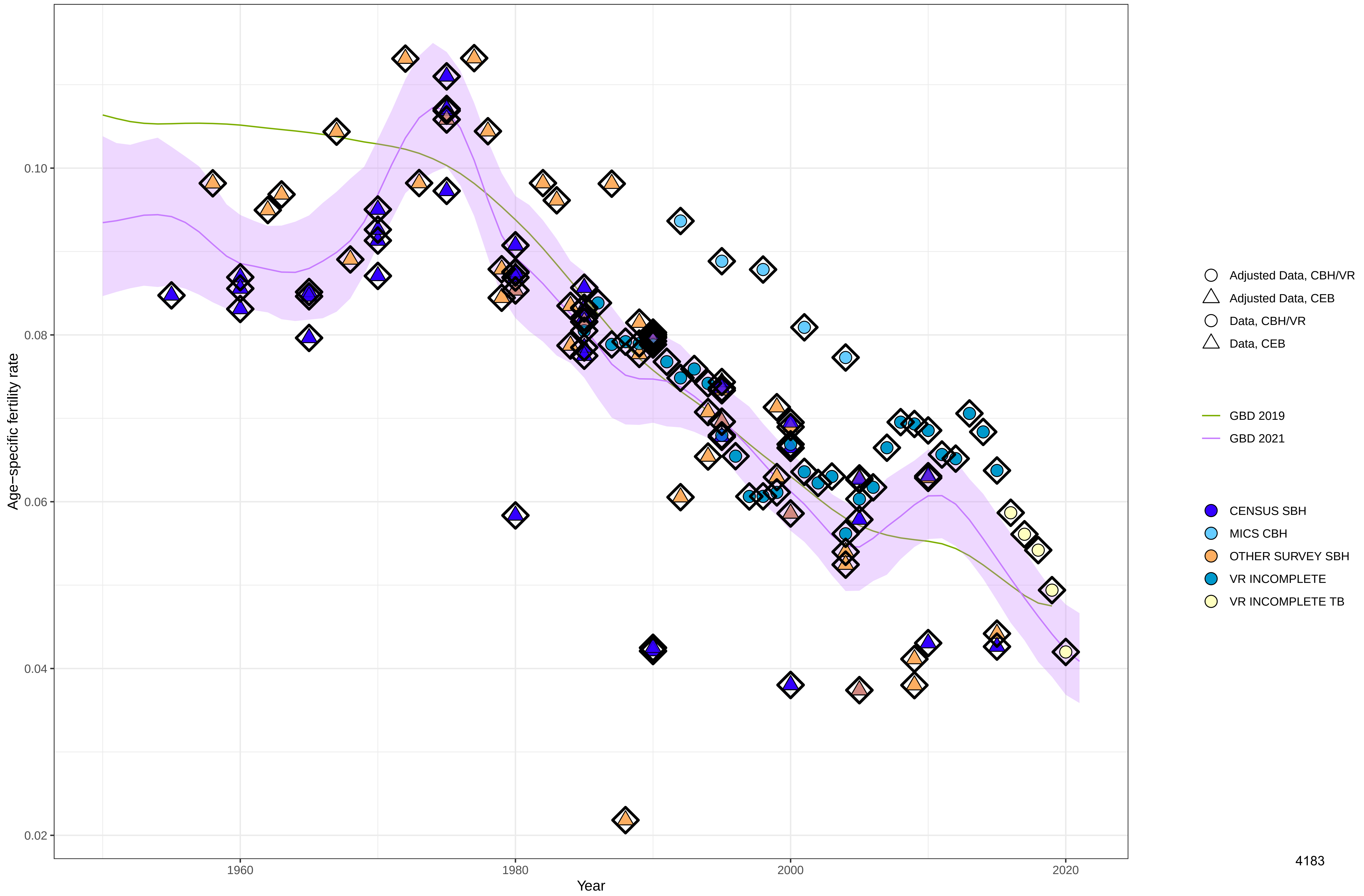

Age−specific fertility fitted trend lines, 1950−2021: Yucatán, Ages: 20−24
Data points enclosed with a diamond are reference sources

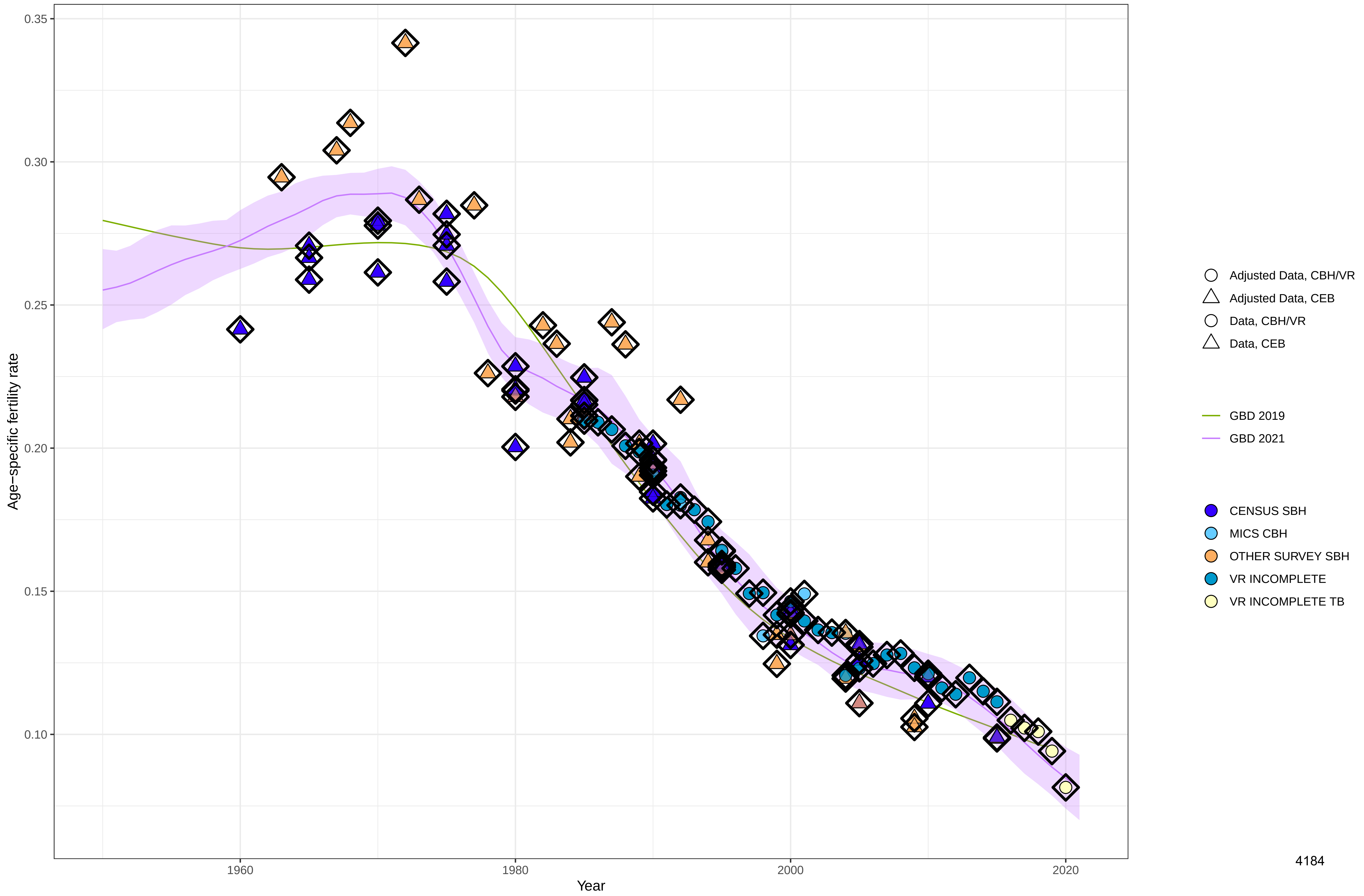

Age−specific fertility fitted trend lines, 1950−2021: Yucatán, Ages: 25−29
Data points enclosed with a diamond are reference sources

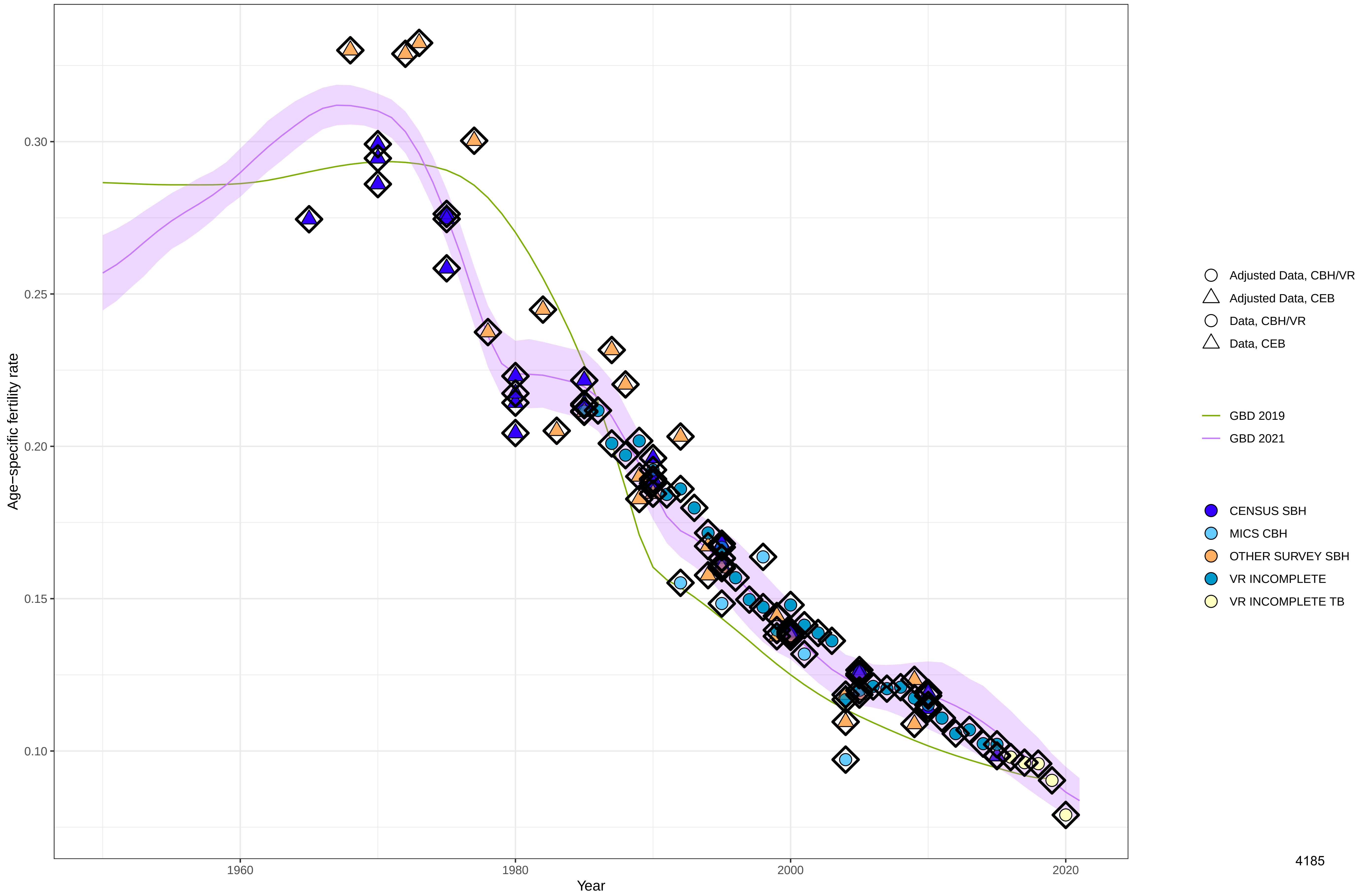

4185

Age−specific fertility fitted trend lines, 1950−2021: Yucatán, Ages: 30−34
Data points enclosed with a diamond are reference sources

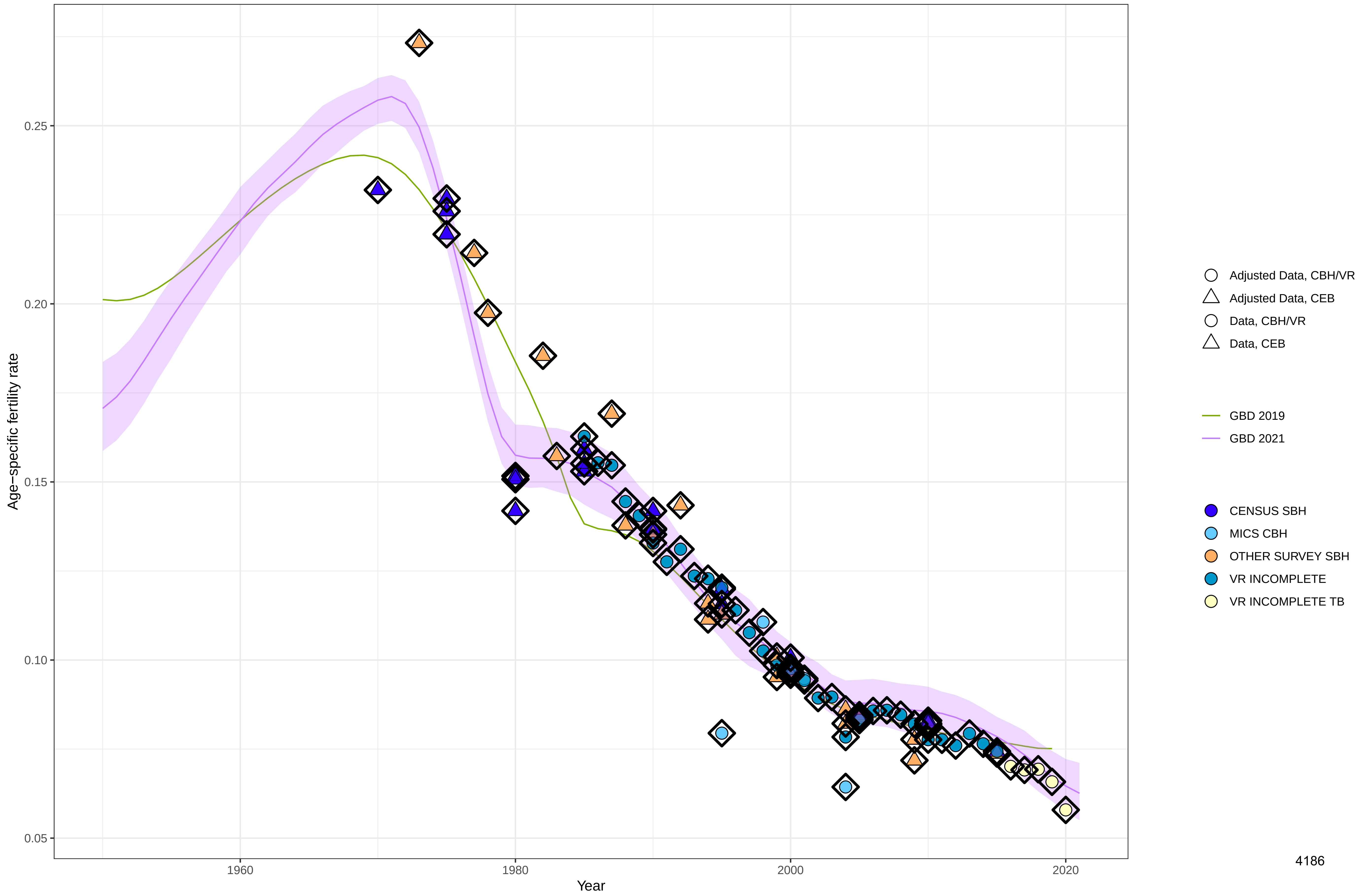

Age−specific fertility fitted trend lines, 1950−2021: Yucatán, Ages: 35−39
Data points enclosed with a diamond are reference sources

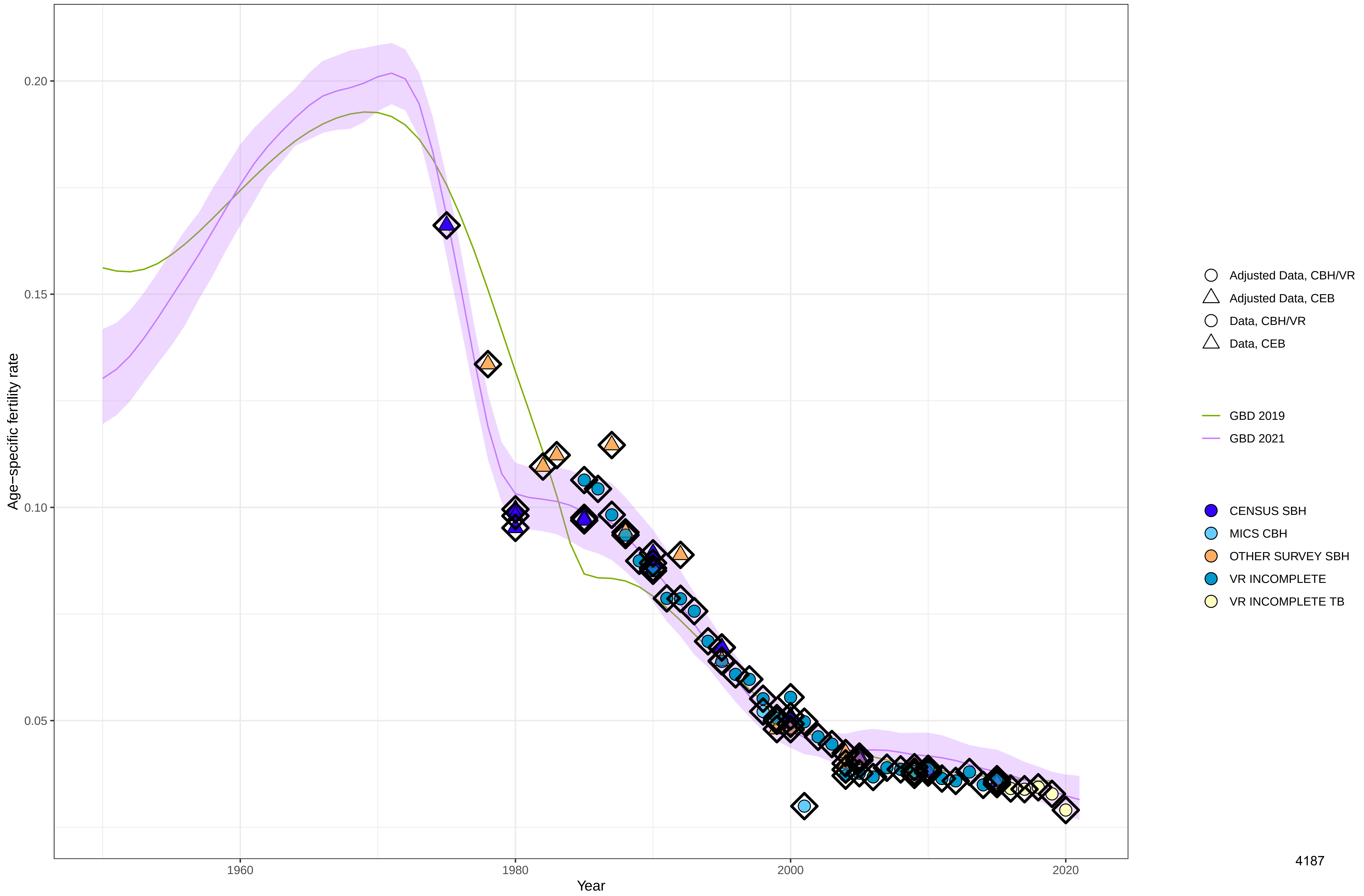

4187

Age−specific fertility fitted trend lines, 1950−2021: Yucatán, Ages: 40−44
Data points enclosed with a diamond are reference sources

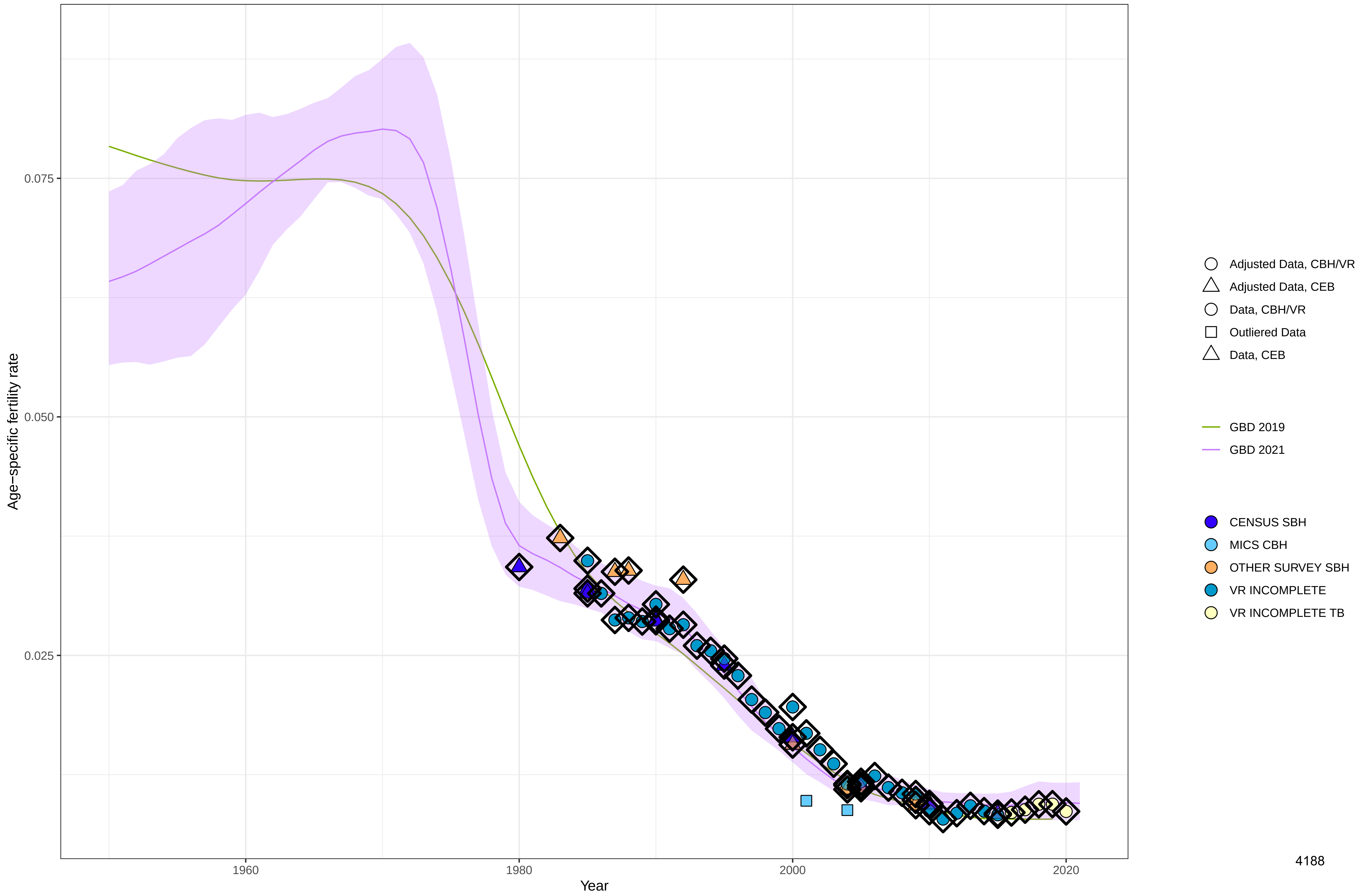

Age−specific fertility fitted trend lines, 1950−2021: Yucatán, Ages: 45−49
Data points enclosed with a diamond are reference sources

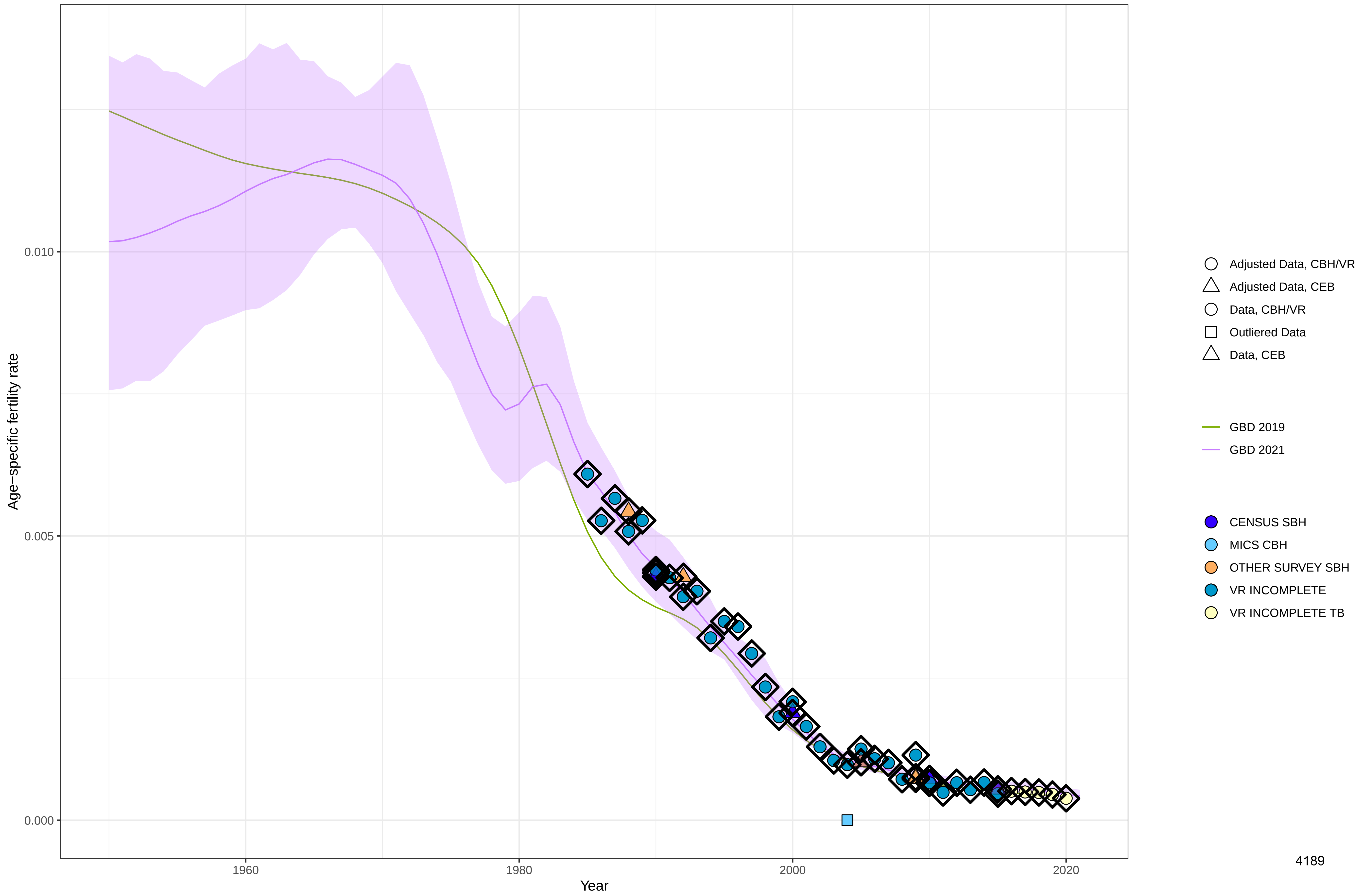

Age−specific fertility fitted trend lines, 1950−2021: Yucatán, Ages: 50−54
Data points enclosed with a diamond are reference sources

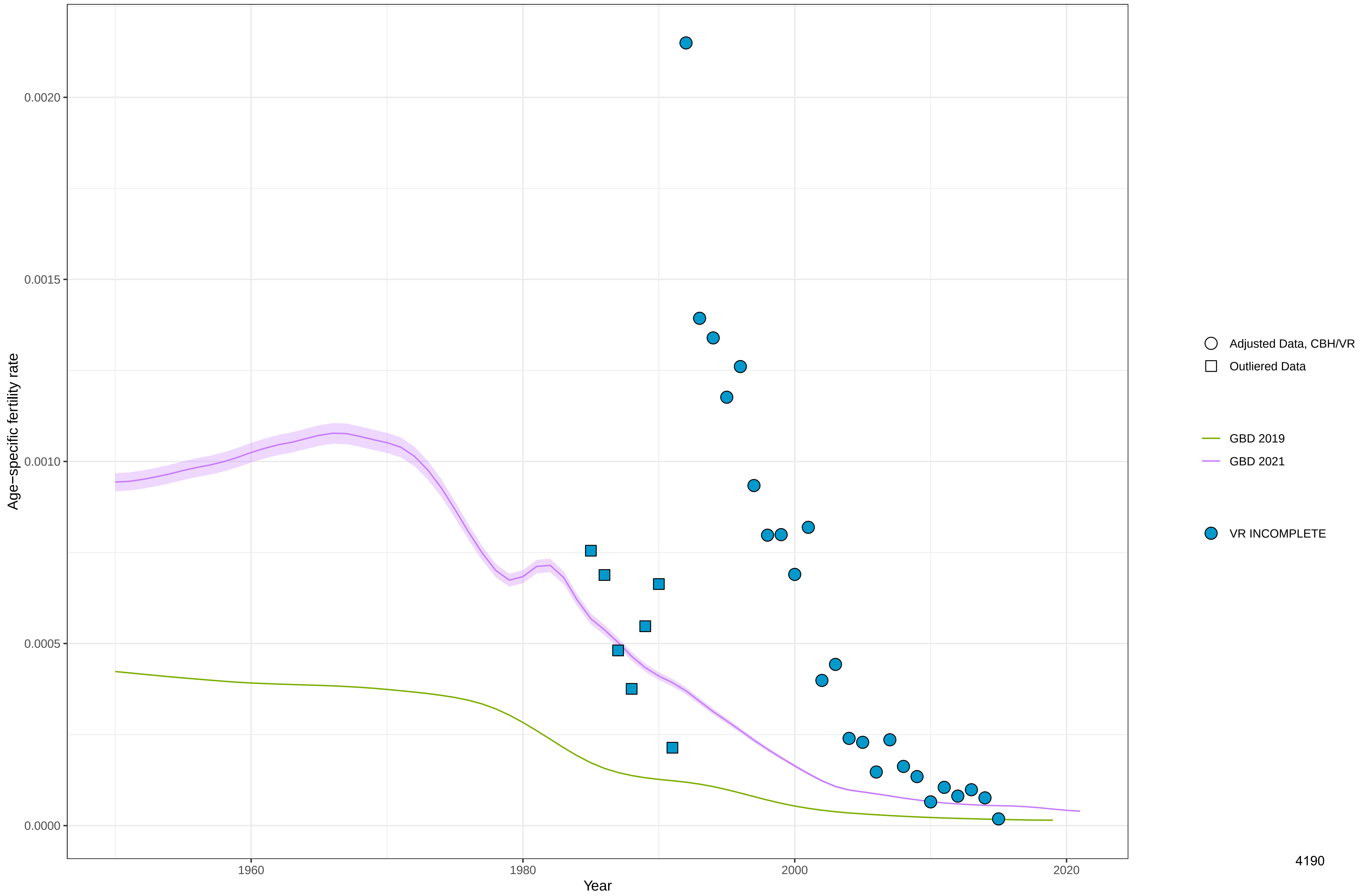

Total fertility rate fitted trend lines, 1950−2021: Yucatán TFR
Data points enclosed with a diamond are reference sources

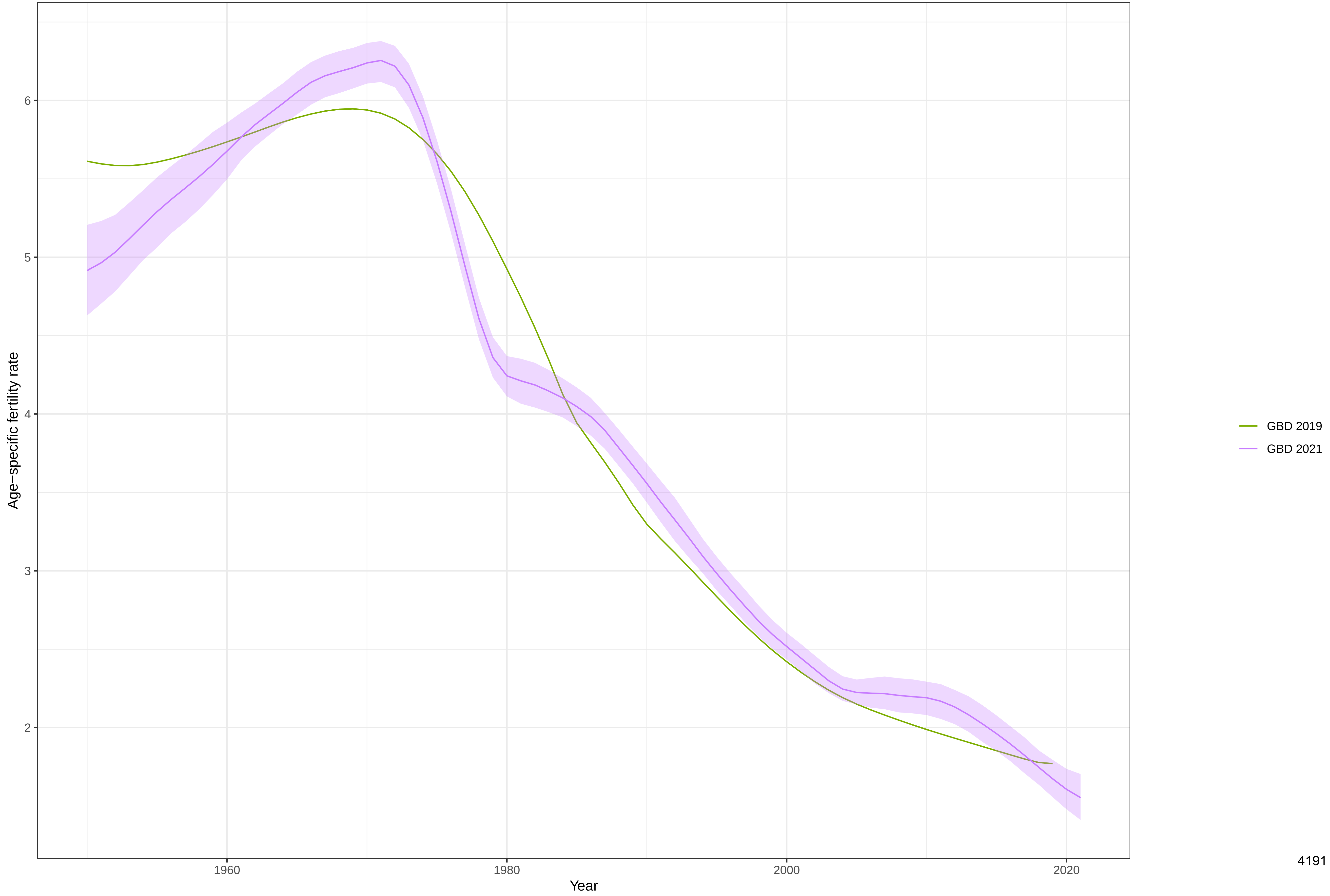

Age−specific fertility fitted trend lines, 1950−2021: Zacatecas, Ages: 10−14
Data points enclosed with a diamond are reference sources

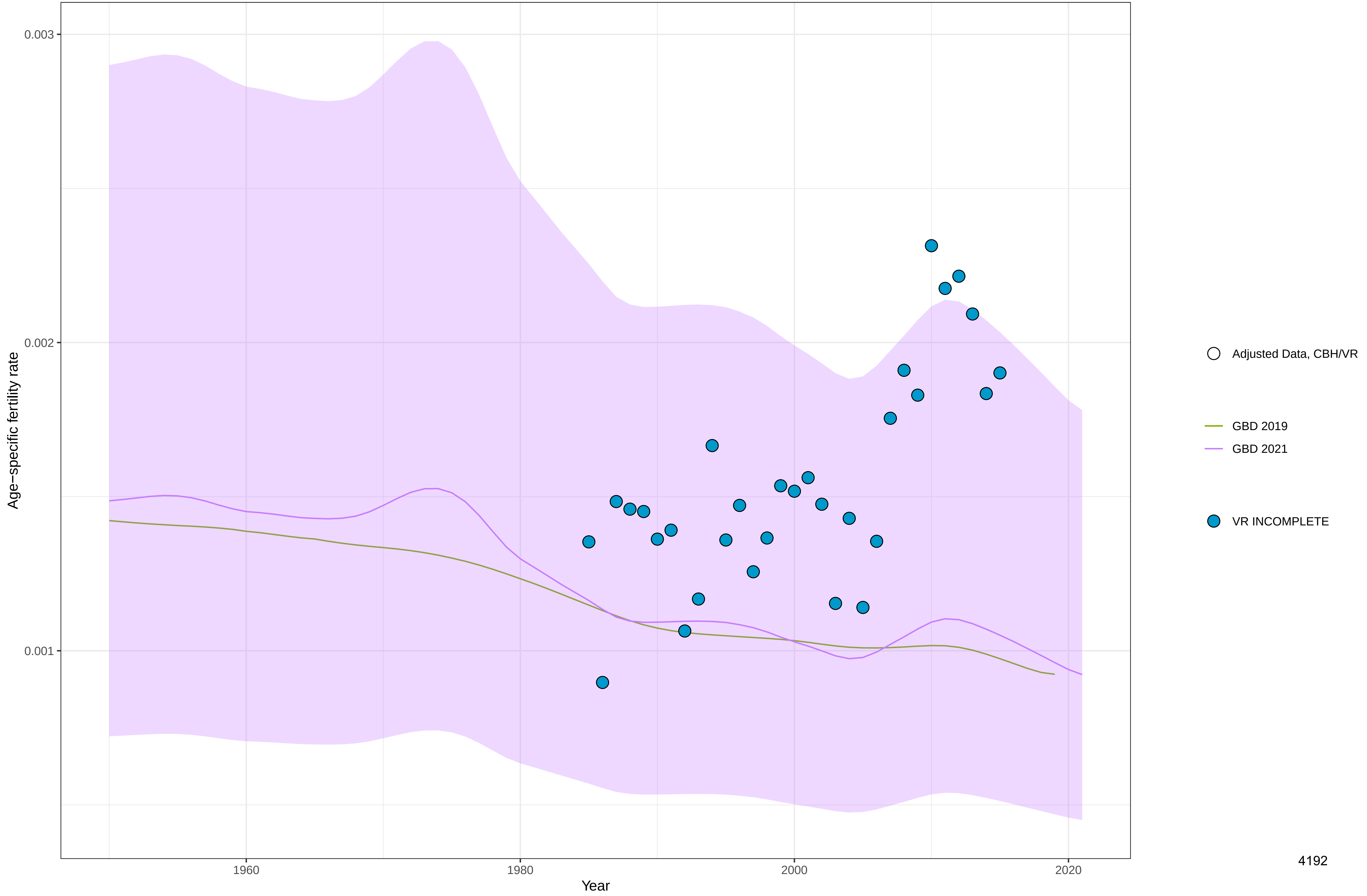

Age−specific fertility fitted trend lines, 1950−2021: Zacatecas, Ages: 15−19
Data points enclosed with a diamond are reference sources

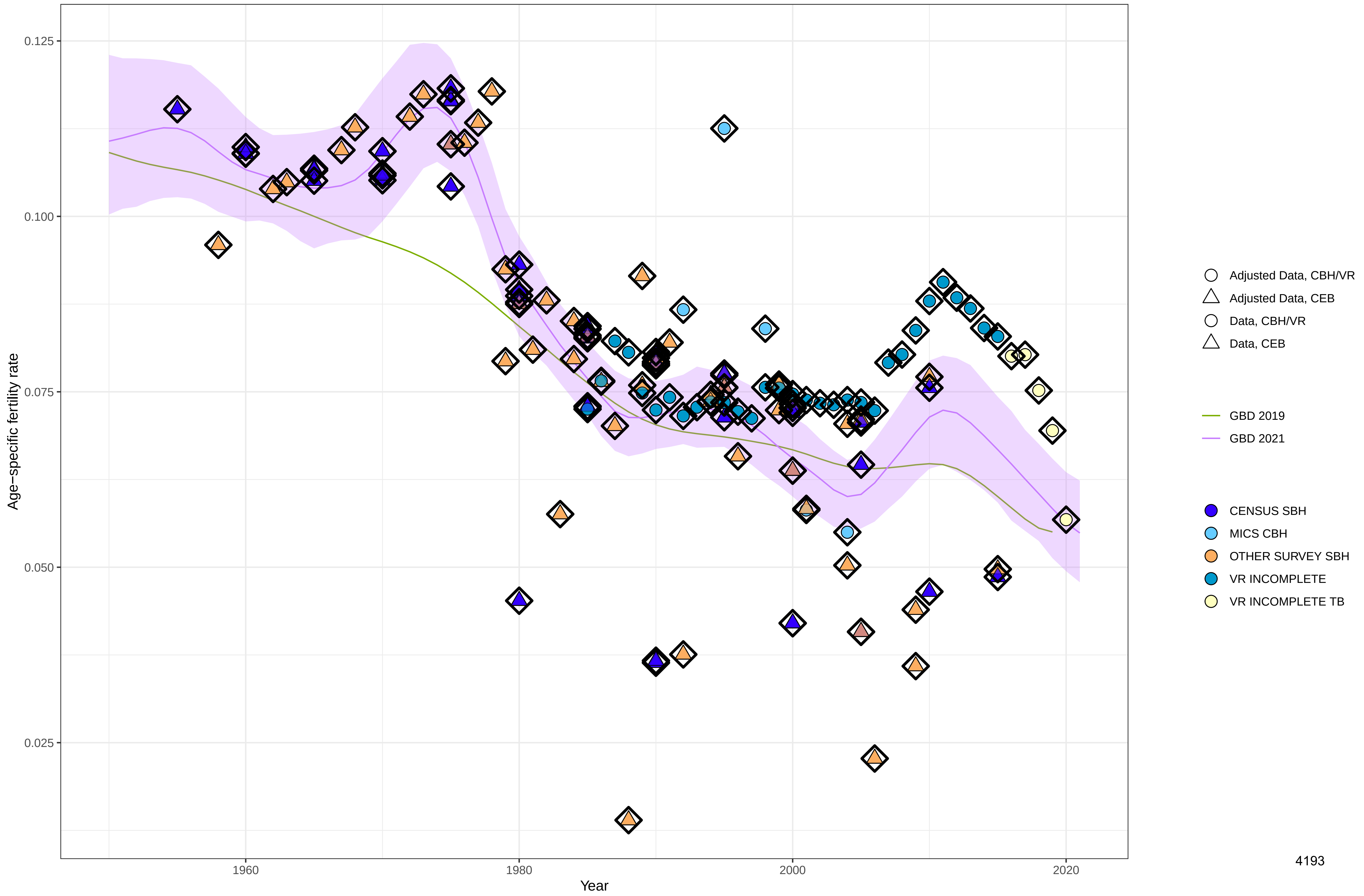

Age−specific fertility fitted trend lines, 1950−2021: Zacatecas, Ages: 20−24
Data points enclosed with a diamond are reference sources

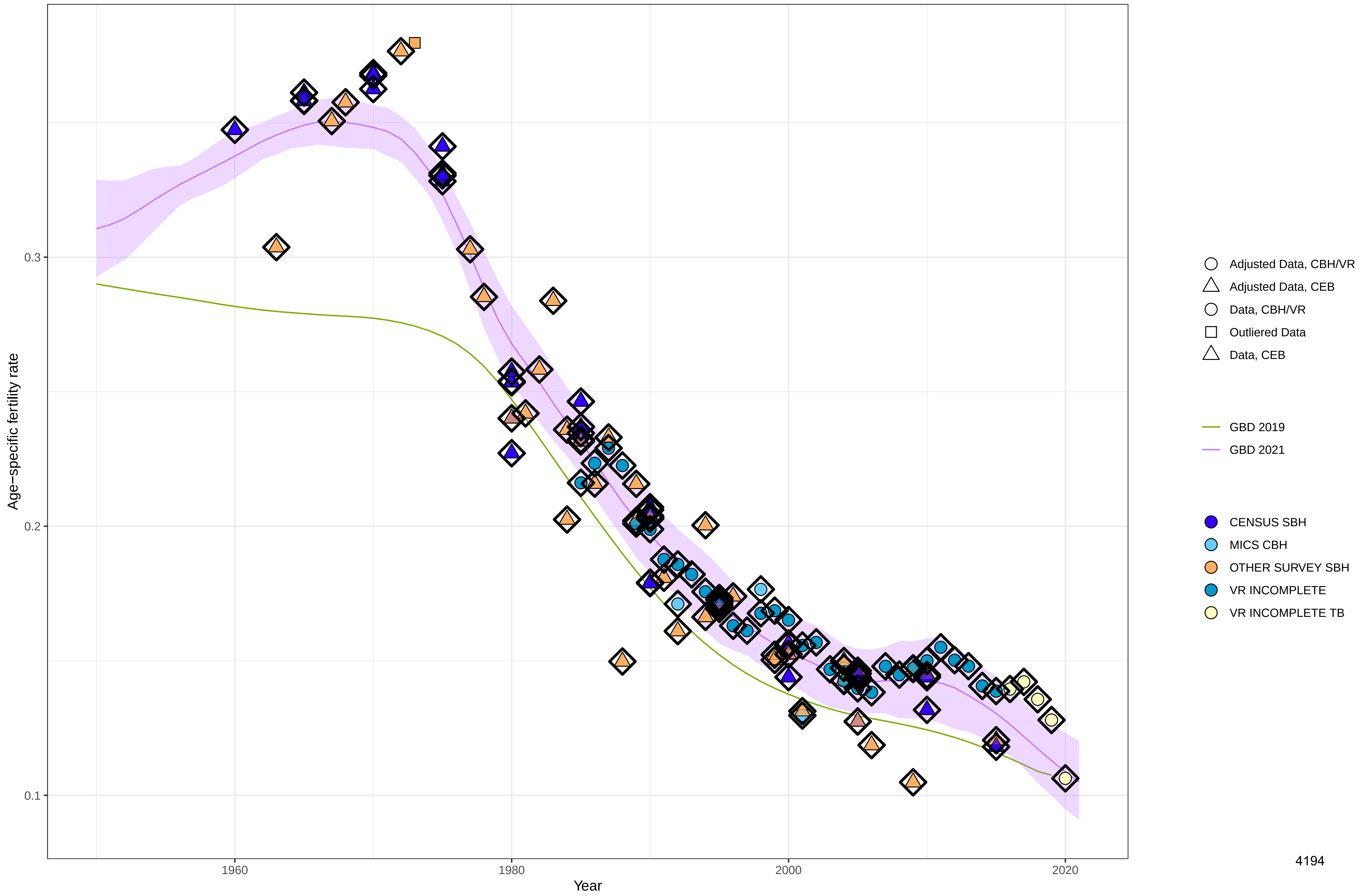

4194

Age−specific fertility fitted trend lines, 1950−2021: Zacatecas, Ages: 25−29
Data points enclosed with a diamond are reference sources

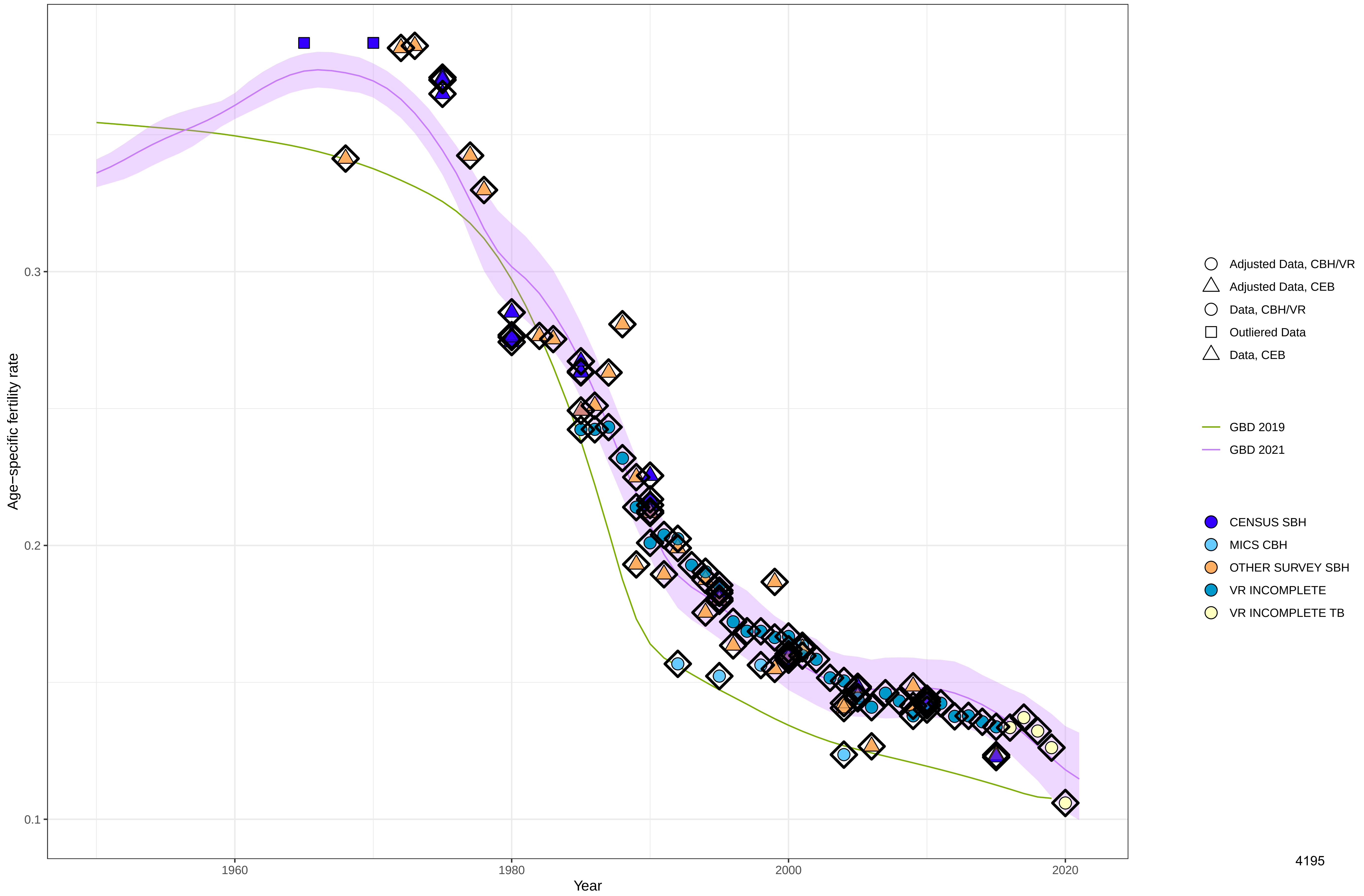

Age−specific fertility fitted trend lines, 1950−2021: Zacatecas, Ages: 30−34
Data points enclosed with a diamond are reference sources

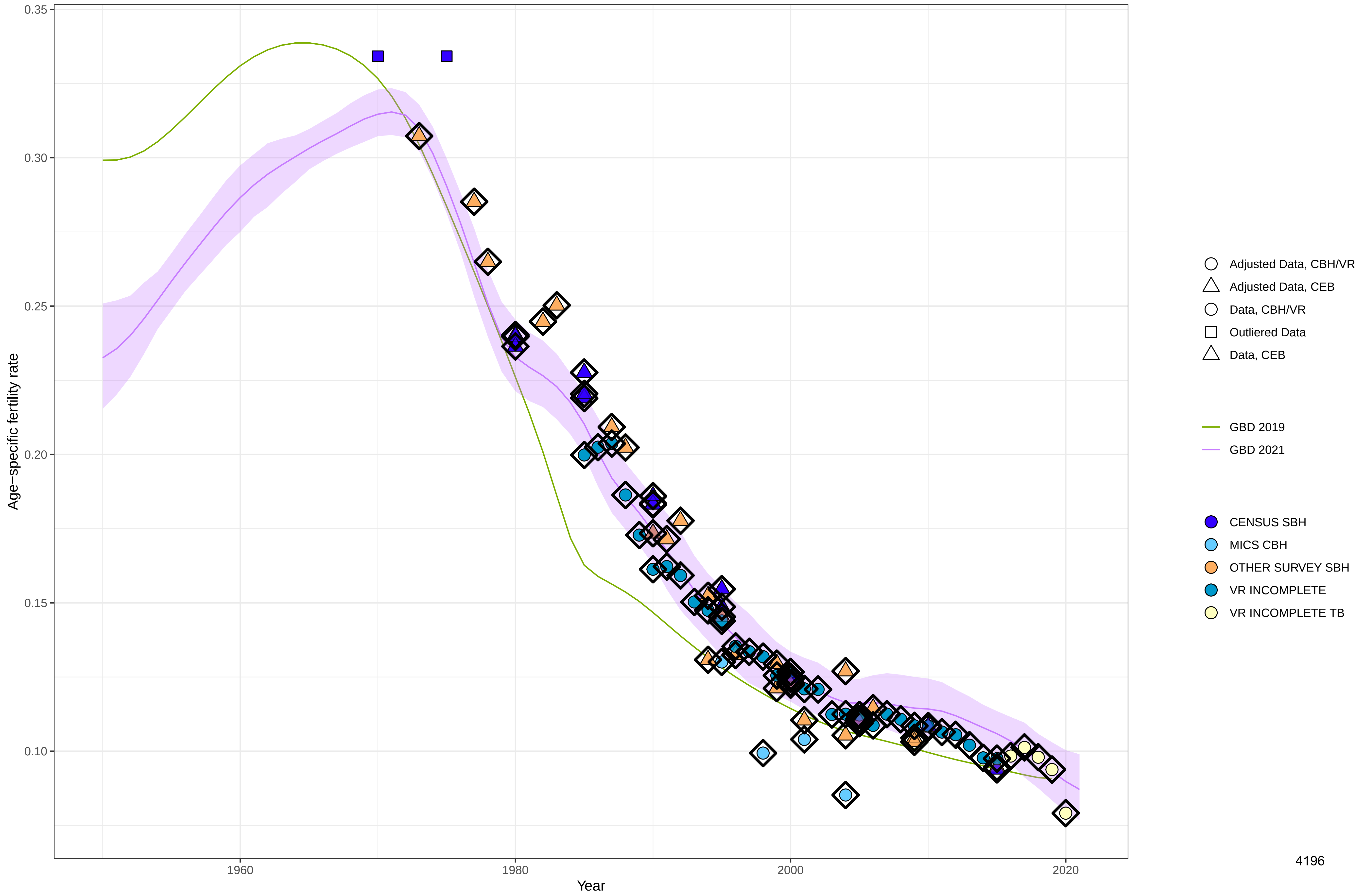

Age−specific fertility fitted trend lines, 1950−2021: Zacatecas, Ages: 35−39
Data points enclosed with a diamond are reference sources

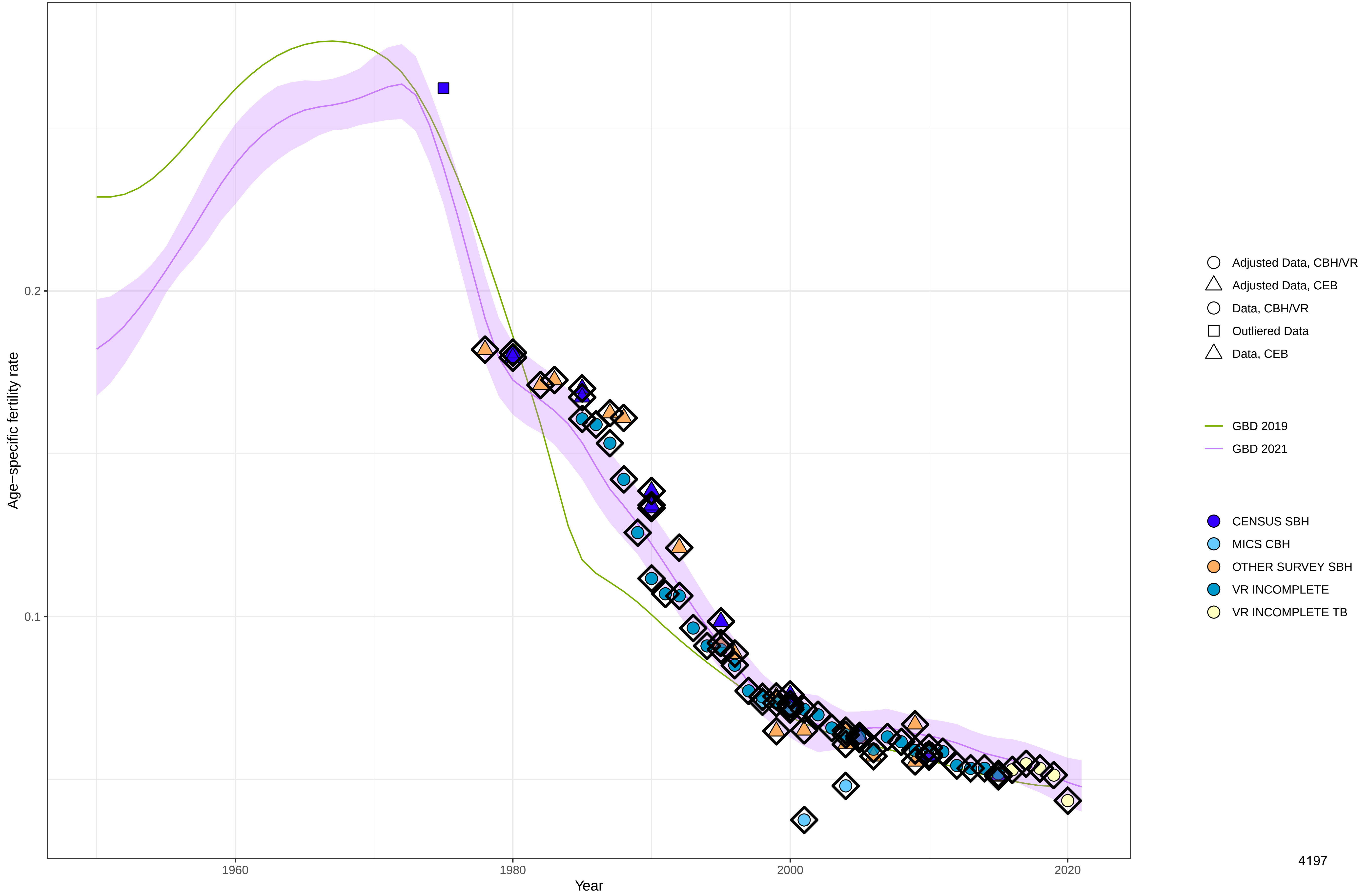

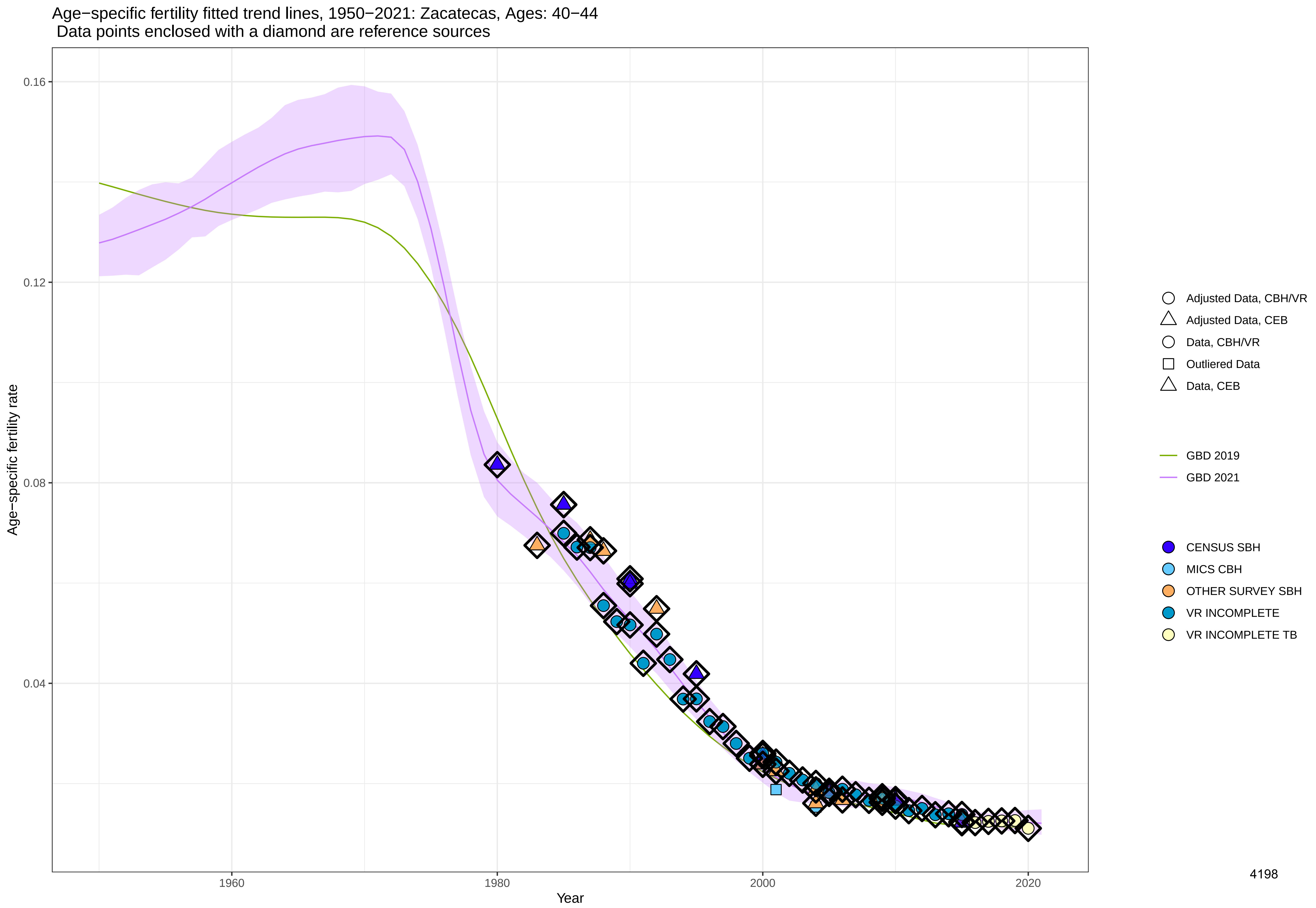

Age−specific fertility fitted trend lines, 1950−2021: Zacatecas, Ages: 40−44
Data points enclosed with a diamond are reference sources
Age−specific fertility rate
0.16
0.12
0.08
0.04
1960
1980
2000
2020
Year
Adjusted Data, CBH/VR
Adjusted Data, CEB
Data, CBH/VR
Outliered Data
Data, CEB
GBD 2019
GBD 2021
CENSUS SBH
MICS CBH
OTHER SURVEY SBH
VR INCOMPLETE
VR INCOMPLETE TB

Age−specific fertility fitted trend lines, 1950−2021: Zacatecas, Ages: 45−49
Data points enclosed with a diamond are reference sources

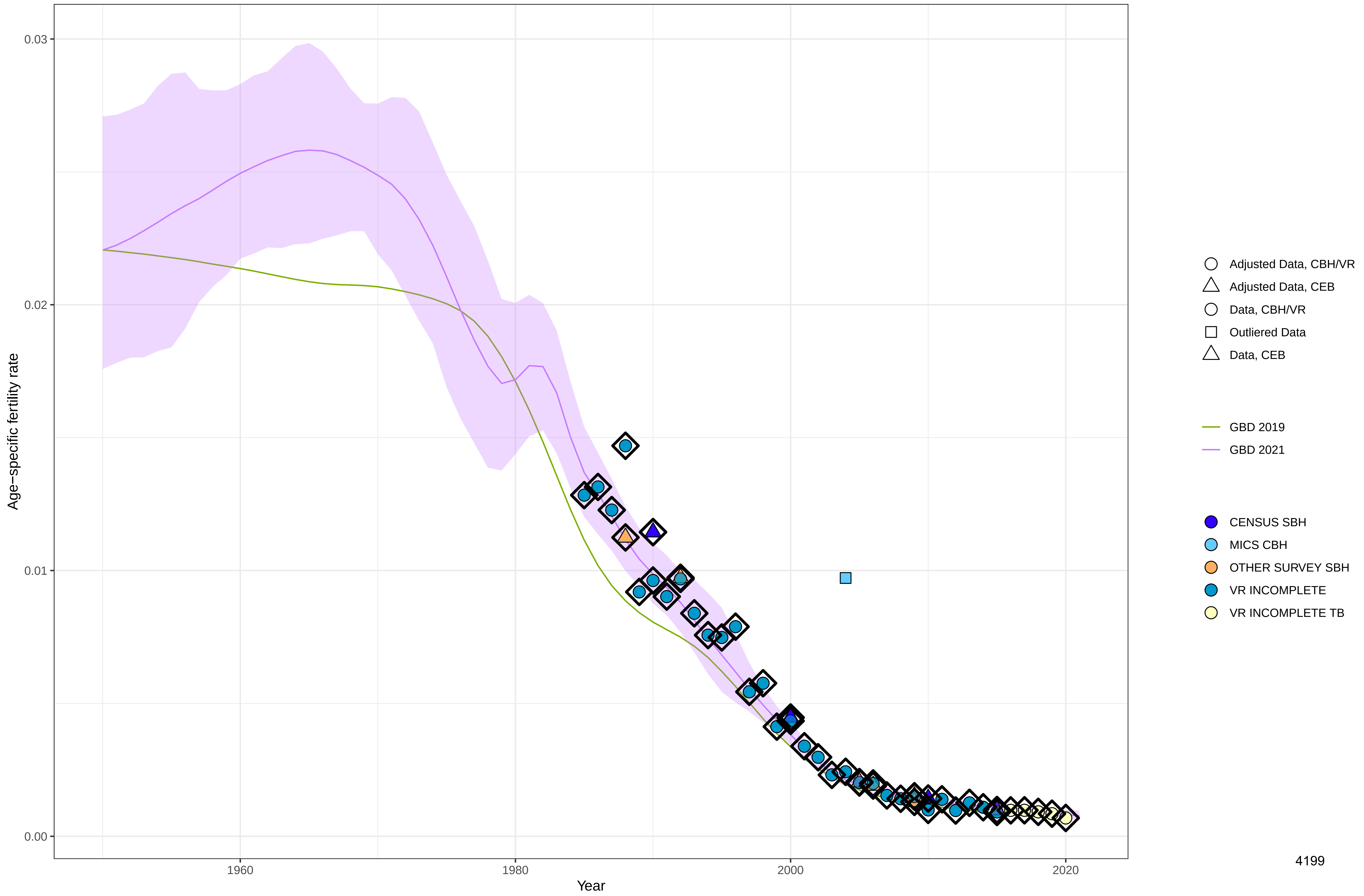

Age−specific fertility fitted trend lines, 1950−2021: Zacatecas, Ages: 50−54
Data points enclosed with a diamond are reference sources

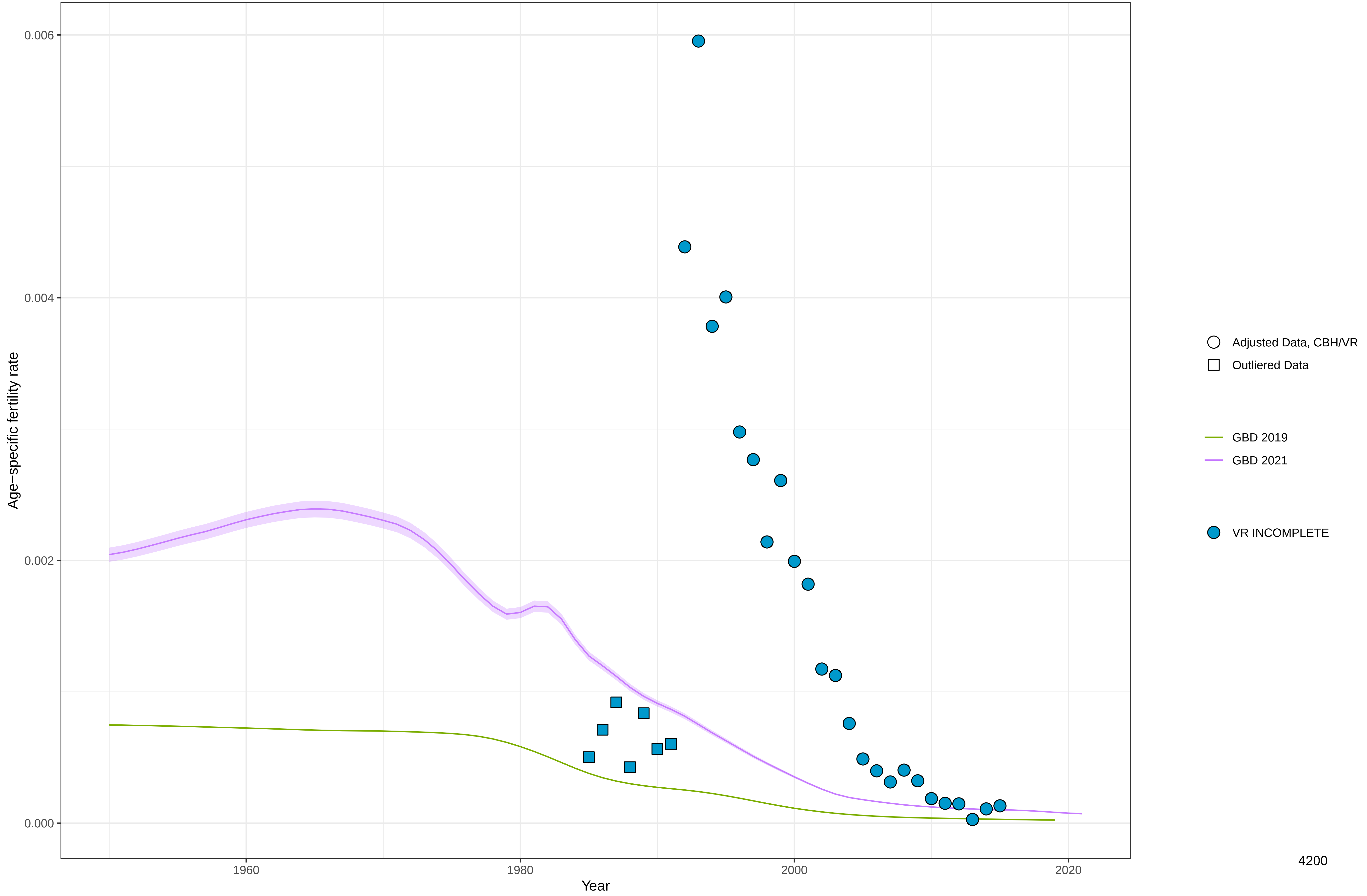

Total fertility rate fitted trend lines, 1950−2021: Zacatecas TFR
Data points enclosed with a diamond are reference sources

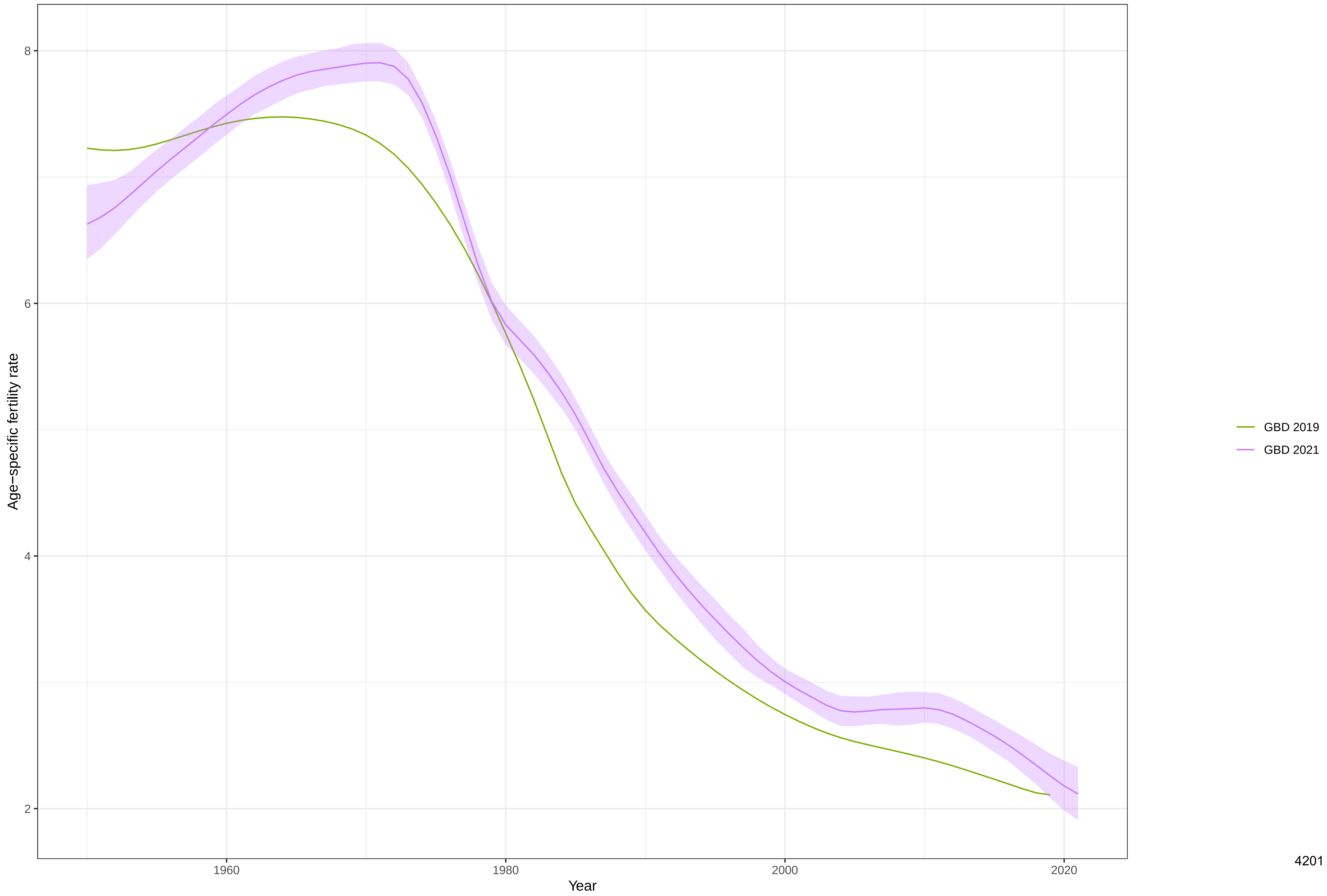

Age−specific fertility fitted trend lines, 1950−2021: Nicaragua, Ages: 10−14
Data points enclosed with a diamond are reference sources

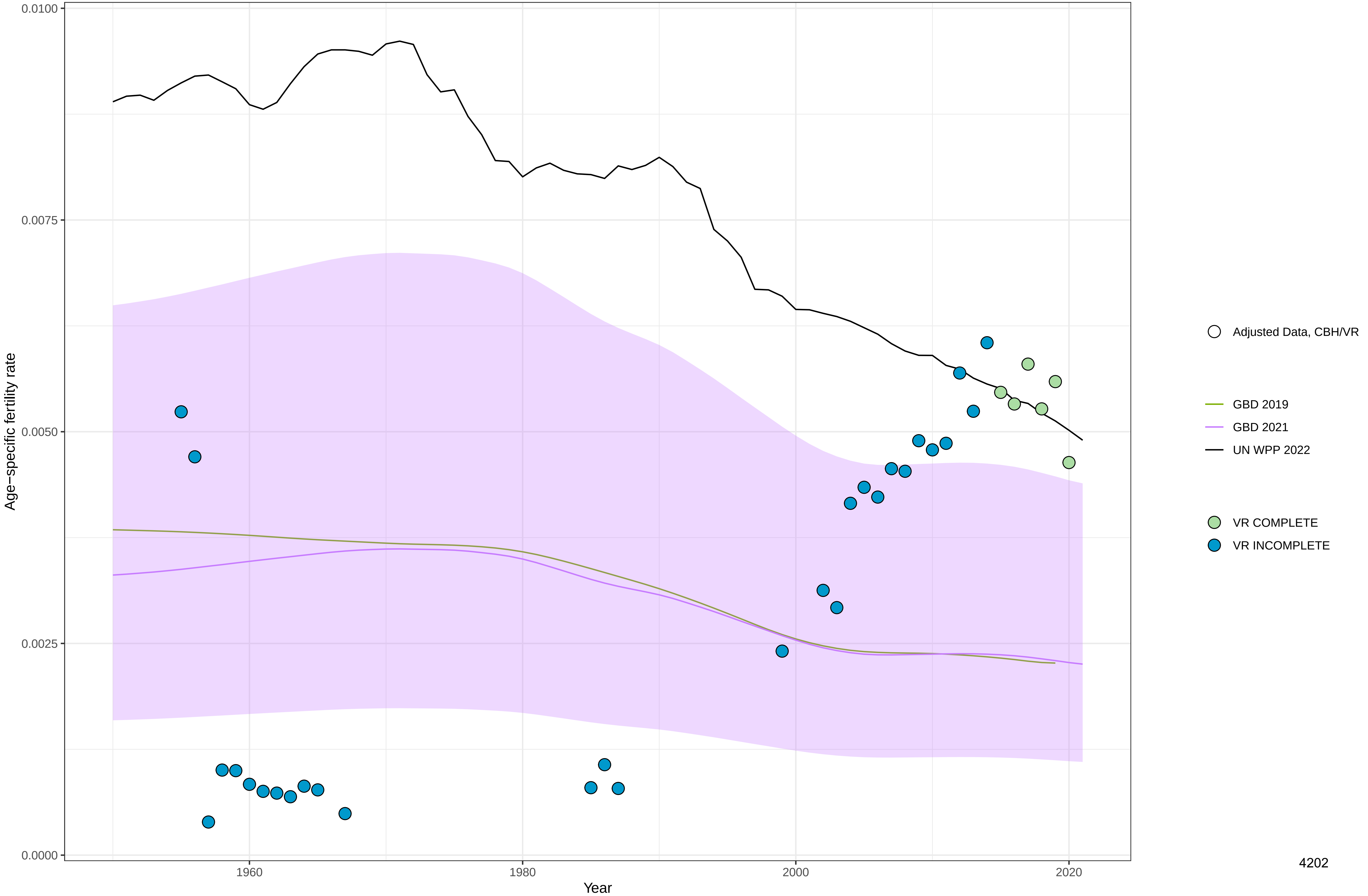

Age−specific fertility fitted trend lines, 1950−2021: Nicaragua, Ages: 15−19
Data points enclosed with a diamond are reference sources

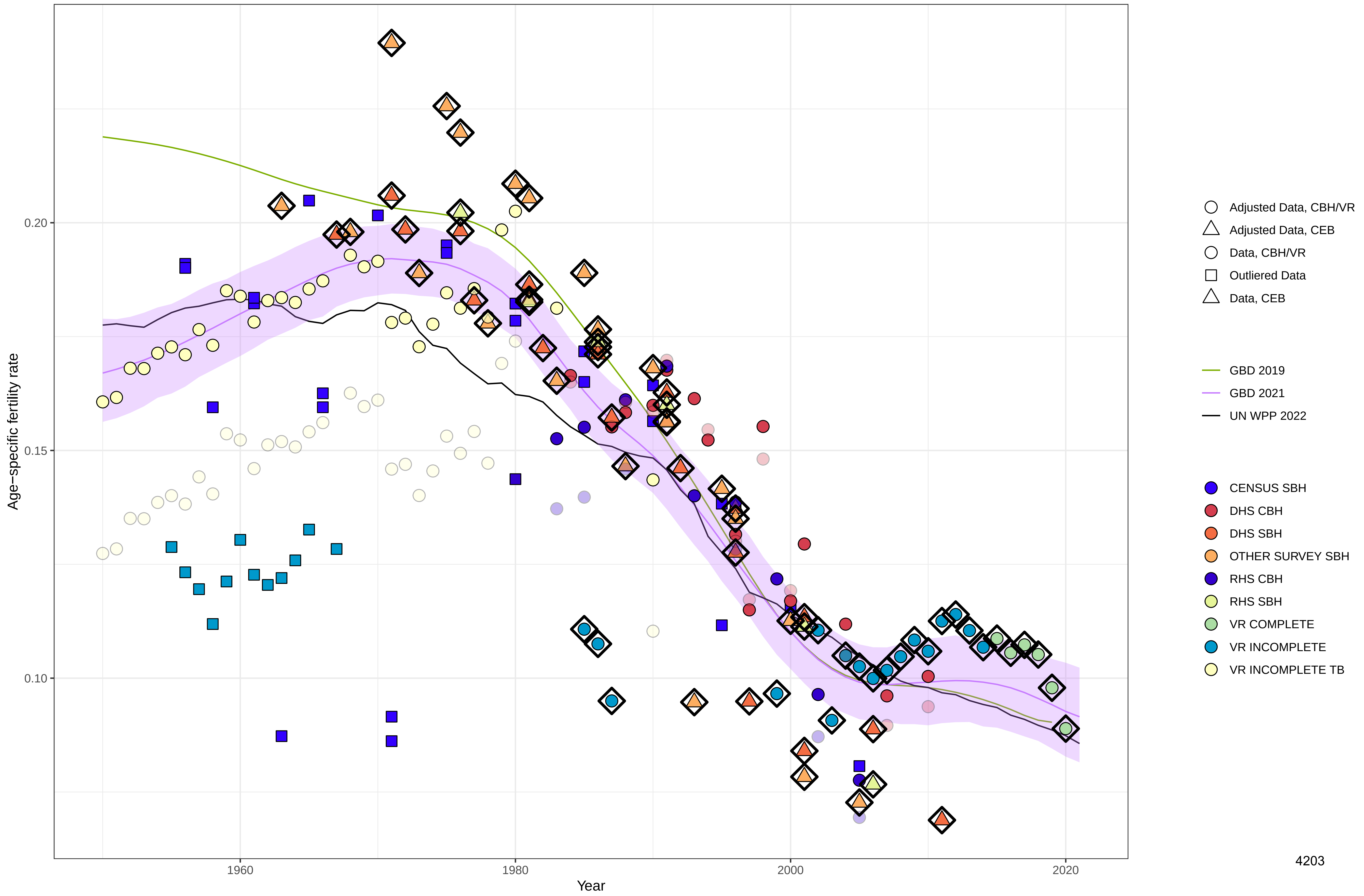

Age−specific fertility fitted trend lines, 1950−2021: Nicaragua, Ages: 20−24
Data points enclosed with a diamond are reference sources

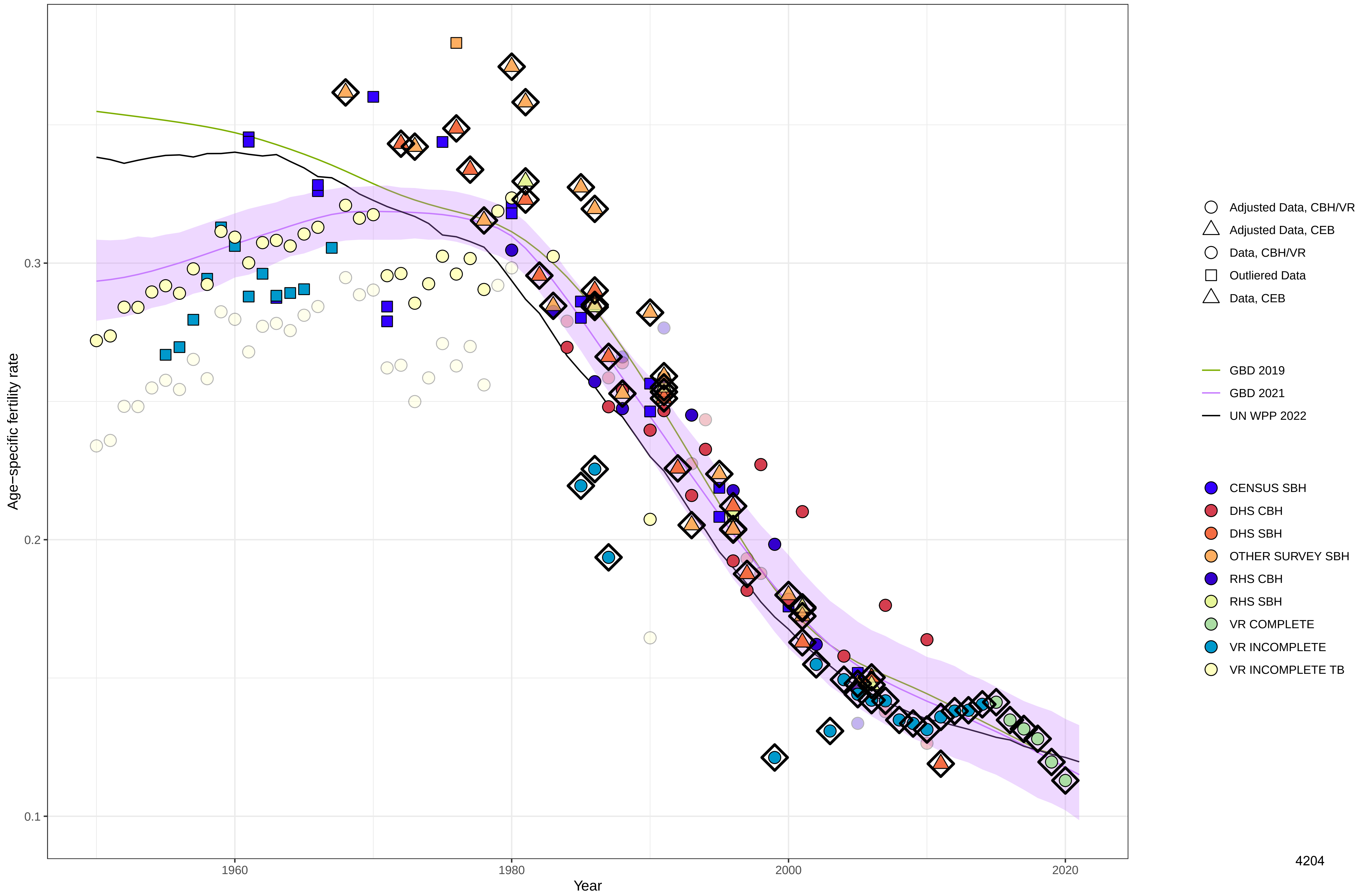

Age−specific fertility fitted trend lines, 1950−2021: Nicaragua, Ages: 25−29
Data points enclosed with a diamond are reference sources

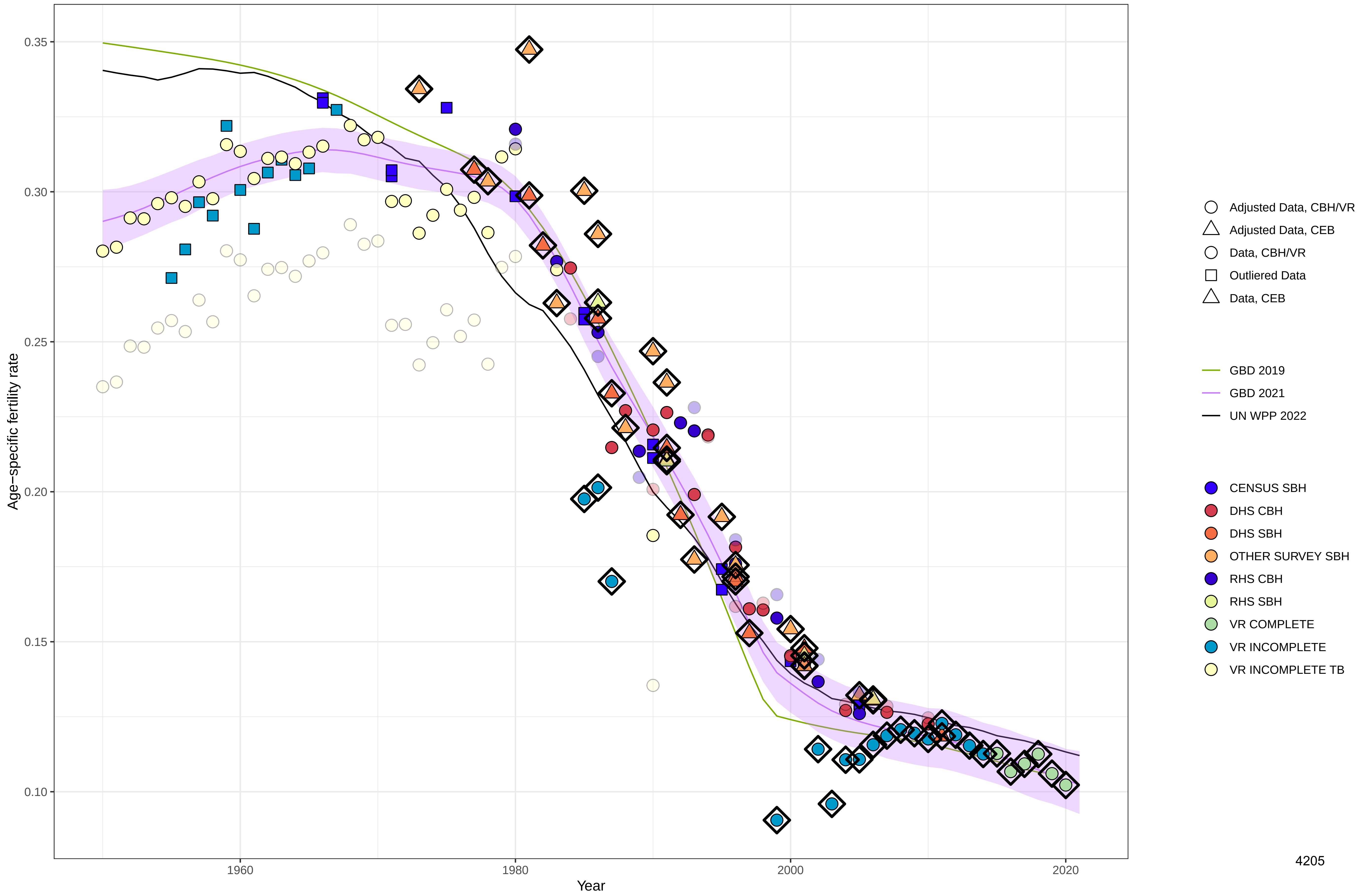

Age−specific fertility fitted trend lines, 1950−2021: Nicaragua, Ages: 30−34
Data points enclosed with a diamond are reference sources

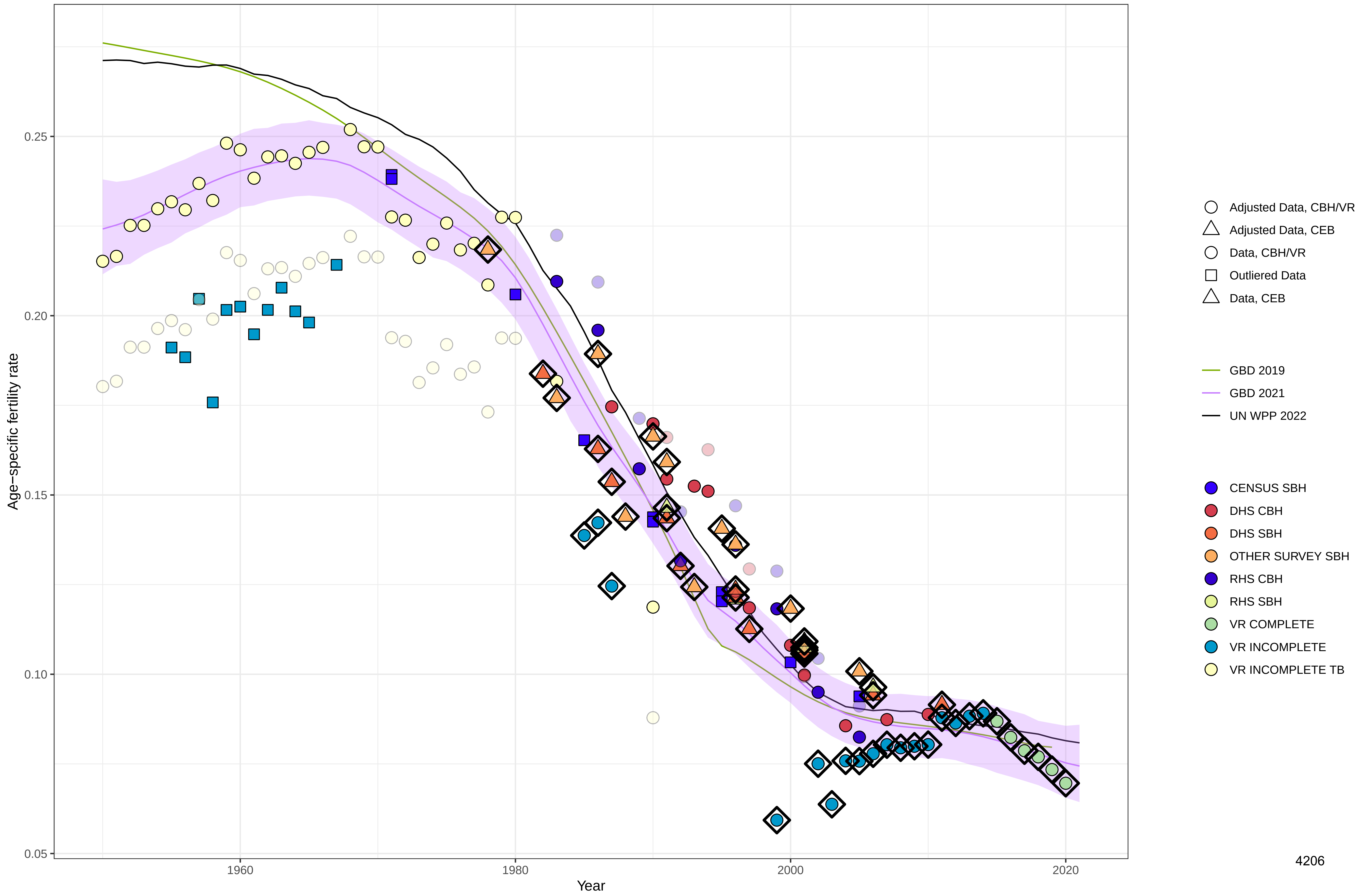

4206

Age−specific fertility fitted trend lines, 1950−2021: Nicaragua, Ages: 35−39
Data points enclosed with a diamond are reference sources

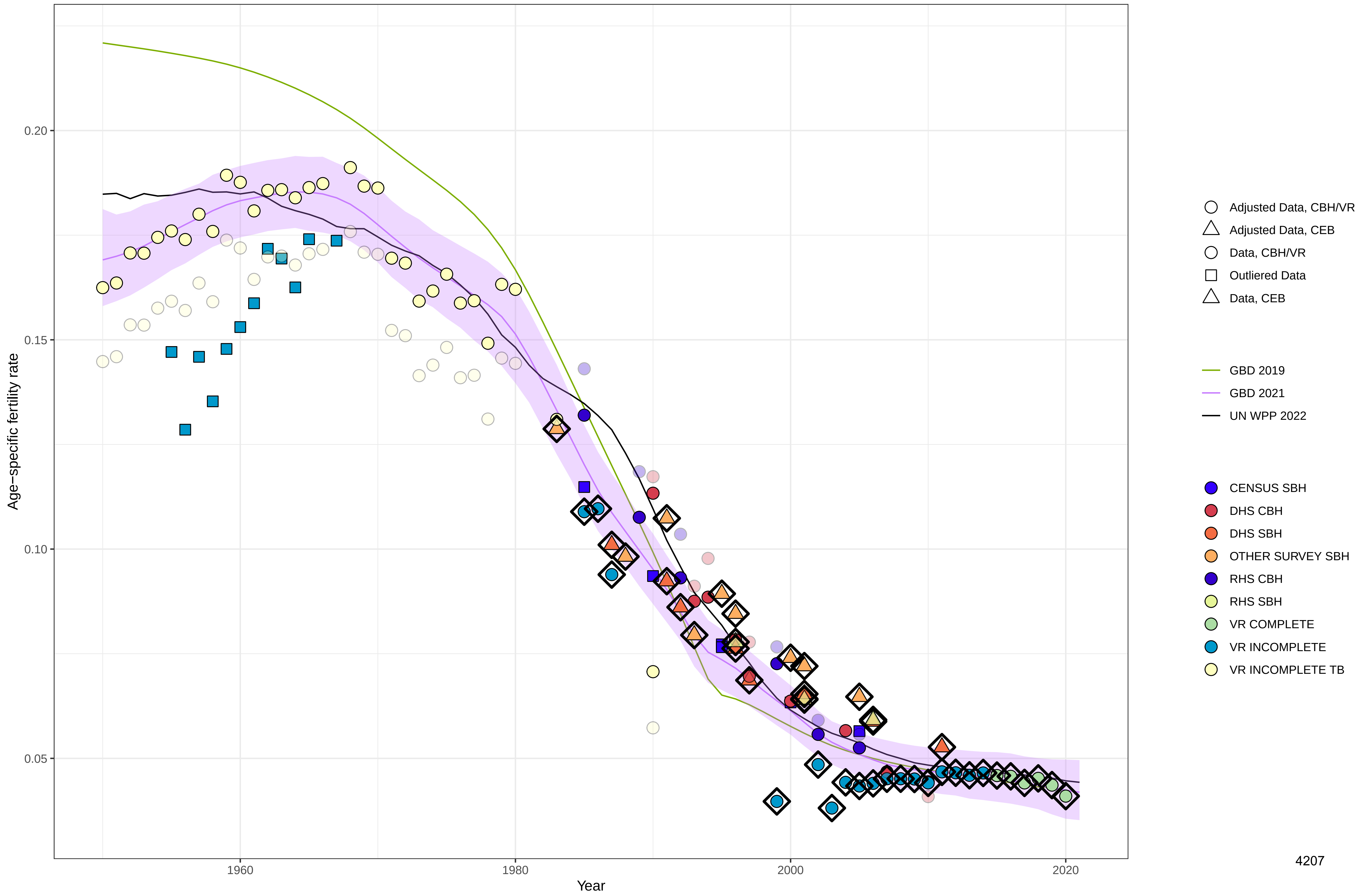

Age−specific fertility fitted trend lines, 1950−2021: Nicaragua, Ages: 40−44
Data points enclosed with a diamond are reference sources

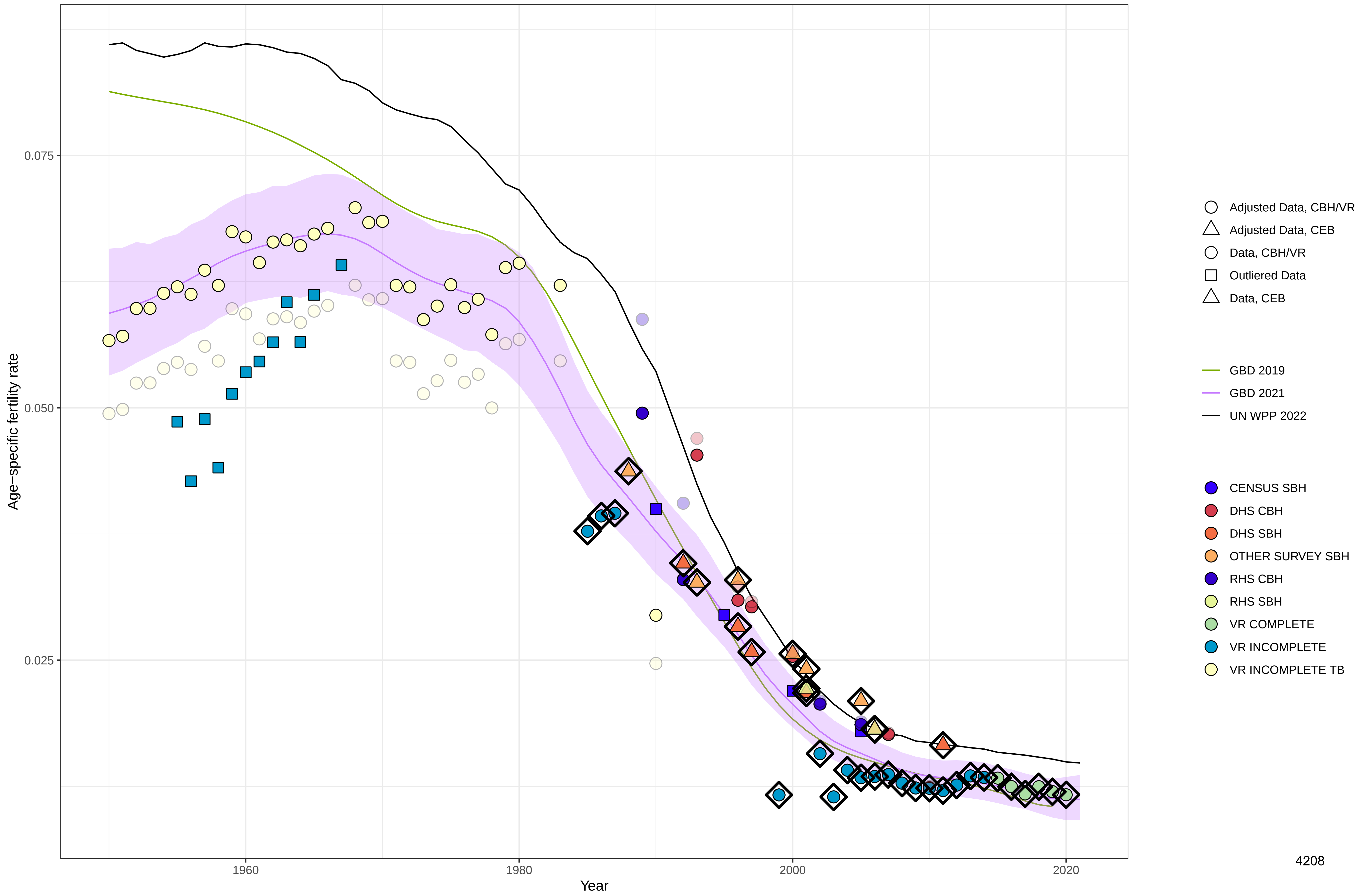

Age−specific fertility fitted trend lines, 1950−2021: Nicaragua, Ages: 45−49
Data points enclosed with a diamond are reference sources

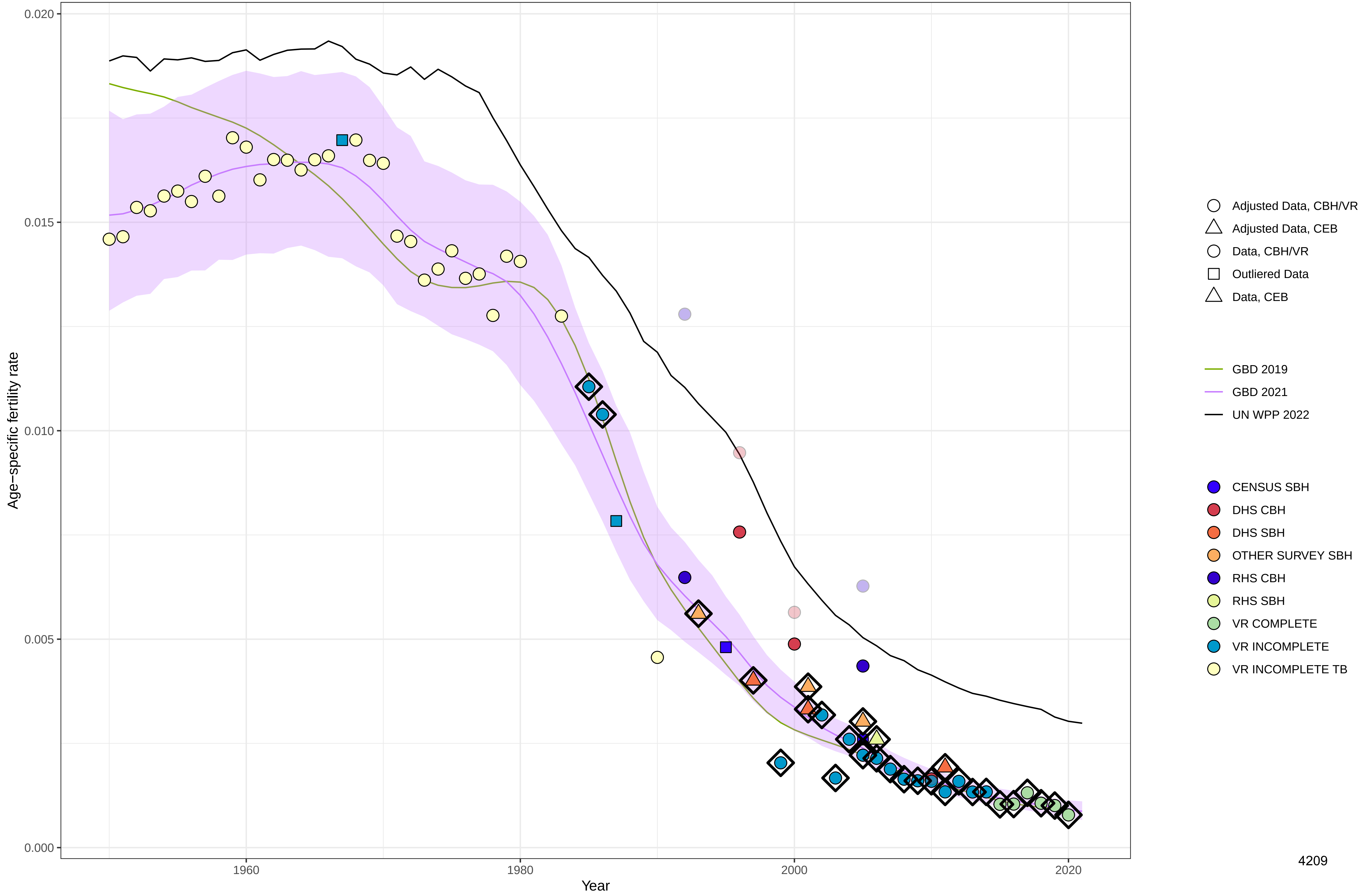

Age−specific fertility fitted trend lines, 1950−2021: Nicaragua, Ages: 50−54
Data points enclosed with a diamond are reference sources

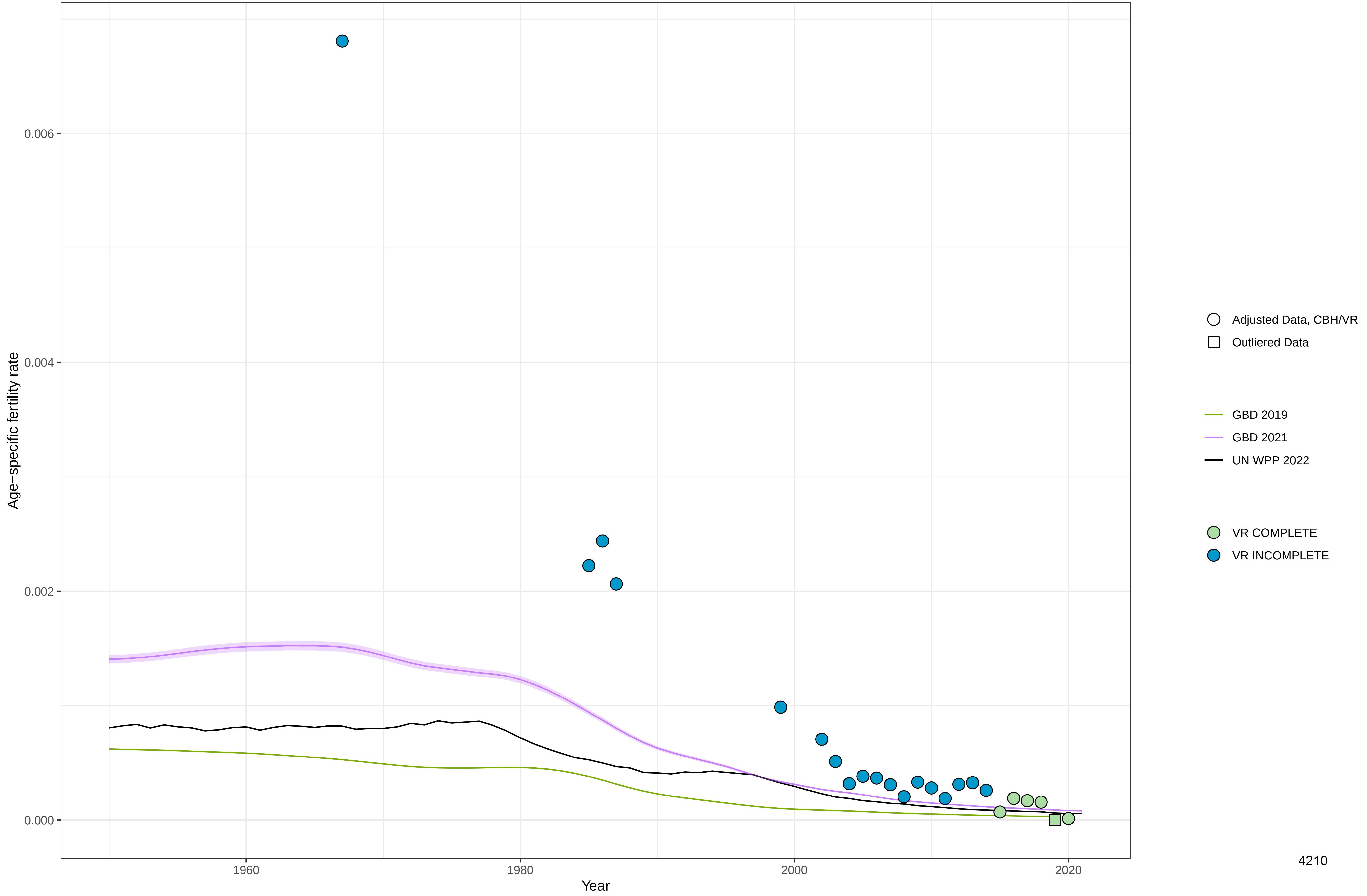

Total fertility rate fitted trend lines, 1950−2021: Nicaragua TFR
Data points enclosed with a diamond are reference sources

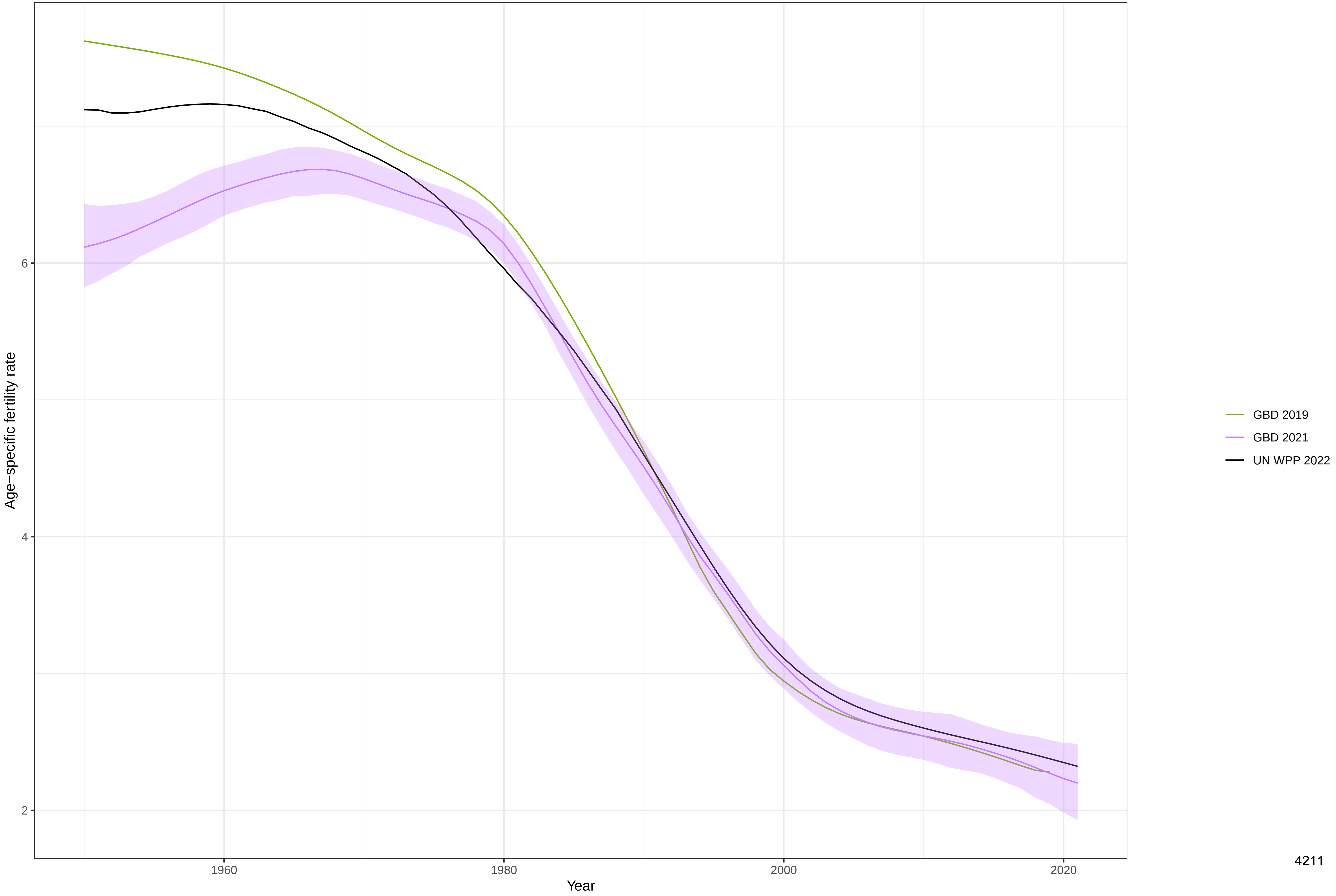

Age−specific fertility fitted trend lines, 1950−2021: Panama, Ages: 10−14
Data points enclosed with a diamond are reference sources

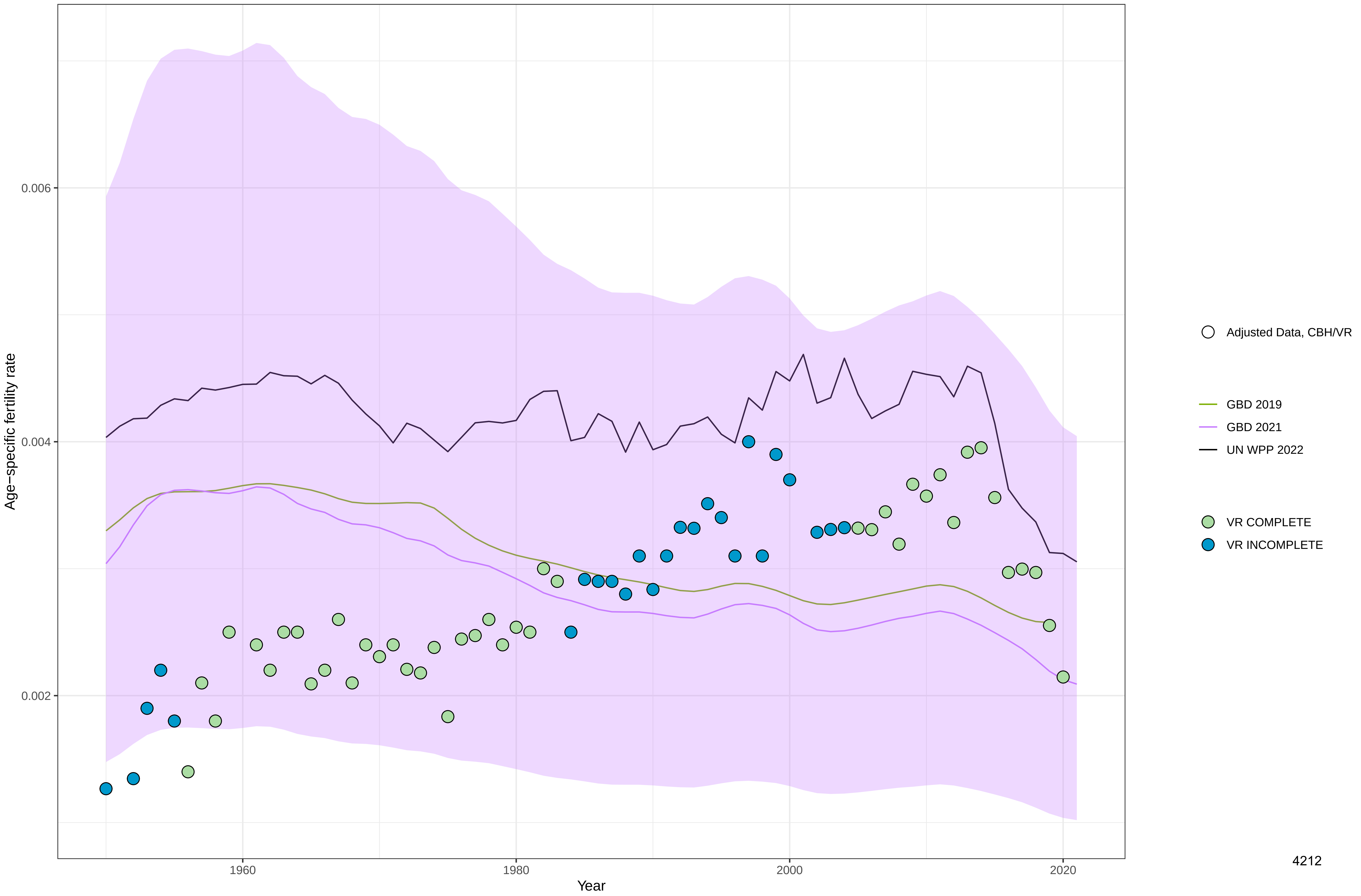

Age−specific fertility fitted trend lines, 1950−2021: Panama, Ages: 15−19
Data points enclosed with a diamond are reference sources

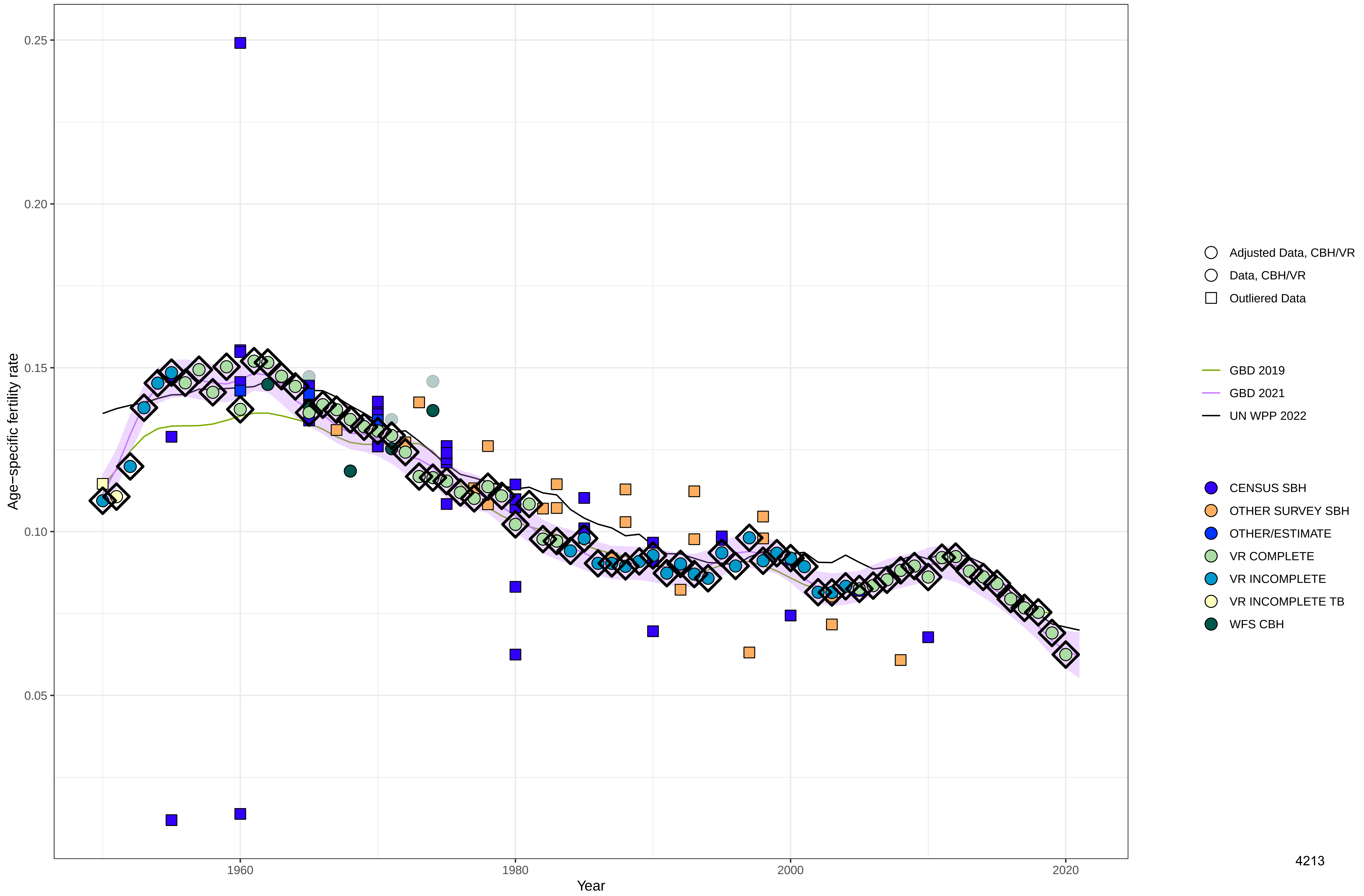

Age−specific fertility fitted trend lines, 1950−2021: Panama, Ages: 20−24
Data points enclosed with a diamond are reference sources

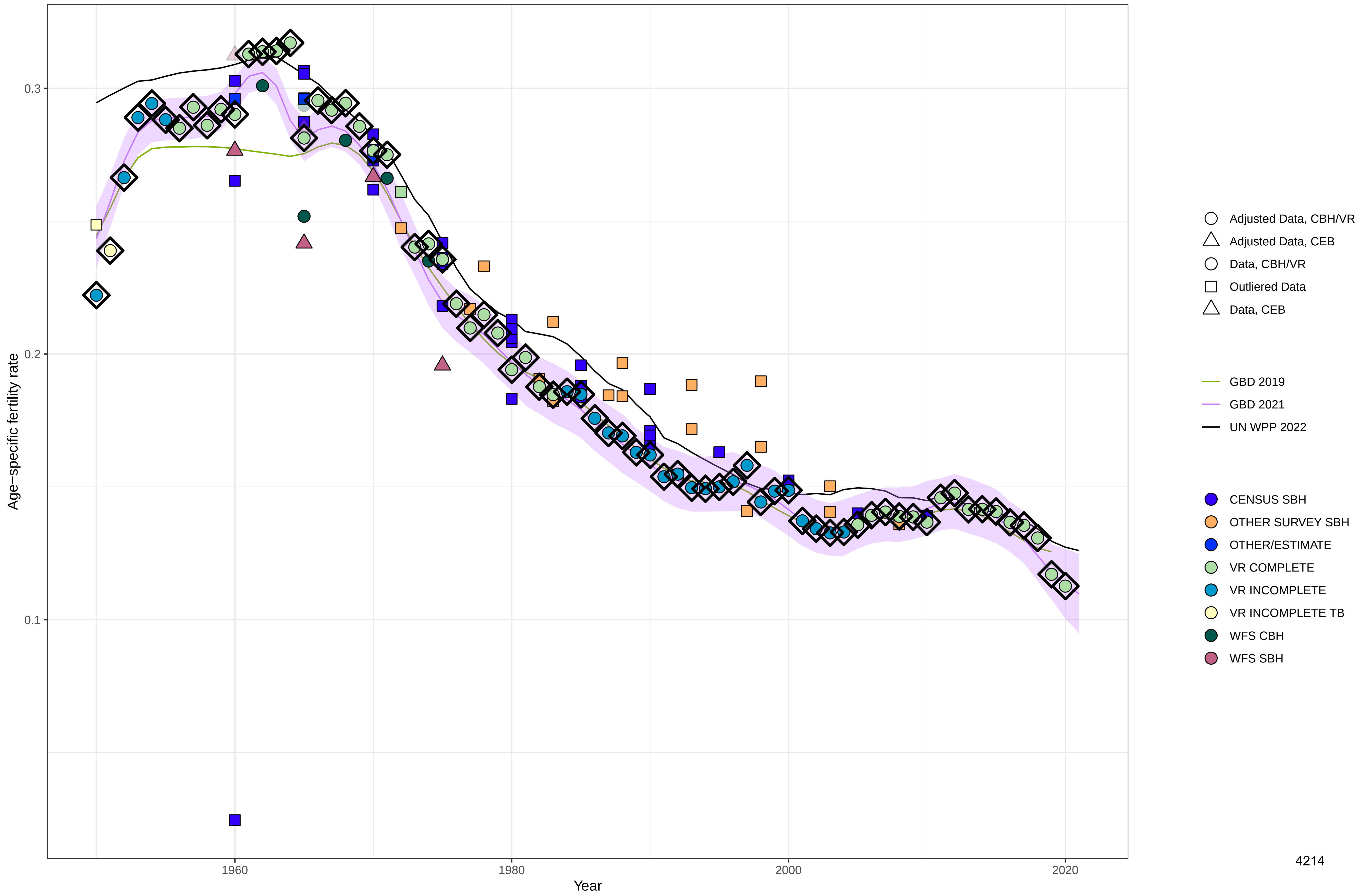

Age−specific fertility fitted trend lines, 1950−2021: Panama, Ages: 25−29
Data points enclosed with a diamond are reference sources

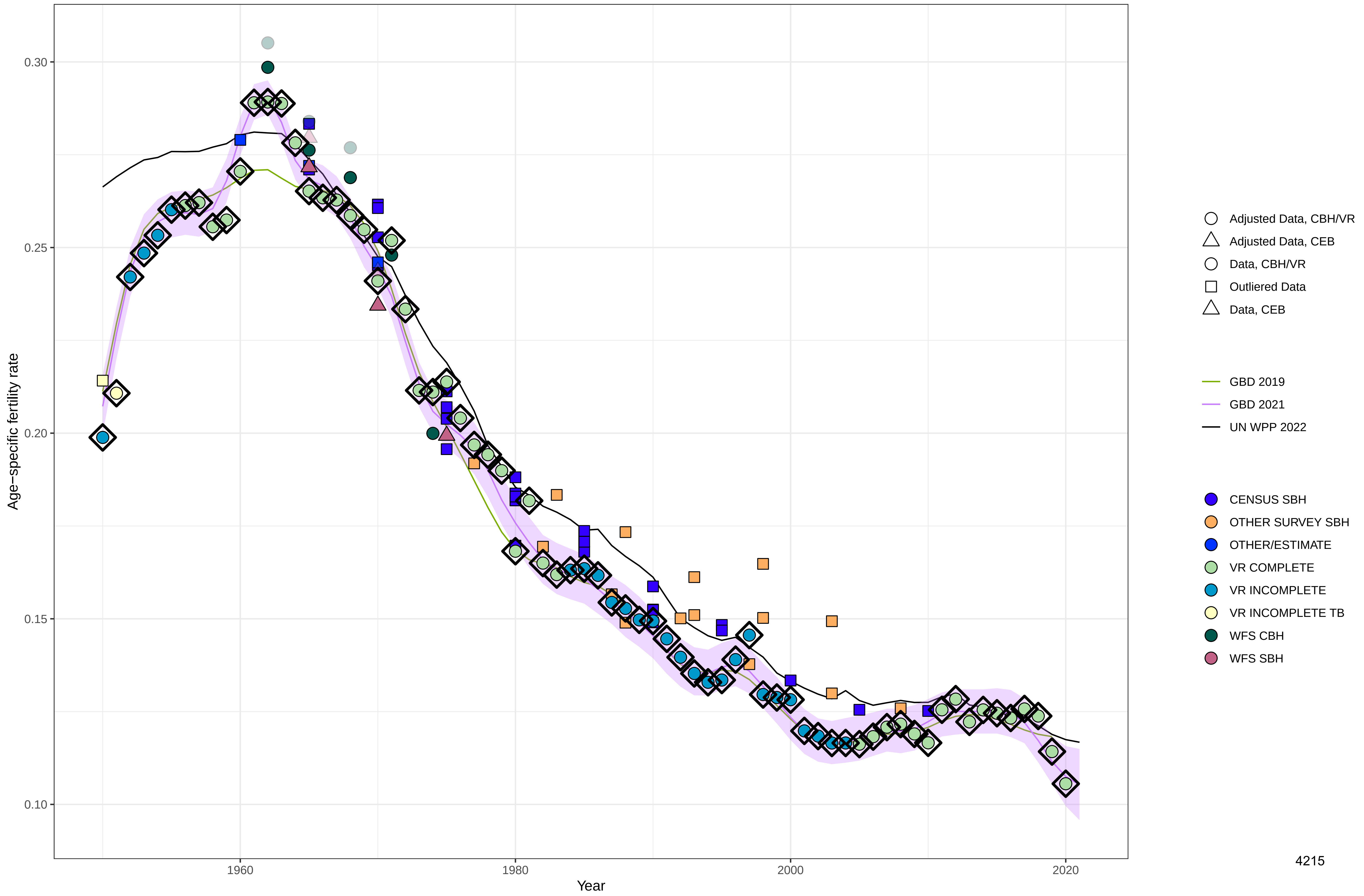

Age−specific fertility fitted trend lines, 1950−2021: Panama, Ages: 30−34
Data points enclosed with a diamond are reference sources

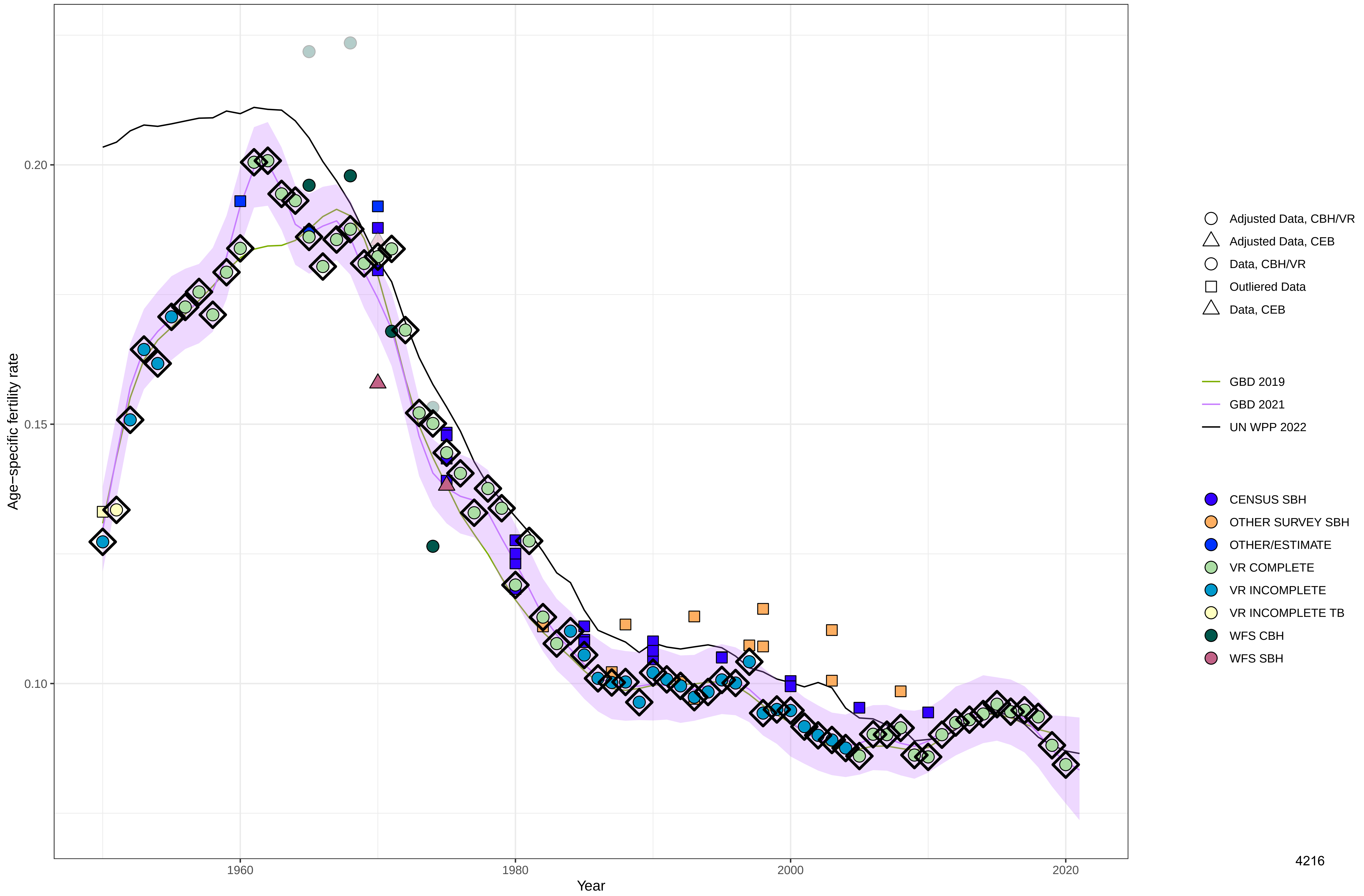

Age−specific fertility fitted trend lines, 1950−2021: Panama, Ages: 35−39
Data points enclosed with a diamond are reference sources

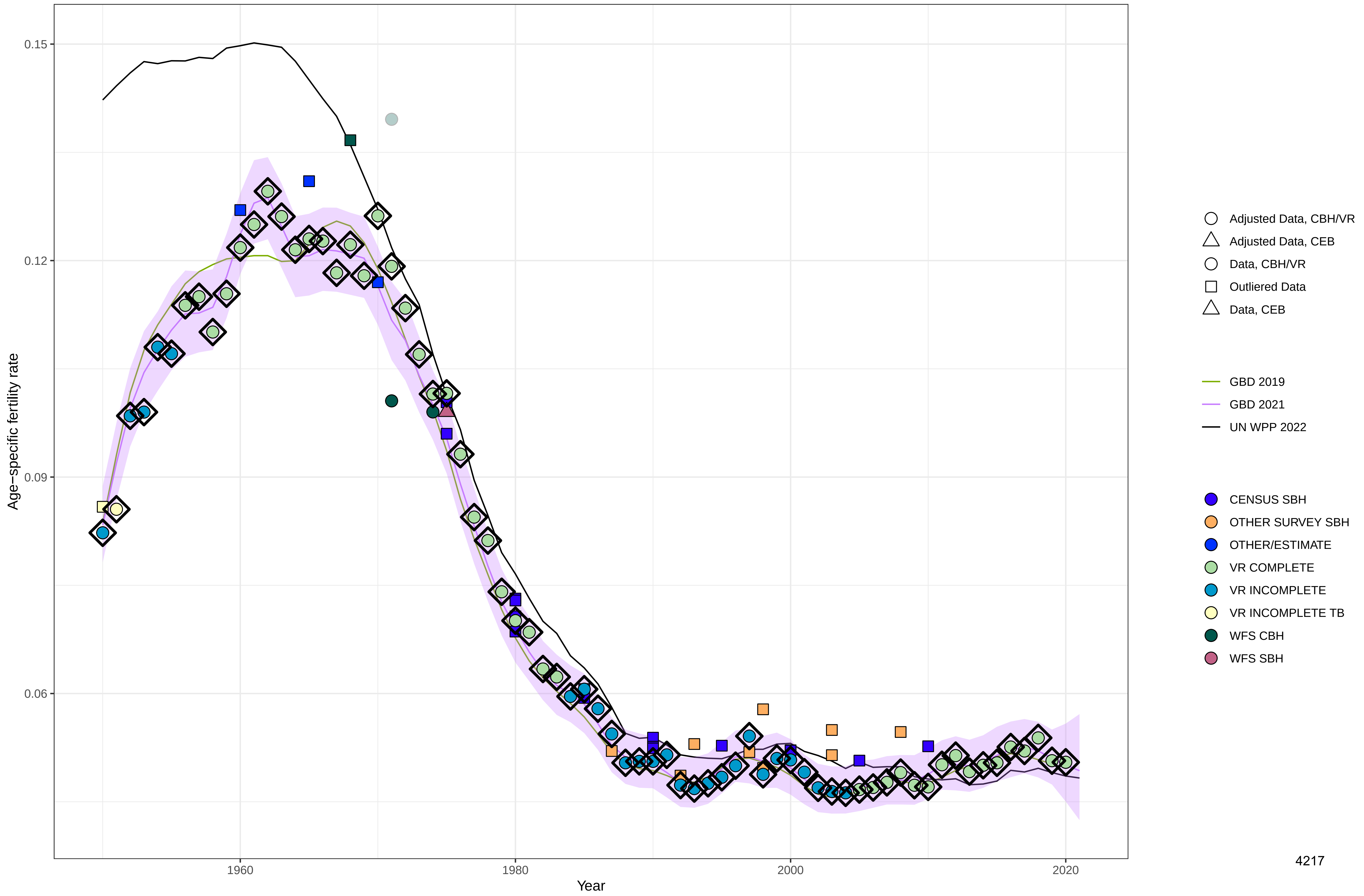

Age−specific fertility fitted trend lines, 1950−2021: Panama, Ages: 40−44
Data points enclosed with a diamond are reference sources

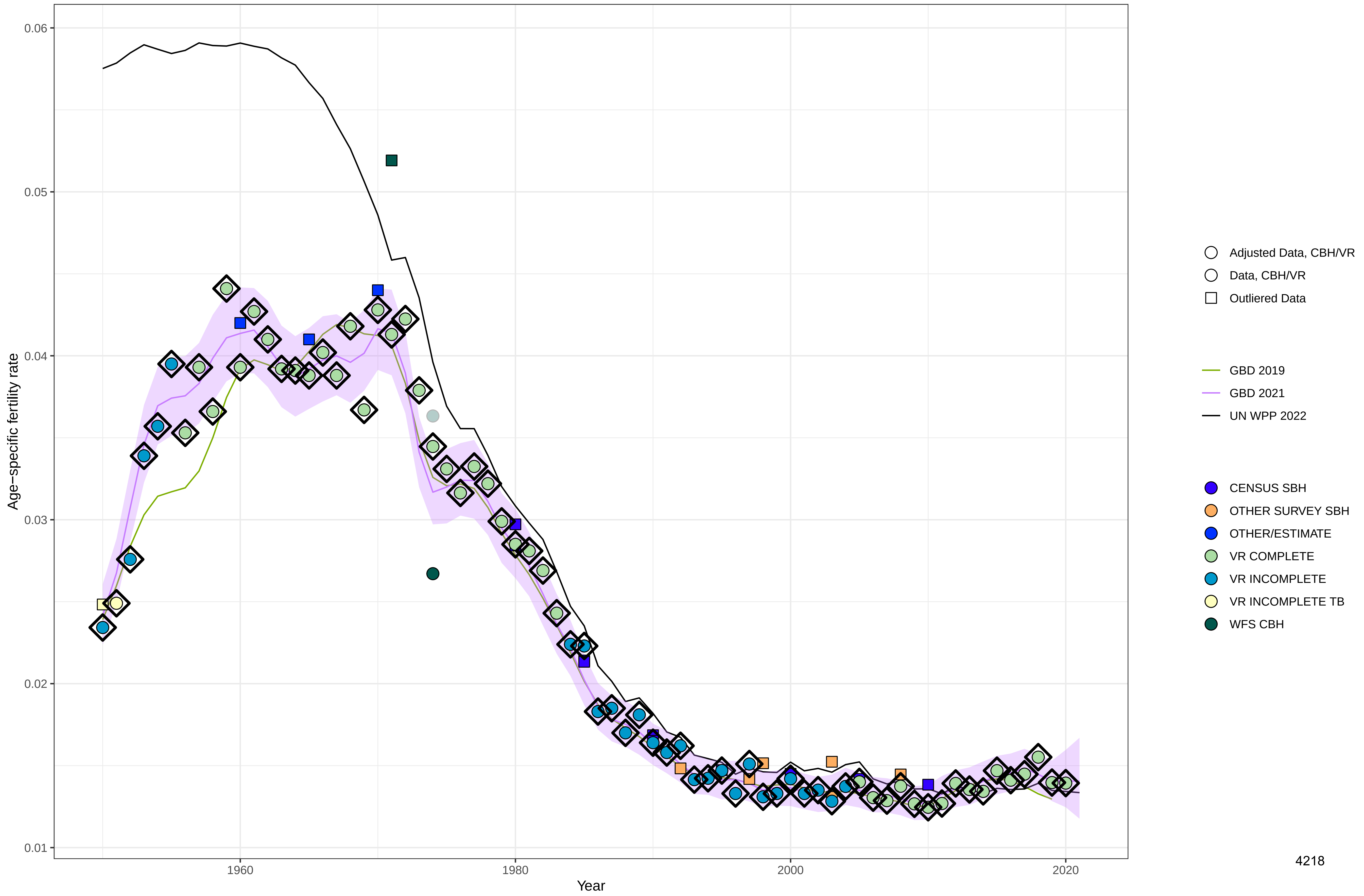

Age−specific fertility fitted trend lines, 1950−2021: Panama, Ages: 45−49
Data points enclosed with a diamond are reference sources

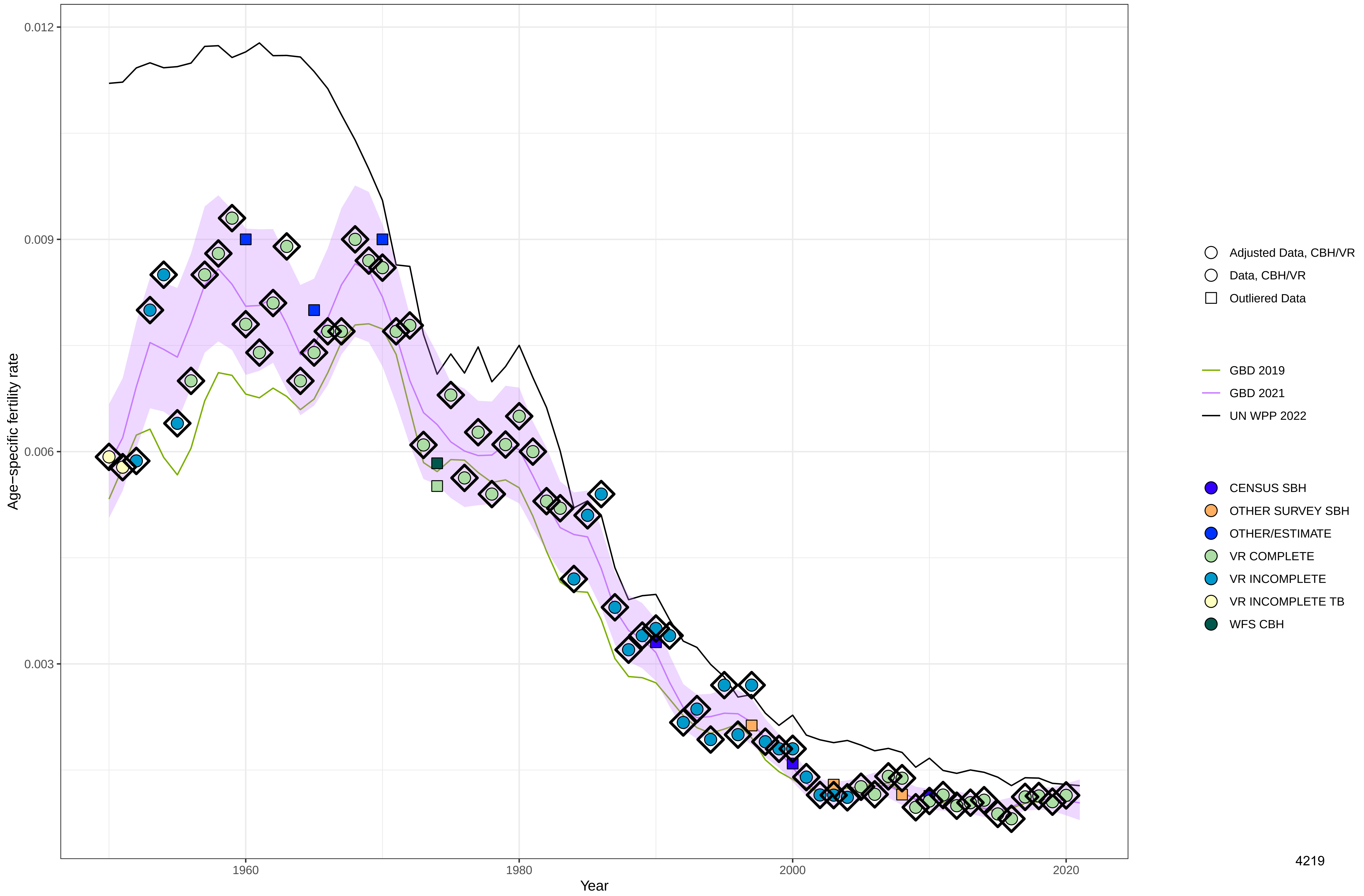

Age−specific fertility fitted trend lines, 1950−2021: Panama, Ages: 50−54
Data points enclosed with a diamond are reference sources

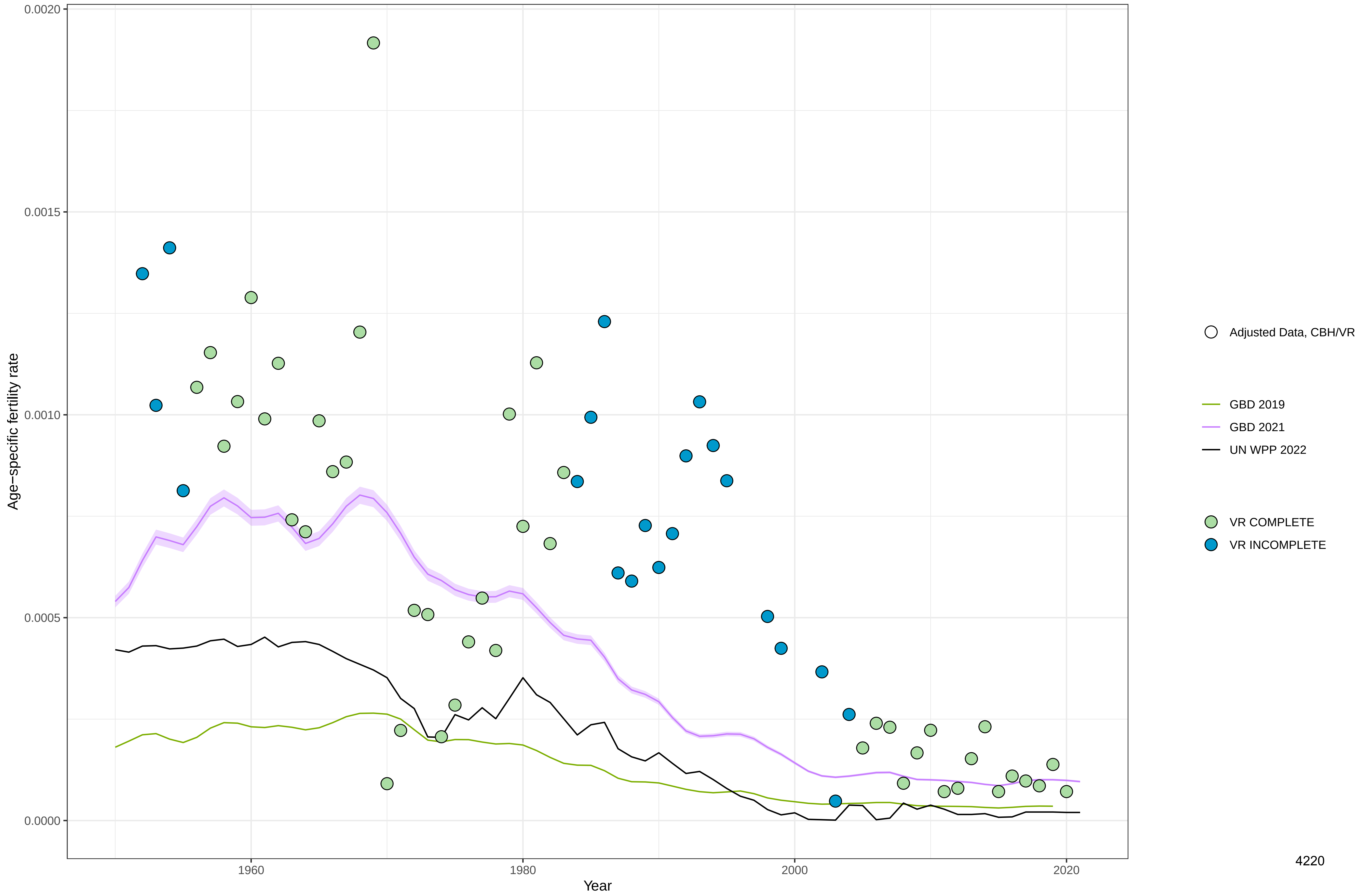

Total fertility rate fitted trend lines, 1950−2021: Panama TFR
Data points enclosed with a diamond are reference sources

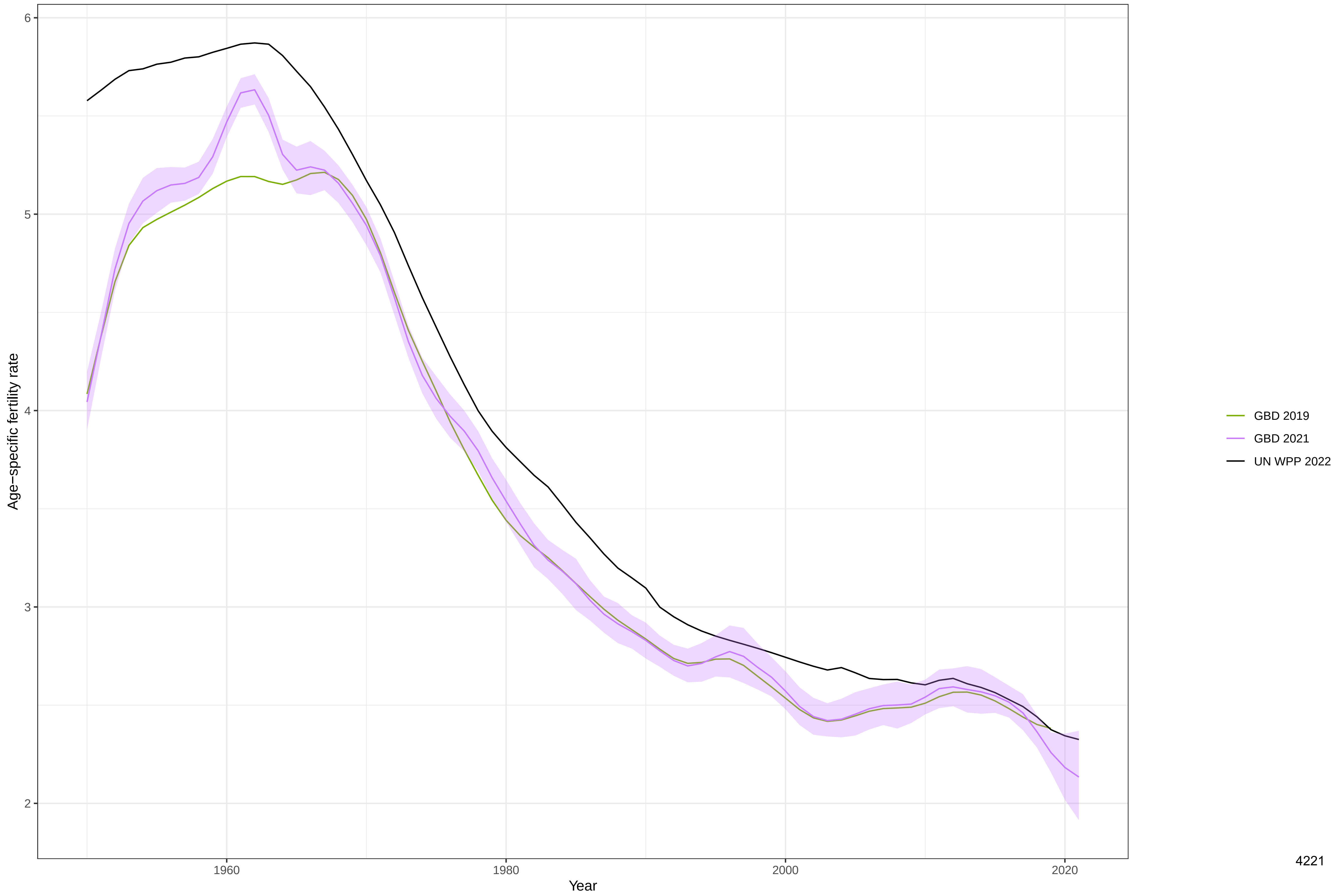

Age−specific fertility fitted trend lines, 1950−2021: Venezuela, Ages: 10−14
Data points enclosed with a diamond are reference sources

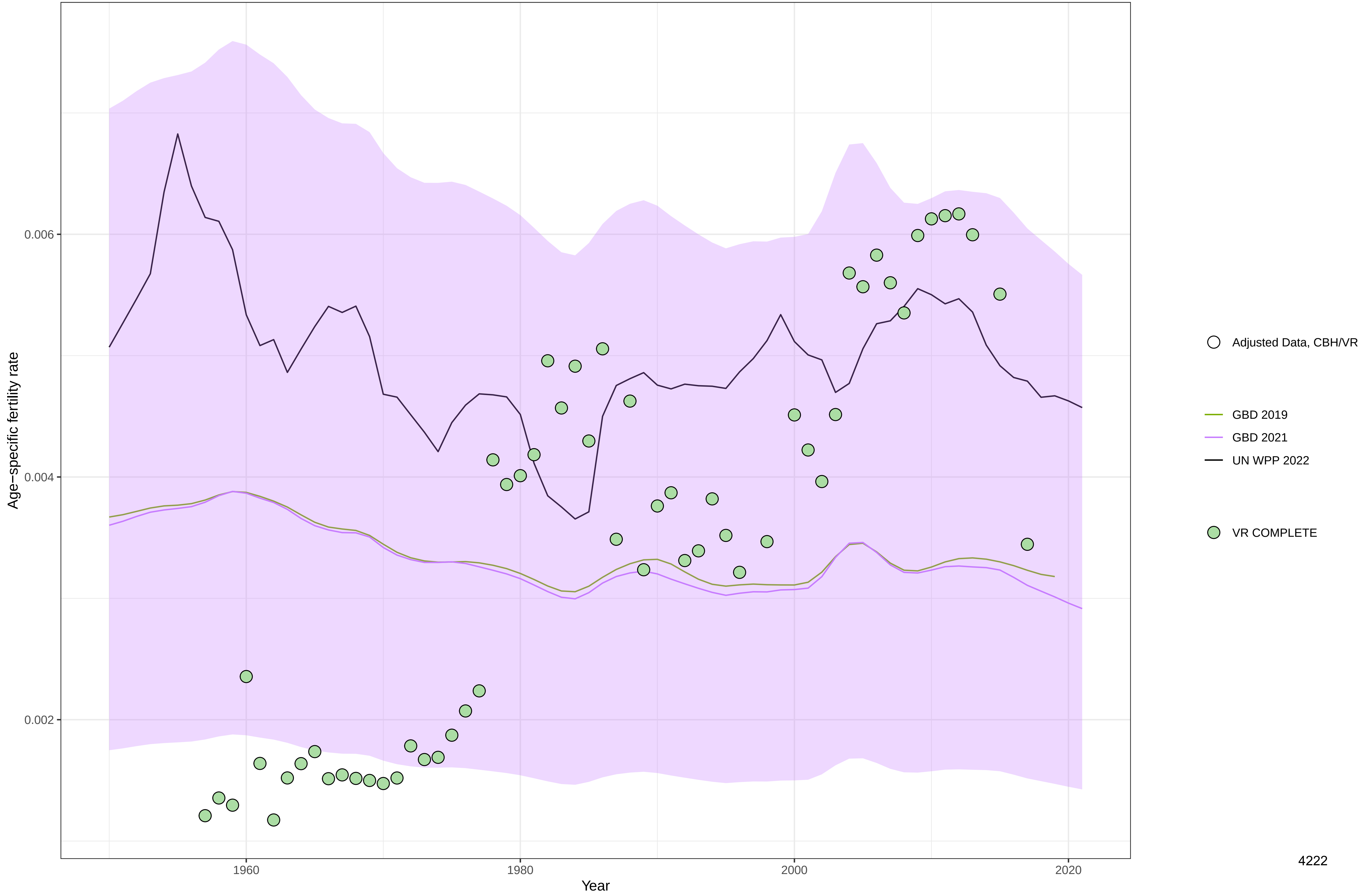

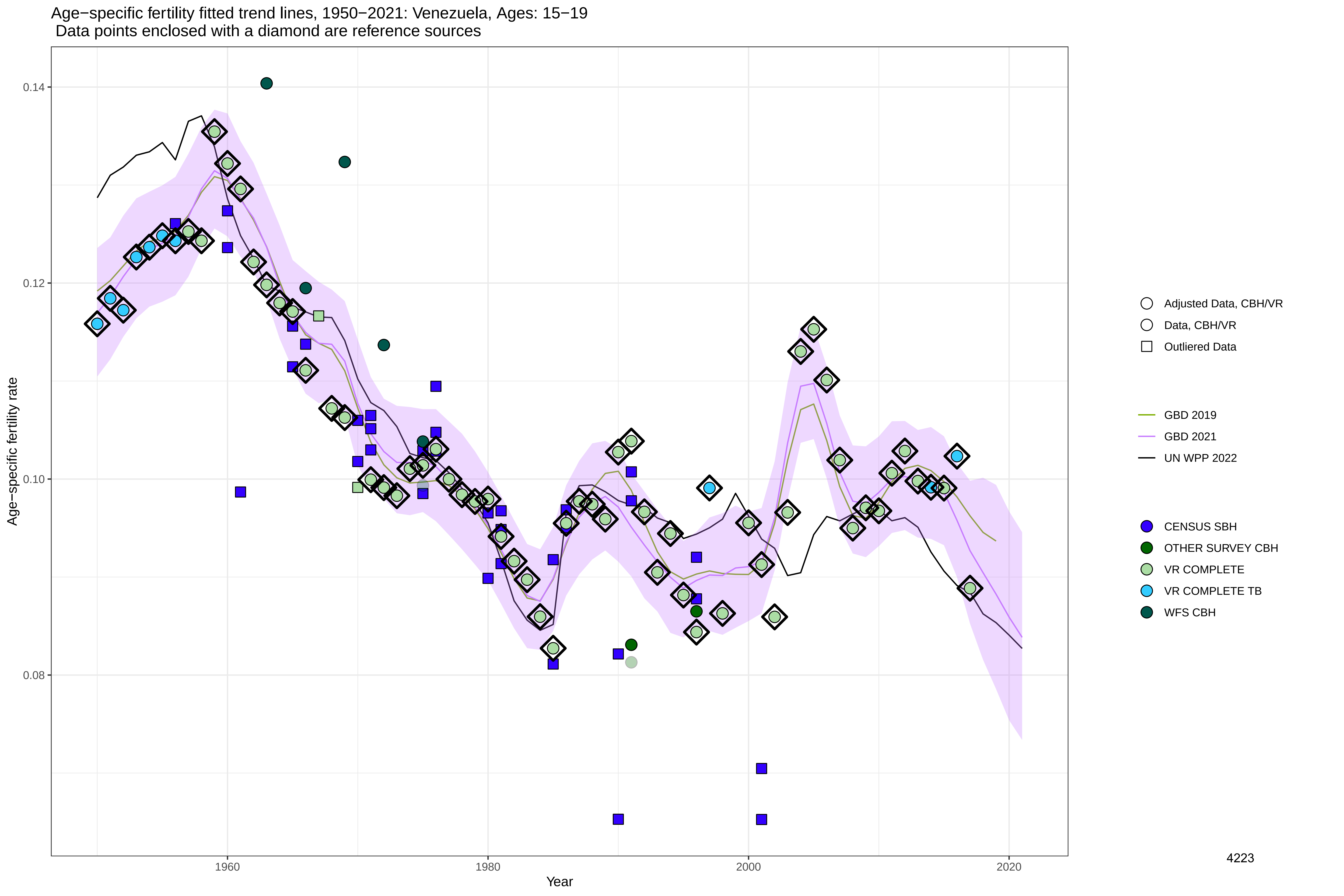

Age−specific fertility fitted trend lines, 1950−2021: Venezuela, Ages: 15−19
Data points enclosed with a diamond are reference sources
0.14
0.12
0.10
0.08
Age−specific fertility rate
1960
1980
2000
2020
Year
Adjusted Data, CBH/VR
Data, CBH/VR
Outliered Data
GBD 2019
GBD 2021
UN WPP 2022
CENSUS SBH
OTHER SURVEY CBH
VR COMPLETE
VR COMPLETE TB
WFS CBH

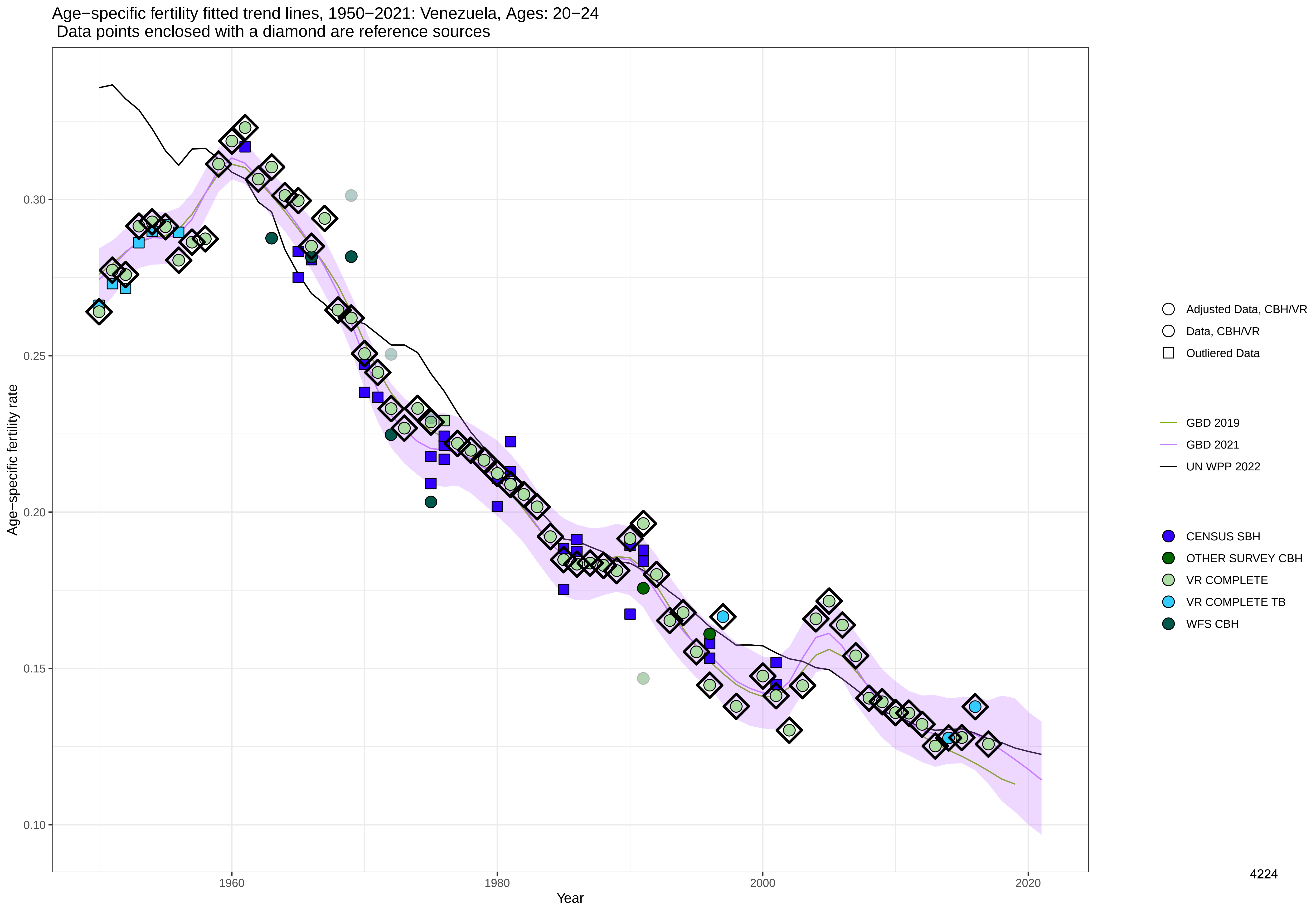

Age−specific fertility fitted trend lines, 1950−2021: Venezuela, Ages: 20−24
Data points enclosed with a diamond are reference sources
Age−specific fertility rate
Year
0.10
0.15
0.20
0.25
0.30
1960
1980
2000
2020
Adjusted Data, CBH/VR
Data, CBH/VR
Outliered Data
GBD 2019
GBD 2021
UN WPP 2022
CENSUS SBH
OTHER SURVEY CBH
VR COMPLETE
VR COMPLETE TB
WFS CBH

Age−specific fertility fitted trend lines, 1950−2021: Venezuela, Ages: 25−29
Data points enclosed with a diamond are reference sources

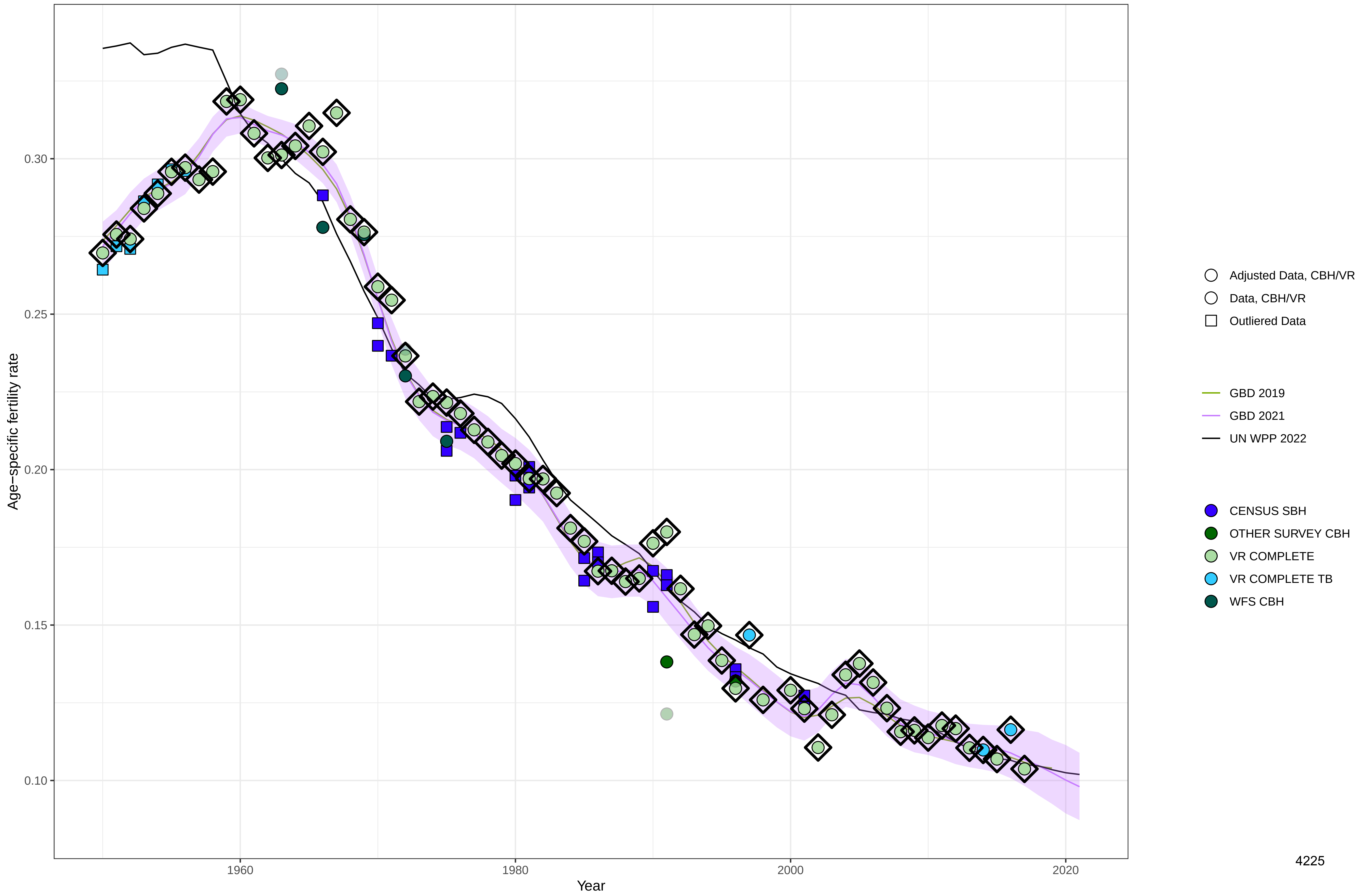

Age−specific fertility fitted trend lines, 1950−2021: Venezuela, Ages: 30−34
Data points enclosed with a diamond are reference sources

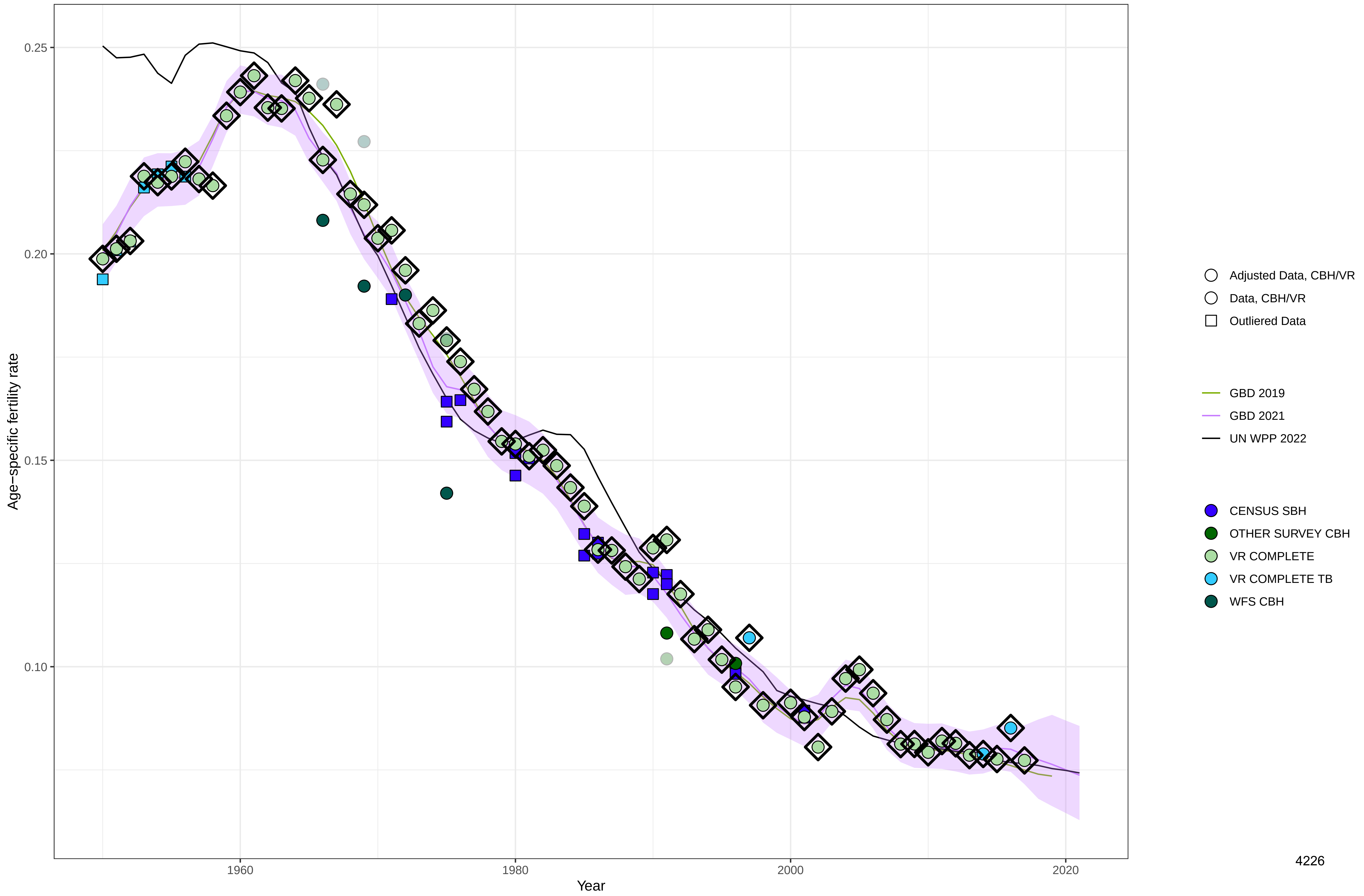

Age−specific fertility fitted trend lines, 1950−2021: Venezuela, Ages: 35−39
Data points enclosed with a diamond are reference sources

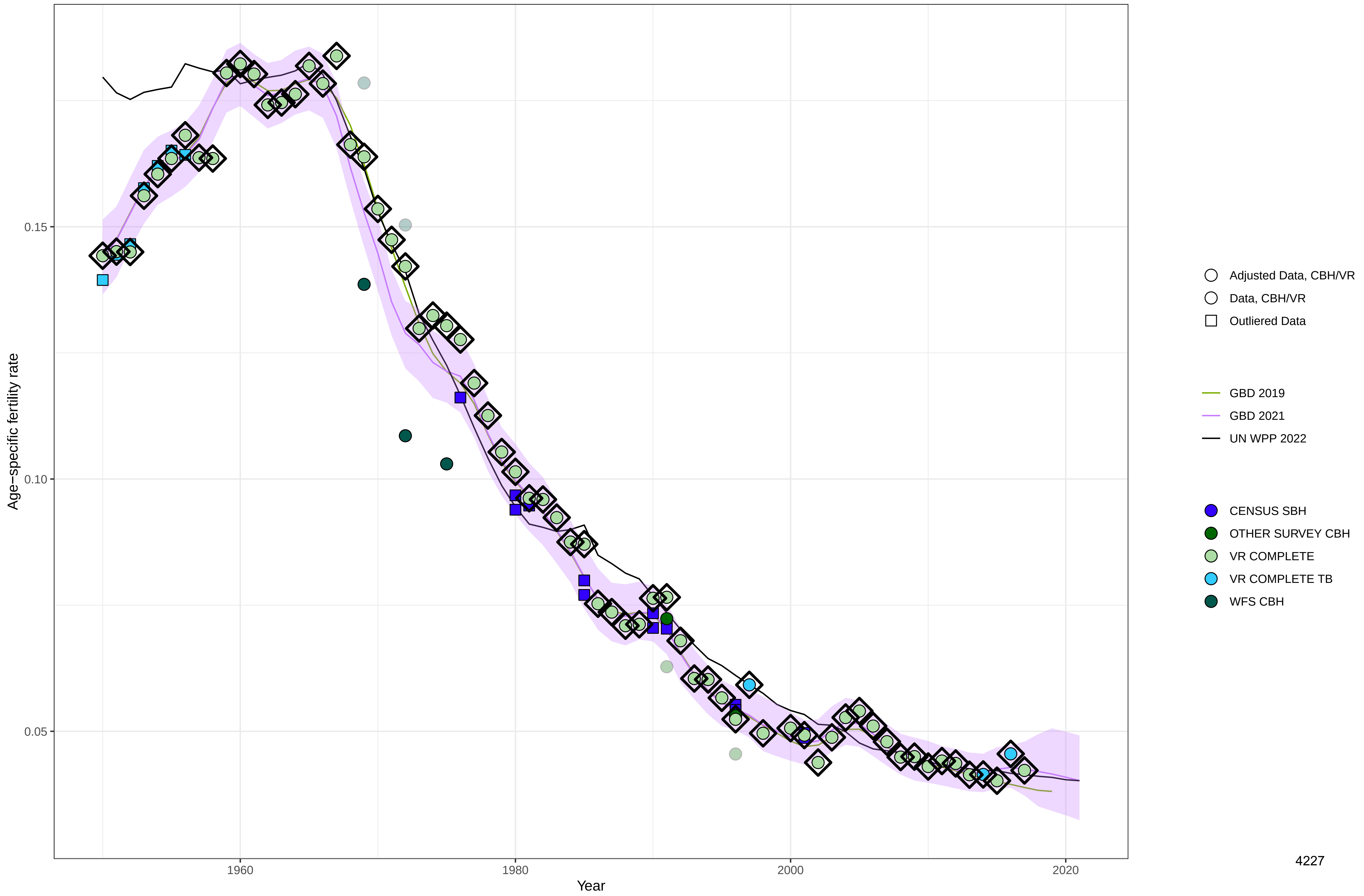

Age−specific fertility fitted trend lines, 1950−2021: Venezuela, Ages: 40−44
Data points enclosed with a diamond are reference sources

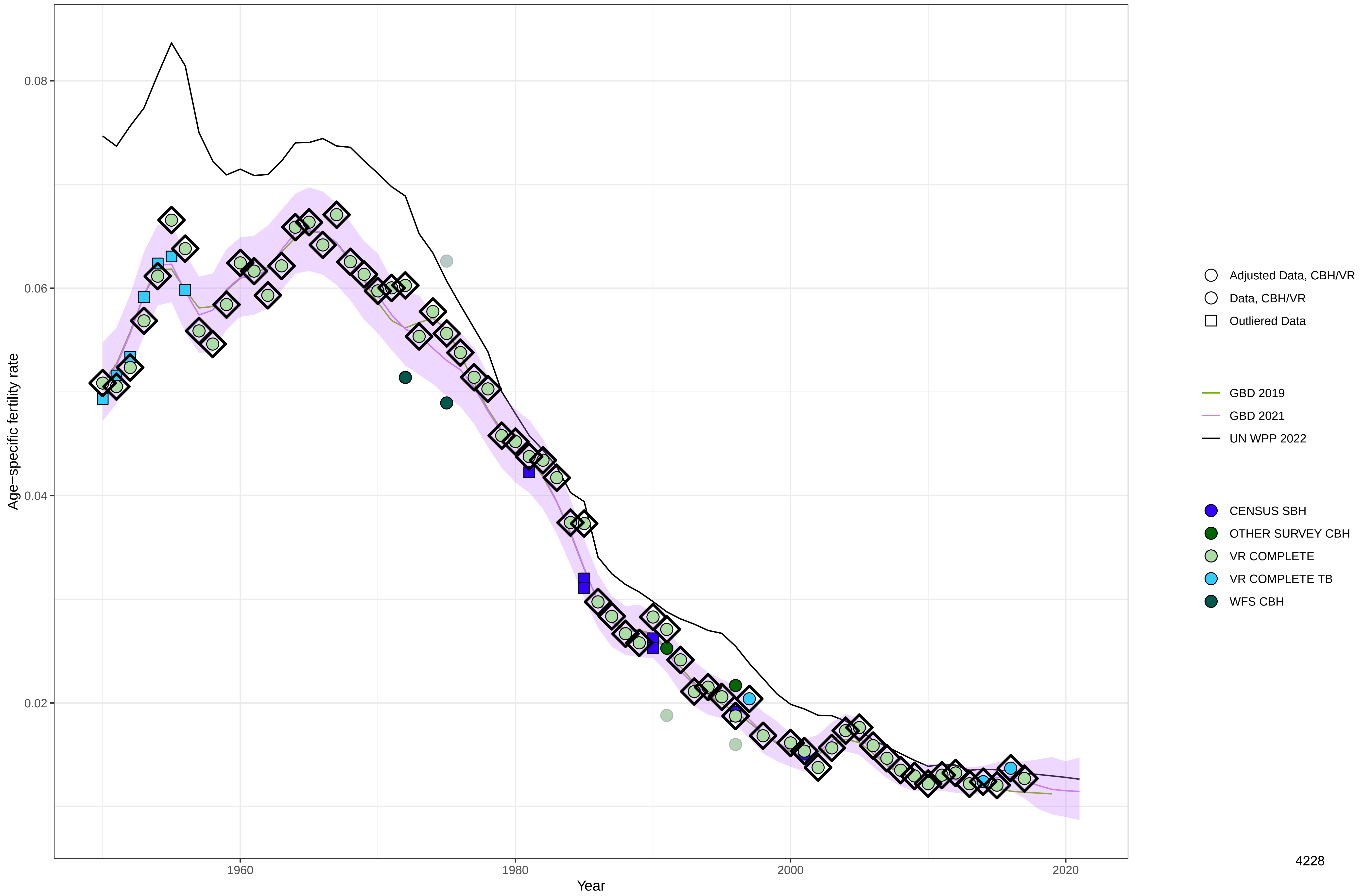

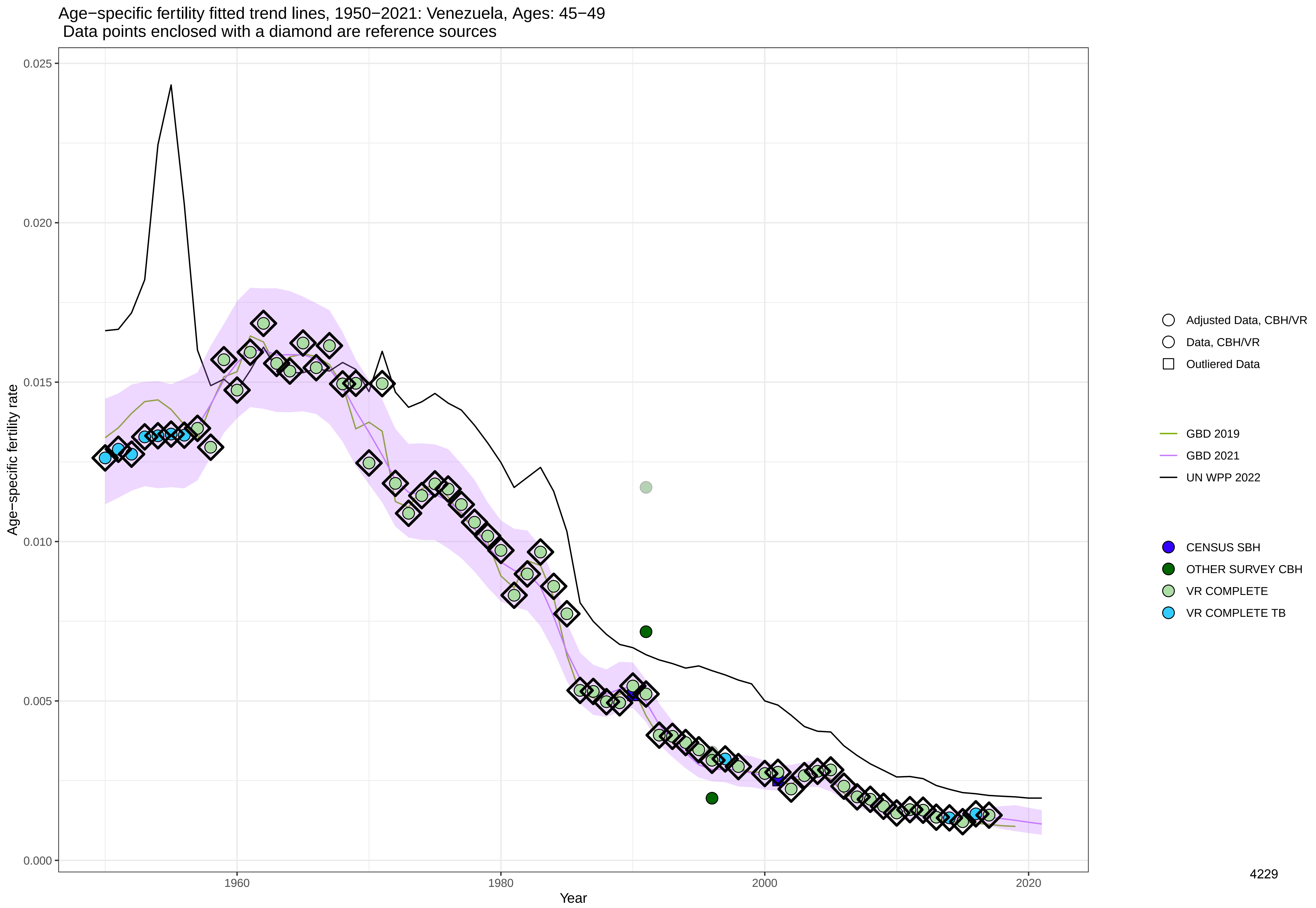

Age−specific fertility fitted trend lines, 1950−2021: Venezuela, Ages: 45−49
Data points enclosed with a diamond are reference sources
Age−specific fertility rate
0.000
0.005
0.010
0.015
0.020
0.025
Year
1960
1980
2000
2020
Adjusted Data, CBH/VR
Data, CBH/VR
Outliered Data
GBD 2019
GBD 2021
UN WPP 2022
CENSUS SBH
OTHER SURVEY CBH
VR COMPLETE
VR COMPLETE TB

4229

Age−specific fertility fitted trend lines, 1950−2021: Venezuela, Ages: 50−54
Data points enclosed with a diamond are reference sources

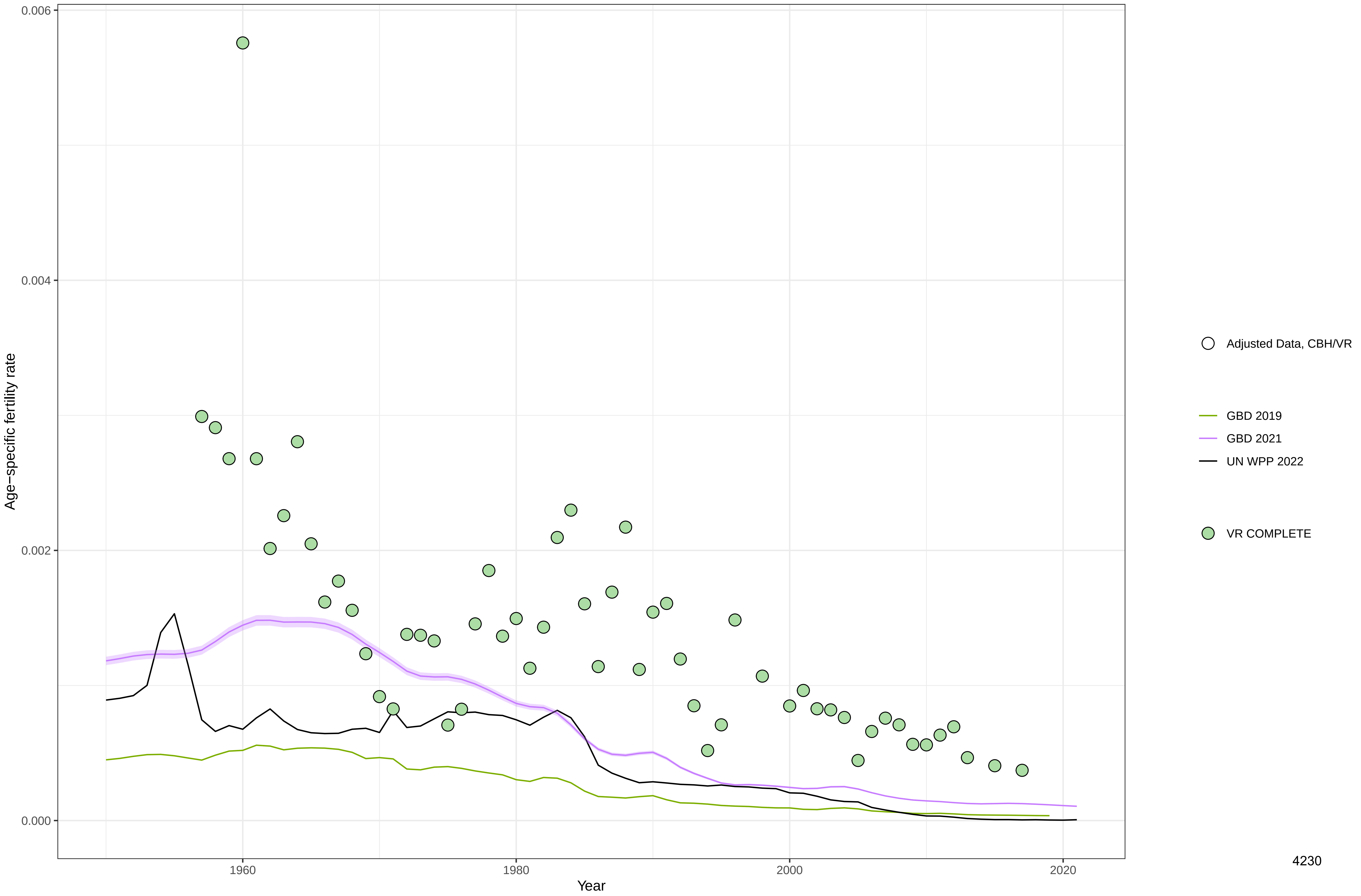

Total fertility rate fitted trend lines, 1950−2021: Venezuela TFR
Data points enclosed with a diamond are reference sources

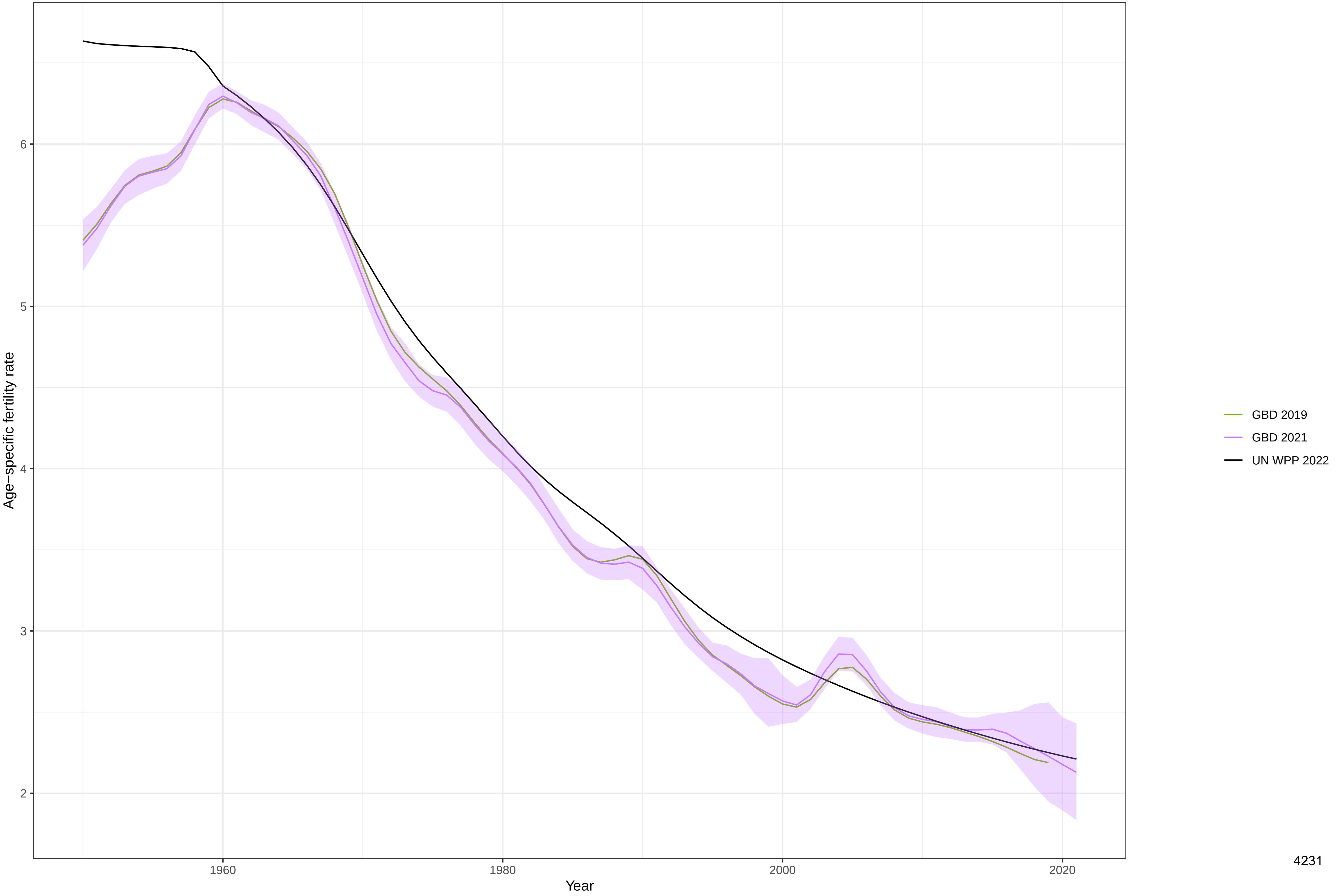

Age−specific fertility fitted trend lines, 1950−2021: Brazil, Ages: 10−14
Data points enclosed with a diamond are reference sources

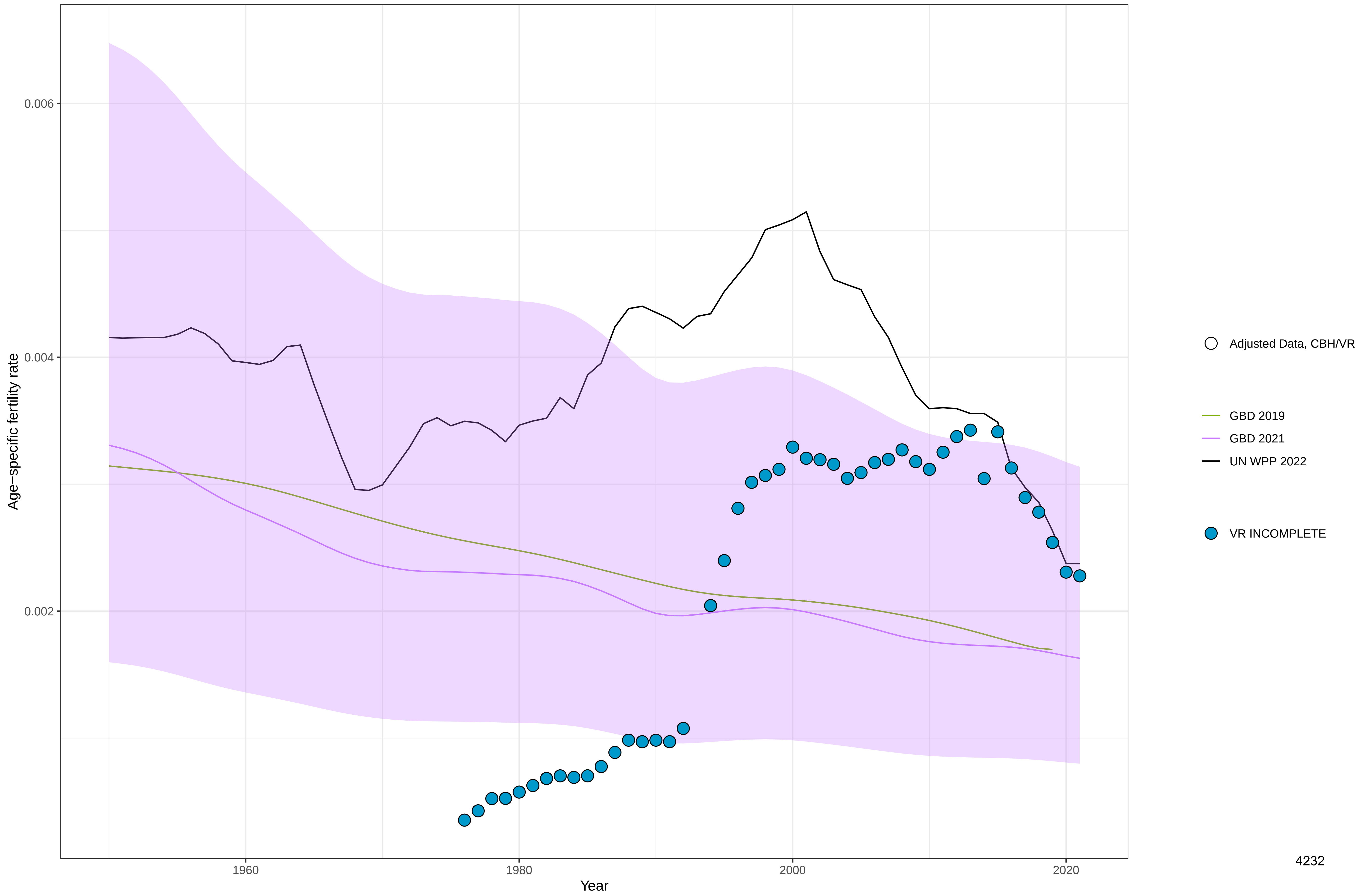

Age−specific fertility fitted trend lines, 1950−2021: Brazil, Ages: 15−19
Data points enclosed with a diamond are reference sources

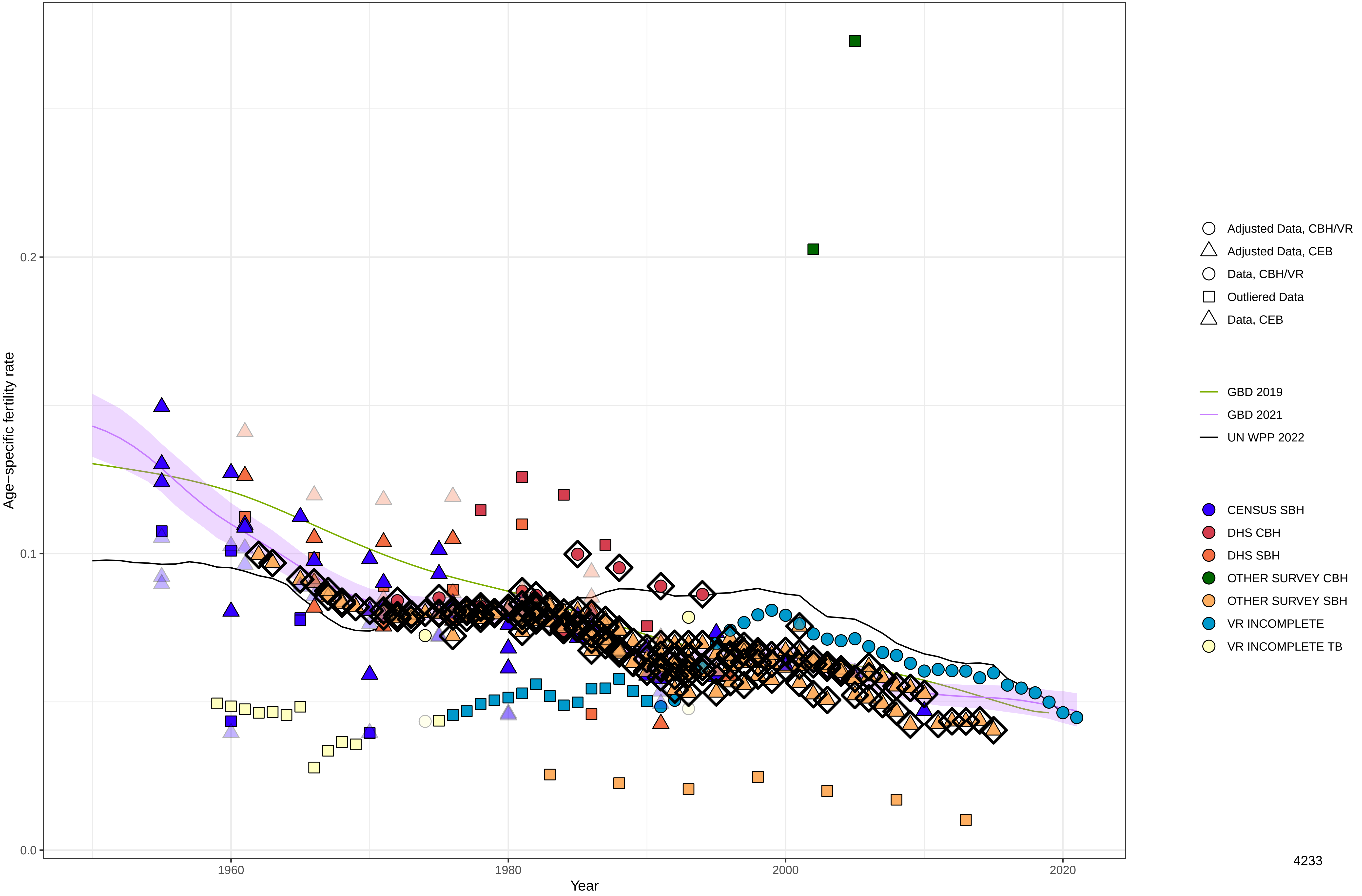

Age−specific fertility fitted trend lines, 1950−2021: Brazil, Ages: 20−24
Data points enclosed with a diamond are reference sources

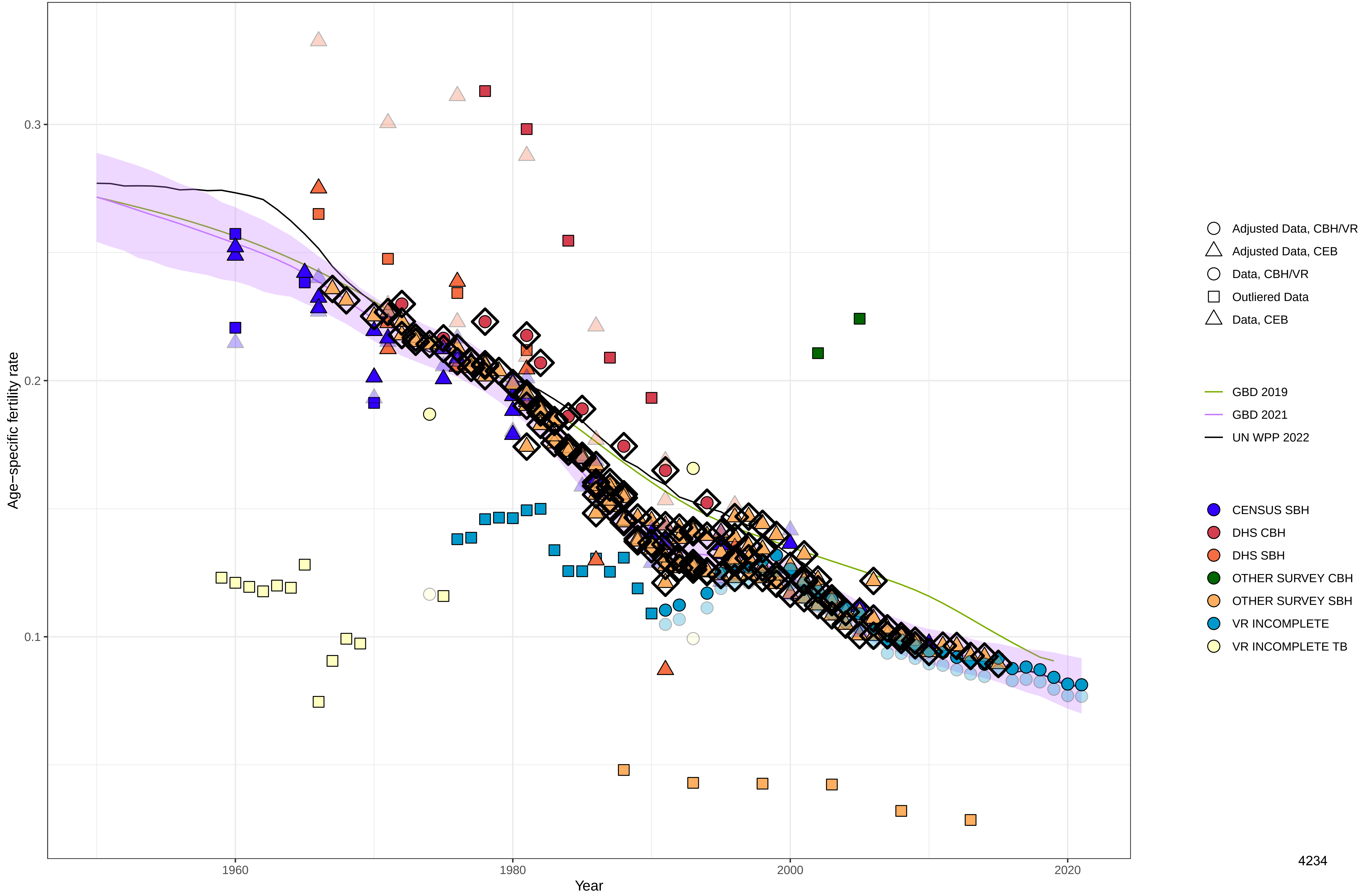

Age−specific fertility fitted trend lines, 1950−2021: Brazil, Ages: 25−29
Data points enclosed with a diamond are reference sources

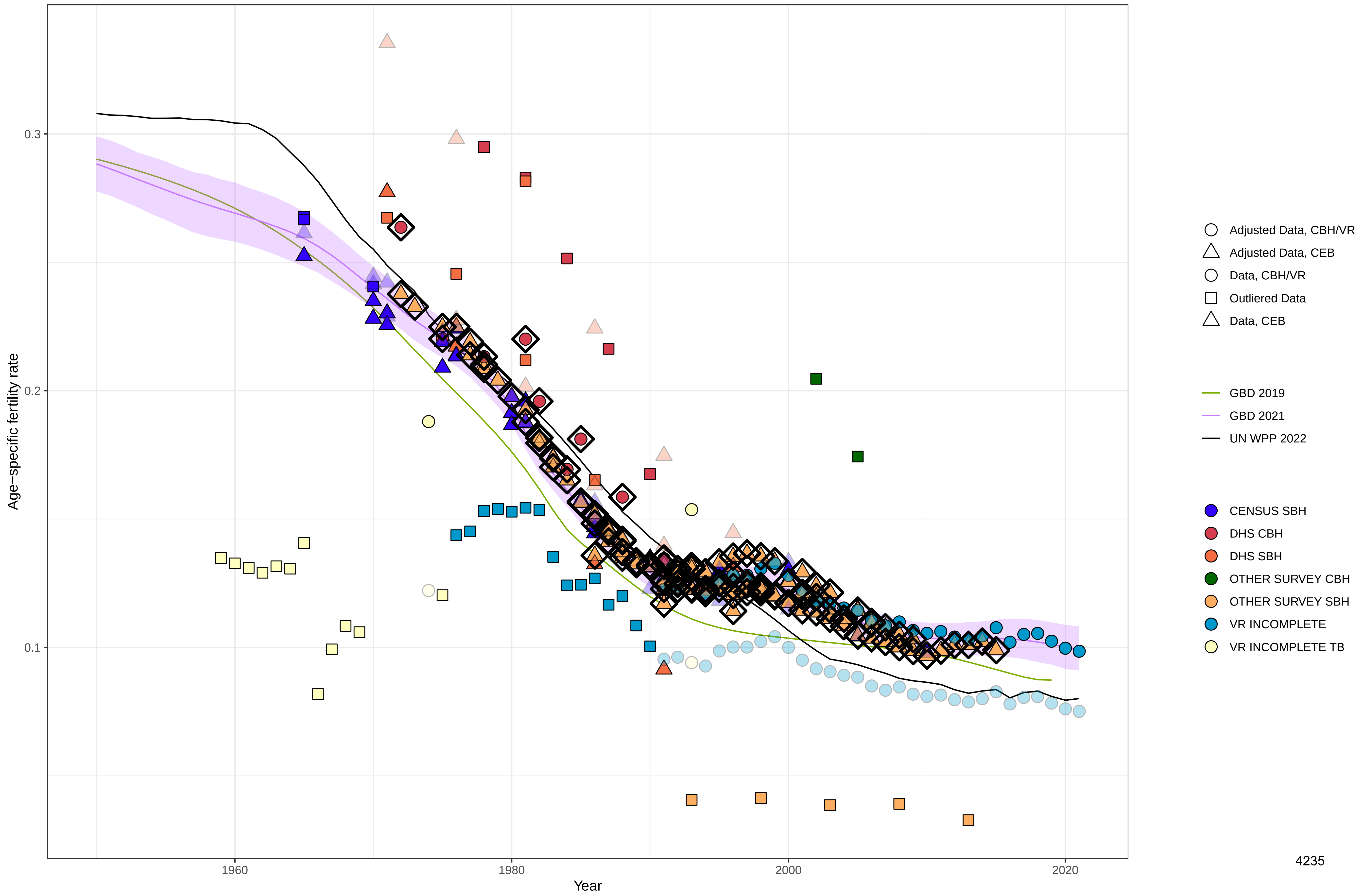

Age−specific fertility fitted trend lines, 1950−2021: Brazil, Ages: 30−34
Data points enclosed with a diamond are reference sources

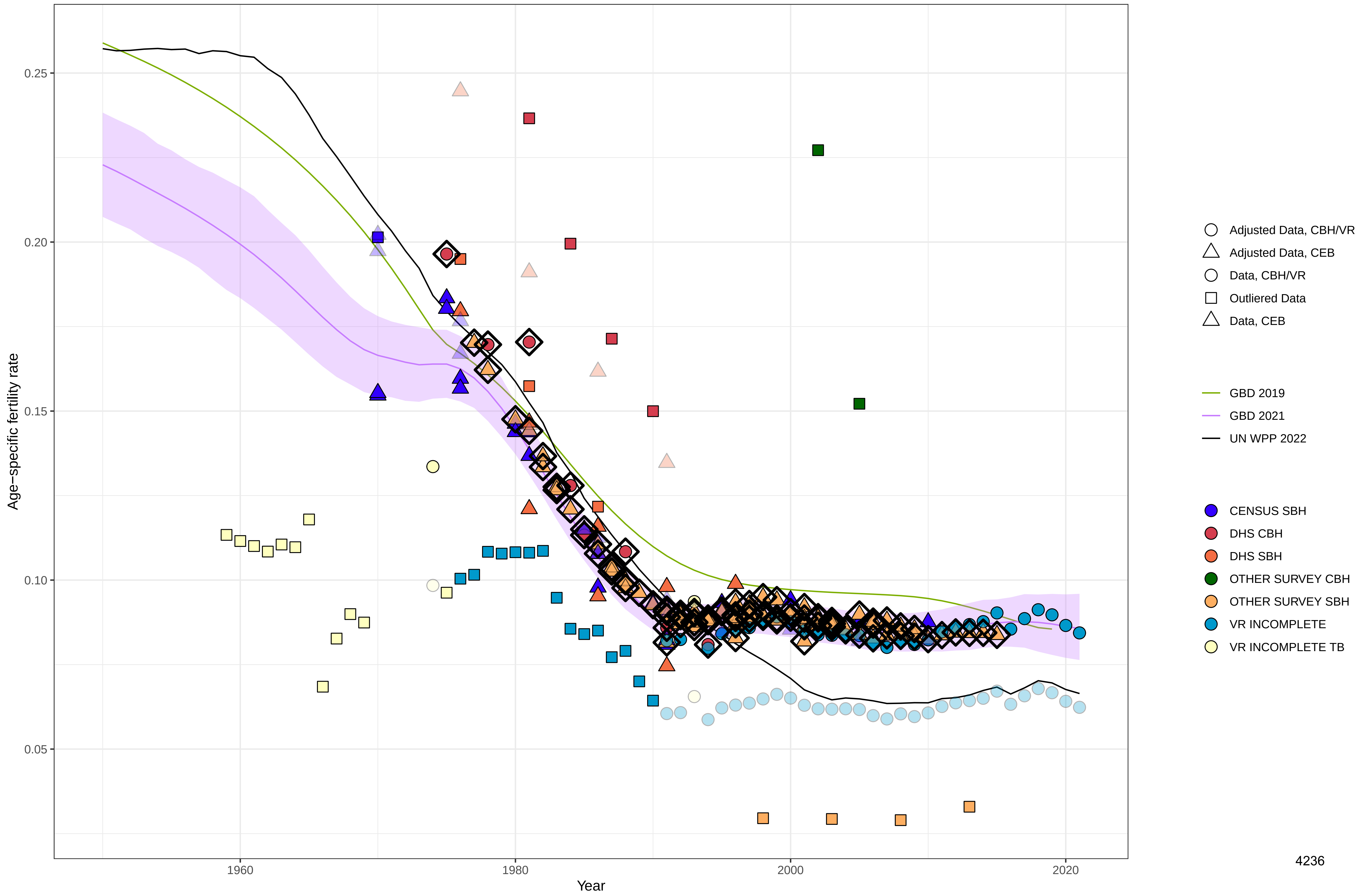

Age−specific fertility fitted trend lines, 1950−2021: Brazil, Ages: 35−39
Data points enclosed with a diamond are reference sources

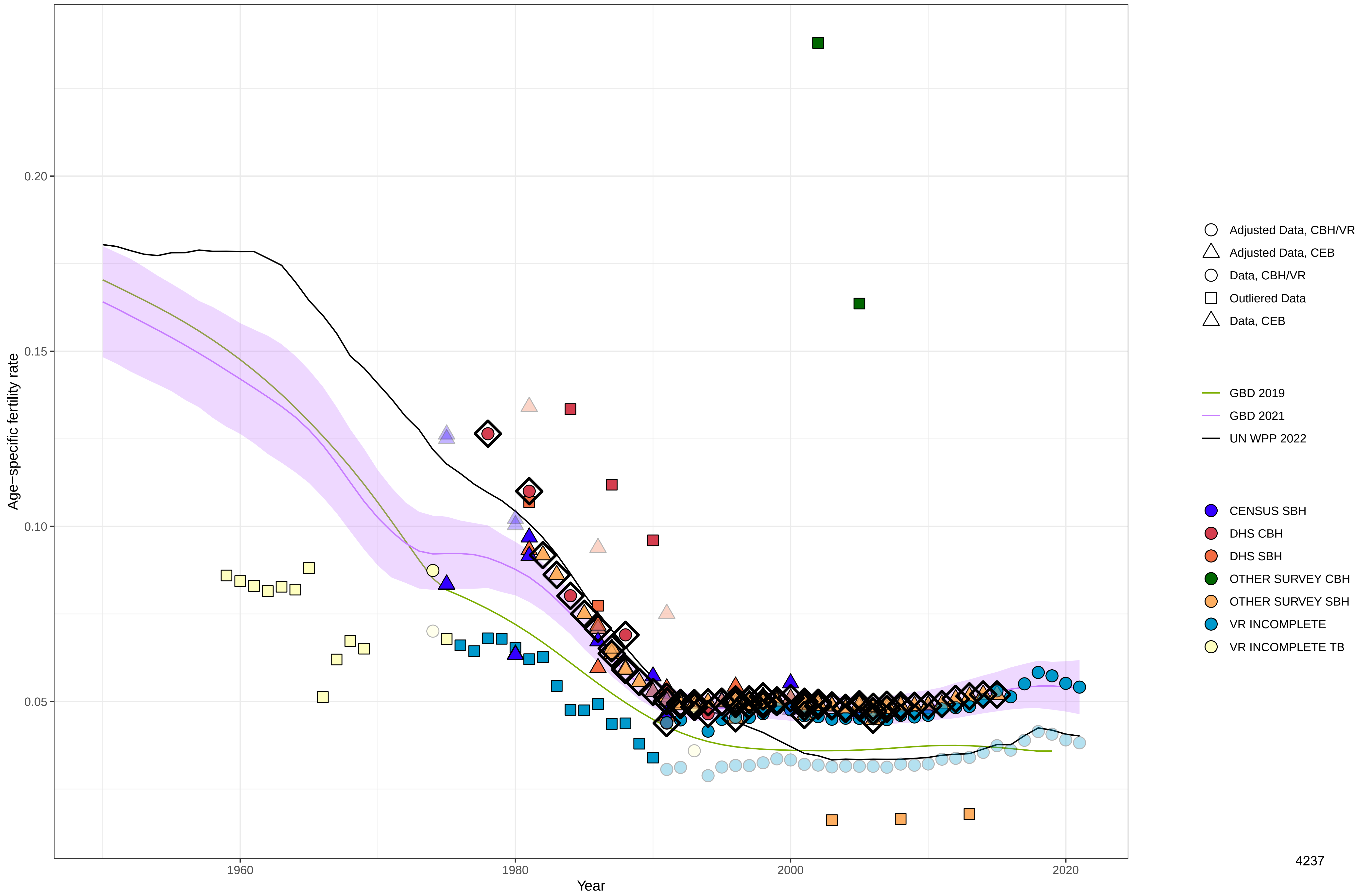

4237

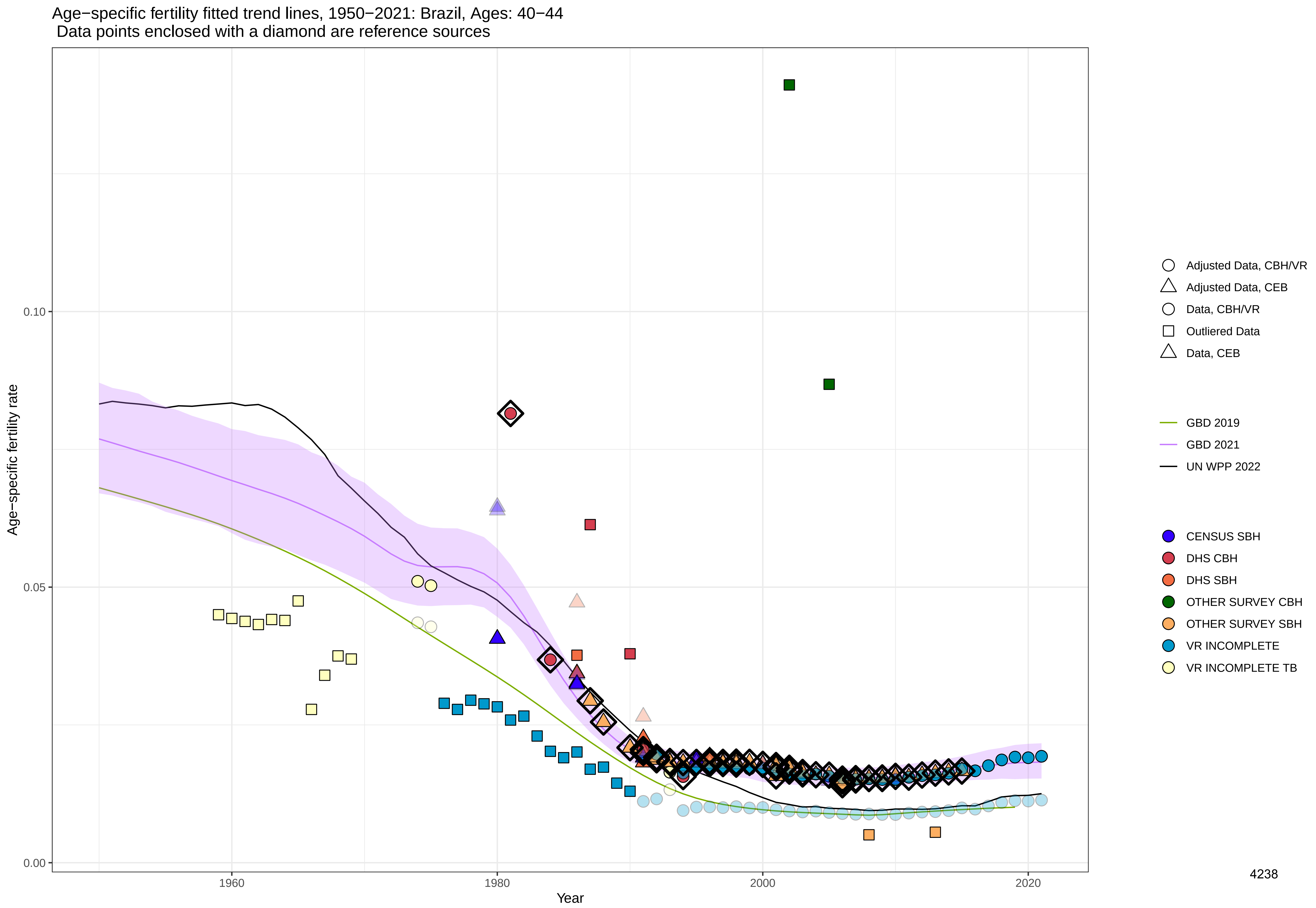

Age−specific fertility fitted trend lines, 1950−2021: Brazil, Ages: 40−44
Data points enclosed with a diamond are reference sources
Age−specific fertility rate
0.00
0.05
0.10
Year
1960
1980
2000
2020
Adjusted Data, CBH/VR
Adjusted Data, CEB
Data, CBH/VR
Outliered Data
Data, CEB
GBD 2019
GBD 2021
UN WPP 2022
CENSUS SBH
DHS CBH
DHS SBH
OTHER SURVEY CBH
OTHER SURVEY SBH
VR INCOMPLETE
VR INCOMPLETE TB

4238

Age−specific fertility fitted trend lines, 1950−2021: Brazil, Ages: 45−49
Data points enclosed with a diamond are reference sources

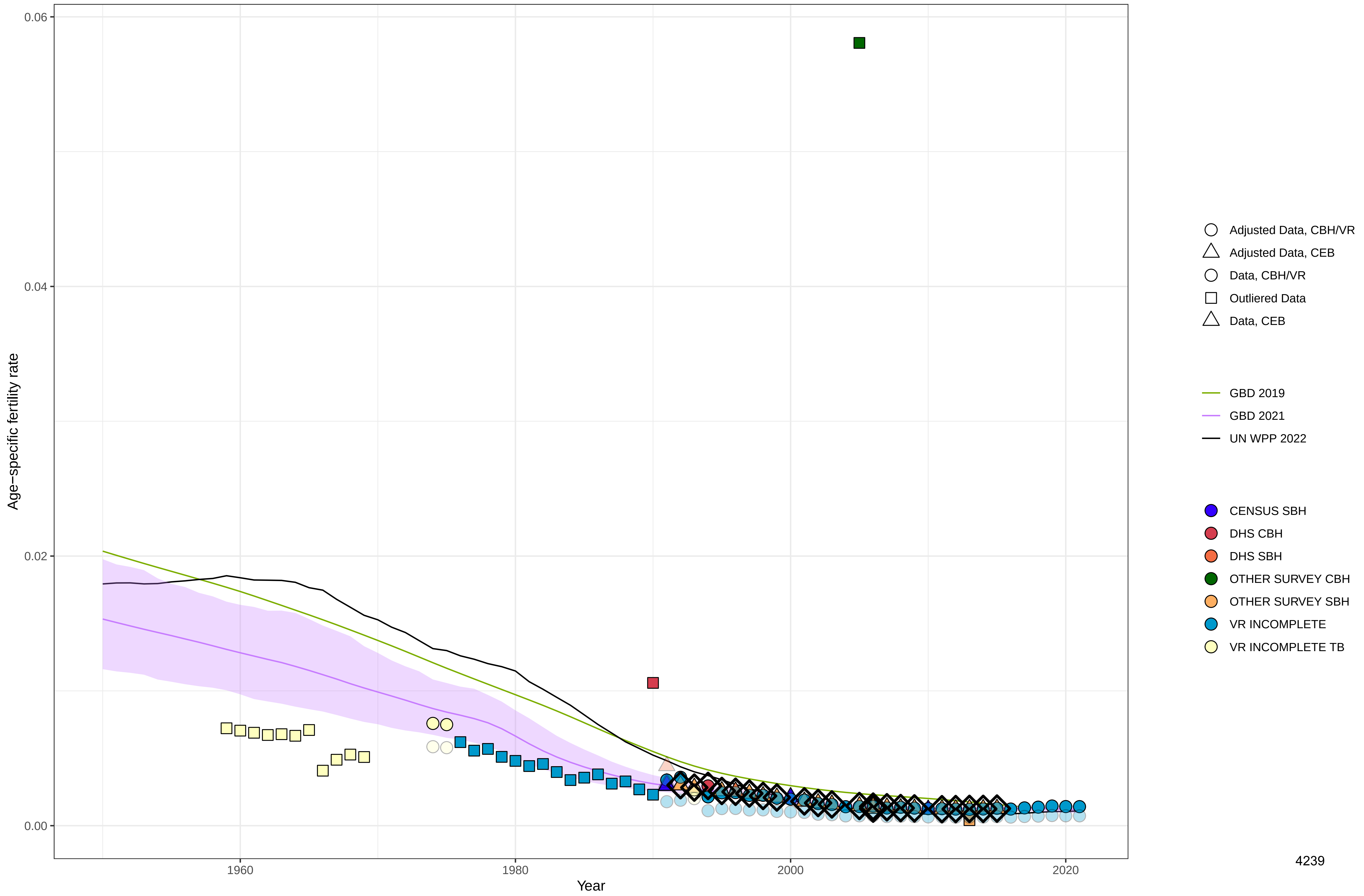

Age−specific fertility fitted trend lines, 1950−2021: Brazil, Ages: 50−54
Data points enclosed with a diamond are reference sources

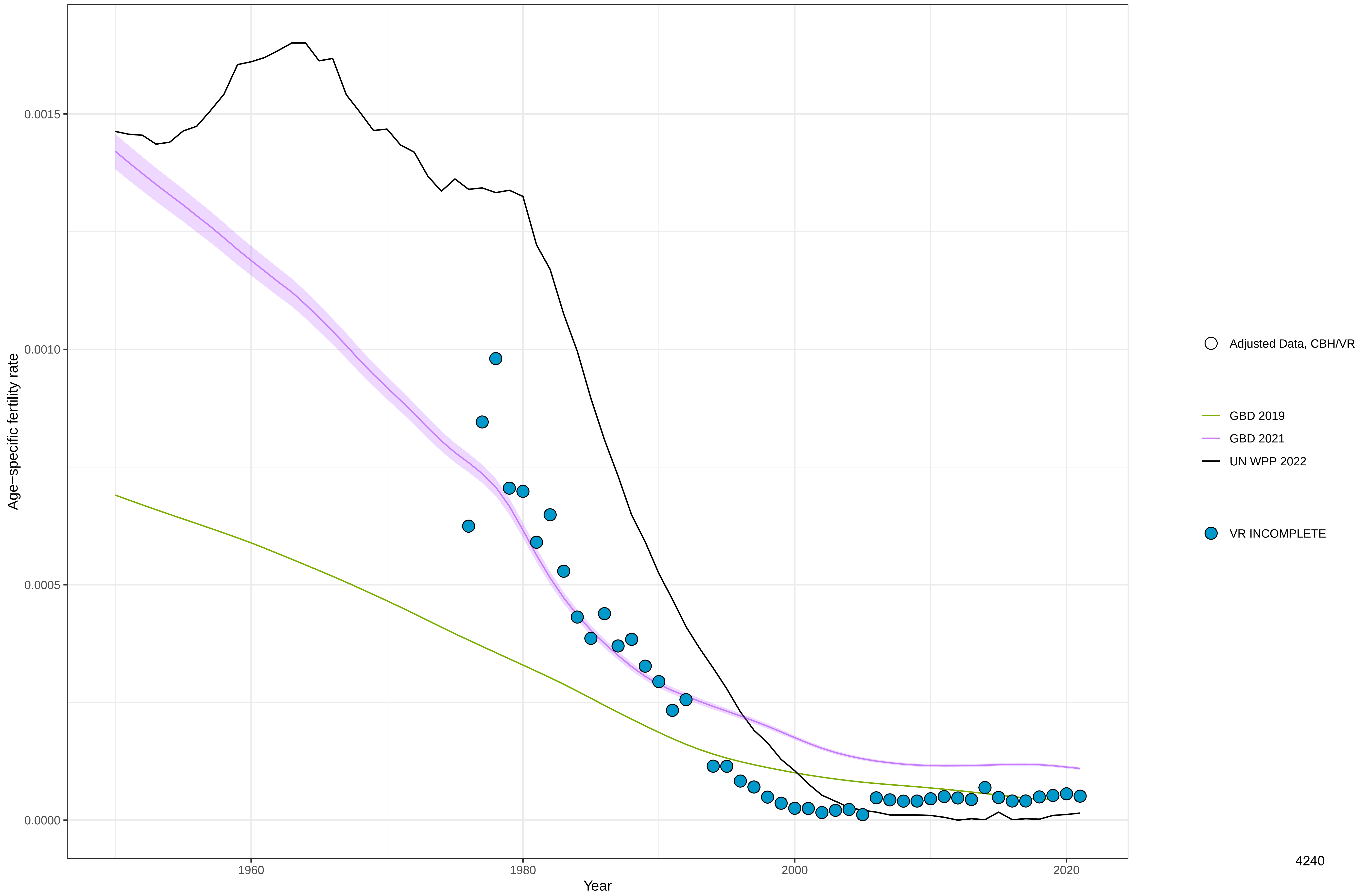

Total fertility rate fitted trend lines, 1950−2021: Brazil TFR
Data points enclosed with a diamond are reference sources

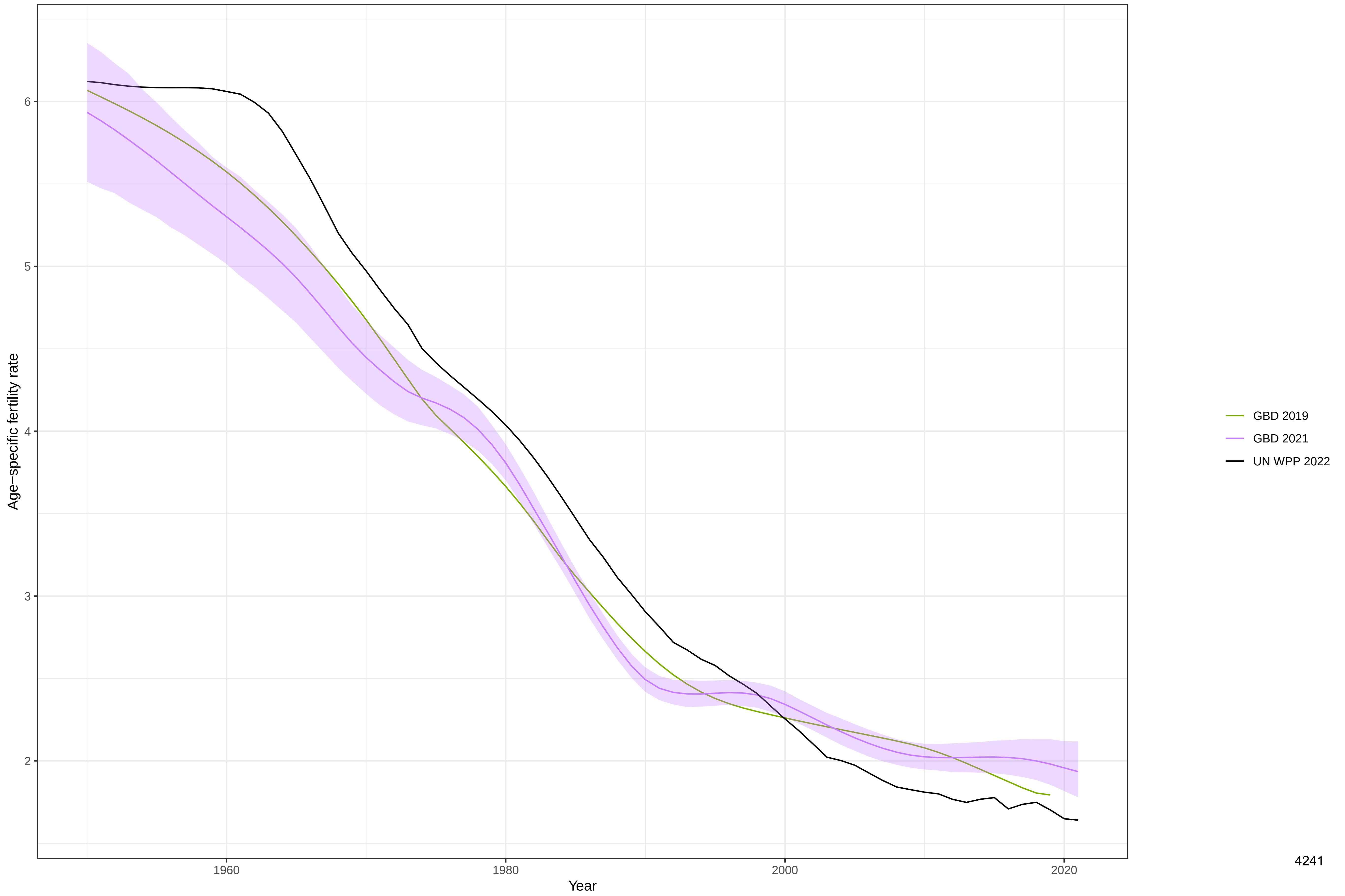

Age−specific fertility fitted trend lines, 1950−2021: Acre, Ages: 10−14
Data points enclosed with a diamond are reference sources

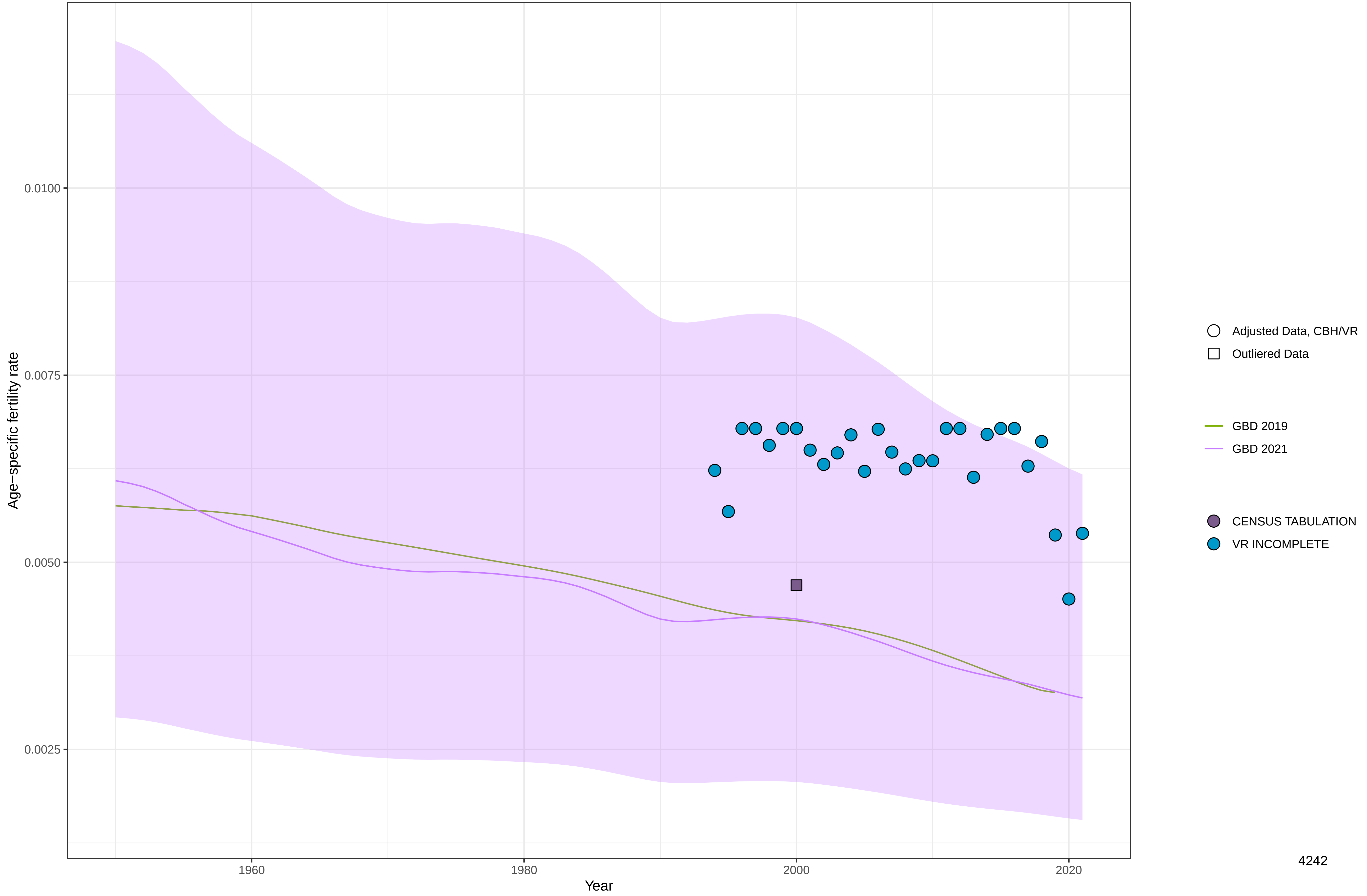

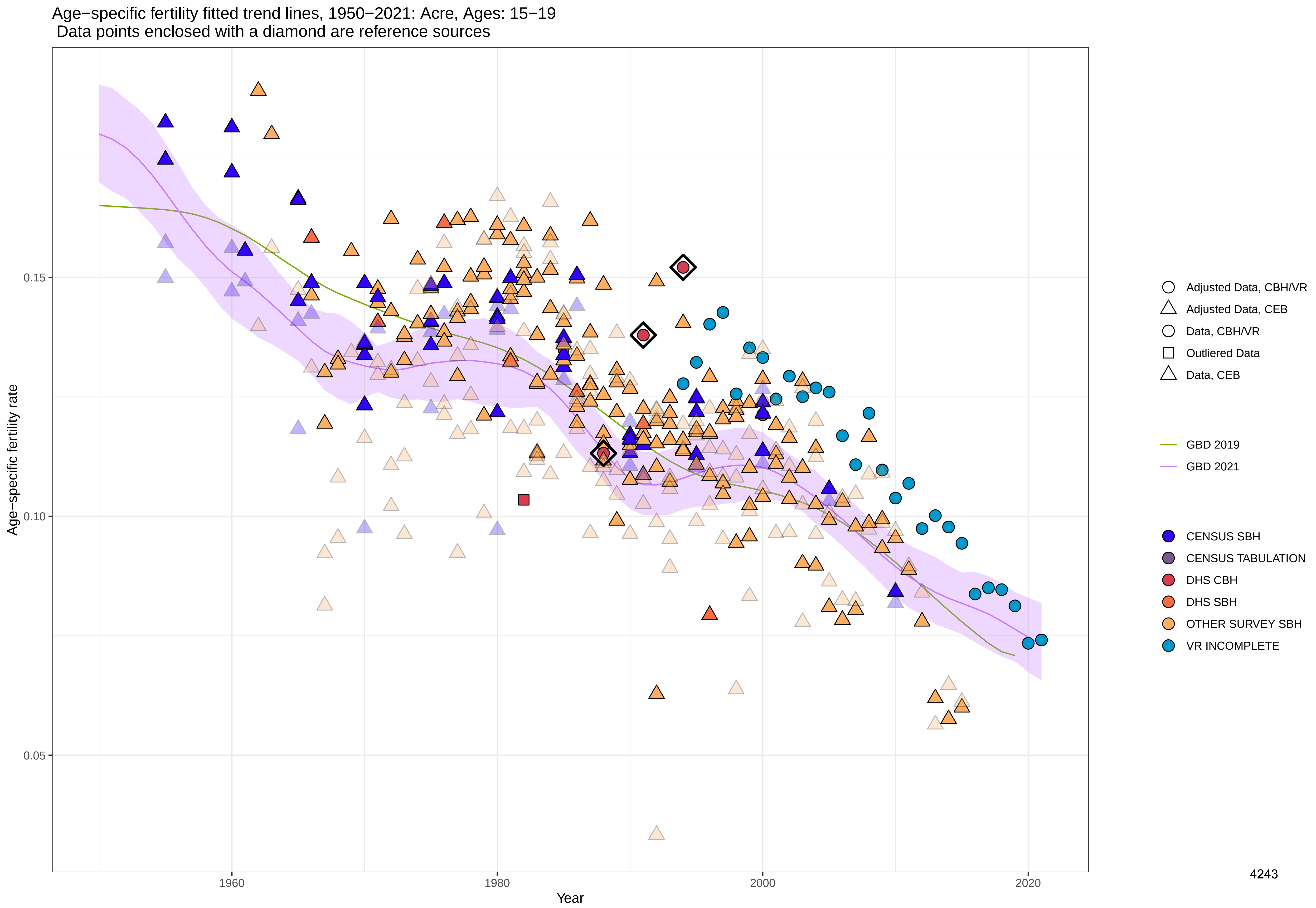
Age−specific fertility fitted trend lines, 1950−2021: Acre, Ages: 15−19
Data points enclosed with a diamond are reference sources
Age−specific fertility rate
0.15
0.10
0.05
1960
1980
2000
2020
Year
Adjusted Data, CBH/VR
Adjusted Data, CEB
Data, CBH/VR
Outliered Data
Data, CEB
GBD 2019
GBD 2021
CENSUS SBH
CENSUS TABULATION
DHS CBH
DHS SBH
OTHER SURVEY SBH
VR INCOMPLETE

Age−specific fertility fitted trend lines, 1950−2021: Acre, Ages: 20−24
Data points enclosed with a diamond are reference sources

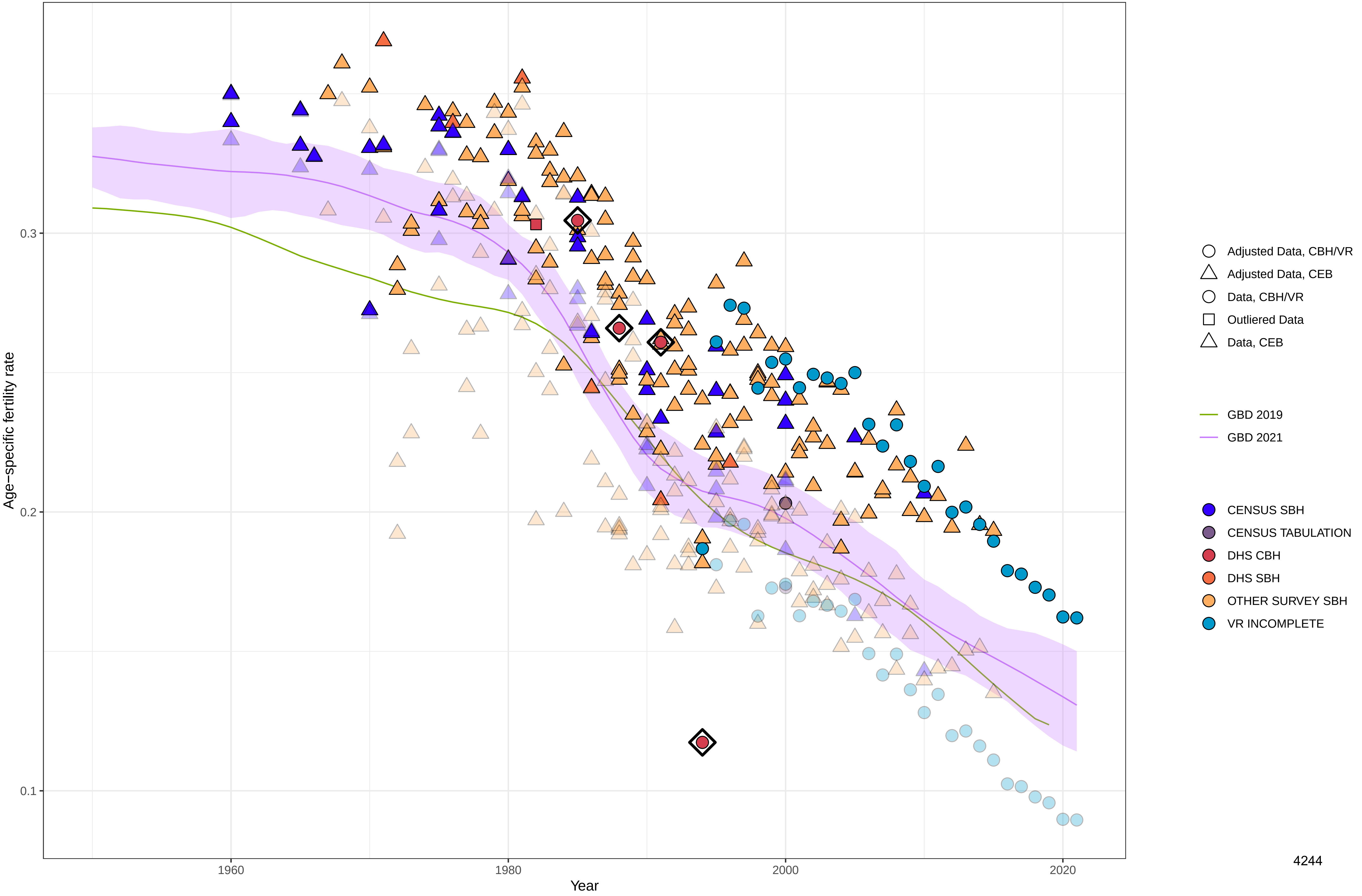

Age−specific fertility fitted trend lines, 1950−2021: Acre, Ages: 25−29
Data points enclosed with a diamond are reference sources

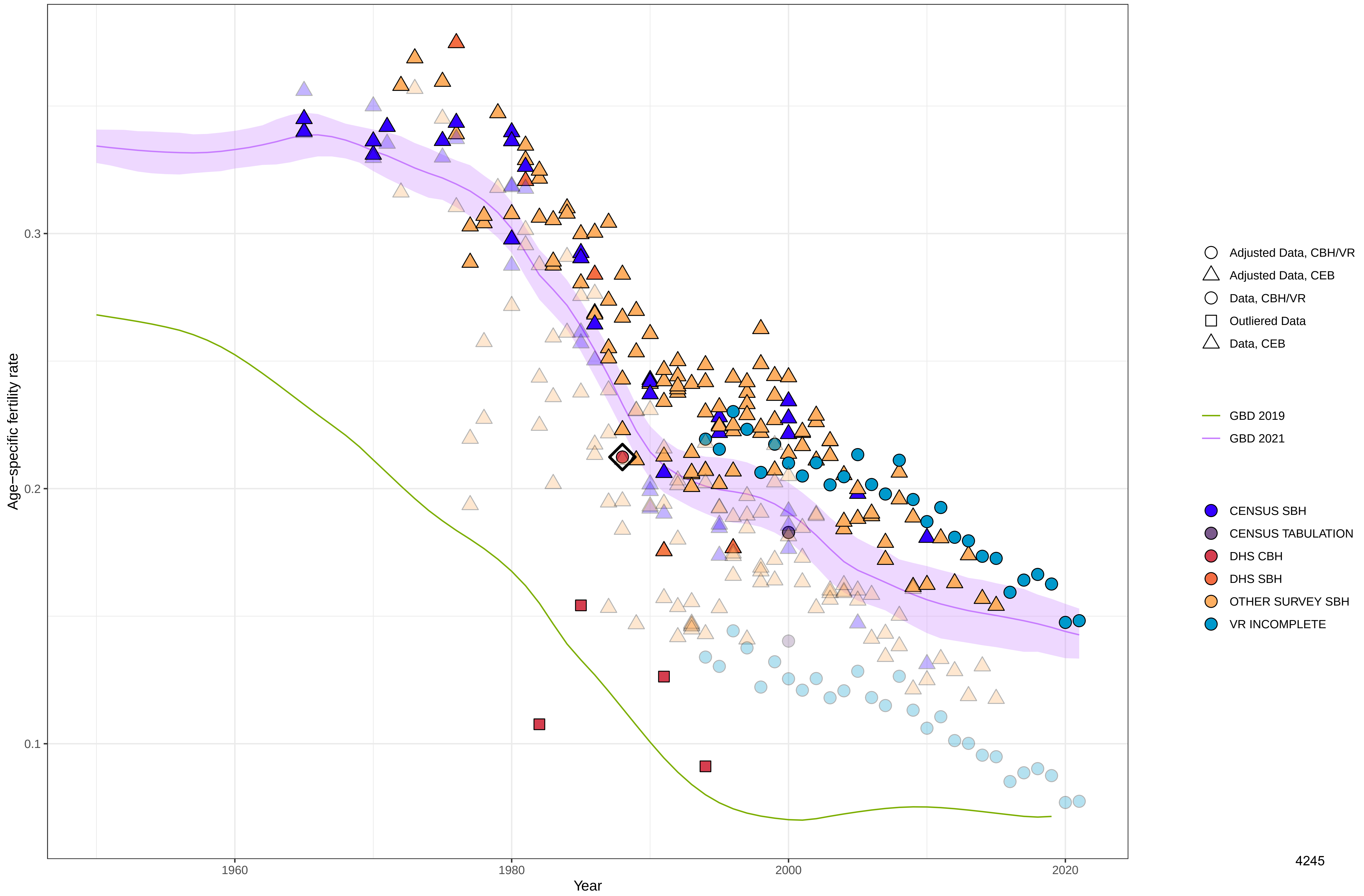

Age−specific fertility fitted trend lines, 1950−2021: Acre, Ages: 30−34
Data points enclosed with a diamond are reference sources

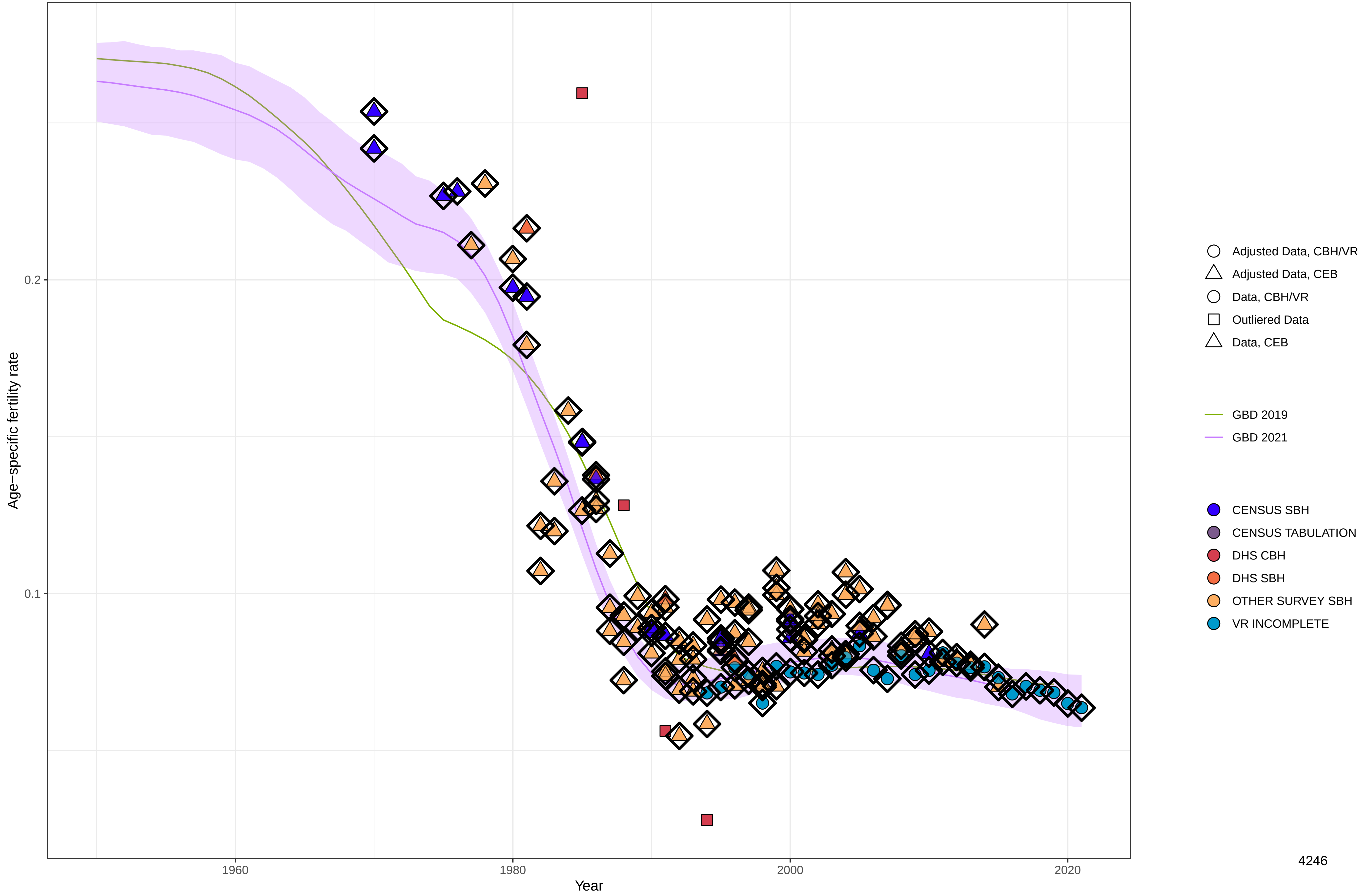

Age−specific fertility fitted trend lines, 1950−2021: Acre, Ages: 35−39
Data points enclosed with a diamond are reference sources

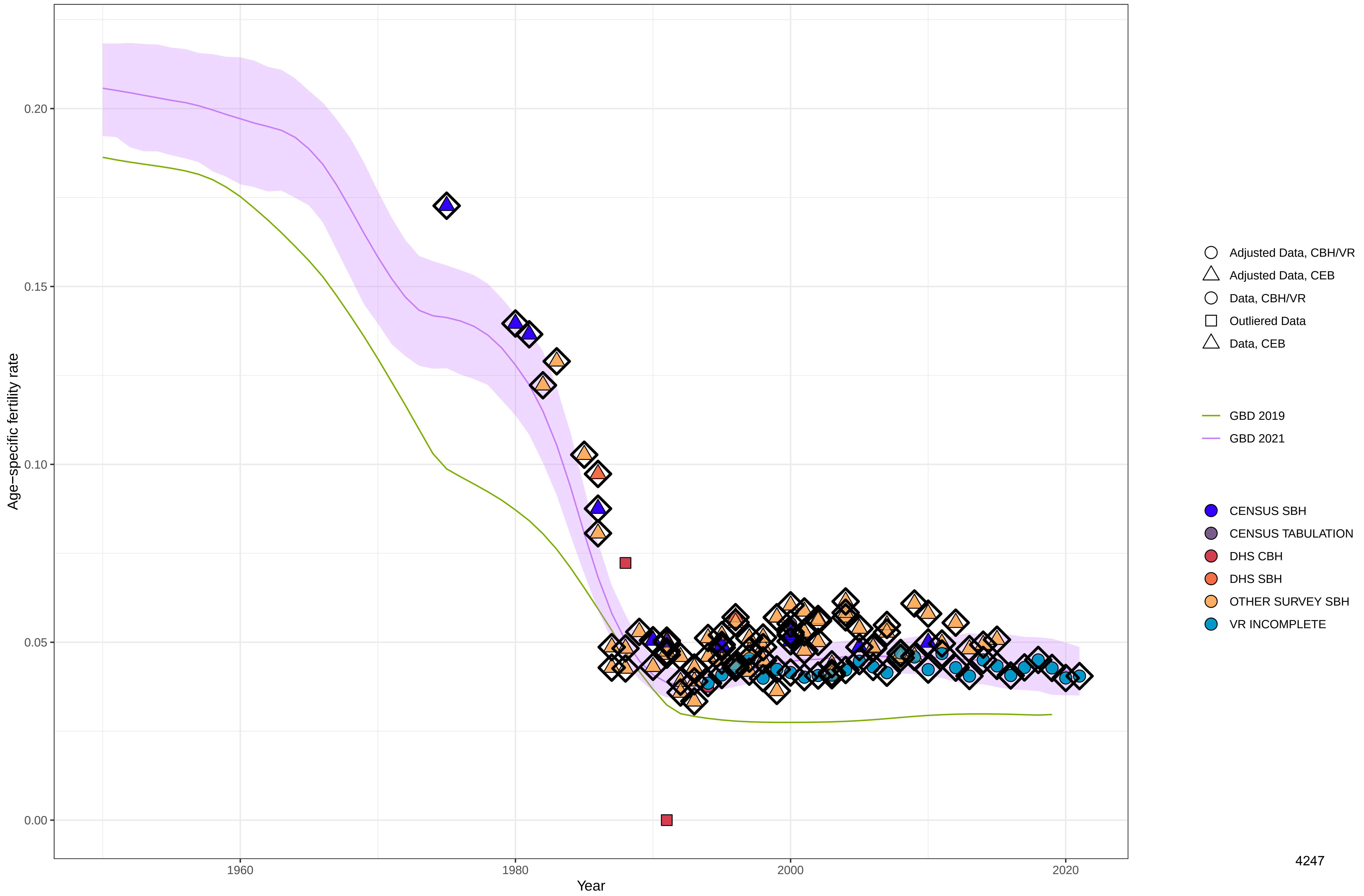

Age−specific fertility fitted trend lines, 1950−2021: Acre, Ages: 40−44
Data points enclosed with a diamond are reference sources

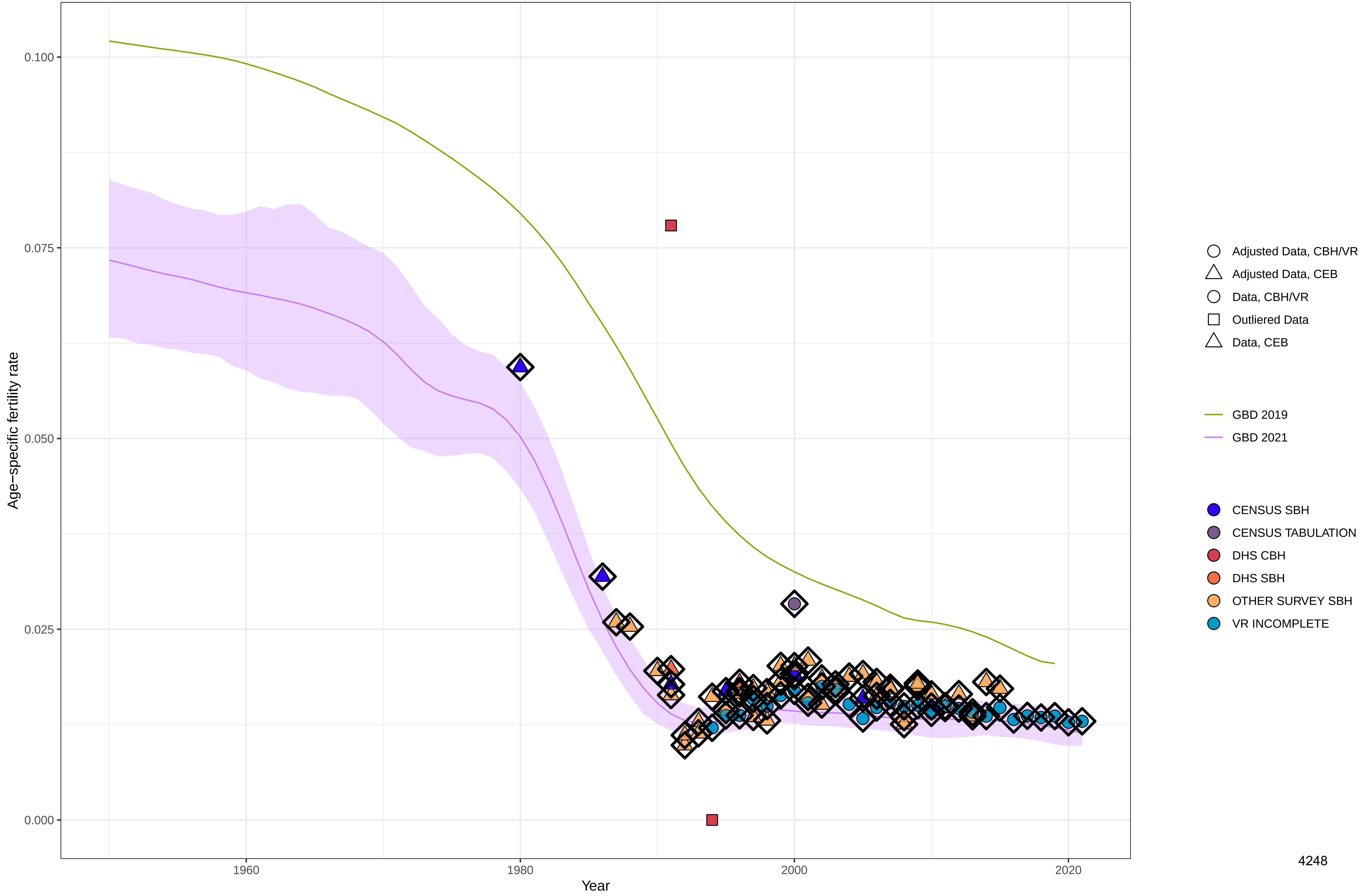

Age−specific fertility fitted trend lines, 1950−2021: Acre, Ages: 45−49
Data points enclosed with a diamond are reference sources

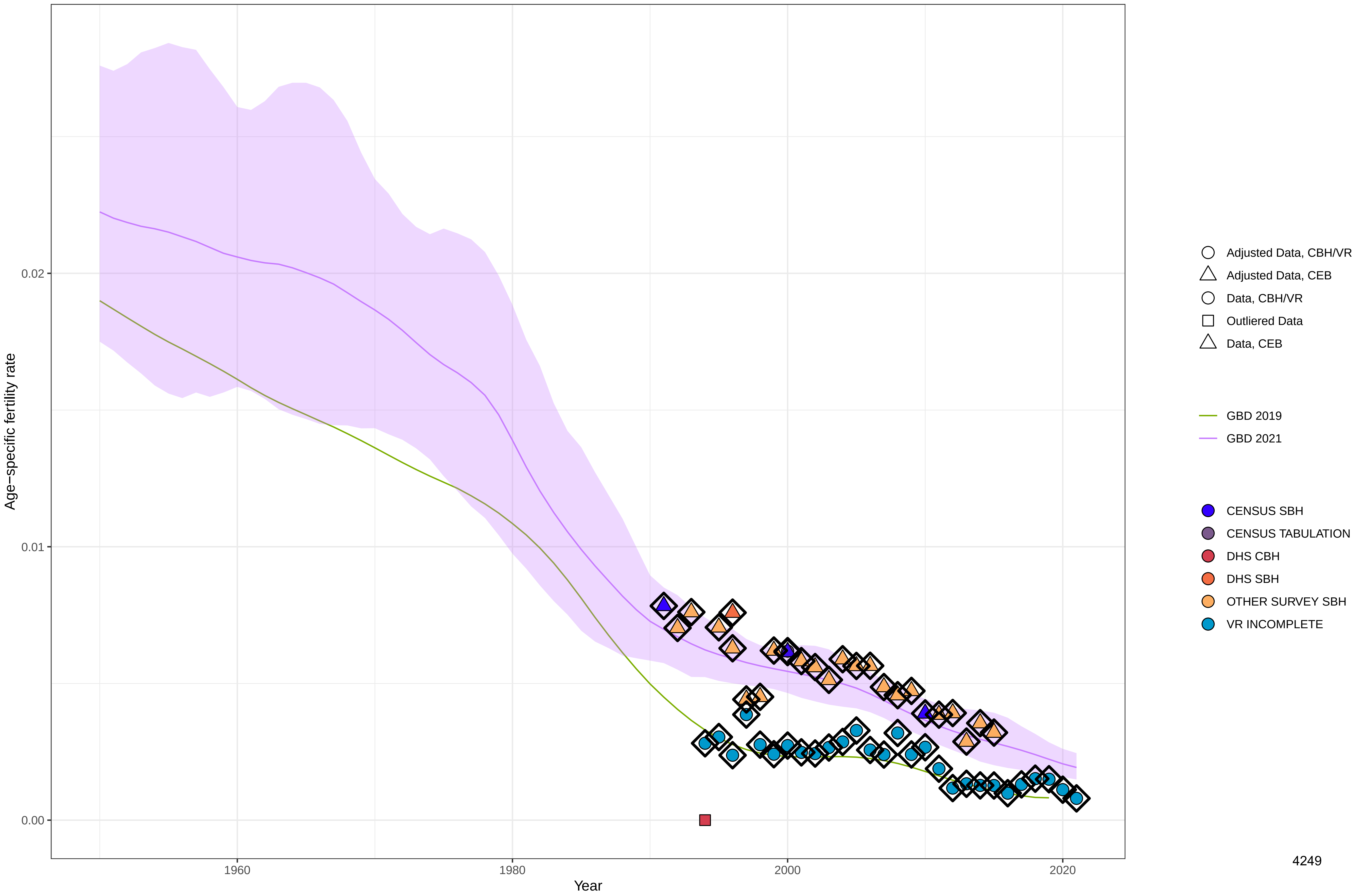

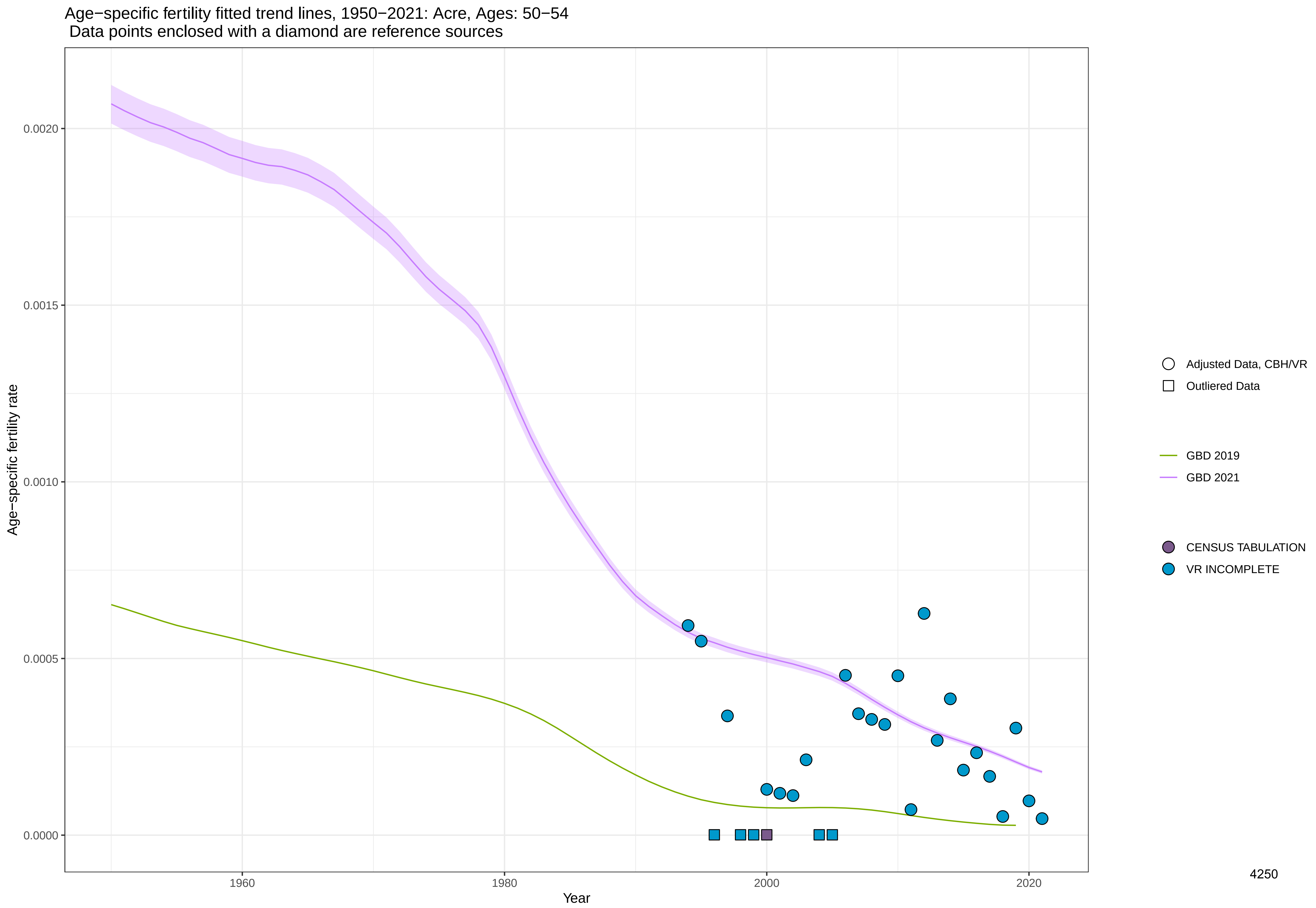

Age−specific fertility fitted trend lines, 1950−2021: Acre, Ages: 50−54
Data points enclosed with a diamond are reference sources
Age−specific fertility rate
0.0020
0.0015
0.0010
0.0005
0.0000
1960
1980
2000
2020
Year
Adjusted Data, CBH/VR
Outliered Data
GBD 2019
GBD 2021
CENSUS TABULATION
VR INCOMPLETE

4250

Total fertility rate fitted trend lines, 1950−2021: Acre TFR
Data points enclosed with a diamond are reference sources

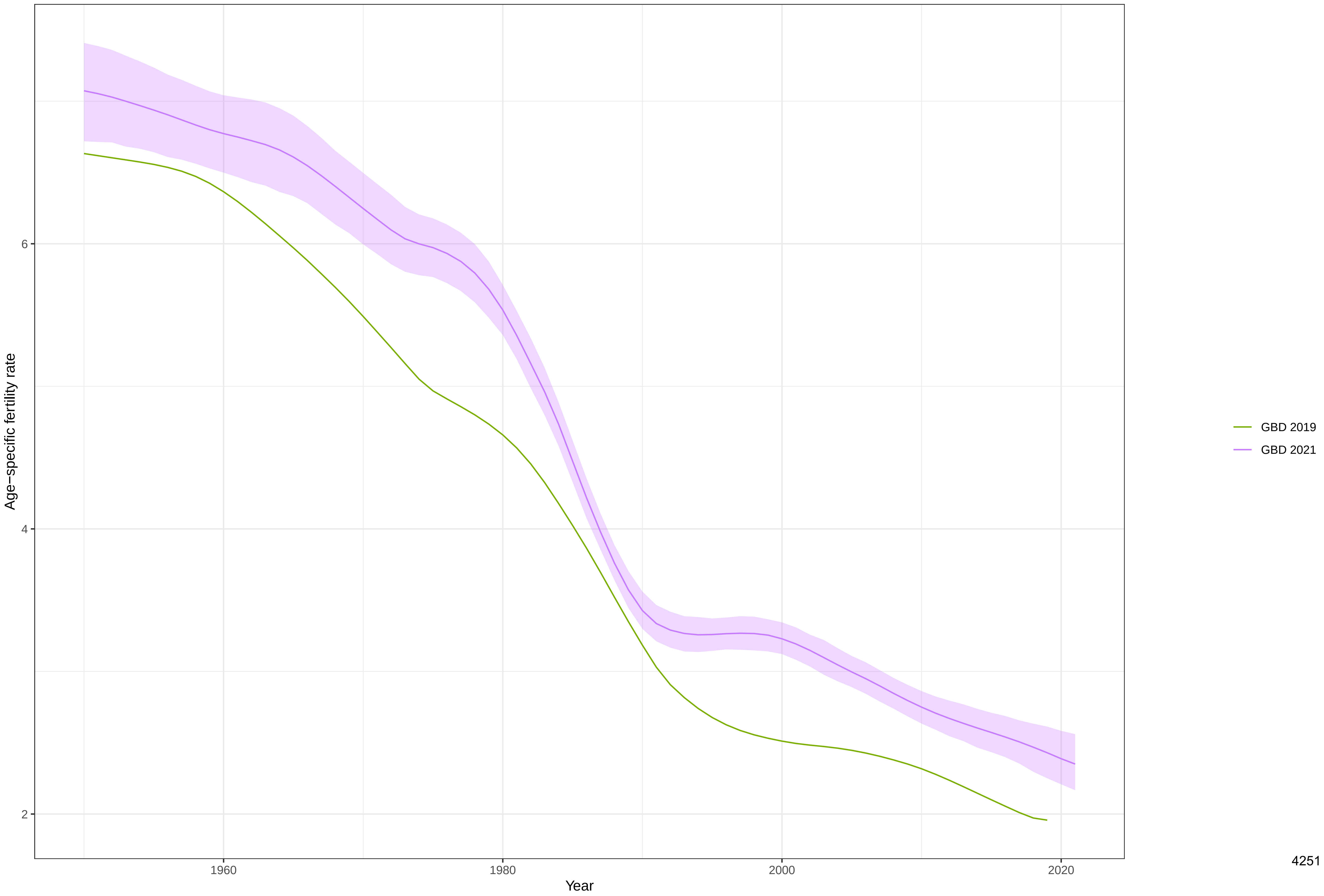

Age−specific fertility fitted trend lines, 1950−2021: Alagoas, Ages: 10−14
Data points enclosed with a diamond are reference sources

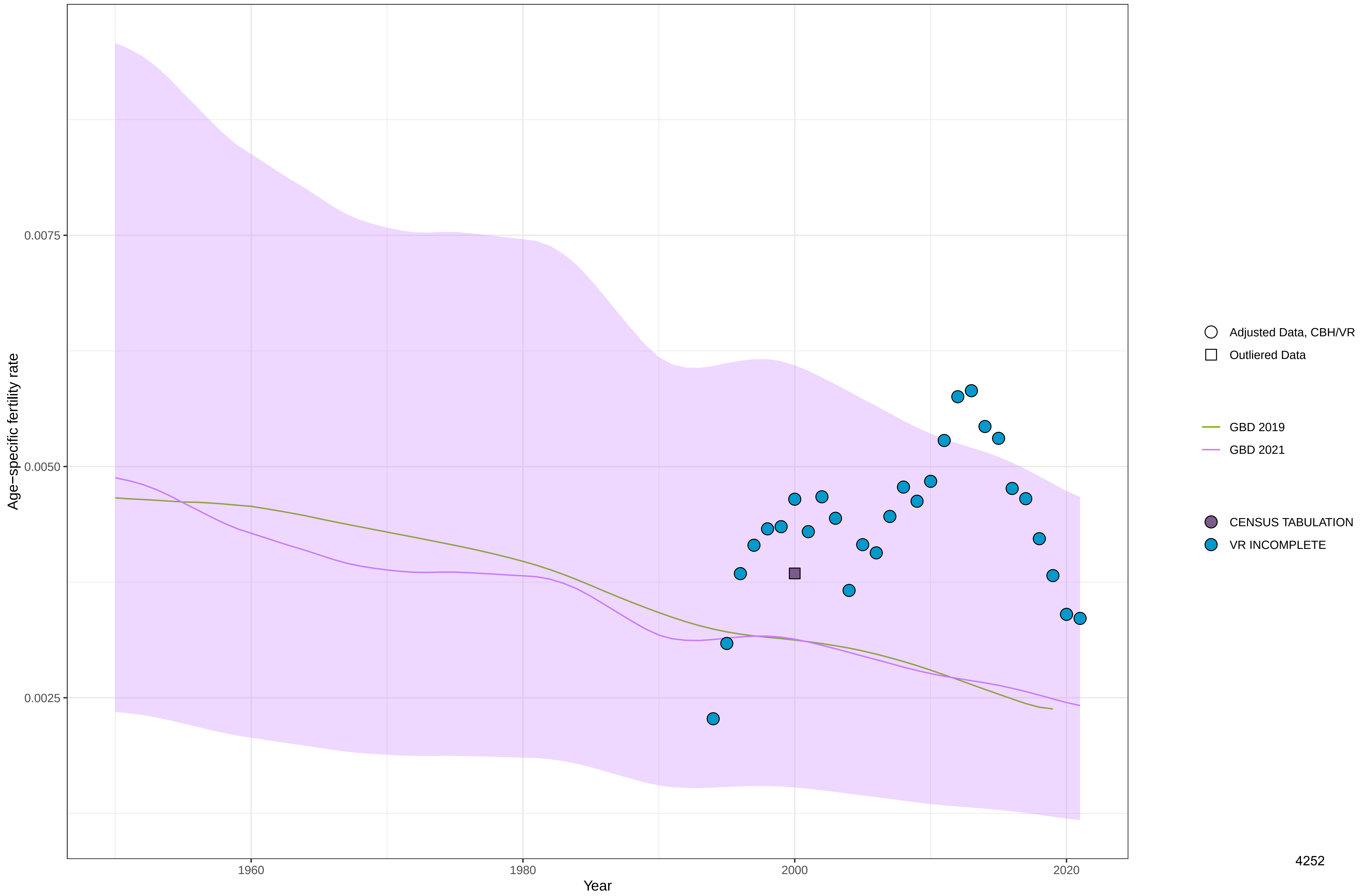

Age−specific fertility fitted trend lines, 1950−2021: Alagoas, Ages: 15−19
Data points enclosed with a diamond are reference sources

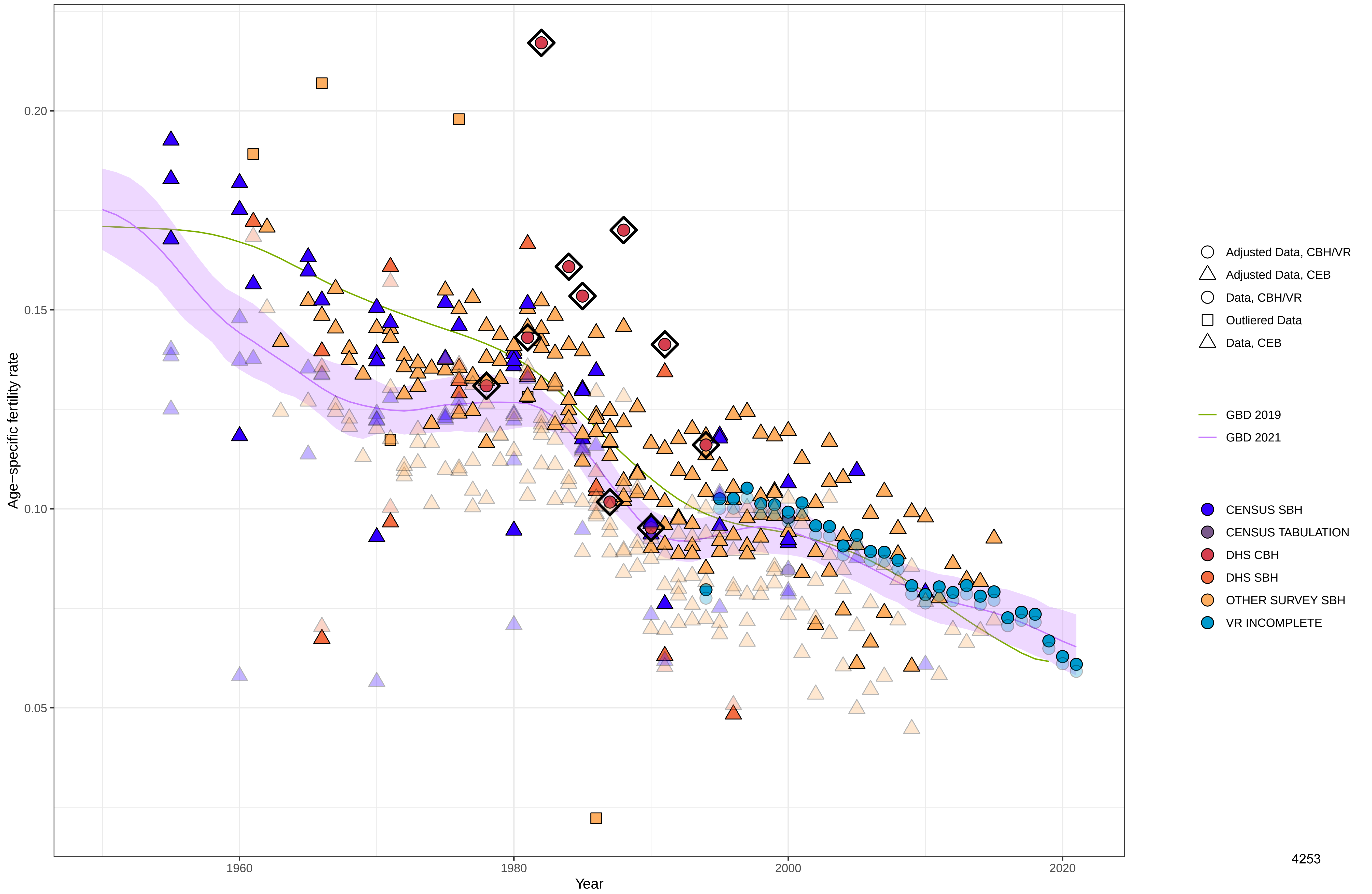

Age−specific fertility fitted trend lines, 1950−2021: Alagoas, Ages: 20−24
Data points enclosed with a diamond are reference sources

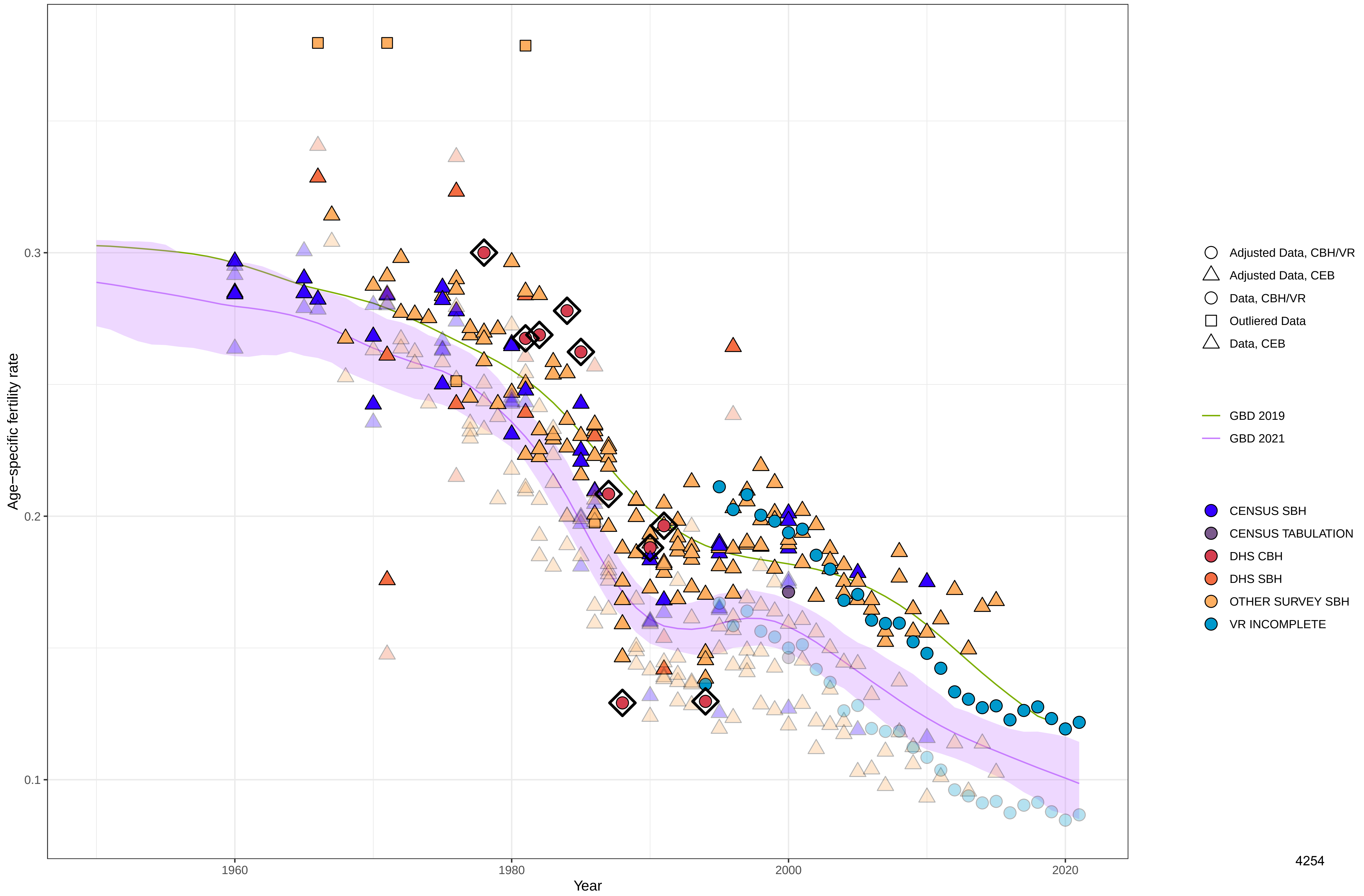

4254

Age−specific fertility fitted trend lines, 1950−2021: Alagoas, Ages: 25−29
Data points enclosed with a diamond are reference sources

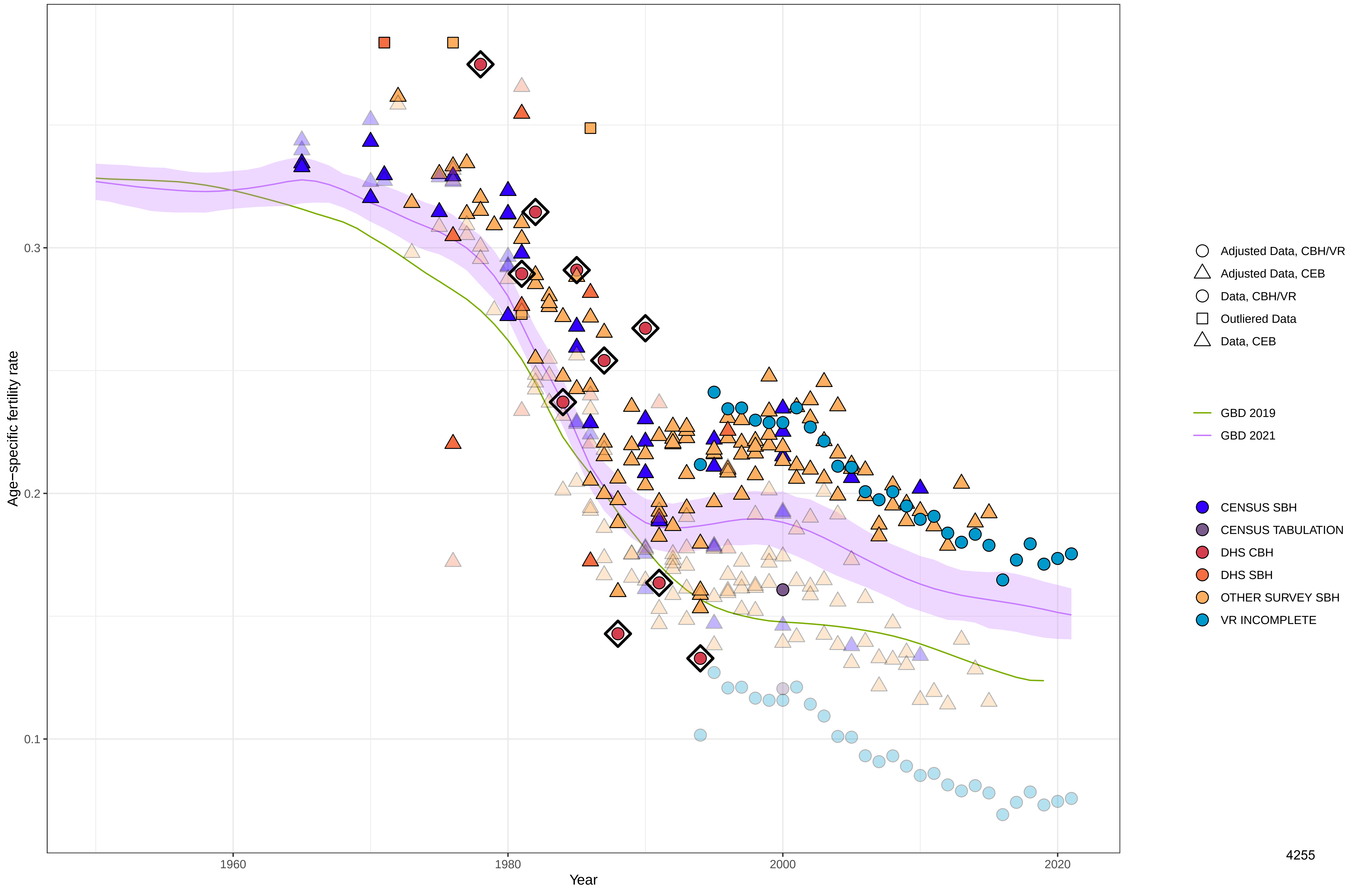

Age−specific fertility fitted trend lines, 1950−2021: Alagoas, Ages: 30−34
Data points enclosed with a diamond are reference sources

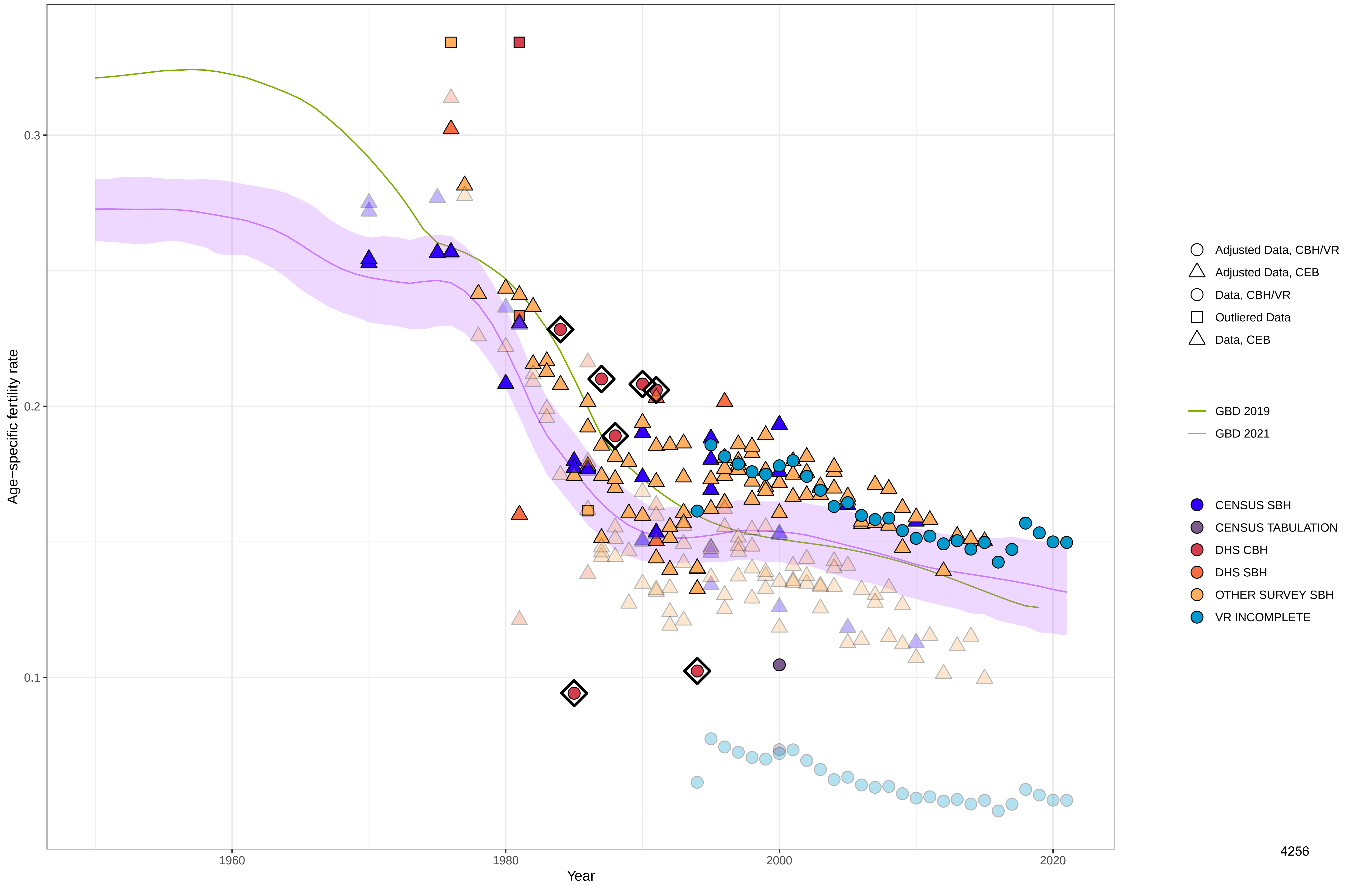

Age−specific fertility fitted trend lines, 1950−2021: Alagoas, Ages: 35−39
Data points enclosed with a diamond are reference sources

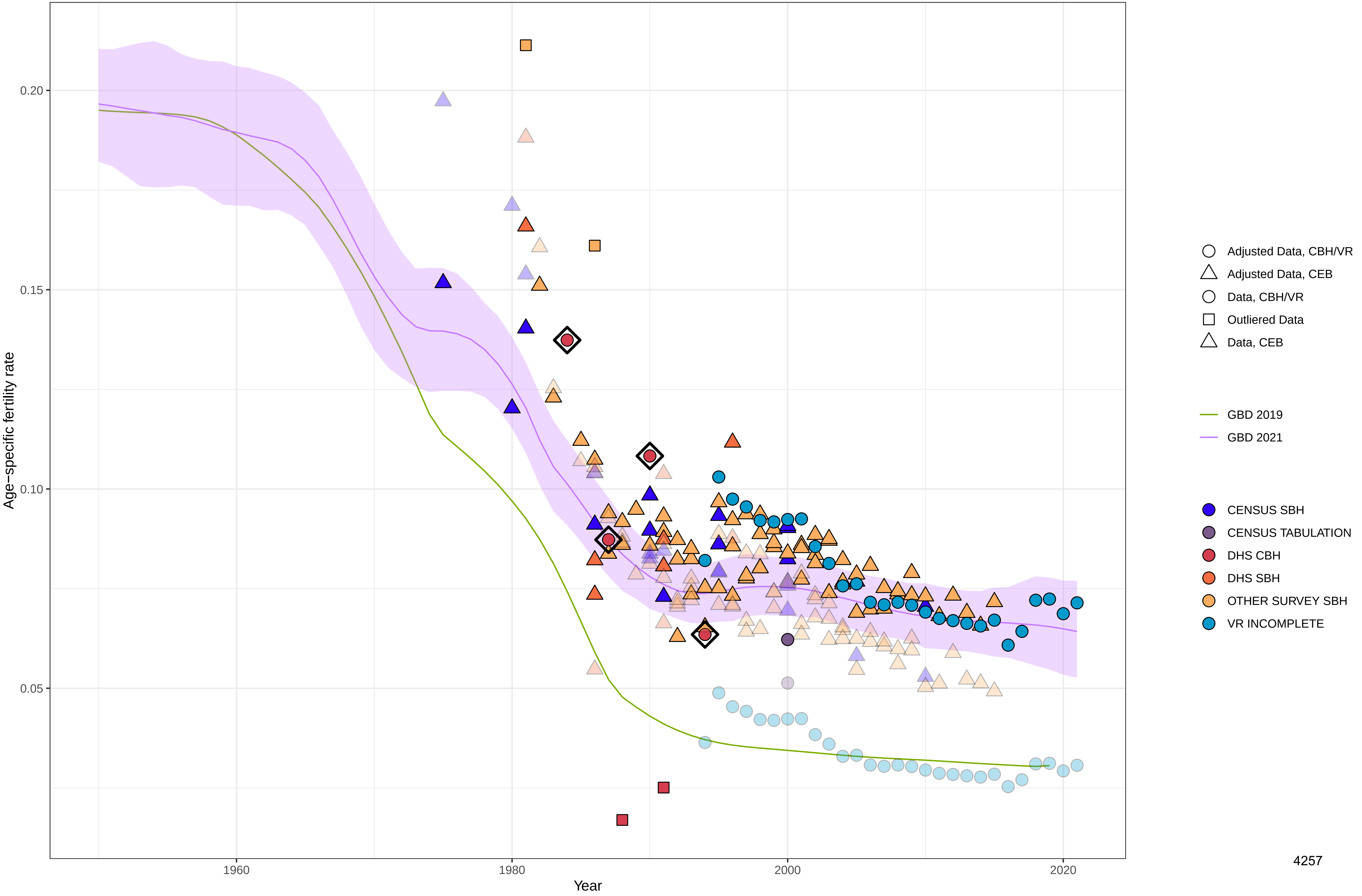

Age−specific fertility fitted trend lines, 1950−2021: Alagoas, Ages: 40−44
Data points enclosed with a diamond are reference sources

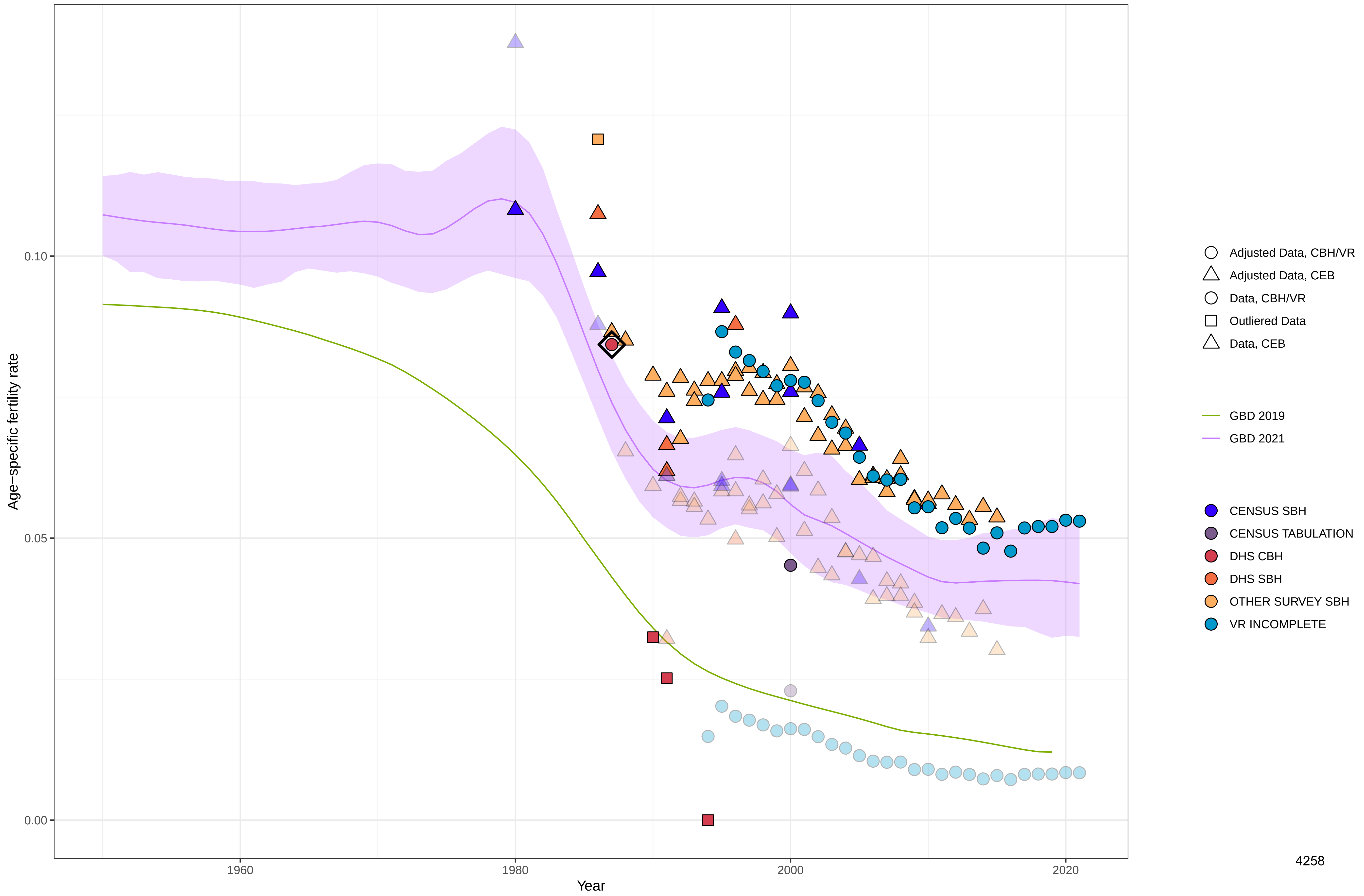

Age−specific fertility fitted trend lines, 1950−2021: Alagoas, Ages: 45−49
Data points enclosed with a diamond are reference sources

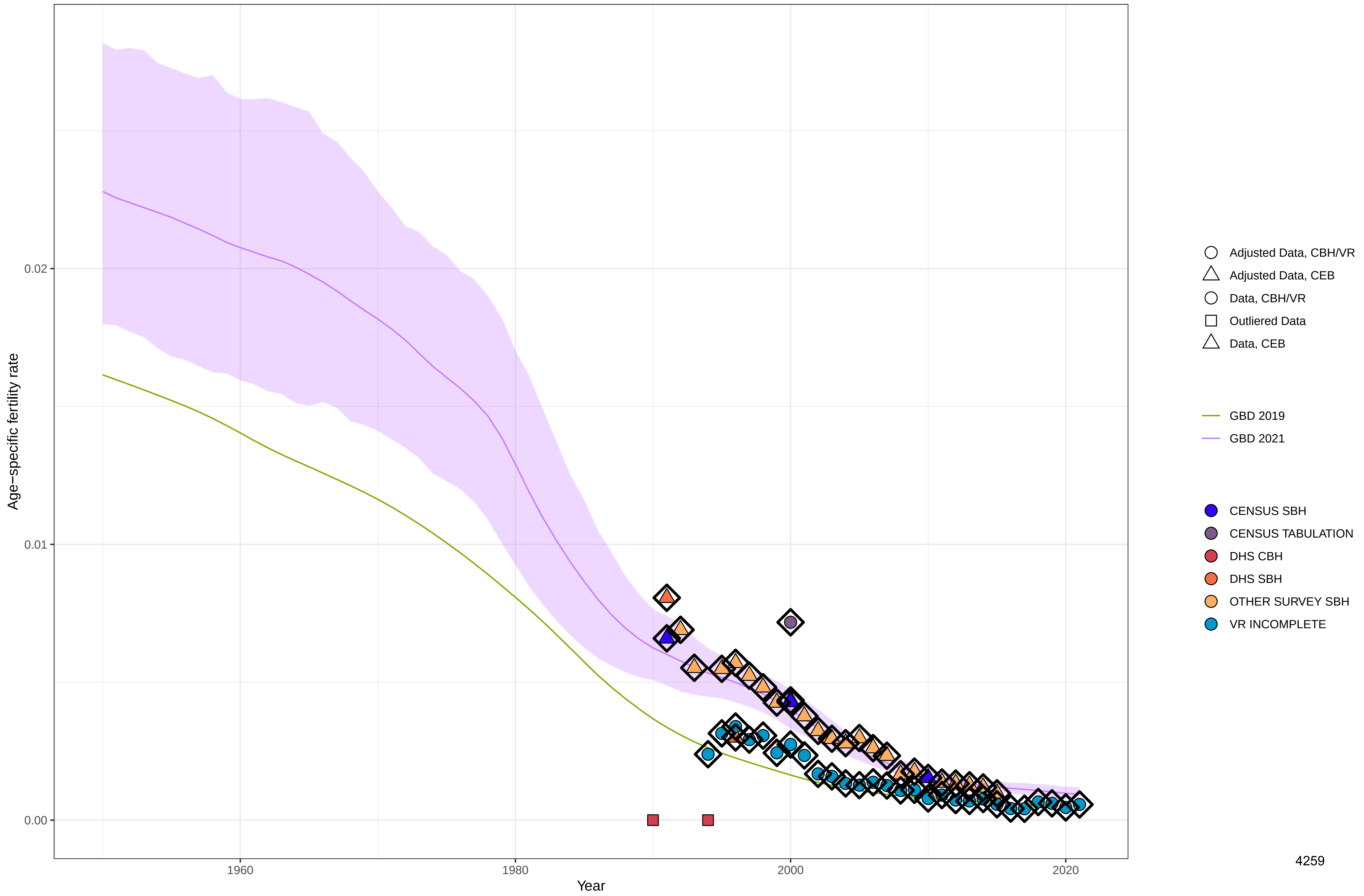

Age−specific fertility fitted trend lines, 1950−2021: Alagoas, Ages: 50−54
Data points enclosed with a diamond are reference sources

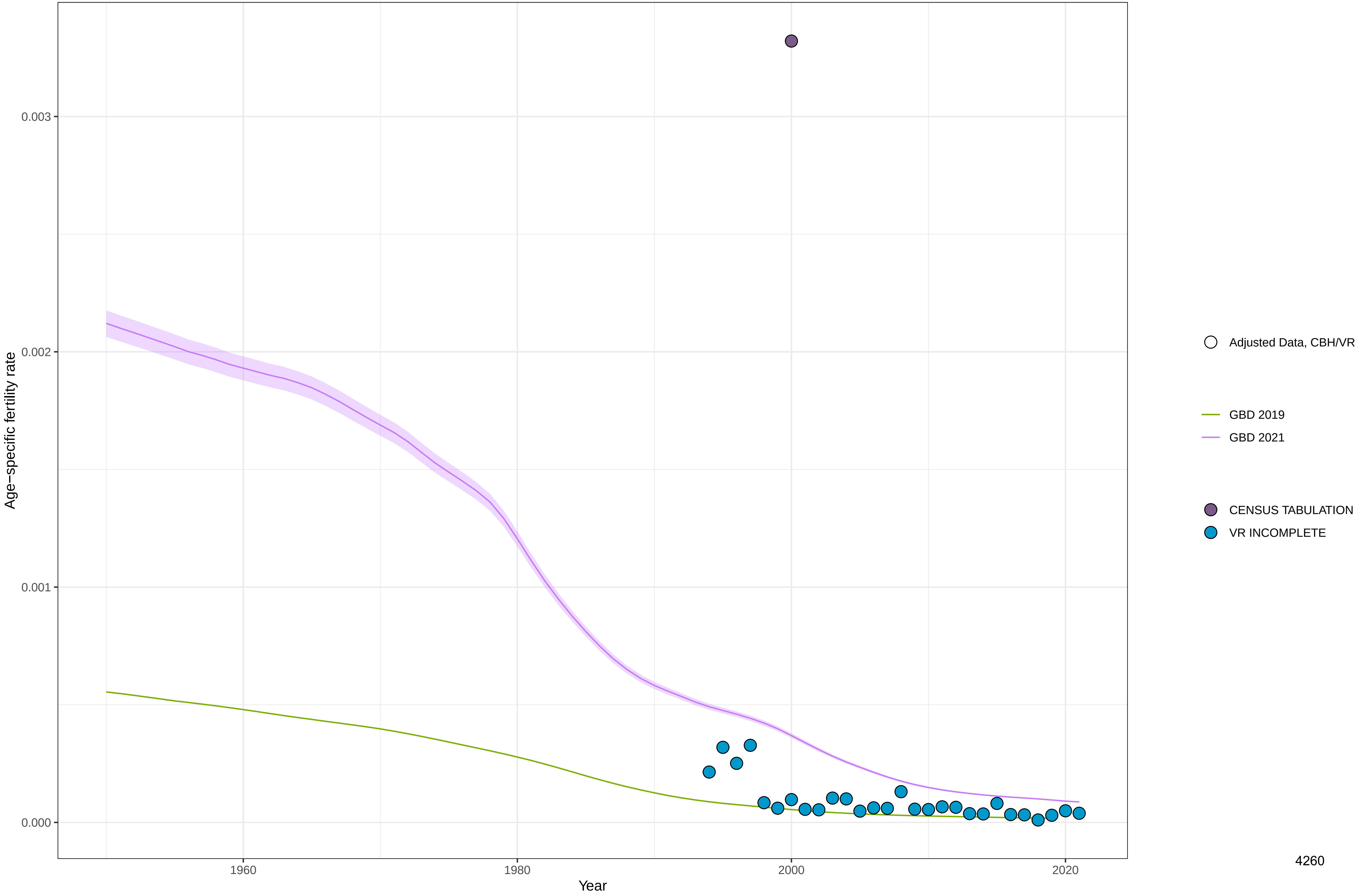

Total fertility rate fitted trend lines, 1950−2021: Alagoas TFR
Data points enclosed with a diamond are reference sources

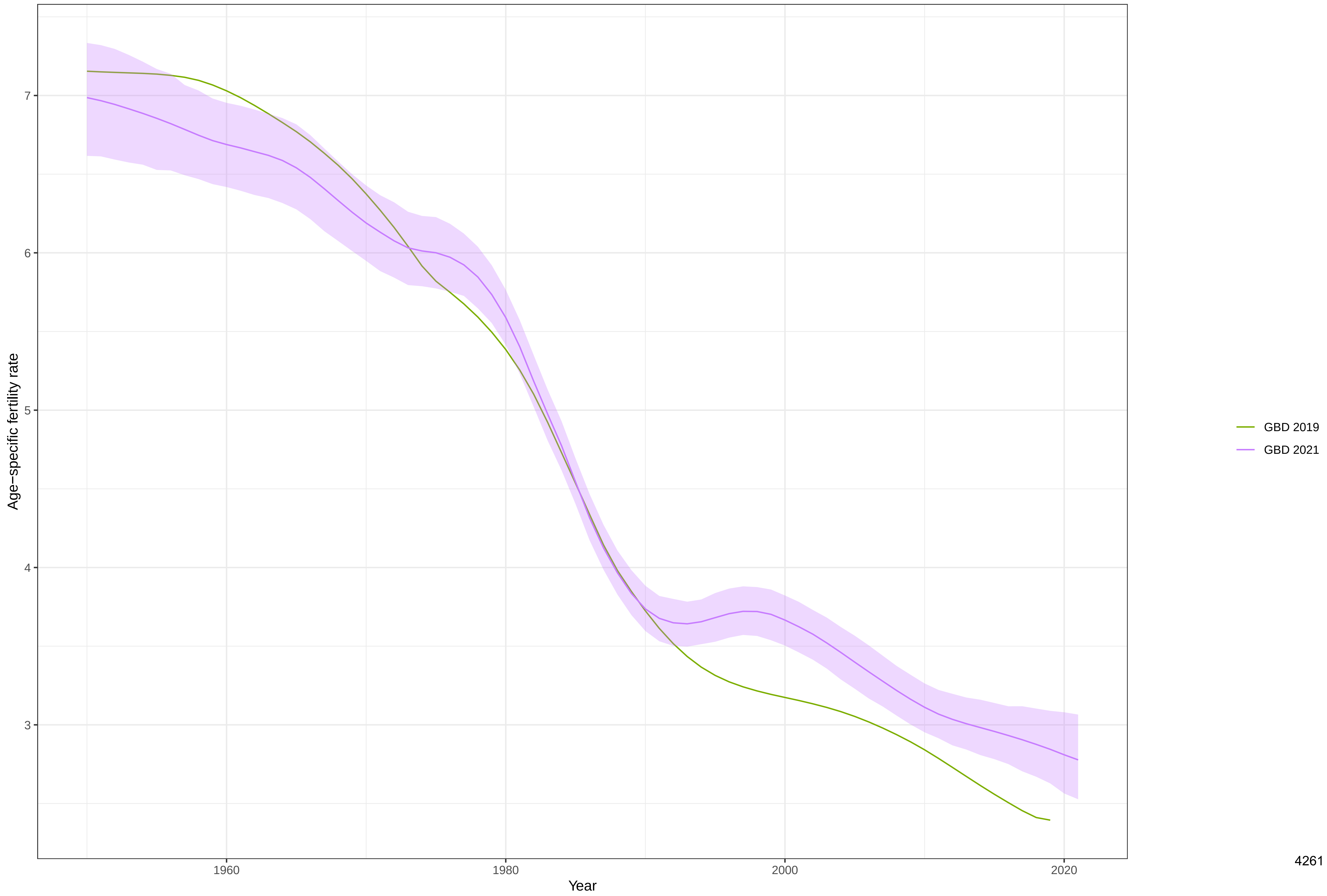

Age−specific fertility fitted trend lines, 1950−2021: Amapá, Ages: 10−14
Data points enclosed with a diamond are reference sources

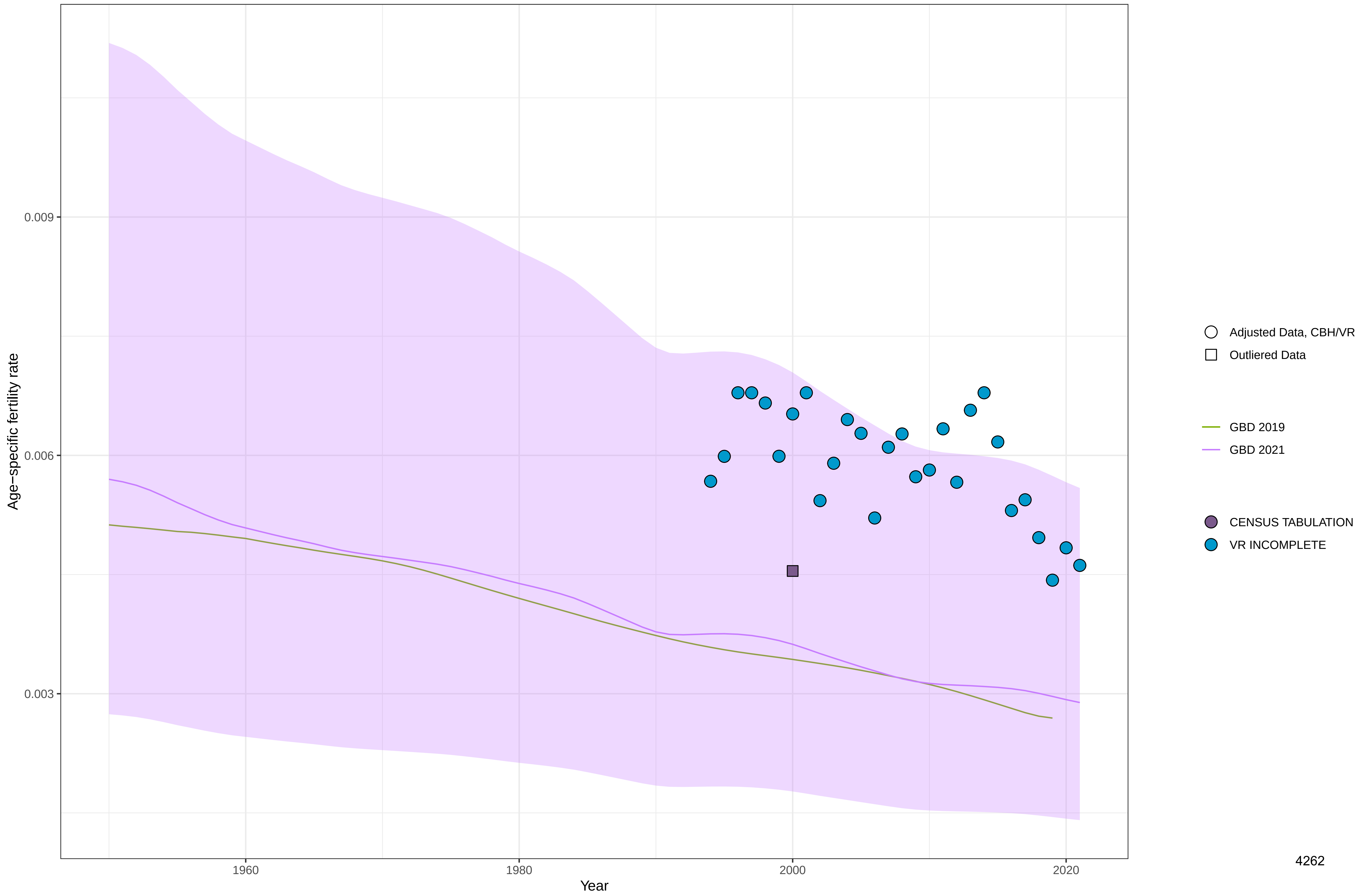

Age−specific fertility fitted trend lines, 1950−2021: Amapá, Ages: 15−19
Data points enclosed with a diamond are reference sources

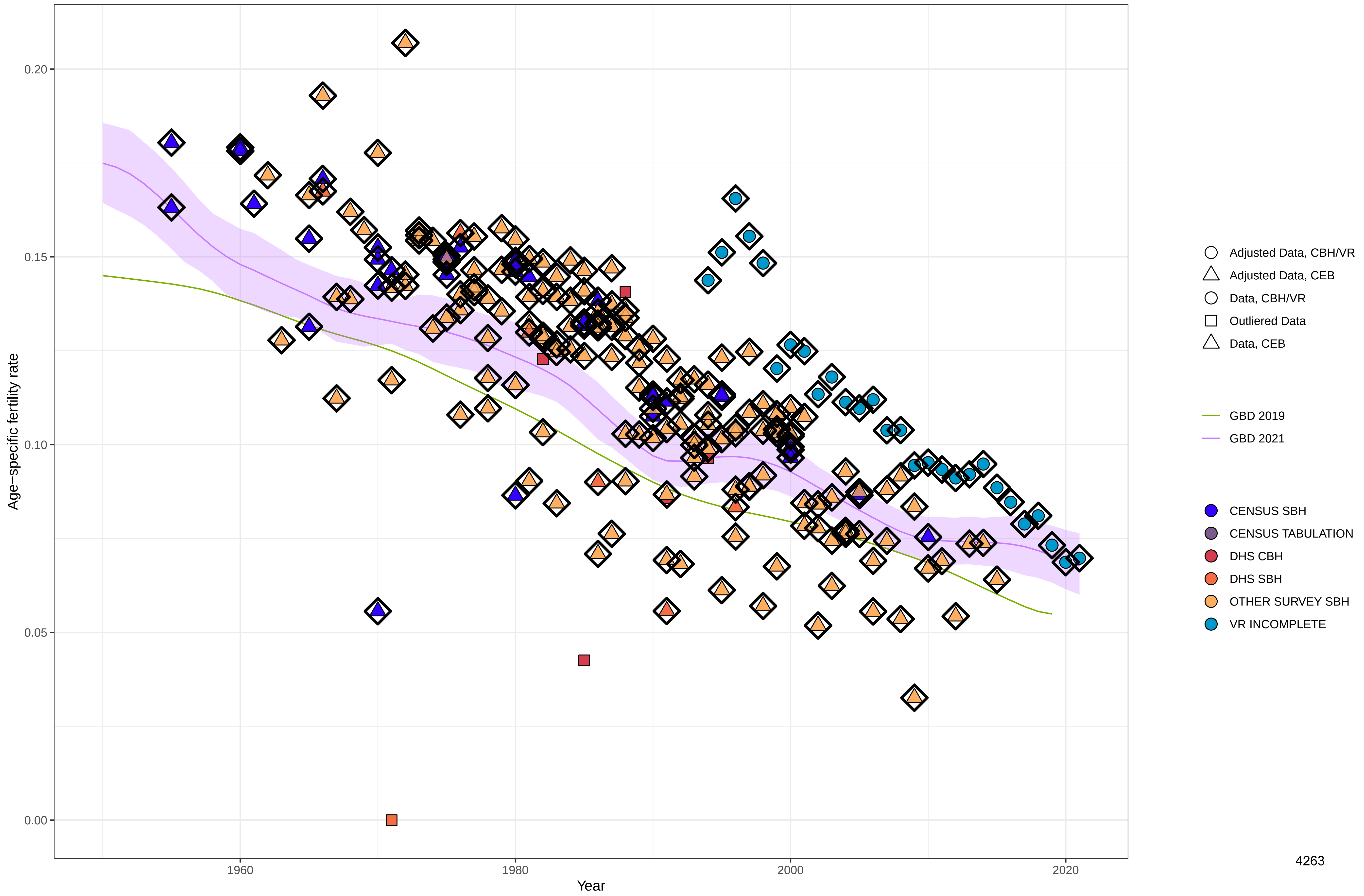

Age−specific fertility fitted trend lines, 1950−2021: Amapá, Ages: 20−24
Data points enclosed with a diamond are reference sources

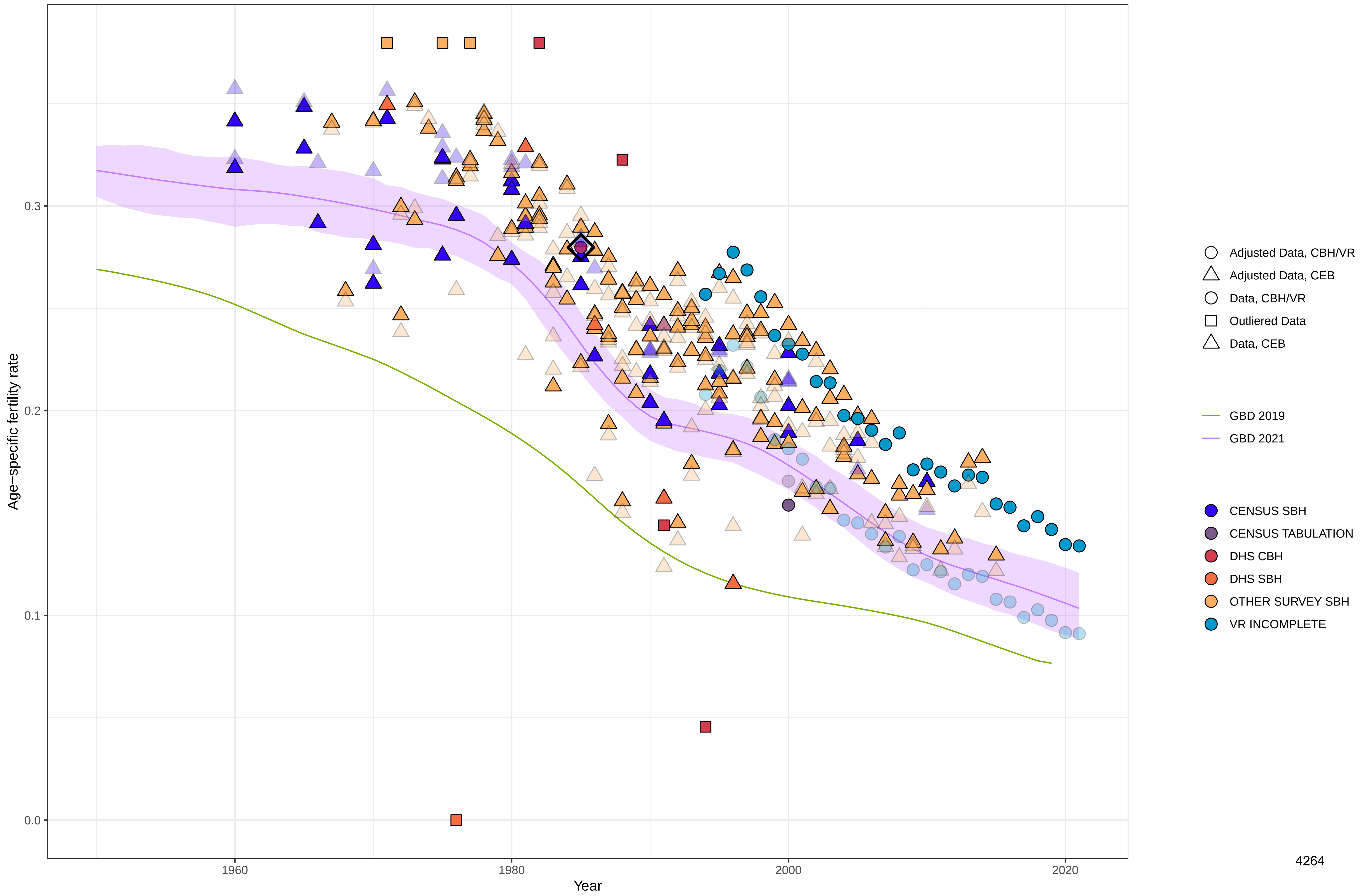

Age−specific fertility fitted trend lines, 1950−2021: Amapá, Ages: 25−29
Data points enclosed with a diamond are reference sources

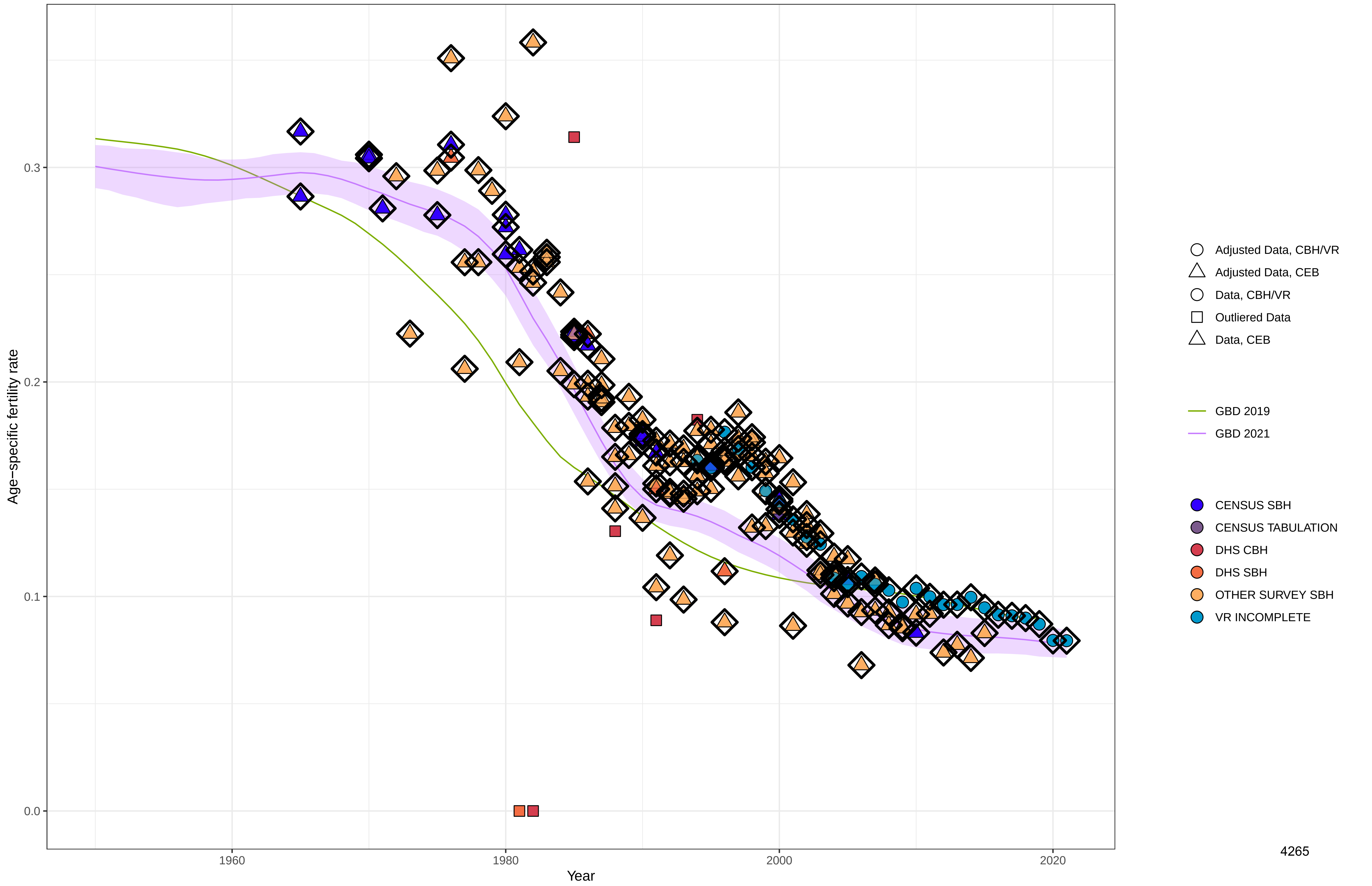

Age−specific fertility fitted trend lines, 1950−2021: Amapá, Ages: 30−34
Data points enclosed with a diamond are reference sources

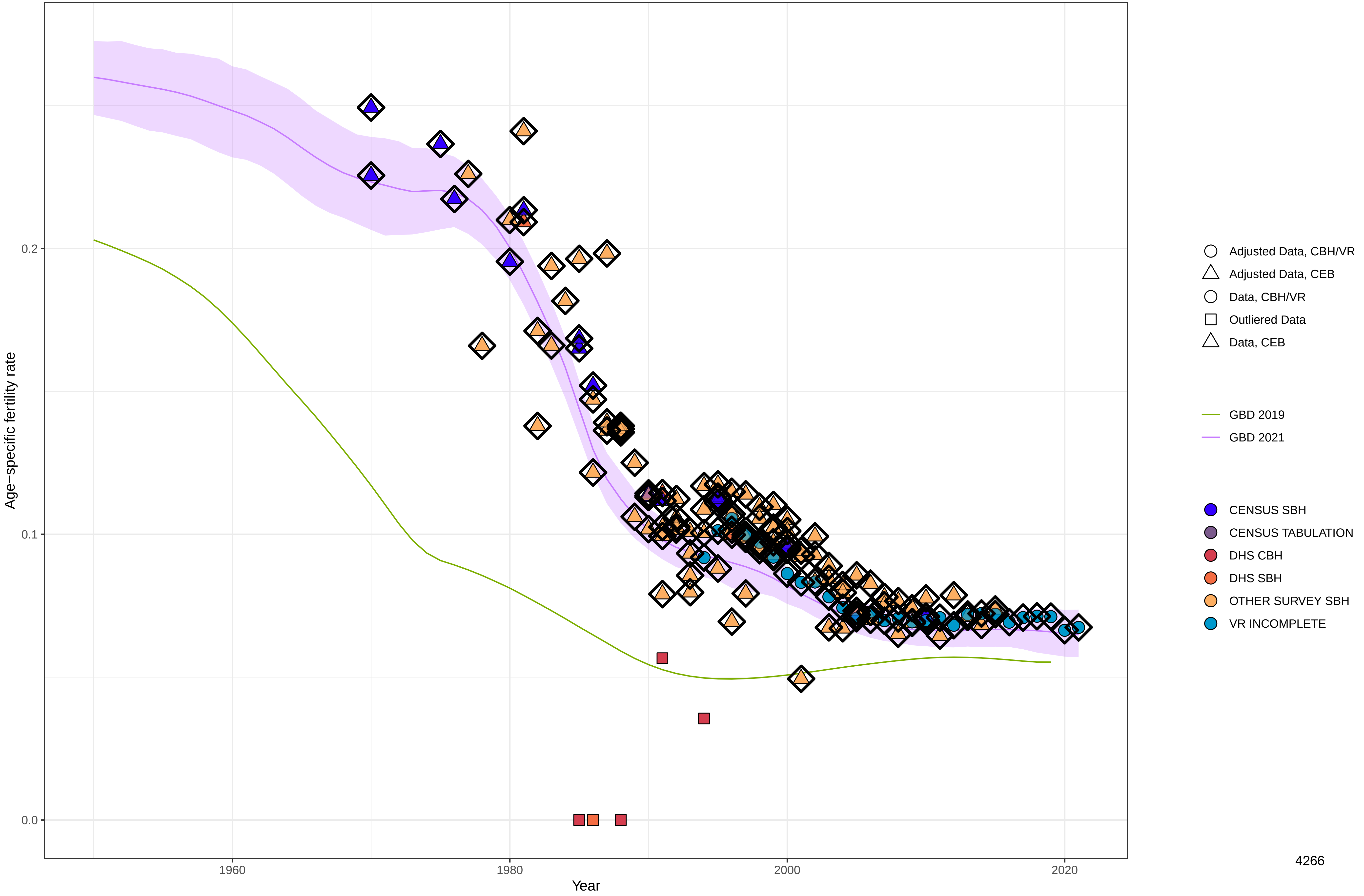

Age−specific fertility fitted trend lines, 1950−2021: Amapá, Ages: 35−39
Data points enclosed with a diamond are reference sources

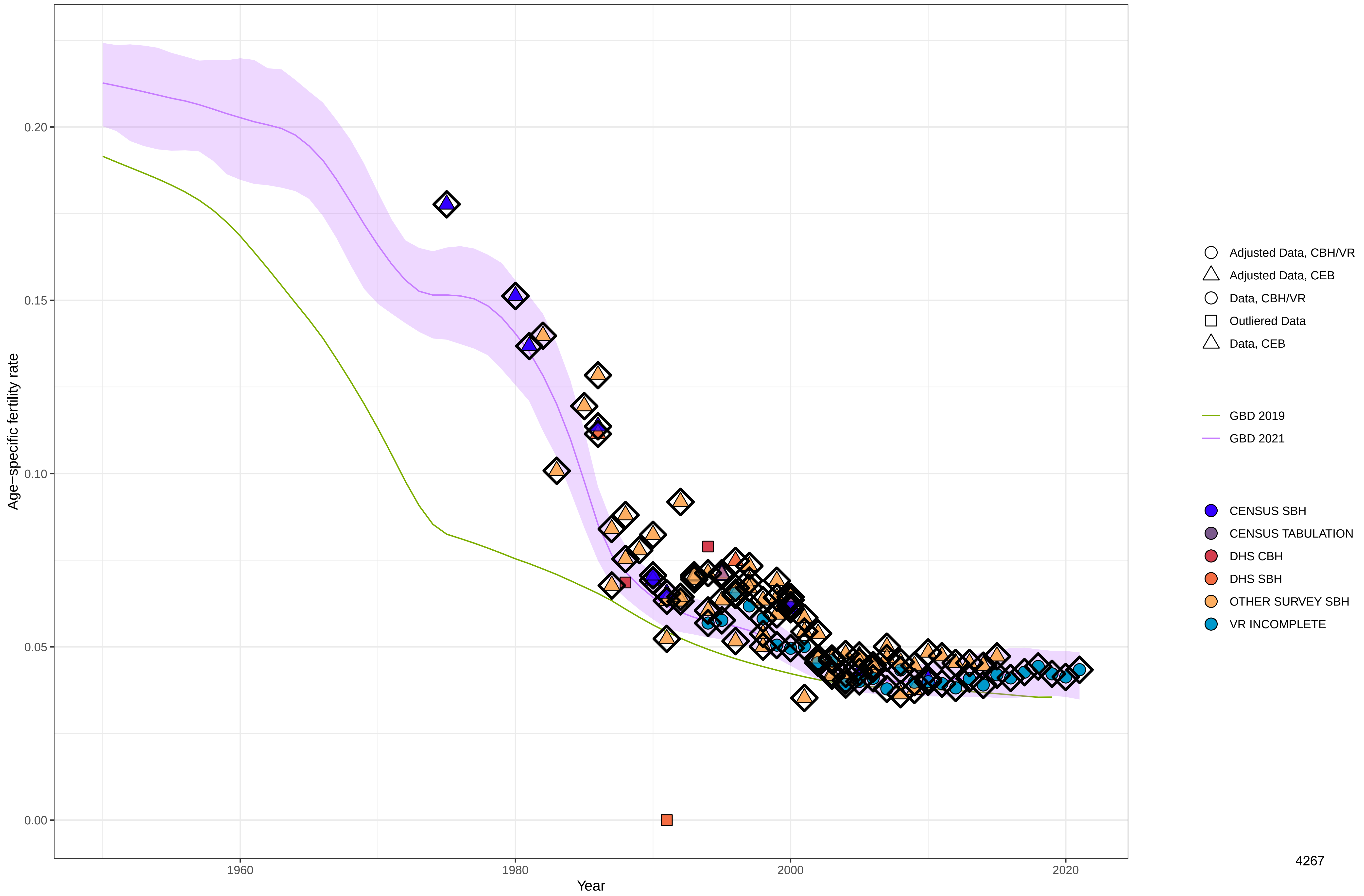

Age−specific fertility fitted trend lines, 1950−2021: Amapá, Ages: 40−44
Data points enclosed with a diamond are reference sources

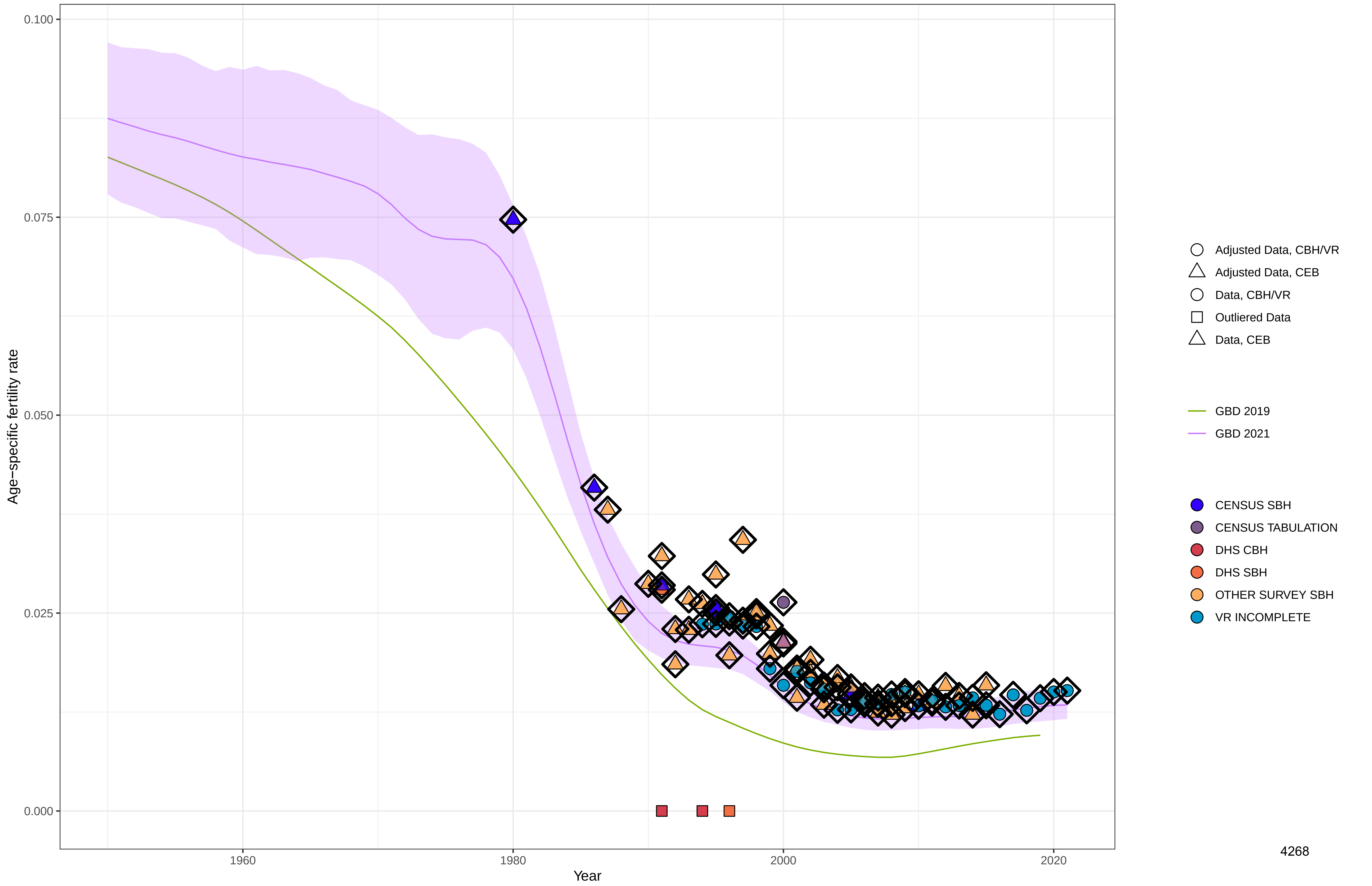

Age−specific fertility fitted trend lines, 1950−2021: Amapá, Ages: 45−49
Data points enclosed with a diamond are reference sources

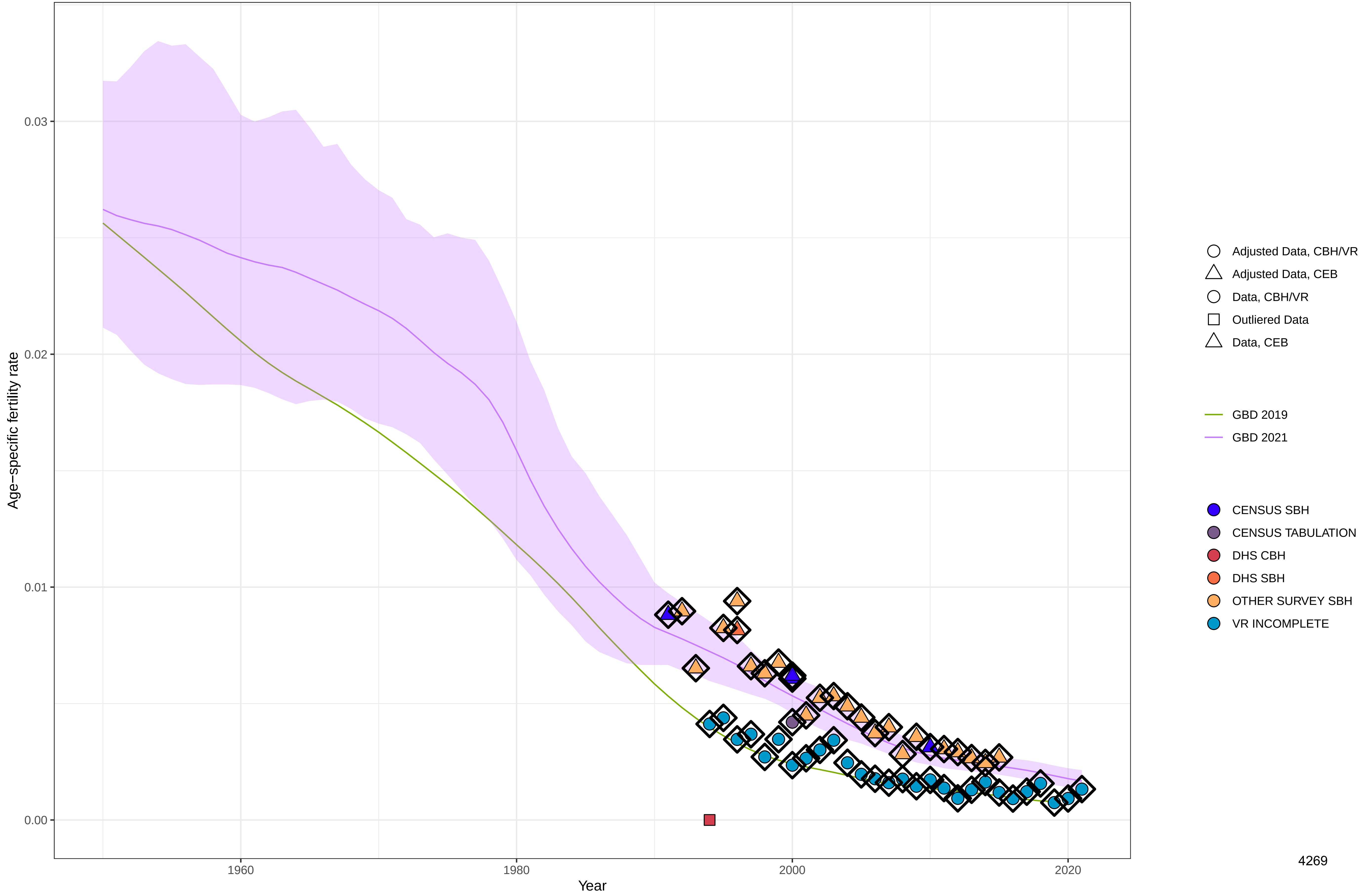

Age−specific fertility fitted trend lines, 1950−2021: Amapá, Ages: 50−54
Data points enclosed with a diamond are reference sources

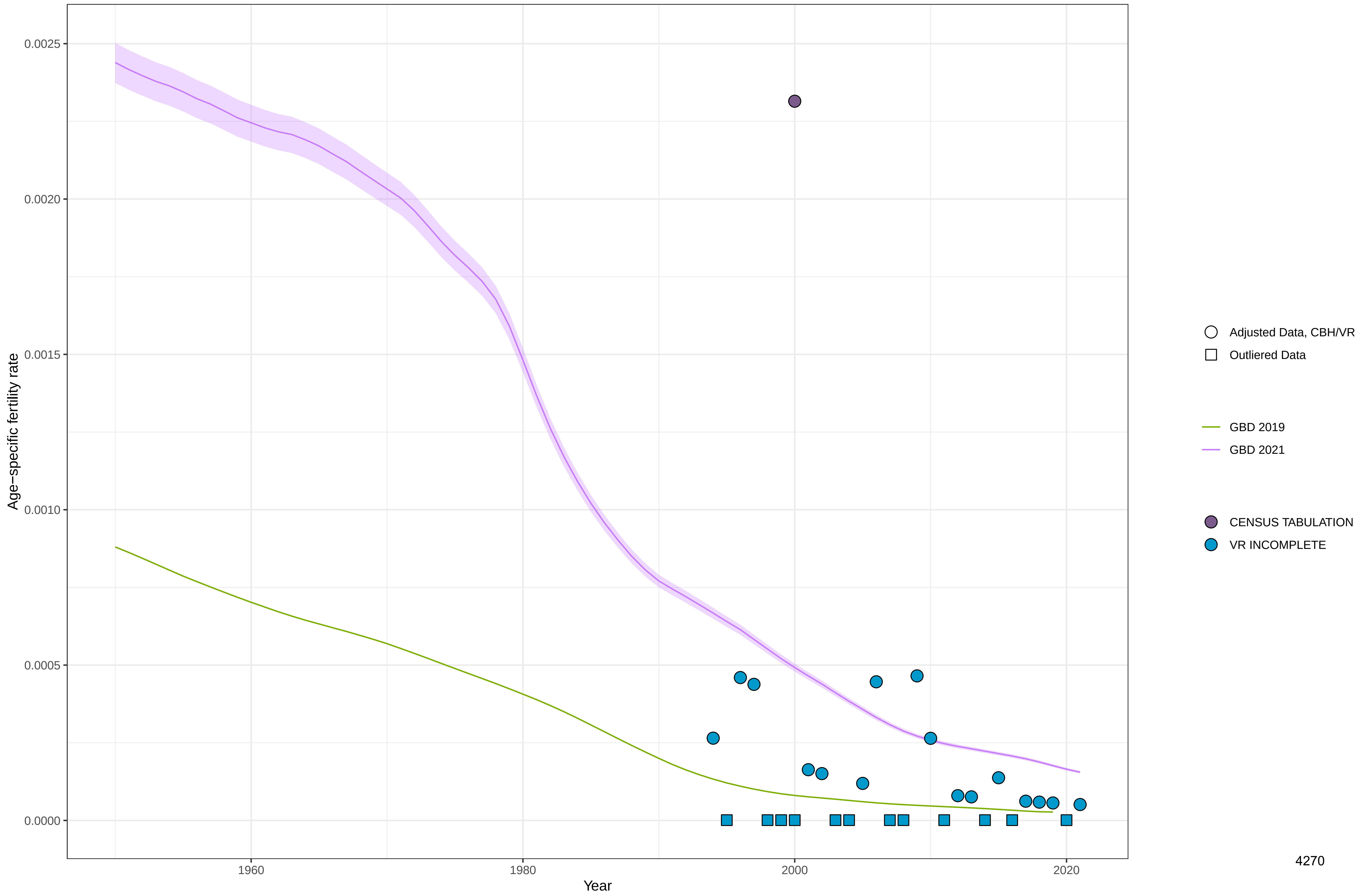

Total fertility rate fitted trend lines, 1950−2021: Amapá TFR
Data points enclosed with a diamond are reference sources

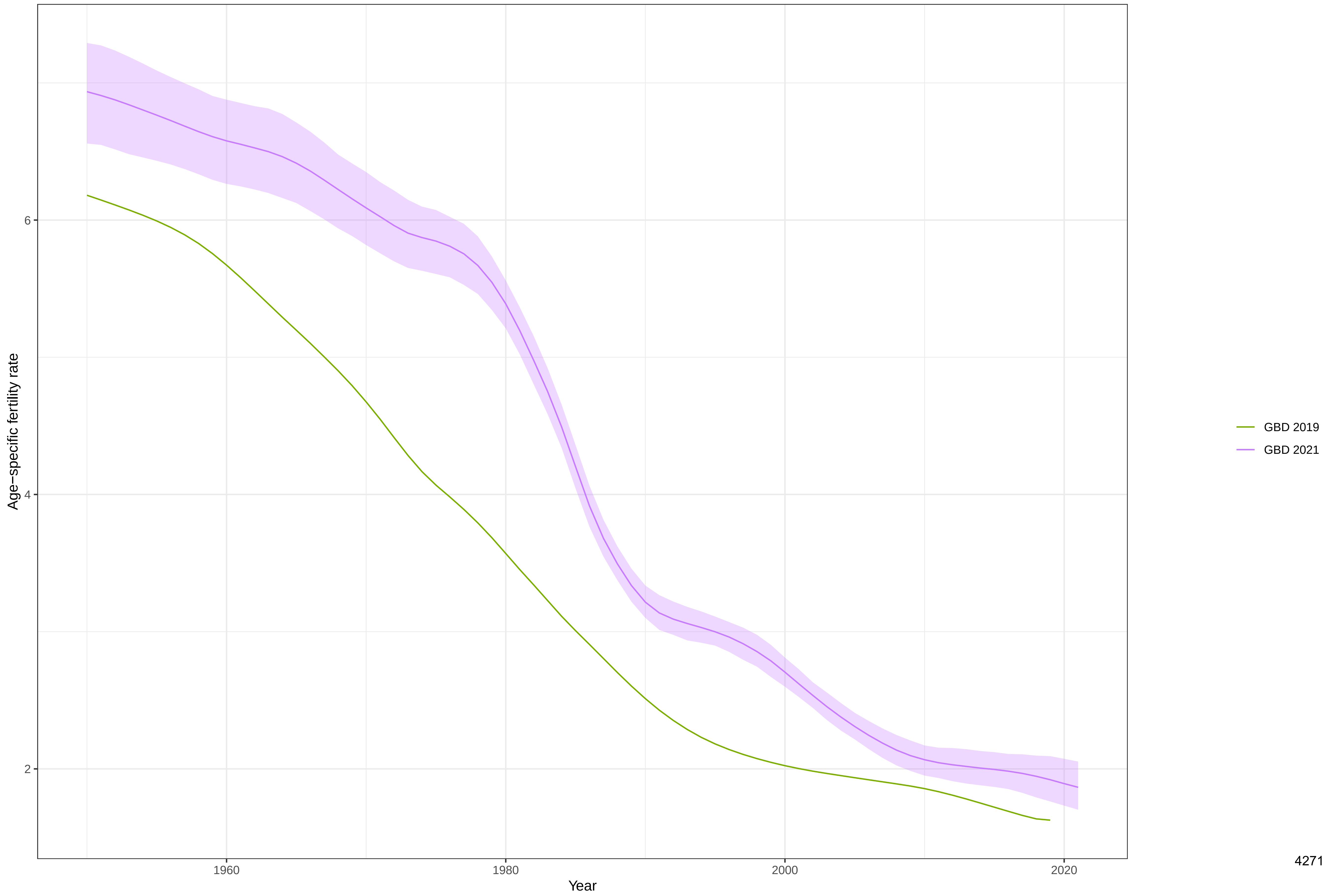

Age−specific fertility fitted trend lines, 1950−2021: Amazonas, Ages: 10−14
Data points enclosed with a diamond are reference sources

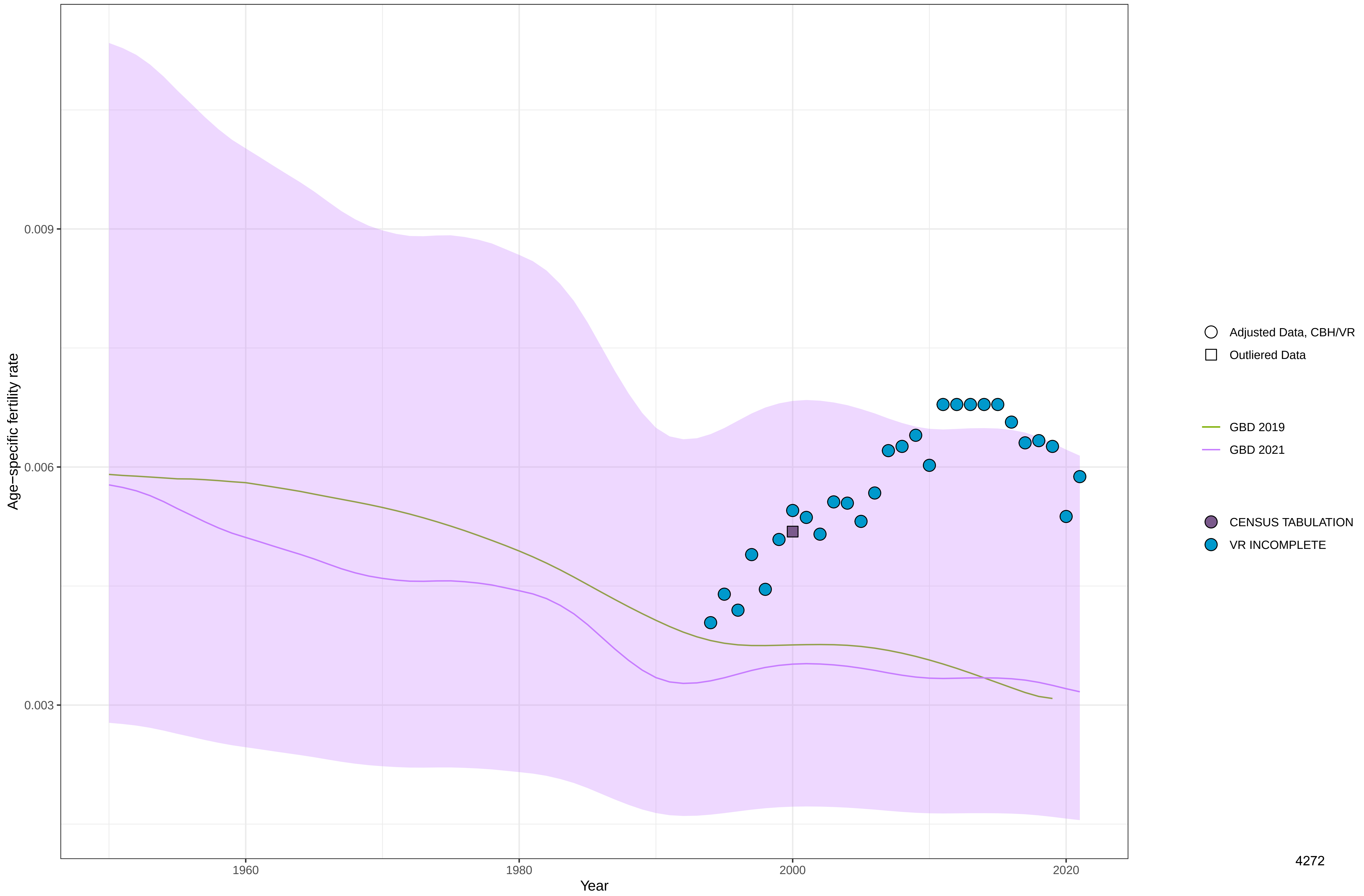

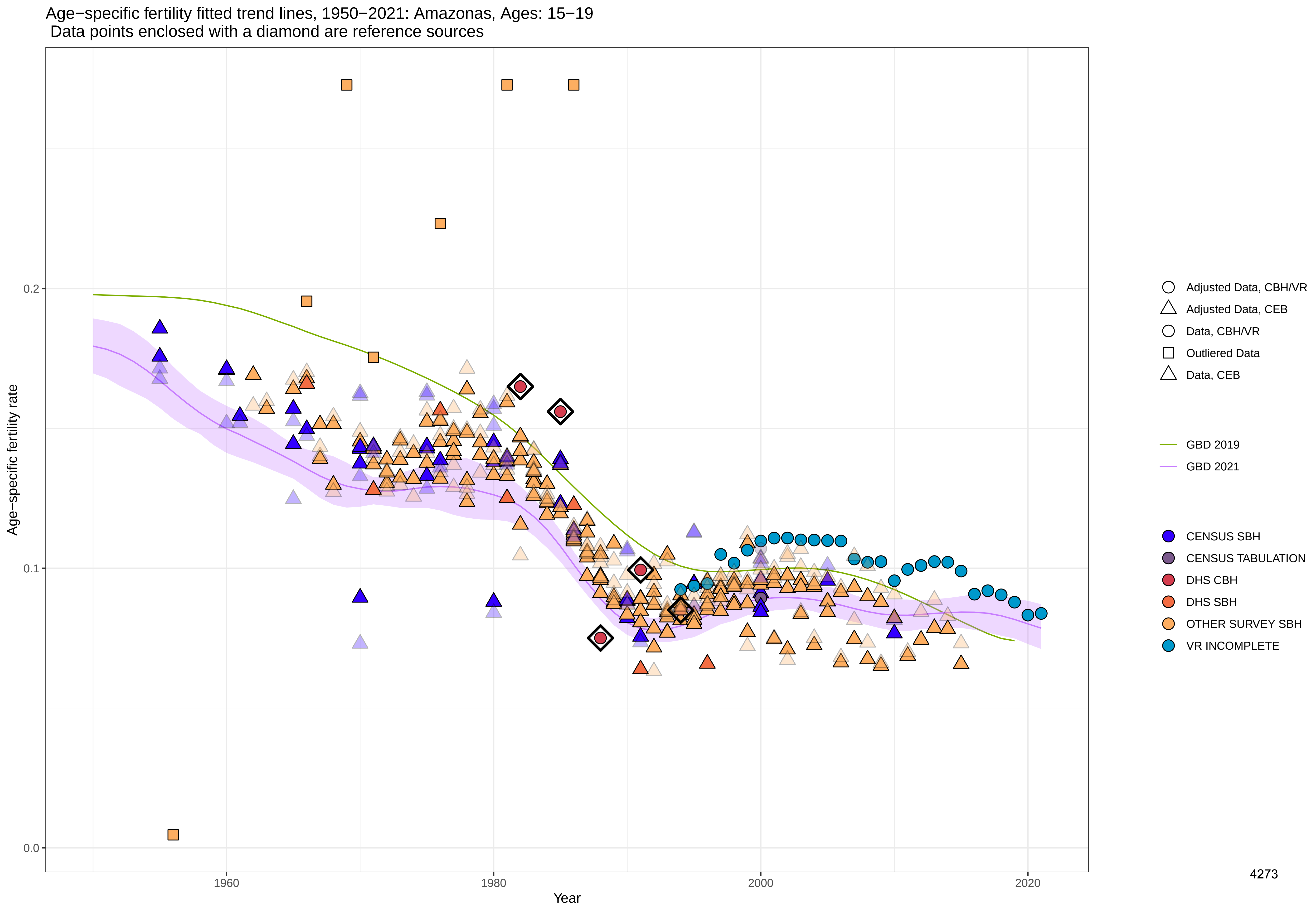

Age−specific fertility fitted trend lines, 1950−2021: Amazonas, Ages: 15−19
Data points enclosed with a diamond are reference sources
Age−specific fertility rate
Year
0.0
0.1
0.2
1960
1980
2000
2020
Adjusted Data, CBH/VR
Adjusted Data, CEB
Data, CBH/VR
Outliered Data
Data, CEB
GBD 2019
GBD 2021
CENSUS SBH
CENSUS TABULATION
DHS CBH
DHS SBH
OTHER SURVEY SBH
VR INCOMPLETE

Age−specific fertility fitted trend lines, 1950−2021: Amazonas, Ages: 20−24
Data points enclosed with a diamond are reference sources

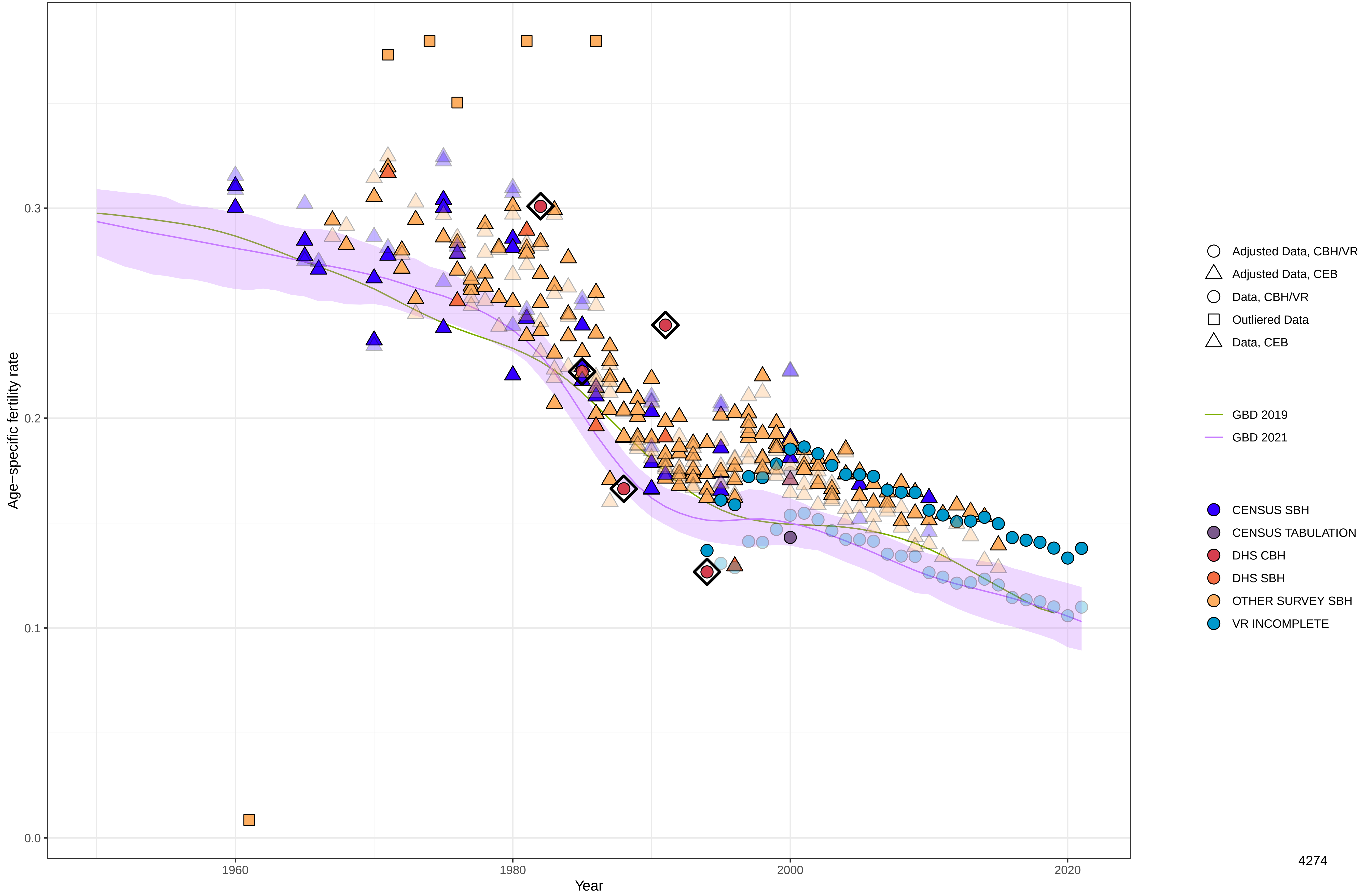

Age−specific fertility fitted trend lines, 1950−2021: Amazonas, Ages: 25−29
Data points enclosed with a diamond are reference sources

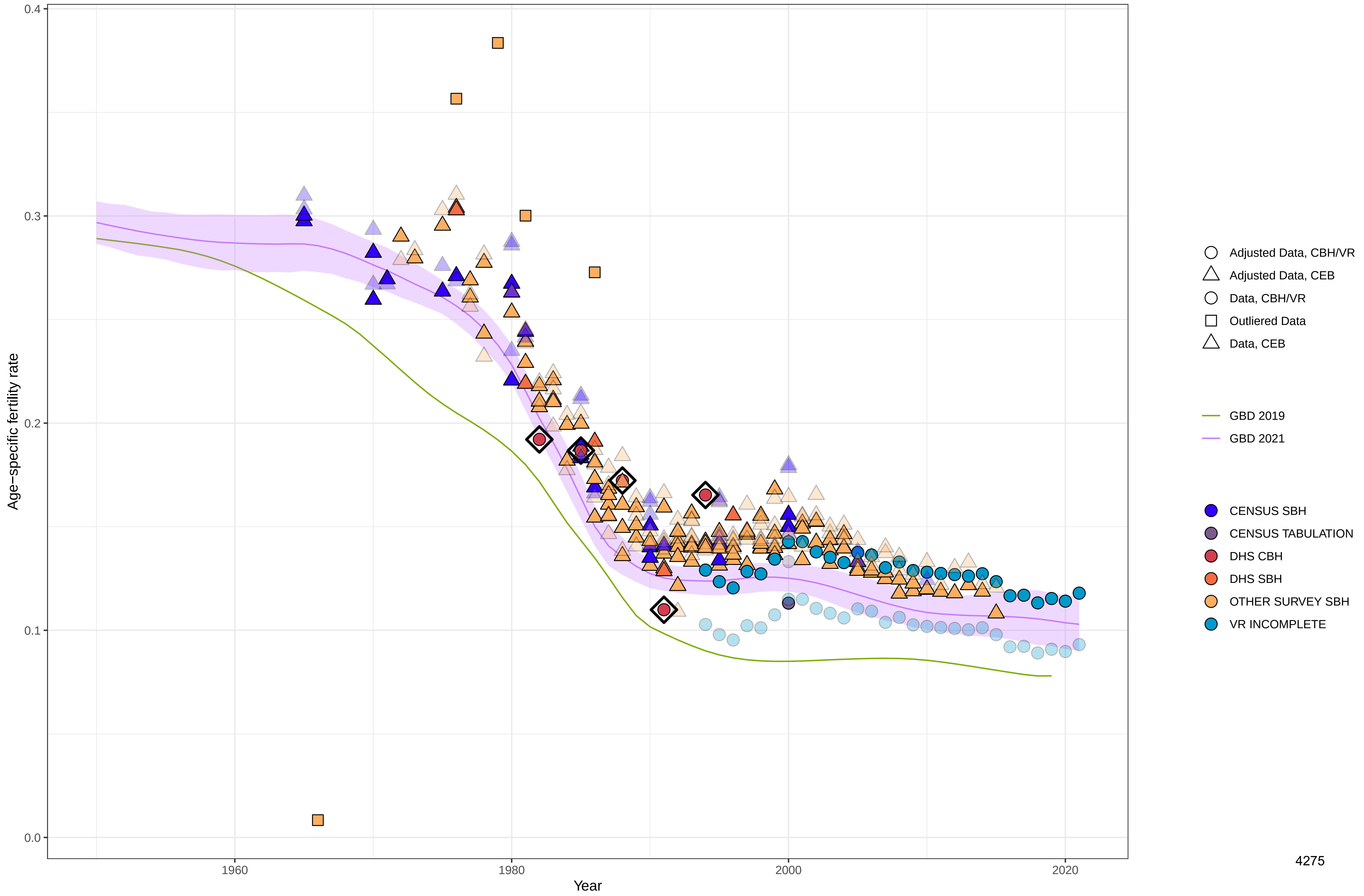

Age−specific fertility fitted trend lines, 1950−2021: Amazonas, Ages: 30−34
Data points enclosed with a diamond are reference sources

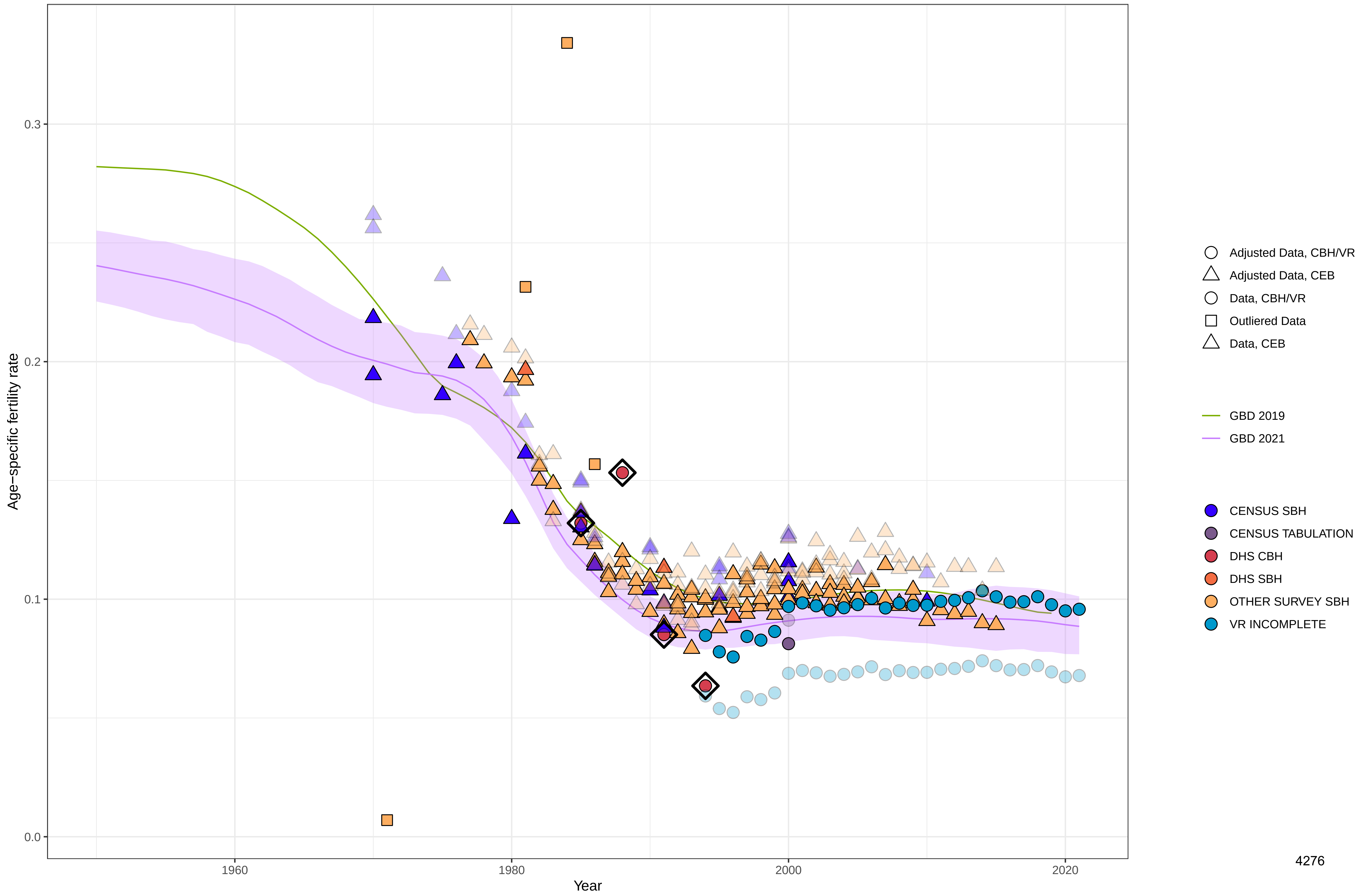

4276

Age−specific fertility fitted trend lines, 1950−2021: Amazonas, Ages: 35−39
Data points enclosed with a diamond are reference sources

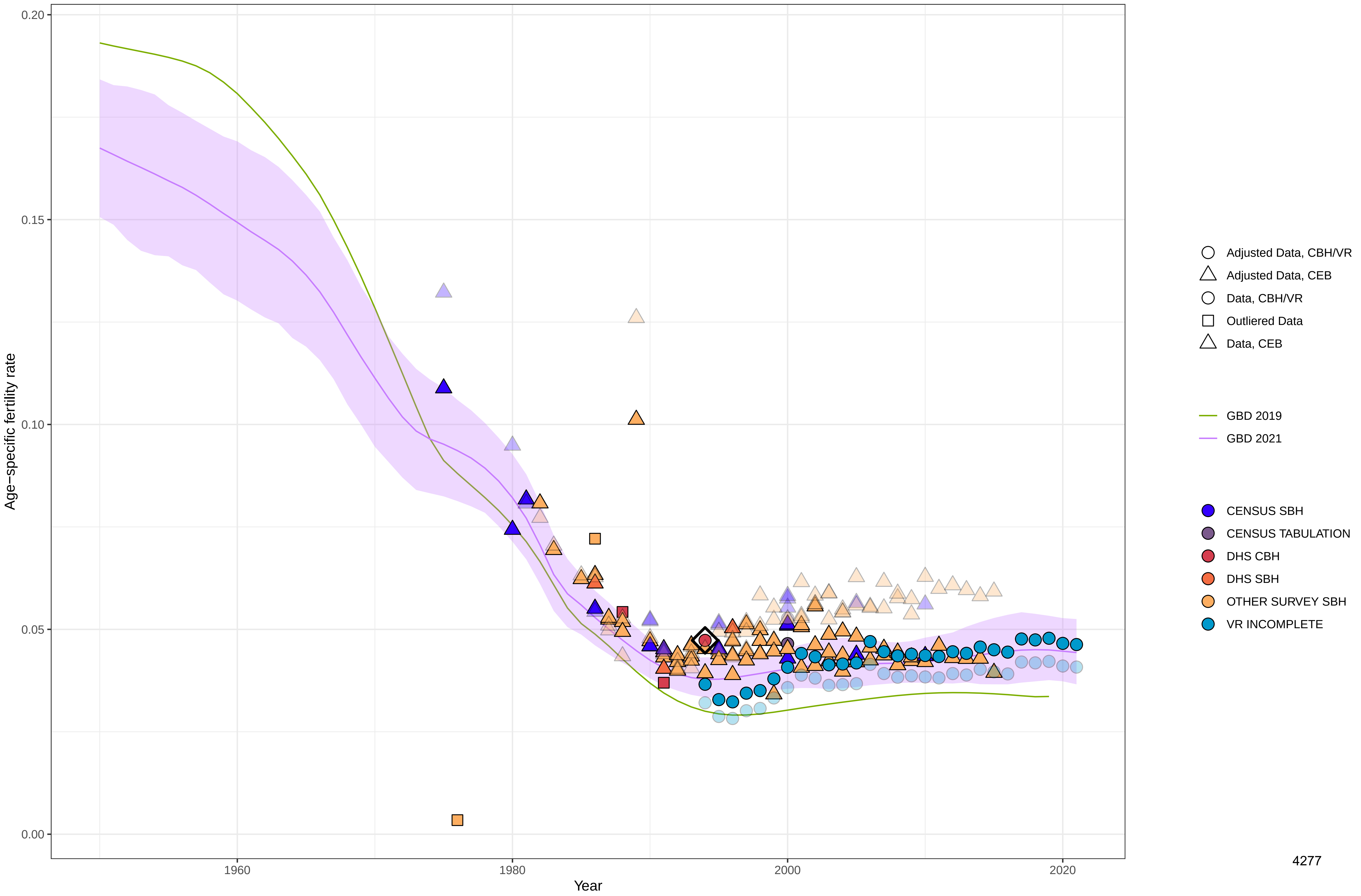

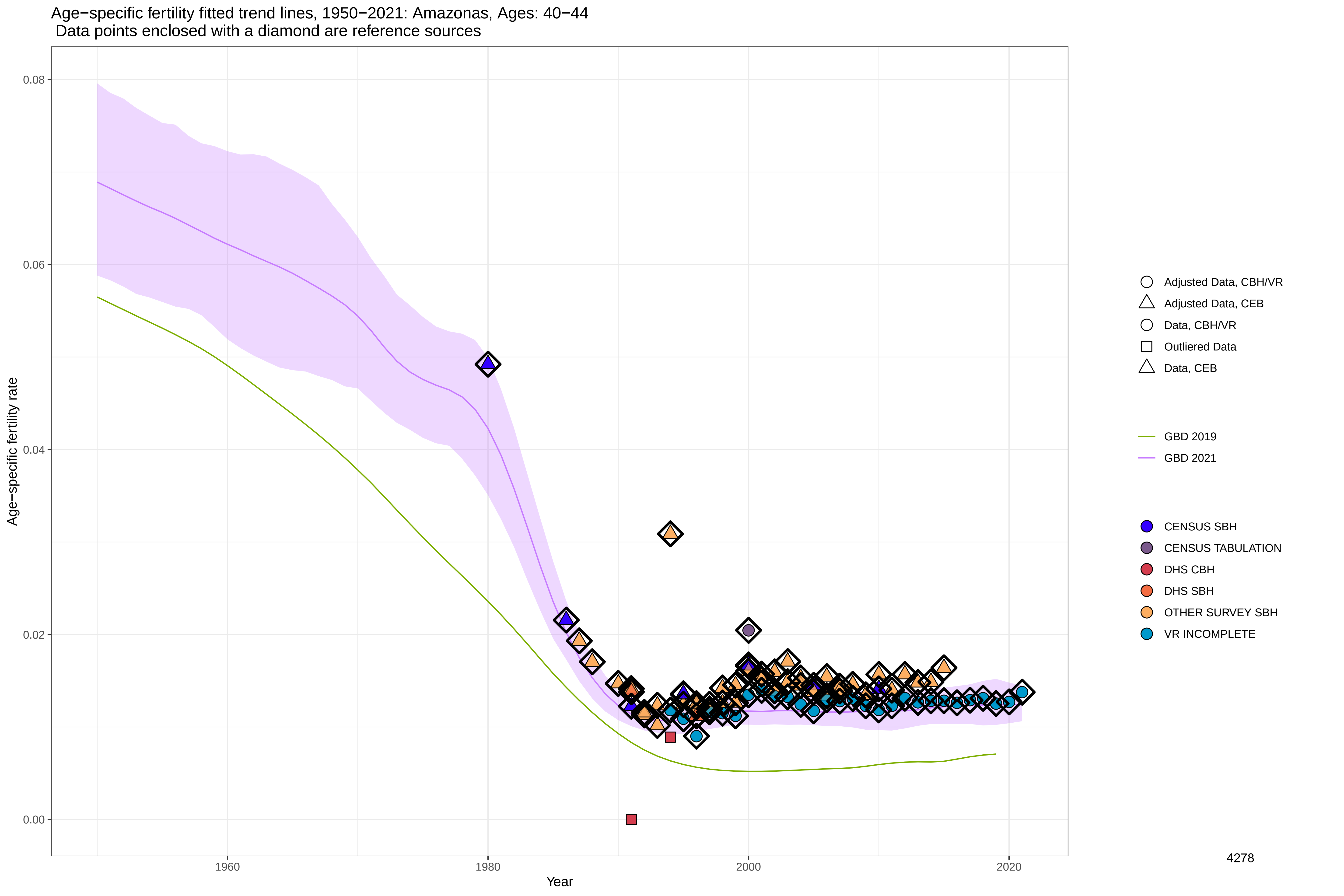

Age−specific fertility fitted trend lines, 1950−2021: Amazonas, Ages: 40−44
Data points enclosed with a diamond are reference sources
Age−specific fertility rate
Year
0.00
0.02
0.04
0.06
0.08
1960
1980
2000
2020
Adjusted Data, CBH/VR
Adjusted Data, CEB
Data, CBH/VR
Outliered Data
Data, CEB
GBD 2019
GBD 2021
CENSUS SBH
CENSUS TABULATION
DHS CBH
DHS SBH
OTHER SURVEY SBH
VR INCOMPLETE

Age−specific fertility fitted trend lines, 1950−2021: Amazonas, Ages: 45−49
Data points enclosed with a diamond are reference sources

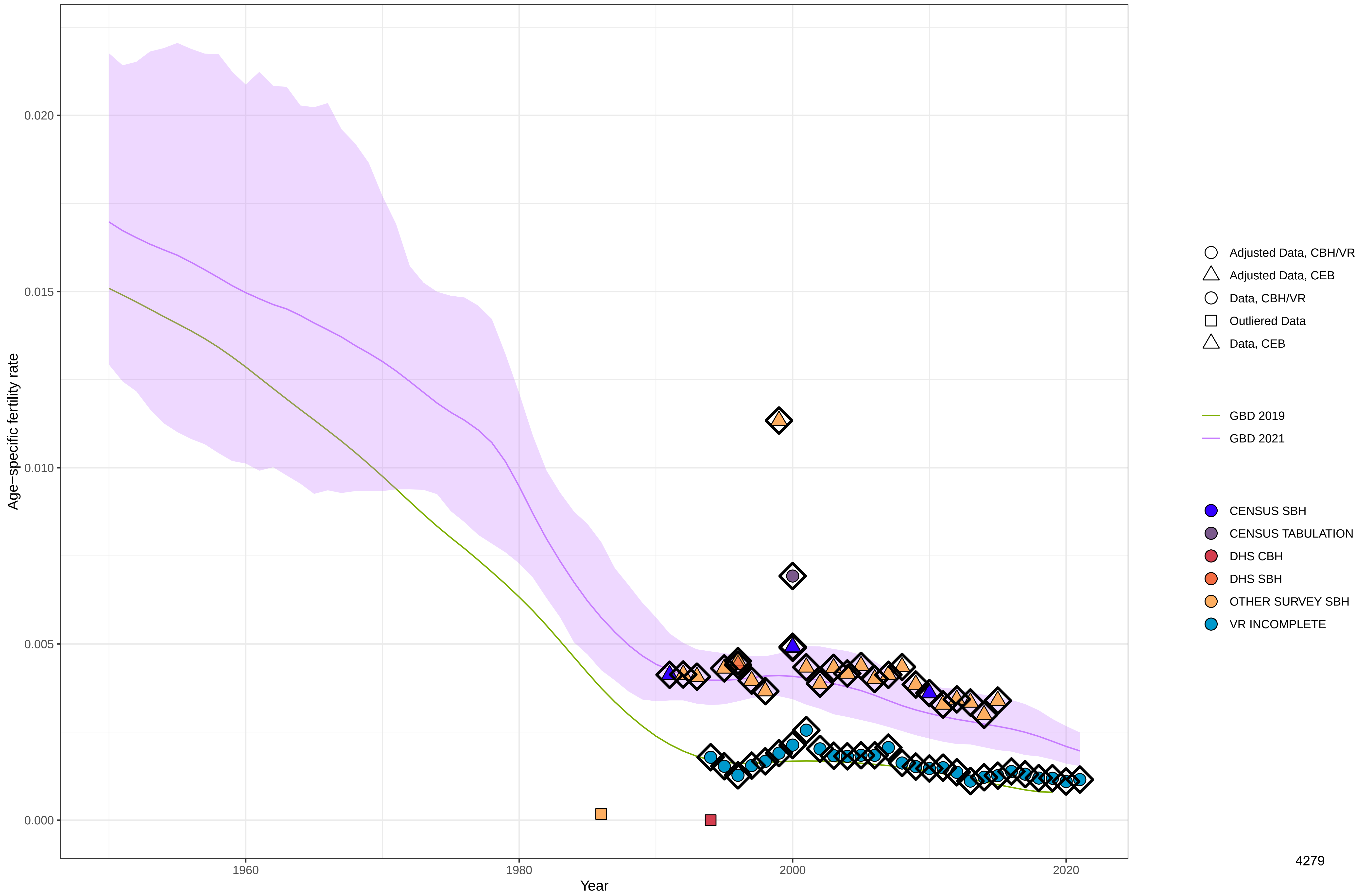

Age−specific fertility fitted trend lines, 1950−2021: Amazonas, Ages: 50−54
Data points enclosed with a diamond are reference sources

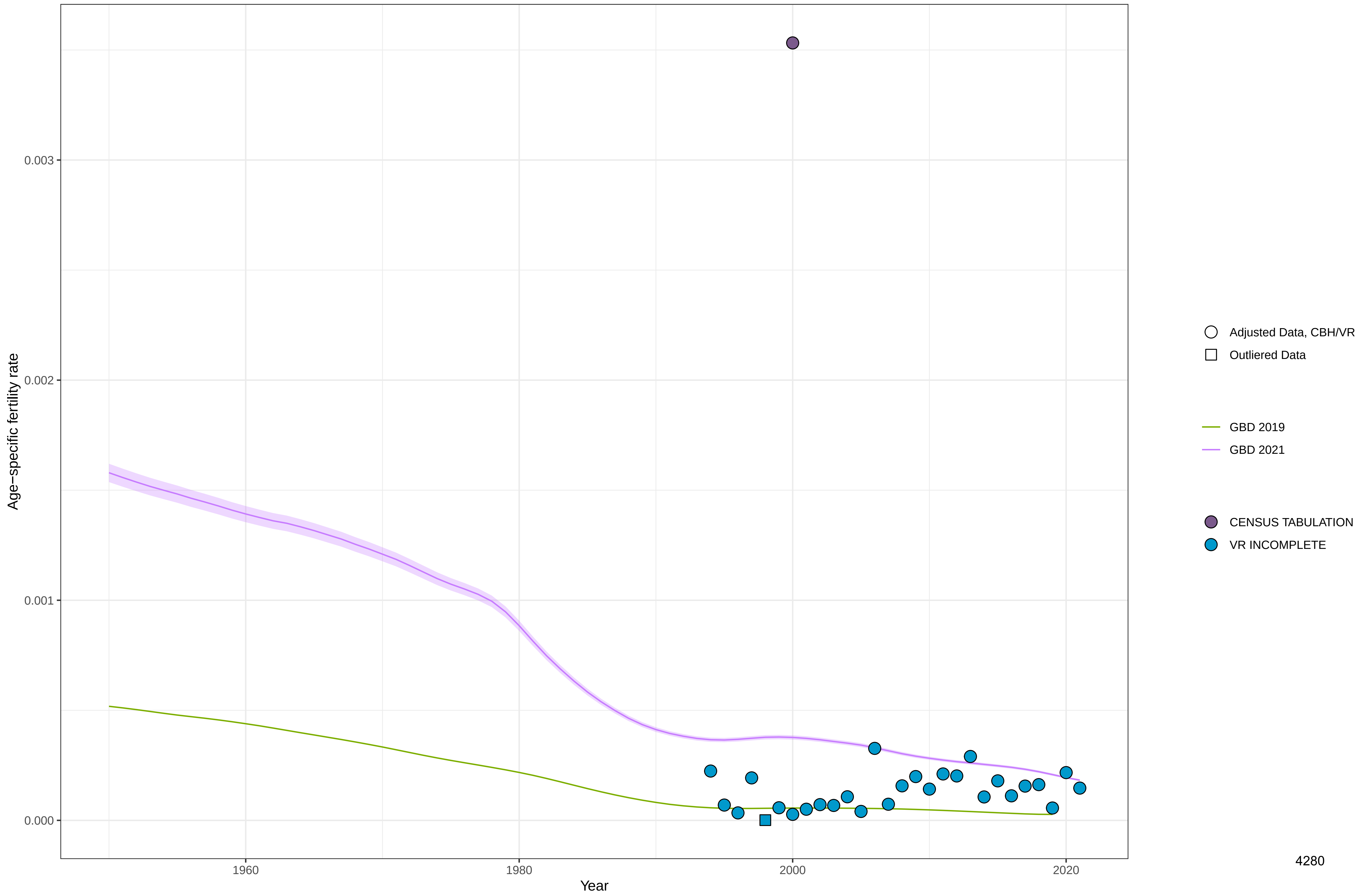

Total fertility rate fitted trend lines, 1950−2021: Amazonas TFR
Data points enclosed with a diamond are reference sources

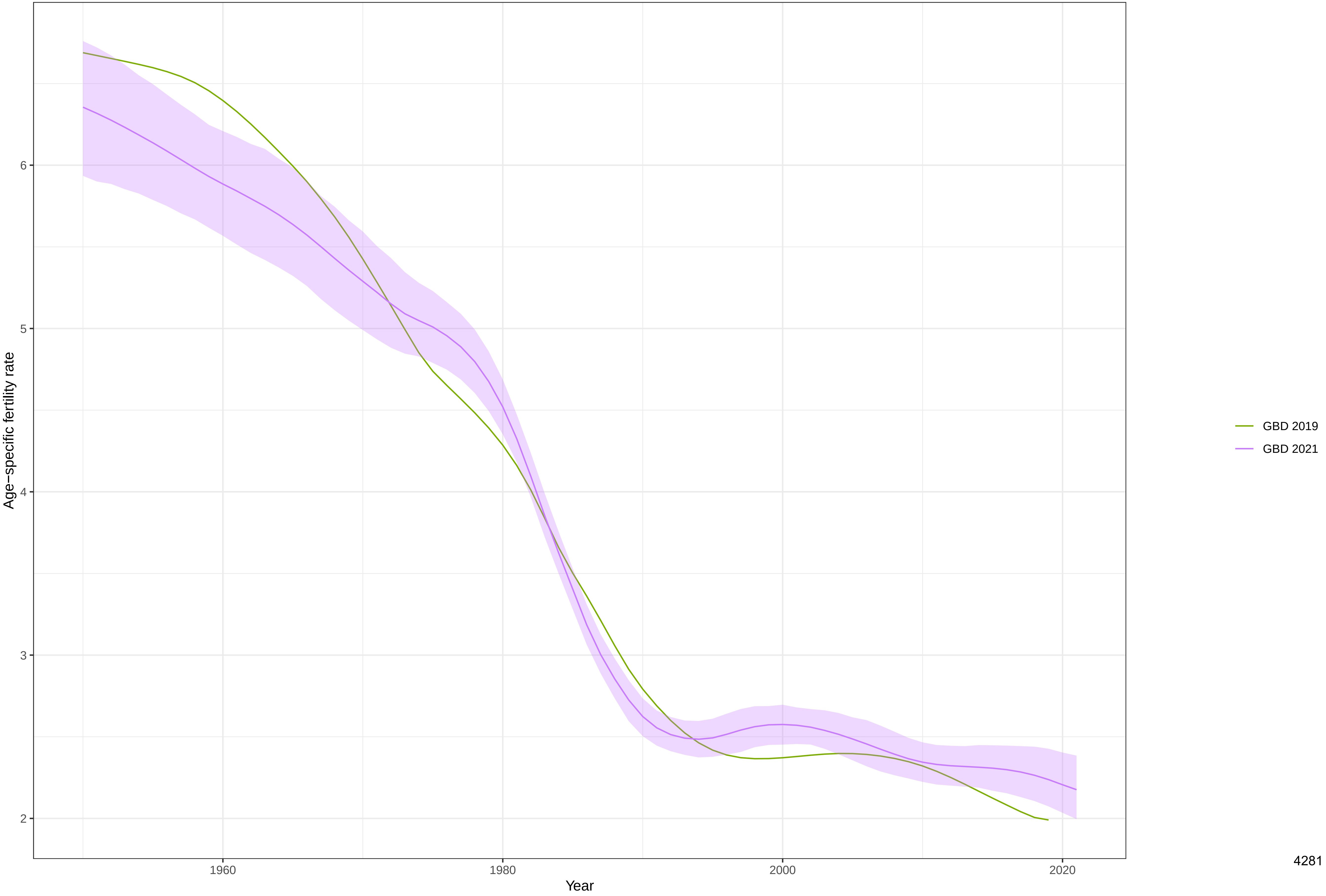

Age−specific fertility fitted trend lines, 1950−2021: Bahia, Ages: 10−14
Data points enclosed with a diamond are reference sources

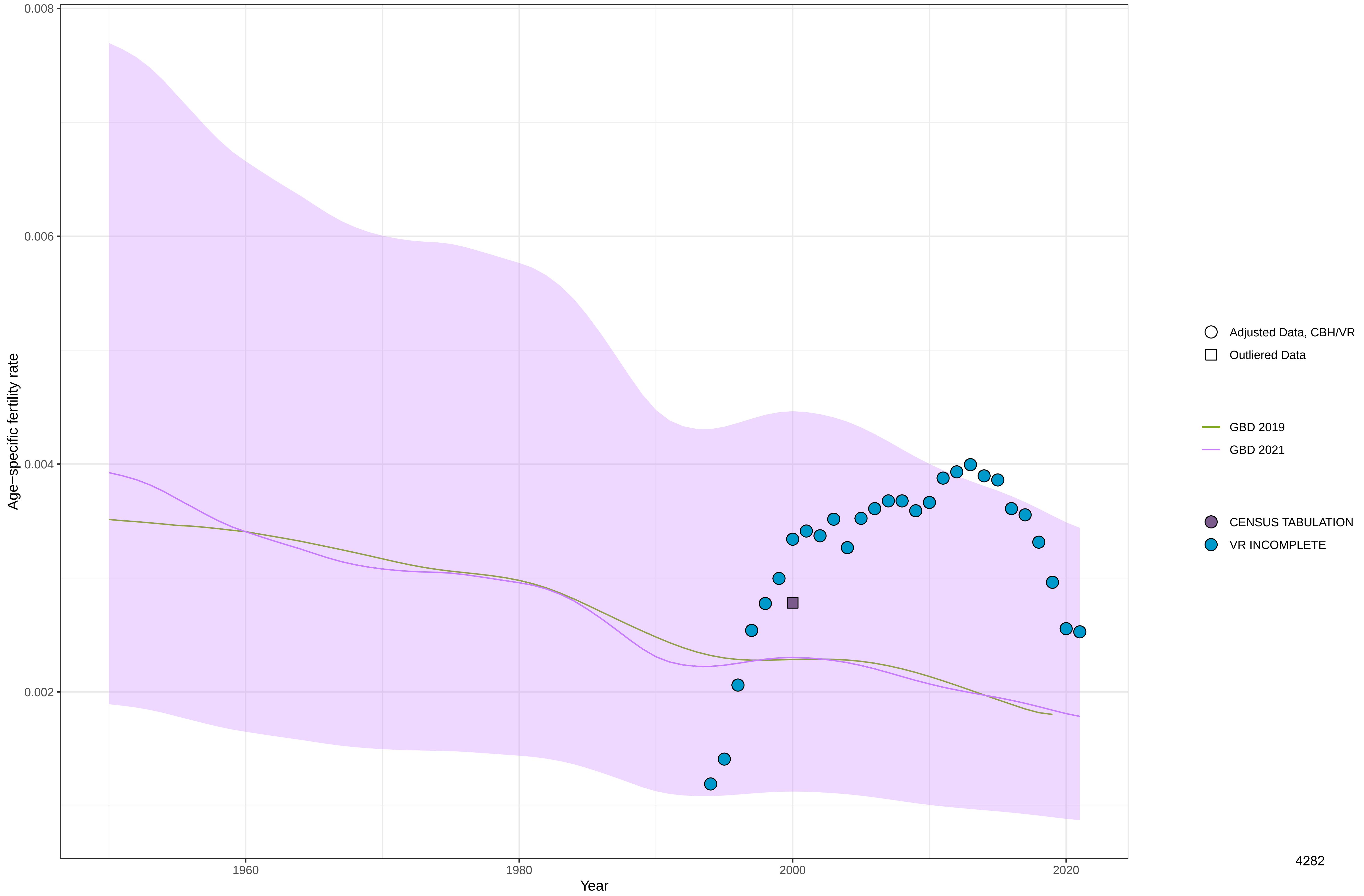

Age−specific fertility fitted trend lines, 1950−2021: Bahia, Ages: 15−19
Data points enclosed with a diamond are reference sources

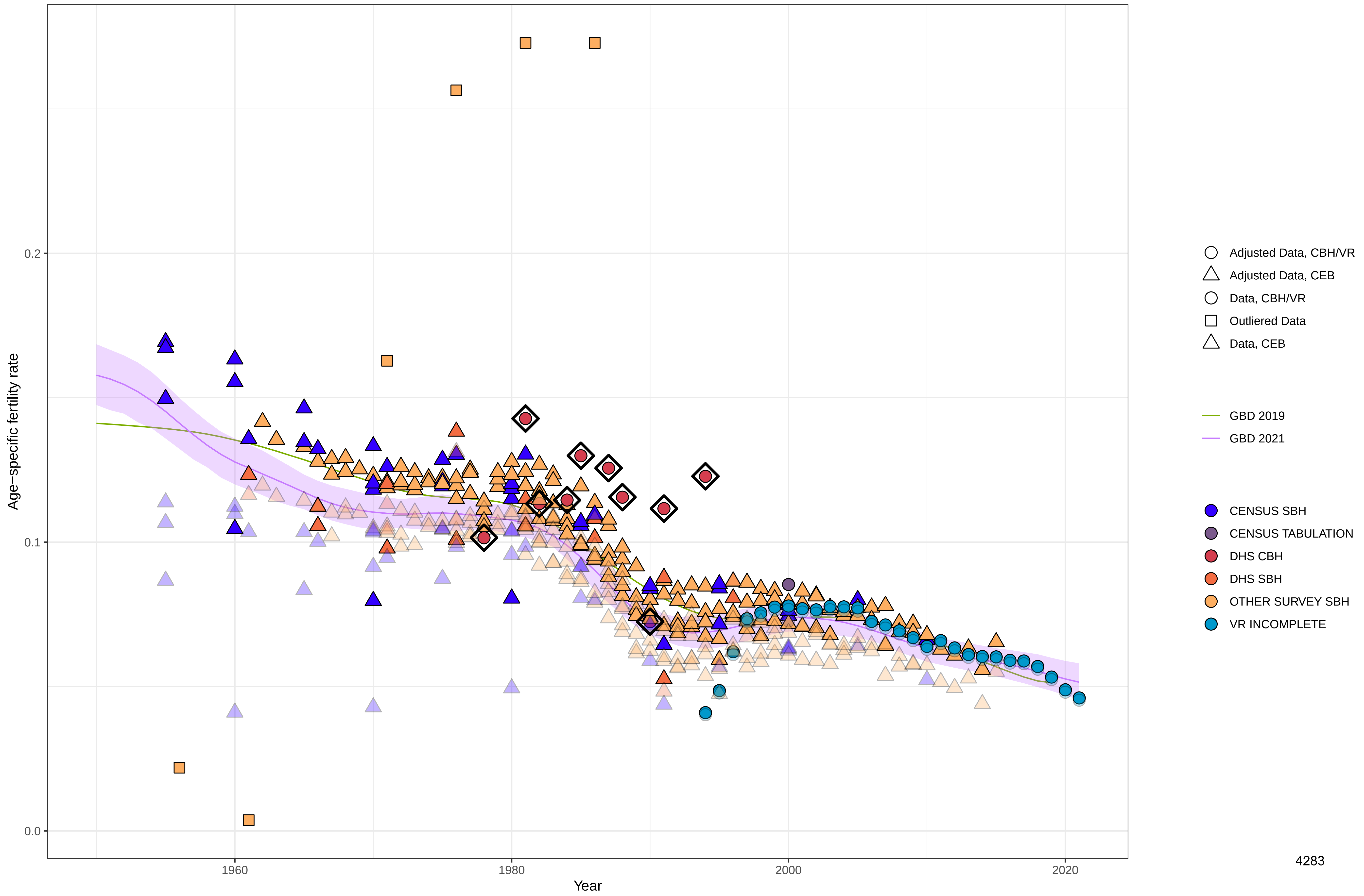

Age−specific fertility fitted trend lines, 1950−2021: Bahia, Ages: 20−24
Data points enclosed with a diamond are reference sources

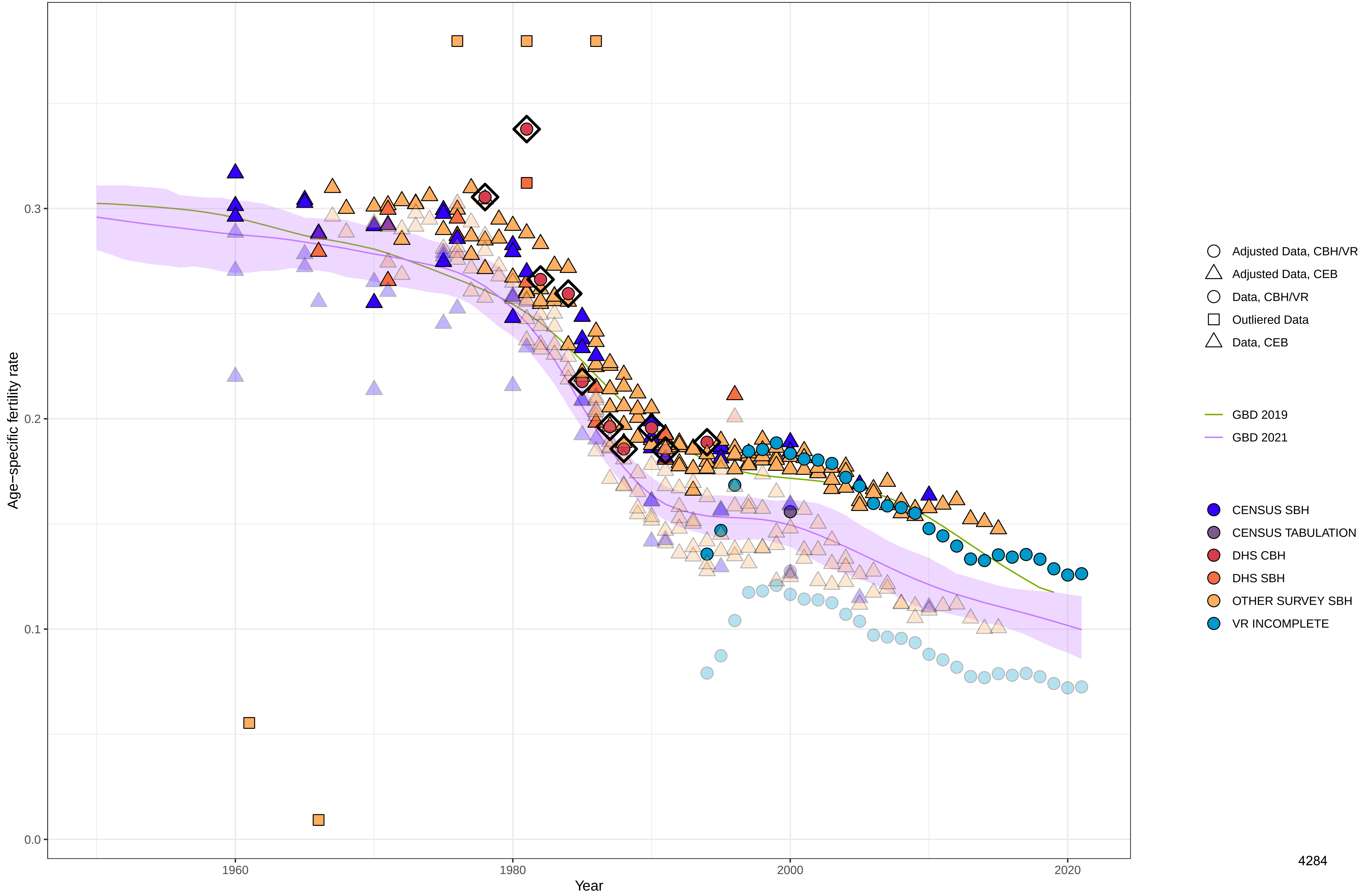

Age−specific fertility fitted trend lines, 1950−2021: Bahia, Ages: 25−29
Data points enclosed with a diamond are reference sources

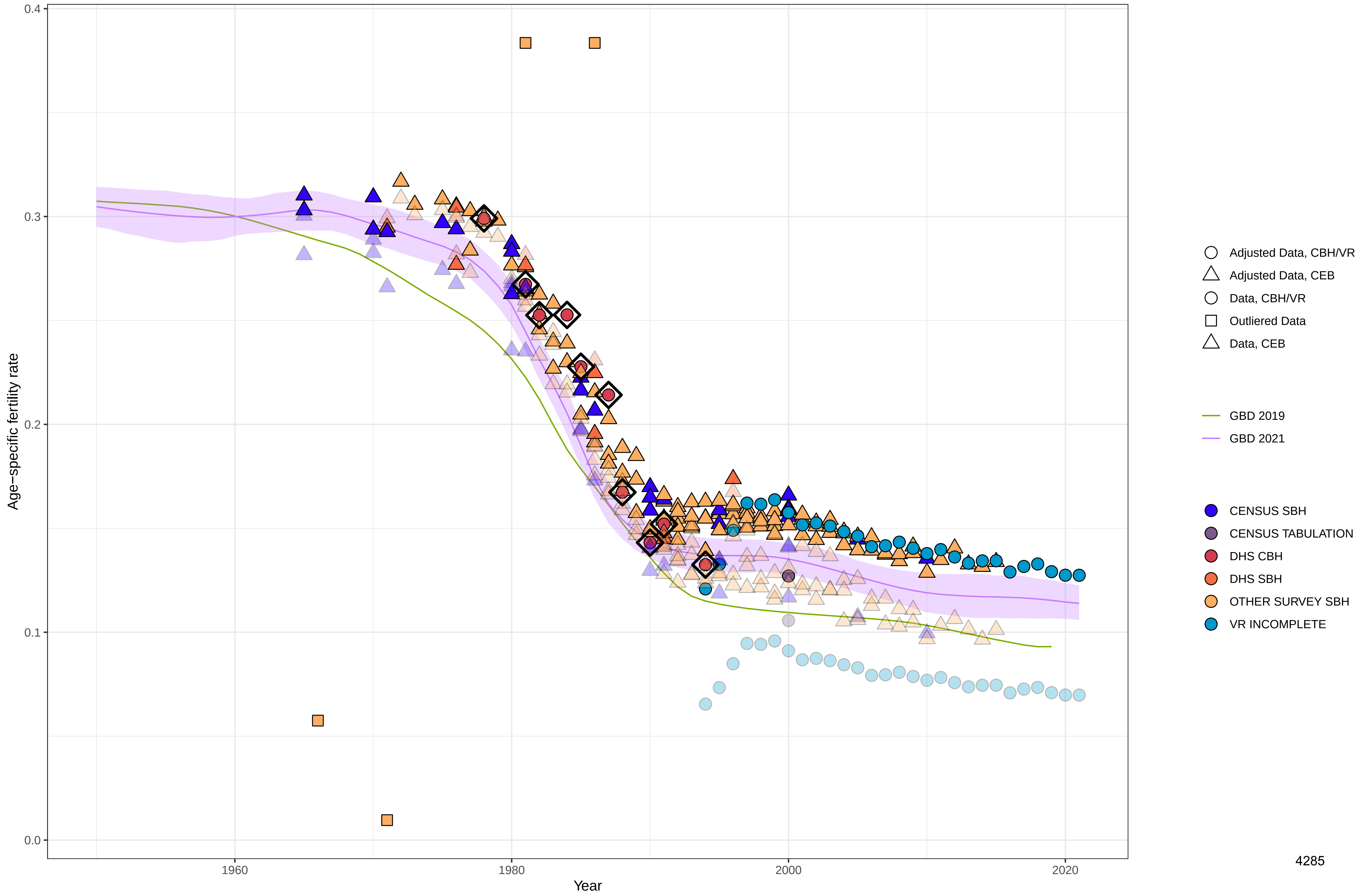

Age−specific fertility fitted trend lines, 1950−2021: Bahia, Ages: 30−34
Data points enclosed with a diamond are reference sources

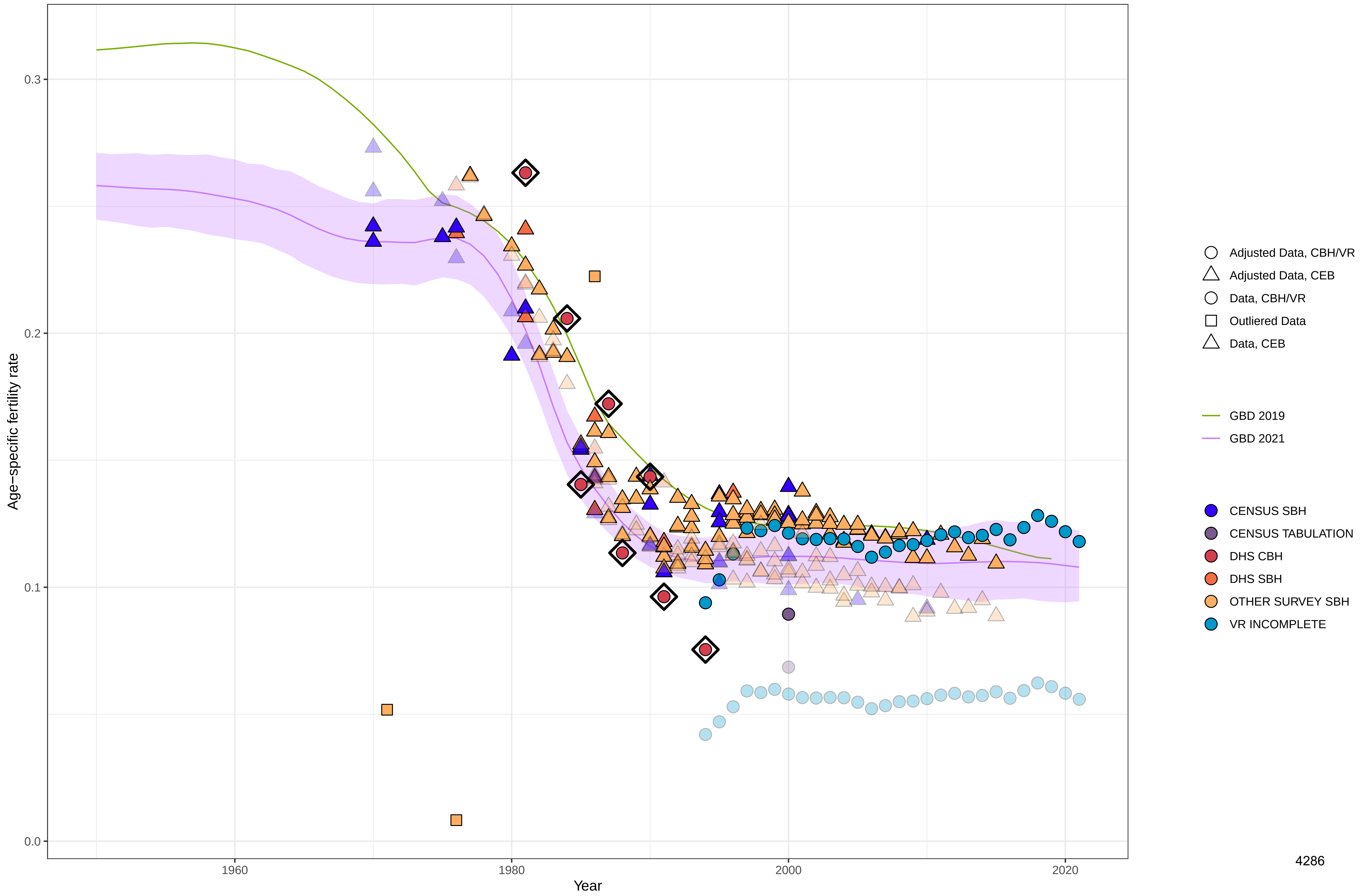

Age−specific fertility fitted trend lines, 1950−2021: Bahia, Ages: 35−39
Data points enclosed with a diamond are reference sources

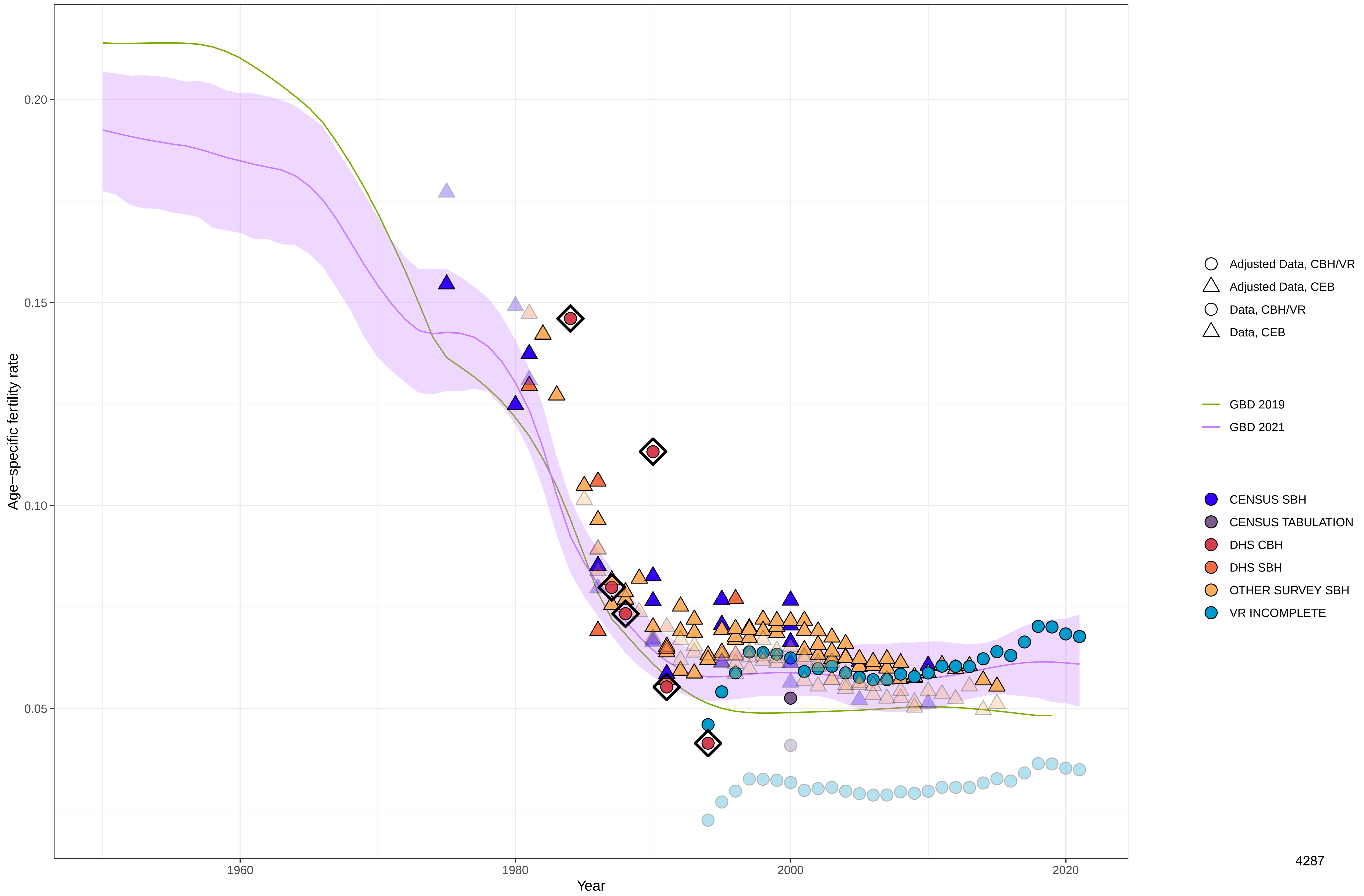

Age−specific fertility fitted trend lines, 1950−2021: Bahia, Ages: 40−44
Data points enclosed with a diamond are reference sources

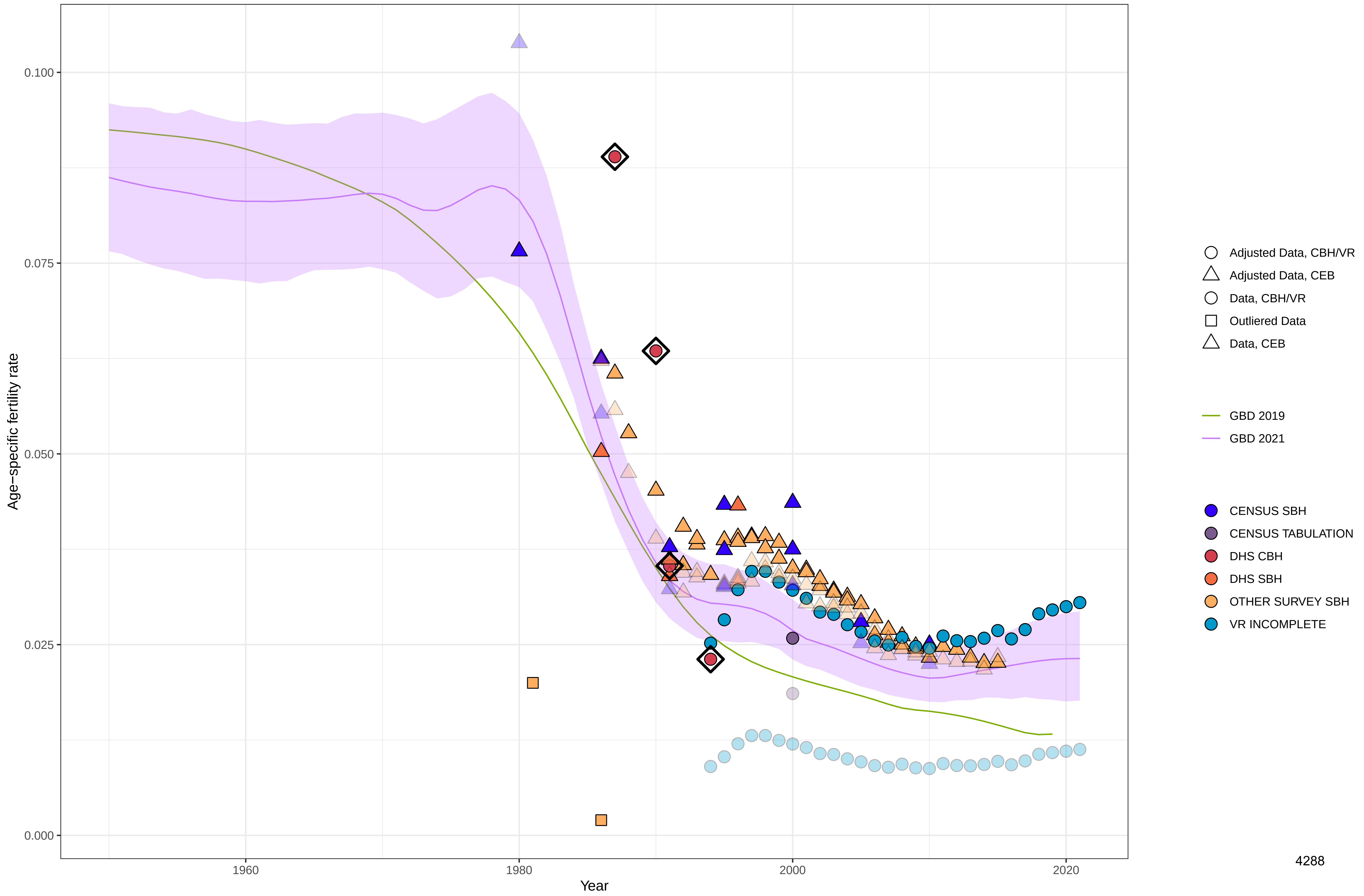

Age−specific fertility fitted trend lines, 1950−2021: Bahia, Ages: 45−49
Data points enclosed with a diamond are reference sources

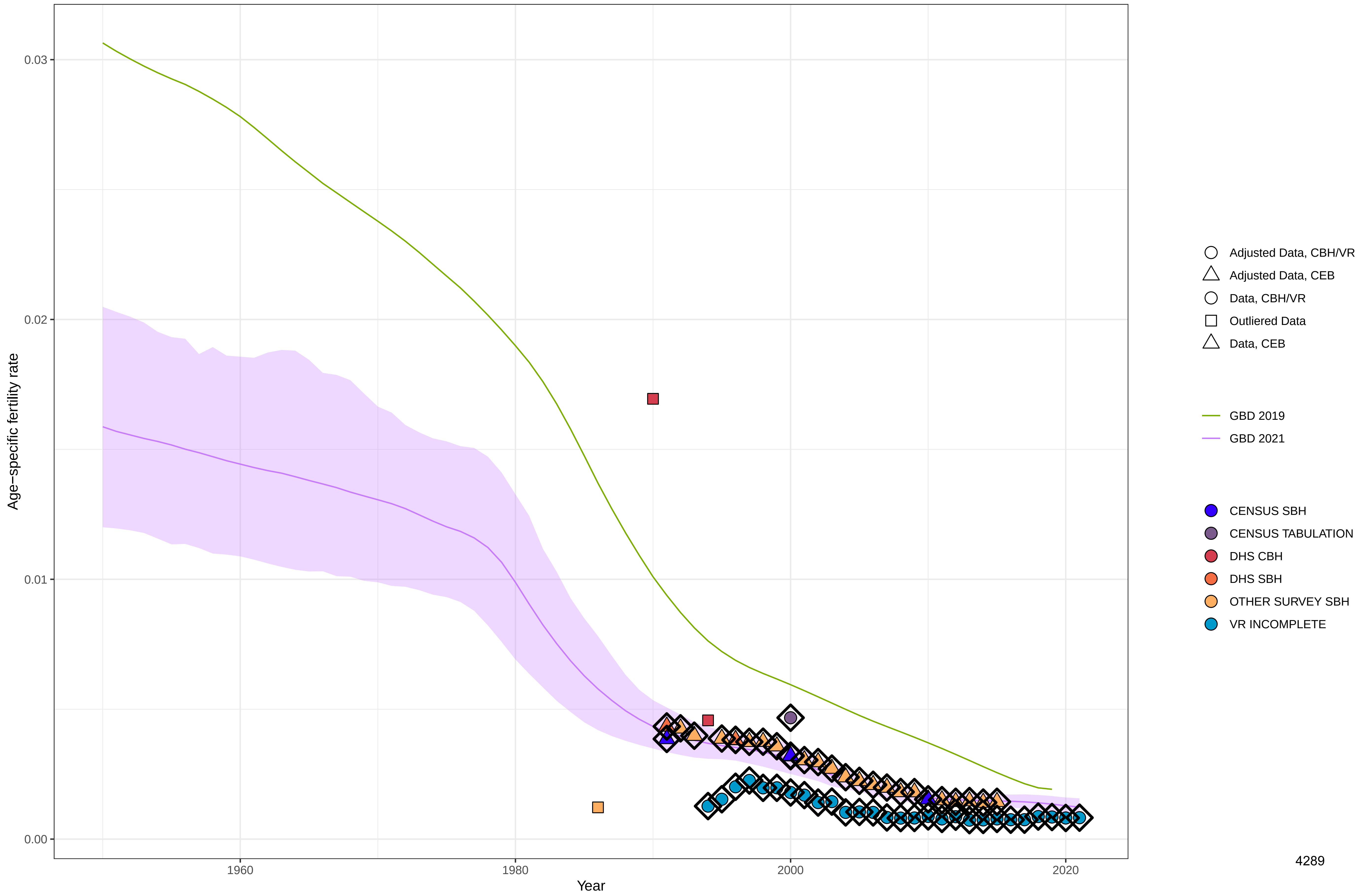

Age−specific fertility fitted trend lines, 1950−2021: Bahia, Ages: 50−54
Data points enclosed with a diamond are reference sources

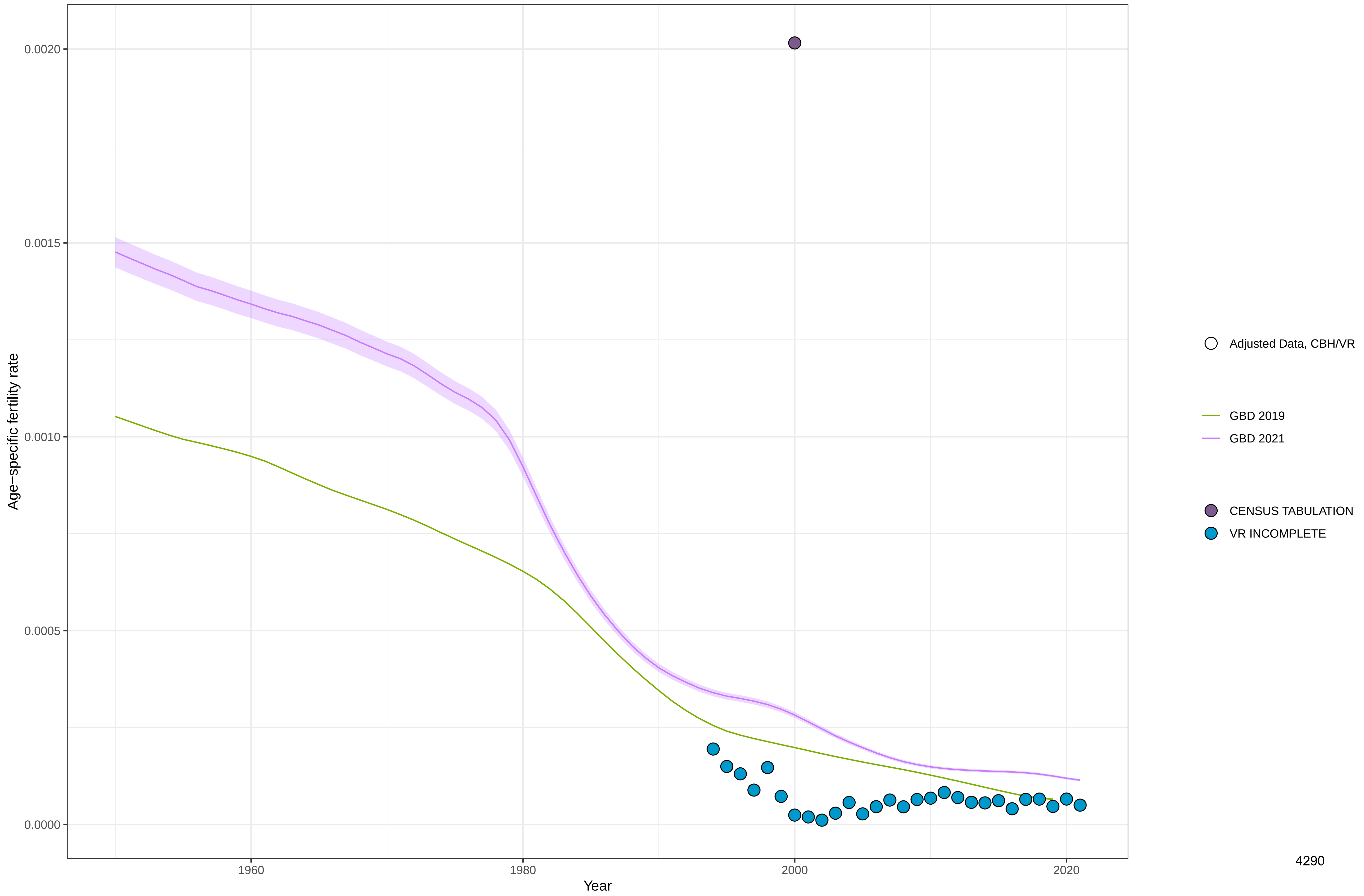

Total fertility rate fitted trend lines, 1950−2021: Bahia TFR
Data points enclosed with a diamond are reference sources

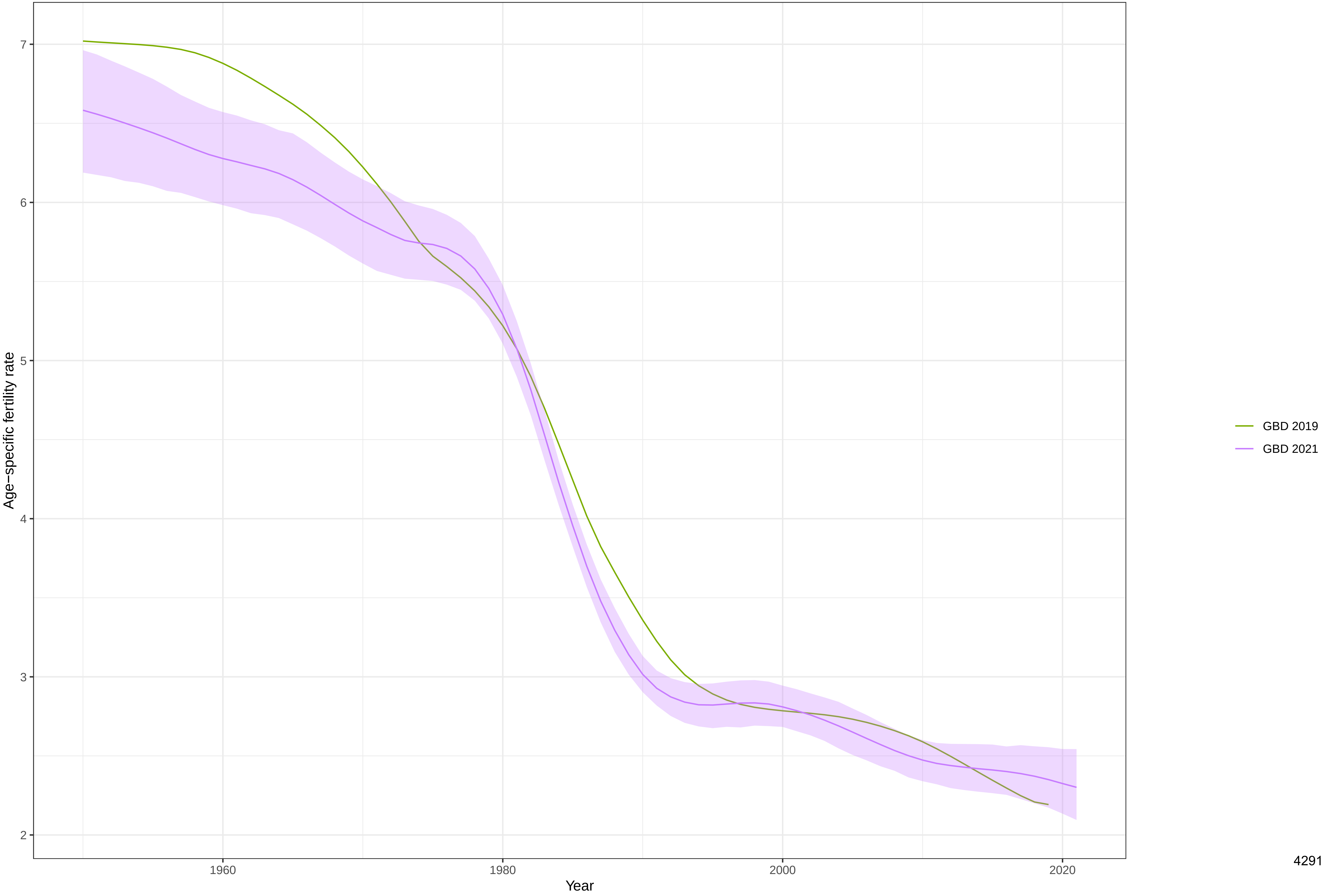

4291

Age−specific fertility fitted trend lines, 1950−2021: Ceará, Ages: 10−14
Data points enclosed with a diamond are reference sources

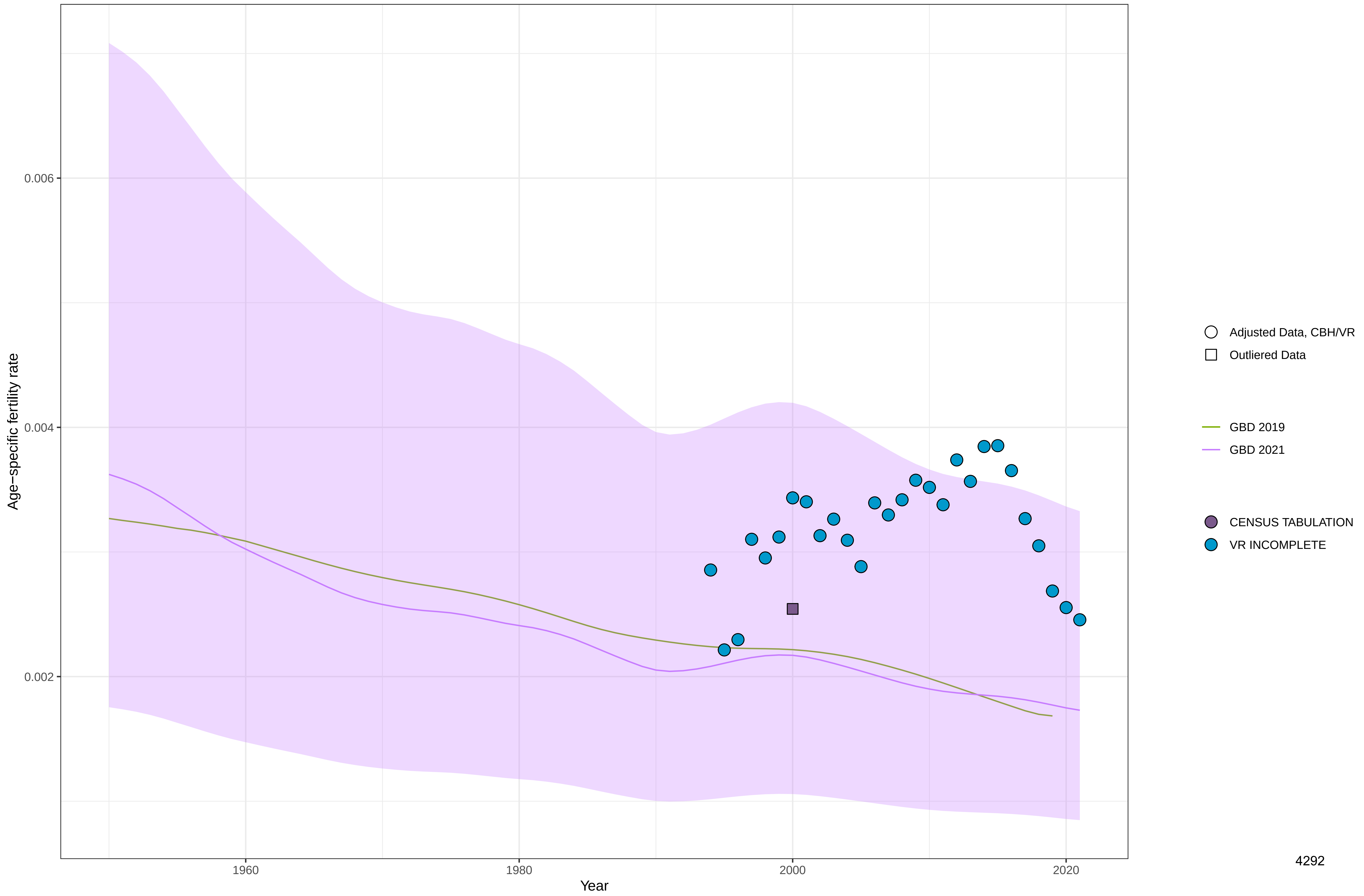

Age−specific fertility fitted trend lines, 1950−2021: Ceará, Ages: 15−19
Data points enclosed with a diamond are reference sources

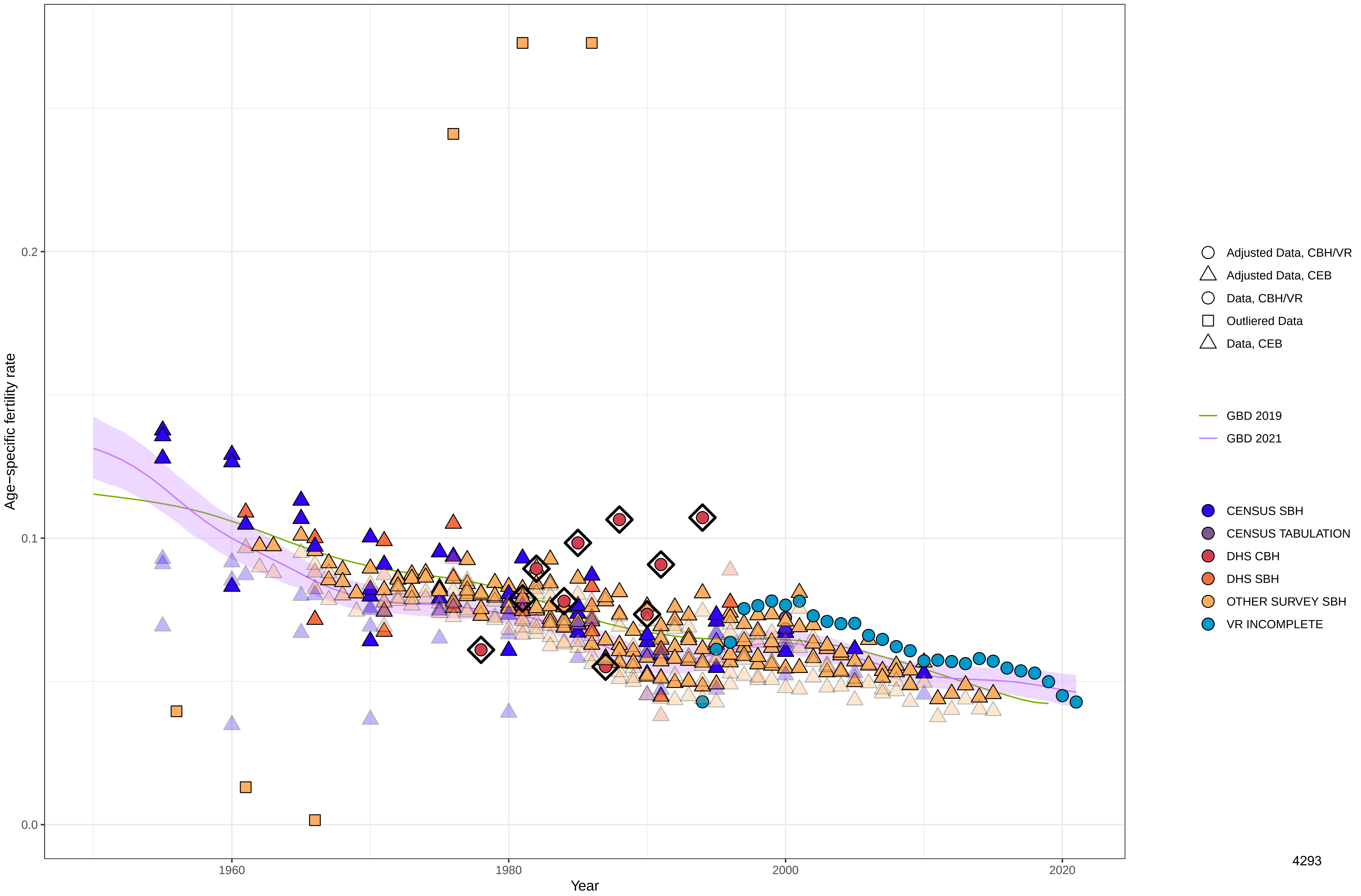

Age−specific fertility fitted trend lines, 1950−2021: Ceará, Ages: 20−24
Data points enclosed with a diamond are reference sources

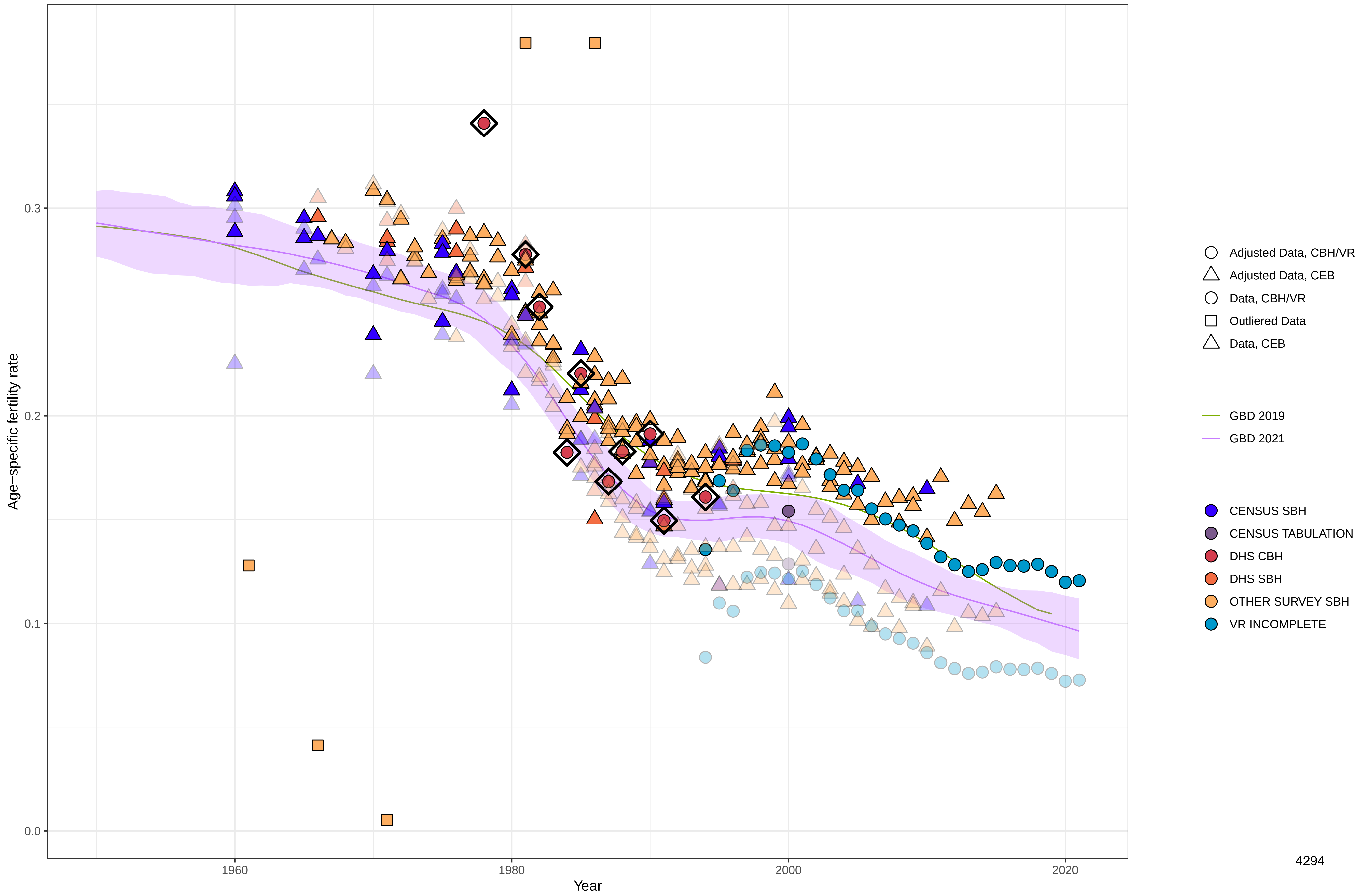

Age−specific fertility fitted trend lines, 1950−2021: Ceará, Ages: 25−29
Data points enclosed with a diamond are reference sources

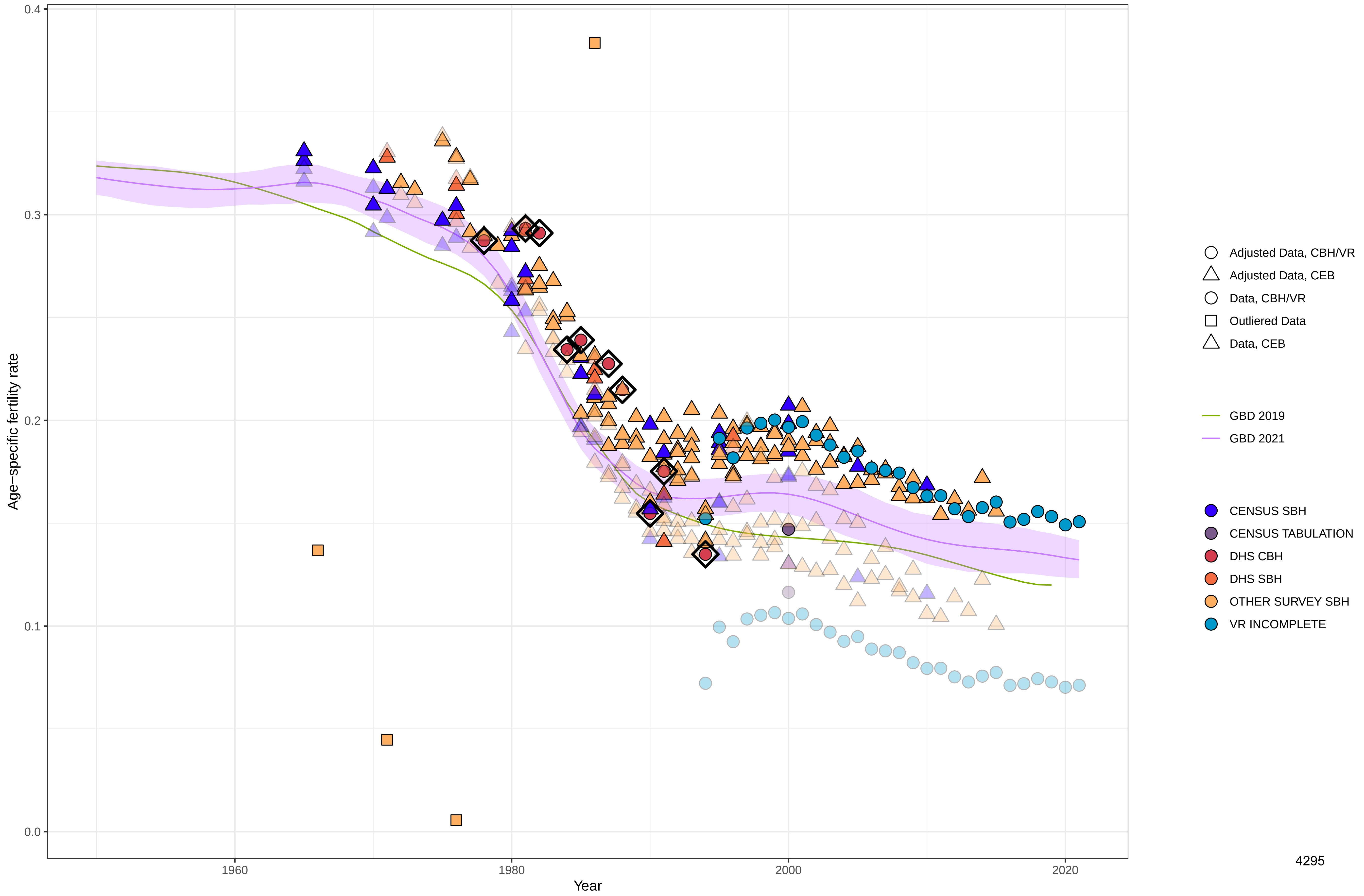

Age−specific fertility fitted trend lines, 1950−2021: Ceará, Ages: 30−34
Data points enclosed with a diamond are reference sources

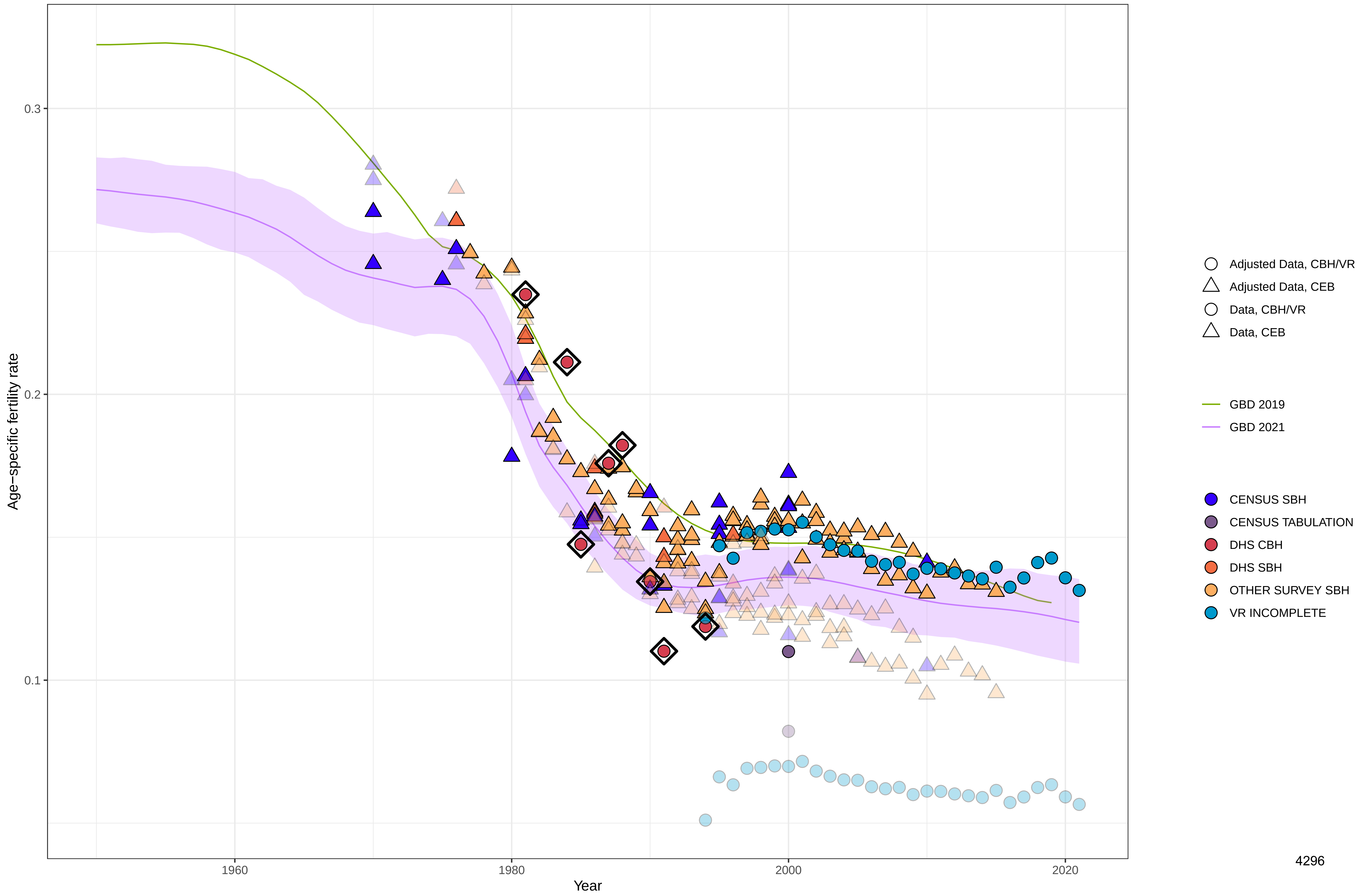

Age−specific fertility fitted trend lines, 1950−2021: Ceará, Ages: 35−39
Data points enclosed with a diamond are reference sources

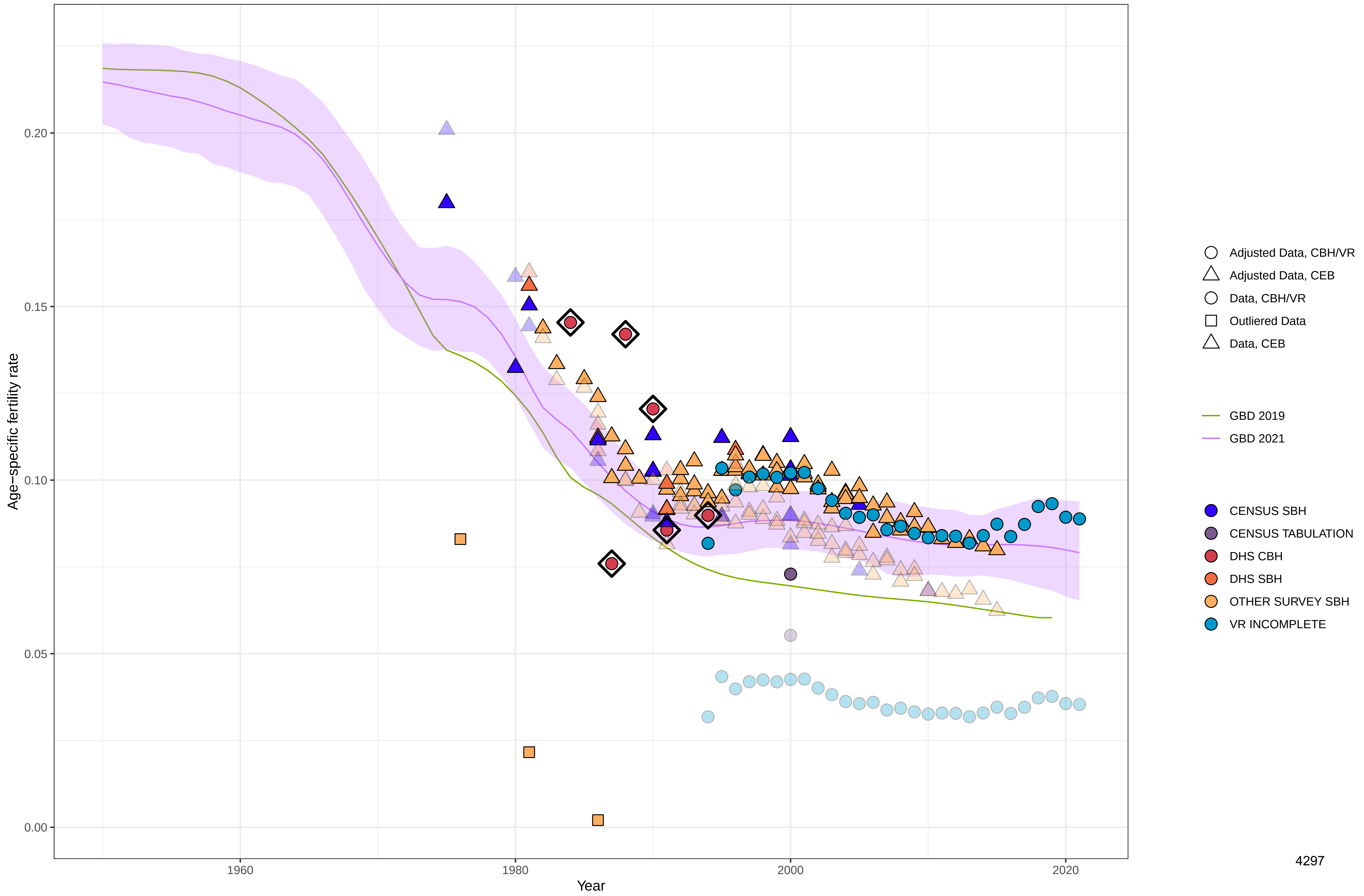

Age−specific fertility fitted trend lines, 1950−2021: Ceará, Ages: 40−44
Data points enclosed with a diamond are reference sources

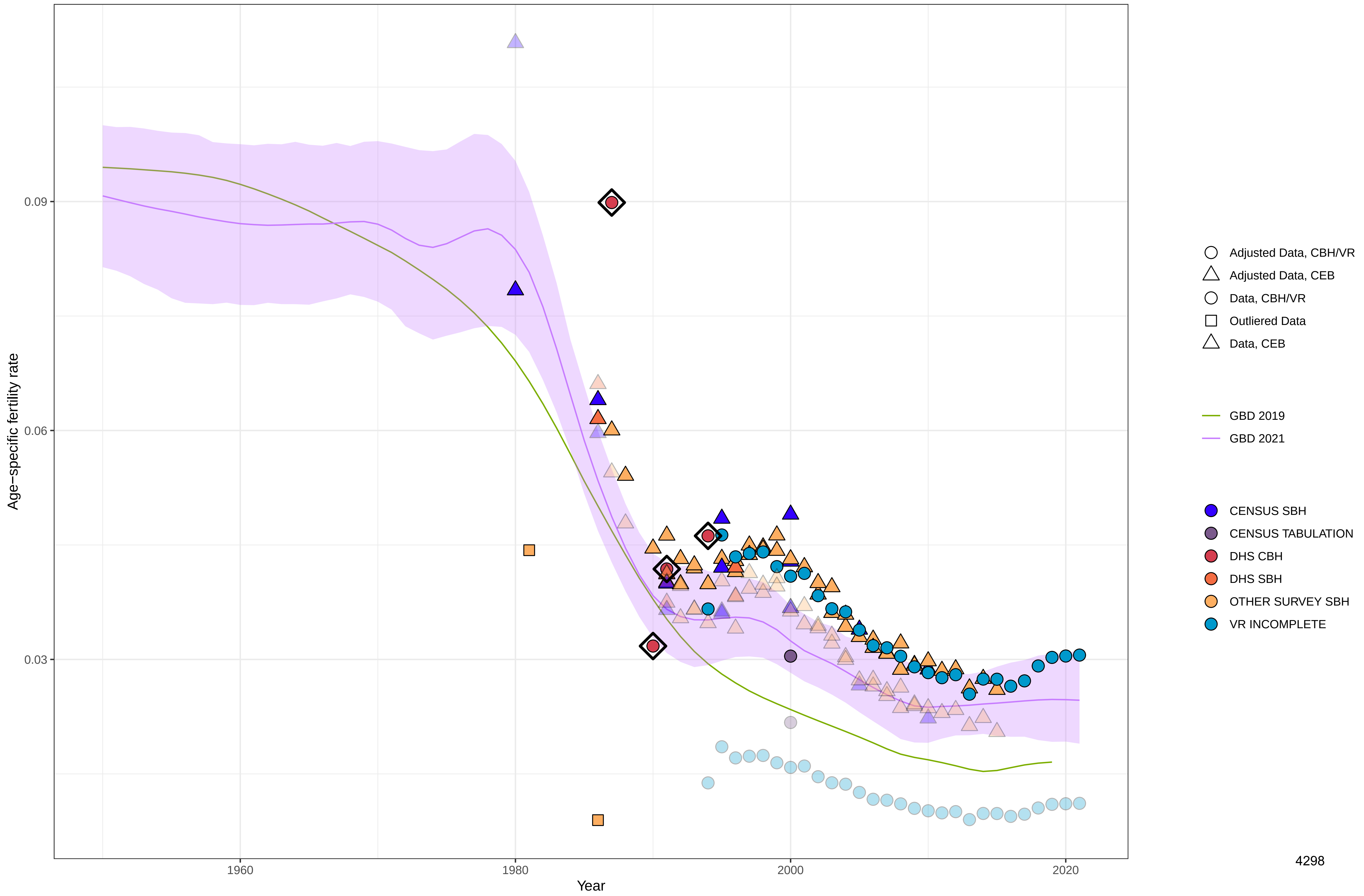

Age−specific fertility fitted trend lines, 1950−2021: Ceará, Ages: 45−49
Data points enclosed with a diamond are reference sources

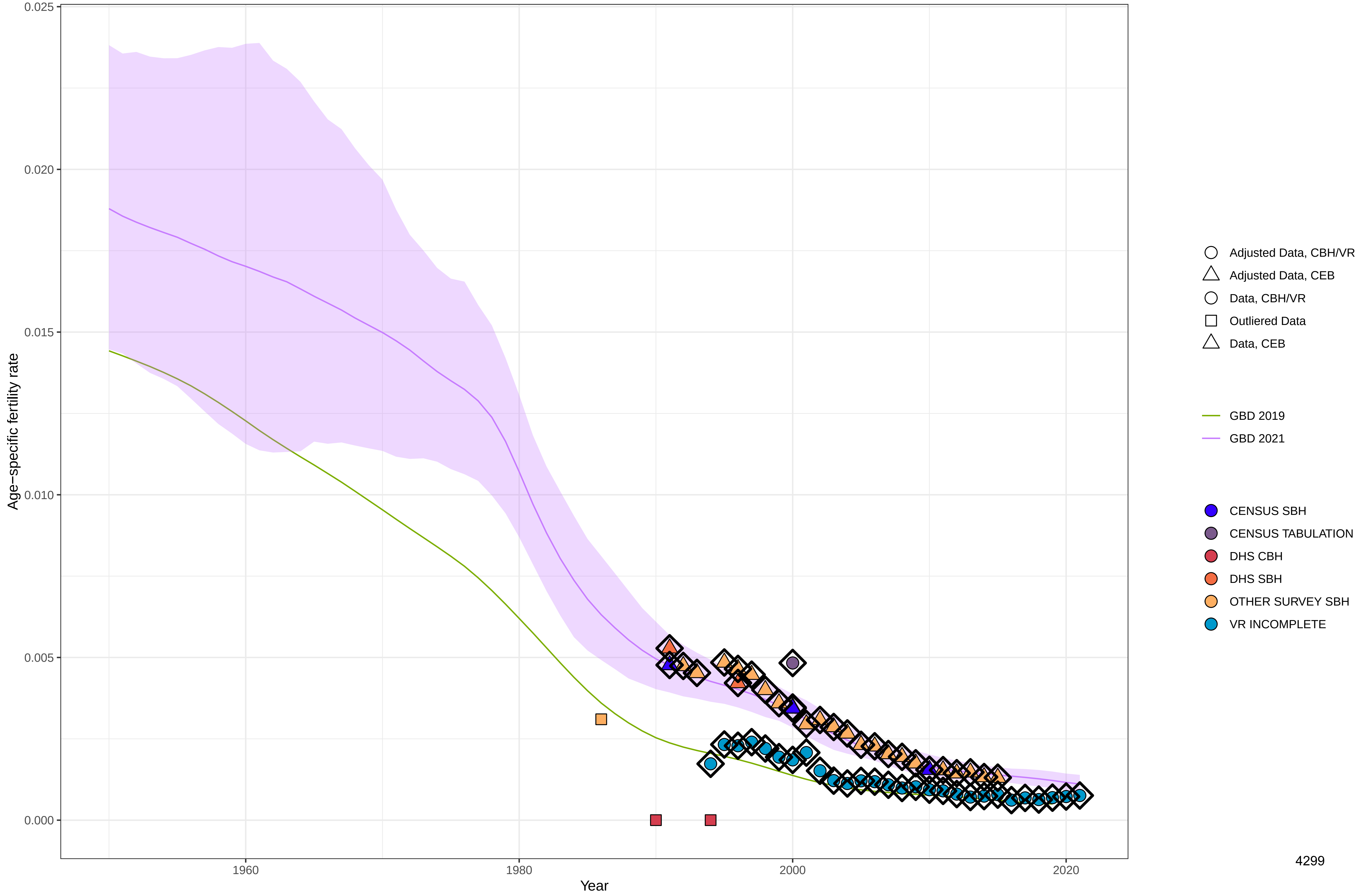

Age−specific fertility fitted trend lines, 1950−2021: Ceará, Ages: 50−54
Data points enclosed with a diamond are reference sources

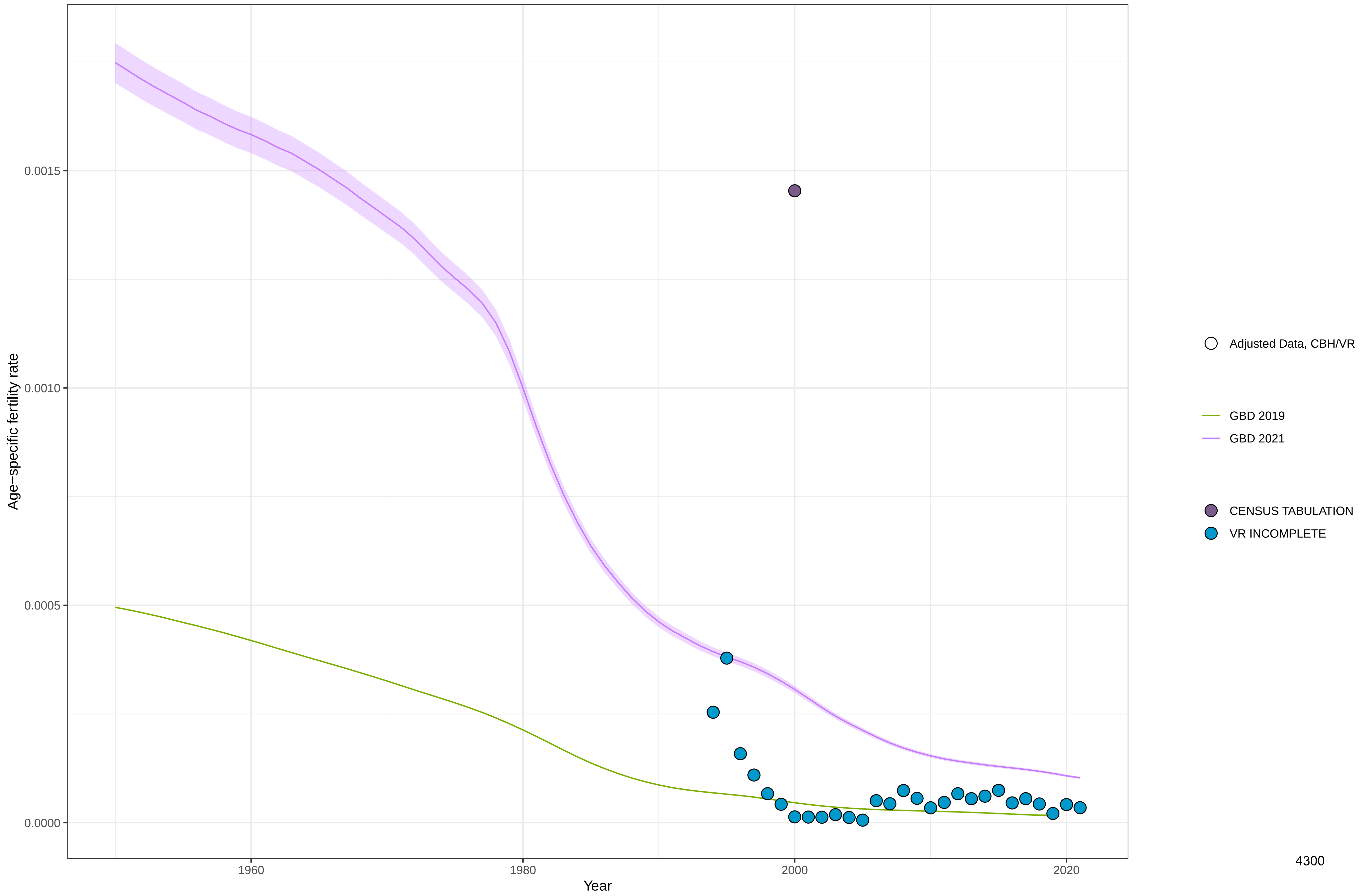

Total fertility rate fitted trend lines, 1950−2021: Ceará TFR
Data points enclosed with a diamond are reference sources

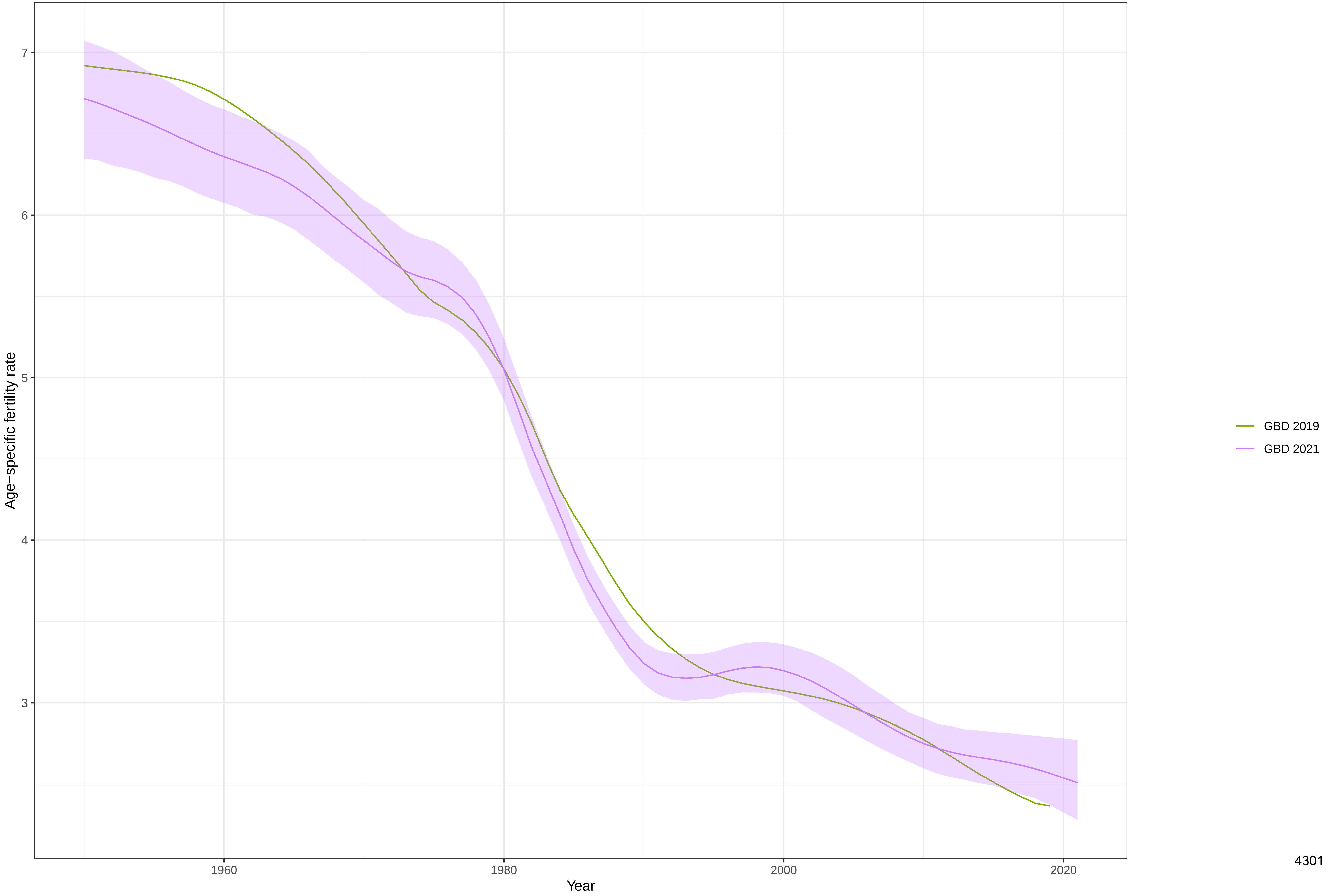

Age−specific fertility fitted trend lines, 1950−2021: Distrito Federal, Ages: 10−14
Data points enclosed with a diamond are reference sources

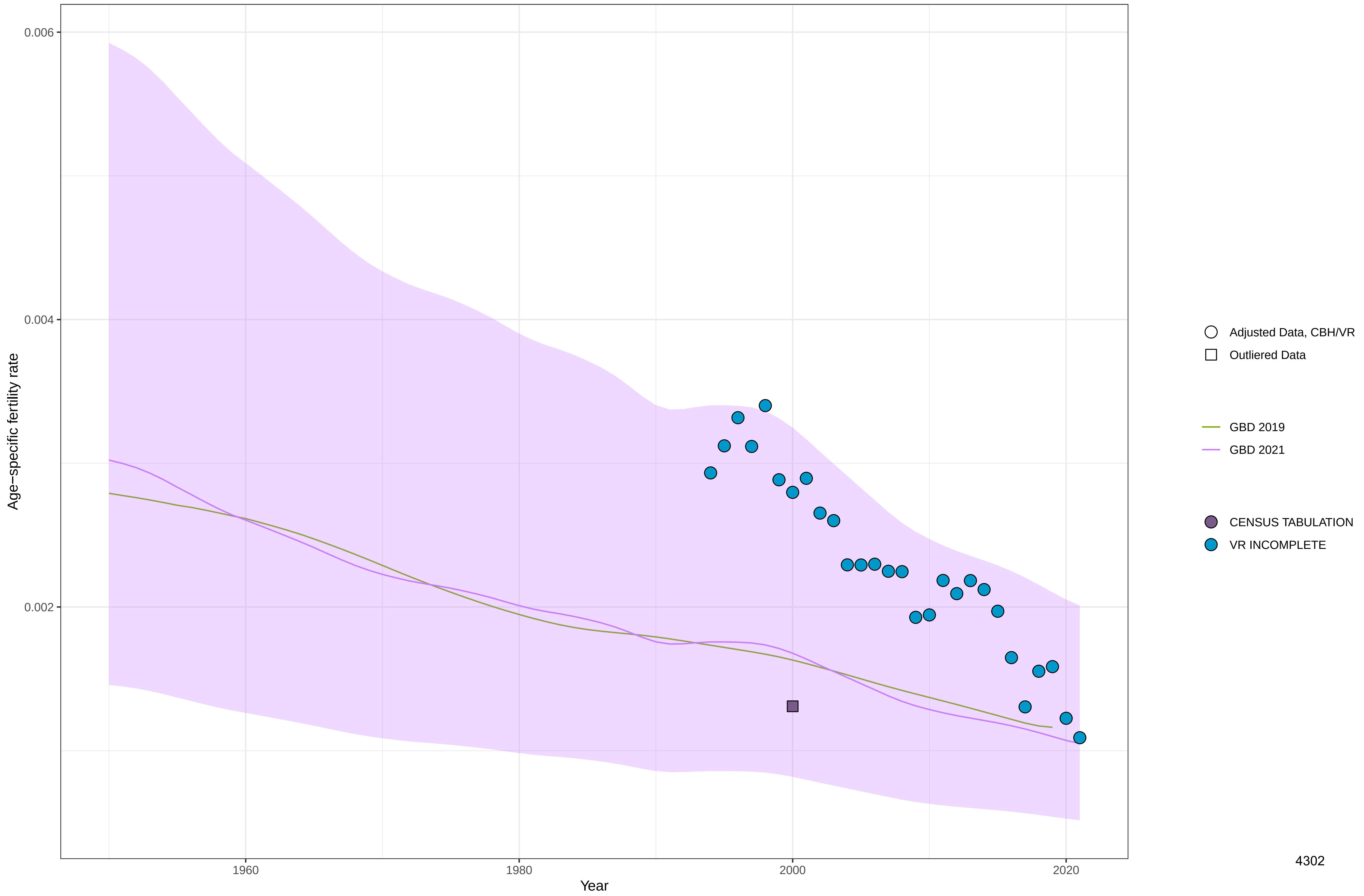

Age−specific fertility fitted trend lines, 1950−2021: Distrito Federal, Ages: 15−19
Data points enclosed with a diamond are reference sources

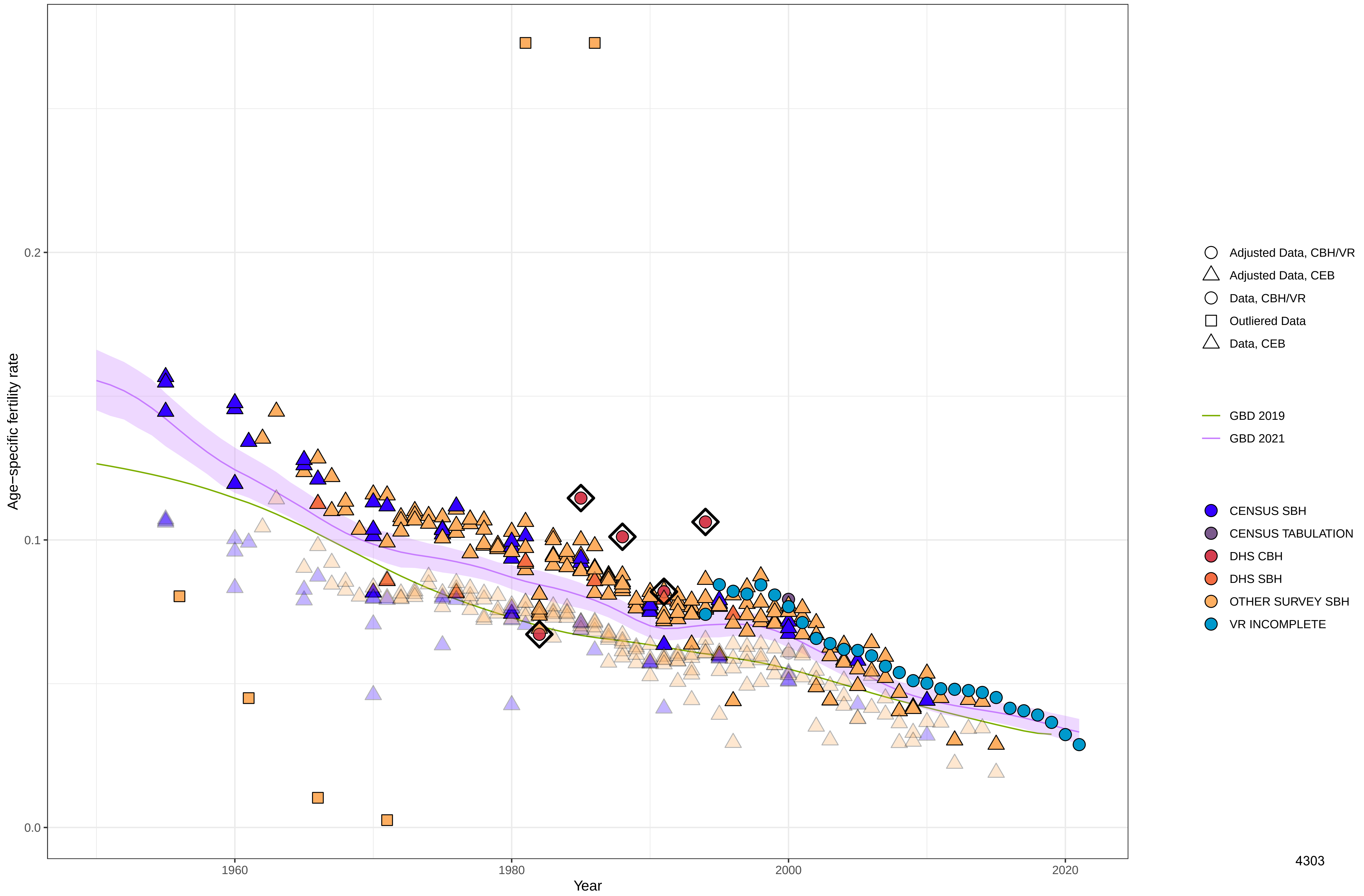

Age−specific fertility fitted trend lines, 1950−2021: Distrito Federal, Ages: 20−24
Data points enclosed with a diamond are reference sources

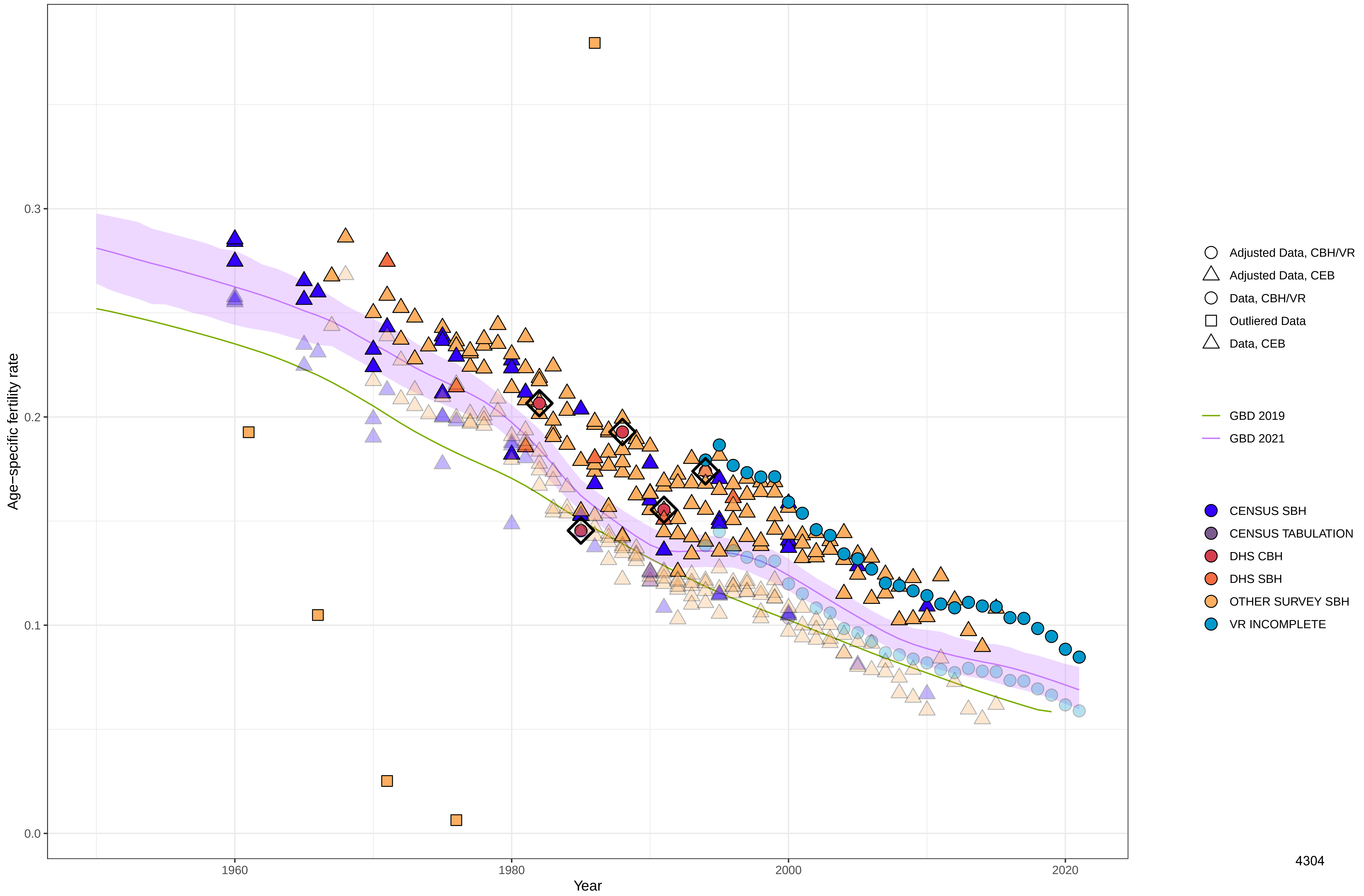

Age−specific fertility fitted trend lines, 1950−2021: Distrito Federal, Ages: 25−29
Data points enclosed with a diamond are reference sources

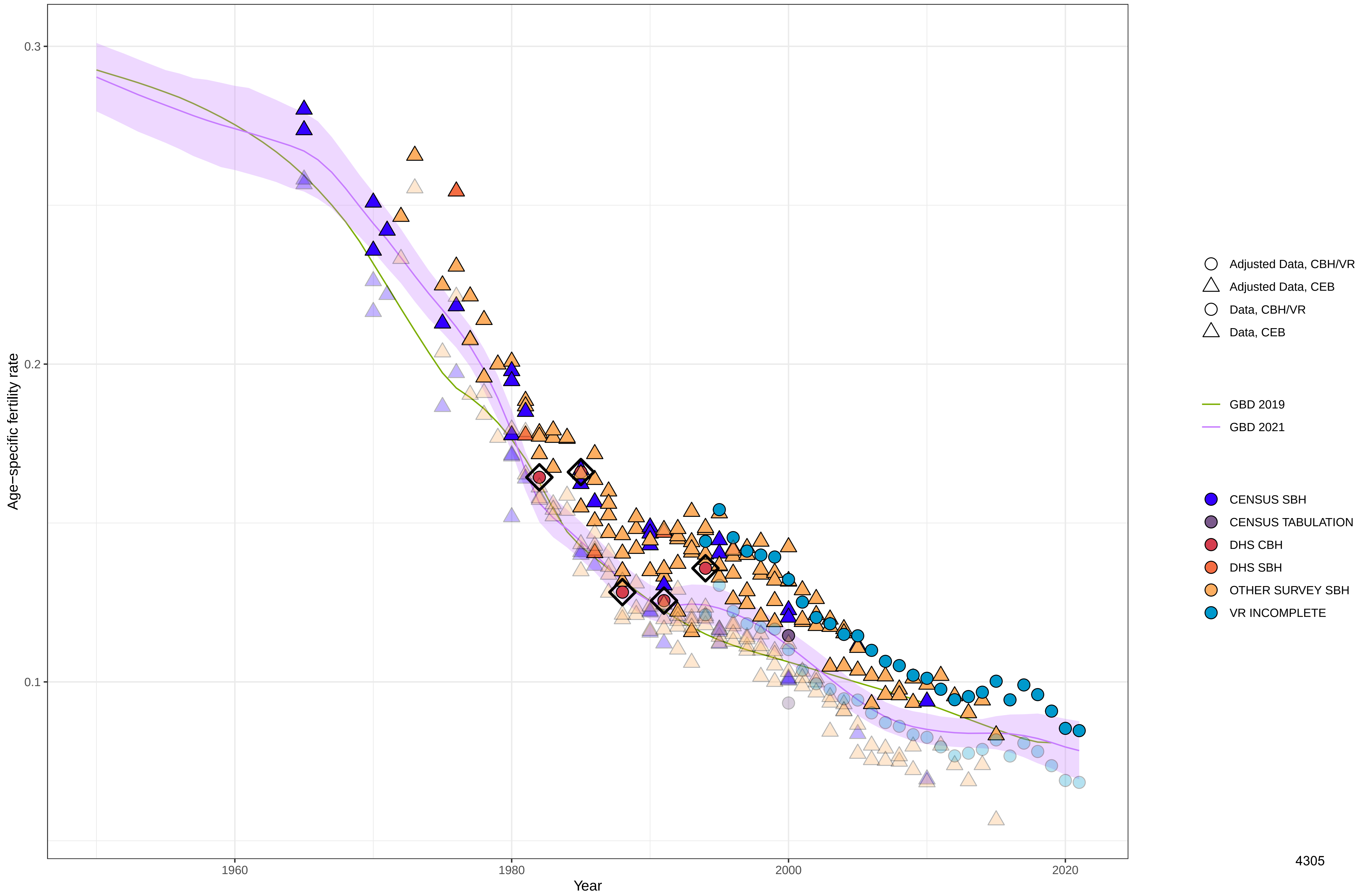

Age−specific fertility fitted trend lines, 1950−2021: Distrito Federal, Ages: 30−34
Data points enclosed with a diamond are reference sources

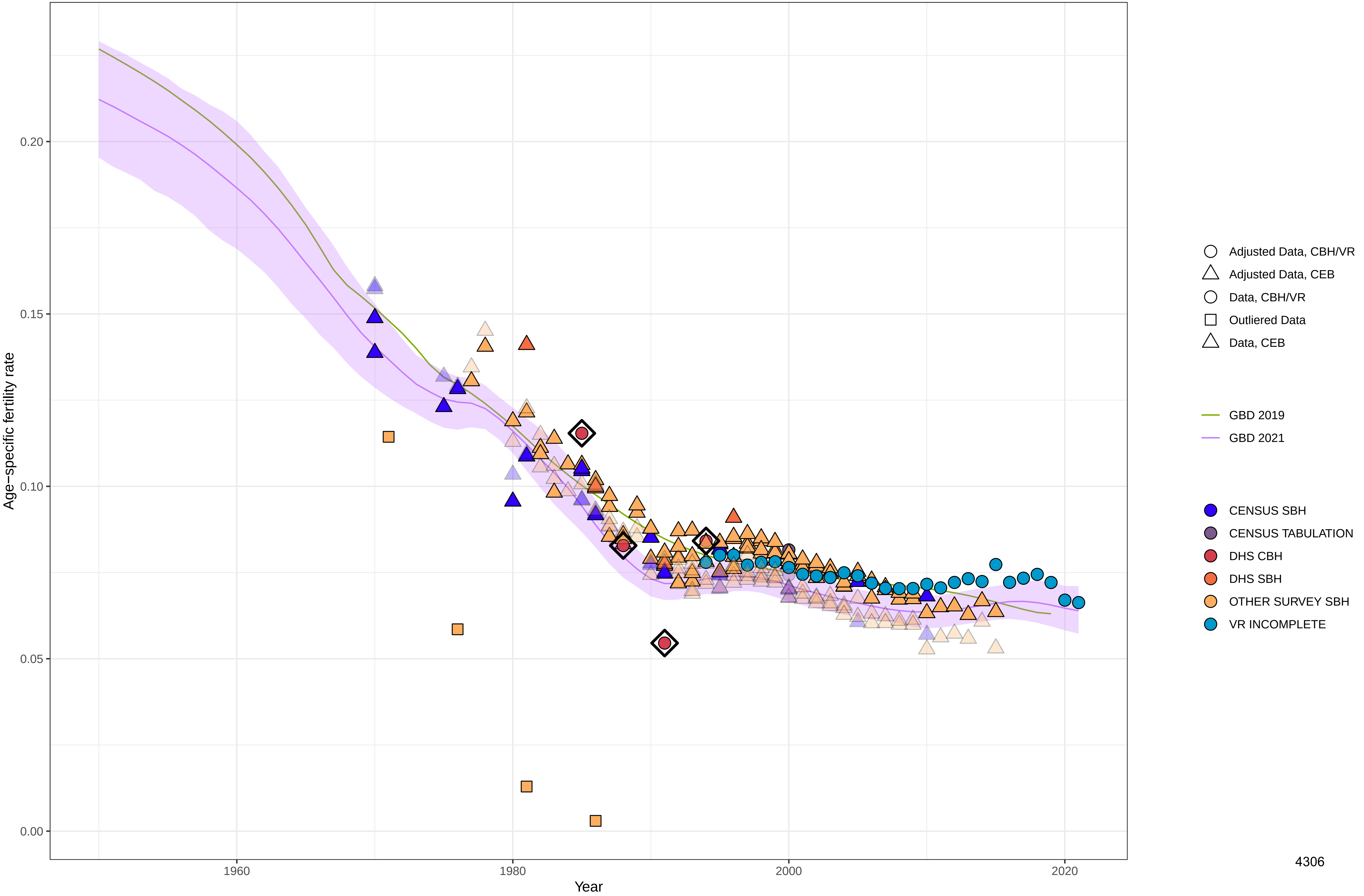

Age−specific fertility fitted trend lines, 1950−2021: Distrito Federal, Ages: 35−39
Data points enclosed with a diamond are reference sources

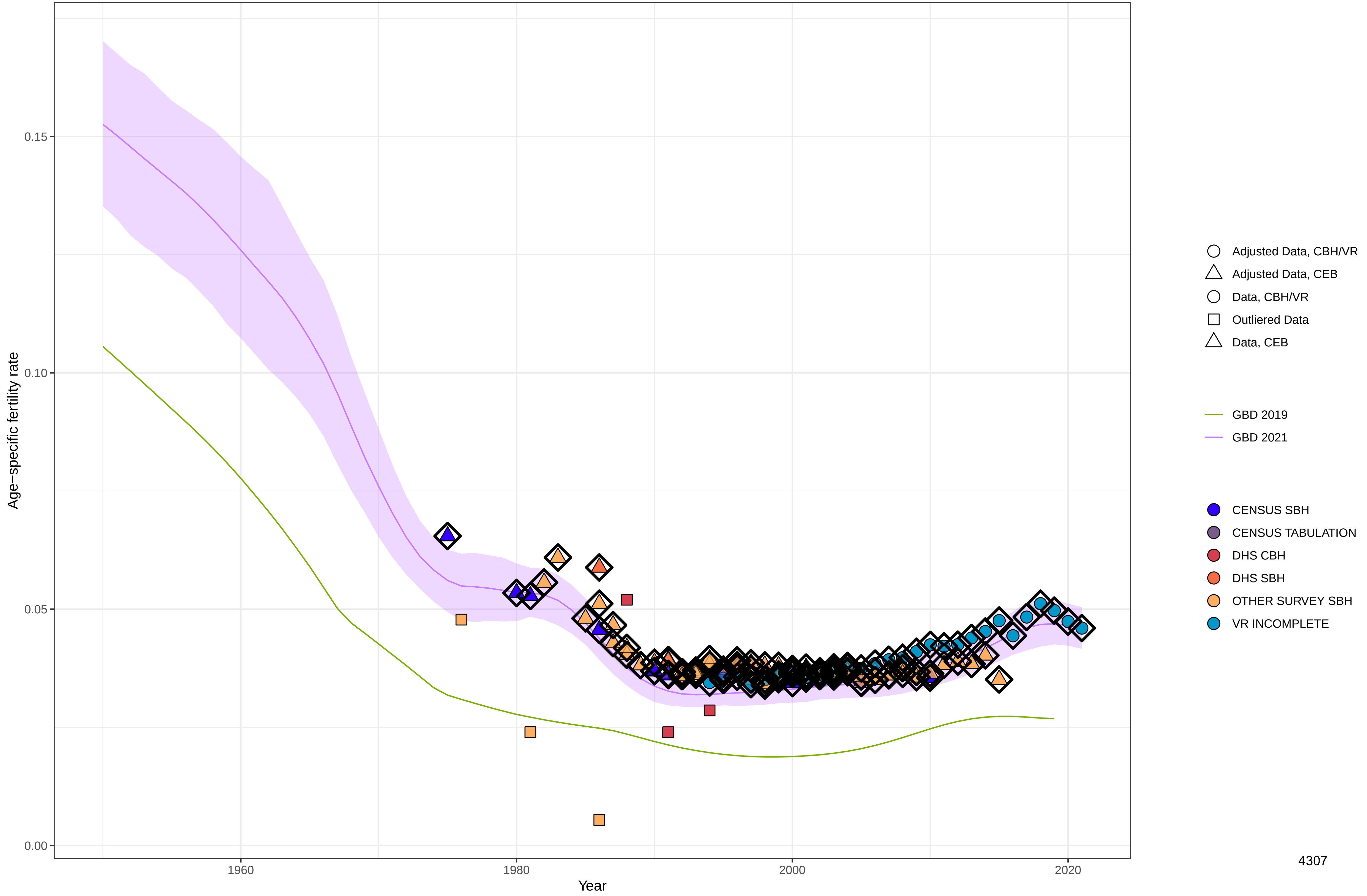

Age−specific fertility fitted trend lines, 1950−2021: Distrito Federal, Ages: 40−44
Data points enclosed with a diamond are reference sources

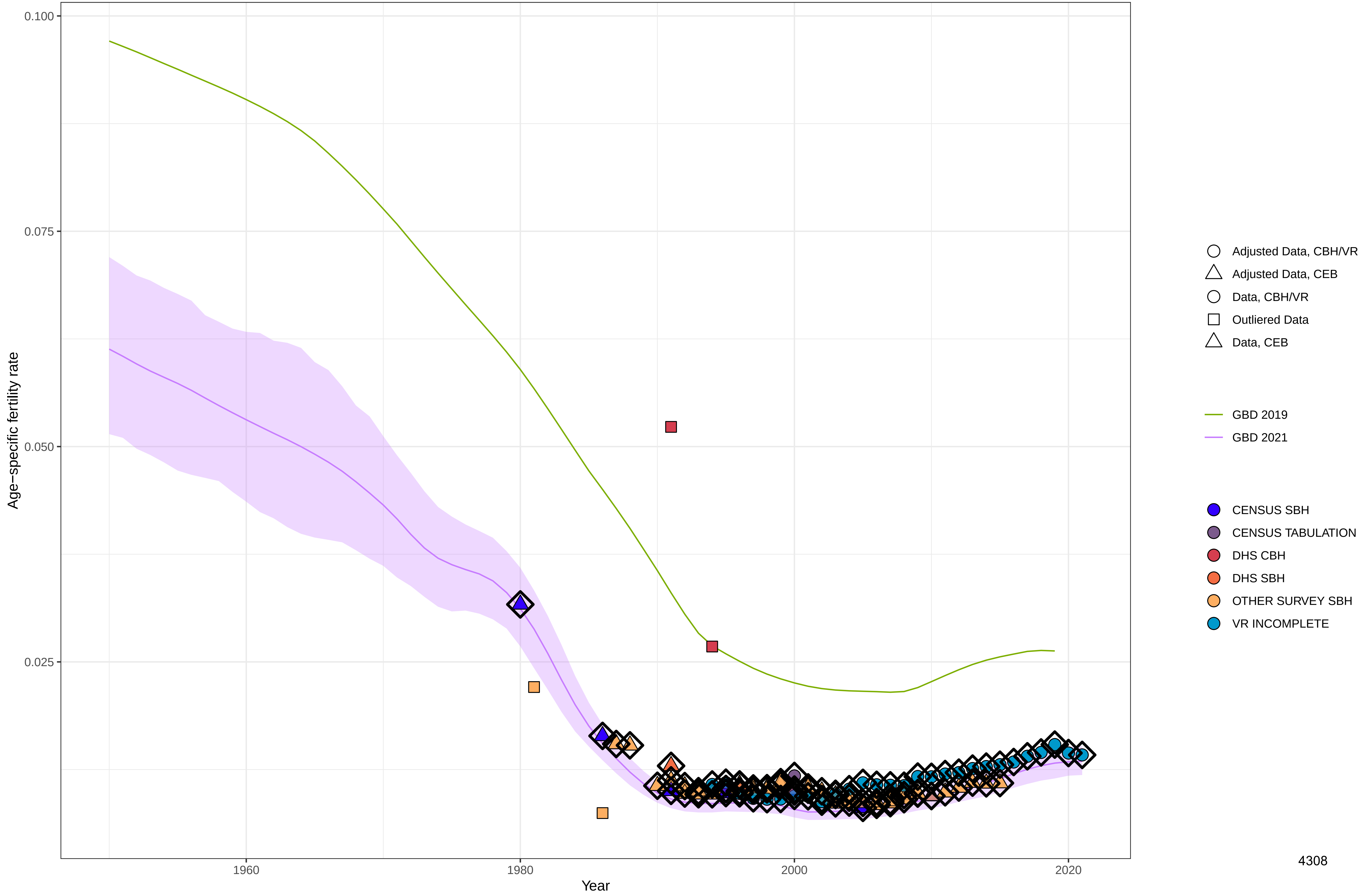

Age−specific fertility fitted trend lines, 1950−2021: Distrito Federal, Ages: 45−49
Data points enclosed with a diamond are reference sources

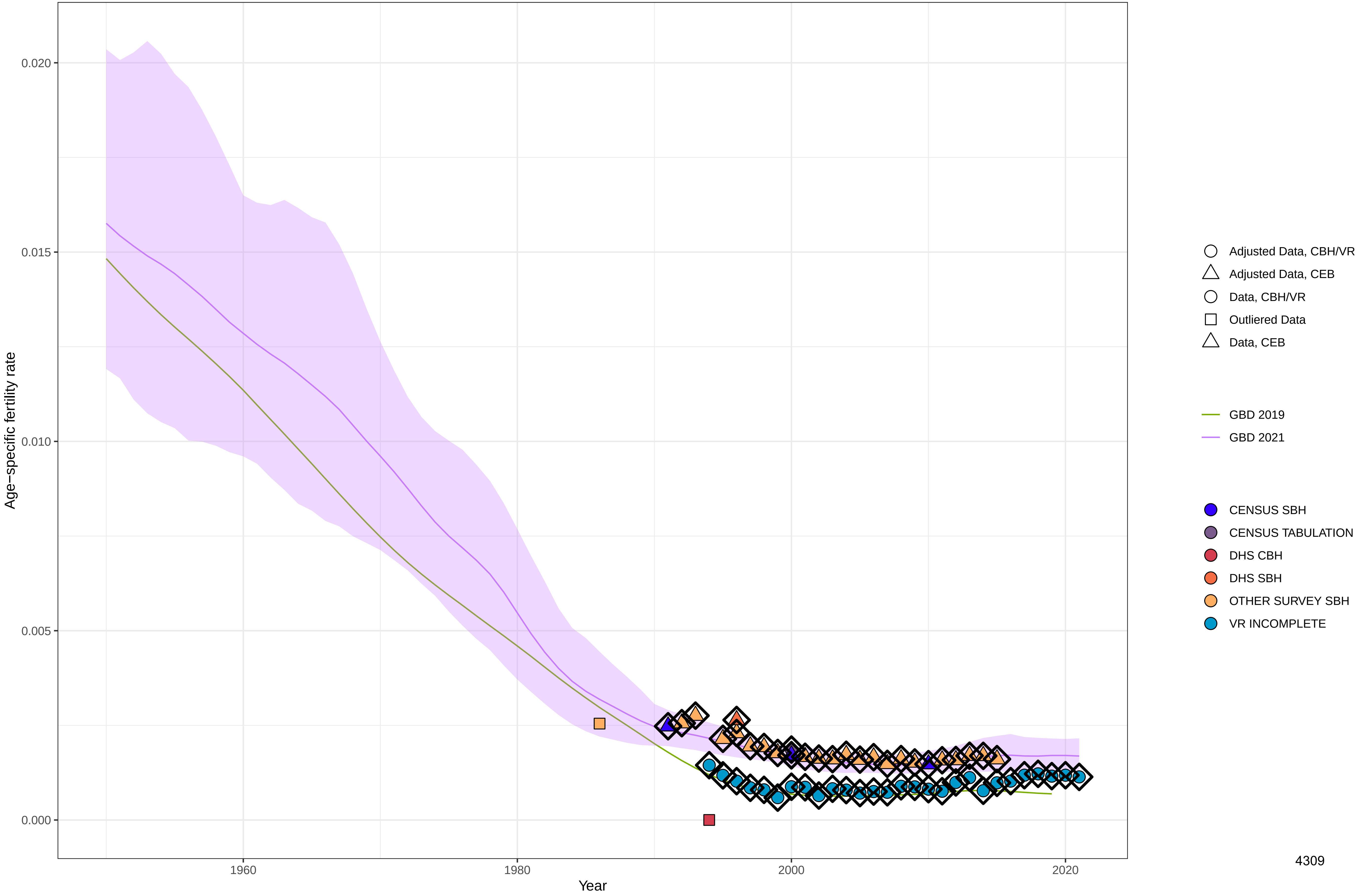

Age−specific fertility fitted trend lines, 1950−2021: Distrito Federal, Ages: 50−54
Data points enclosed with a diamond are reference sources

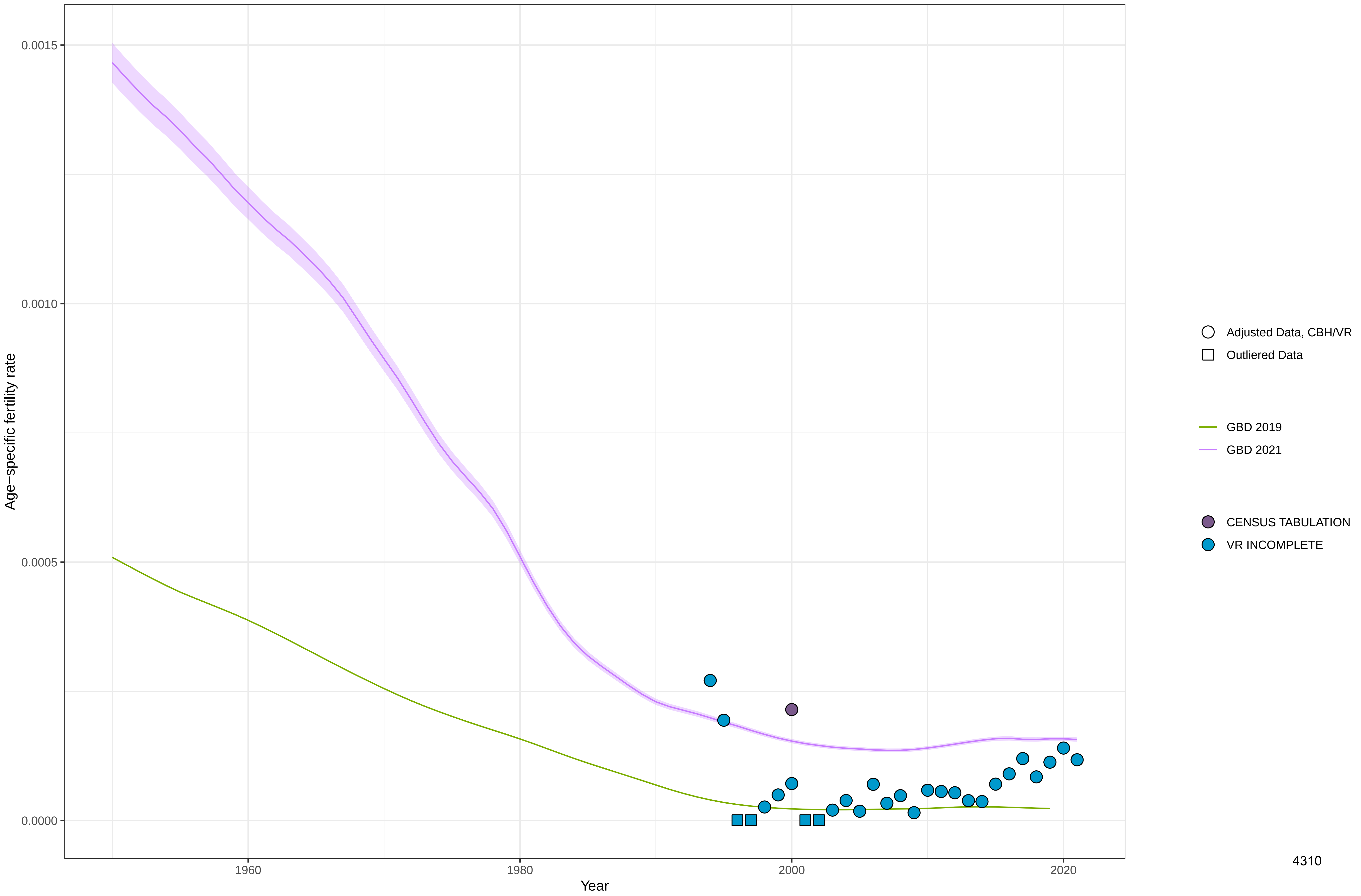

Total fertility rate fitted trend lines, 1950−2021: Distrito Federal TFR
Data points enclosed with a diamond are reference sources

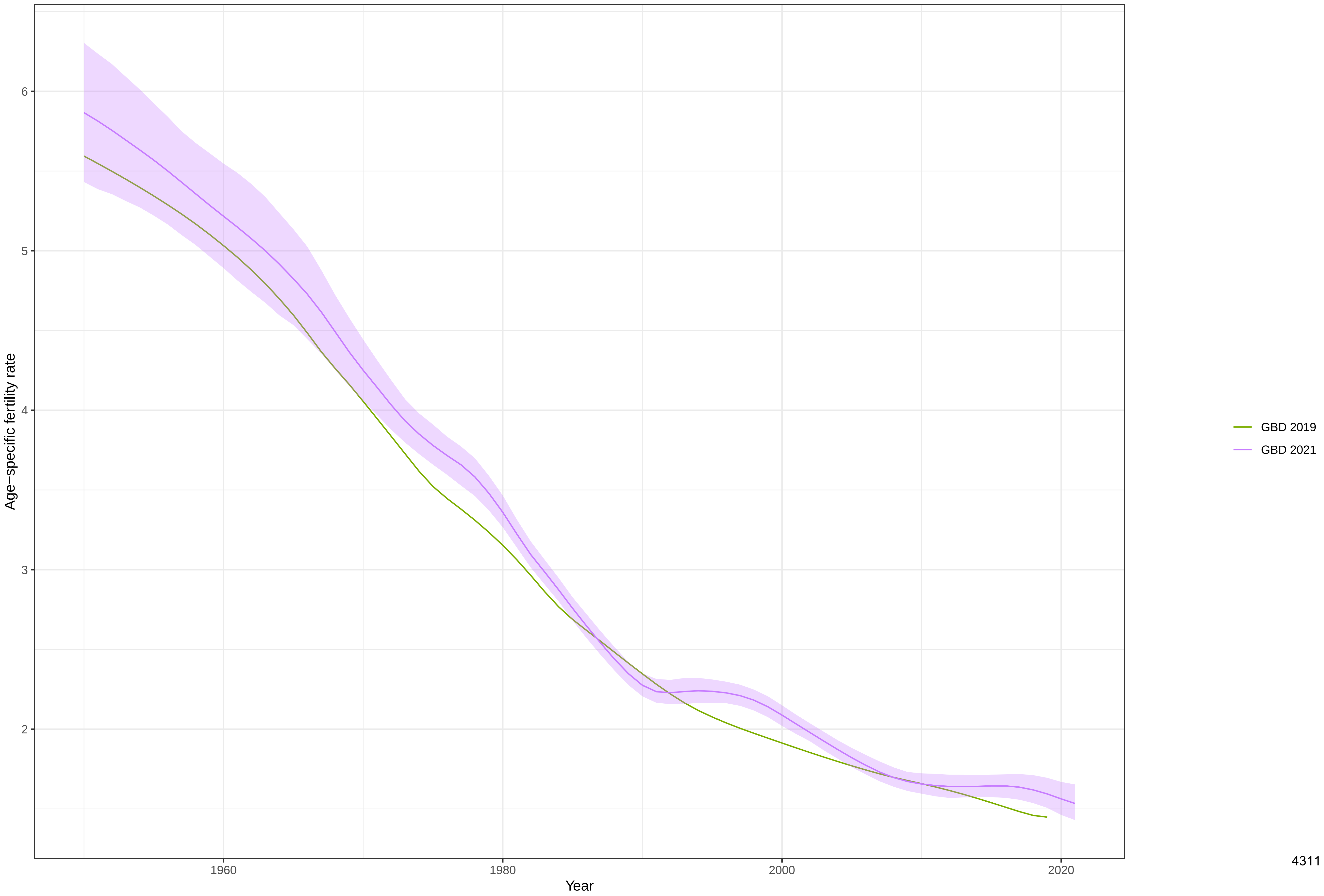

Age−specific fertility fitted trend lines, 1950−2021: Espírito Santo, Ages: 10−14
Data points enclosed with a diamond are reference sources

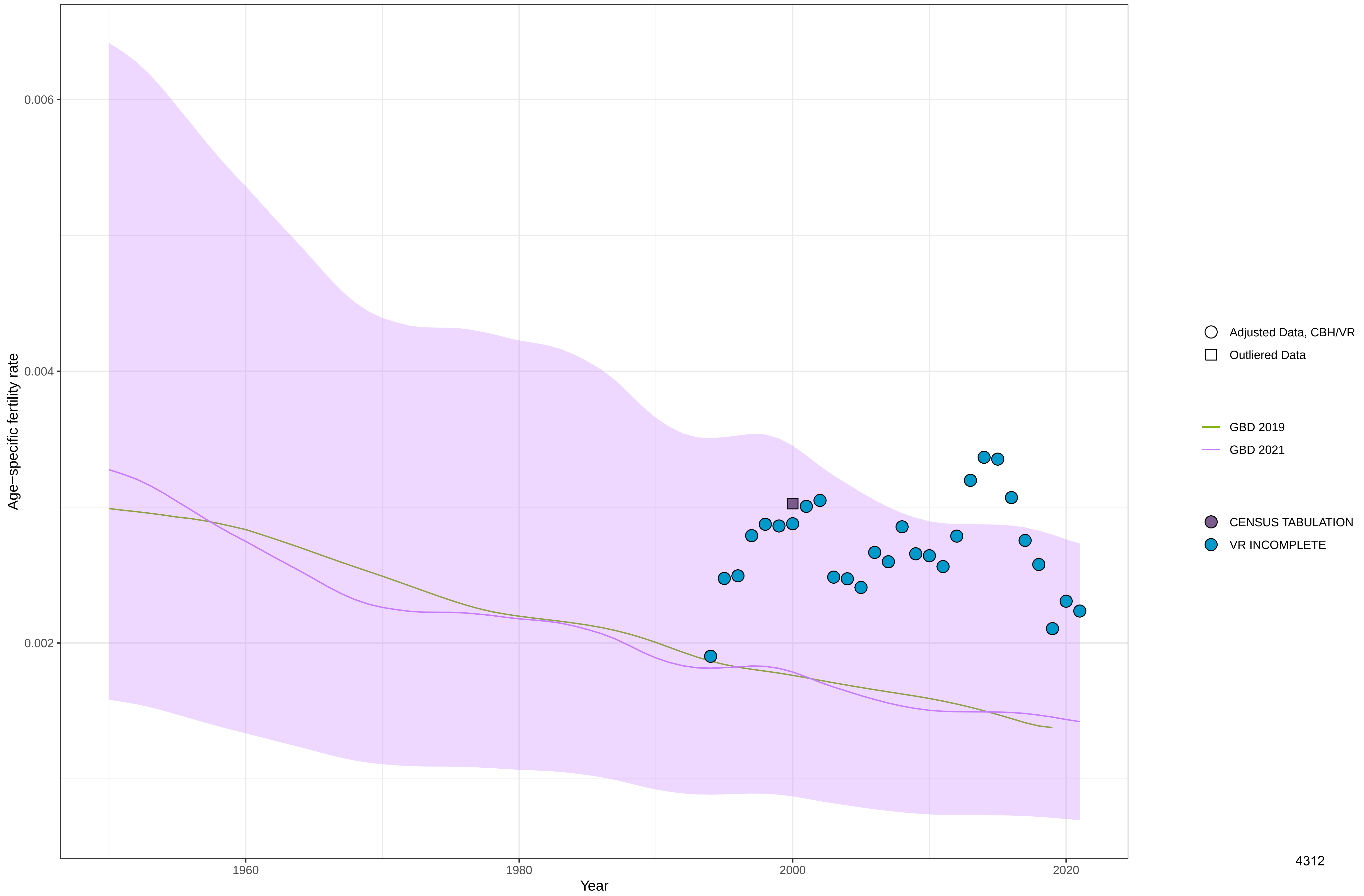

4312

Age−specific fertility fitted trend lines, 1950−2021: Espírito Santo, Ages: 15−19
Data points enclosed with a diamond are reference sources

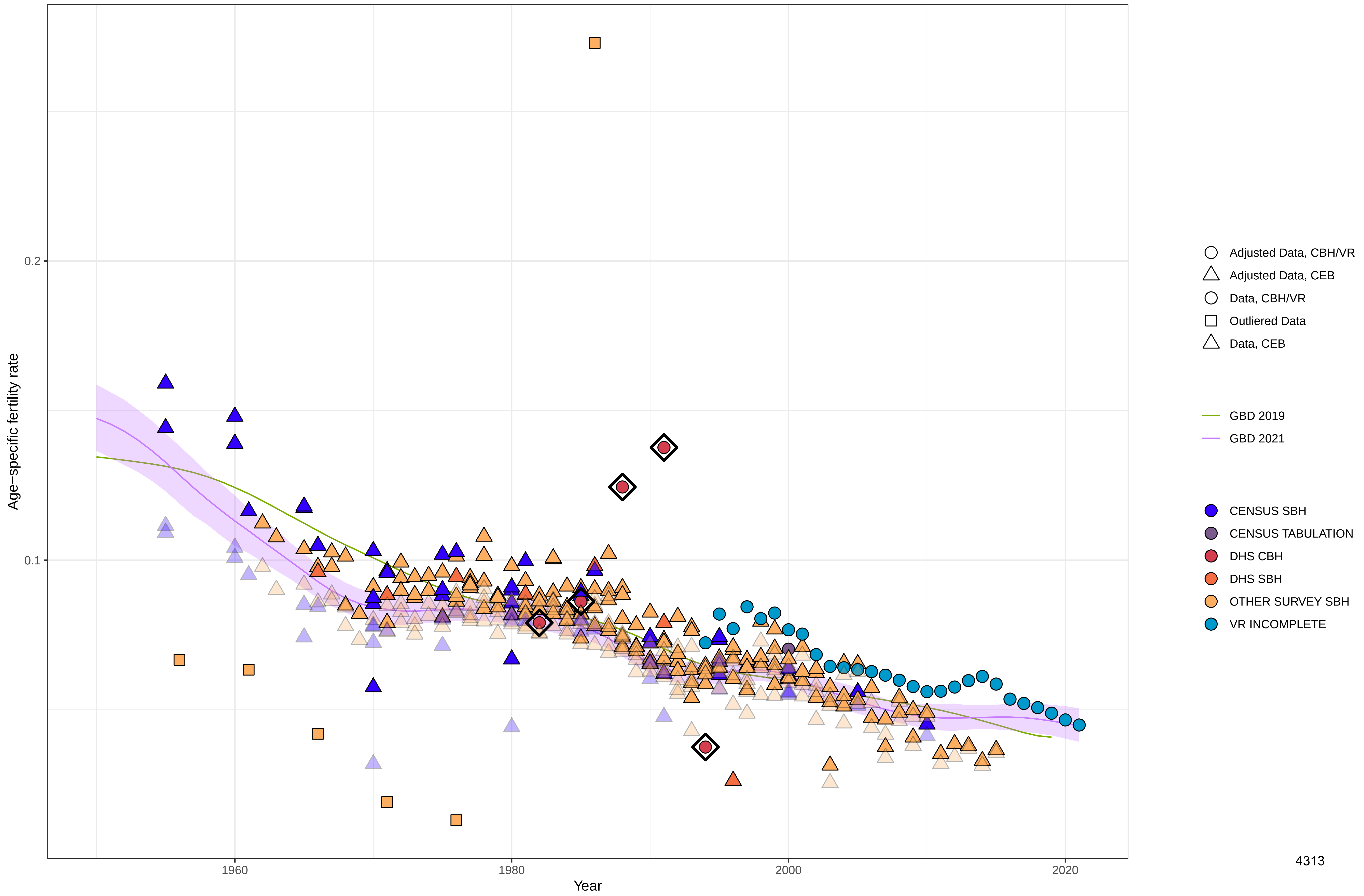

Age−specific fertility fitted trend lines, 1950−2021: Espírito Santo, Ages: 20−24
Data points enclosed with a diamond are reference sources

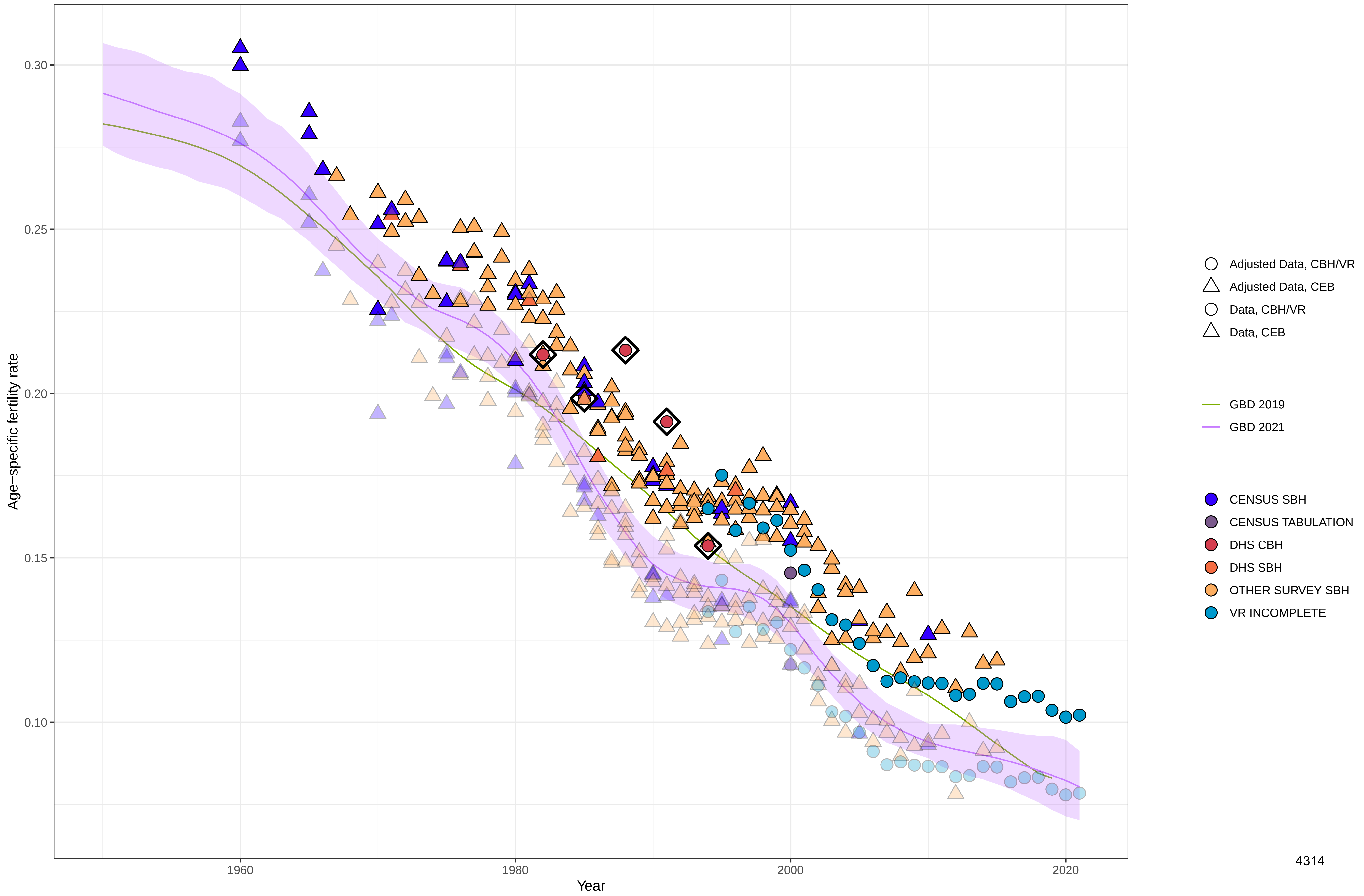

Age−specific fertility fitted trend lines, 1950−2021: Espírito Santo, Ages: 25−29
Data points enclosed with a diamond are reference sources

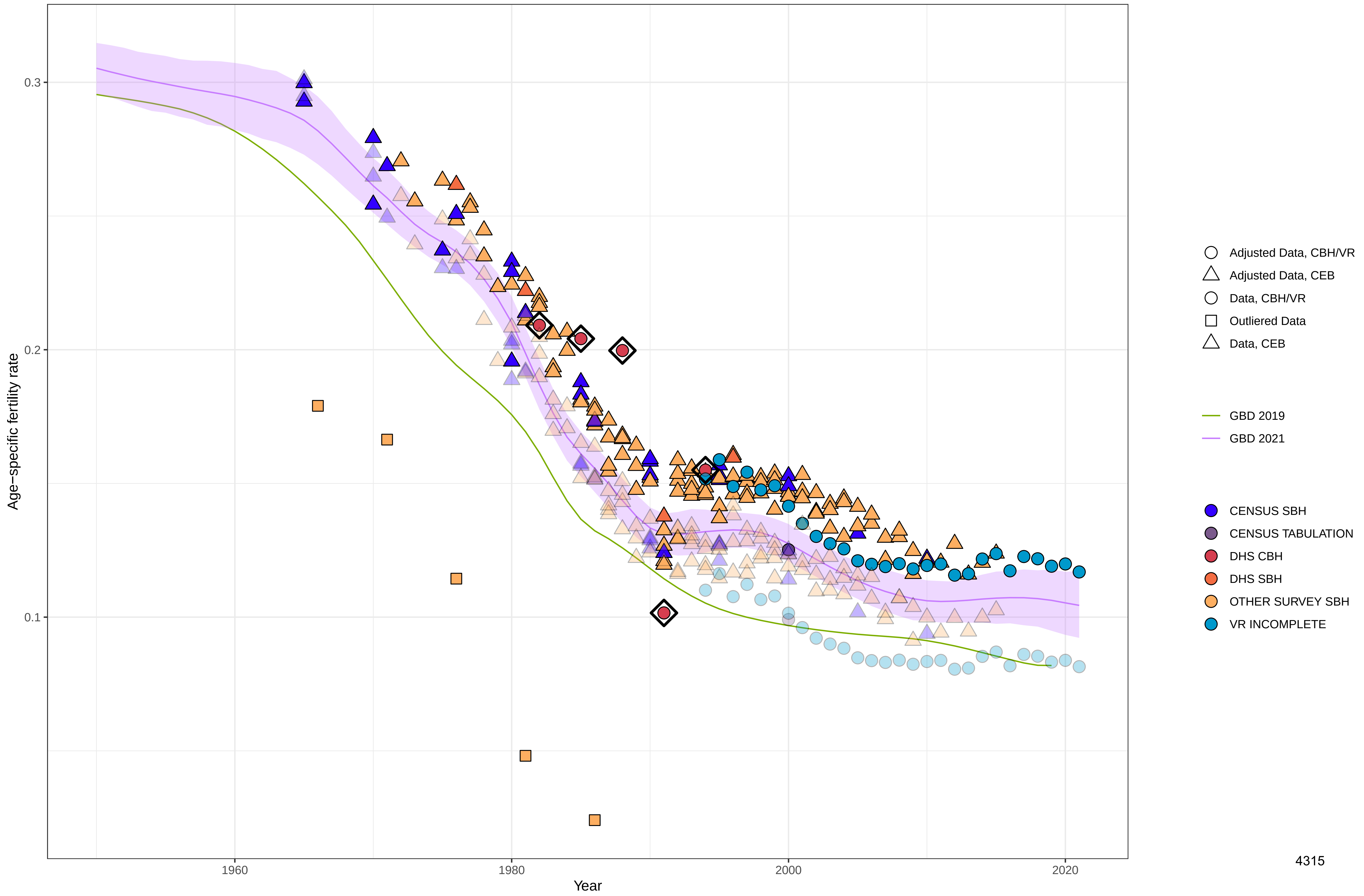

Age−specific fertility fitted trend lines, 1950−2021: Espírito Santo, Ages: 30−34
Data points enclosed with a diamond are reference sources

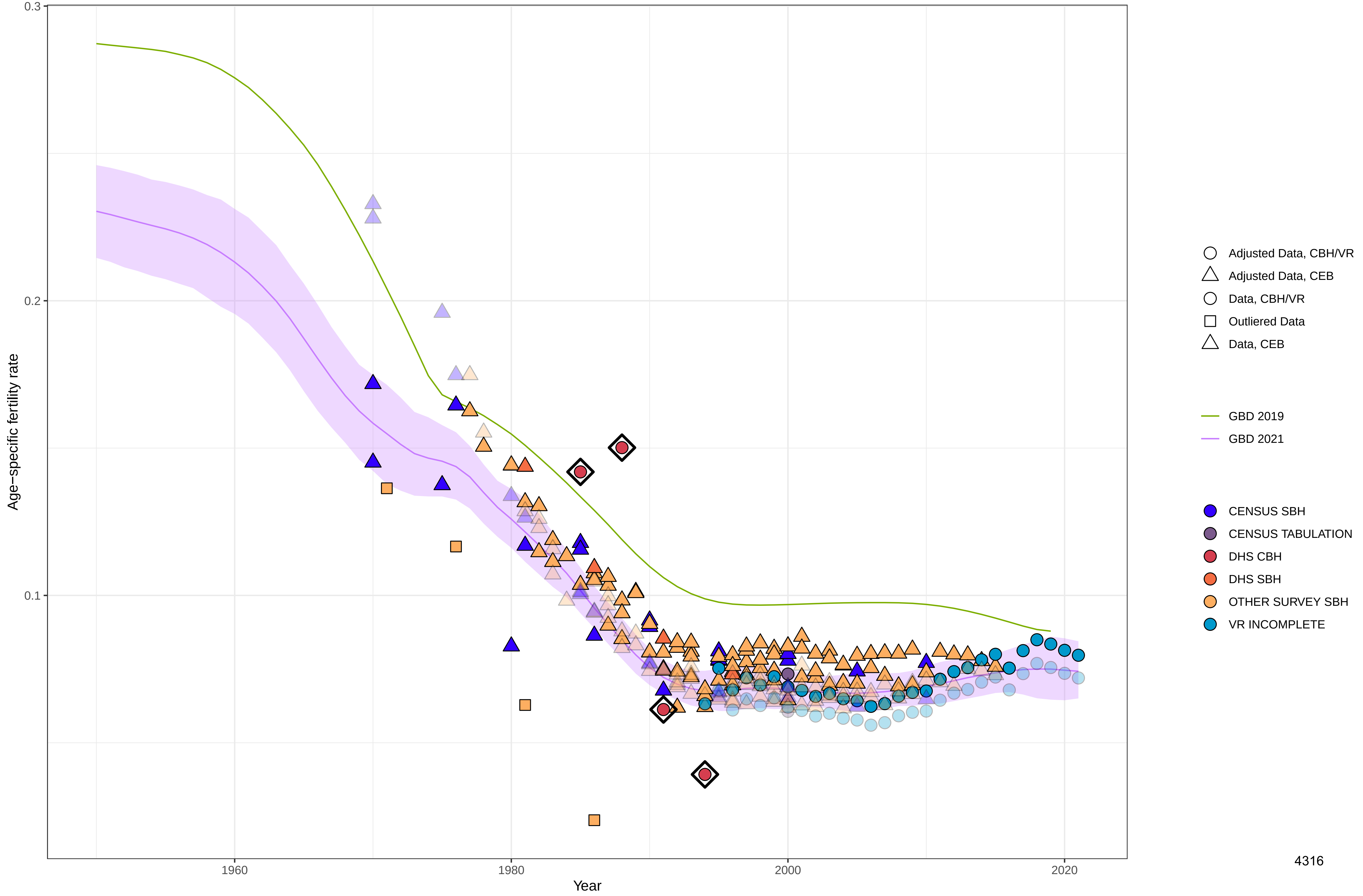

Age−specific fertility fitted trend lines, 1950−2021: Espírito Santo, Ages: 35−39
Data points enclosed with a diamond are reference sources

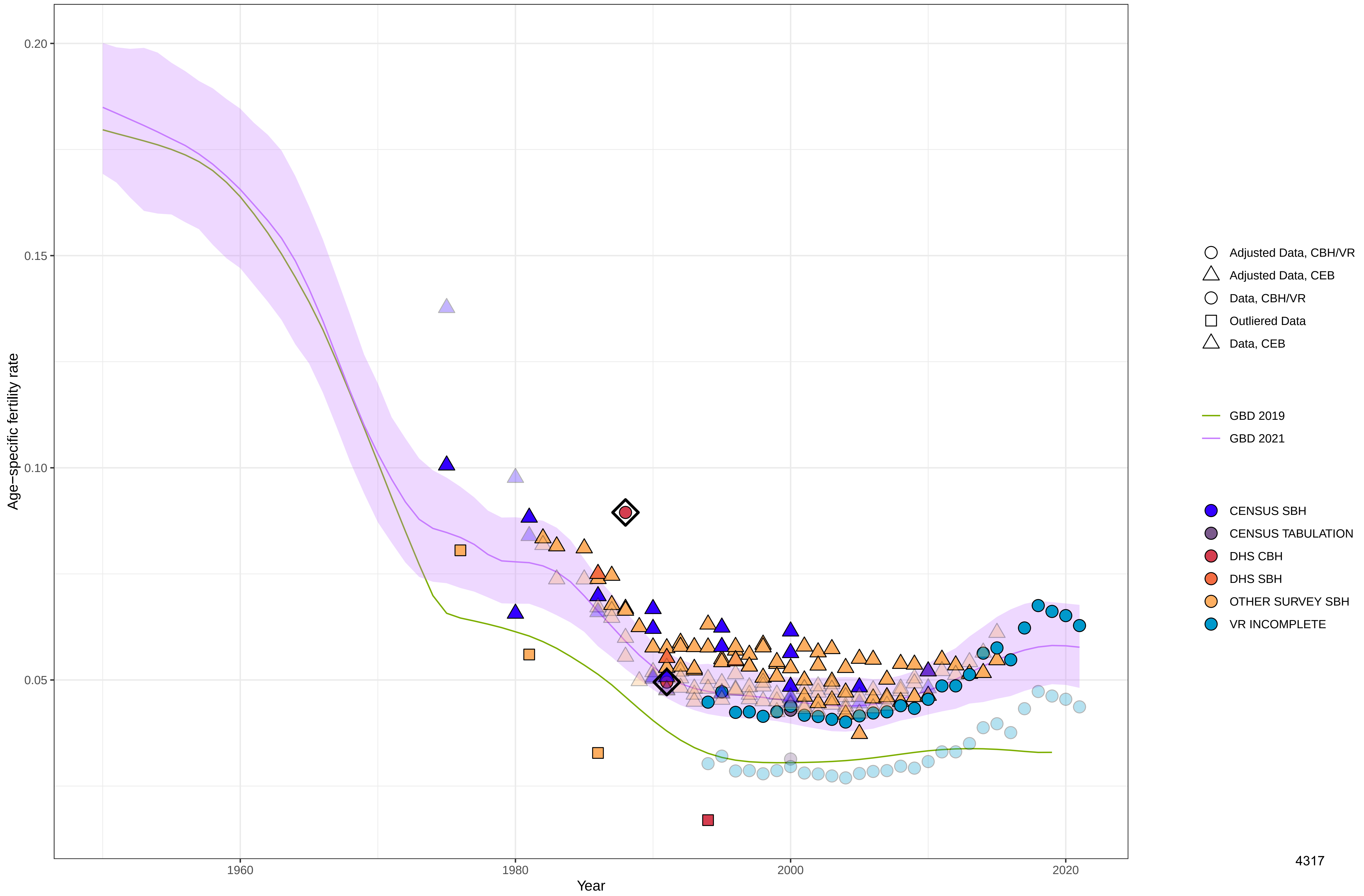

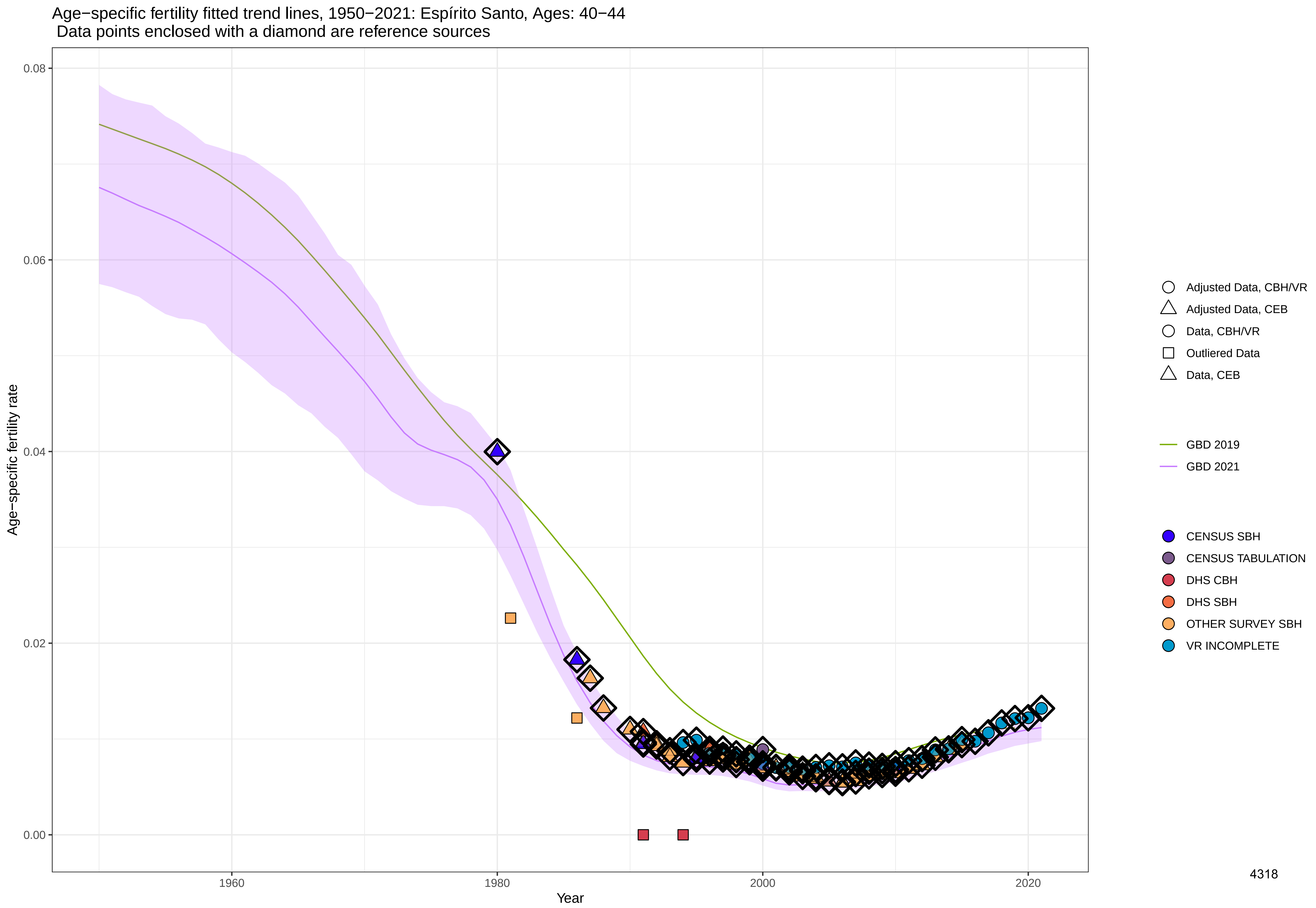
Age−specific fertility fitted trend lines, 1950−2021: Espírito Santo, Ages: 40−44
Data points enclosed with a diamond are reference sources
Age−specific fertility rate
0.08
0.06
0.04
0.02
0.00
1960
1980
2000
2020
Year
Adjusted Data, CBH/VR
Adjusted Data, CEB
Data, CBH/VR
Outliered Data
Data, CEB
GBD 2019
GBD 2021
CENSUS SBH
CENSUS TABULATION
DHS CBH
DHS SBH
OTHER SURVEY SBH
VR INCOMPLETE

4318

Age−specific fertility fitted trend lines, 1950−2021: Espírito Santo, Ages: 45−49
Data points enclosed with a diamond are reference sources

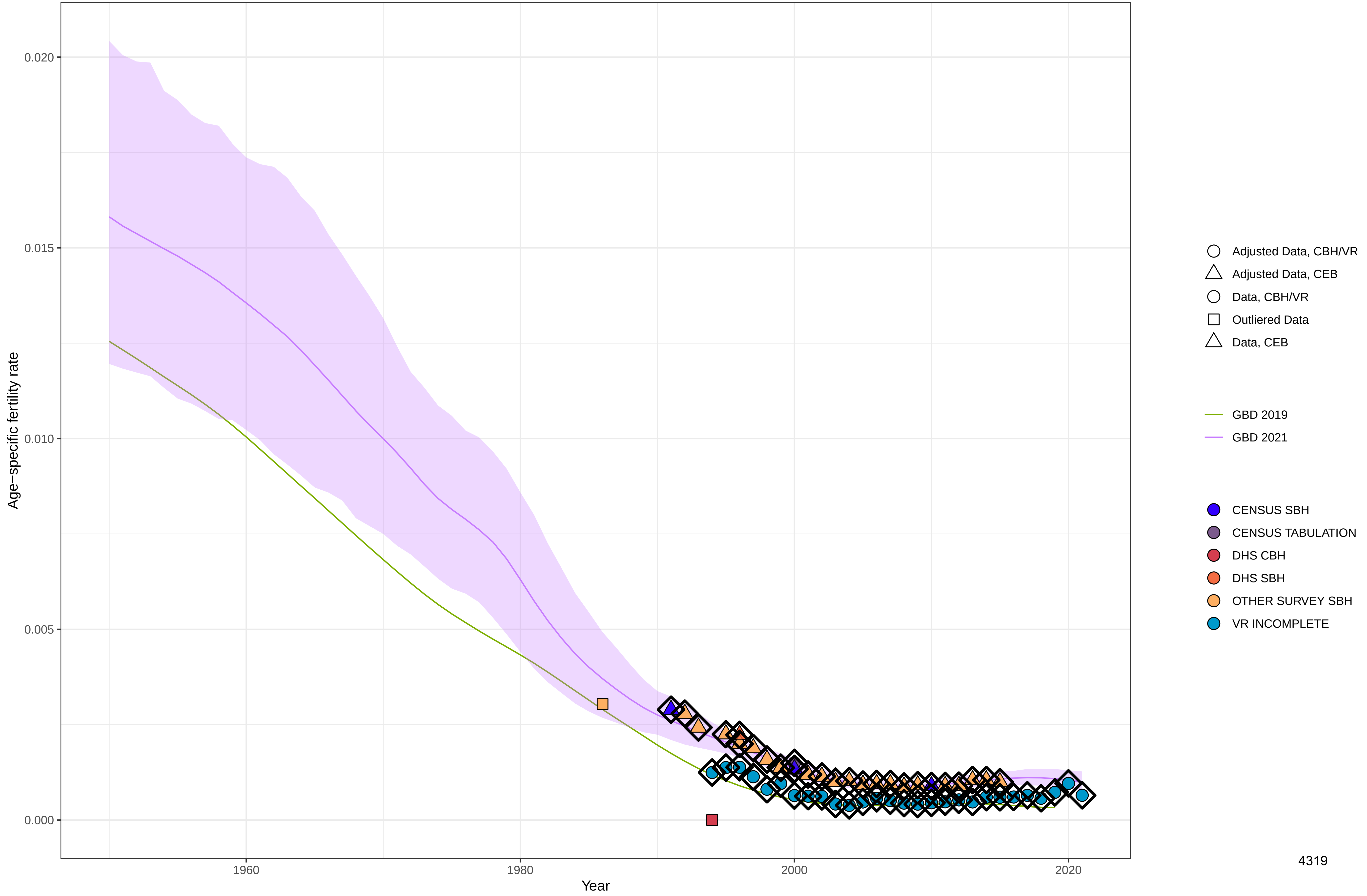

Age−specific fertility fitted trend lines, 1950−2021: Espírito Santo, Ages: 50−54
Data points enclosed with a diamond are reference sources

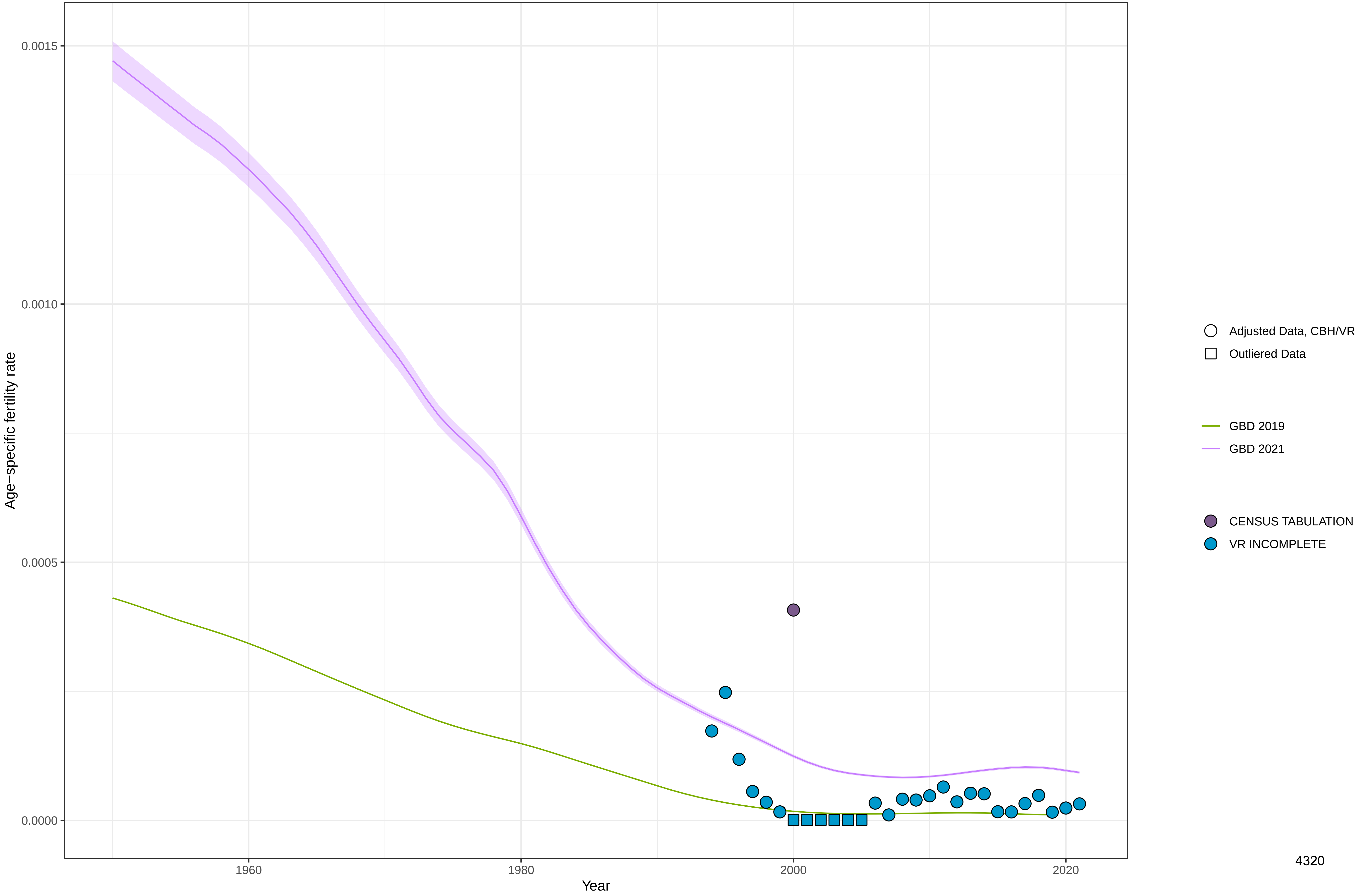

Total fertility rate fitted trend lines, 1950−2021: Espírito Santo TFR
Data points enclosed with a diamond are reference sources

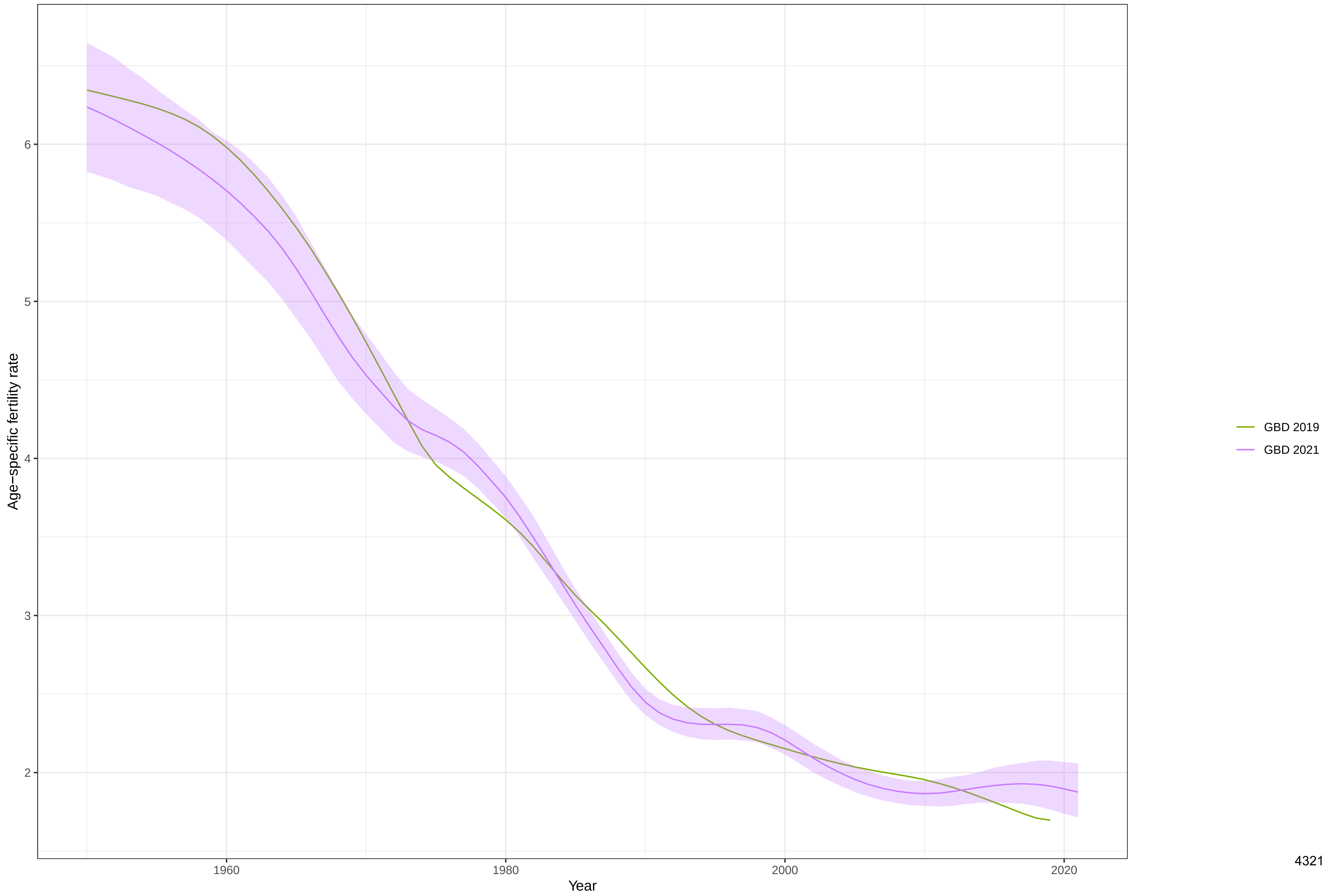

Age−specific fertility fitted trend lines, 1950−2021: Goiás, Ages: 10−14
Data points enclosed with a diamond are reference sources

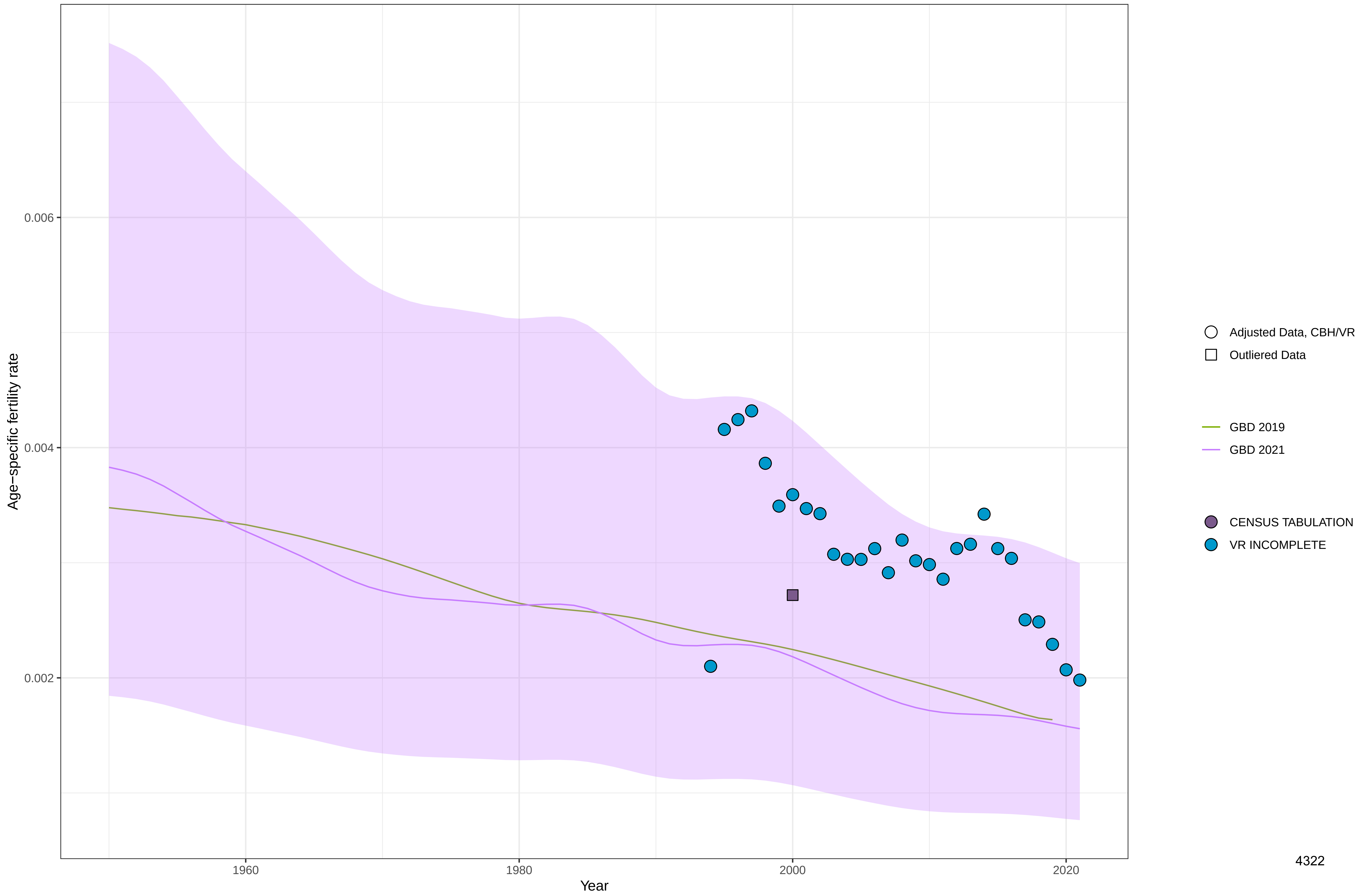

Age−specific fertility fitted trend lines, 1950−2021: Goiás, Ages: 15−19
Data points enclosed with a diamond are reference sources

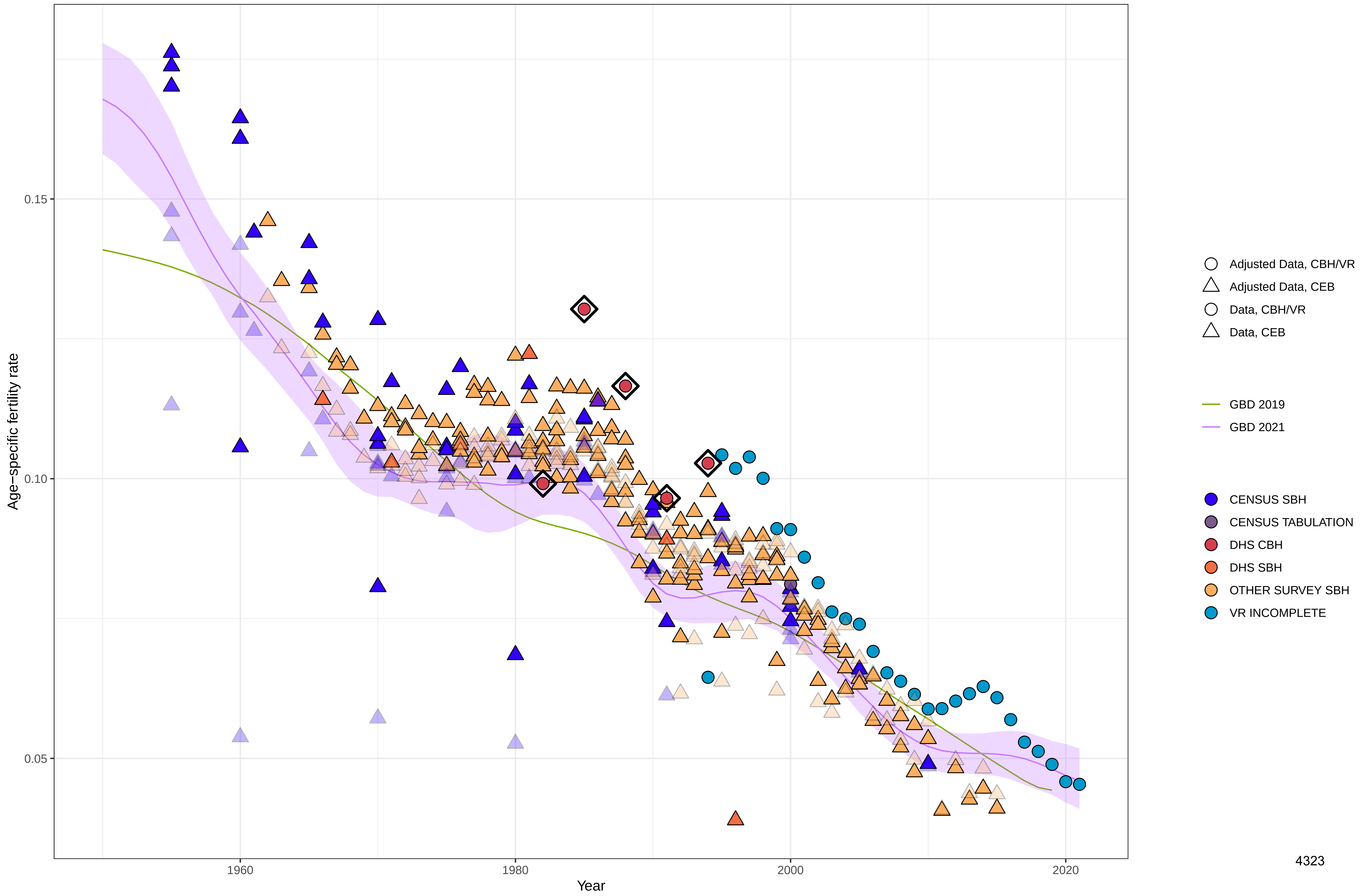

Age−specific fertility fitted trend lines, 1950−2021: Goiás, Ages: 20−24
Data points enclosed with a diamond are reference sources

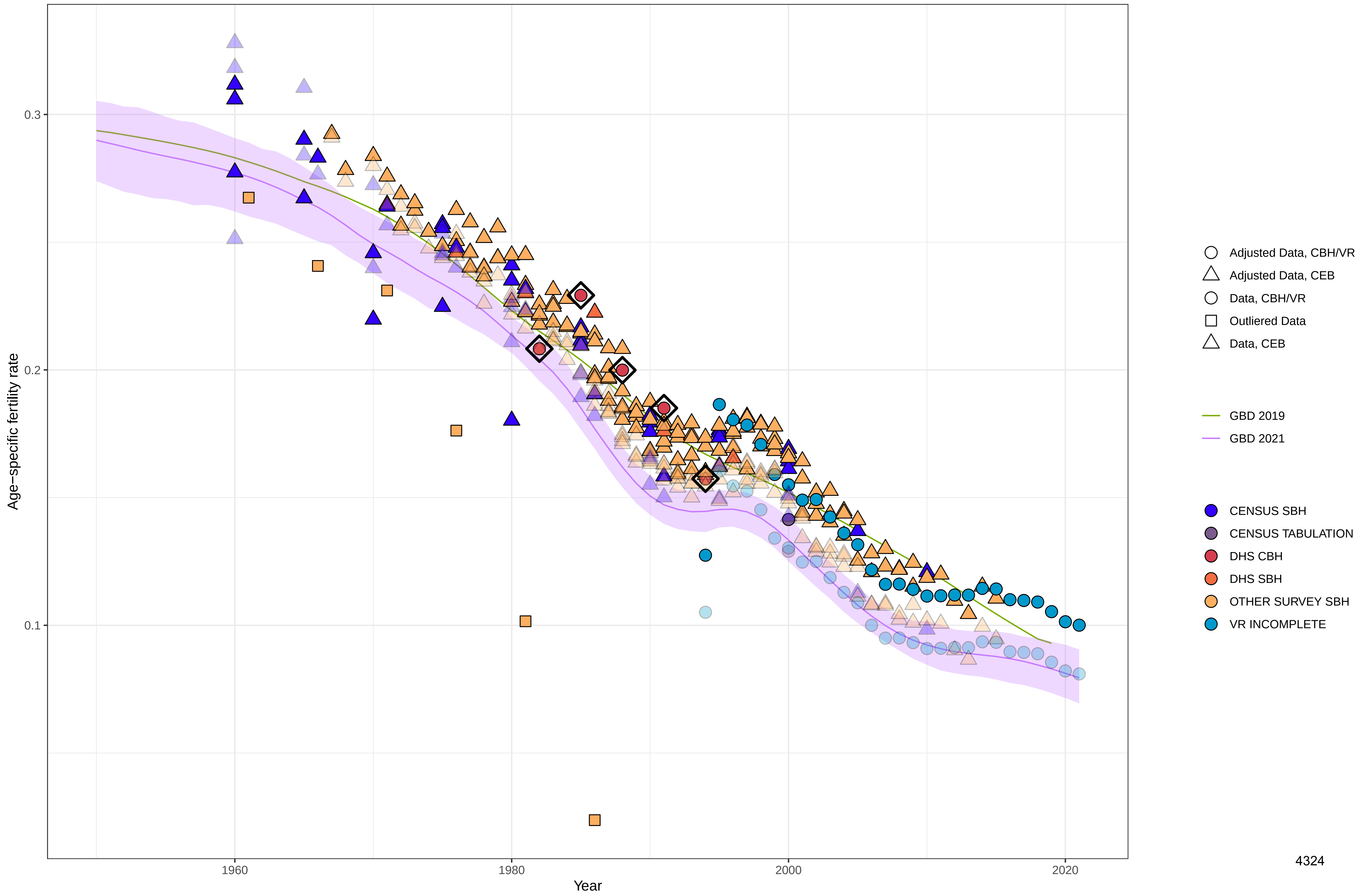

Age−specific fertility fitted trend lines, 1950−2021: Goiás, Ages: 25−29
Data points enclosed with a diamond are reference sources

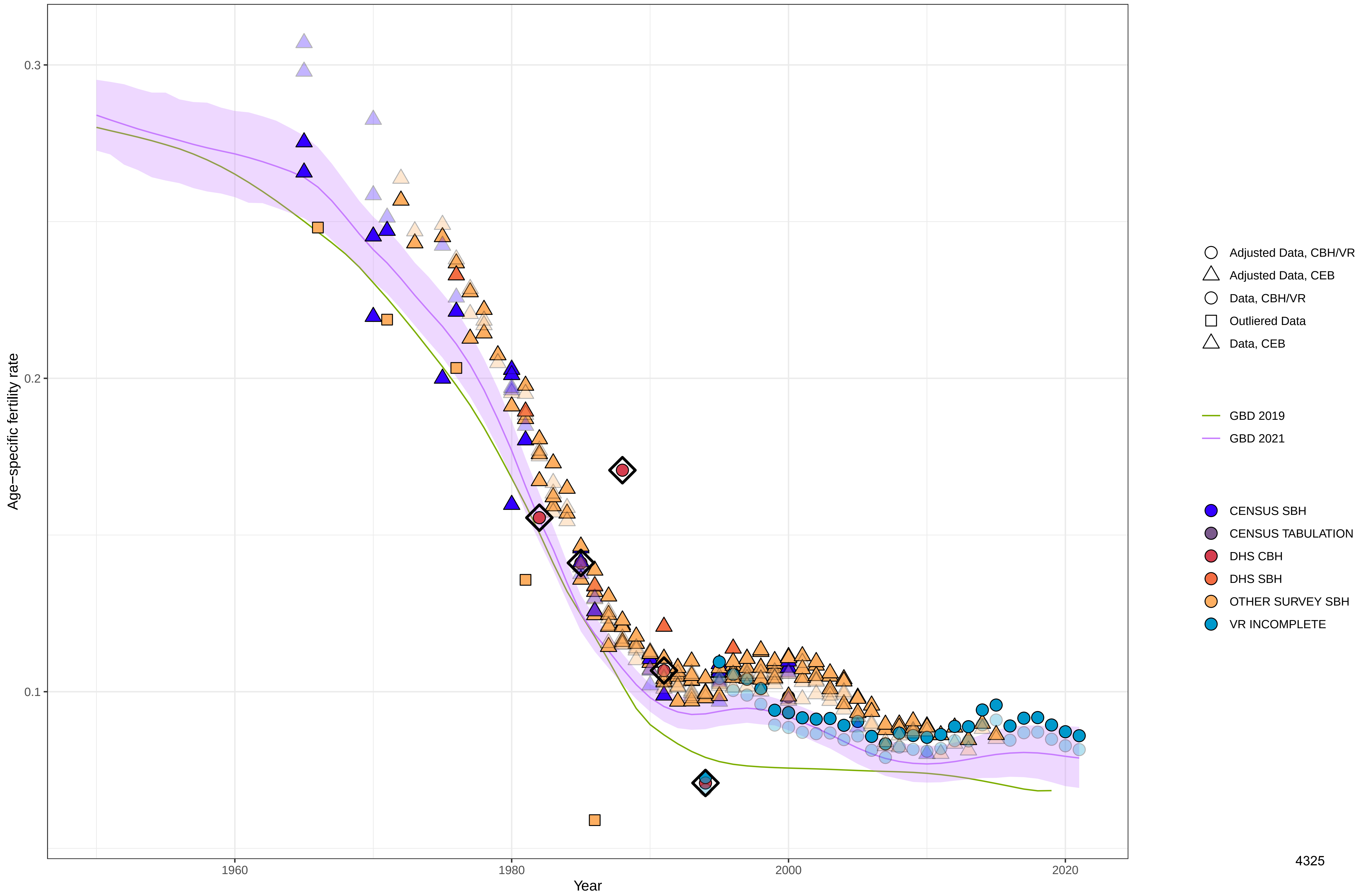

Age−specific fertility fitted trend lines, 1950−2021: Goiás, Ages: 30−34
Data points enclosed with a diamond are reference sources

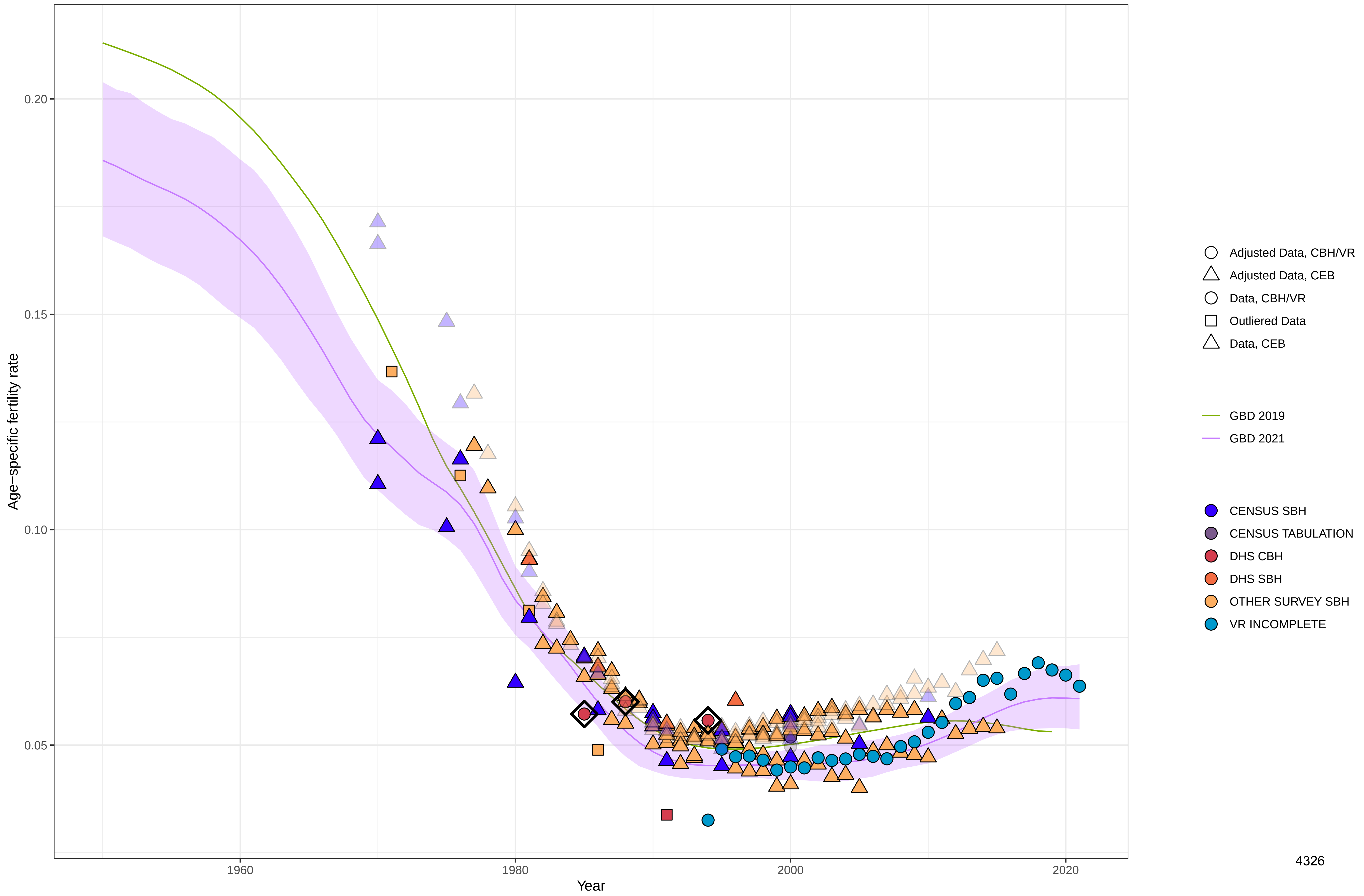

Age−specific fertility fitted trend lines, 1950−2021: Goiás, Ages: 35−39
Data points enclosed with a diamond are reference sources

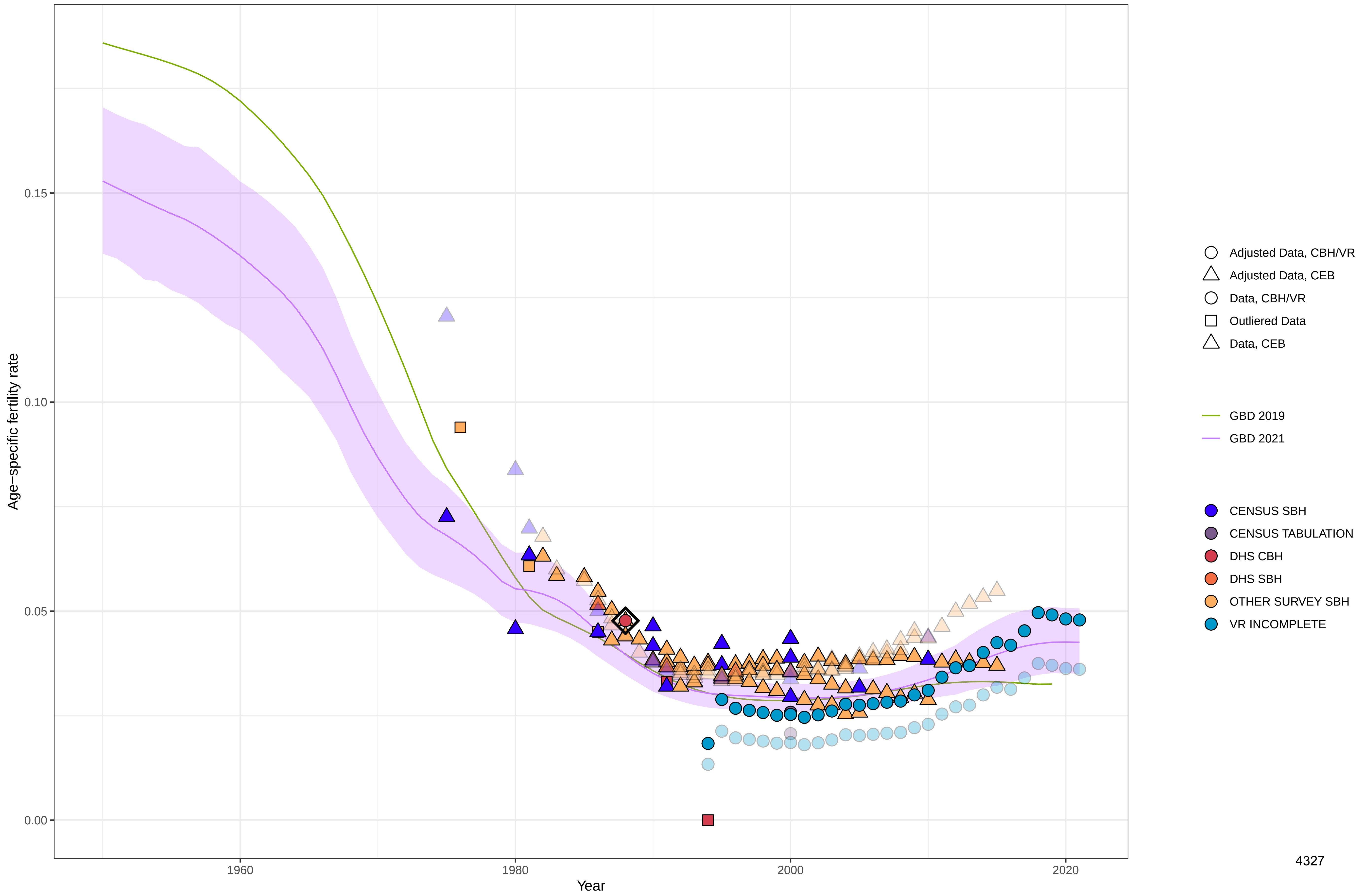

Age−specific fertility fitted trend lines, 1950−2021: Goiás, Ages: 40−44
Data points enclosed with a diamond are reference sources

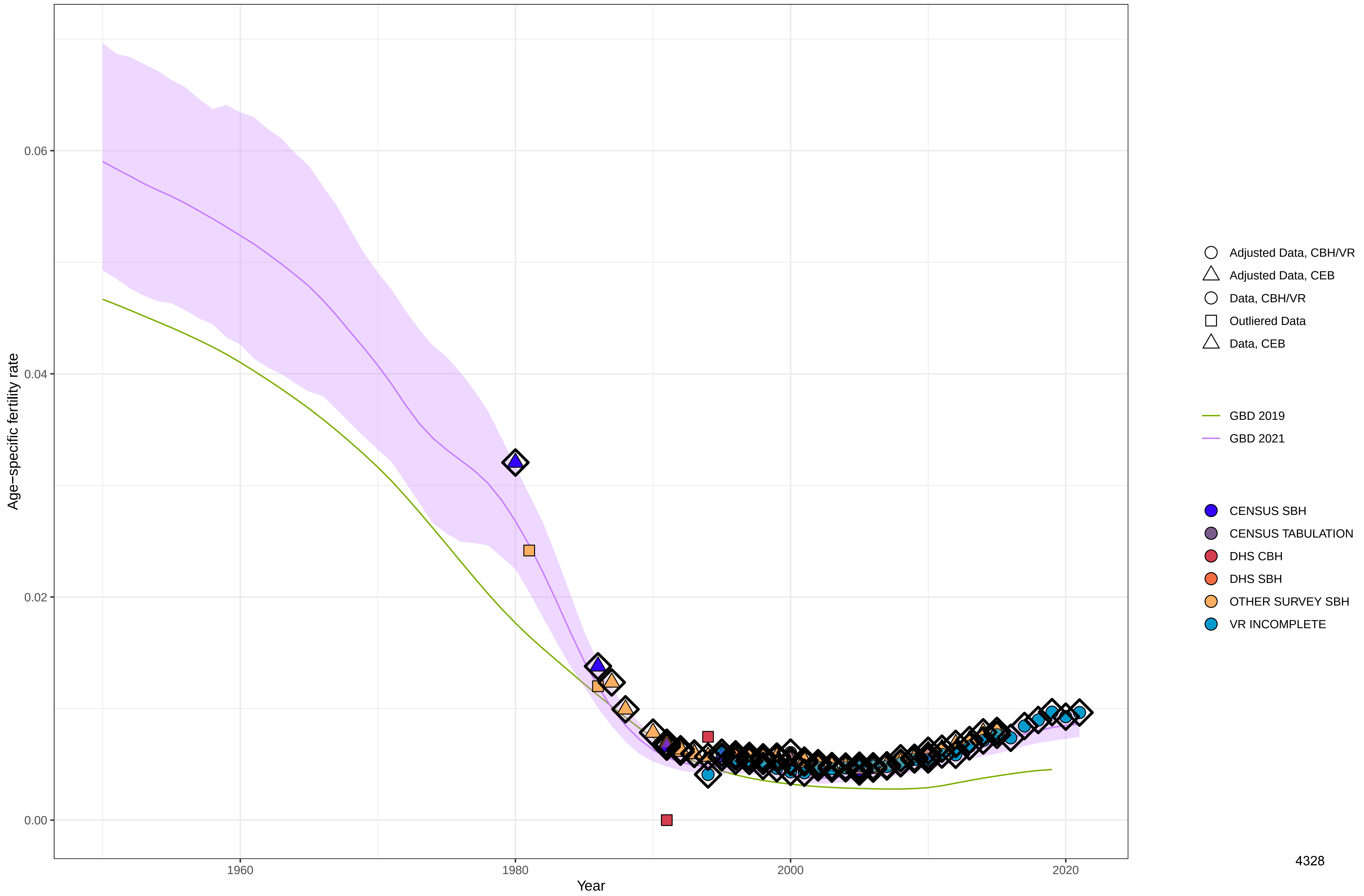

Age−specific fertility fitted trend lines, 1950−2021: Goiás, Ages: 45−49
Data points enclosed with a diamond are reference sources

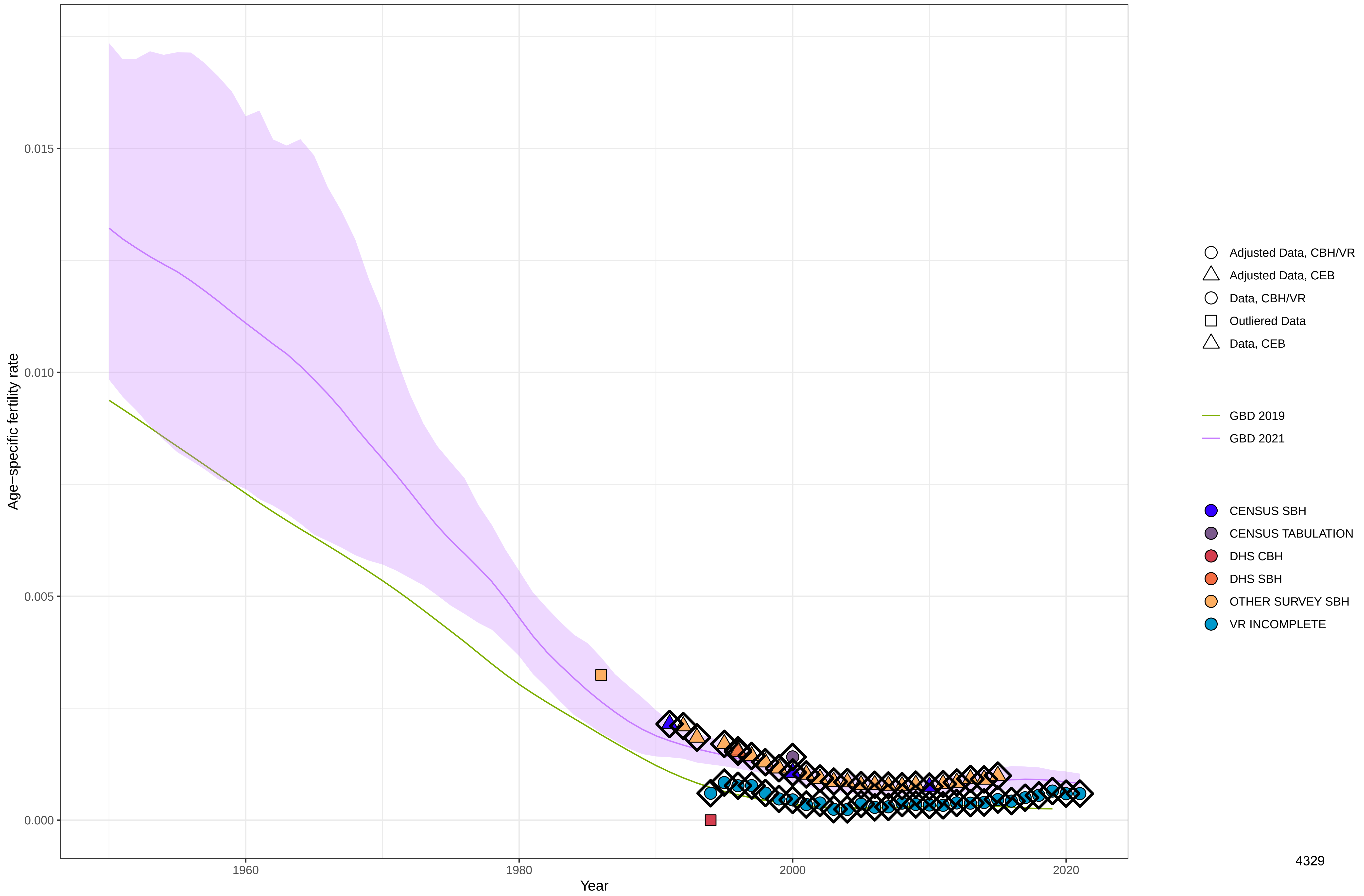

Age−specific fertility fitted trend lines, 1950−2021: Goiás, Ages: 50−54
Data points enclosed with a diamond are reference sources

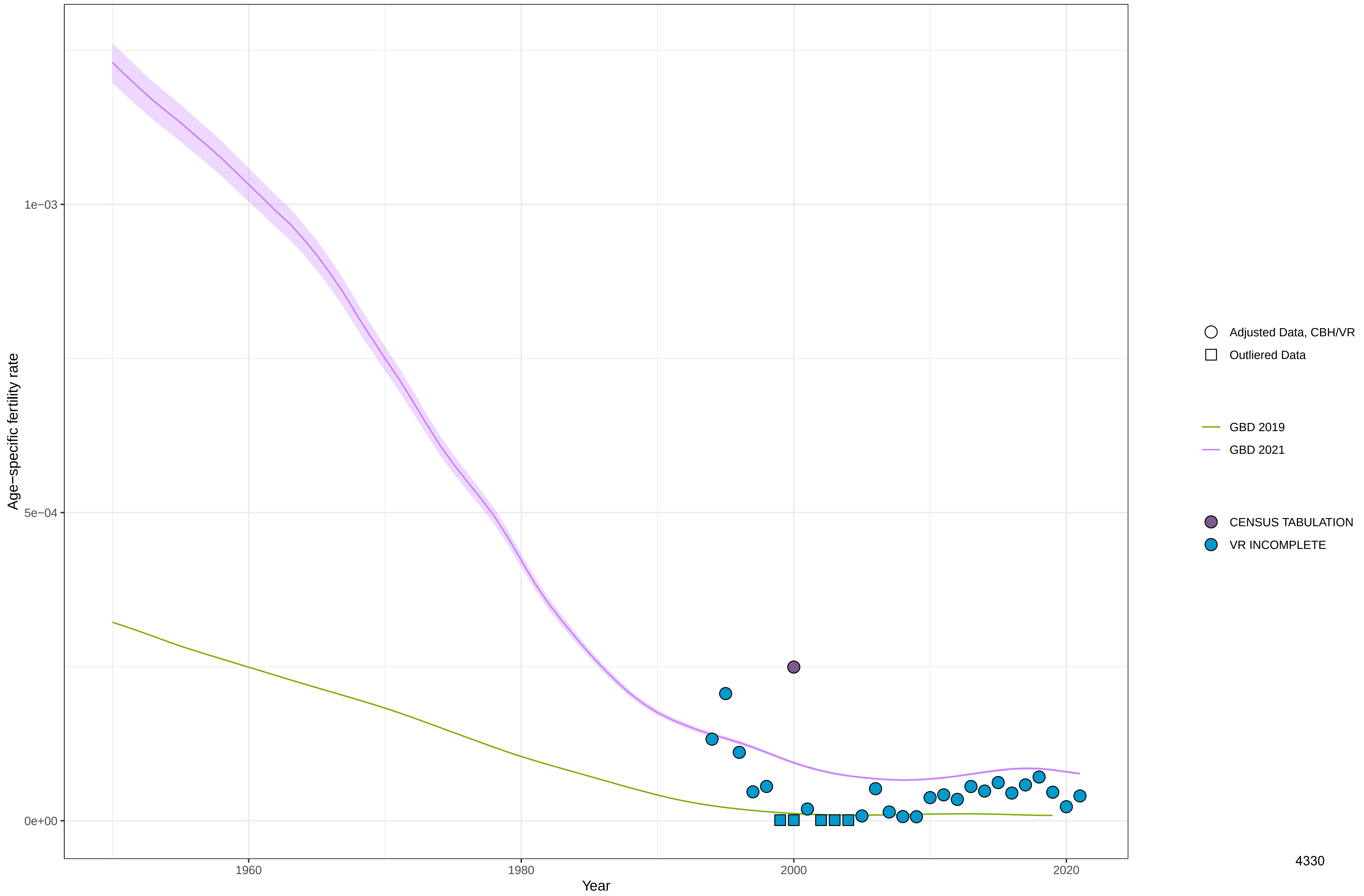

Total fertility rate fitted trend lines, 1950−2021: Goiás TFR
Data points enclosed with a diamond are reference sources

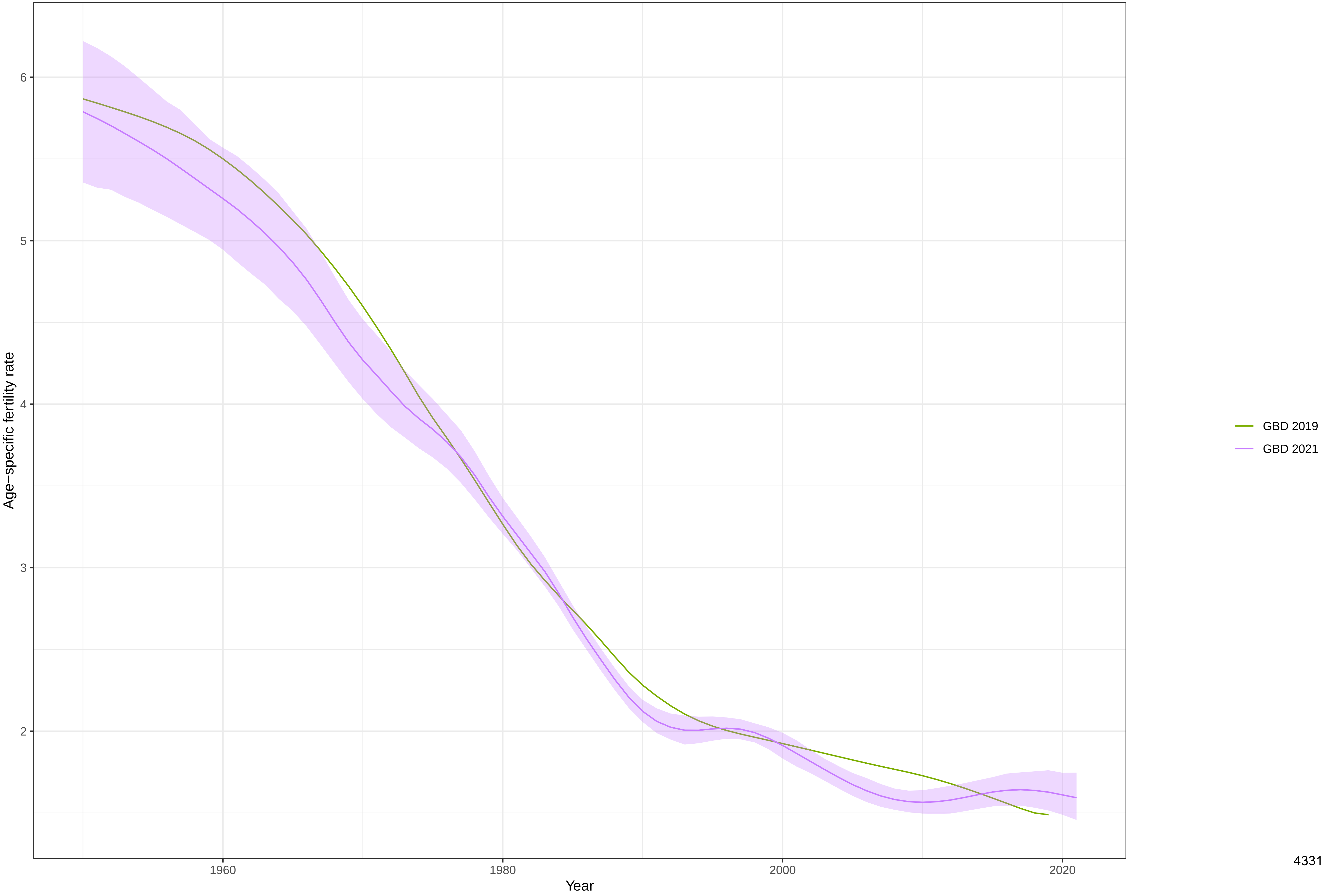

Age−specific fertility fitted trend lines, 1950−2021: Maranhão, Ages: 10−14
Data points enclosed with a diamond are reference sources

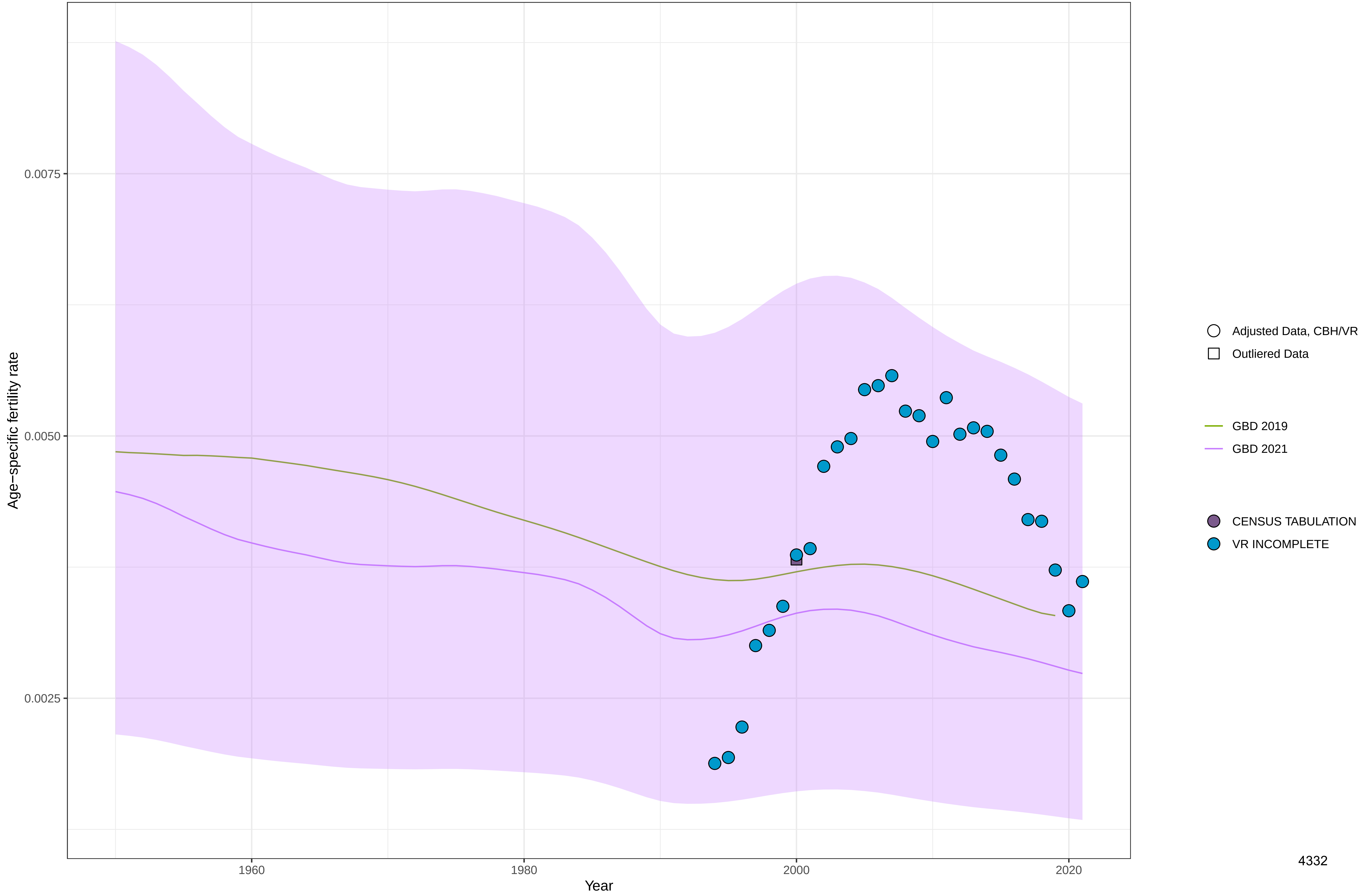

4332

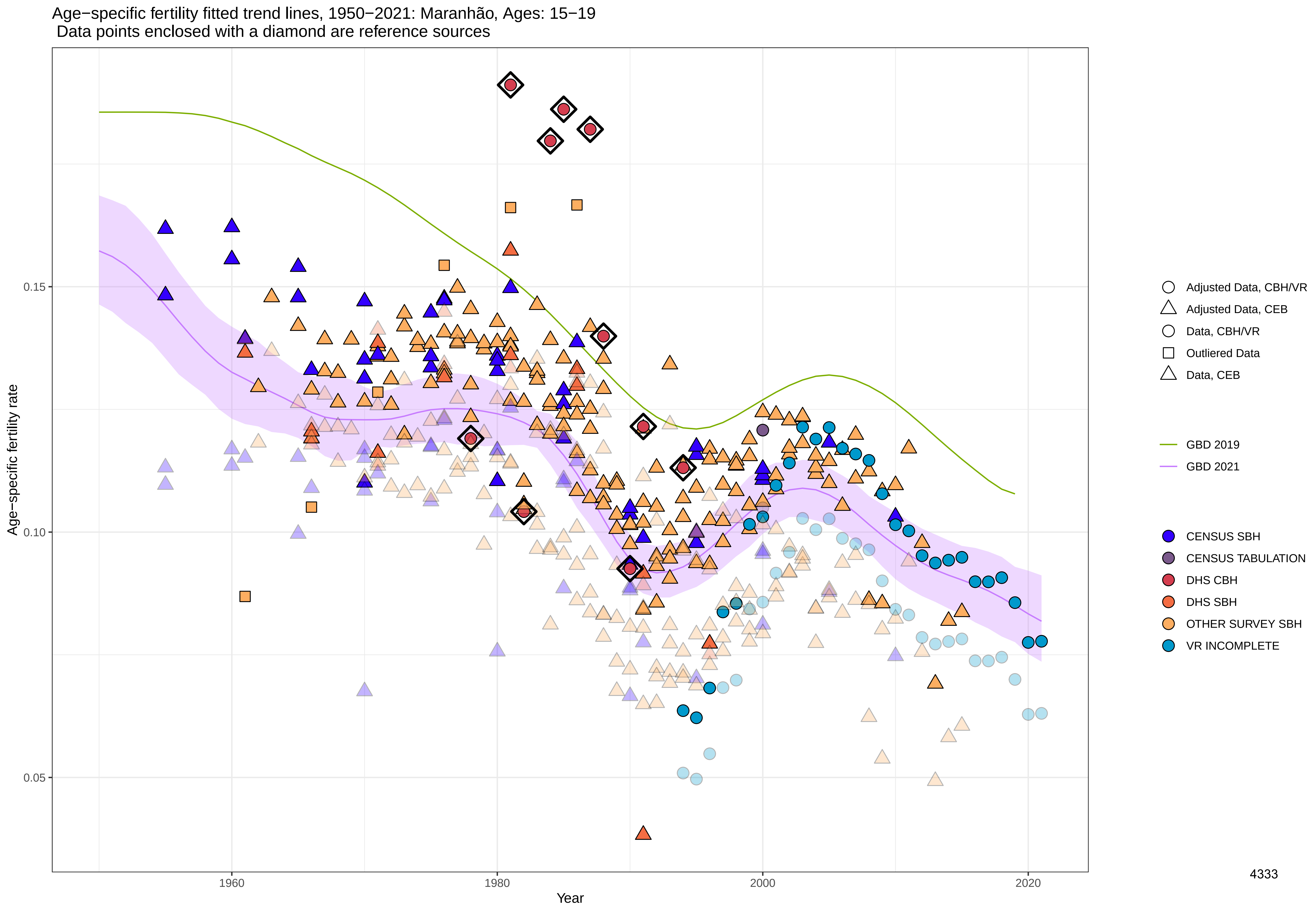

Age−specific fertility fitted trend lines, 1950−2021: Maranhão, Ages: 15−19
Data points enclosed with a diamond are reference sources
Age−specific fertility rate
0.05
0.10
0.15
Year
1960
1980
2000
2020
Adjusted Data, CBH/VR
Adjusted Data, CEB
Data, CBH/VR
Outliered Data
Data, CEB
GBD 2019
GBD 2021
CENSUS SBH
CENSUS TABULATION
DHS CBH
DHS SBH
OTHER SURVEY SBH
VR INCOMPLETE

Age−specific fertility fitted trend lines, 1950−2021: Maranhão, Ages: 20−24
Data points enclosed with a diamond are reference sources

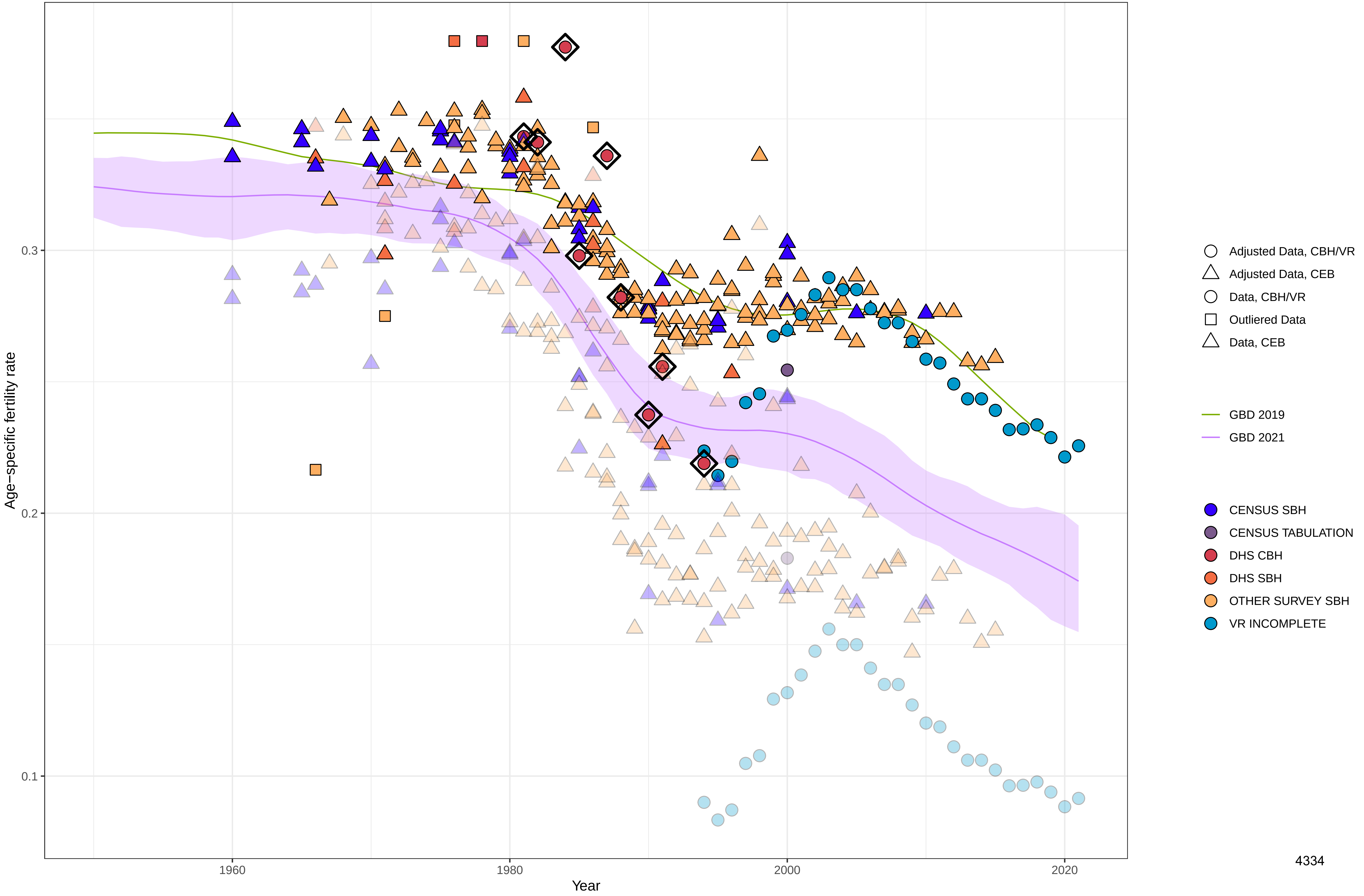

Age−specific fertility fitted trend lines, 1950−2021: Maranhão, Ages: 25−29
Data points enclosed with a diamond are reference sources

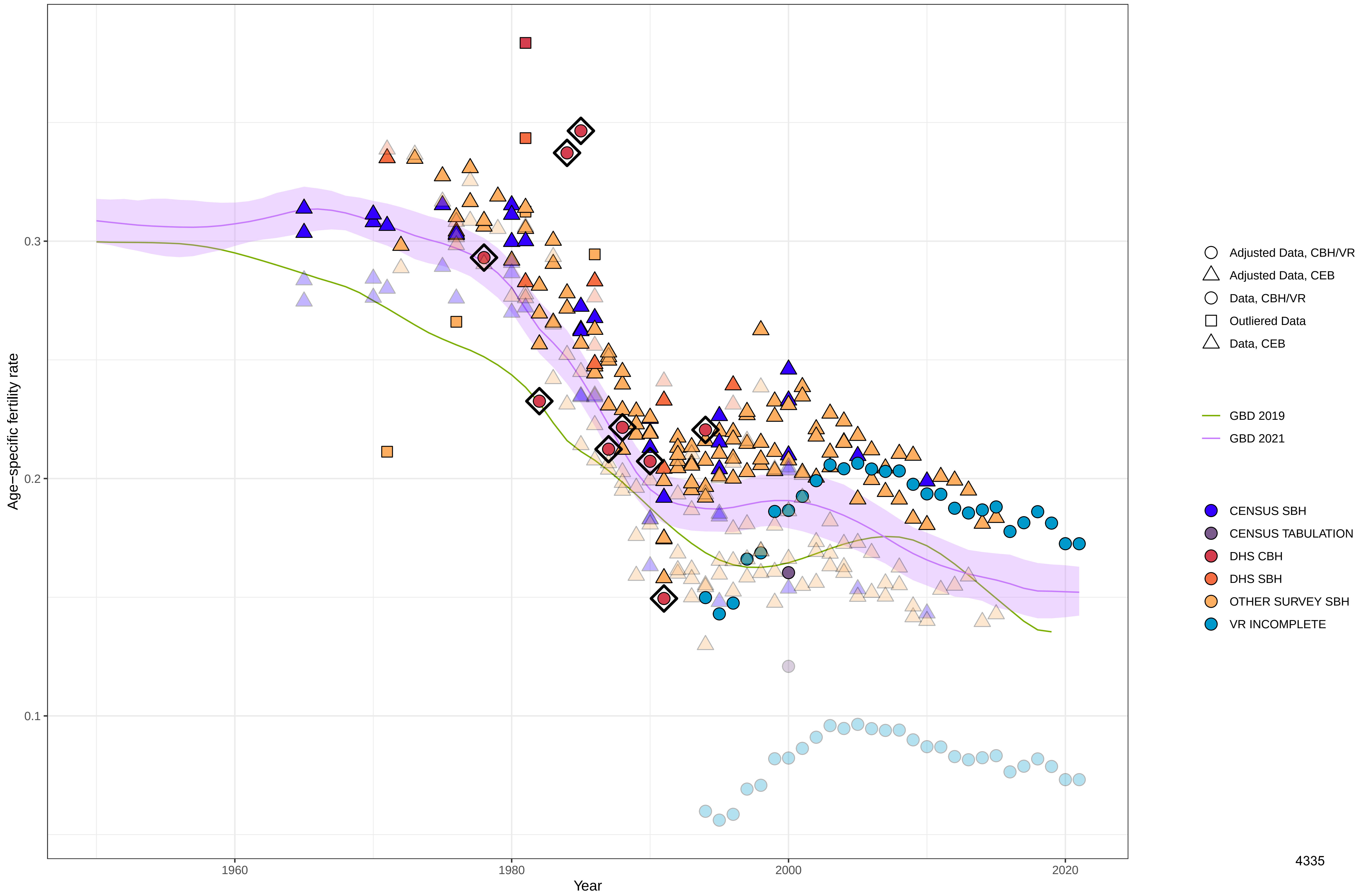

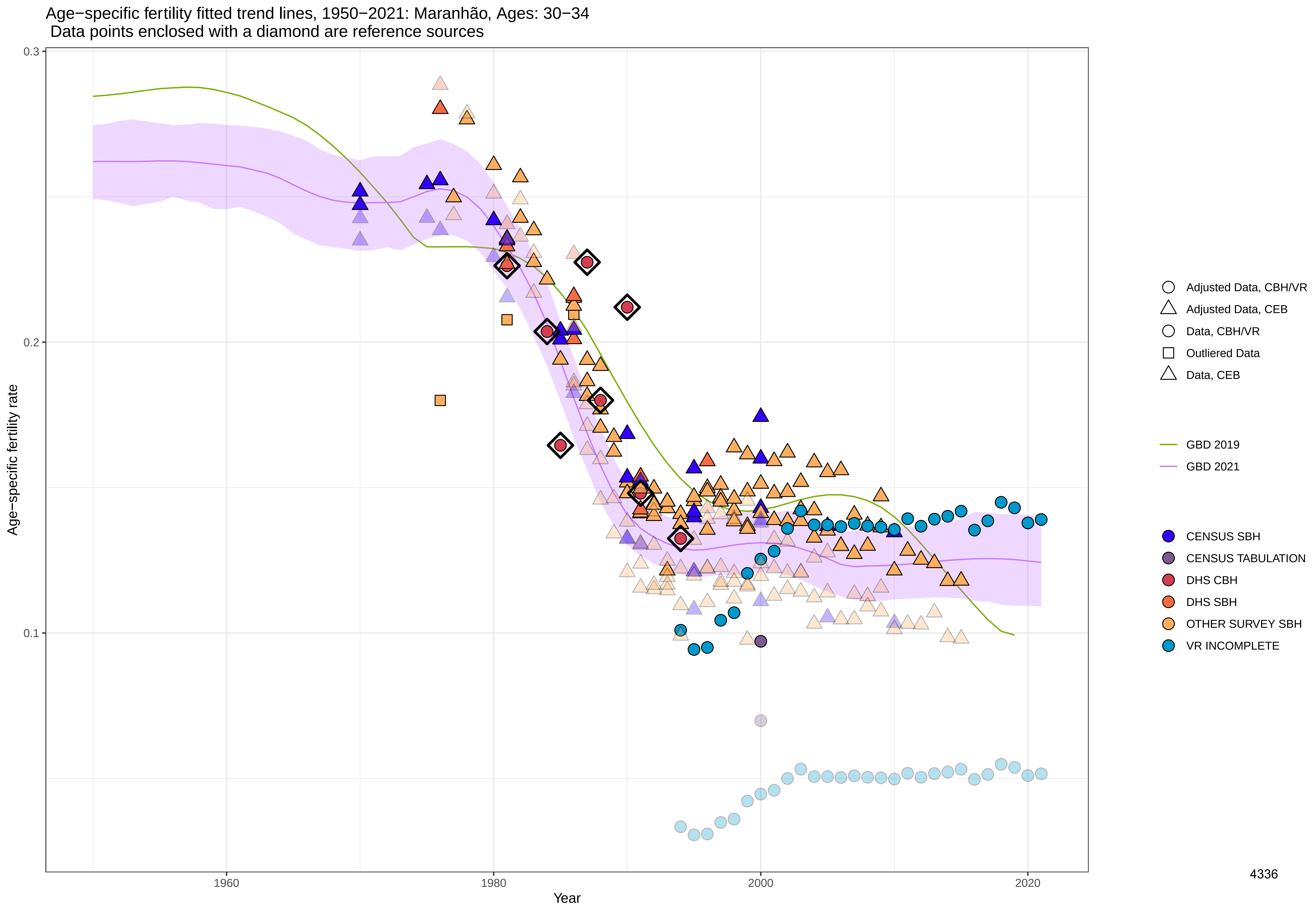

Age−specific fertility fitted trend lines, 1950−2021: Maranhão, Ages: 30−34
Data points enclosed with a diamond are reference sources
Age−specific fertility rate
Year
0.3
0.2
0.1
1960
1980
2000
2020
Adjusted Data, CBH/VR
Adjusted Data, CEB
Data, CBH/VR
Outliered Data
Data, CEB
GBD 2019
GBD 2021
CENSUS SBH
CENSUS TABULATION
DHS CBH
DHS SBH
OTHER SURVEY SBH
VR INCOMPLETE

Age−specific fertility fitted trend lines, 1950−2021: Maranhão, Ages: 35−39
Data points enclosed with a diamond are reference sources

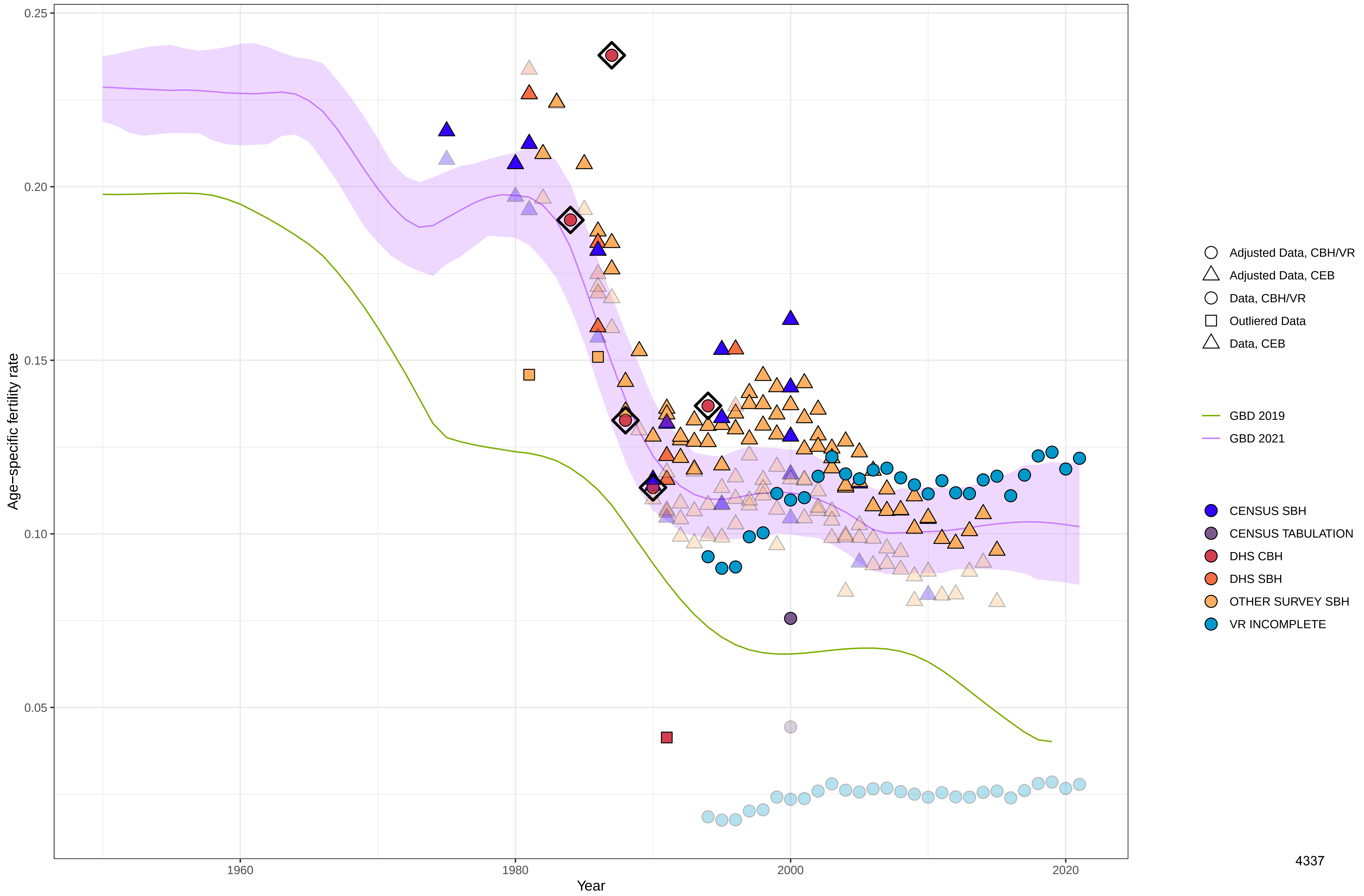

Age−specific fertility fitted trend lines, 1950−2021: Maranhão, Ages: 40−44
Data points enclosed with a diamond are reference sources

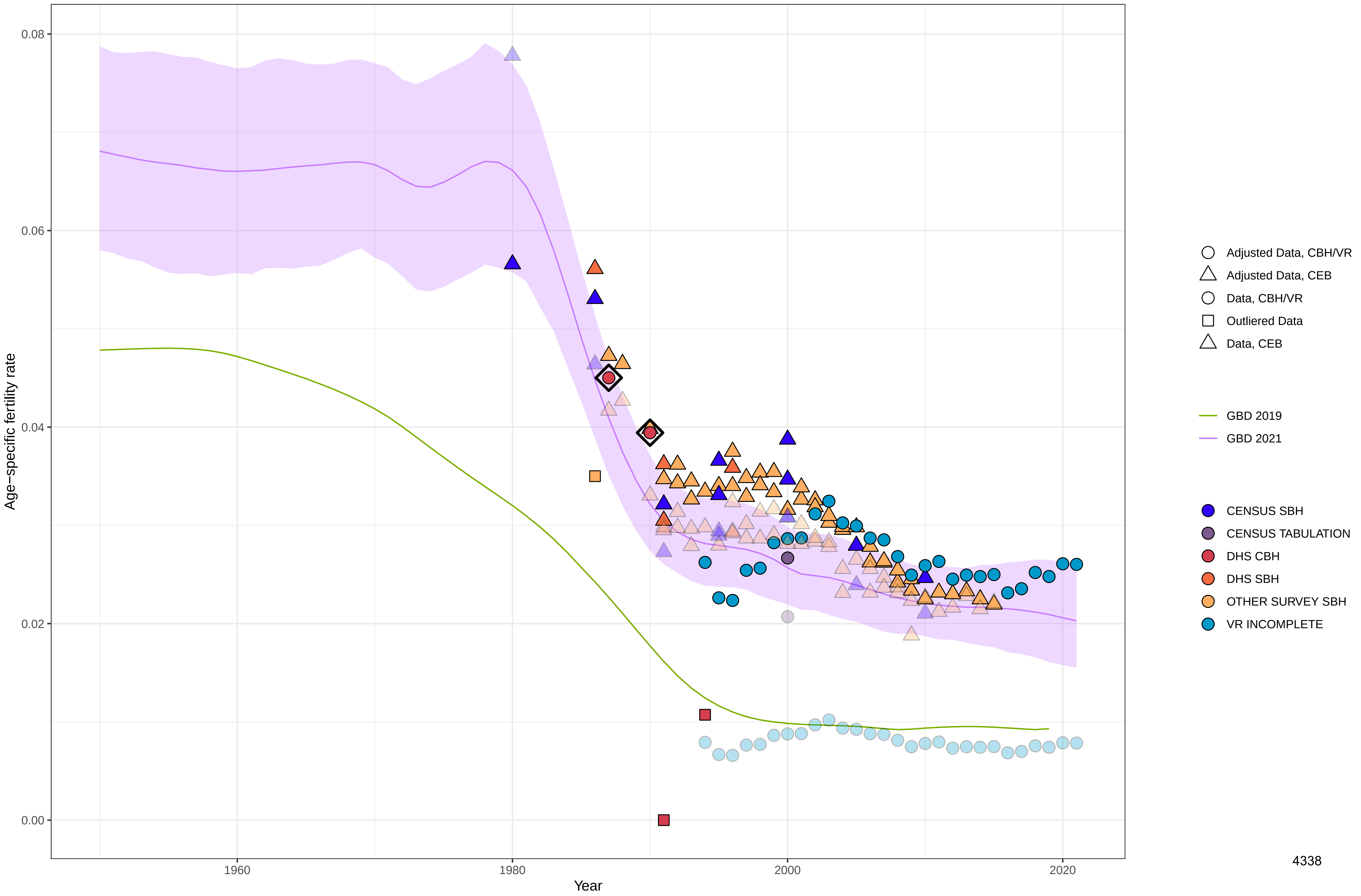

4338

Age−specific fertility fitted trend lines, 1950−2021: Maranhão, Ages: 45−49
Data points enclosed with a diamond are reference sources

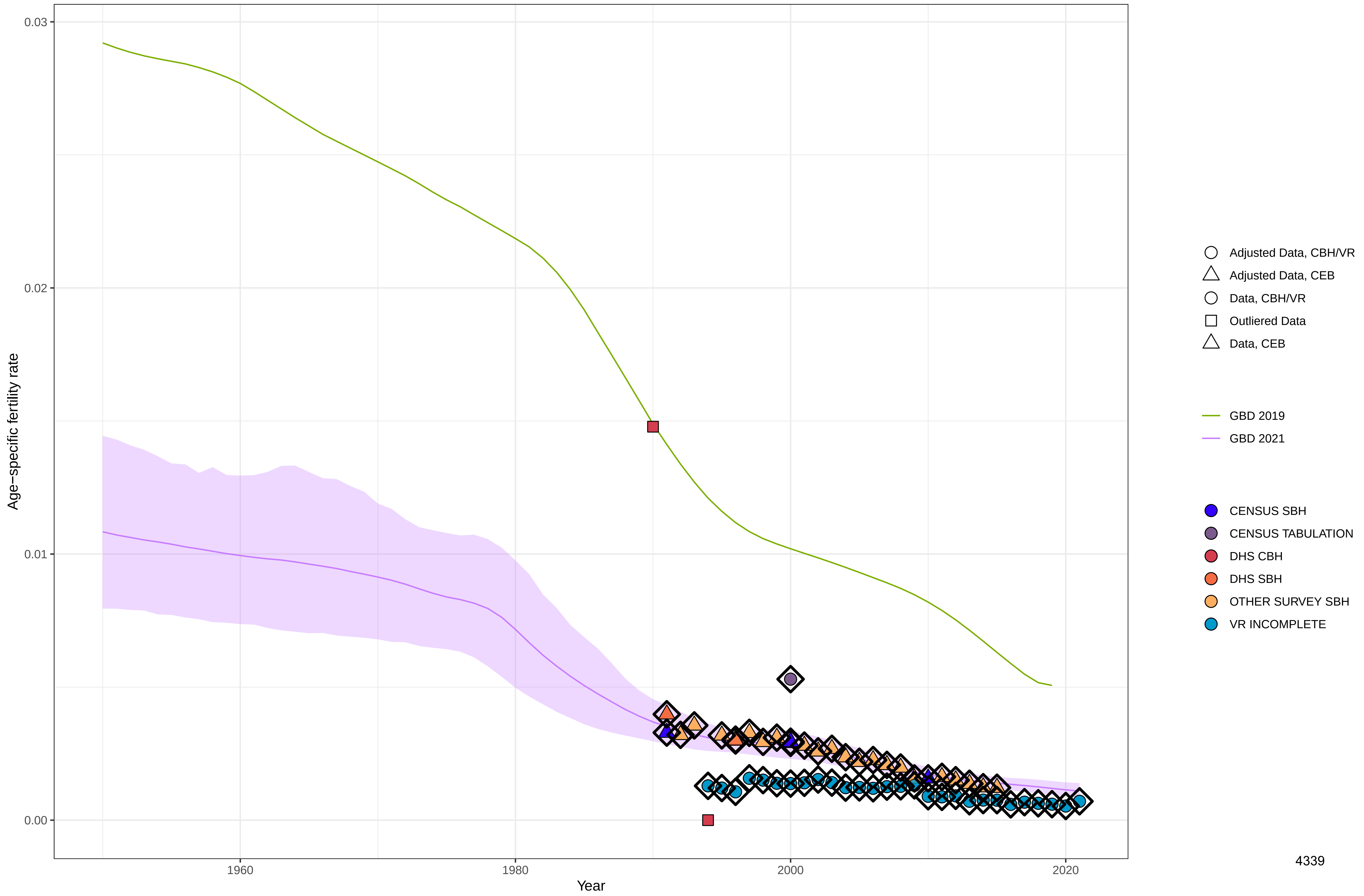

Age−specific fertility fitted trend lines, 1950−2021: Maranhão, Ages: 50−54
Data points enclosed with a diamond are reference sources

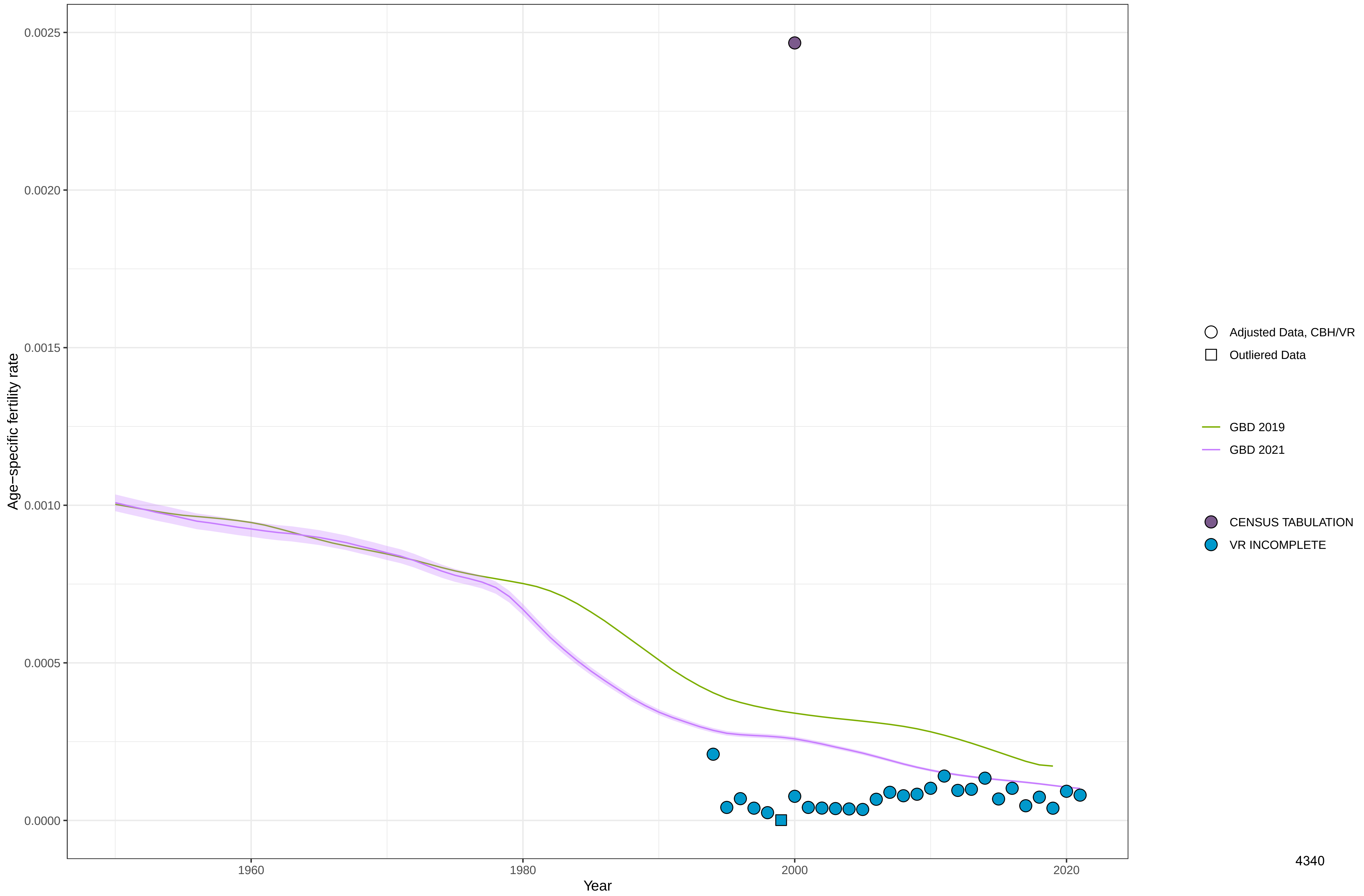

Total fertility rate fitted trend lines, 1950−2021: Maranhão TFR
Data points enclosed with a diamond are reference sources

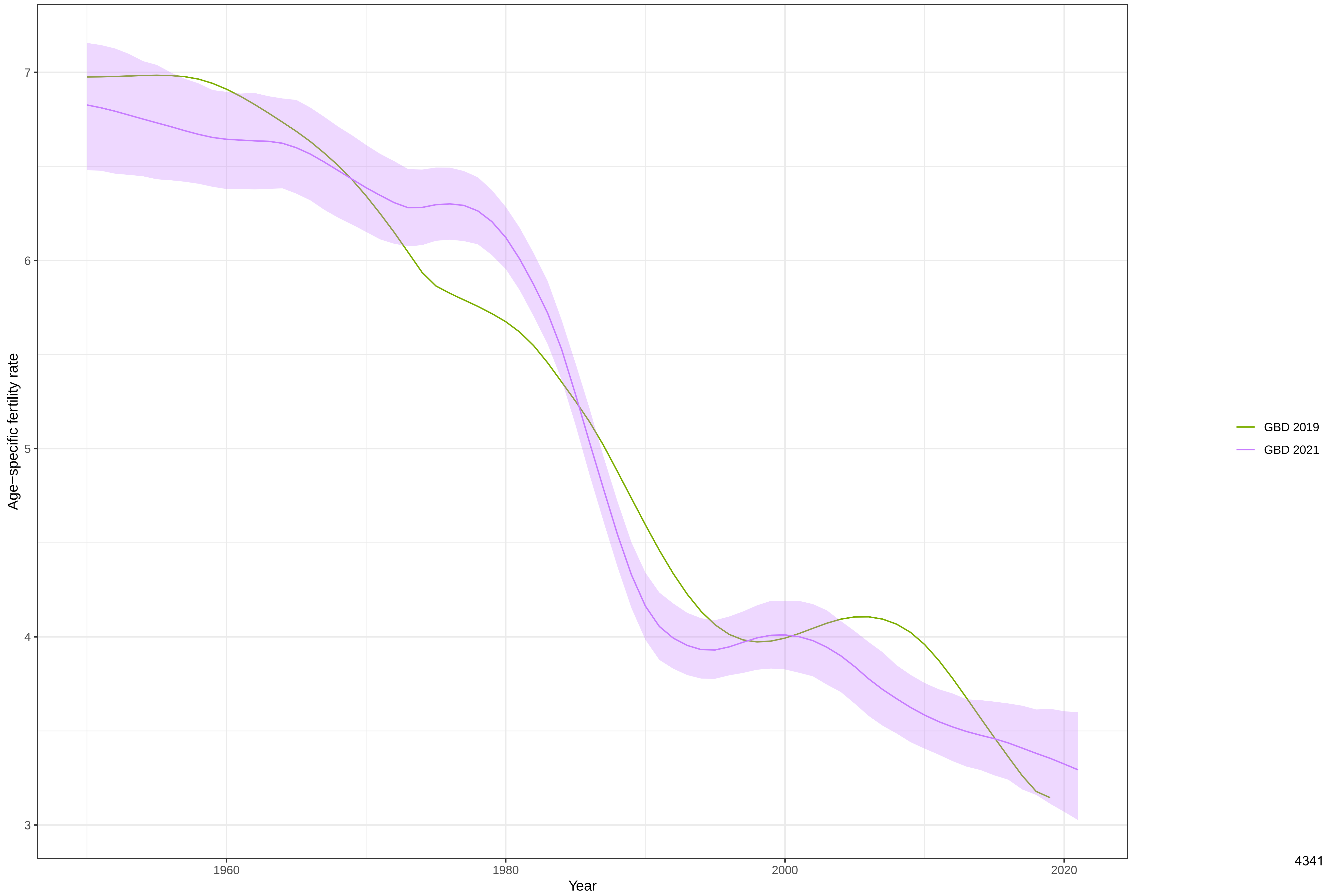

Age−specific fertility fitted trend lines, 1950−2021: Mato Grosso, Ages: 10−14
Data points enclosed with a diamond are reference sources

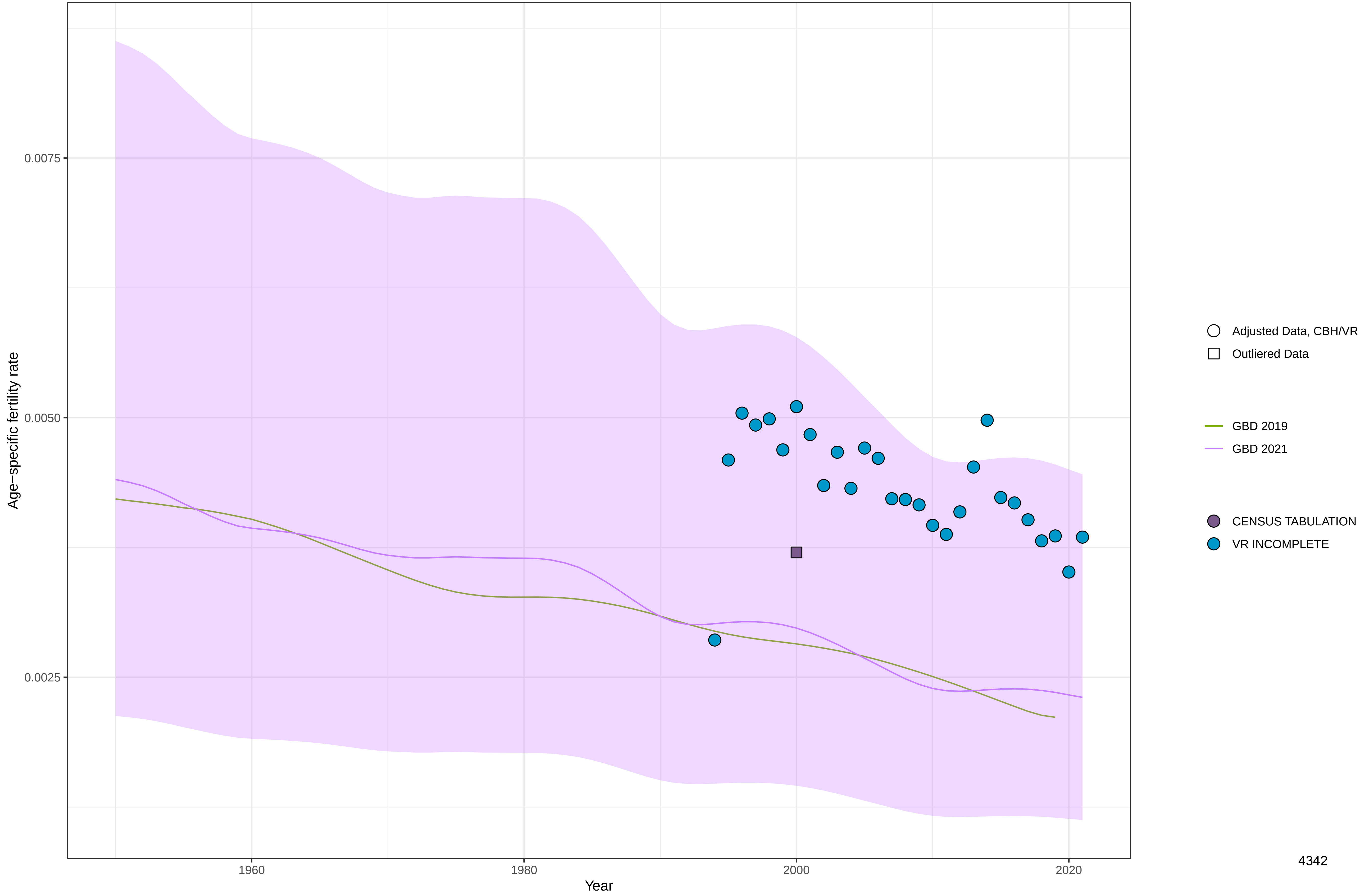

Age−specific fertility fitted trend lines, 1950−2021: Mato Grosso, Ages: 15−19
Data points enclosed with a diamond are reference sources

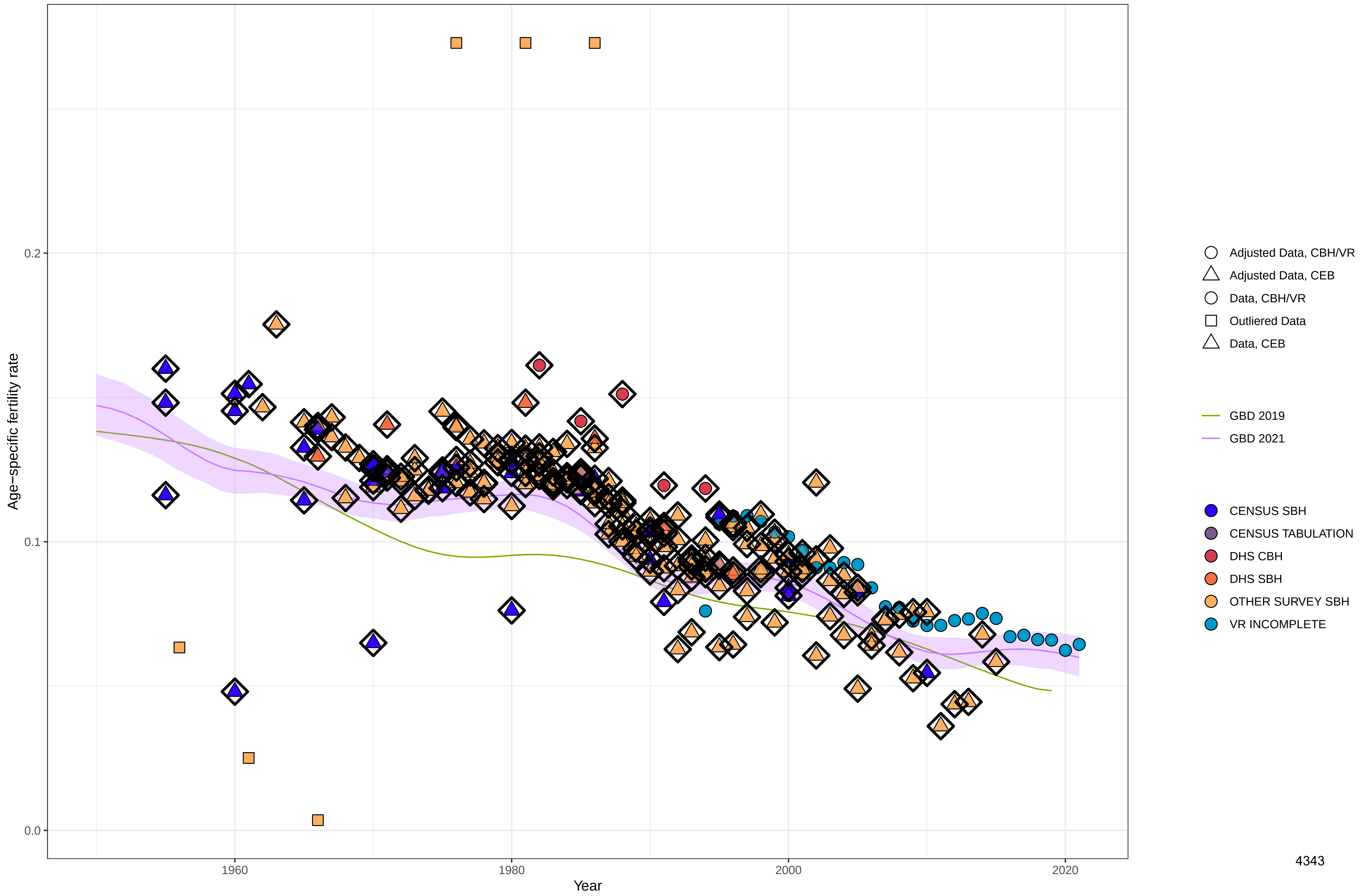

Age−specific fertility fitted trend lines, 1950−2021: Mato Grosso, Ages: 20−24
Data points enclosed with a diamond are reference sources

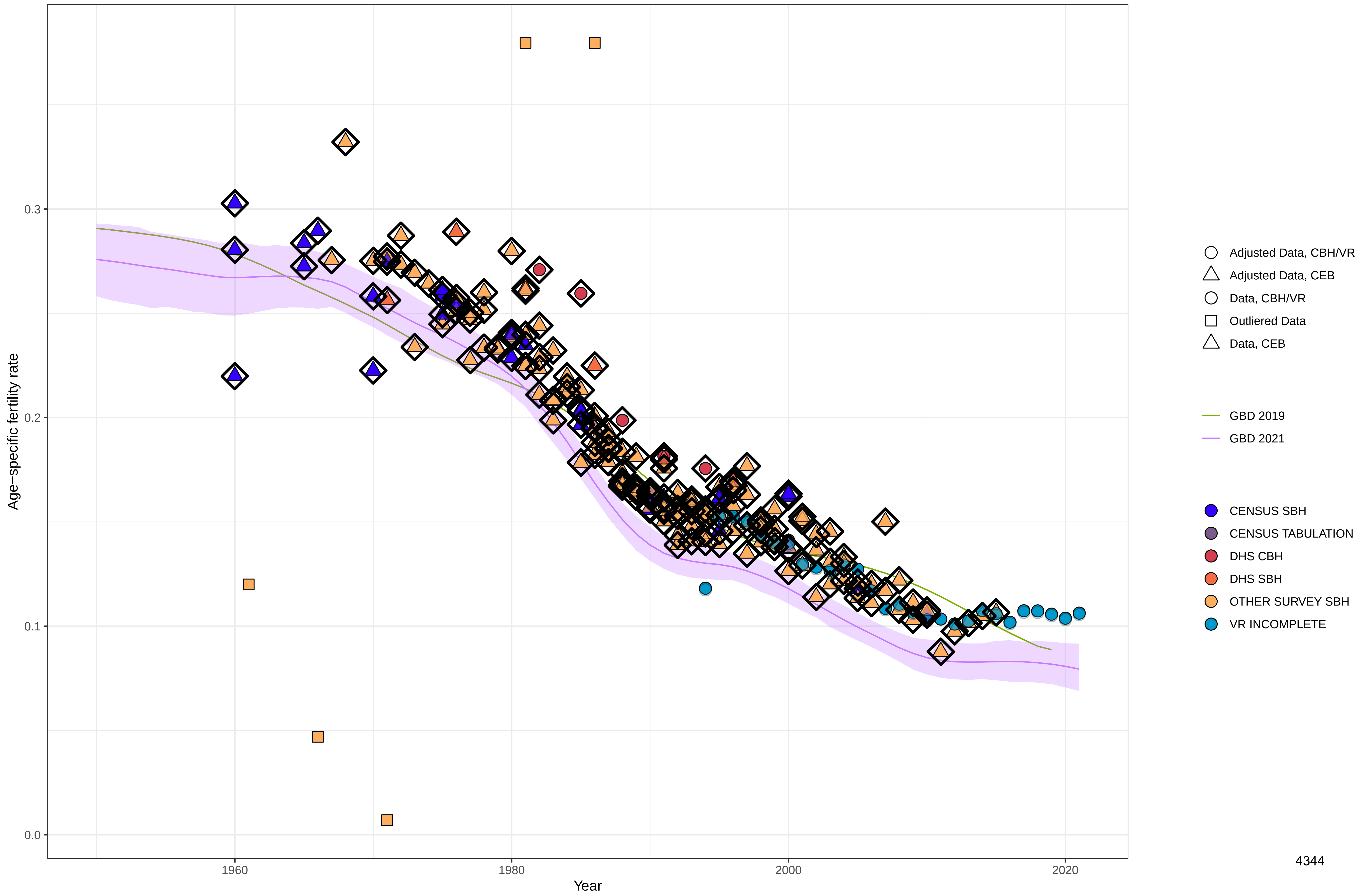

Age−specific fertility fitted trend lines, 1950−2021: Mato Grosso, Ages: 25−29
Data points enclosed with a diamond are reference sources

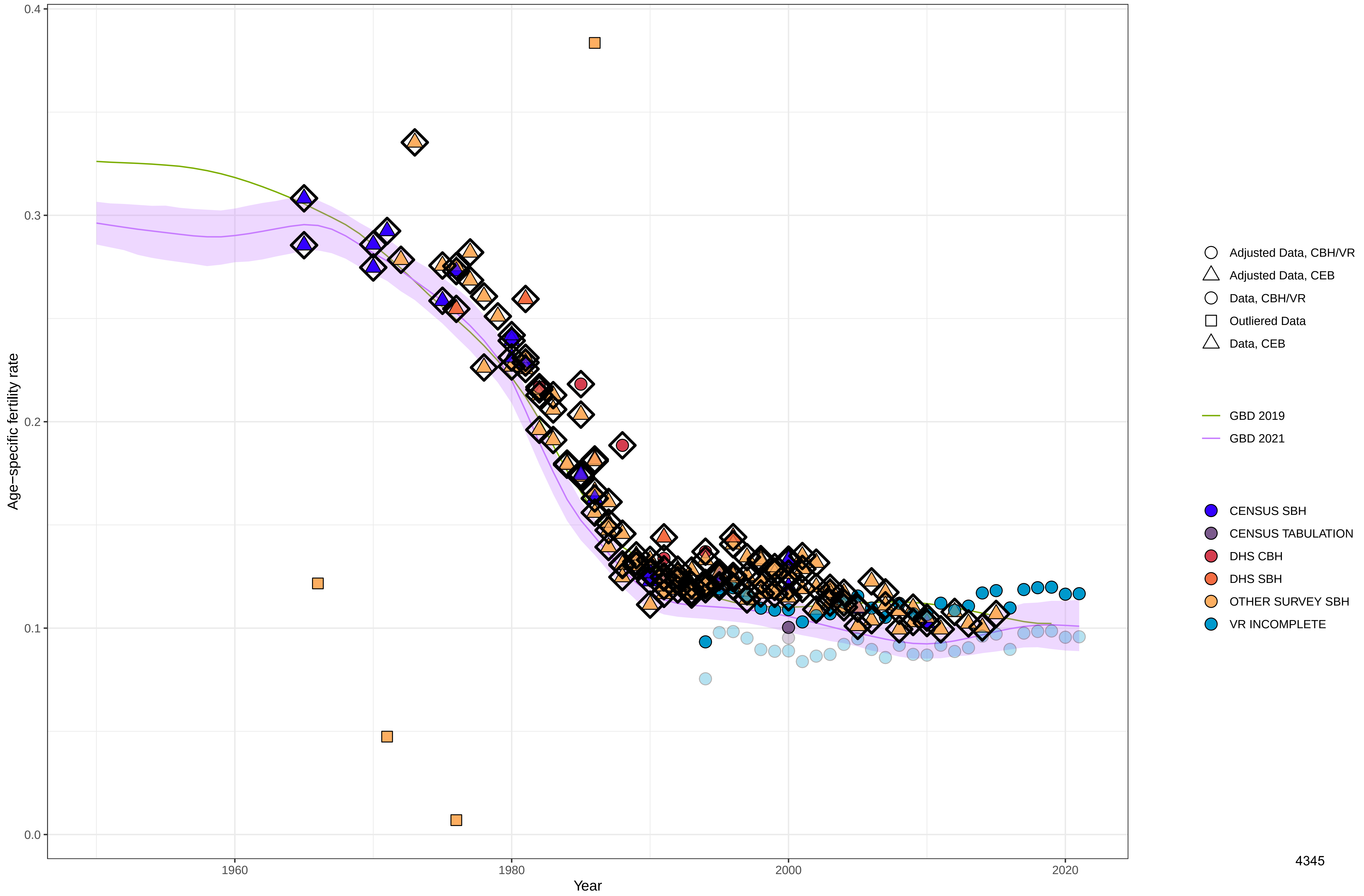

Age−specific fertility fitted trend lines, 1950−2021: Mato Grosso, Ages: 30−34
Data points enclosed with a diamond are reference sources

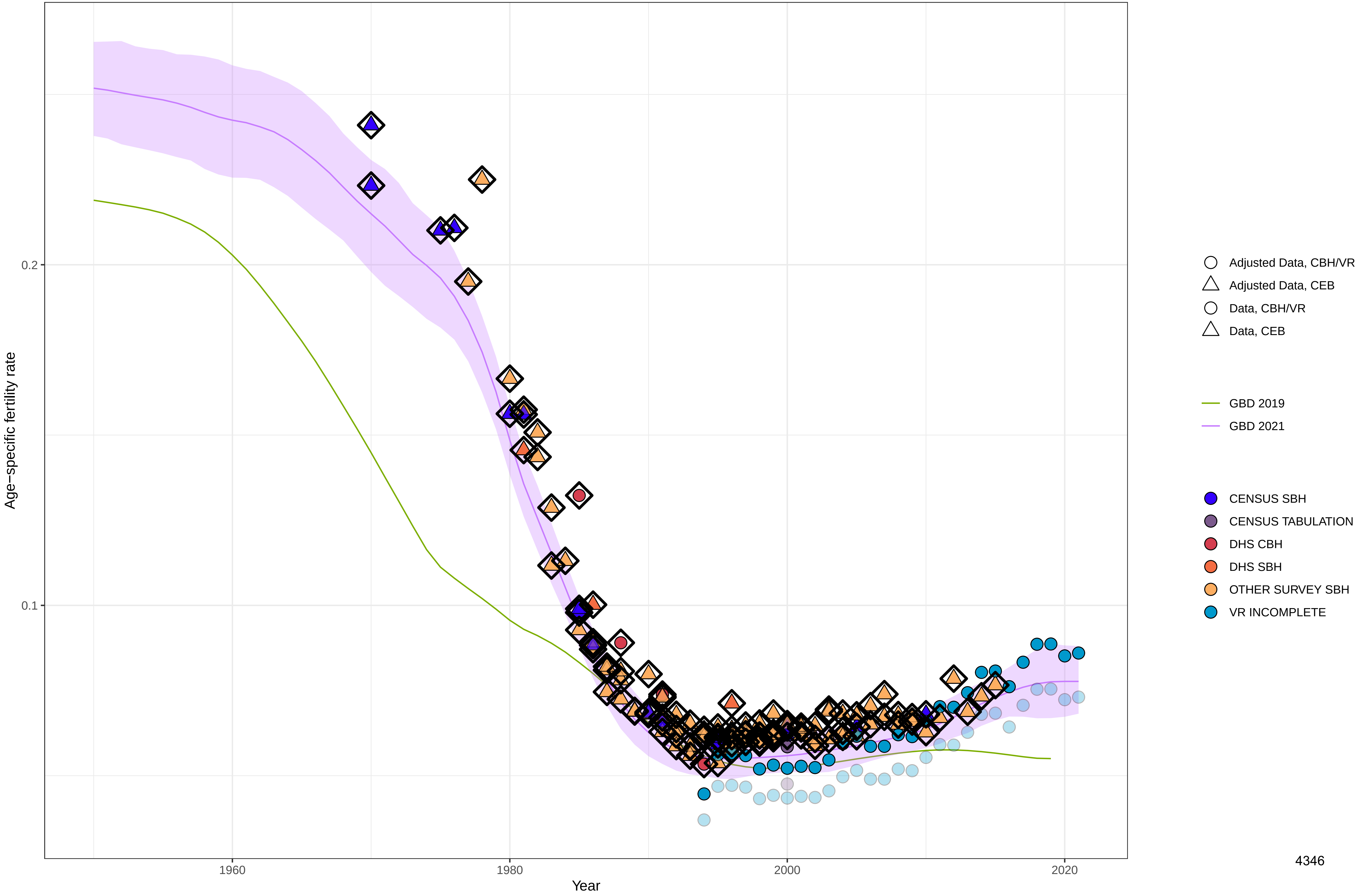

Age−specific fertility fitted trend lines, 1950−2021: Mato Grosso, Ages: 35−39
Data points enclosed with a diamond are reference sources

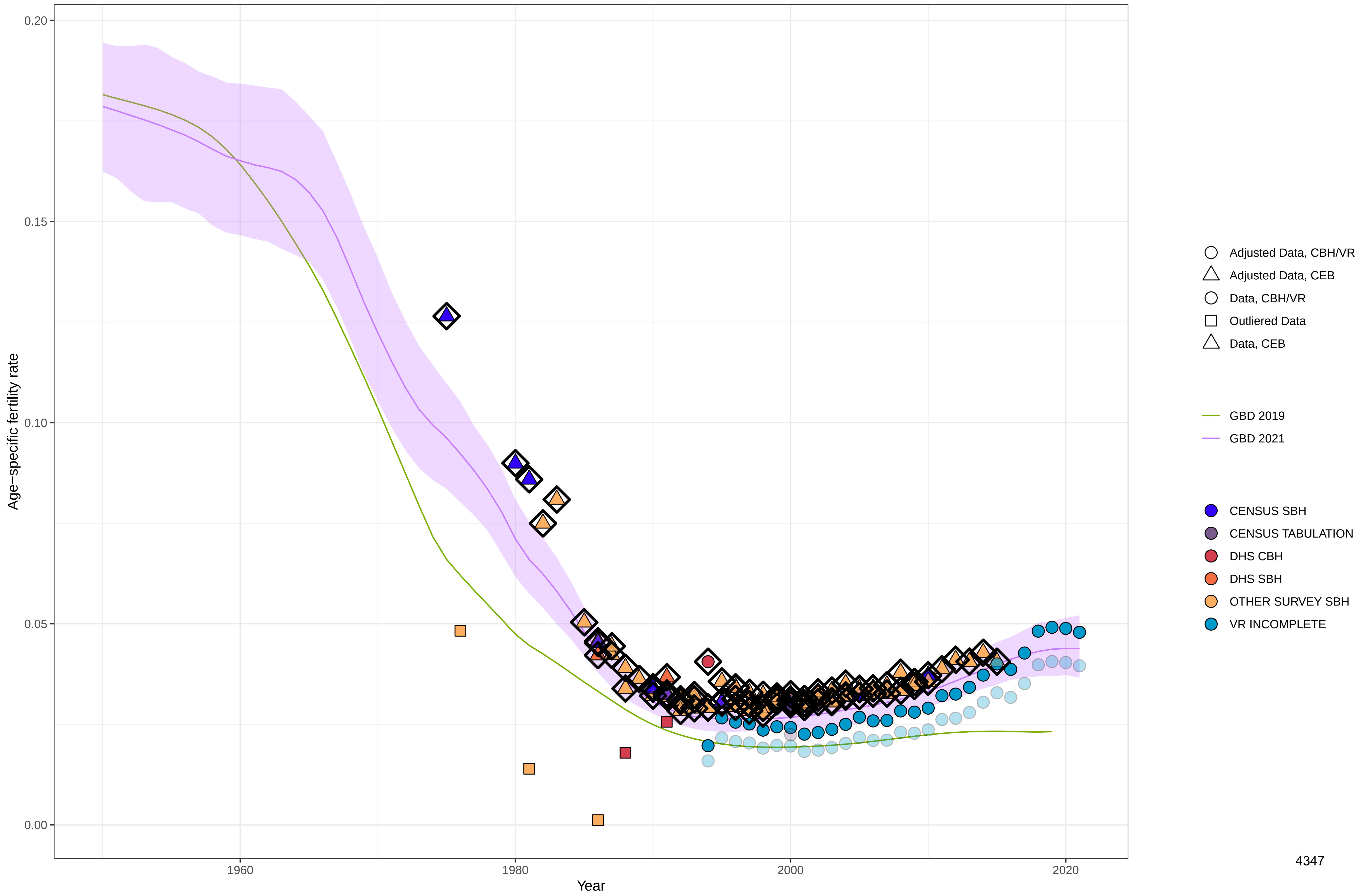

Age−specific fertility fitted trend lines, 1950−2021: Mato Grosso, Ages: 40−44
Data points enclosed with a diamond are reference sources

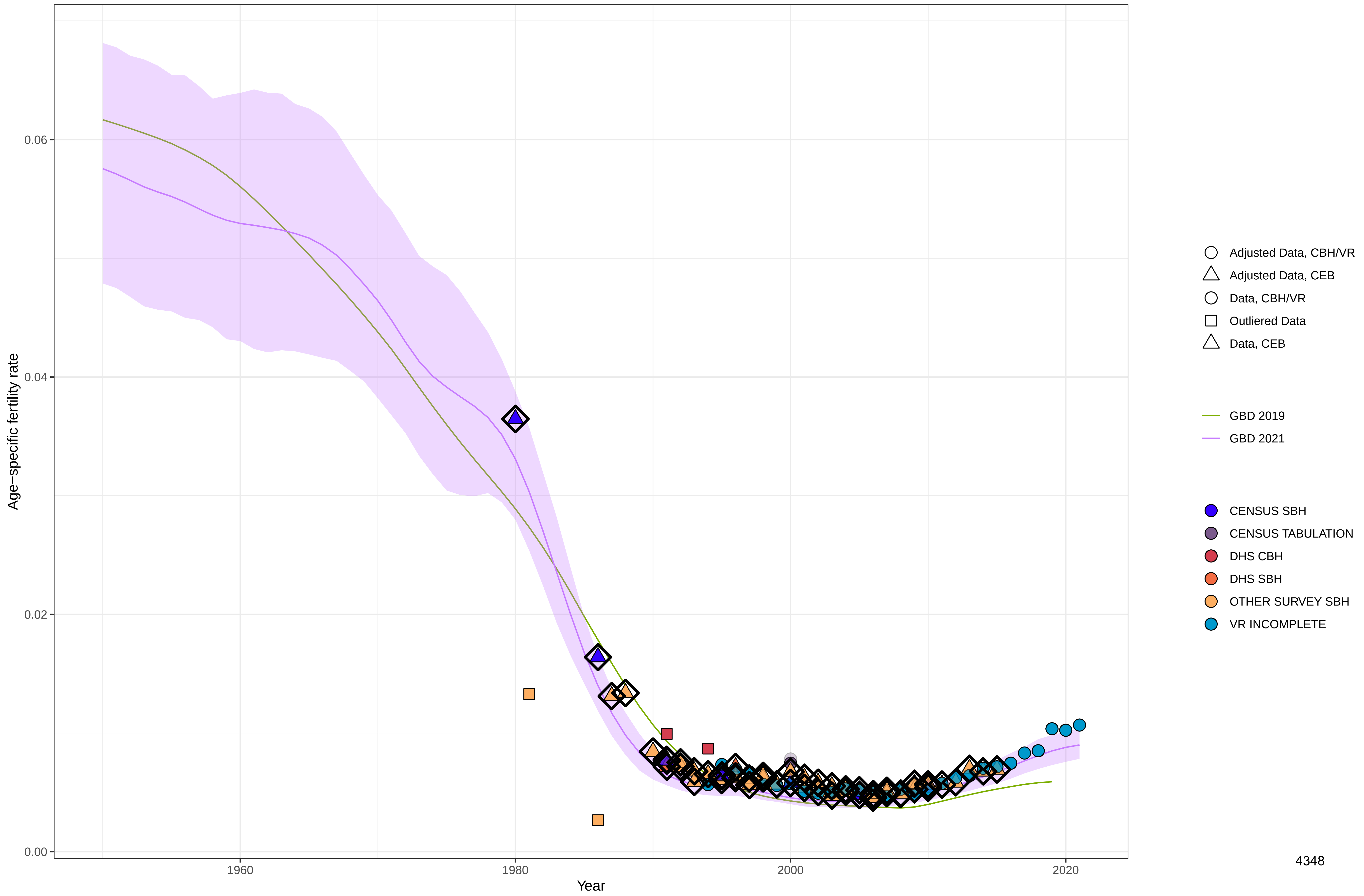

Age−specific fertility fitted trend lines, 1950−2021: Mato Grosso, Ages: 45−49
Data points enclosed with a diamond are reference sources

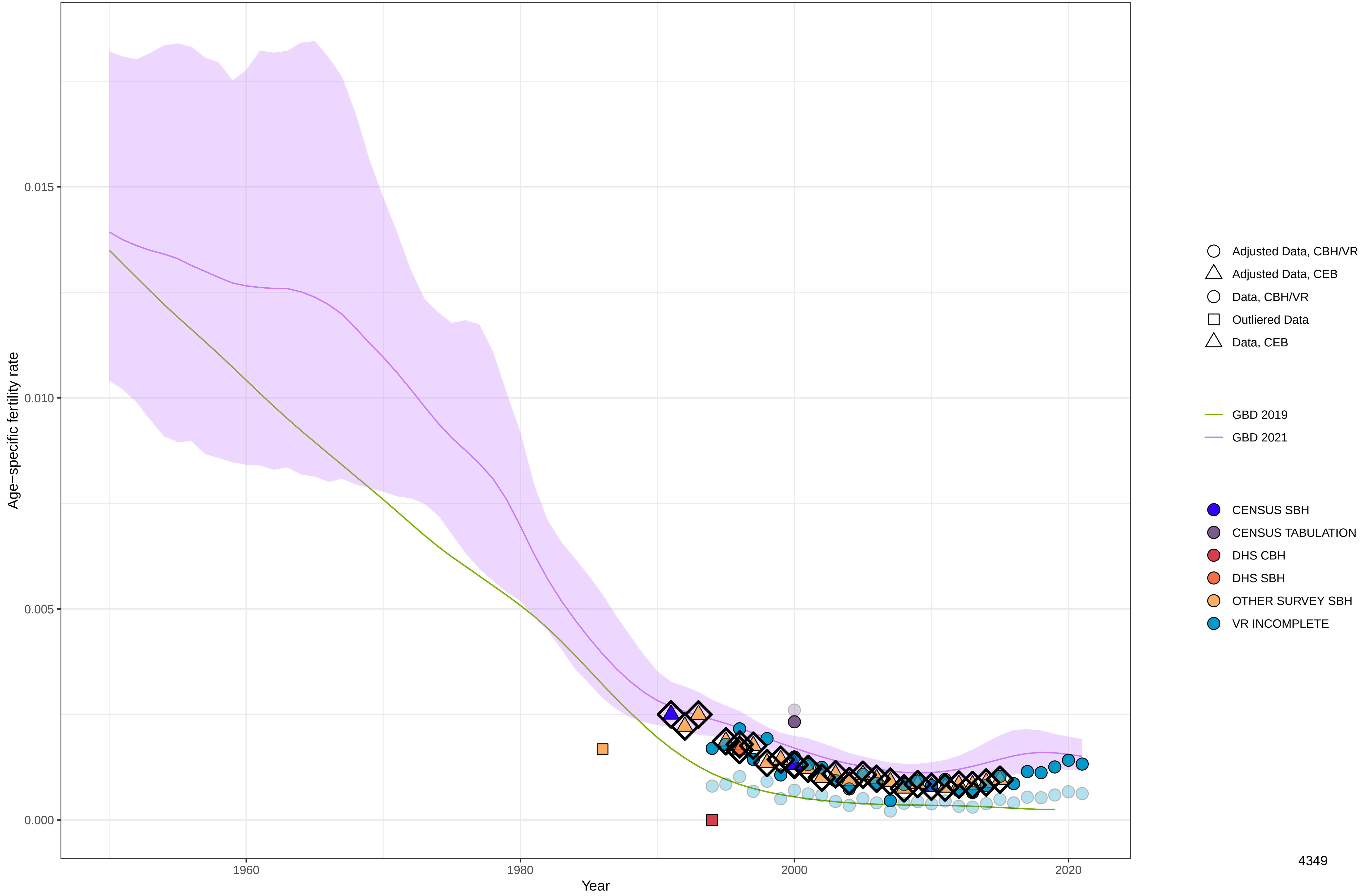

Age−specific fertility fitted trend lines, 1950−2021: Mato Grosso, Ages: 50−54
Data points enclosed with a diamond are reference sources

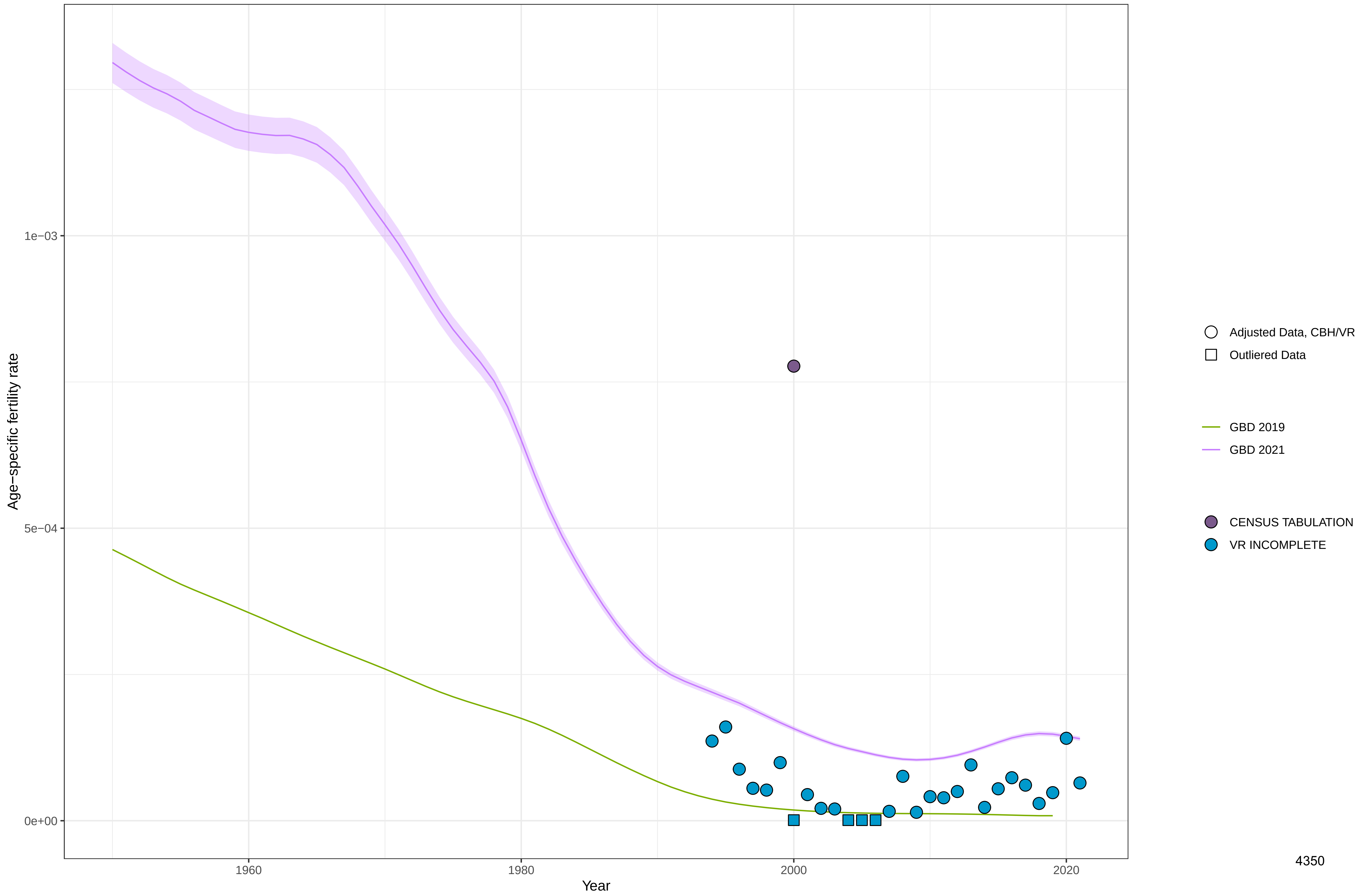

Total fertility rate fitted trend lines, 1950−2021: Mato Grosso TFR
Data points enclosed with a diamond are reference sources

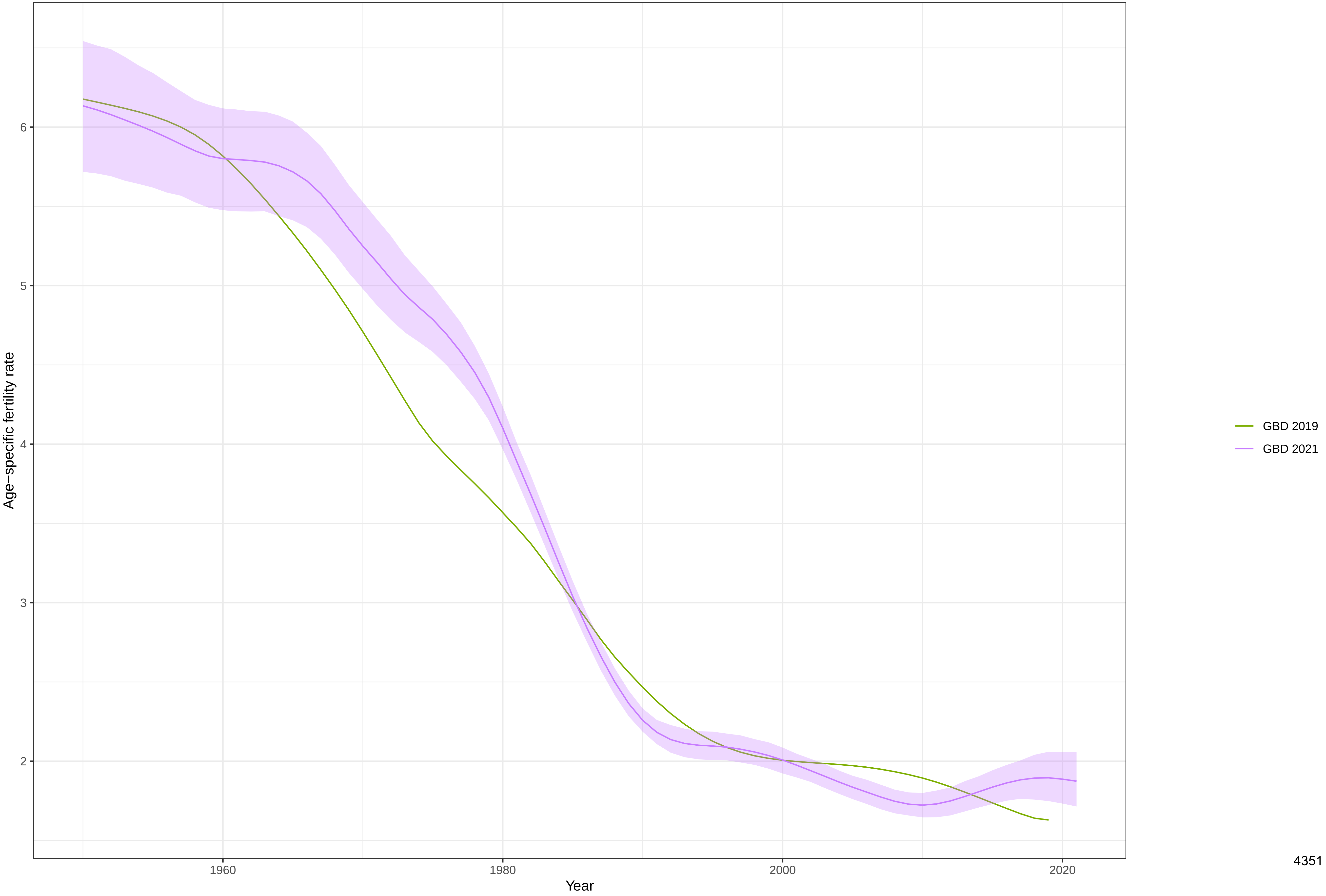

Age−specific fertility fitted trend lines, 1950−2021: Mato Grosso do Sul, Ages: 10−14
Data points enclosed with a diamond are reference sources

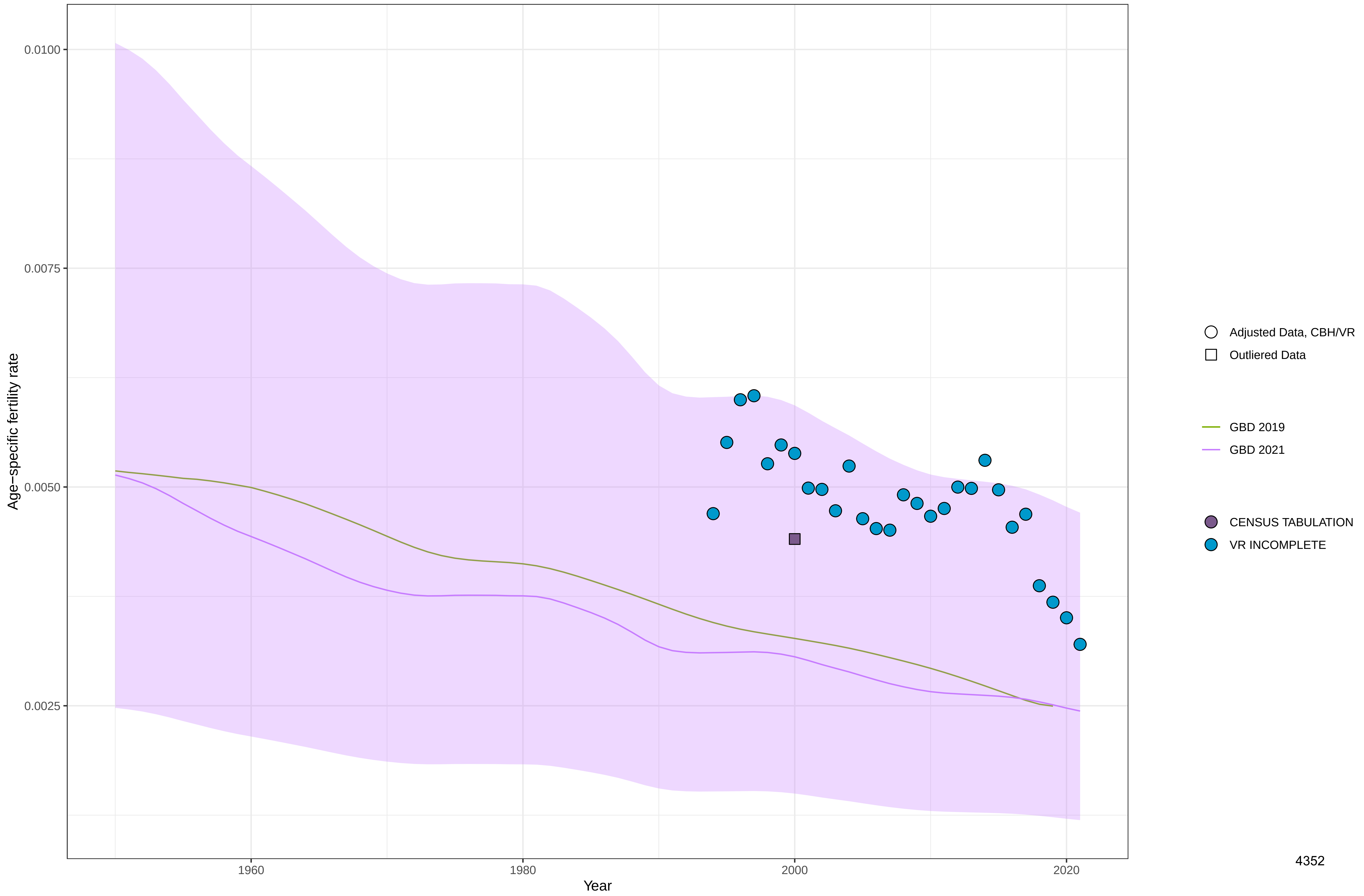

Age−specific fertility fitted trend lines, 1950−2021: Mato Grosso do Sul, Ages: 15−19
Data points enclosed with a diamond are reference sources

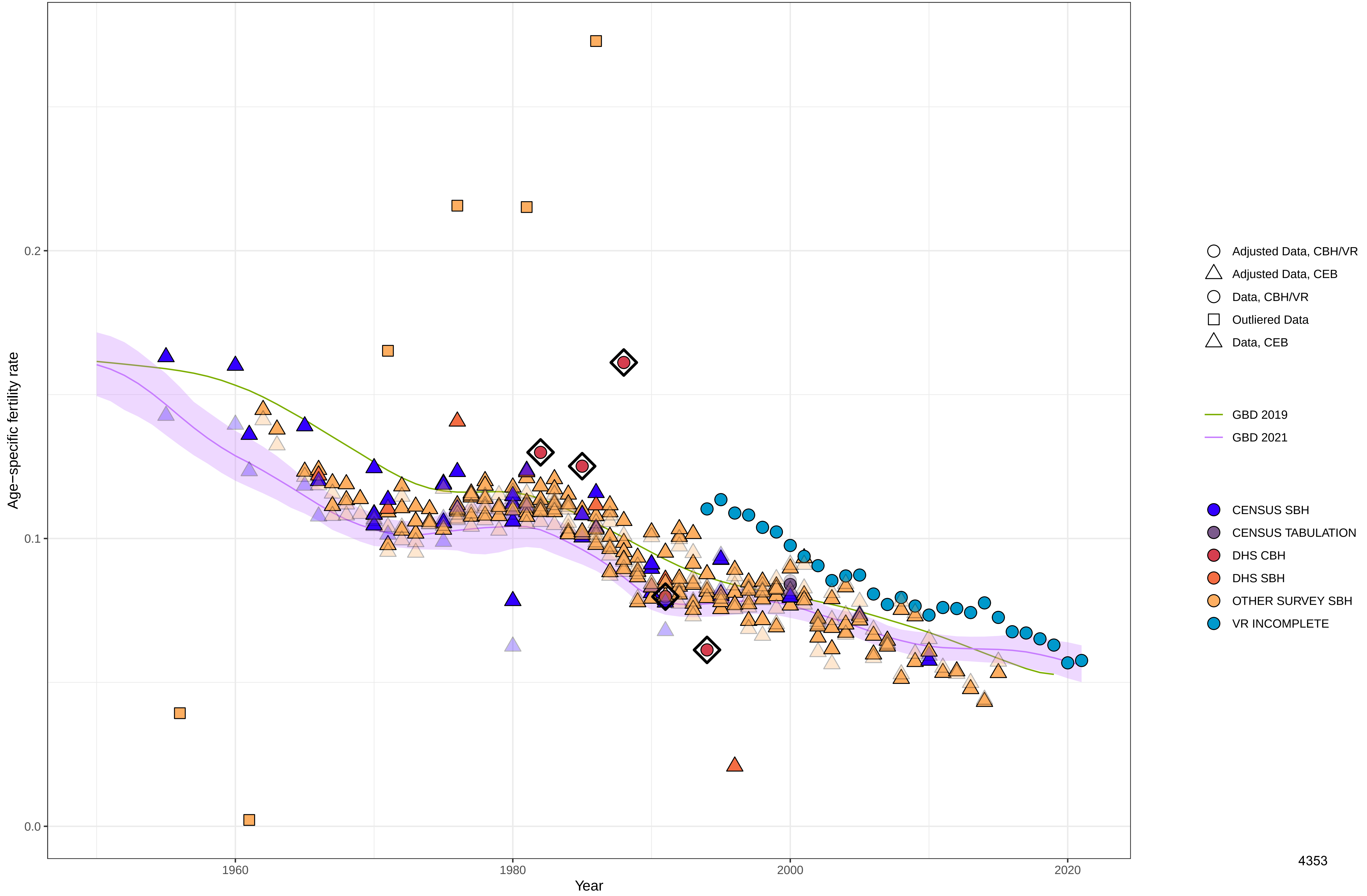

Age−specific fertility fitted trend lines, 1950−2021: Mato Grosso do Sul, Ages: 20−24
Data points enclosed with a diamond are reference sources

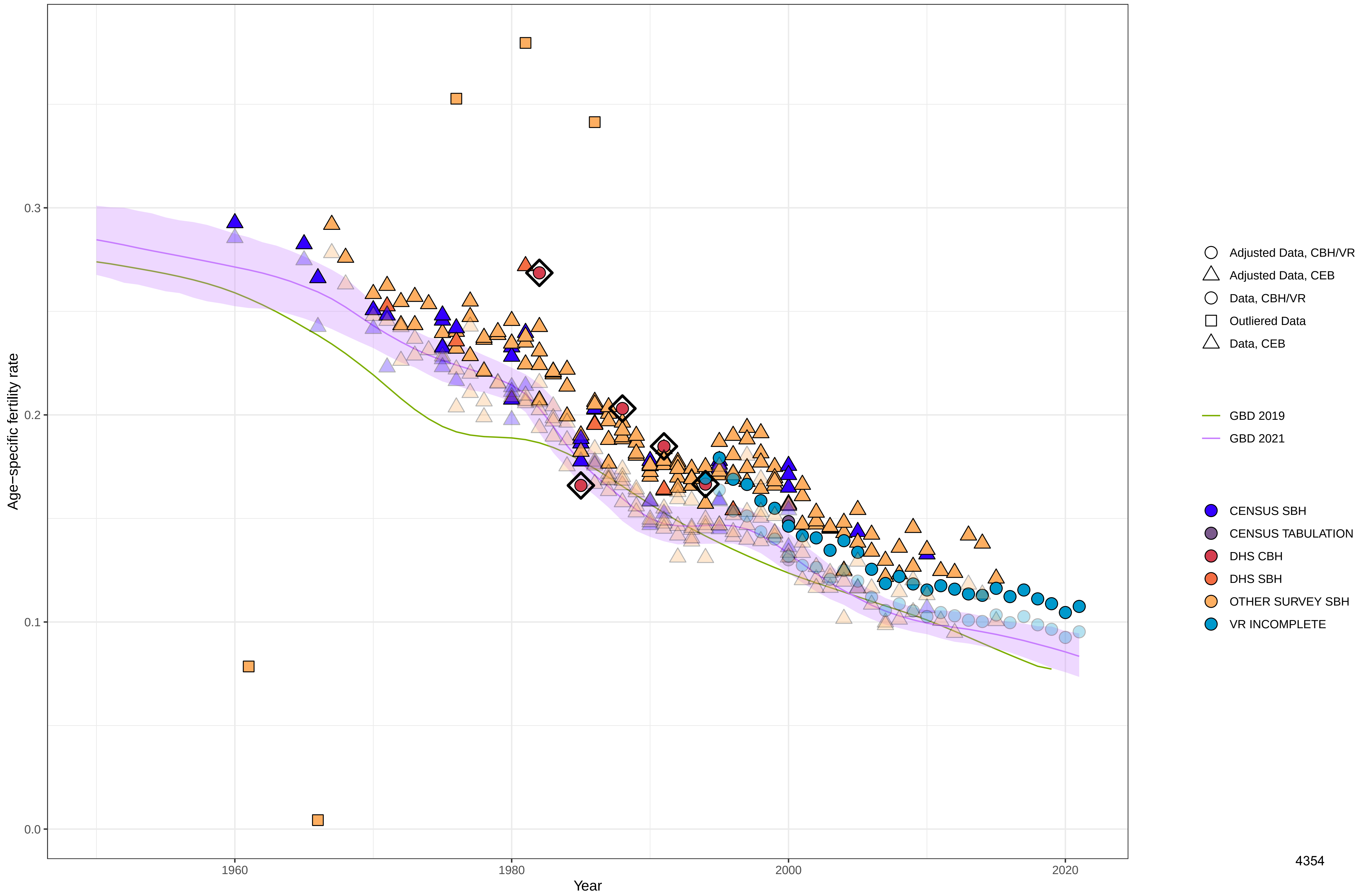

Age−specific fertility fitted trend lines, 1950−2021: Mato Grosso do Sul, Ages: 25−29
Data points enclosed with a diamond are reference sources

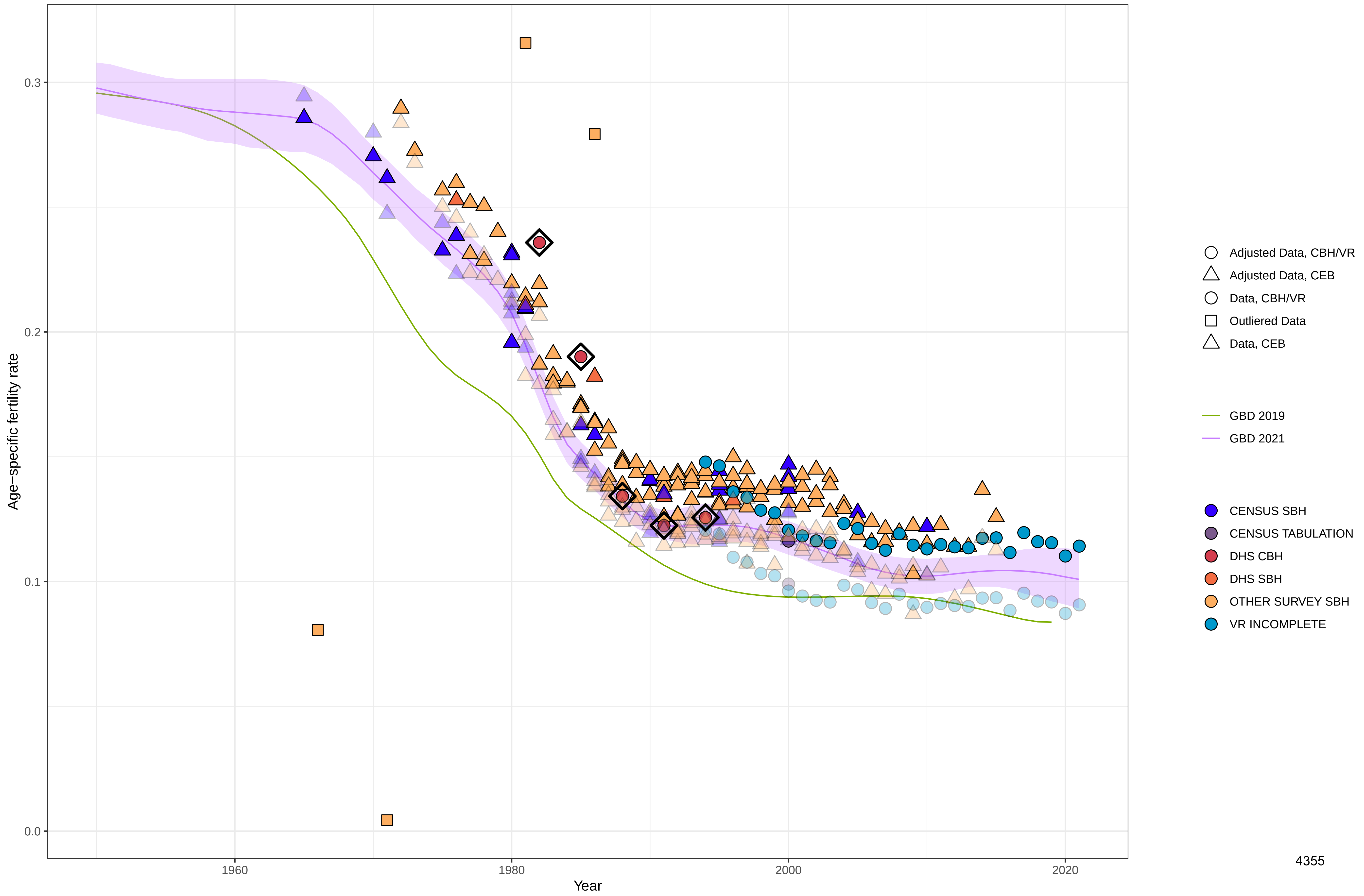

Age−specific fertility fitted trend lines, 1950−2021: Mato Grosso do Sul, Ages: 30−34
Data points enclosed with a diamond are reference sources

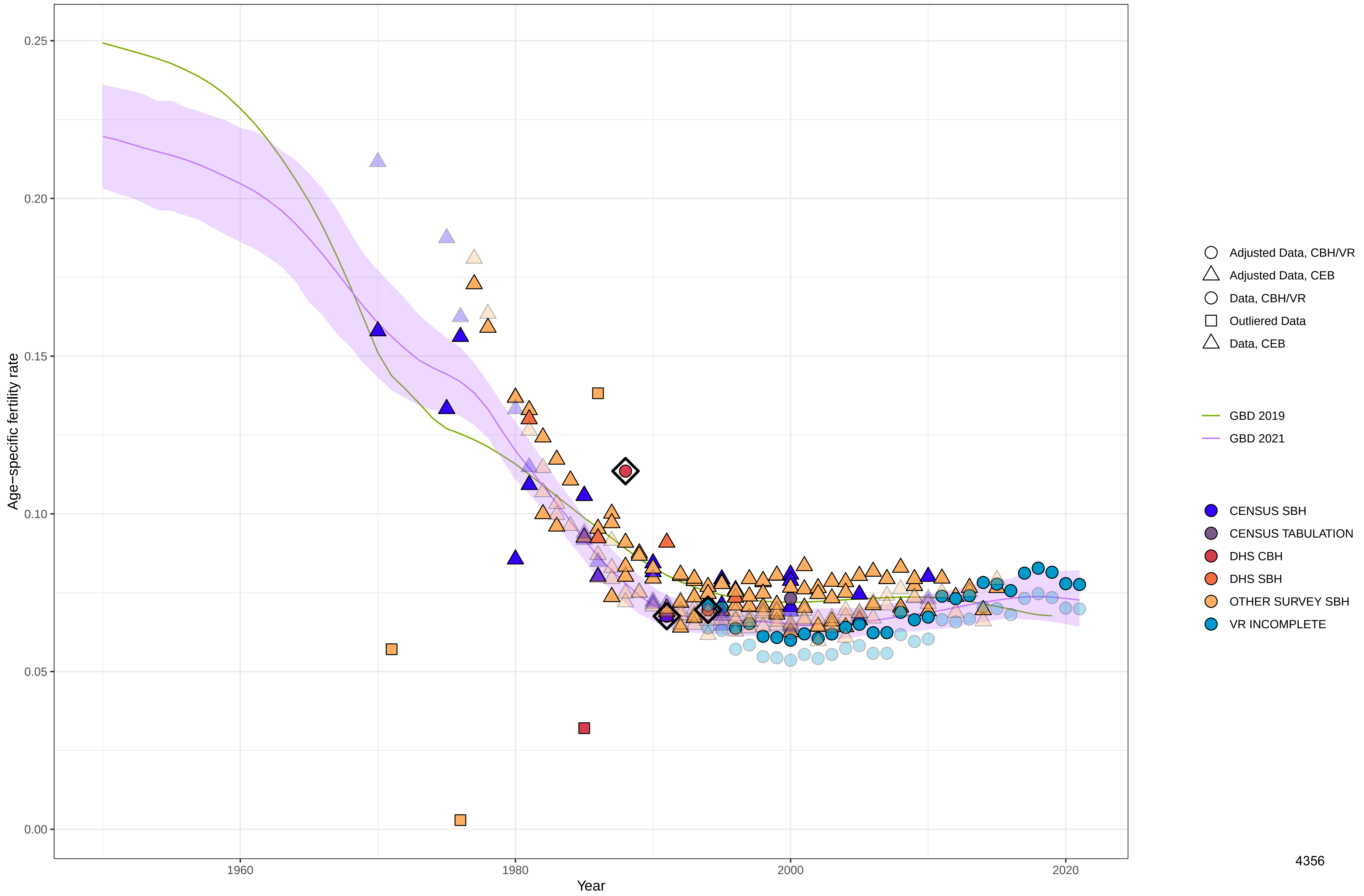

Age−specific fertility fitted trend lines, 1950−2021: Mato Grosso do Sul, Ages: 35−39
Data points enclosed with a diamond are reference sources

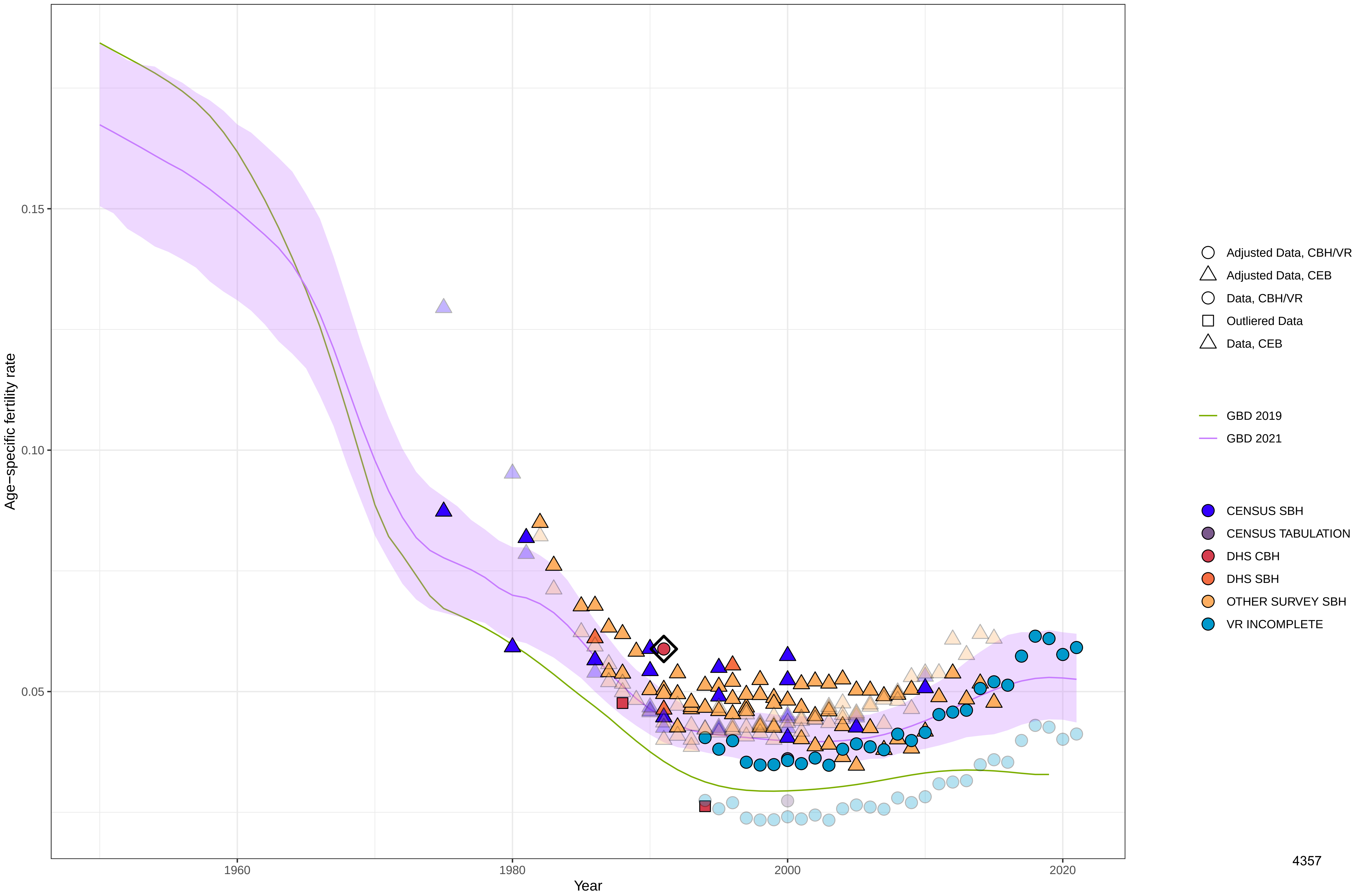

Age−specific fertility fitted trend lines, 1950−2021: Mato Grosso do Sul, Ages: 40−44
Data points enclosed with a diamond are reference sources

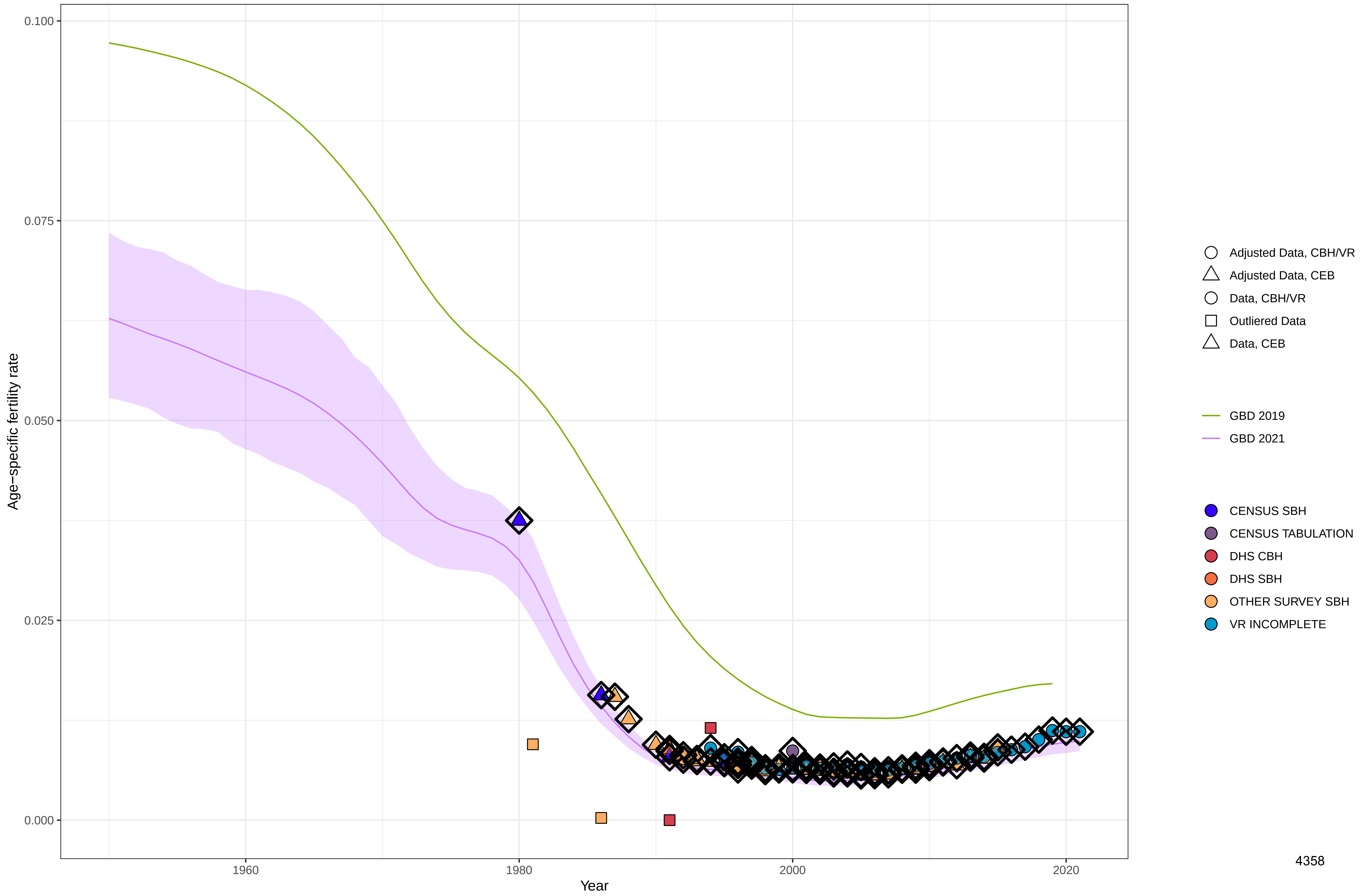

Age−specific fertility fitted trend lines, 1950−2021: Mato Grosso do Sul, Ages: 45−49
Data points enclosed with a diamond are reference sources

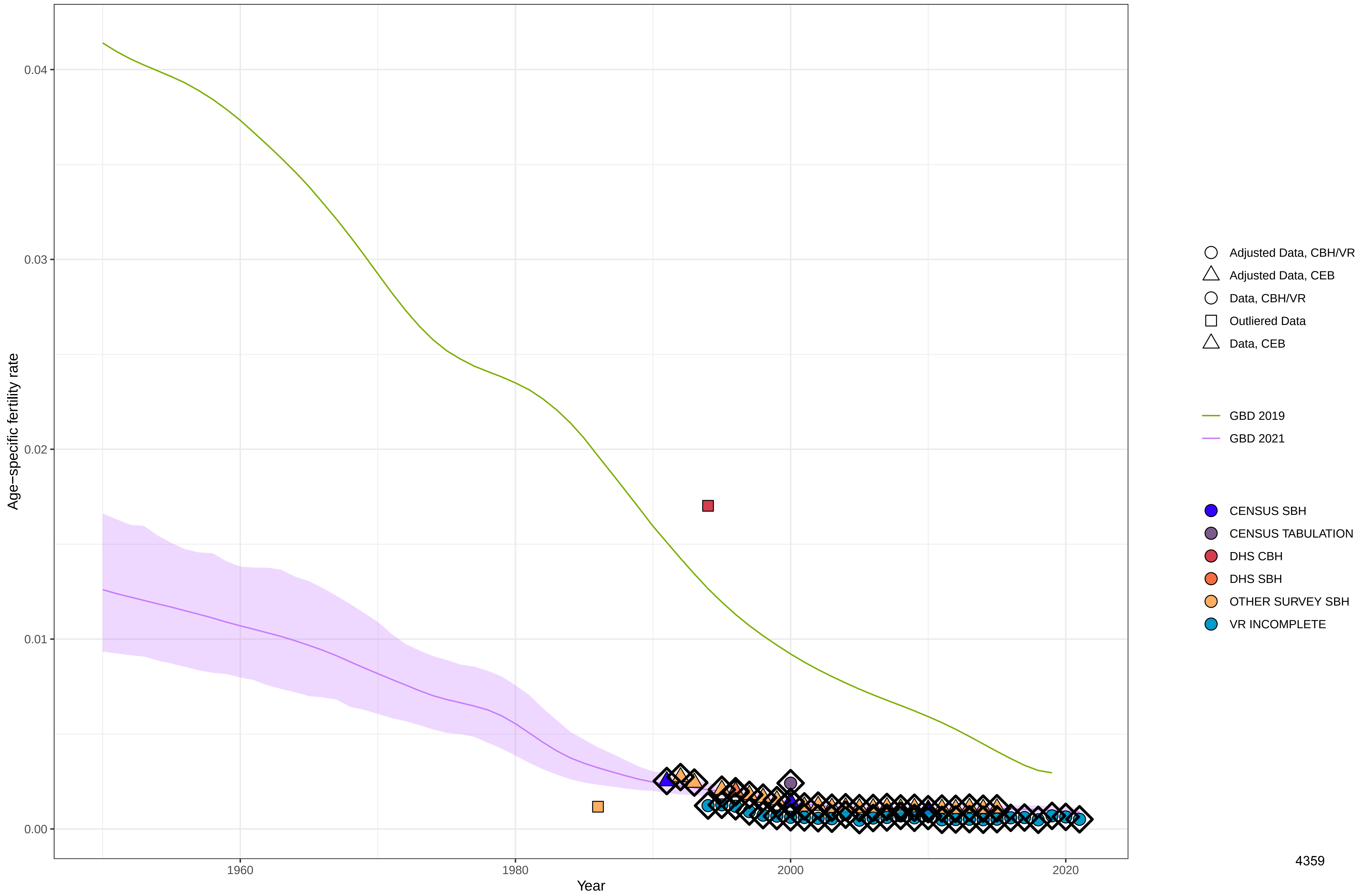

Age−specific fertility fitted trend lines, 1950−2021: Mato Grosso do Sul, Ages: 50−54
Data points enclosed with a diamond are reference sources

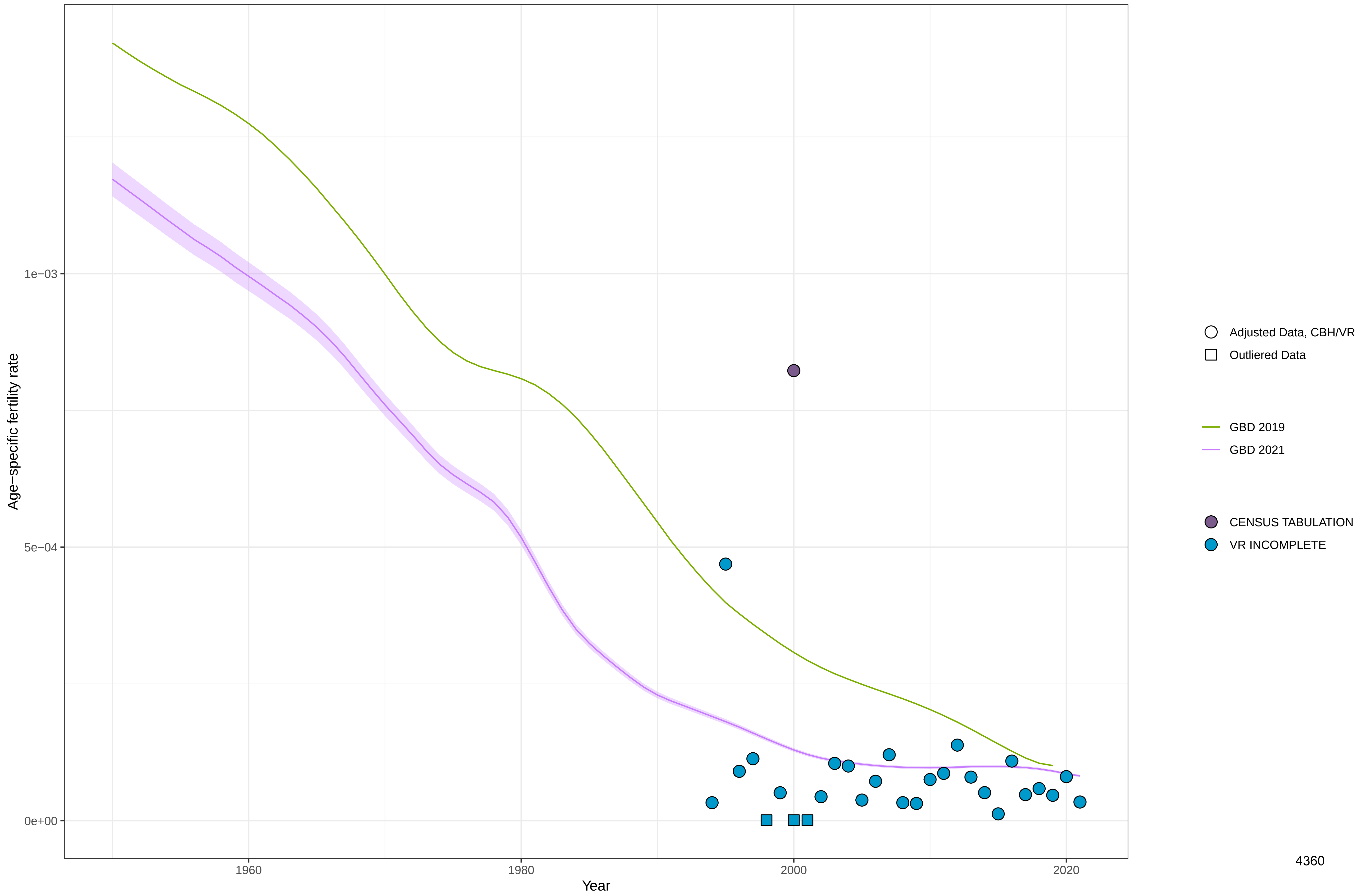

Total fertility rate fitted trend lines, 1950−2021: Mato Grosso do Sul TFR
Data points enclosed with a diamond are reference sources

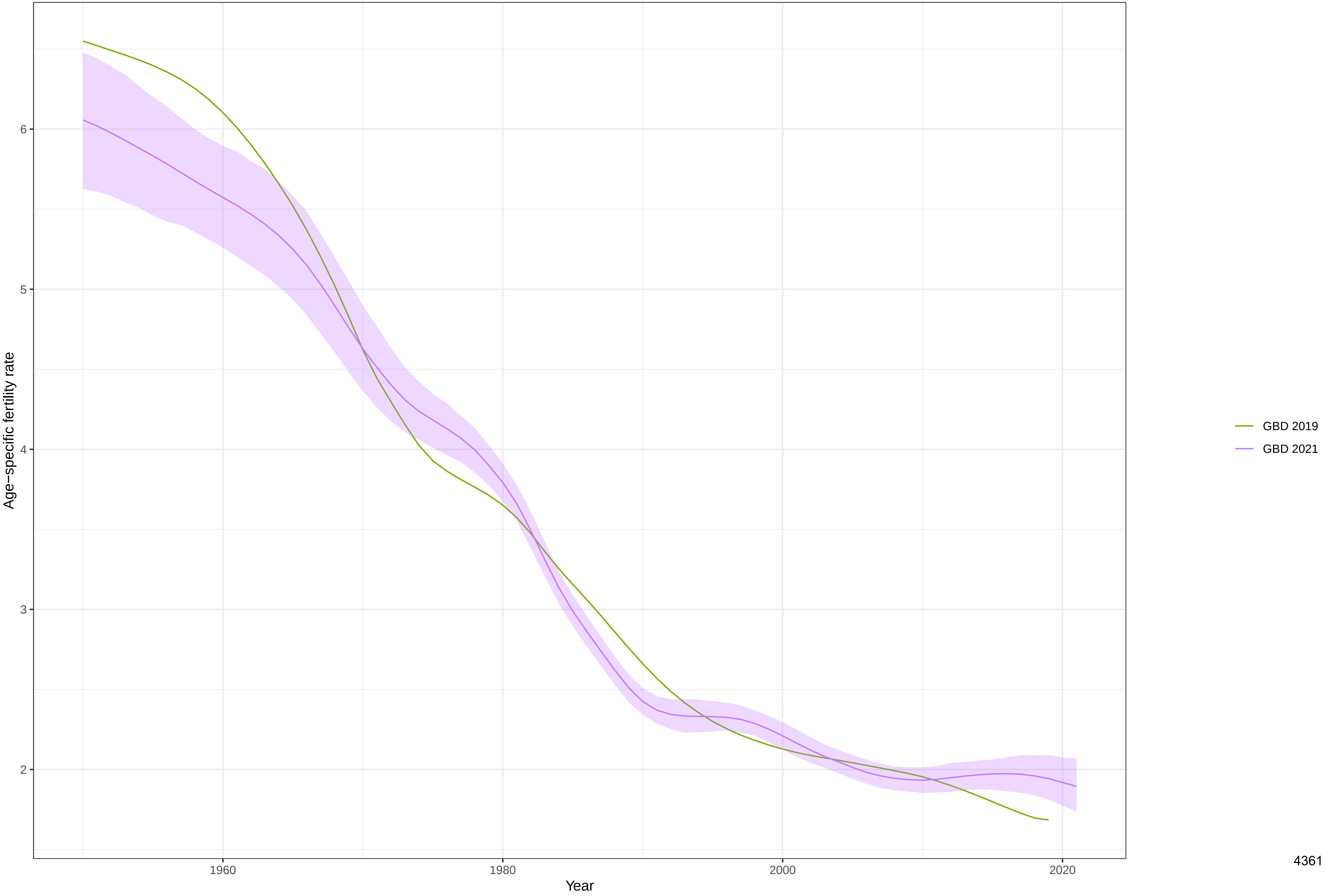

Age−specific fertility fitted trend lines, 1950−2021: Minas Gerais, Ages: 10−14
Data points enclosed with a diamond are reference sources

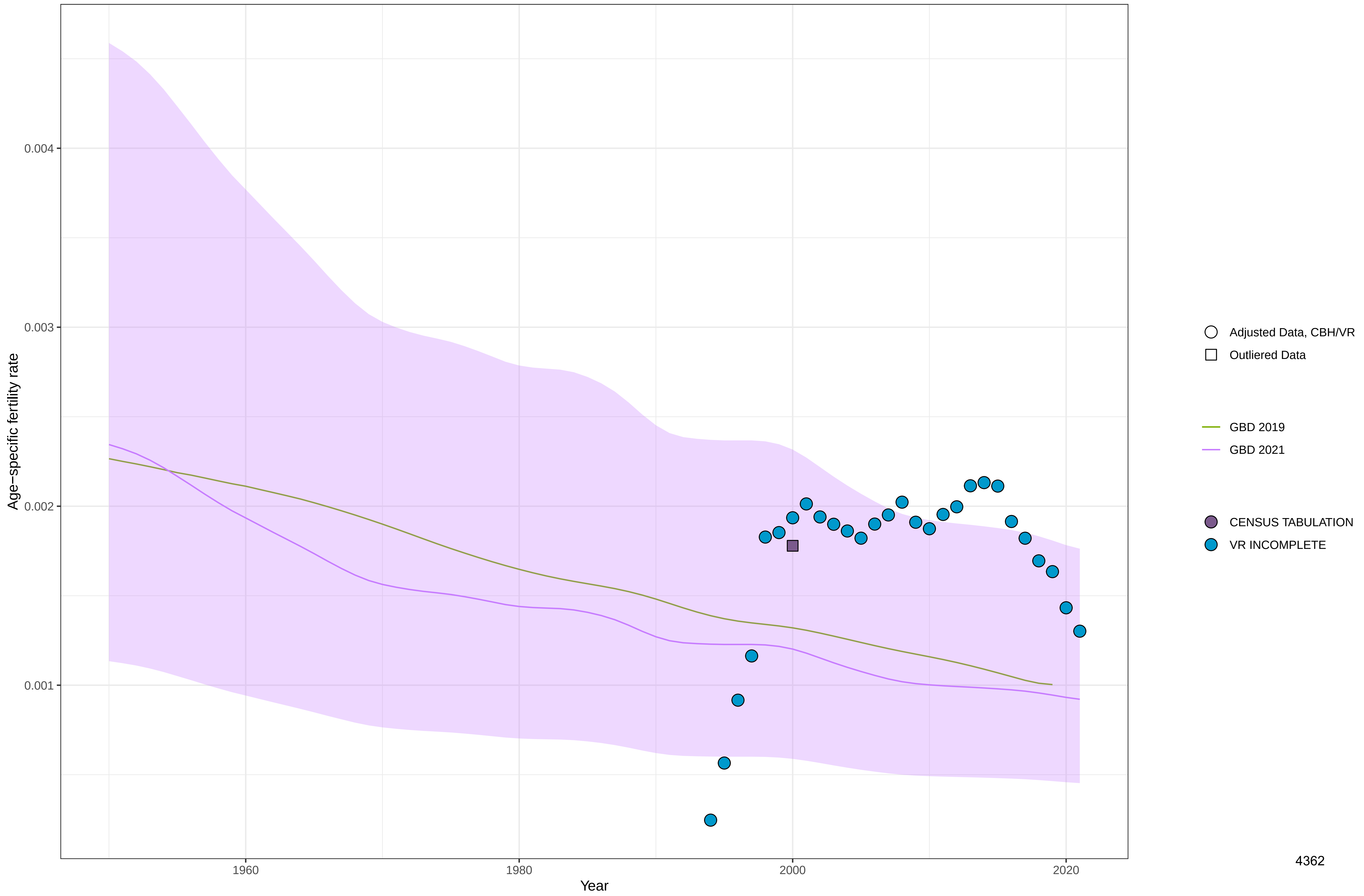

Age−specific fertility fitted trend lines, 1950−2021: Minas Gerais, Ages: 15−19
Data points enclosed with a diamond are reference sources

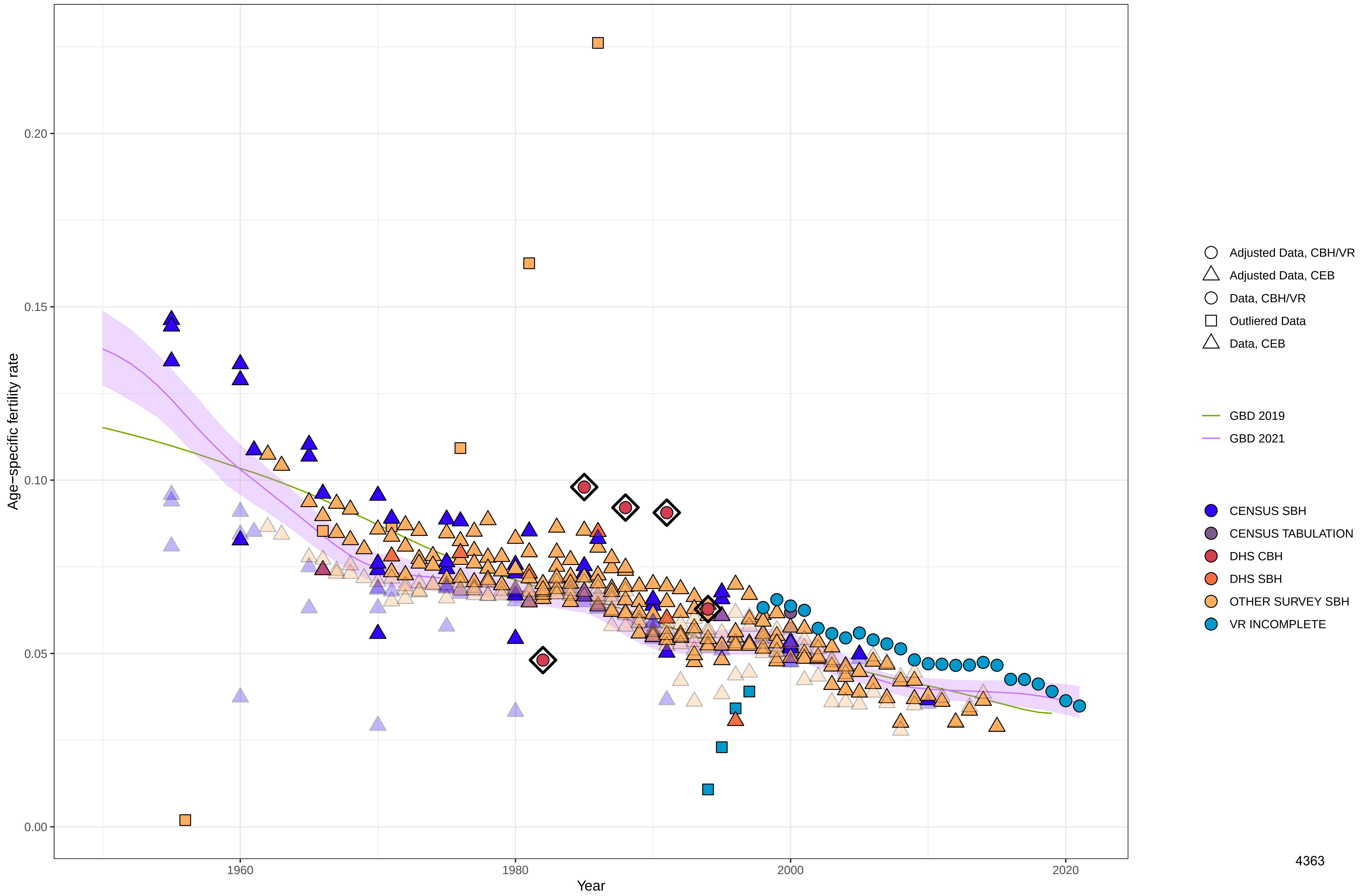

Age−specific fertility fitted trend lines, 1950−2021: Minas Gerais, Ages: 20−24
Data points enclosed with a diamond are reference sources

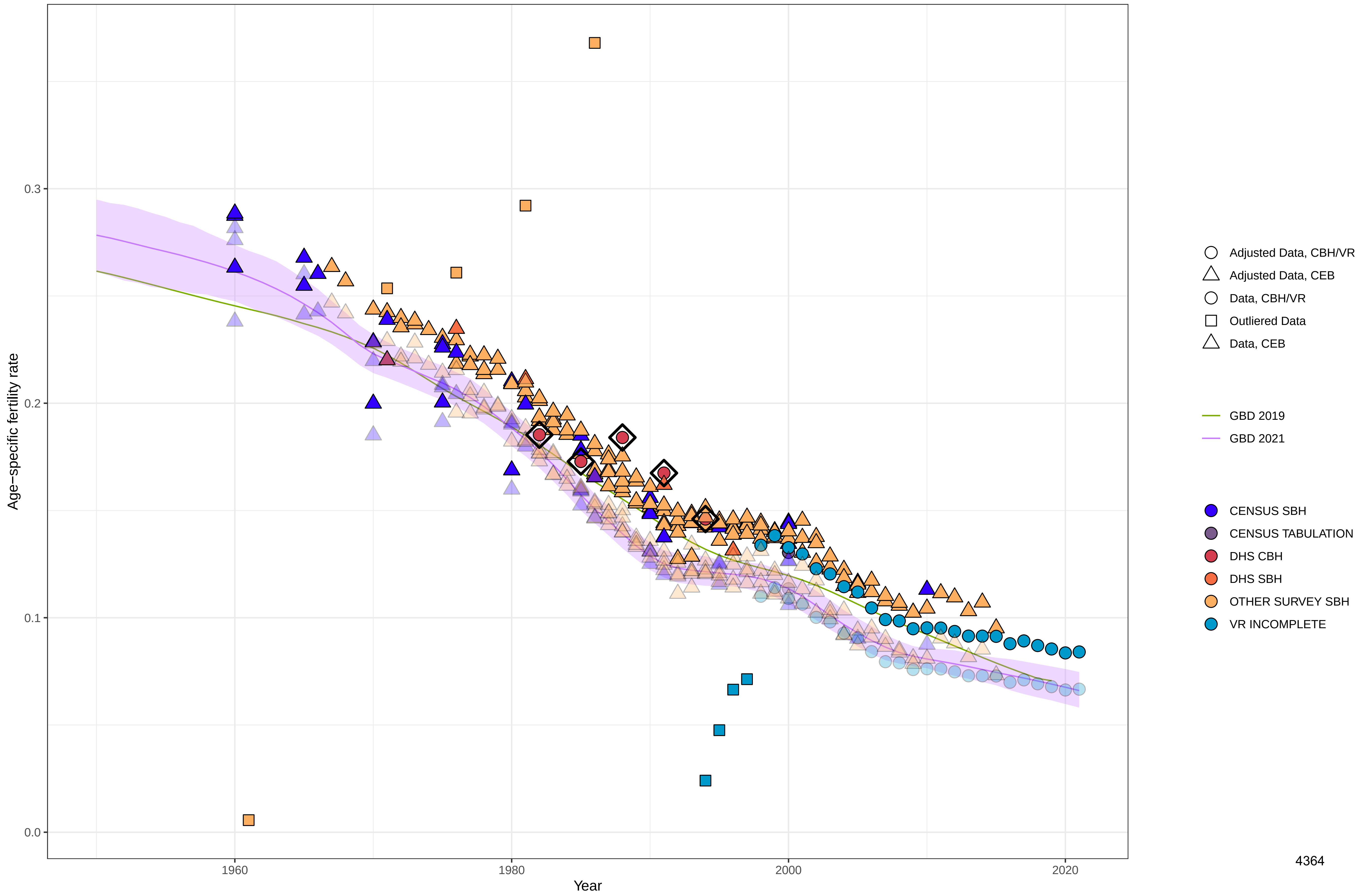

Age−specific fertility fitted trend lines, 1950−2021: Minas Gerais, Ages: 25−29
Data points enclosed with a diamond are reference sources

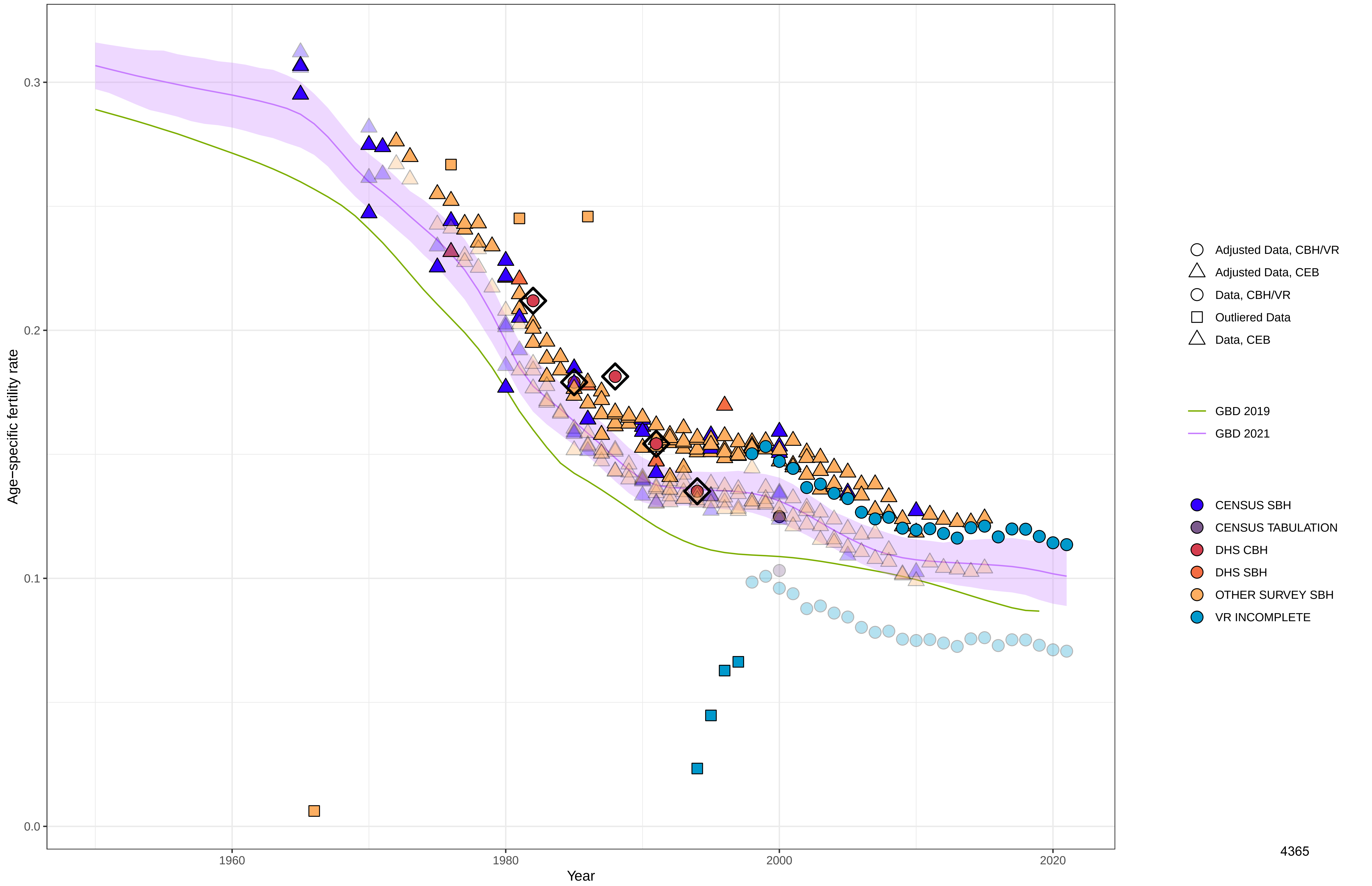

Age−specific fertility fitted trend lines, 1950−2021: Minas Gerais, Ages: 30−34
Data points enclosed with a diamond are reference sources

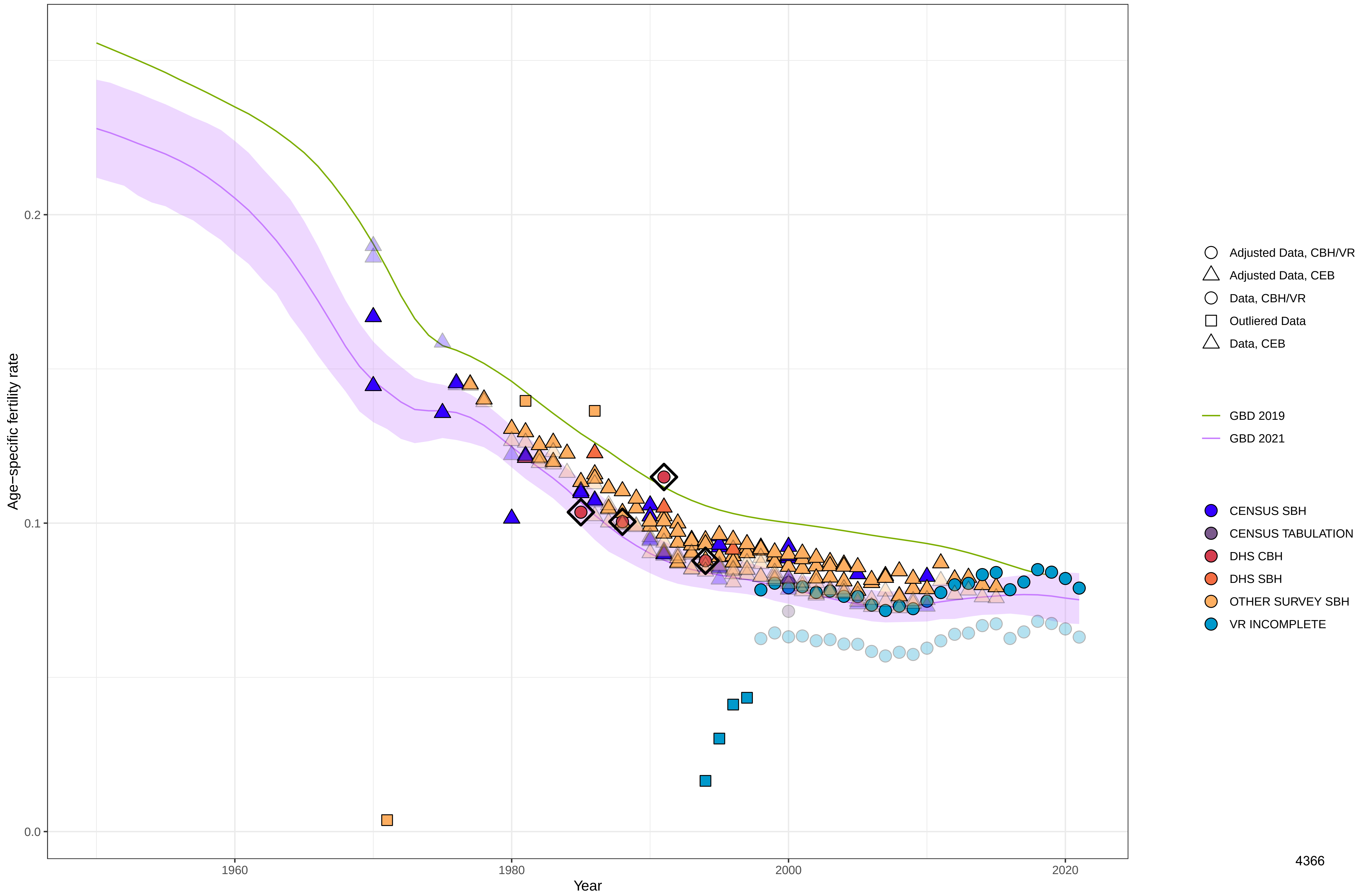

Age−specific fertility fitted trend lines, 1950−2021: Minas Gerais, Ages: 35−39
Data points enclosed with a diamond are reference sources

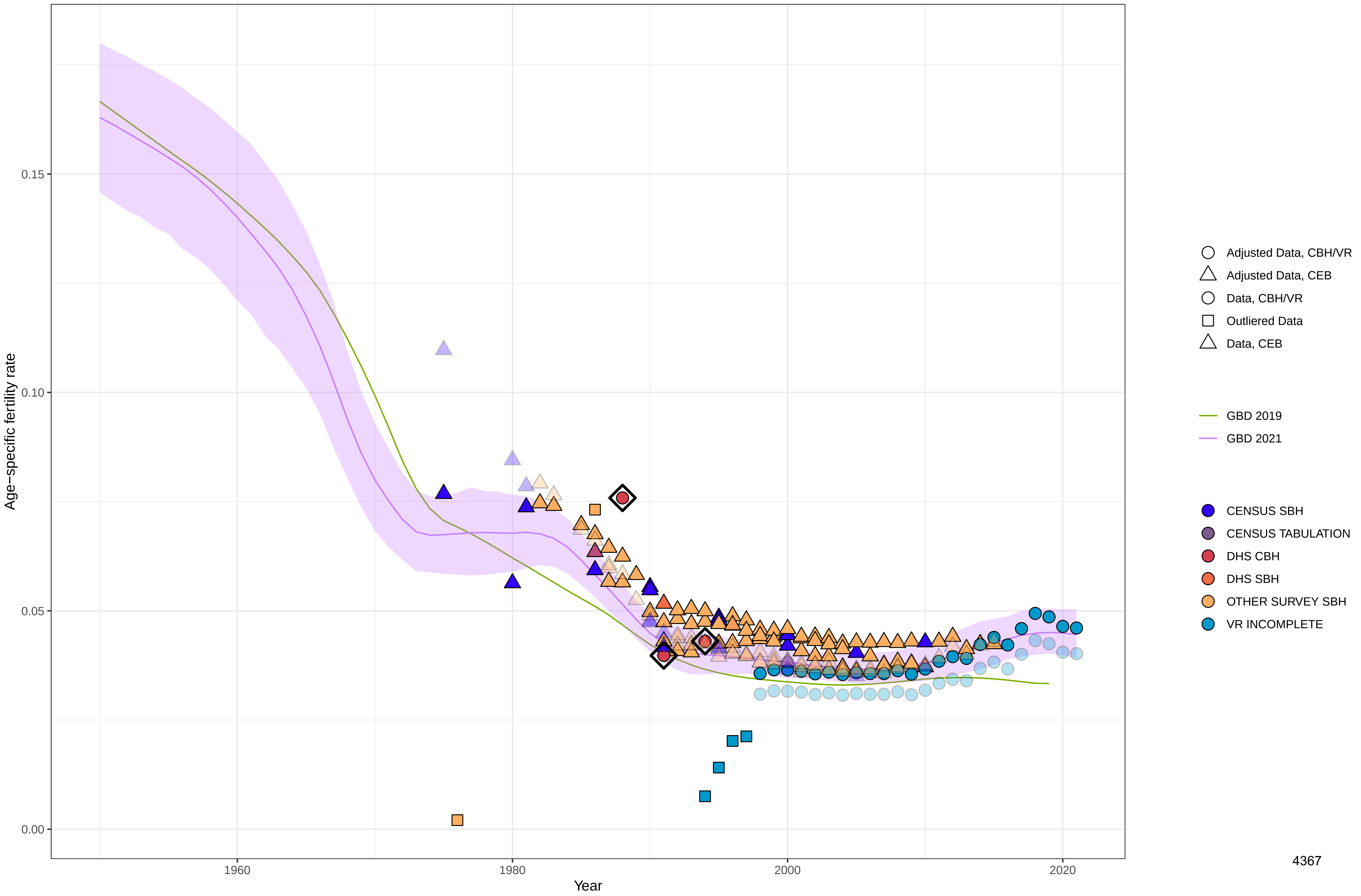

Age−specific fertility fitted trend lines, 1950−2021: Minas Gerais, Ages: 40−44
Data points enclosed with a diamond are reference sources

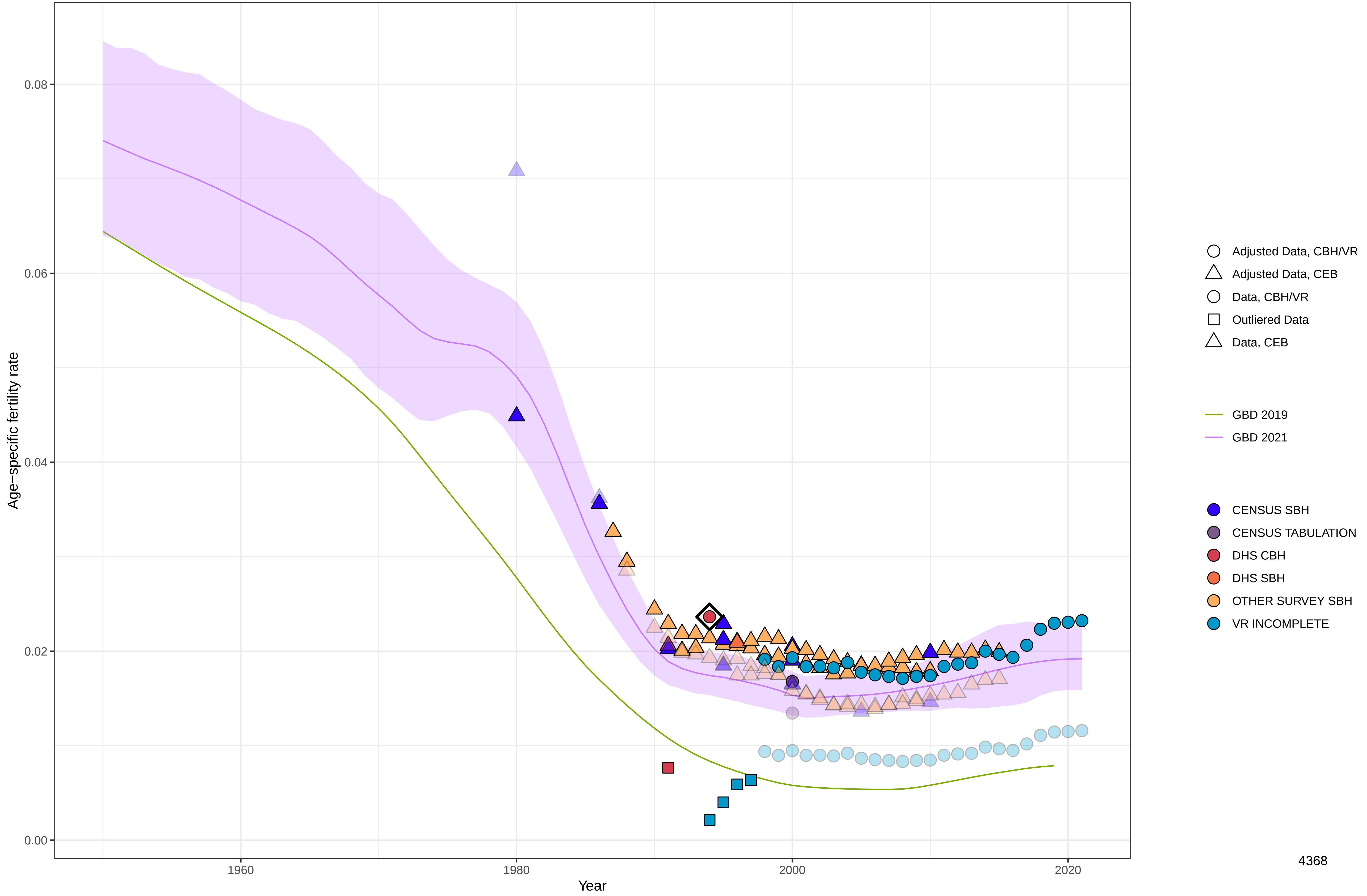

Age−specific fertility fitted trend lines, 1950−2021: Minas Gerais, Ages: 45−49
Data points enclosed with a diamond are reference sources

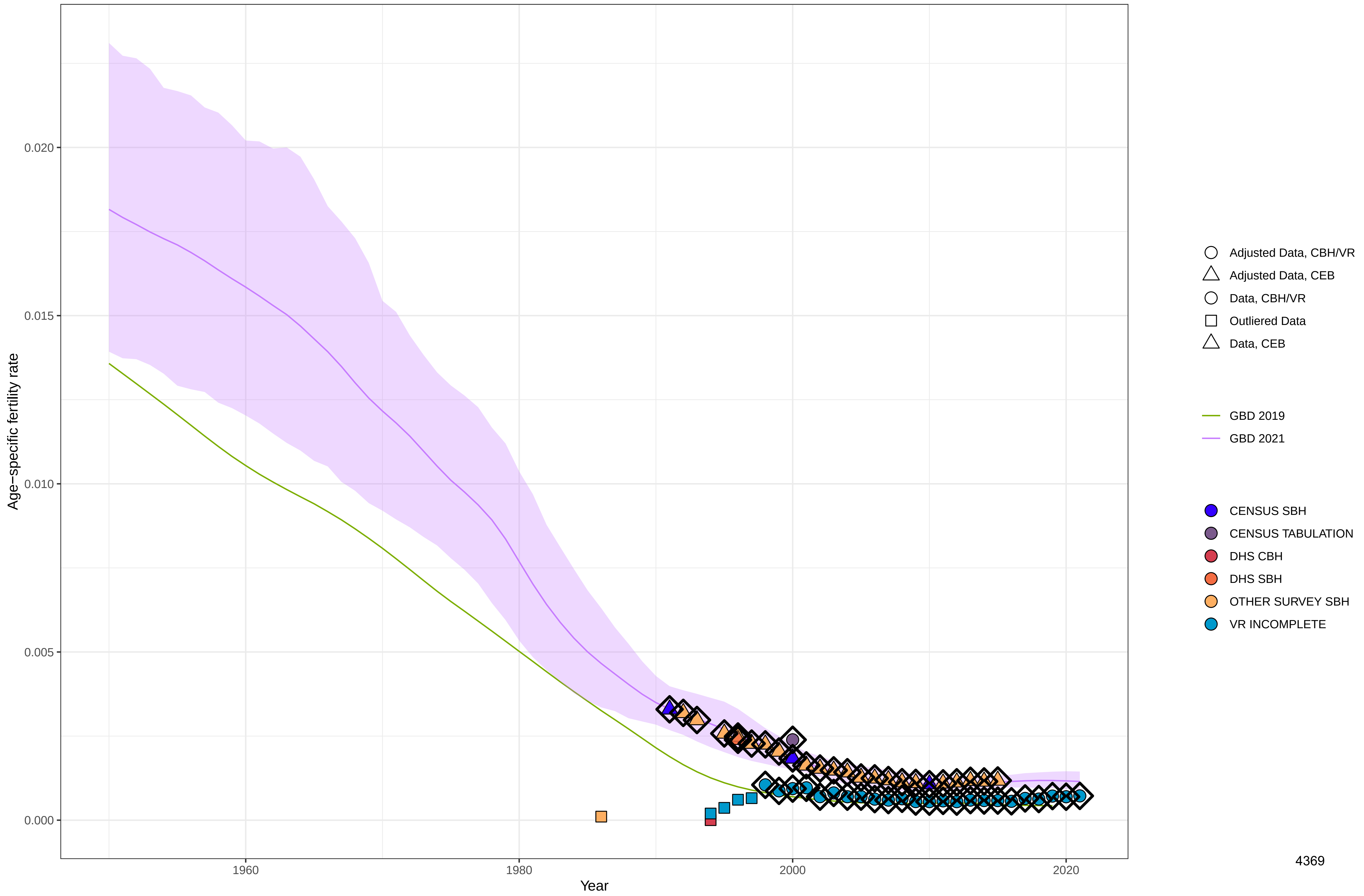

4369

Age−specific fertility fitted trend lines, 1950−2021: Minas Gerais, Ages: 50−54
Data points enclosed with a diamond are reference sources

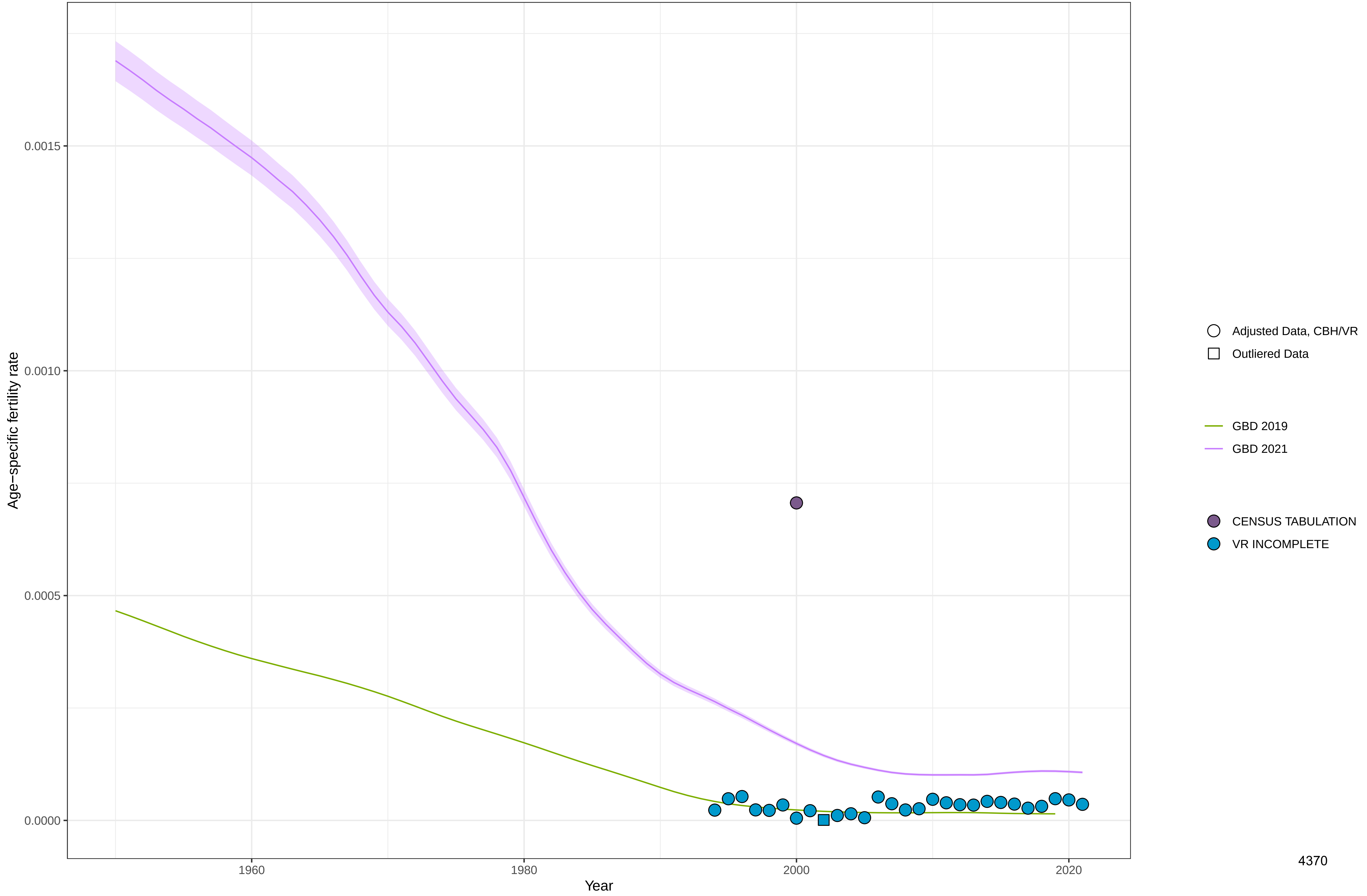

Total fertility rate fitted trend lines, 1950−2021: Minas Gerais TFR
Data points enclosed with a diamond are reference sources

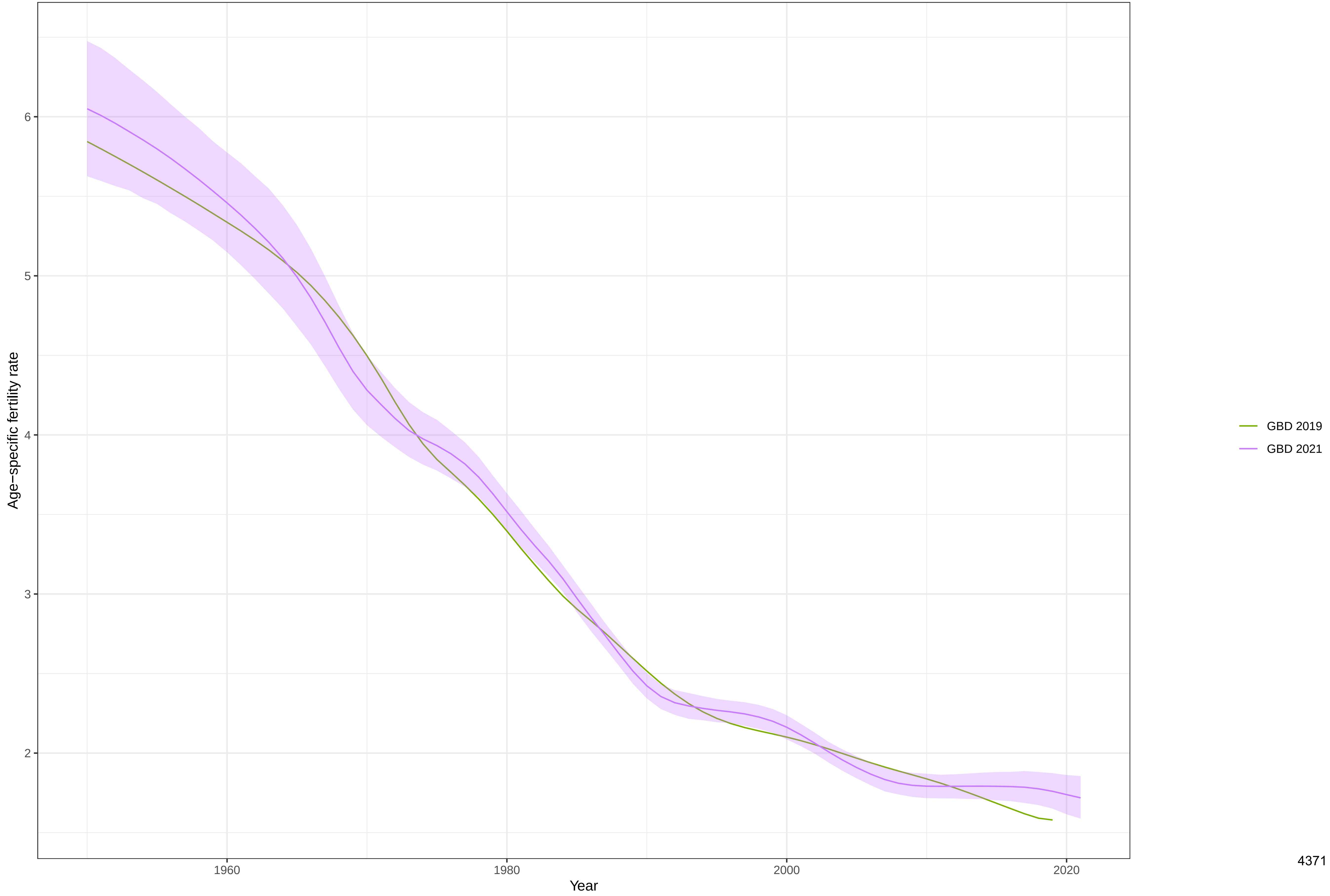

Age−specific fertility fitted trend lines, 1950−2021: Pará, Ages: 10−14
Data points enclosed with a diamond are reference sources

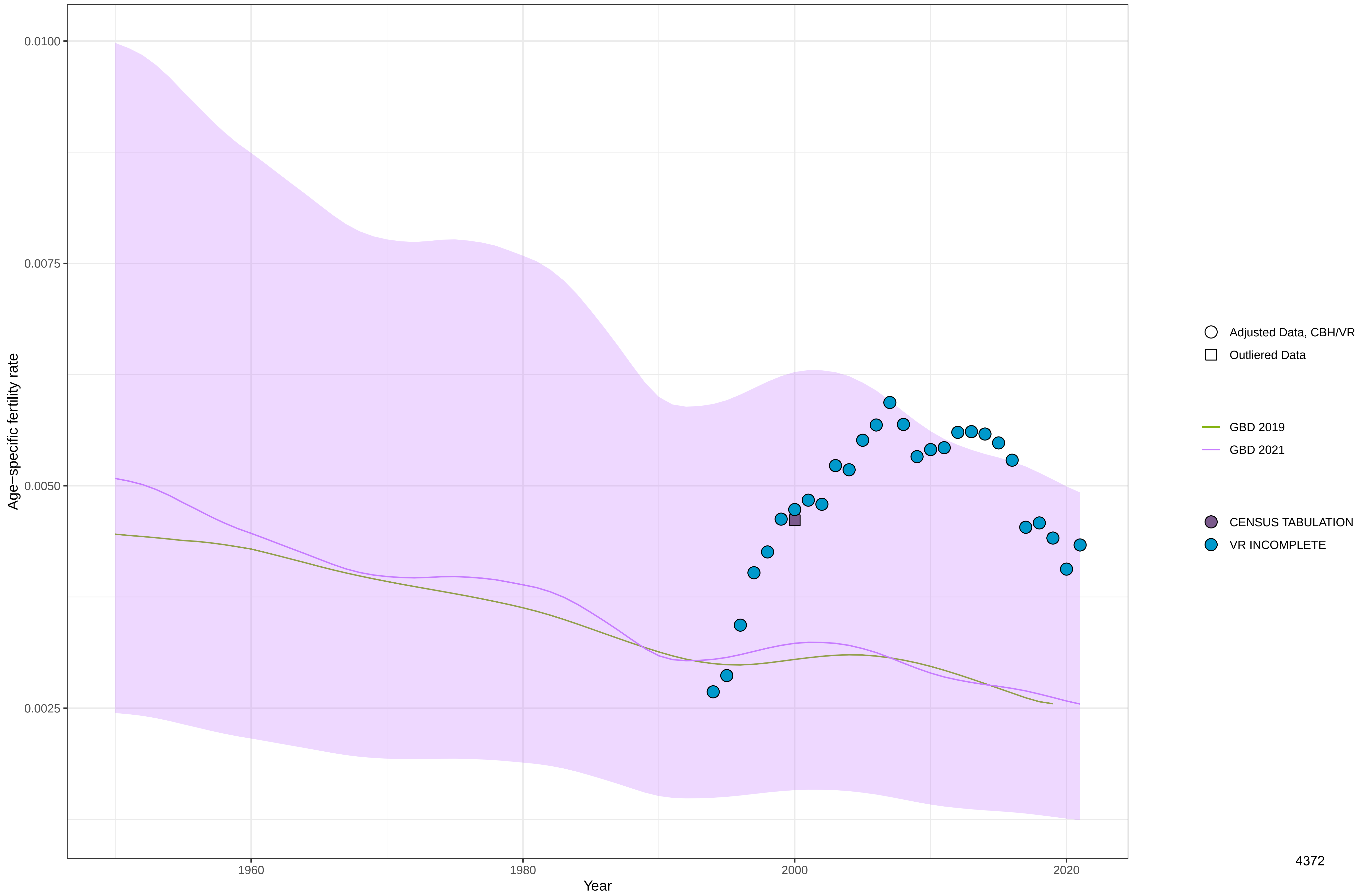

Age−specific fertility fitted trend lines, 1950−2021: Pará, Ages: 15−19
Data points enclosed with a diamond are reference sources

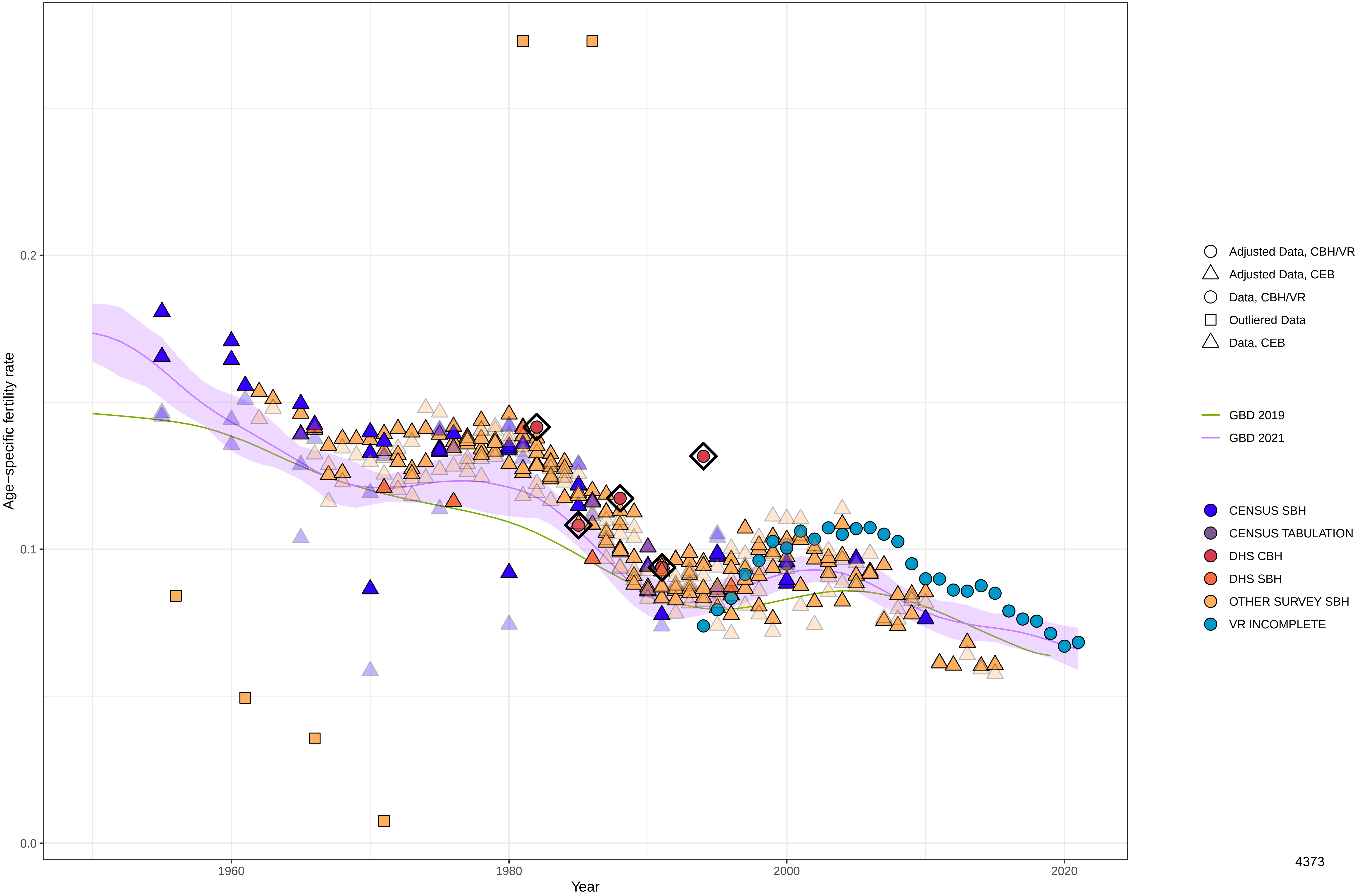

Age−specific fertility fitted trend lines, 1950−2021: Pará, Ages: 20−24
Data points enclosed with a diamond are reference sources

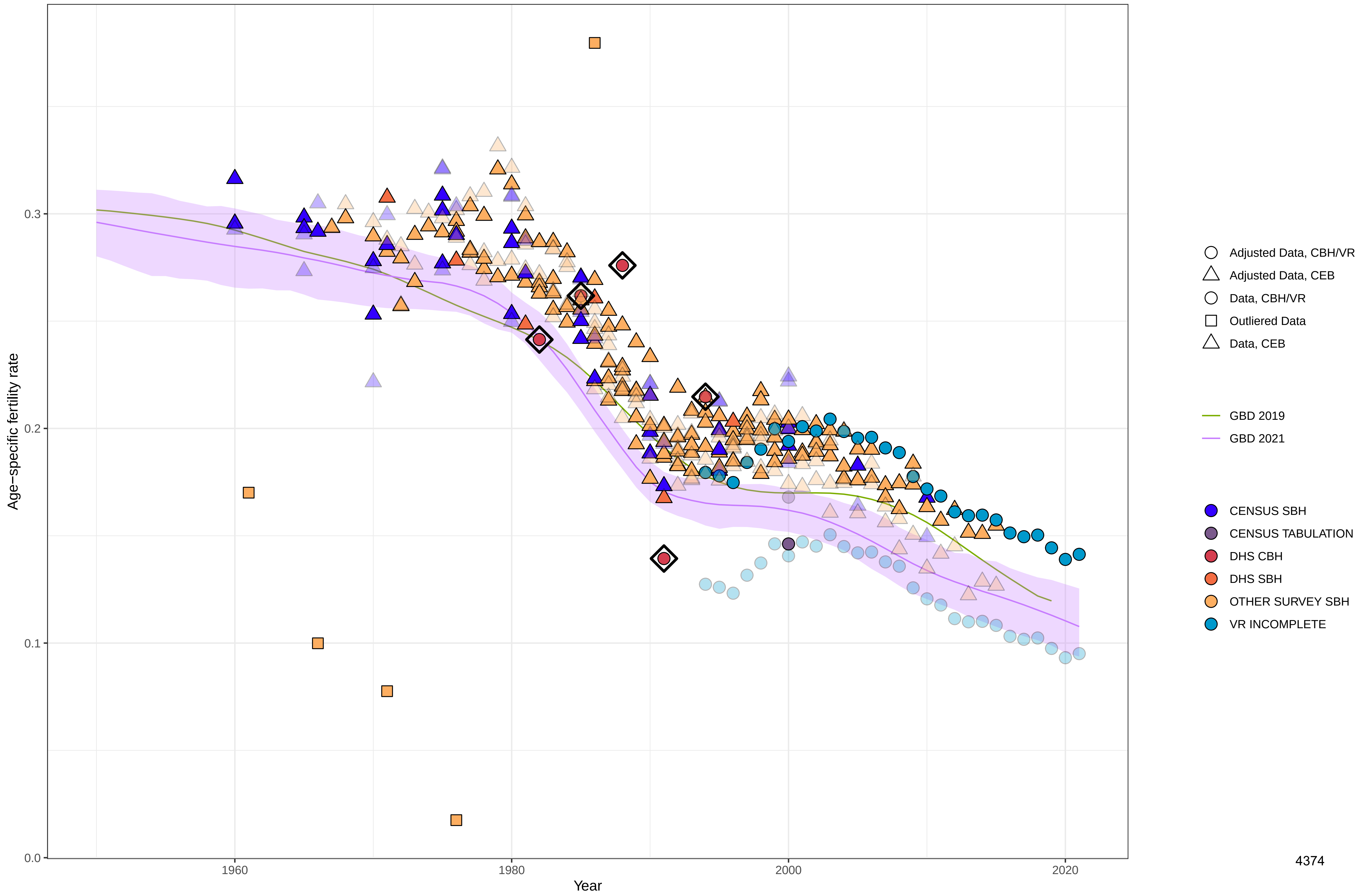

Age−specific fertility fitted trend lines, 1950−2021: Pará, Ages: 25−29
Data points enclosed with a diamond are reference sources

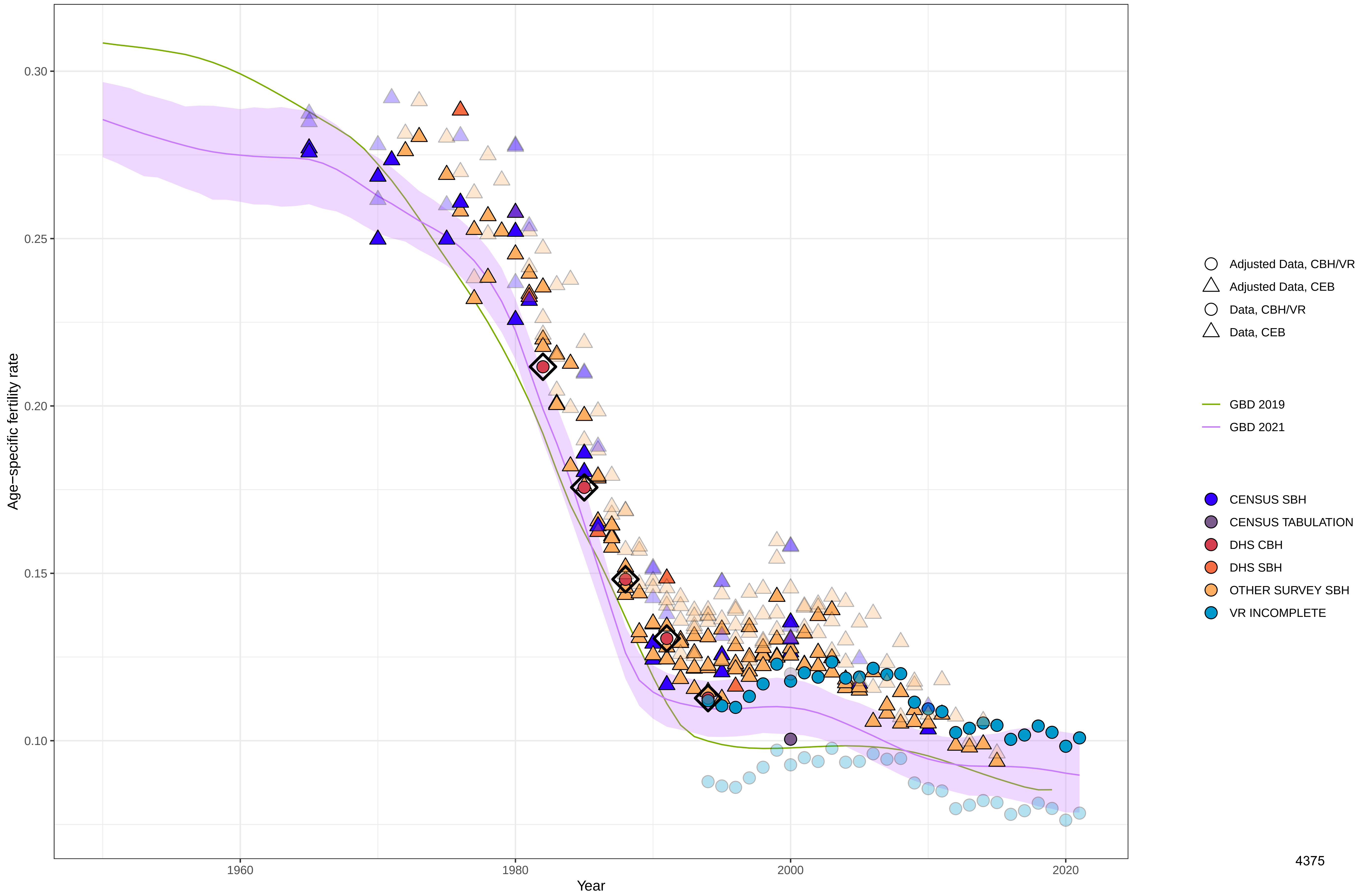

Age−specific fertility fitted trend lines, 1950−2021: Pará, Ages: 30−34
Data points enclosed with a diamond are reference sources

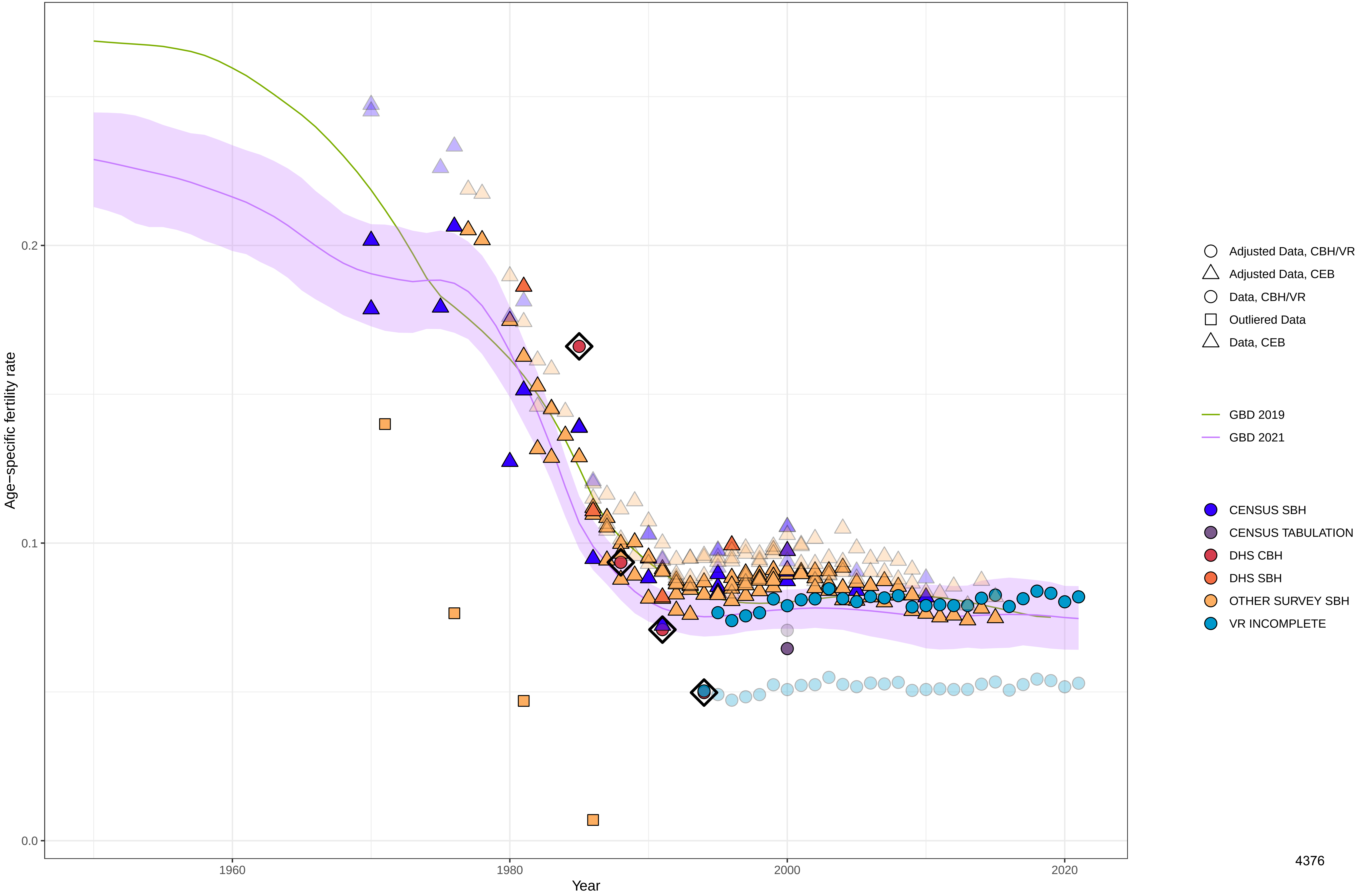

Age−specific fertility fitted trend lines, 1950−2021: Pará, Ages: 35−39
Data points enclosed with a diamond are reference sources

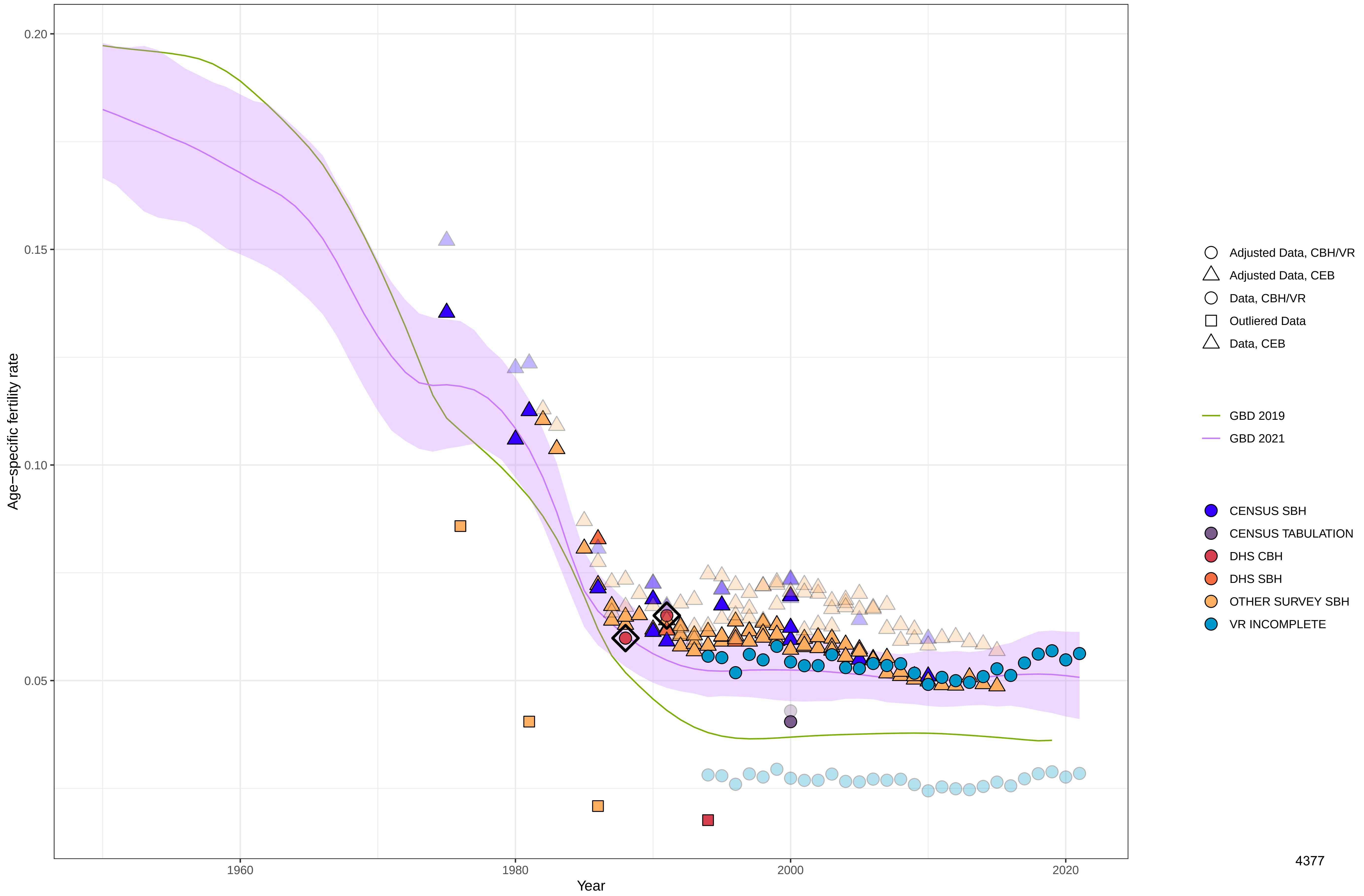

Age−specific fertility fitted trend lines, 1950−2021: Pará, Ages: 40−44
Data points enclosed with a diamond are reference sources

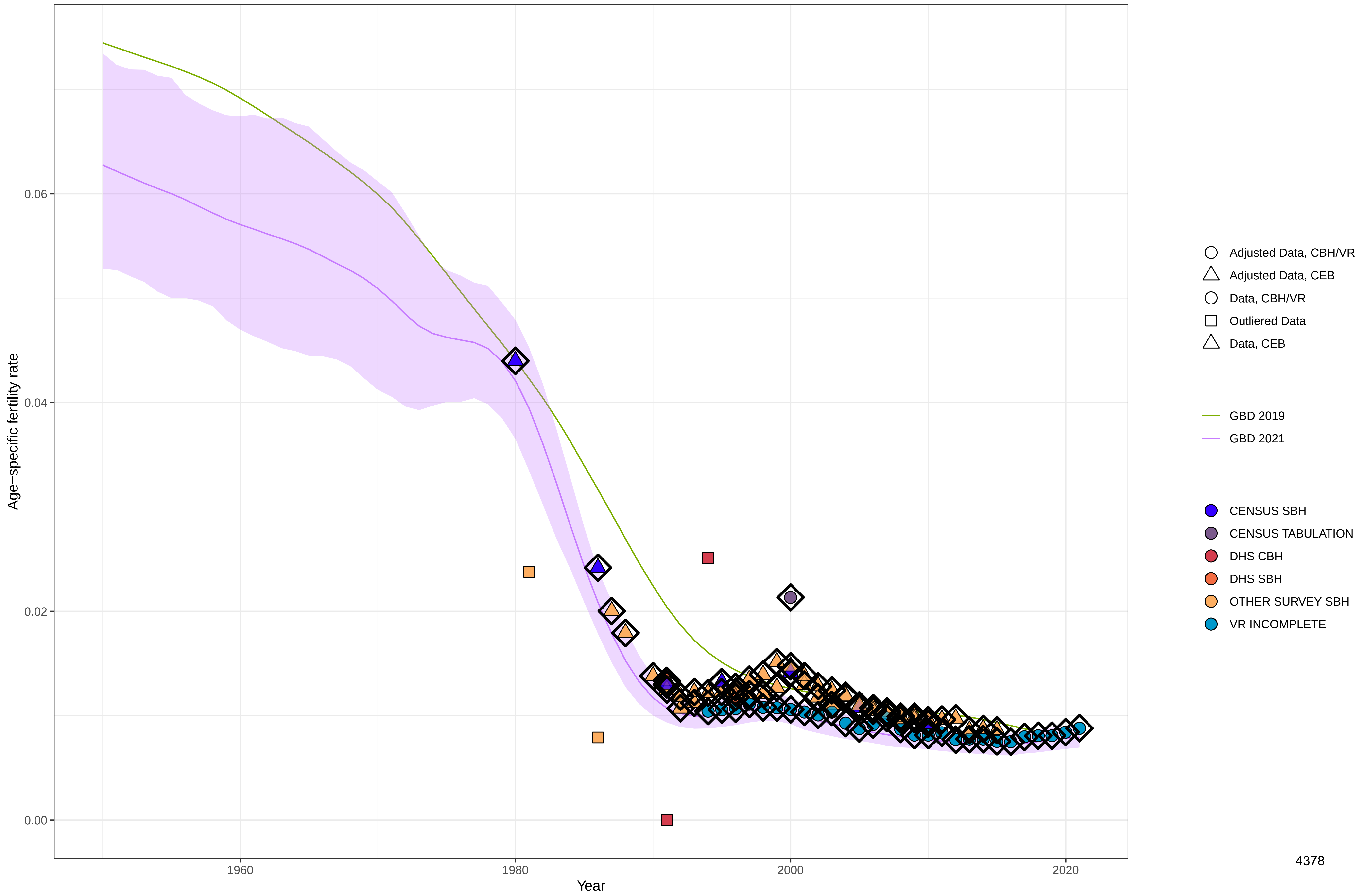

Age−specific fertility fitted trend lines, 1950−2021: Pará, Ages: 45−49
Data points enclosed with a diamond are reference sources

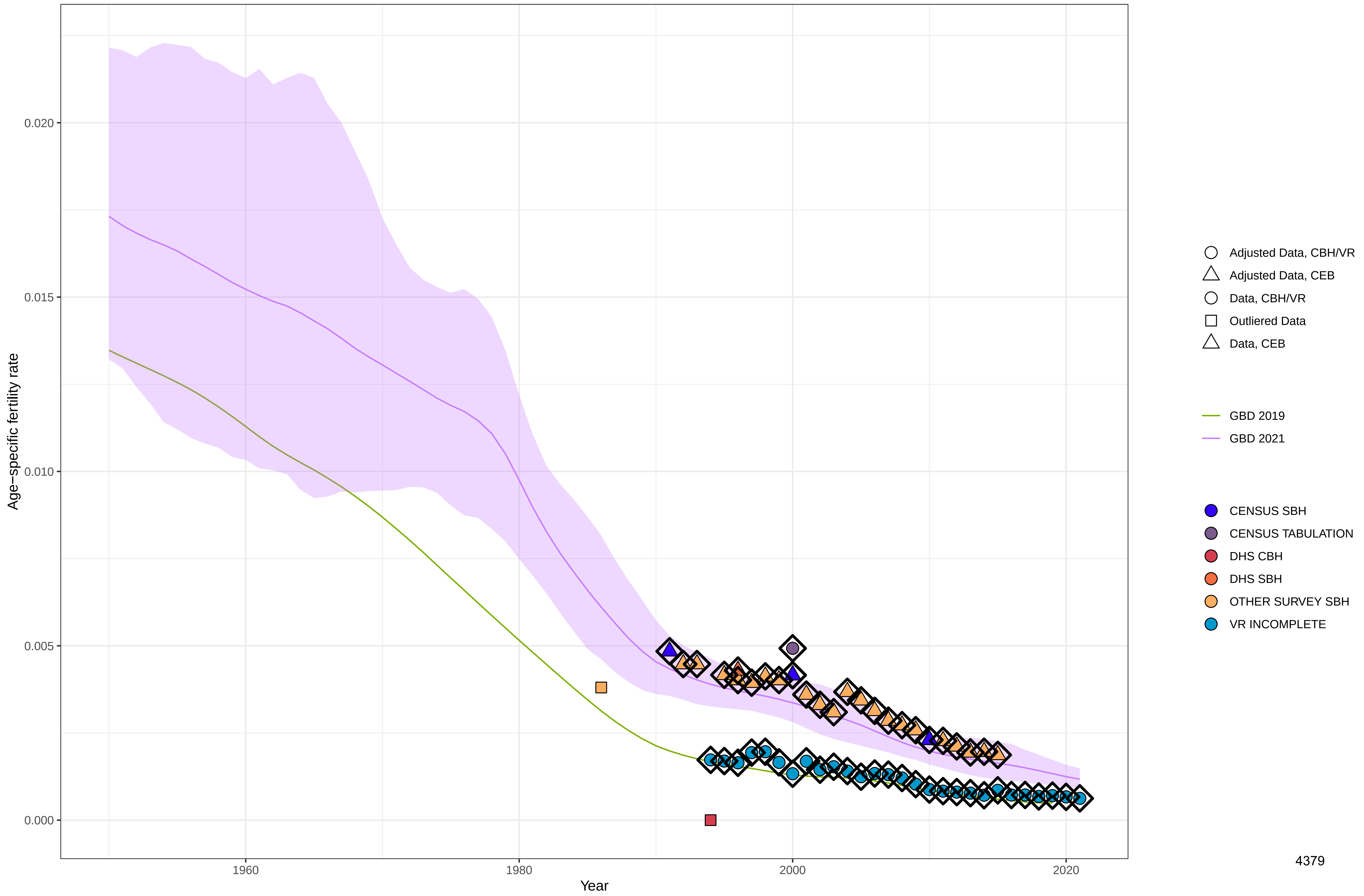

Age−specific fertility fitted trend lines, 1950−2021: Pará, Ages: 50−54
Data points enclosed with a diamond are reference sources

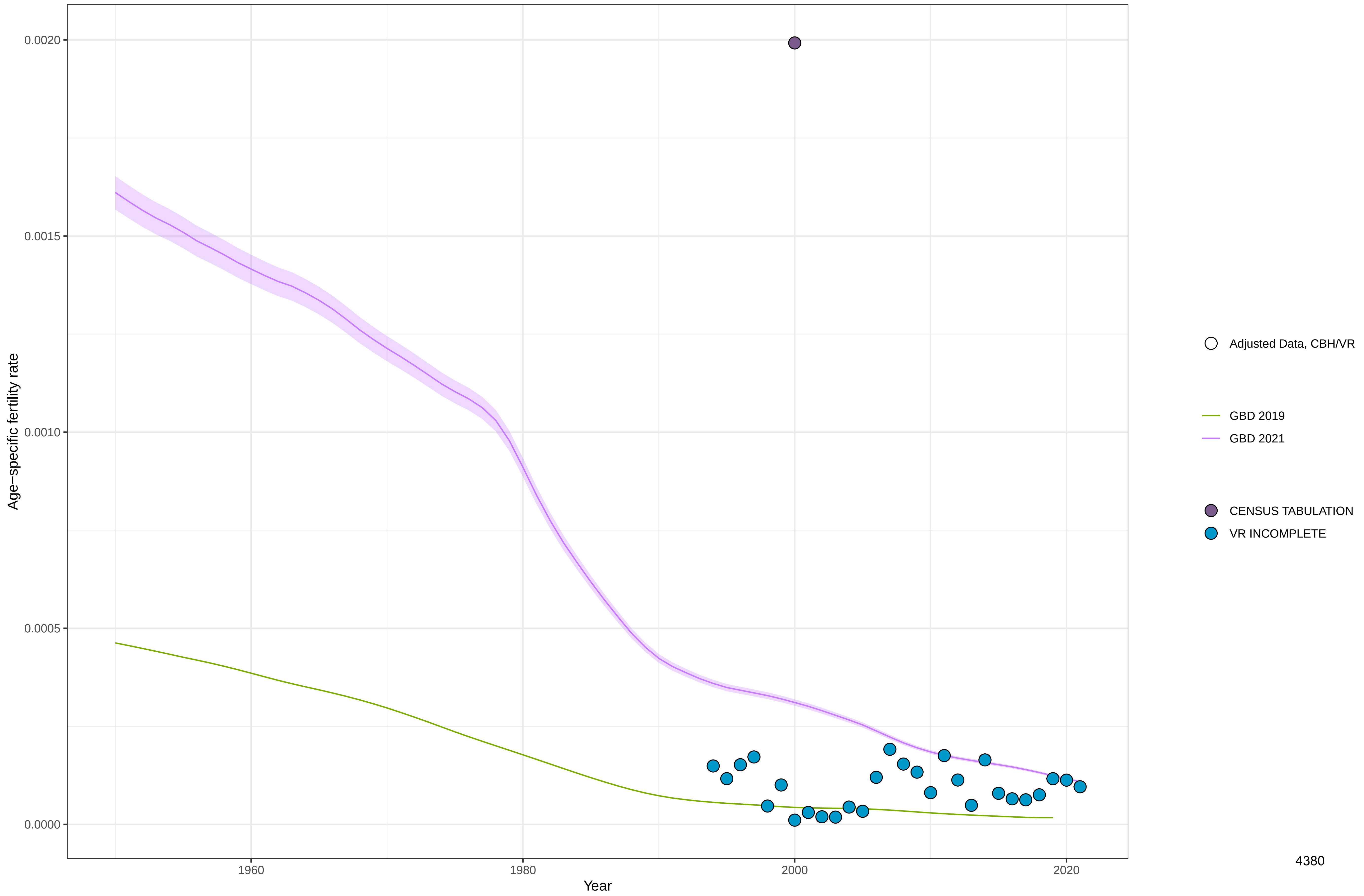

Total fertility rate fitted trend lines, 1950−2021: Pará TFR
Data points enclosed with a diamond are reference sources

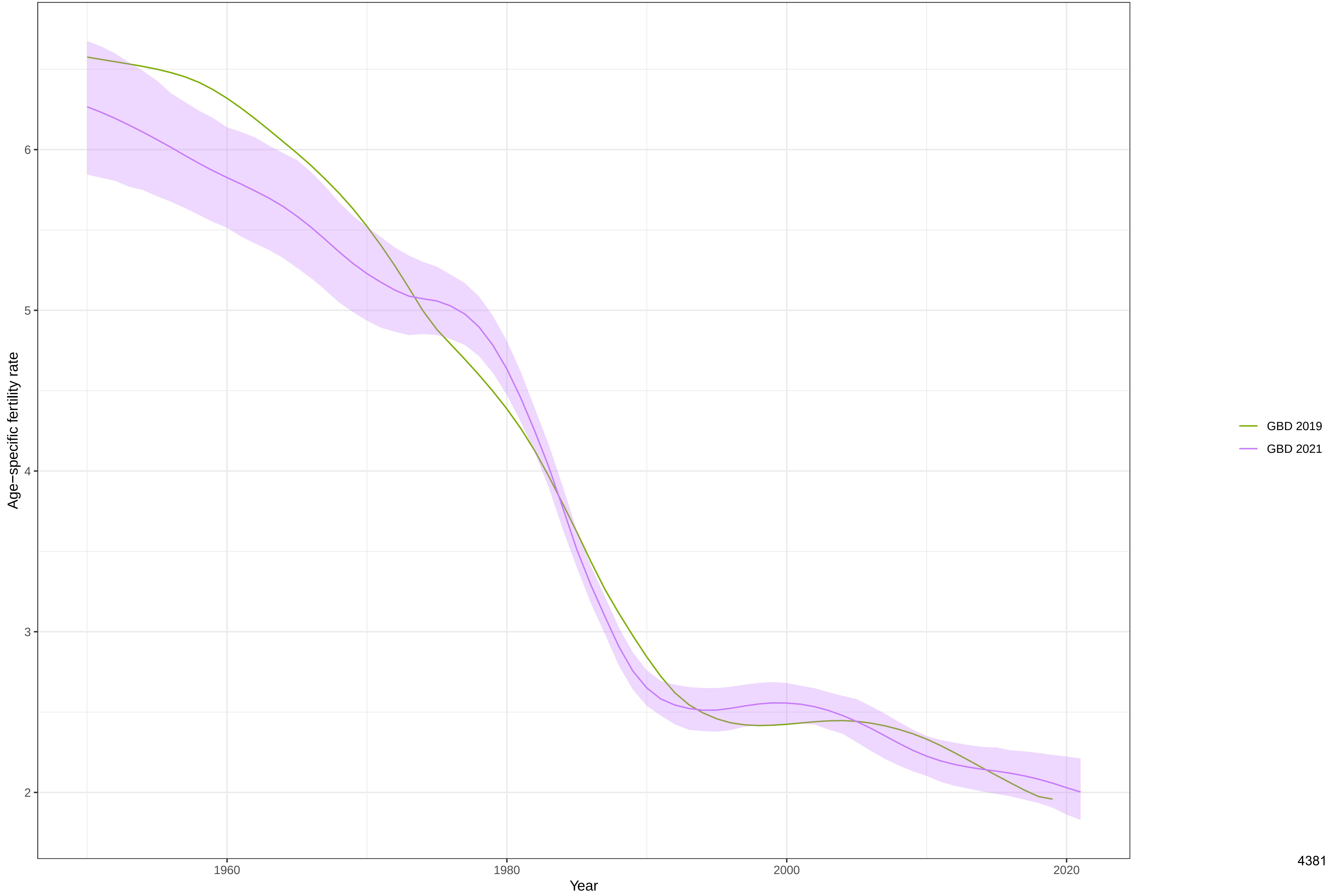

Age−specific fertility fitted trend lines, 1950−2021: Paraíba, Ages: 10−14
Data points enclosed with a diamond are reference sources

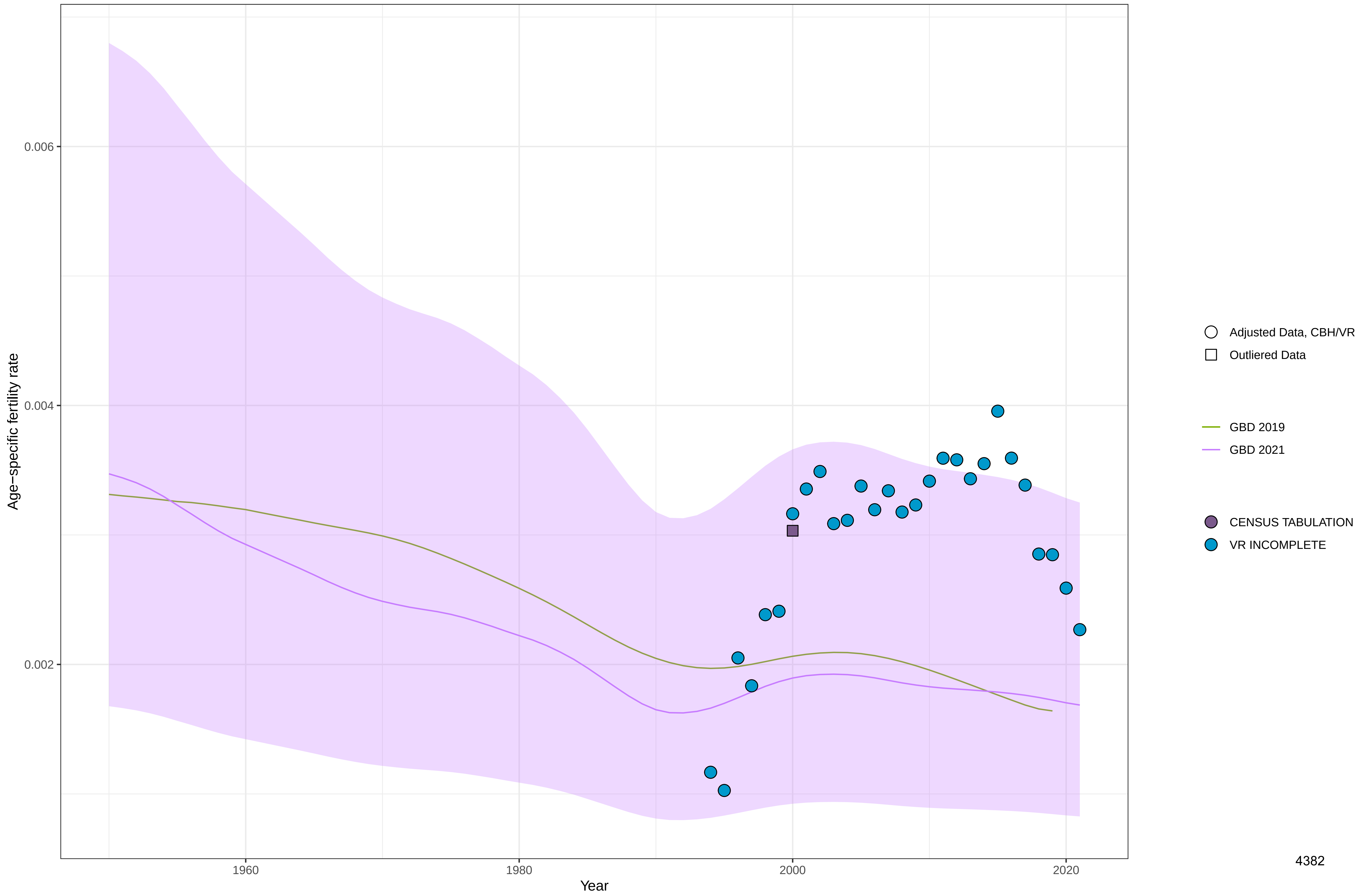

Age−specific fertility fitted trend lines, 1950−2021: Paraíba, Ages: 15−19
Data points enclosed with a diamond are reference sources

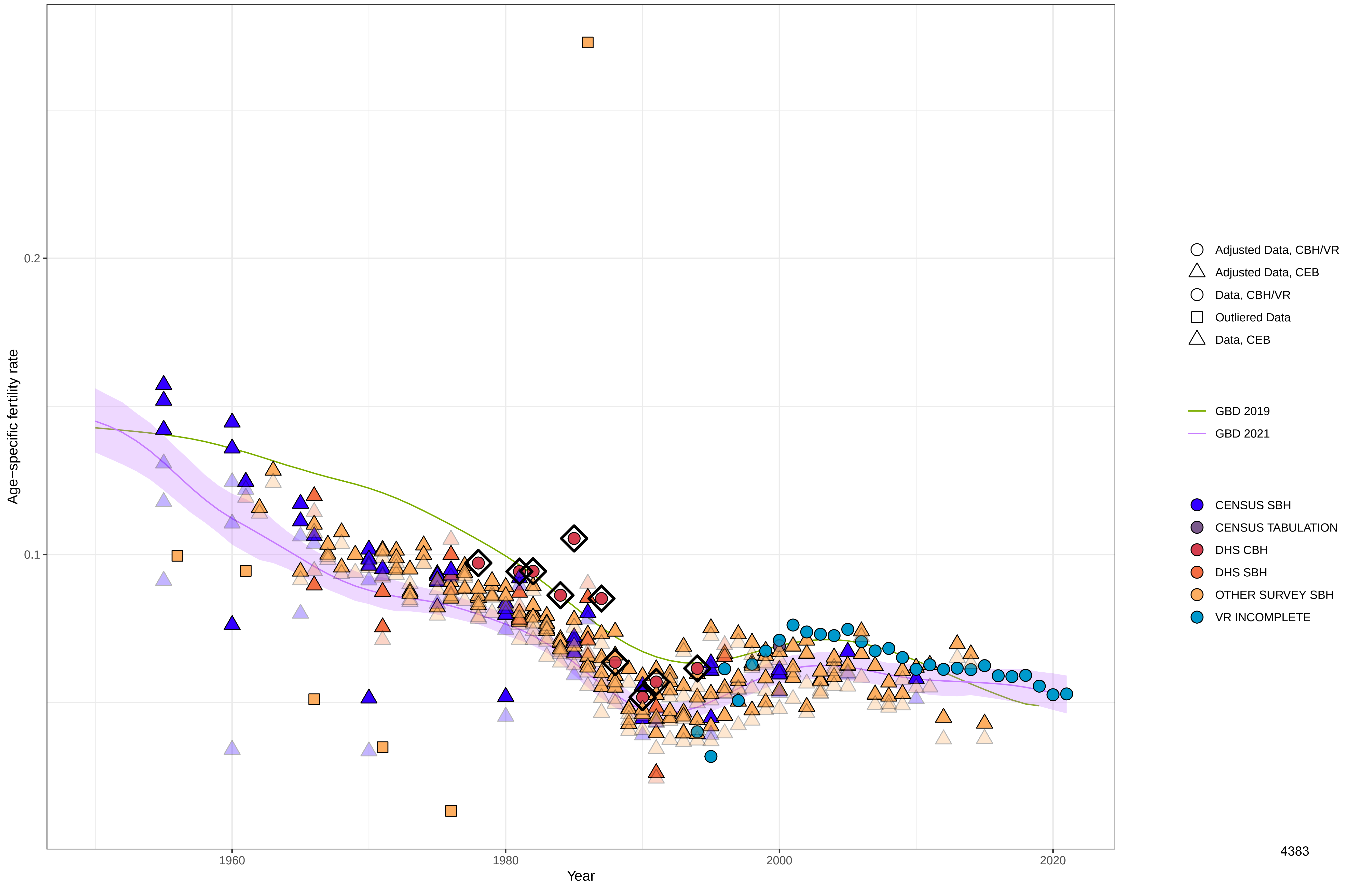

Age−specific fertility fitted trend lines, 1950−2021: Paraíba, Ages: 20−24
Data points enclosed with a diamond are reference sources

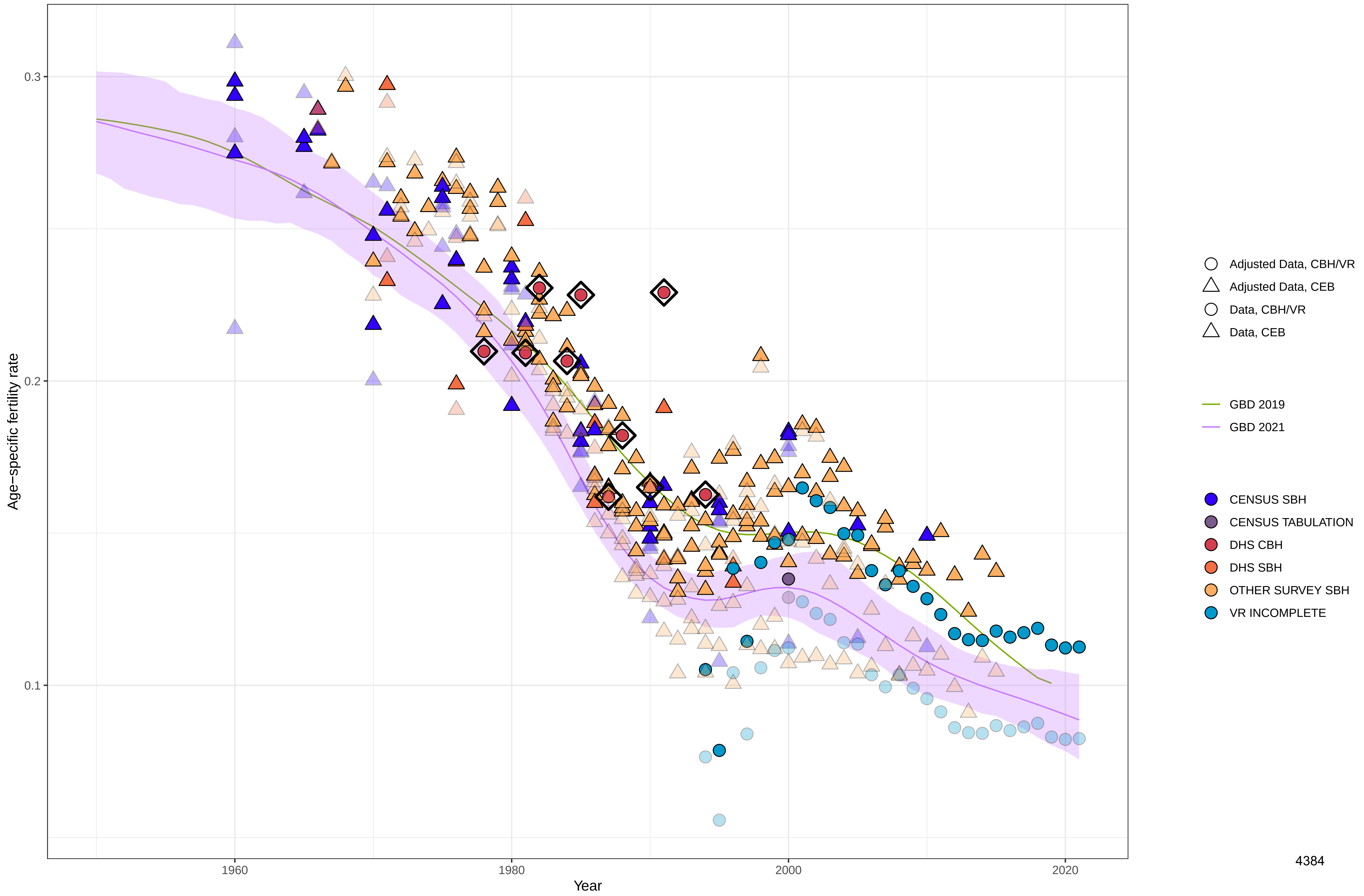

Age−specific fertility fitted trend lines, 1950−2021: Paraíba, Ages: 25−29
Data points enclosed with a diamond are reference sources

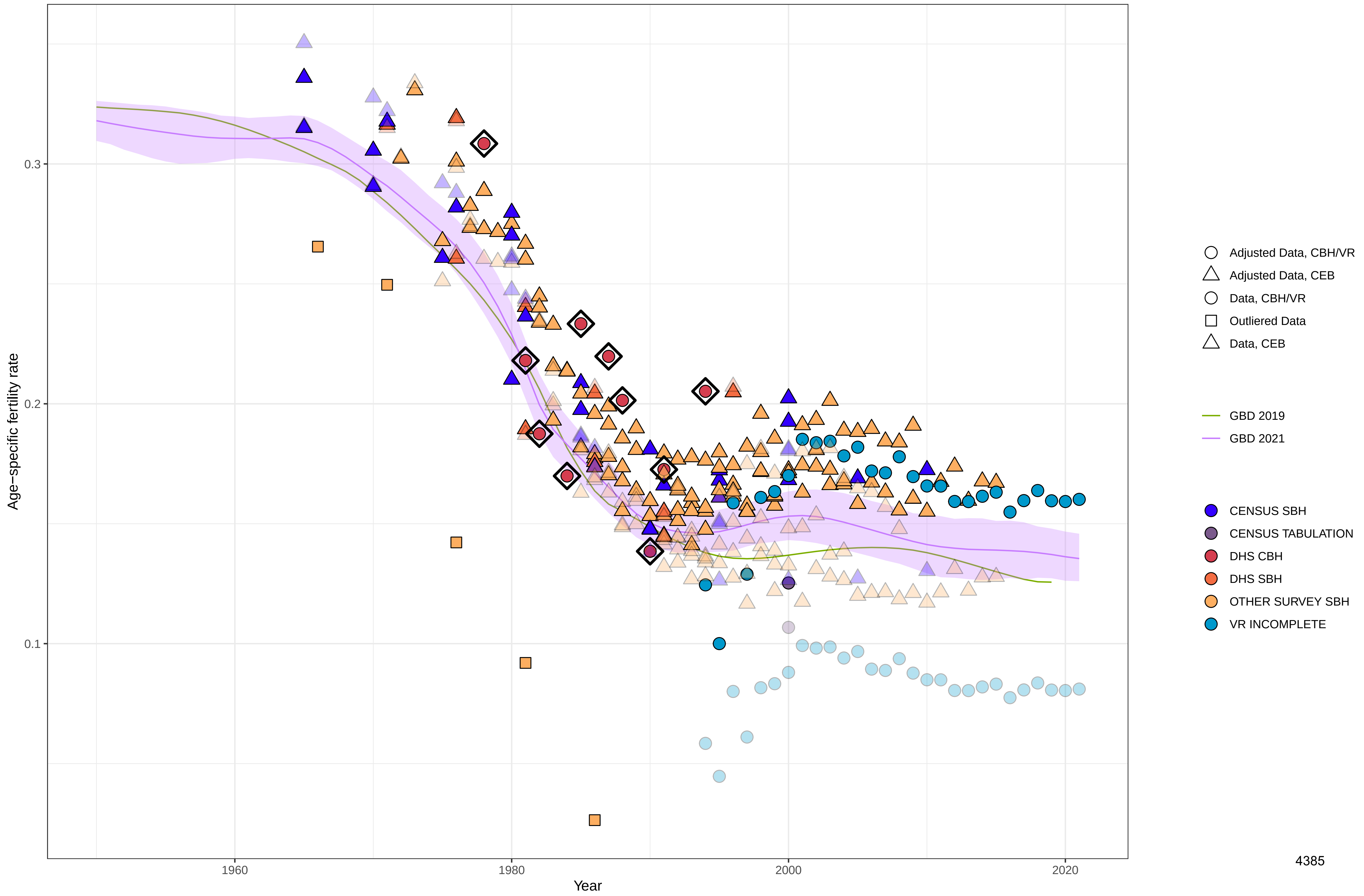

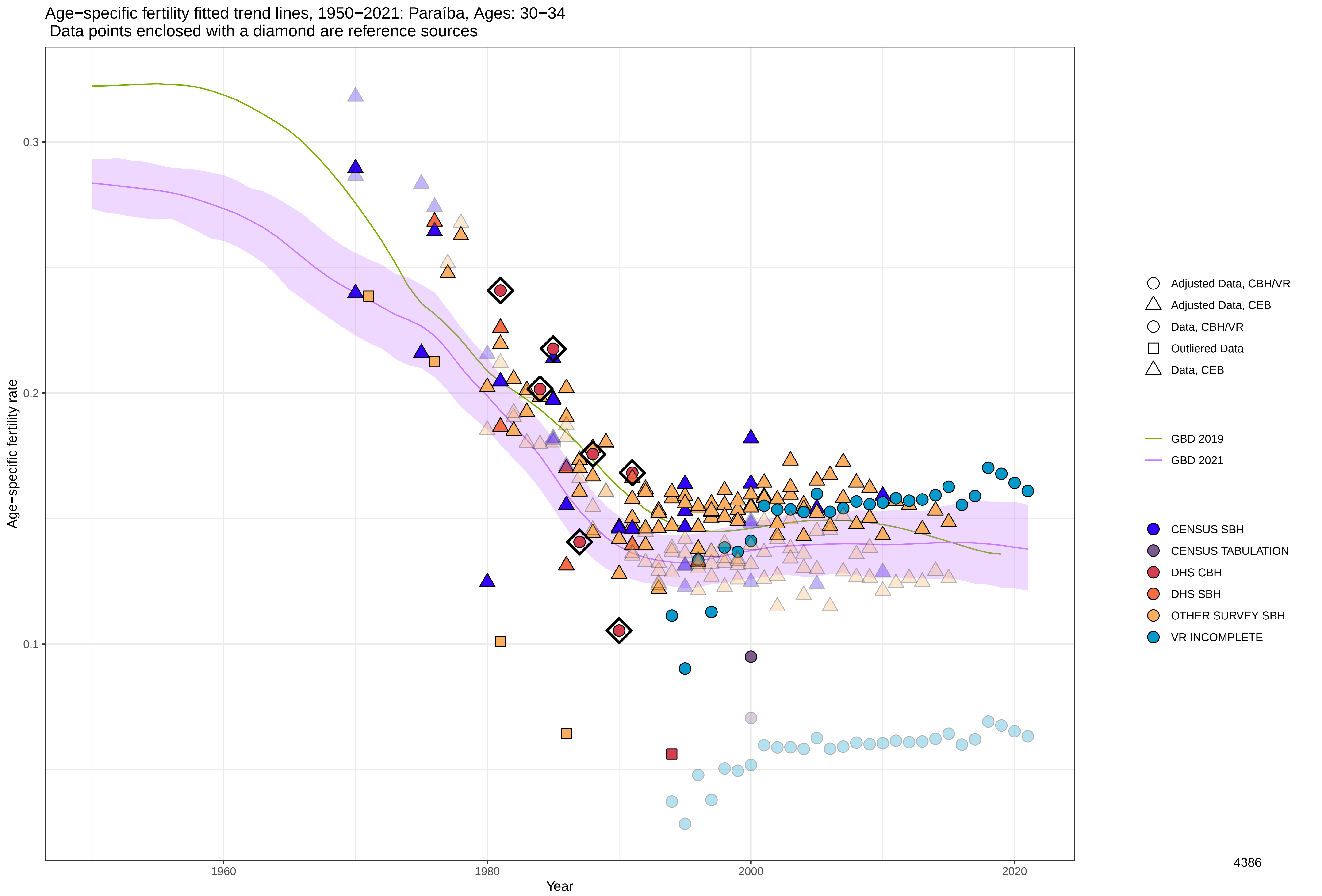

Age−specific fertility fitted trend lines, 1950−2021: Paraíba, Ages: 30−34
Data points enclosed with a diamond are reference sources
Age−specific fertility rate
Year
0.1
0.2
0.3
1960
1980
2000
2020
Adjusted Data, CBH/VR
Adjusted Data, CEB
Data, CBH/VR
Outliered Data
Data, CEB
GBD 2019
GBD 2021
CENSUS SBH
CENSUS TABULATION
DHS CBH
DHS SBH
OTHER SURVEY SBH
VR INCOMPLETE

4386

Age−specific fertility fitted trend lines, 1950−2021: Paraíba, Ages: 35−39
Data points enclosed with a diamond are reference sources

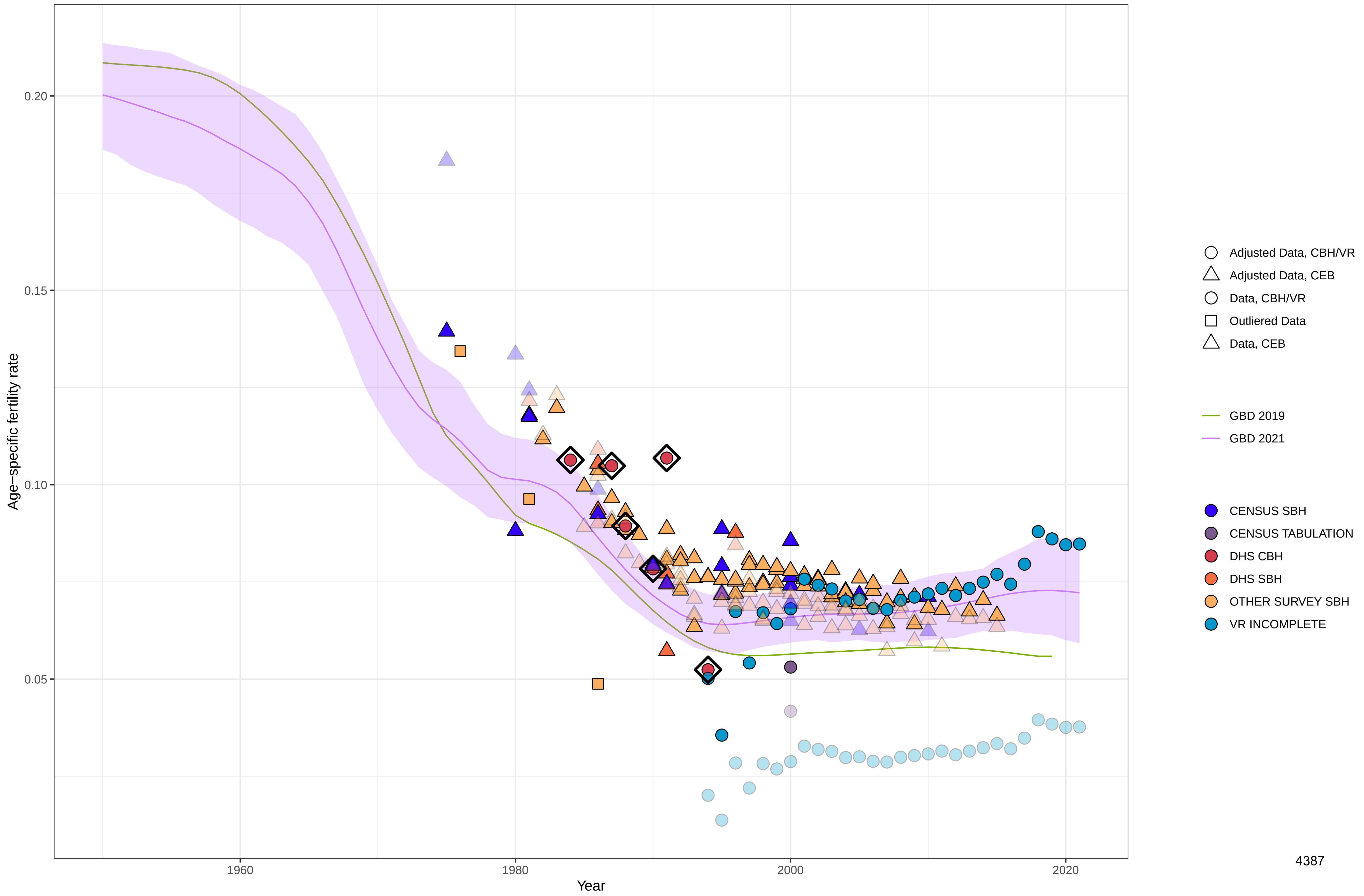

Age−specific fertility fitted trend lines, 1950−2021: Paraíba, Ages: 40−44
Data points enclosed with a diamond are reference sources

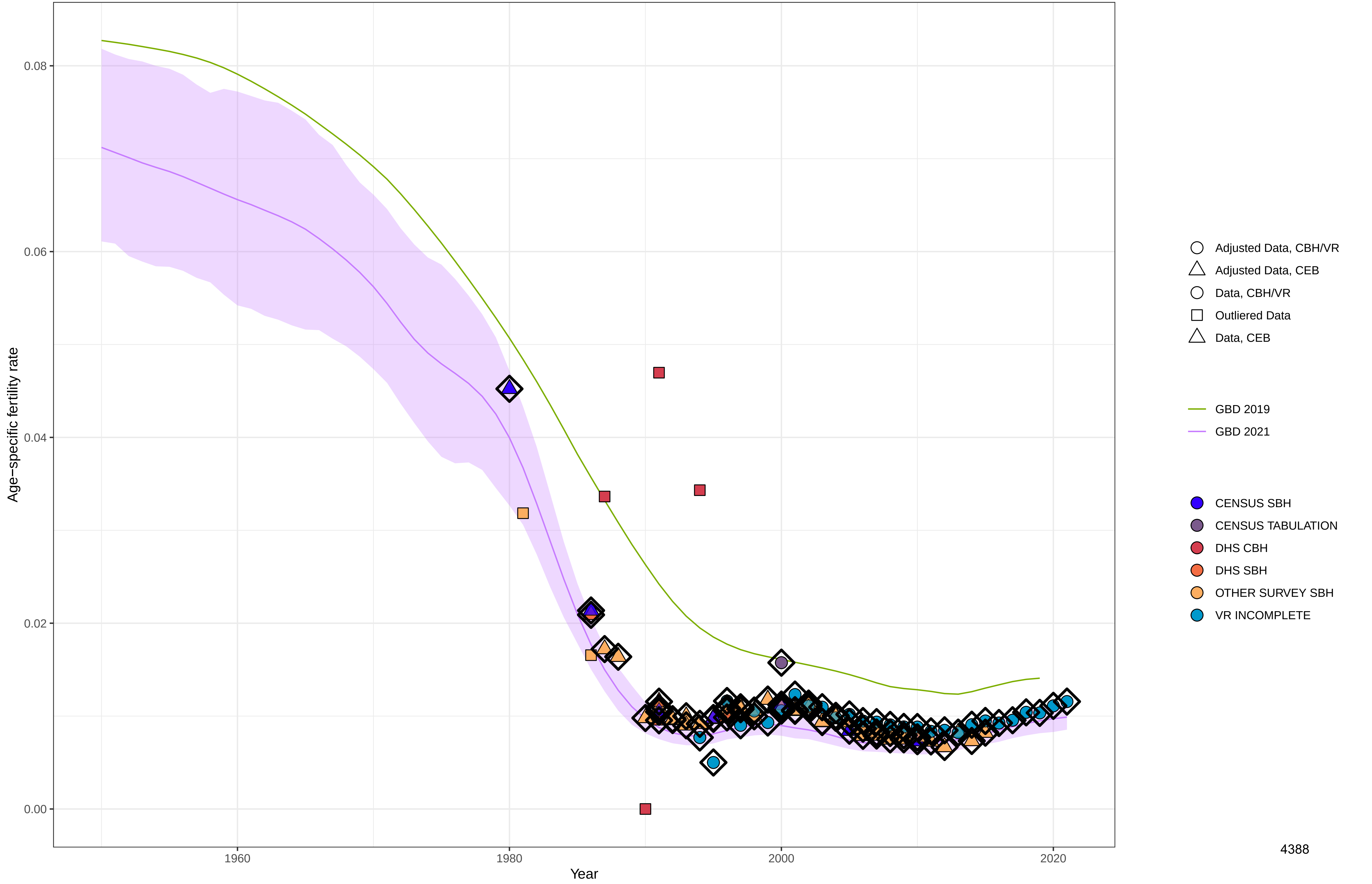

Age−specific fertility fitted trend lines, 1950−2021: Paraíba, Ages: 45−49
Data points enclosed with a diamond are reference sources

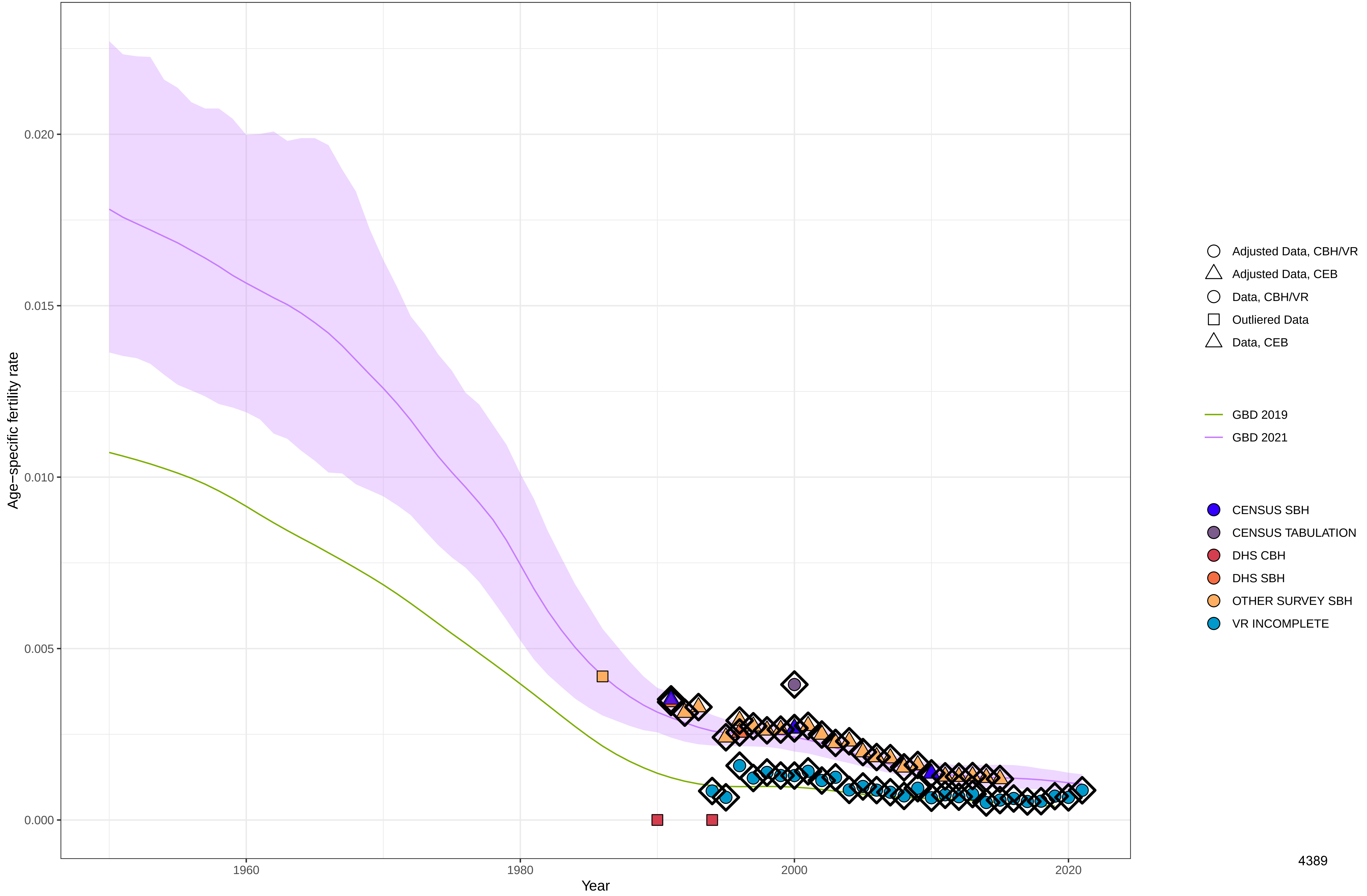

Age−specific fertility fitted trend lines, 1950−2021: Paraíba, Ages: 50−54
Data points enclosed with a diamond are reference sources

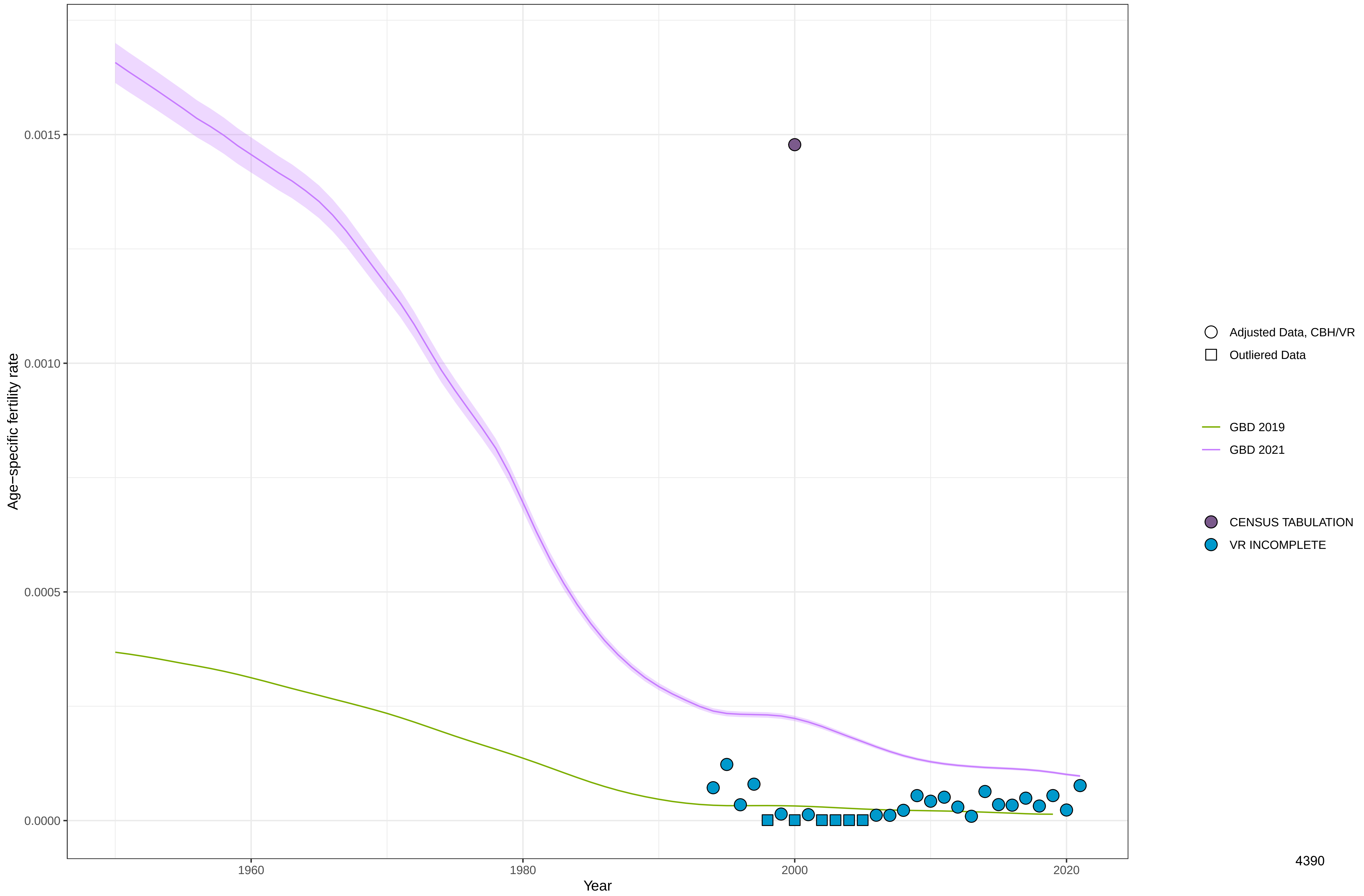

Total fertility rate fitted trend lines, 1950−2021: Paraíba TFR
Data points enclosed with a diamond are reference sources

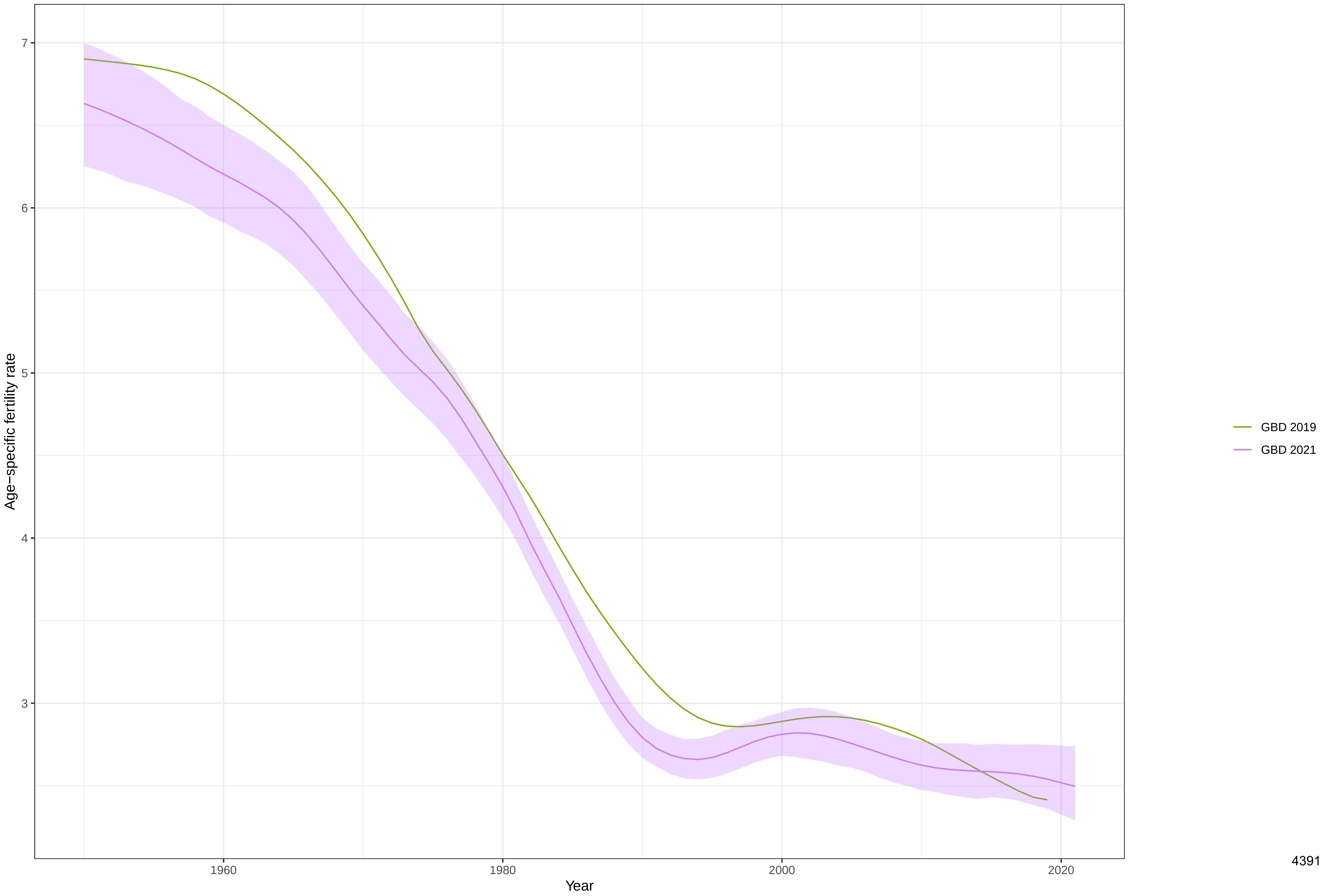

Age−specific fertility fitted trend lines, 1950−2021: Paraná, Ages: 10−14
Data points enclosed with a diamond are reference sources

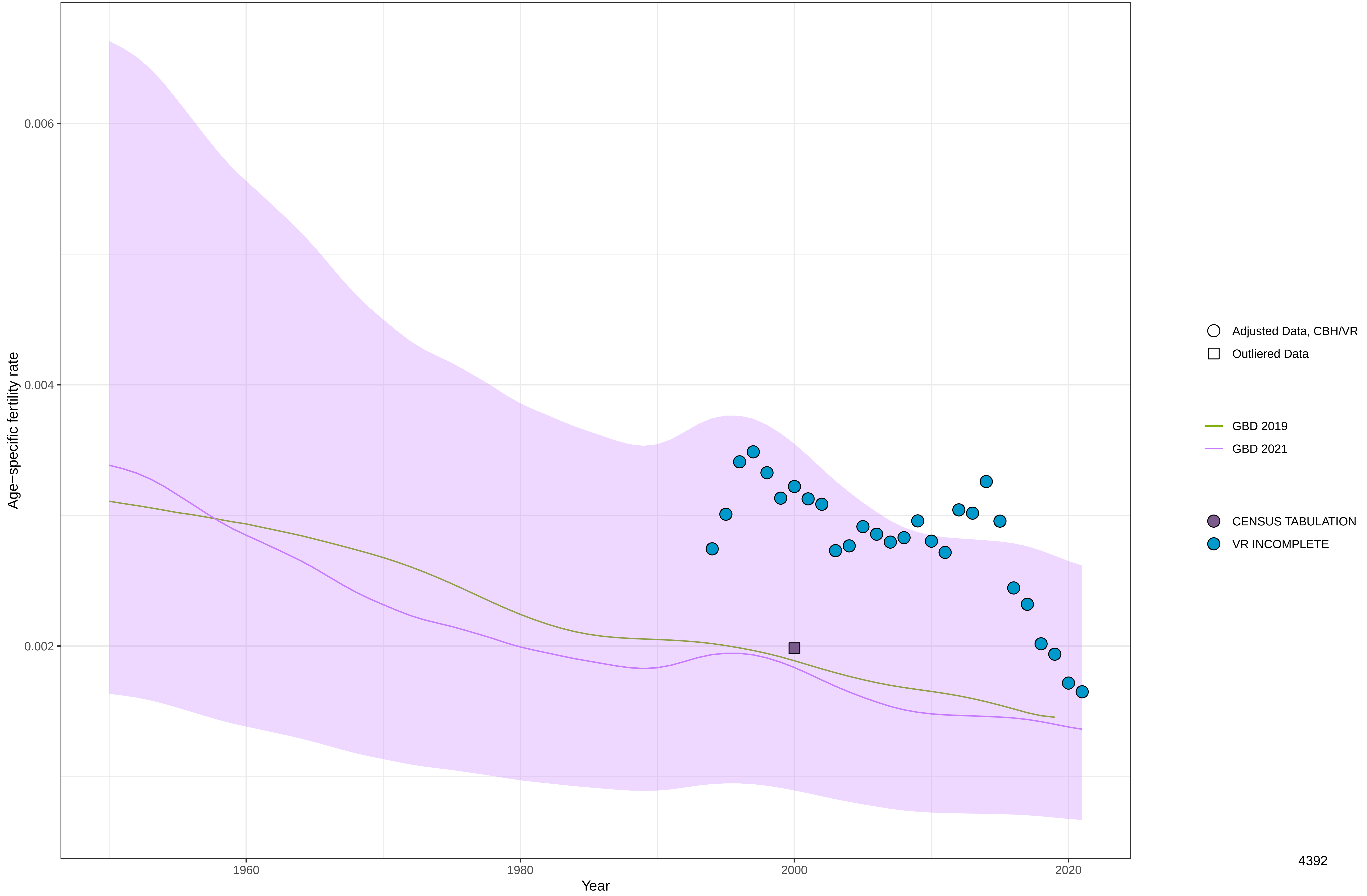

4392

Age−specific fertility fitted trend lines, 1950−2021: Paraná, Ages: 15−19
Data points enclosed with a diamond are reference sources

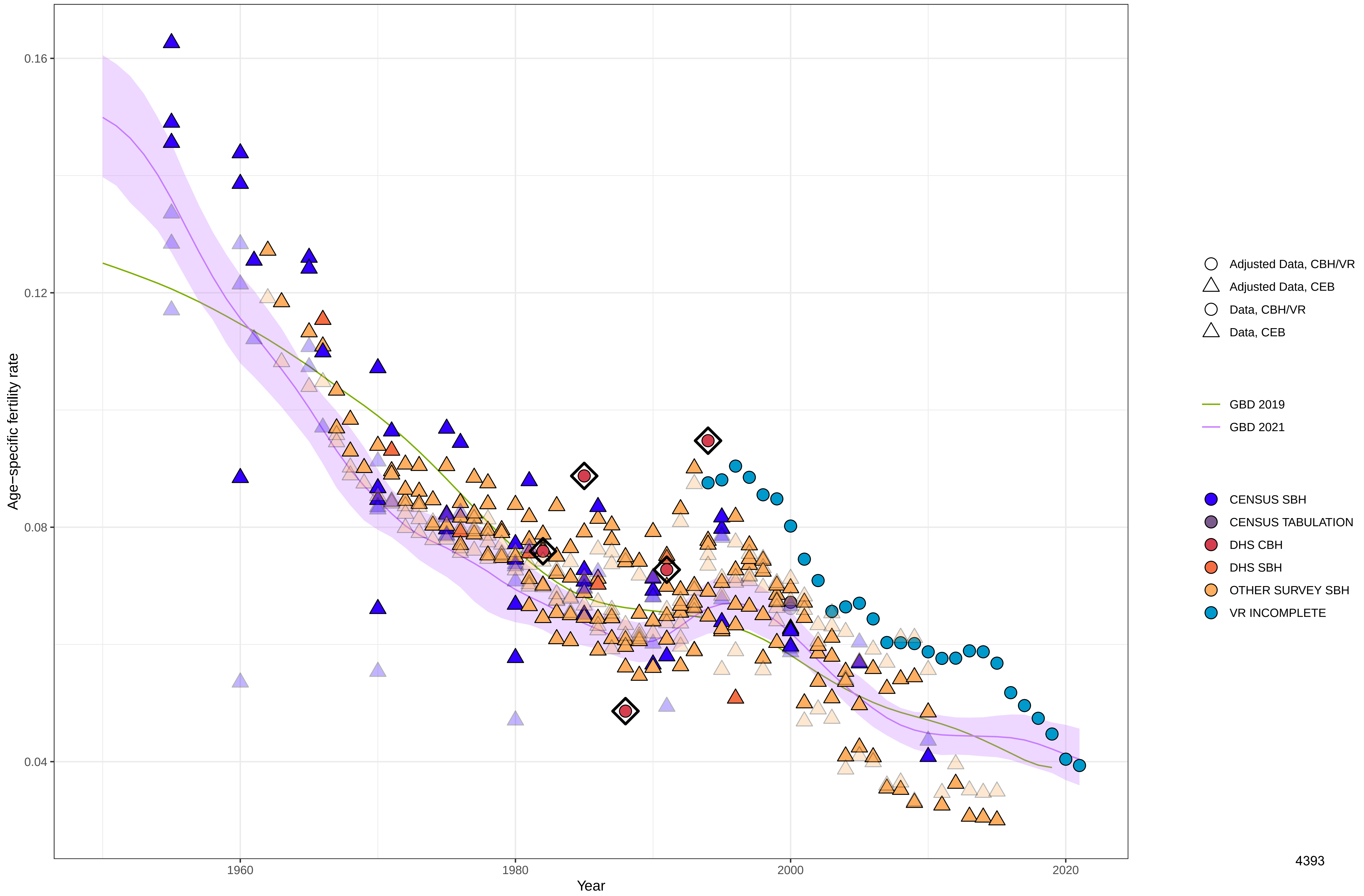

Age−specific fertility fitted trend lines, 1950−2021: Paraná, Ages: 20−24
Data points enclosed with a diamond are reference sources

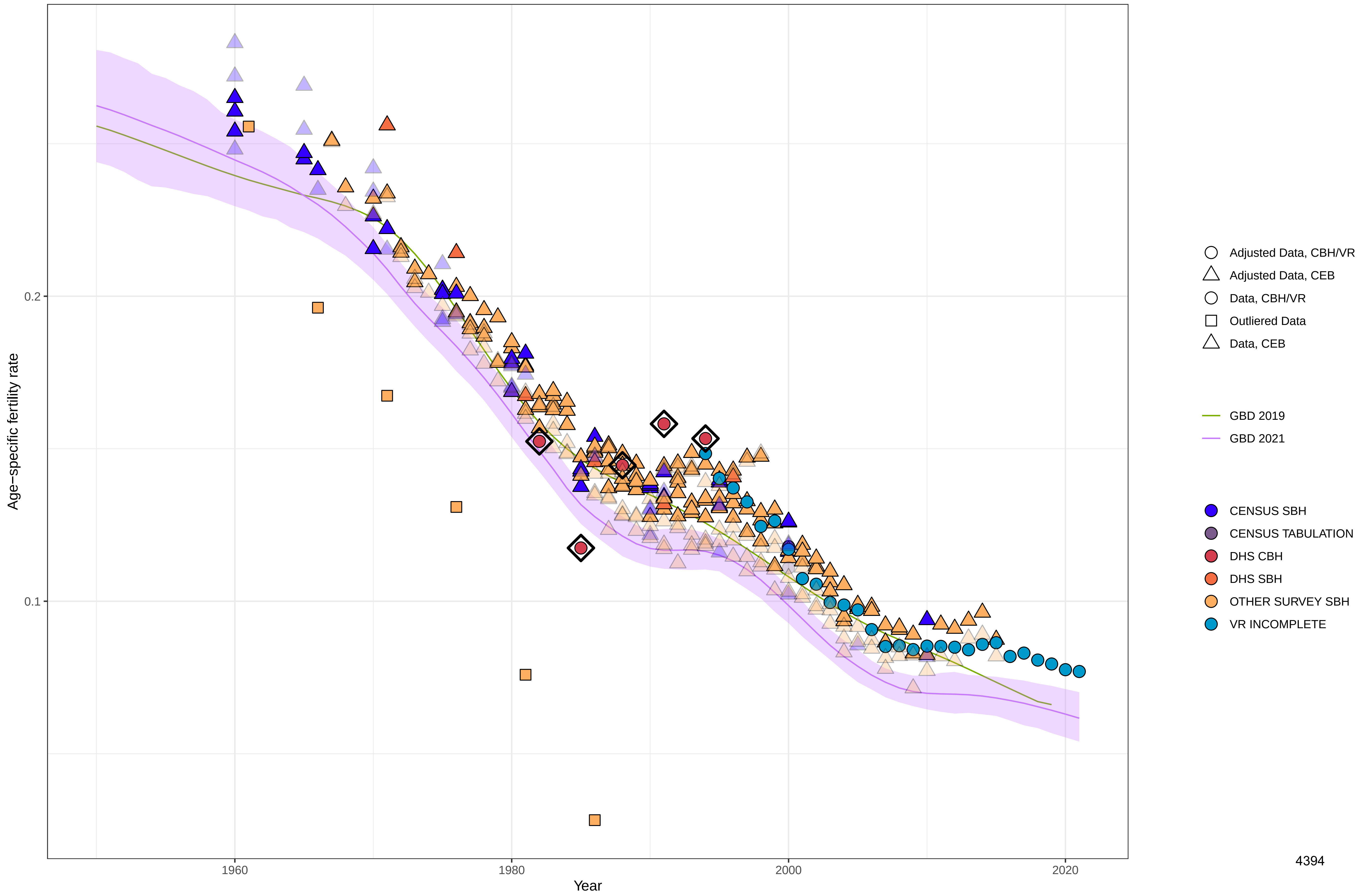

Age−specific fertility fitted trend lines, 1950−2021: Paraná, Ages: 25−29
Data points enclosed with a diamond are reference sources

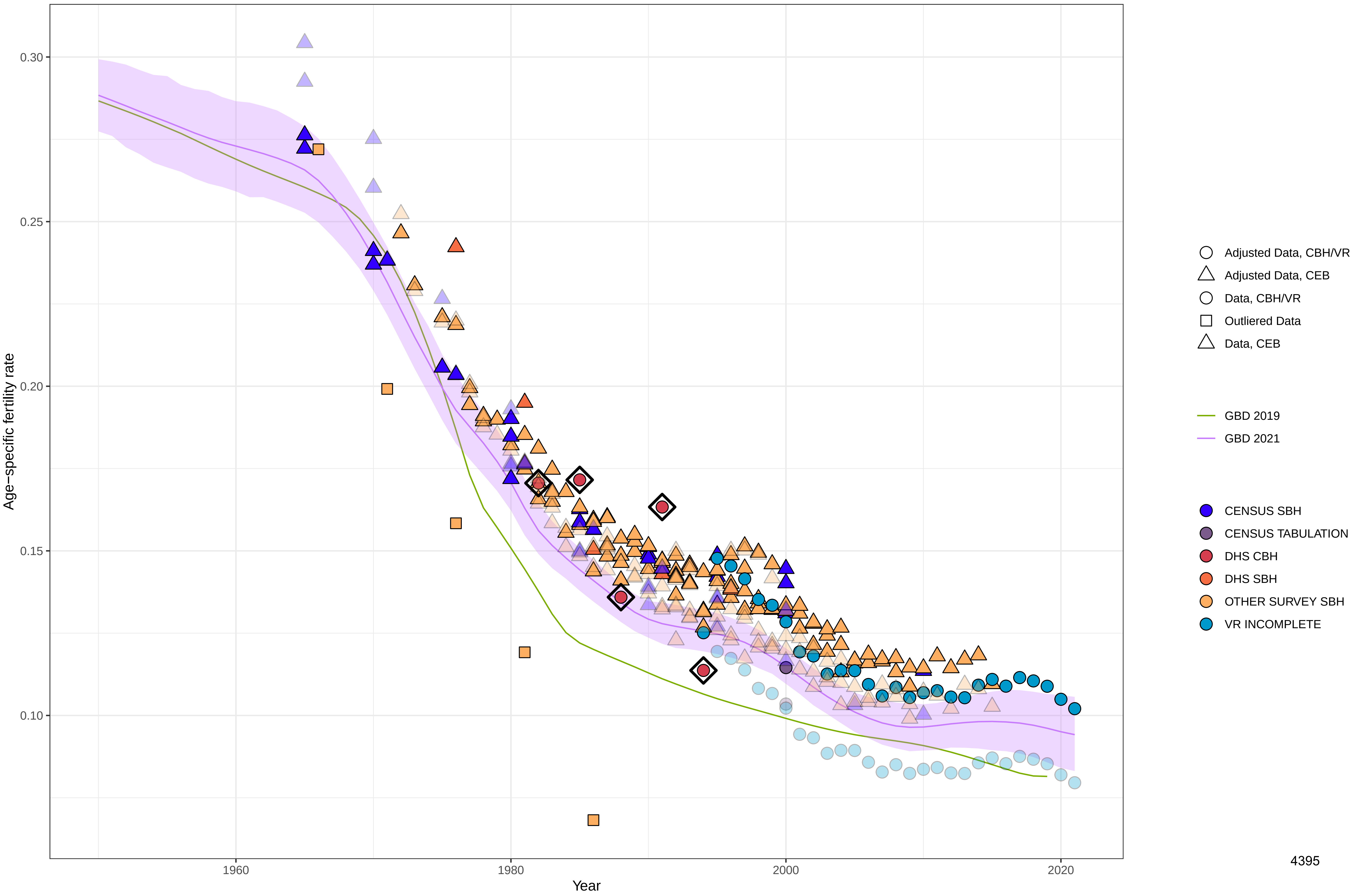

Age−specific fertility fitted trend lines, 1950−2021: Paraná, Ages: 30−34
Data points enclosed with a diamond are reference sources

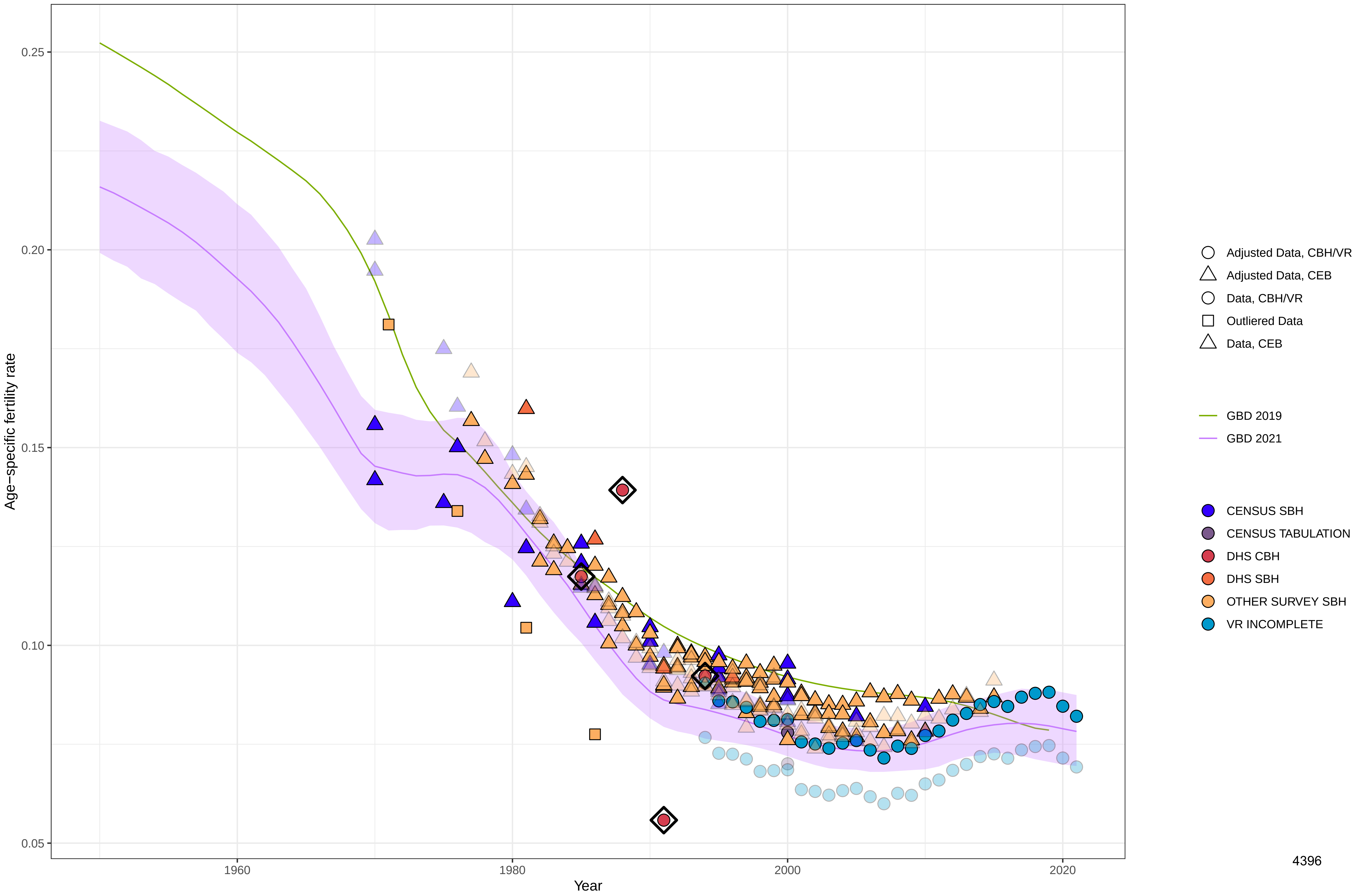

Age−specific fertility fitted trend lines, 1950−2021: Paraná, Ages: 35−39
Data points enclosed with a diamond are reference sources

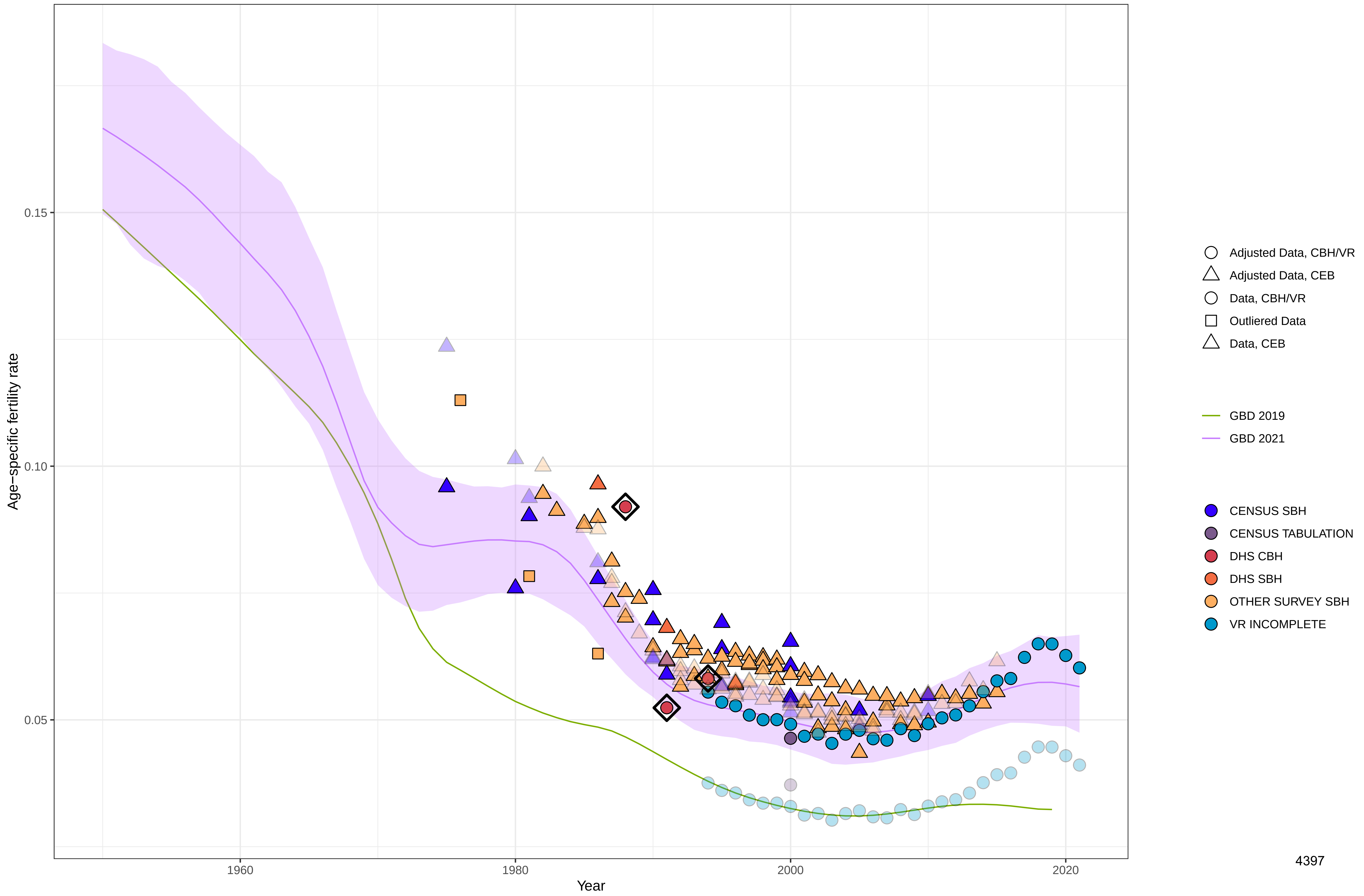

Age−specific fertility fitted trend lines, 1950−2021: Paraná, Ages: 40−44
Data points enclosed with a diamond are reference sources

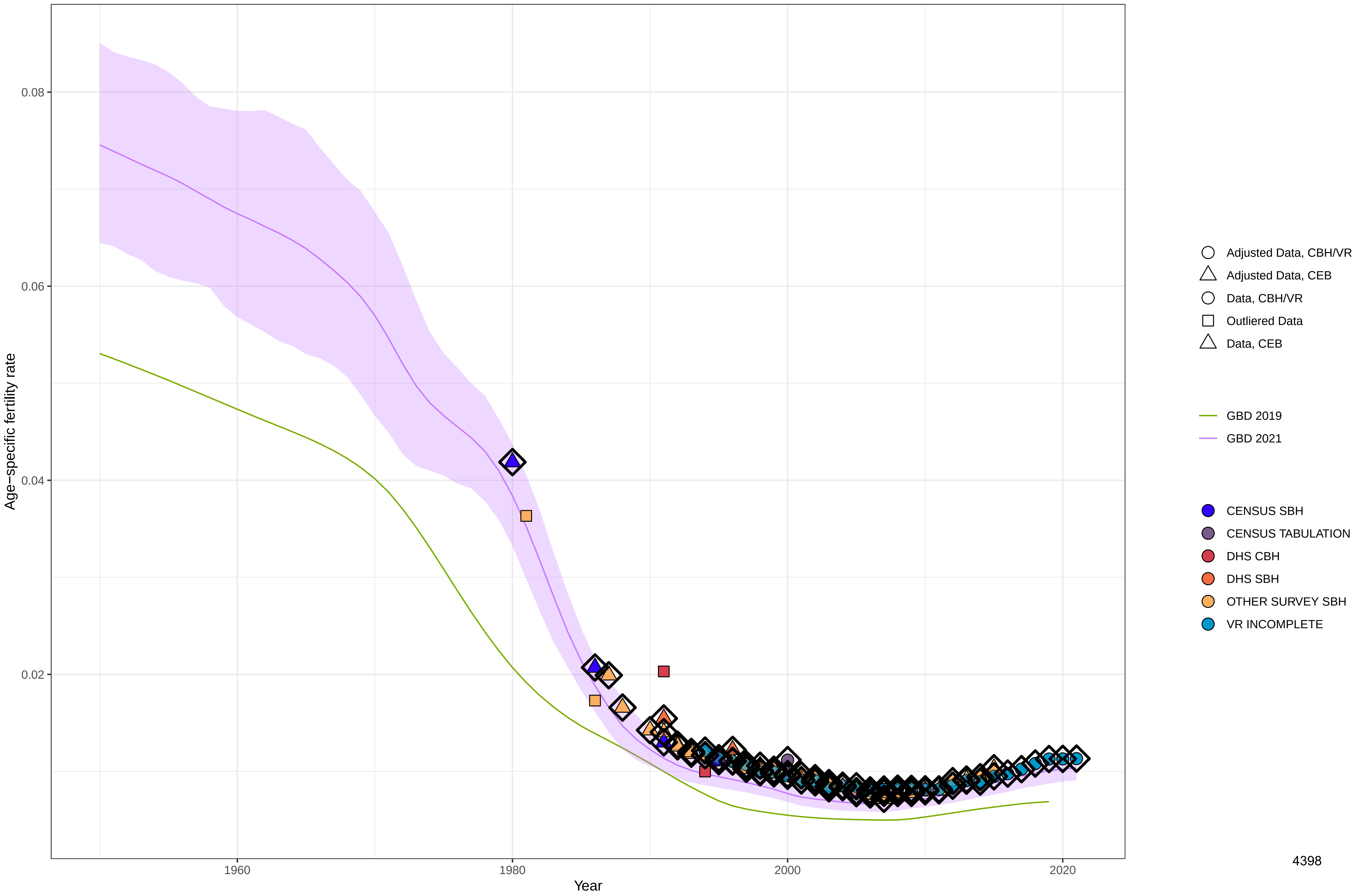

4398

Age−specific fertility fitted trend lines, 1950−2021: Paraná, Ages: 45−49
Data points enclosed with a diamond are reference sources

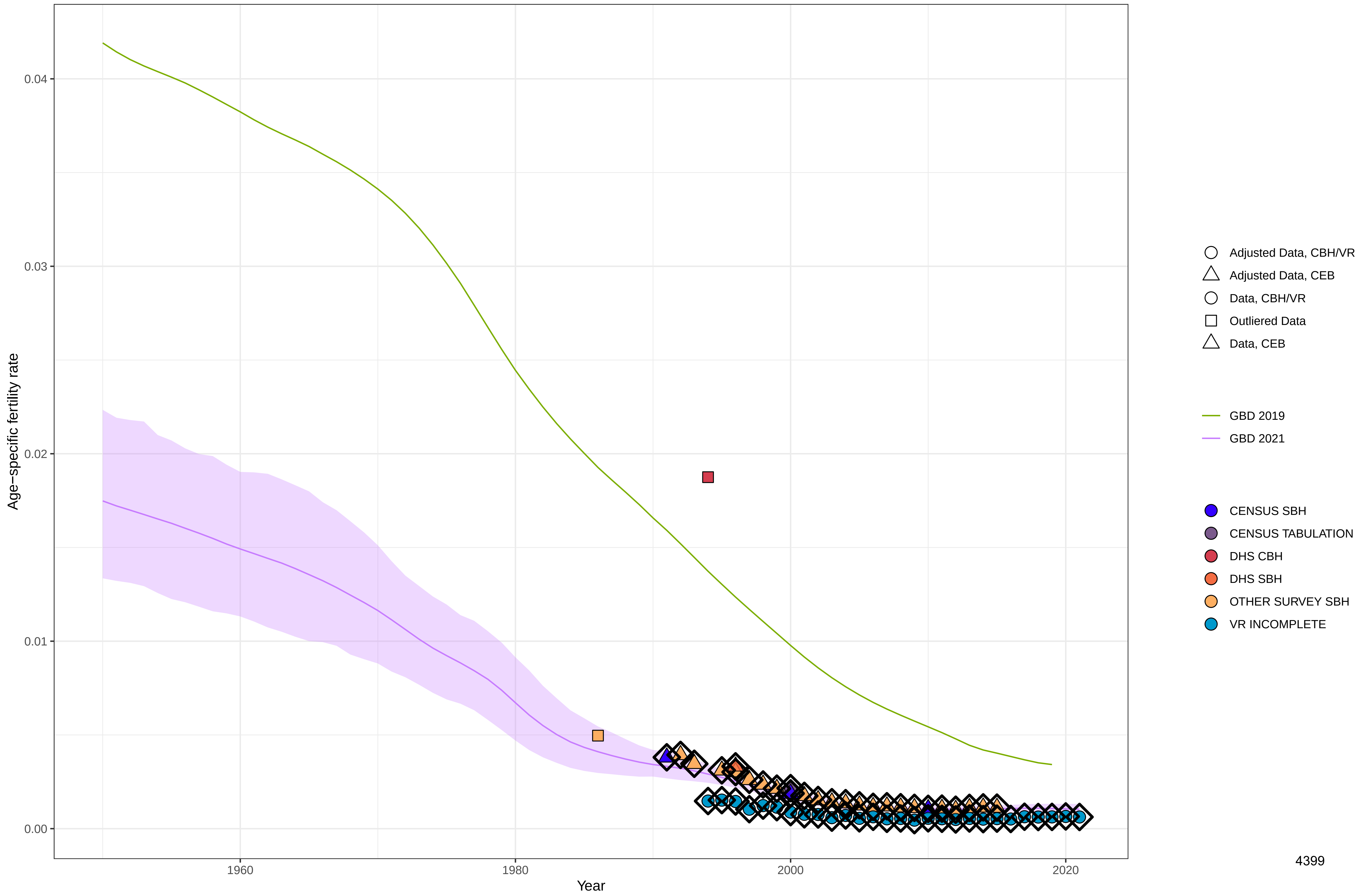

Age−specific fertility fitted trend lines, 1950−2021: Paraná, Ages: 50−54
Data points enclosed with a diamond are reference sources

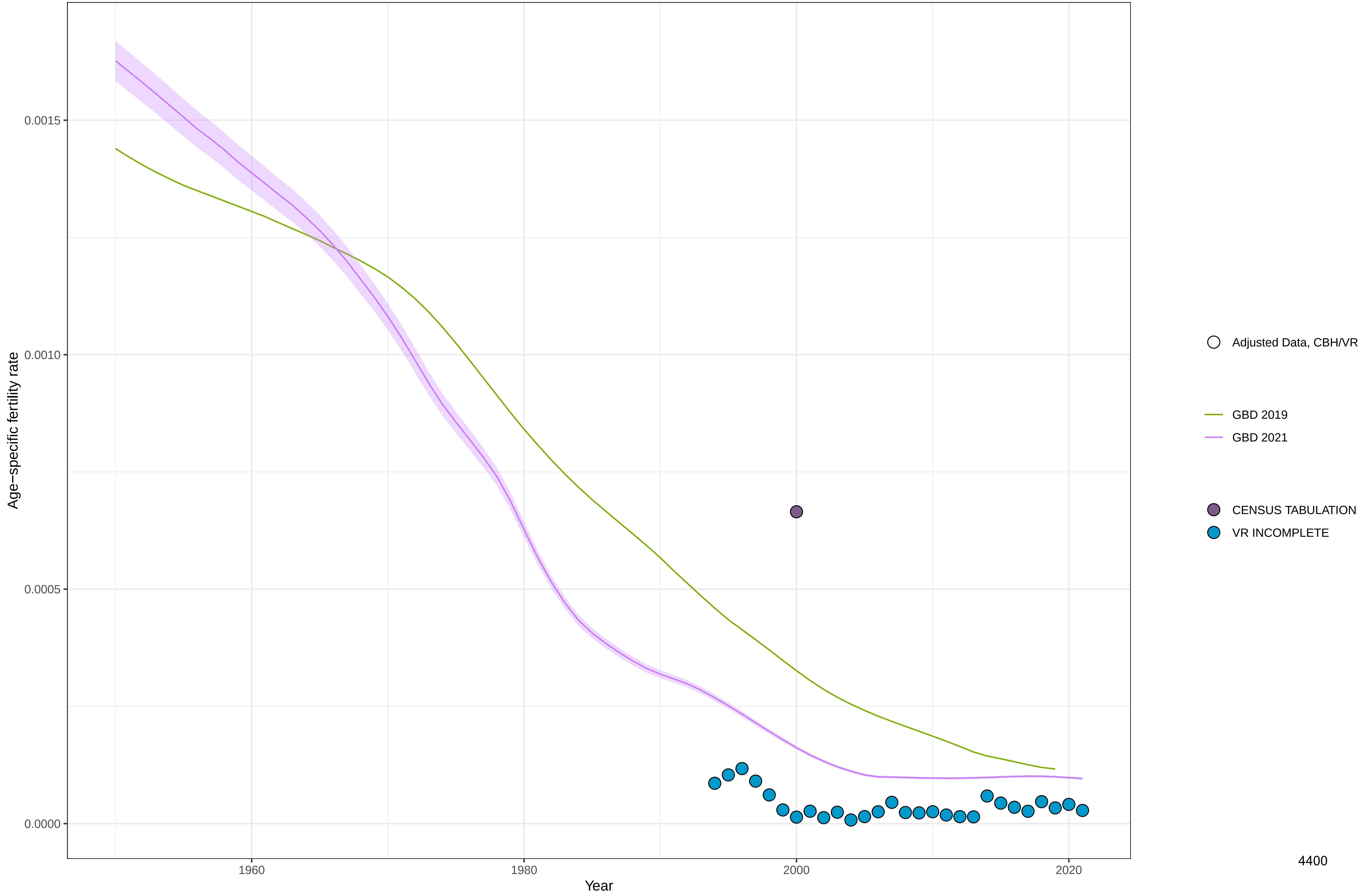

Total fertility rate fitted trend lines, 1950−2021: Paraná TFR
Data points enclosed with a diamond are reference sources

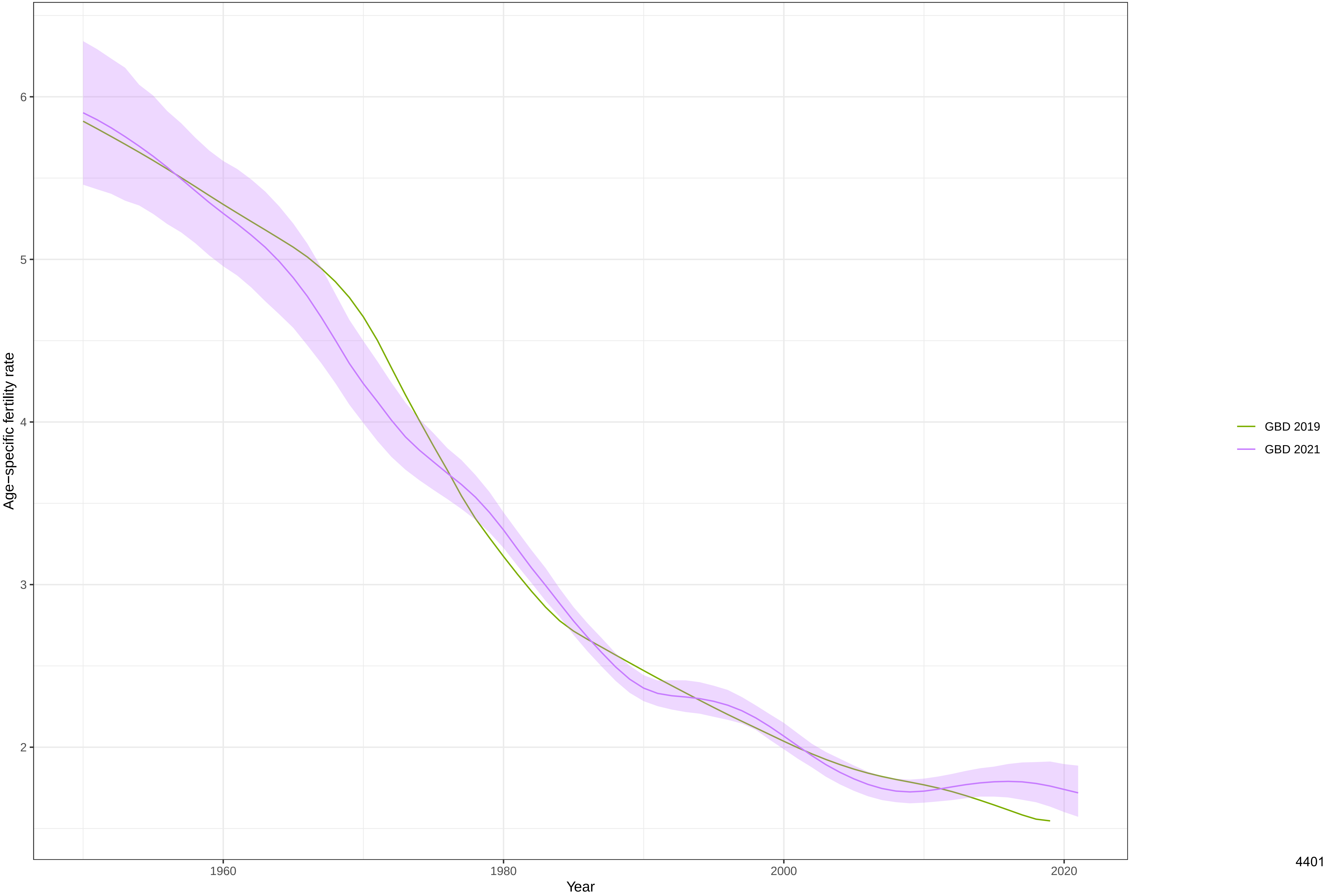

Age−specific fertility fitted trend lines, 1950−2021: Pernambuco, Ages: 10−14
Data points enclosed with a diamond are reference sources

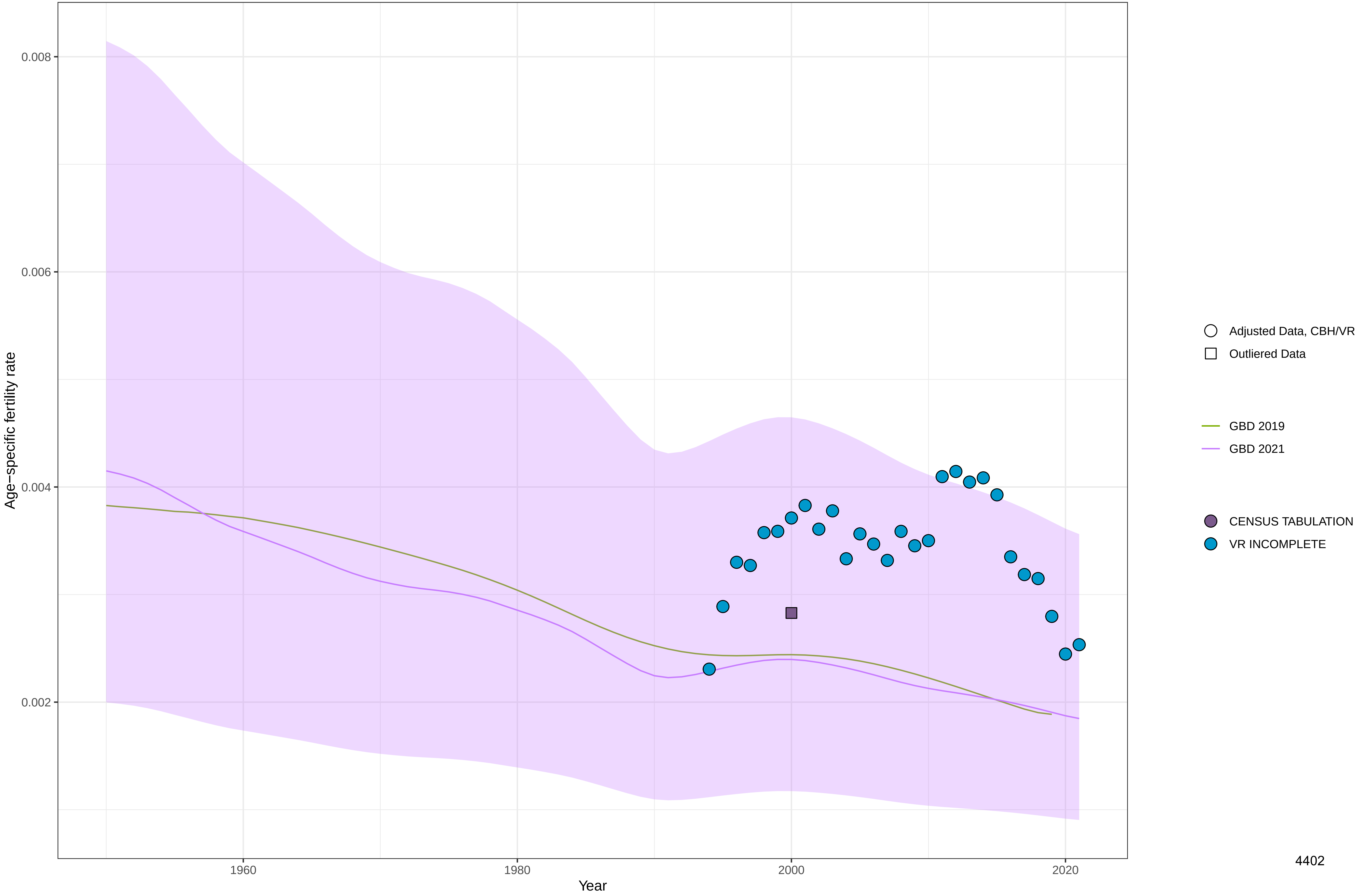

Age−specific fertility fitted trend lines, 1950−2021: Pernambuco, Ages: 15−19
Data points enclosed with a diamond are reference sources

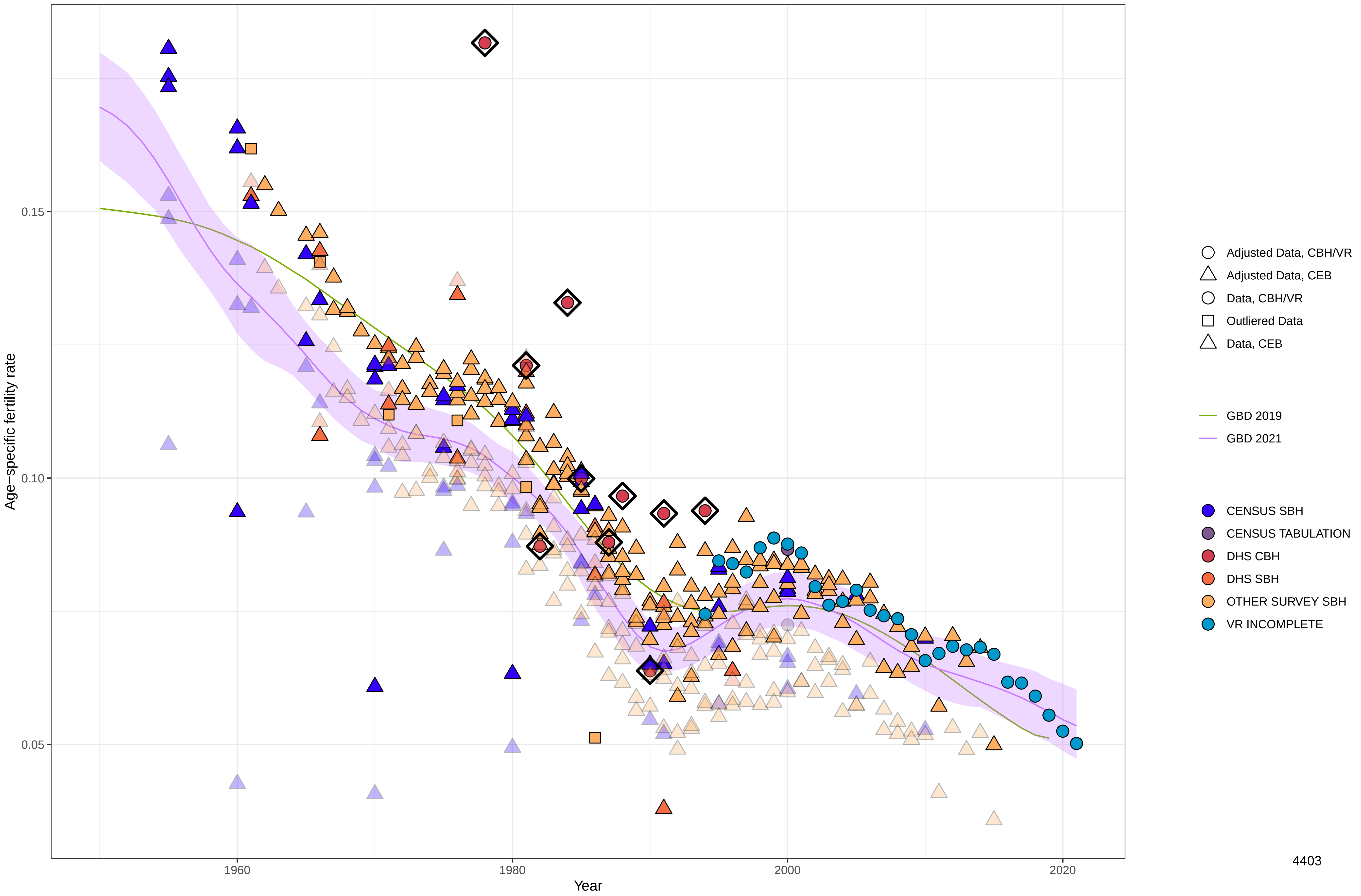

Age−specific fertility fitted trend lines, 1950−2021: Pernambuco, Ages: 20−24
Data points enclosed with a diamond are reference sources

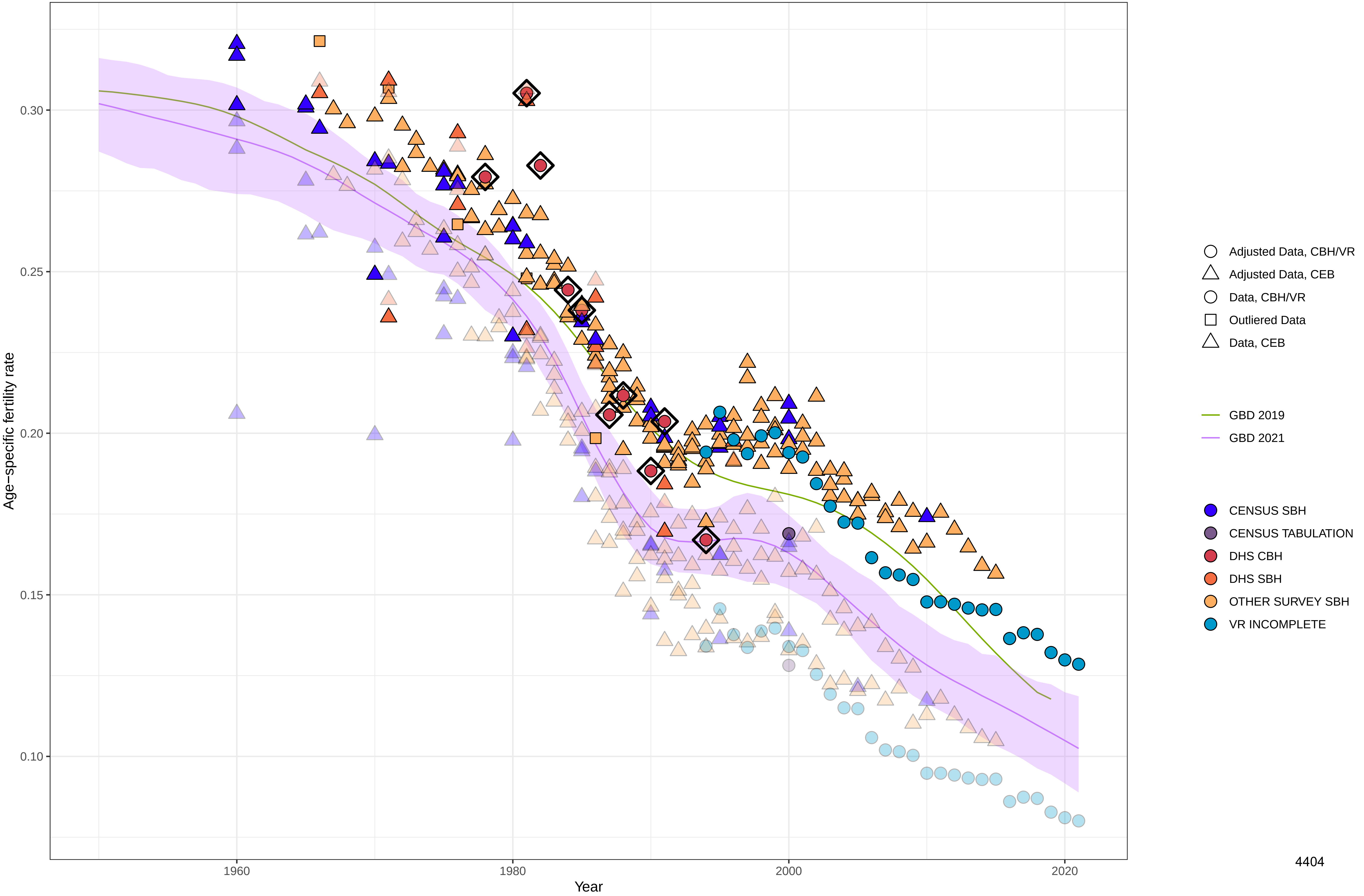

Age−specific fertility fitted trend lines, 1950−2021: Pernambuco, Ages: 25−29
Data points enclosed with a diamond are reference sources

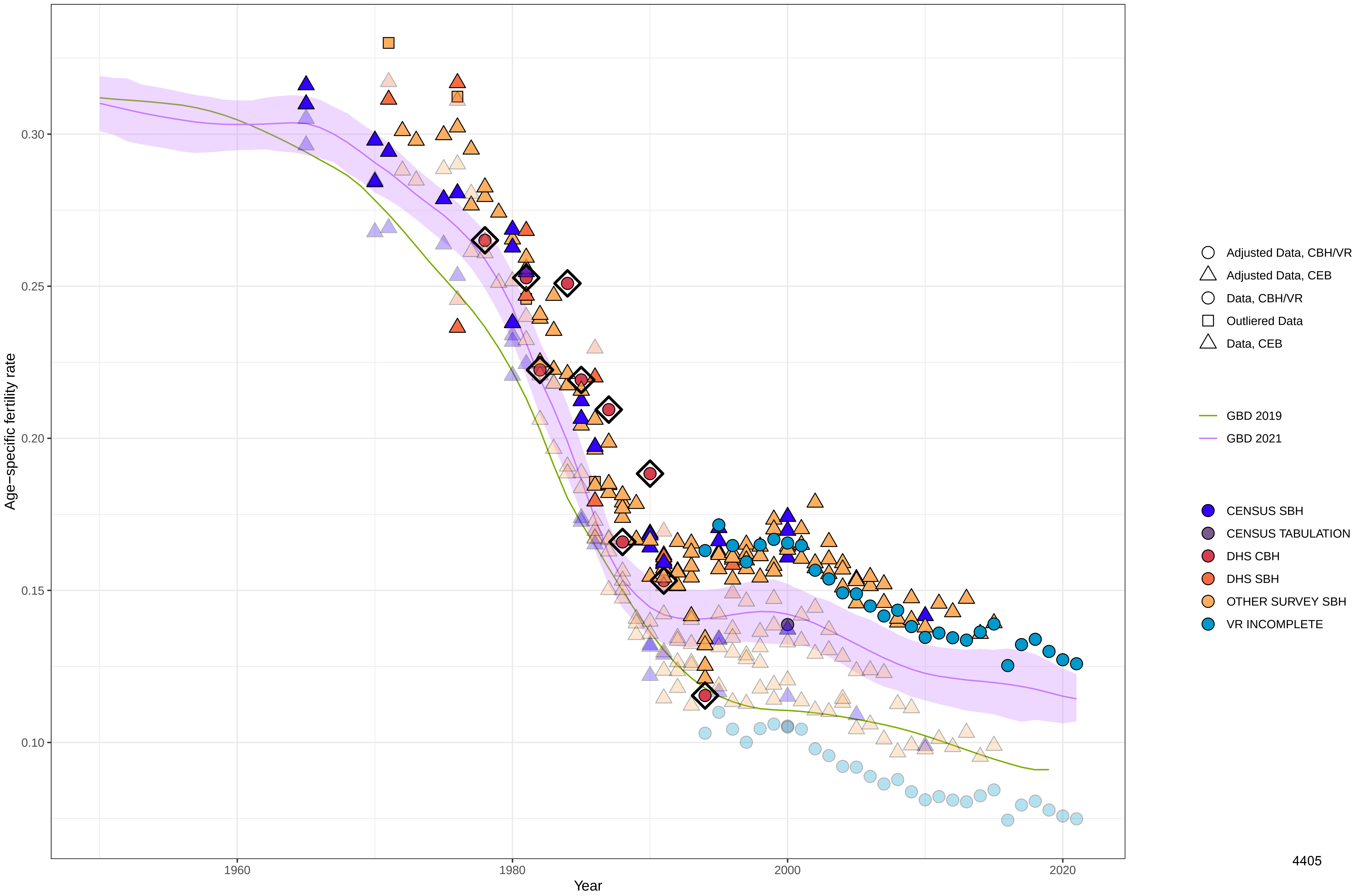

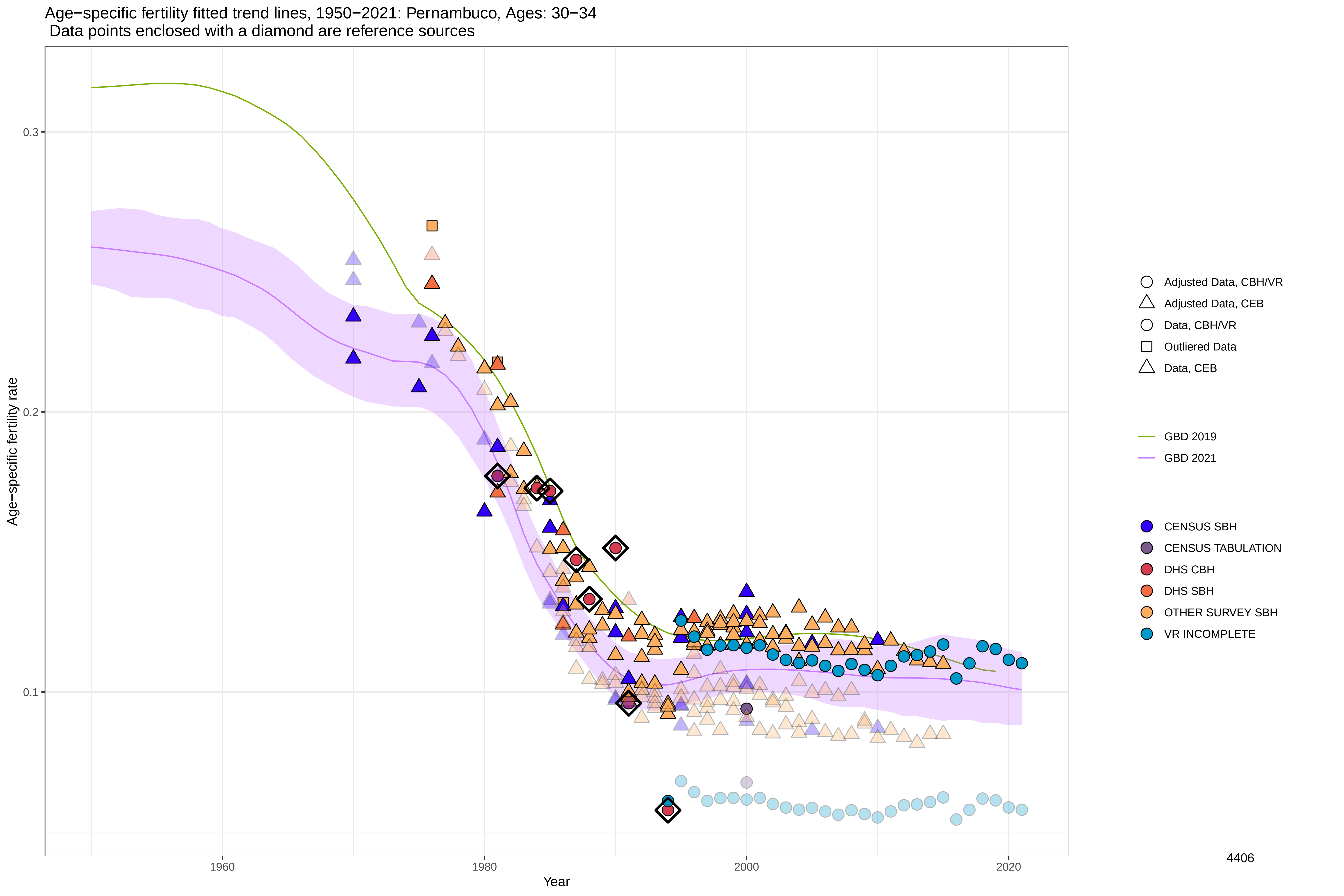

Age−specific fertility fitted trend lines, 1950−2021: Pernambuco, Ages: 30−34
Data points enclosed with a diamond are reference sources
Age−specific fertility rate
0.3
0.2
0.1
Year
1960
1980
2000
2020
Adjusted Data, CBH/VR
Adjusted Data, CEB
Data, CBH/VR
Outliered Data
Data, CEB
GBD 2019
GBD 2021
CENSUS SBH
CENSUS TABULATION
DHS CBH
DHS SBH
OTHER SURVEY SBH
VR INCOMPLETE

Age−specific fertility fitted trend lines, 1950−2021: Pernambuco, Ages: 35−39
Data points enclosed with a diamond are reference sources

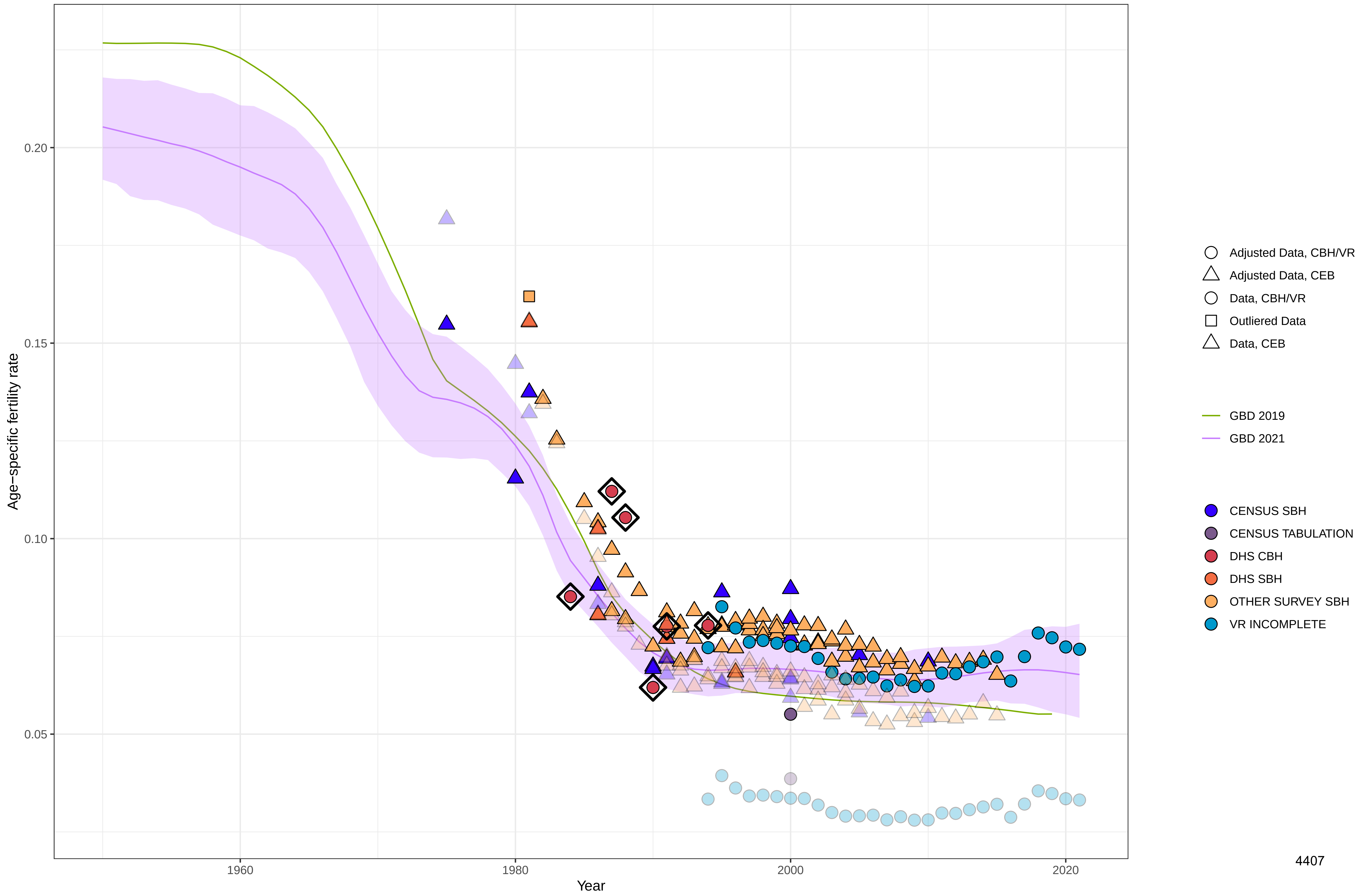

Age−specific fertility fitted trend lines, 1950−2021: Pernambuco, Ages: 40−44
Data points enclosed with a diamond are reference sources

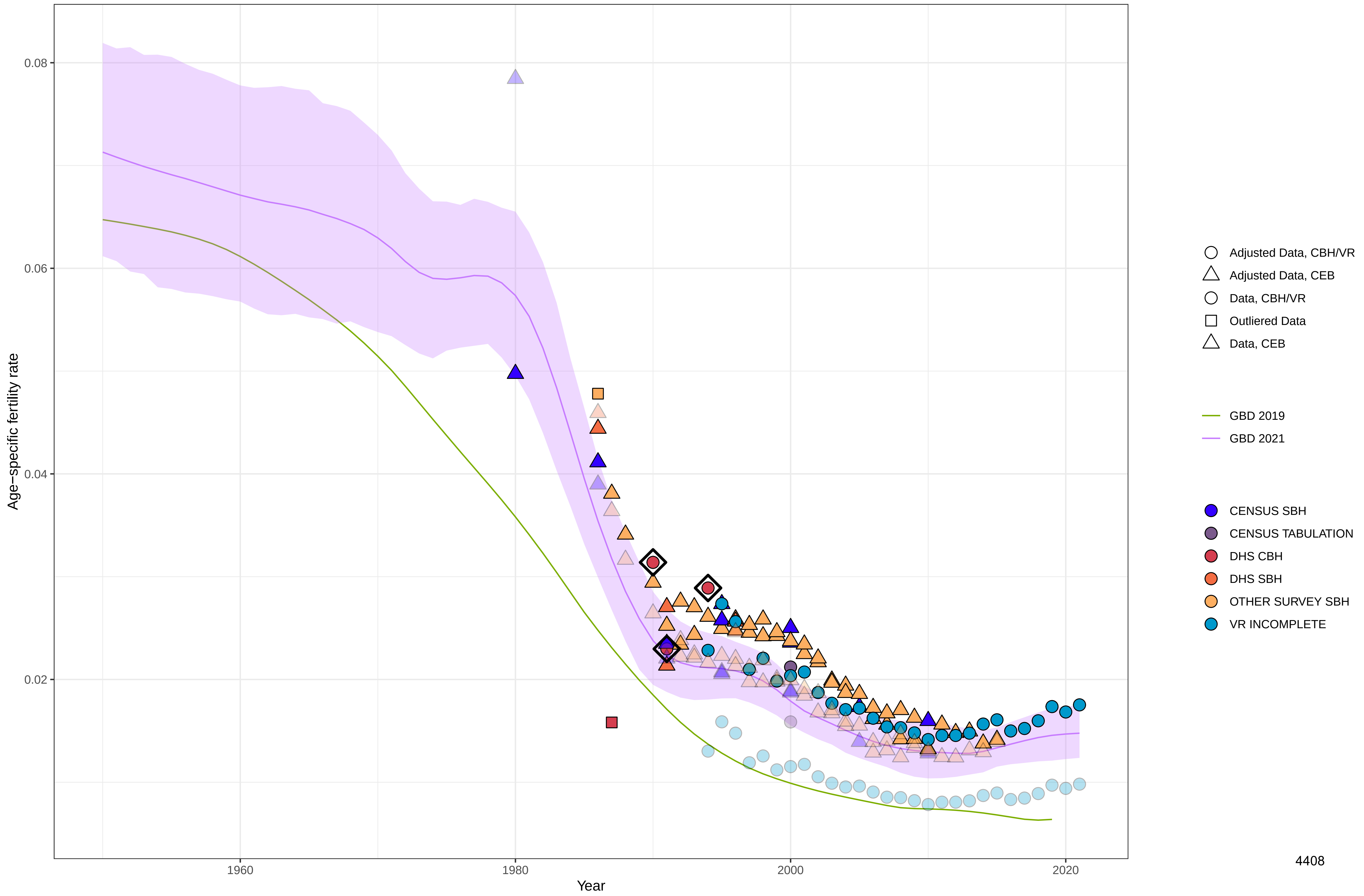

Age−specific fertility fitted trend lines, 1950−2021: Pernambuco, Ages: 45−49
Data points enclosed with a diamond are reference sources

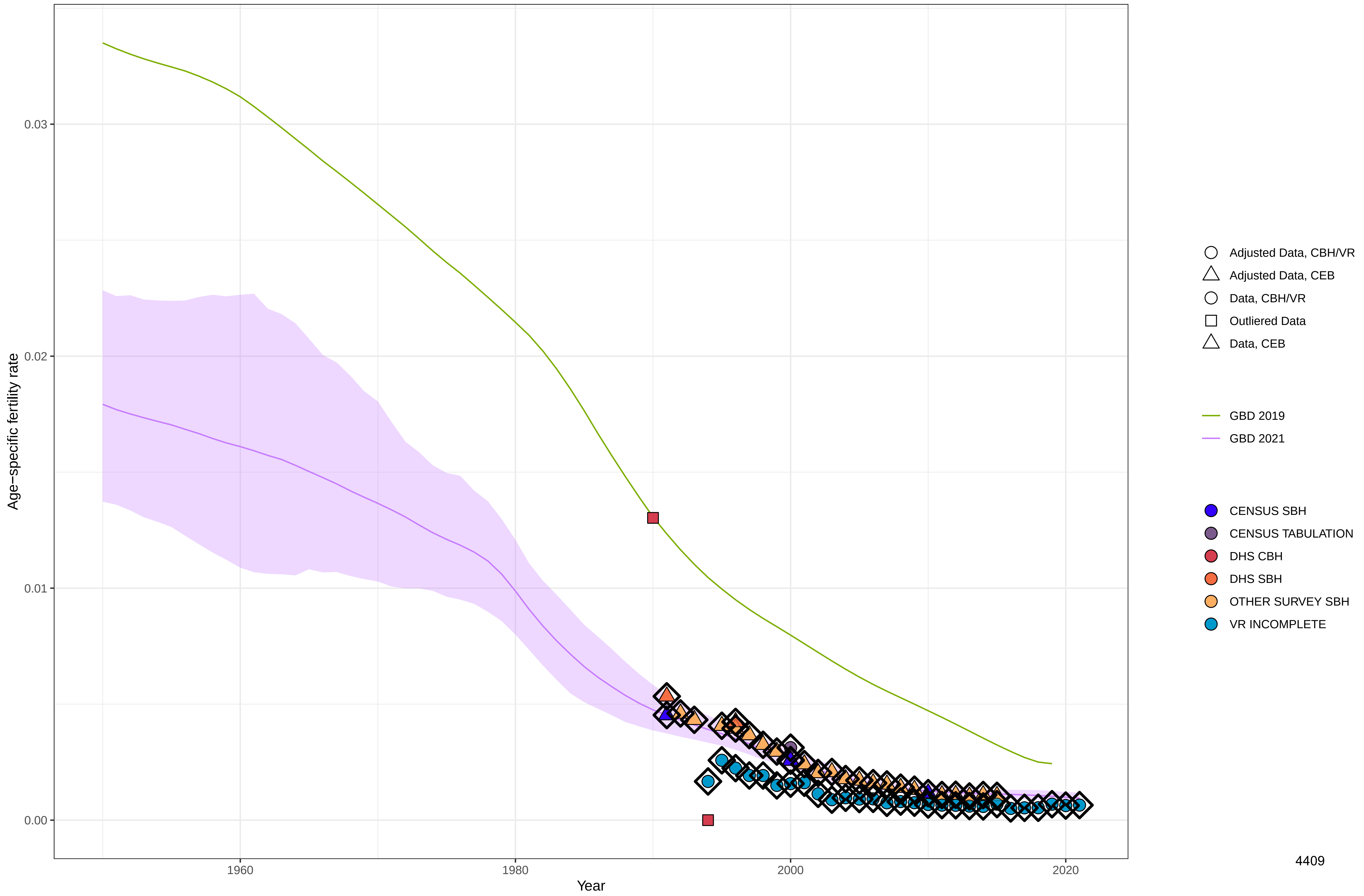

Age−specific fertility fitted trend lines, 1950−2021: Pernambuco, Ages: 50−54
Data points enclosed with a diamond are reference sources

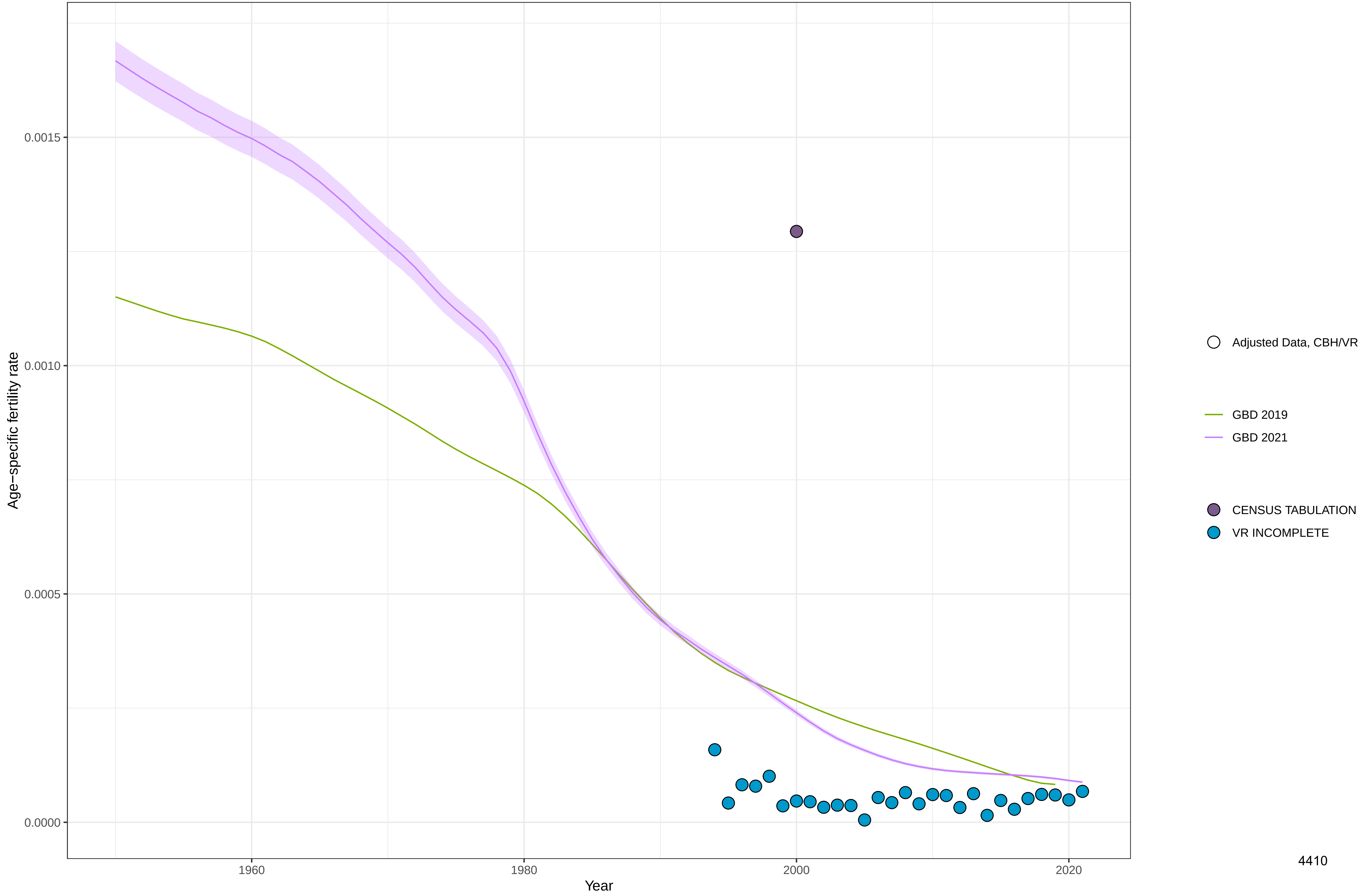

Total fertility rate fitted trend lines, 1950−2021: Pernambuco TFR
Data points enclosed with a diamond are reference sources

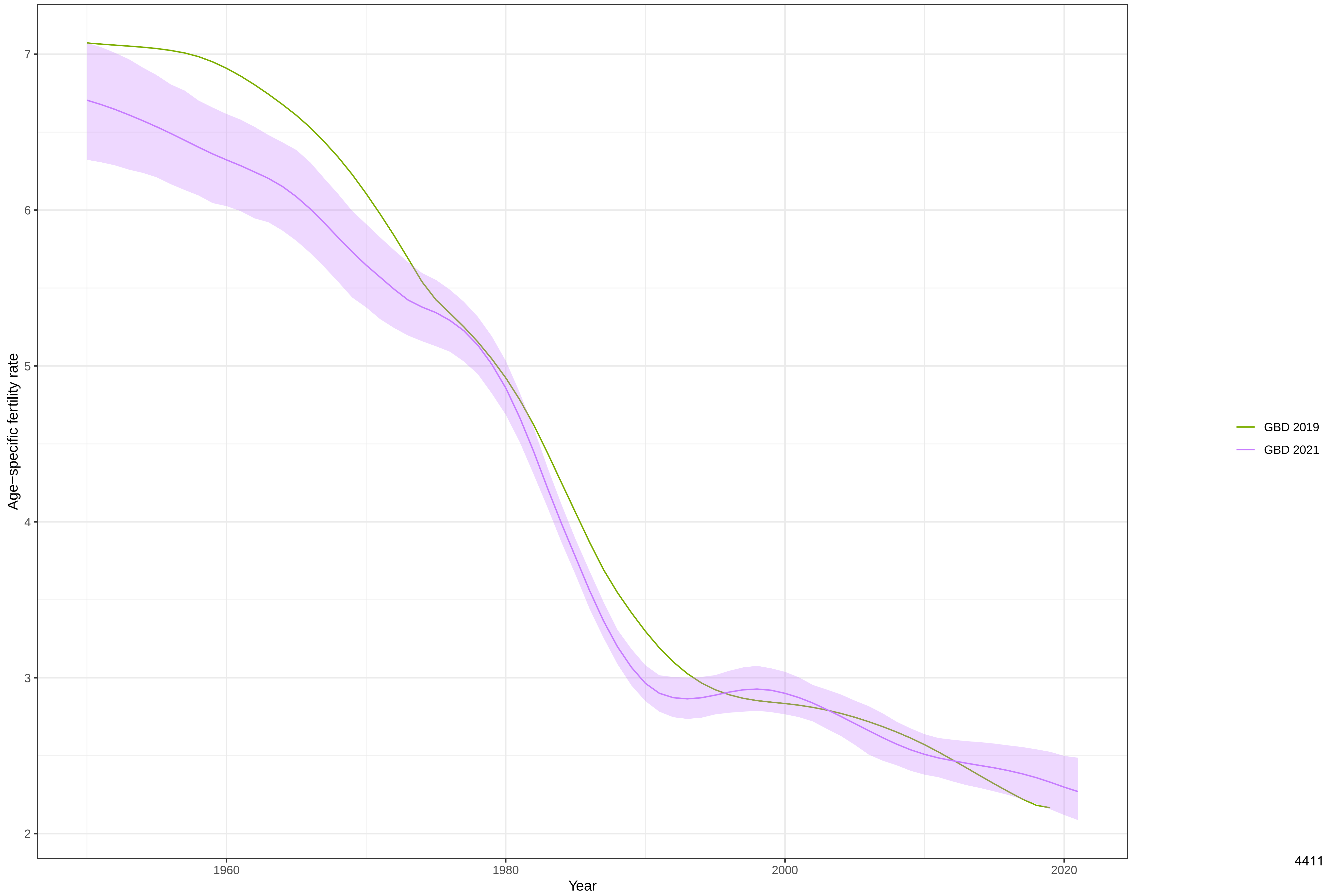

4411

Age−specific fertility fitted trend lines, 1950−2021: Piauí, Ages: 10−14
Data points enclosed with a diamond are reference sources

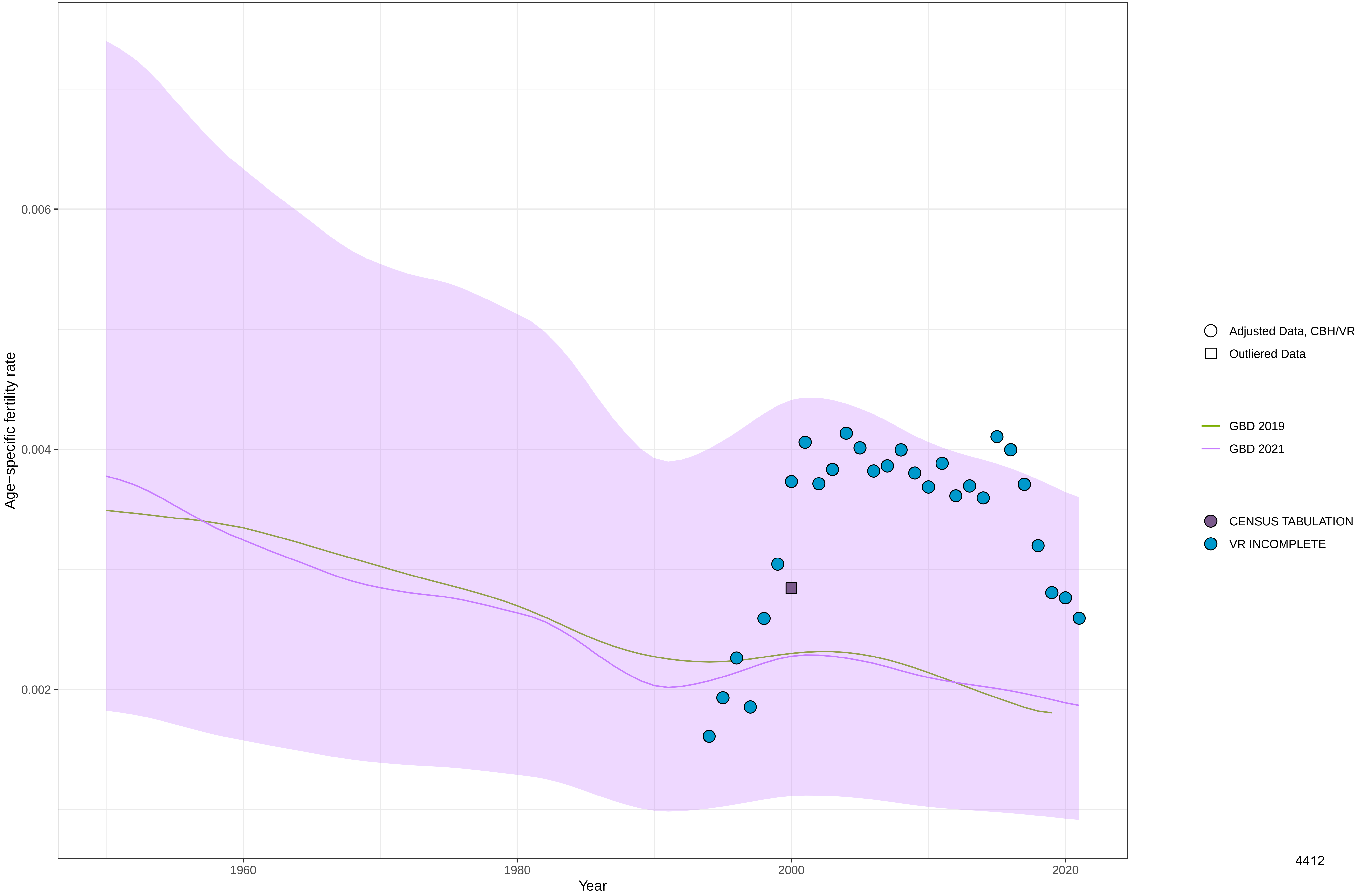

Age−specific fertility fitted trend lines, 1950−2021: Piauí, Ages: 15−19
Data points enclosed with a diamond are reference sources

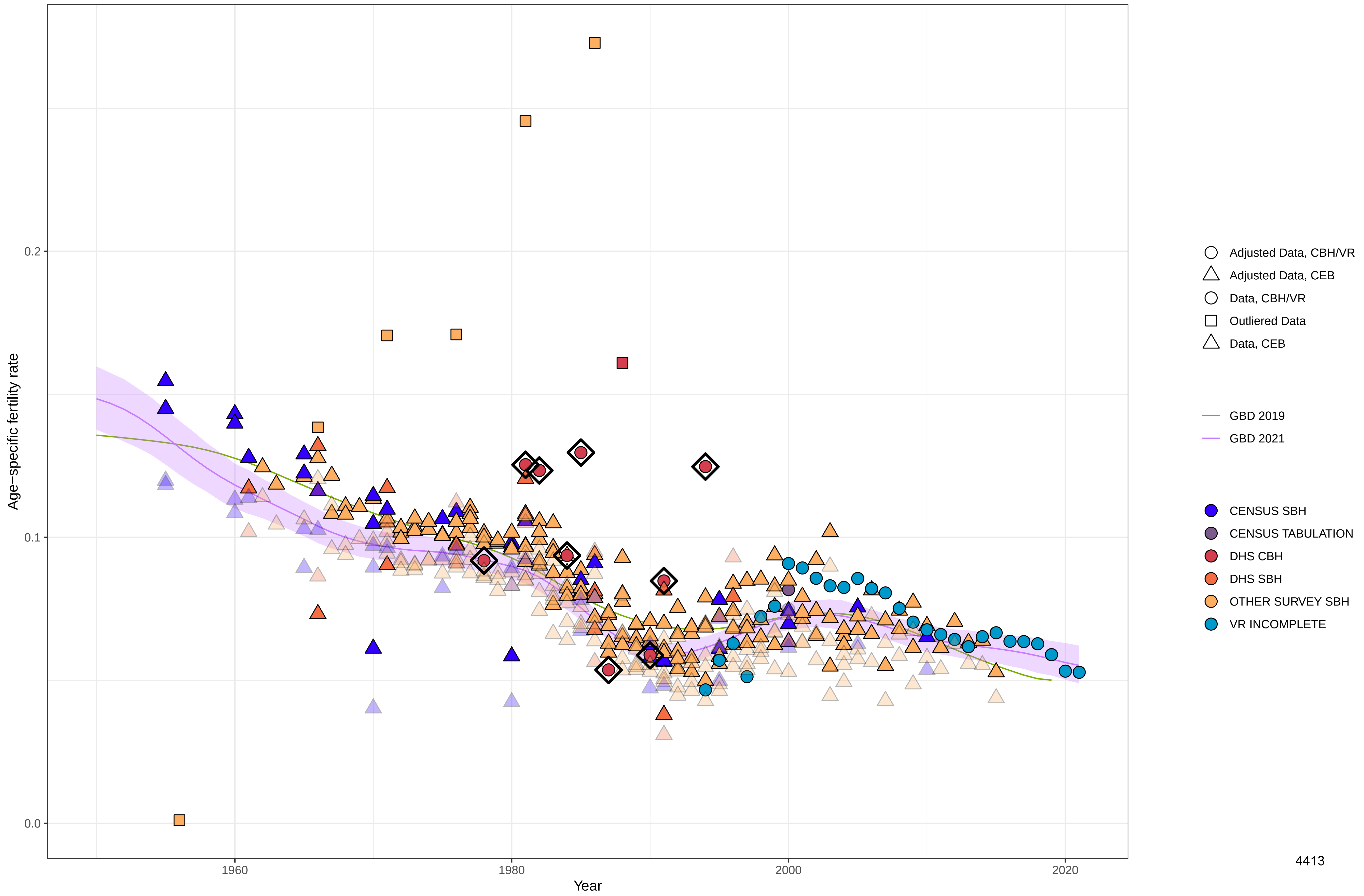

Age−specific fertility fitted trend lines, 1950−2021: Piauí, Ages: 20−24
Data points enclosed with a diamond are reference sources

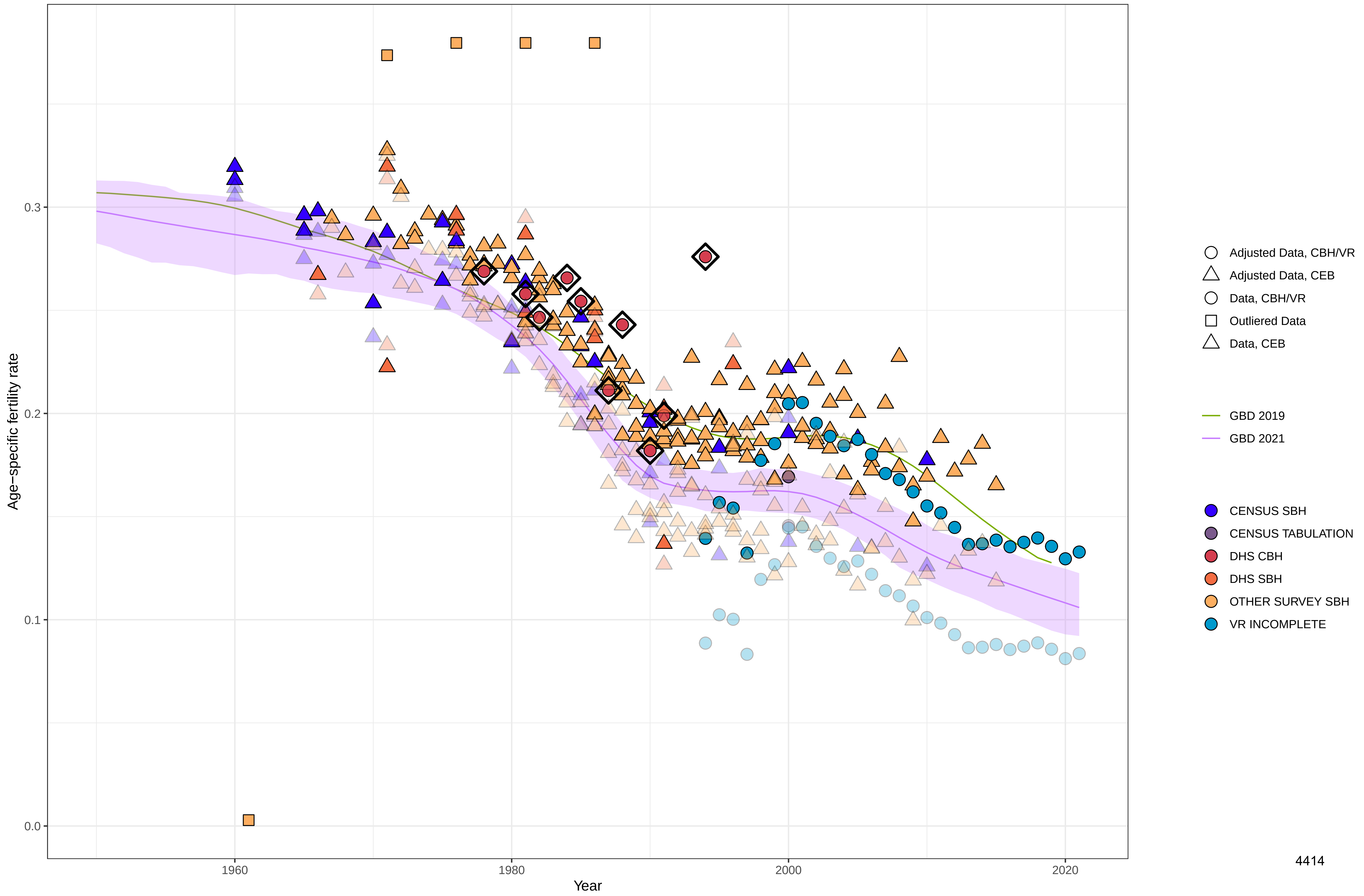

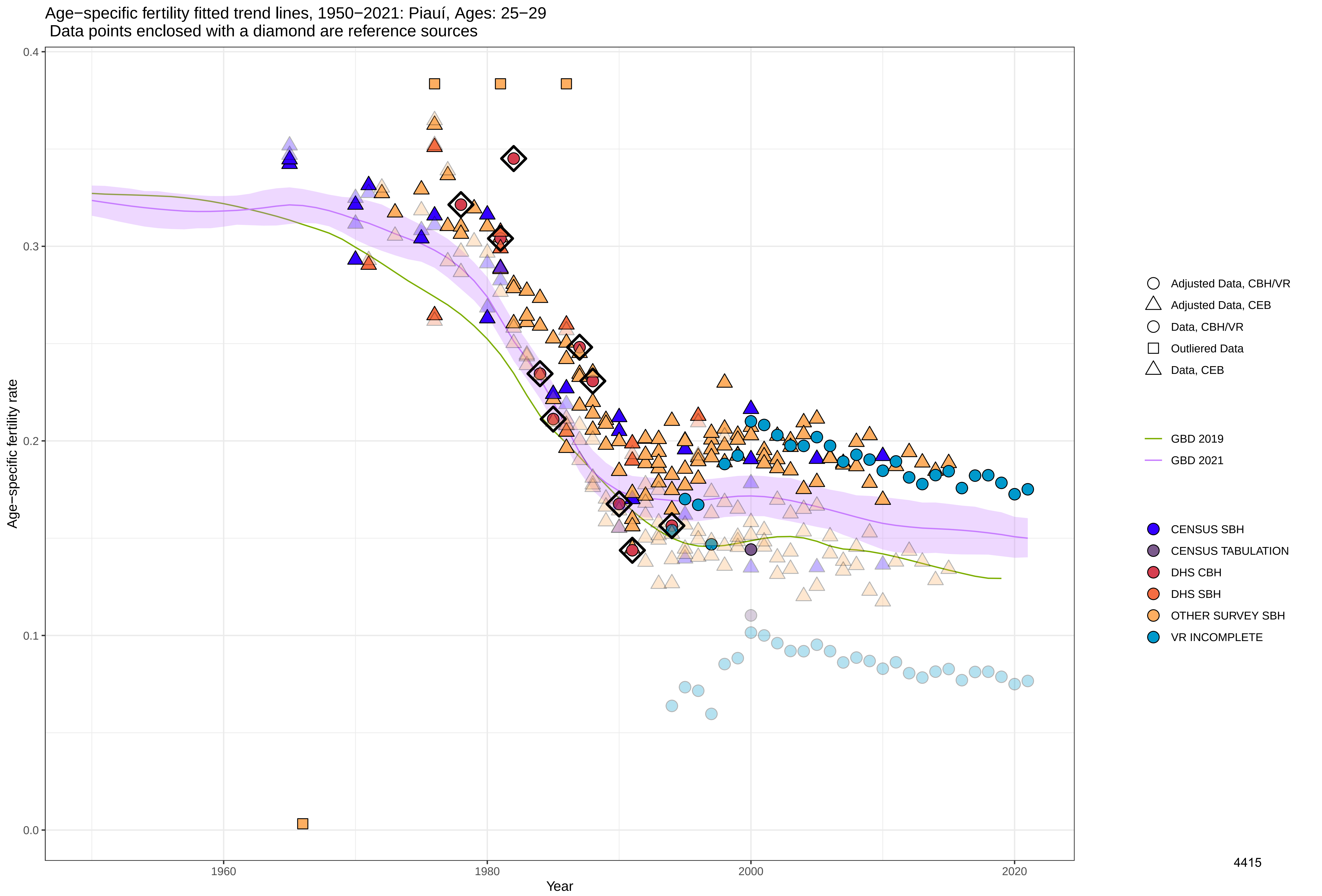

Age−specific fertility fitted trend lines, 1950−2021: Piauí, Ages: 25−29
Data points enclosed with a diamond are reference sources
Age−specific fertility rate
Year
0.0
0.1
0.2
0.3
0.4
1960
1980
2000
2020
Adjusted Data, CBH/VR
Adjusted Data, CEB
Data, CBH/VR
Outliered Data
Data, CEB
GBD 2019
GBD 2021
CENSUS SBH
CENSUS TABULATION
DHS CBH
DHS SBH
OTHER SURVEY SBH
VR INCOMPLETE

Age−specific fertility fitted trend lines, 1950−2021: Piauí, Ages: 30−34
Data points enclosed with a diamond are reference sources

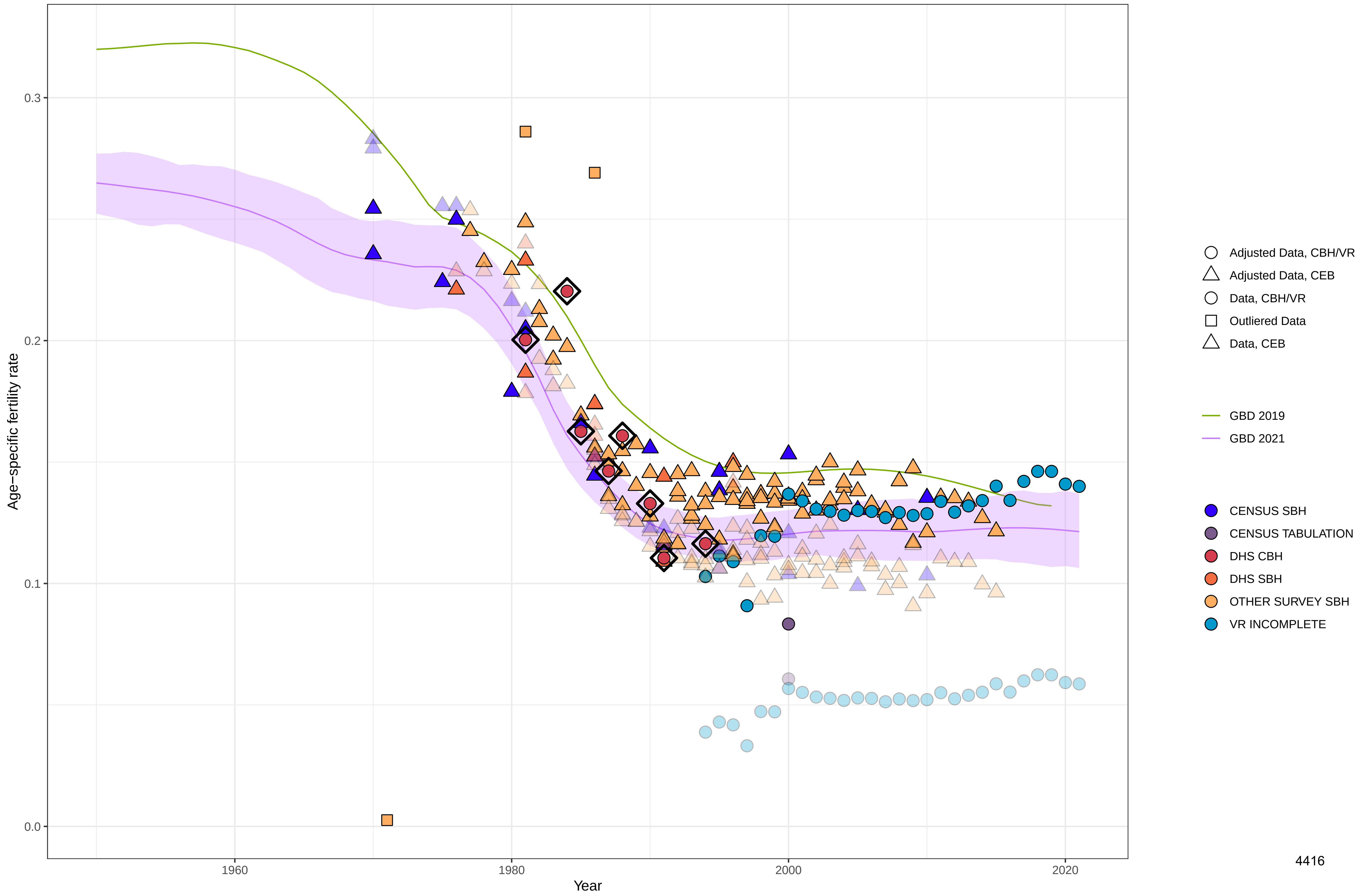

Age−specific fertility fitted trend lines, 1950−2021: Piauí, Ages: 35−39
Data points enclosed with a diamond are reference sources

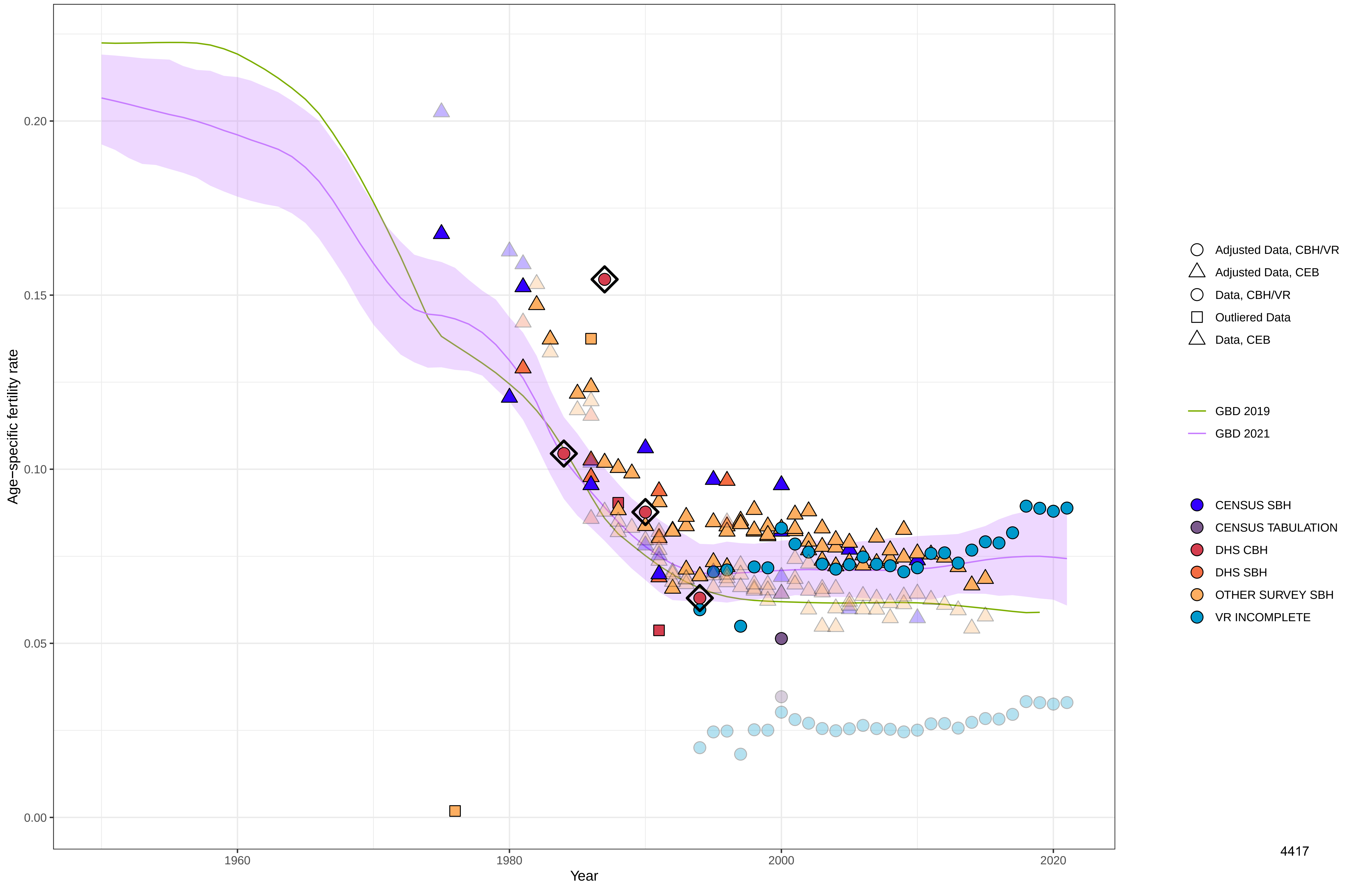

4417

Age−specific fertility fitted trend lines, 1950−2021: Piauí, Ages: 40−44
Data points enclosed with a diamond are reference sources

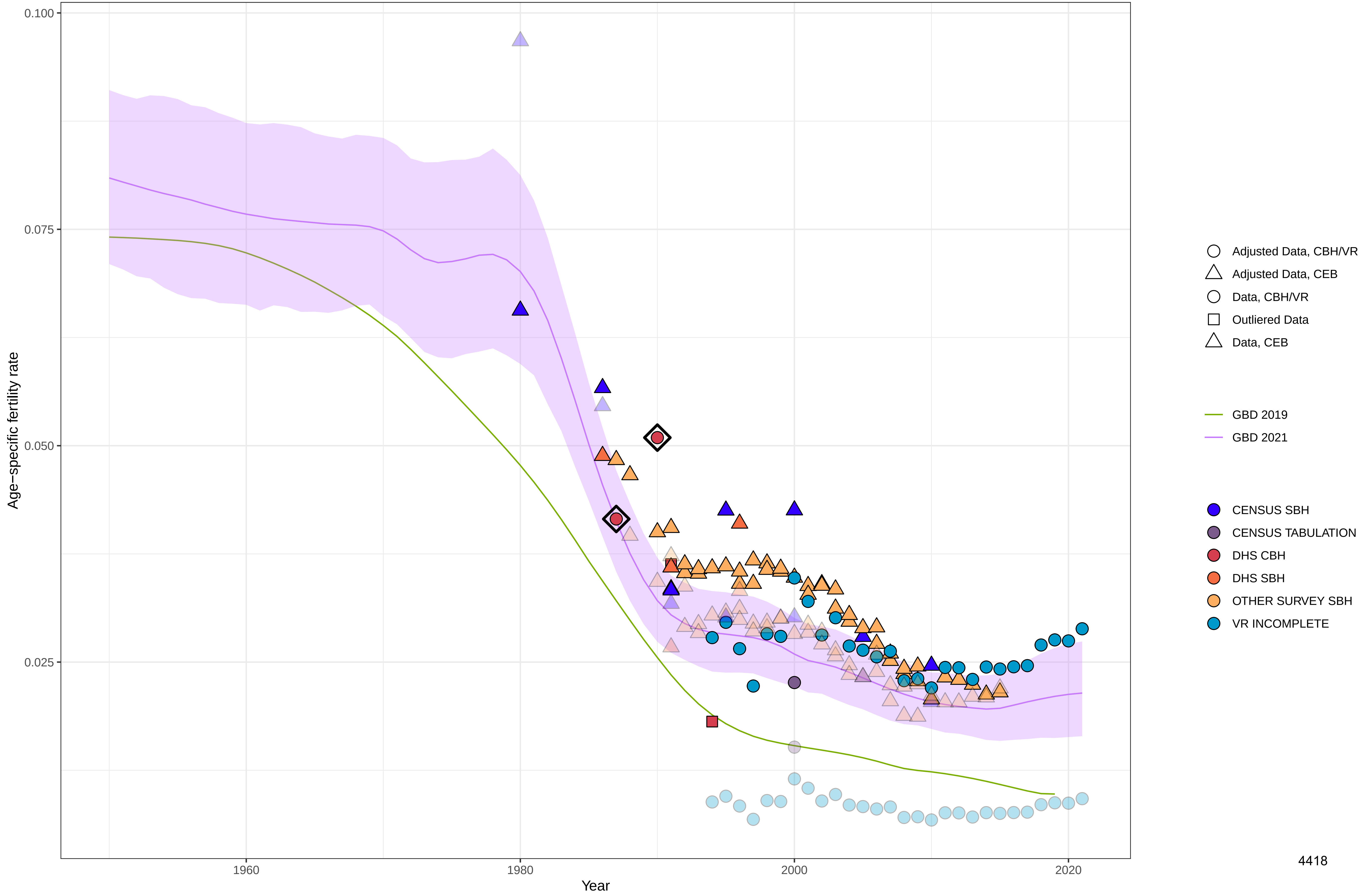

4418

Age−specific fertility fitted trend lines, 1950−2021: Piauí, Ages: 45−49
Data points enclosed with a diamond are reference sources

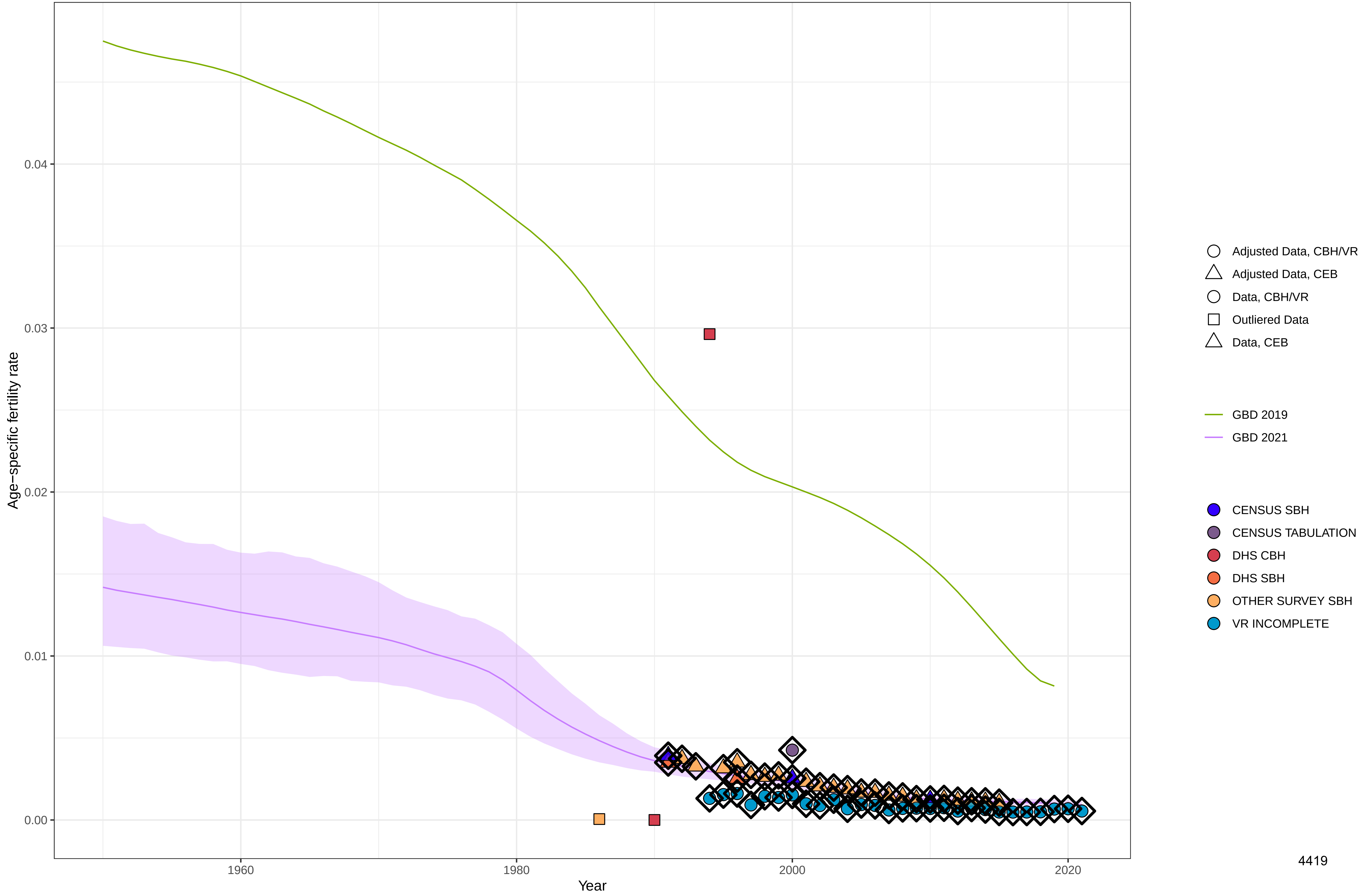

Age−specific fertility fitted trend lines, 1950−2021: Piauí, Ages: 50−54
Data points enclosed with a diamond are reference sources

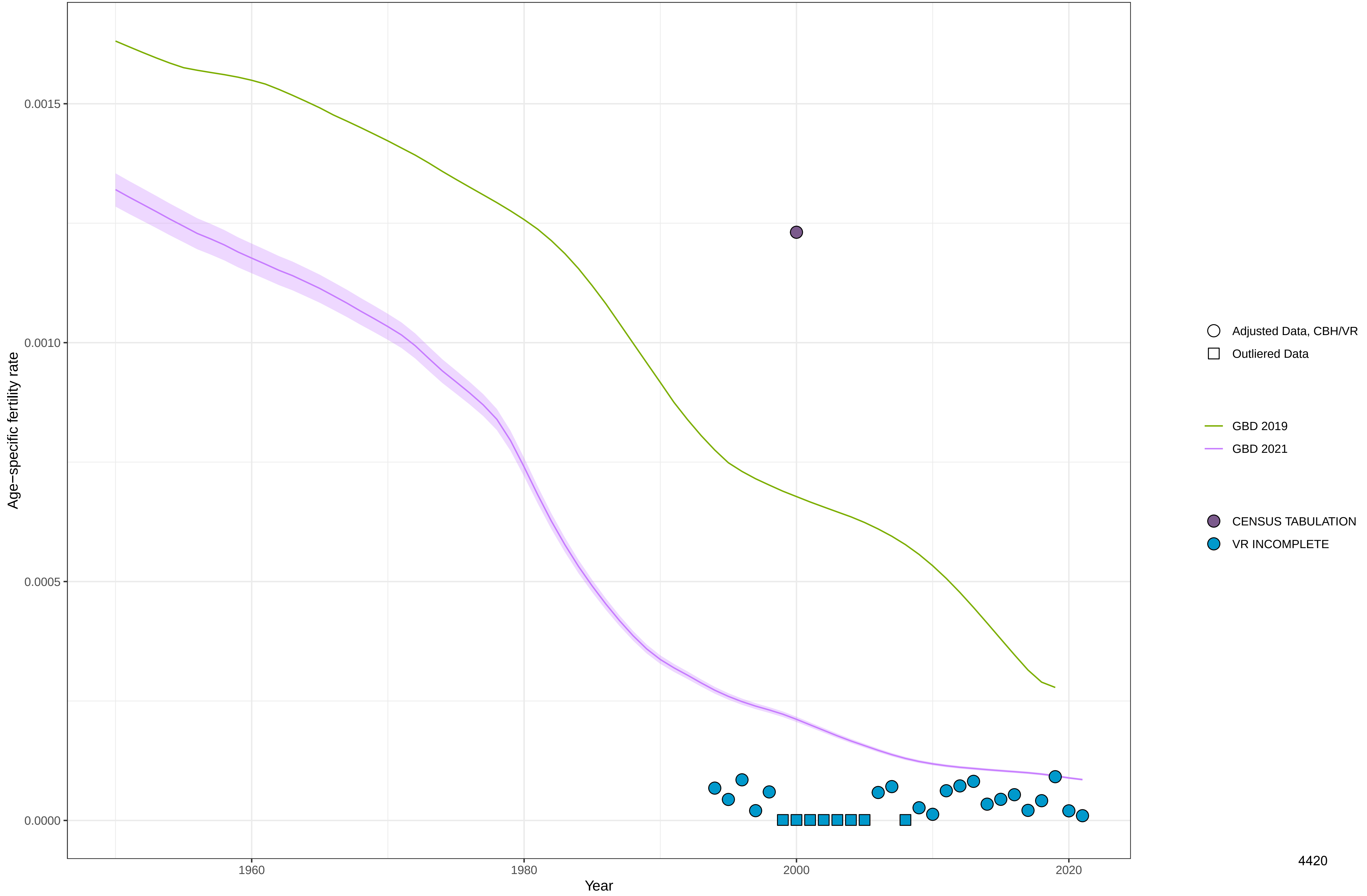

Total fertility rate fitted trend lines, 1950−2021: Piauí TFR
Data points enclosed with a diamond are reference sources

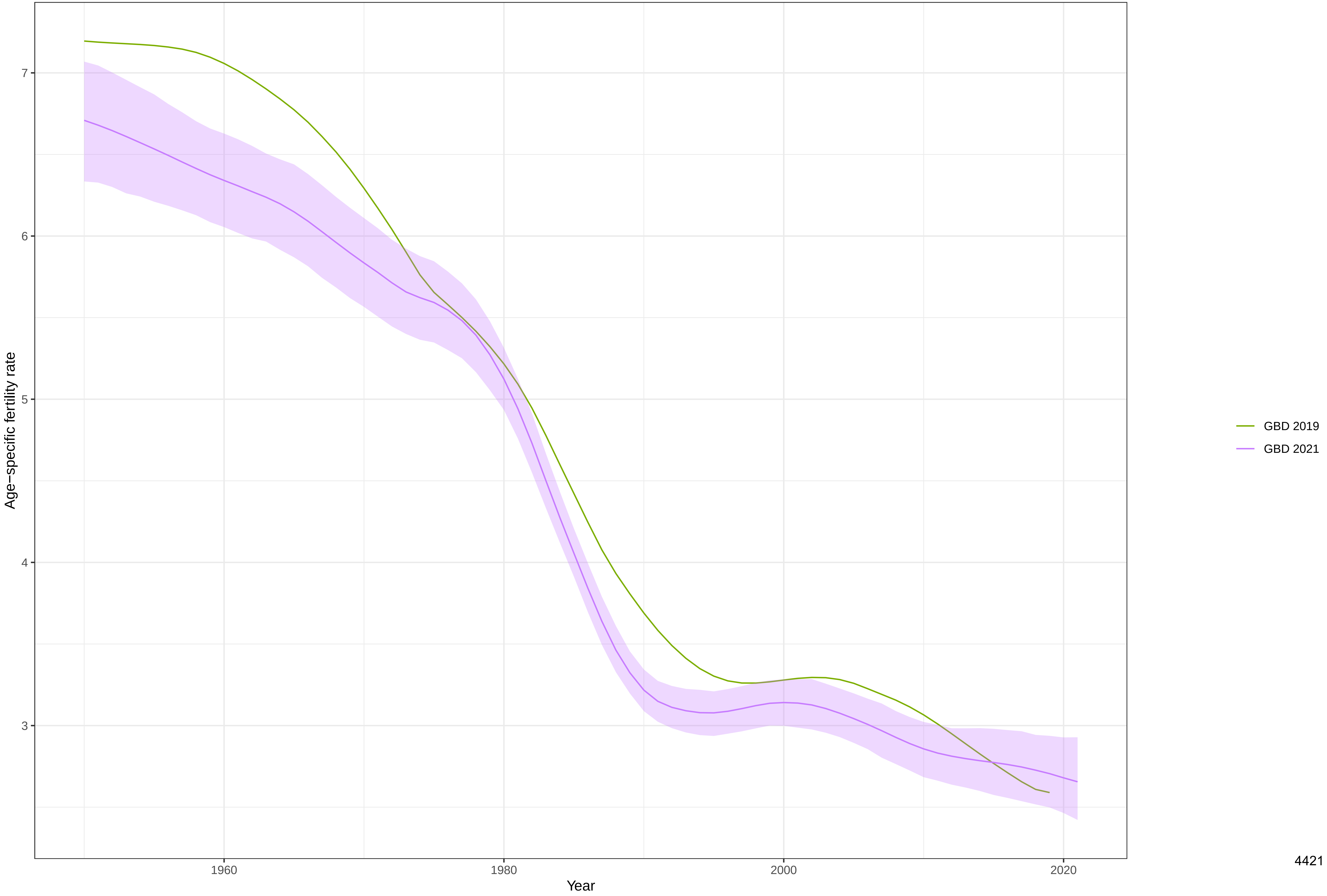

Age−specific fertility fitted trend lines, 1950−2021: Rio de Janeiro, Ages: 10−14
Data points enclosed with a diamond are reference sources

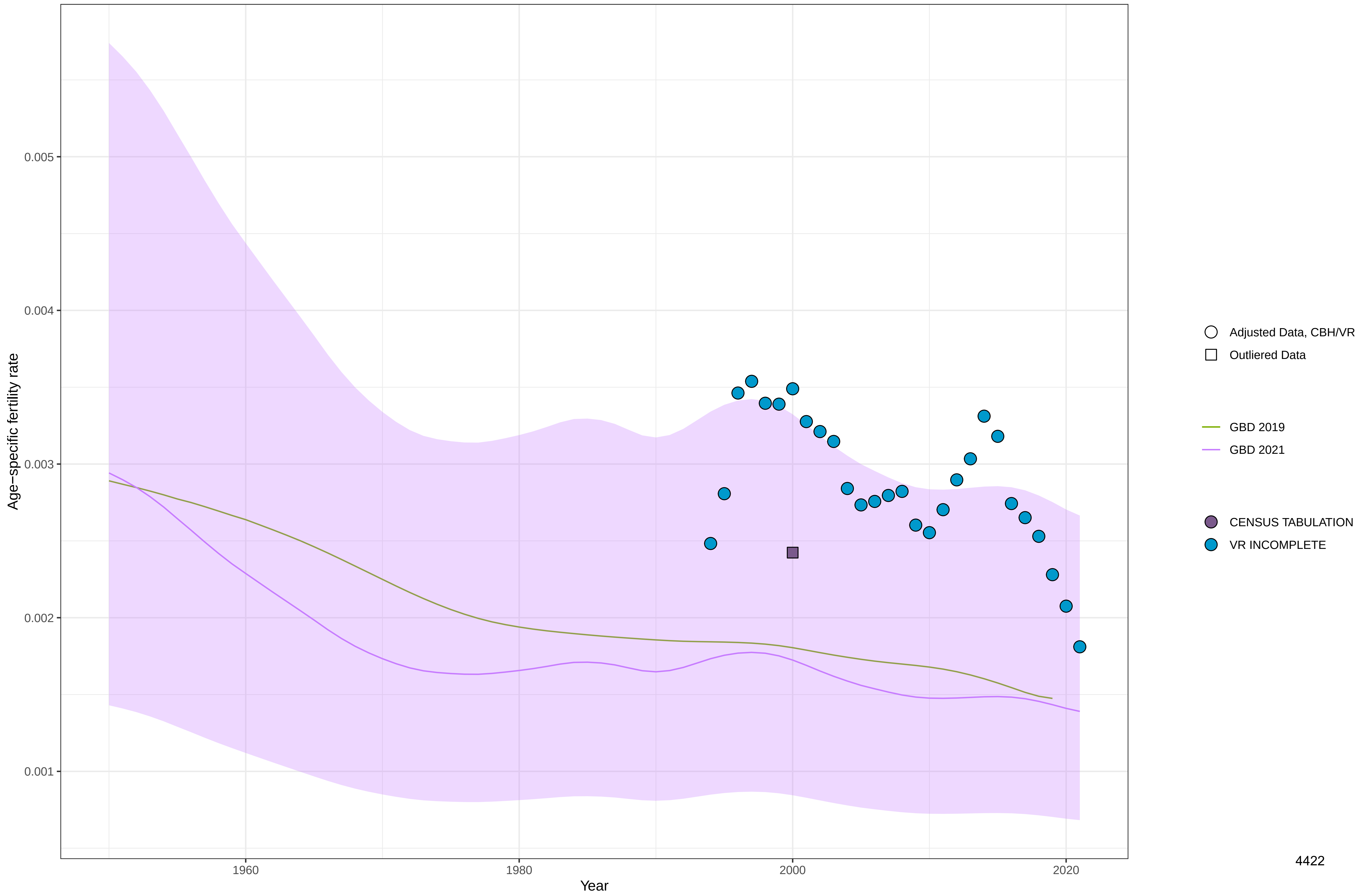

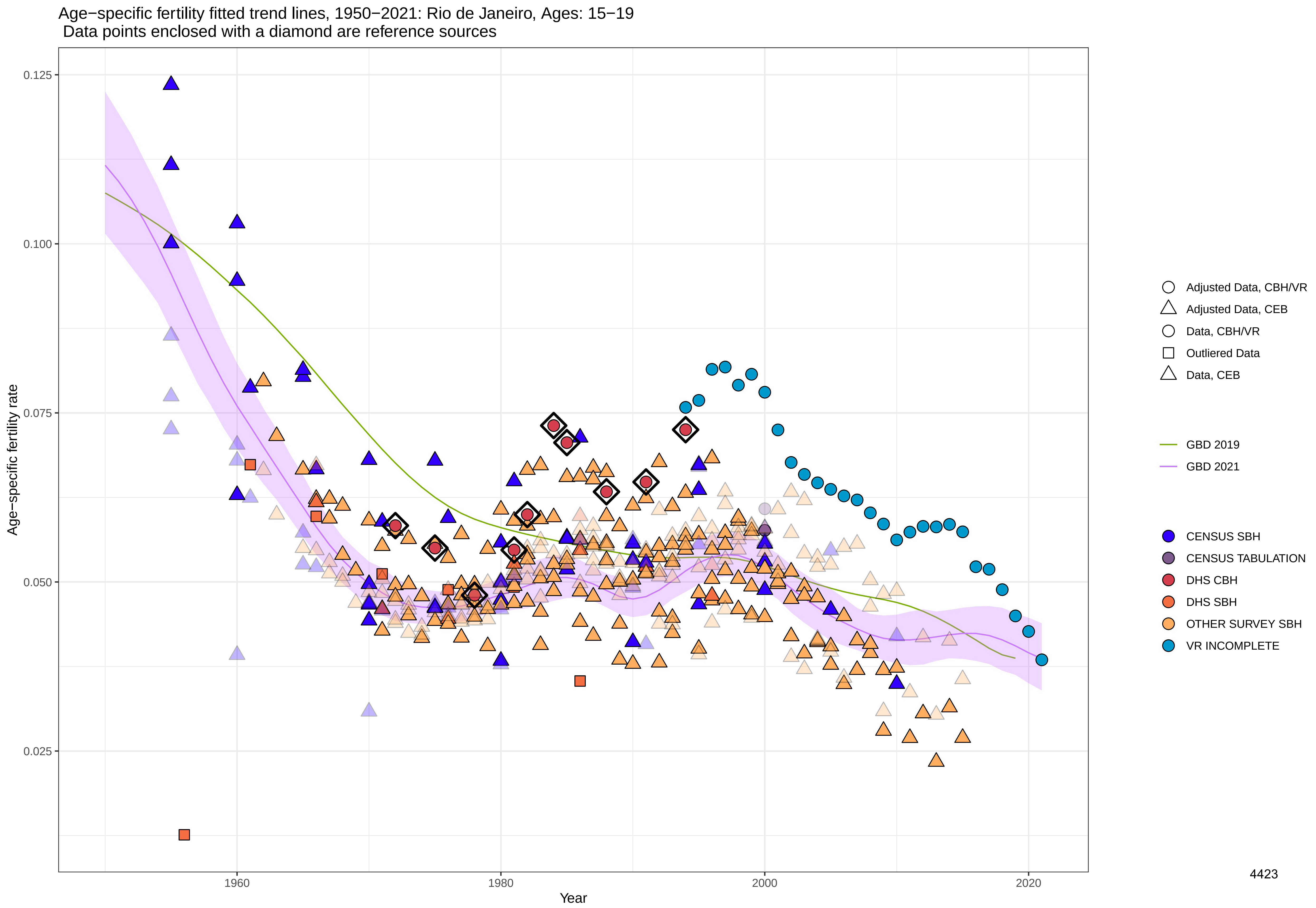

Age−specific fertility fitted trend lines, 1950−2021: Rio de Janeiro, Ages: 15−19
Data points enclosed with a diamond are reference sources
Age−specific fertility rate
0.125
0.100
0.075
0.050
0.025
1960
1980
2000
2020
Year
Adjusted Data, CBH/VR
Adjusted Data, CEB
Data, CBH/VR
Outliered Data
Data, CEB
GBD 2019
GBD 2021
CENSUS SBH
CENSUS TABULATION
DHS CBH
DHS SBH
OTHER SURVEY SBH
VR INCOMPLETE

4423

Age−specific fertility fitted trend lines, 1950−2021: Rio de Janeiro, Ages: 20−24
Data points enclosed with a diamond are reference sources

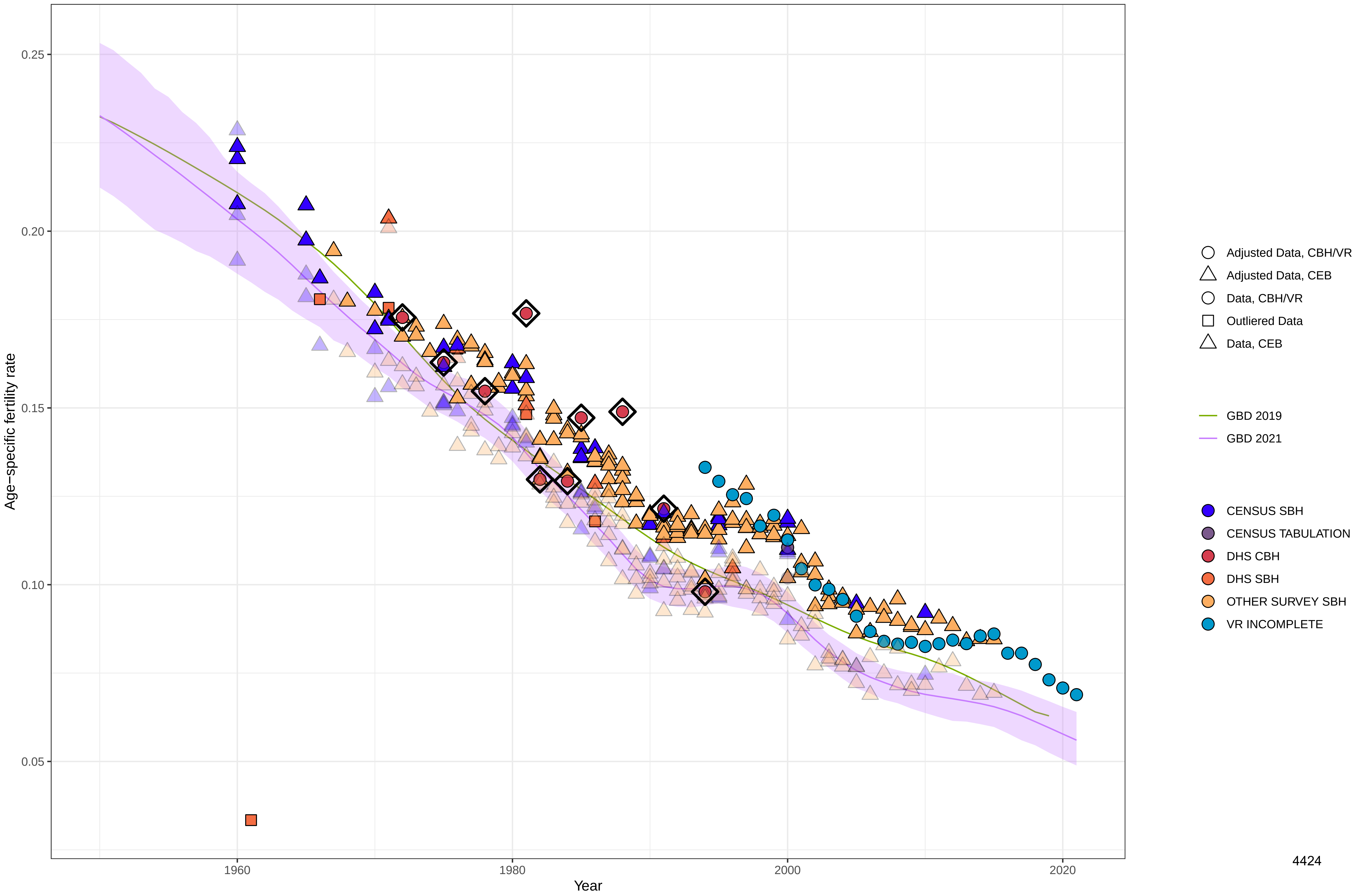

Age−specific fertility fitted trend lines, 1950−2021: Rio de Janeiro, Ages: 25−29
Data points enclosed with a diamond are reference sources

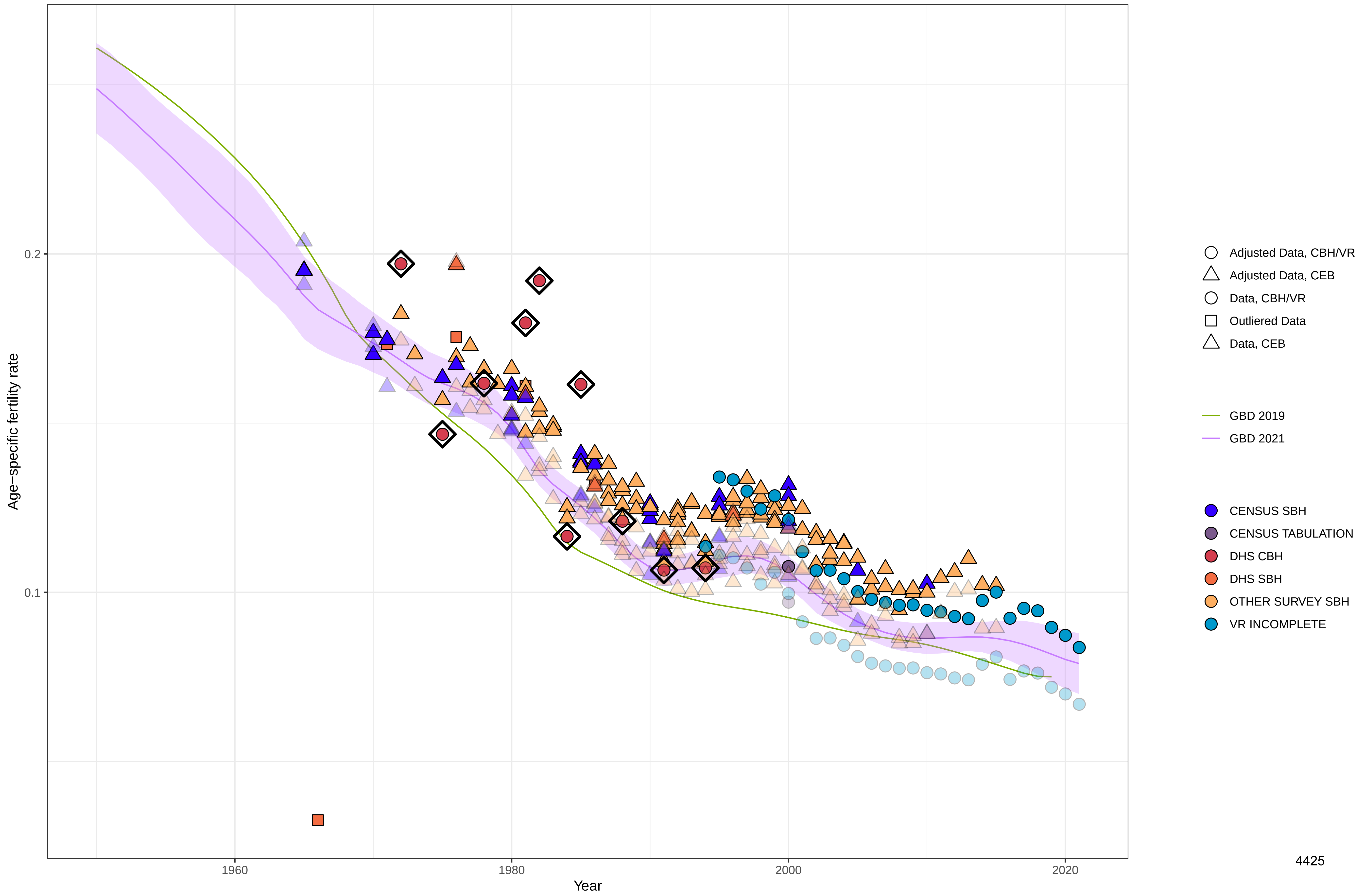

Age−specific fertility fitted trend lines, 1950−2021: Rio de Janeiro, Ages: 30−34
Data points enclosed with a diamond are reference sources

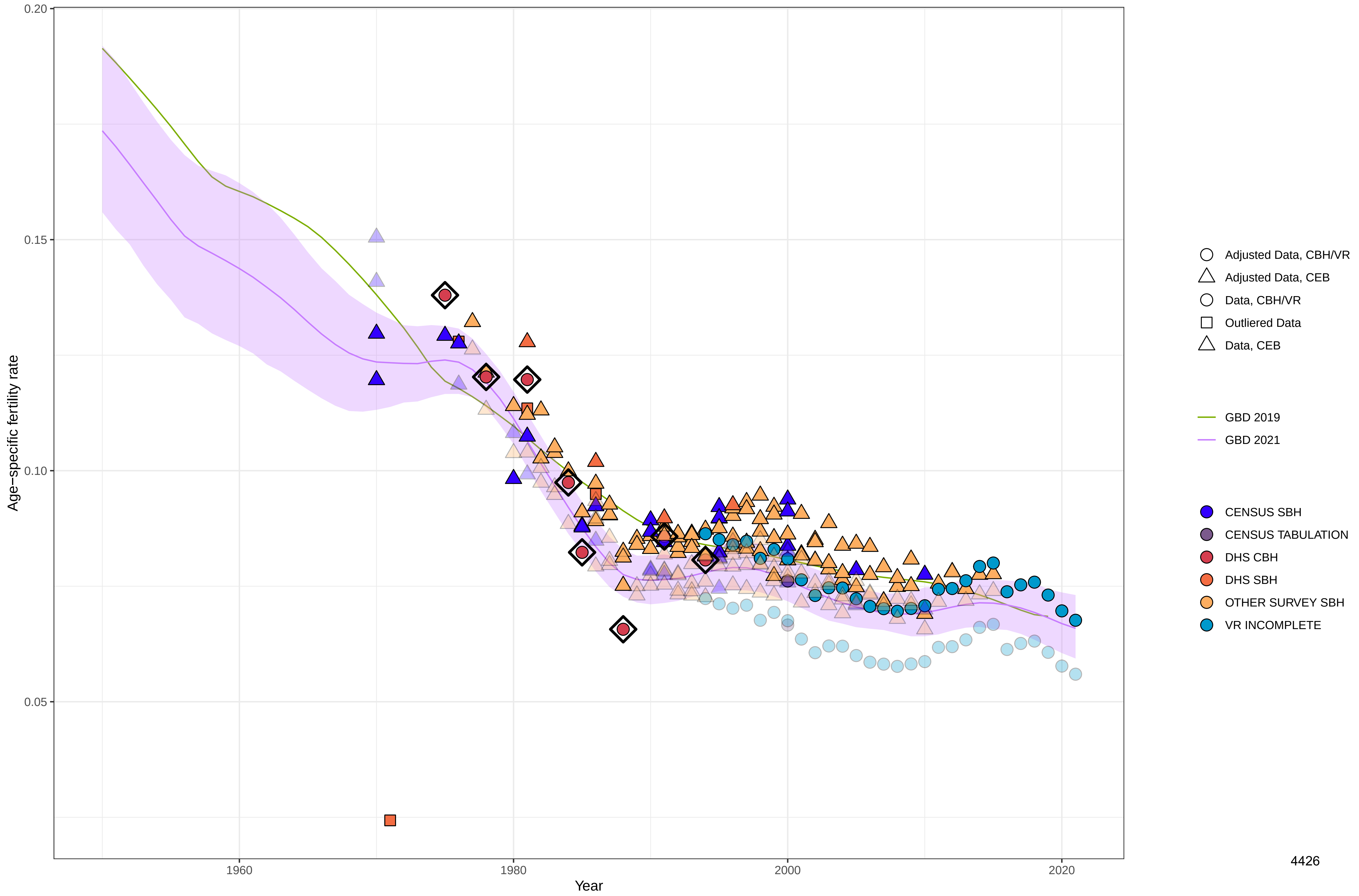

Age−specific fertility fitted trend lines, 1950−2021: Rio de Janeiro, Ages: 35−39
Data points enclosed with a diamond are reference sources

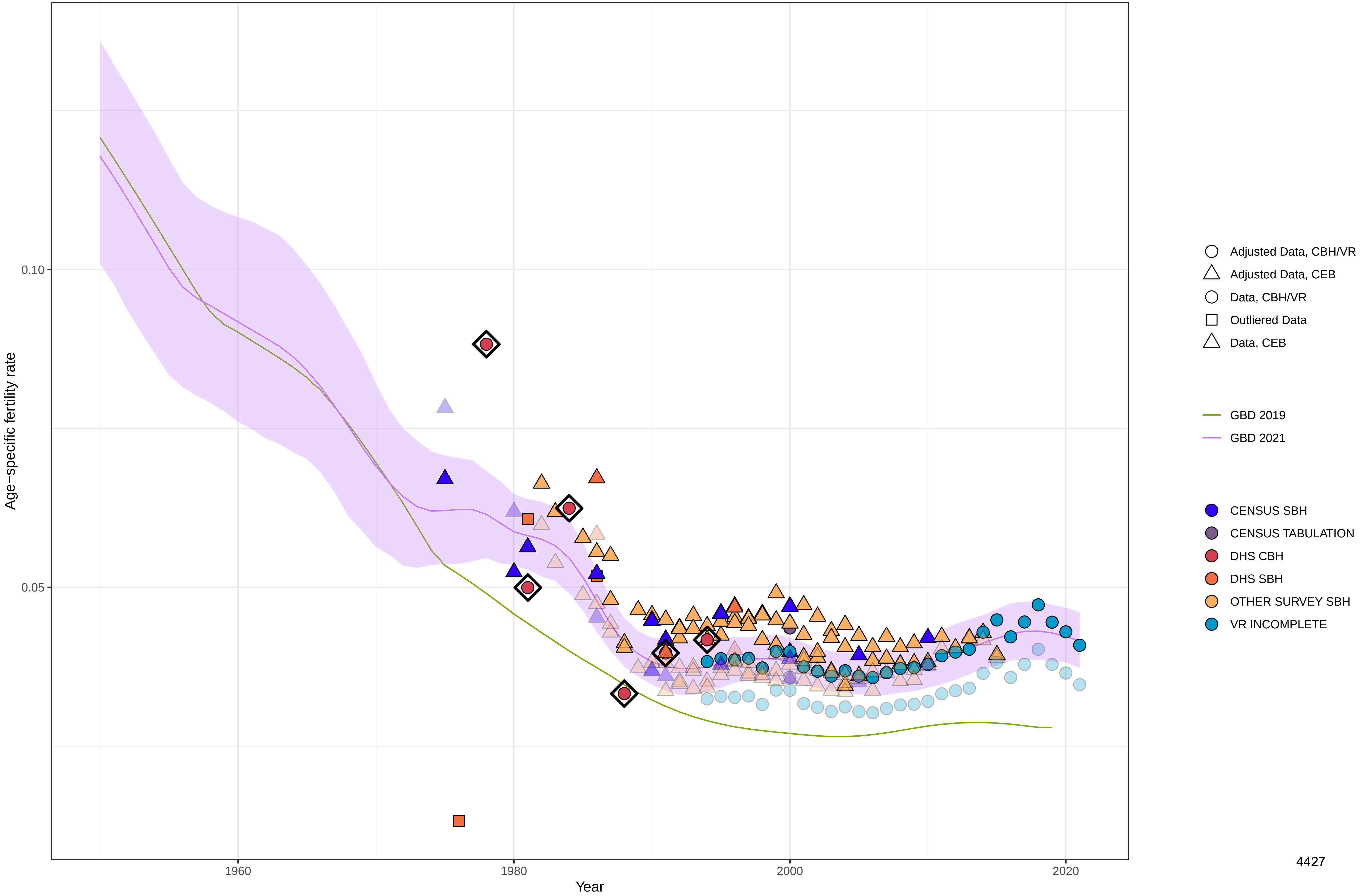

Age−specific fertility fitted trend lines, 1950−2021: Rio de Janeiro, Ages: 40−44
Data points enclosed with a diamond are reference sources

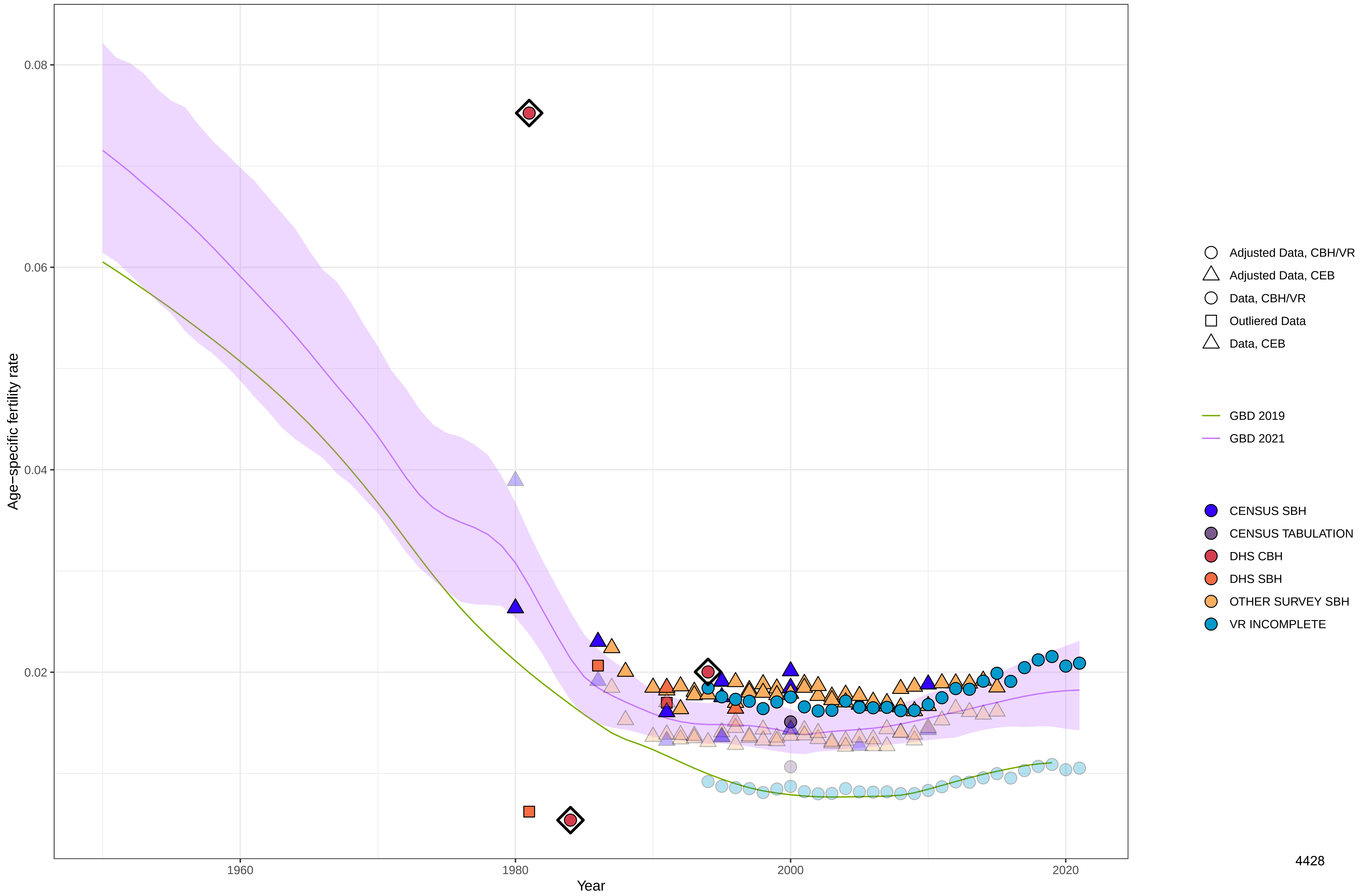

Age−specific fertility fitted trend lines, 1950−2021: Rio de Janeiro, Ages: 45−49
Data points enclosed with a diamond are reference sources

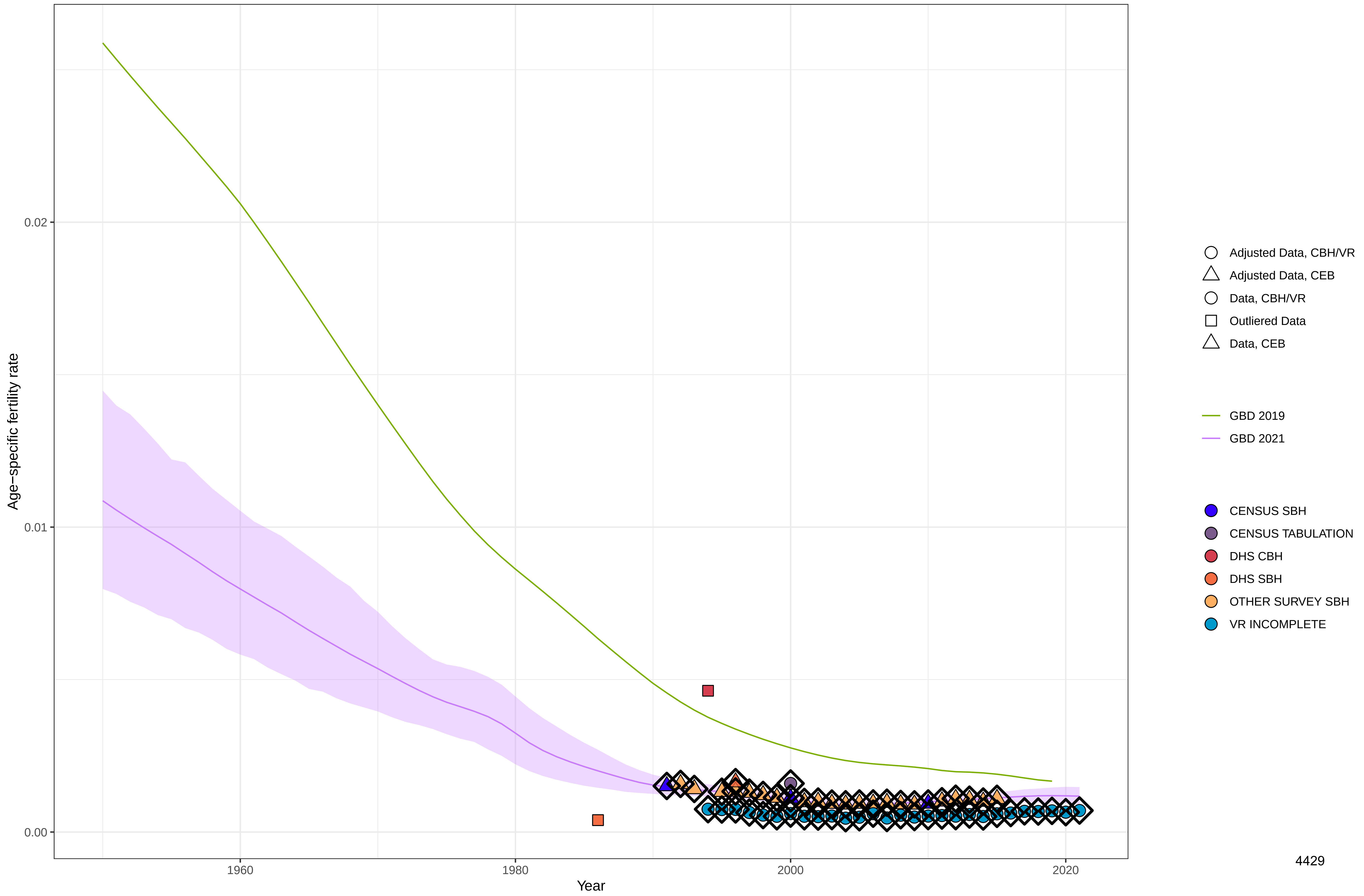

Age−specific fertility fitted trend lines, 1950−2021: Rio de Janeiro, Ages: 50−54
Data points enclosed with a diamond are reference sources

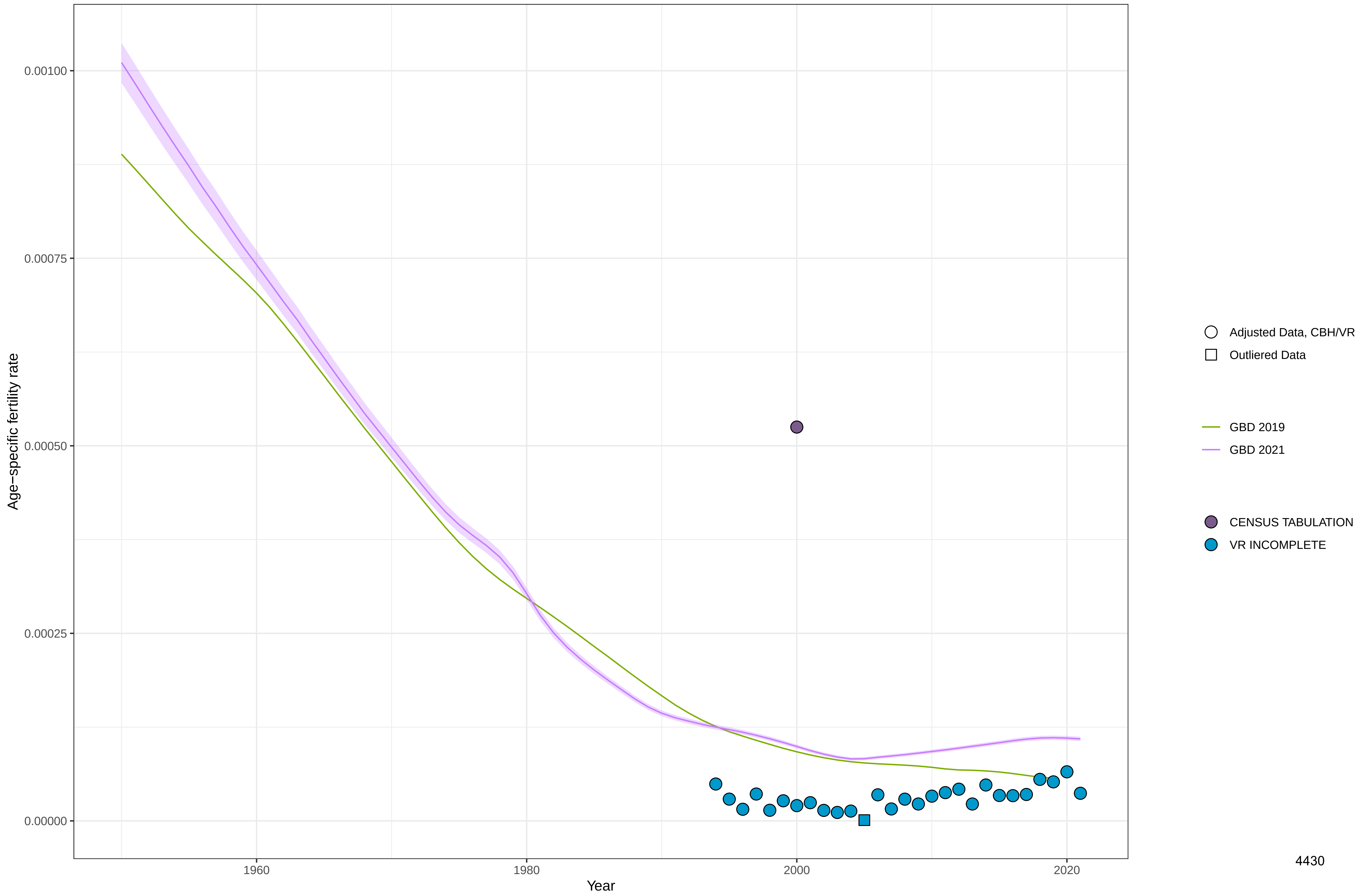

Total fertility rate fitted trend lines, 1950−2021: Rio de Janeiro TFR
Data points enclosed with a diamond are reference sources

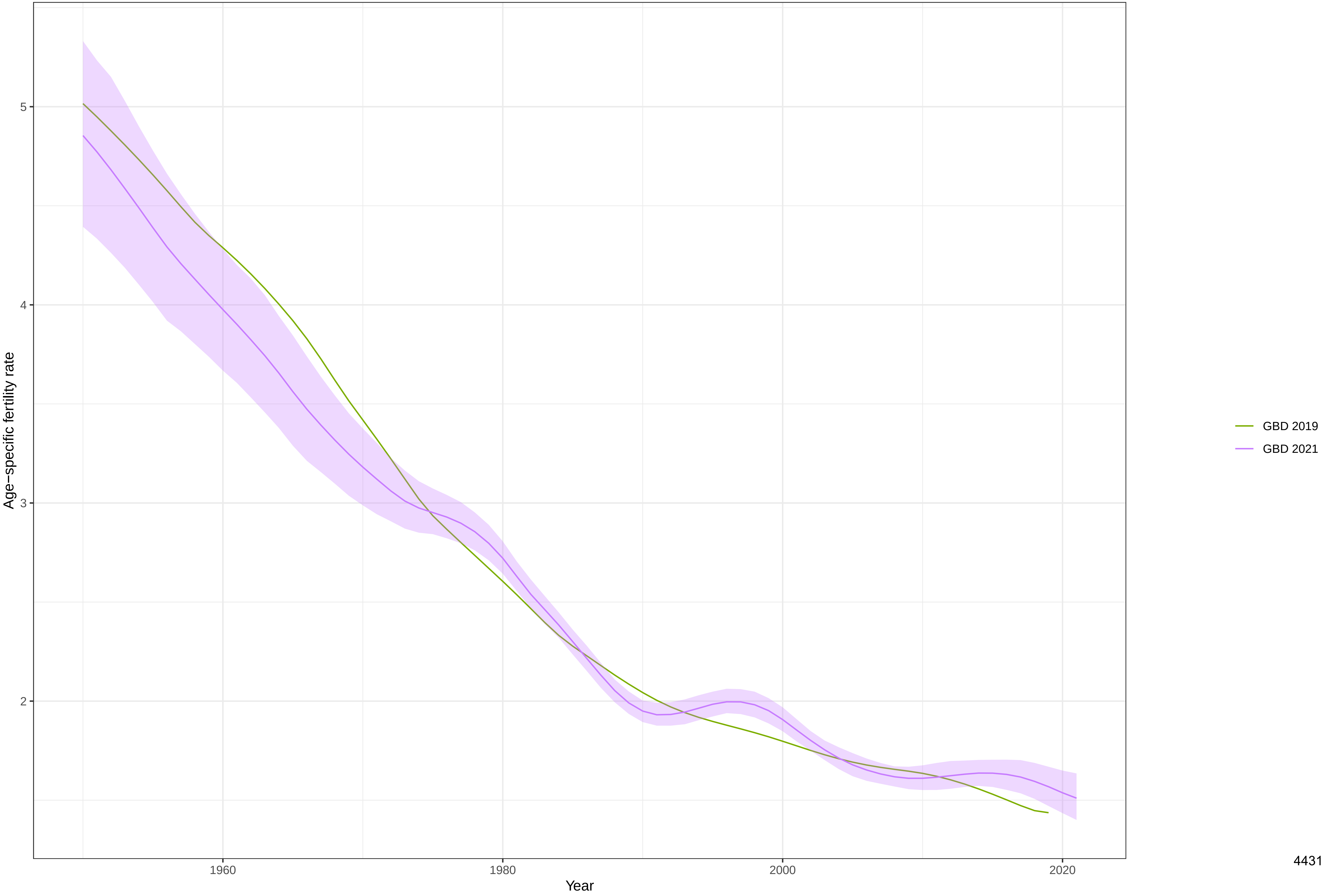

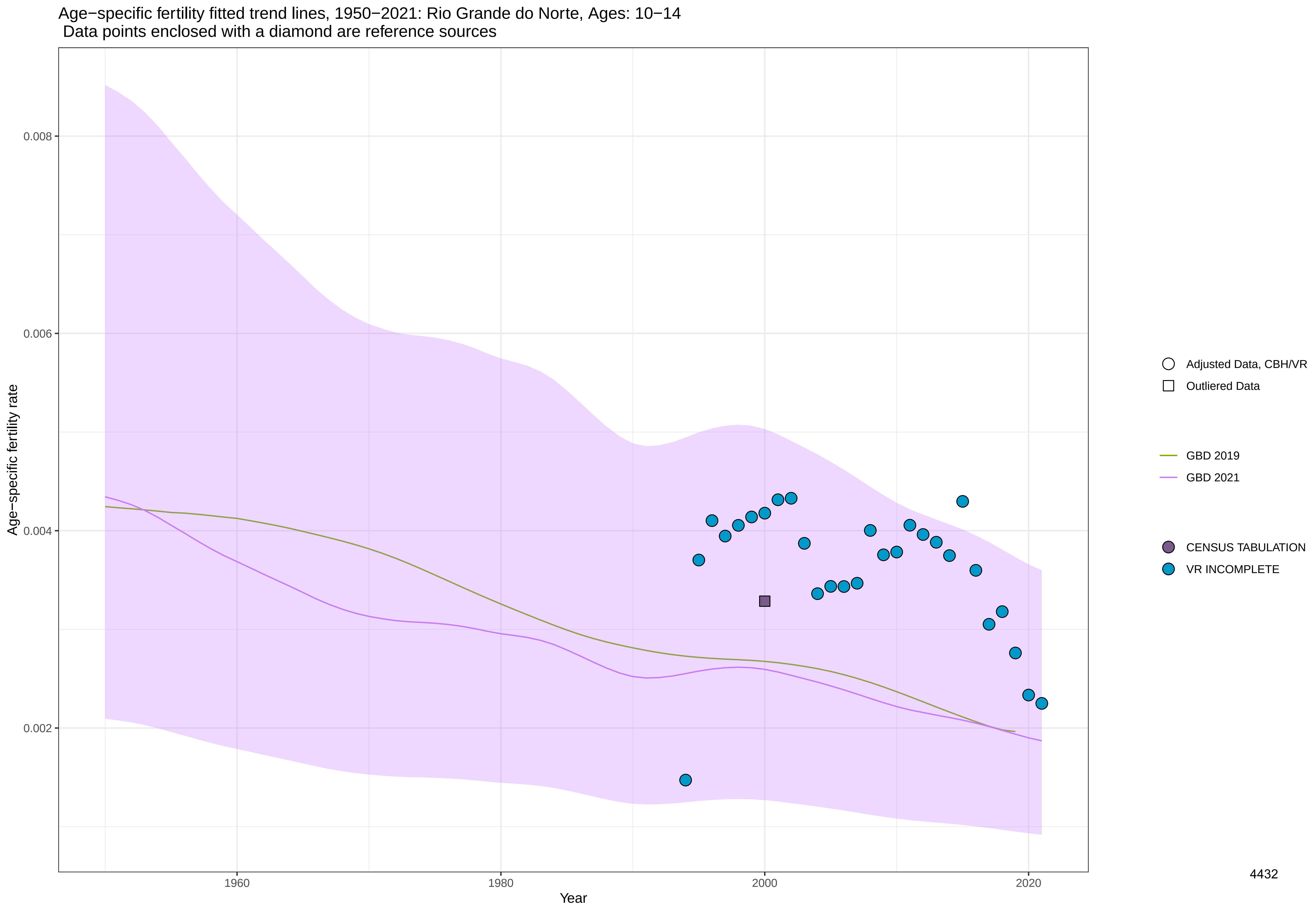

Age−specific fertility fitted trend lines, 1950−2021: Rio Grande do Norte, Ages: 10−14
Data points enclosed with a diamond are reference sources
Age−specific fertility rate
0.008
0.006
0.004
0.002
1960
1980
2000
2020
Year
Adjusted Data, CBH/VR
Outliered Data
GBD 2019
GBD 2021
CENSUS TABULATION
VR INCOMPLETE

Age−specific fertility fitted trend lines, 1950−2021: Rio Grande do Norte, Ages: 15−19
Data points enclosed with a diamond are reference sources

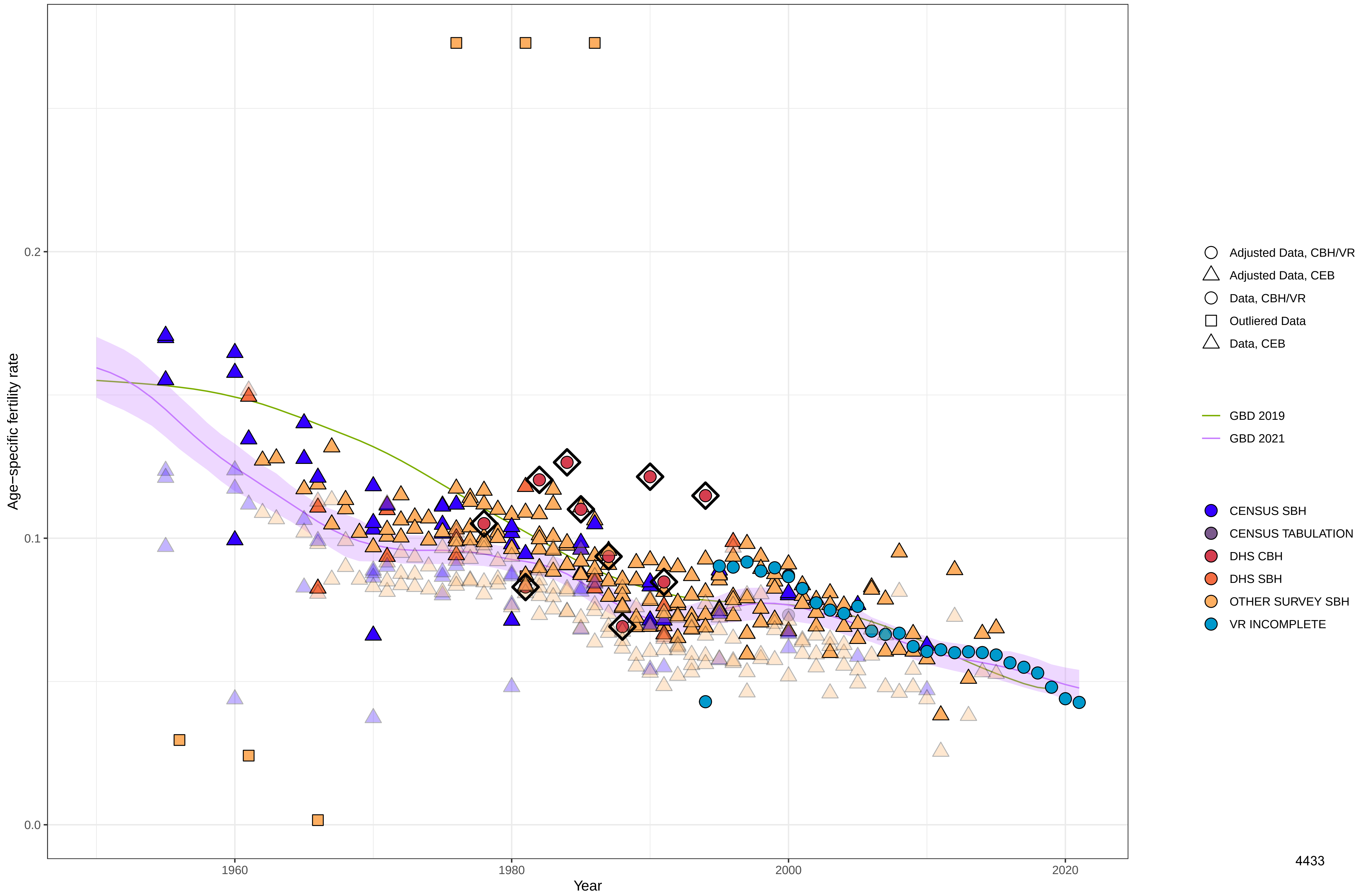

Age−specific fertility fitted trend lines, 1950−2021: Rio Grande do Norte, Ages: 20−24
Data points enclosed with a diamond are reference sources

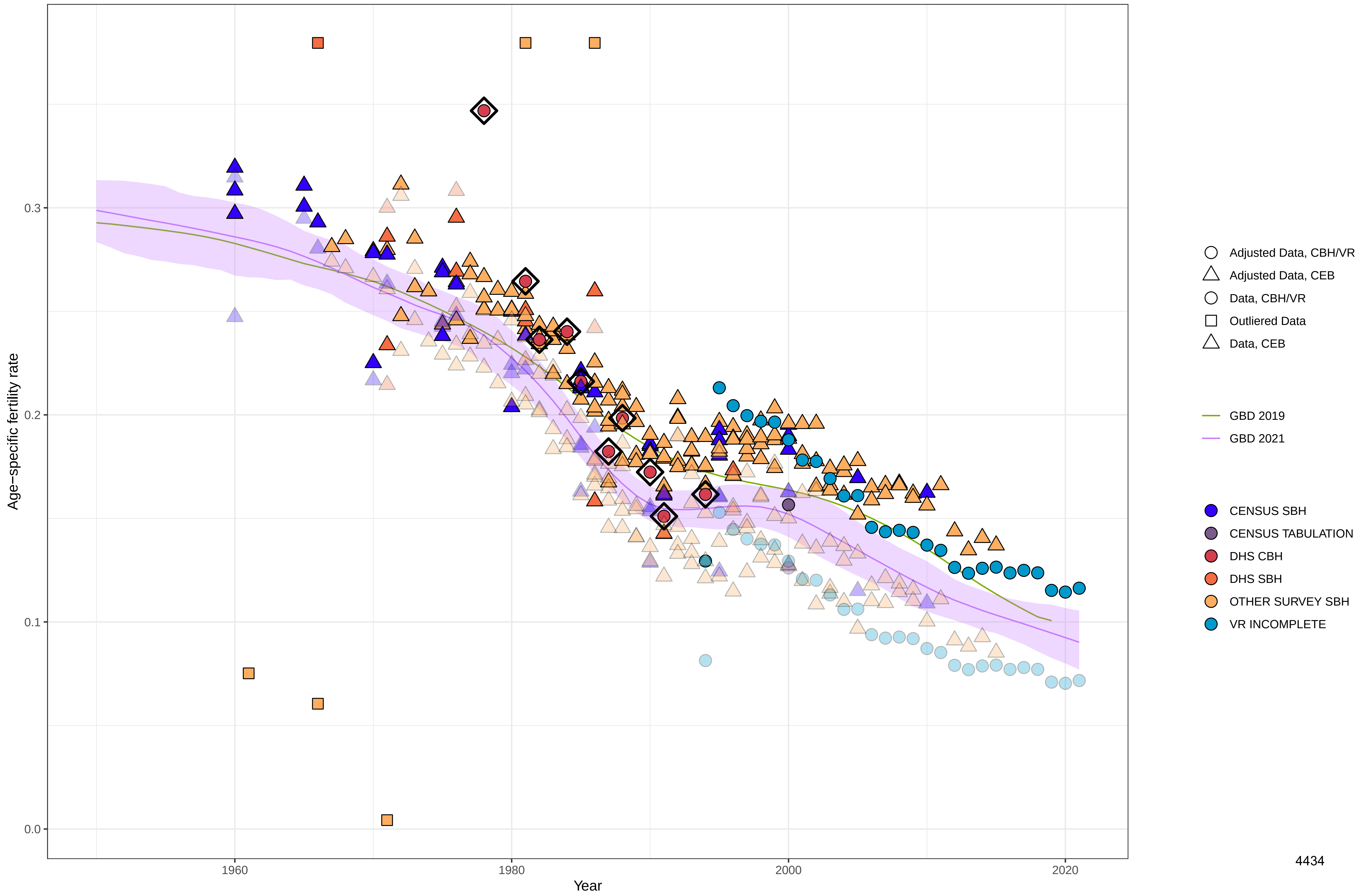

Age−specific fertility fitted trend lines, 1950−2021: Rio Grande do Norte, Ages: 25−29
Data points enclosed with a diamond are reference sources

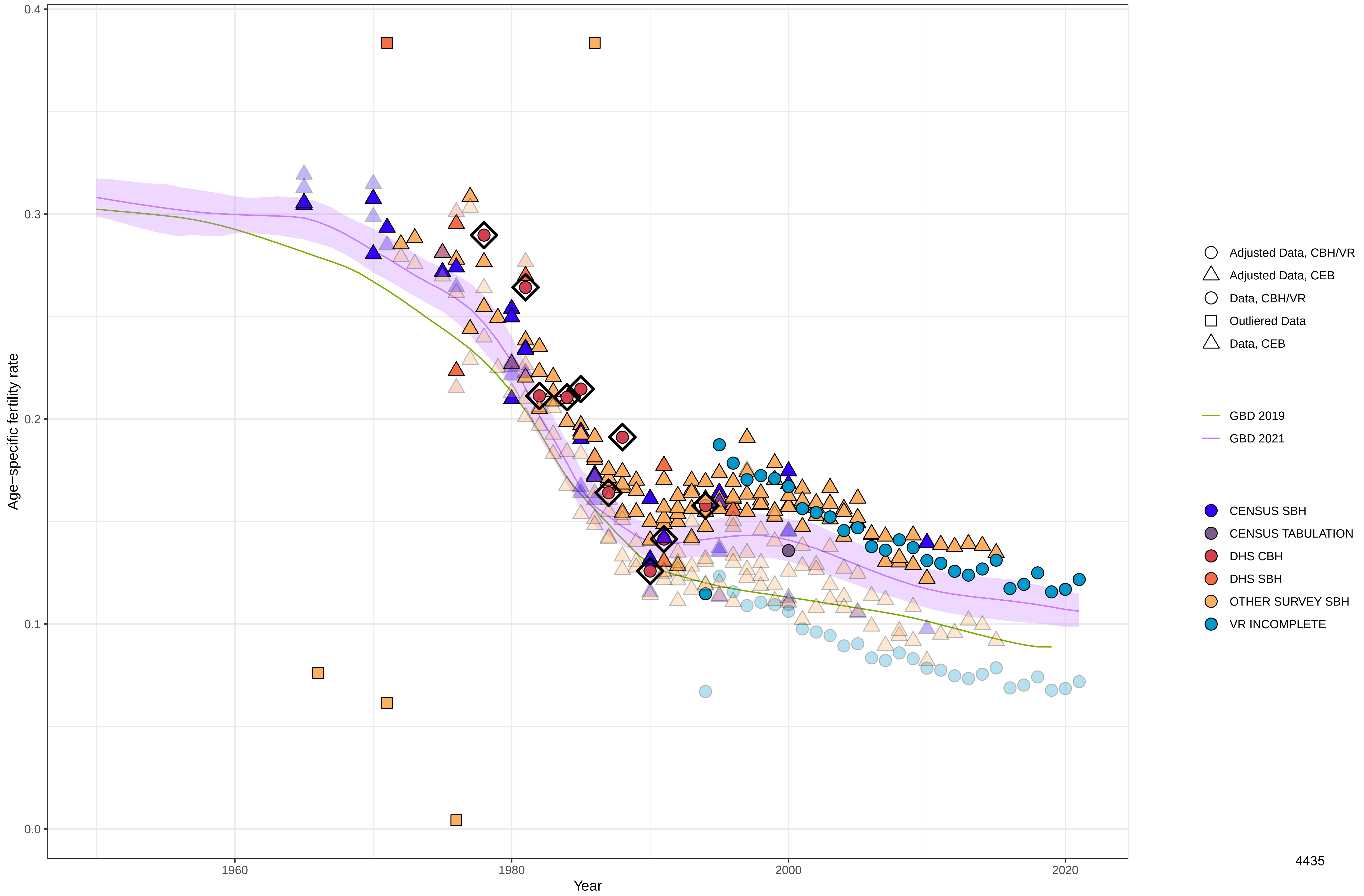

Age−specific fertility fitted trend lines, 1950−2021: Rio Grande do Norte, Ages: 30−34
Data points enclosed with a diamond are reference sources

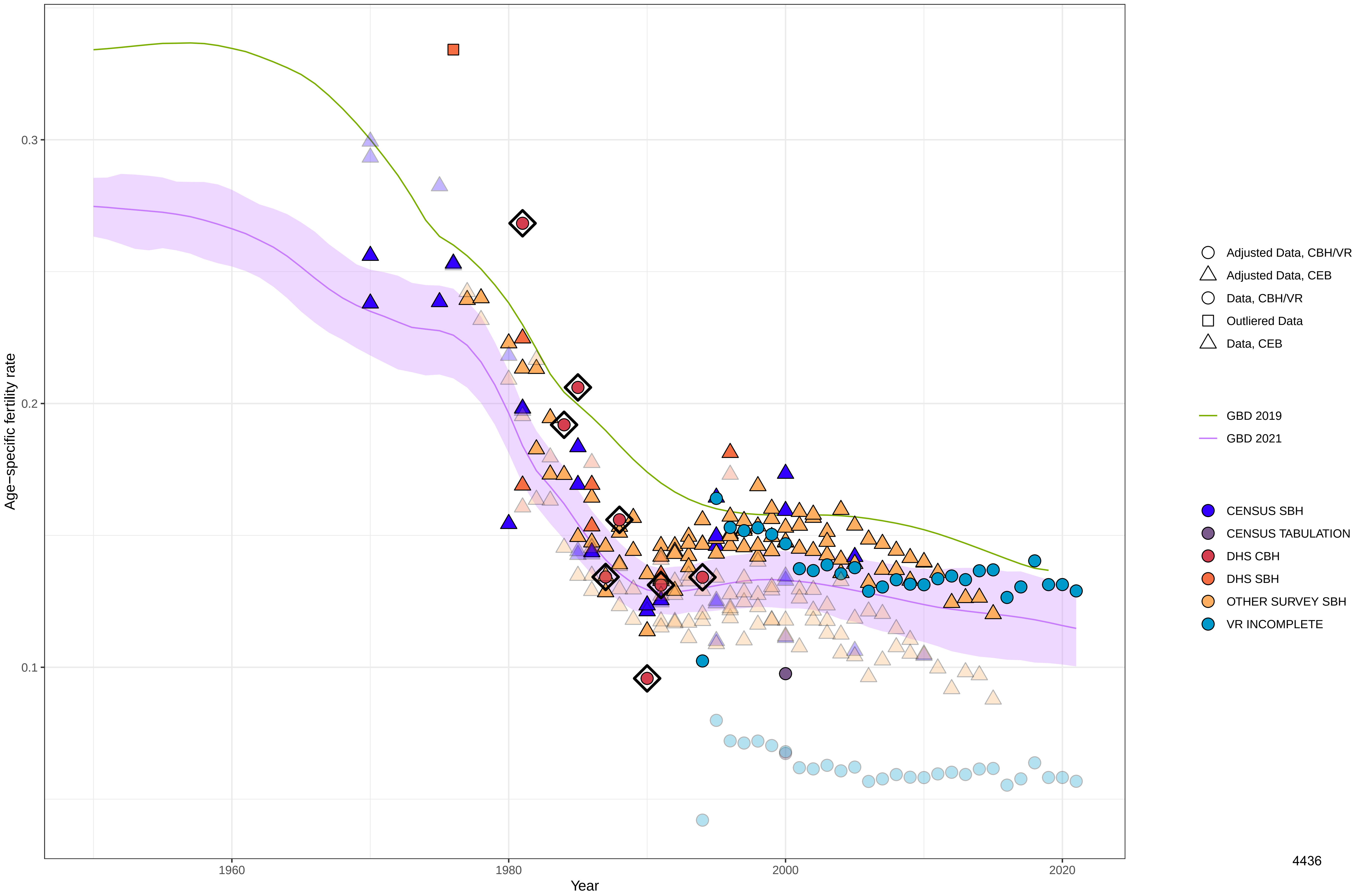

Age−specific fertility fitted trend lines, 1950−2021: Rio Grande do Norte, Ages: 35−39
Data points enclosed with a diamond are reference sources

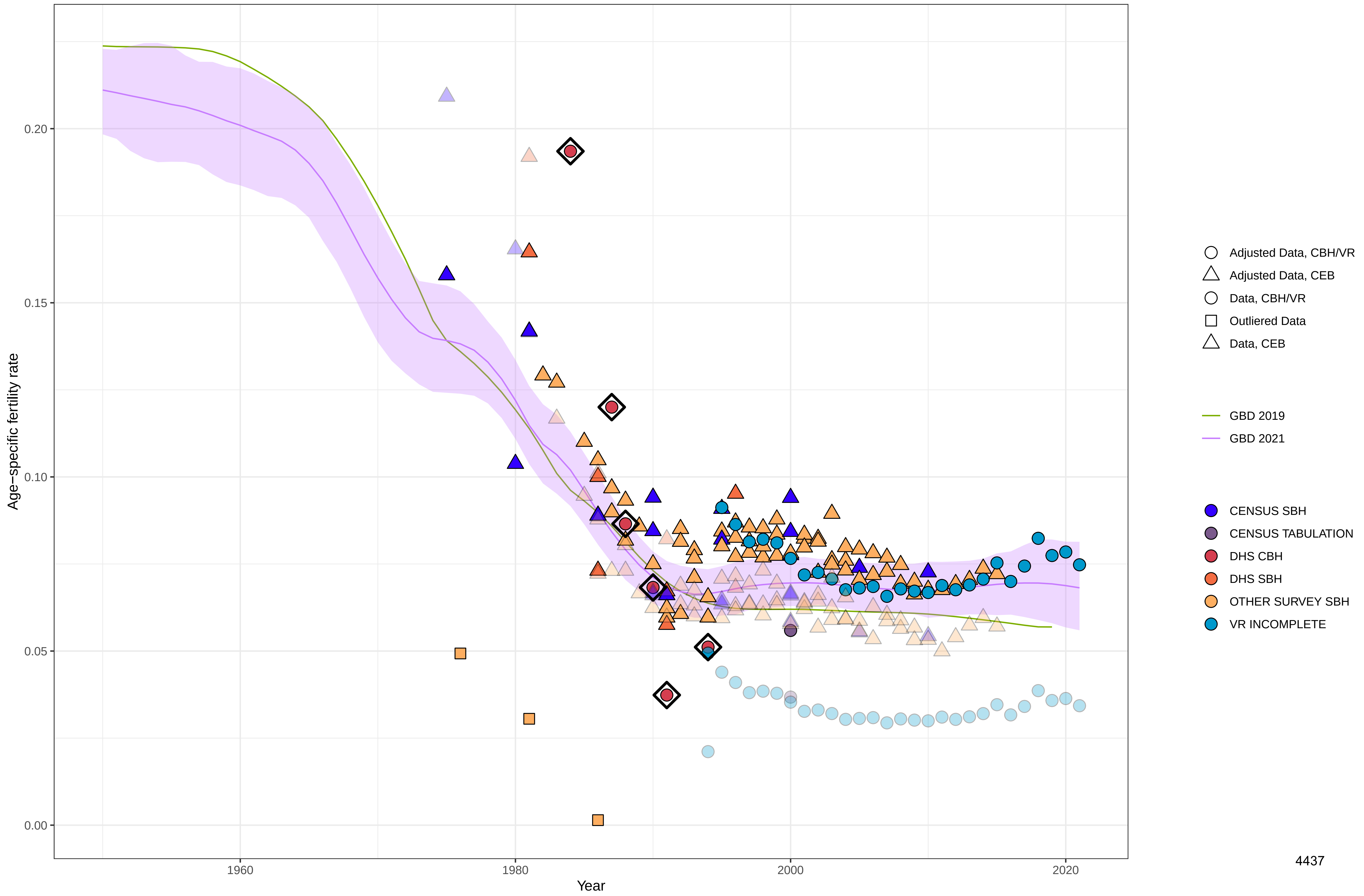

4437

Age−specific fertility fitted trend lines, 1950−2021: Rio Grande do Norte, Ages: 40−44
Data points enclosed with a diamond are reference sources

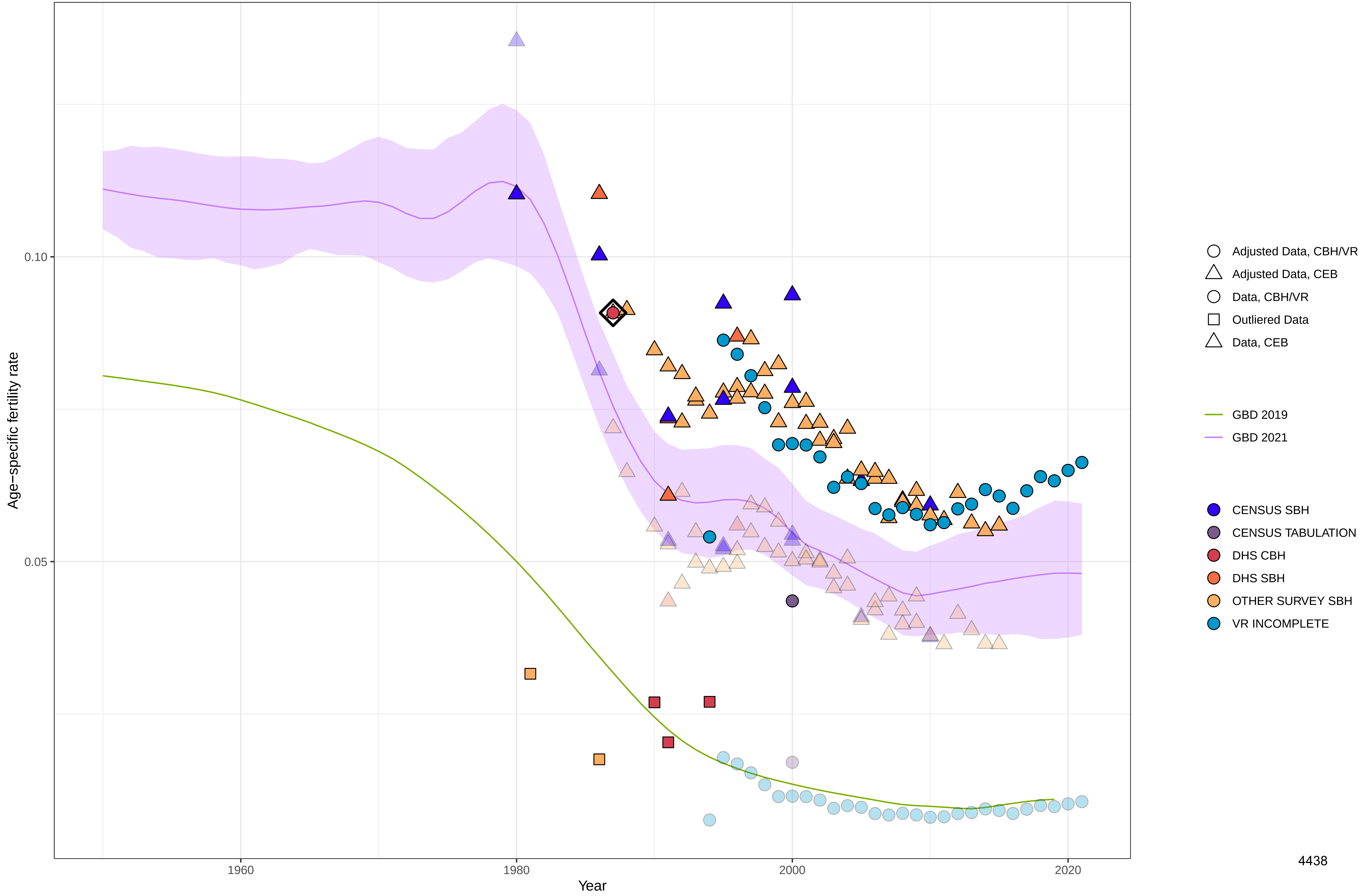

4438

Age−specific fertility fitted trend lines, 1950−2021: Rio Grande do Norte, Ages: 45−49
Data points enclosed with a diamond are reference sources

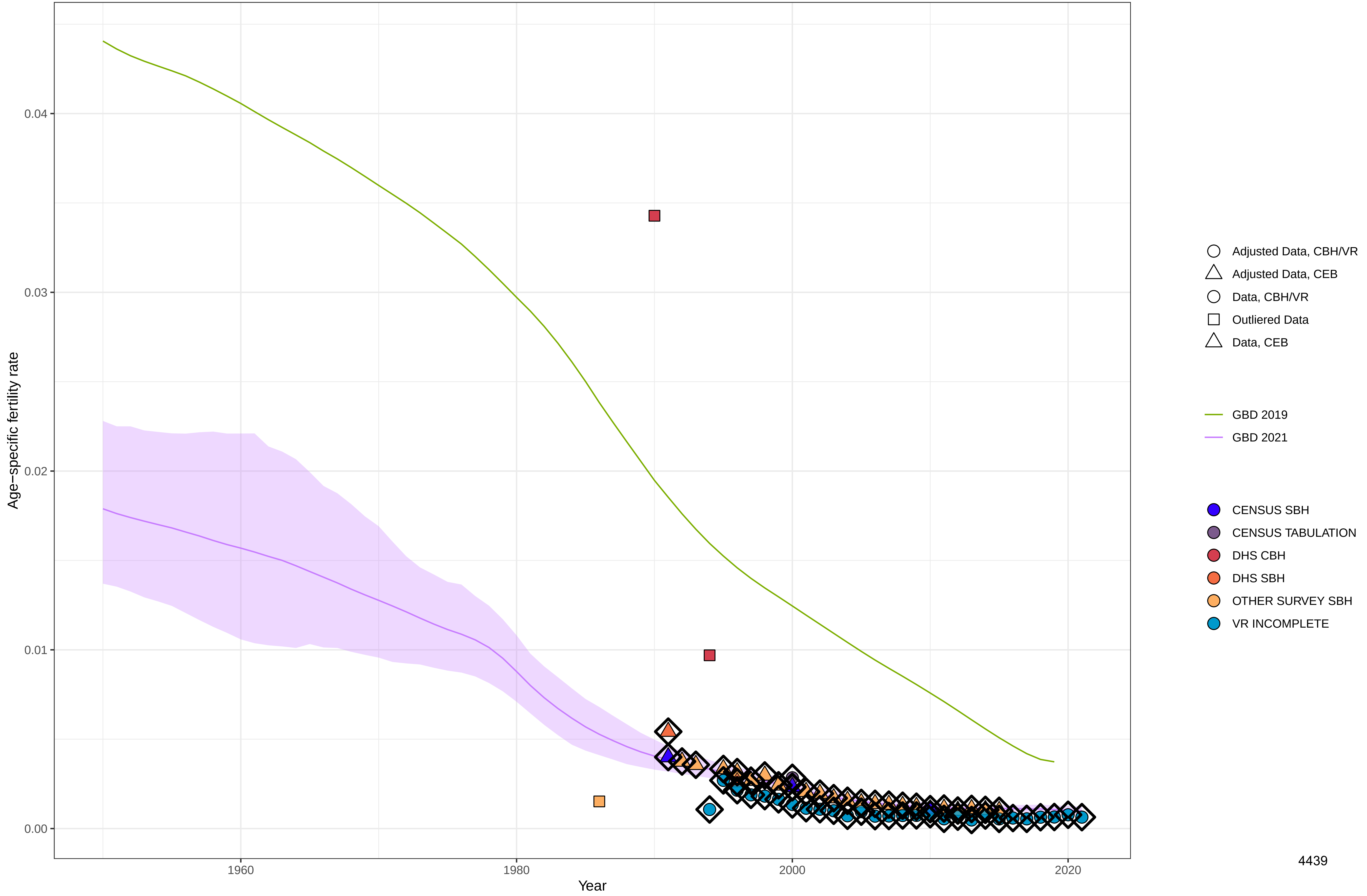

Age−specific fertility fitted trend lines, 1950−2021: Rio Grande do Norte, Ages: 50−54
Data points enclosed with a diamond are reference sources

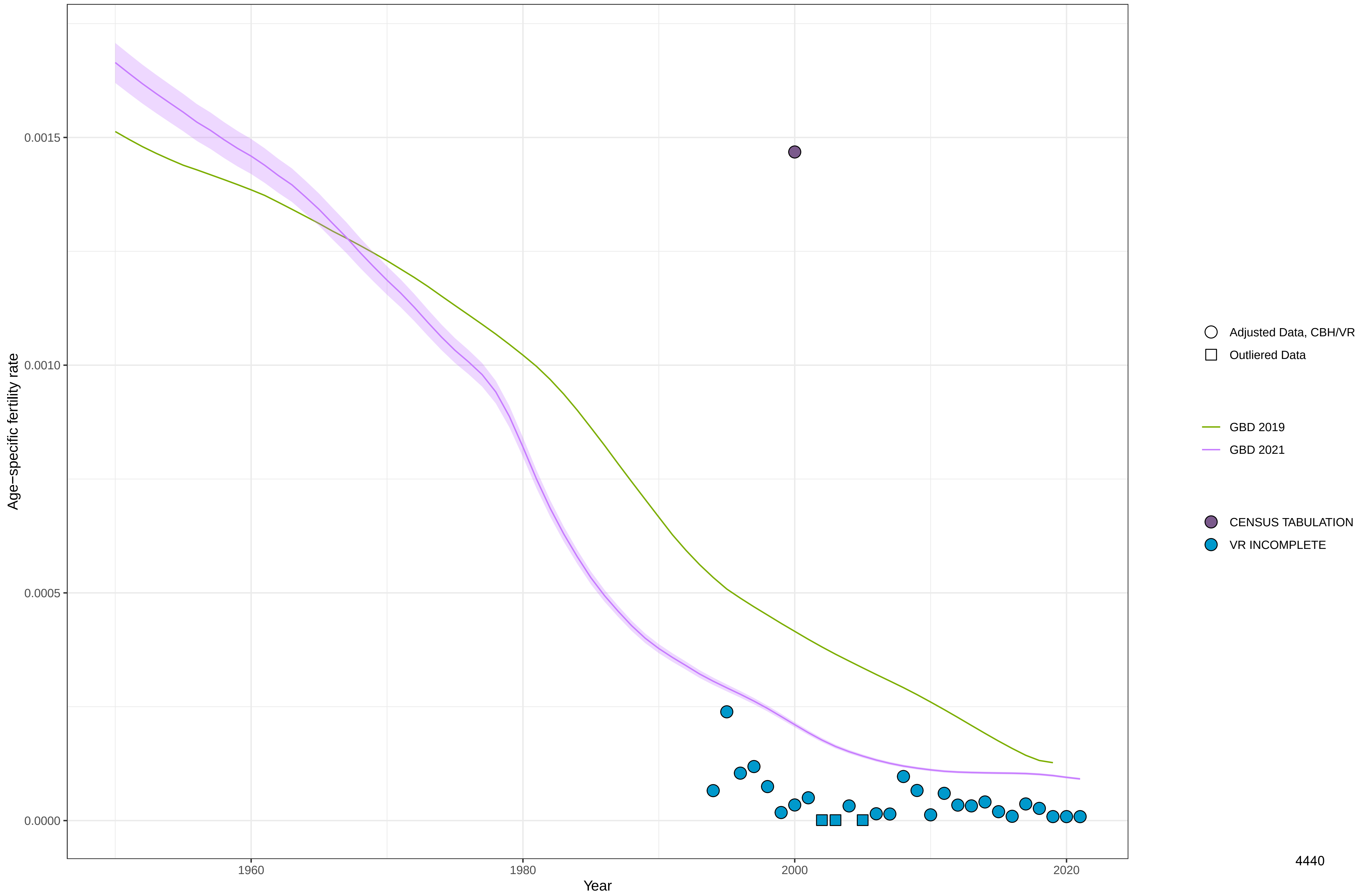

Total fertility rate fitted trend lines, 1950−2021: Rio Grande do Norte TFR
Data points enclosed with a diamond are reference sources

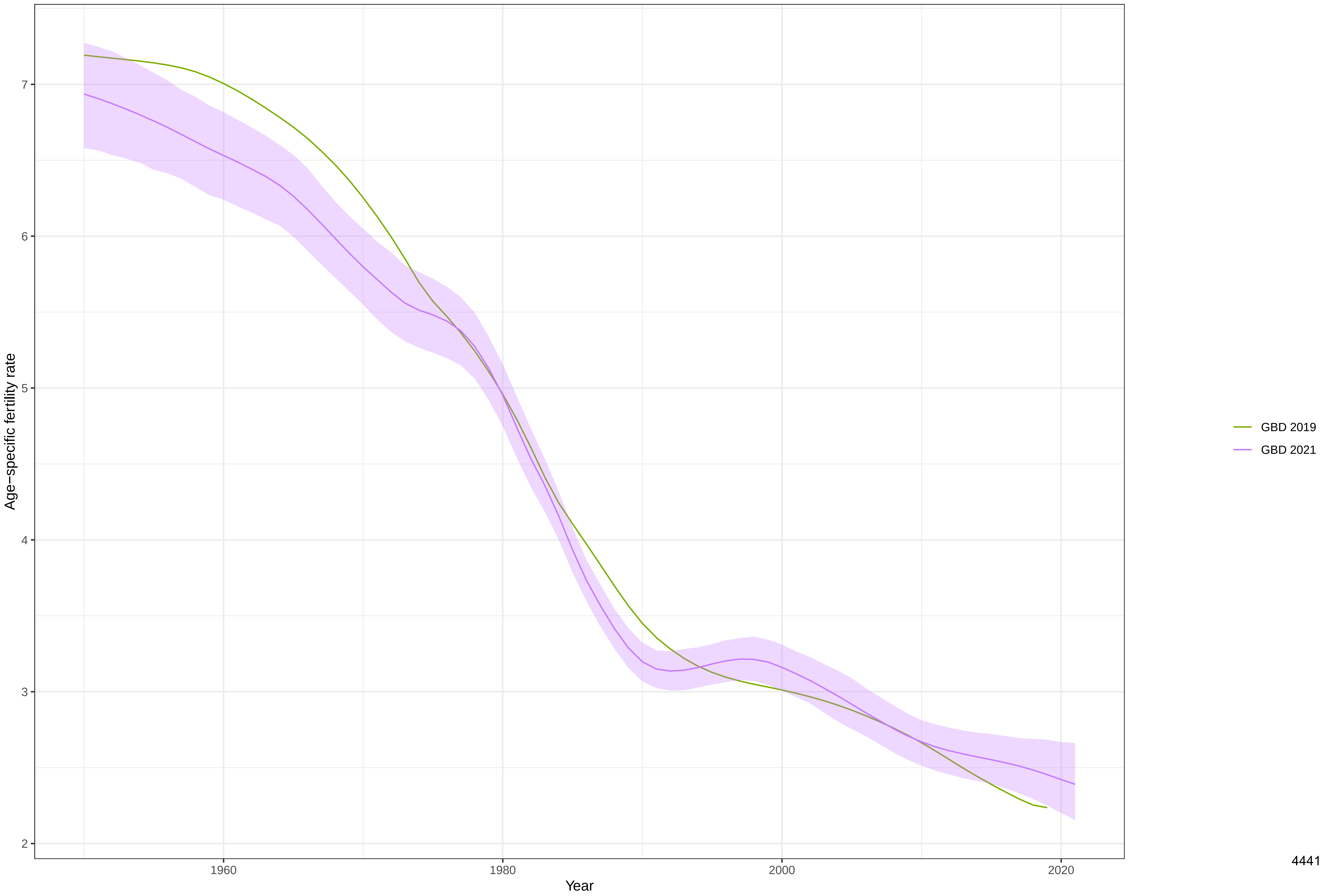

Age−specific fertility fitted trend lines, 1950−2021: Rio Grande do Sul, Ages: 10−14
Data points enclosed with a diamond are reference sources

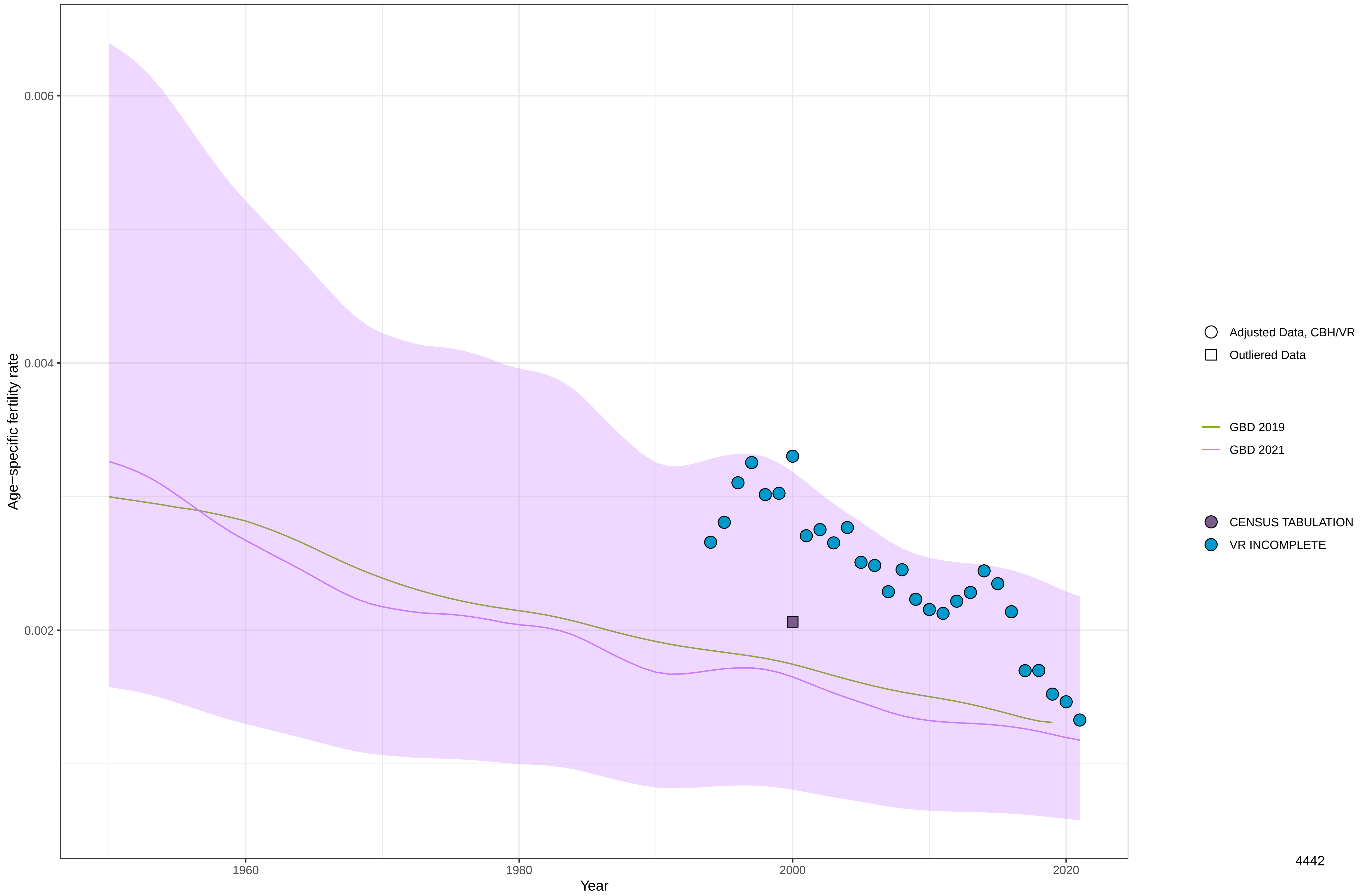

Age−specific fertility fitted trend lines, 1950−2021: Rio Grande do Sul, Ages: 15−19
Data points enclosed with a diamond are reference sources

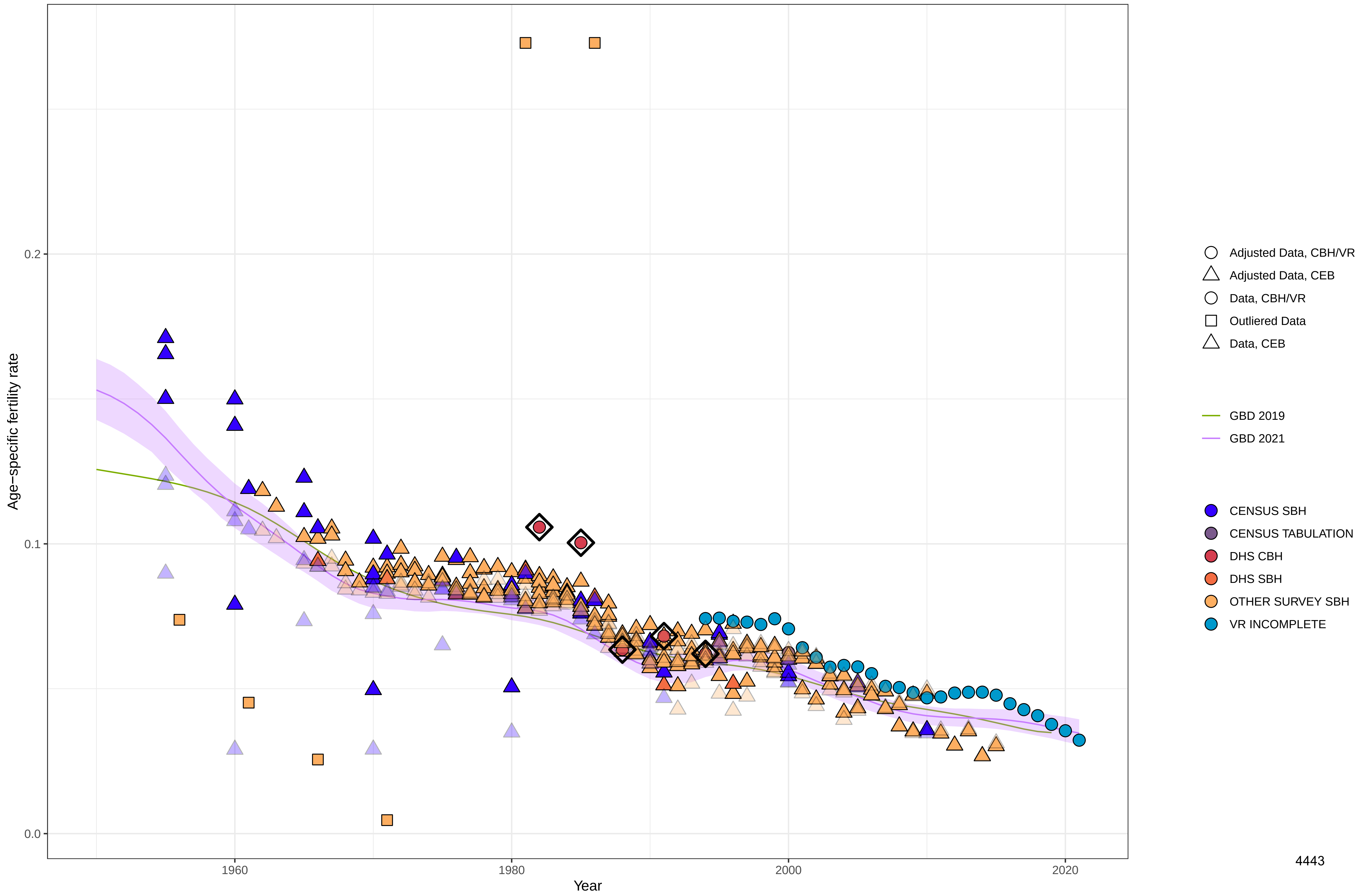

Age−specific fertility fitted trend lines, 1950−2021: Rio Grande do Sul, Ages: 20−24
Data points enclosed with a diamond are reference sources

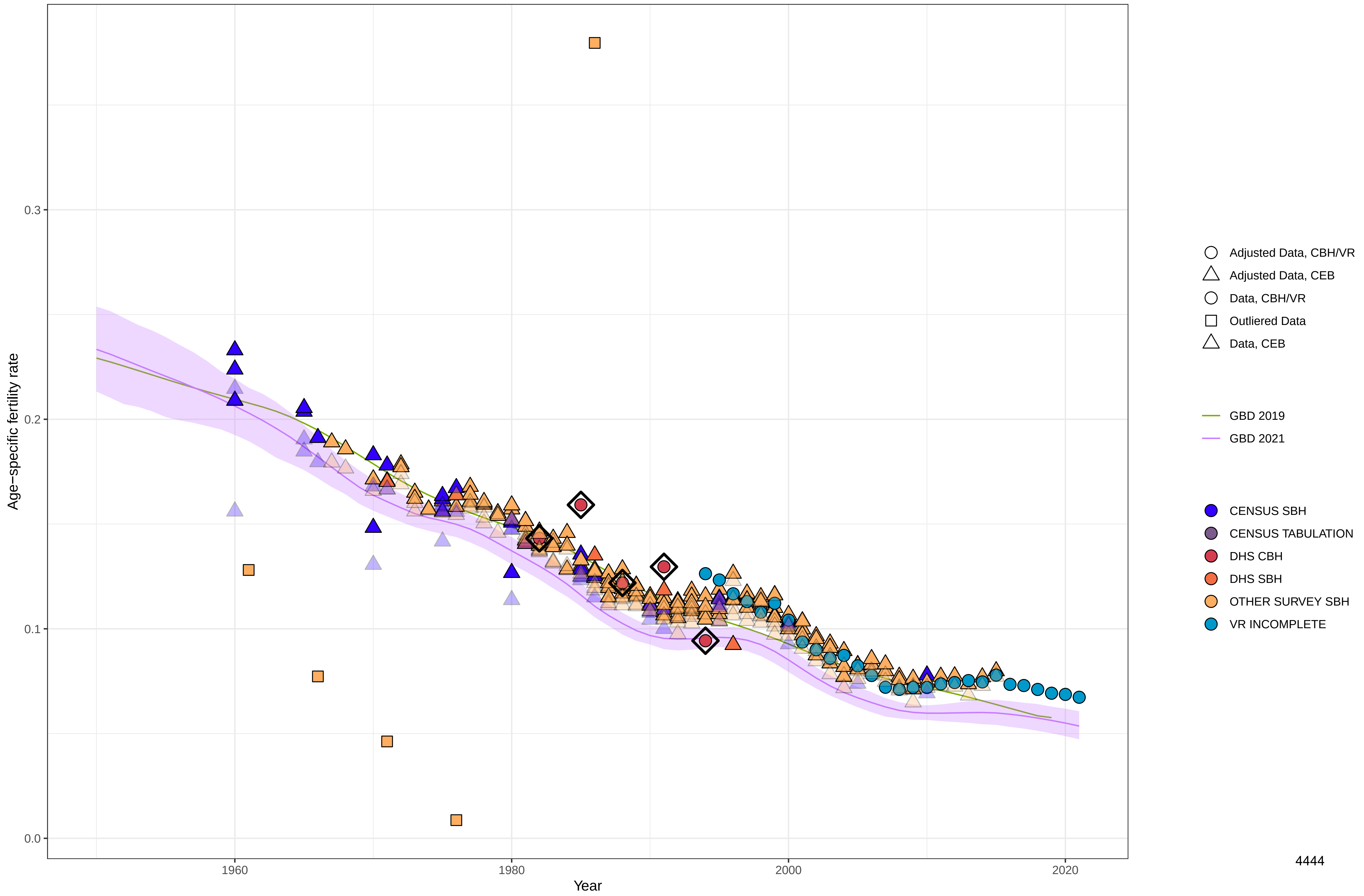

Age−specific fertility fitted trend lines, 1950−2021: Rio Grande do Sul, Ages: 25−29
Data points enclosed with a diamond are reference sources

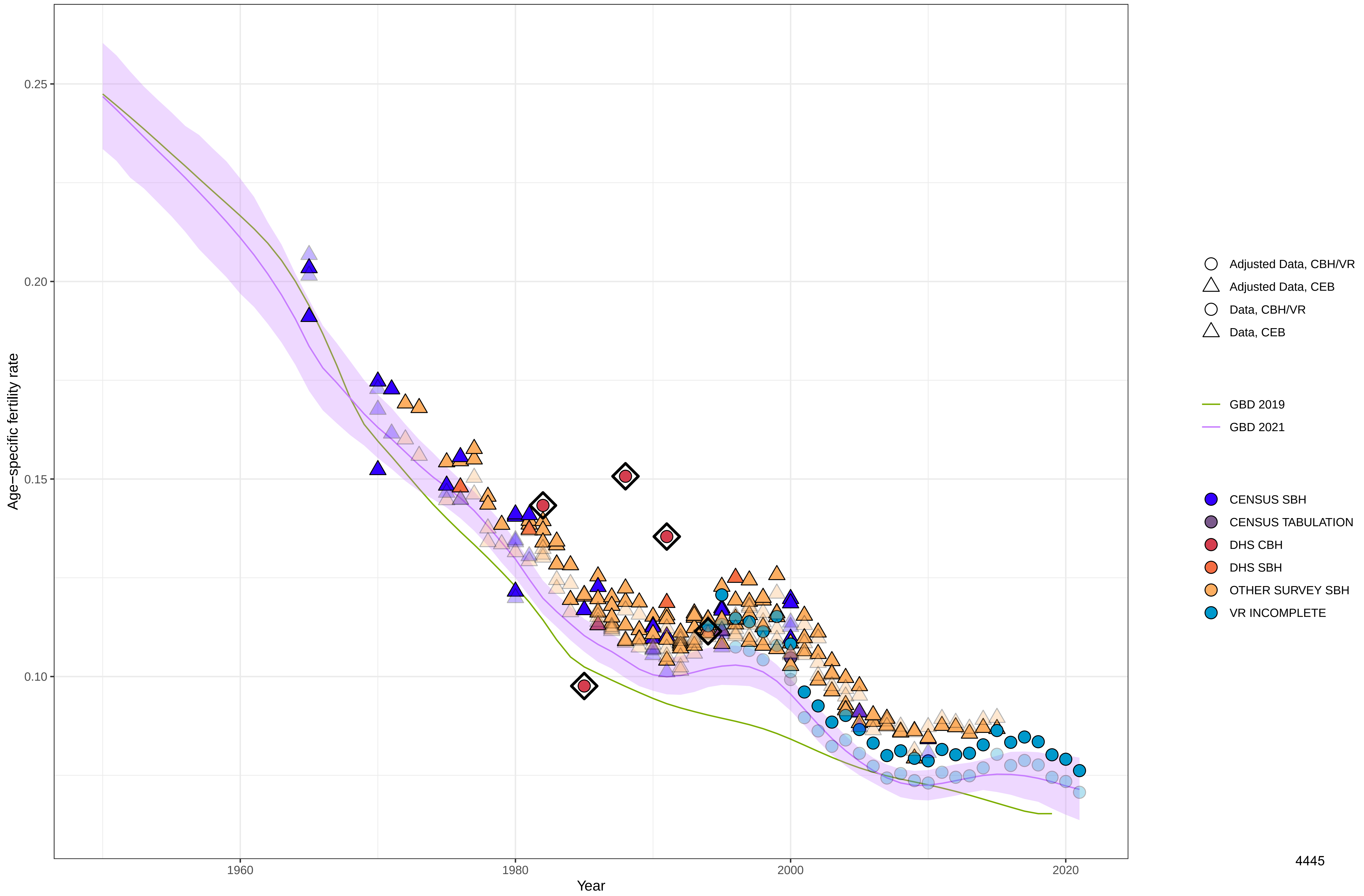

Age−specific fertility fitted trend lines, 1950−2021: Rio Grande do Sul, Ages: 30−34
Data points enclosed with a diamond are reference sources

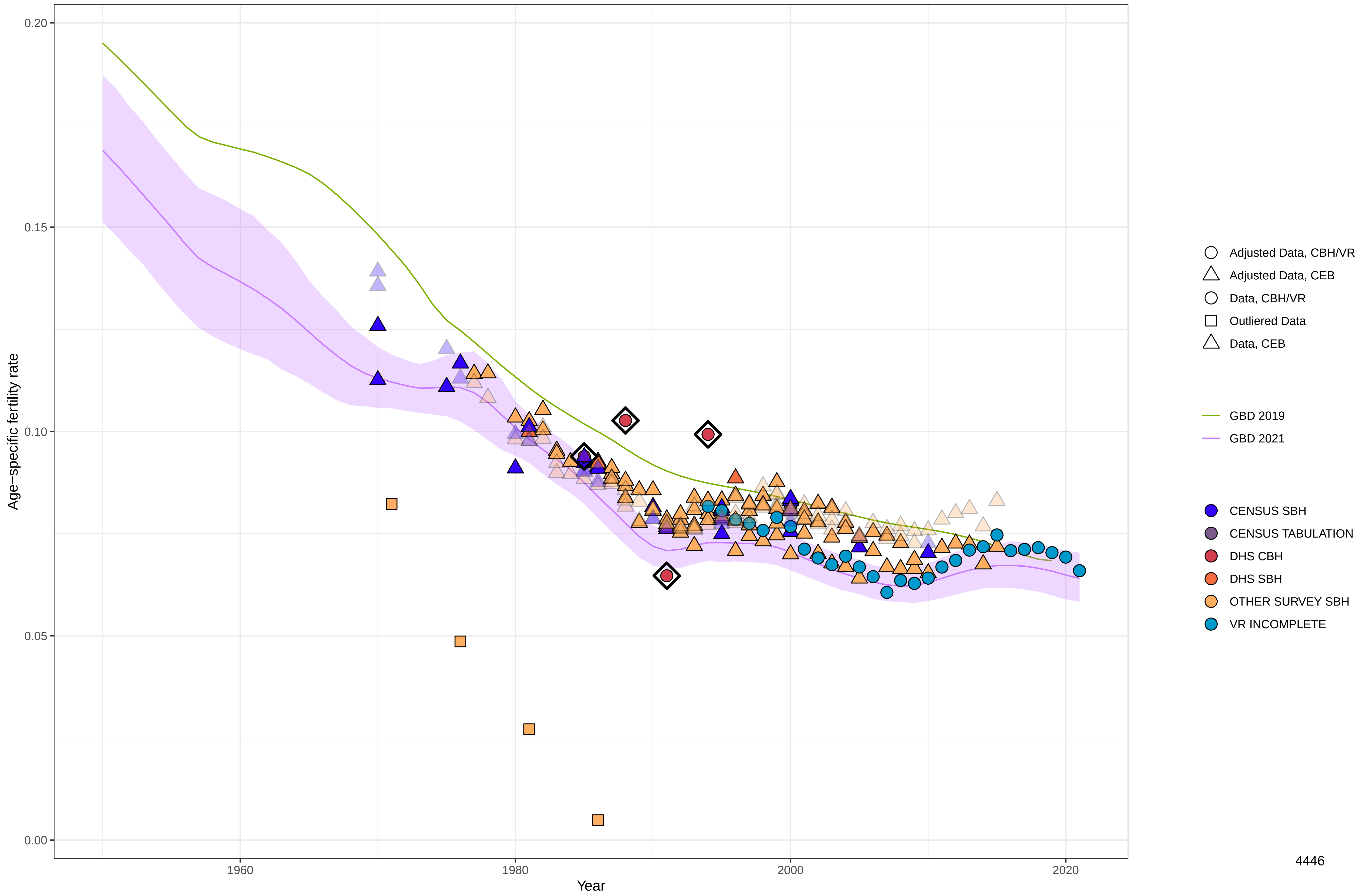

Age−specific fertility fitted trend lines, 1950−2021: Rio Grande do Sul, Ages: 35−39
Data points enclosed with a diamond are reference sources

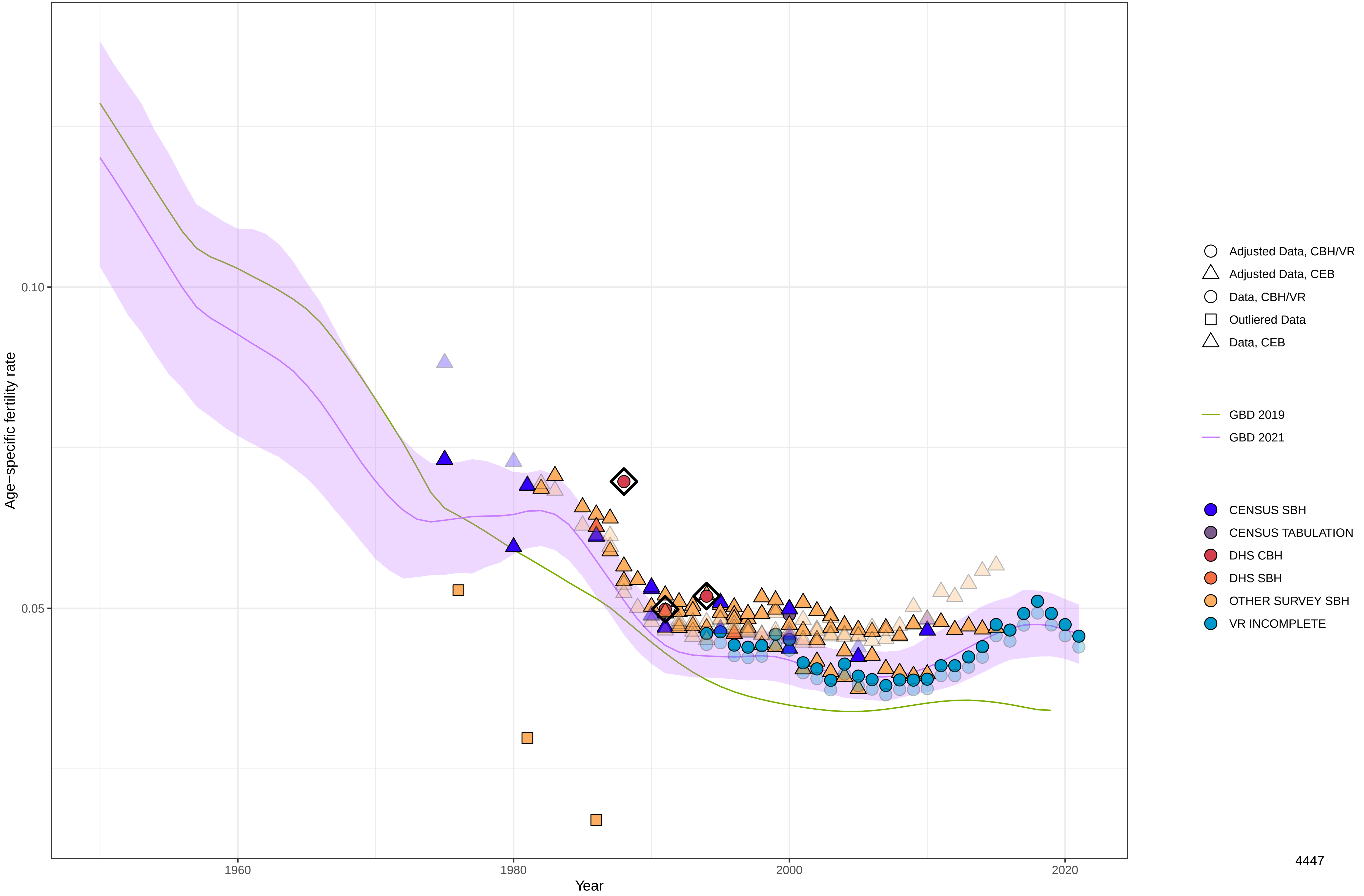

4447

Age−specific fertility fitted trend lines, 1950−2021: Rio Grande do Sul, Ages: 40−44
Data points enclosed with a diamond are reference sources

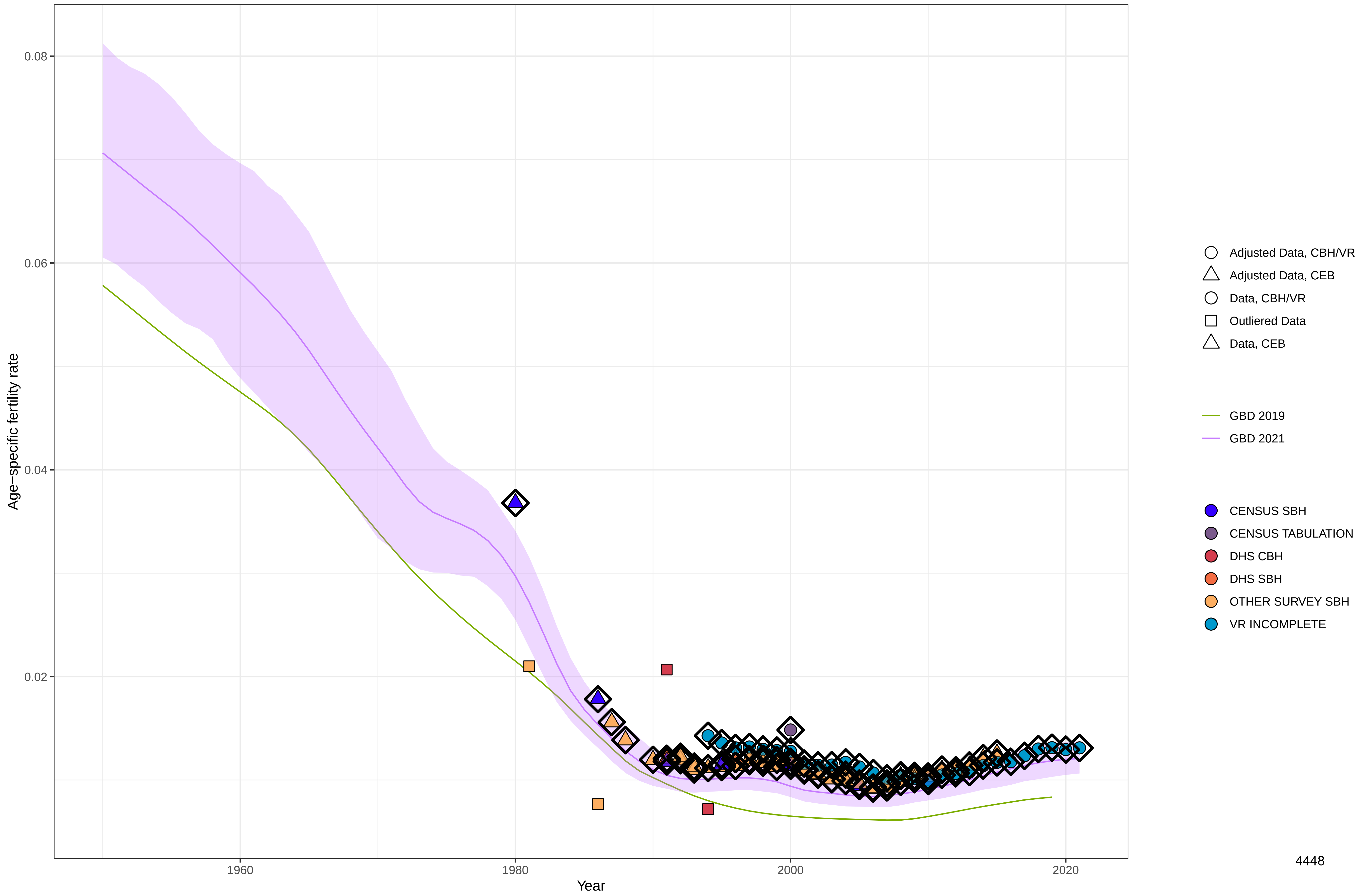

Age−specific fertility fitted trend lines, 1950−2021: Rio Grande do Sul, Ages: 45−49
Data points enclosed with a diamond are reference sources

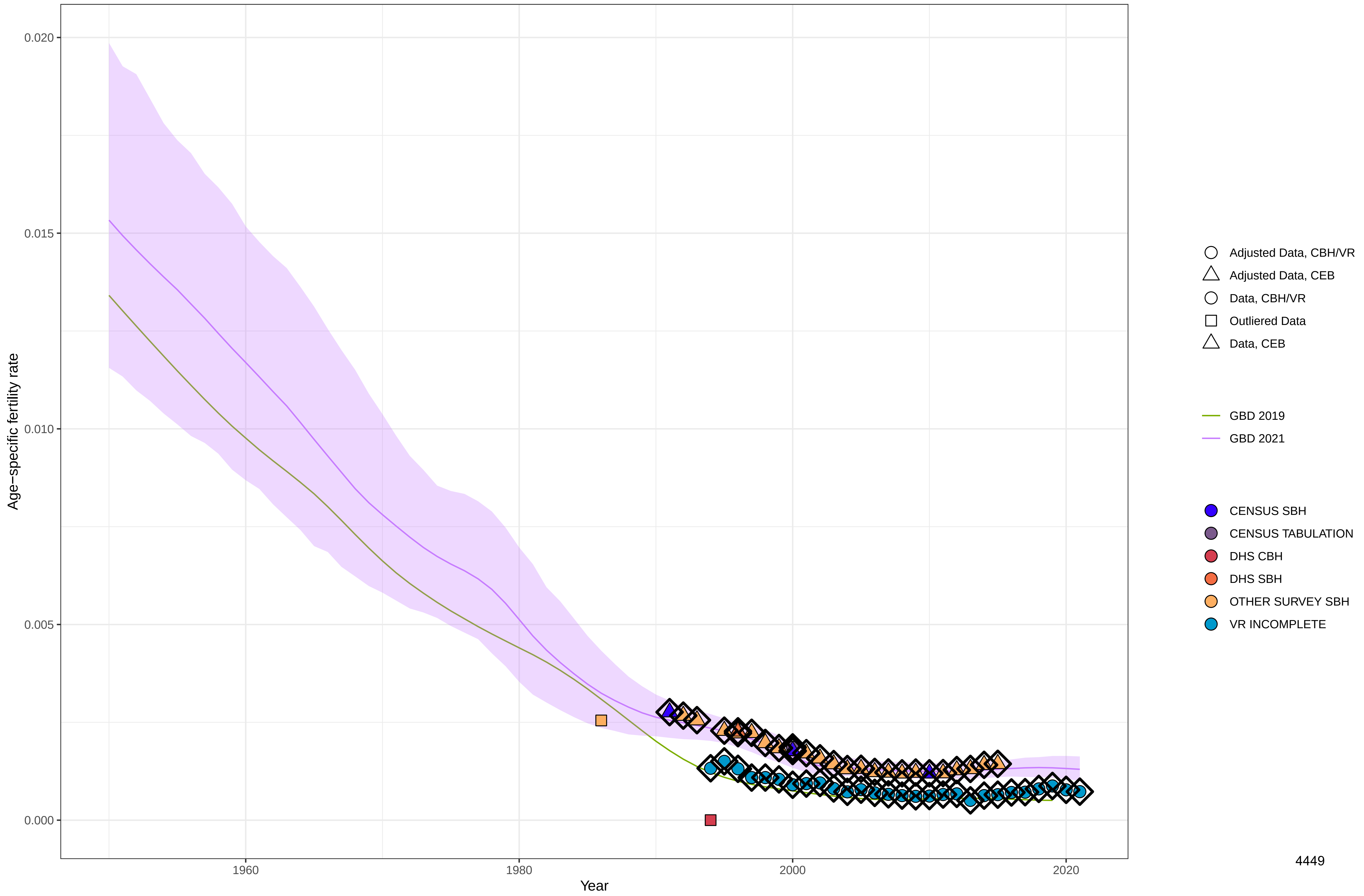

Age−specific fertility fitted trend lines, 1950−2021: Rio Grande do Sul, Ages: 50−54
Data points enclosed with a diamond are reference sources

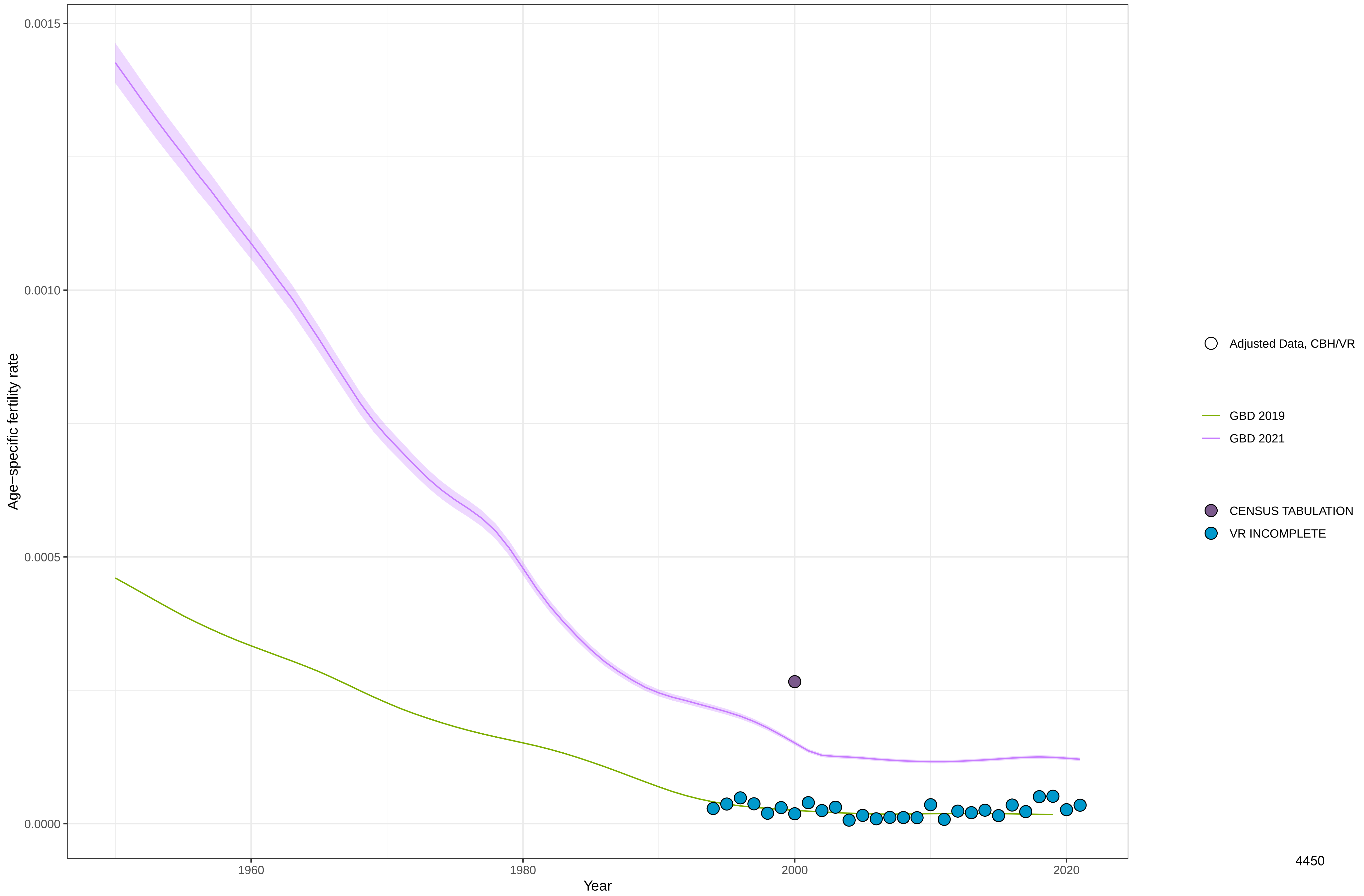

Total fertility rate fitted trend lines, 1950−2021: Rio Grande do Sul TFR
Data points enclosed with a diamond are reference sources

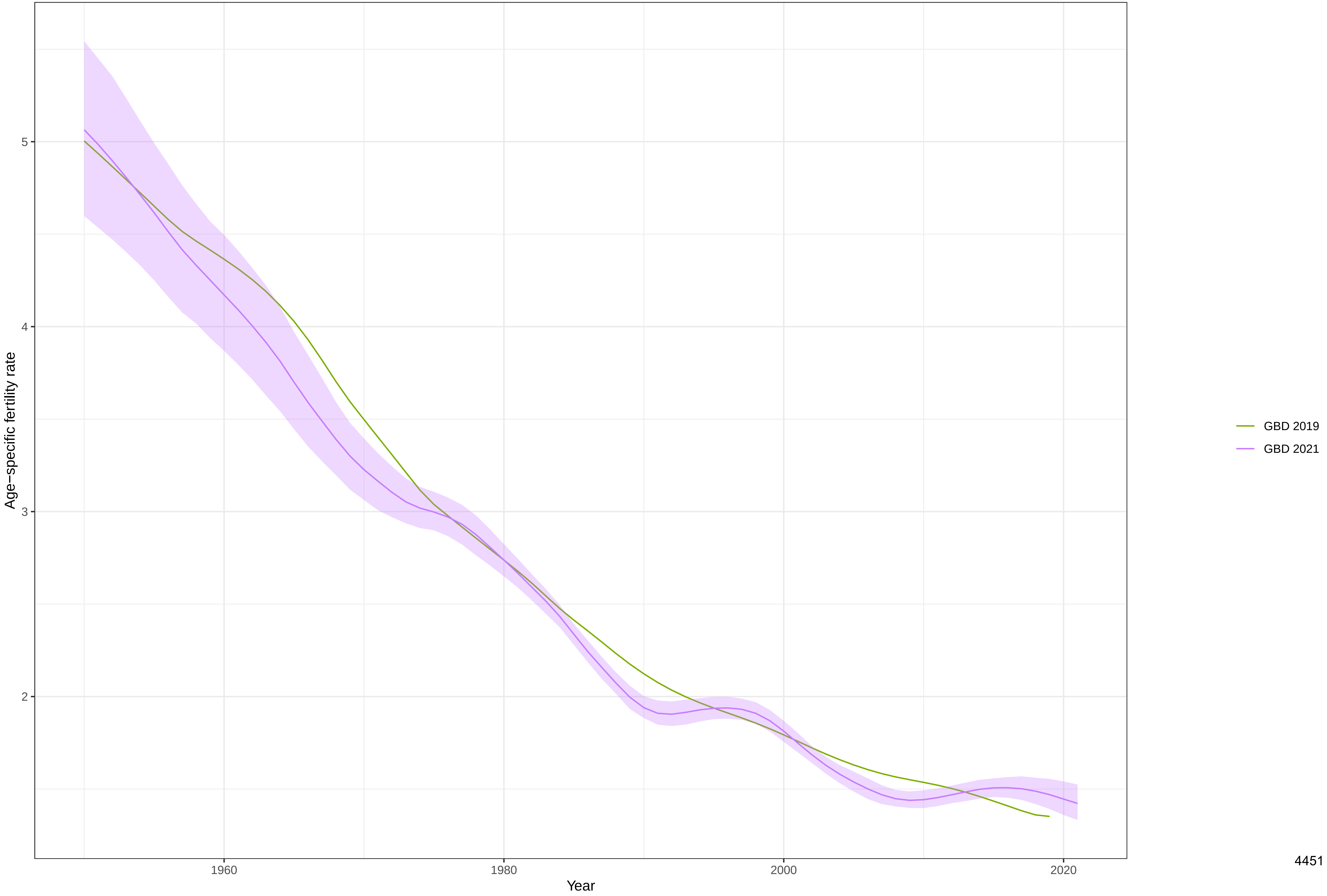

Age−specific fertility fitted trend lines, 1950−2021: Rondônia, Ages: 10−14
Data points enclosed with a diamond are reference sources

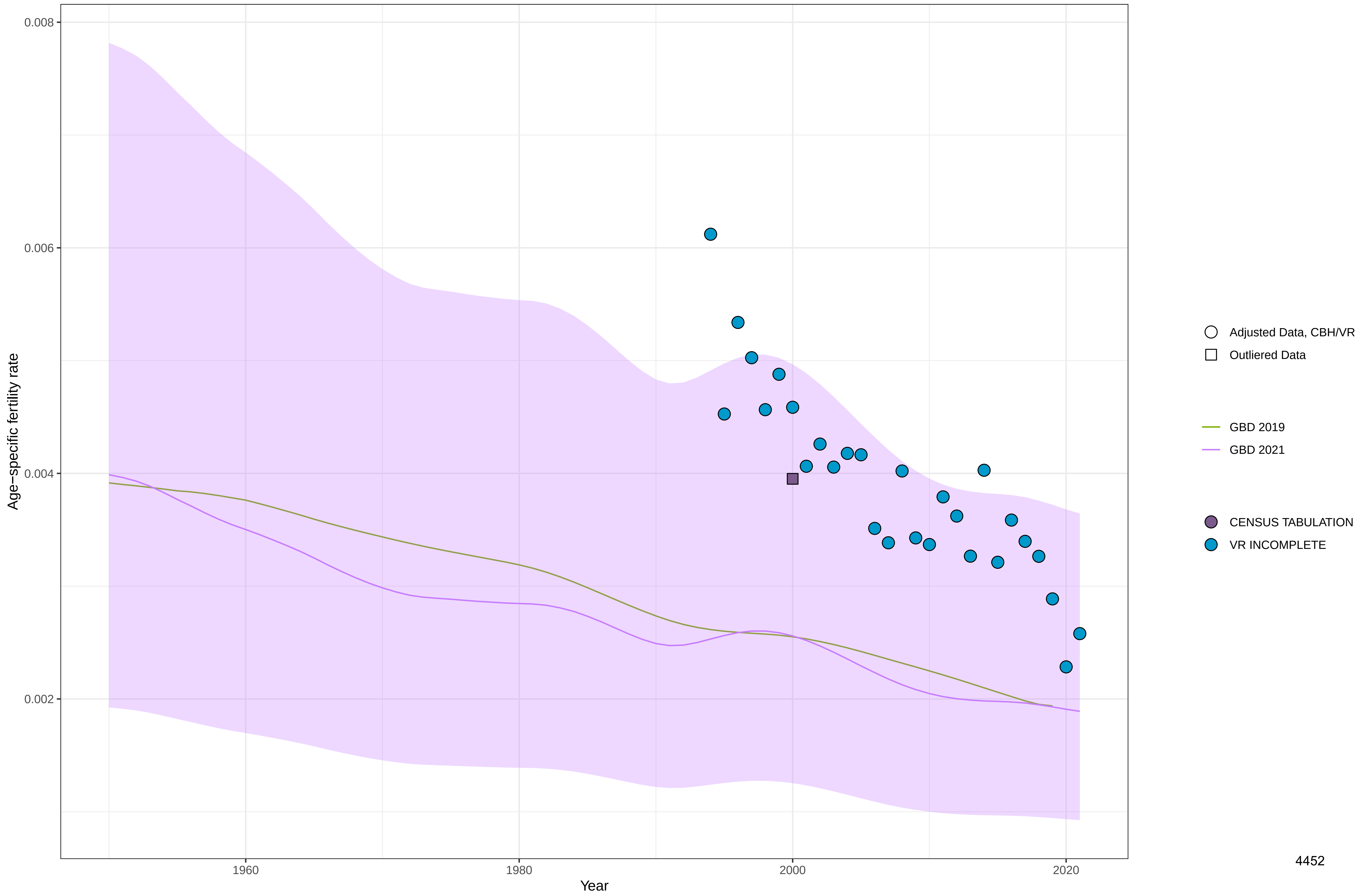

Age−specific fertility fitted trend lines, 1950−2021: Rondônia, Ages: 15−19
Data points enclosed with a diamond are reference sources

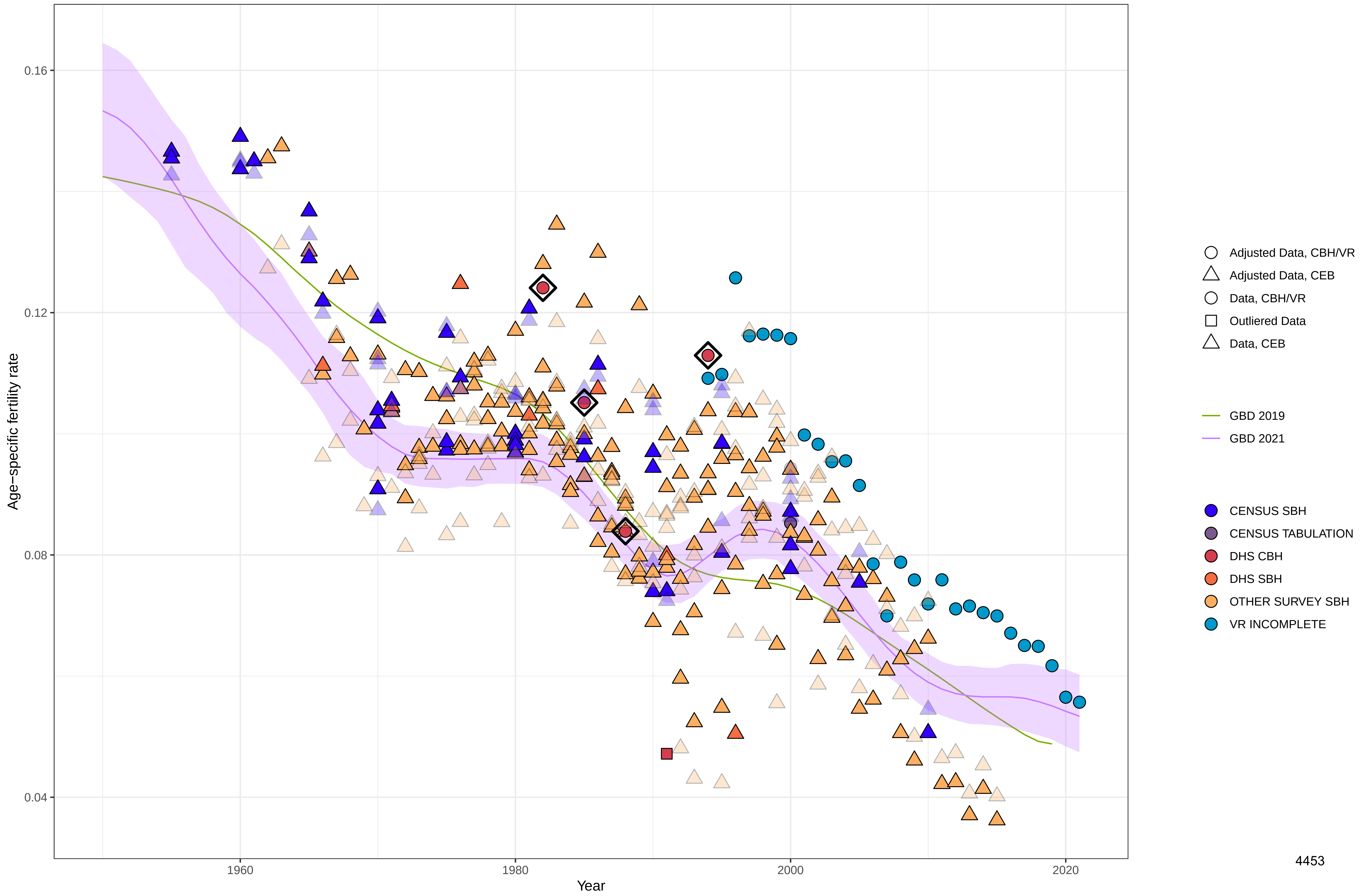

Age−specific fertility fitted trend lines, 1950−2021: Rondônia, Ages: 20−24
Data points enclosed with a diamond are reference sources

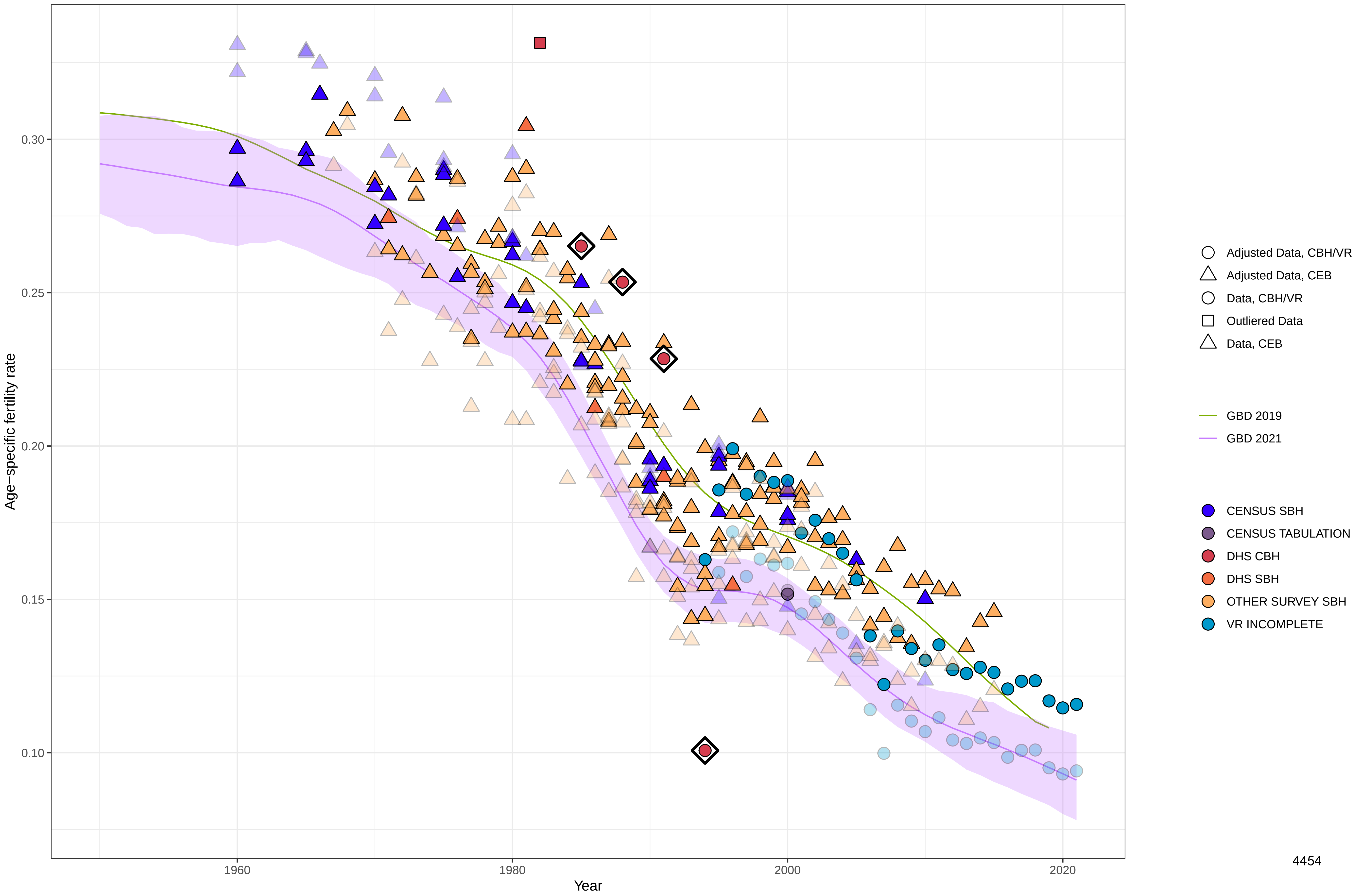

Age−specific fertility fitted trend lines, 1950−2021: Rondônia, Ages: 25−29
Data points enclosed with a diamond are reference sources

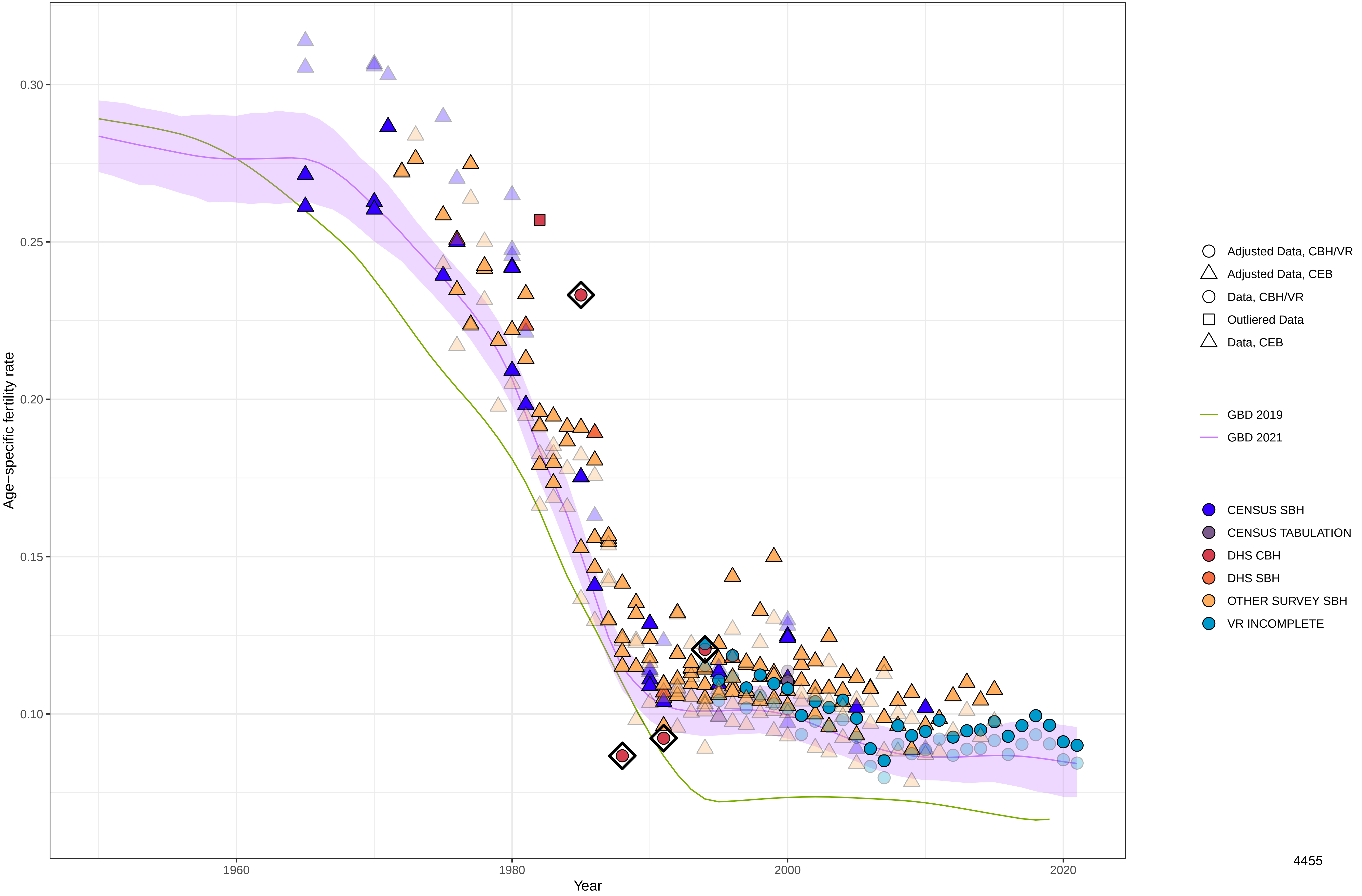

Age−specific fertility fitted trend lines, 1950−2021: Rondônia, Ages: 30−34
Data points enclosed with a diamond are reference sources

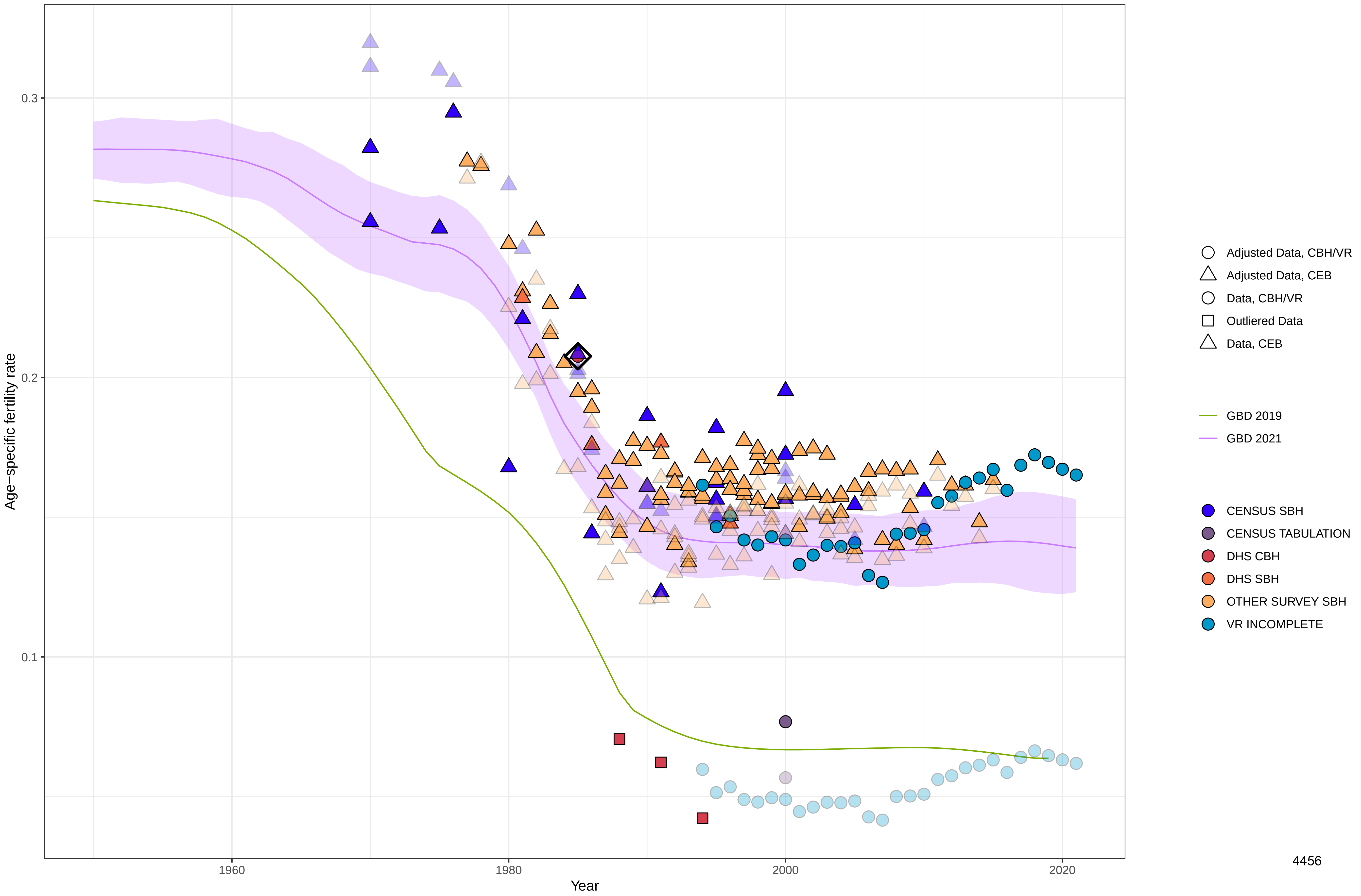

Age−specific fertility fitted trend lines, 1950−2021: Rondônia, Ages: 35−39
Data points enclosed with a diamond are reference sources

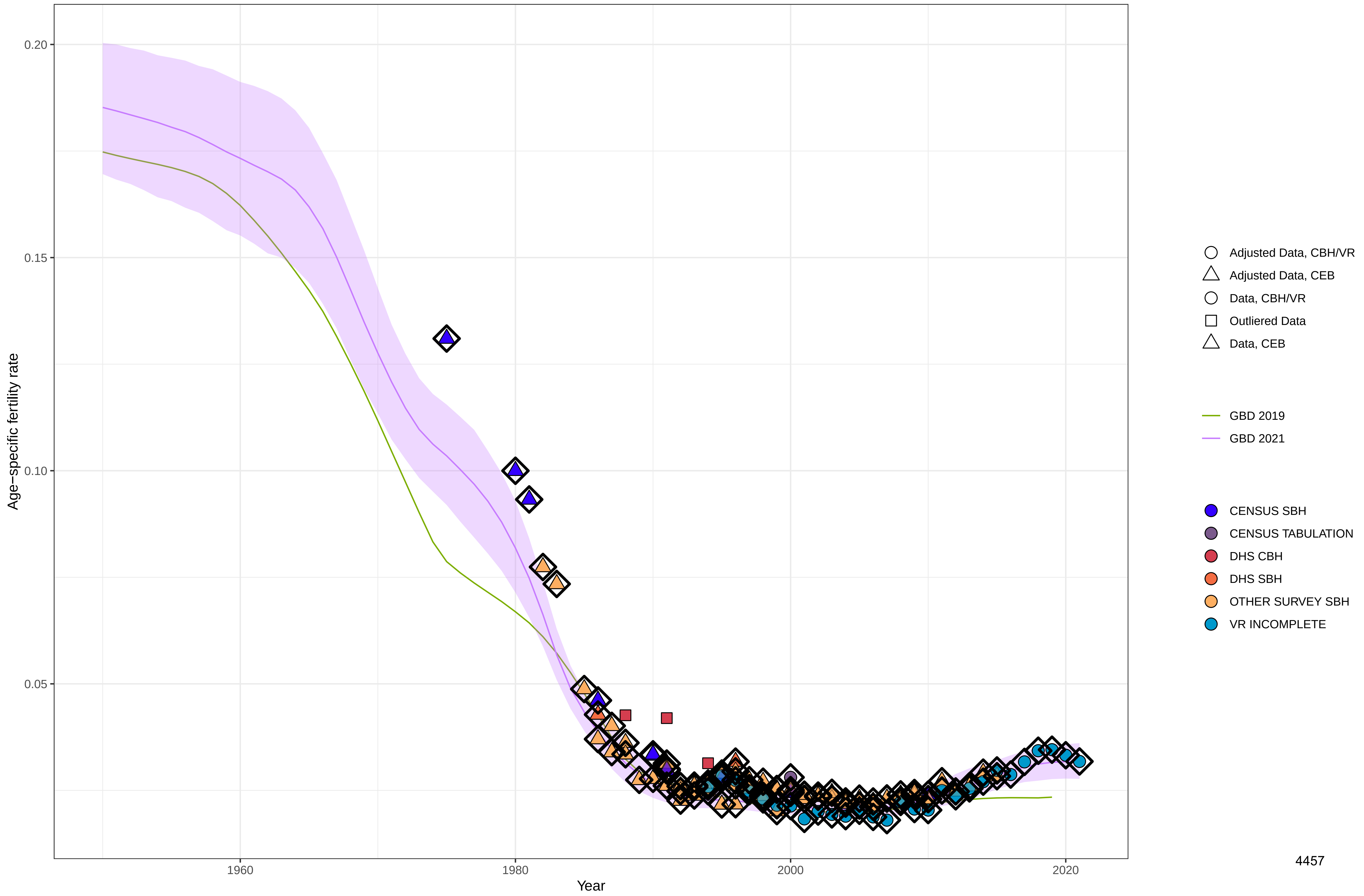

Age−specific fertility fitted trend lines, 1950−2021: Rondônia, Ages: 40−44
Data points enclosed with a diamond are reference sources

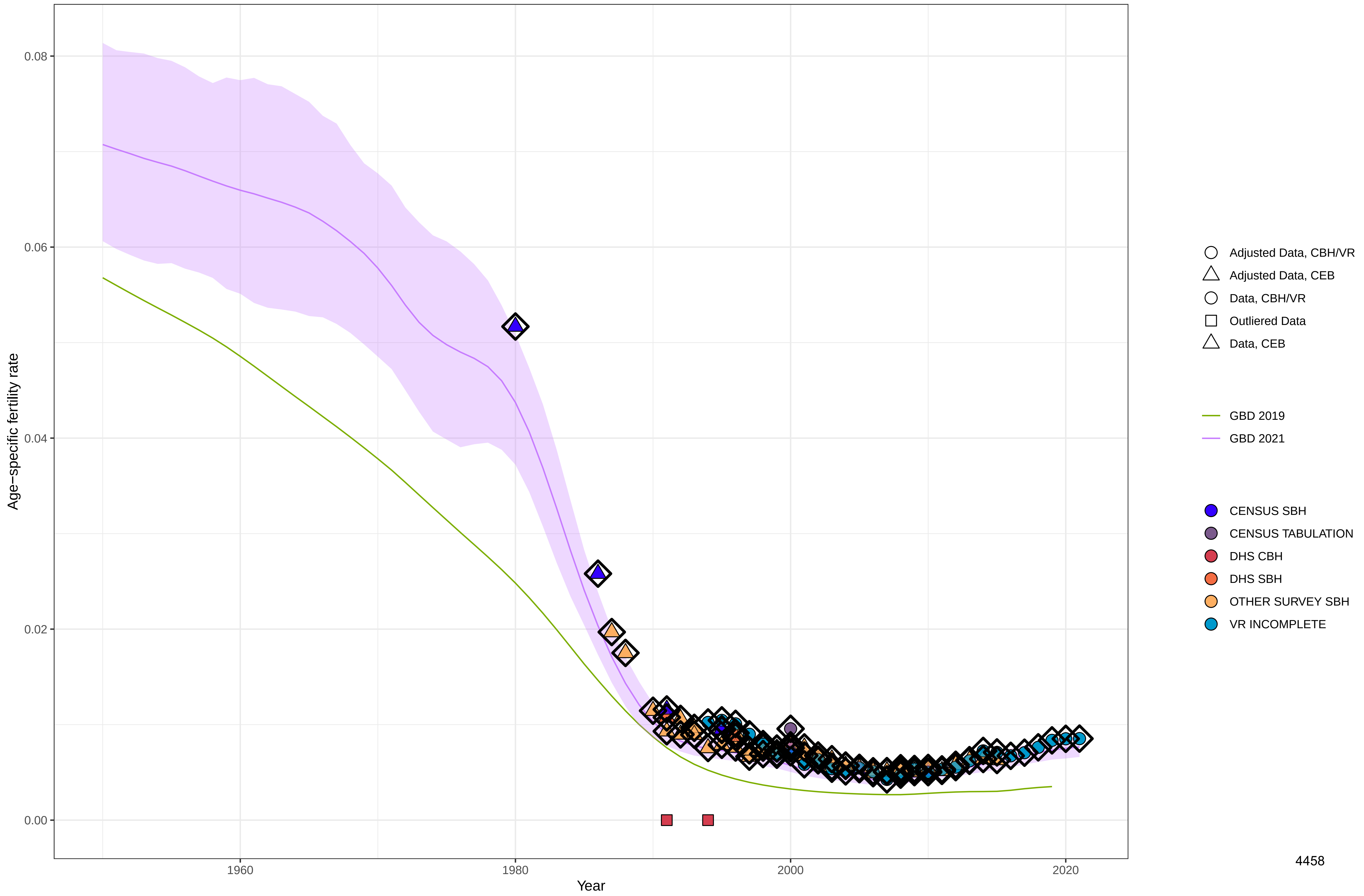

Age−specific fertility fitted trend lines, 1950−2021: Rondônia, Ages: 45−49
Data points enclosed with a diamond are reference sources

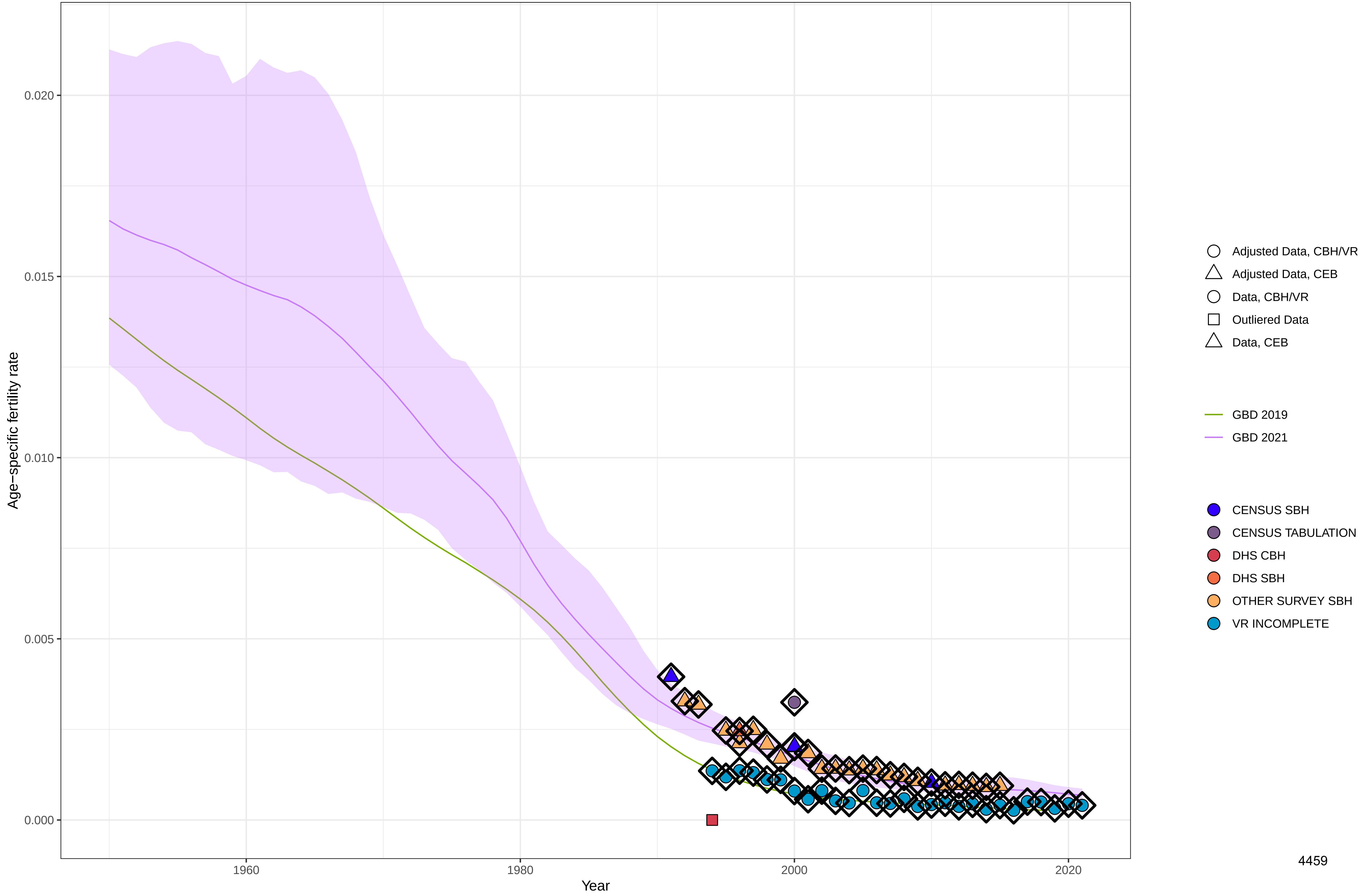

Age−specific fertility fitted trend lines, 1950−2021: Rondônia, Ages: 50−54
Data points enclosed with a diamond are reference sources

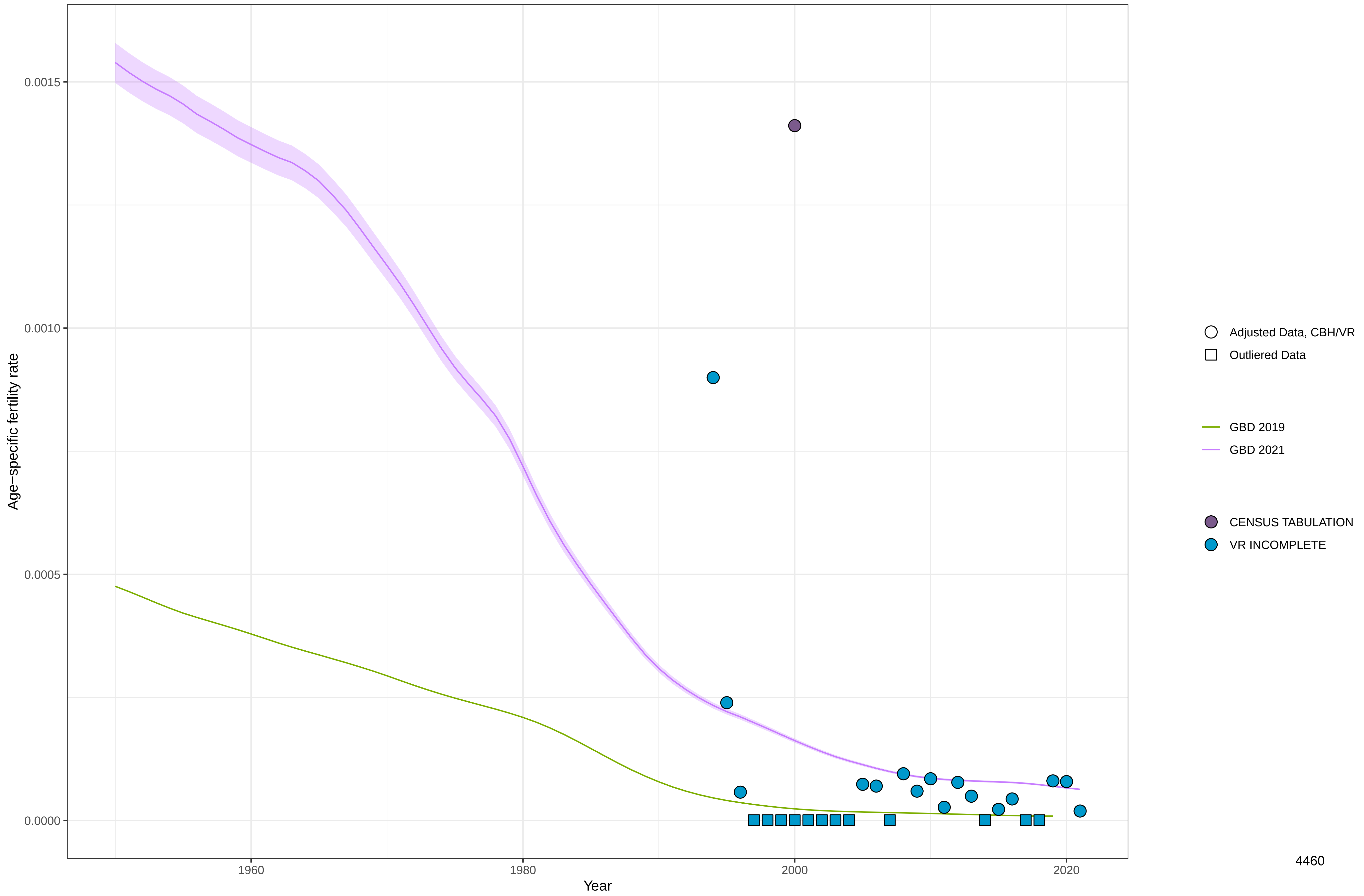

Total fertility rate fitted trend lines, 1950−2021: Rondônia TFR
Data points enclosed with a diamond are reference sources

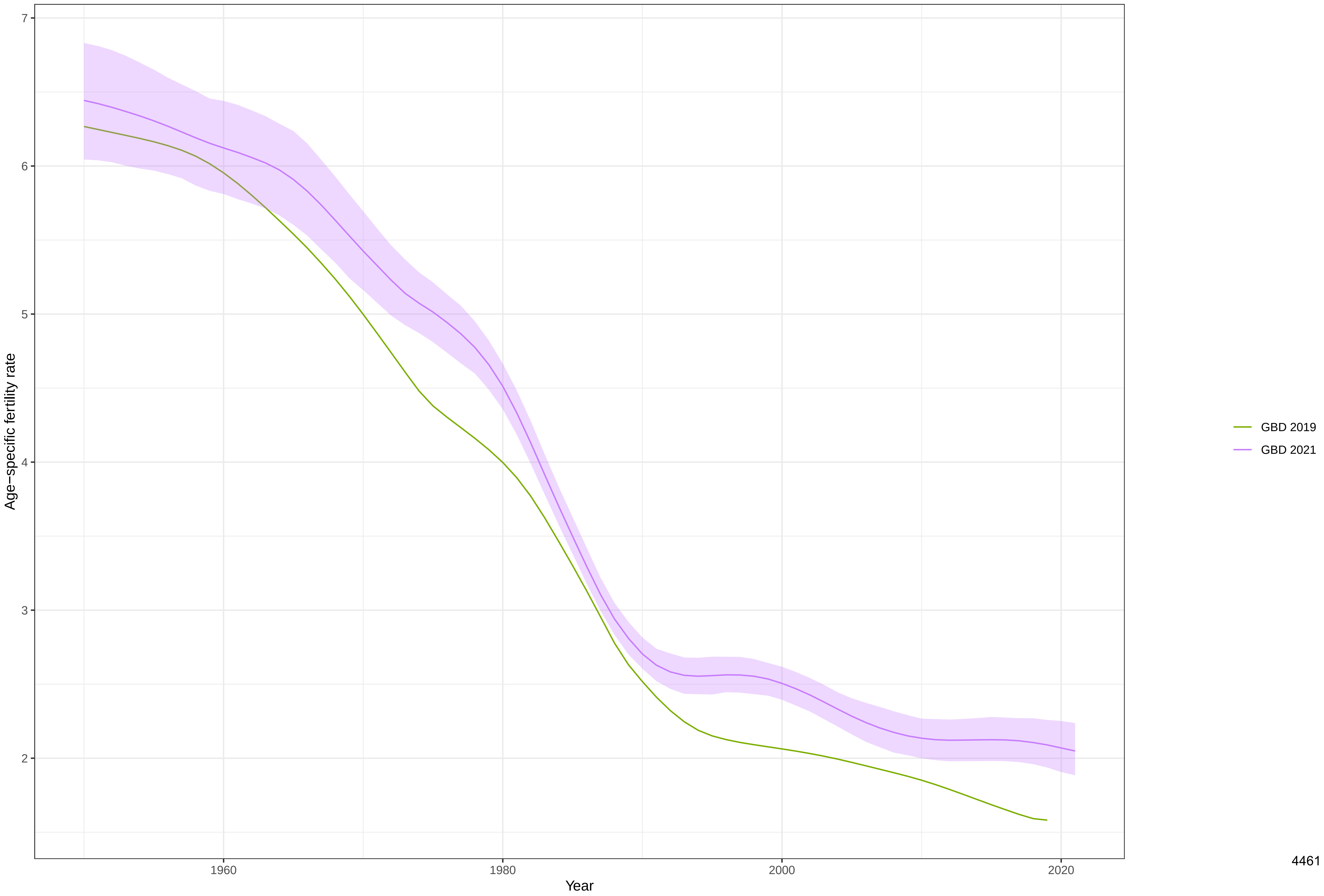

4461

Age−specific fertility fitted trend lines, 1950−2021: Roraima, Ages: 10−14
Data points enclosed with a diamond are reference sources

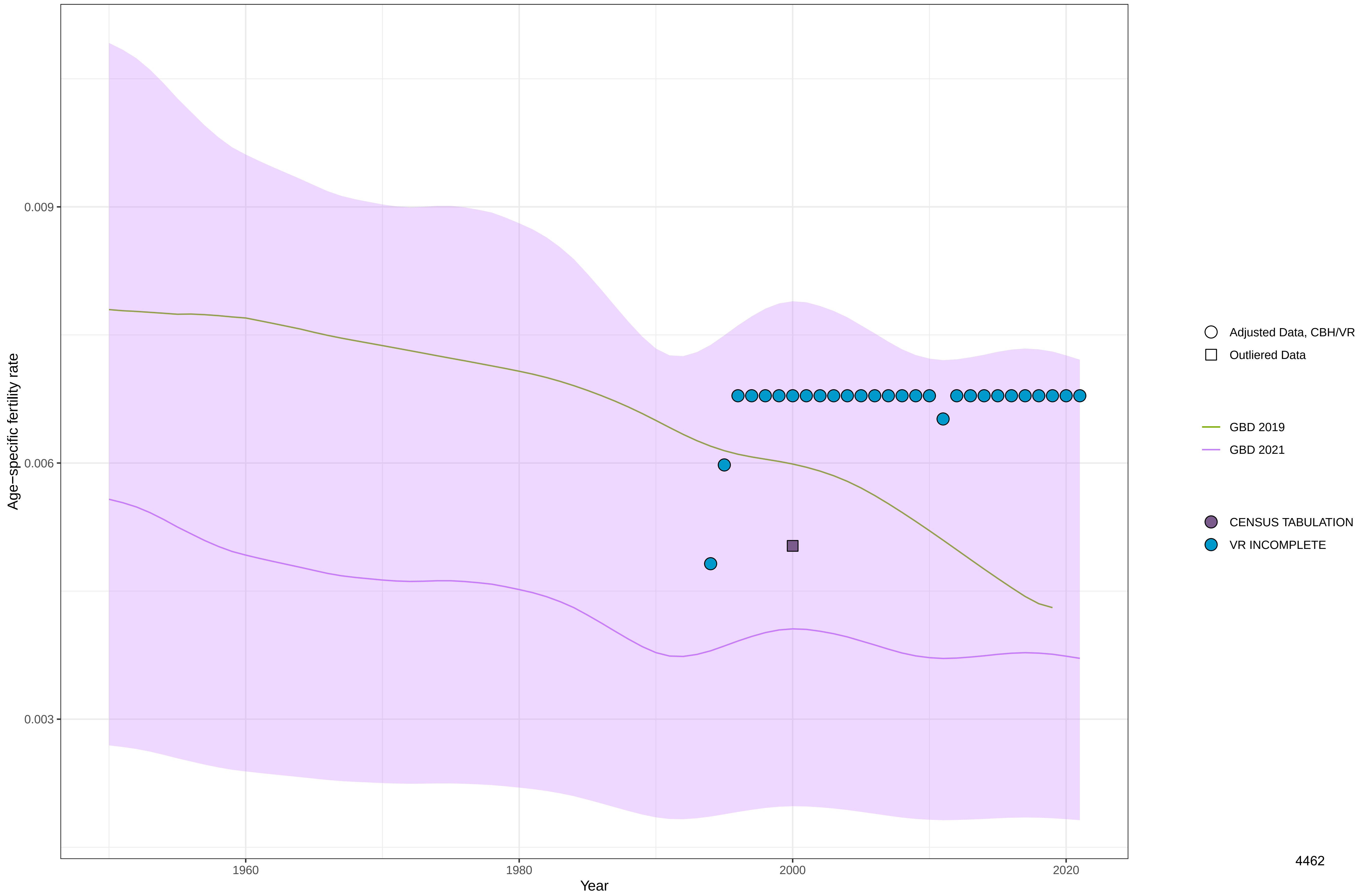

Age−specific fertility fitted trend lines, 1950−2021: Roraima, Ages: 15−19
Data points enclosed with a diamond are reference sources

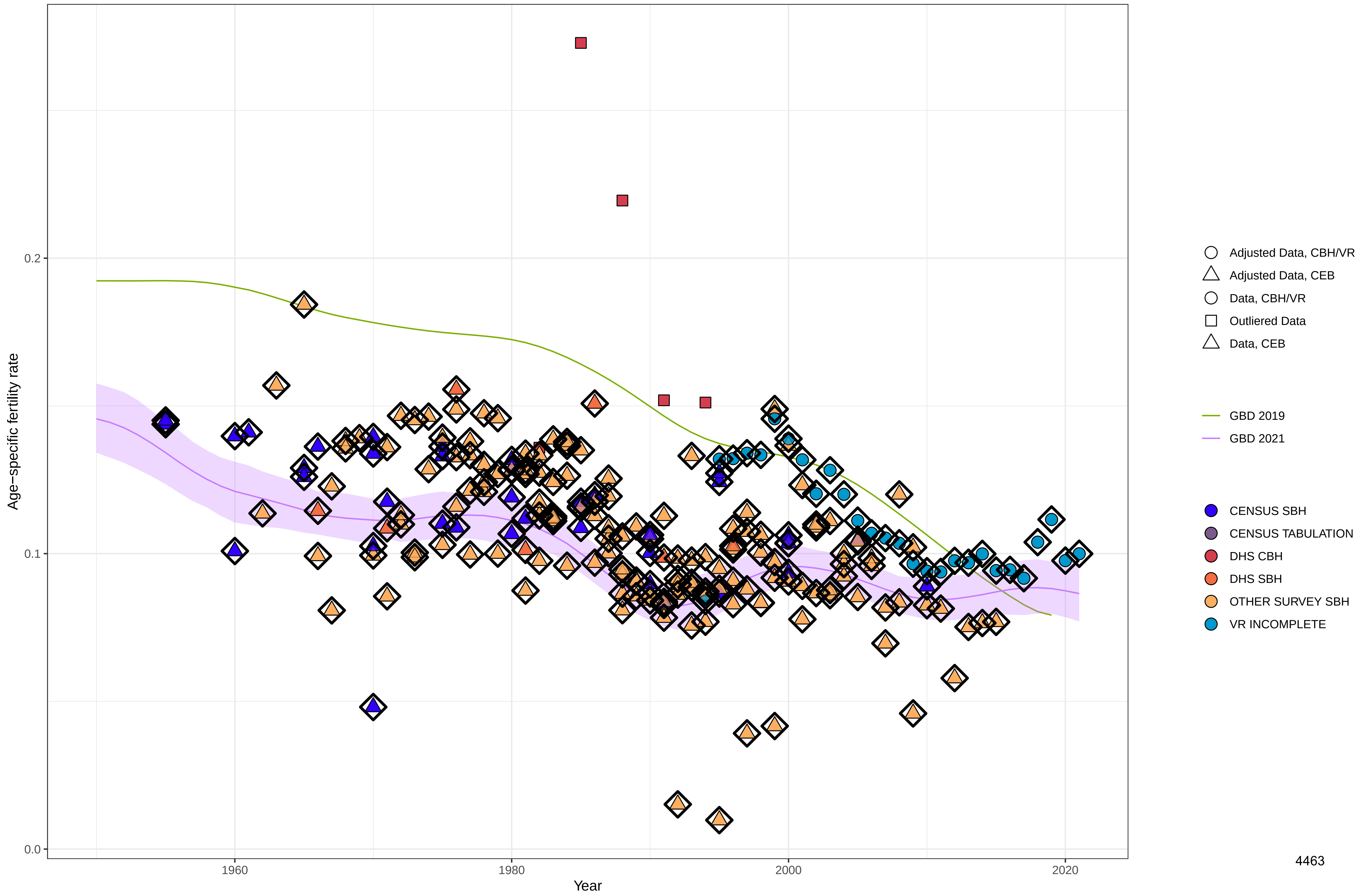

Age−specific fertility fitted trend lines, 1950−2021: Roraima, Ages: 20−24
Data points enclosed with a diamond are reference sources

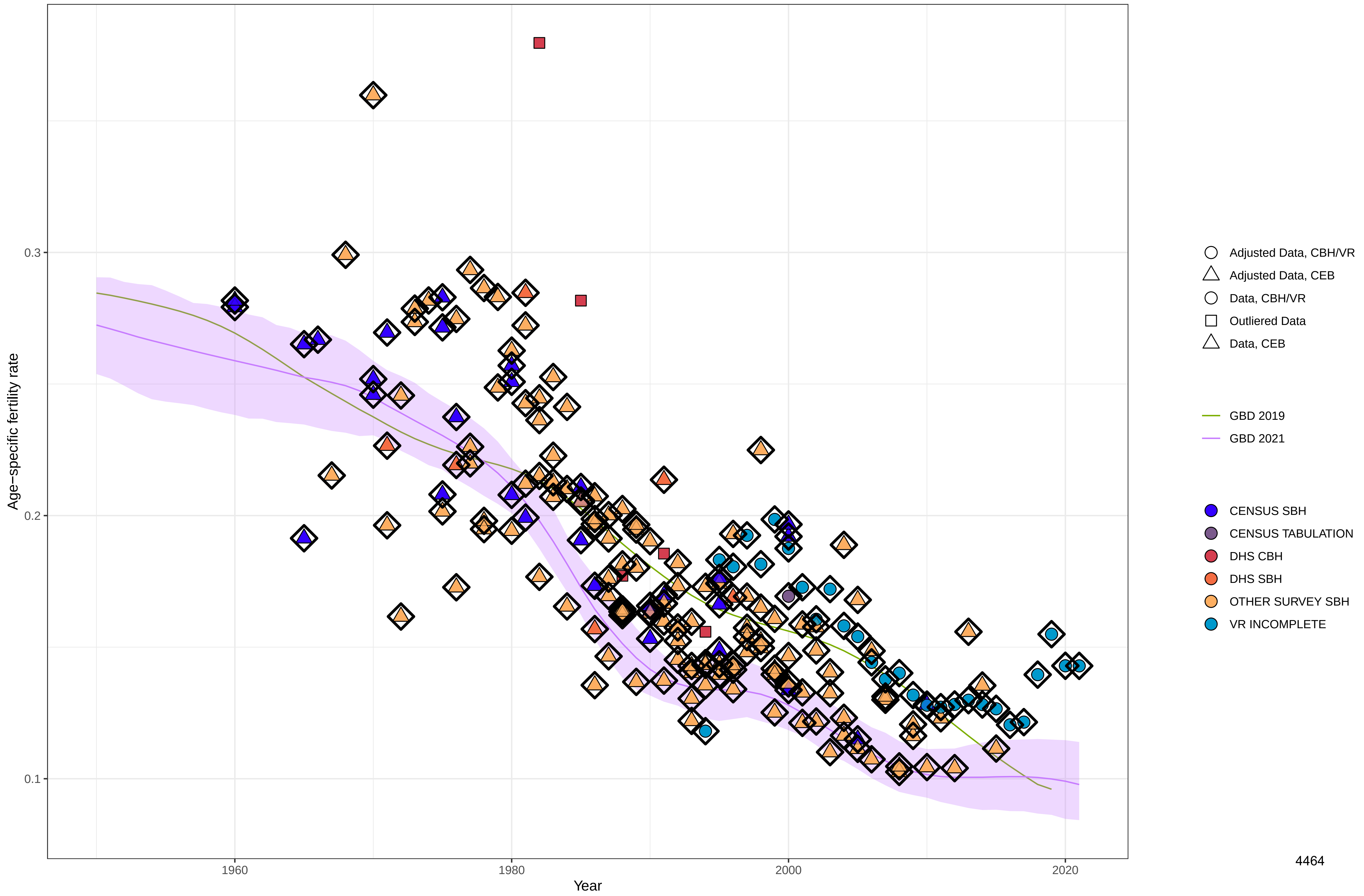

4464

Age−specific fertility fitted trend lines, 1950−2021: Roraima, Ages: 25−29
Data points enclosed with a diamond are reference sources

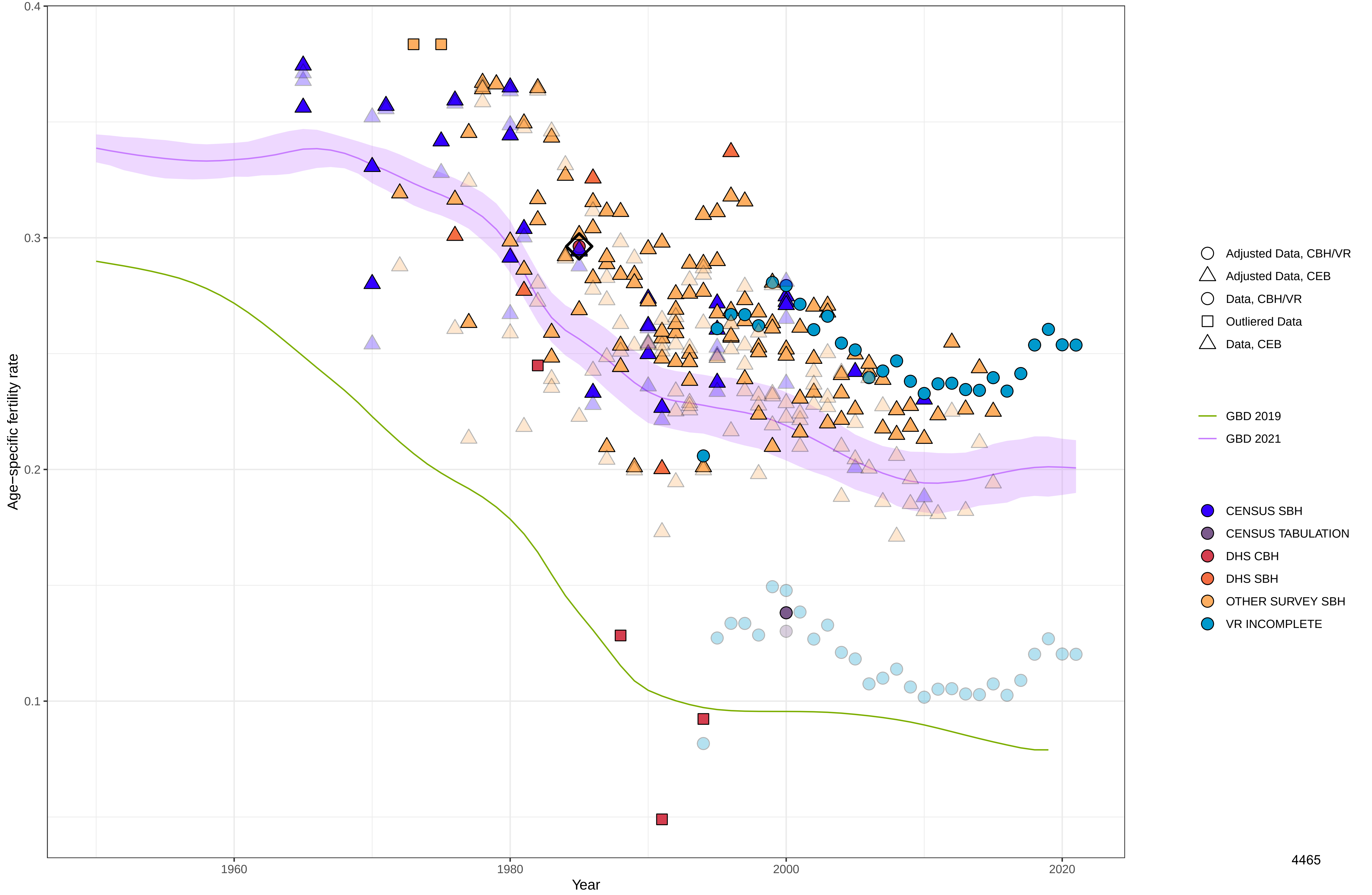

Age−specific fertility fitted trend lines, 1950−2021: Roraima, Ages: 30−34
Data points enclosed with a diamond are reference sources

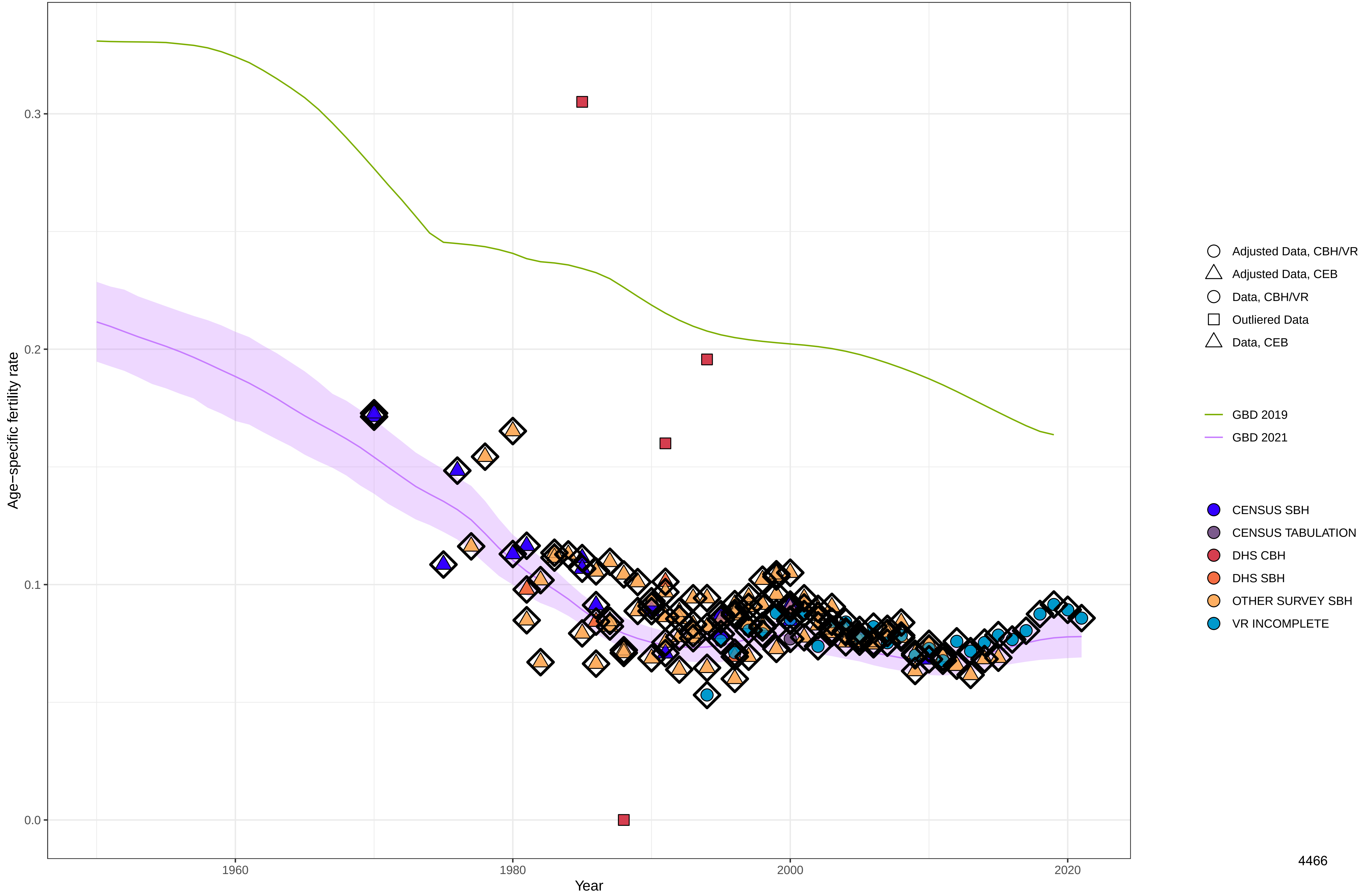

Age−specific fertility fitted trend lines, 1950−2021: Roraima, Ages: 35−39
Data points enclosed with a diamond are reference sources

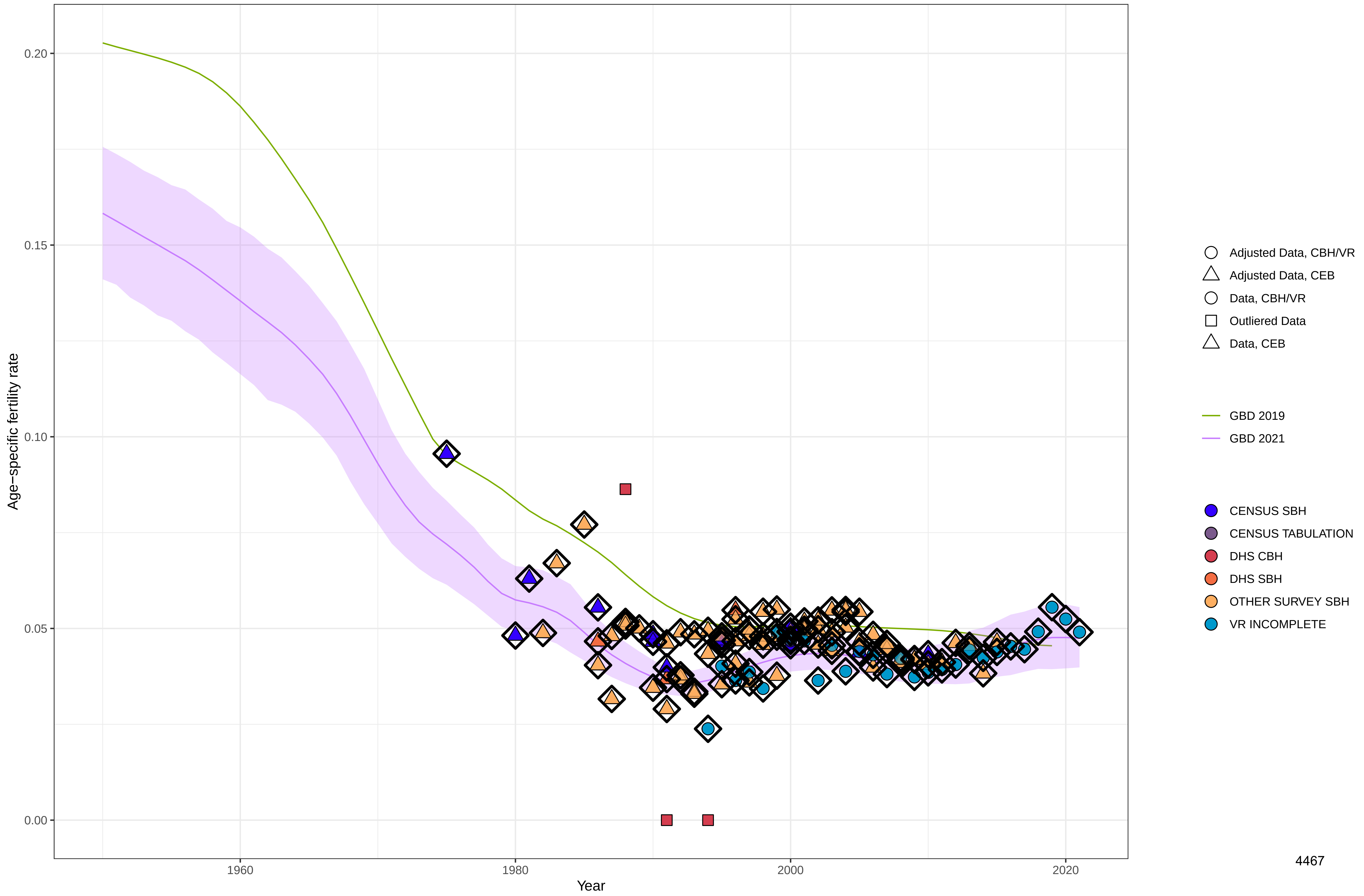

Age−specific fertility fitted trend lines, 1950−2021: Roraima, Ages: 40−44
Data points enclosed with a diamond are reference sources

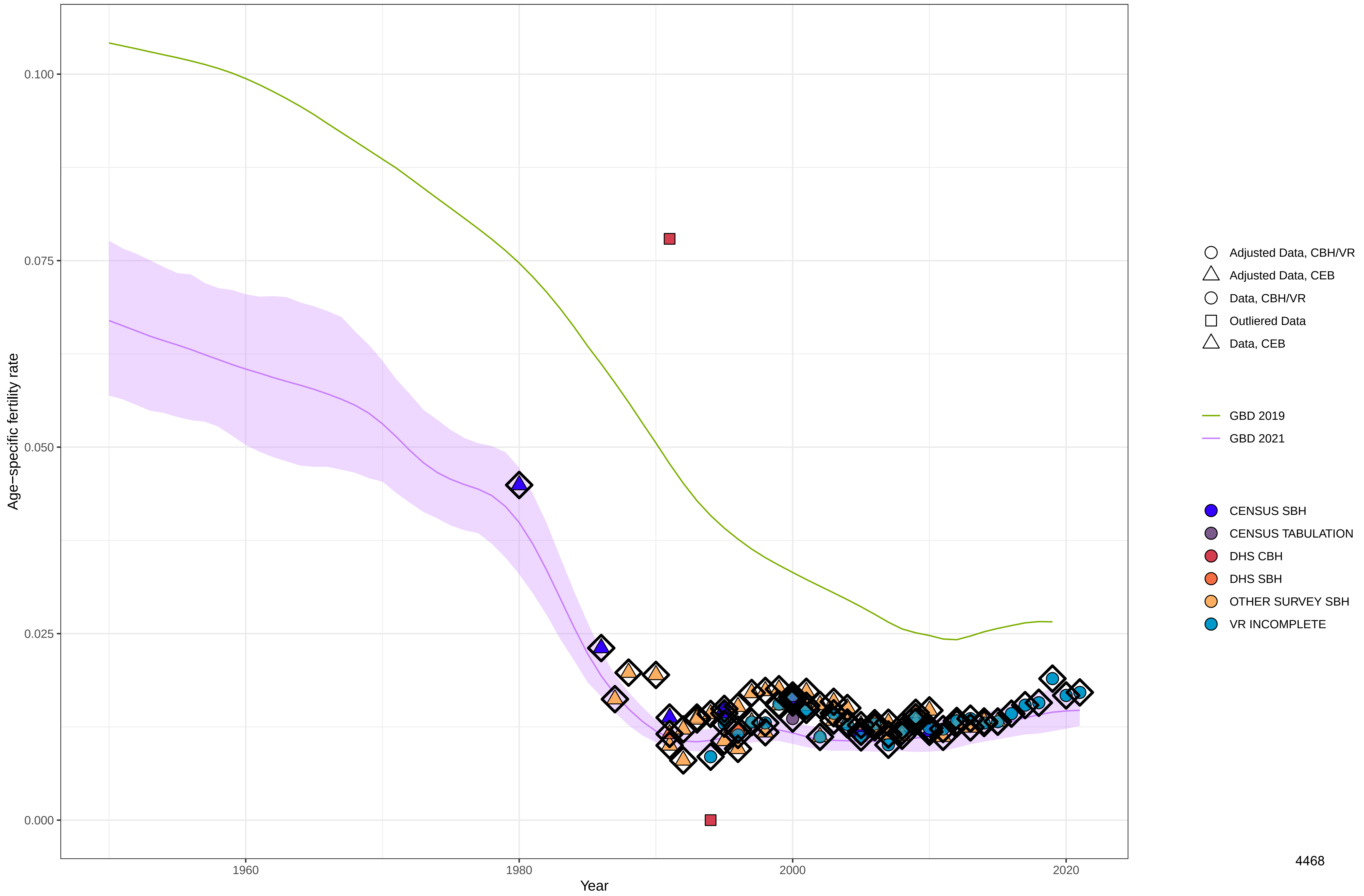

Age−specific fertility fitted trend lines, 1950−2021: Roraima, Ages: 45−49
Data points enclosed with a diamond are reference sources

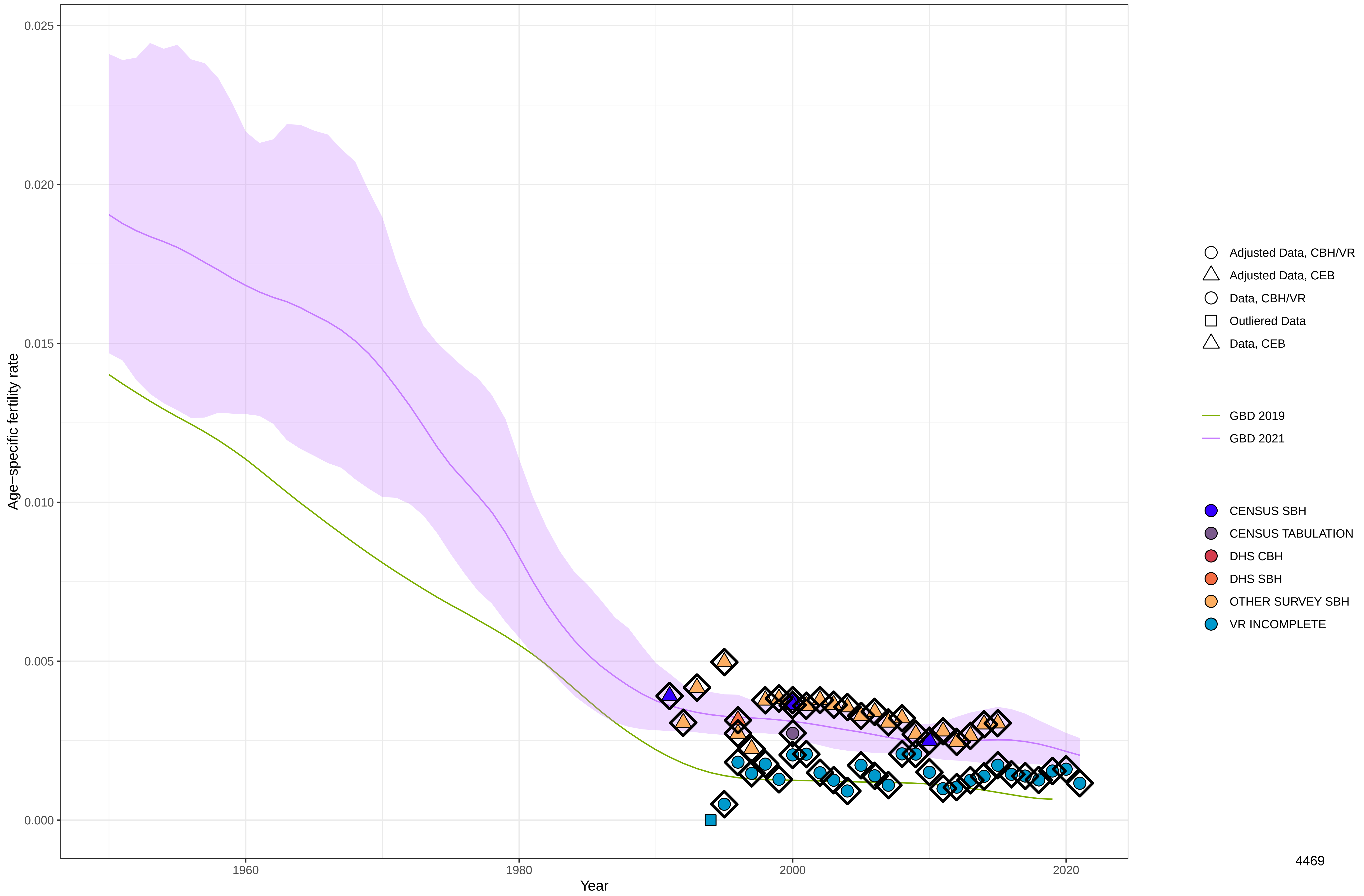

Age−specific fertility fitted trend lines, 1950−2021: Roraima, Ages: 50−54
Data points enclosed with a diamond are reference sources

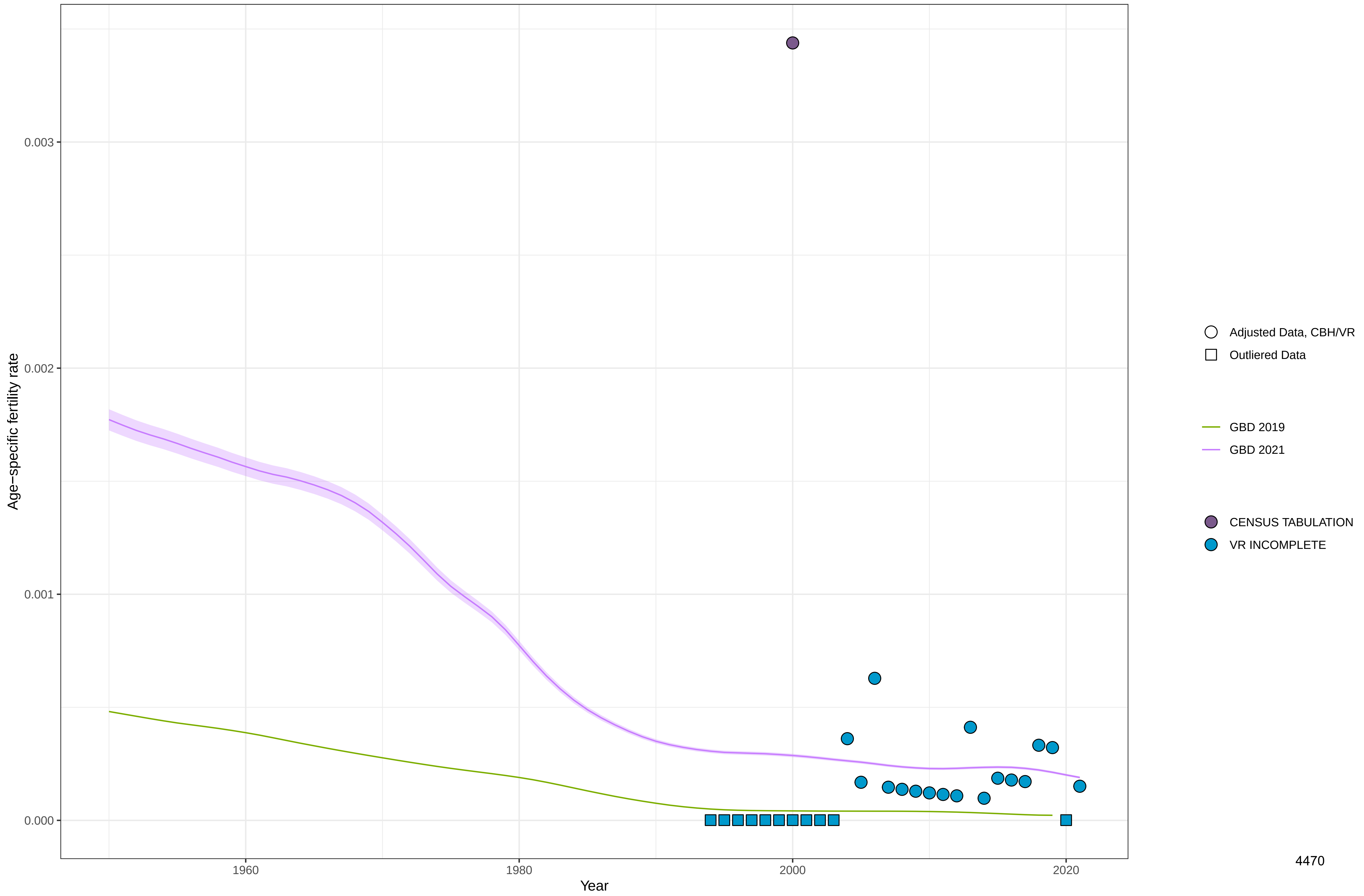

Total fertility rate fitted trend lines, 1950−2021: Roraima TFR
Data points enclosed with a diamond are reference sources

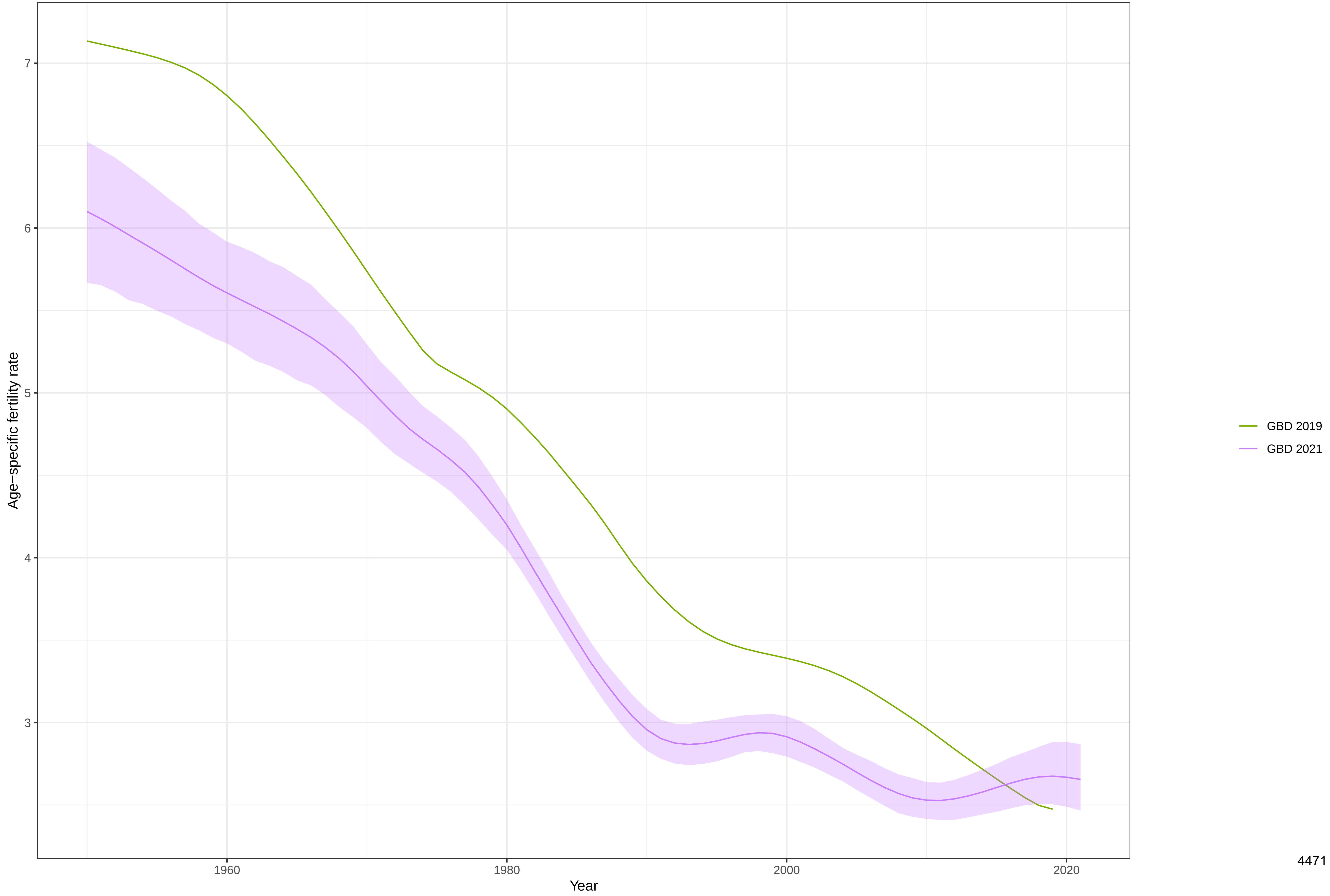

Age−specific fertility fitted trend lines, 1950−2021: Santa Catarina, Ages: 10−14
Data points enclosed with a diamond are reference sources

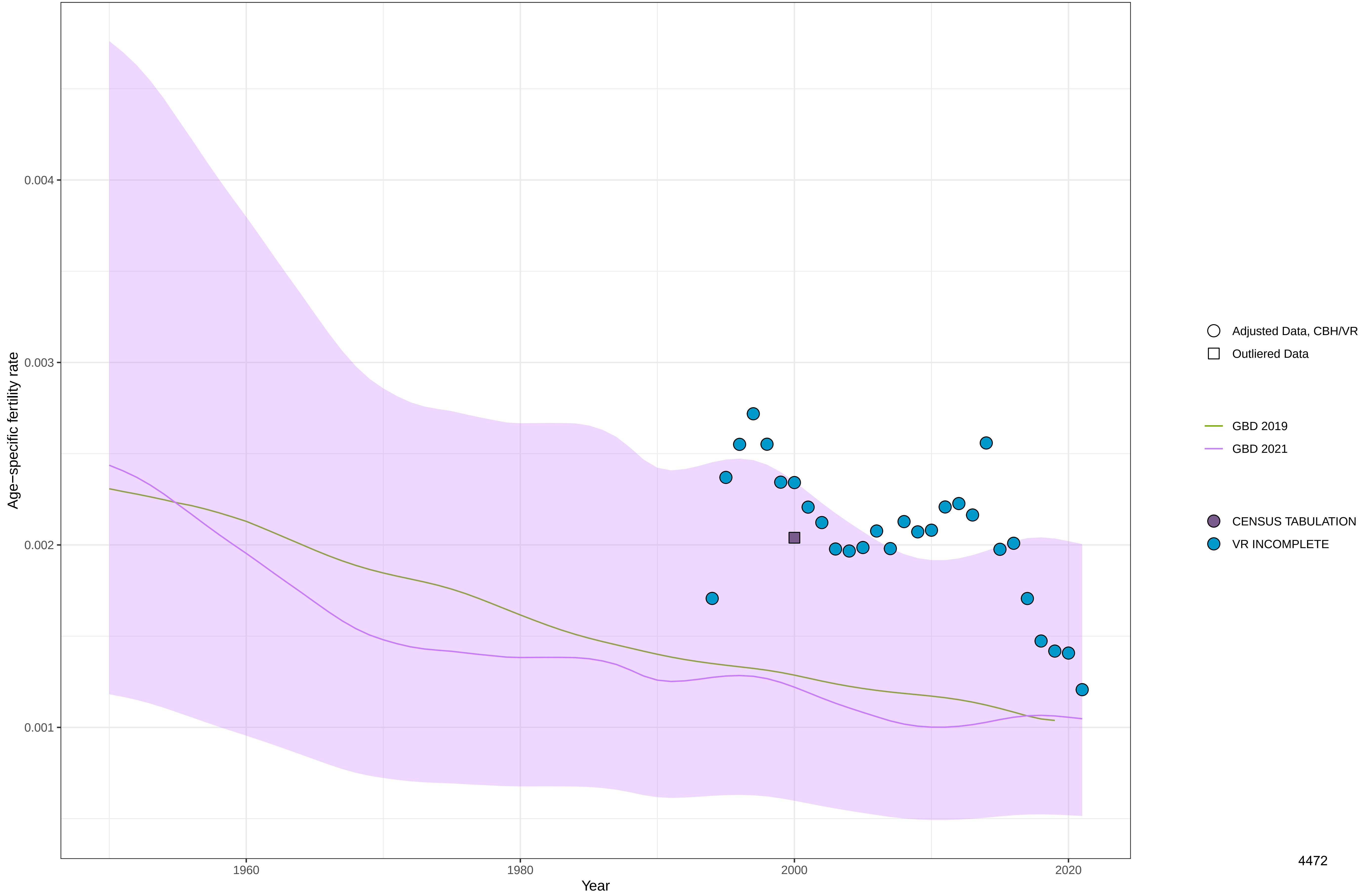

Age−specific fertility fitted trend lines, 1950−2021: Santa Catarina, Ages: 15−19
Data points enclosed with a diamond are reference sources

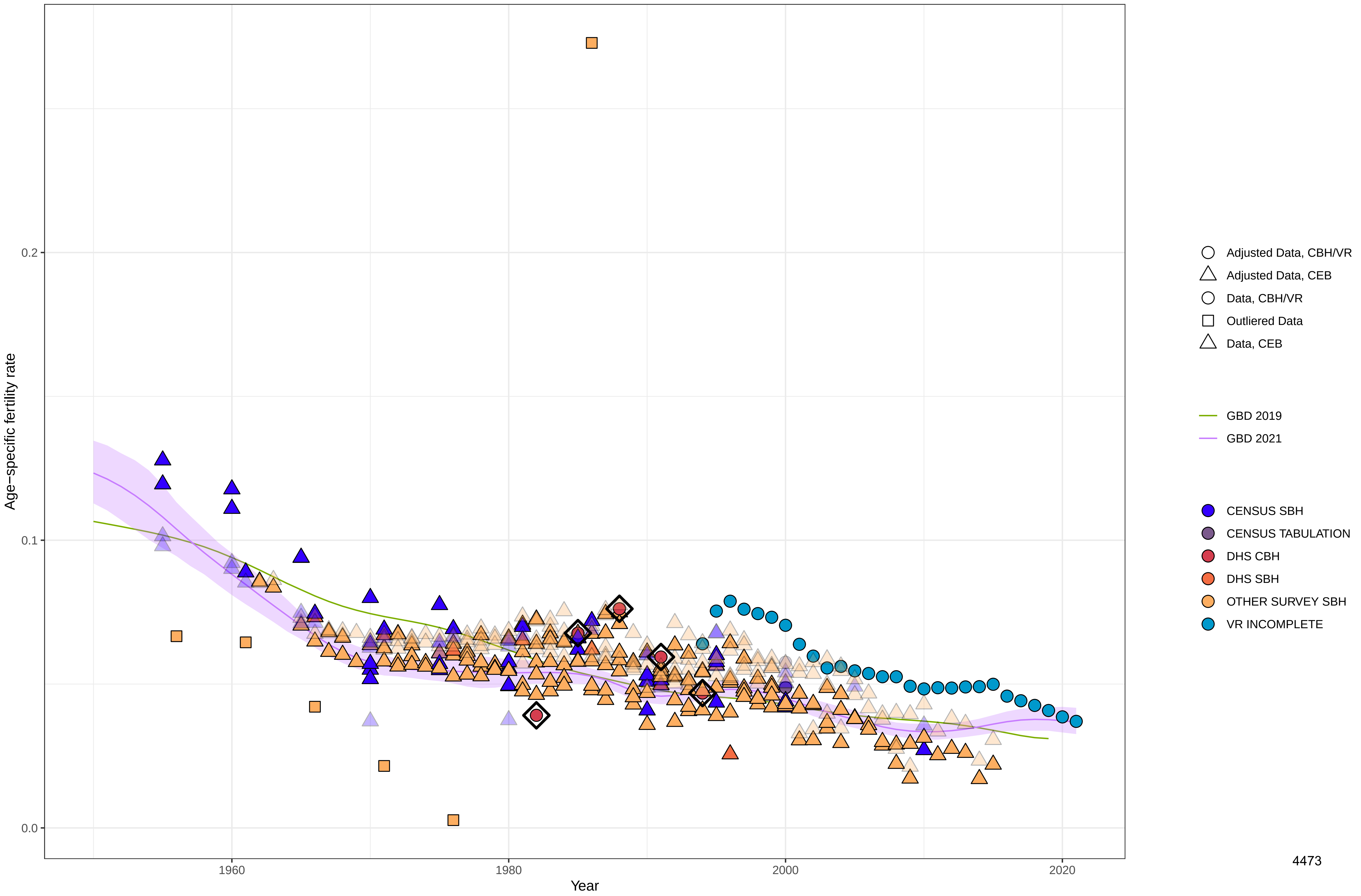

Age−specific fertility fitted trend lines, 1950−2021: Santa Catarina, Ages: 20−24
Data points enclosed with a diamond are reference sources

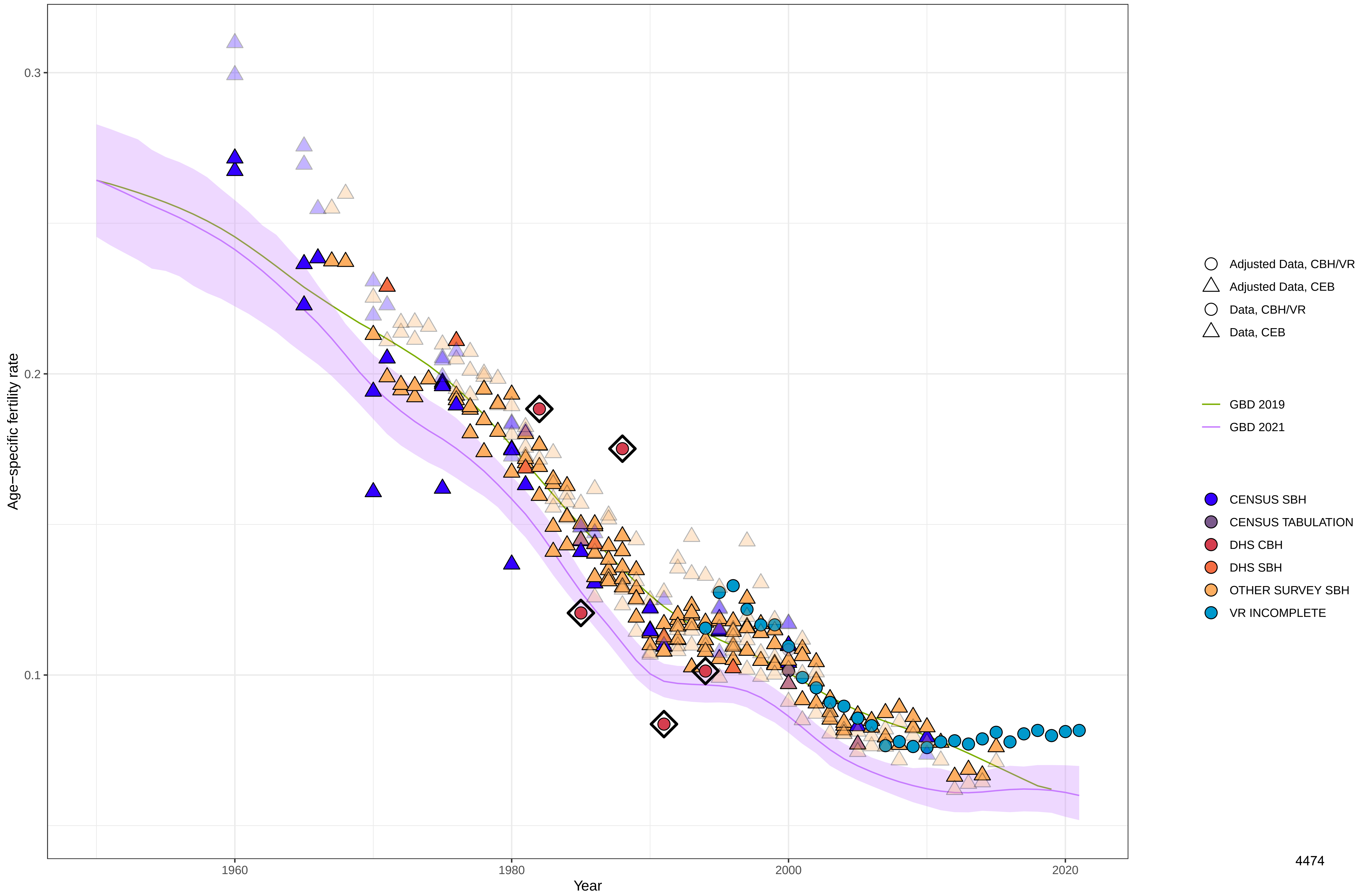

Age−specific fertility fitted trend lines, 1950−2021: Santa Catarina, Ages: 25−29
Data points enclosed with a diamond are reference sources

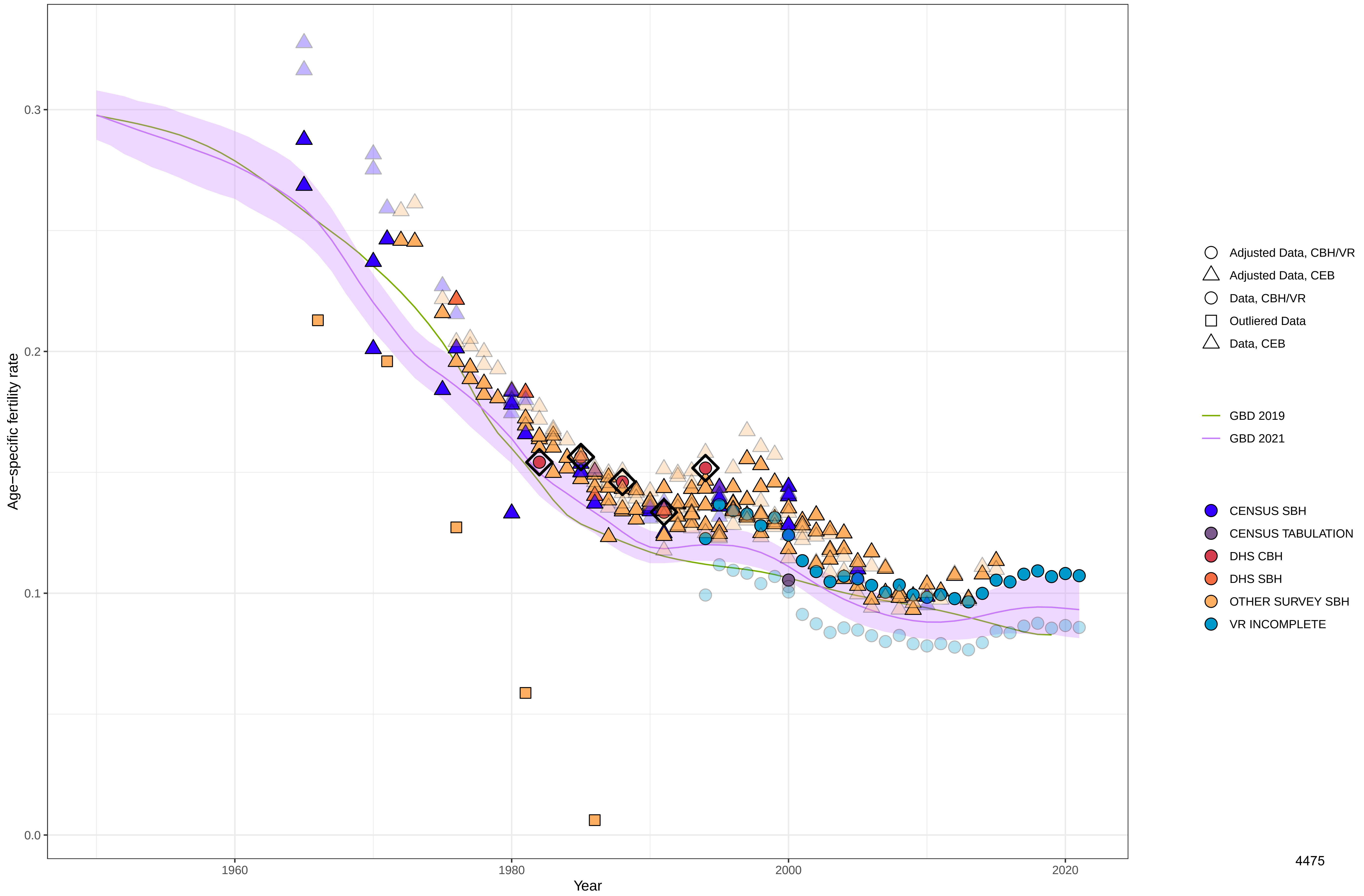

Age−specific fertility fitted trend lines, 1950−2021: Santa Catarina, Ages: 30−34
Data points enclosed with a diamond are reference sources

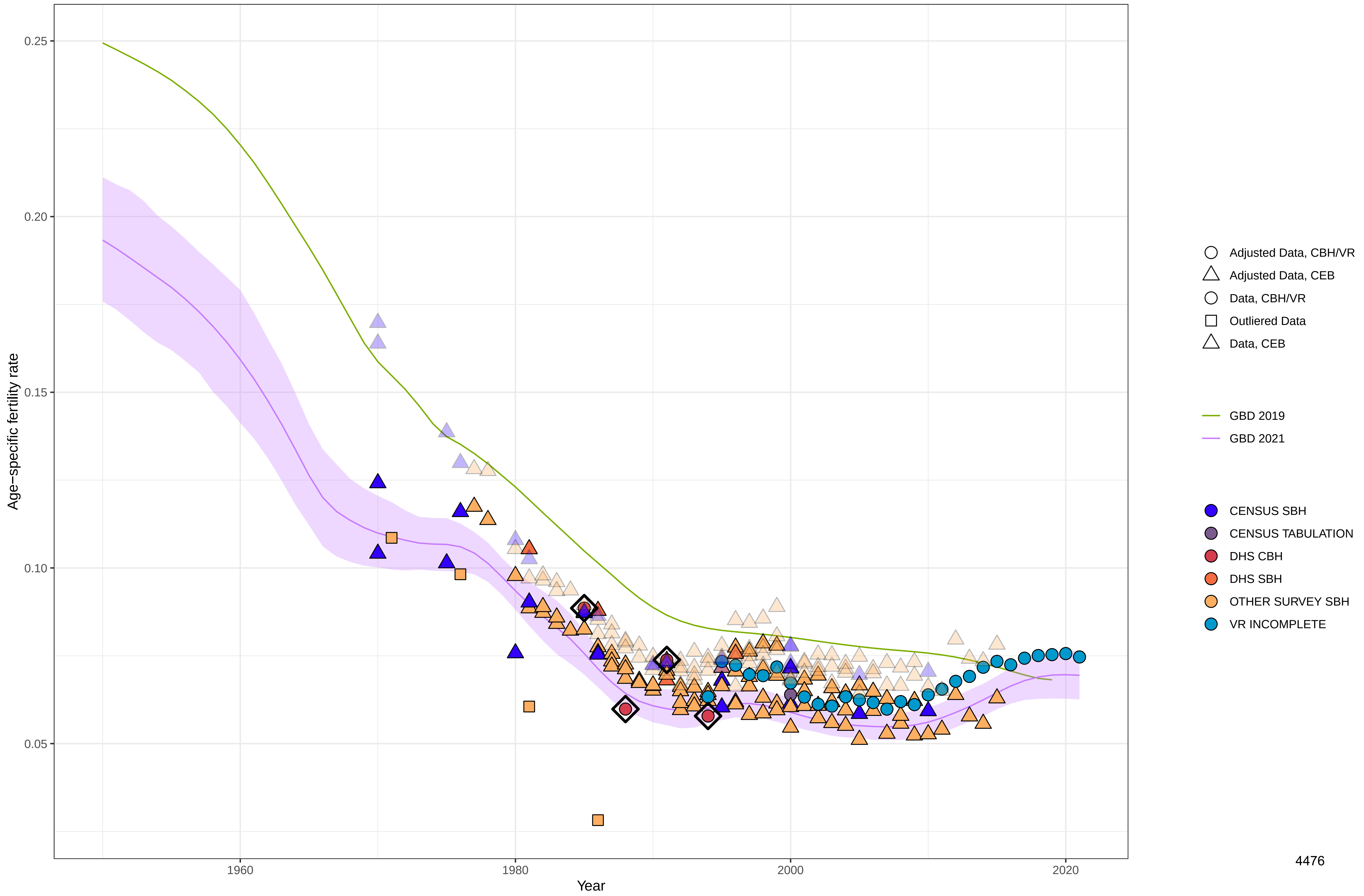

Age−specific fertility fitted trend lines, 1950−2021: Santa Catarina, Ages: 35−39
Data points enclosed with a diamond are reference sources

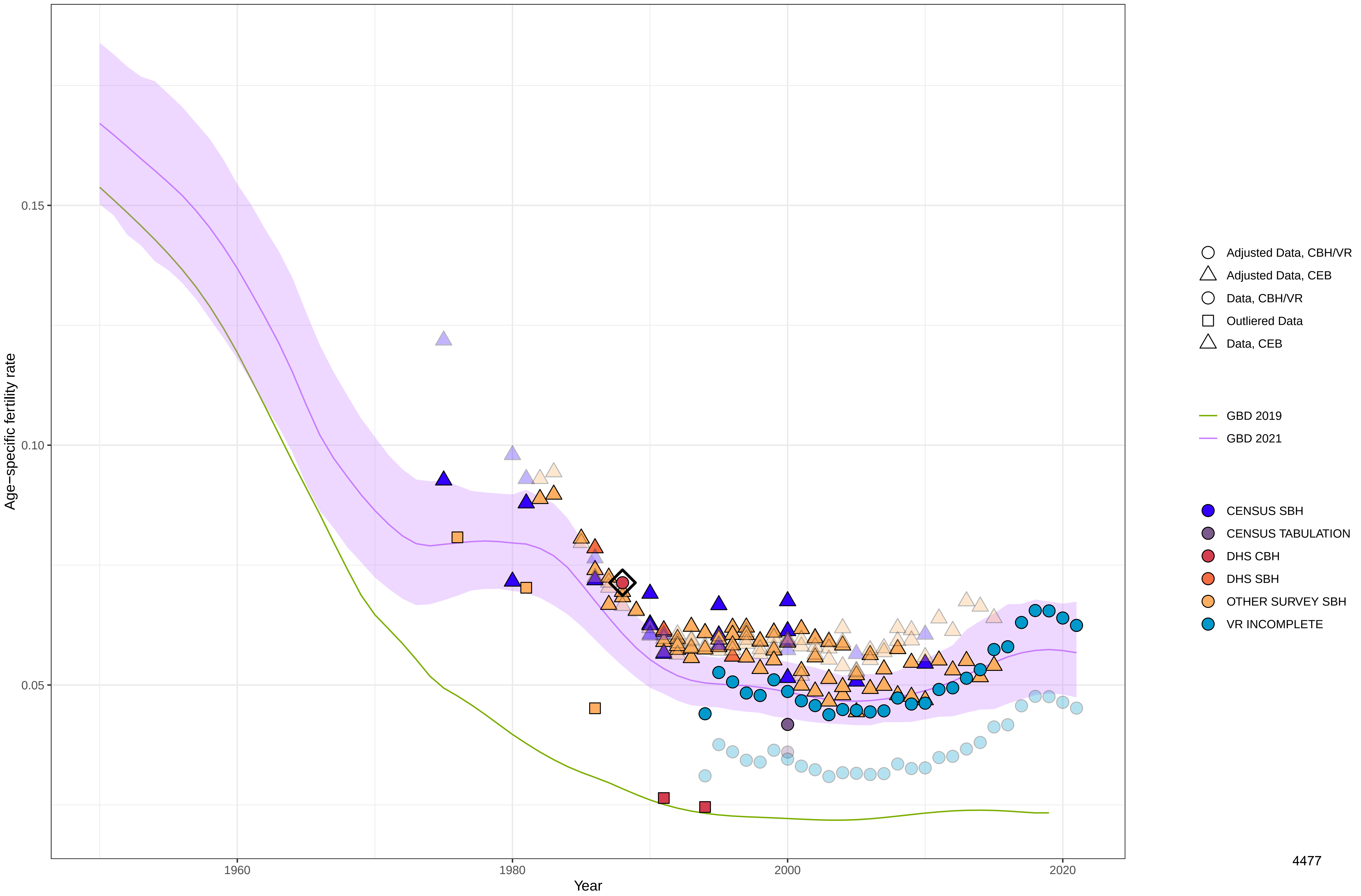

Age−specific fertility fitted trend lines, 1950−2021: Santa Catarina, Ages: 40−44
Data points enclosed with a diamond are reference sources

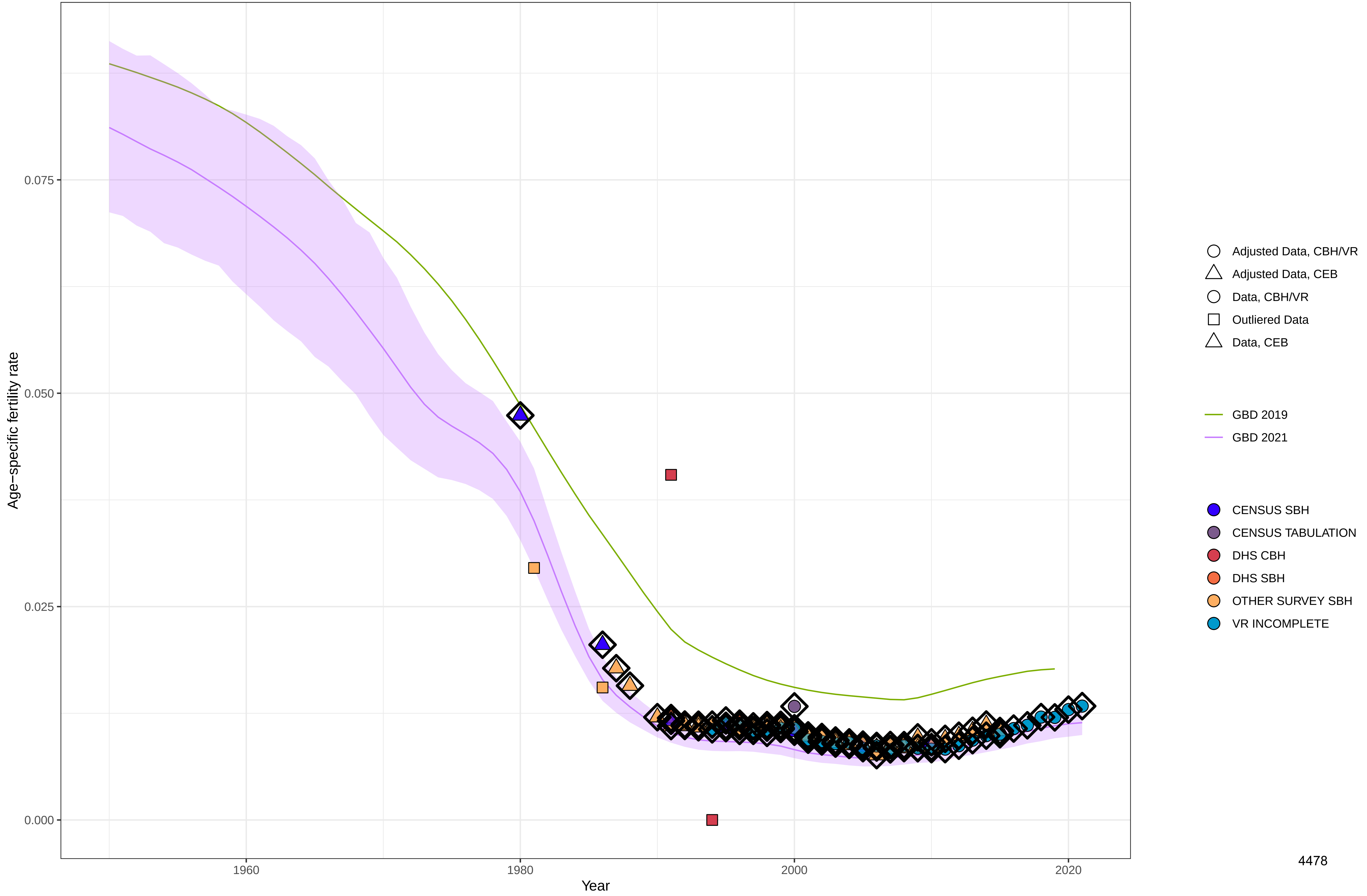

Age−specific fertility fitted trend lines, 1950−2021: Santa Catarina, Ages: 45−49
Data points enclosed with a diamond are reference sources

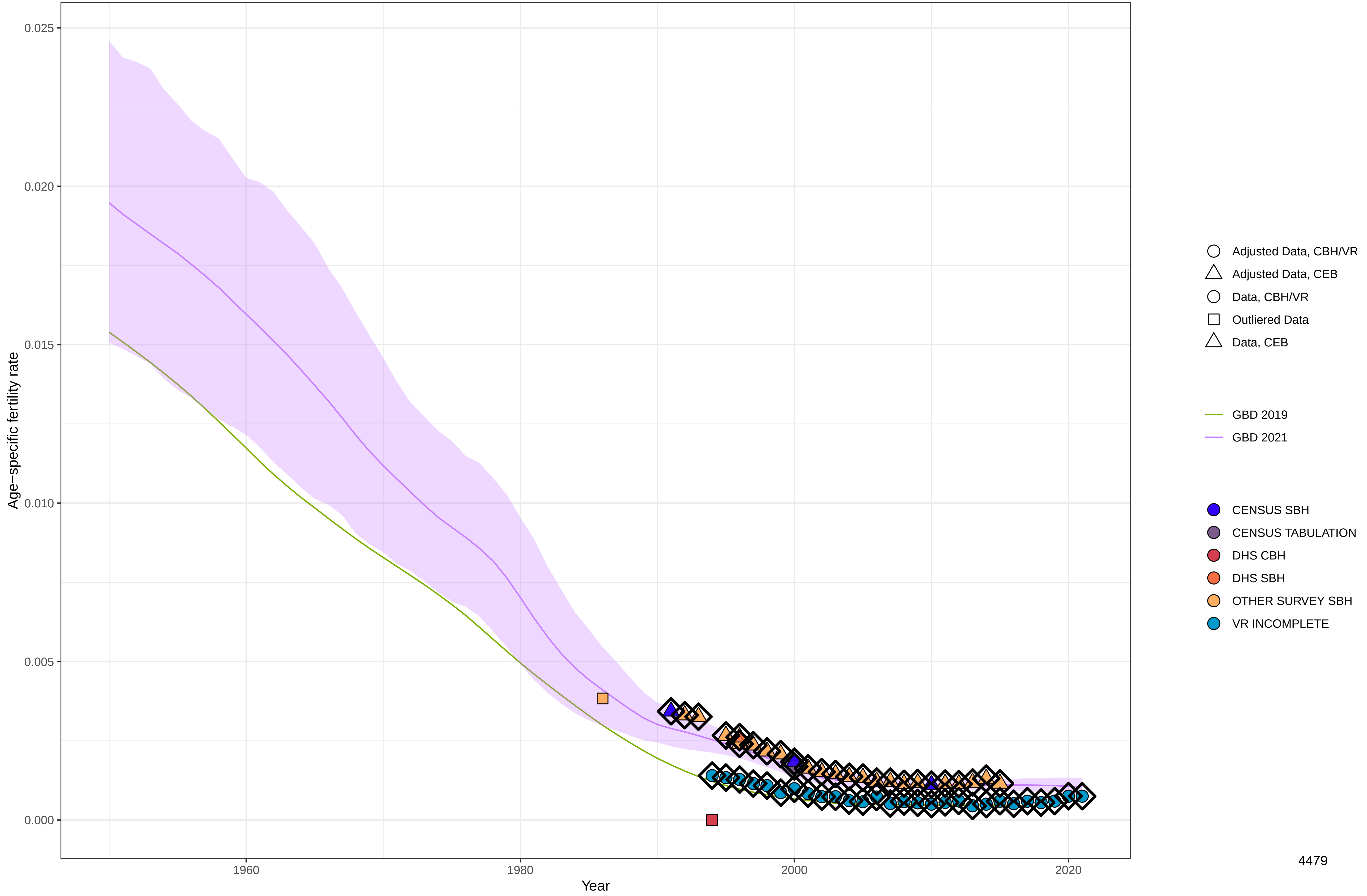

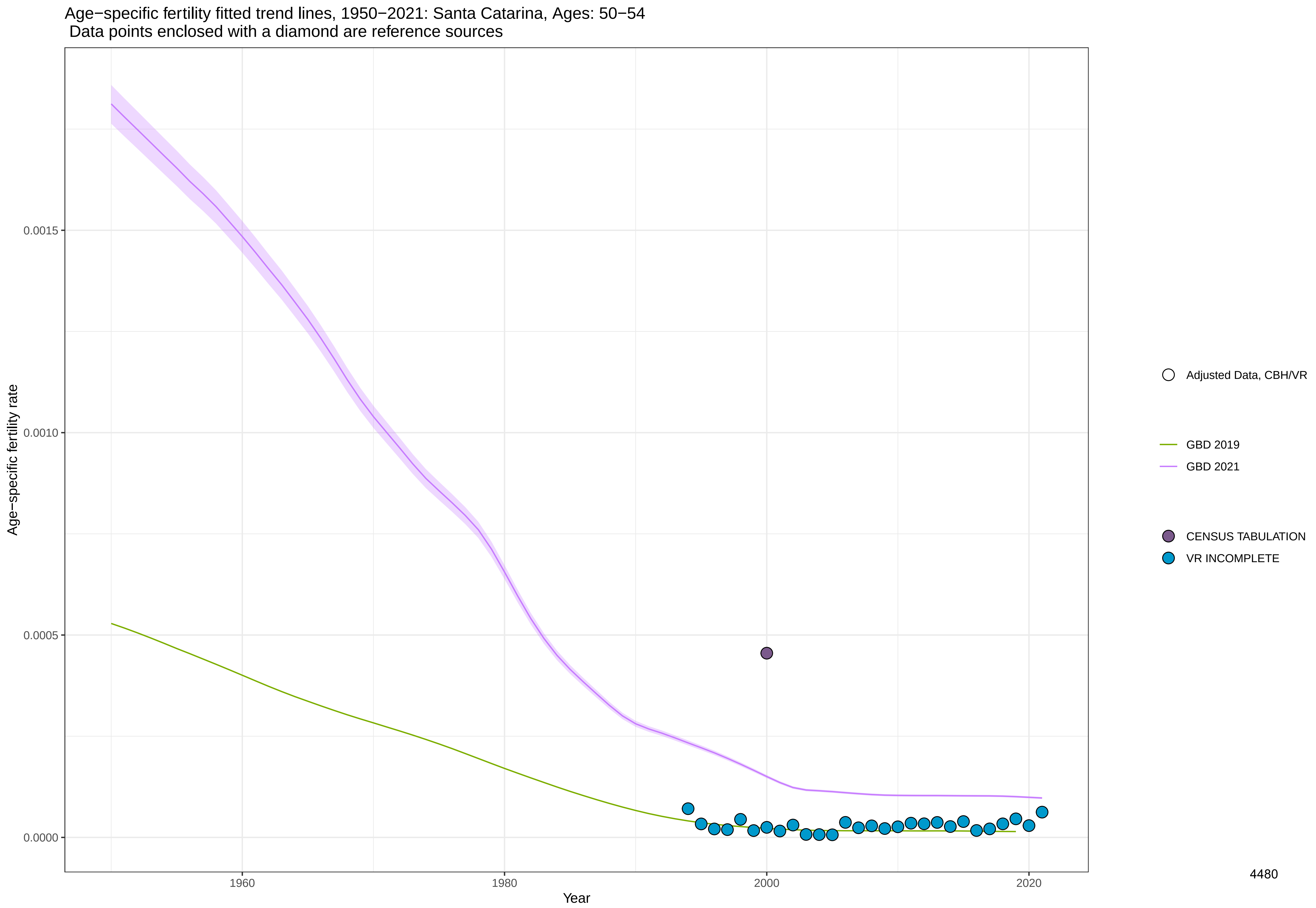

Age−specific fertility fitted trend lines, 1950−2021: Santa Catarina, Ages: 50−54
Data points enclosed with a diamond are reference sources
Age−specific fertility rate
0.0015
0.0010
0.0005
0.0000
1960
1980
2000
2020
Year
Adjusted Data, CBH/VR
GBD 2019
GBD 2021
CENSUS TABULATION
VR INCOMPLETE

Total fertility rate fitted trend lines, 1950−2021: Santa Catarina TFR
Data points enclosed with a diamond are reference sources

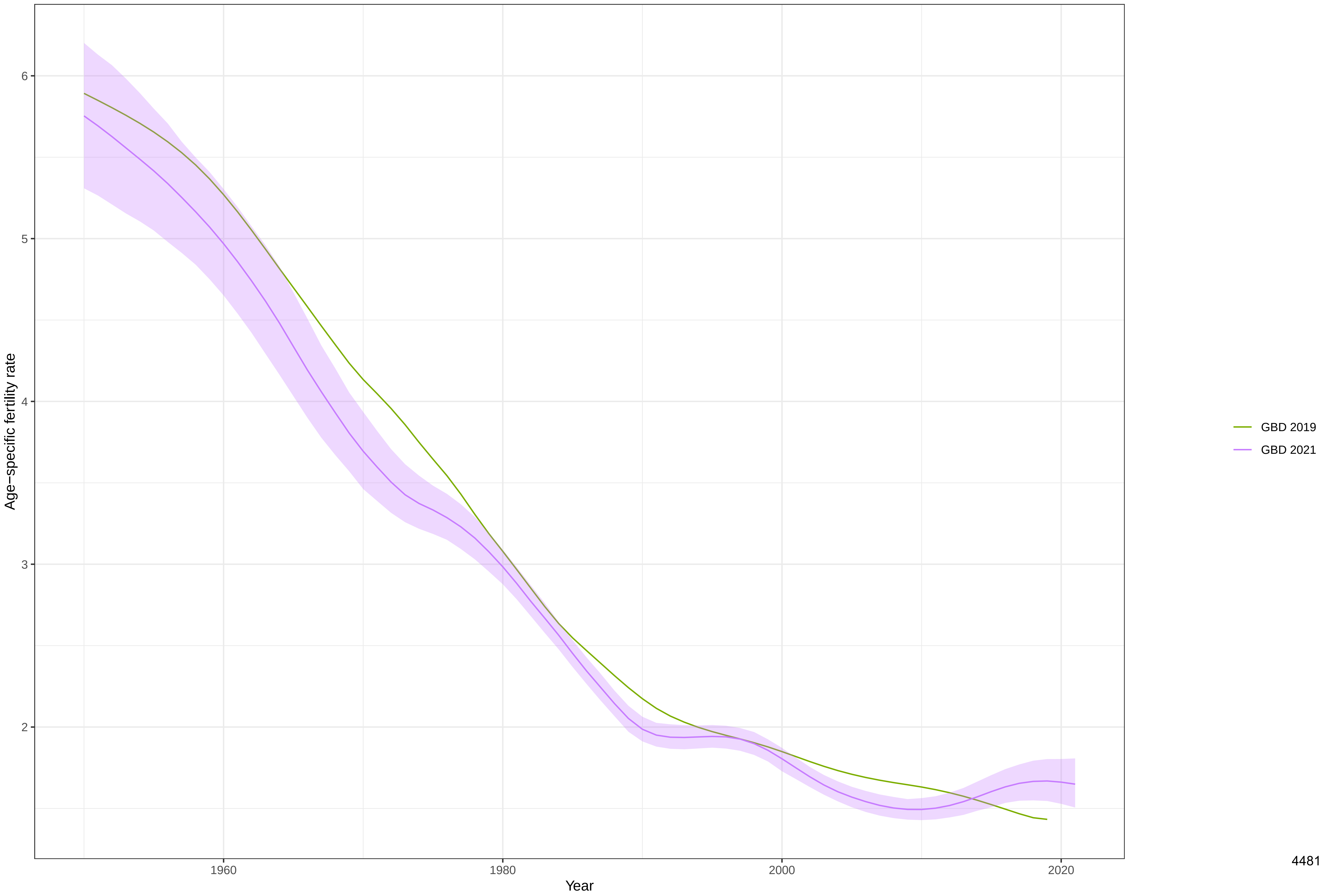

Age−specific fertility fitted trend lines, 1950−2021: São Paulo, Ages: 10−14
Data points enclosed with a diamond are reference sources

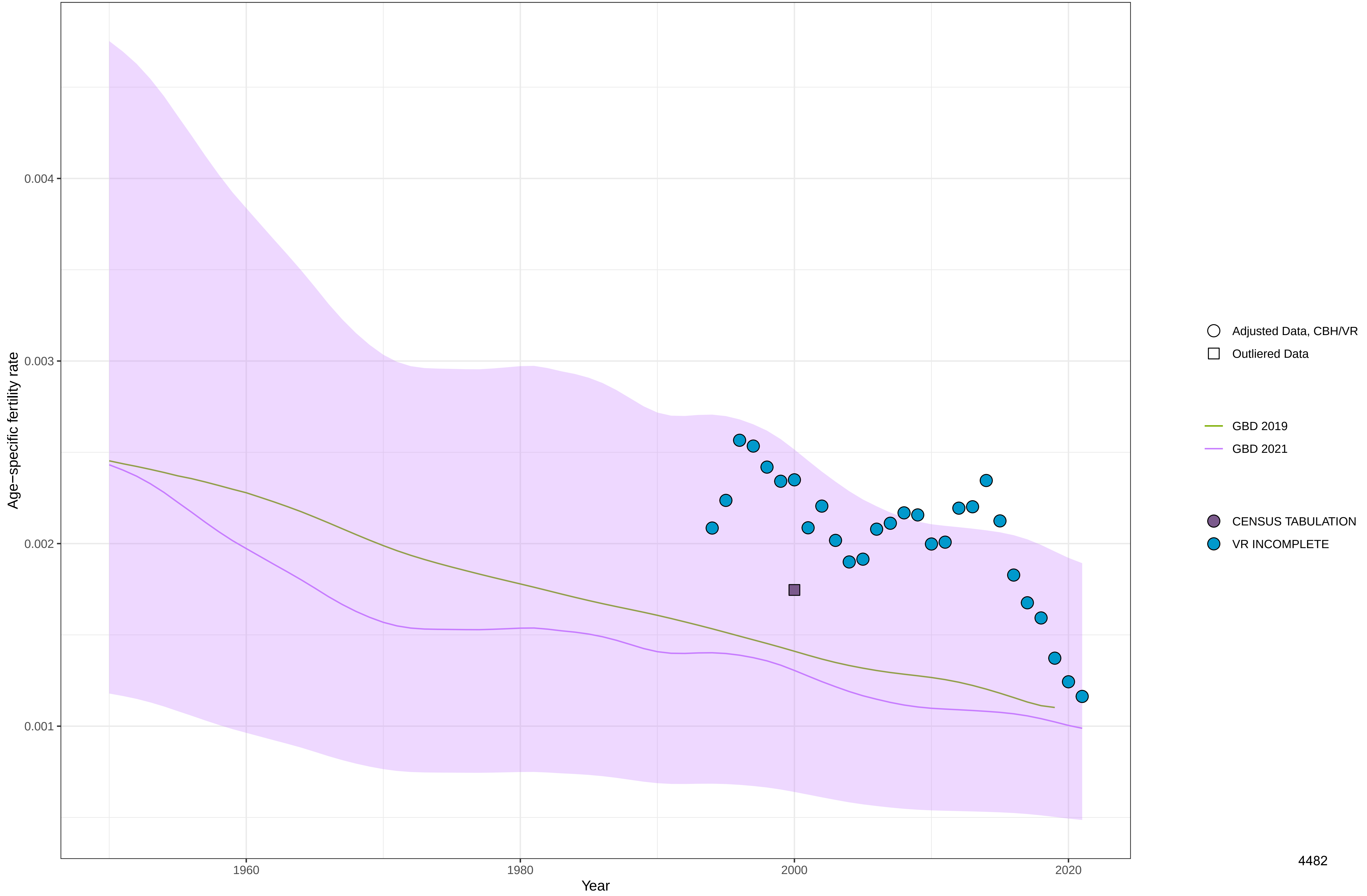

4482

Age−specific fertility fitted trend lines, 1950−2021: São Paulo, Ages: 15−19
Data points enclosed with a diamond are reference sources

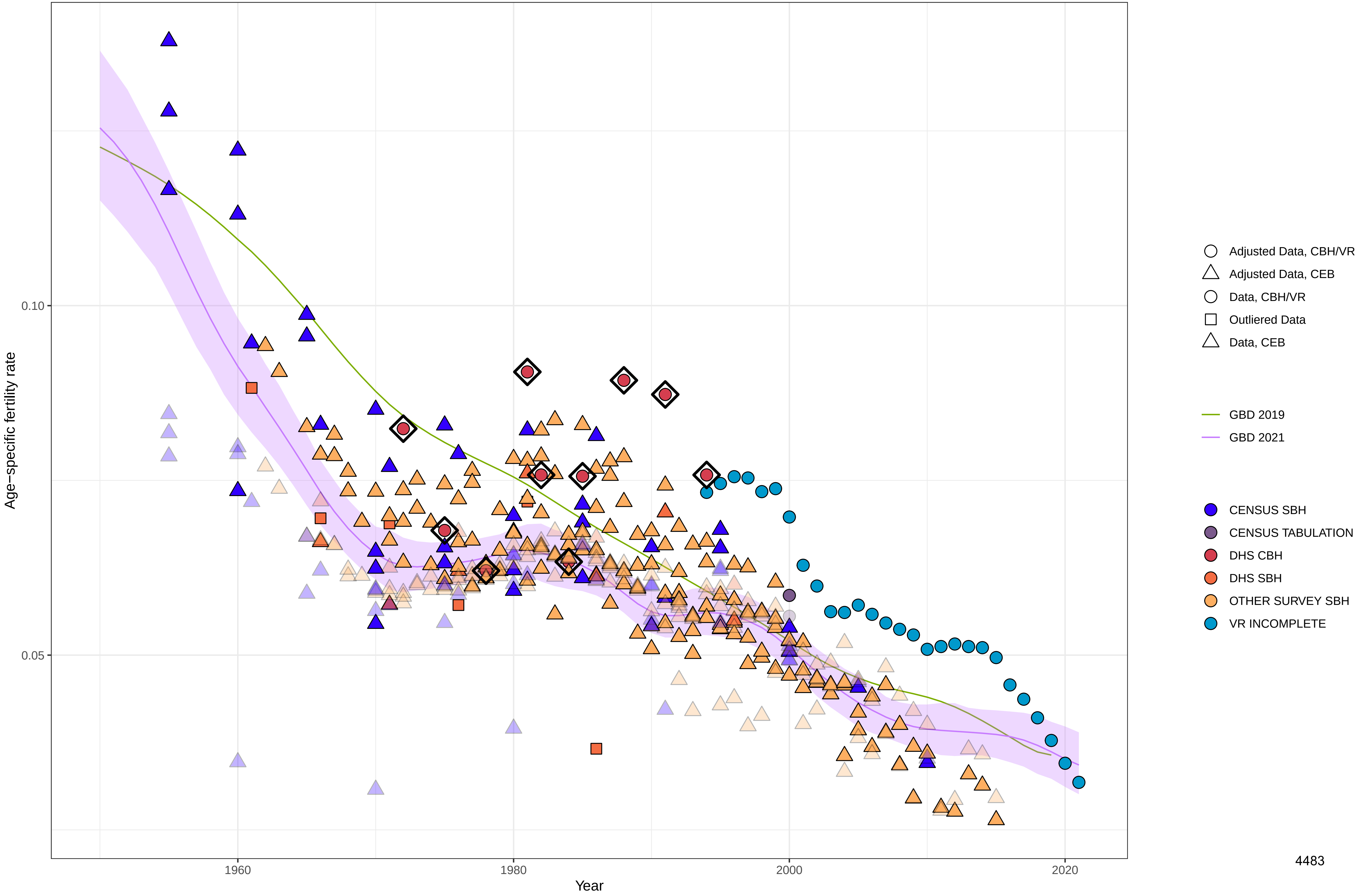

Age−specific fertility fitted trend lines, 1950−2021: São Paulo, Ages: 20−24
Data points enclosed with a diamond are reference sources

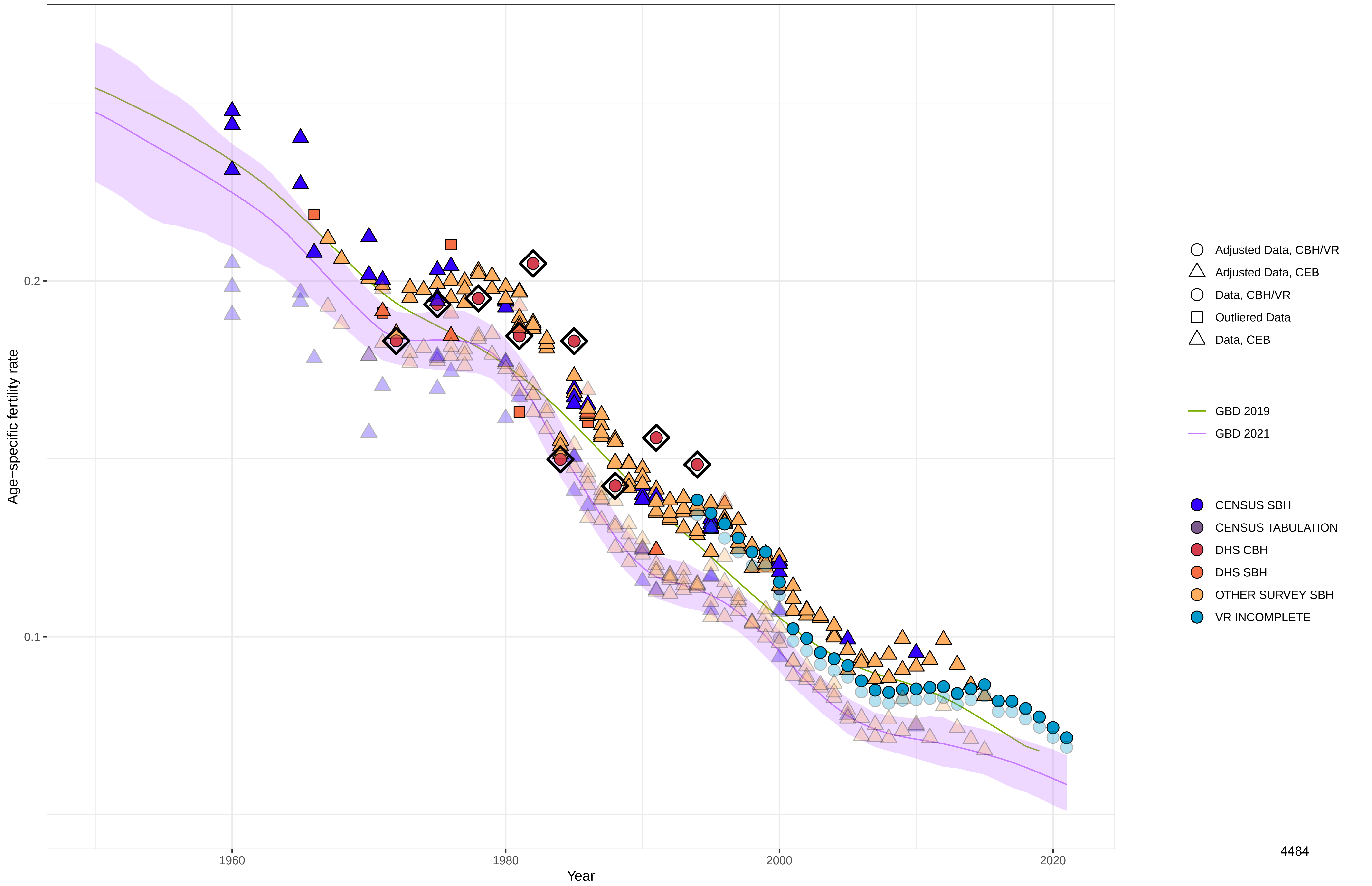

Age−specific fertility fitted trend lines, 1950−2021: São Paulo, Ages: 25−29
Data points enclosed with a diamond are reference sources

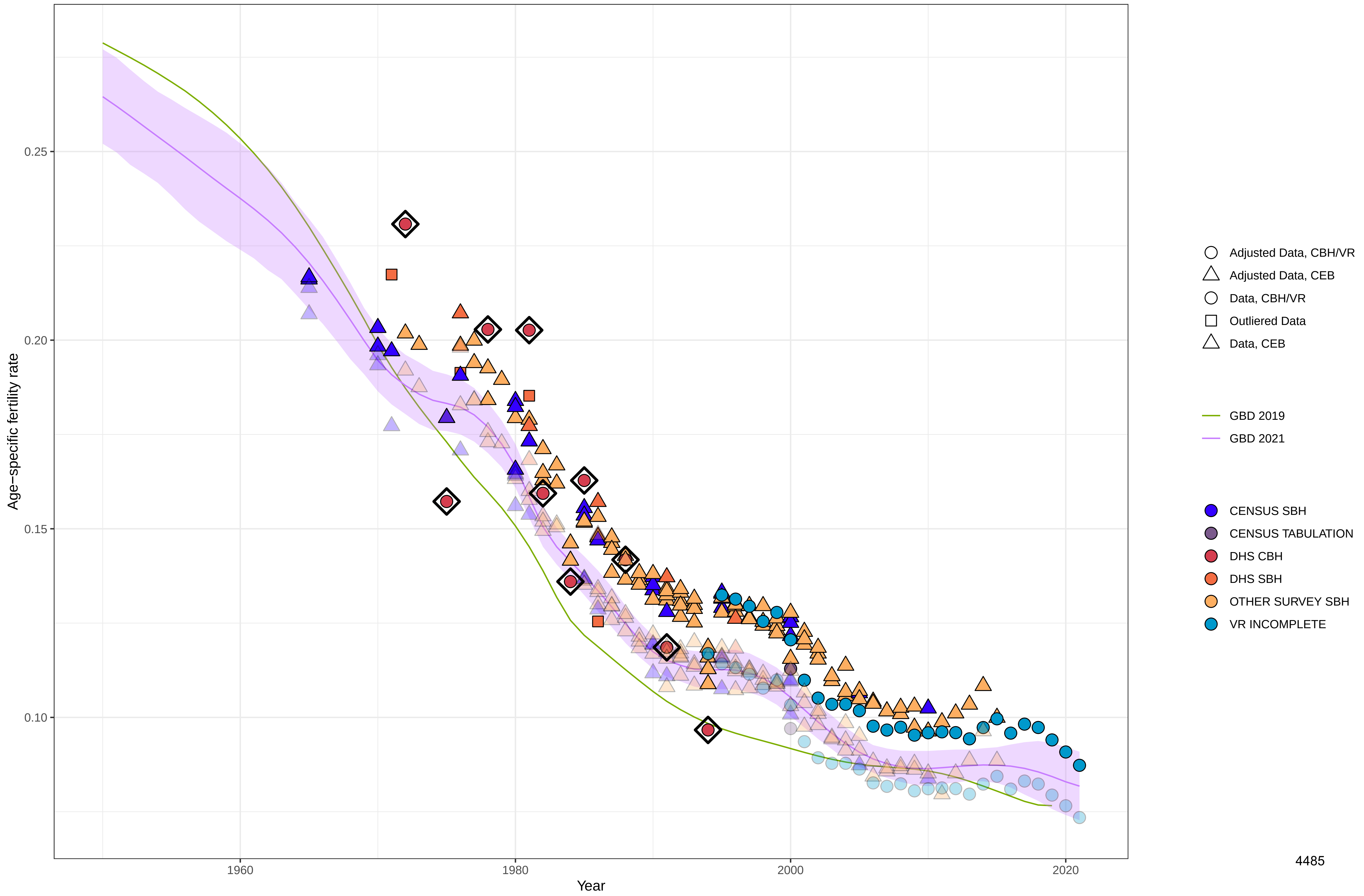

Age−specific fertility fitted trend lines, 1950−2021: São Paulo, Ages: 30−34
Data points enclosed with a diamond are reference sources

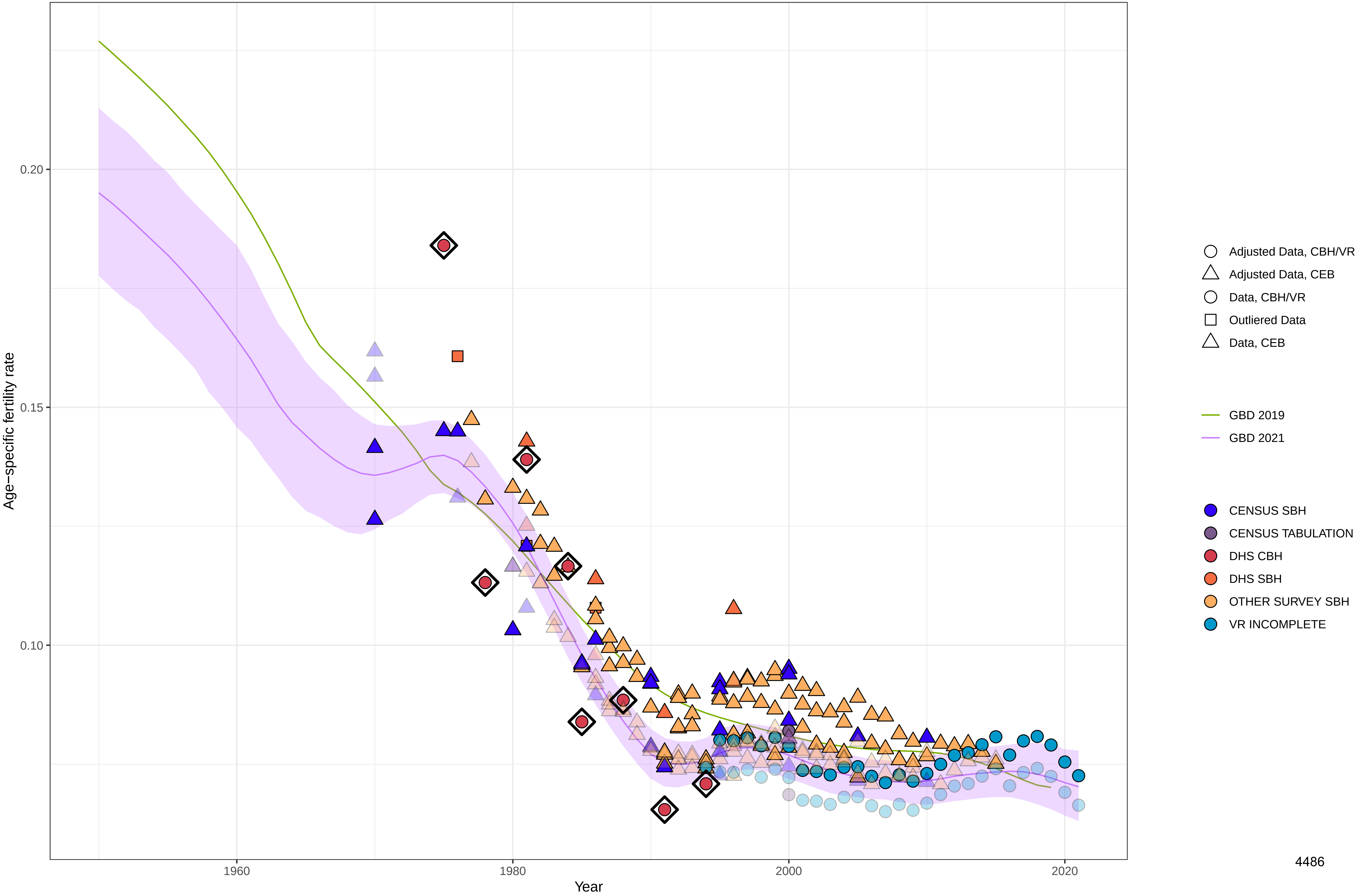

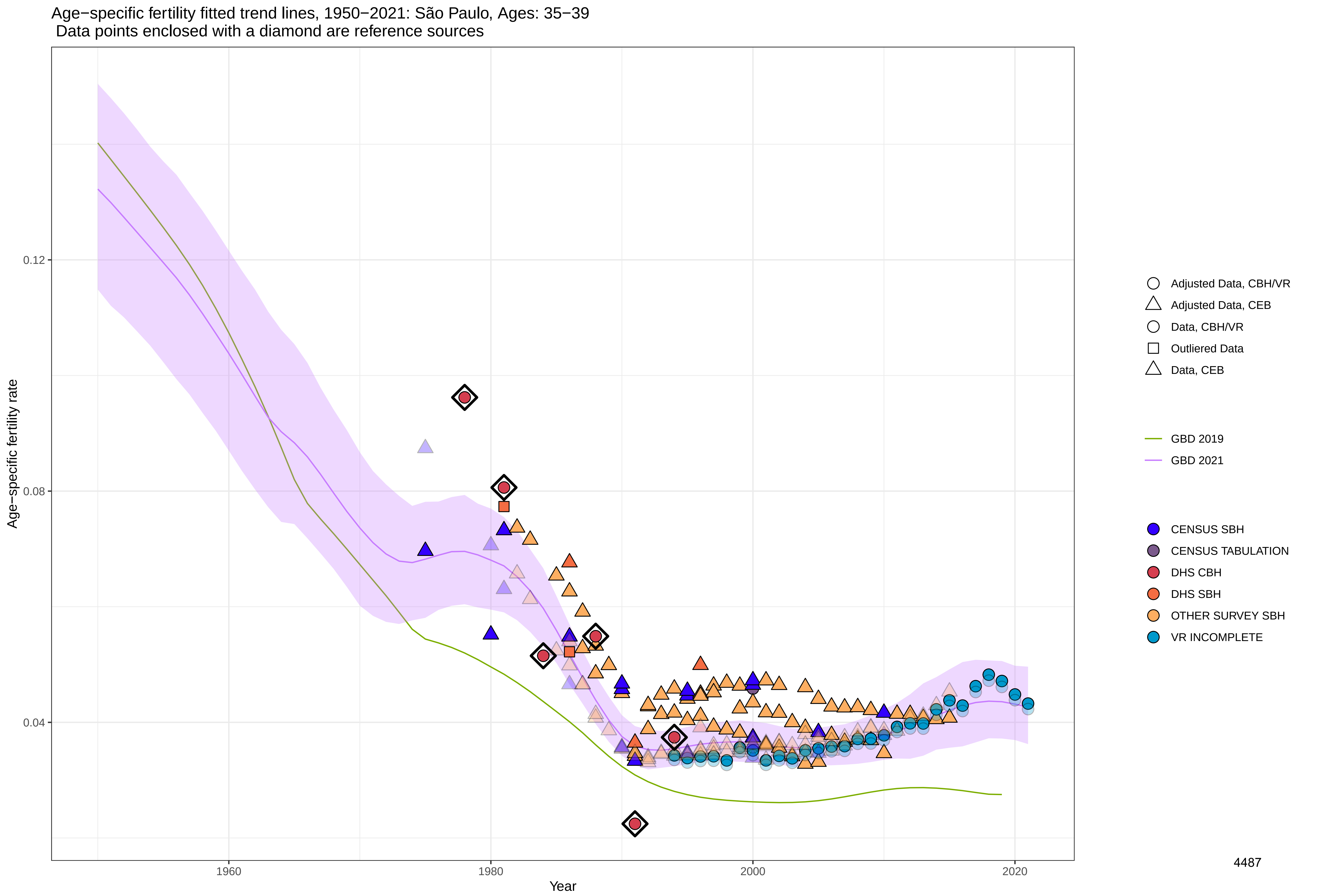

Age−specific fertility fitted trend lines, 1950−2021: São Paulo, Ages: 35−39
Data points enclosed with a diamond are reference sources
Age−specific fertility rate
0.12
0.08
0.04
1960
1980
2000
2020
Year
Adjusted Data, CBH/VR
Adjusted Data, CEB
Data, CBH/VR
Outliered Data
Data, CEB
GBD 2019
GBD 2021
CENSUS SBH
CENSUS TABULATION
DHS CBH
DHS SBH
OTHER SURVEY SBH
VR INCOMPLETE

Age−specific fertility fitted trend lines, 1950−2021: São Paulo, Ages: 40−44
Data points enclosed with a diamond are reference sources

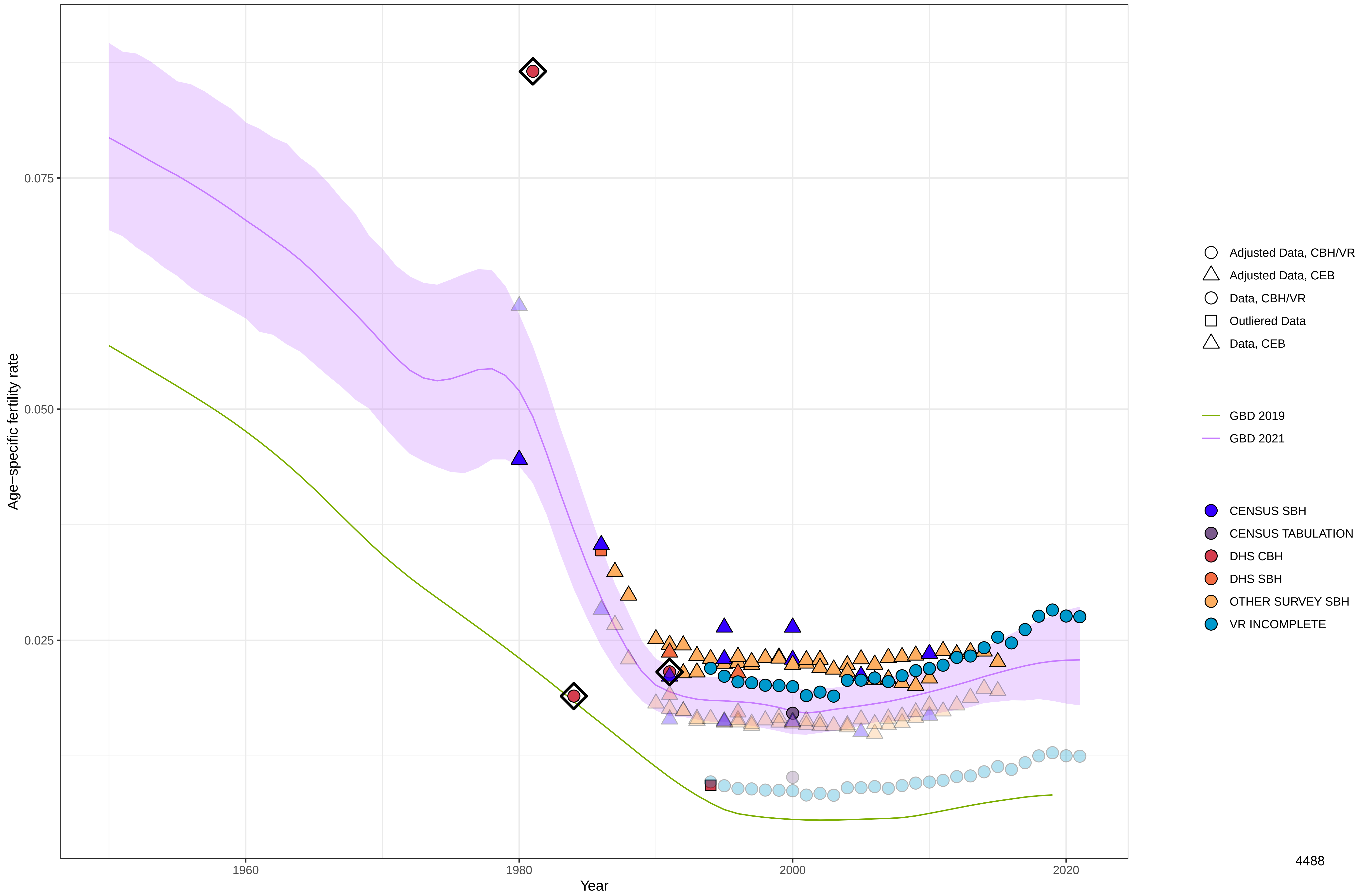

4488

Age−specific fertility fitted trend lines, 1950−2021: São Paulo, Ages: 45−49
Data points enclosed with a diamond are reference sources

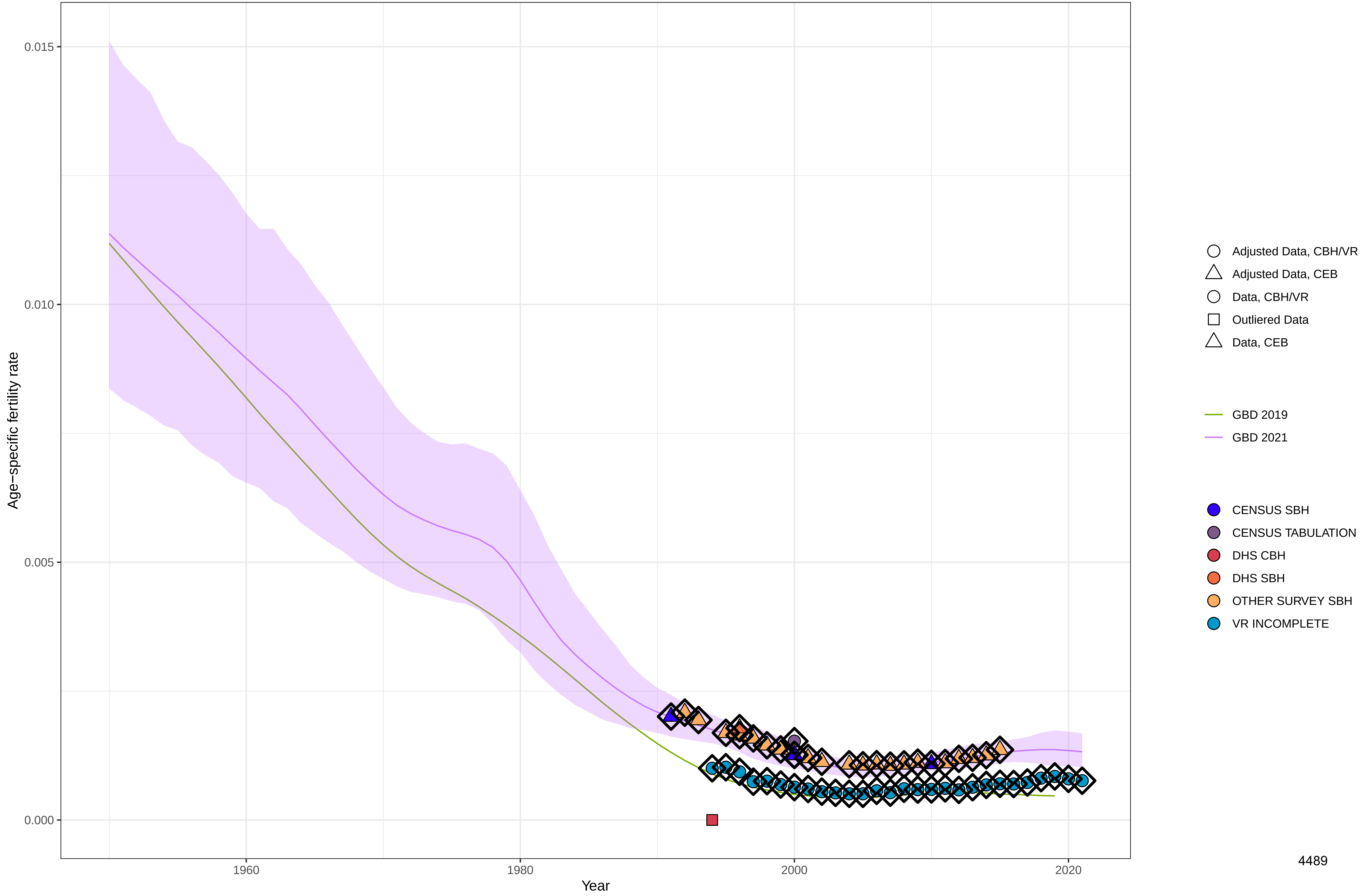

Age−specific fertility fitted trend lines, 1950−2021: São Paulo, Ages: 50−54
Data points enclosed with a diamond are reference sources

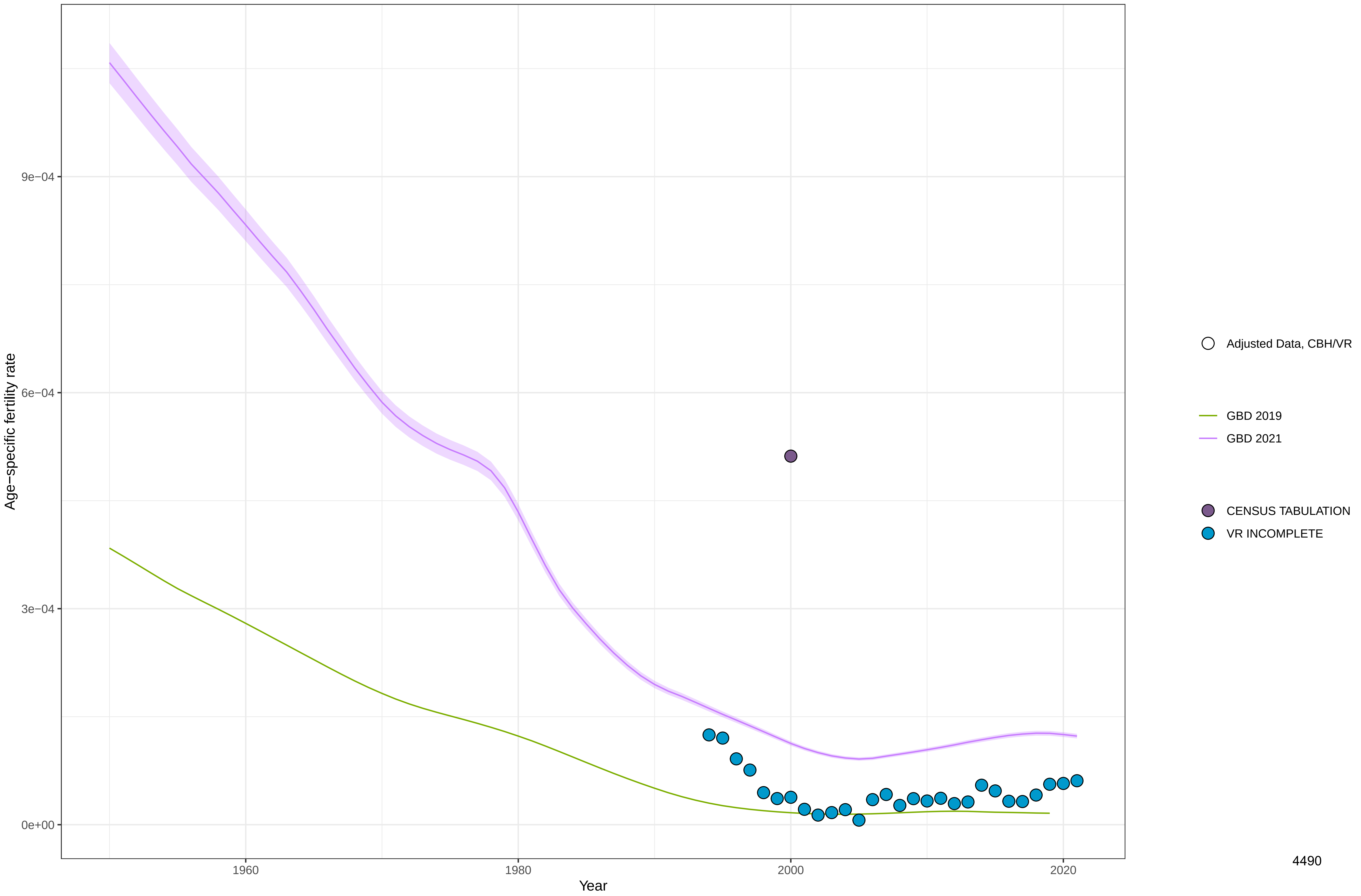

Total fertility rate fitted trend lines, 1950−2021: São Paulo TFR
Data points enclosed with a diamond are reference sources

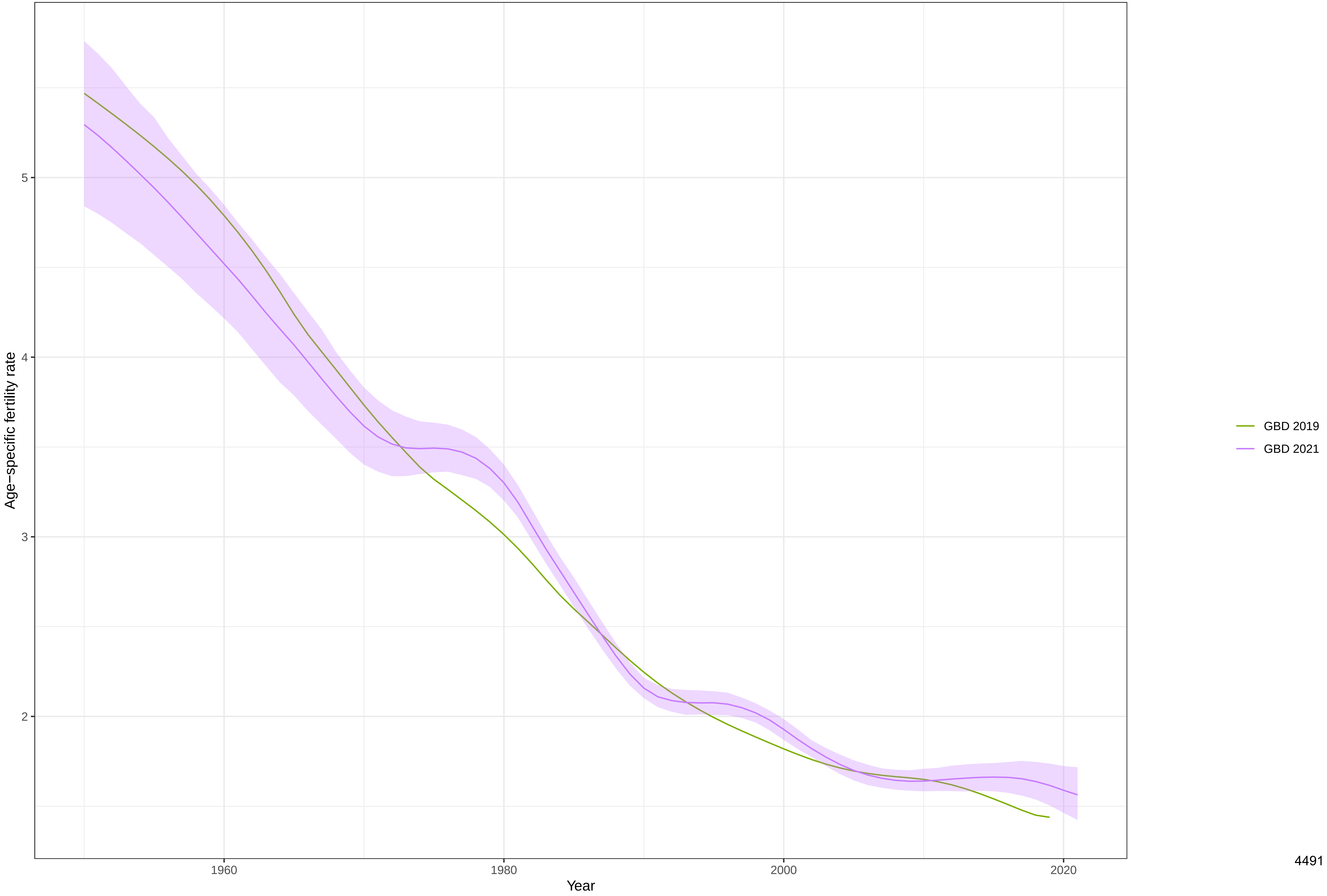

Age−specific fertility fitted trend lines, 1950−2021: Sergipe, Ages: 10−14
Data points enclosed with a diamond are reference sources

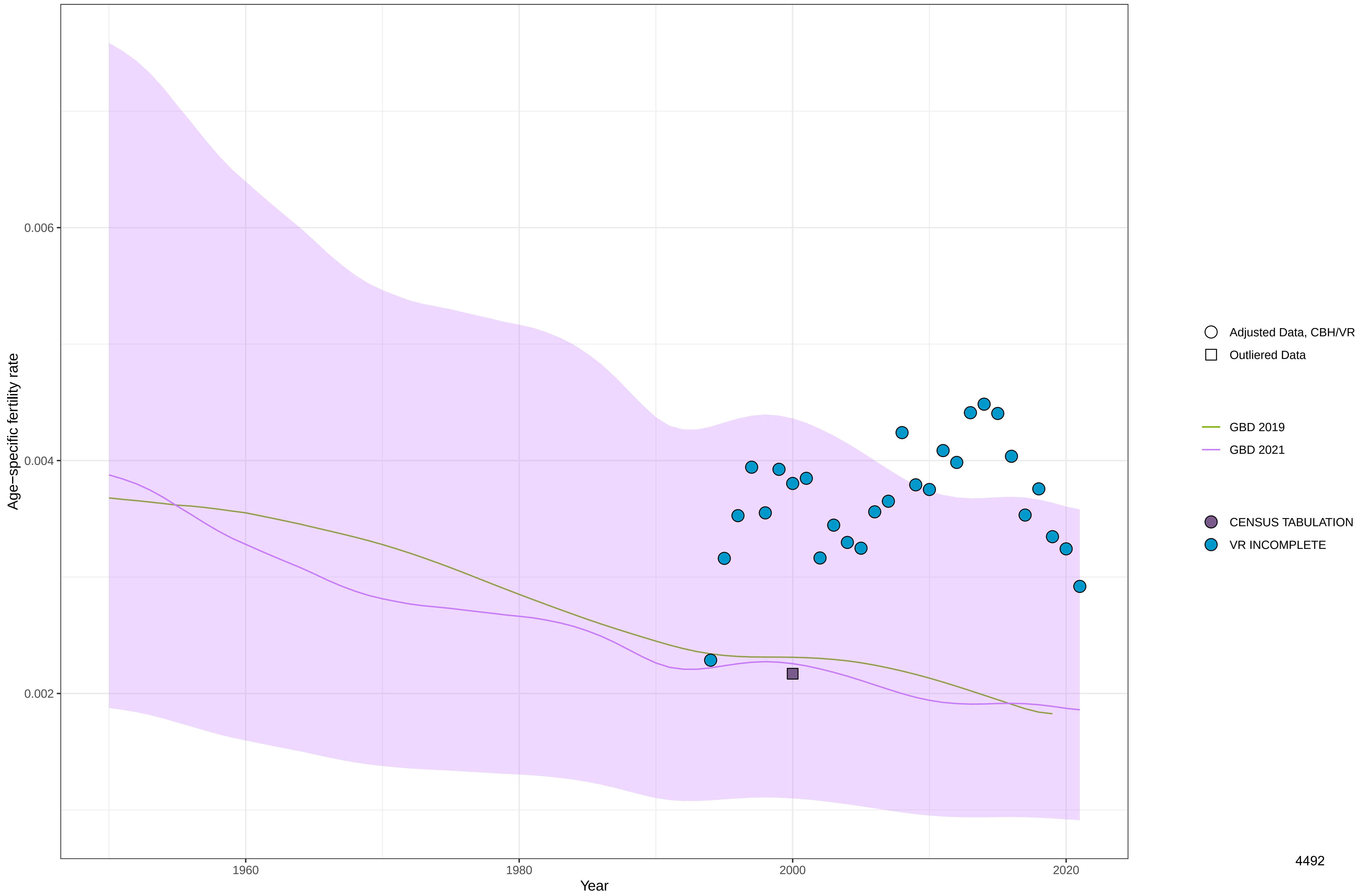

Age−specific fertility fitted trend lines, 1950−2021: Sergipe, Ages: 15−19
Data points enclosed with a diamond are reference sources

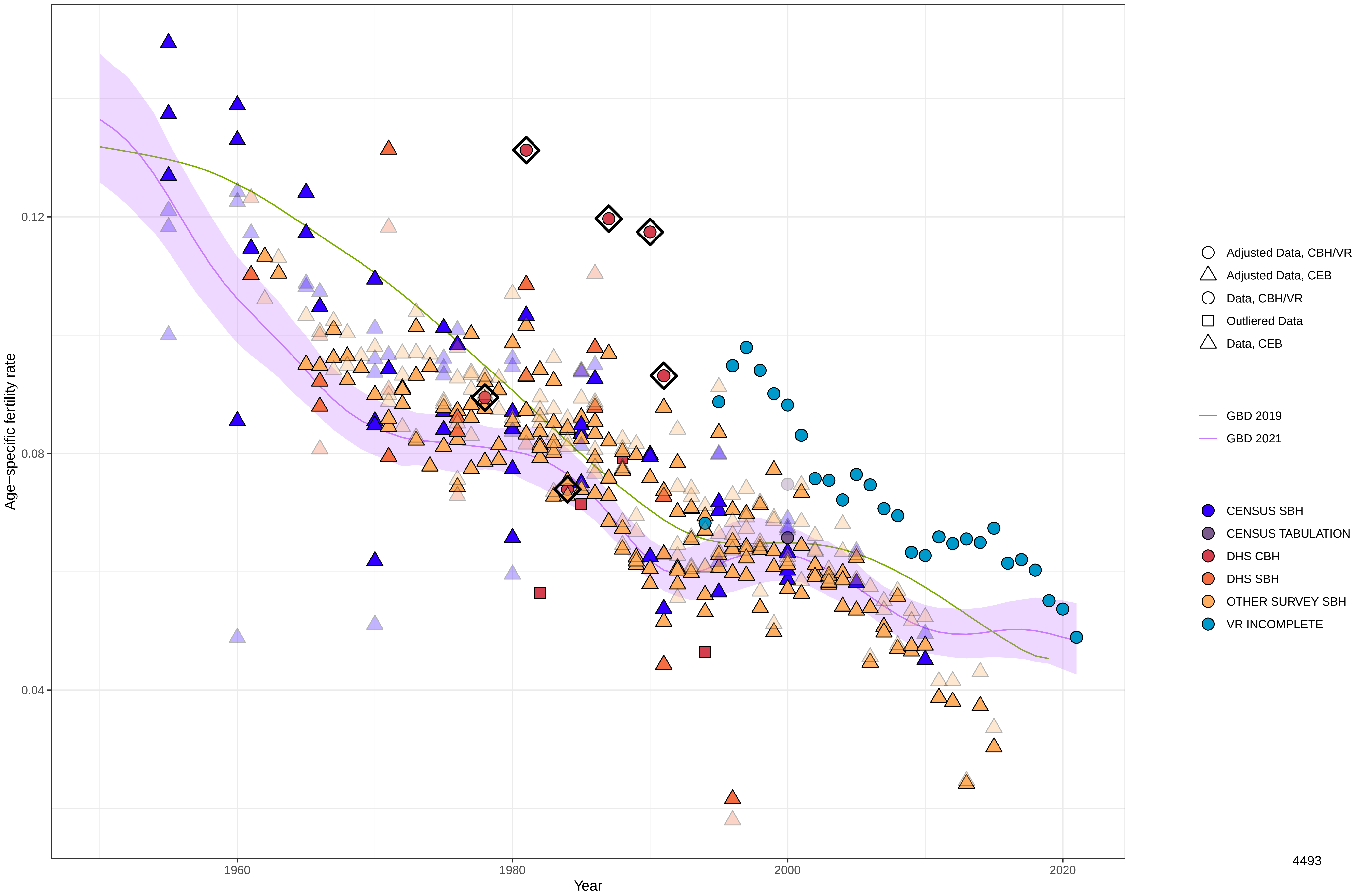

Age−specific fertility fitted trend lines, 1950−2021: Sergipe, Ages: 20−24
Data points enclosed with a diamond are reference sources

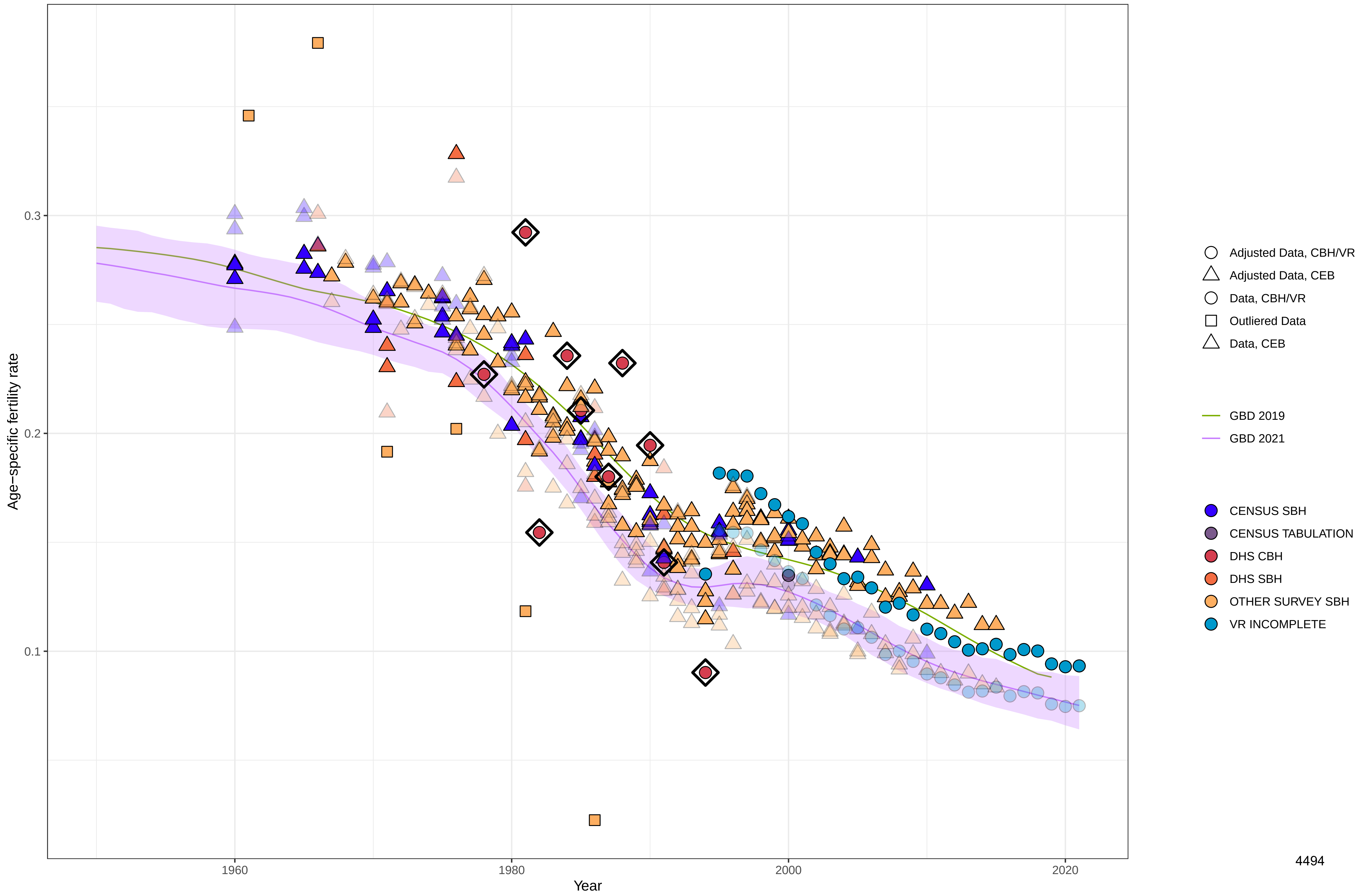

Age−specific fertility fitted trend lines, 1950−2021: Sergipe, Ages: 25−29
Data points enclosed with a diamond are reference sources

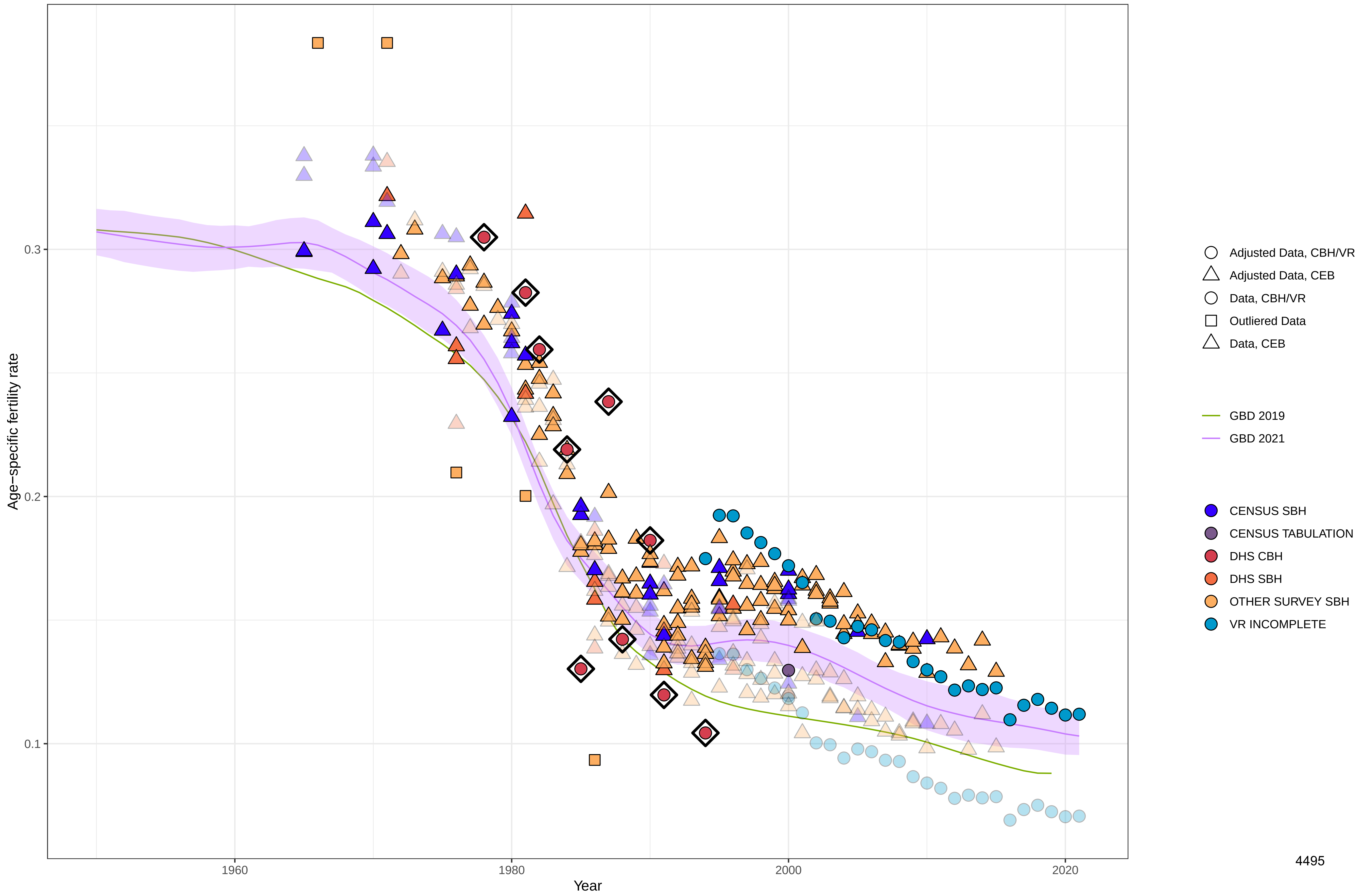

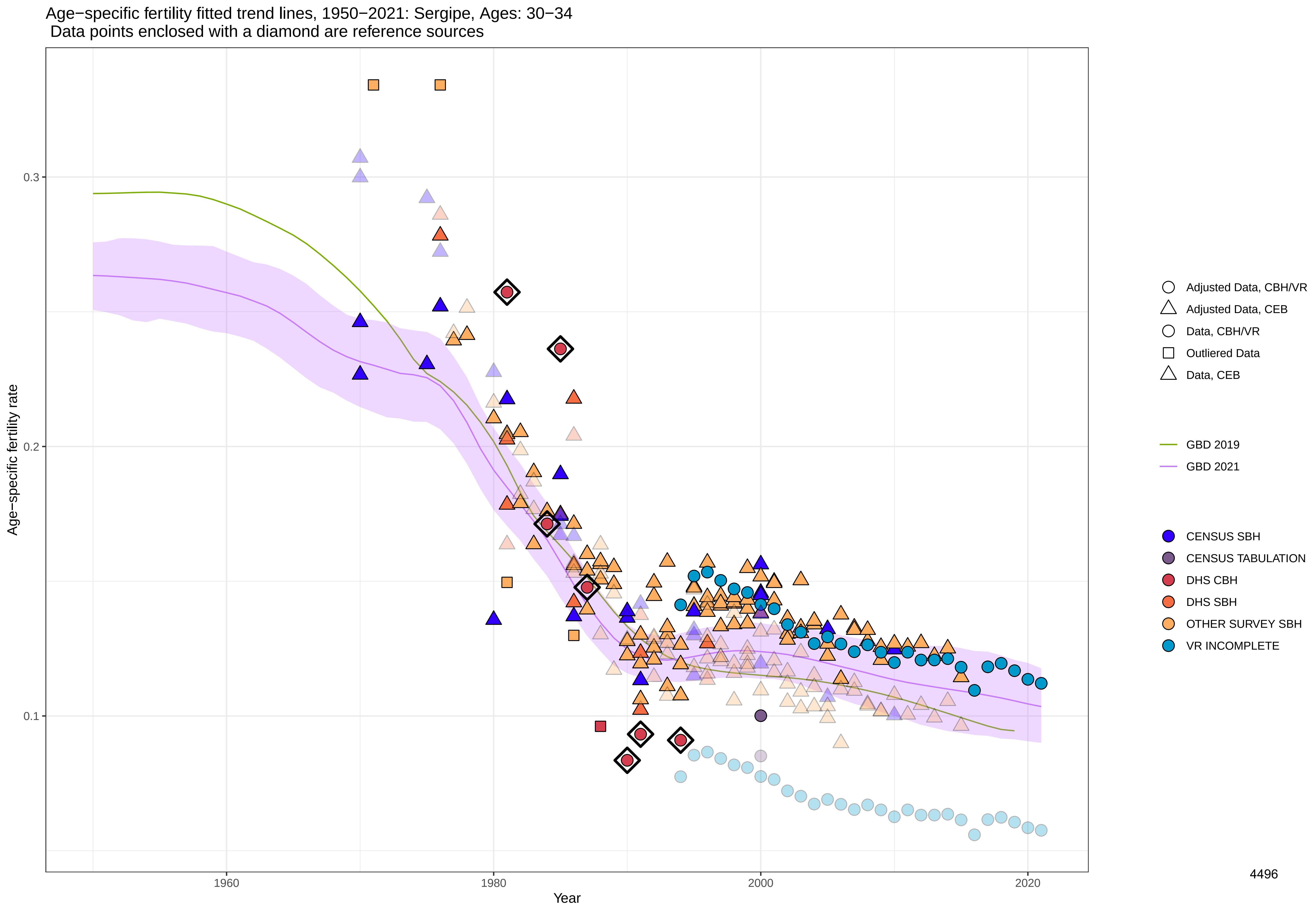

Age−specific fertility fitted trend lines, 1950−2021: Sergipe, Ages: 30−34
Data points enclosed with a diamond are reference sources
Age−specific fertility rate
0.3
0.2
0.1
Year
1960
1980
2000
2020
Adjusted Data, CBH/VR
Adjusted Data, CEB
Data, CBH/VR
Outliered Data
Data, CEB
GBD 2019
GBD 2021
CENSUS SBH
CENSUS TABULATION
DHS CBH
DHS SBH
OTHER SURVEY SBH
VR INCOMPLETE

4496

Age−specific fertility fitted trend lines, 1950−2021: Sergipe, Ages: 35−39
Data points enclosed with a diamond are reference sources

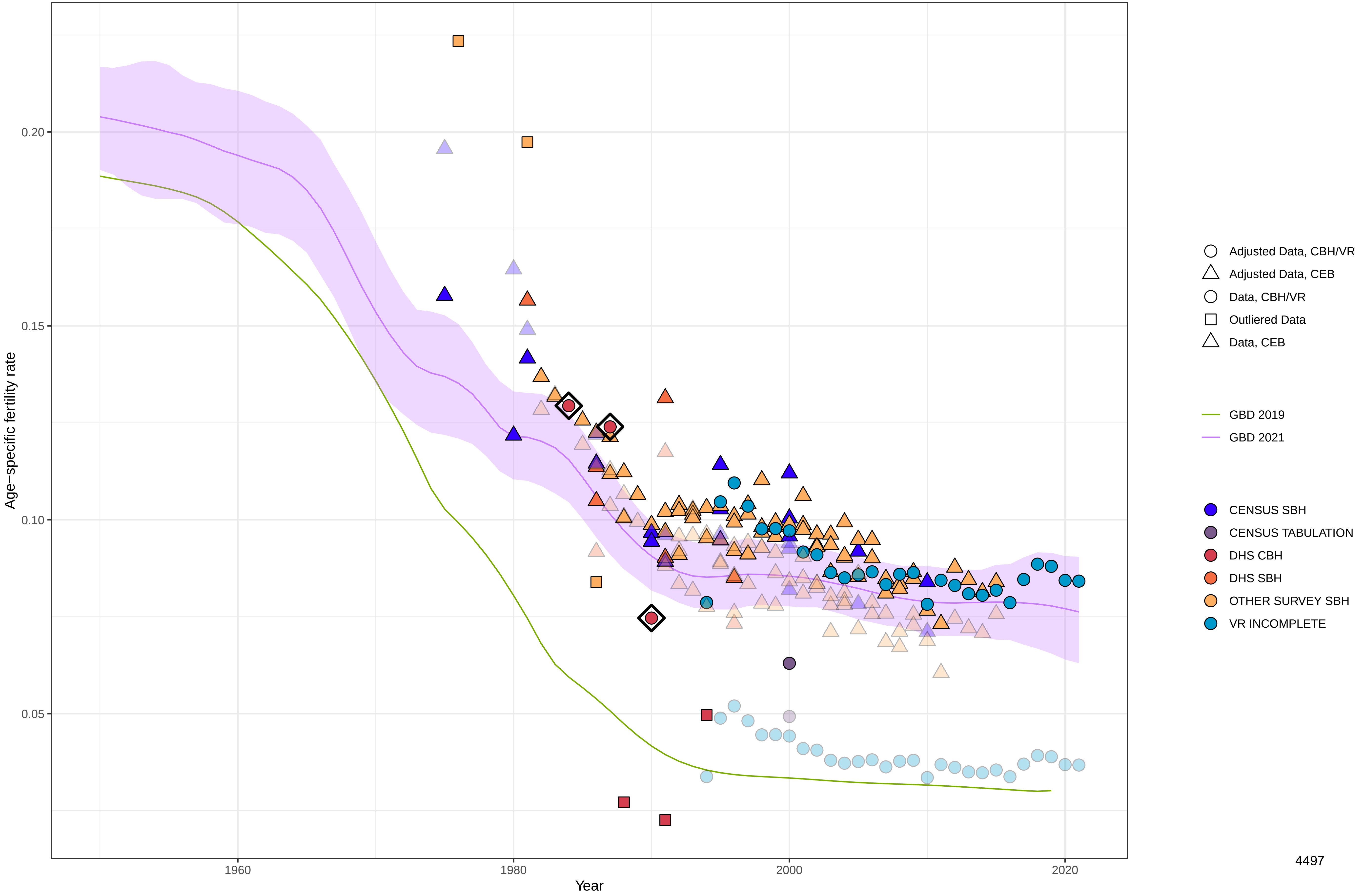

Age−specific fertility fitted trend lines, 1950−2021: Sergipe, Ages: 40−44
Data points enclosed with a diamond are reference sources

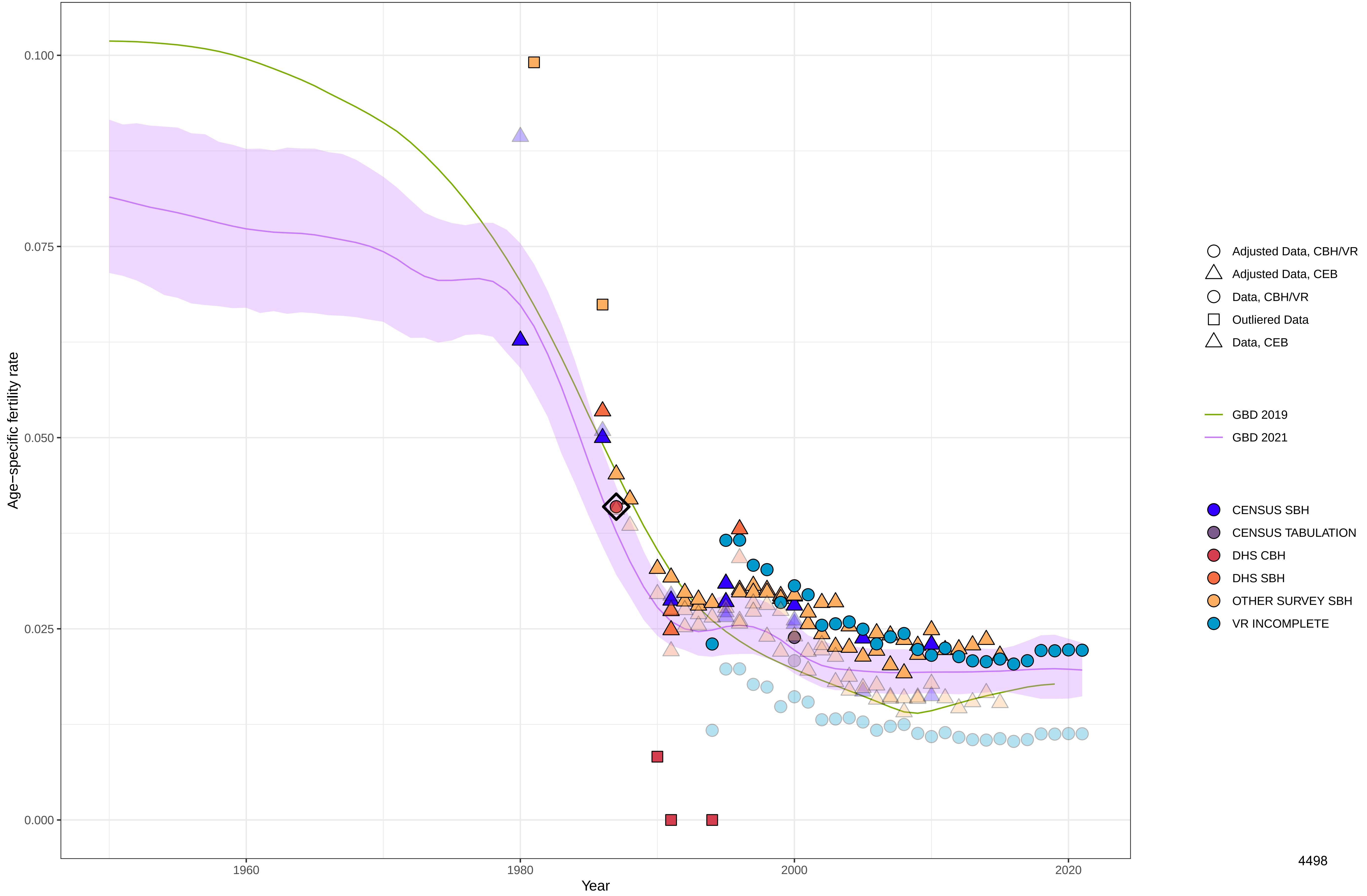

Age−specific fertility fitted trend lines, 1950−2021: Sergipe, Ages: 45−49
Data points enclosed with a diamond are reference sources

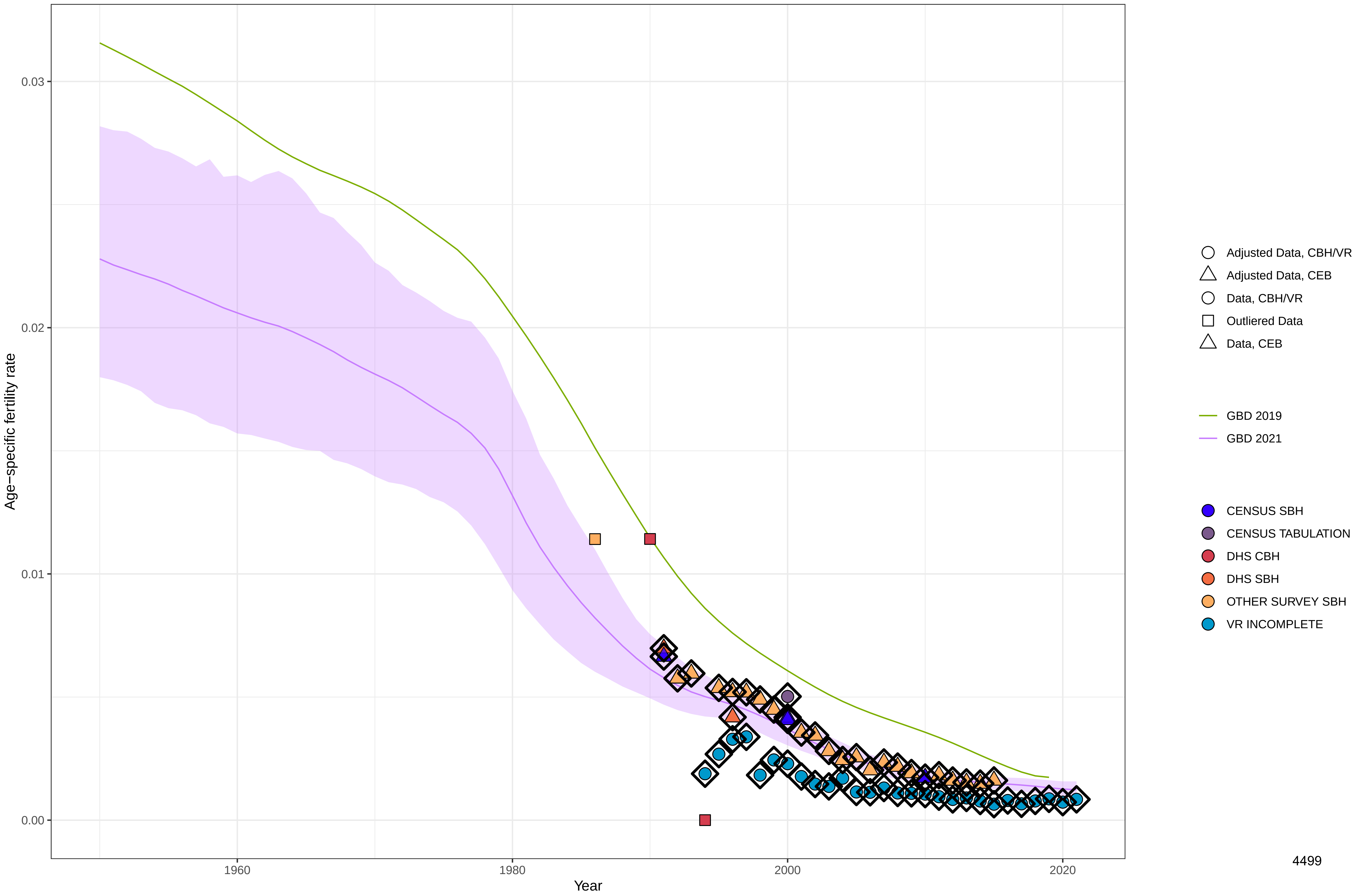

Age−specific fertility fitted trend lines, 1950−2021: Sergipe, Ages: 50−54
Data points enclosed with a diamond are reference sources

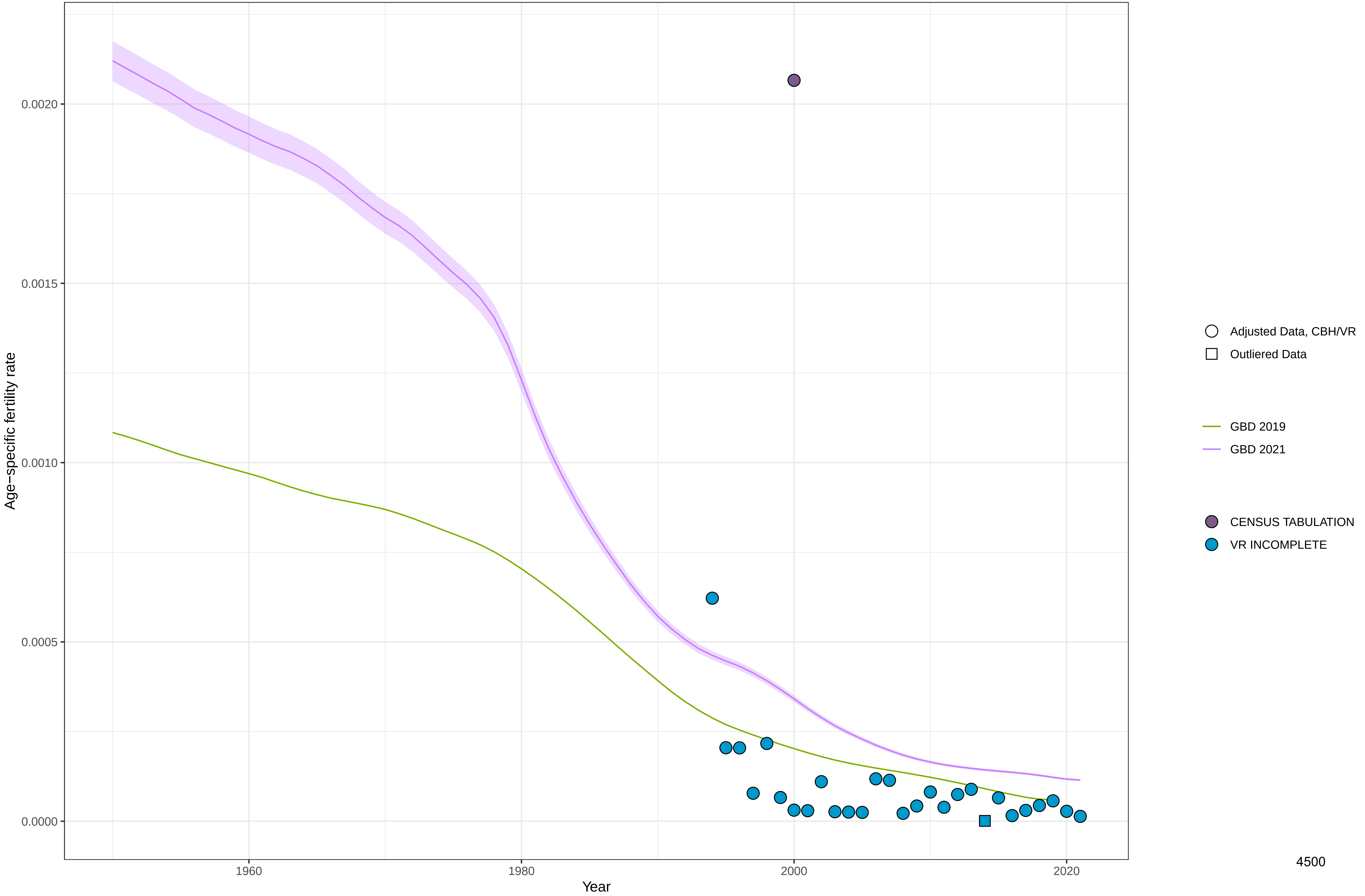

Total fertility rate fitted trend lines, 1950−2021: Sergipe TFR
Data points enclosed with a diamond are reference sources

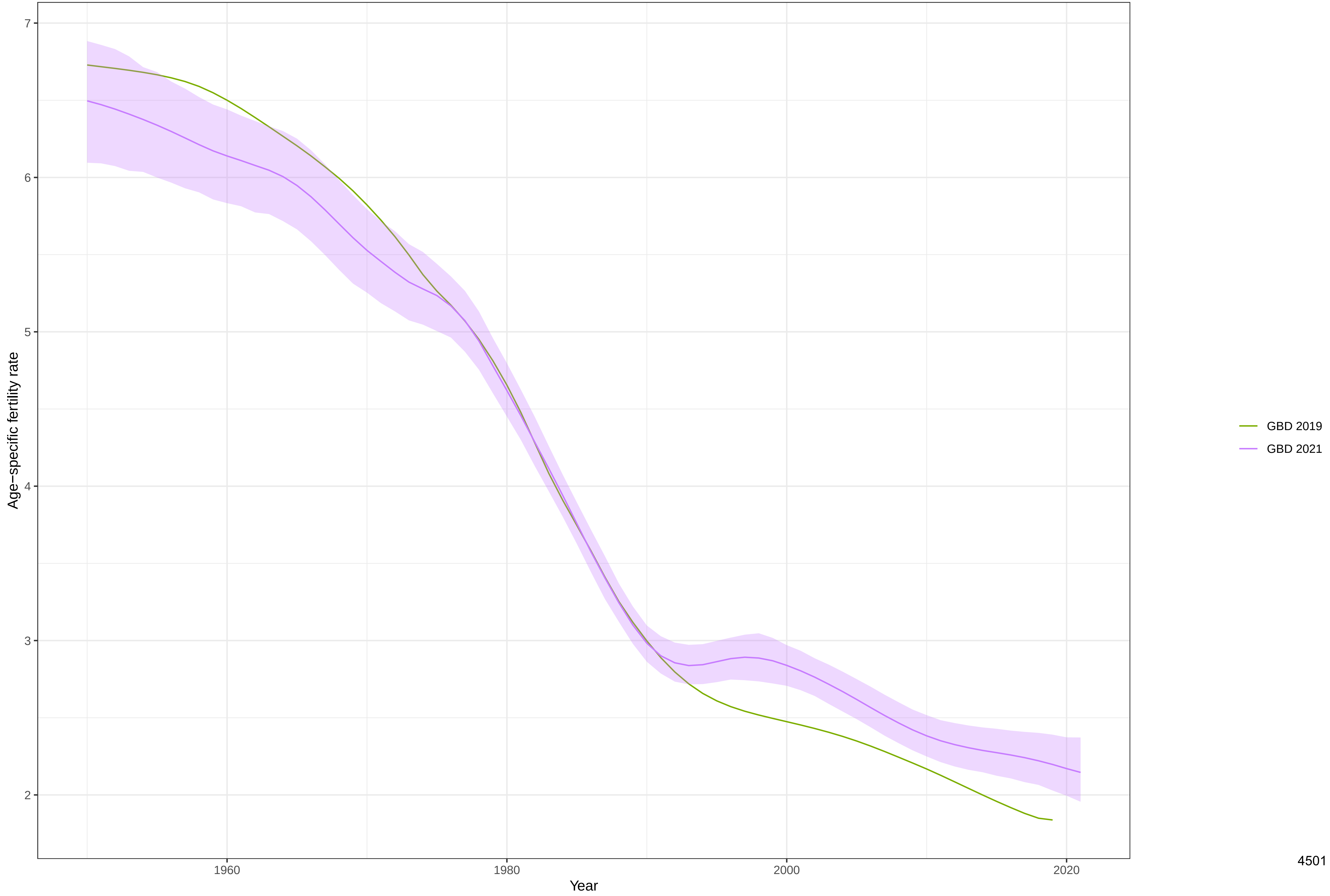

Age−specific fertility fitted trend lines, 1950−2021: Tocantins, Ages: 10−14
Data points enclosed with a diamond are reference sources

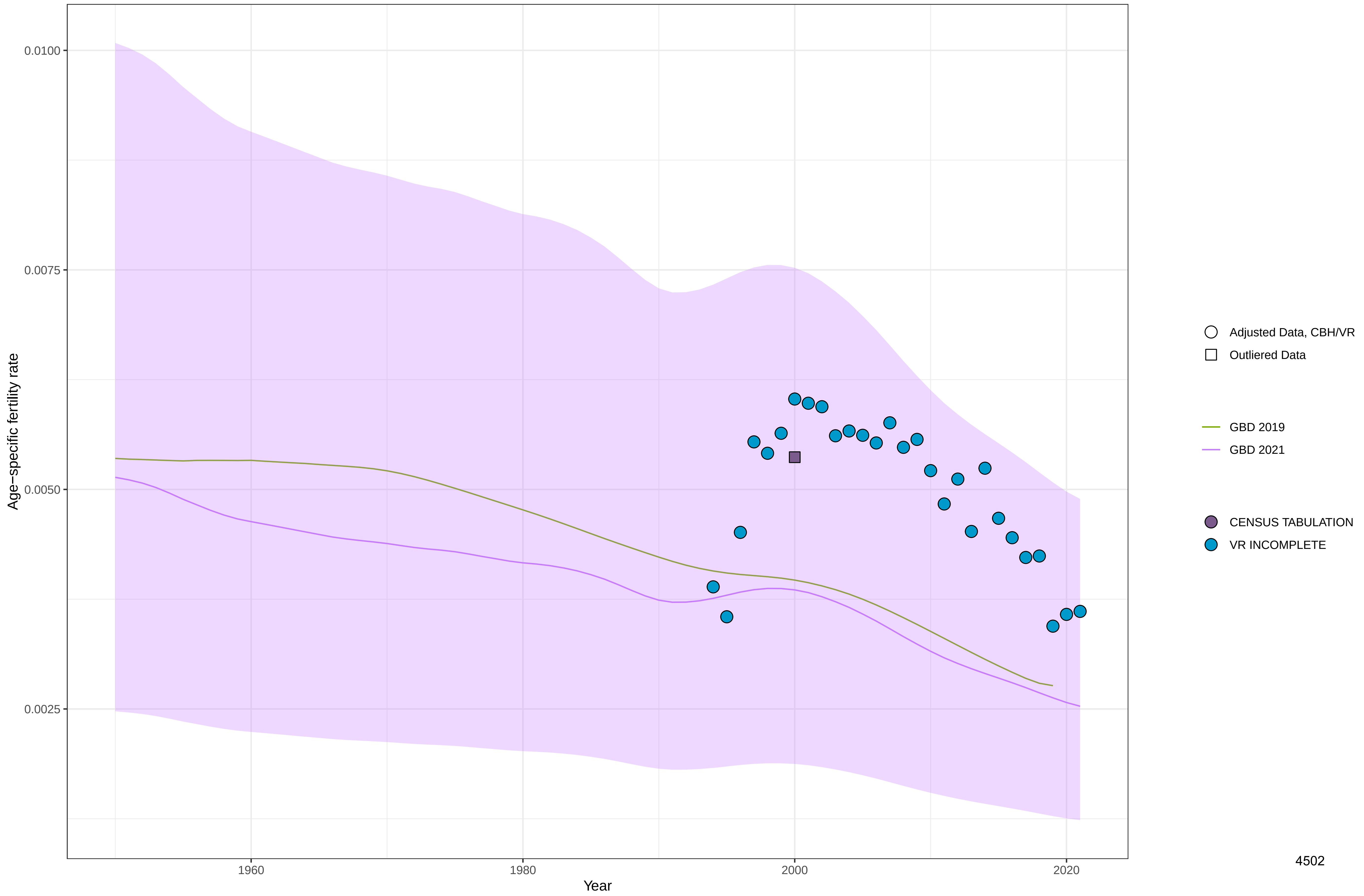

Age−specific fertility fitted trend lines, 1950−2021: Tocantins, Ages: 15−19
Data points enclosed with a diamond are reference sources

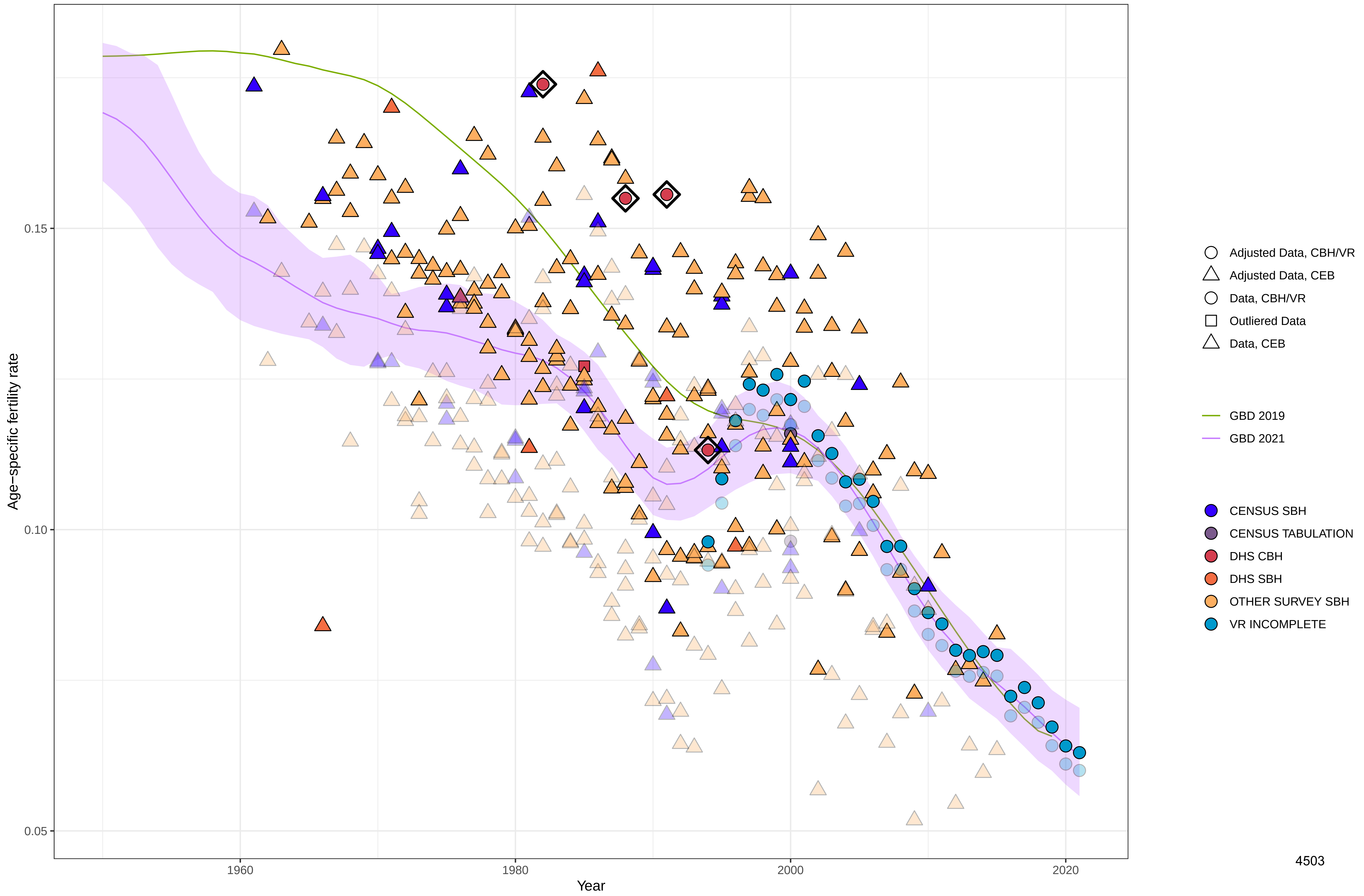

Age−specific fertility fitted trend lines, 1950−2021: Tocantins, Ages: 20−24
Data points enclosed with a diamond are reference sources

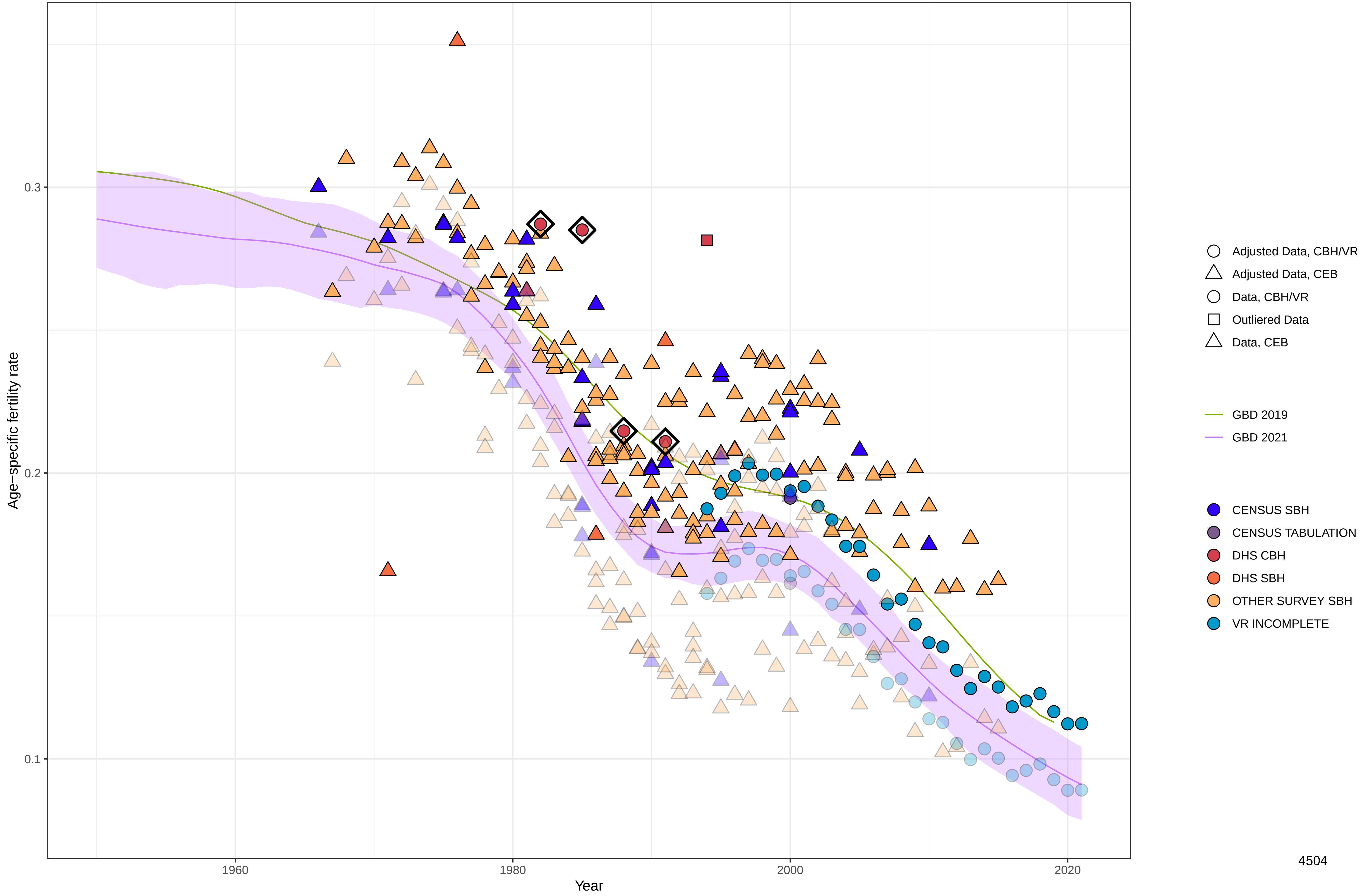

Age−specific fertility fitted trend lines, 1950−2021: Tocantins, Ages: 25−29
Data points enclosed with a diamond are reference sources

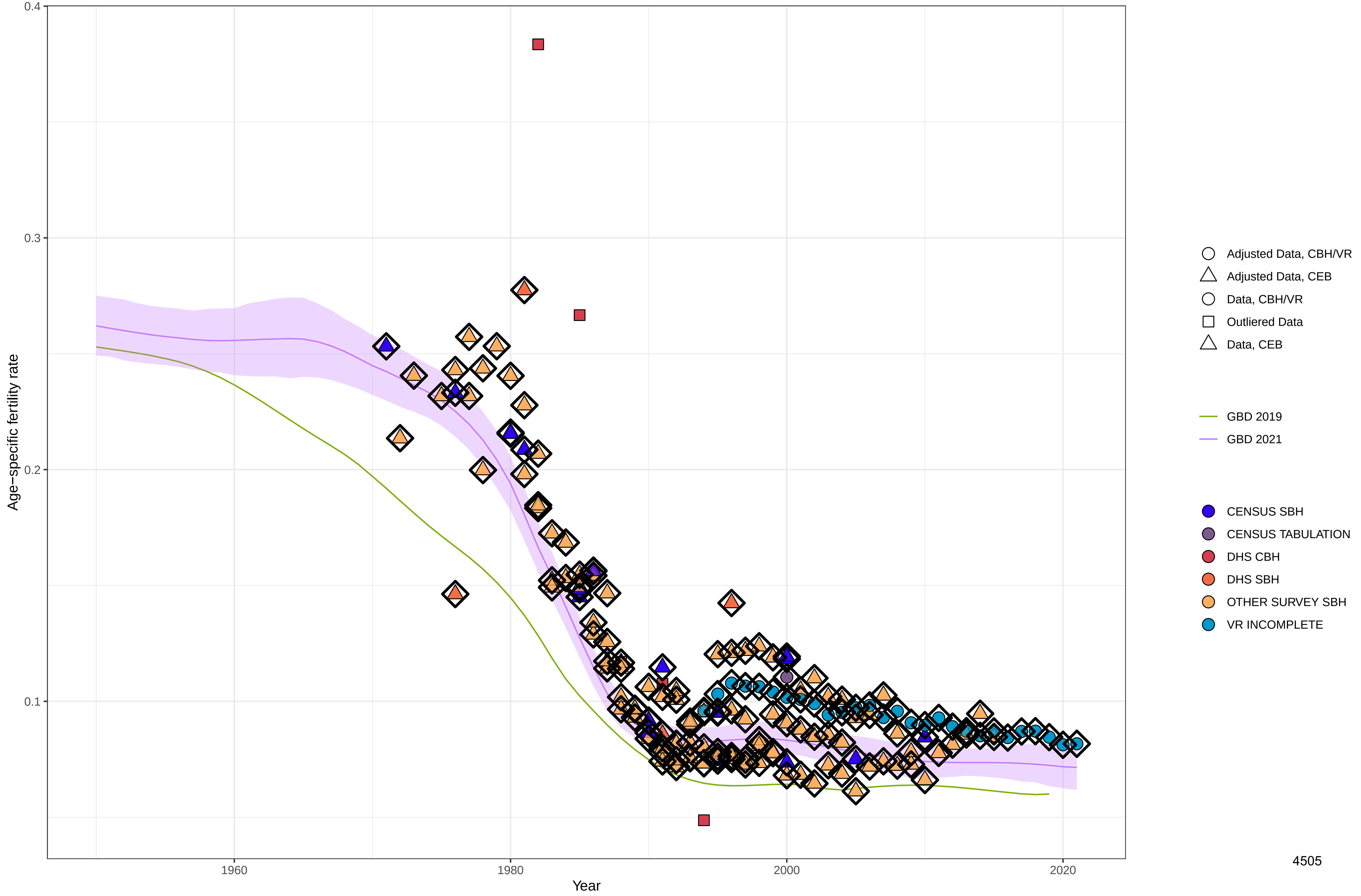

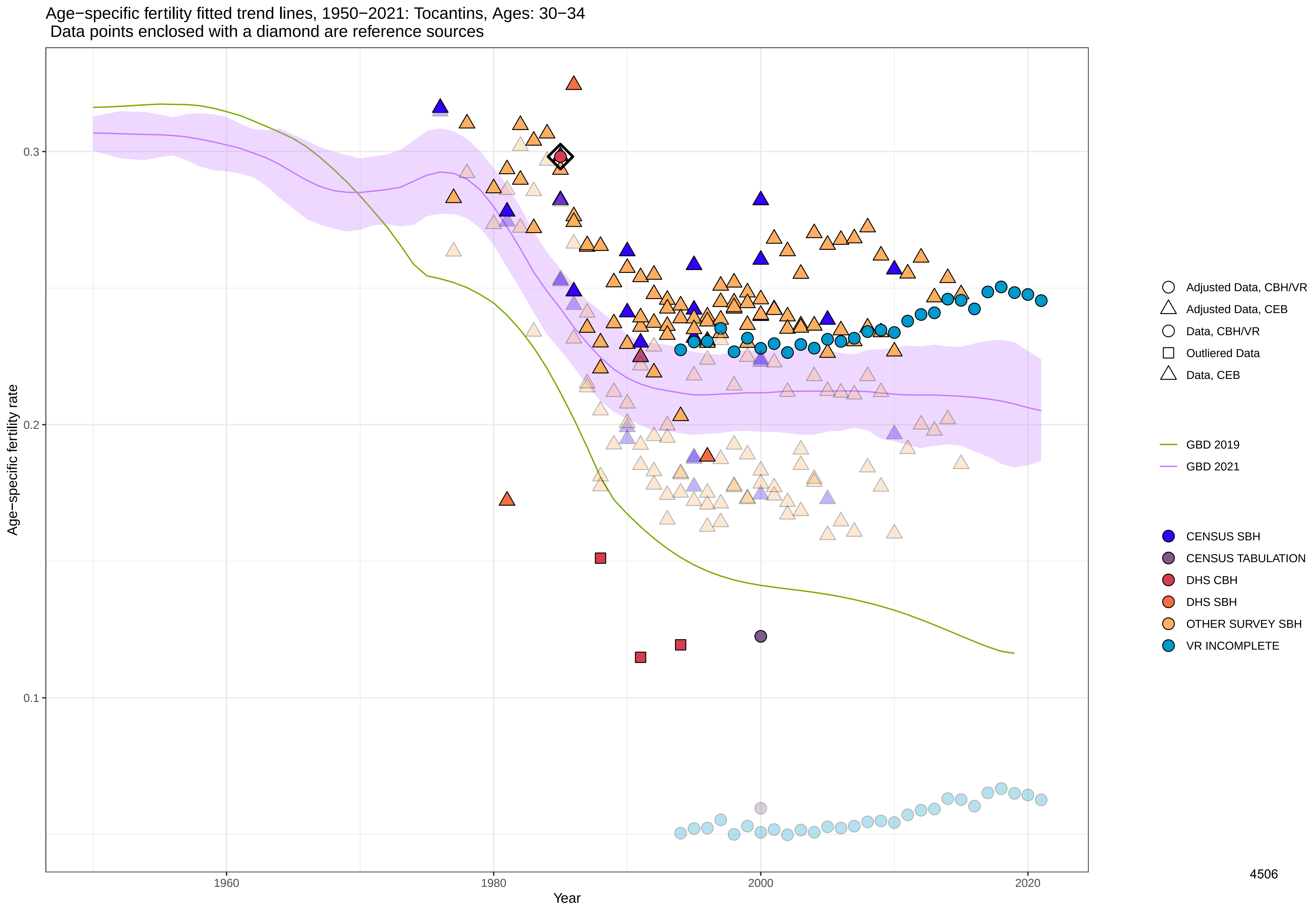

Age−specific fertility fitted trend lines, 1950−2021: Tocantins, Ages: 30−34
Data points enclosed with a diamond are reference sources
Age−specific fertility rate
Year
0.1
0.2
0.3
1960
1980
2000
2020
Adjusted Data, CBH/VR
Adjusted Data, CEB
Data, CBH/VR
Outliered Data
Data, CEB
GBD 2019
GBD 2021
CENSUS SBH
CENSUS TABULATION
DHS CBH
DHS SBH
OTHER SURVEY SBH
VR INCOMPLETE

4506

Age−specific fertility fitted trend lines, 1950−2021: Tocantins, Ages: 35−39
Data points enclosed with a diamond are reference sources

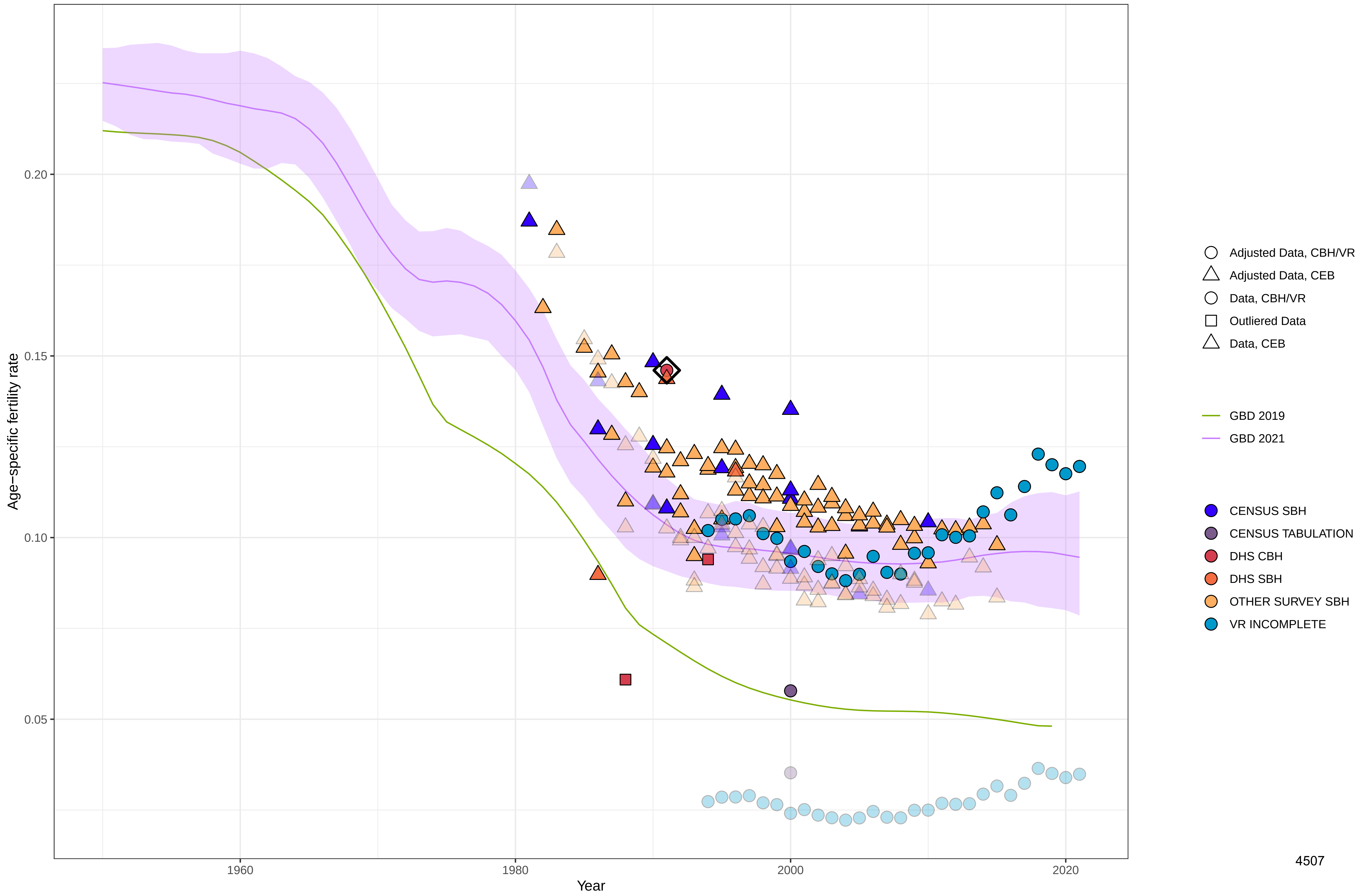

Age−specific fertility fitted trend lines, 1950−2021: Tocantins, Ages: 40−44
Data points enclosed with a diamond are reference sources

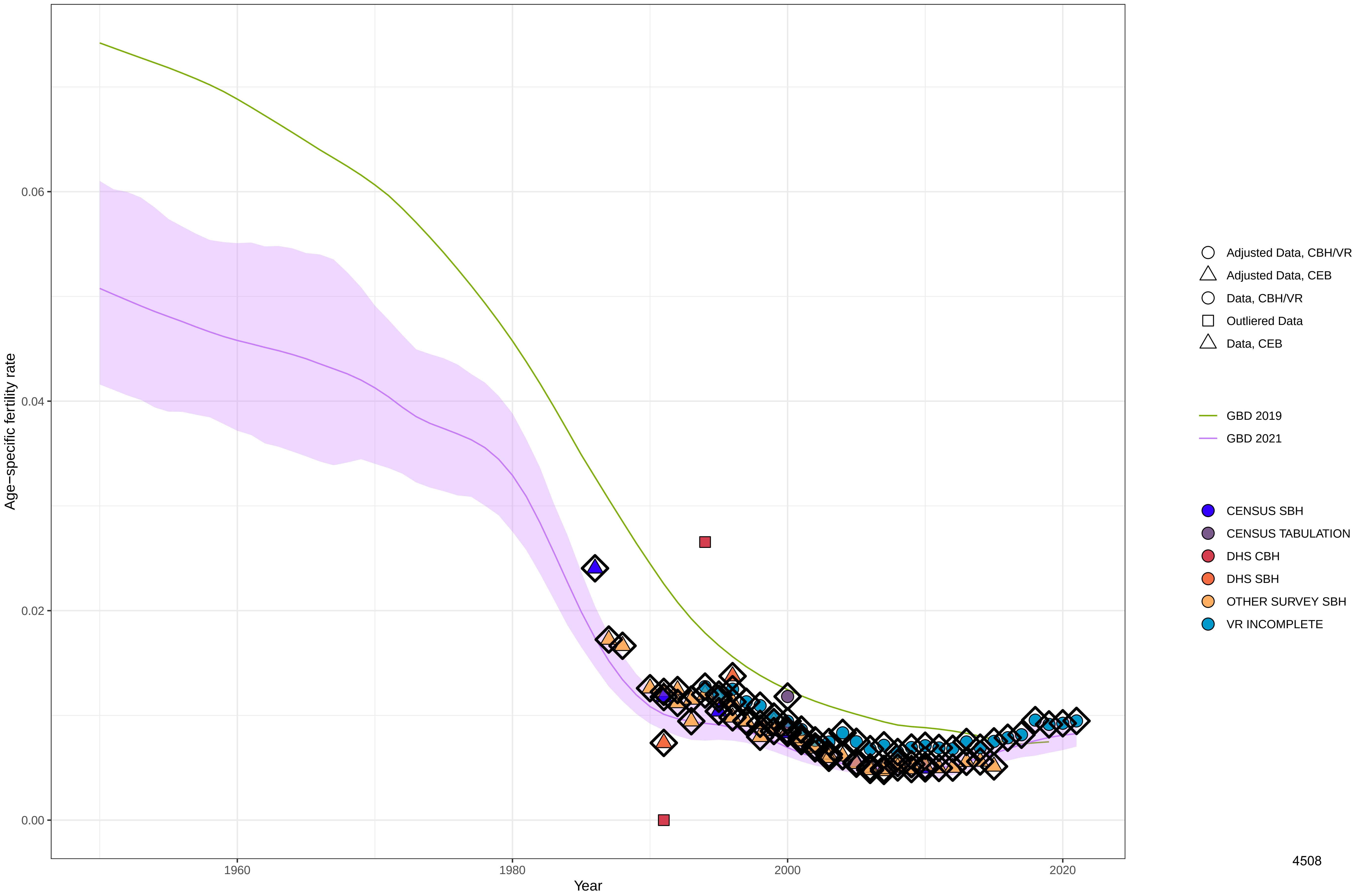

Age−specific fertility fitted trend lines, 1950−2021: Tocantins, Ages: 45−49
Data points enclosed with a diamond are reference sources

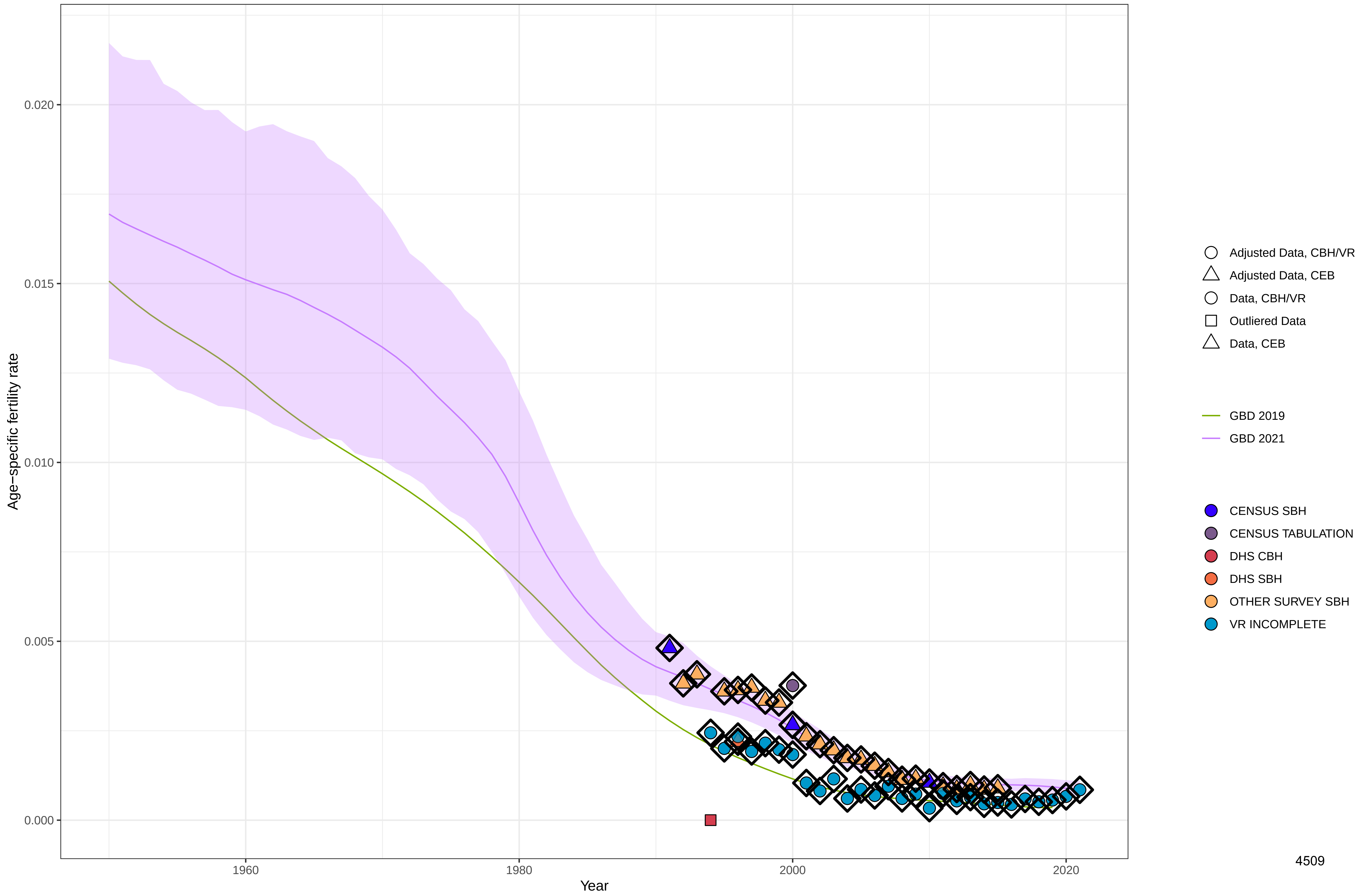

Age−specific fertility fitted trend lines, 1950−2021: Tocantins, Ages: 50−54
Data points enclosed with a diamond are reference sources

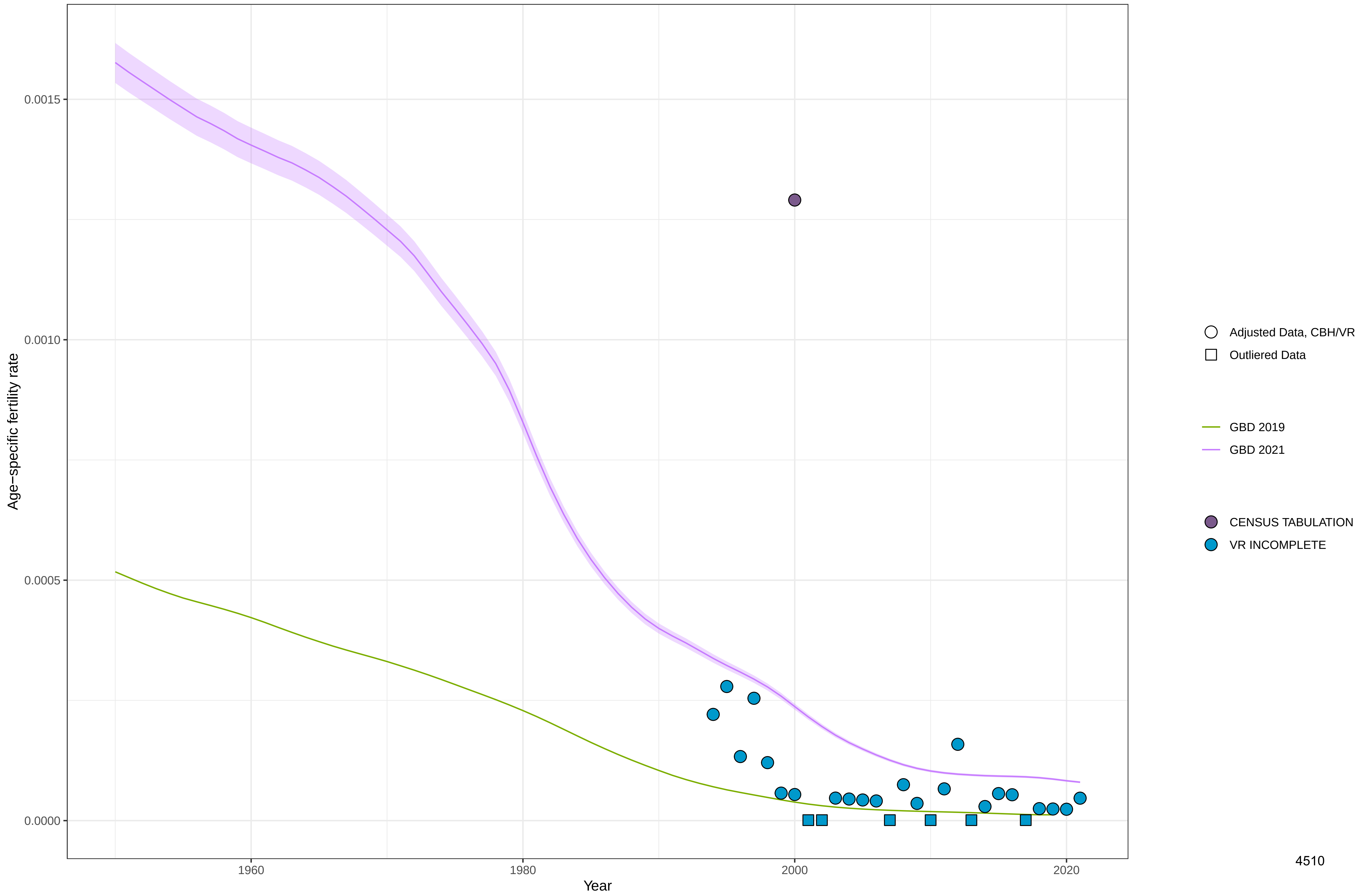

Total fertility rate fitted trend lines, 1950−2021: Tocantins TFR
Data points enclosed with a diamond are reference sources

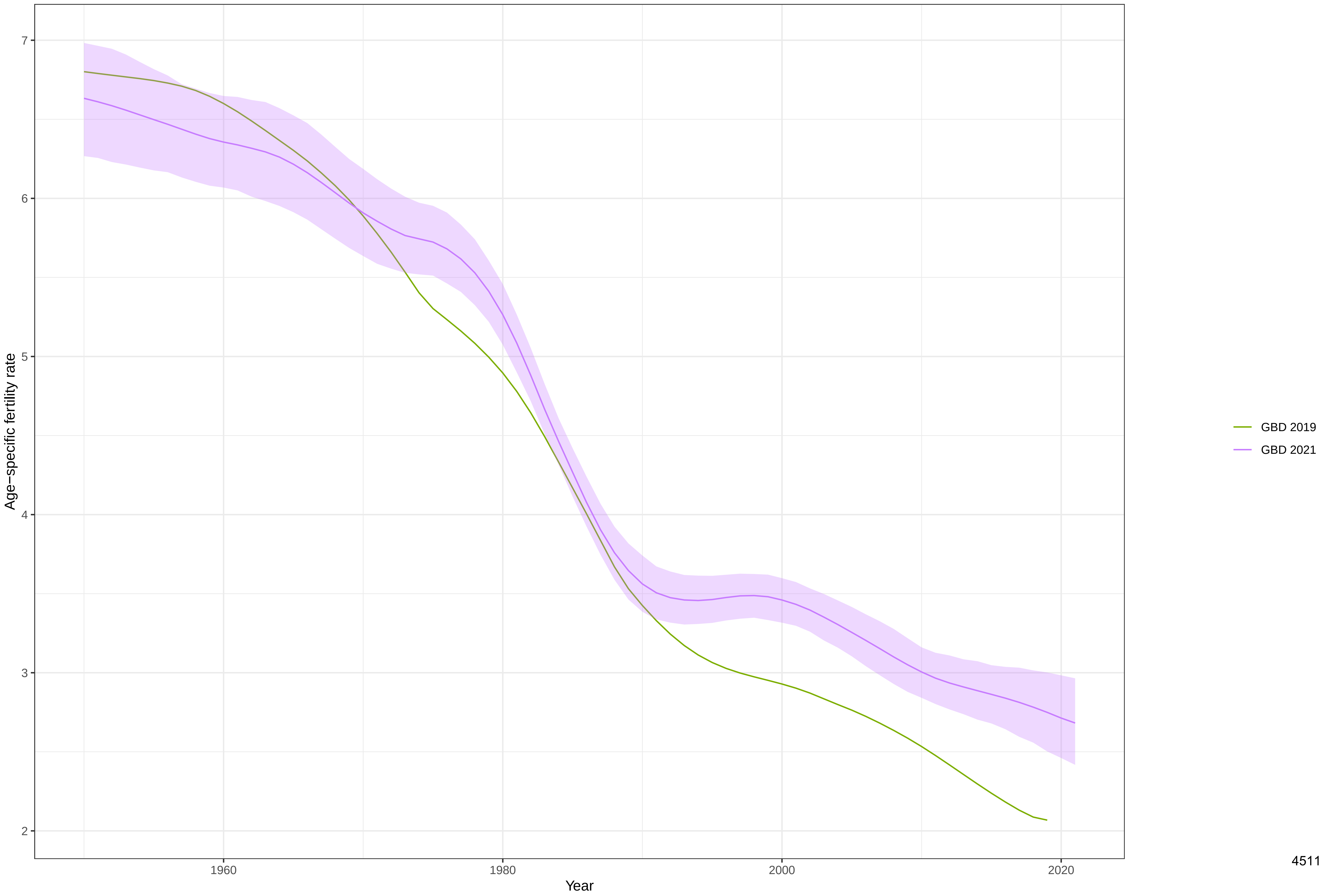

4511

Age−specific fertility fitted trend lines, 1950−2021: Paraguay, Ages: 10−14
Data points enclosed with a diamond are reference sources

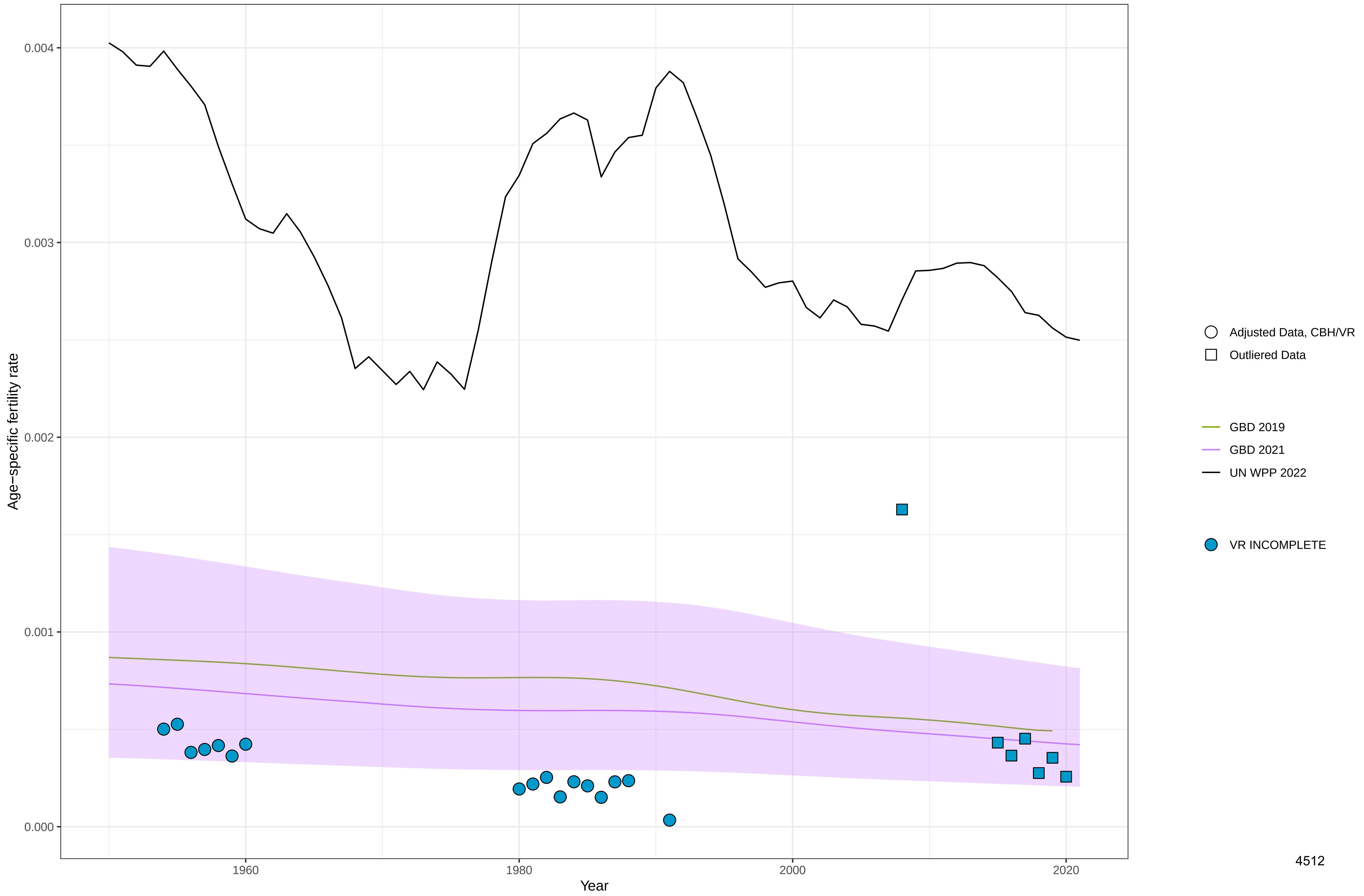

Age−specific fertility fitted trend lines, 1950−2021: Paraguay, Ages: 15−19
Data points enclosed with a diamond are reference sources

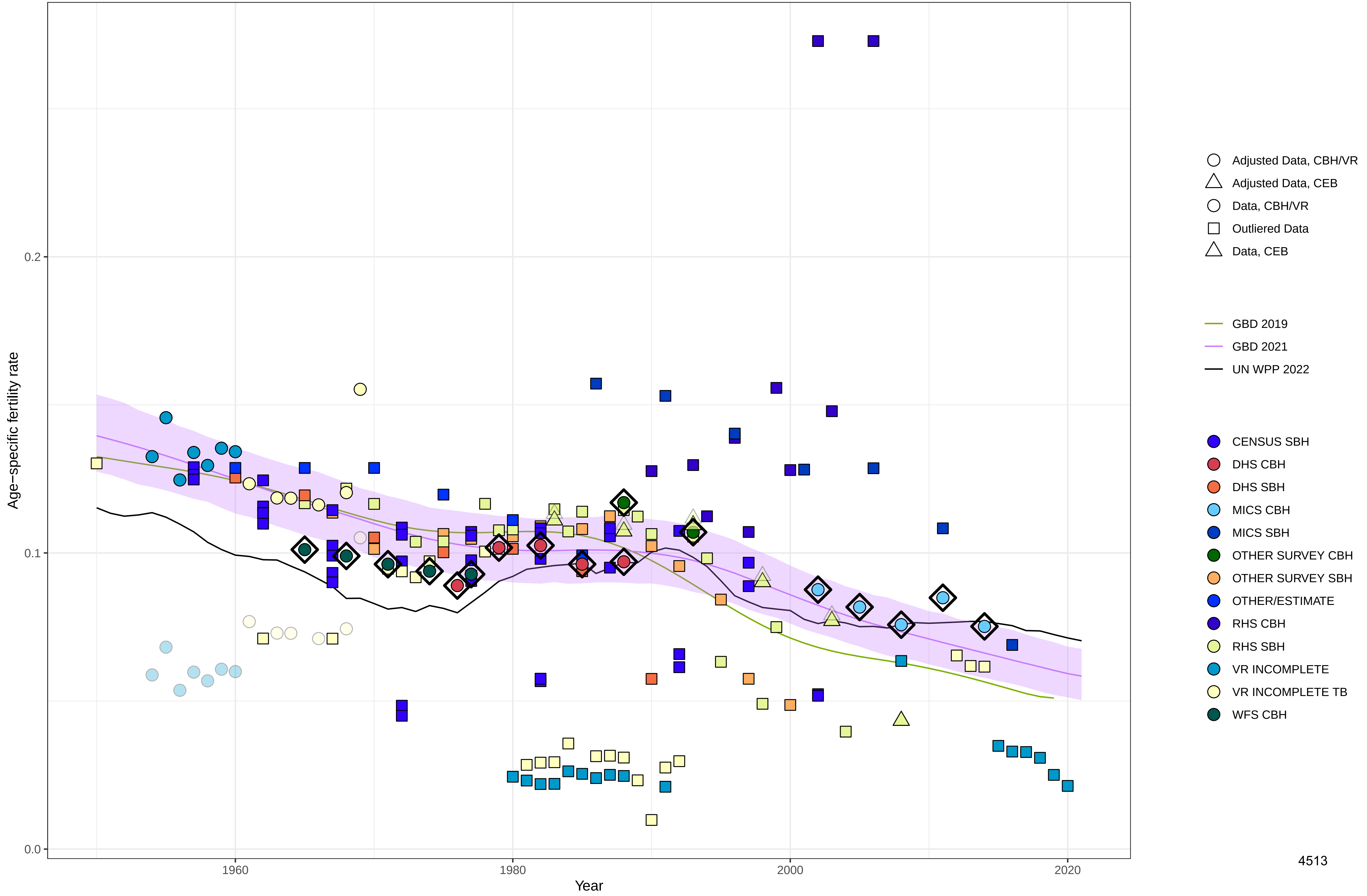

Age−specific fertility fitted trend lines, 1950−2021: Paraguay, Ages: 20−24
Data points enclosed with a diamond are reference sources

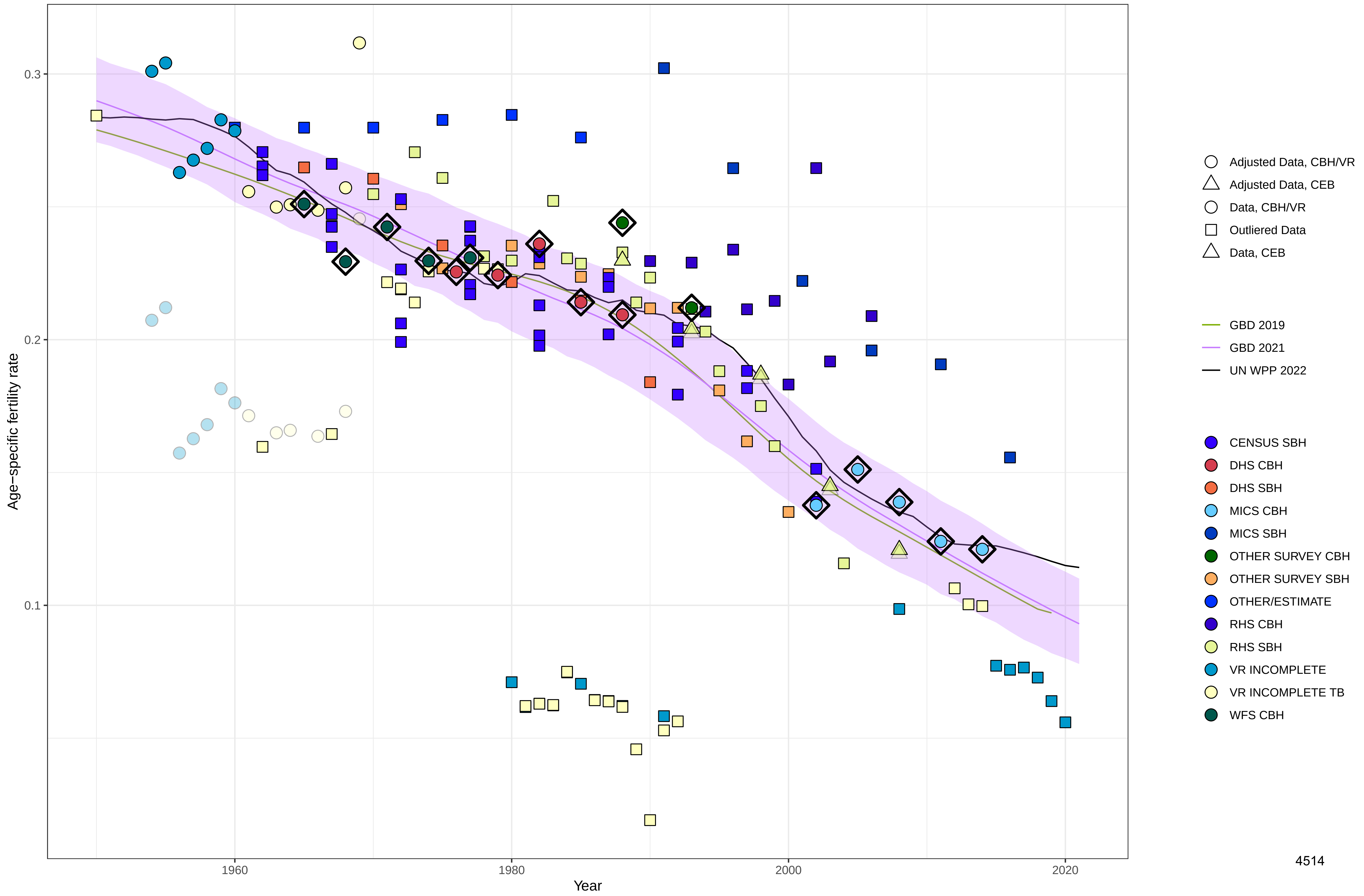

Age−specific fertility fitted trend lines, 1950−2021: Paraguay, Ages: 25−29
Data points enclosed with a diamond are reference sources

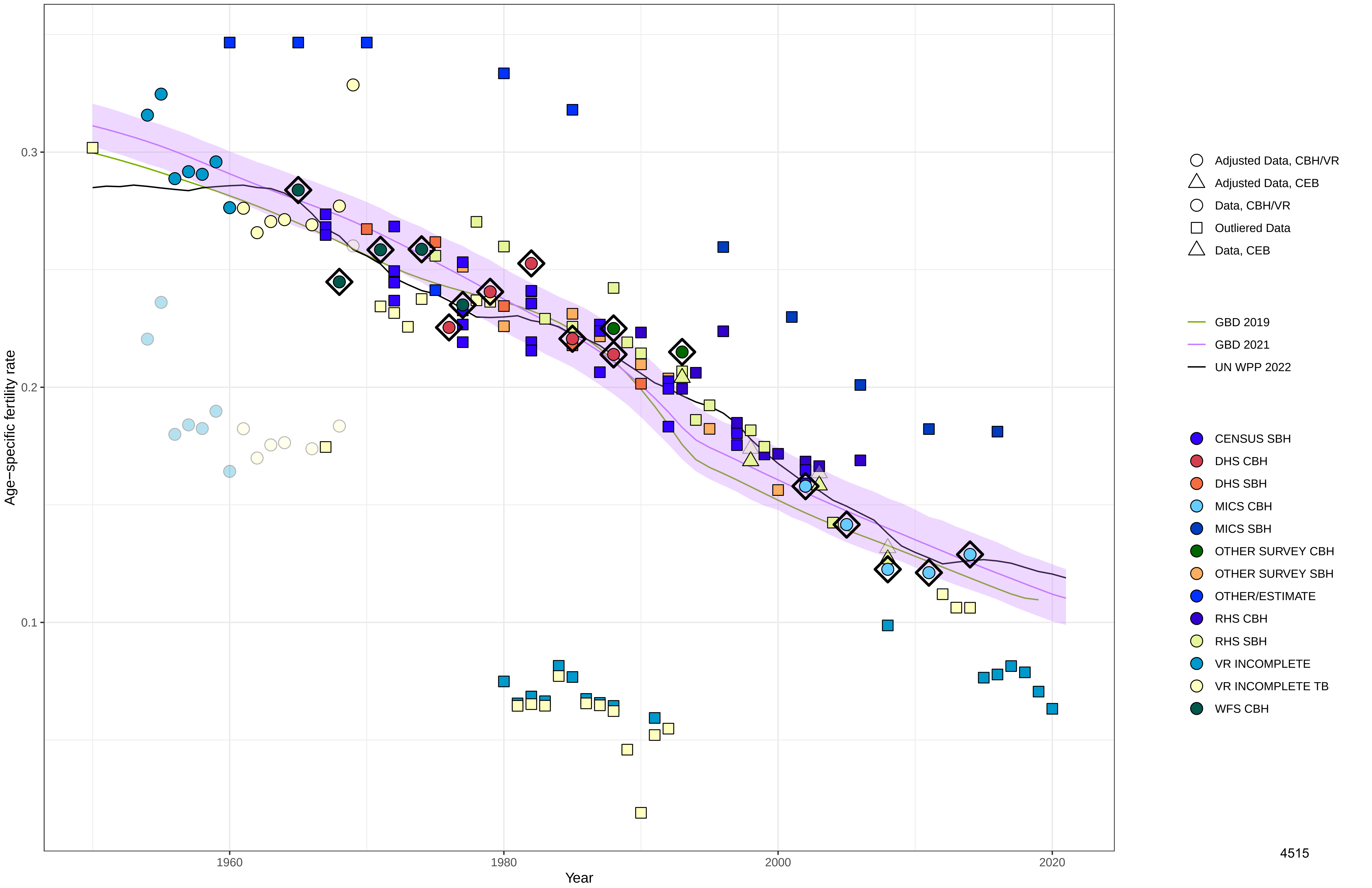

Age−specific fertility fitted trend lines, 1950−2021: Paraguay, Ages: 30−34
Data points enclosed with a diamond are reference sources

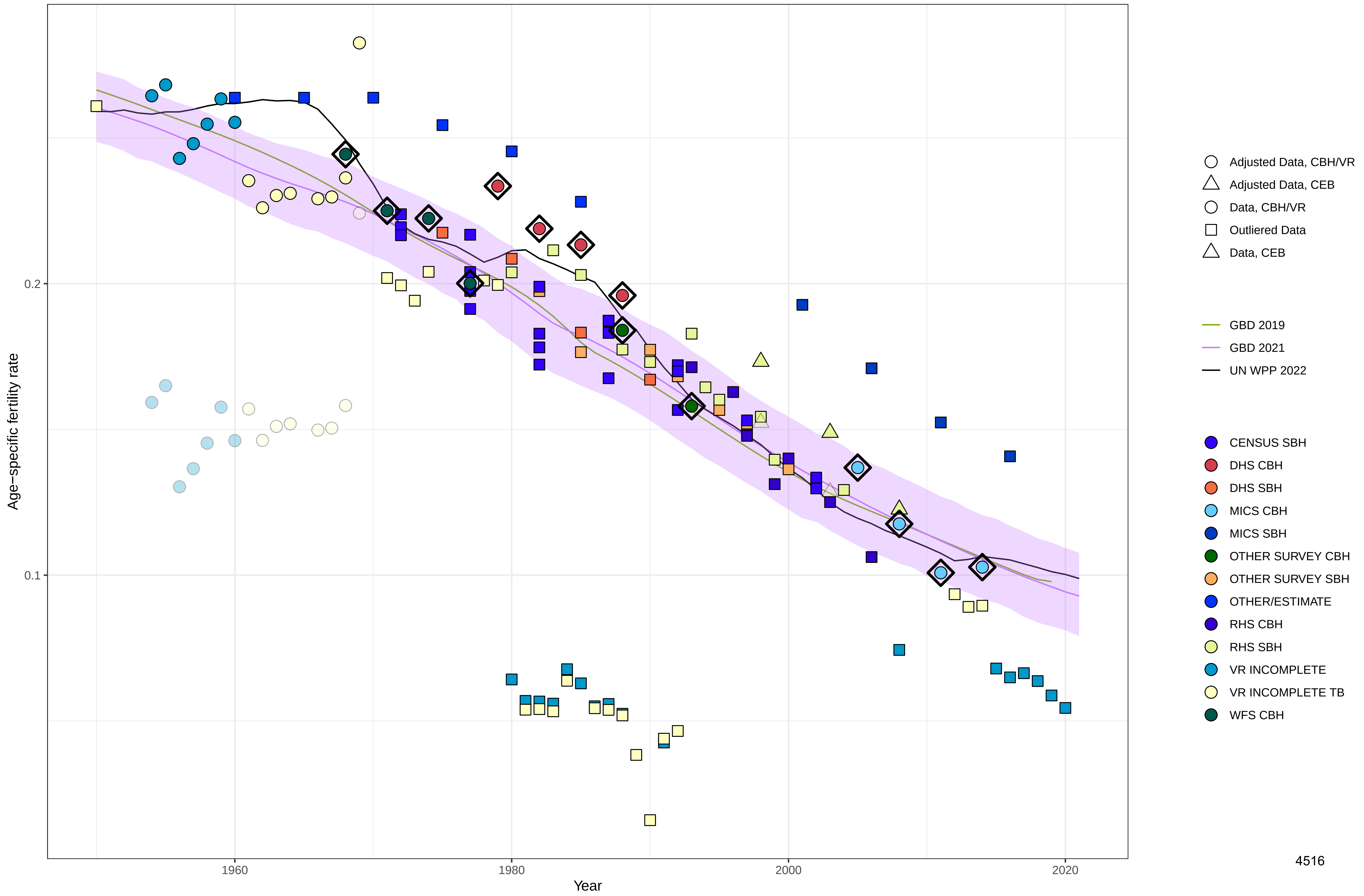

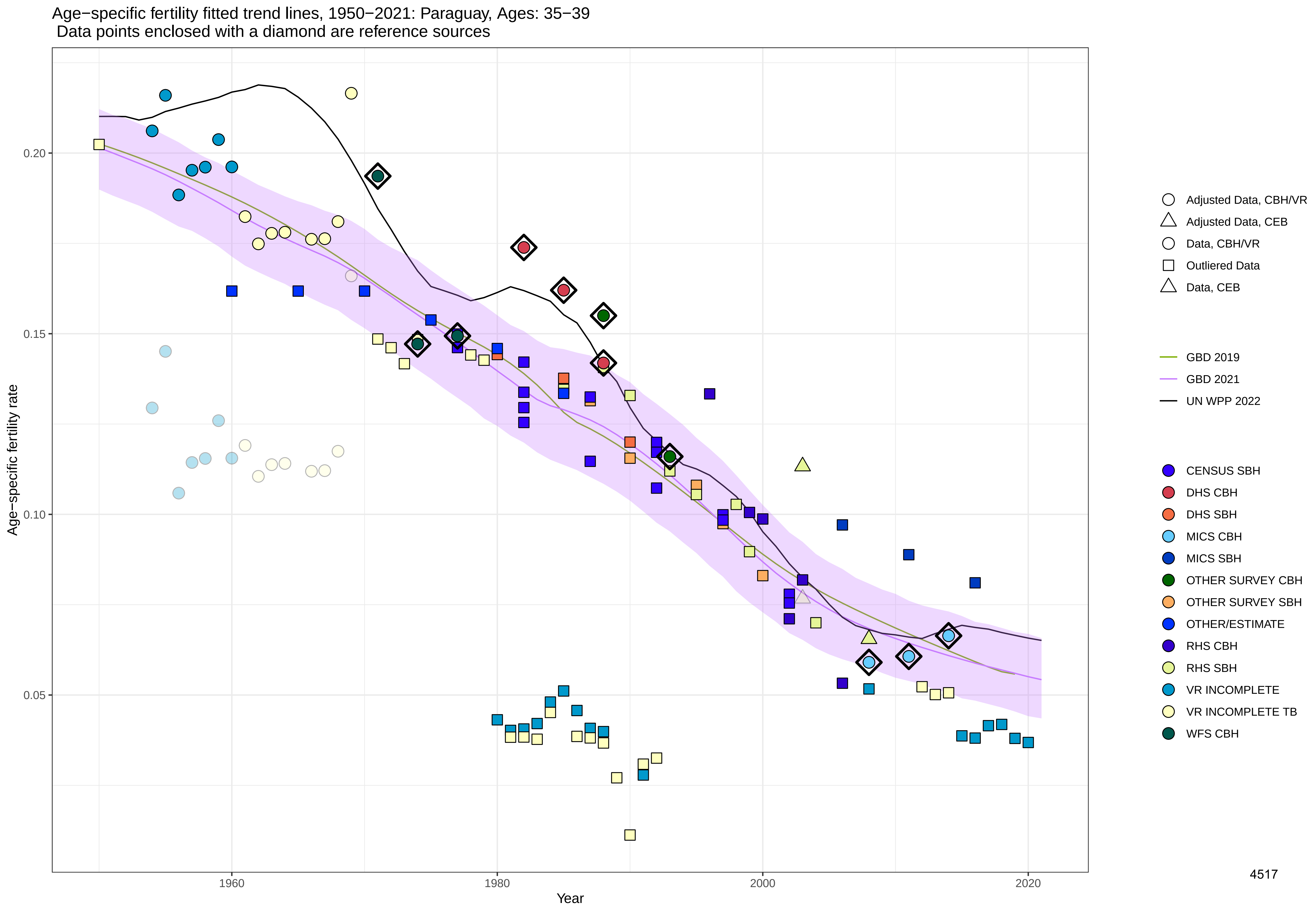

Age−specific fertility fitted trend lines, 1950−2021: Paraguay, Ages: 35−39
Data points enclosed with a diamond are reference sources
Age−specific fertility rate
Year
0.20
0.15
0.10
0.05
1960
1980
2000
2020
Adjusted Data, CBH/VR
Adjusted Data, CEB
Data, CBH/VR
Outliered Data
Data, CEB
GBD 2019
GBD 2021
UN WPP 2022
CENSUS SBH
DHS CBH
DHS SBH
MICS CBH
MICS SBH
OTHER SURVEY CBH
OTHER SURVEY SBH
OTHER/ESTIMATE
RHS CBH
RHS SBH
VR INCOMPLETE
VR INCOMPLETE TB
WFS CBH

4517

Age−specific fertility fitted trend lines, 1950−2021: Paraguay, Ages: 40−44
Data points enclosed with a diamond are reference sources

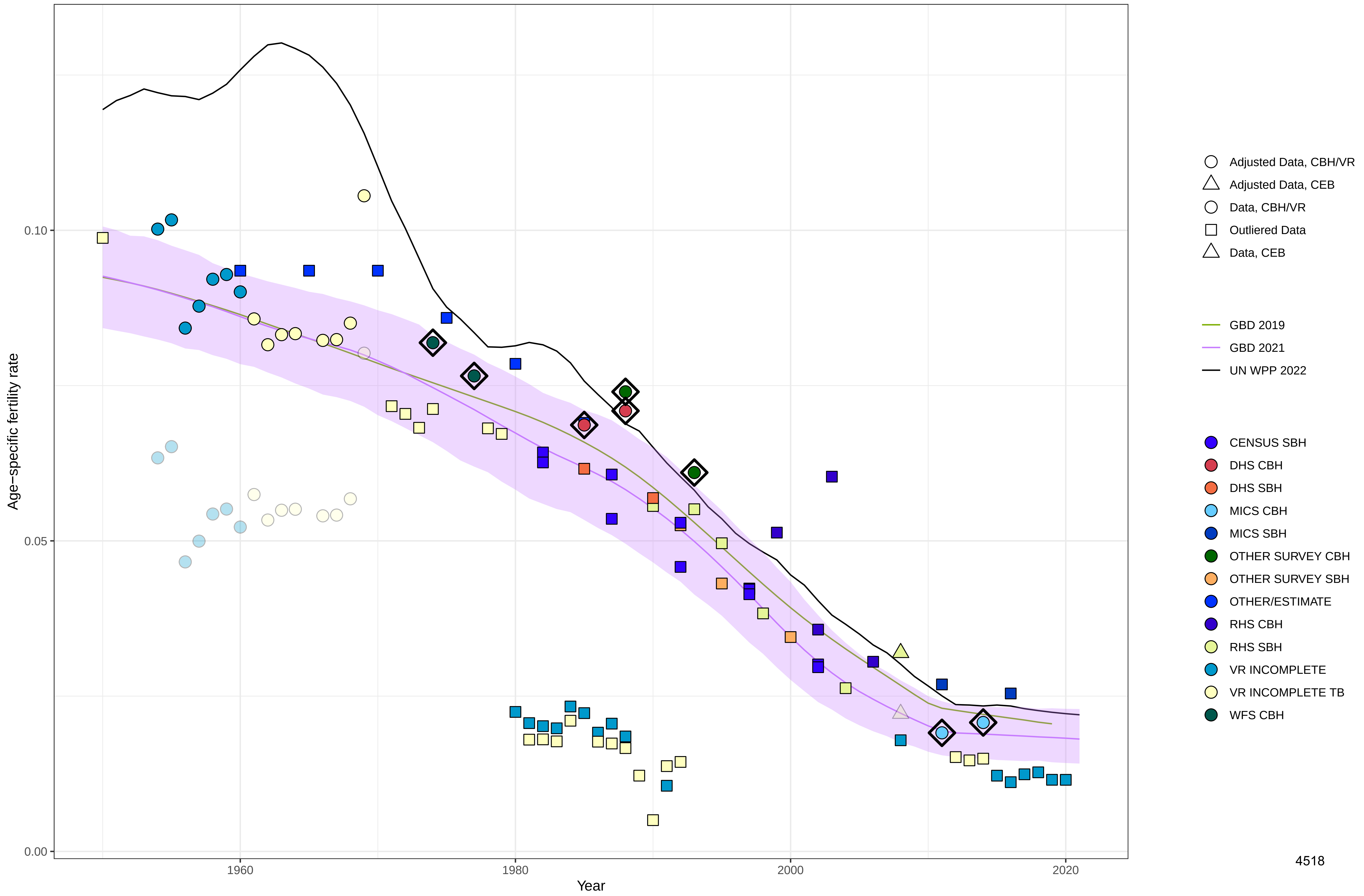

Age−specific fertility fitted trend lines, 1950−2021: Paraguay, Ages: 45−49
Data points enclosed with a diamond are reference sources

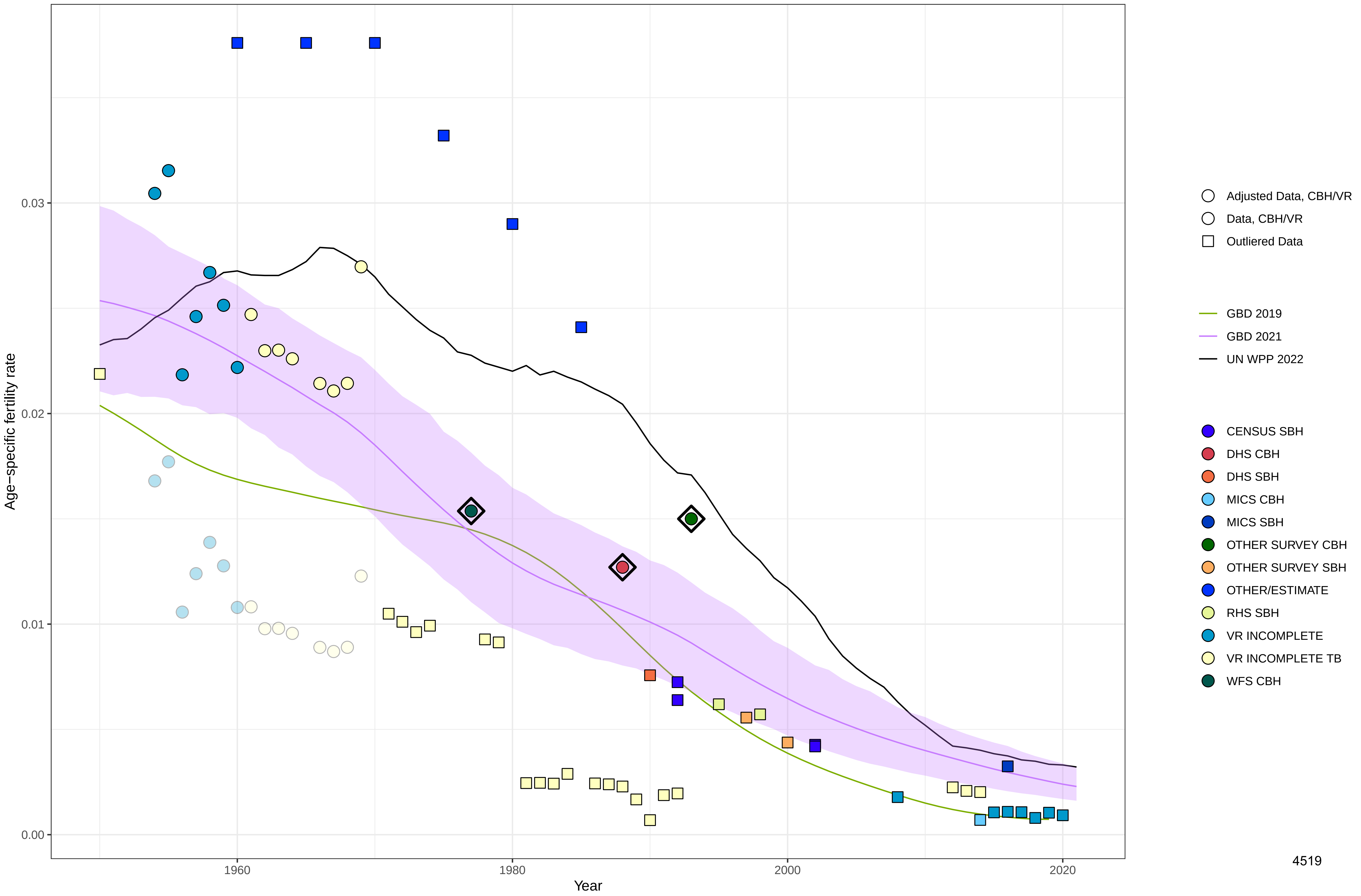

Age−specific fertility fitted trend lines, 1950−2021: Paraguay, Ages: 50−54
Data points enclosed with a diamond are reference sources

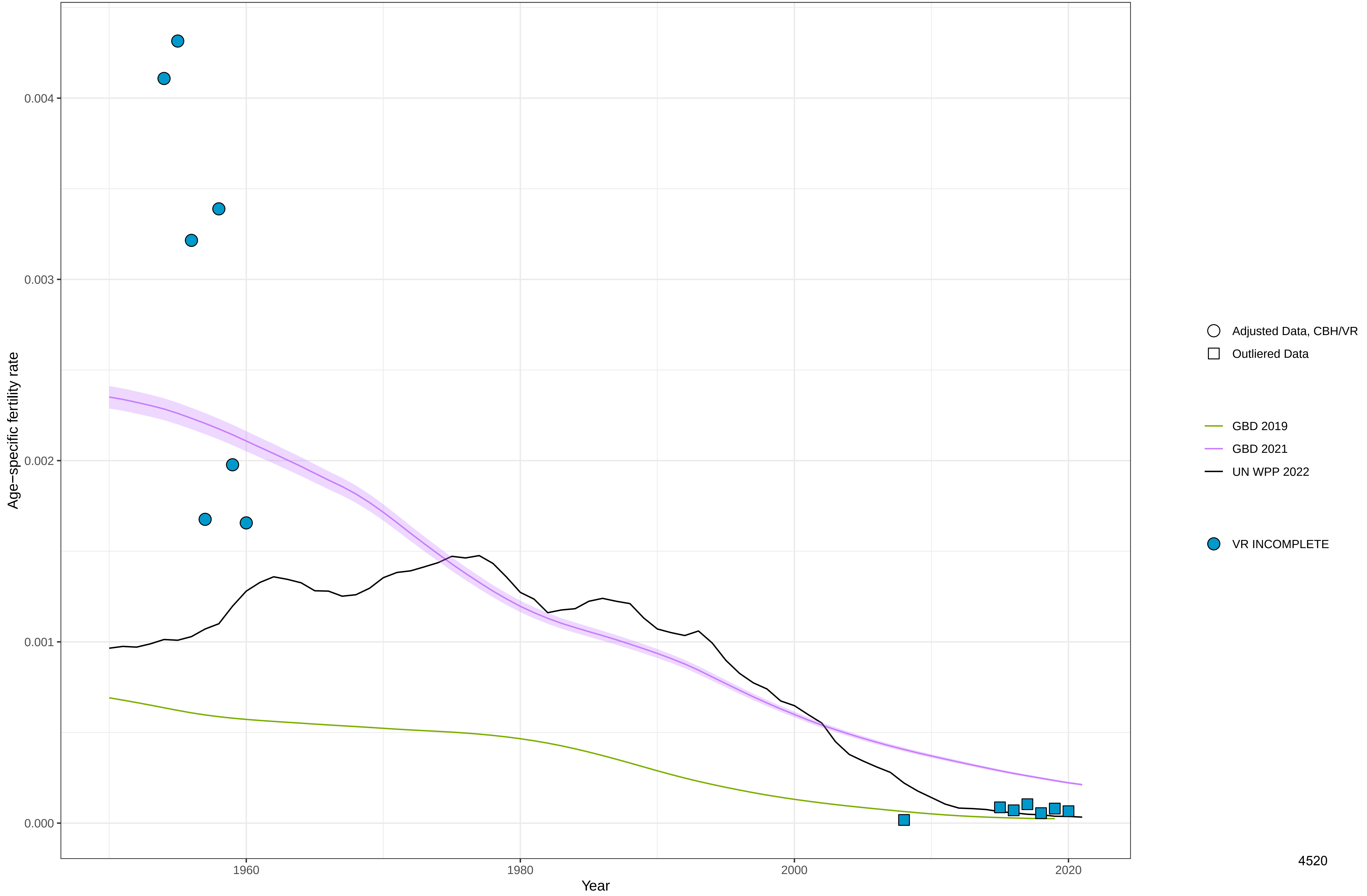

Total fertility rate fitted trend lines, 1950−2021: Paraguay TFR
Data points enclosed with a diamond are reference sources

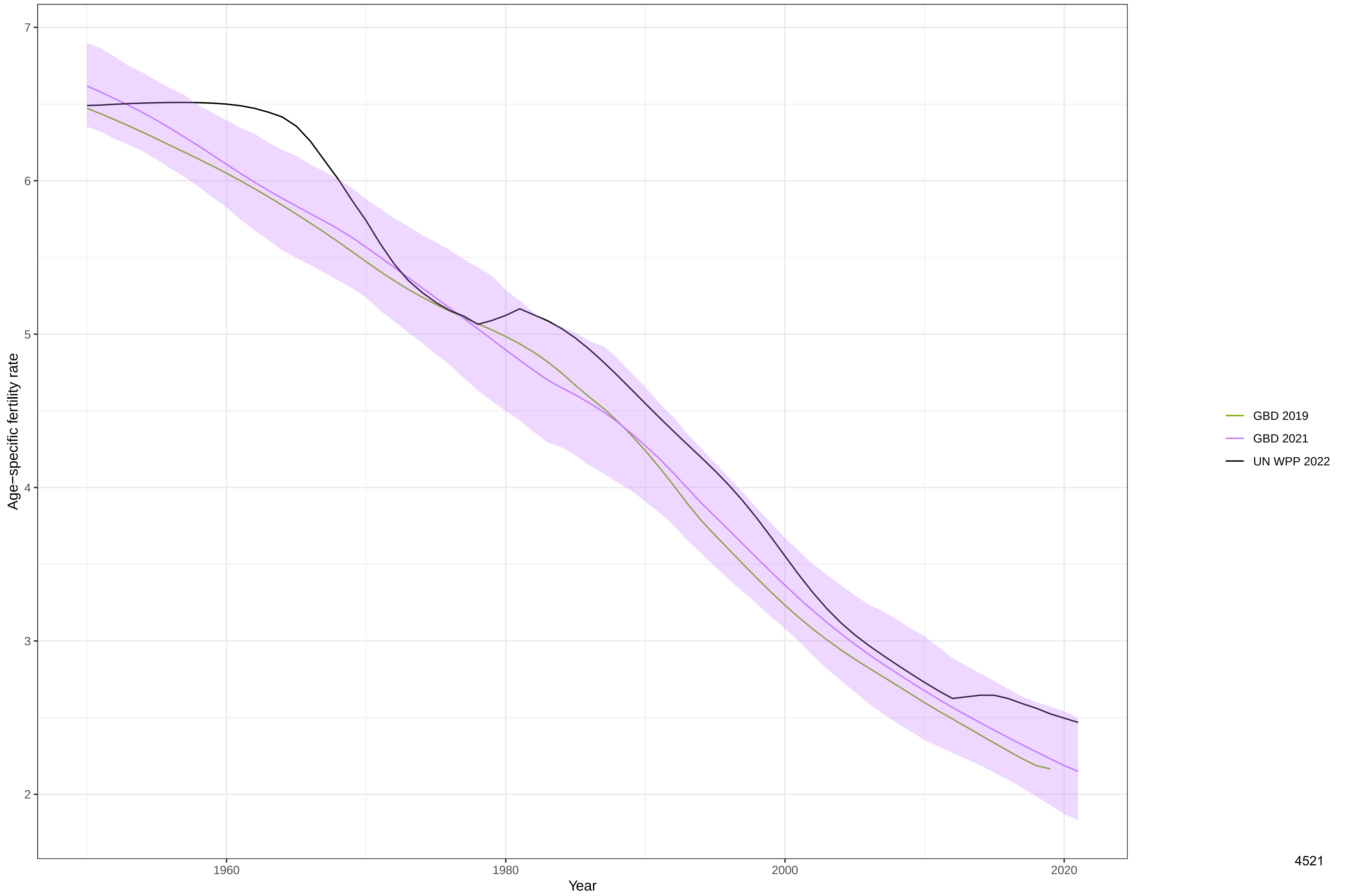

Age−specific fertility fitted trend lines, 1950−2021: Afghanistan, Ages: 10−14
Data points enclosed with a diamond are reference sources

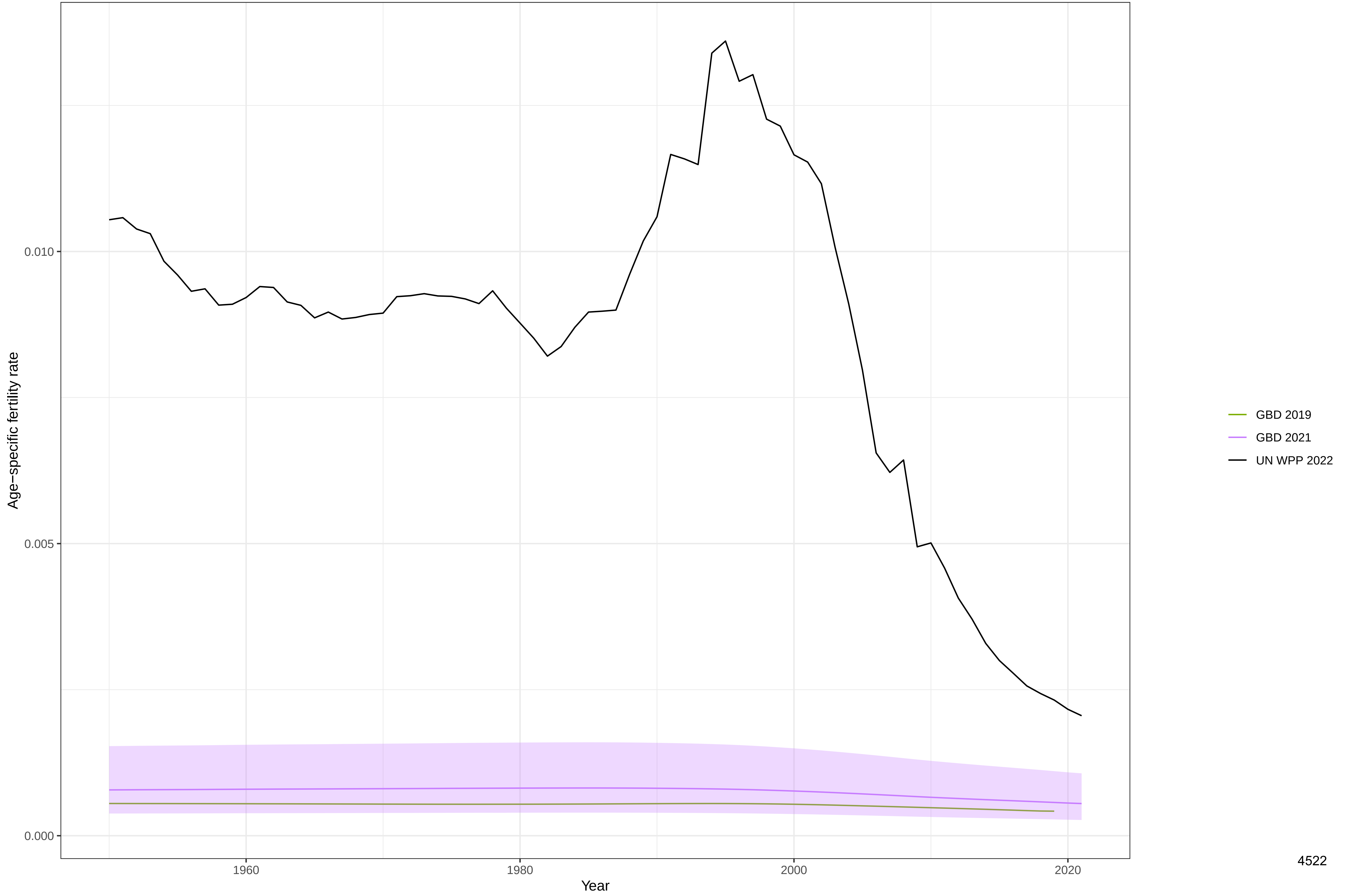

Age−specific fertility fitted trend lines, 1950−2021: Afghanistan, Ages: 15−19
Data points enclosed with a diamond are reference sources

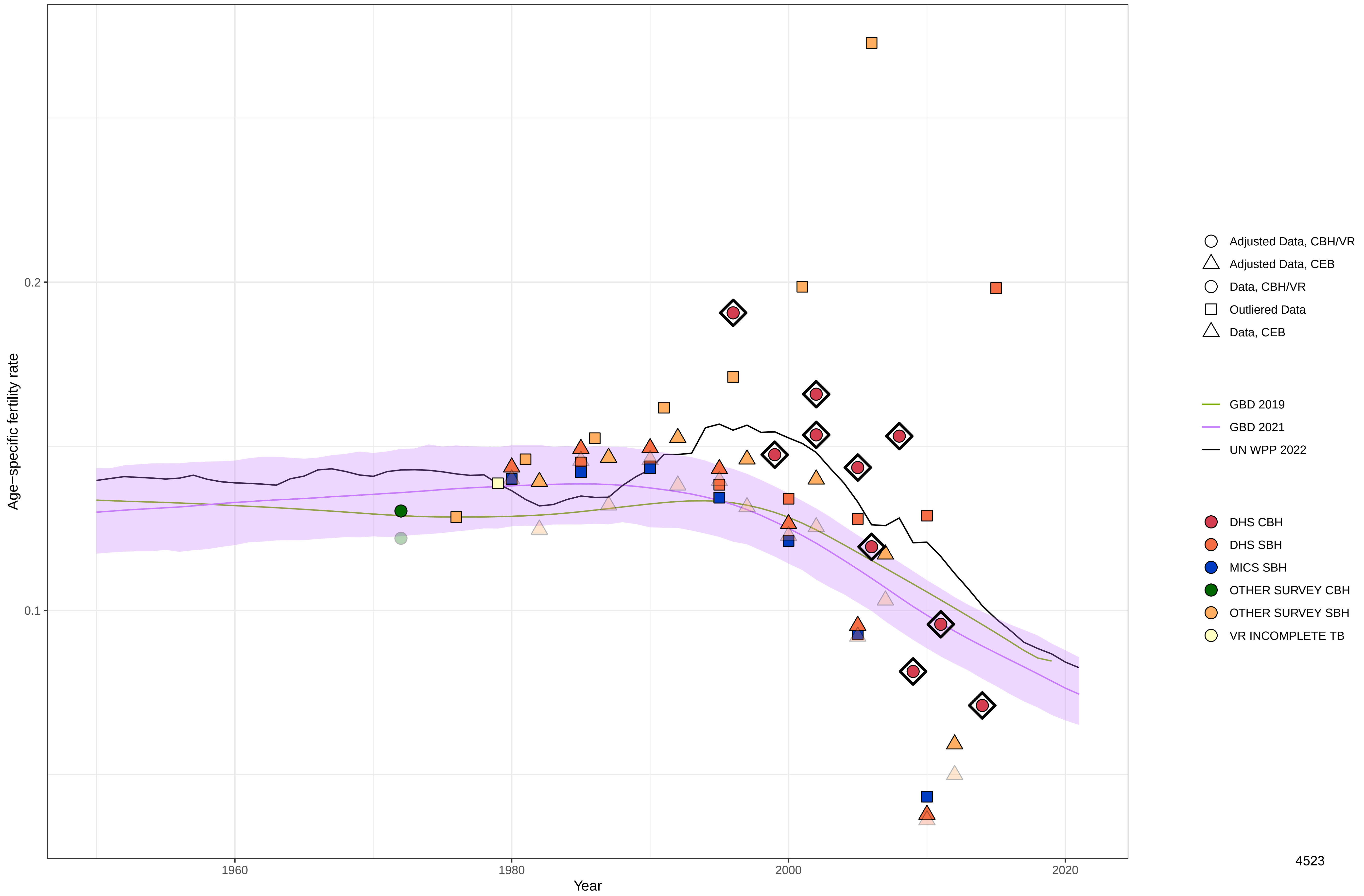

Age−specific fertility fitted trend lines, 1950−2021: Afghanistan, Ages: 20−24
Data points enclosed with a diamond are reference sources

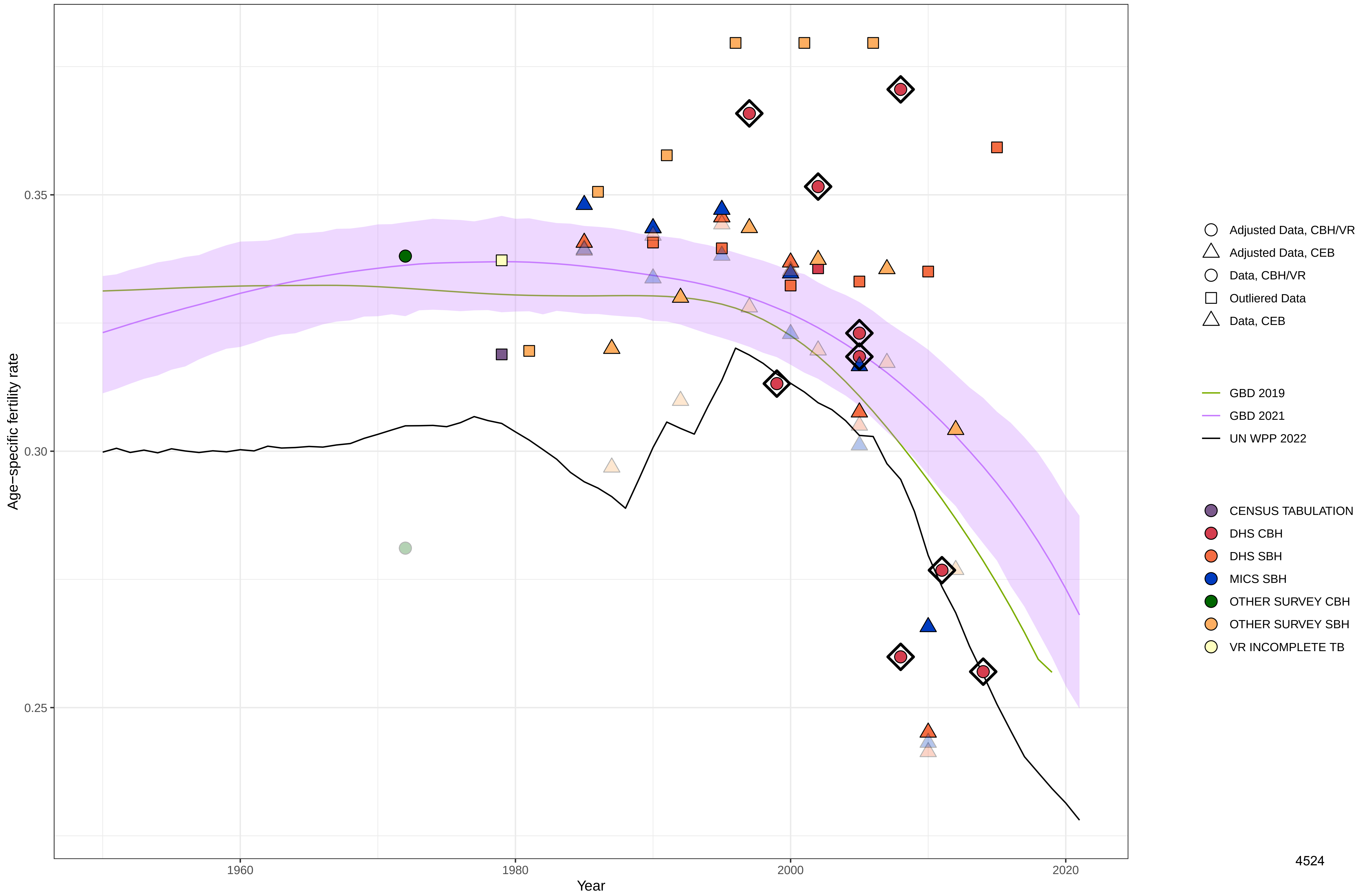

Age−specific fertility fitted trend lines, 1950−2021: Afghanistan, Ages: 25−29
Data points enclosed with a diamond are reference sources

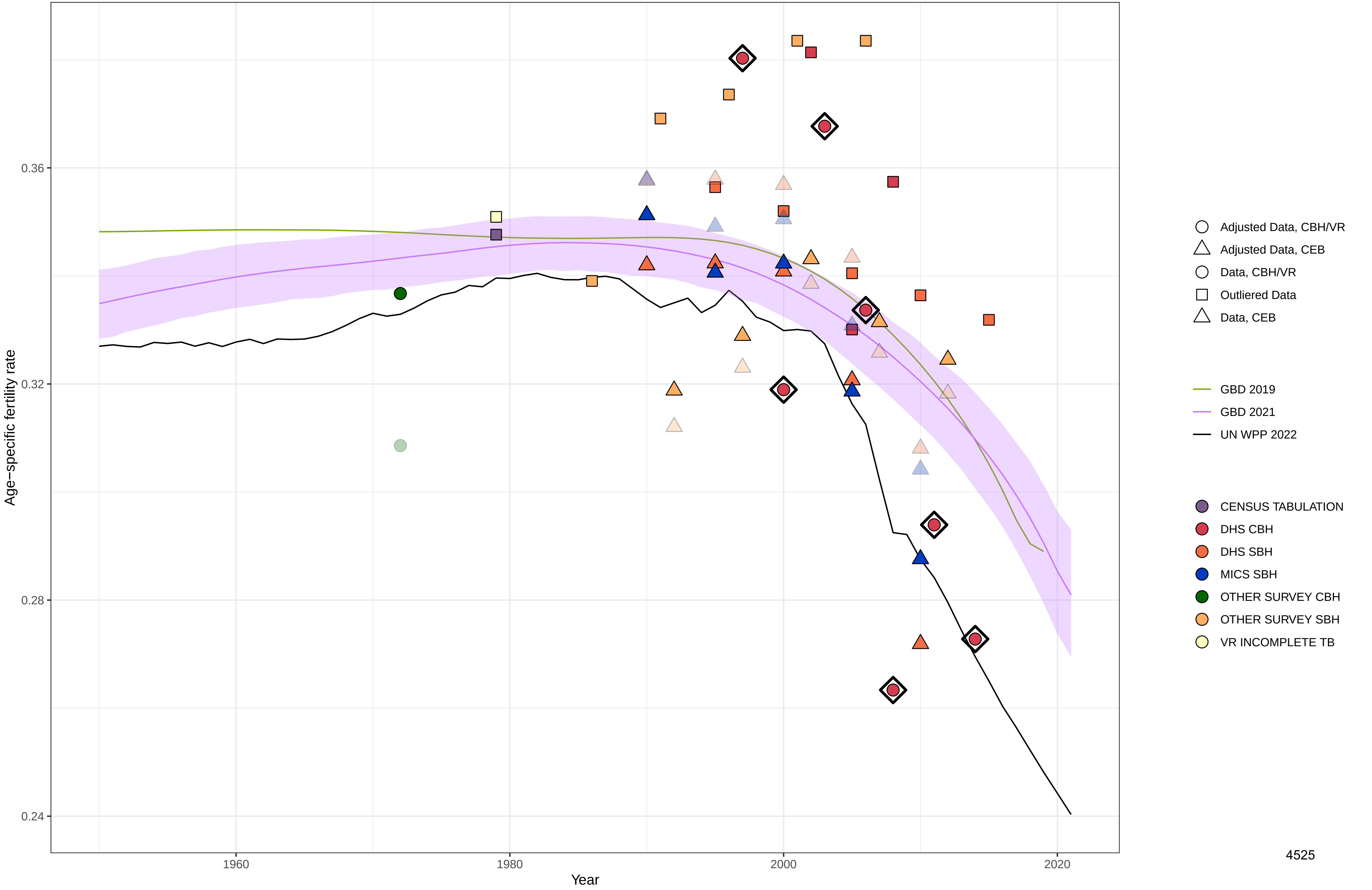

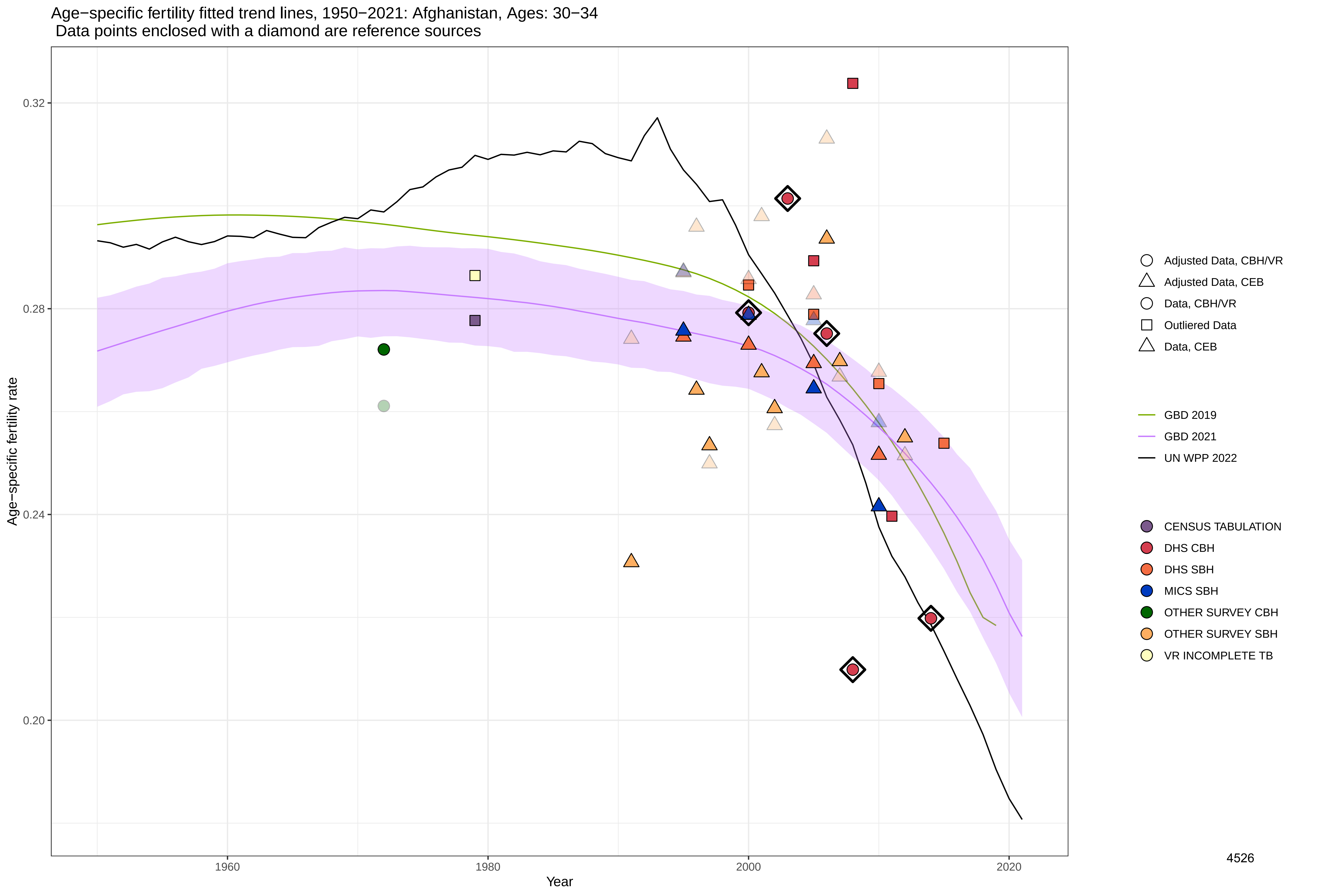

Age−specific fertility fitted trend lines, 1950−2021: Afghanistan, Ages: 30−34
Data points enclosed with a diamond are reference sources
Age−specific fertility rate
0.32
0.28
0.24
0.20
1960
1980
2000
2020
Year
Adjusted Data, CBH/VR
Adjusted Data, CEB
Data, CBH/VR
Outliered Data
Data, CEB
GBD 2019
GBD 2021
UN WPP 2022
CENSUS TABULATION
DHS CBH
DHS SBH
MICS SBH
OTHER SURVEY CBH
OTHER SURVEY SBH
VR INCOMPLETE TB

Age−specific fertility fitted trend lines, 1950−2021: Afghanistan, Ages: 35−39
Data points enclosed with a diamond are reference sources

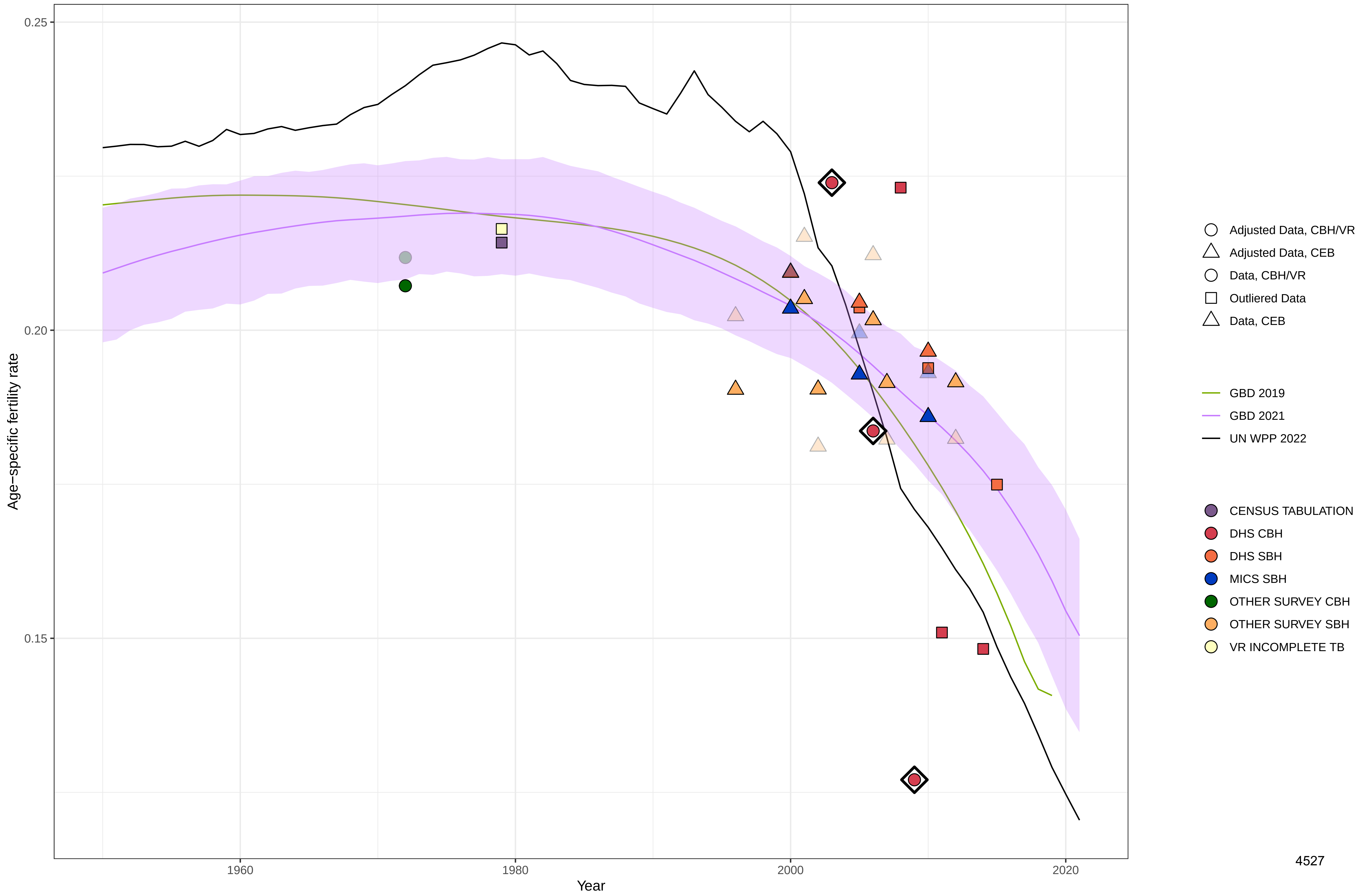

Age−specific fertility fitted trend lines, 1950−2021: Afghanistan, Ages: 40−44
Data points enclosed with a diamond are reference sources

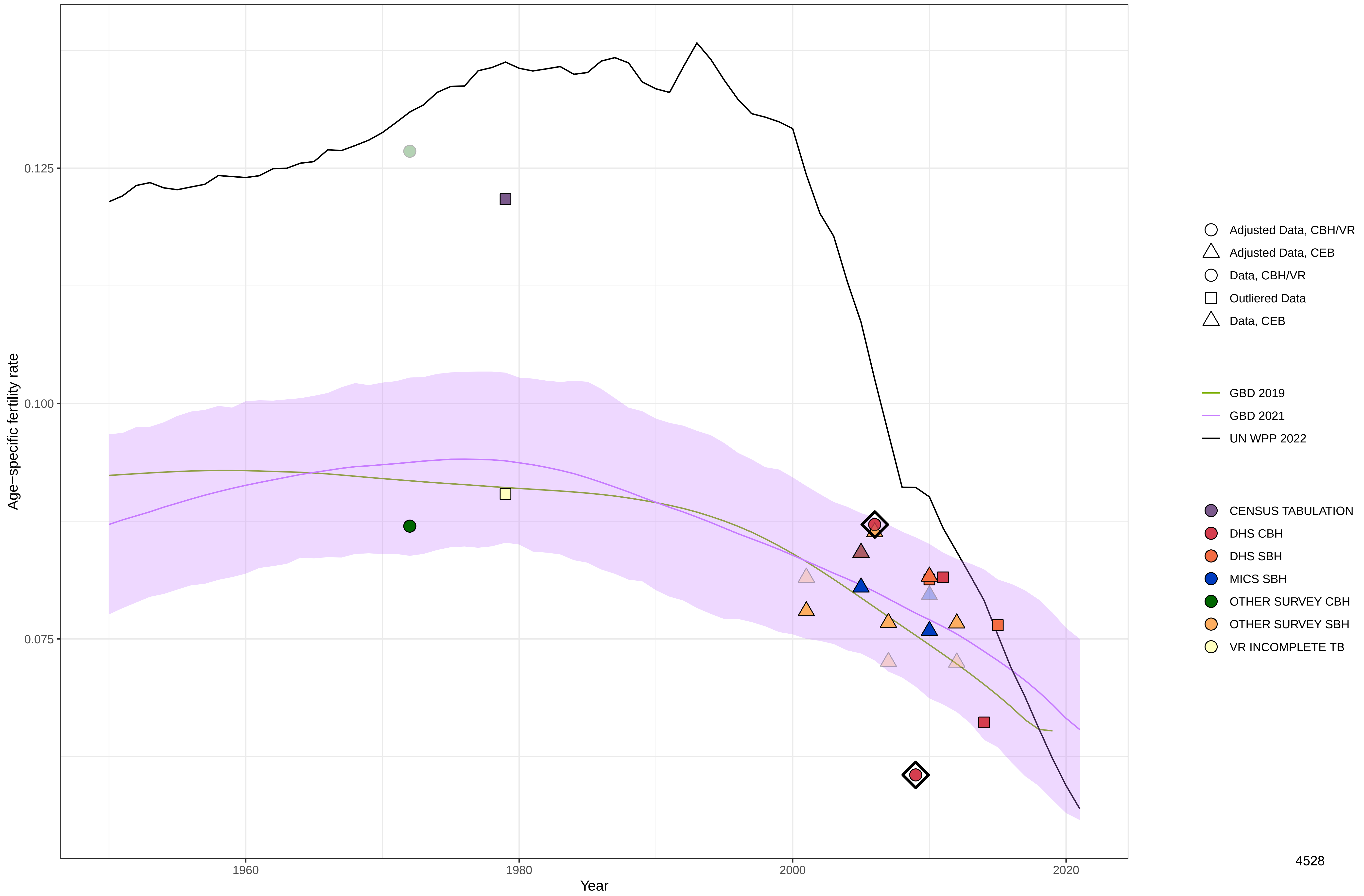

Age−specific fertility fitted trend lines, 1950−2021: Afghanistan, Ages: 45−49
Data points enclosed with a diamond are reference sources

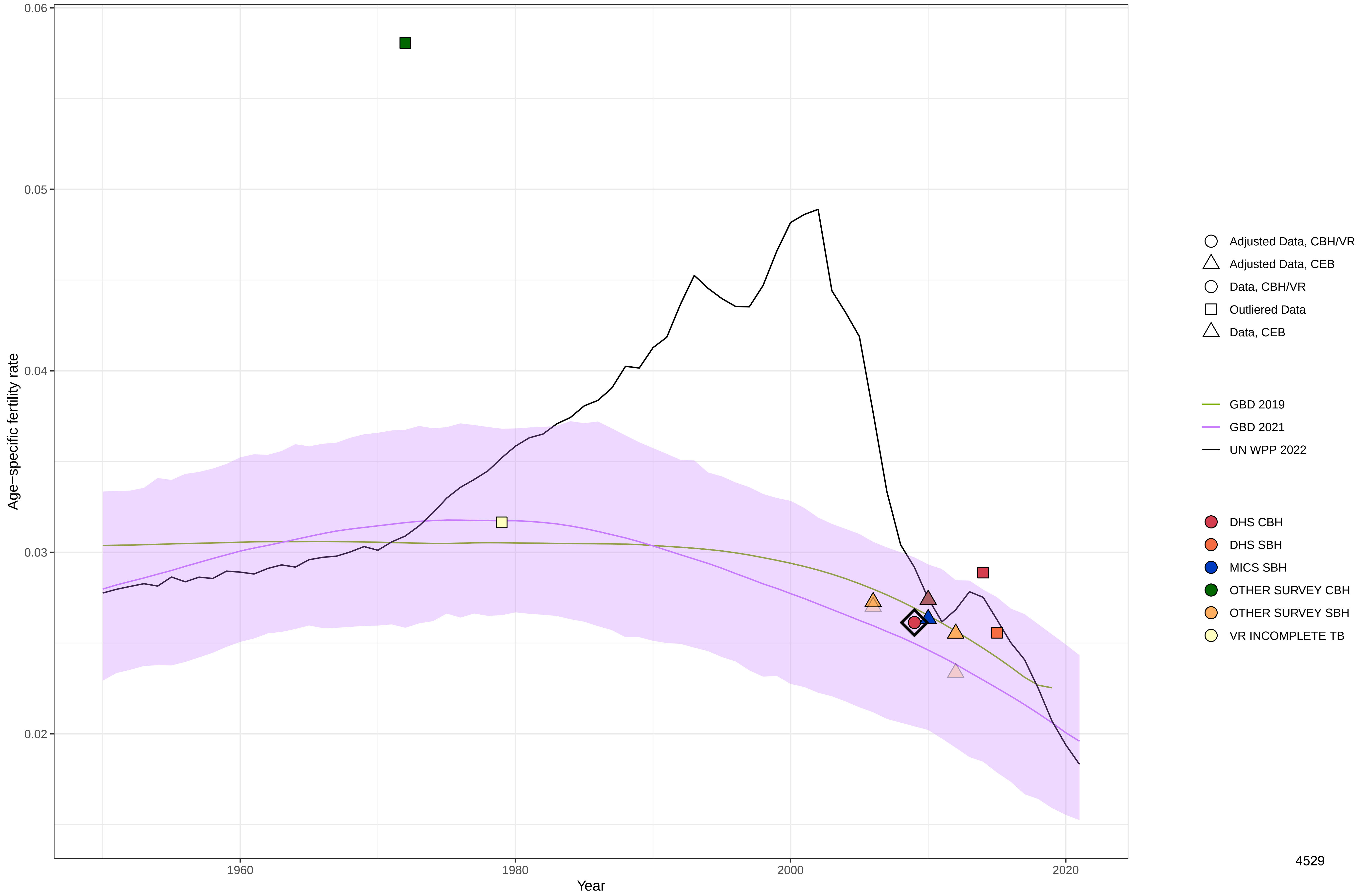

Age−specific fertility fitted trend lines, 1950−2021: Afghanistan, Ages: 50−54
Data points enclosed with a diamond are reference sources

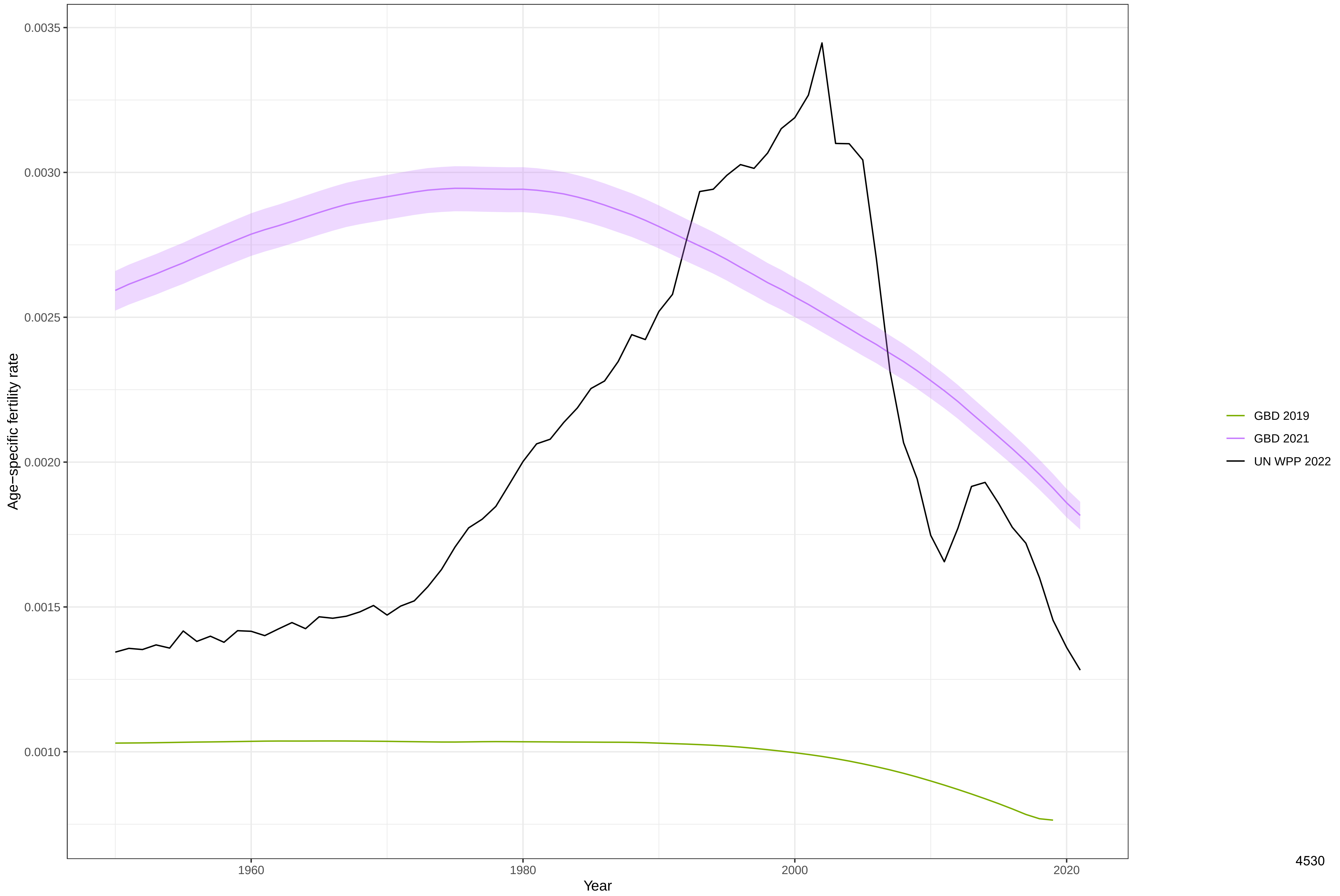

Total fertility rate fitted trend lines, 1950−2021: Afghanistan TFR
Data points enclosed with a diamond are reference sources

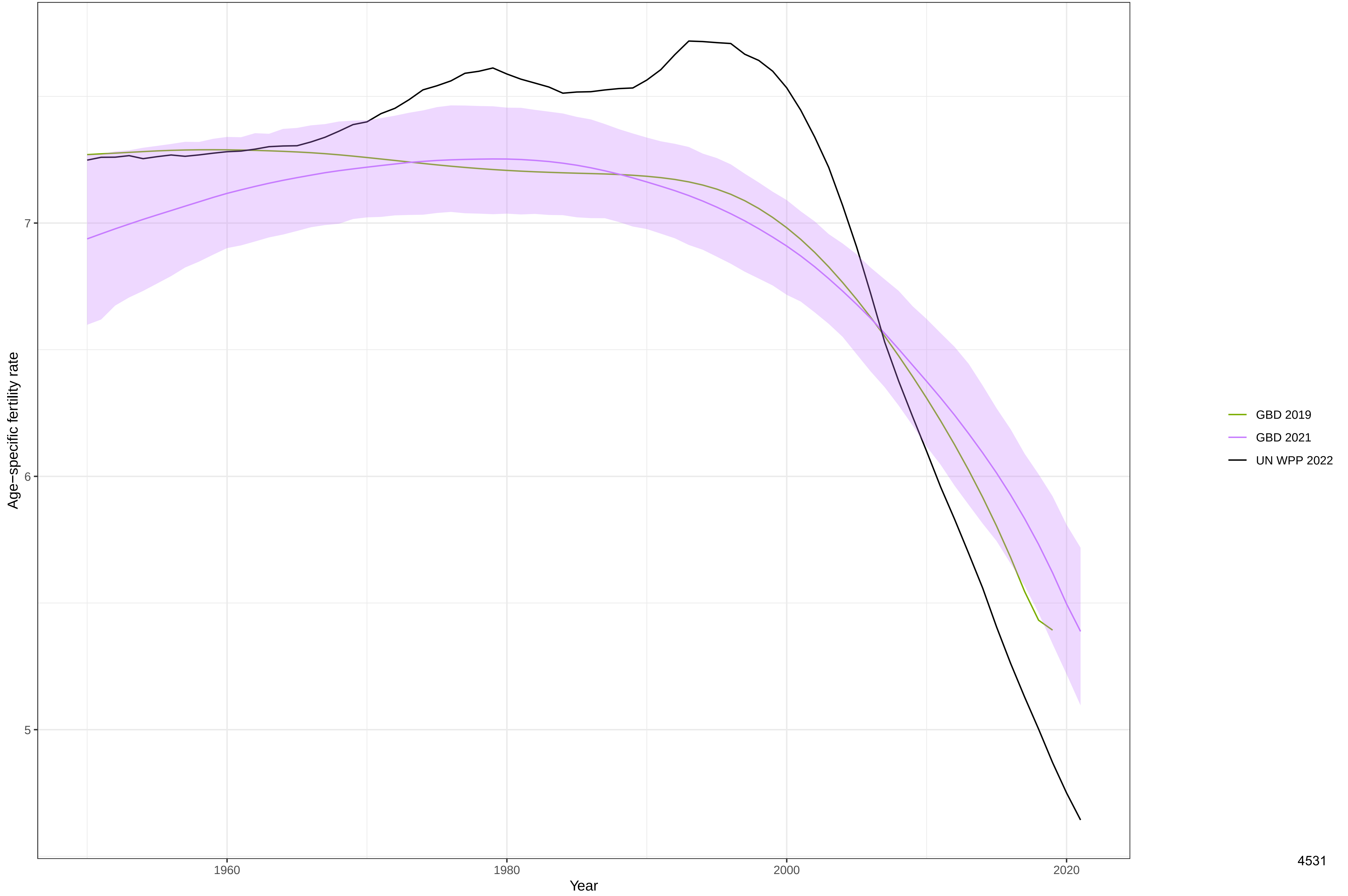

Age−specific fertility fitted trend lines, 1950−2021: Algeria, Ages: 10−14
Data points enclosed with a diamond are reference sources

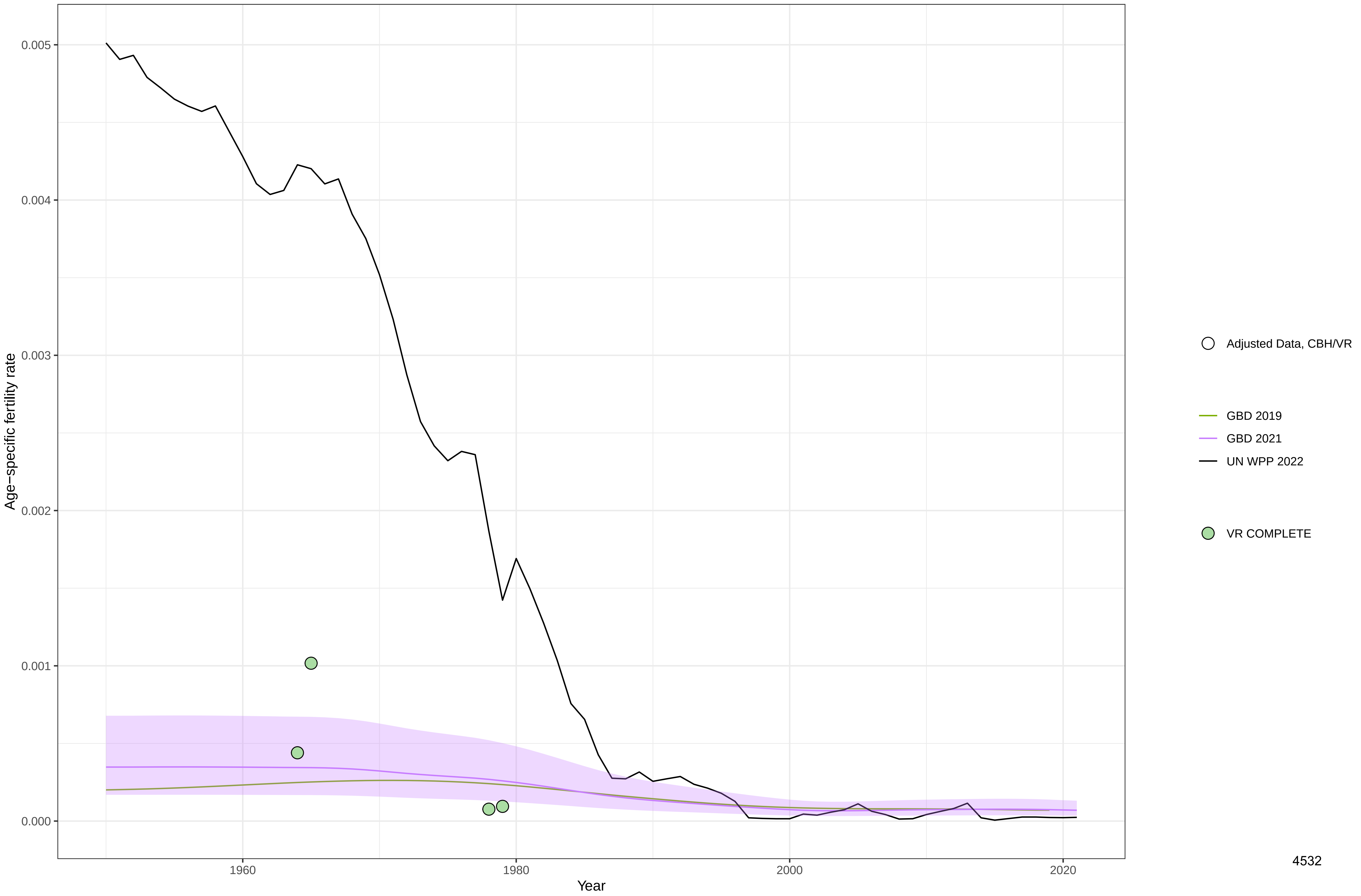

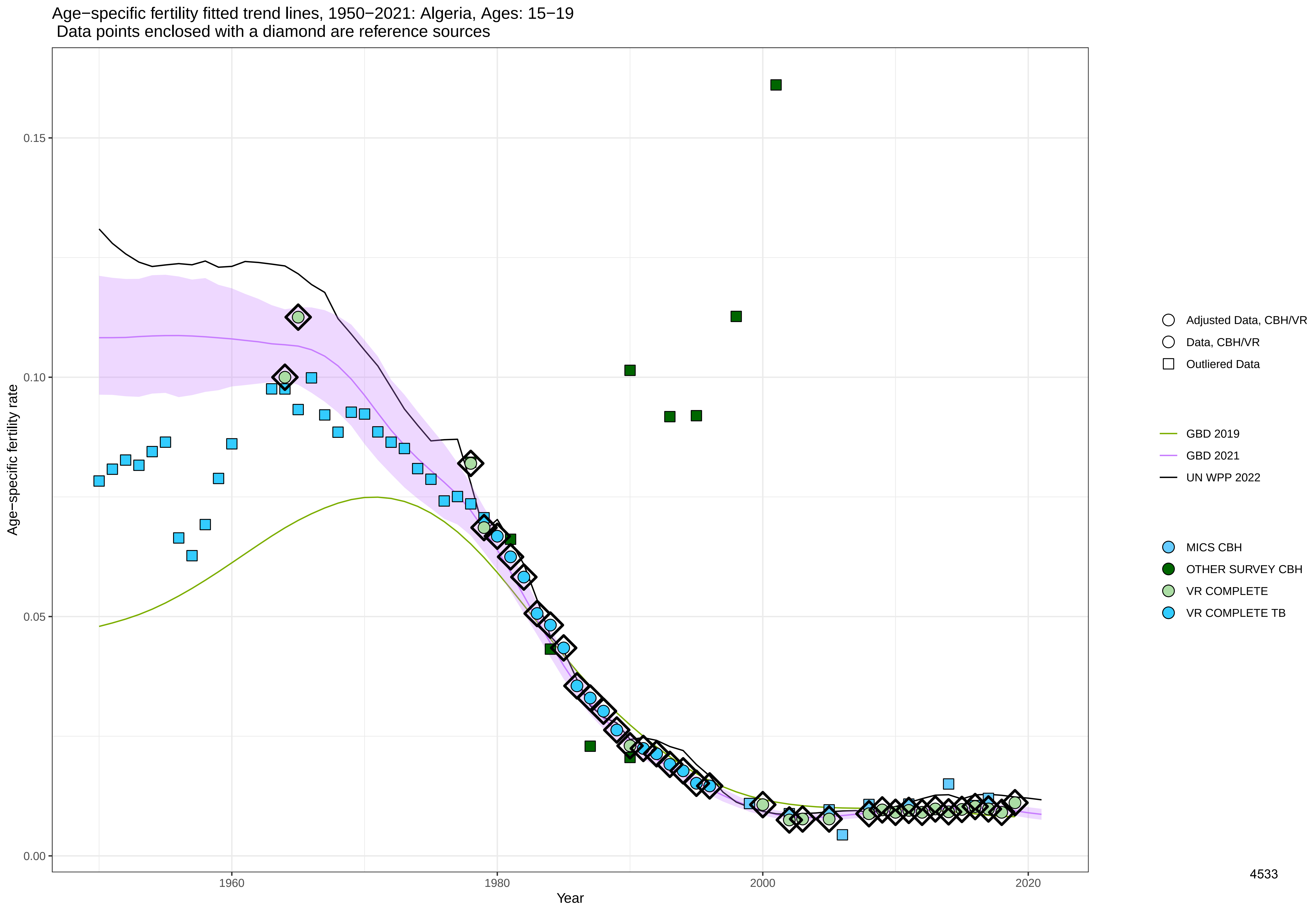
Age−specific fertility fitted trend lines, 1950−2021: Algeria, Ages: 15−19
Data points enclosed with a diamond are reference sources
Age−specific fertility rate
0.15
0.10
0.05
0.00
1960
1980
2000
2020
Year
Adjusted Data, CBH/VR
Data, CBH/VR
Outliered Data
GBD 2019
GBD 2021
UN WPP 2022
MICS CBH
OTHER SURVEY CBH
VR COMPLETE
VR COMPLETE TB

Age−specific fertility fitted trend lines, 1950−2021: Algeria, Ages: 20−24
Data points enclosed with a diamond are reference sources

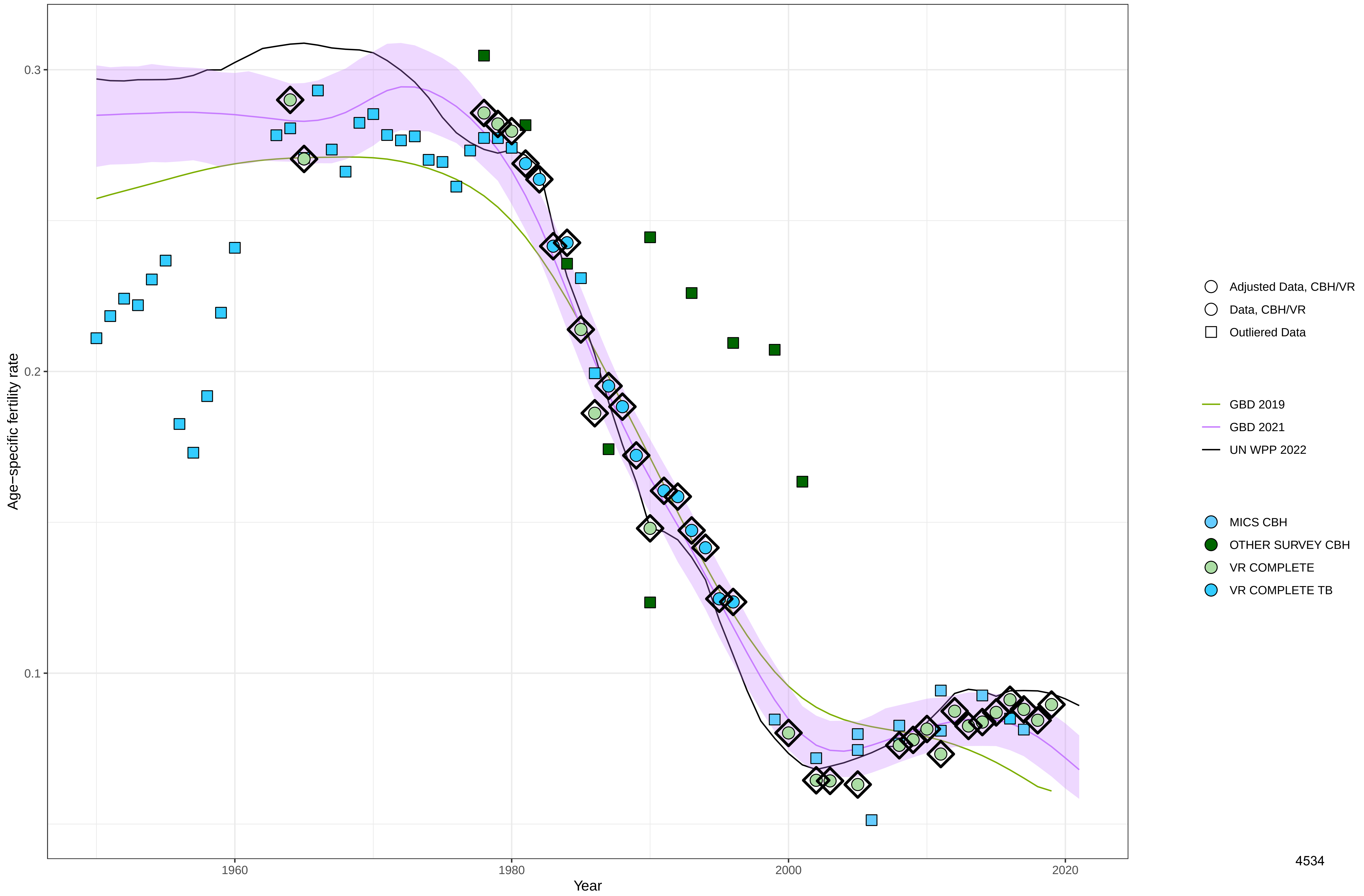

Age−specific fertility fitted trend lines, 1950−2021: Algeria, Ages: 25−29
Data points enclosed with a diamond are reference sources

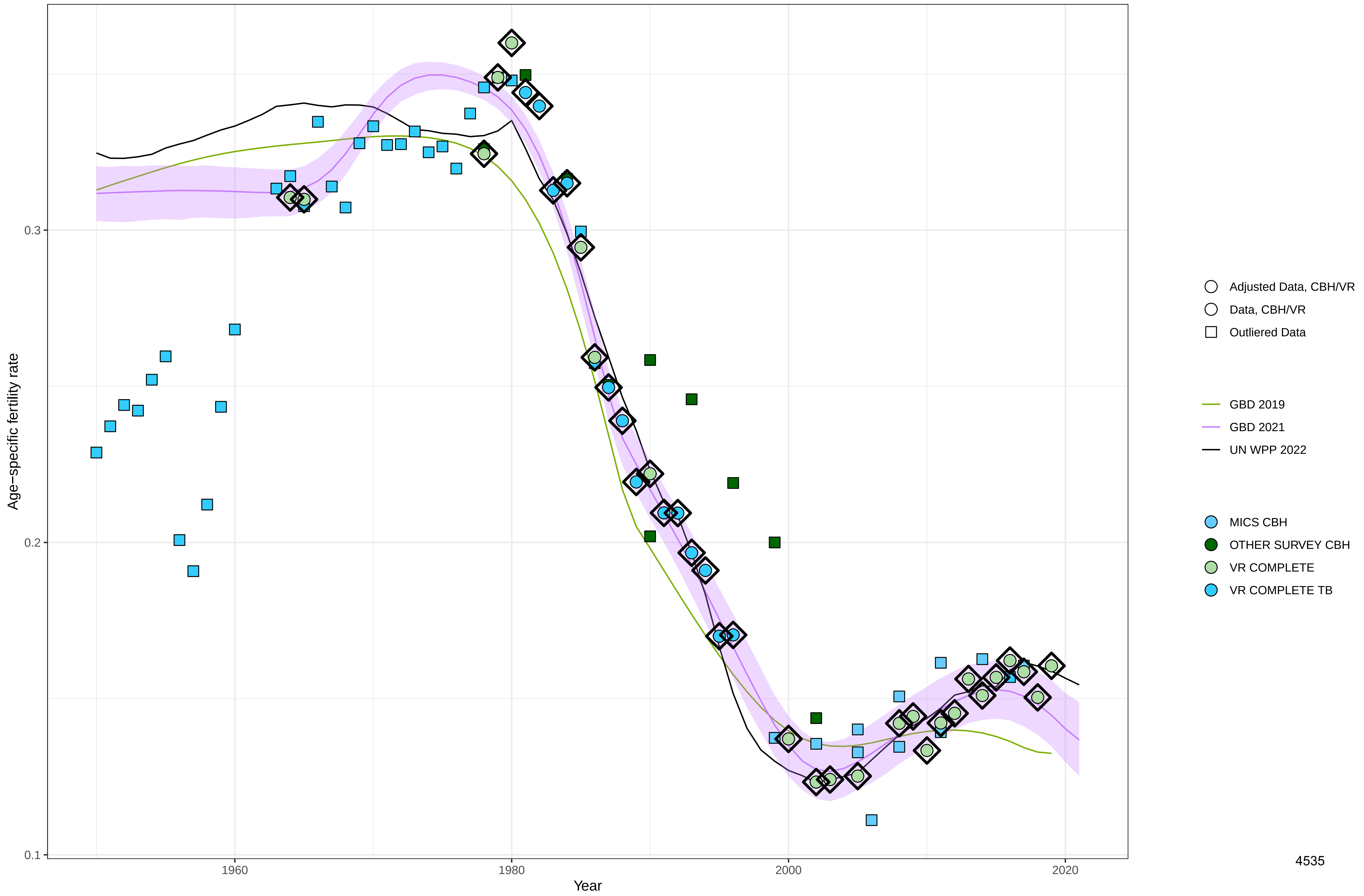

Age−specific fertility fitted trend lines, 1950−2021: Algeria, Ages: 30−34
Data points enclosed with a diamond are reference sources

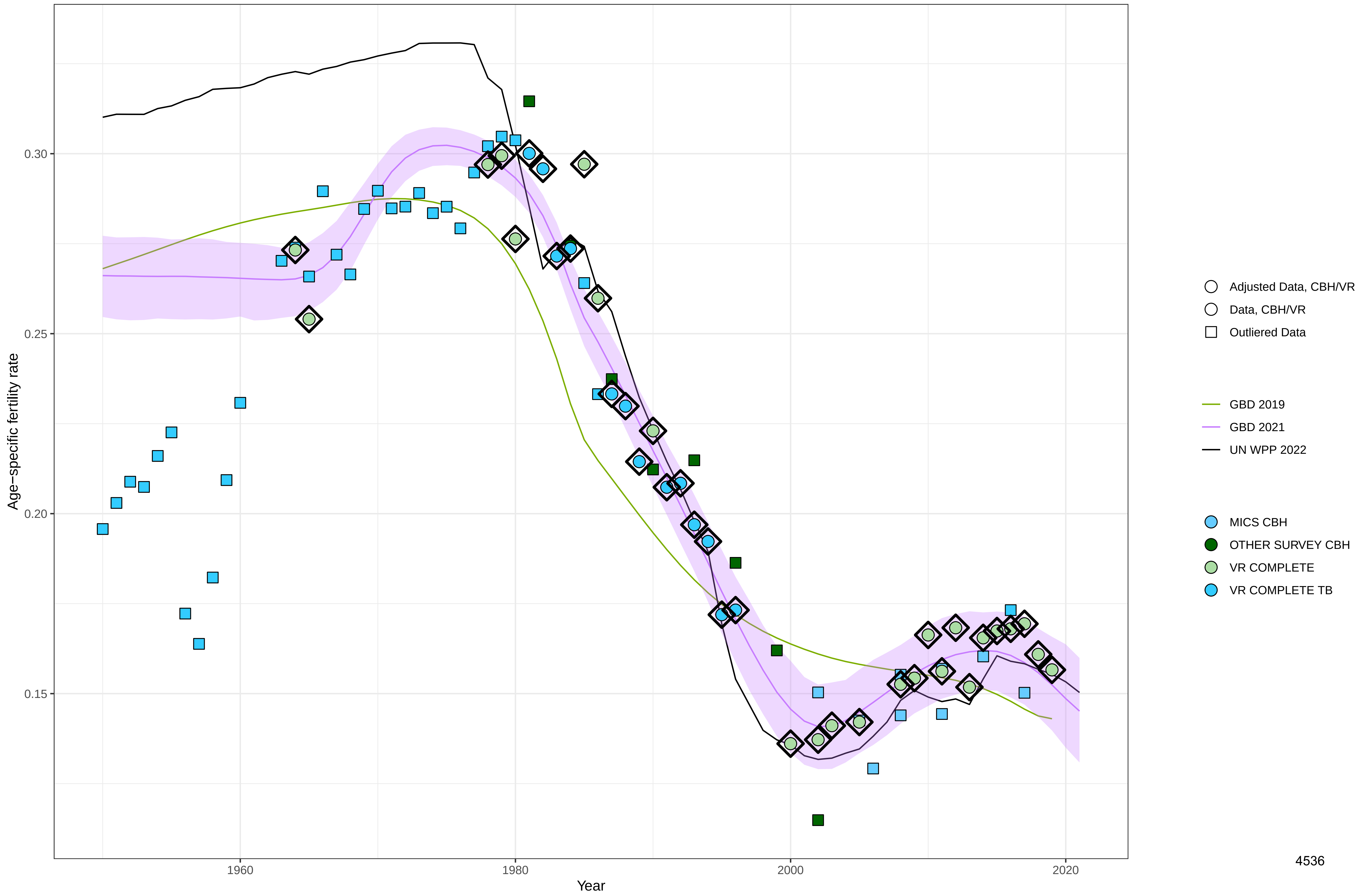

Age−specific fertility fitted trend lines, 1950−2021: Algeria, Ages: 35−39
Data points enclosed with a diamond are reference sources

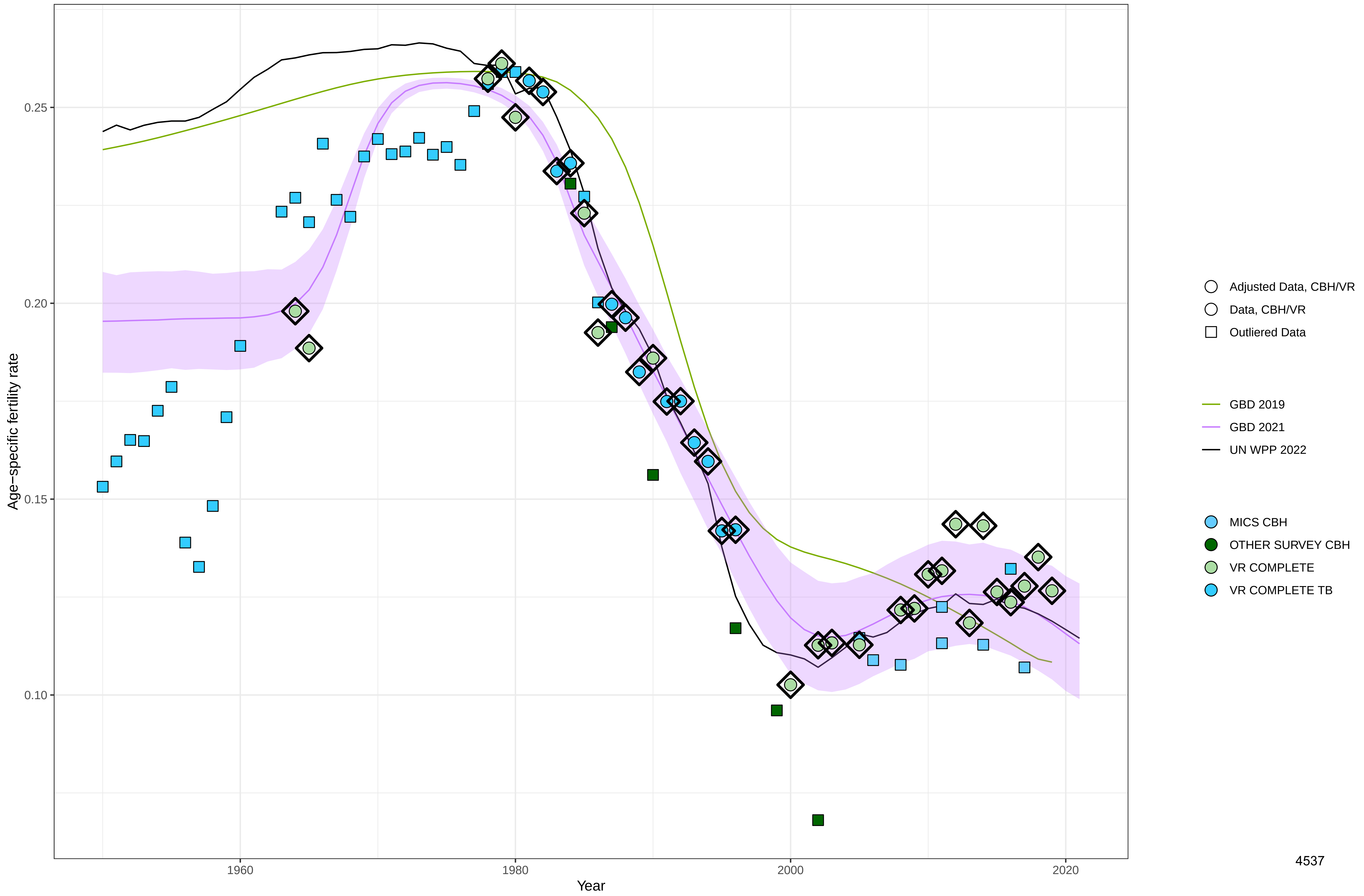

Age−specific fertility fitted trend lines, 1950−2021: Algeria, Ages: 40−44
Data points enclosed with a diamond are reference sources

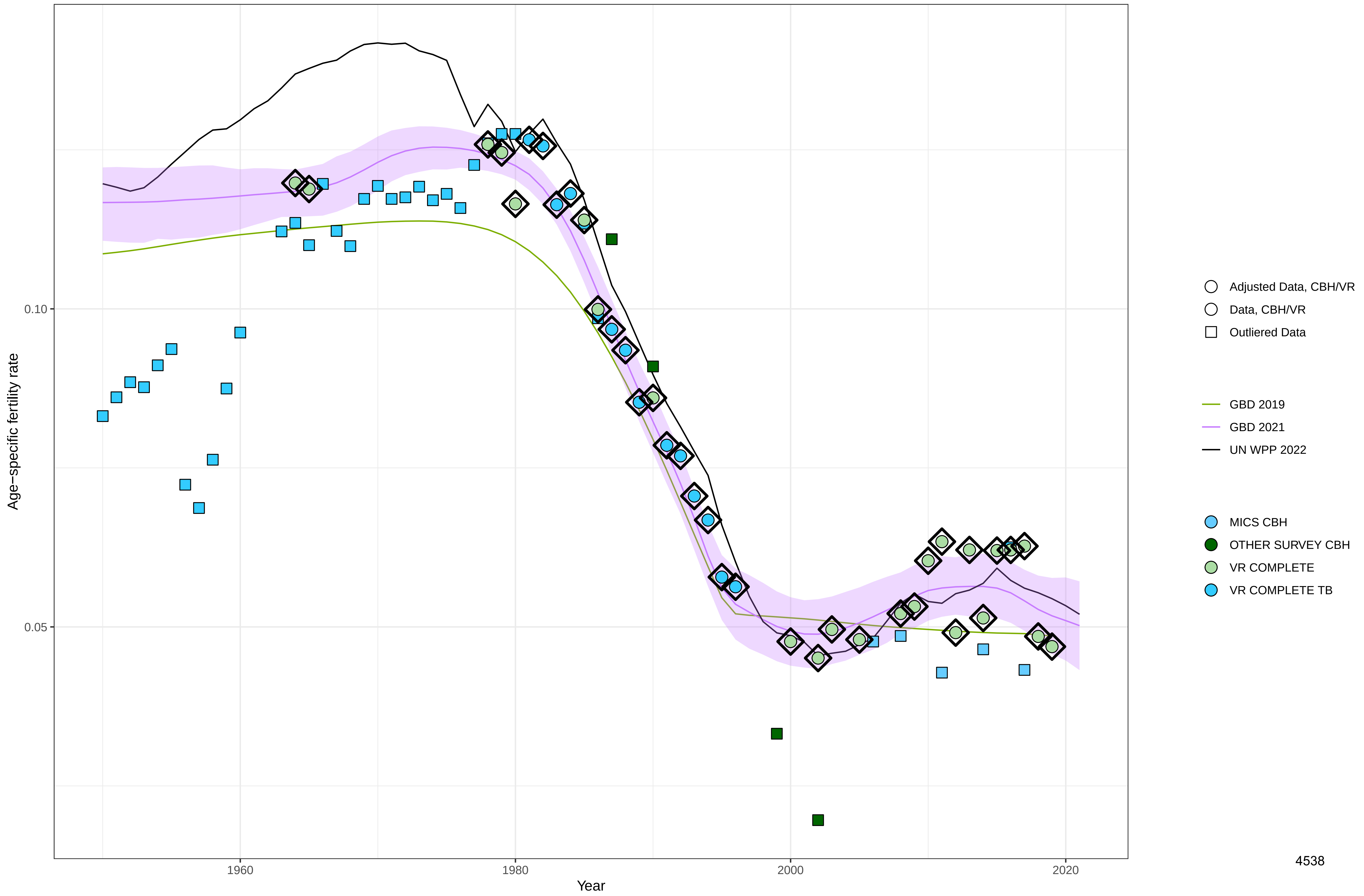

Age−specific fertility fitted trend lines, 1950−2021: Algeria, Ages: 45−49
Data points enclosed with a diamond are reference sources

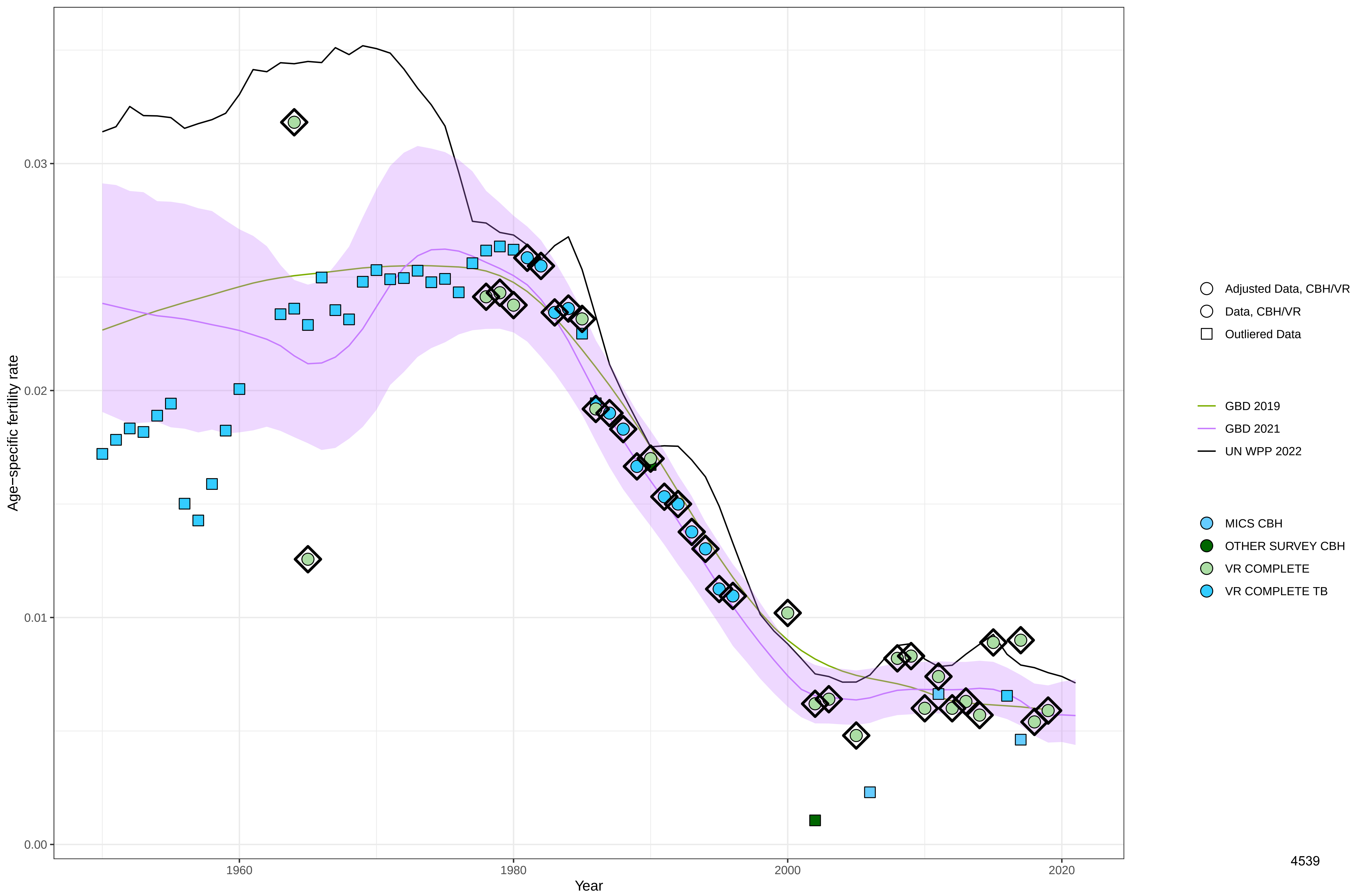

Age−specific fertility fitted trend lines, 1950−2021: Algeria, Ages: 50−54
Data points enclosed with a diamond are reference sources

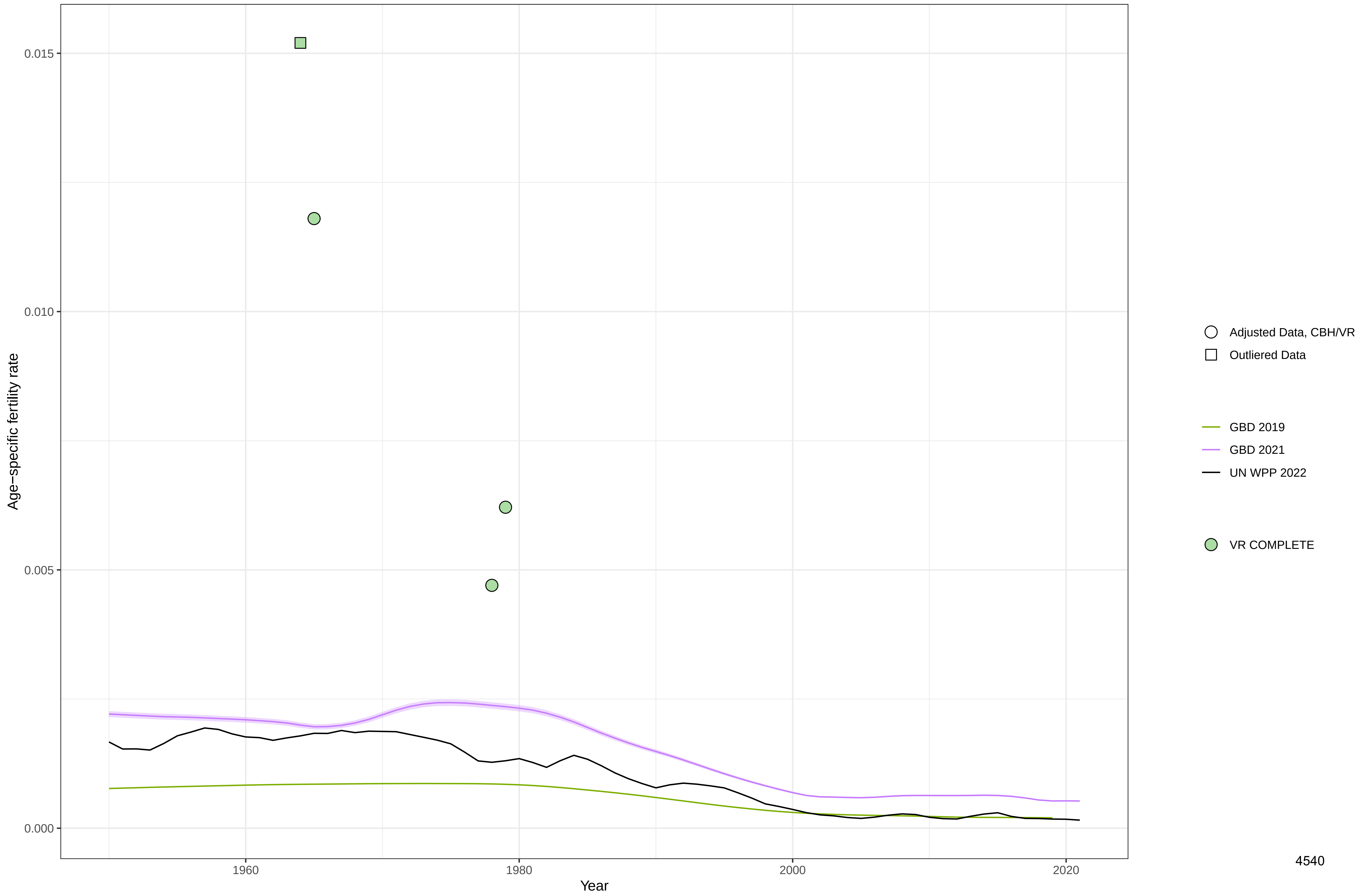

Total fertility rate fitted trend lines, 1950−2021: Algeria TFR
Data points enclosed with a diamond are reference sources

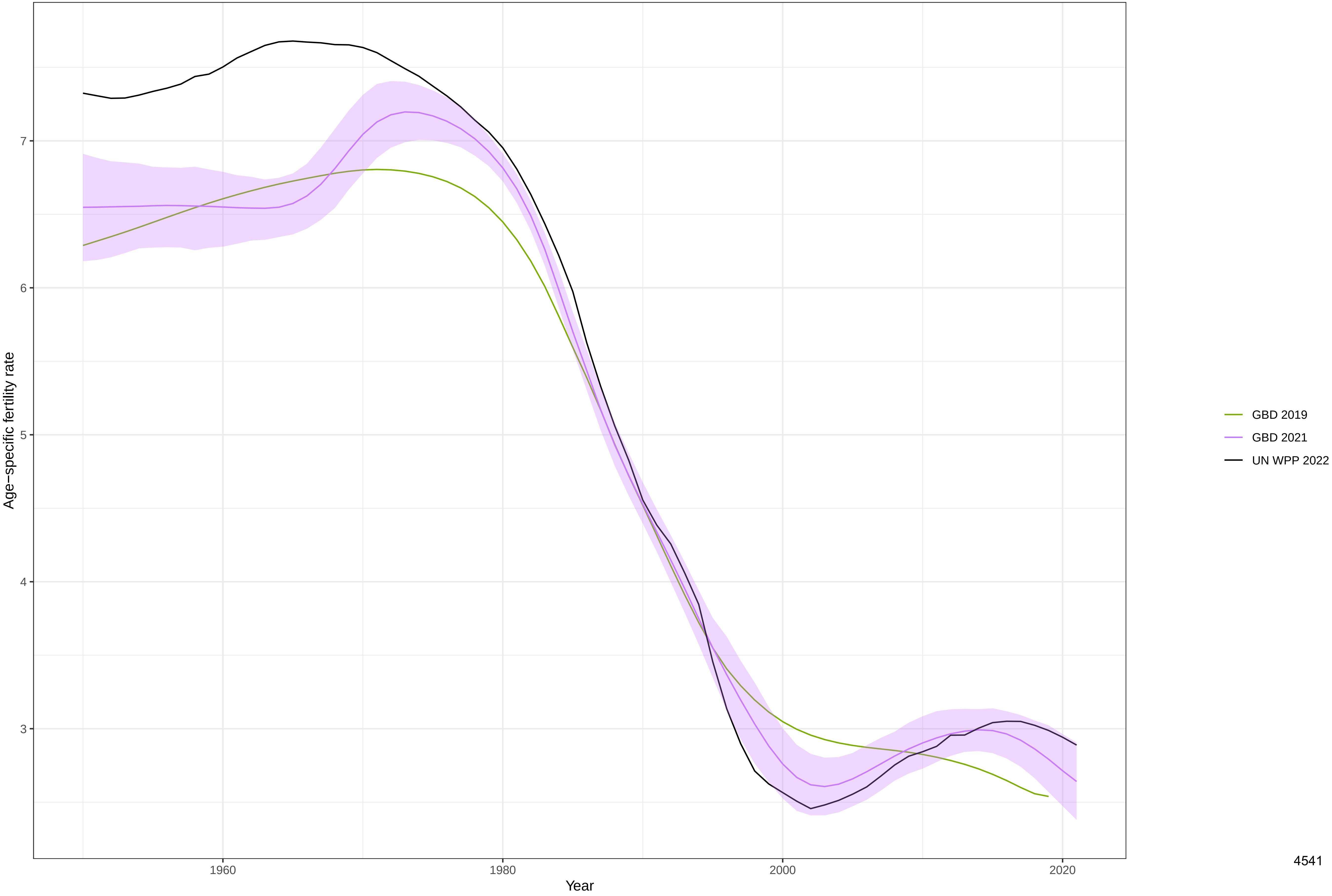

Age−specific fertility fitted trend lines, 1950−2021: Bahrain, Ages: 10−14
Data points enclosed with a diamond are reference sources

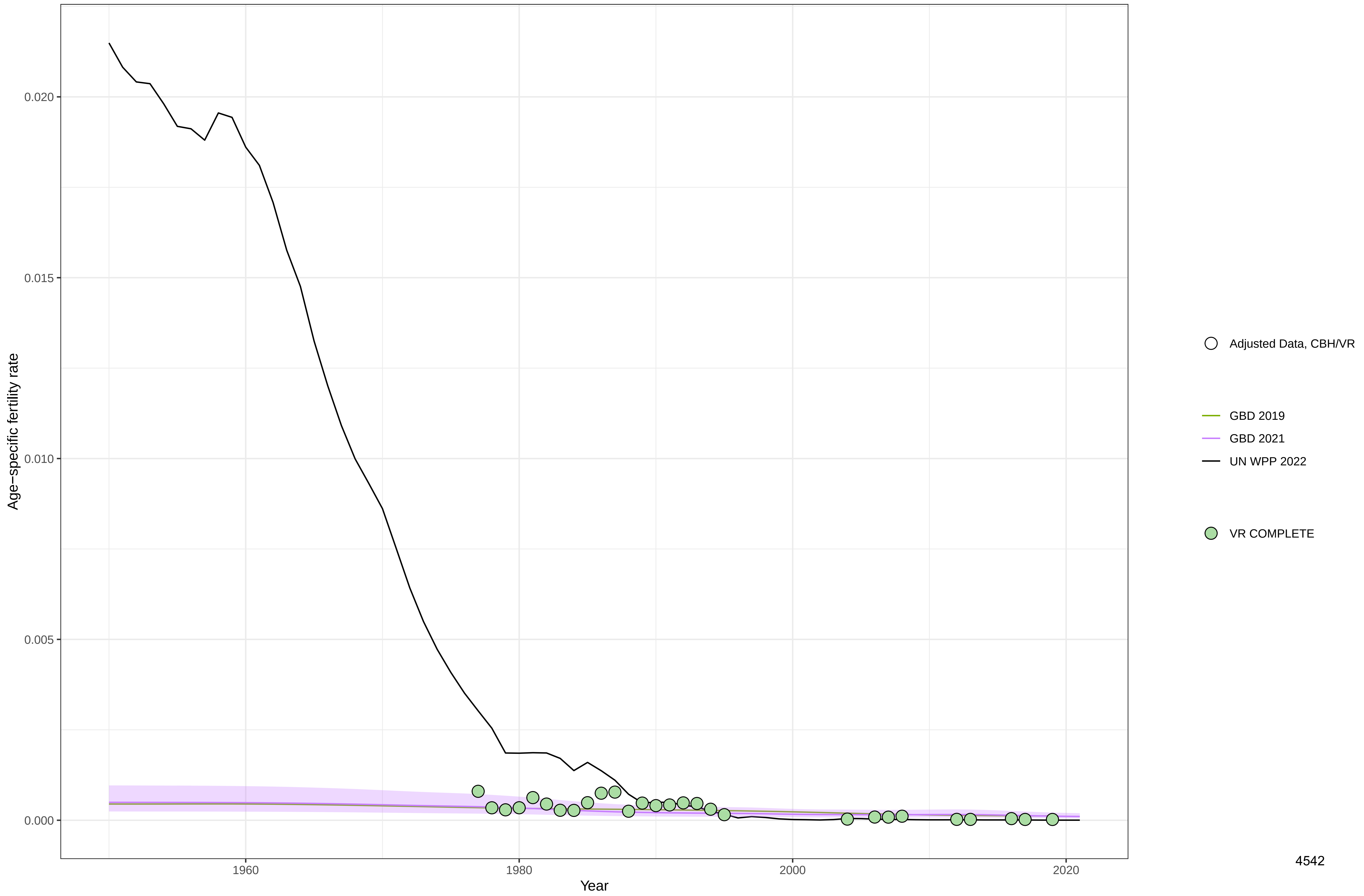

Age−specific fertility fitted trend lines, 1950−2021: Bahrain, Ages: 15−19
Data points enclosed with a diamond are reference sources

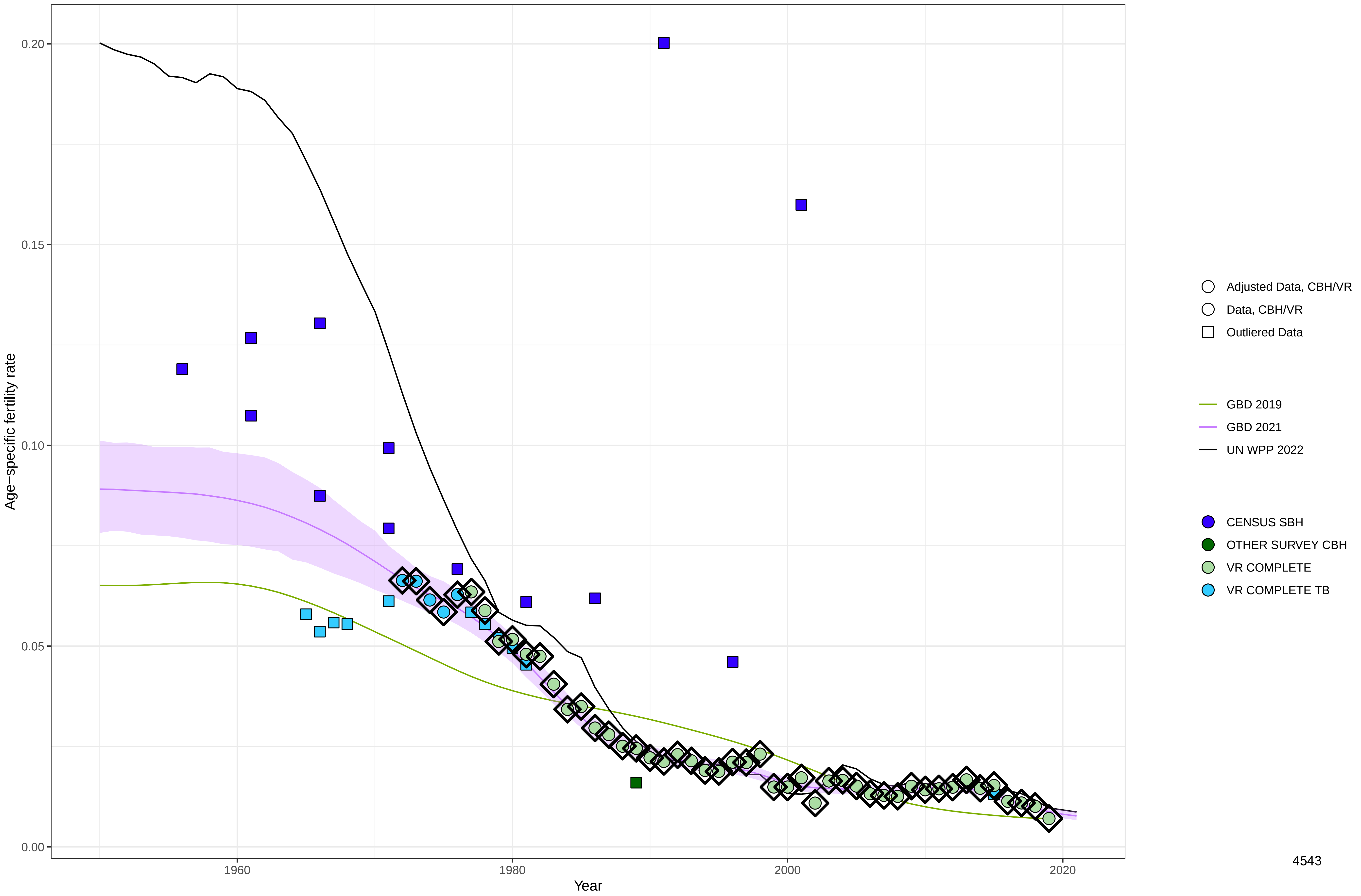

Age−specific fertility fitted trend lines, 1950−2021: Bahrain, Ages: 20−24
Data points enclosed with a diamond are reference sources

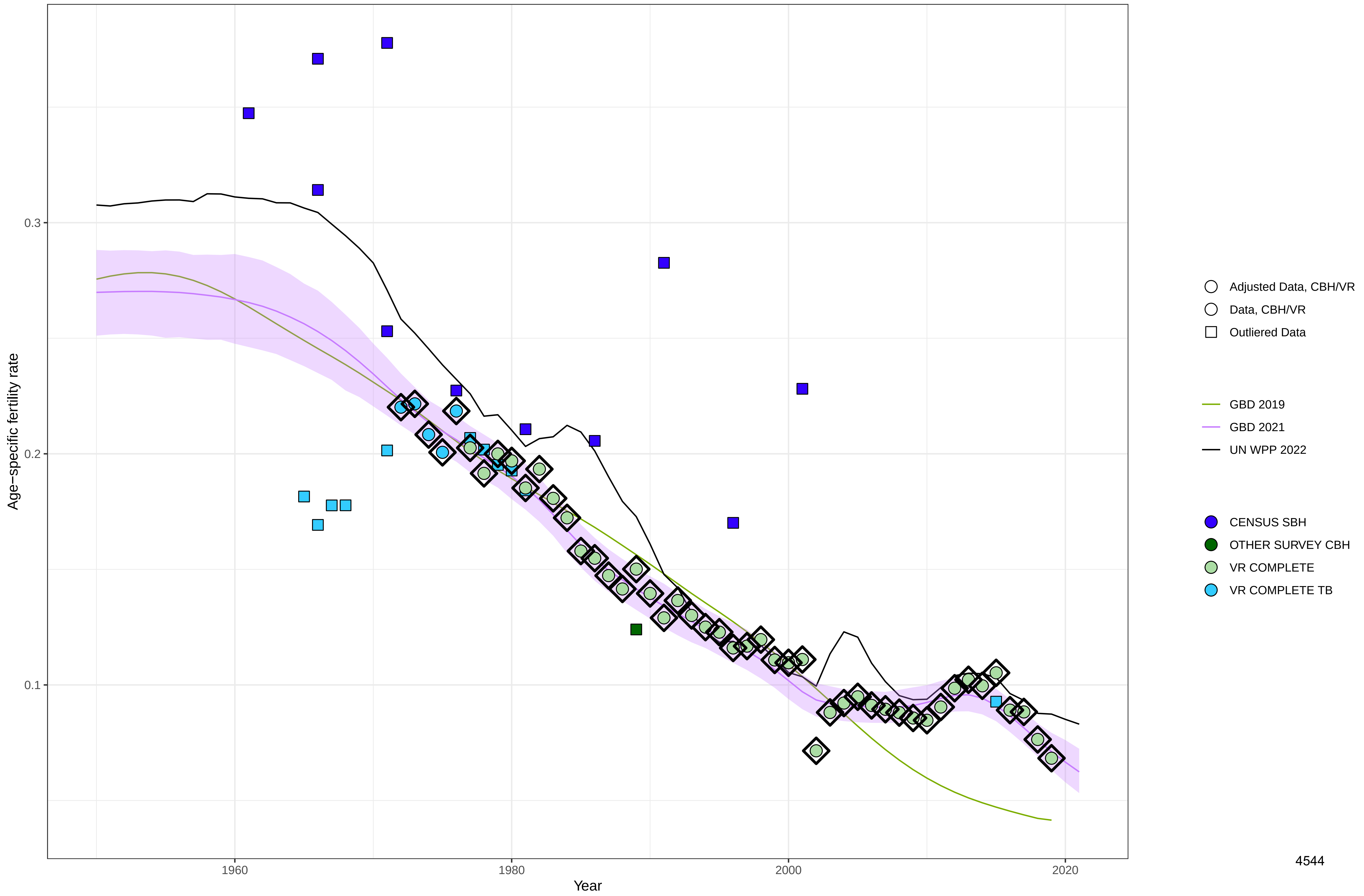

Age−specific fertility fitted trend lines, 1950−2021: Bahrain, Ages: 25−29
Data points enclosed with a diamond are reference sources

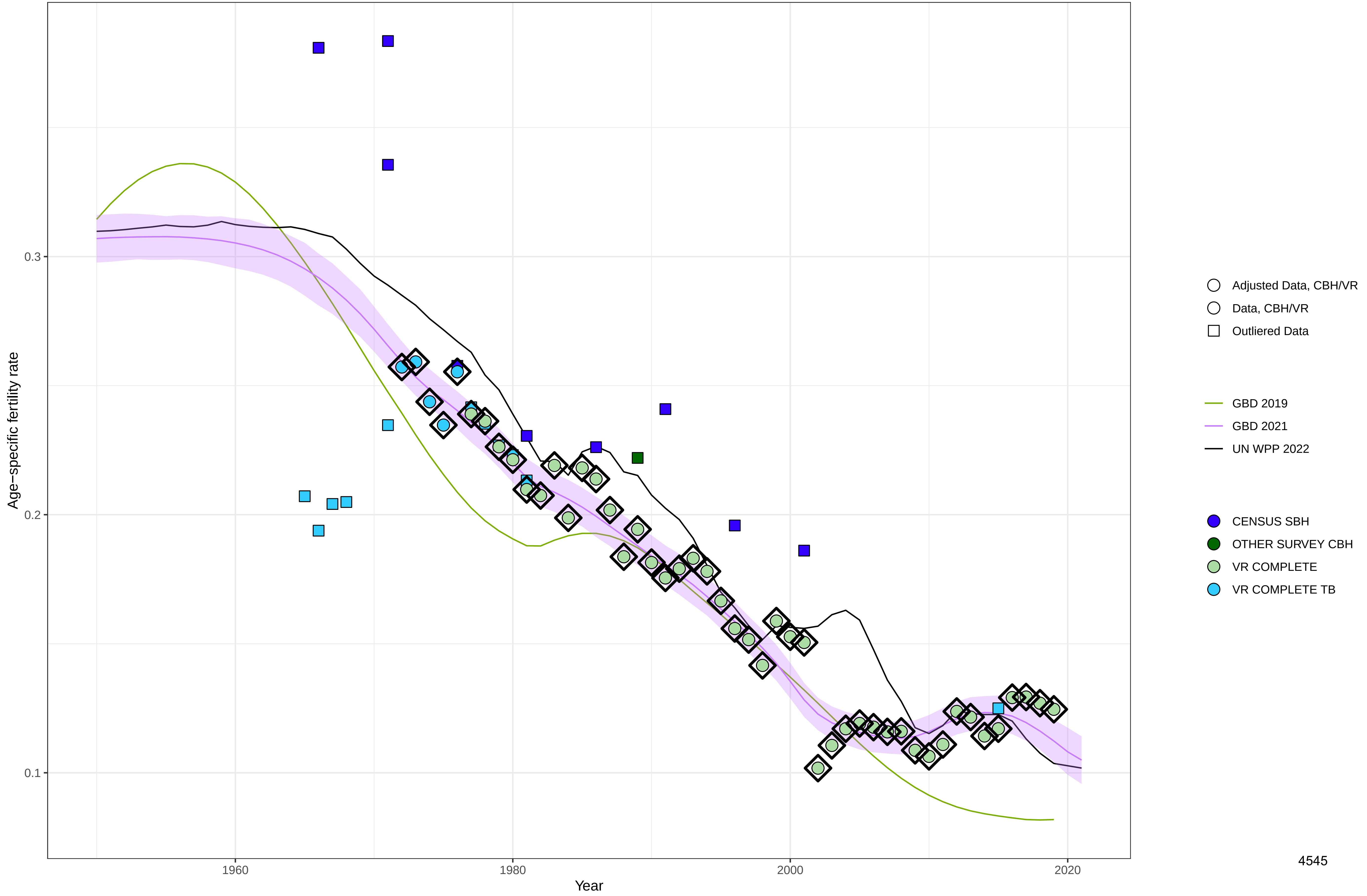

Age−specific fertility fitted trend lines, 1950−2021: Bahrain, Ages: 30−34
Data points enclosed with a diamond are reference sources

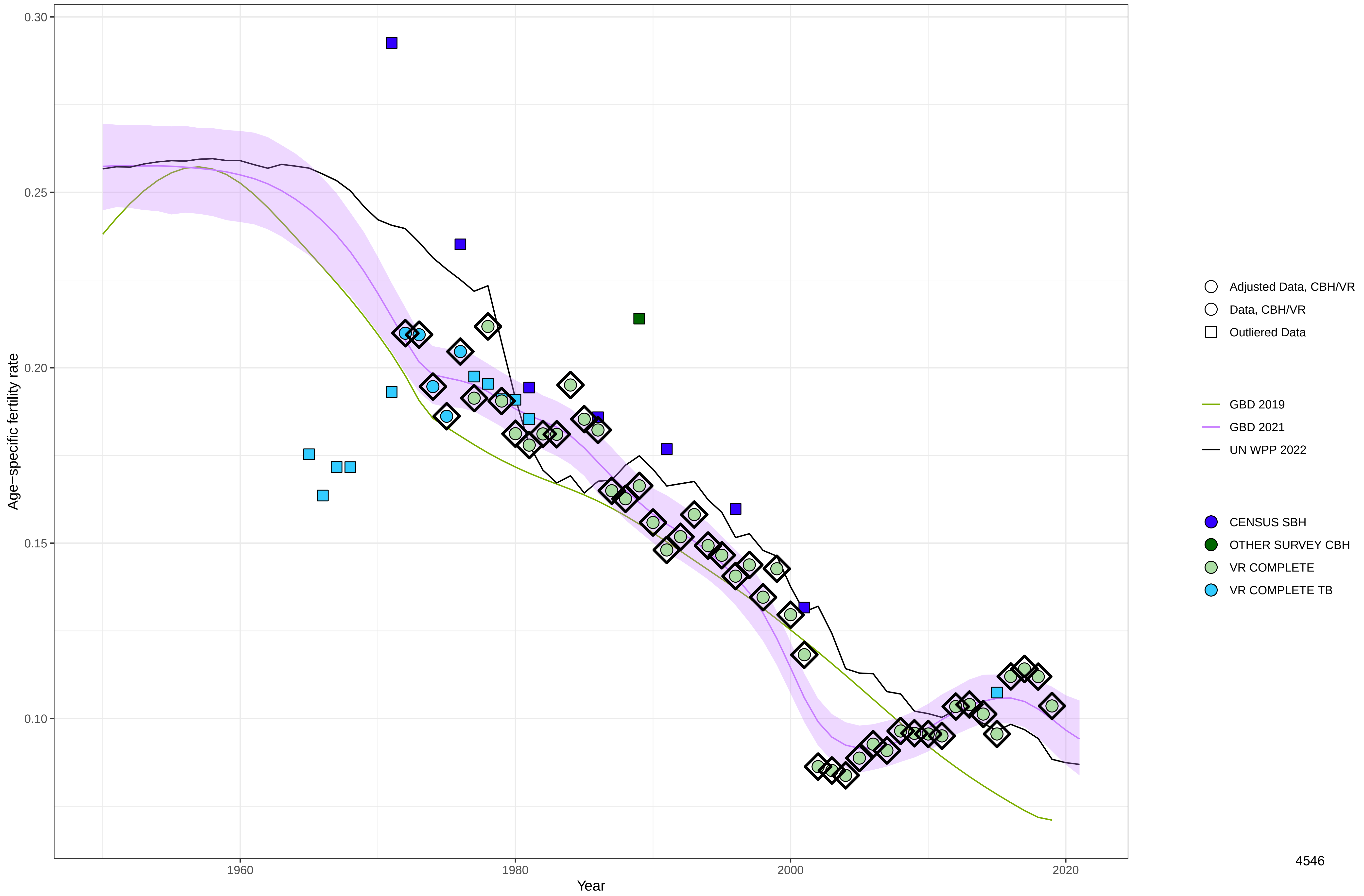

Age−specific fertility fitted trend lines, 1950−2021: Bahrain, Ages: 35−39
Data points enclosed with a diamond are reference sources

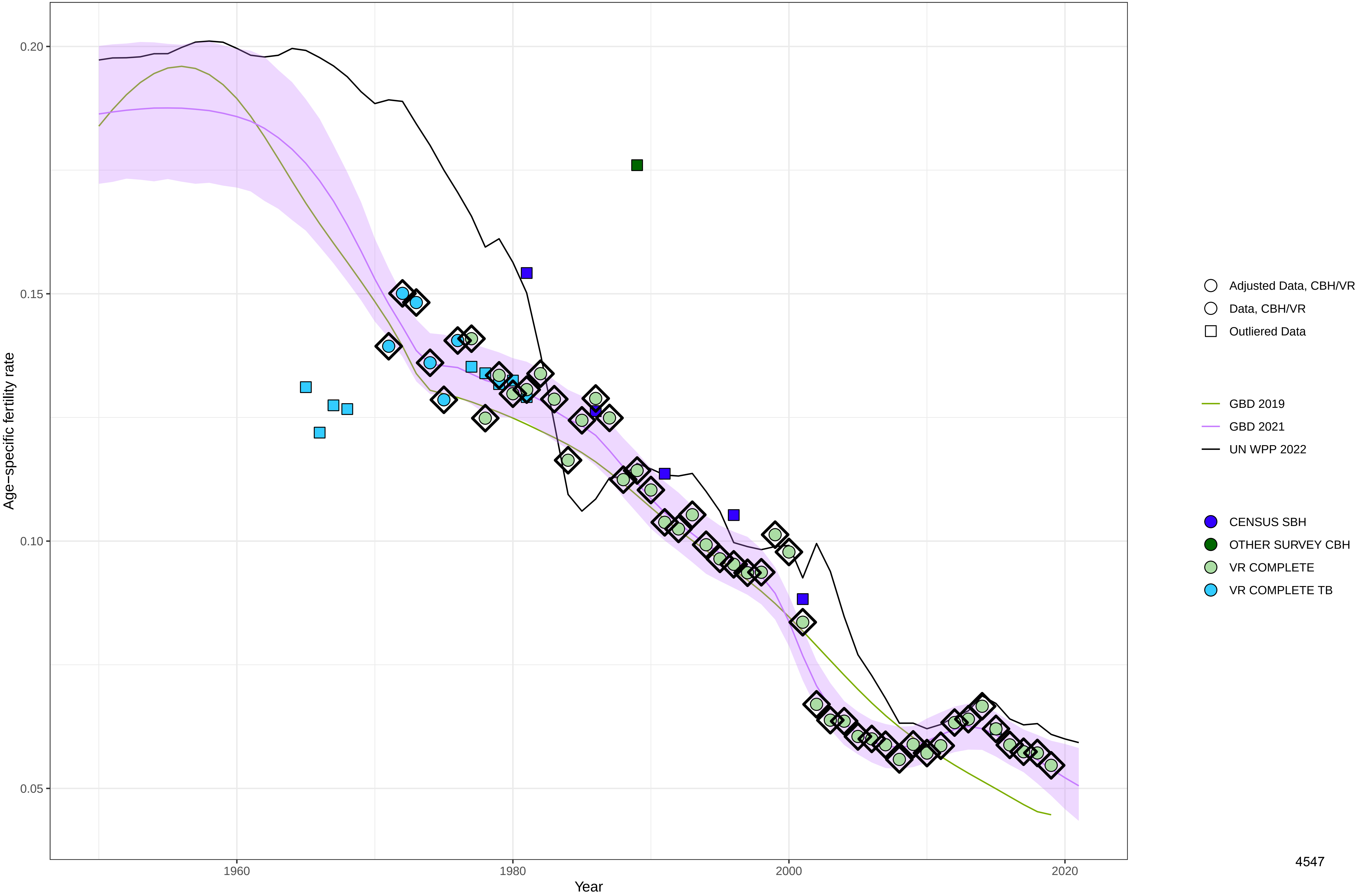

Age−specific fertility fitted trend lines, 1950−2021: Bahrain, Ages: 40−44
Data points enclosed with a diamond are reference sources

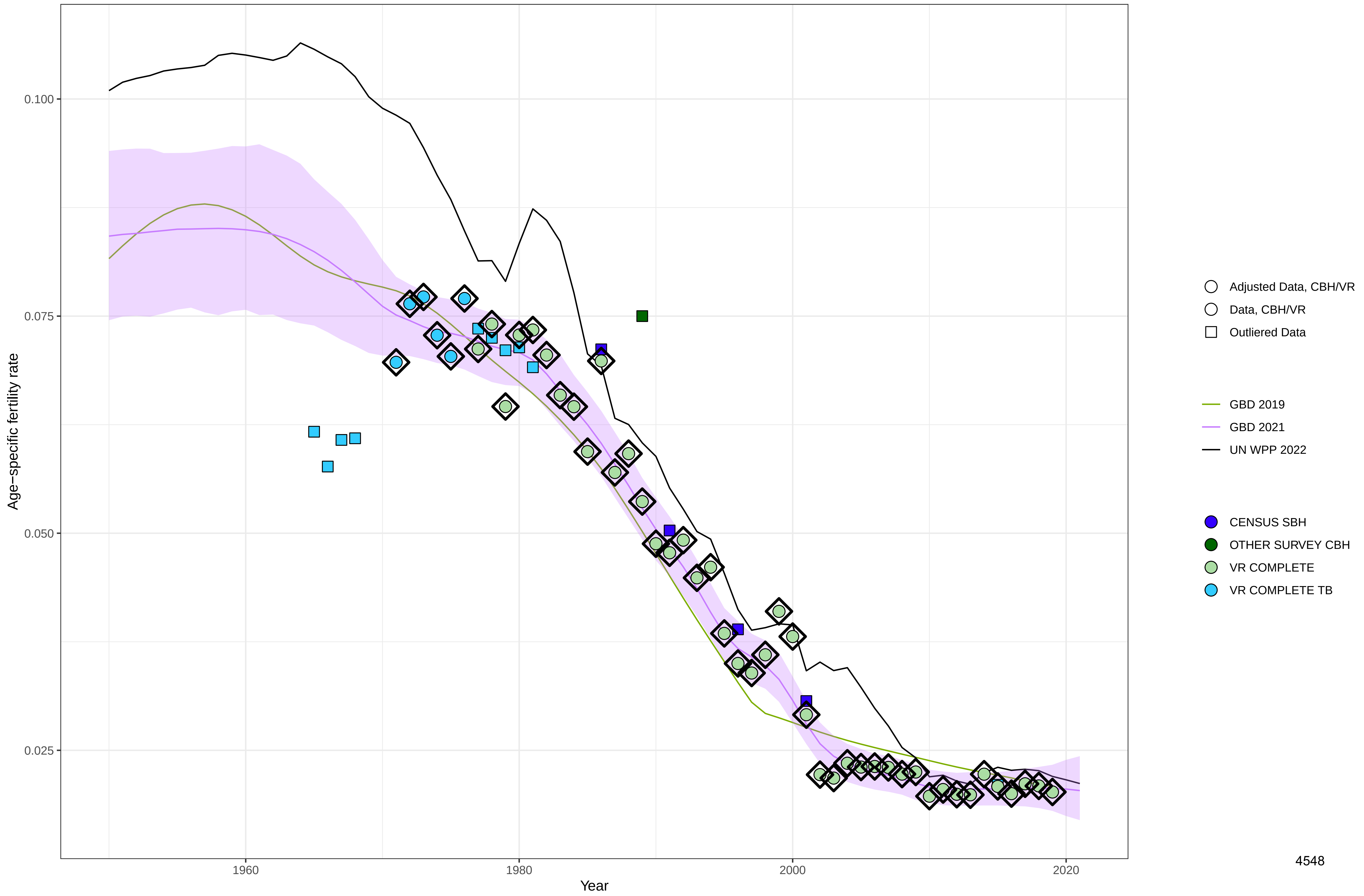

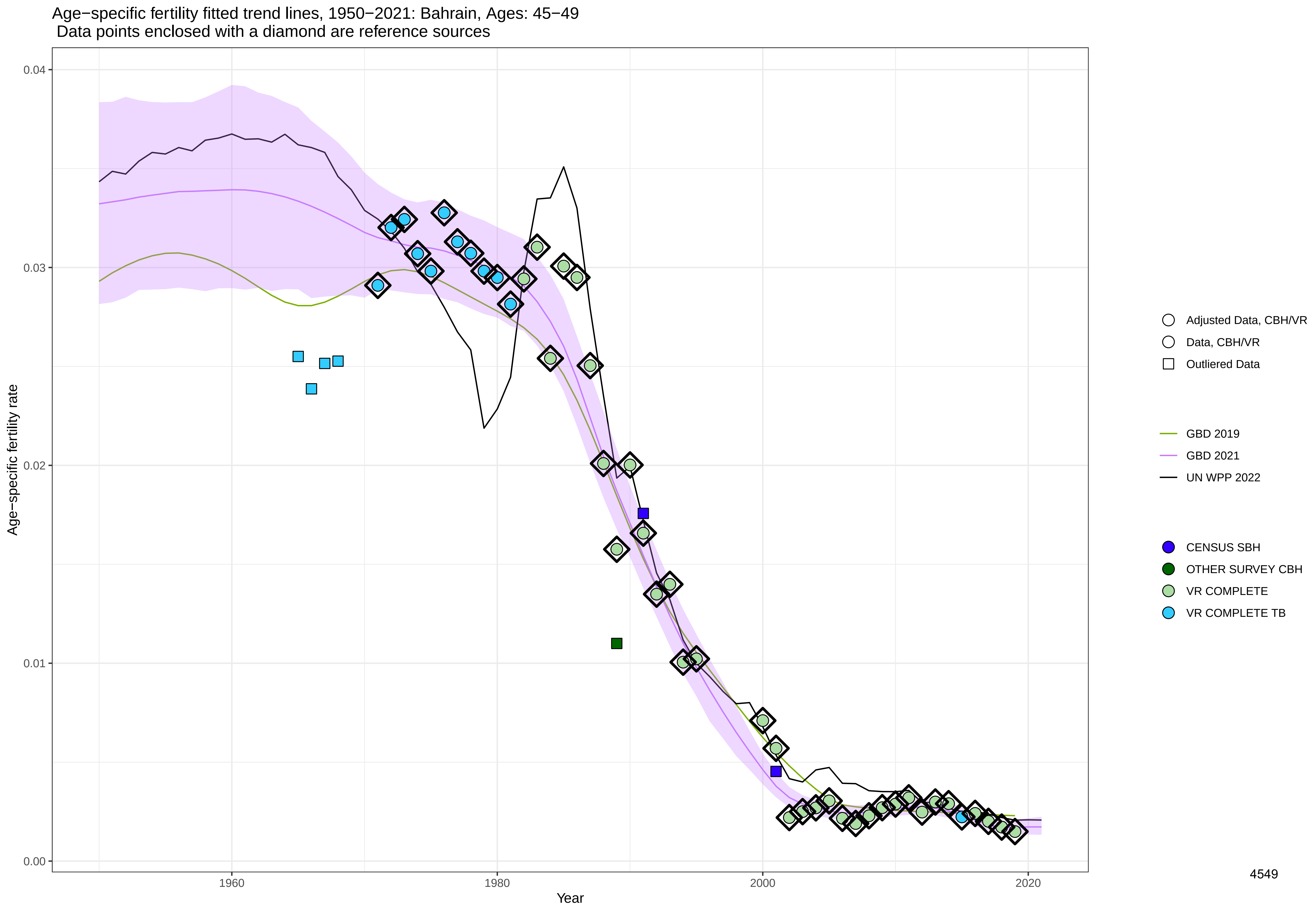

Age−specific fertility fitted trend lines, 1950−2021: Bahrain, Ages: 45−49
Data points enclosed with a diamond are reference sources
Age−specific fertility rate
Year
0.00
0.01
0.02
0.03
0.04
1960
1980
2000
2020
Adjusted Data, CBH/VR
Data, CBH/VR
Outliered Data
GBD 2019
GBD 2021
UN WPP 2022
CENSUS SBH
OTHER SURVEY CBH
VR COMPLETE
VR COMPLETE TB

Age−specific fertility fitted trend lines, 1950−2021: Bahrain, Ages: 50−54
Data points enclosed with a diamond are reference sources

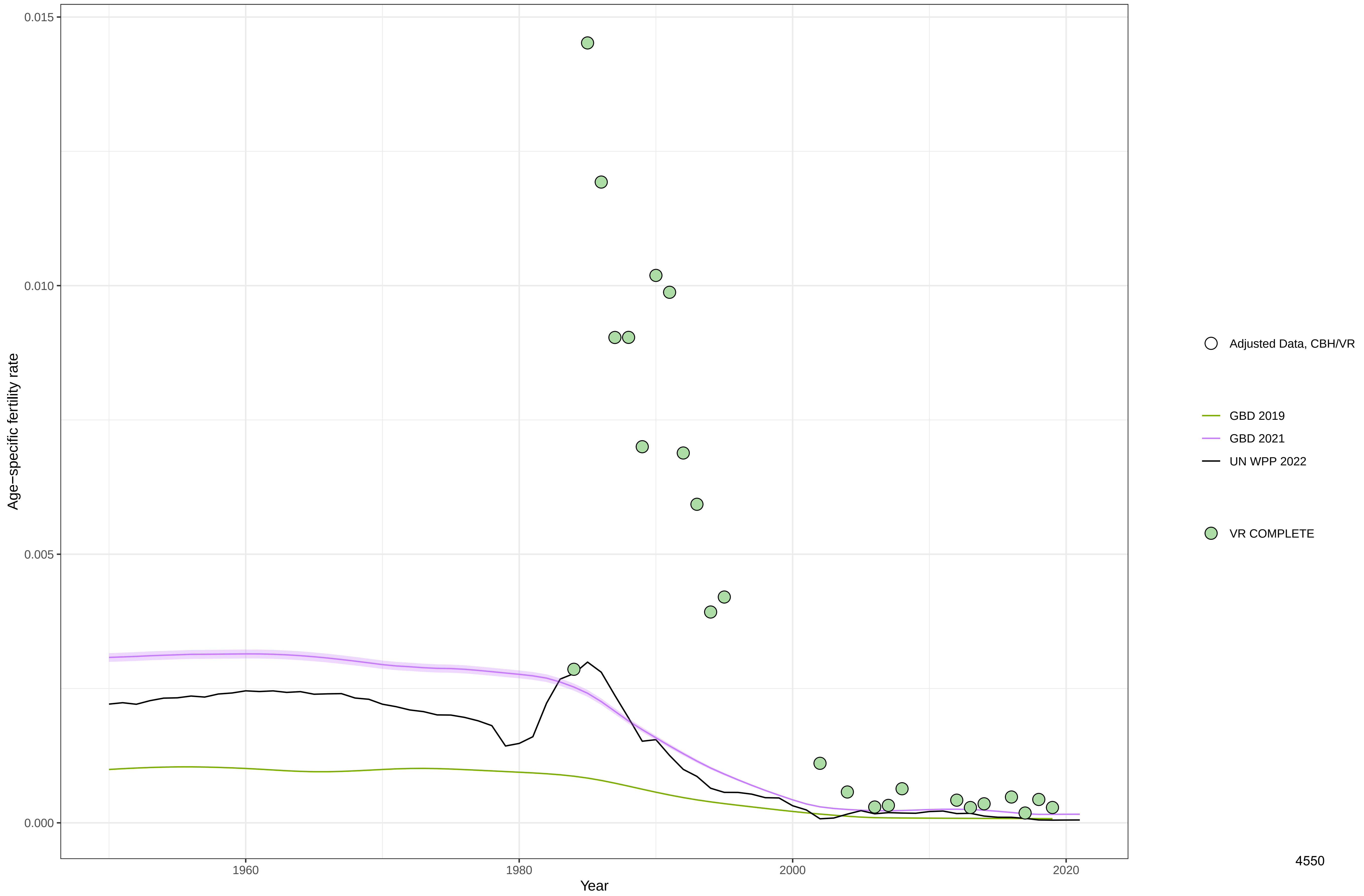

Total fertility rate fitted trend lines, 1950−2021: Bahrain TFR
Data points enclosed with a diamond are reference sources

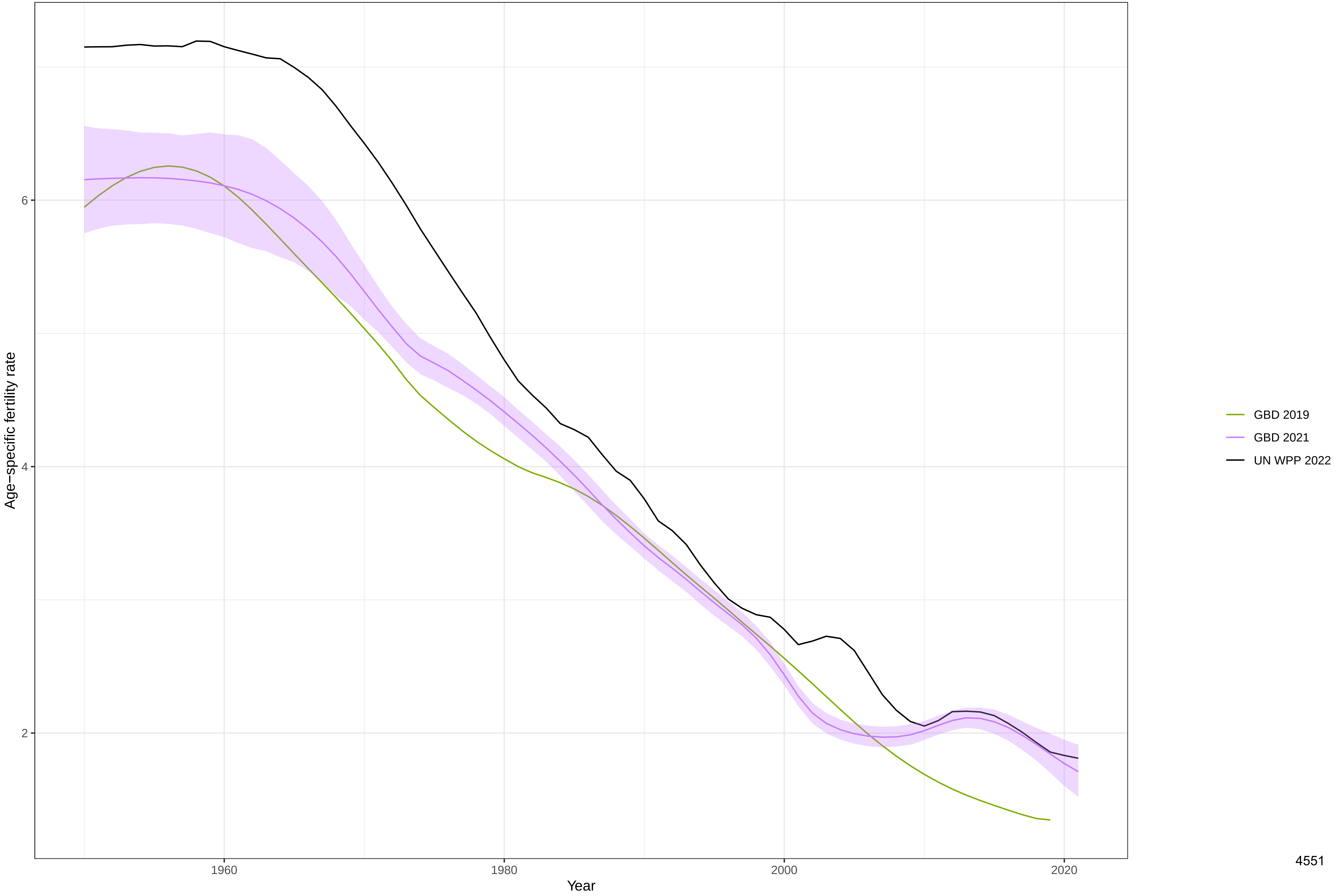

Age−specific fertility fitted trend lines, 1950−2021: Egypt, Ages: 10−14
Data points enclosed with a diamond are reference sources

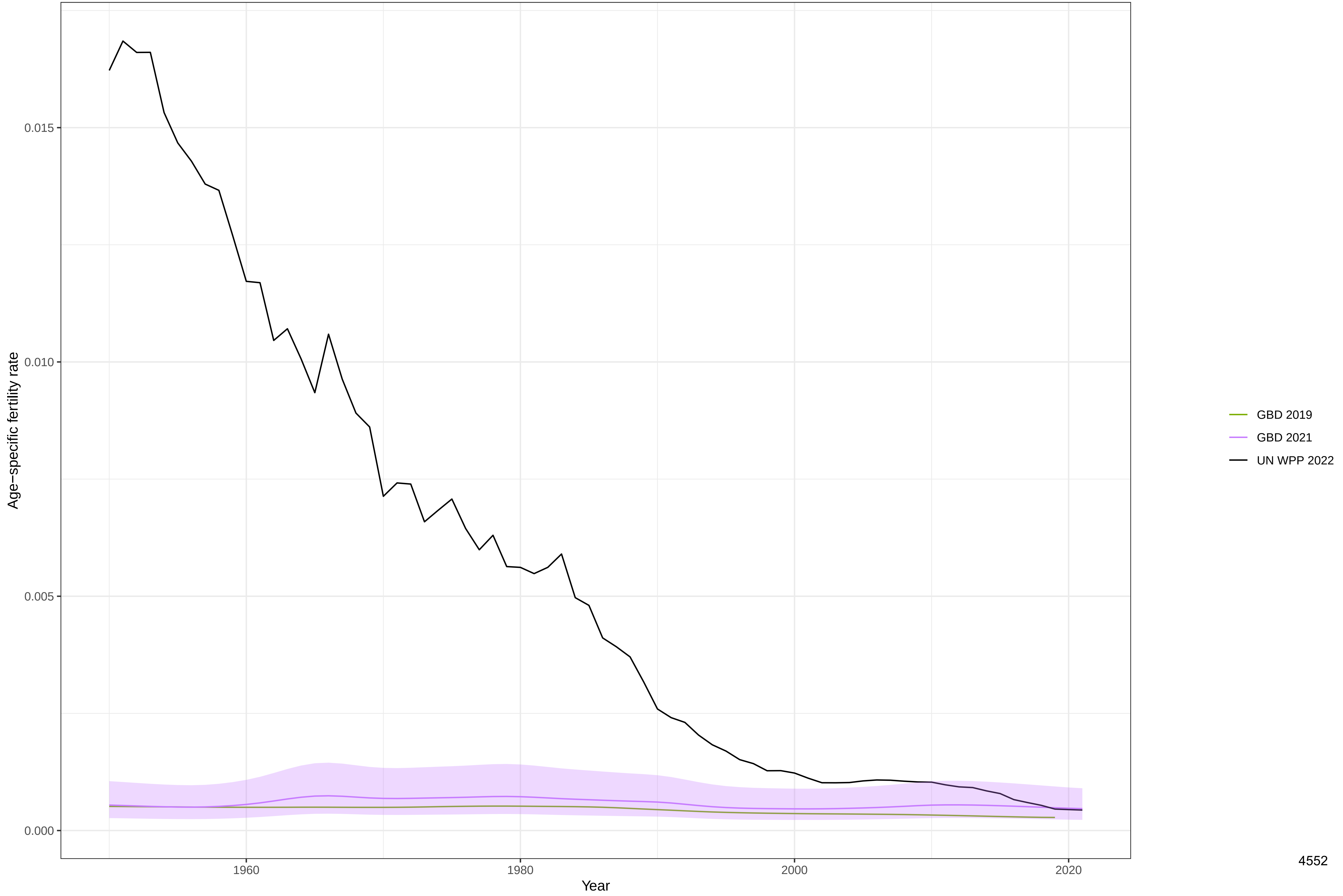

Age−specific fertility fitted trend lines, 1950−2021: Egypt, Ages: 15−19
Data points enclosed with a diamond are reference sources

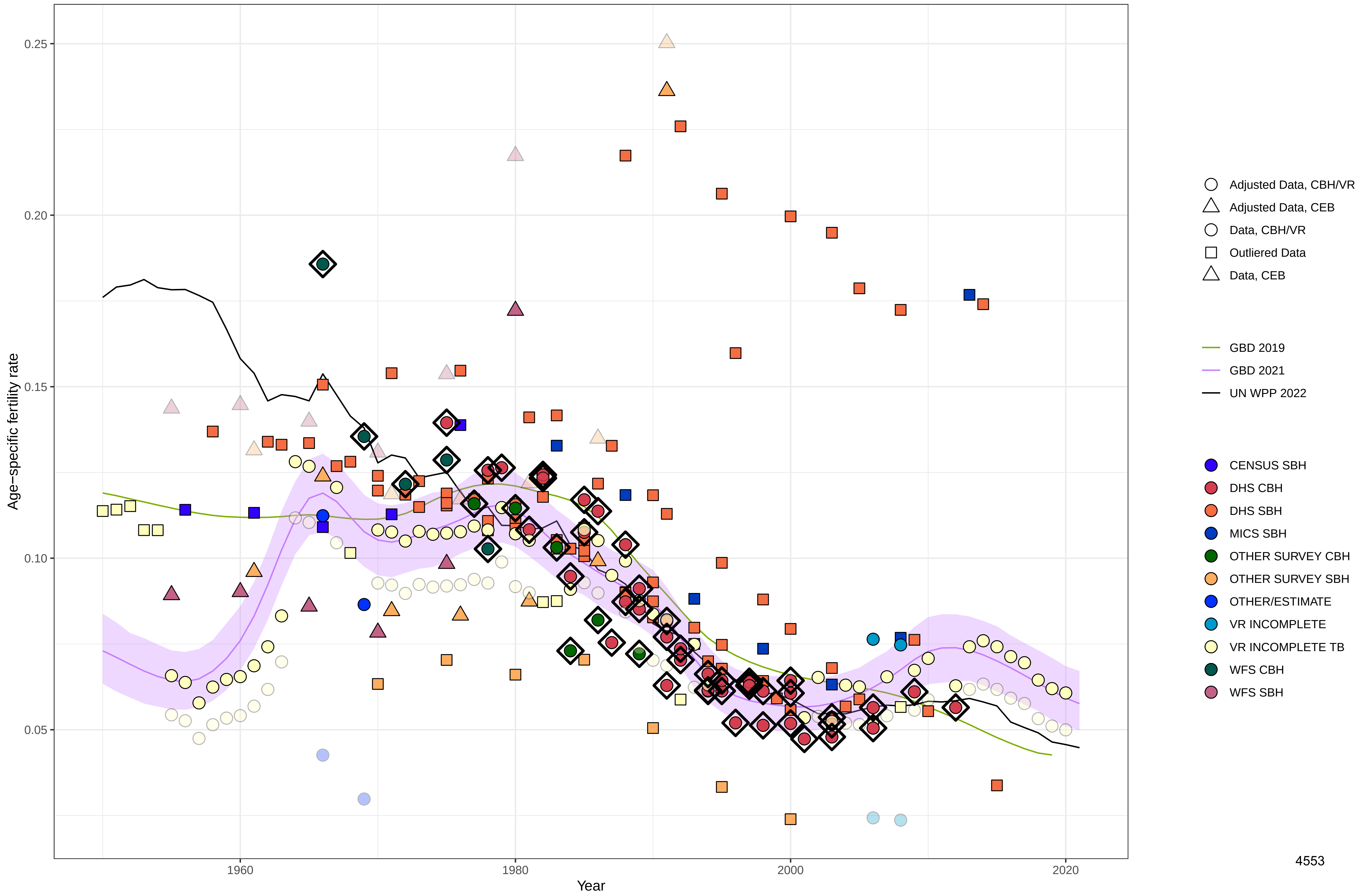

Age−specific fertility fitted trend lines, 1950−2021: Egypt, Ages: 20−24
Data points enclosed with a diamond are reference sources

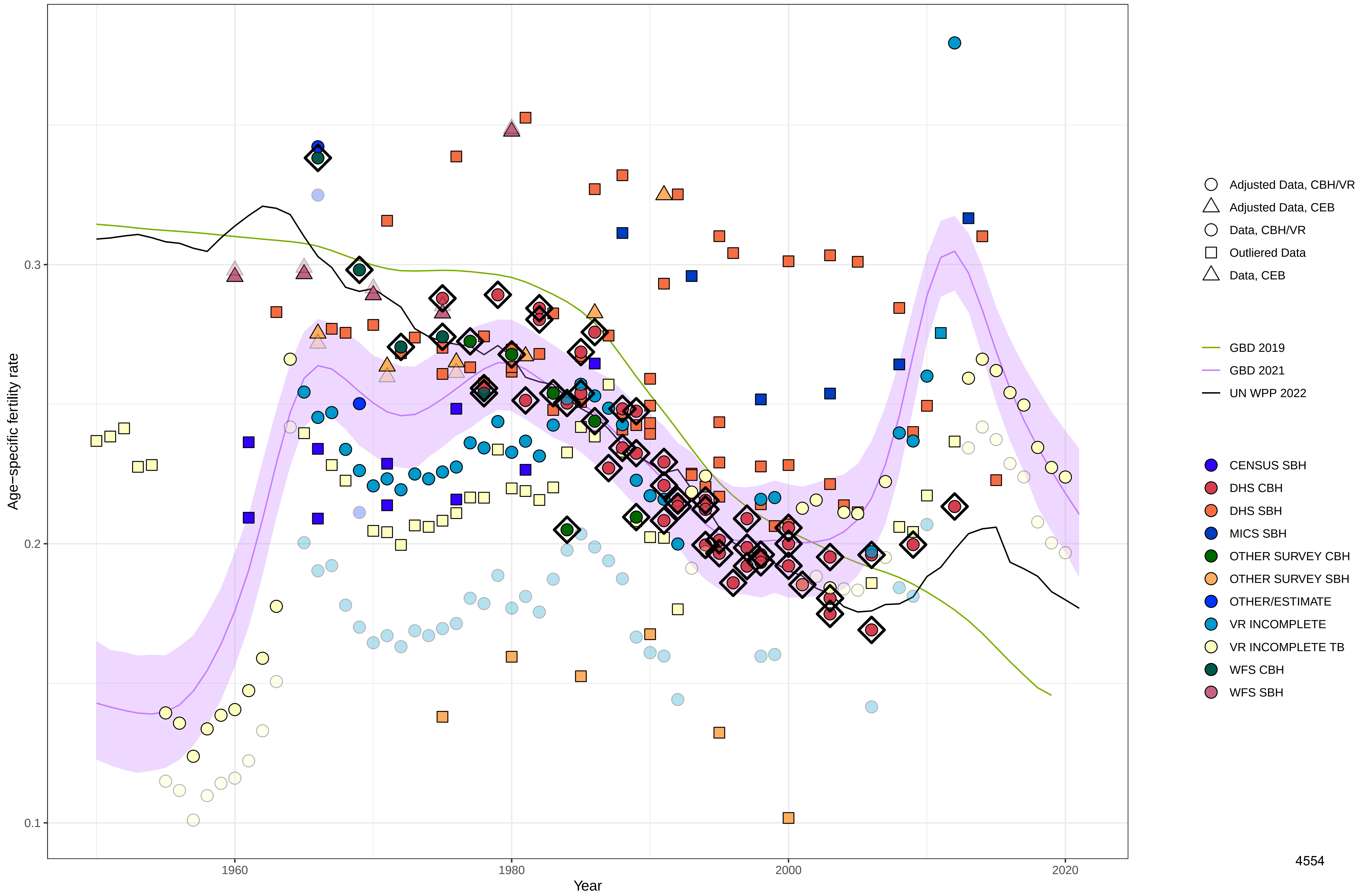

Age−specific fertility fitted trend lines, 1950−2021: Egypt, Ages: 25−29
Data points enclosed with a diamond are reference sources

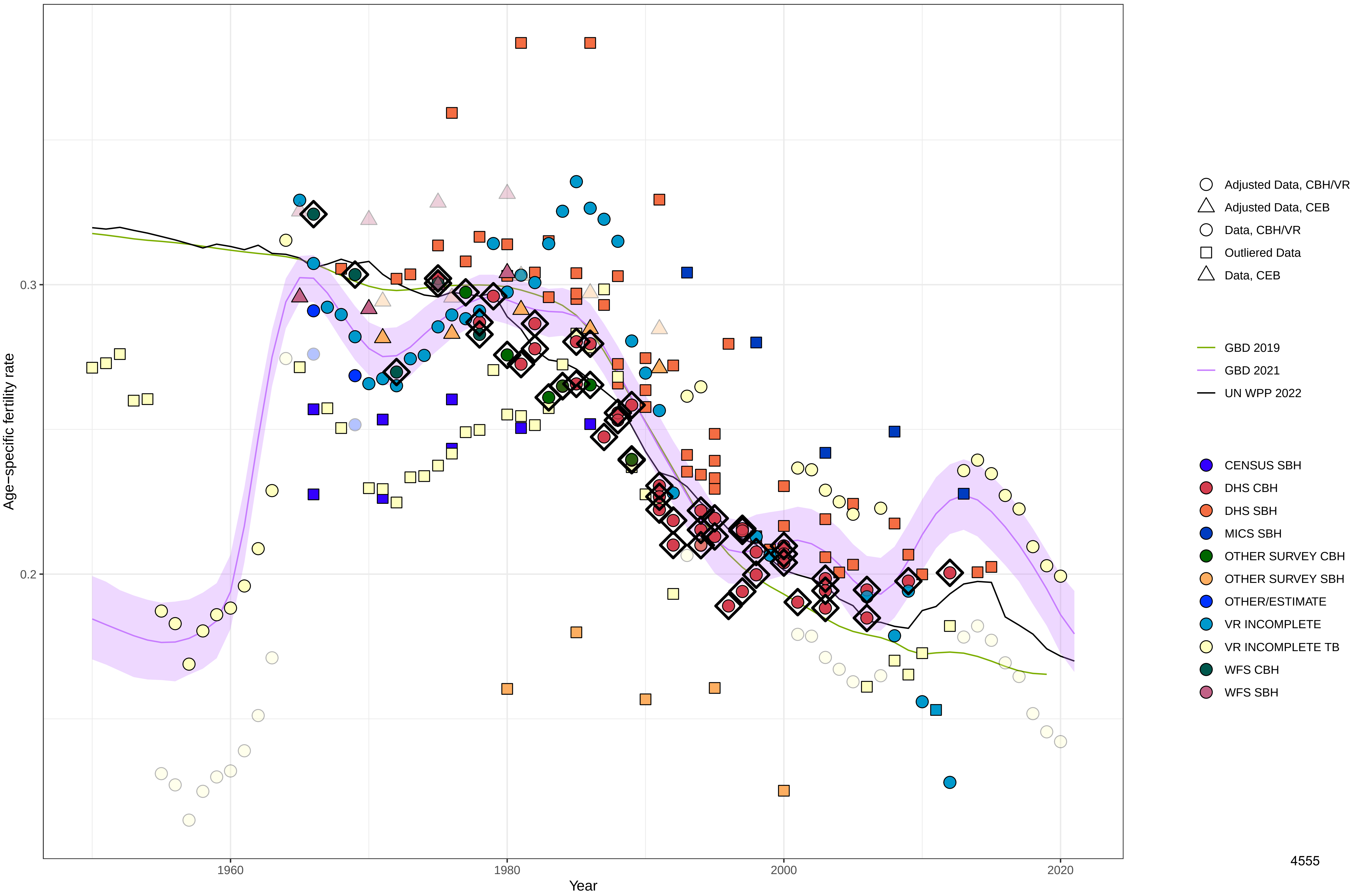

Age−specific fertility fitted trend lines, 1950−2021: Egypt, Ages: 30−34
Data points enclosed with a diamond are reference sources

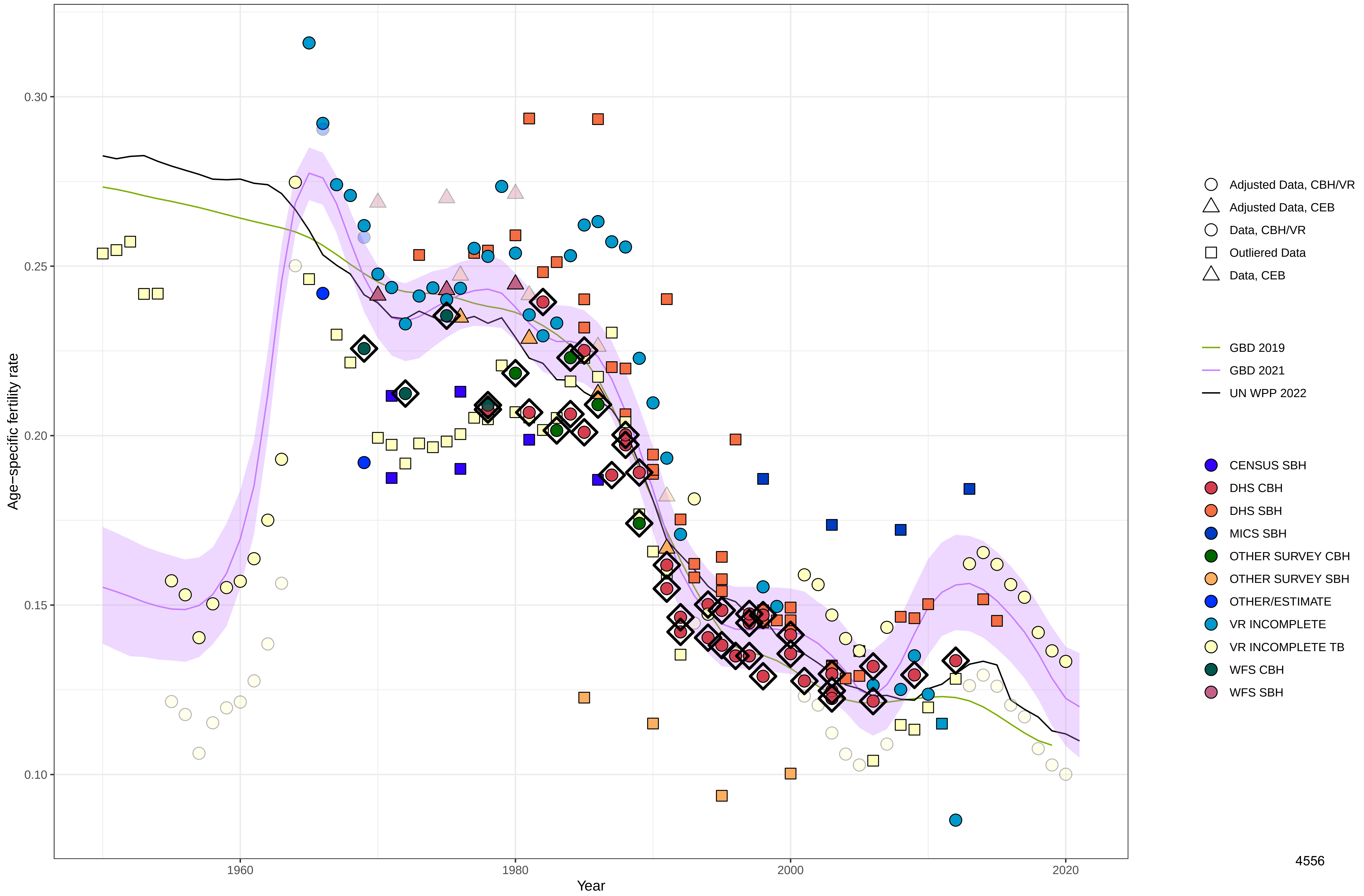

Age−specific fertility fitted trend lines, 1950−2021: Egypt, Ages: 35−39
Data points enclosed with a diamond are reference sources

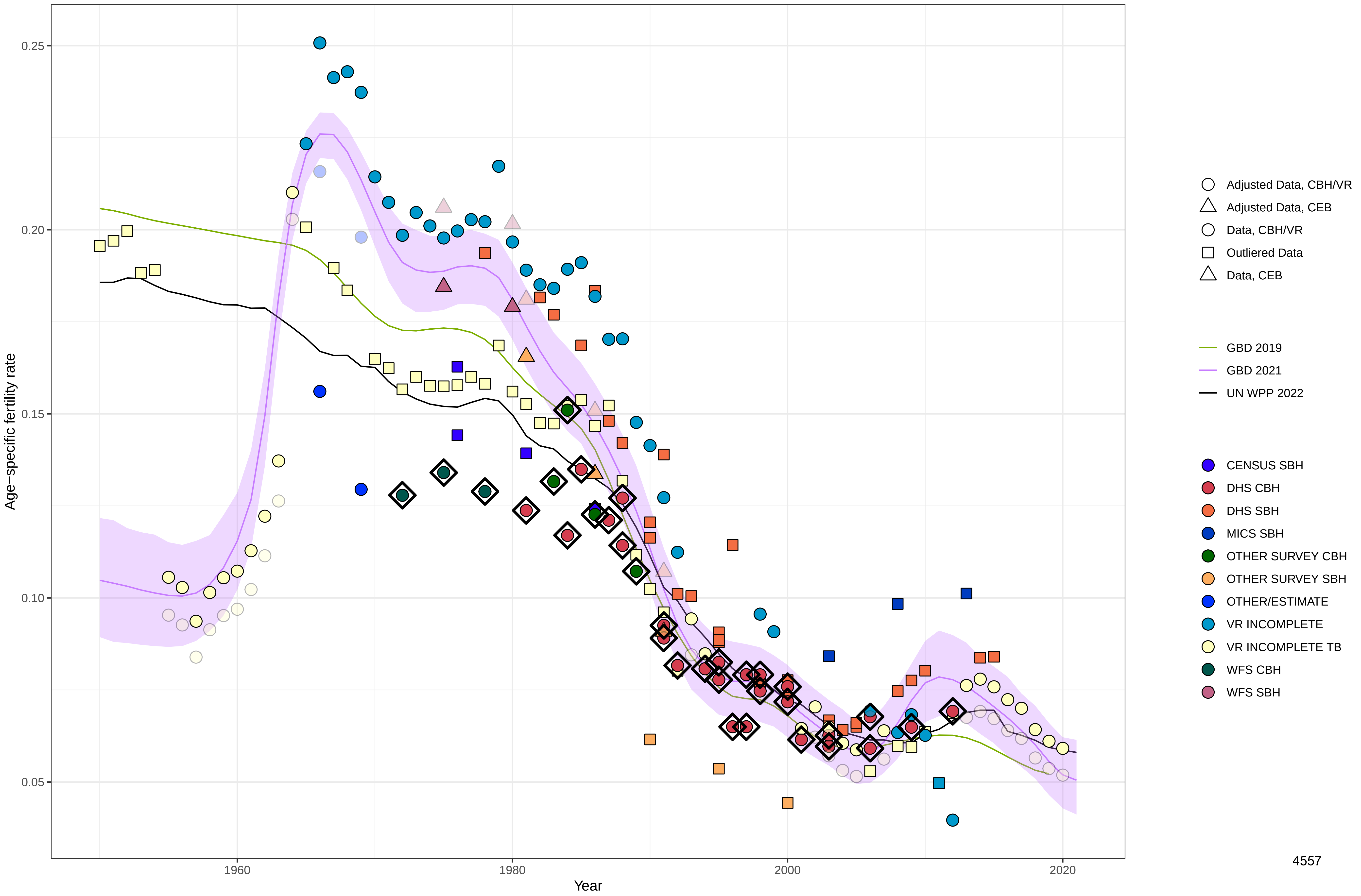

Age−specific fertility fitted trend lines, 1950−2021: Egypt, Ages: 40−44
Data points enclosed with a diamond are reference sources

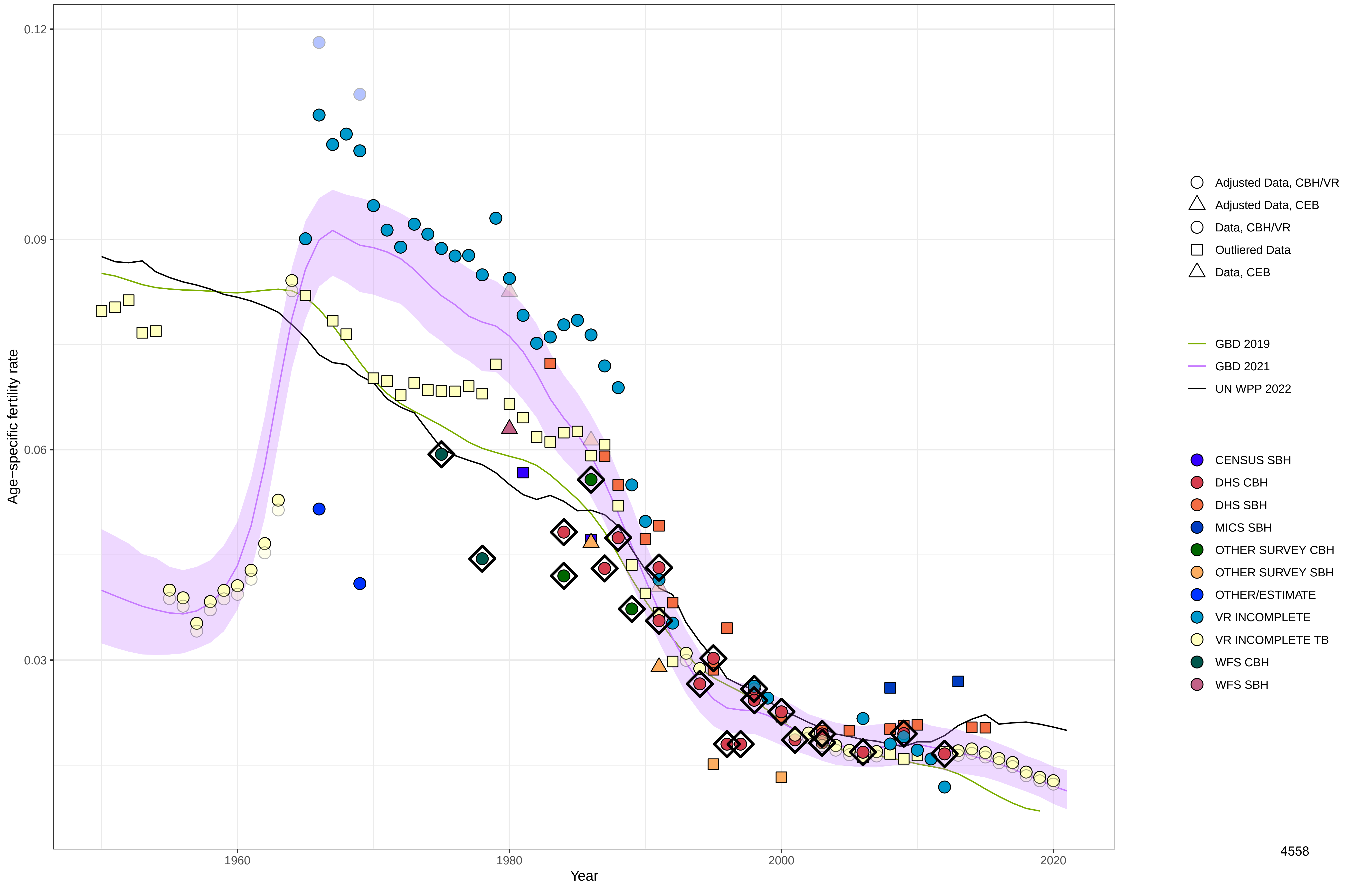

Age−specific fertility fitted trend lines, 1950−2021: Egypt, Ages: 45−49
Data points enclosed with a diamond are reference sources

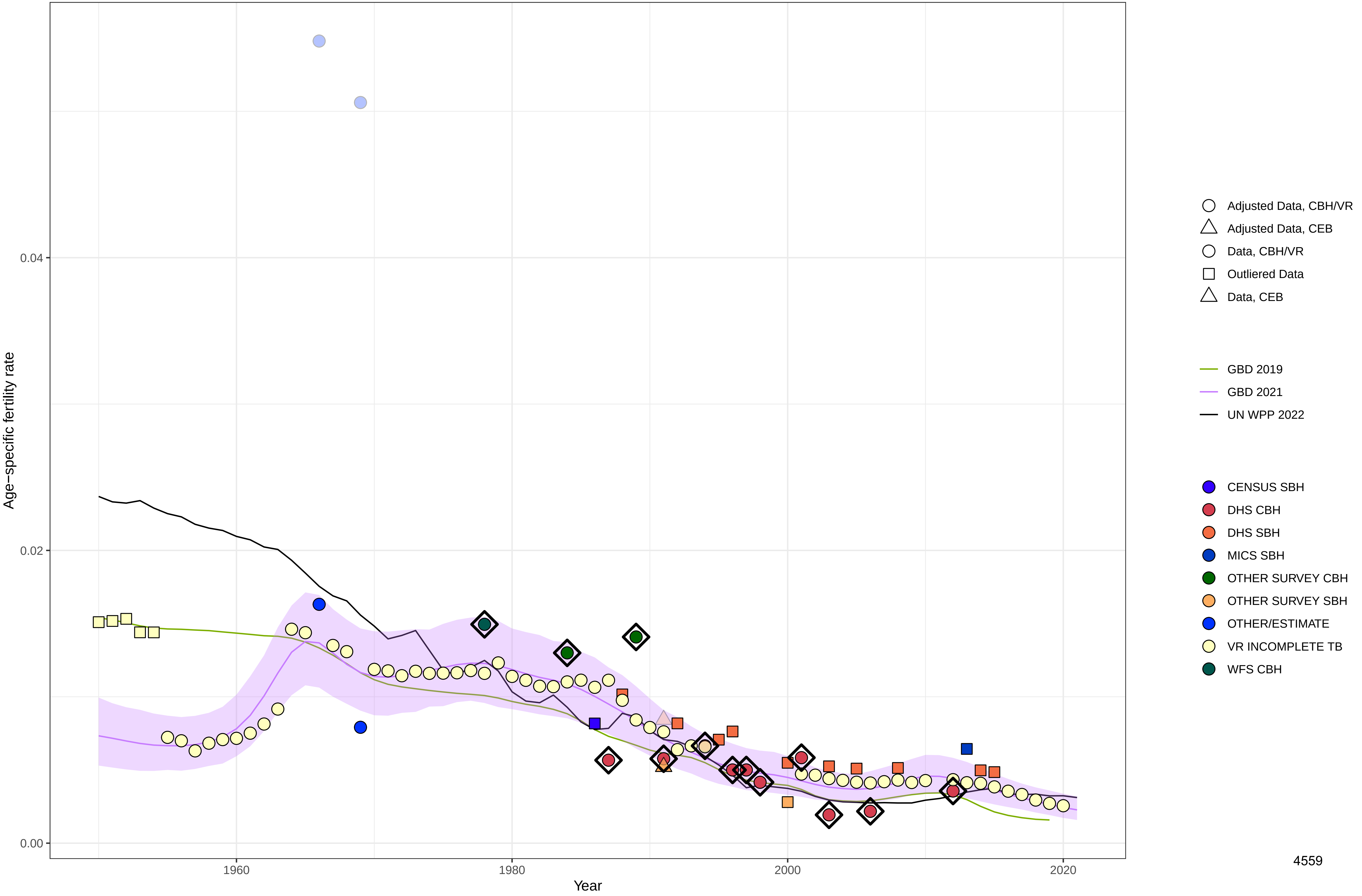

Age−specific fertility fitted trend lines, 1950−2021: Egypt, Ages: 50−54
Data points enclosed with a diamond are reference sources

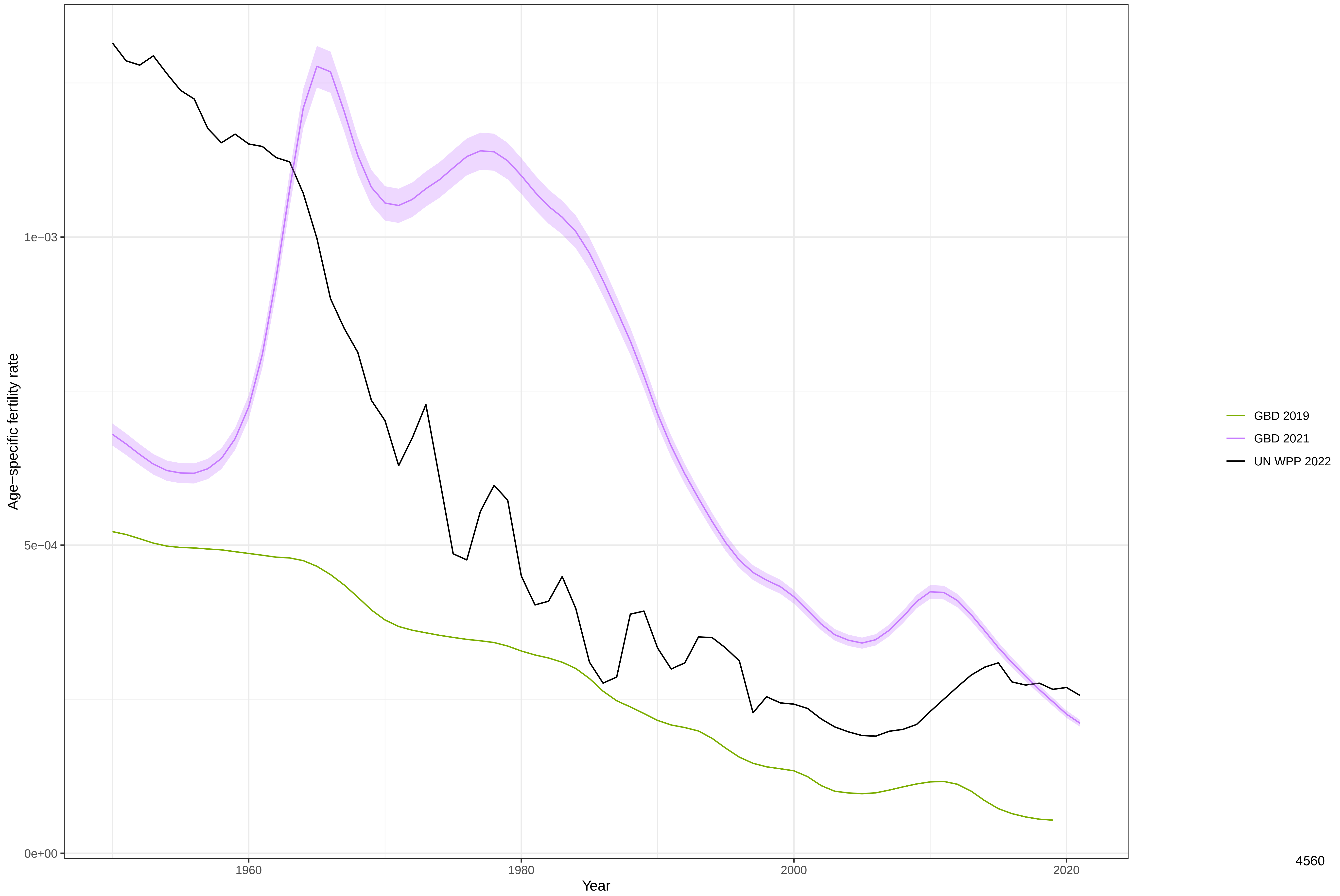

Total fertility rate fitted trend lines, 1950−2021: Egypt TFR
Data points enclosed with a diamond are reference sources

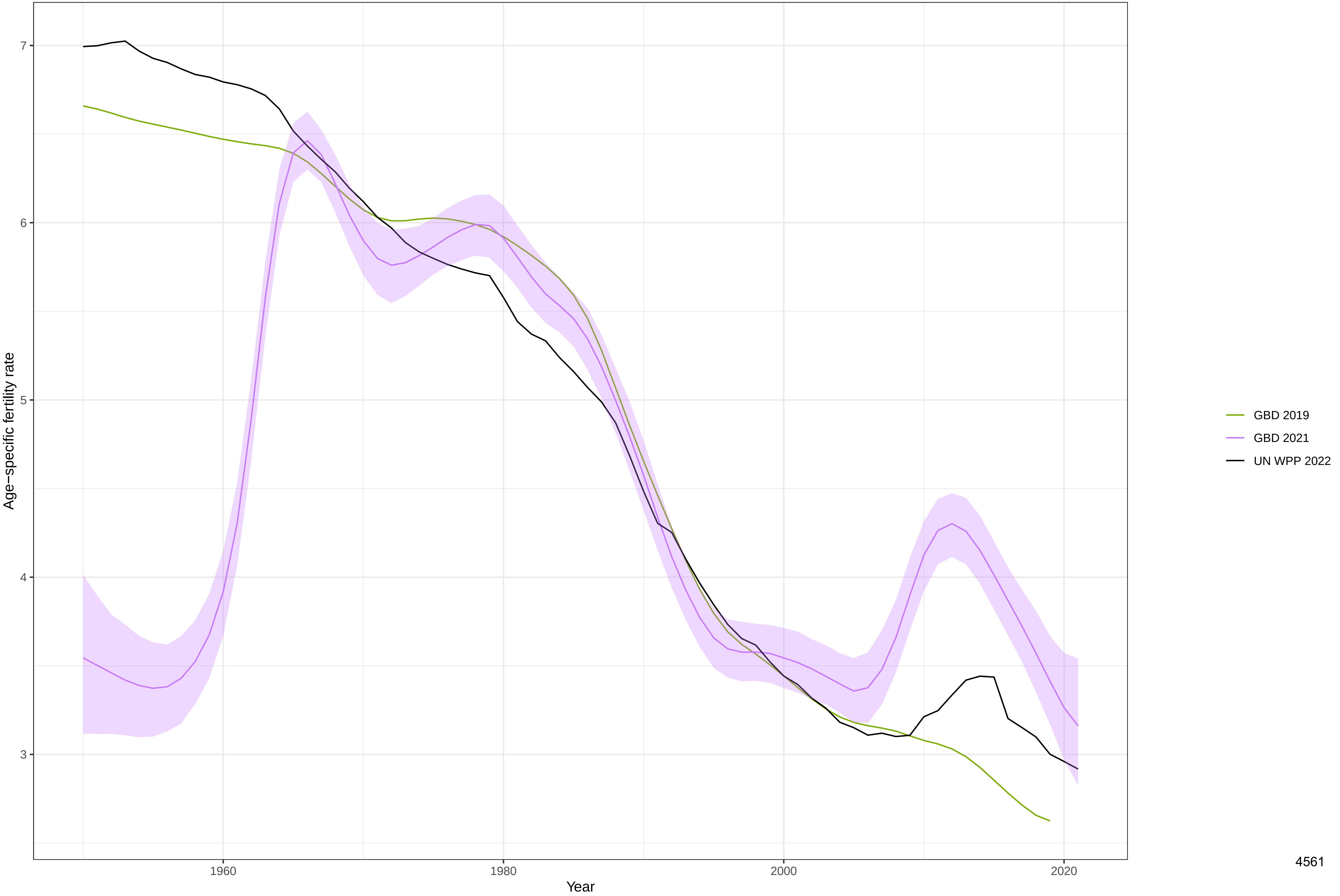

Age−specific fertility fitted trend lines, 1950−2021: Iran, Ages: 10−14
Data points enclosed with a diamond are reference sources

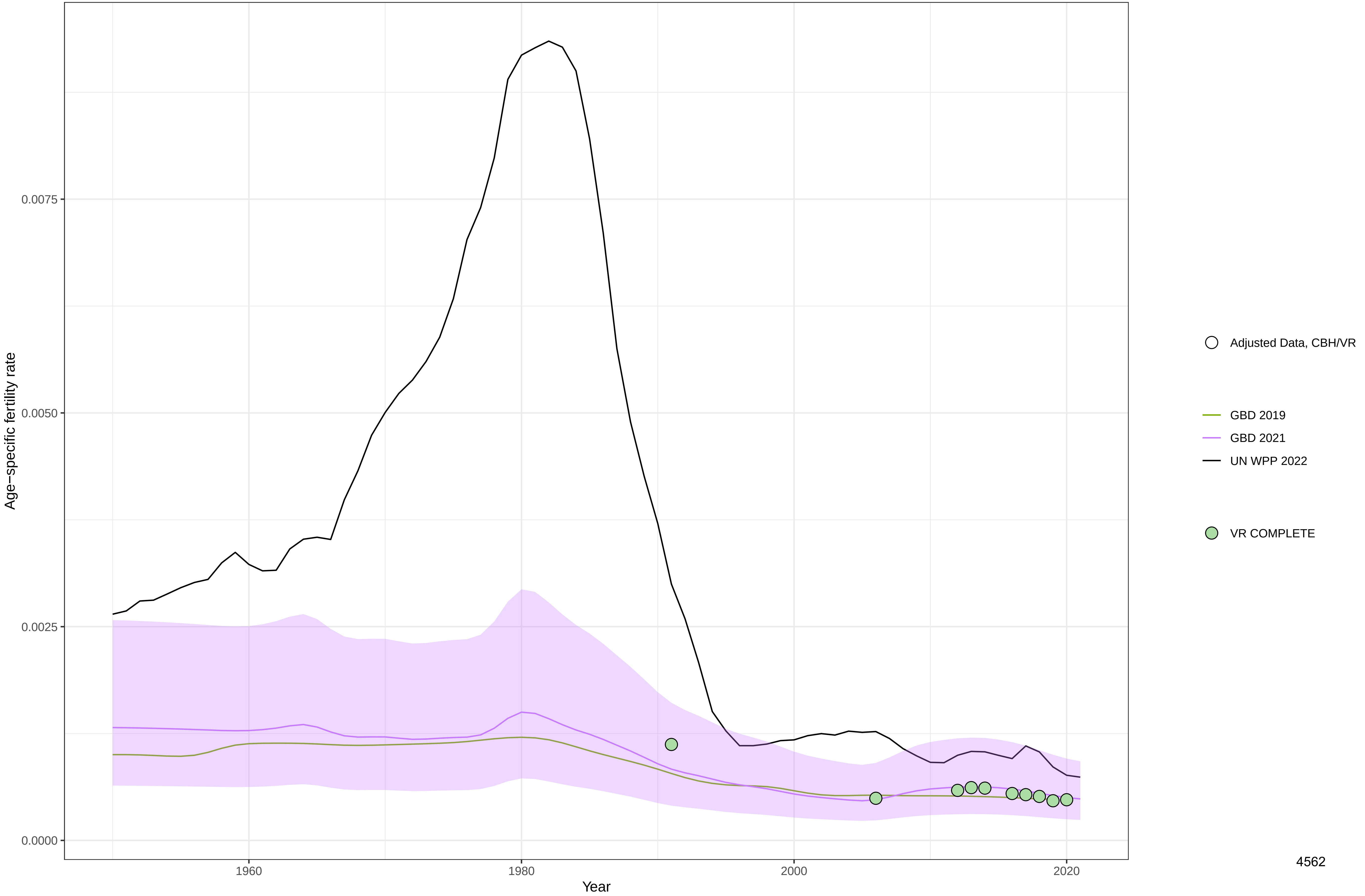

Age−specific fertility fitted trend lines, 1950−2021: Iran, Ages: 15−19
Data points enclosed with a diamond are reference sources

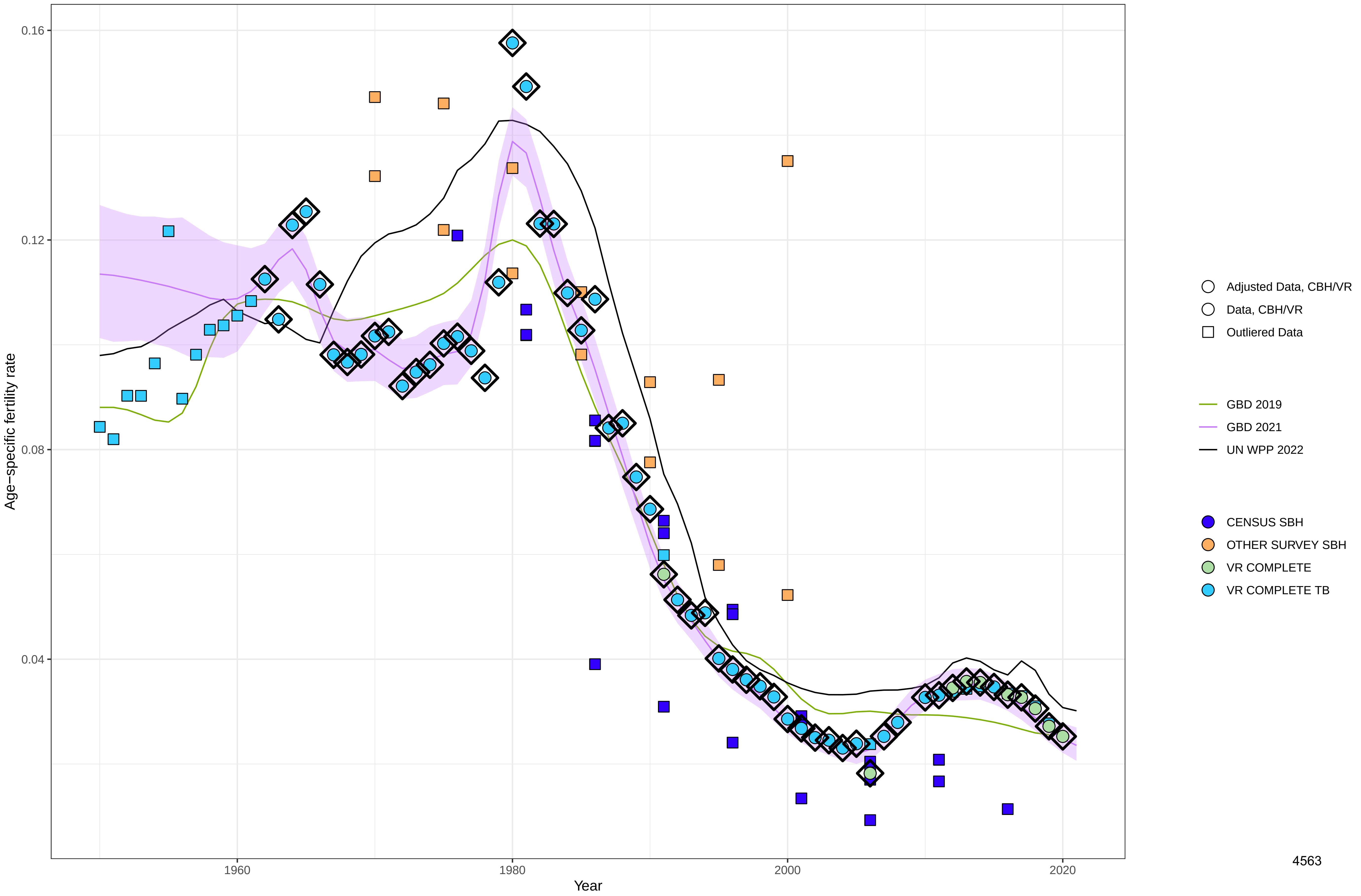

Age−specific fertility fitted trend lines, 1950−2021: Iran, Ages: 20−24
Data points enclosed with a diamond are reference sources

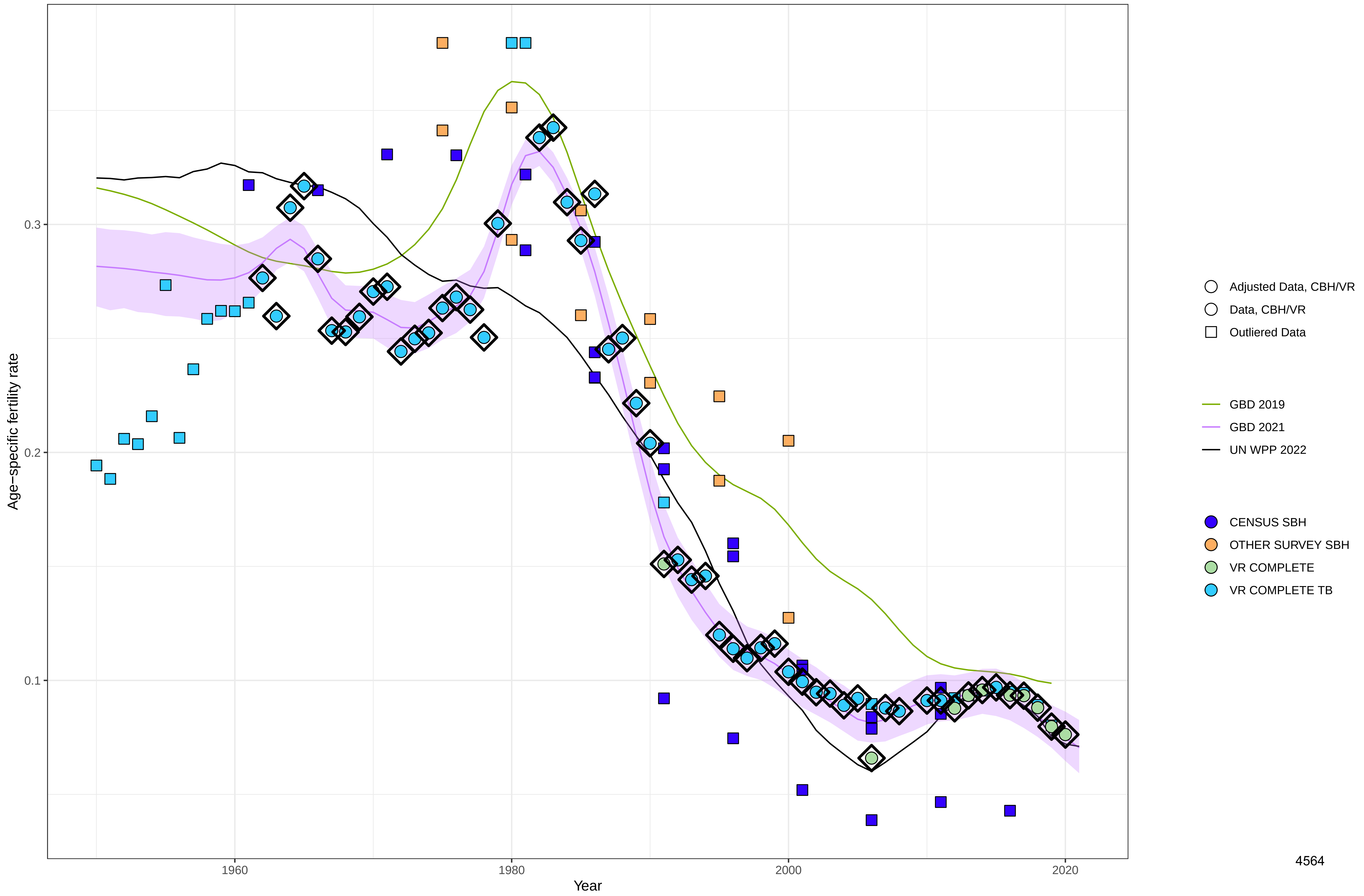

Age−specific fertility fitted trend lines, 1950−2021: Iran, Ages: 25−29
Data points enclosed with a diamond are reference sources

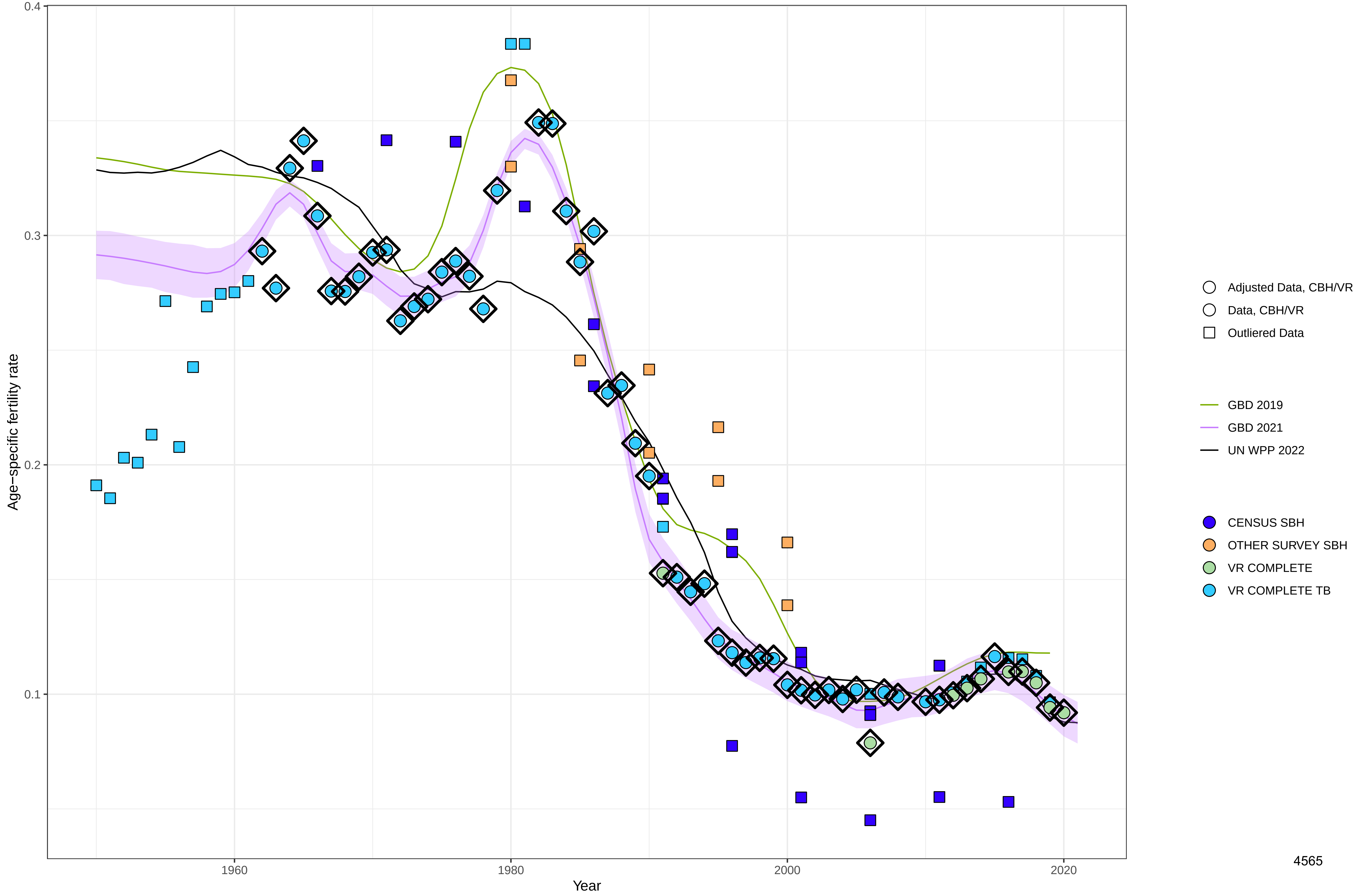

Age−specific fertility fitted trend lines, 1950−2021: Iran, Ages: 30−34
Data points enclosed with a diamond are reference sources

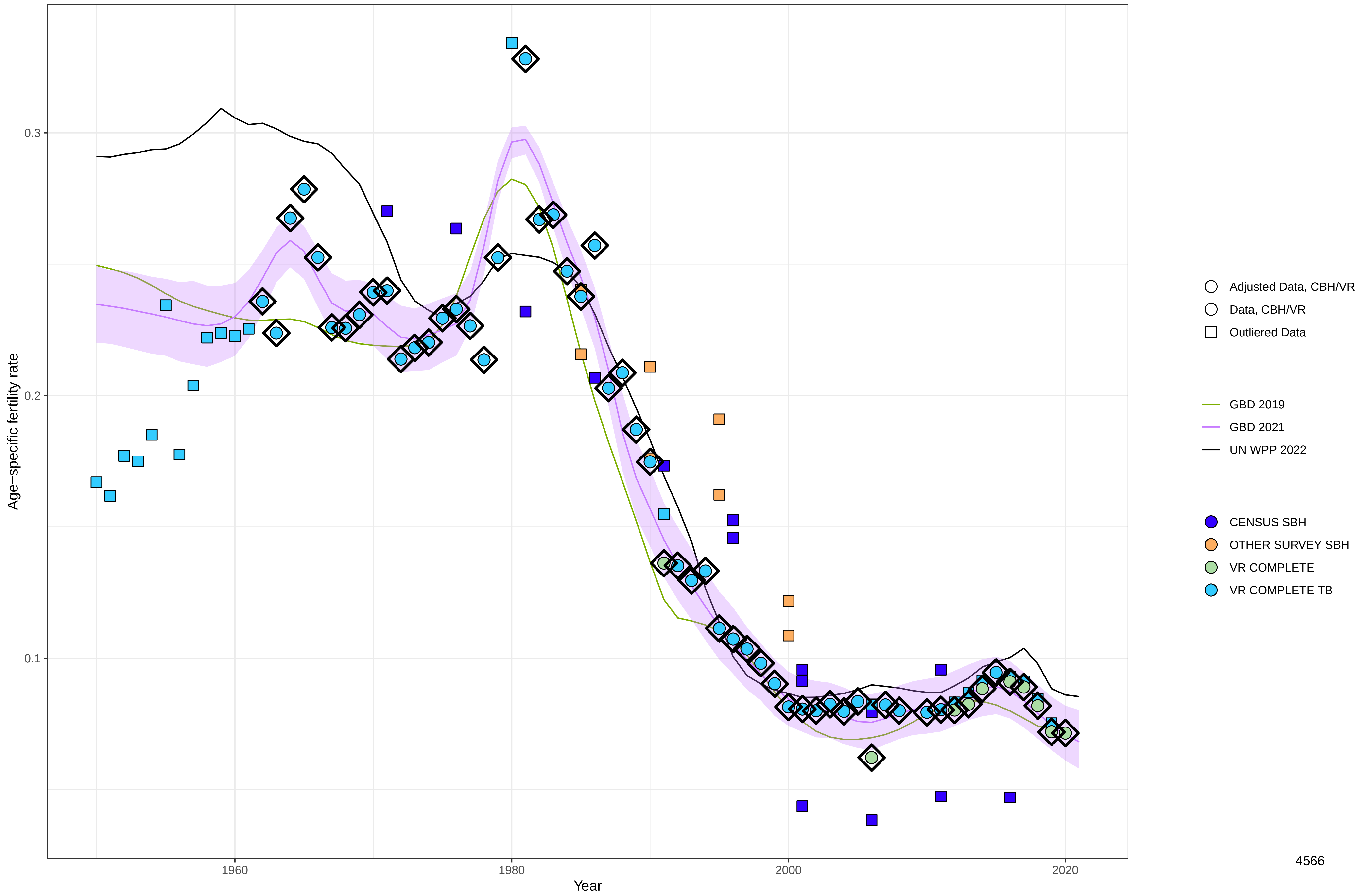

Age−specific fertility fitted trend lines, 1950−2021: Iran, Ages: 35−39
Data points enclosed with a diamond are reference sources

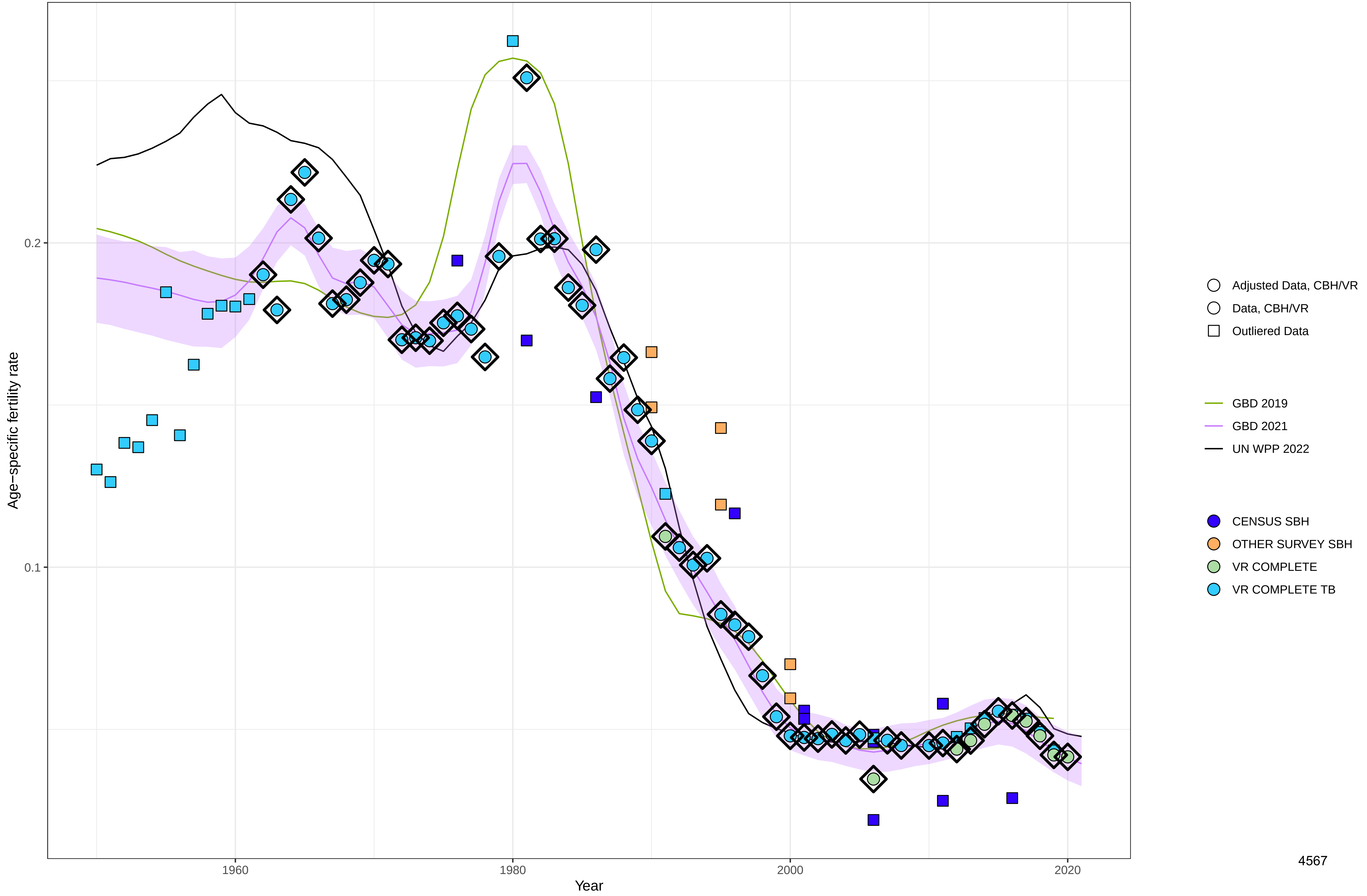

Age−specific fertility fitted trend lines, 1950−2021: Iran, Ages: 40−44
Data points enclosed with a diamond are reference sources

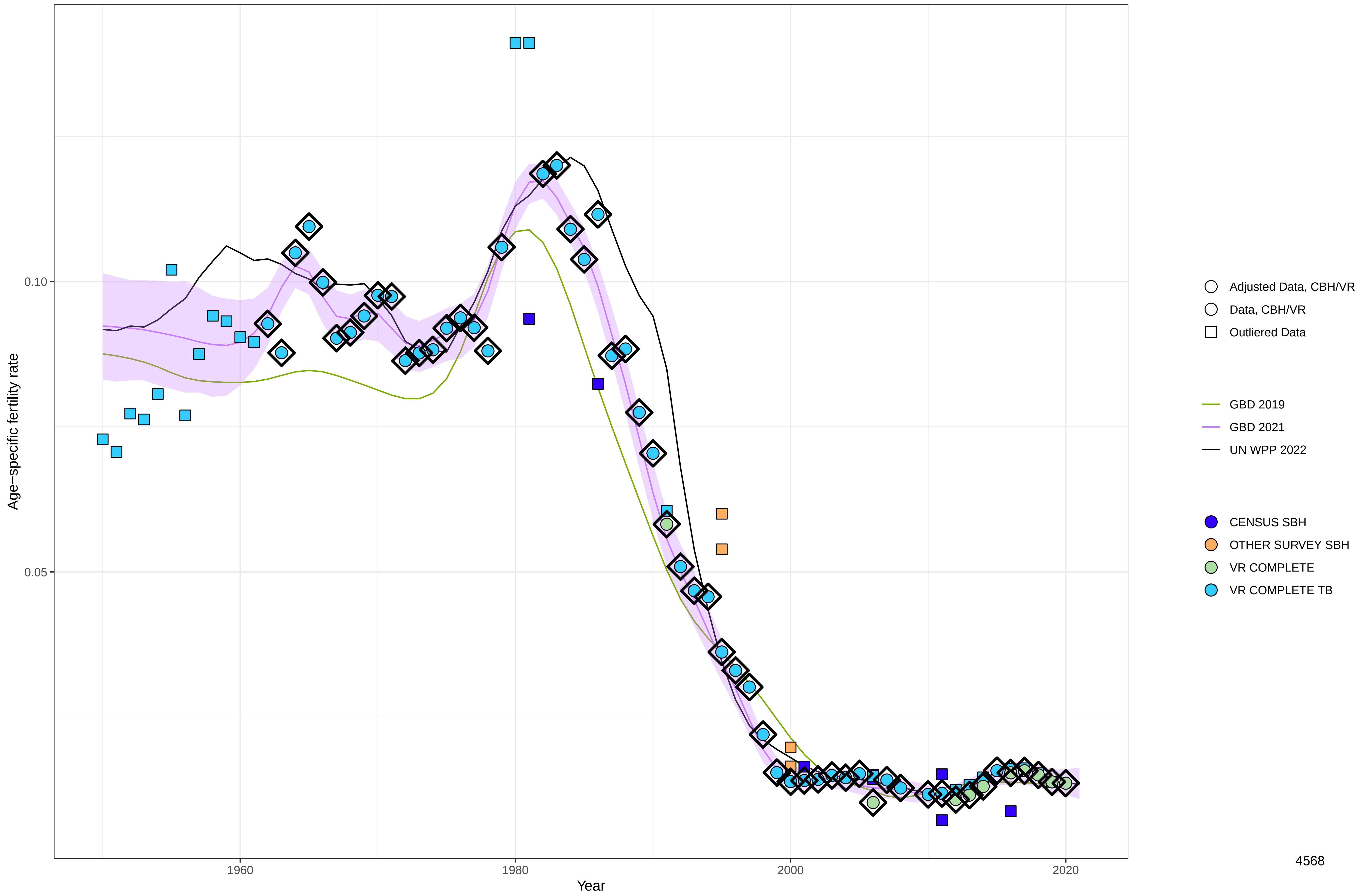

Age−specific fertility fitted trend lines, 1950−2021: Iran, Ages: 45−49
Data points enclosed with a diamond are reference sources

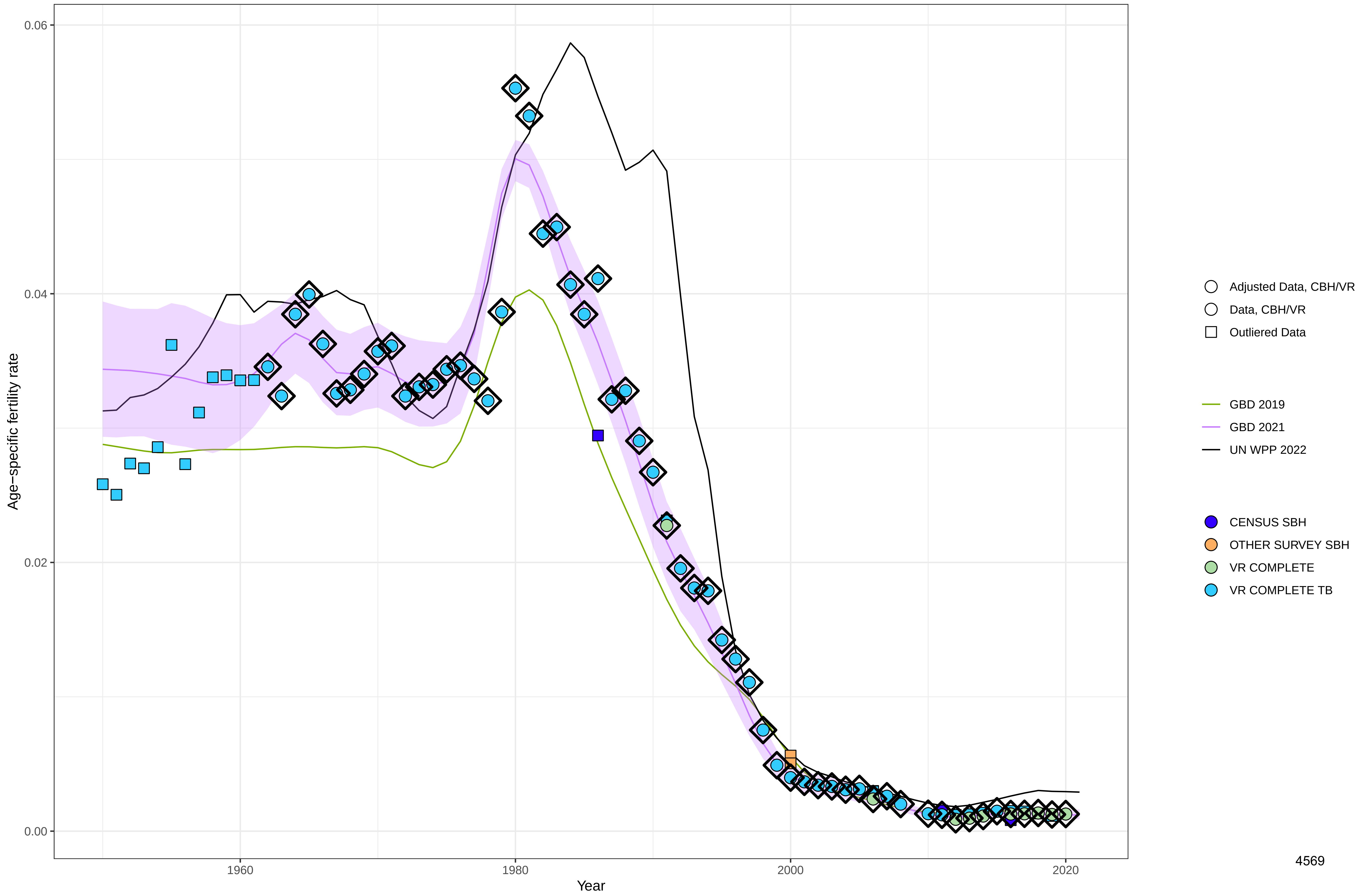

Age−specific fertility fitted trend lines, 1950−2021: Iran, Ages: 50−54
Data points enclosed with a diamond are reference sources

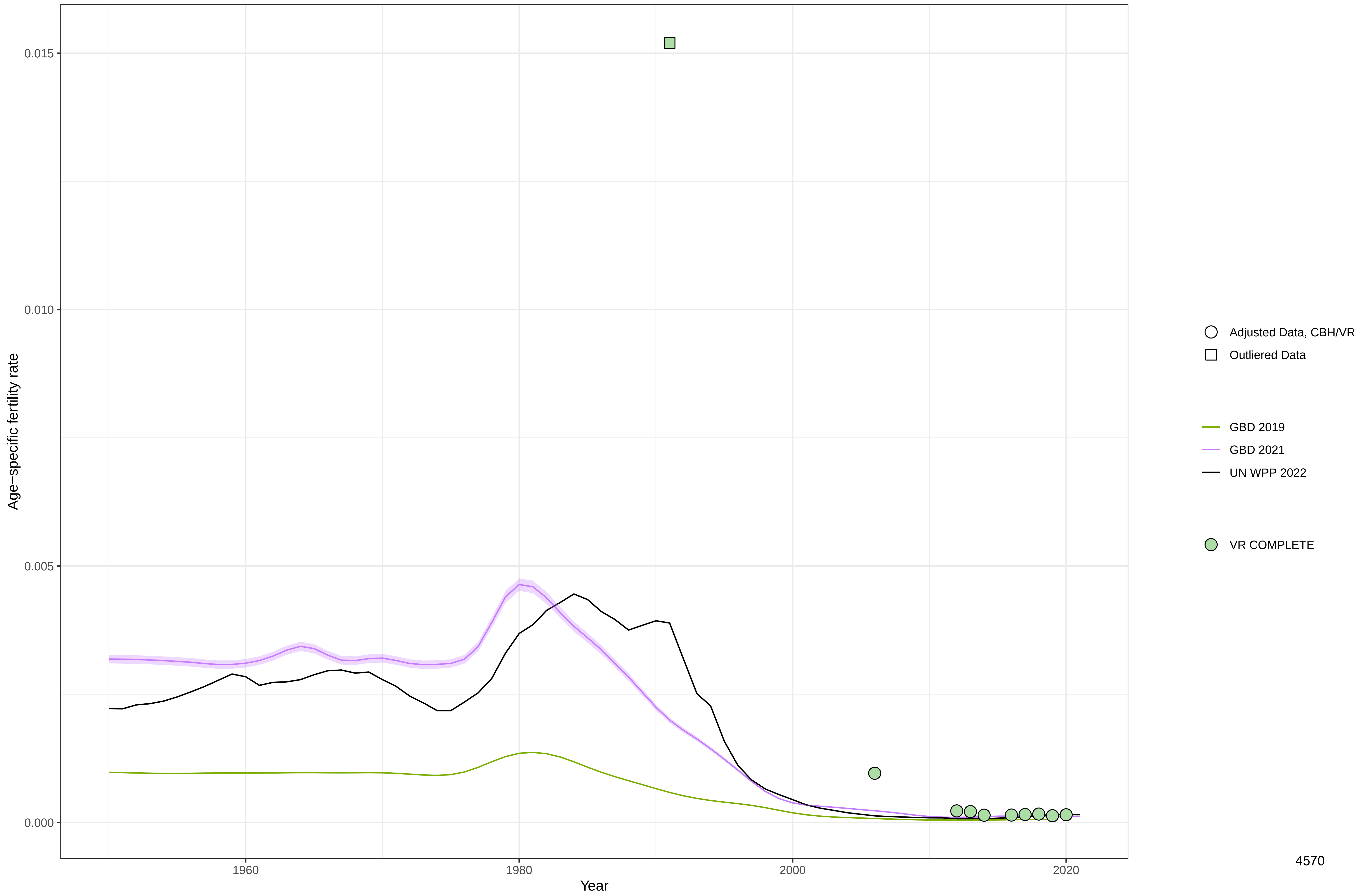

Total fertility rate fitted trend lines, 1950−2021: Iran TFR
Data points enclosed with a diamond are reference sources

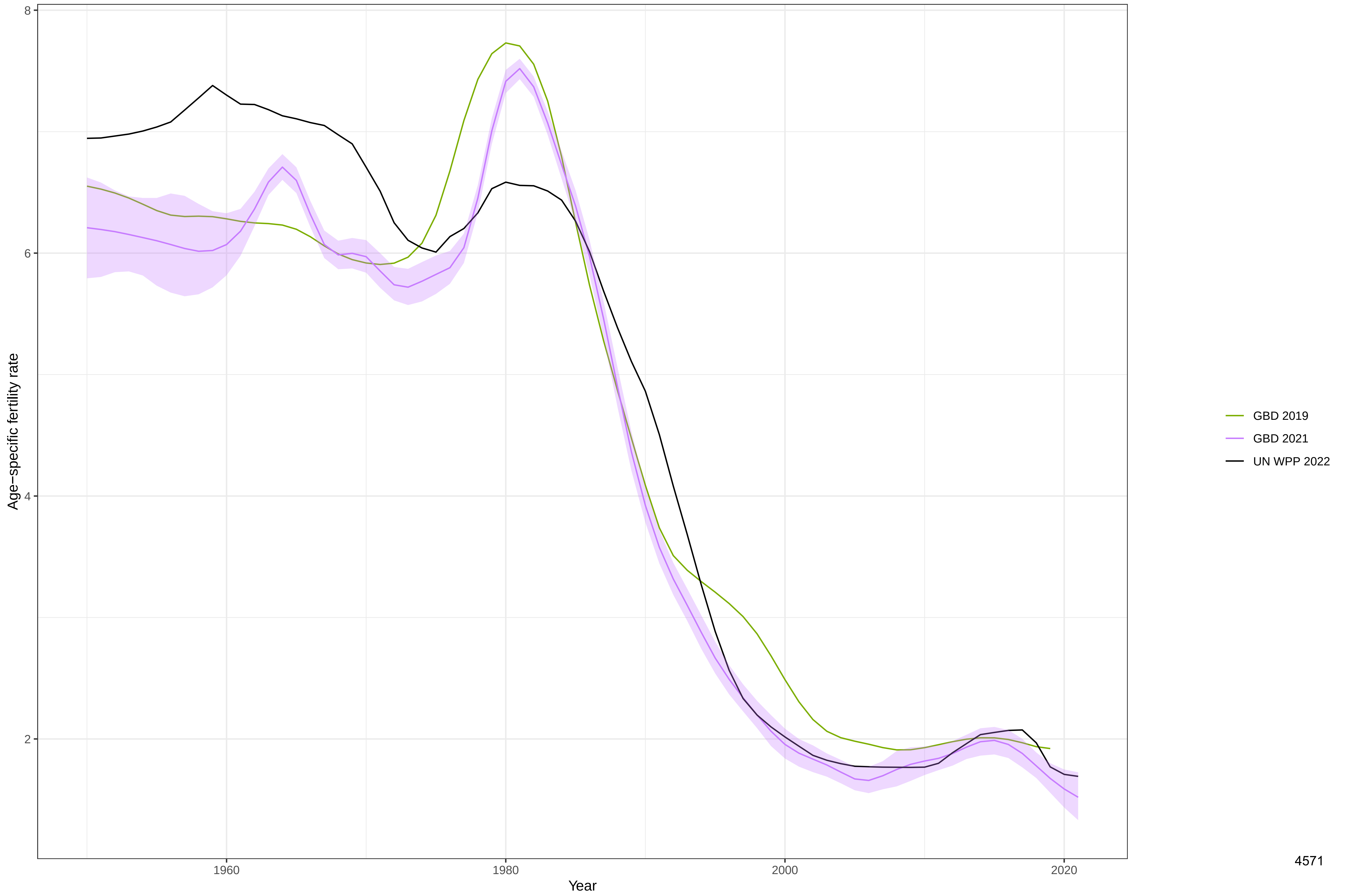

Age−specific fertility fitted trend lines, 1950−2021: Alborz, Ages: 10−14
Data points enclosed with a diamond are reference sources

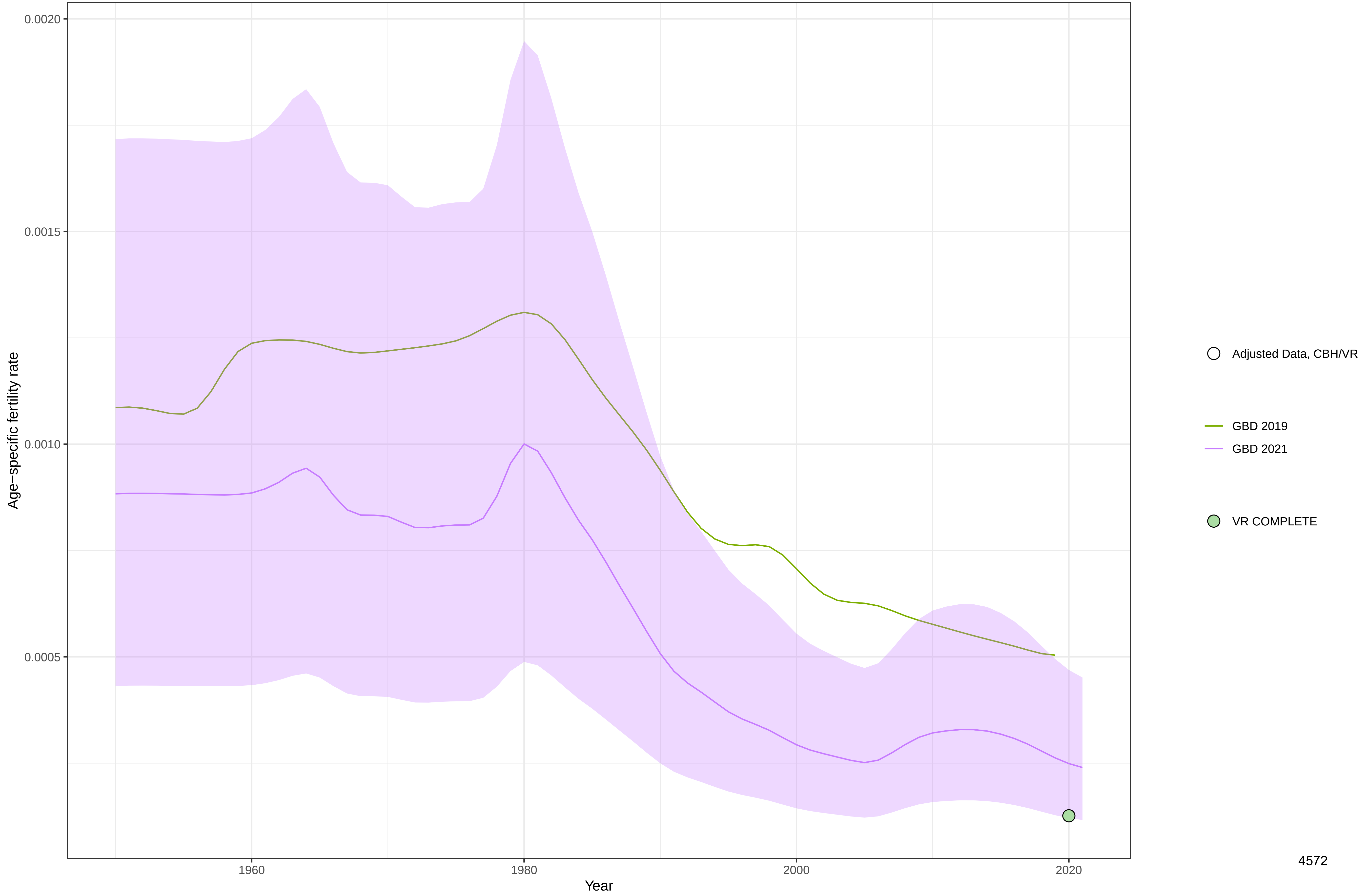

Age−specific fertility fitted trend lines, 1950−2021: Alborz, Ages: 15−19
Data points enclosed with a diamond are reference sources

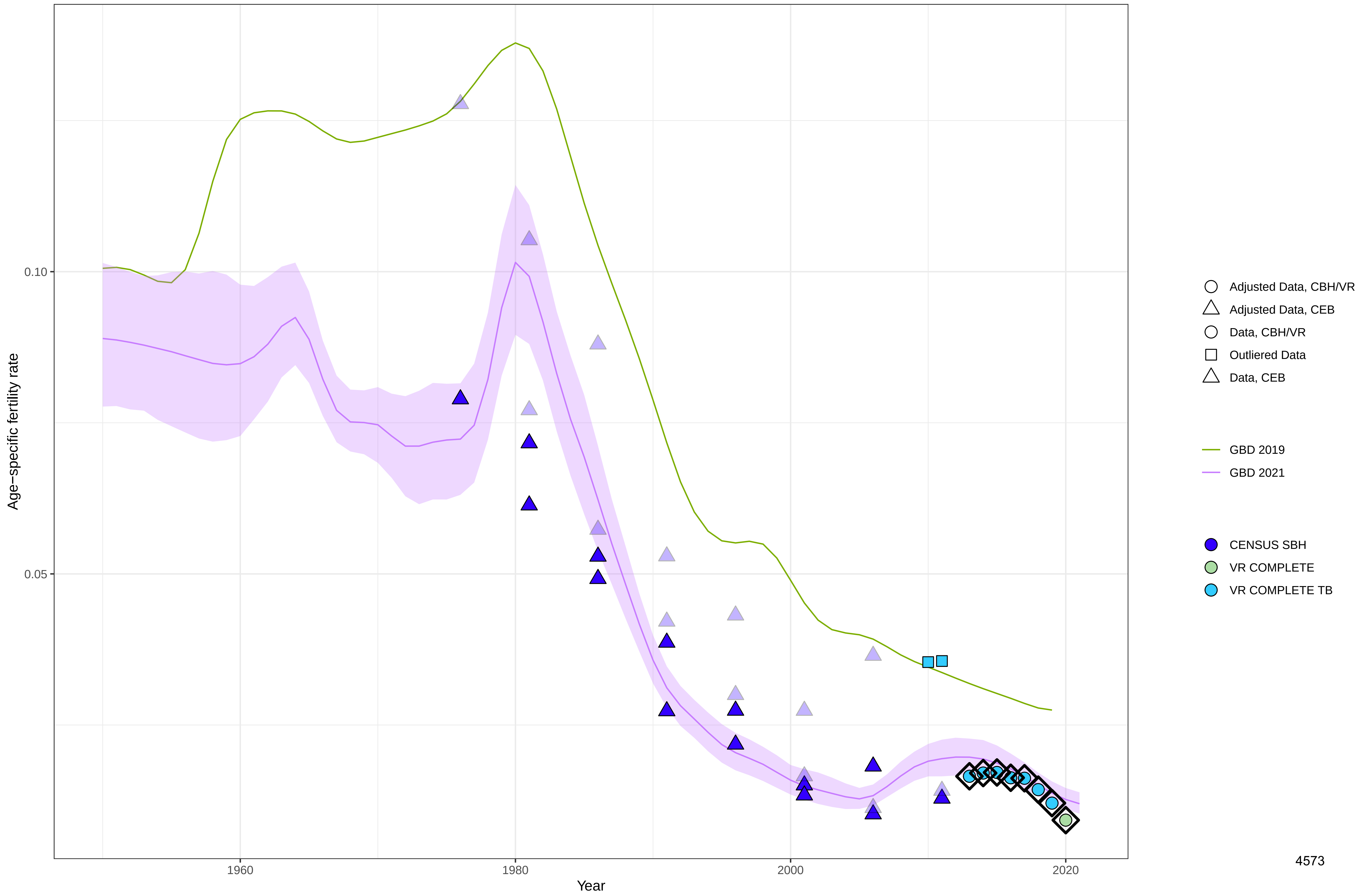

Age−specific fertility fitted trend lines, 1950−2021: Alborz, Ages: 20−24
Data points enclosed with a diamond are reference sources

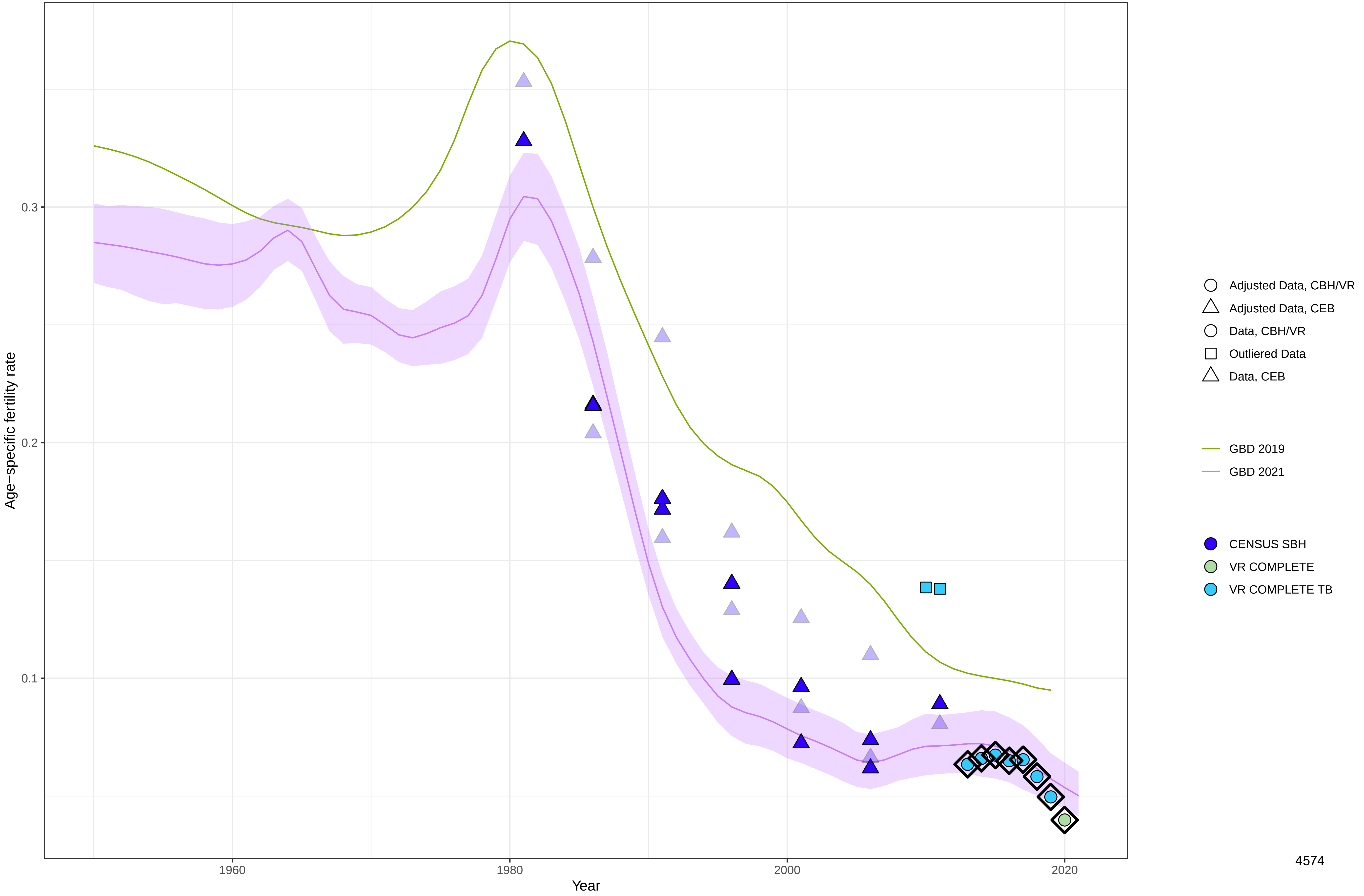

Age−specific fertility fitted trend lines, 1950−2021: Alborz, Ages: 25−29
Data points enclosed with a diamond are reference sources

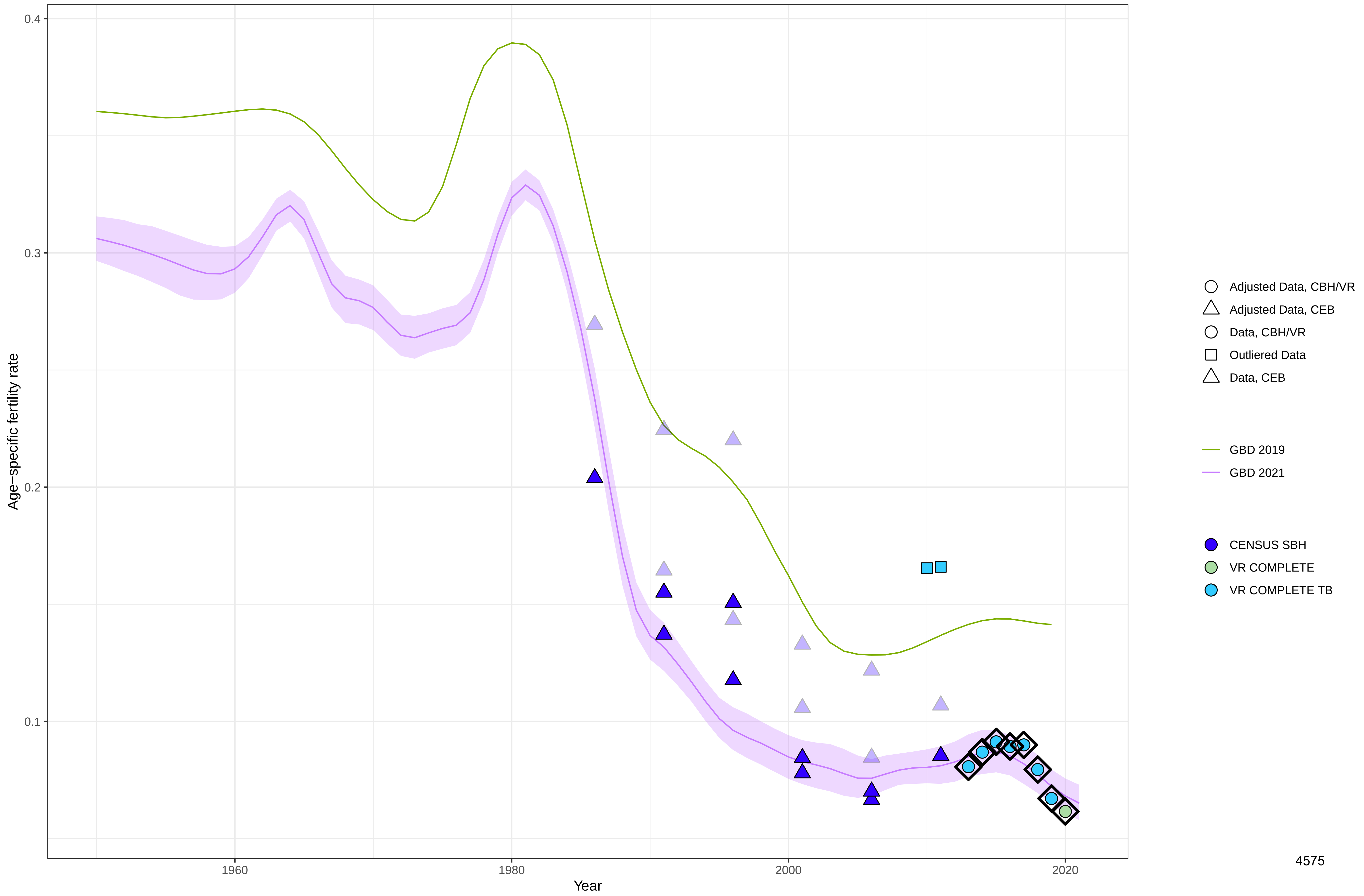

Age−specific fertility fitted trend lines, 1950−2021: Alborz, Ages: 30−34
Data points enclosed with a diamond are reference sources

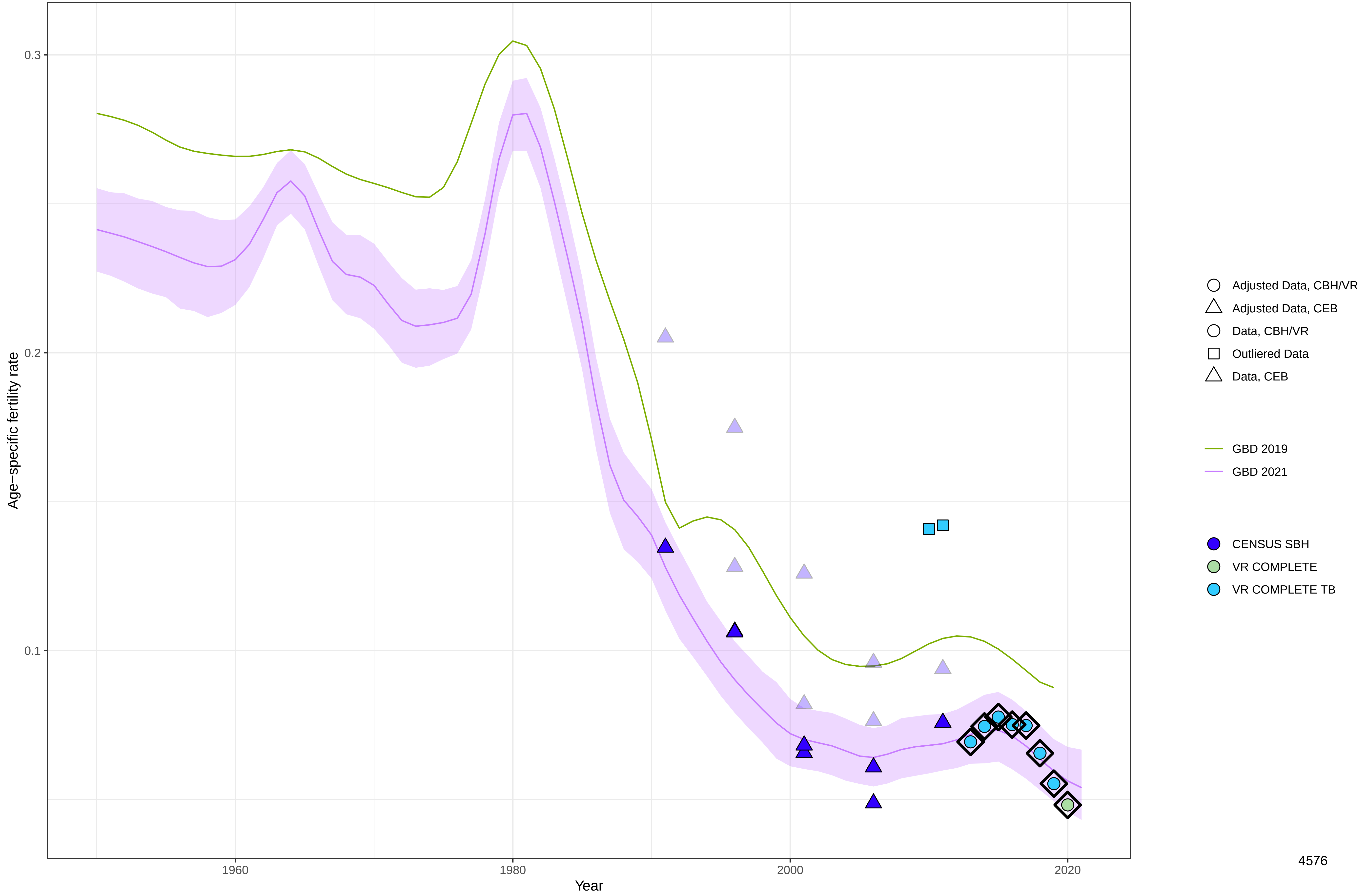

Age−specific fertility fitted trend lines, 1950−2021: Alborz, Ages: 35−39
Data points enclosed with a diamond are reference sources

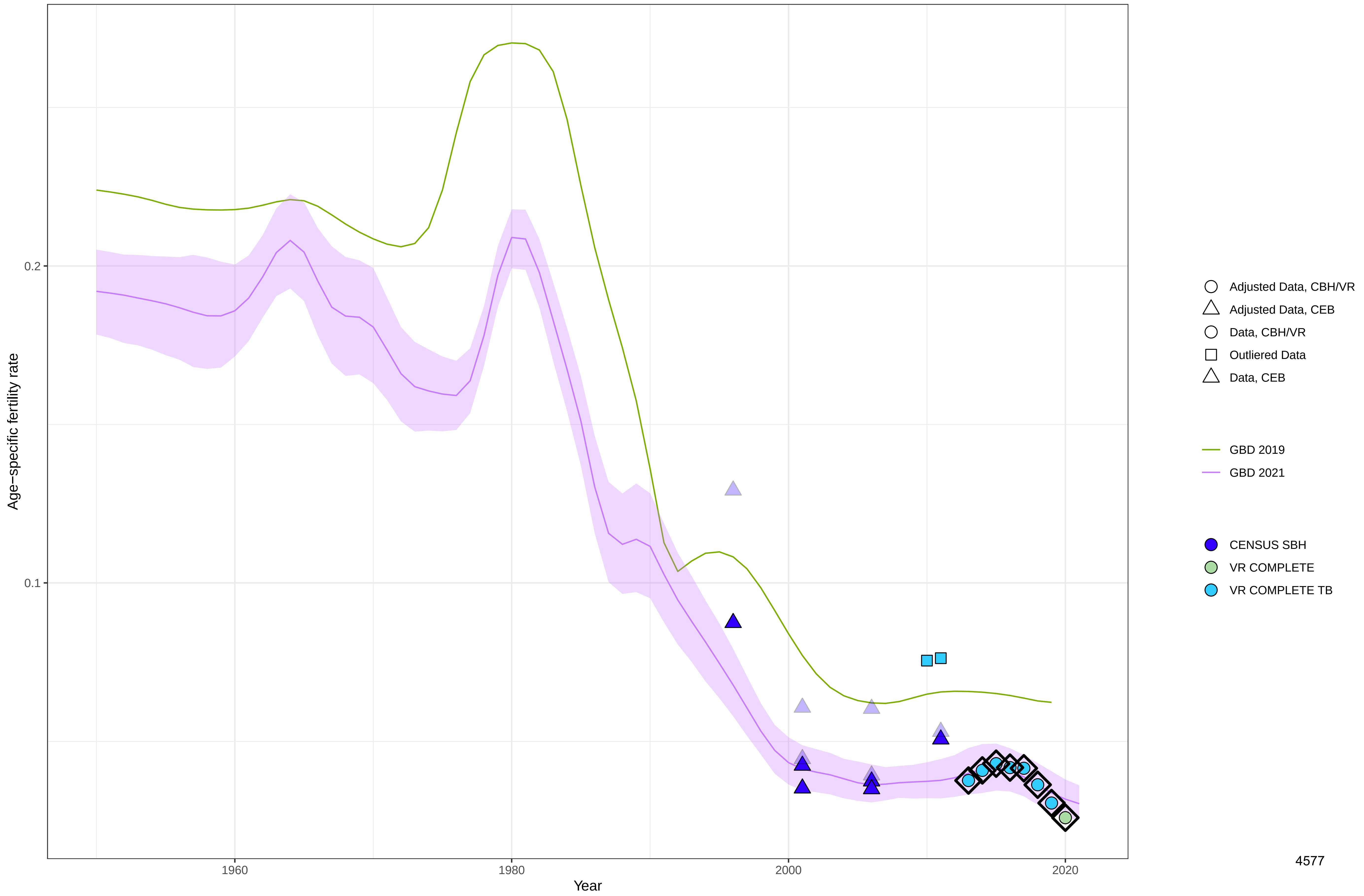

Age−specific fertility fitted trend lines, 1950−2021: Alborz, Ages: 40−44
Data points enclosed with a diamond are reference sources

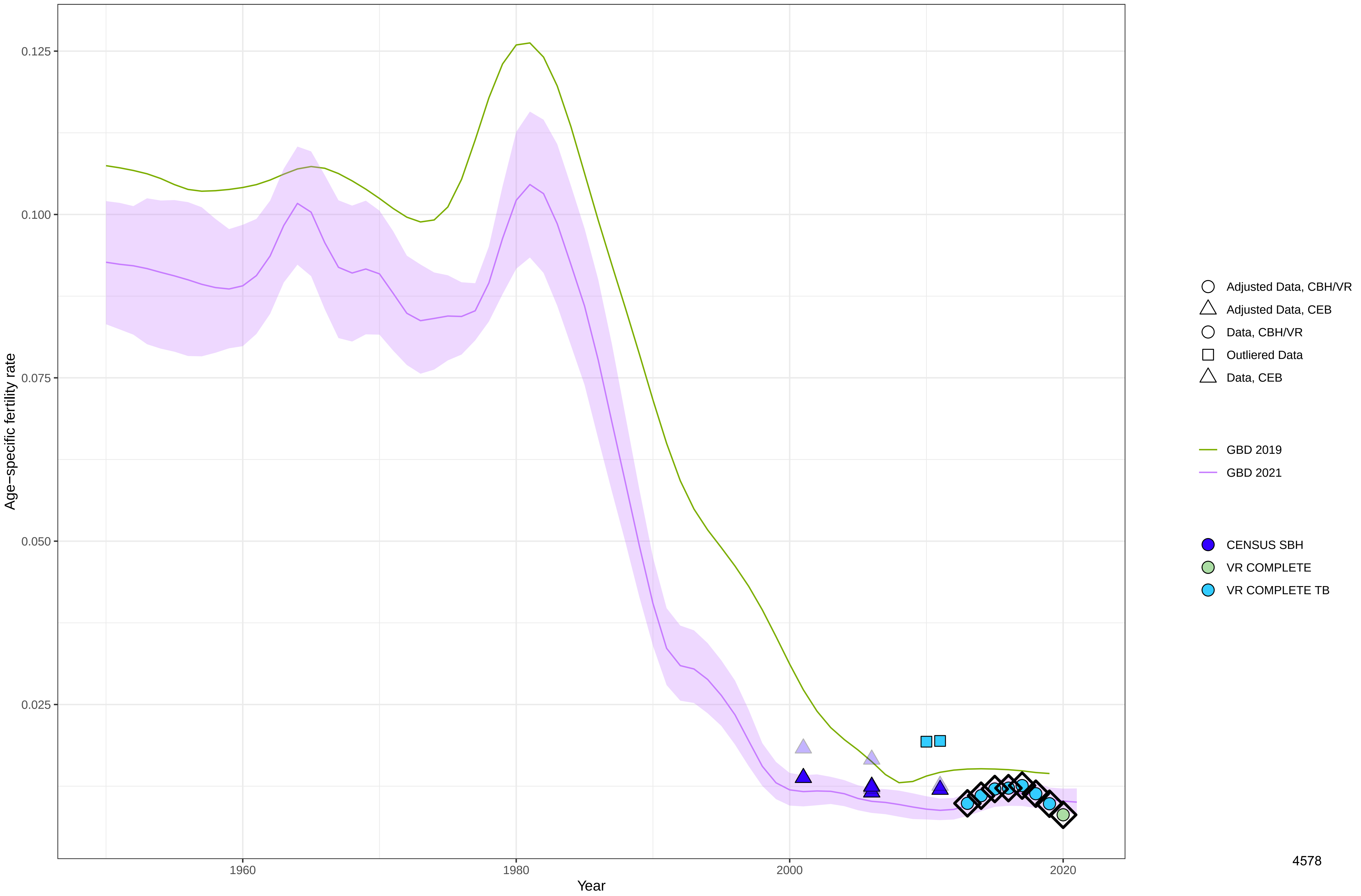

Age−specific fertility fitted trend lines, 1950−2021: Alborz, Ages: 45−49
Data points enclosed with a diamond are reference sources

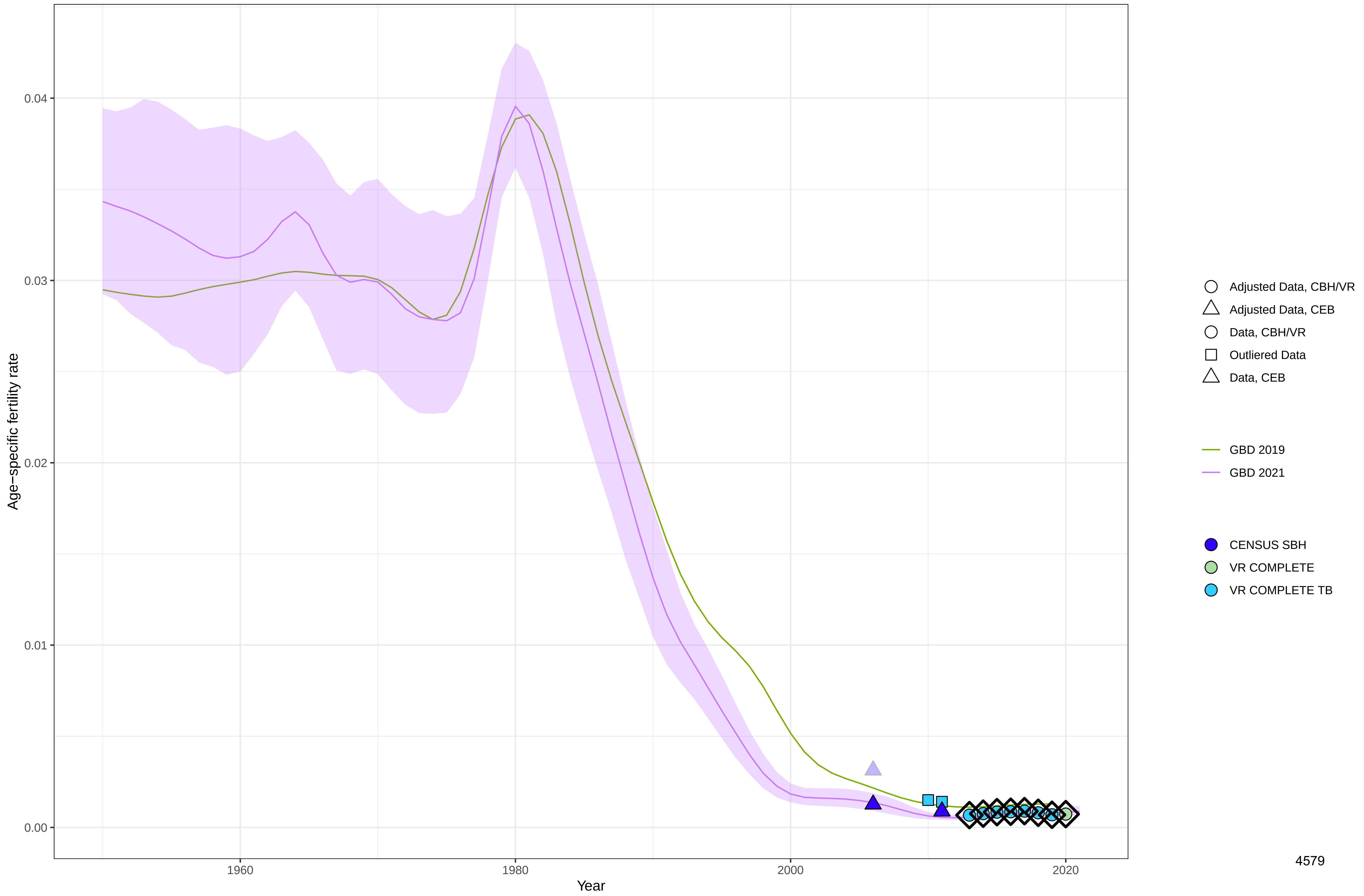

Age−specific fertility fitted trend lines, 1950−2021: Alborz, Ages: 50−54
Data points enclosed with a diamond are reference sources

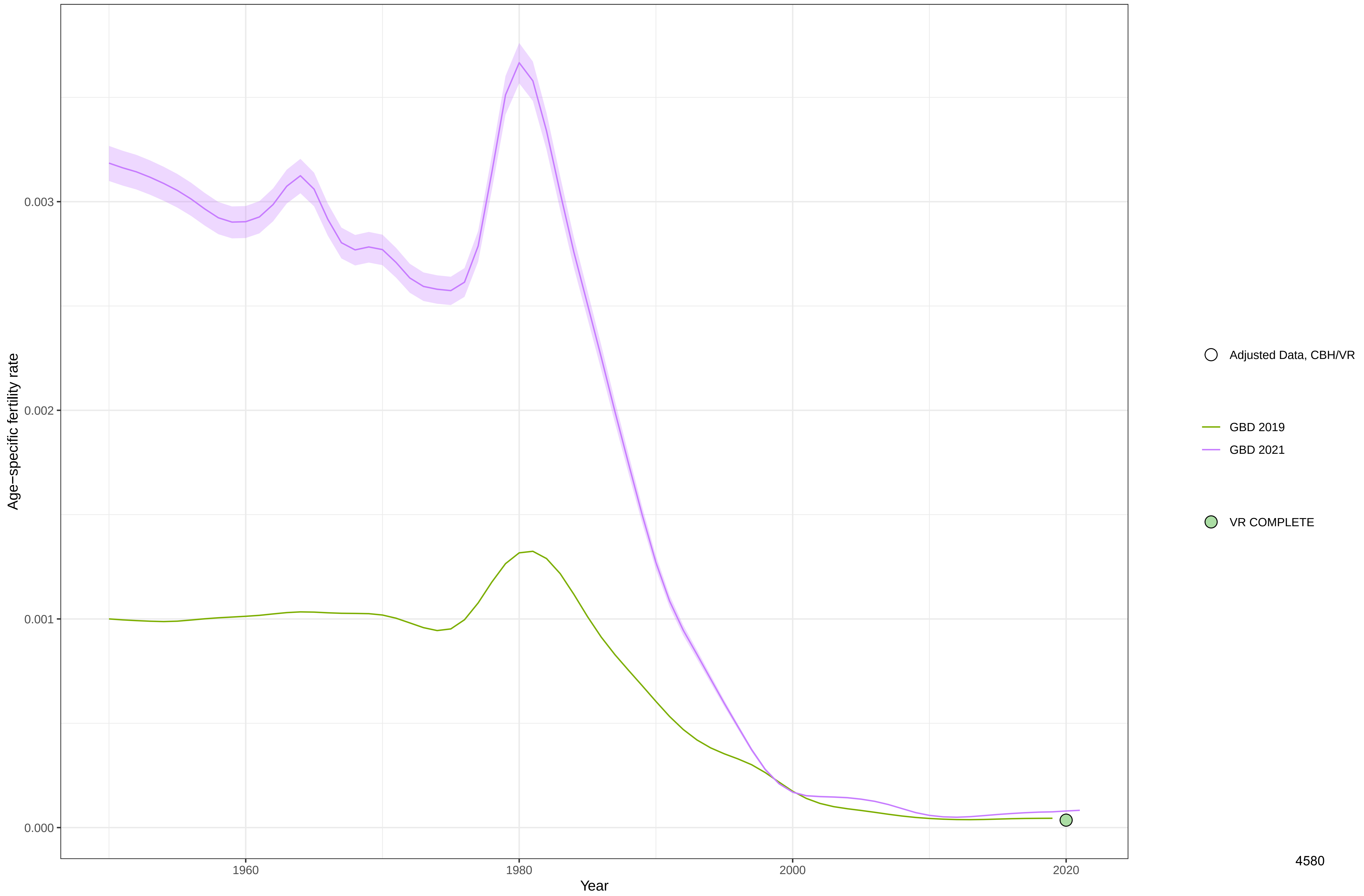

Total fertility rate fitted trend lines, 1950−2021: Alborz TFR
Data points enclosed with a diamond are reference sources

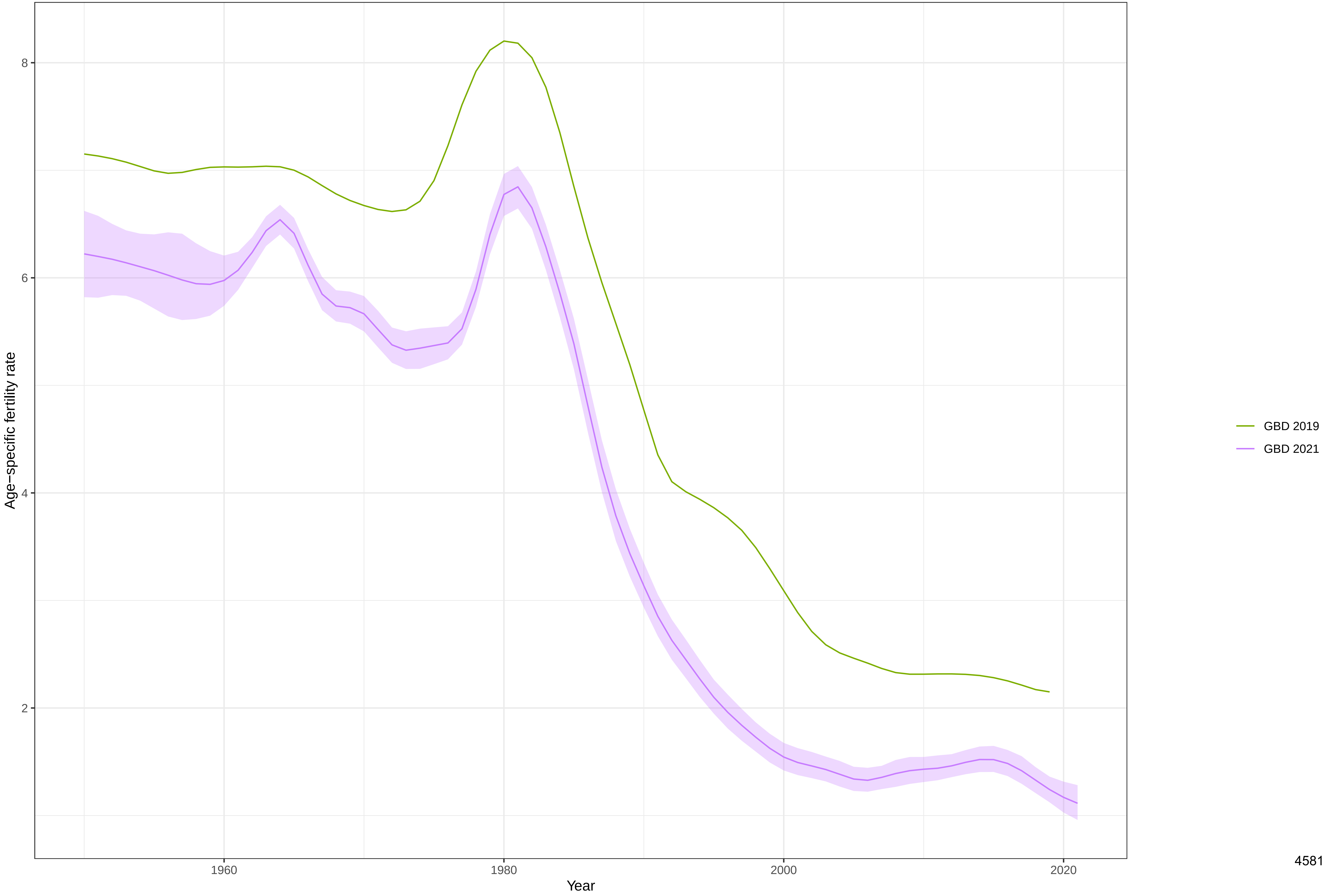

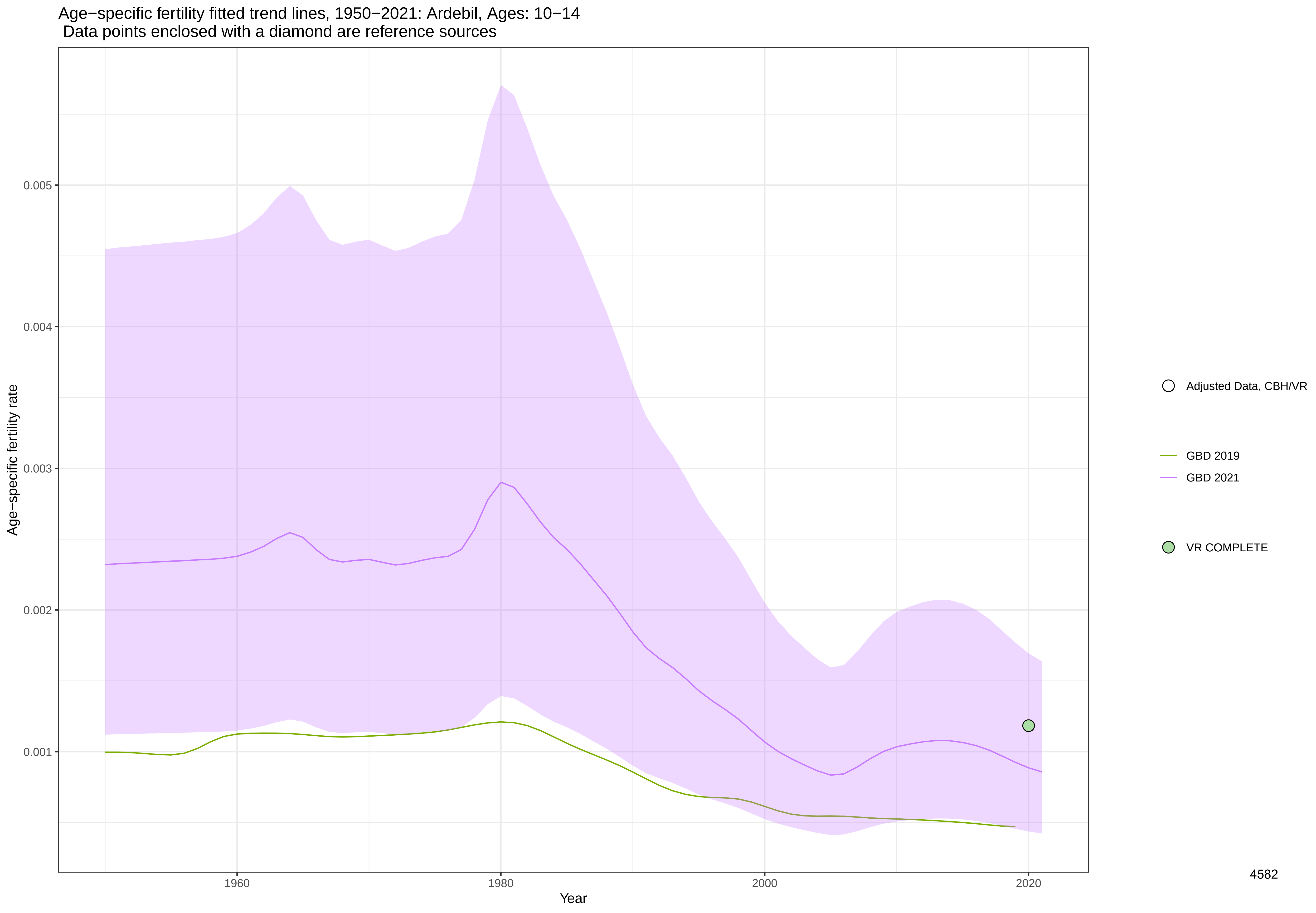

Age−specific fertility fitted trend lines, 1950−2021: Ardebil, Ages: 10−14
Data points enclosed with a diamond are reference sources
Age−specific fertility rate
0.005
0.004
0.003
0.002
0.001
1960
1980
2000
2020
Year
Adjusted Data, CBH/VR
GBD 2019
GBD 2021
VR COMPLETE

Age−specific fertility fitted trend lines, 1950−2021: Ardebil, Ages: 15−19
Data points enclosed with a diamond are reference sources

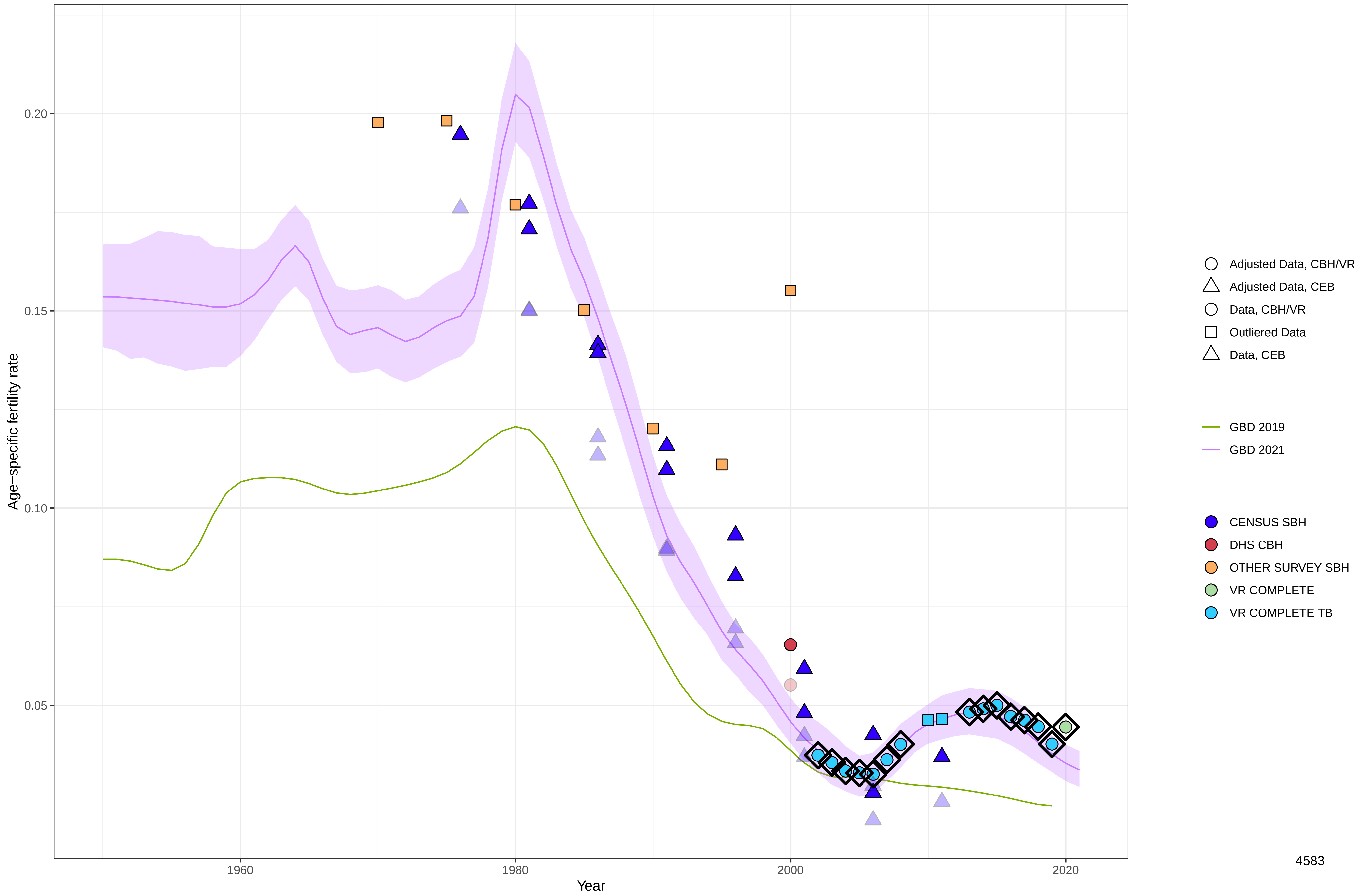

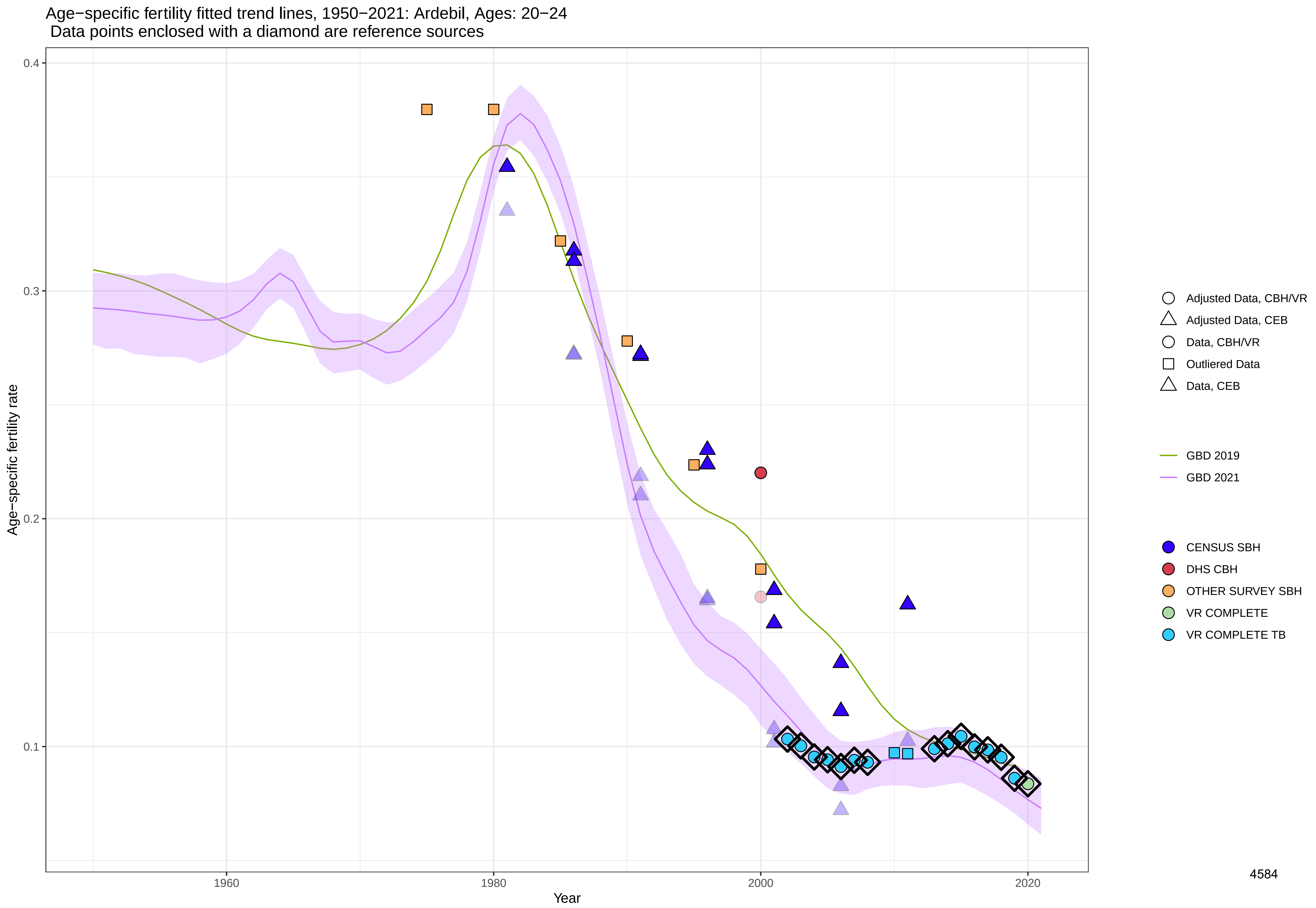

Age−specific fertility fitted trend lines, 1950−2021: Ardebil, Ages: 20−24
Data points enclosed with a diamond are reference sources
Age−specific fertility rate
Year
0.1
0.2
0.3
0.4
1960
1980
2000
2020
Adjusted Data, CBH/VR
Adjusted Data, CEB
Data, CBH/VR
Outliered Data
Data, CEB
GBD 2019
GBD 2021
CENSUS SBH
DHS CBH
OTHER SURVEY SBH
VR COMPLETE
VR COMPLETE TB

Age−specific fertility fitted trend lines, 1950−2021: Ardebil, Ages: 25−29
Data points enclosed with a diamond are reference sources

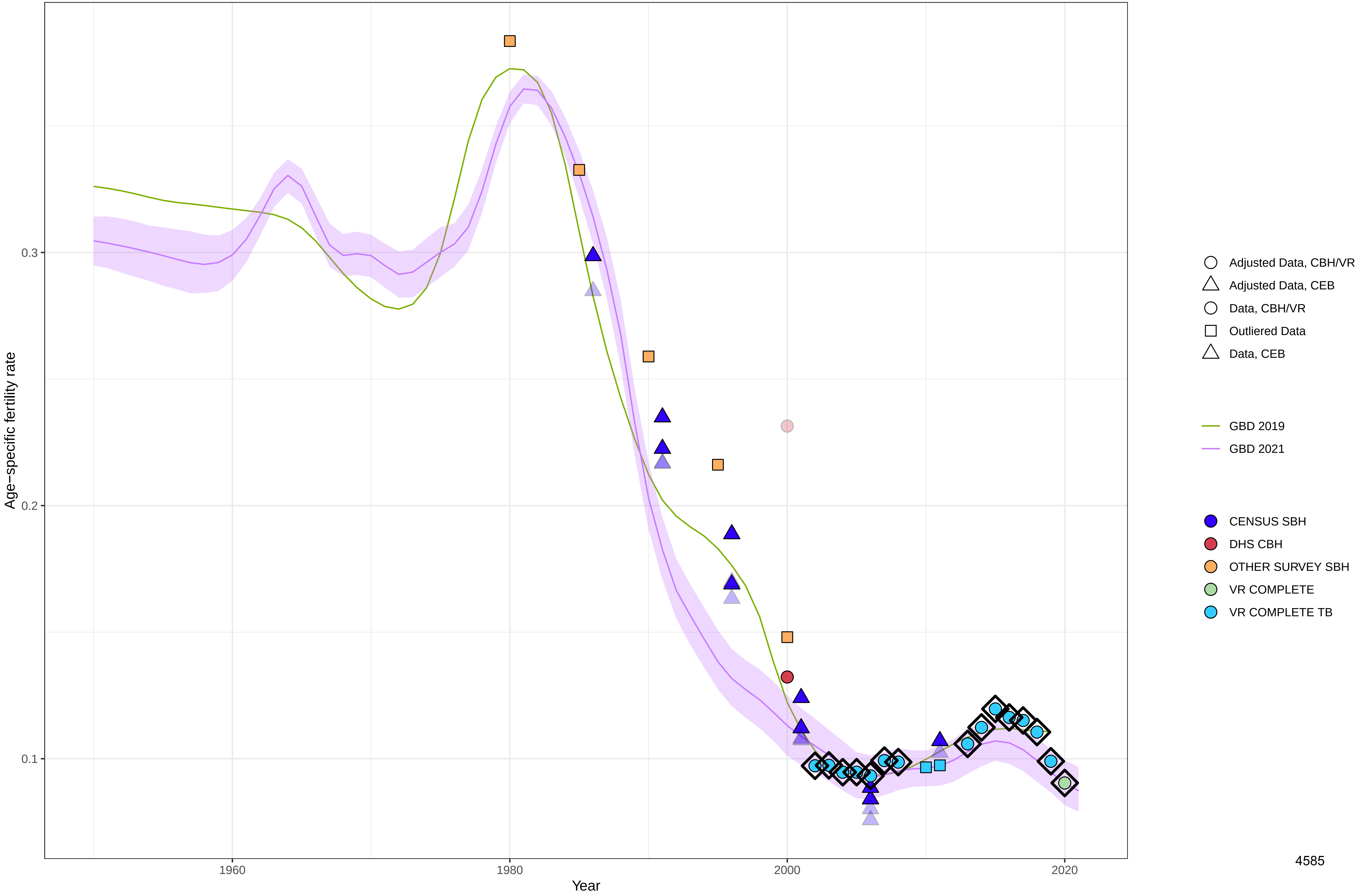

Age−specific fertility fitted trend lines, 1950−2021: Ardebil, Ages: 30−34
Data points enclosed with a diamond are reference sources

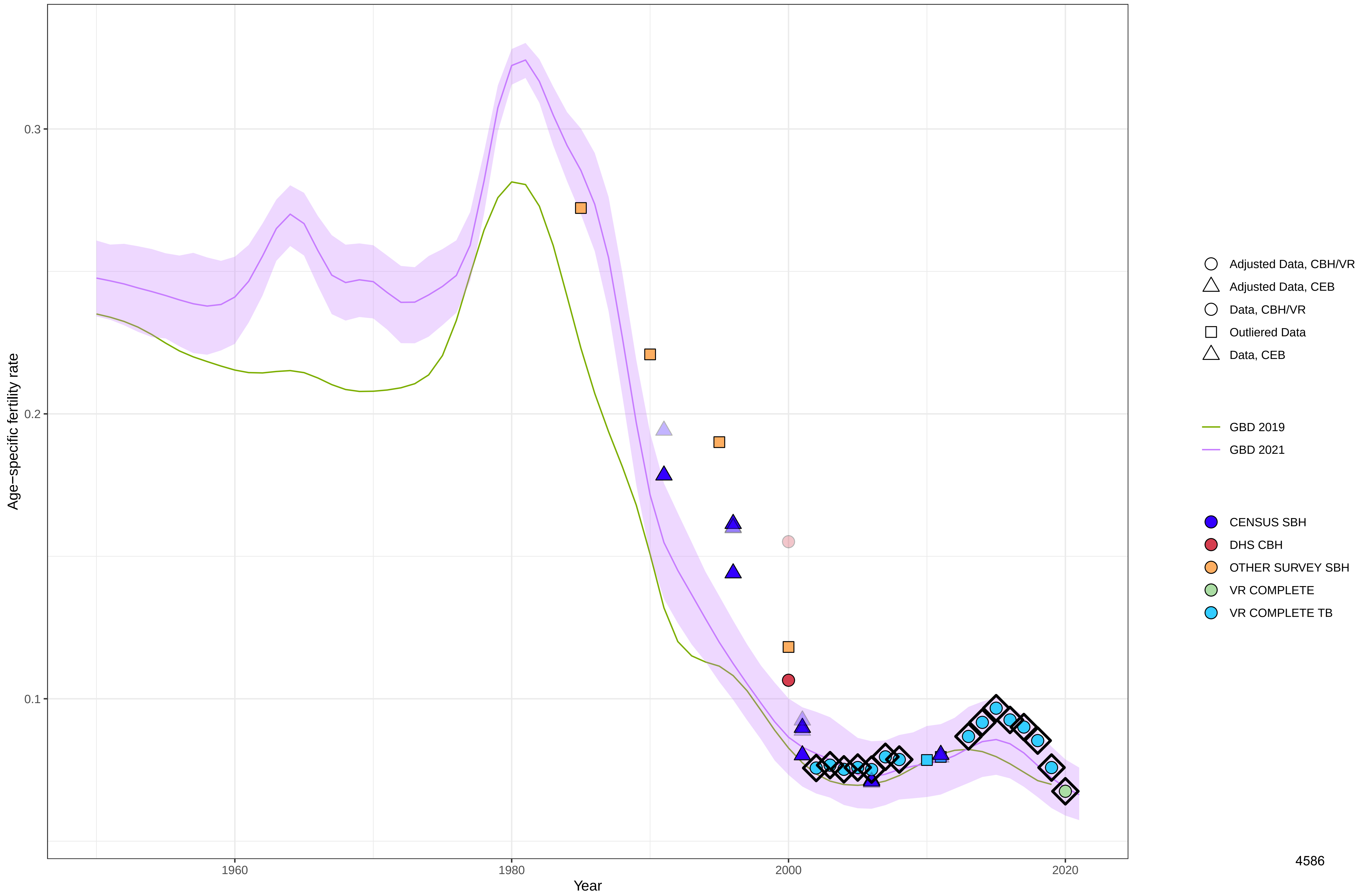

Age−specific fertility fitted trend lines, 1950−2021: Ardebil, Ages: 35−39
Data points enclosed with a diamond are reference sources

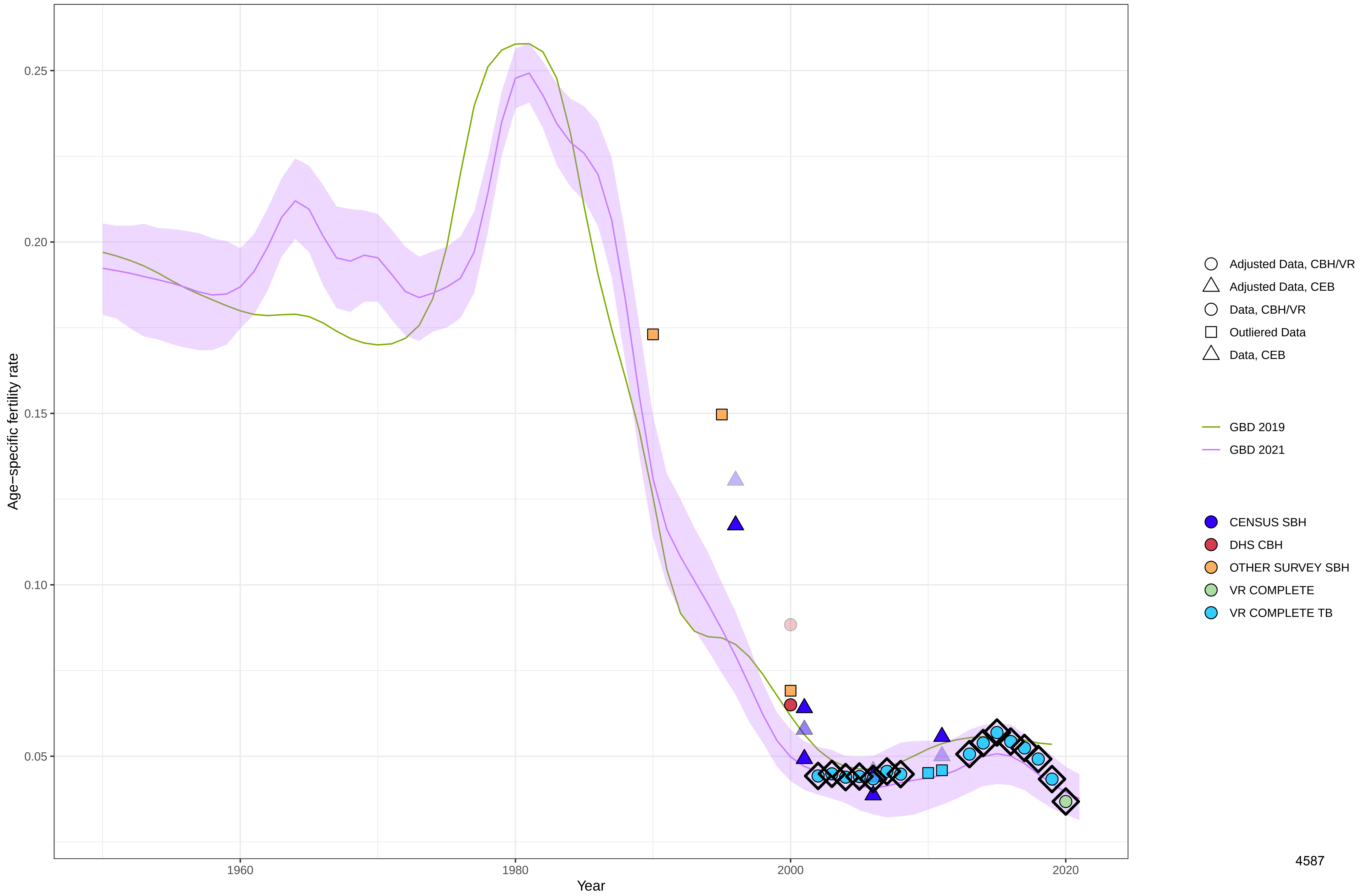

Age−specific fertility fitted trend lines, 1950−2021: Ardebil, Ages: 40−44
Data points enclosed with a diamond are reference sources

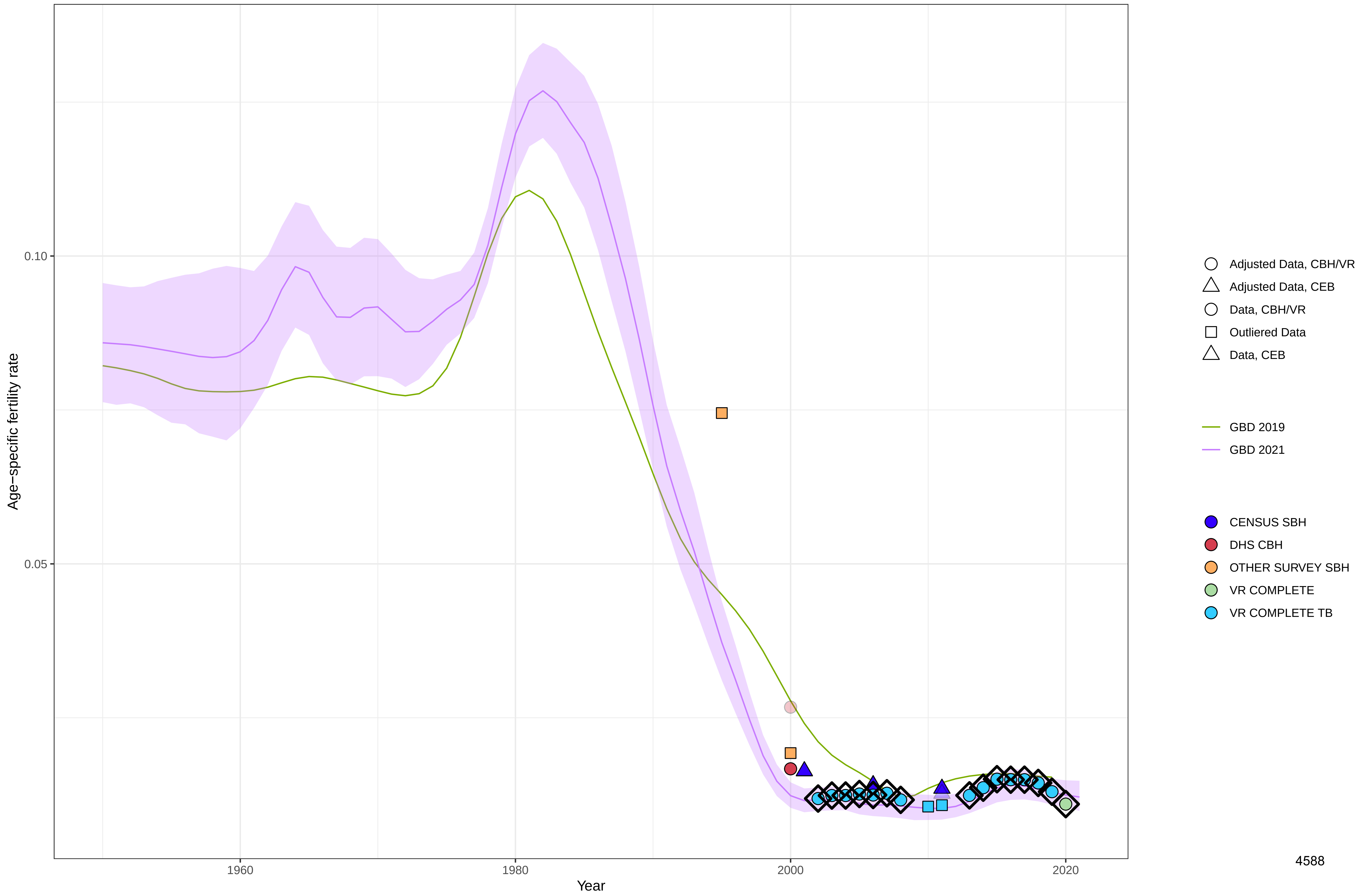

Age−specific fertility fitted trend lines, 1950−2021: Ardebil, Ages: 45−49
Data points enclosed with a diamond are reference sources

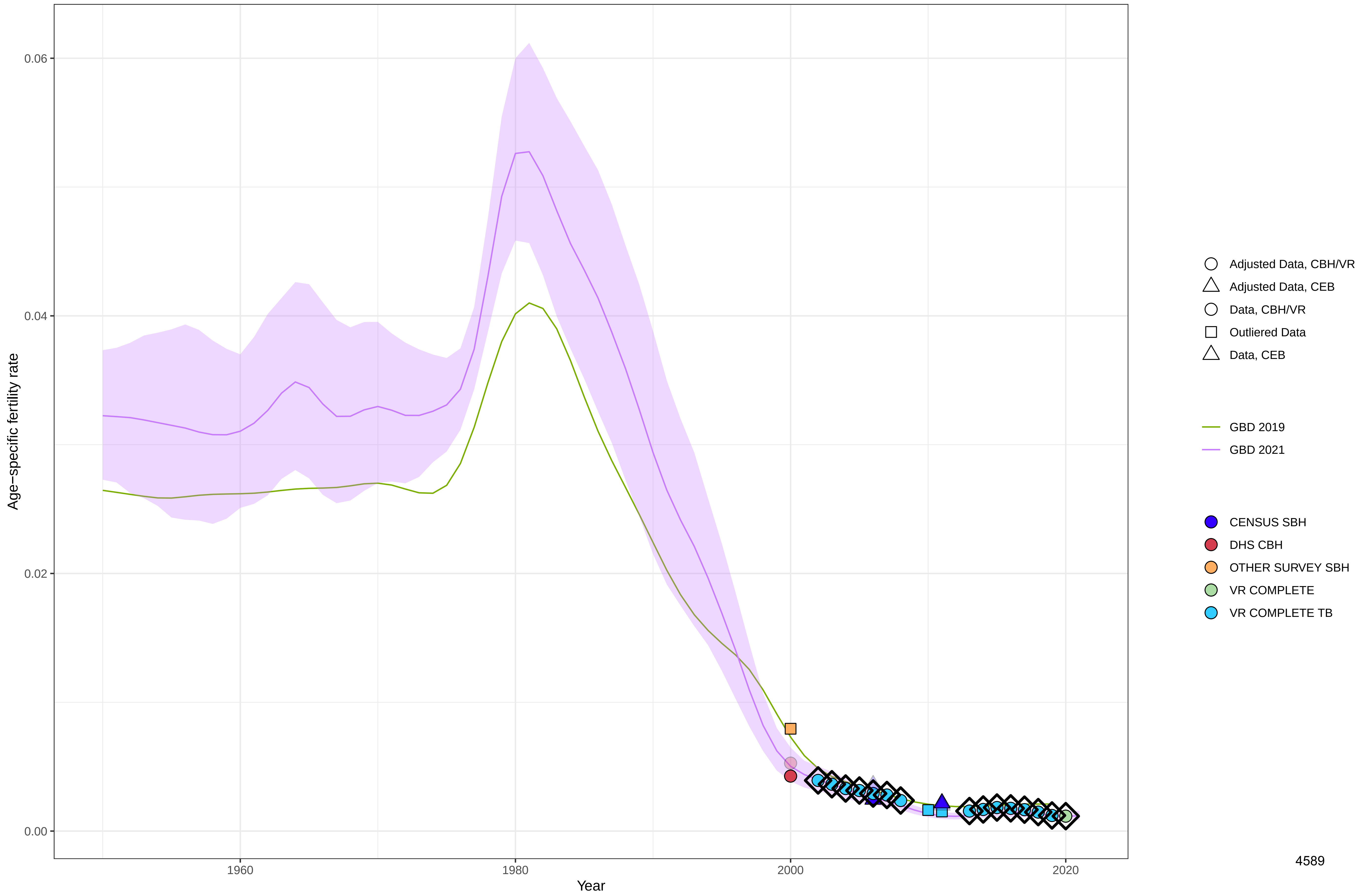

Age−specific fertility fitted trend lines, 1950−2021: Ardebil, Ages: 50−54
Data points enclosed with a diamond are reference sources

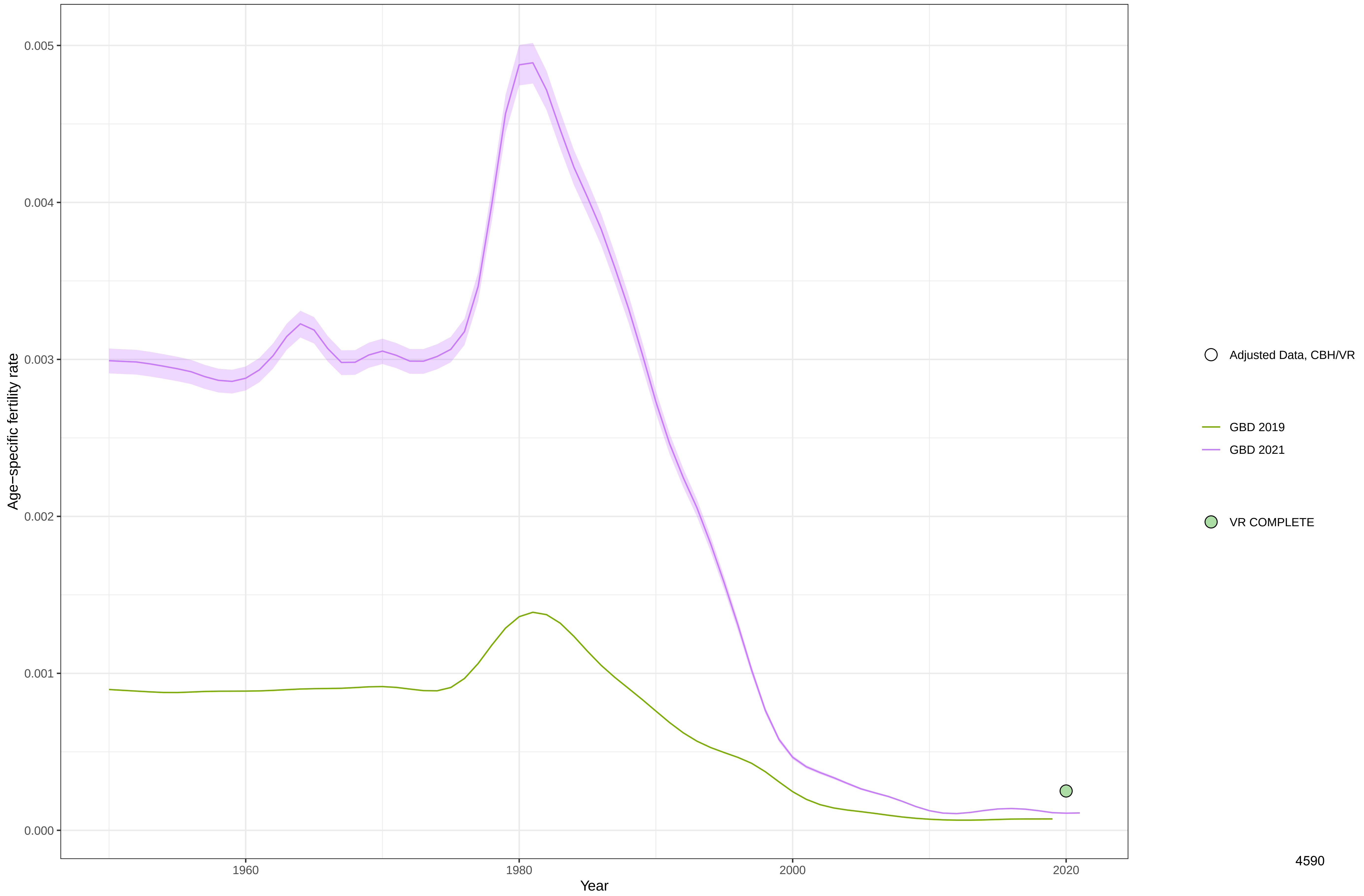

Total fertility rate fitted trend lines, 1950−2021: Ardebil TFR
Data points enclosed with a diamond are reference sources

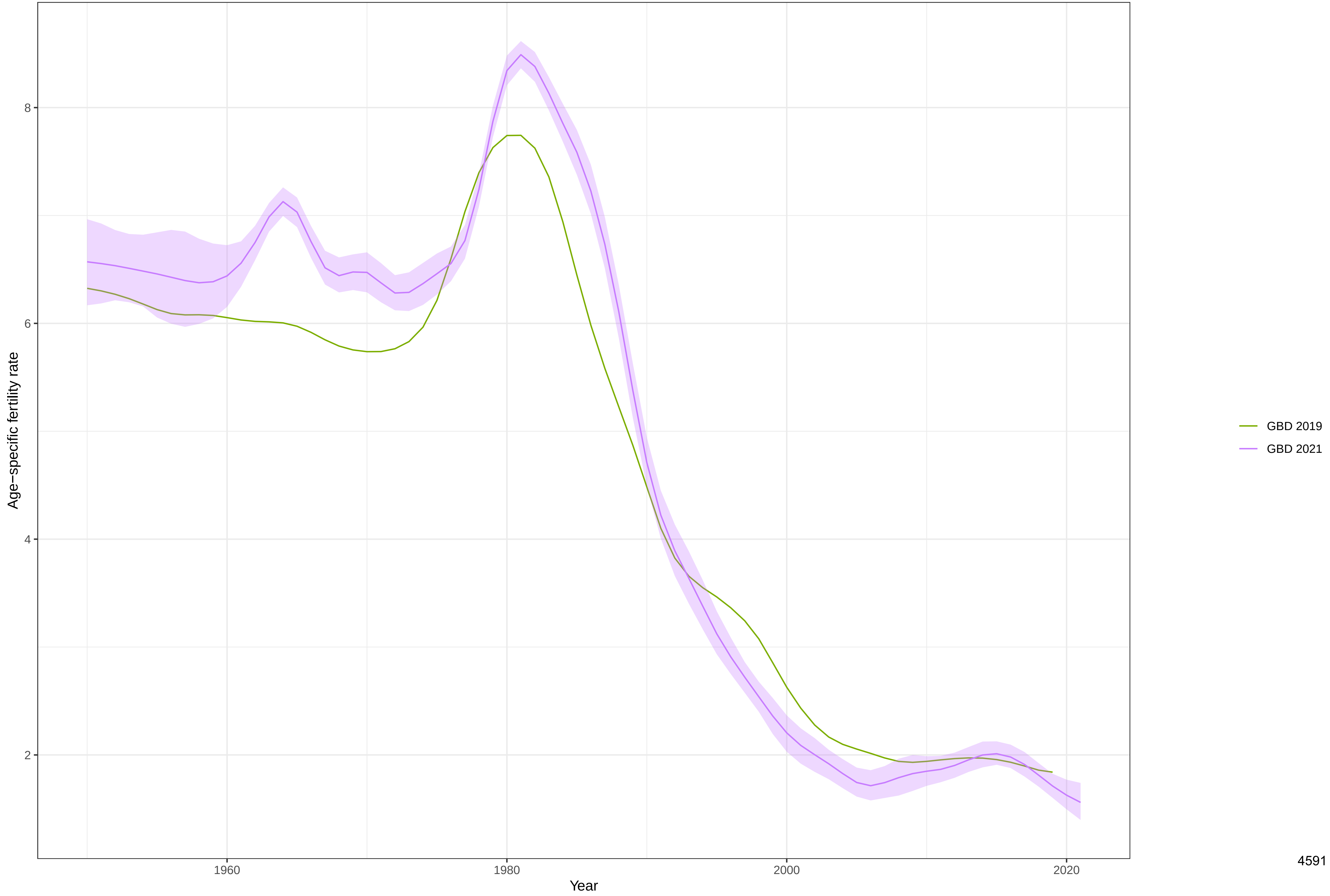

Age−specific fertility fitted trend lines, 1950−2021: Bushehr, Ages: 10−14
Data points enclosed with a diamond are reference sources

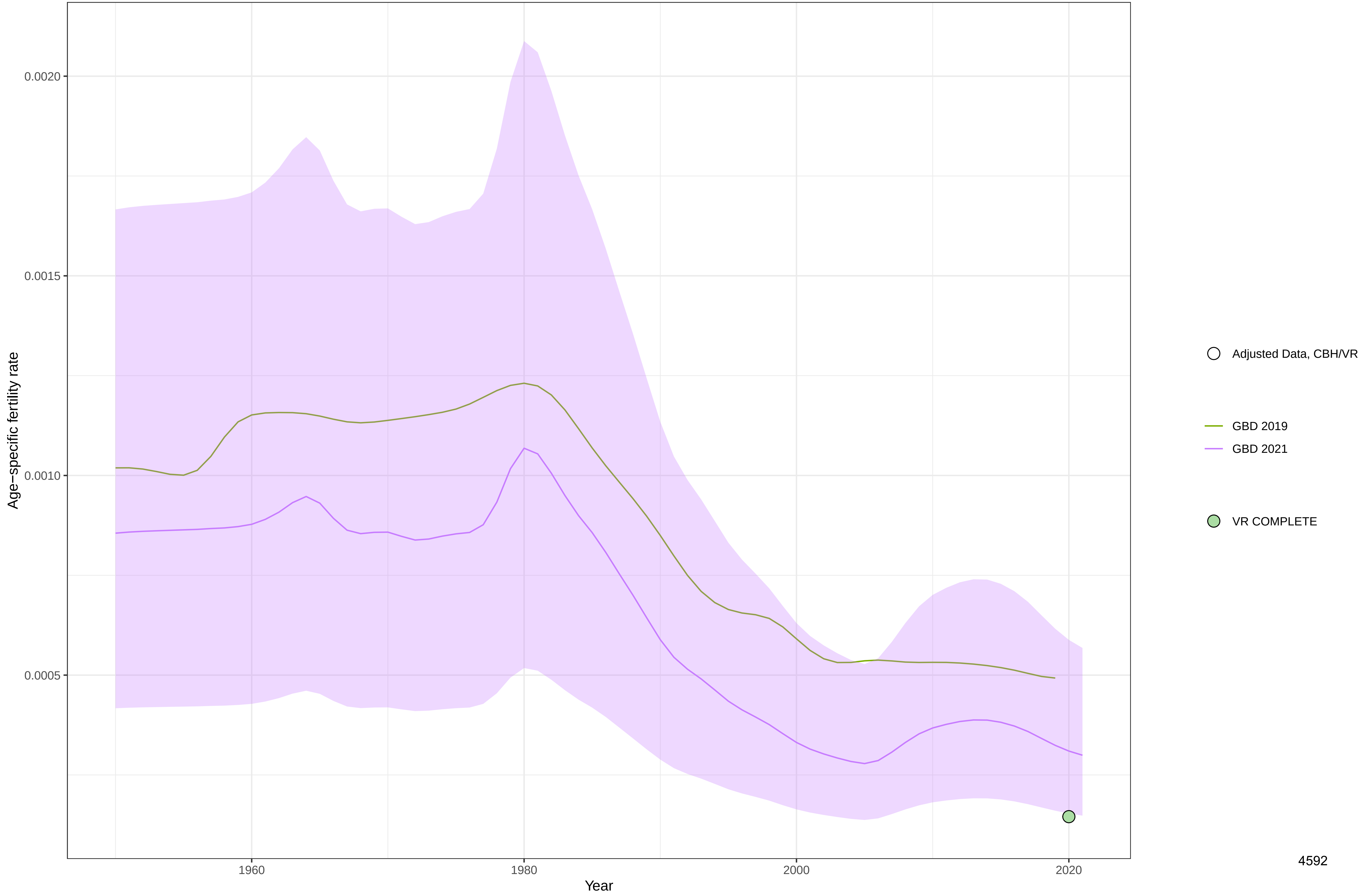

Age−specific fertility fitted trend lines, 1950−2021: Bushehr, Ages: 15−19
Data points enclosed with a diamond are reference sources

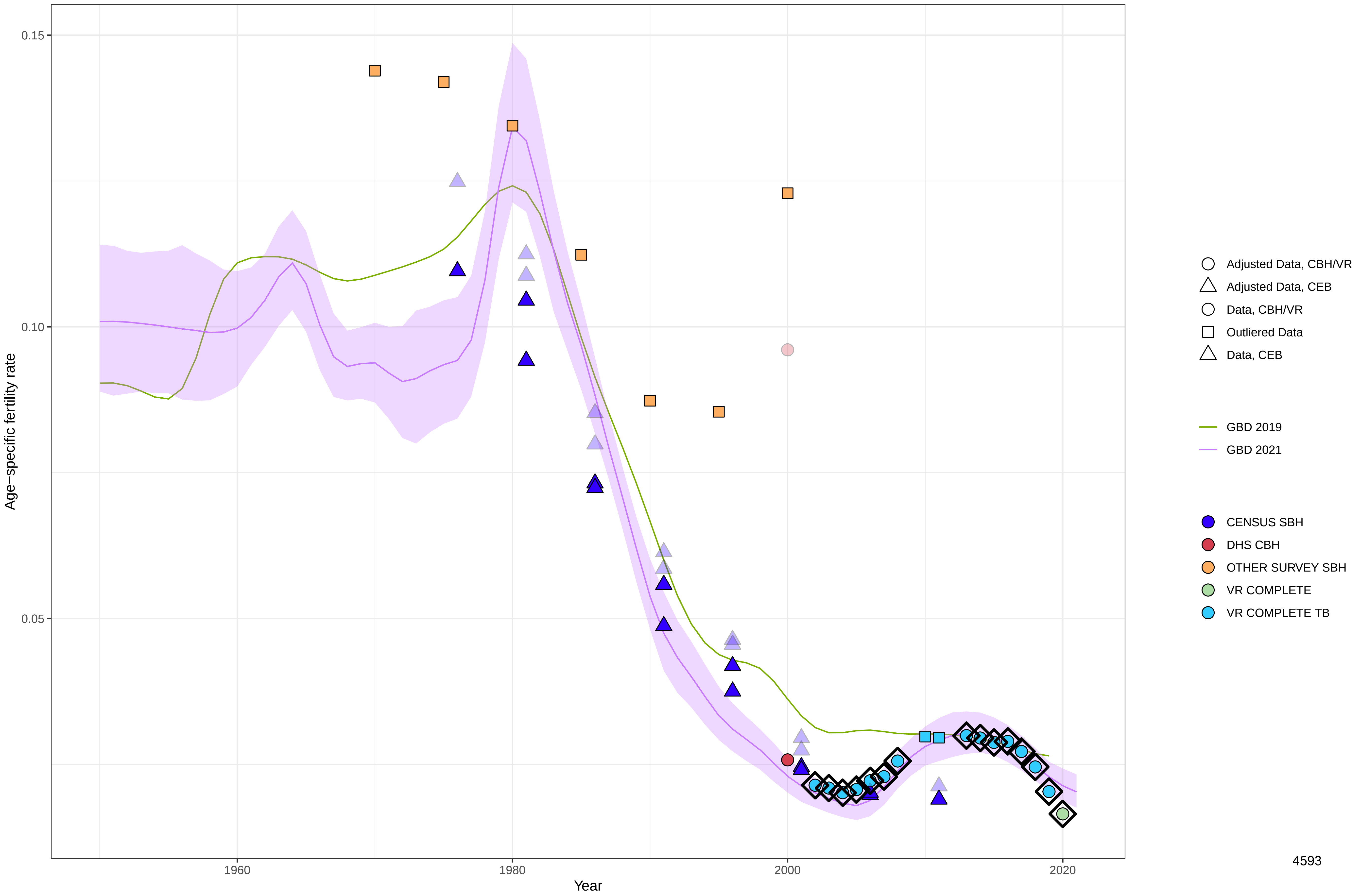

Age−specific fertility fitted trend lines, 1950−2021: Bushehr, Ages: 20−24
Data points enclosed with a diamond are reference sources

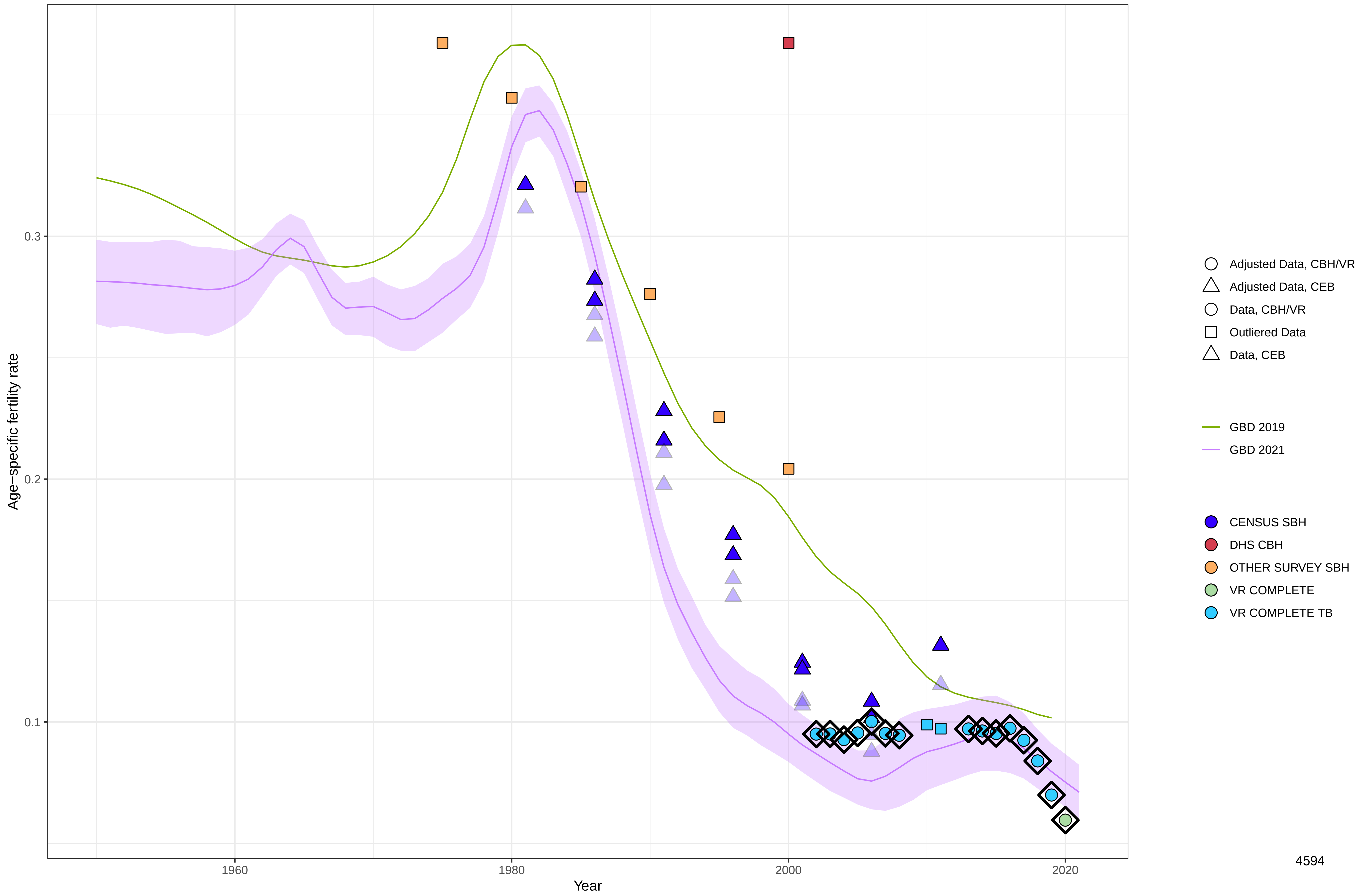

Age−specific fertility fitted trend lines, 1950−2021: Bushehr, Ages: 25−29
Data points enclosed with a diamond are reference sources

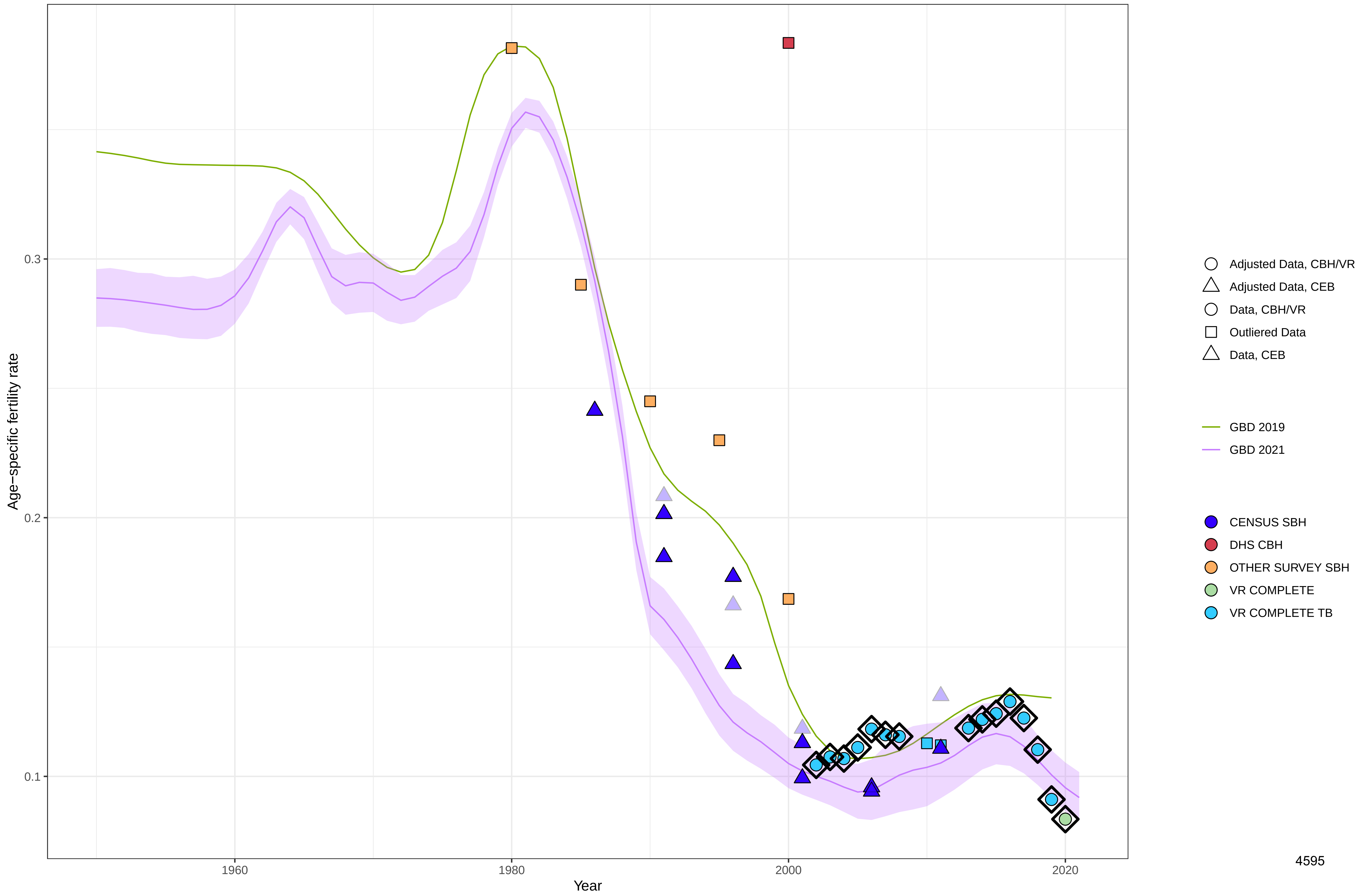

Age−specific fertility fitted trend lines, 1950−2021: Bushehr, Ages: 30−34
Data points enclosed with a diamond are reference sources

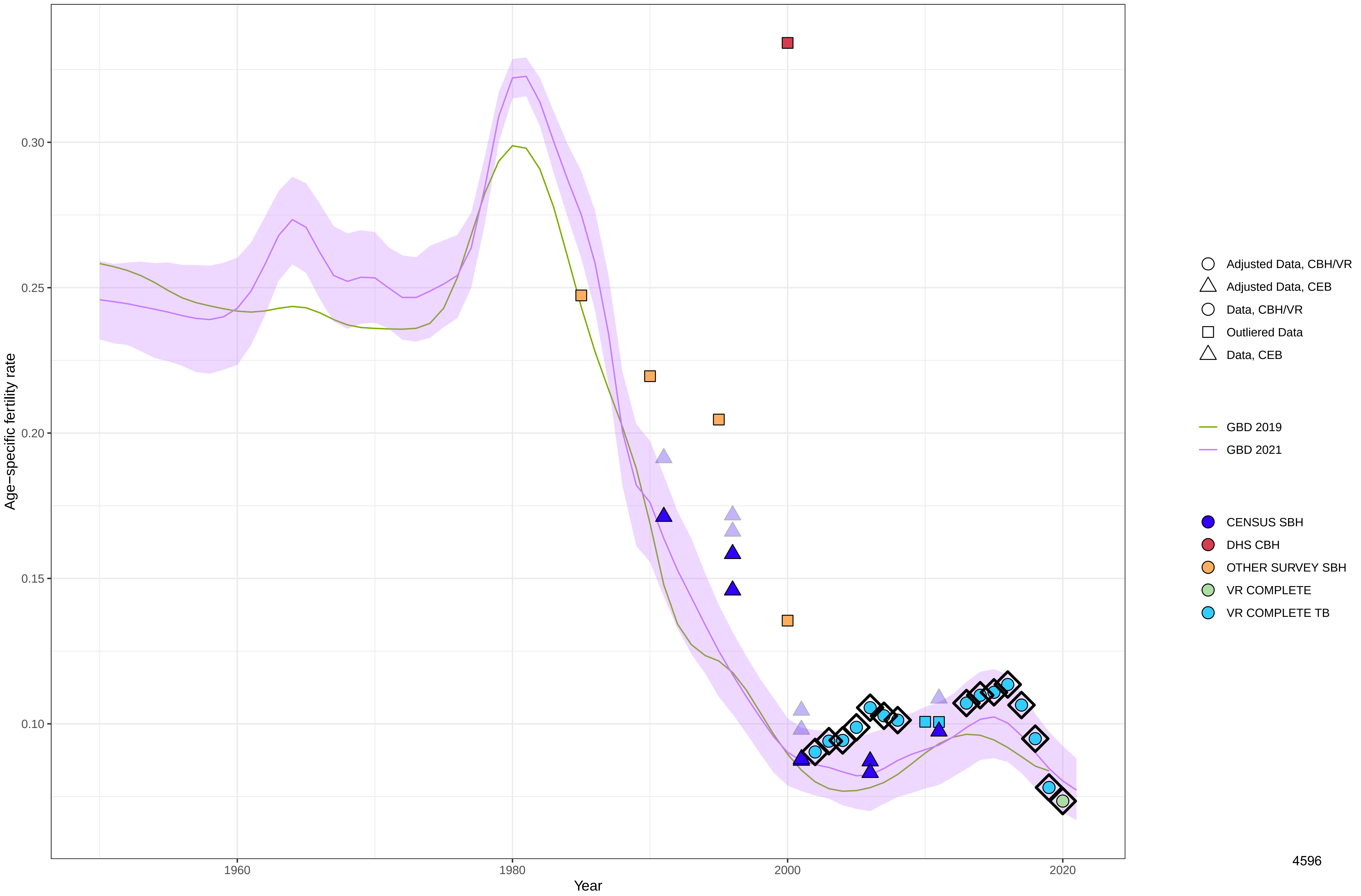

Age−specific fertility fitted trend lines, 1950−2021: Bushehr, Ages: 35−39
Data points enclosed with a diamond are reference sources

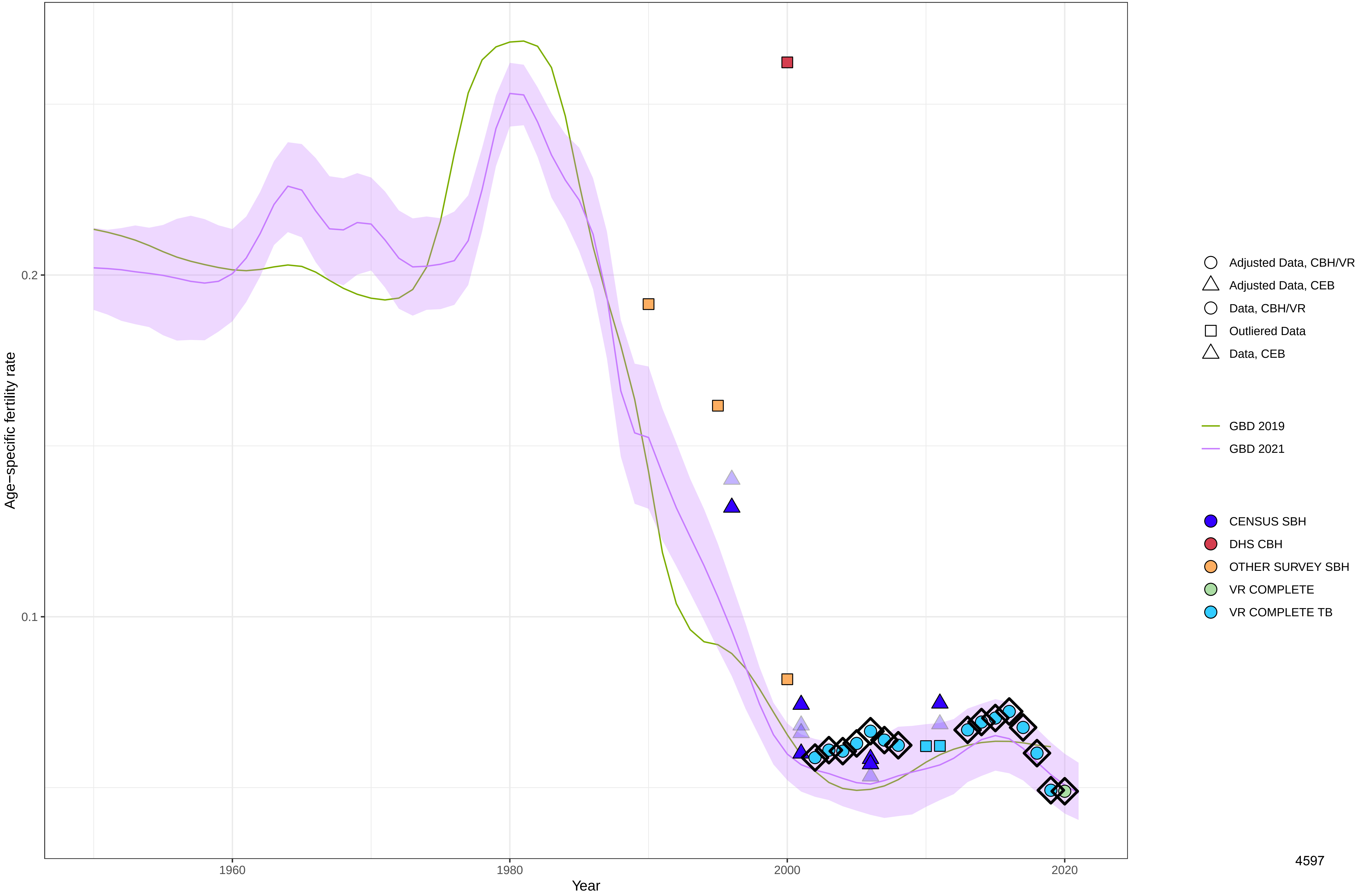

Age−specific fertility fitted trend lines, 1950−2021: Bushehr, Ages: 40−44
Data points enclosed with a diamond are reference sources

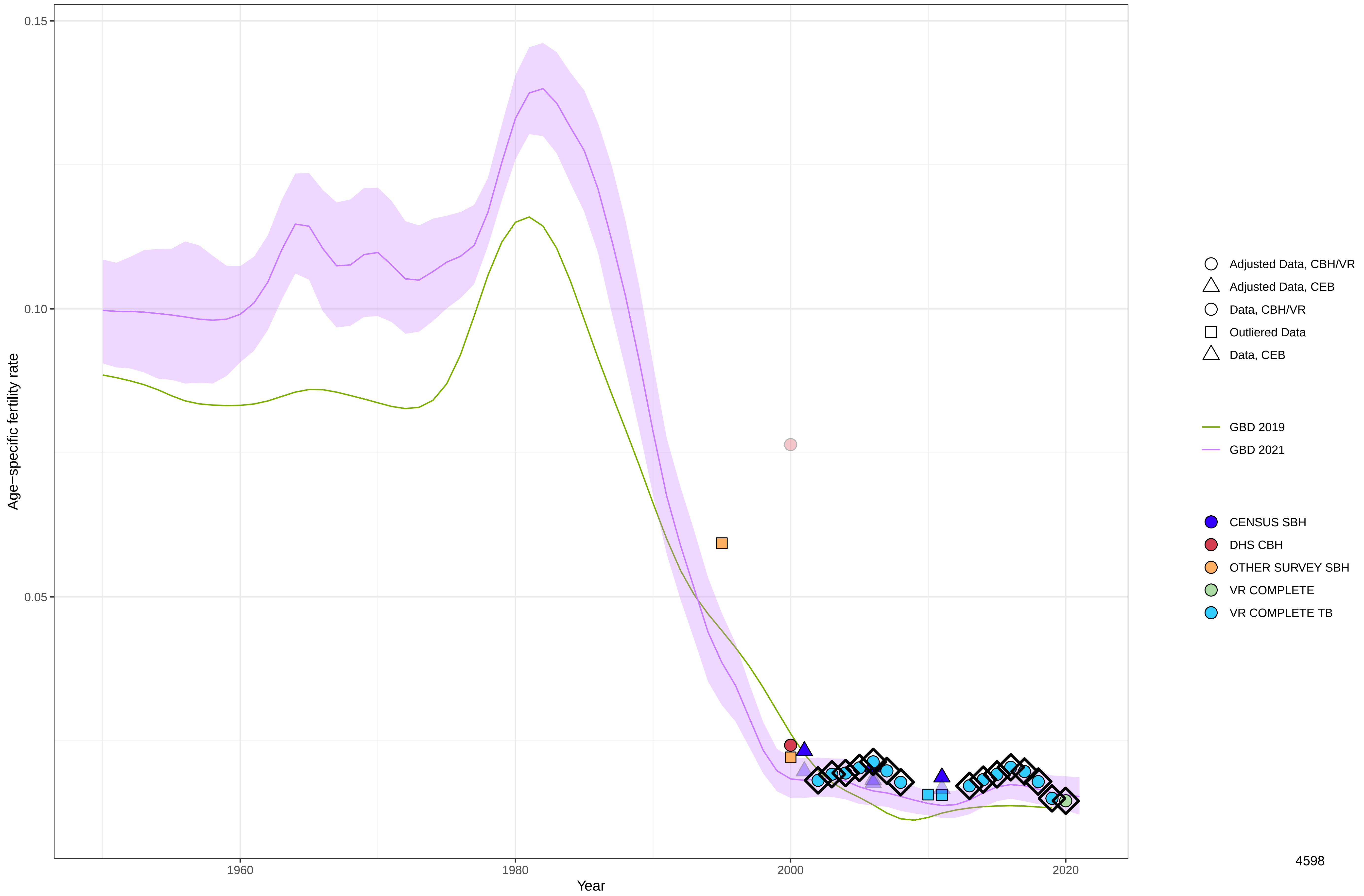

Age−specific fertility fitted trend lines, 1950−2021: Bushehr, Ages: 45−49
Data points enclosed with a diamond are reference sources

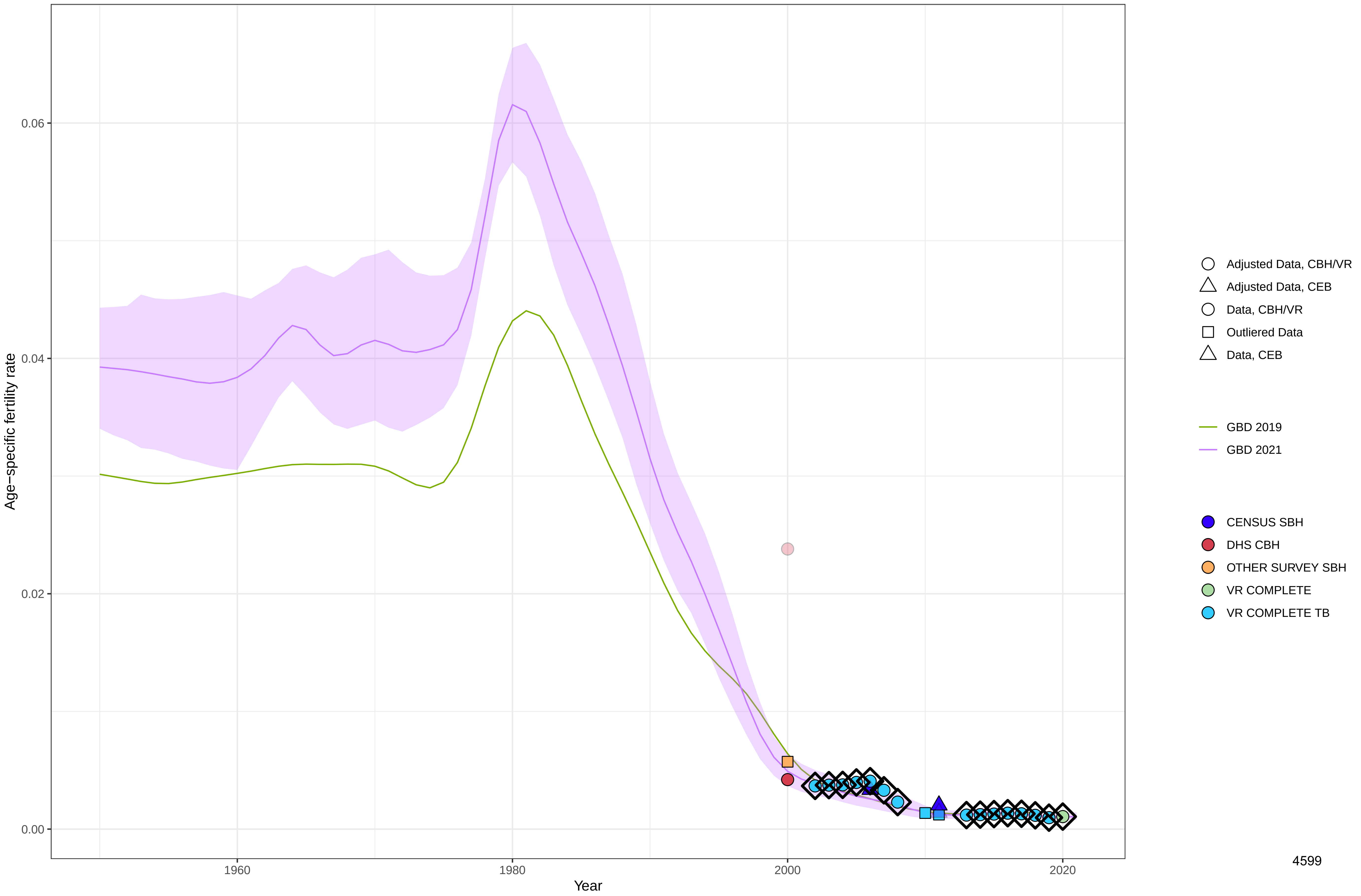

Age−specific fertility fitted trend lines, 1950−2021: Bushehr, Ages: 50−54
Data points enclosed with a diamond are reference sources

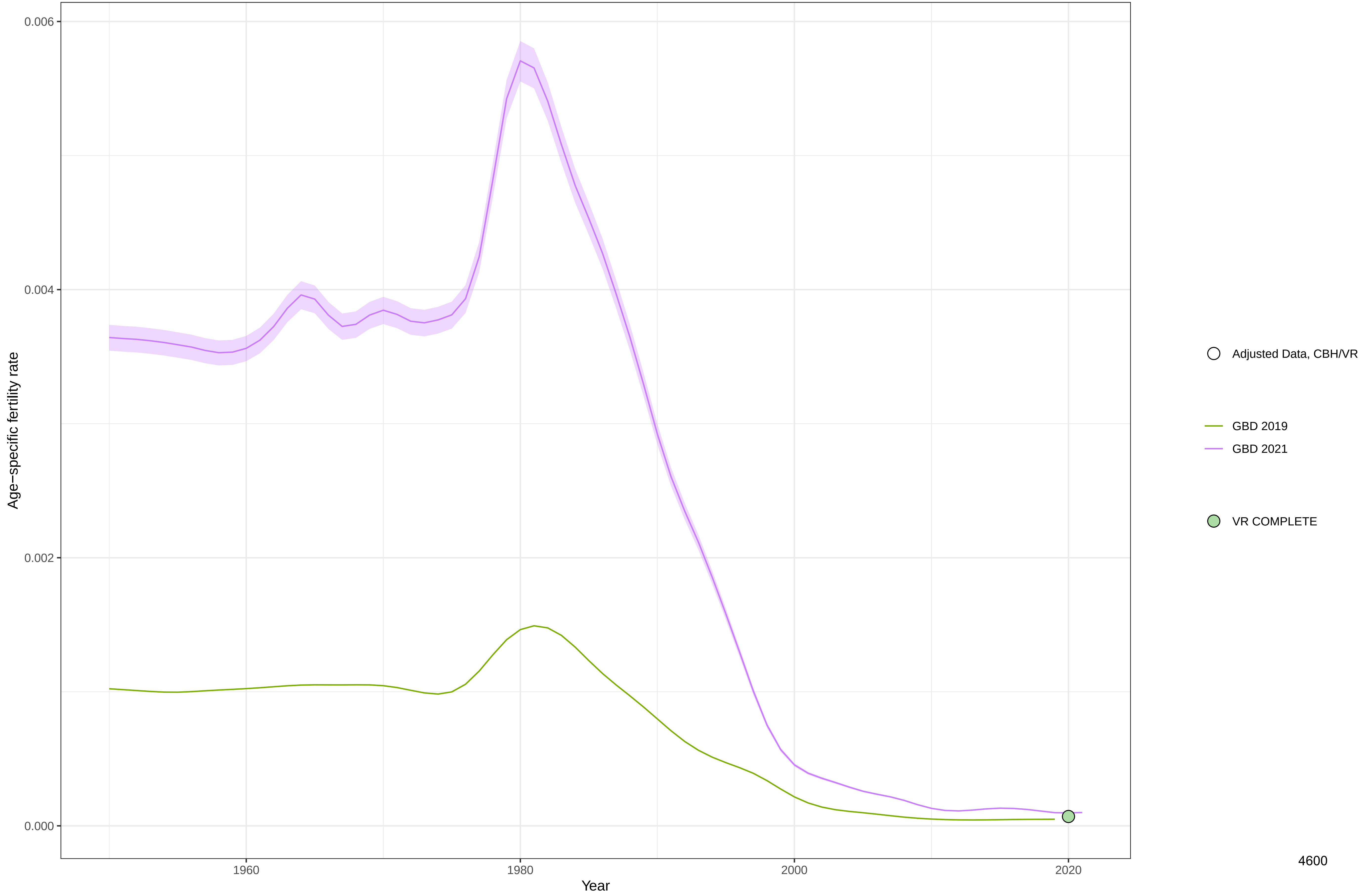

Total fertility rate fitted trend lines, 1950−2021: Bushehr TFR
Data points enclosed with a diamond are reference sources

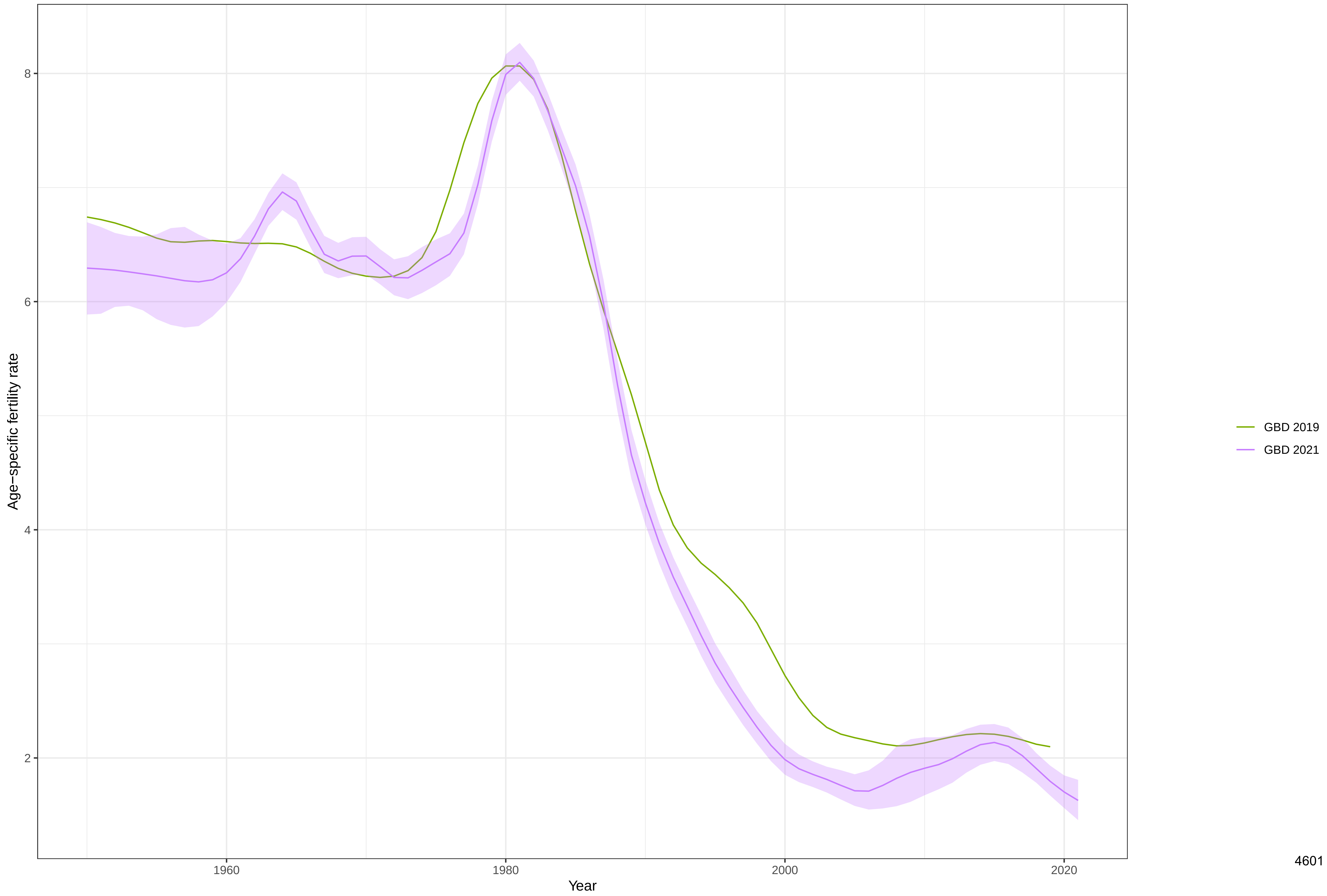

Age−specific fertility fitted trend lines, 1950−2021: Chahar Mahaal and Bakhtiari, Ages: 10−14
Data points enclosed with a diamond are reference sources

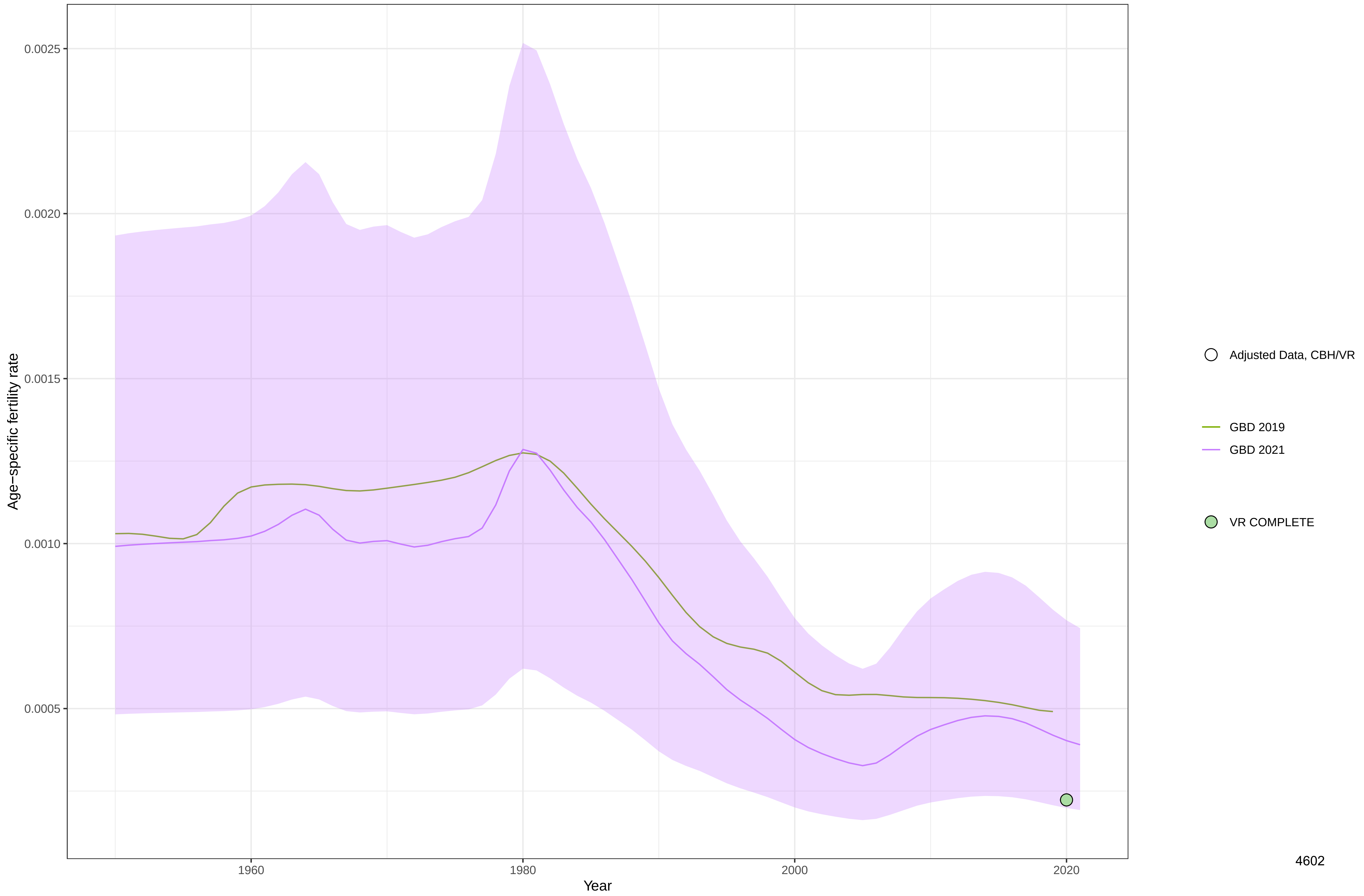

Age−specific fertility fitted trend lines, 1950−2021: Chahar Mahaal and Bakhtiari, Ages: 15−19
Data points enclosed with a diamond are reference sources

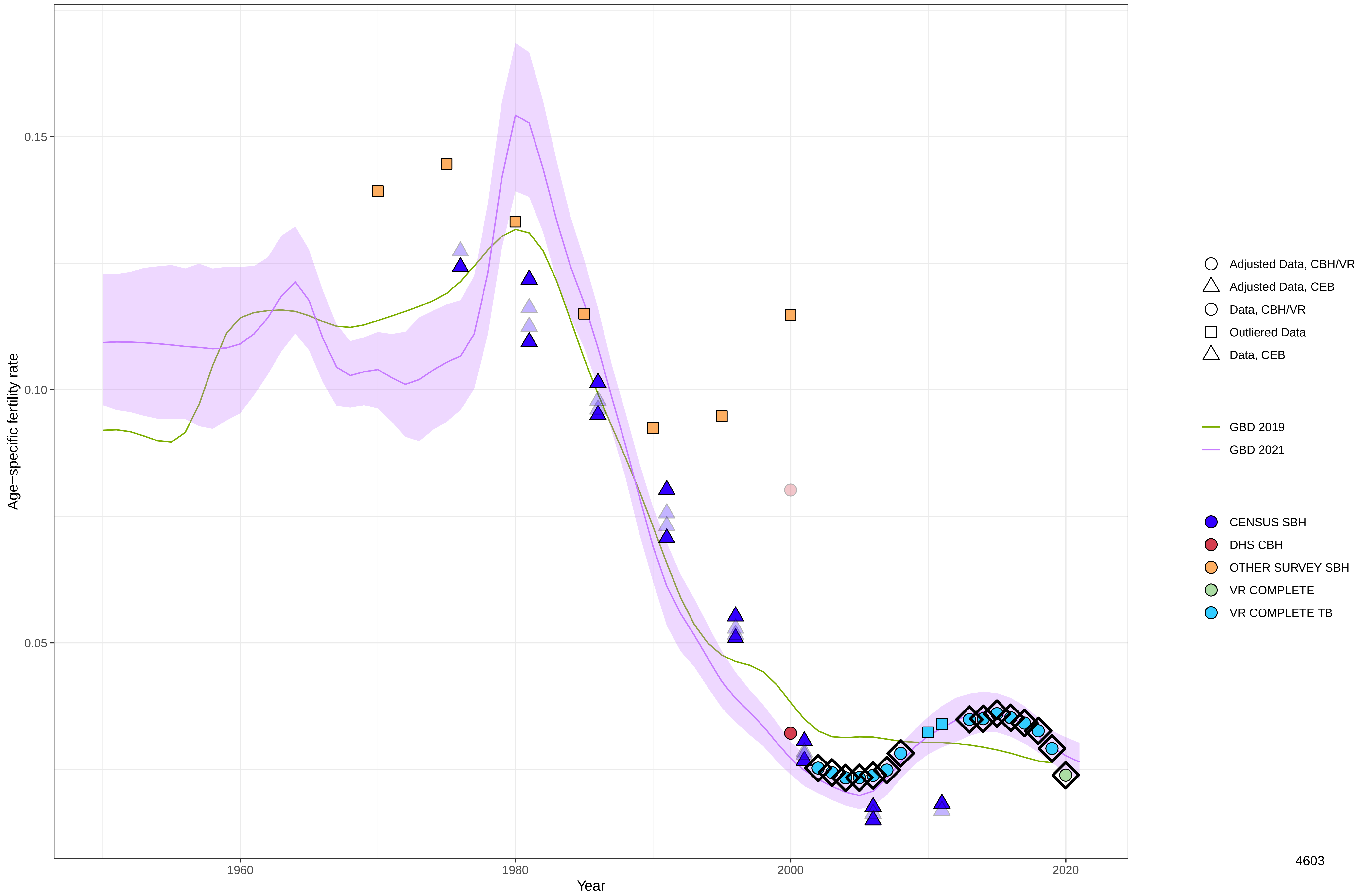

Age−specific fertility fitted trend lines, 1950−2021: Chahar Mahaal and Bakhtiari, Ages: 20−24
Data points enclosed with a diamond are reference sources

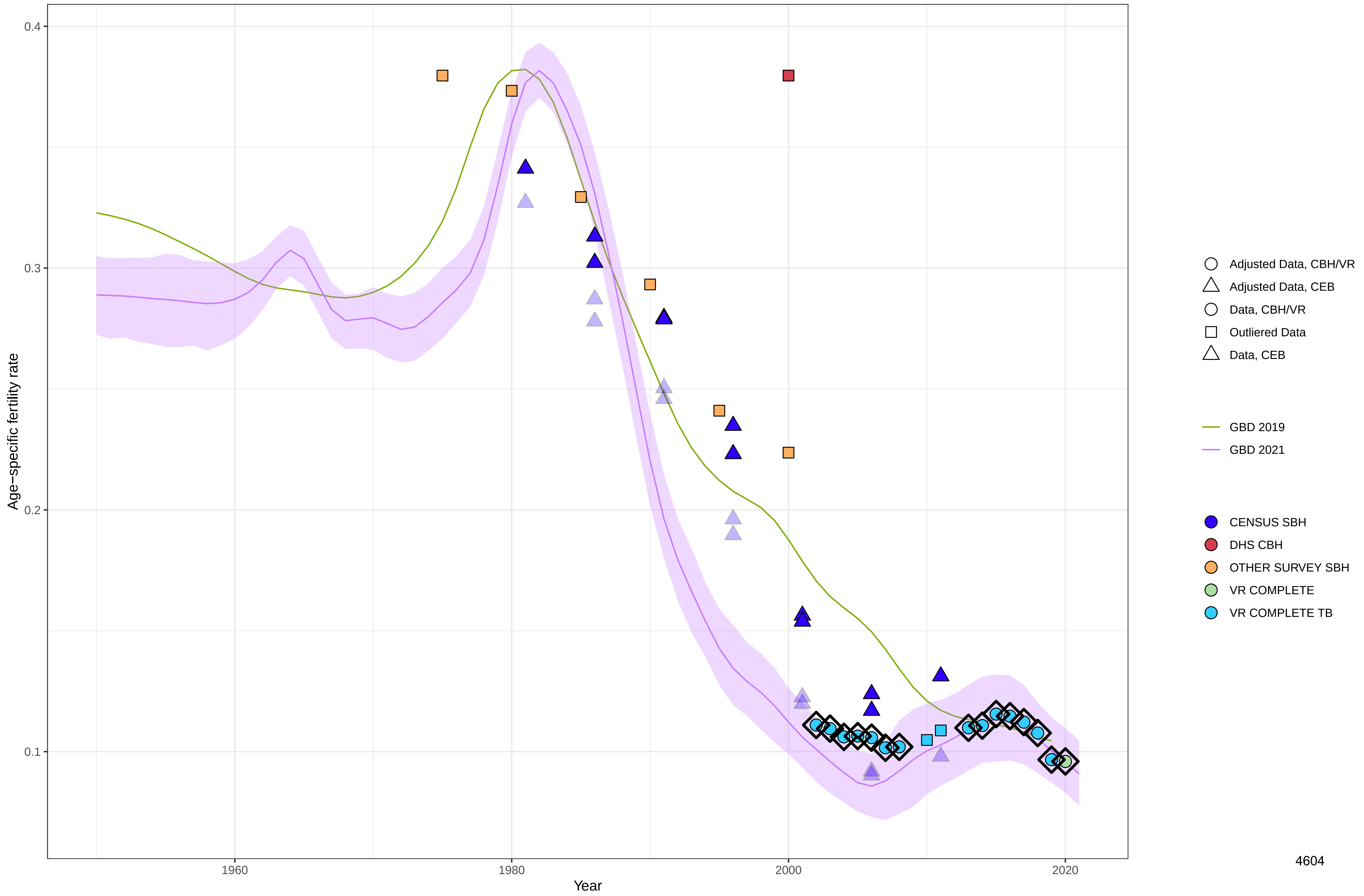

Age−specific fertility fitted trend lines, 1950−2021: Chahar Mahaal and Bakhtiari, Ages: 25−29
Data points enclosed with a diamond are reference sources

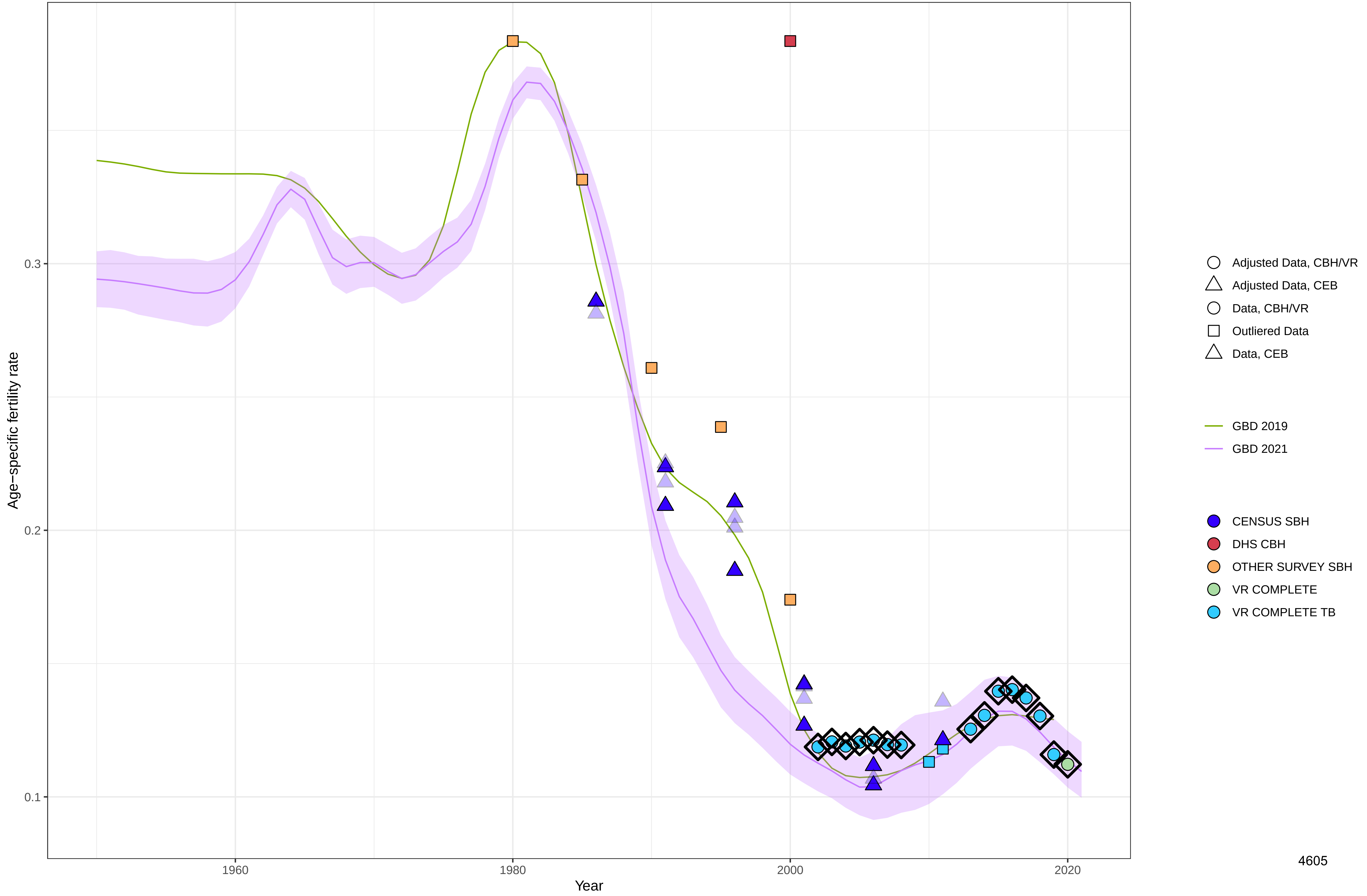

Age−specific fertility fitted trend lines, 1950−2021: Chahar Mahaal and Bakhtiari, Ages: 30−34
Data points enclosed with a diamond are reference sources

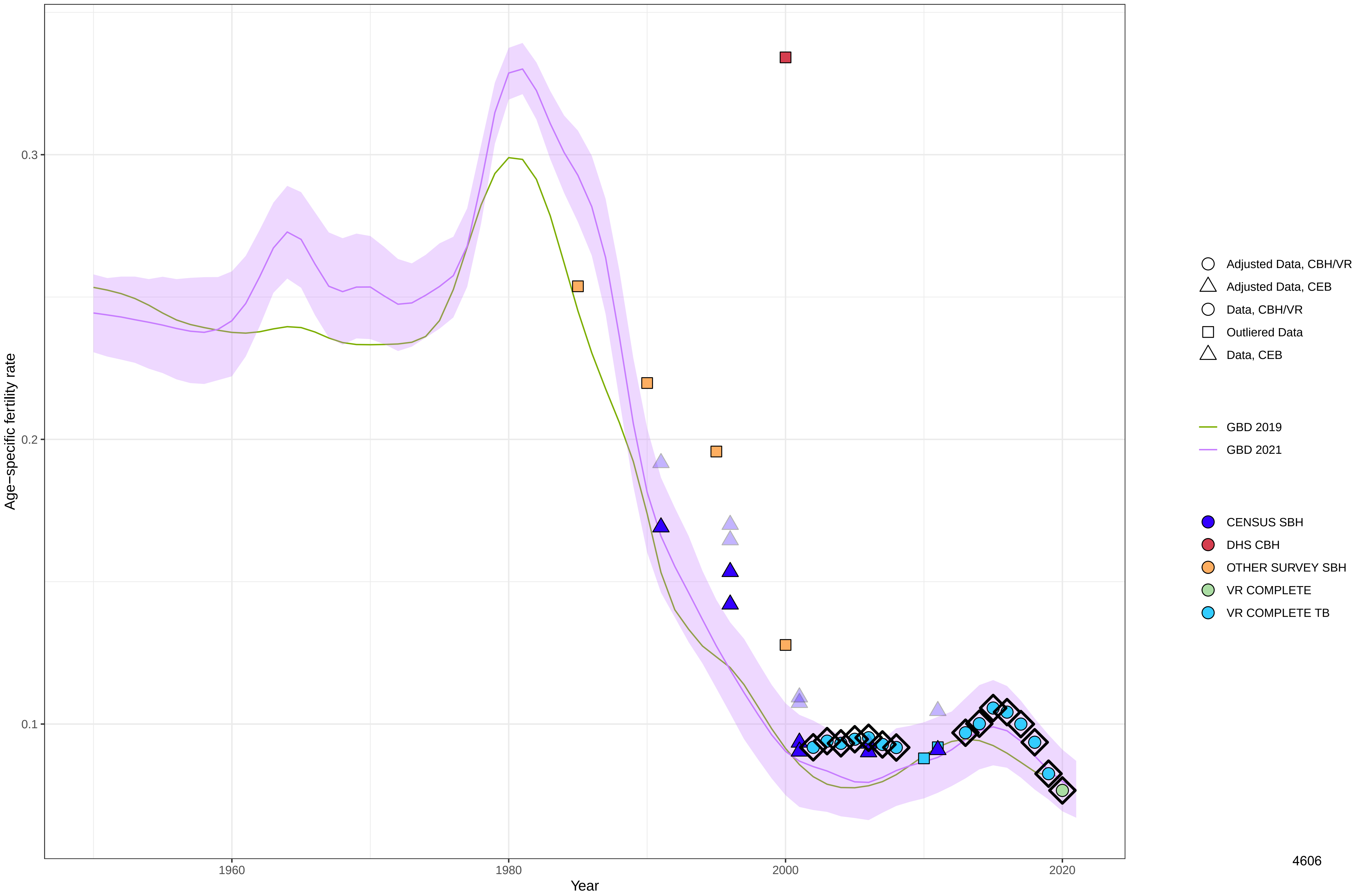

Age−specific fertility fitted trend lines, 1950−2021: Chahar Mahaal and Bakhtiari, Ages: 35−39
Data points enclosed with a diamond are reference sources

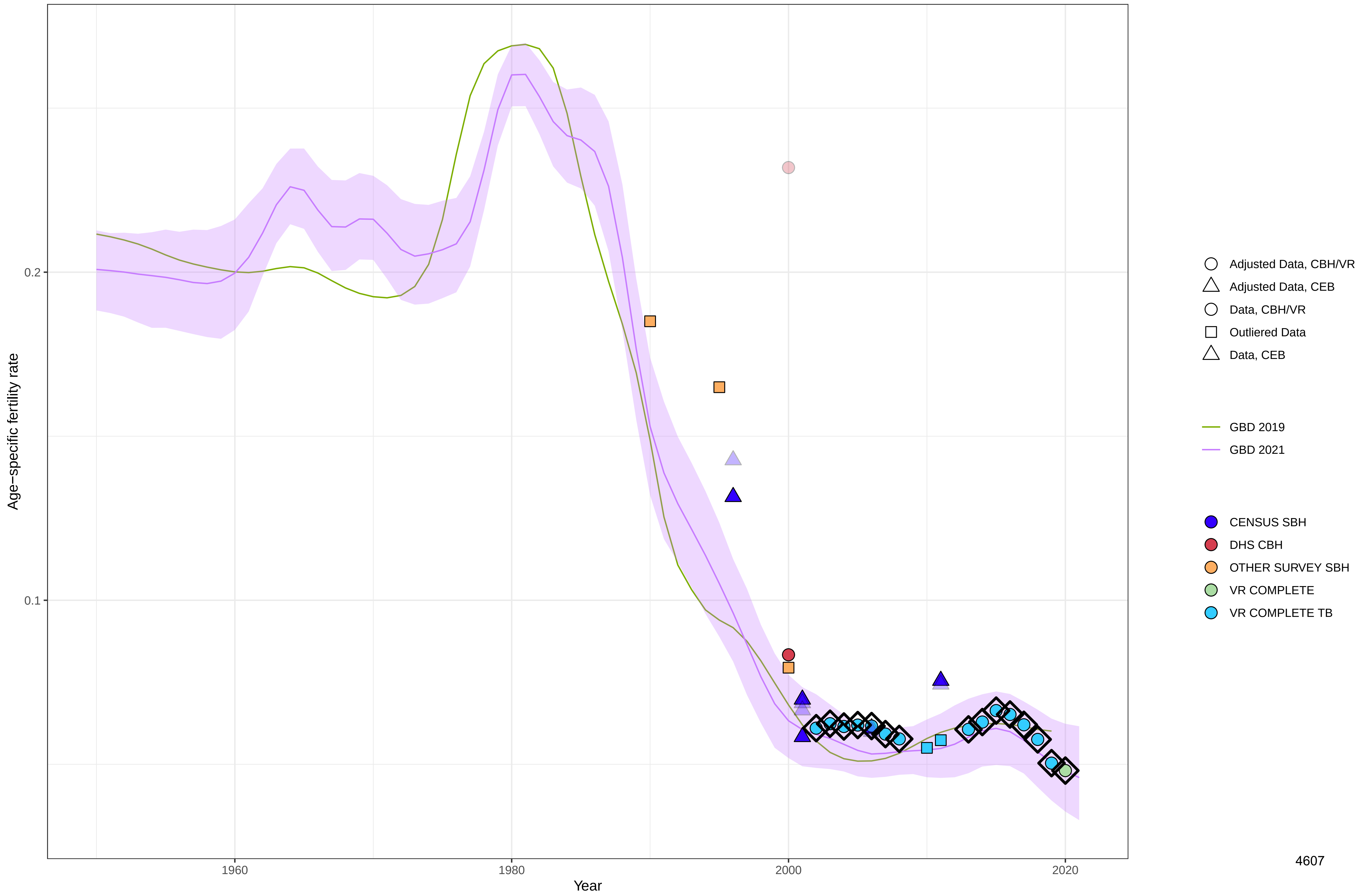

Age−specific fertility fitted trend lines, 1950−2021: Chahar Mahaal and Bakhtiari, Ages: 40−44
Data points enclosed with a diamond are reference sources

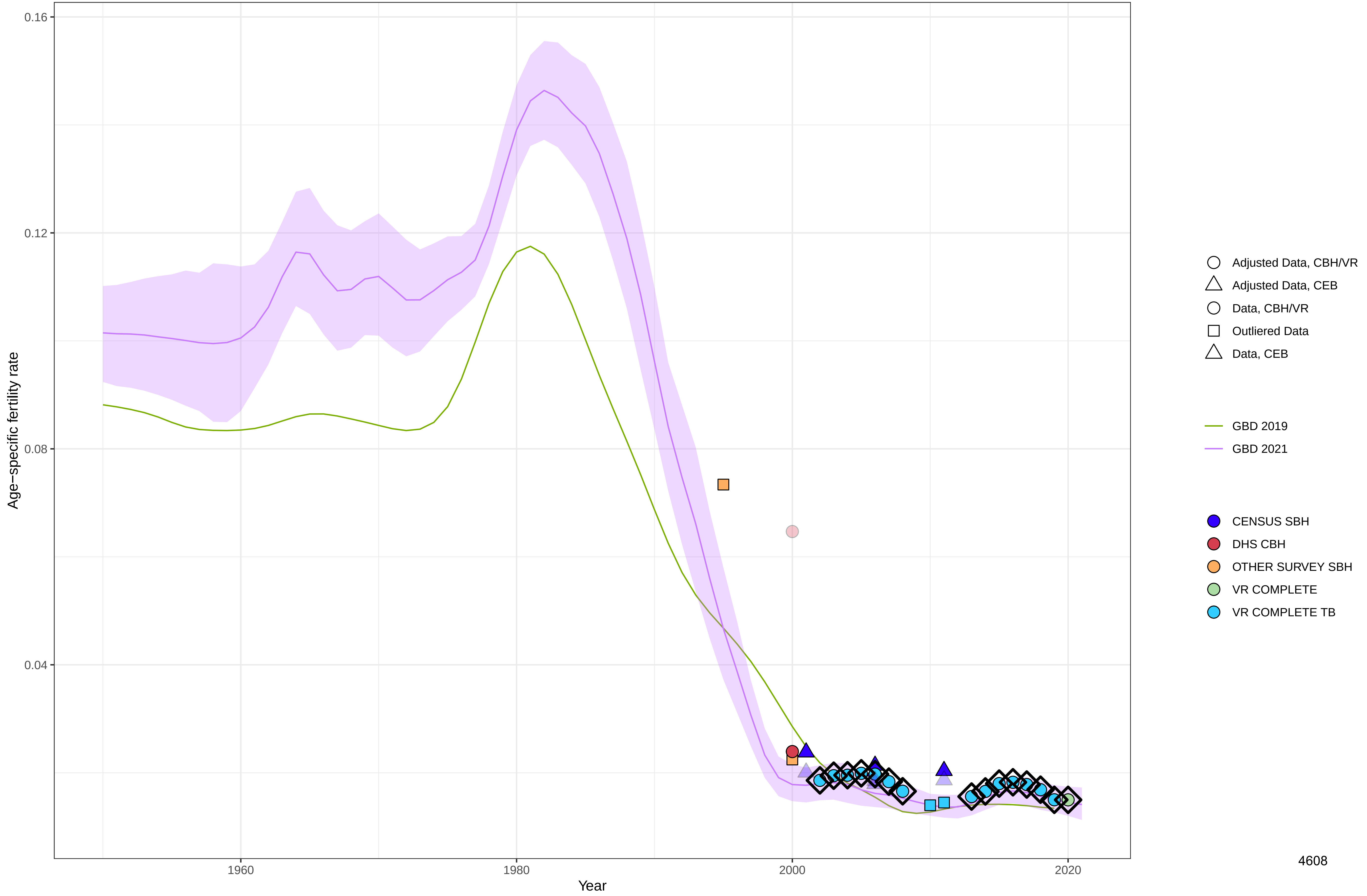

Age−specific fertility fitted trend lines, 1950−2021: Chahar Mahaal and Bakhtiari, Ages: 45−49
Data points enclosed with a diamond are reference sources

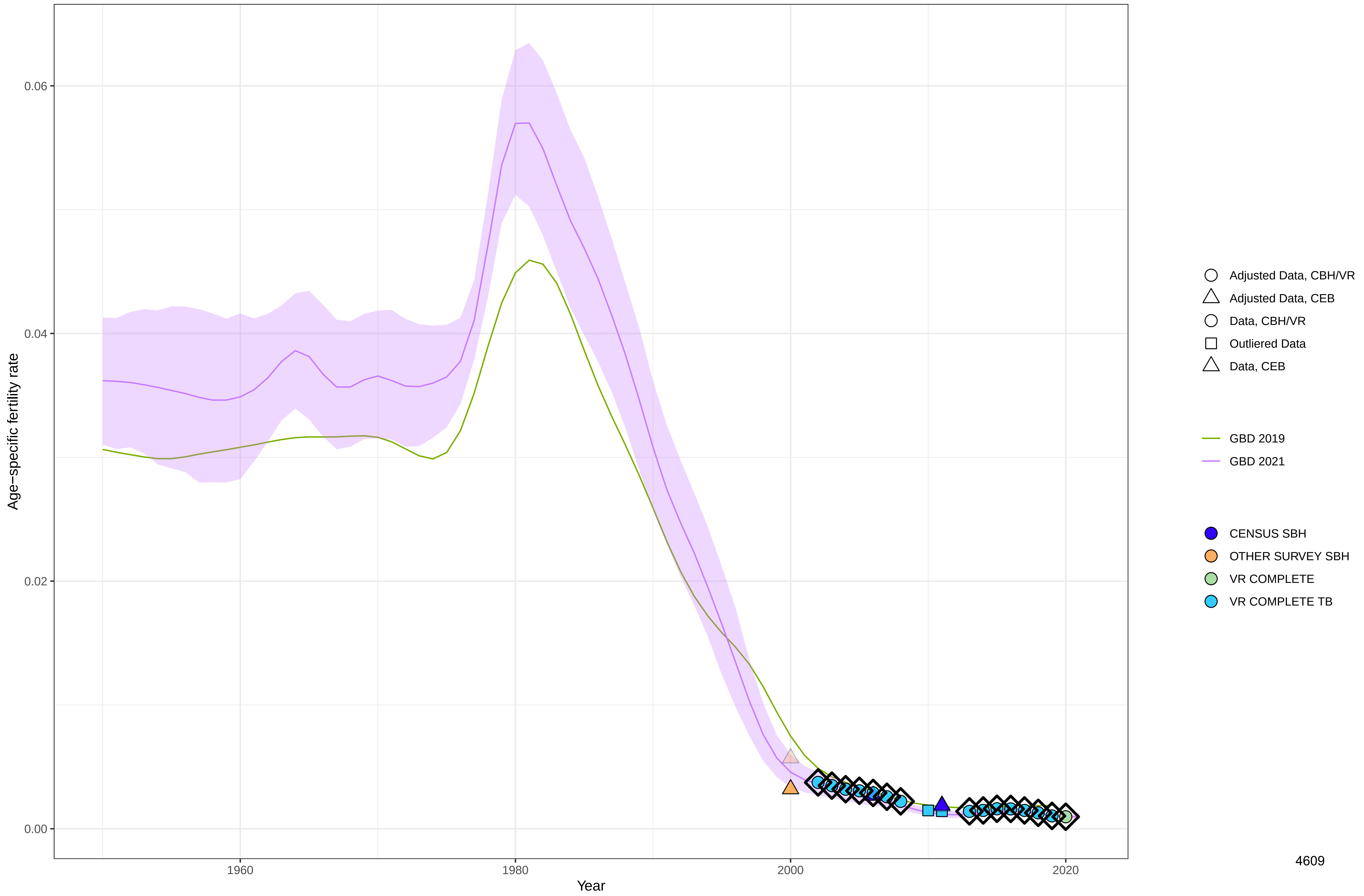

Age−specific fertility fitted trend lines, 1950−2021: Chahar Mahaal and Bakhtiari, Ages: 50−54
Data points enclosed with a diamond are reference sources

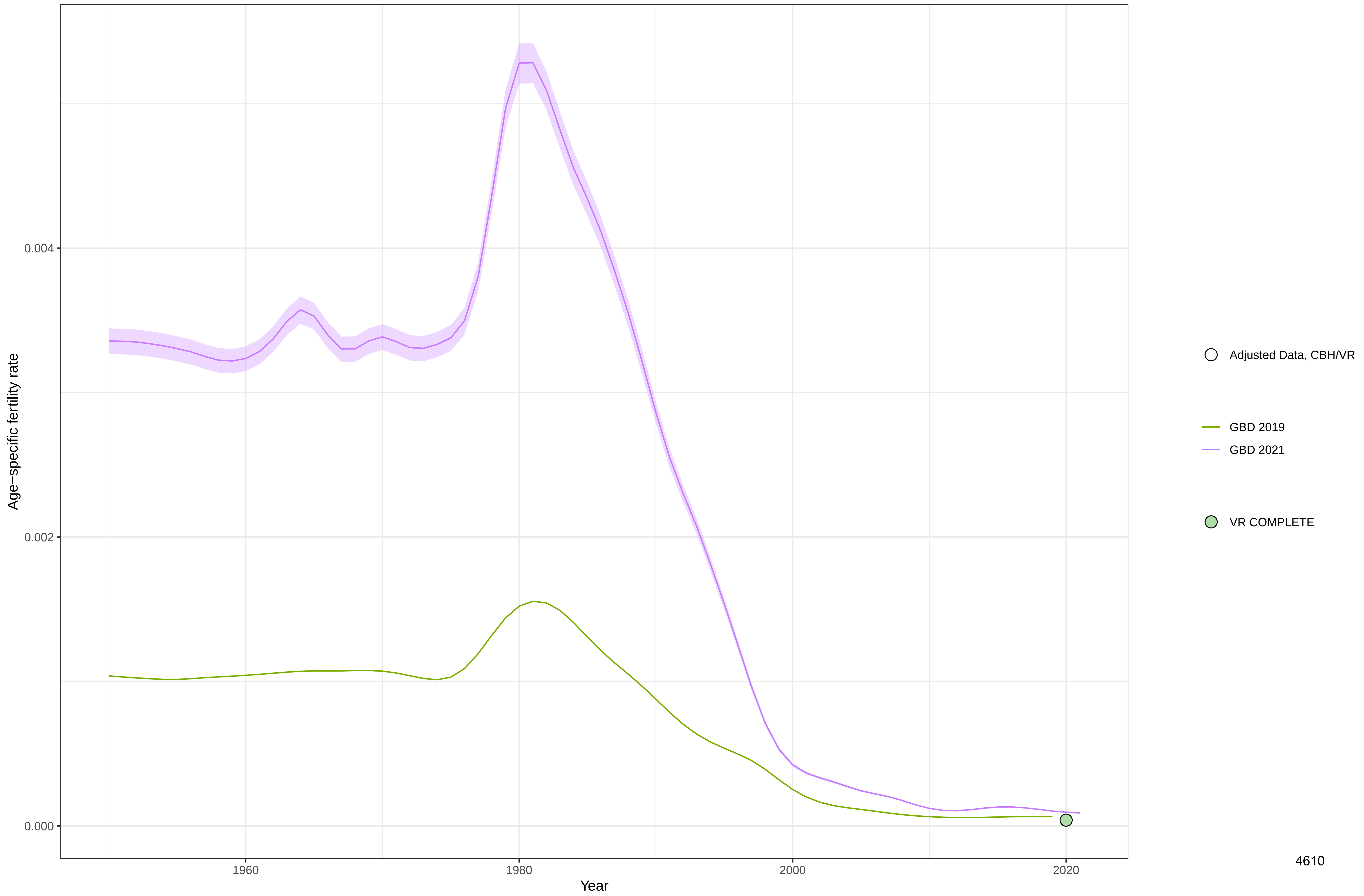

Total fertility rate fitted trend lines, 1950−2021: Chahar Mahaal and Bakhtiari TFR
Data points enclosed with a diamond are reference sources

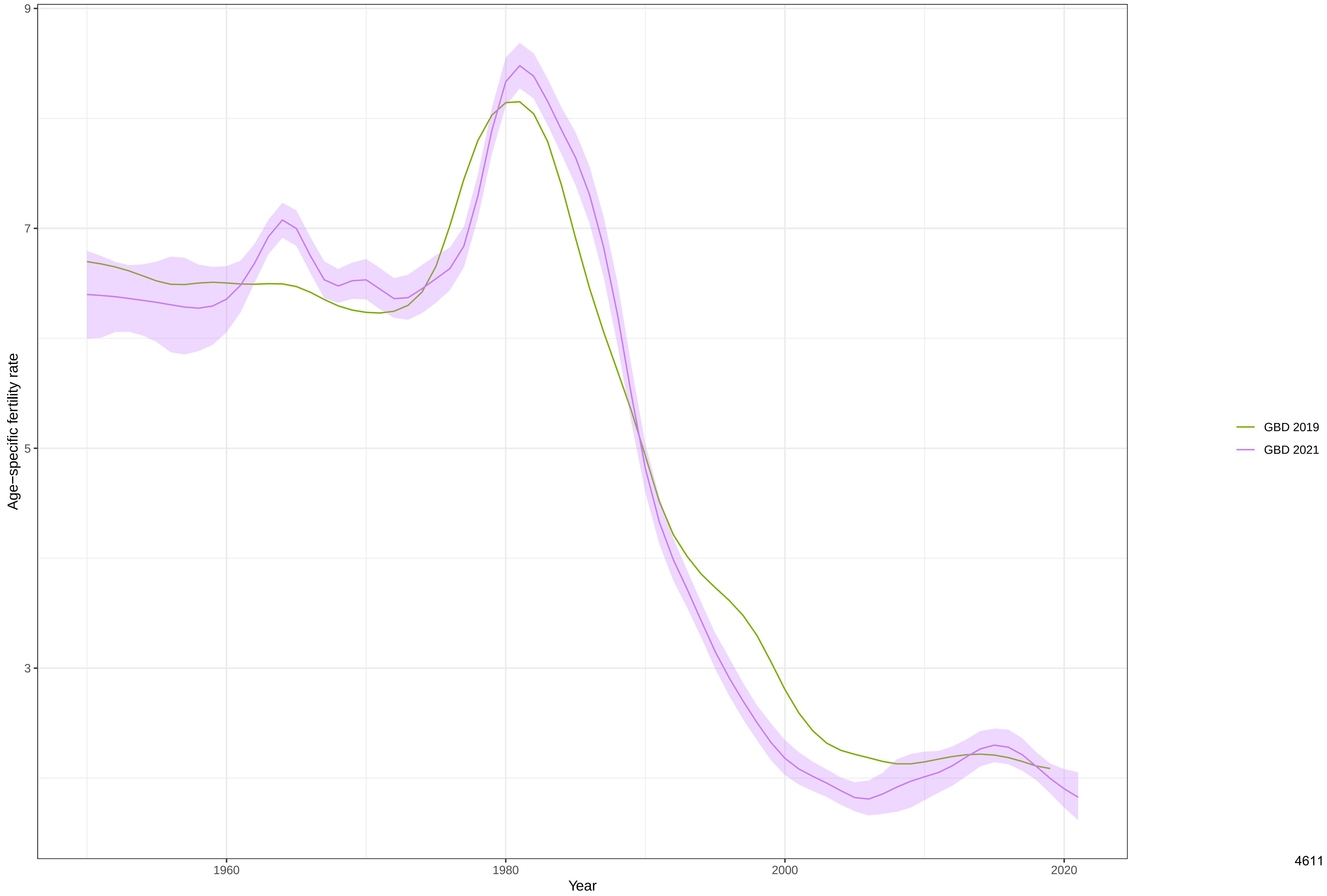

Age−specific fertility fitted trend lines, 1950−2021: East Azarbayejan, Ages: 10−14
Data points enclosed with a diamond are reference sources

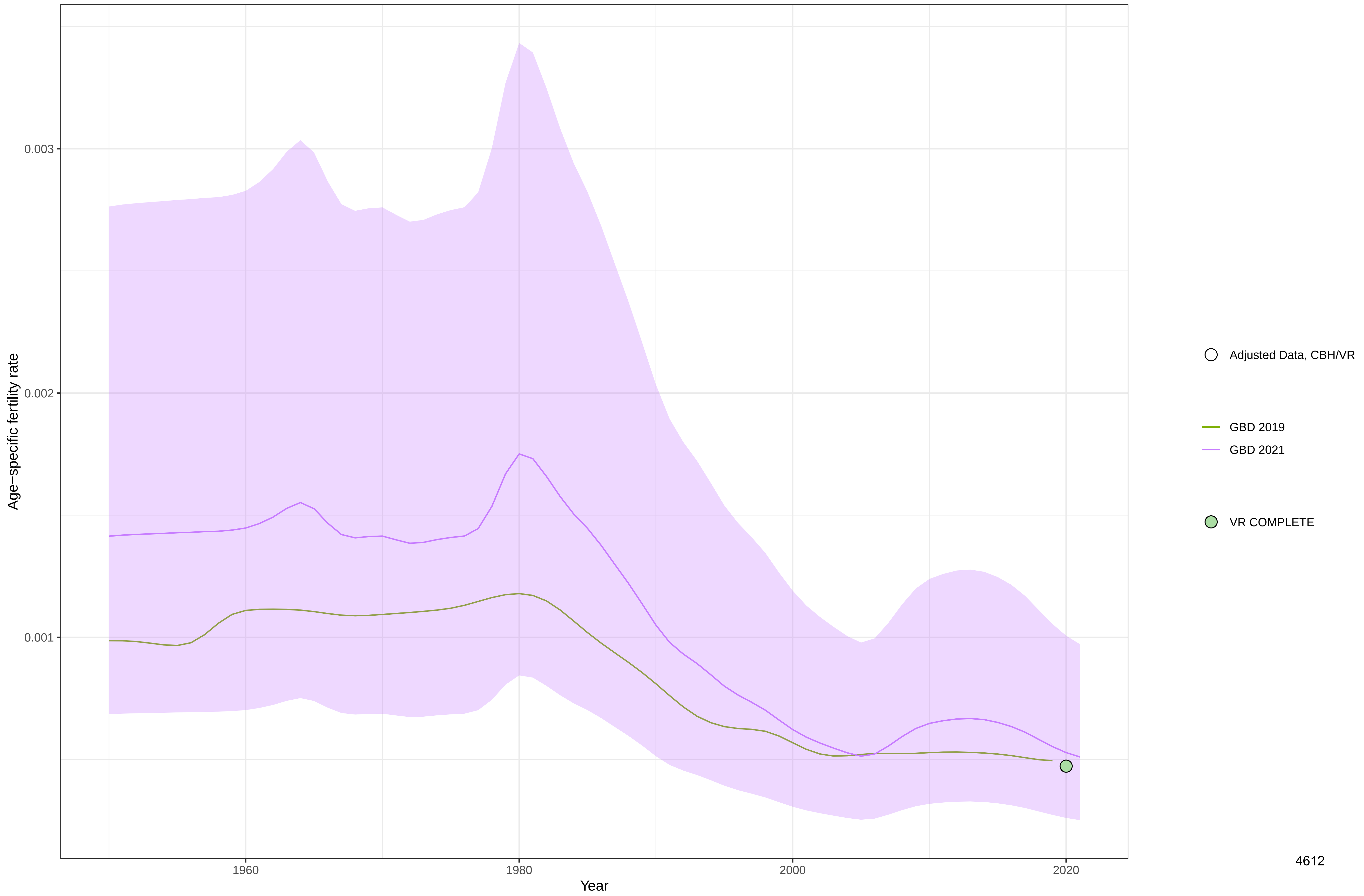

Age−specific fertility fitted trend lines, 1950−2021: East Azarbayejan, Ages: 15−19
Data points enclosed with a diamond are reference sources

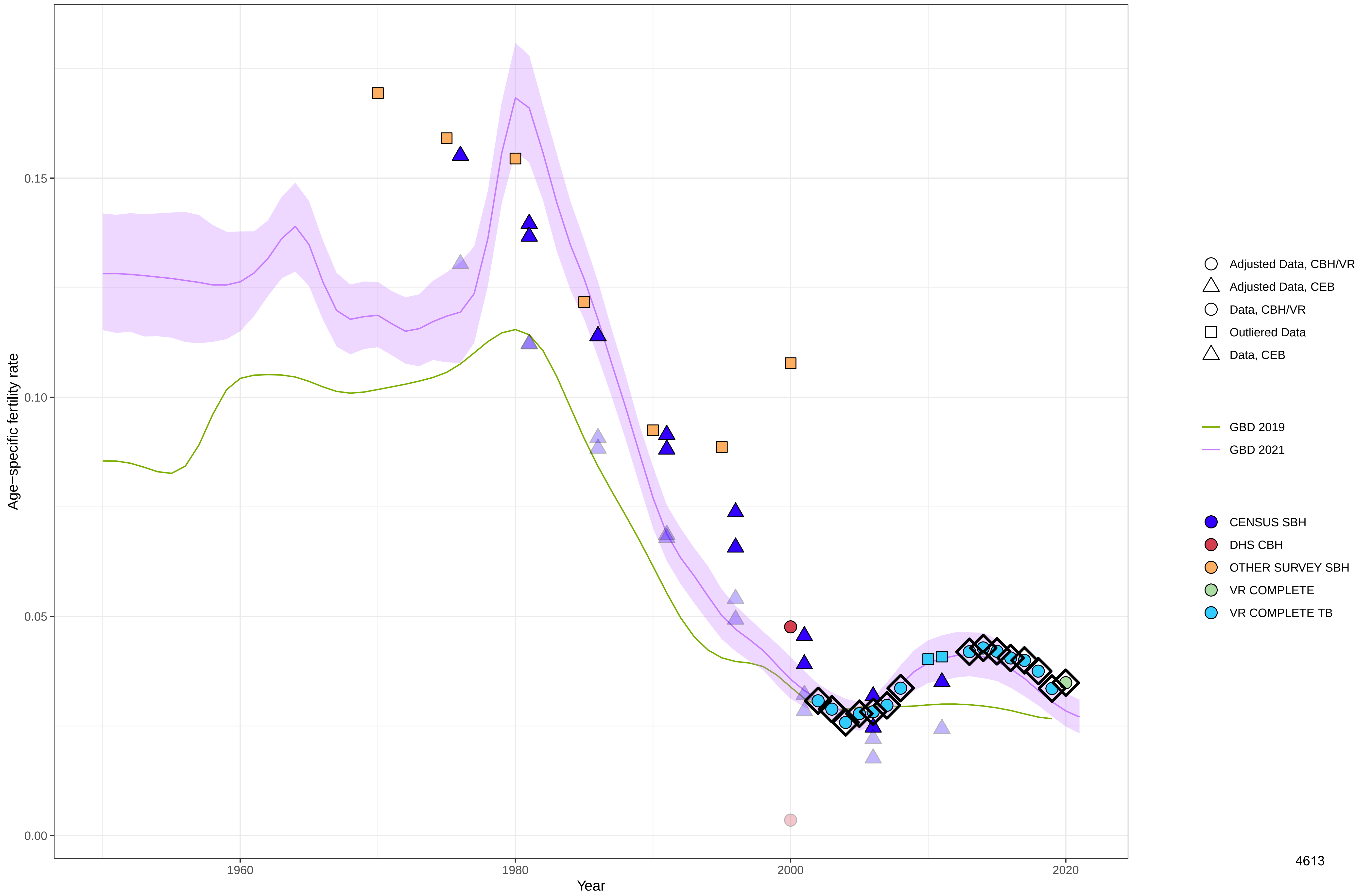

Age−specific fertility fitted trend lines, 1950−2021: East Azarbayejan, Ages: 20−24
Data points enclosed with a diamond are reference sources

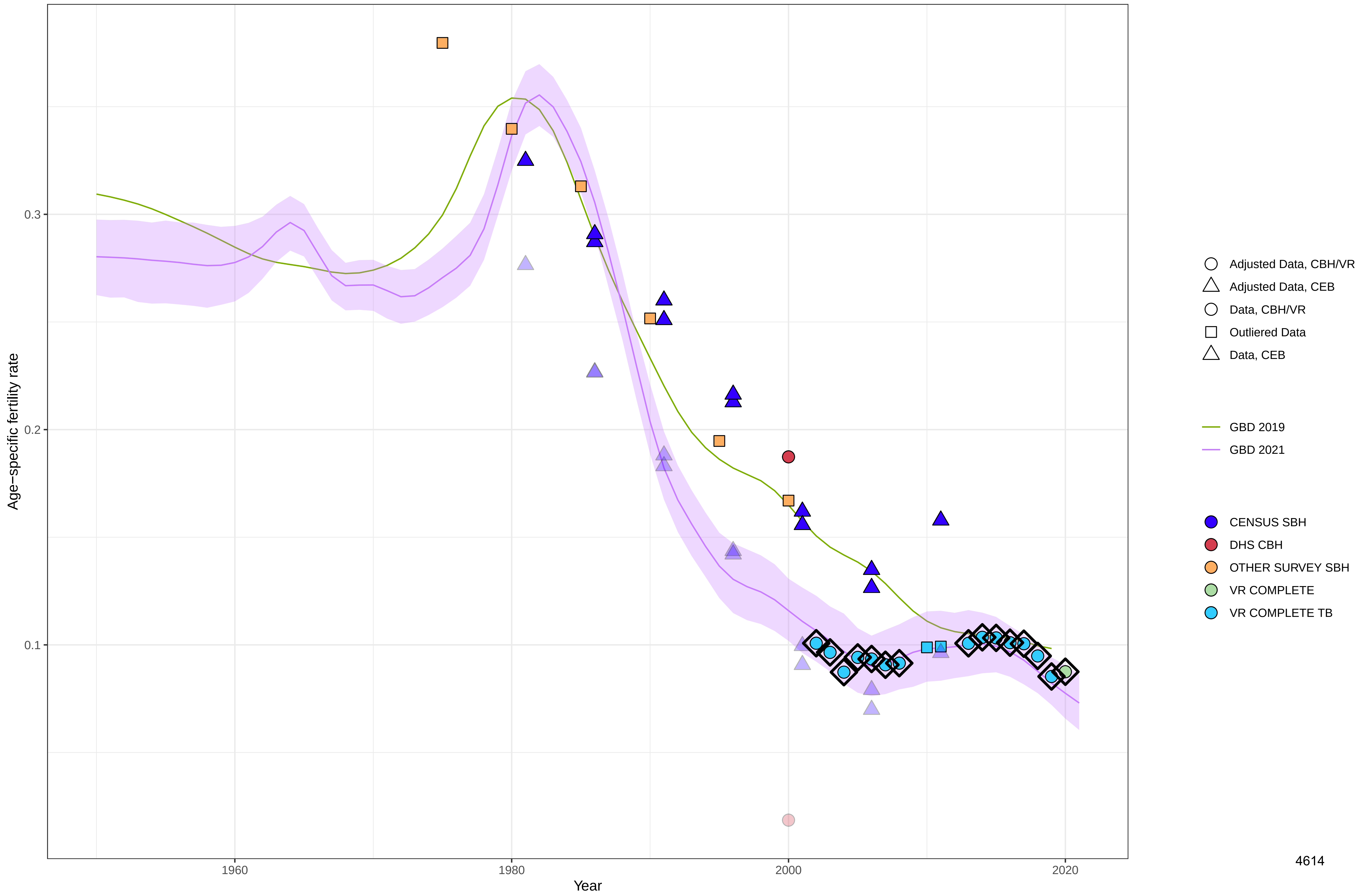

Age−specific fertility fitted trend lines, 1950−2021: East Azarbayejan, Ages: 25−29
Data points enclosed with a diamond are reference sources

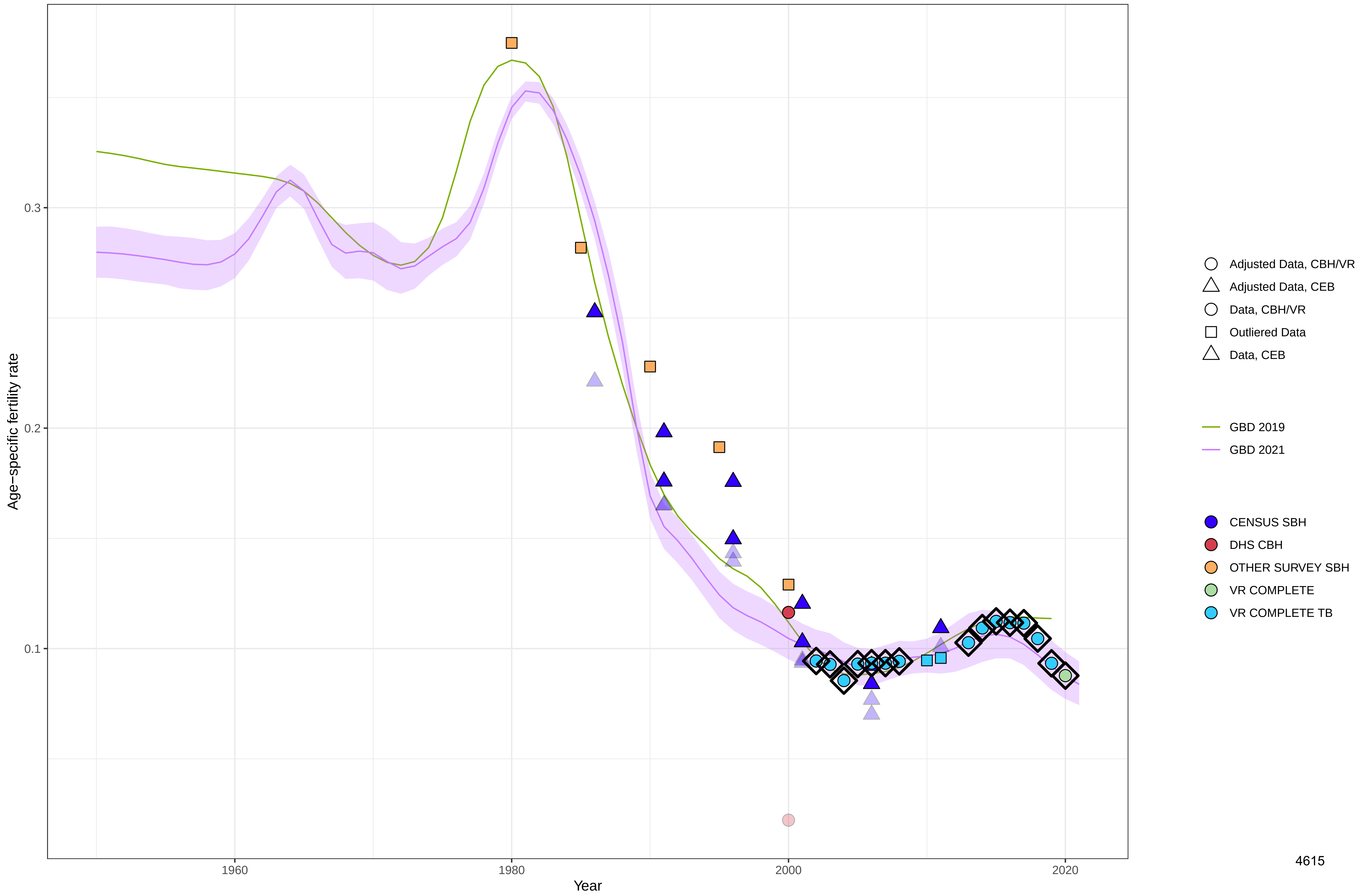

Age−specific fertility fitted trend lines, 1950−2021: East Azarbayejan, Ages: 30−34
Data points enclosed with a diamond are reference sources

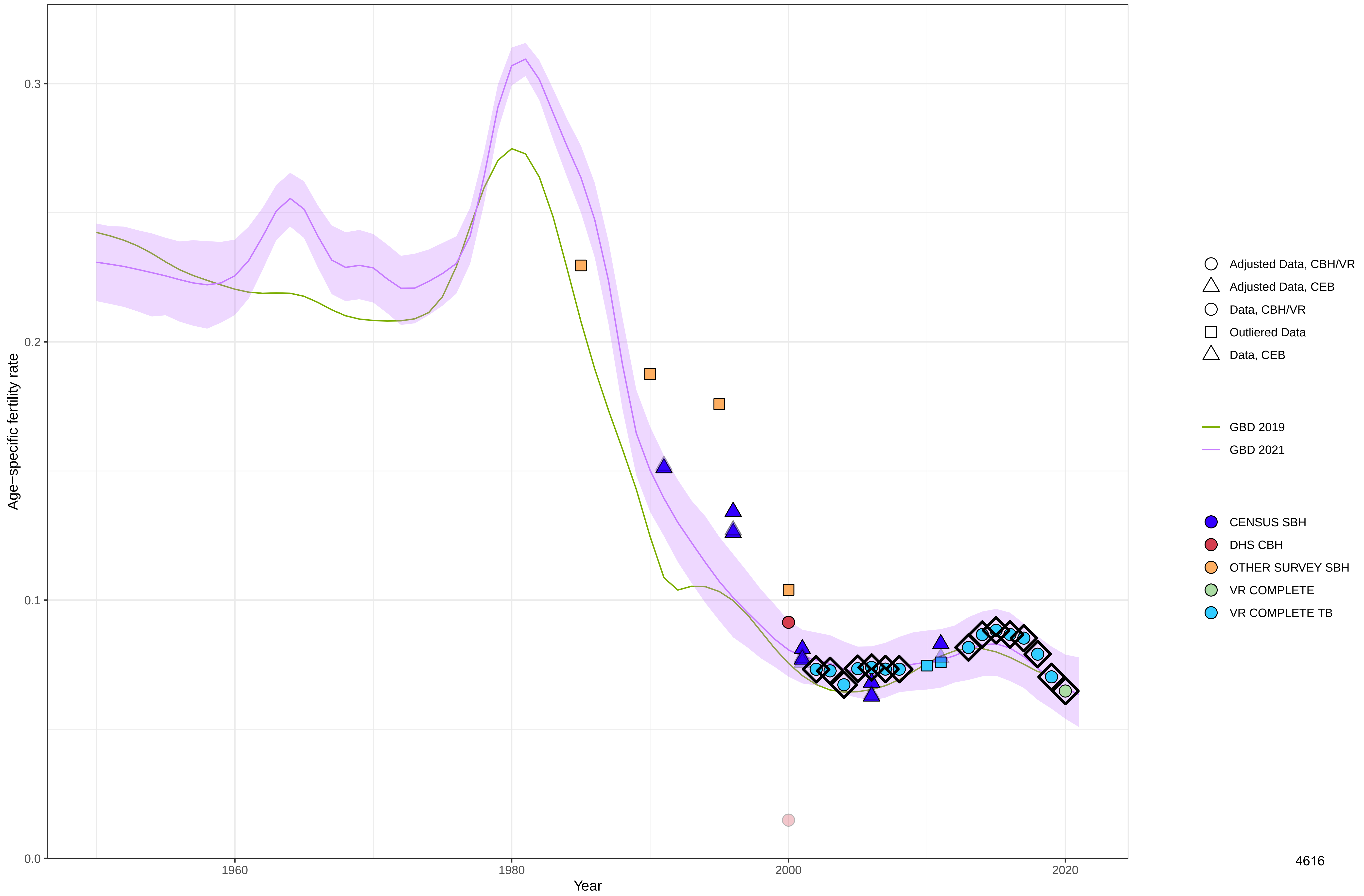

Age−specific fertility fitted trend lines, 1950−2021: East Azarbayejan, Ages: 35−39
Data points enclosed with a diamond are reference sources

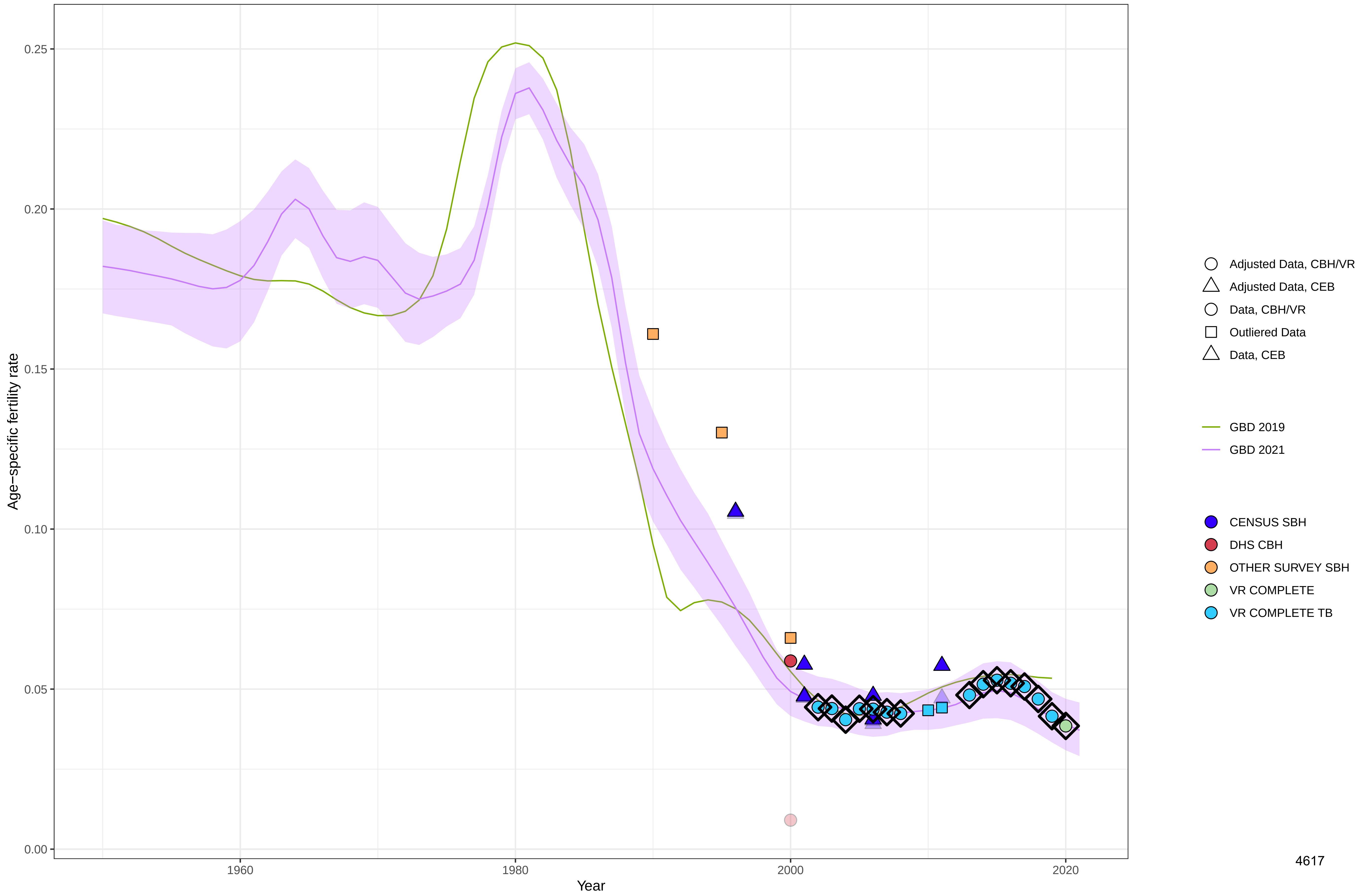

Age−specific fertility fitted trend lines, 1950−2021: East Azarbayejan, Ages: 40−44
Data points enclosed with a diamond are reference sources

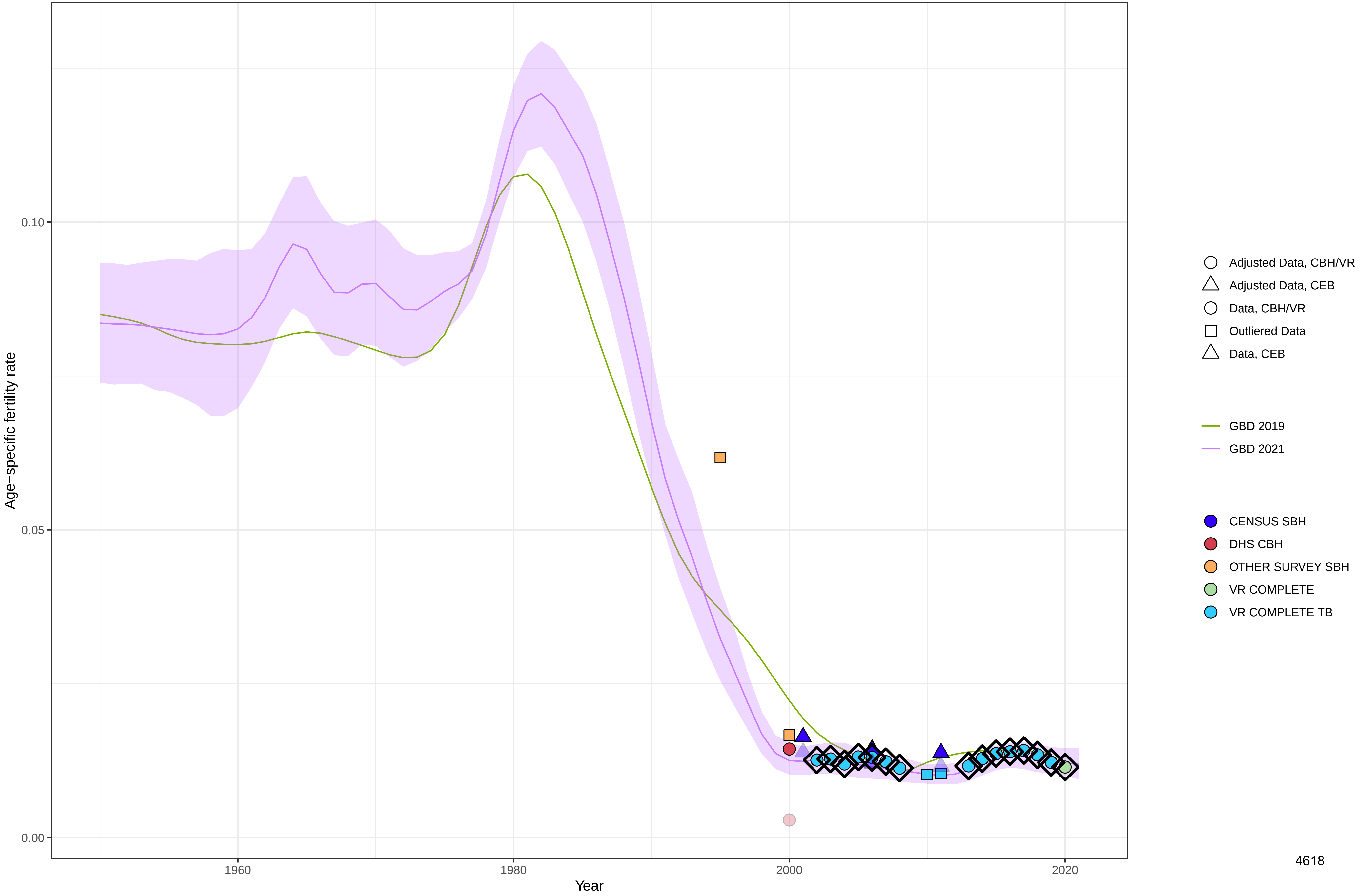

Age−specific fertility fitted trend lines, 1950−2021: East Azarbayejan, Ages: 45−49
Data points enclosed with a diamond are reference sources

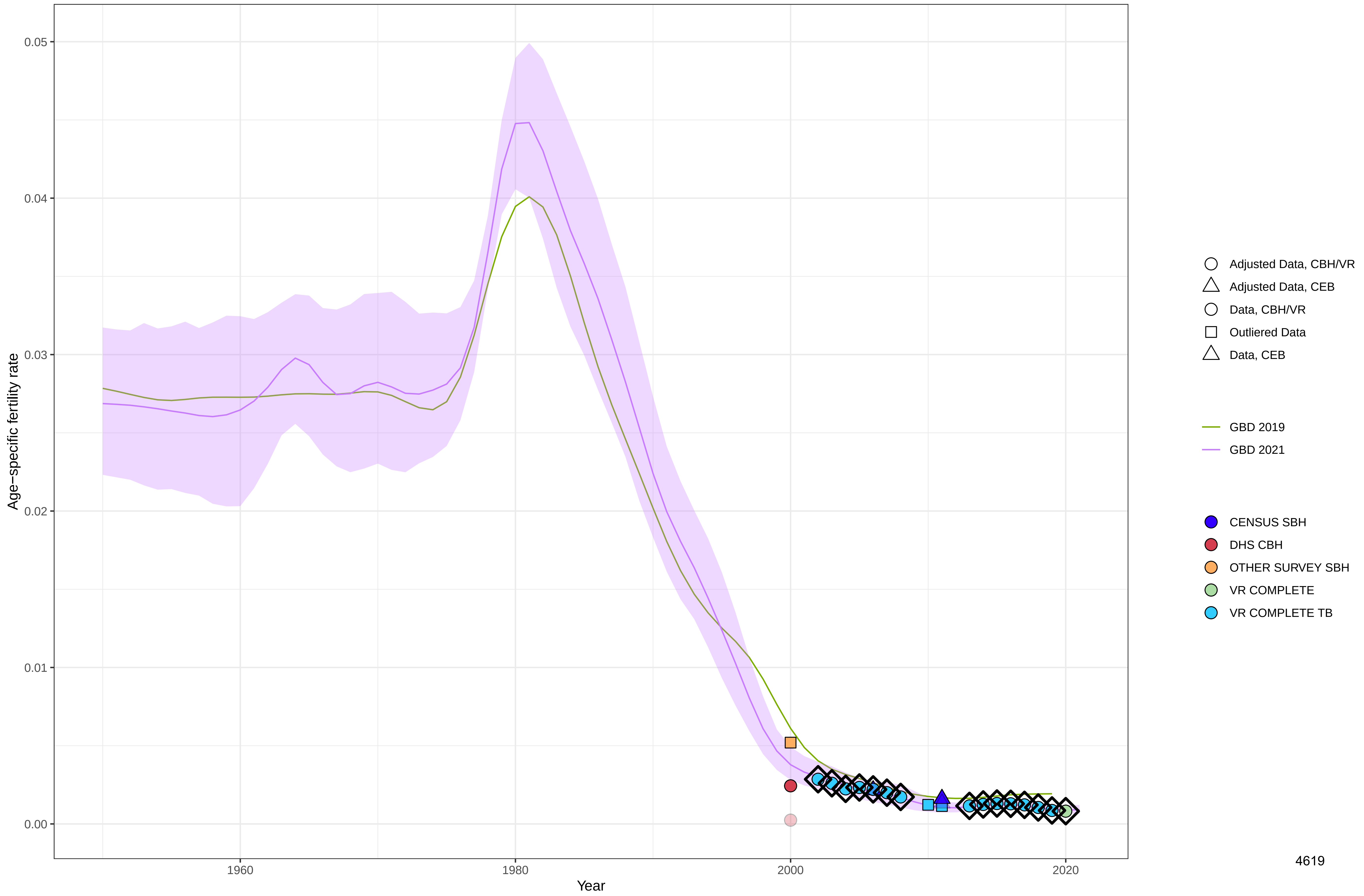

Age−specific fertility fitted trend lines, 1950−2021: East Azarbayejan, Ages: 50−54
Data points enclosed with a diamond are reference sources

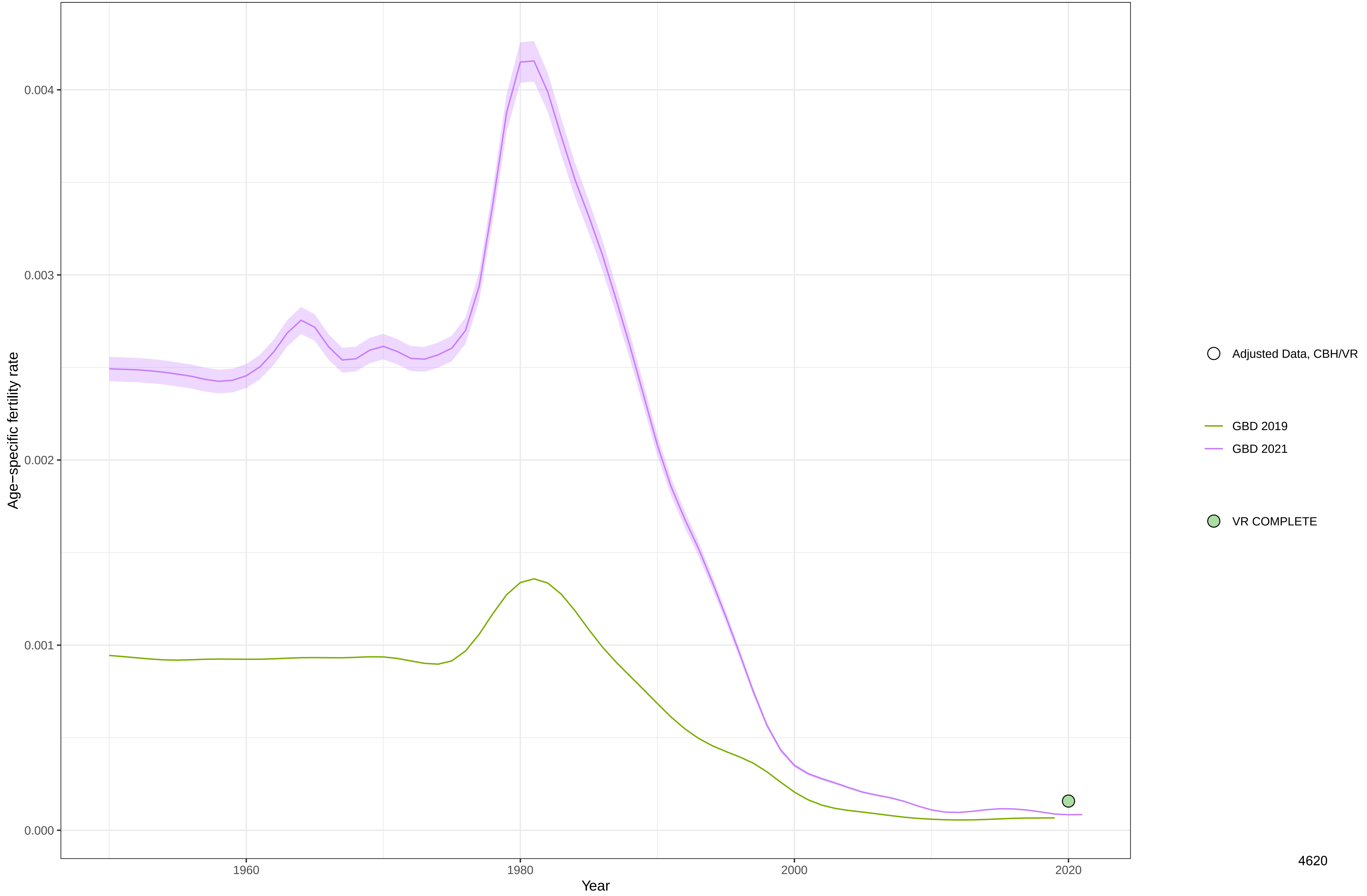

Total fertility rate fitted trend lines, 1950−2021: East Azarbayejan TFR
Data points enclosed with a diamond are reference sources

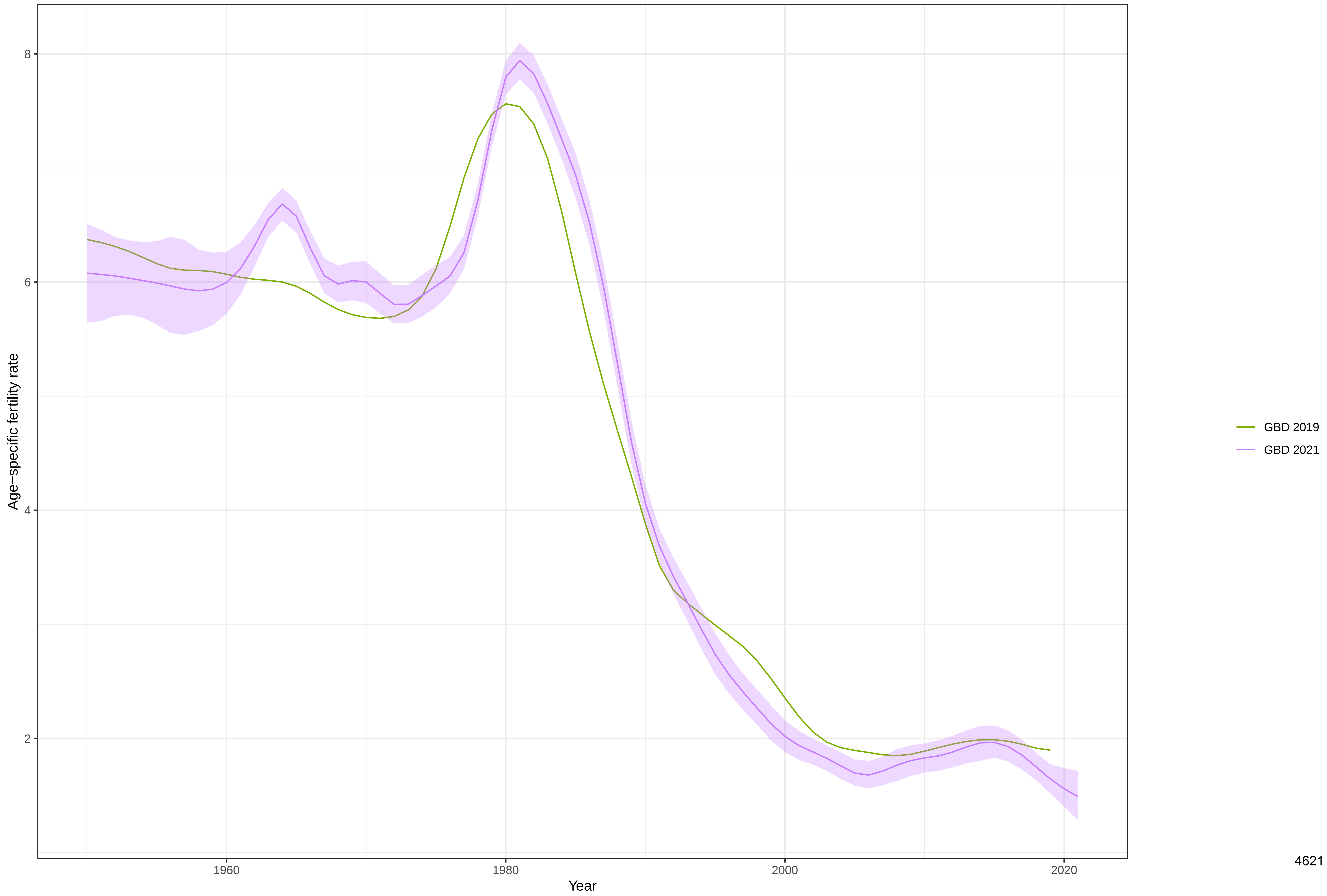

Age−specific fertility fitted trend lines, 1950−2021: Fars, Ages: 10−14
Data points enclosed with a diamond are reference sources

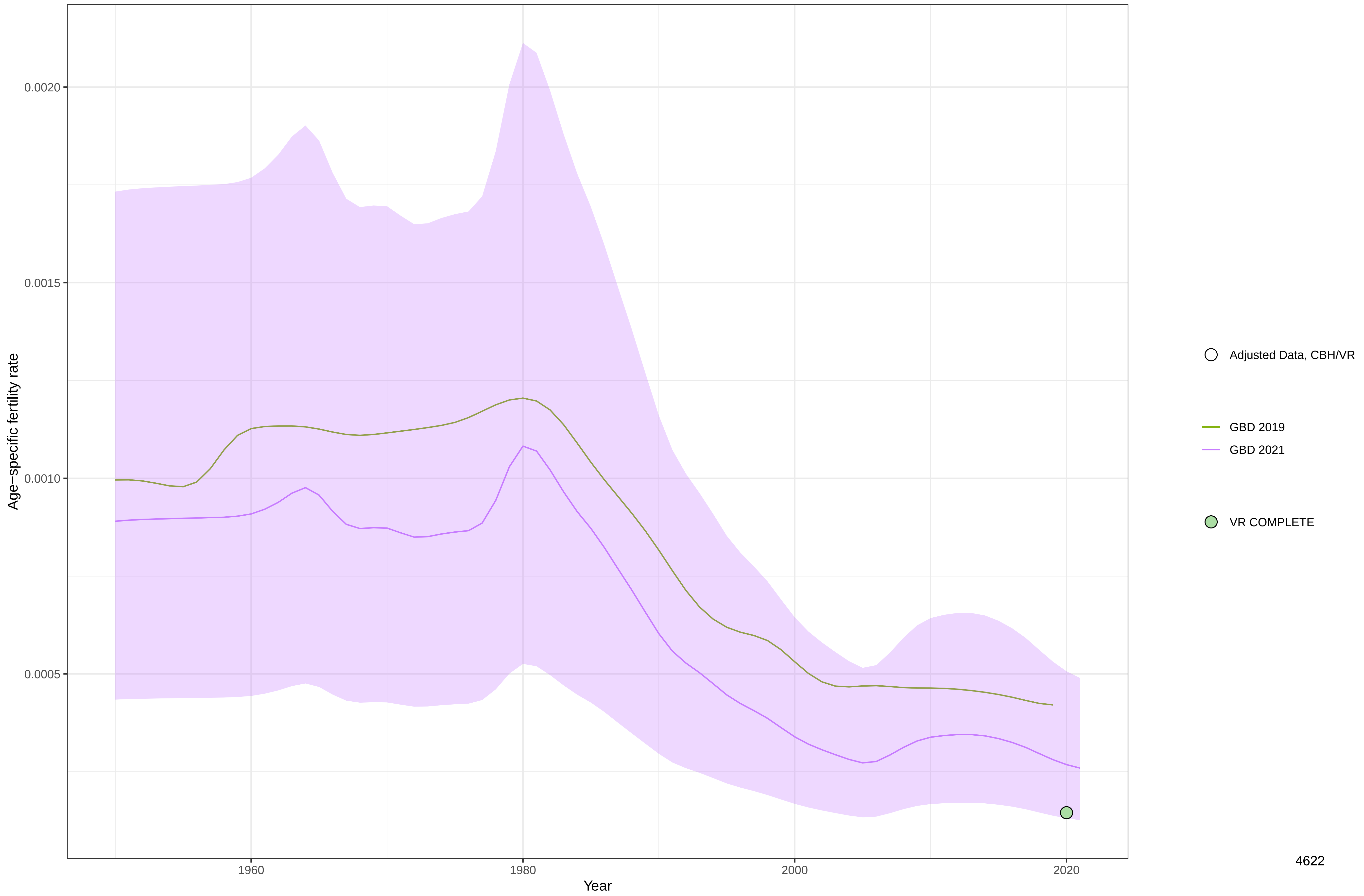

Age−specific fertility fitted trend lines, 1950−2021: Fars, Ages: 15−19
Data points enclosed with a diamond are reference sources

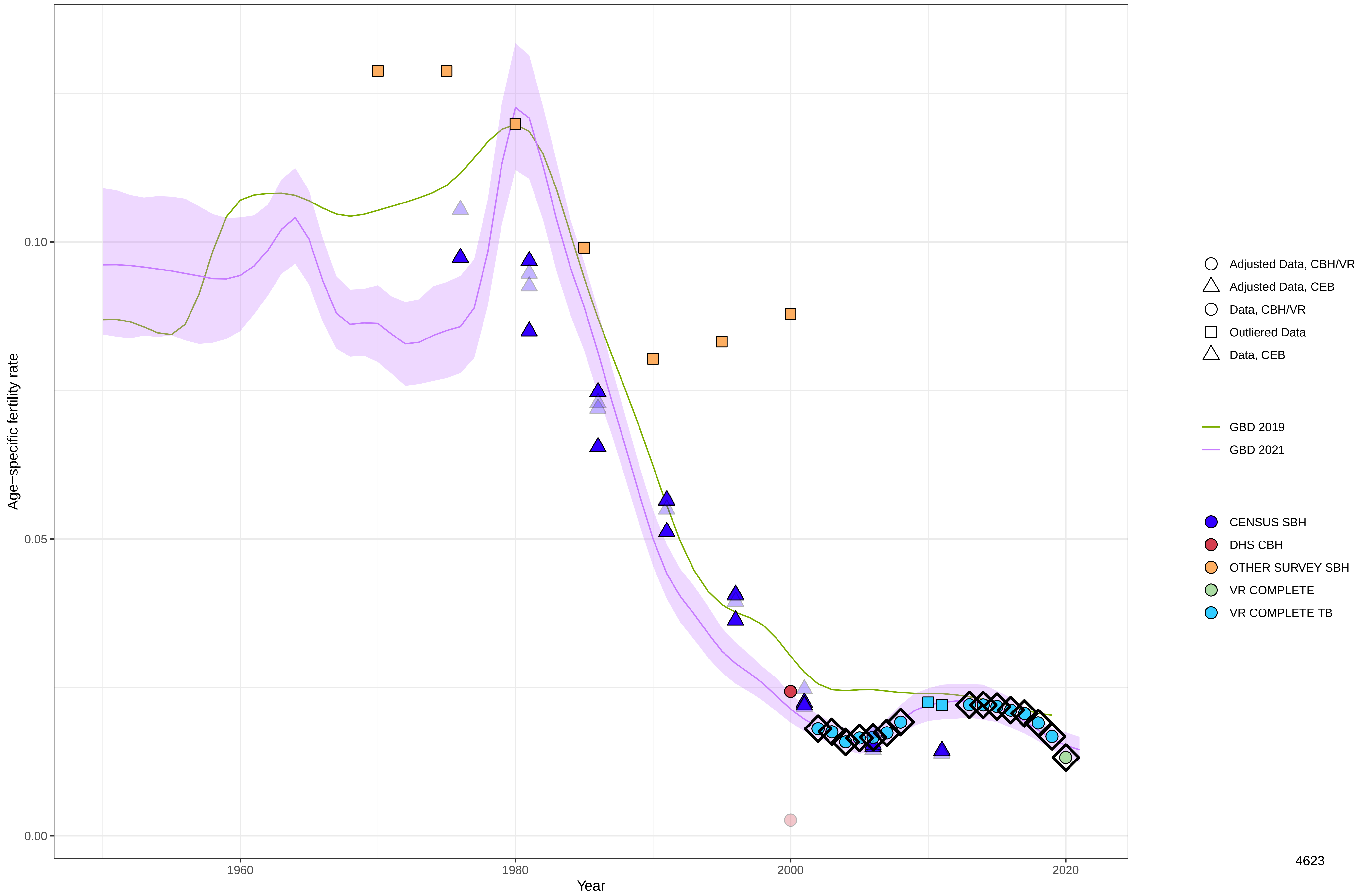

Age−specific fertility fitted trend lines, 1950−2021: Fars, Ages: 20−24
Data points enclosed with a diamond are reference sources

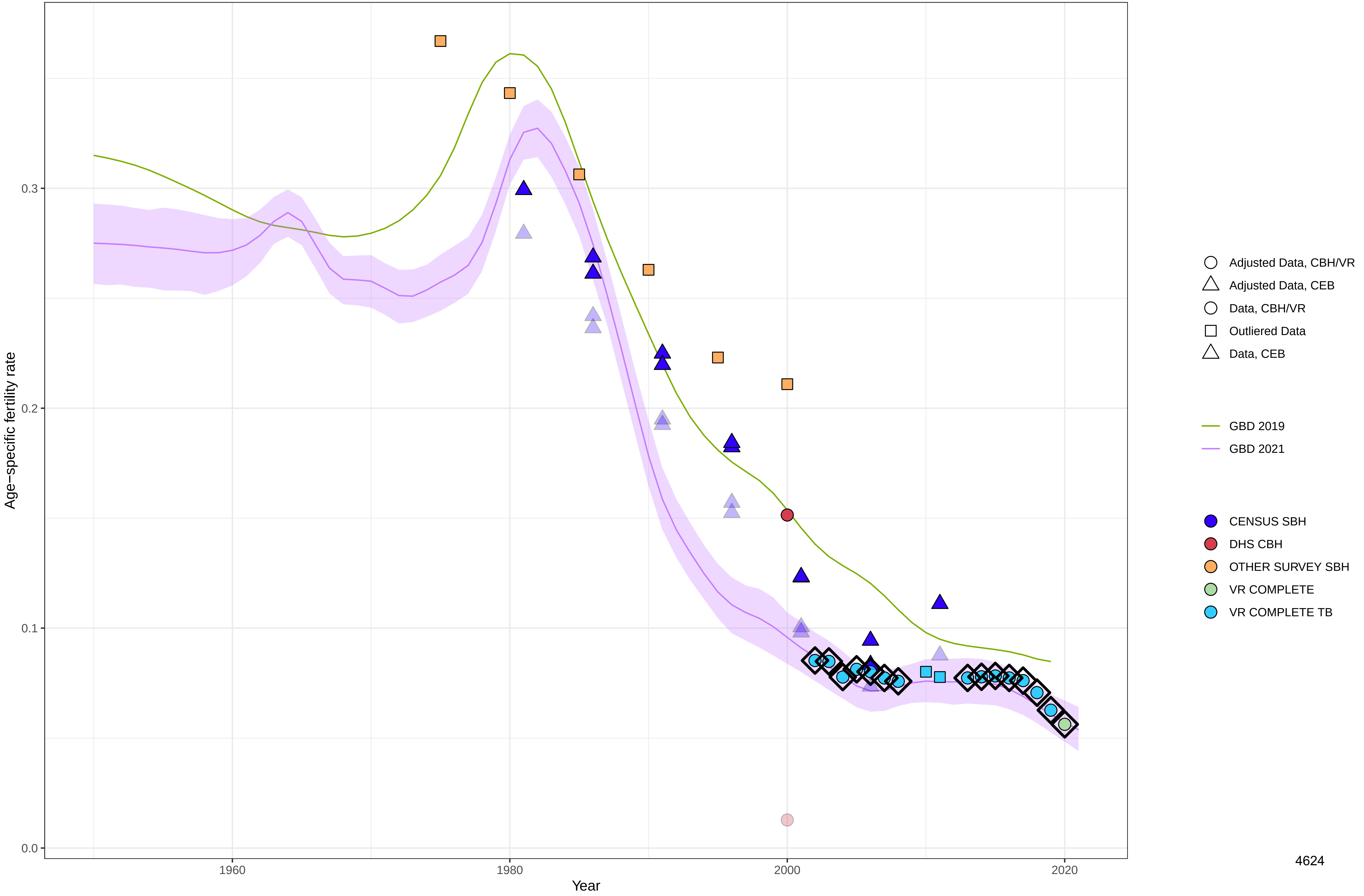

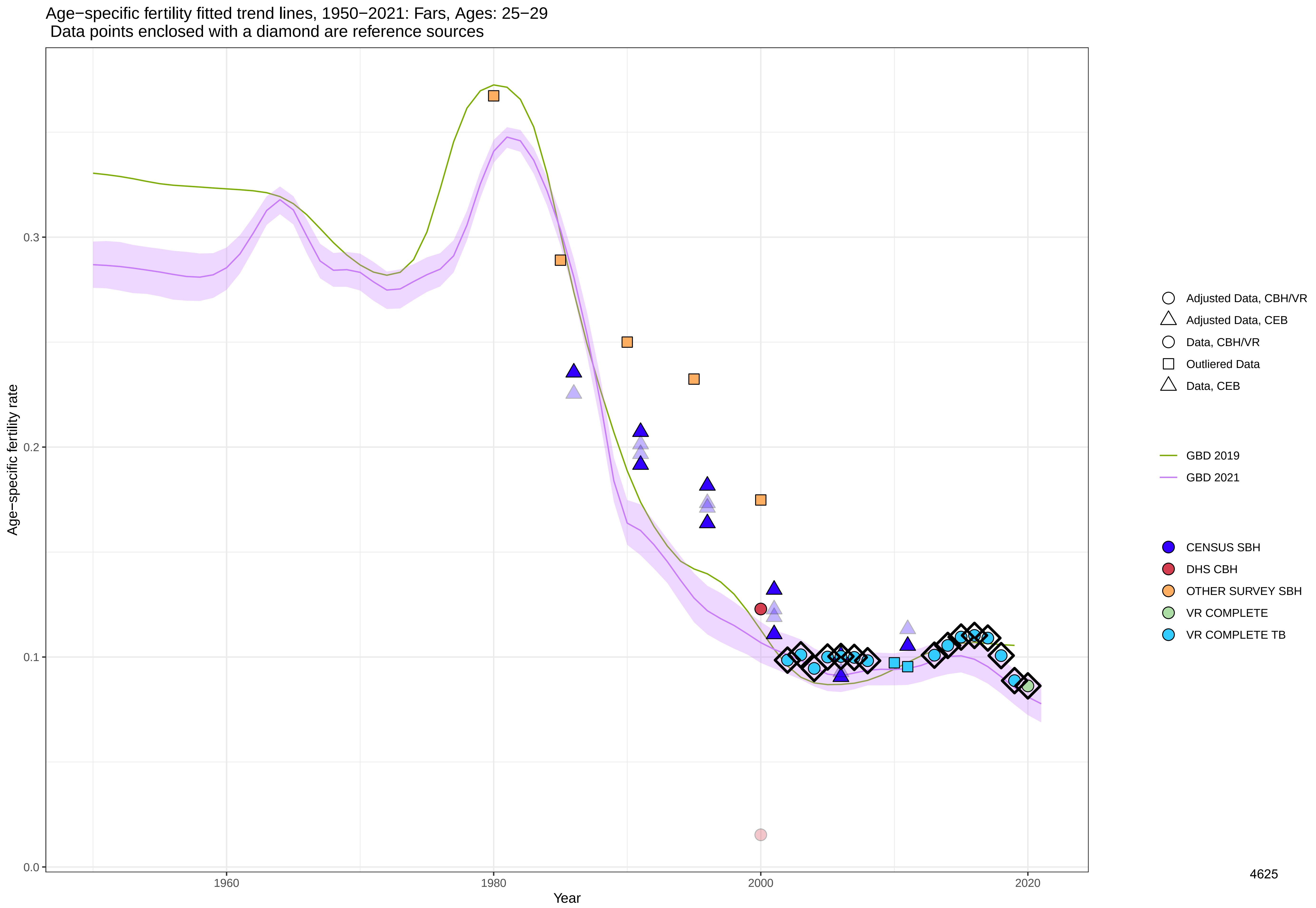

Age−specific fertility fitted trend lines, 1950−2021: Fars, Ages: 25−29
Data points enclosed with a diamond are reference sources
Age−specific fertility rate
0.0
0.1
0.2
0.3
Year
1960
1980
2000
2020
Adjusted Data, CBH/VR
Adjusted Data, CEB
Data, CBH/VR
Outliered Data
Data, CEB
GBD 2019
GBD 2021
CENSUS SBH
DHS CBH
OTHER SURVEY SBH
VR COMPLETE
VR COMPLETE TB

Age−specific fertility fitted trend lines, 1950−2021: Fars, Ages: 30−34
Data points enclosed with a diamond are reference sources

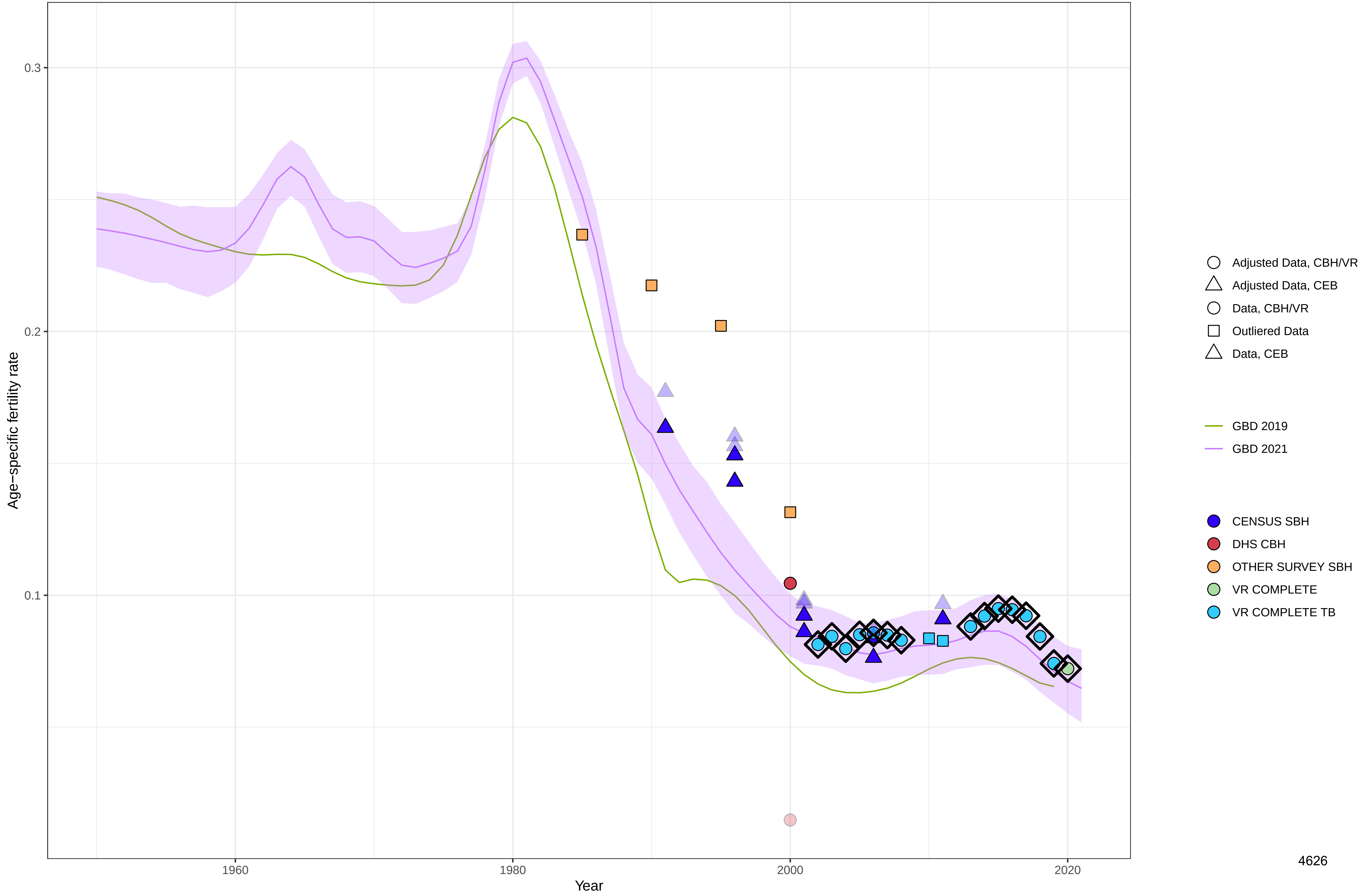

Age−specific fertility fitted trend lines, 1950−2021: Fars, Ages: 35−39
Data points enclosed with a diamond are reference sources

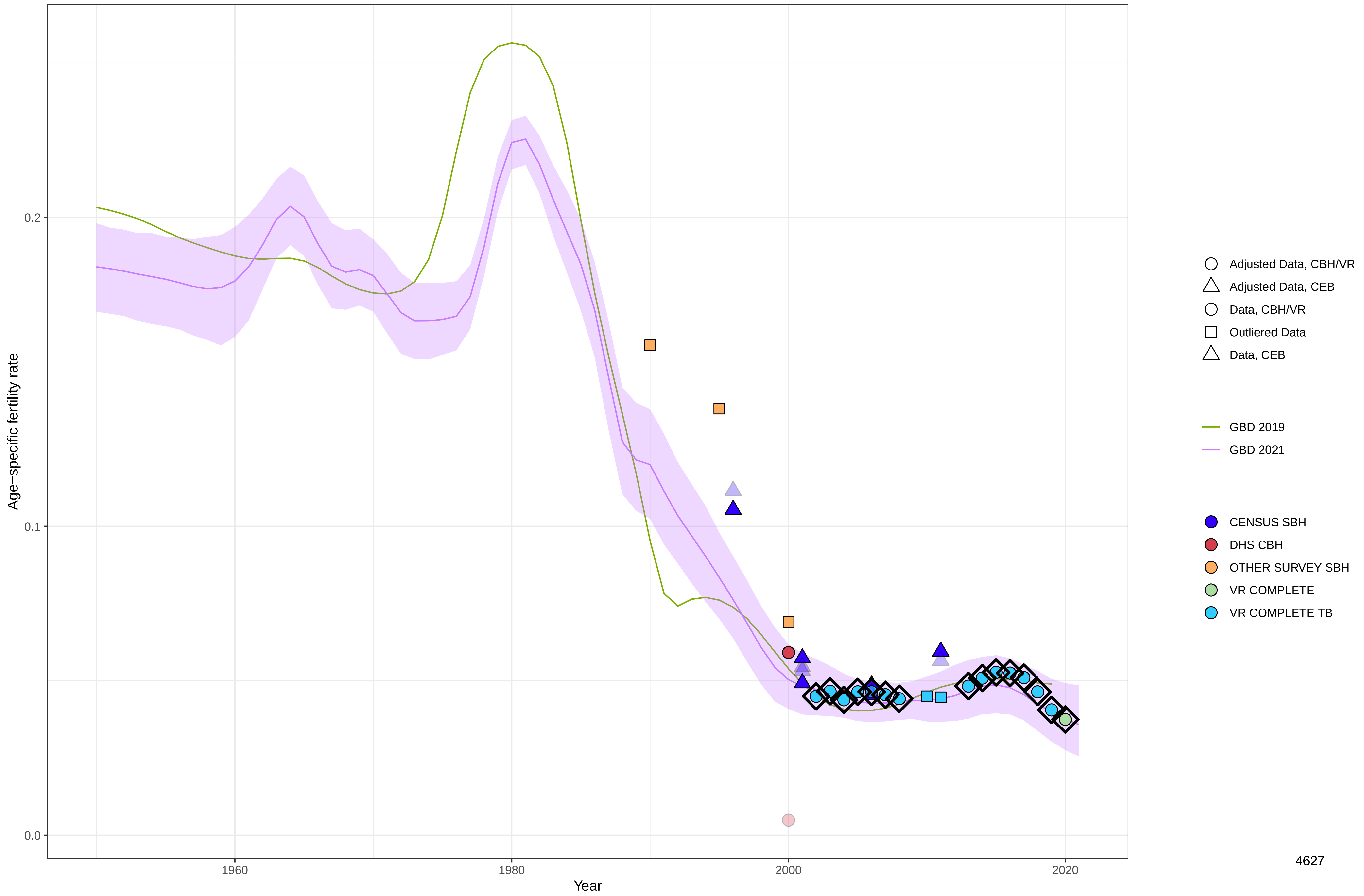

Age−specific fertility fitted trend lines, 1950−2021: Fars, Ages: 40−44
Data points enclosed with a diamond are reference sources

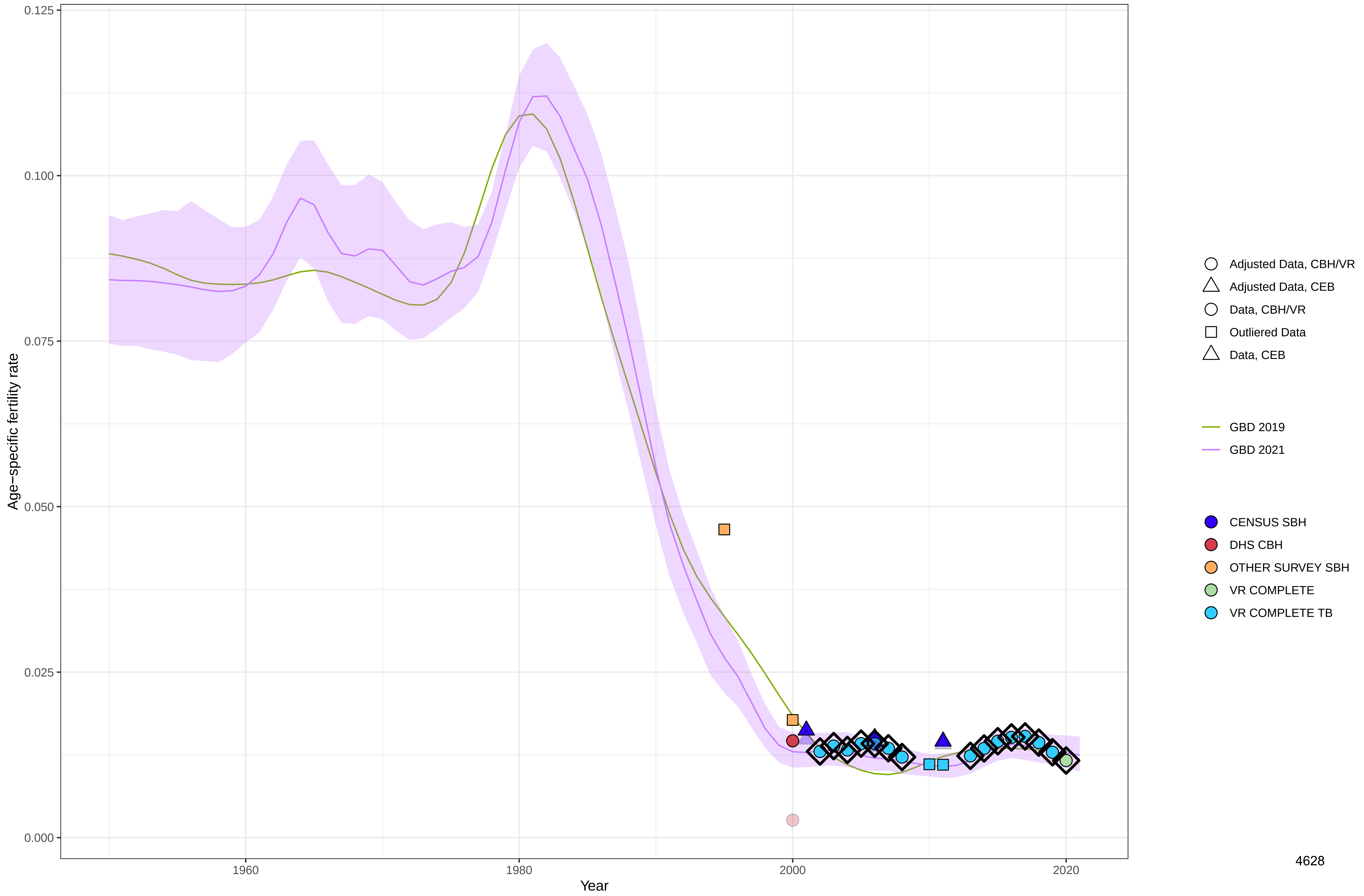

Age−specific fertility fitted trend lines, 1950−2021: Fars, Ages: 45−49
Data points enclosed with a diamond are reference sources

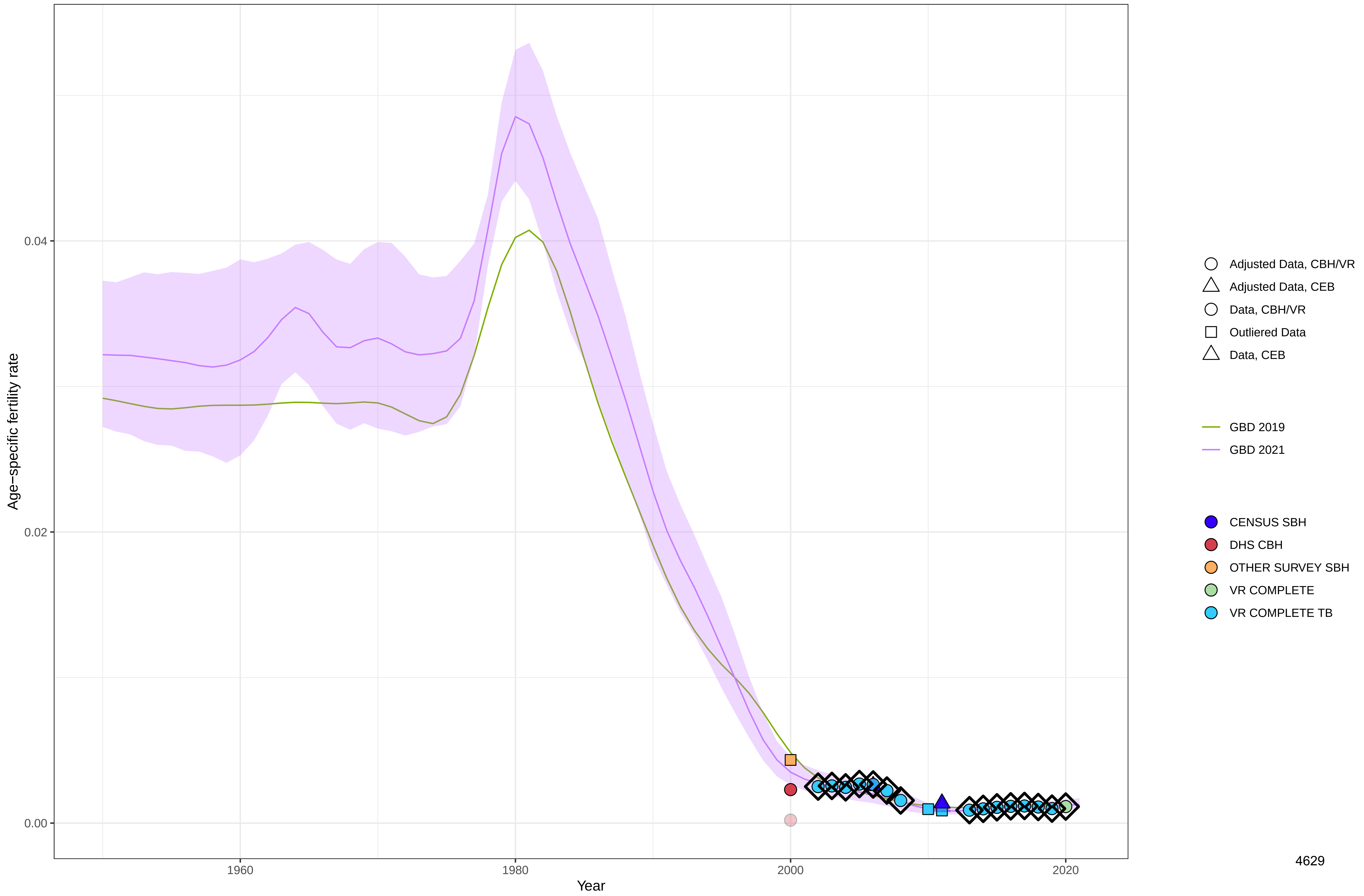

Age−specific fertility fitted trend lines, 1950−2021: Fars, Ages: 50−54
Data points enclosed with a diamond are reference sources

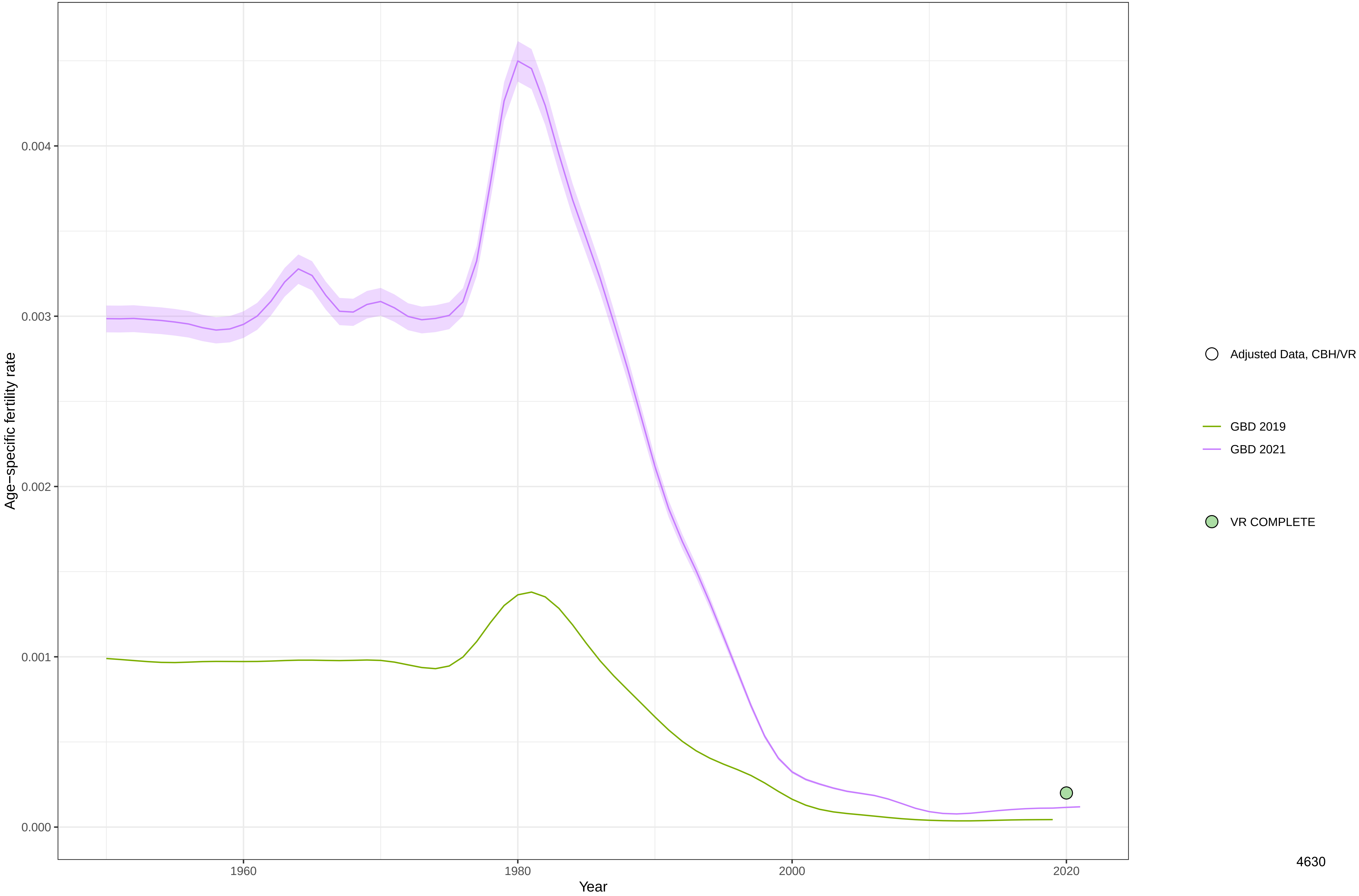

Total fertility rate fitted trend lines, 1950−2021: Fars TFR
Data points enclosed with a diamond are reference sources

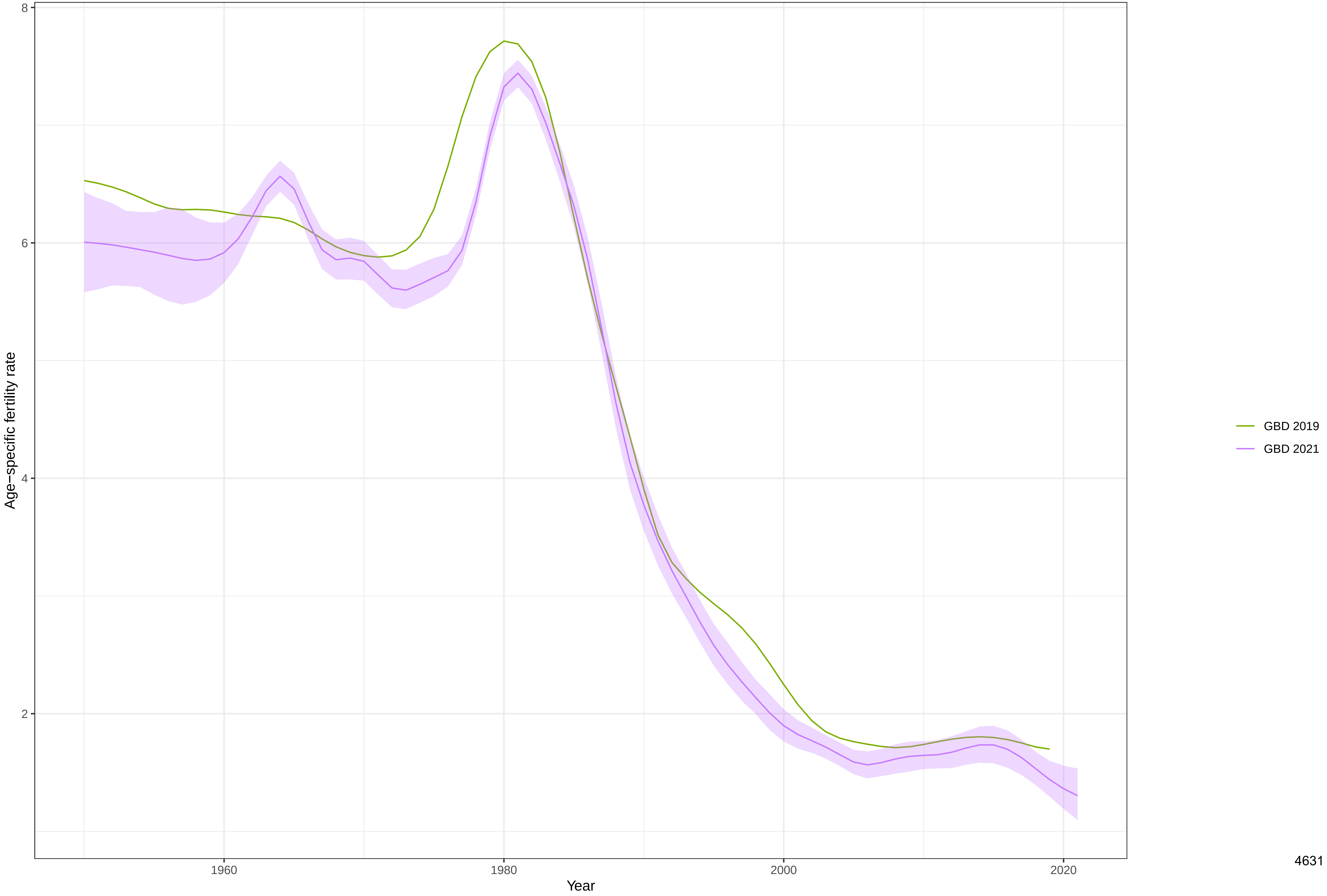

4631

Age−specific fertility fitted trend lines, 1950−2021: Gilan, Ages: 10−14
Data points enclosed with a diamond are reference sources

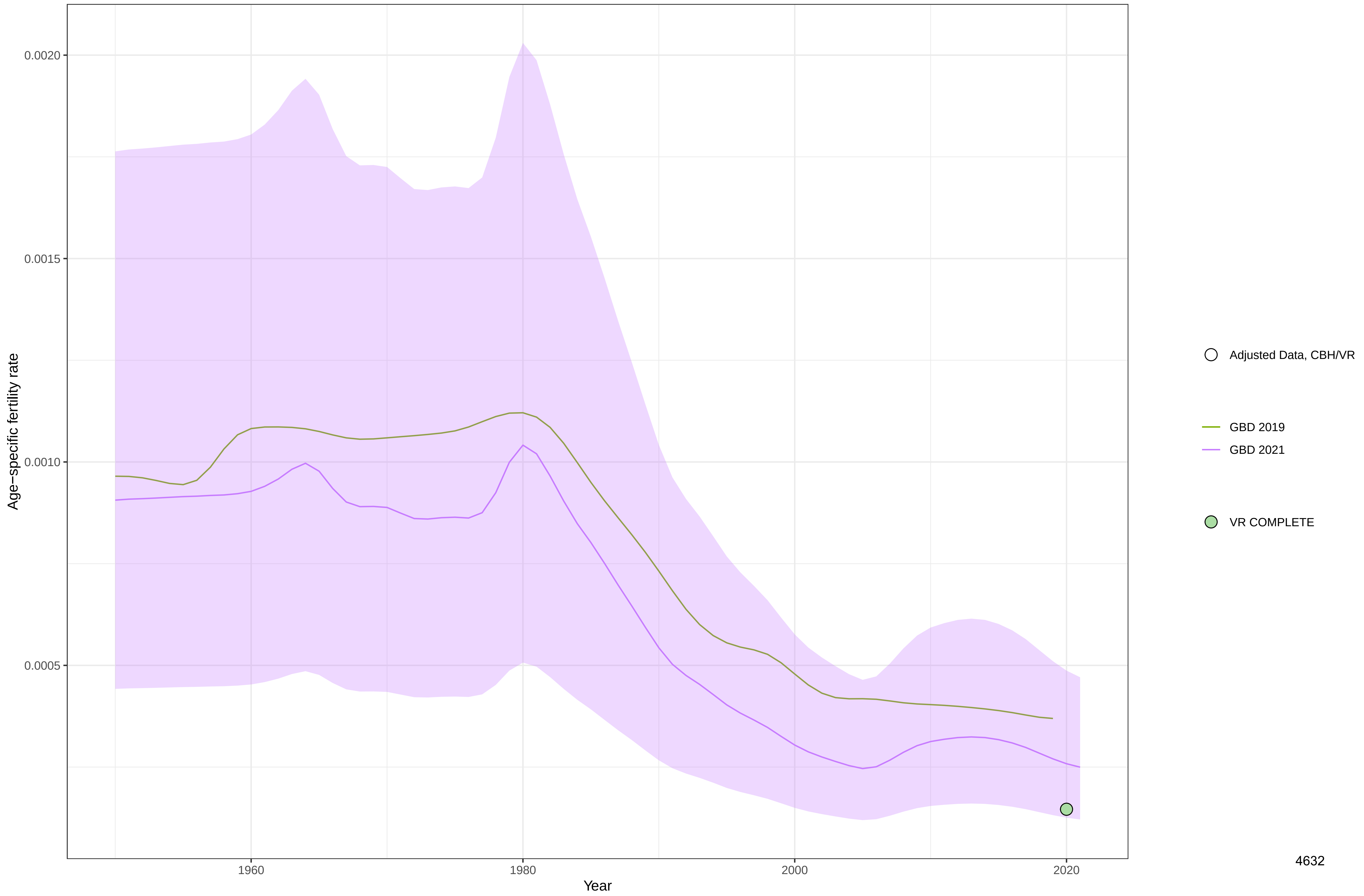

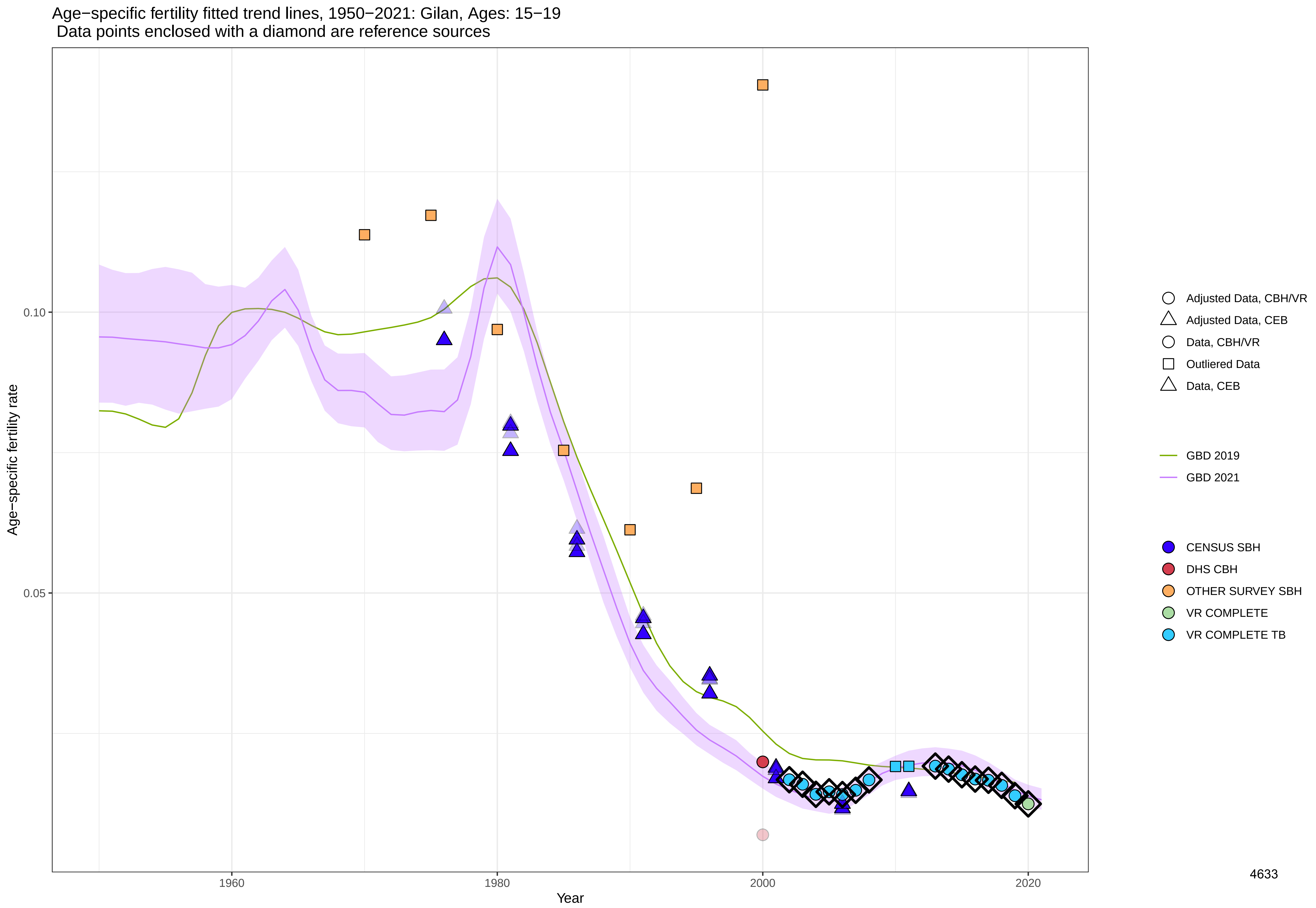

Age−specific fertility fitted trend lines, 1950−2021: Gilan, Ages: 15−19
Data points enclosed with a diamond are reference sources
Age−specific fertility rate
0.10
0.05
1960
1980
2000
2020
Year
Adjusted Data, CBH/VR
Adjusted Data, CEB
Data, CBH/VR
Outliered Data
Data, CEB
GBD 2019
GBD 2021
CENSUS SBH
DHS CBH
OTHER SURVEY SBH
VR COMPLETE
VR COMPLETE TB

Age−specific fertility fitted trend lines, 1950−2021: Gilan, Ages: 20−24
Data points enclosed with a diamond are reference sources

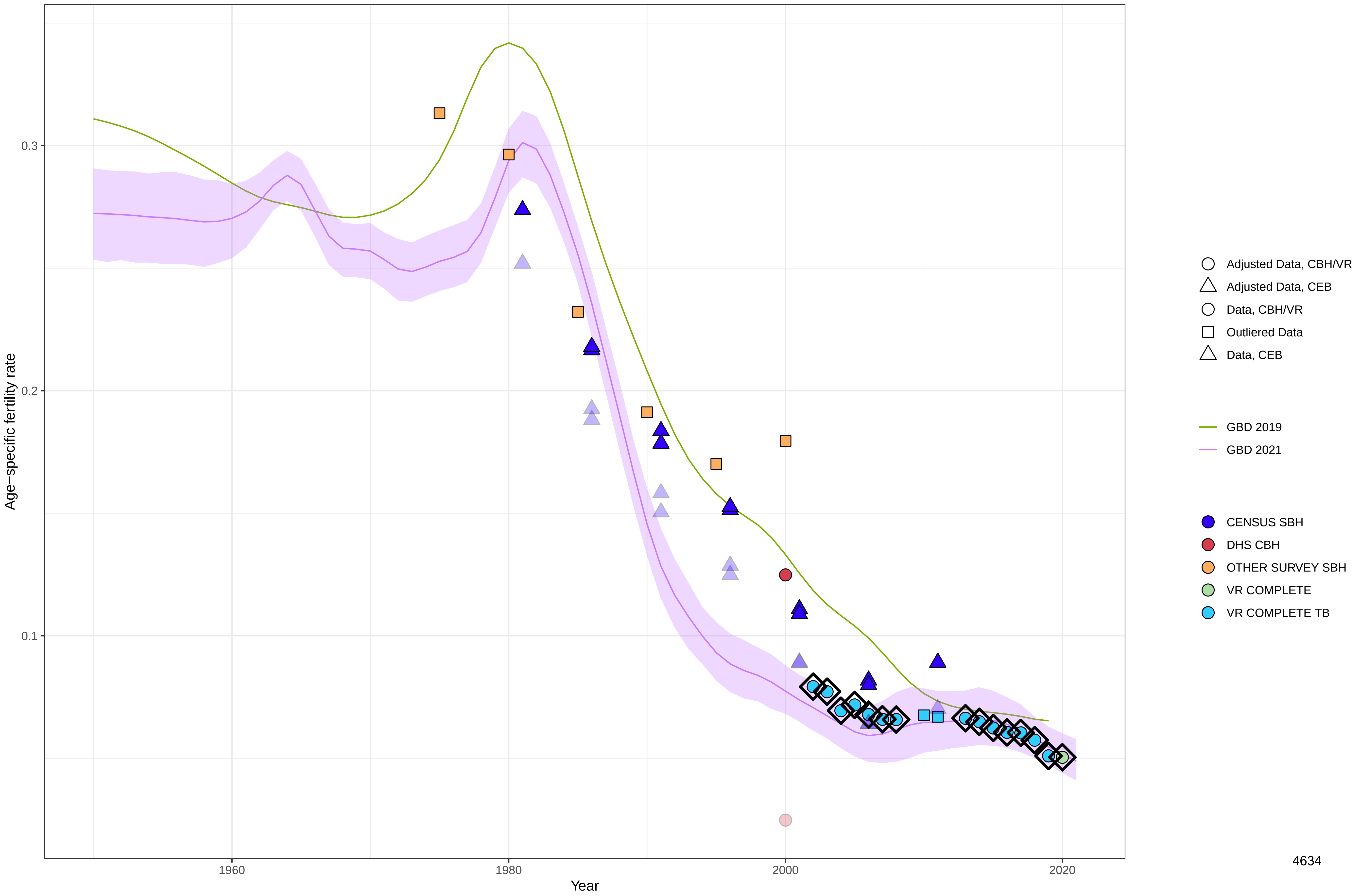

Age−specific fertility fitted trend lines, 1950−2021: Gilan, Ages: 25−29
Data points enclosed with a diamond are reference sources

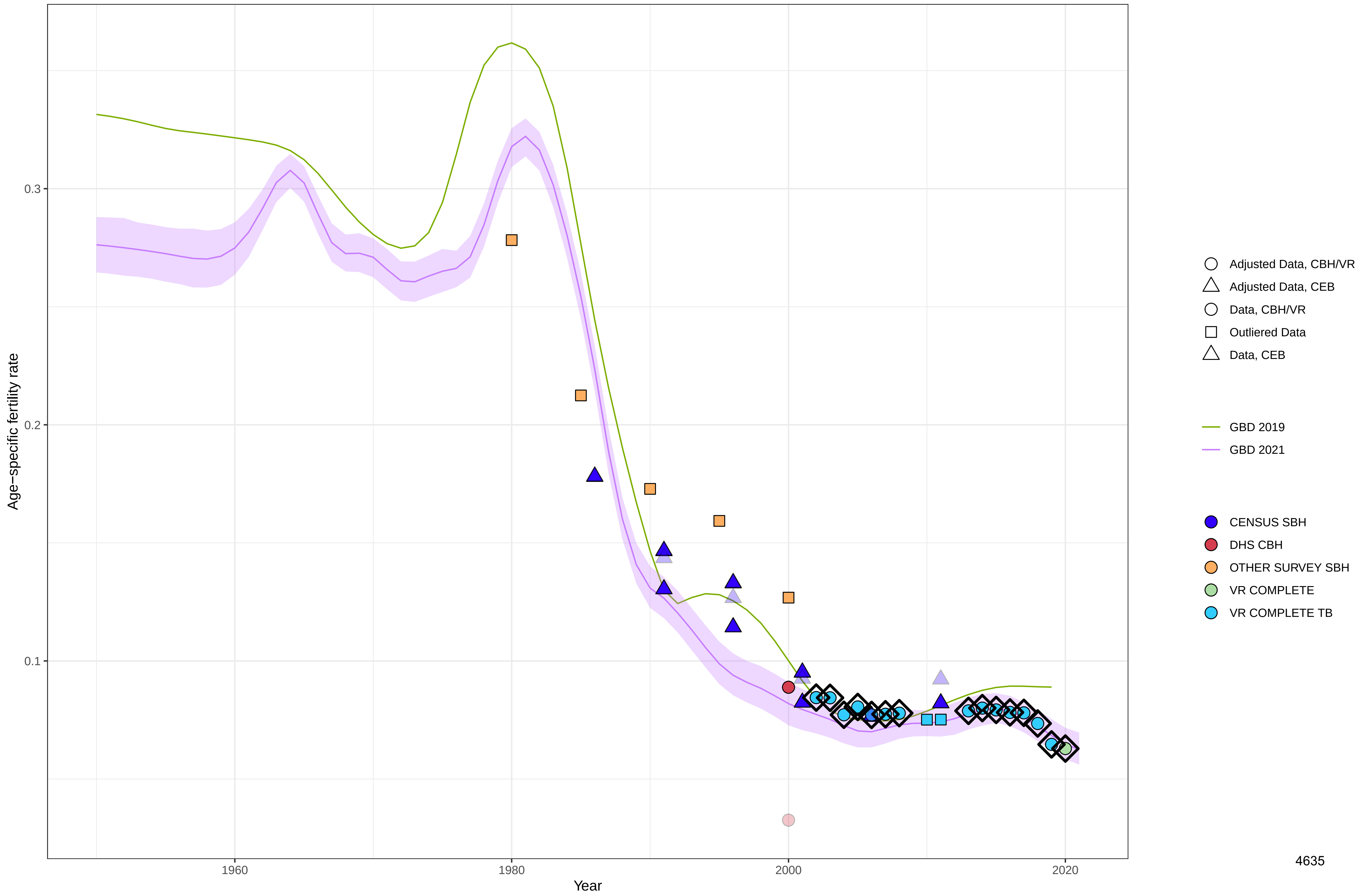

Age−specific fertility fitted trend lines, 1950−2021: Gilan, Ages: 30−34
Data points enclosed with a diamond are reference sources

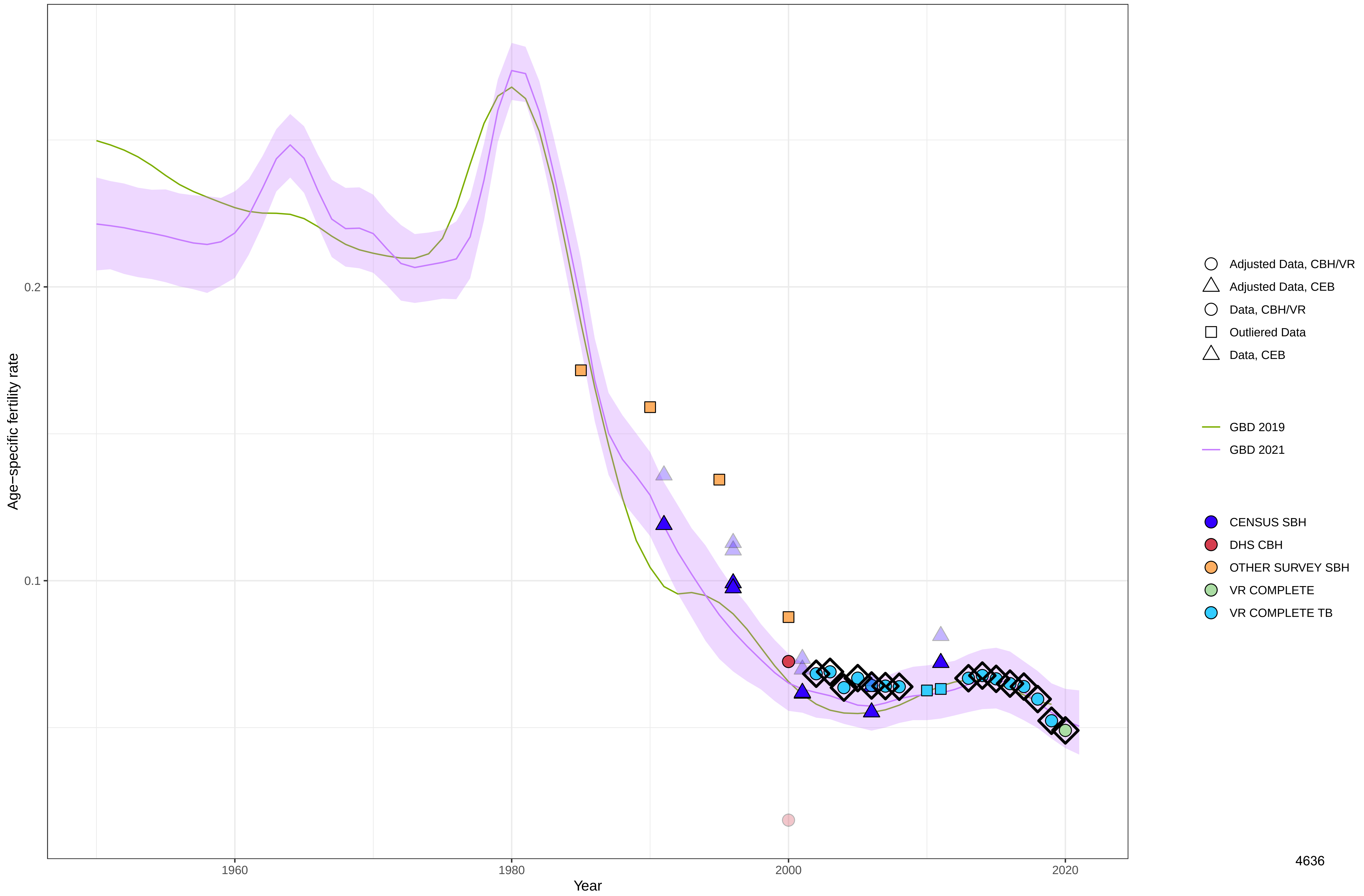

Age−specific fertility fitted trend lines, 1950−2021: Gilan, Ages: 35−39
Data points enclosed with a diamond are reference sources

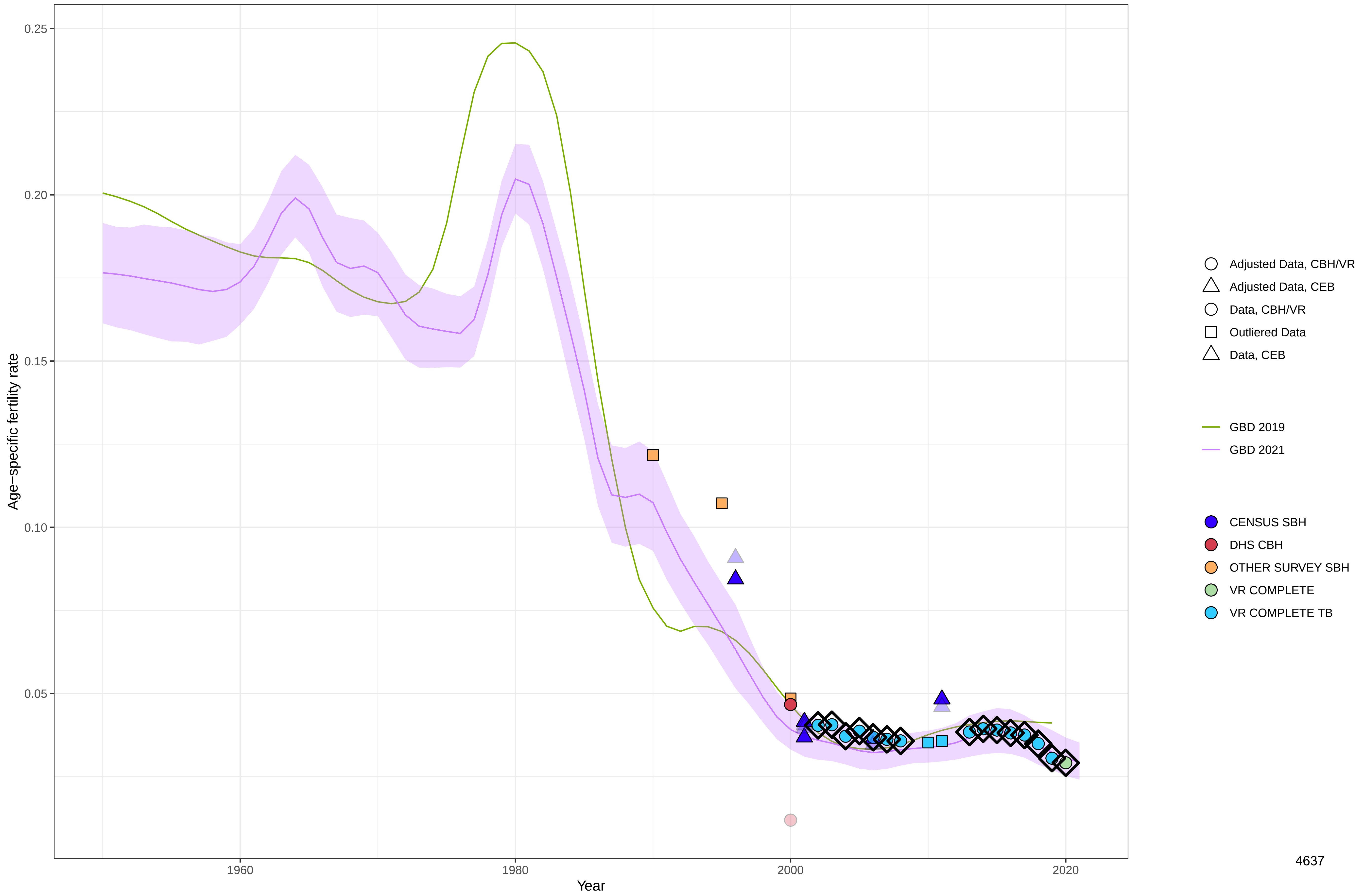

Age−specific fertility fitted trend lines, 1950−2021: Gilan, Ages: 40−44
Data points enclosed with a diamond are reference sources

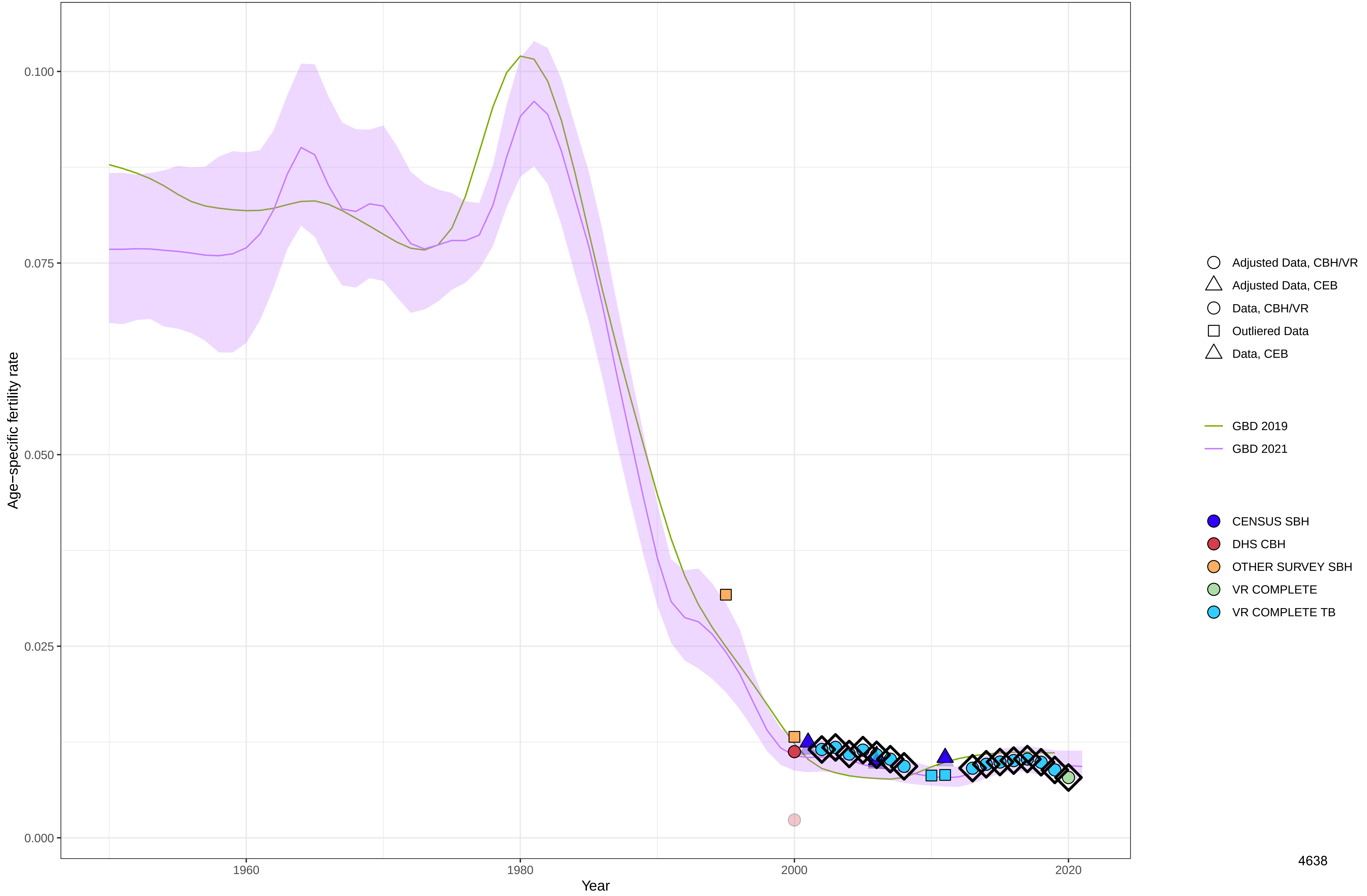

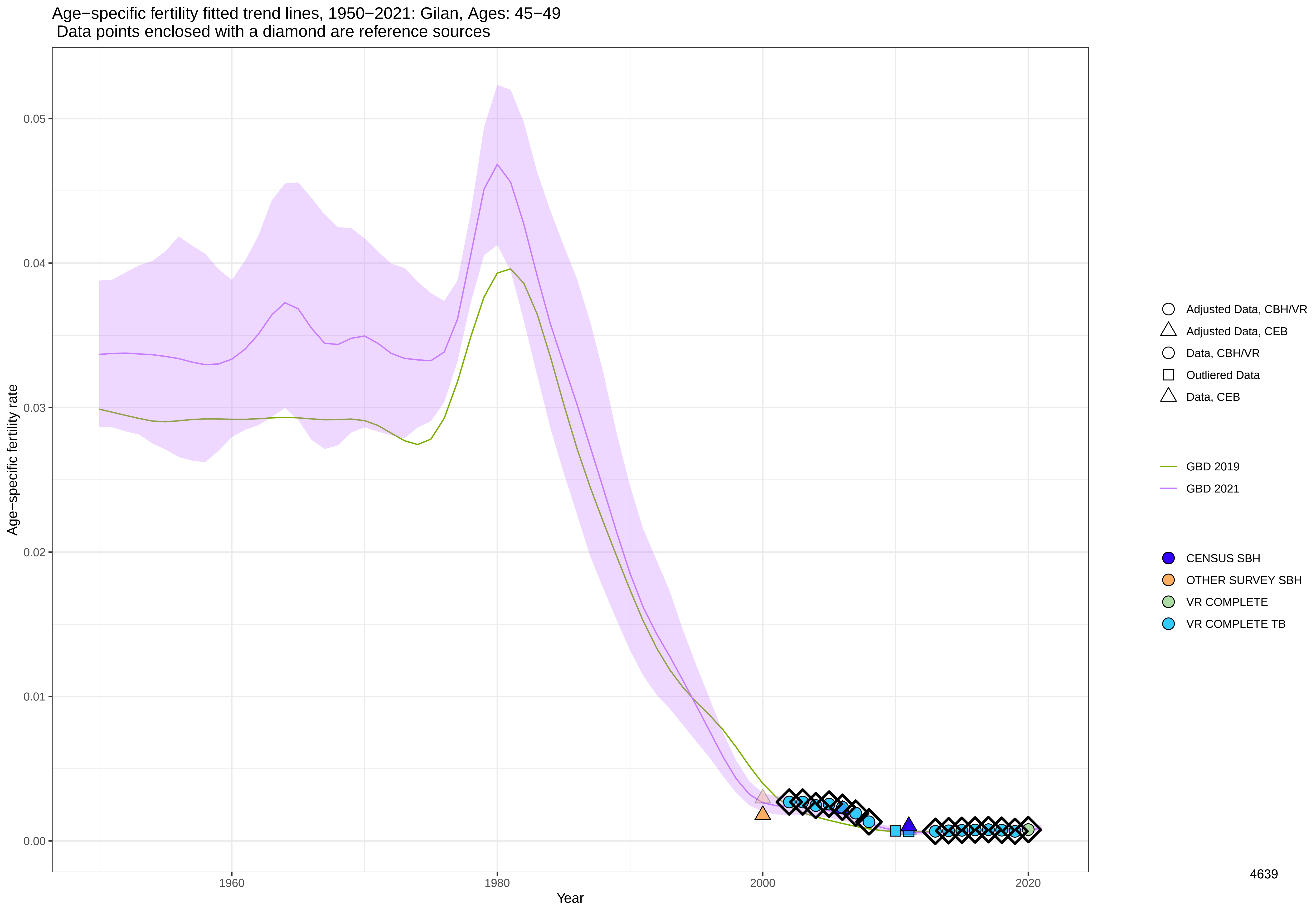

Age−specific fertility fitted trend lines, 1950−2021: Gilan, Ages: 45−49
Data points enclosed with a diamond are reference sources
Age−specific fertility rate
Year
0.00
0.01
0.02
0.03
0.04
0.05
1960
1980
2000
2020
Adjusted Data, CBH/VR
Adjusted Data, CEB
Data, CBH/VR
Outliered Data
Data, CEB
GBD 2019
GBD 2021
CENSUS SBH
OTHER SURVEY SBH
VR COMPLETE
VR COMPLETE TB

4639

Age−specific fertility fitted trend lines, 1950−2021: Gilan, Ages: 50−54
Data points enclosed with a diamond are reference sources

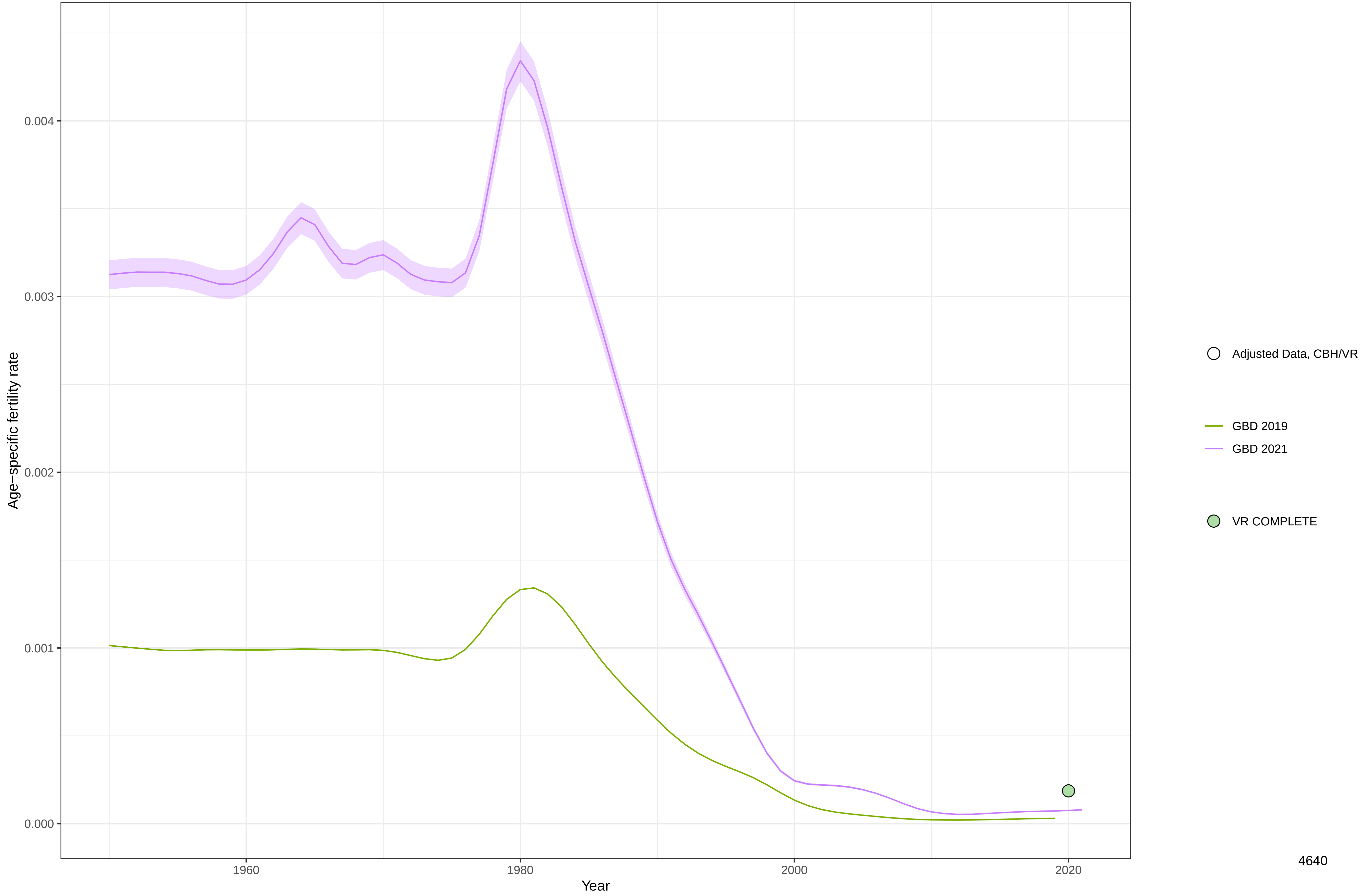

Total fertility rate fitted trend lines, 1950−2021: Gilan TFR
Data points enclosed with a diamond are reference sources

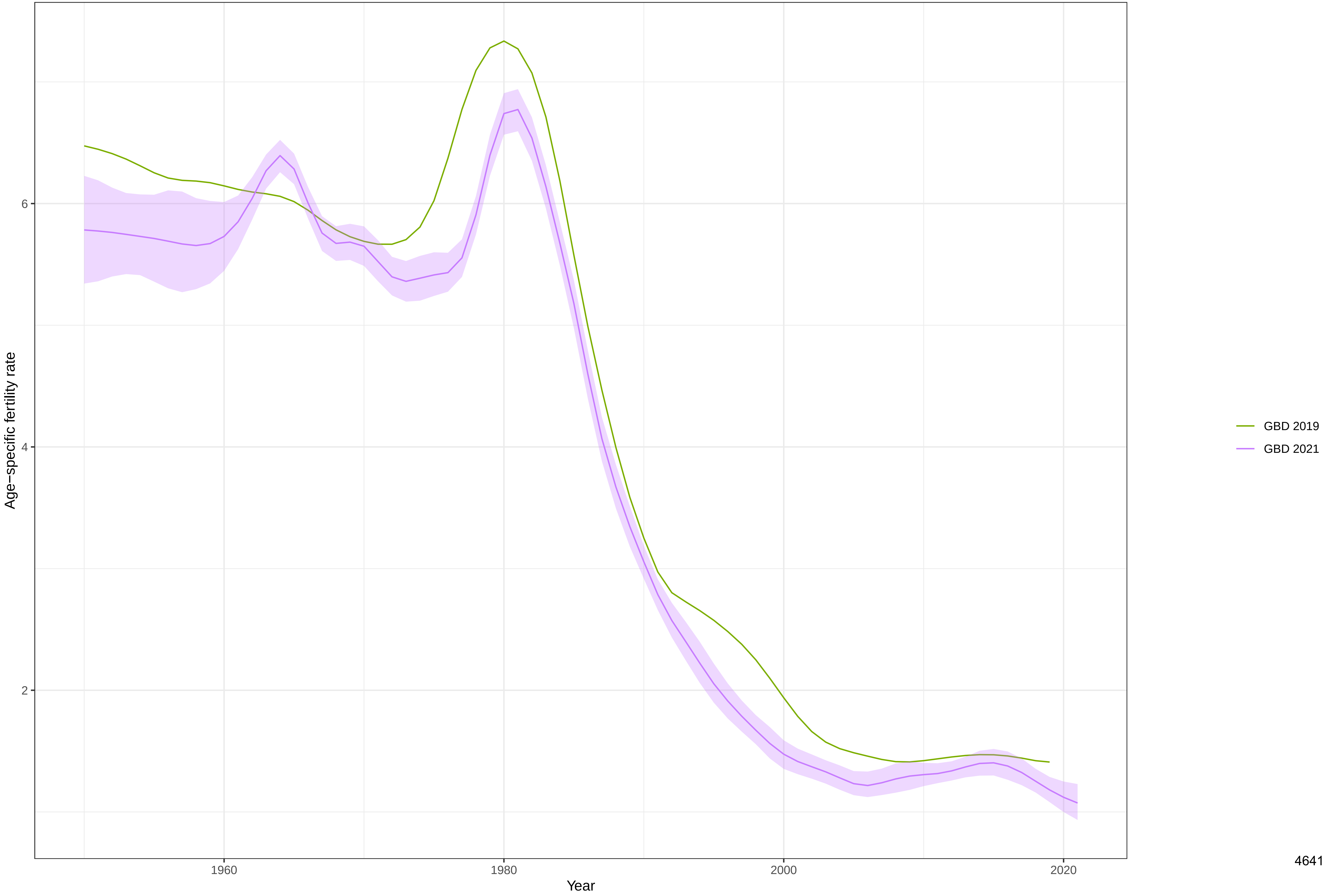

Age−specific fertility fitted trend lines, 1950−2021: Golestan, Ages: 10−14
Data points enclosed with a diamond are reference sources

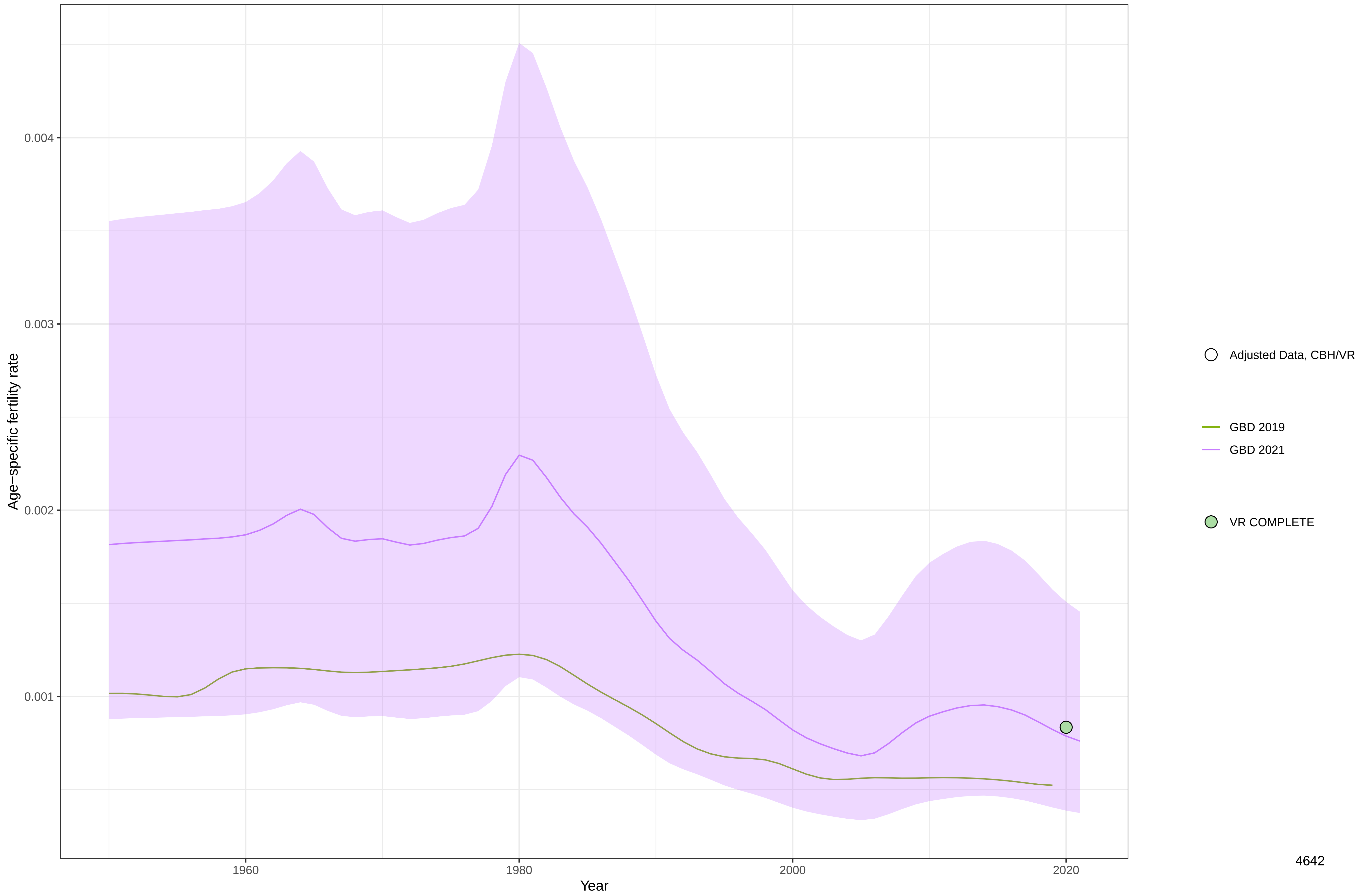

Age−specific fertility fitted trend lines, 1950−2021: Golestan, Ages: 15−19
Data points enclosed with a diamond are reference sources

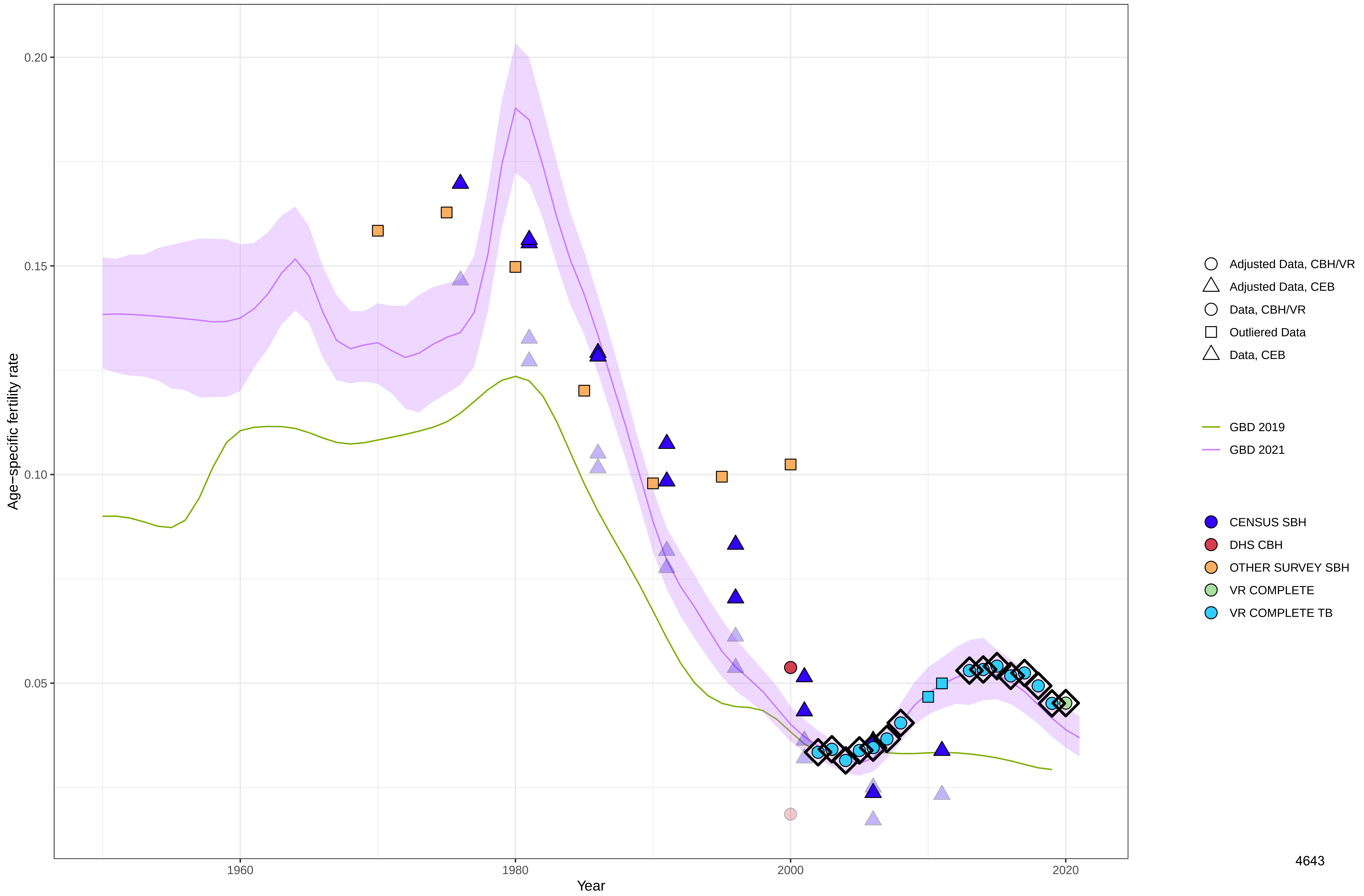

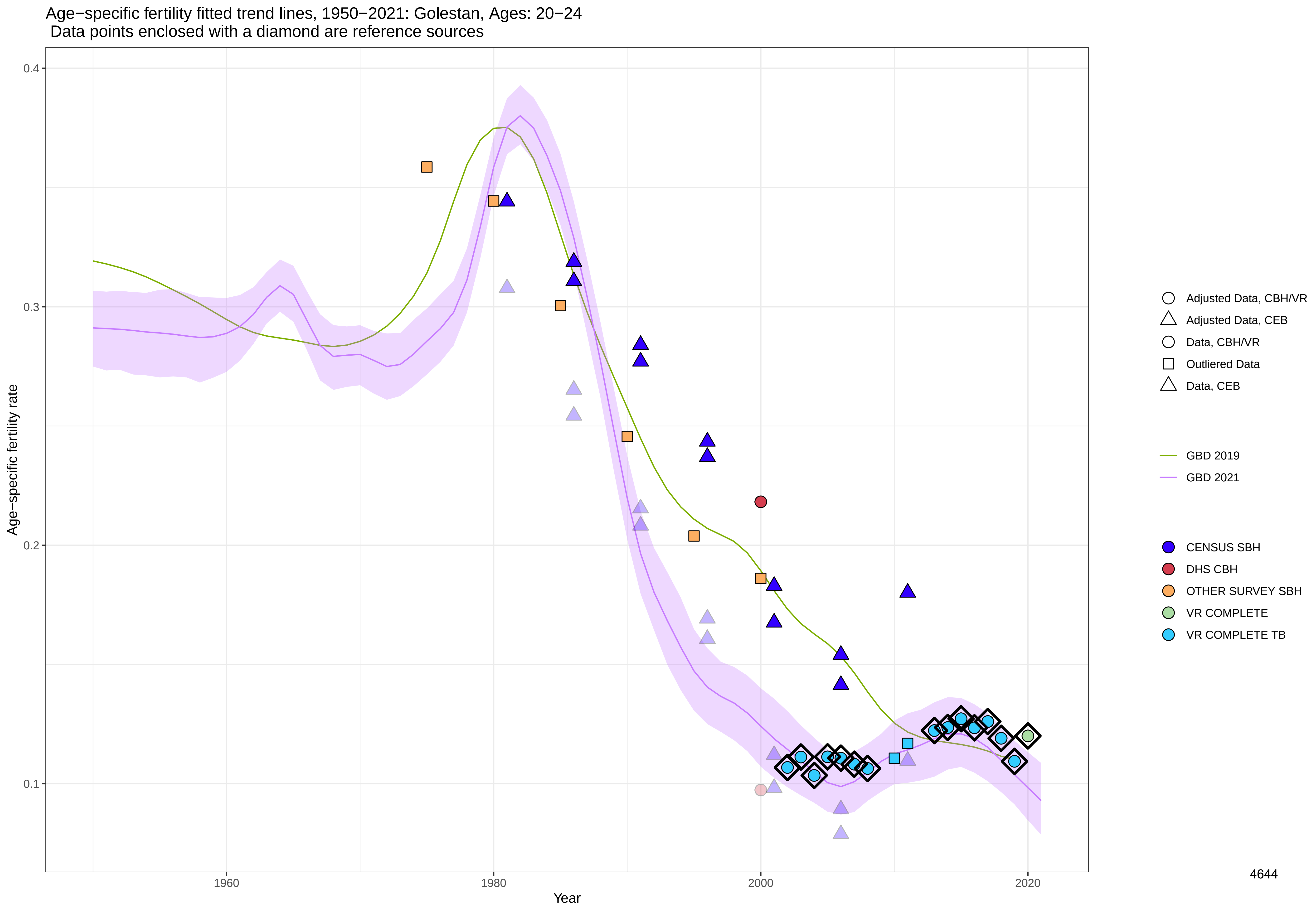

Age−specific fertility fitted trend lines, 1950−2021: Golestan, Ages: 20−24
Data points enclosed with a diamond are reference sources
Age−specific fertility rate
0.1
0.2
0.3
0.4
1960
1980
2000
2020
Year
Adjusted Data, CBH/VR
Adjusted Data, CEB
Data, CBH/VR
Outliered Data
Data, CEB
GBD 2019
GBD 2021
CENSUS SBH
DHS CBH
OTHER SURVEY SBH
VR COMPLETE
VR COMPLETE TB

Age−specific fertility fitted trend lines, 1950−2021: Golestan, Ages: 25−29
Data points enclosed with a diamond are reference sources

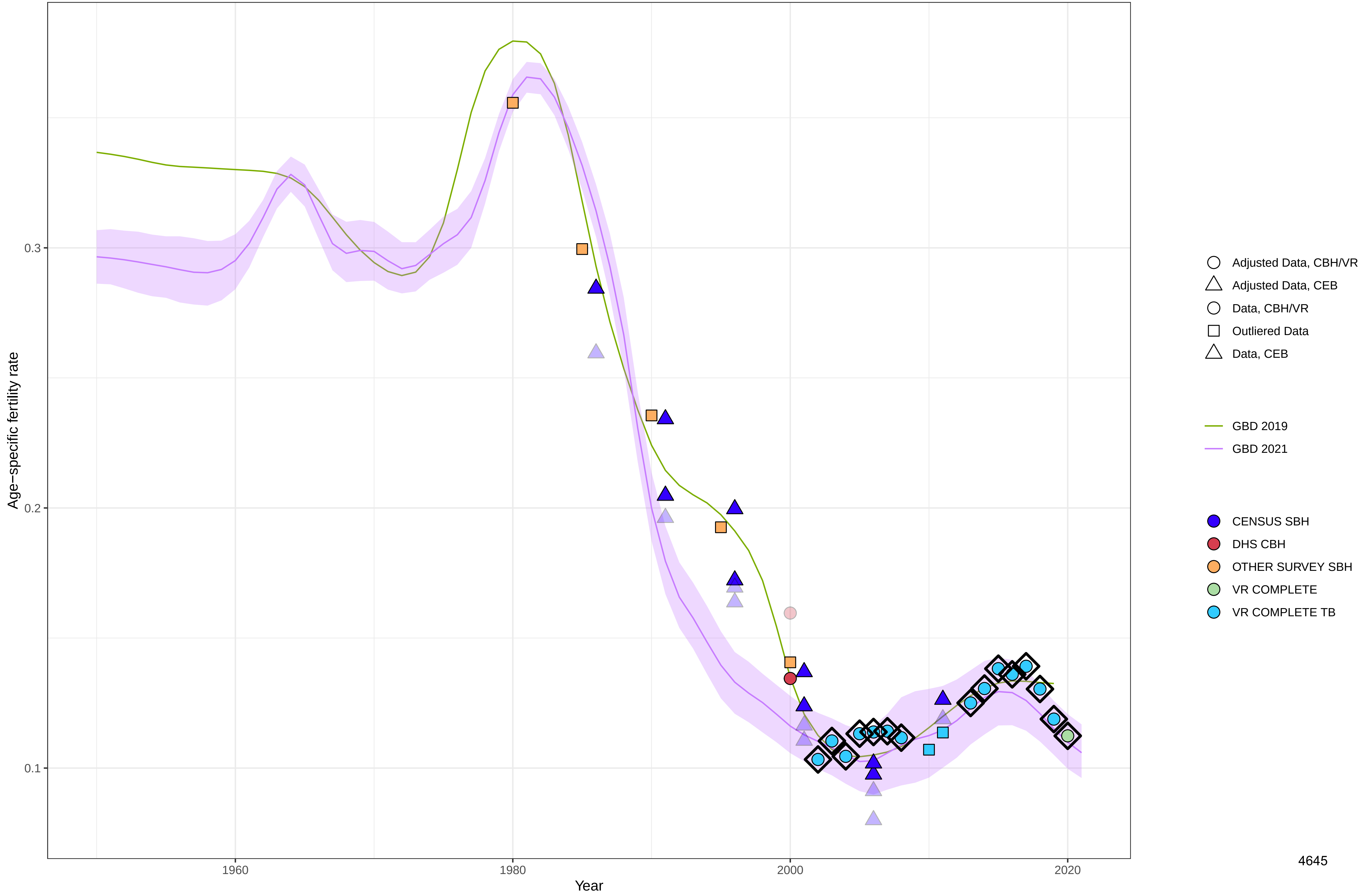

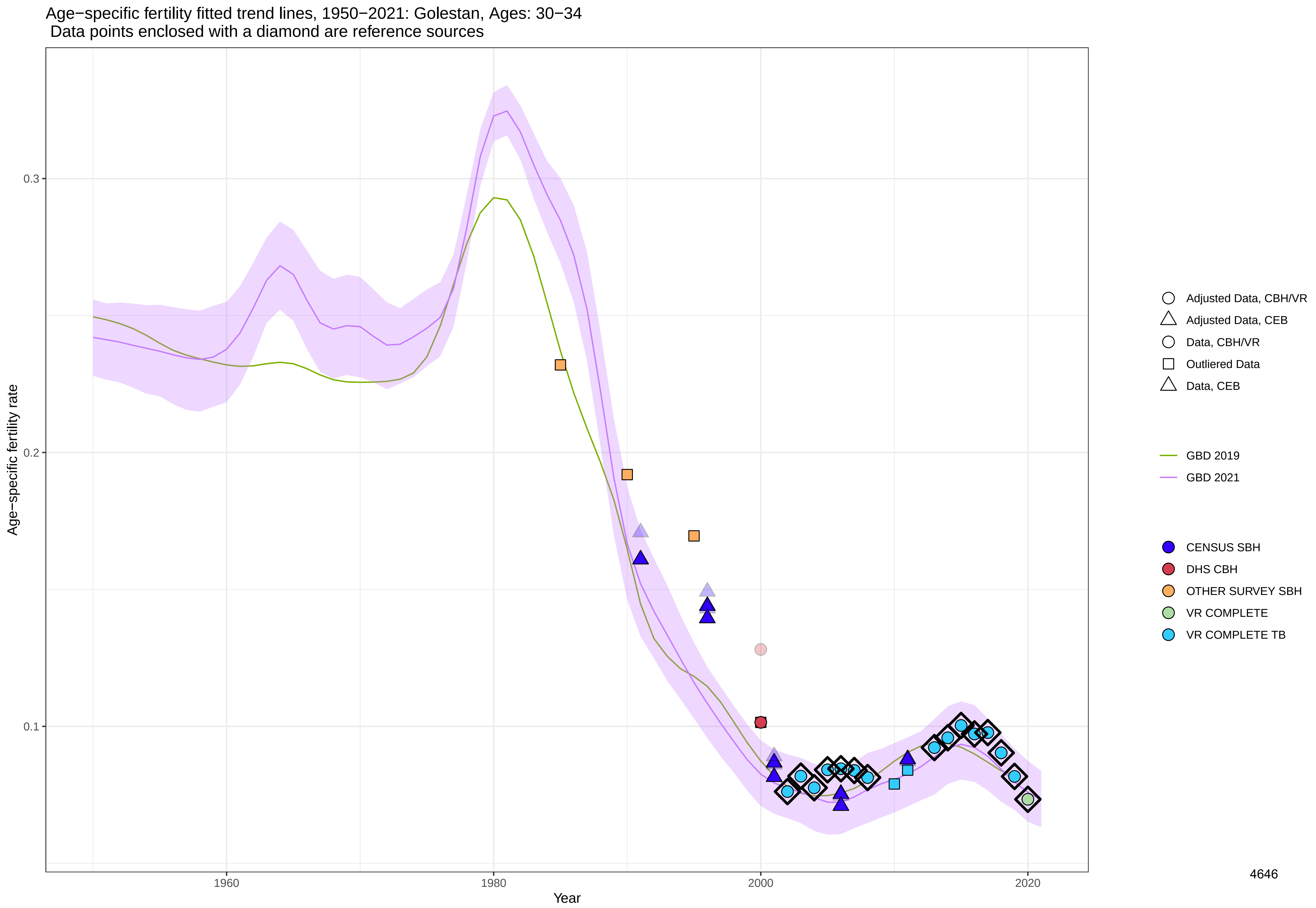

Age−specific fertility fitted trend lines, 1950−2021: Golestan, Ages: 30−34
Data points enclosed with a diamond are reference sources
Age−specific fertility rate
Year
0.1
0.2
0.3
1960
1980
2000
2020
Adjusted Data, CBH/VR
Adjusted Data, CEB
Data, CBH/VR
Outliered Data
Data, CEB
GBD 2019
GBD 2021
CENSUS SBH
DHS CBH
OTHER SURVEY SBH
VR COMPLETE
VR COMPLETE TB

Age−specific fertility fitted trend lines, 1950−2021: Golestan, Ages: 35−39
Data points enclosed with a diamond are reference sources

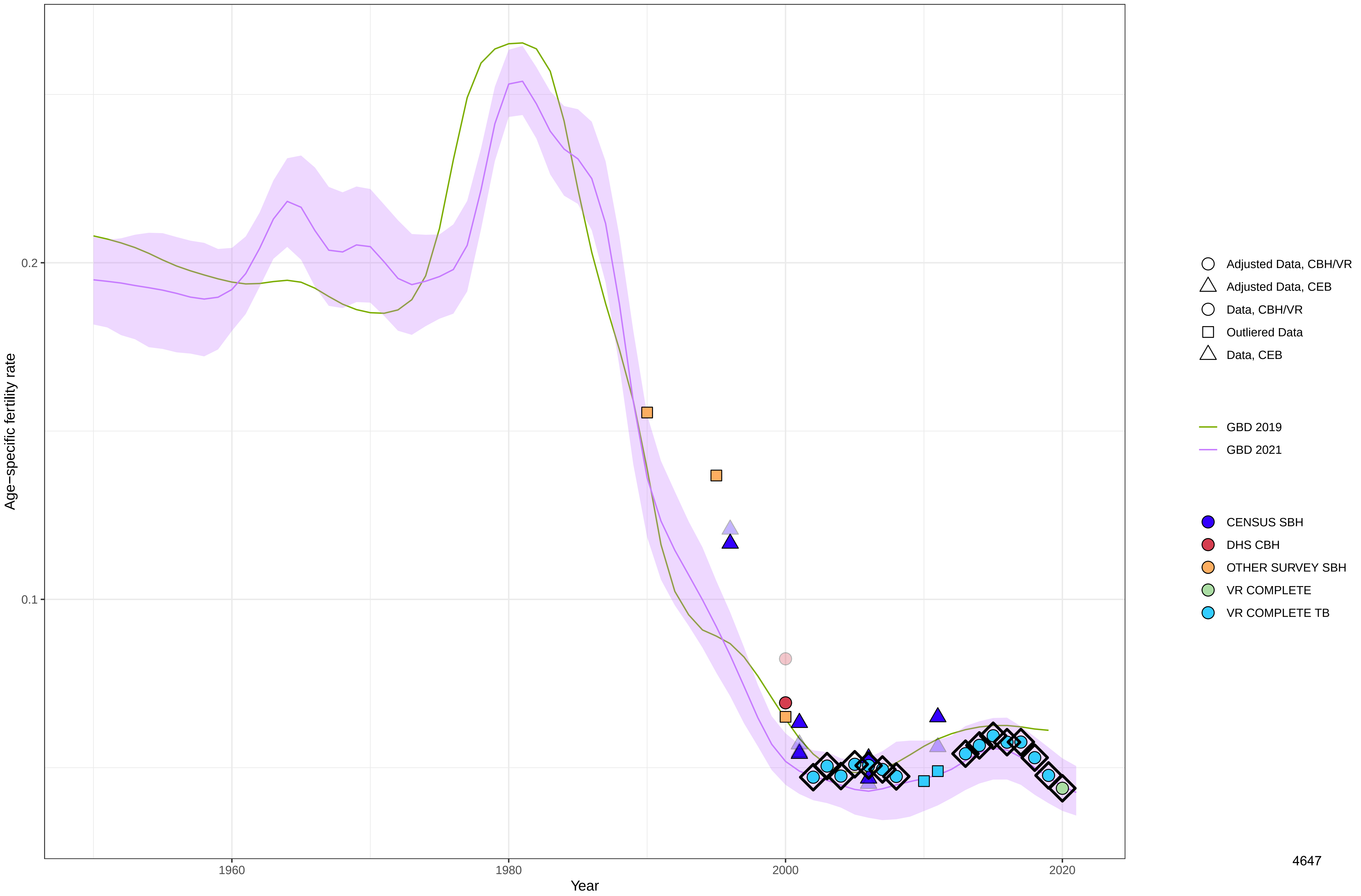

Age−specific fertility fitted trend lines, 1950−2021: Golestan, Ages: 40−44
Data points enclosed with a diamond are reference sources

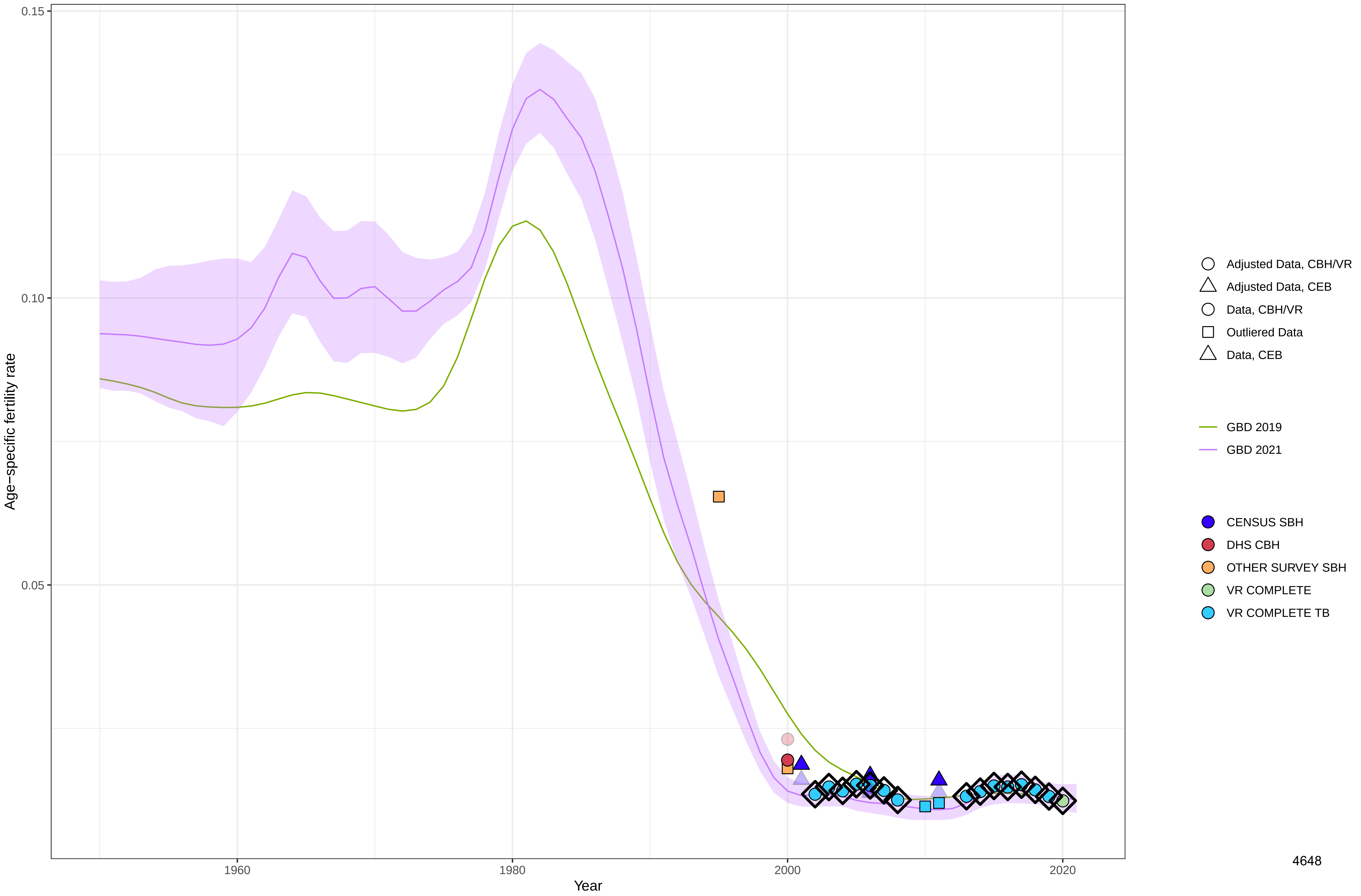

Age−specific fertility fitted trend lines, 1950−2021: Golestan, Ages: 45−49
Data points enclosed with a diamond are reference sources

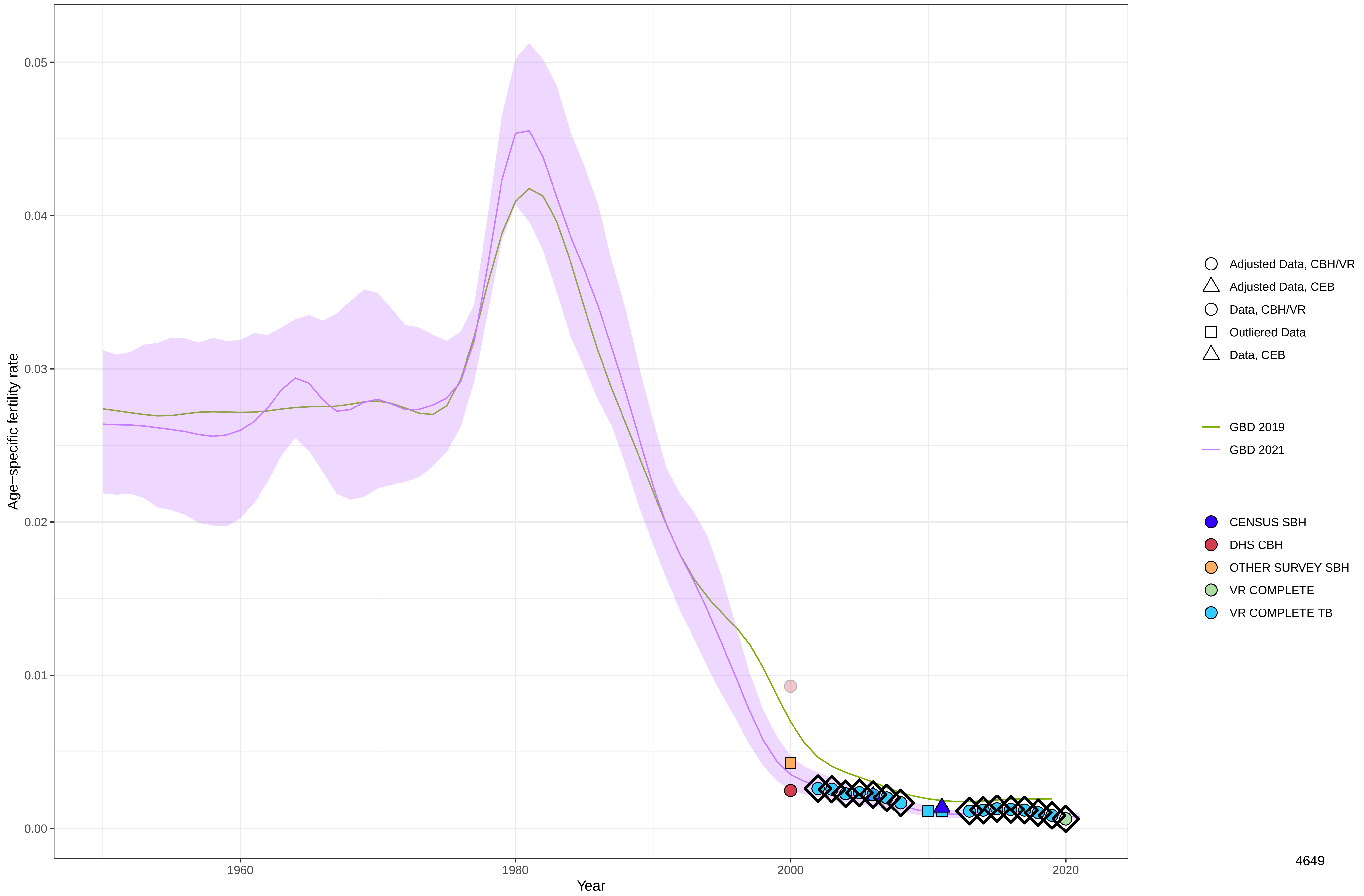

Age−specific fertility fitted trend lines, 1950−2021: Golestan, Ages: 50−54
Data points enclosed with a diamond are reference sources

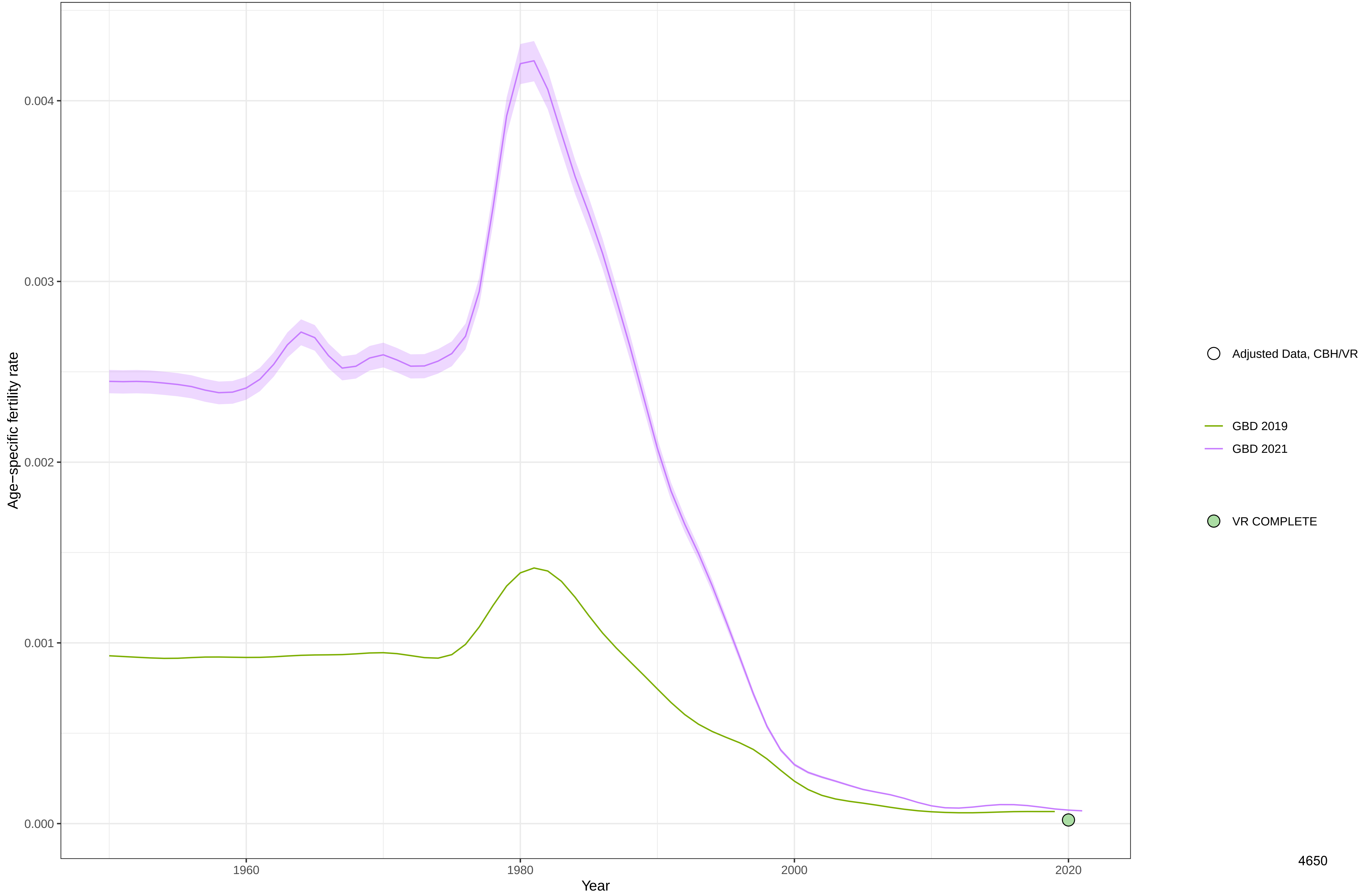

Total fertility rate fitted trend lines, 1950−2021: Golestan TFR
Data points enclosed with a diamond are reference sources

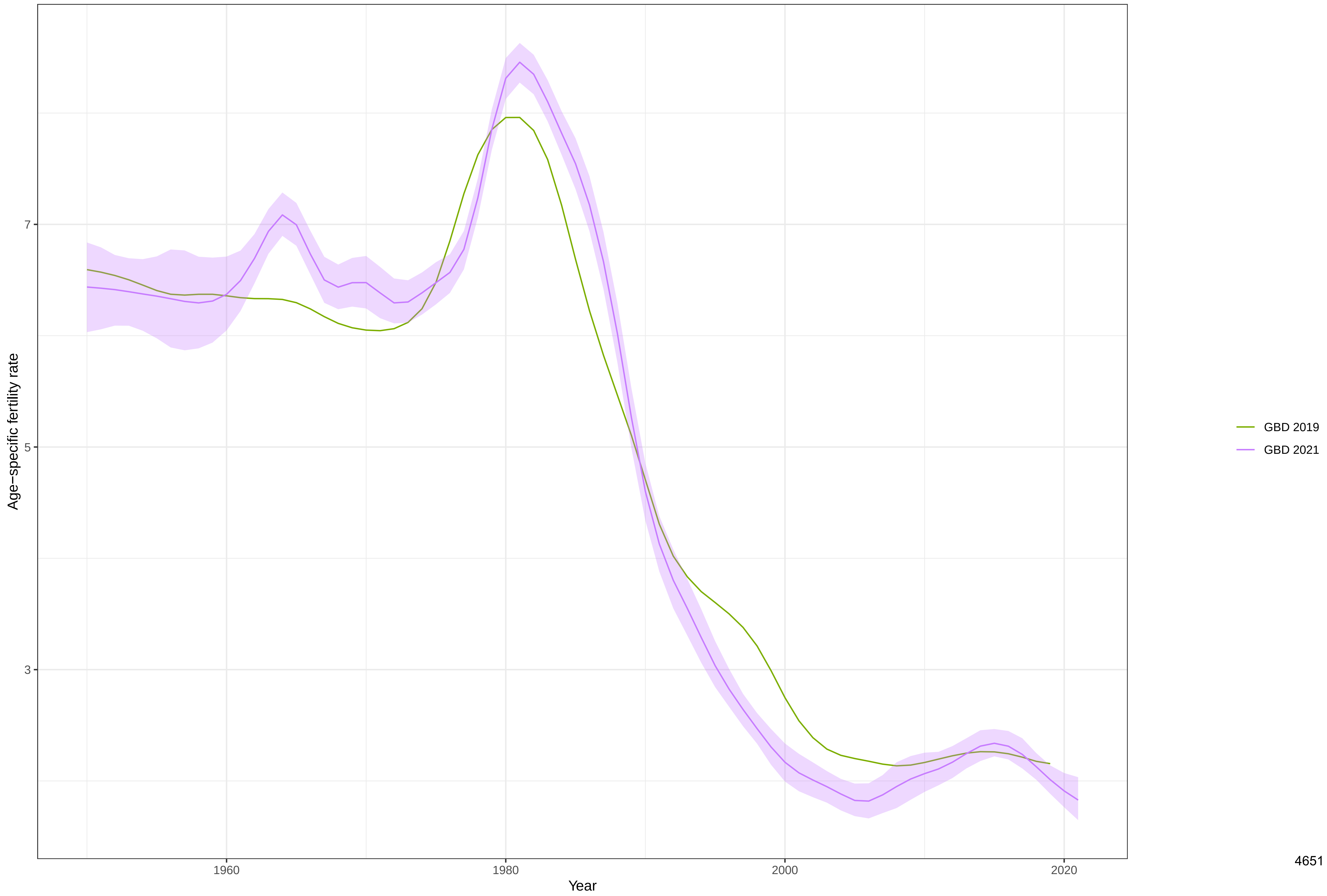

Age−specific fertility fitted trend lines, 1950−2021: Hamadan, Ages: 10−14
Data points enclosed with a diamond are reference sources

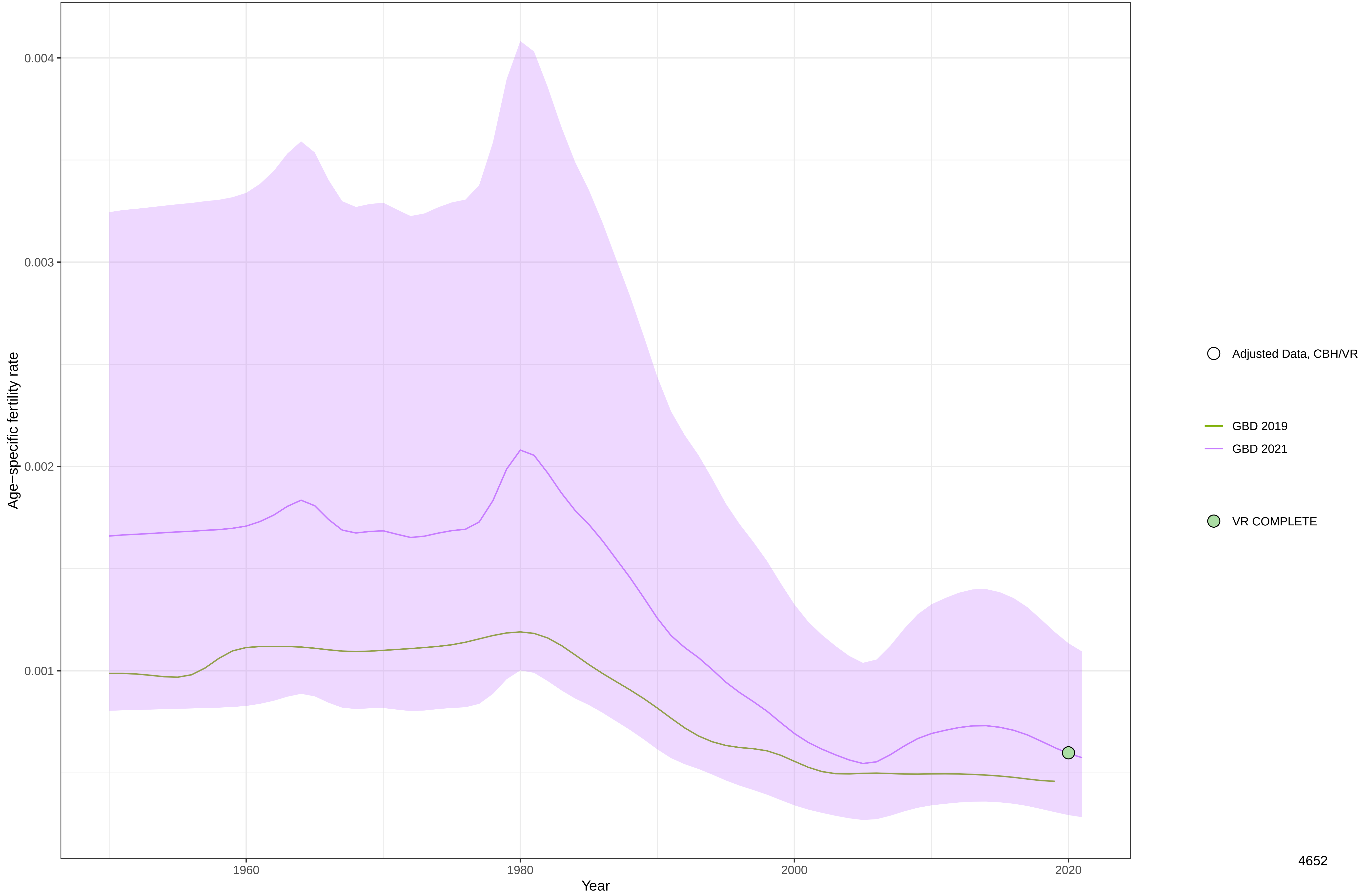

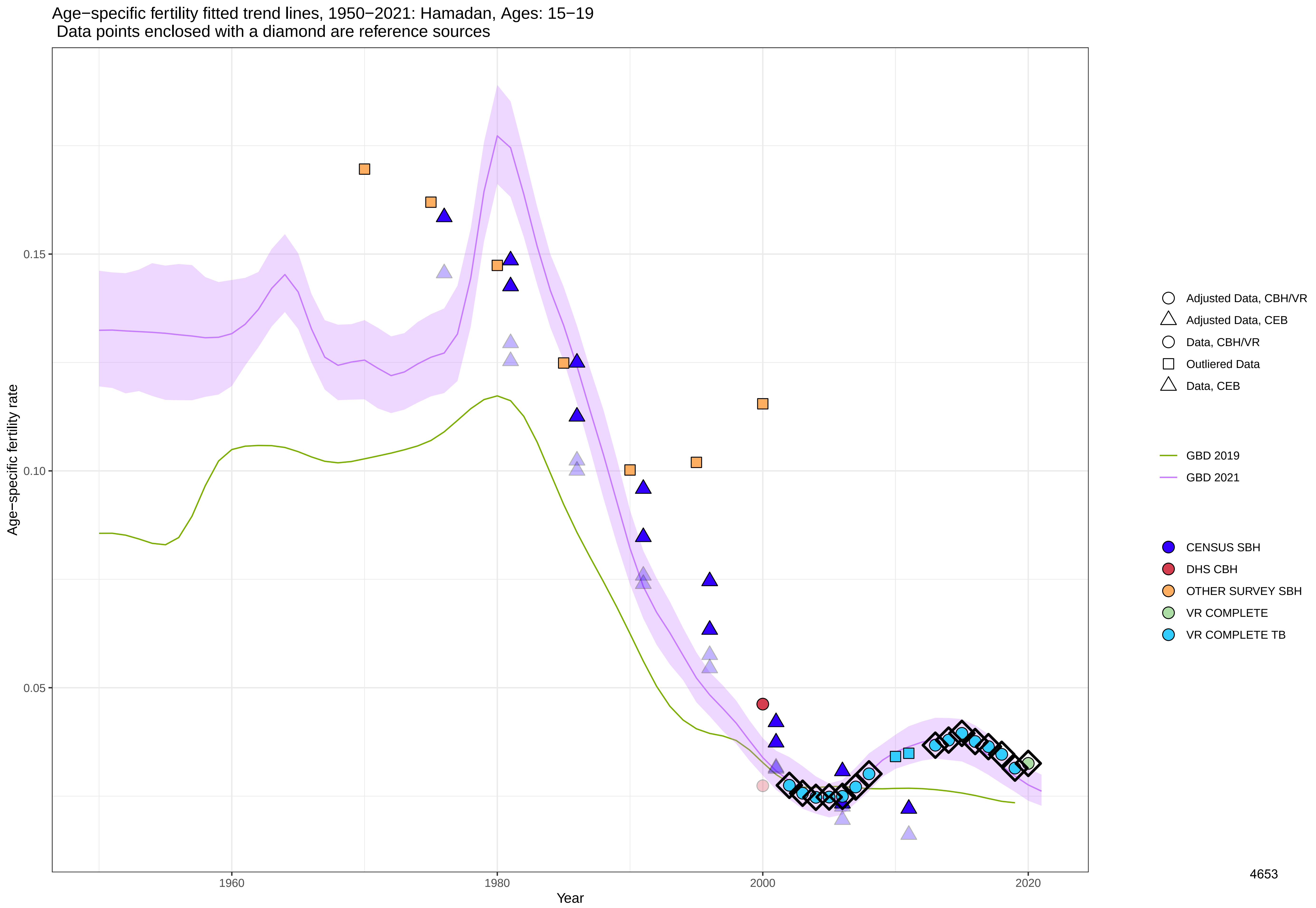

Age−specific fertility fitted trend lines, 1950−2021: Hamadan, Ages: 15−19
Data points enclosed with a diamond are reference sources
Age−specific fertility rate
0.15
0.10
0.05
Year
1960
1980
2000
2020
Adjusted Data, CBH/VR
Adjusted Data, CEB
Data, CBH/VR
Outliered Data
Data, CEB
GBD 2019
GBD 2021
CENSUS SBH
DHS CBH
OTHER SURVEY SBH
VR COMPLETE
VR COMPLETE TB

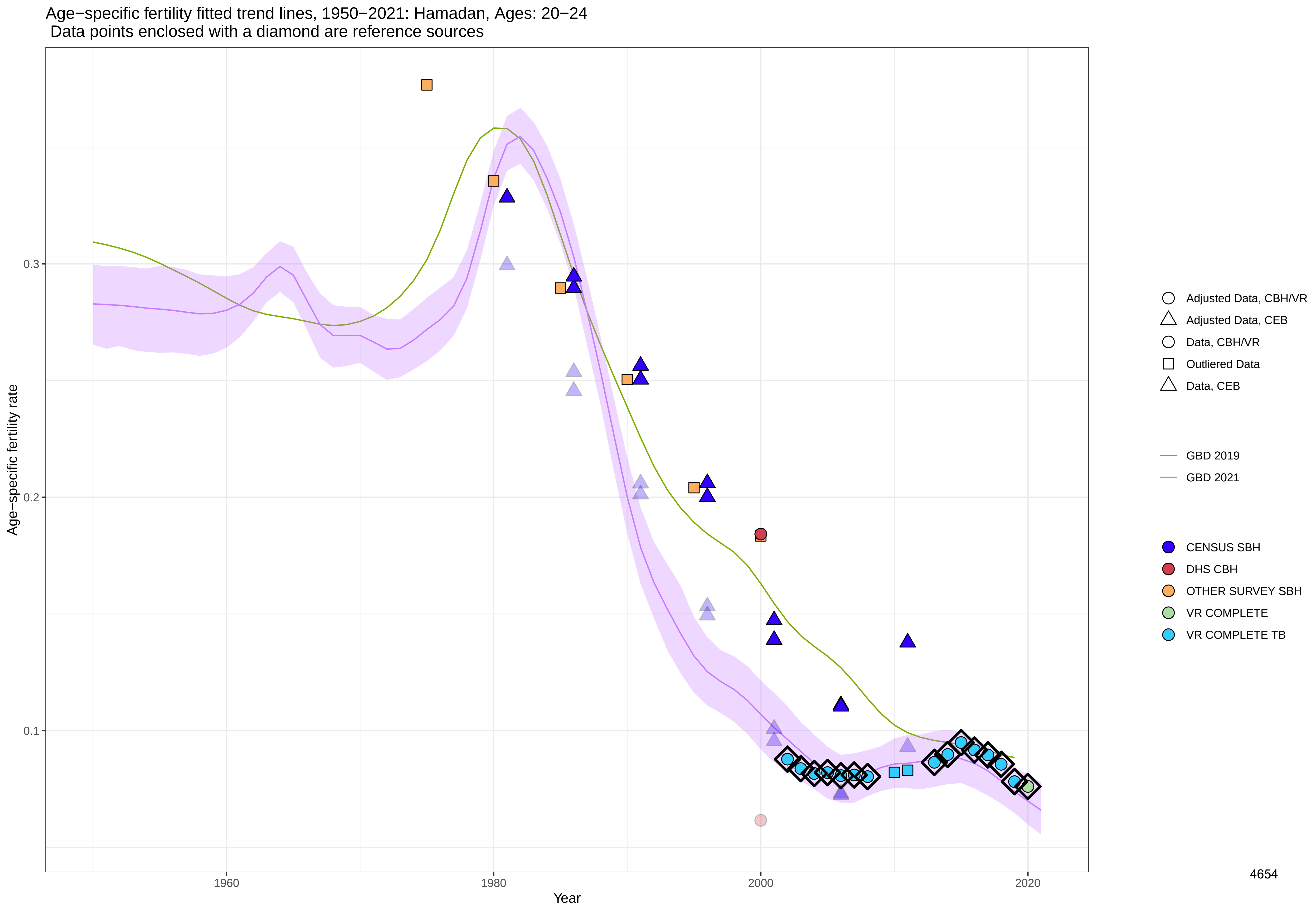

Age−specific fertility fitted trend lines, 1950−2021: Hamadan, Ages: 20−24
Data points enclosed with a diamond are reference sources
Age−specific fertility rate
Year
0.1
0.2
0.3
1960
1980
2000
2020
Adjusted Data, CBH/VR
Adjusted Data, CEB
Data, CBH/VR
Outliered Data
Data, CEB
GBD 2019
GBD 2021
CENSUS SBH
DHS CBH
OTHER SURVEY SBH
VR COMPLETE
VR COMPLETE TB

Age−specific fertility fitted trend lines, 1950−2021: Hamadan, Ages: 25−29
Data points enclosed with a diamond are reference sources

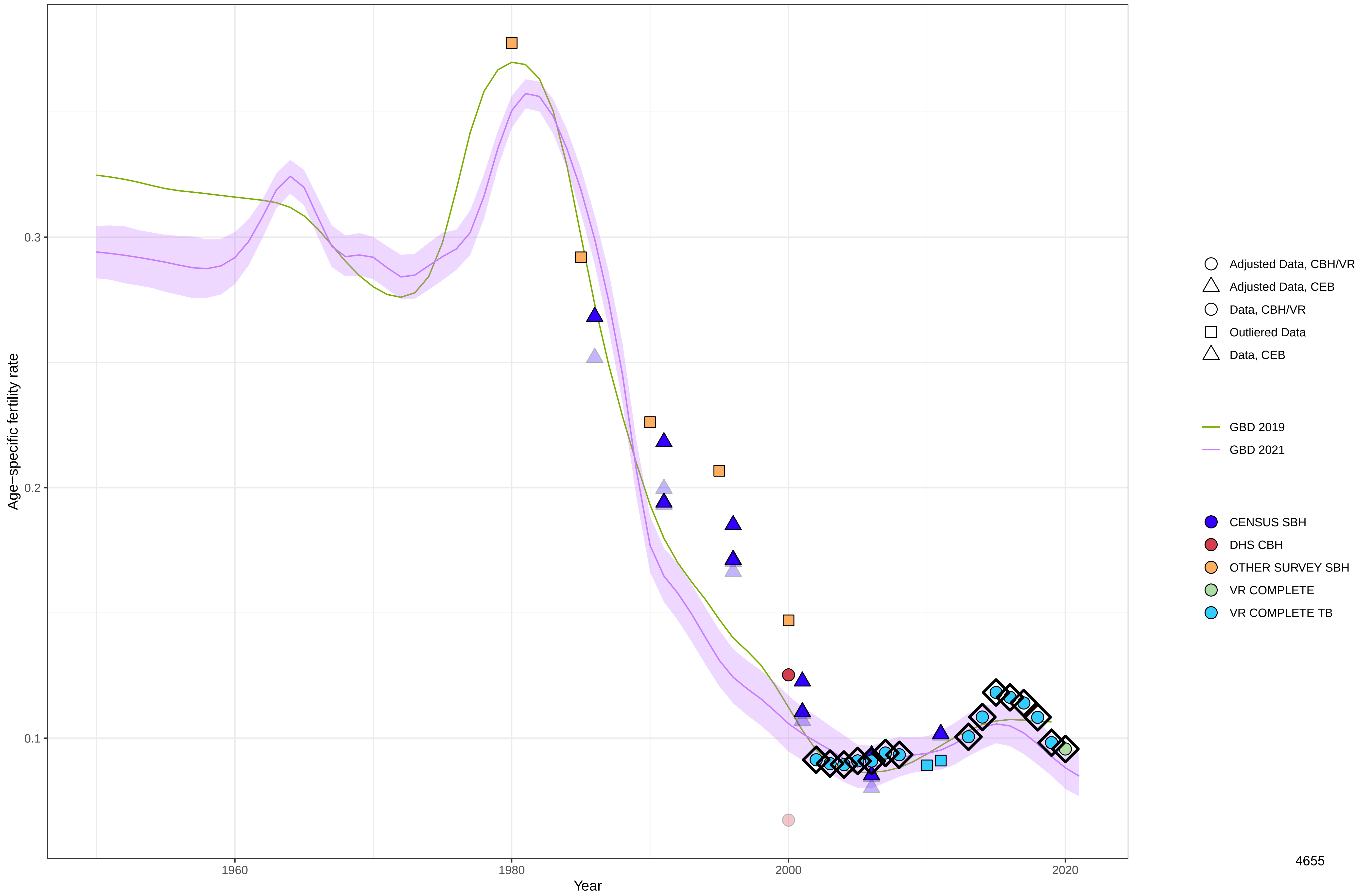

Age−specific fertility fitted trend lines, 1950−2021: Hamadan, Ages: 30−34
Data points enclosed with a diamond are reference sources

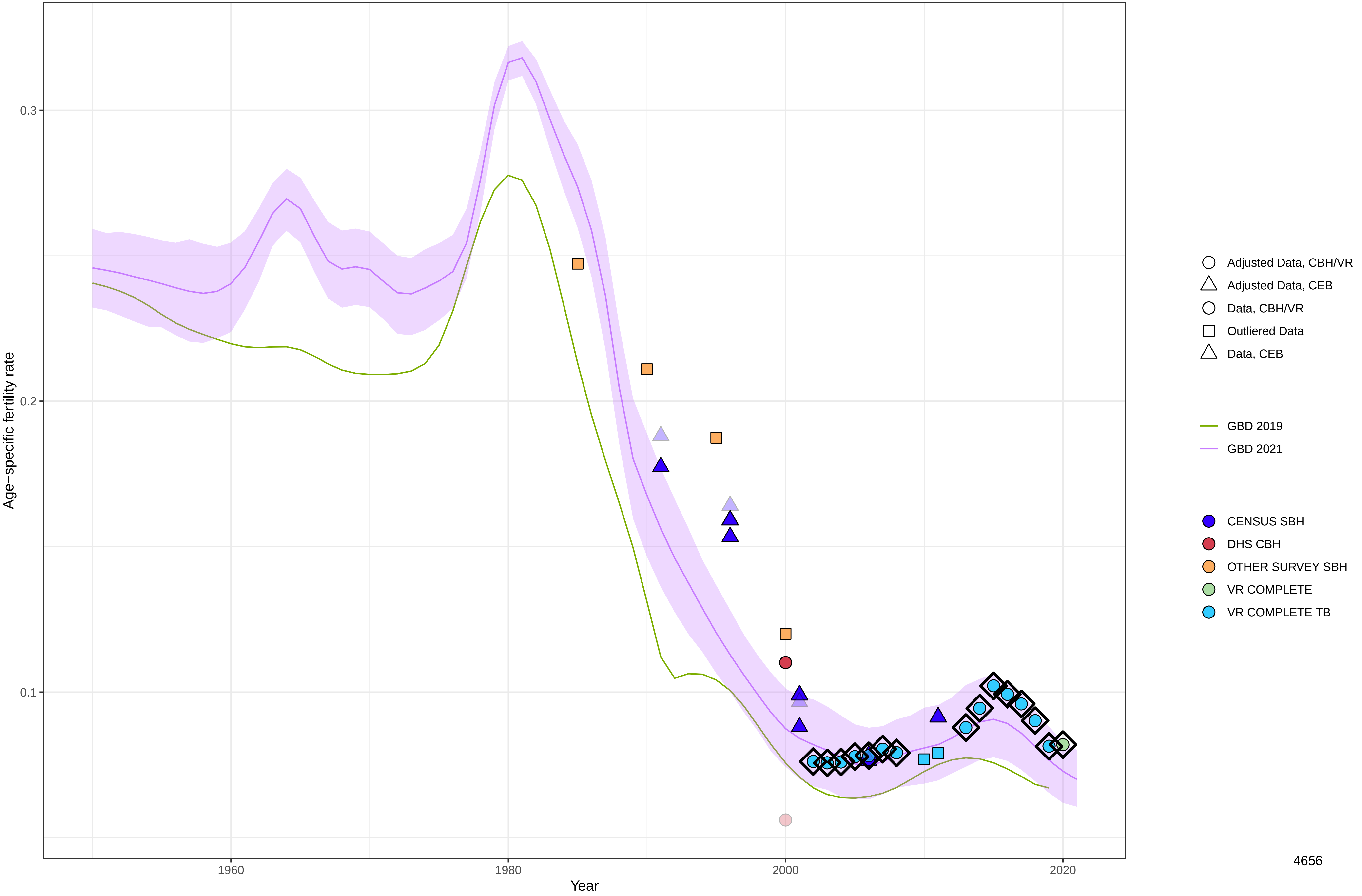

Age−specific fertility fitted trend lines, 1950−2021: Hamadan, Ages: 35−39
Data points enclosed with a diamond are reference sources

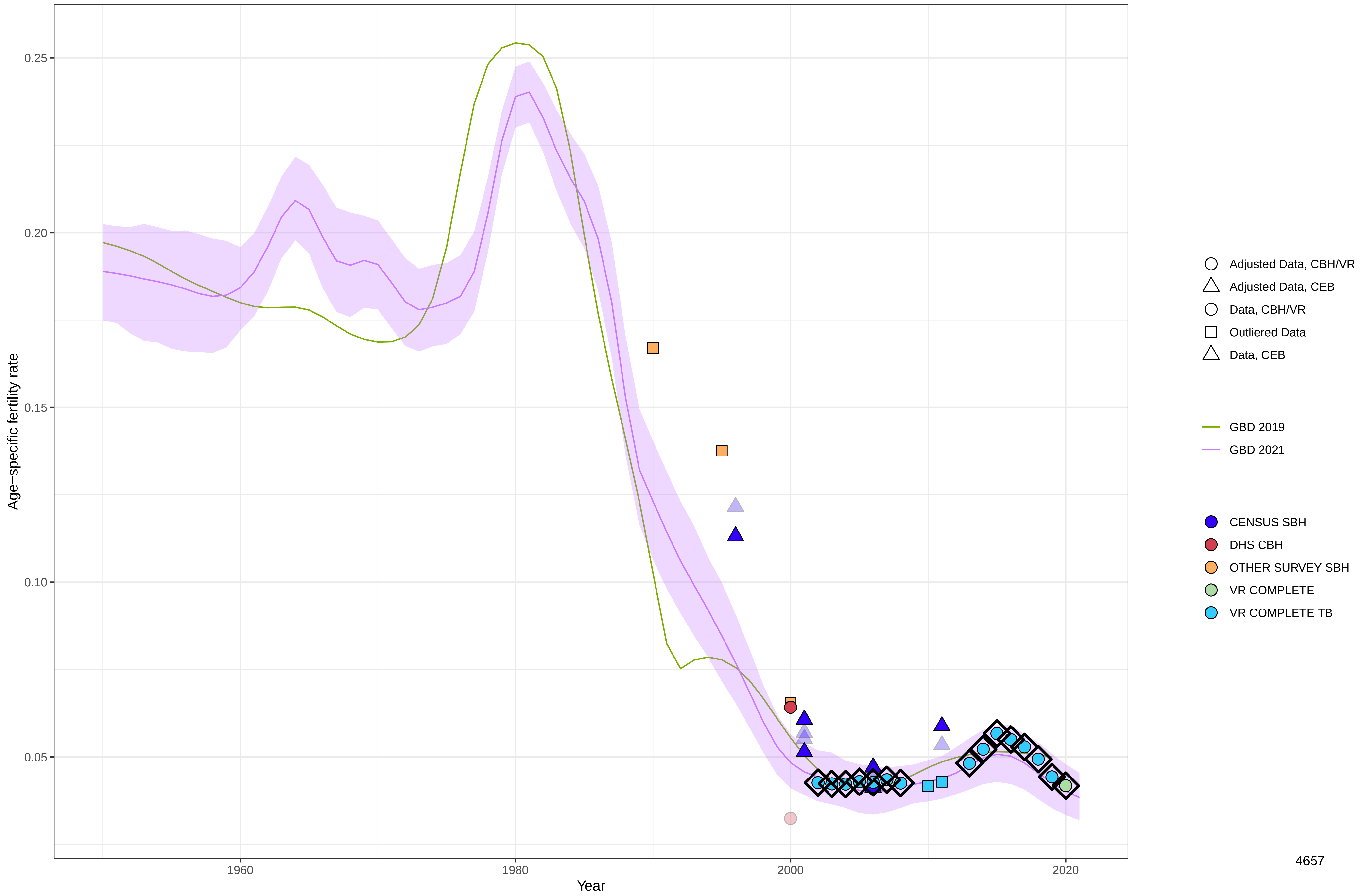

Age−specific fertility fitted trend lines, 1950−2021: Hamadan, Ages: 40−44
Data points enclosed with a diamond are reference sources

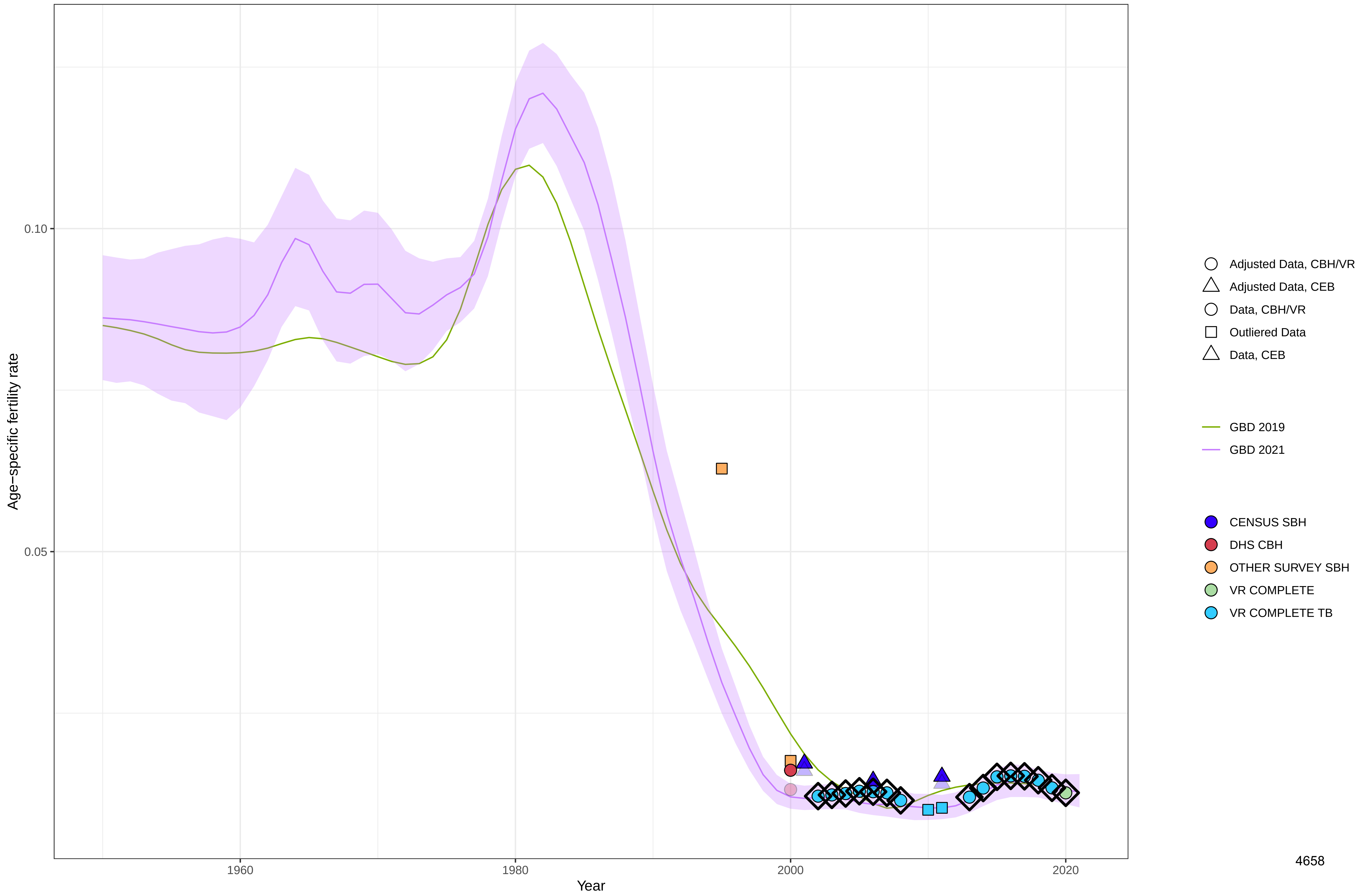

Age−specific fertility fitted trend lines, 1950−2021: Hamadan, Ages: 45−49
Data points enclosed with a diamond are reference sources

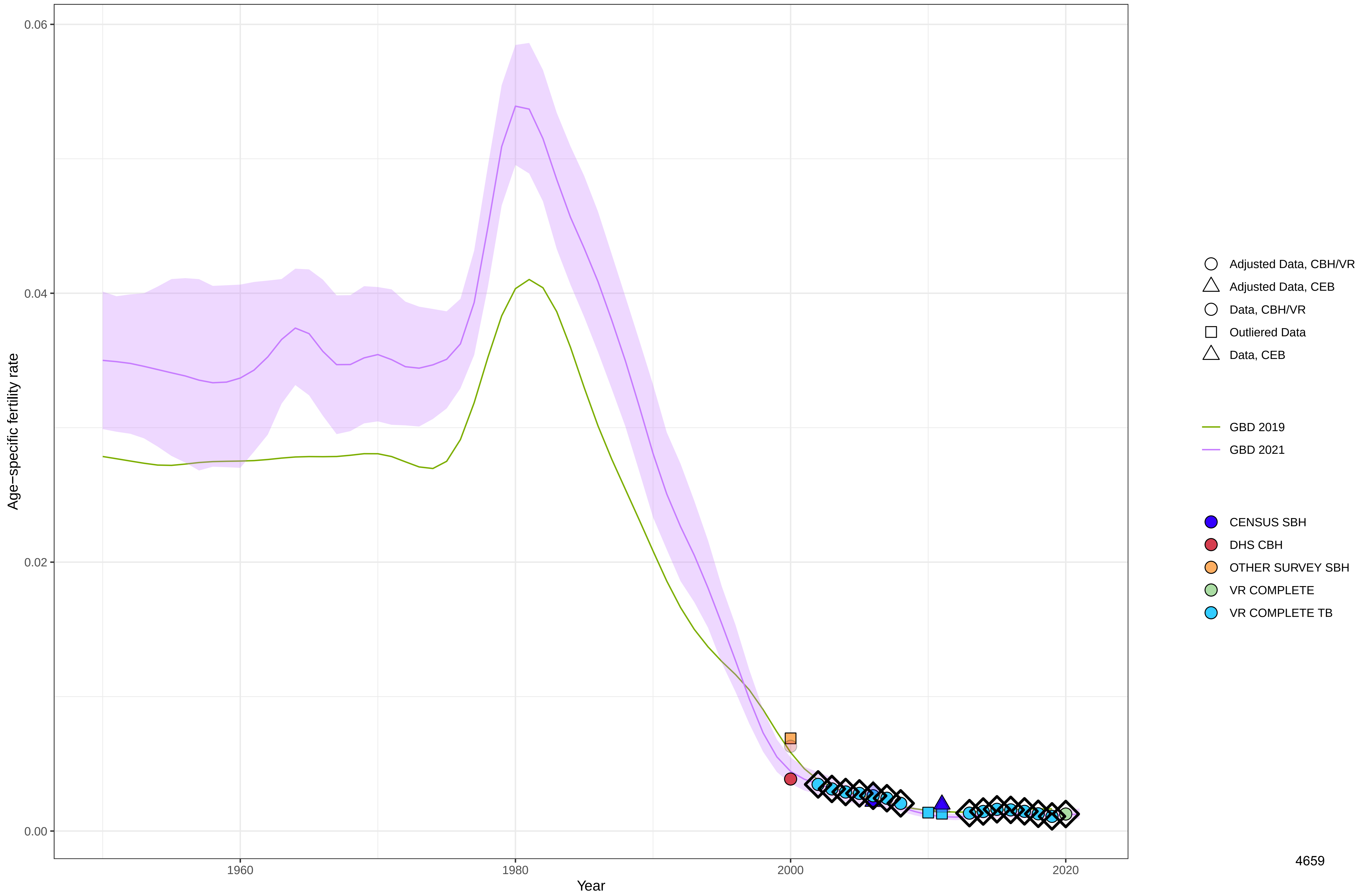

Age−specific fertility fitted trend lines, 1950−2021: Hamadan, Ages: 50−54
Data points enclosed with a diamond are reference sources

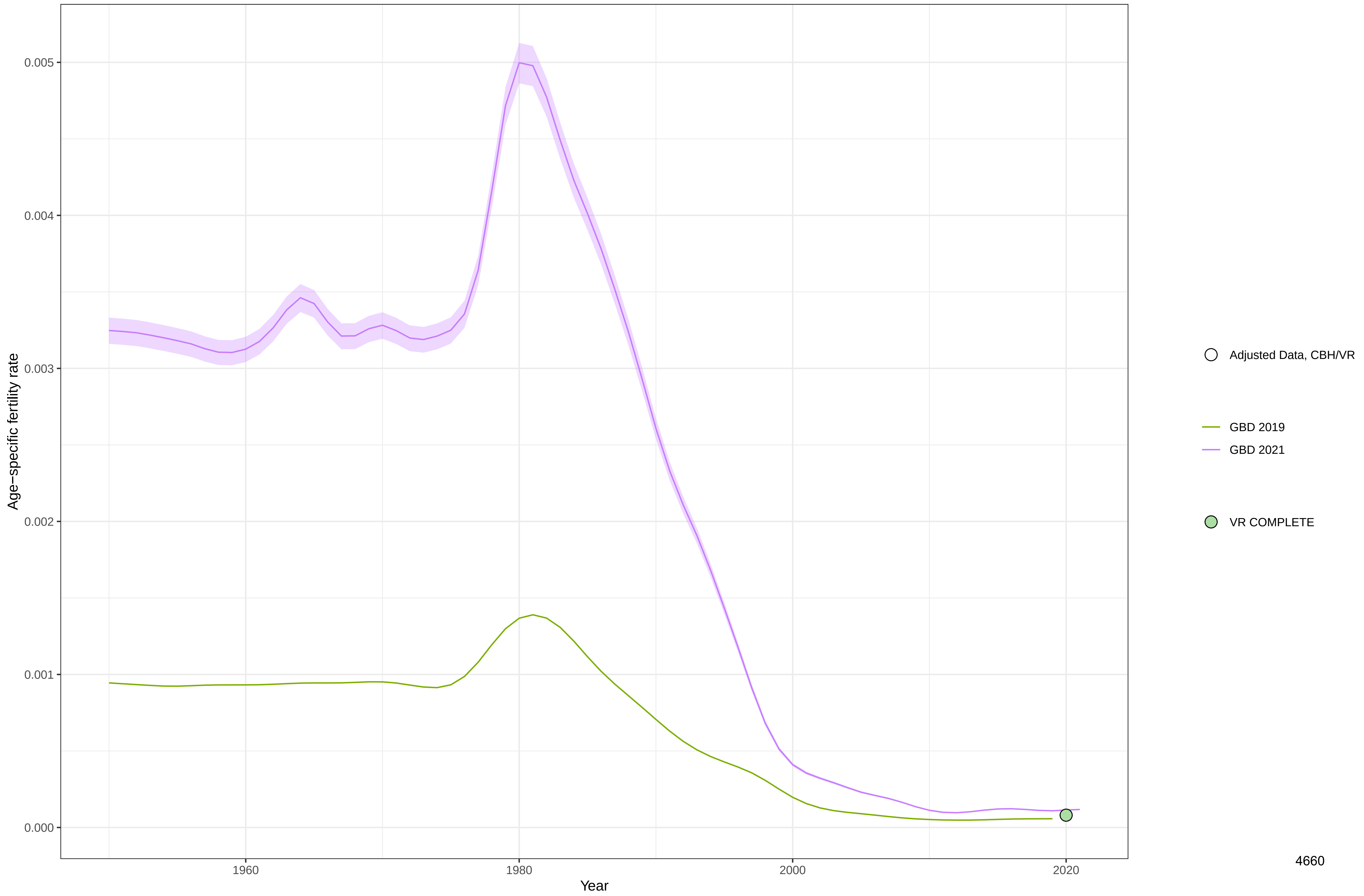

Total fertility rate fitted trend lines, 1950−2021: Hamadan TFR
Data points enclosed with a diamond are reference sources

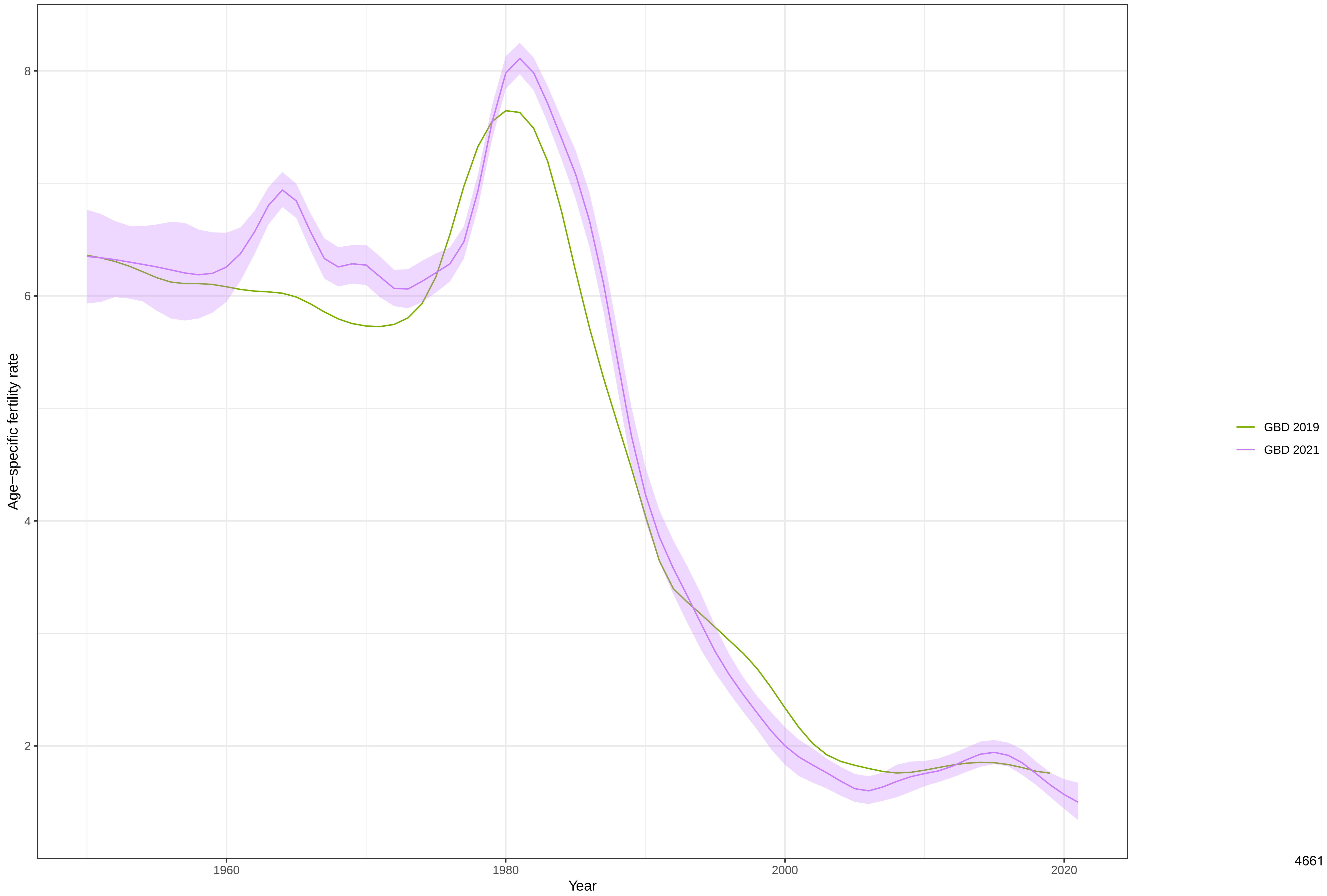

Age−specific fertility fitted trend lines, 1950−2021: Hormozgan, Ages: 10−14
Data points enclosed with a diamond are reference sources

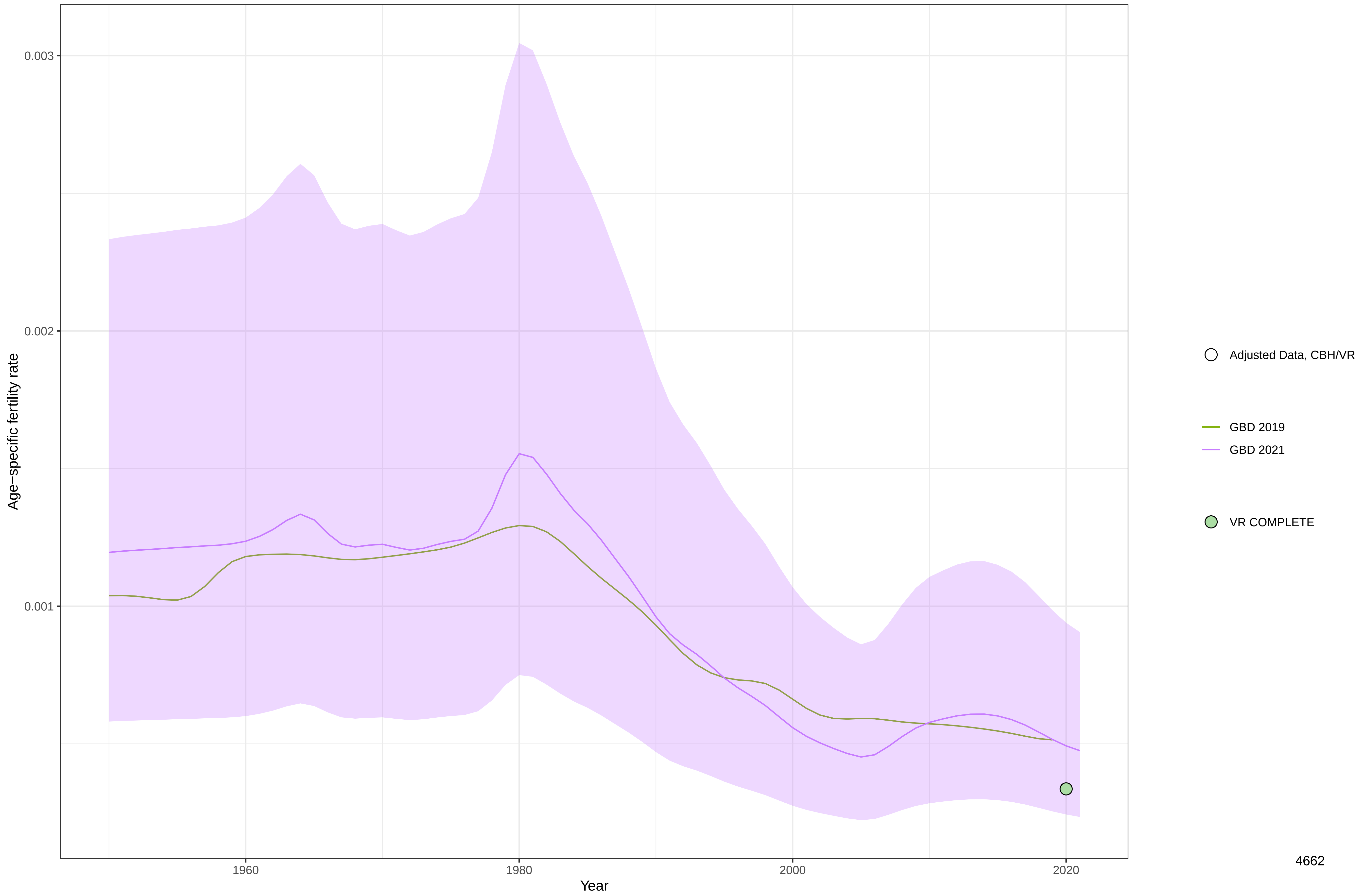

Age−specific fertility fitted trend lines, 1950−2021: Hormozgan, Ages: 15−19
Data points enclosed with a diamond are reference sources

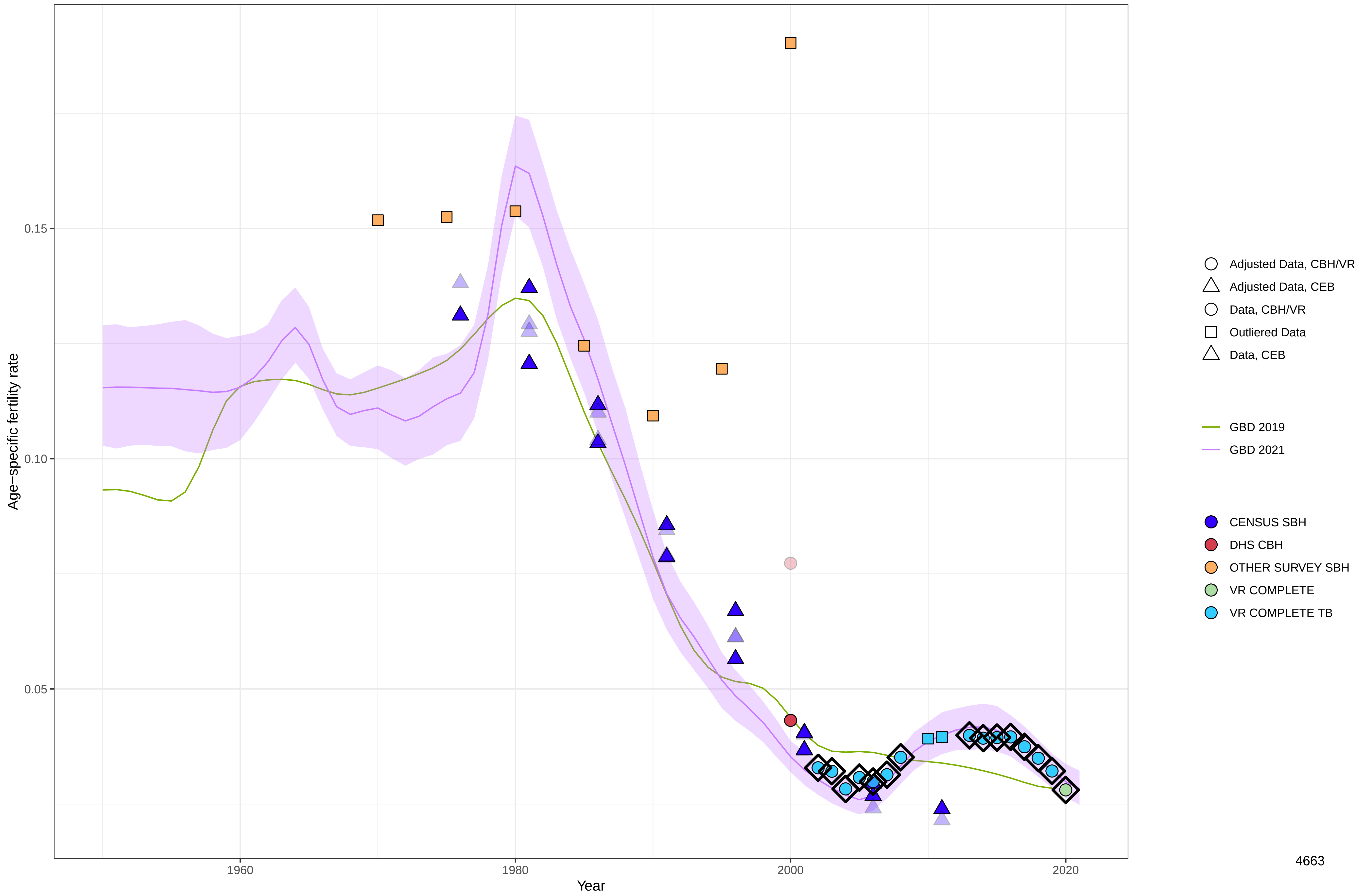

Age−specific fertility fitted trend lines, 1950−2021: Hormozgan, Ages: 20−24
Data points enclosed with a diamond are reference sources

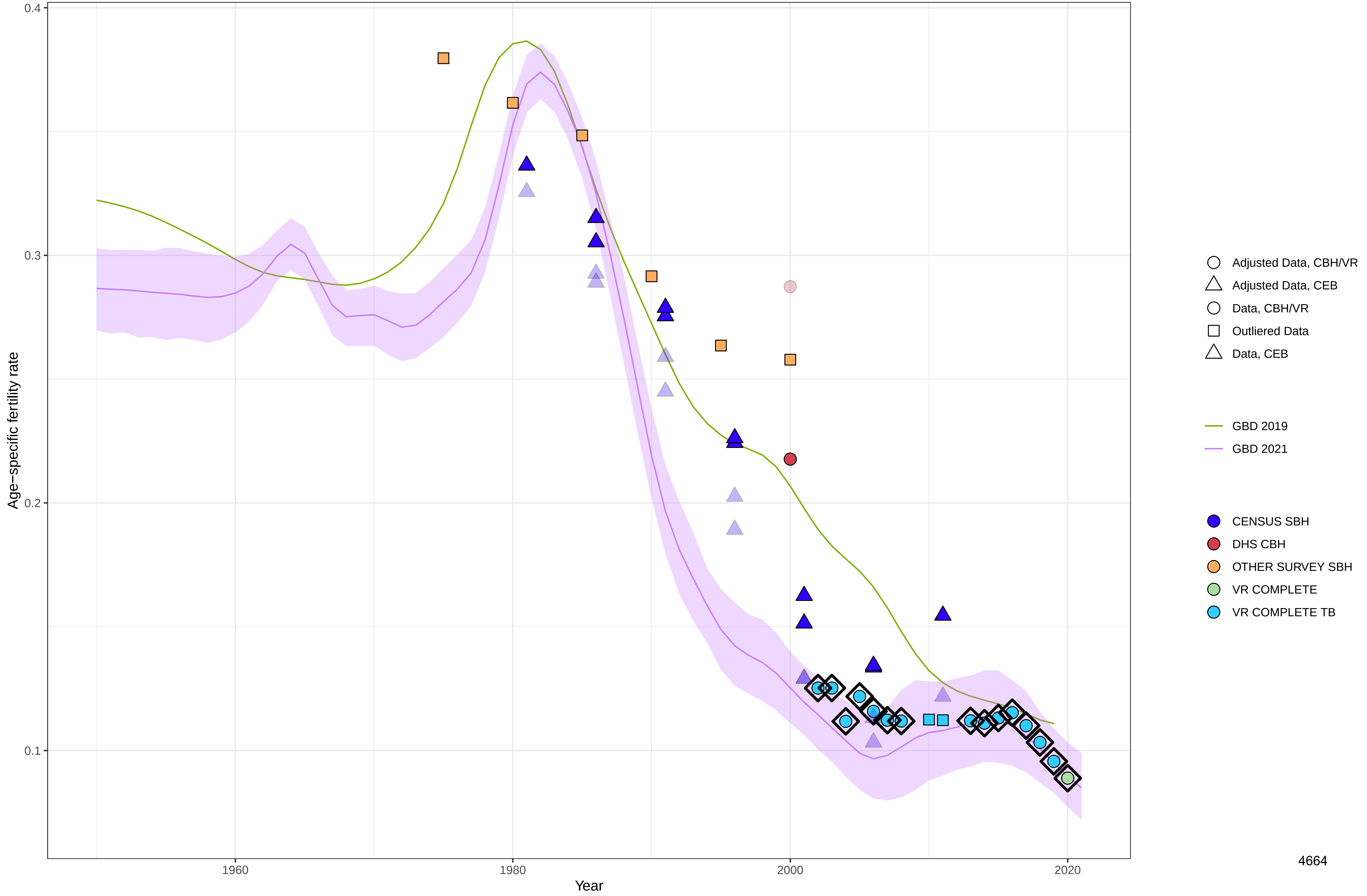

Age−specific fertility fitted trend lines, 1950−2021: Hormozgan, Ages: 25−29
Data points enclosed with a diamond are reference sources

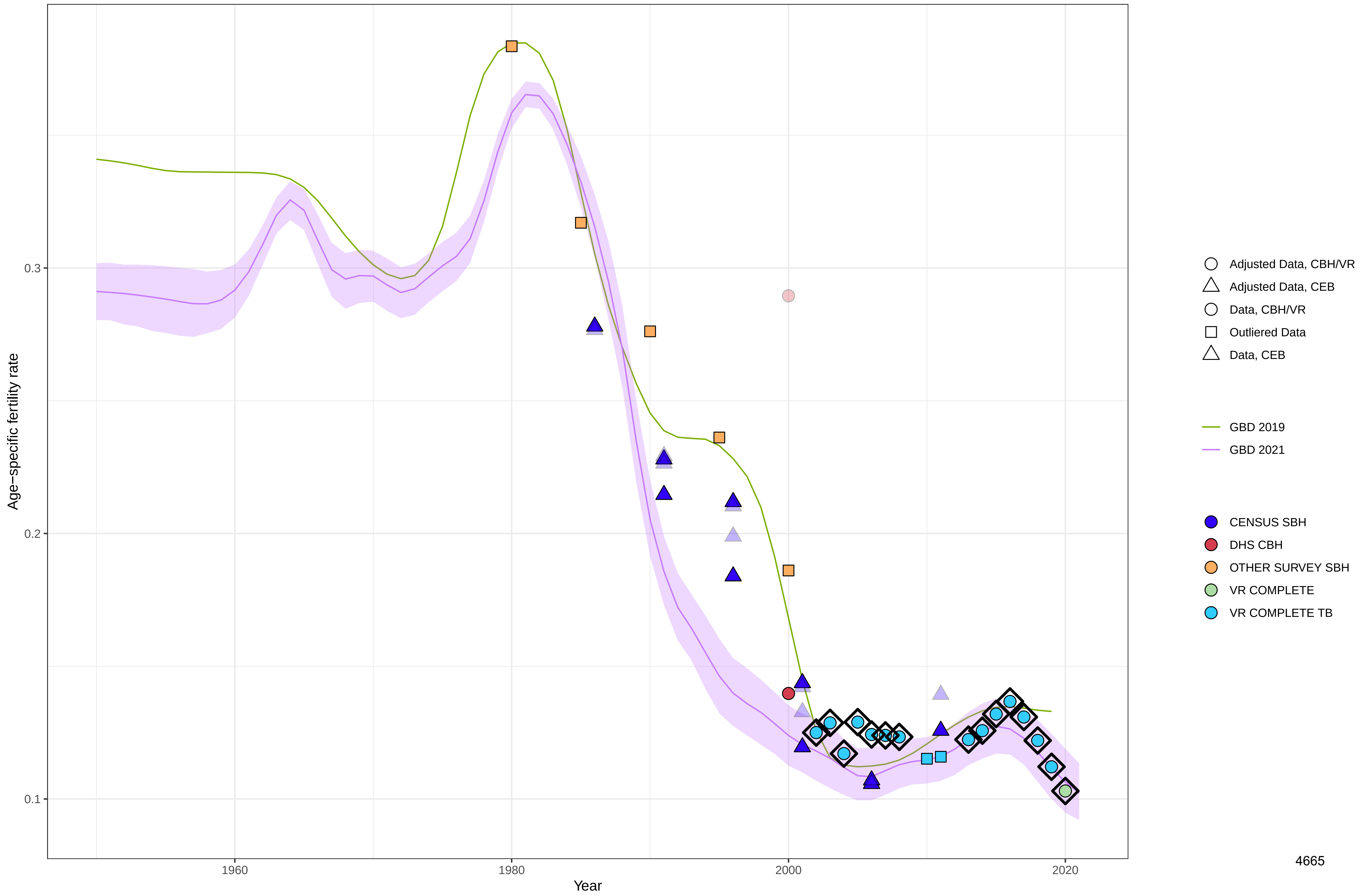

Age−specific fertility fitted trend lines, 1950−2021: Hormozgan, Ages: 30−34
Data points enclosed with a diamond are reference sources

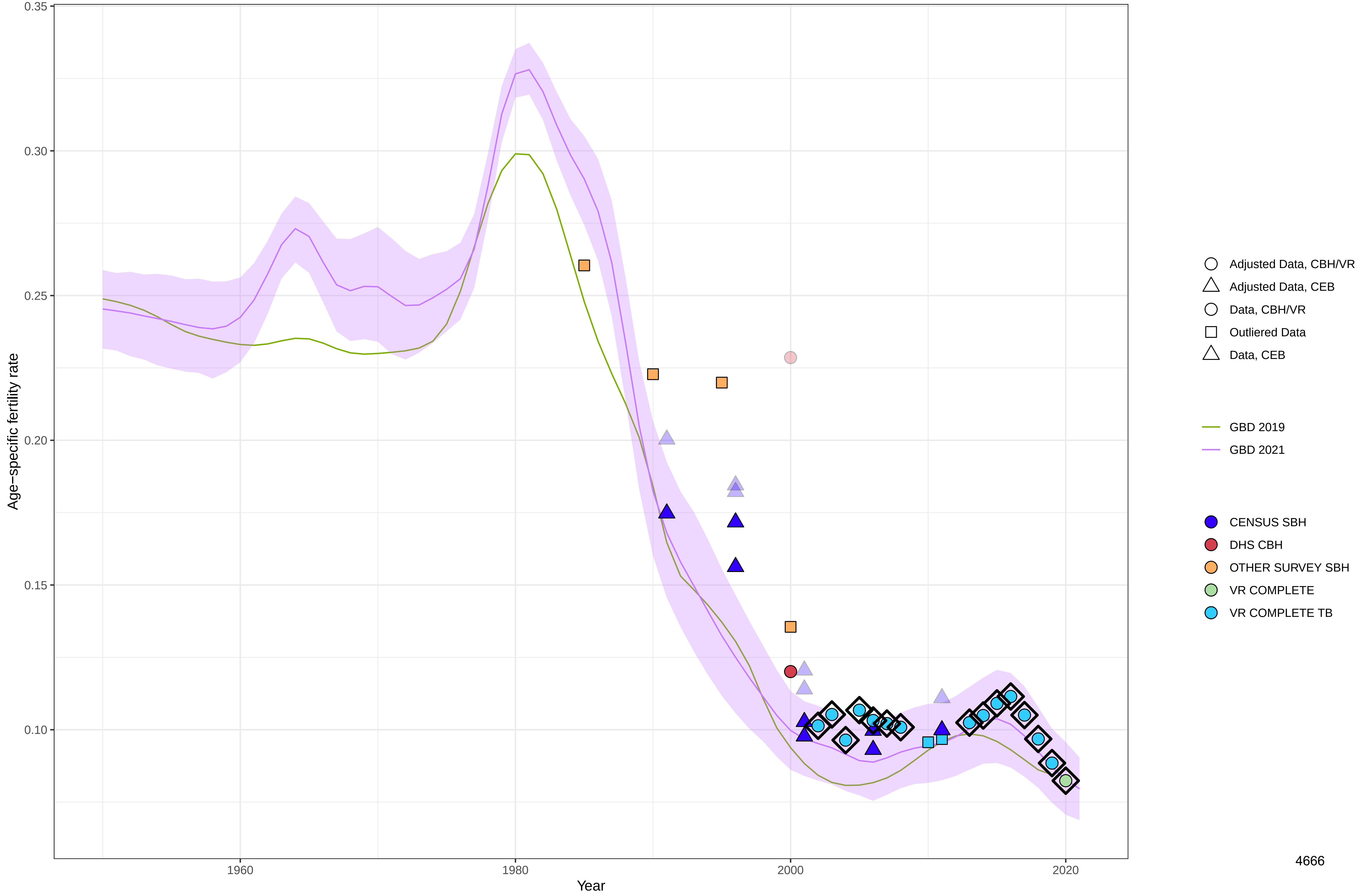

Age−specific fertility fitted trend lines, 1950−2021: Hormozgan, Ages: 35−39
Data points enclosed with a diamond are reference sources

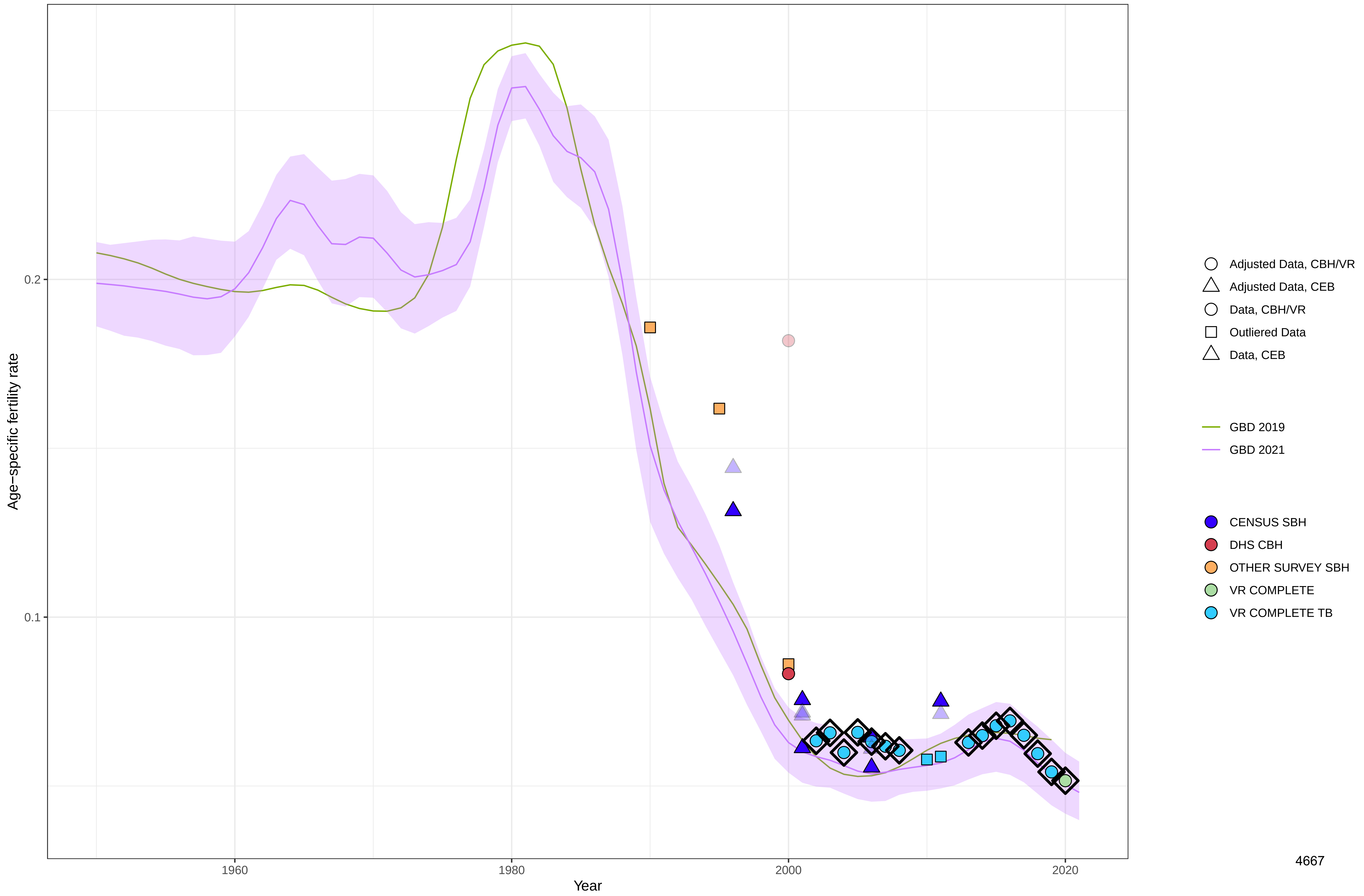

Age−specific fertility fitted trend lines, 1950−2021: Hormozgan, Ages: 40−44
Data points enclosed with a diamond are reference sources

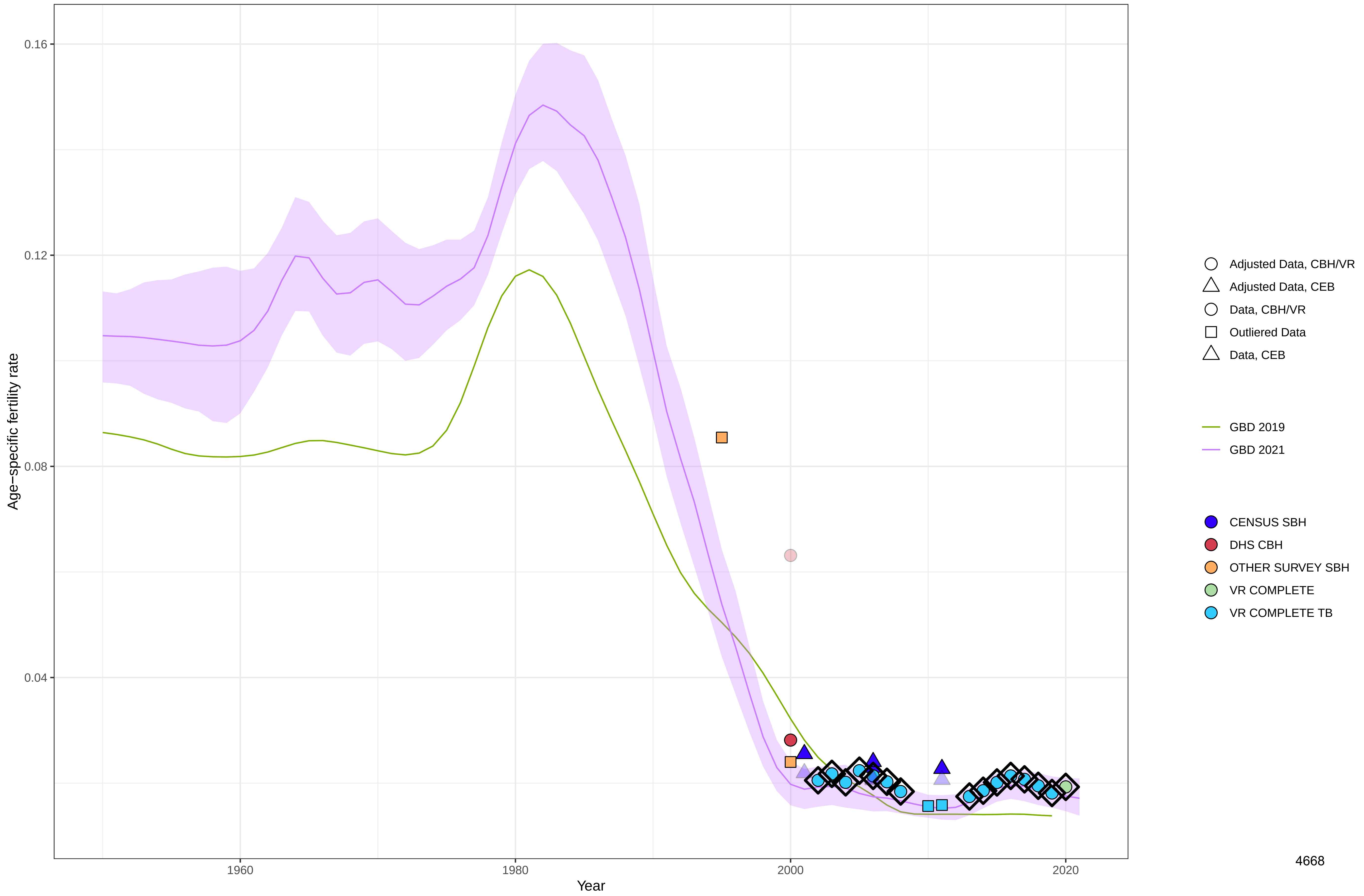

Age−specific fertility fitted trend lines, 1950−2021: Hormozgan, Ages: 45−49
Data points enclosed with a diamond are reference sources

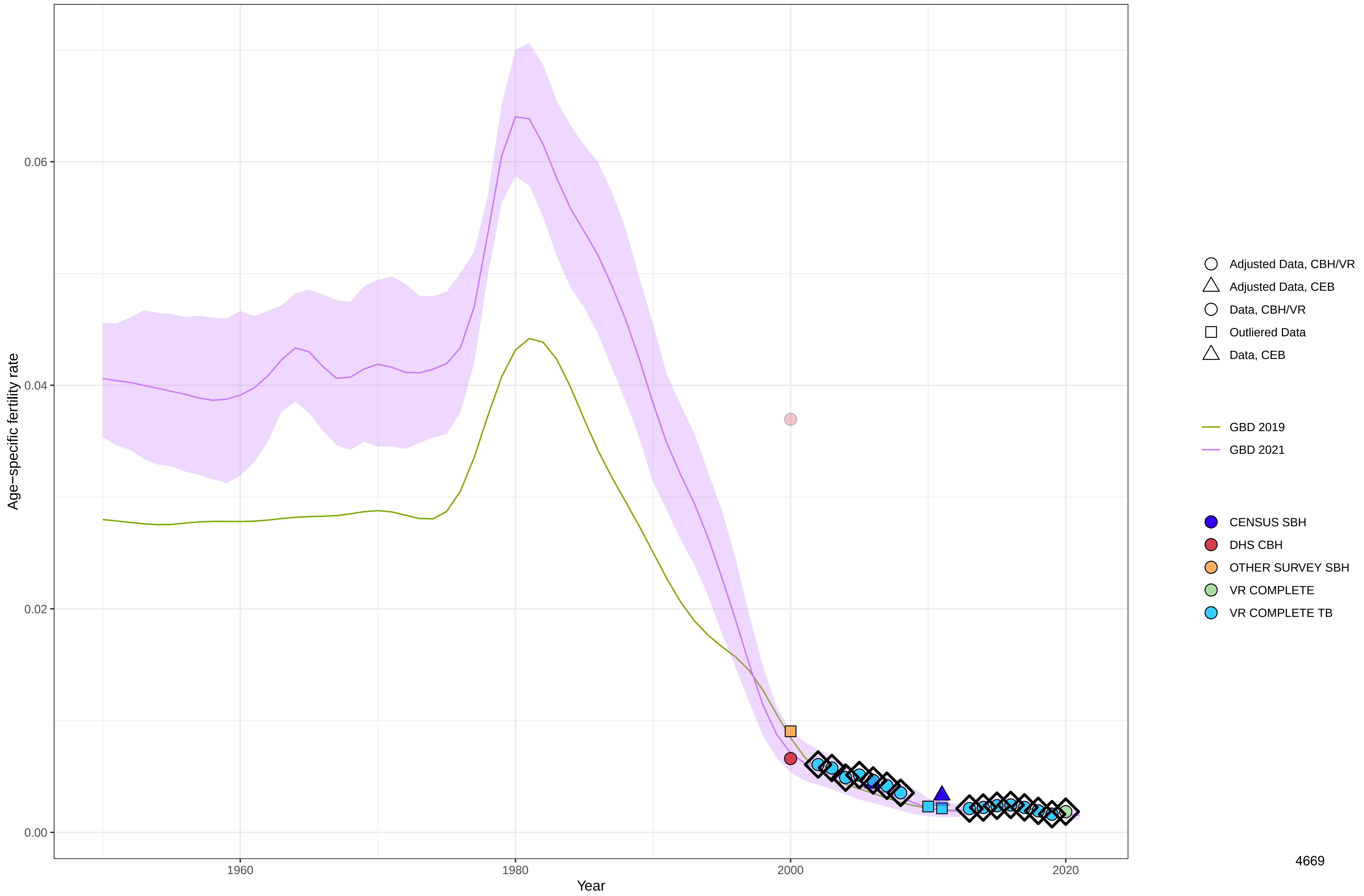

Age−specific fertility fitted trend lines, 1950−2021: Hormozgan, Ages: 50−54
Data points enclosed with a diamond are reference sources

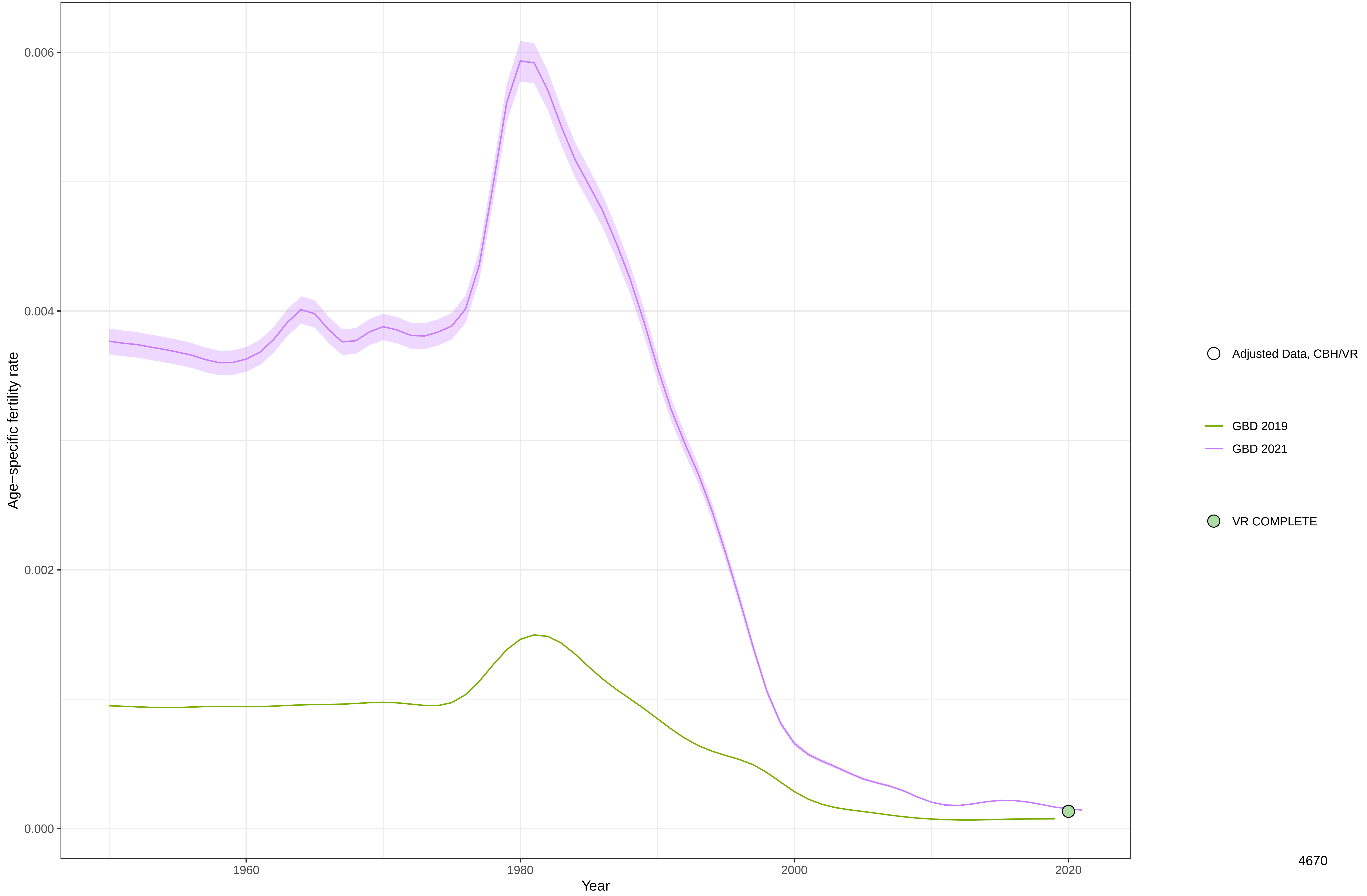

Total fertility rate fitted trend lines, 1950−2021: Hormozgan TFR
Data points enclosed with a diamond are reference sources

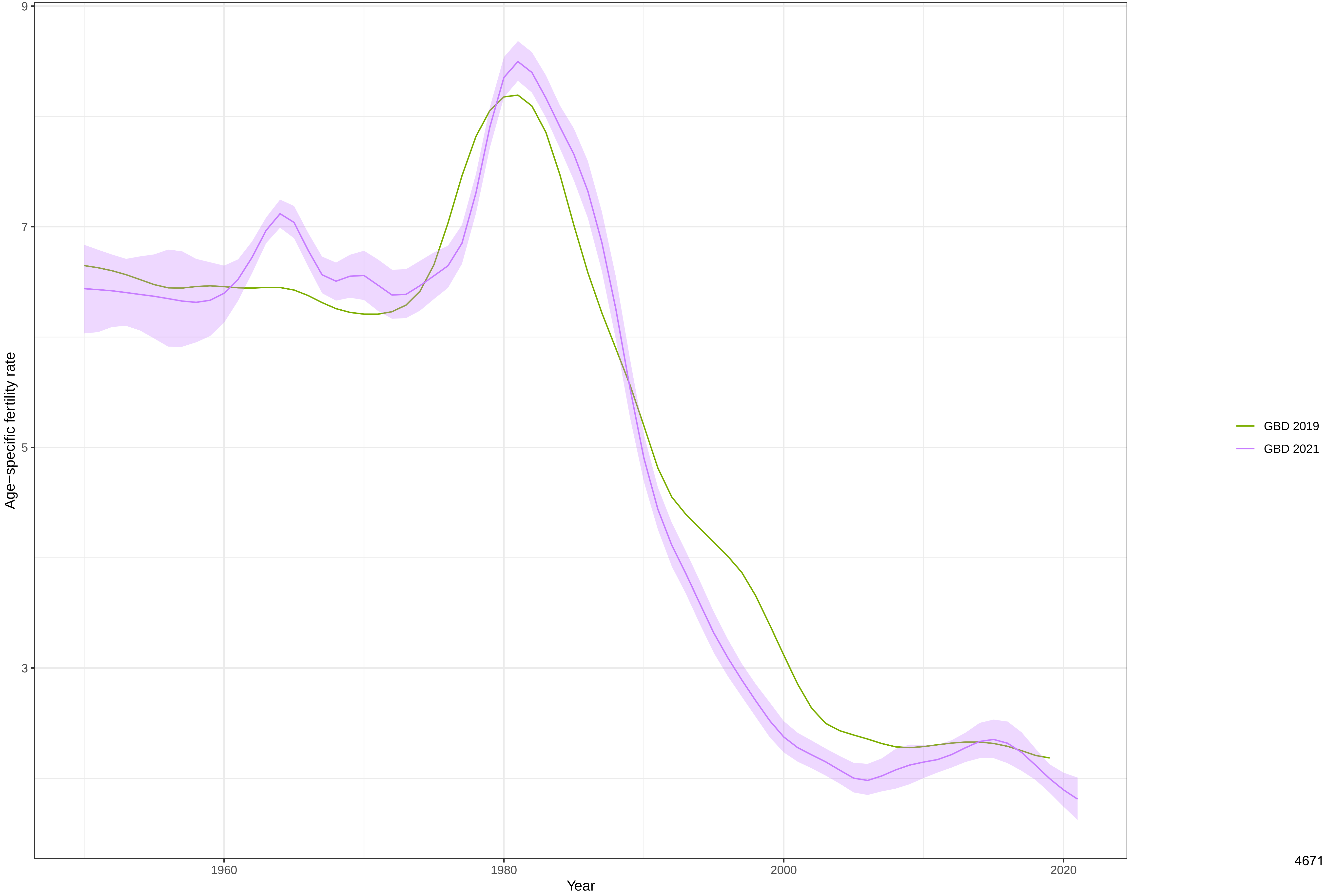

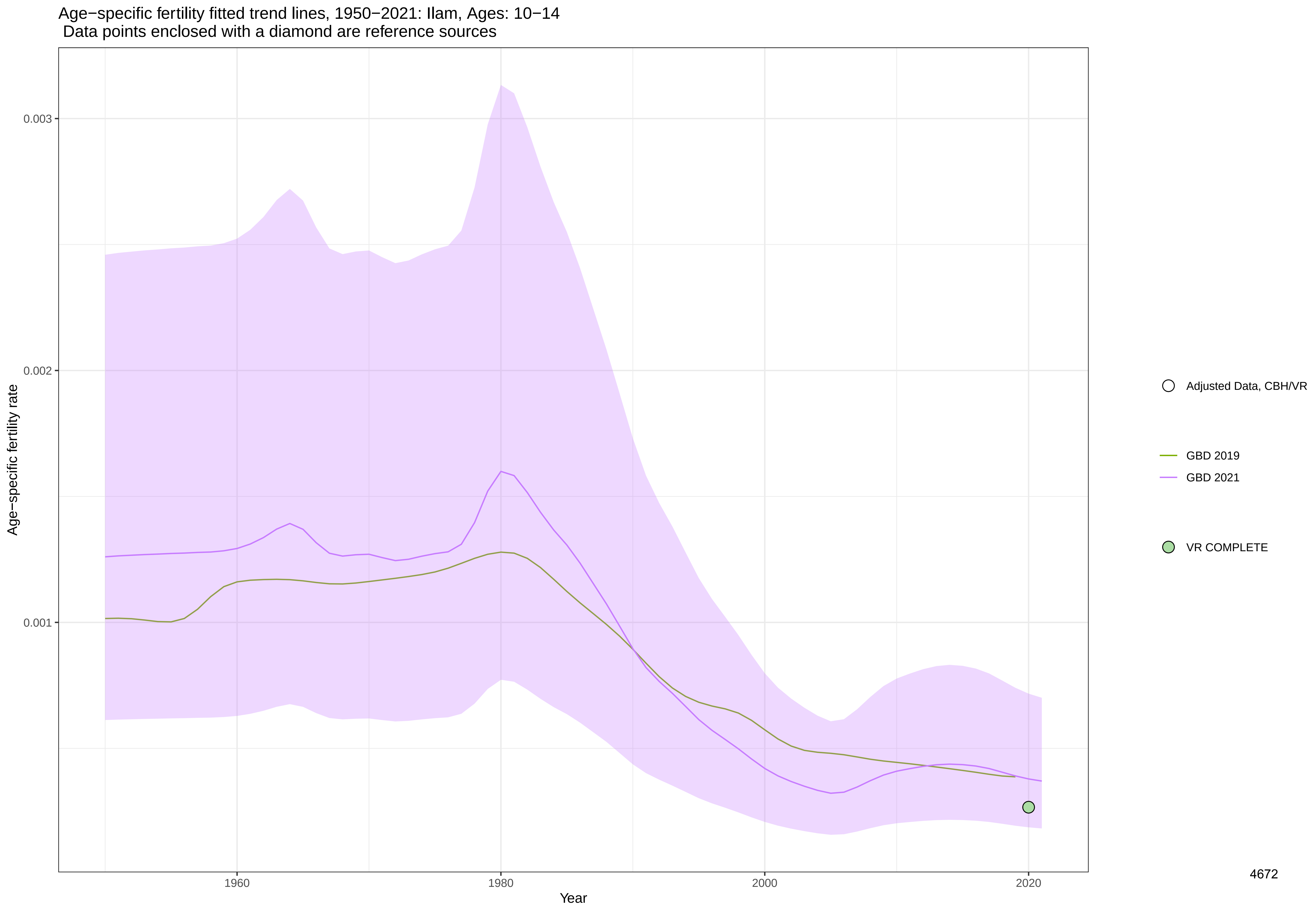

Age−specific fertility fitted trend lines, 1950−2021: Ilam, Ages: 10−14
Data points enclosed with a diamond are reference sources
0.003
0.002
0.001
Age−specific fertility rate
1960
1980
2000
2020
Year
Adjusted Data, CBH/VR
GBD 2019
GBD 2021
VR COMPLETE

4672

Age−specific fertility fitted trend lines, 1950−2021: Ilam, Ages: 15−19
Data points enclosed with a diamond are reference sources

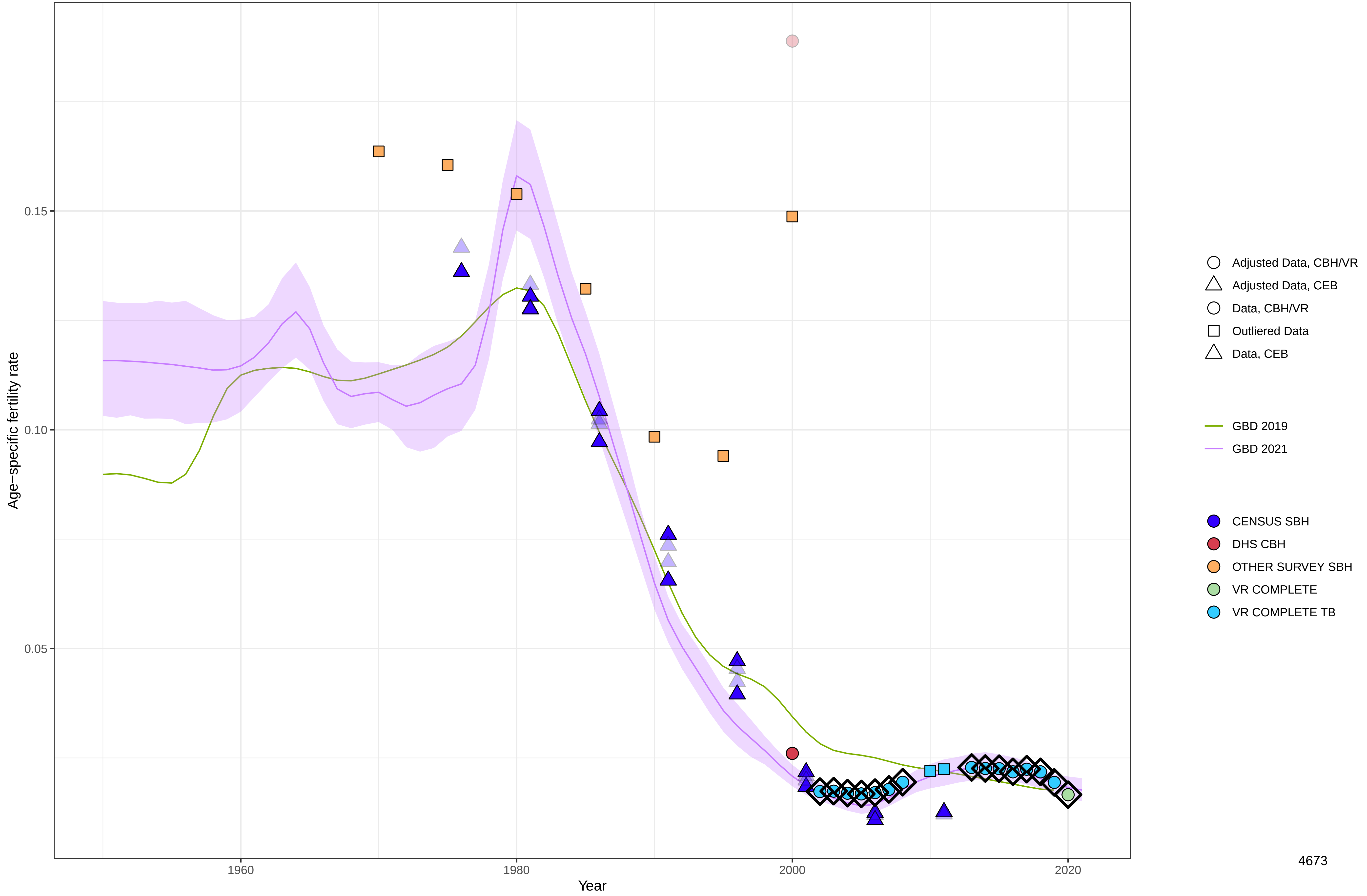

Age−specific fertility fitted trend lines, 1950−2021: Ilam, Ages: 20−24
Data points enclosed with a diamond are reference sources

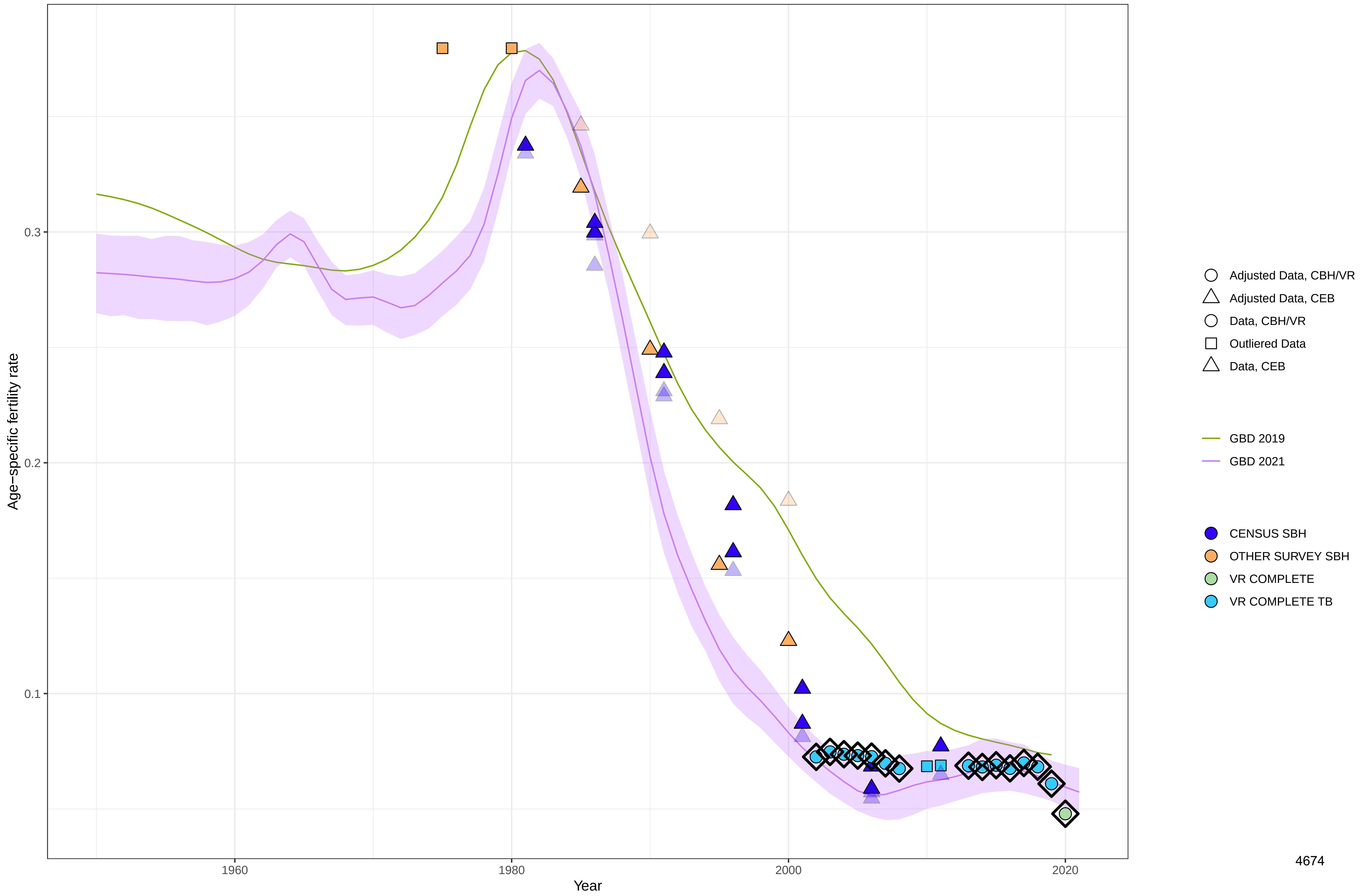

Age−specific fertility fitted trend lines, 1950−2021: Ilam, Ages: 25−29
Data points enclosed with a diamond are reference sources

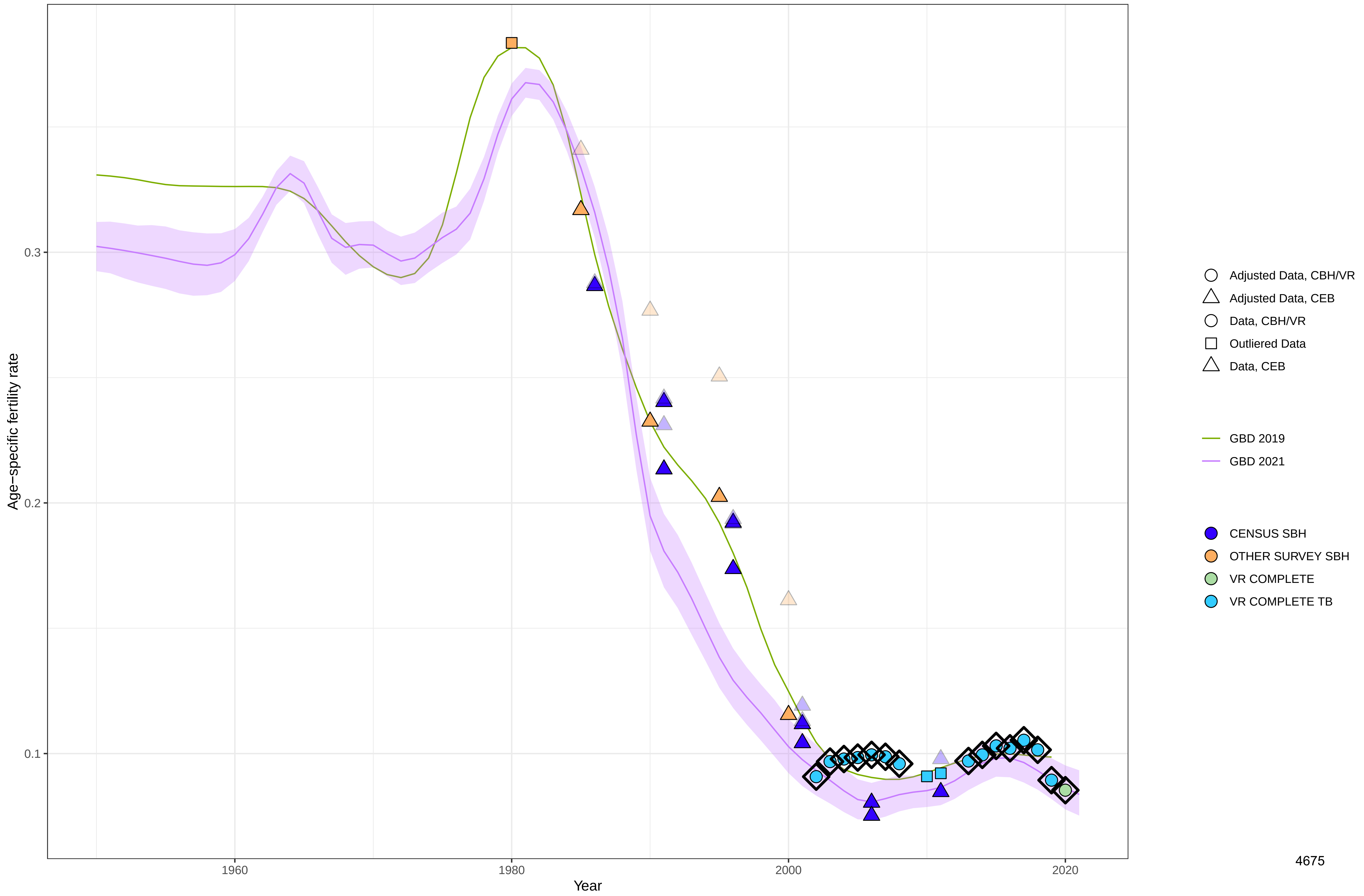

Age−specific fertility fitted trend lines, 1950−2021: Ilam, Ages: 30−34
Data points enclosed with a diamond are reference sources

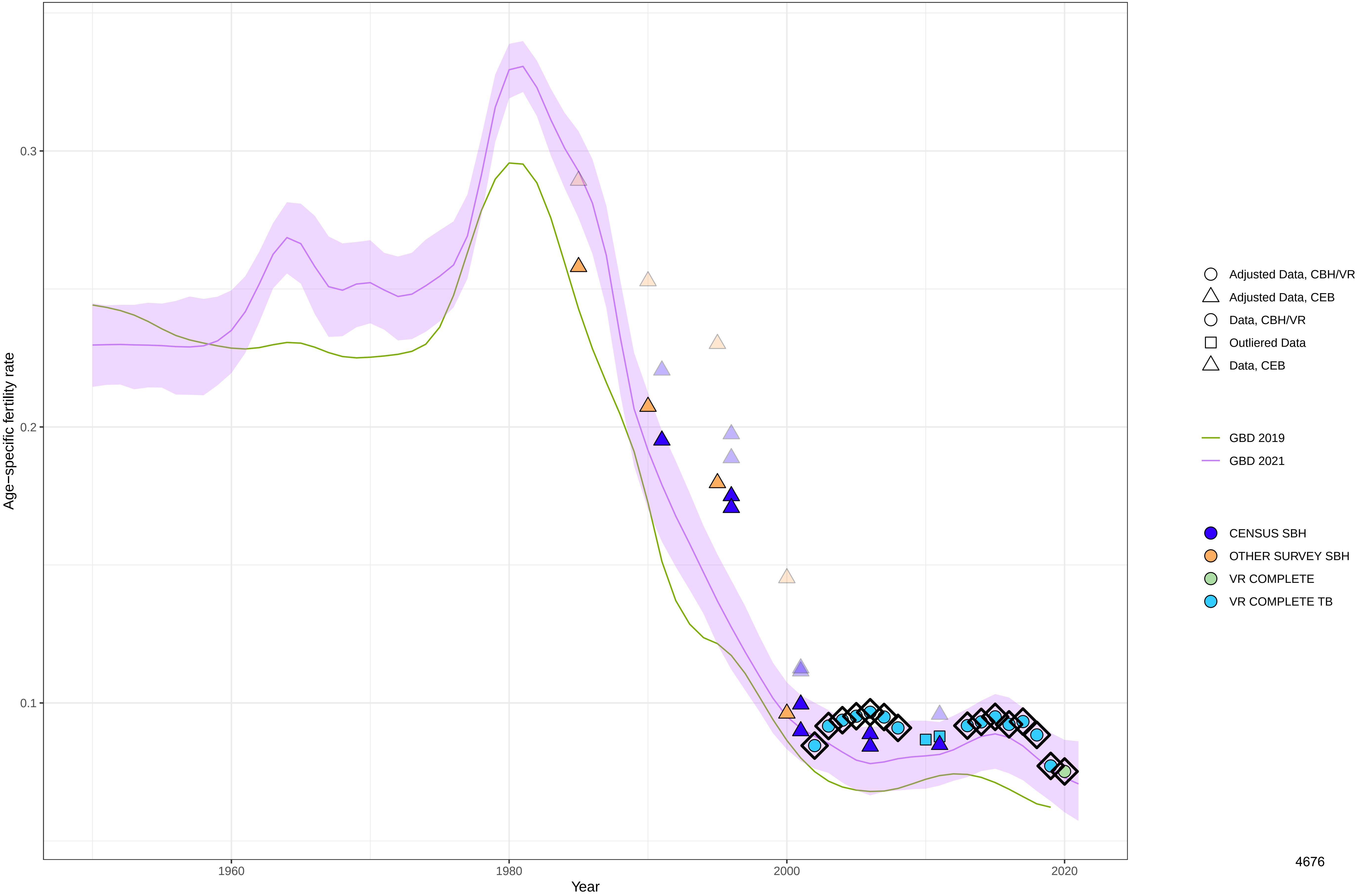

Age−specific fertility fitted trend lines, 1950−2021: Ilam, Ages: 35−39
Data points enclosed with a diamond are reference sources

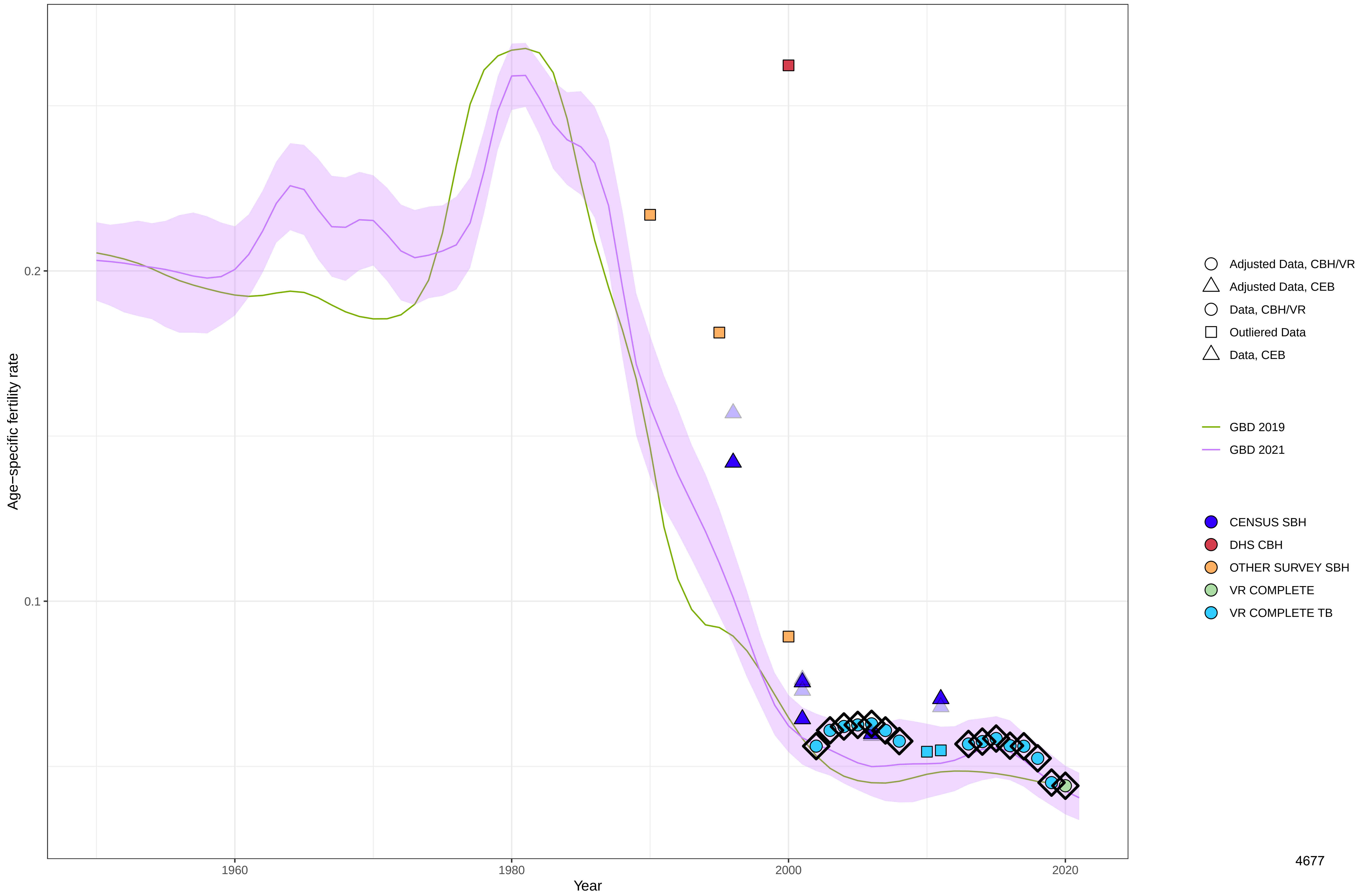

Age−specific fertility fitted trend lines, 1950−2021: Ilam, Ages: 40−44
Data points enclosed with a diamond are reference sources

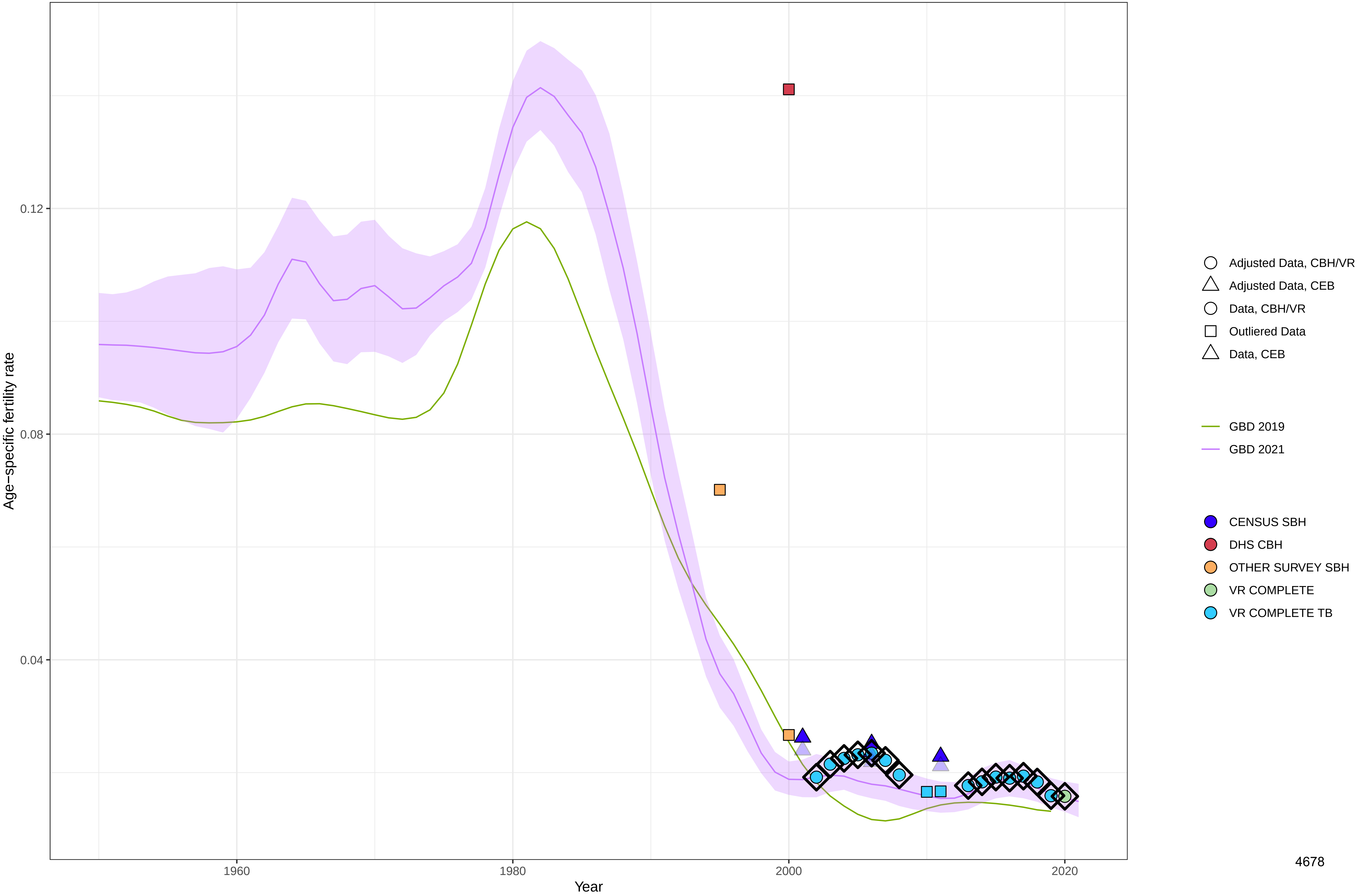

Age−specific fertility fitted trend lines, 1950−2021: Ilam, Ages: 45−49
Data points enclosed with a diamond are reference sources

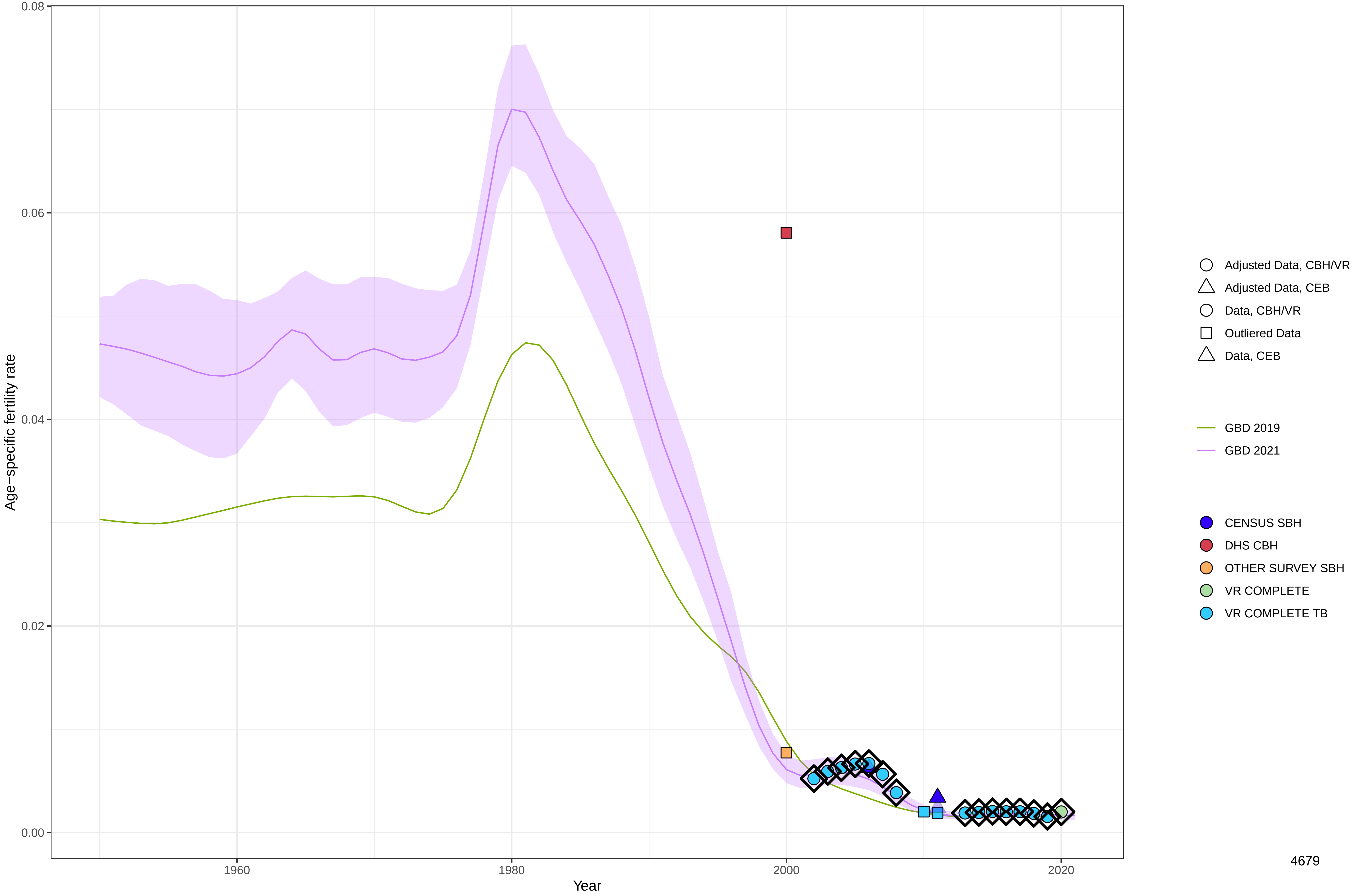

Age−specific fertility fitted trend lines, 1950−2021: Ilam, Ages: 50−54
Data points enclosed with a diamond are reference sources

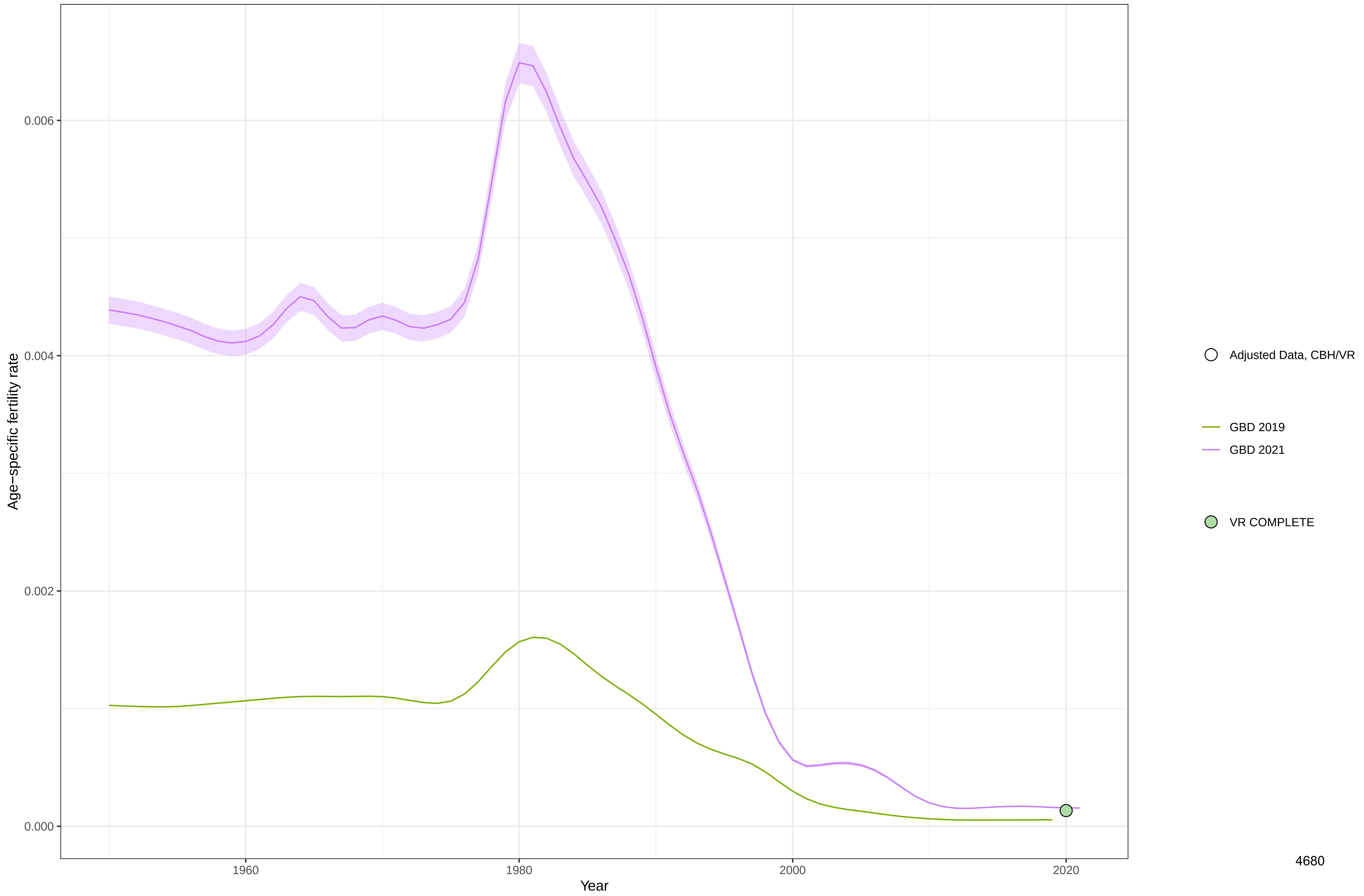

Total fertility rate fitted trend lines, 1950−2021: Ilam TFR
Data points enclosed with a diamond are reference sources

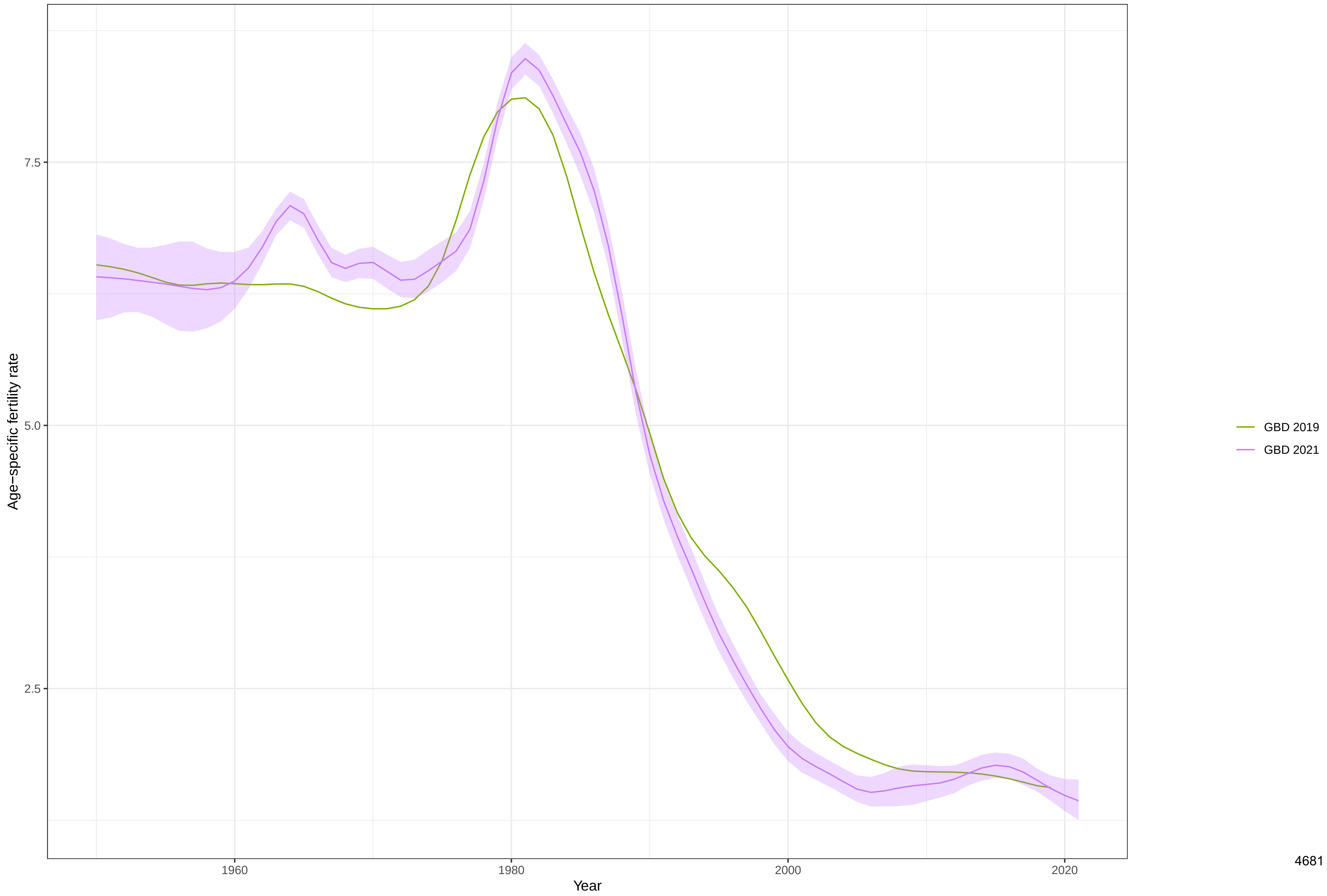

Age−specific fertility fitted trend lines, 1950−2021: Isfahan, Ages: 10−14
Data points enclosed with a diamond are reference sources

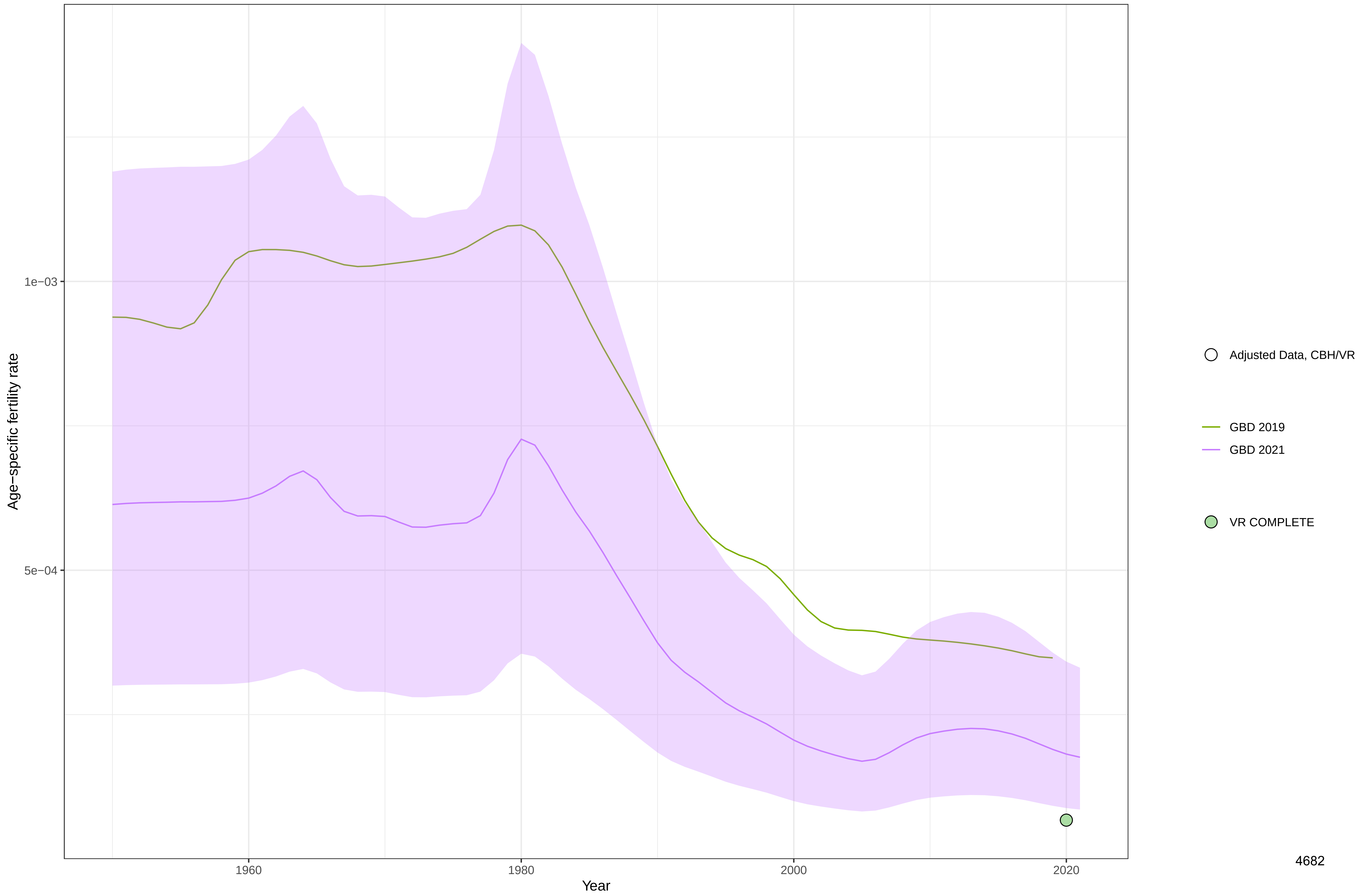

Age−specific fertility fitted trend lines, 1950−2021: Isfahan, Ages: 15−19
Data points enclosed with a diamond are reference sources

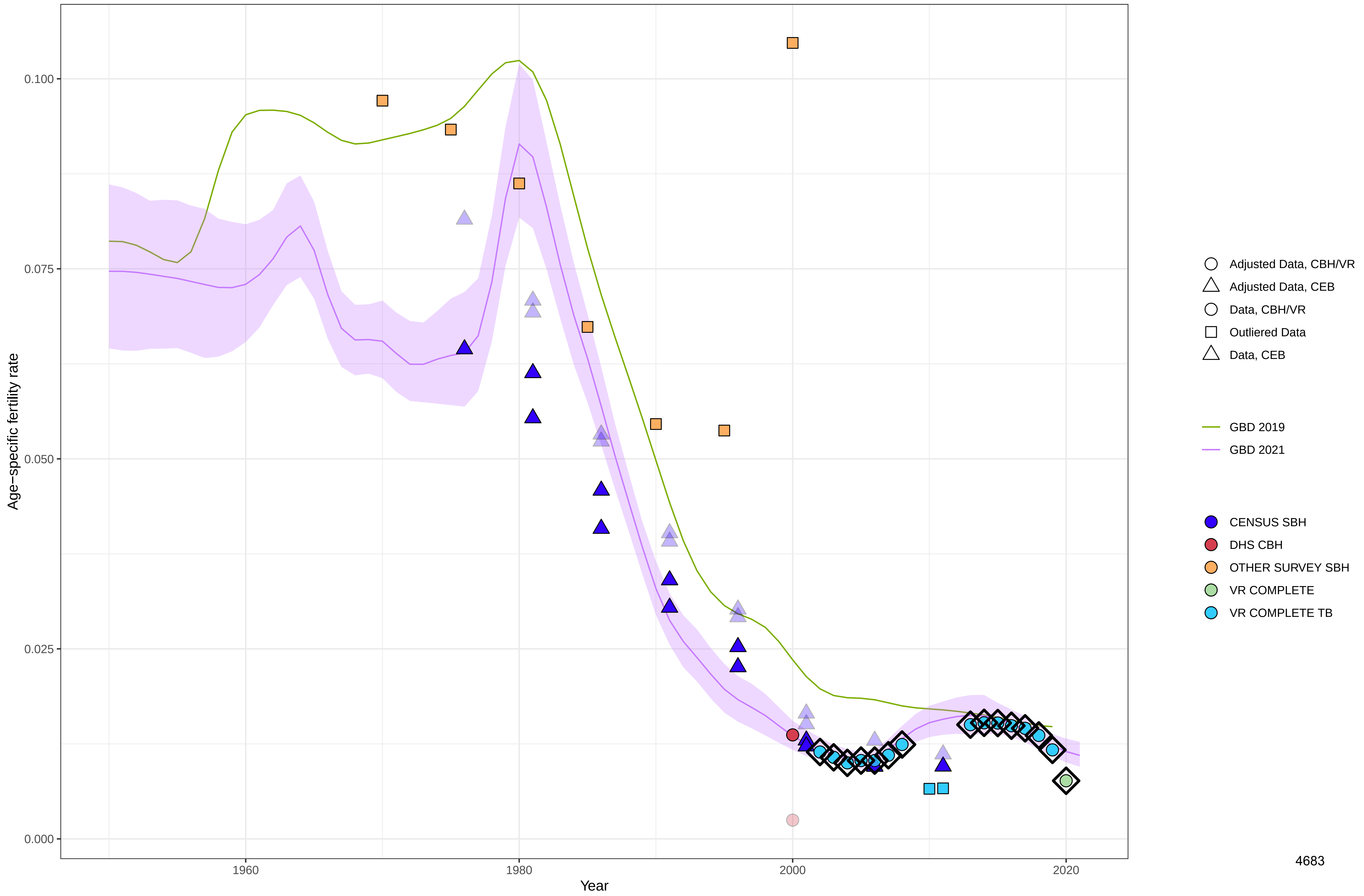

Age−specific fertility fitted trend lines, 1950−2021: Isfahan, Ages: 20−24
Data points enclosed with a diamond are reference sources

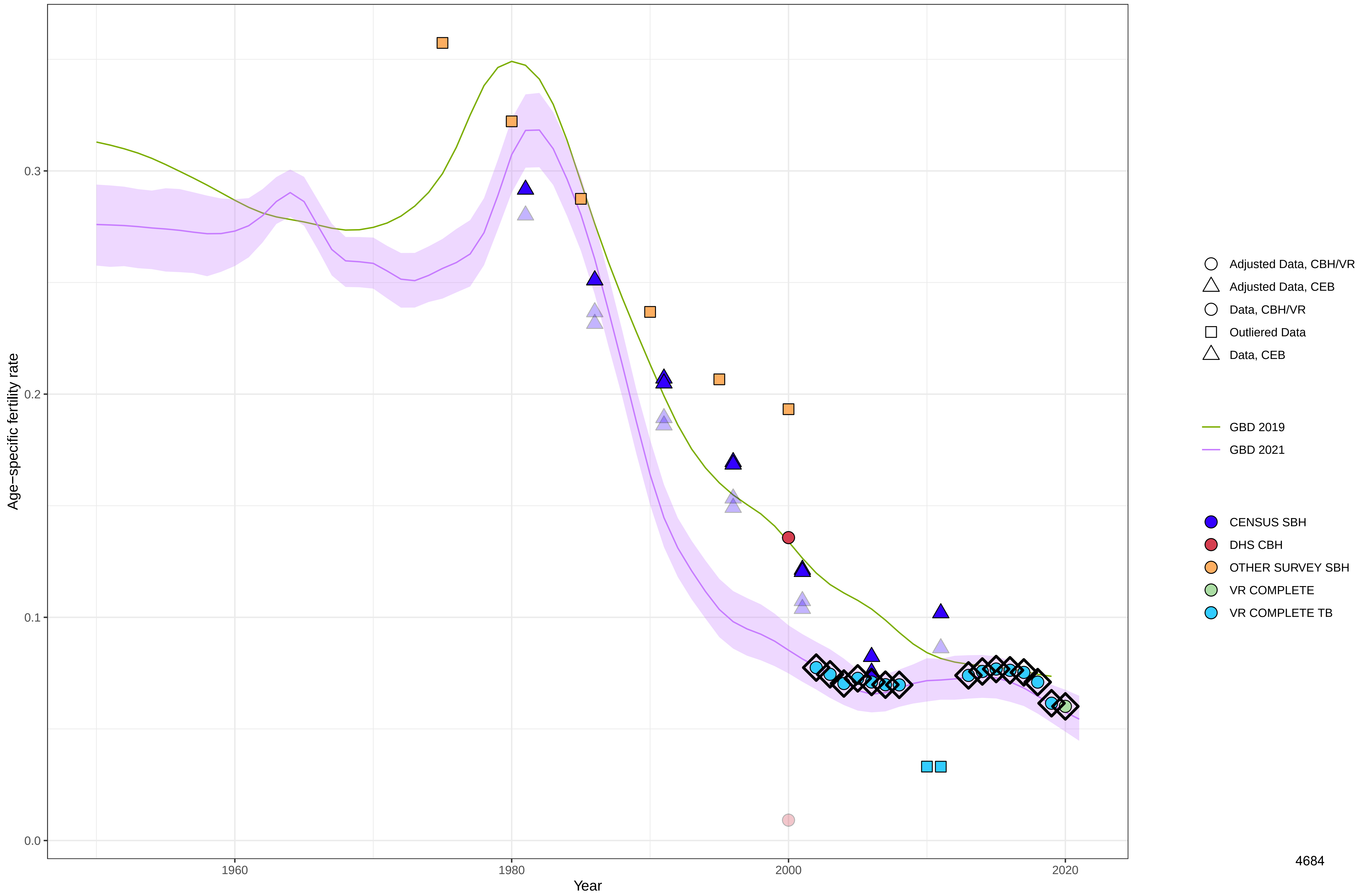

Age−specific fertility fitted trend lines, 1950−2021: Isfahan, Ages: 25−29
Data points enclosed with a diamond are reference sources

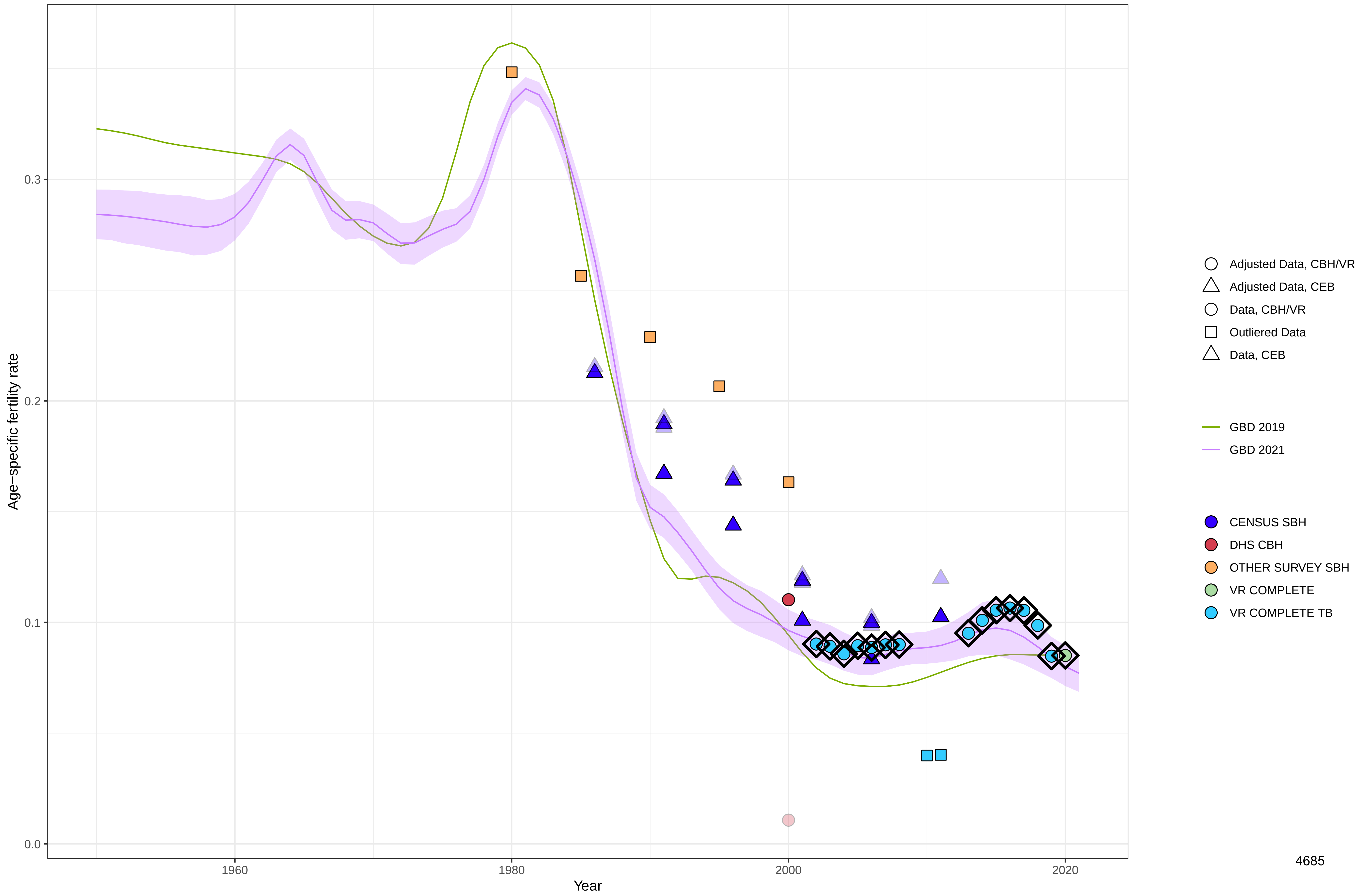

Age−specific fertility fitted trend lines, 1950−2021: Isfahan, Ages: 30−34
Data points enclosed with a diamond are reference sources

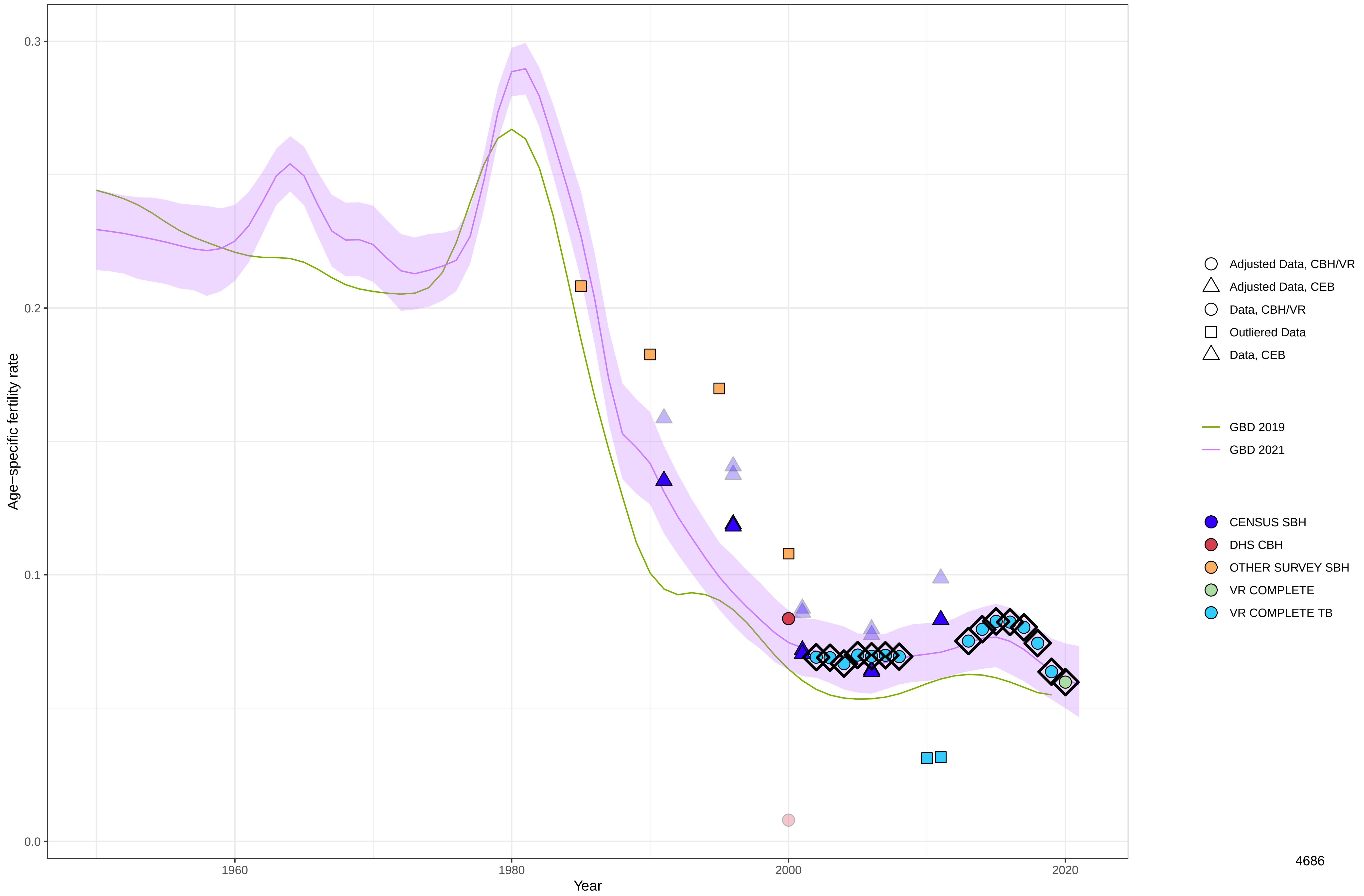

Age−specific fertility fitted trend lines, 1950−2021: Isfahan, Ages: 35−39
Data points enclosed with a diamond are reference sources

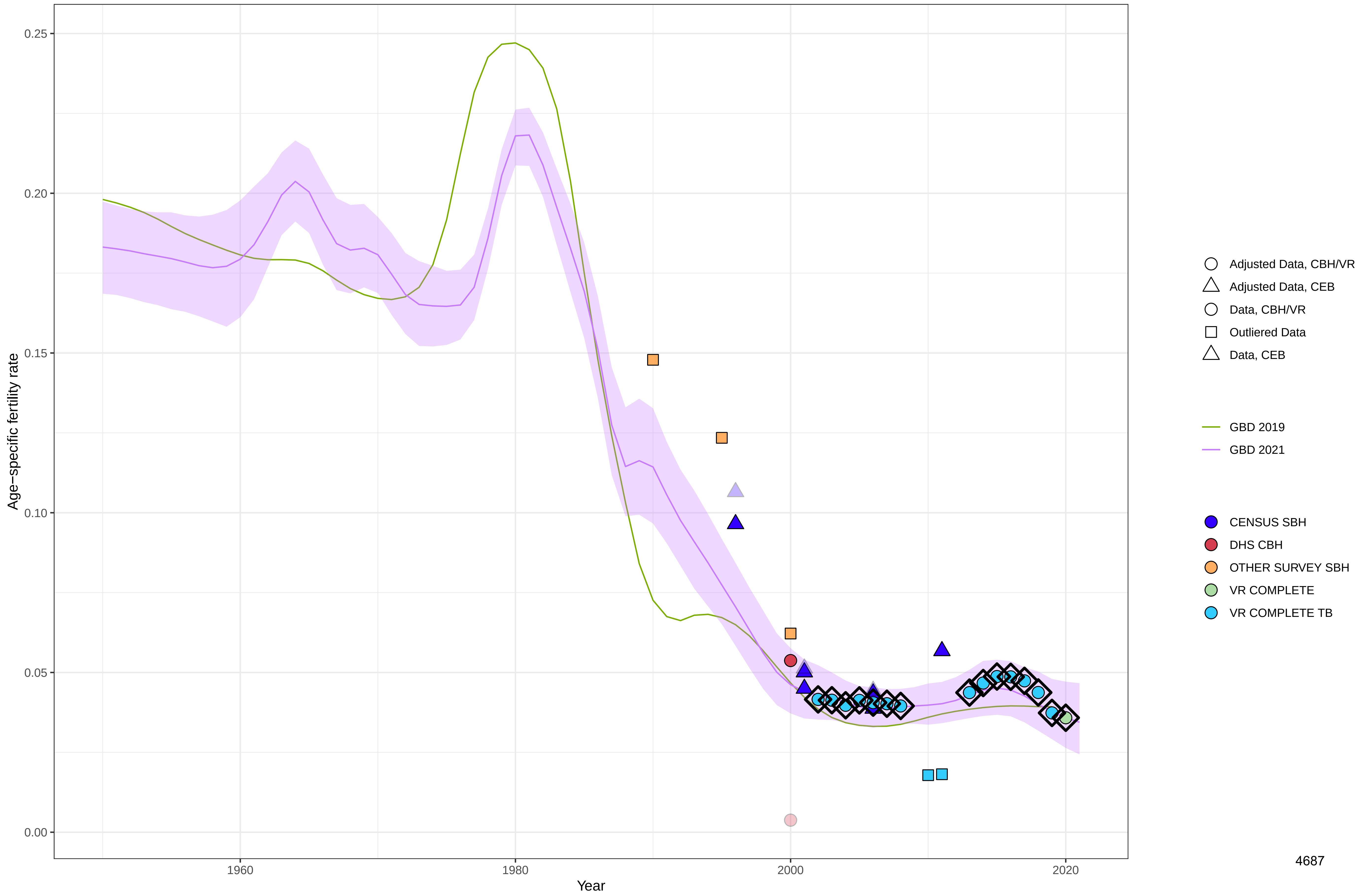

Age−specific fertility fitted trend lines, 1950−2021: Isfahan, Ages: 40−44
Data points enclosed with a diamond are reference sources

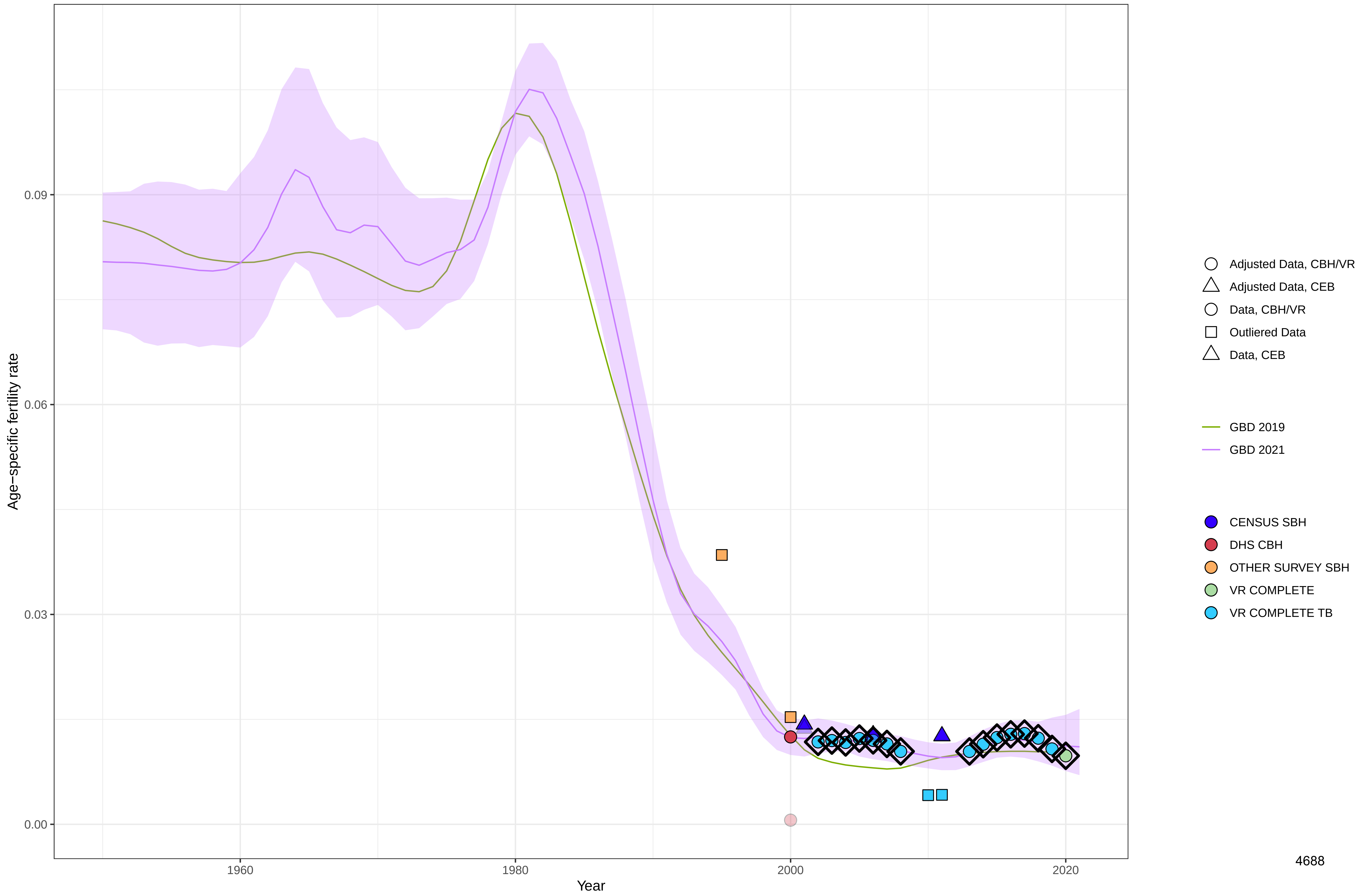

Age−specific fertility fitted trend lines, 1950−2021: Isfahan, Ages: 45−49
Data points enclosed with a diamond are reference sources

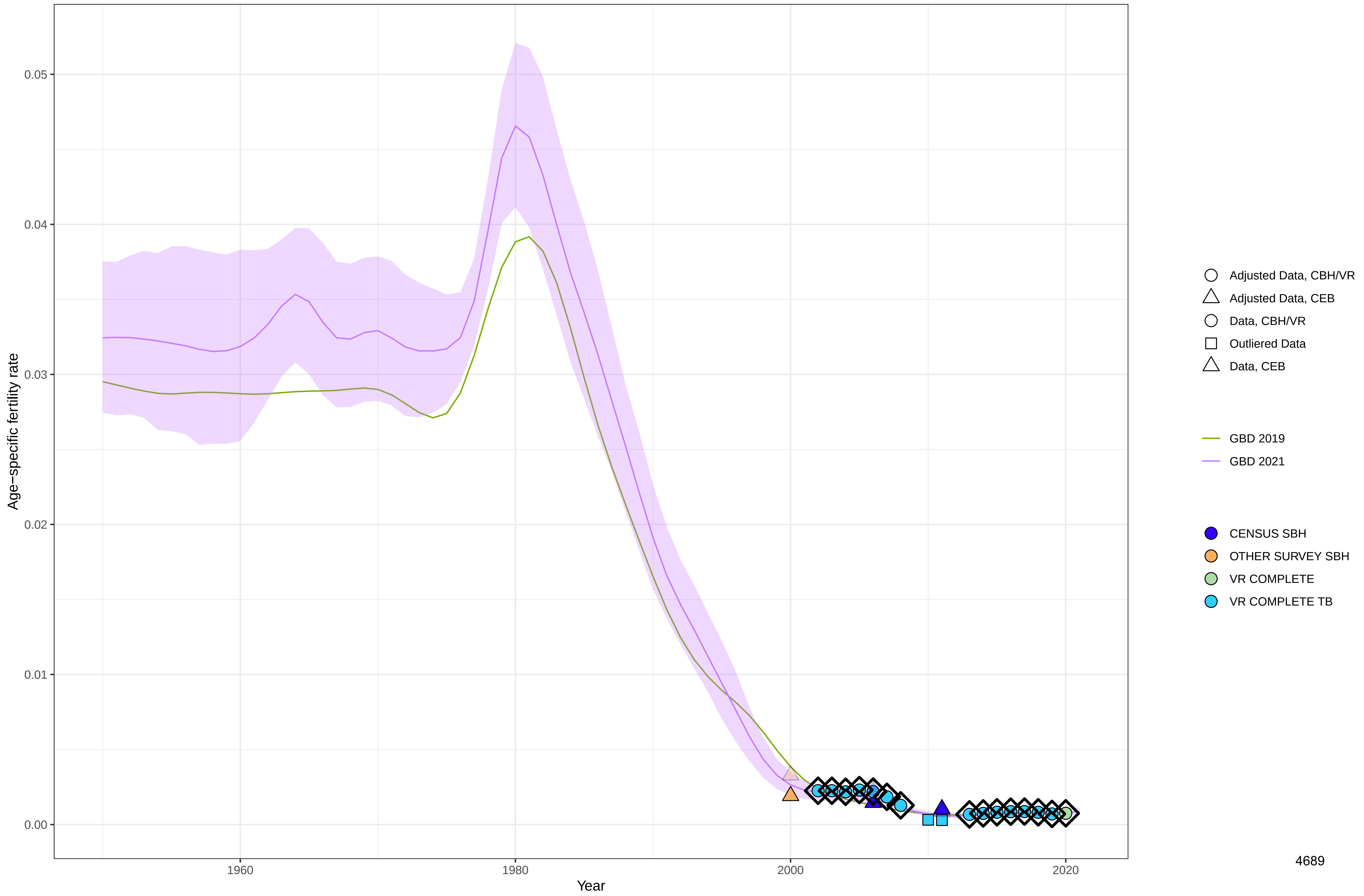

Age−specific fertility fitted trend lines, 1950−2021: Isfahan, Ages: 50−54
Data points enclosed with a diamond are reference sources

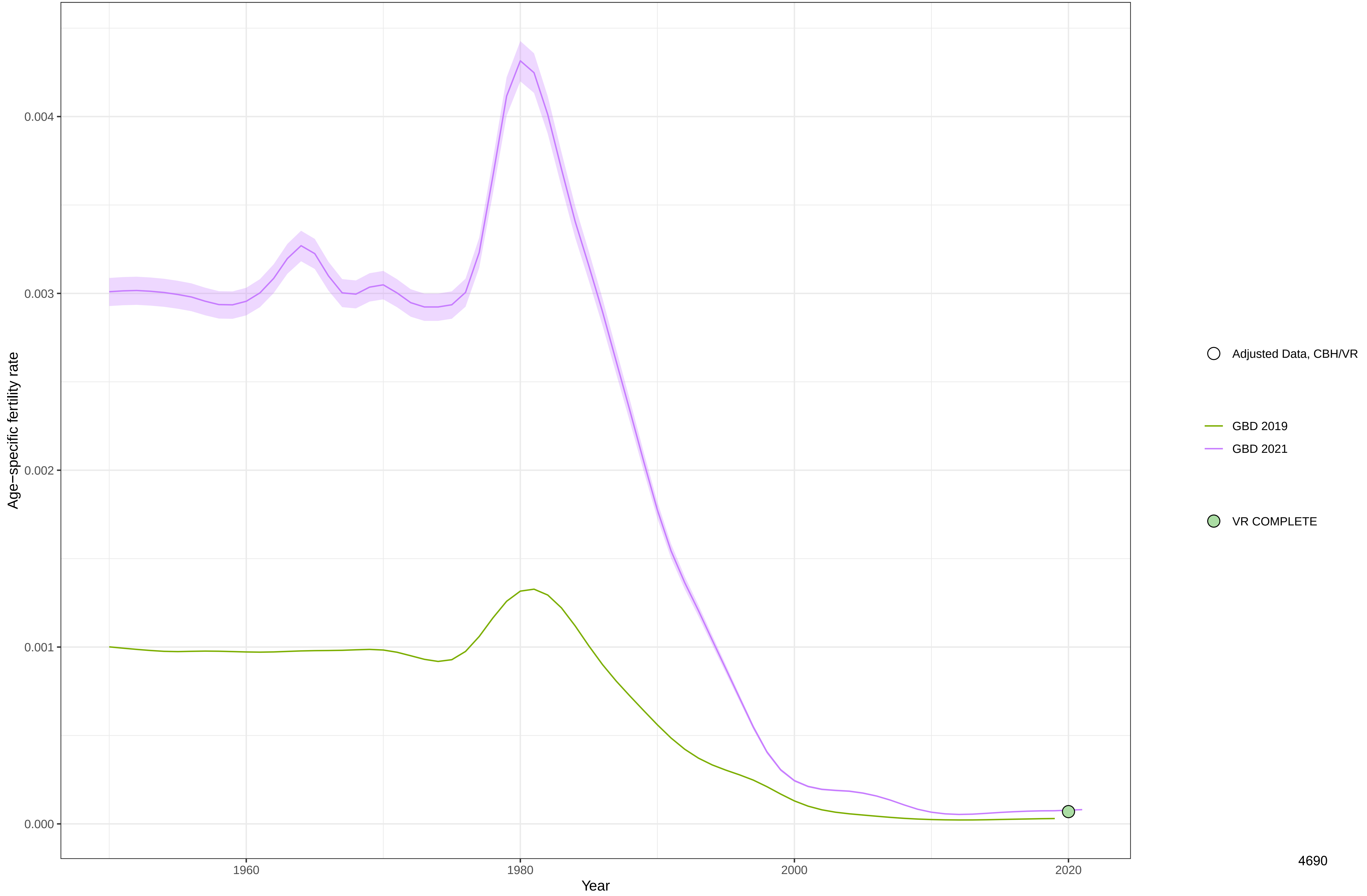

Total fertility rate fitted trend lines, 1950−2021: Isfahan TFR
Data points enclosed with a diamond are reference sources

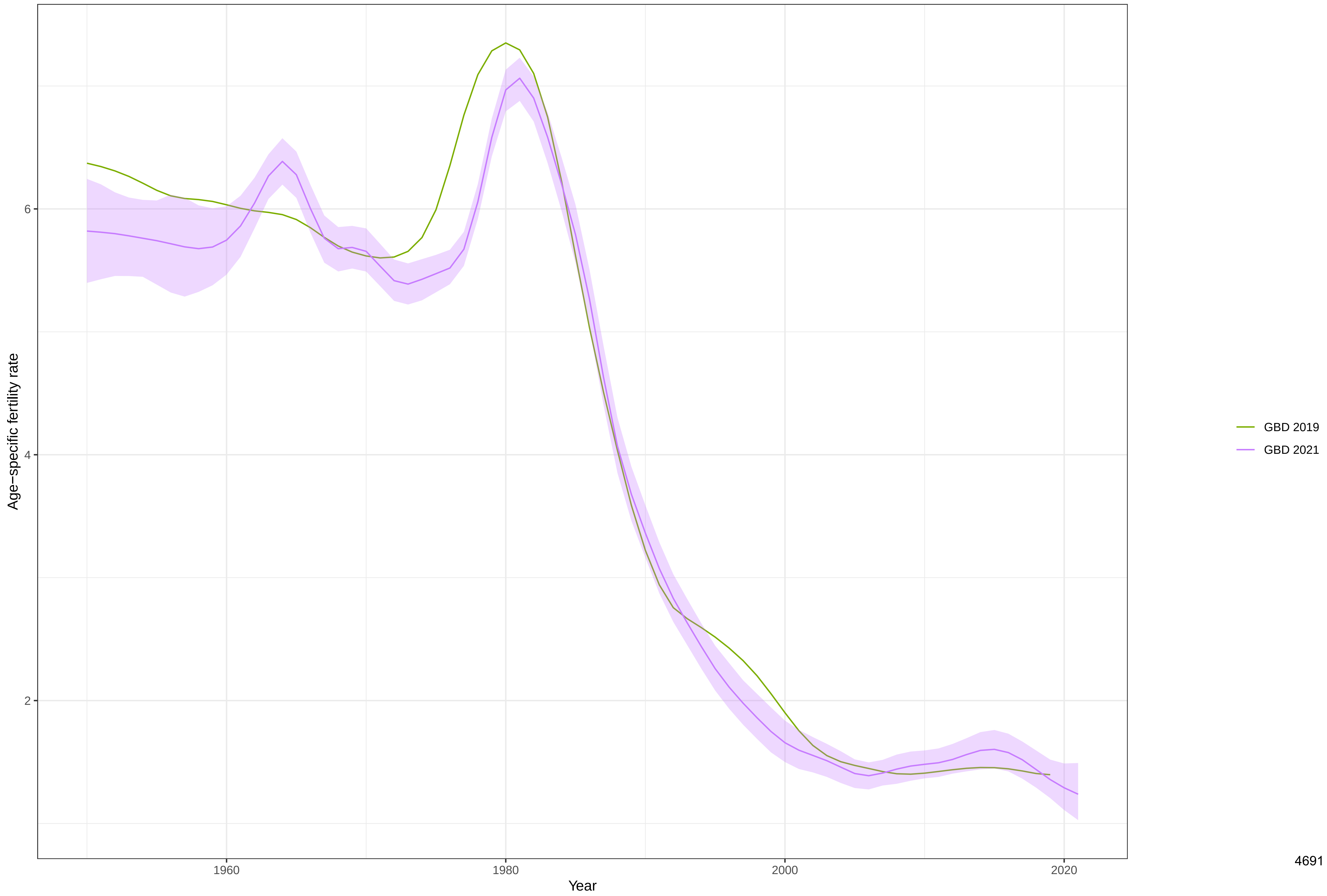

Age−specific fertility fitted trend lines, 1950−2021: Kerman, Ages: 10−14
Data points enclosed with a diamond are reference sources

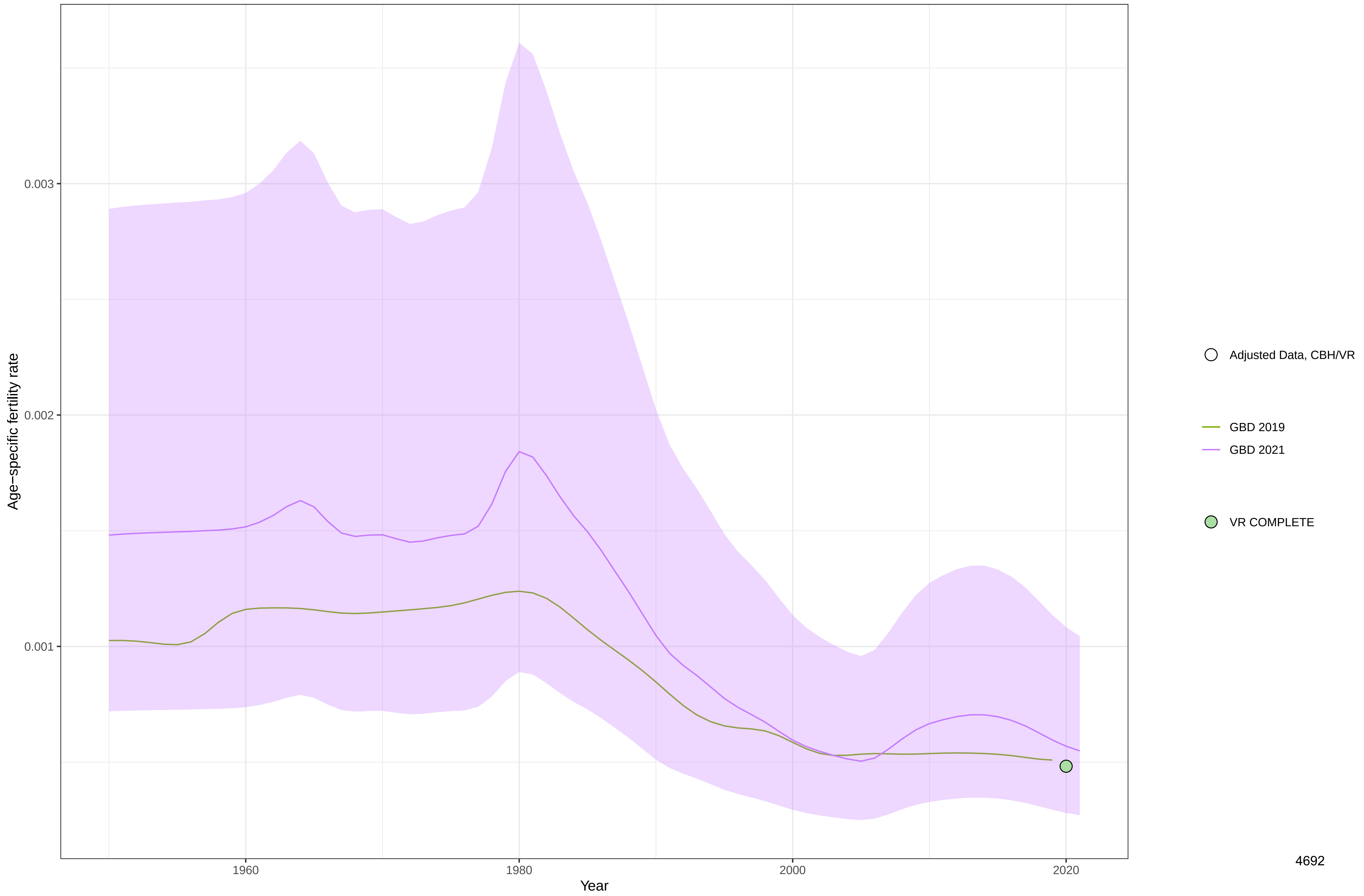

Age−specific fertility fitted trend lines, 1950−2021: Kerman, Ages: 15−19
Data points enclosed with a diamond are reference sources

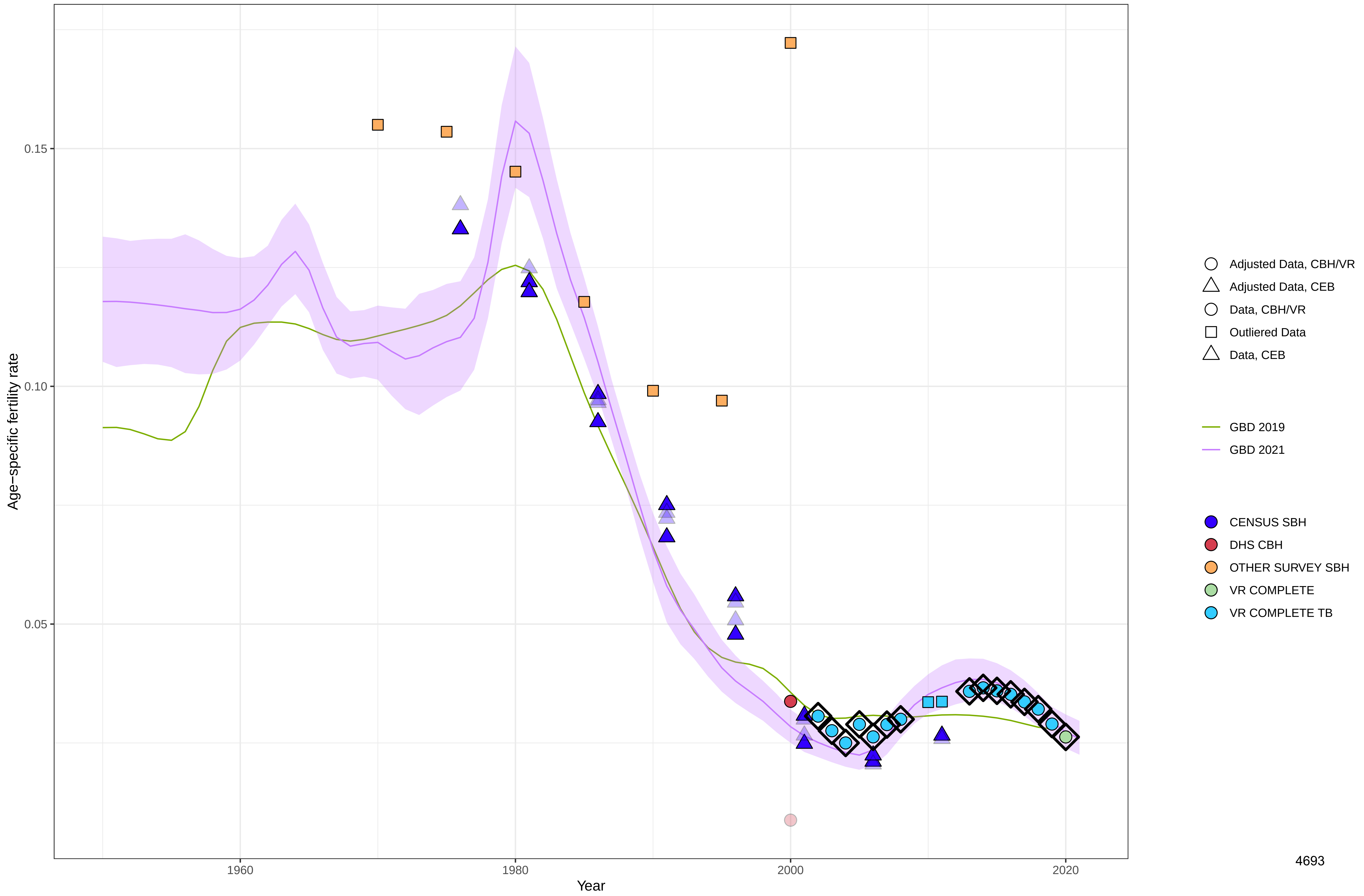

Age−specific fertility fitted trend lines, 1950−2021: Kerman, Ages: 20−24
Data points enclosed with a diamond are reference sources

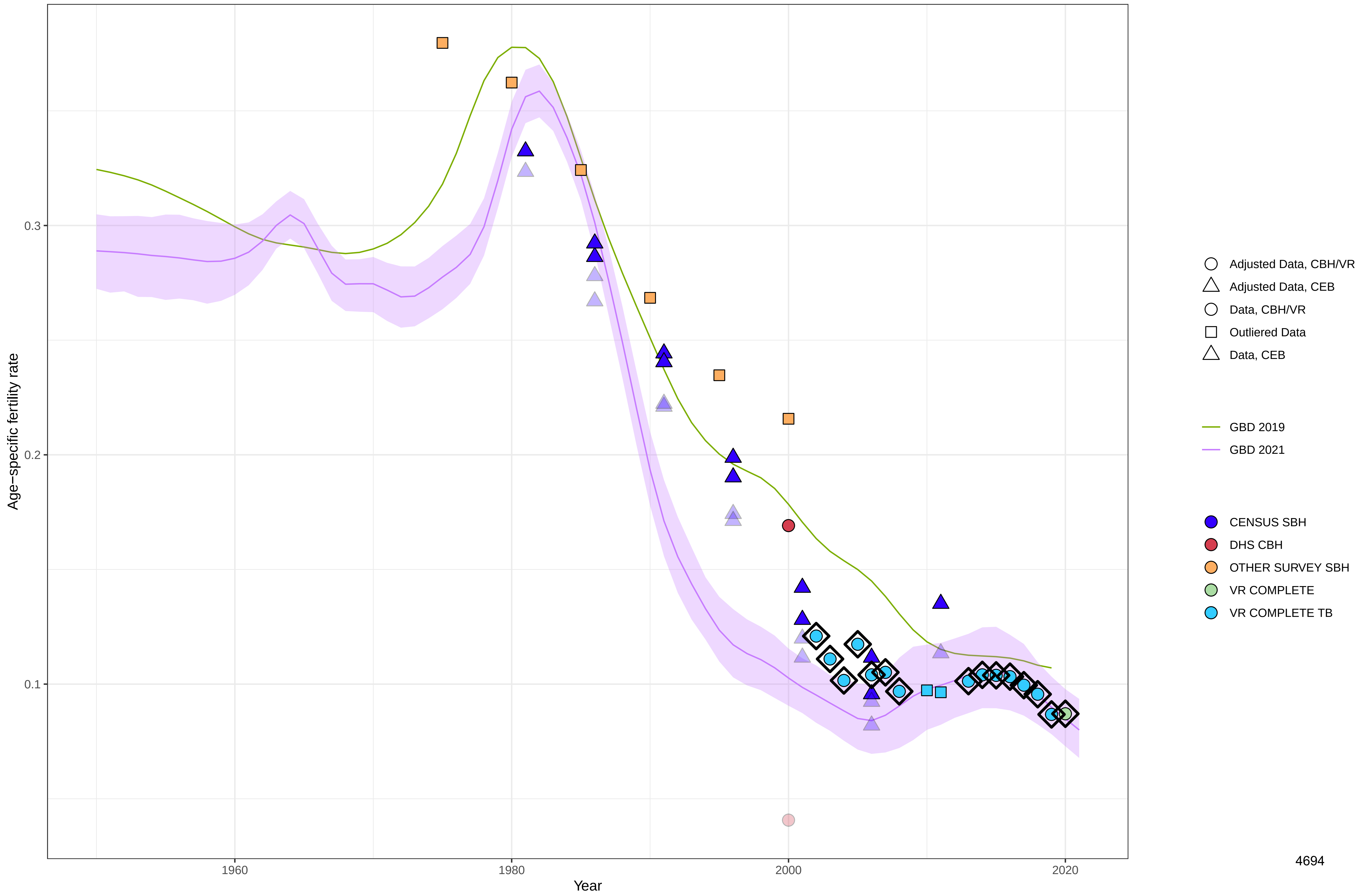

Age−specific fertility fitted trend lines, 1950−2021: Kerman, Ages: 25−29
Data points enclosed with a diamond are reference sources

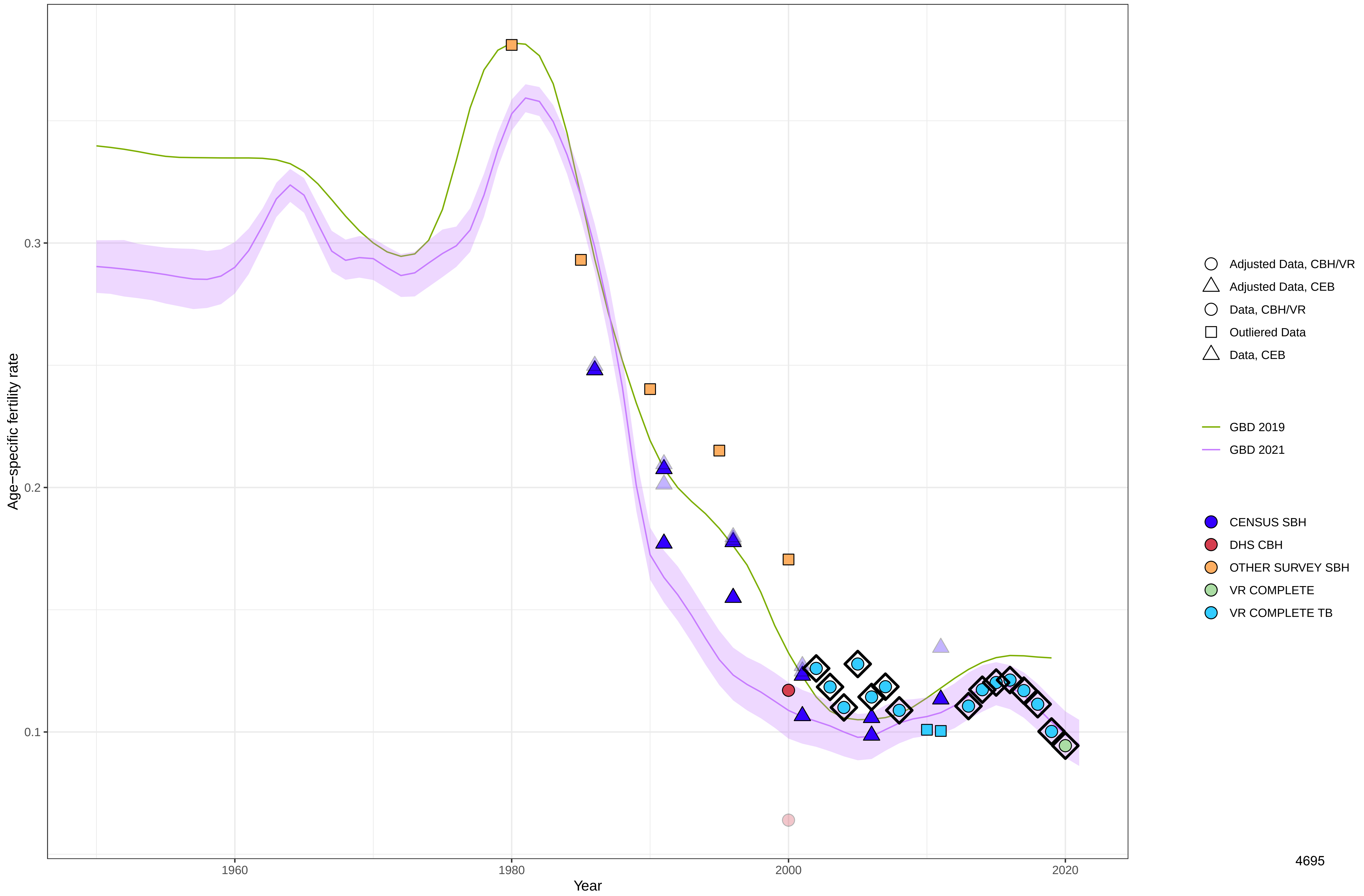

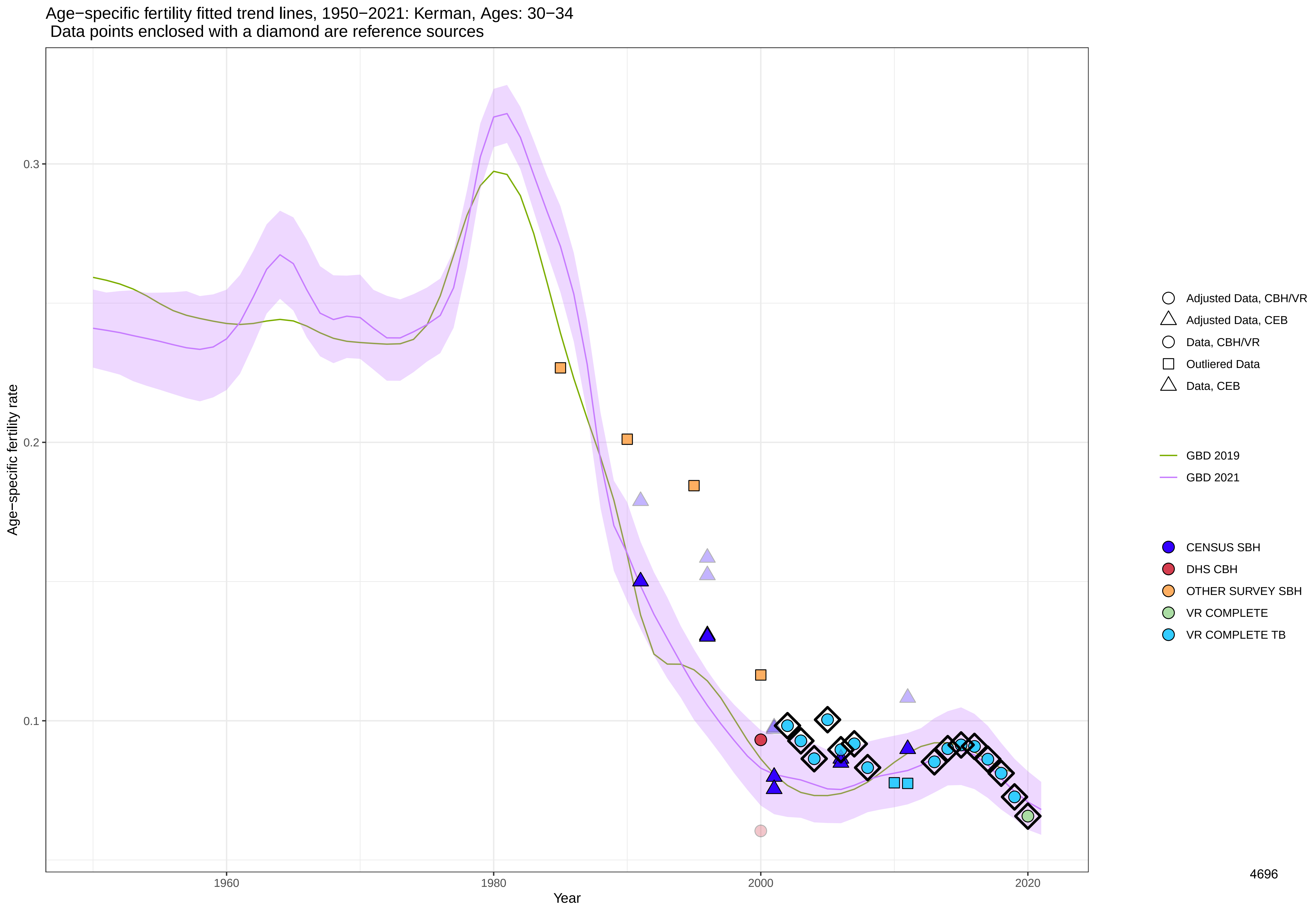

Age−specific fertility fitted trend lines, 1950−2021: Kerman, Ages: 30−34
Data points enclosed with a diamond are reference sources
Age−specific fertility rate
Year
0.1
0.2
0.3
1960
1980
2000
2020
Adjusted Data, CBH/VR
Adjusted Data, CEB
Data, CBH/VR
Outliered Data
Data, CEB
GBD 2019
GBD 2021
CENSUS SBH
DHS CBH
OTHER SURVEY SBH
VR COMPLETE
VR COMPLETE TB

4696

Age−specific fertility fitted trend lines, 1950−2021: Kerman, Ages: 35−39
Data points enclosed with a diamond are reference sources

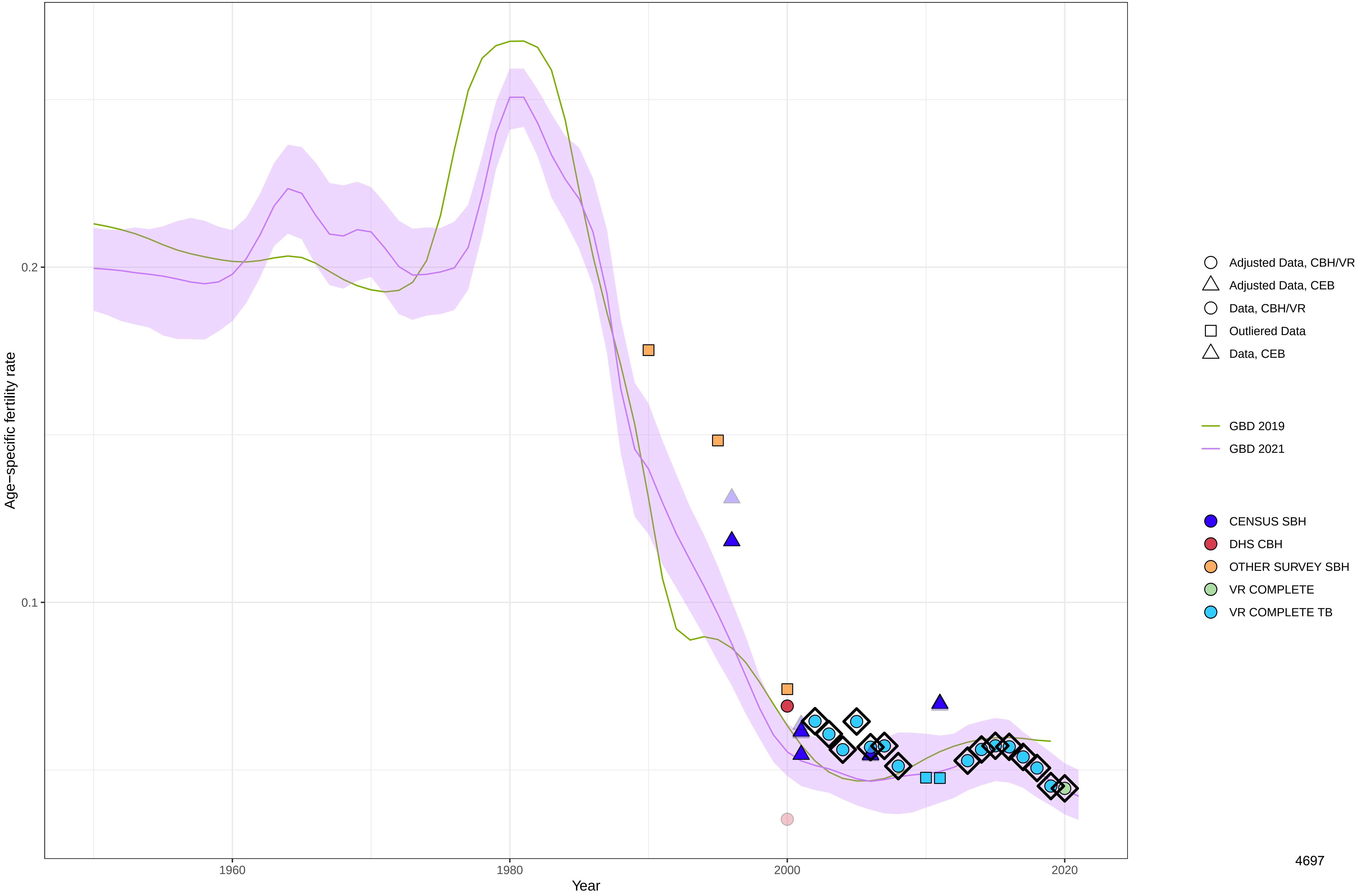

Age−specific fertility fitted trend lines, 1950−2021: Kerman, Ages: 40−44
Data points enclosed with a diamond are reference sources

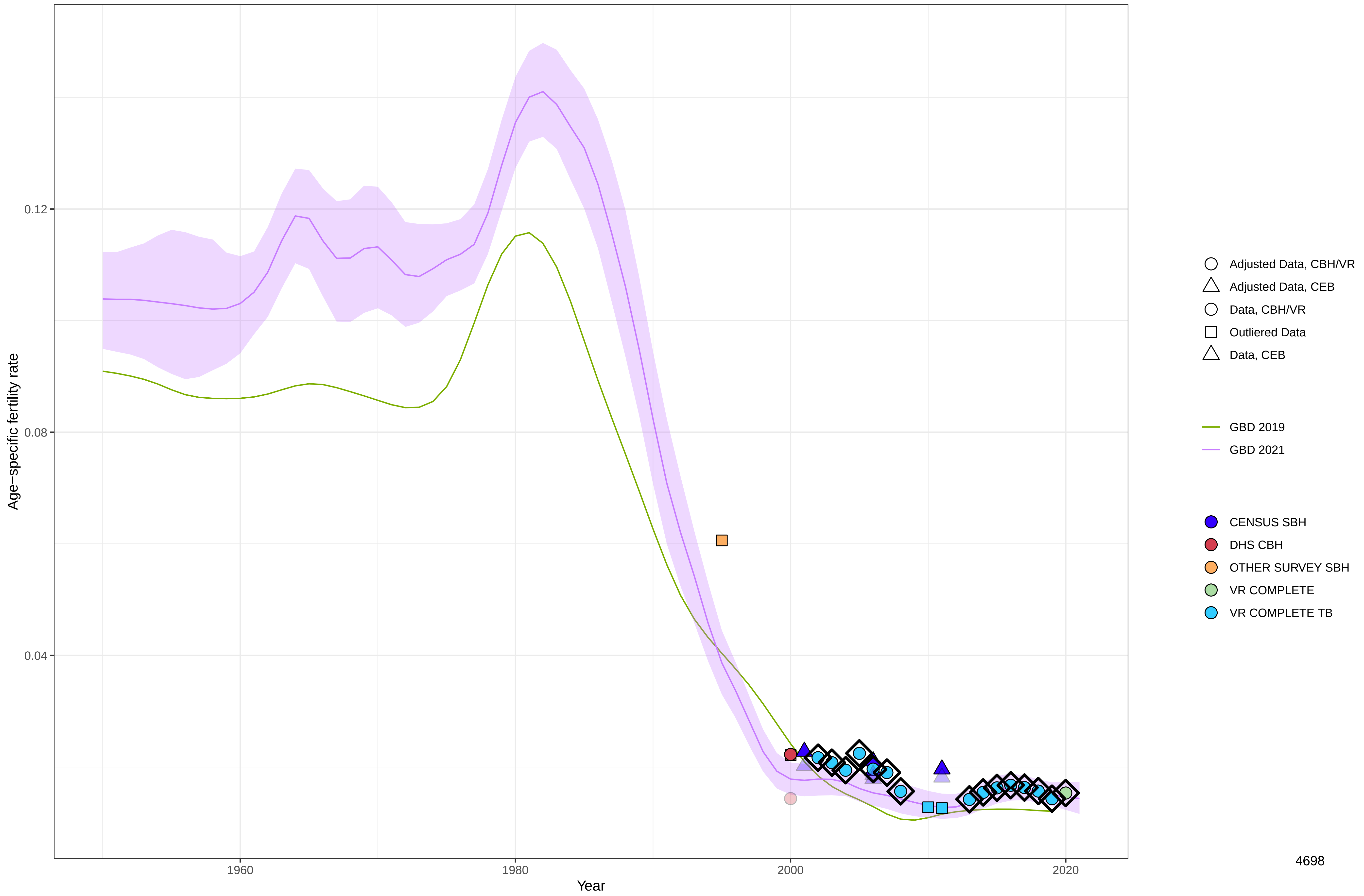

Age−specific fertility fitted trend lines, 1950−2021: Kerman, Ages: 45−49
Data points enclosed with a diamond are reference sources

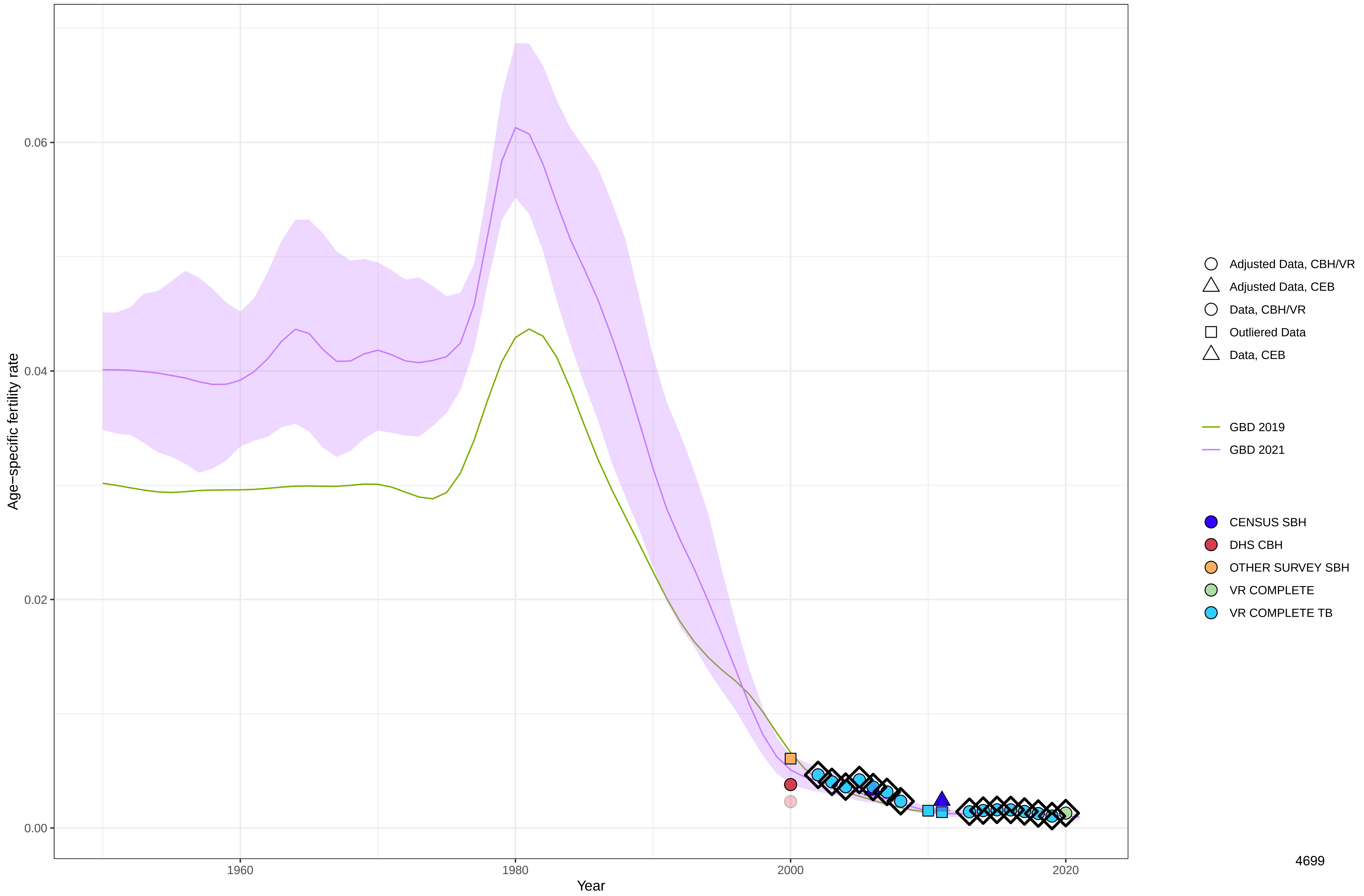

Age−specific fertility fitted trend lines, 1950−2021: Kerman, Ages: 50−54
Data points enclosed with a diamond are reference sources

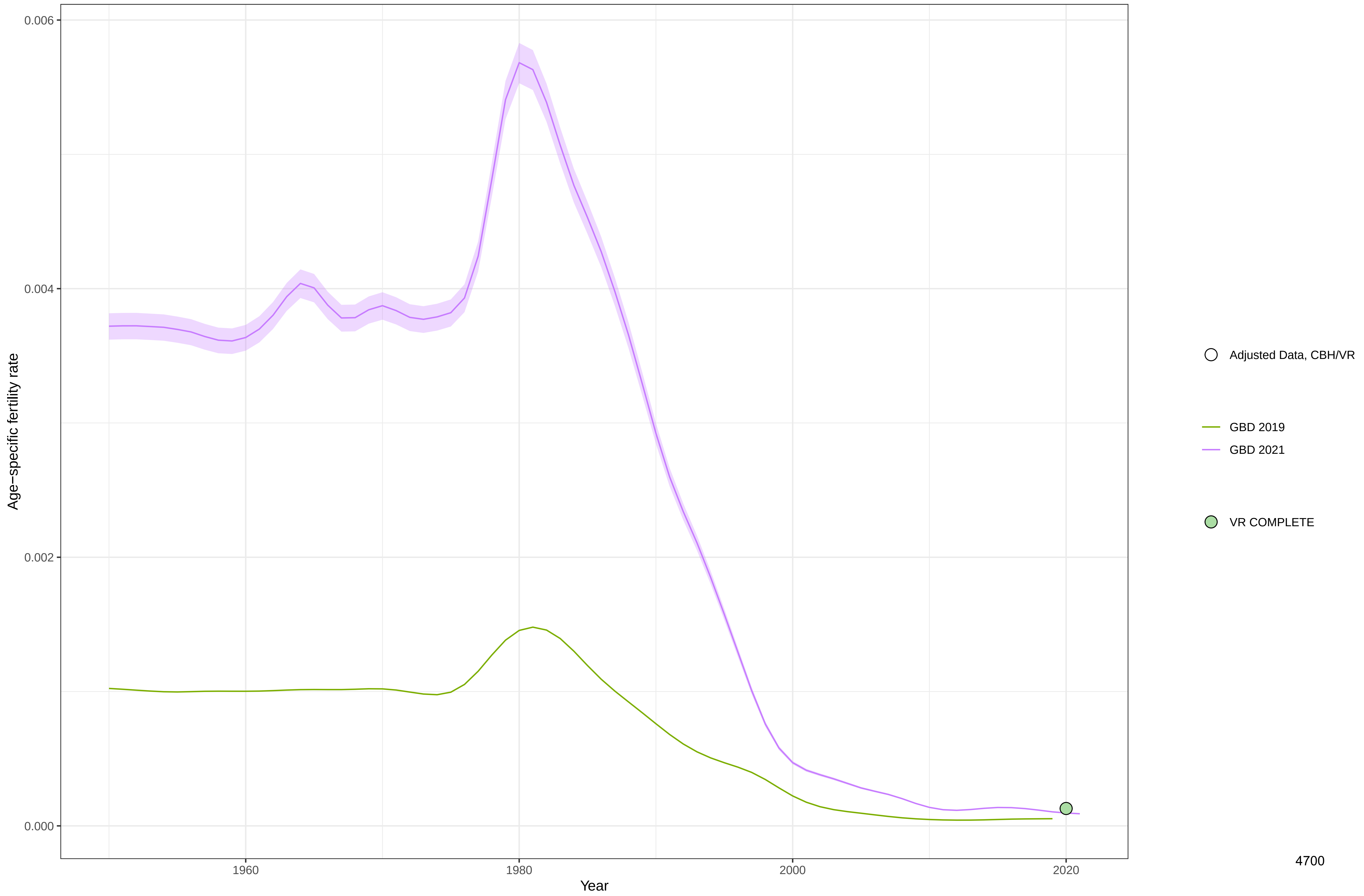

Total fertility rate fitted trend lines, 1950−2021: Kerman TFR
Data points enclosed with a diamond are reference sources

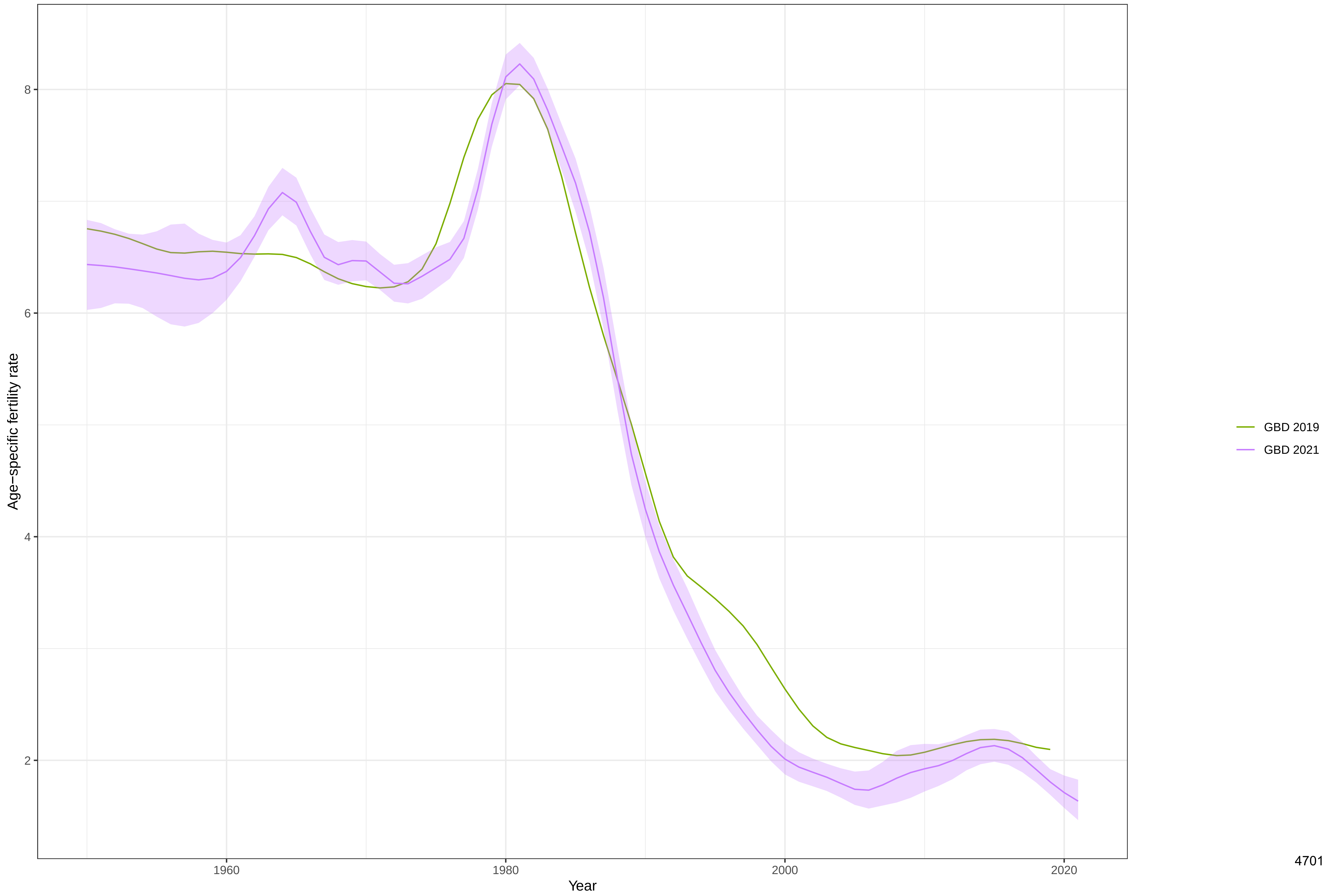

Age−specific fertility fitted trend lines, 1950−2021: Kermanshah, Ages: 10−14
Data points enclosed with a diamond are reference sources

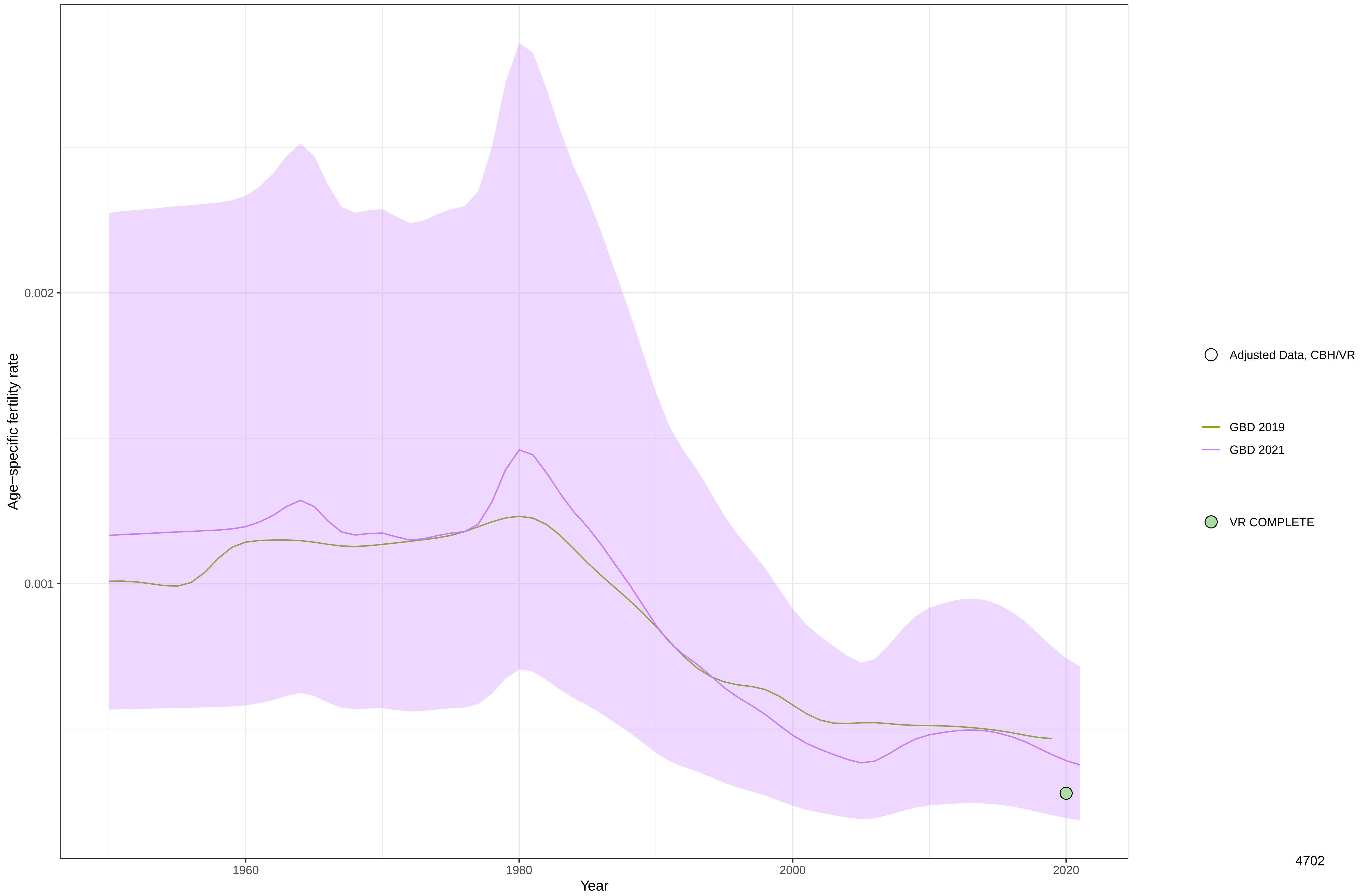

Age−specific fertility fitted trend lines, 1950−2021: Kermanshah, Ages: 15−19
Data points enclosed with a diamond are reference sources

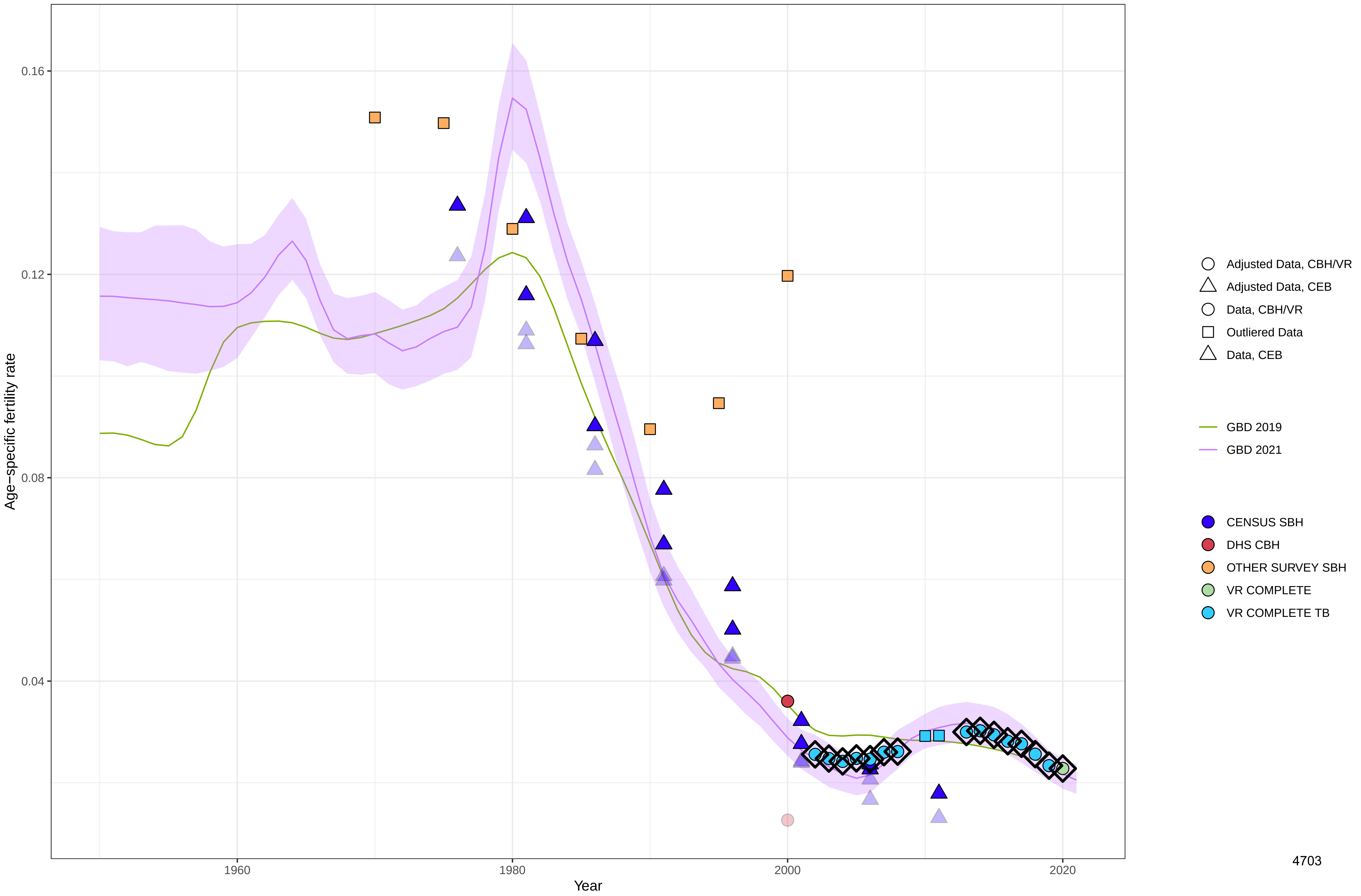

Age−specific fertility fitted trend lines, 1950−2021: Kermanshah, Ages: 20−24
Data points enclosed with a diamond are reference sources

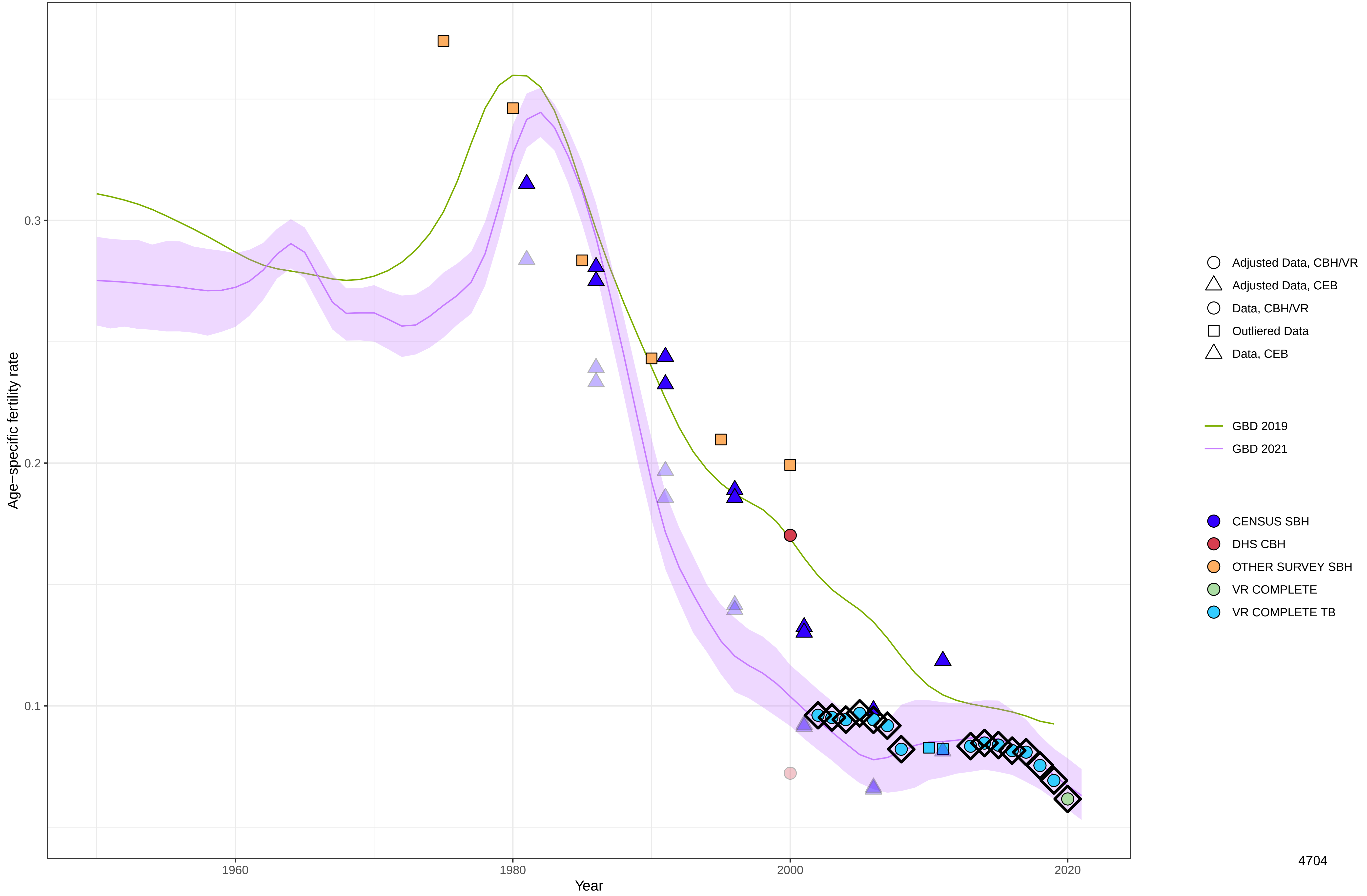

Age−specific fertility fitted trend lines, 1950−2021: Kermanshah, Ages: 25−29
Data points enclosed with a diamond are reference sources

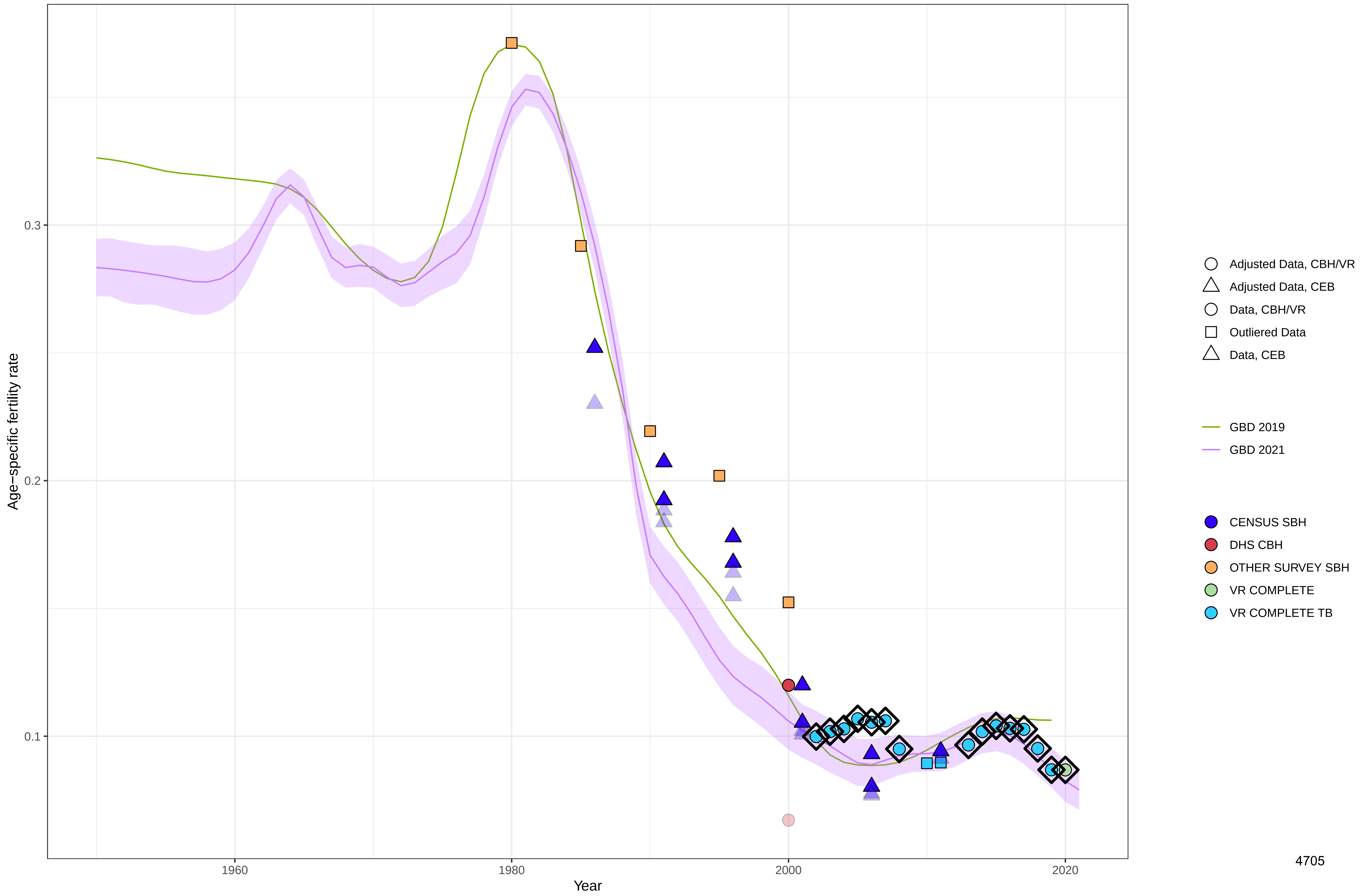

Age−specific fertility fitted trend lines, 1950−2021: Kermanshah, Ages: 30−34
Data points enclosed with a diamond are reference sources

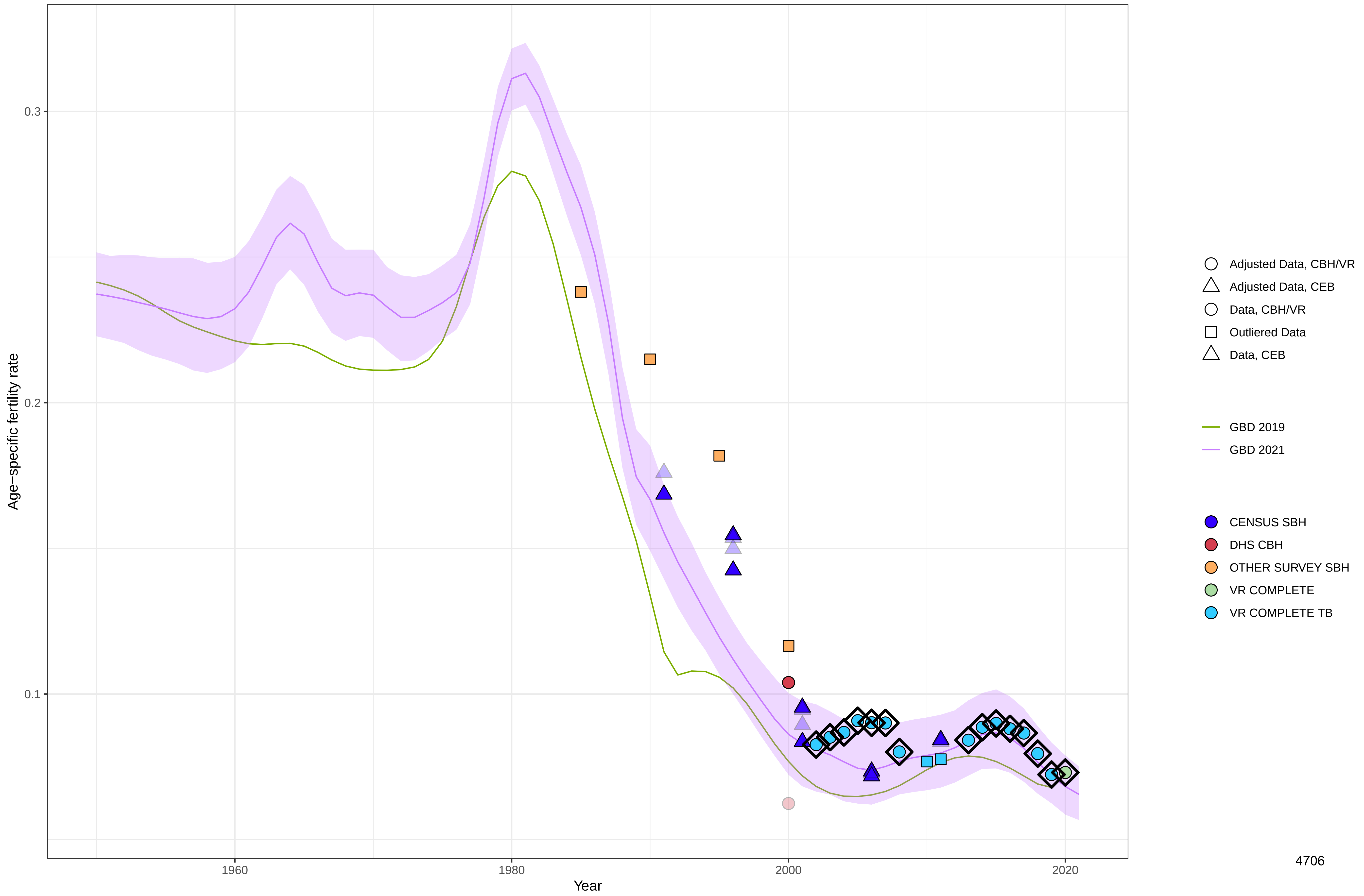

Age−specific fertility fitted trend lines, 1950−2021: Kermanshah, Ages: 35−39
Data points enclosed with a diamond are reference sources

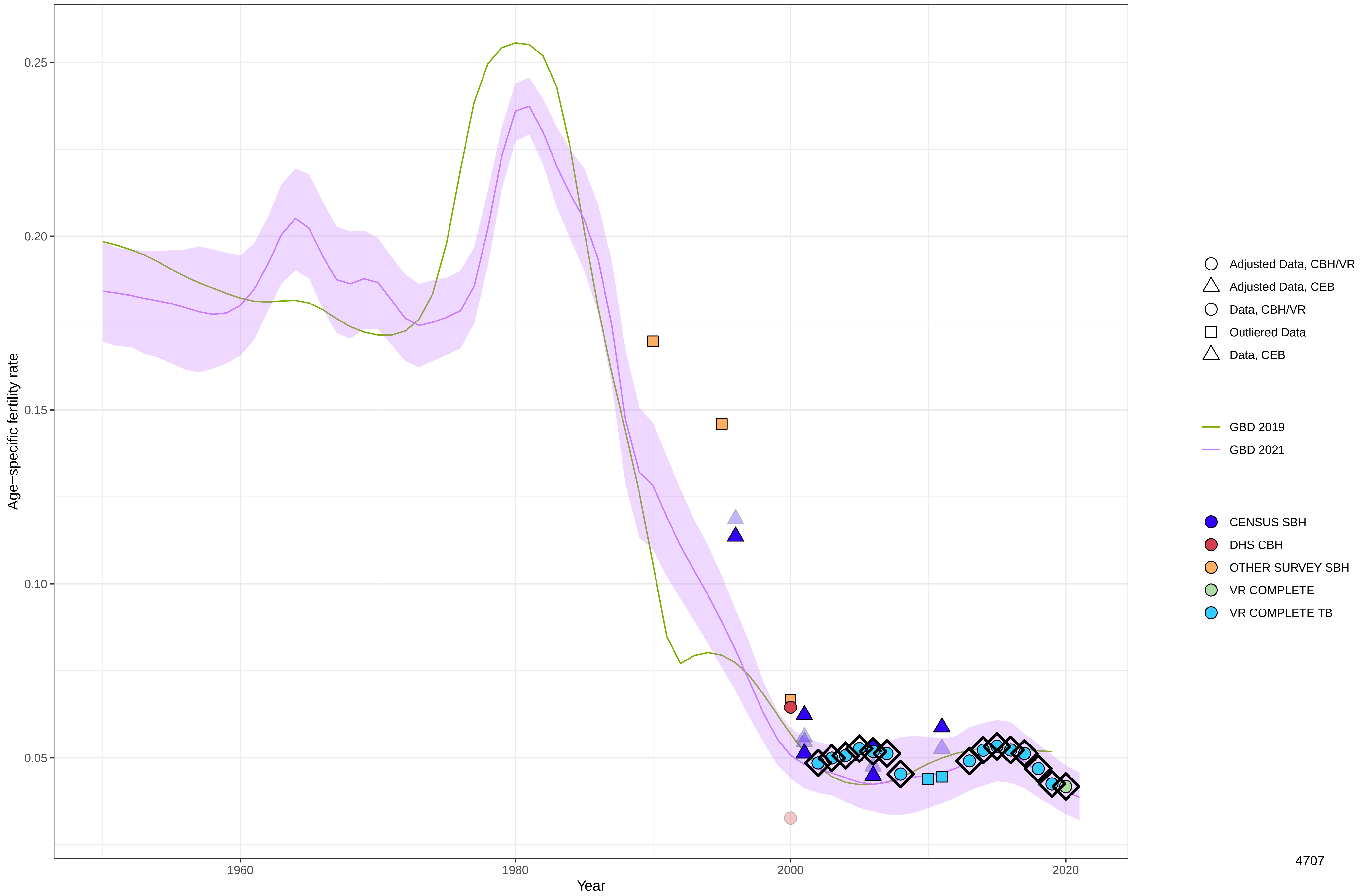

Age−specific fertility fitted trend lines, 1950−2021: Kermanshah, Ages: 40−44
Data points enclosed with a diamond are reference sources

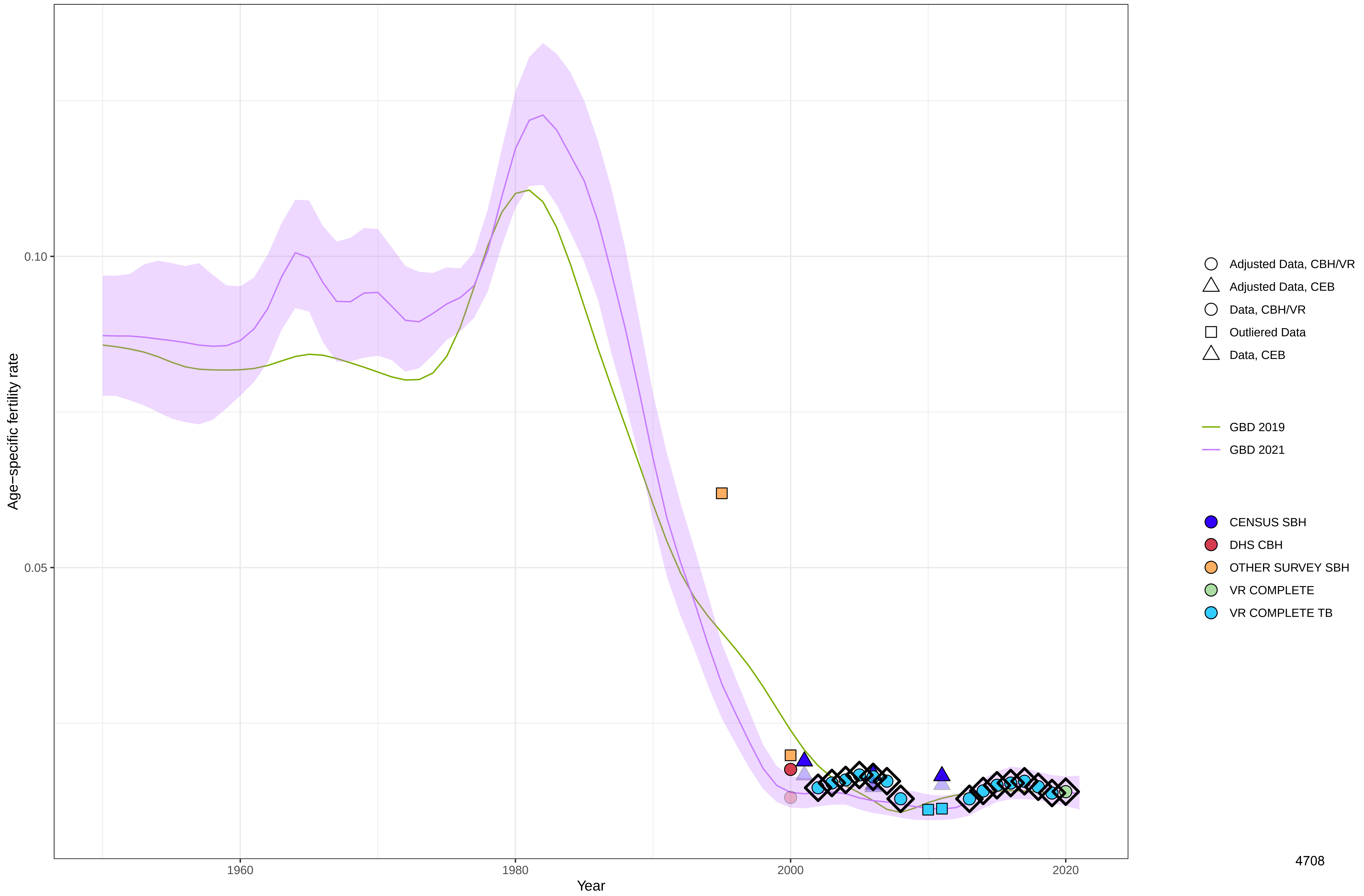

Age−specific fertility fitted trend lines, 1950−2021: Kermanshah, Ages: 45−49
Data points enclosed with a diamond are reference sources

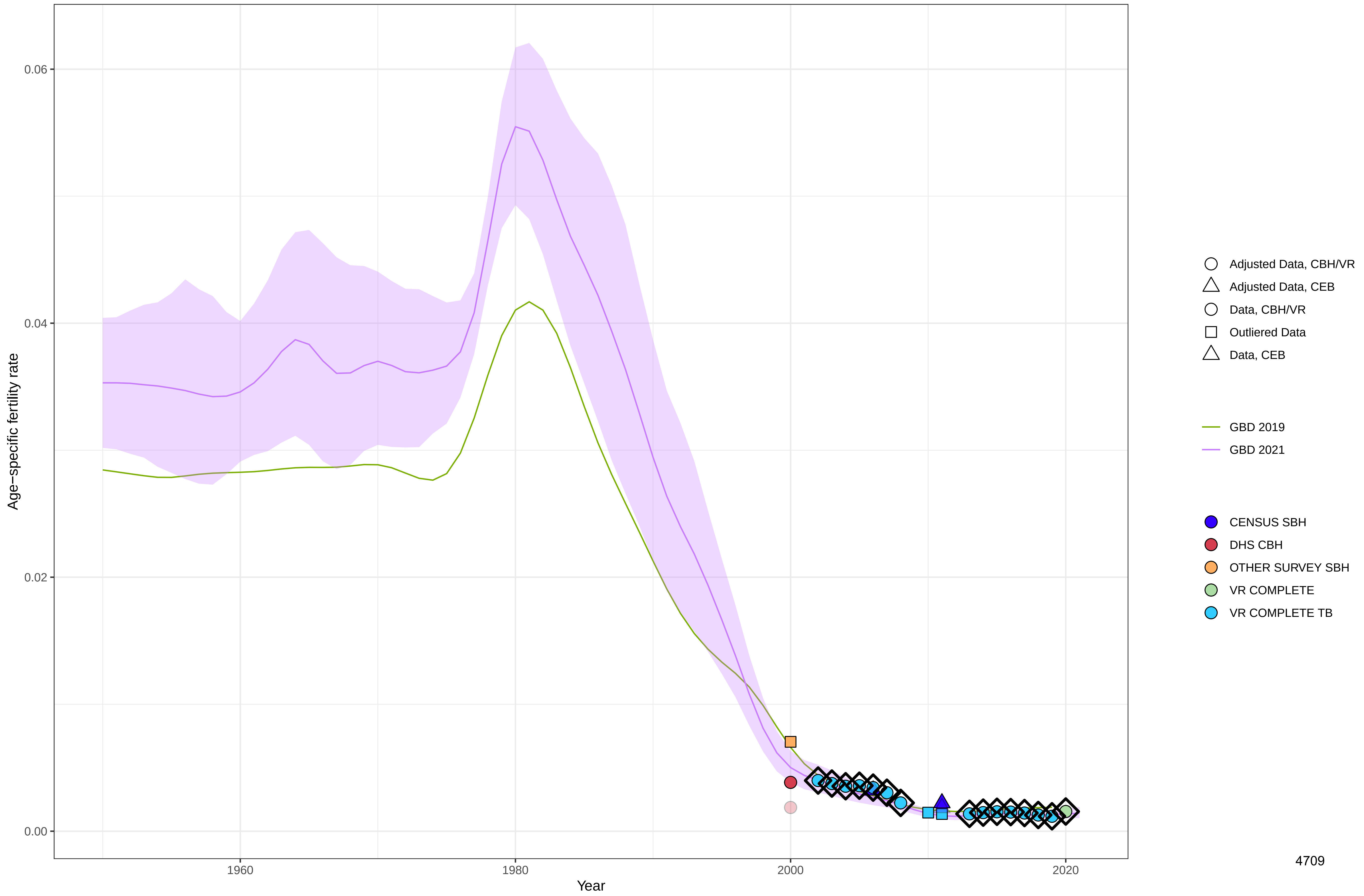

Age−specific fertility fitted trend lines, 1950−2021: Kermanshah, Ages: 50−54
Data points enclosed with a diamond are reference sources

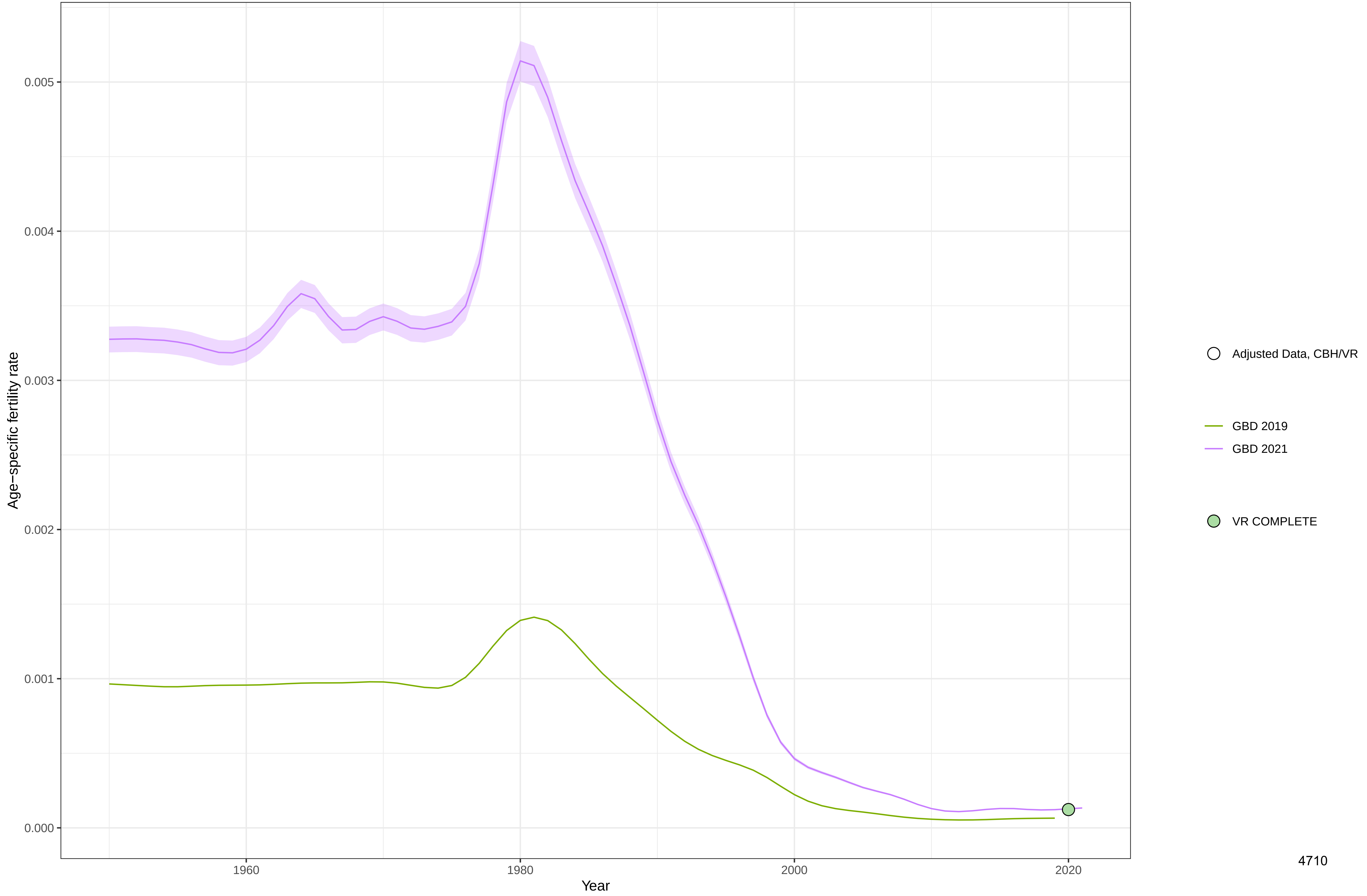

Total fertility rate fitted trend lines, 1950−2021: Kermanshah TFR
Data points enclosed with a diamond are reference sources

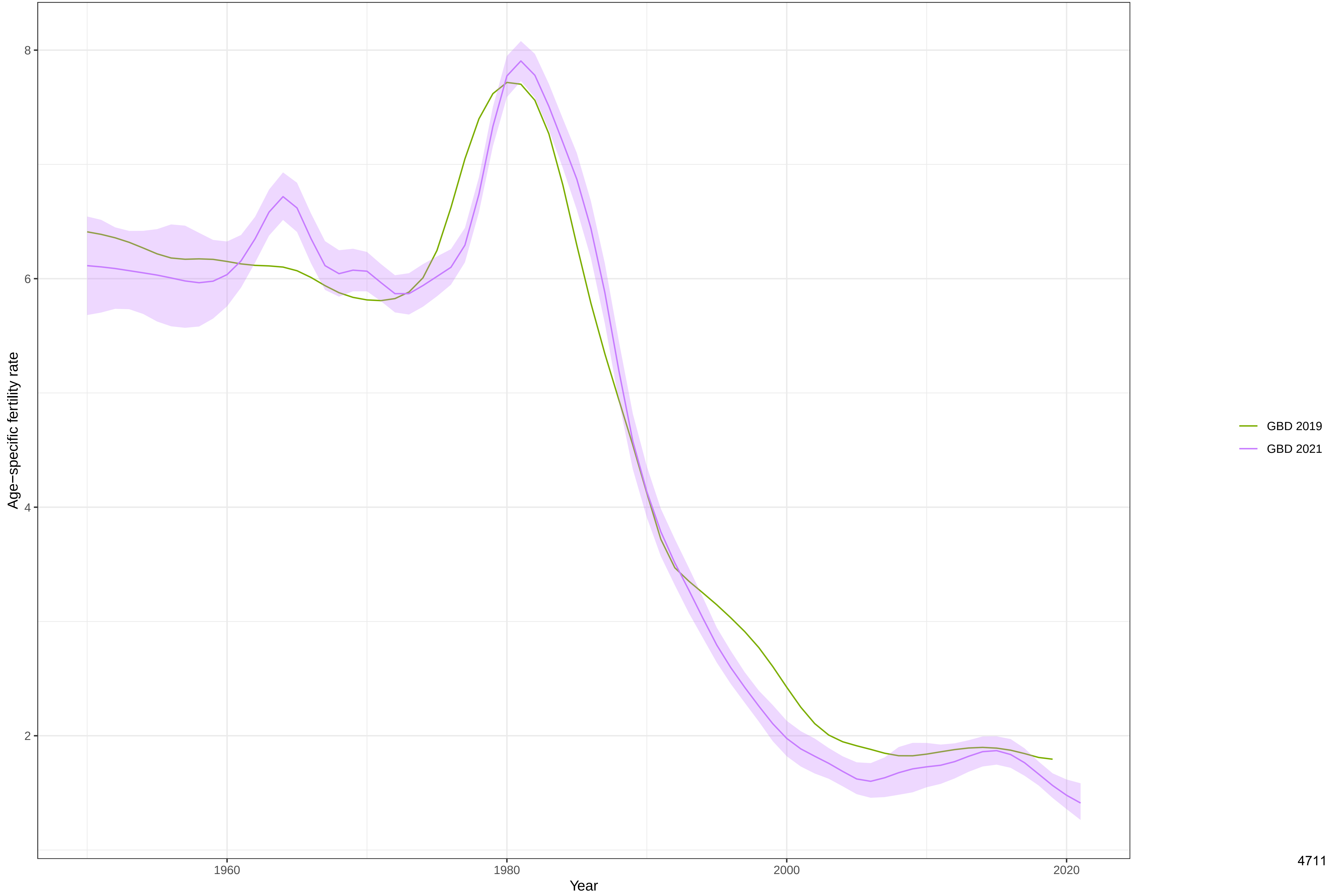

Age−specific fertility fitted trend lines, 1950−2021: Khorasan−e−Razavi, Ages: 10−14
Data points enclosed with a diamond are reference sources

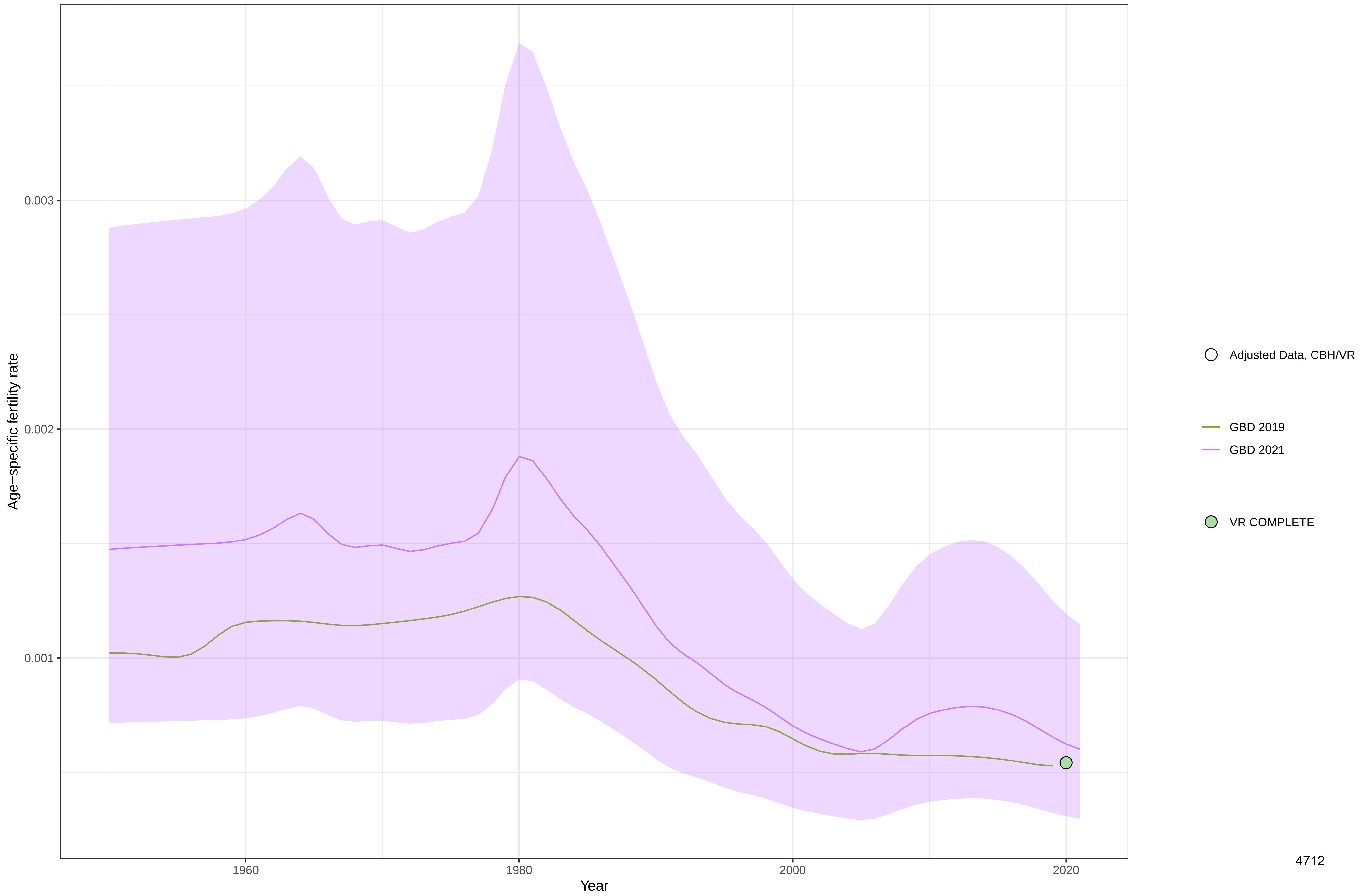

Age−specific fertility fitted trend lines, 1950−2021: Khorasan−e−Razavi, Ages: 15−19
Data points enclosed with a diamond are reference sources

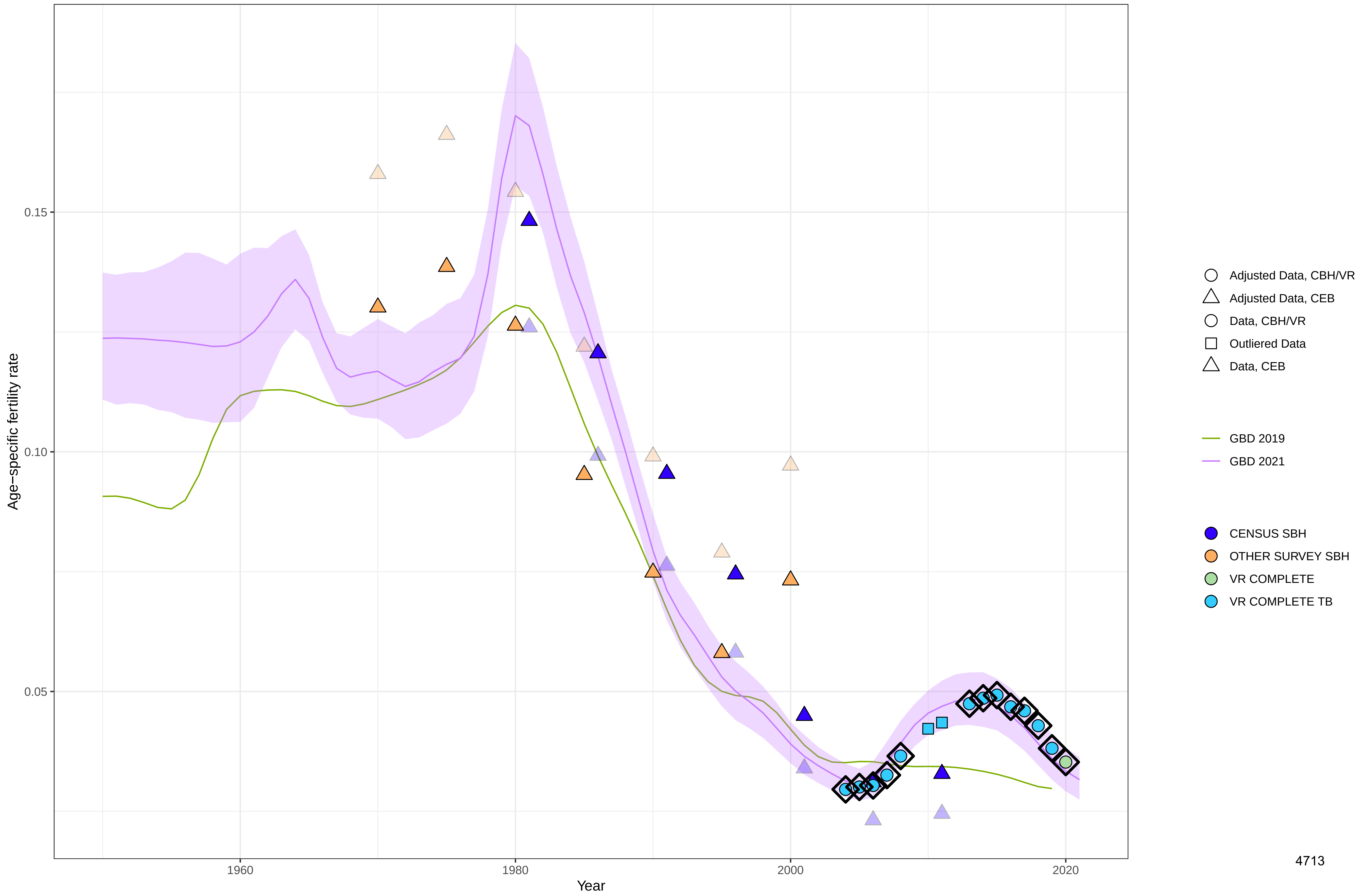

Age−specific fertility fitted trend lines, 1950−2021: Khorasan−e−Razavi, Ages: 20−24
 Data points enclosed with a diamond are reference sources

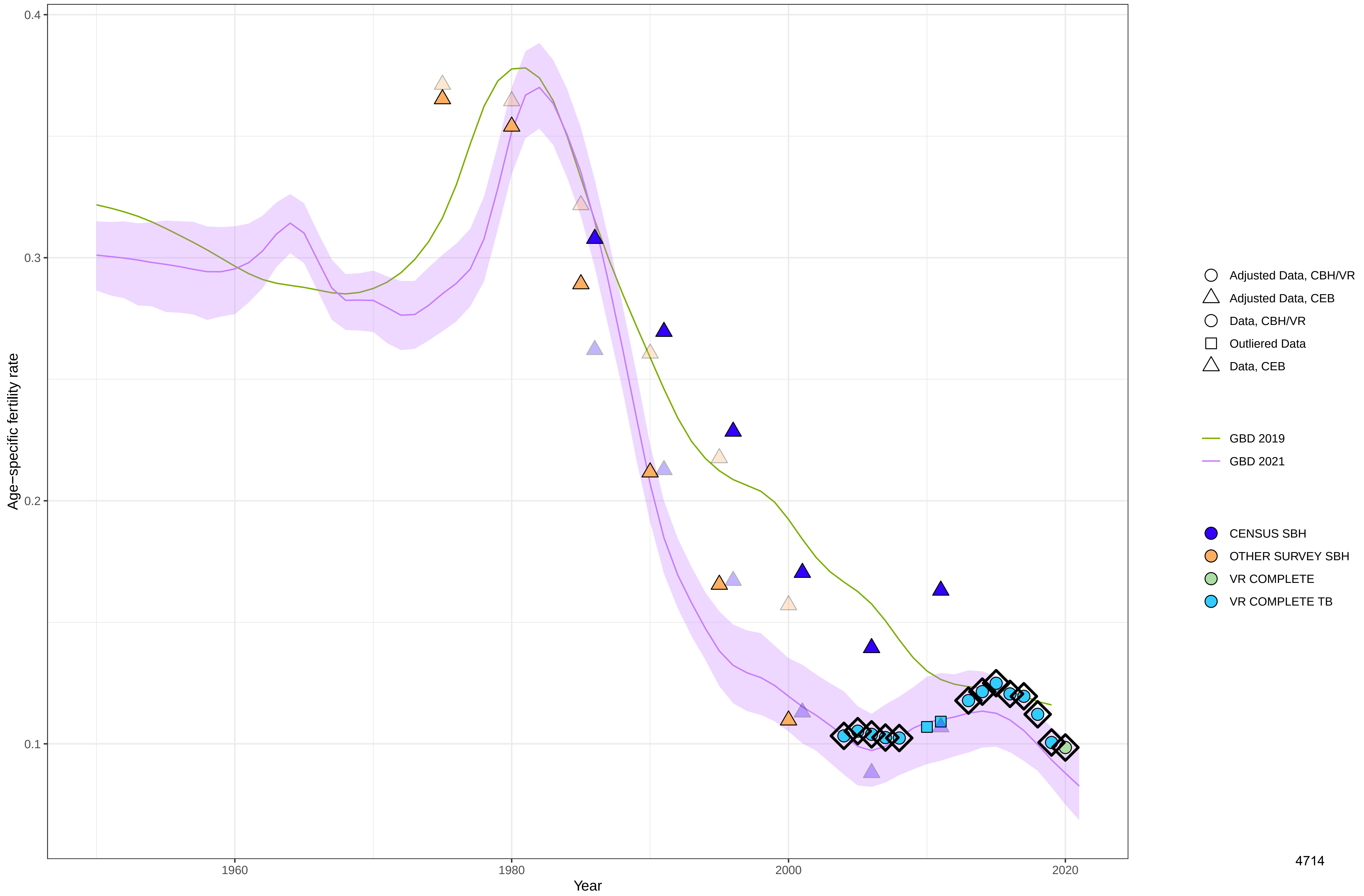

Age−specific fertility fitted trend lines, 1950−2021: Khorasan−e−Razavi, Ages: 25−29
Data points enclosed with a diamond are reference sources

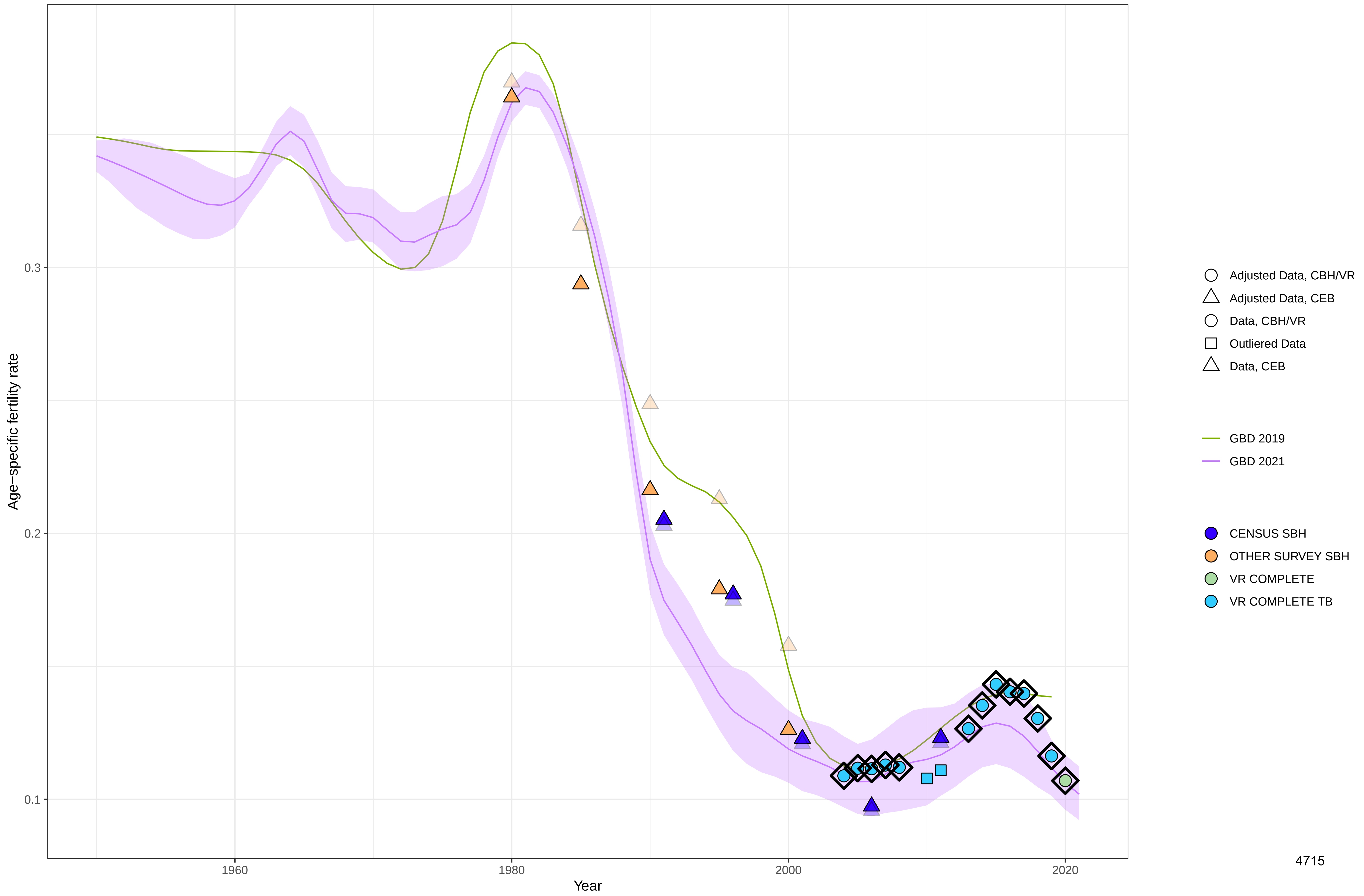

Age−specific fertility fitted trend lines, 1950−2021: Khorasan−e−Razavi, Ages: 30−34
Data points enclosed with a diamond are reference sources

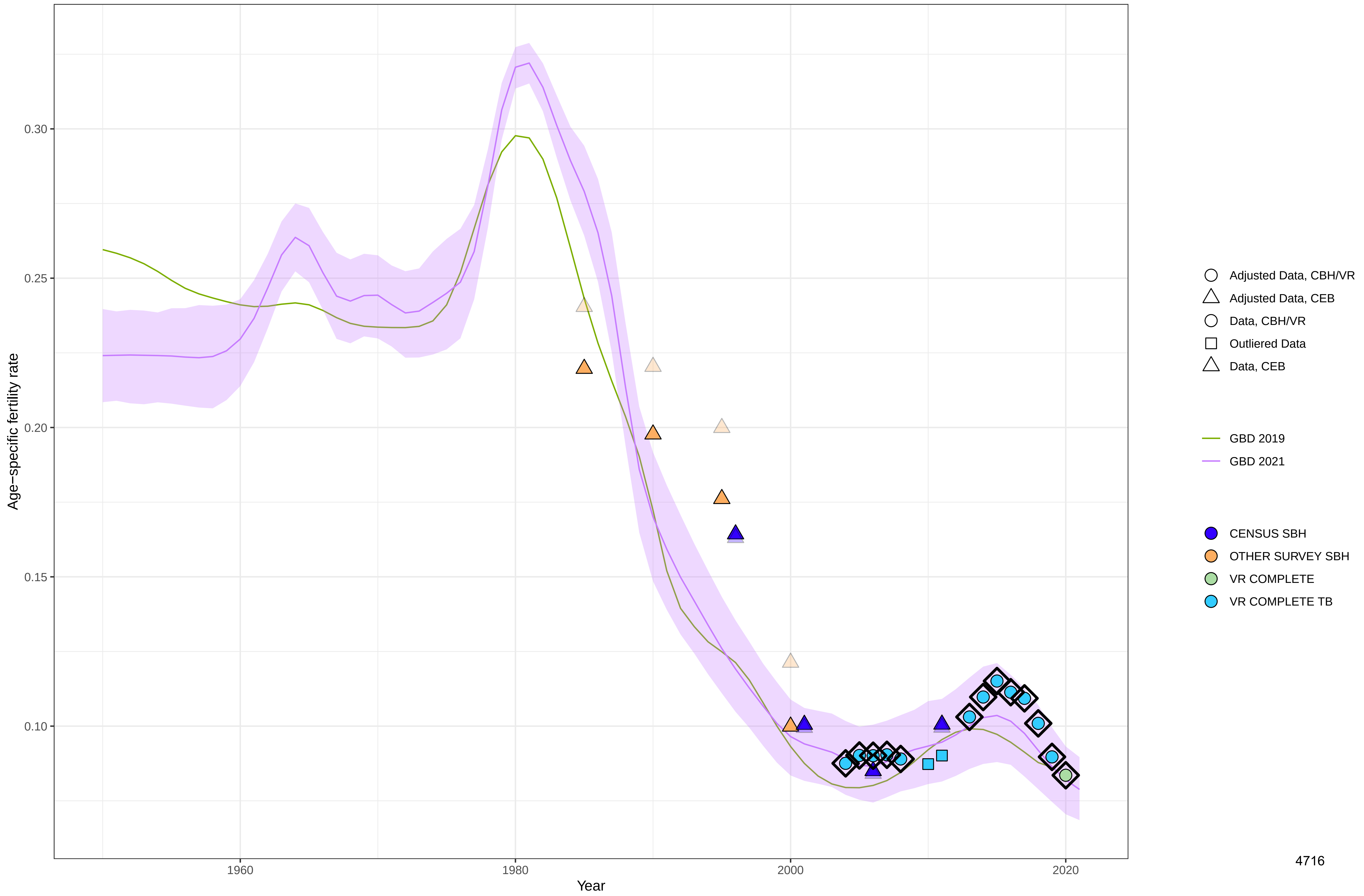

Age−specific fertility fitted trend lines, 1950−2021: Khorasan−e−Razavi, Ages: 35−39
Data points enclosed with a diamond are reference sources

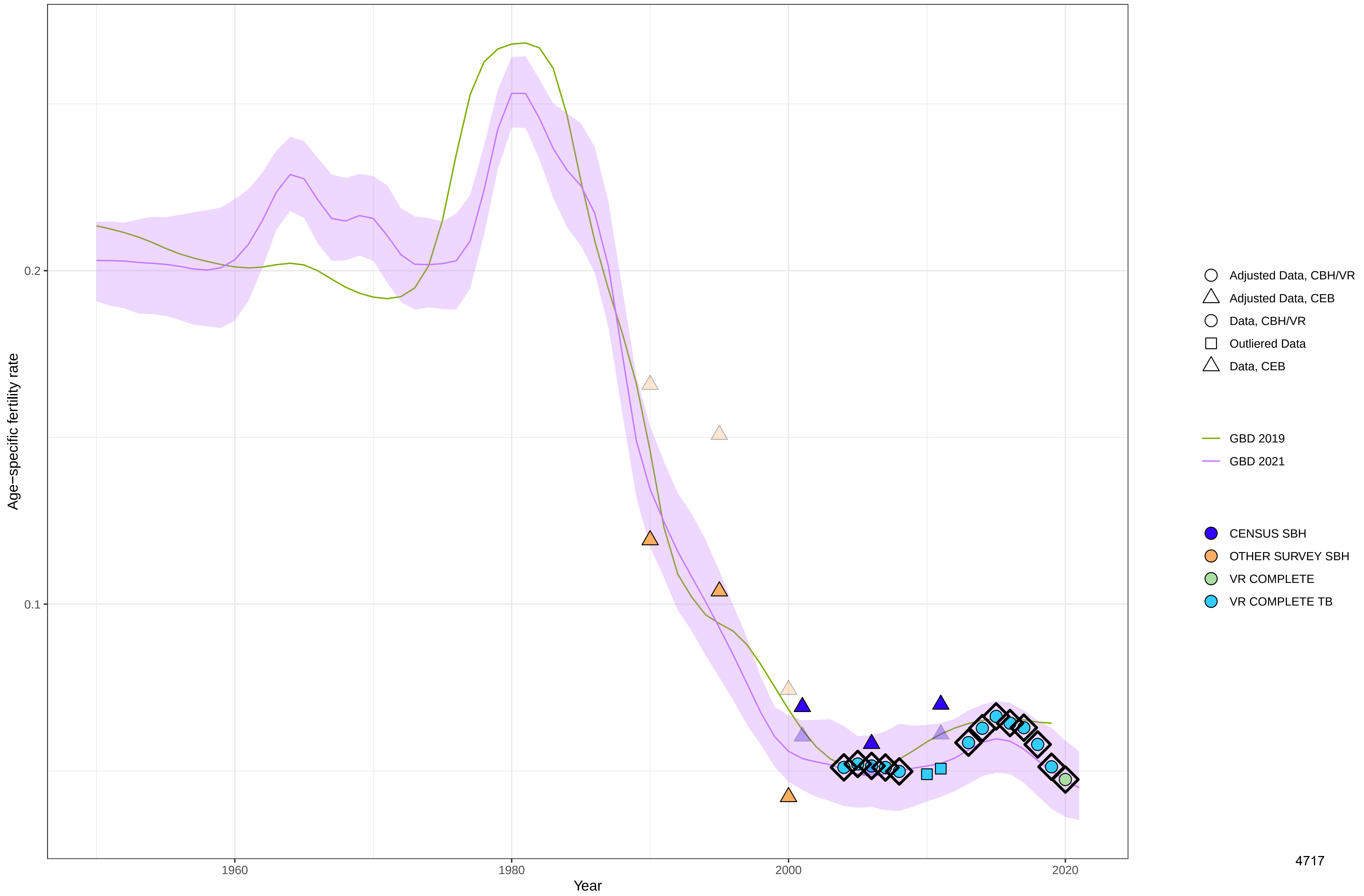

Age−specific fertility fitted trend lines, 1950−2021: Khorasan−e−Razavi, Ages: 40−44
Data points enclosed with a diamond are reference sources

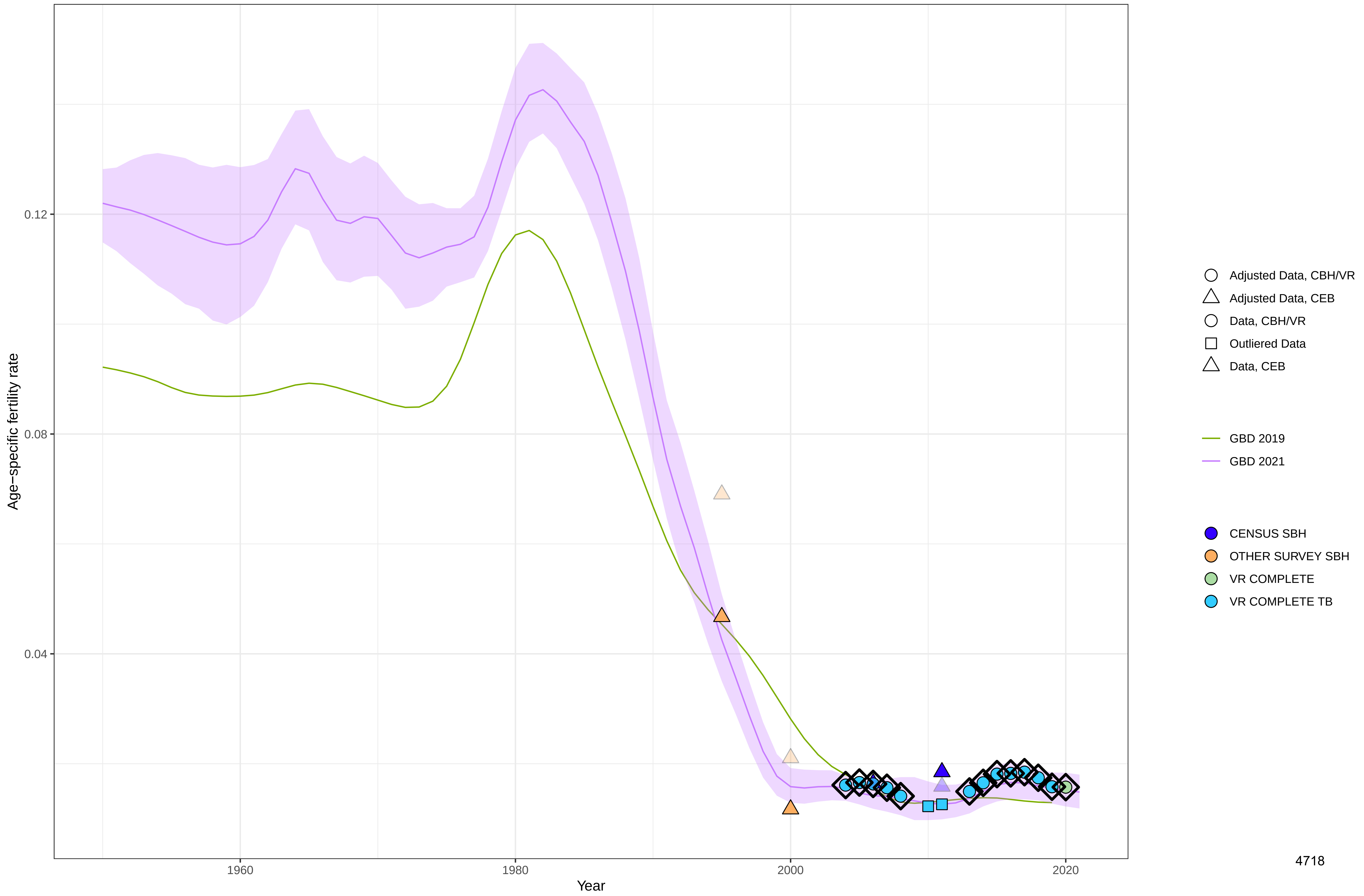

Age−specific fertility fitted trend lines, 1950−2021: Khorasan−e−Razavi, Ages: 45−49
Data points enclosed with a diamond are reference sources

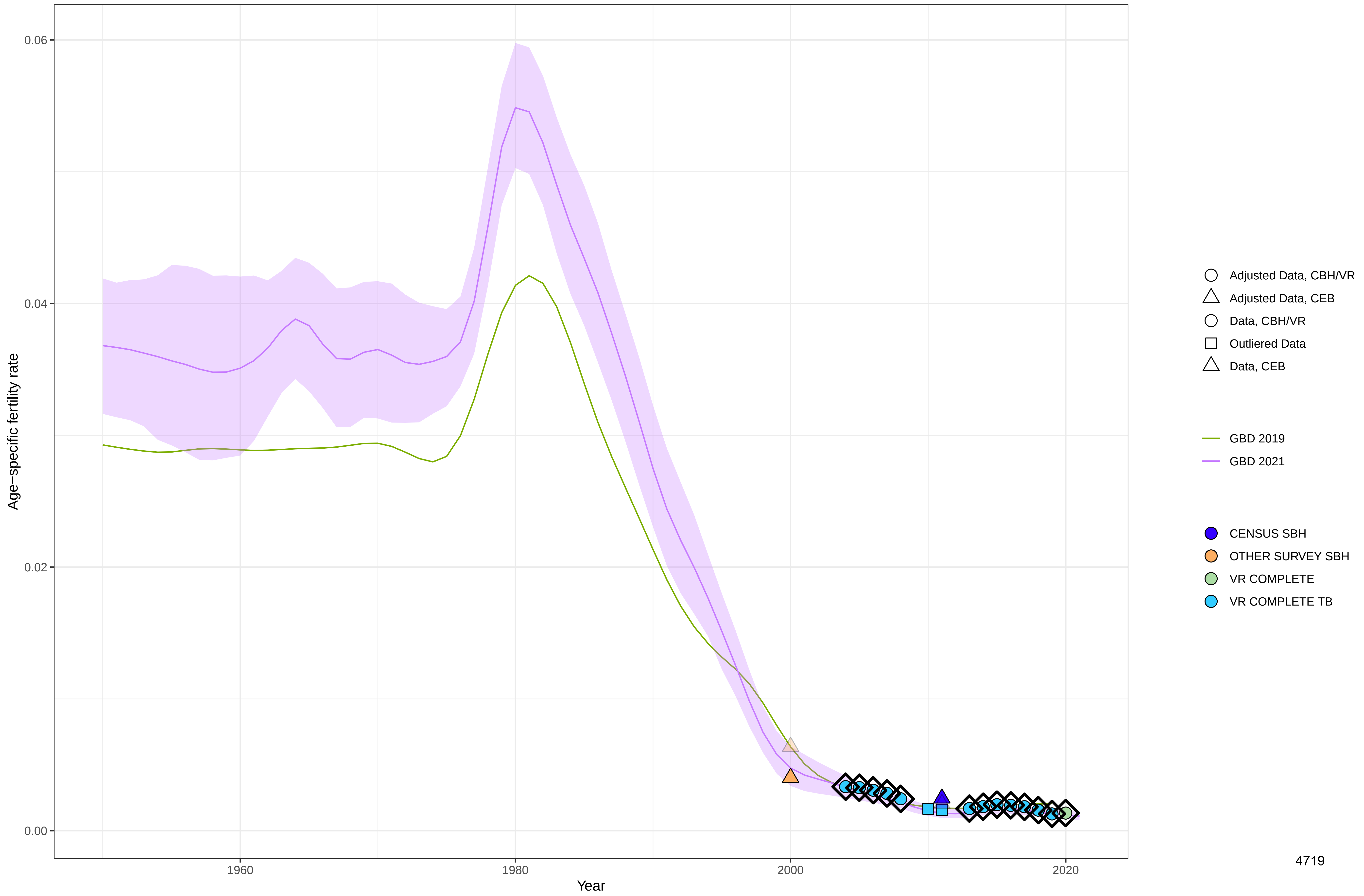

Age−specific fertility fitted trend lines, 1950−2021: Khorasan−e−Razavi, Ages: 50−54
Data points enclosed with a diamond are reference sources

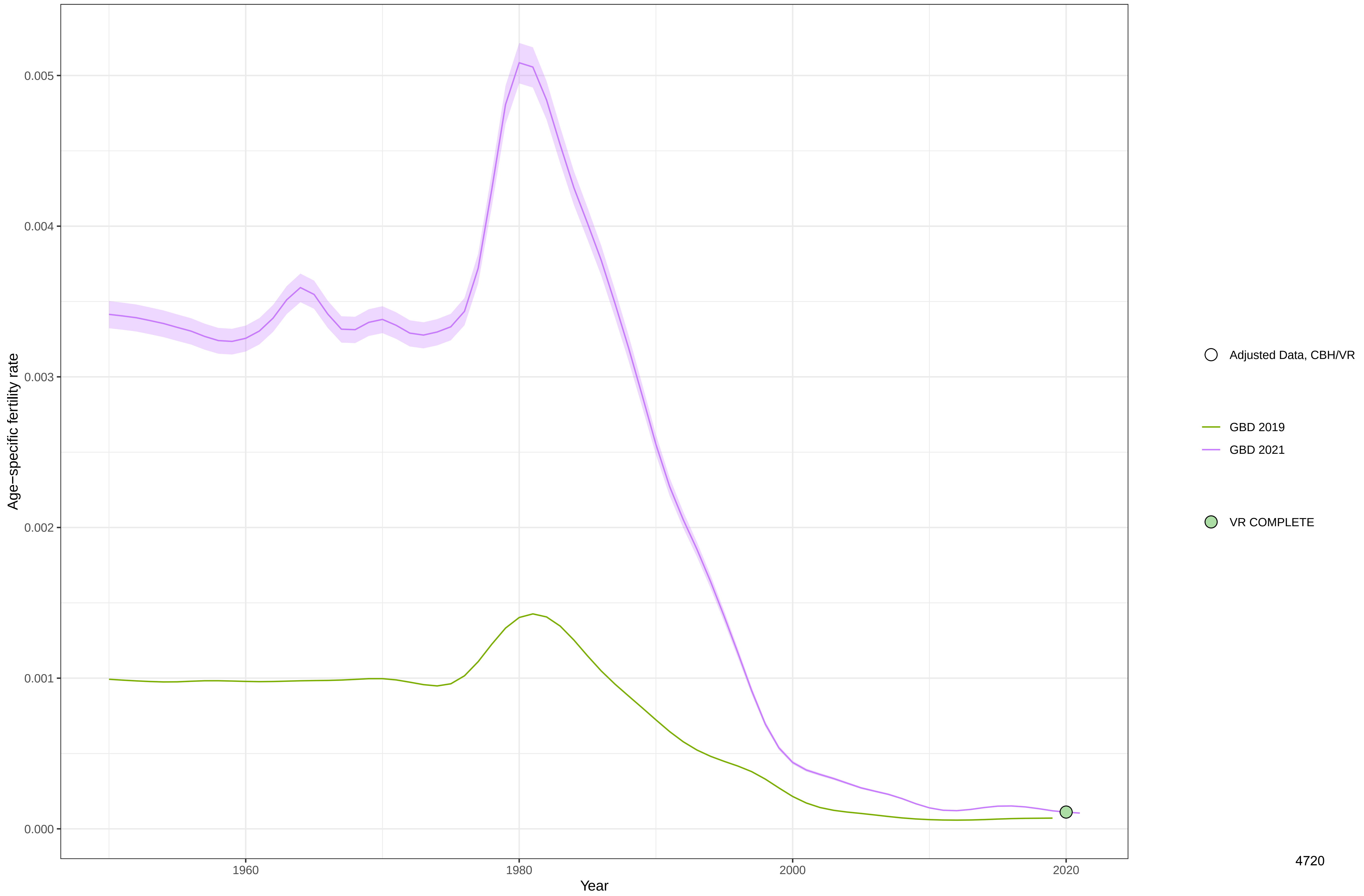

Total fertility rate fitted trend lines, 1950−2021: Khorasan−e−Razavi TFR
Data points enclosed with a diamond are reference sources

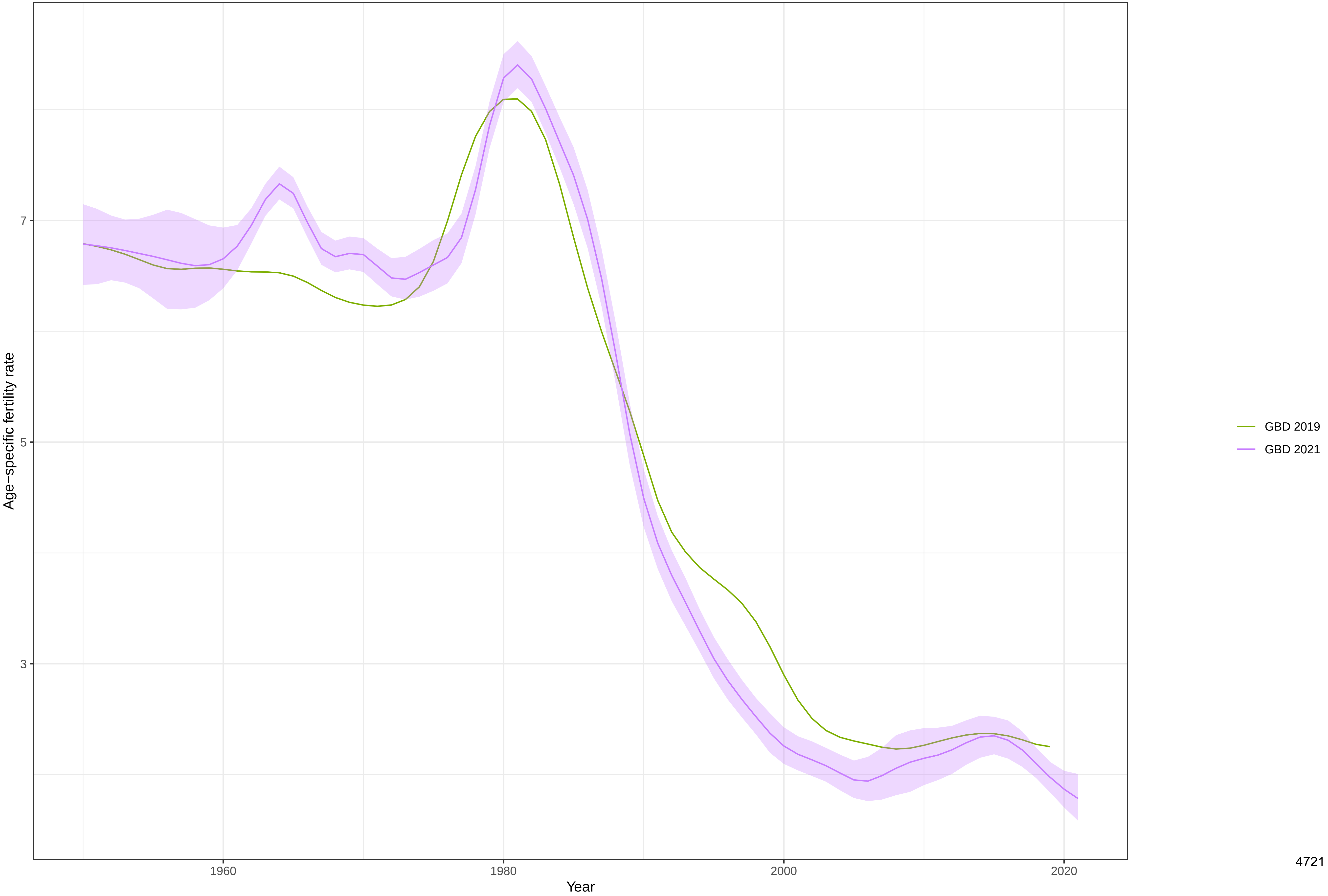

4721

Age−specific fertility fitted trend lines, 1950−2021: Khuzestan, Ages: 10−14
Data points enclosed with a diamond are reference sources

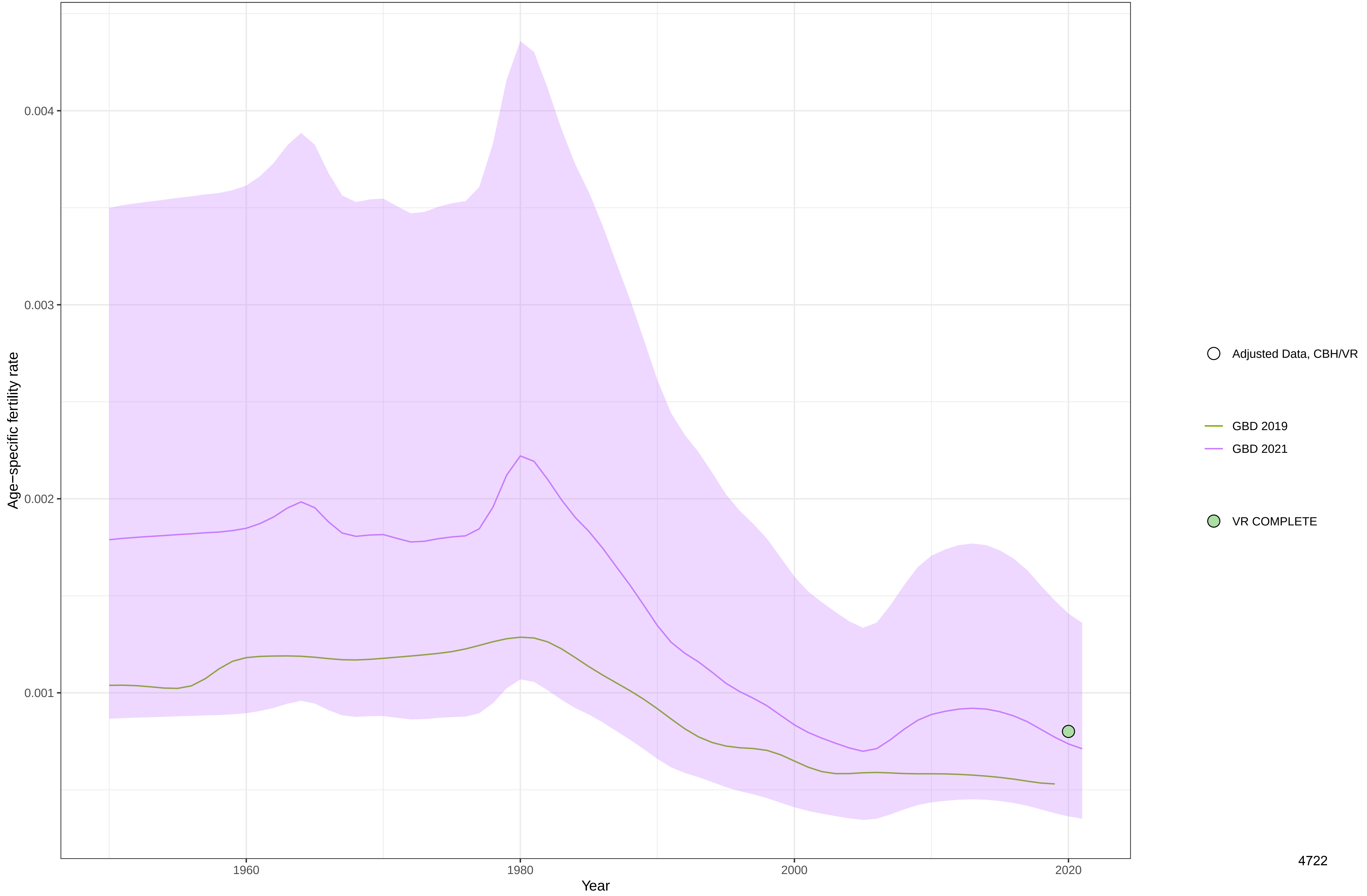

4722

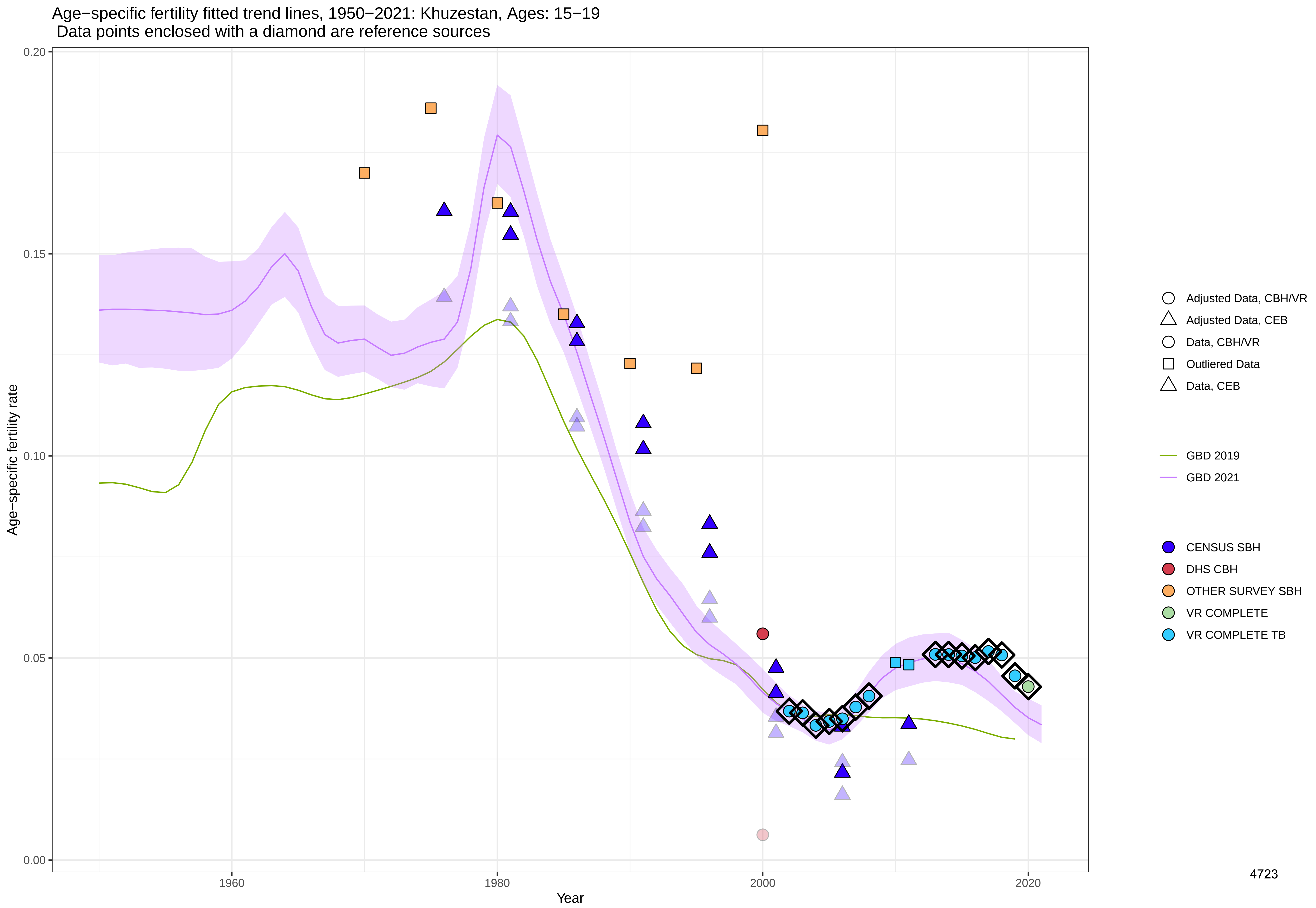
Age−specific fertility fitted trend lines, 1950−2021: Khuzestan, Ages: 15−19
Data points enclosed with a diamond are reference sources
0.20
0.15
0.10
0.05
0.00
Age−specific fertility rate
1960
1980
2000
2020
Year
Adjusted Data, CBH/VR
Adjusted Data, CEB
Data, CBH/VR
Outliered Data
Data, CEB
GBD 2019
GBD 2021
CENSUS SBH
DHS CBH
OTHER SURVEY SBH
VR COMPLETE
VR COMPLETE TB

Age−specific fertility fitted trend lines, 1950−2021: Khuzestan, Ages: 20−24
Data points enclosed with a diamond are reference sources

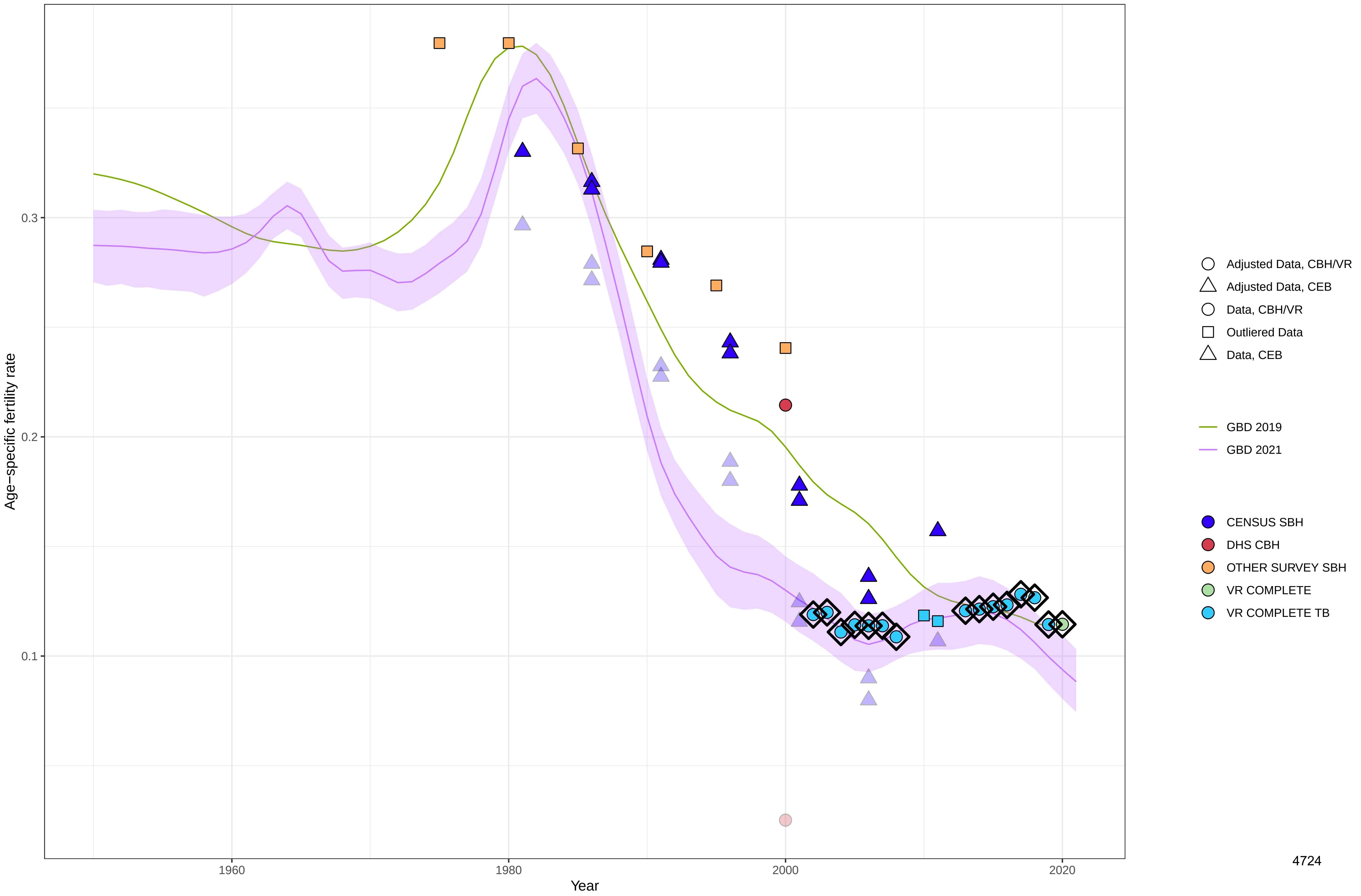

Age−specific fertility fitted trend lines, 1950−2021: Khuzestan, Ages: 25−29
Data points enclosed with a diamond are reference sources

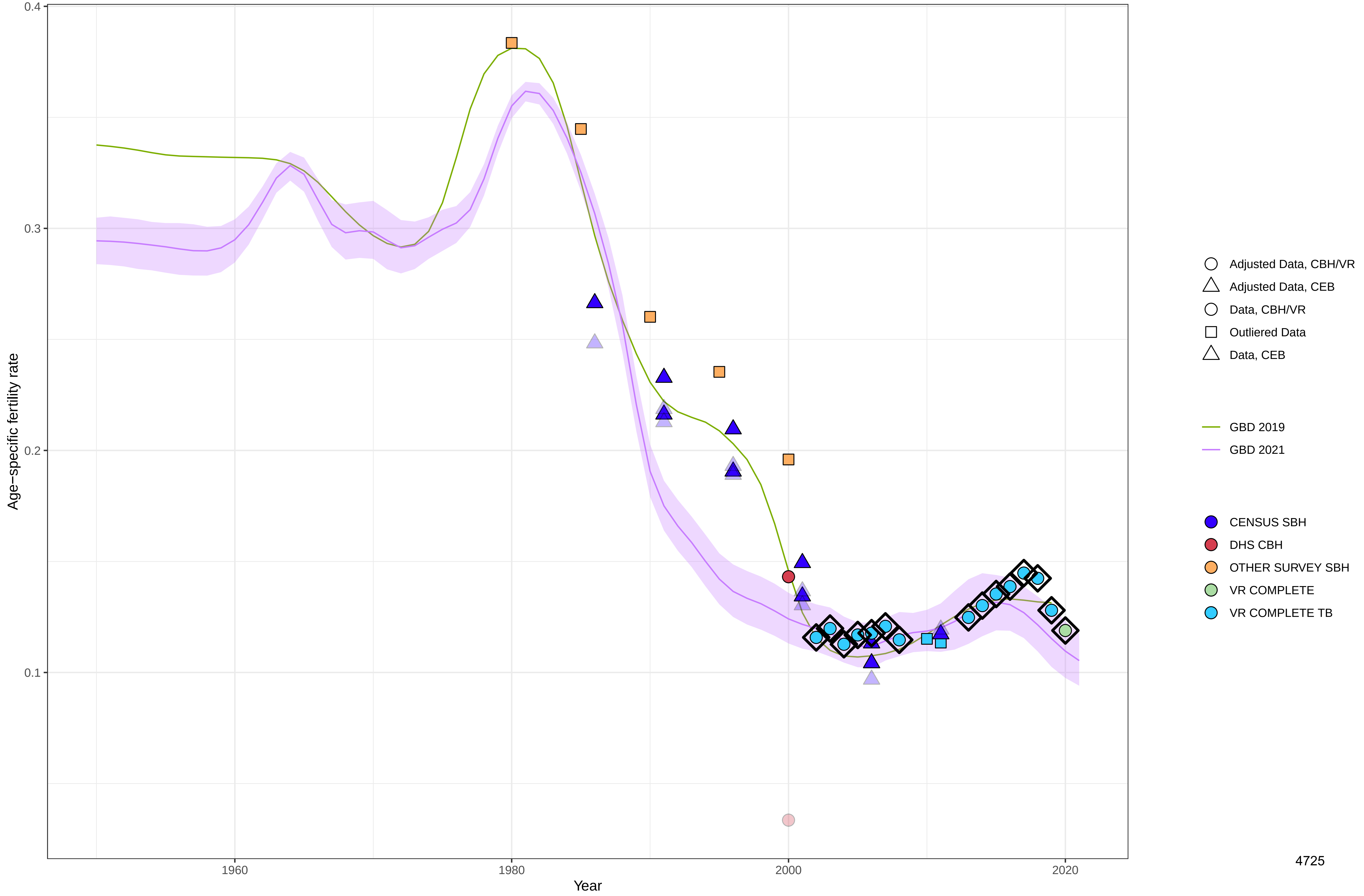

Age−specific fertility fitted trend lines, 1950−2021: Khuzestan, Ages: 30−34
Data points enclosed with a diamond are reference sources

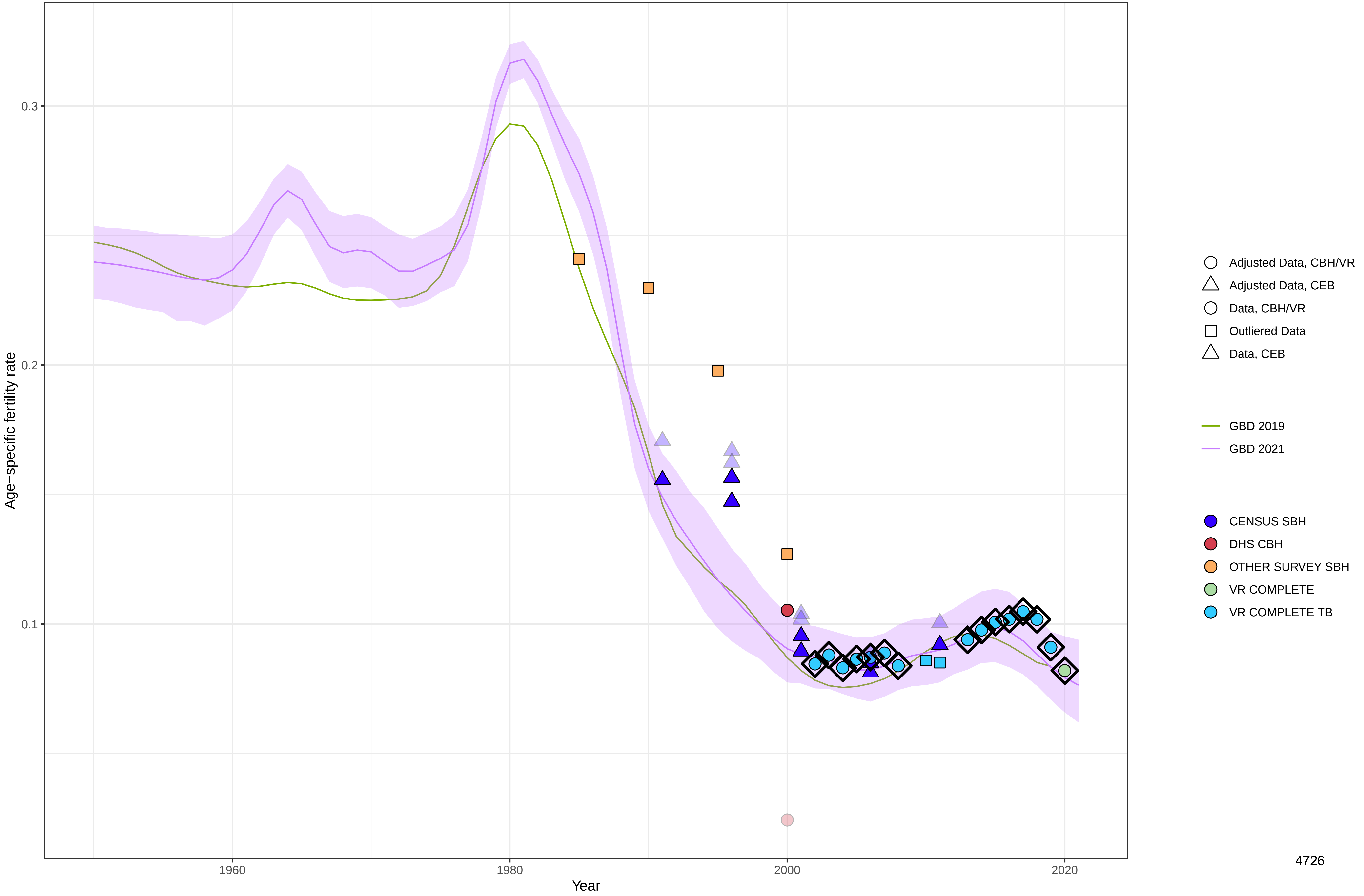

4726

Age−specific fertility fitted trend lines, 1950−2021: Khuzestan, Ages: 35−39
Data points enclosed with a diamond are reference sources

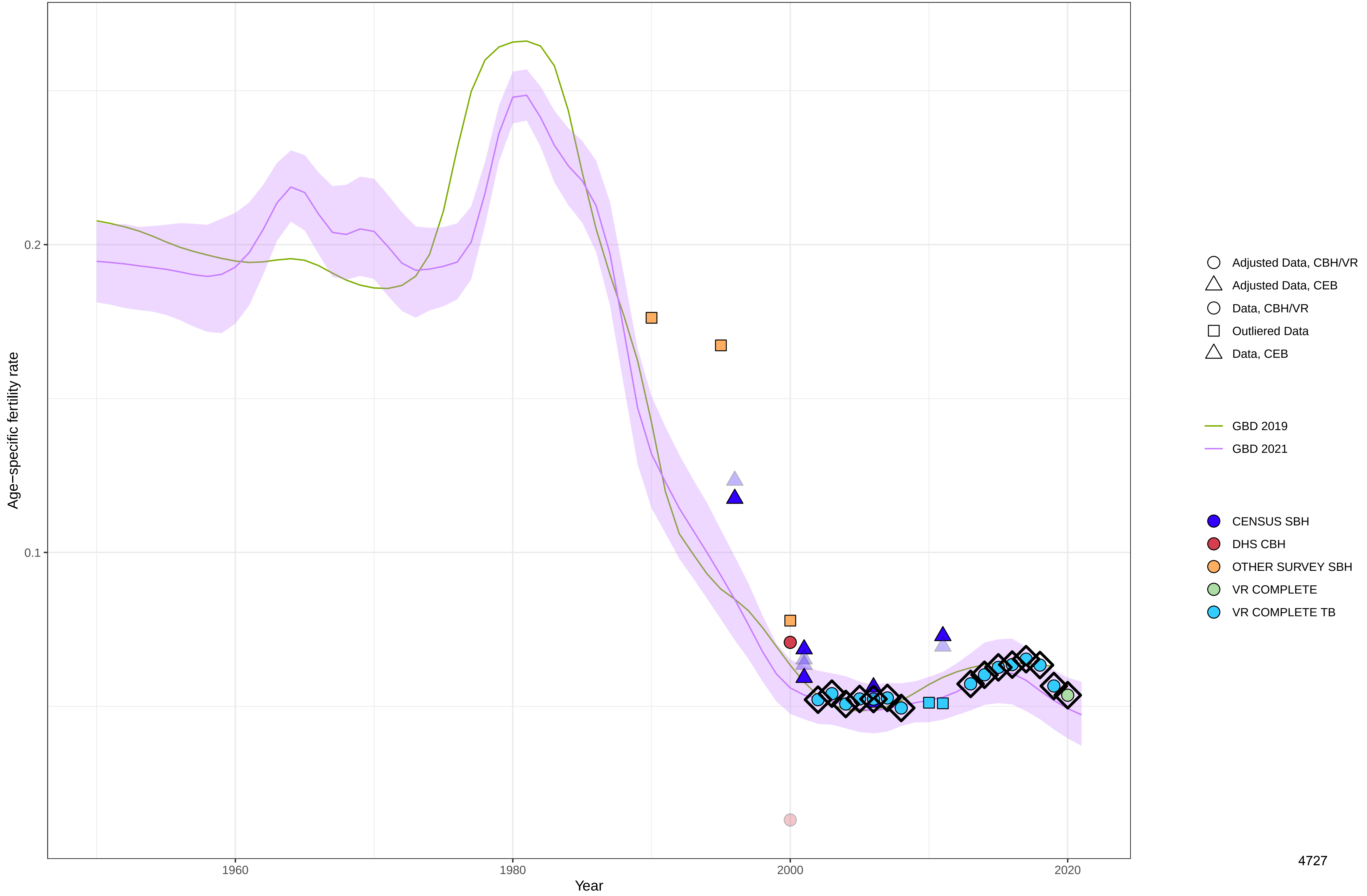

Age−specific fertility fitted trend lines, 1950−2021: Khuzestan, Ages: 40−44
Data points enclosed with a diamond are reference sources

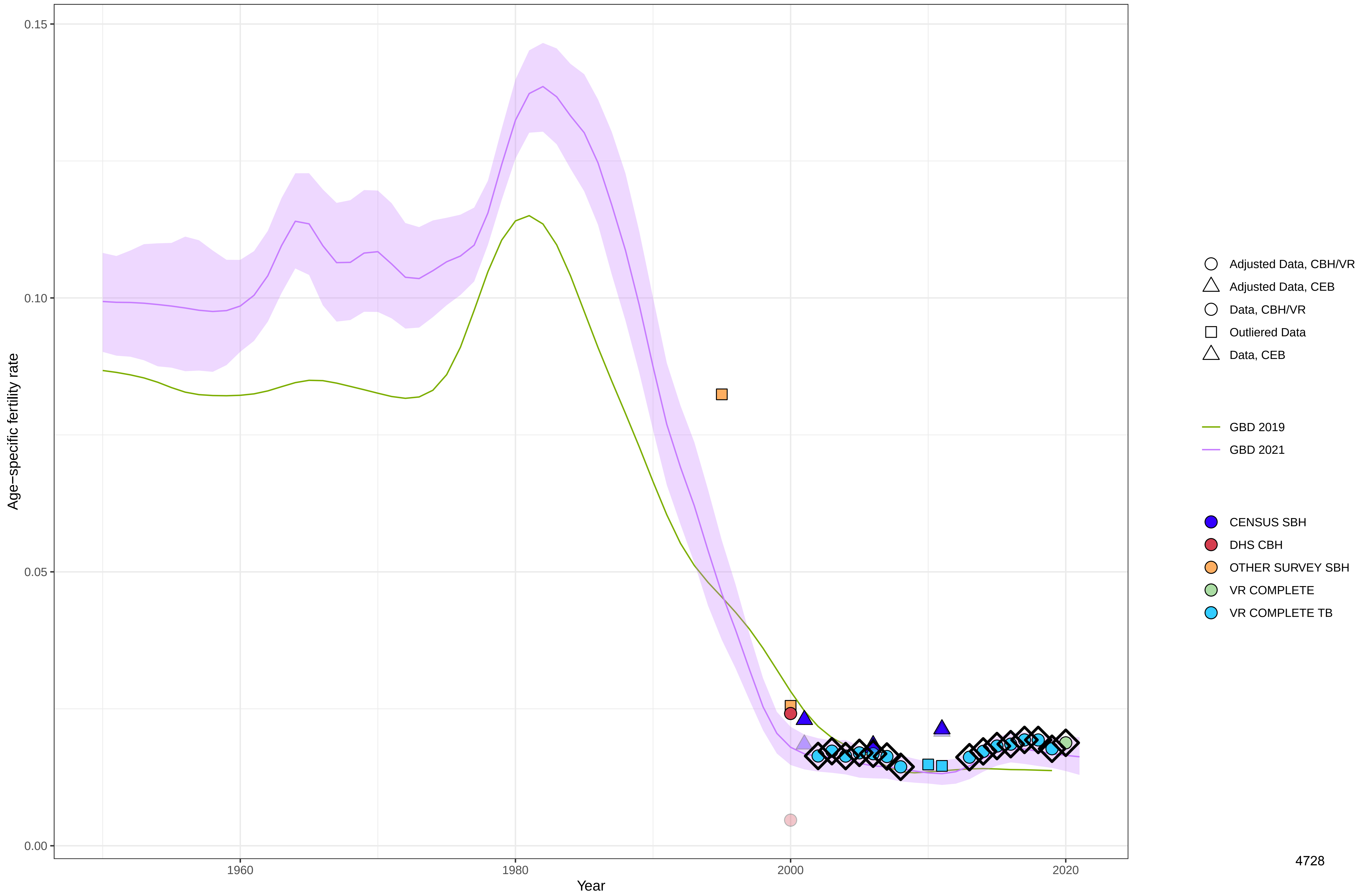

Age−specific fertility fitted trend lines, 1950−2021: Khuzestan, Ages: 45−49
Data points enclosed with a diamond are reference sources

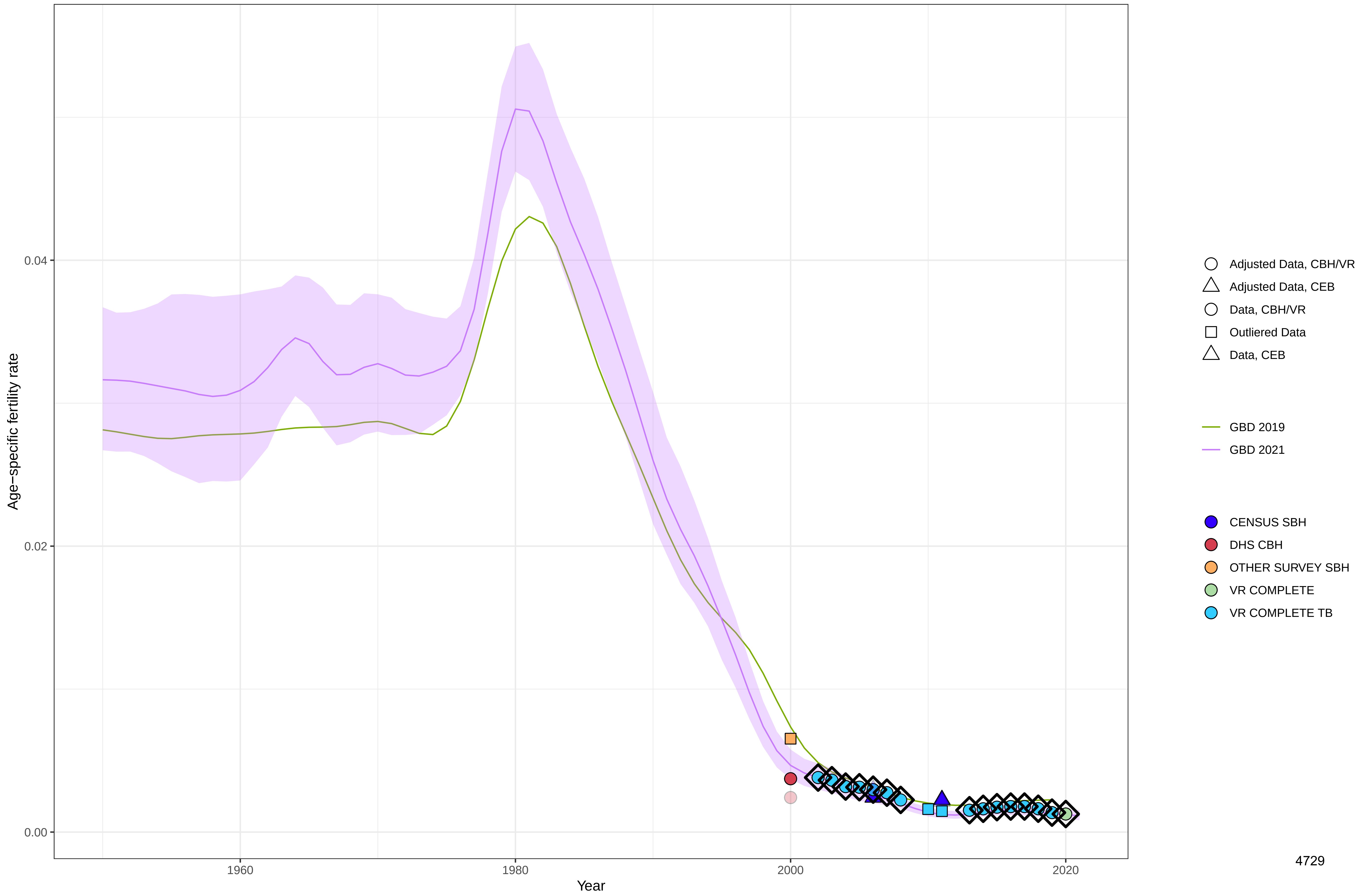

Age−specific fertility fitted trend lines, 1950−2021: Khuzestan, Ages: 50−54
Data points enclosed with a diamond are reference sources

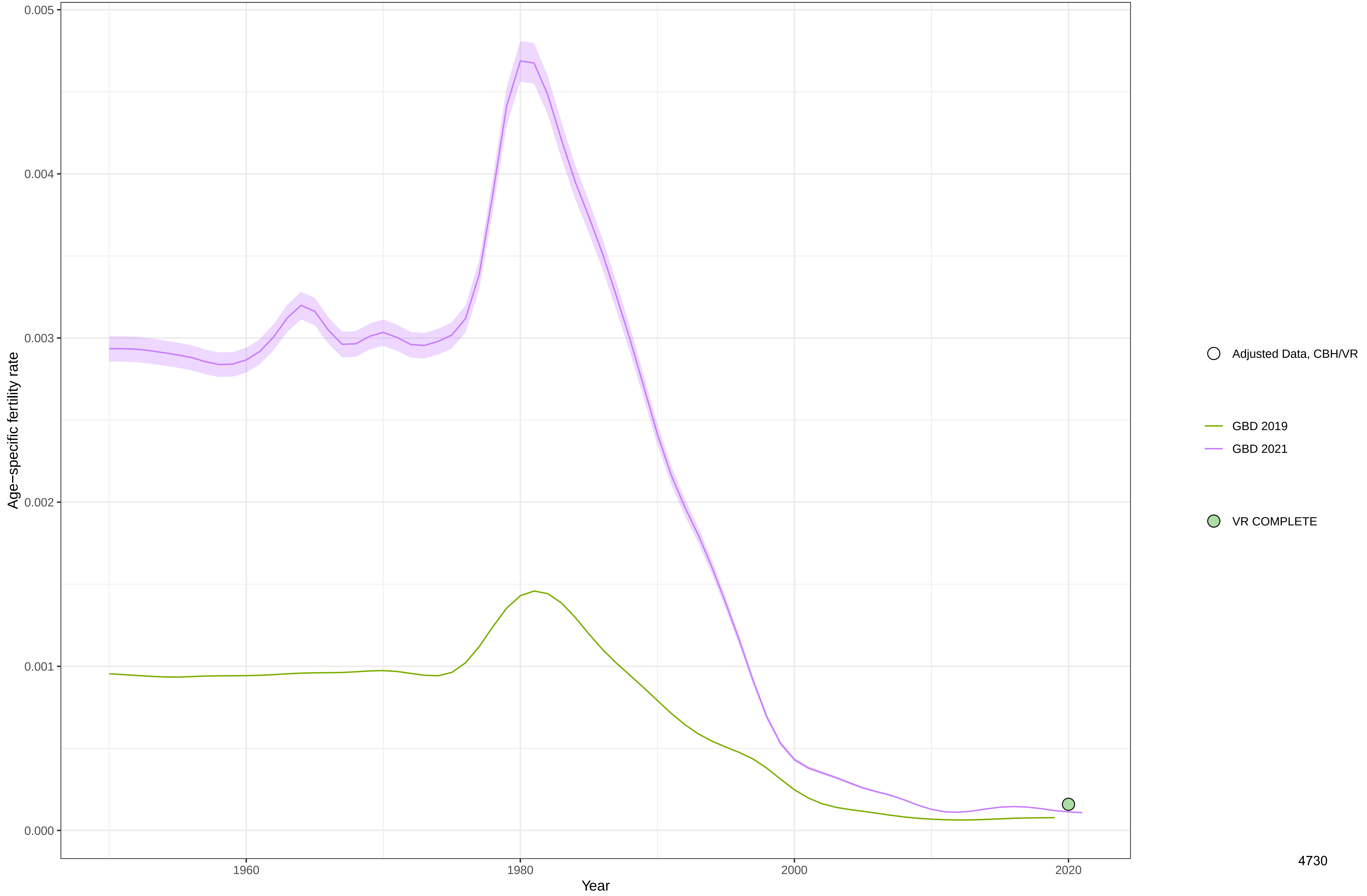

Total fertility rate fitted trend lines, 1950−2021: Khuzestan TFR
Data points enclosed with a diamond are reference sources

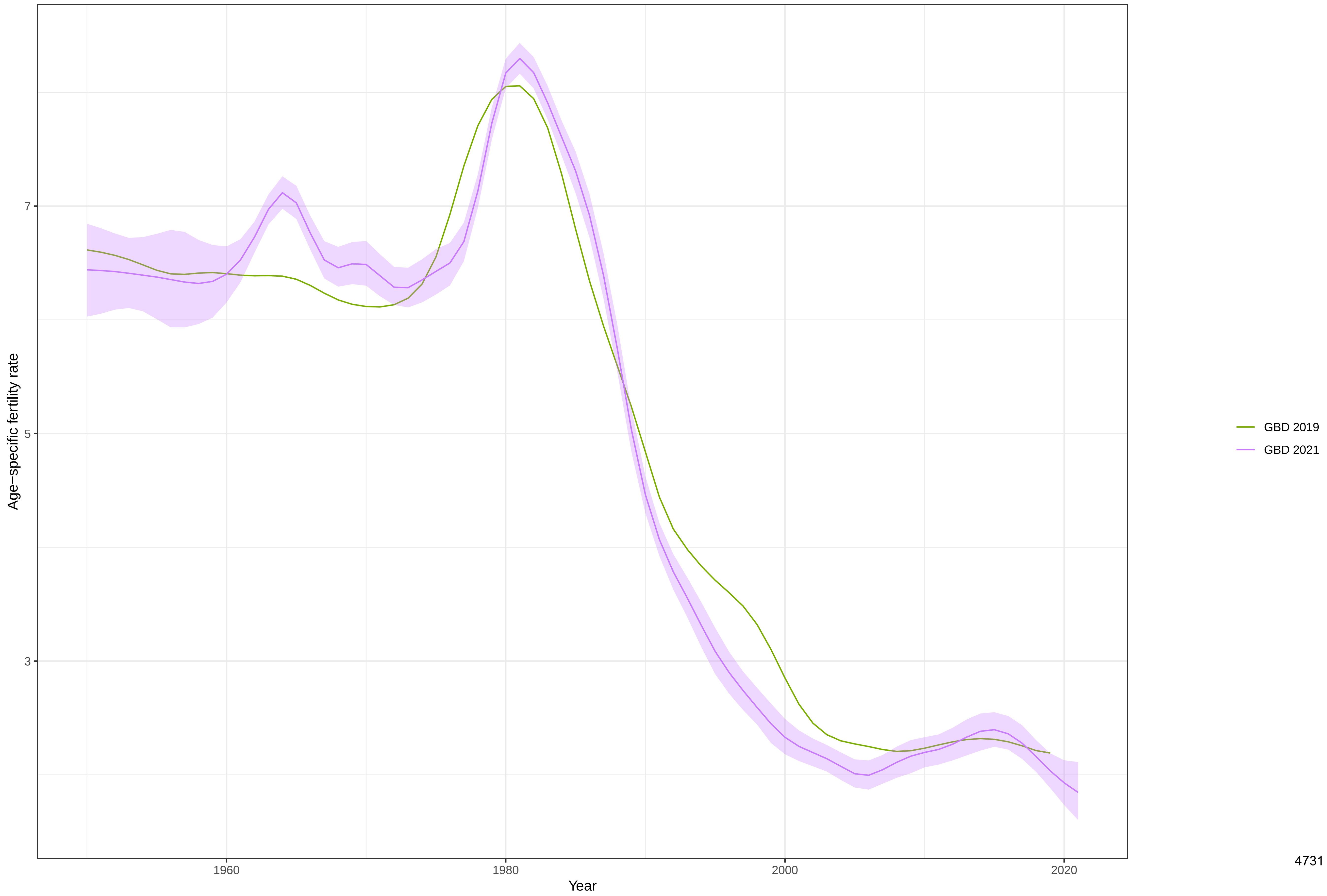

Age−specific fertility fitted trend lines, 1950−2021: Kohgiluyeh and Boyer−Ahmad, Ages: 10−14
Data points enclosed with a diamond are reference sources

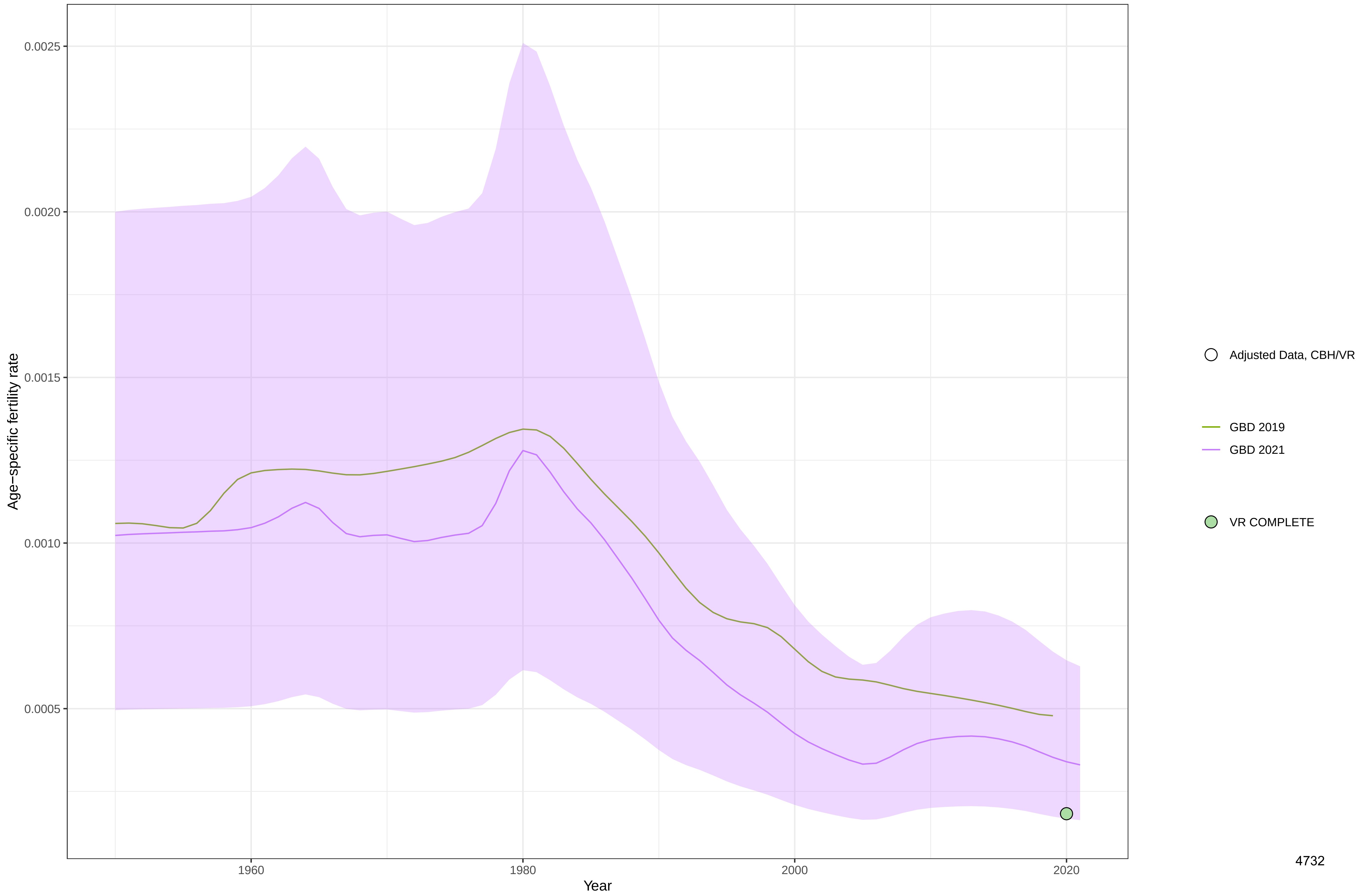

Age−specific fertility fitted trend lines, 1950−2021: Kohgiluyeh and Boyer−Ahmad, Ages: 15−19
Data points enclosed with a diamond are reference sources

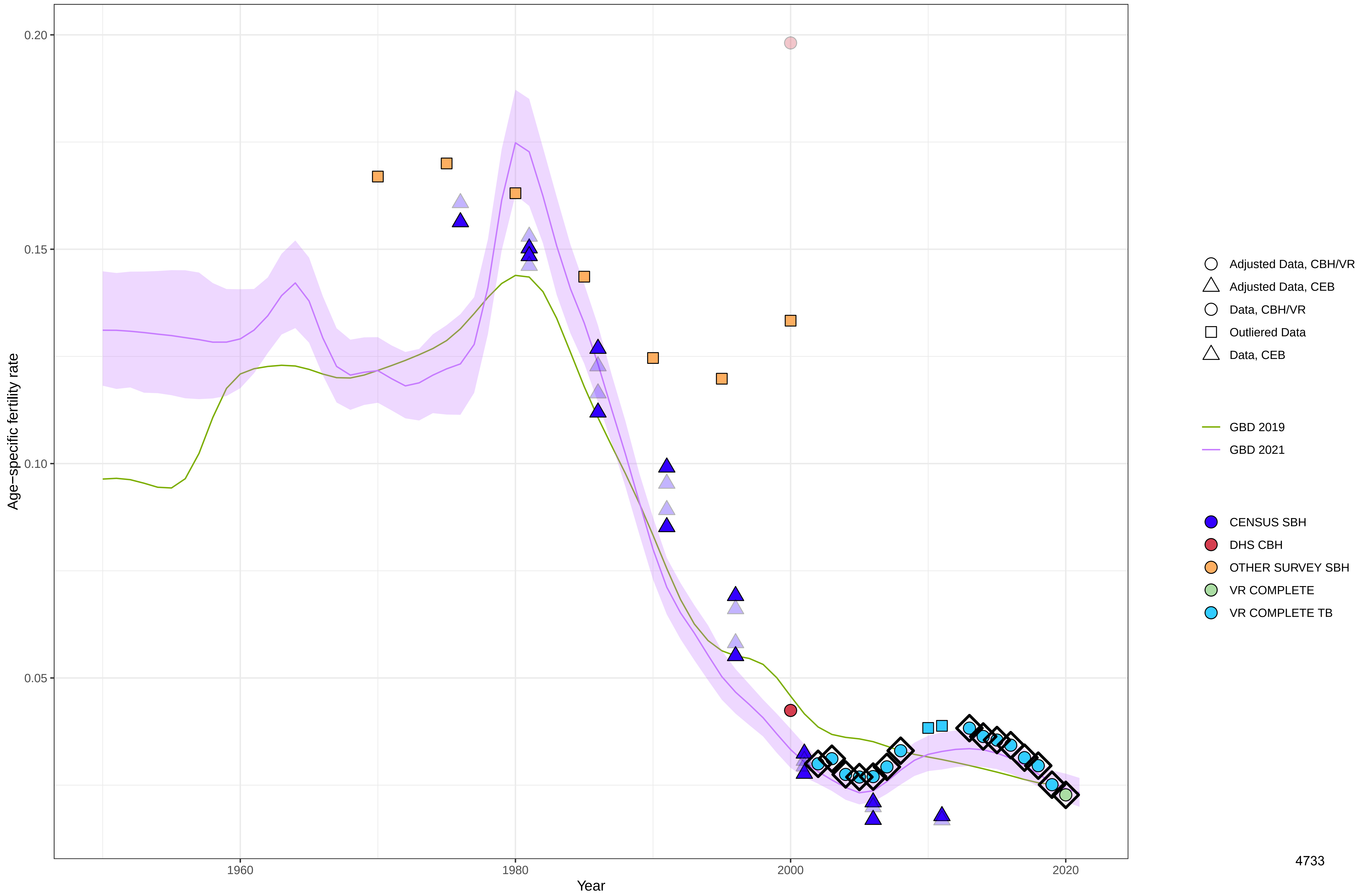

4733

Age−specific fertility fitted trend lines, 1950−2021: Kohgiluyeh and Boyer−Ahmad, Ages: 20−24
Data points enclosed with a diamond are reference sources

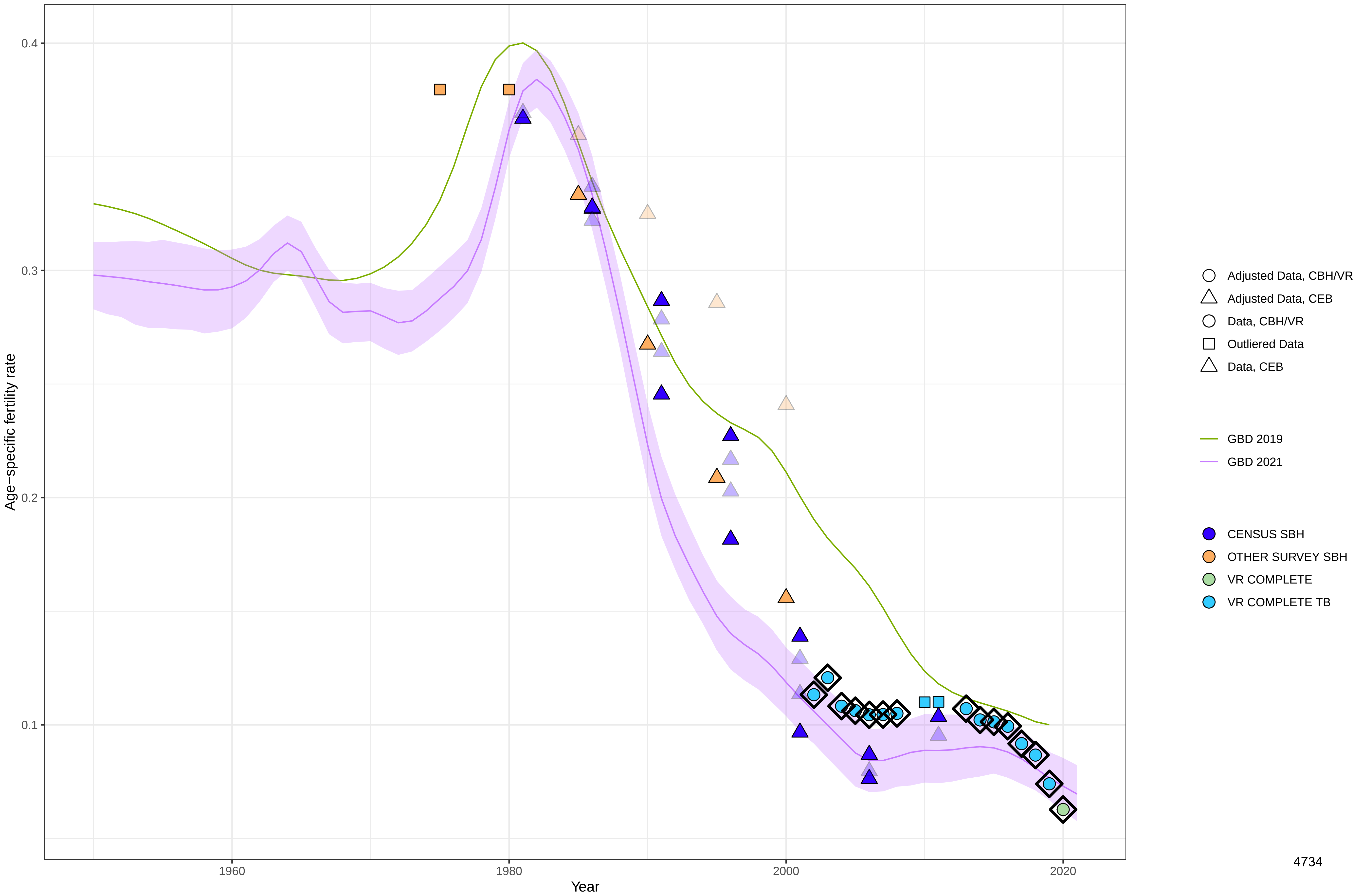

Age−specific fertility fitted trend lines, 1950−2021: Kohgiluyeh and Boyer−Ahmad, Ages: 25−29
Data points enclosed with a diamond are reference sources

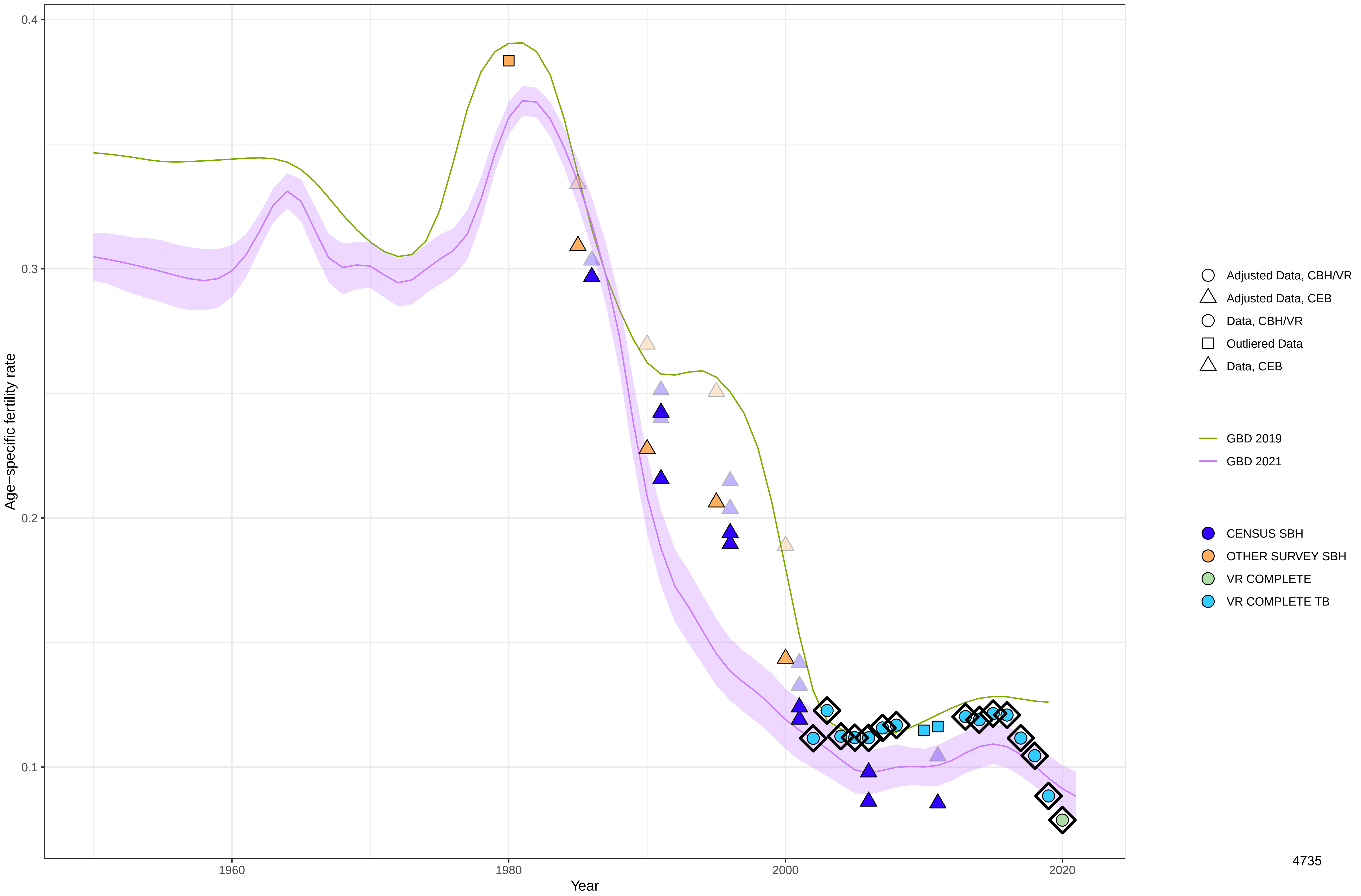

Age−specific fertility fitted trend lines, 1950−2021: Kohgiluyeh and Boyer−Ahmad, Ages: 30−34
Data points enclosed with a diamond are reference sources

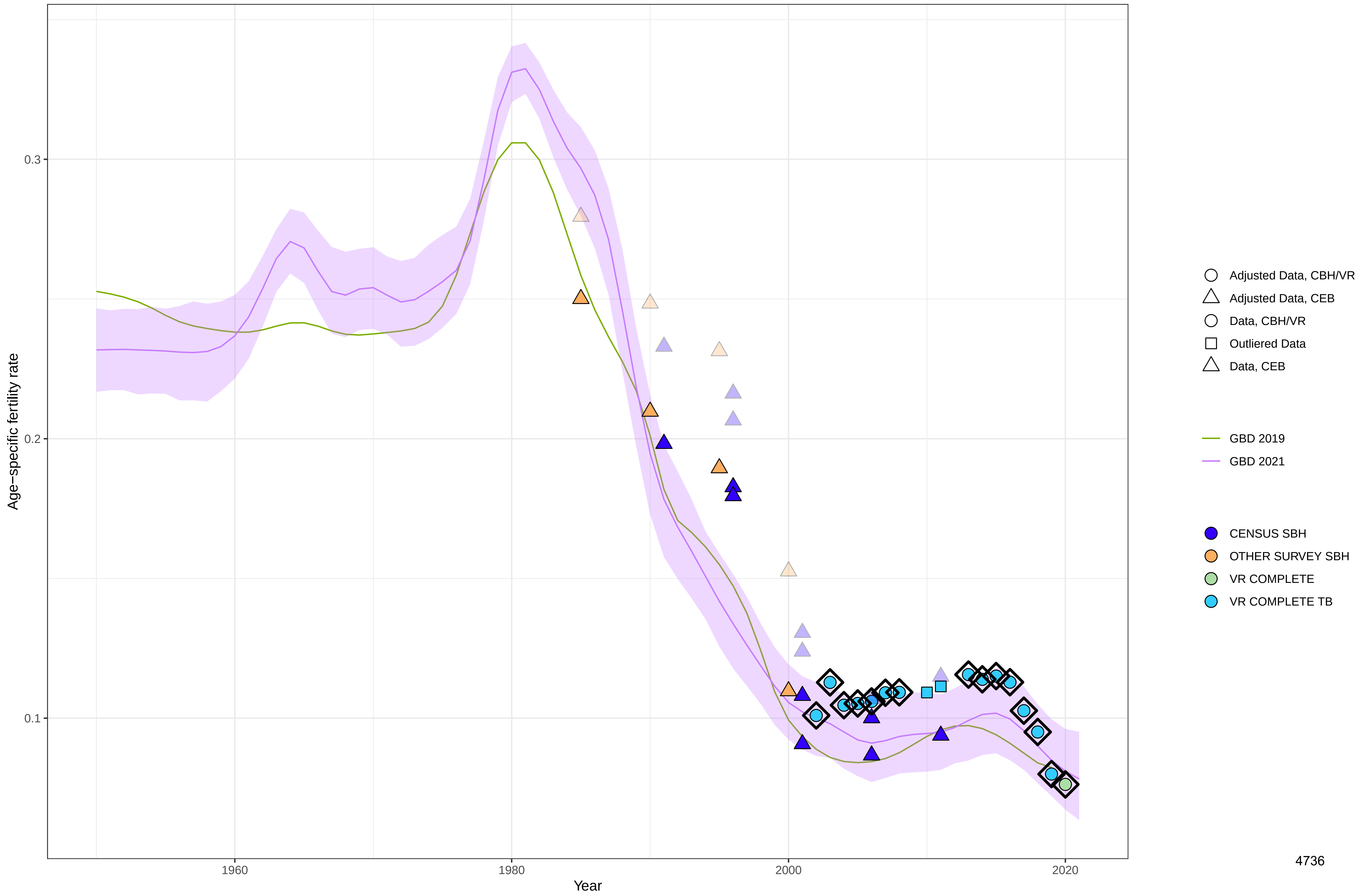

Age−specific fertility fitted trend lines, 1950−2021: Kohgiluyeh and Boyer−Ahmad, Ages: 35−39
Data points enclosed with a diamond are reference sources

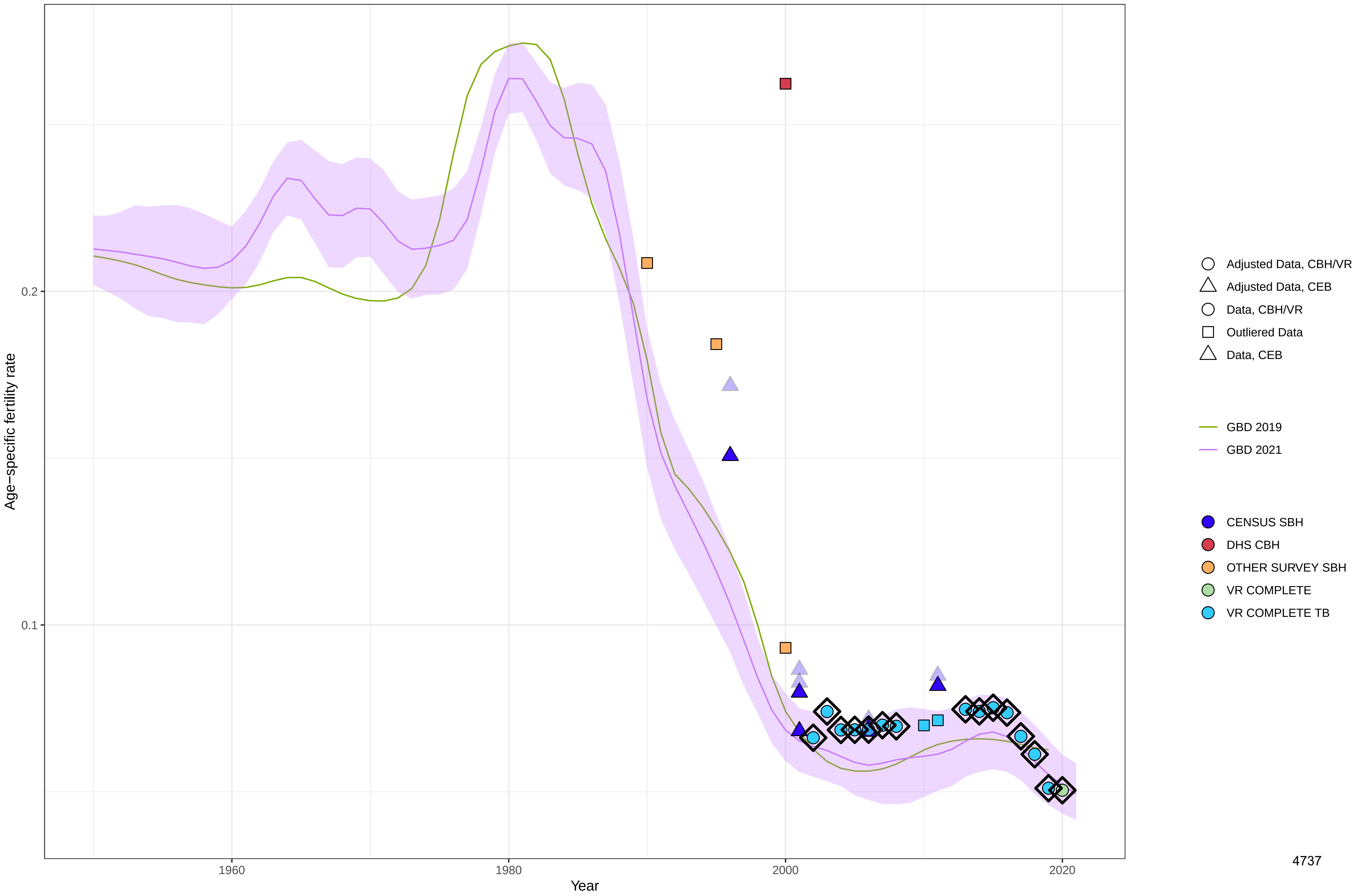

Age−specific fertility fitted trend lines, 1950−2021: Kohgiluyeh and Boyer−Ahmad, Ages: 40−44
Data points enclosed with a diamond are reference sources

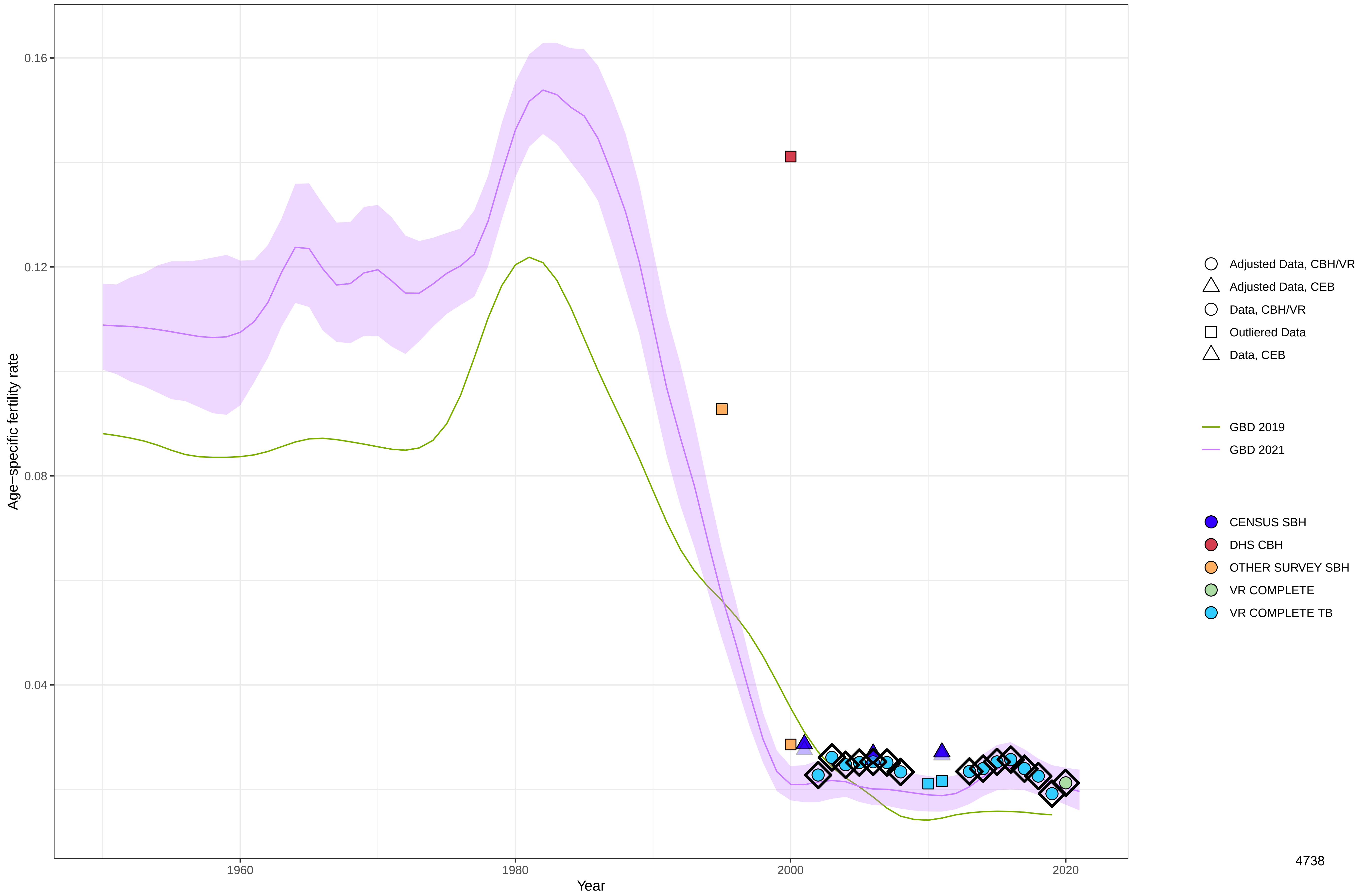

Age−specific fertility fitted trend lines, 1950−2021: Kohgiluyeh and Boyer−Ahmad, Ages: 45−49
Data points enclosed with a diamond are reference sources

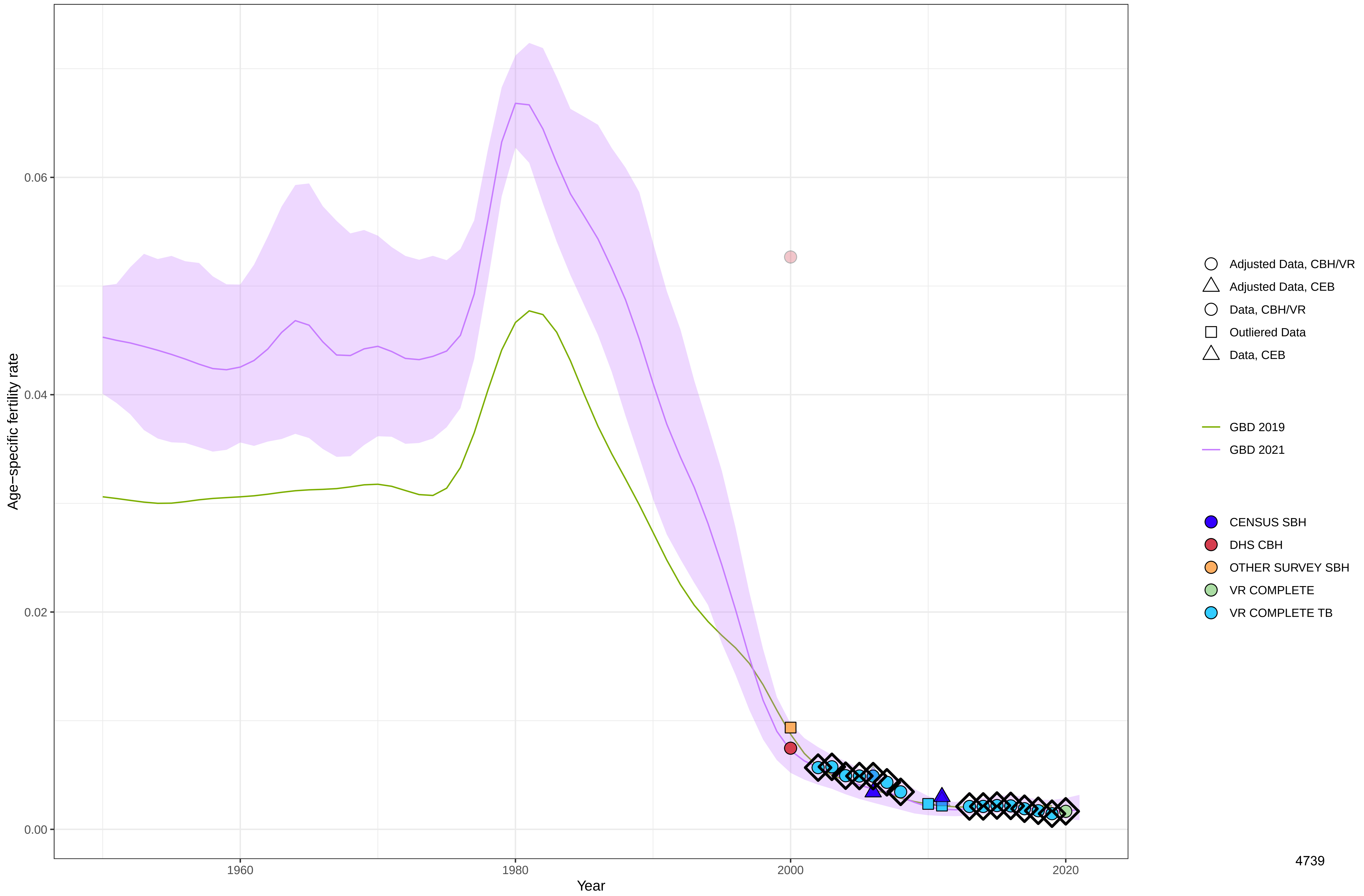

Age−specific fertility fitted trend lines, 1950−2021: Kohgiluyeh and Boyer−Ahmad, Ages: 50−54
Data points enclosed with a diamond are reference sources

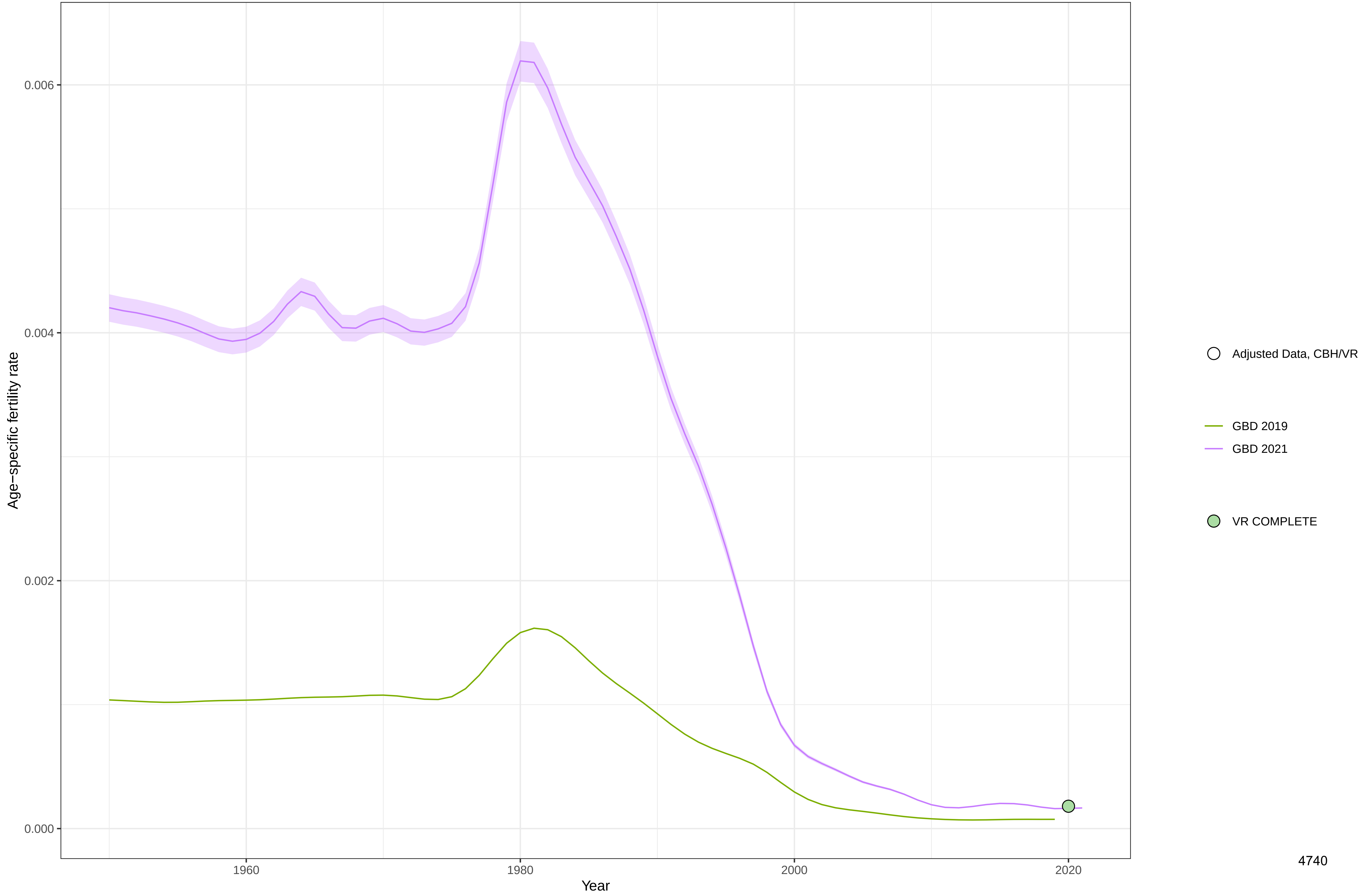

Total fertility rate fitted trend lines, 1950−2021: Kohgiluyeh and Boyer−Ahmad TFR
Data points enclosed with a diamond are reference sources

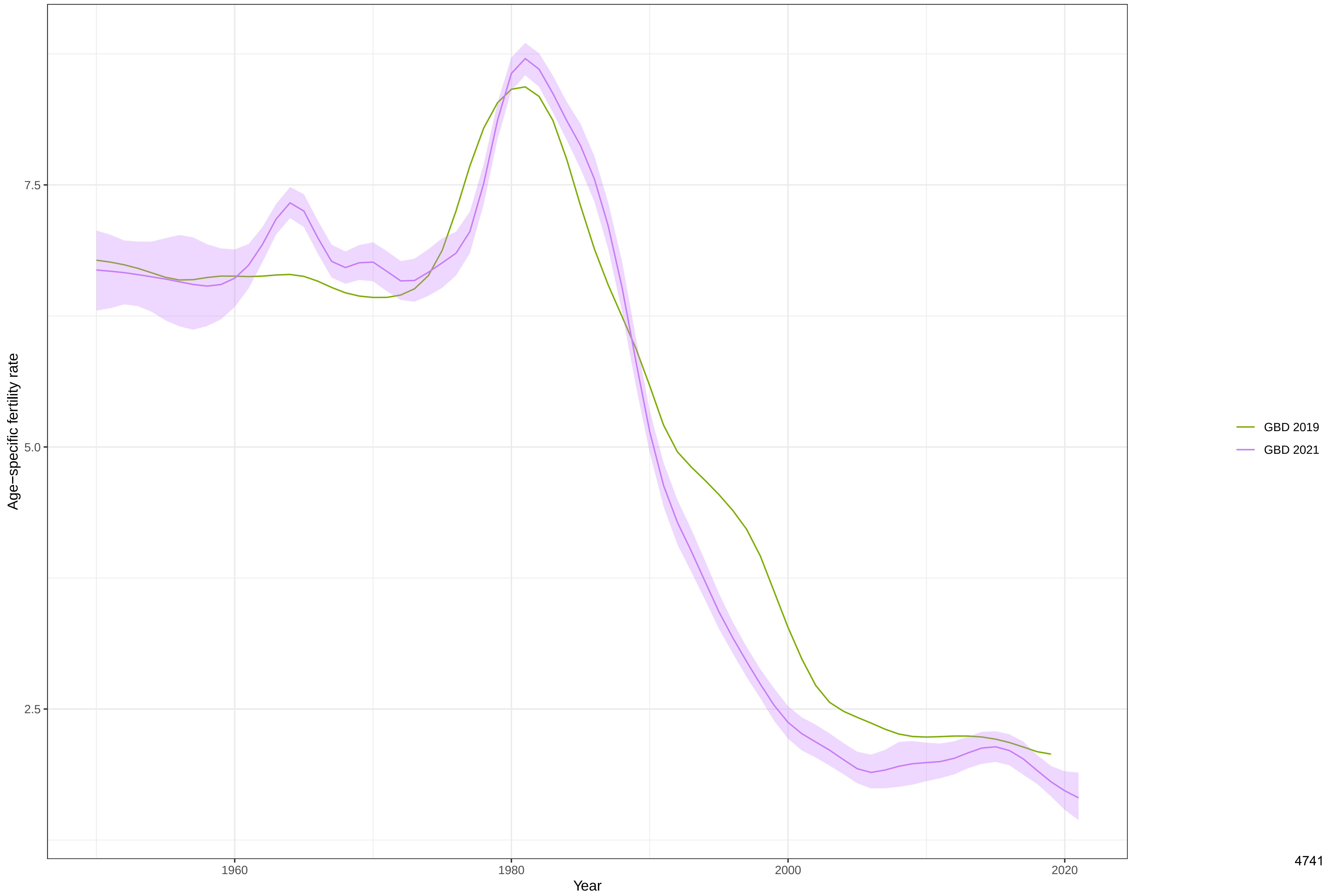

Age−specific fertility fitted trend lines, 1950−2021: Kurdistan, Ages: 10−14
Data points enclosed with a diamond are reference sources

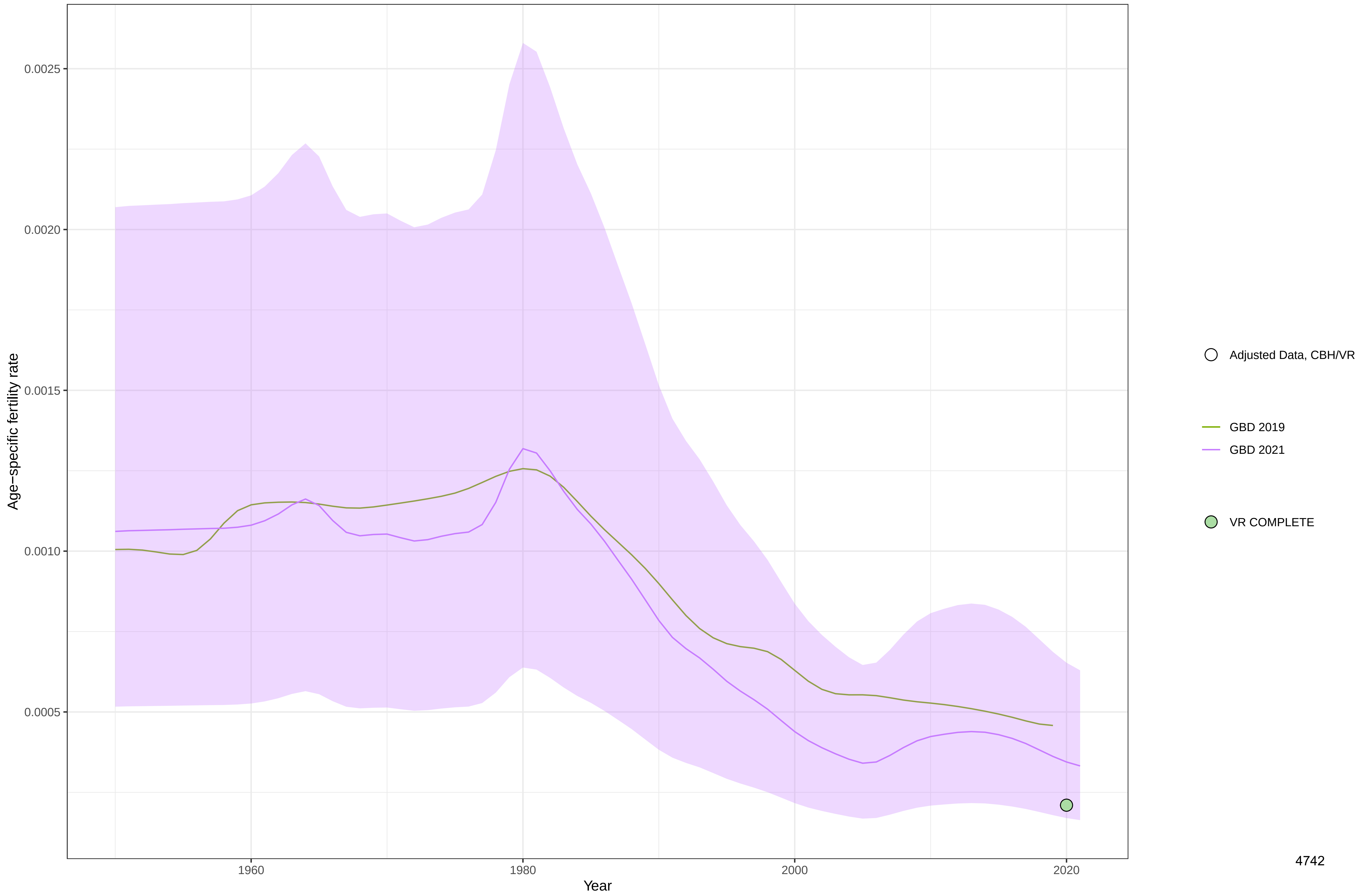

4742

Age−specific fertility fitted trend lines, 1950−2021: Kurdistan, Ages: 15−19
Data points enclosed with a diamond are reference sources

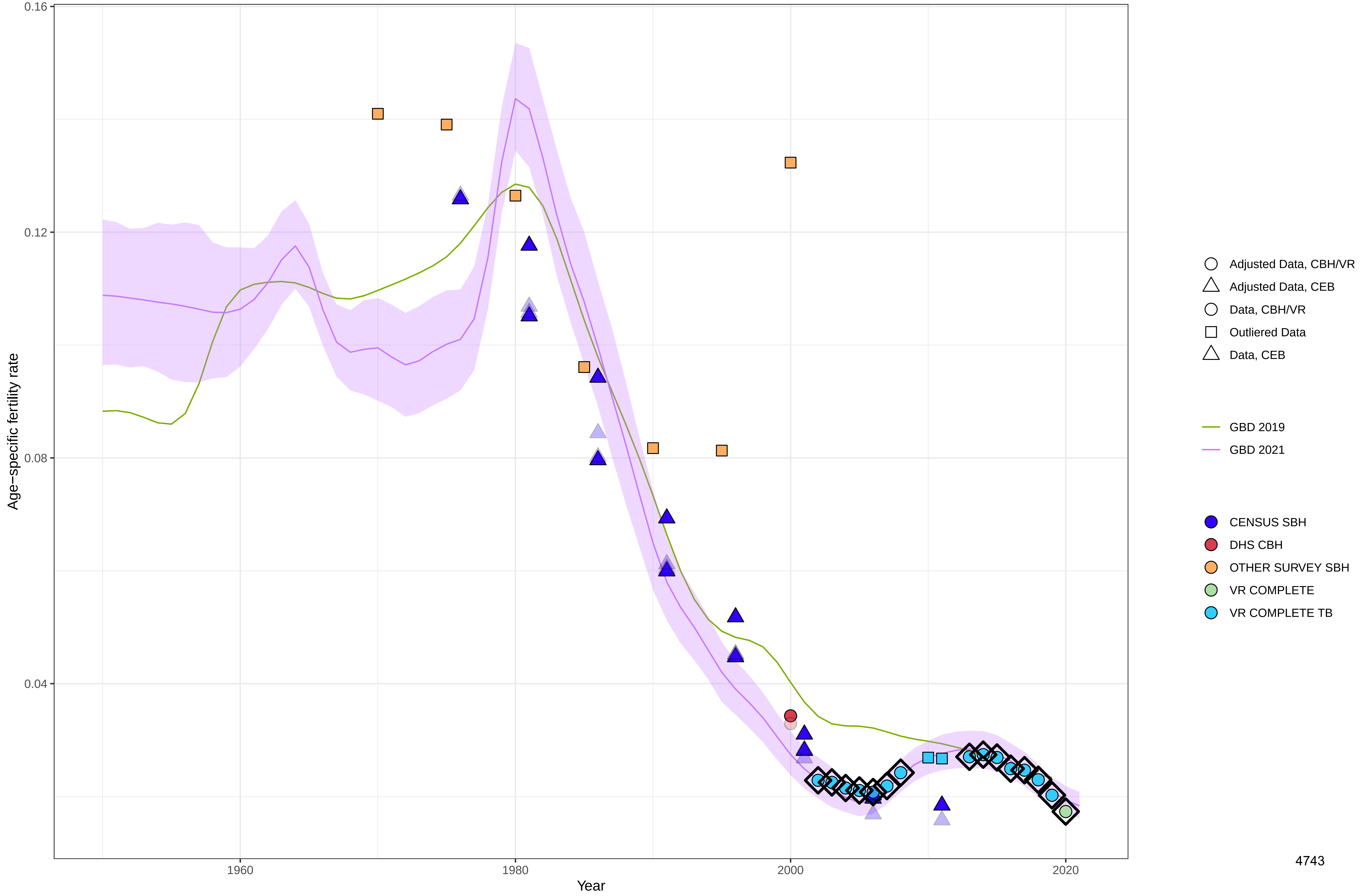

Age−specific fertility fitted trend lines, 1950−2021: Kurdistan, Ages: 20−24
Data points enclosed with a diamond are reference sources

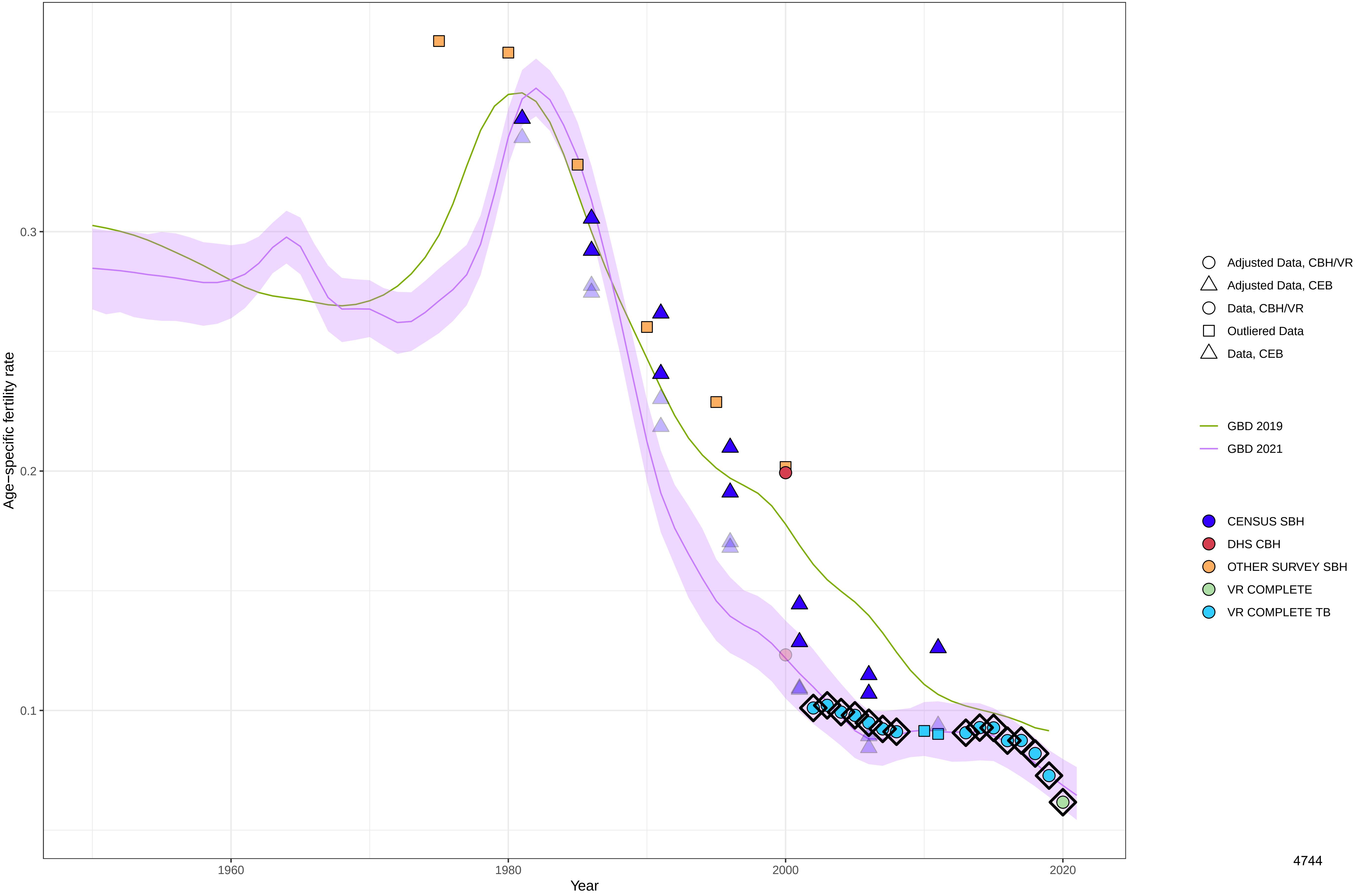

Age−specific fertility fitted trend lines, 1950−2021: Kurdistan, Ages: 25−29
Data points enclosed with a diamond are reference sources

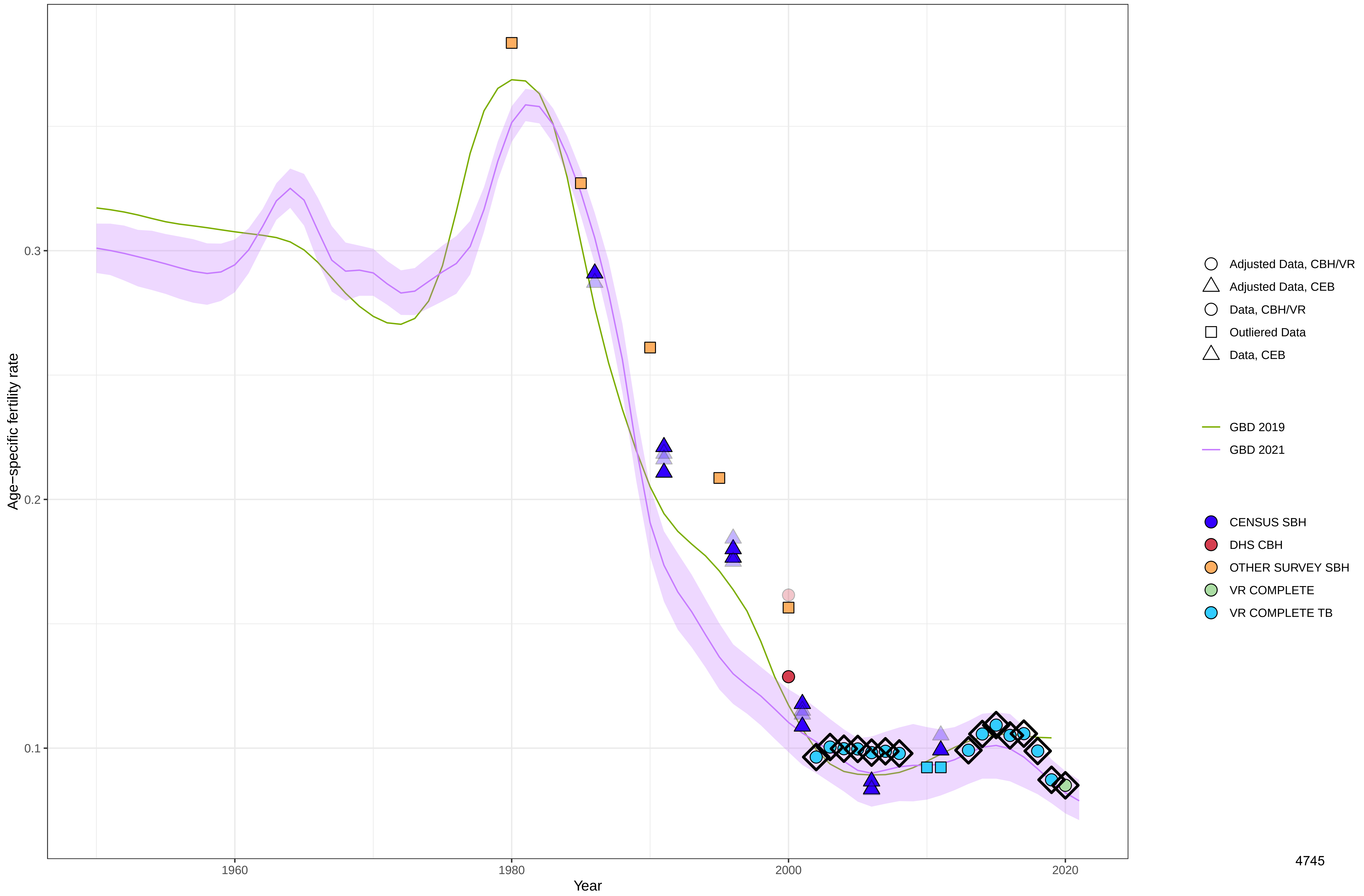

Age−specific fertility fitted trend lines, 1950−2021: Kurdistan, Ages: 30−34
Data points enclosed with a diamond are reference sources

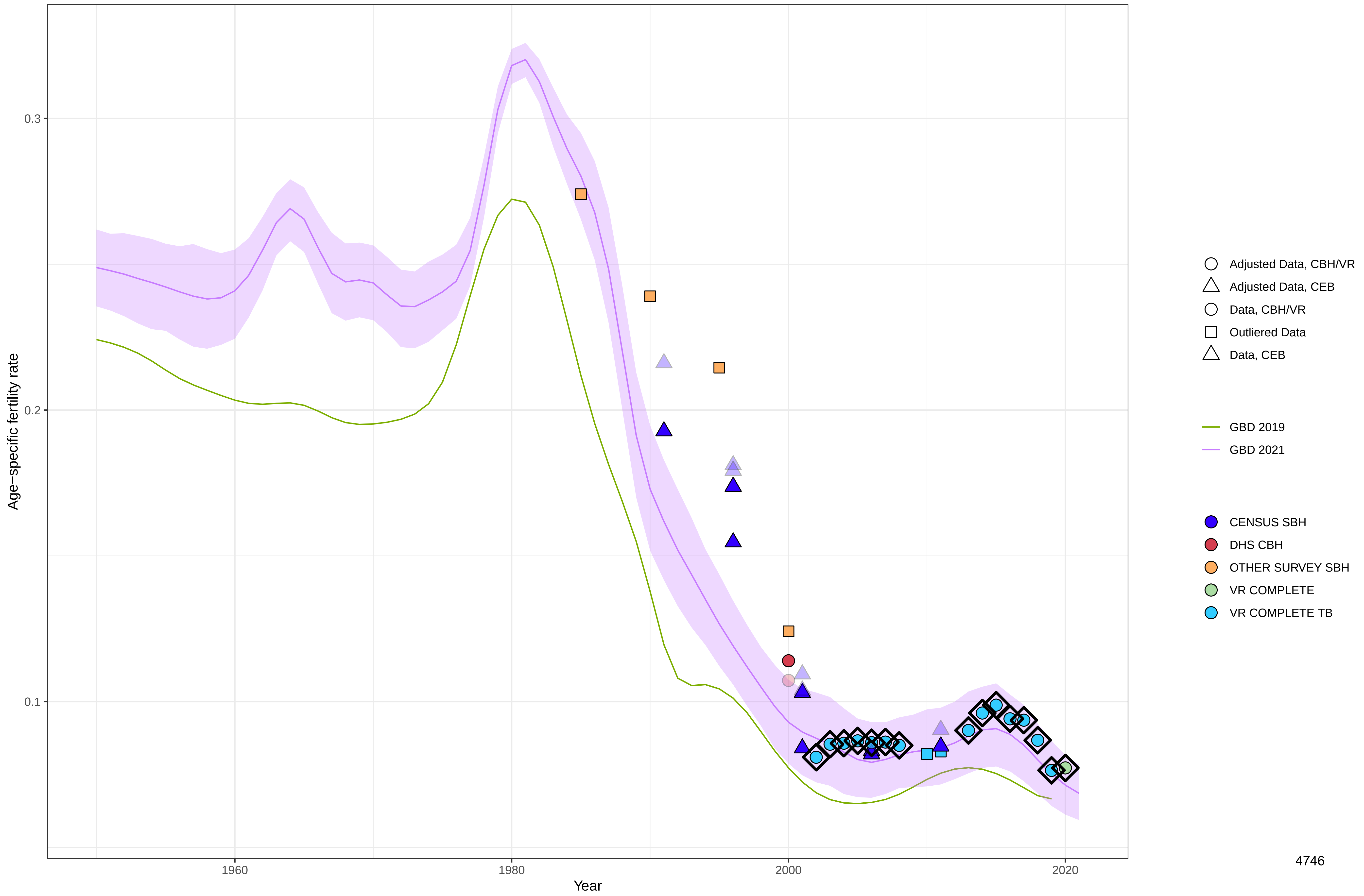

Age−specific fertility fitted trend lines, 1950−2021: Kurdistan, Ages: 35−39
Data points enclosed with a diamond are reference sources

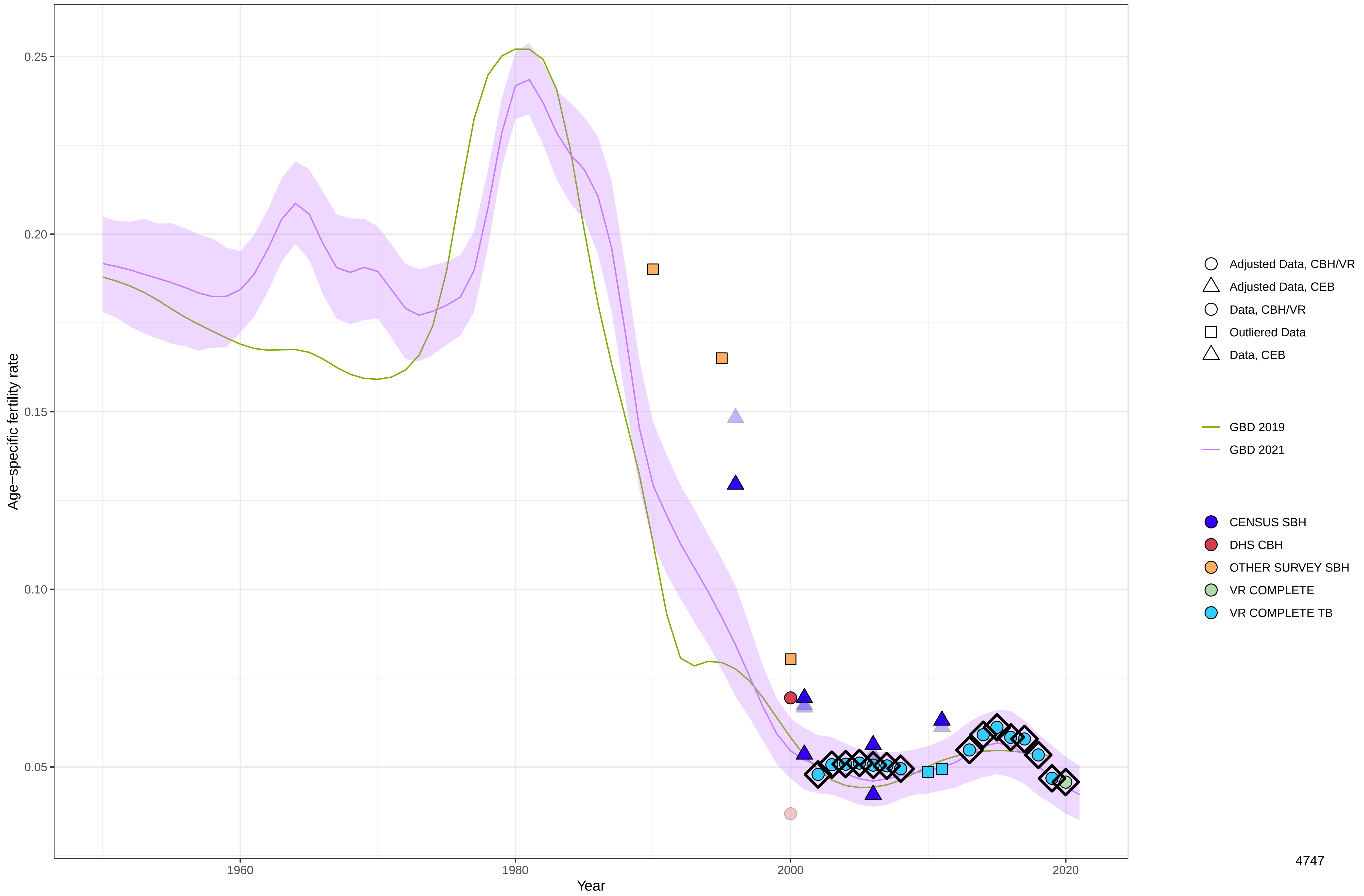

Age−specific fertility fitted trend lines, 1950−2021: Kurdistan, Ages: 40−44
Data points enclosed with a diamond are reference sources

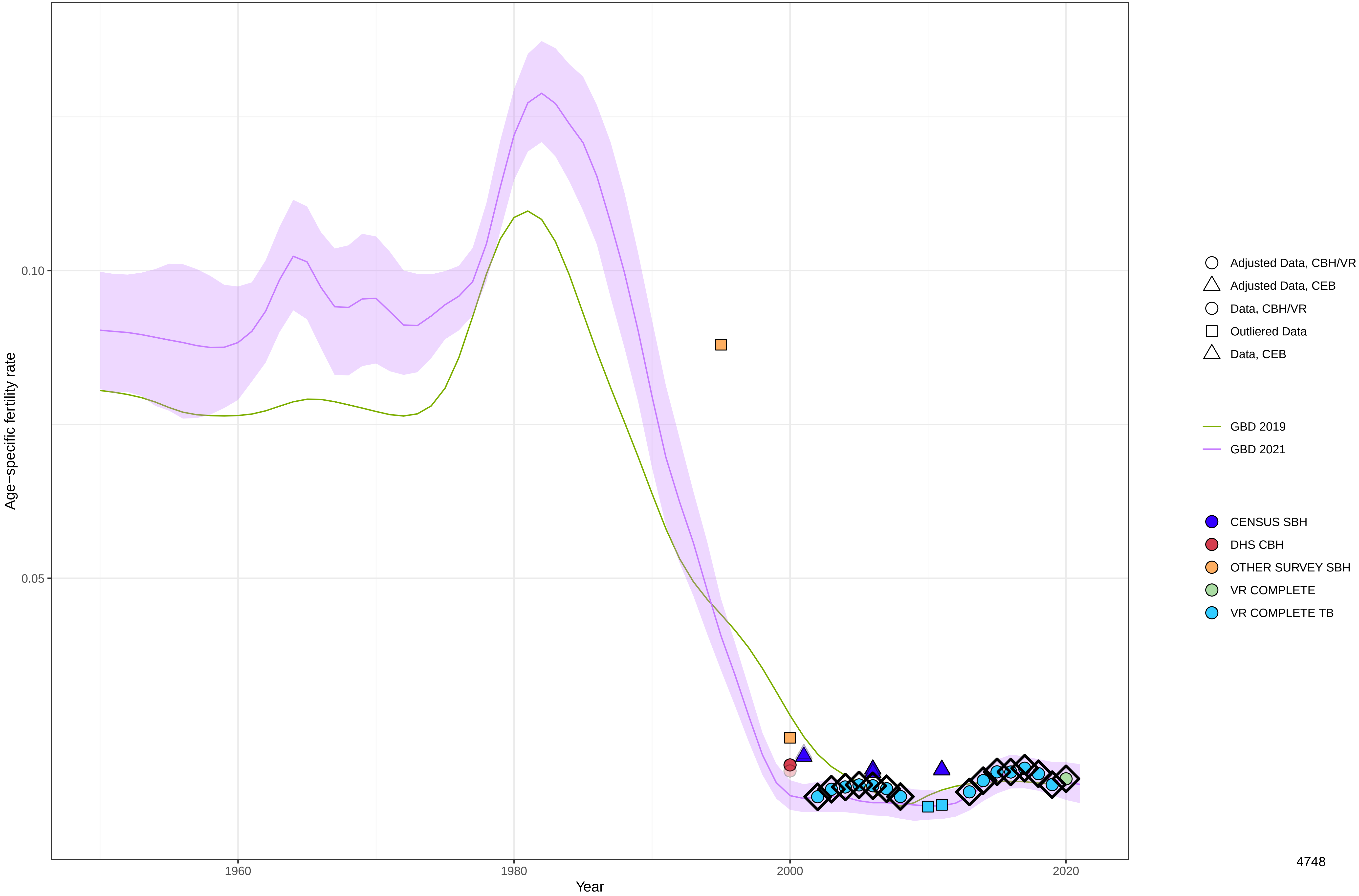

Age−specific fertility fitted trend lines, 1950−2021: Kurdistan, Ages: 45−49
Data points enclosed with a diamond are reference sources

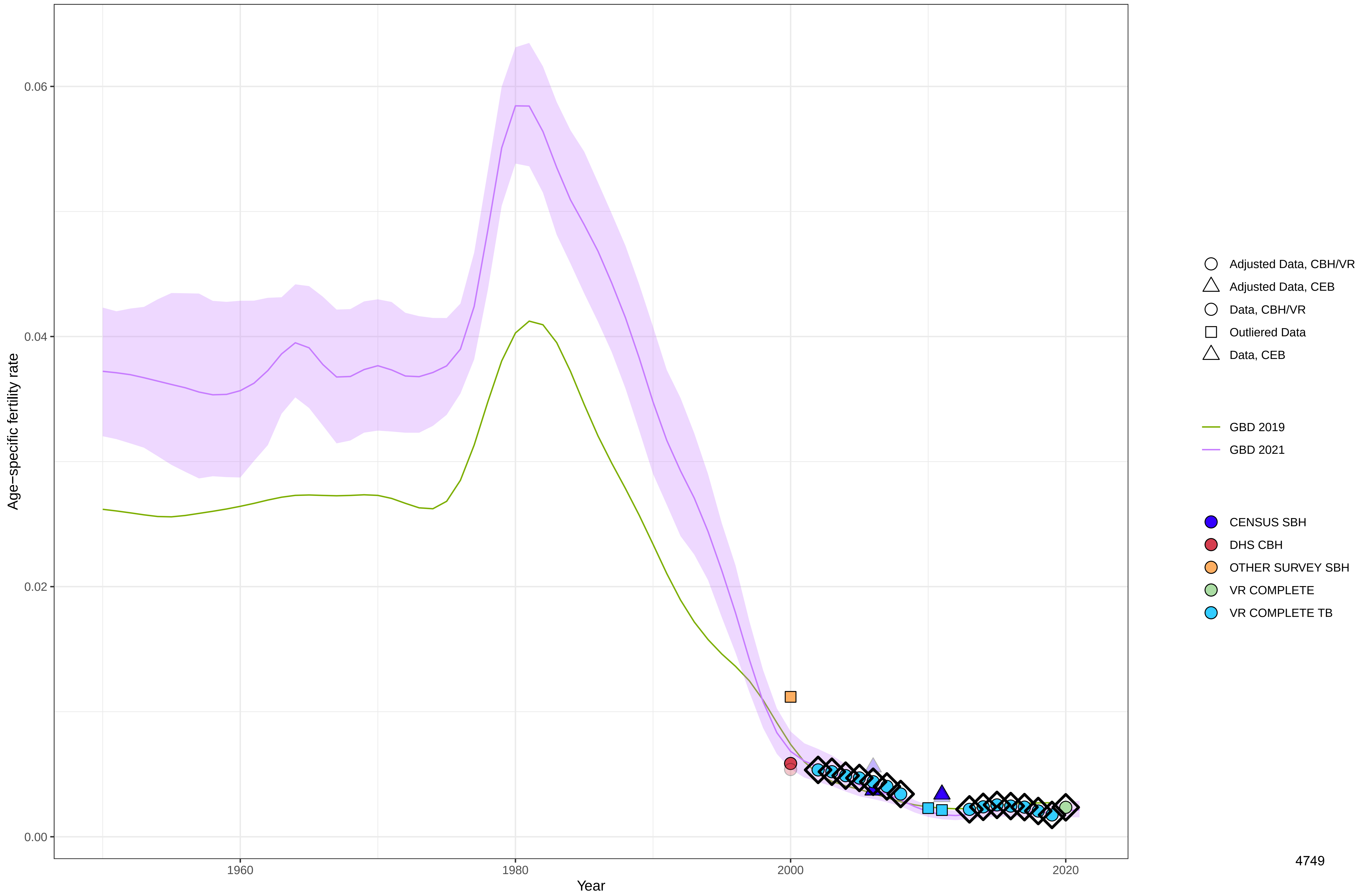

Age−specific fertility fitted trend lines, 1950−2021: Kurdistan, Ages: 50−54
Data points enclosed with a diamond are reference sources

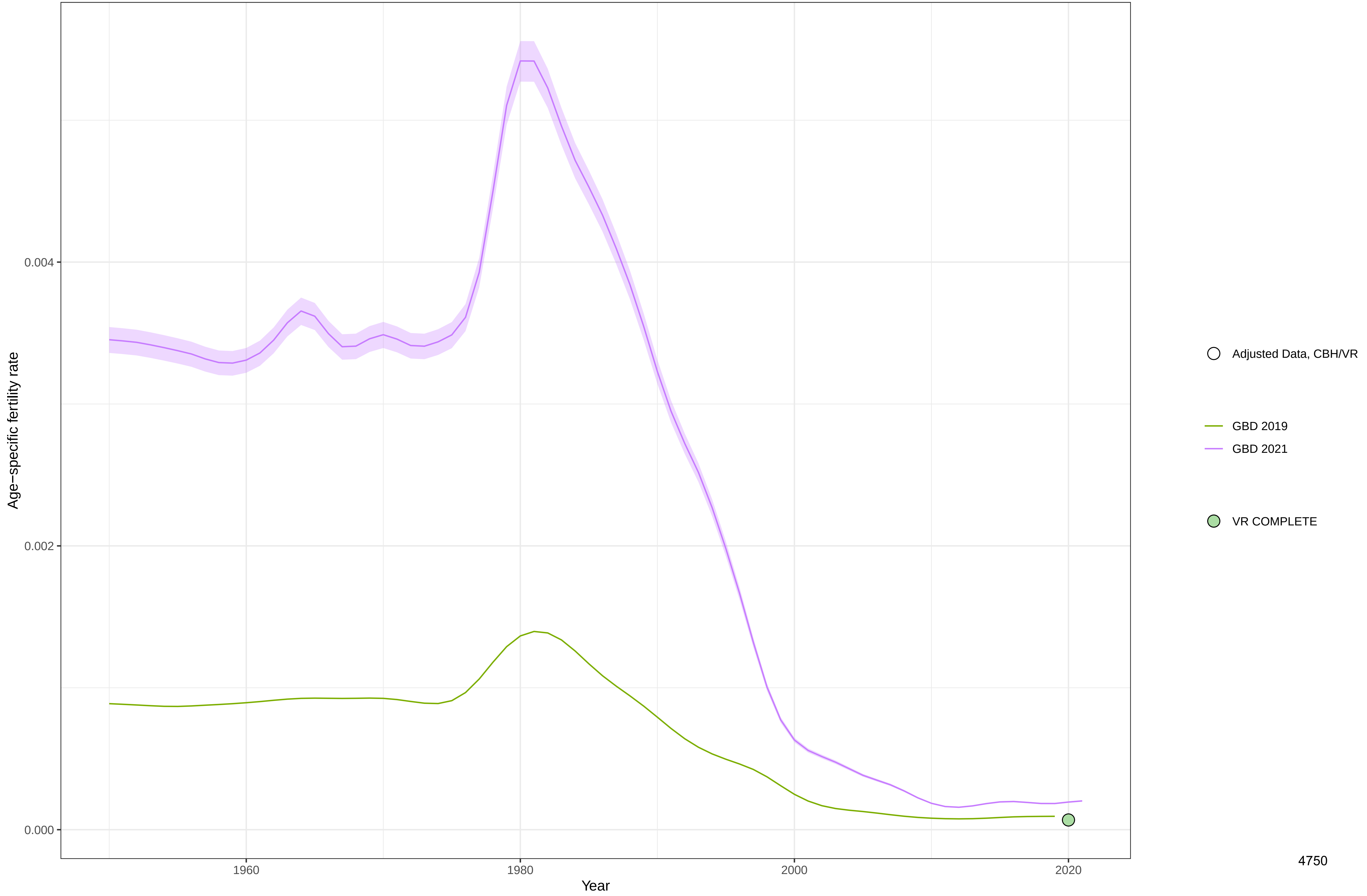

Total fertility rate fitted trend lines, 1950−2021: Kurdistan TFR
Data points enclosed with a diamond are reference sources

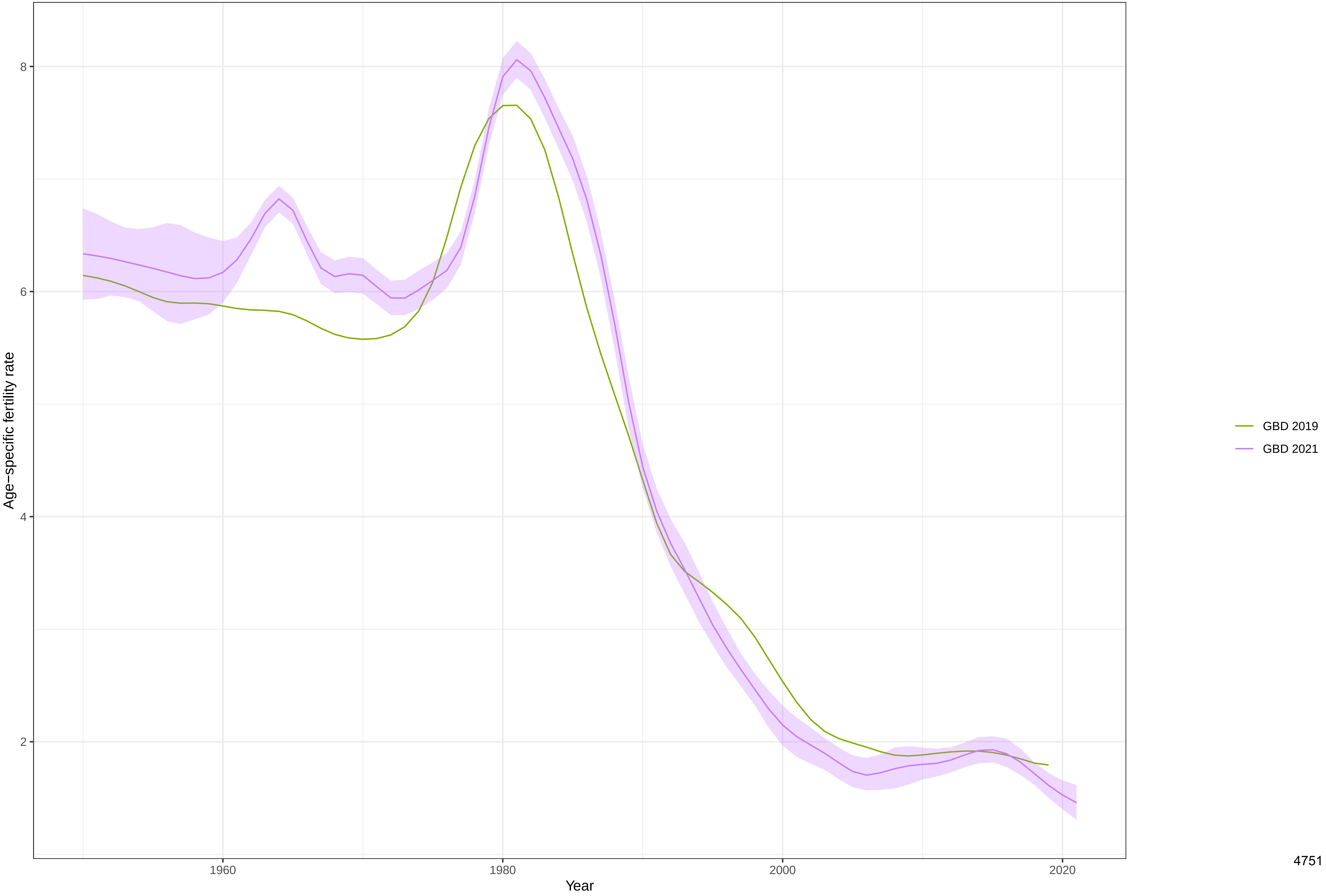

Age−specific fertility fitted trend lines, 1950−2021: Lorestan, Ages: 10−14
Data points enclosed with a diamond are reference sources

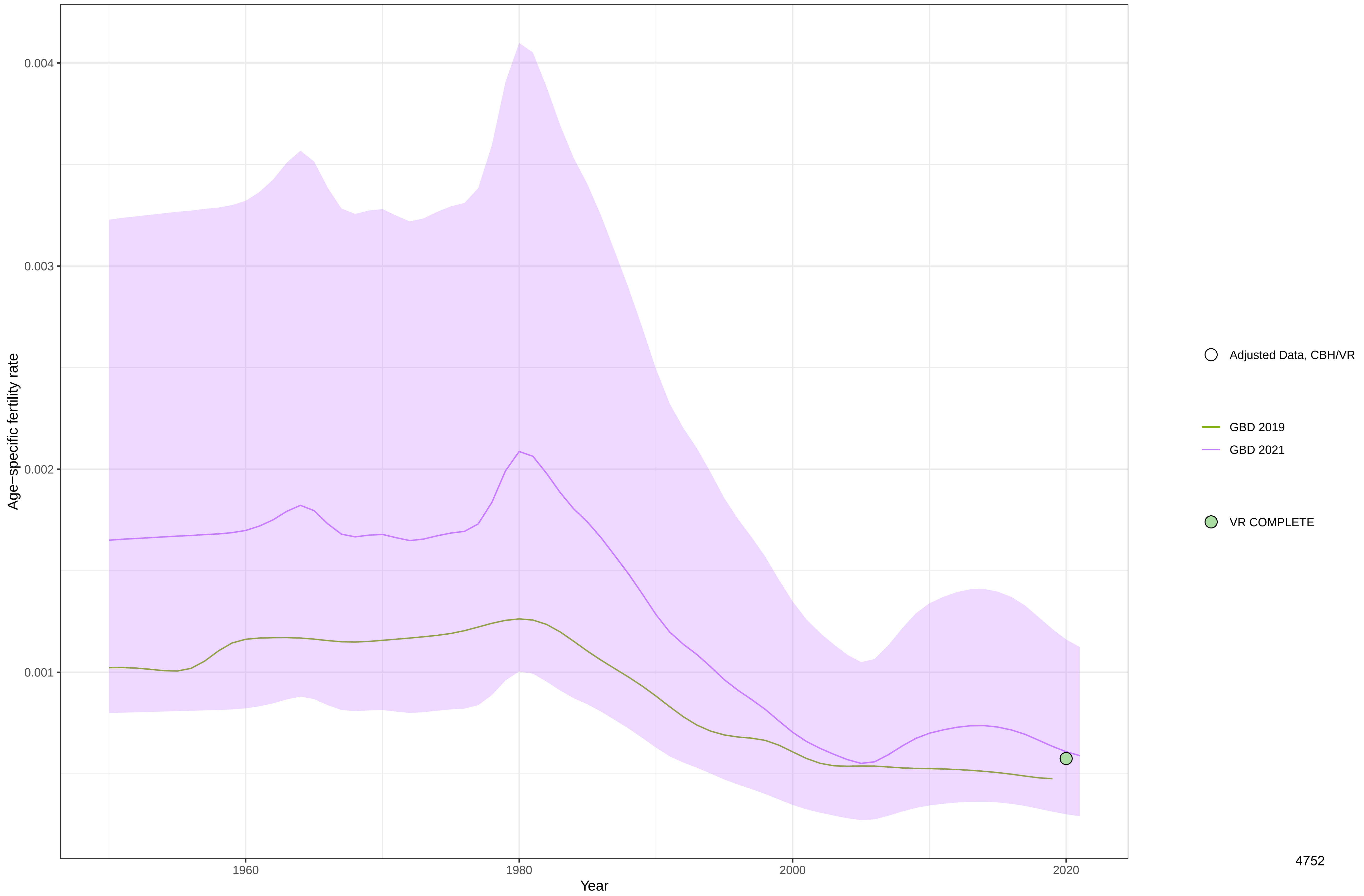

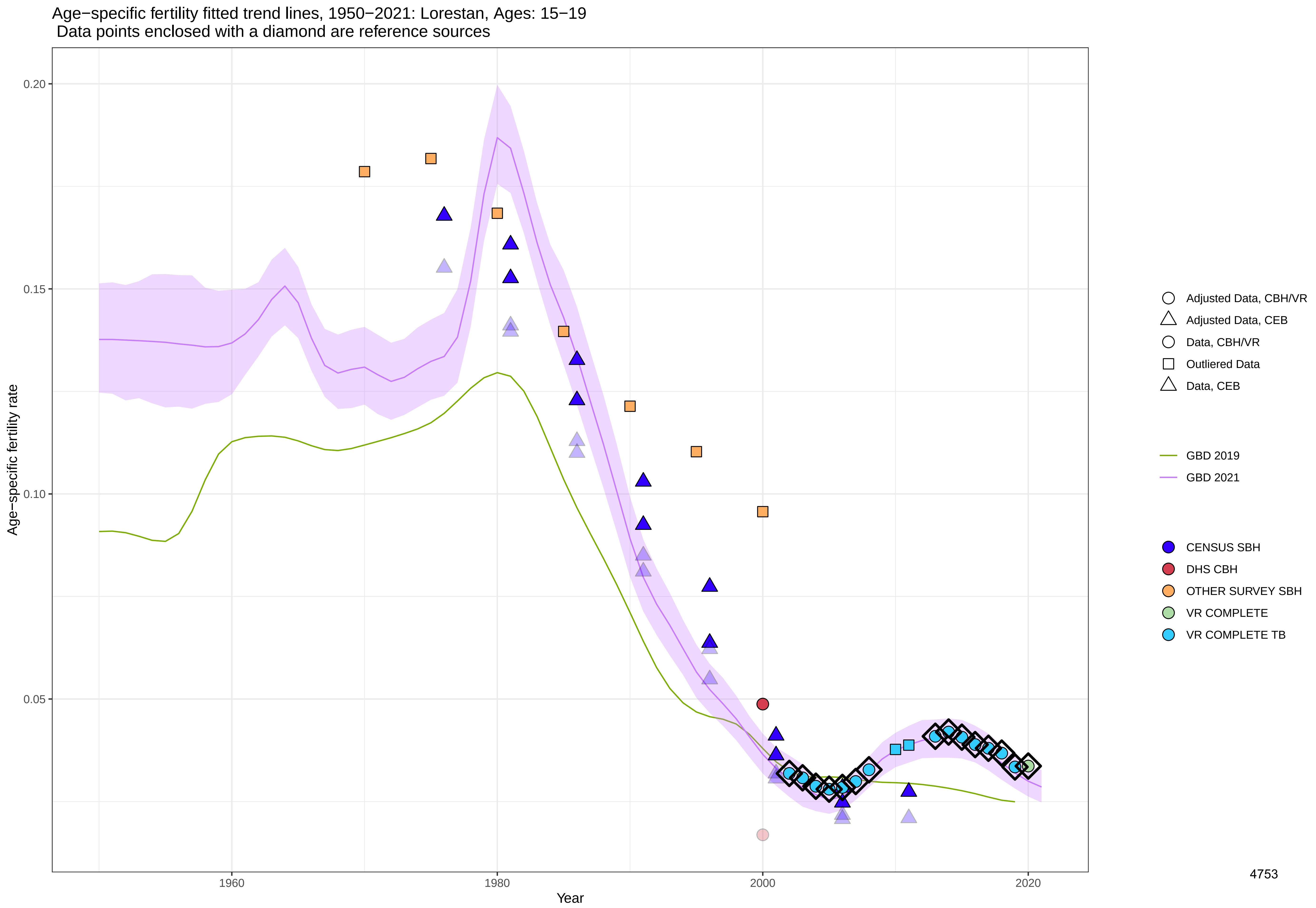

Age−specific fertility fitted trend lines, 1950−2021: Lorestan, Ages: 15−19
Data points enclosed with a diamond are reference sources
0.20
0.15
0.10
0.05
Age−specific fertility rate
1960
1980
2000
2020
Year
Adjusted Data, CBH/VR
Adjusted Data, CEB
Data, CBH/VR
Outliered Data
Data, CEB
GBD 2019
GBD 2021
CENSUS SBH
DHS CBH
OTHER SURVEY SBH
VR COMPLETE
VR COMPLETE TB

Age−specific fertility fitted trend lines, 1950−2021: Lorestan, Ages: 20−24
Data points enclosed with a diamond are reference sources

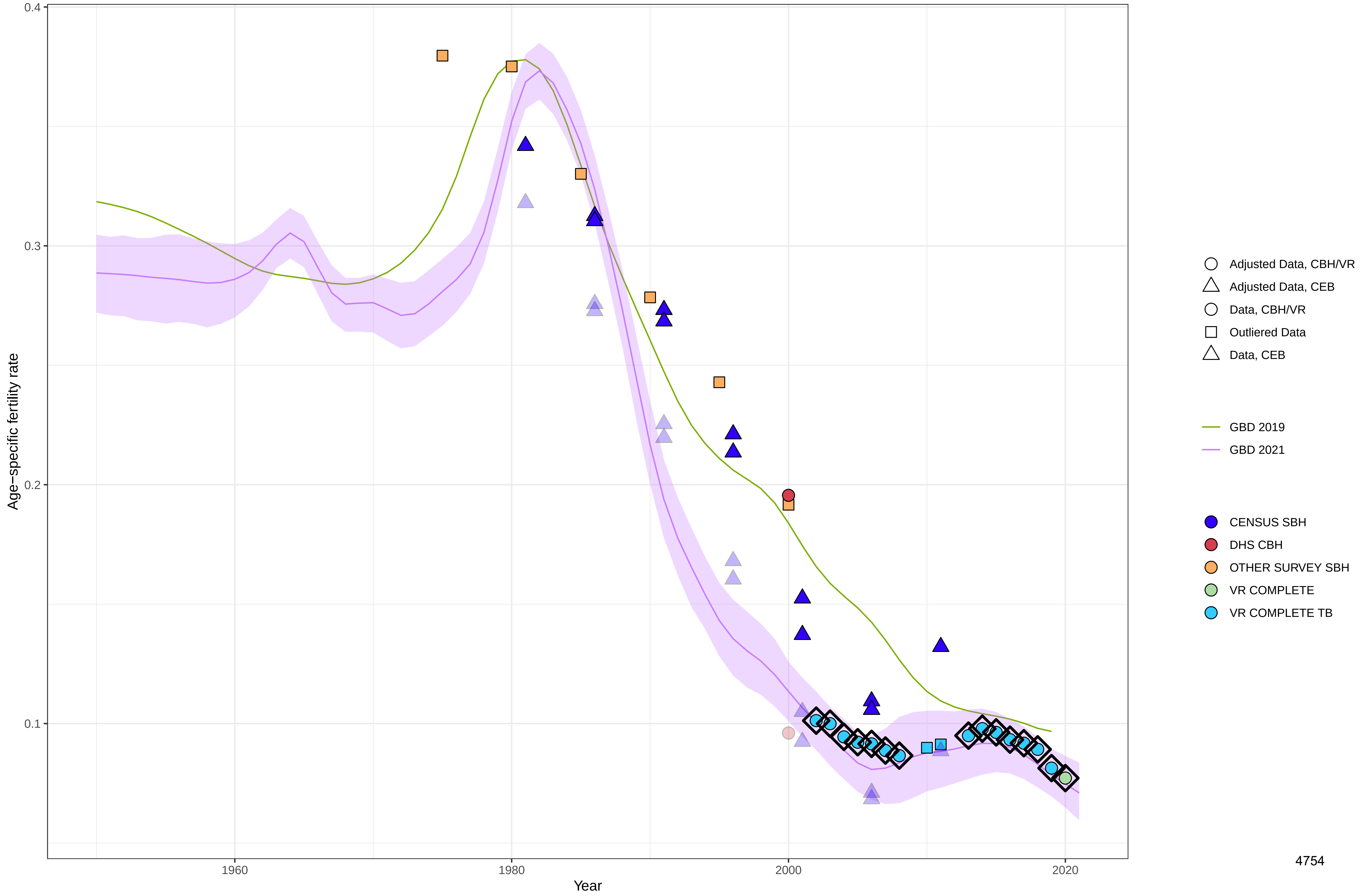

Age−specific fertility fitted trend lines, 1950−2021: Lorestan, Ages: 25−29
Data points enclosed with a diamond are reference sources

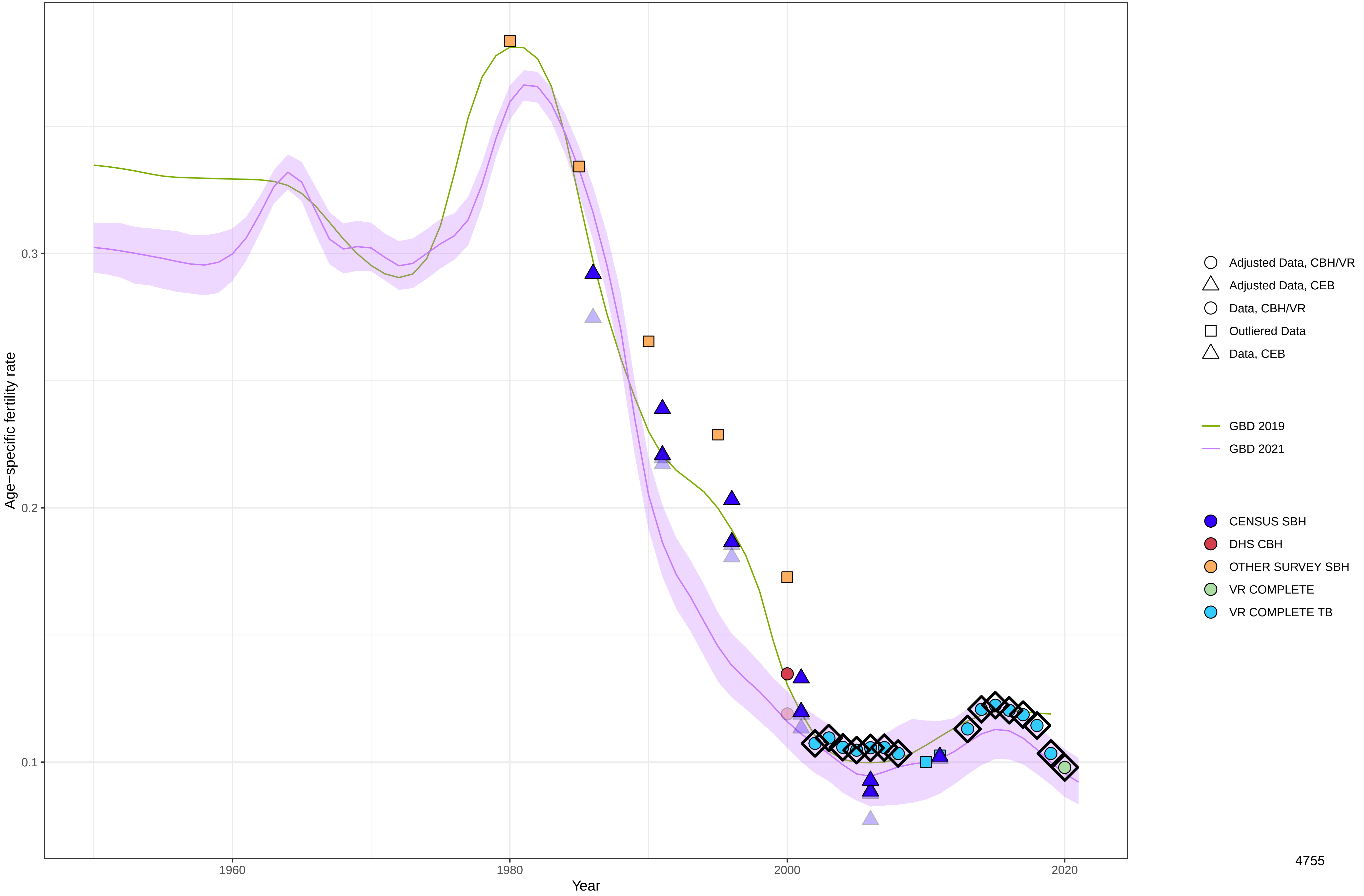

Age−specific fertility fitted trend lines, 1950−2021: Lorestan, Ages: 30−34
Data points enclosed with a diamond are reference sources

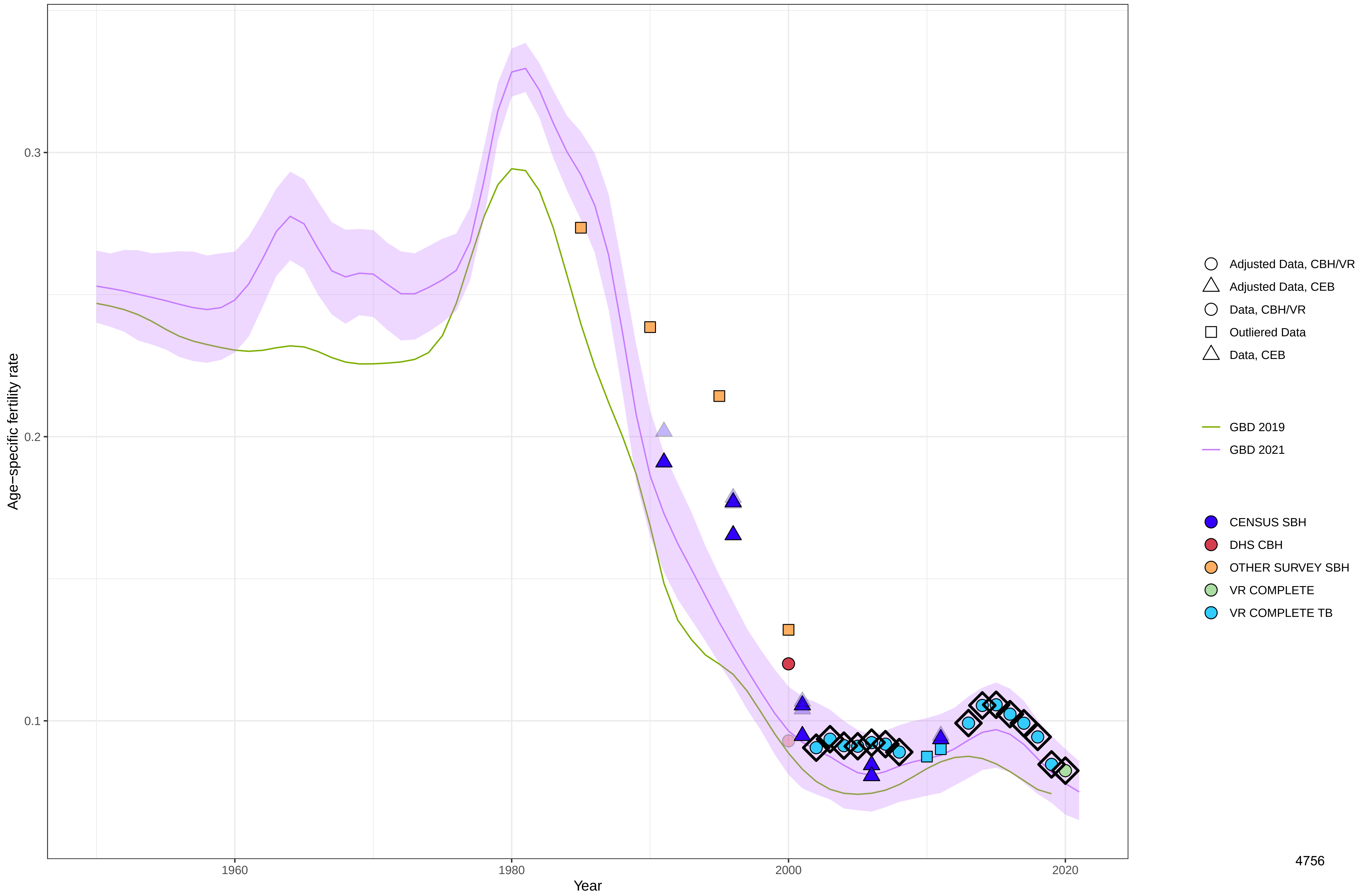

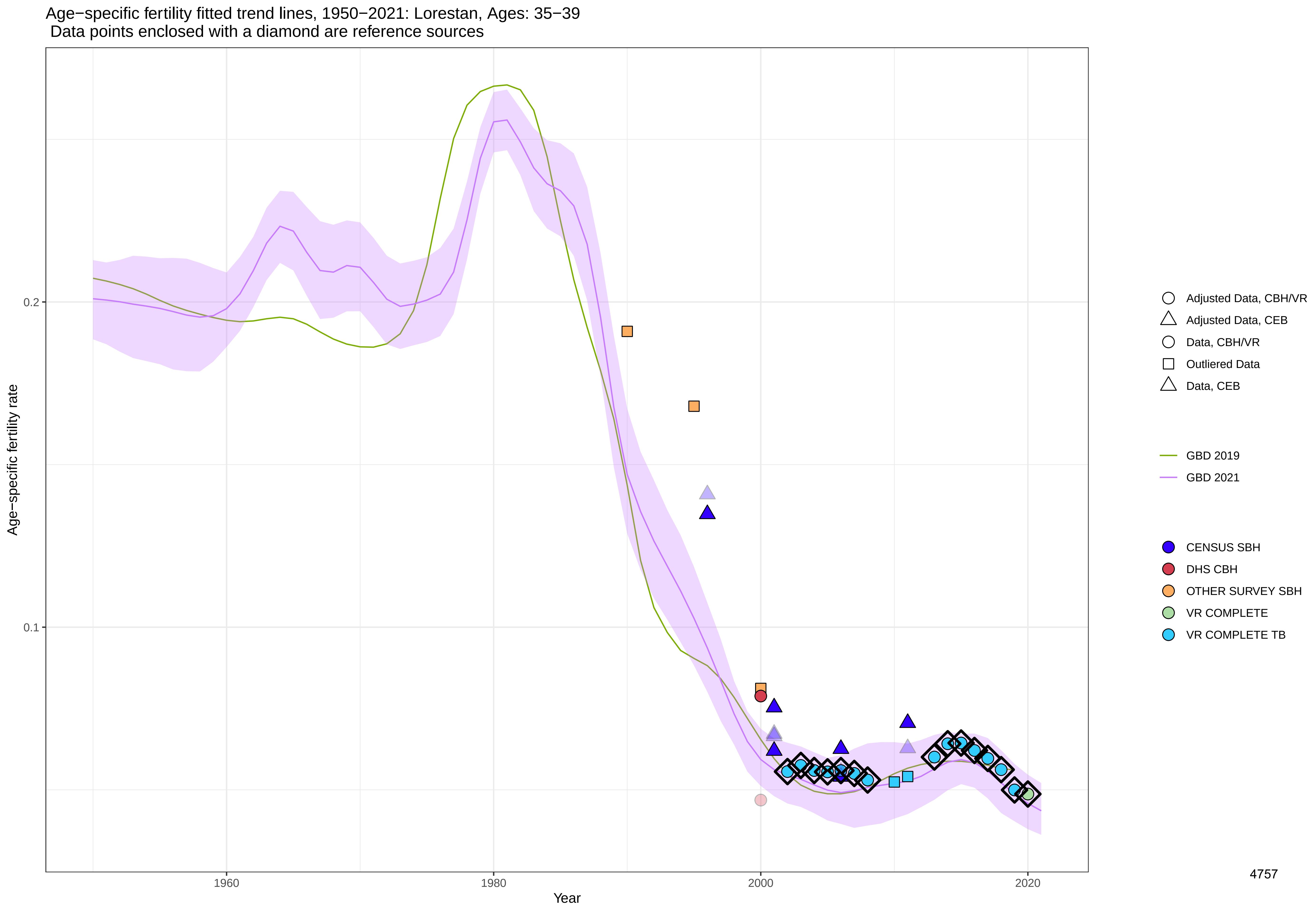

Age−specific fertility fitted trend lines, 1950−2021: Lorestan, Ages: 35−39
Data points enclosed with a diamond are reference sources
Age−specific fertility rate
Year
0.2
0.1
1960
1980
2000
2020
Adjusted Data, CBH/VR
Adjusted Data, CEB
Data, CBH/VR
Outliered Data
Data, CEB
GBD 2019
GBD 2021
CENSUS SBH
DHS CBH
OTHER SURVEY SBH
VR COMPLETE
VR COMPLETE TB

Age−specific fertility fitted trend lines, 1950−2021: Lorestan, Ages: 40−44
Data points enclosed with a diamond are reference sources

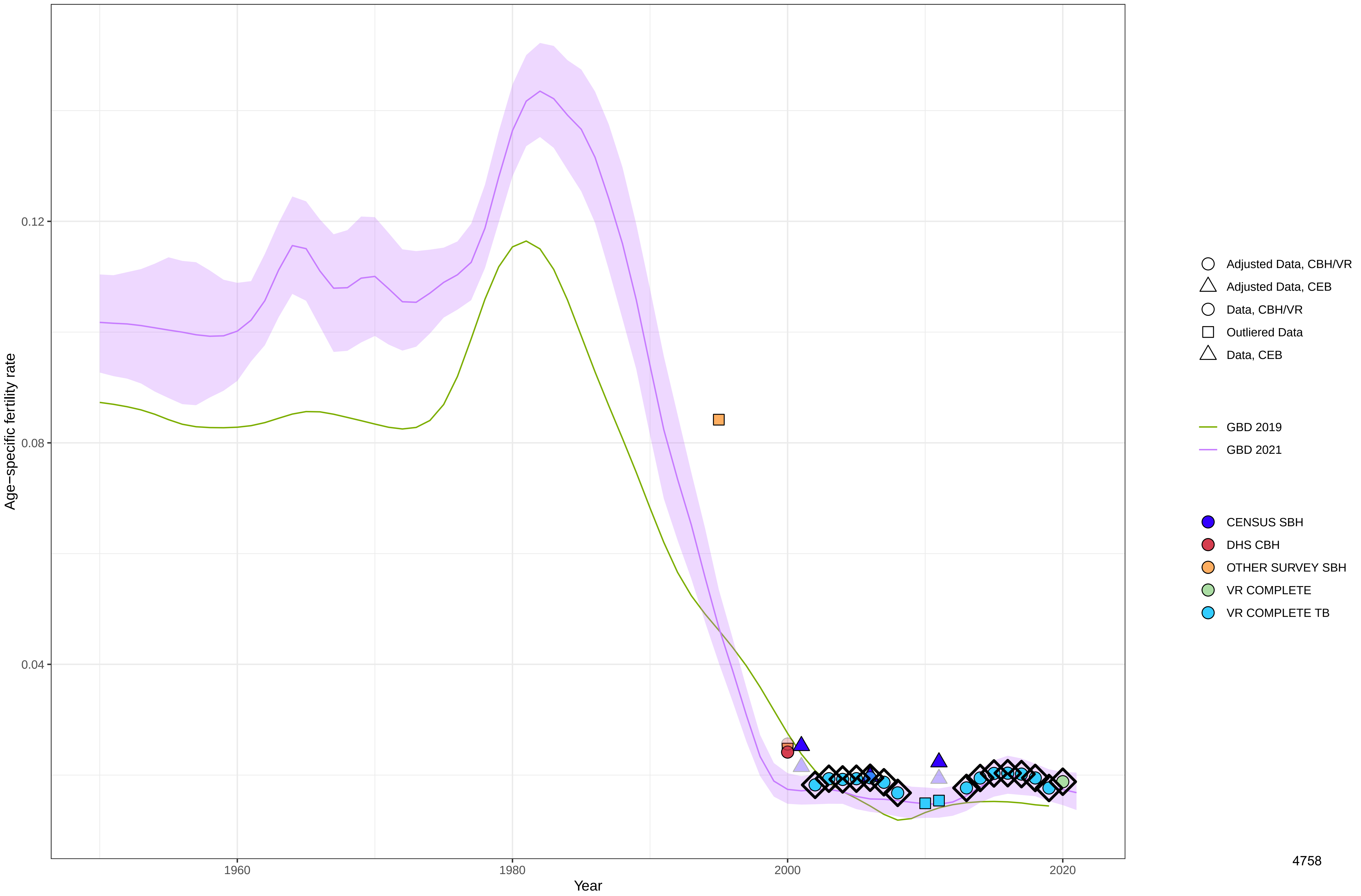

Age−specific fertility fitted trend lines, 1950−2021: Lorestan, Ages: 45−49
Data points enclosed with a diamond are reference sources

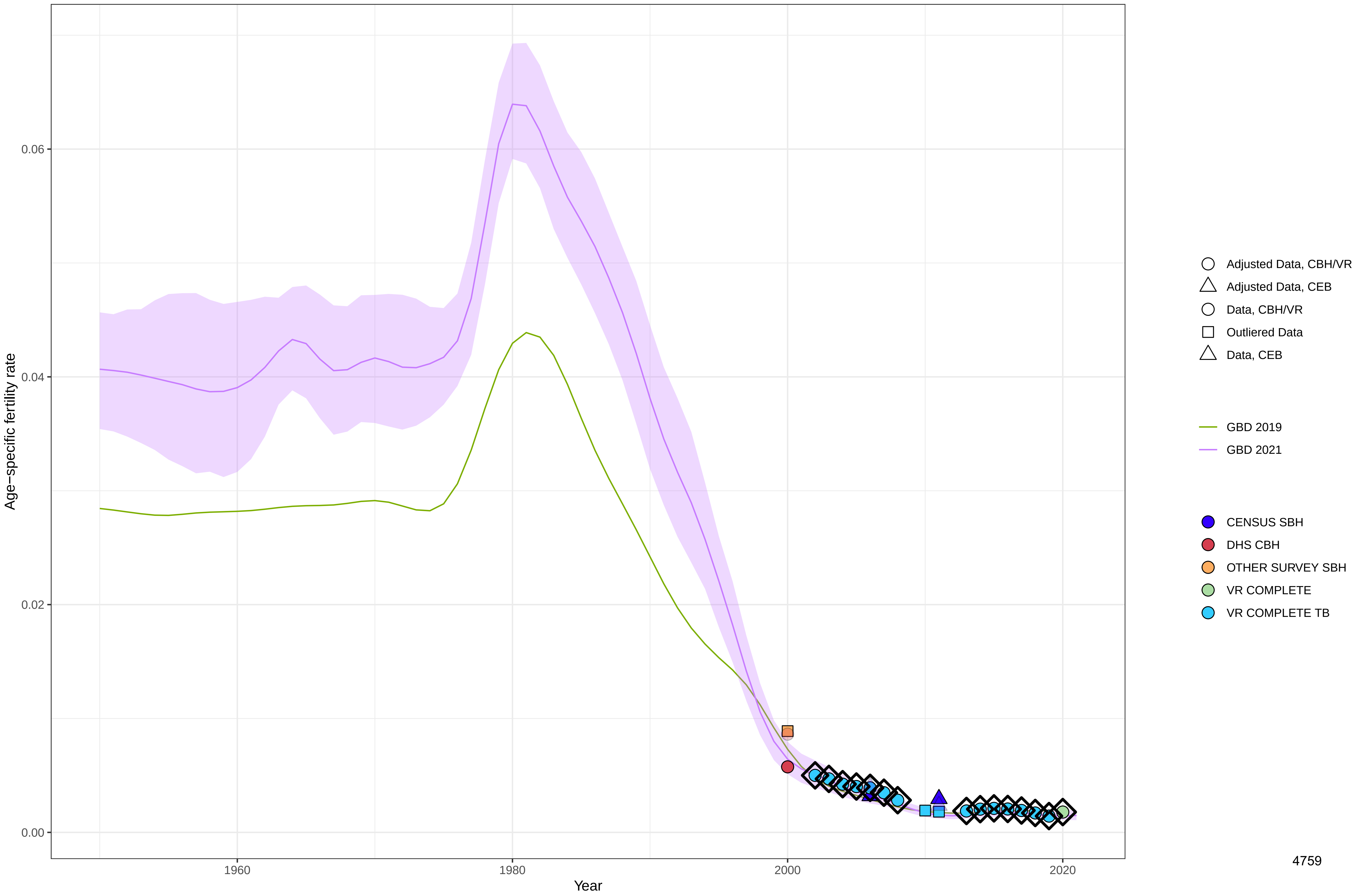

Age−specific fertility fitted trend lines, 1950−2021: Lorestan, Ages: 50−54
Data points enclosed with a diamond are reference sources

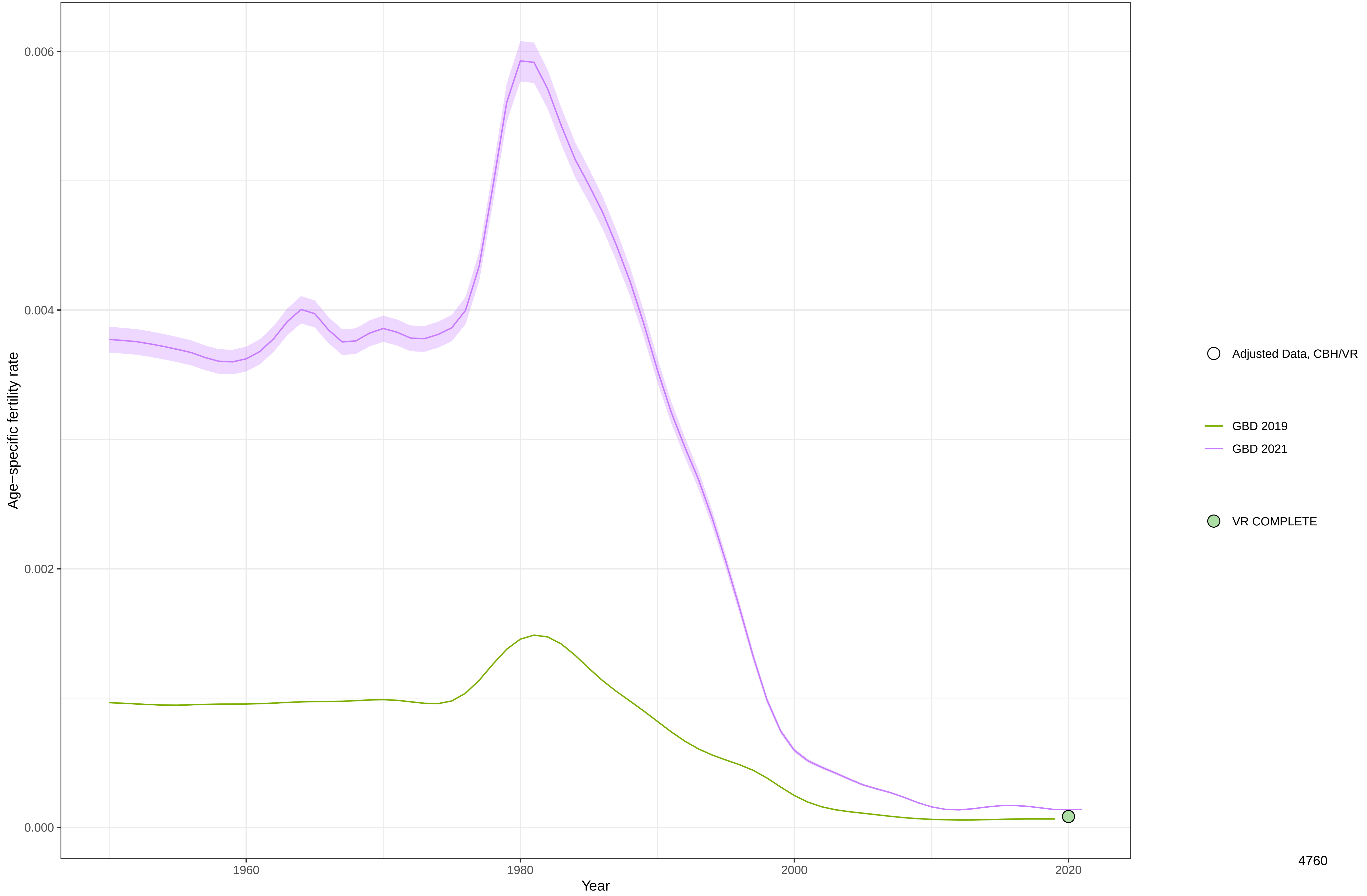

Total fertility rate fitted trend lines, 1950−2021: Lorestan TFR
Data points enclosed with a diamond are reference sources

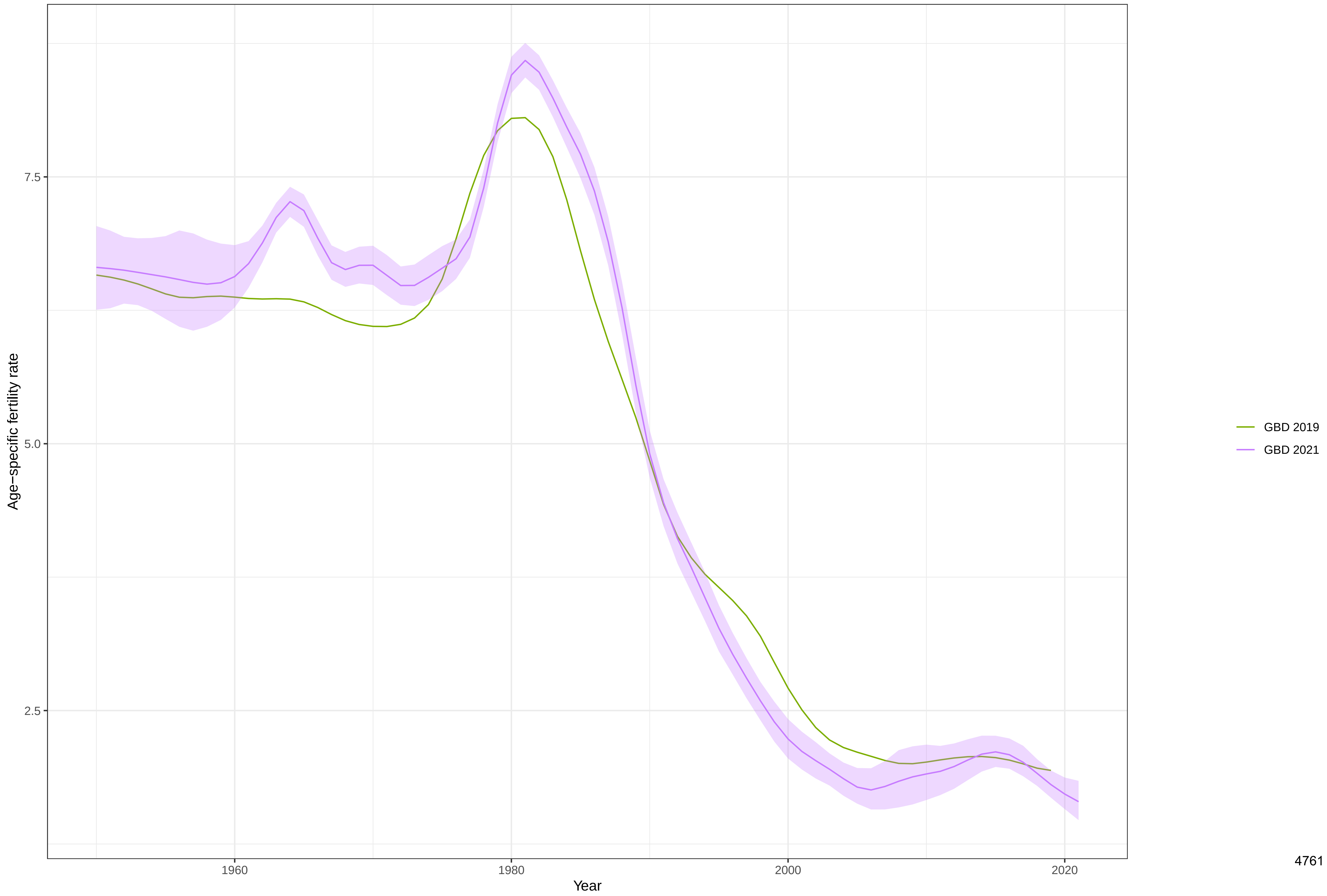

Age−specific fertility fitted trend lines, 1950−2021: Markazi, Ages: 10−14
Data points enclosed with a diamond are reference sources

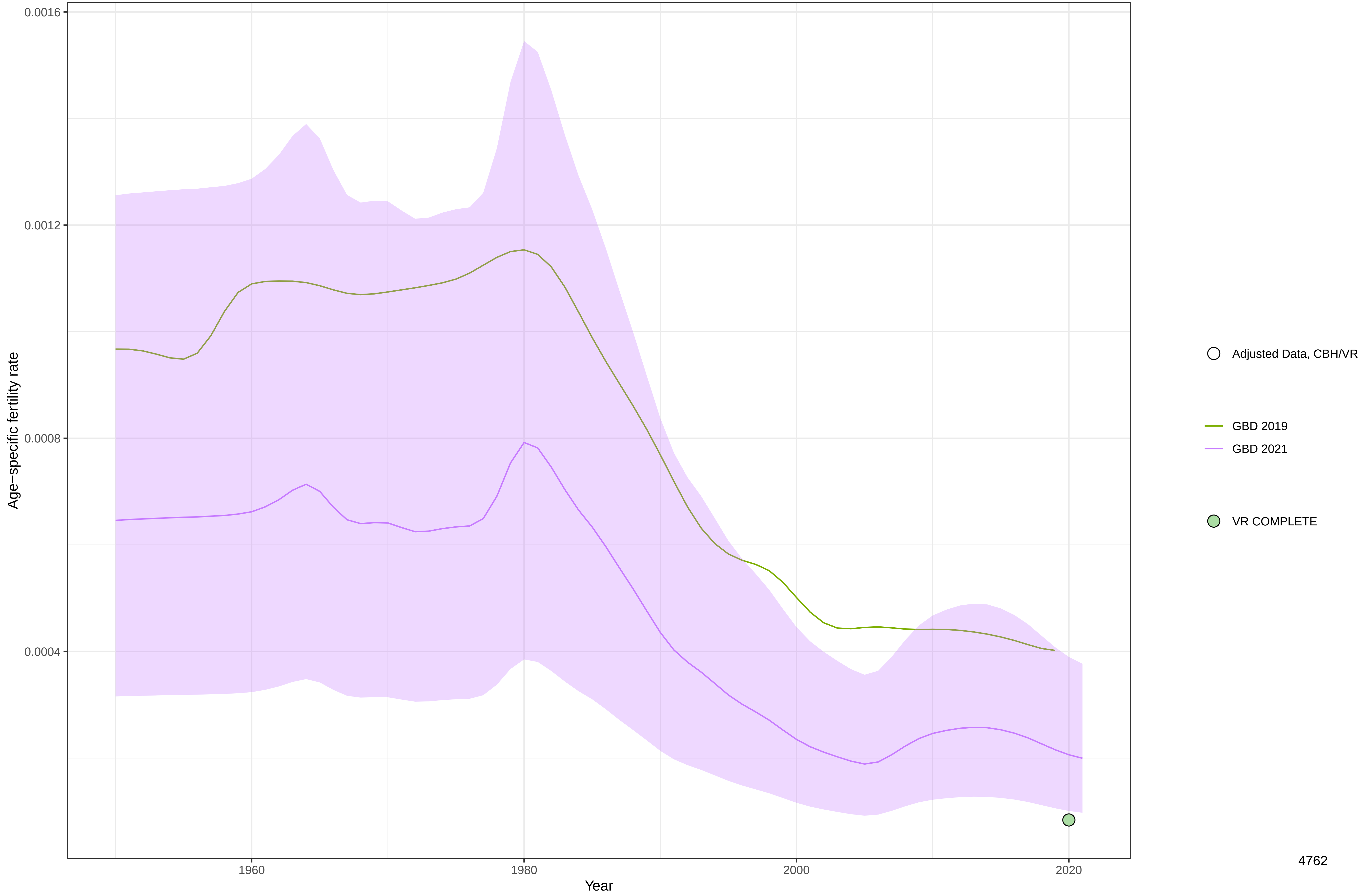

Age−specific fertility fitted trend lines, 1950−2021: Markazi, Ages: 15−19
Data points enclosed with a diamond are reference sources

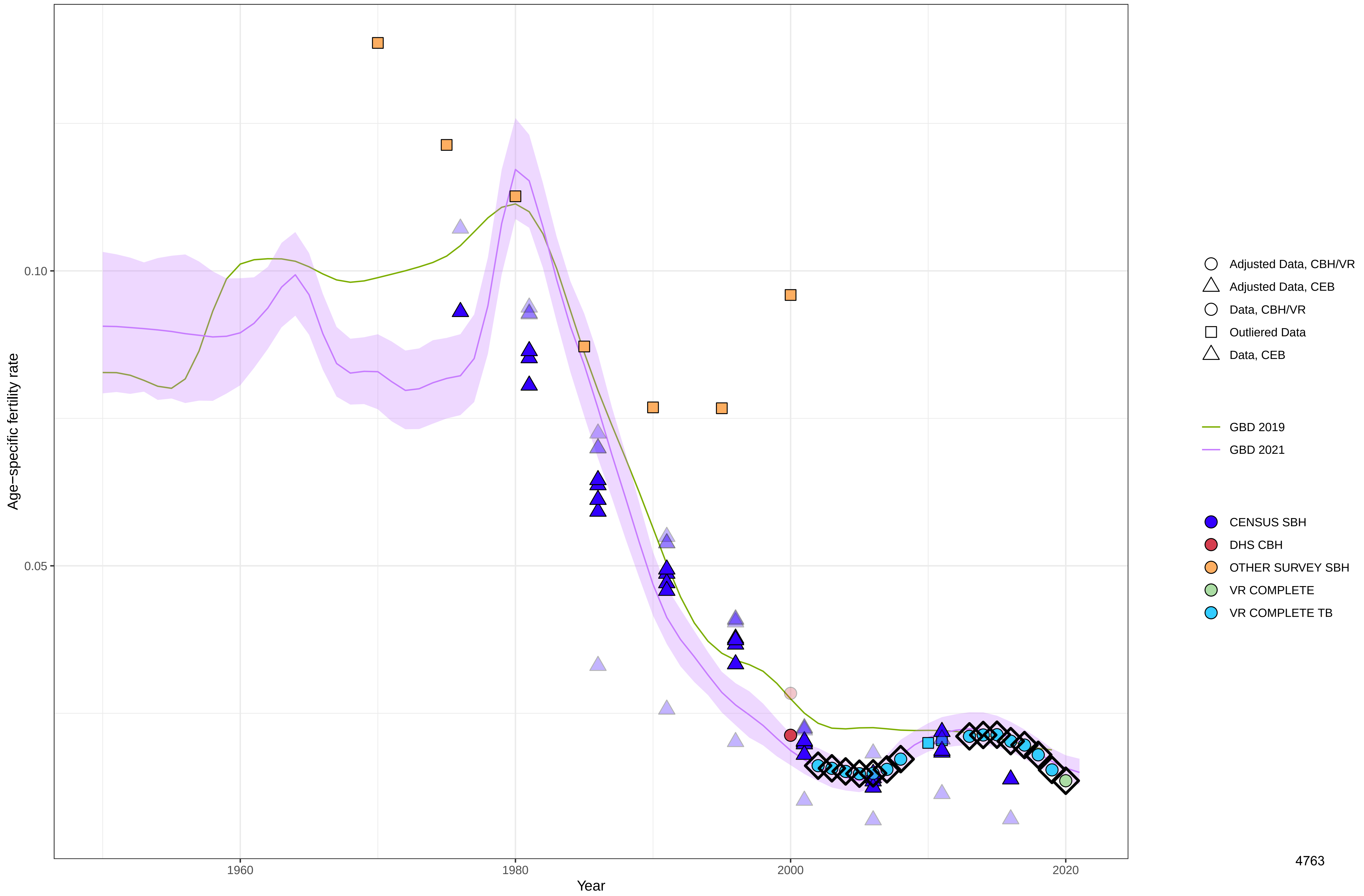

Age−specific fertility fitted trend lines, 1950−2021: Markazi, Ages: 20−24
Data points enclosed with a diamond are reference sources

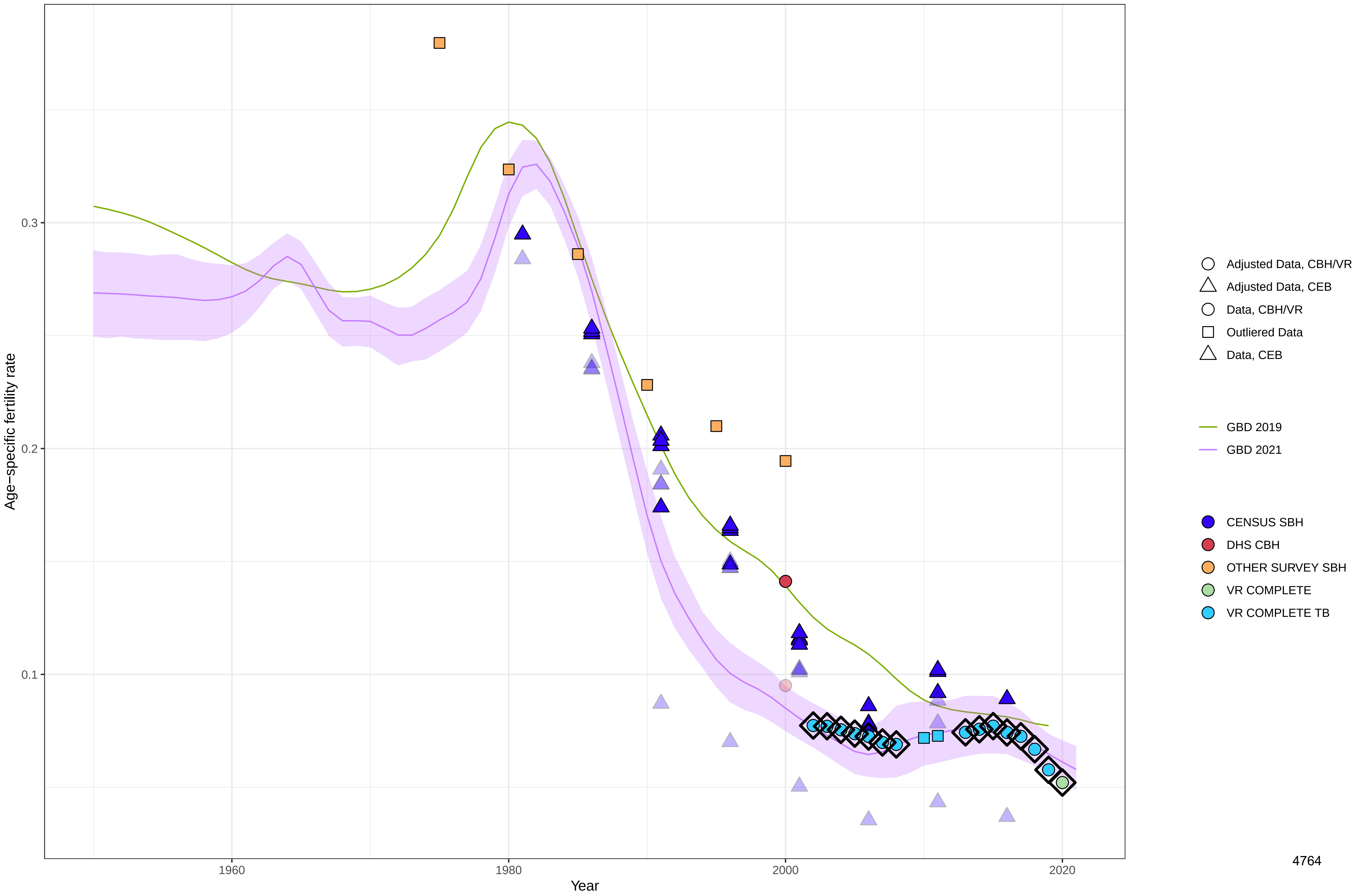

Age−specific fertility fitted trend lines, 1950−2021: Markazi, Ages: 25−29
Data points enclosed with a diamond are reference sources

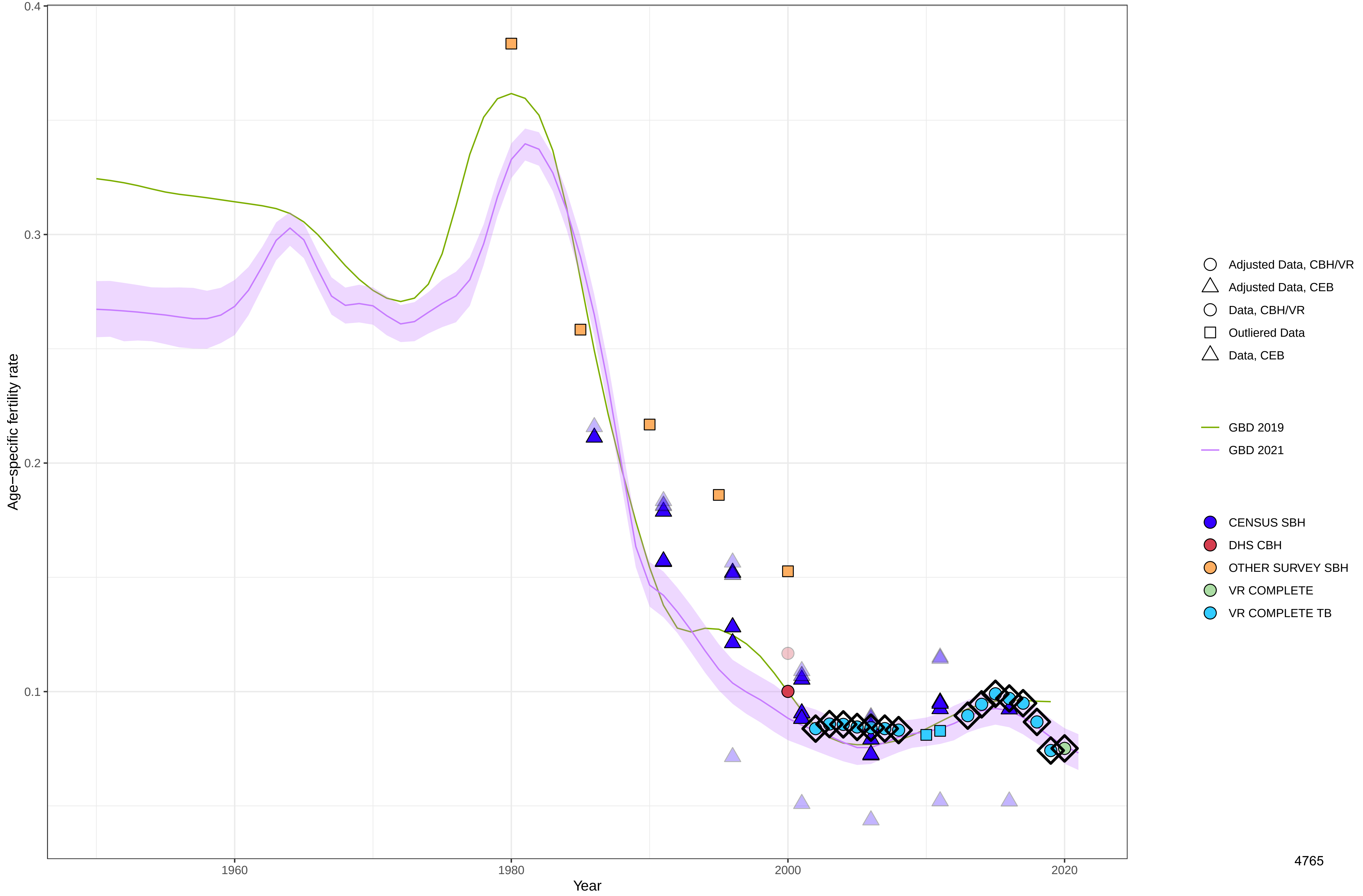

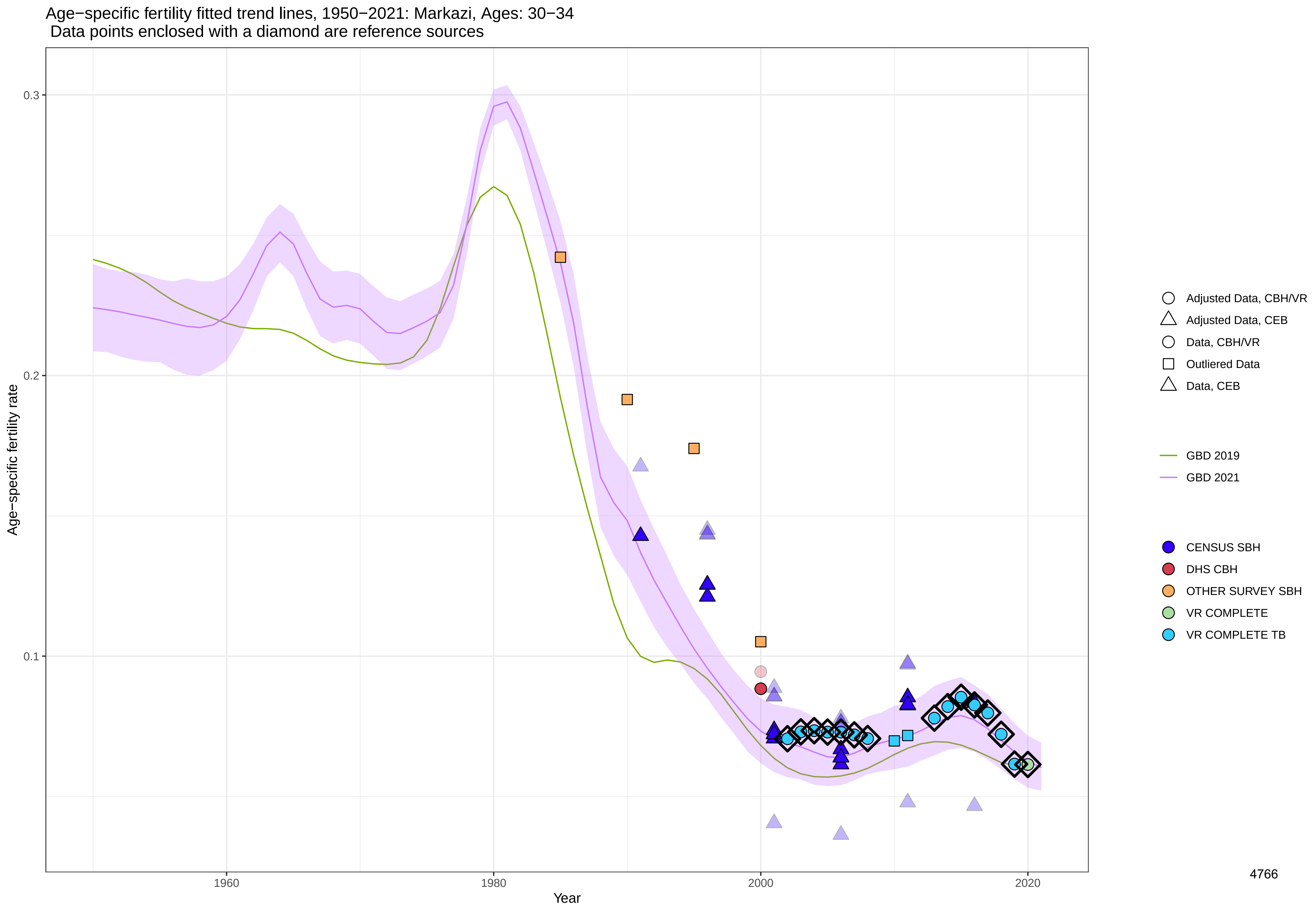

Age−specific fertility fitted trend lines, 1950−2021: Markazi, Ages: 30−34
Data points enclosed with a diamond are reference sources
0.3
0.2
0.1
Age−specific fertility rate
1960
1980
2000
2020
Year
Adjusted Data, CBH/VR
Adjusted Data, CEB
Data, CBH/VR
Outliered Data
Data, CEB
GBD 2019
GBD 2021
CENSUS SBH
DHS CBH
OTHER SURVEY SBH
VR COMPLETE
VR COMPLETE TB

Age−specific fertility fitted trend lines, 1950−2021: Markazi, Ages: 35−39
Data points enclosed with a diamond are reference sources

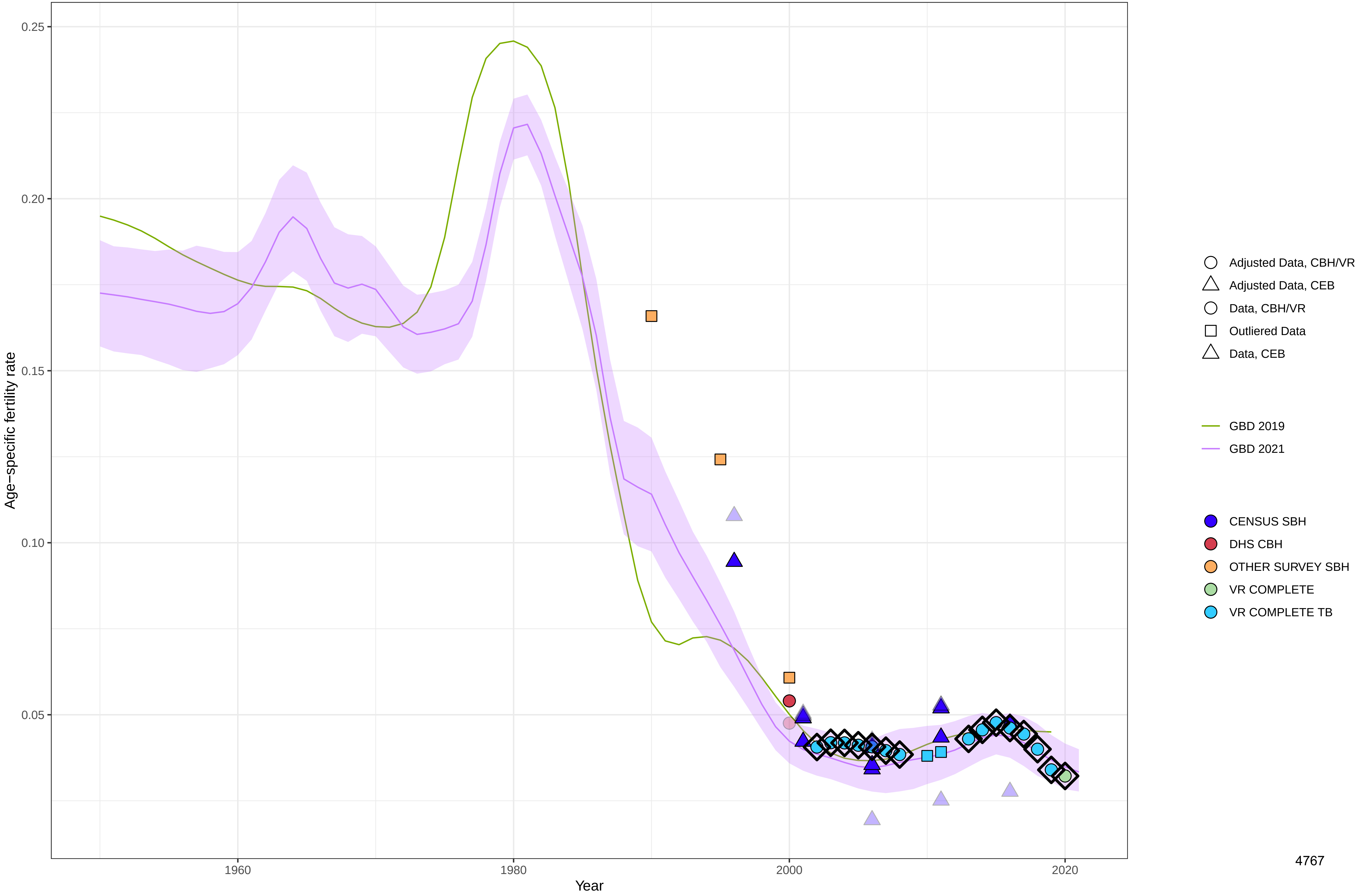

Age−specific fertility fitted trend lines, 1950−2021: Markazi, Ages: 40−44
Data points enclosed with a diamond are reference sources

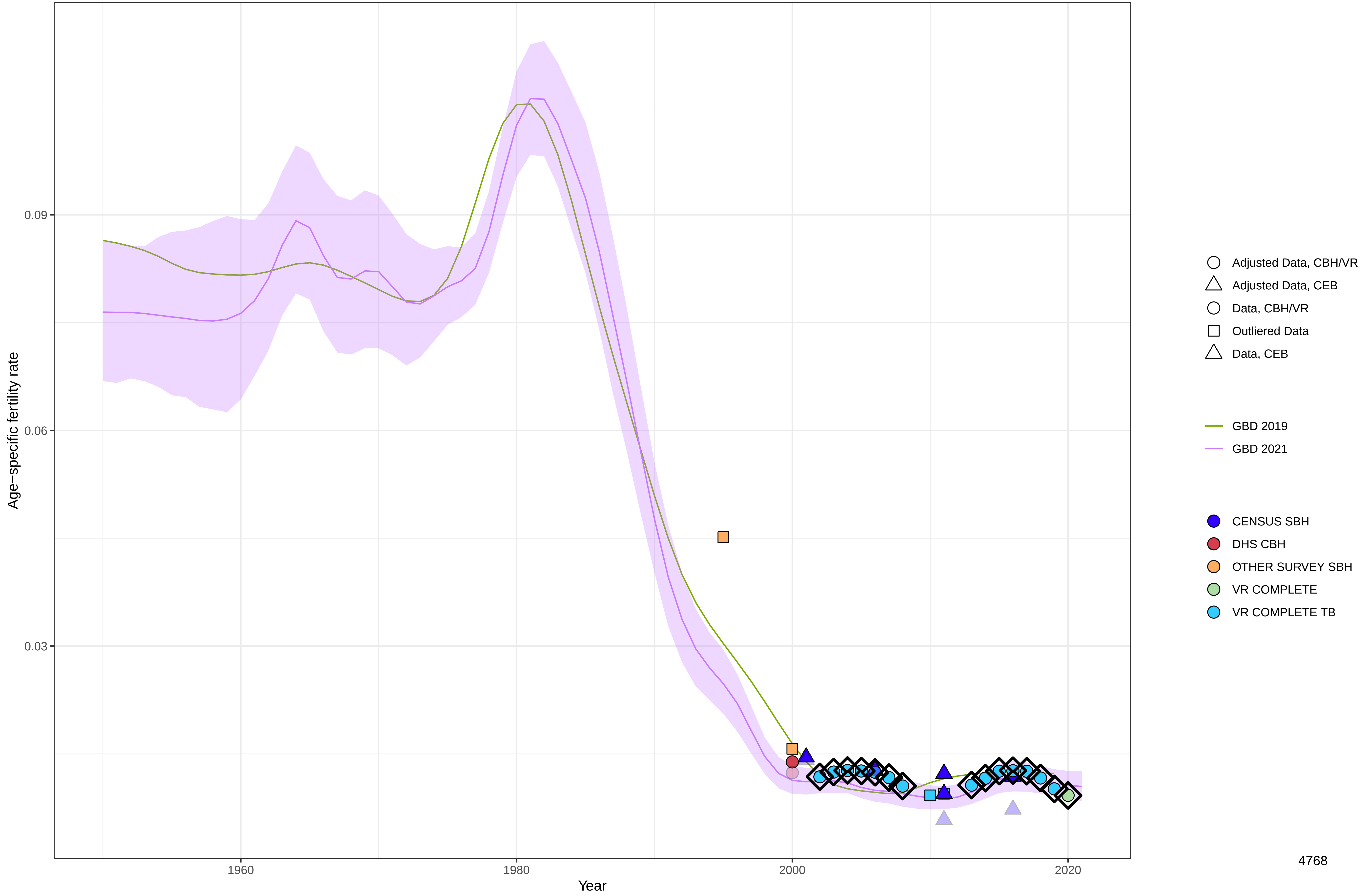

Age−specific fertility fitted trend lines, 1950−2021: Markazi, Ages: 45−49
Data points enclosed with a diamond are reference sources

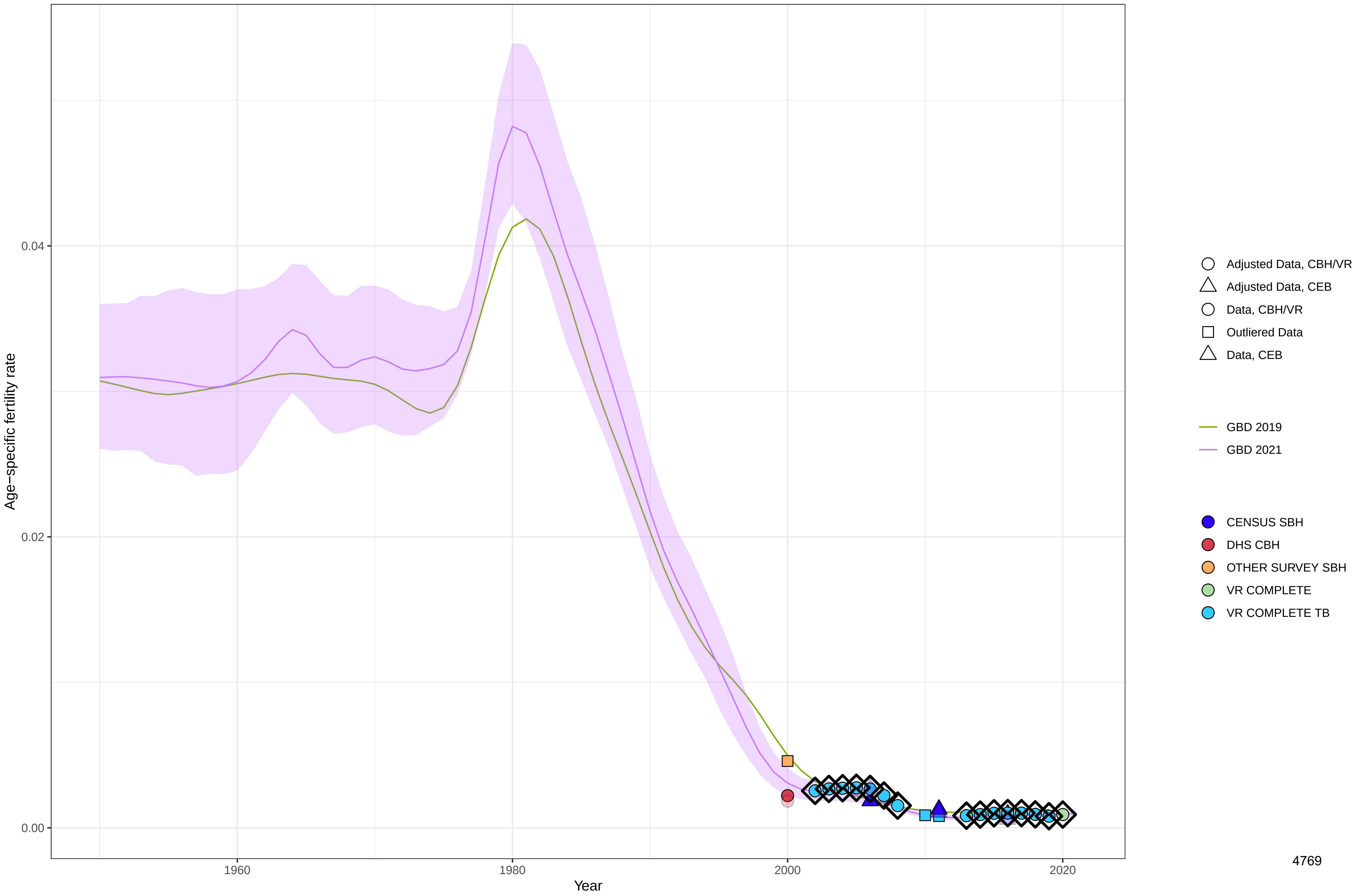

Age−specific fertility fitted trend lines, 1950−2021: Markazi, Ages: 50−54
Data points enclosed with a diamond are reference sources

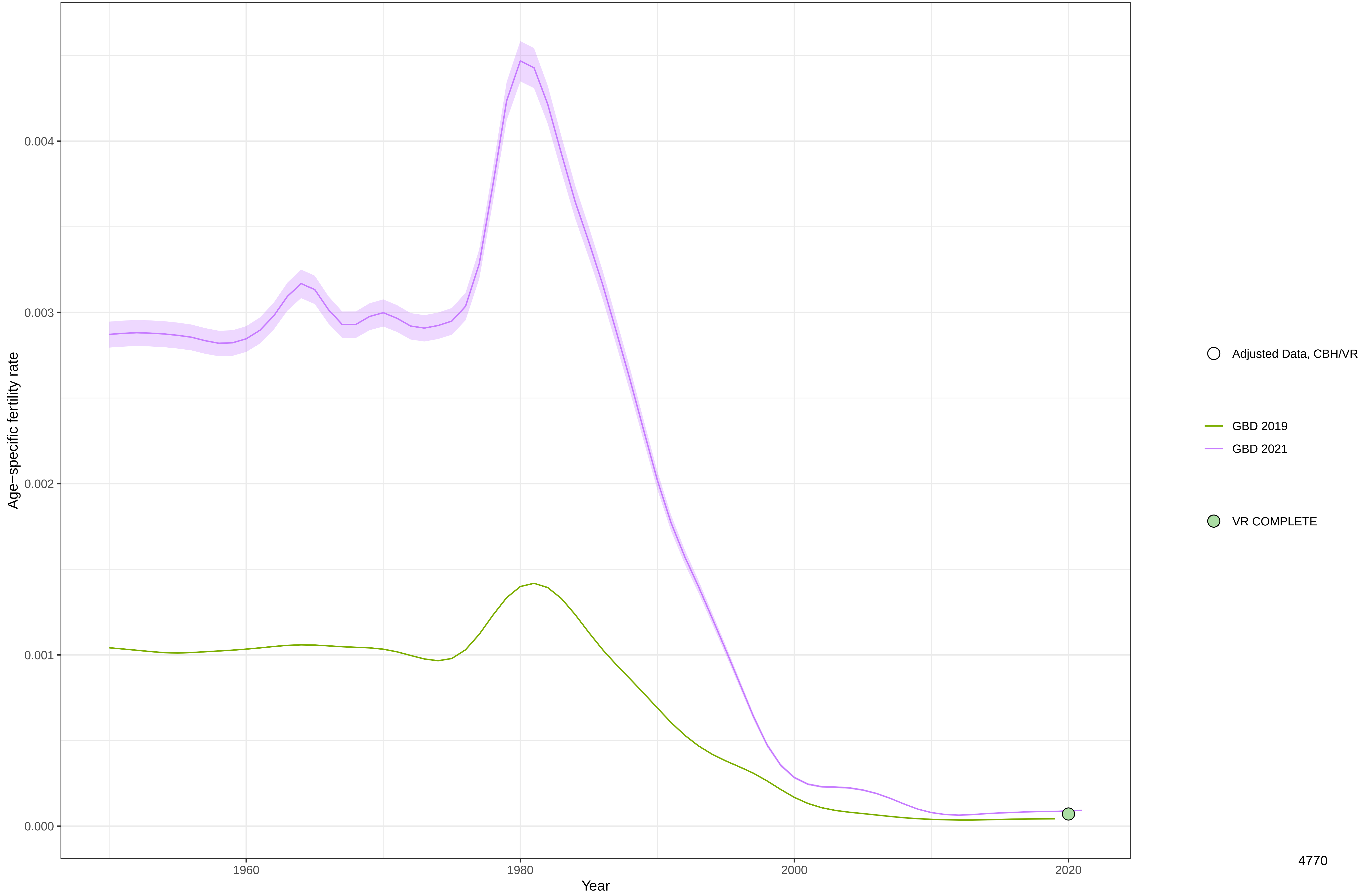

Total fertility rate fitted trend lines, 1950−2021: Markazi TFR
Data points enclosed with a diamond are reference sources

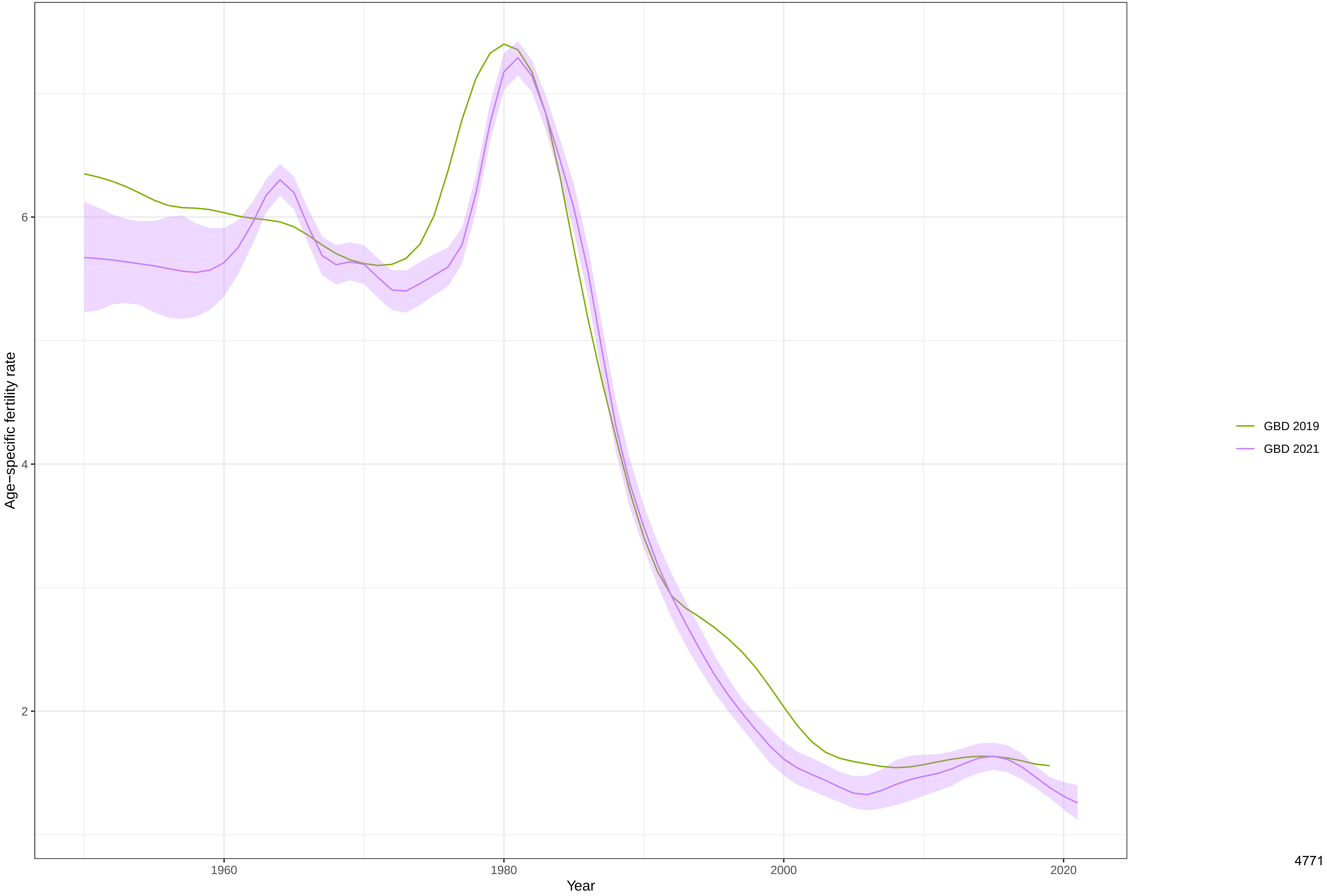

Age−specific fertility fitted trend lines, 1950−2021: Mazandaran, Ages: 10−14
Data points enclosed with a diamond are reference sources

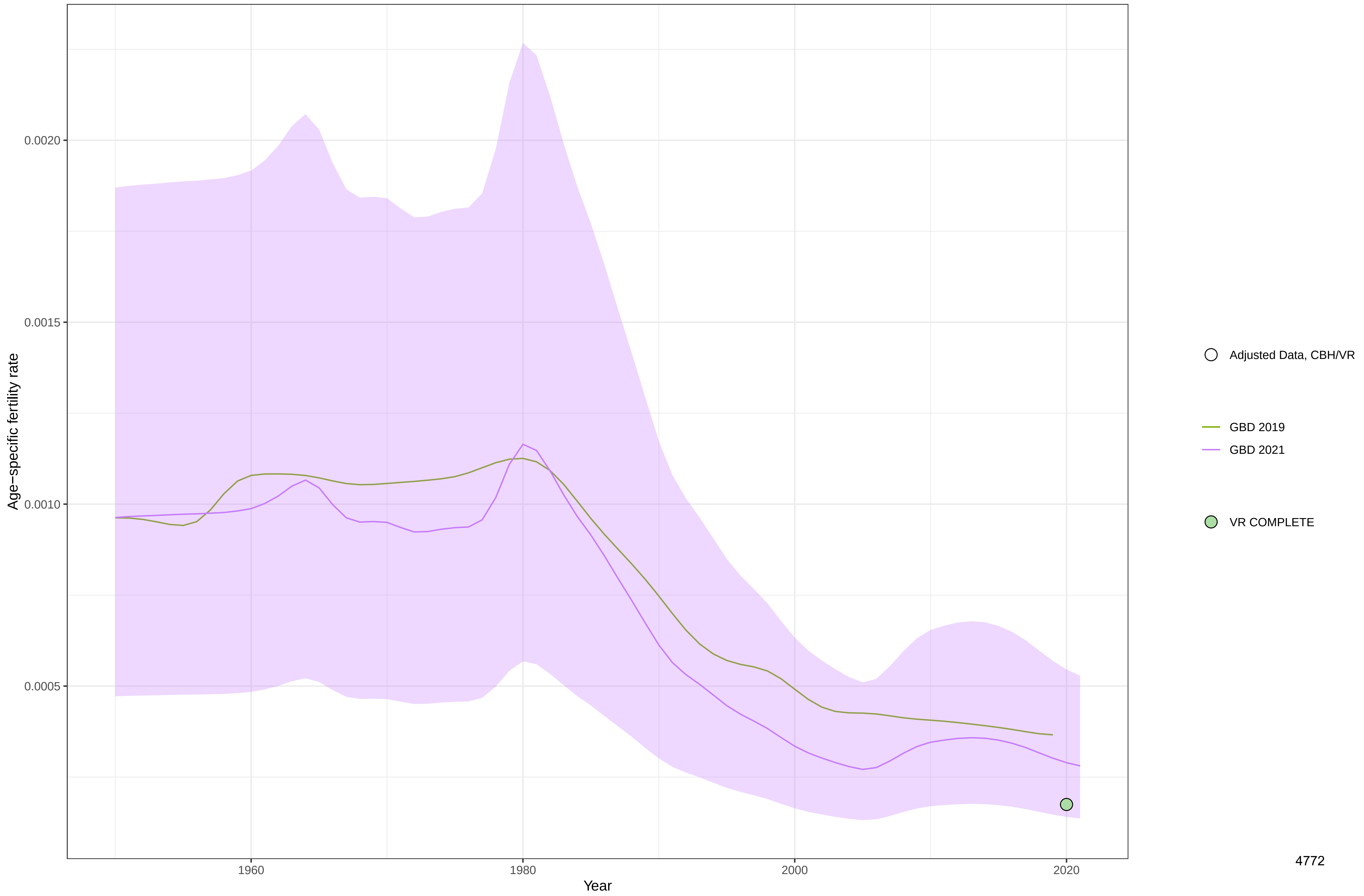

4772

Age−specific fertility fitted trend lines, 1950−2021: Mazandaran, Ages: 15−19
Data points enclosed with a diamond are reference sources

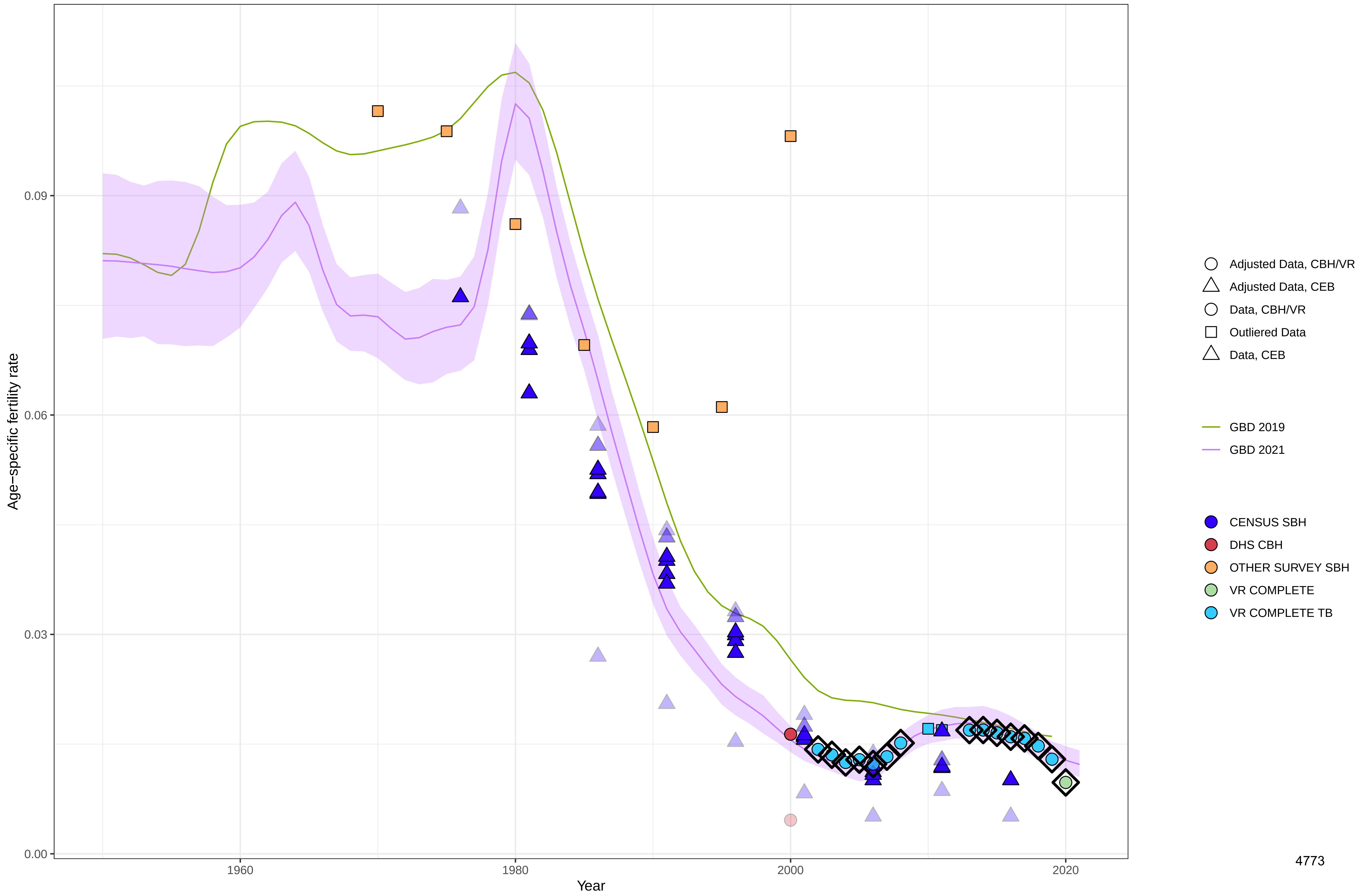

Age−specific fertility fitted trend lines, 1950−2021: Mazandaran, Ages: 20−24
Data points enclosed with a diamond are reference sources

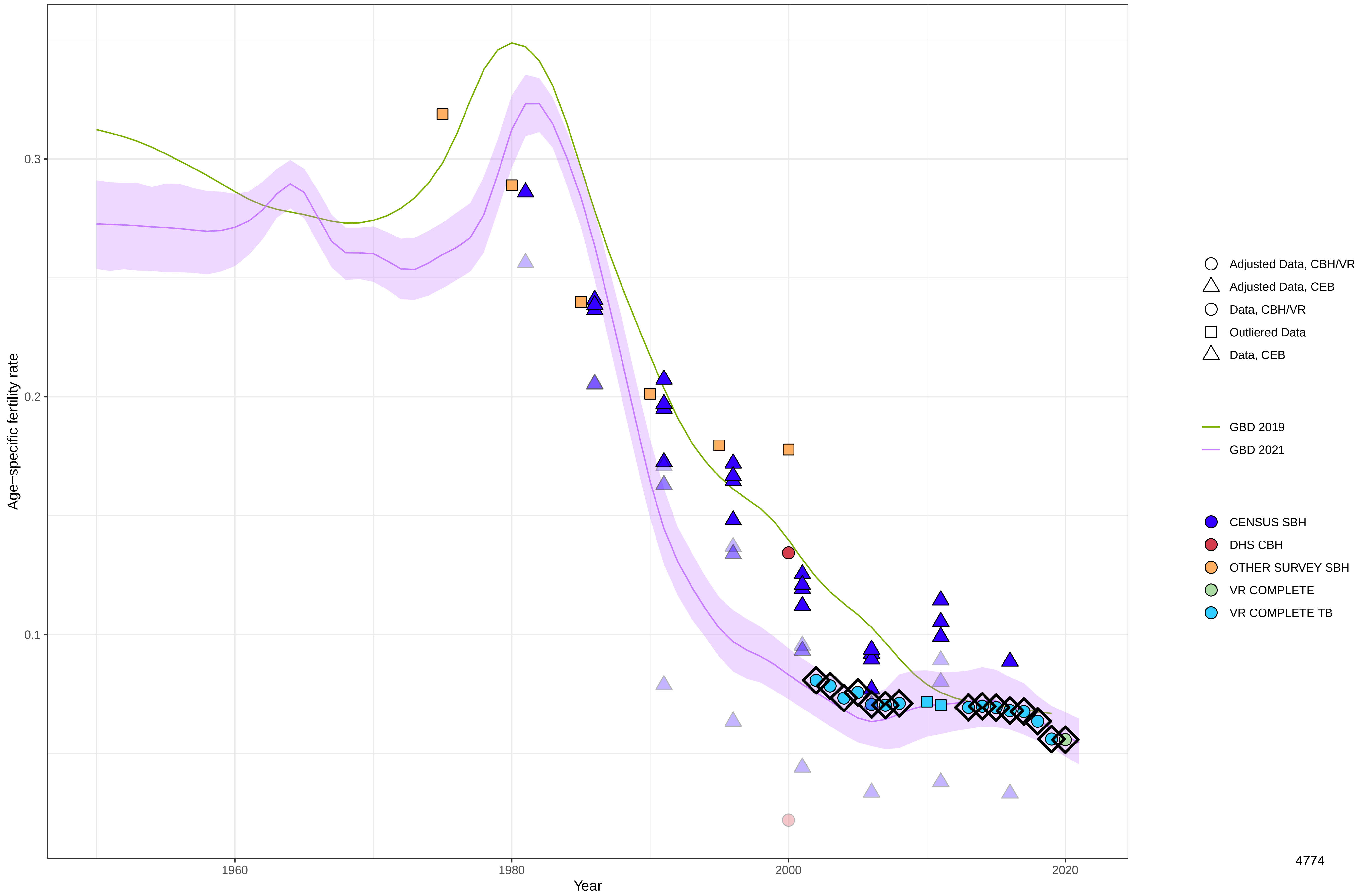

Age−specific fertility fitted trend lines, 1950−2021: Mazandaran, Ages: 25−29
Data points enclosed with a diamond are reference sources

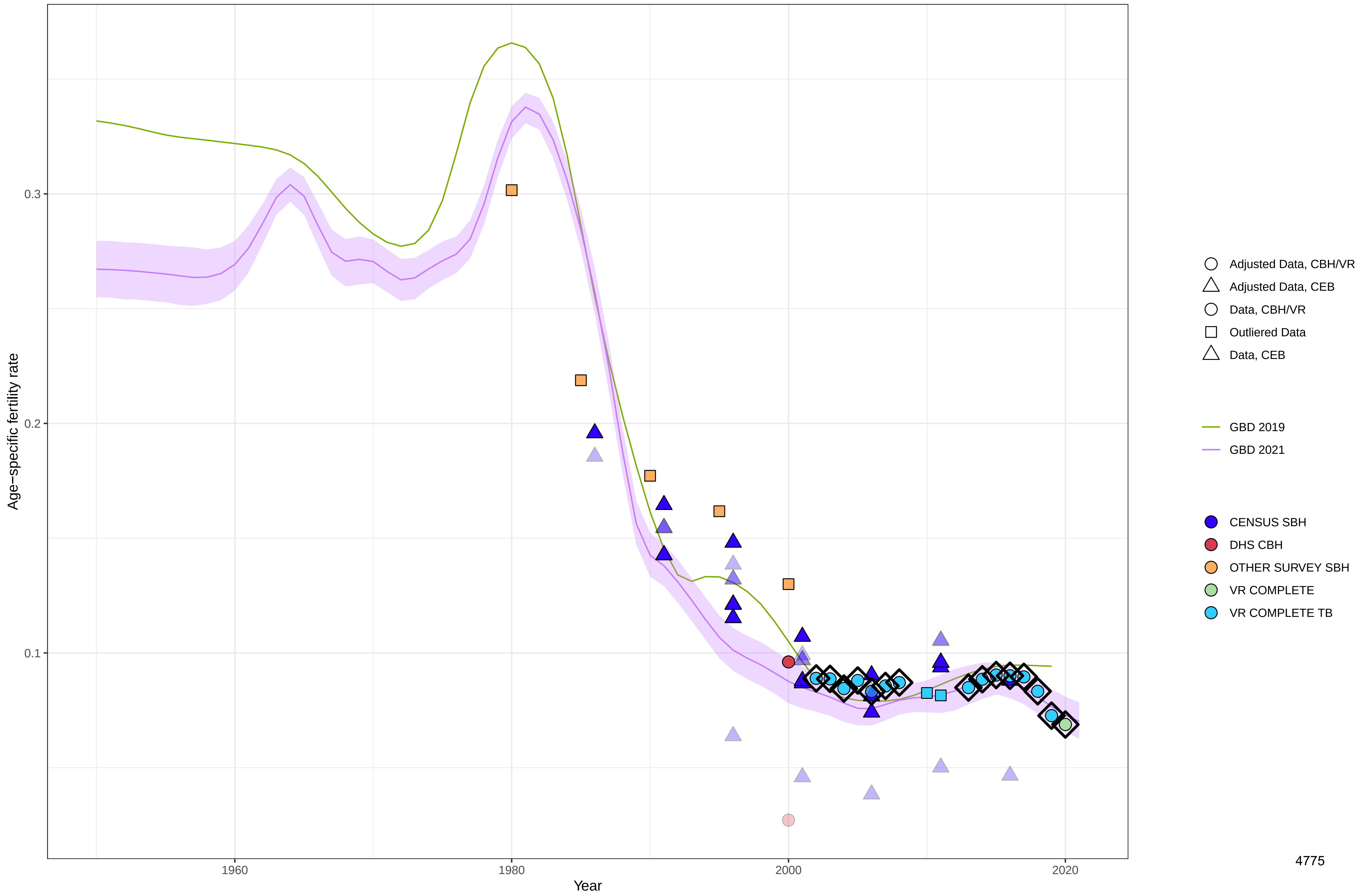

Age−specific fertility fitted trend lines, 1950−2021: Mazandaran, Ages: 30−34
Data points enclosed with a diamond are reference sources

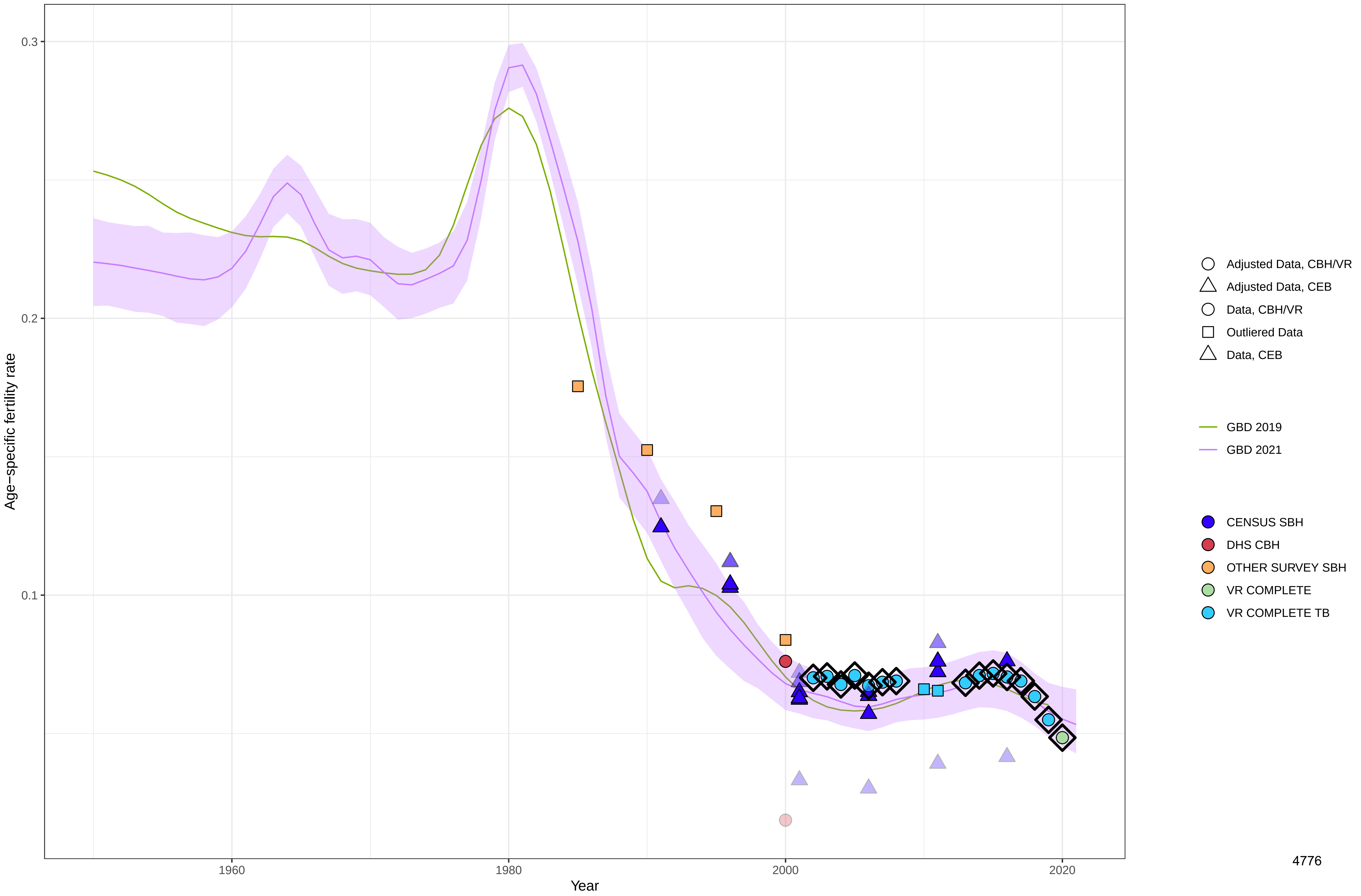

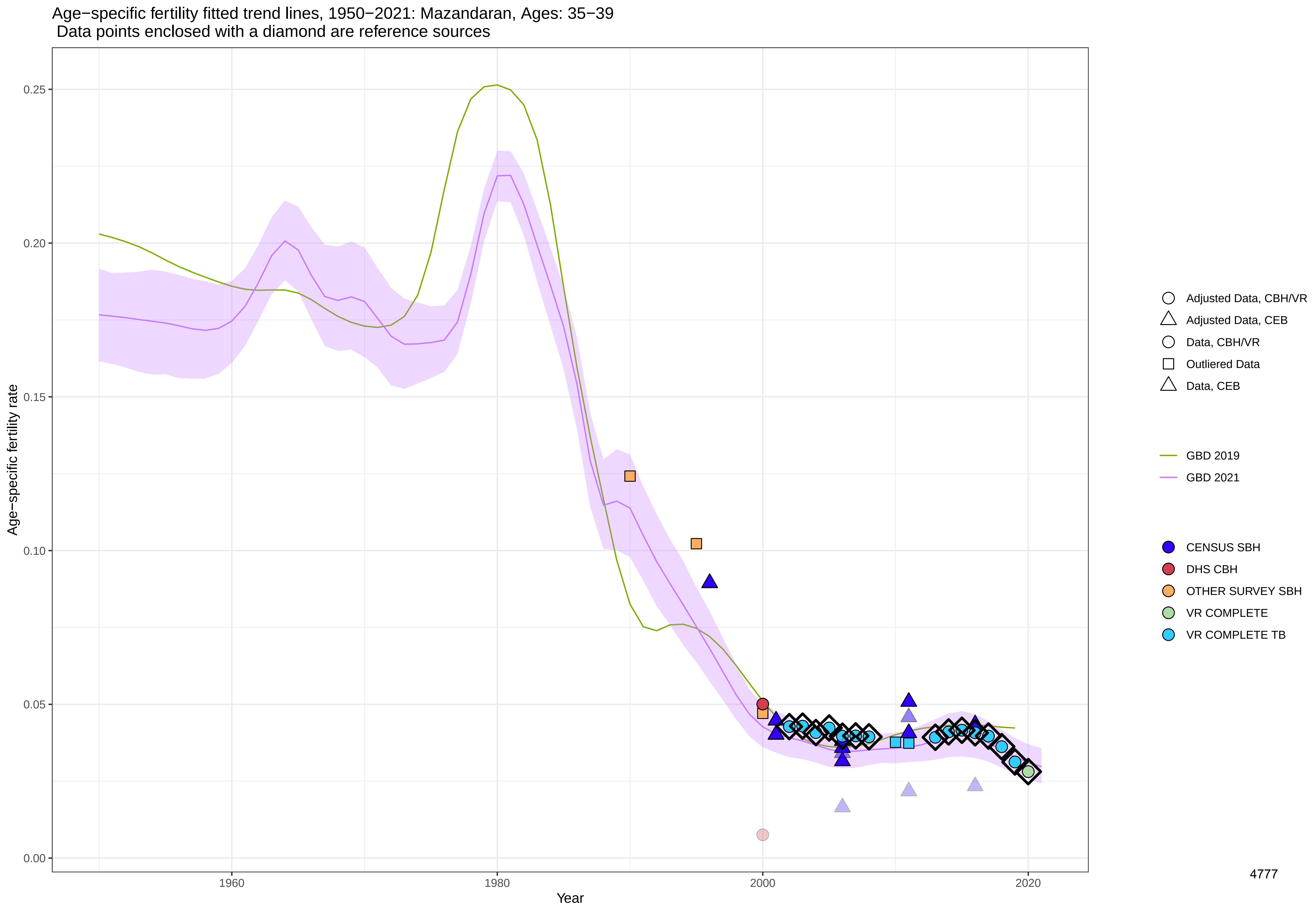

Age−specific fertility fitted trend lines, 1950−2021: Mazandaran, Ages: 35−39
Data points enclosed with a diamond are reference sources
Age−specific fertility rate
0.25
0.20
0.15
0.10
0.05
0.00
1960
1980
2000
2020
Year
Adjusted Data, CBH/VR
Adjusted Data, CEB
Data, CBH/VR
Outliered Data
Data, CEB
GBD 2019
GBD 2021
CENSUS SBH
DHS CBH
OTHER SURVEY SBH
VR COMPLETE
VR COMPLETE TB

4777

Age−specific fertility fitted trend lines, 1950−2021: Mazandaran, Ages: 40−44
Data points enclosed with a diamond are reference sources

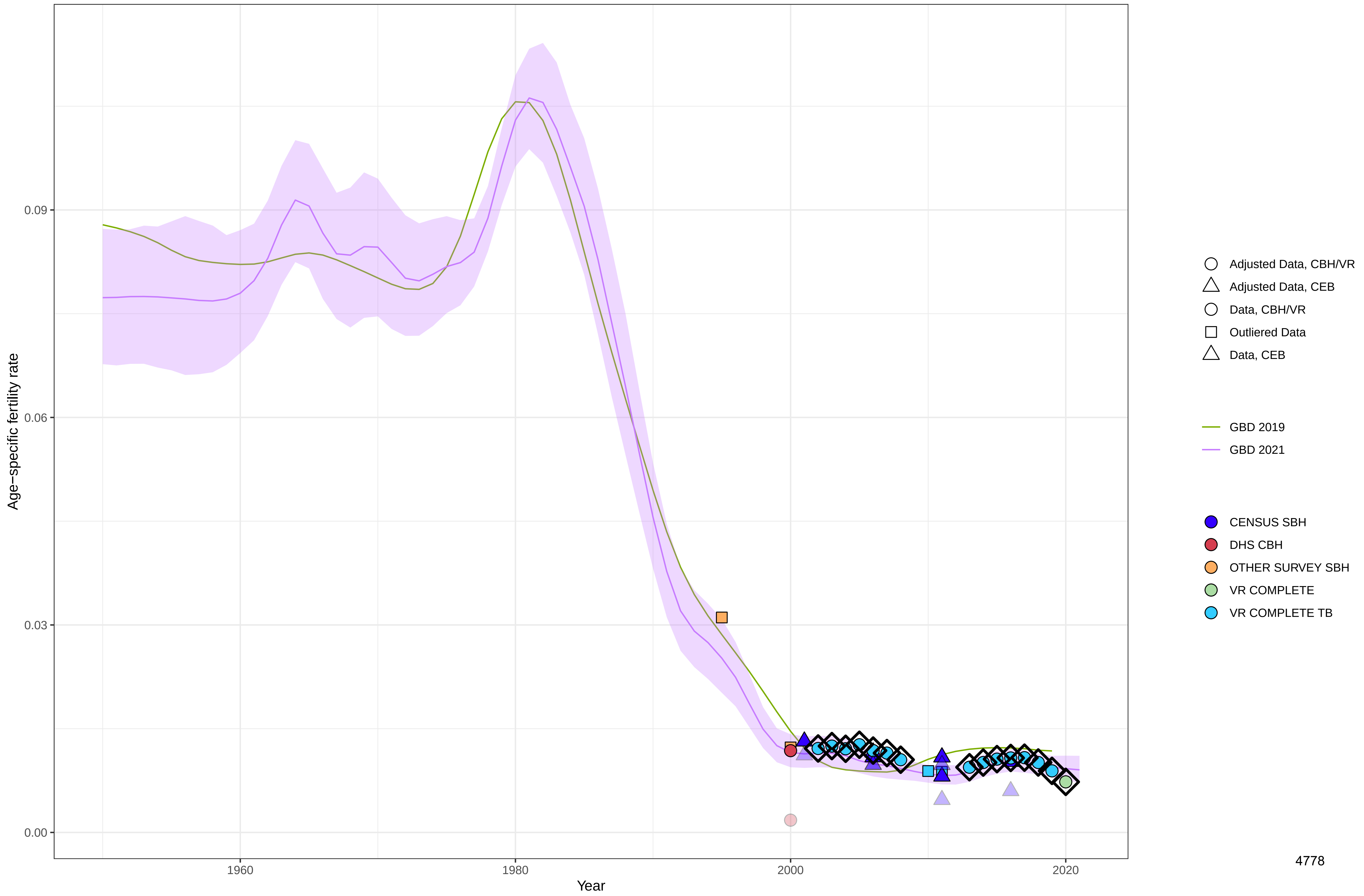

Age−specific fertility fitted trend lines, 1950−2021: Mazandaran, Ages: 45−49
Data points enclosed with a diamond are reference sources

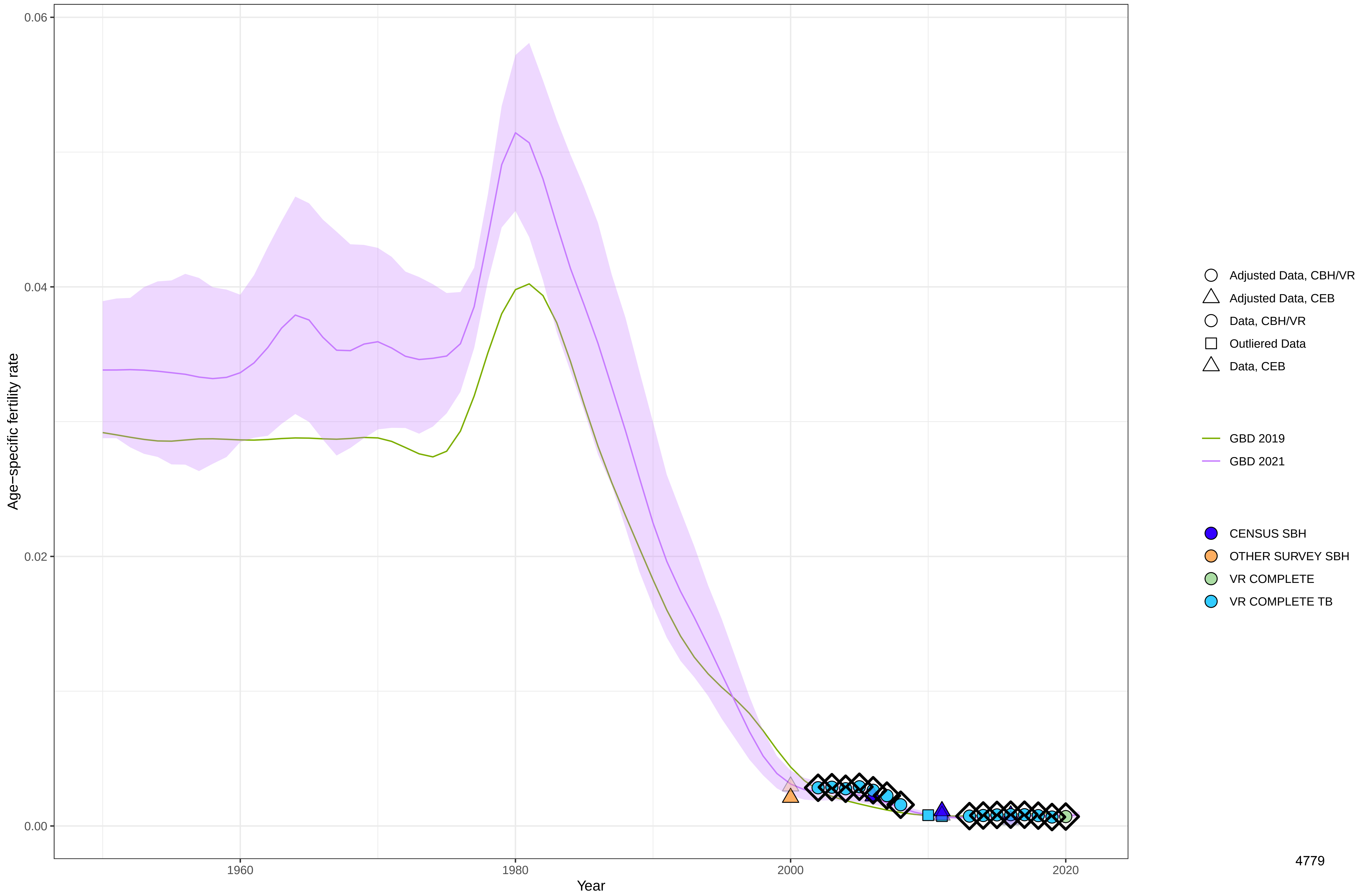

Age−specific fertility fitted trend lines, 1950−2021: Mazandaran, Ages: 50−54
Data points enclosed with a diamond are reference sources

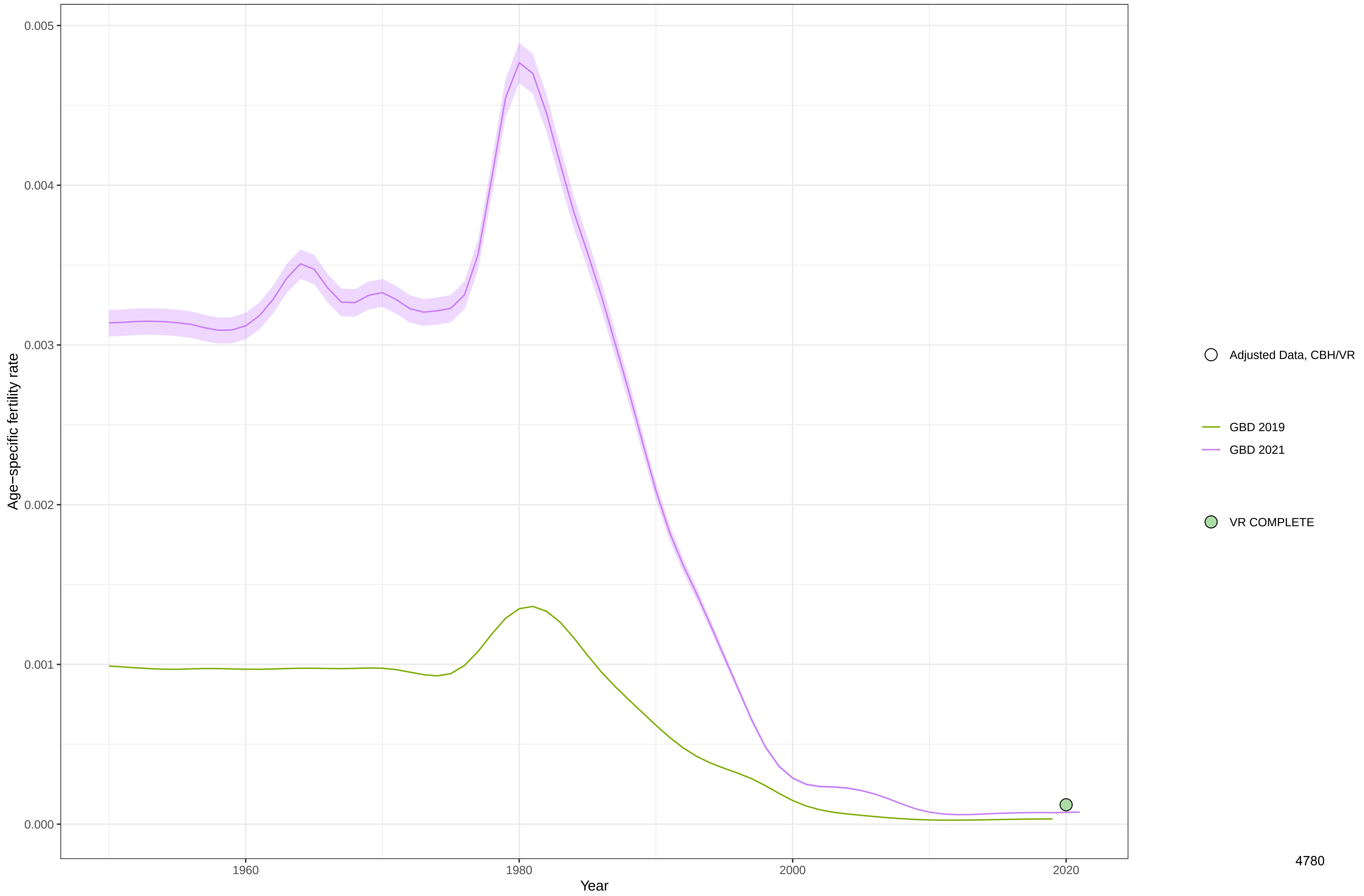

Total fertility rate fitted trend lines, 1950−2021: Mazandaran TFR
Data points enclosed with a diamond are reference sources

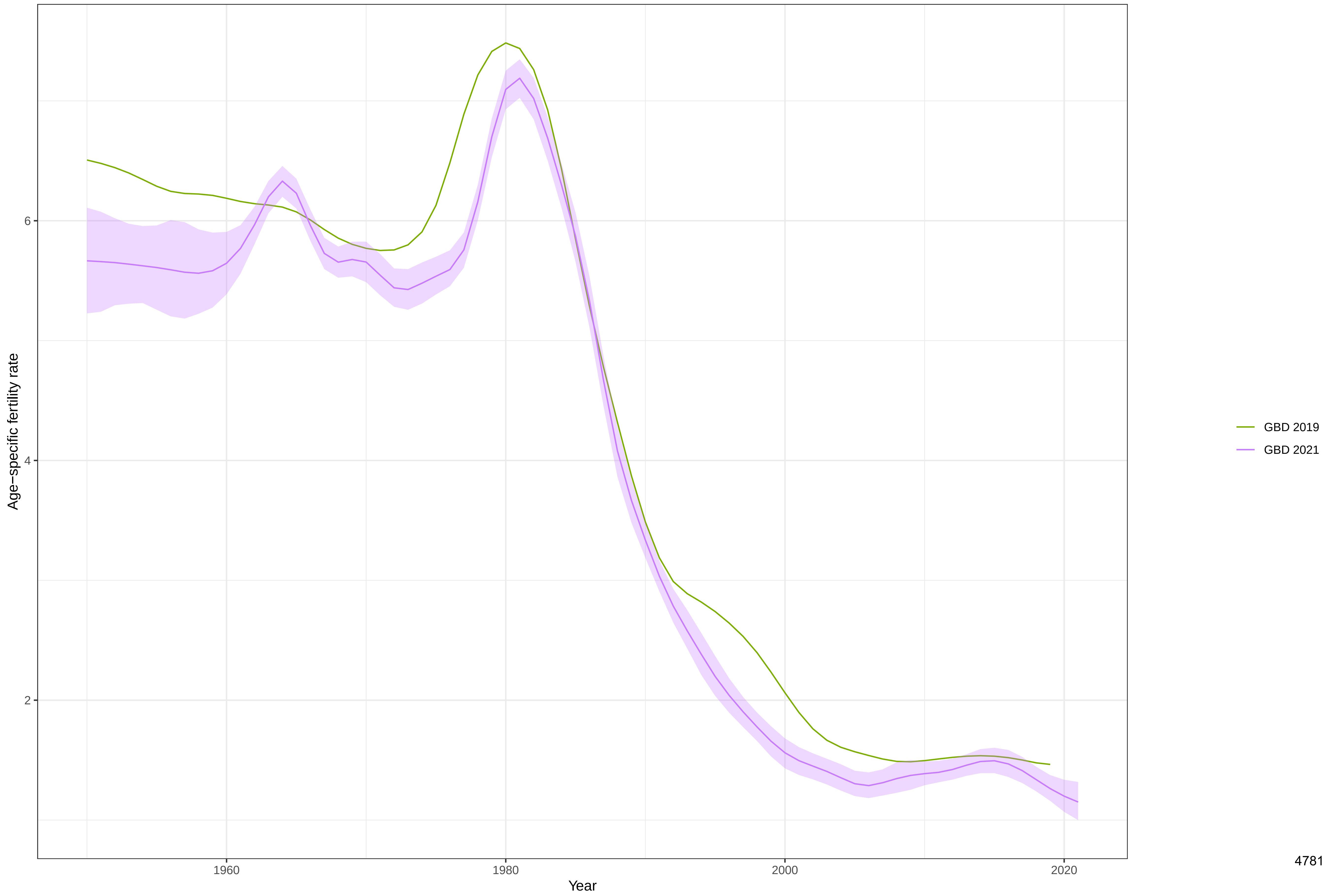

Age−specific fertility fitted trend lines, 1950−2021: North Khorasan, Ages: 10−14
Data points enclosed with a diamond are reference sources

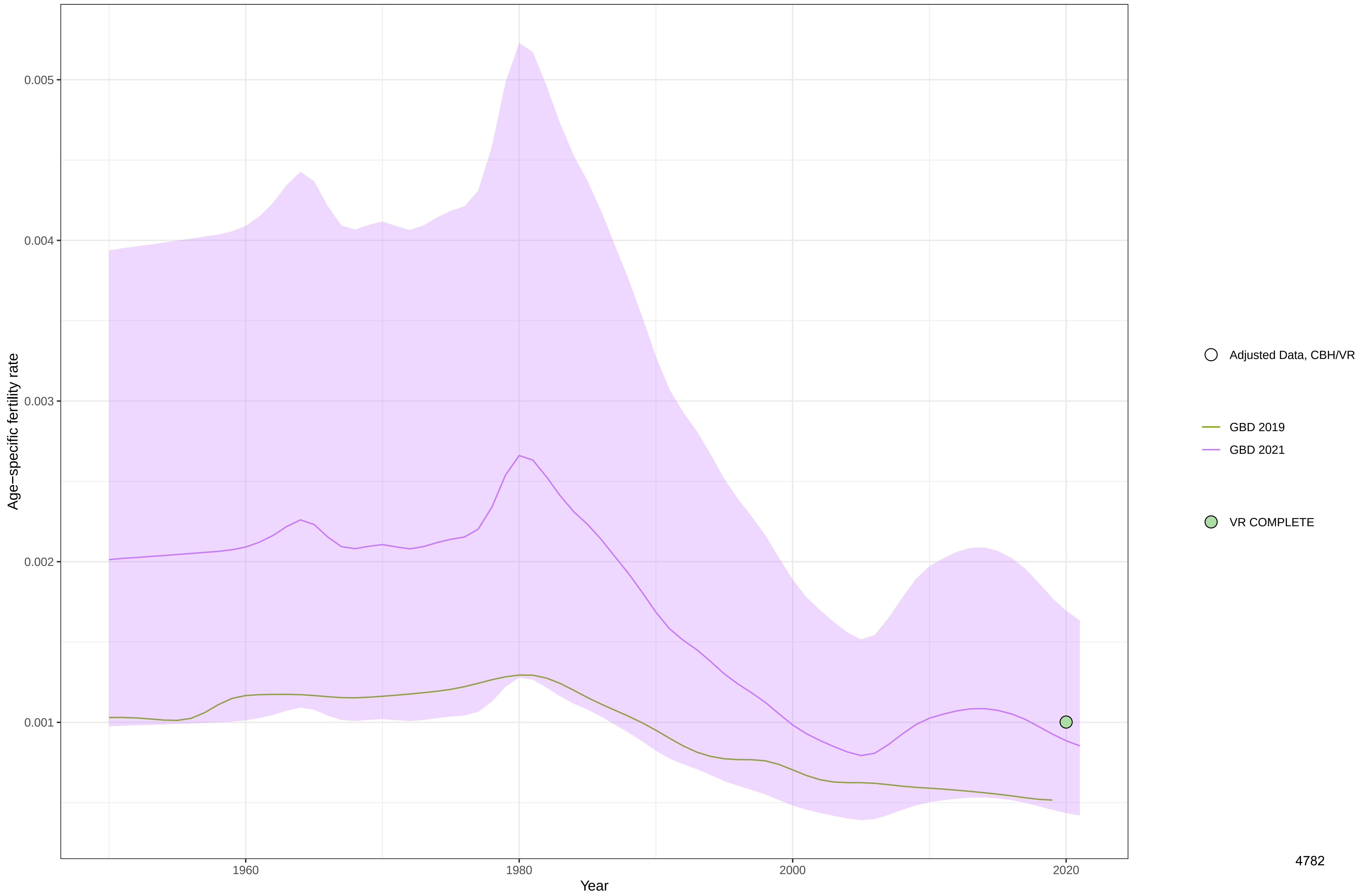

Age−specific fertility fitted trend lines, 1950−2021: North Khorasan, Ages: 15−19
Data points enclosed with a diamond are reference sources

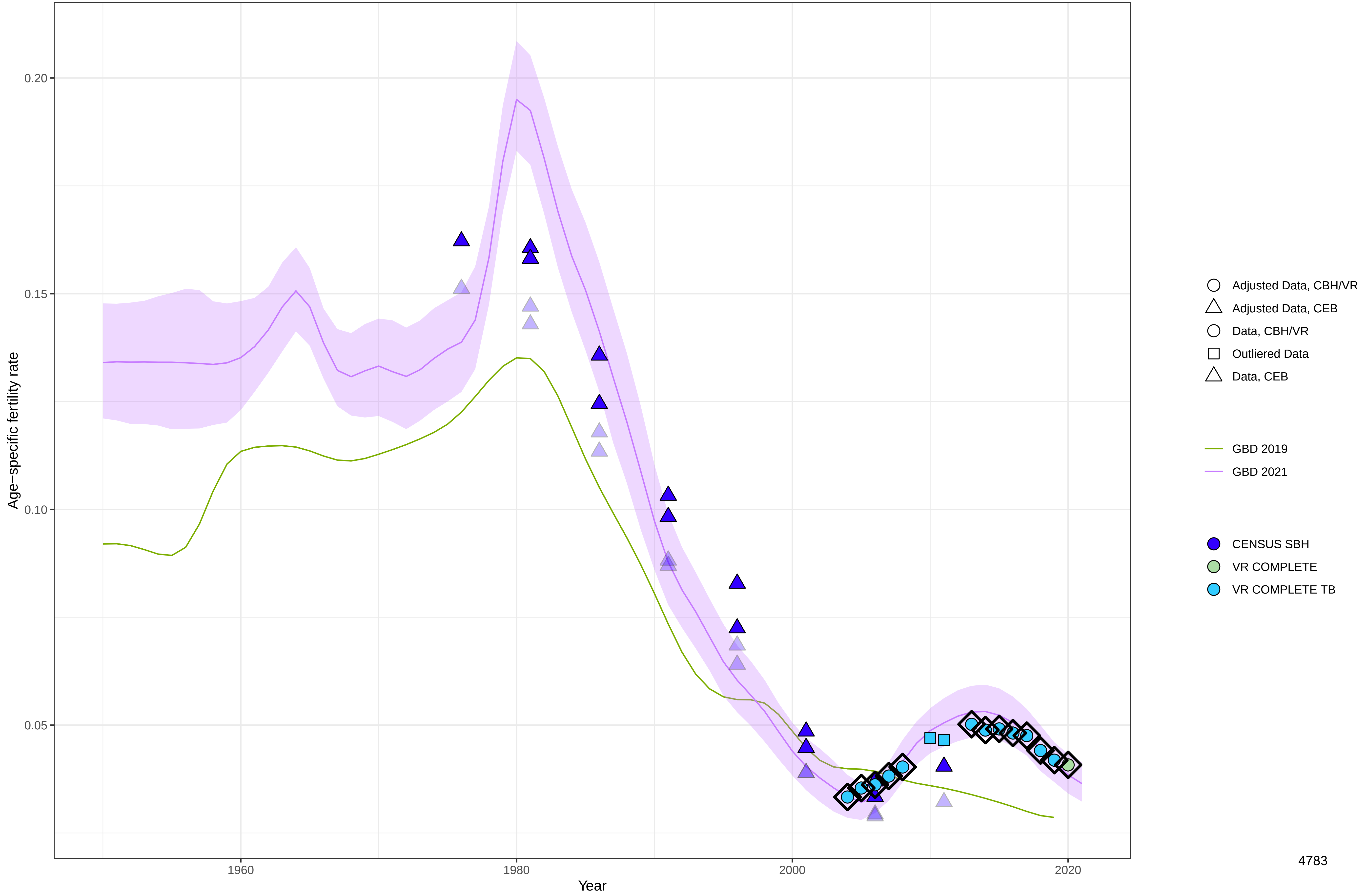

Age−specific fertility fitted trend lines, 1950−2021: North Khorasan, Ages: 20−24
Data points enclosed with a diamond are reference sources

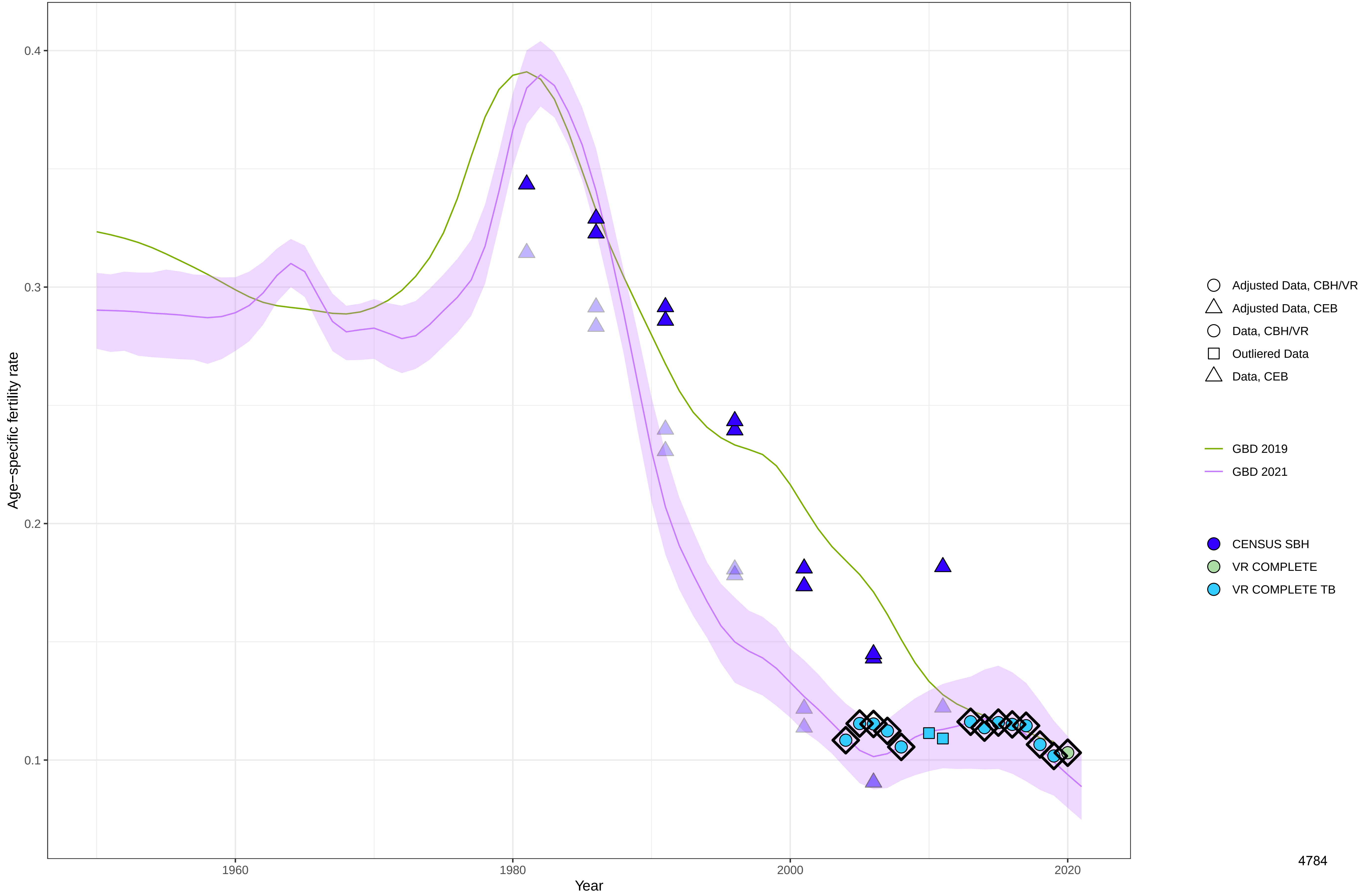

4784

Age−specific fertility fitted trend lines, 1950−2021: North Khorasan, Ages: 25−29
Data points enclosed with a diamond are reference sources

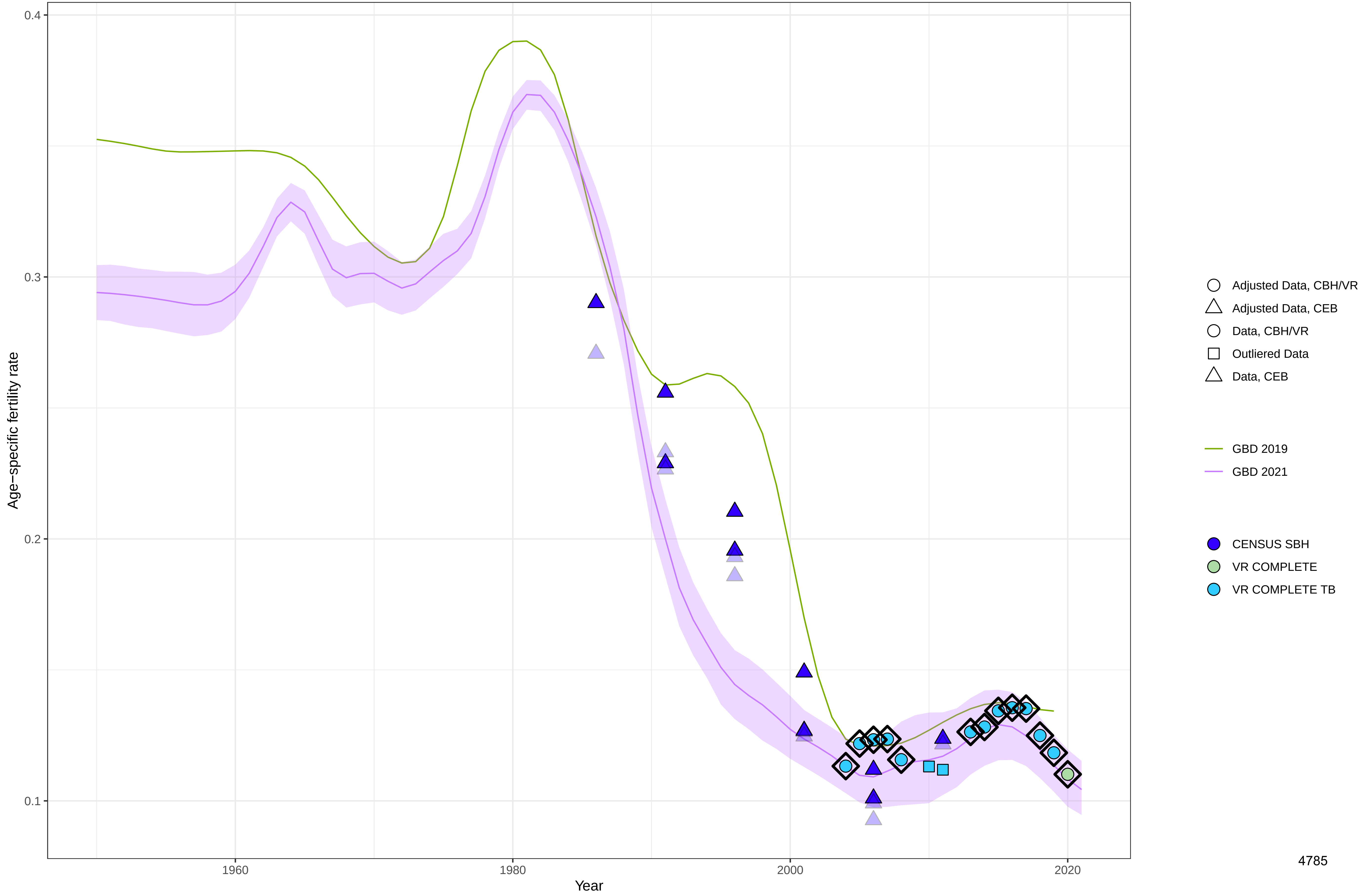

Age−specific fertility fitted trend lines, 1950−2021: North Khorasan, Ages: 30−34
Data points enclosed with a diamond are reference sources

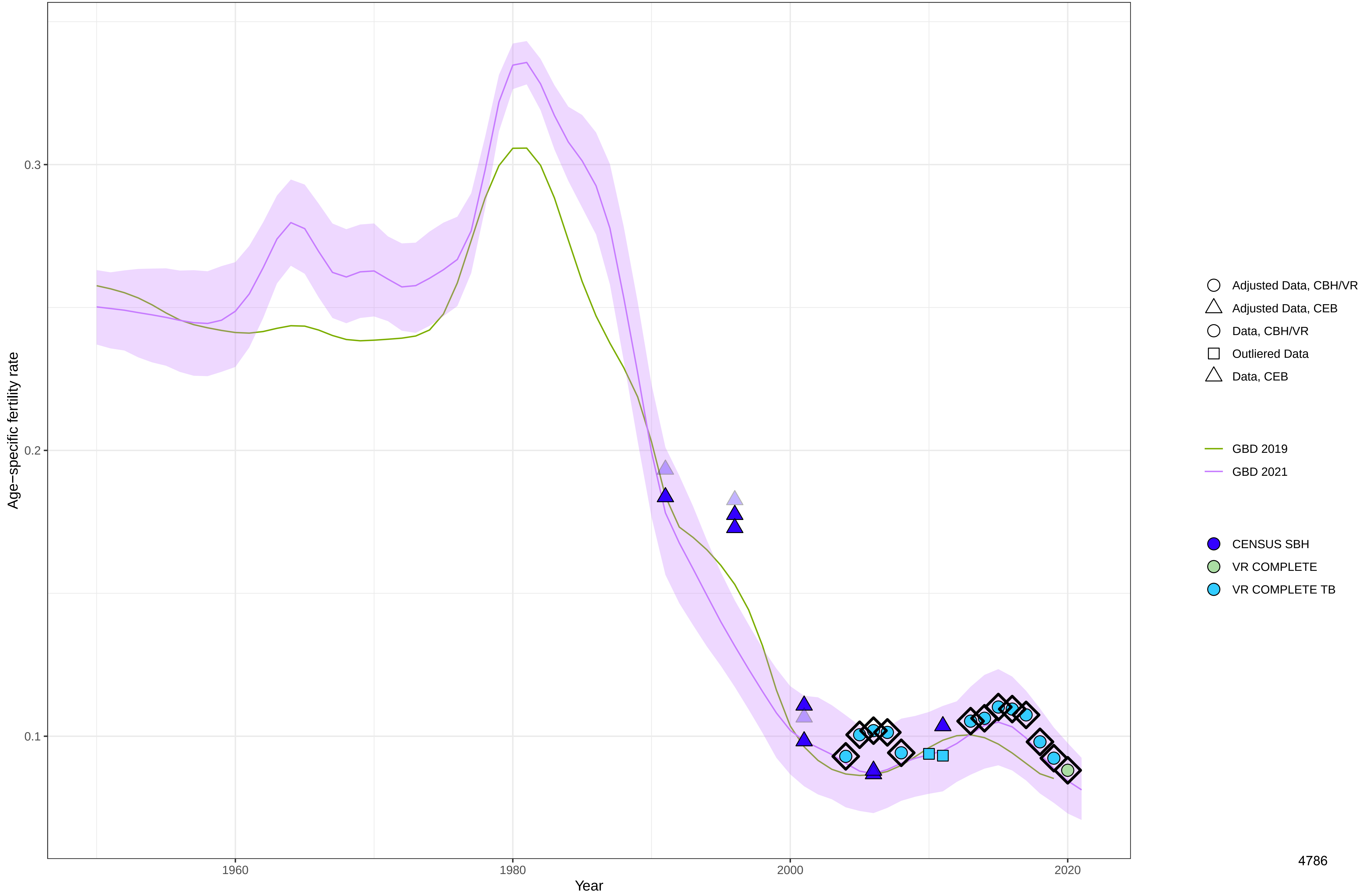

Age−specific fertility fitted trend lines, 1950−2021: North Khorasan, Ages: 35−39
Data points enclosed with a diamond are reference sources

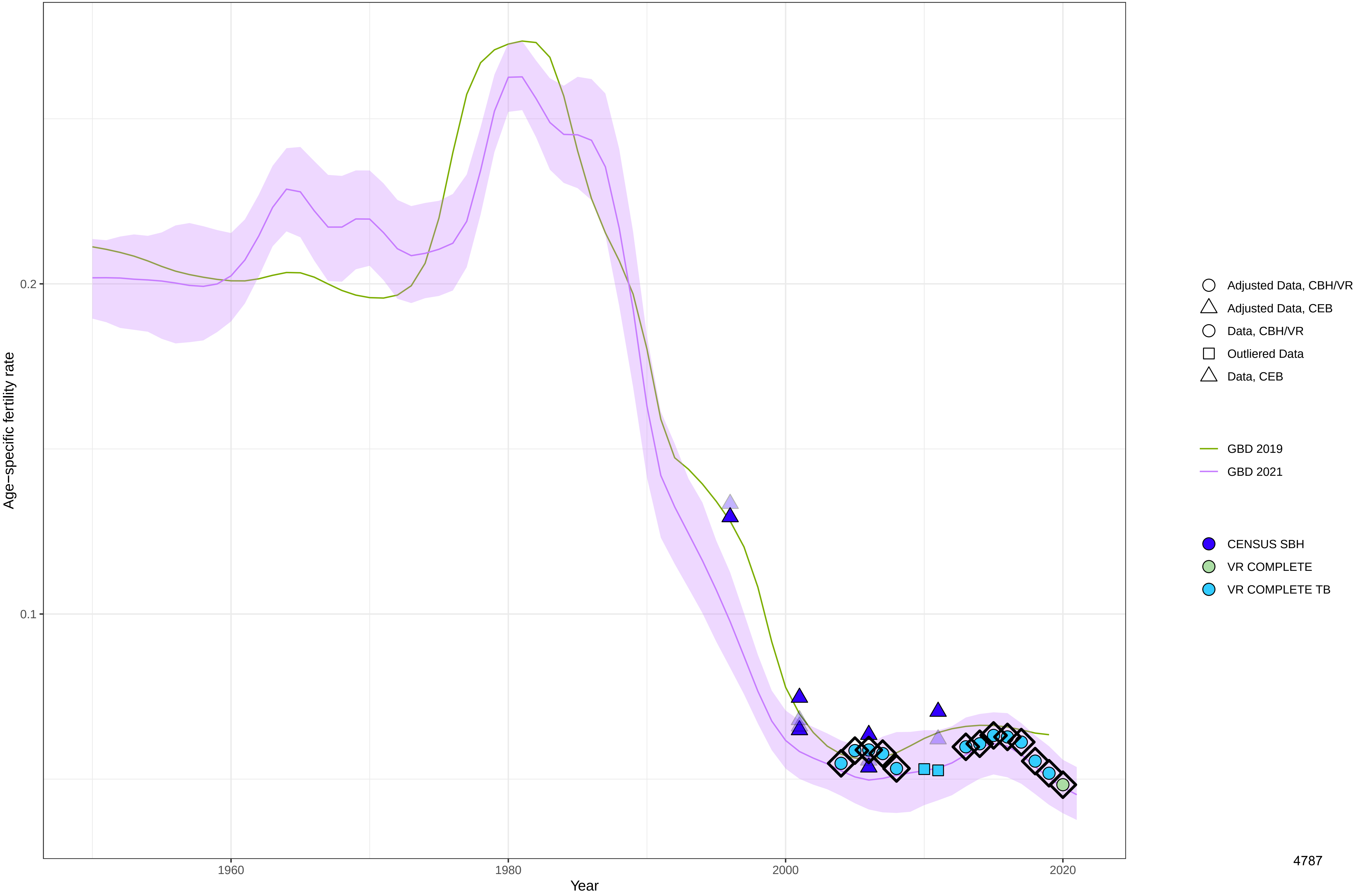

Age−specific fertility fitted trend lines, 1950−2021: North Khorasan, Ages: 40−44
Data points enclosed with a diamond are reference sources

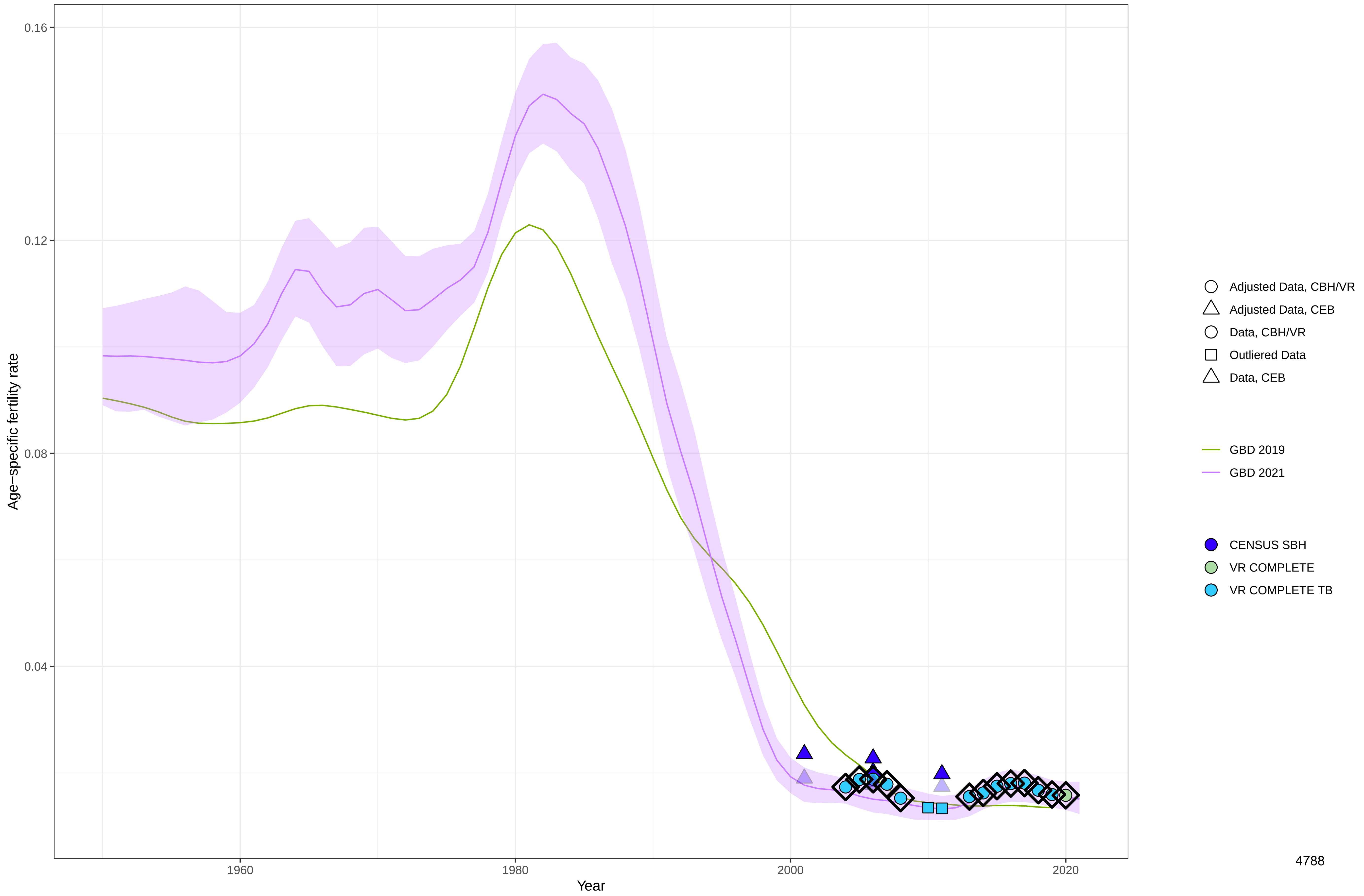

Age−specific fertility fitted trend lines, 1950−2021: North Khorasan, Ages: 45−49
Data points enclosed with a diamond are reference sources

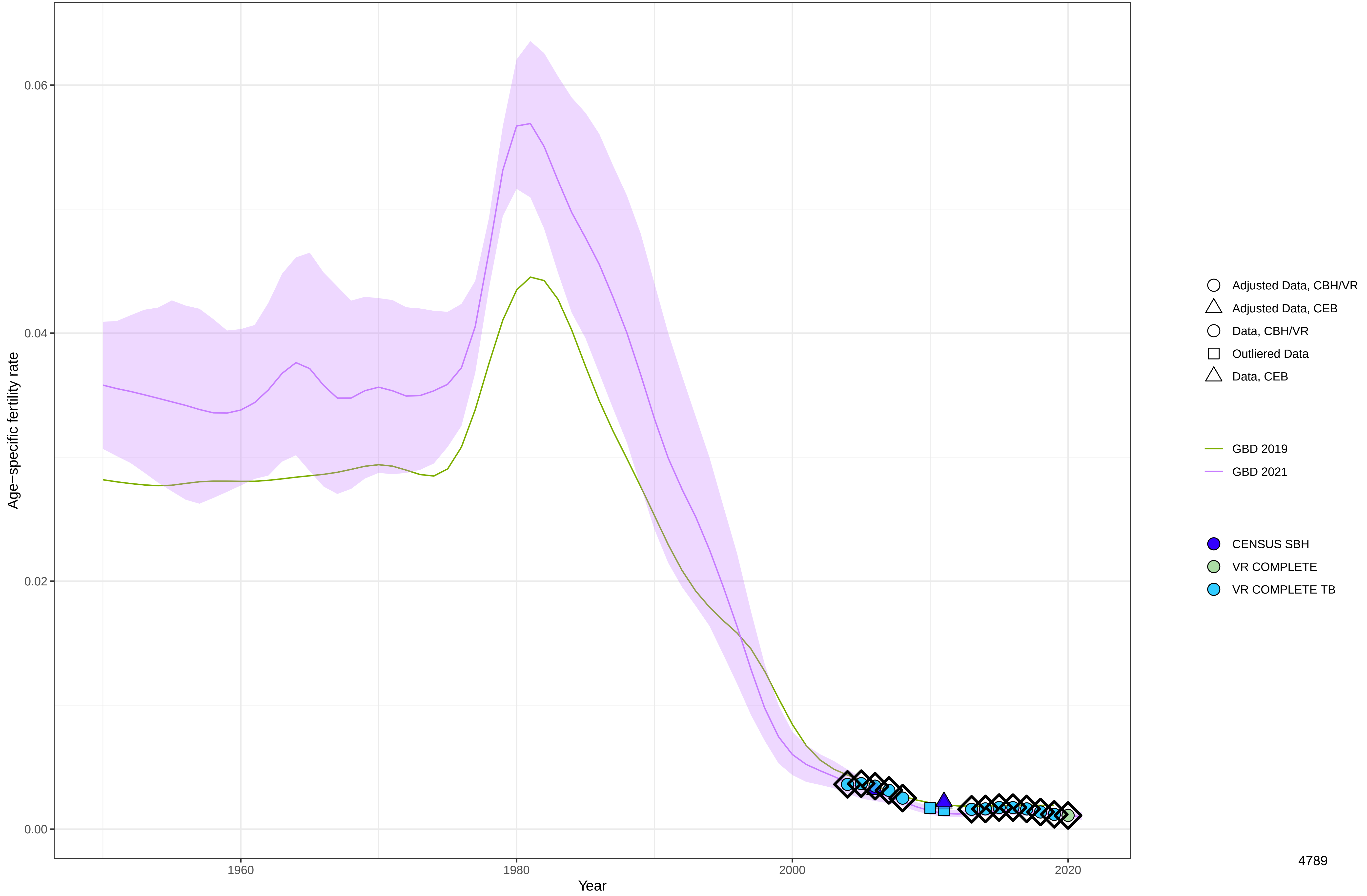

Age−specific fertility fitted trend lines, 1950−2021: North Khorasan, Ages: 50−54
Data points enclosed with a diamond are reference sources

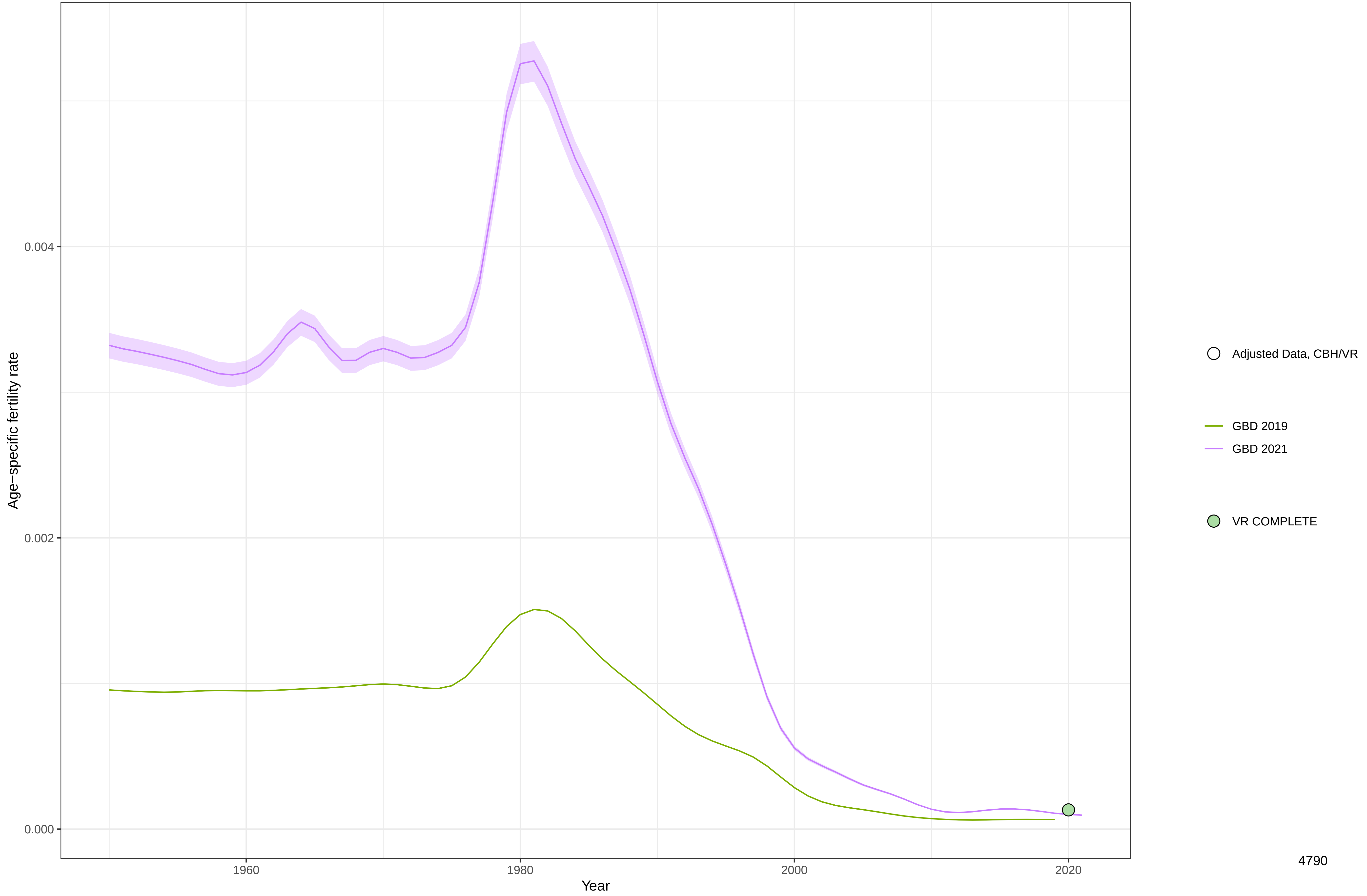

Total fertility rate fitted trend lines, 1950−2021: North Khorasan TFR
Data points enclosed with a diamond are reference sources

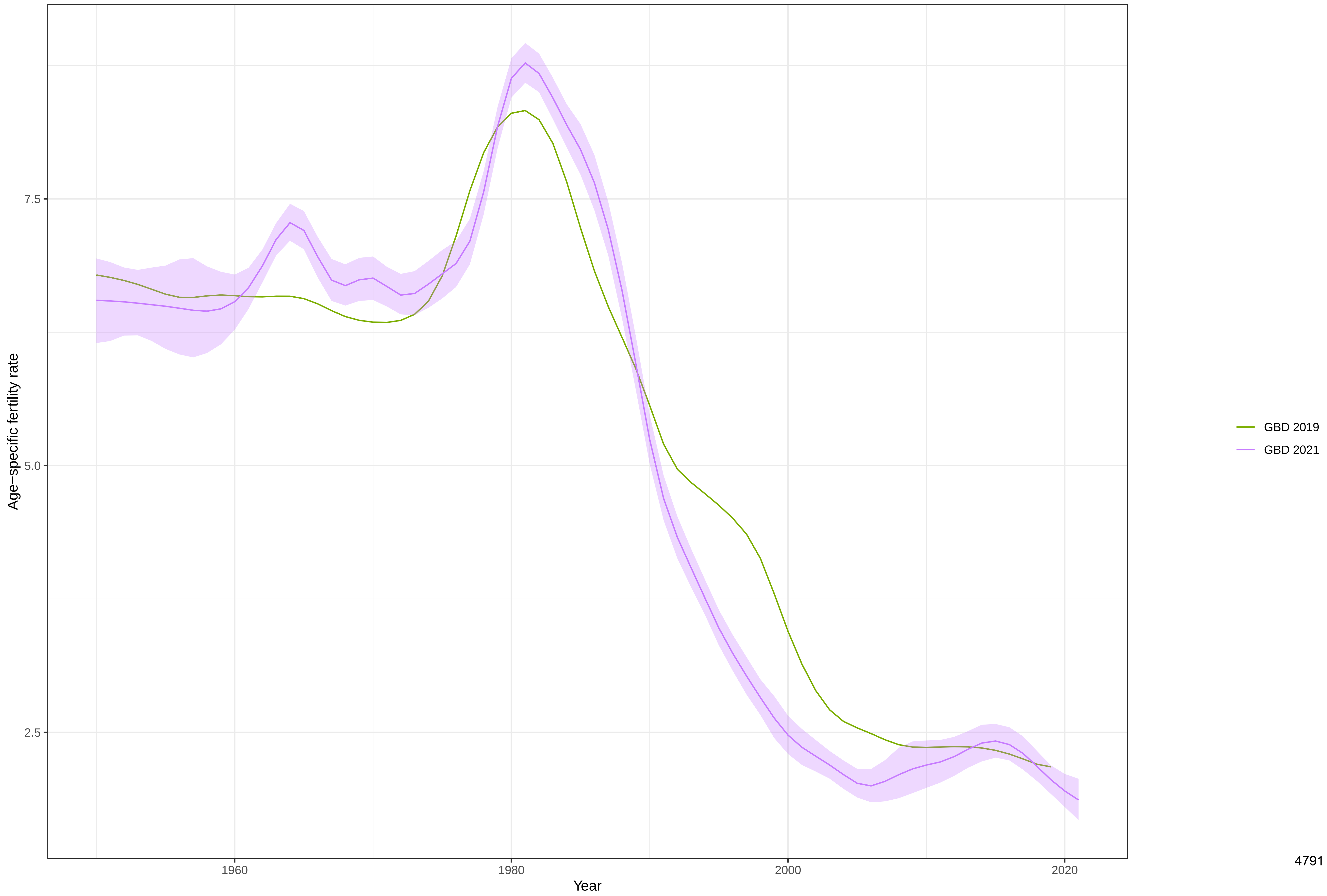

Age−specific fertility fitted trend lines, 1950−2021: Qazvin, Ages: 10−14
Data points enclosed with a diamond are reference sources

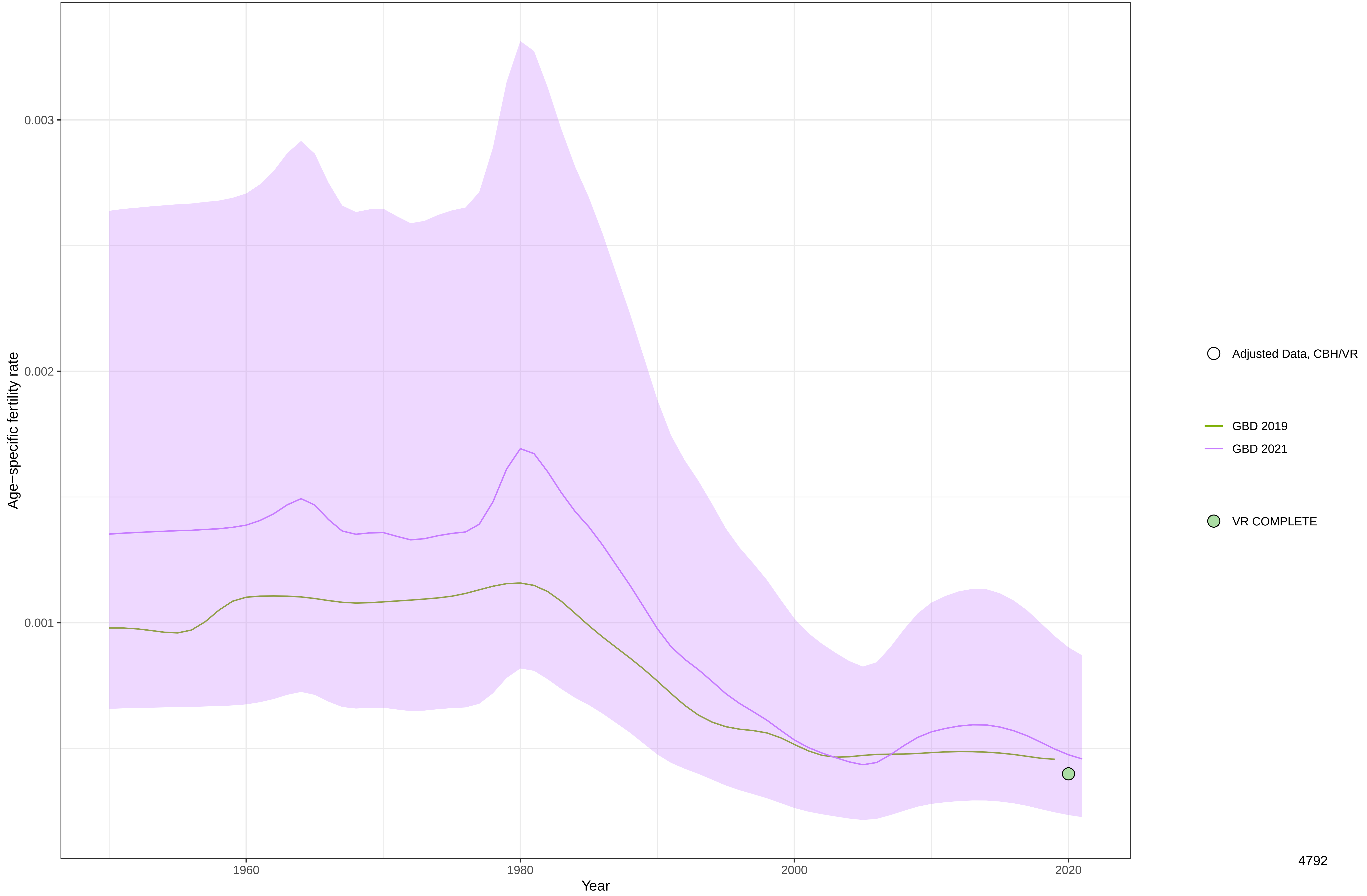

Age−specific fertility fitted trend lines, 1950−2021: Qazvin, Ages: 15−19
Data points enclosed with a diamond are reference sources

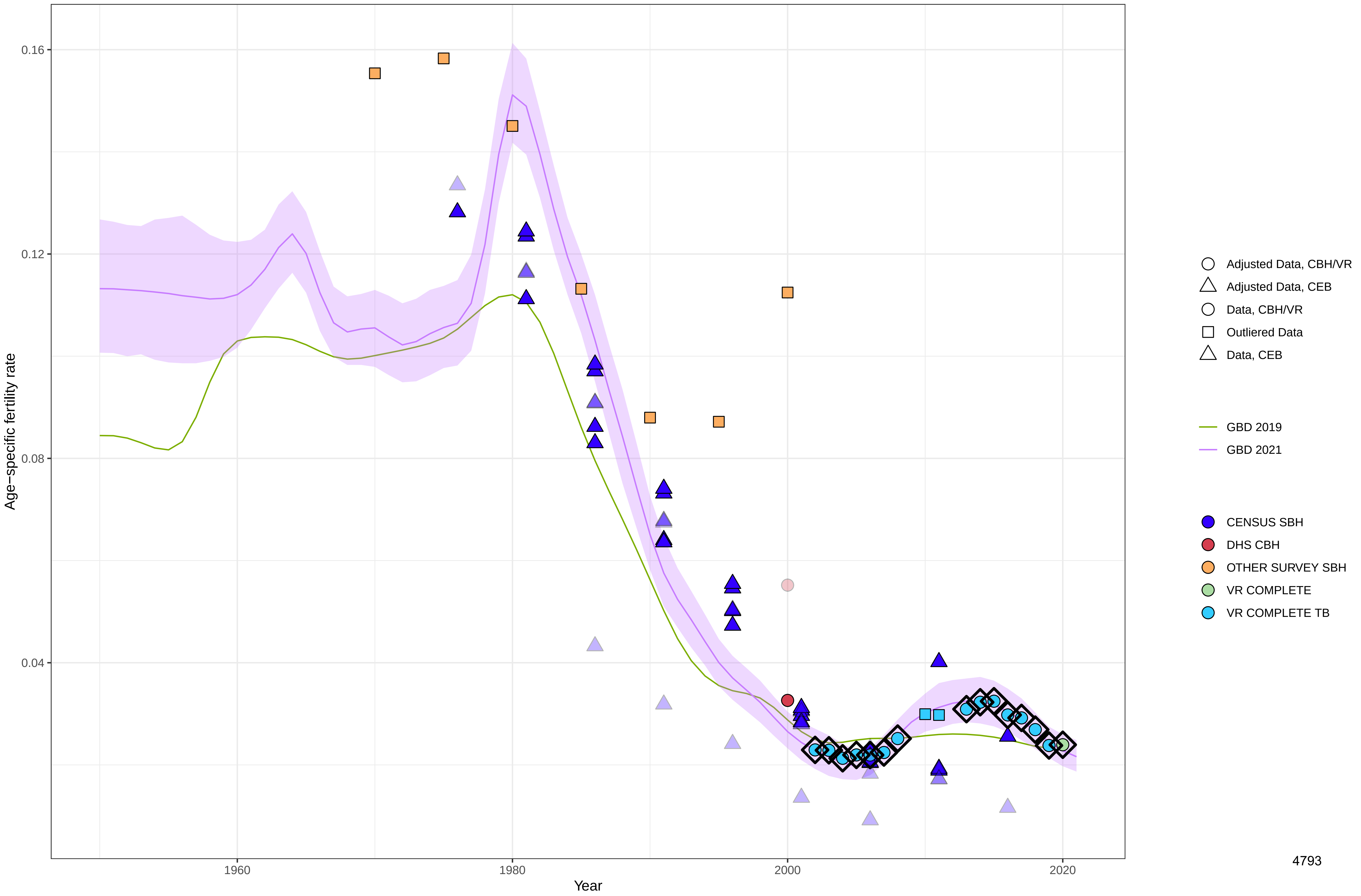

Age−specific fertility fitted trend lines, 1950−2021: Qazvin, Ages: 20−24
Data points enclosed with a diamond are reference sources

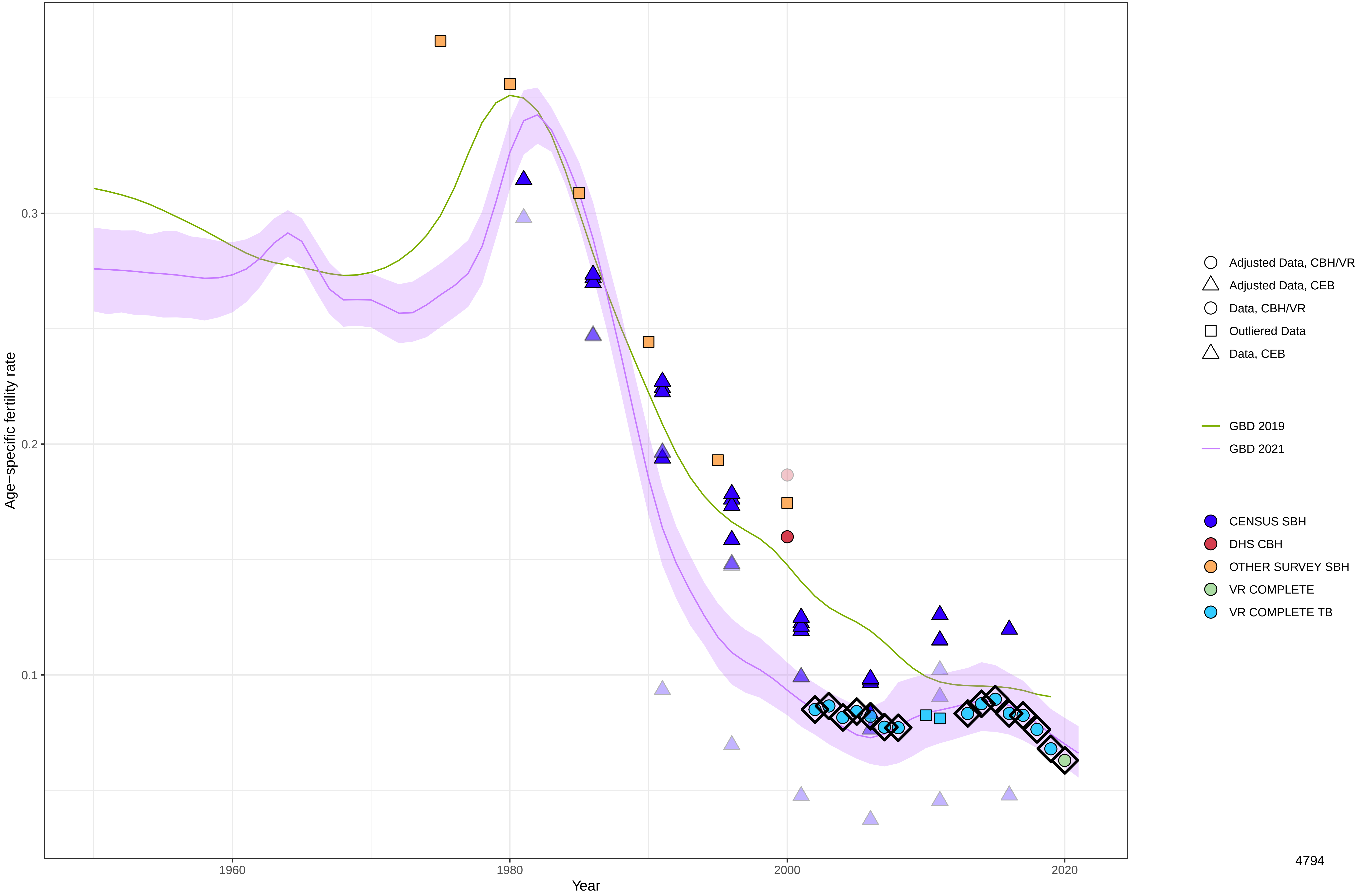

Age−specific fertility fitted trend lines, 1950−2021: Qazvin, Ages: 25−29
Data points enclosed with a diamond are reference sources

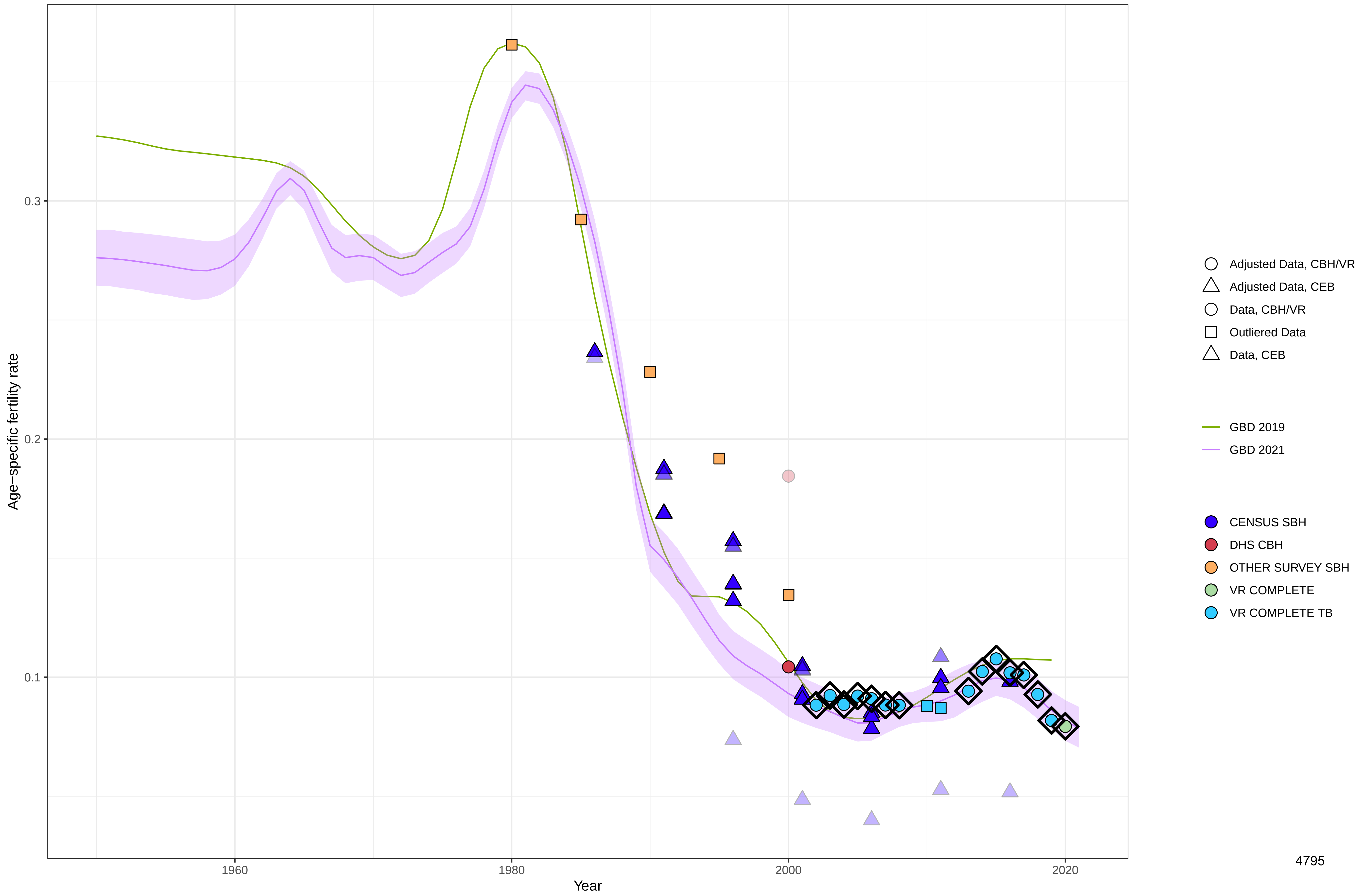

Age−specific fertility fitted trend lines, 1950−2021: Qazvin, Ages: 30−34
Data points enclosed with a diamond are reference sources

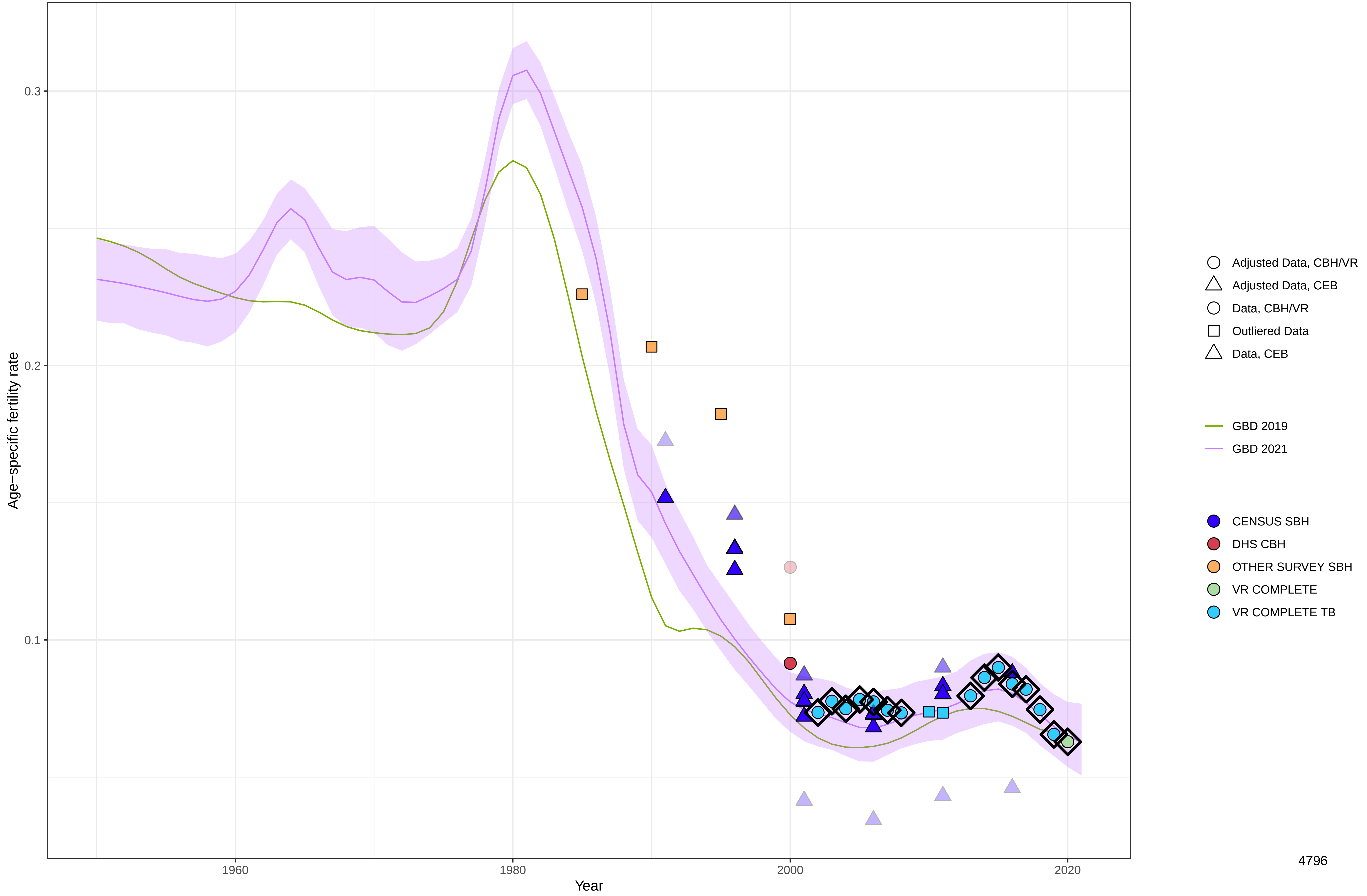

Age−specific fertility fitted trend lines, 1950−2021: Qazvin, Ages: 35−39
Data points enclosed with a diamond are reference sources

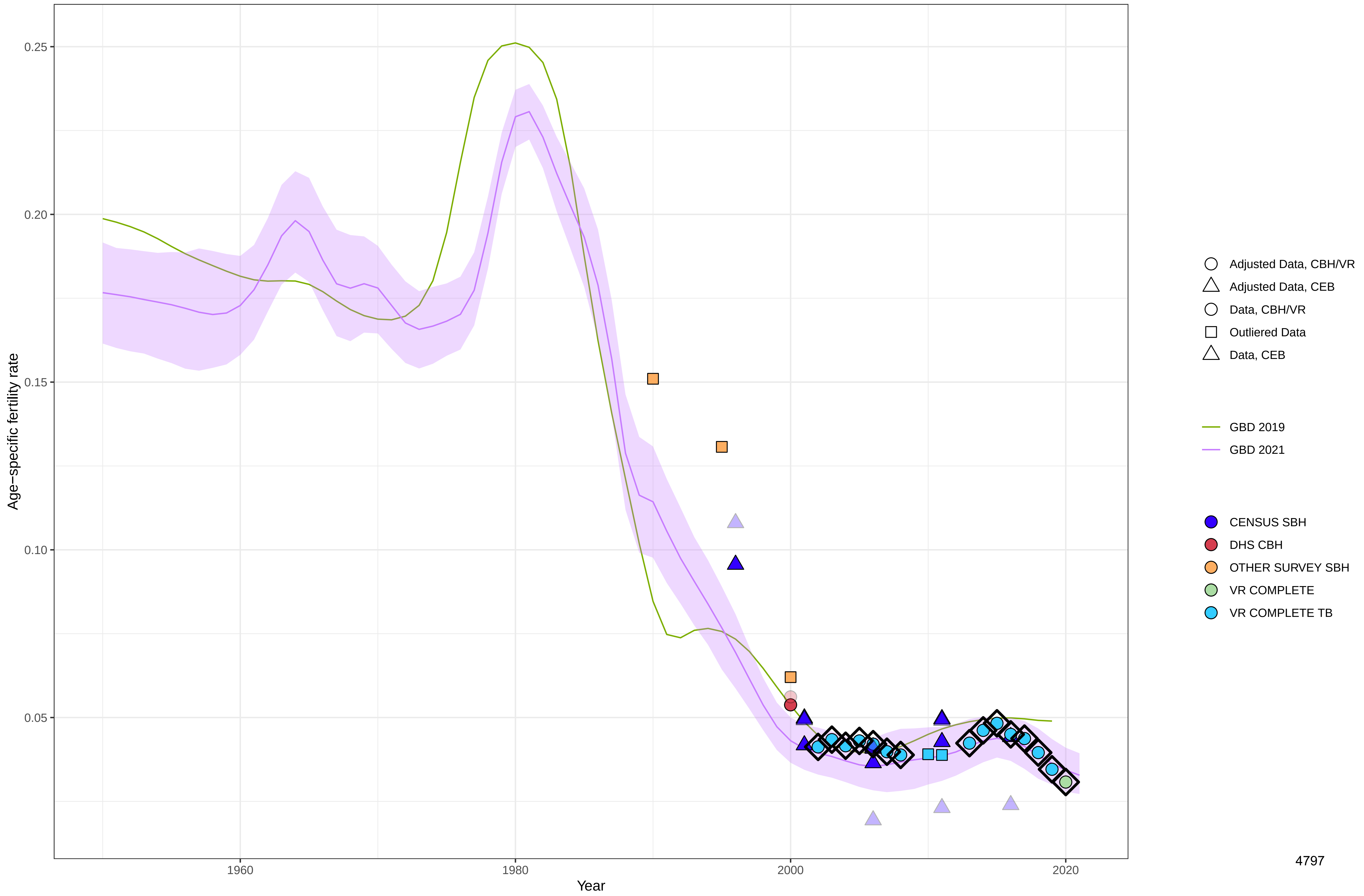

Age−specific fertility fitted trend lines, 1950−2021: Qazvin, Ages: 40−44
Data points enclosed with a diamond are reference sources

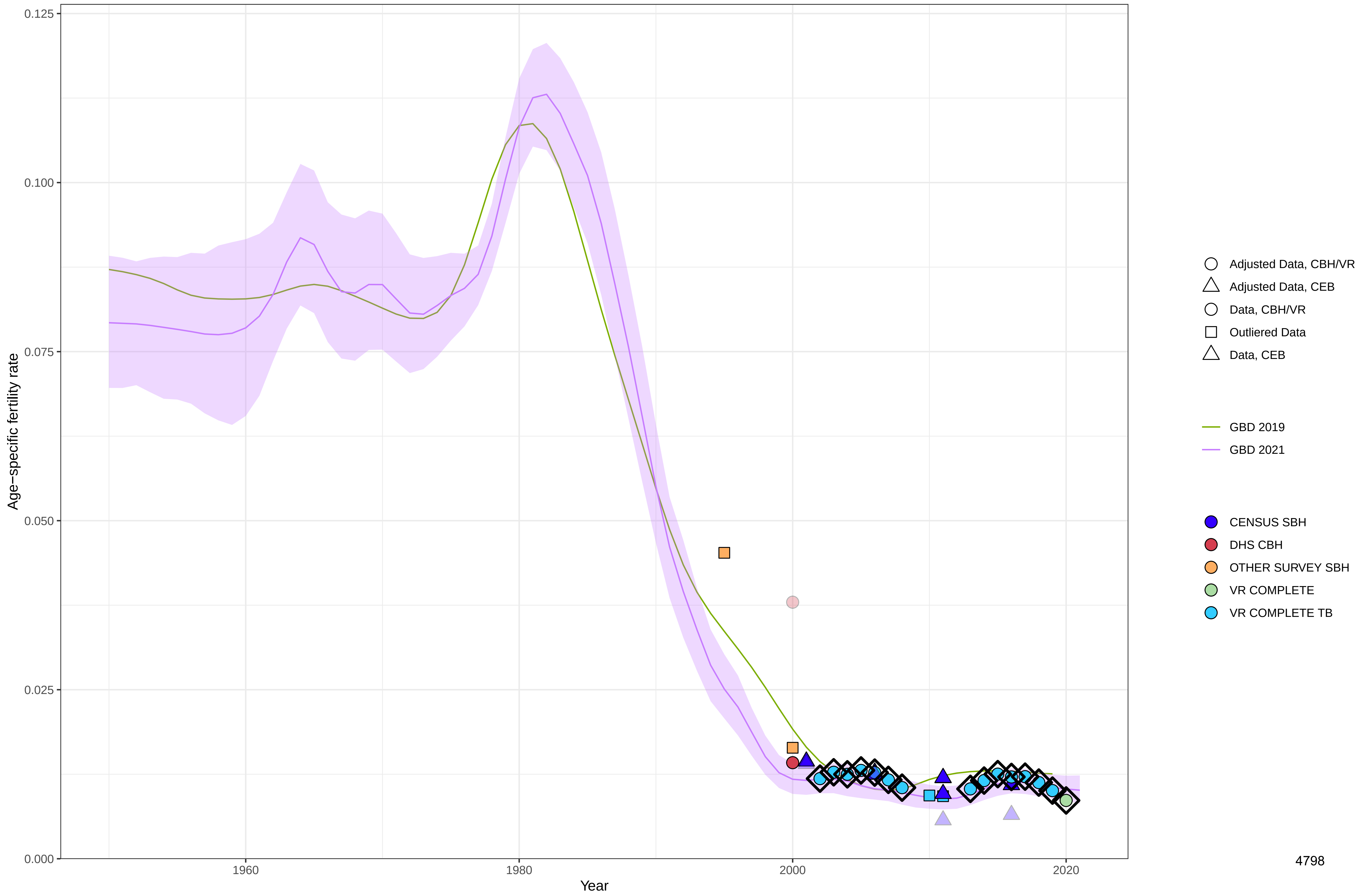

Age−specific fertility fitted trend lines, 1950−2021: Qazvin, Ages: 45−49
Data points enclosed with a diamond are reference sources

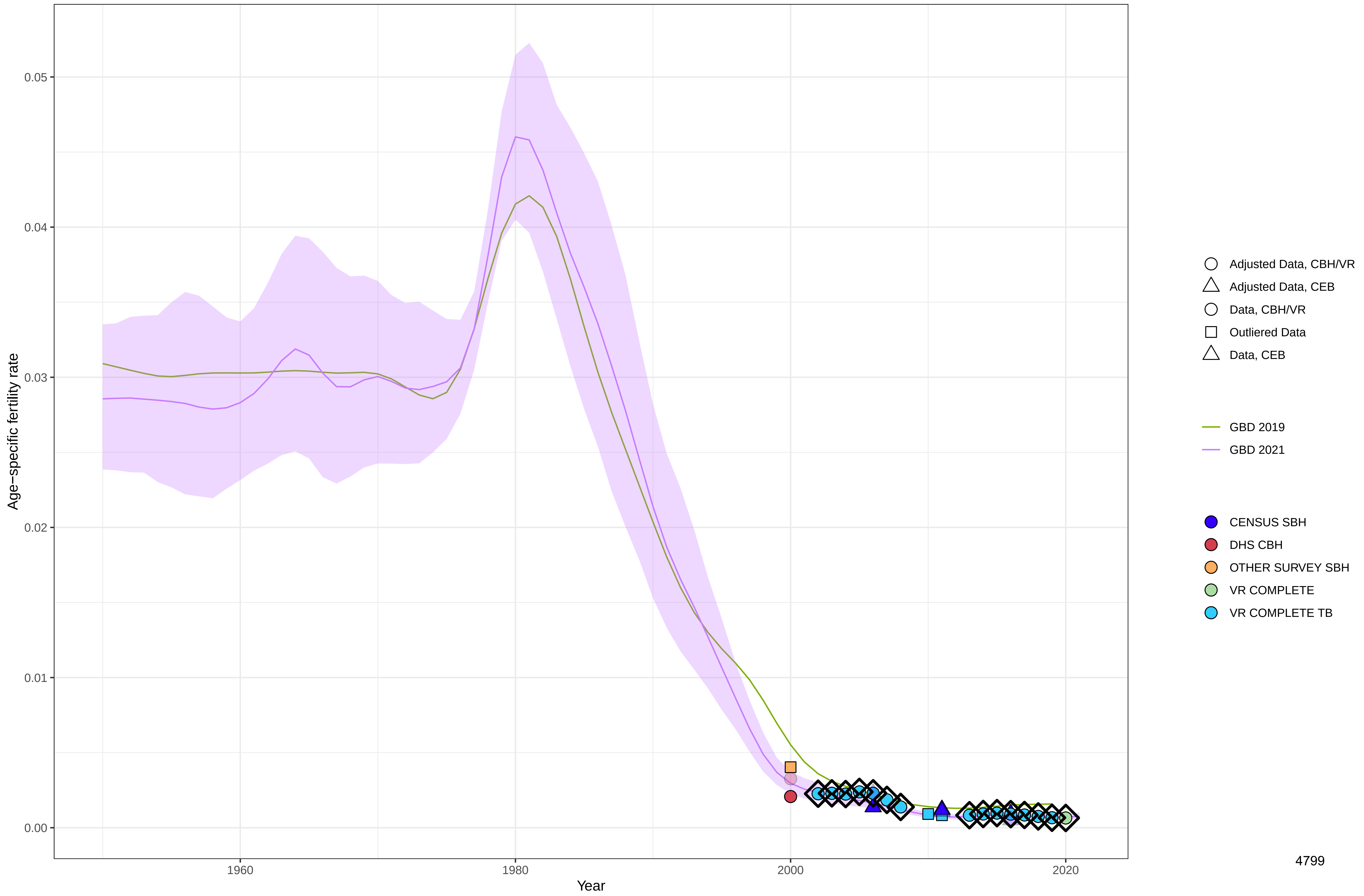

4799

Age−specific fertility fitted trend lines, 1950−2021: Qazvin, Ages: 50−54
Data points enclosed with a diamond are reference sources

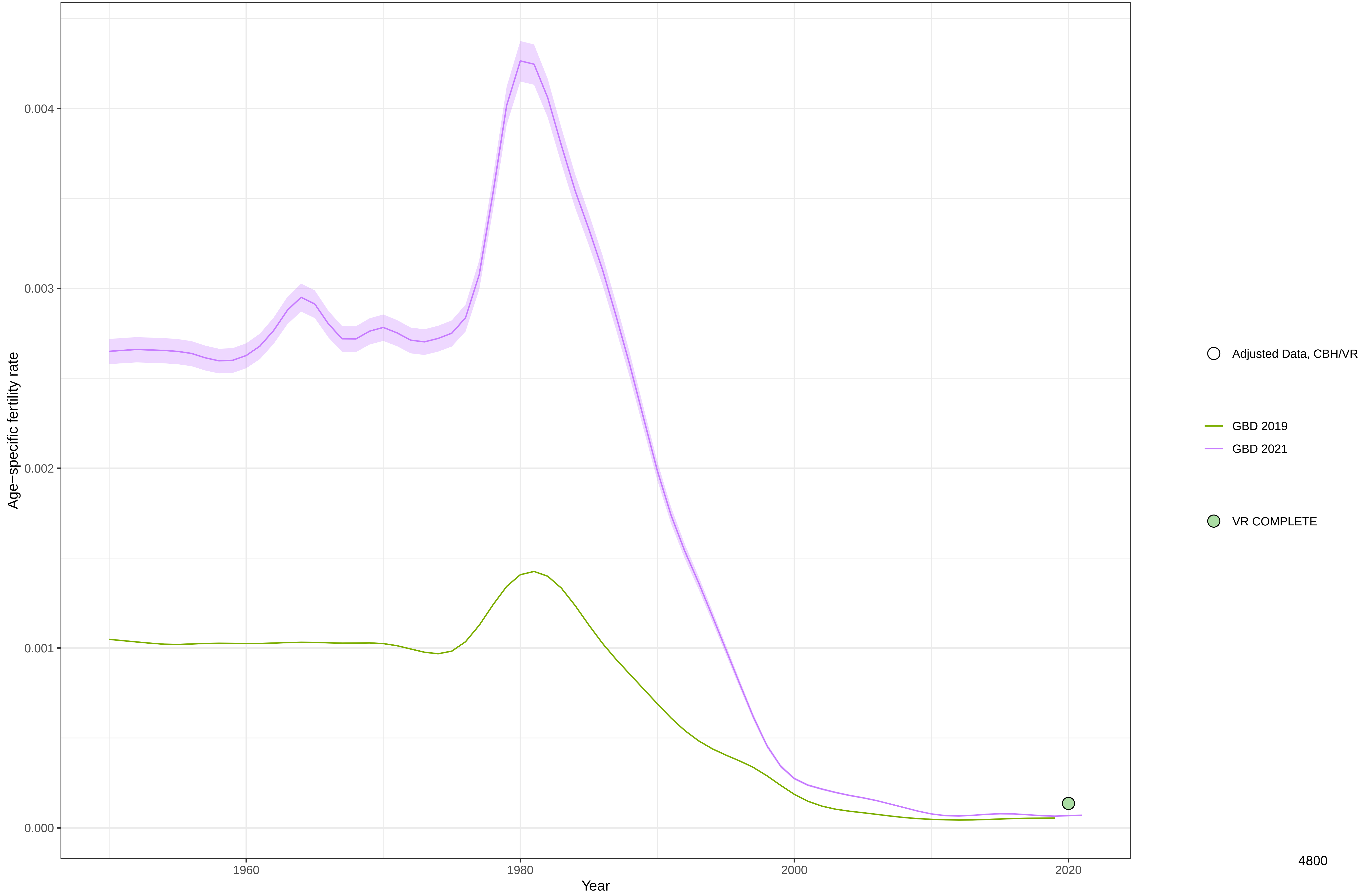

Total fertility rate fitted trend lines, 1950−2021: Qazvin TFR
Data points enclosed with a diamond are reference sources

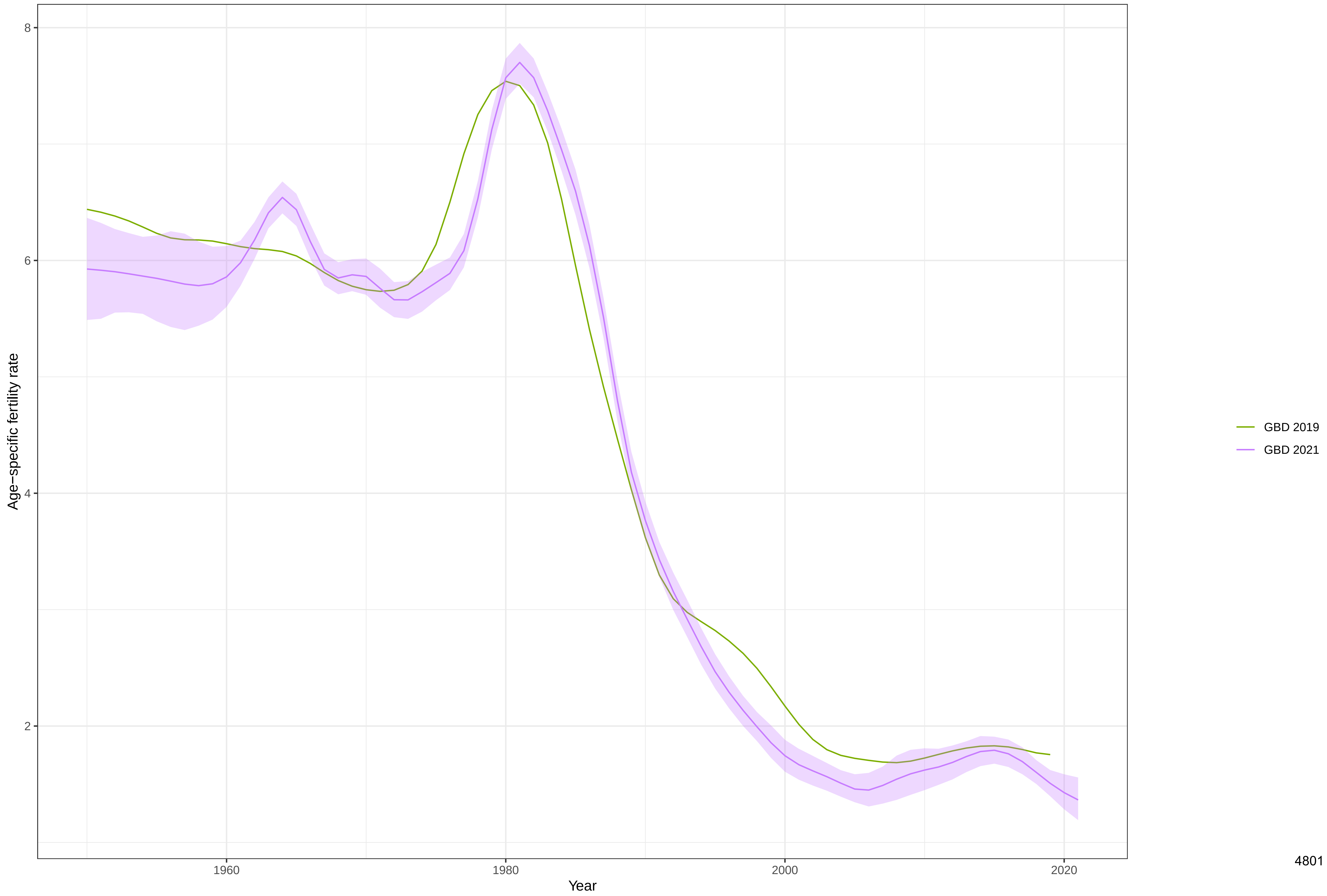

Age−specific fertility fitted trend lines, 1950−2021: Qom, Ages: 10−14
Data points enclosed with a diamond are reference sources

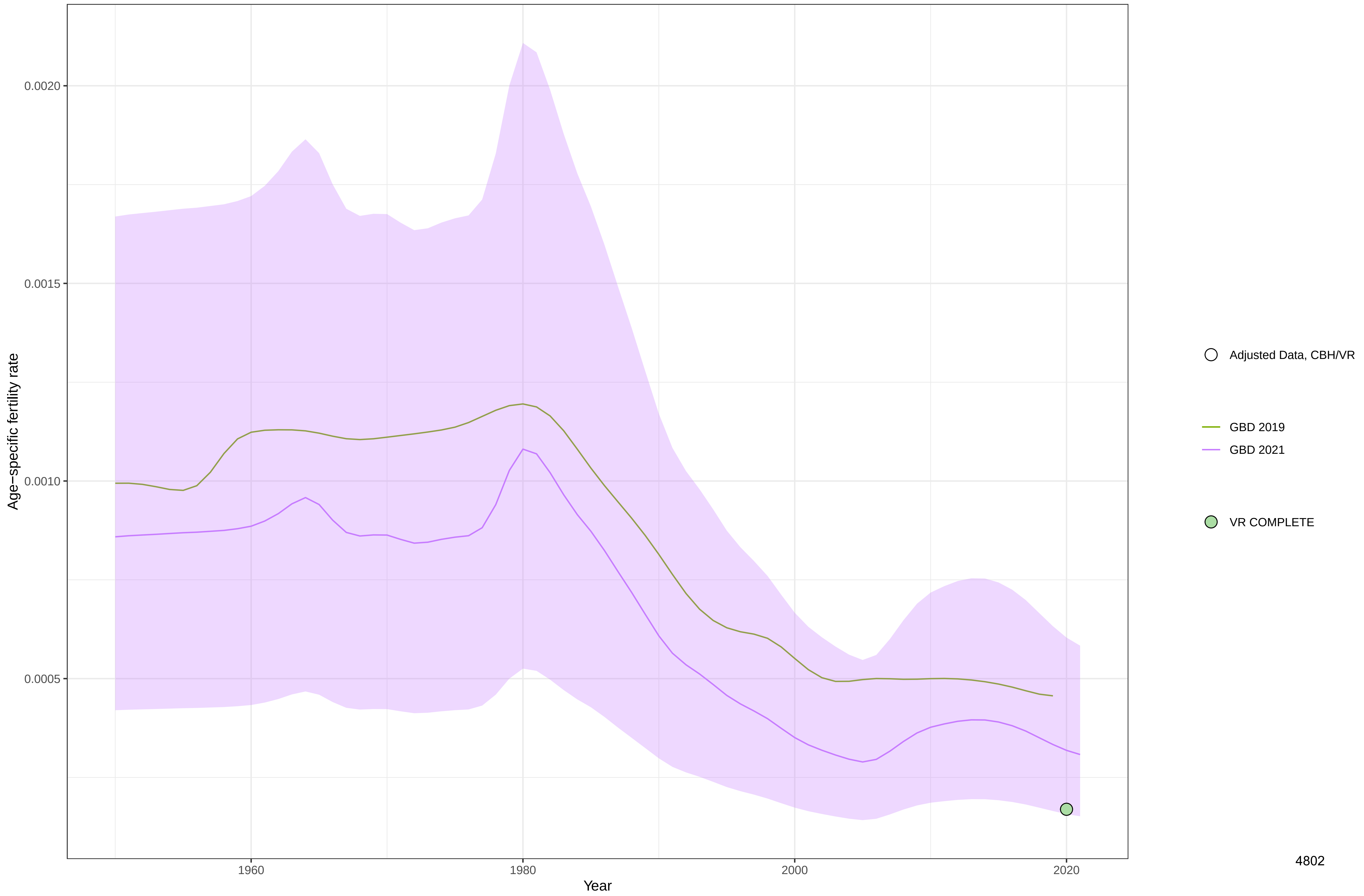

Age−specific fertility fitted trend lines, 1950−2021: Qom, Ages: 15−19
Data points enclosed with a diamond are reference sources

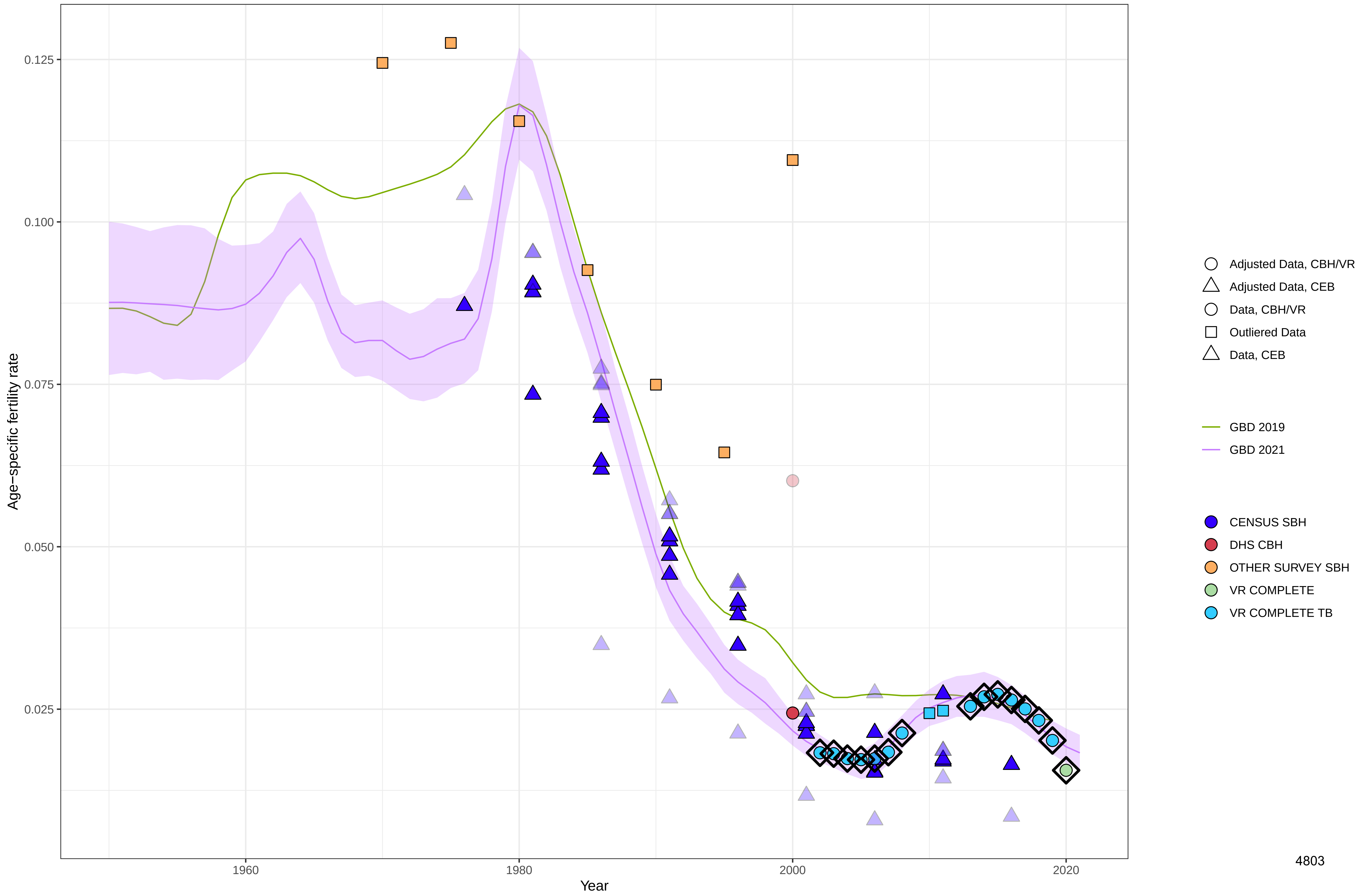

Age−specific fertility fitted trend lines, 1950−2021: Qom, Ages: 20−24
Data points enclosed with a diamond are reference sources

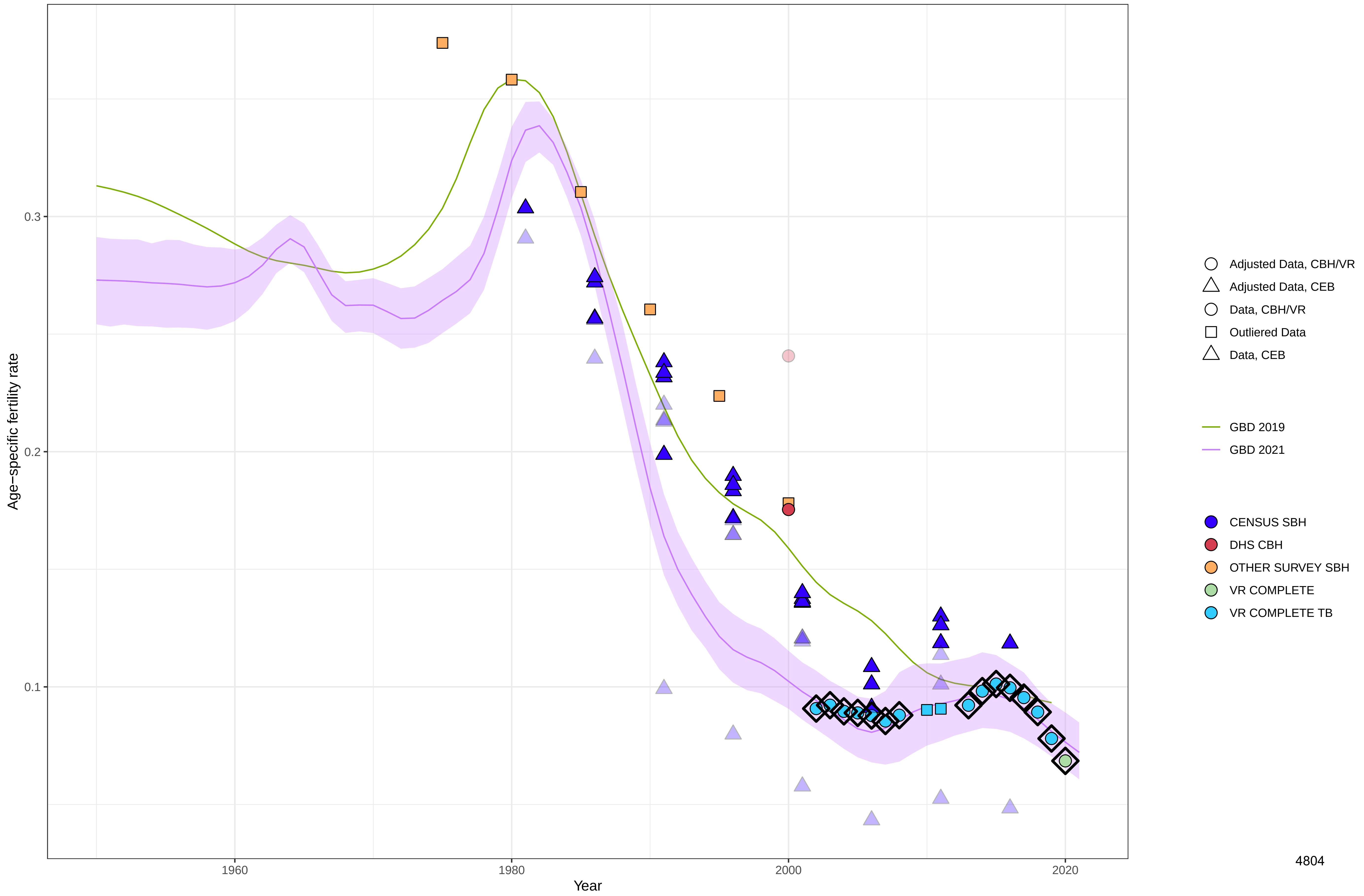

Age−specific fertility fitted trend lines, 1950−2021: Qom, Ages: 25−29
Data points enclosed with a diamond are reference sources

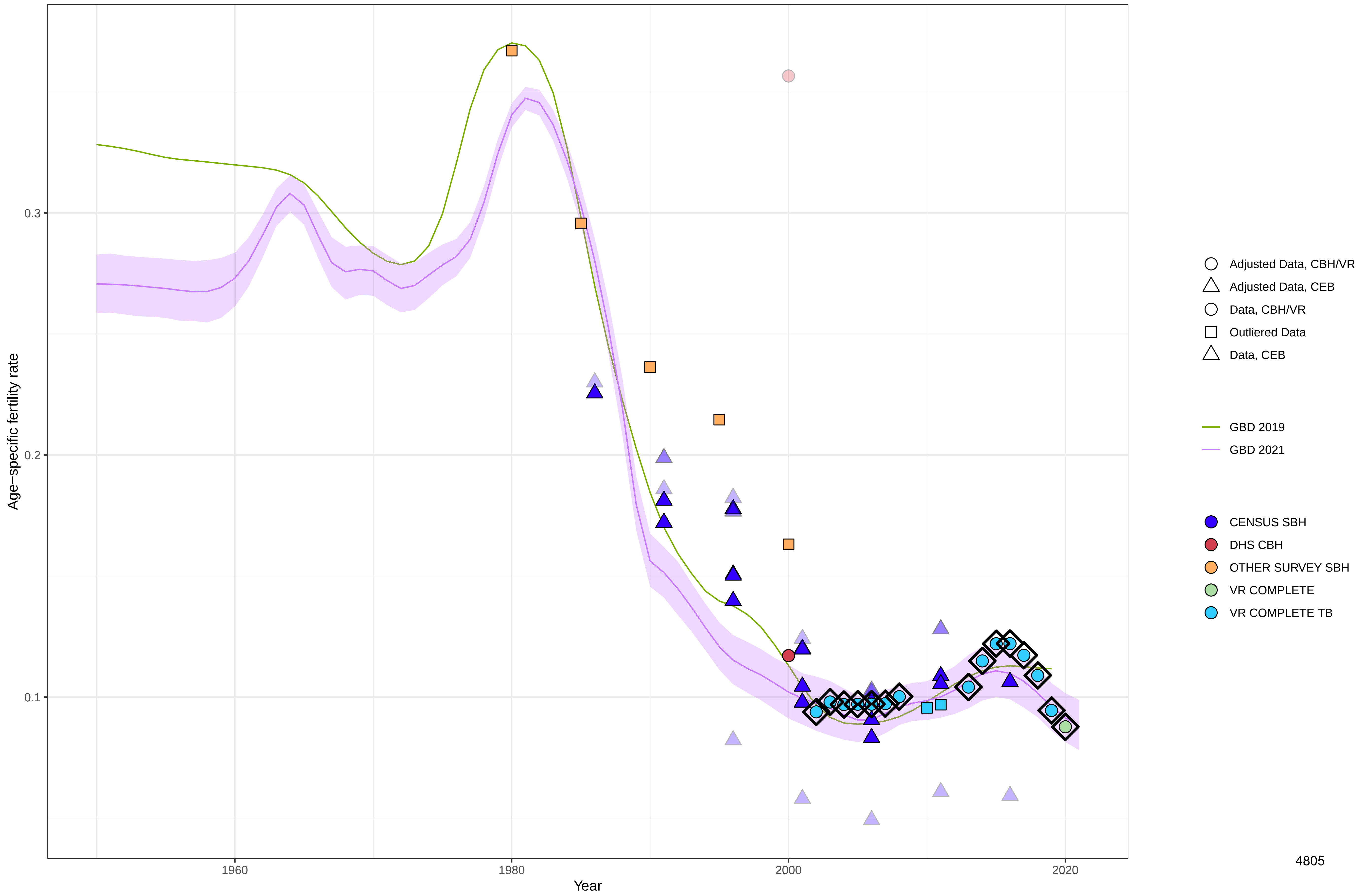

Age−specific fertility fitted trend lines, 1950−2021: Qom, Ages: 30−34
Data points enclosed with a diamond are reference sources

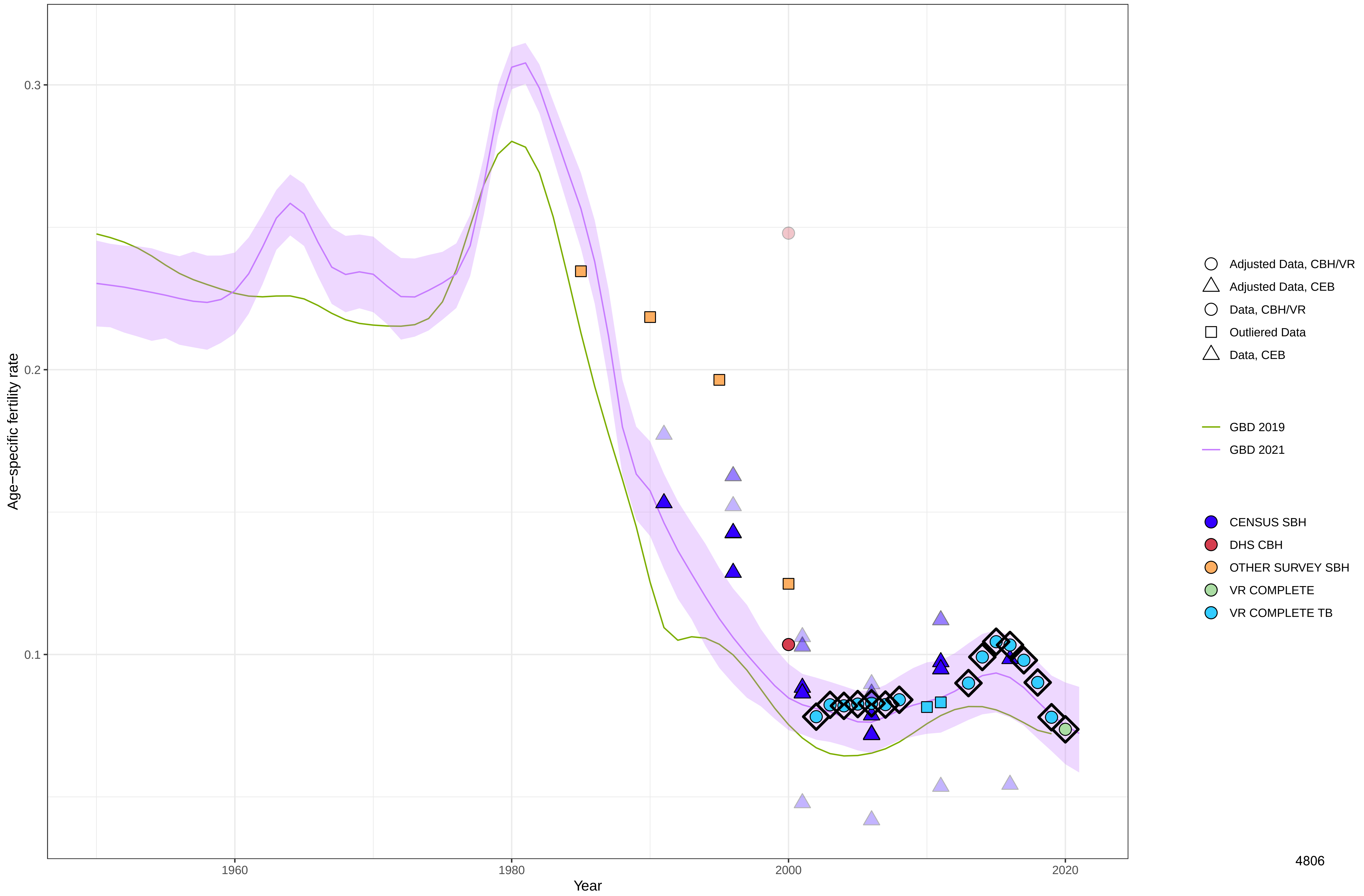

Age−specific fertility fitted trend lines, 1950−2021: Qom, Ages: 35−39
Data points enclosed with a diamond are reference sources

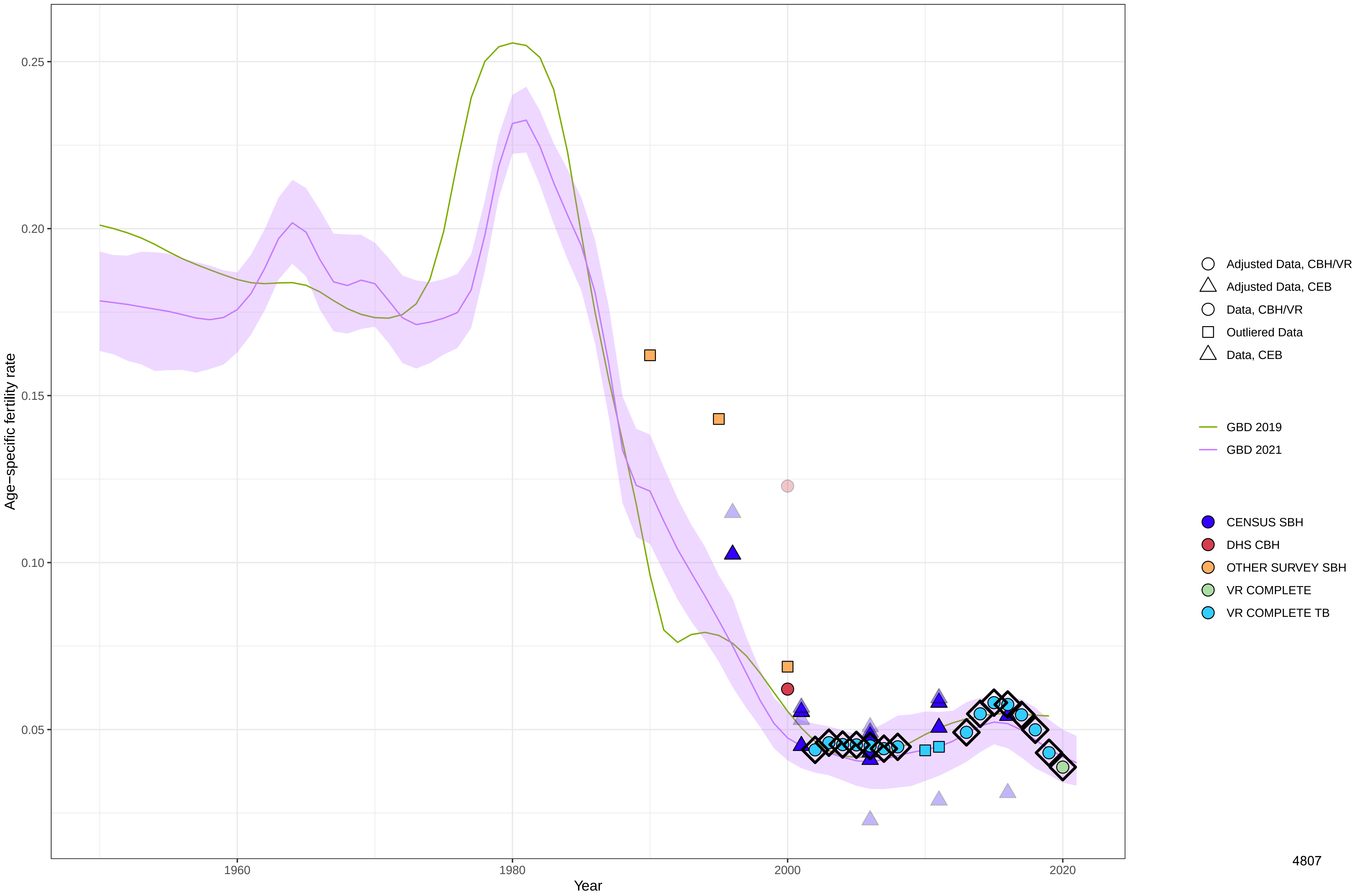

Age−specific fertility fitted trend lines, 1950−2021: Qom, Ages: 40−44
Data points enclosed with a diamond are reference sources

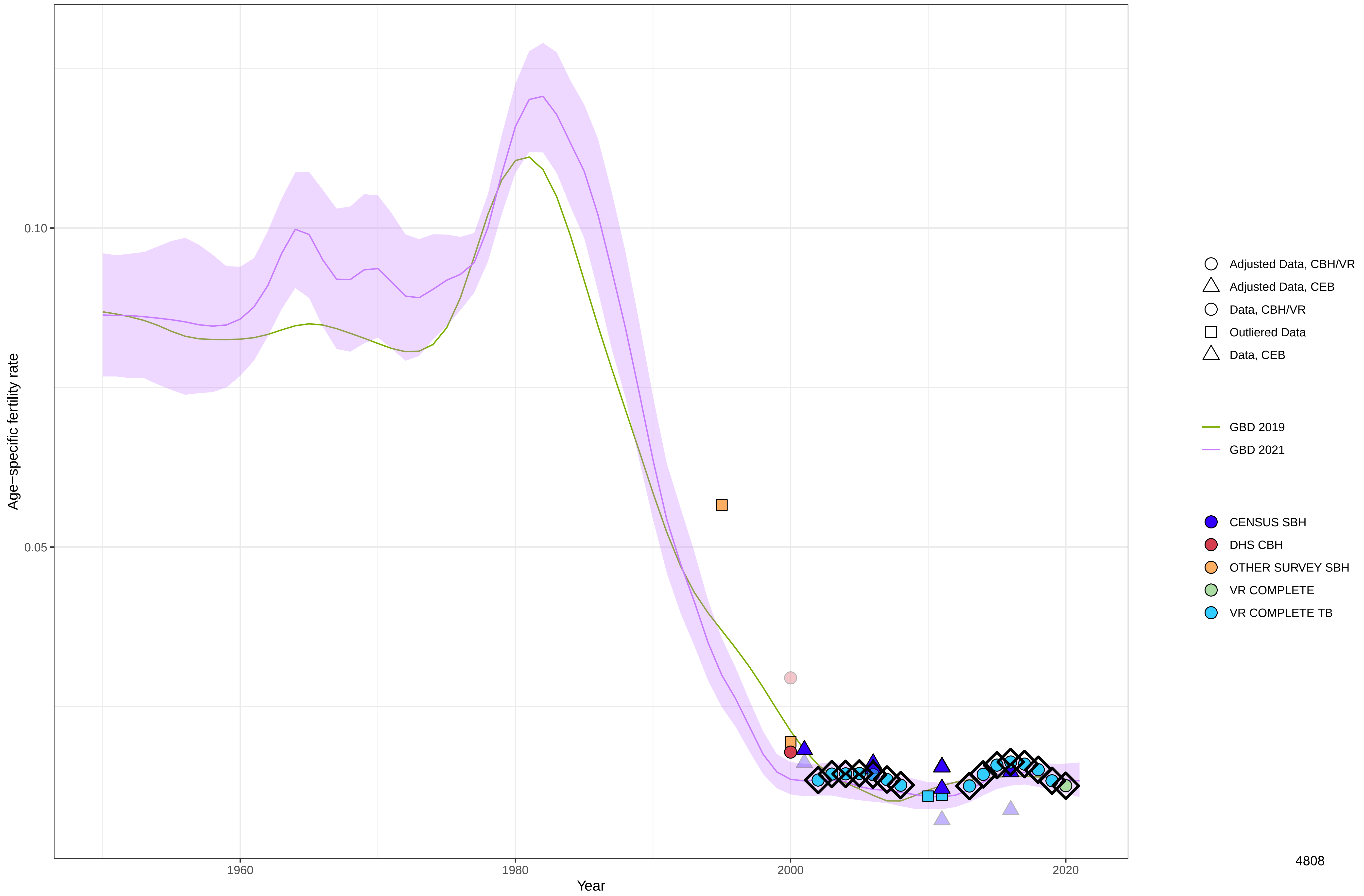

Age−specific fertility fitted trend lines, 1950−2021: Qom, Ages: 45−49
Data points enclosed with a diamond are reference sources

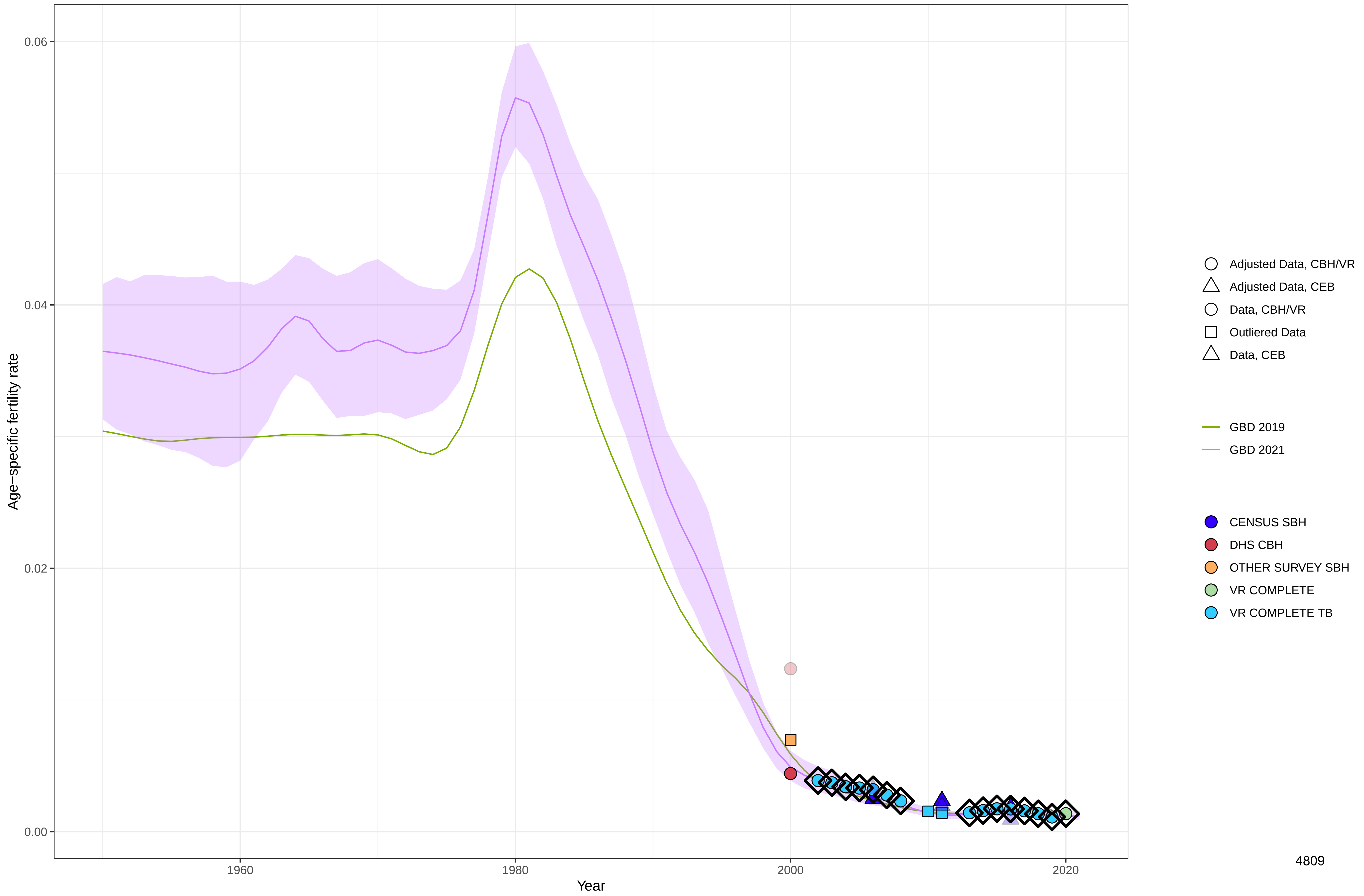

4809

Age−specific fertility fitted trend lines, 1950−2021: Qom, Ages: 50−54
Data points enclosed with a diamond are reference sources

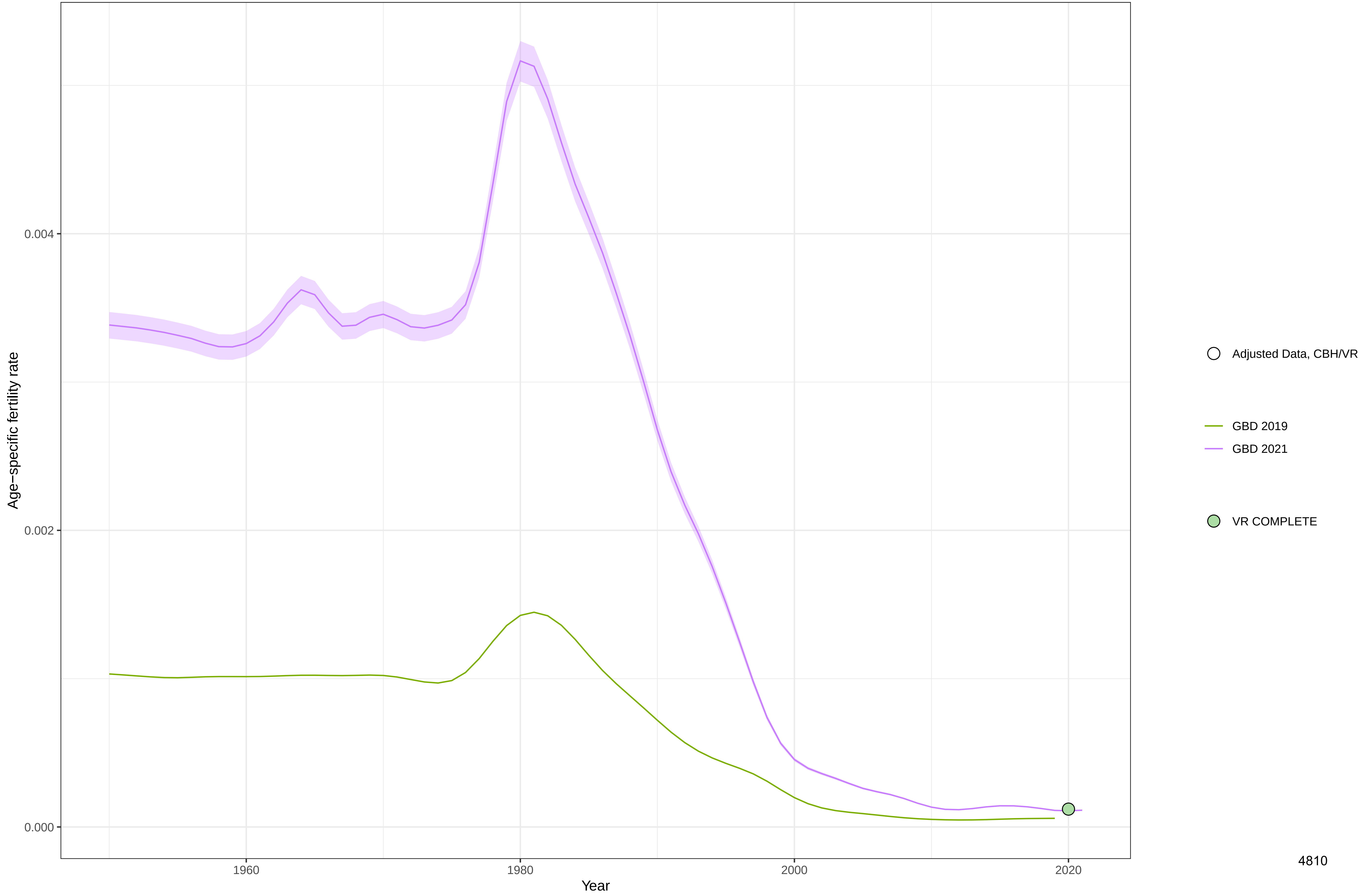

Total fertility rate fitted trend lines, 1950−2021: Qom TFR
Data points enclosed with a diamond are reference sources

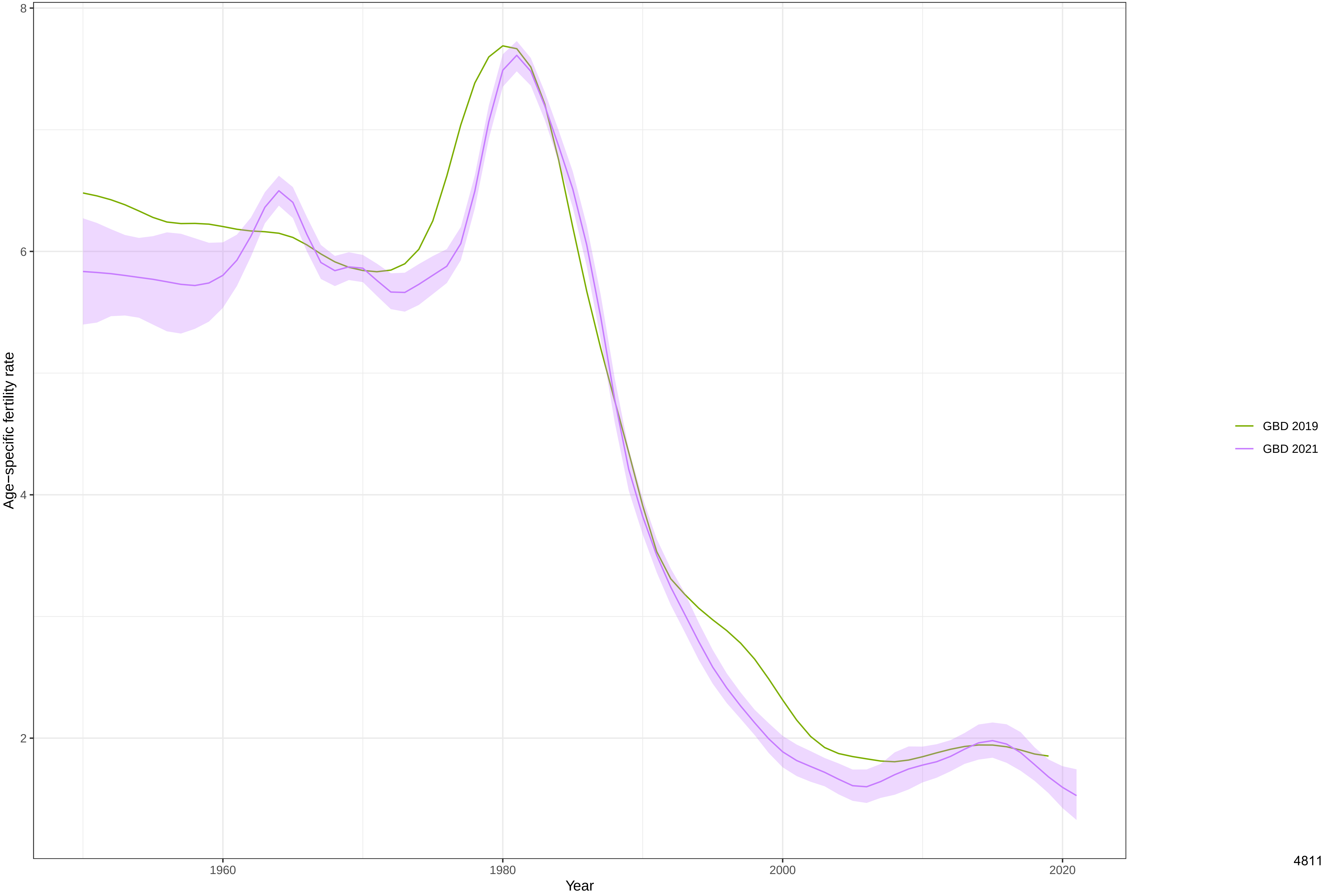

Age−specific fertility fitted trend lines, 1950−2021: Semnan, Ages: 10−14
Data points enclosed with a diamond are reference sources

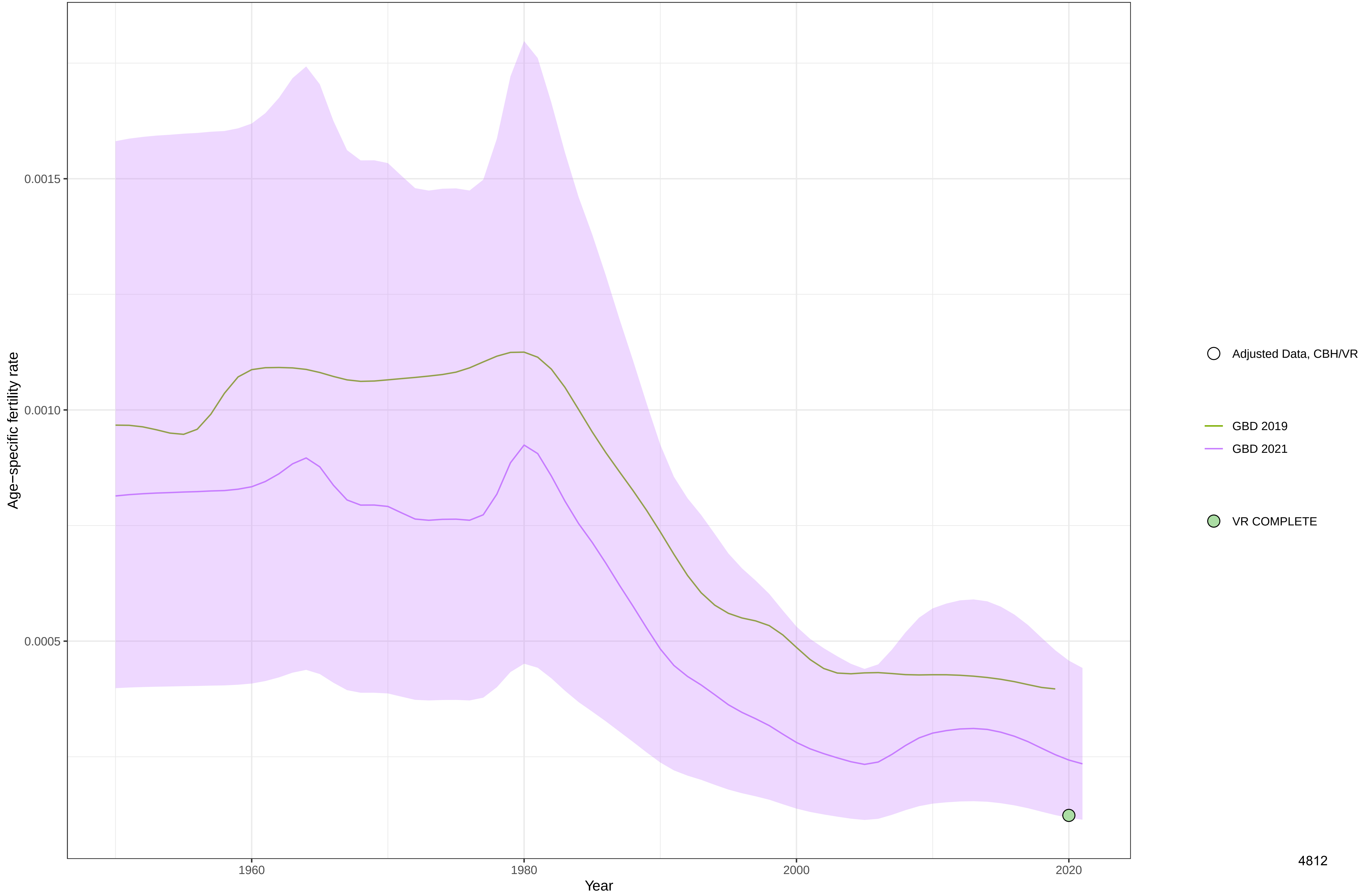

Age−specific fertility fitted trend lines, 1950−2021: Semnan, Ages: 15−19
Data points enclosed with a diamond are reference sources

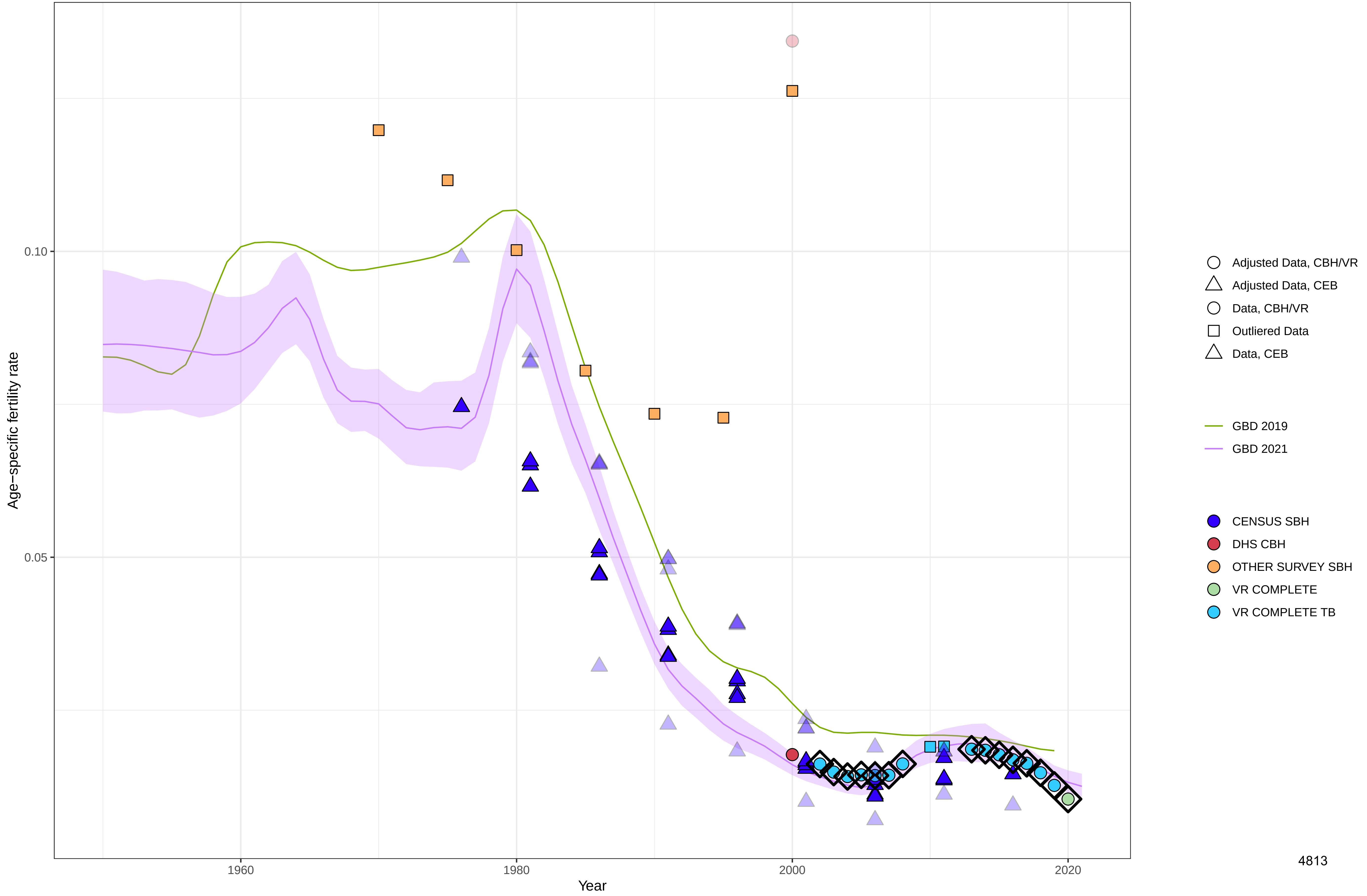

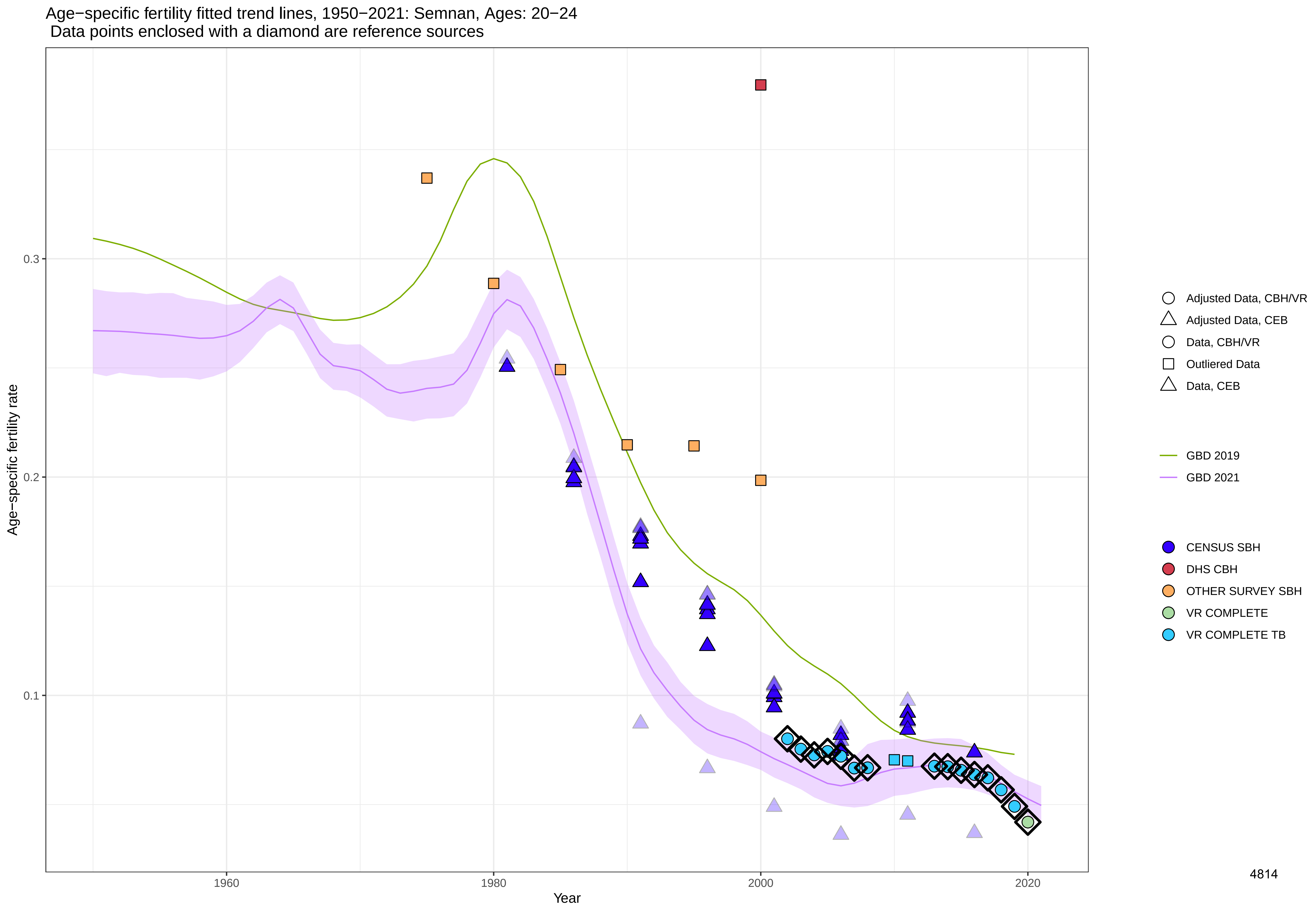

Age−specific fertility fitted trend lines, 1950−2021: Semnan, Ages: 20−24
Data points enclosed with a diamond are reference sources
Age−specific fertility rate
0.1
0.2
0.3
Year
1960
1980
2000
2020
Adjusted Data, CBH/VR
Adjusted Data, CEB
Data, CBH/VR
Outliered Data
Data, CEB
GBD 2019
GBD 2021
CENSUS SBH
DHS CBH
OTHER SURVEY SBH
VR COMPLETE
VR COMPLETE TB

Age−specific fertility fitted trend lines, 1950−2021: Semnan, Ages: 25−29
Data points enclosed with a diamond are reference sources

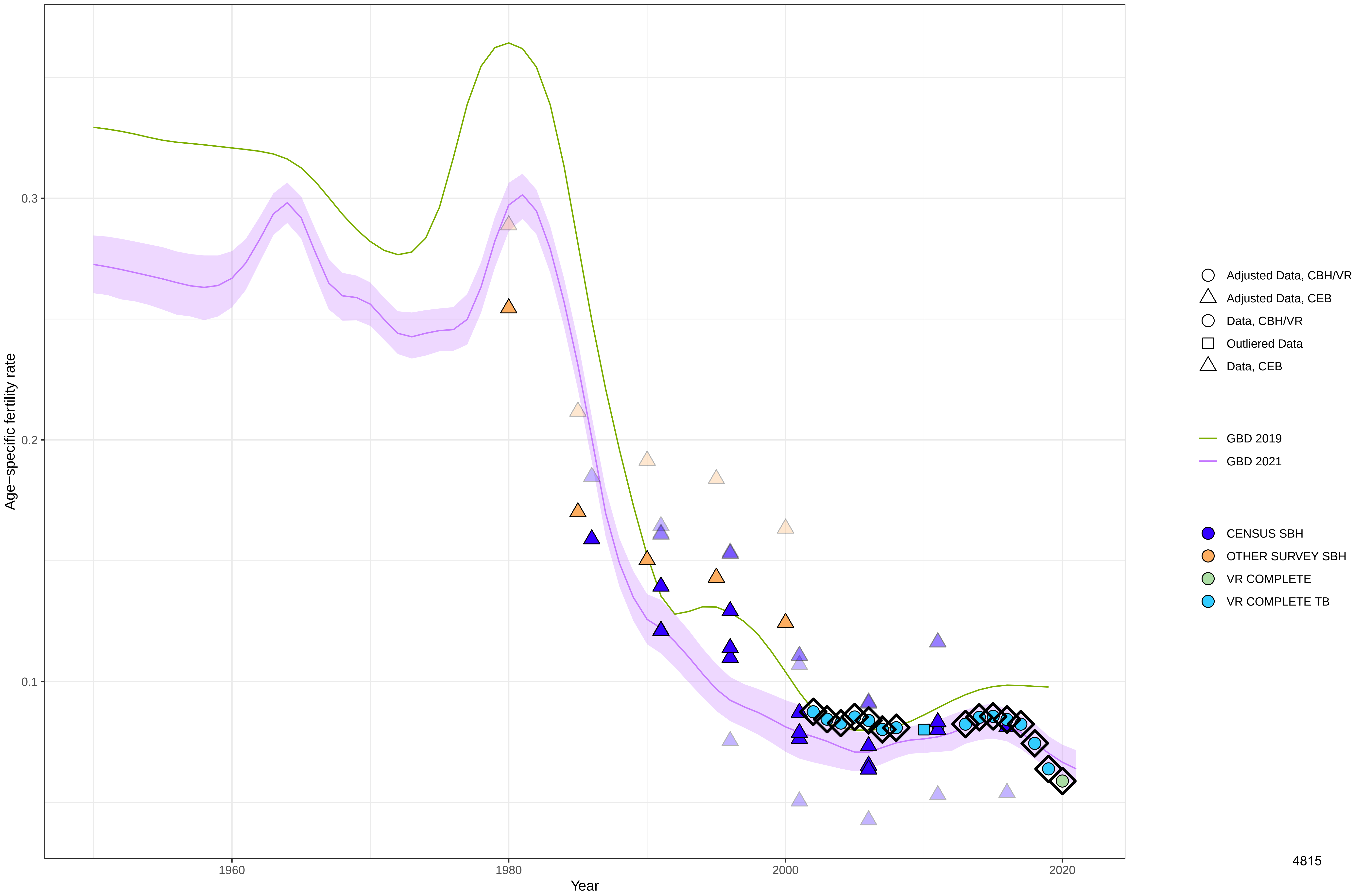

Age−specific fertility fitted trend lines, 1950−2021: Semnan, Ages: 30−34
Data points enclosed with a diamond are reference sources

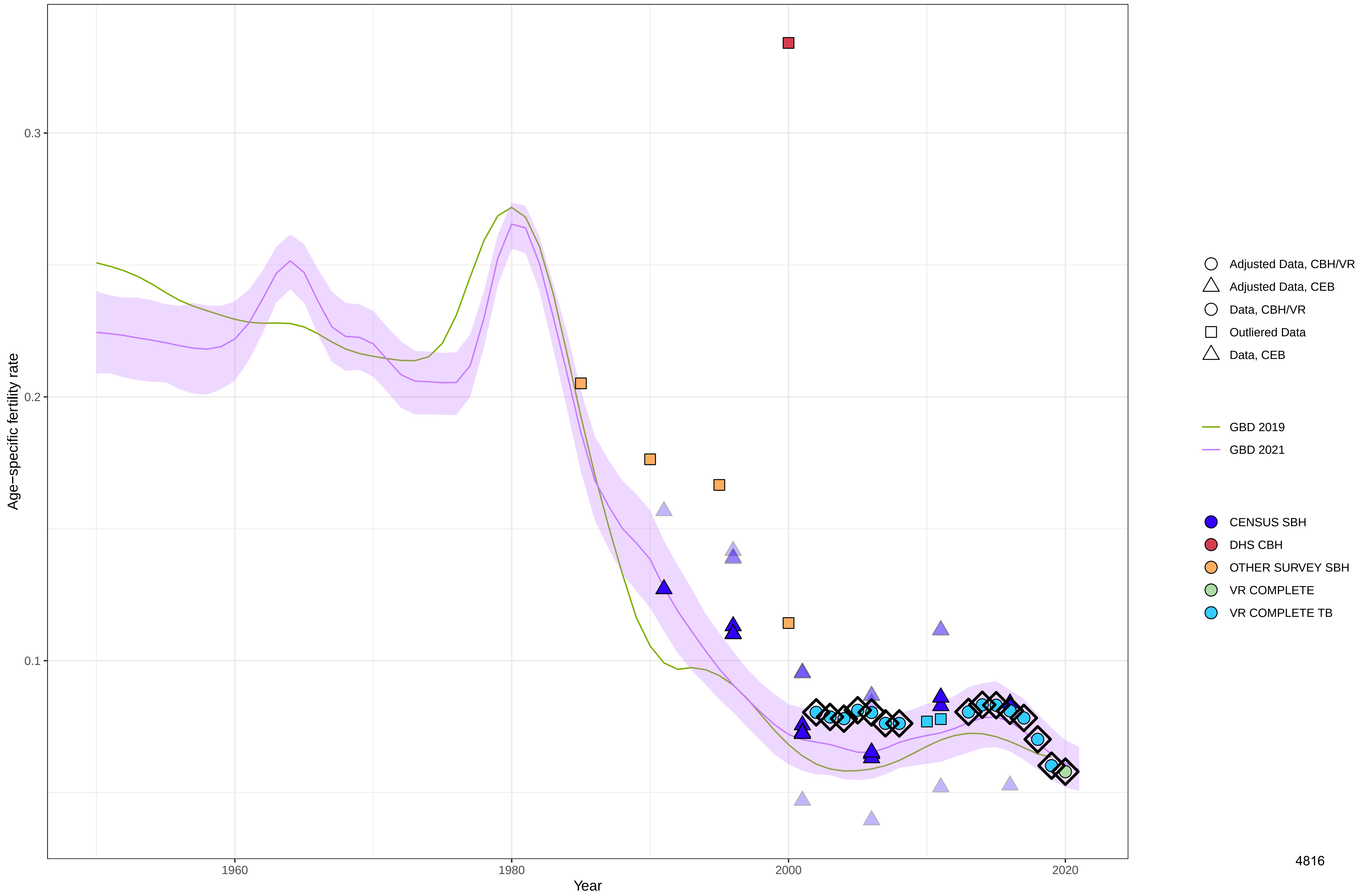

4816

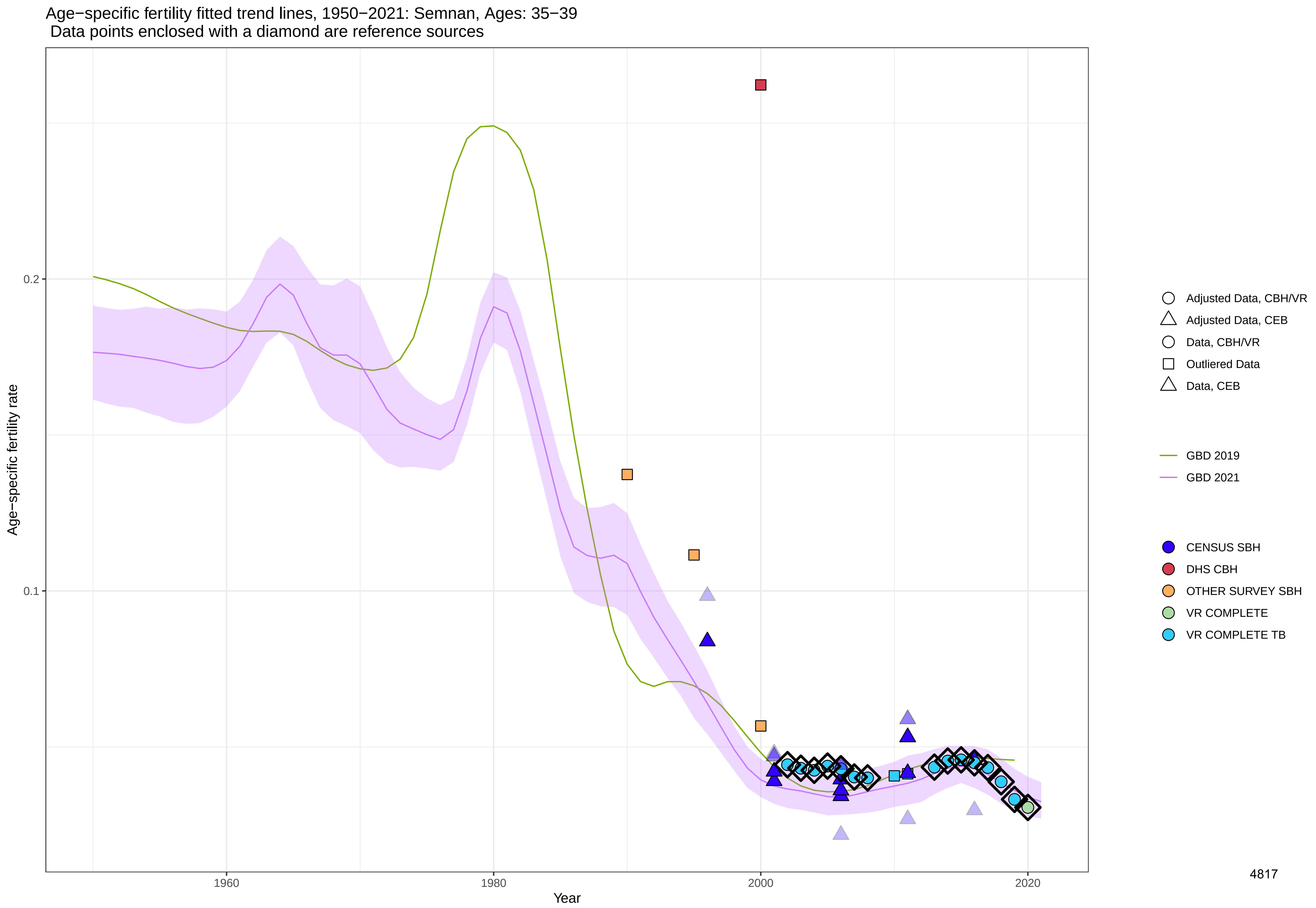

Age−specific fertility fitted trend lines, 1950−2021: Semnan, Ages: 35−39
Data points enclosed with a diamond are reference sources
Age−specific fertility rate
0.2
0.1
1960
1980
2000
2020
Year
Adjusted Data, CBH/VR
Adjusted Data, CEB
Data, CBH/VR
Outliered Data
Data, CEB
GBD 2019
GBD 2021
CENSUS SBH
DHS CBH
OTHER SURVEY SBH
VR COMPLETE
VR COMPLETE TB

Age−specific fertility fitted trend lines, 1950−2021: Semnan, Ages: 40−44
 Data points enclosed with a diamond are reference sources

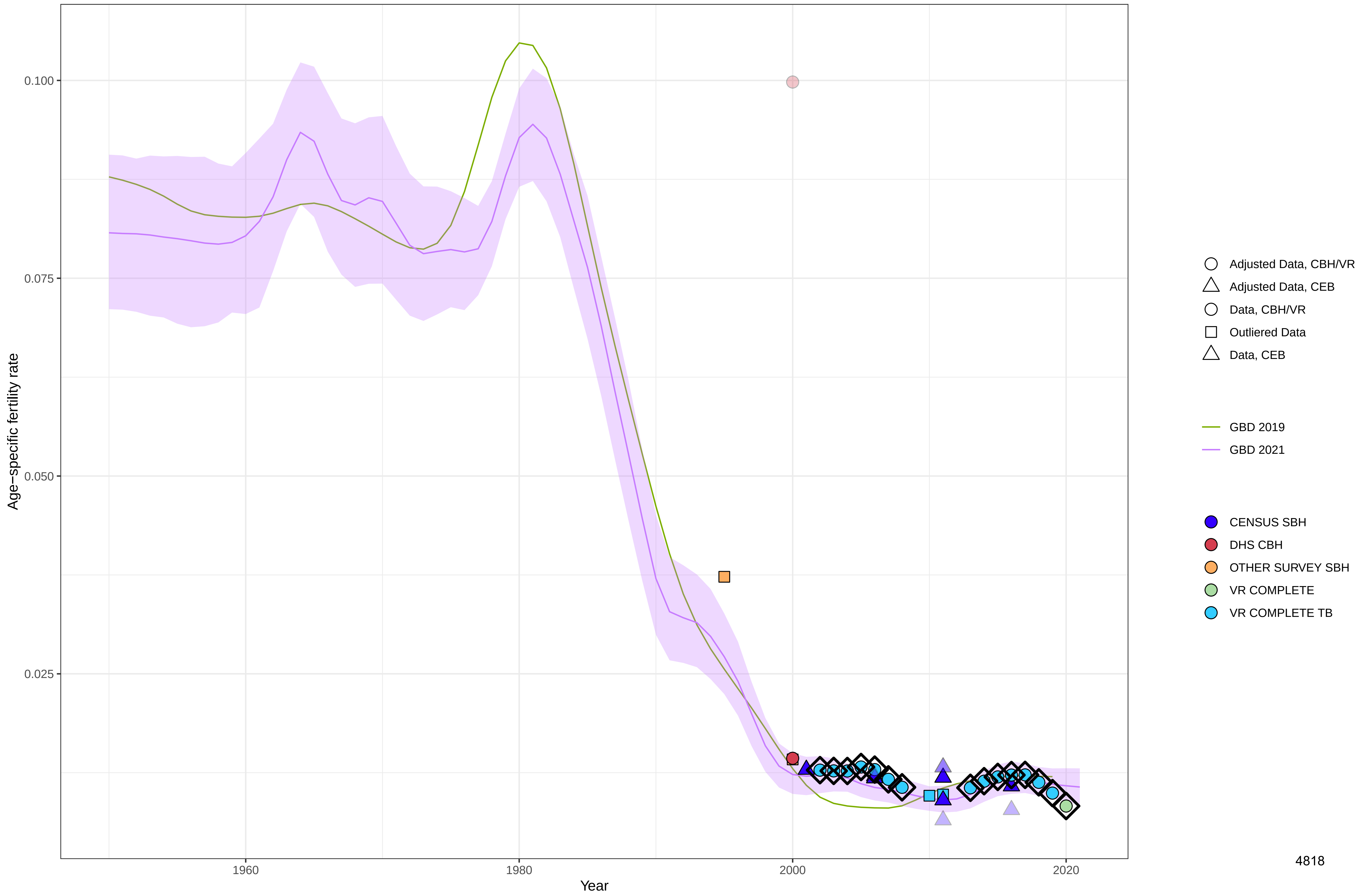

Age−specific fertility fitted trend lines, 1950−2021: Semnan, Ages: 45−49
Data points enclosed with a diamond are reference sources

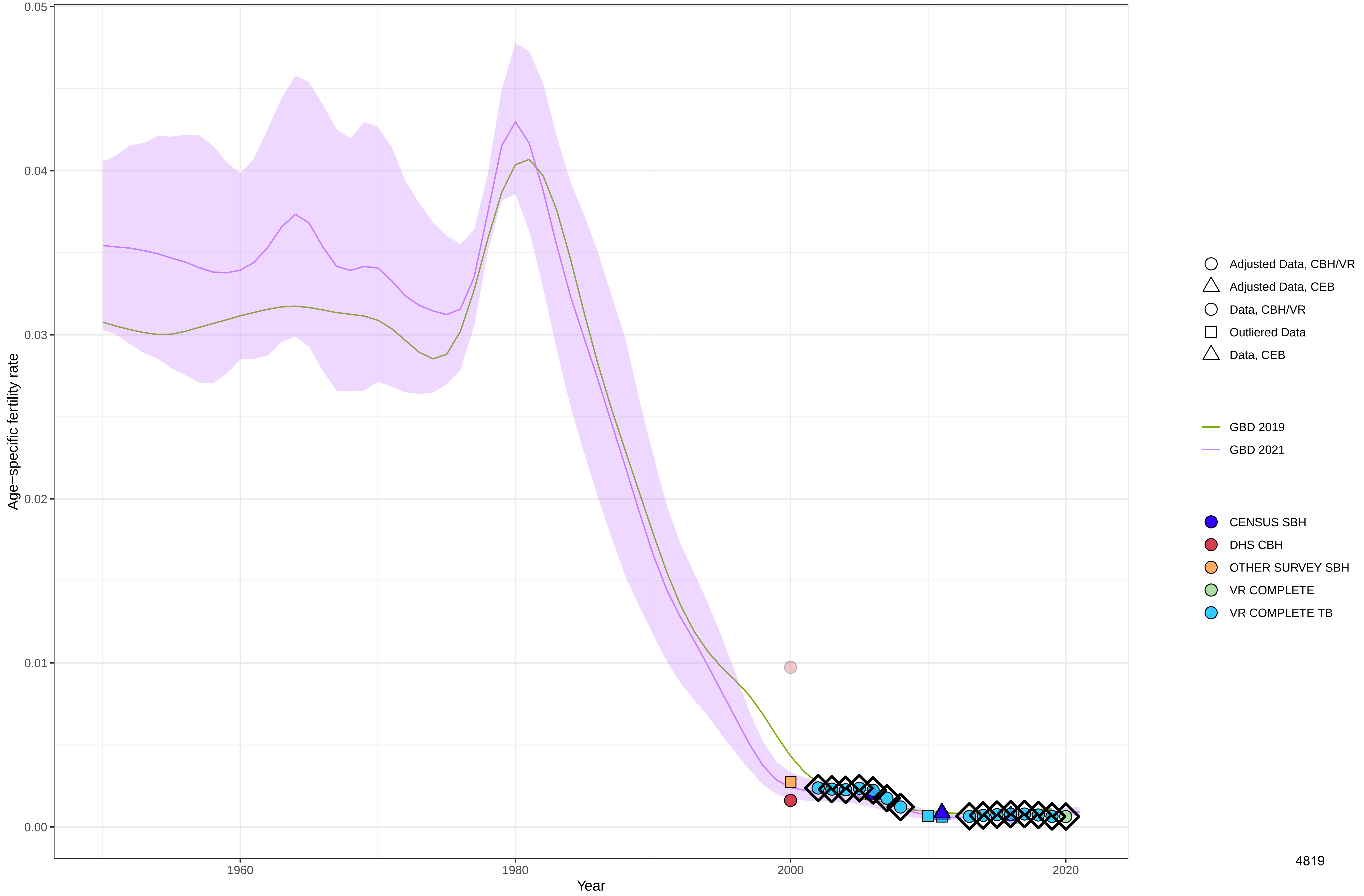

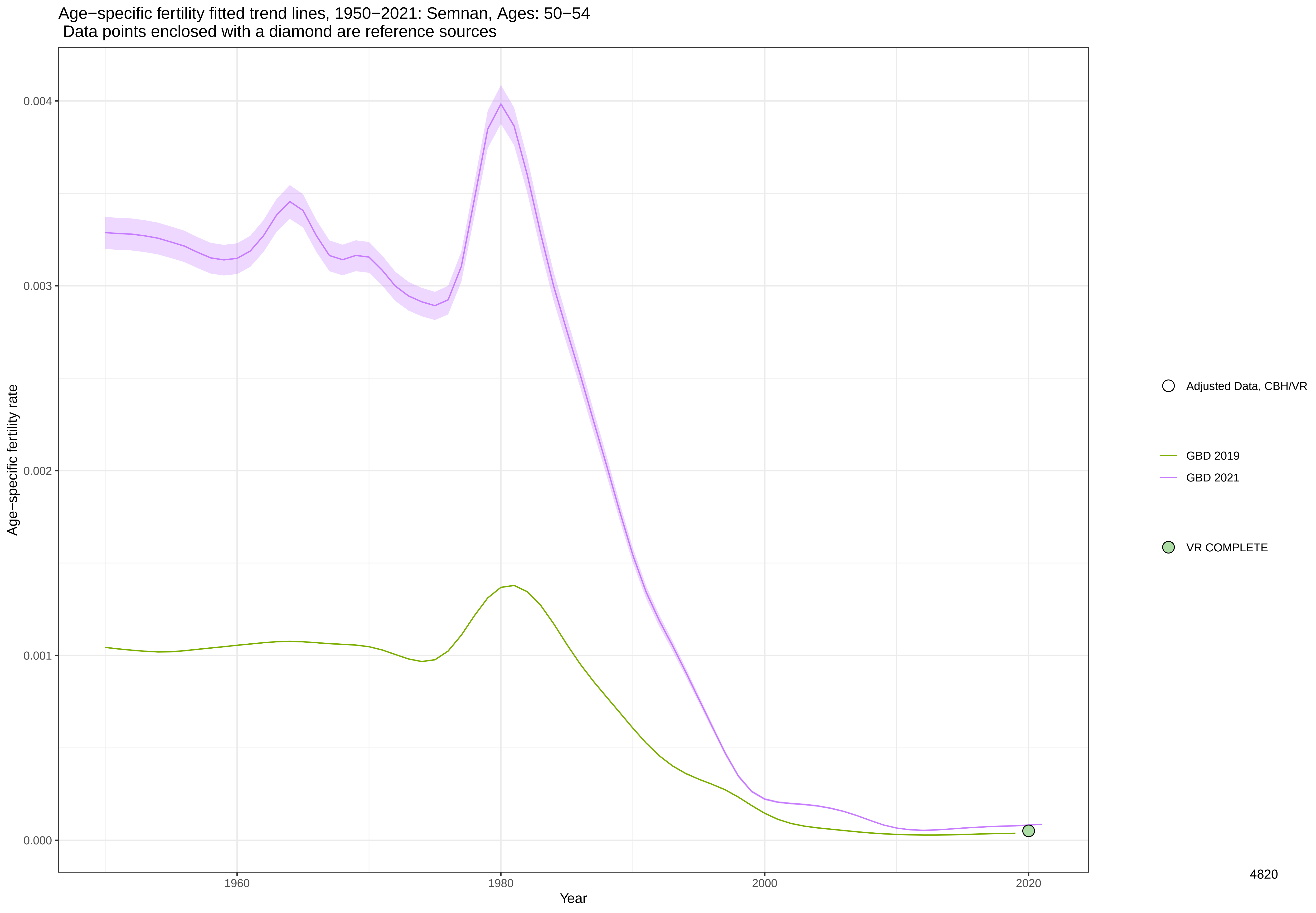

Age−specific fertility fitted trend lines, 1950−2021: Semnan, Ages: 50−54
Data points enclosed with a diamond are reference sources
Age−specific fertility rate
0.000
0.001
0.002
0.003
0.004
Year
1960
1980
2000
2020
Adjusted Data, CBH/VR
GBD 2019
GBD 2021
VR COMPLETE

Total fertility rate fitted trend lines, 1950−2021: Semnan TFR
Data points enclosed with a diamond are reference sources

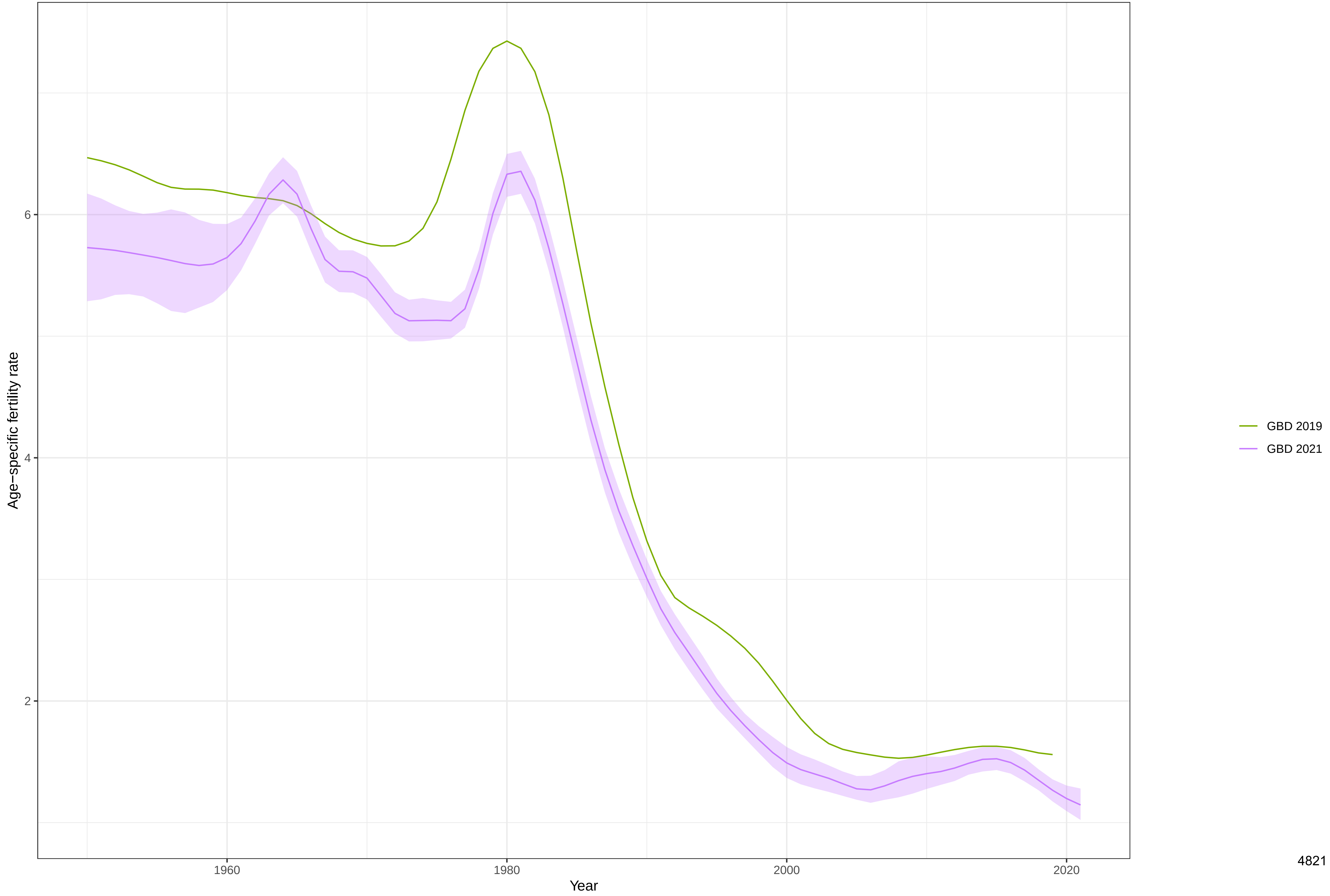

Age−specific fertility fitted trend lines, 1950−2021: Sistan and Baluchistan, Ages: 10−14
Data points enclosed with a diamond are reference sources

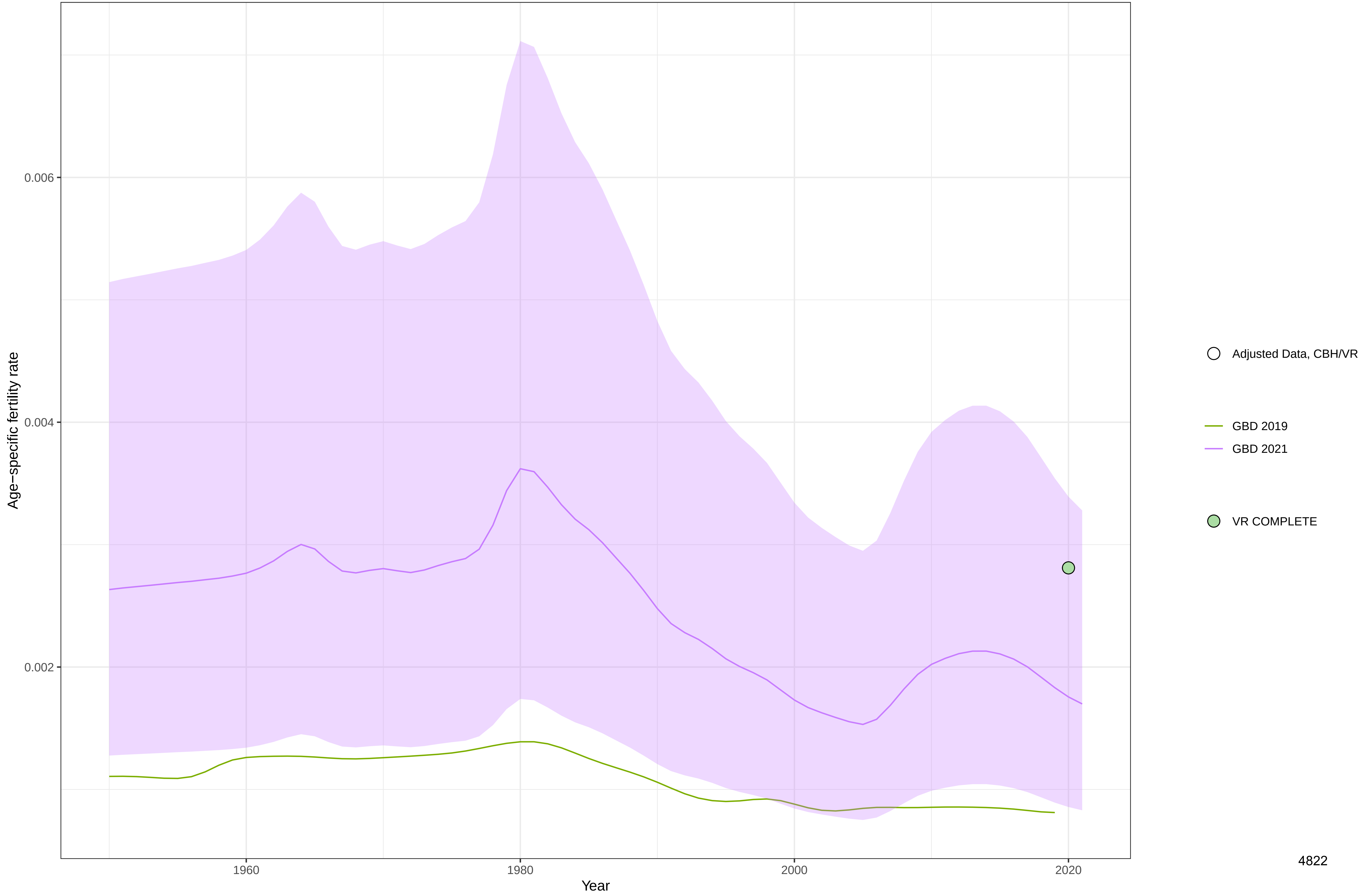

Age−specific fertility fitted trend lines, 1950−2021: Sistan and Baluchistan, Ages: 15−19
Data points enclosed with a diamond are reference sources

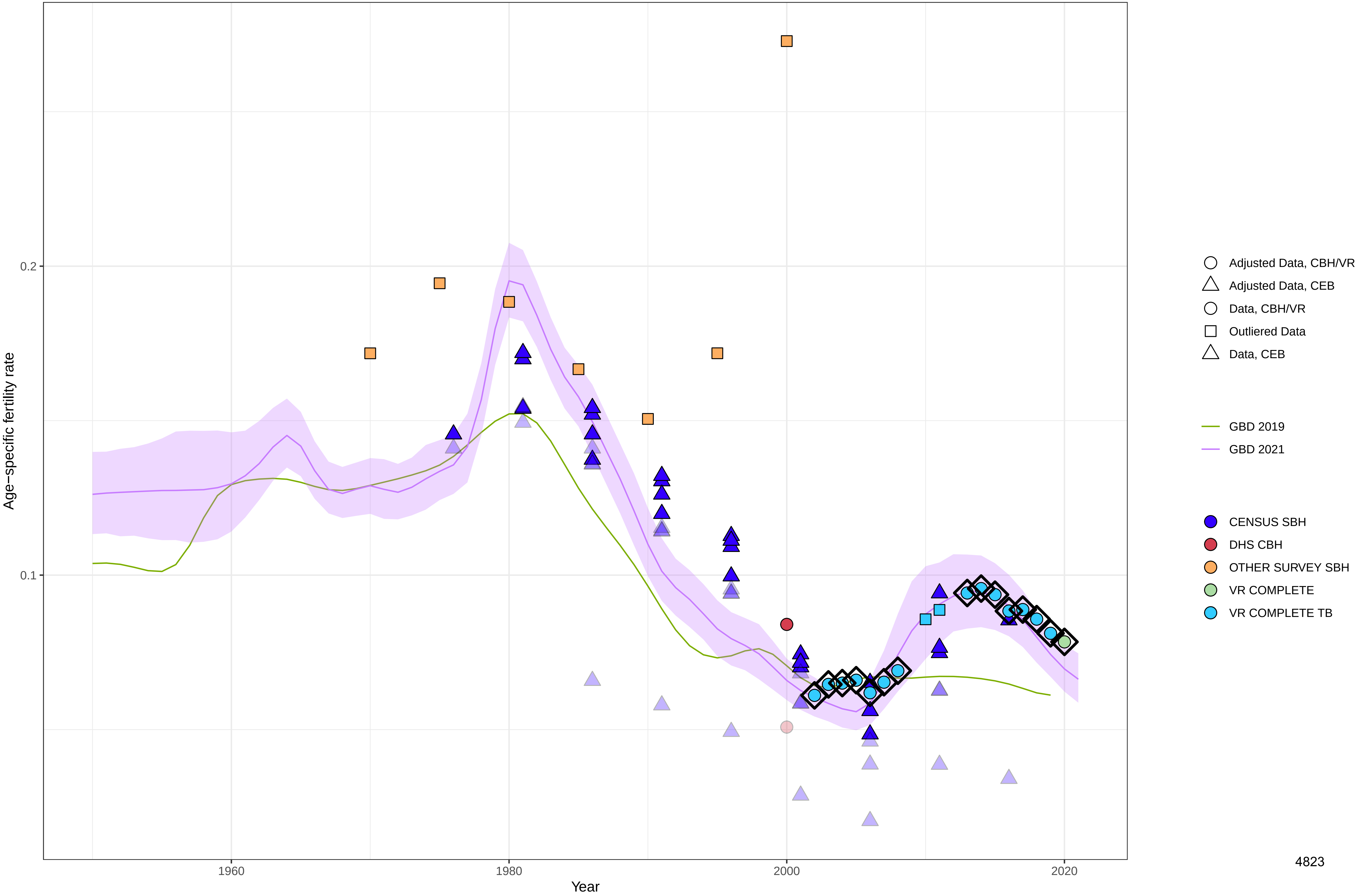

Age−specific fertility fitted trend lines, 1950−2021: Sistan and Baluchistan, Ages: 20−24
Data points enclosed with a diamond are reference sources

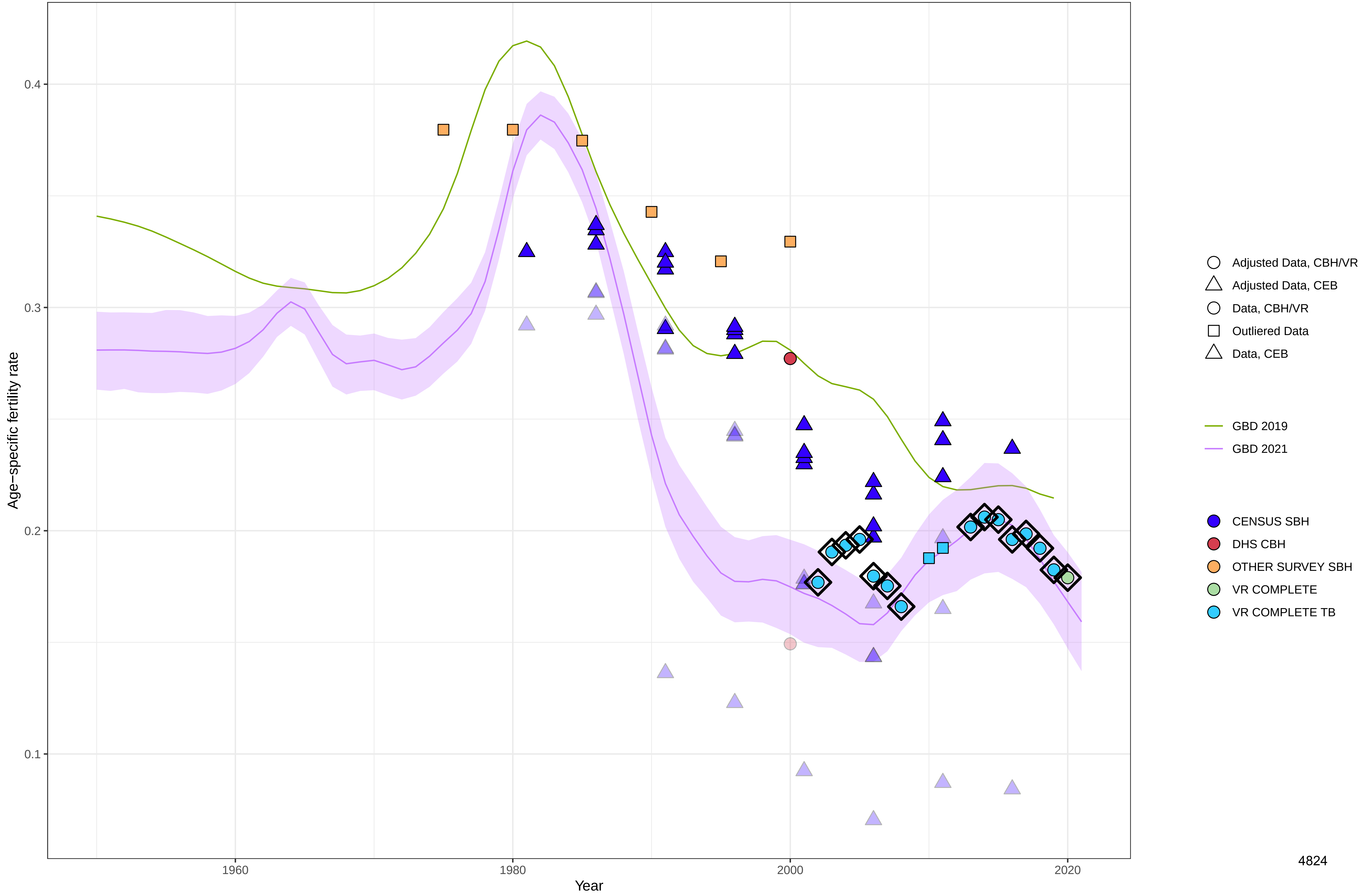

Age−specific fertility fitted trend lines, 1950−2021: Sistan and Baluchistan, Ages: 25−29
Data points enclosed with a diamond are reference sources

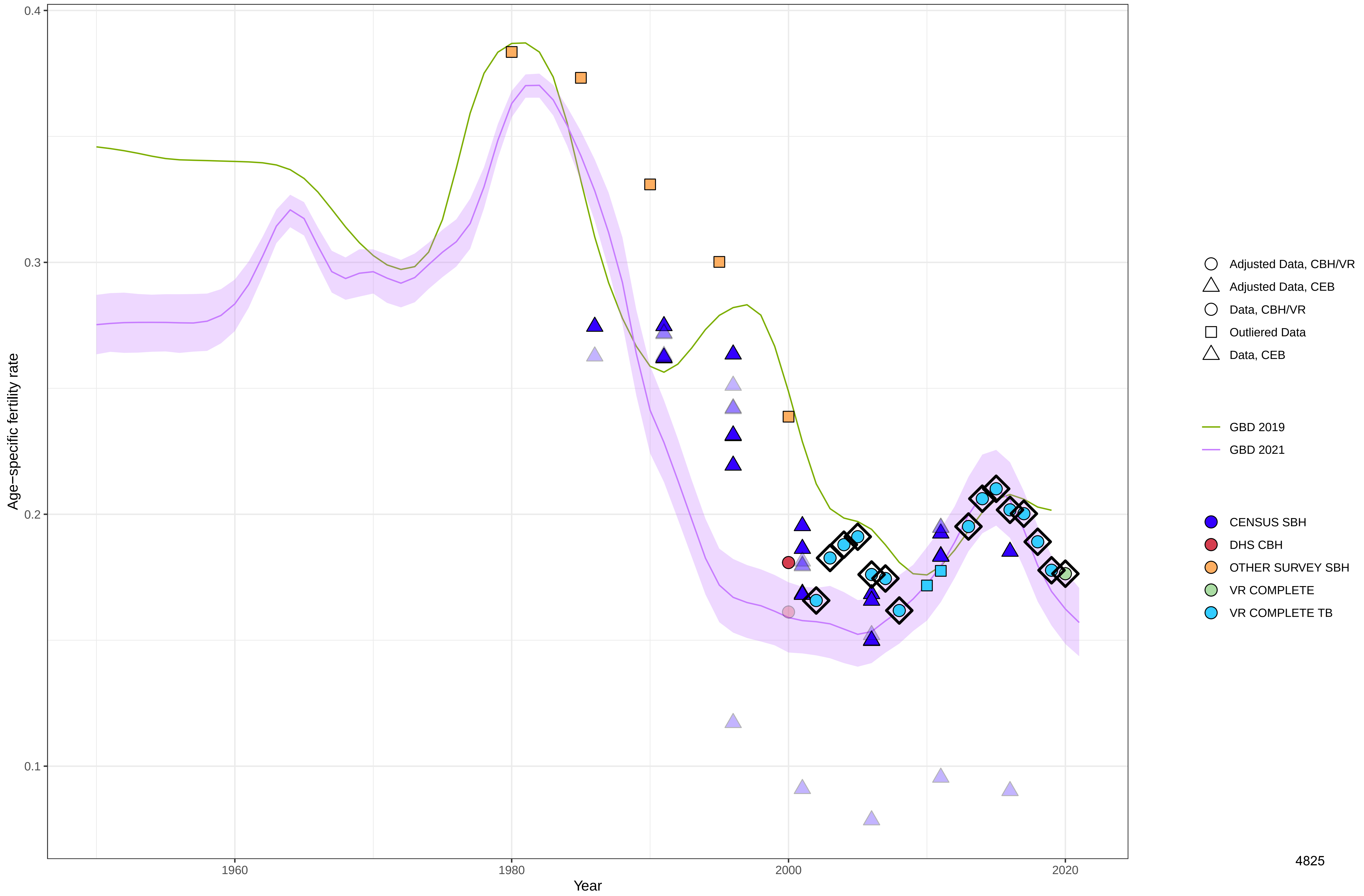

Age−specific fertility fitted trend lines, 1950−2021: Sistan and Baluchistan, Ages: 30−34
Data points enclosed with a diamond are reference sources

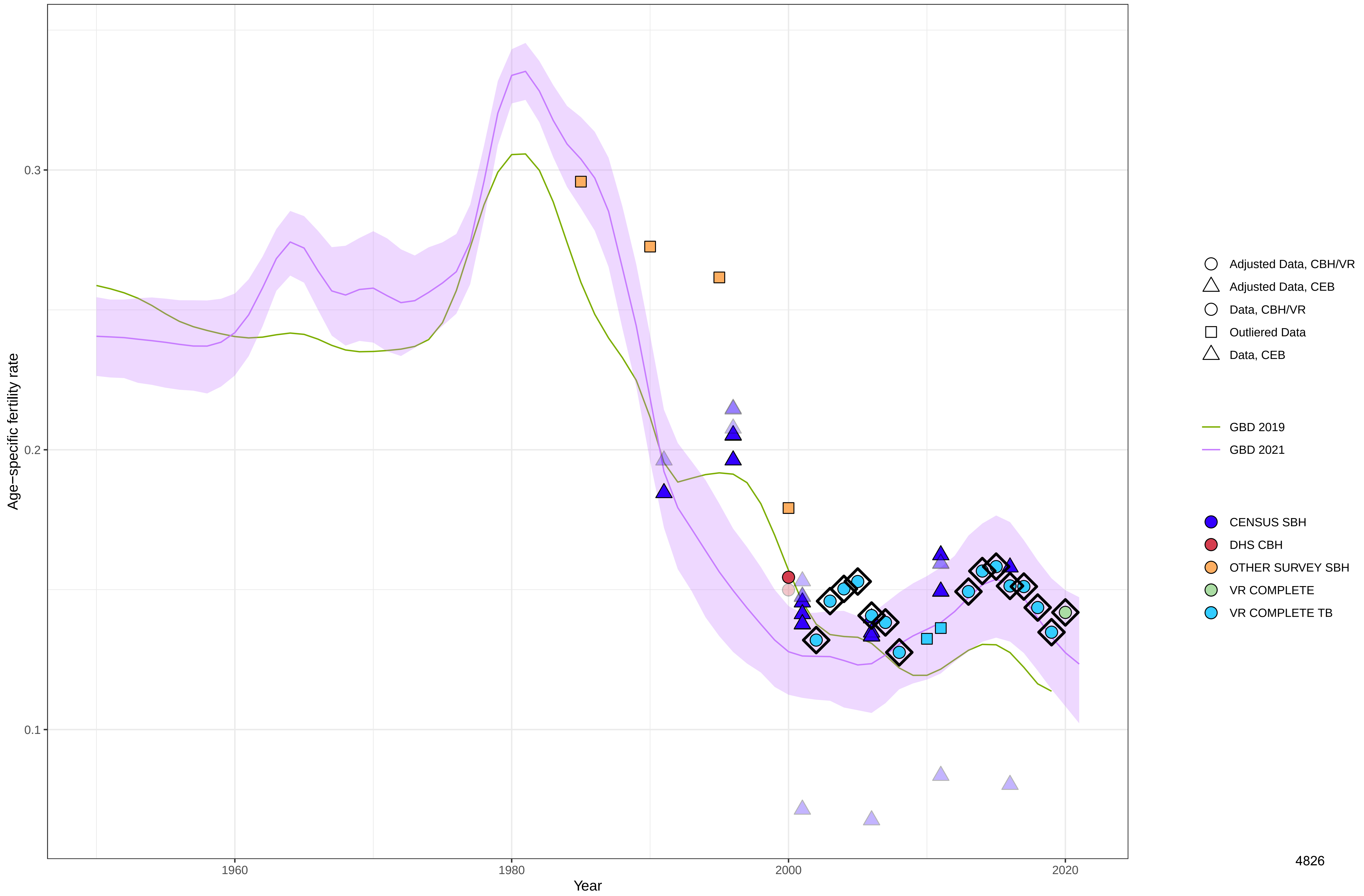

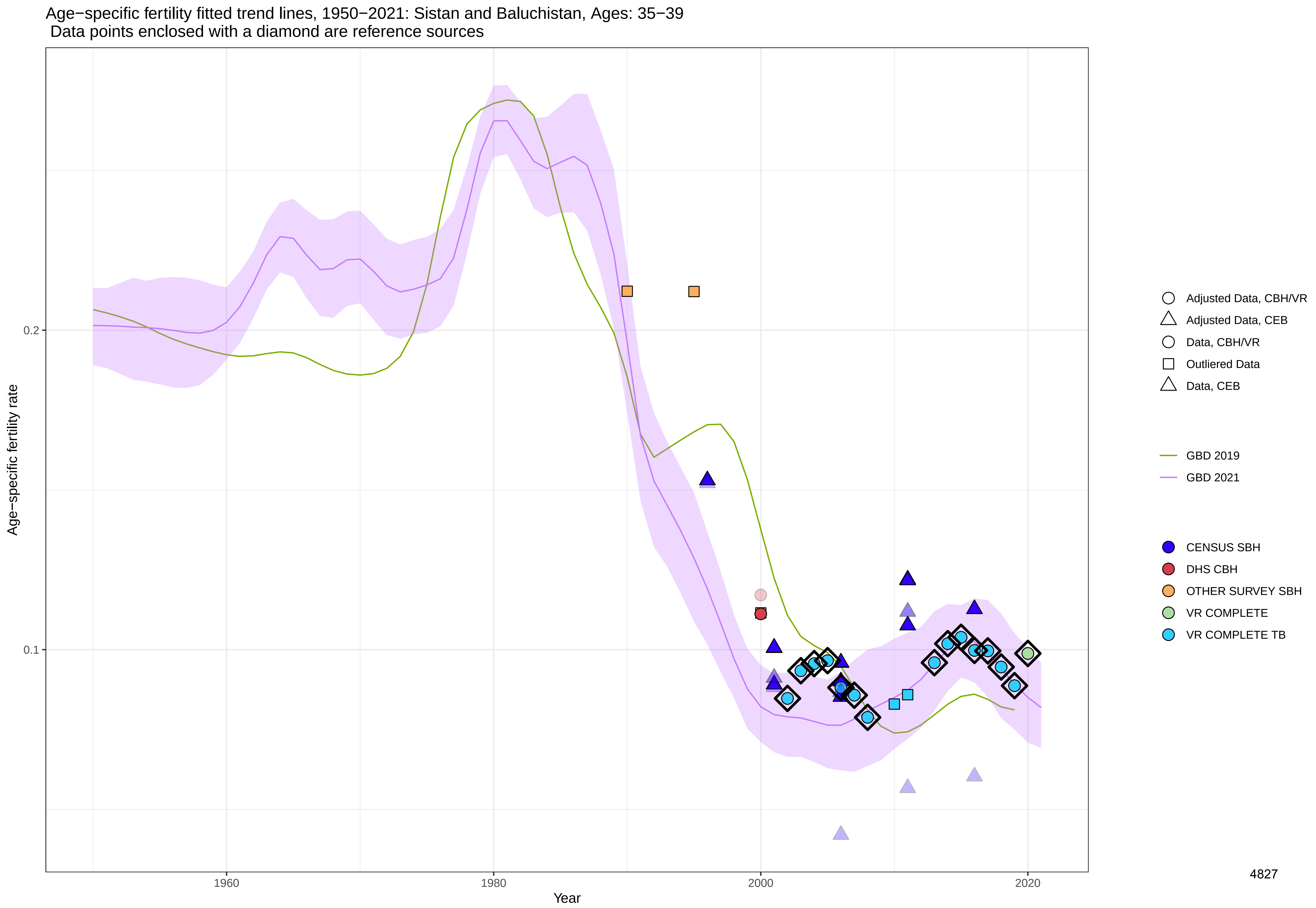
Age−specific fertility fitted trend lines, 1950−2021: Sistan and Baluchistan, Ages: 35−39
Data points enclosed with a diamond are reference sources
Age−specific fertility rate
0.2
0.1
1960
1980
2000
2020
Year
Adjusted Data, CBH/VR
Adjusted Data, CEB
Data, CBH/VR
Outliered Data
Data, CEB
GBD 2019
GBD 2021
CENSUS SBH
DHS CBH
OTHER SURVEY SBH
VR COMPLETE
VR COMPLETE TB

4827

Age−specific fertility fitted trend lines, 1950−2021: Sistan and Baluchistan, Ages: 40−44
Data points enclosed with a diamond are reference sources

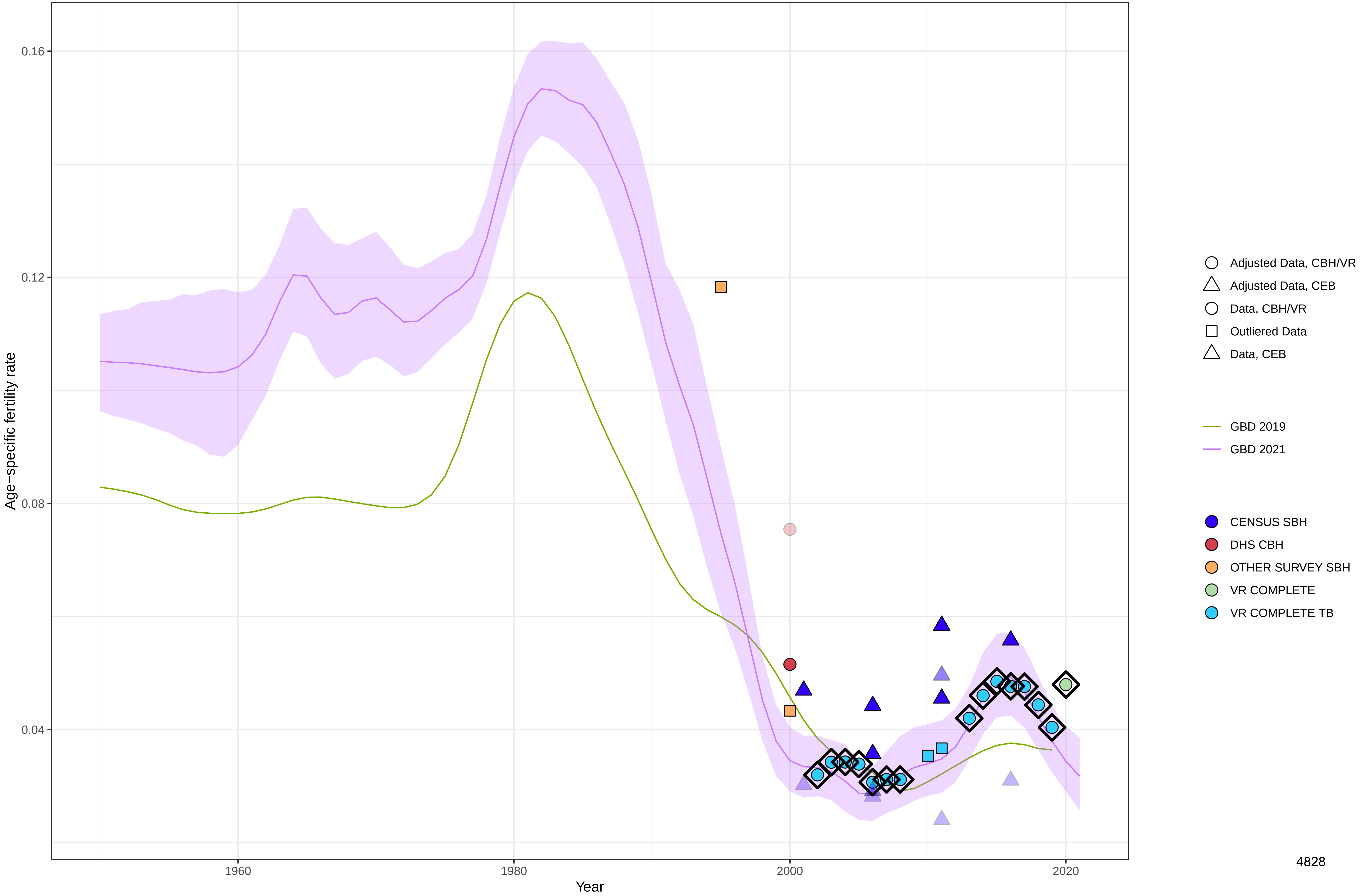

Age−specific fertility fitted trend lines, 1950−2021: Sistan and Baluchistan, Ages: 45−49
Data points enclosed with a diamond are reference sources

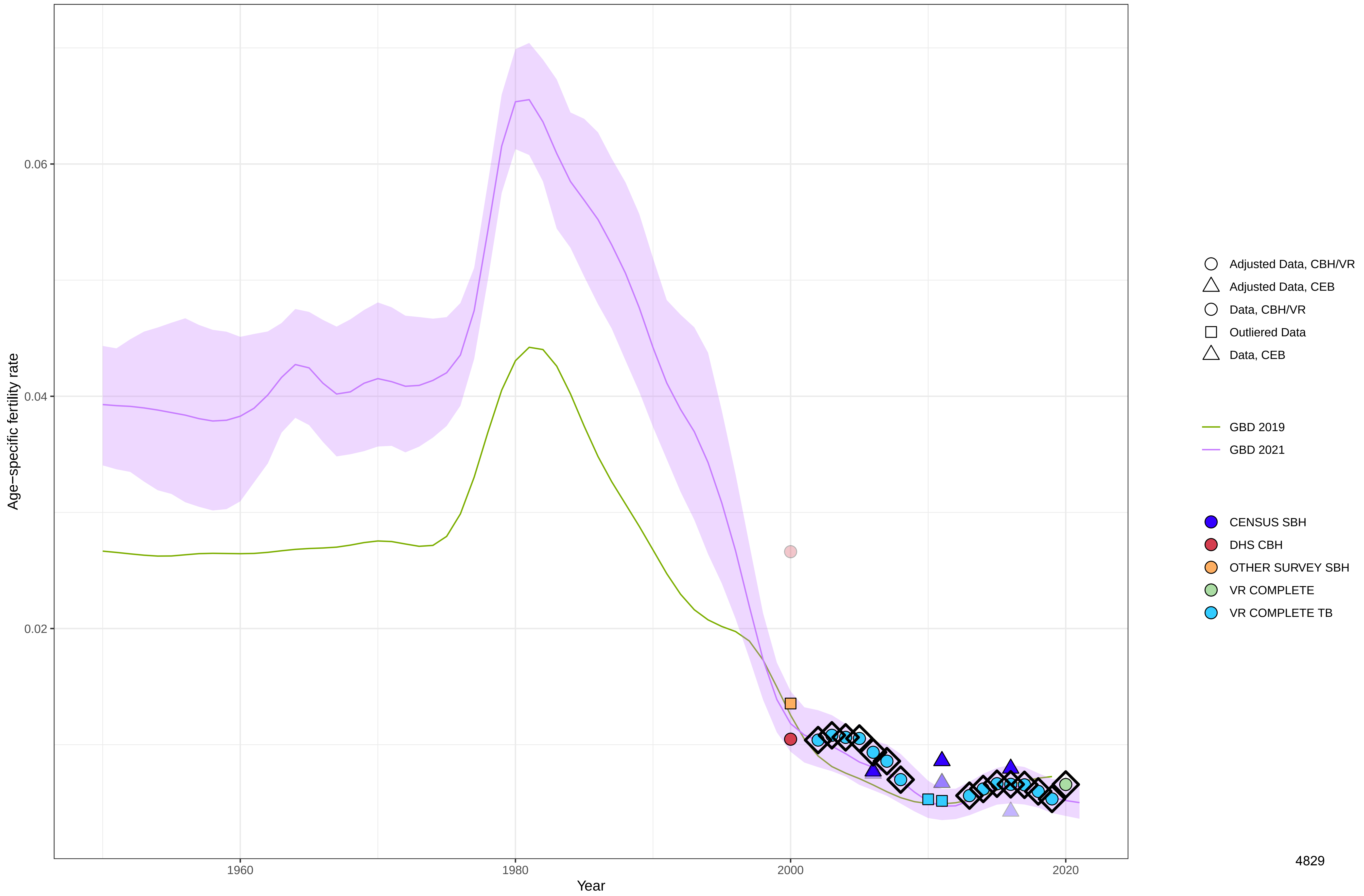

Age−specific fertility fitted trend lines, 1950−2021: Sistan and Baluchistan, Ages: 50−54
Data points enclosed with a diamond are reference sources

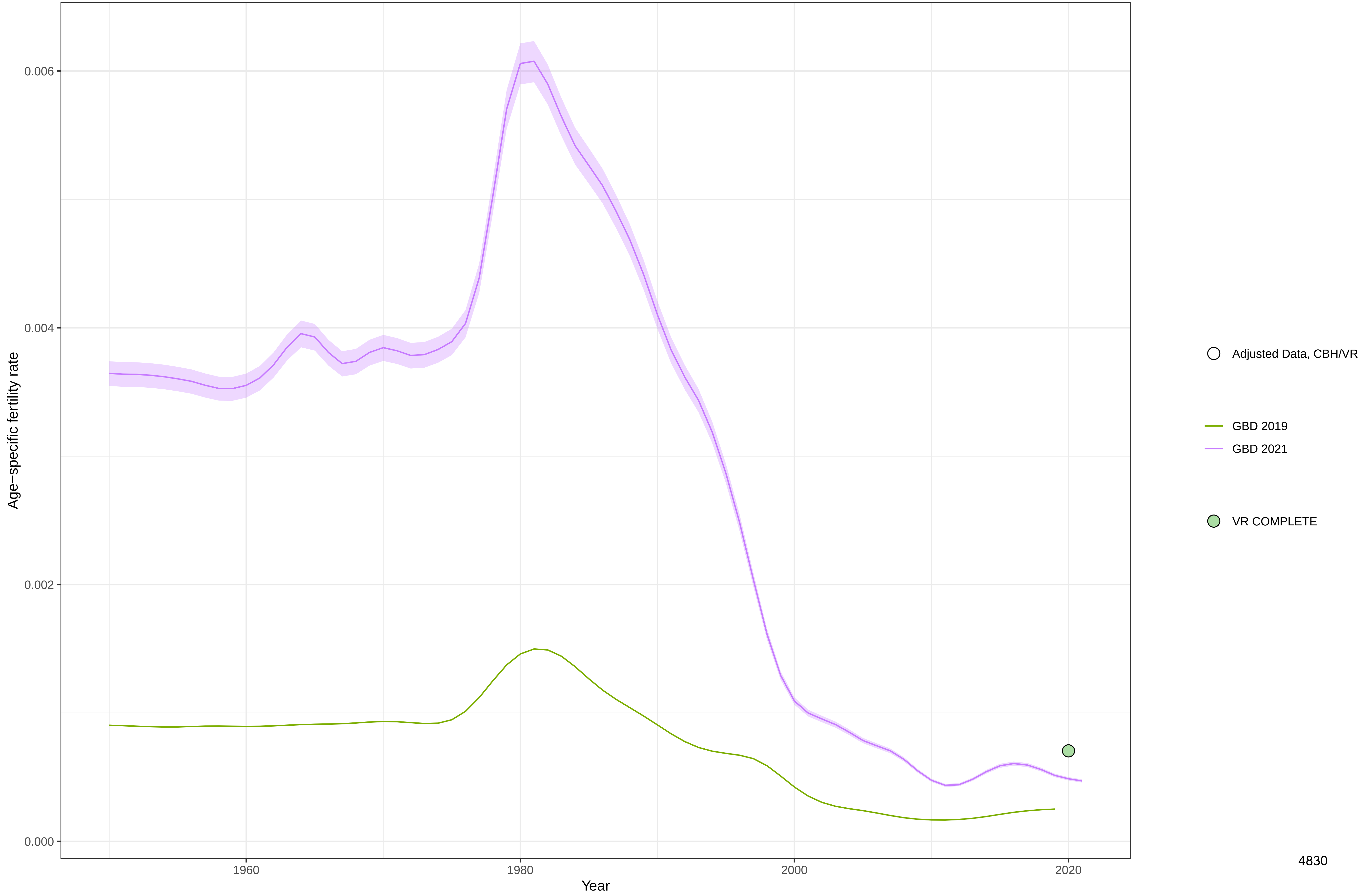

Total fertility rate fitted trend lines, 1950−2021: Sistan and Baluchistan TFR
Data points enclosed with a diamond are reference sources

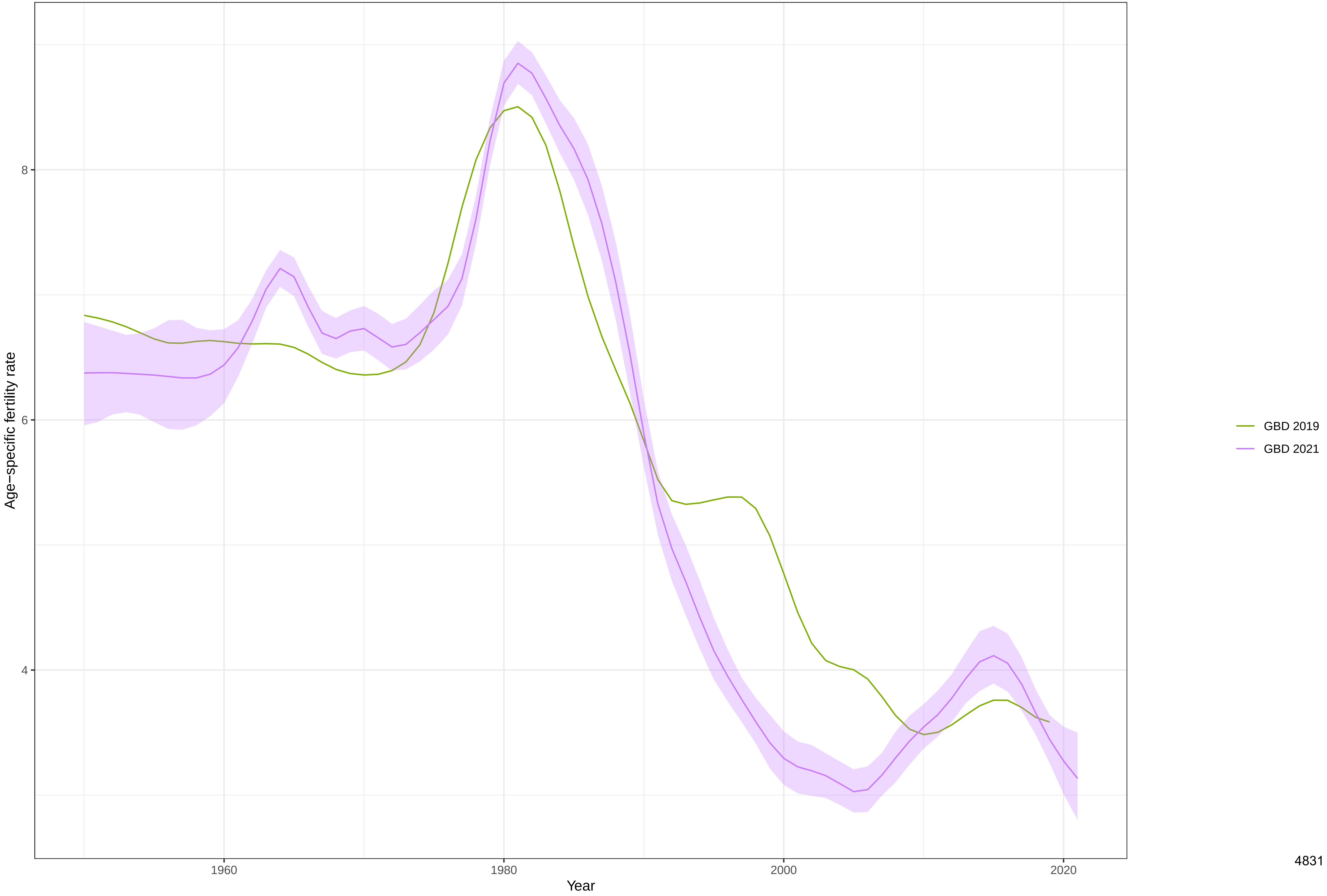

Age−specific fertility fitted trend lines, 1950−2021: South Khorasan, Ages: 10−14
Data points enclosed with a diamond are reference sources

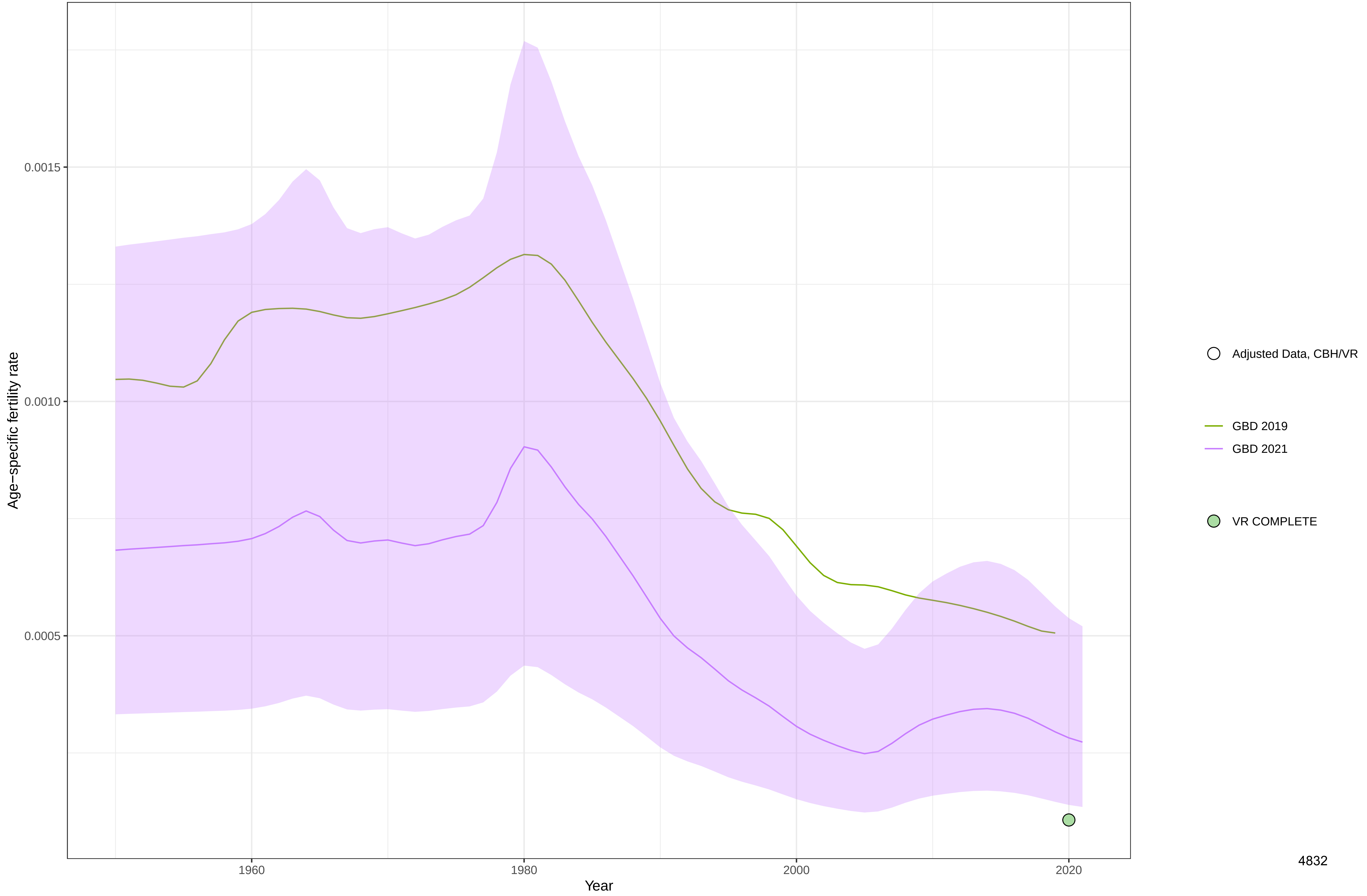

Age−specific fertility fitted trend lines, 1950−2021: South Khorasan, Ages: 15−19
Data points enclosed with a diamond are reference sources

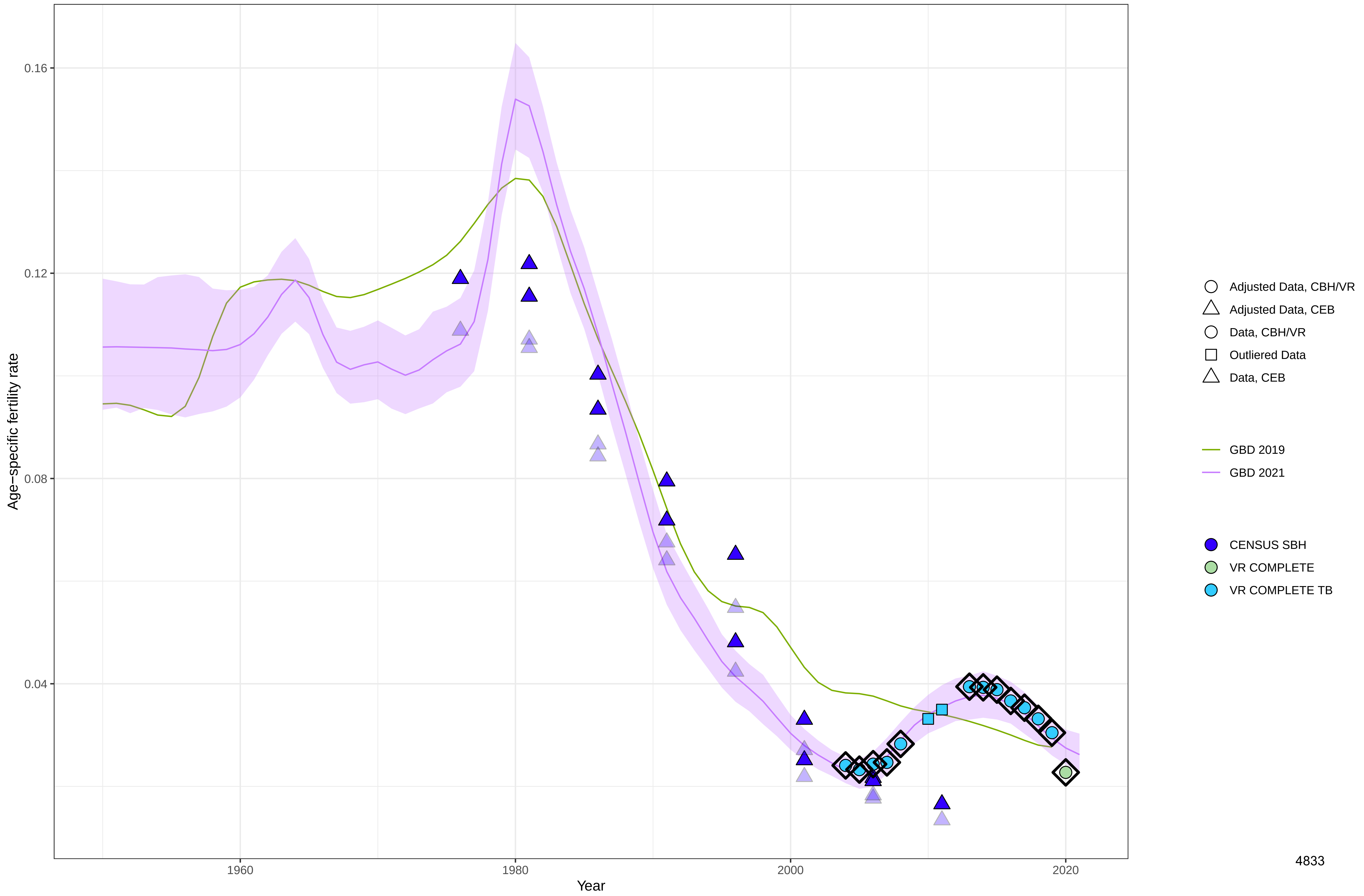

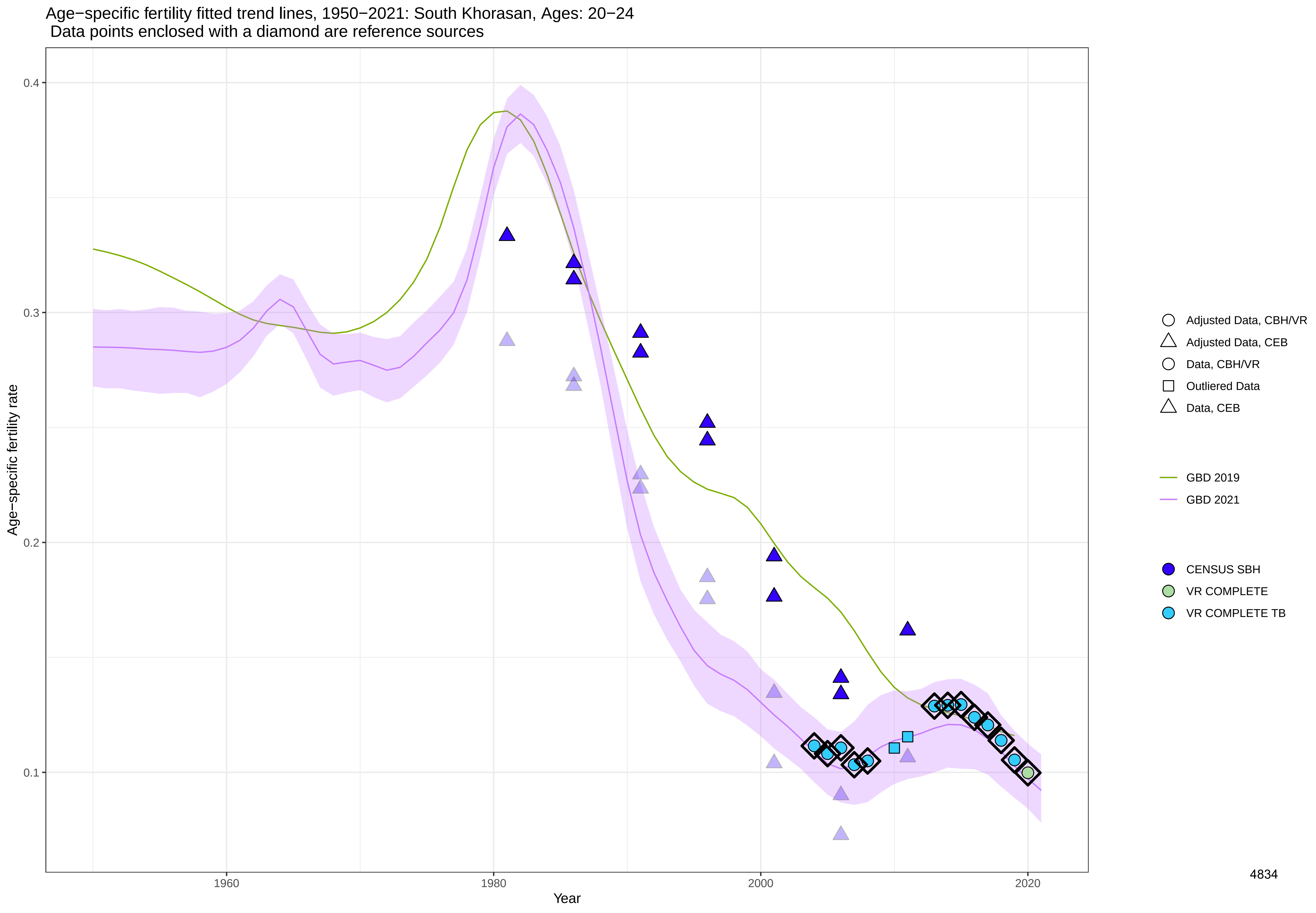
Age−specific fertility fitted trend lines, 1950−2021: South Khorasan, Ages: 20−24
Data points enclosed with a diamond are reference sources
Age−specific fertility rate
0.4
0.3
0.2
0.1
1960
1980
2000
2020
Year
Adjusted Data, CBH/VR
Adjusted Data, CEB
Data, CBH/VR
Outliered Data
Data, CEB
GBD 2019
GBD 2021
CENSUS SBH
VR COMPLETE
VR COMPLETE TB

4834

Age−specific fertility fitted trend lines, 1950−2021: South Khorasan, Ages: 25−29
Data points enclosed with a diamond are reference sources

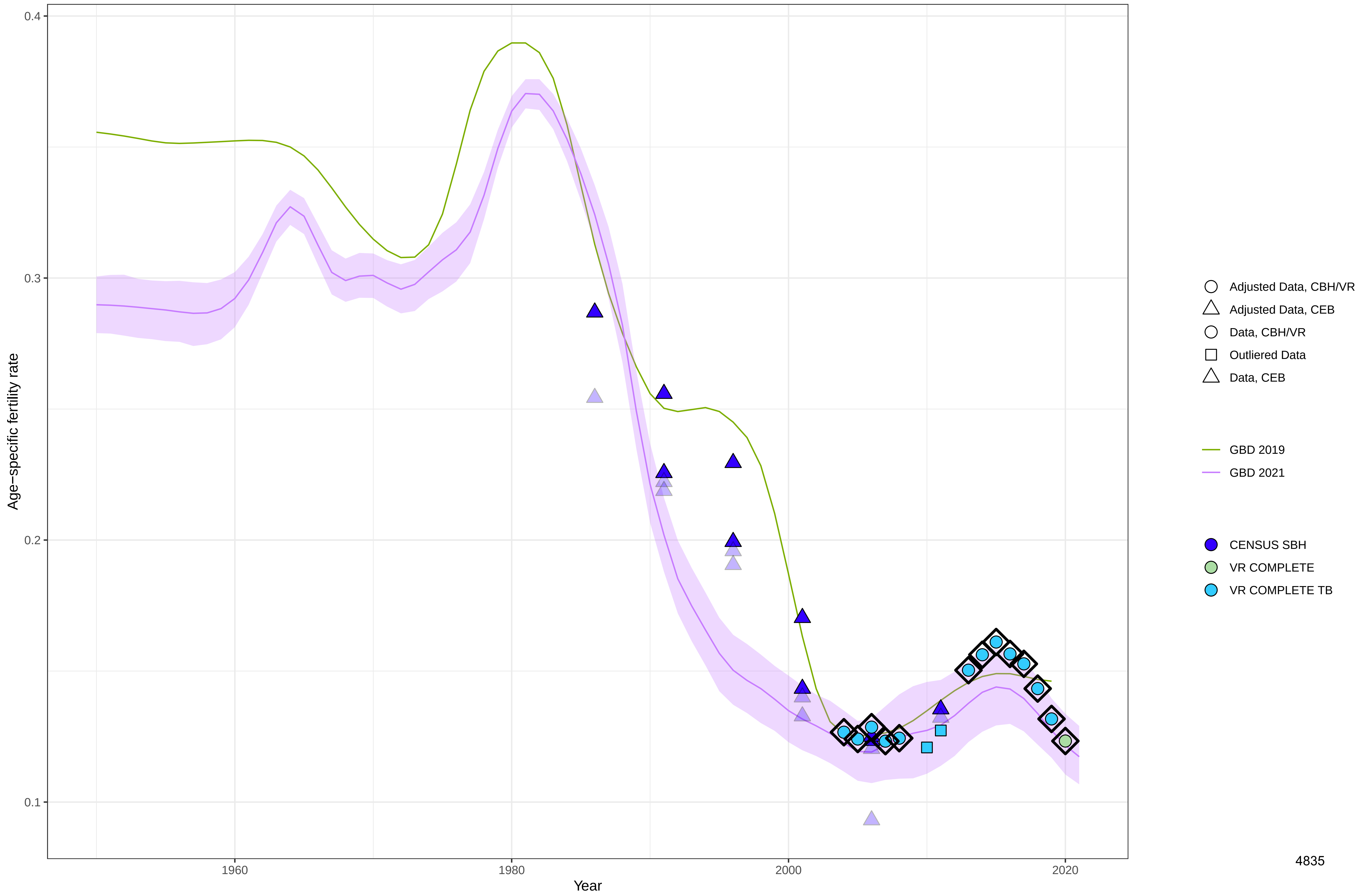

Age−specific fertility fitted trend lines, 1950−2021: South Khorasan, Ages: 30−34
Data points enclosed with a diamond are reference sources

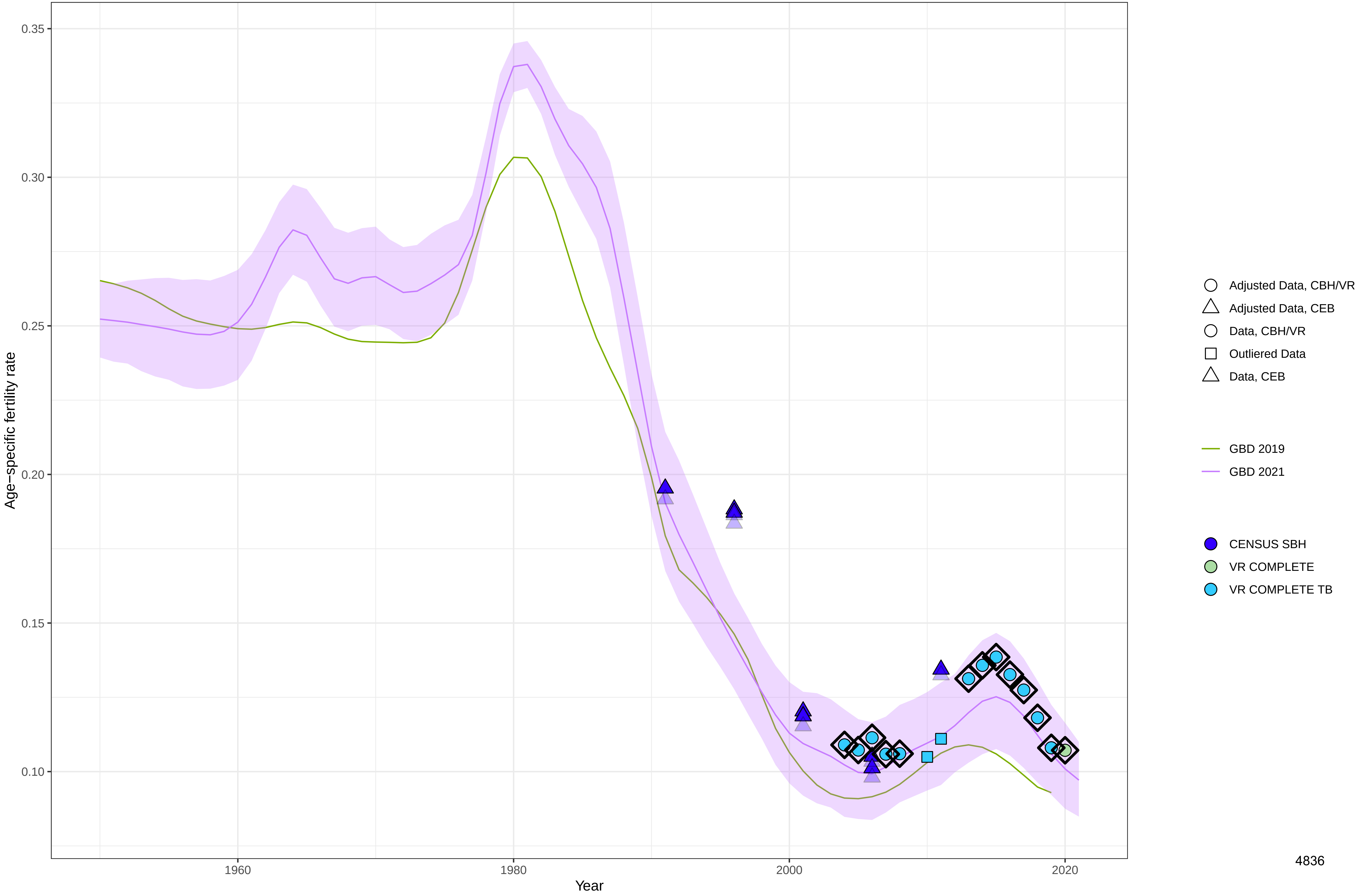

Age−specific fertility fitted trend lines, 1950−2021: South Khorasan, Ages: 35−39
Data points enclosed with a diamond are reference sources

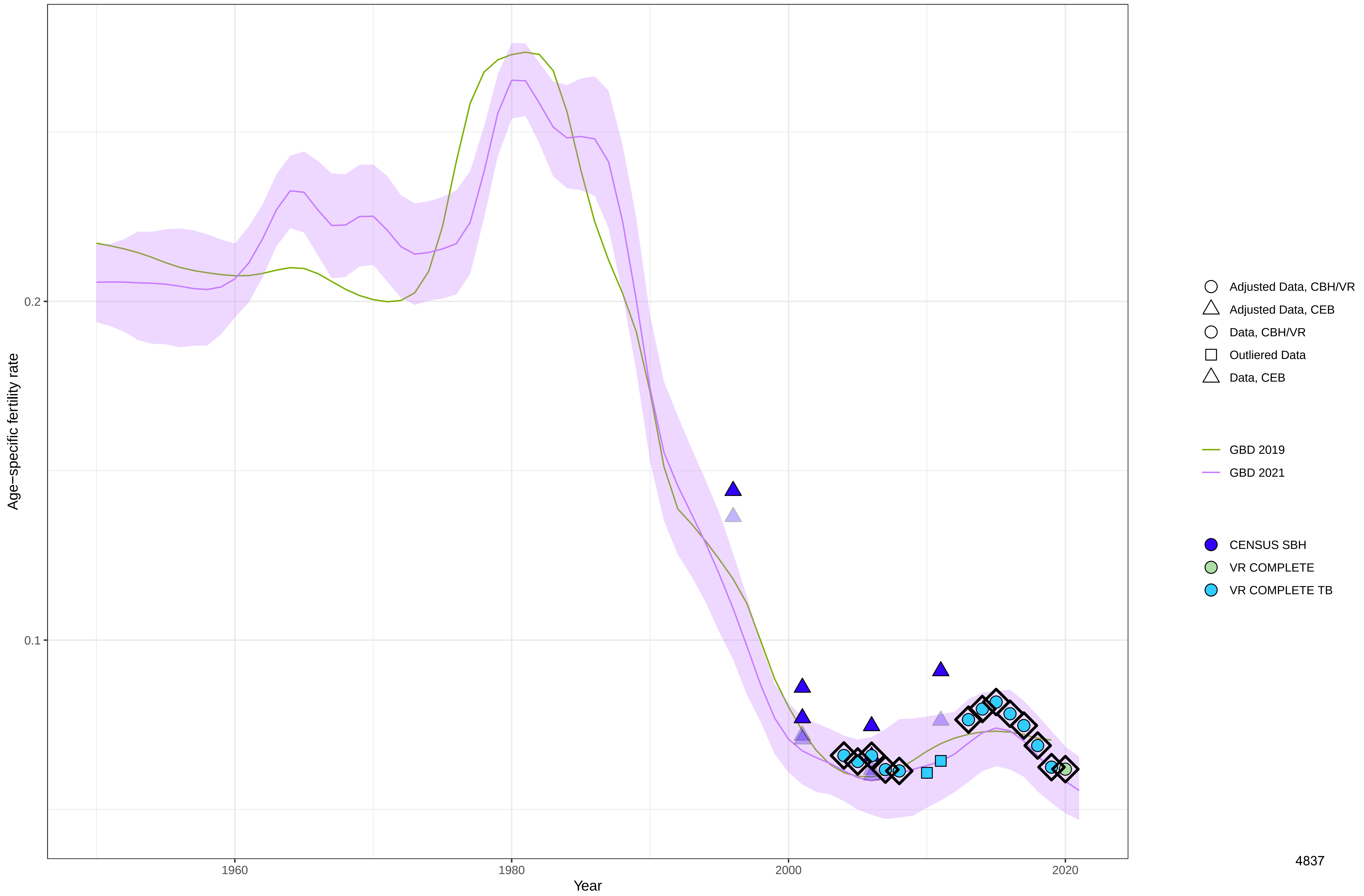

Age−specific fertility fitted trend lines, 1950−2021: South Khorasan, Ages: 40−44
Data points enclosed with a diamond are reference sources

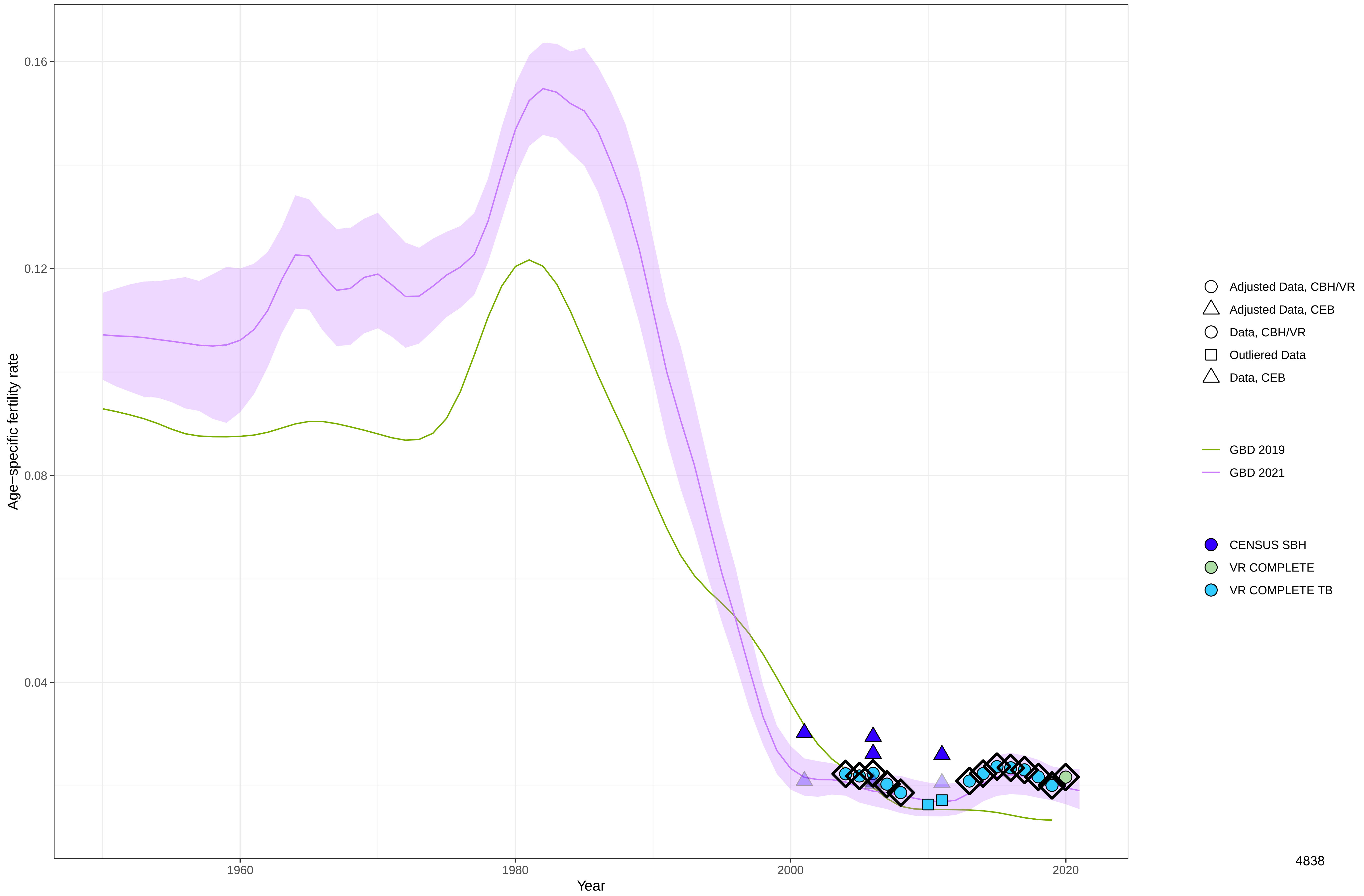

Age−specific fertility fitted trend lines, 1950−2021: South Khorasan, Ages: 45−49
Data points enclosed with a diamond are reference sources

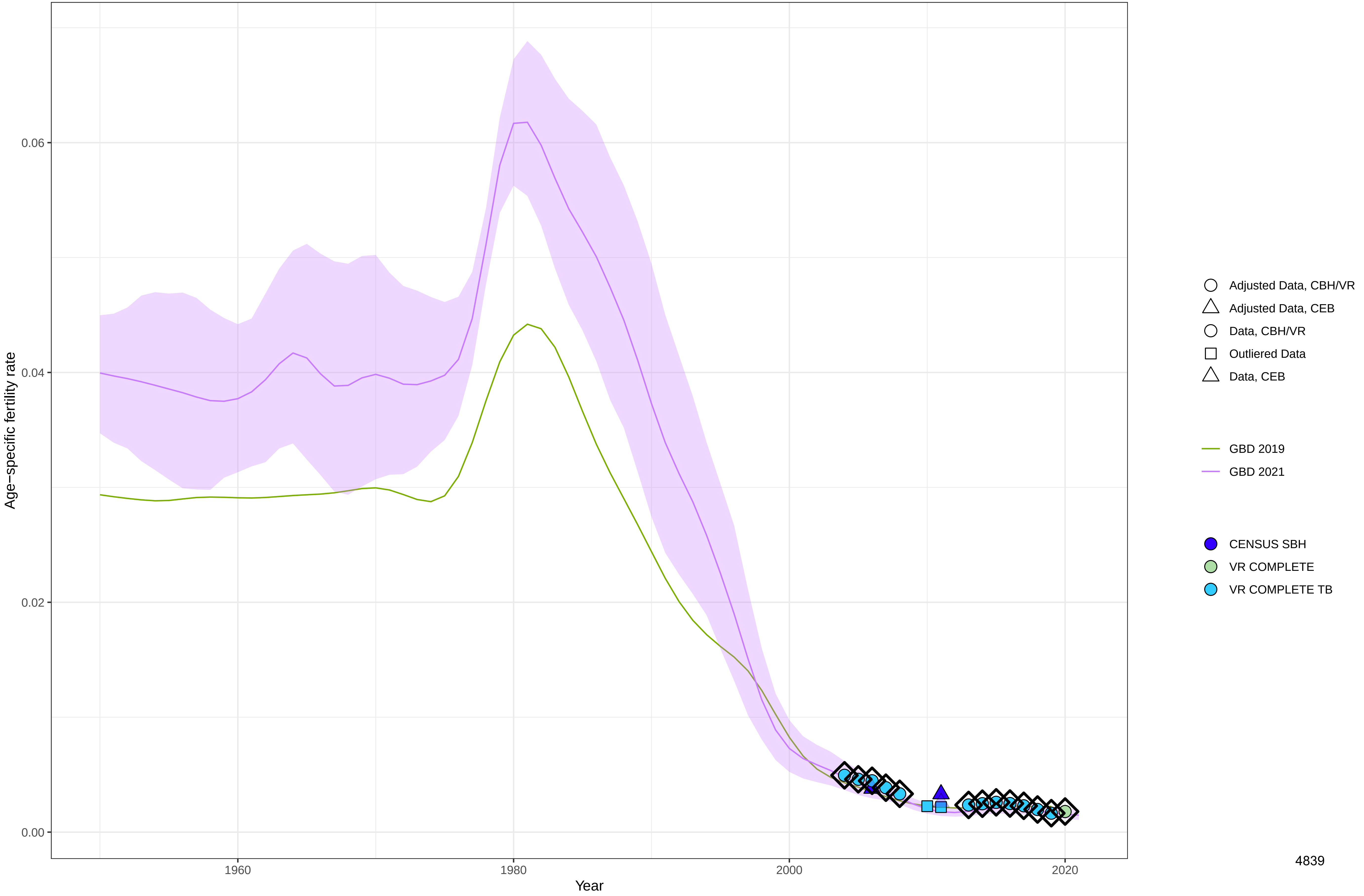

Age−specific fertility fitted trend lines, 1950−2021: South Khorasan, Ages: 50−54
Data points enclosed with a diamond are reference sources

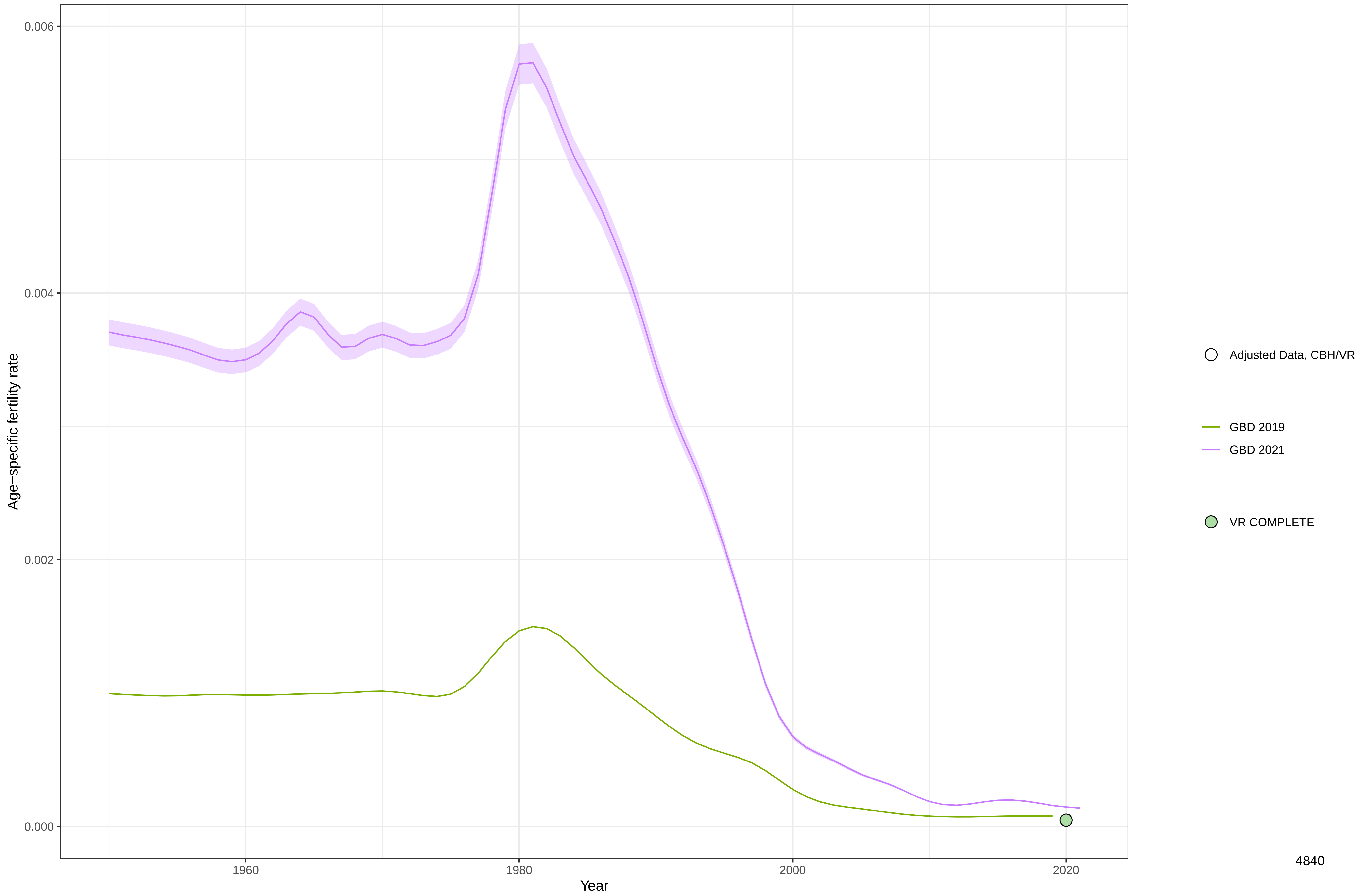

Total fertility rate fitted trend lines, 1950−2021: South Khorasan TFR
Data points enclosed with a diamond are reference sources

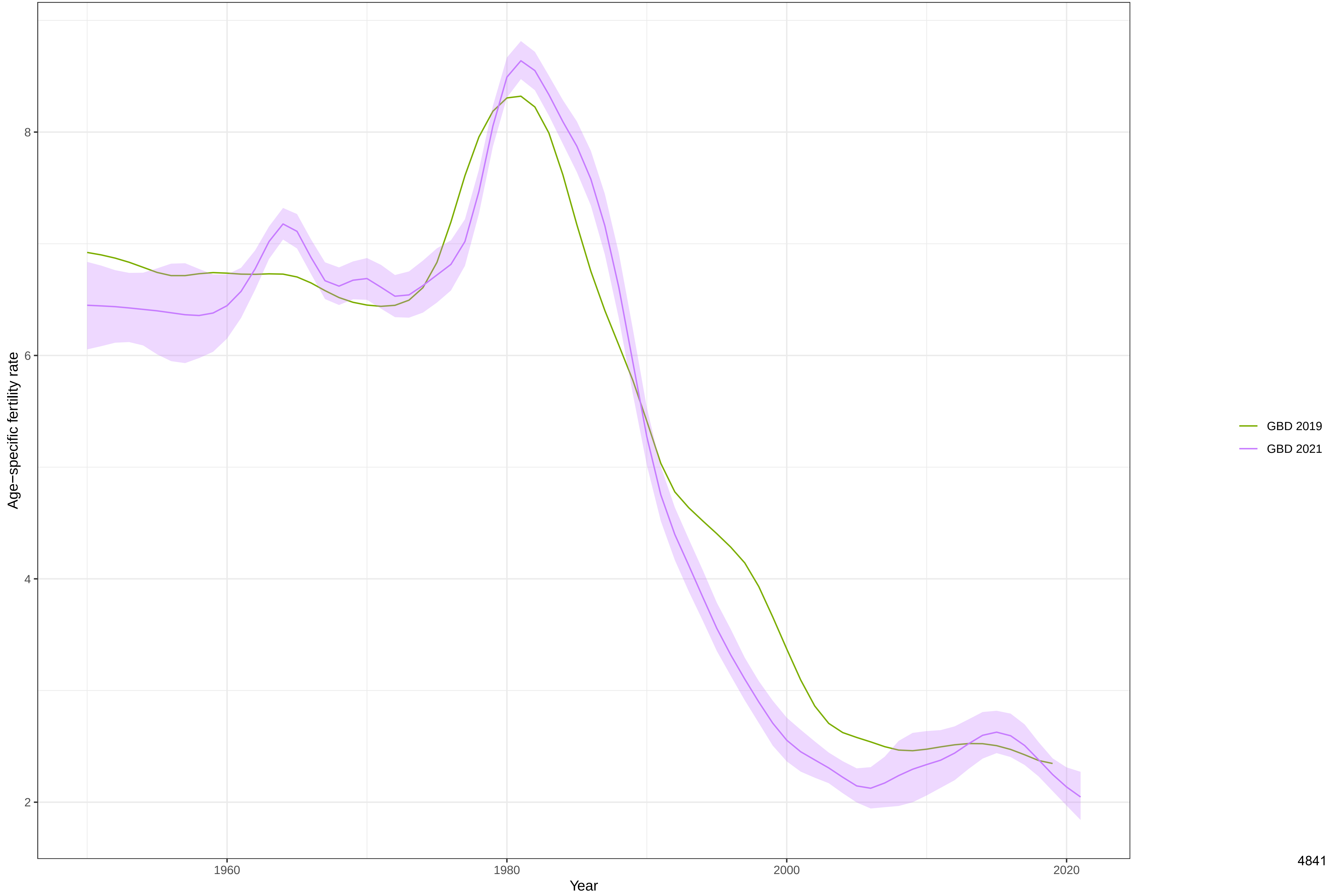

Age−specific fertility fitted trend lines, 1950−2021: Tehran, Ages: 10−14
Data points enclosed with a diamond are reference sources

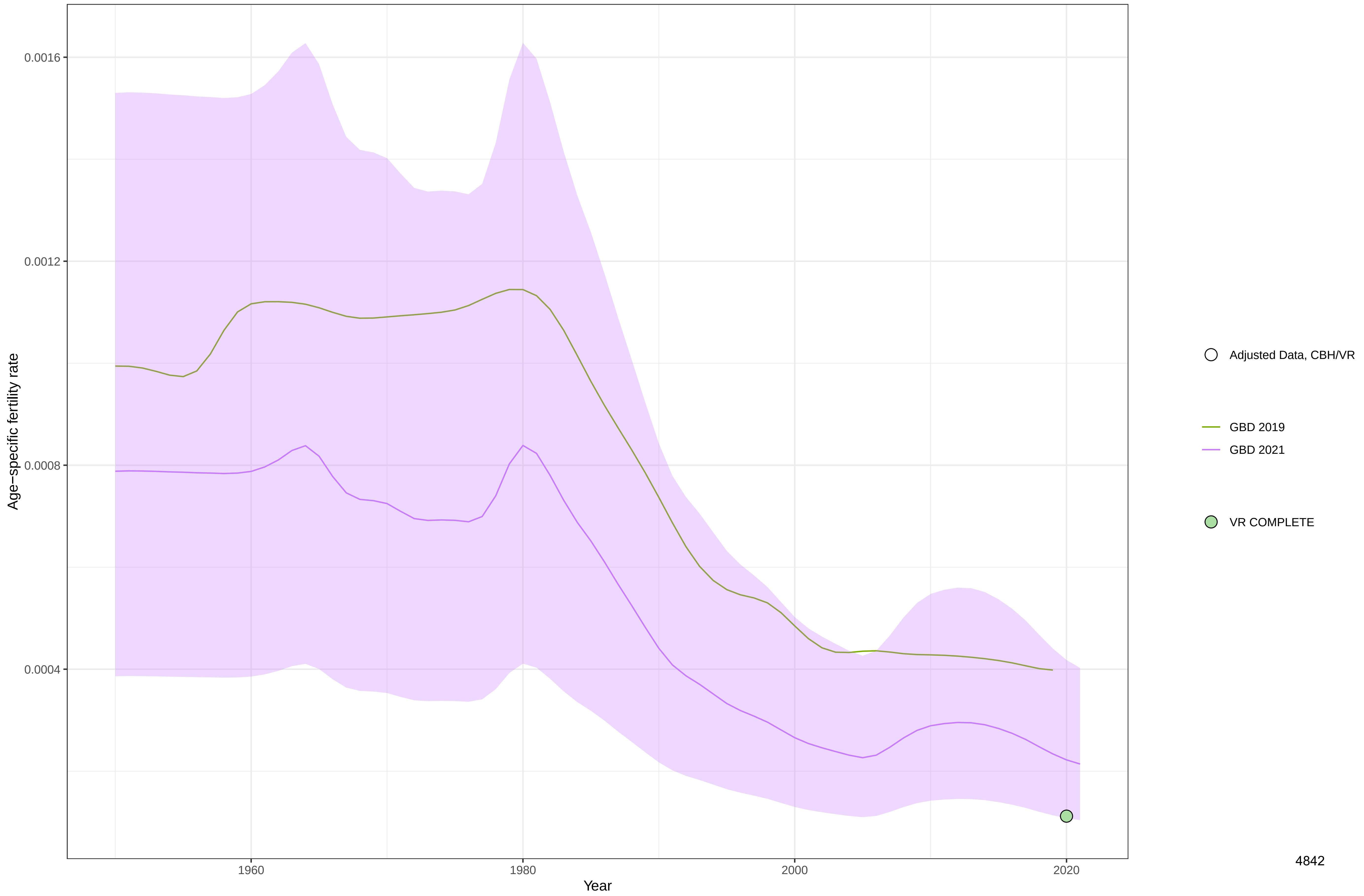

Age−specific fertility fitted trend lines, 1950−2021: Tehran, Ages: 15−19
Data points enclosed with a diamond are reference sources

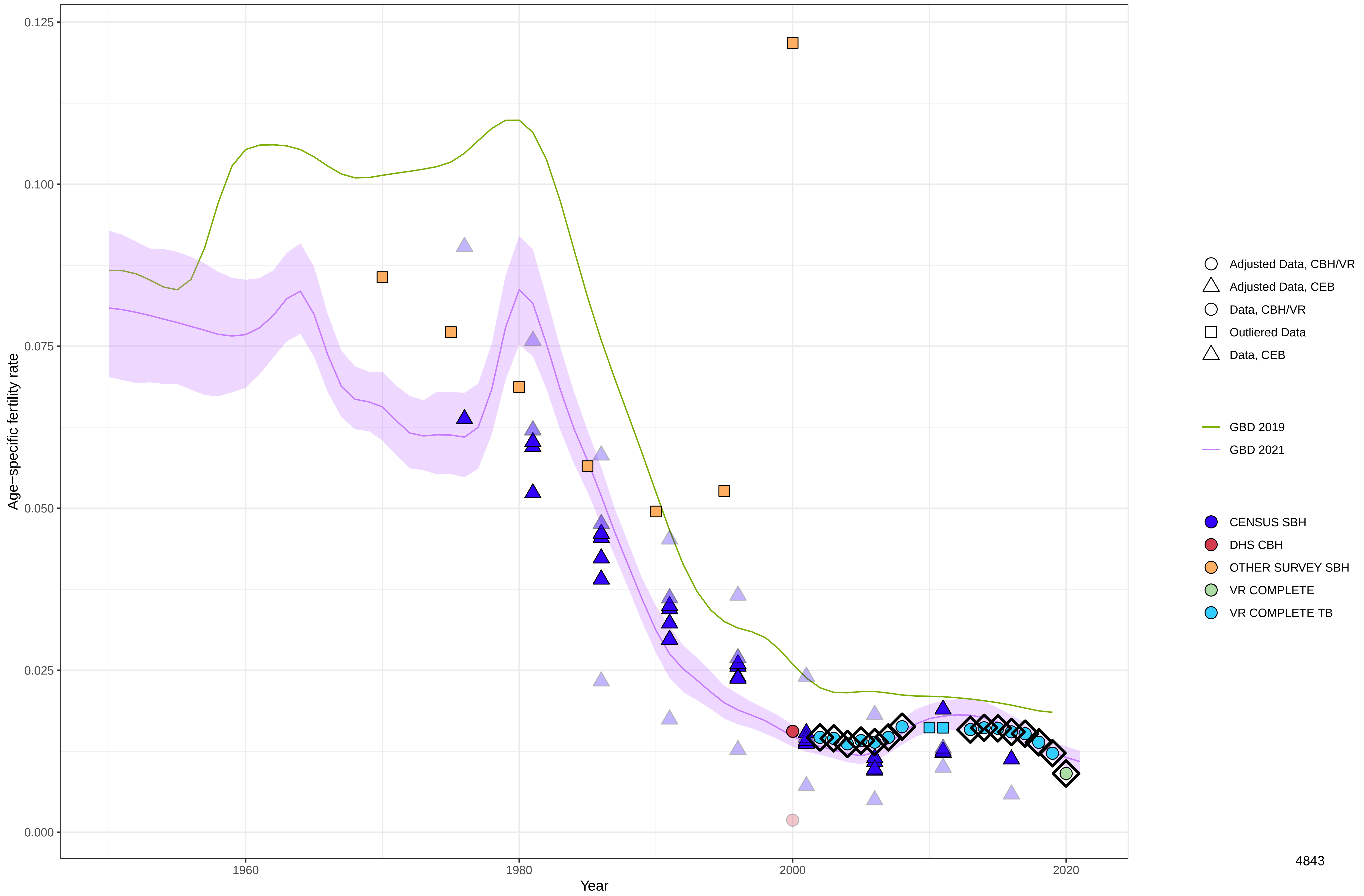

Age−specific fertility fitted trend lines, 1950−2021: Tehran, Ages: 20−24
Data points enclosed with a diamond are reference sources

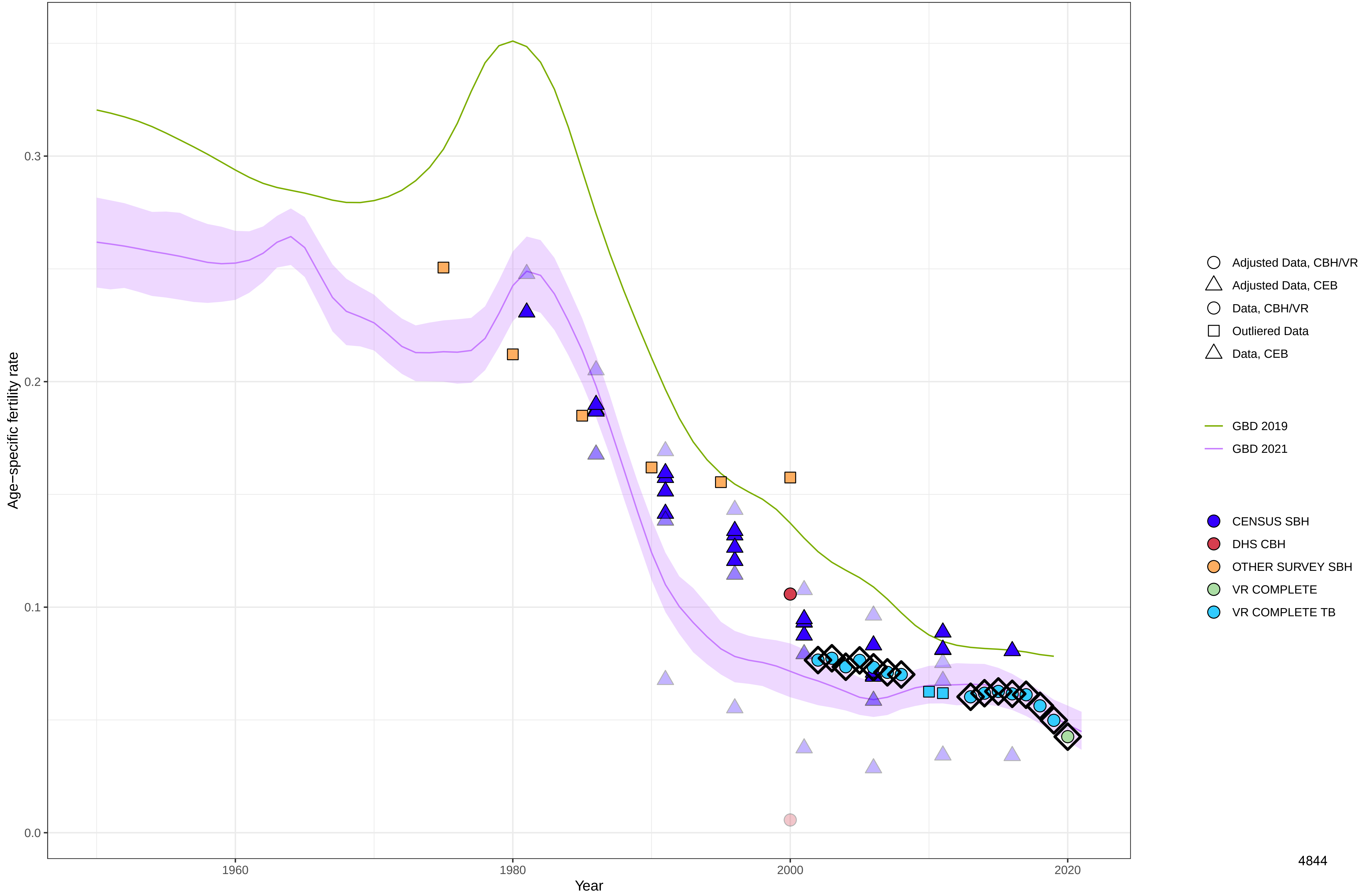

Age−specific fertility fitted trend lines, 1950−2021: Tehran, Ages: 25−29
Data points enclosed with a diamond are reference sources

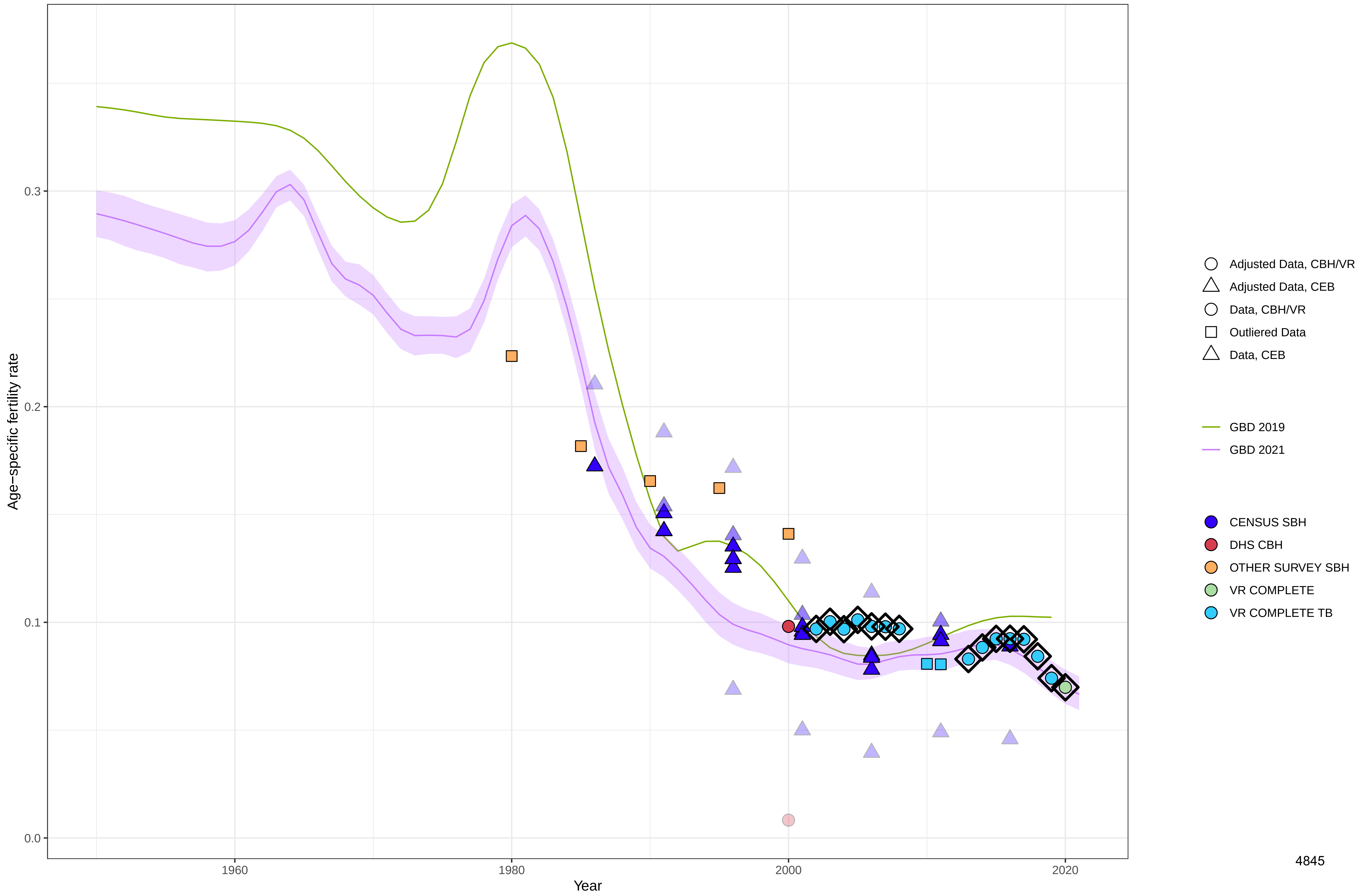

Age−specific fertility fitted trend lines, 1950−2021: Tehran, Ages: 30−34
Data points enclosed with a diamond are reference sources

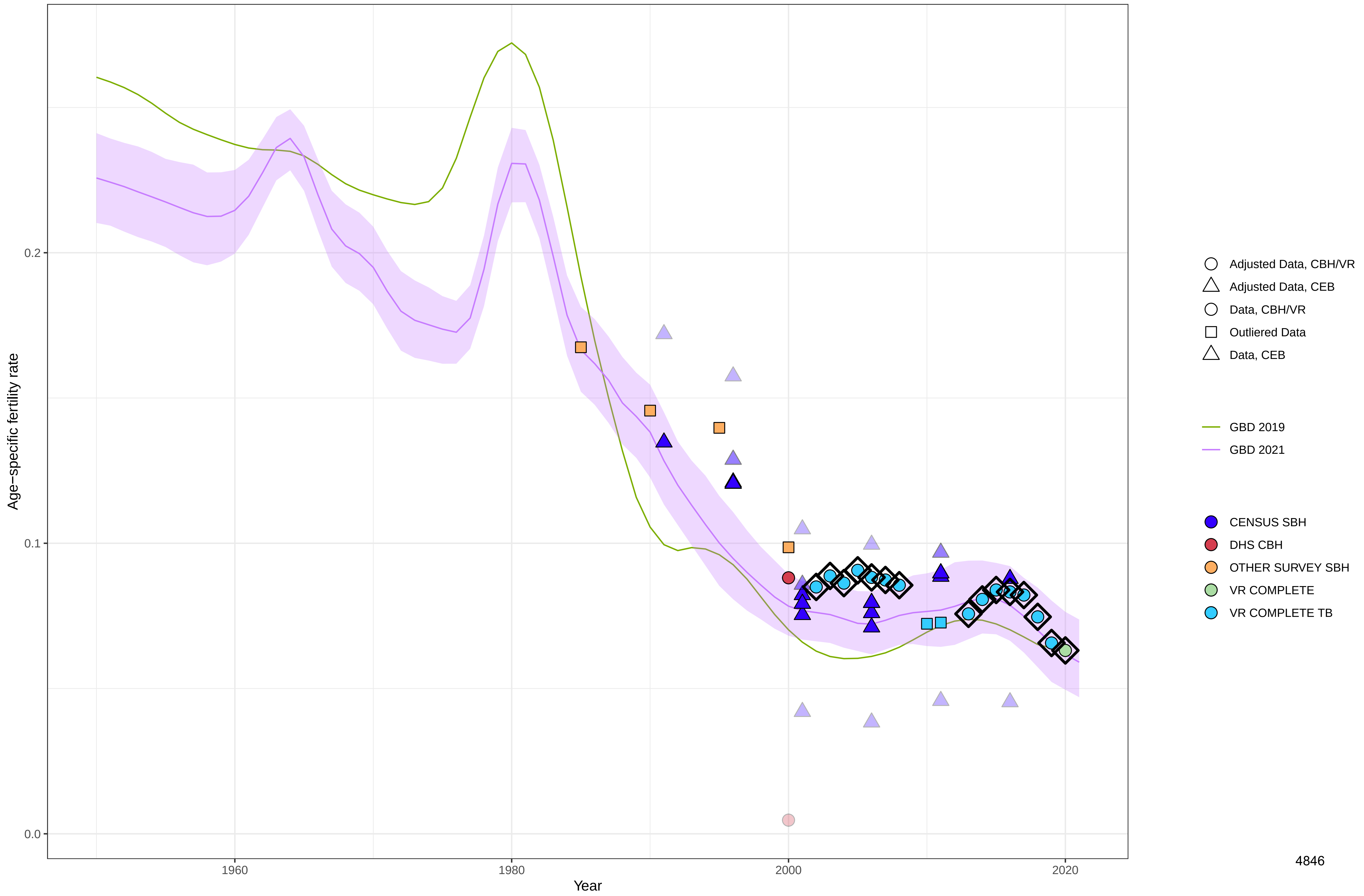

Age−specific fertility fitted trend lines, 1950−2021: Tehran, Ages: 35−39
Data points enclosed with a diamond are reference sources

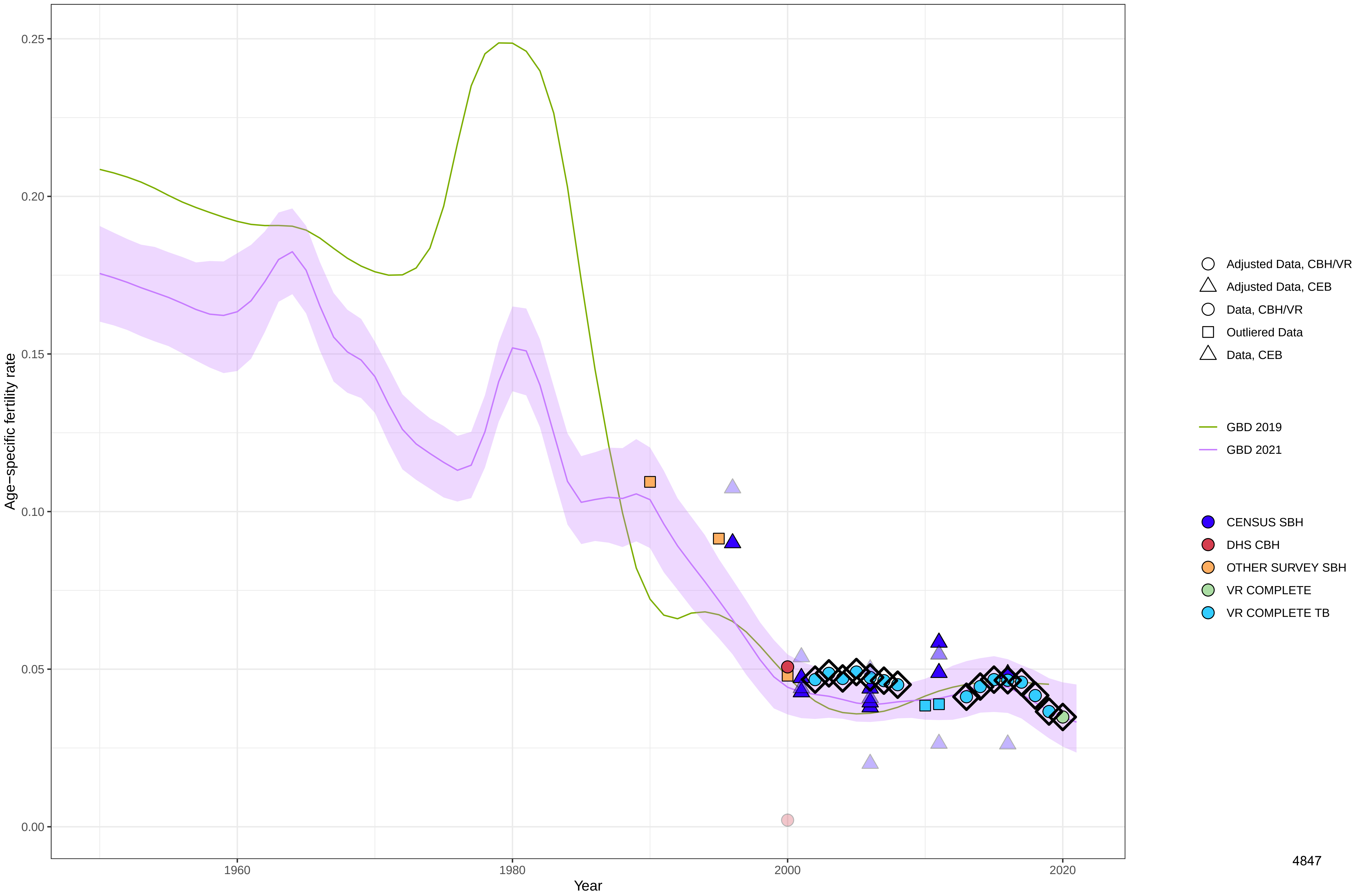

Age−specific fertility fitted trend lines, 1950−2021: Tehran, Ages: 40−44
Data points enclosed with a diamond are reference sources

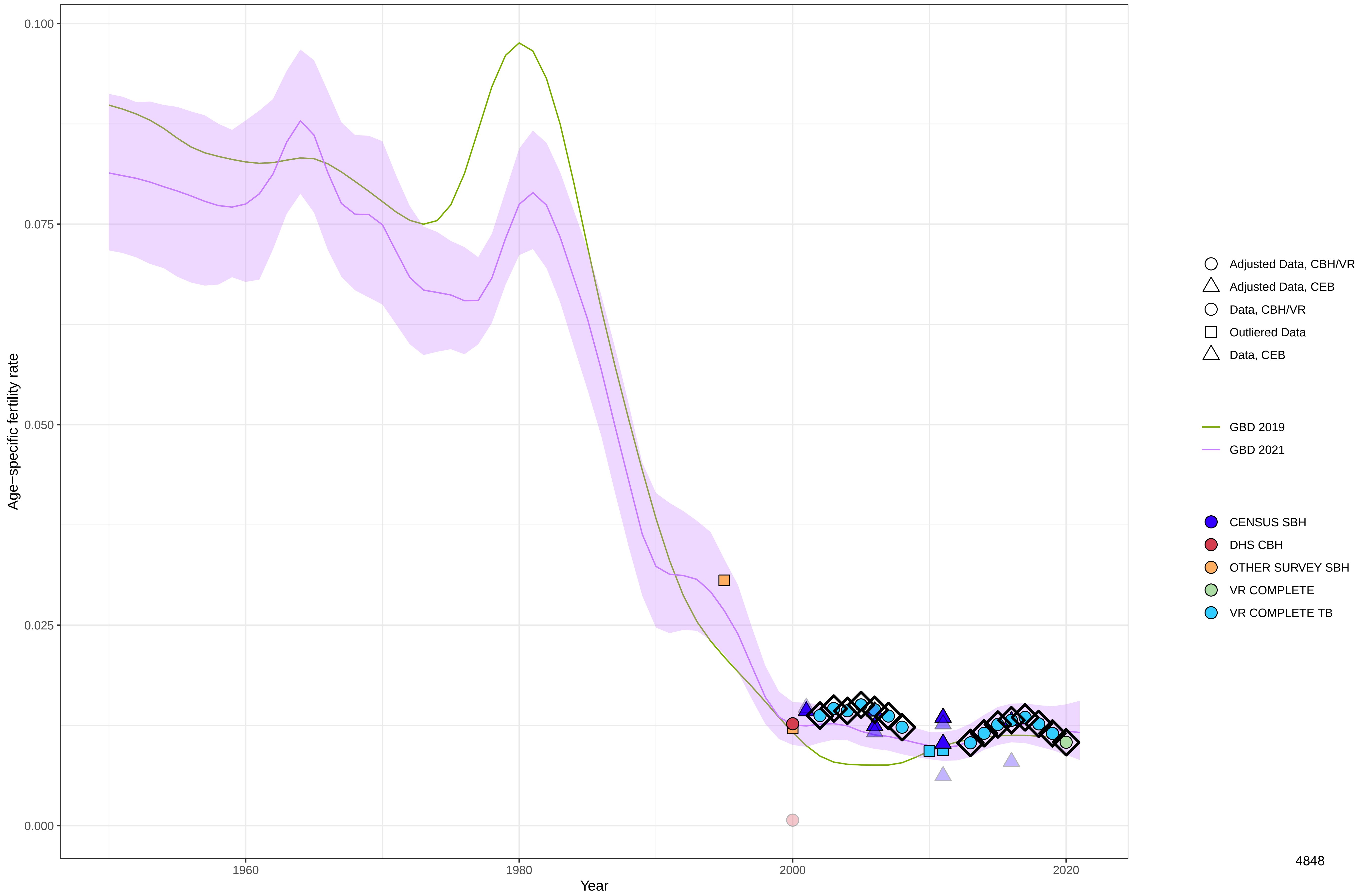

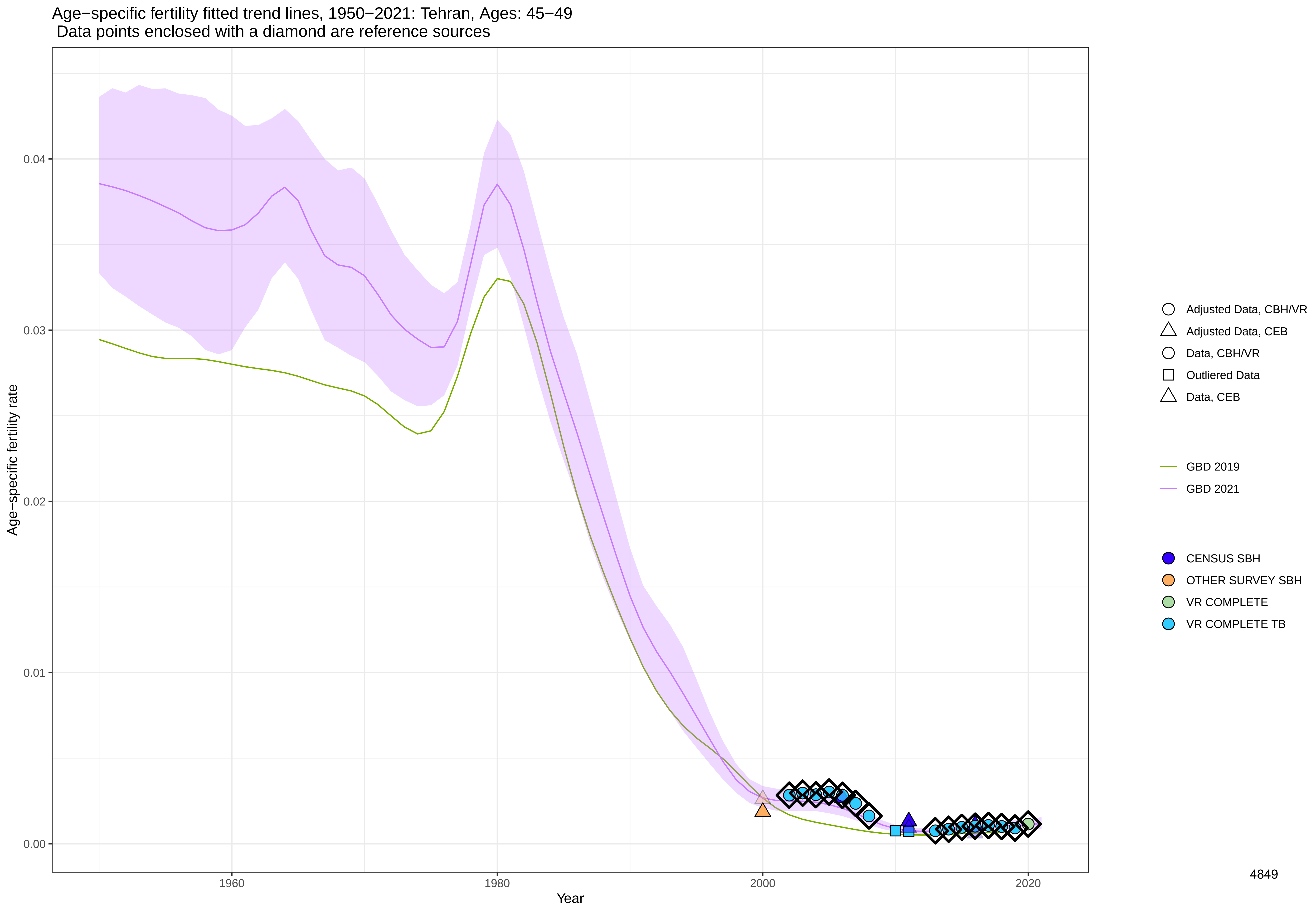

Age−specific fertility fitted trend lines, 1950−2021: Tehran, Ages: 45−49
Data points enclosed with a diamond are reference sources
Age−specific fertility rate
0.00
0.01
0.02
0.03
0.04
Year
1960
1980
2000
2020
Adjusted Data, CBH/VR
Adjusted Data, CEB
Data, CBH/VR
Outliered Data
Data, CEB
GBD 2019
GBD 2021
CENSUS SBH
OTHER SURVEY SBH
VR COMPLETE
VR COMPLETE TB

Age−specific fertility fitted trend lines, 1950−2021: Tehran, Ages: 50−54
Data points enclosed with a diamond are reference sources

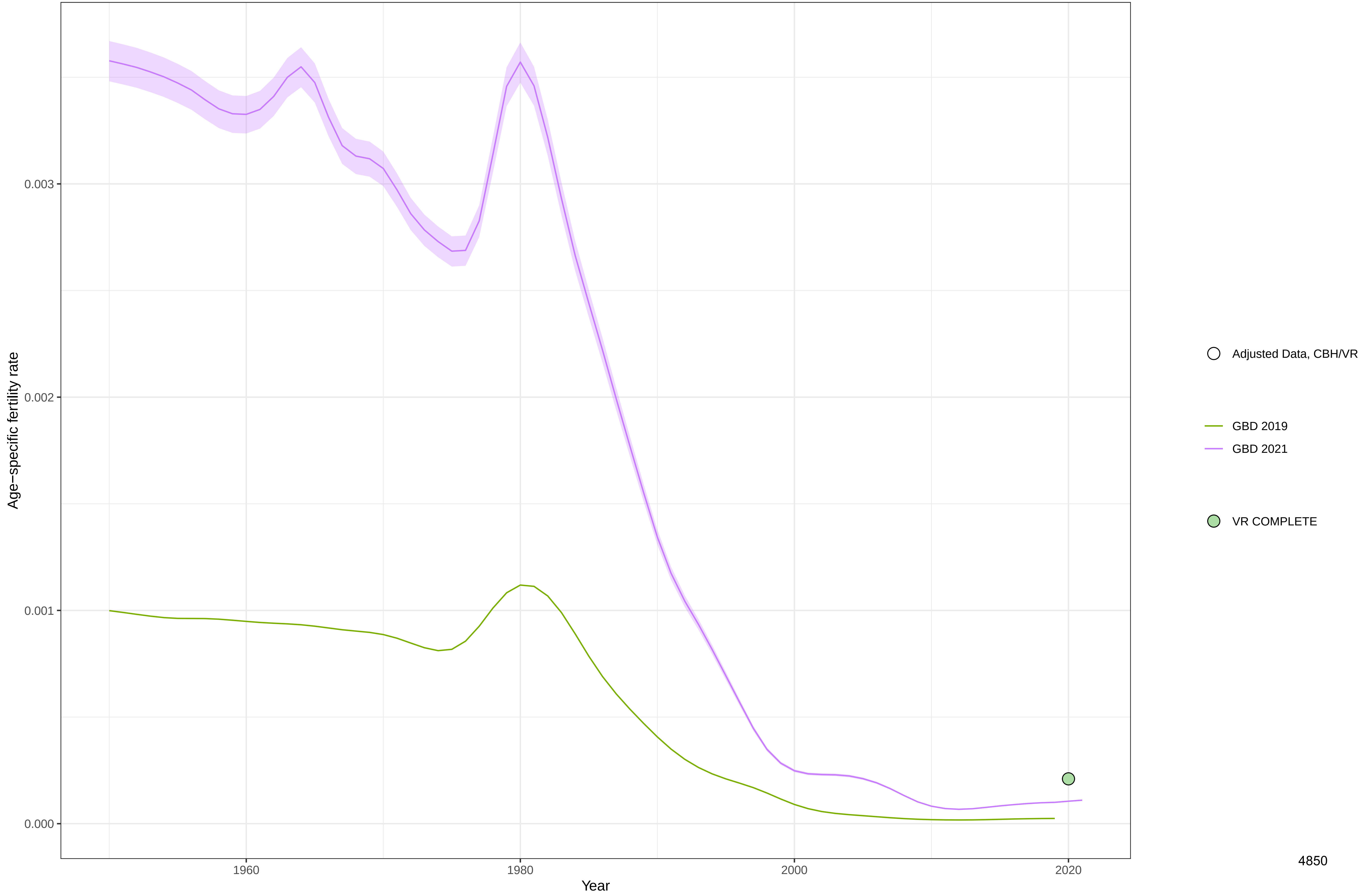

Total fertility rate fitted trend lines, 1950−2021: Tehran TFR
Data points enclosed with a diamond are reference sources

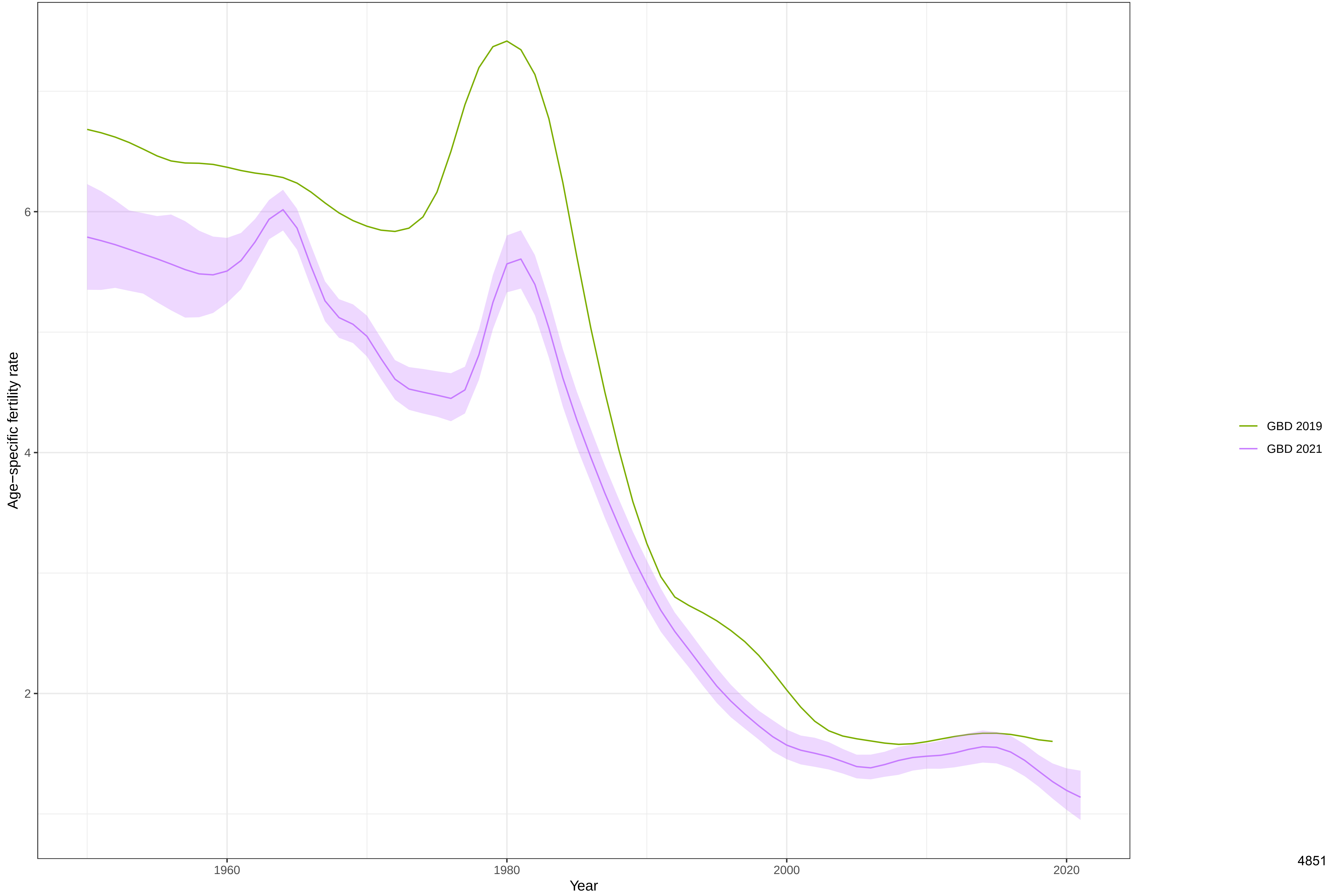

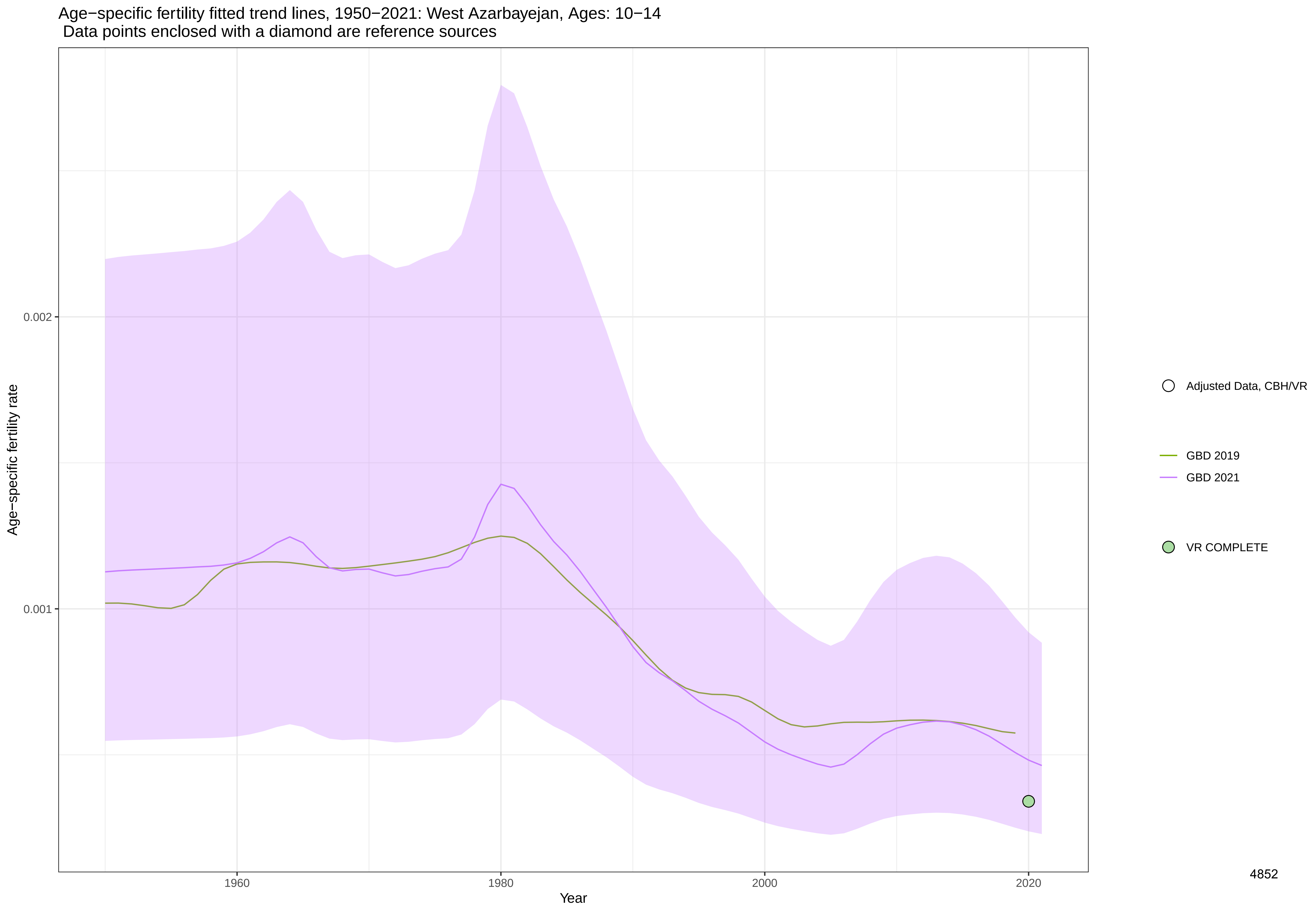

Age−specific fertility fitted trend lines, 1950−2021: West Azarbayejan, Ages: 10−14
Data points enclosed with a diamond are reference sources
Age−specific fertility rate
0.002
0.001
1960
1980
2000
2020
Year
Adjusted Data, CBH/VR
GBD 2019
GBD 2021
VR COMPLETE

Age−specific fertility fitted trend lines, 1950−2021: West Azarbayejan, Ages: 15−19
Data points enclosed with a diamond are reference sources

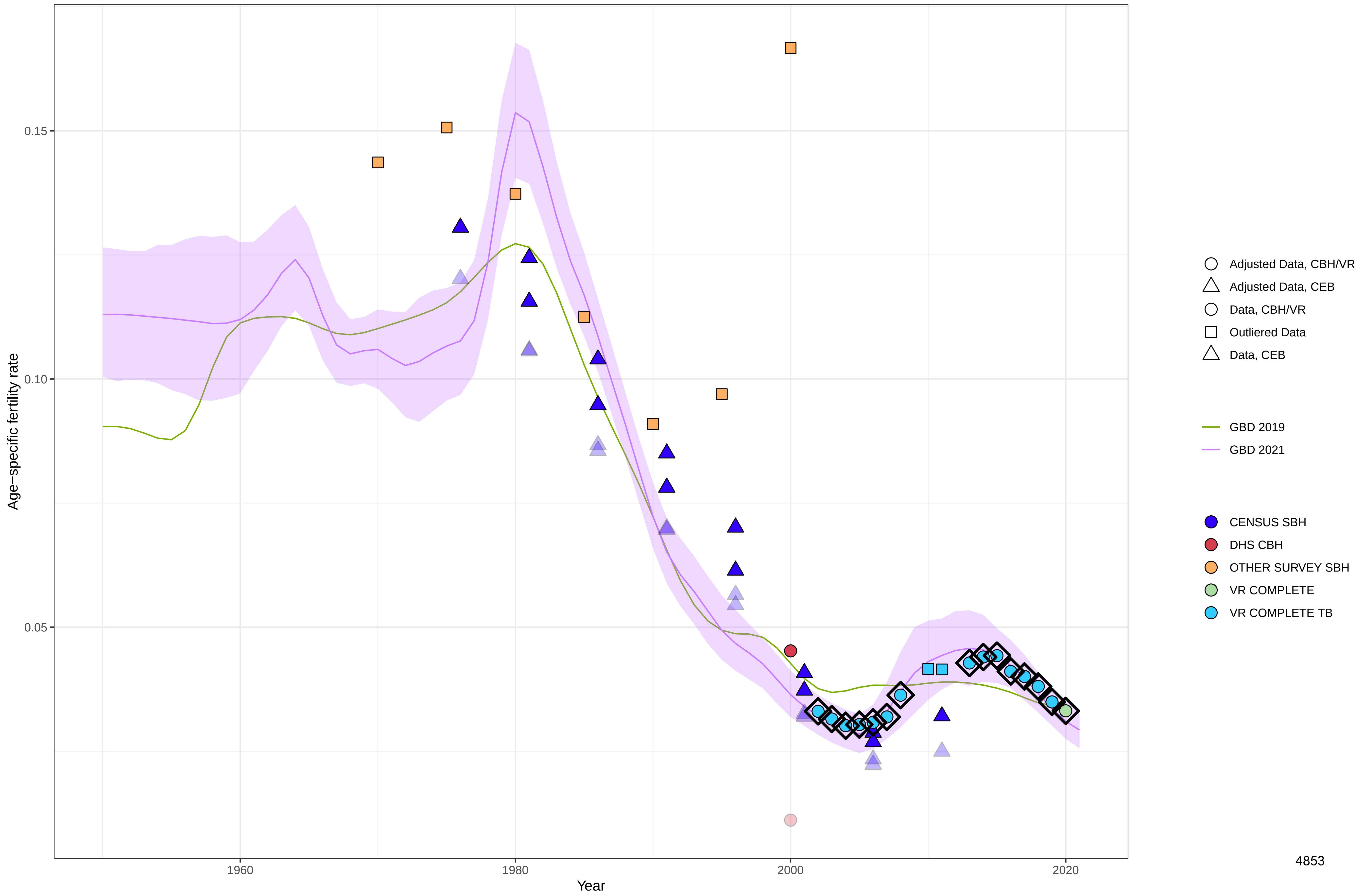

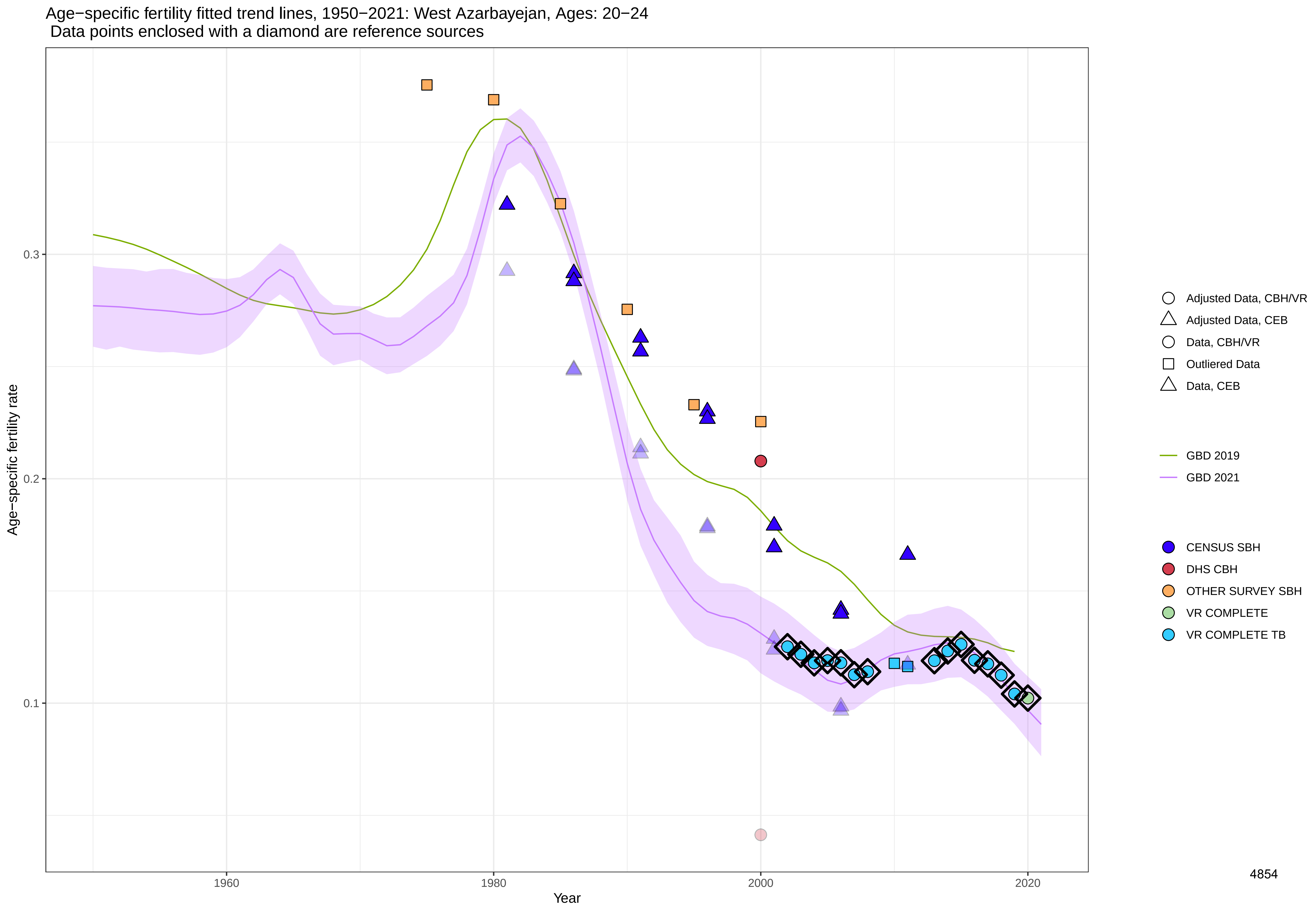
Age−specific fertility fitted trend lines, 1950−2021: West Azarbayejan, Ages: 20−24
Data points enclosed with a diamond are reference sources
Age−specific fertility rate
0.3
0.2
0.1
1960
1980
2000
2020
Year
Adjusted Data, CBH/VR
Adjusted Data, CEB
Data, CBH/VR
Outliered Data
Data, CEB
GBD 2019
GBD 2021
CENSUS SBH
DHS CBH
OTHER SURVEY SBH
VR COMPLETE
VR COMPLETE TB

4854

Age−specific fertility fitted trend lines, 1950−2021: West Azarbayejan, Ages: 25−29
Data points enclosed with a diamond are reference sources

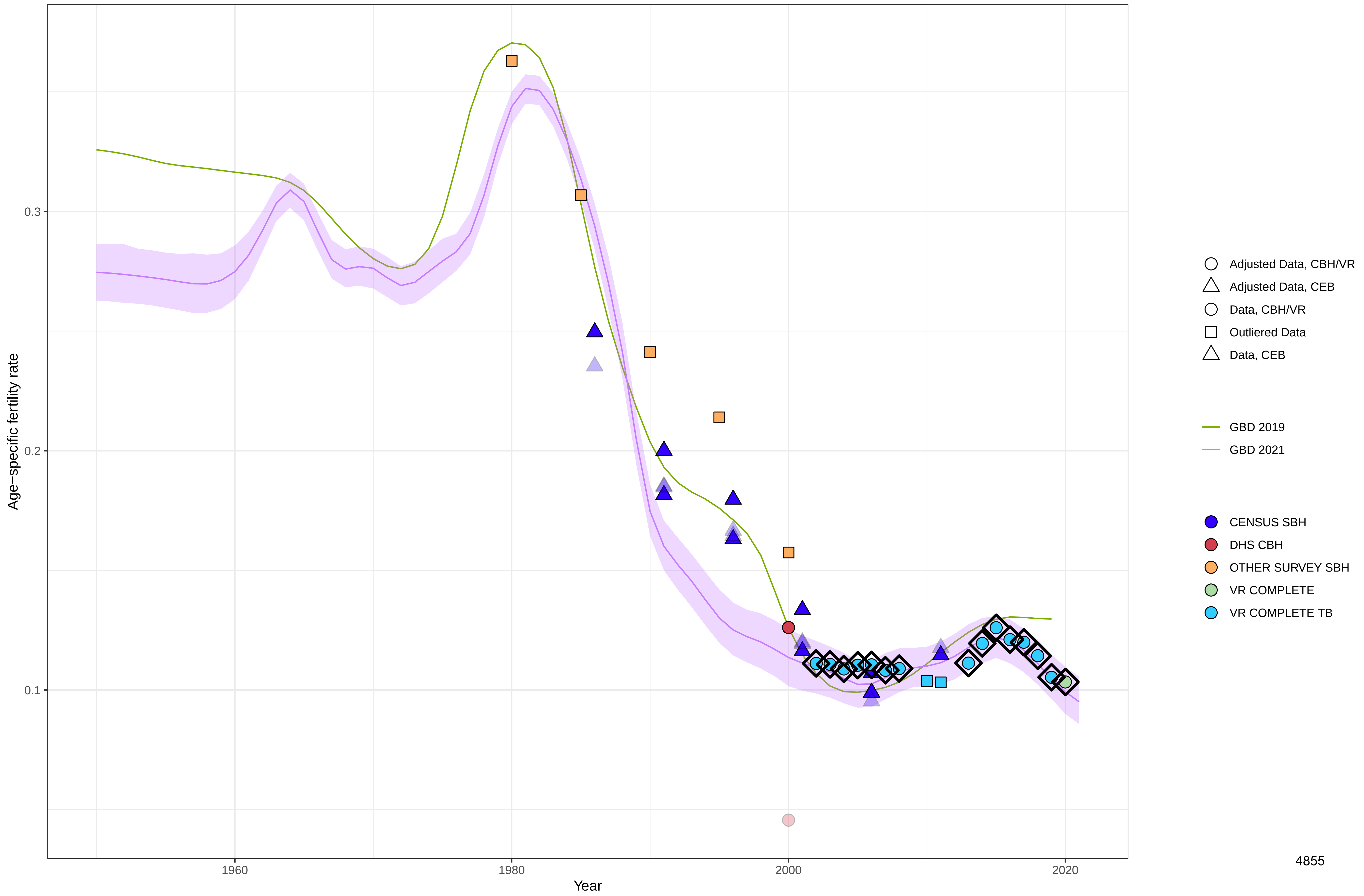

Age−specific fertility fitted trend lines, 1950−2021: West Azarbayejan, Ages: 30−34
Data points enclosed with a diamond are reference sources

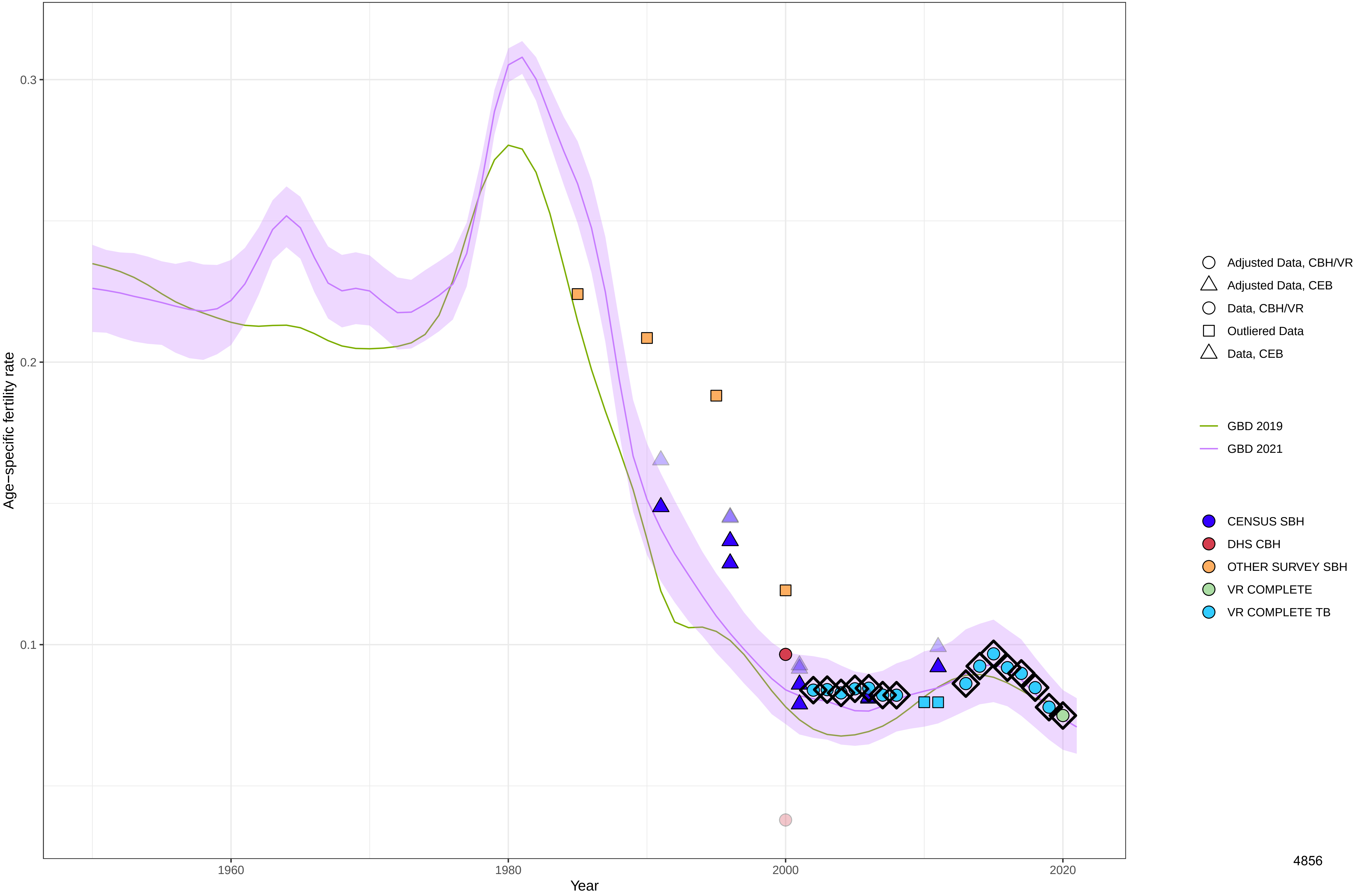

Age−specific fertility fitted trend lines, 1950−2021: West Azarbayejan, Ages: 35−39
Data points enclosed with a diamond are reference sources

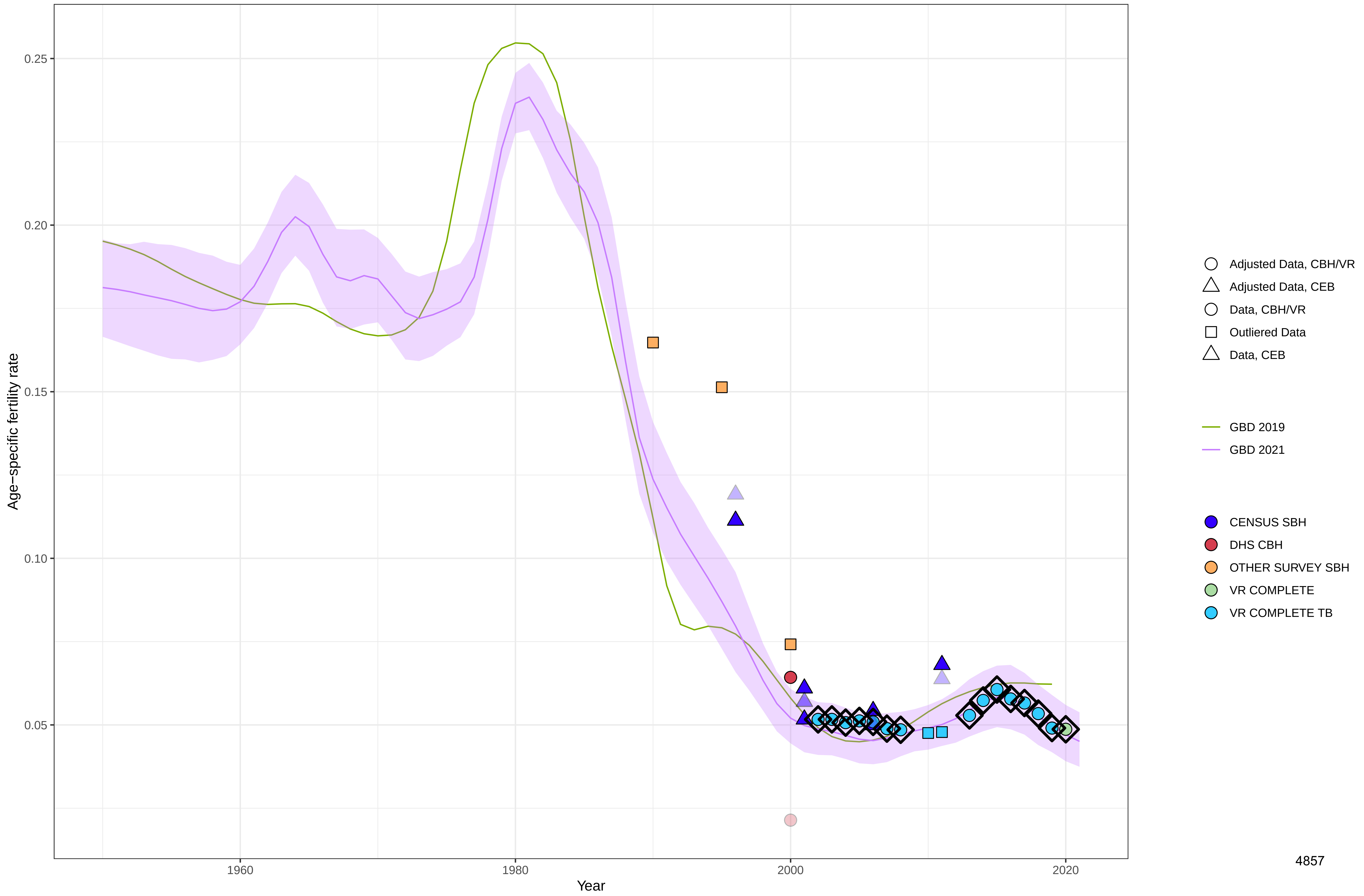

Age−specific fertility fitted trend lines, 1950−2021: West Azarbayejan, Ages: 40−44
Data points enclosed with a diamond are reference sources

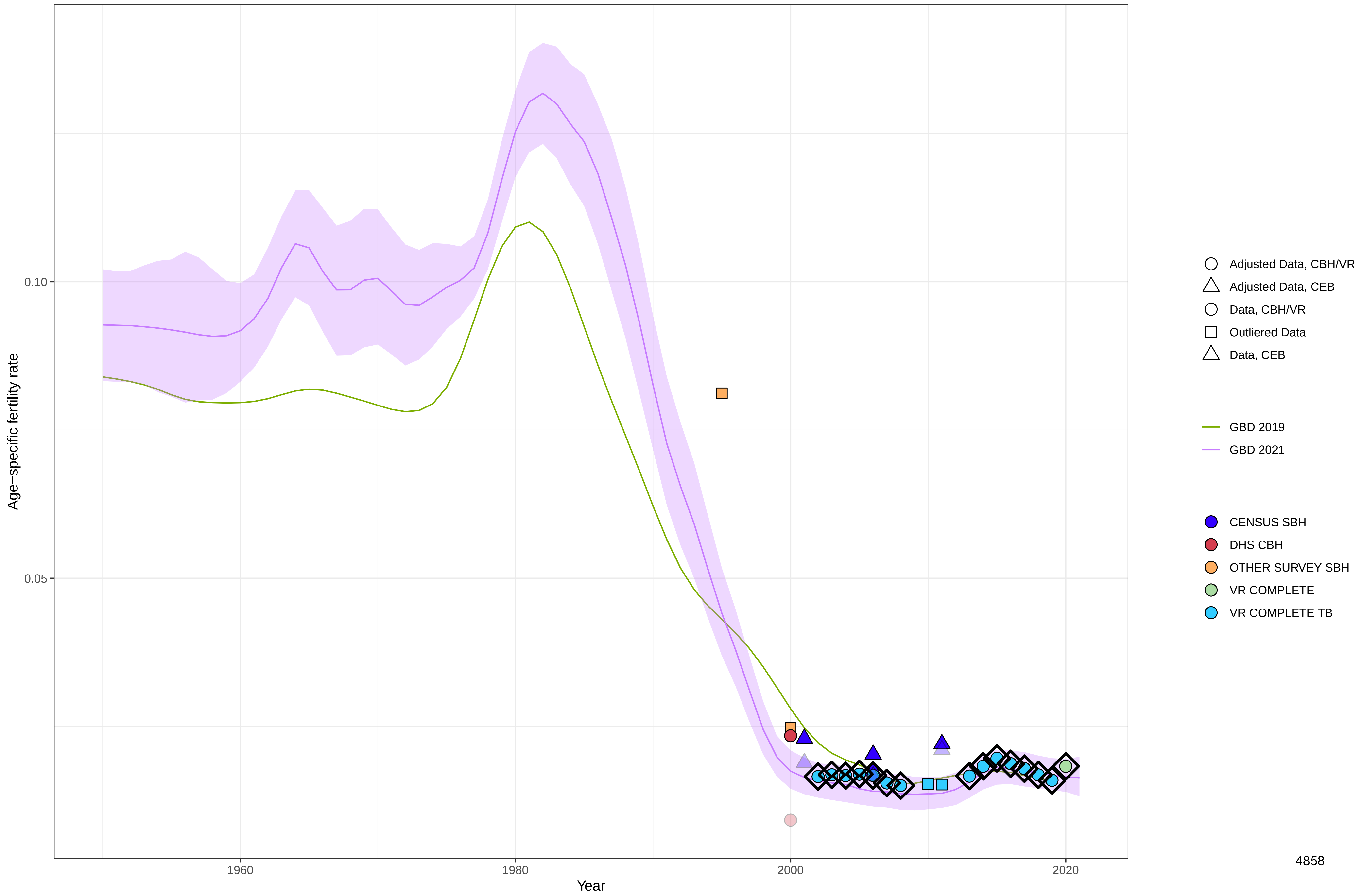

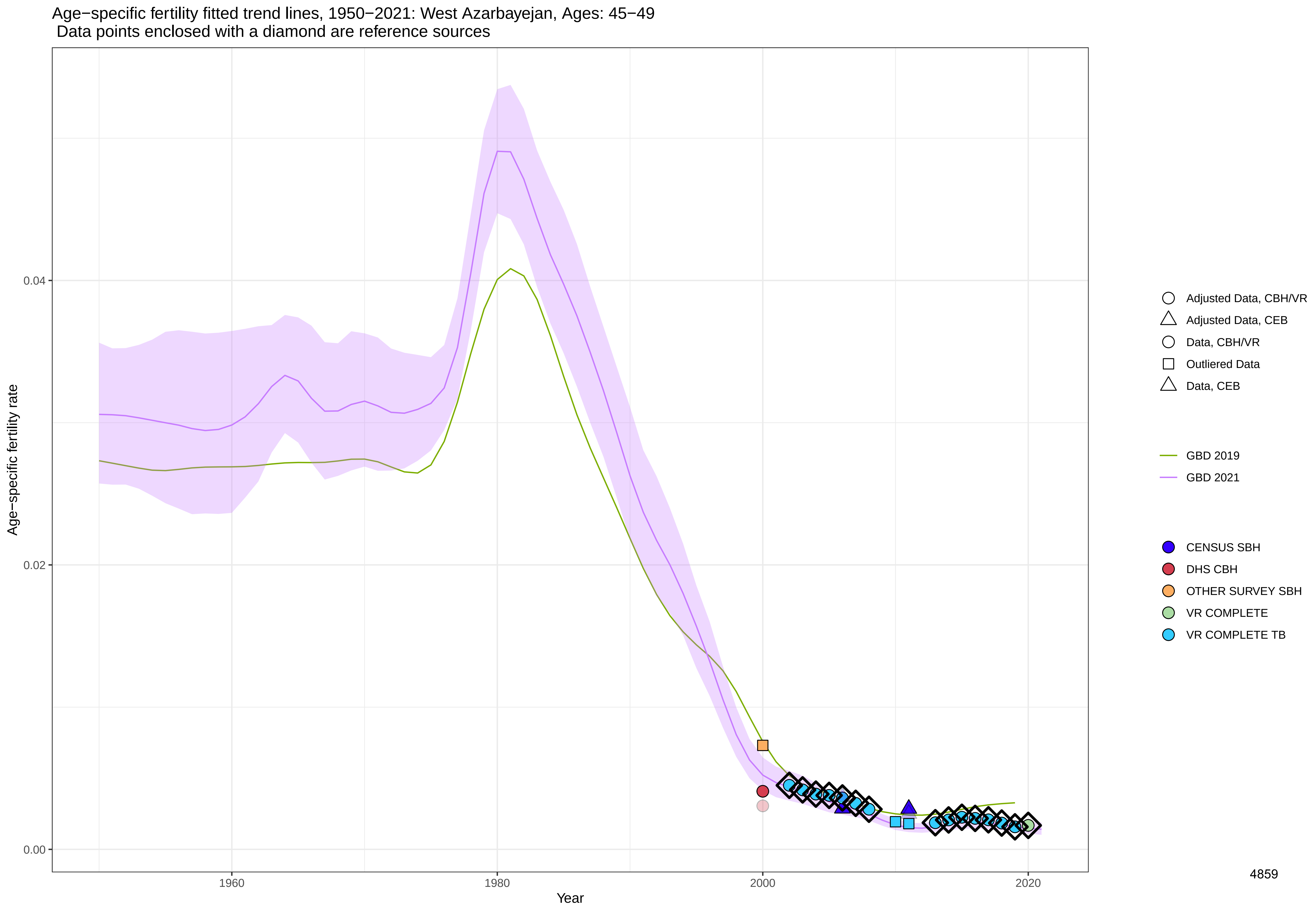
Age−specific fertility fitted trend lines, 1950−2021: West Azarbayejan, Ages: 45−49
Data points enclosed with a diamond are reference sources
Age−specific fertility rate
0.00
0.02
0.04
1960
1980
2000
2020
Year
Adjusted Data, CBH/VR
Adjusted Data, CEB
Data, CBH/VR
Outliered Data
Data, CEB
GBD 2019
GBD 2021
CENSUS SBH
DHS CBH
OTHER SURVEY SBH
VR COMPLETE
VR COMPLETE TB

4859

Age−specific fertility fitted trend lines, 1950−2021: West Azarbayejan, Ages: 50−54
Data points enclosed with a diamond are reference sources

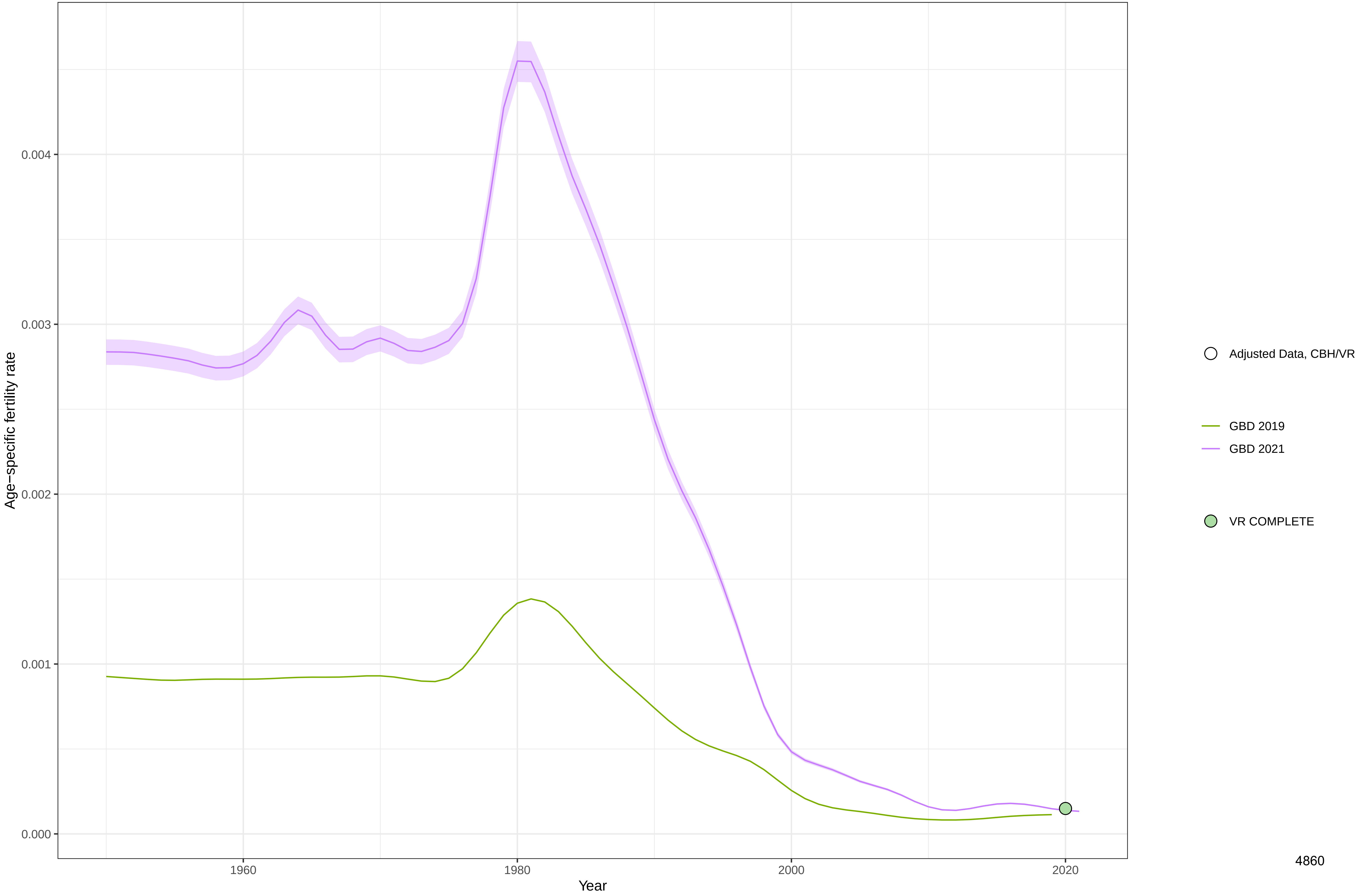

Total fertility rate fitted trend lines, 1950−2021: West Azarbayejan TFR
Data points enclosed with a diamond are reference sources

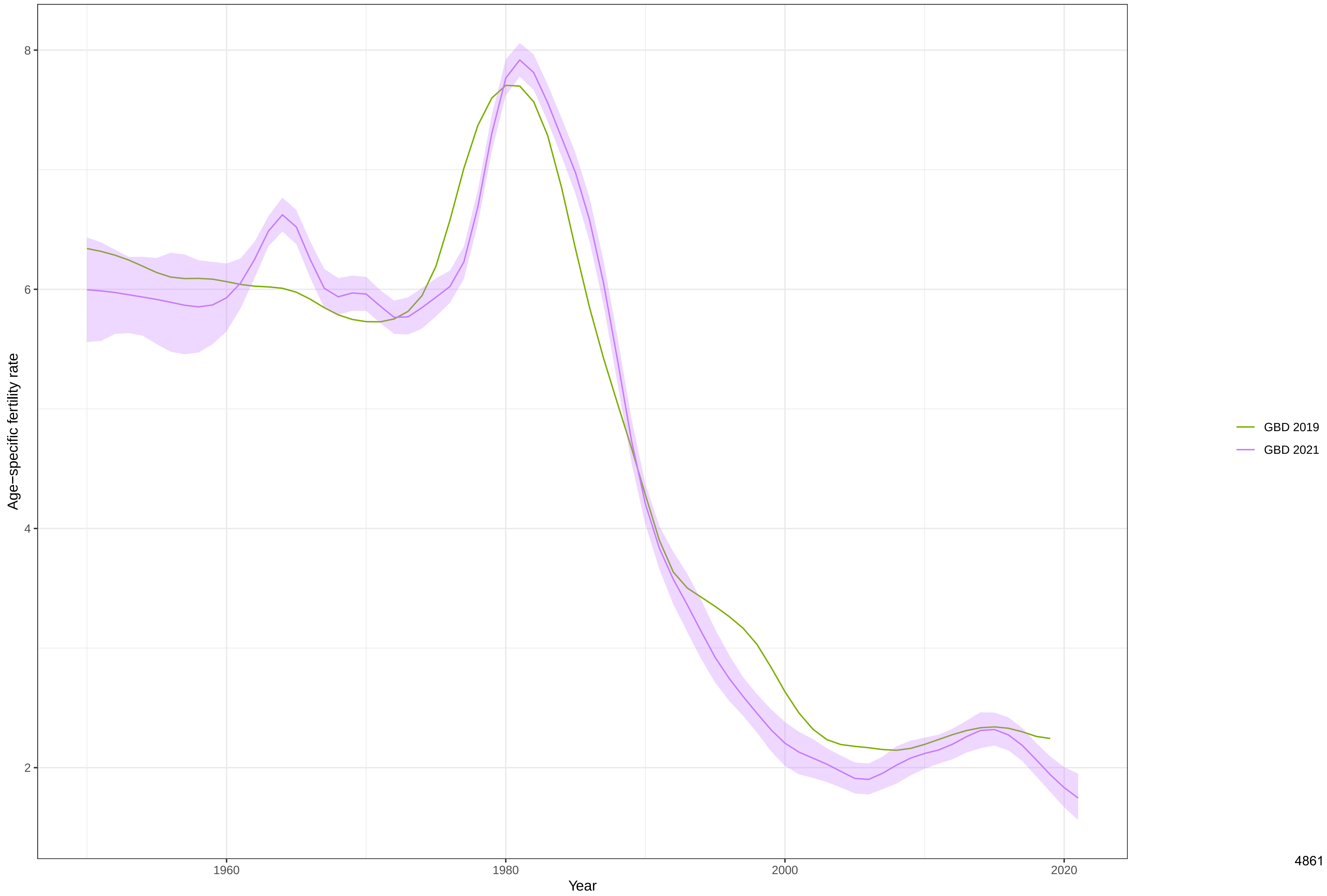

Age−specific fertility fitted trend lines, 1950−2021: Yazd, Ages: 10−14
Data points enclosed with a diamond are reference sources

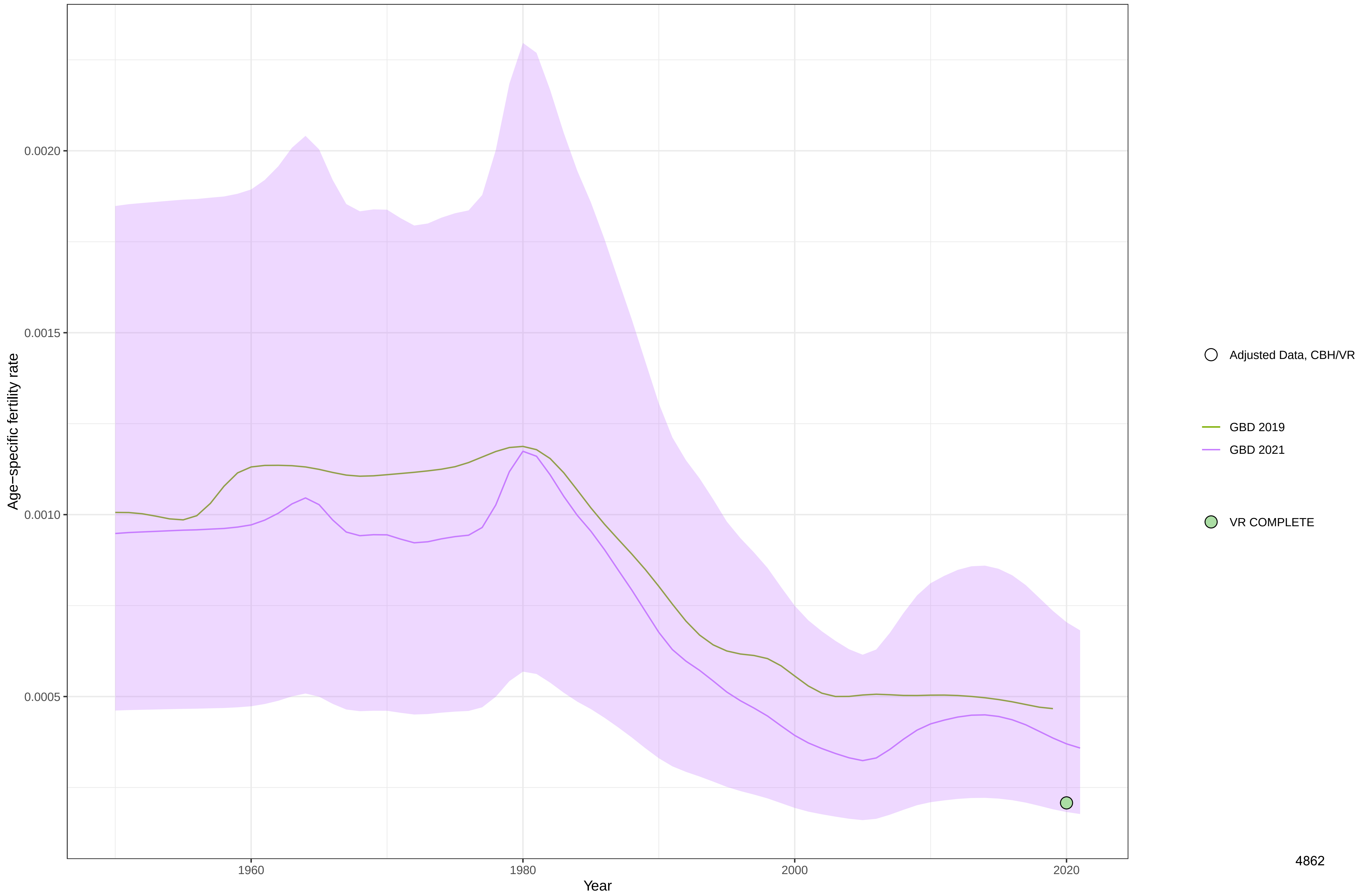

Age−specific fertility fitted trend lines, 1950−2021: Yazd, Ages: 15−19
Data points enclosed with a diamond are reference sources

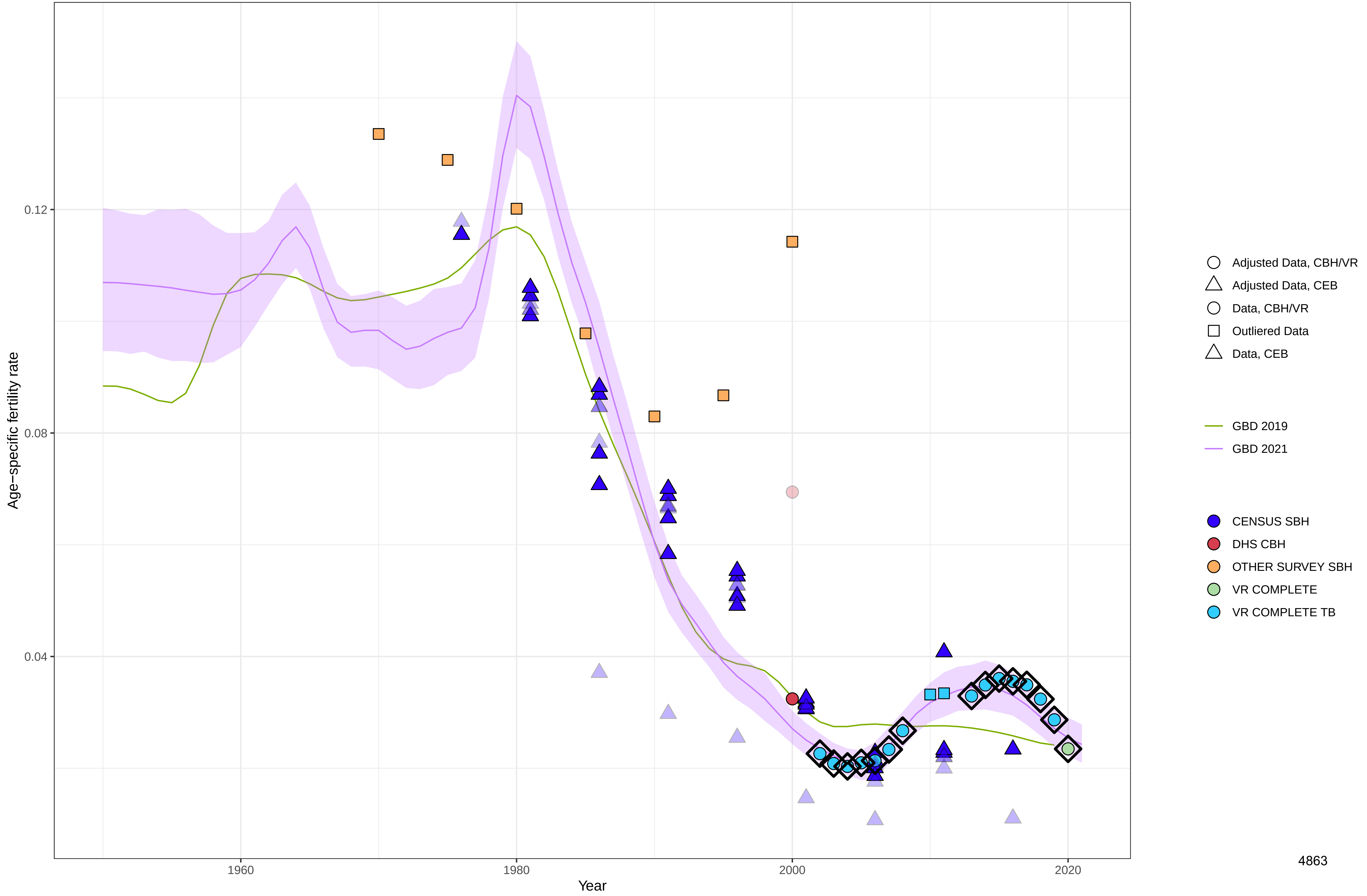

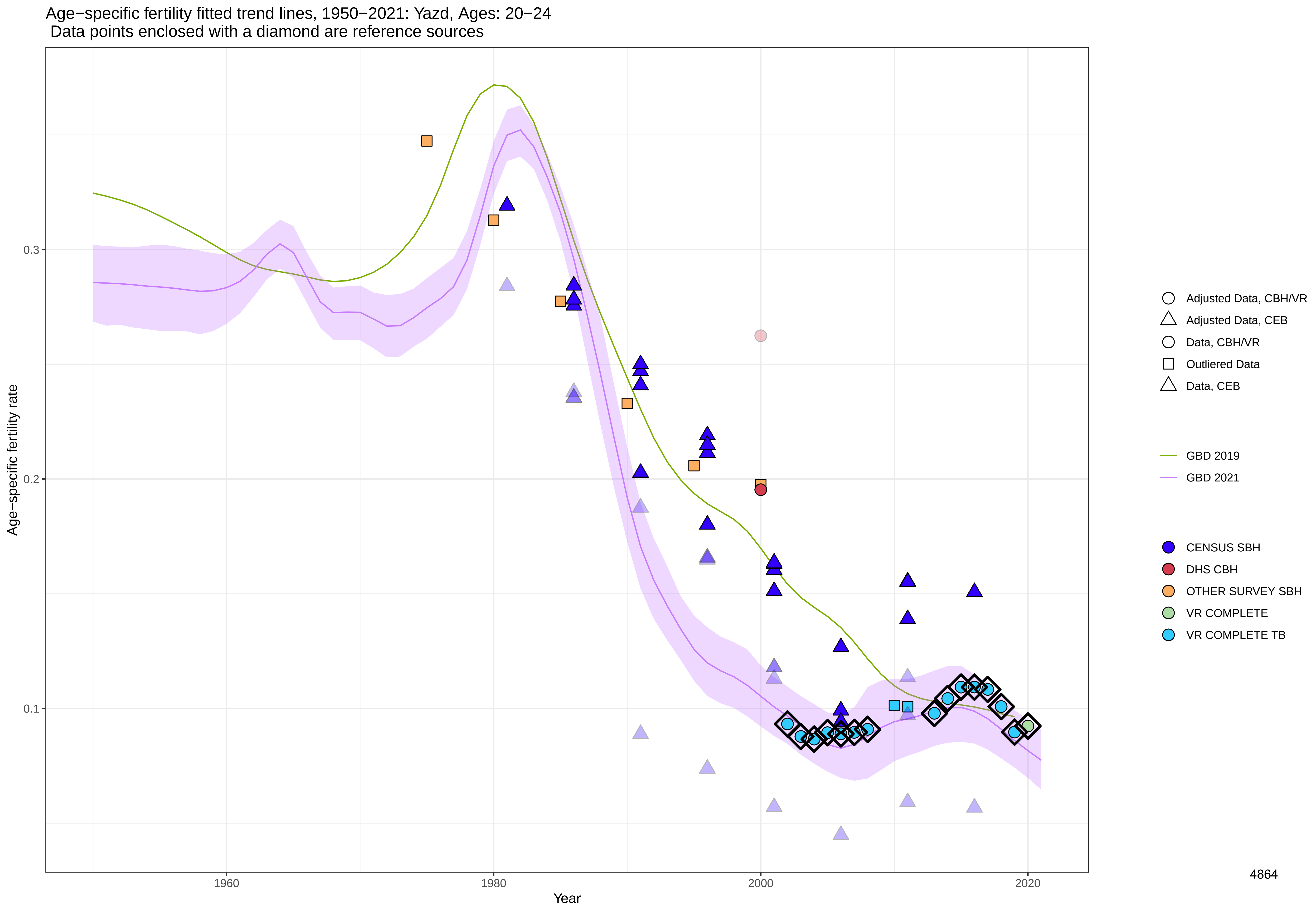

Age−specific fertility fitted trend lines, 1950−2021: Yazd, Ages: 20−24
Data points enclosed with a diamond are reference sources
Age−specific fertility rate
0.3
0.2
0.1
1960
1980
2000
2020
Year
Adjusted Data, CBH/VR
Adjusted Data, CEB
Data, CBH/VR
Outliered Data
Data, CEB
GBD 2019
GBD 2021
CENSUS SBH
DHS CBH
OTHER SURVEY SBH
VR COMPLETE
VR COMPLETE TB

Age−specific fertility fitted trend lines, 1950−2021: Yazd, Ages: 25−29
Data points enclosed with a diamond are reference sources

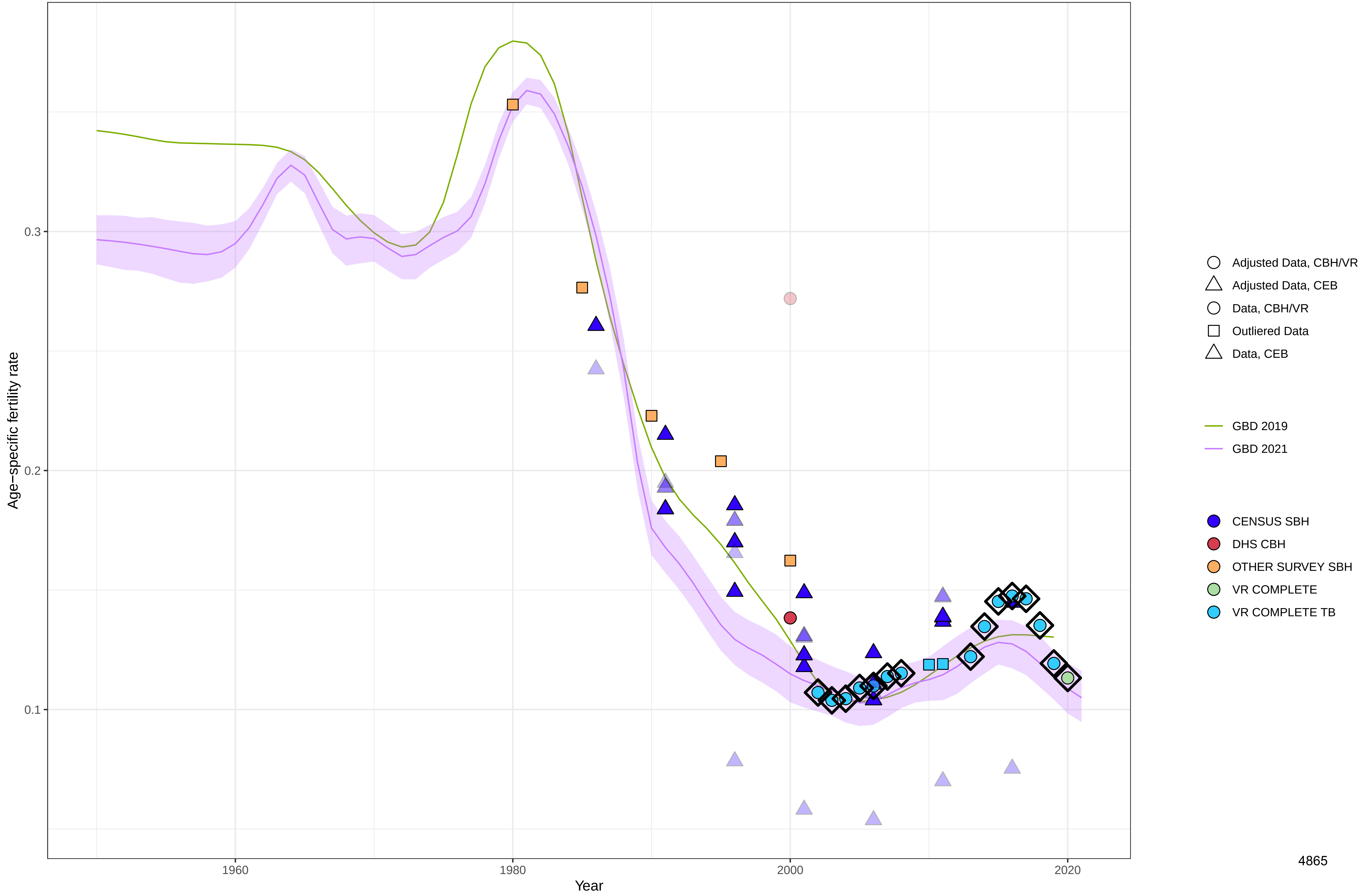

Age−specific fertility fitted trend lines, 1950−2021: Yazd, Ages: 30−34
Data points enclosed with a diamond are reference sources

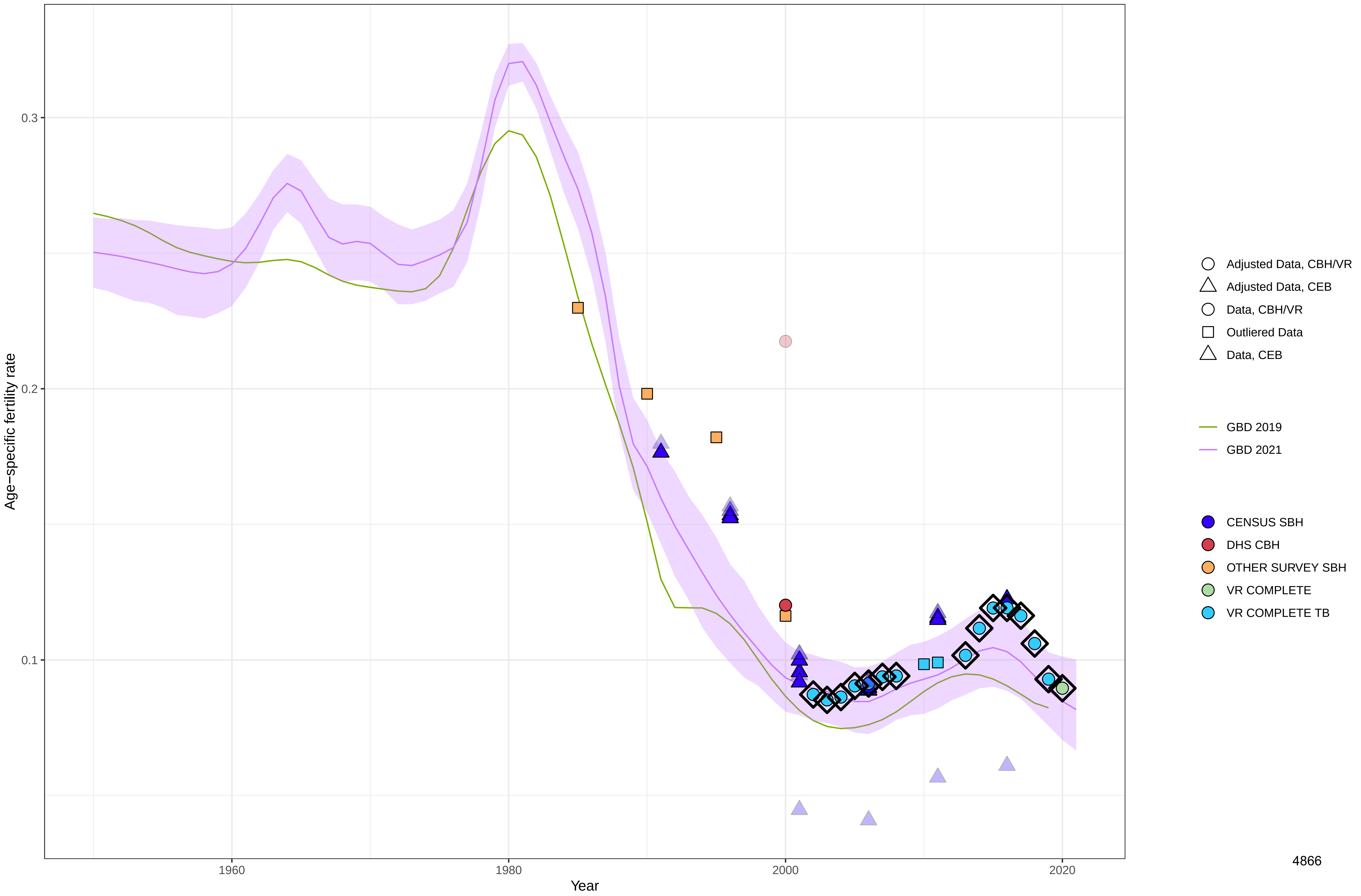

Age−specific fertility fitted trend lines, 1950−2021: Yazd, Ages: 35−39
Data points enclosed with a diamond are reference sources

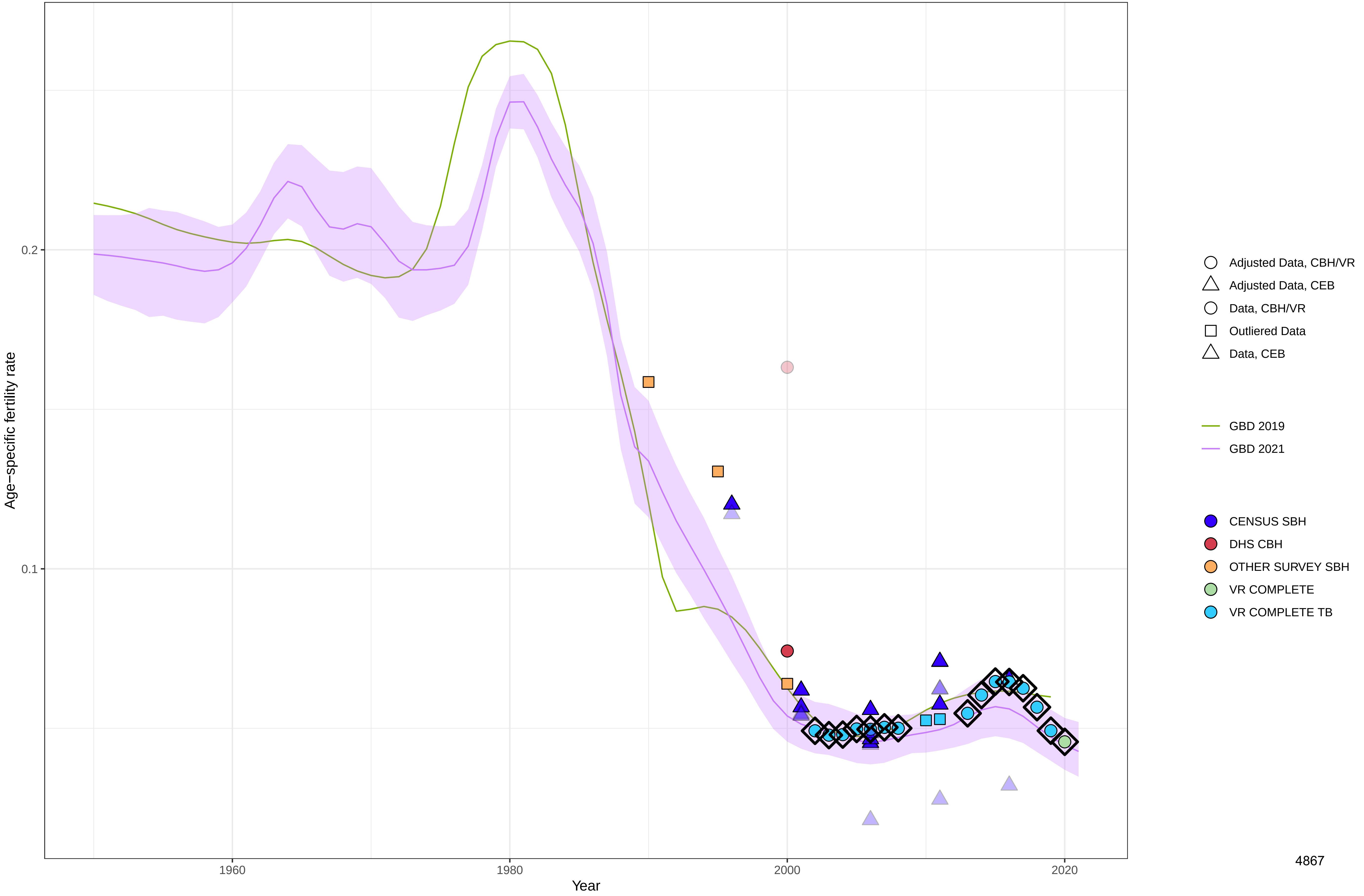

Age−specific fertility fitted trend lines, 1950−2021: Yazd, Ages: 40−44
Data points enclosed with a diamond are reference sources

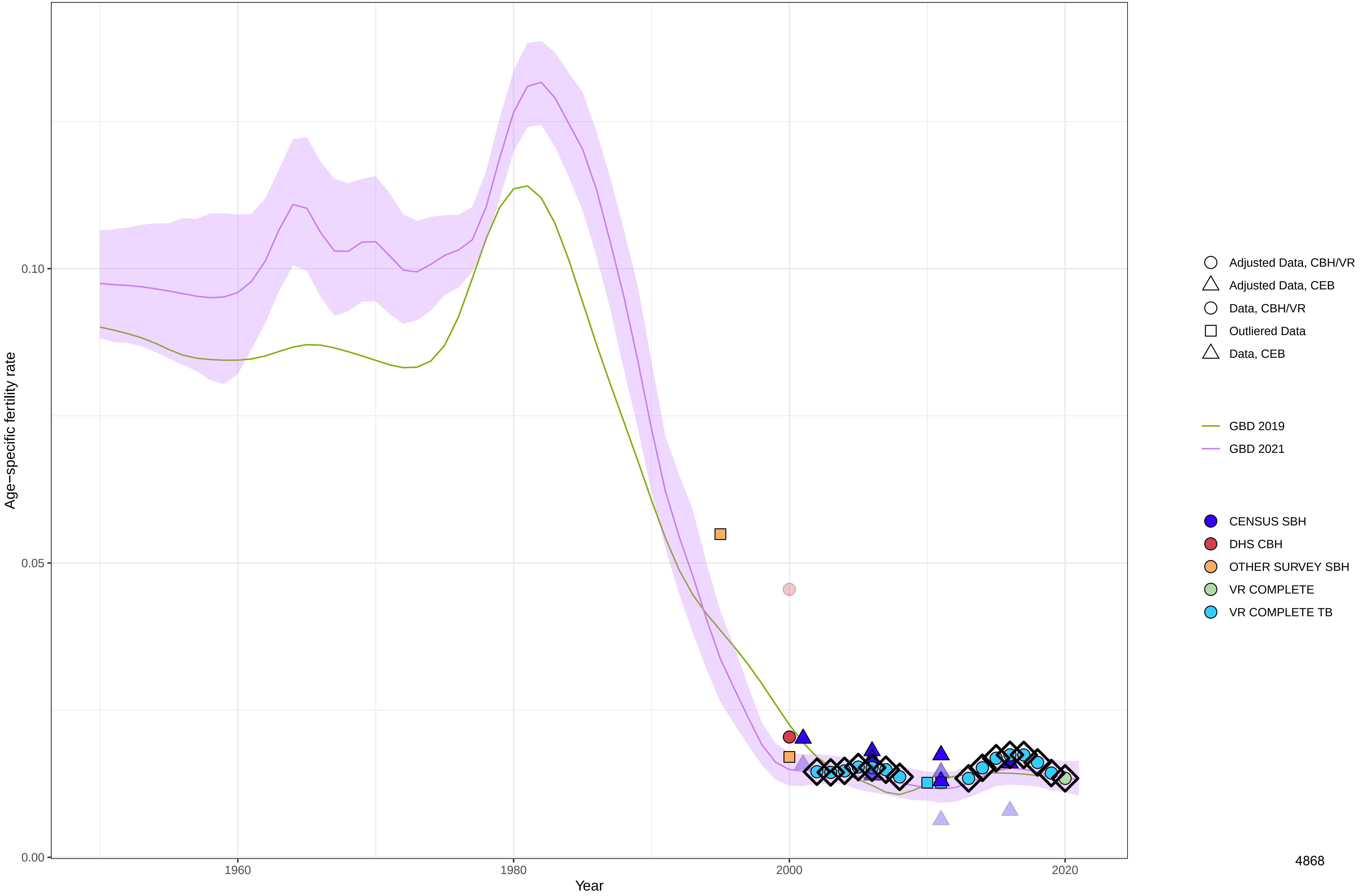

Age−specific fertility fitted trend lines, 1950−2021: Yazd, Ages: 45−49
Data points enclosed with a diamond are reference sources

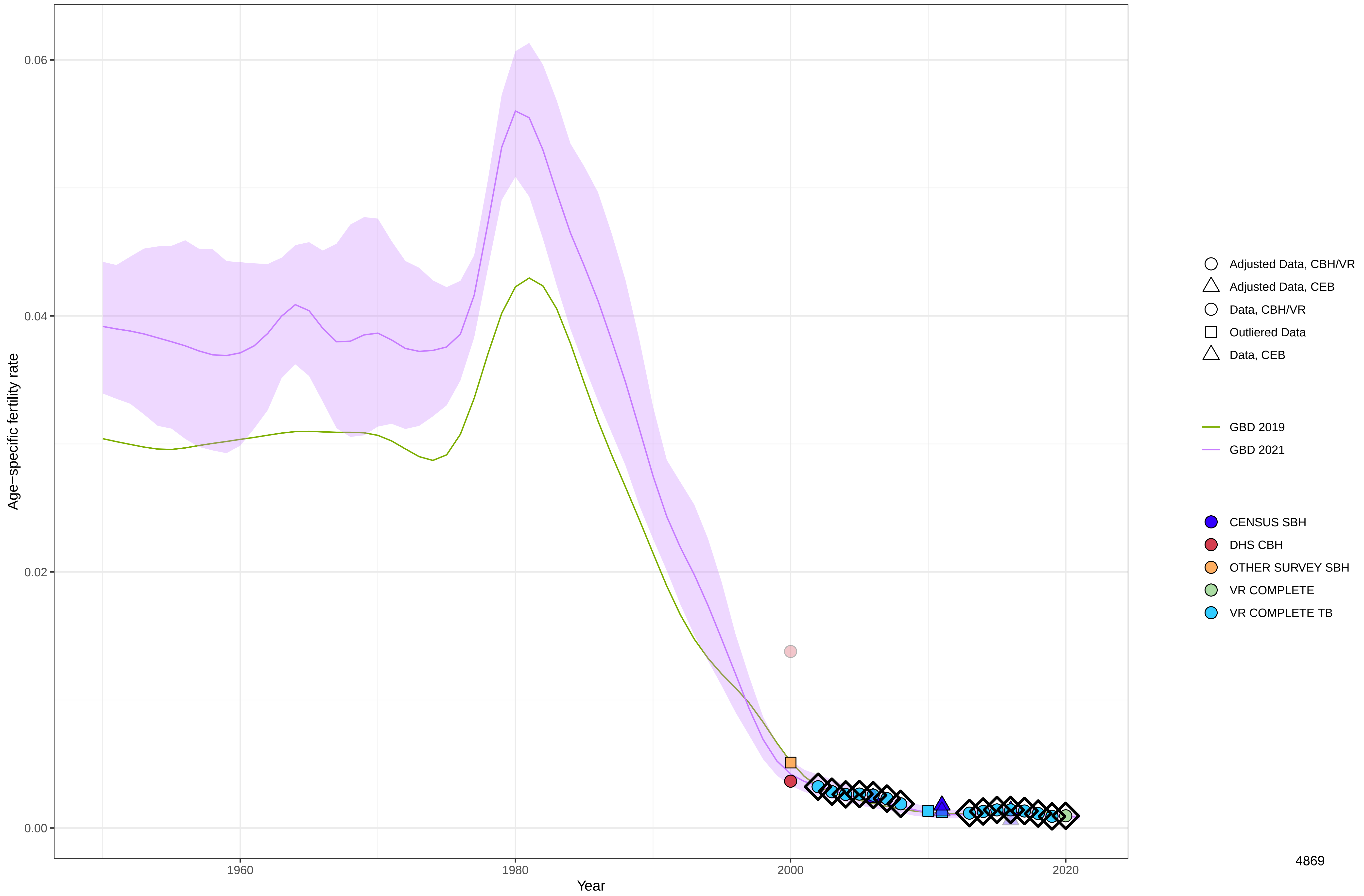

Age−specific fertility fitted trend lines, 1950−2021: Yazd, Ages: 50−54
Data points enclosed with a diamond are reference sources

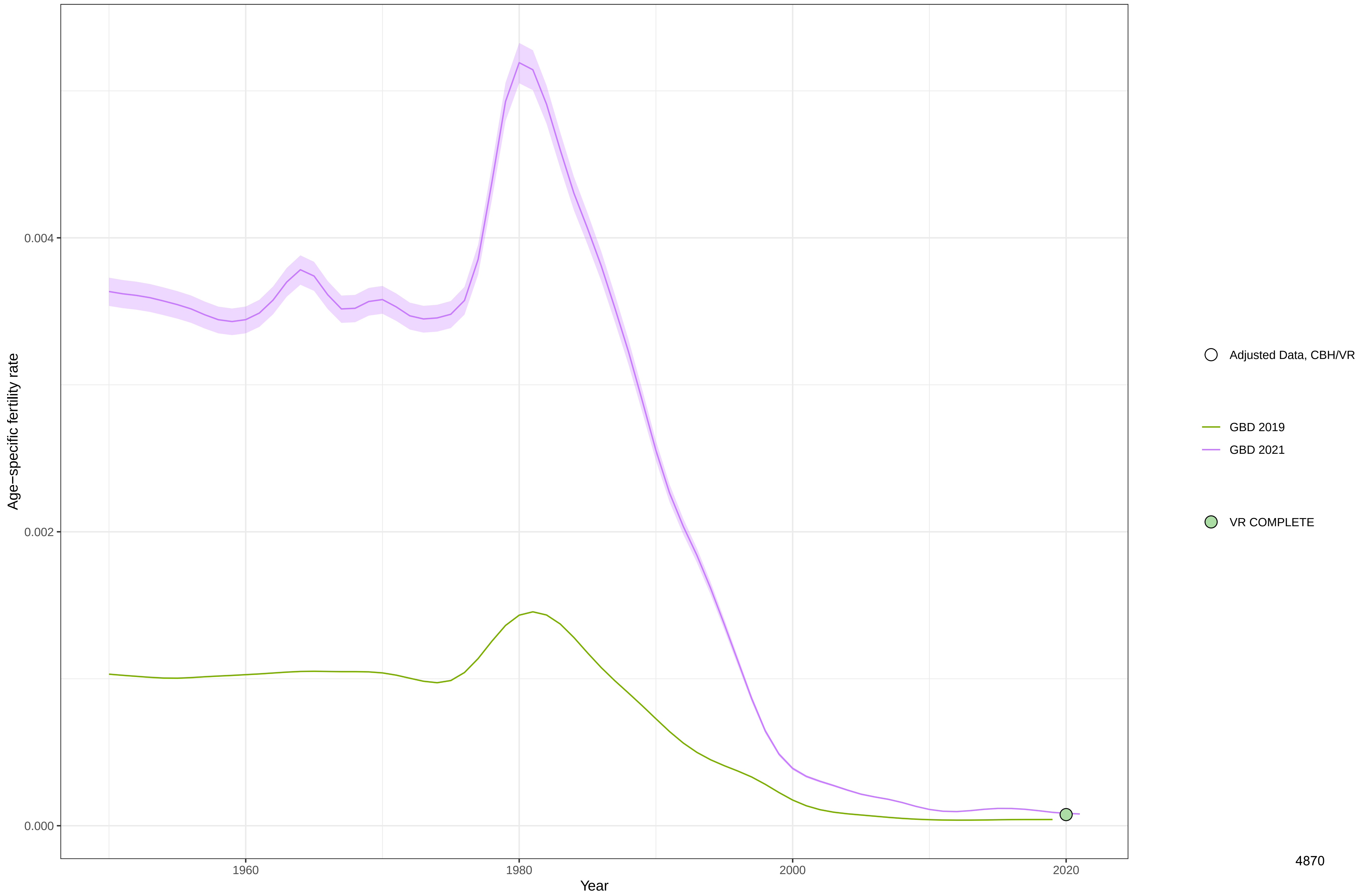

Total fertility rate fitted trend lines, 1950−2021: Yazd TFR
Data points enclosed with a diamond are reference sources

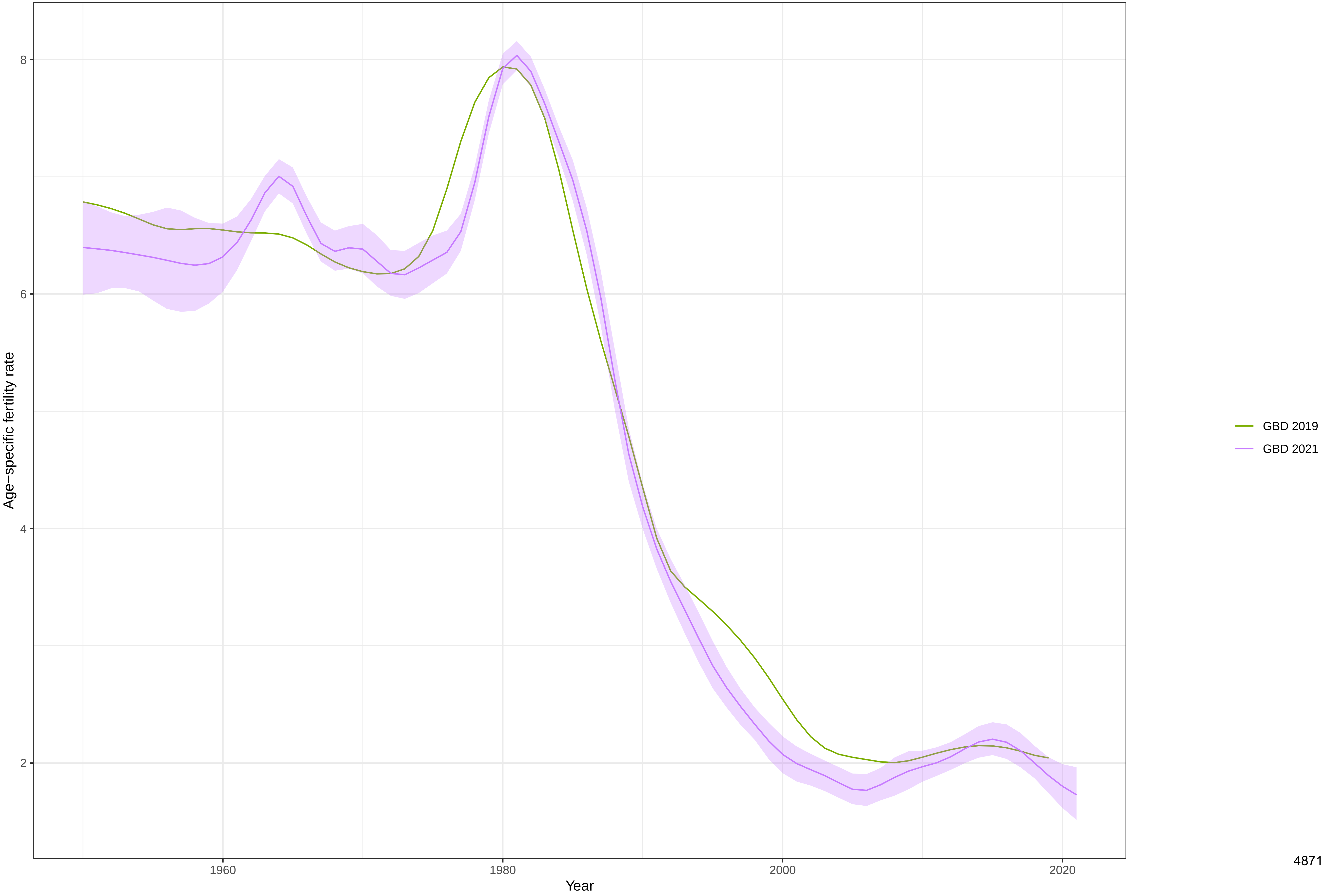

Age−specific fertility fitted trend lines, 1950−2021: Zanjan, Ages: 10−14
Data points enclosed with a diamond are reference sources

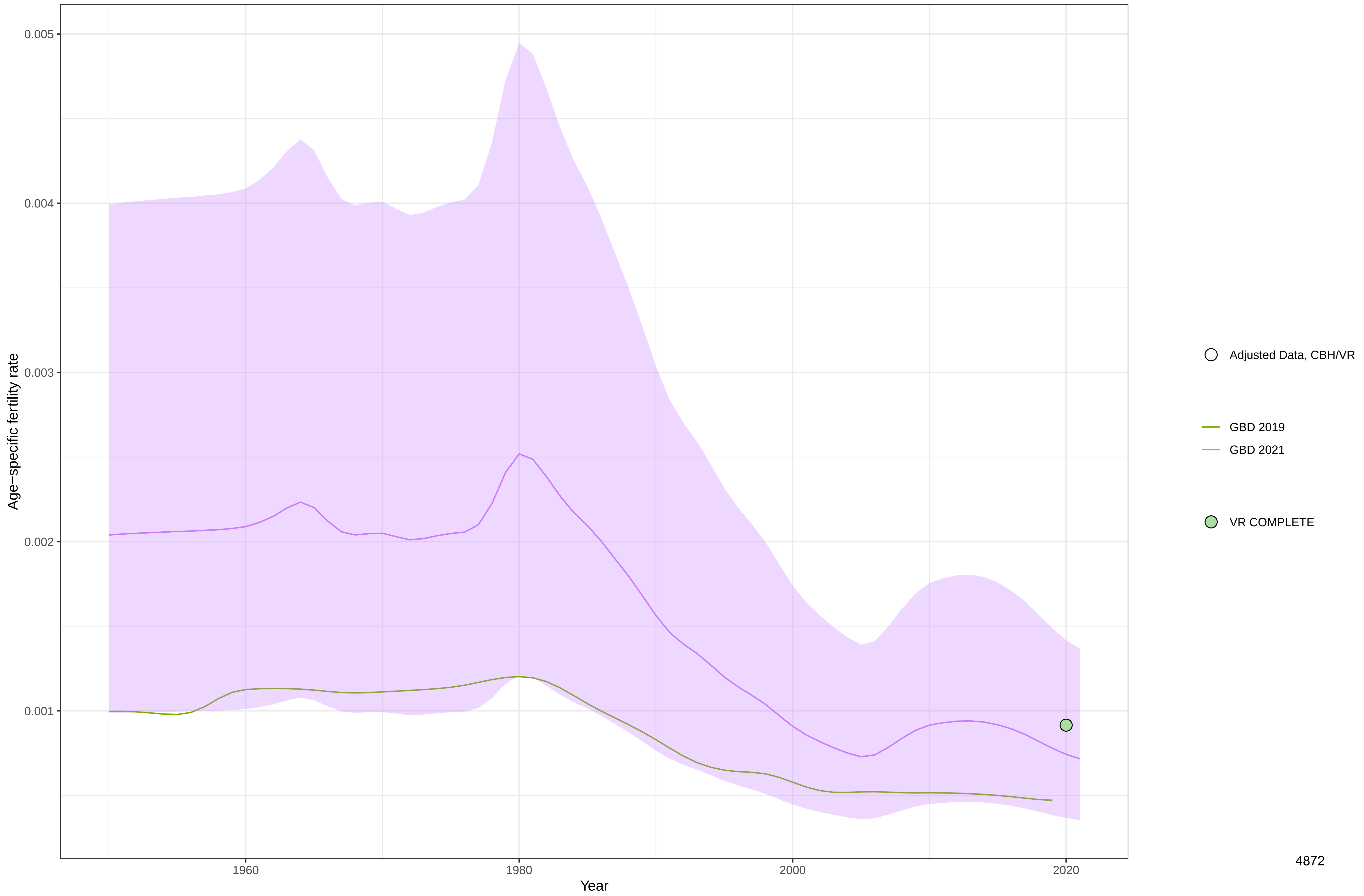

Age−specific fertility fitted trend lines, 1950−2021: Zanjan, Ages: 15−19
Data points enclosed with a diamond are reference sources

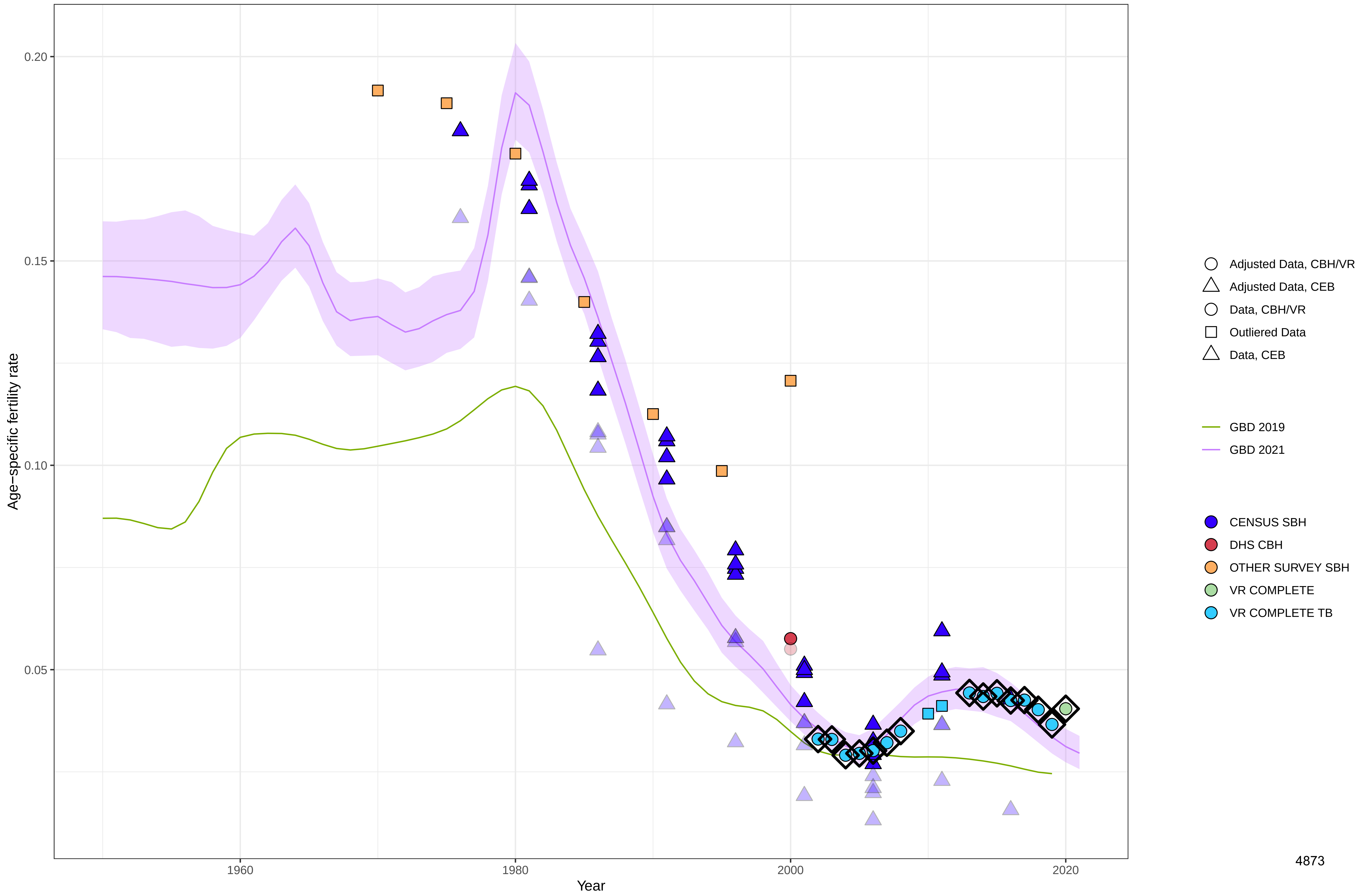

Age−specific fertility fitted trend lines, 1950−2021: Zanjan, Ages: 20−24
Data points enclosed with a diamond are reference sources

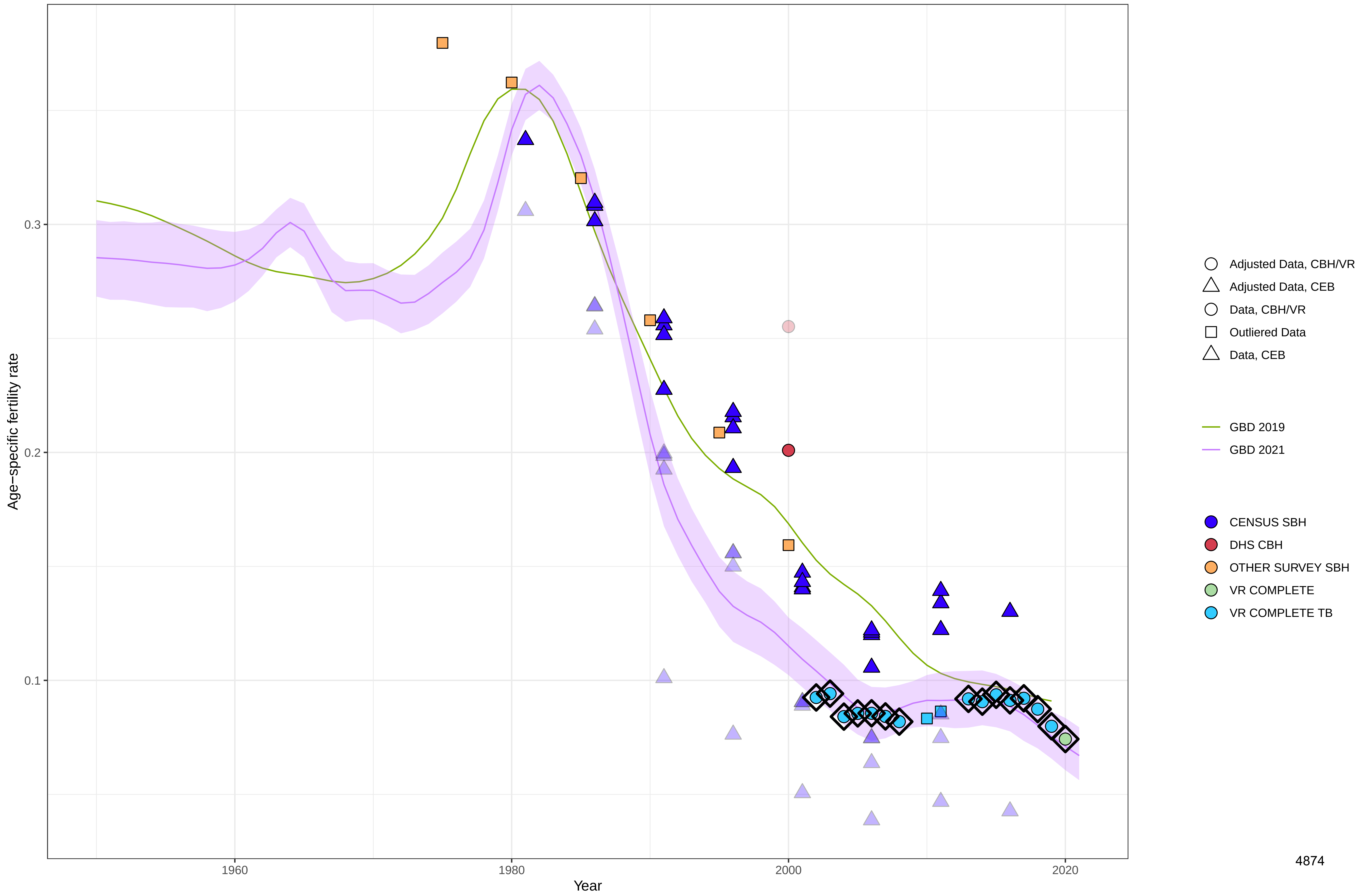

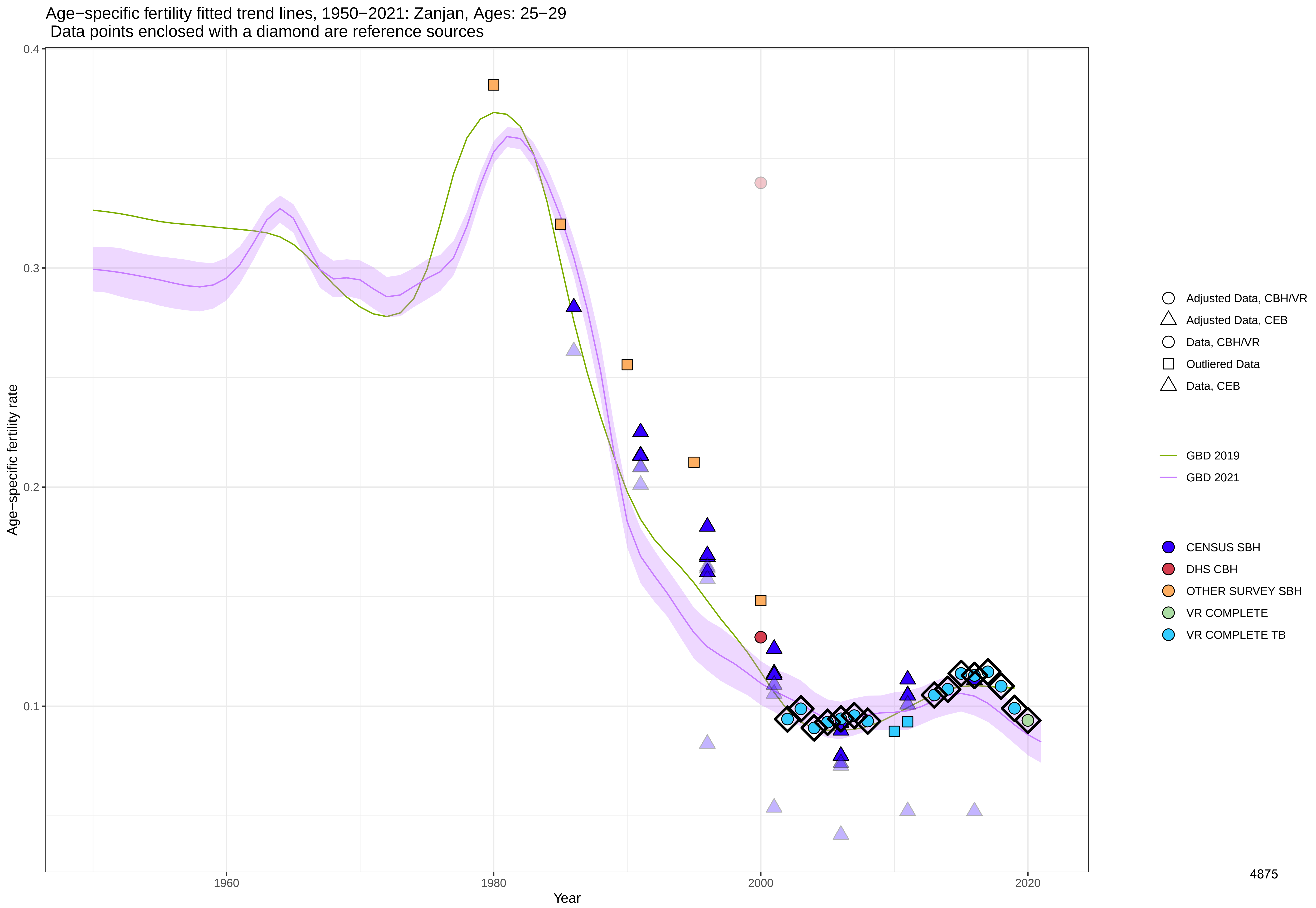

Age−specific fertility fitted trend lines, 1950−2021: Zanjan, Ages: 25−29
Data points enclosed with a diamond are reference sources
Age−specific fertility rate
0.4
0.3
0.2
0.1
Year
1960
1980
2000
2020
Adjusted Data, CBH/VR
Adjusted Data, CEB
Data, CBH/VR
Outliered Data
Data, CEB
GBD 2019
GBD 2021
CENSUS SBH
DHS CBH
OTHER SURVEY SBH
VR COMPLETE
VR COMPLETE TB

Age−specific fertility fitted trend lines, 1950−2021: Zanjan, Ages: 30−34
Data points enclosed with a diamond are reference sources

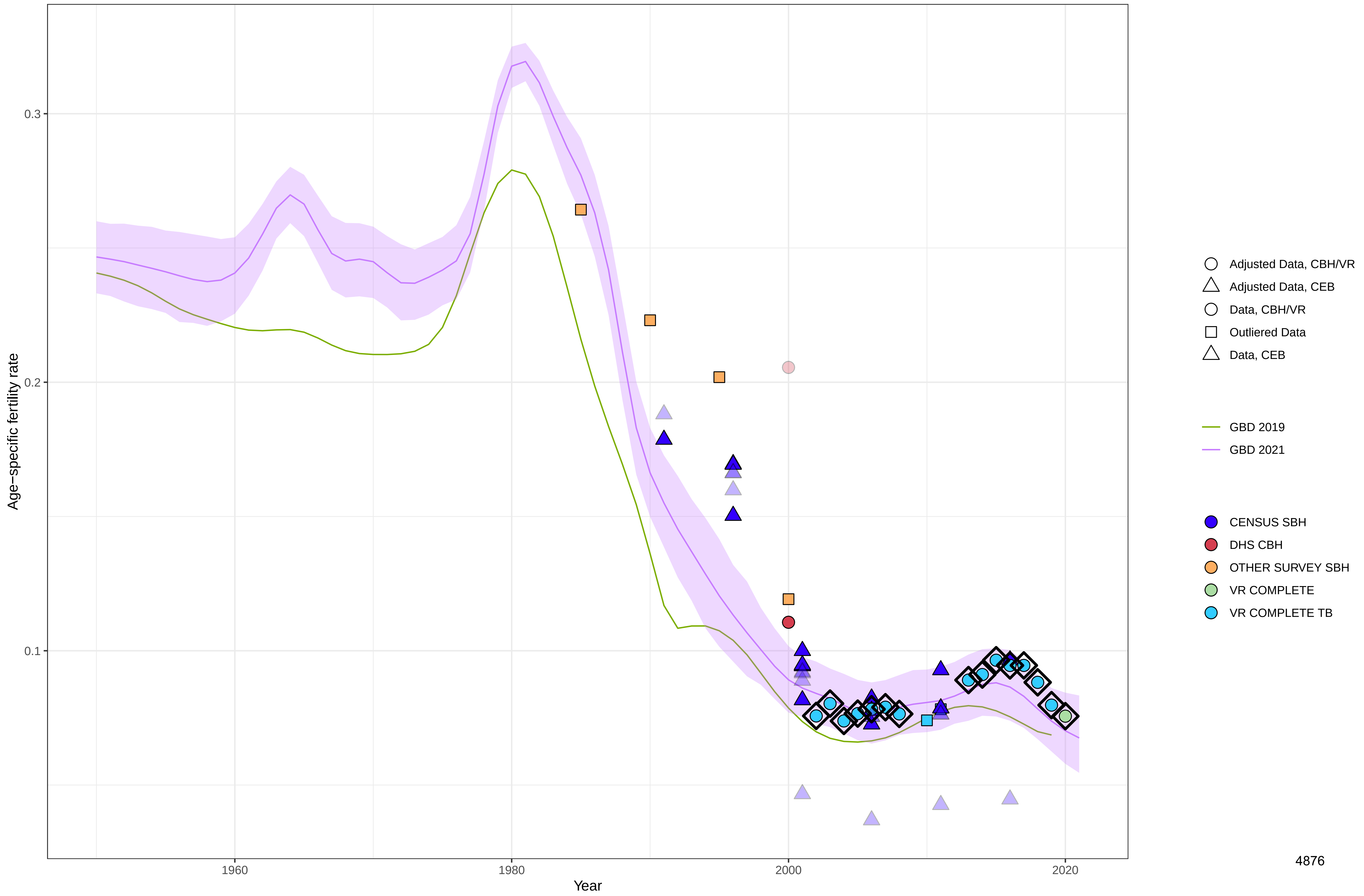

Age−specific fertility fitted trend lines, 1950−2021: Zanjan, Ages: 35−39
Data points enclosed with a diamond are reference sources

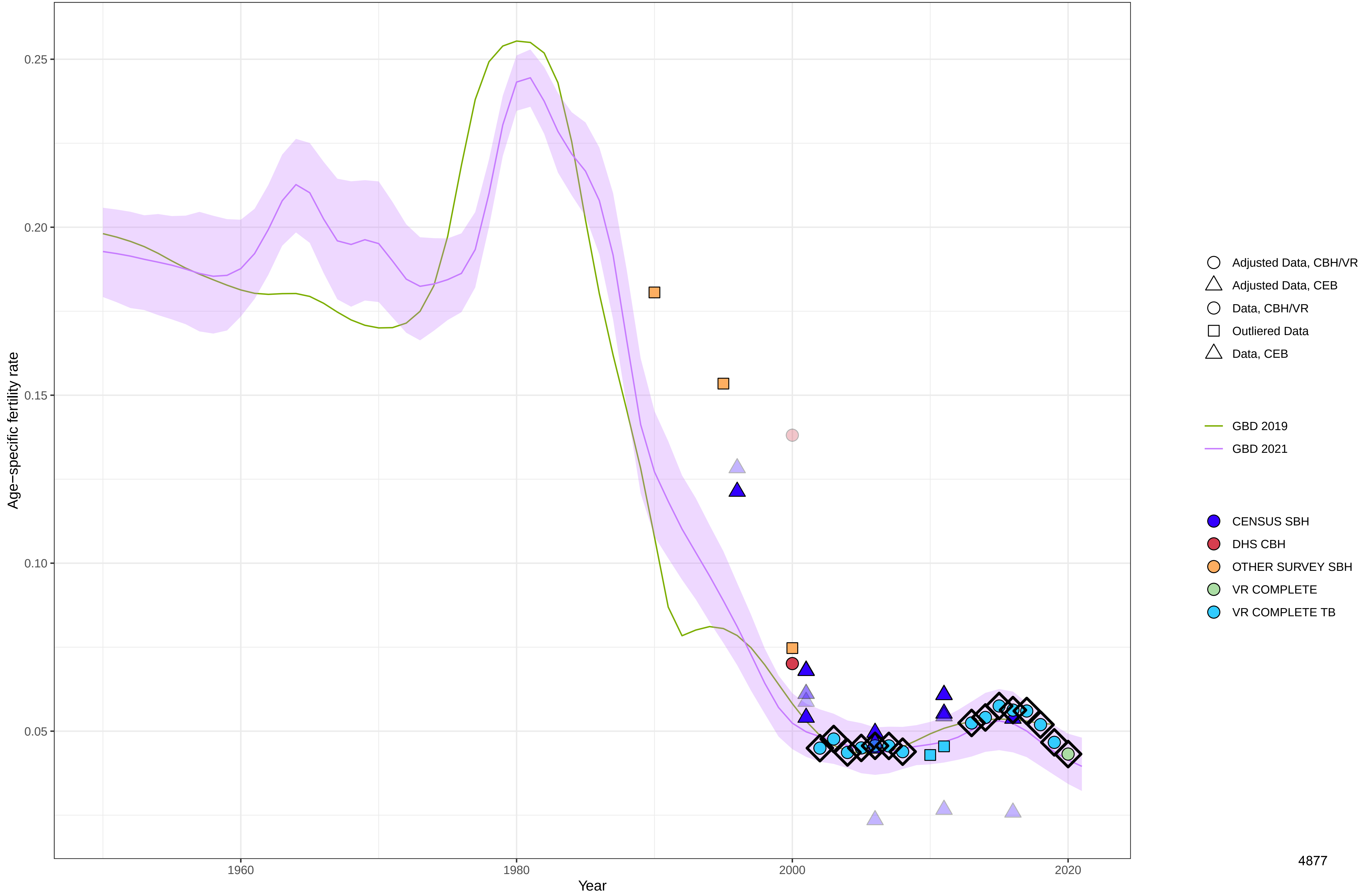

Age−specific fertility fitted trend lines, 1950−2021: Zanjan, Ages: 40−44
Data points enclosed with a diamond are reference sources

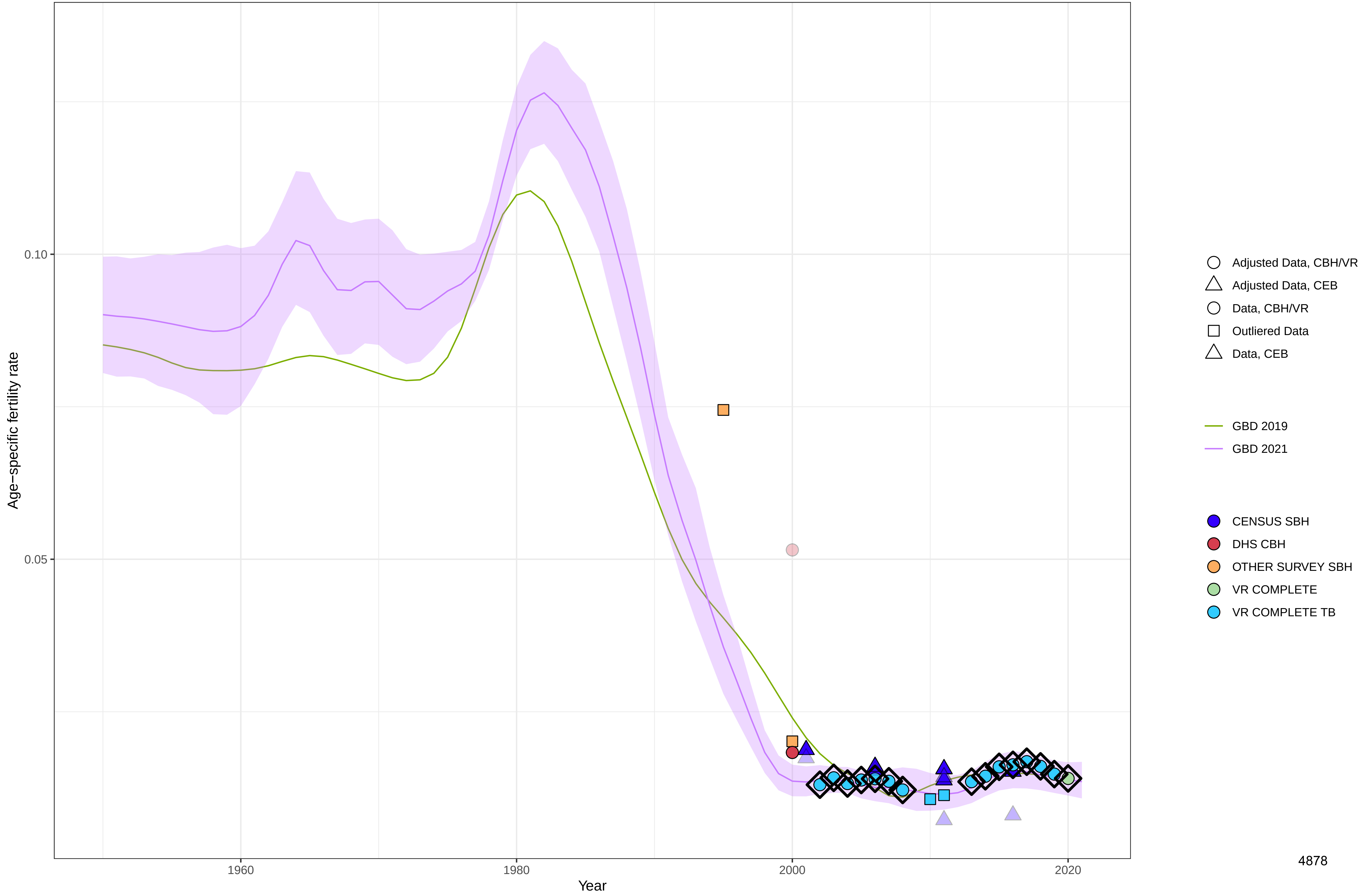

Age−specific fertility fitted trend lines, 1950−2021: Zanjan, Ages: 45−49
Data points enclosed with a diamond are reference sources

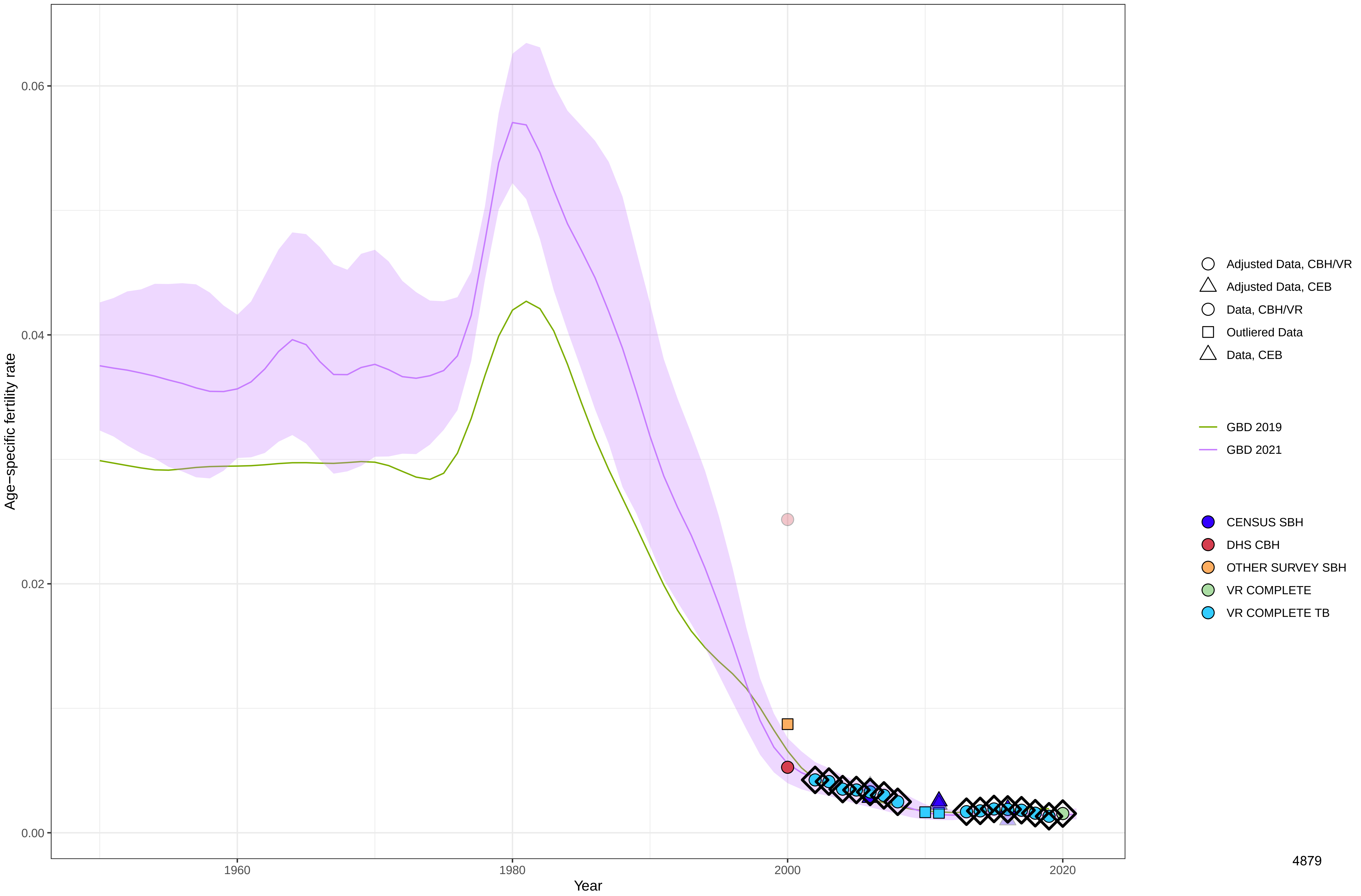

Age−specific fertility fitted trend lines, 1950−2021: Zanjan, Ages: 50−54
Data points enclosed with a diamond are reference sources

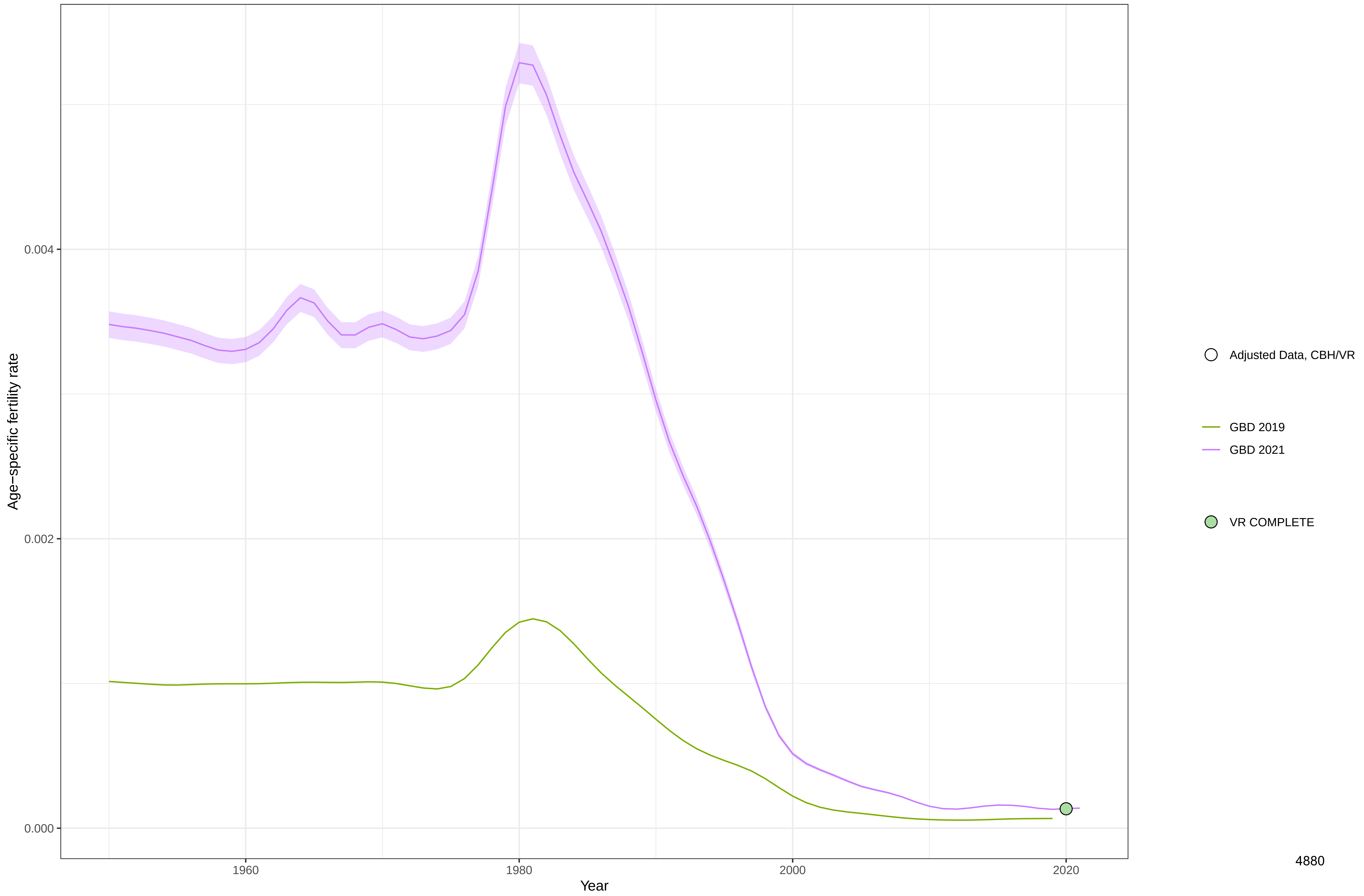

Total fertility rate fitted trend lines, 1950−2021: Zanjan TFR
Data points enclosed with a diamond are reference sources

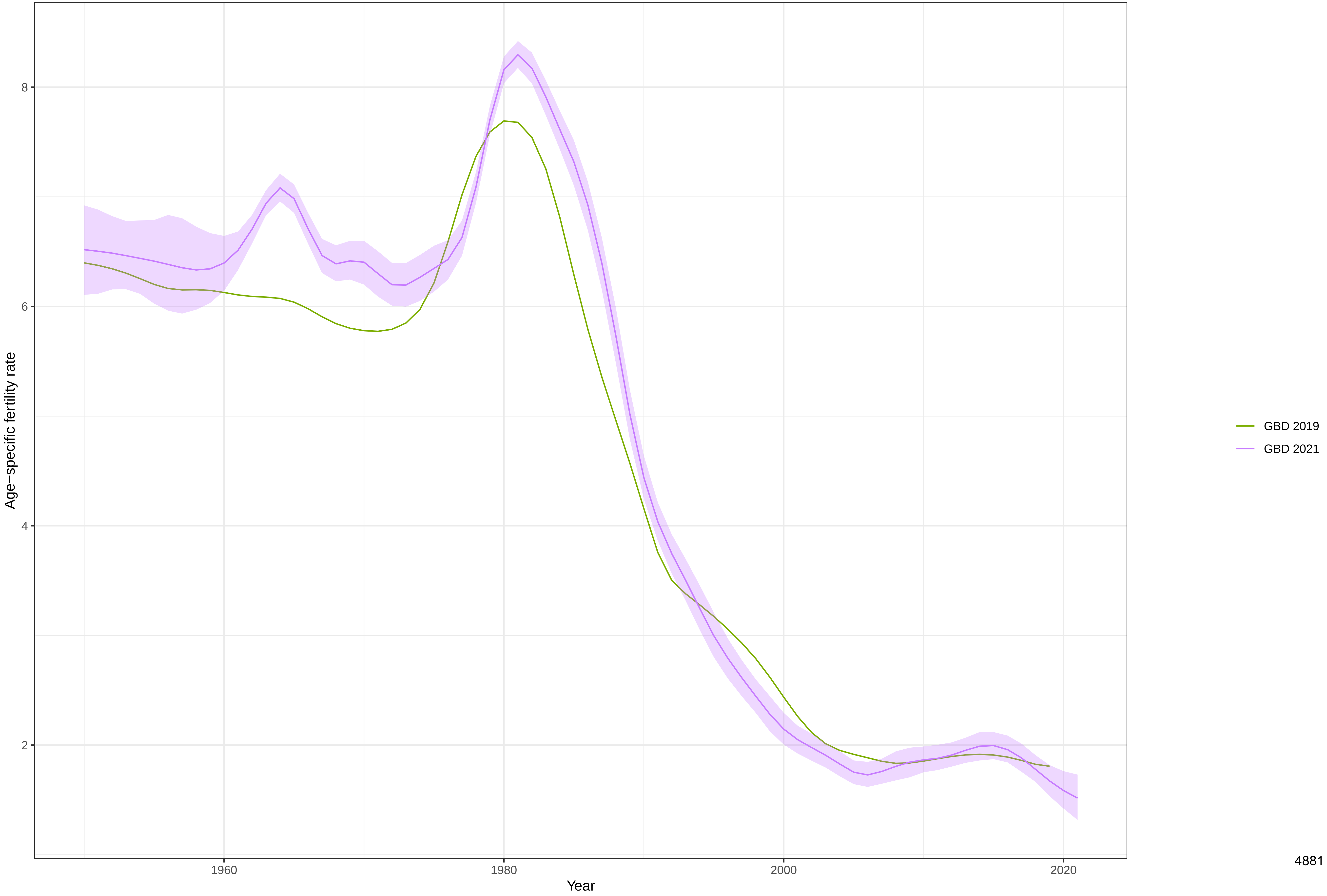

Age−specific fertility fitted trend lines, 1950−2021: Iraq, Ages: 10−14
Data points enclosed with a diamond are reference sources

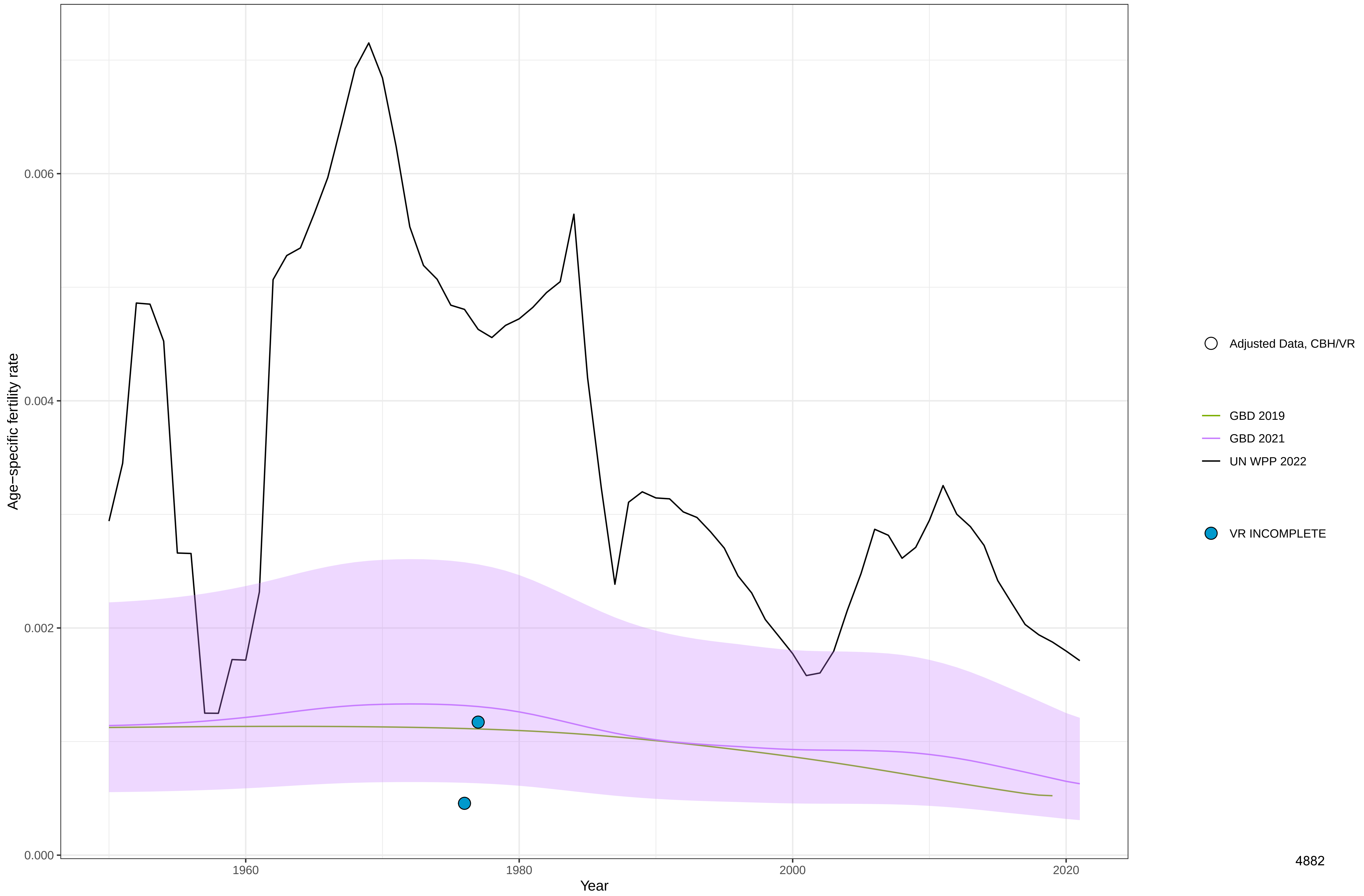

Age−specific fertility fitted trend lines, 1950−2021: Iraq, Ages: 15−19
Data points enclosed with a diamond are reference sources

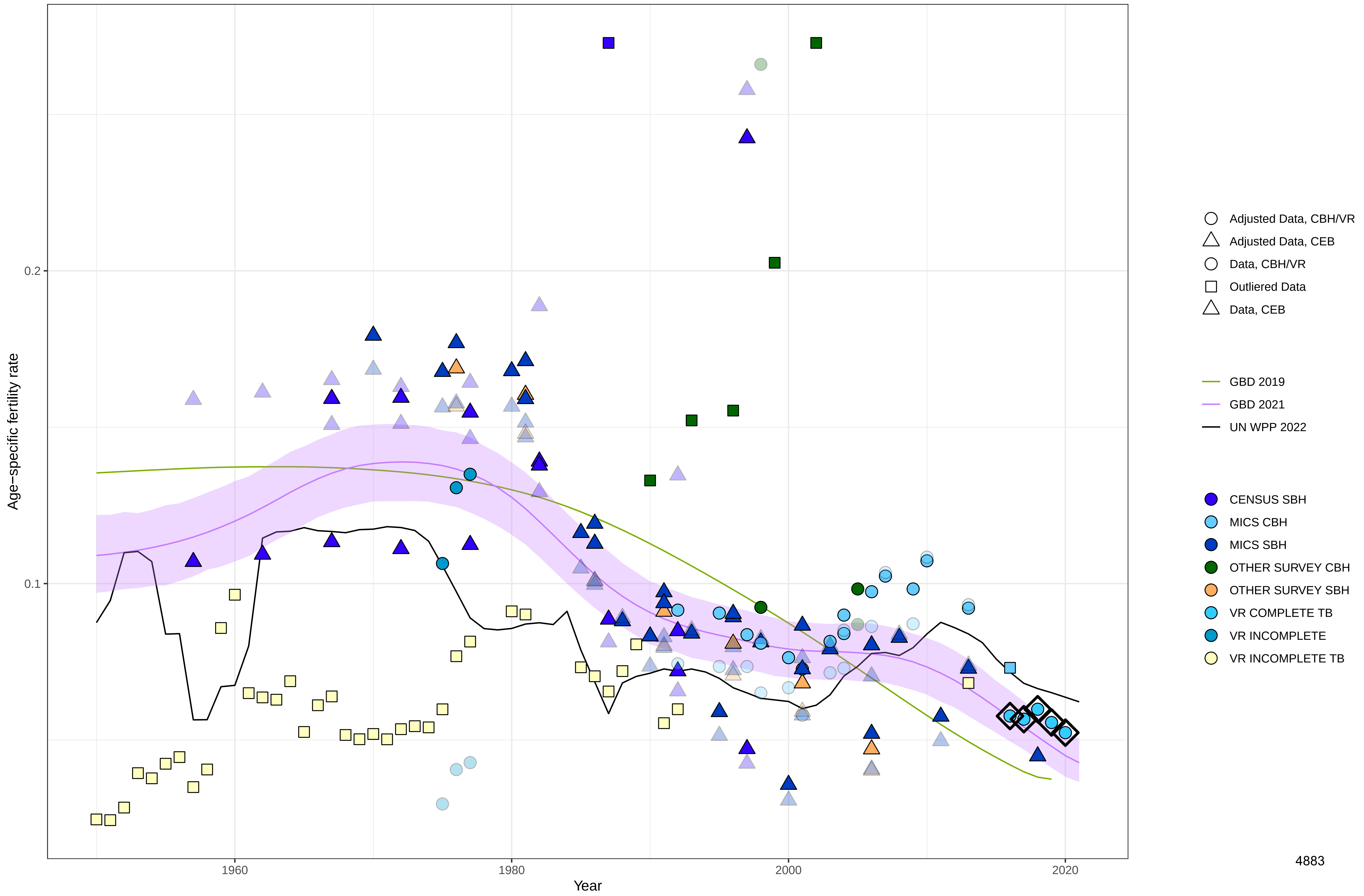

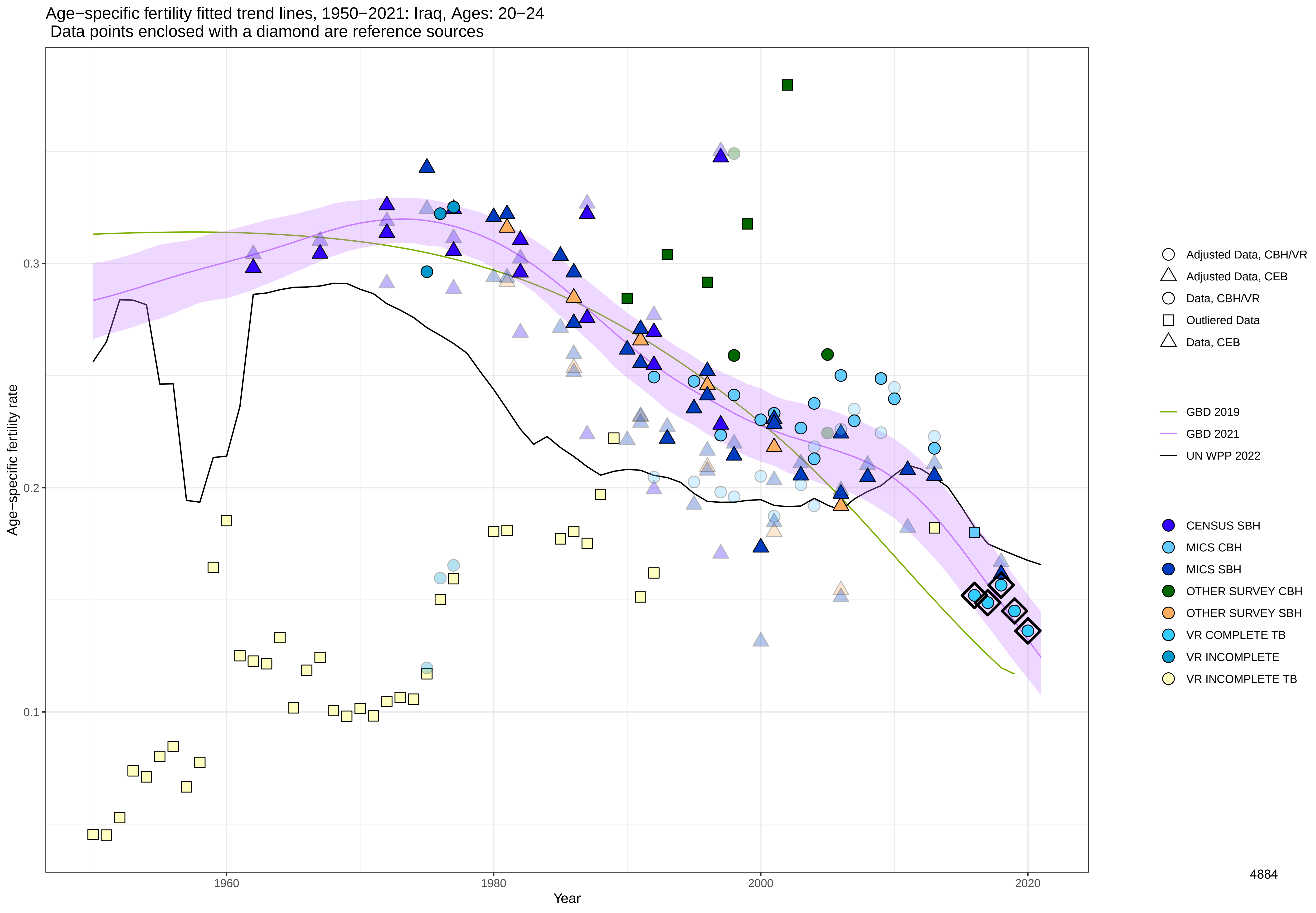

Age−specific fertility fitted trend lines, 1950−2021: Iraq, Ages: 20−24
Data points enclosed with a diamond are reference sources
Age−specific fertility rate
Year
0.1
0.2
0.3
1960
1980
2000
2020
Adjusted Data, CBH/VR
Adjusted Data, CEB
Data, CBH/VR
Outliered Data
Data, CEB
GBD 2019
GBD 2021
UN WPP 2022
CENSUS SBH
MICS CBH
MICS SBH
OTHER SURVEY CBH
OTHER SURVEY SBH
VR COMPLETE TB
VR INCOMPLETE
VR INCOMPLETE TB

Age−specific fertility fitted trend lines, 1950−2021: Iraq, Ages: 25−29
Data points enclosed with a diamond are reference sources

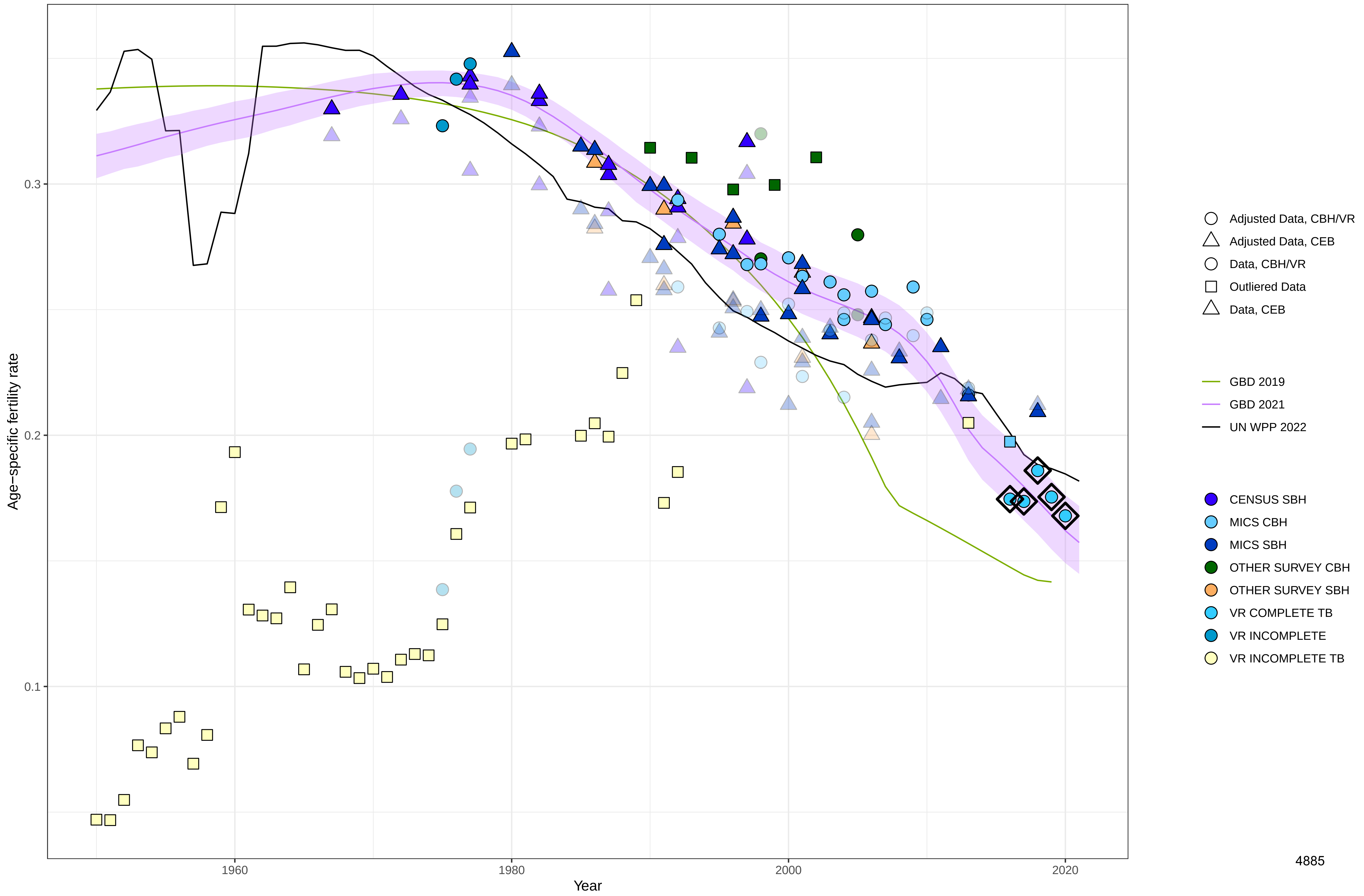

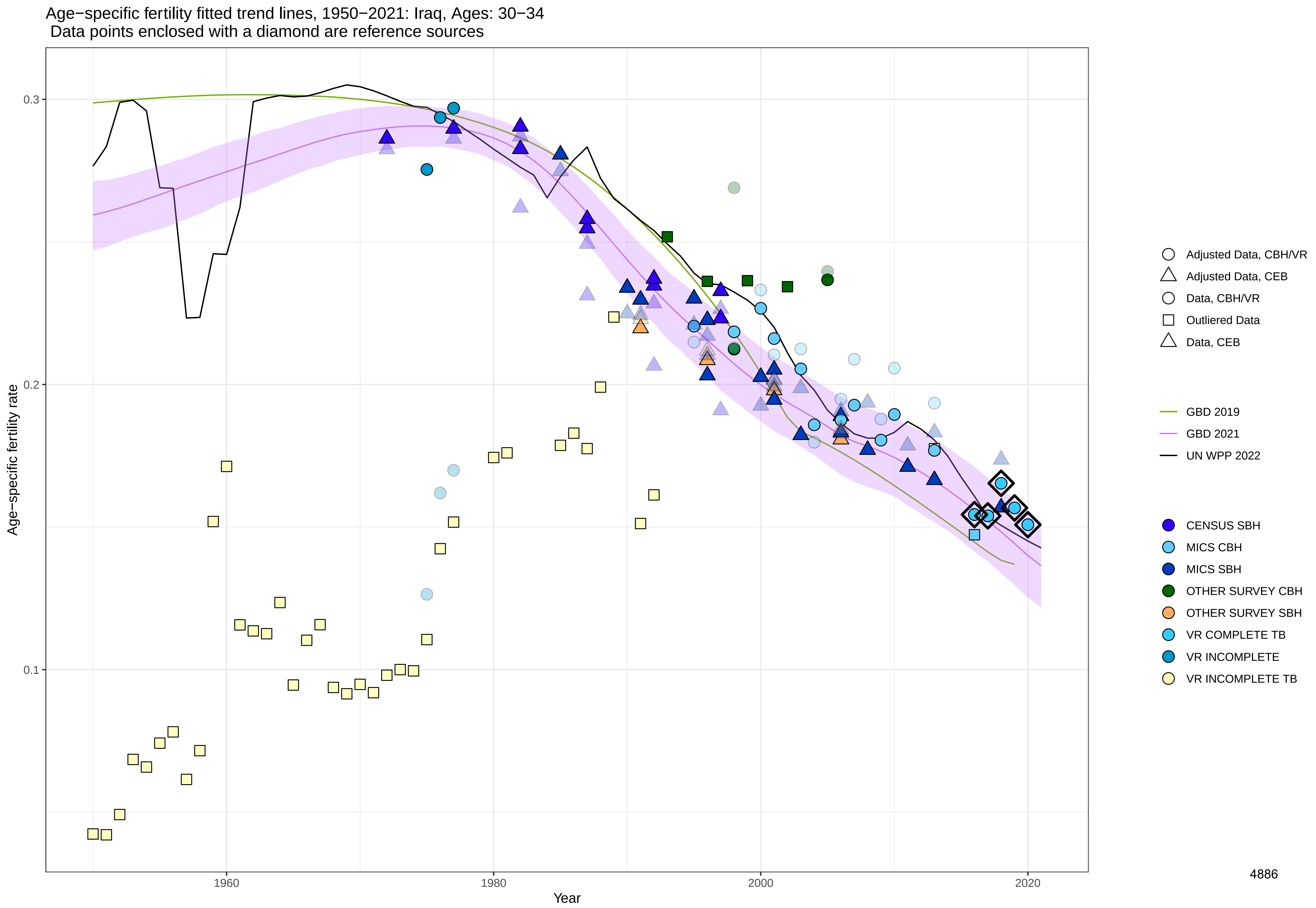
Age−specific fertility fitted trend lines, 1950−2021: Iraq, Ages: 30−34
Data points enclosed with a diamond are reference sources
Age−specific fertility rate
Year
0.1
0.2
0.3
1960
1980
2000
2020
Adjusted Data, CBH/VR
Adjusted Data, CEB
Data, CBH/VR
Outliered Data
Data, CEB
GBD 2019
GBD 2021
UN WPP 2022
CENSUS SBH
MICS CBH
MICS SBH
OTHER SURVEY CBH
OTHER SURVEY SBH
VR COMPLETE TB
VR INCOMPLETE
VR INCOMPLETE TB

Age−specific fertility fitted trend lines, 1950−2021: Iraq, Ages: 35−39
Data points enclosed with a diamond are reference sources

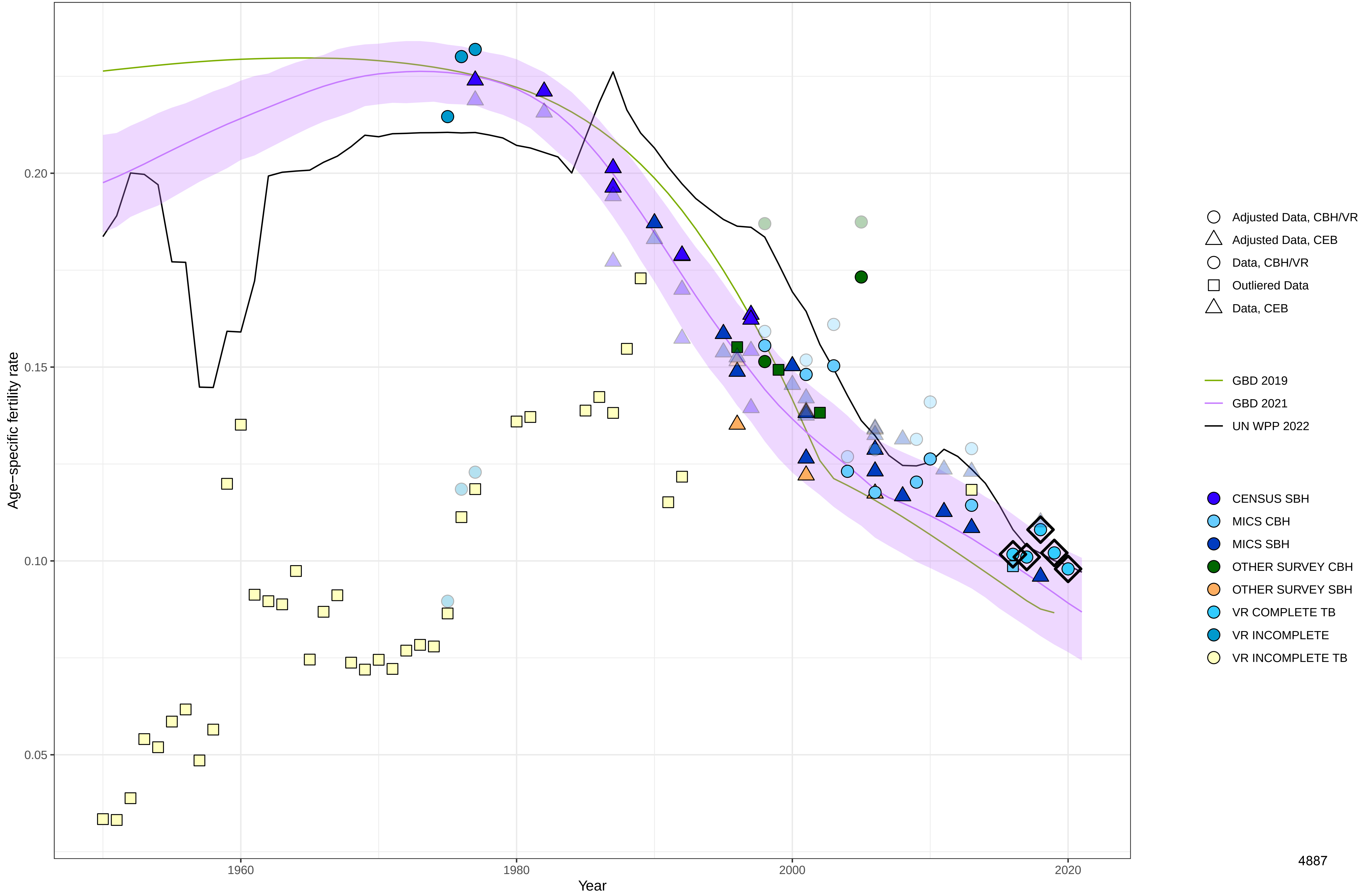

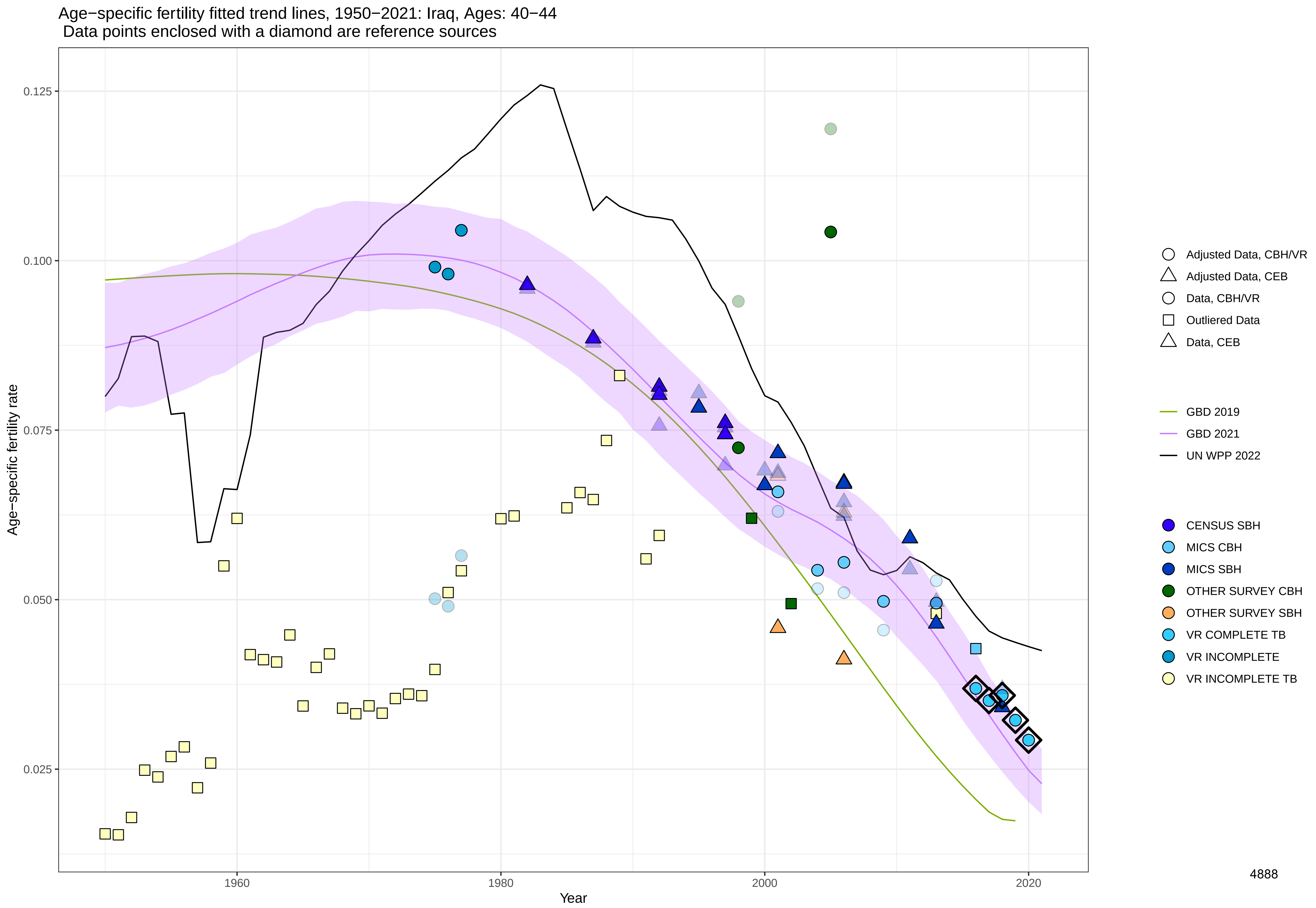

Age−specific fertility fitted trend lines, 1950−2021: Iraq, Ages: 40−44
Data points enclosed with a diamond are reference sources
Age−specific fertility rate
0.125
0.100
0.075
0.050
0.025
1960
1980
2000
2020
Year
Adjusted Data, CBH/VR
Adjusted Data, CEB
Data, CBH/VR
Outliered Data
Data, CEB
GBD 2019
GBD 2021
UN WPP 2022
CENSUS SBH
MICS CBH
MICS SBH
OTHER SURVEY CBH
OTHER SURVEY SBH
VR COMPLETE TB
VR INCOMPLETE
VR INCOMPLETE TB

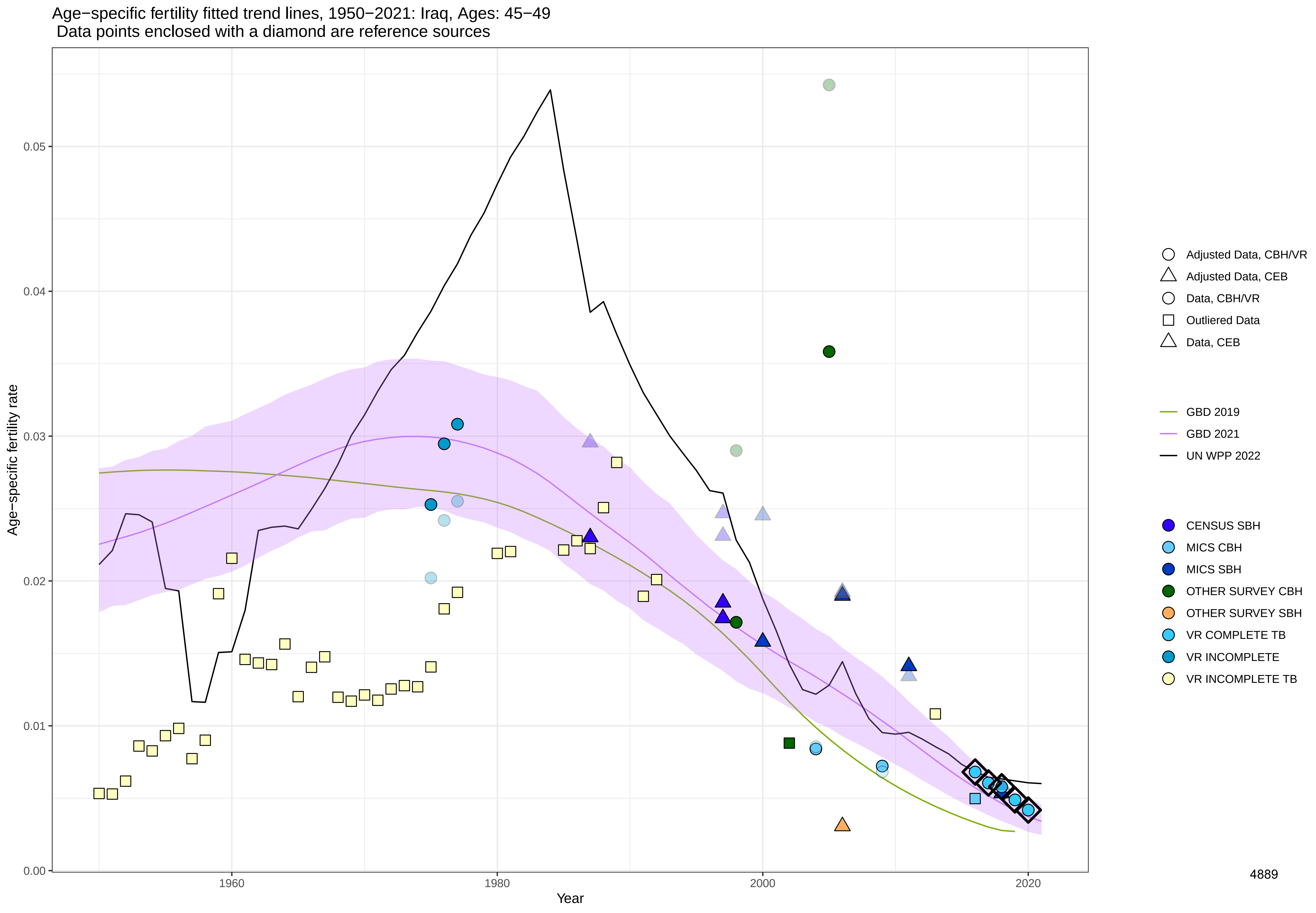

Age−specific fertility fitted trend lines, 1950−2021: Iraq, Ages: 45−49
Data points enclosed with a diamond are reference sources
Age−specific fertility rate
0.00
0.01
0.02
0.03
0.04
0.05
Year
1960
1980
2000
2020
Adjusted Data, CBH/VR
Adjusted Data, CEB
Data, CBH/VR
Outliered Data
Data, CEB
GBD 2019
GBD 2021
UN WPP 2022
CENSUS SBH
MICS CBH
MICS SBH
OTHER SURVEY CBH
OTHER SURVEY SBH
VR COMPLETE TB
VR INCOMPLETE
VR INCOMPLETE TB

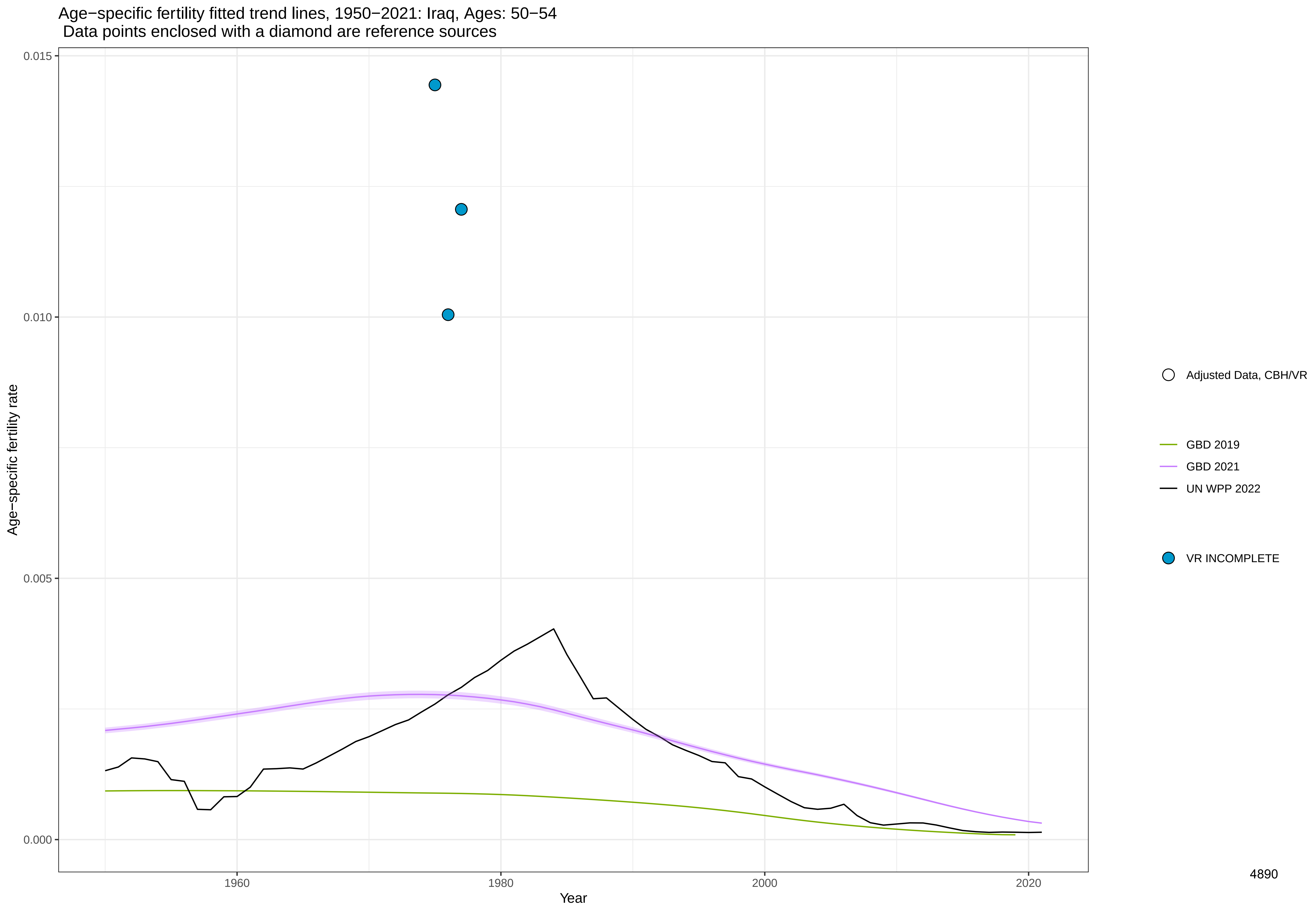

Age−specific fertility fitted trend lines, 1950−2021: Iraq, Ages: 50−54
Data points enclosed with a diamond are reference sources
0.015
0.010
0.005
0.000
Age−specific fertility rate
1960
1980
2000
2020
Year
Adjusted Data, CBH/VR
GBD 2019
GBD 2021
UN WPP 2022
VR INCOMPLETE

Total fertility rate fitted trend lines, 1950−2021: Iraq TFR
Data points enclosed with a diamond are reference sources

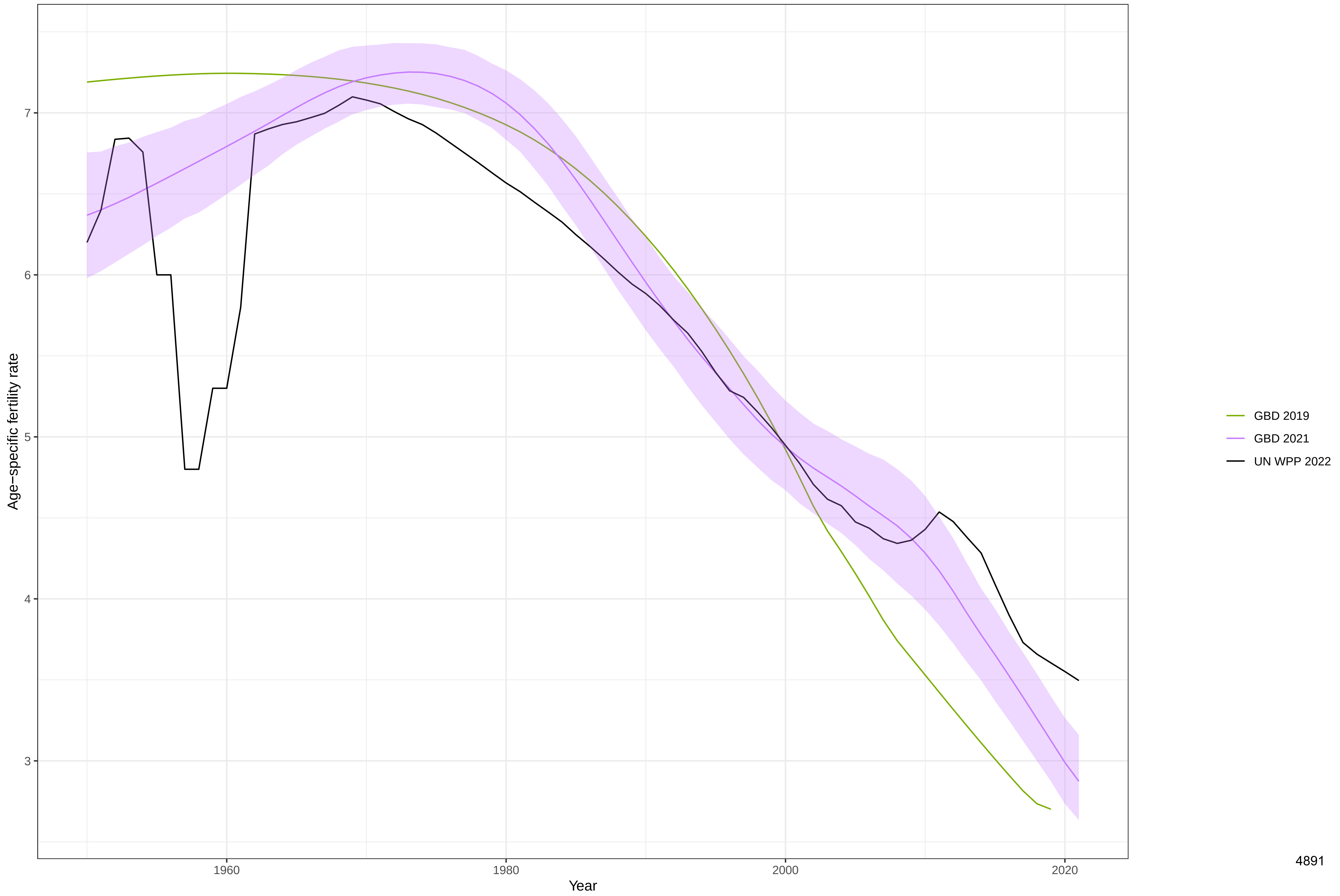

4891

Age−specific fertility fitted trend lines, 1950−2021: Jordan, Ages: 10−14
Data points enclosed with a diamond are reference sources

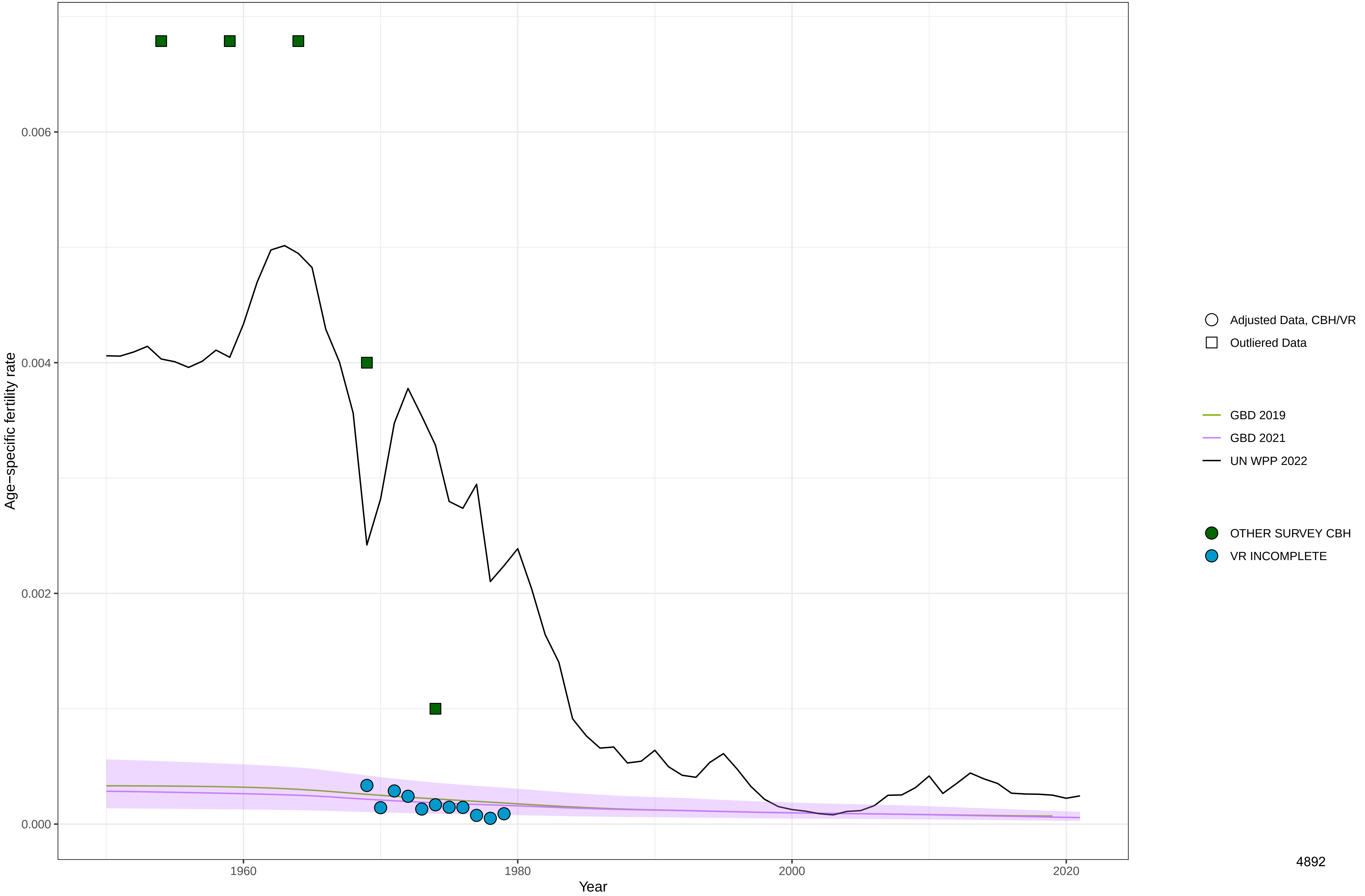

Age−specific fertility fitted trend lines, 1950−2021: Jordan, Ages: 15−19
Data points enclosed with a diamond are reference sources

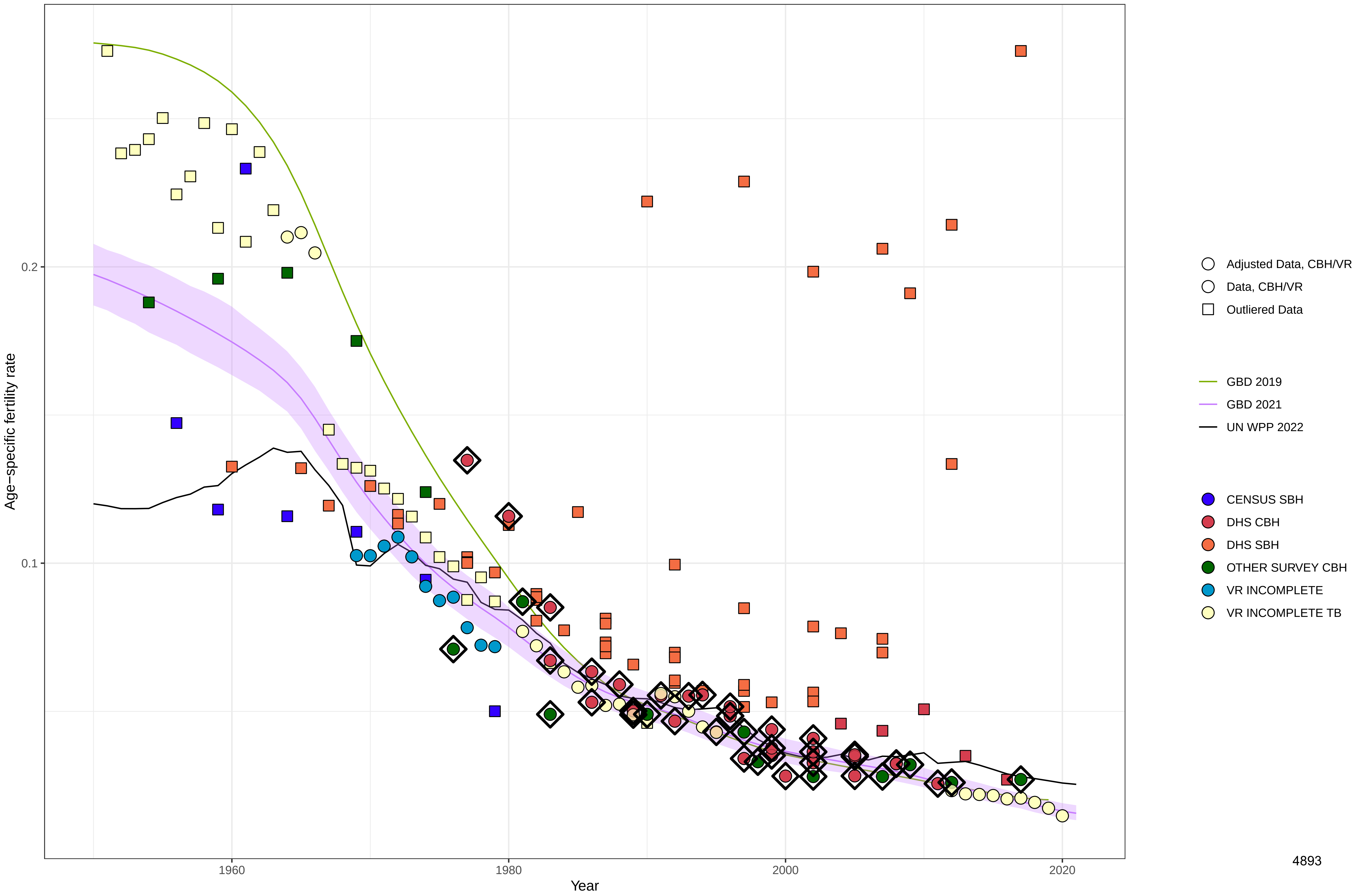

Age−specific fertility fitted trend lines, 1950−2021: Jordan, Ages: 20−24
Data points enclosed with a diamond are reference sources

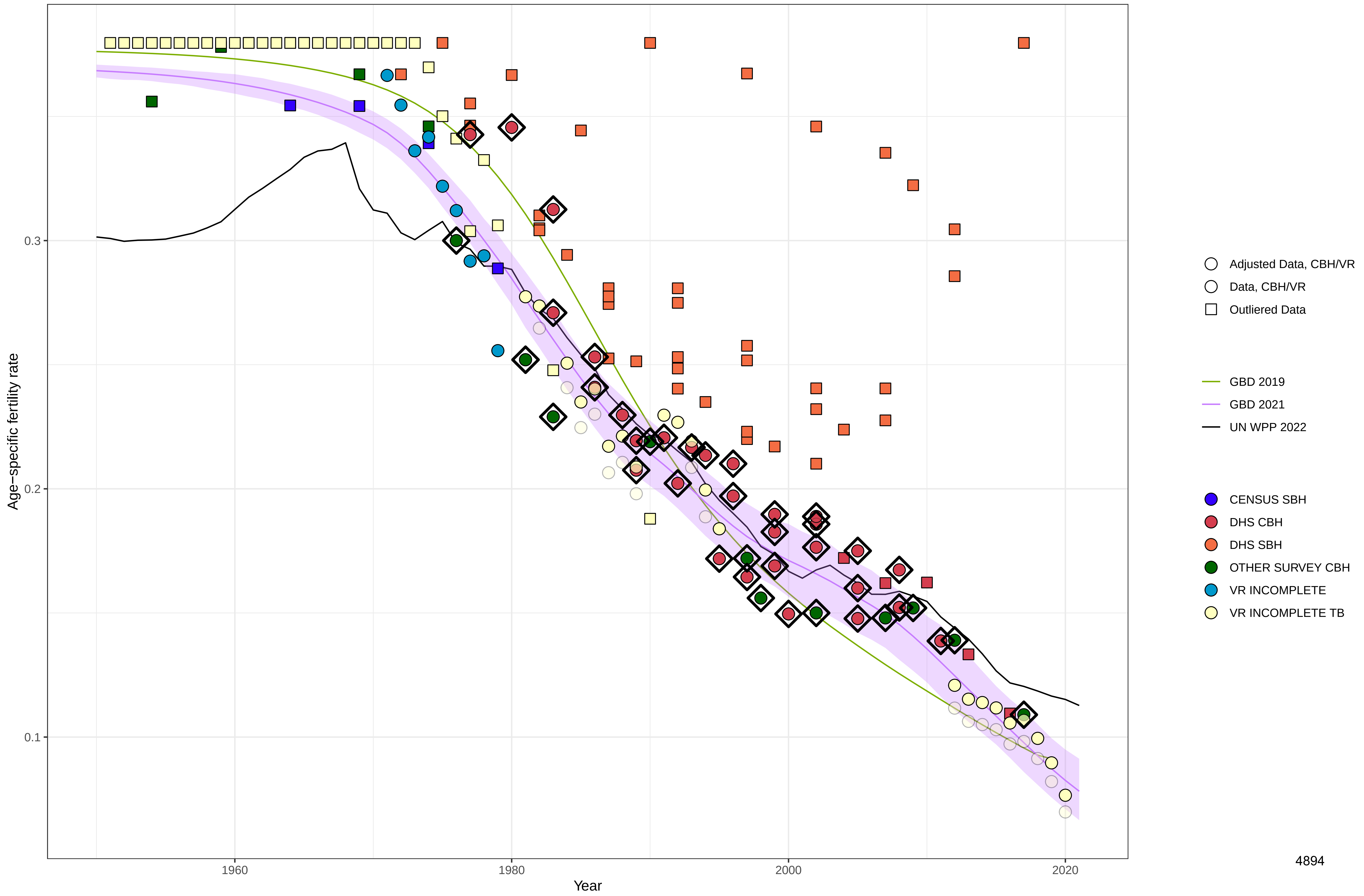

Age−specific fertility fitted trend lines, 1950−2021: Jordan, Ages: 25−29
Data points enclosed with a diamond are reference sources

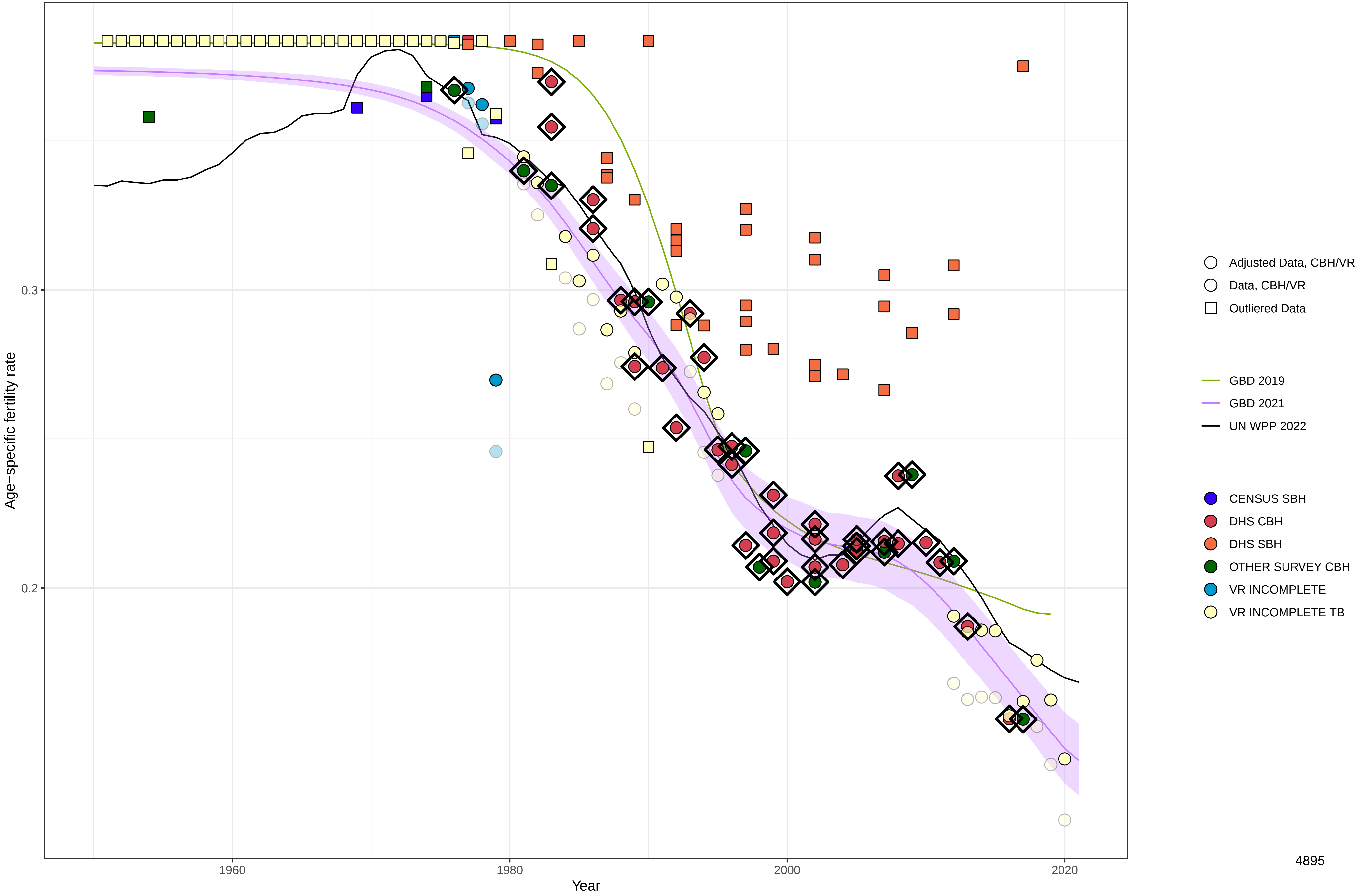

Age−specific fertility fitted trend lines, 1950−2021: Jordan, Ages: 30−34
Data points enclosed with a diamond are reference sources

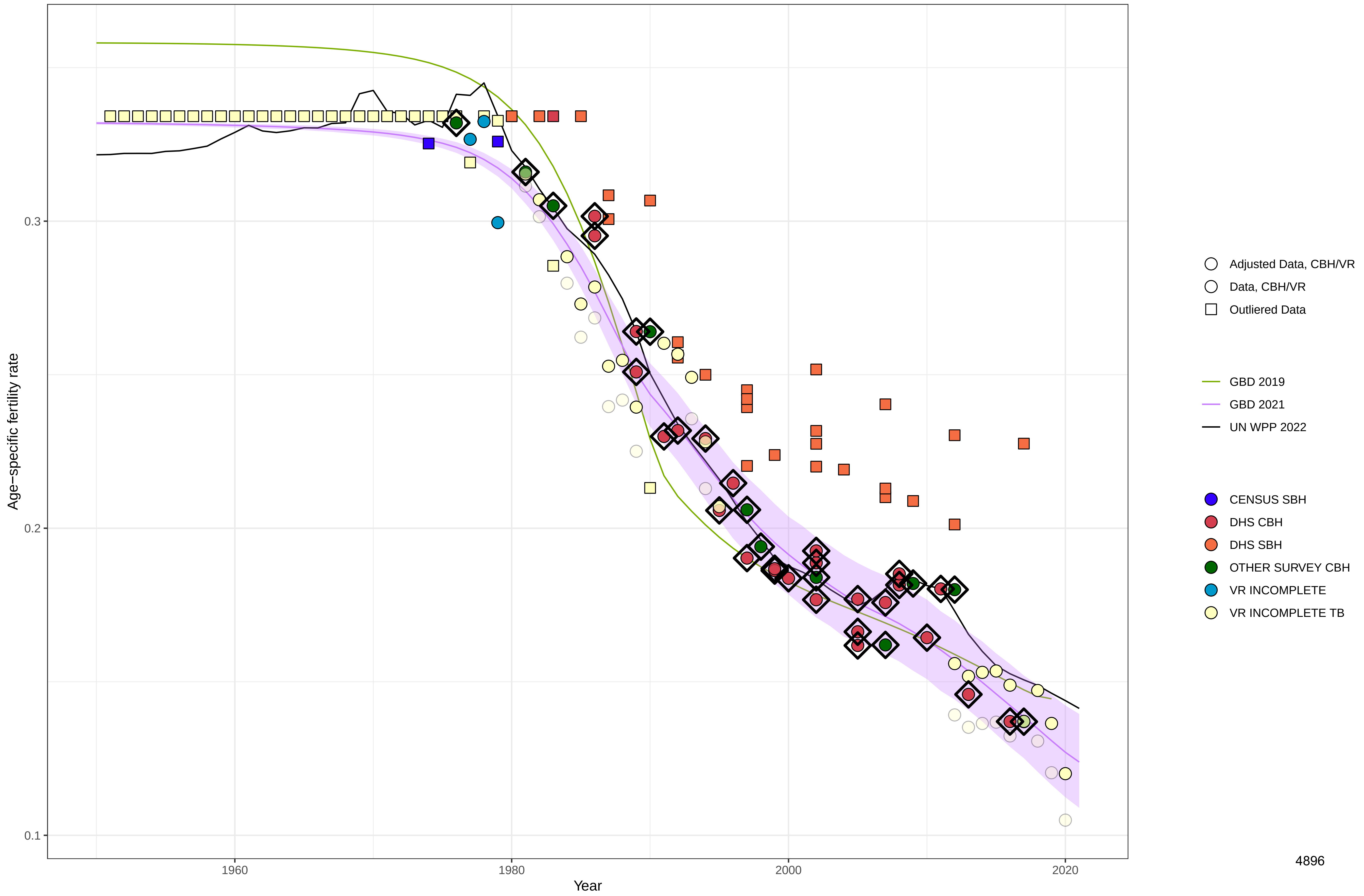

Age−specific fertility fitted trend lines, 1950−2021: Jordan, Ages: 35−39
Data points enclosed with a diamond are reference sources

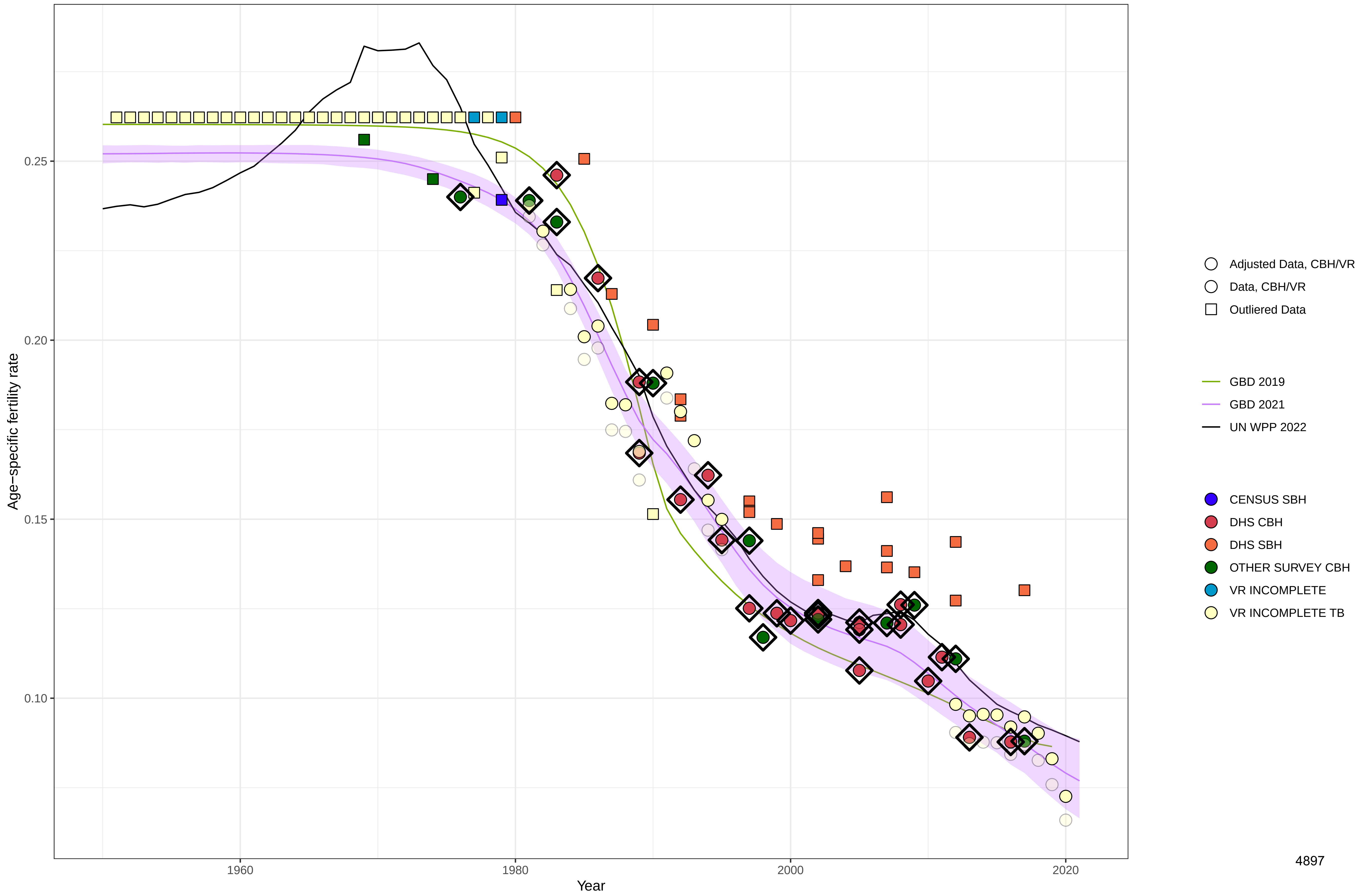

Age−specific fertility fitted trend lines, 1950−2021: Jordan, Ages: 40−44
Data points enclosed with a diamond are reference sources

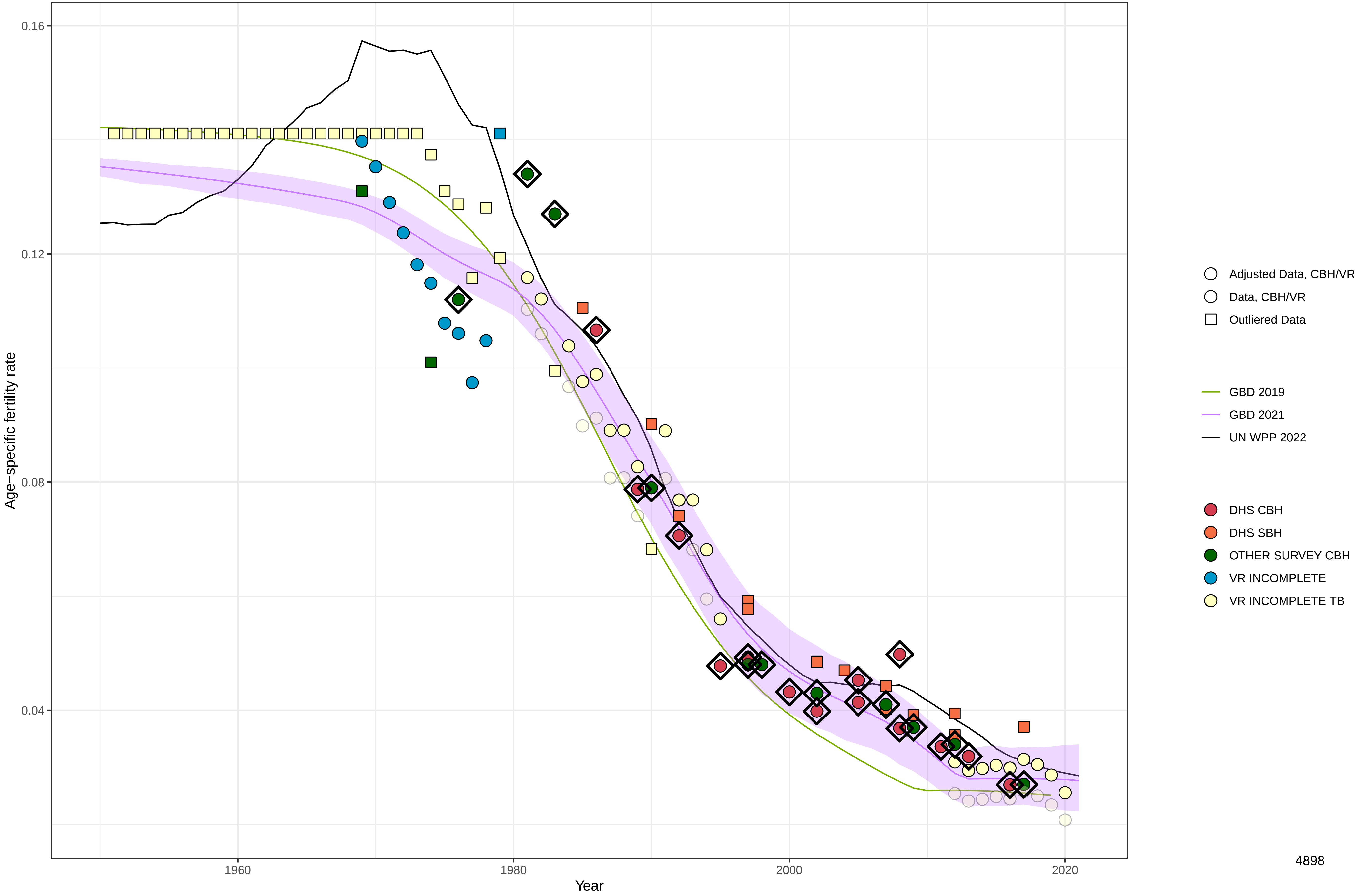

Age−specific fertility fitted trend lines, 1950−2021: Jordan, Ages: 45−49
Data points enclosed with a diamond are reference sources

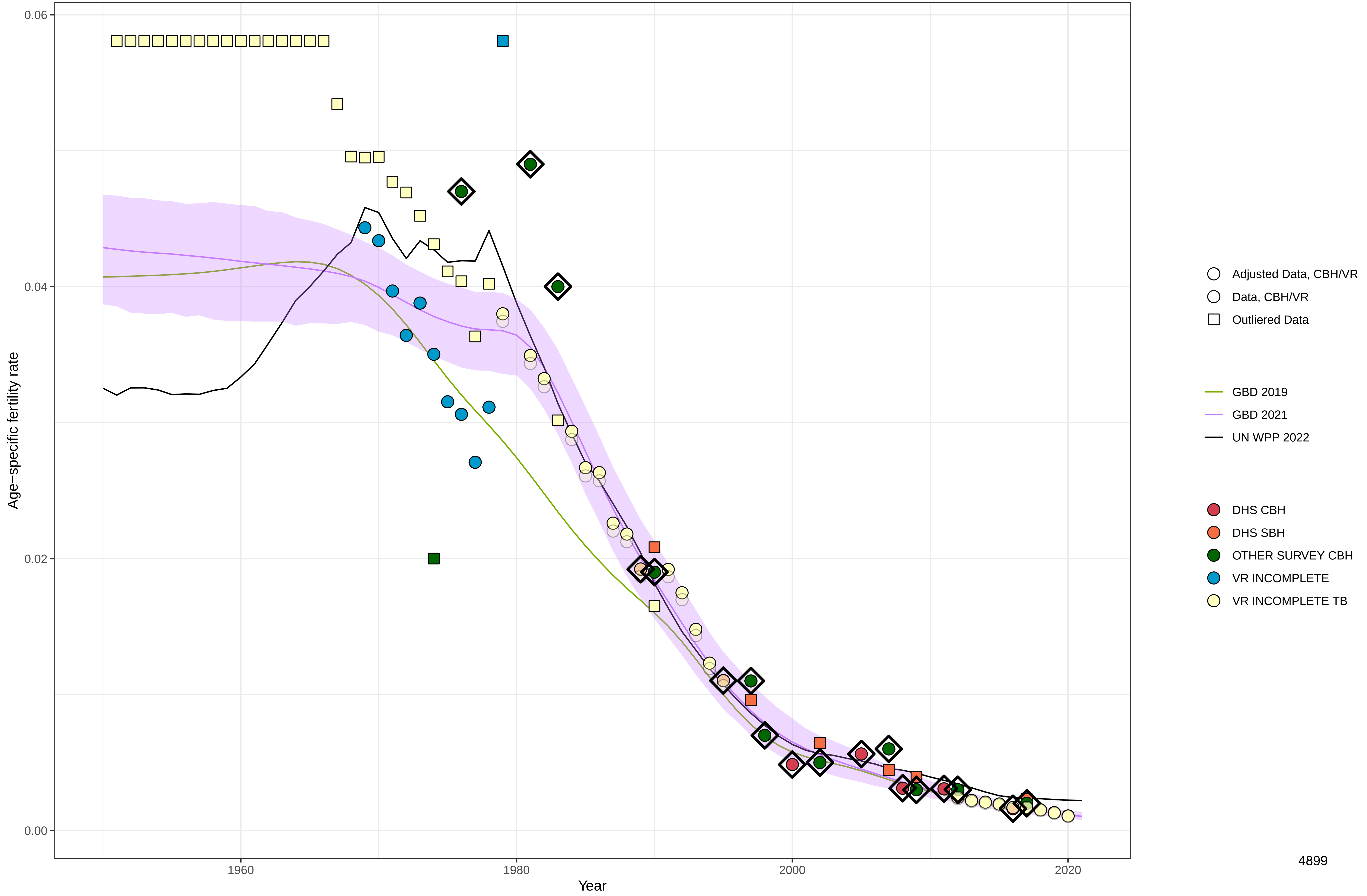

Age−specific fertility fitted trend lines, 1950−2021: Jordan, Ages: 50−54
Data points enclosed with a diamond are reference sources

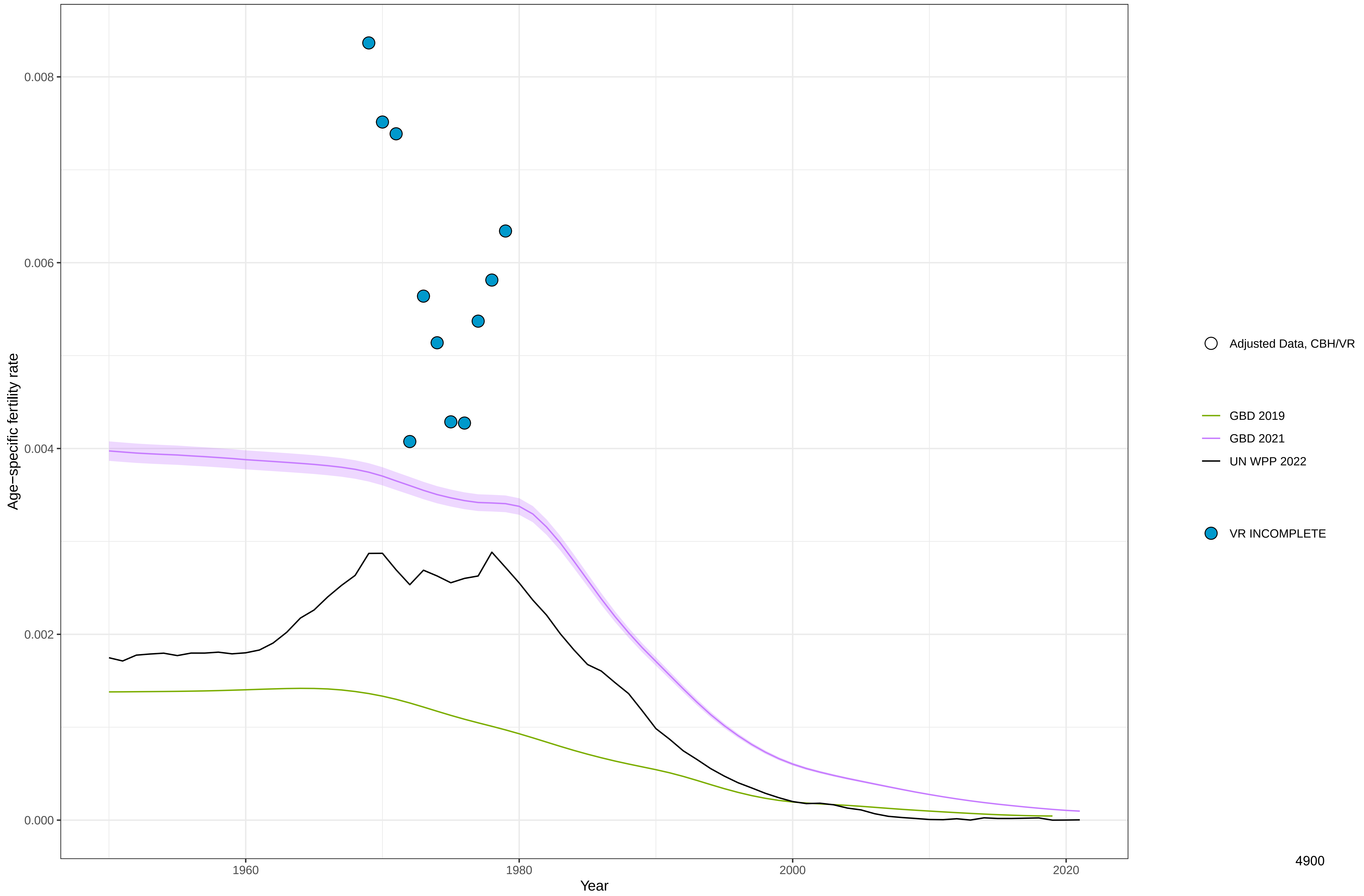

Total fertility rate fitted trend lines, 1950−2021: Jordan TFR
Data points enclosed with a diamond are reference sources

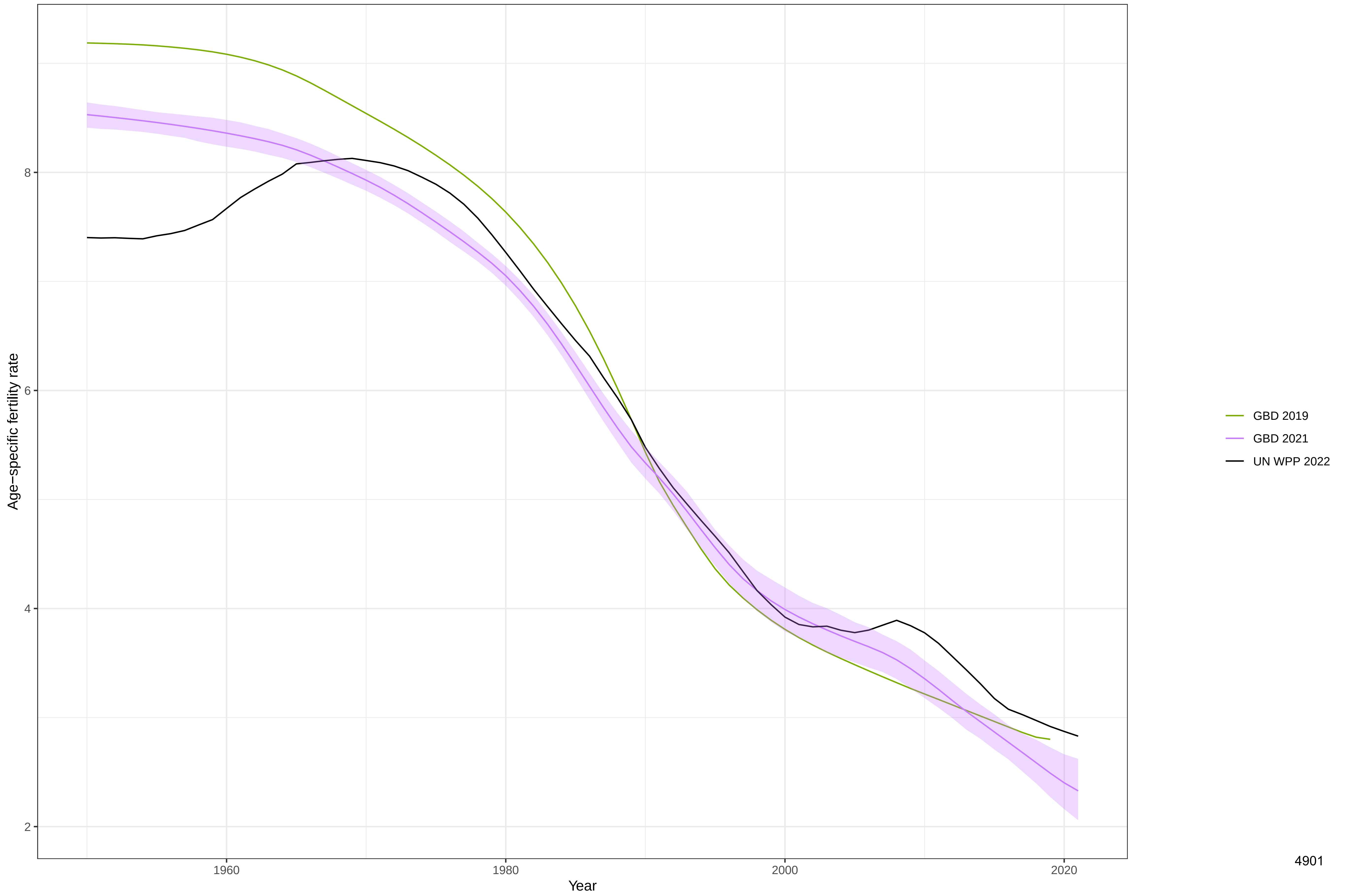

Age−specific fertility fitted trend lines, 1950−2021: Kuwait, Ages: 10−14
Data points enclosed with a diamond are reference sources

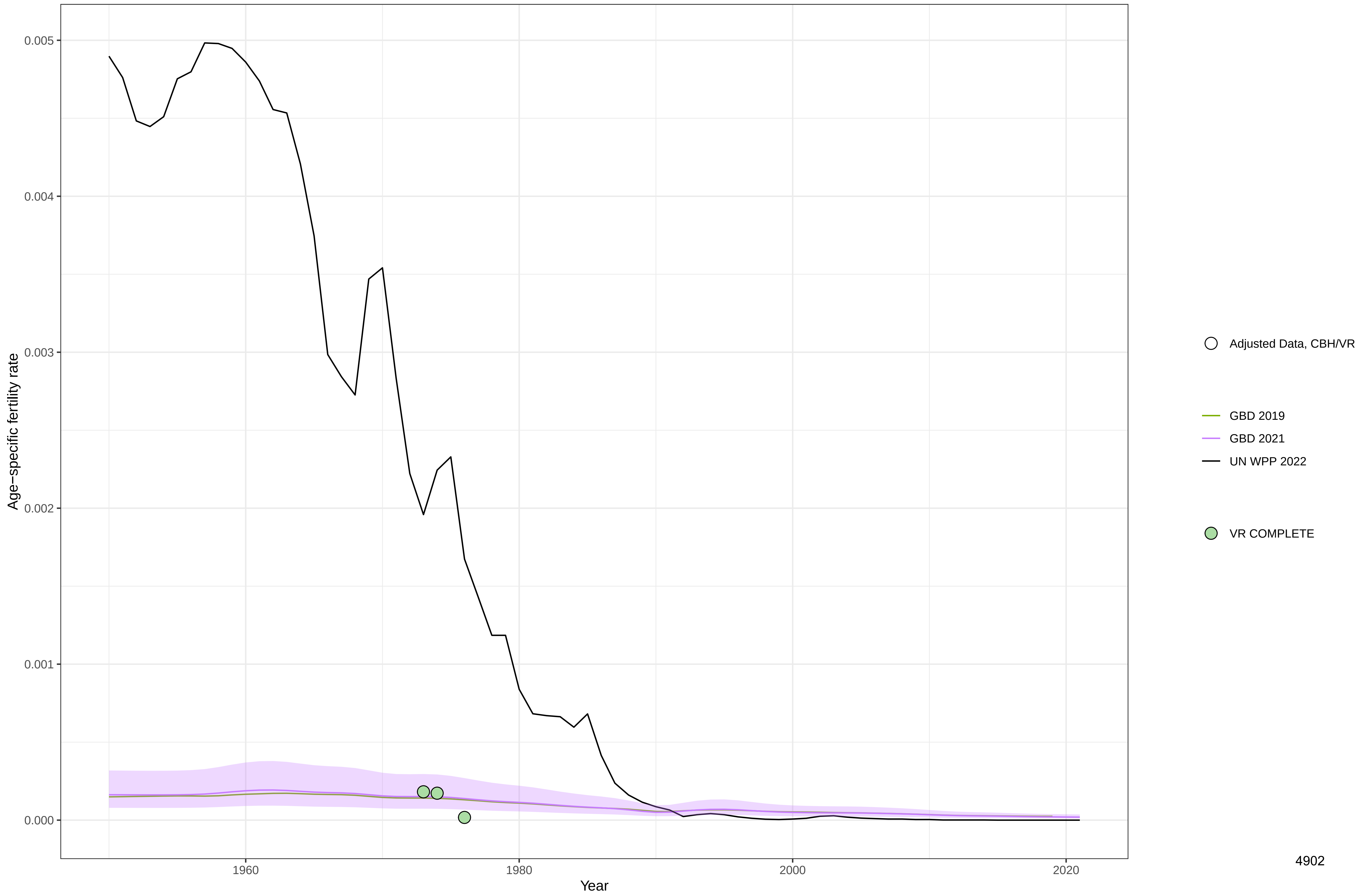

Age−specific fertility fitted trend lines, 1950−2021: Kuwait, Ages: 15−19
Data points enclosed with a diamond are reference sources

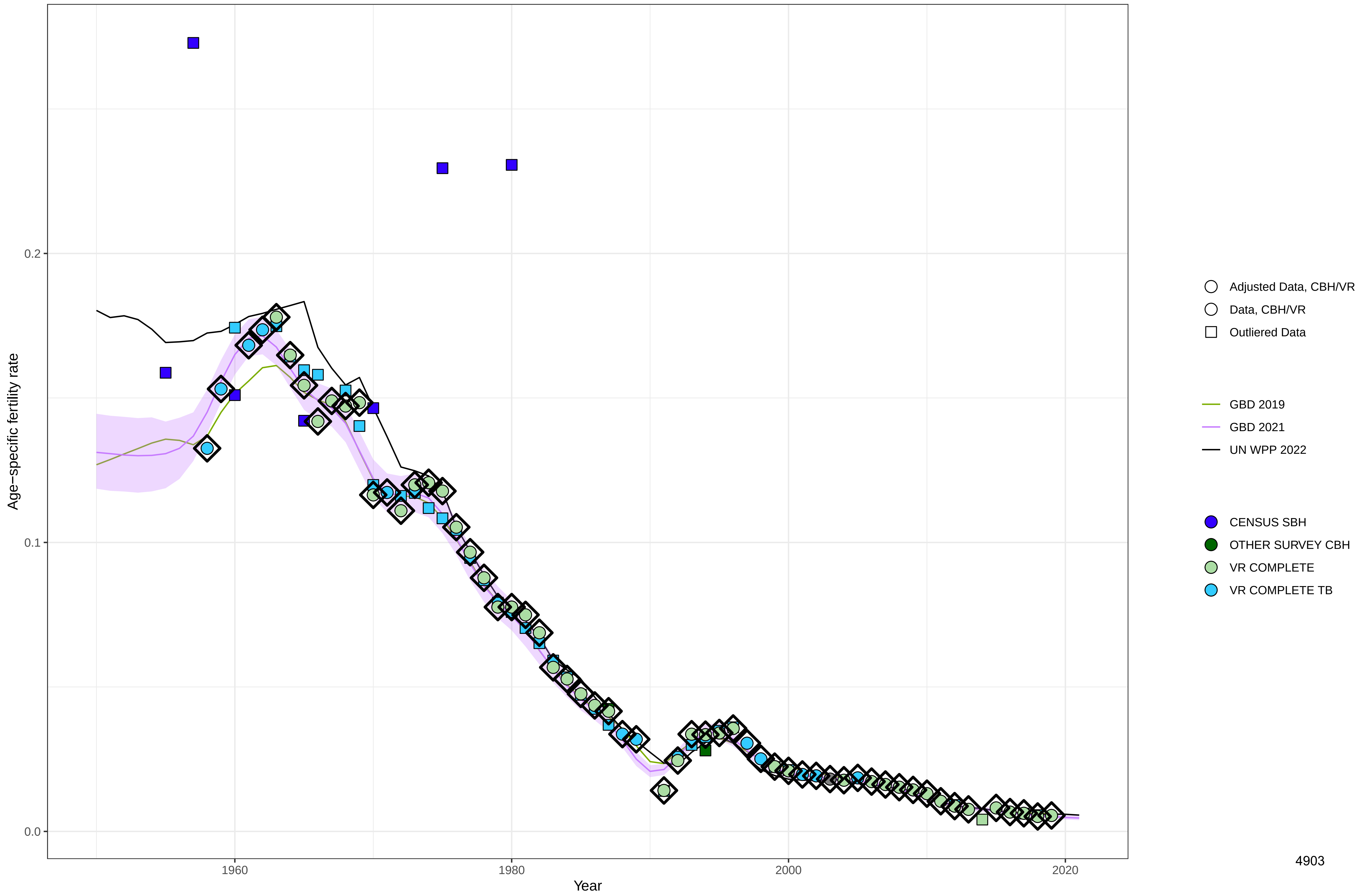

Age−specific fertility fitted trend lines, 1950−2021: Kuwait, Ages: 20−24
Data points enclosed with a diamond are reference sources

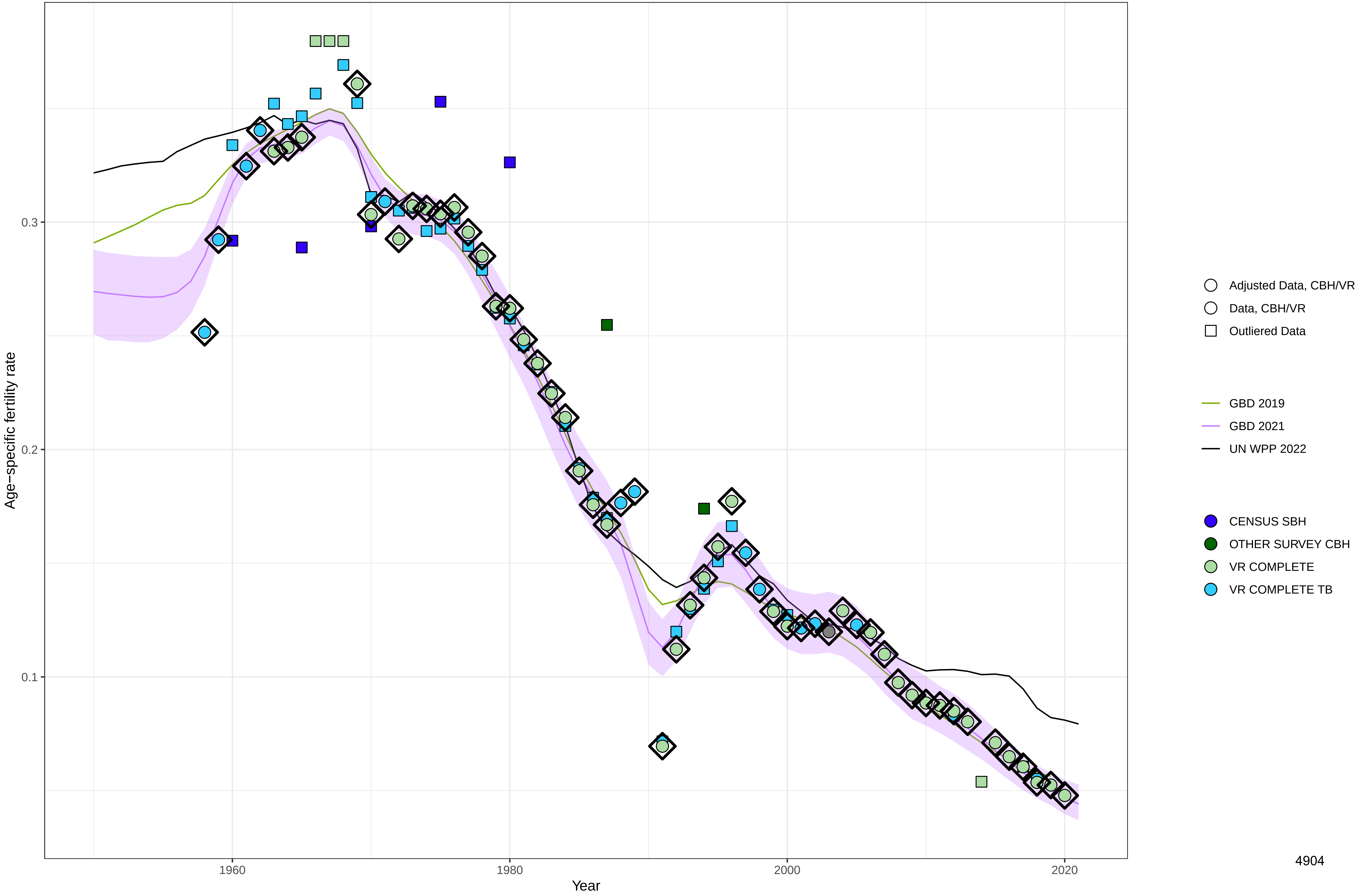

Age−specific fertility fitted trend lines, 1950−2021: Kuwait, Ages: 25−29
Data points enclosed with a diamond are reference sources

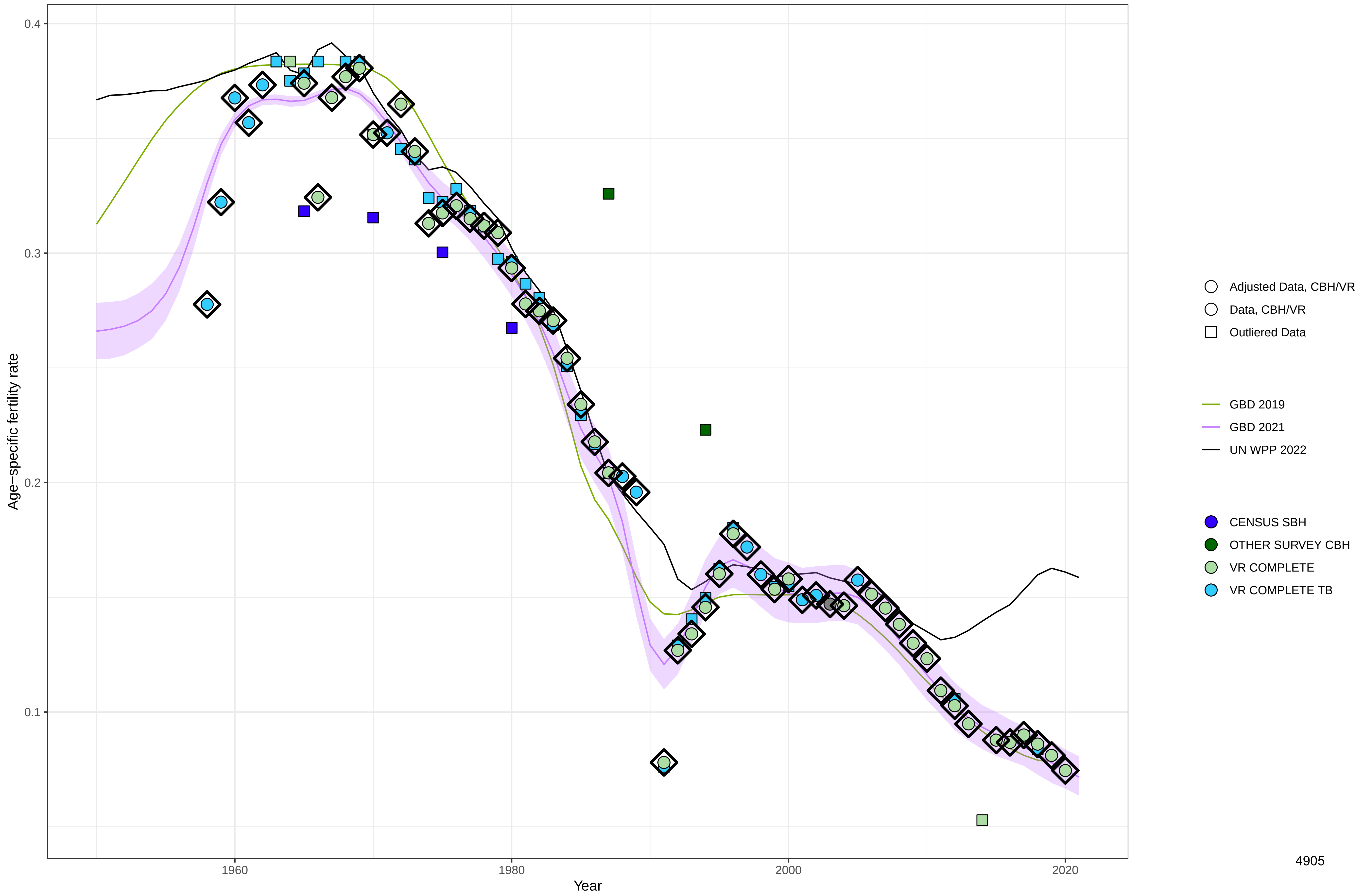

Age−specific fertility fitted trend lines, 1950−2021: Kuwait, Ages: 30−34
Data points enclosed with a diamond are reference sources

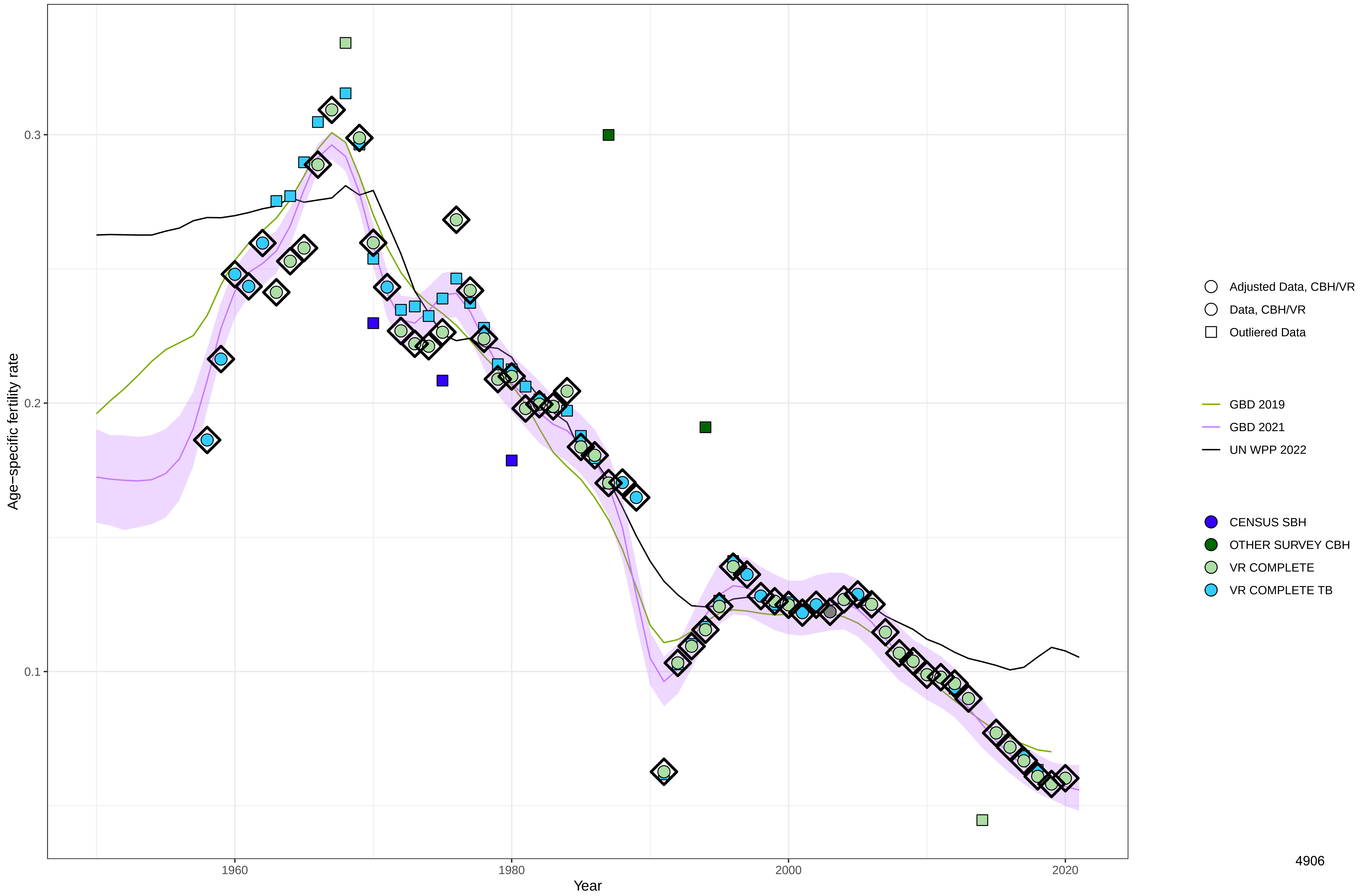

Age−specific fertility fitted trend lines, 1950−2021: Kuwait, Ages: 35−39
Data points enclosed with a diamond are reference sources

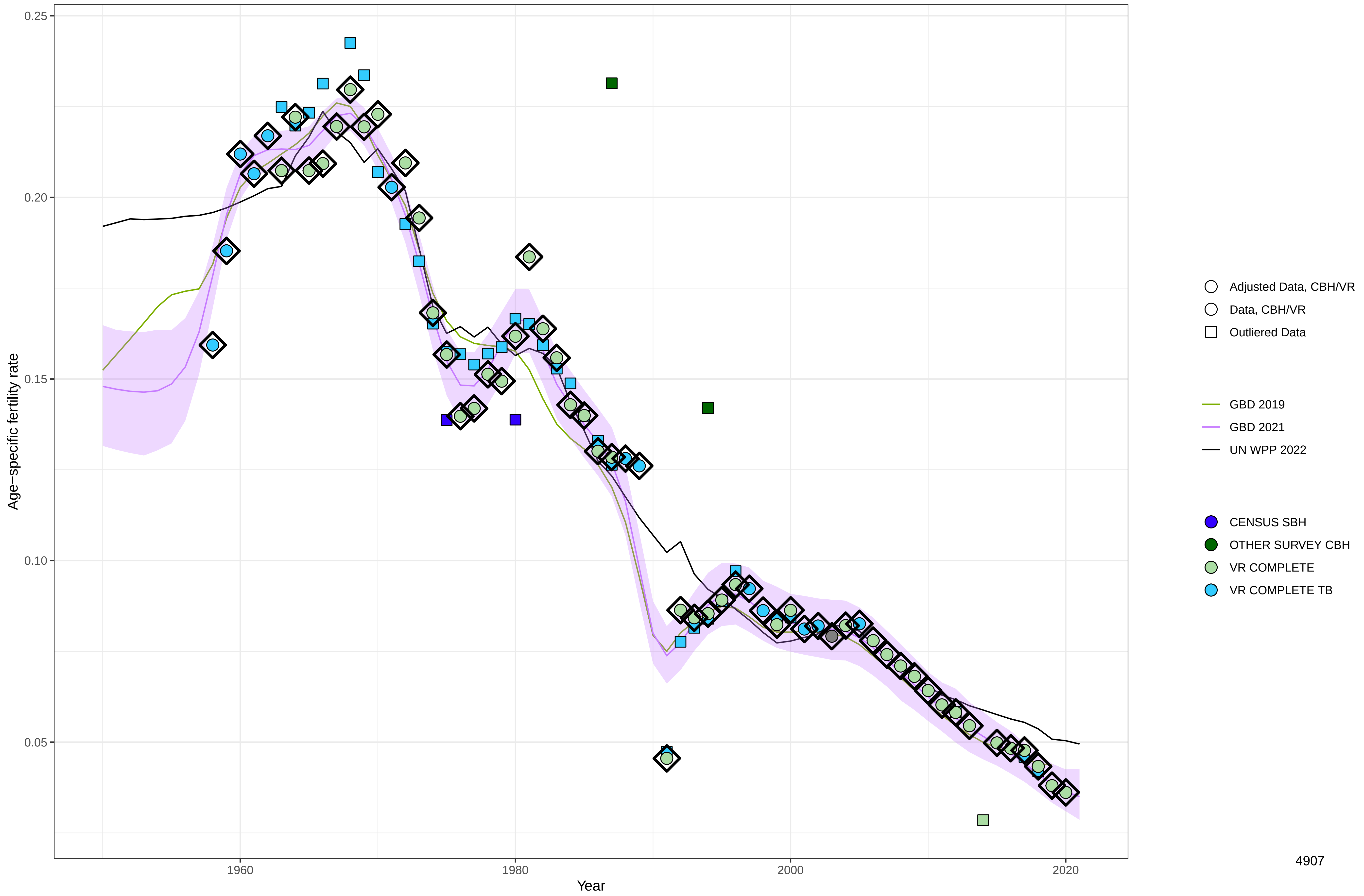

Age−specific fertility fitted trend lines, 1950−2021: Kuwait, Ages: 40−44
Data points enclosed with a diamond are reference sources

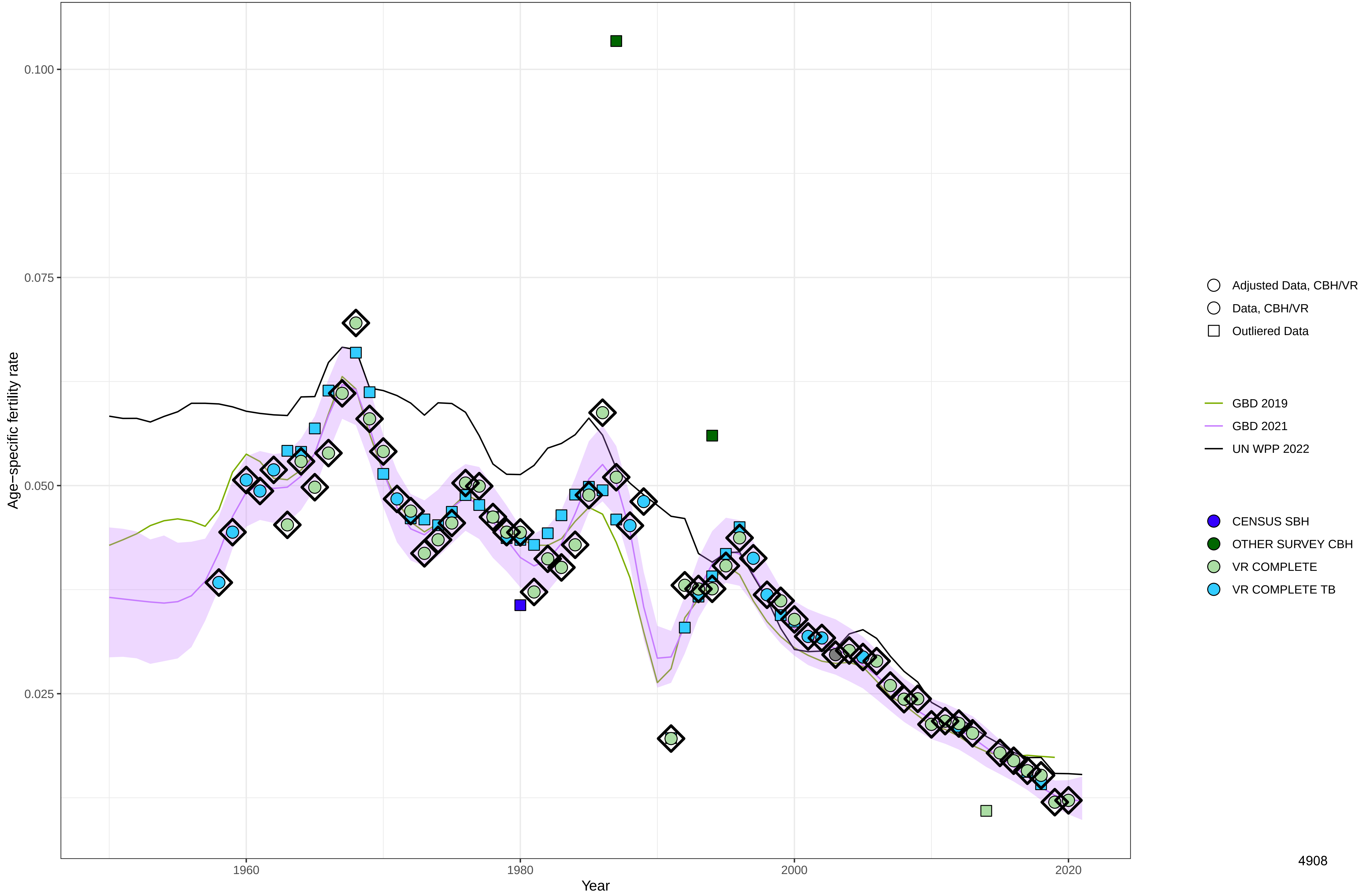

Age−specific fertility fitted trend lines, 1950−2021: Kuwait, Ages: 45−49
Data points enclosed with a diamond are reference sources

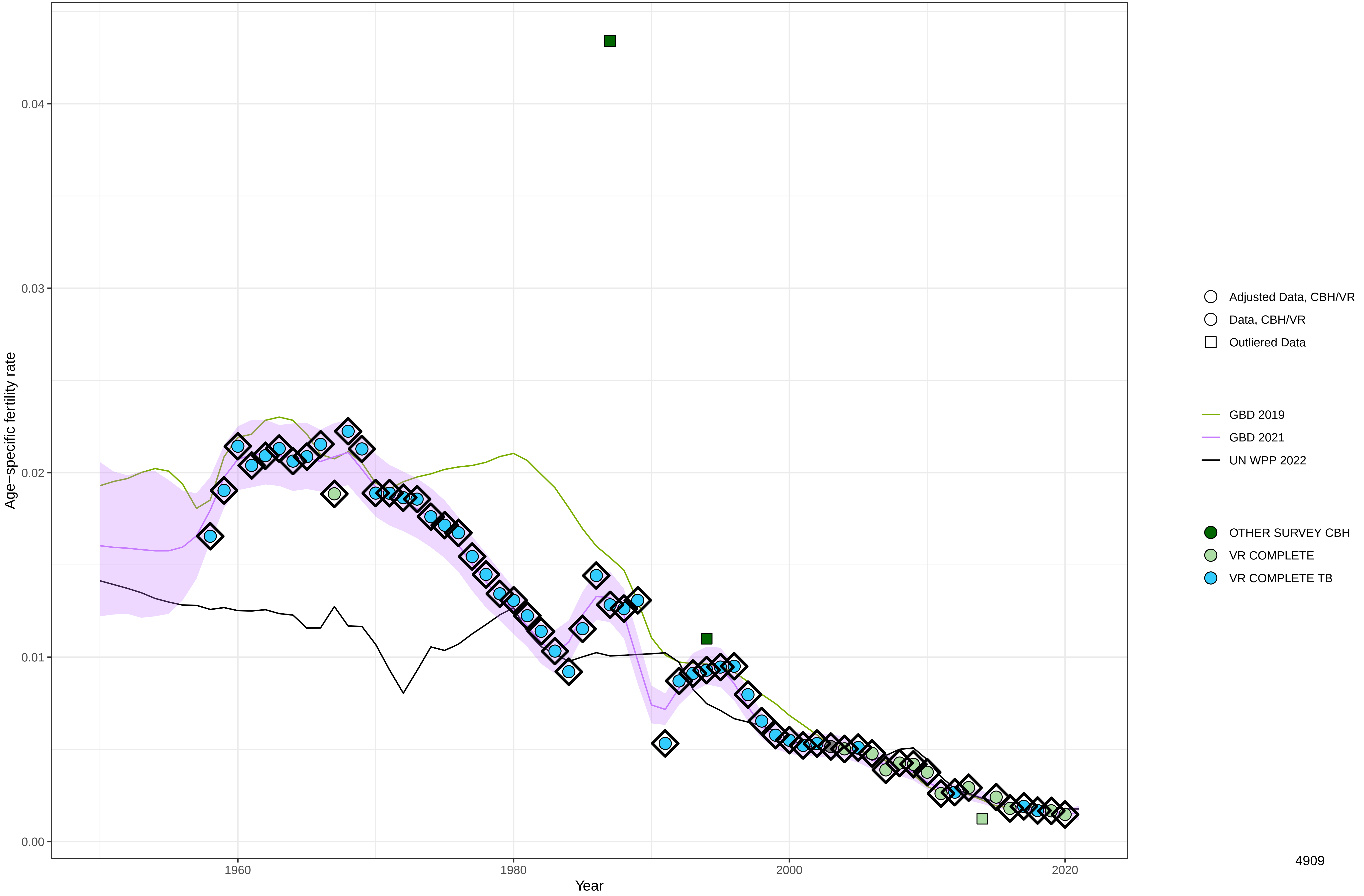

Age−specific fertility fitted trend lines, 1950−2021: Kuwait, Ages: 50−54
Data points enclosed with a diamond are reference sources

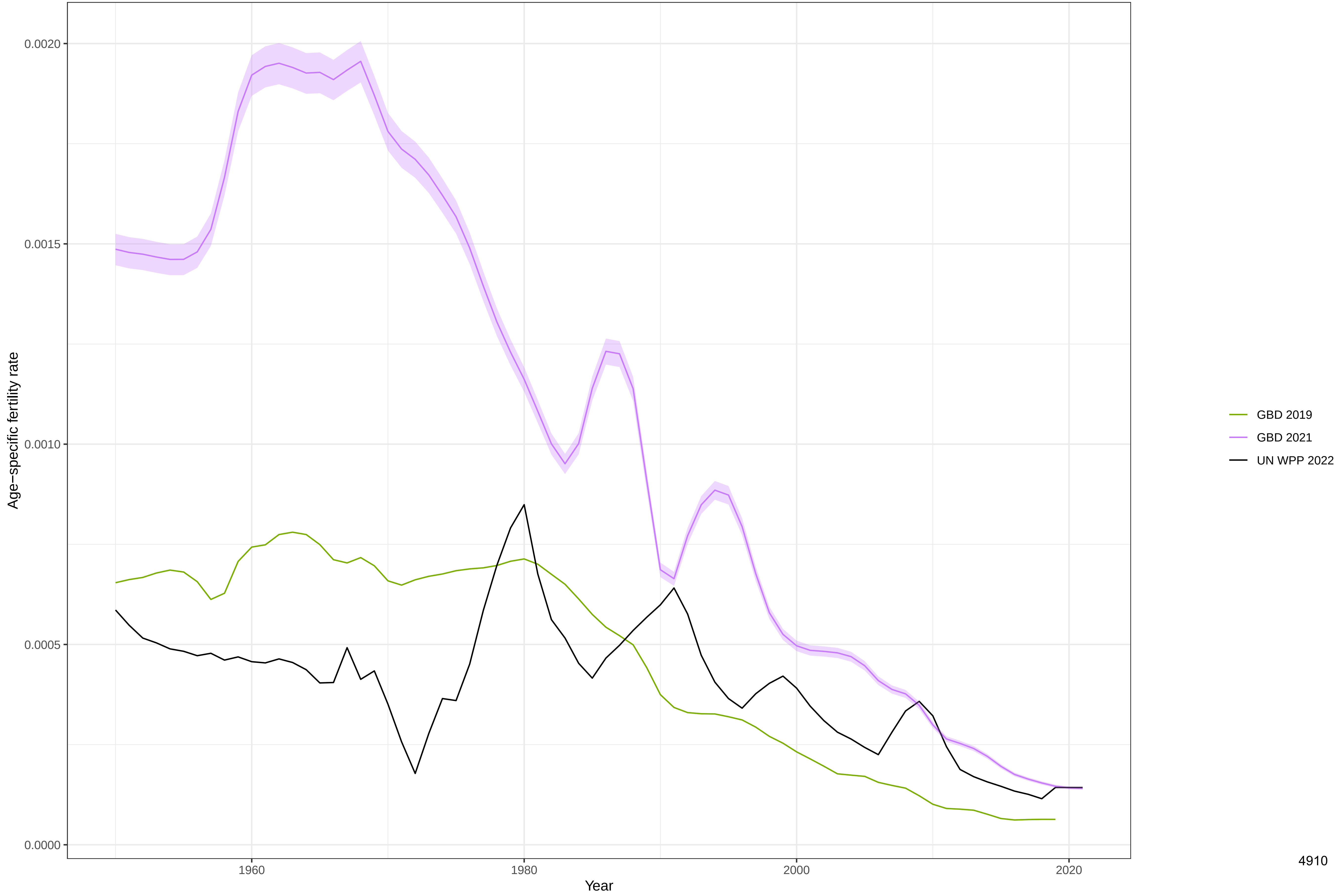

Total fertility rate fitted trend lines, 1950−2021: Kuwait TFR
Data points enclosed with a diamond are reference sources

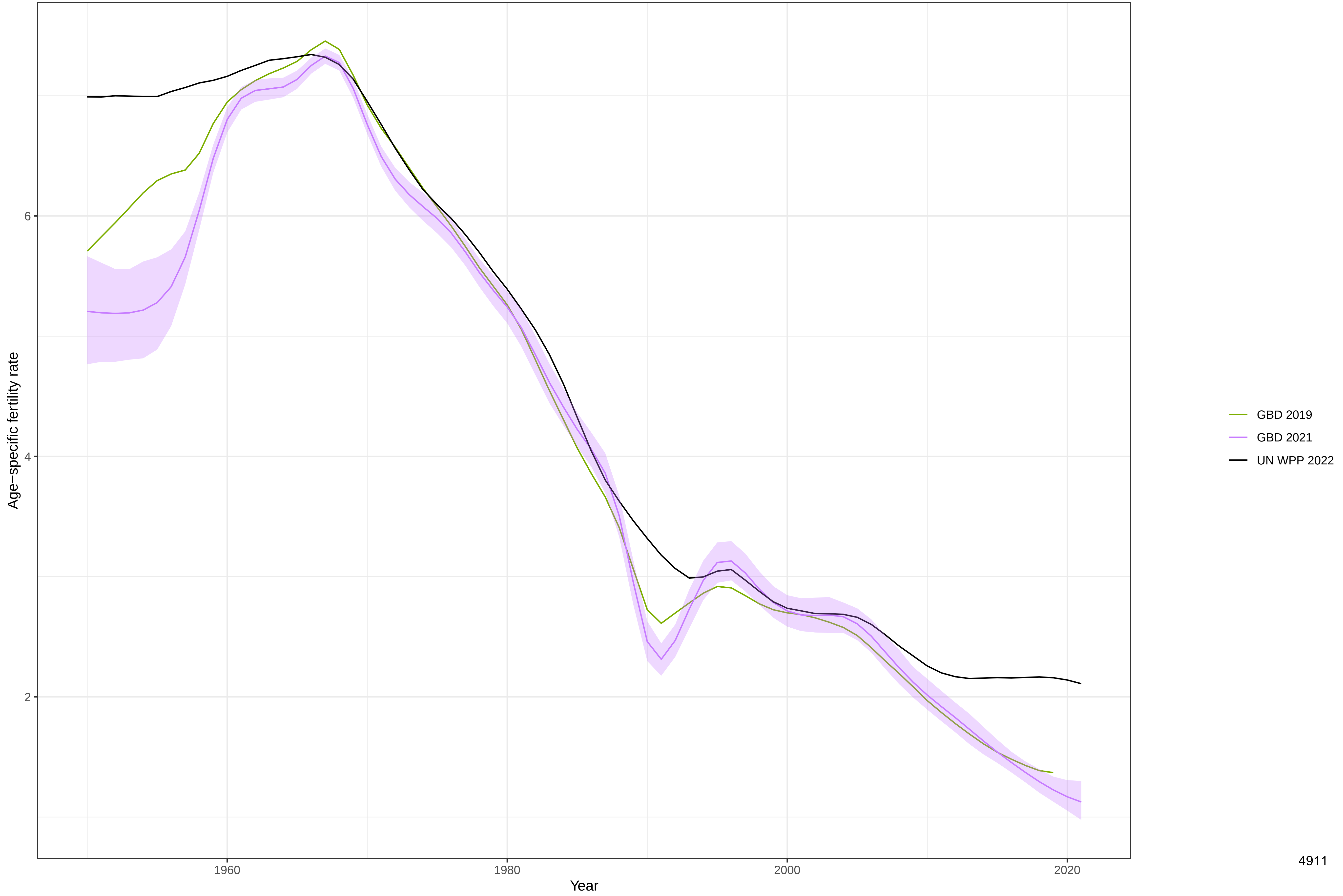

Age−specific fertility fitted trend lines, 1950−2021: Lebanon, Ages: 10−14
Data points enclosed with a diamond are reference sources

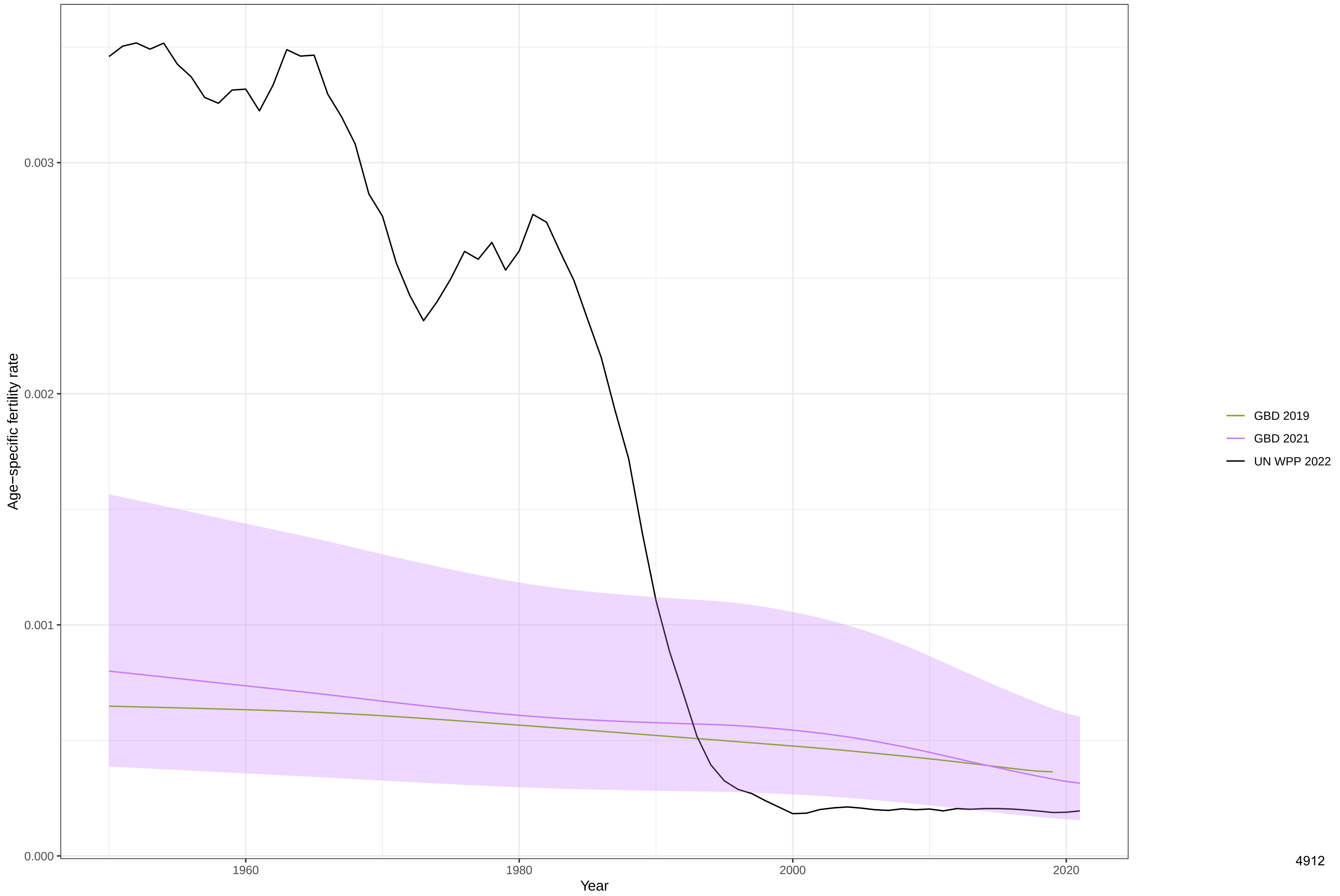

Age−specific fertility fitted trend lines, 1950−2021: Lebanon, Ages: 15−19
Data points enclosed with a diamond are reference sources

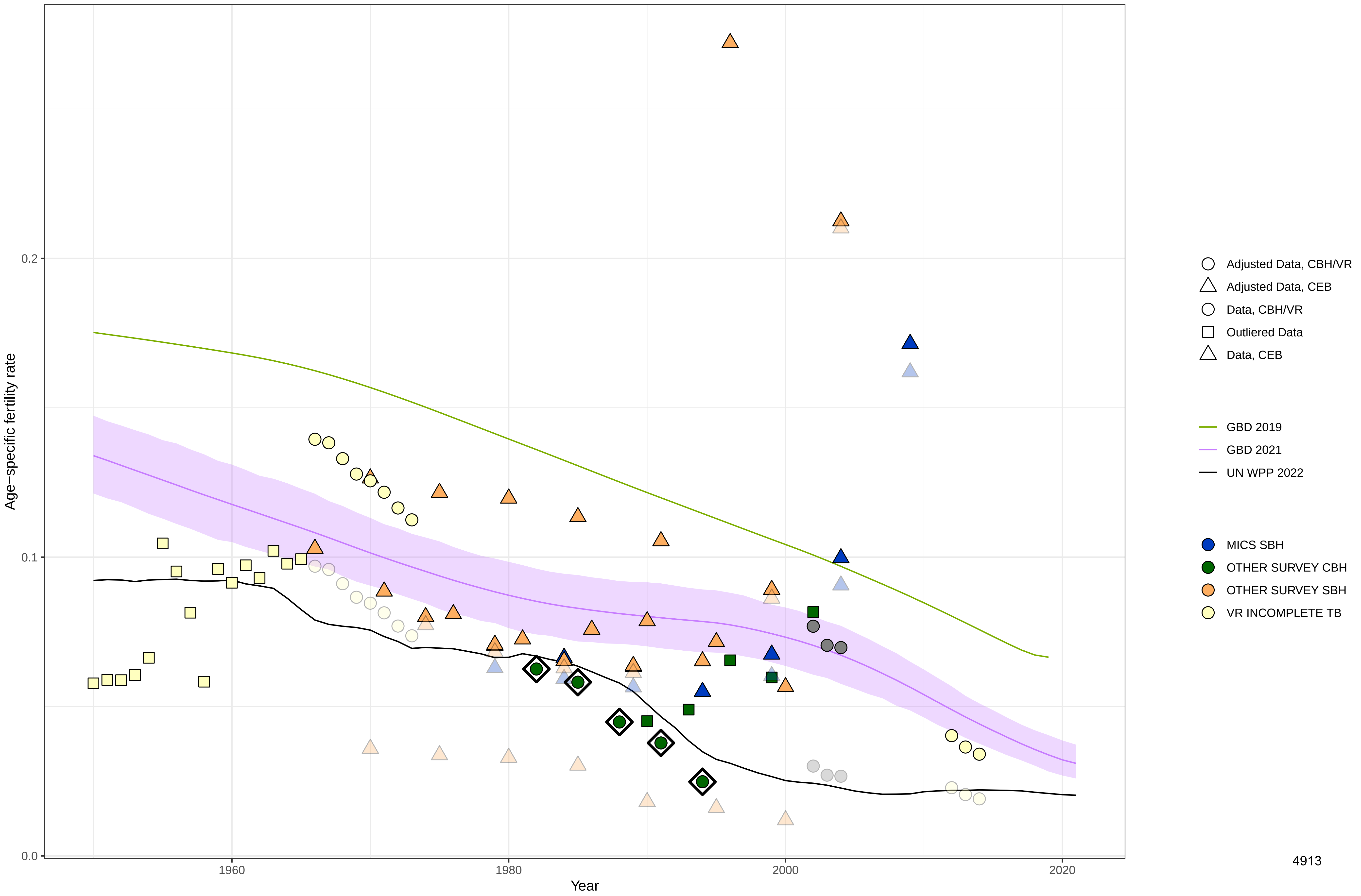

Age−specific fertility fitted trend lines, 1950−2021: Lebanon, Ages: 20−24
Data points enclosed with a diamond are reference sources

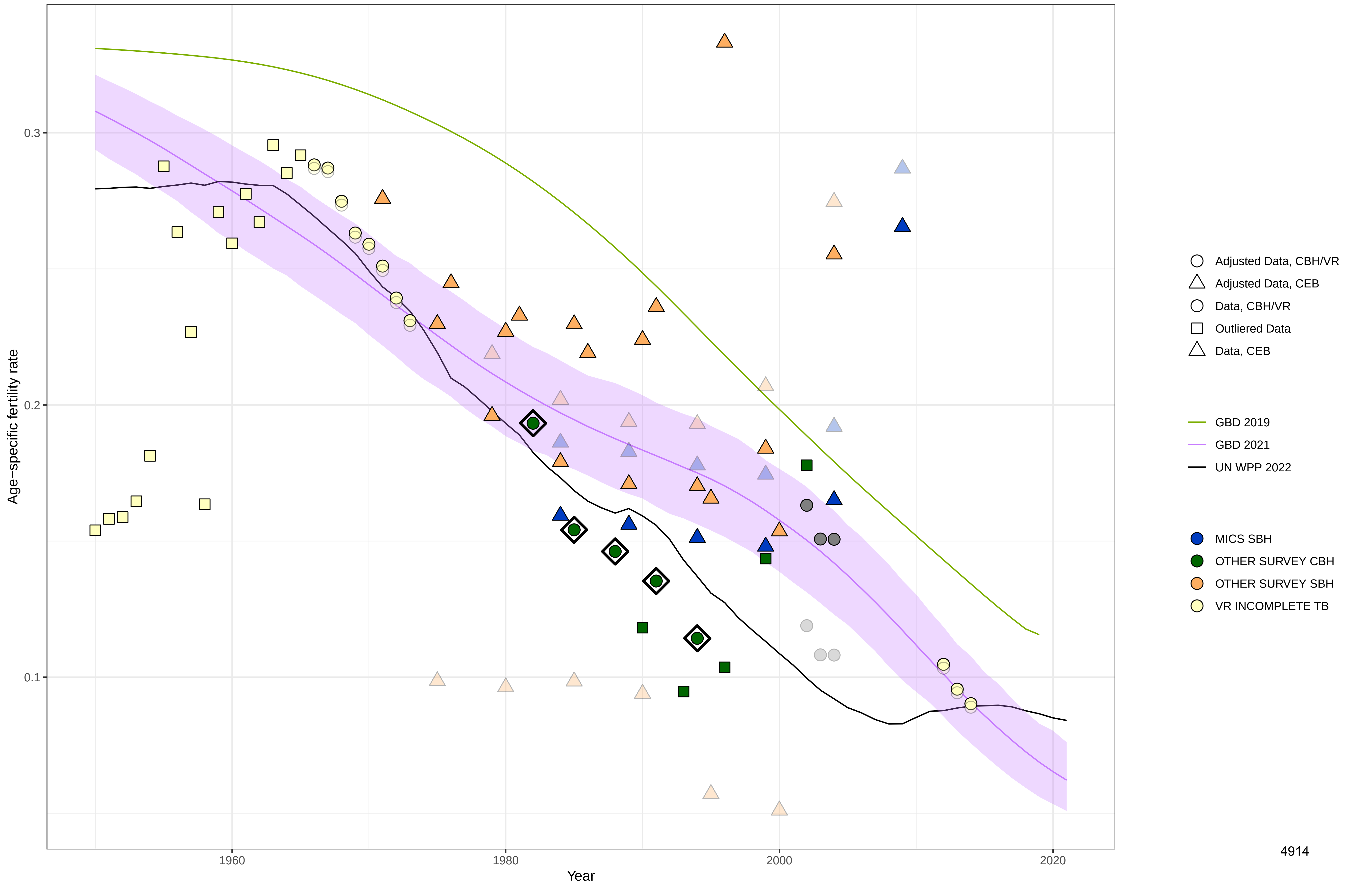

Age−specific fertility fitted trend lines, 1950−2021: Lebanon, Ages: 25−29
Data points enclosed with a diamond are reference sources

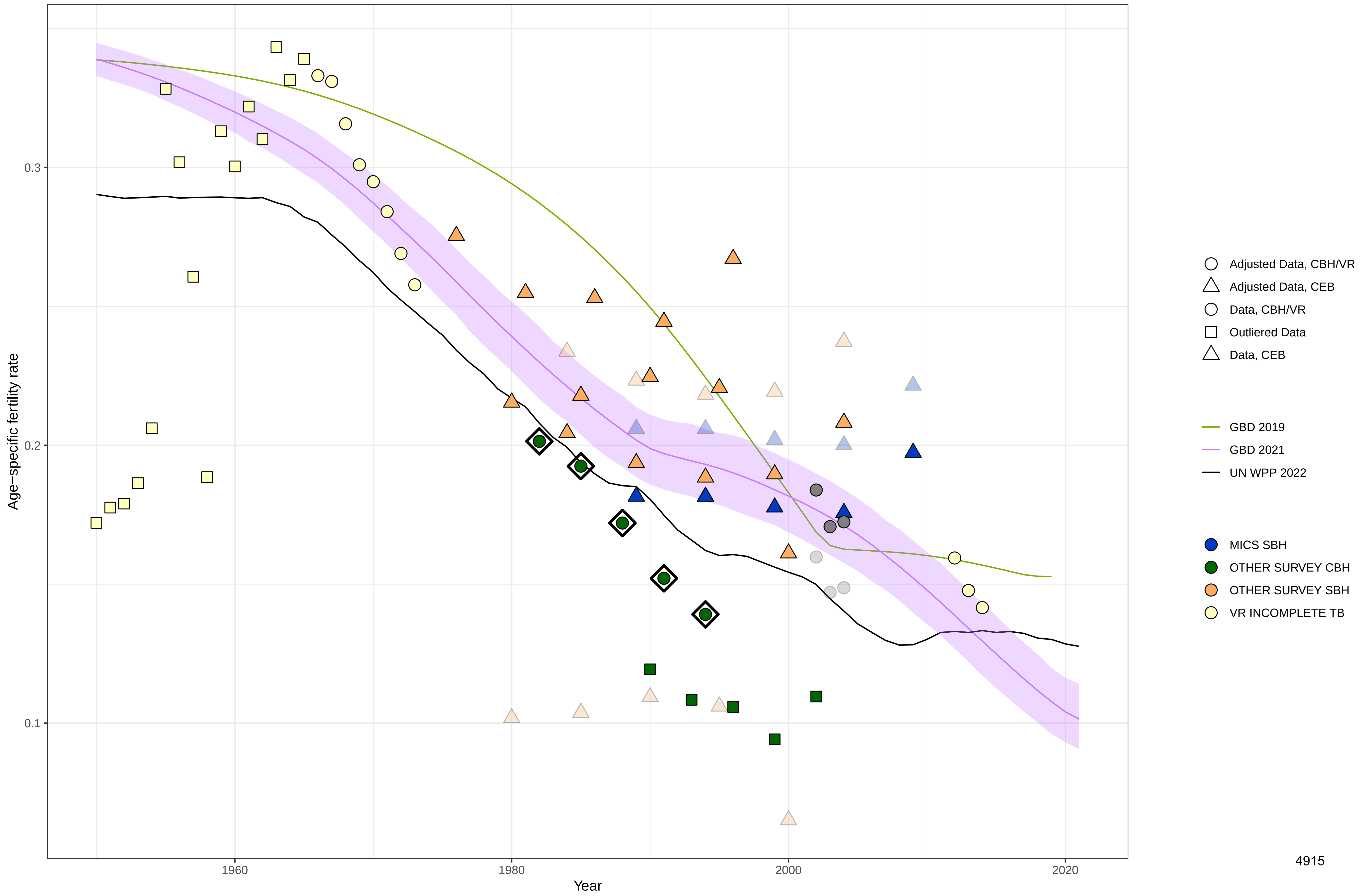

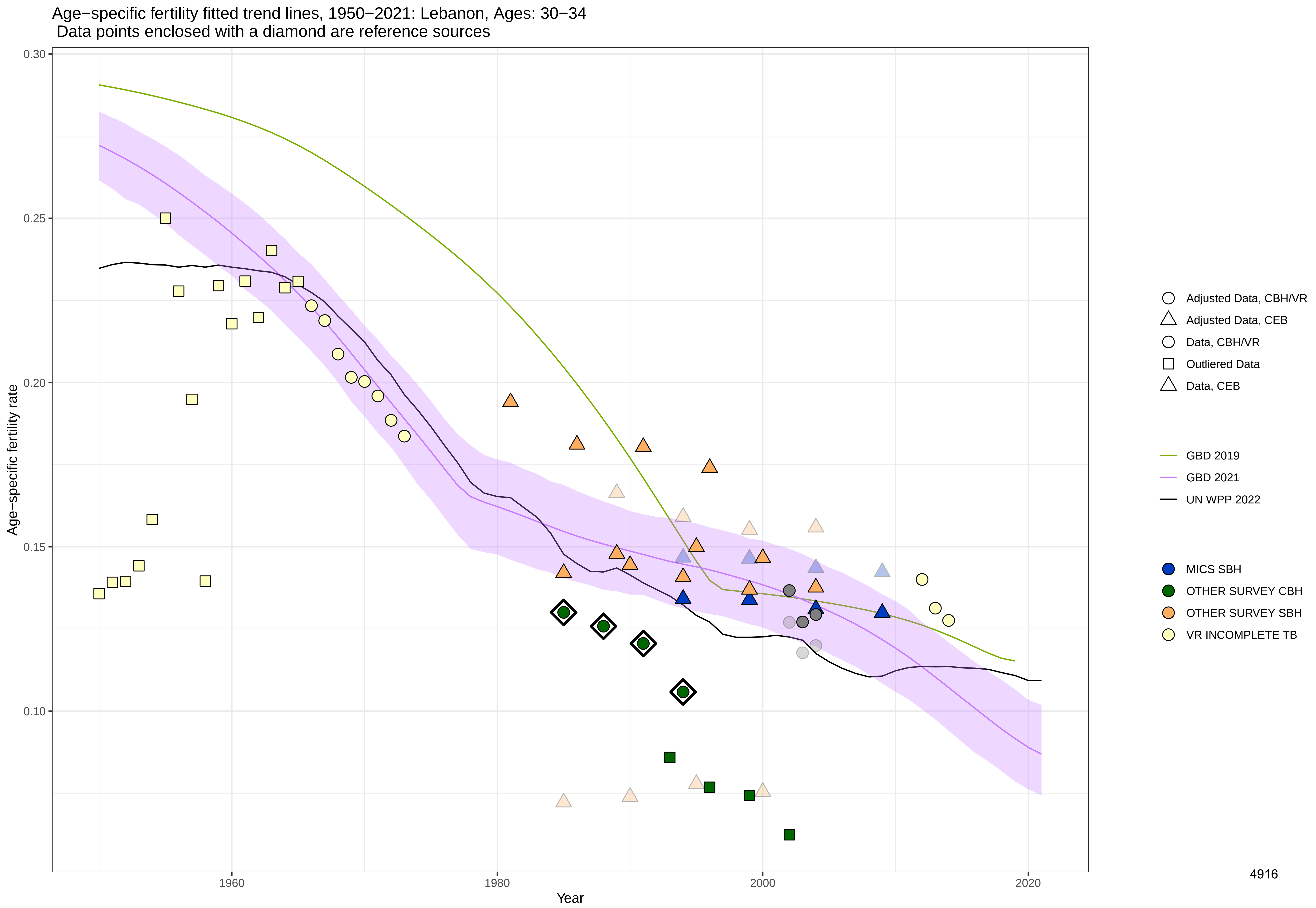

Age−specific fertility fitted trend lines, 1950−2021: Lebanon, Ages: 30−34
Data points enclosed with a diamond are reference sources
Age−specific fertility rate
Year
0.30
0.25
0.20
0.15
0.10
1960
1980
2000
2020
Adjusted Data, CBH/VR
Adjusted Data, CEB
Data, CBH/VR
Outliered Data
Data, CEB
GBD 2019
GBD 2021
UN WPP 2022
MICS SBH
OTHER SURVEY CBH
OTHER SURVEY SBH
VR INCOMPLETE TB

Age−specific fertility fitted trend lines, 1950−2021: Lebanon, Ages: 35−39
Data points enclosed with a diamond are reference sources

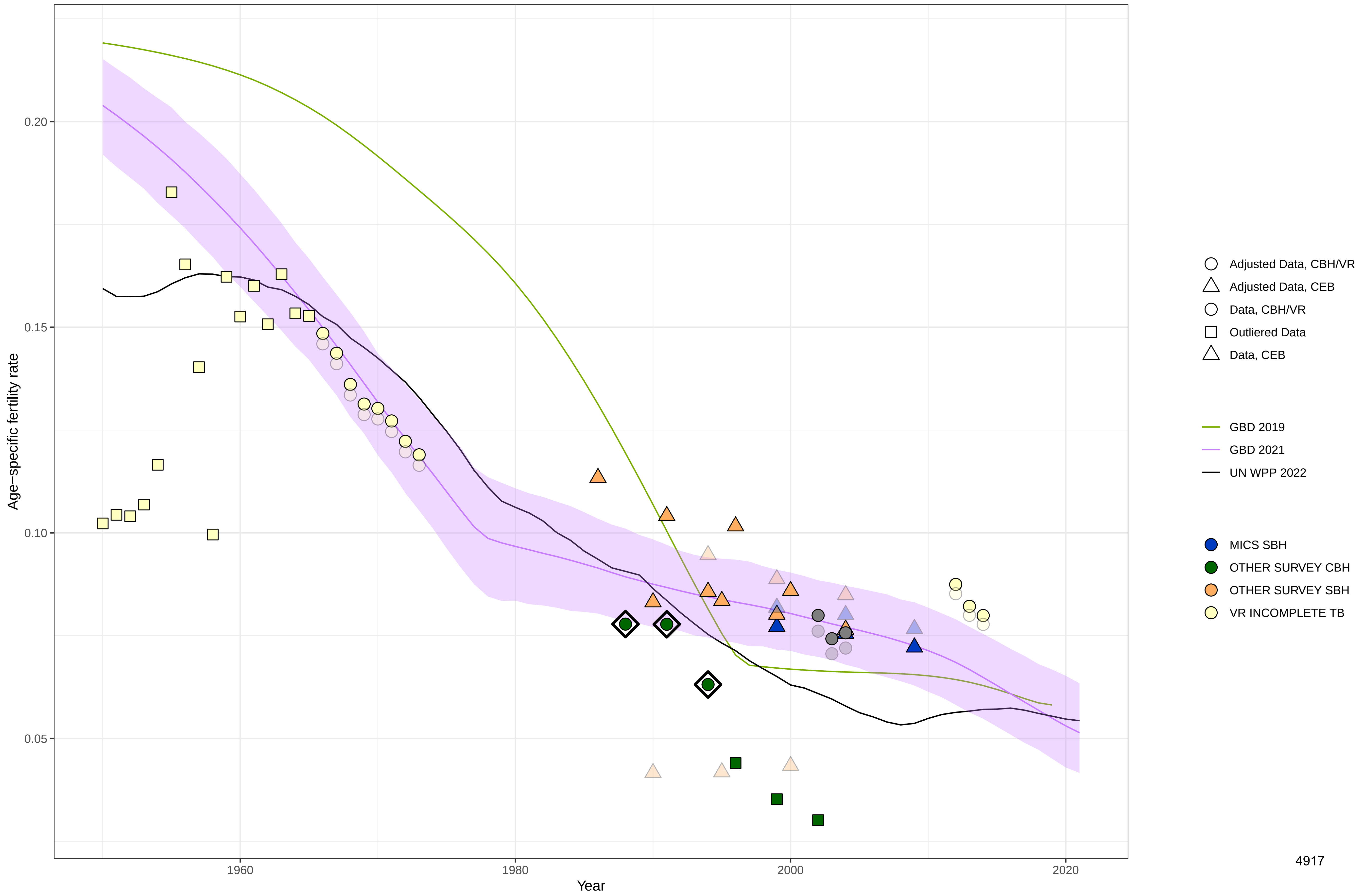

Age−specific fertility fitted trend lines, 1950−2021: Lebanon, Ages: 40−44
Data points enclosed with a diamond are reference sources

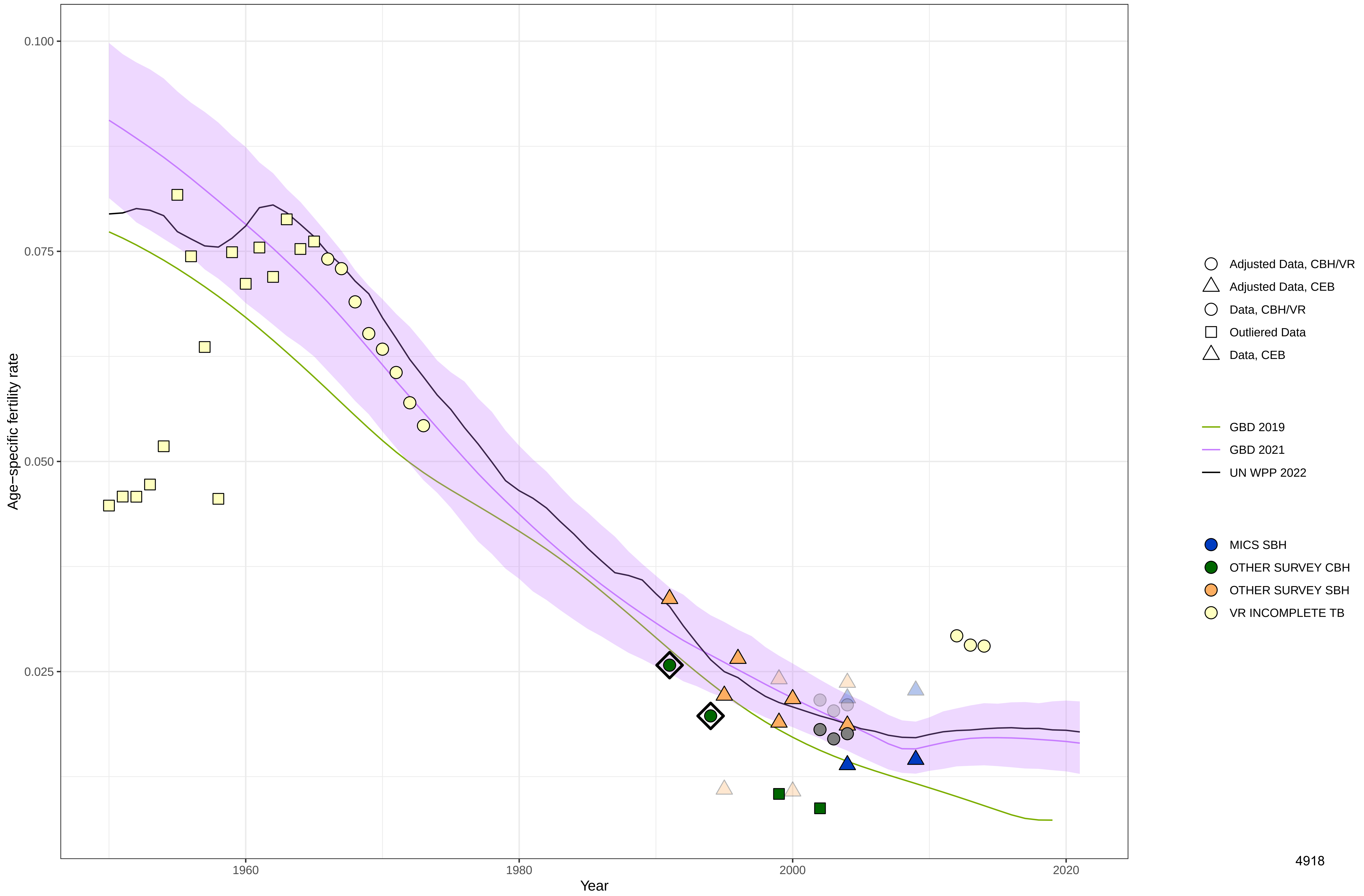

Age−specific fertility fitted trend lines, 1950−2021: Lebanon, Ages: 45−49
 Data points enclosed with a diamond are reference sources

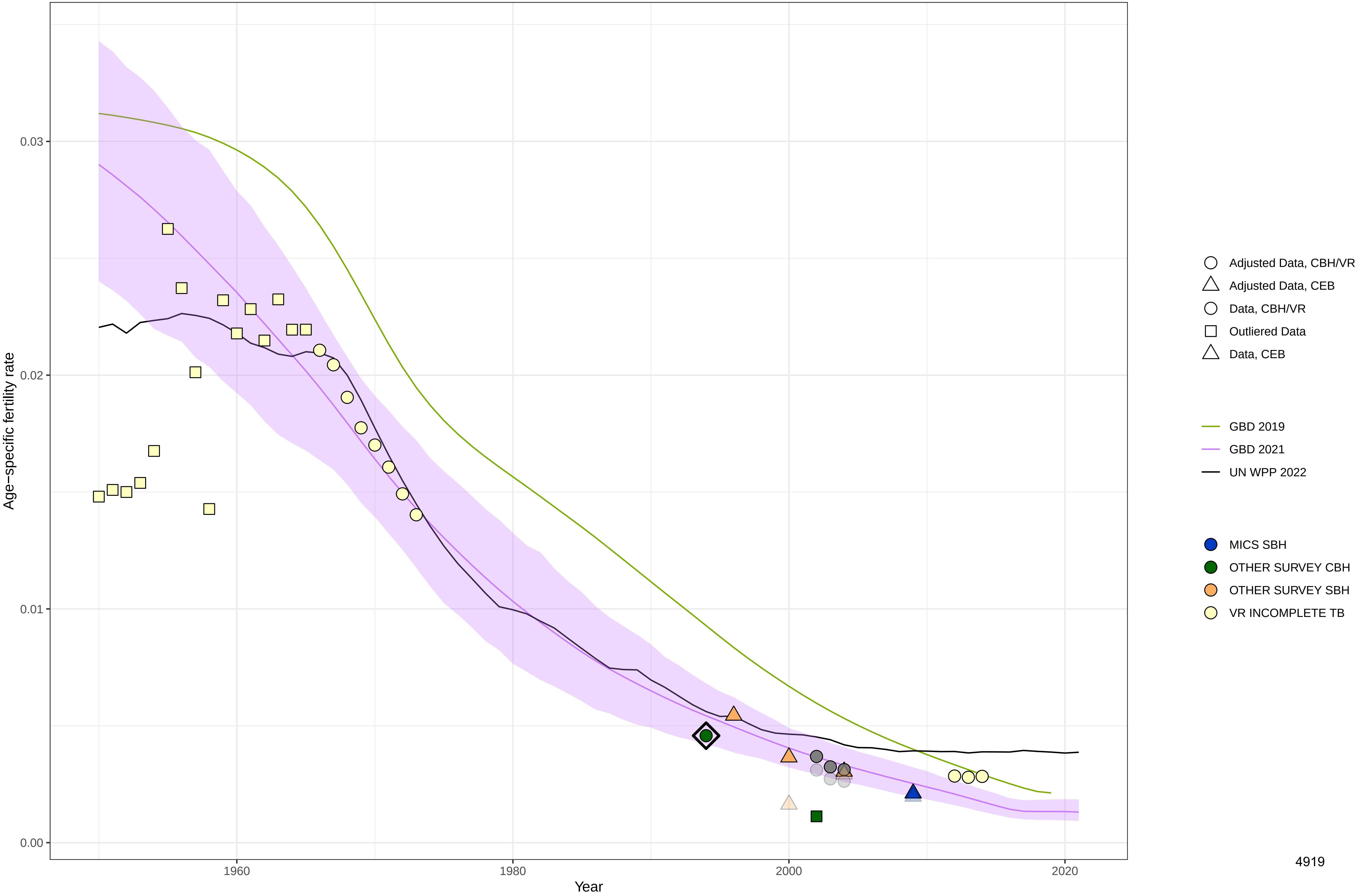

Age−specific fertility fitted trend lines, 1950−2021: Lebanon, Ages: 50−54
Data points enclosed with a diamond are reference sources

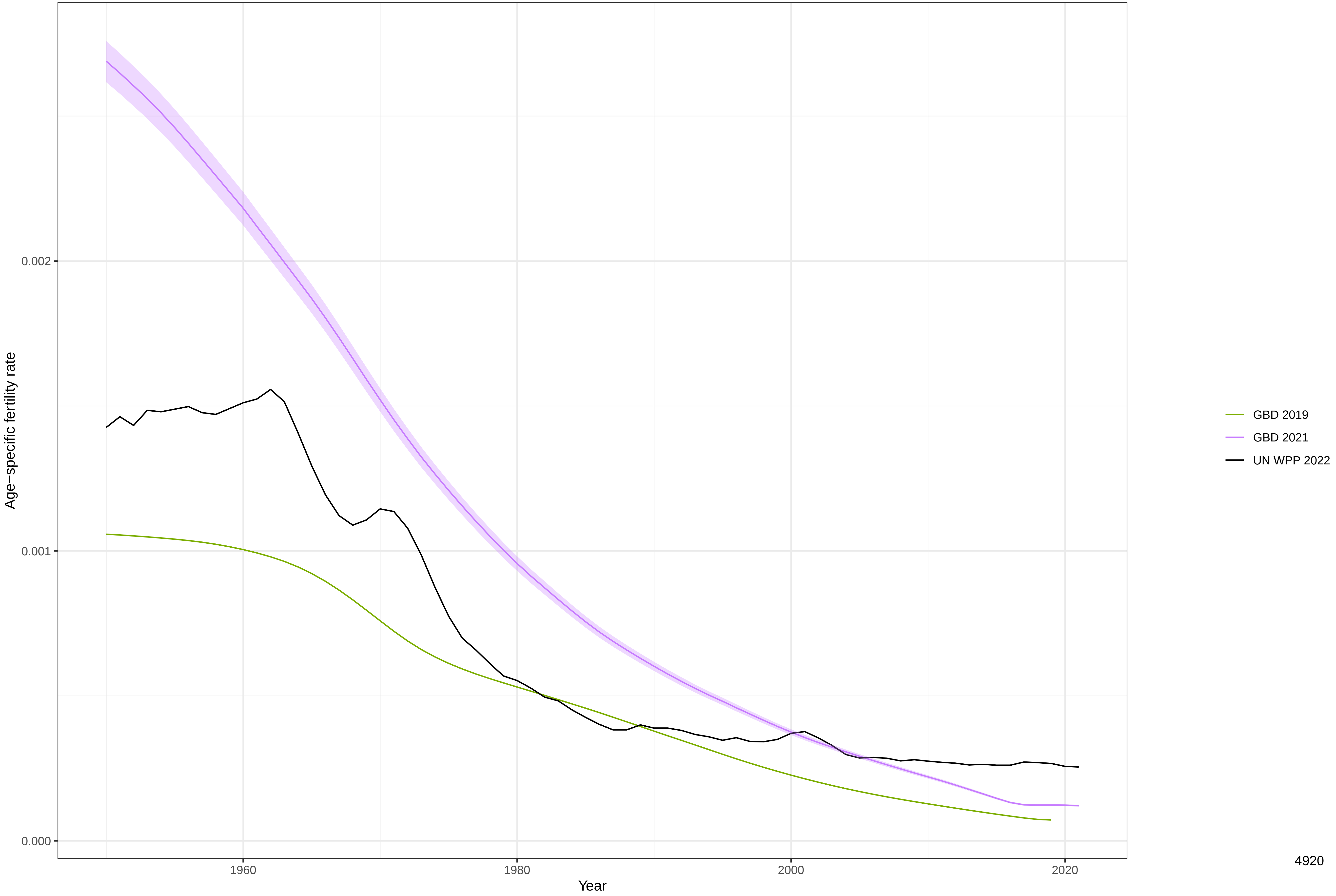

Total fertility rate fitted trend lines, 1950−2021: Lebanon TFR
Data points enclosed with a diamond are reference sources

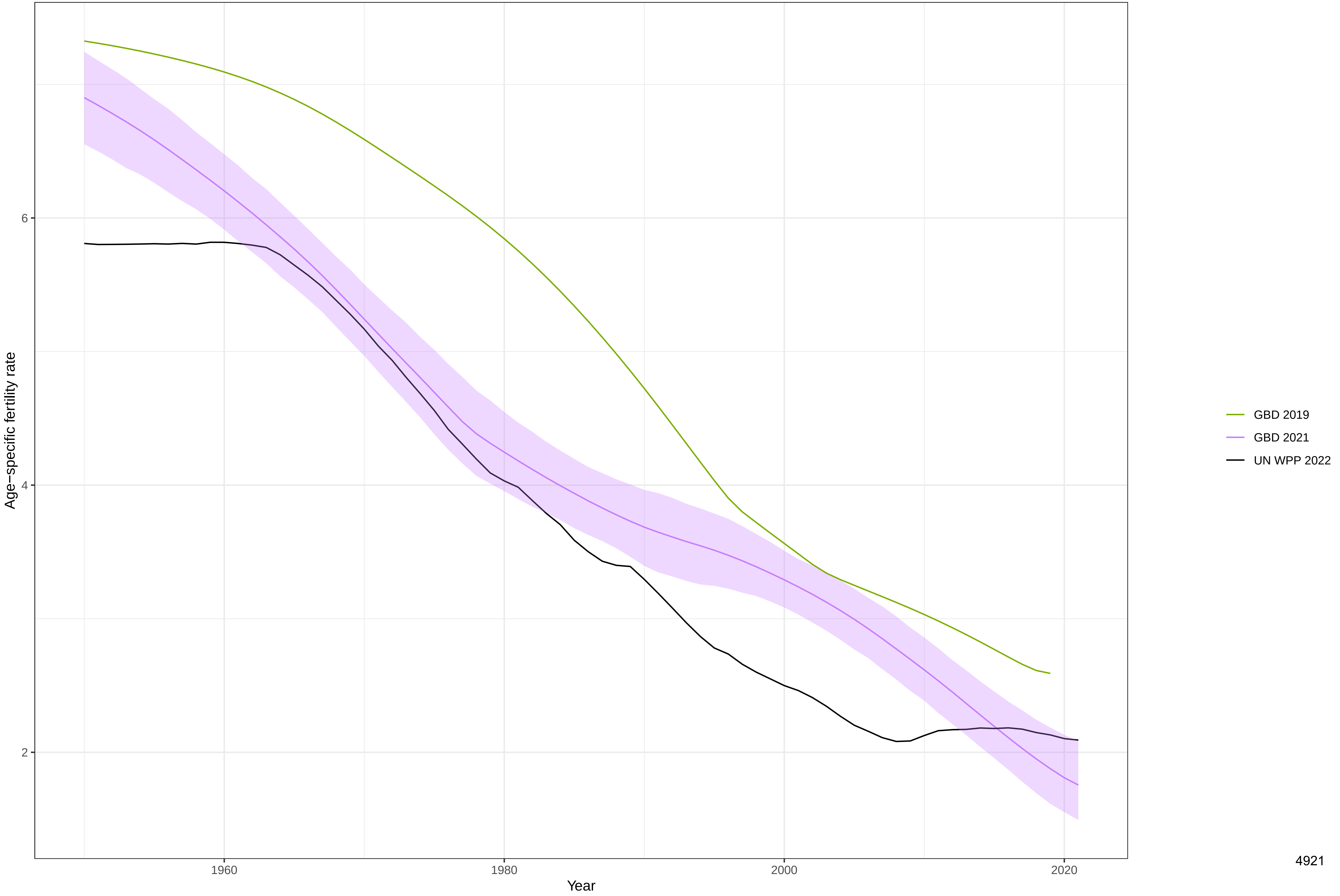

Age−specific fertility fitted trend lines, 1950−2021: Libya, Ages: 10−14
Data points enclosed with a diamond are reference sources

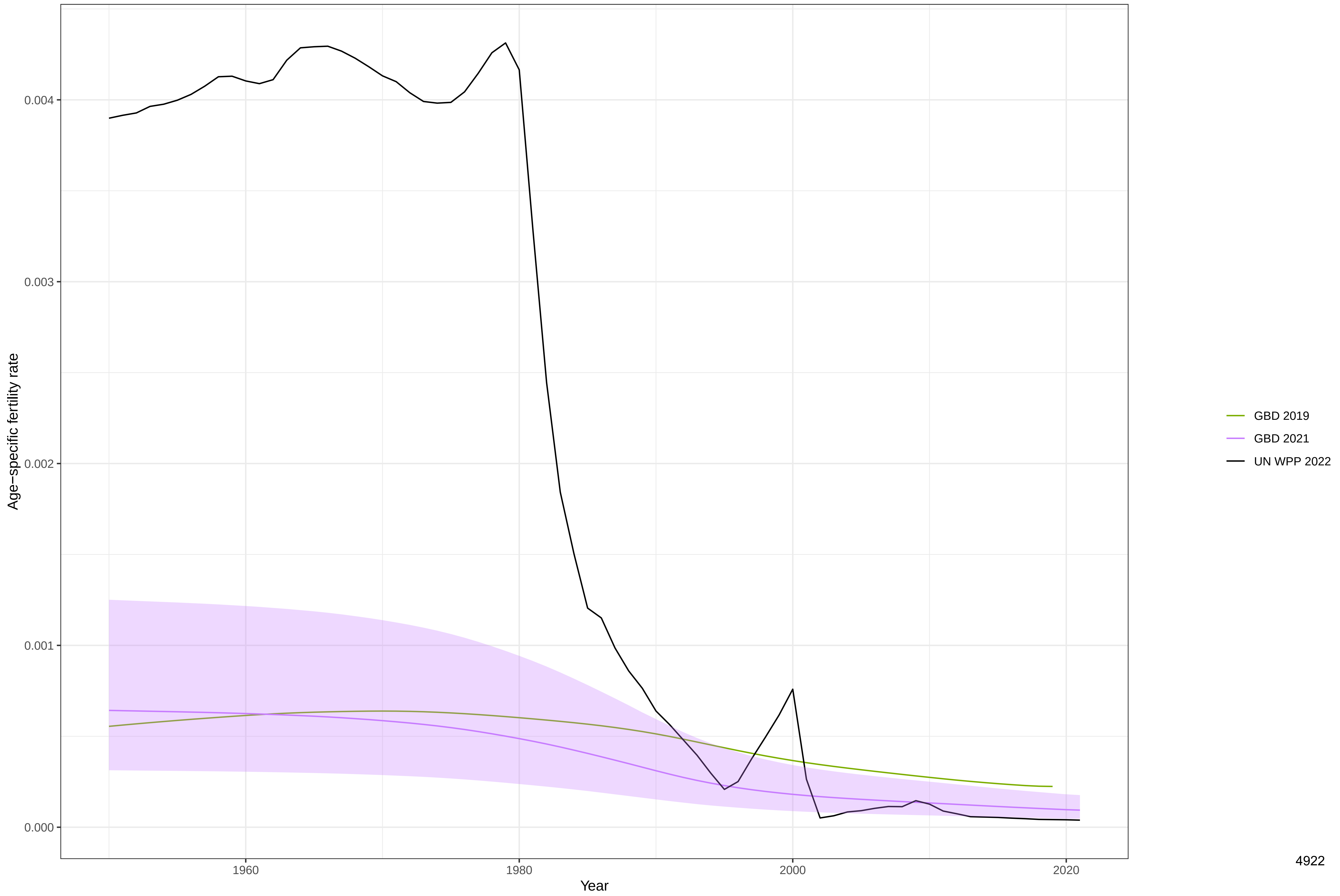

Age−specific fertility fitted trend lines, 1950−2021: Libya, Ages: 15−19
Data points enclosed with a diamond are reference sources

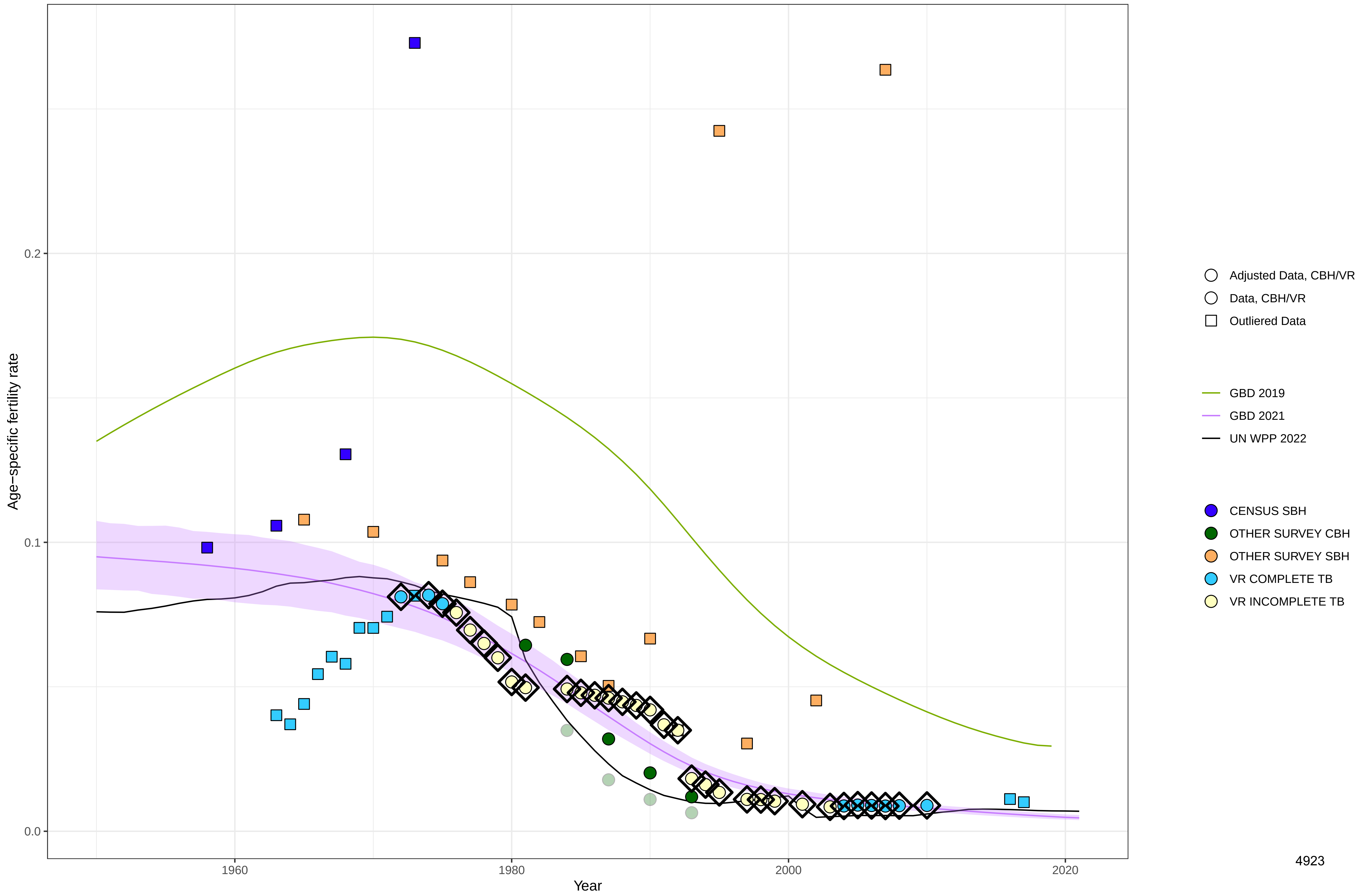

Age−specific fertility fitted trend lines, 1950−2021: Libya, Ages: 20−24
Data points enclosed with a diamond are reference sources

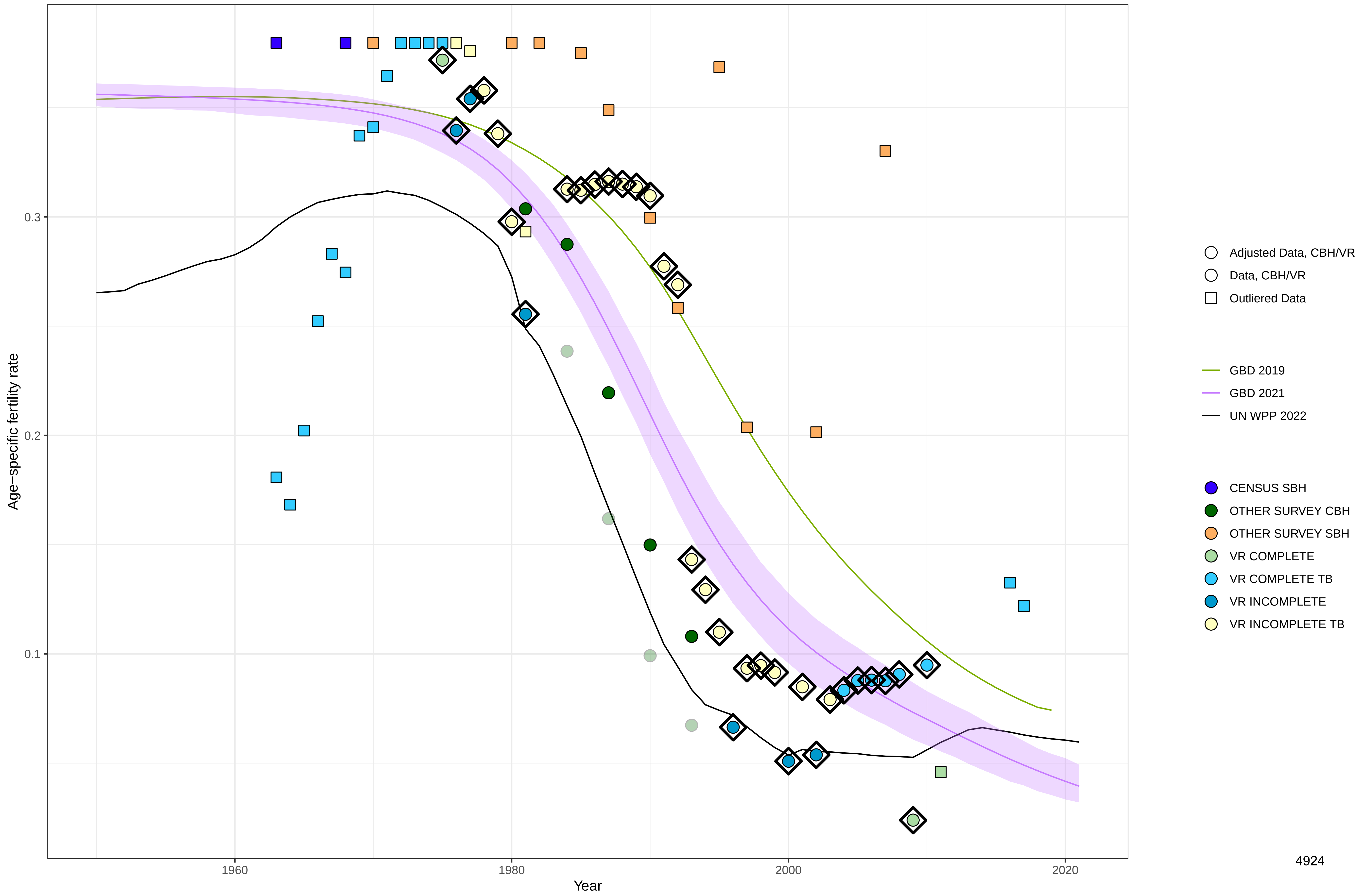

Age−specific fertility fitted trend lines, 1950−2021: Libya, Ages: 25−29
Data points enclosed with a diamond are reference sources

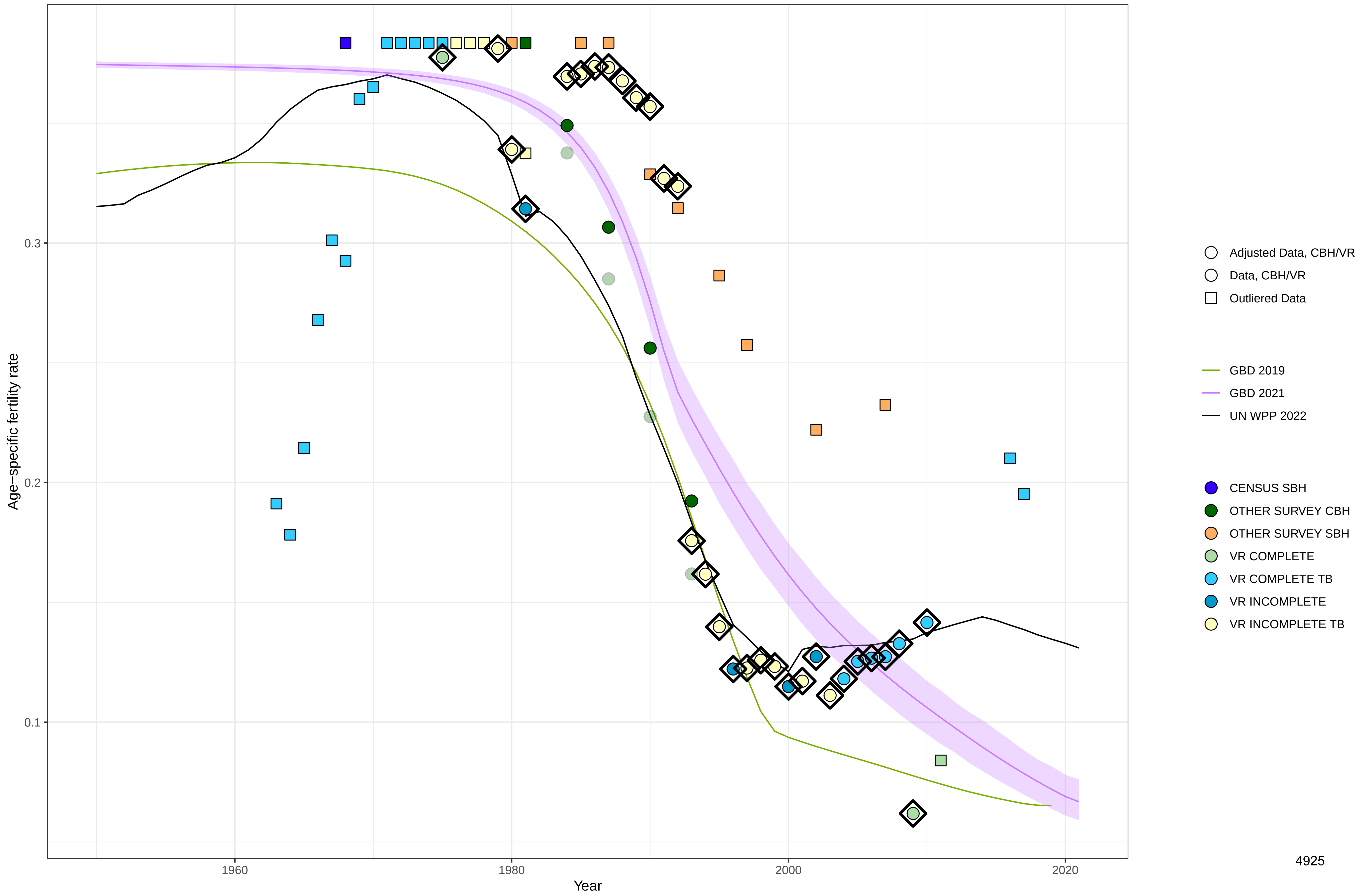

Age−specific fertility fitted trend lines, 1950−2021: Libya, Ages: 30−34
Data points enclosed with a diamond are reference sources

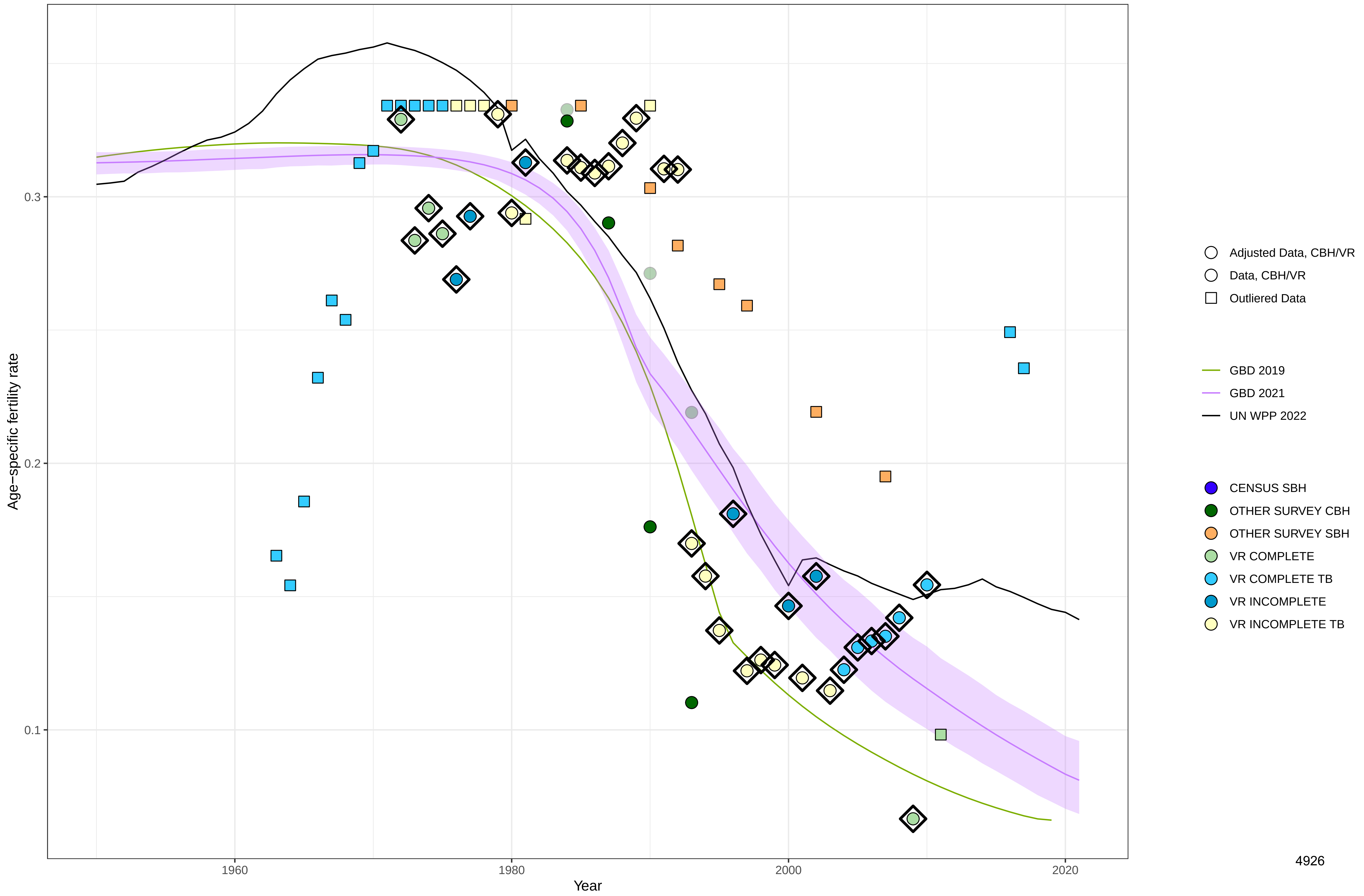

Age−specific fertility fitted trend lines, 1950−2021: Libya, Ages: 35−39
Data points enclosed with a diamond are reference sources

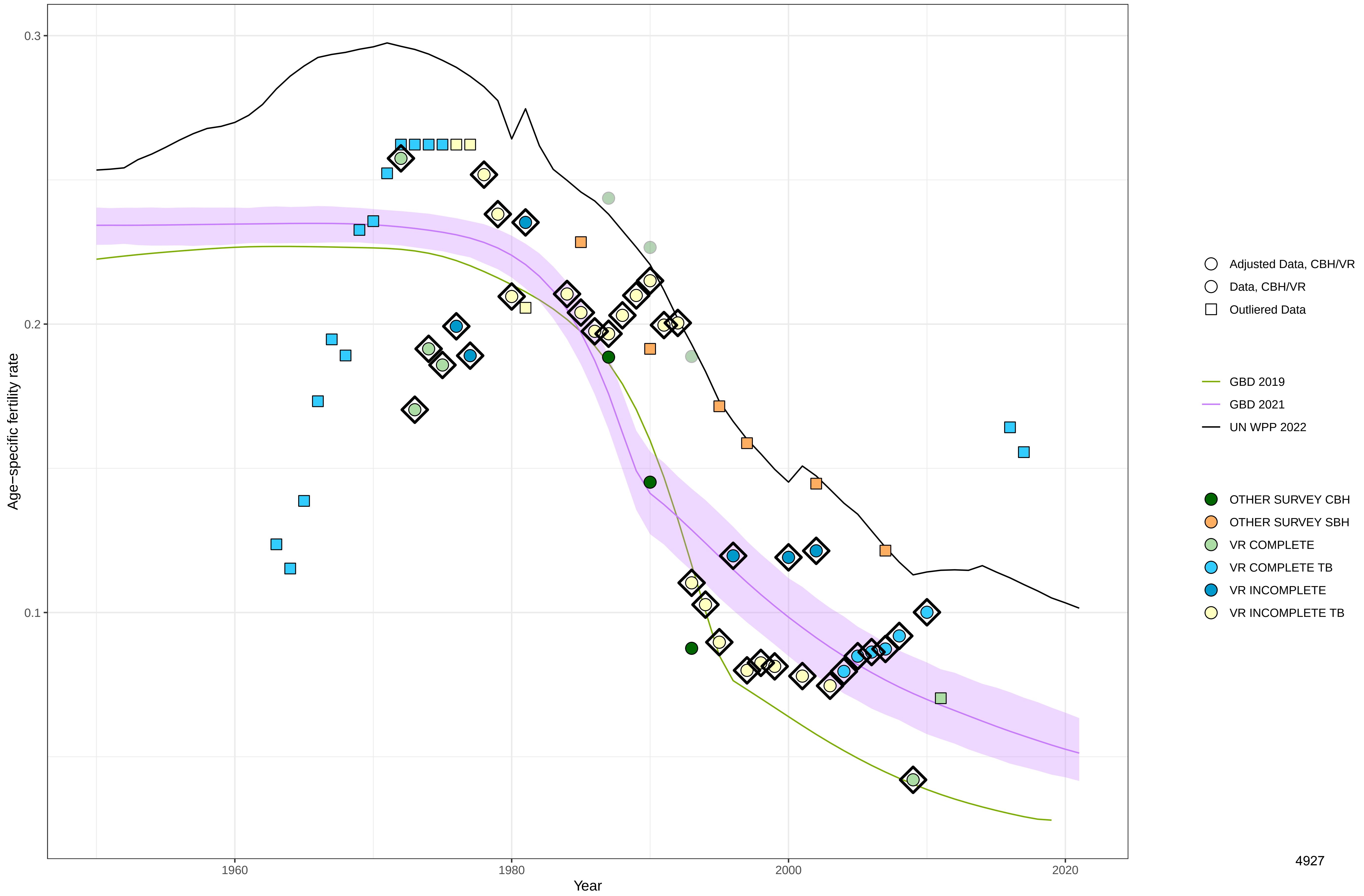

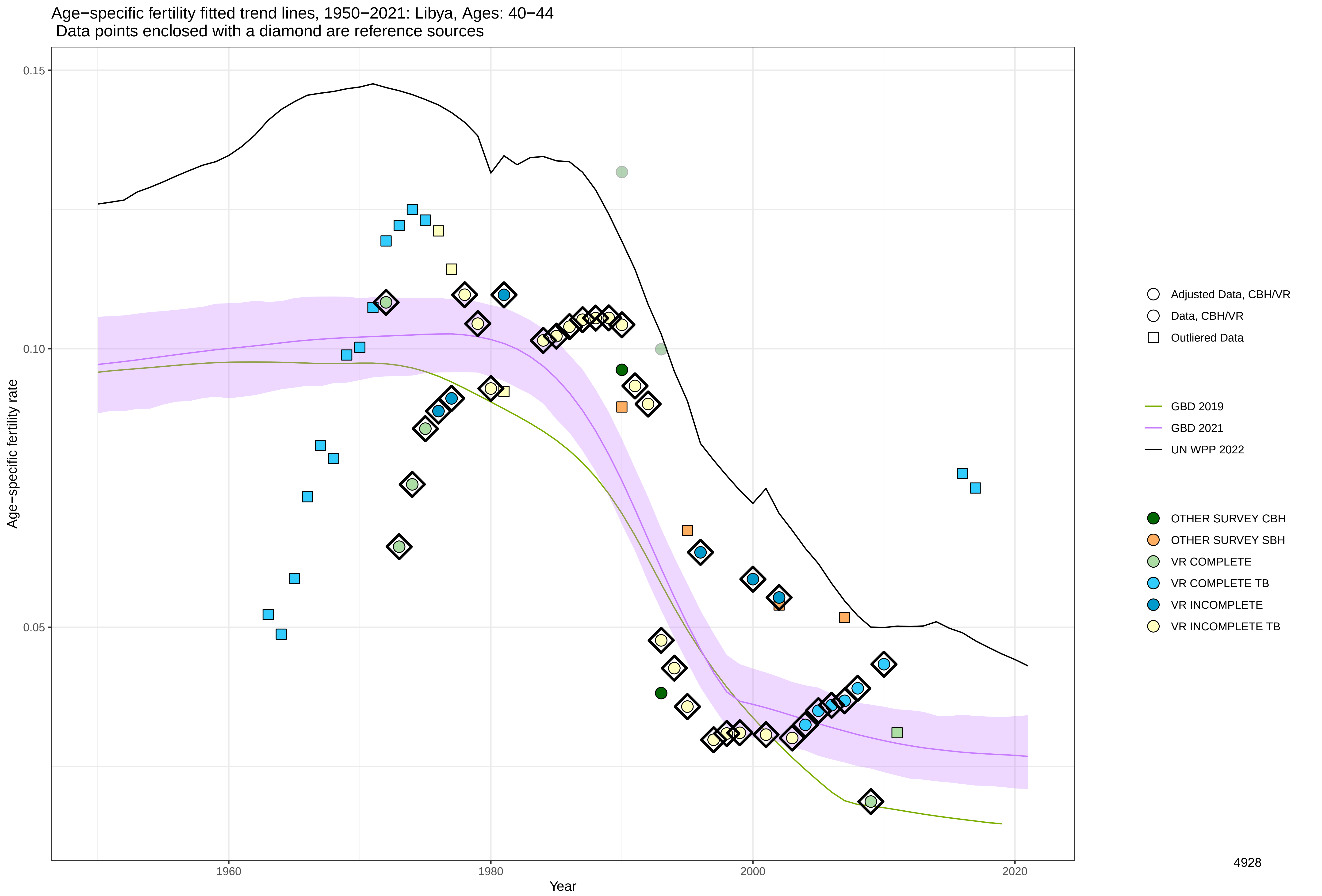

Age−specific fertility fitted trend lines, 1950−2021: Libya, Ages: 40−44
Data points enclosed with a diamond are reference sources
0.15
0.10
0.05
Age−specific fertility rate
1960
1980
2000
2020
Year
Adjusted Data, CBH/VR
Data, CBH/VR
Outliered Data
GBD 2019
GBD 2021
UN WPP 2022
OTHER SURVEY CBH
OTHER SURVEY SBH
VR COMPLETE
VR COMPLETE TB
VR INCOMPLETE
VR INCOMPLETE TB

4928

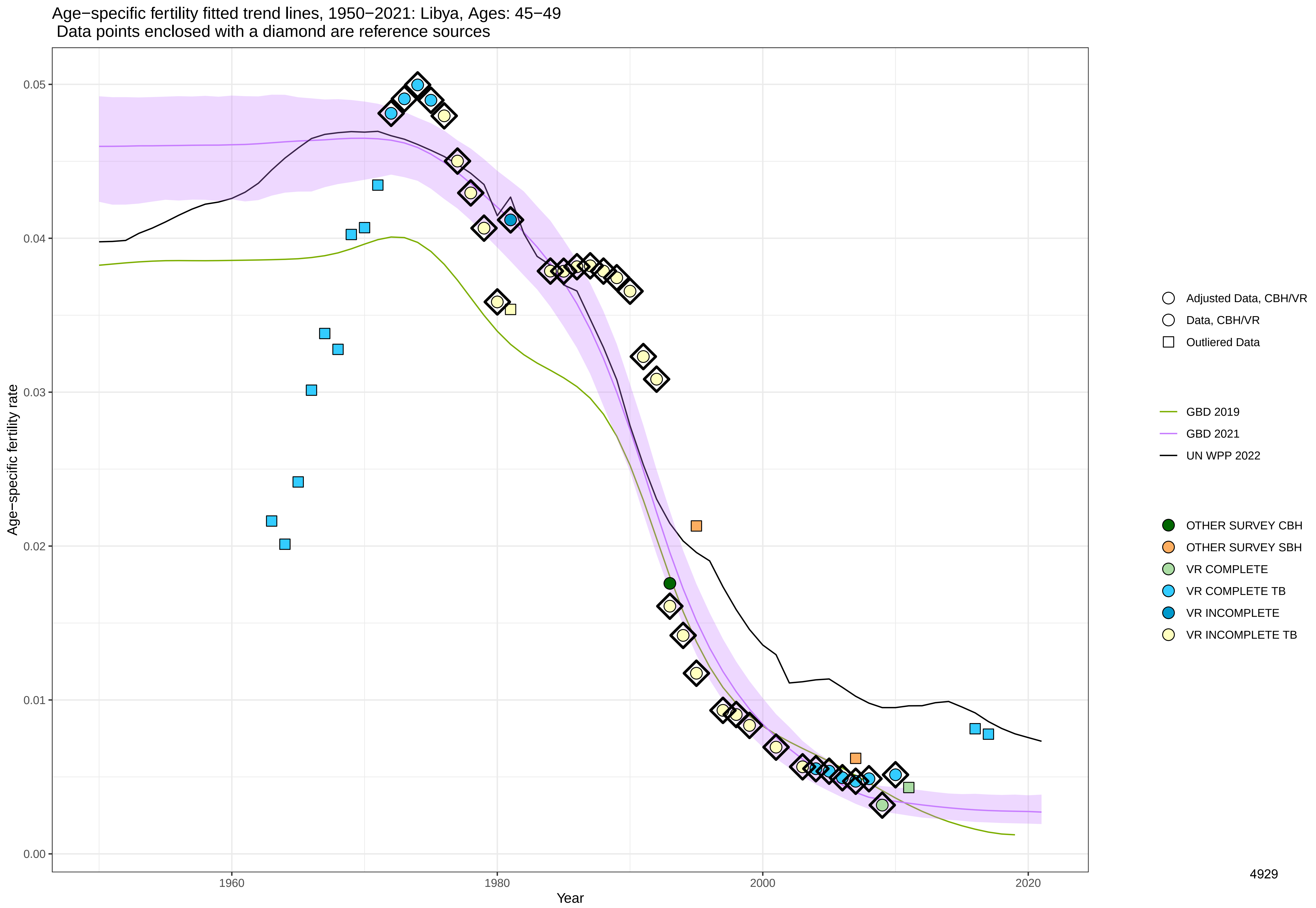
Age−specific fertility fitted trend lines, 1950−2021: Libya, Ages: 45−49
Data points enclosed with a diamond are reference sources
Age−specific fertility rate
Year
0.00
0.01
0.02
0.03
0.04
0.05
1960
1980
2000
2020
Adjusted Data, CBH/VR
Data, CBH/VR
Outliered Data
GBD 2019
GBD 2021
UN WPP 2022
OTHER SURVEY CBH
OTHER SURVEY SBH
VR COMPLETE
VR COMPLETE TB
VR INCOMPLETE
VR INCOMPLETE TB

4929

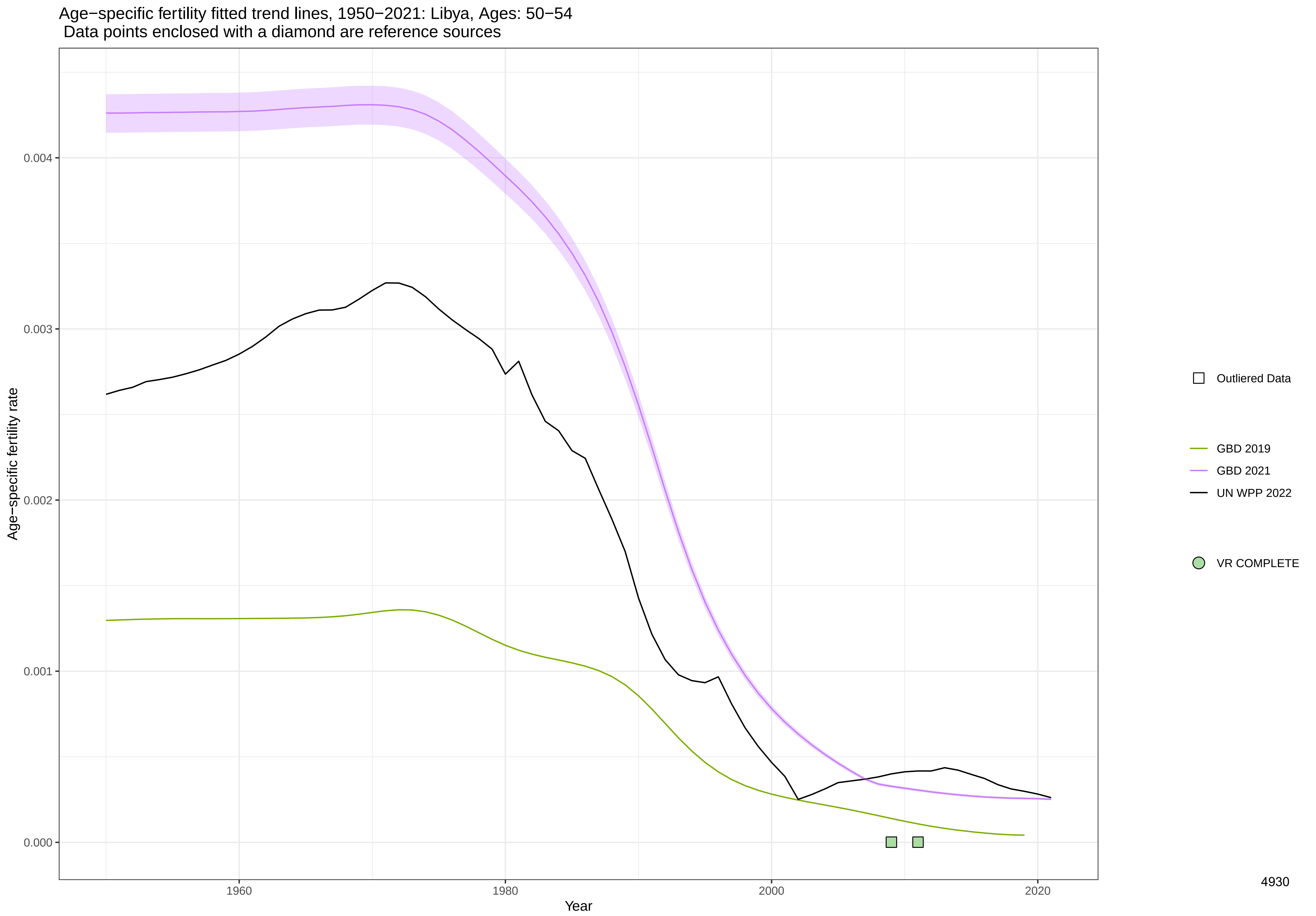

Age−specific fertility fitted trend lines, 1950−2021: Libya, Ages: 50−54
Data points enclosed with a diamond are reference sources
Age−specific fertility rate
0.000
0.001
0.002
0.003
0.004
1960
1980
2000
2020
Year
Outliered Data
GBD 2019
GBD 2021
UN WPP 2022
VR COMPLETE

Total fertility rate fitted trend lines, 1950−2021: Libya TFR
Data points enclosed with a diamond are reference sources

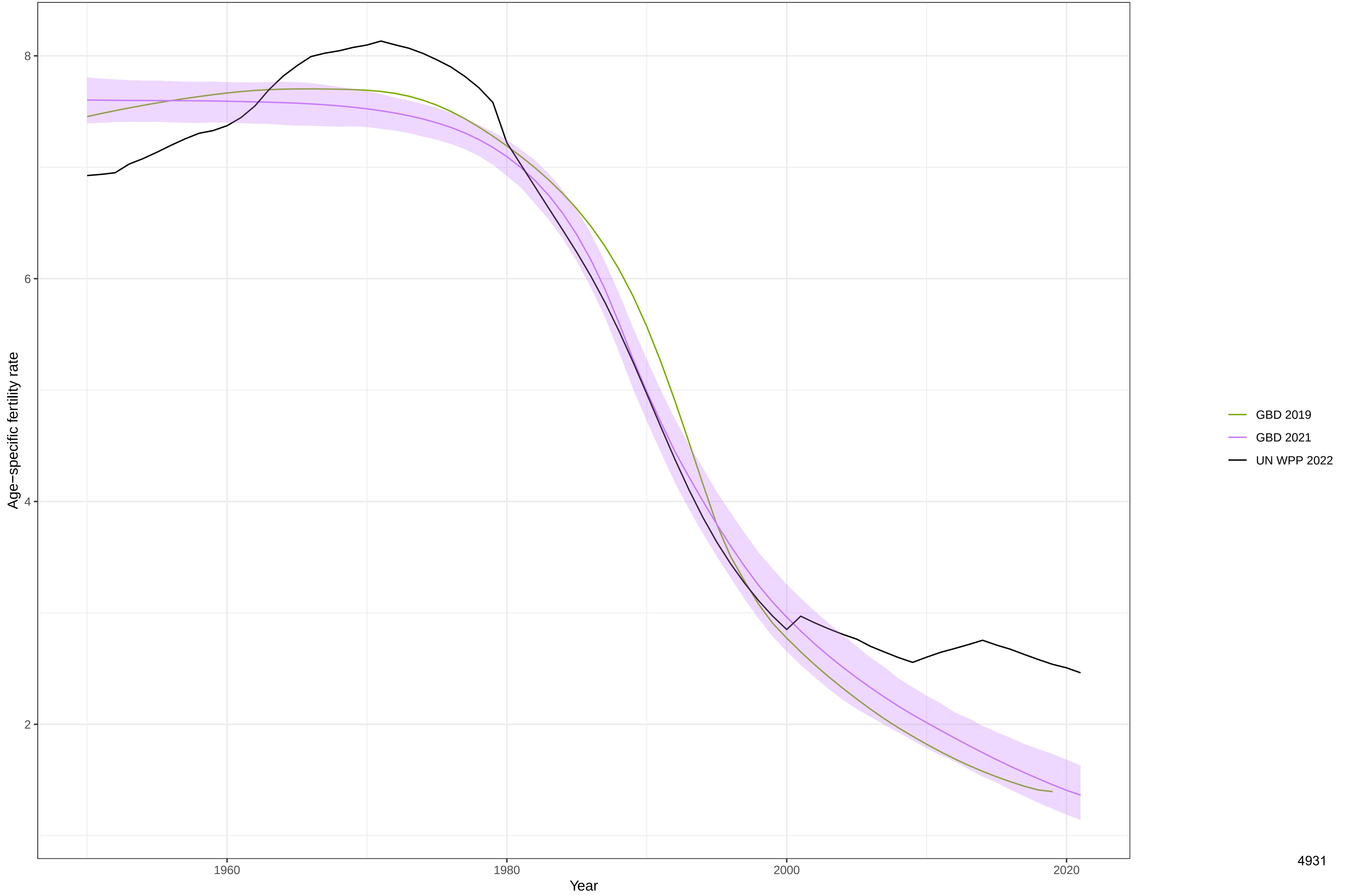

Age−specific fertility fitted trend lines, 1950−2021: Morocco, Ages: 10−14
Data points enclosed with a diamond are reference sources

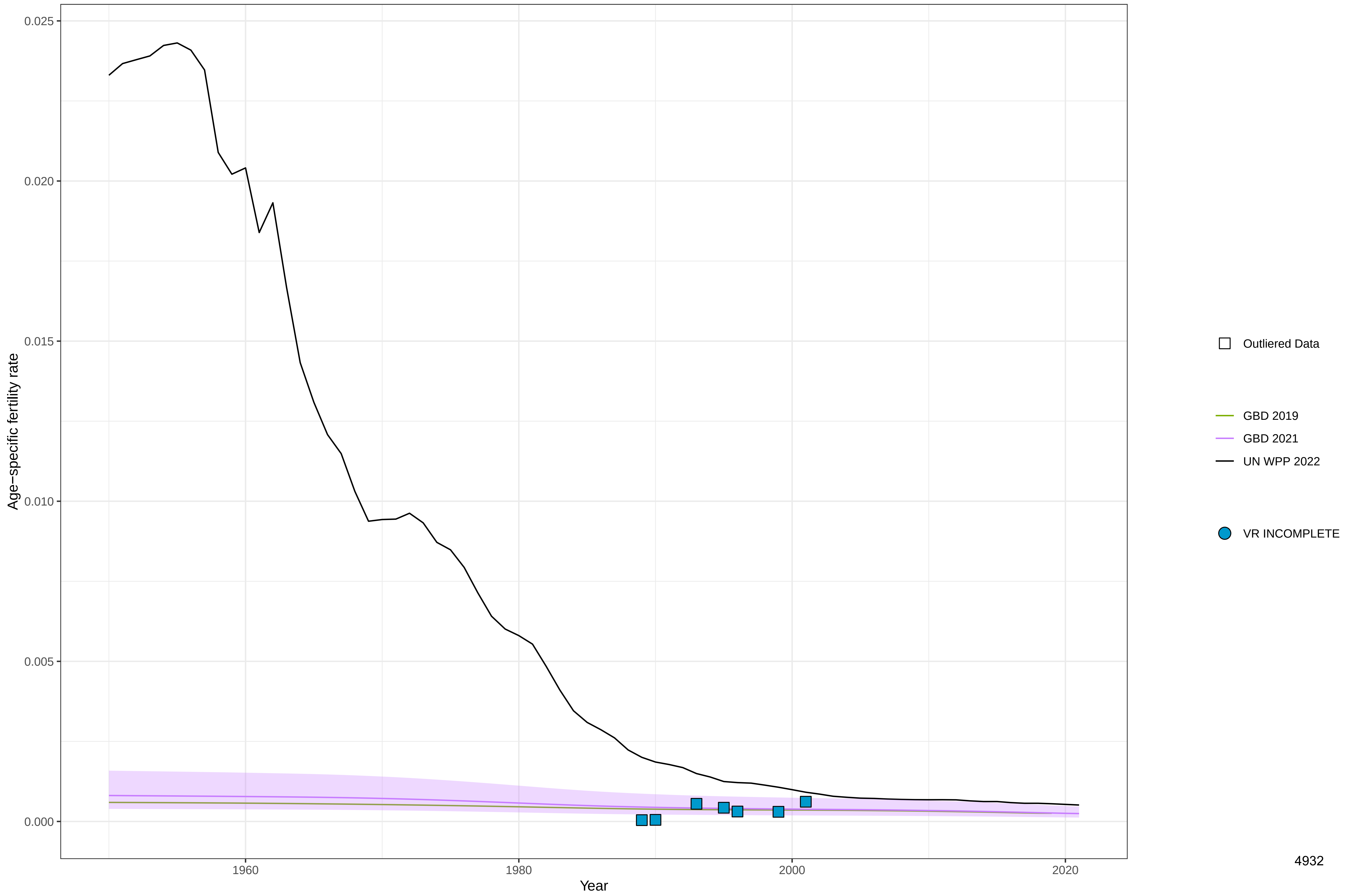

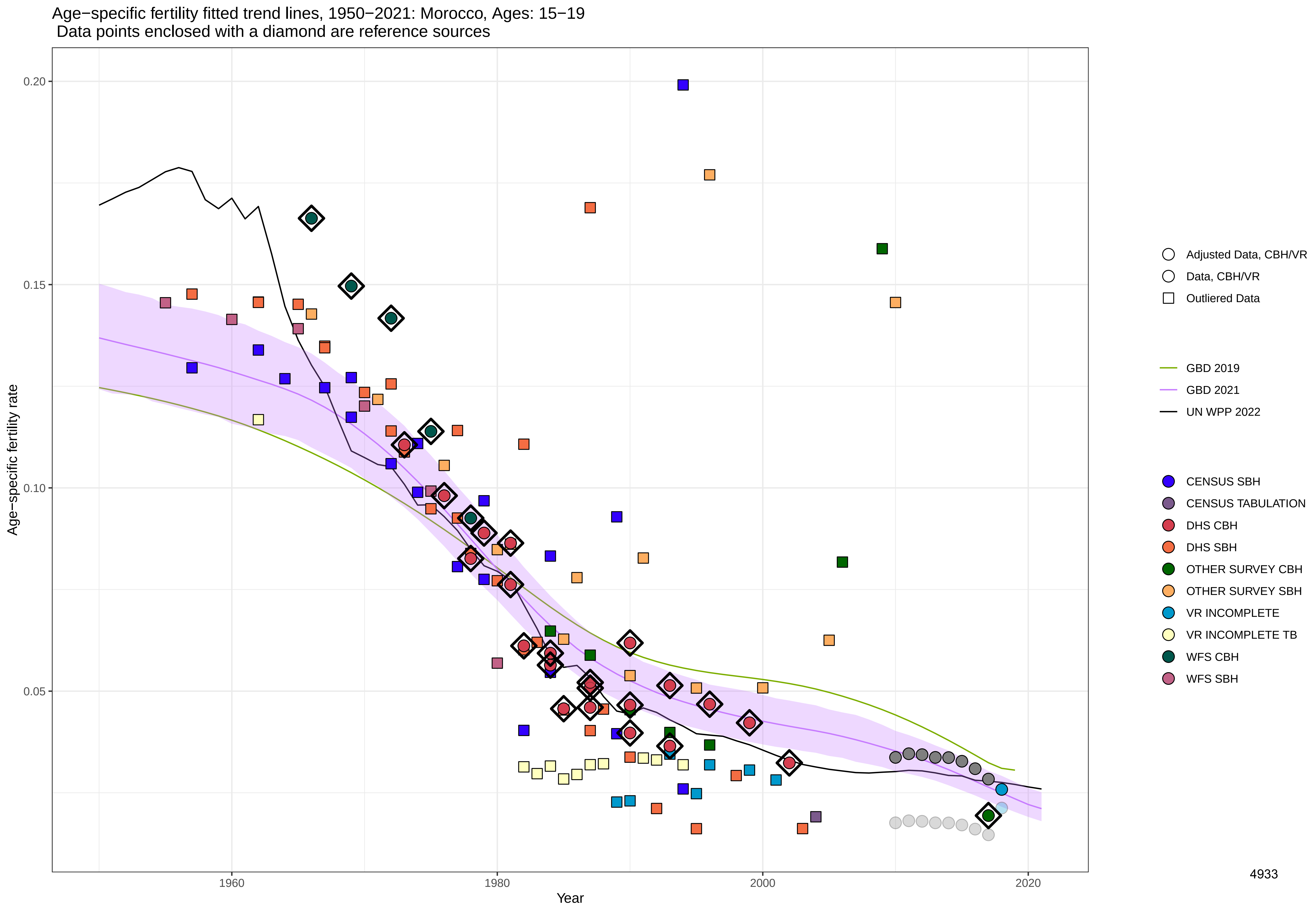
Age−specific fertility fitted trend lines, 1950−2021: Morocco, Ages: 15−19
Data points enclosed with a diamond are reference sources
Age−specific fertility rate
0.20
0.15
0.10
0.05
1960
1980
2000
2020
Year
Adjusted Data, CBH/VR
Data, CBH/VR
Outliered Data
GBD 2019
GBD 2021
UN WPP 2022
CENSUS SBH
CENSUS TABULATION
DHS CBH
DHS SBH
OTHER SURVEY CBH
OTHER SURVEY SBH
VR INCOMPLETE
VR INCOMPLETE TB
WFS CBH
WFS SBH

Age−specific fertility fitted trend lines, 1950−2021: Morocco, Ages: 20−24
Data points enclosed with a diamond are reference sources

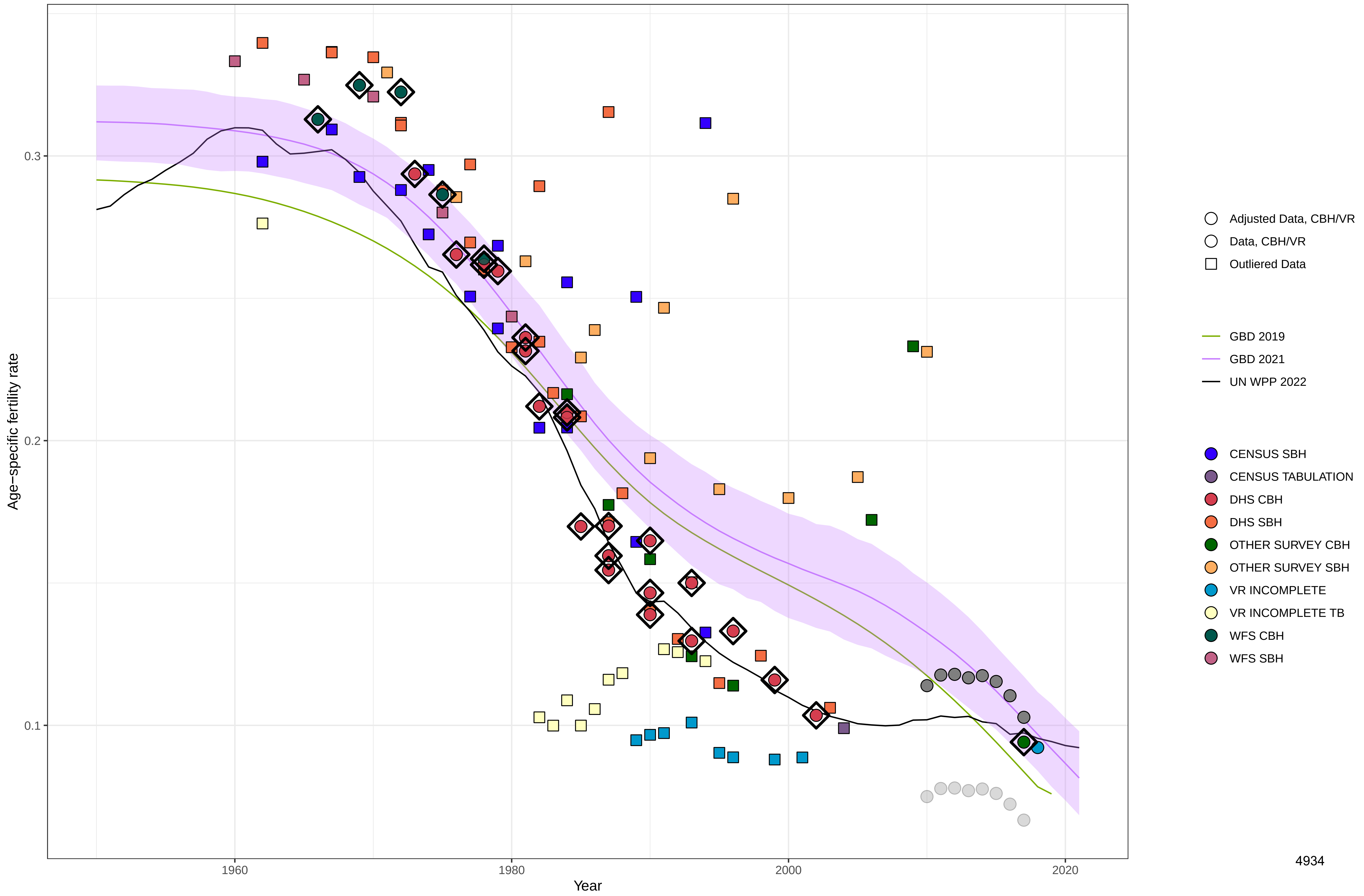

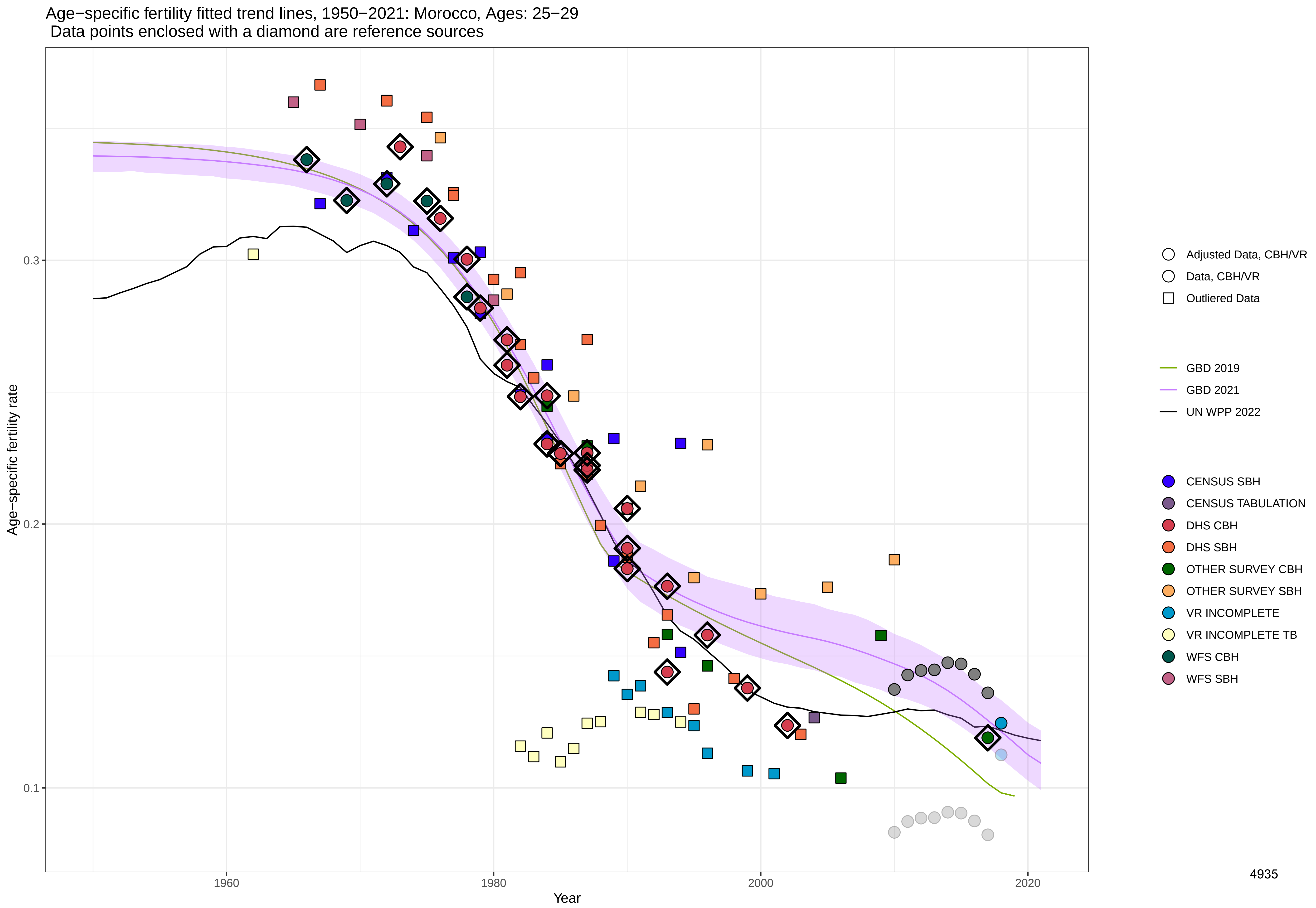

Age−specific fertility fitted trend lines, 1950−2021: Morocco, Ages: 25−29
Data points enclosed with a diamond are reference sources
Age−specific fertility rate
Year
0.1
0.2
0.3
1960
1980
2000
2020
Adjusted Data, CBH/VR
Data, CBH/VR
Outliered Data
GBD 2019
GBD 2021
UN WPP 2022
CENSUS SBH
CENSUS TABULATION
DHS CBH
DHS SBH
OTHER SURVEY CBH
OTHER SURVEY SBH
VR INCOMPLETE
VR INCOMPLETE TB
WFS CBH
WFS SBH

4935

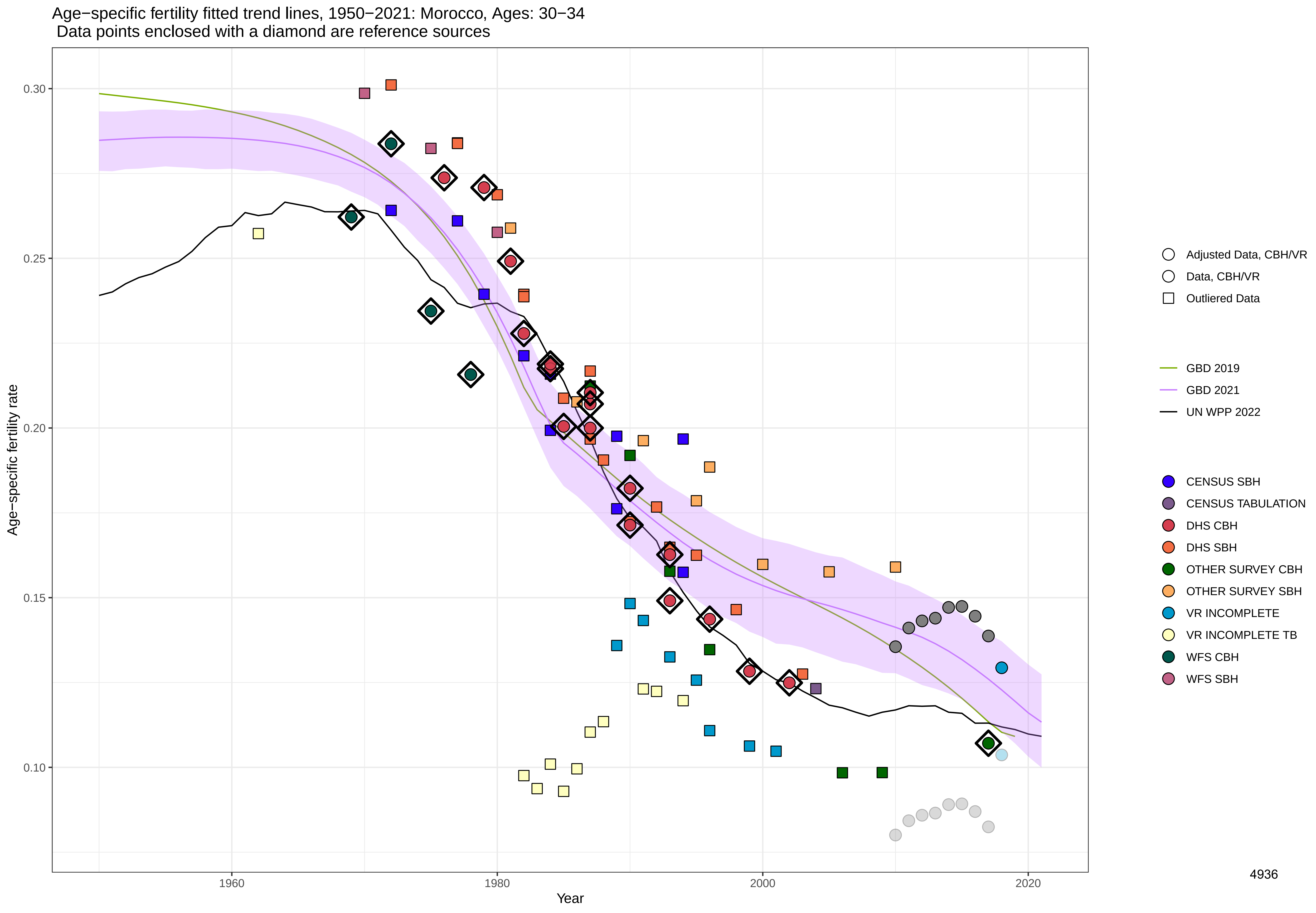

Age−specific fertility fitted trend lines, 1950−2021: Morocco, Ages: 30−34
Data points enclosed with a diamond are reference sources
Age−specific fertility rate
Year
0.30
0.25
0.20
0.15
0.10
1960
1980
2000
2020
Adjusted Data, CBH/VR
Data, CBH/VR
Outliered Data
GBD 2019
GBD 2021
UN WPP 2022
CENSUS SBH
CENSUS TABULATION
DHS CBH
DHS SBH
OTHER SURVEY CBH
OTHER SURVEY SBH
VR INCOMPLETE
VR INCOMPLETE TB
WFS CBH
WFS SBH

Age−specific fertility fitted trend lines, 1950−2021: Morocco, Ages: 35−39
Data points enclosed with a diamond are reference sources

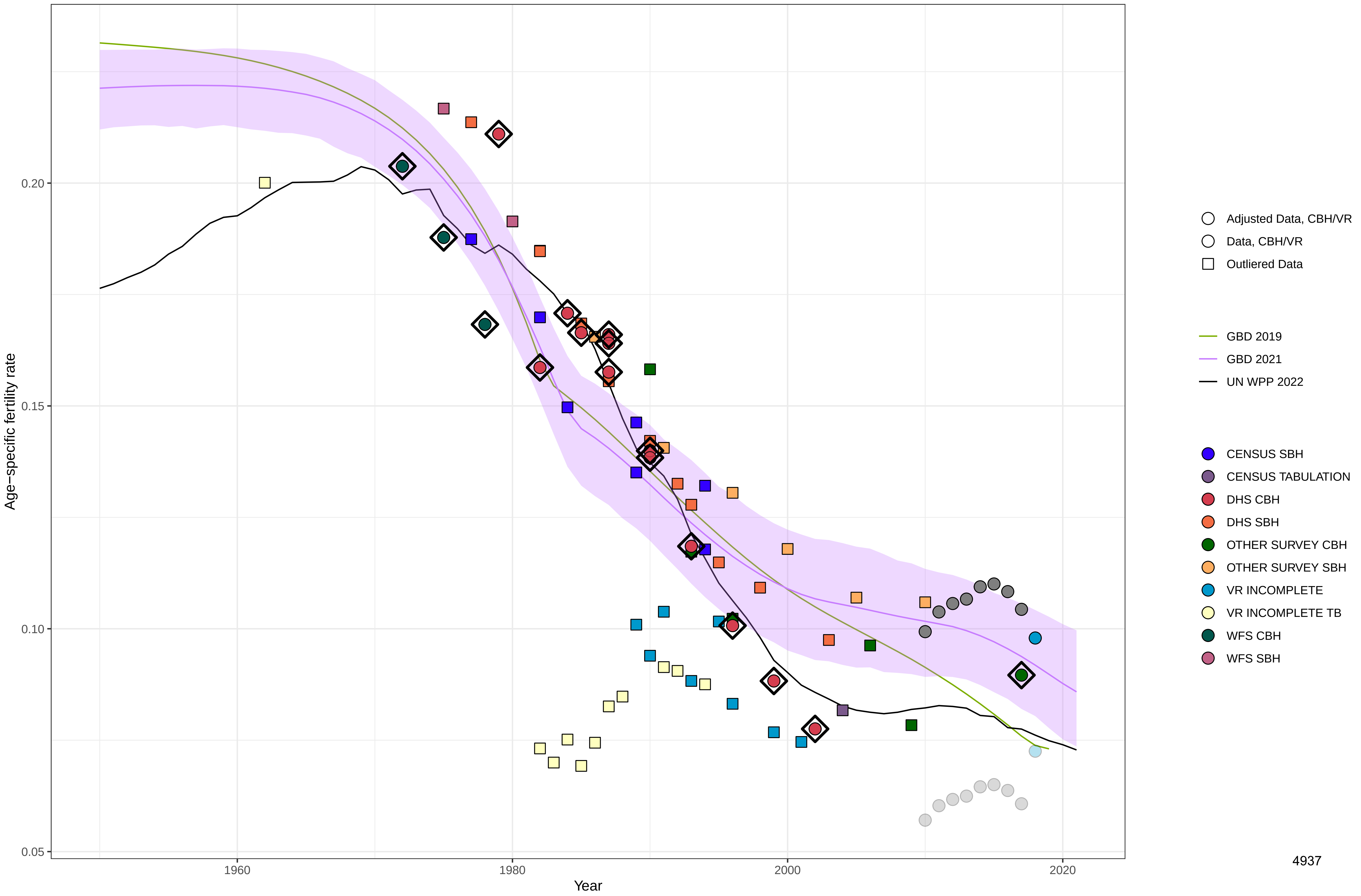

Age−specific fertility fitted trend lines, 1950−2021: Morocco, Ages: 40−44
Data points enclosed with a diamond are reference sources

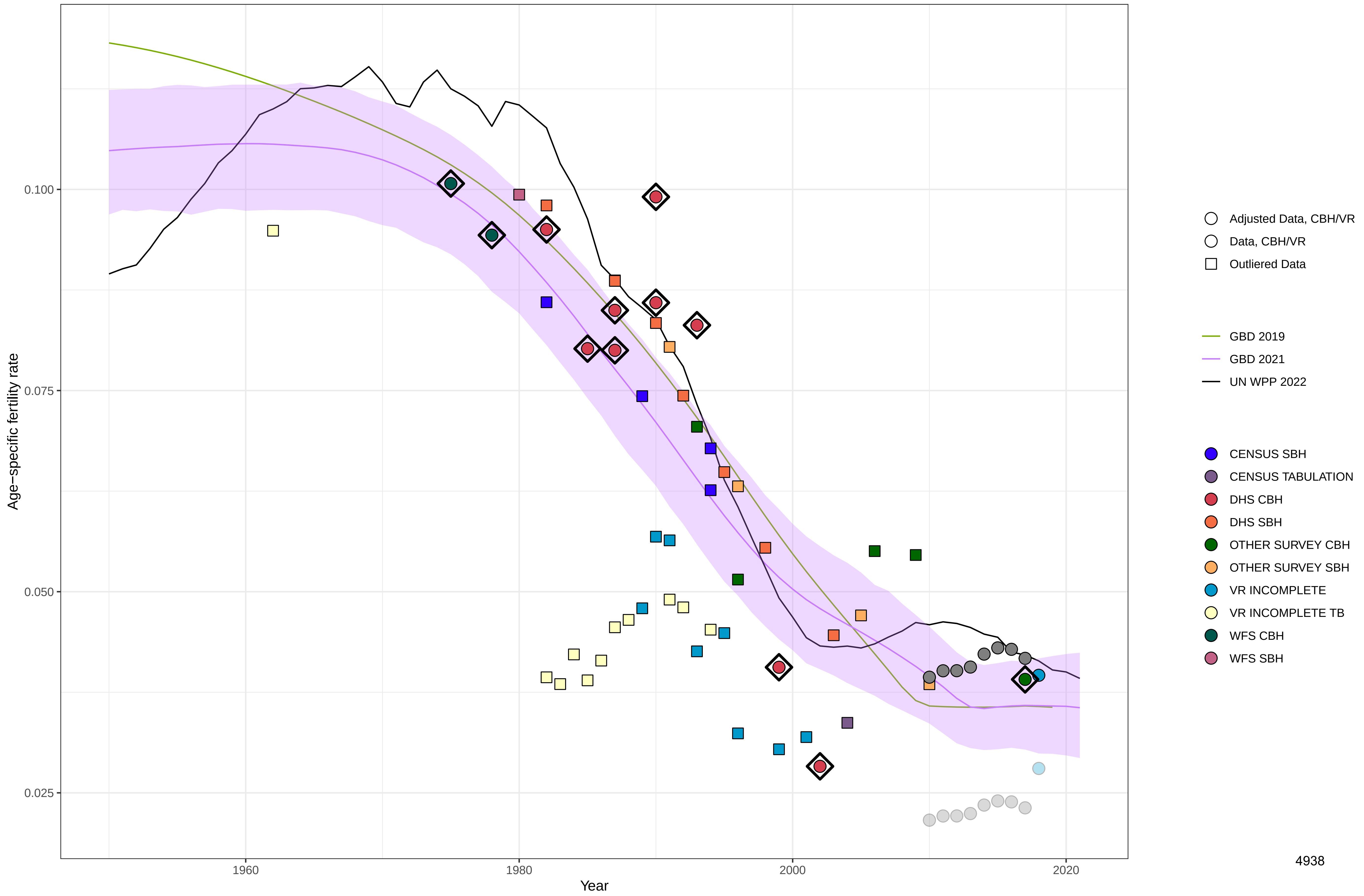

4938

Age−specific fertility fitted trend lines, 1950−2021: Morocco, Ages: 45−49
Data points enclosed with a diamond are reference sources

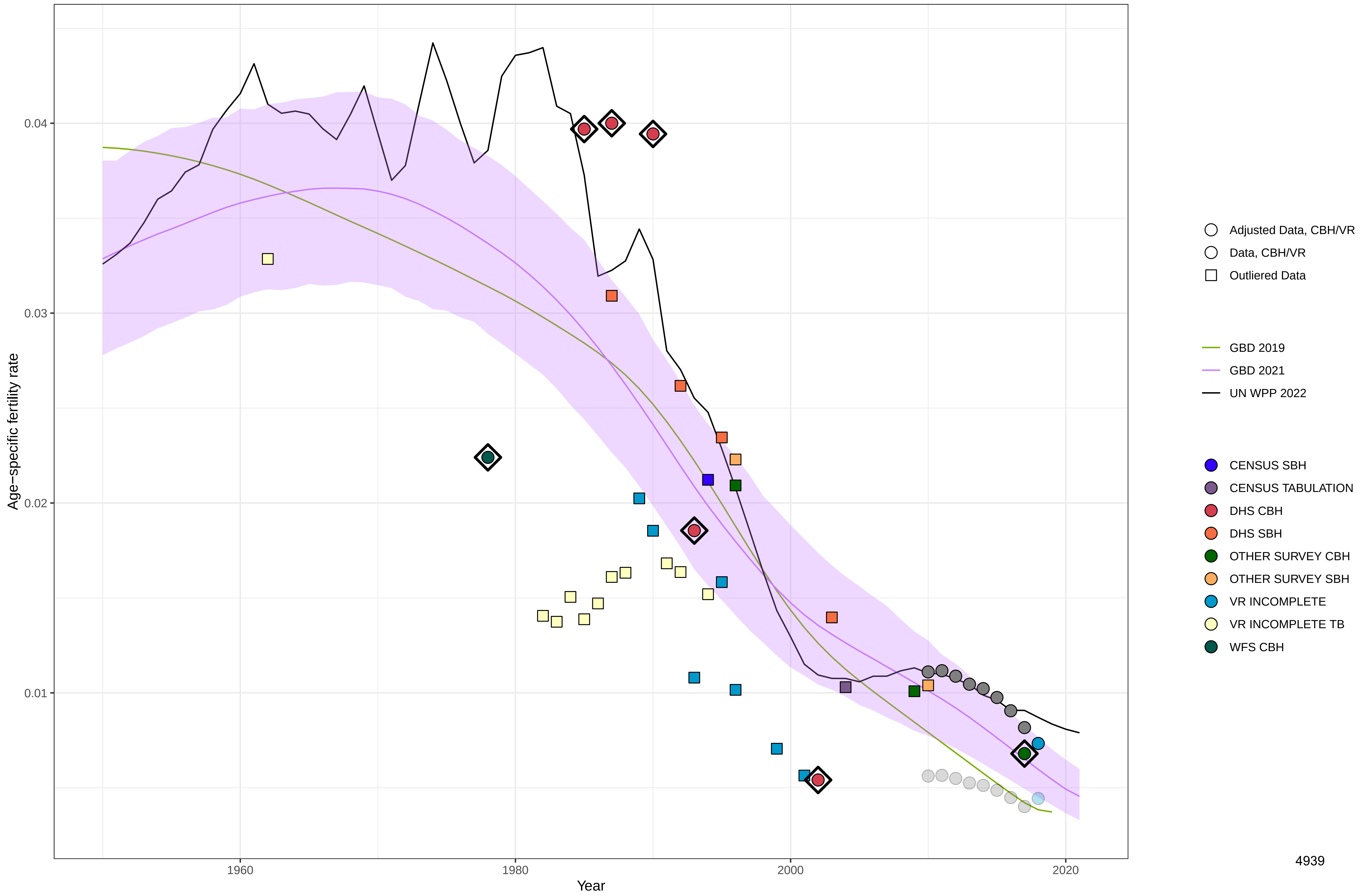

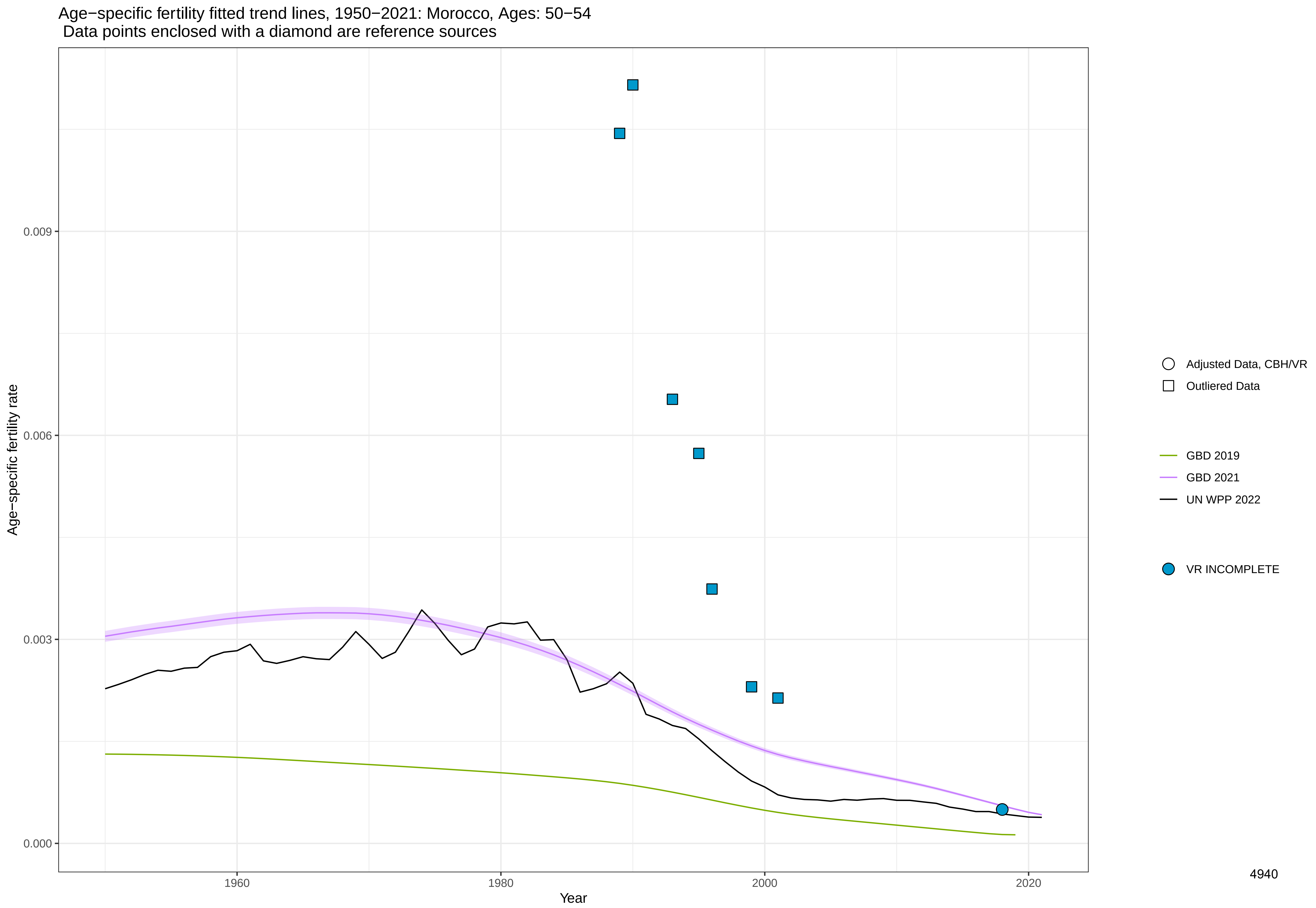
Age−specific fertility fitted trend lines, 1950−2021: Morocco, Ages: 50−54
Data points enclosed with a diamond are reference sources
Age−specific fertility rate
0.000
0.003
0.006
0.009
Year
1960
1980
2000
2020
Adjusted Data, CBH/VR
Outliered Data
GBD 2019
GBD 2021
UN WPP 2022
VR INCOMPLETE

Total fertility rate fitted trend lines, 1950−2021: Morocco TFR
Data points enclosed with a diamond are reference sources

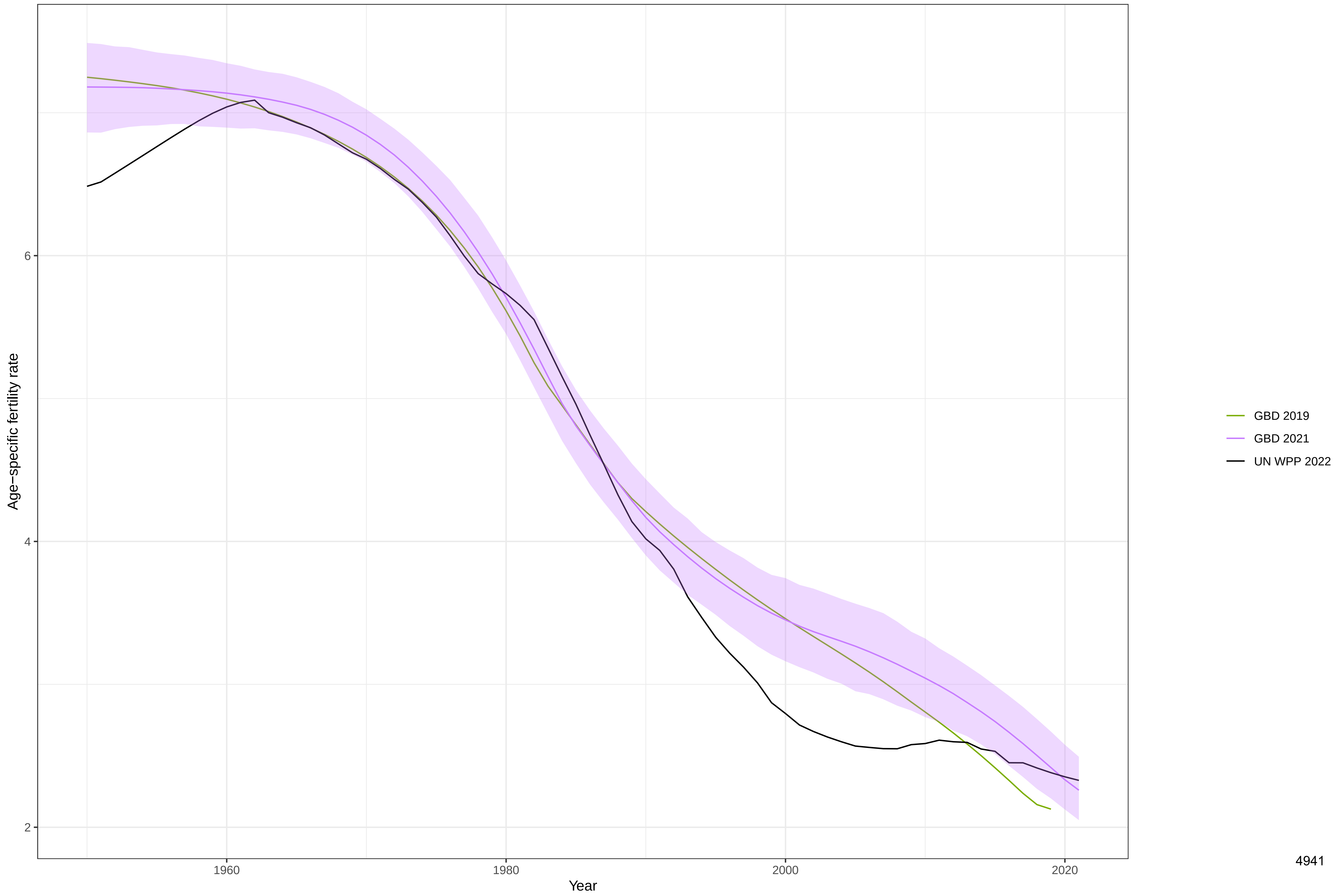

4941

Age−specific fertility fitted trend lines, 1950−2021: Oman, Ages: 10−14
Data points enclosed with a diamond are reference sources

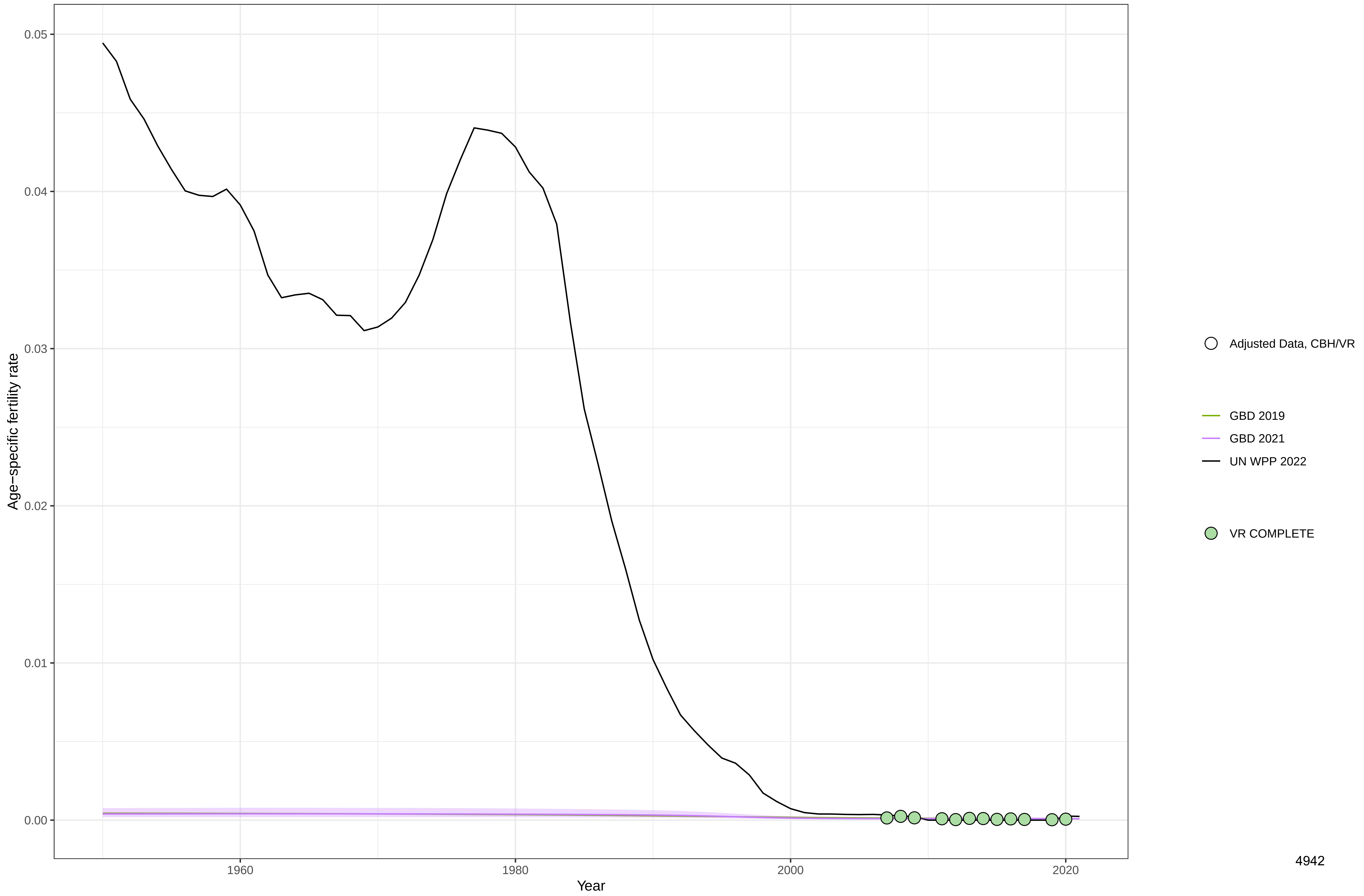

Age−specific fertility fitted trend lines, 1950−2021: Oman, Ages: 15−19
Data points enclosed with a diamond are reference sources

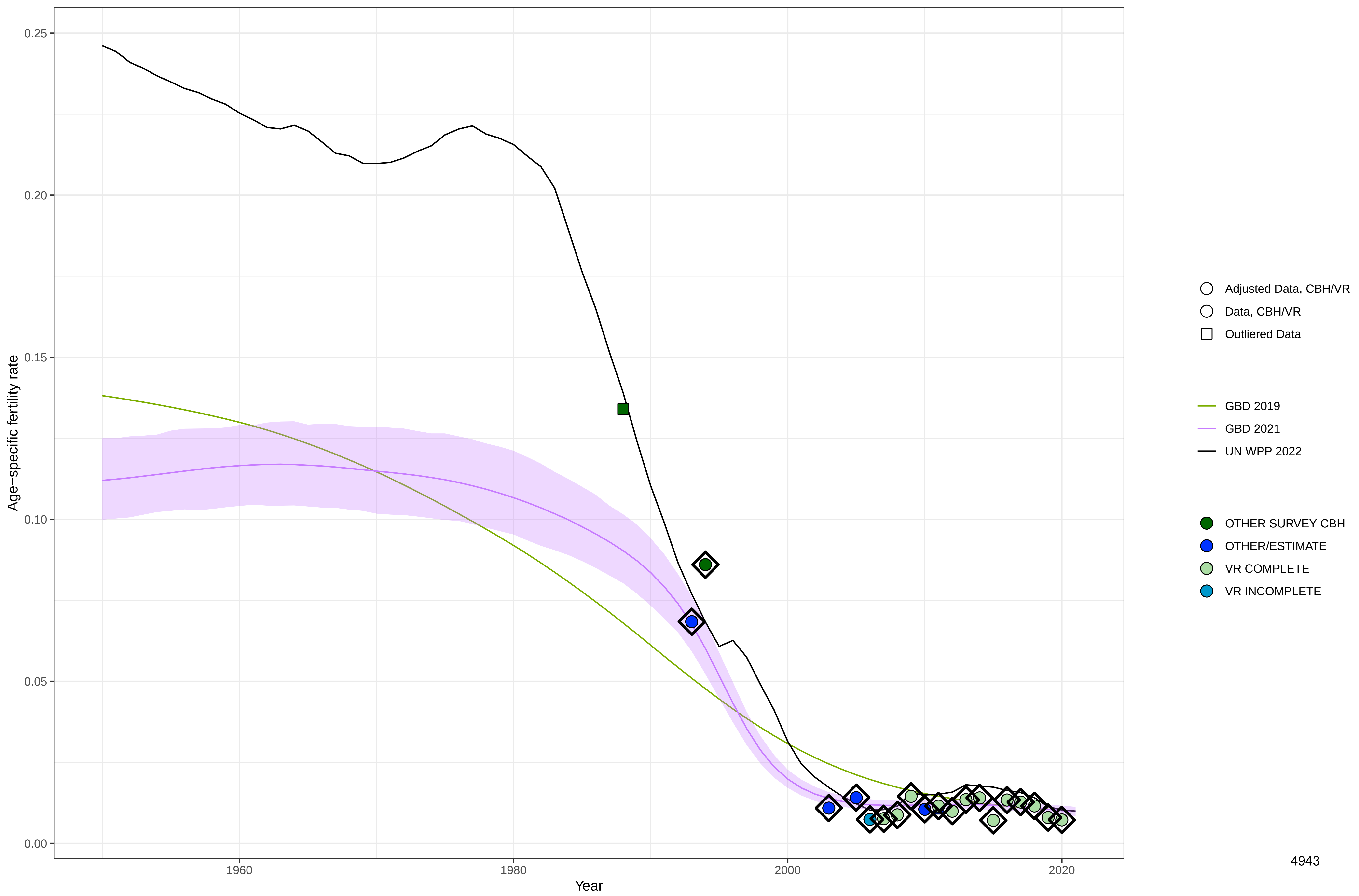

Age−specific fertility fitted trend lines, 1950−2021: Oman, Ages: 20−24
Data points enclosed with a diamond are reference sources

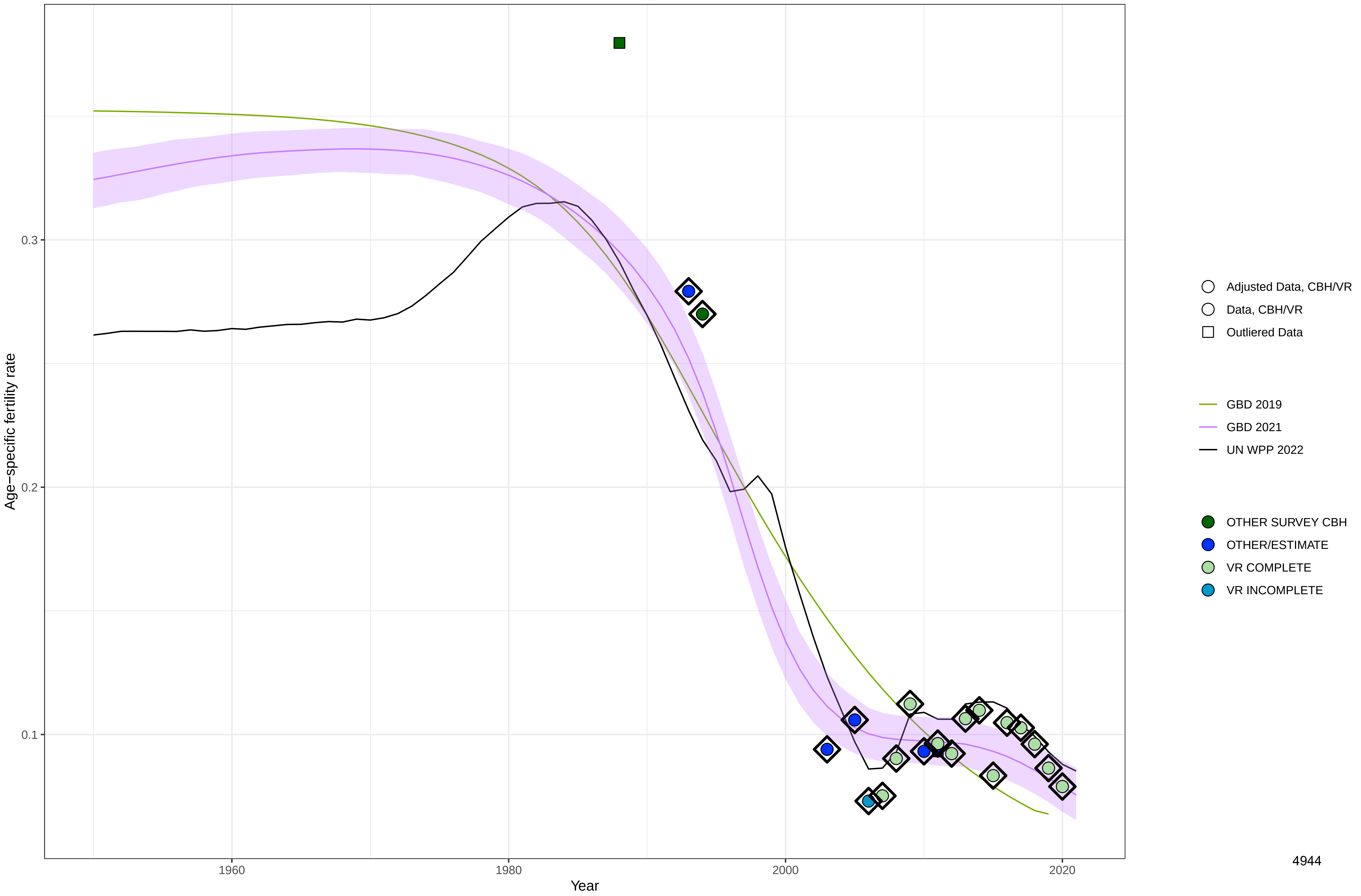

Age−specific fertility fitted trend lines, 1950−2021: Oman, Ages: 25−29
Data points enclosed with a diamond are reference sources

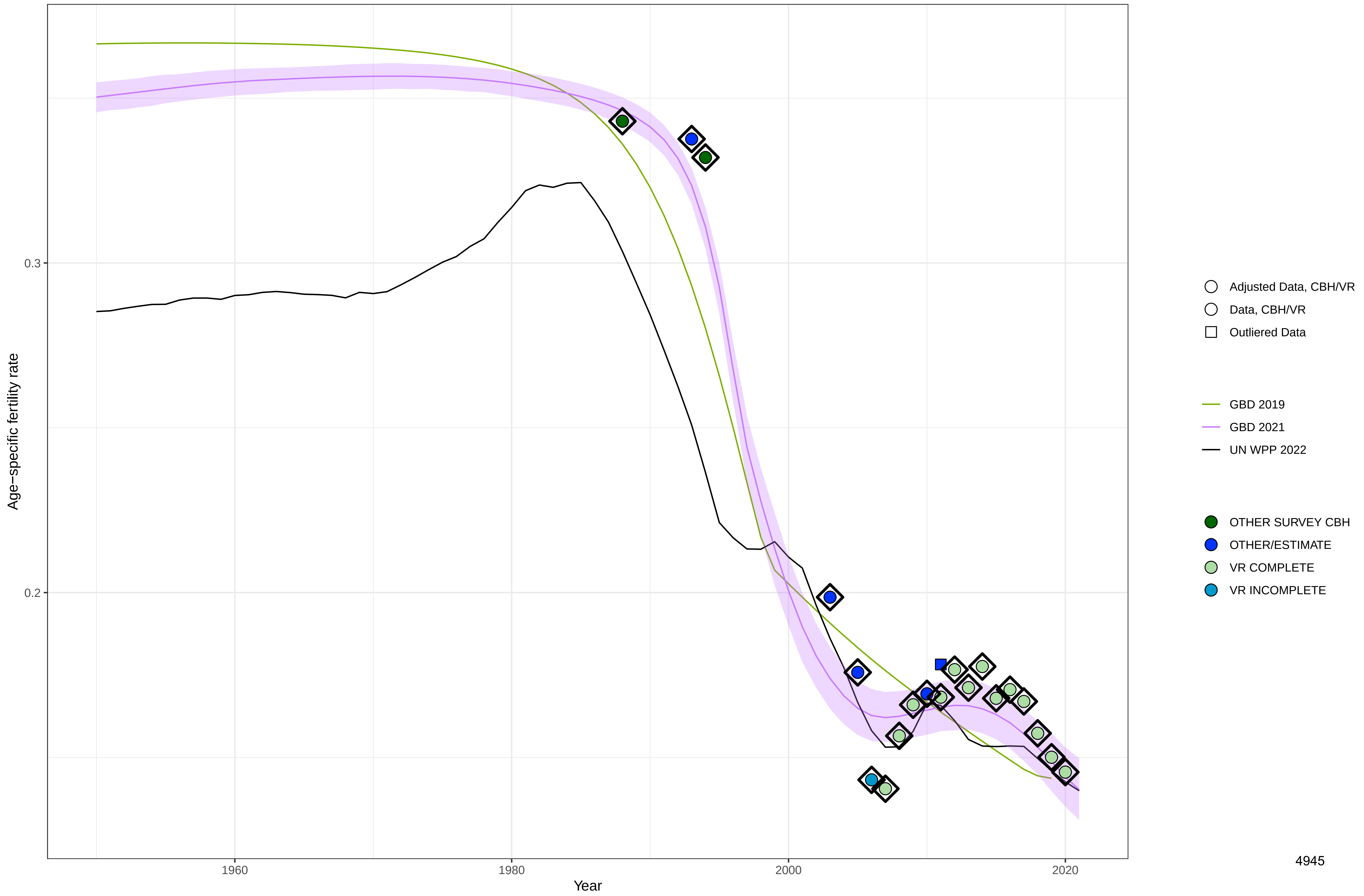

Age−specific fertility fitted trend lines, 1950−2021: Oman, Ages: 30−34
Data points enclosed with a diamond are reference sources

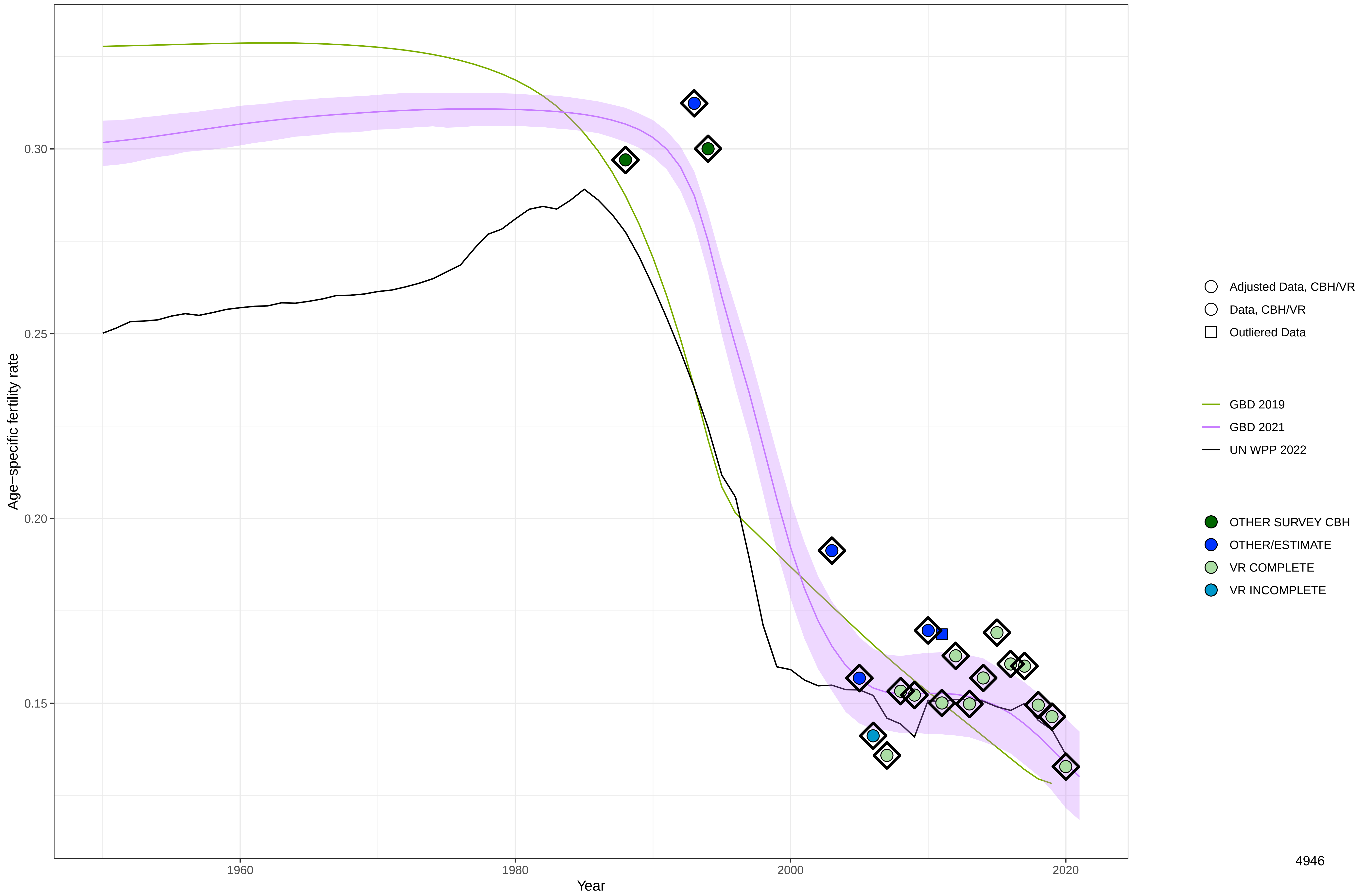

4946

Age−specific fertility fitted trend lines, 1950−2021: Oman, Ages: 35−39
Data points enclosed with a diamond are reference sources

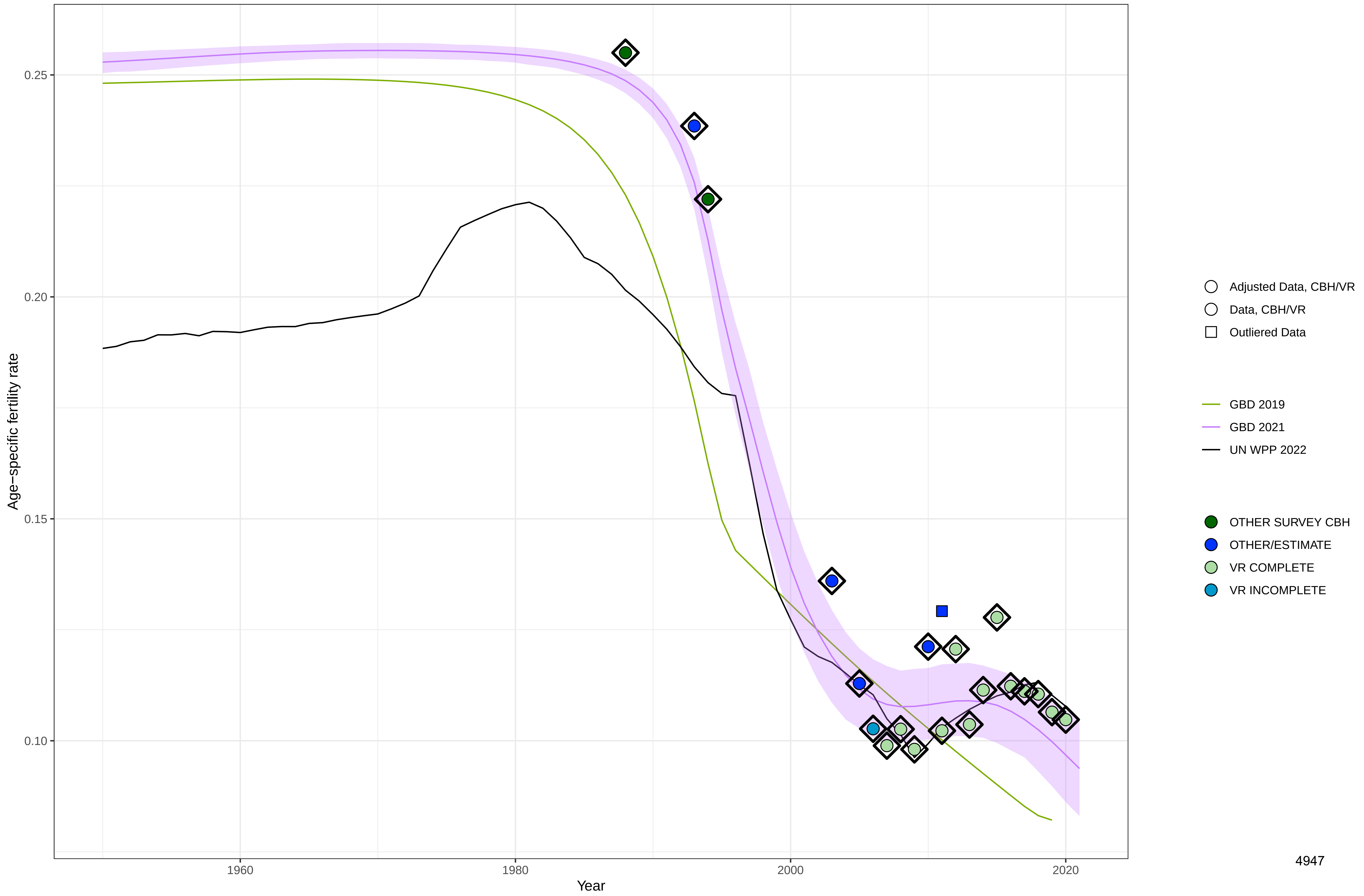

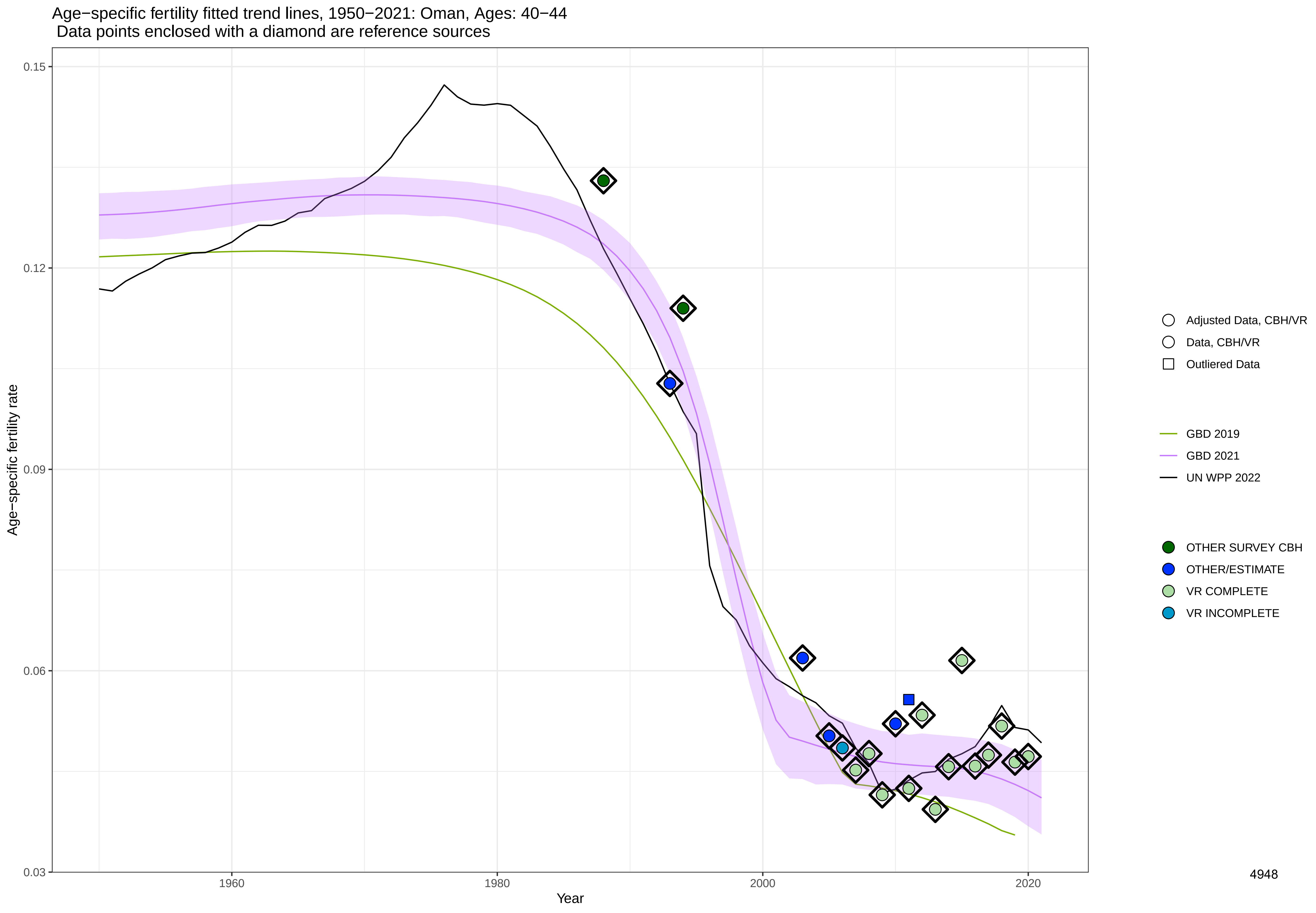

Age−specific fertility fitted trend lines, 1950−2021: Oman, Ages: 40−44
Data points enclosed with a diamond are reference sources
Age−specific fertility rate
Year
0.15
0.12
0.09
0.06
0.03
1960
1980
2000
2020
Adjusted Data, CBH/VR
Data, CBH/VR
Outliered Data
GBD 2019
GBD 2021
UN WPP 2022
OTHER SURVEY CBH
OTHER/ESTIMATE
VR COMPLETE
VR INCOMPLETE

Age−specific fertility fitted trend lines, 1950−2021: Oman, Ages: 45−49
Data points enclosed with a diamond are reference sources

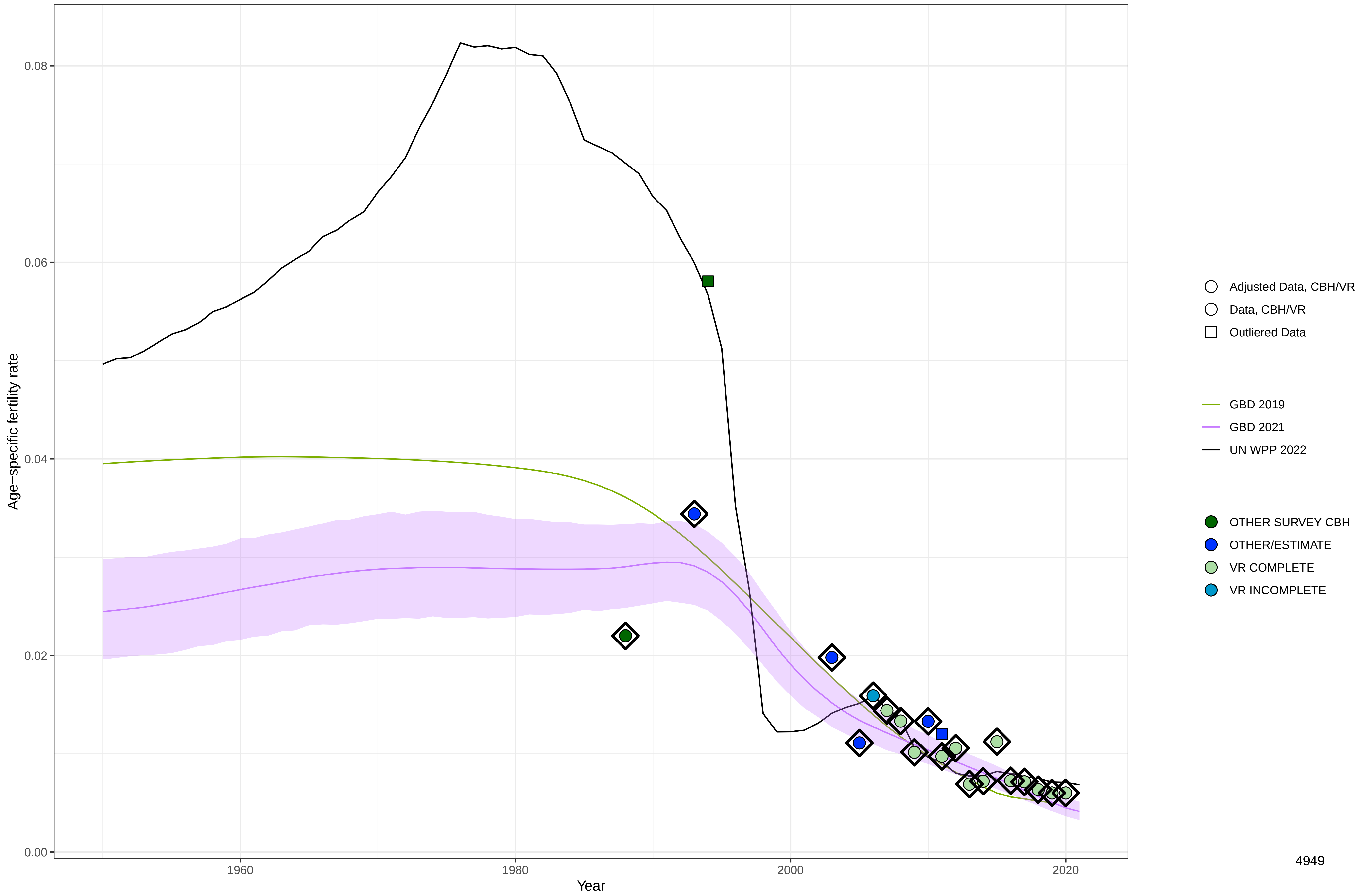

Age−specific fertility fitted trend lines, 1950−2021: Oman, Ages: 50−54
Data points enclosed with a diamond are reference sources

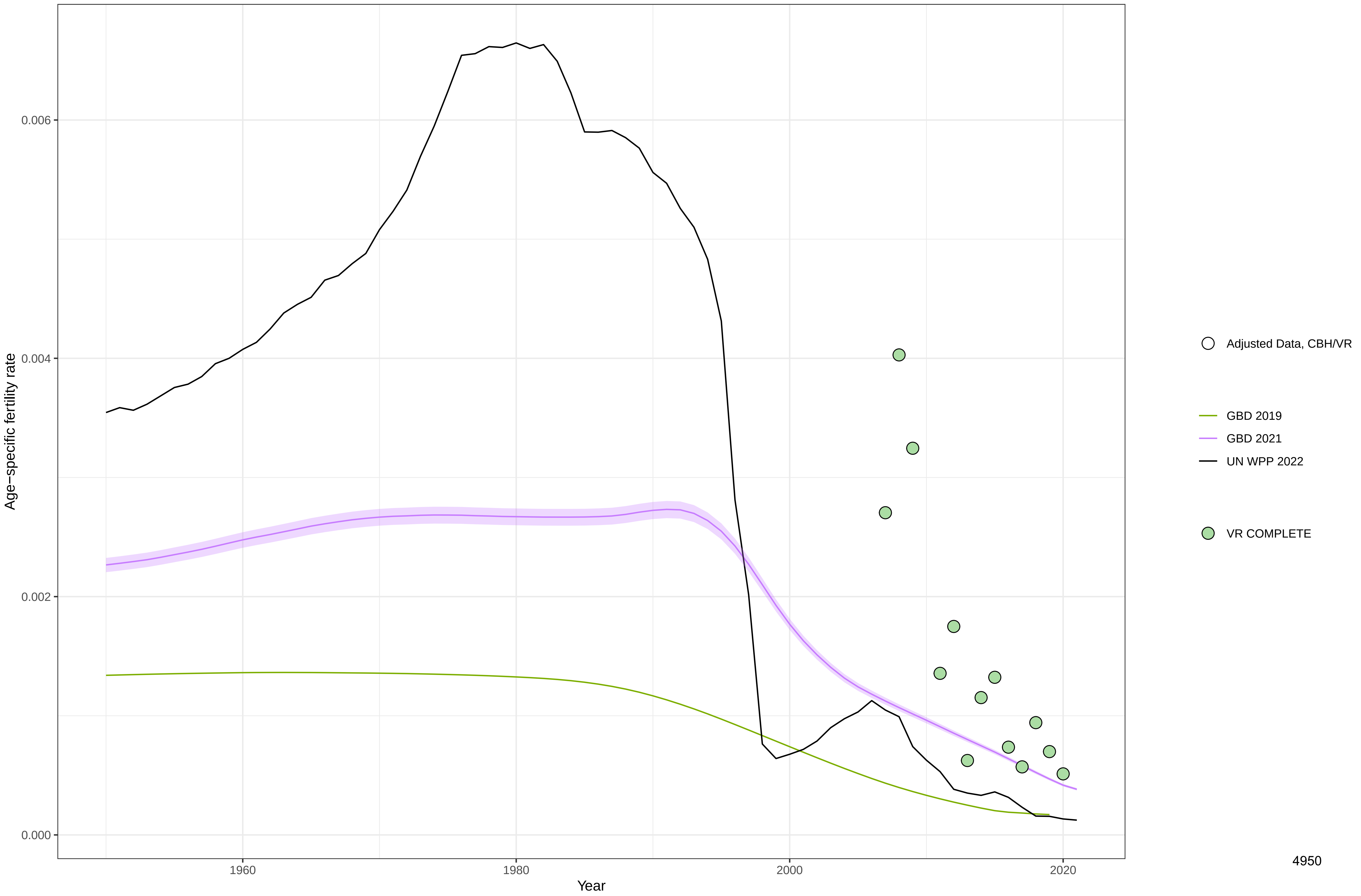

Total fertility rate fitted trend lines, 1950−2021: Oman TFR
Data points enclosed with a diamond are reference sources

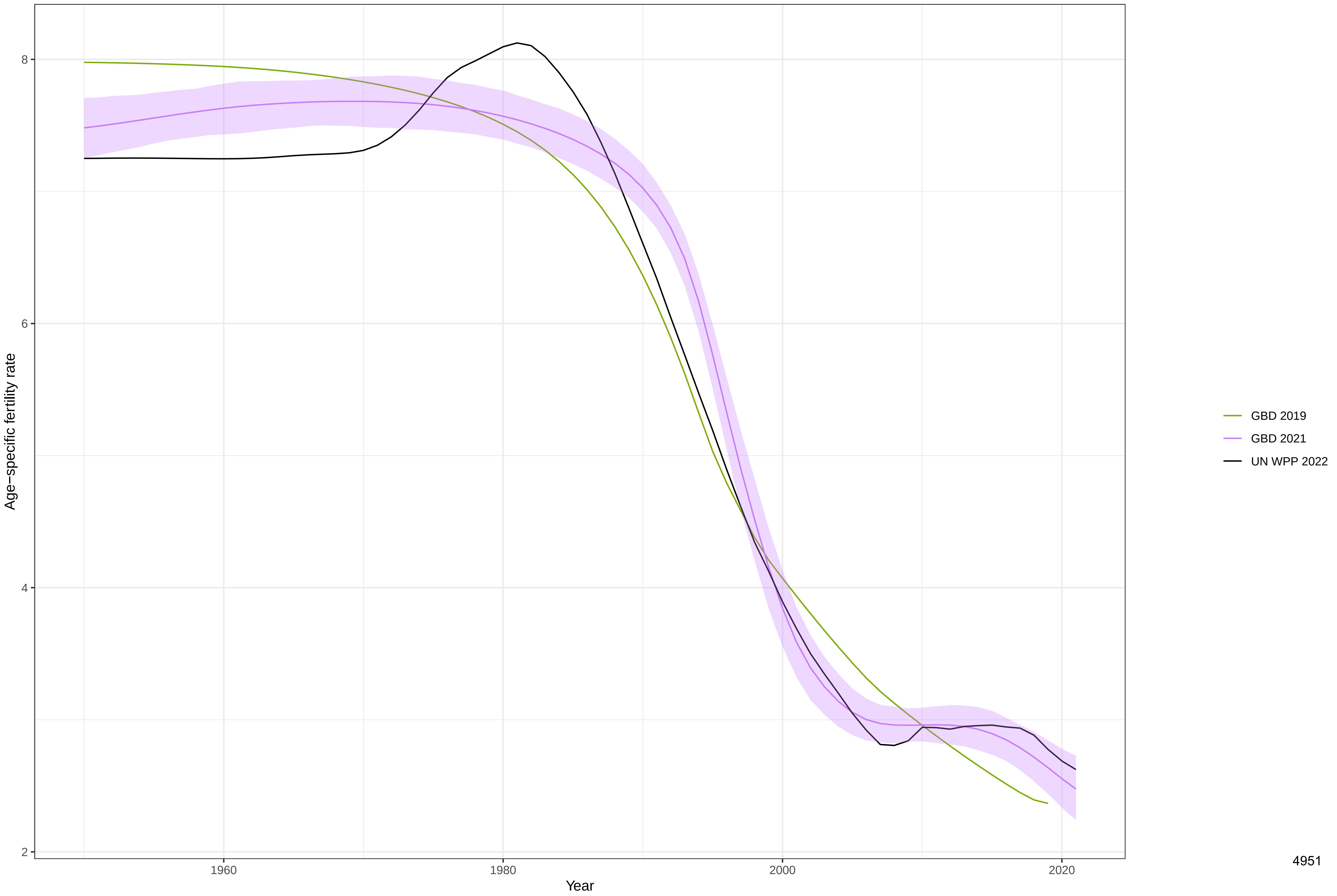

Age−specific fertility fitted trend lines, 1950−2021: Palestine, Ages: 10−14
Data points enclosed with a diamond are reference sources

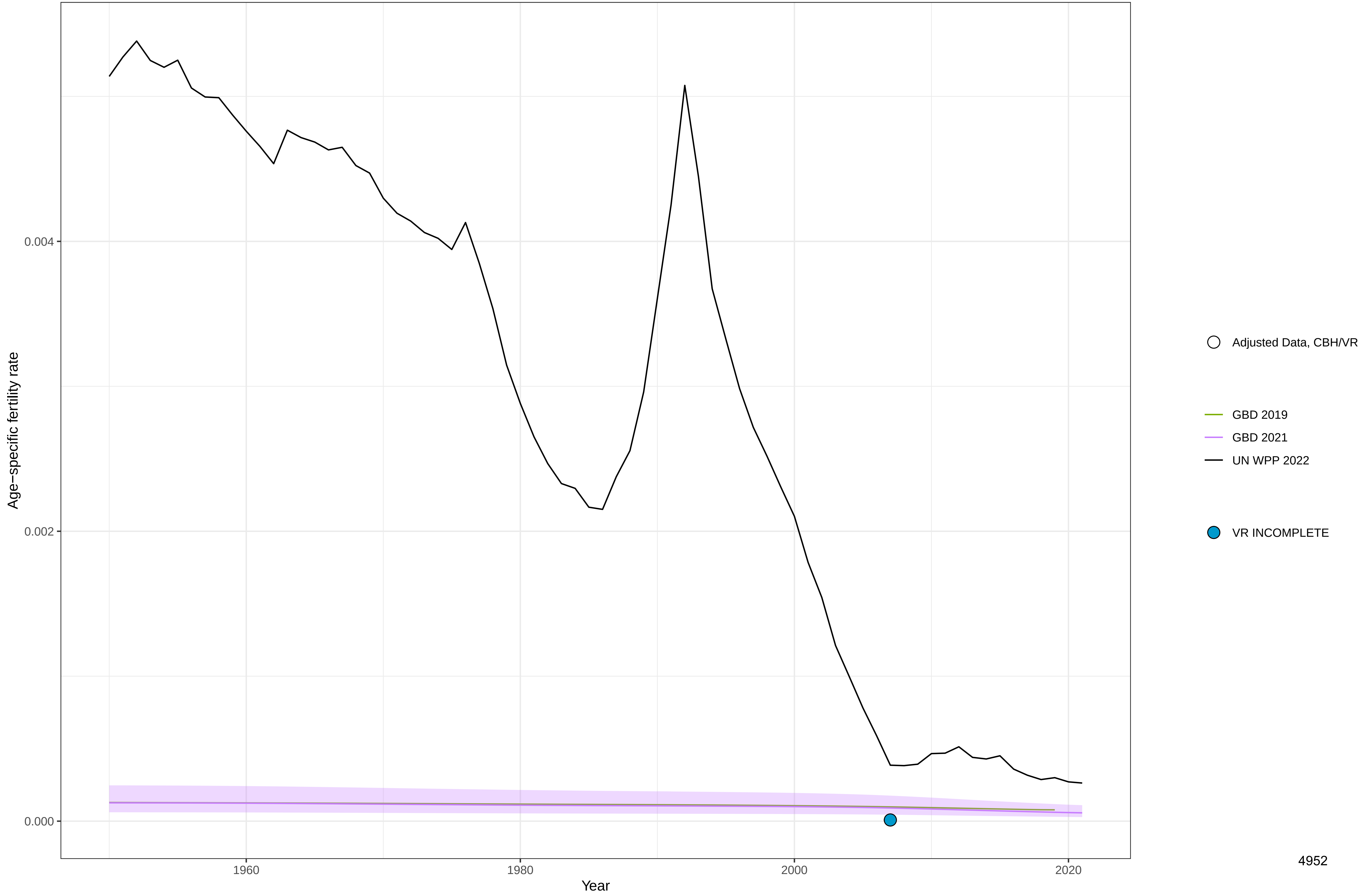

Age−specific fertility fitted trend lines, 1950−2021: Palestine, Ages: 15−19
Data points enclosed with a diamond are reference sources

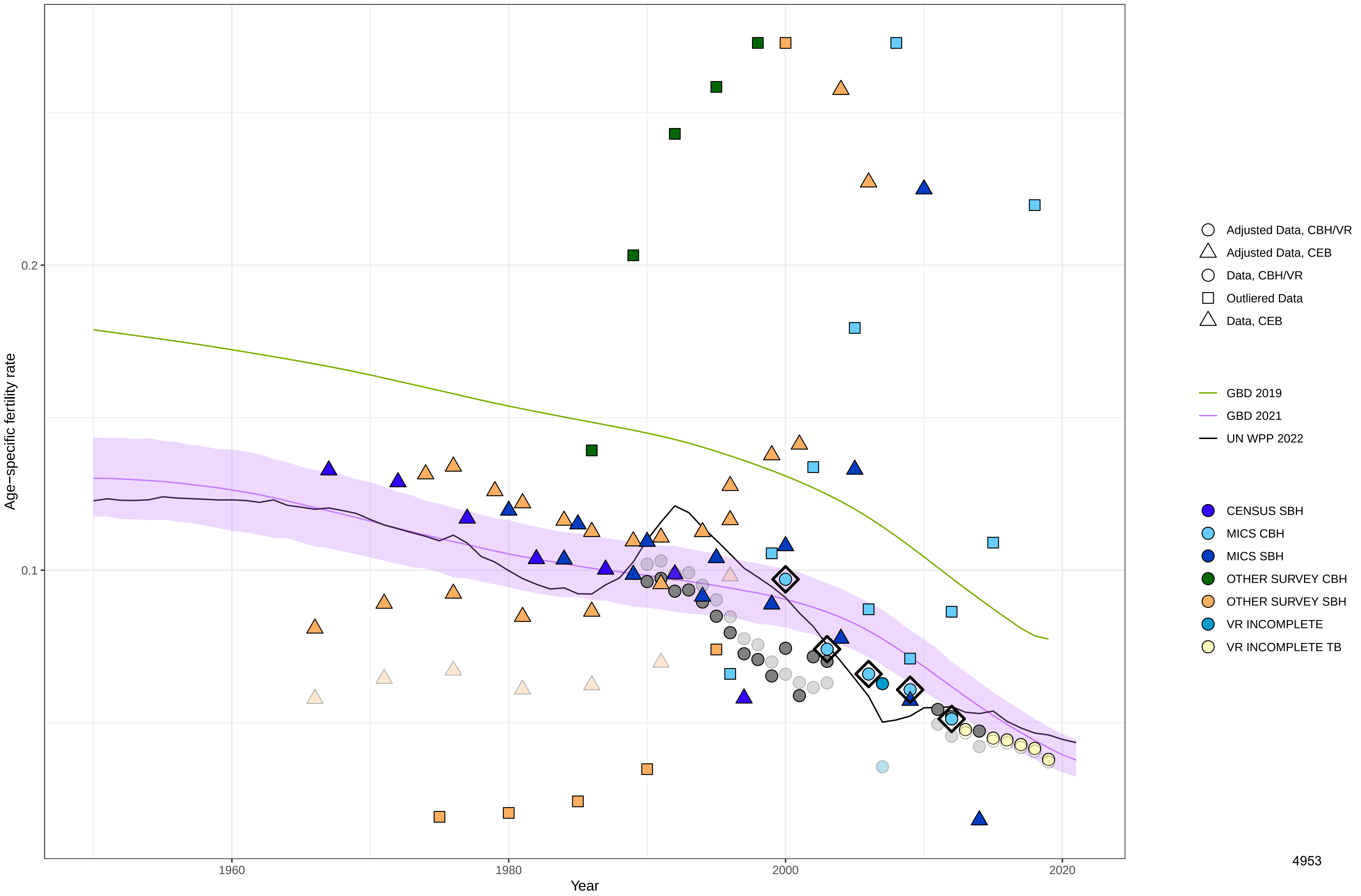

Age−specific fertility fitted trend lines, 1950−2021: Palestine, Ages: 20−24
Data points enclosed with a diamond are reference sources

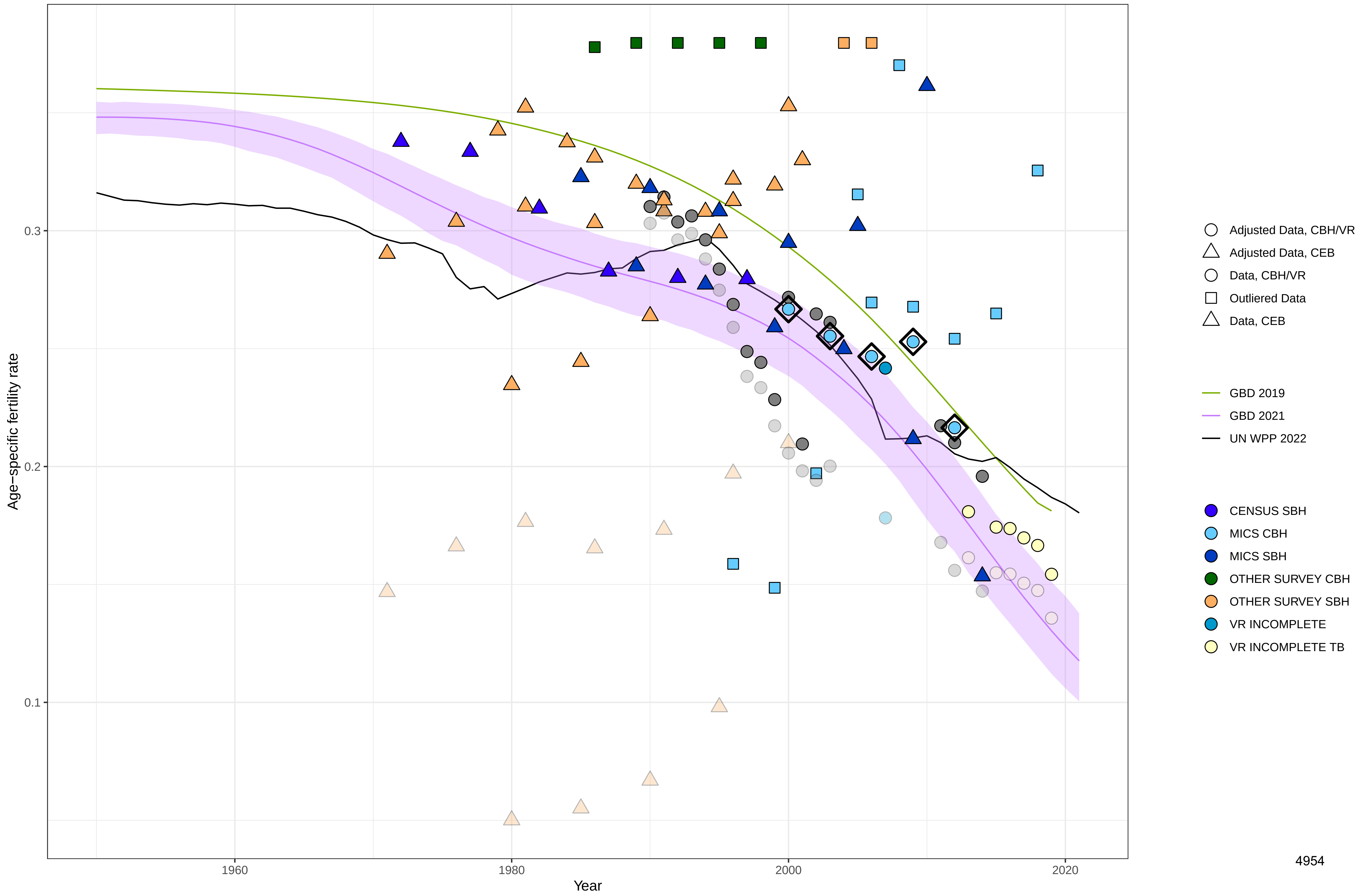

Age−specific fertility fitted trend lines, 1950−2021: Palestine, Ages: 25−29
Data points enclosed with a diamond are reference sources

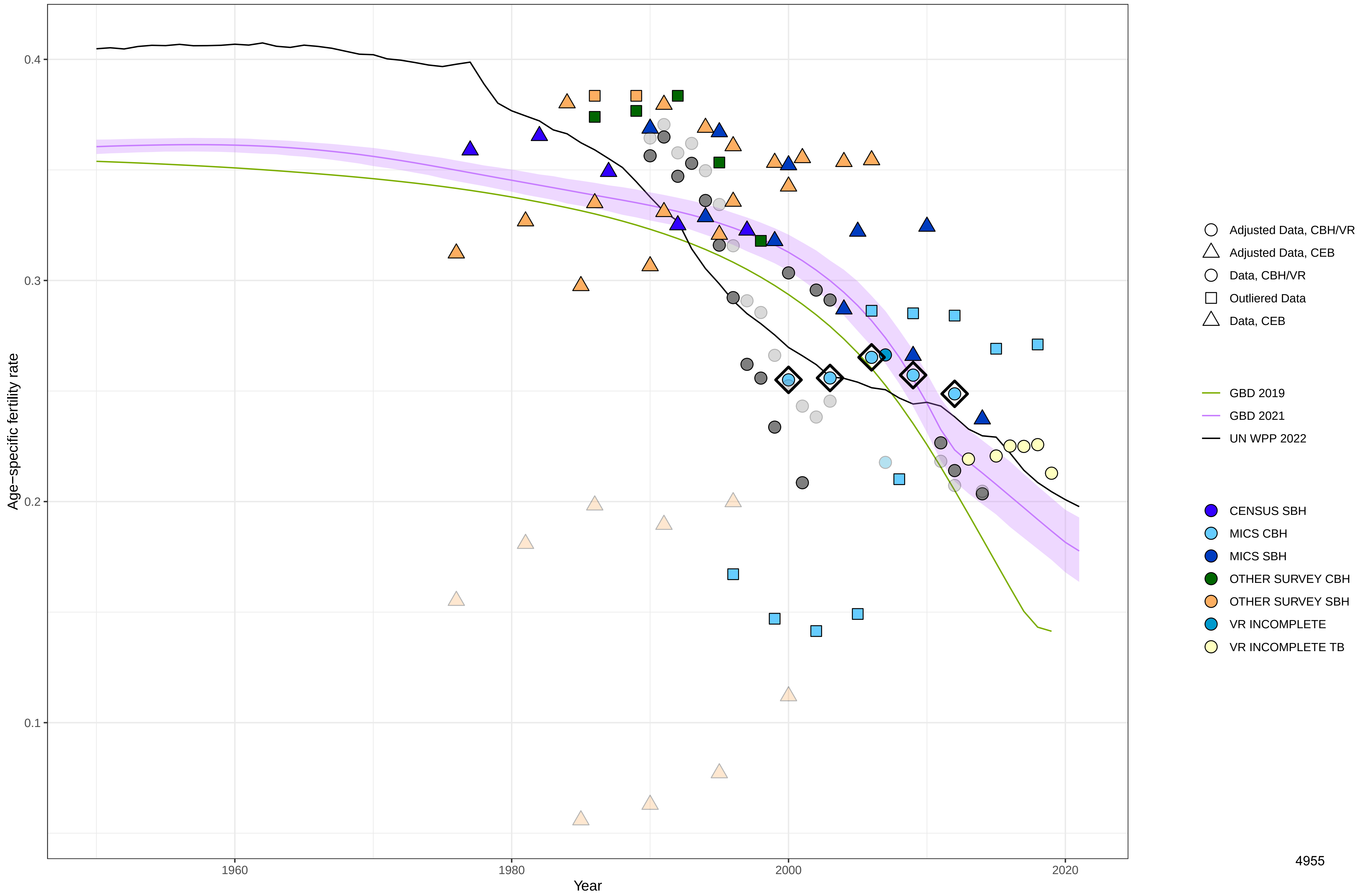

Age−specific fertility fitted trend lines, 1950−2021: Palestine, Ages: 30−34
Data points enclosed with a diamond are reference sources

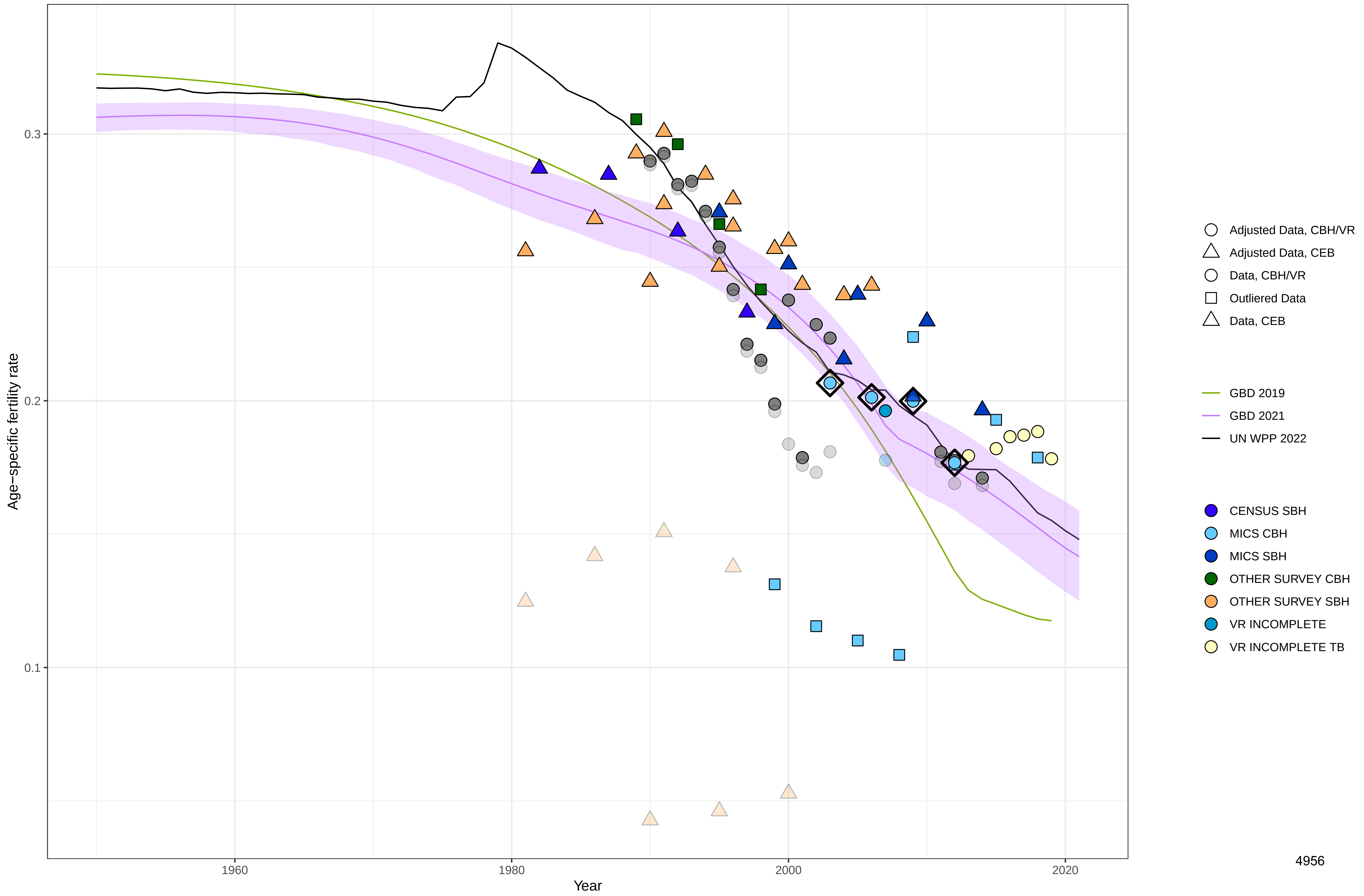

Age−specific fertility fitted trend lines, 1950−2021: Palestine, Ages: 35−39
Data points enclosed with a diamond are reference sources

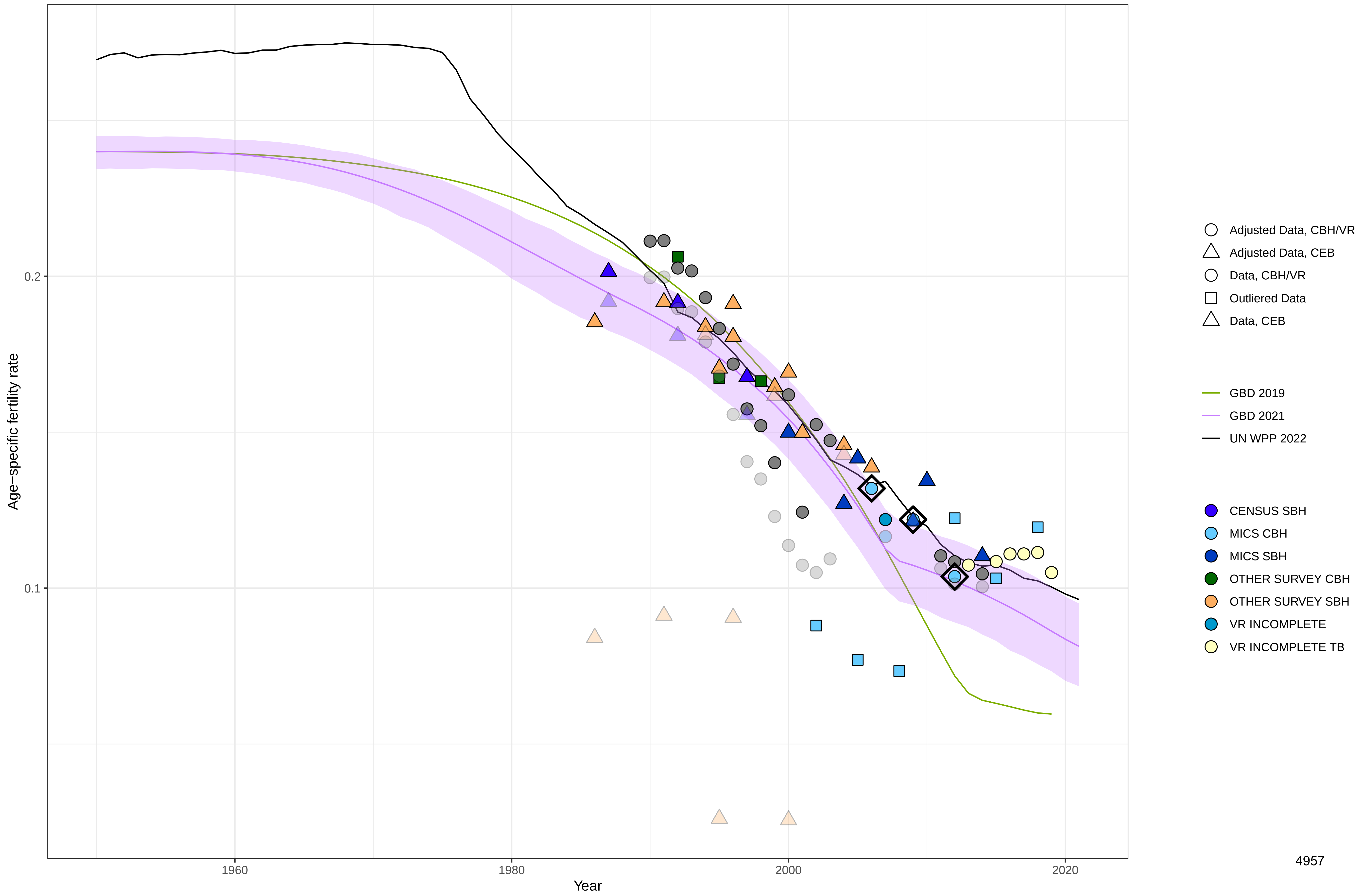

4957

Age−specific fertility fitted trend lines, 1950−2021: Palestine, Ages: 40−44
Data points enclosed with a diamond are reference sources

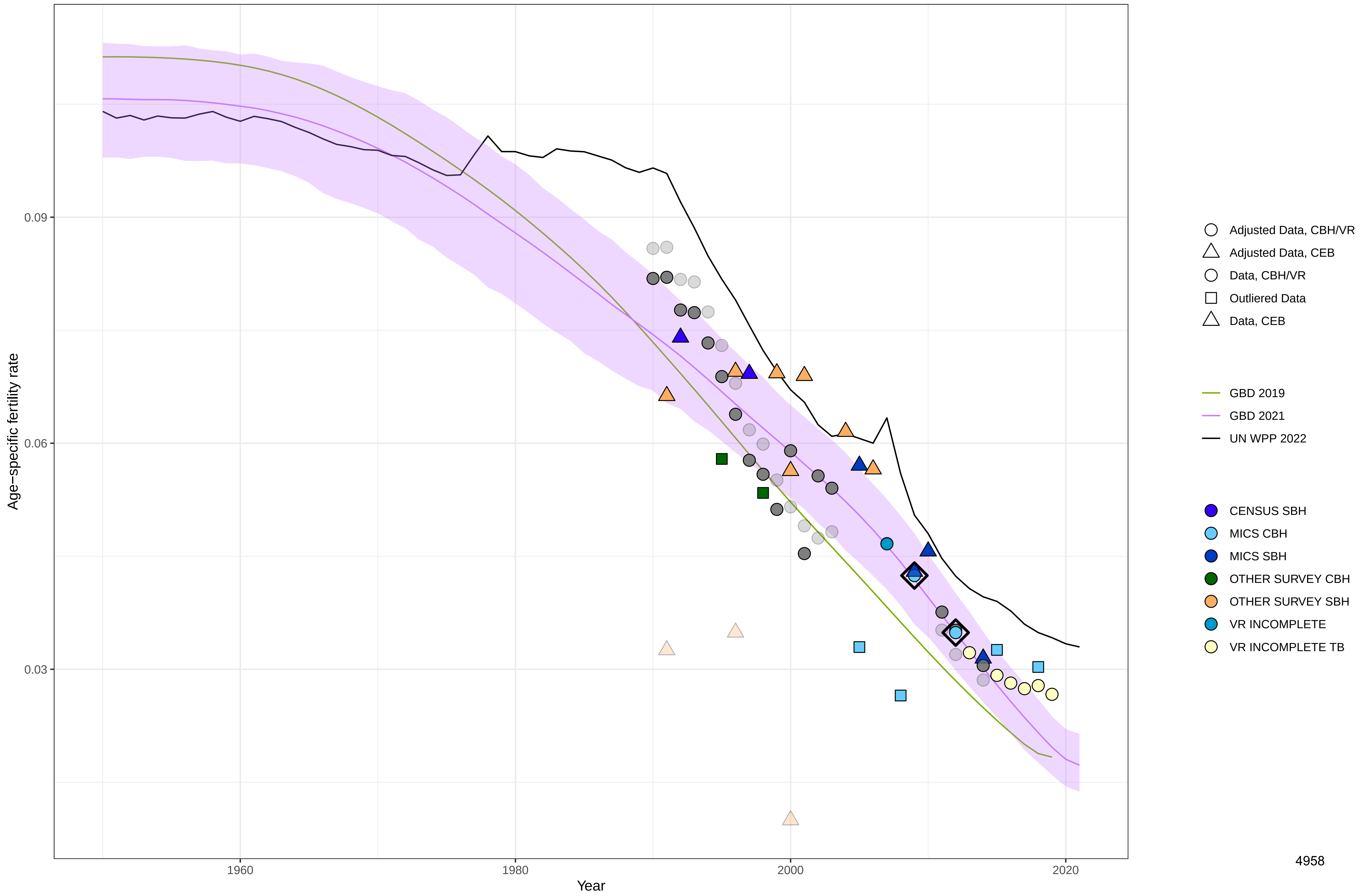

Age−specific fertility fitted trend lines, 1950−2021: Palestine, Ages: 45−49
Data points enclosed with a diamond are reference sources

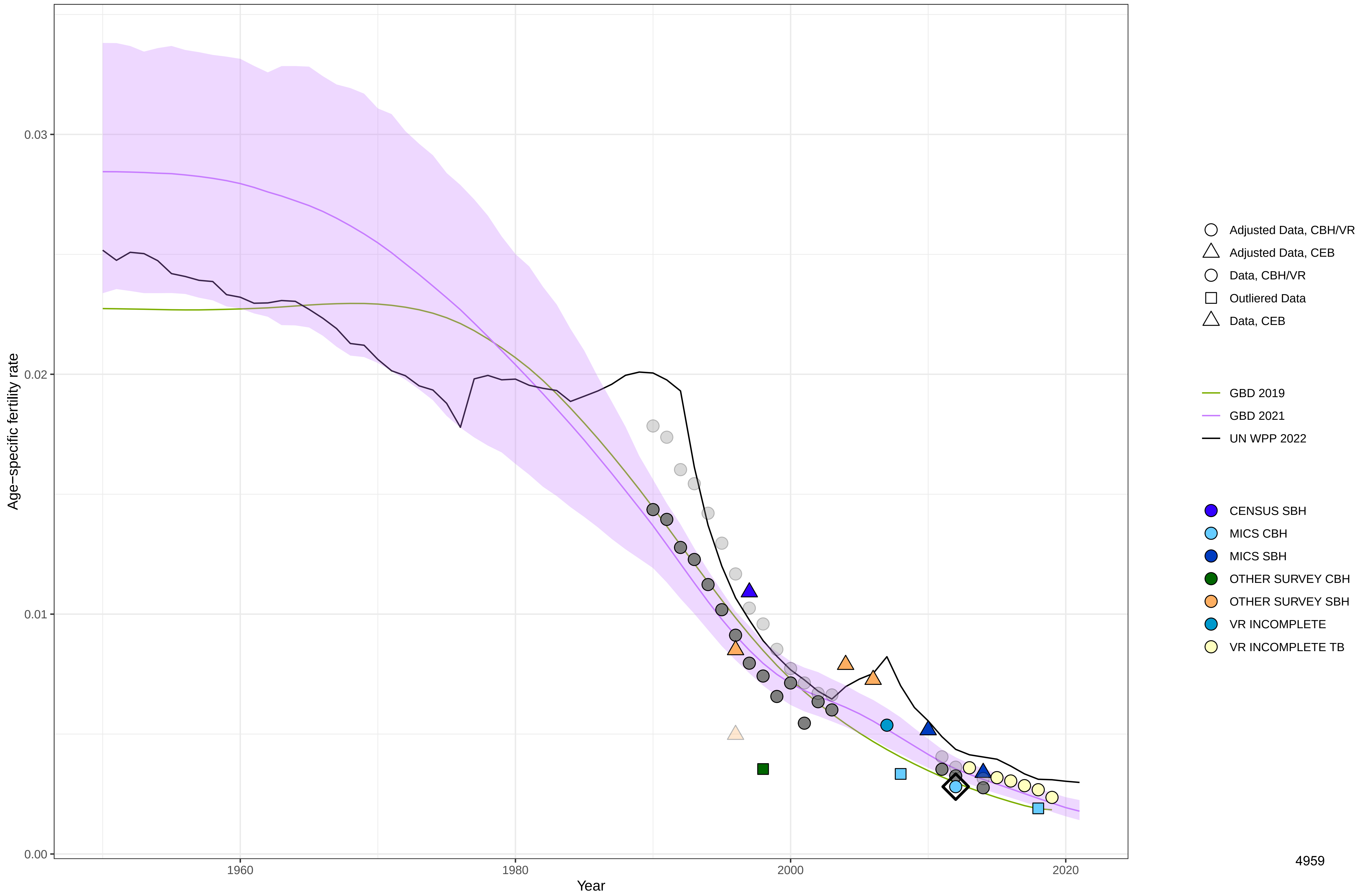

Age−specific fertility fitted trend lines, 1950−2021: Palestine, Ages: 50−54
Data points enclosed with a diamond are reference sources

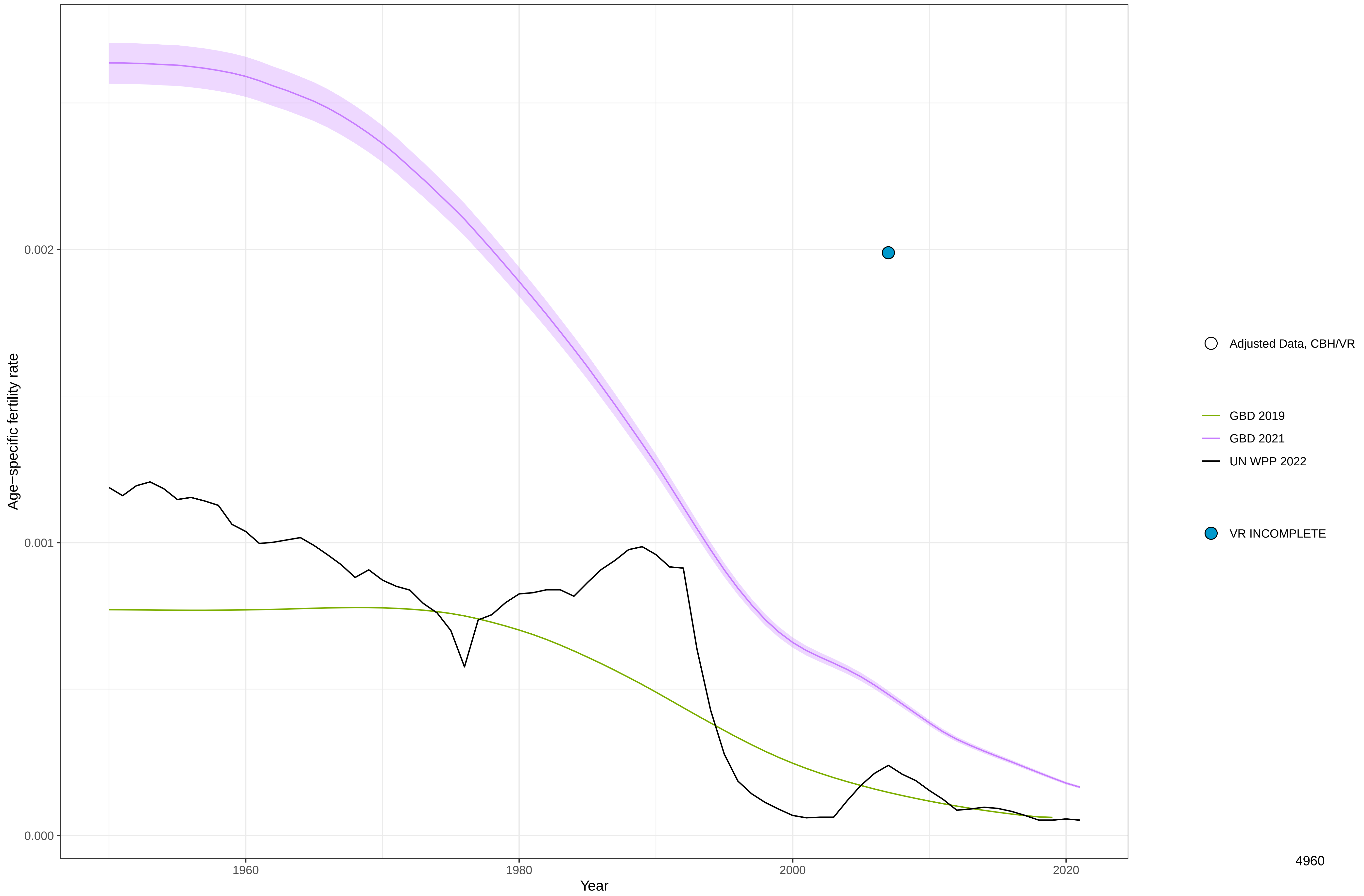

4960

Total fertility rate fitted trend lines, 1950−2021: Palestine TFR
Data points enclosed with a diamond are reference sources

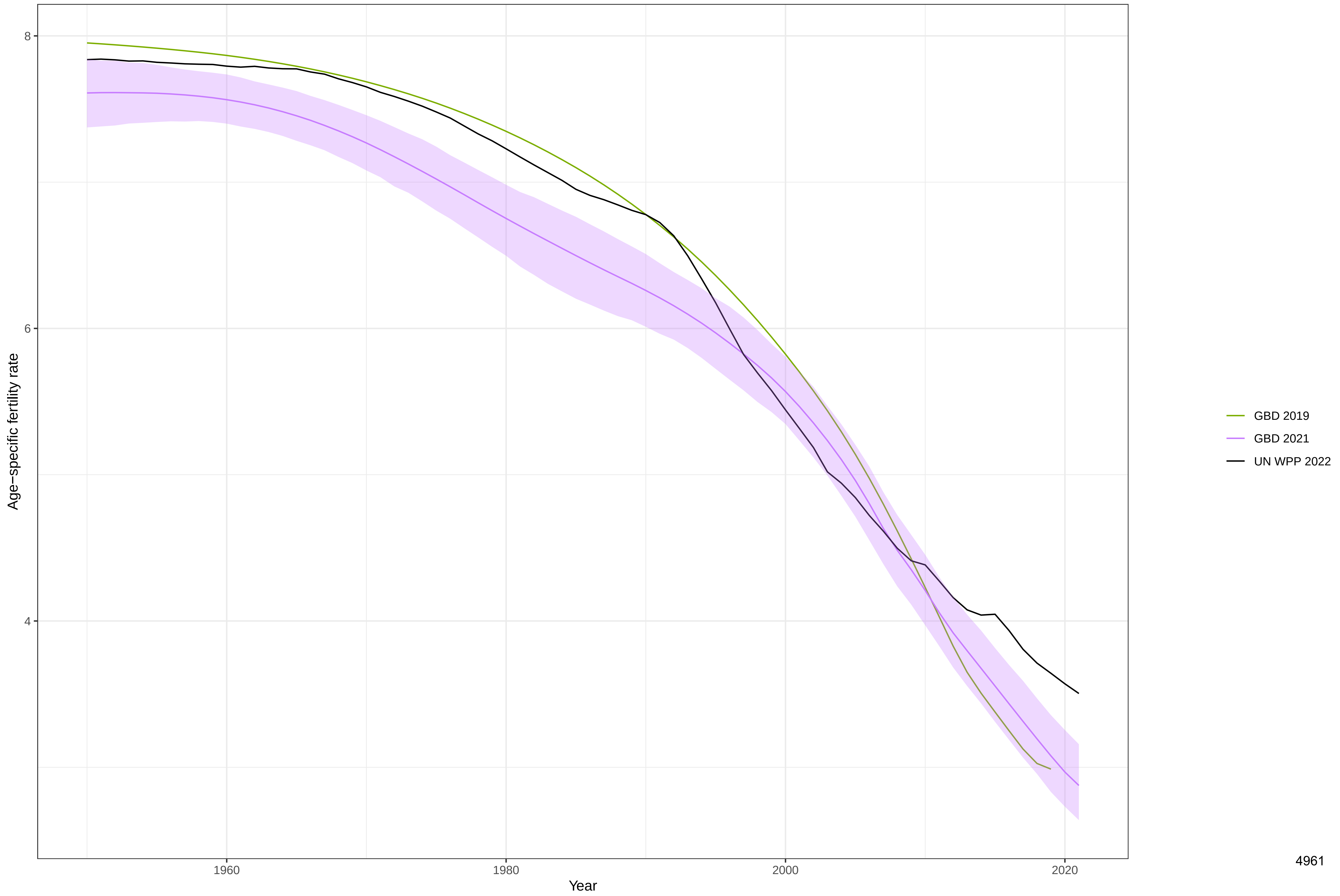

Age−specific fertility fitted trend lines, 1950−2021: Qatar, Ages: 10−14
Data points enclosed with a diamond are reference sources

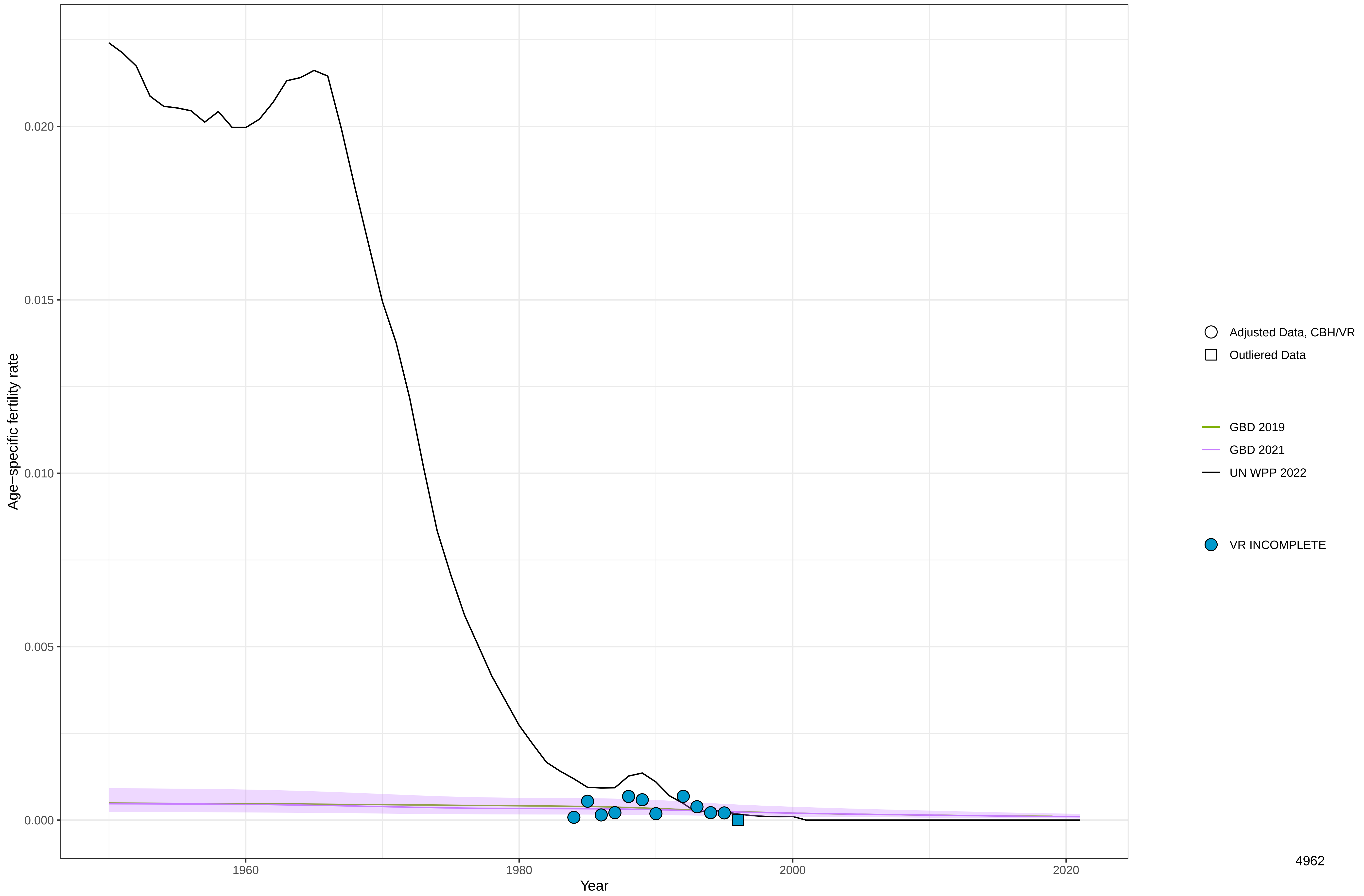

Age−specific fertility fitted trend lines, 1950−2021: Qatar, Ages: 15−19
Data points enclosed with a diamond are reference sources

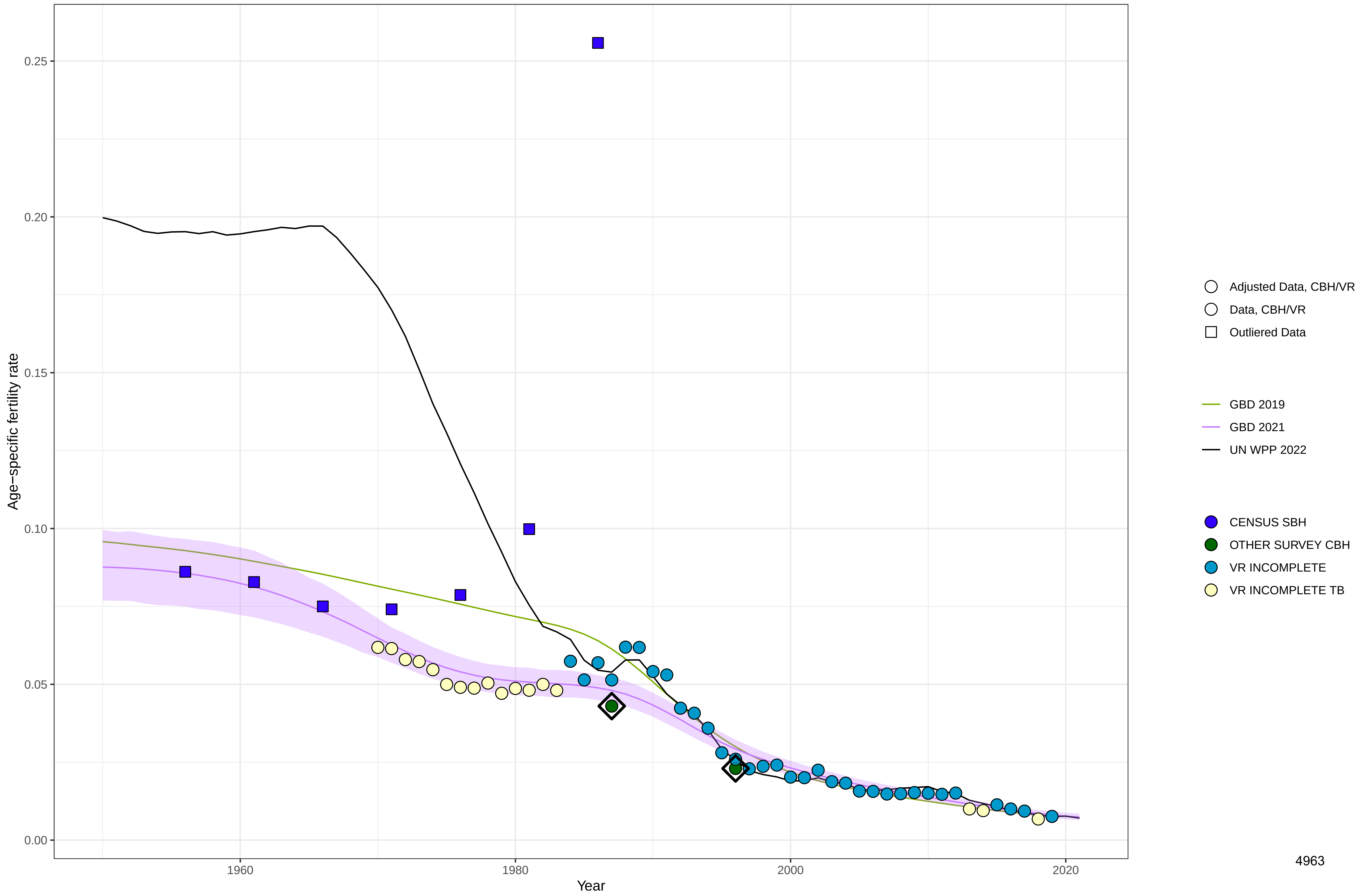

Age−specific fertility fitted trend lines, 1950−2021: Qatar, Ages: 20−24
Data points enclosed with a diamond are reference sources

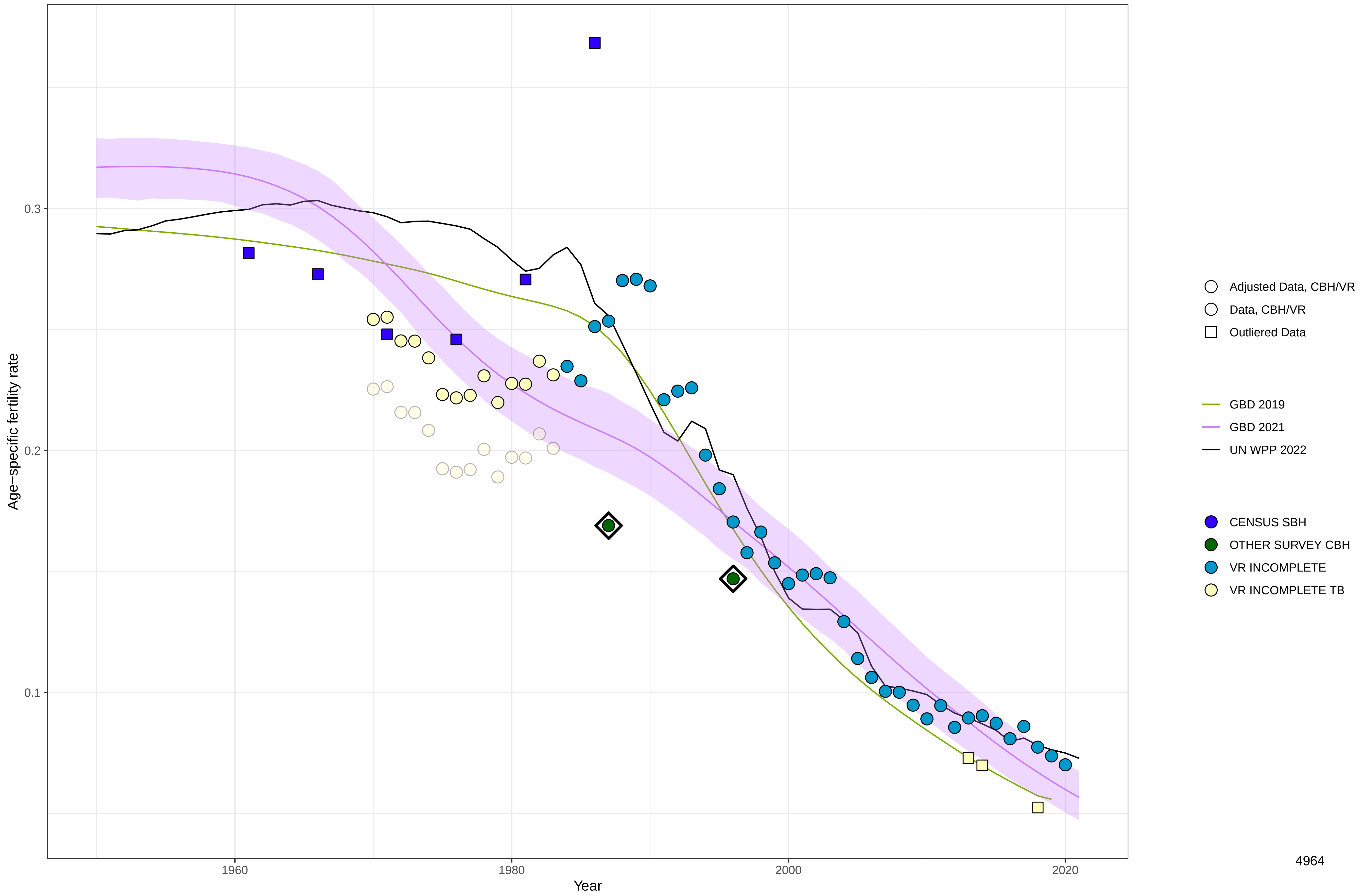

Age−specific fertility fitted trend lines, 1950−2021: Qatar, Ages: 25−29
Data points enclosed with a diamond are reference sources

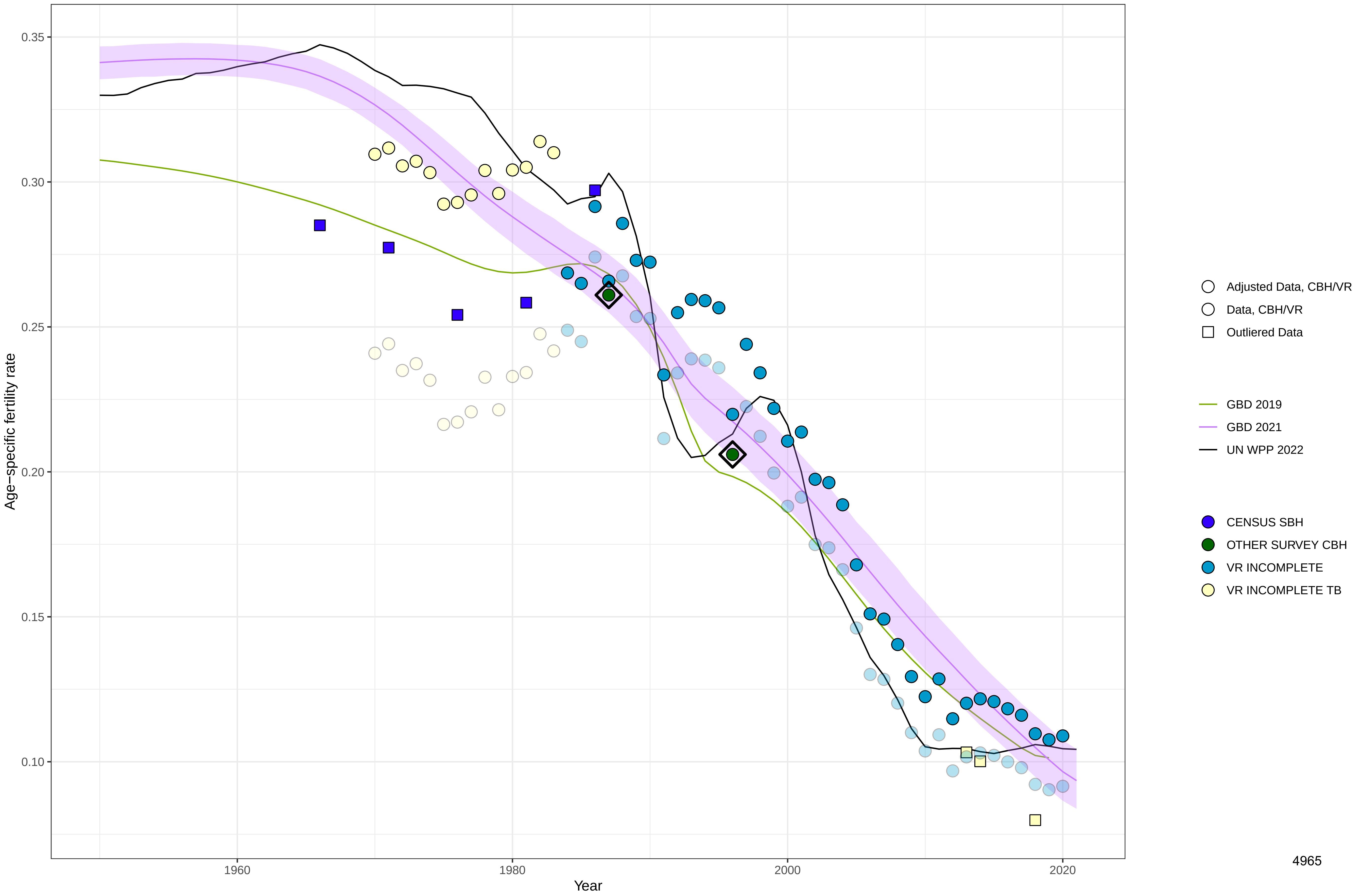

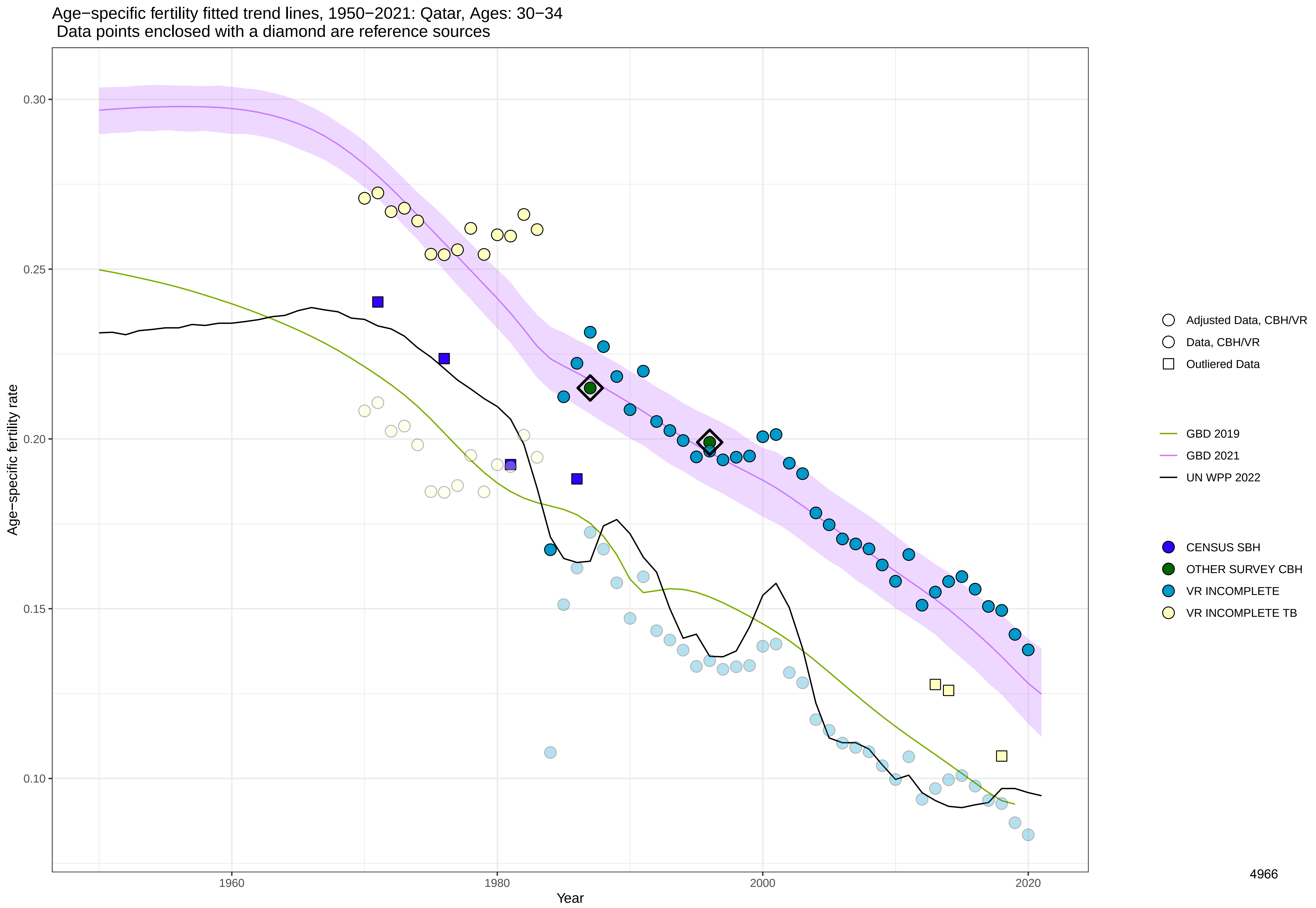
Age−specific fertility fitted trend lines, 1950−2021: Qatar, Ages: 30−34
Data points enclosed with a diamond are reference sources
Age−specific fertility rate
Year
0.30
0.25
0.20
0.15
0.10
1960
1980
2000
2020
Adjusted Data, CBH/VR
Data, CBH/VR
Outliered Data
GBD 2019
GBD 2021
UN WPP 2022
CENSUS SBH
OTHER SURVEY CBH
VR INCOMPLETE
VR INCOMPLETE TB

4966

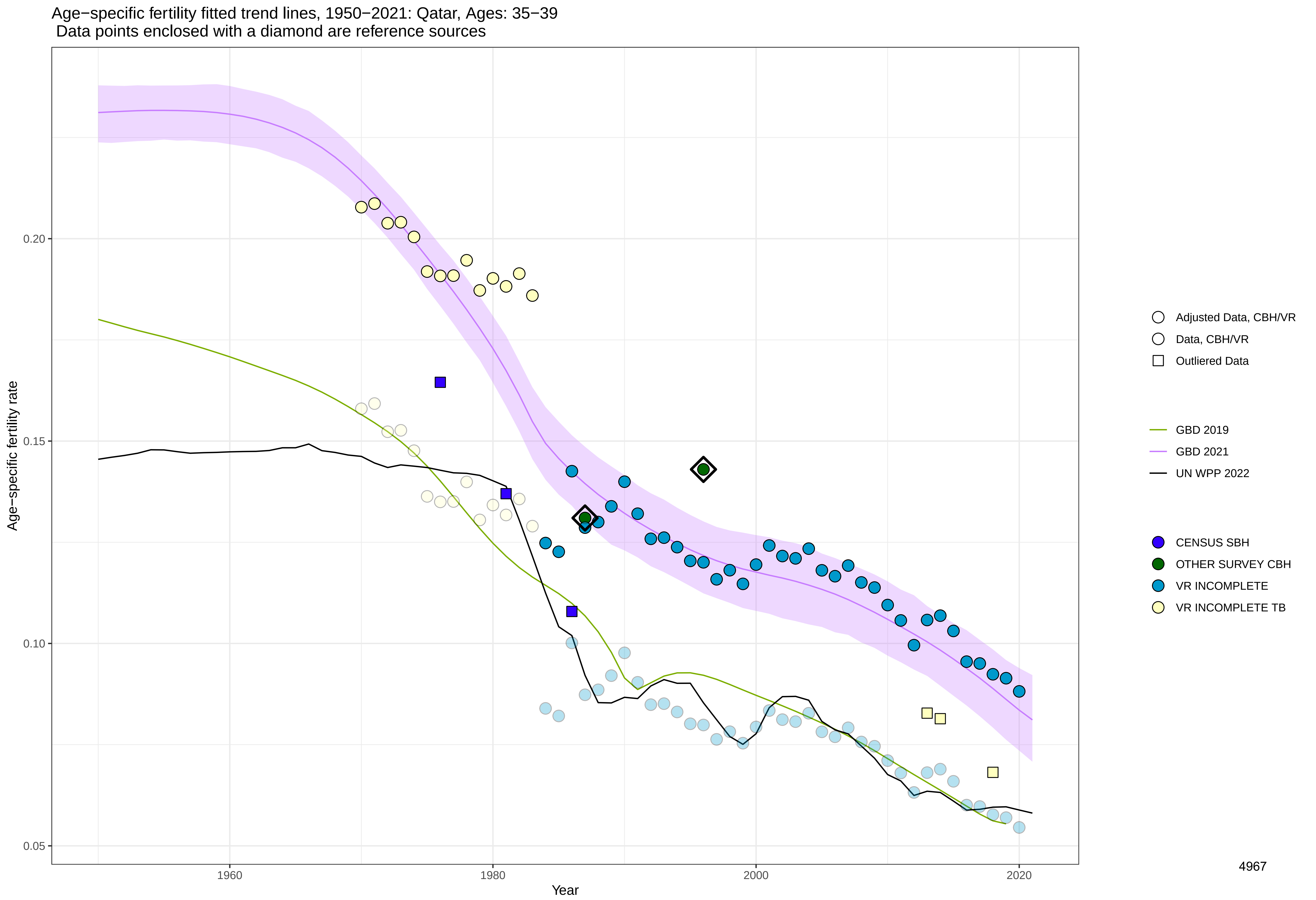

Age−specific fertility fitted trend lines, 1950−2021: Qatar, Ages: 35−39
Data points enclosed with a diamond are reference sources
Age−specific fertility rate
0.20
0.15
0.10
0.05
1960
1980
2000
2020
Year
Adjusted Data, CBH/VR
Data, CBH/VR
Outliered Data
GBD 2019
GBD 2021
UN WPP 2022
CENSUS SBH
OTHER SURVEY CBH
VR INCOMPLETE
VR INCOMPLETE TB

4967

Age−specific fertility fitted trend lines, 1950−2021: Qatar, Ages: 40−44
Data points enclosed with a diamond are reference sources

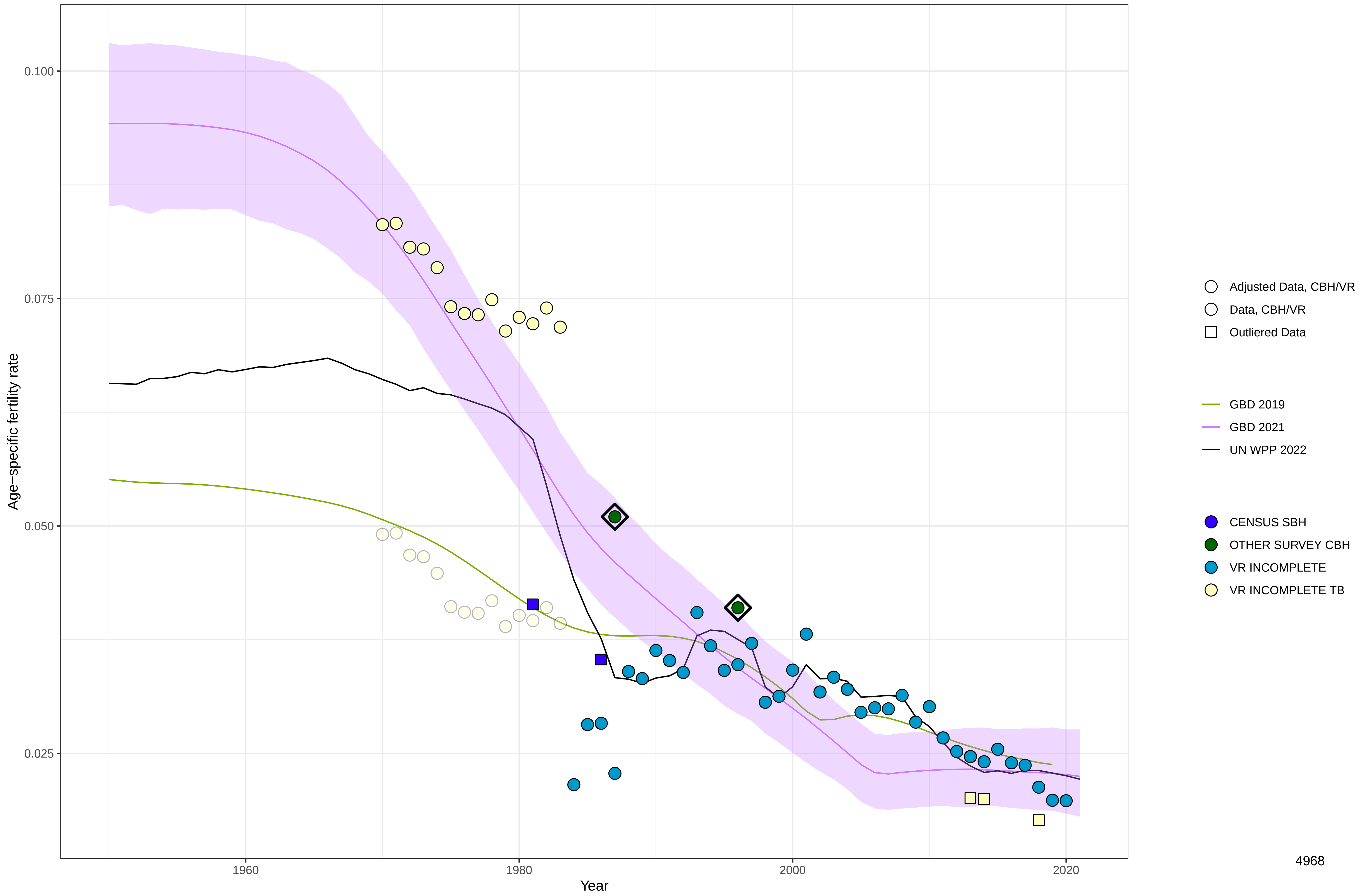

Age−specific fertility fitted trend lines, 1950−2021: Qatar, Ages: 45−49
Data points enclosed with a diamond are reference sources

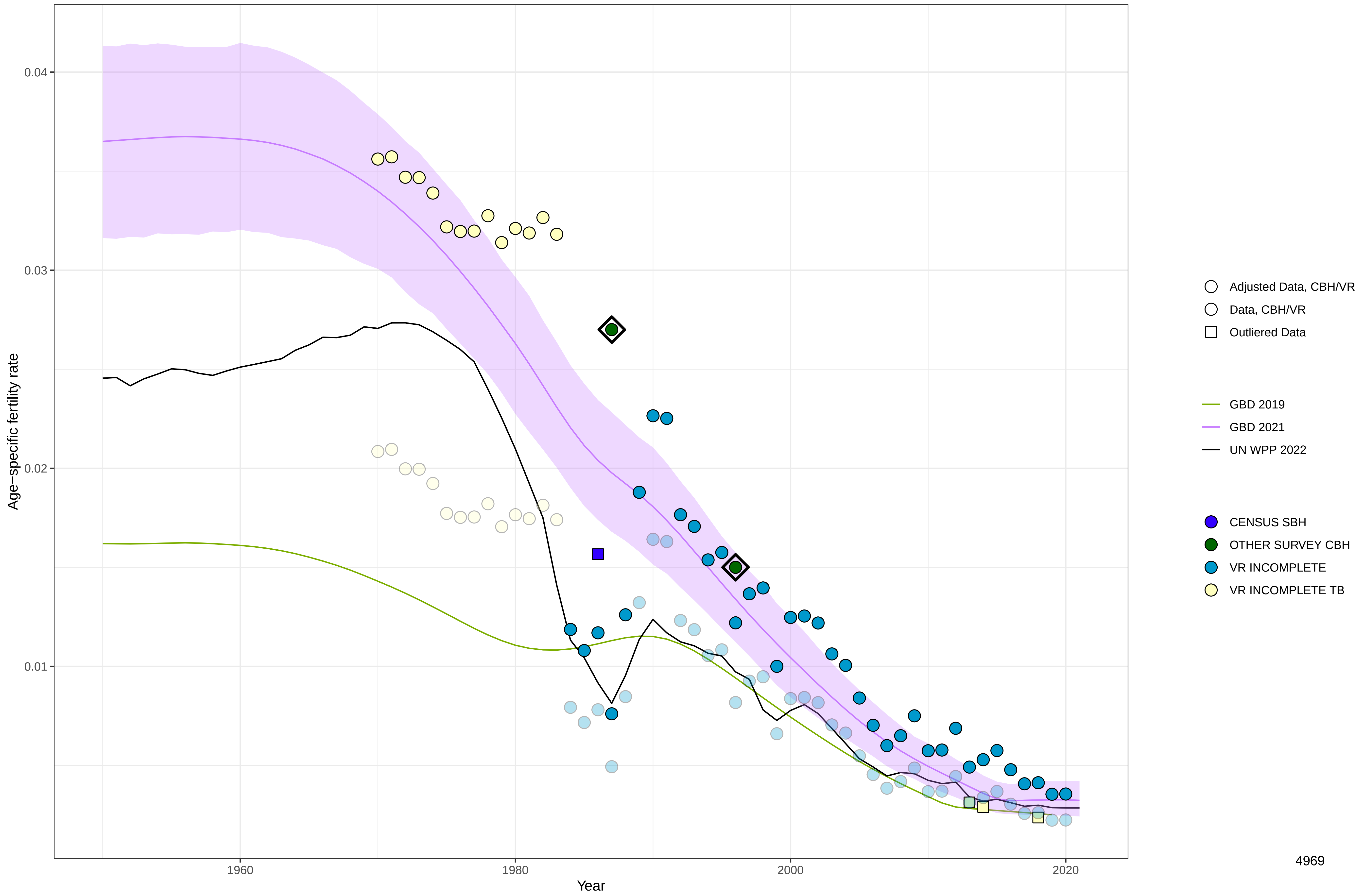

Age−specific fertility fitted trend lines, 1950−2021: Qatar, Ages: 50−54
Data points enclosed with a diamond are reference sources

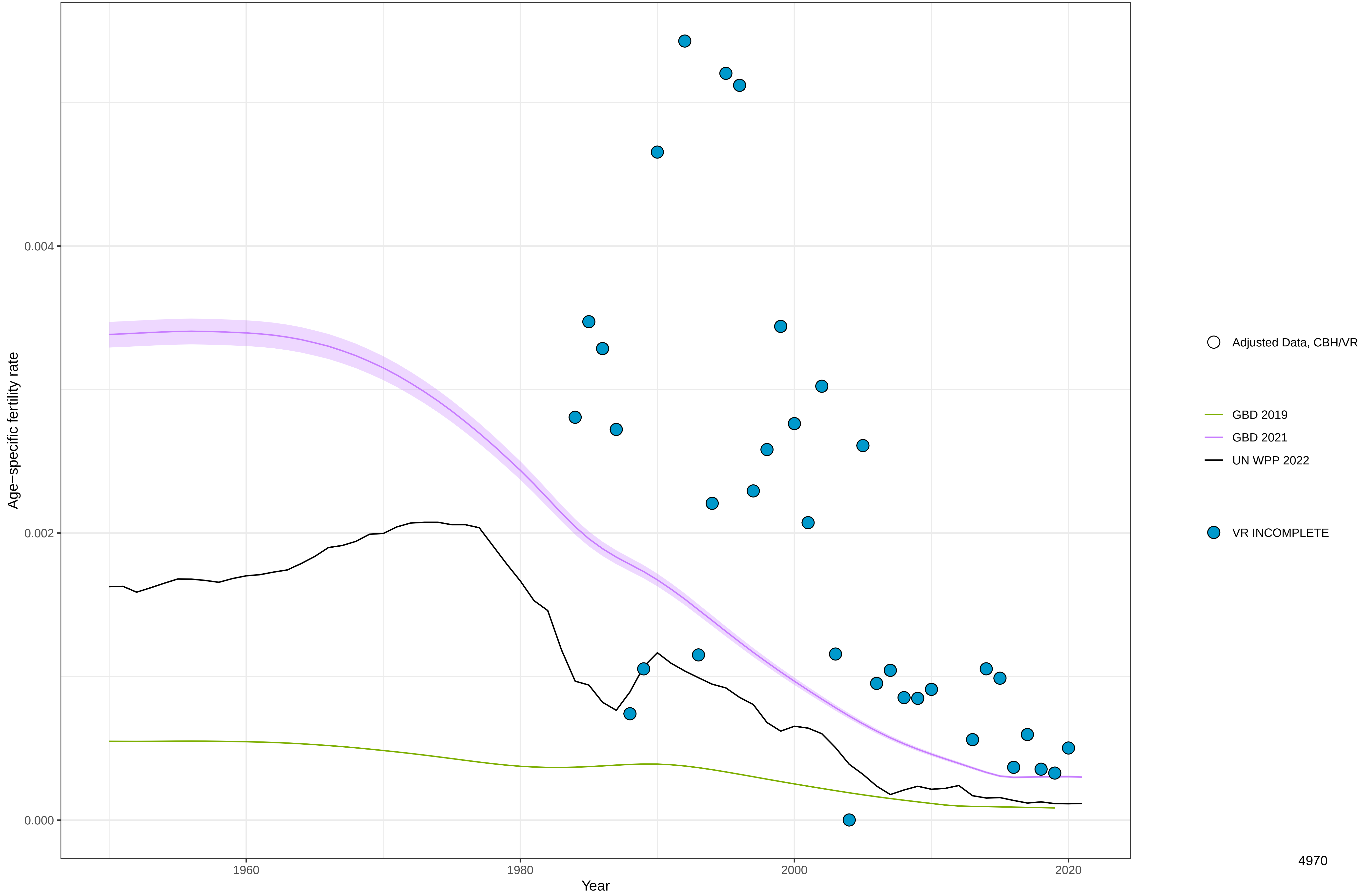

Total fertility rate fitted trend lines, 1950−2021: Qatar TFR
Data points enclosed with a diamond are reference sources

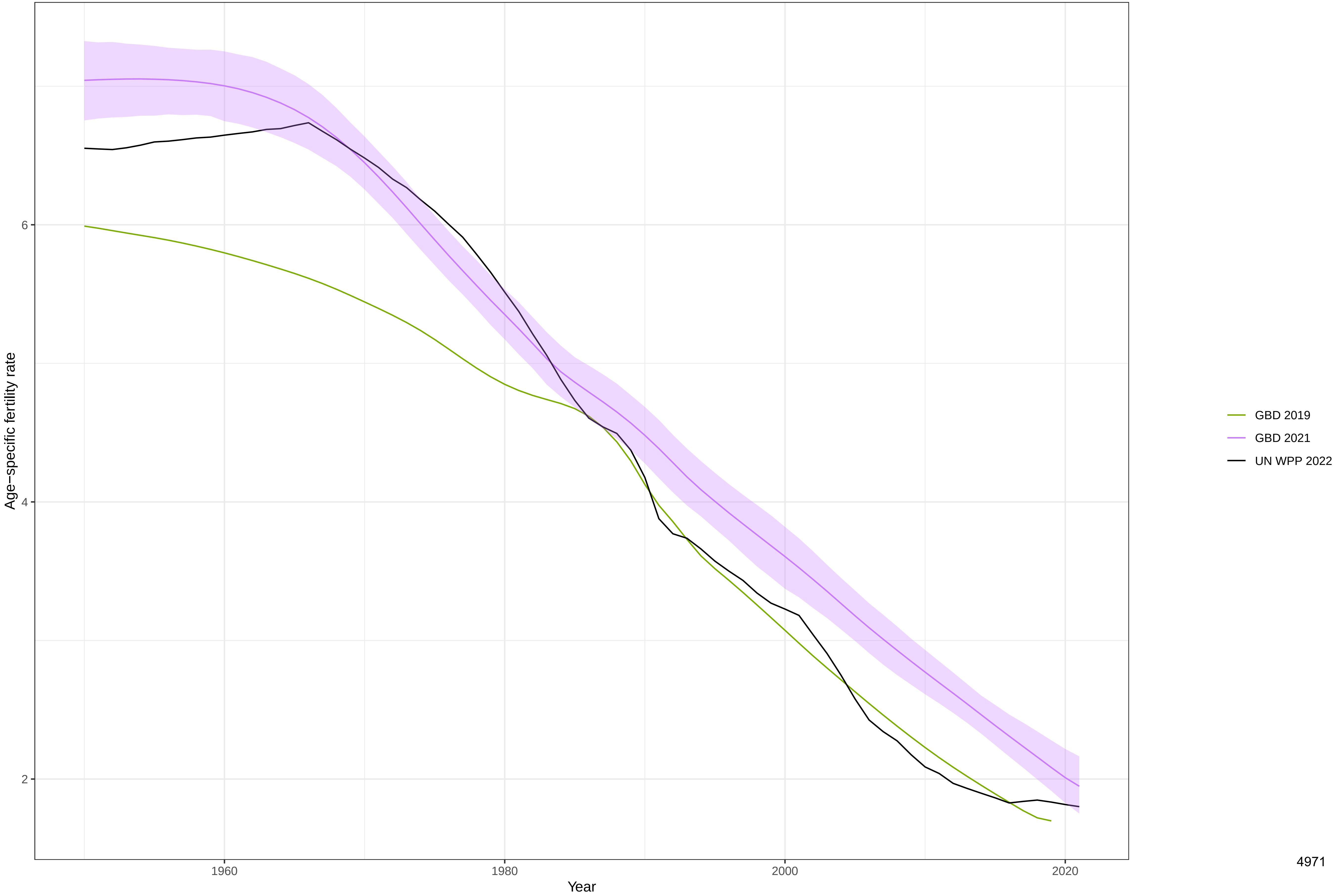

Age−specific fertility fitted trend lines, 1950−2021: Saudi Arabia, Ages: 10−14
Data points enclosed with a diamond are reference sources

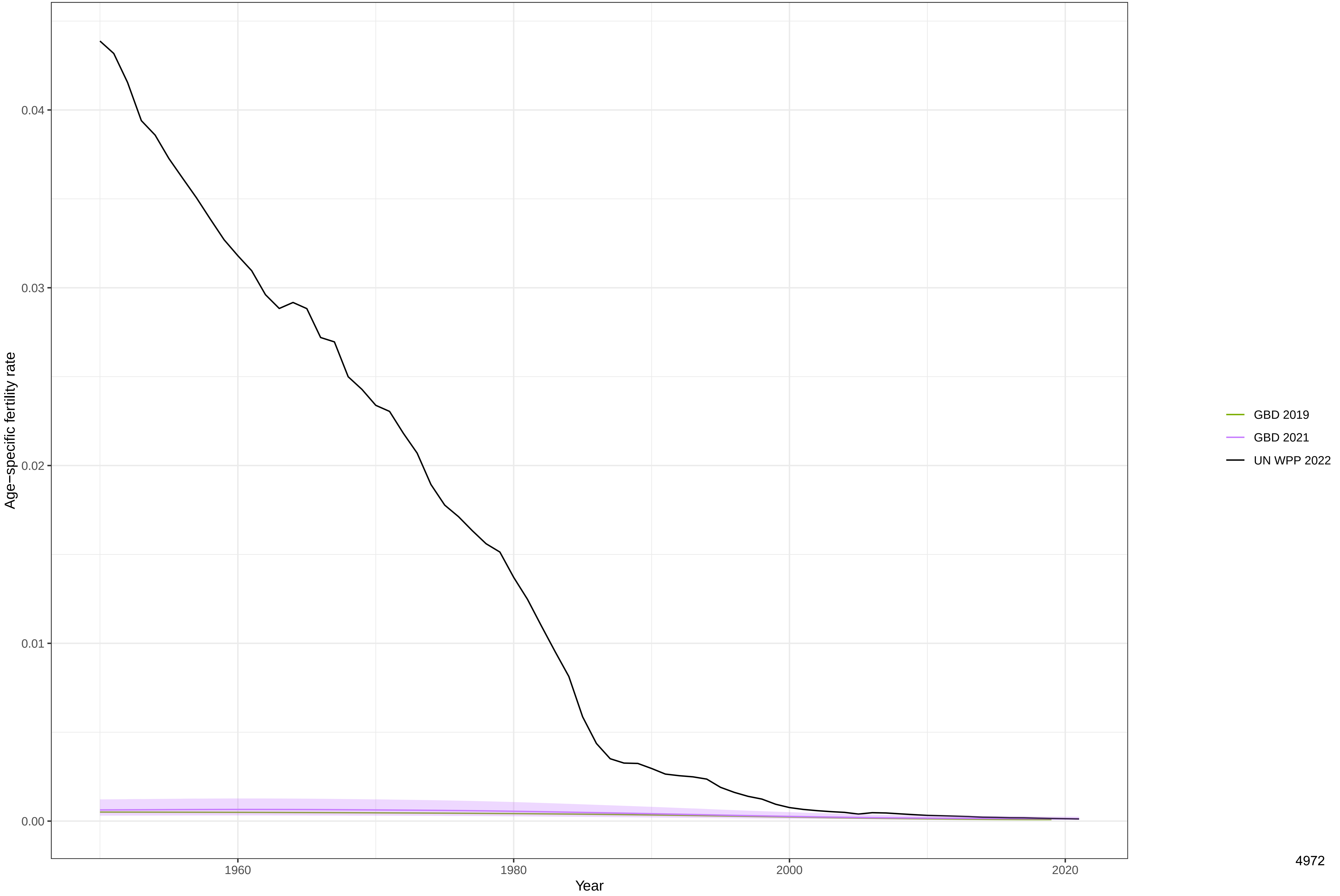

Age−specific fertility fitted trend lines, 1950−2021: Saudi Arabia, Ages: 15−19
Data points enclosed with a diamond are reference sources

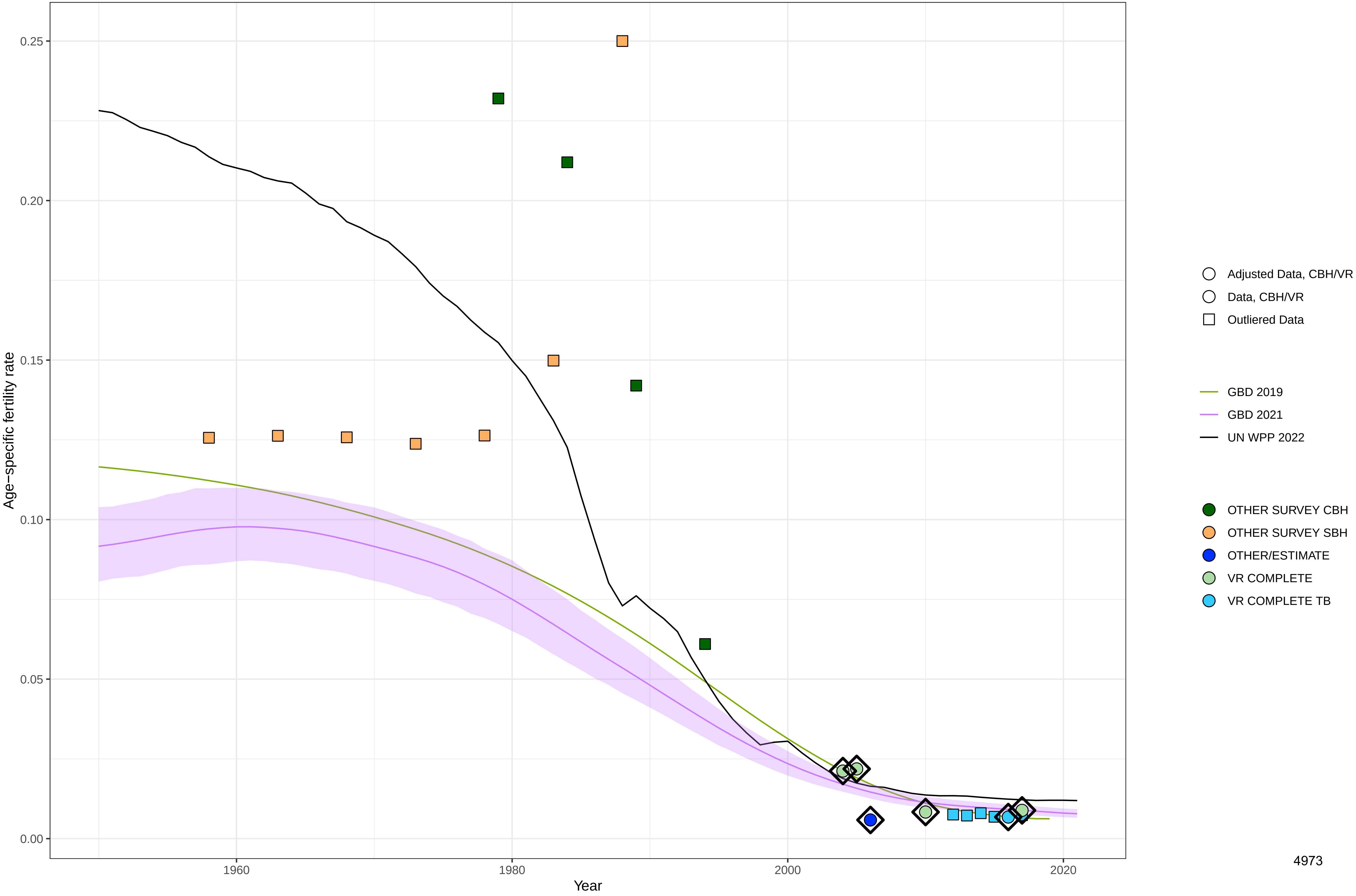

Age−specific fertility fitted trend lines, 1950−2021: Saudi Arabia, Ages: 20−24
Data points enclosed with a diamond are reference sources

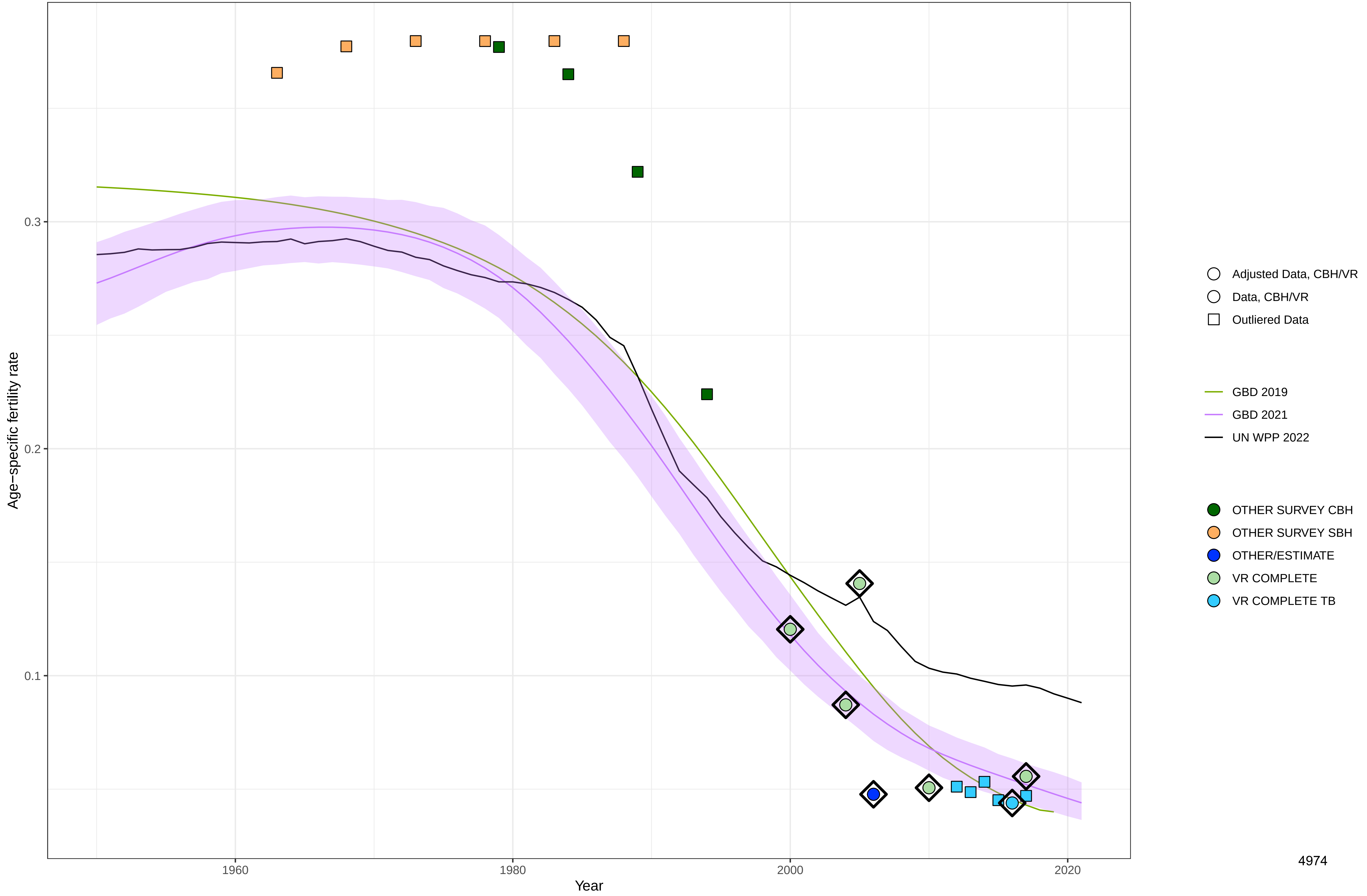

Age−specific fertility fitted trend lines, 1950−2021: Saudi Arabia, Ages: 25−29
Data points enclosed with a diamond are reference sources

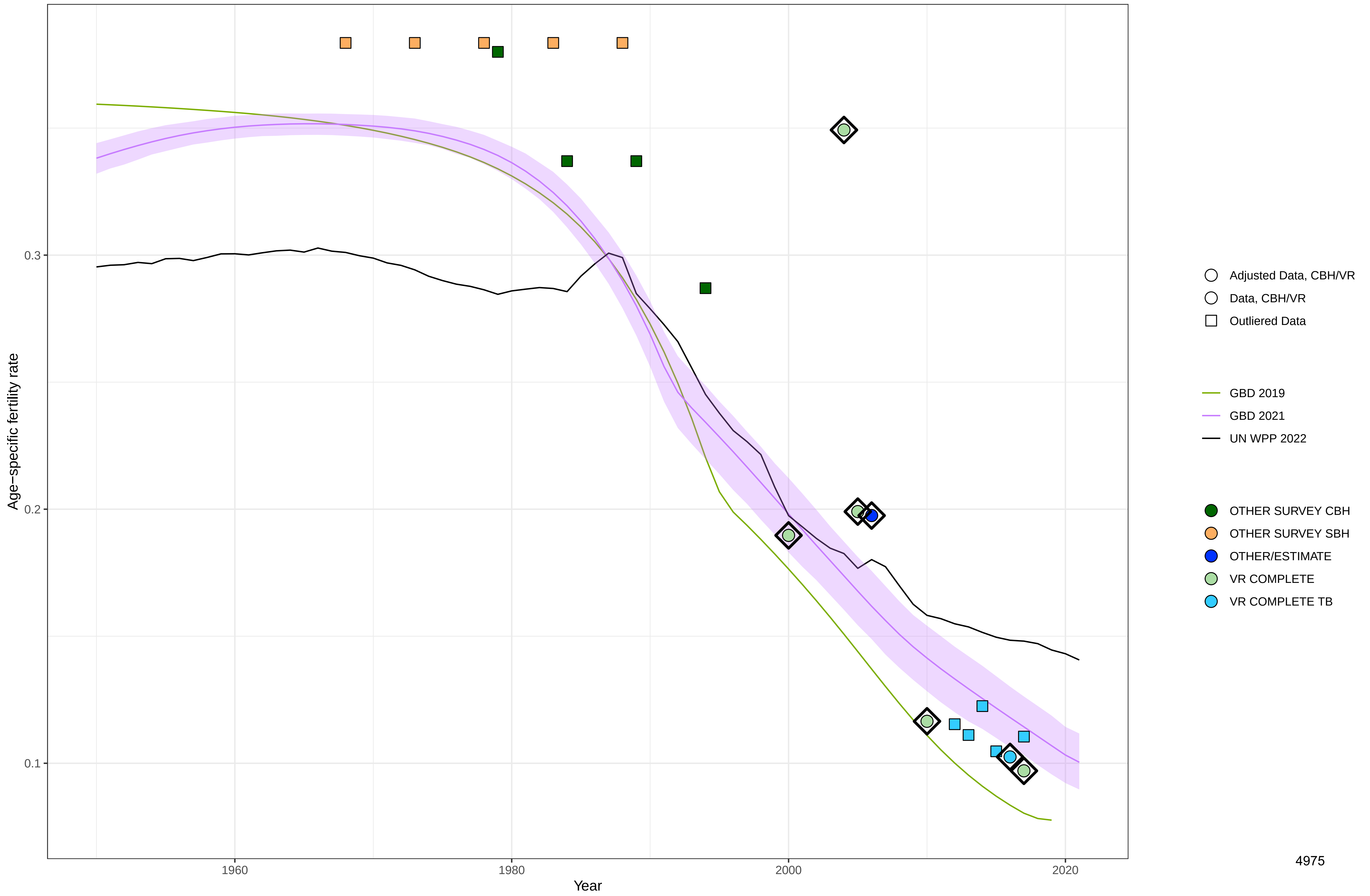

Age−specific fertility fitted trend lines, 1950−2021: Saudi Arabia, Ages: 30−34
Data points enclosed with a diamond are reference sources

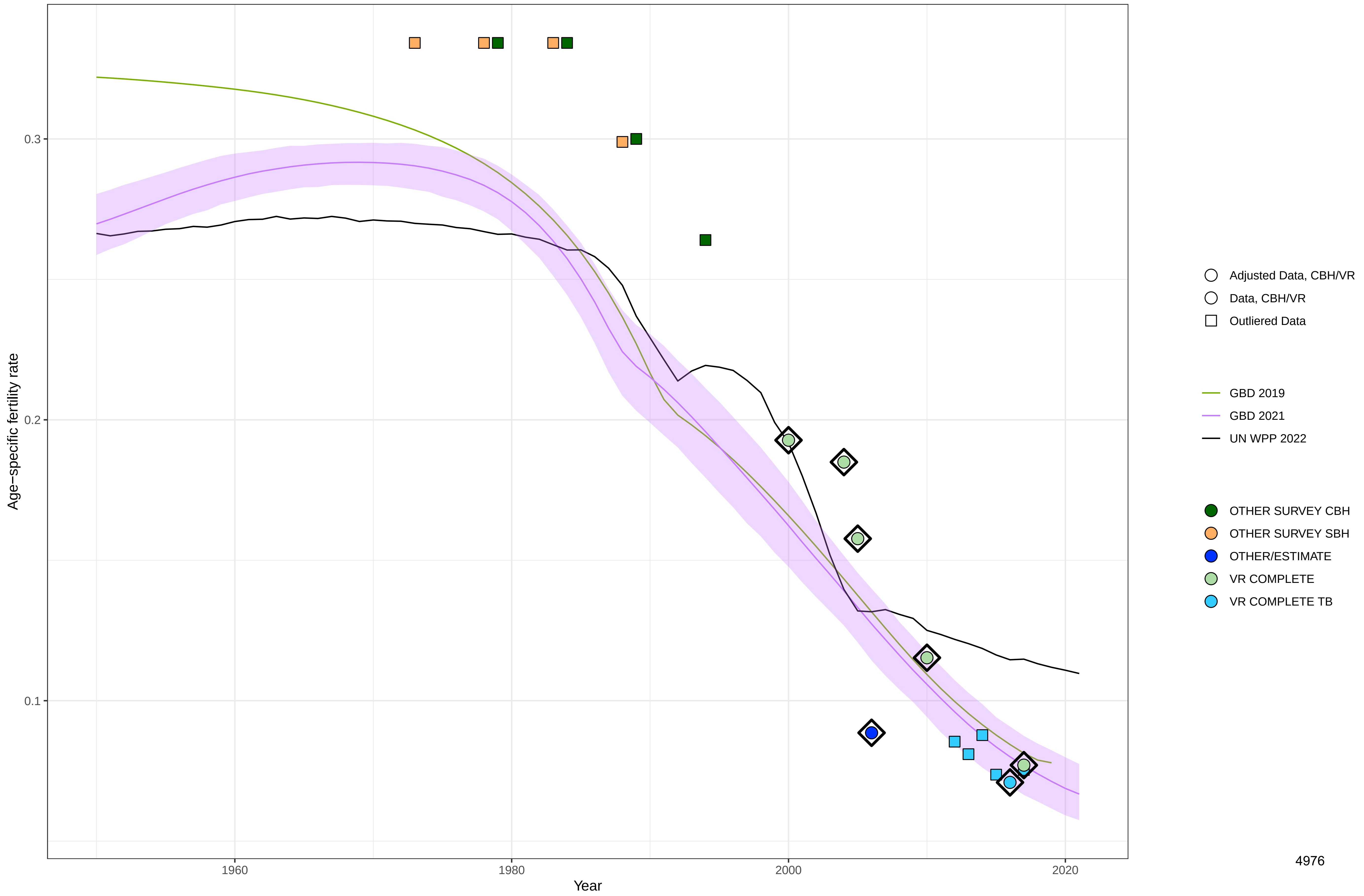

4976

Age−specific fertility fitted trend lines, 1950−2021: Saudi Arabia, Ages: 35−39
Data points enclosed with a diamond are reference sources

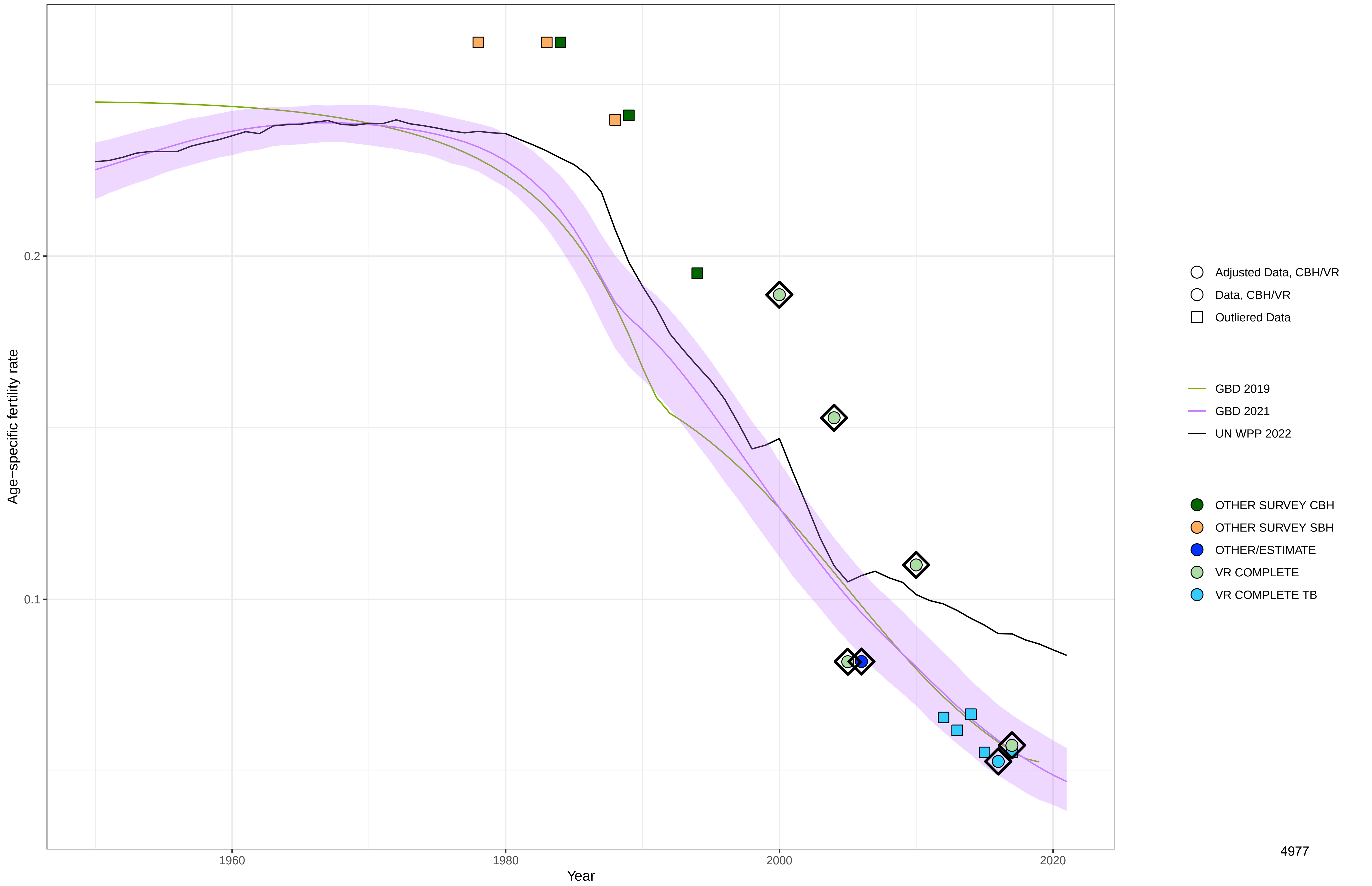

Age−specific fertility fitted trend lines, 1950−2021: Saudi Arabia, Ages: 40−44
Data points enclosed with a diamond are reference sources

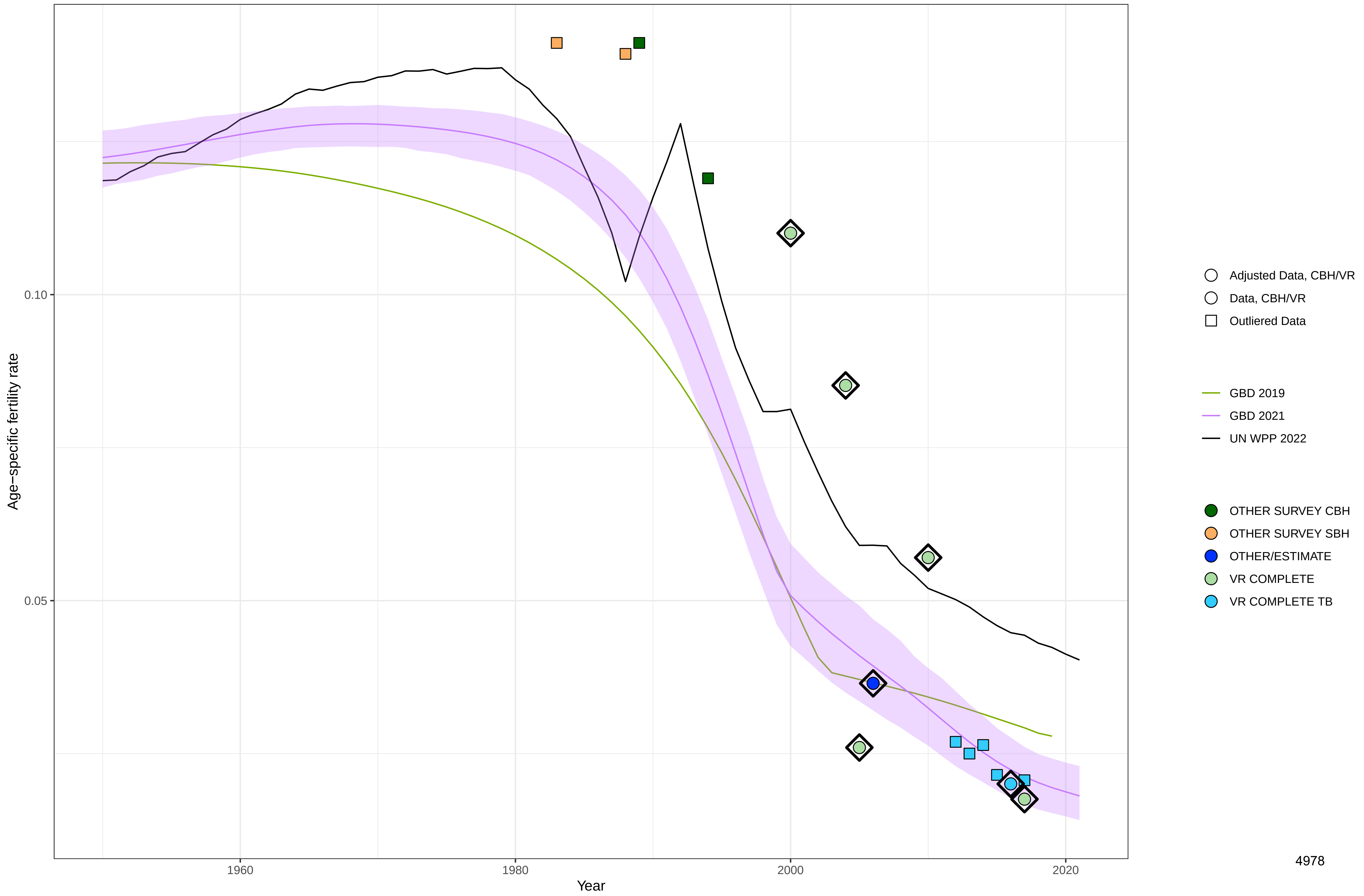

4978

Age−specific fertility fitted trend lines, 1950−2021: Saudi Arabia, Ages: 45−49
Data points enclosed with a diamond are reference sources

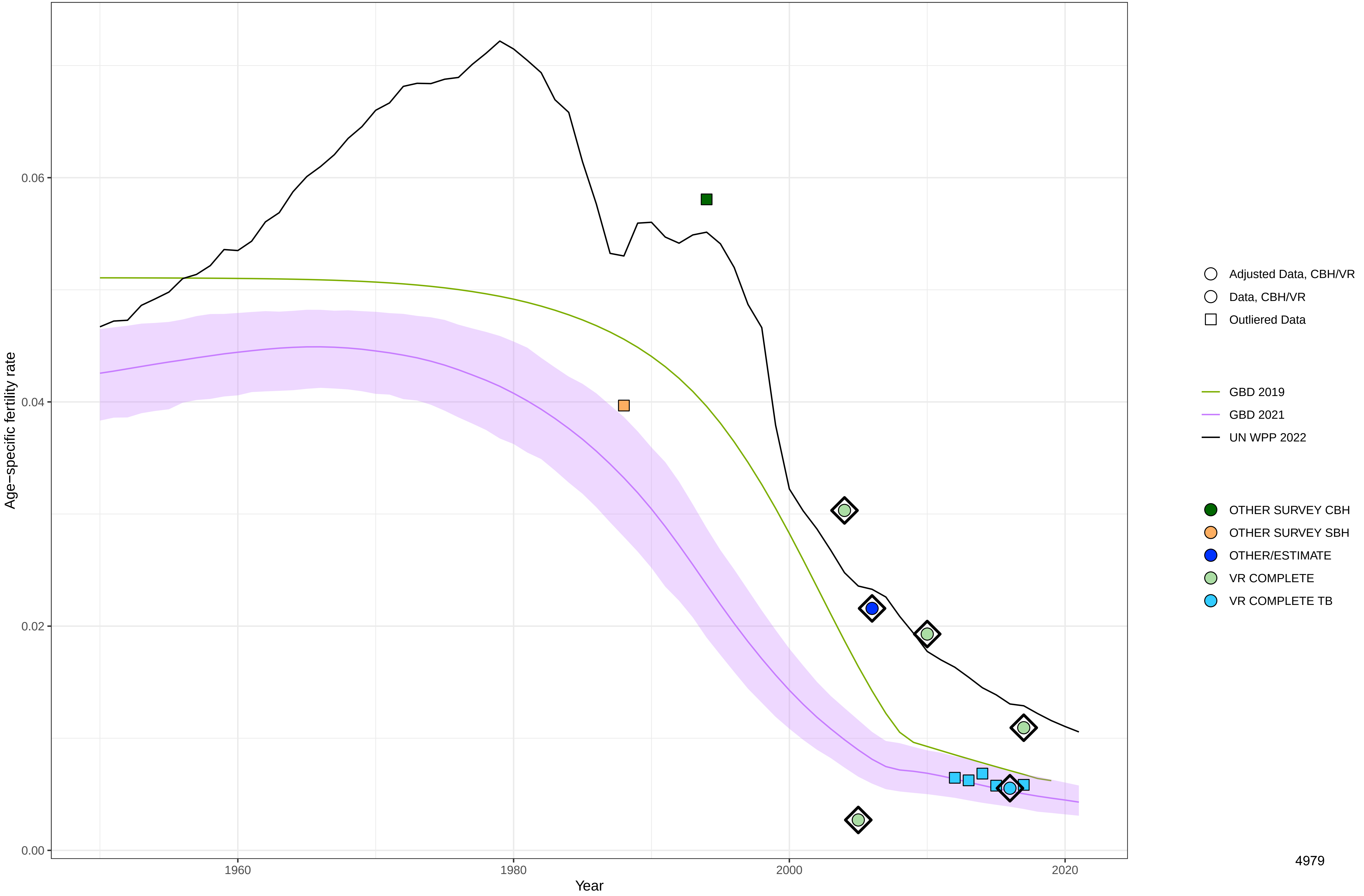

Age−specific fertility fitted trend lines, 1950−2021: Saudi Arabia, Ages: 50−54
Data points enclosed with a diamond are reference sources

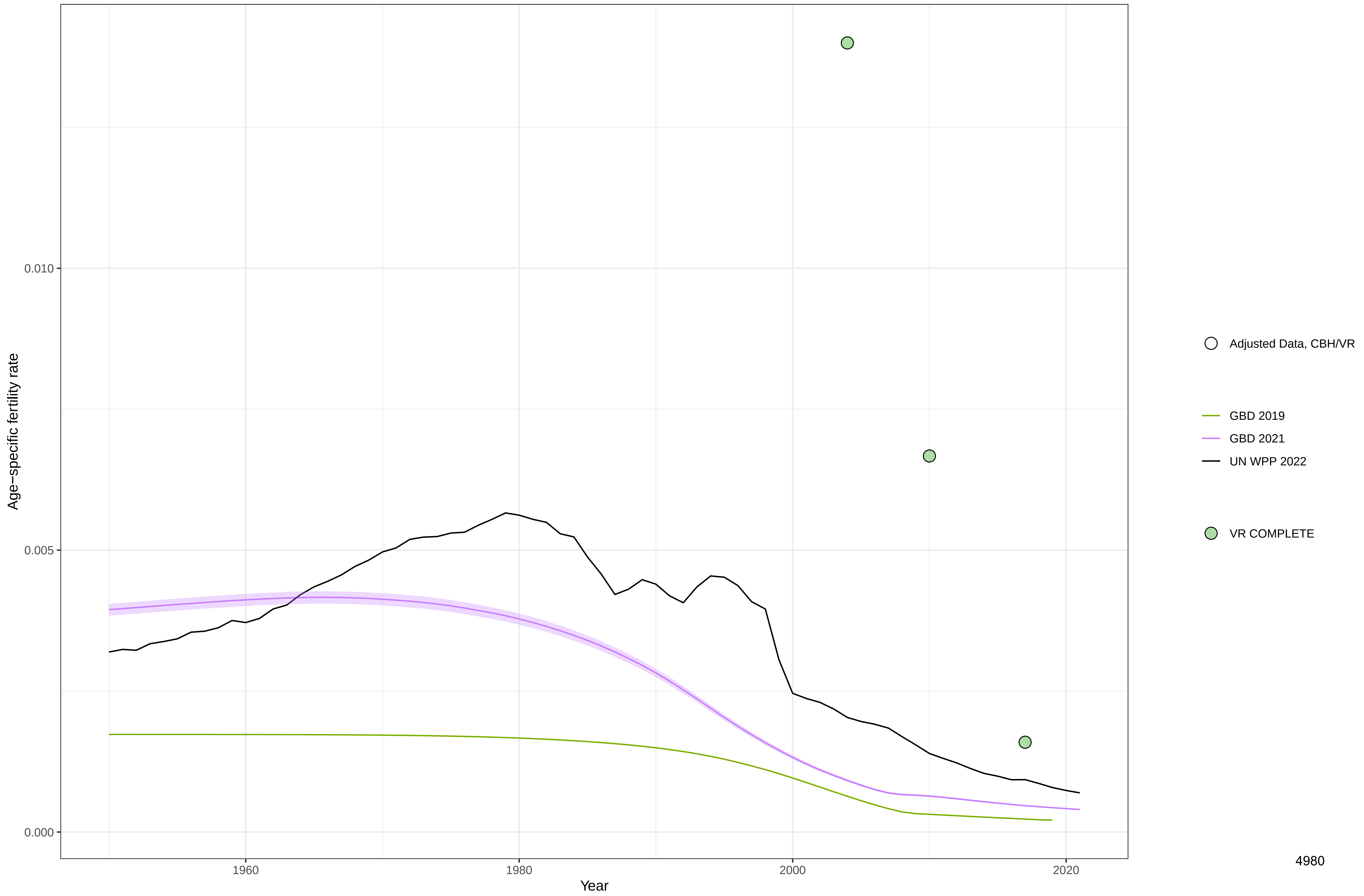

Total fertility rate fitted trend lines, 1950−2021: Saudi Arabia TFR
Data points enclosed with a diamond are reference sources

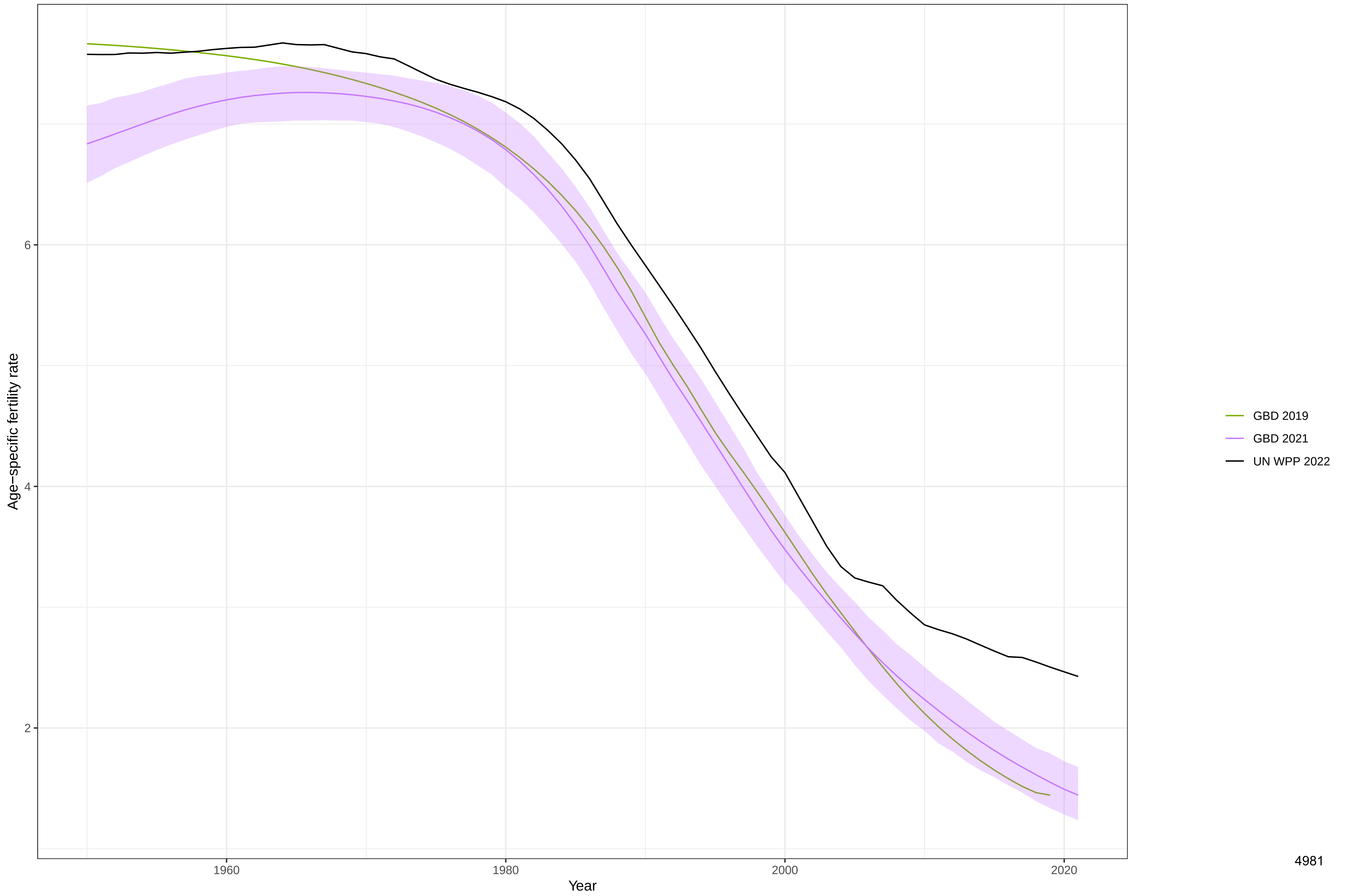

Age−specific fertility fitted trend lines, 1950−2021: Sudan, Ages: 10−14
Data points enclosed with a diamond are reference sources

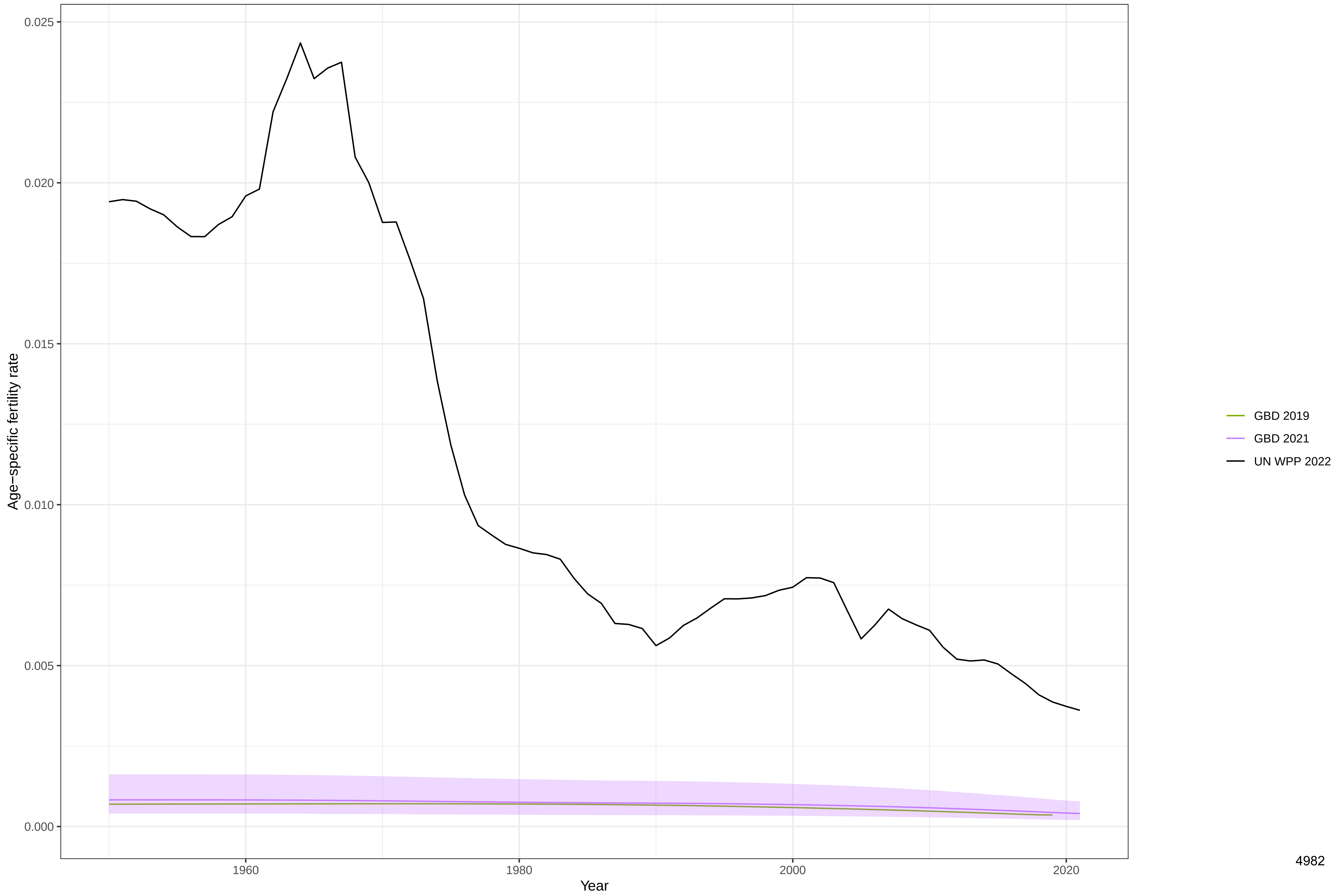

Age−specific fertility fitted trend lines, 1950−2021: Sudan, Ages: 15−19
Data points enclosed with a diamond are reference sources

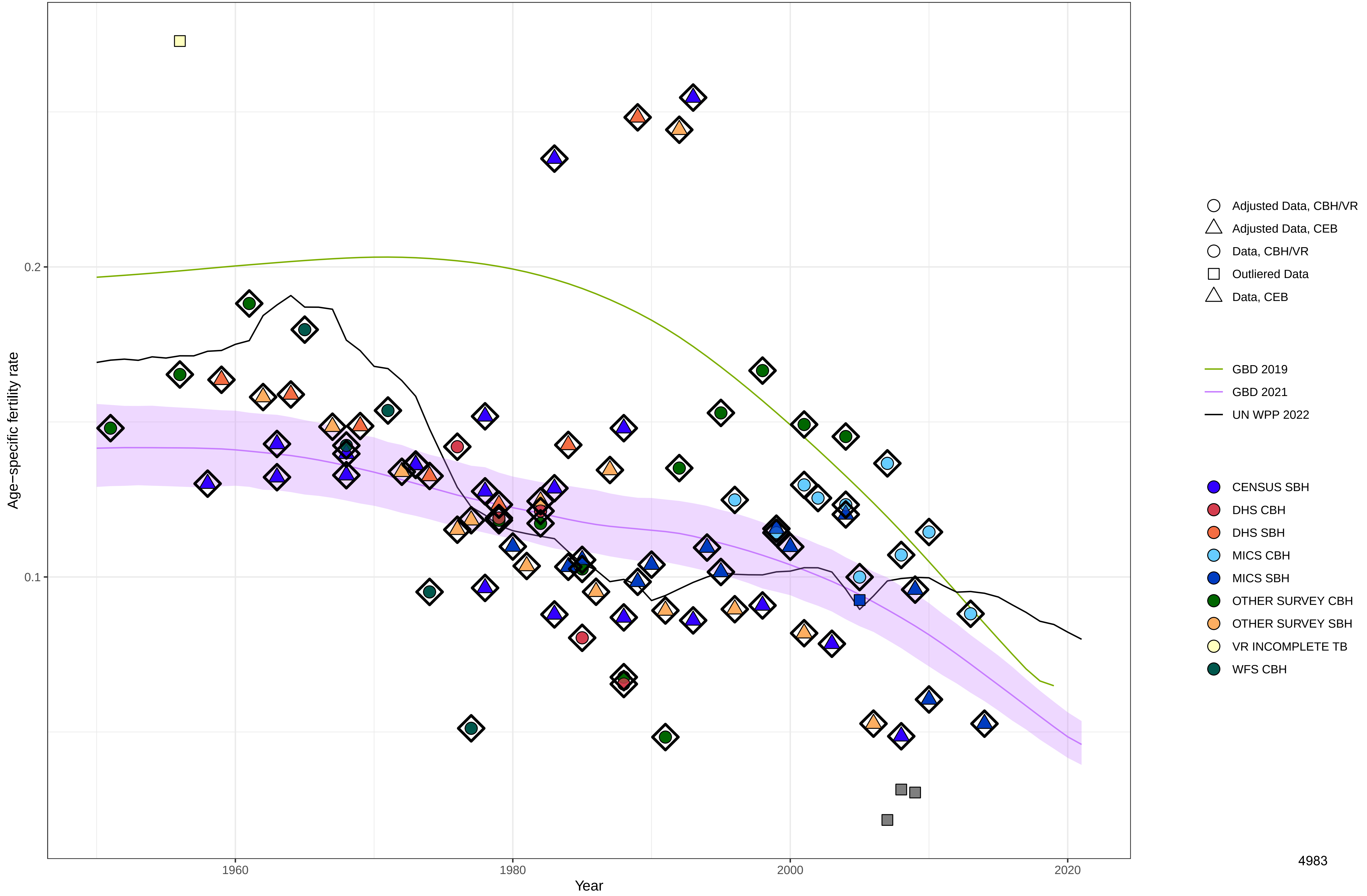

Age−specific fertility fitted trend lines, 1950−2021: Sudan, Ages: 20−24
Data points enclosed with a diamond are reference sources

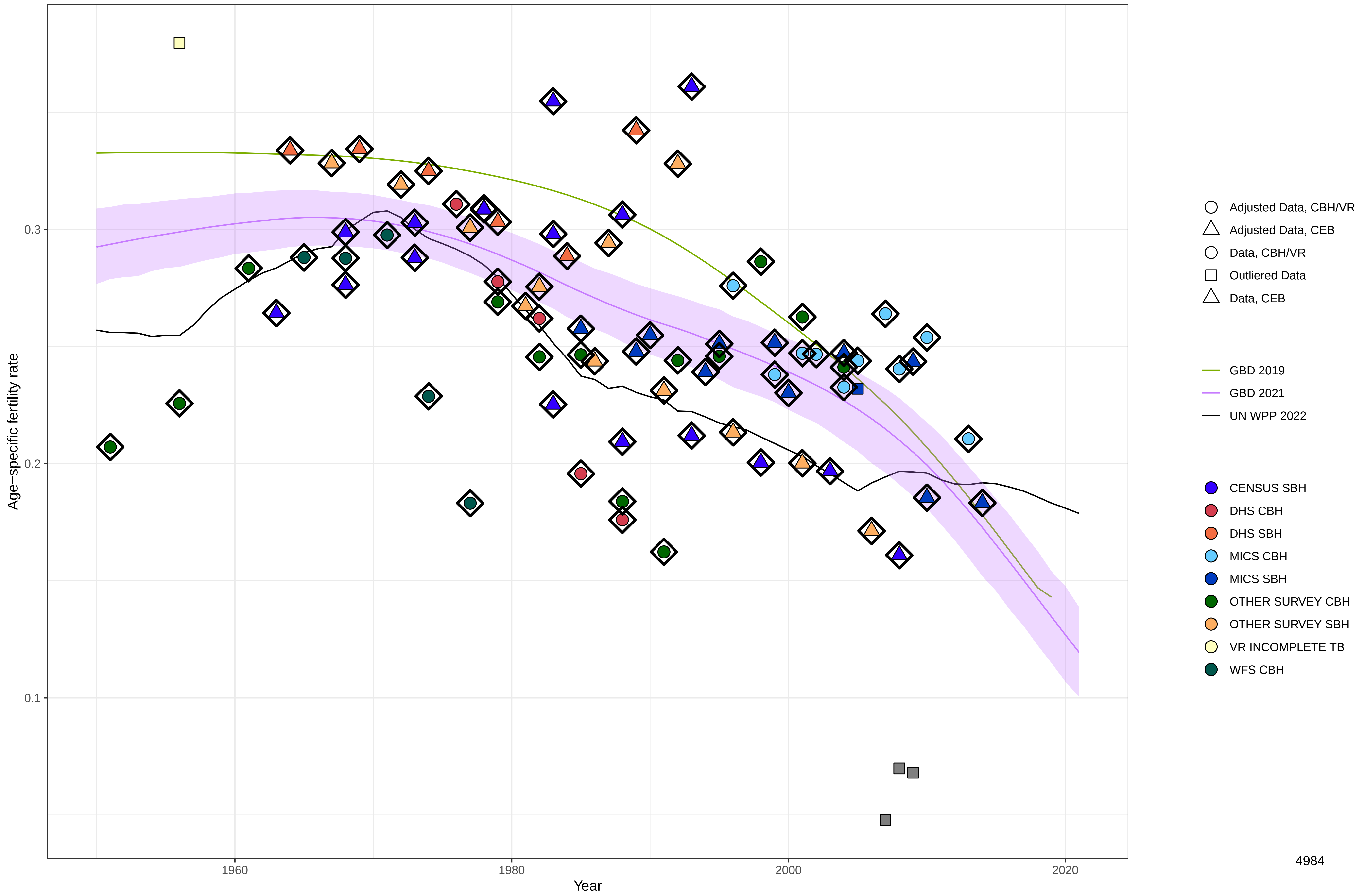

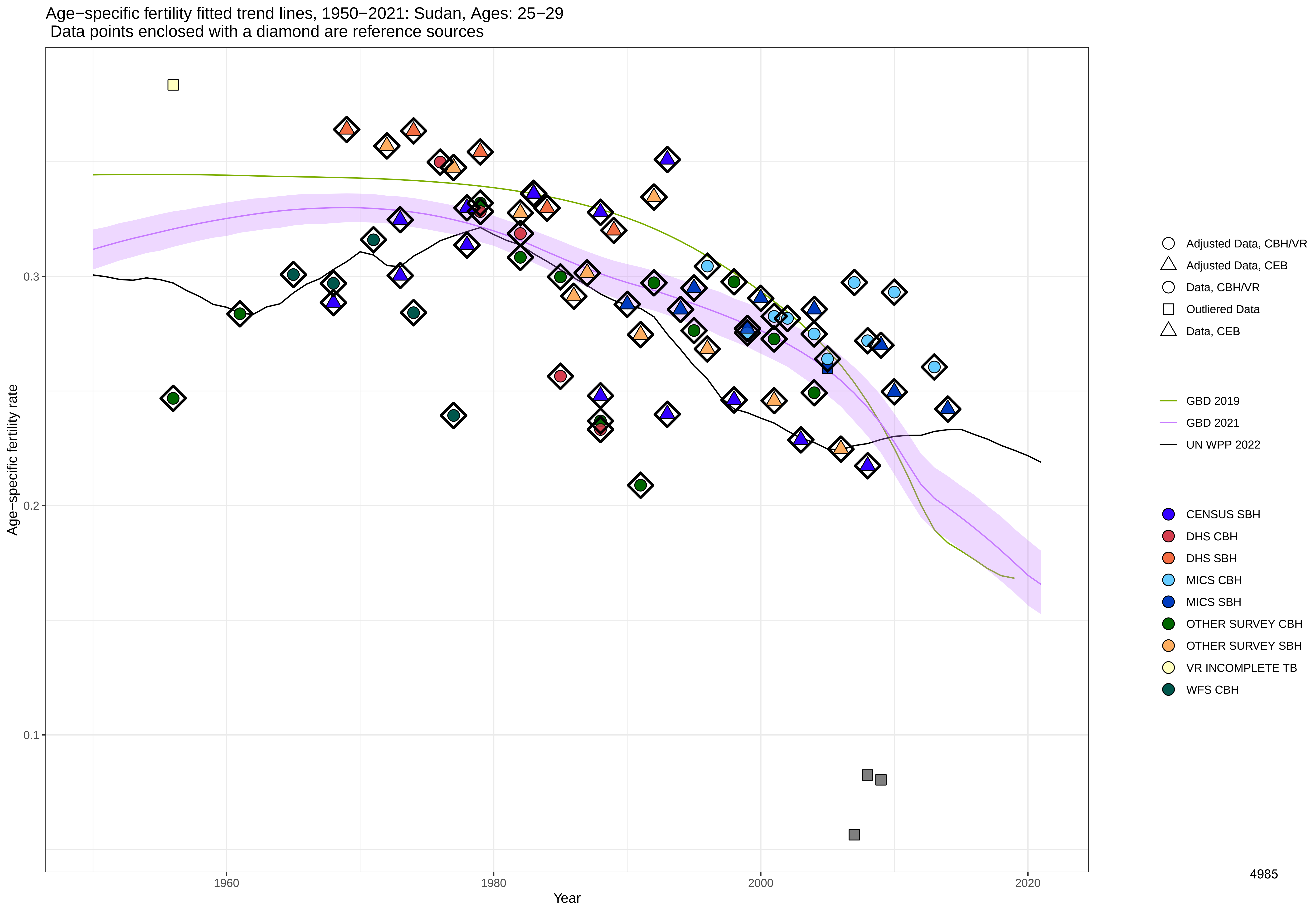

Age−specific fertility fitted trend lines, 1950−2021: Sudan, Ages: 25−29
Data points enclosed with a diamond are reference sources
Age−specific fertility rate
0.3
0.2
0.1
1960
1980
2000
2020
Year
Adjusted Data, CBH/VR
Adjusted Data, CEB
Data, CBH/VR
Outliered Data
Data, CEB
GBD 2019
GBD 2021
UN WPP 2022
CENSUS SBH
DHS CBH
DHS SBH
MICS CBH
MICS SBH
OTHER SURVEY CBH
OTHER SURVEY SBH
VR INCOMPLETE TB
WFS CBH

Age−specific fertility fitted trend lines, 1950−2021: Sudan, Ages: 30−34
Data points enclosed with a diamond are reference sources

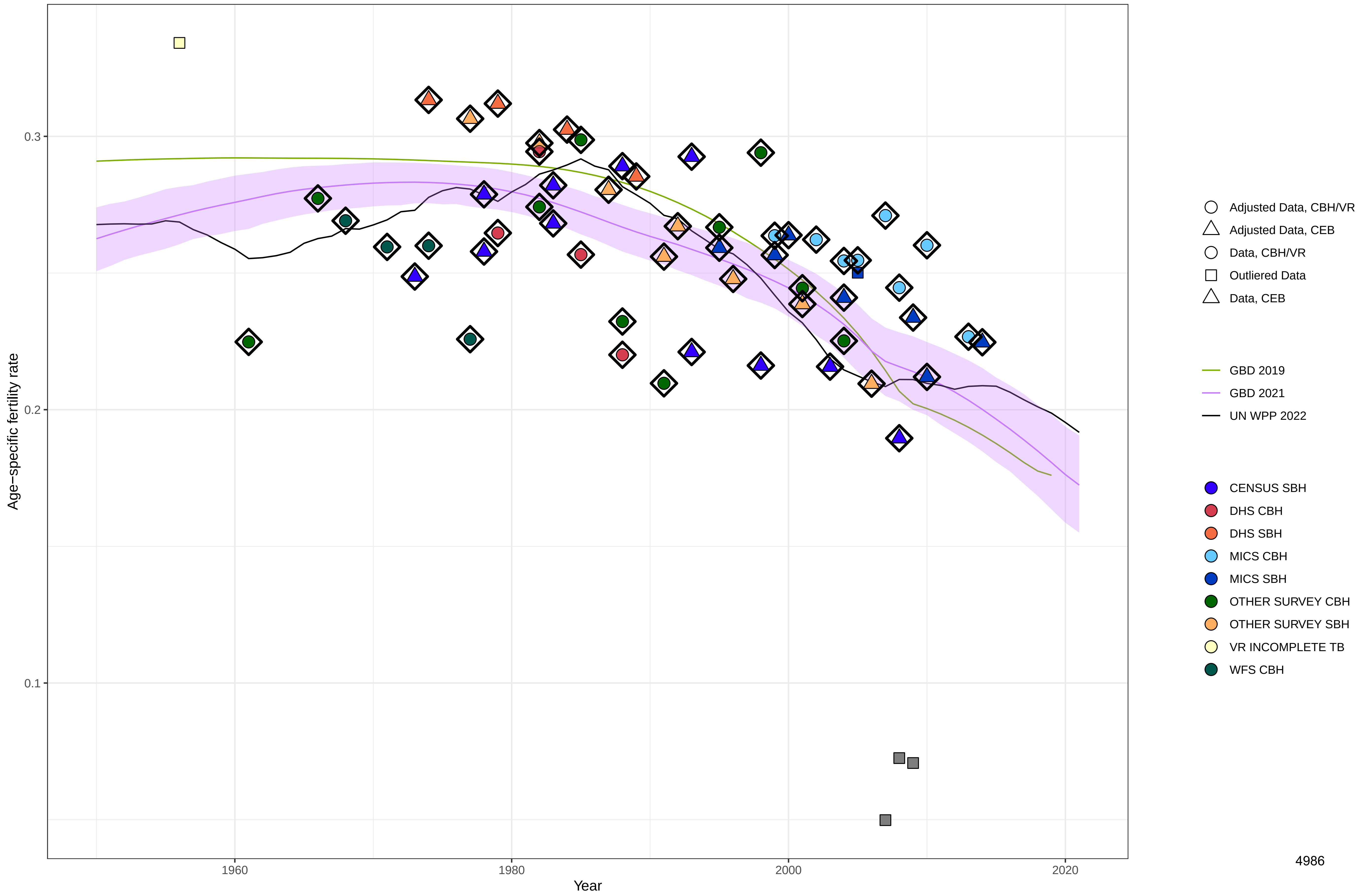

Age−specific fertility fitted trend lines, 1950−2021: Sudan, Ages: 35−39
Data points enclosed with a diamond are reference sources

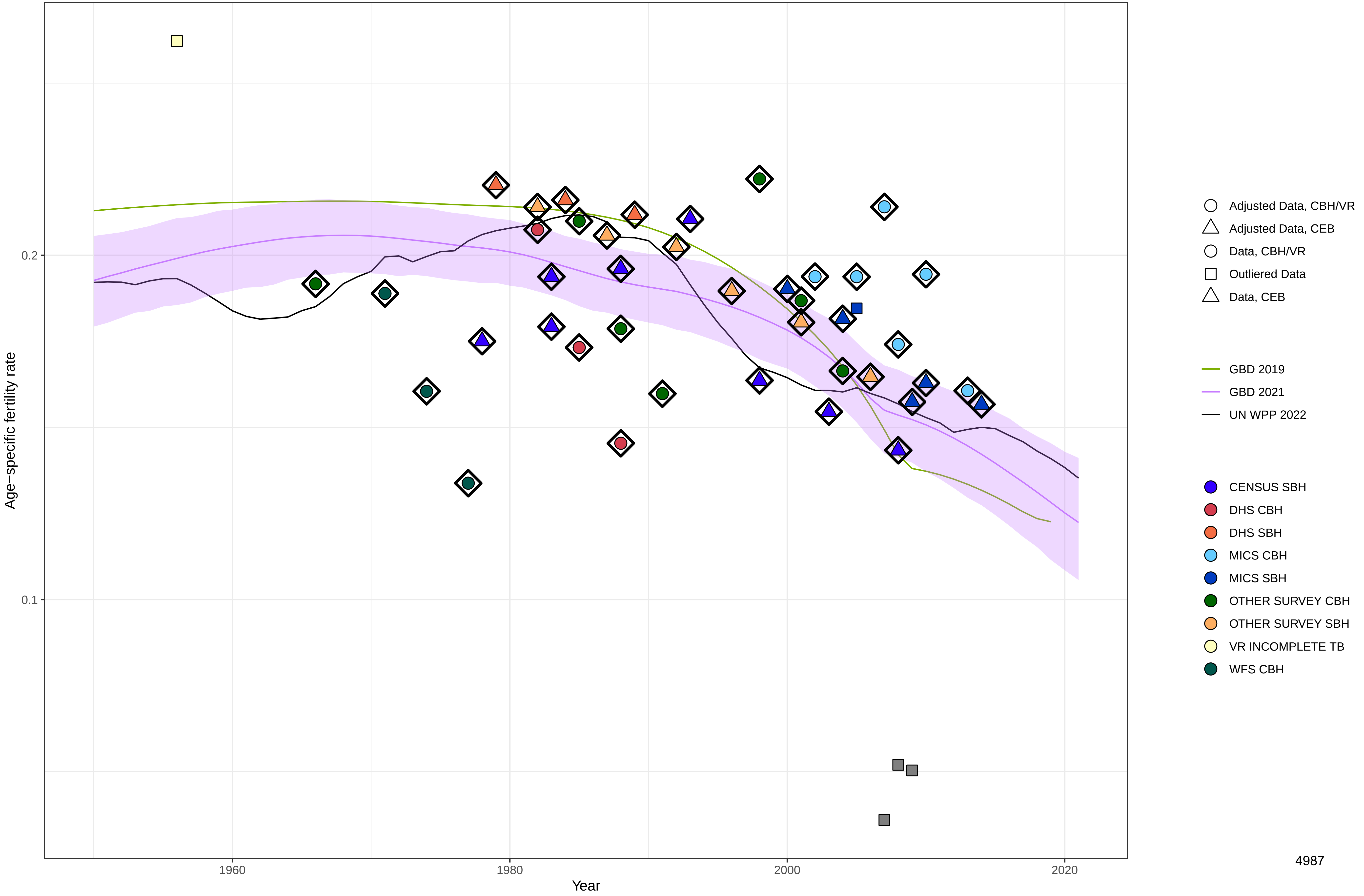

Age−specific fertility fitted trend lines, 1950−2021: Sudan, Ages: 40−44
Data points enclosed with a diamond are reference sources

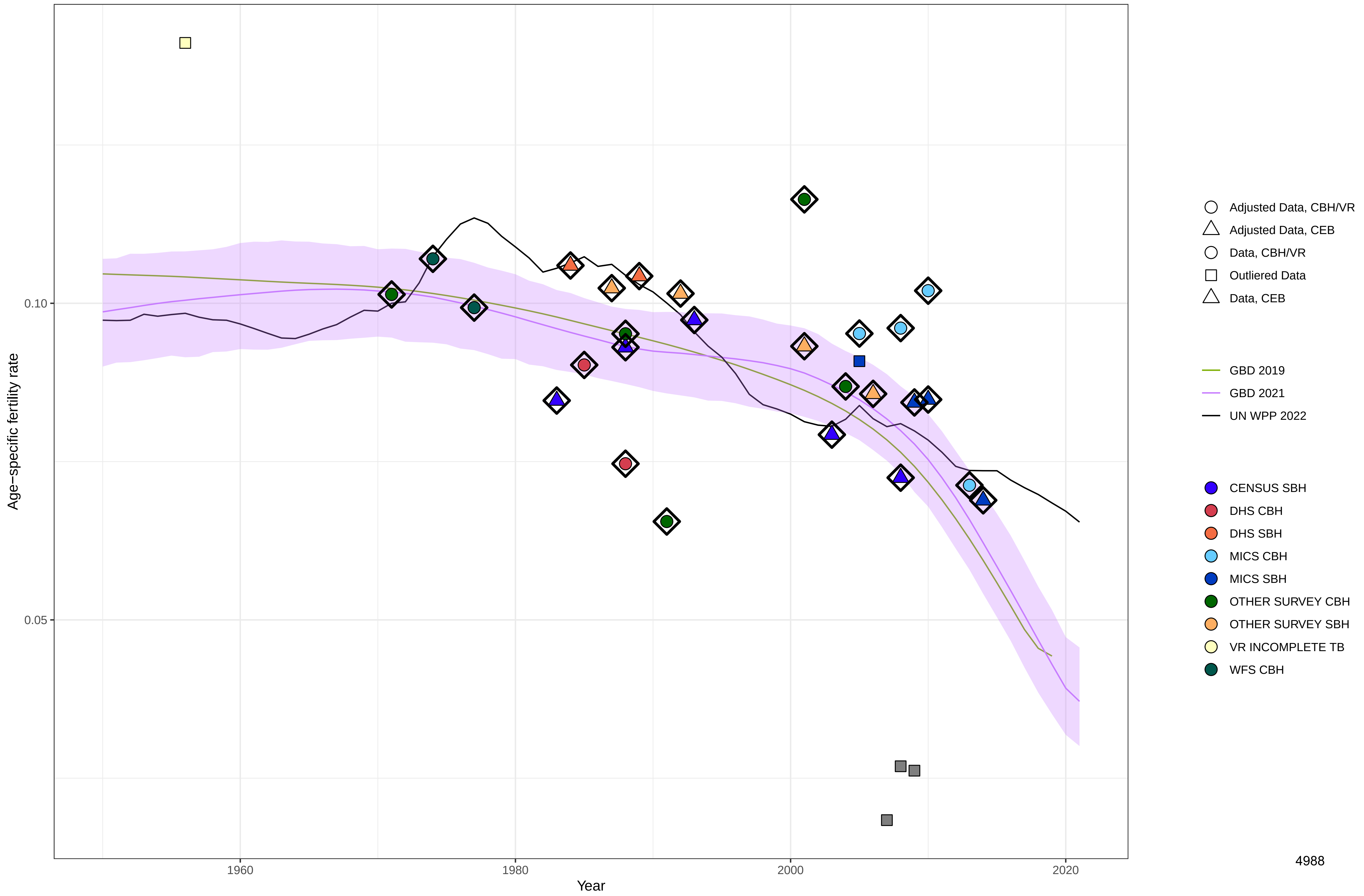

Age−specific fertility fitted trend lines, 1950−2021: Sudan, Ages: 45−49
Data points enclosed with a diamond are reference sources

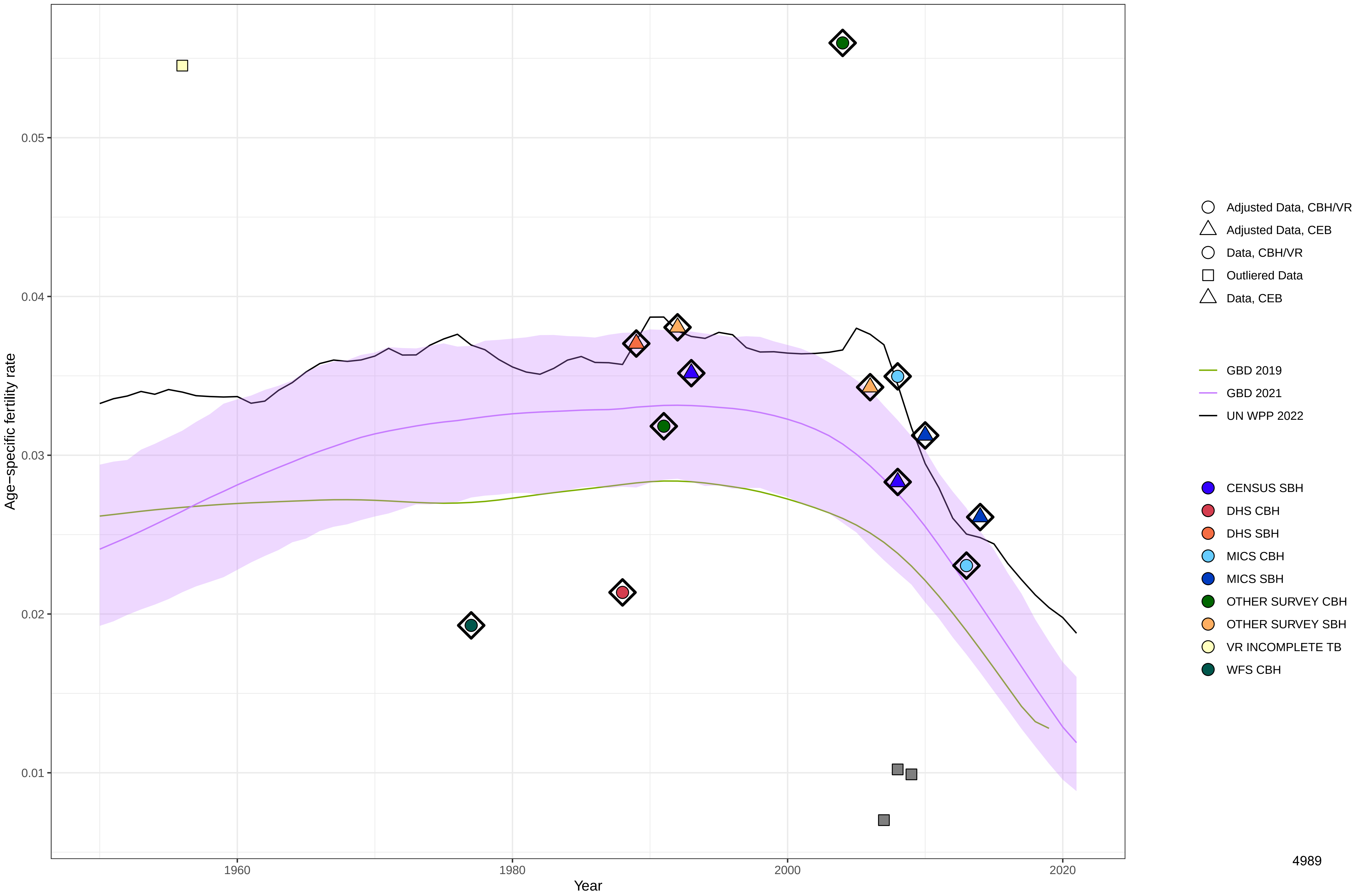

4989

Age−specific fertility fitted trend lines, 1950−2021: Sudan, Ages: 50−54
Data points enclosed with a diamond are reference sources

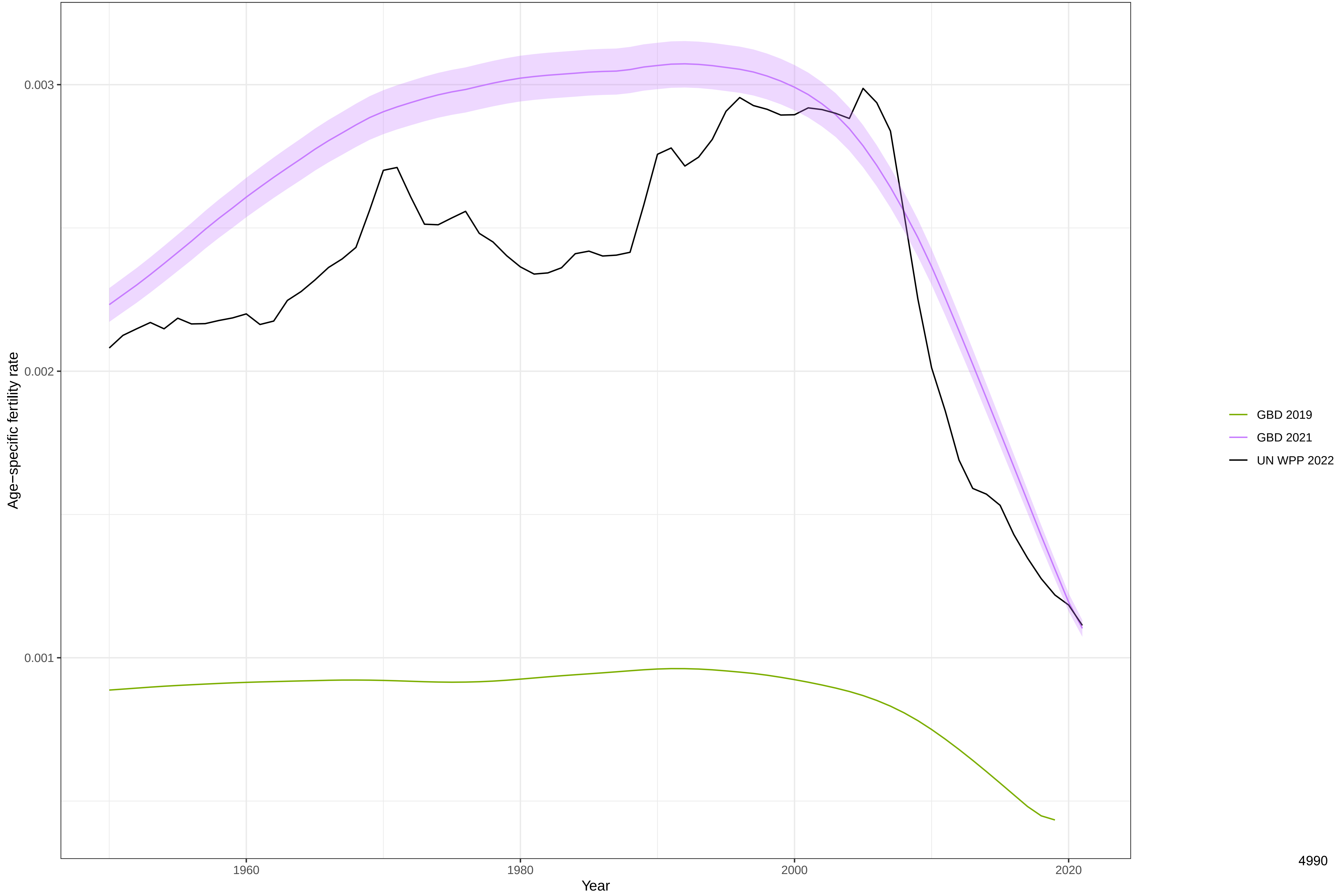

Total fertility rate fitted trend lines, 1950−2021: Sudan TFR
Data points enclosed with a diamond are reference sources

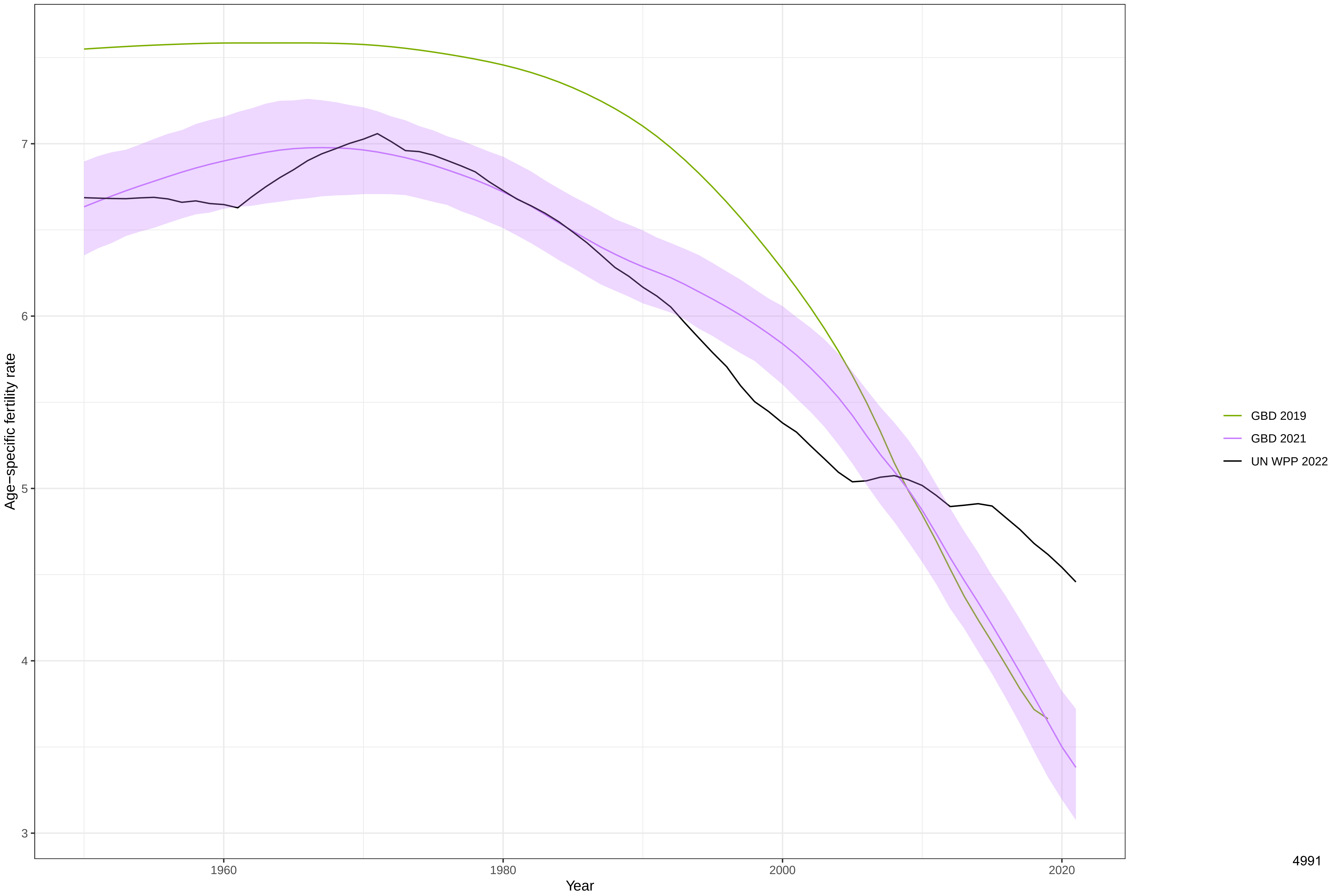

Age−specific fertility fitted trend lines, 1950−2021: Syria, Ages: 10−14
Data points enclosed with a diamond are reference sources

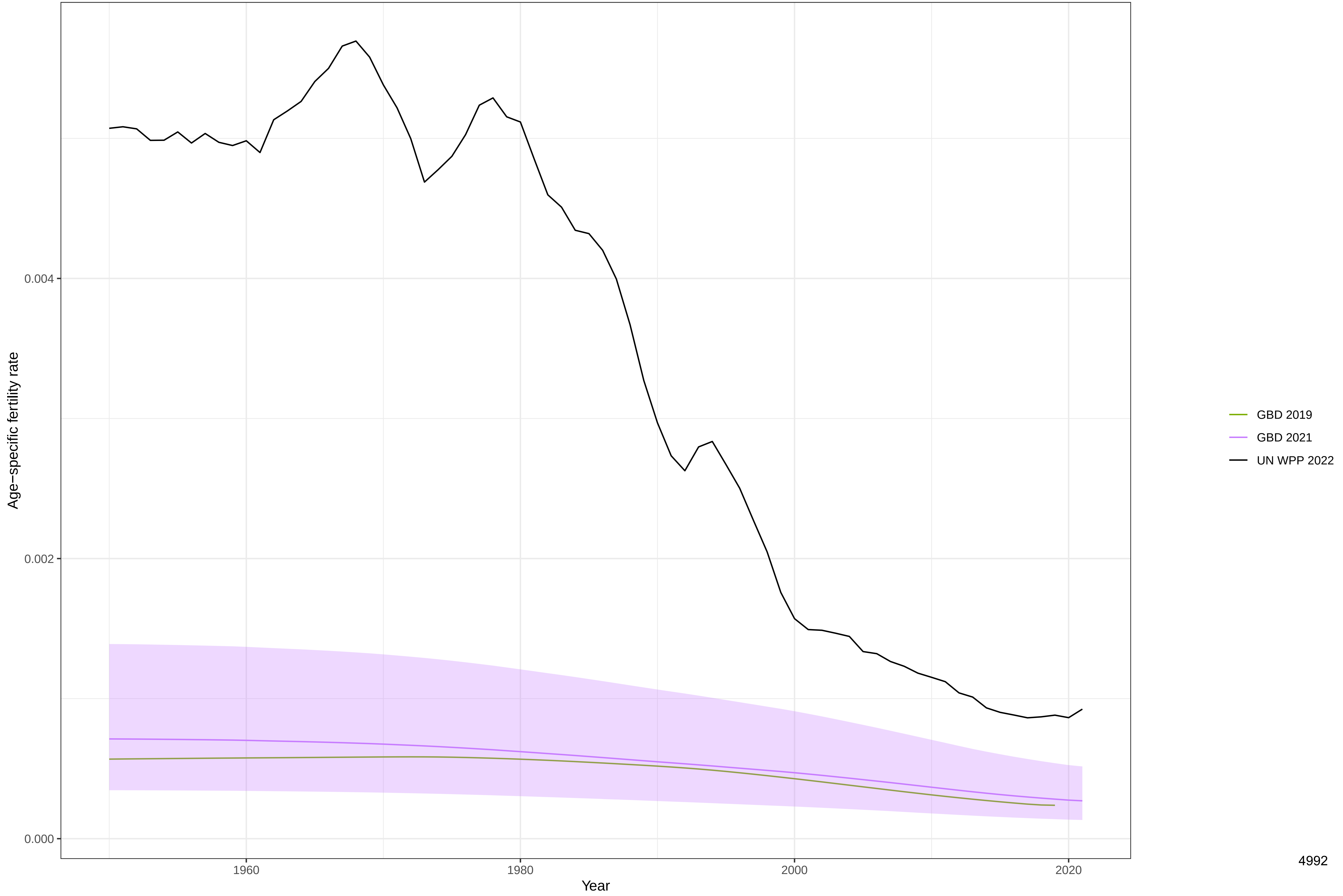

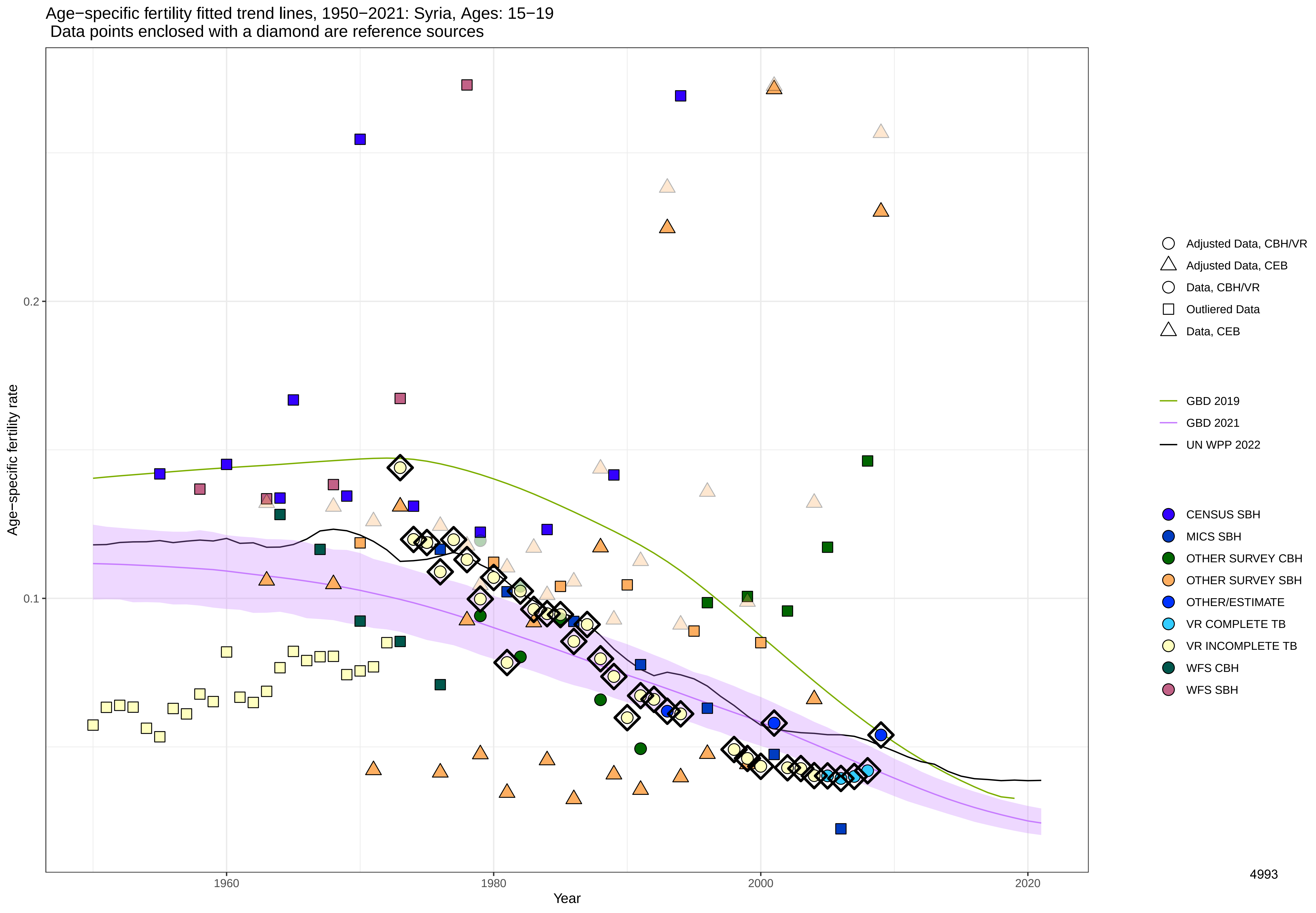

Age−specific fertility fitted trend lines, 1950−2021: Syria, Ages: 15−19
Data points enclosed with a diamond are reference sources
Age−specific fertility rate
Year
0.1
0.2
1960
1980
2000
2020
Adjusted Data, CBH/VR
Adjusted Data, CEB
Data, CBH/VR
Outliered Data
Data, CEB
GBD 2019
GBD 2021
UN WPP 2022
CENSUS SBH
MICS SBH
OTHER SURVEY CBH
OTHER SURVEY SBH
OTHER/ESTIMATE
VR COMPLETE TB
VR INCOMPLETE TB
WFS CBH
WFS SBH

4993

Age−specific fertility fitted trend lines, 1950−2021: Syria, Ages: 20−24
Data points enclosed with a diamond are reference sources

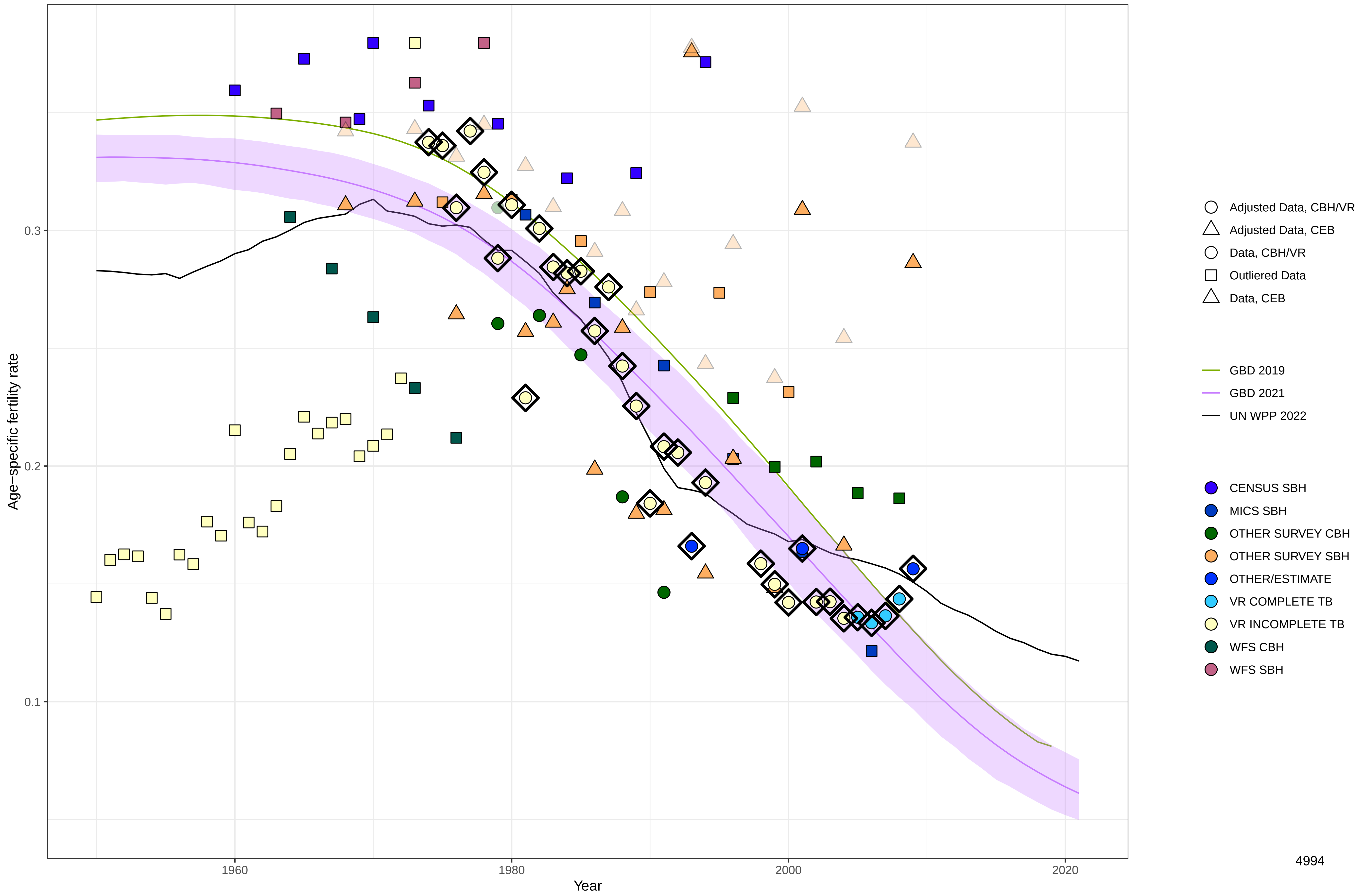

Age−specific fertility fitted trend lines, 1950−2021: Syria, Ages: 25−29
Data points enclosed with a diamond are reference sources

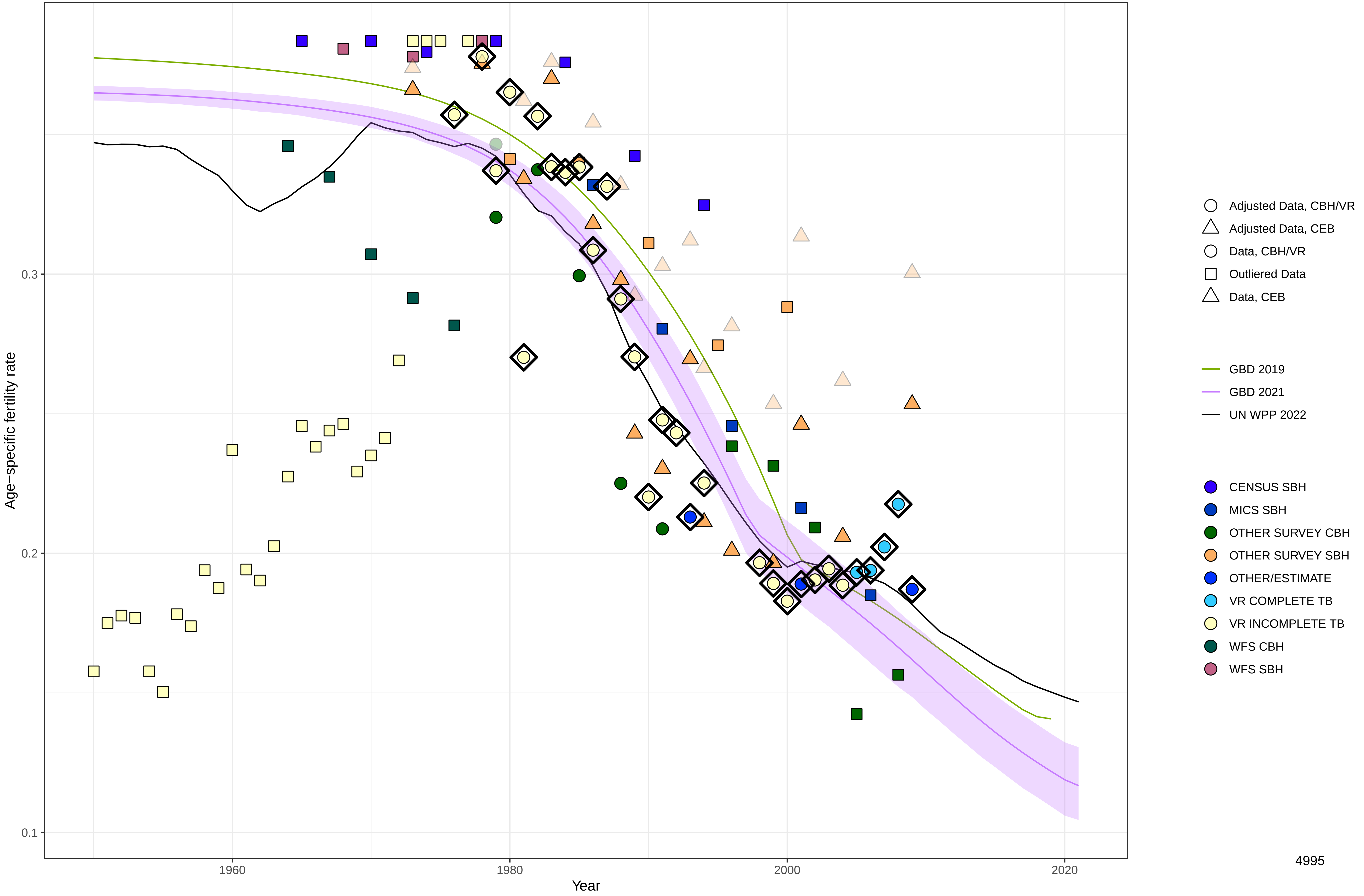

Age−specific fertility fitted trend lines, 1950−2021: Syria, Ages: 30−34
Data points enclosed with a diamond are reference sources

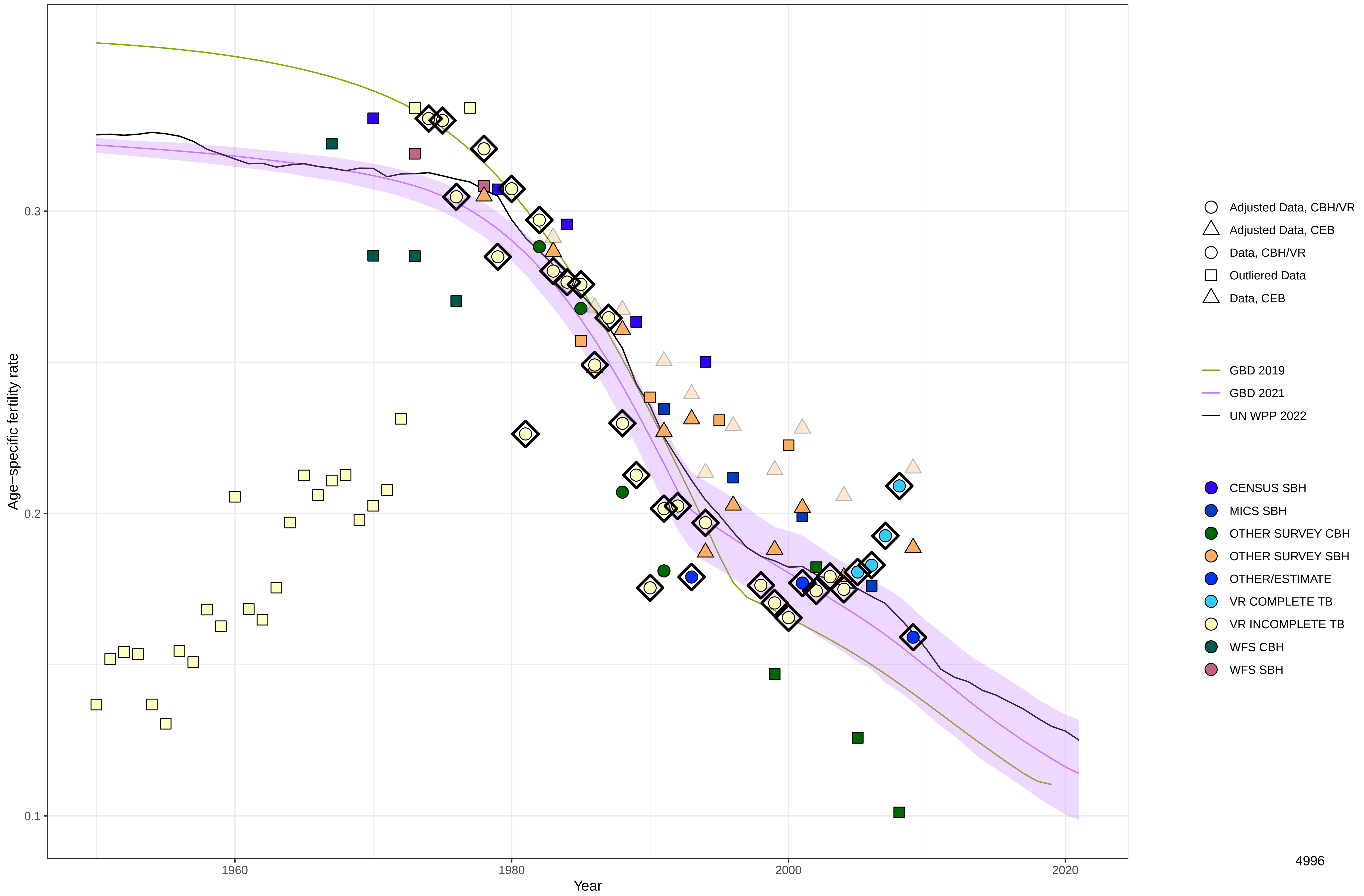

Age−specific fertility fitted trend lines, 1950−2021: Syria, Ages: 35−39
Data points enclosed with a diamond are reference sources

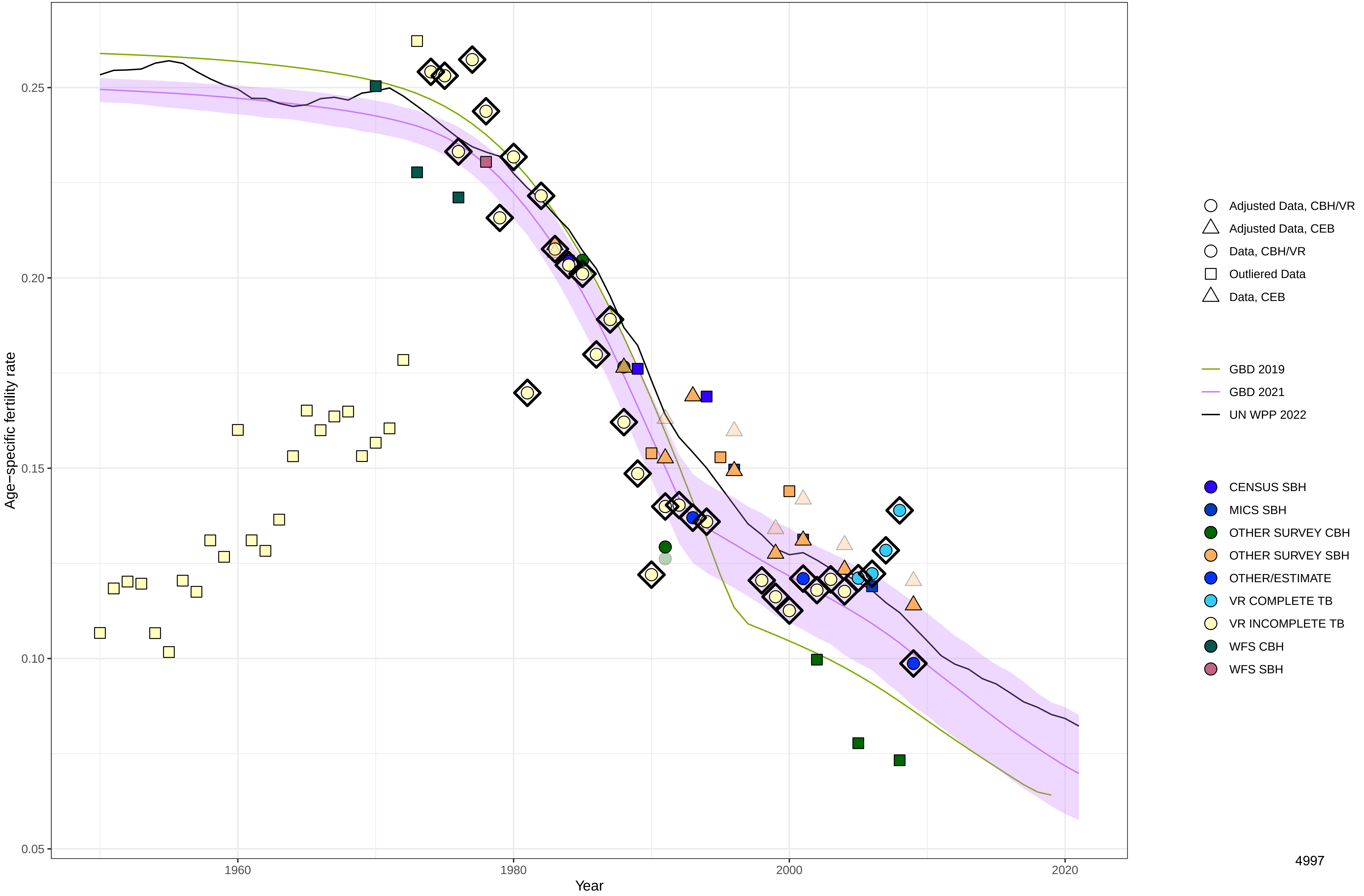

Age−specific fertility fitted trend lines, 1950−2021: Syria, Ages: 40−44
Data points enclosed with a diamond are reference sources

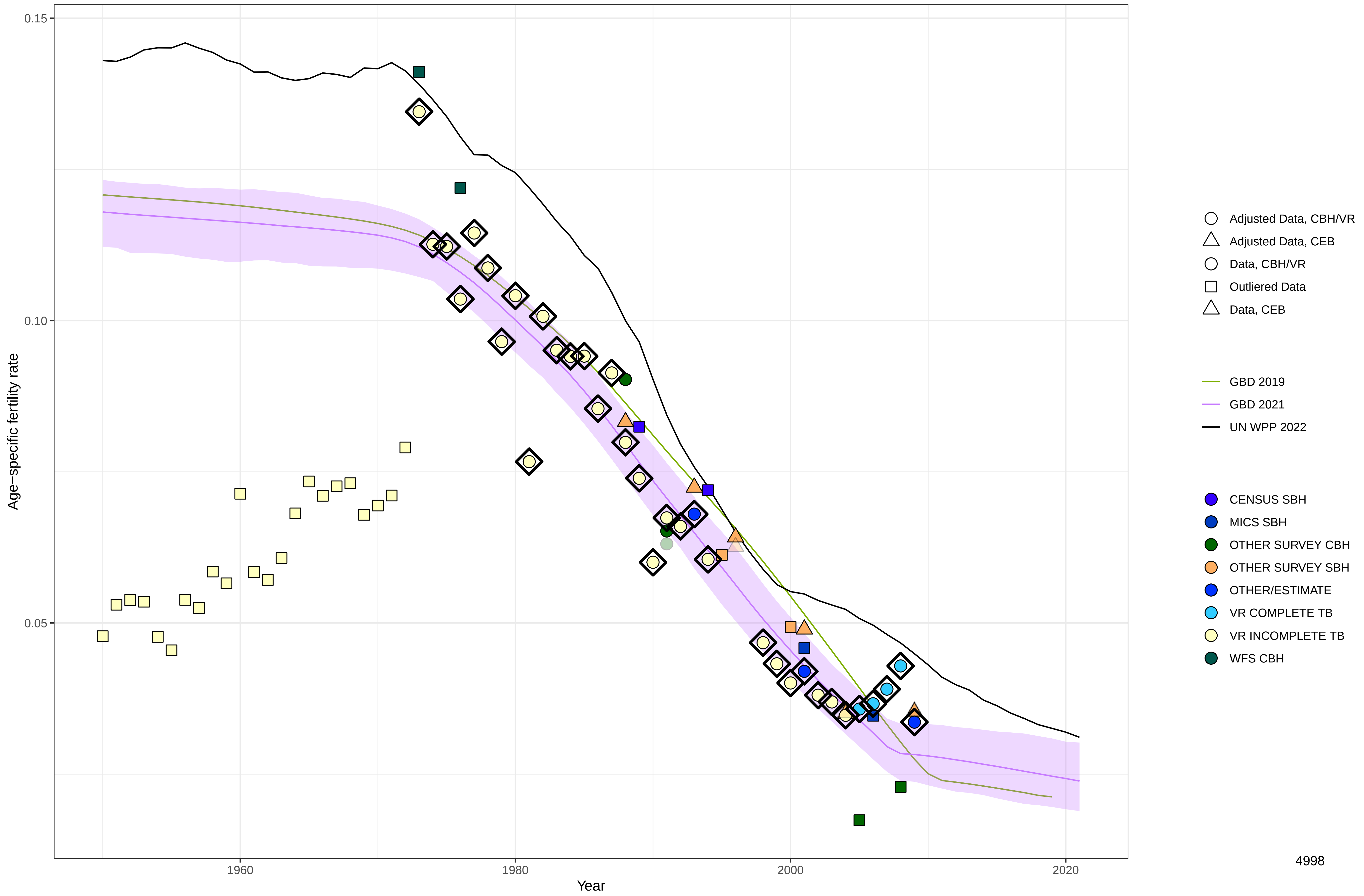

Age−specific fertility fitted trend lines, 1950−2021: Syria, Ages: 45−49
Data points enclosed with a diamond are reference sources

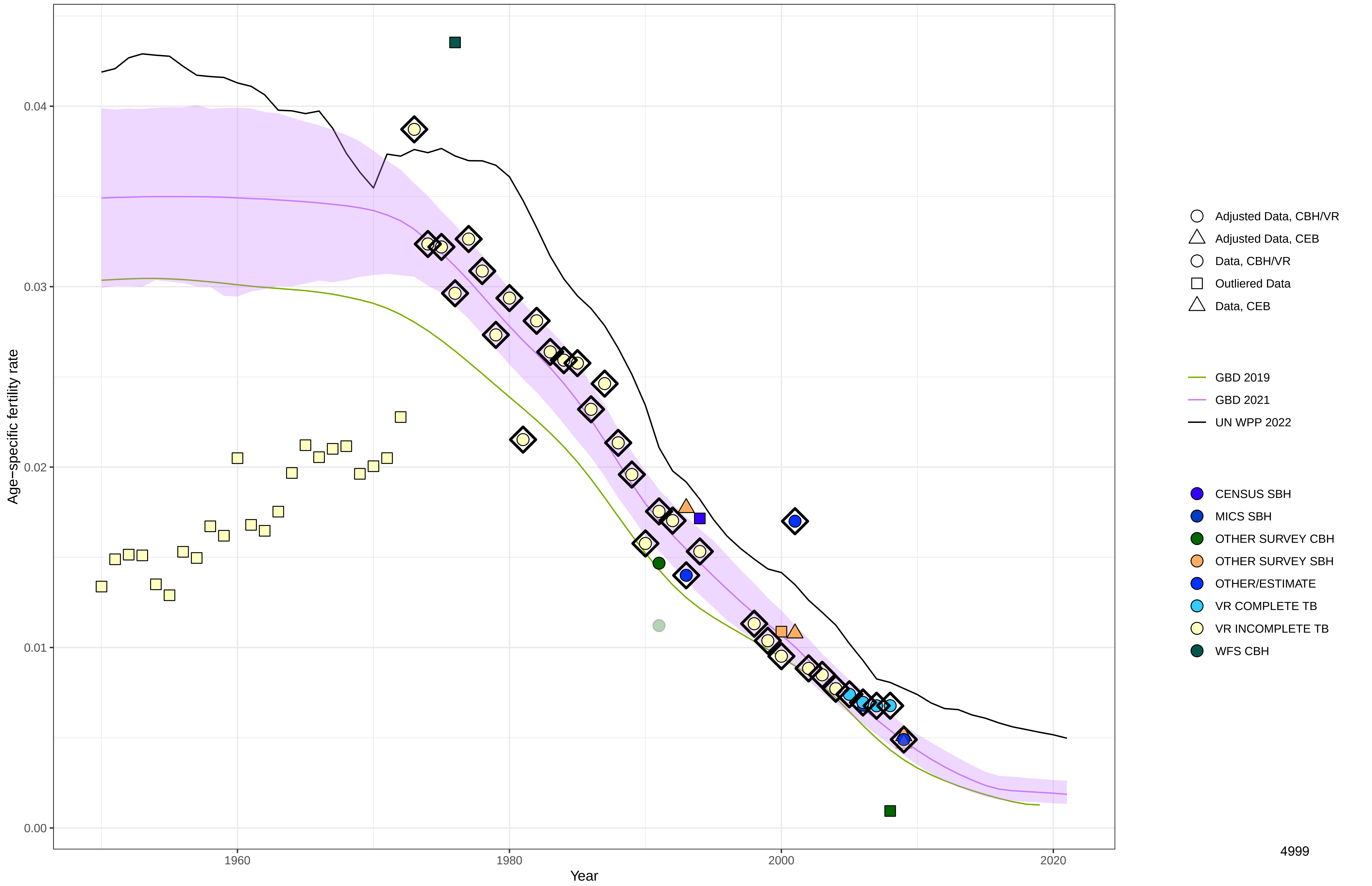

Age−specific fertility fitted trend lines, 1950−2021: Syria, Ages: 50−54
Data points enclosed with a diamond are reference sources

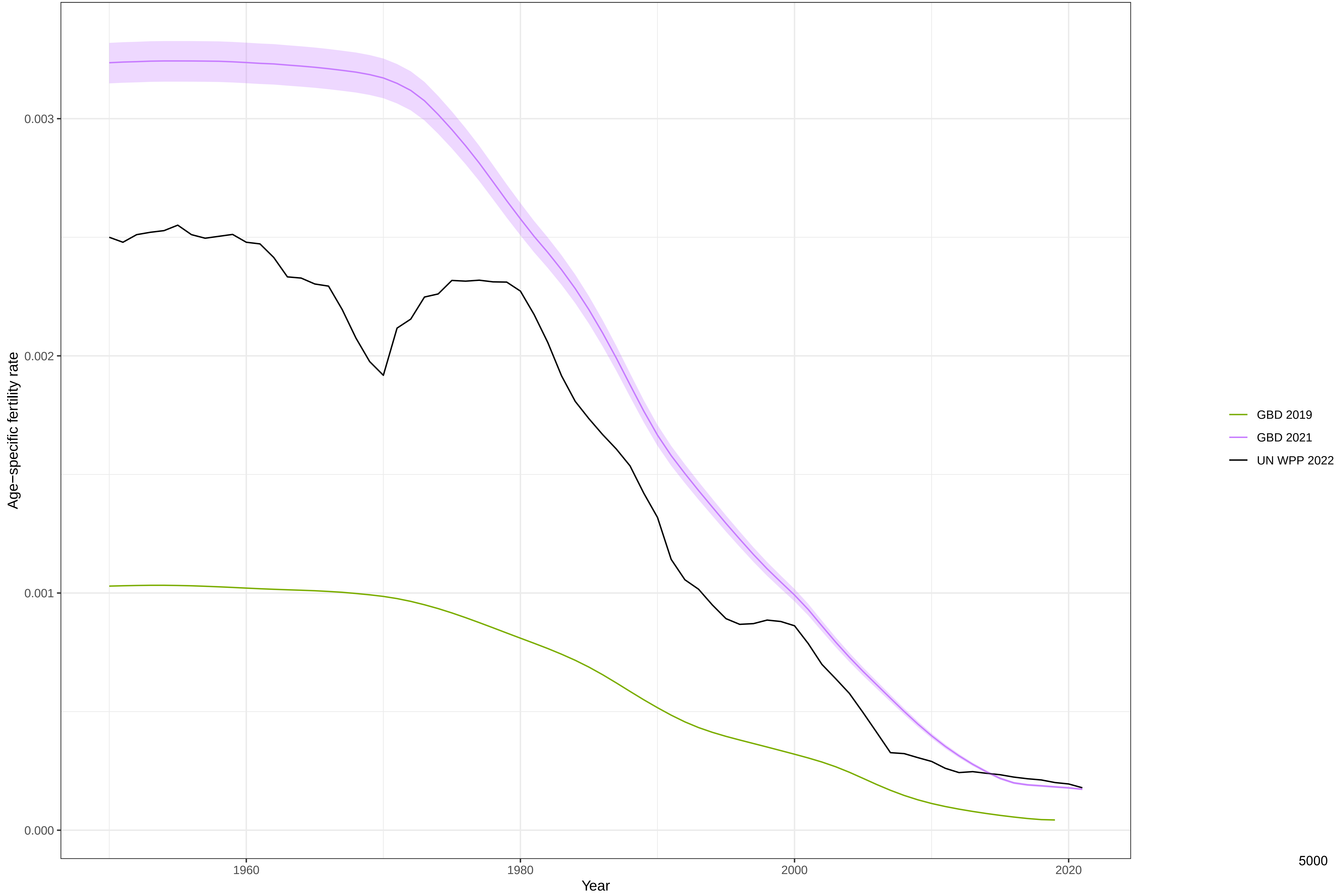

Total fertility rate fitted trend lines, 1950−2021: Syria TFR
Data points enclosed with a diamond are reference sources

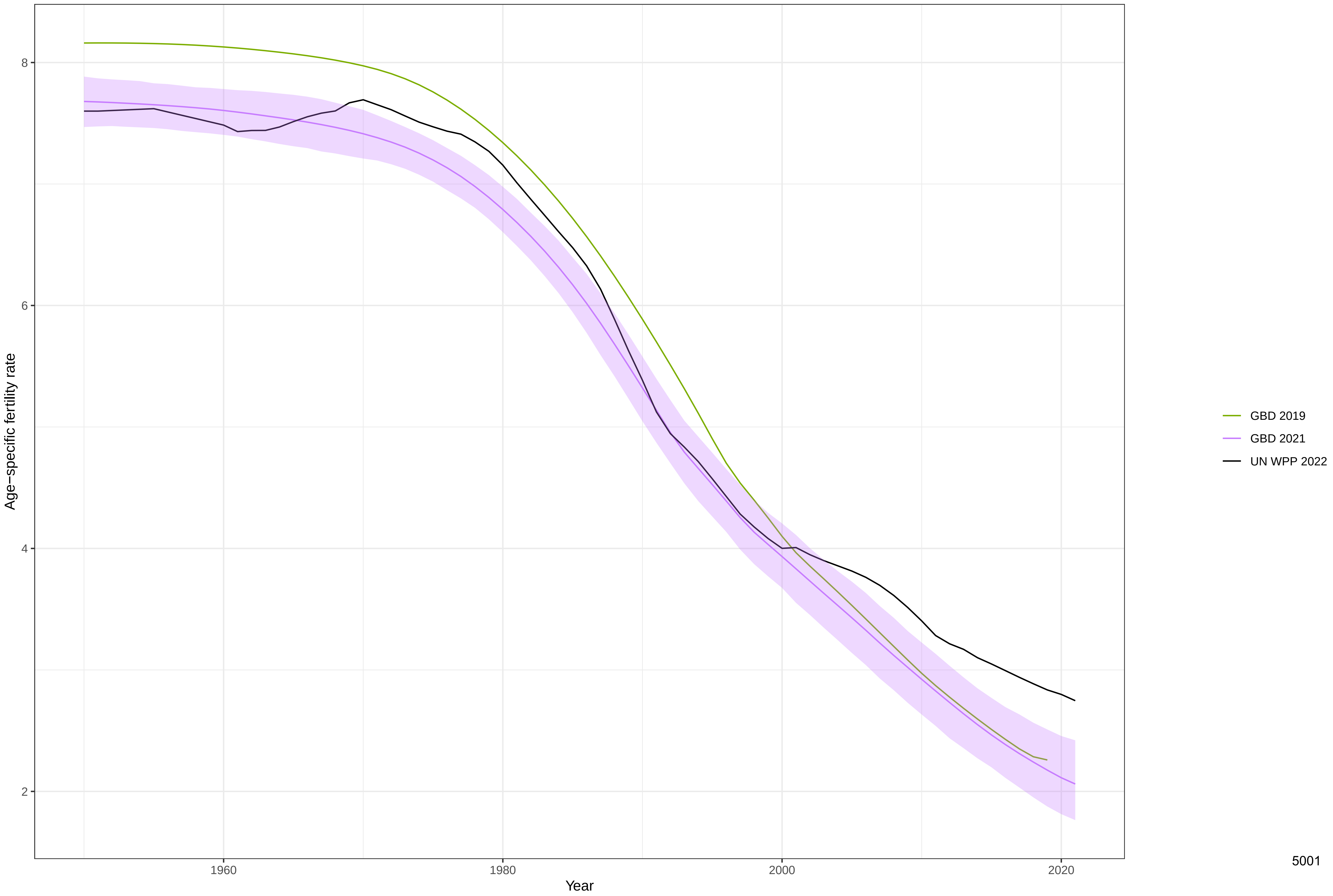

Age−specific fertility fitted trend lines, 1950−2021: Tunisia, Ages: 10−14
Data points enclosed with a diamond are reference sources

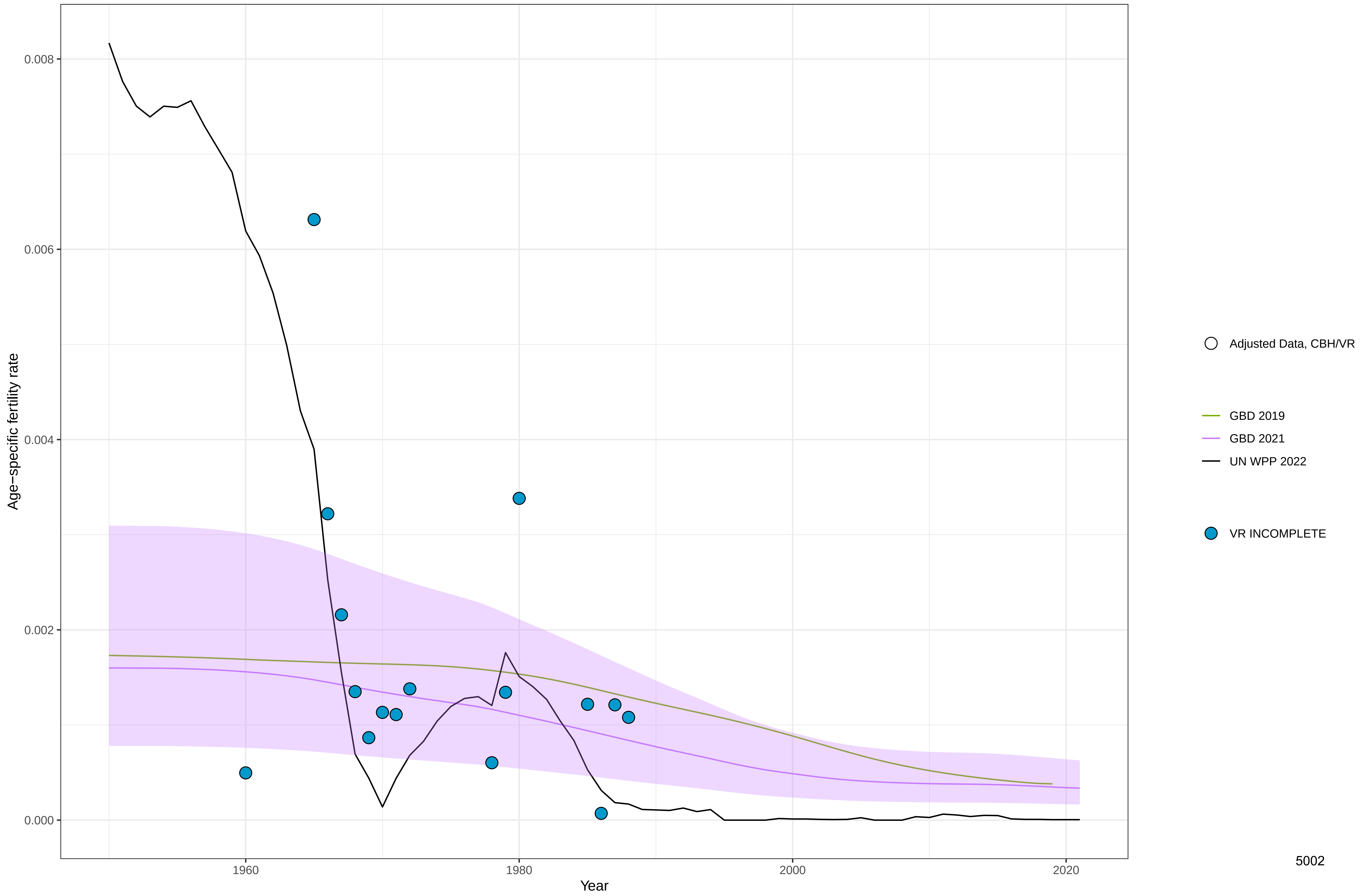

Age−specific fertility fitted trend lines, 1950−2021: Tunisia, Ages: 15−19
Data points enclosed with a diamond are reference sources

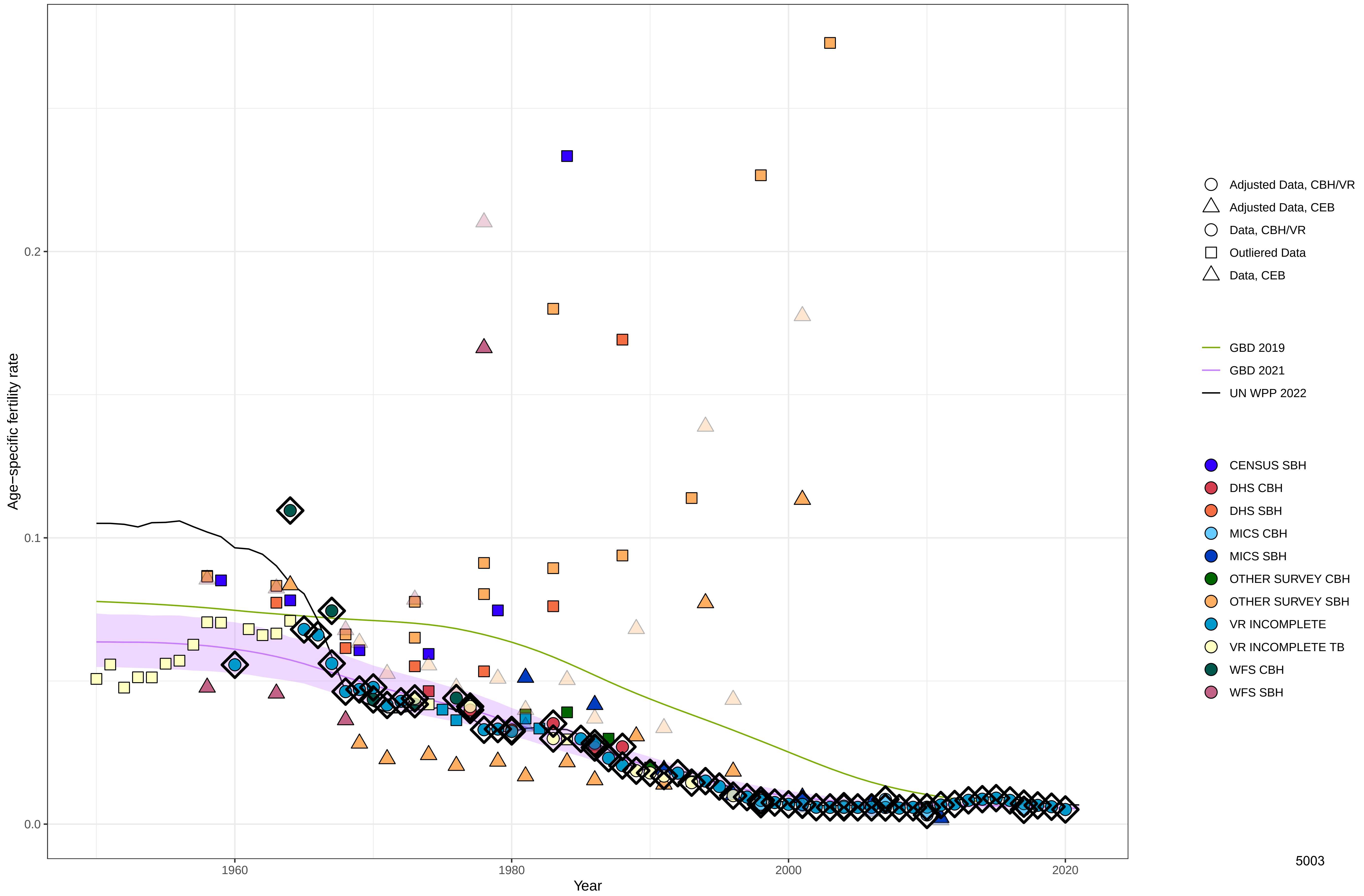

Age−specific fertility fitted trend lines, 1950−2021: Tunisia, Ages: 20−24
Data points enclosed with a diamond are reference sources

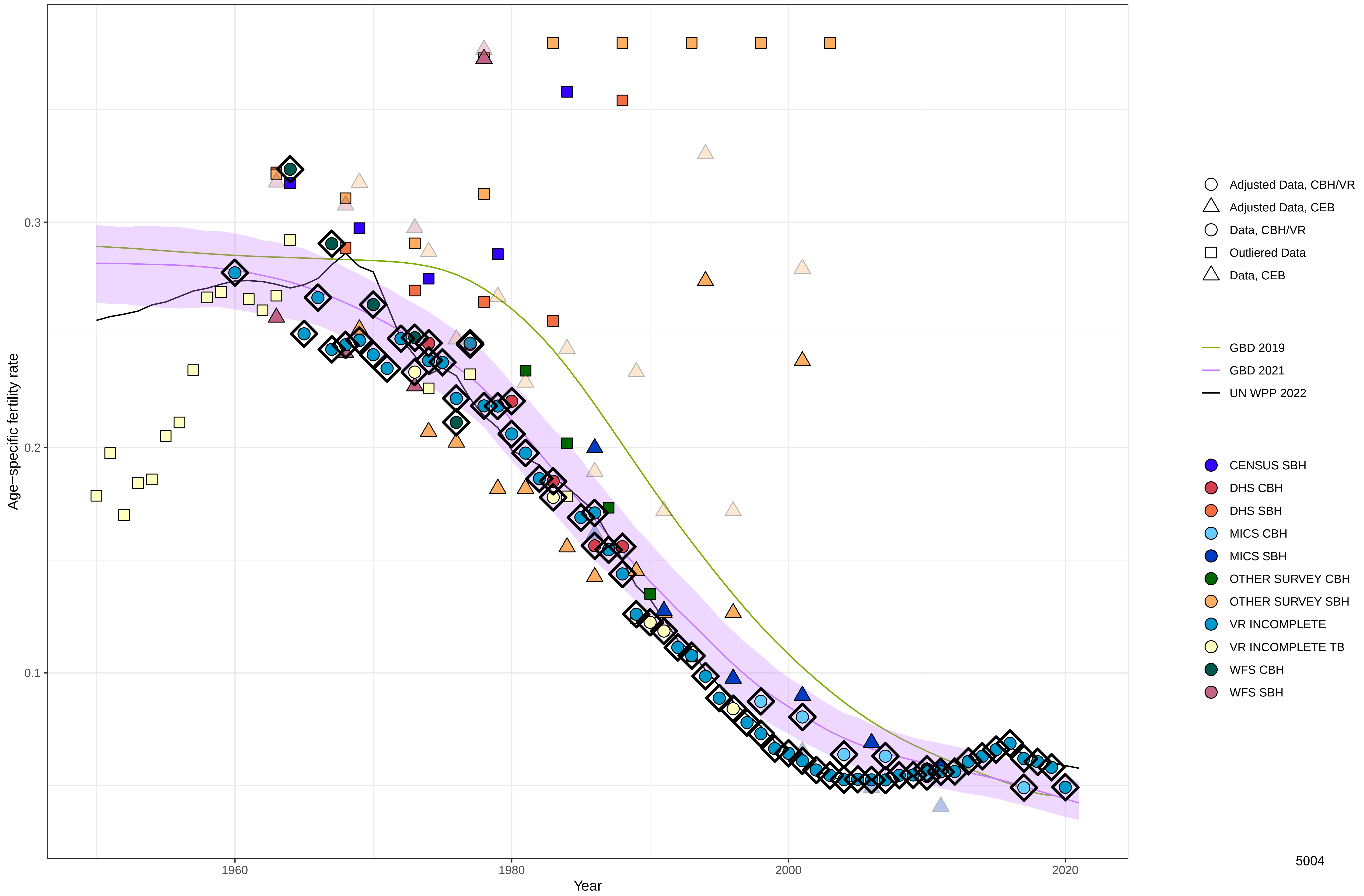

Age−specific fertility fitted trend lines, 1950−2021: Tunisia, Ages: 25−29
Data points enclosed with a diamond are reference sources

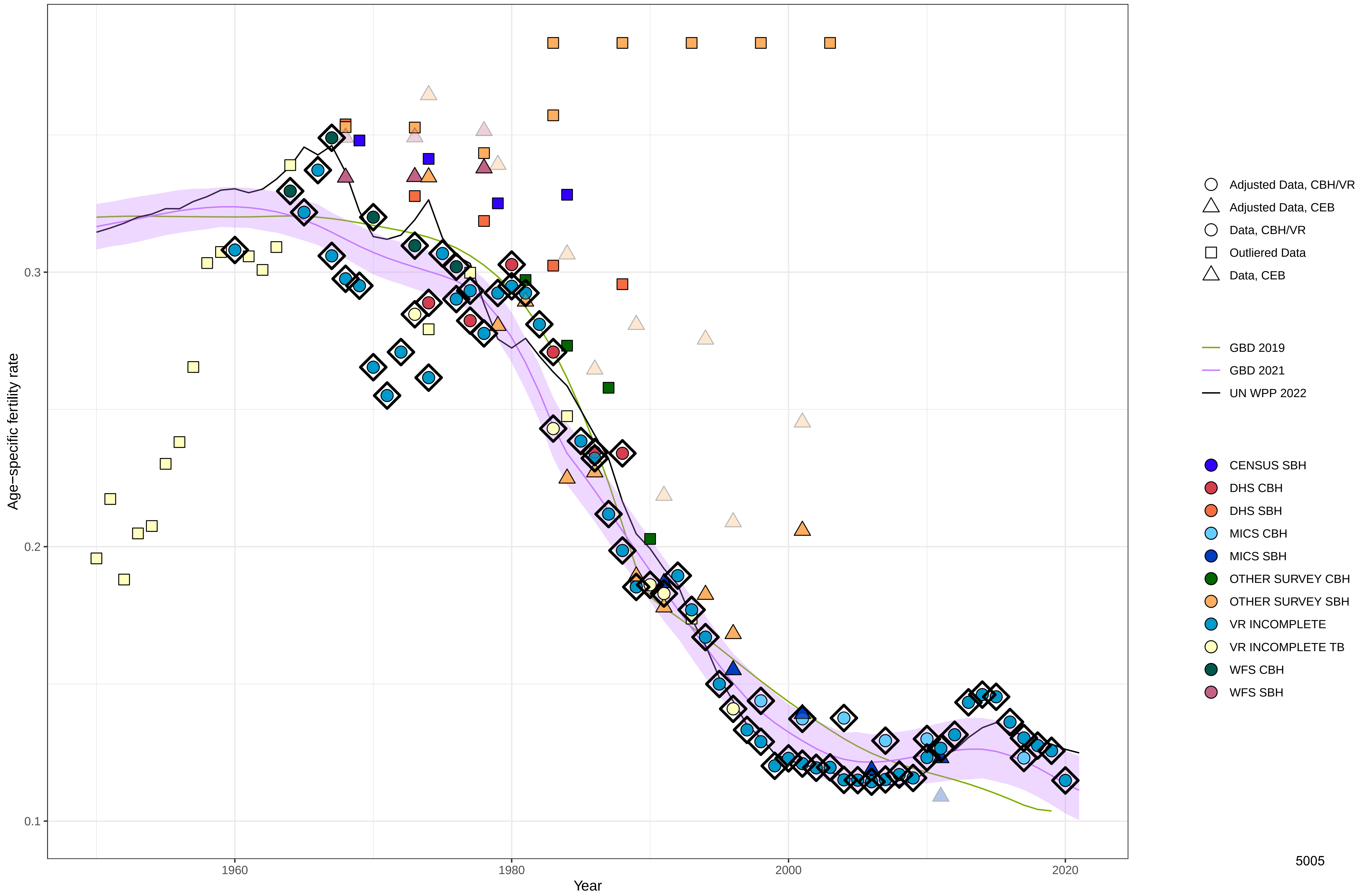

Age−specific fertility fitted trend lines, 1950−2021: Tunisia, Ages: 30−34
Data points enclosed with a diamond are reference sources

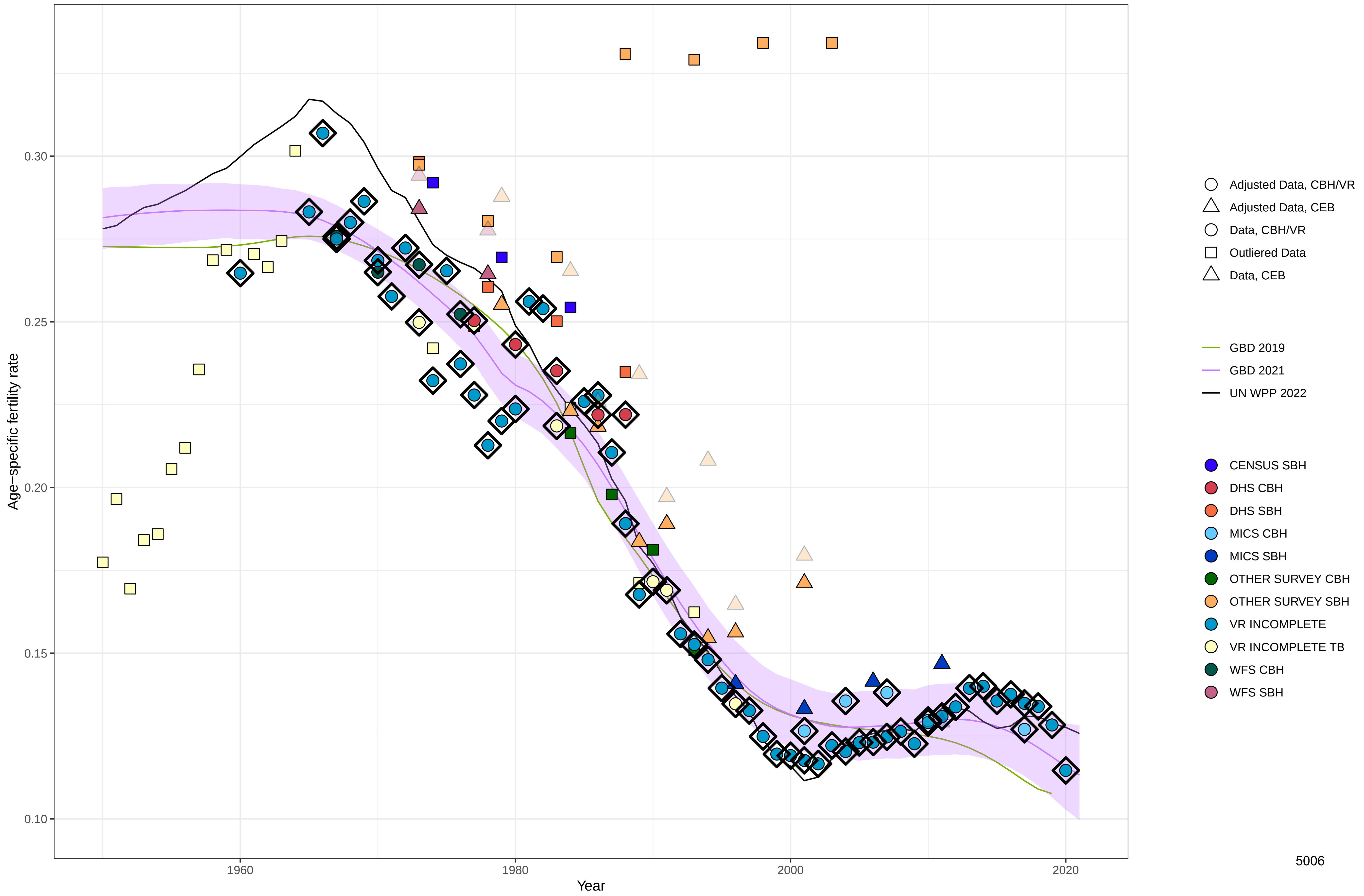

Age−specific fertility fitted trend lines, 1950−2021: Tunisia, Ages: 35−39
Data points enclosed with a diamond are reference sources

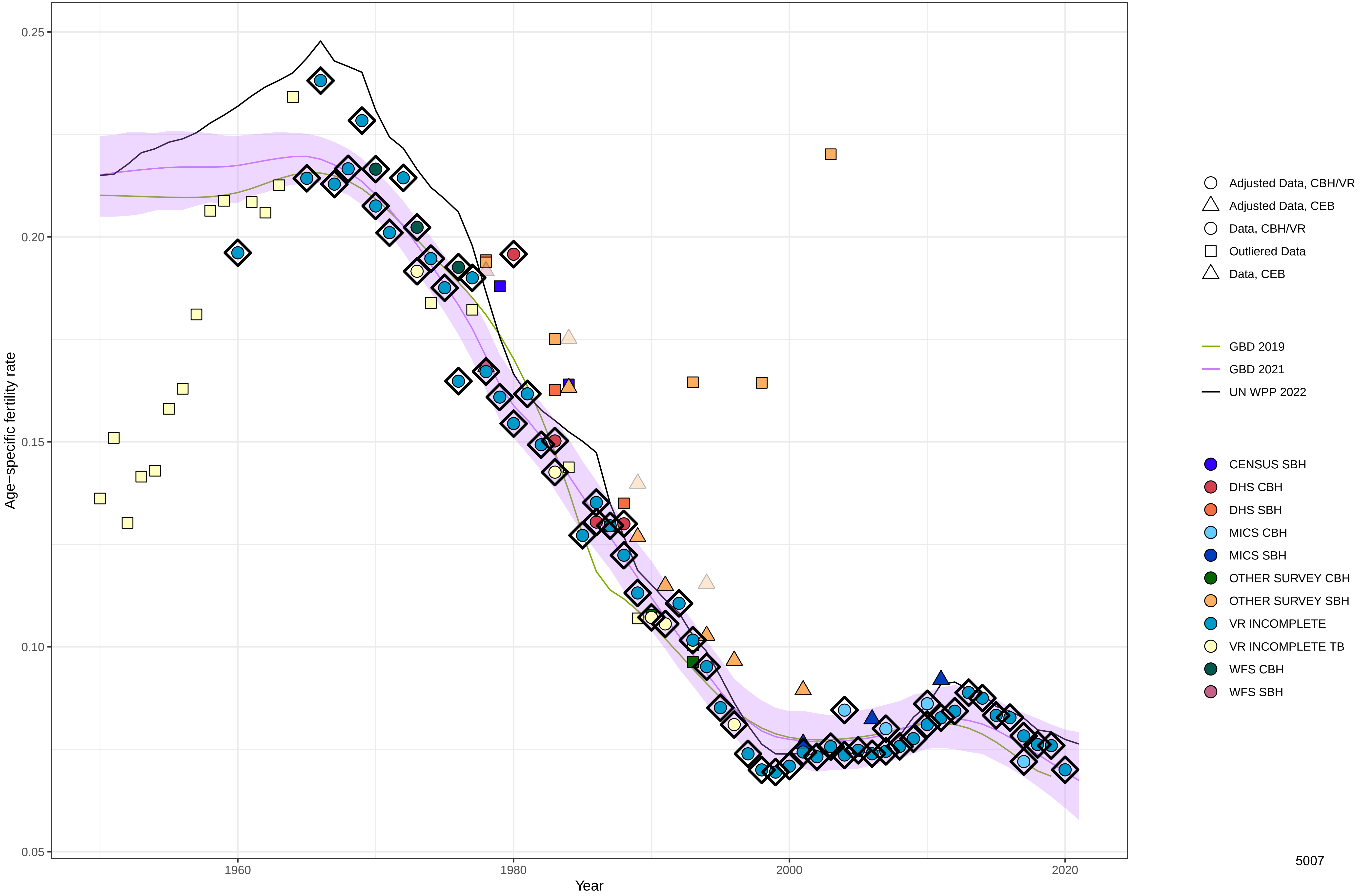

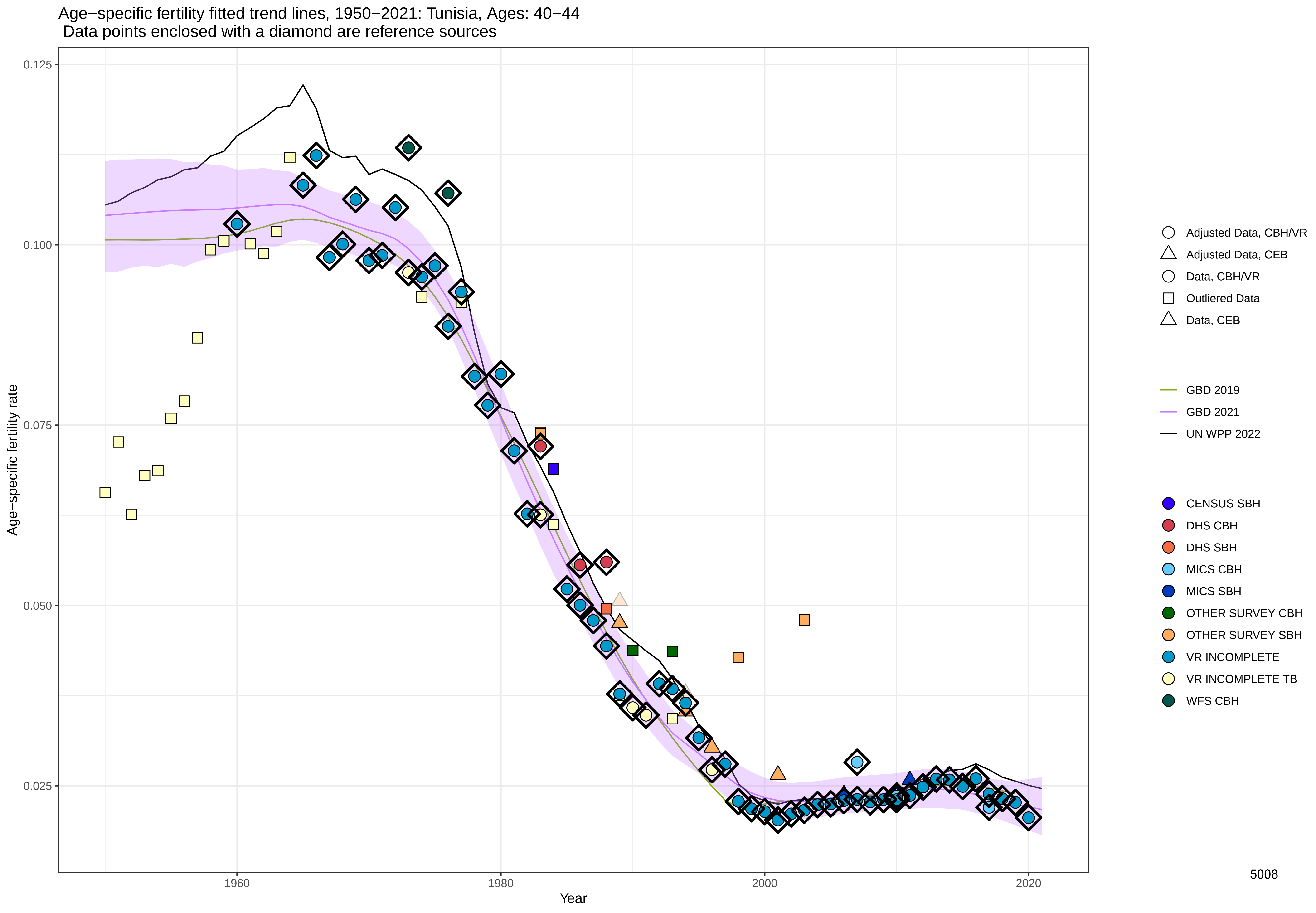
Age−specific fertility fitted trend lines, 1950−2021: Tunisia, Ages: 40−44
Data points enclosed with a diamond are reference sources
Age−specific fertility rate
Year
0.125
0.100
0.075
0.050
0.025
1960
1980
2000
2020
Adjusted Data, CBH/VR
Adjusted Data, CEB
Data, CBH/VR
Outliered Data
Data, CEB
GBD 2019
GBD 2021
UN WPP 2022
CENSUS SBH
DHS CBH
DHS SBH
MICS CBH
MICS SBH
OTHER SURVEY CBH
OTHER SURVEY SBH
VR INCOMPLETE
VR INCOMPLETE TB
WFS CBH

Age−specific fertility fitted trend lines, 1950−2021: Tunisia, Ages: 45−49
Data points enclosed with a diamond are reference sources

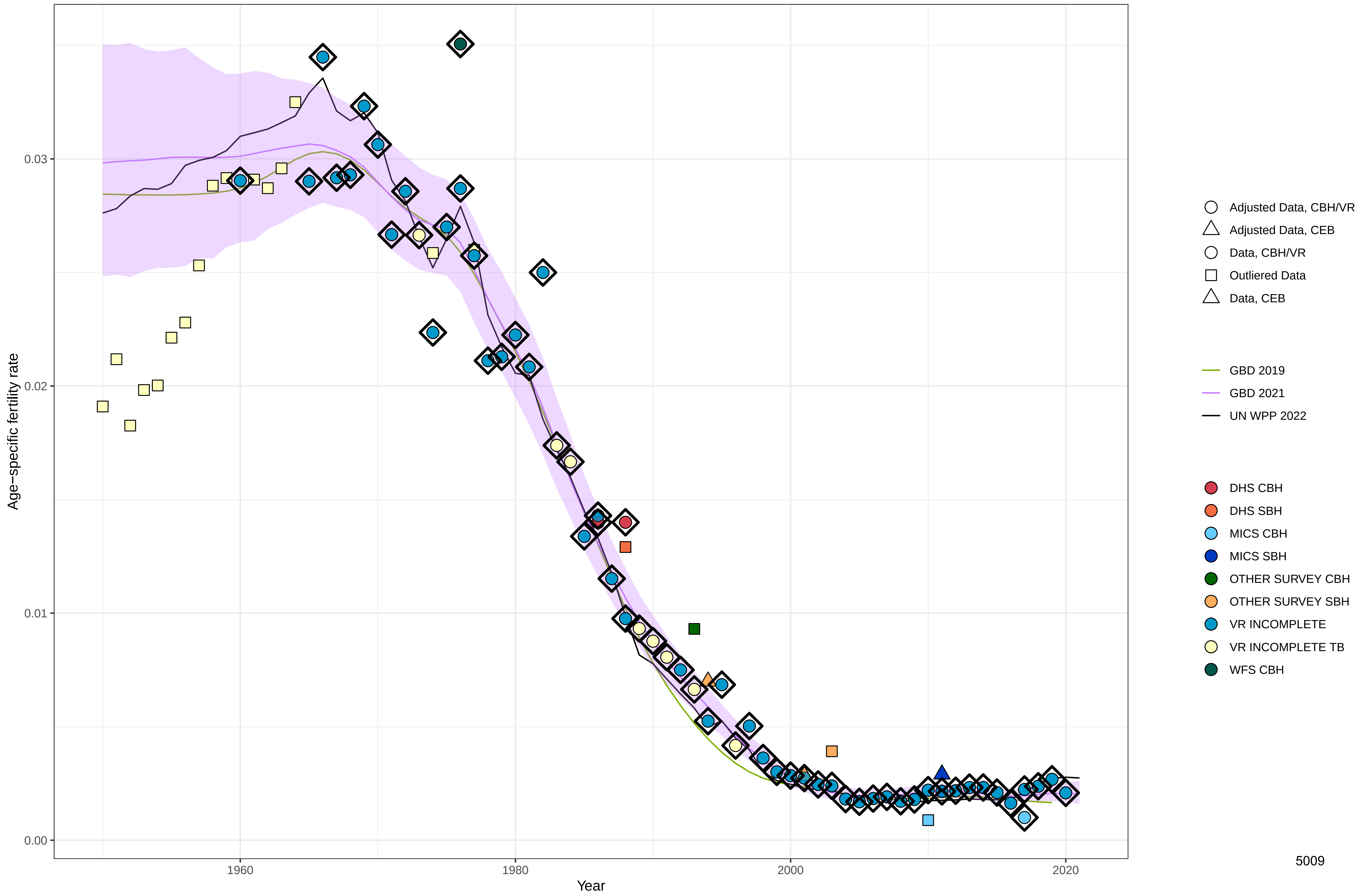

Age−specific fertility fitted trend lines, 1950−2021: Tunisia, Ages: 50−54
Data points enclosed with a diamond are reference sources

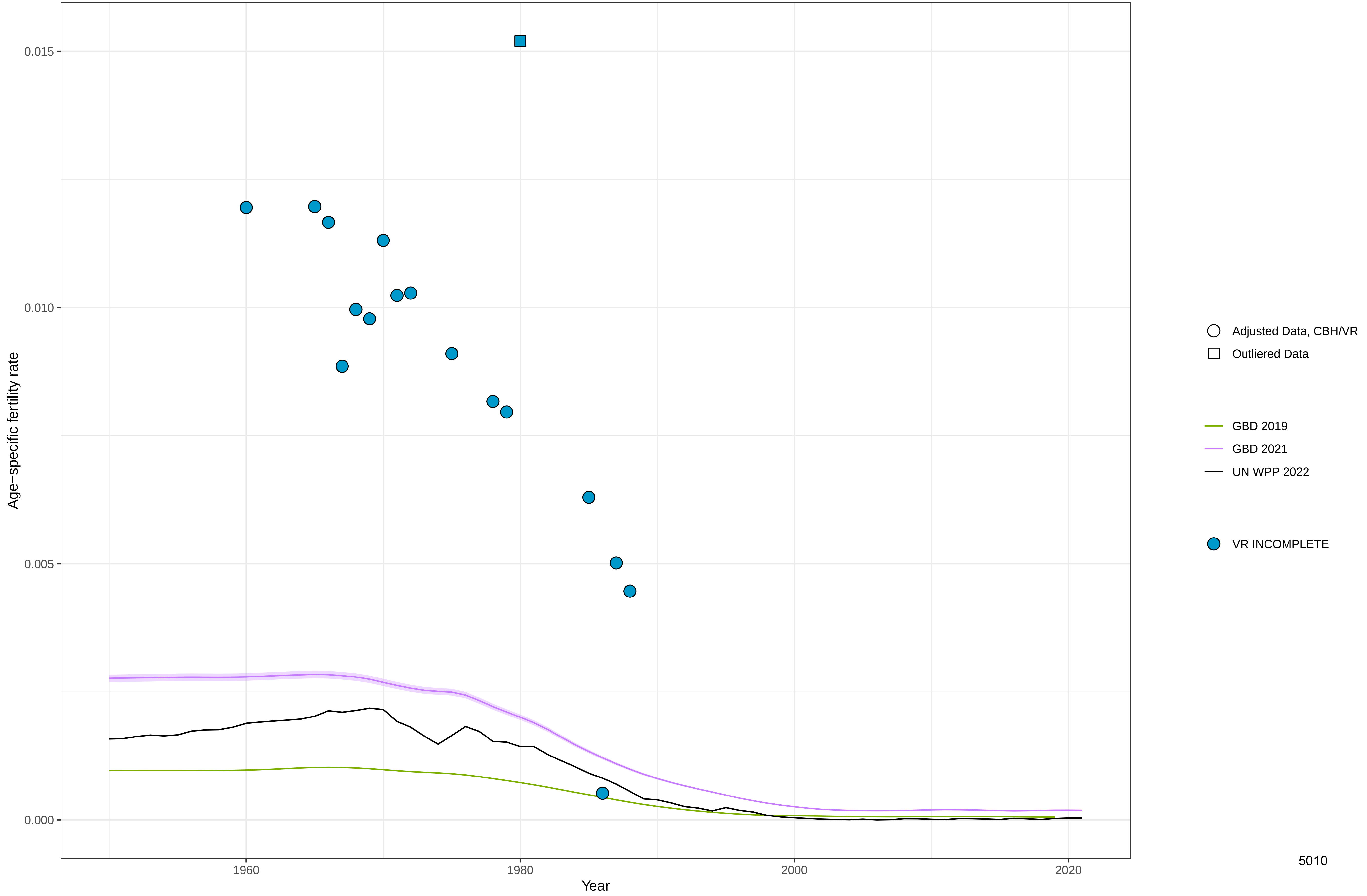

Total fertility rate fitted trend lines, 1950−2021: Tunisia TFR
Data points enclosed with a diamond are reference sources

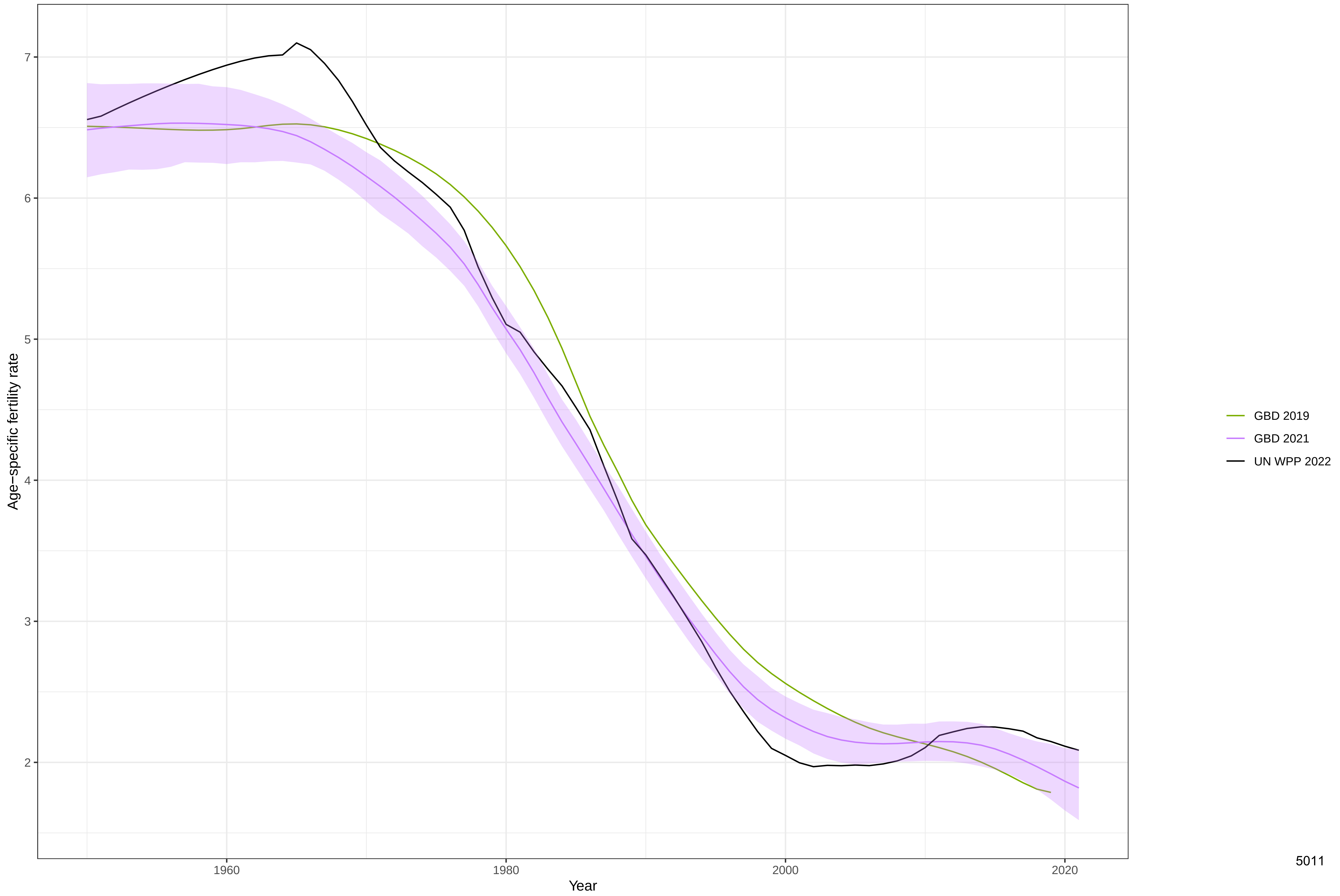

Age−specific fertility fitted trend lines, 1950−2021: Türkiye, Ages: 10−14
Data points enclosed with a diamond are reference sources

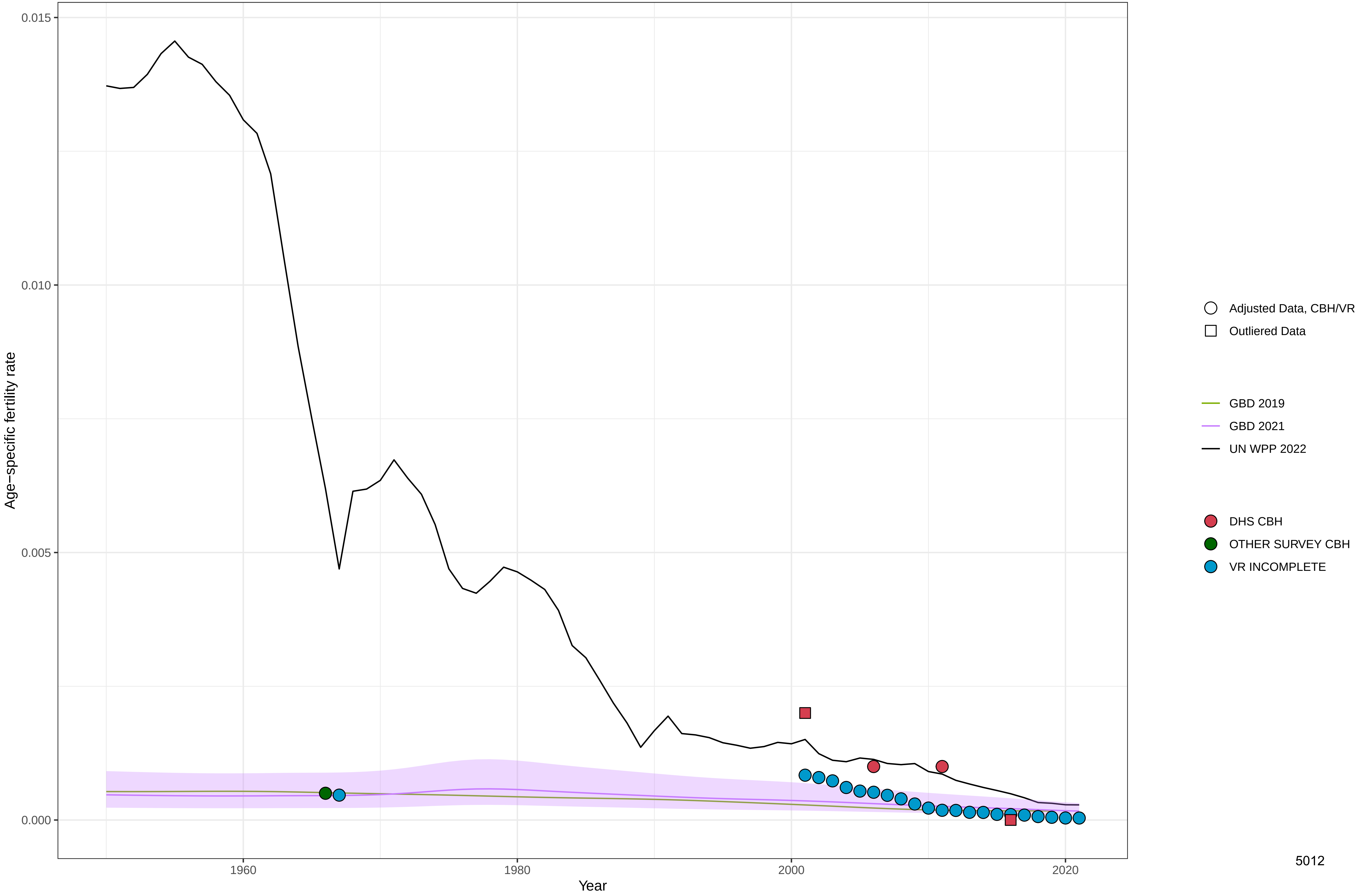

Age−specific fertility fitted trend lines, 1950−2021: Türkiye, Ages: 15−19
Data points enclosed with a diamond are reference sources

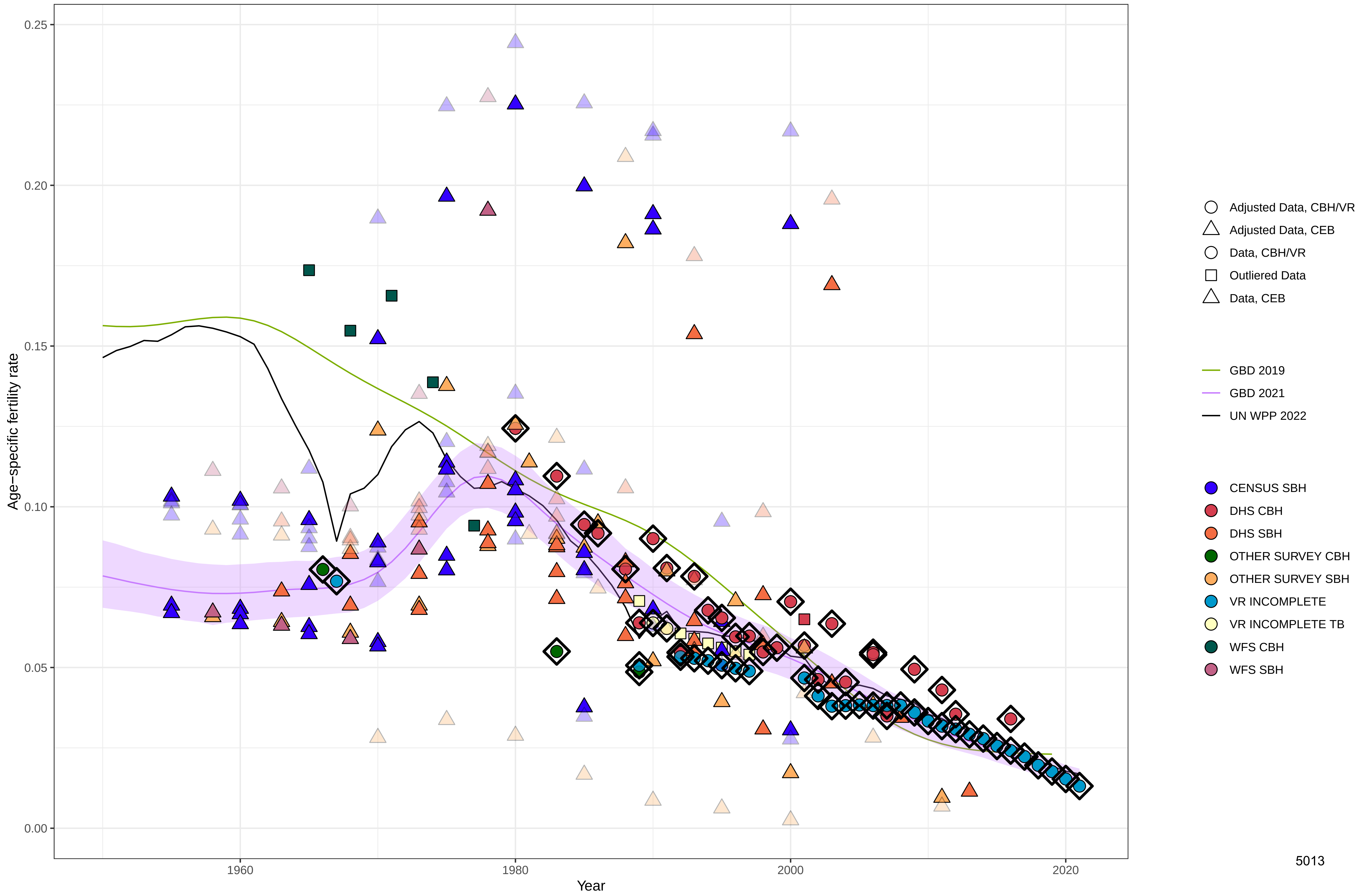

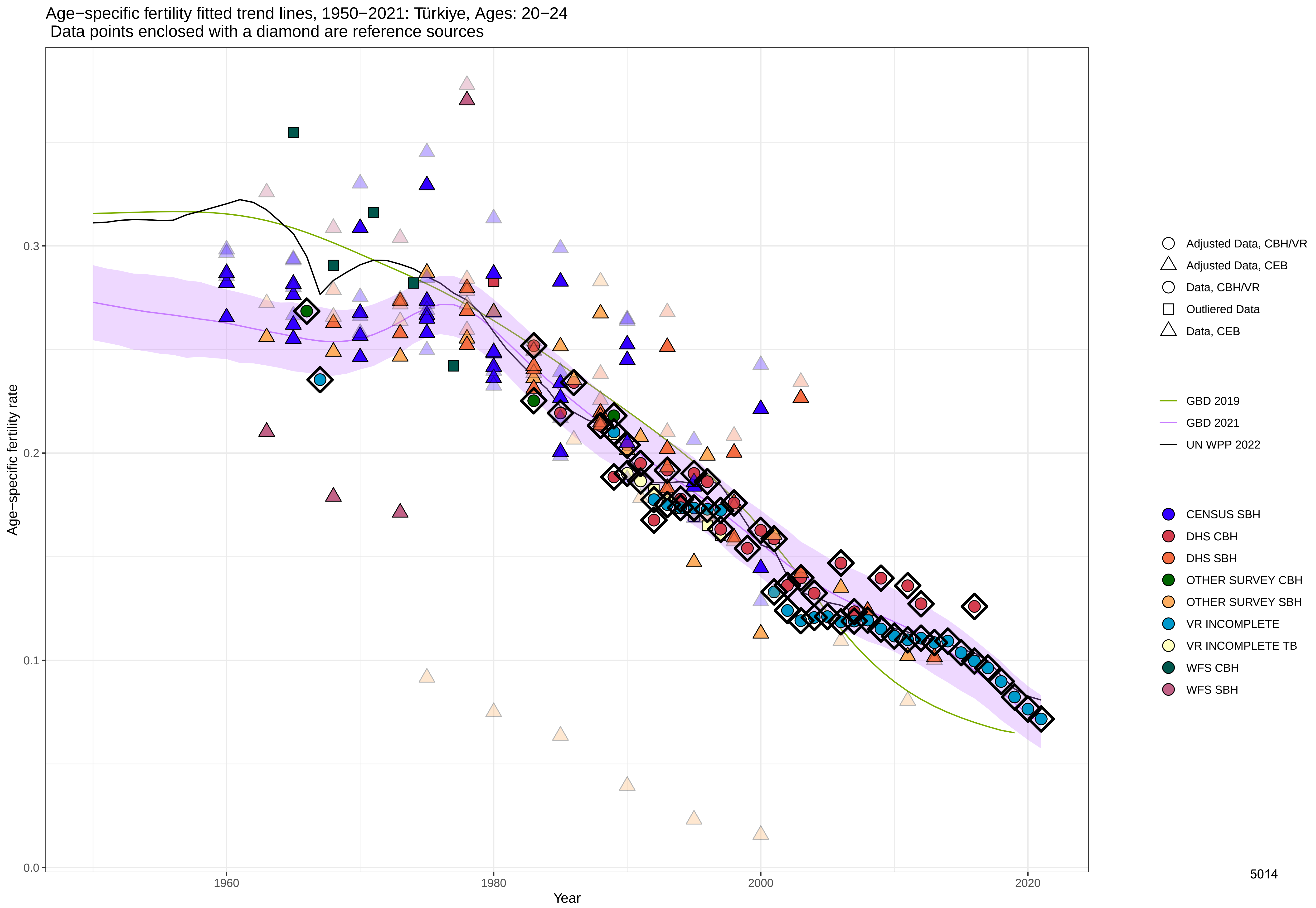

Age−specific fertility fitted trend lines, 1950−2021: Türkiye, Ages: 20−24
Data points enclosed with a diamond are reference sources
Age−specific fertility rate
Year
0.0
0.1
0.2
0.3
1960
1980
2000
2020
Adjusted Data, CBH/VR
Adjusted Data, CEB
Data, CBH/VR
Outliered Data
Data, CEB
GBD 2019
GBD 2021
UN WPP 2022
CENSUS SBH
DHS CBH
DHS SBH
OTHER SURVEY CBH
OTHER SURVEY SBH
VR INCOMPLETE
VR INCOMPLETE TB
WFS CBH
WFS SBH

Age−specific fertility fitted trend lines, 1950−2021: Türkiye, Ages: 25−29
Data points enclosed with a diamond are reference sources

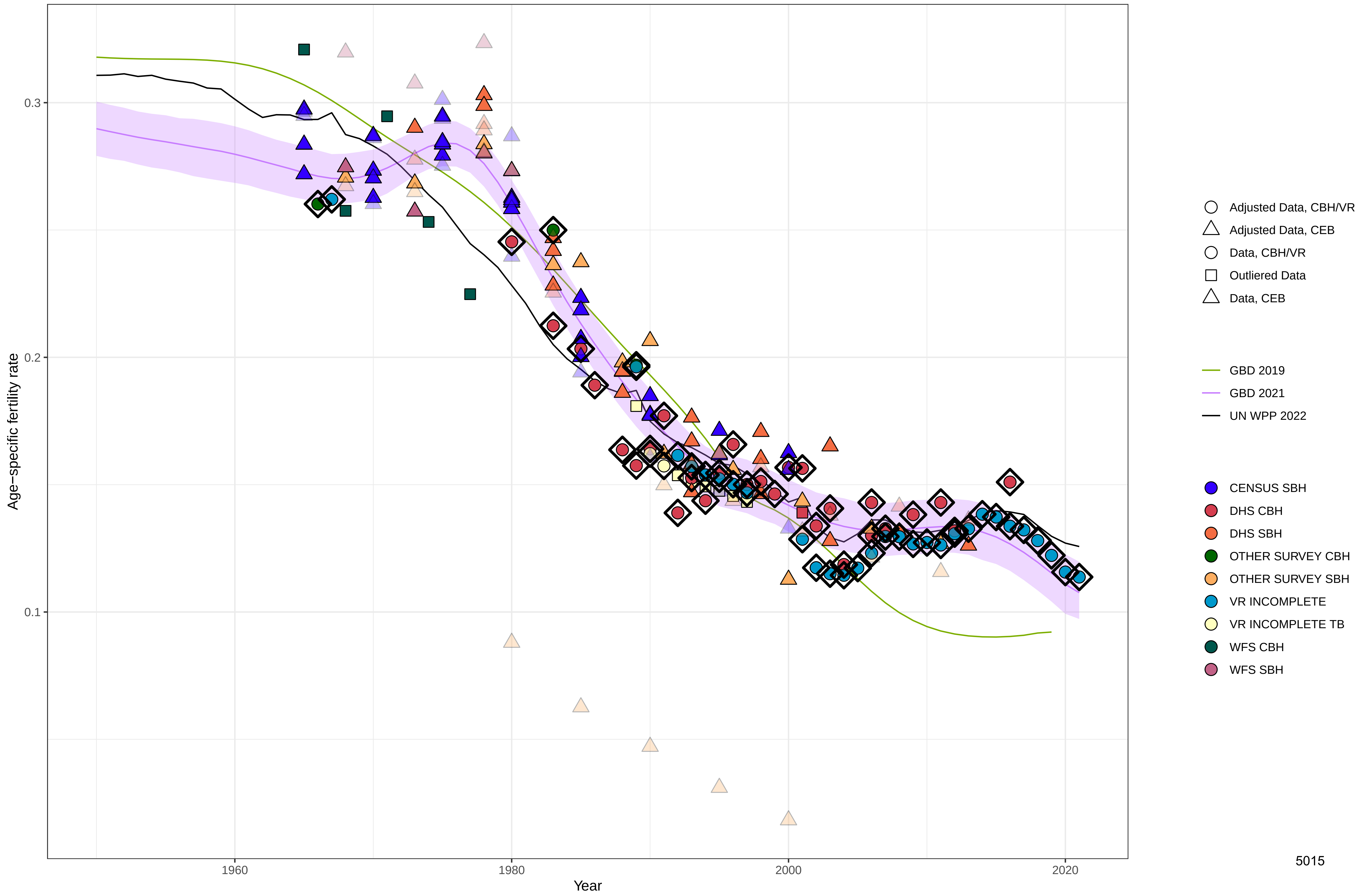

Age−specific fertility fitted trend lines, 1950−2021: Türkiye, Ages: 30−34
Data points enclosed with a diamond are reference sources

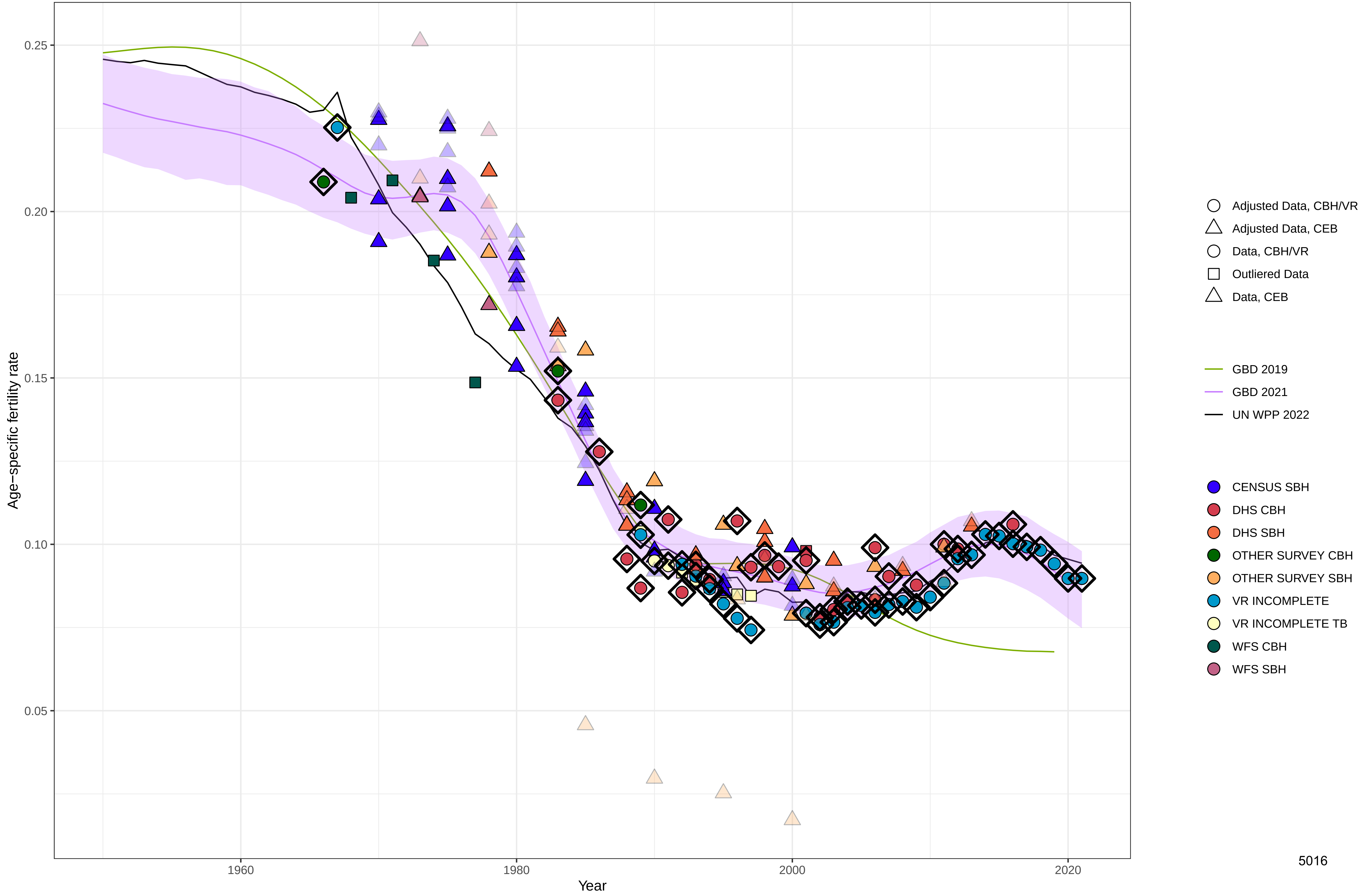

Age−specific fertility fitted trend lines, 1950−2021: Türkiye, Ages: 35−39
Data points enclosed with a diamond are reference sources

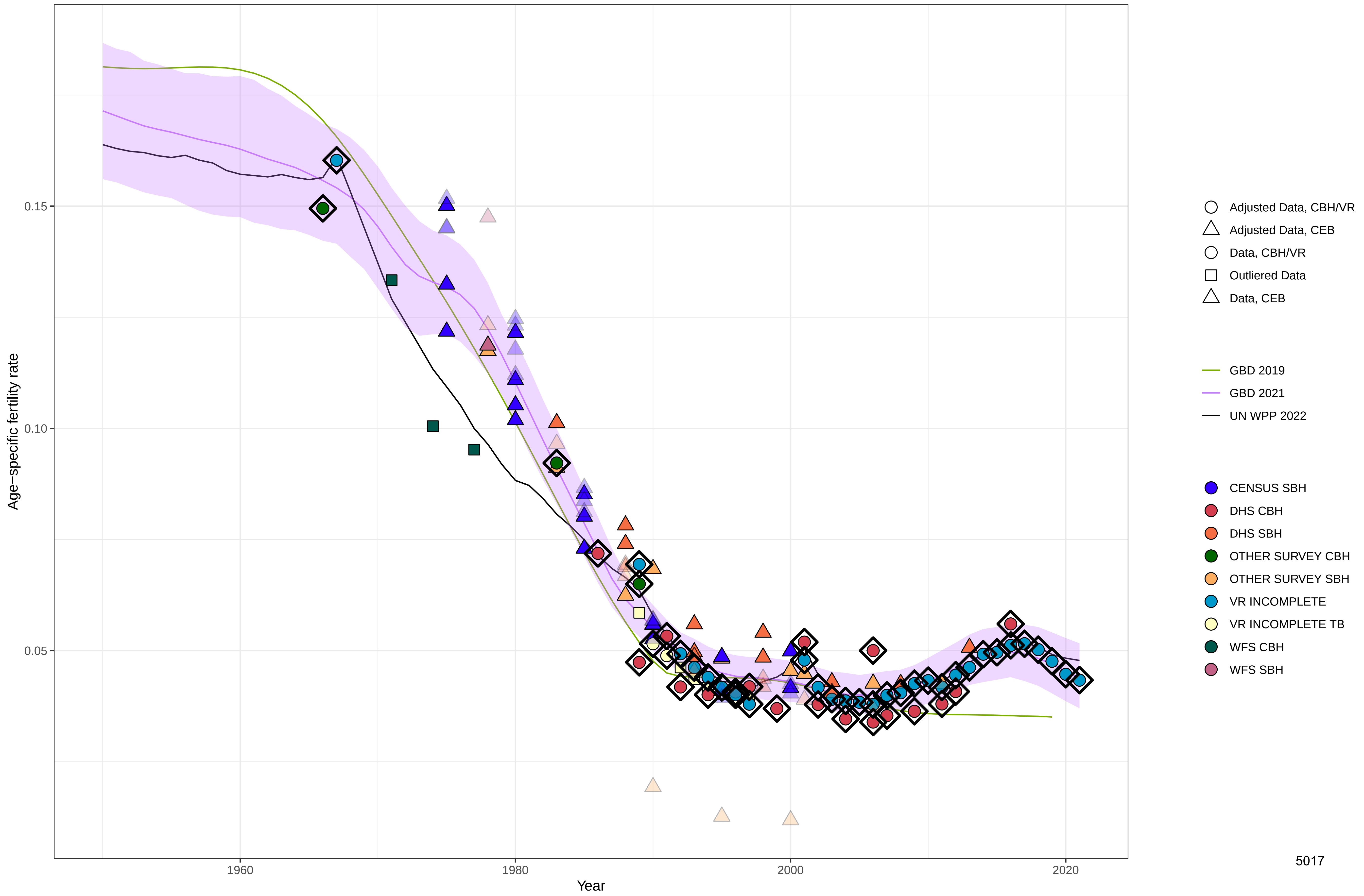

Age−specific fertility fitted trend lines, 1950−2021: Türkiye, Ages: 40−44
Data points enclosed with a diamond are reference sources

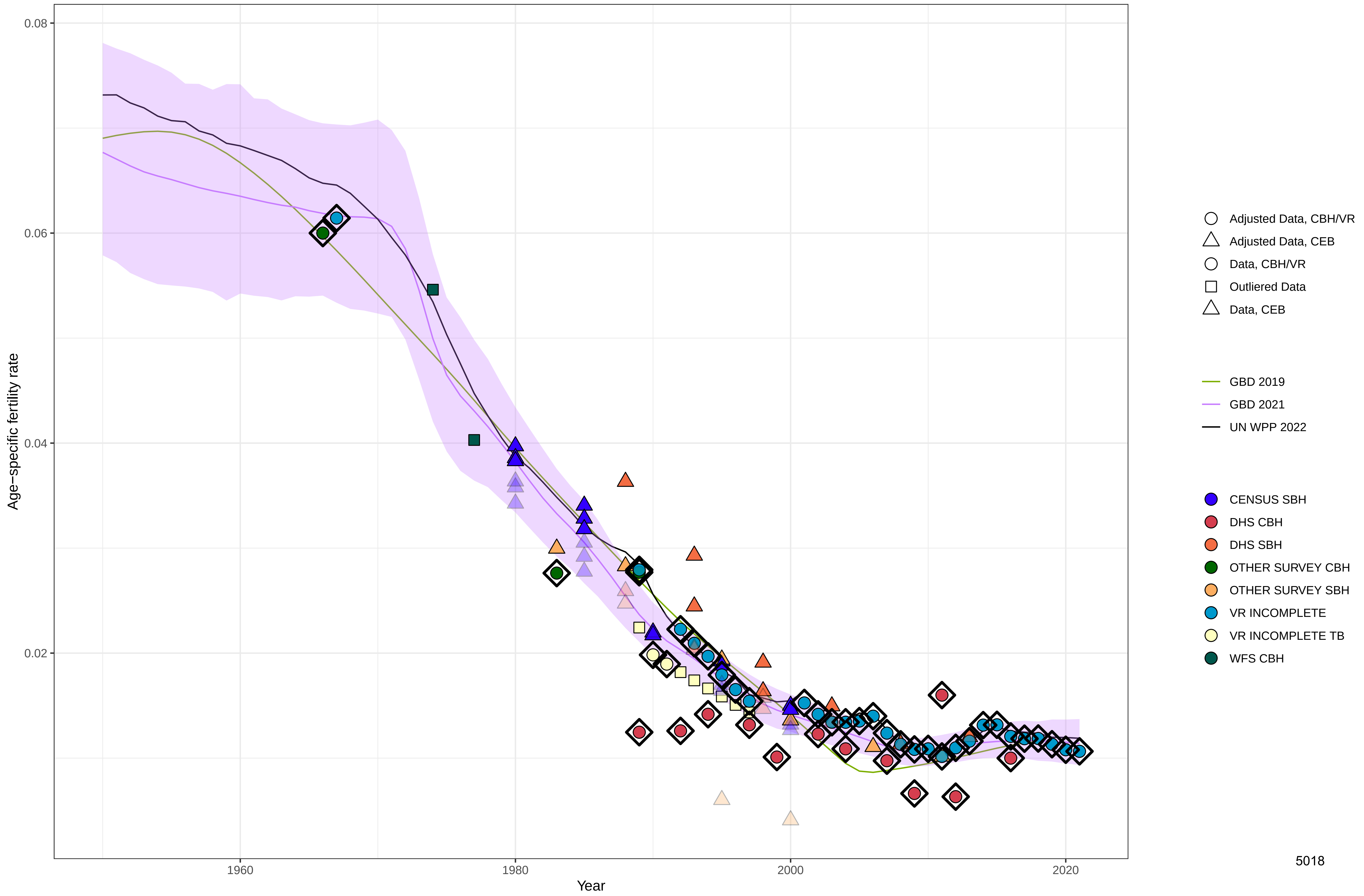

Age−specific fertility fitted trend lines, 1950−2021: Türkiye, Ages: 45−49
Data points enclosed with a diamond are reference sources

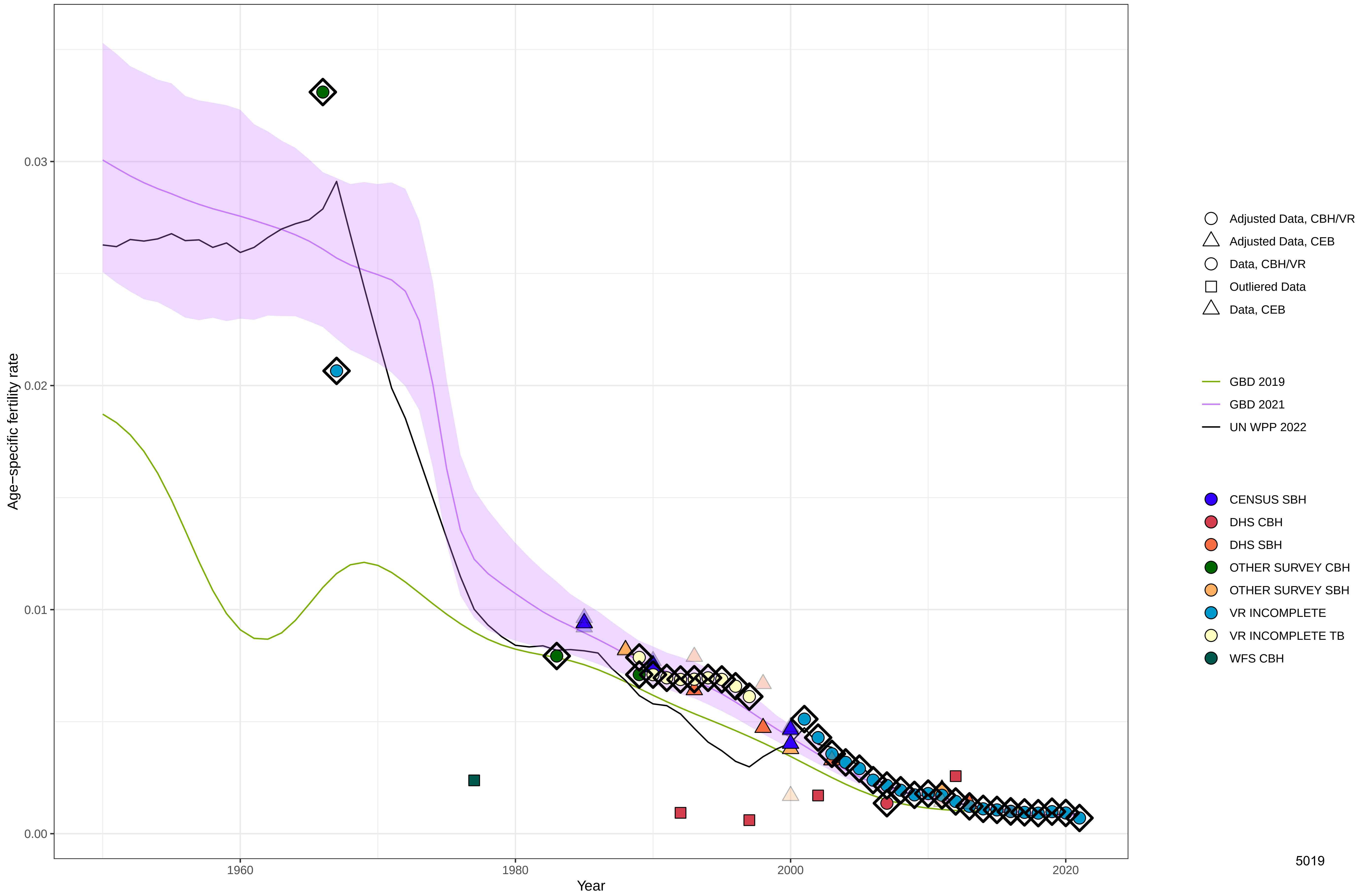

Age−specific fertility fitted trend lines, 1950−2021: Türkiye, Ages: 50−54
Data points enclosed with a diamond are reference sources

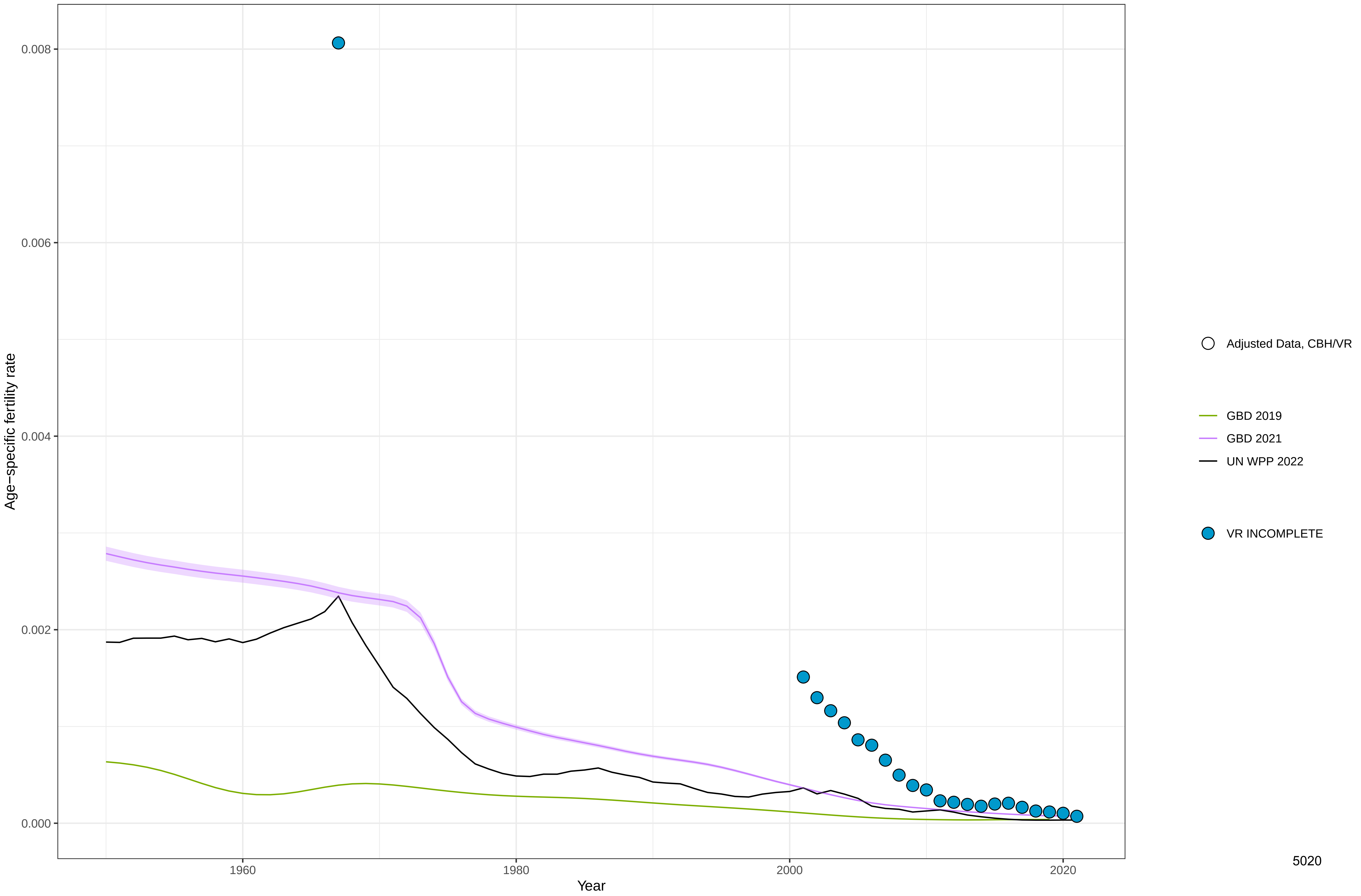

Total fertility rate fitted trend lines, 1950−2021: Türkiye TFR
Data points enclosed with a diamond are reference sources

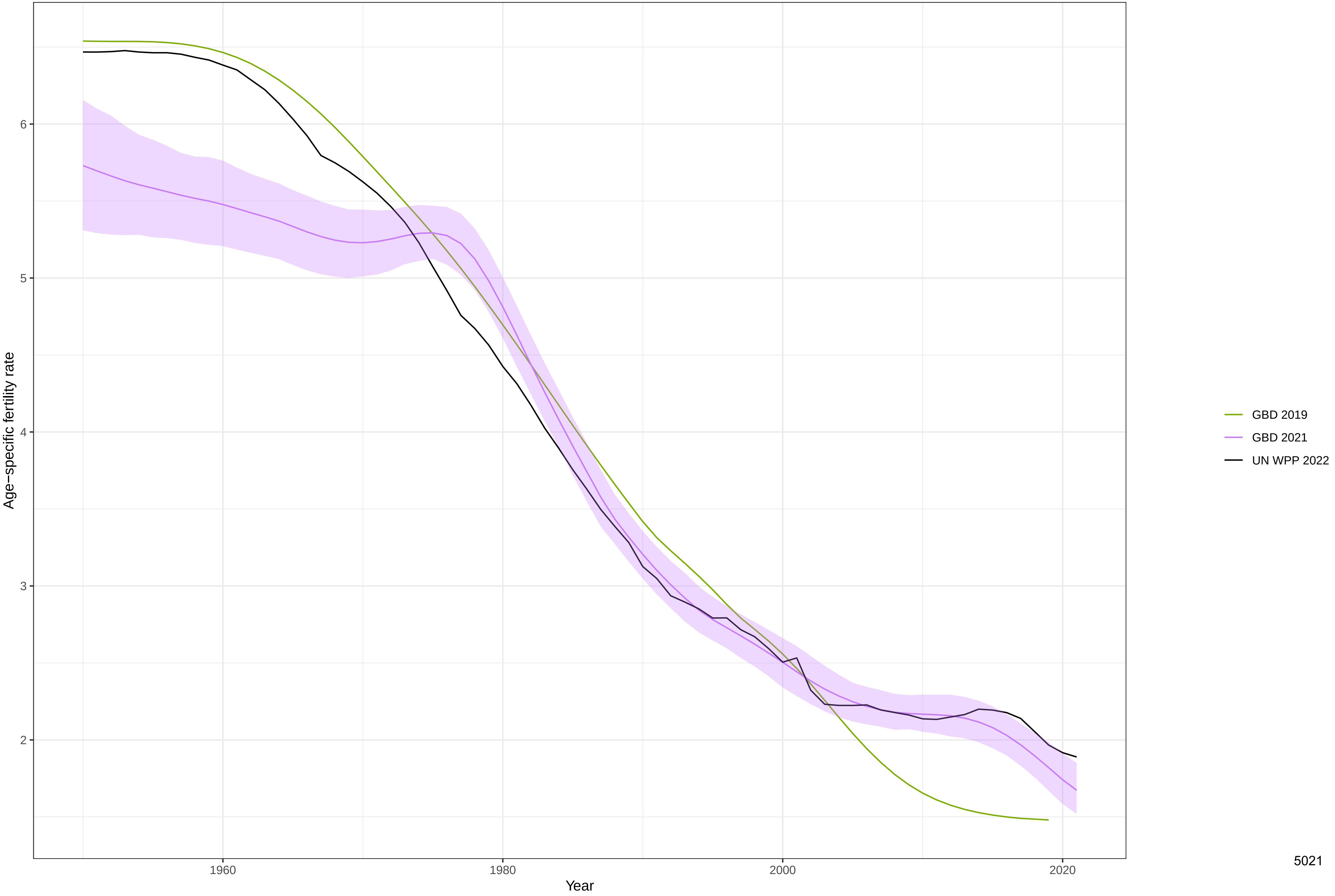

Age−specific fertility fitted trend lines, 1950−2021: United Arab Emirates, Ages: 10−14
Data points enclosed with a diamond are reference sources

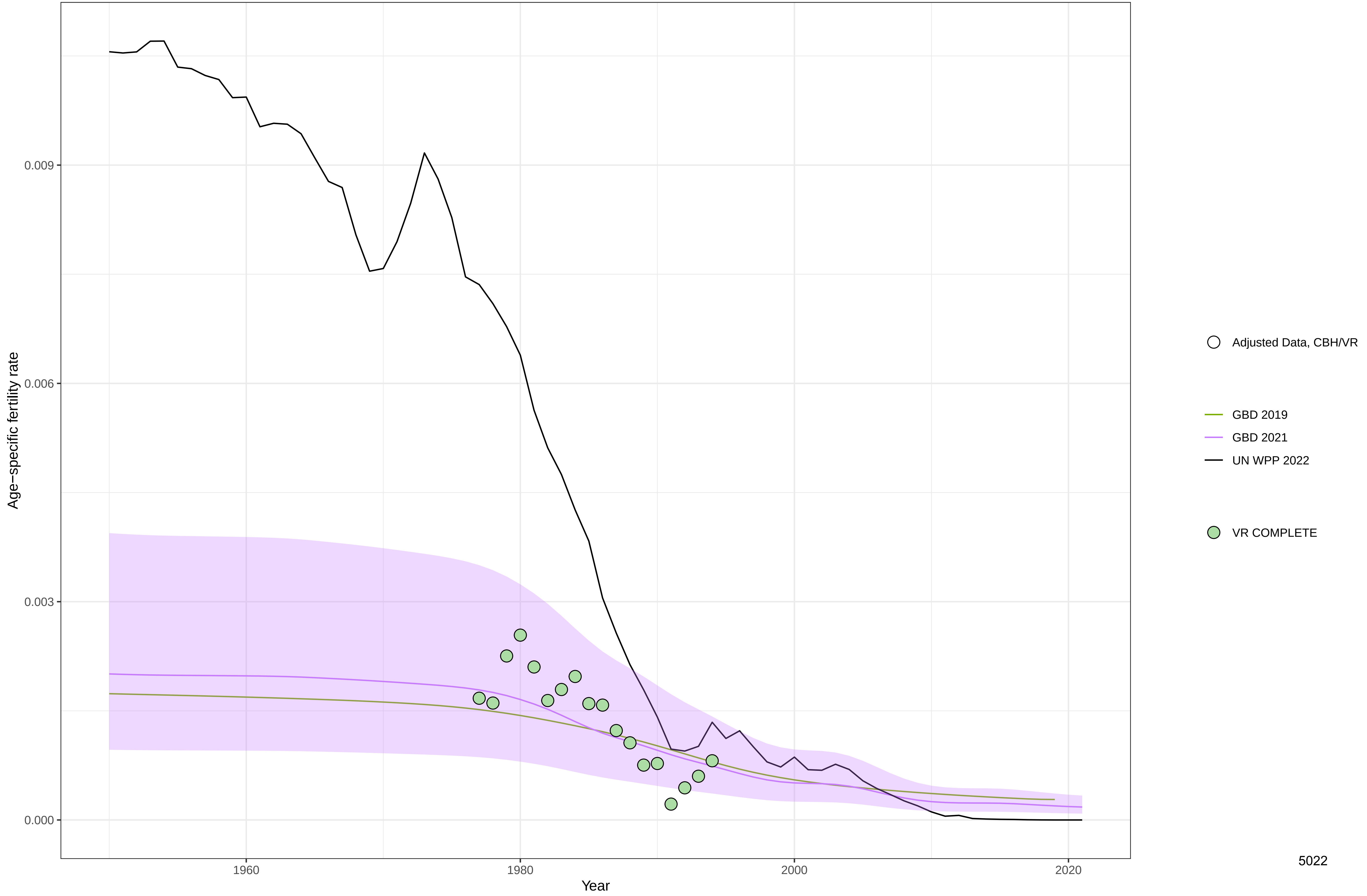

Age−specific fertility fitted trend lines, 1950−2021: United Arab Emirates, Ages: 15−19
Data points enclosed with a diamond are reference sources

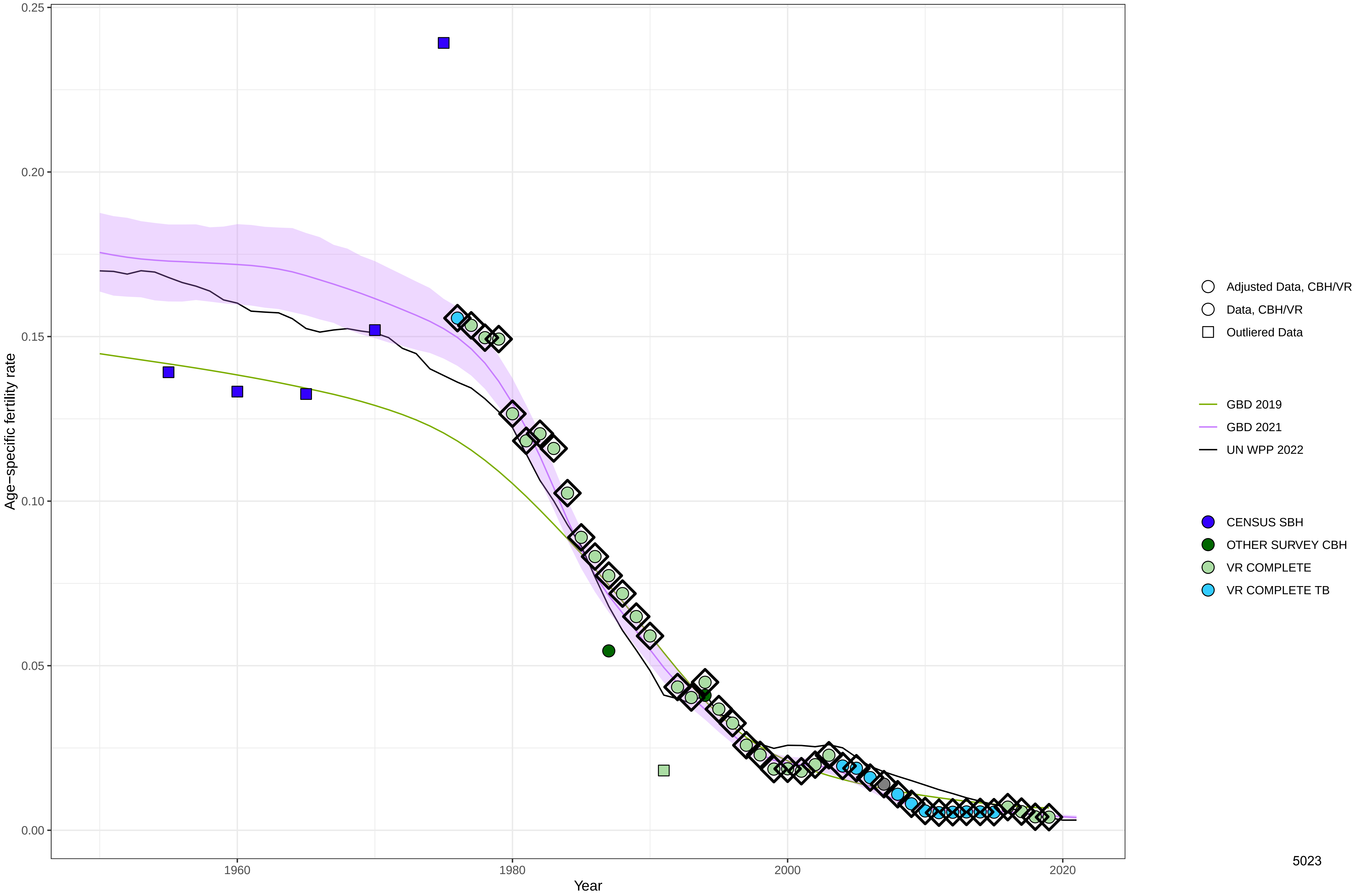

Age−specific fertility fitted trend lines, 1950−2021: United Arab Emirates, Ages: 20−24
Data points enclosed with a diamond are reference sources

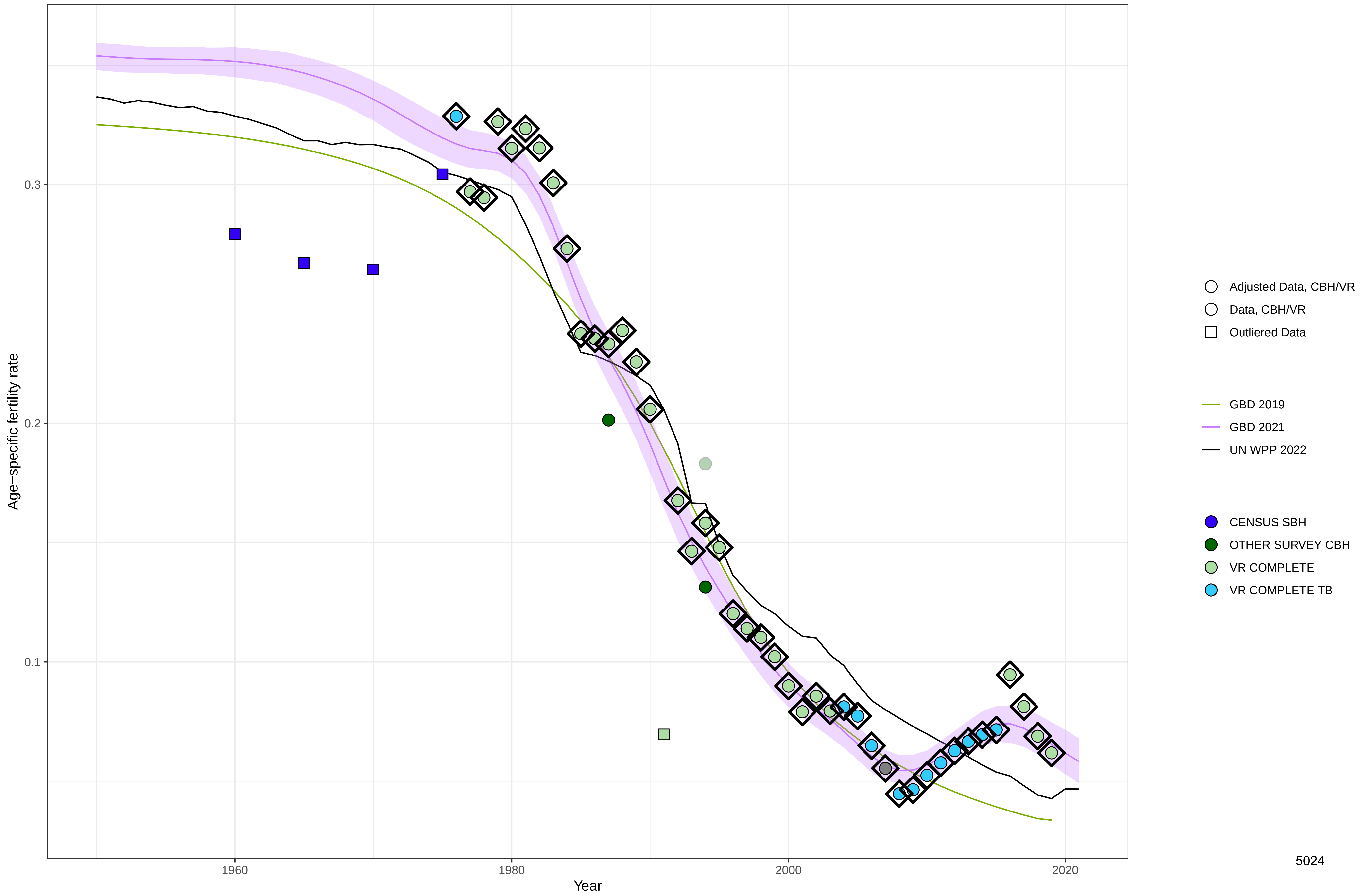

Age−specific fertility fitted trend lines, 1950−2021: United Arab Emirates, Ages: 25−29
Data points enclosed with a diamond are reference sources

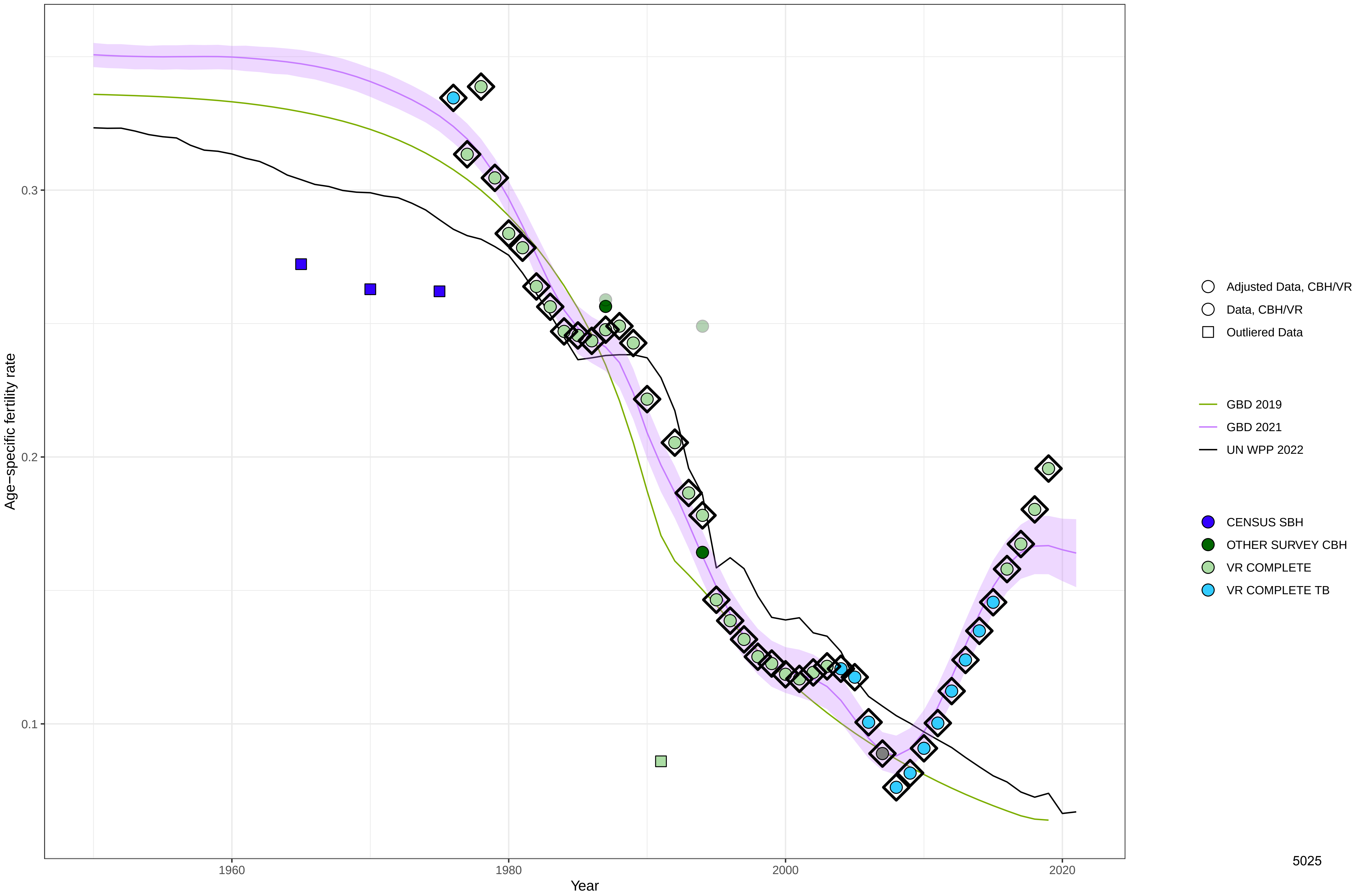

Age−specific fertility fitted trend lines, 1950−2021: United Arab Emirates, Ages: 30−34
Data points enclosed with a diamond are reference sources

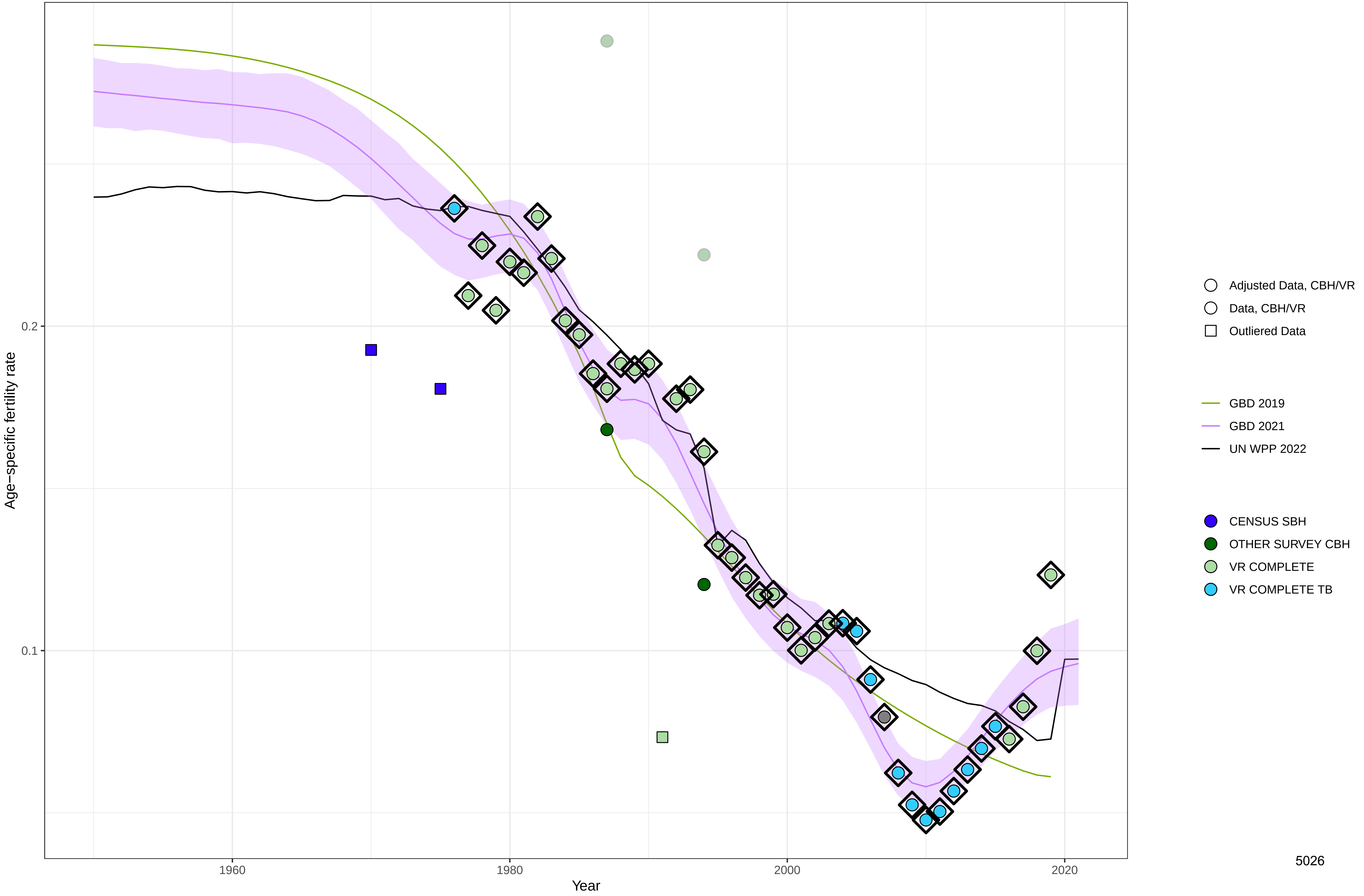

Age−specific fertility fitted trend lines, 1950−2021: United Arab Emirates, Ages: 35−39
Data points enclosed with a diamond are reference sources

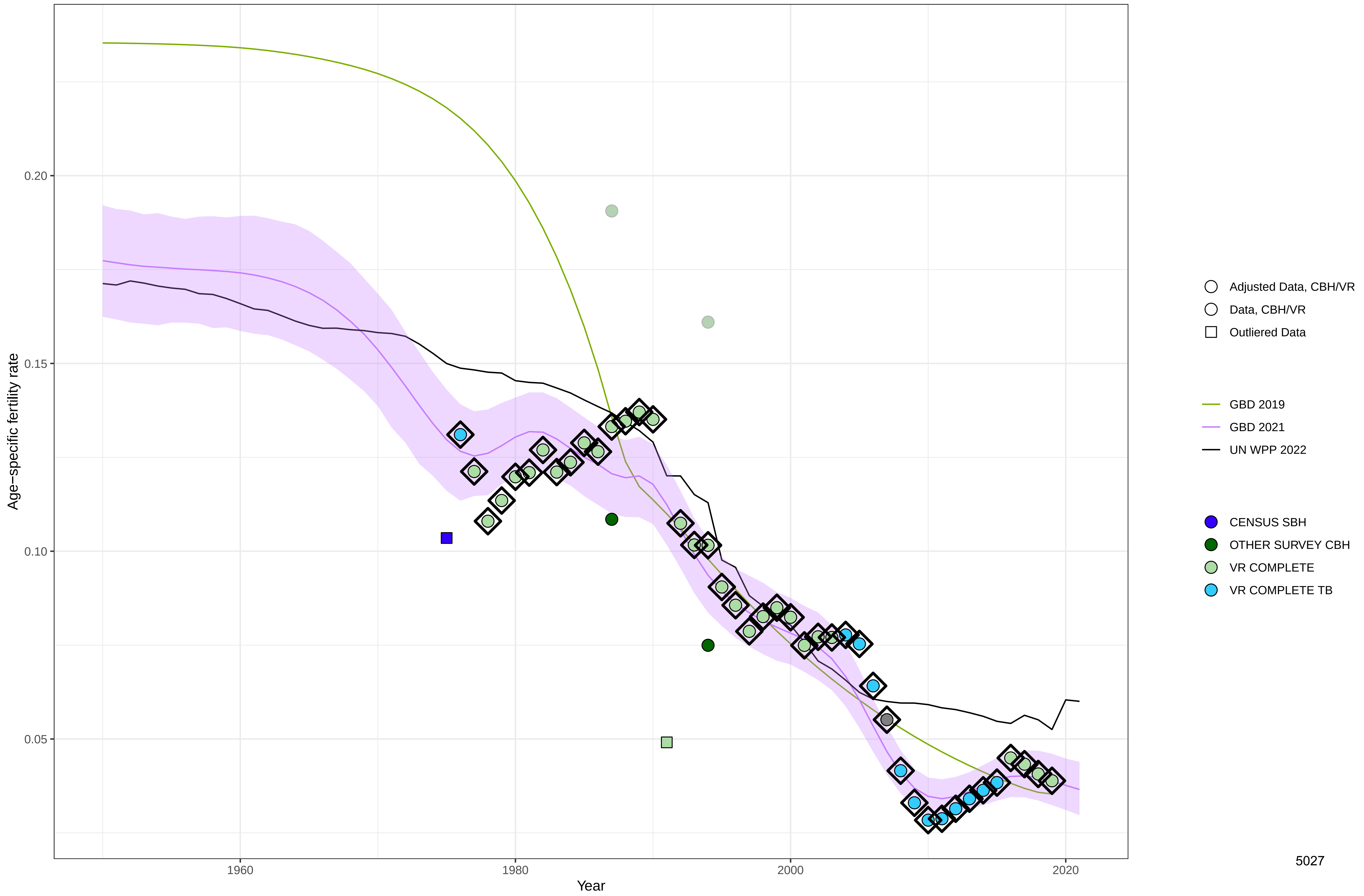

Age−specific fertility fitted trend lines, 1950−2021: United Arab Emirates, Ages: 40−44
Data points enclosed with a diamond are reference sources

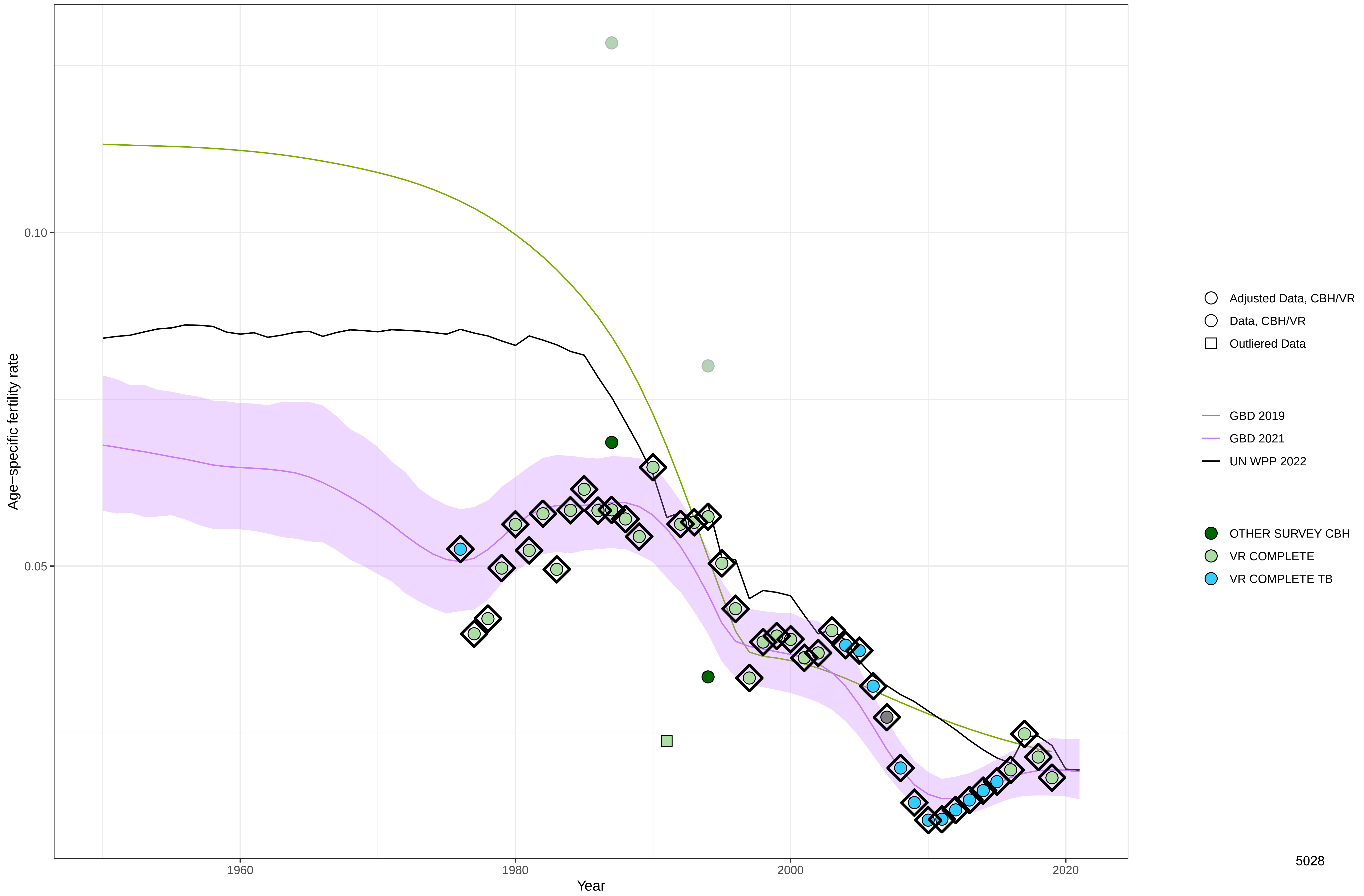

Age−specific fertility fitted trend lines, 1950−2021: United Arab Emirates, Ages: 45−49
Data points enclosed with a diamond are reference sources

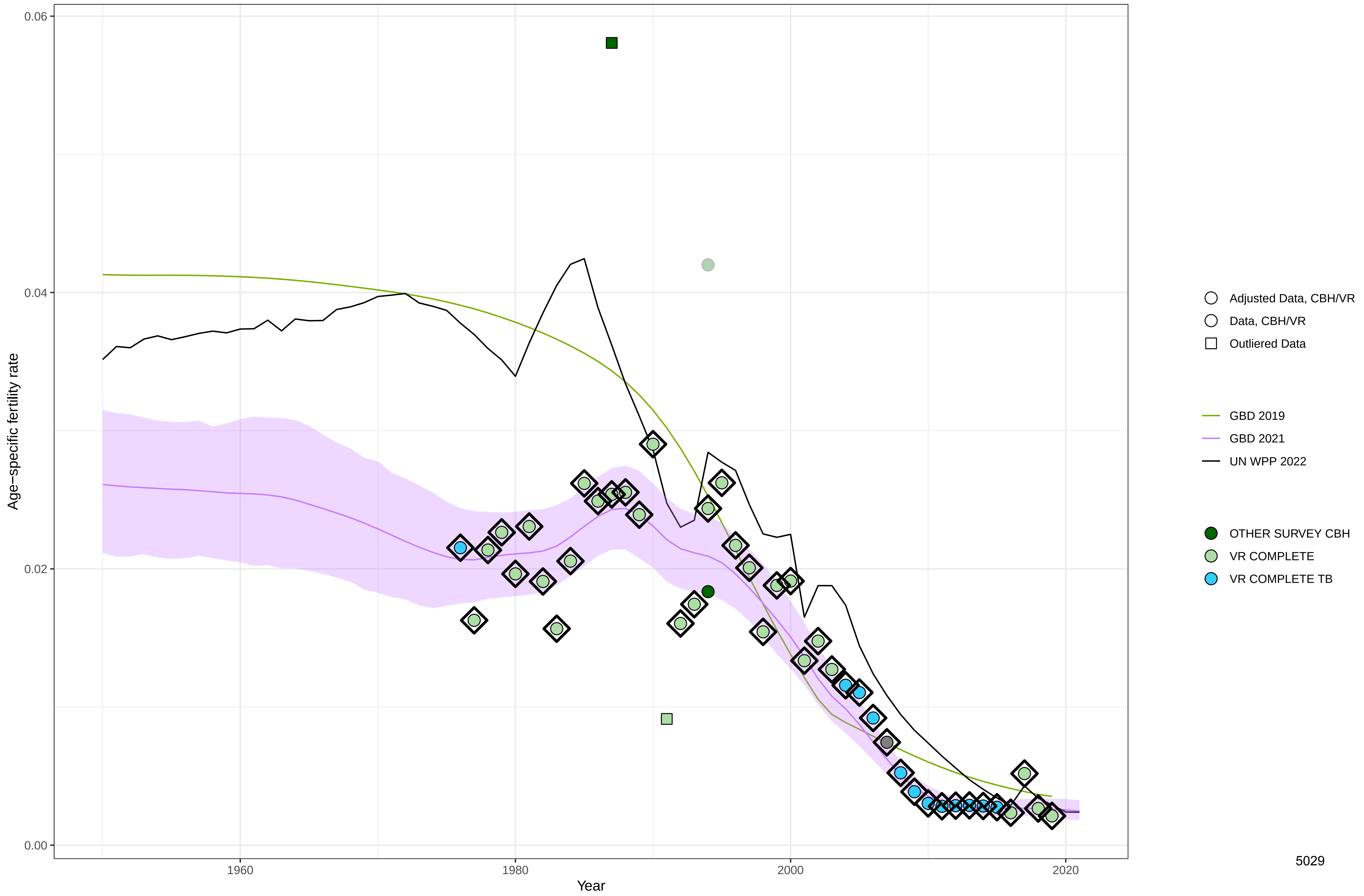

Age−specific fertility fitted trend lines, 1950−2021: United Arab Emirates, Ages: 50−54
Data points enclosed with a diamond are reference sources

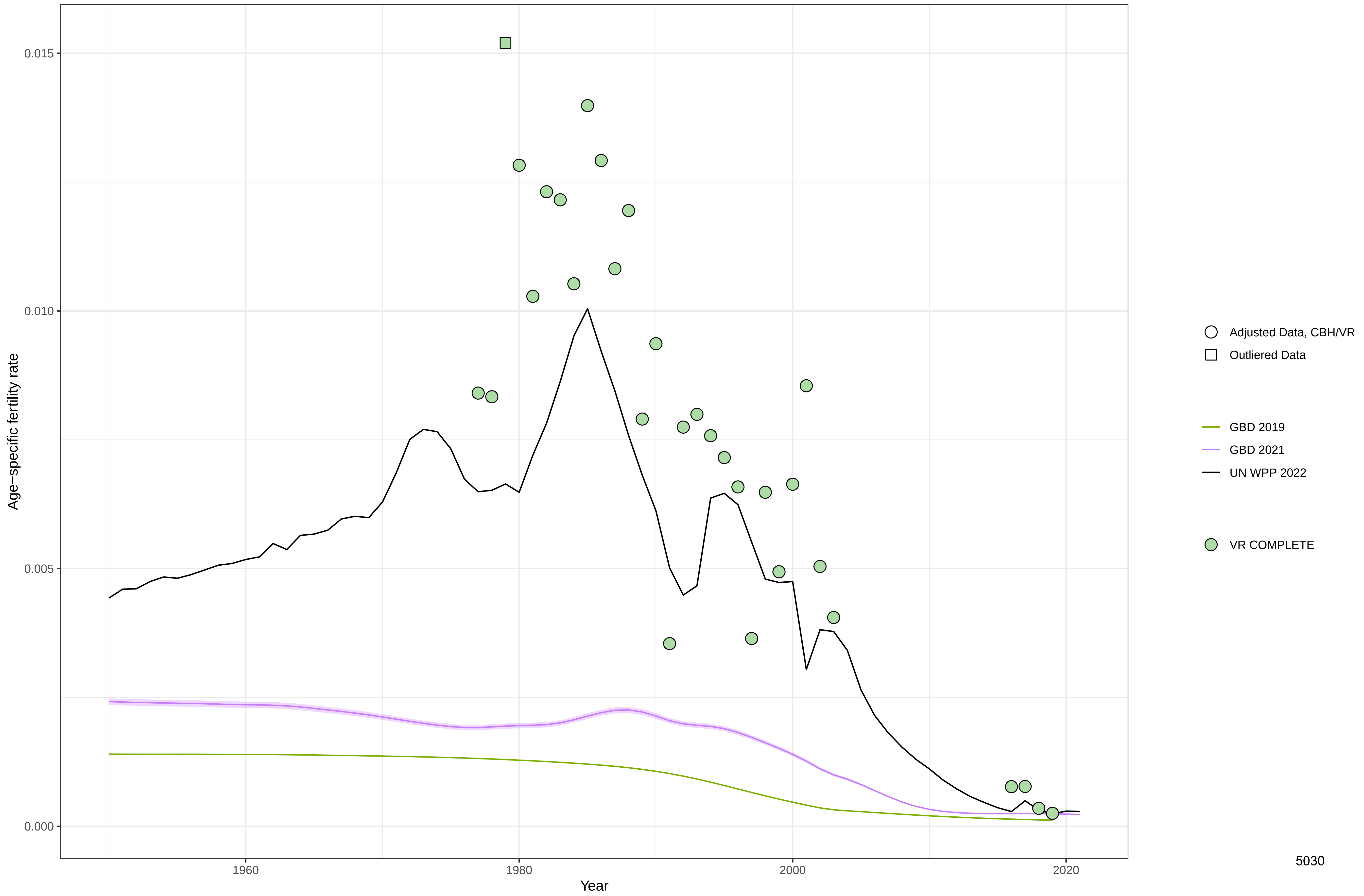

Total fertility rate fitted trend lines, 1950−2021: United Arab Emirates TFR
Data points enclosed with a diamond are reference sources

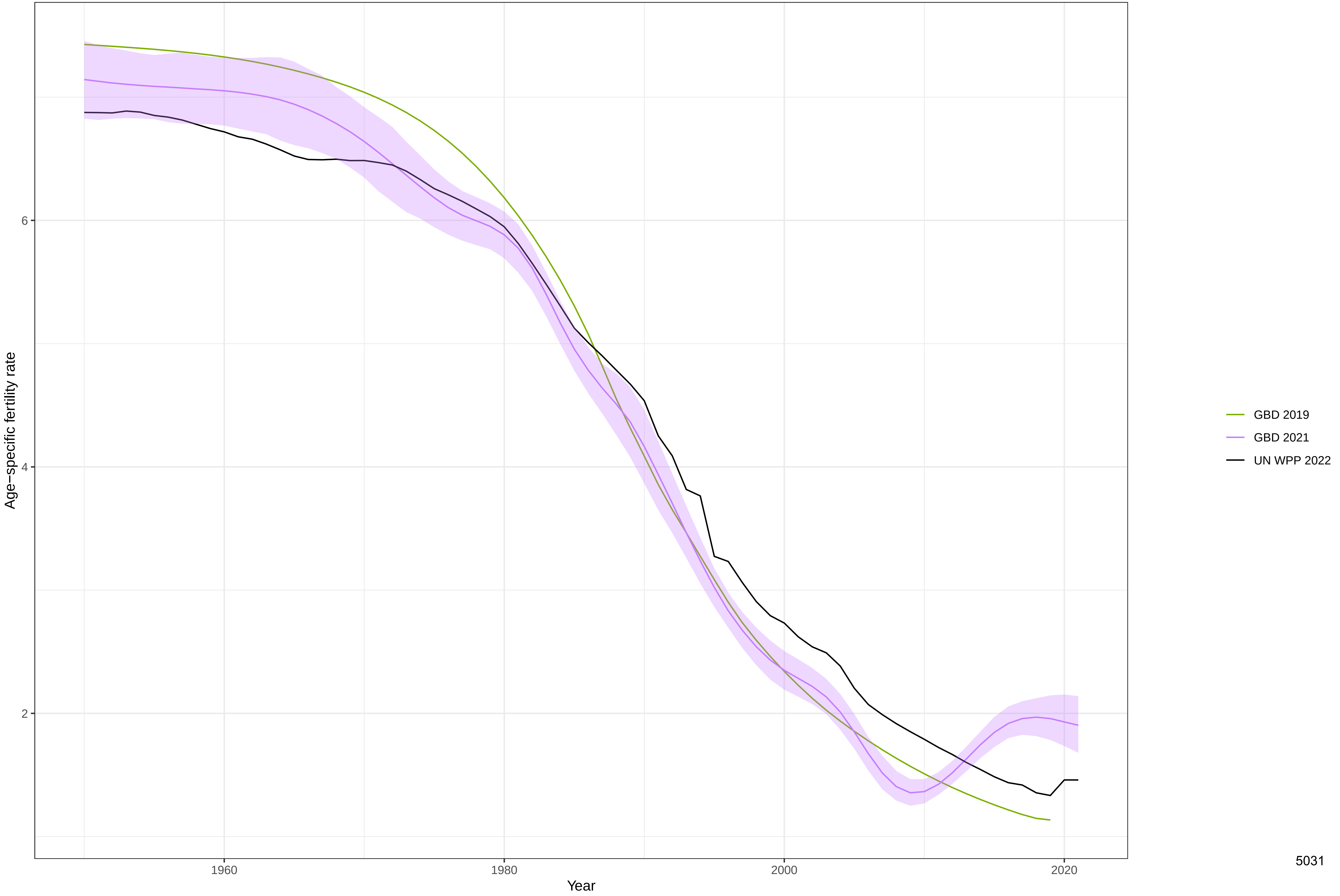

Age−specific fertility fitted trend lines, 1950−2021: Yemen, Ages: 10−14
Data points enclosed with a diamond are reference sources

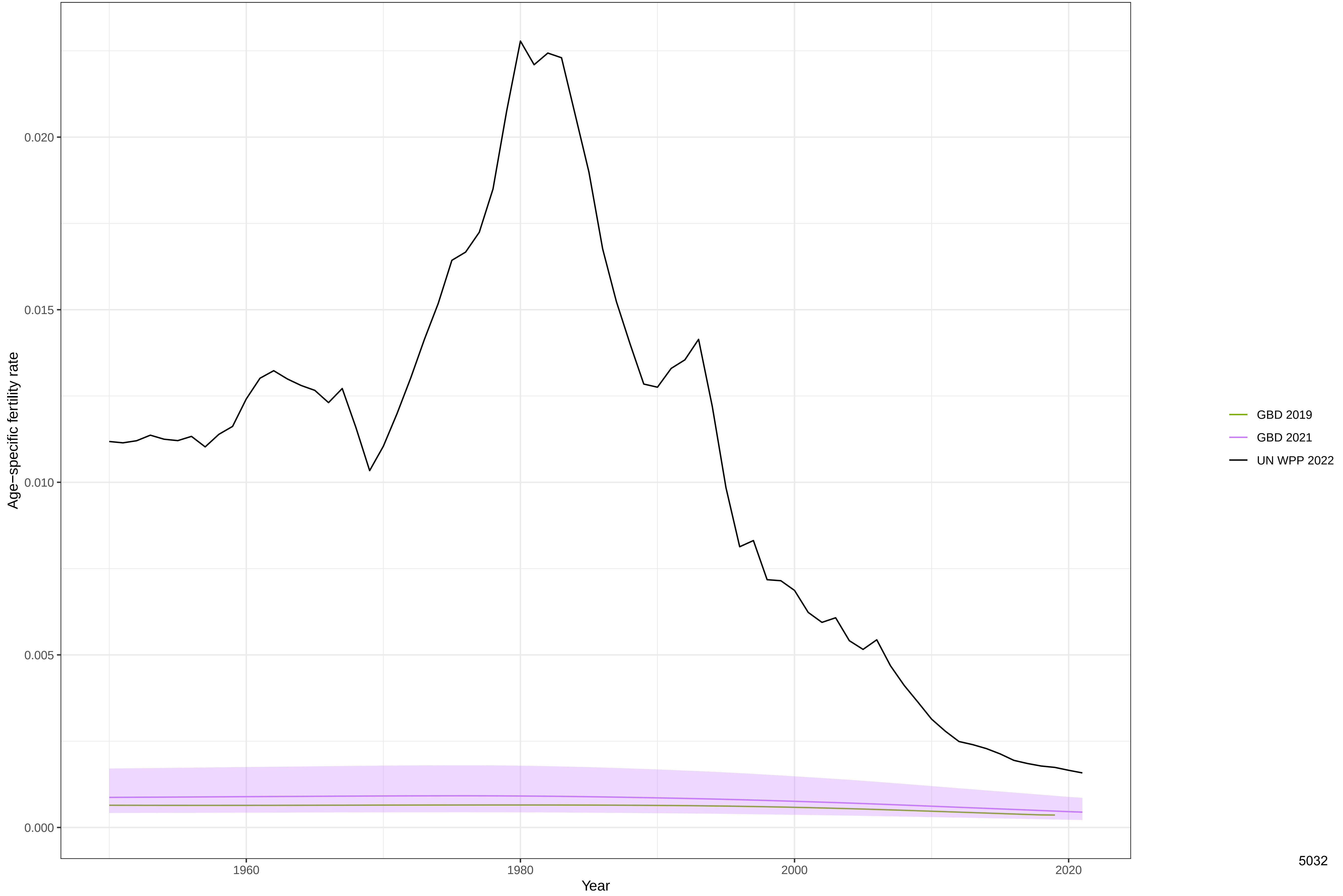

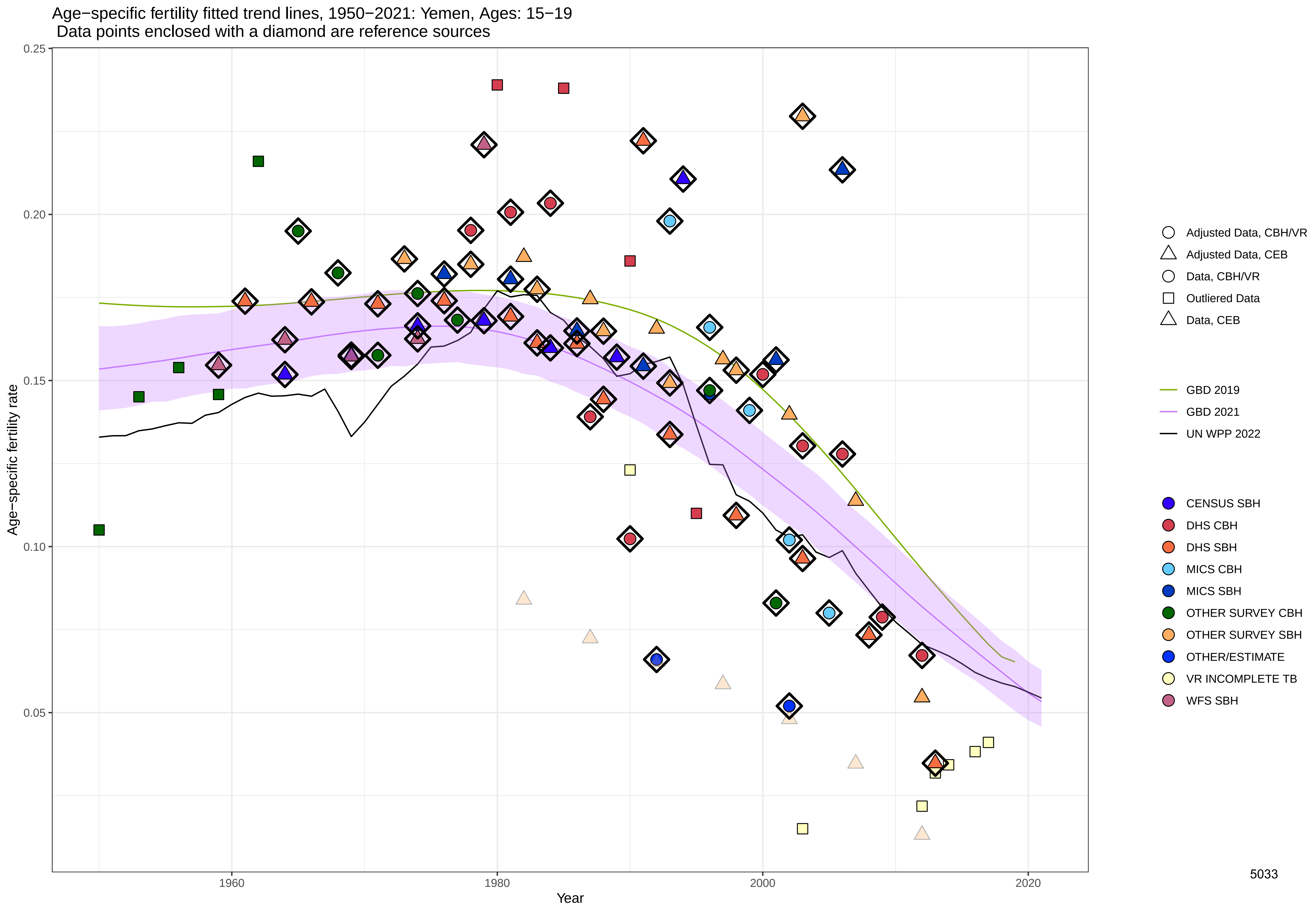

Age−specific fertility fitted trend lines, 1950−2021: Yemen, Ages: 15−19
Data points enclosed with a diamond are reference sources
Age−specific fertility rate
Year
0.25
0.20
0.15
0.10
0.05
1960
1980
2000
2020
Adjusted Data, CBH/VR
Adjusted Data, CEB
Data, CBH/VR
Outliered Data
Data, CEB
GBD 2019
GBD 2021
UN WPP 2022
CENSUS SBH
DHS CBH
DHS SBH
MICS CBH
MICS SBH
OTHER SURVEY CBH
OTHER SURVEY SBH
OTHER/ESTIMATE
VR INCOMPLETE TB
WFS SBH

5033

Age−specific fertility fitted trend lines, 1950−2021: Yemen, Ages: 20−24
Data points enclosed with a diamond are reference sources

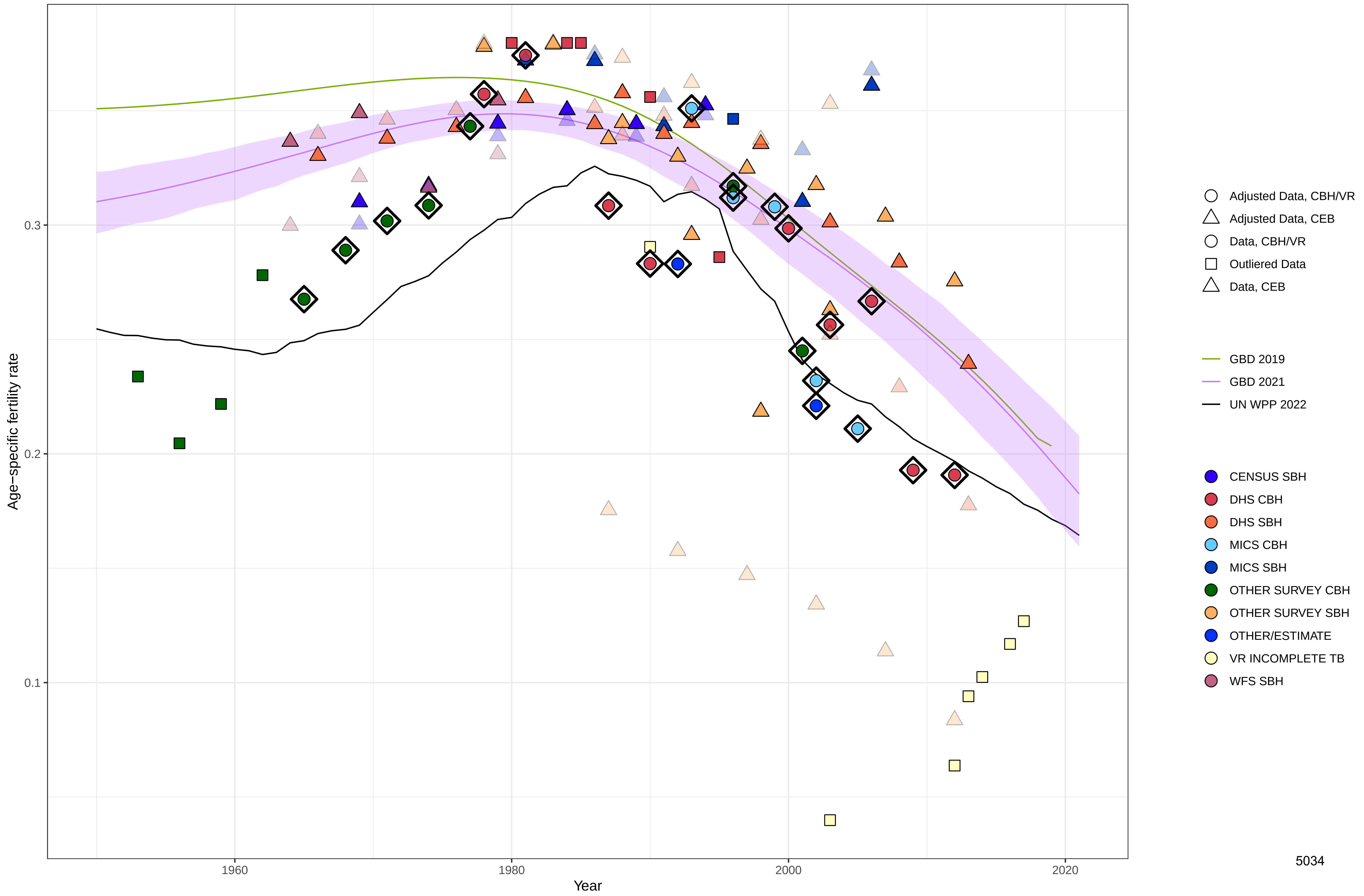

Age−specific fertility fitted trend lines, 1950−2021: Yemen, Ages: 25−29
Data points enclosed with a diamond are reference sources

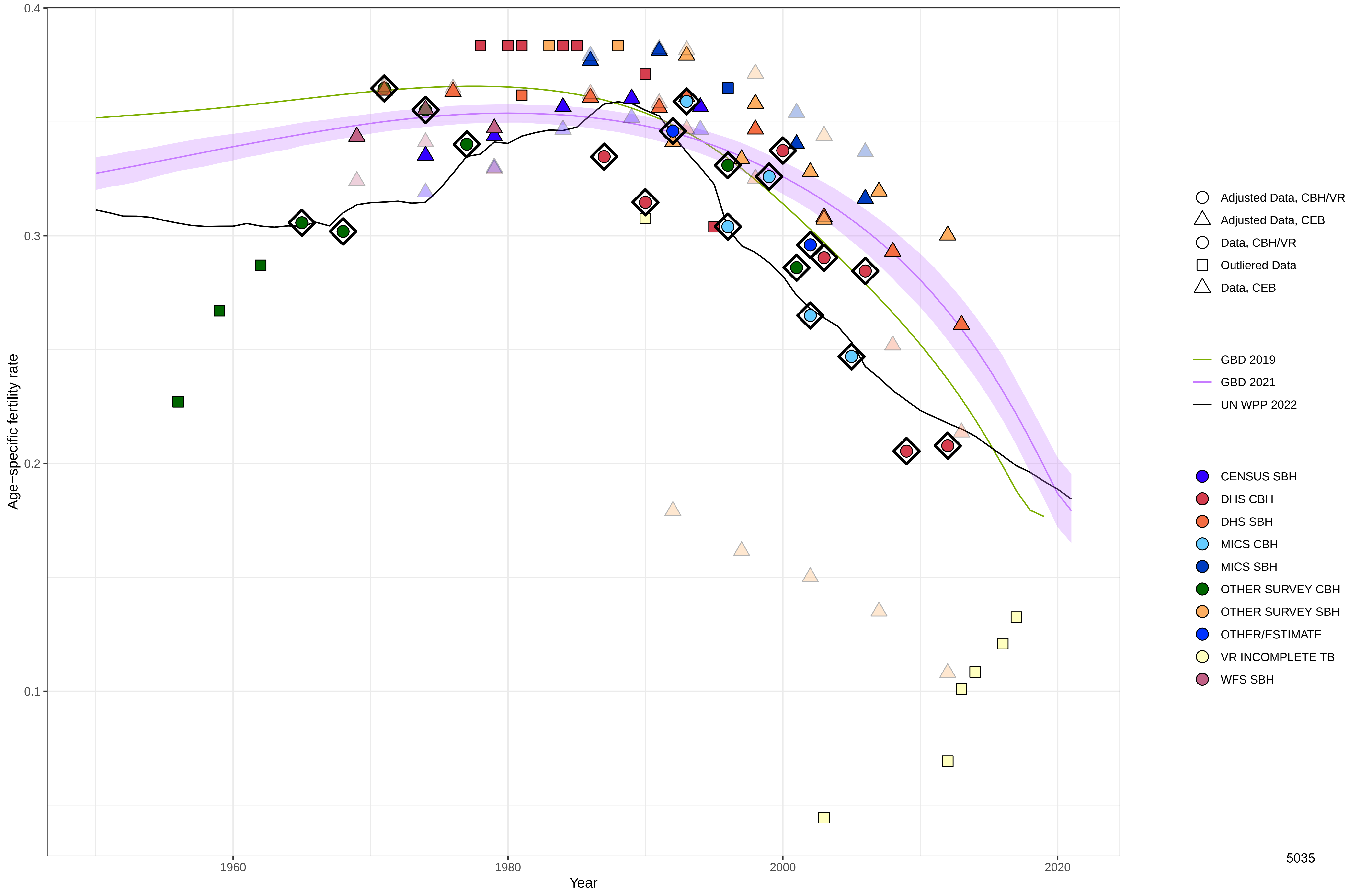

Age−specific fertility fitted trend lines, 1950−2021: Yemen, Ages: 30−34
Data points enclosed with a diamond are reference sources

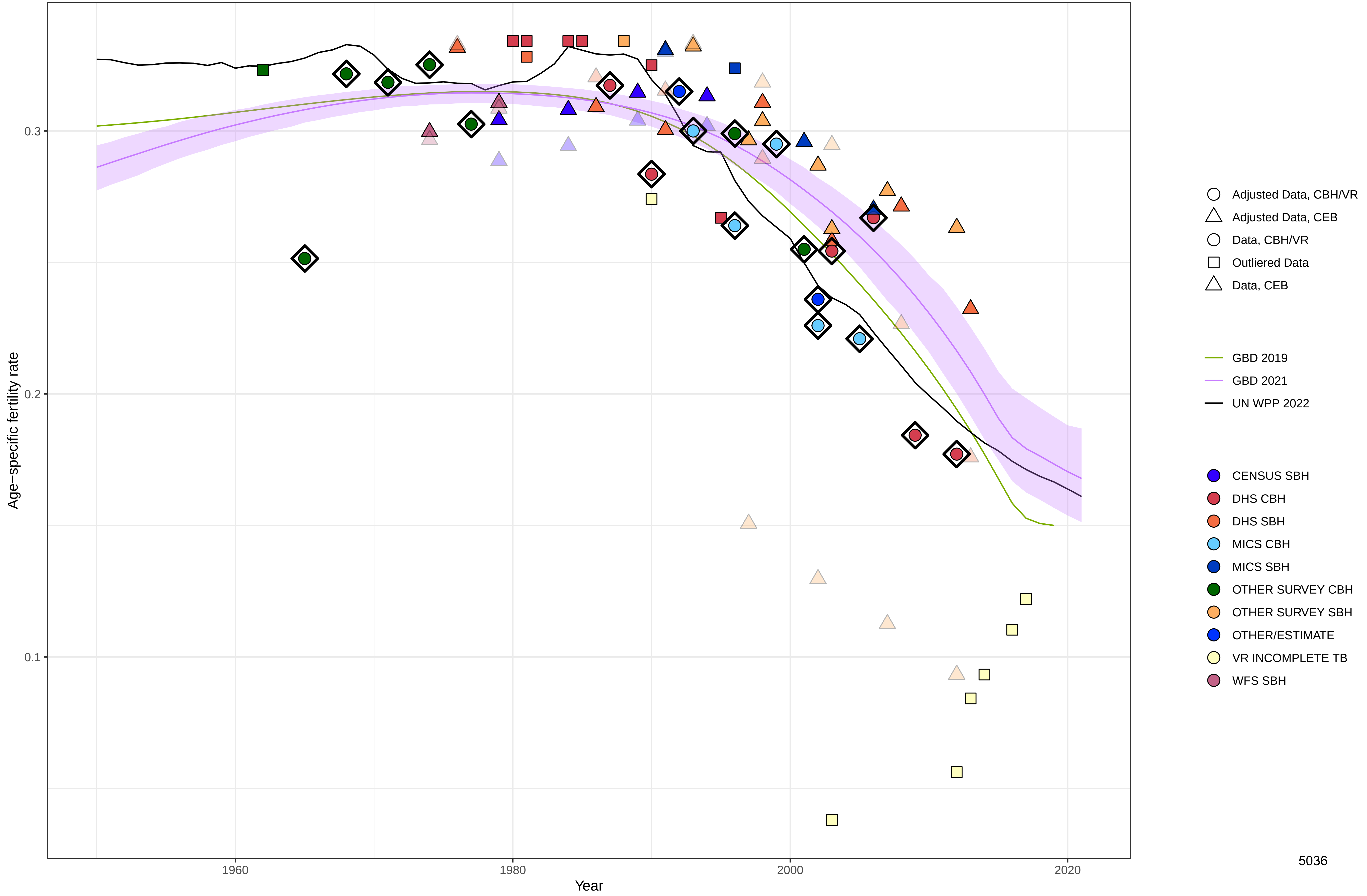

Age−specific fertility fitted trend lines, 1950−2021: Yemen, Ages: 35−39
Data points enclosed with a diamond are reference sources

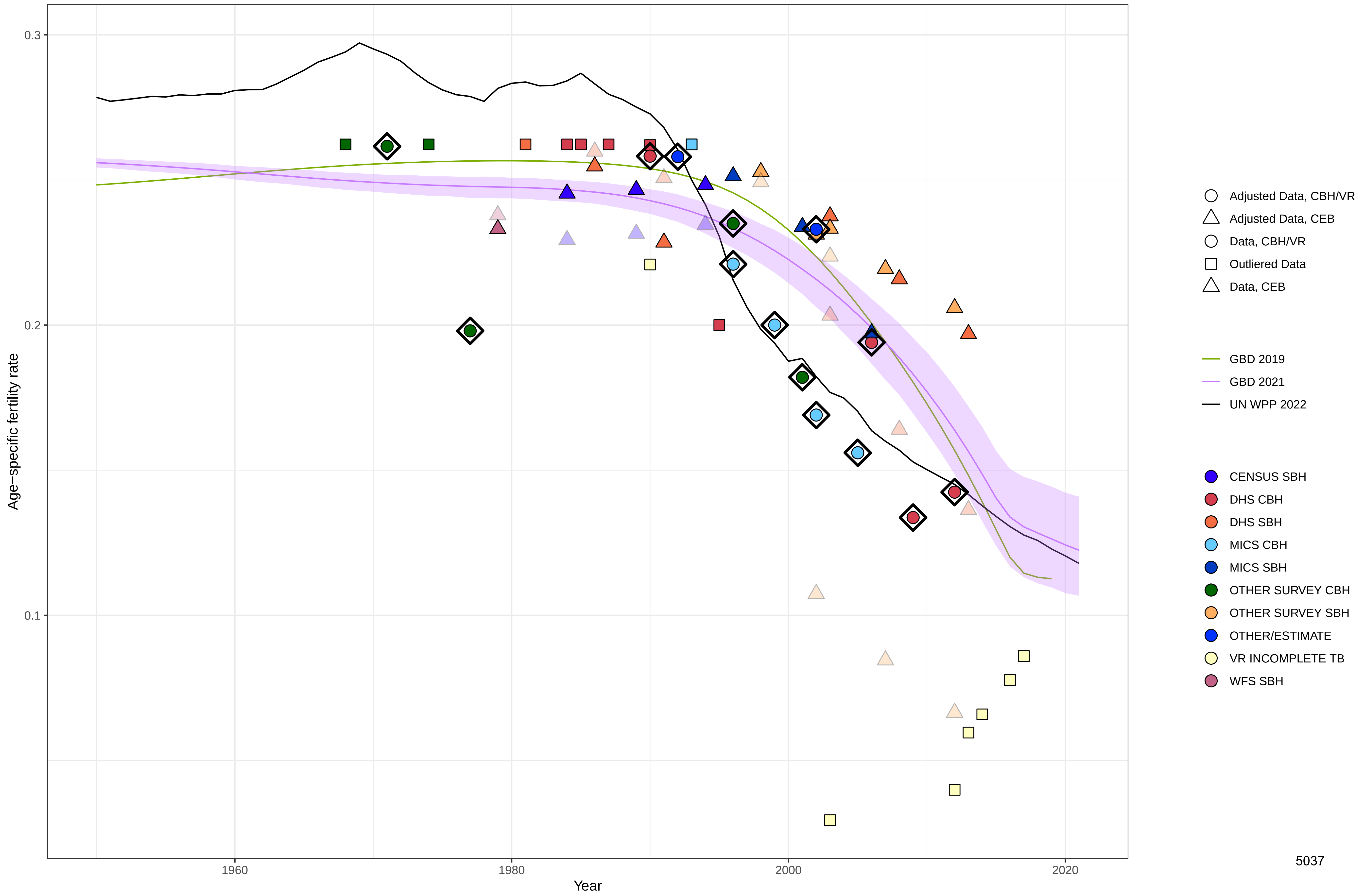

Age−specific fertility fitted trend lines, 1950−2021: Yemen, Ages: 40−44
Data points enclosed with a diamond are reference sources

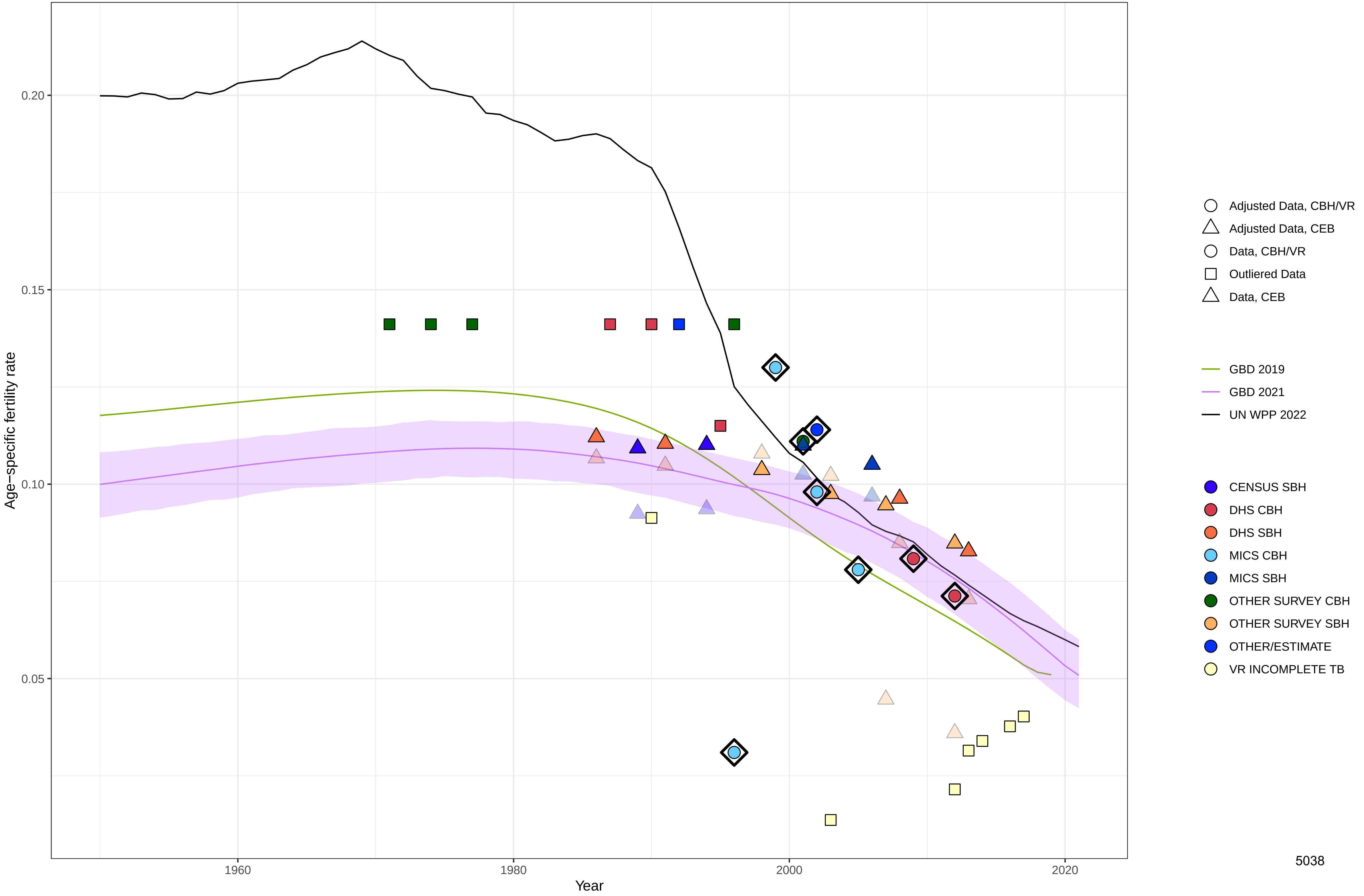

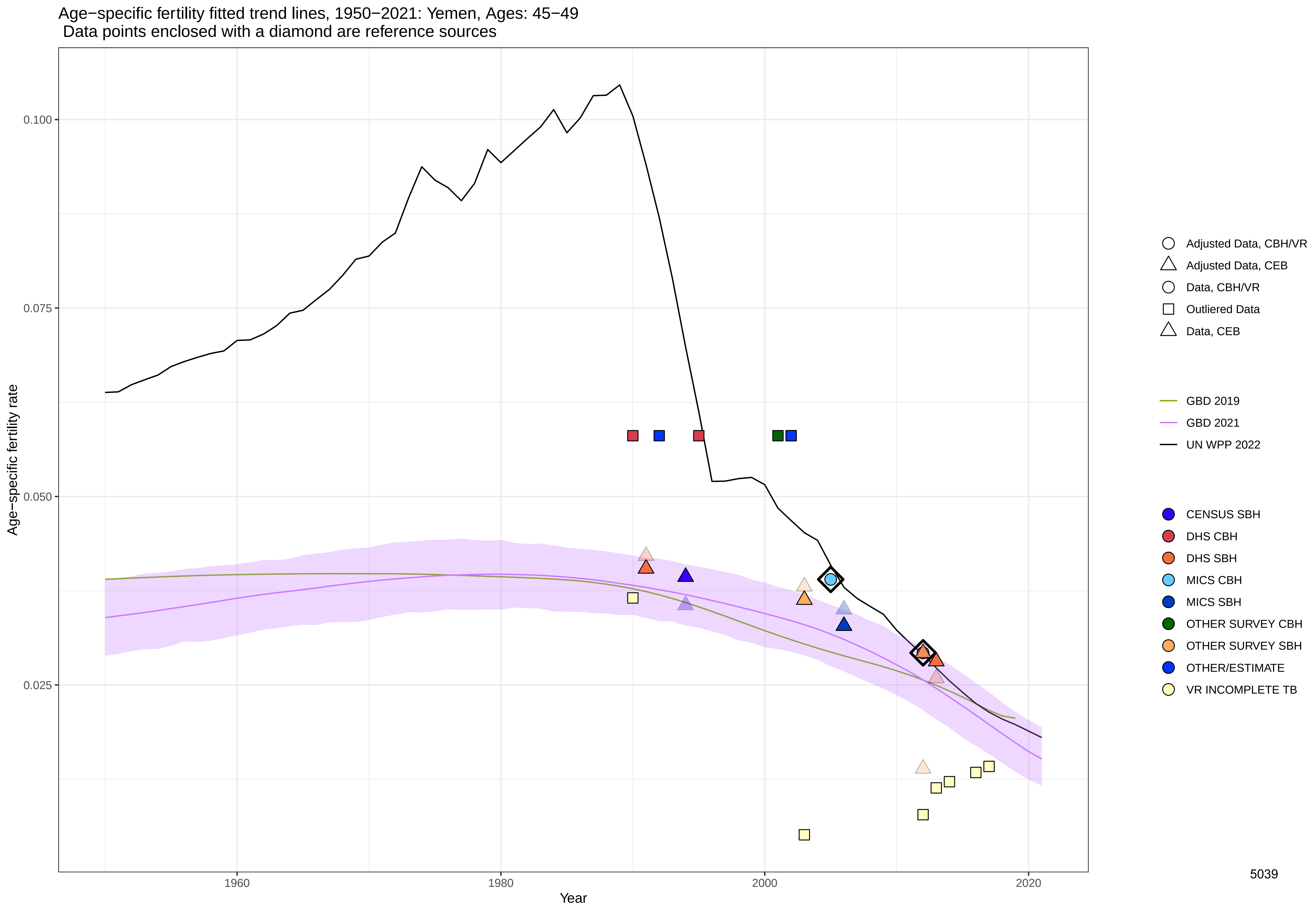

Age−specific fertility fitted trend lines, 1950−2021: Yemen, Ages: 45−49
Data points enclosed with a diamond are reference sources
Age−specific fertility rate
0.100
0.075
0.050
0.025
1960
1980
2000
2020
Year
Adjusted Data, CBH/VR
Adjusted Data, CEB
Data, CBH/VR
Outliered Data
Data, CEB
GBD 2019
GBD 2021
UN WPP 2022
CENSUS SBH
DHS CBH
DHS SBH
MICS CBH
MICS SBH
OTHER SURVEY CBH
OTHER SURVEY SBH
OTHER/ESTIMATE
VR INCOMPLETE TB

Age−specific fertility fitted trend lines, 1950−2021: Yemen, Ages: 50−54
Data points enclosed with a diamond are reference sources

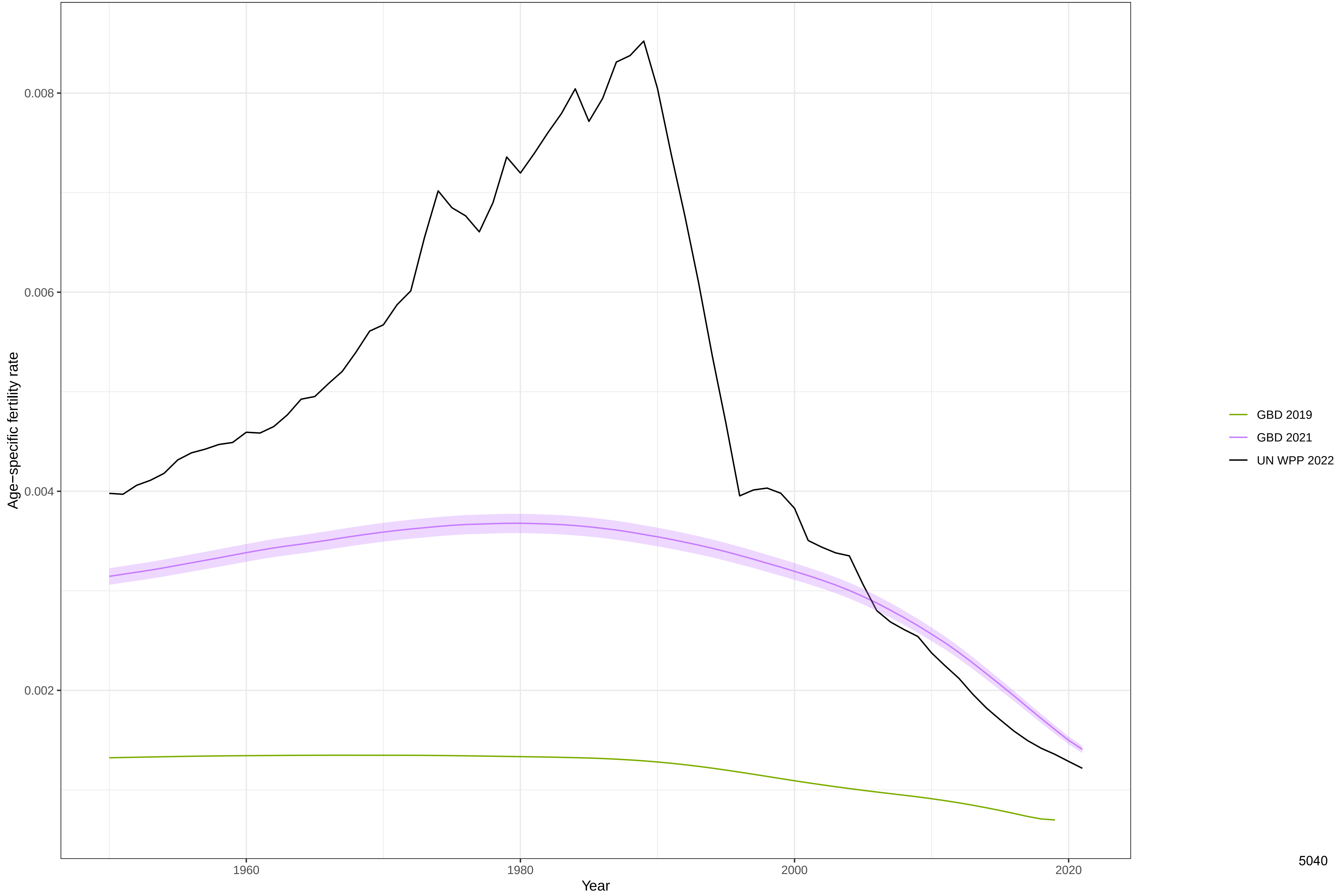

Total fertility rate fitted trend lines, 1950−2021: Yemen TFR
Data points enclosed with a diamond are reference sources

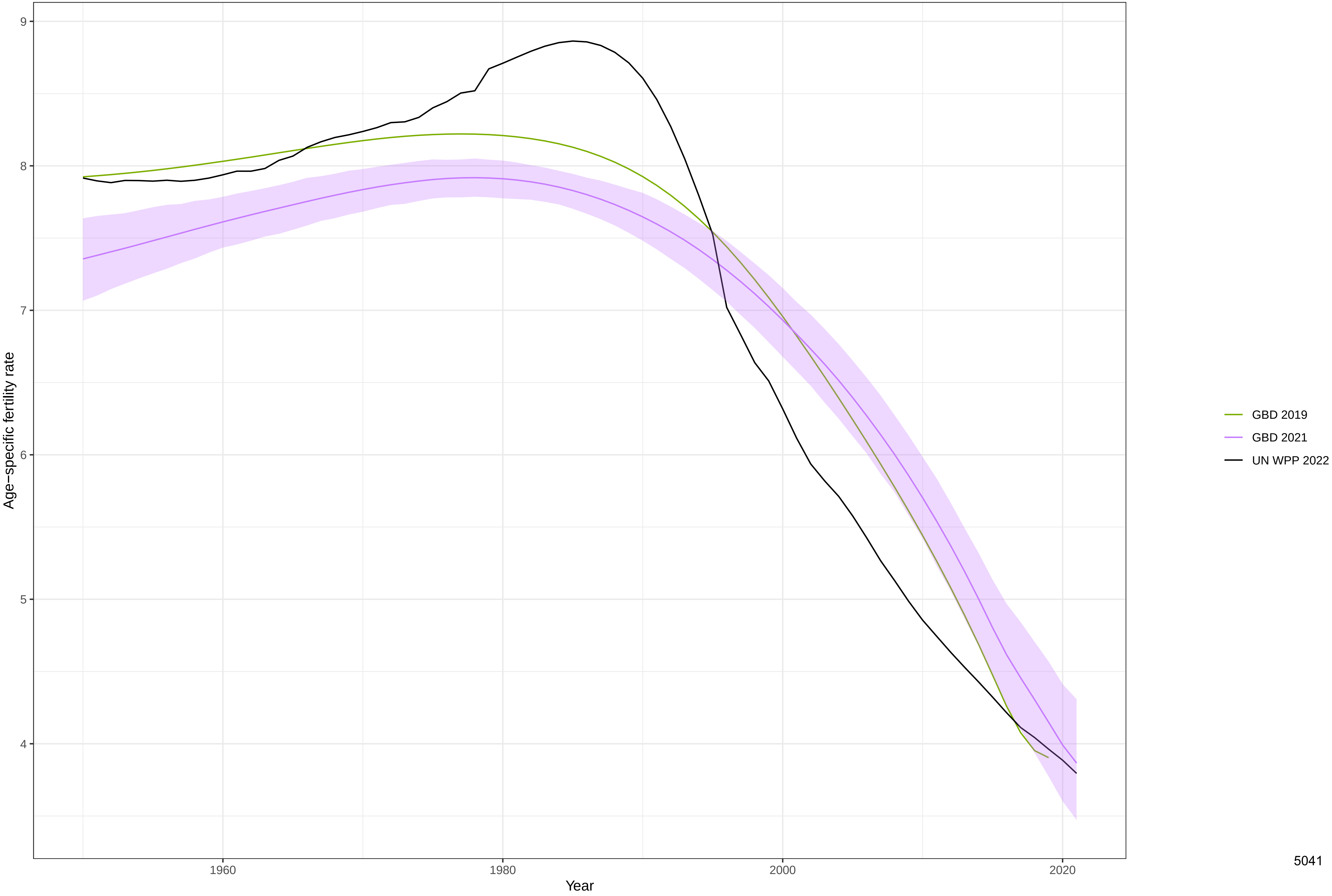

Age−specific fertility fitted trend lines, 1950−2021: Bangladesh, Ages: 10−14
Data points enclosed with a diamond are reference sources

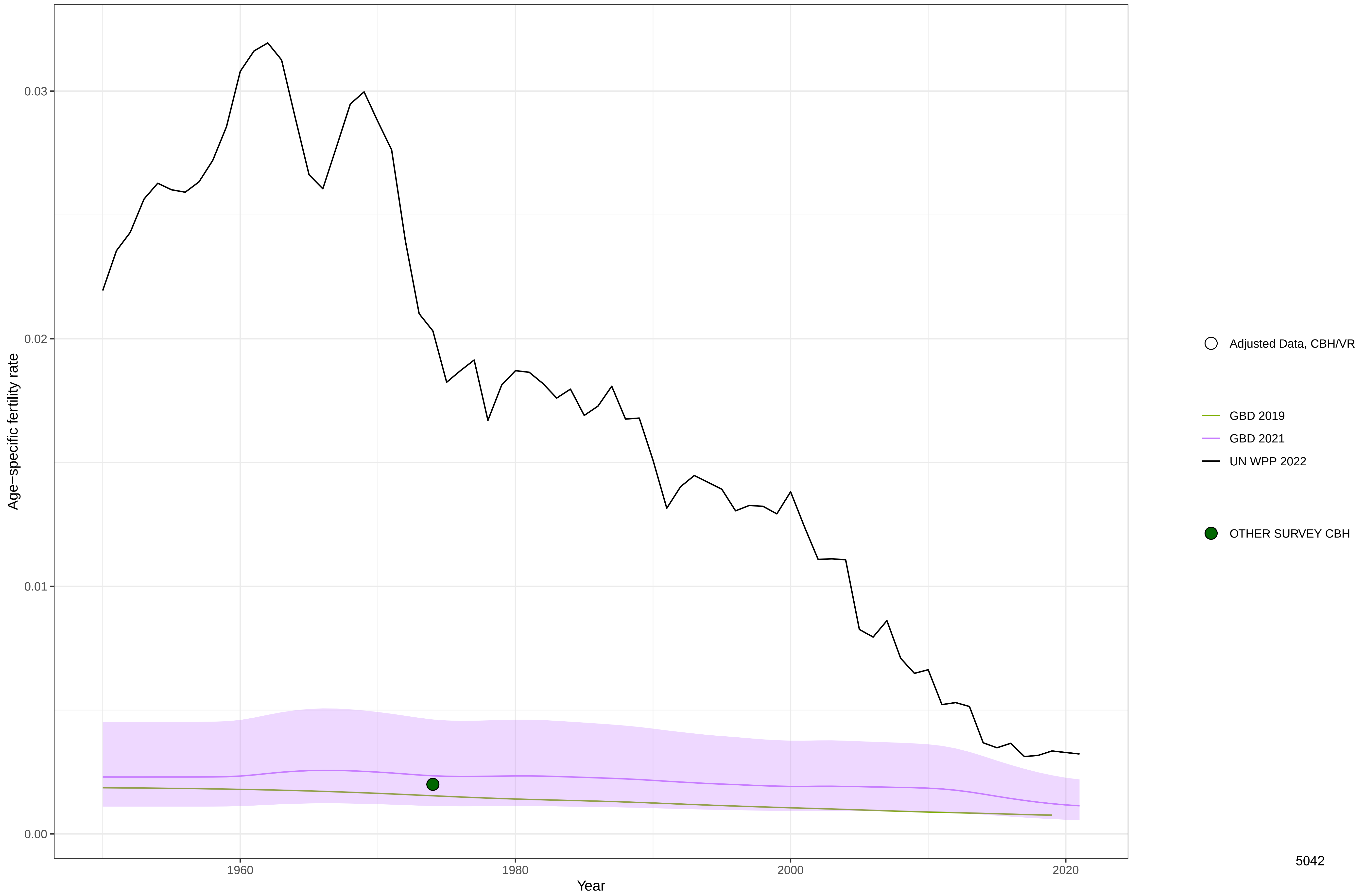

Age−specific fertility fitted trend lines, 1950−2021: Bangladesh, Ages: 15−19
Data points enclosed with a diamond are reference sources

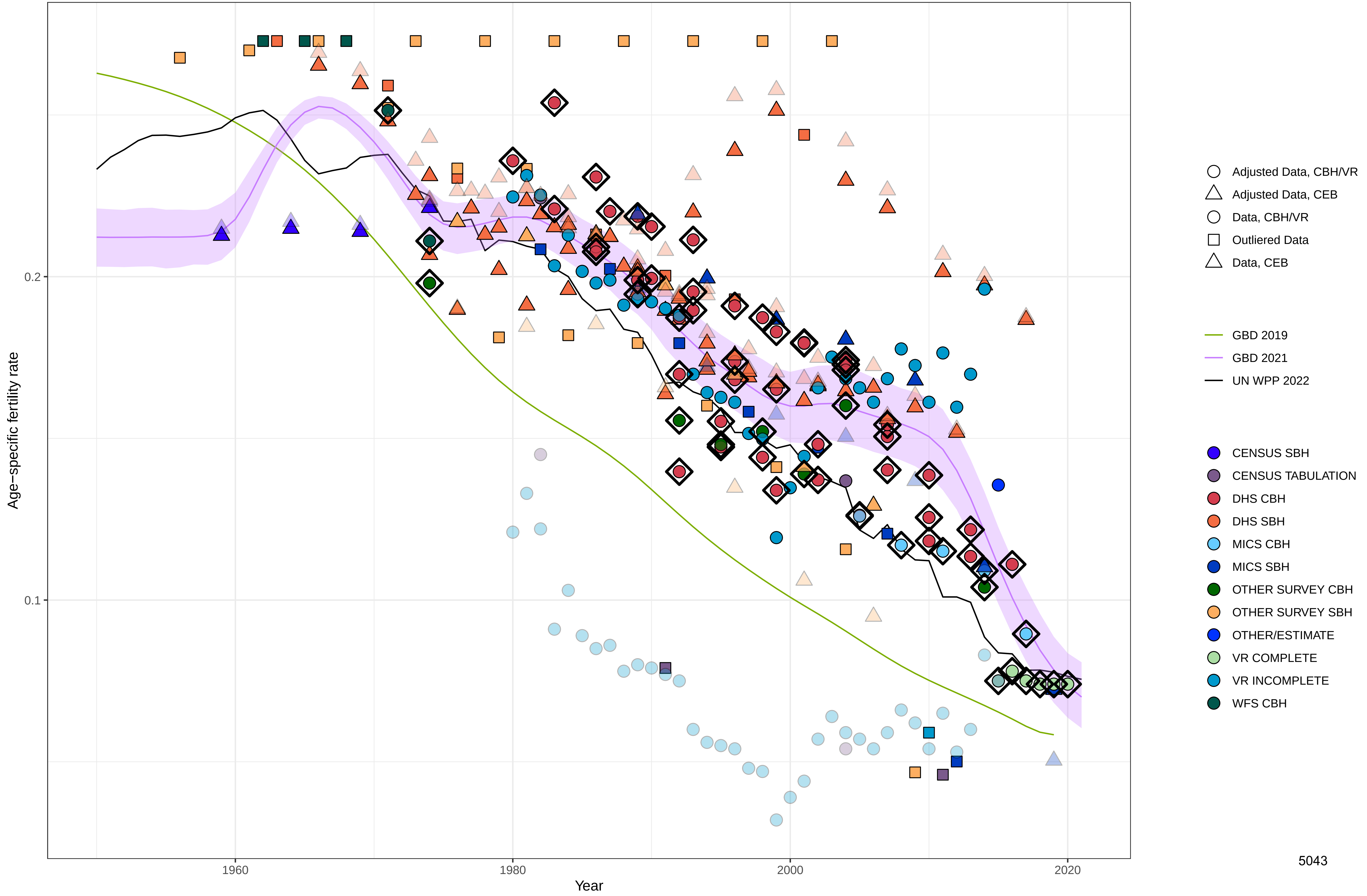

Age−specific fertility fitted trend lines, 1950−2021: Bangladesh, Ages: 20−24
Data points enclosed with a diamond are reference sources

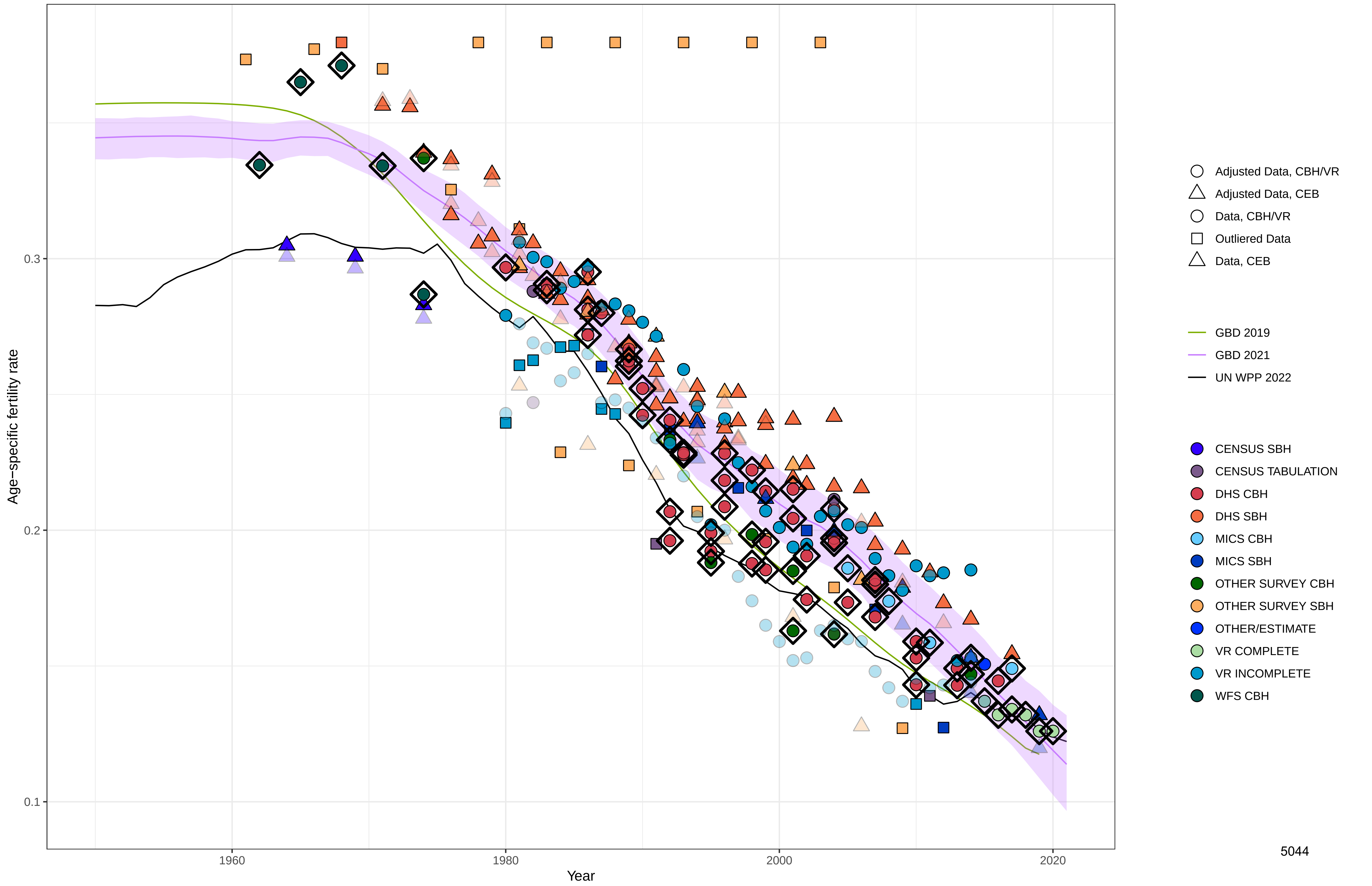

Age−specific fertility fitted trend lines, 1950−2021: Bangladesh, Ages: 25−29
Data points enclosed with a diamond are reference sources

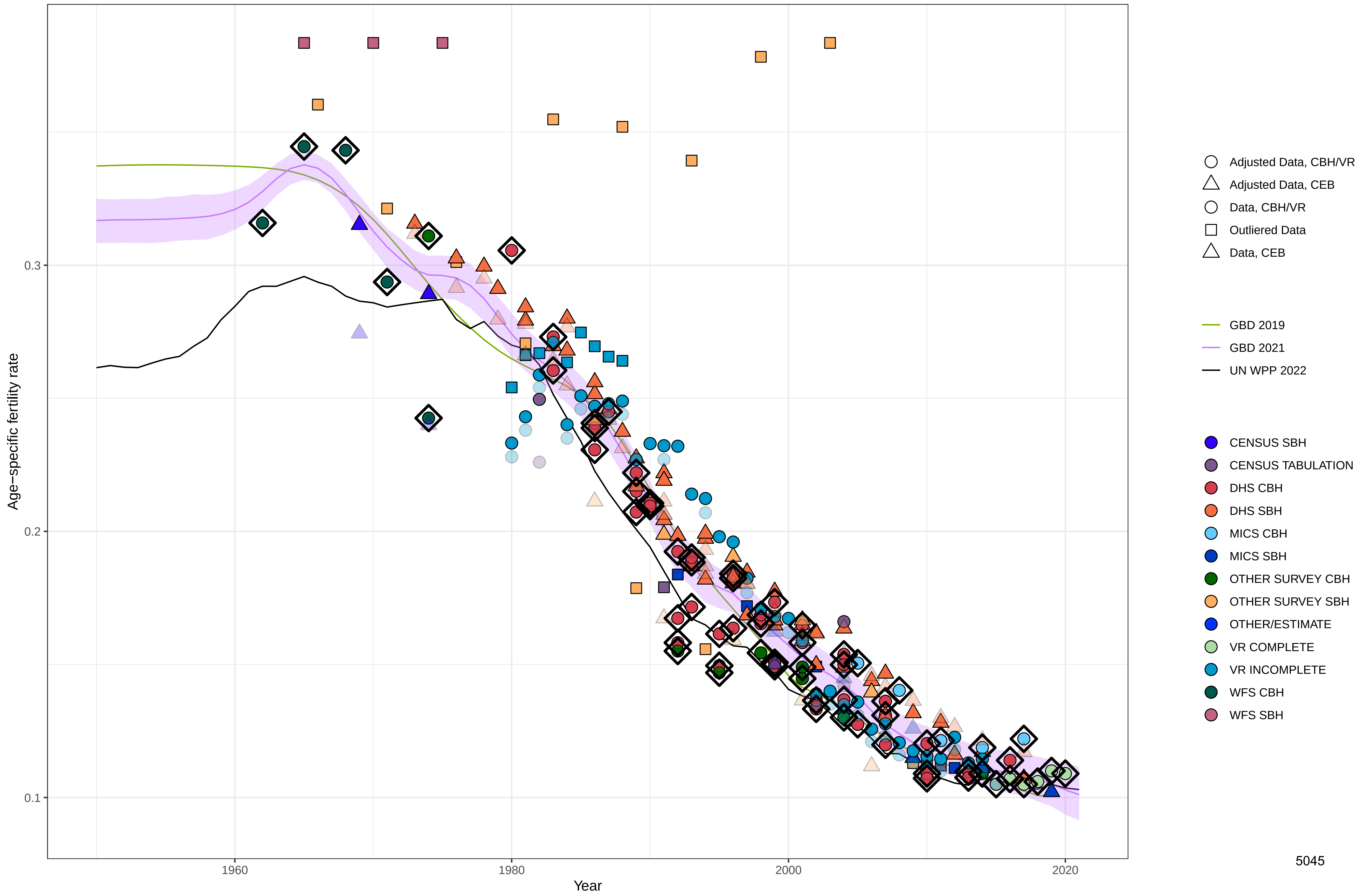

Age−specific fertility fitted trend lines, 1950−2021: Bangladesh, Ages: 30−34
Data points enclosed with a diamond are reference sources

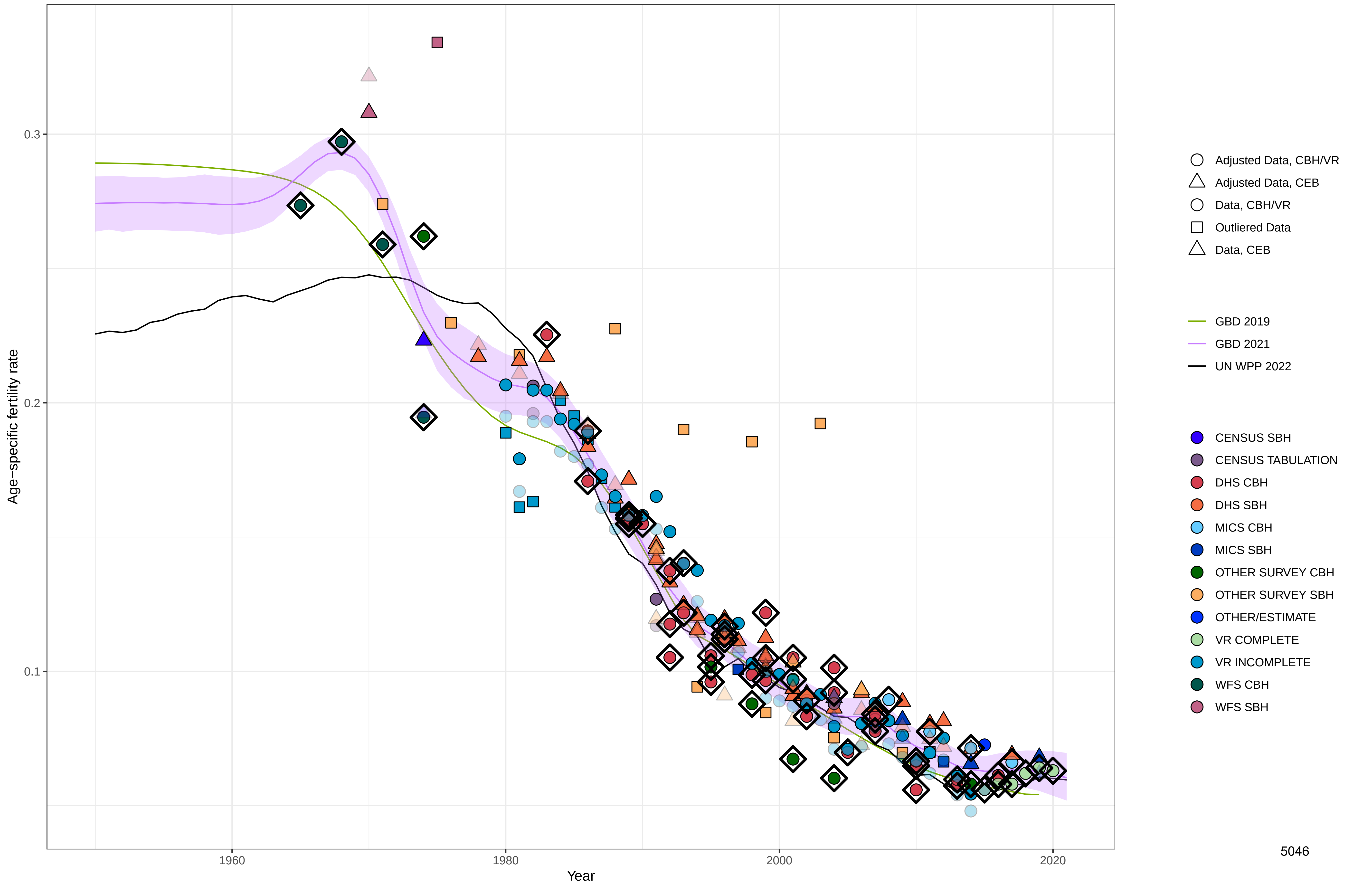

Age−specific fertility fitted trend lines, 1950−2021: Bangladesh, Ages: 35−39
Data points enclosed with a diamond are reference sources

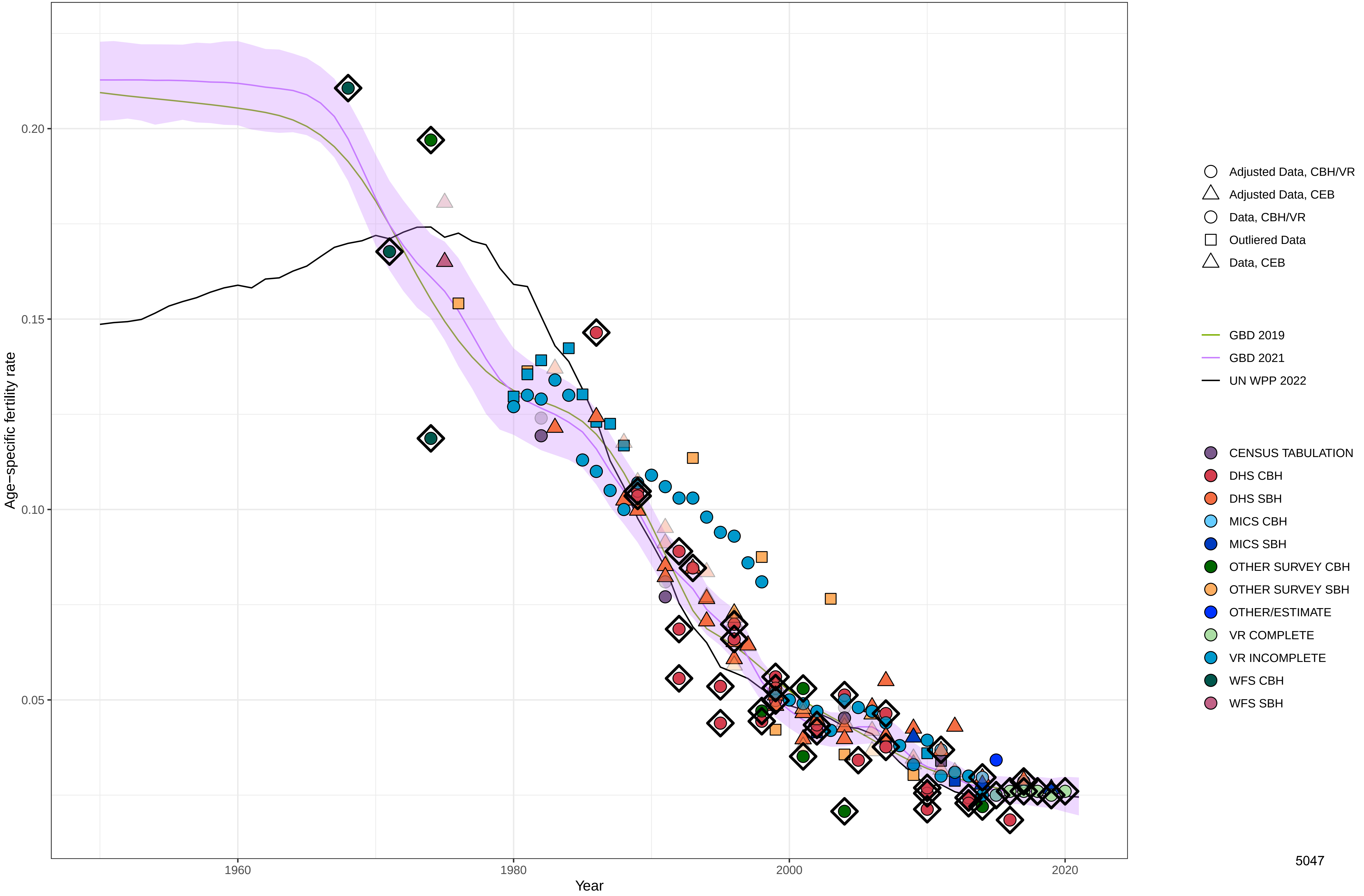

Age−specific fertility fitted trend lines, 1950−2021: Bangladesh, Ages: 40−44
Data points enclosed with a diamond are reference sources

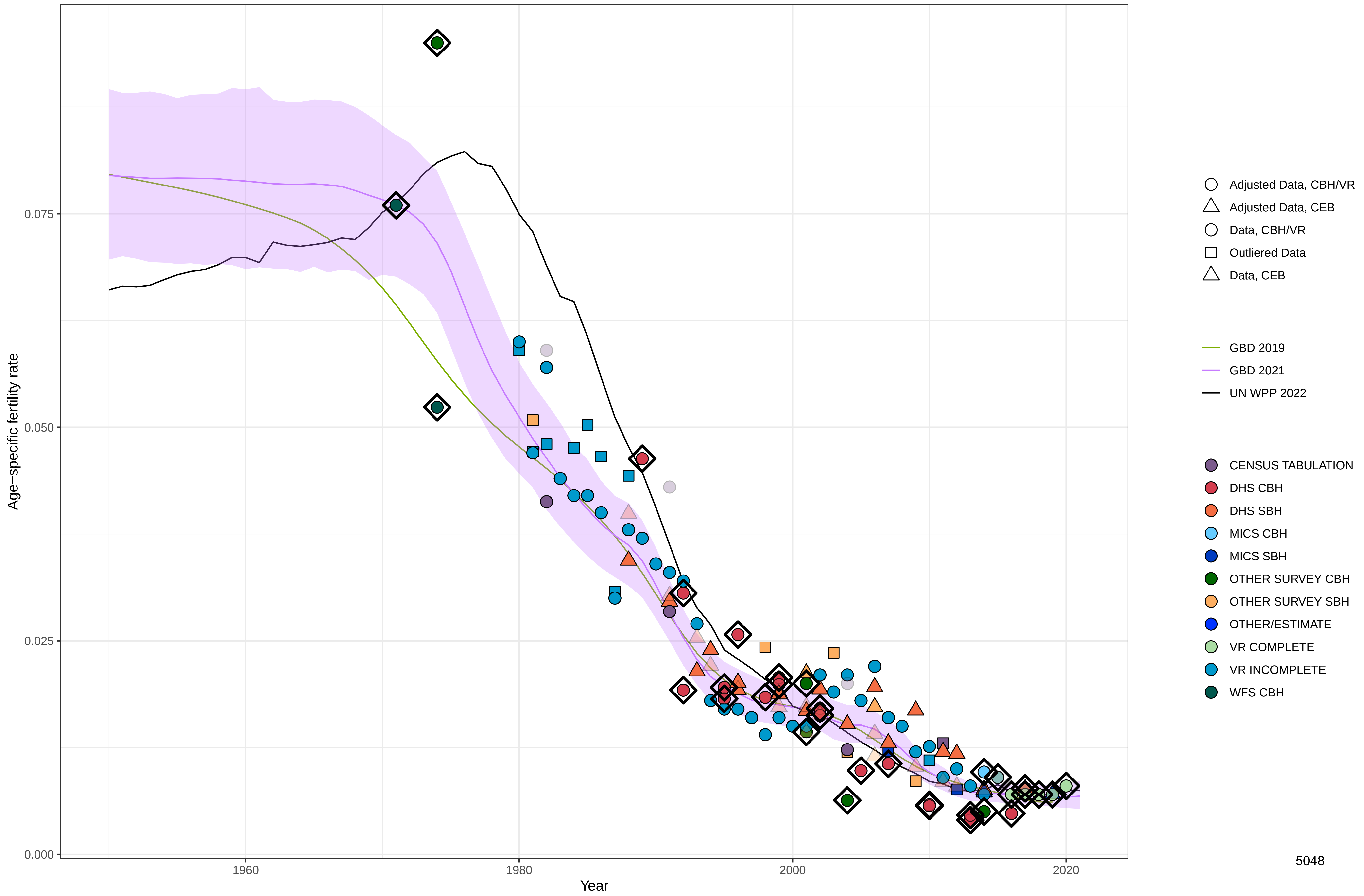

Age−specific fertility fitted trend lines, 1950−2021: Bangladesh, Ages: 45−49
Data points enclosed with a diamond are reference sources

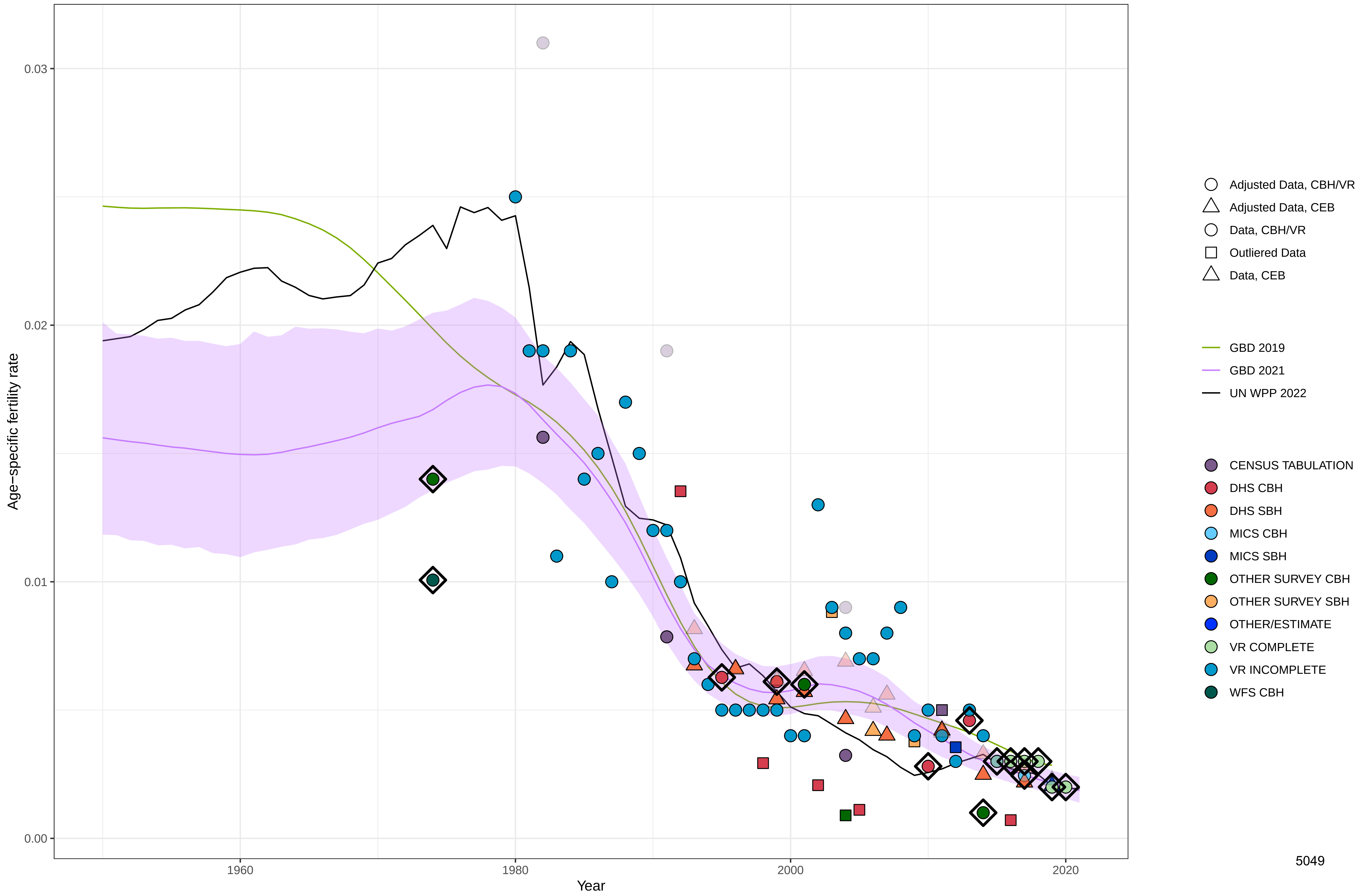

Age−specific fertility fitted trend lines, 1950−2021: Bangladesh, Ages: 50−54
Data points enclosed with a diamond are reference sources

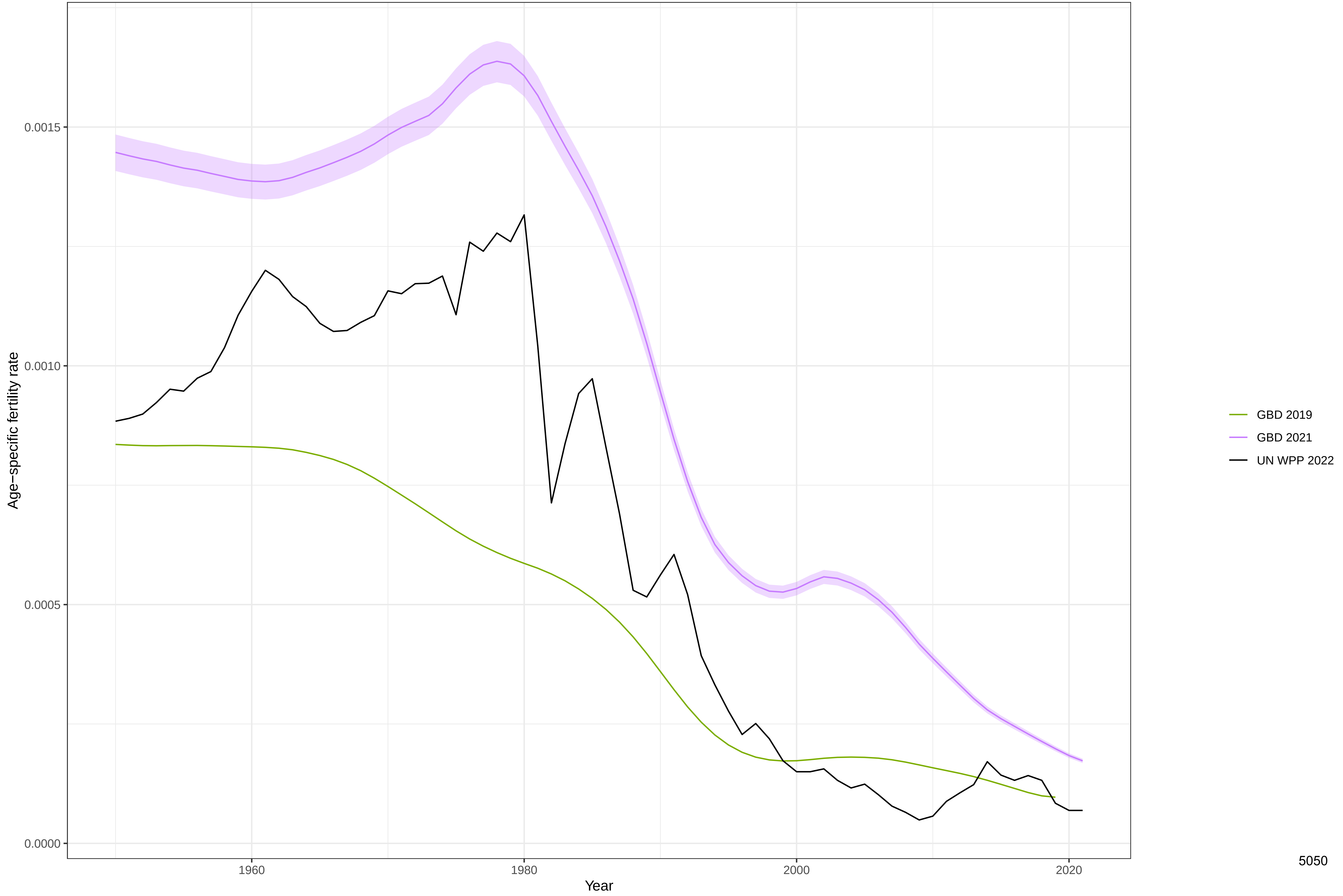

Total fertility rate fitted trend lines, 1950−2021: Bangladesh TFR
Data points enclosed with a diamond are reference sources

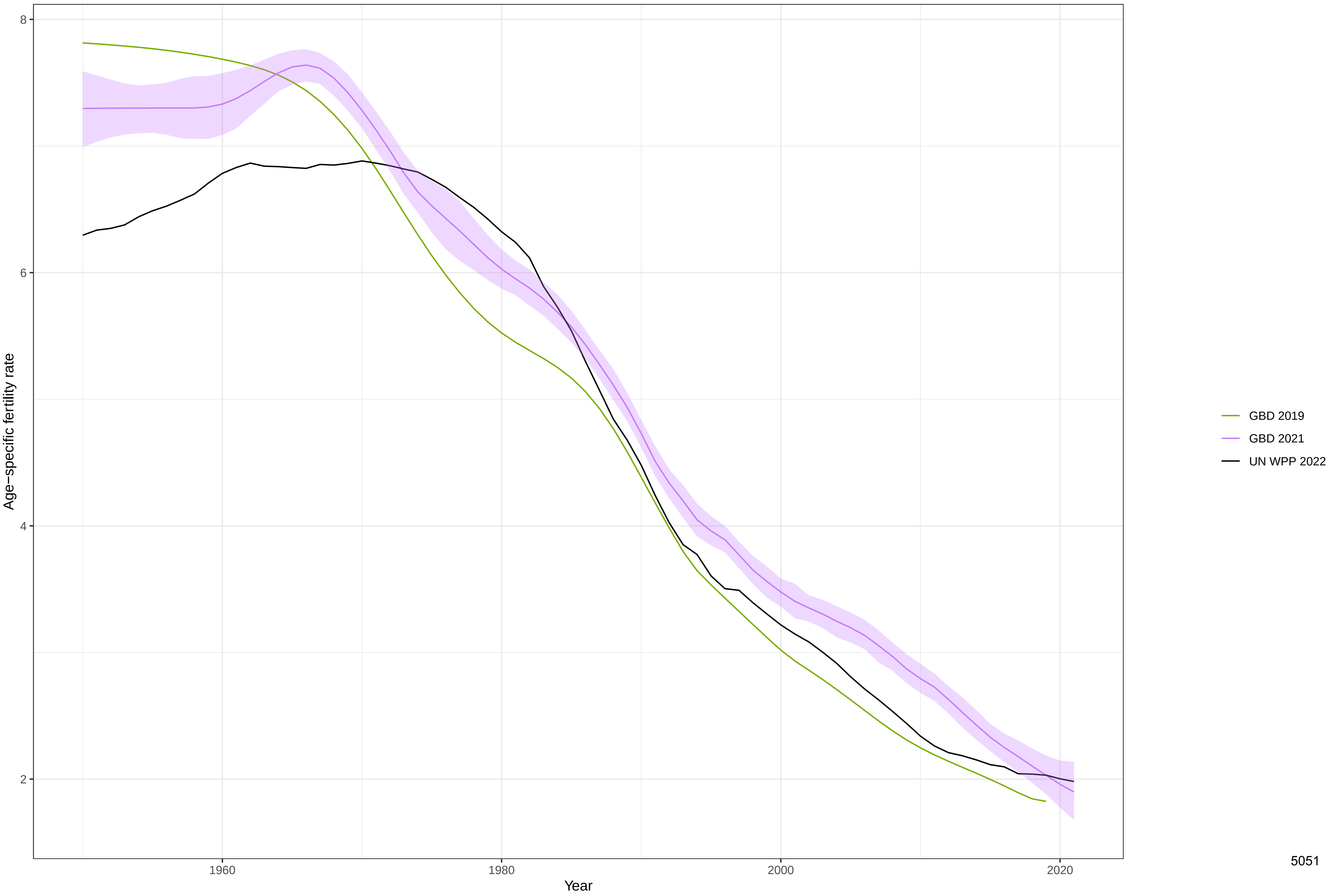

Age−specific fertility fitted trend lines, 1950−2021: Bhutan, Ages: 10−14
Data points enclosed with a diamond are reference sources

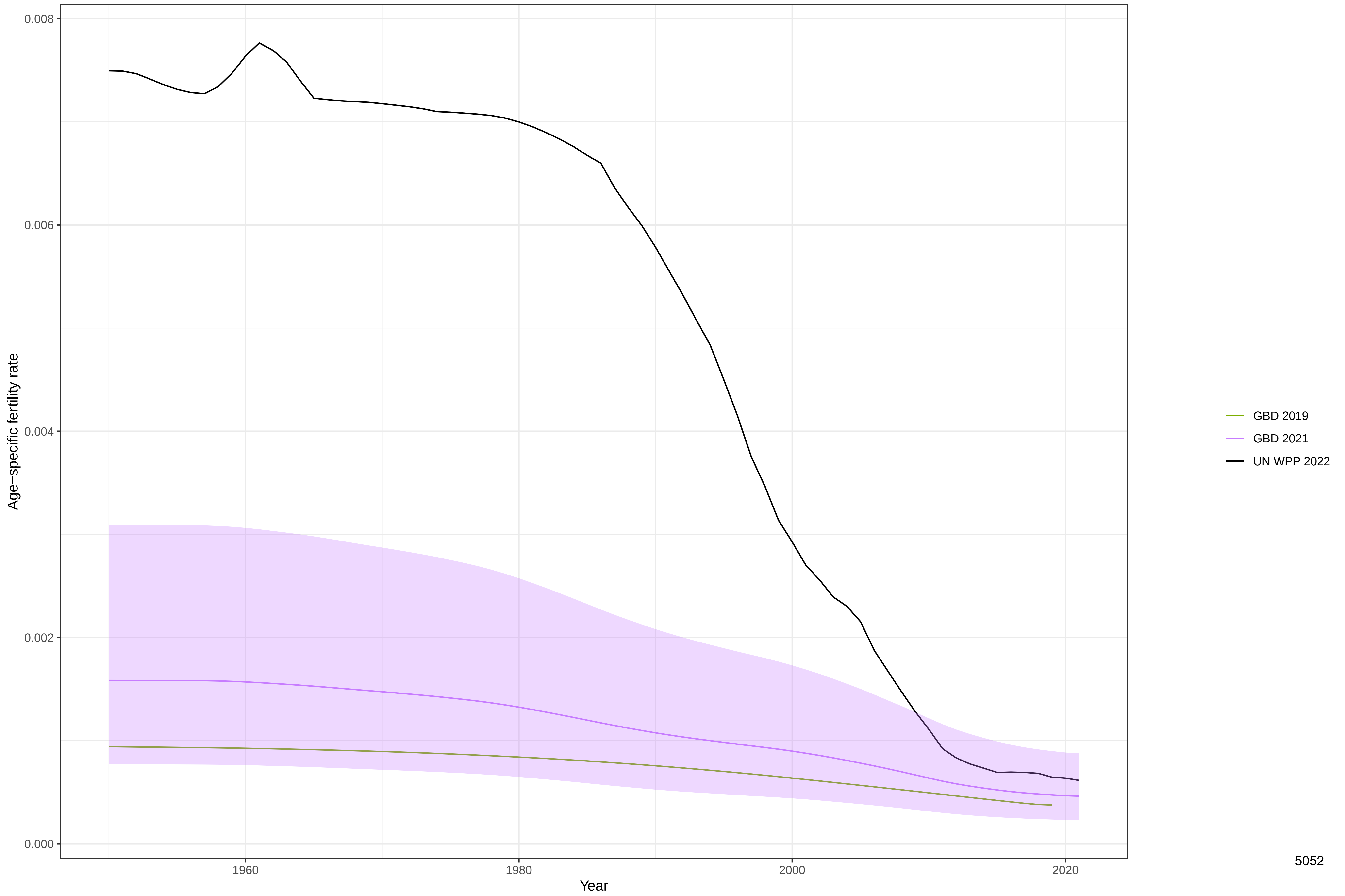

Age−specific fertility fitted trend lines, 1950−2021: Bhutan, Ages: 15−19
Data points enclosed with a diamond are reference sources

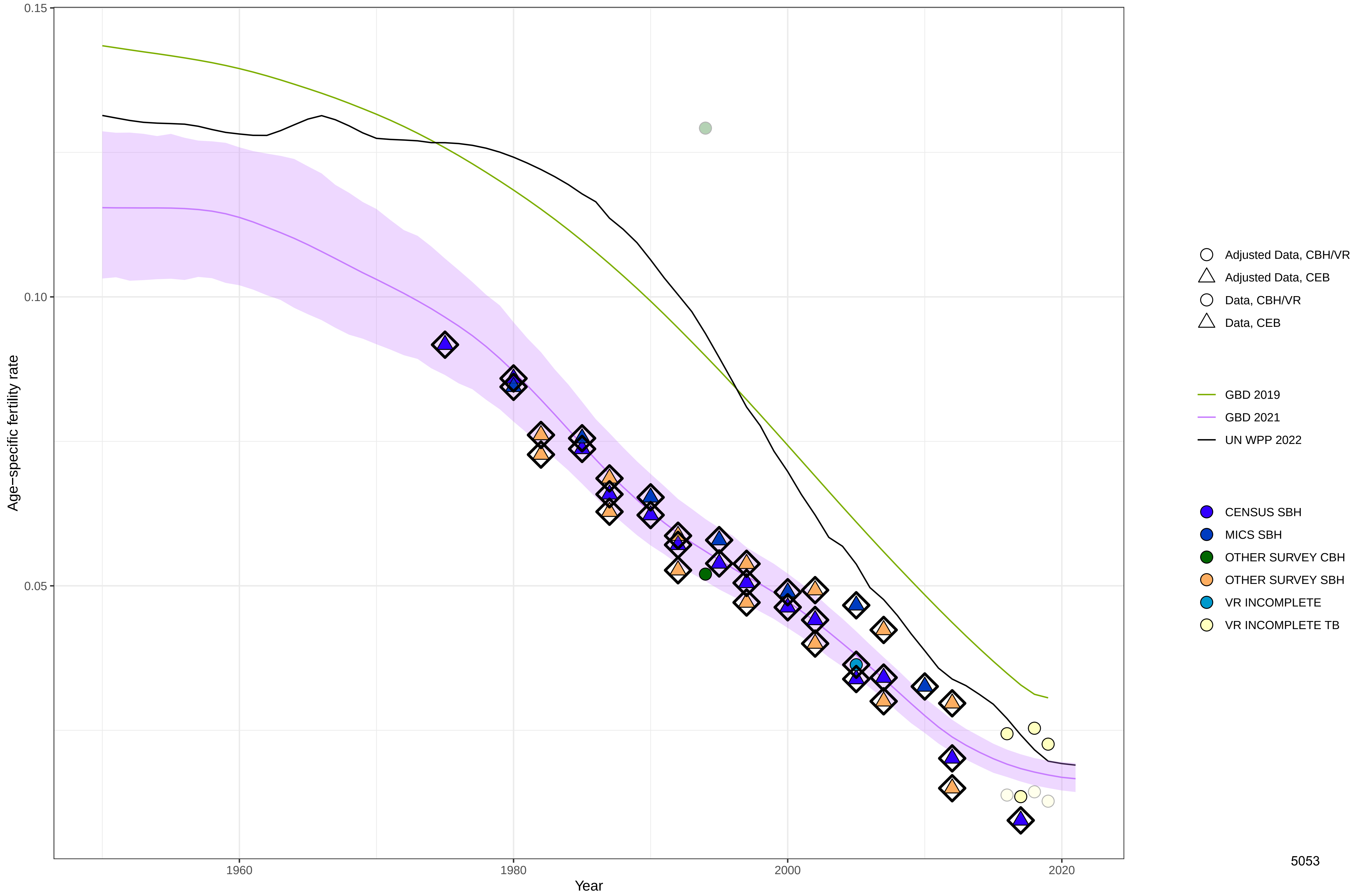

Age−specific fertility fitted trend lines, 1950−2021: Bhutan, Ages: 20−24
Data points enclosed with a diamond are reference sources

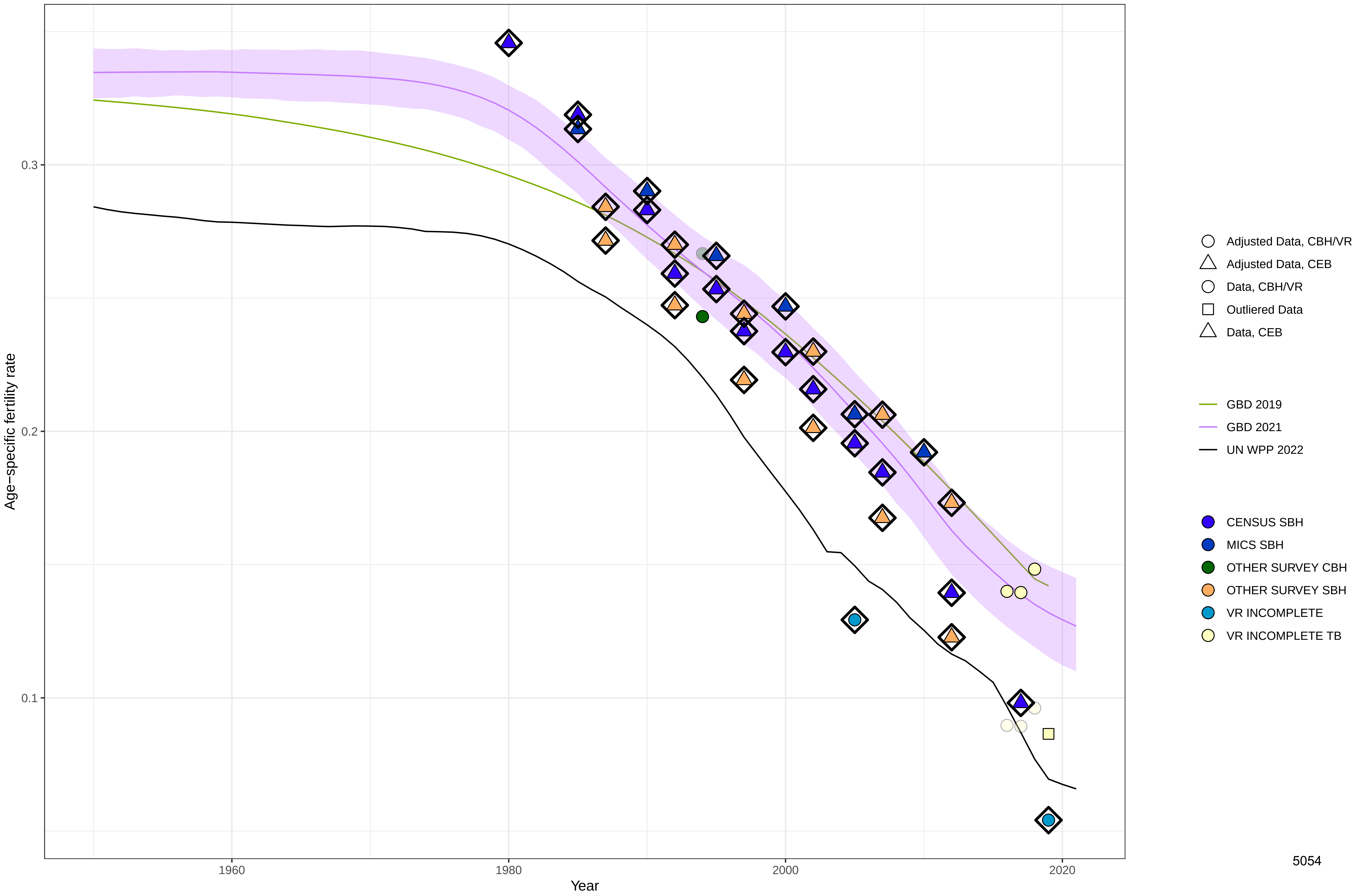

Age−specific fertility fitted trend lines, 1950−2021: Bhutan, Ages: 25−29
Data points enclosed with a diamond are reference sources

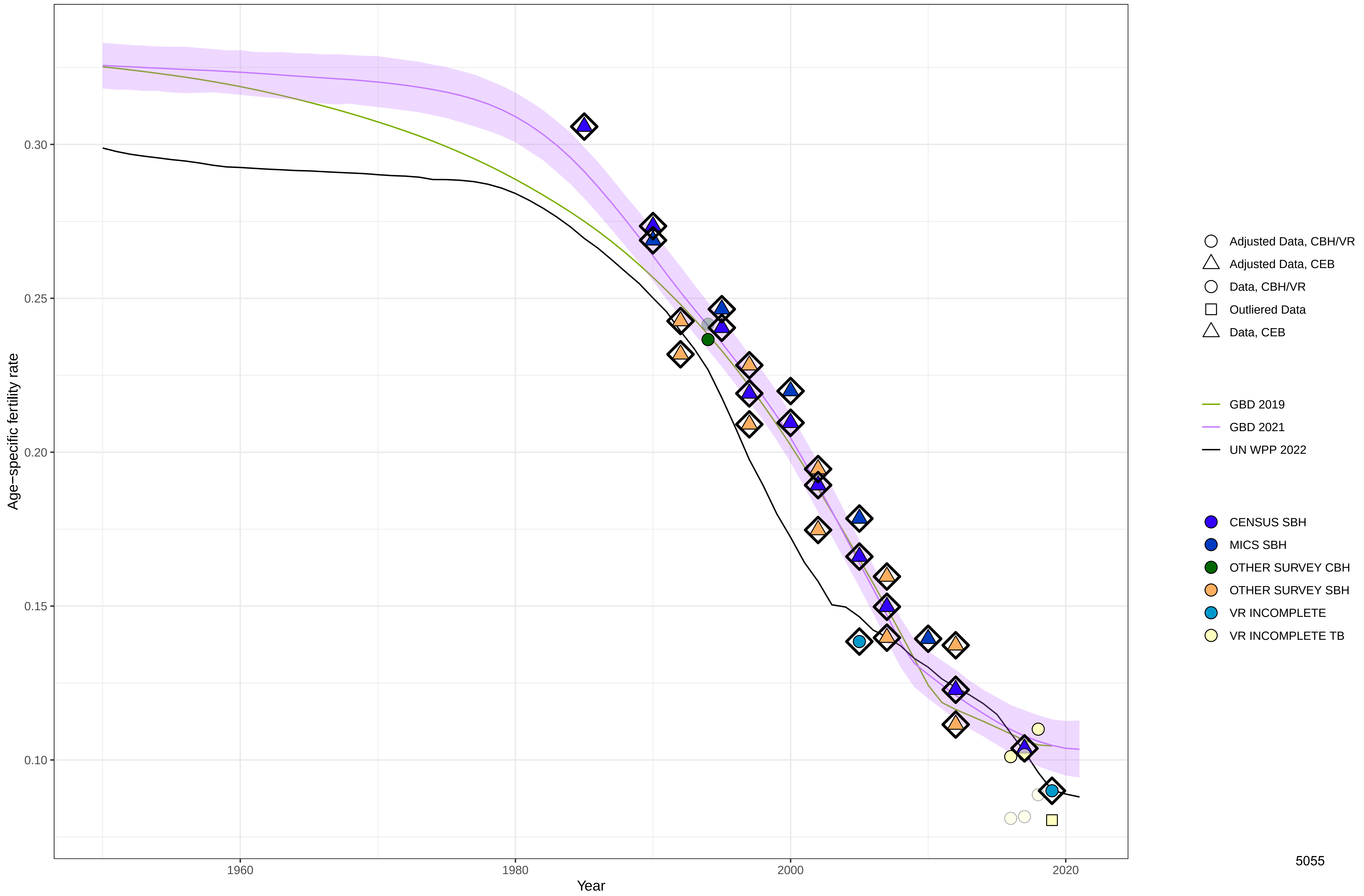

Age−specific fertility fitted trend lines, 1950−2021: Bhutan, Ages: 30−34
Data points enclosed with a diamond are reference sources

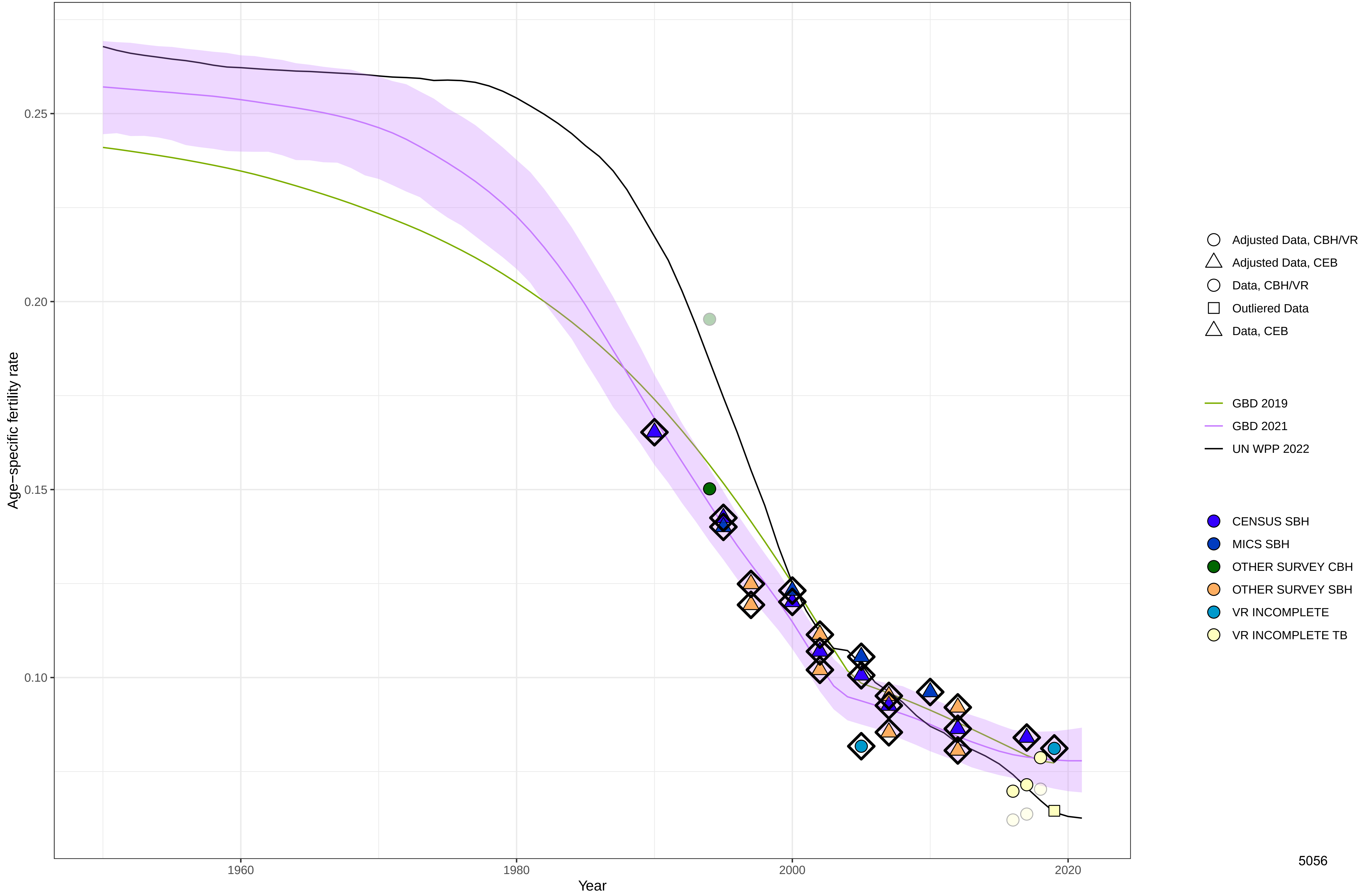

Age−specific fertility fitted trend lines, 1950−2021: Bhutan, Ages: 35−39
Data points enclosed with a diamond are reference sources

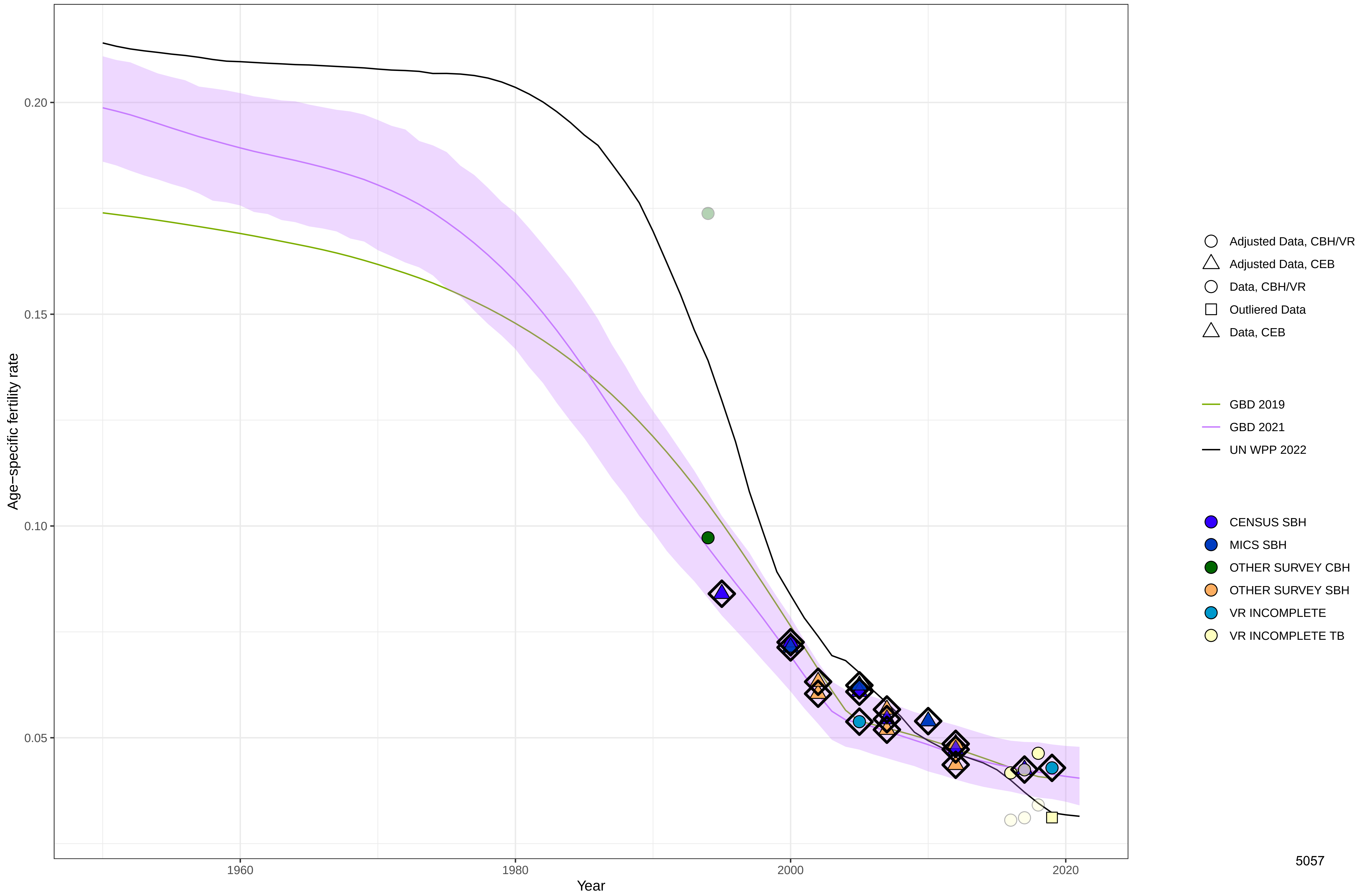

Age−specific fertility fitted trend lines, 1950−2021: Bhutan, Ages: 40−44
Data points enclosed with a diamond are reference sources

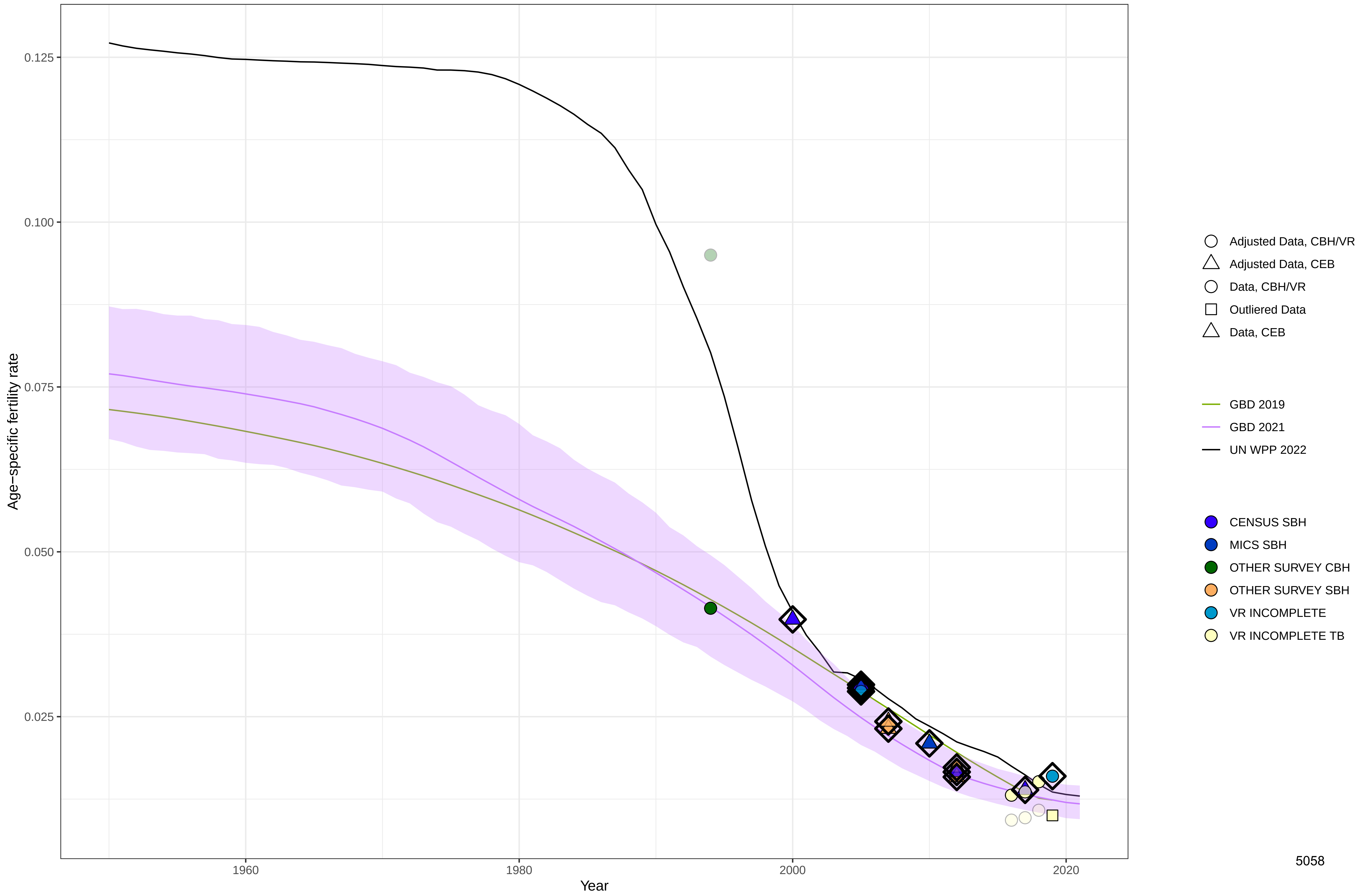

Age−specific fertility fitted trend lines, 1950−2021: Bhutan, Ages: 45−49
Data points enclosed with a diamond are reference sources

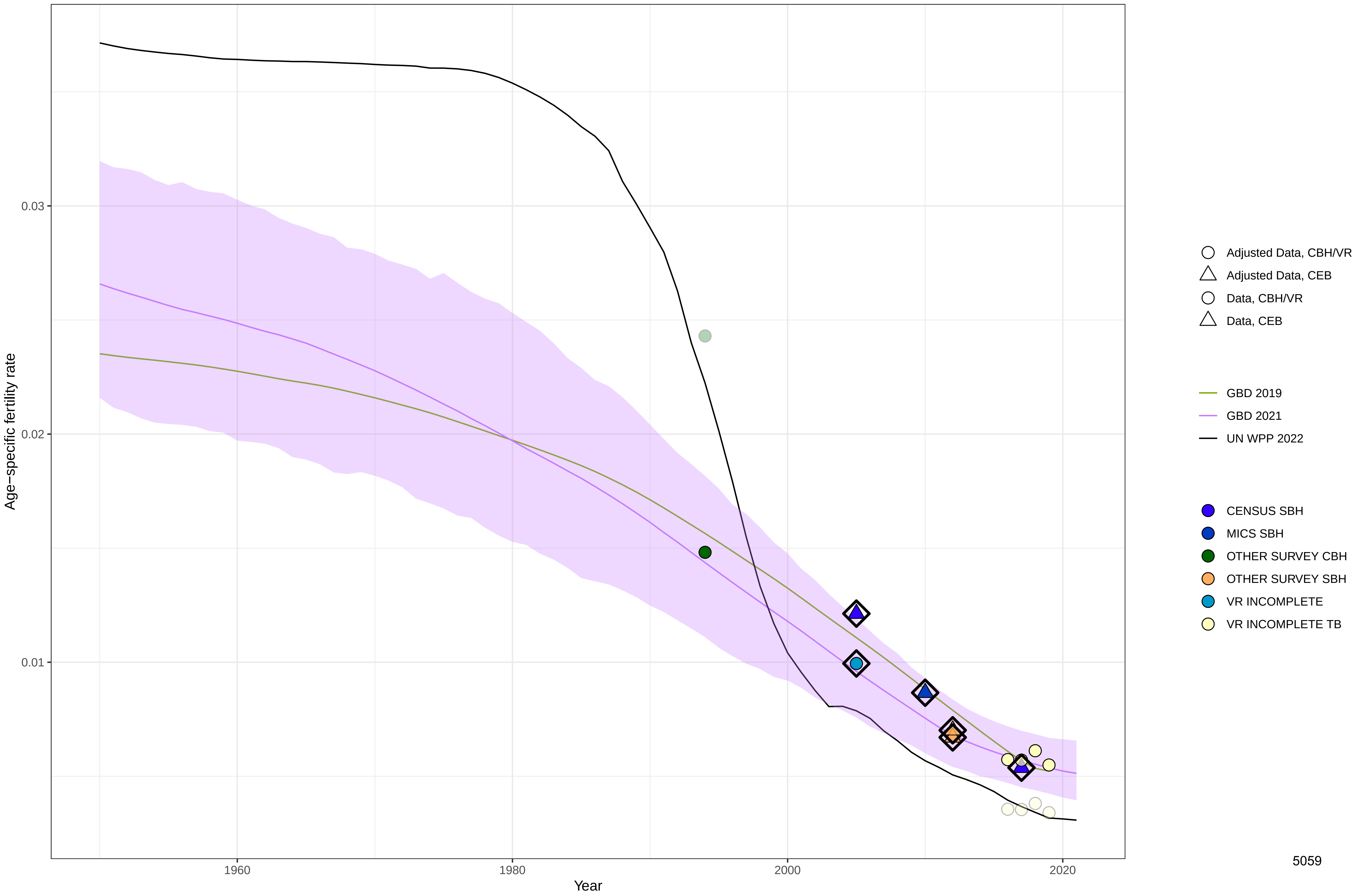

Age−specific fertility fitted trend lines, 1950−2021: Bhutan, Ages: 50−54
Data points enclosed with a diamond are reference sources

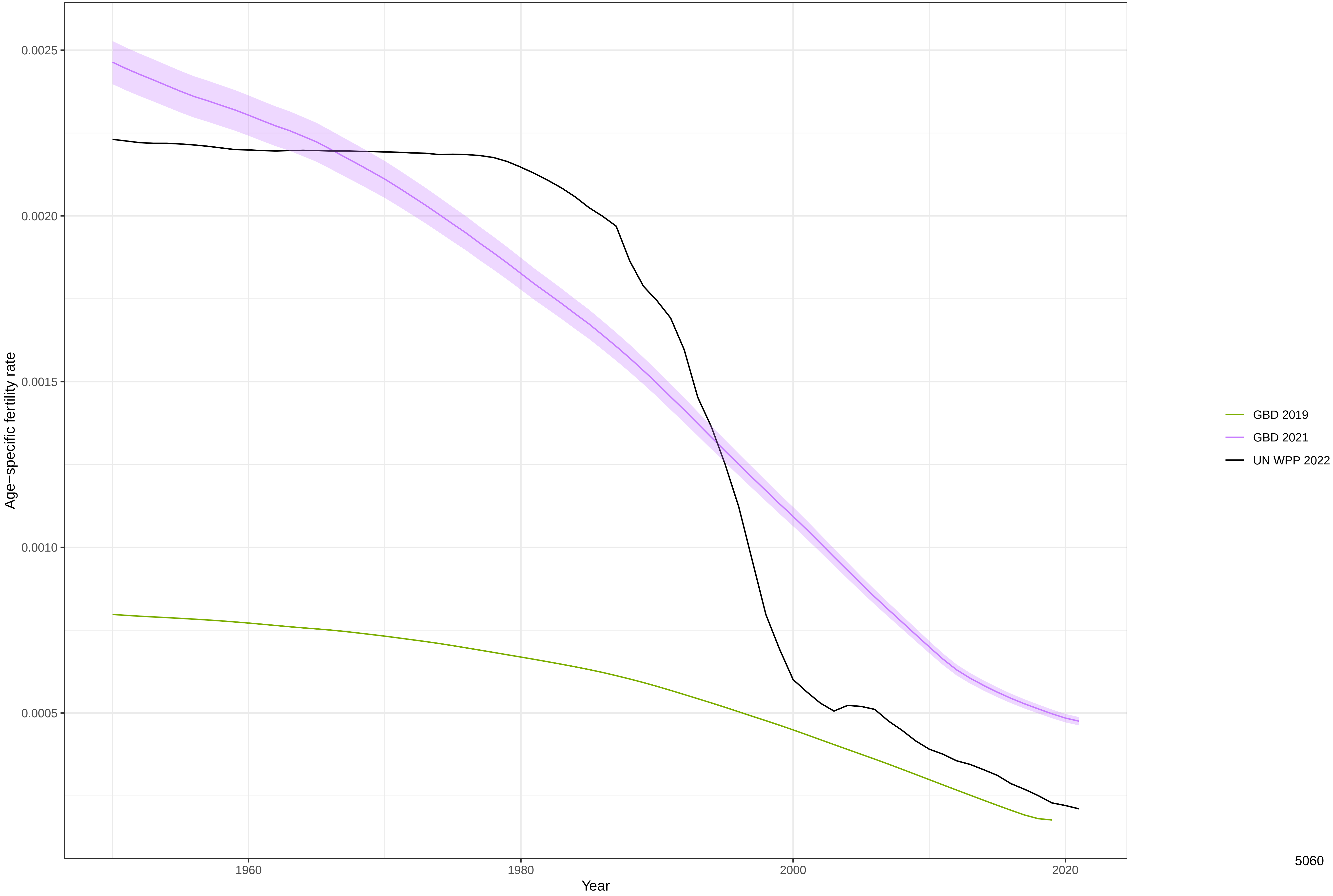

Total fertility rate fitted trend lines, 1950−2021: Bhutan TFR
Data points enclosed with a diamond are reference sources

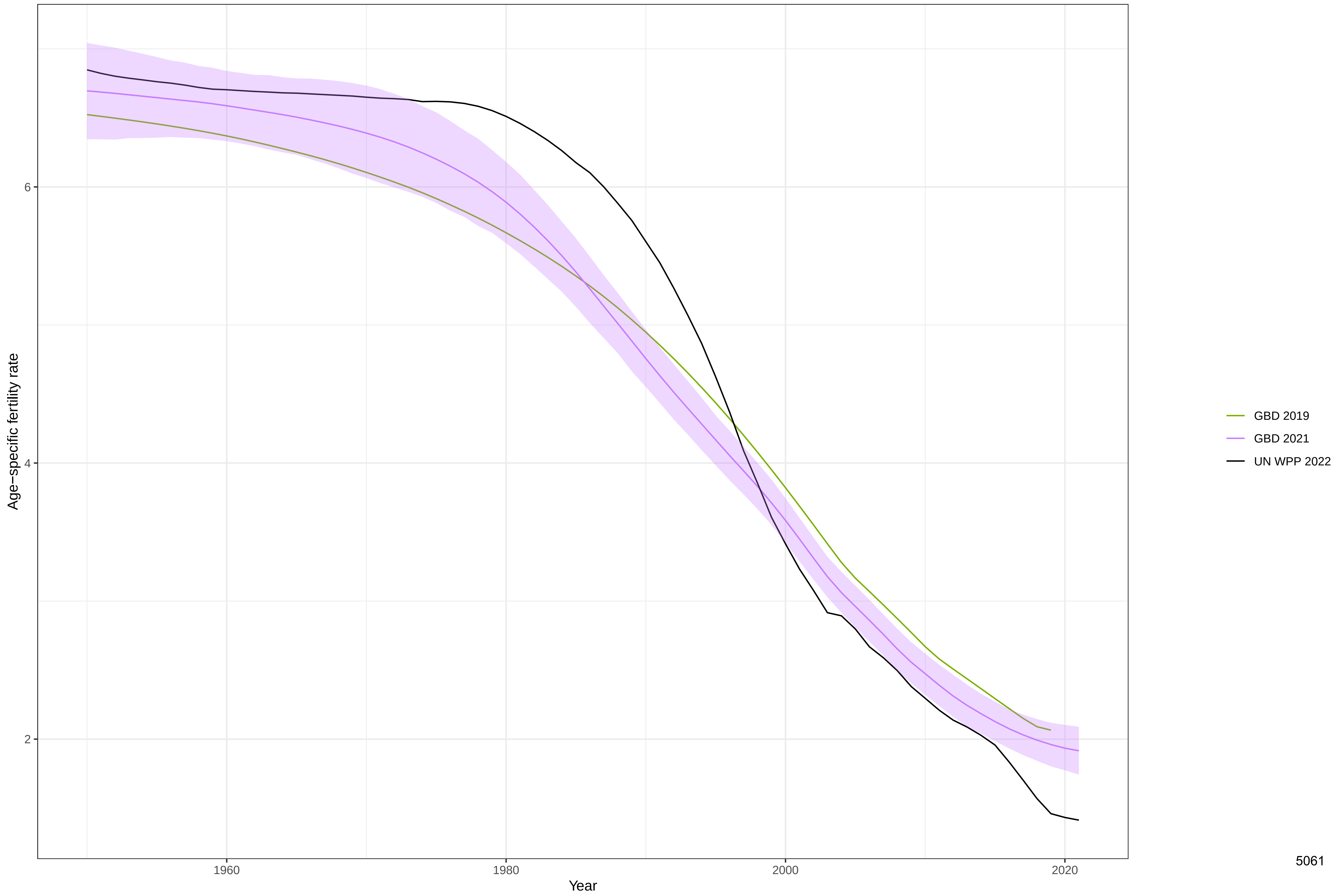

Age−specific fertility fitted trend lines, 1950−2021: India, Ages: 10−14
Data points enclosed with a diamond are reference sources

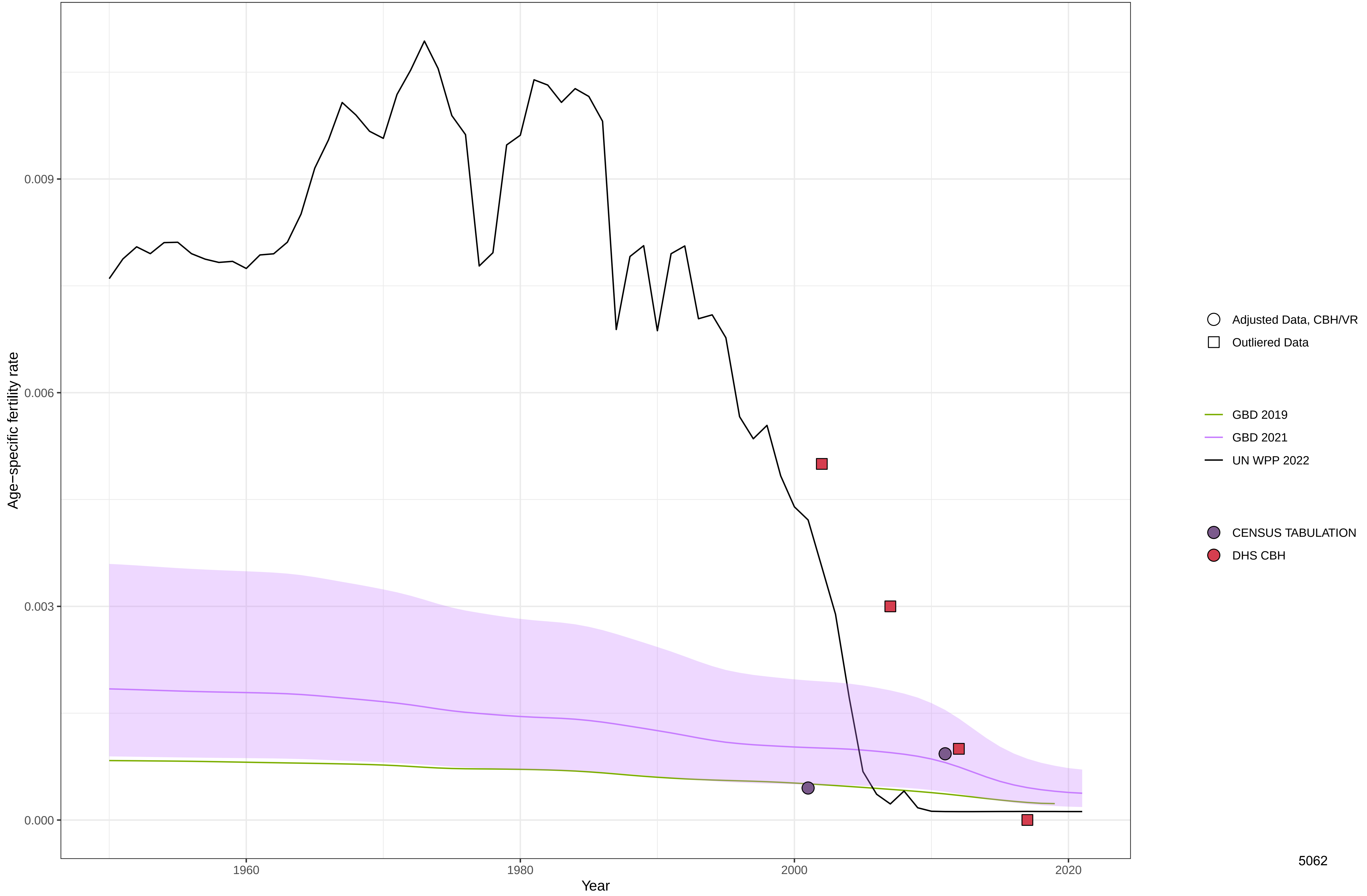

Age−specific fertility fitted trend lines, 1950−2021: India, Ages: 15−19
Data points enclosed with a diamond are reference sources

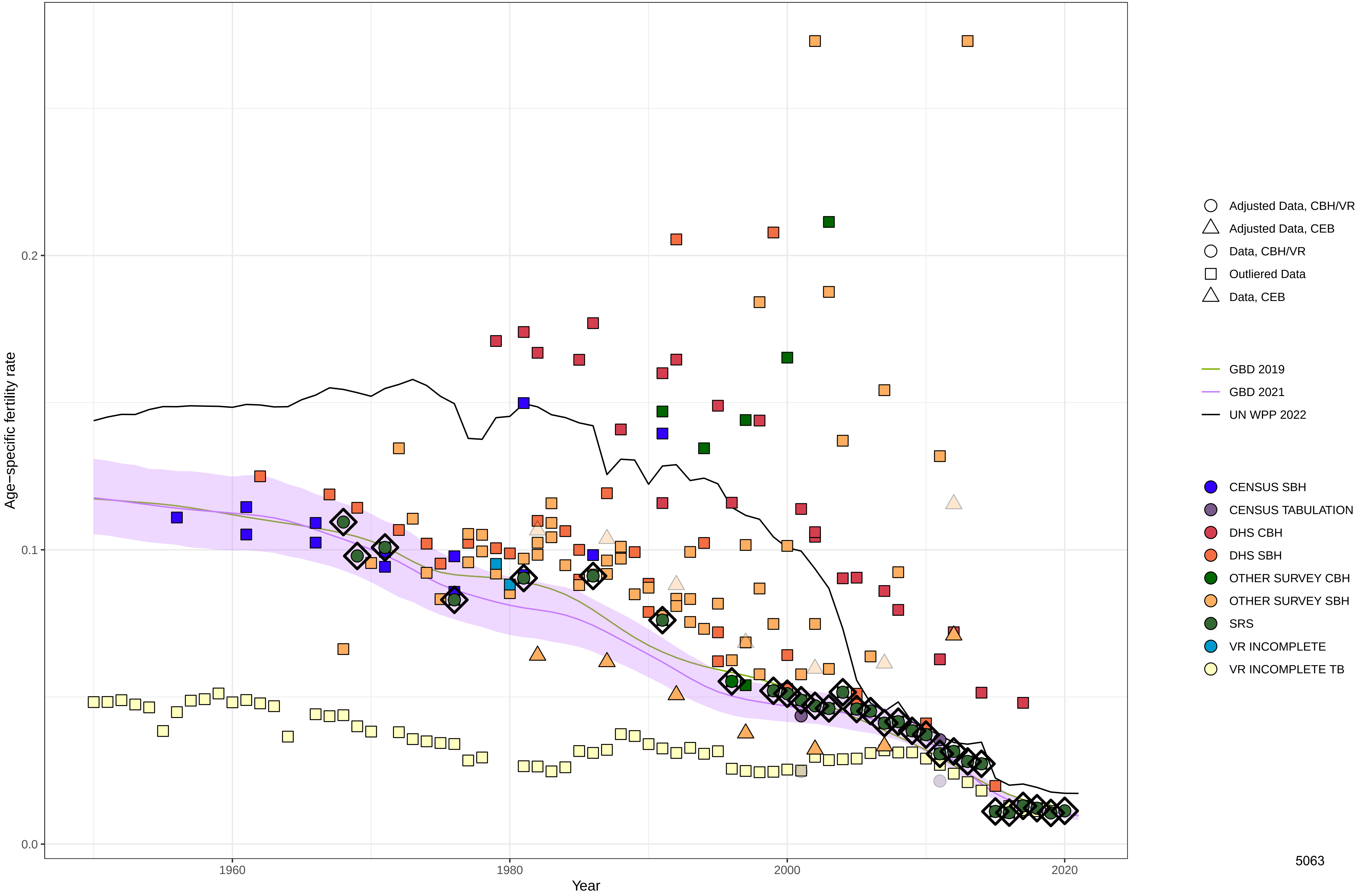

Age−specific fertility fitted trend lines, 1950−2021: India, Ages: 20−24
Data points enclosed with a diamond are reference sources

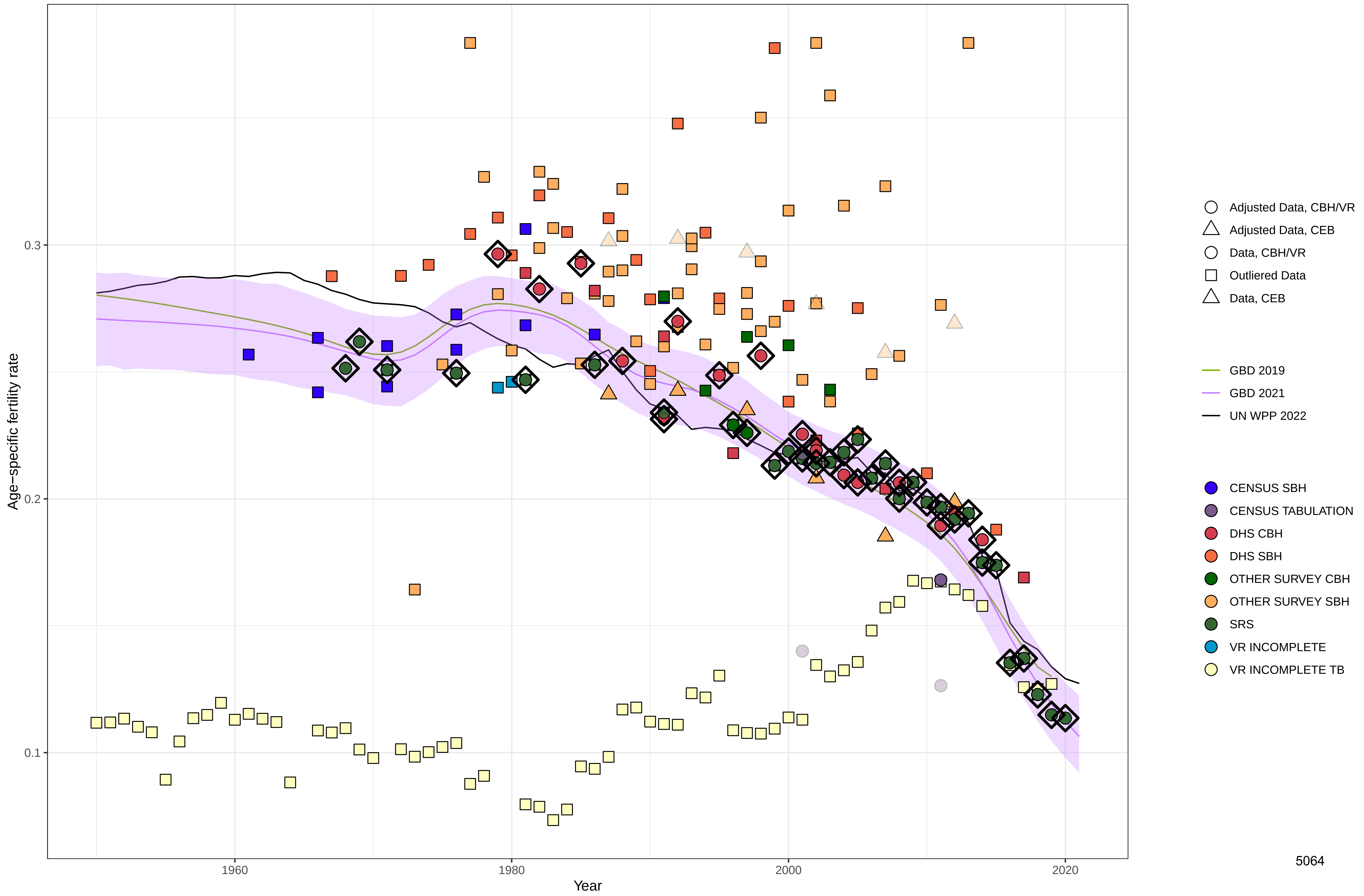

Age−specific fertility fitted trend lines, 1950−2021: India, Ages: 25−29
Data points enclosed with a diamond are reference sources

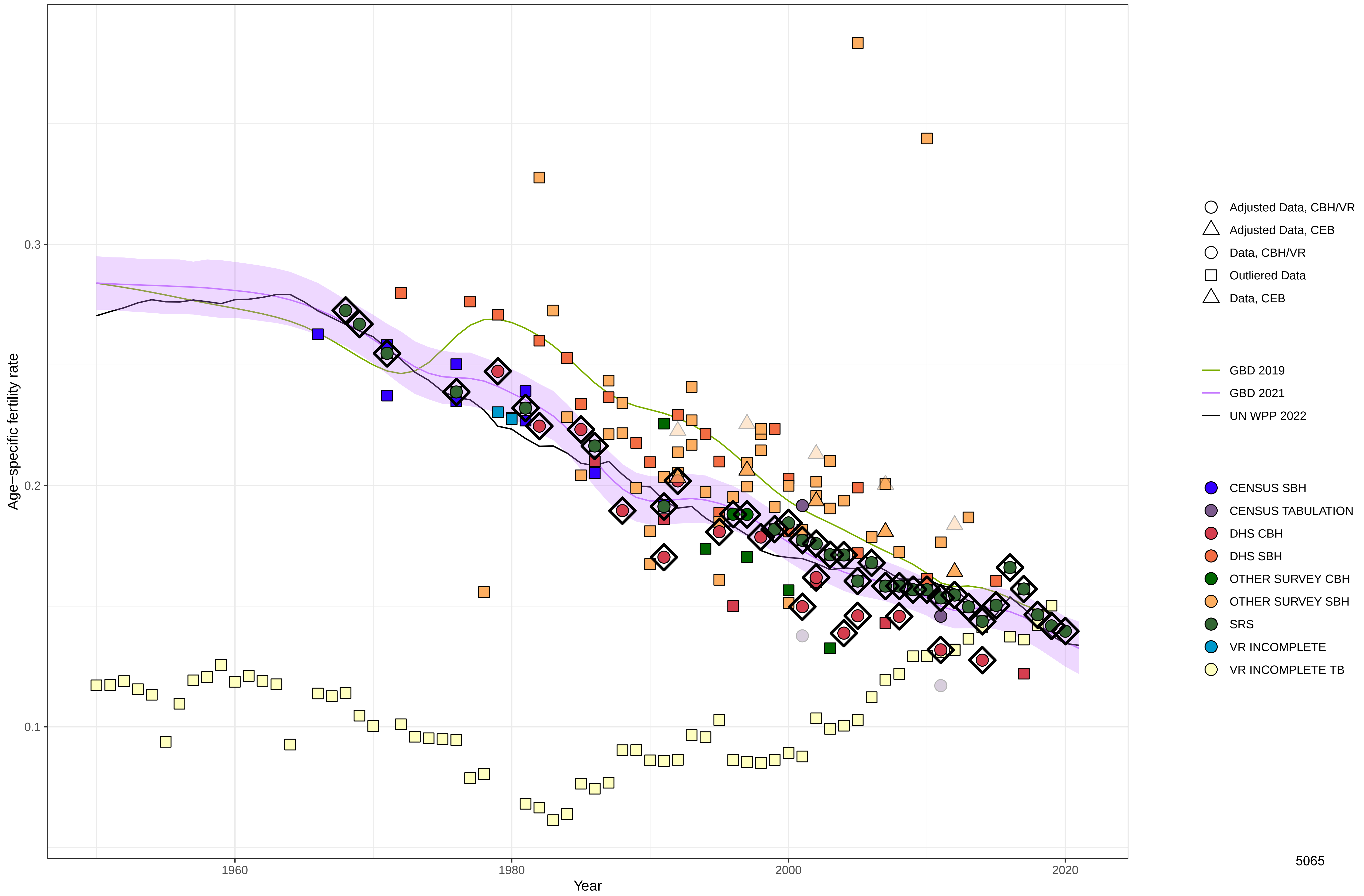

Age−specific fertility fitted trend lines, 1950−2021: India, Ages: 30−34
Data points enclosed with a diamond are reference sources

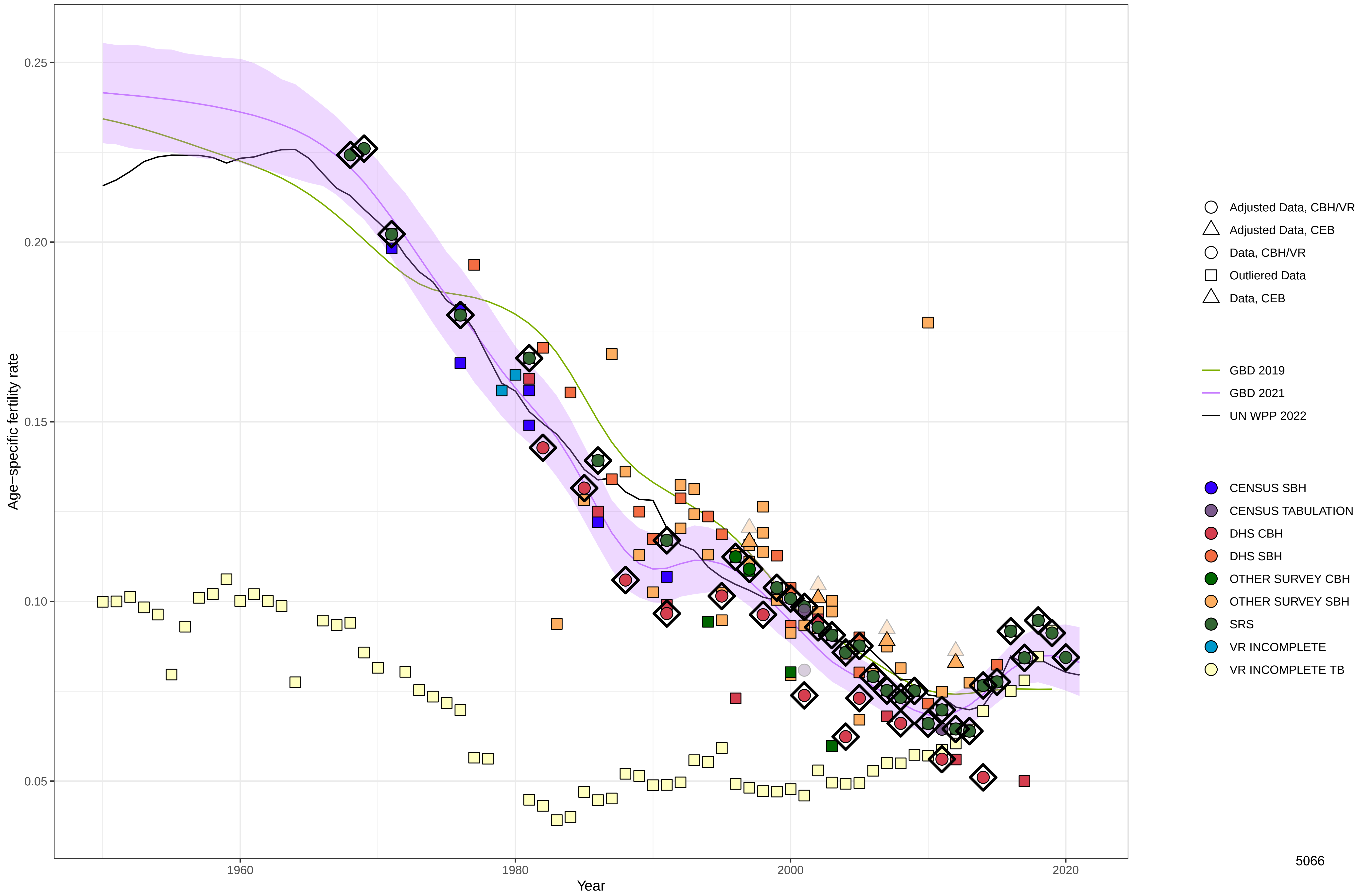

Age−specific fertility fitted trend lines, 1950−2021: India, Ages: 35−39
Data points enclosed with a diamond are reference sources

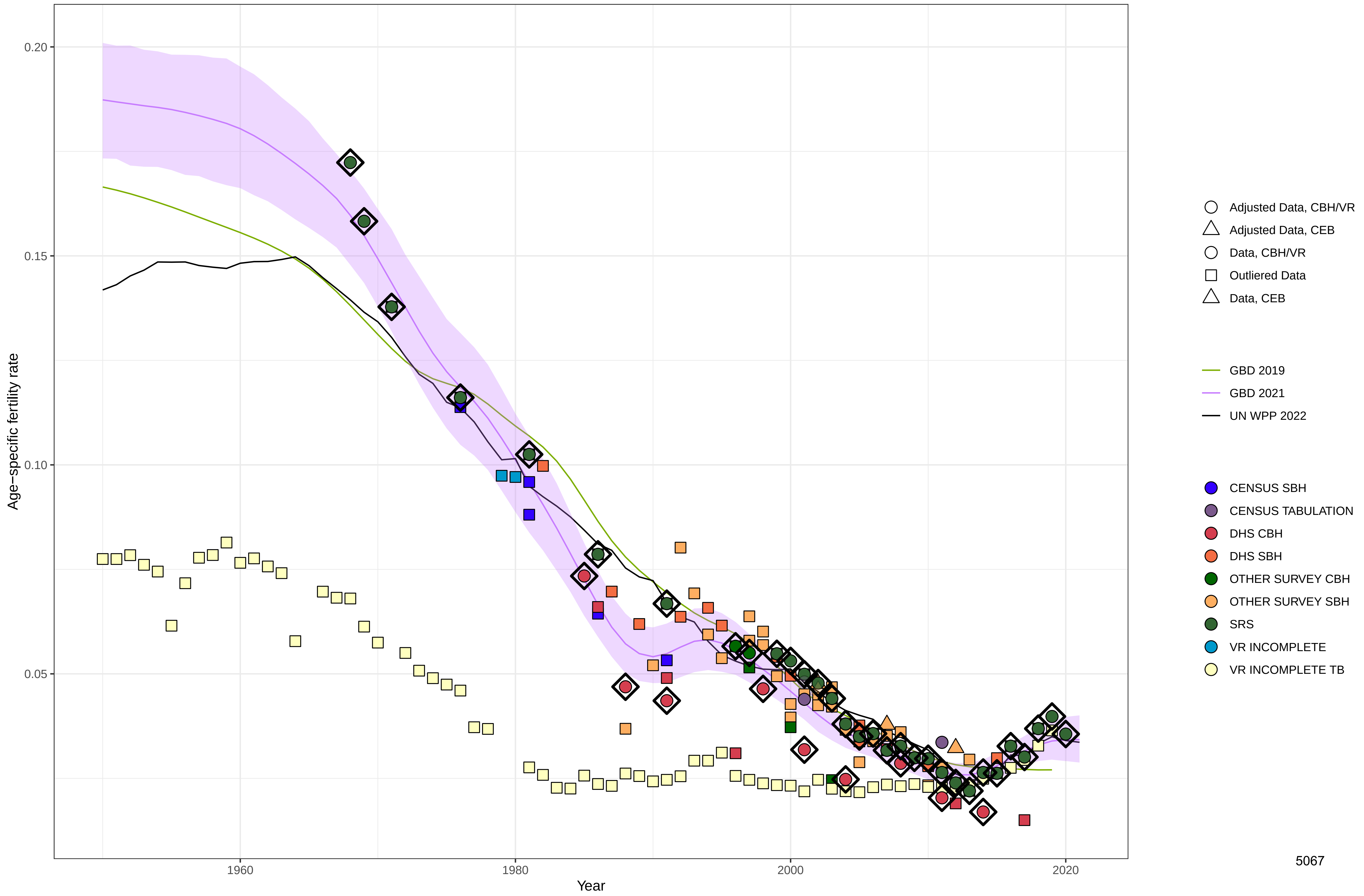

Age−specific fertility fitted trend lines, 1950−2021: India, Ages: 40−44
Data points enclosed with a diamond are reference sources

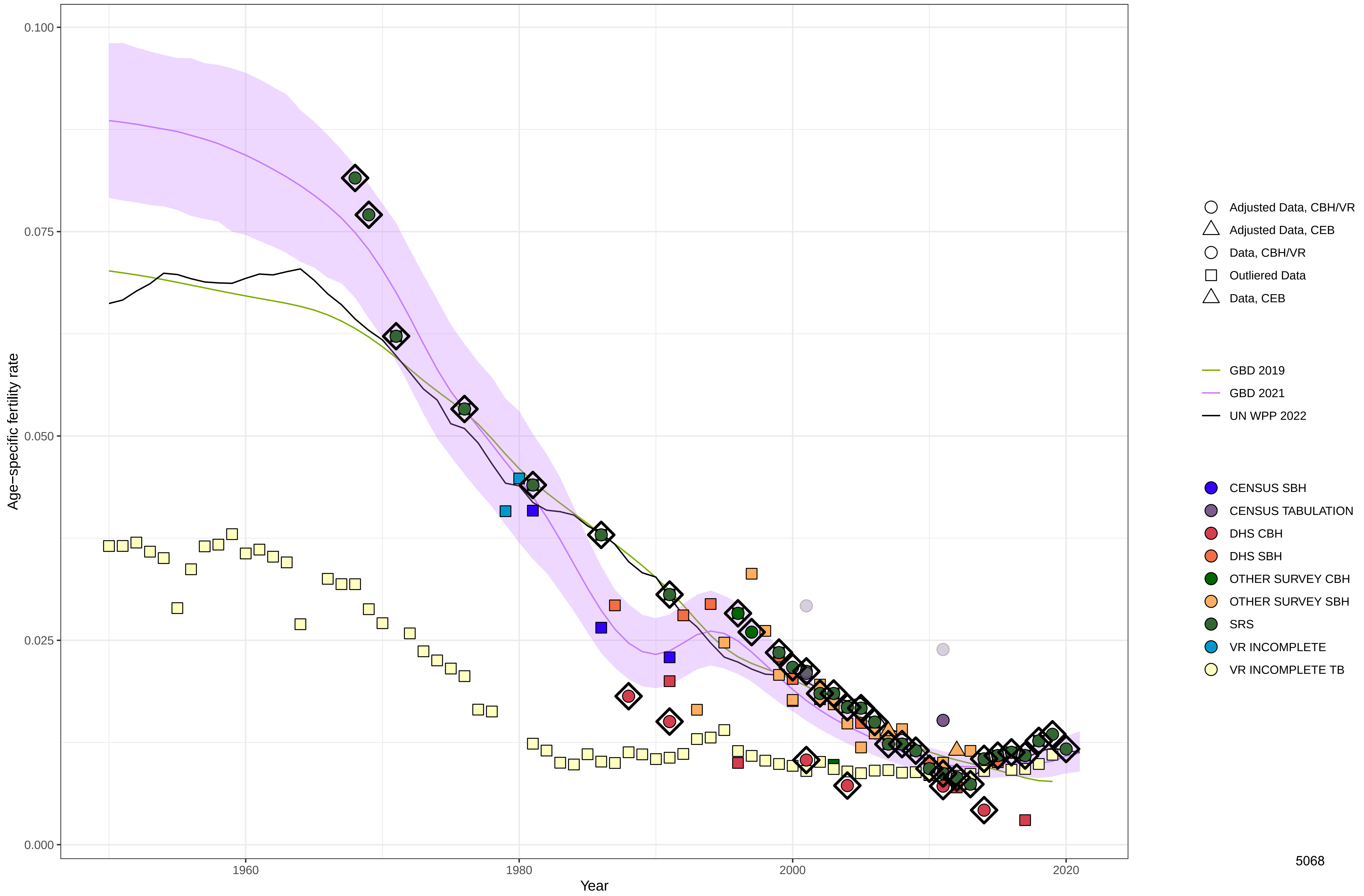

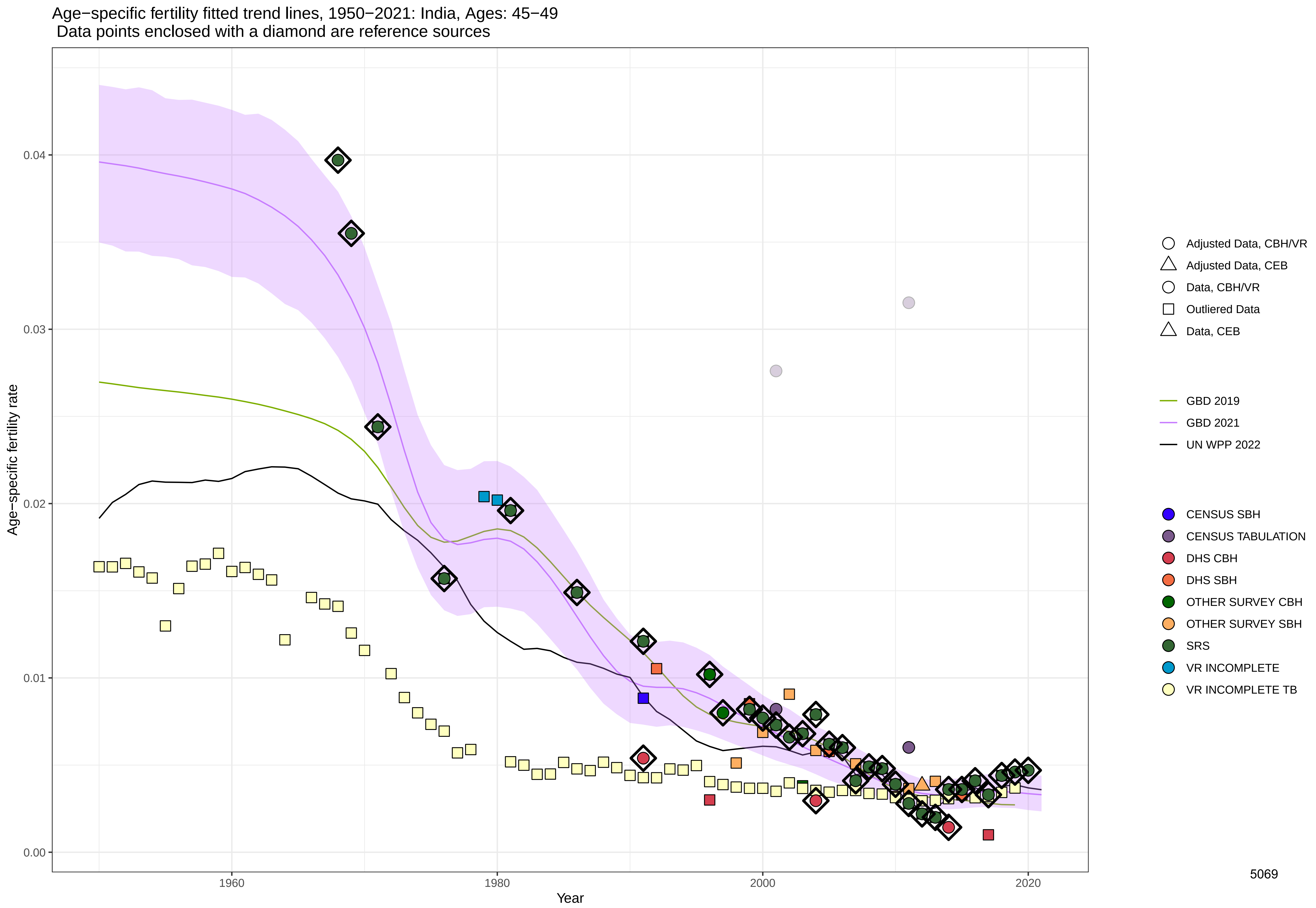

Age−specific fertility fitted trend lines, 1950−2021: India, Ages: 45−49
Data points enclosed with a diamond are reference sources
Age−specific fertility rate
Year
0.00
0.01
0.02
0.03
0.04
1960
1980
2000
2020
Adjusted Data, CBH/VR
Adjusted Data, CEB
Data, CBH/VR
Outliered Data
Data, CEB
GBD 2019
GBD 2021
UN WPP 2022
CENSUS SBH
CENSUS TABULATION
DHS CBH
DHS SBH
OTHER SURVEY CBH
OTHER SURVEY SBH
SRS
VR INCOMPLETE
VR INCOMPLETE TB

Age−specific fertility fitted trend lines, 1950−2021: India, Ages: 50−54
Data points enclosed with a diamond are reference sources

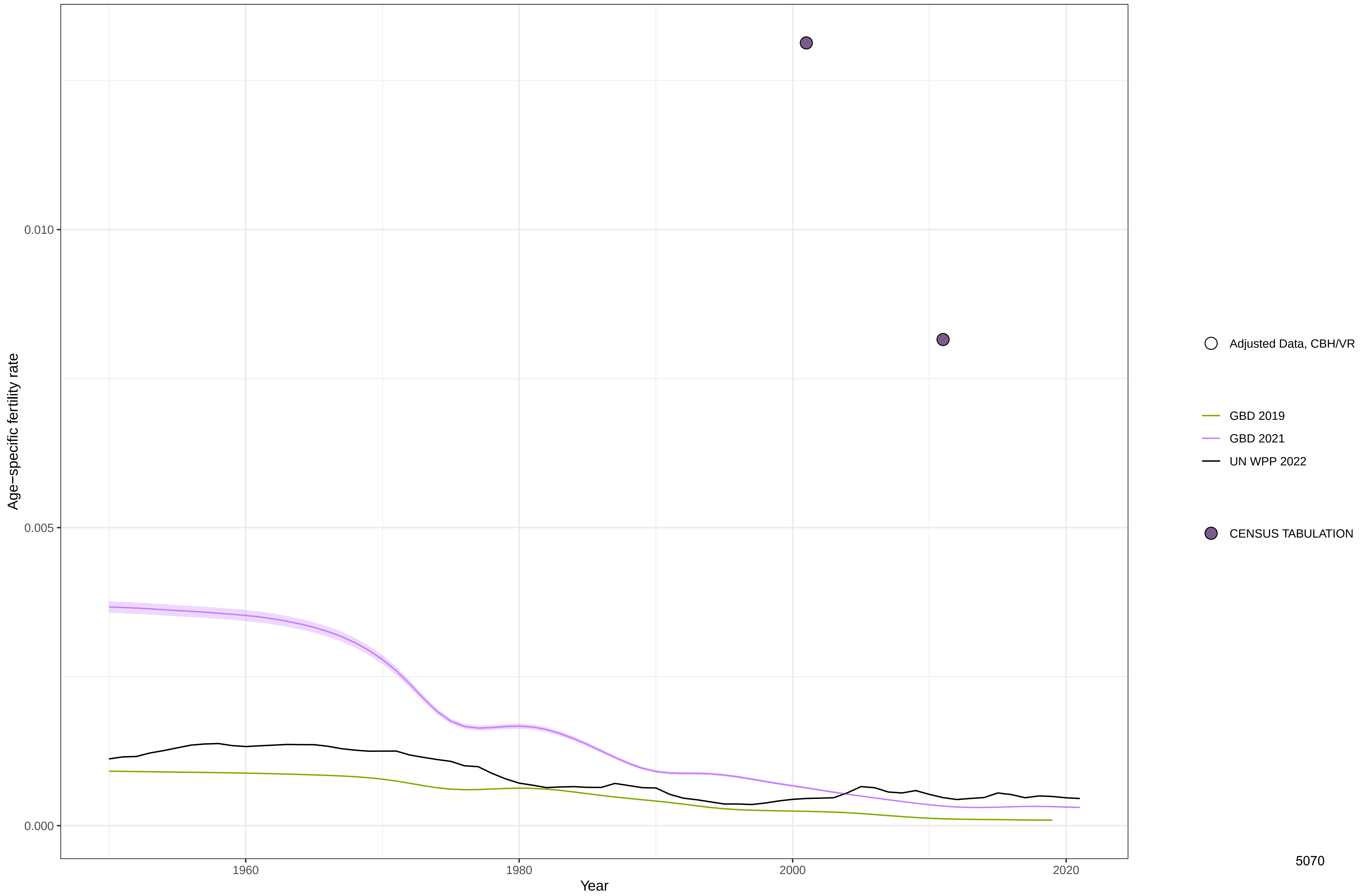

Total fertility rate fitted trend lines, 1950−2021: India TFR
Data points enclosed with a diamond are reference sources

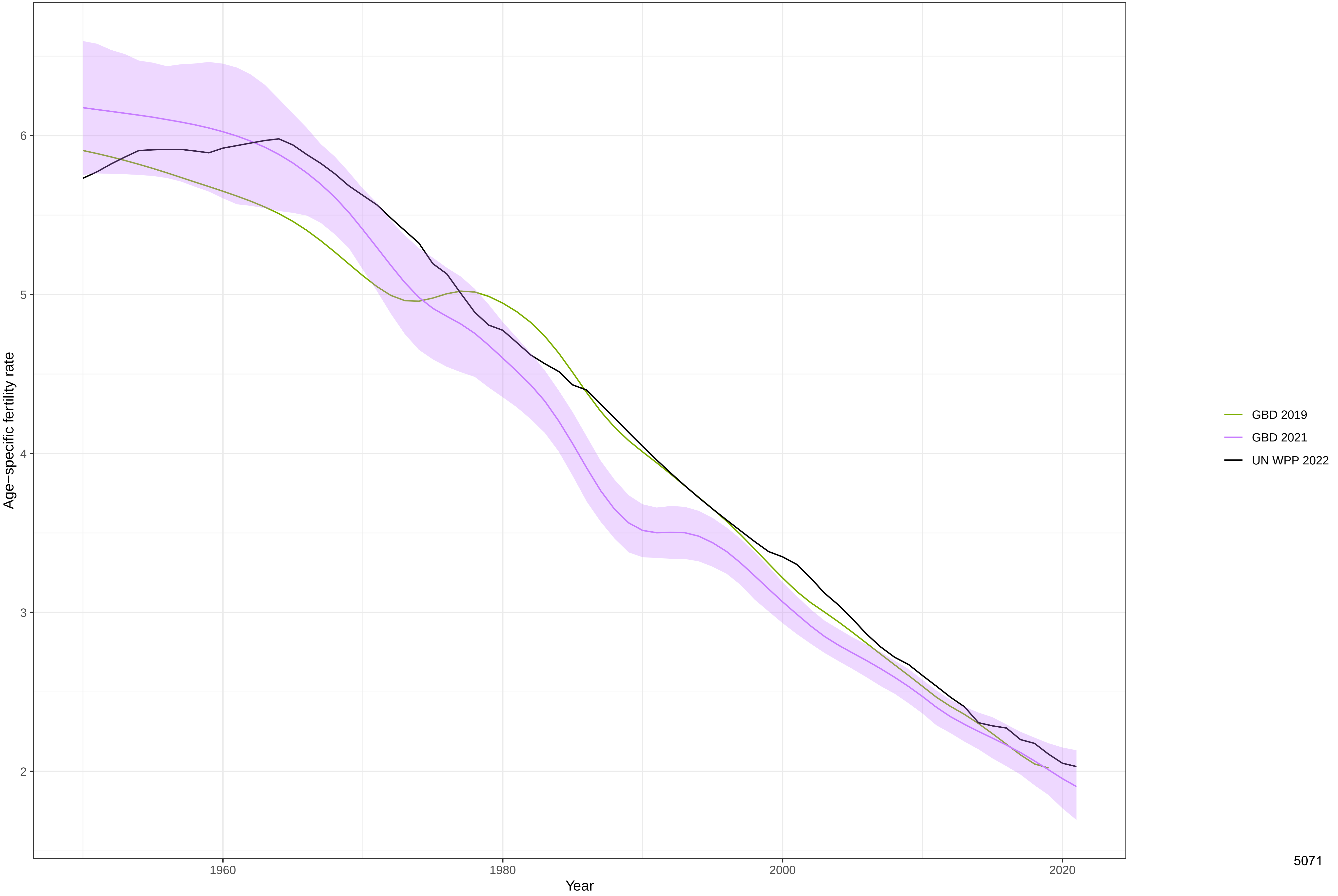

Age−specific fertility fitted trend lines, 1950−2021: Nepal, Ages: 10−14
Data points enclosed with a diamond are reference sources

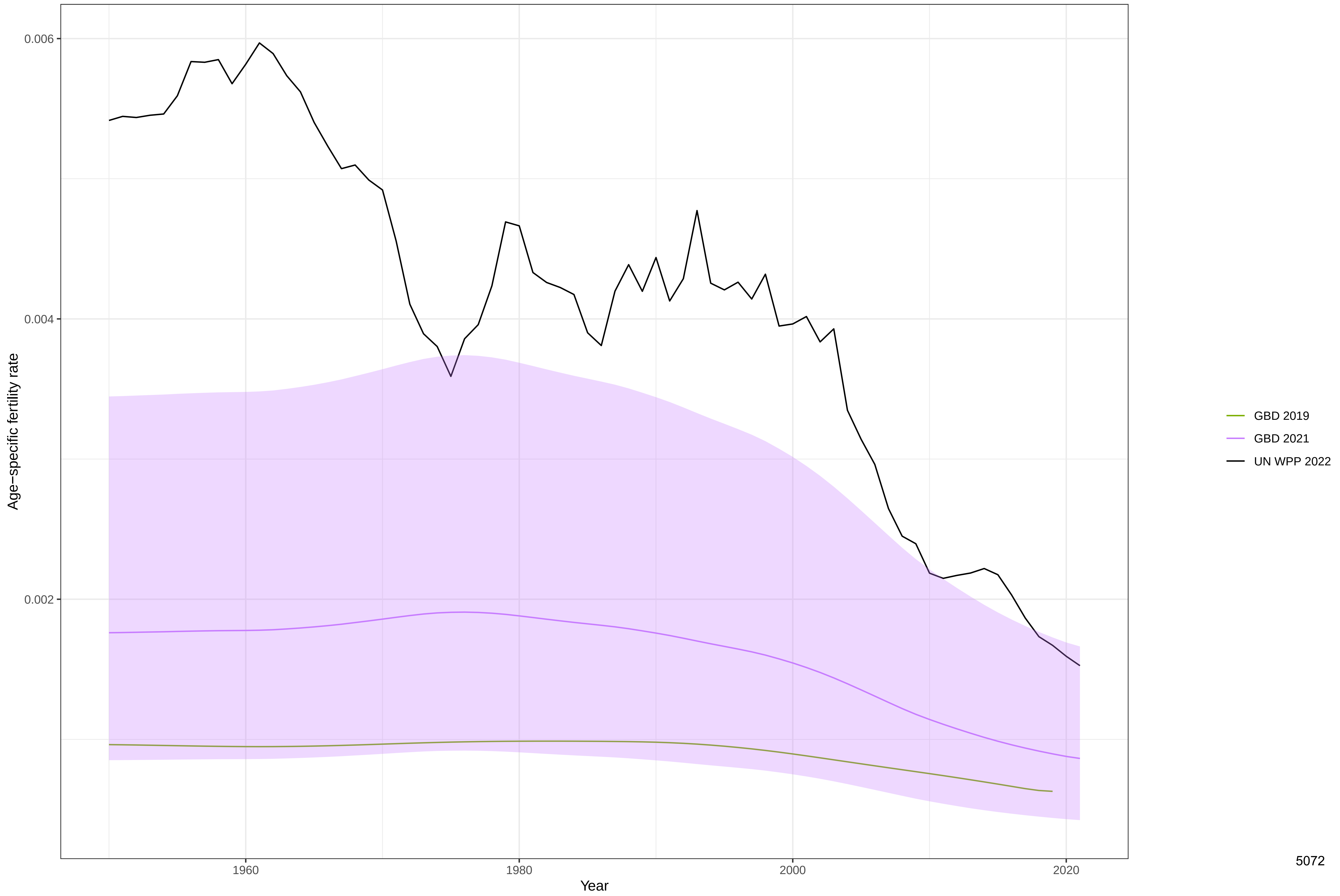

Age−specific fertility fitted trend lines, 1950−2021: Nepal, Ages: 15−19
Data points enclosed with a diamond are reference sources

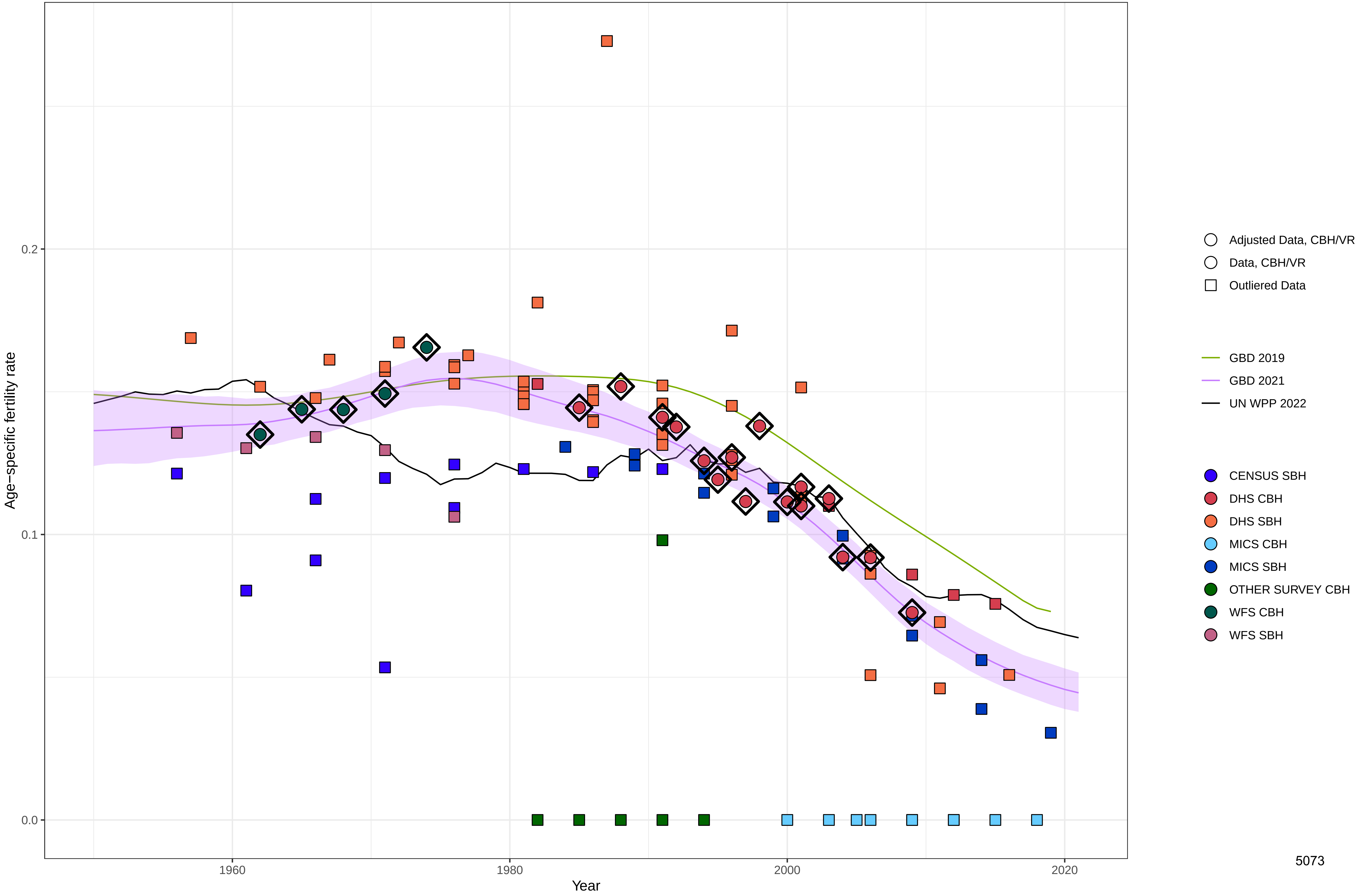

Age−specific fertility fitted trend lines, 1950−2021: Nepal, Ages: 20−24
Data points enclosed with a diamond are reference sources

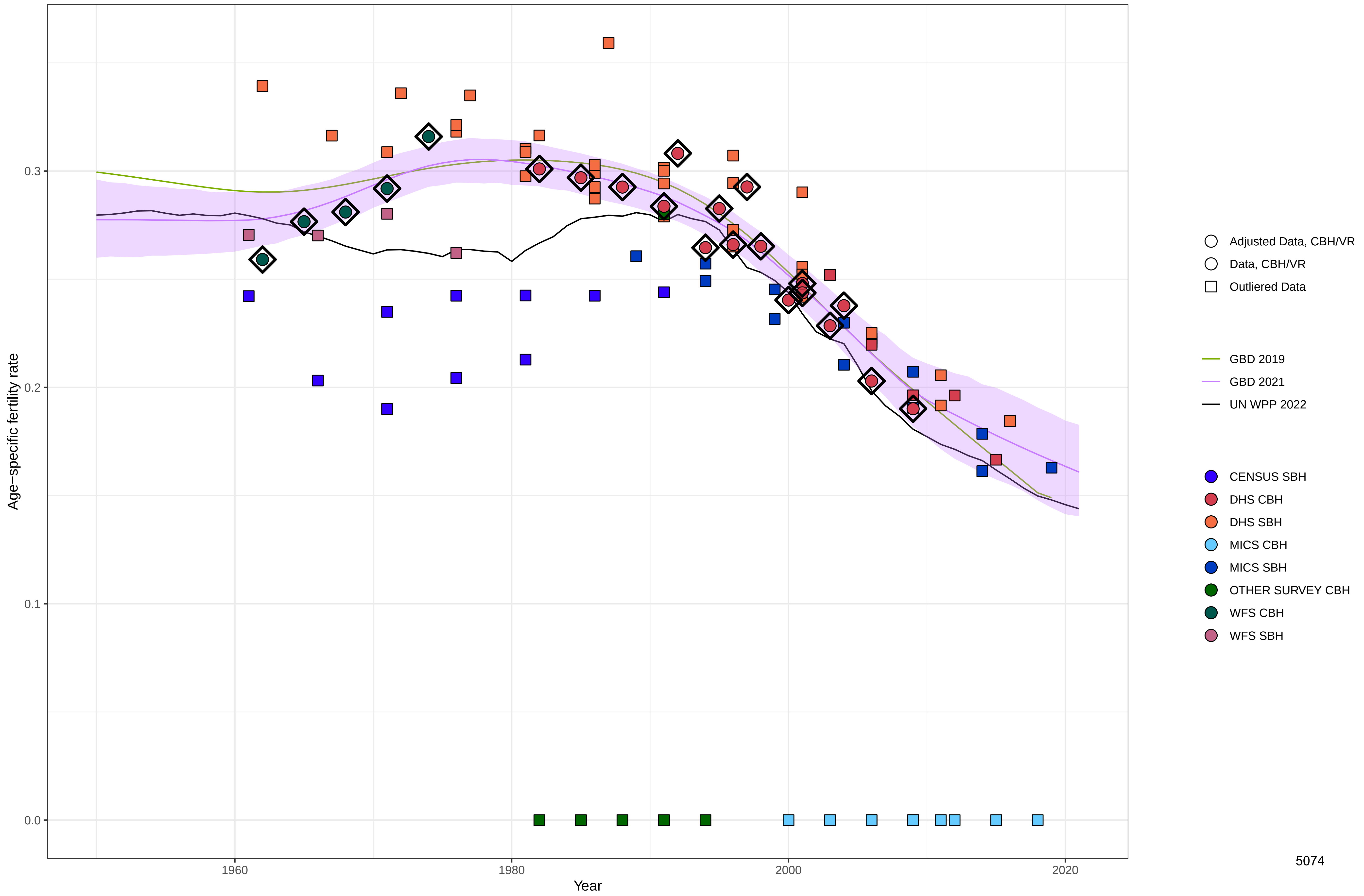

Age−specific fertility fitted trend lines, 1950−2021: Nepal, Ages: 25−29
Data points enclosed with a diamond are reference sources

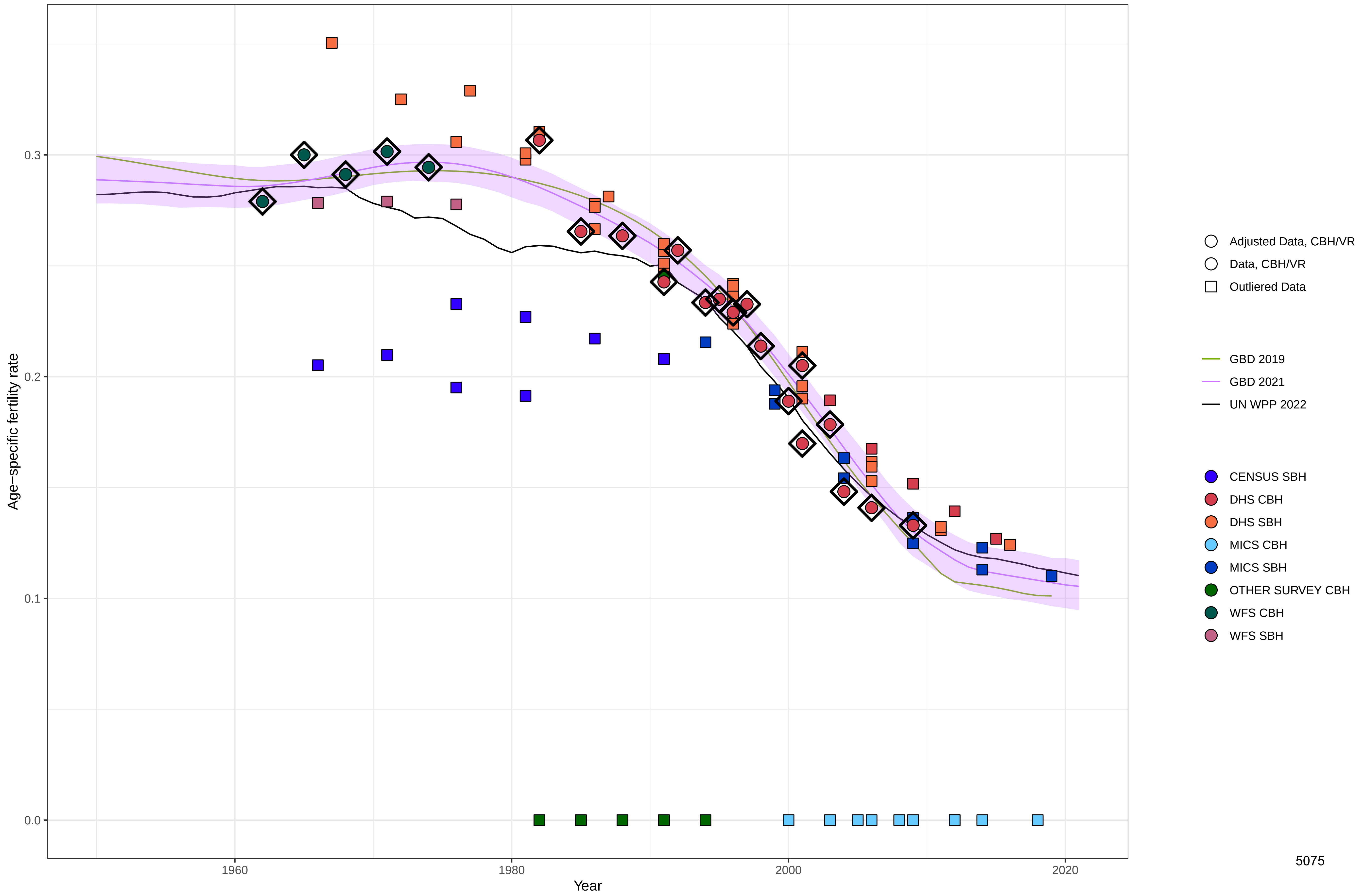

Age−specific fertility fitted trend lines, 1950−2021: Nepal, Ages: 30−34
Data points enclosed with a diamond are reference sources

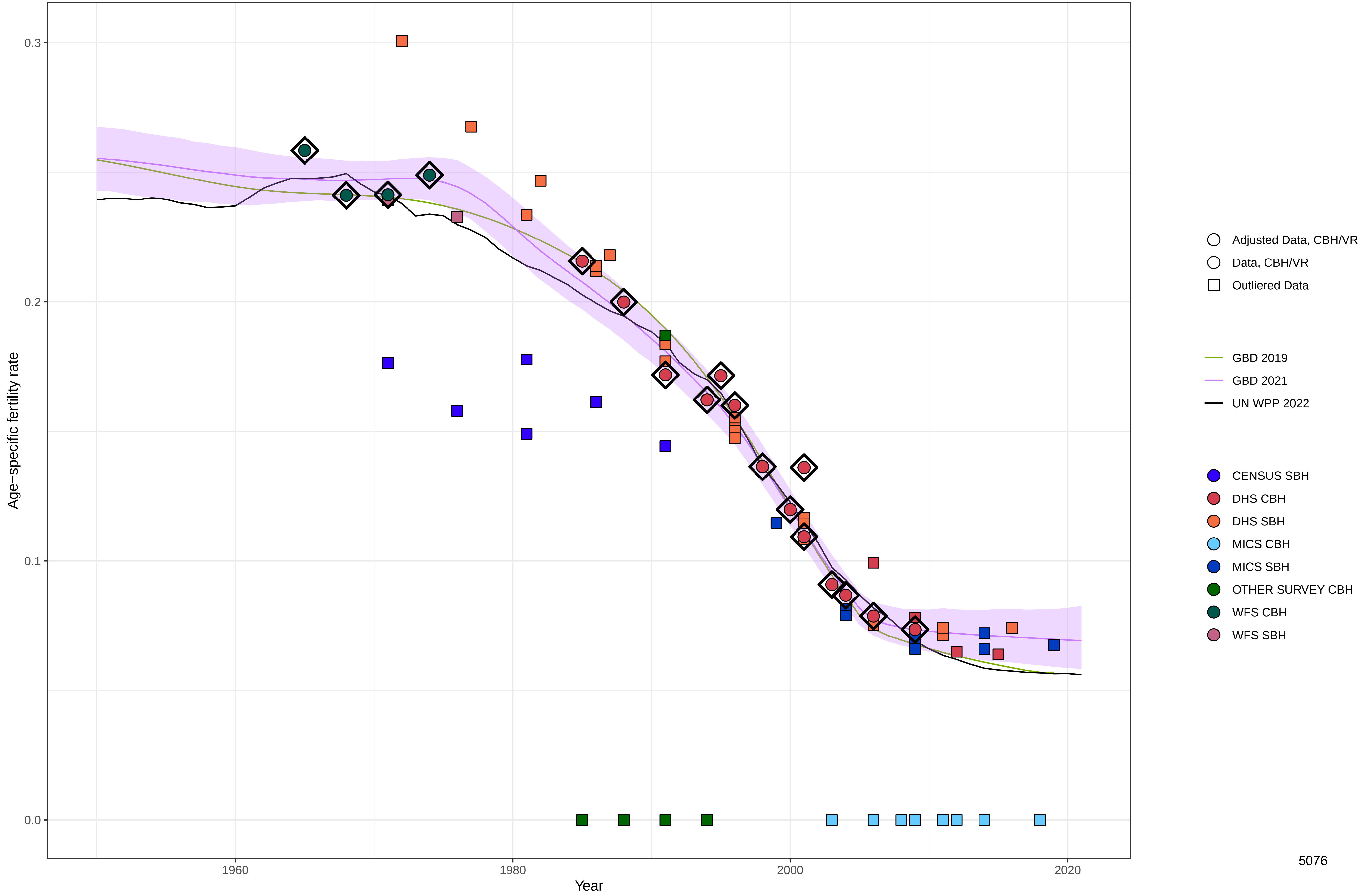

Age−specific fertility fitted trend lines, 1950−2021: Nepal, Ages: 35−39
Data points enclosed with a diamond are reference sources

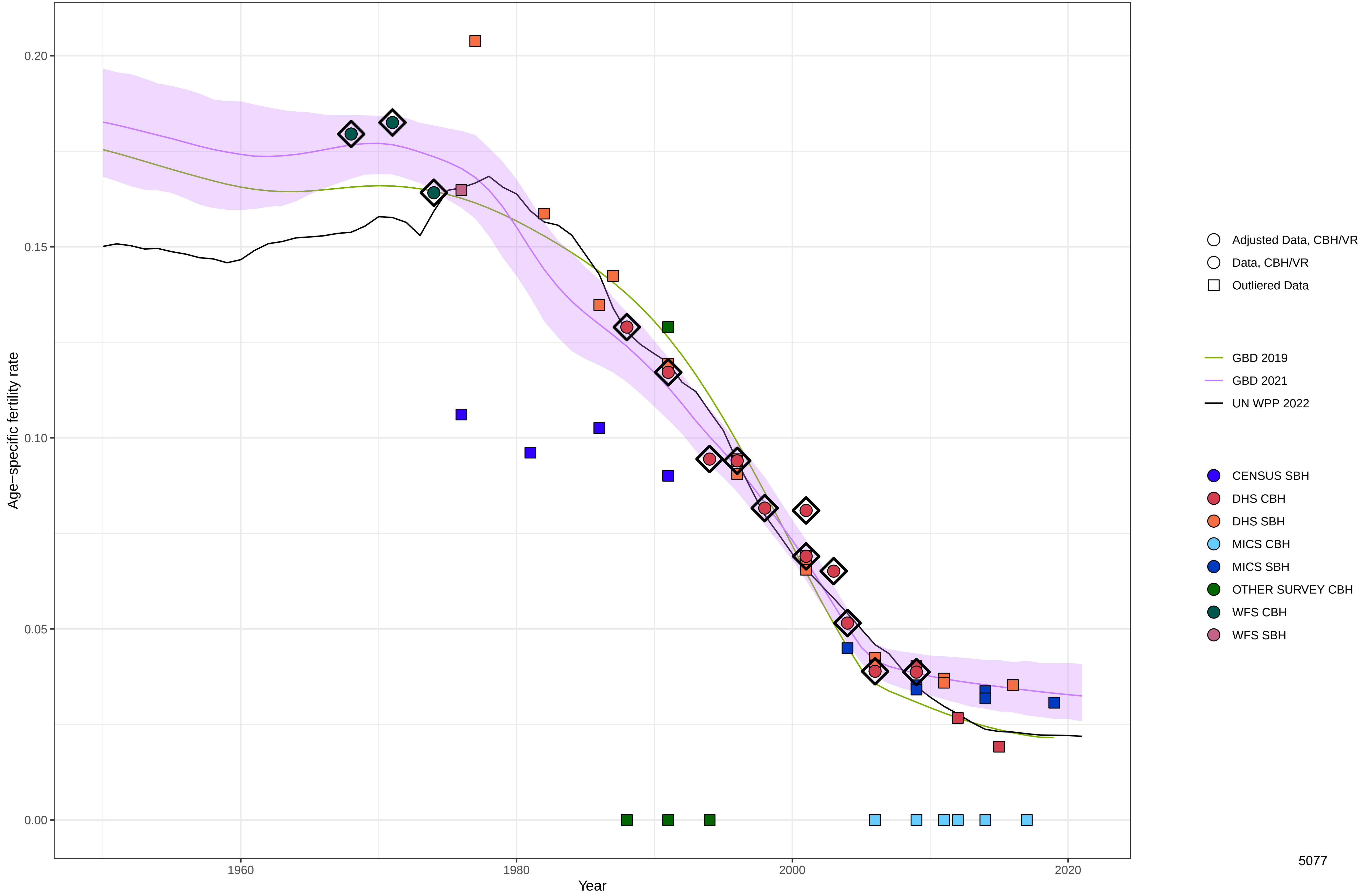

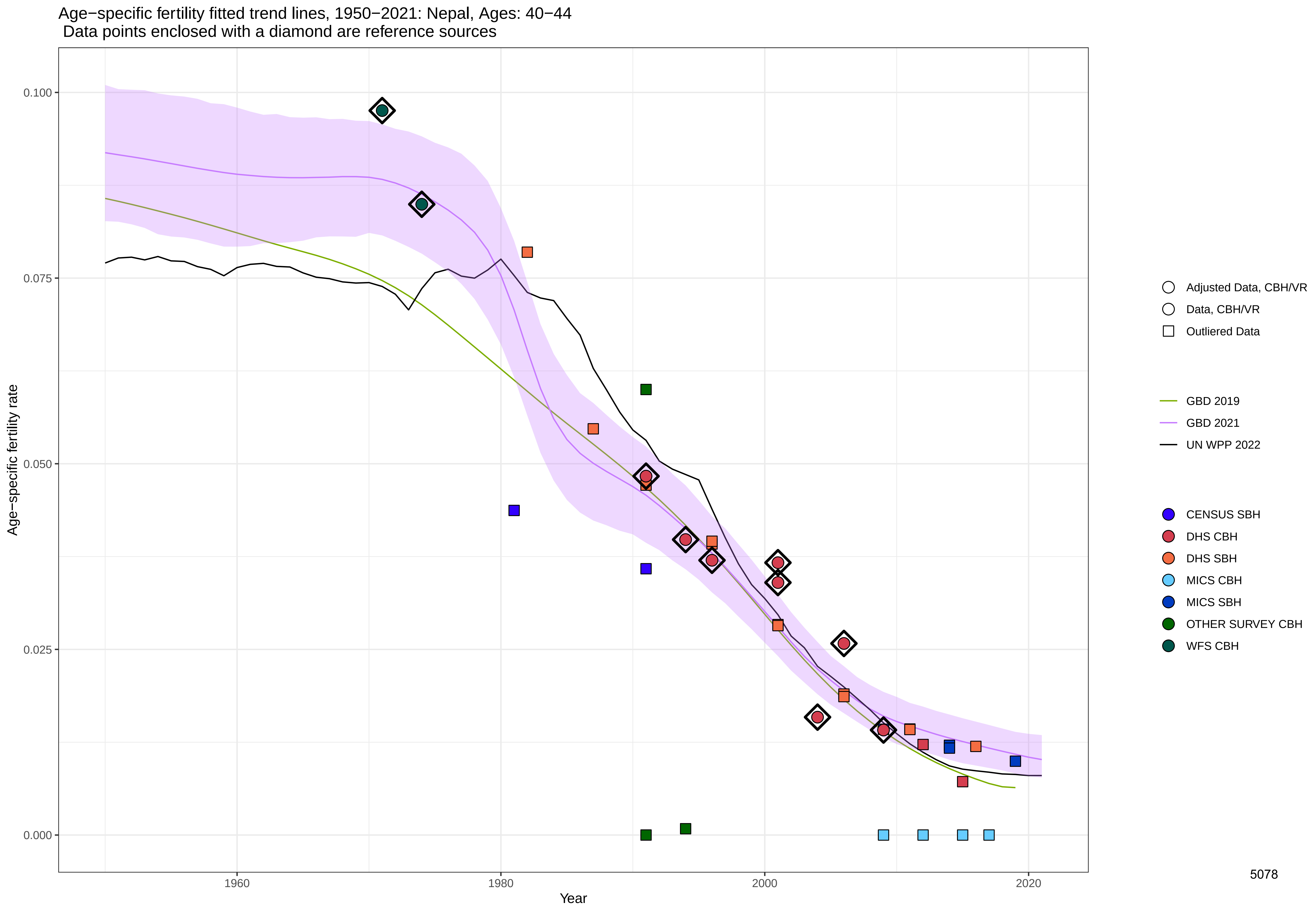

Age−specific fertility fitted trend lines, 1950−2021: Nepal, Ages: 40−44
Data points enclosed with a diamond are reference sources
0.100
0.075
0.050
0.025
0.000
Age−specific fertility rate
1960
1980
2000
2020
Year
Adjusted Data, CBH/VR
Data, CBH/VR
Outliered Data
GBD 2019
GBD 2021
UN WPP 2022
CENSUS SBH
DHS CBH
DHS SBH
MICS CBH
MICS SBH
OTHER SURVEY CBH
WFS CBH

Age−specific fertility fitted trend lines, 1950−2021: Nepal, Ages: 45−49
Data points enclosed with a diamond are reference sources

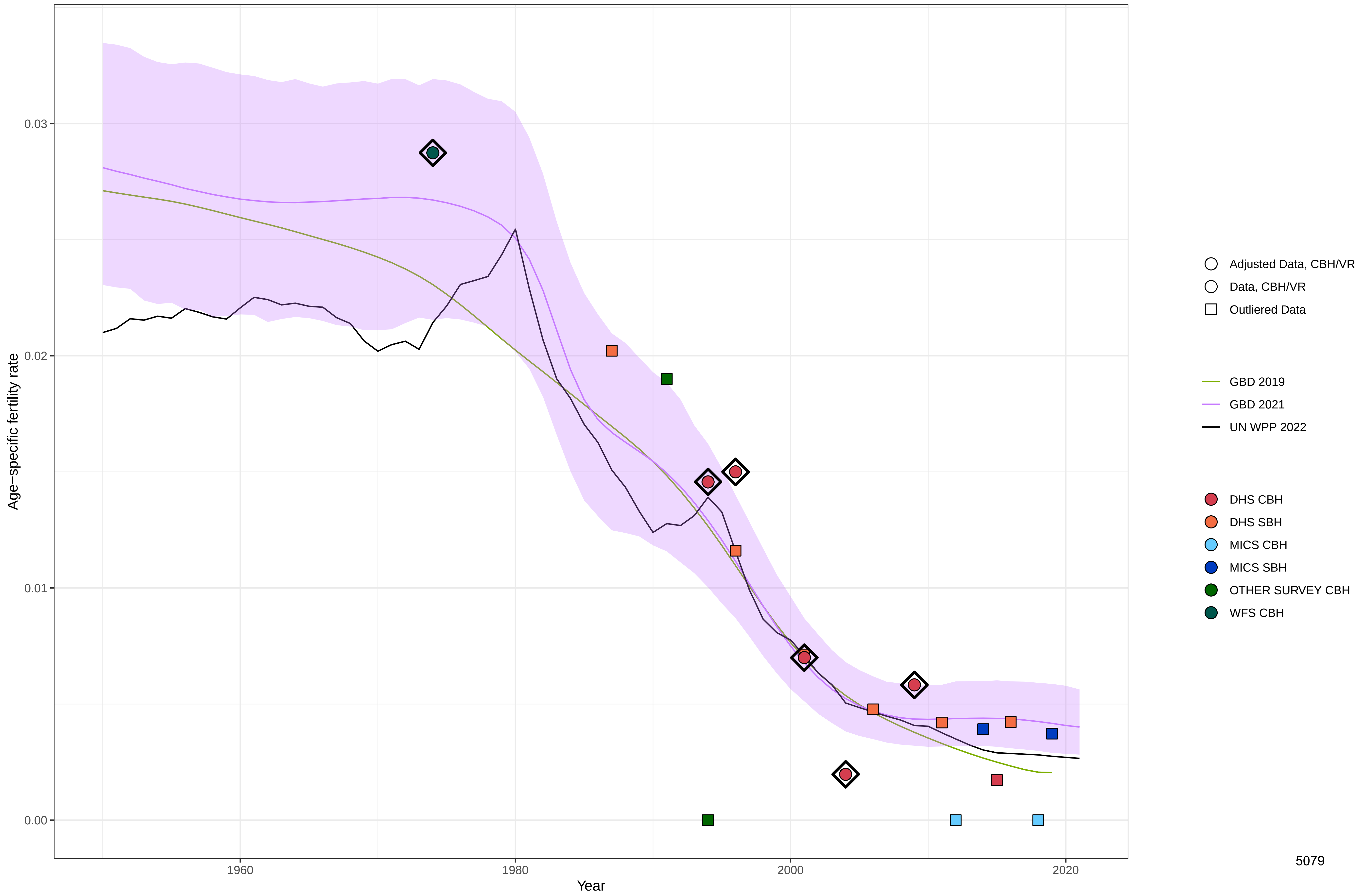

Age−specific fertility fitted trend lines, 1950−2021: Nepal, Ages: 50−54
Data points enclosed with a diamond are reference sources

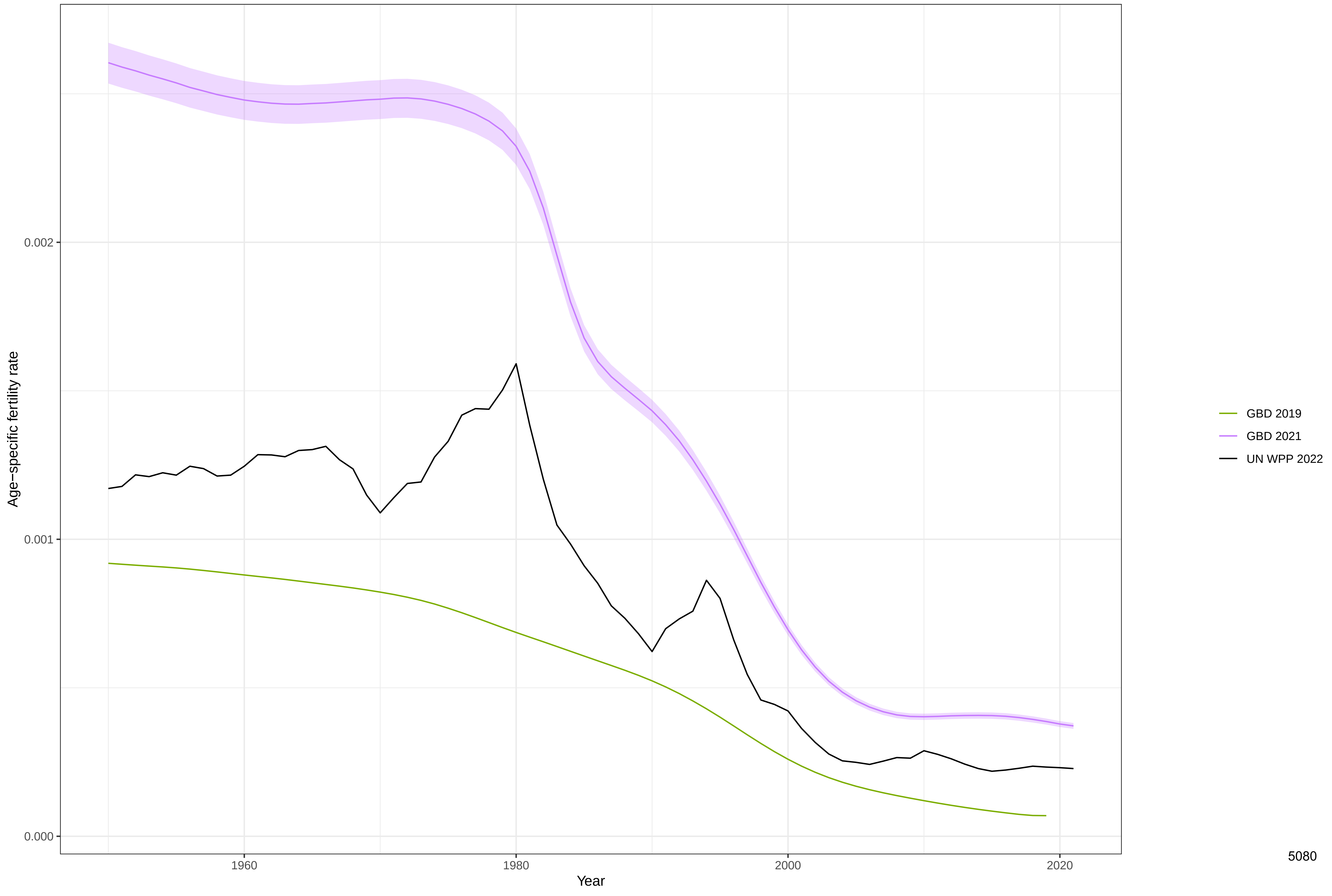

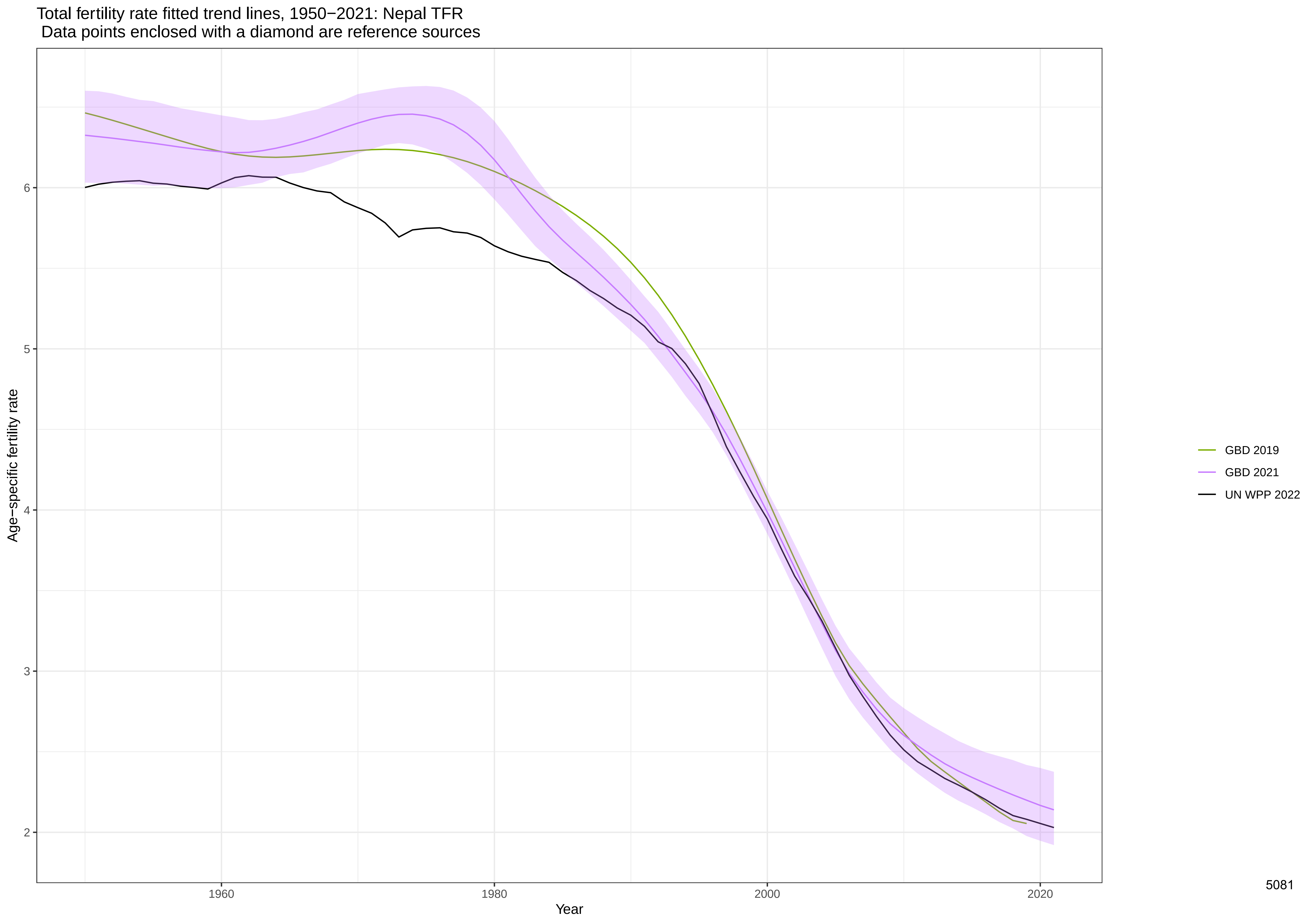
Total fertility rate fitted trend lines, 1950−2021: Nepal TFR
Data points enclosed with a diamond are reference sources
Age−specific fertility rate
Year
6
5
4
3
2
1960
1980
2000
2020
GBD 2019
GBD 2021
UN WPP 2022

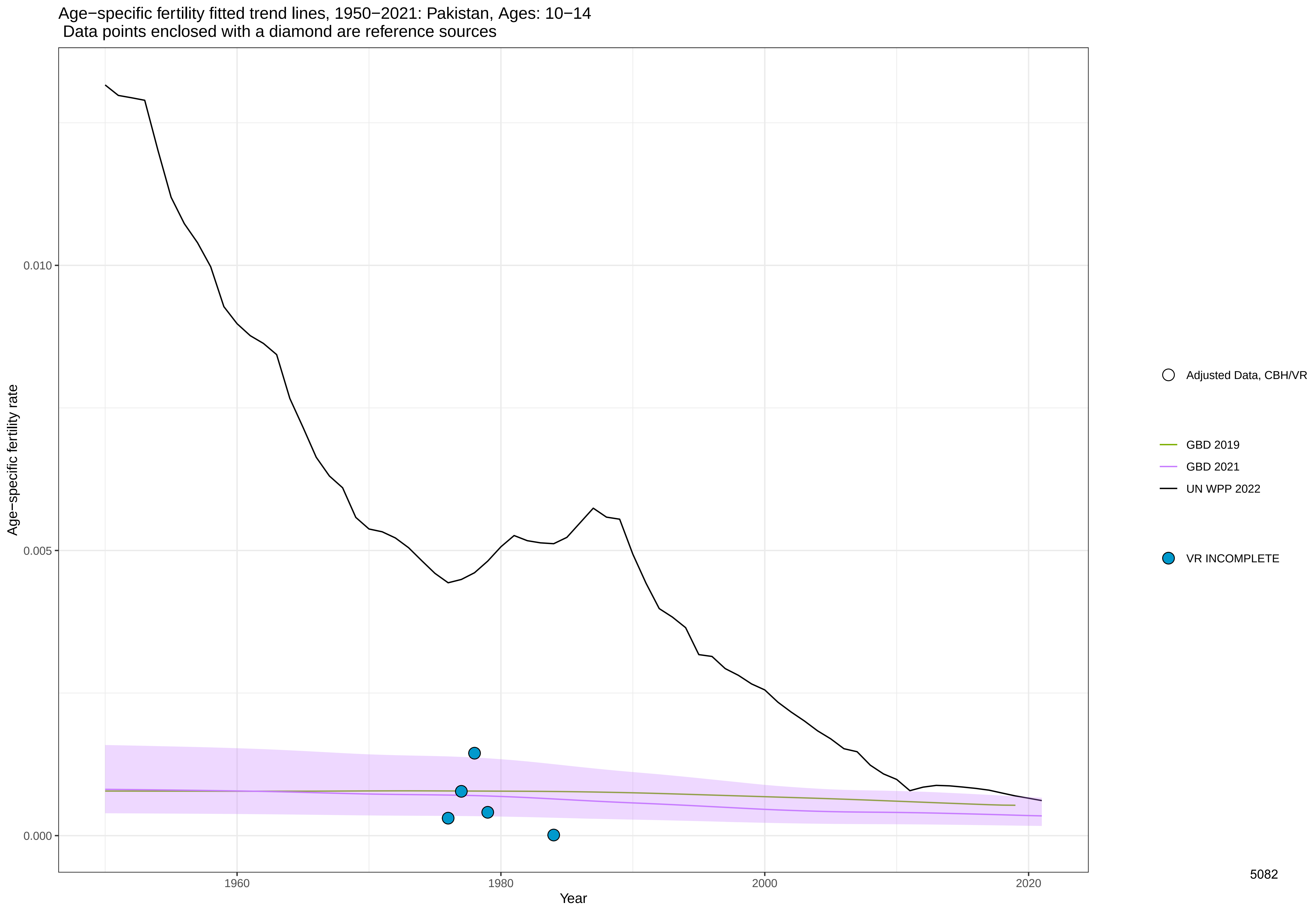

Age−specific fertility fitted trend lines, 1950−2021: Pakistan, Ages: 10−14
Data points enclosed with a diamond are reference sources
0.010
0.005
0.000
Age−specific fertility rate
1960
1980
2000
2020
Year
Adjusted Data, CBH/VR
GBD 2019
GBD 2021
UN WPP 2022
VR INCOMPLETE

Age−specific fertility fitted trend lines, 1950−2021: Pakistan, Ages: 15−19
Data points enclosed with a diamond are reference sources

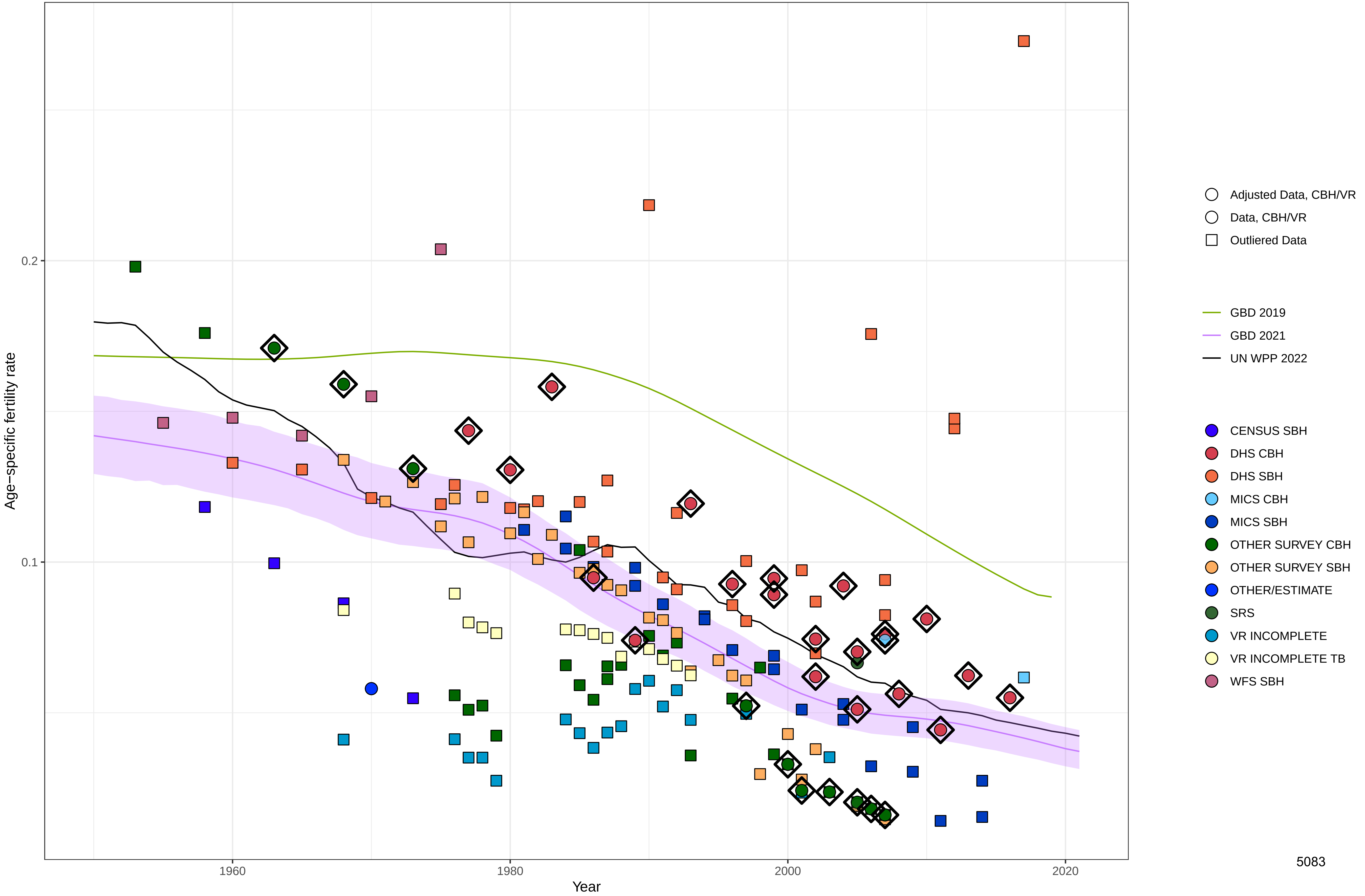

Age−specific fertility fitted trend lines, 1950−2021: Pakistan, Ages: 20−24
Data points enclosed with a diamond are reference sources

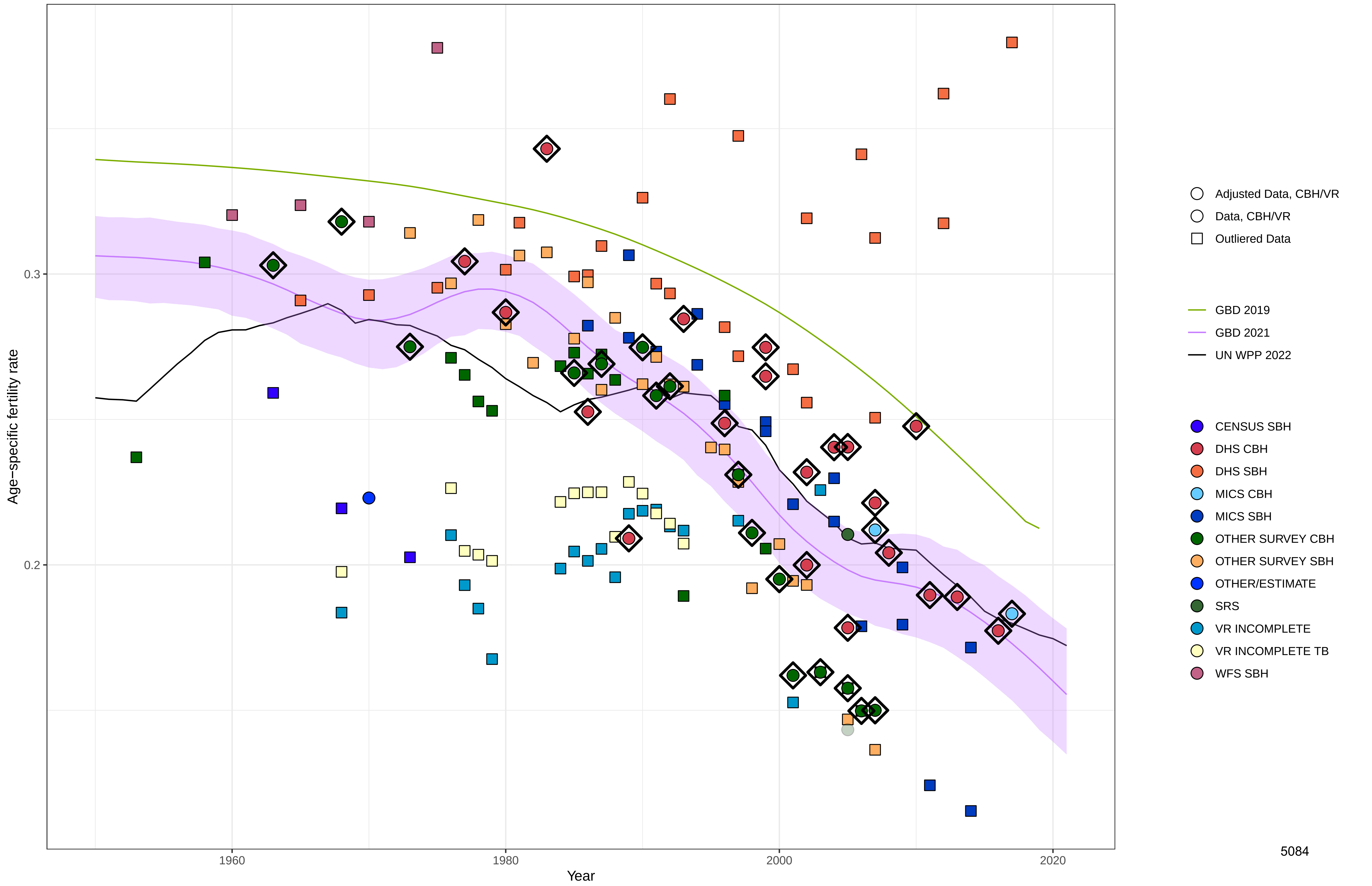

Age−specific fertility fitted trend lines, 1950−2021: Pakistan, Ages: 25−29
Data points enclosed with a diamond are reference sources

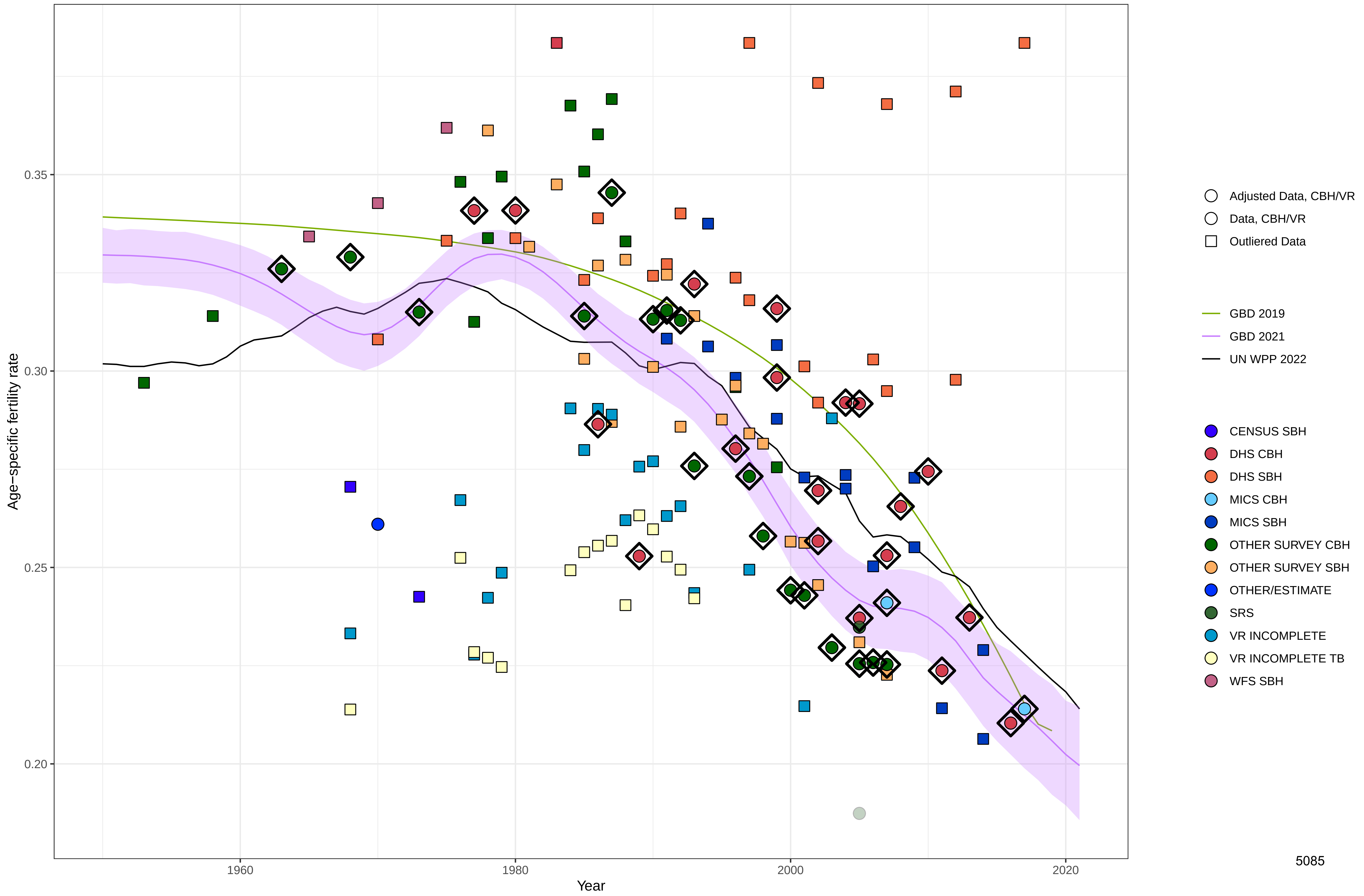

Age−specific fertility fitted trend lines, 1950−2021: Pakistan, Ages: 30−34
Data points enclosed with a diamond are reference sources

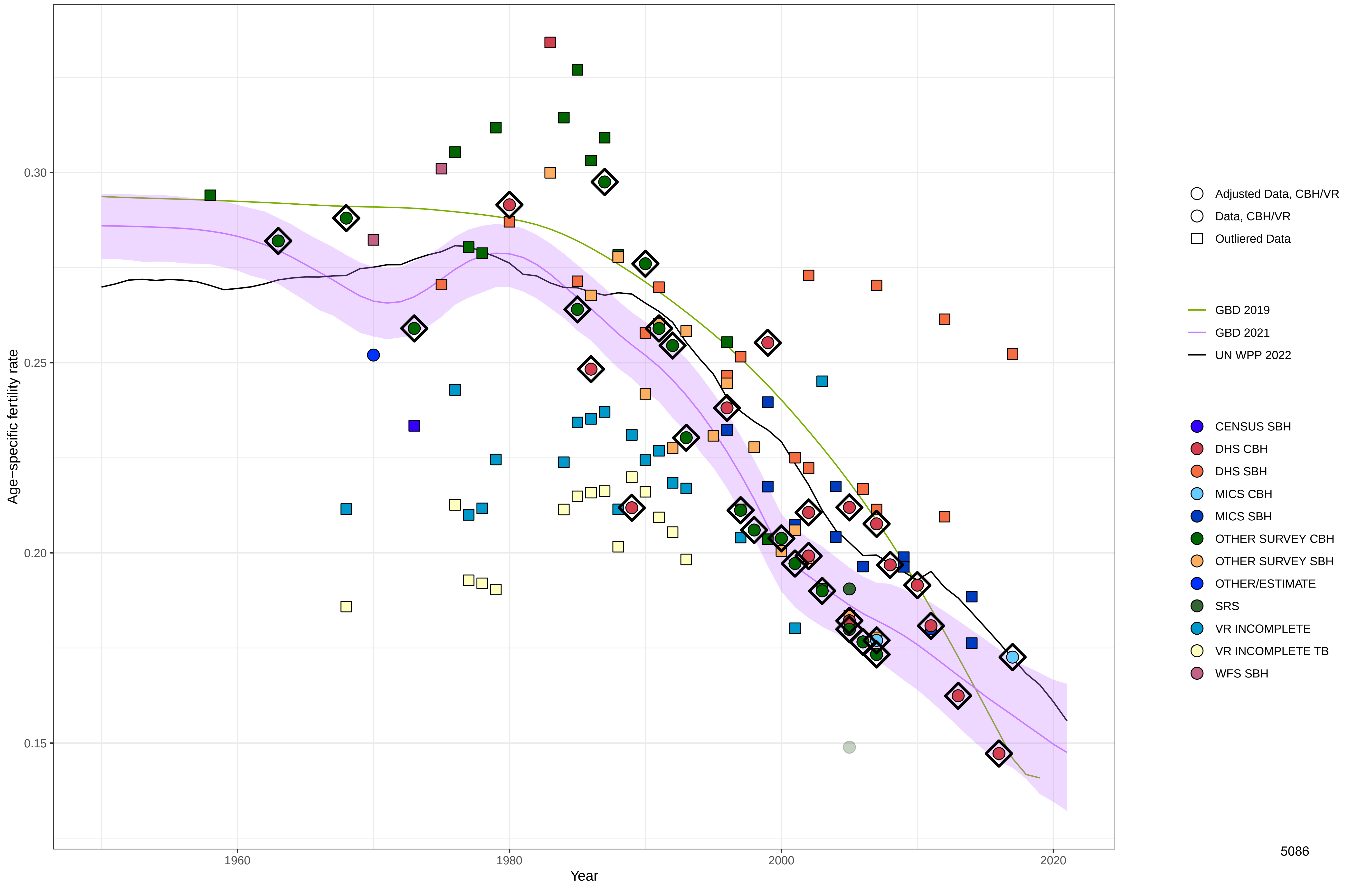

Age−specific fertility fitted trend lines, 1950−2021: Pakistan, Ages: 35−39
Data points enclosed with a diamond are reference sources

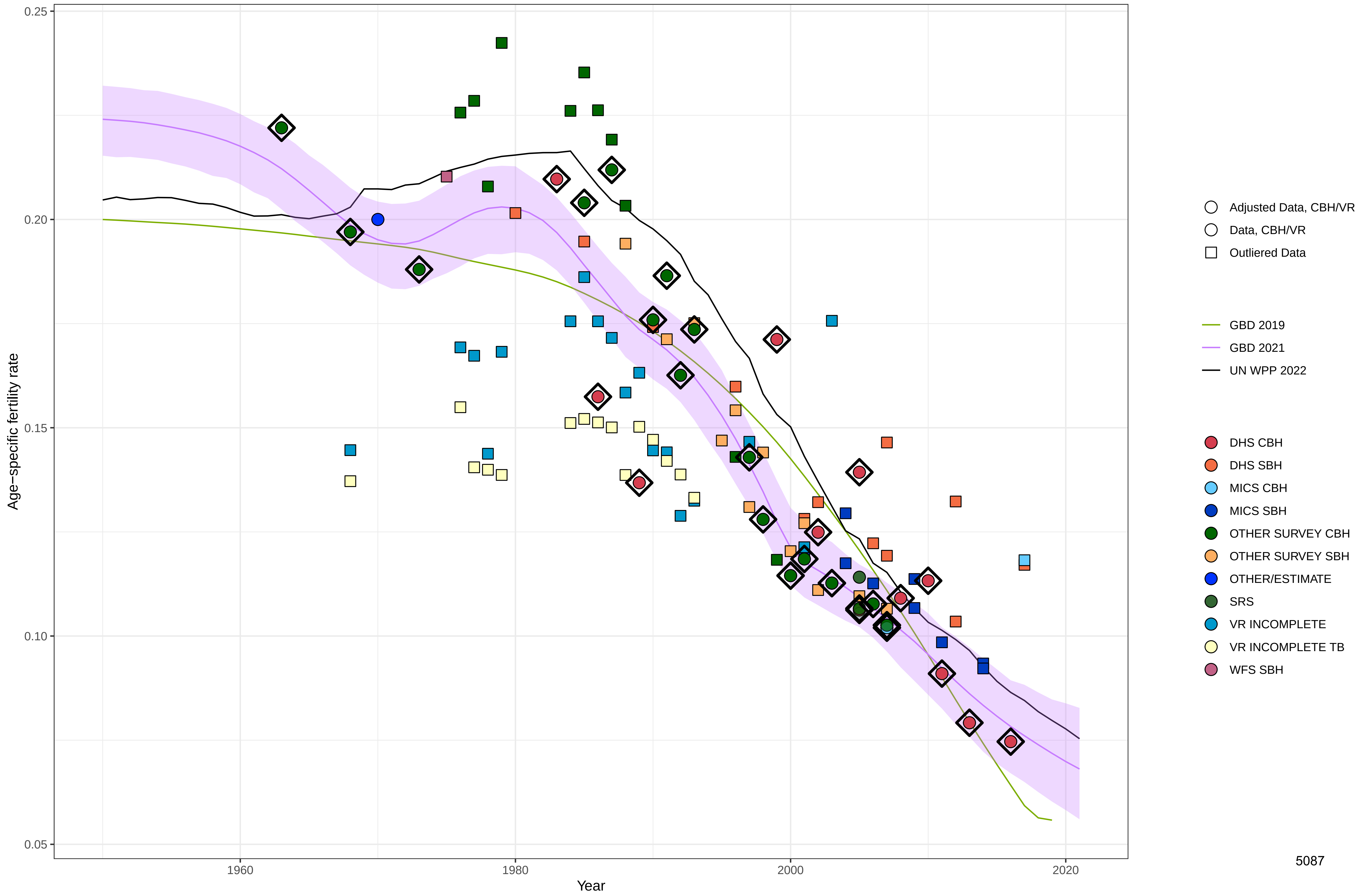

Age−specific fertility fitted trend lines, 1950−2021: Pakistan, Ages: 40−44
Data points enclosed with a diamond are reference sources

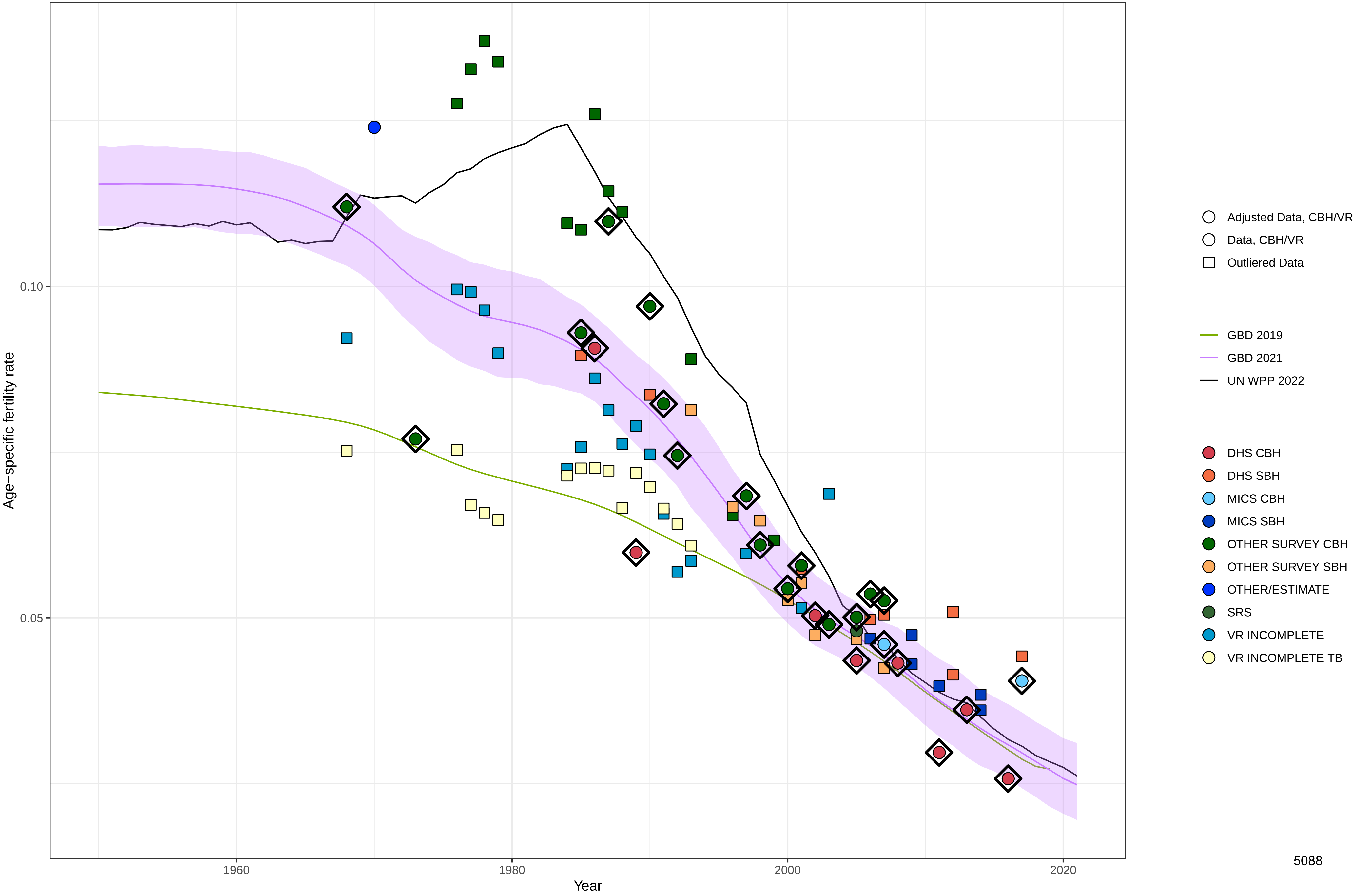

Age−specific fertility fitted trend lines, 1950−2021: Pakistan, Ages: 45−49
Data points enclosed with a diamond are reference sources

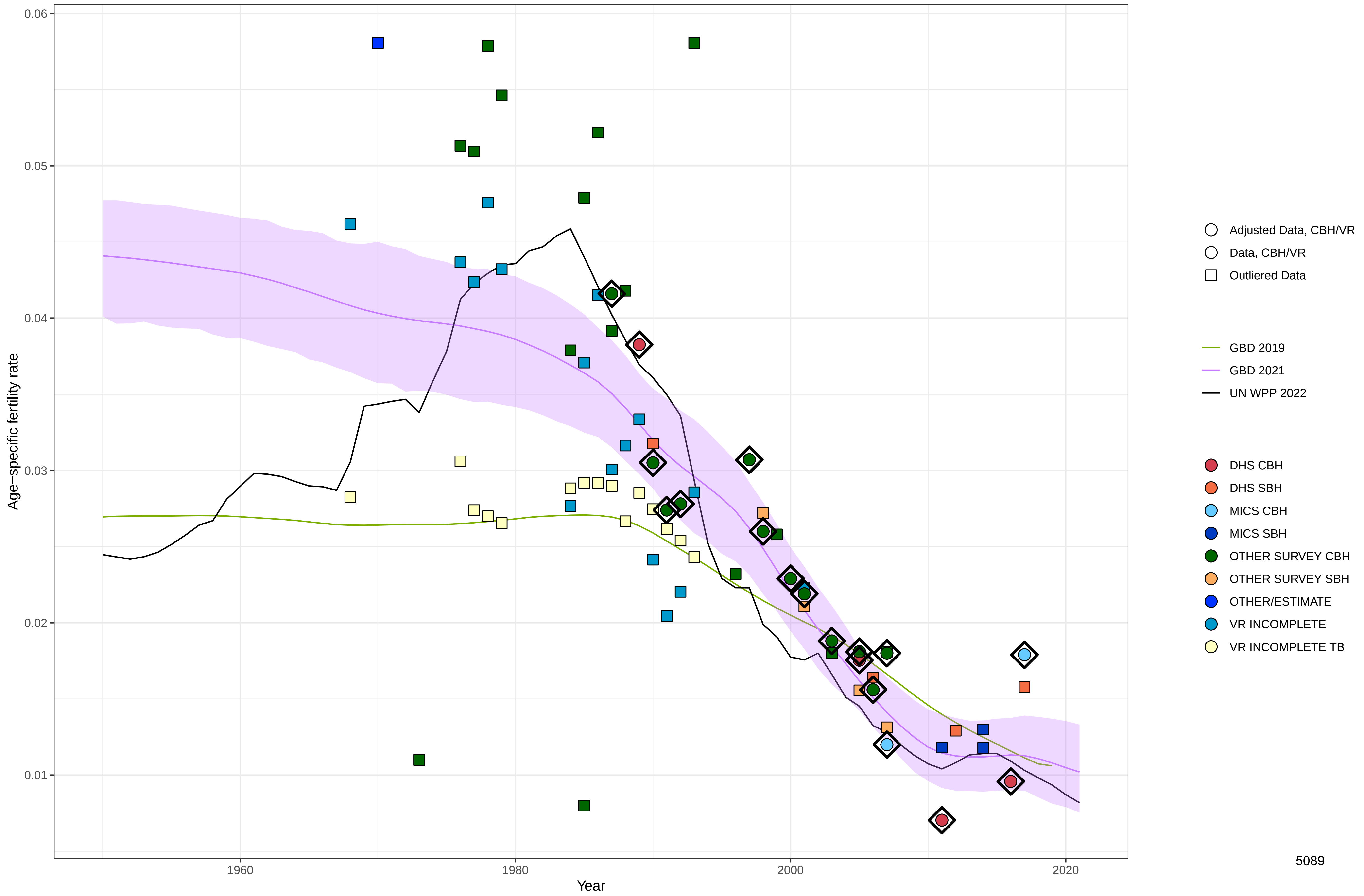

Age−specific fertility fitted trend lines, 1950−2021: Pakistan, Ages: 50−54
Data points enclosed with a diamond are reference sources

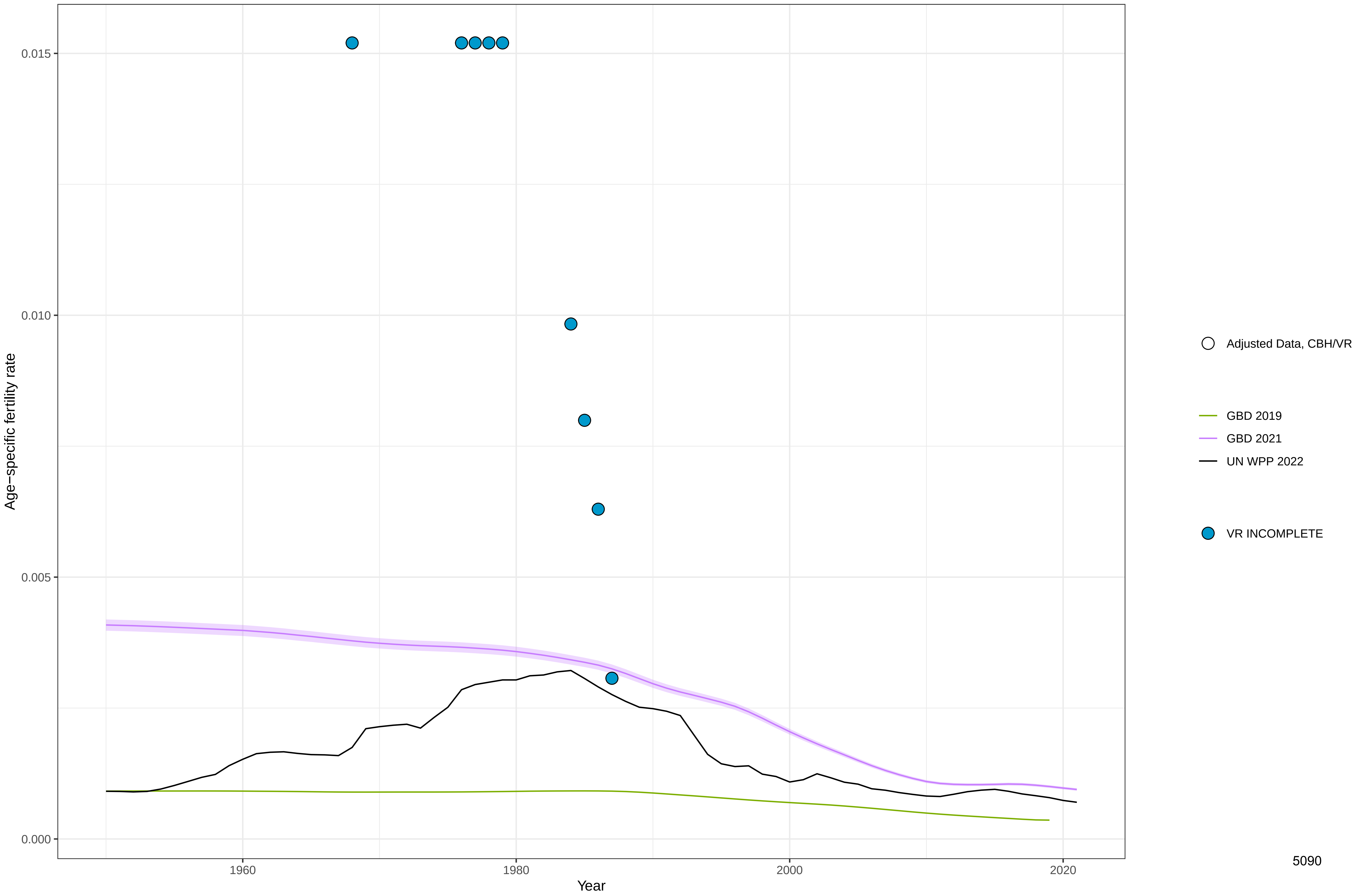

Total fertility rate fitted trend lines, 1950−2021: Pakistan TFR
Data points enclosed with a diamond are reference sources

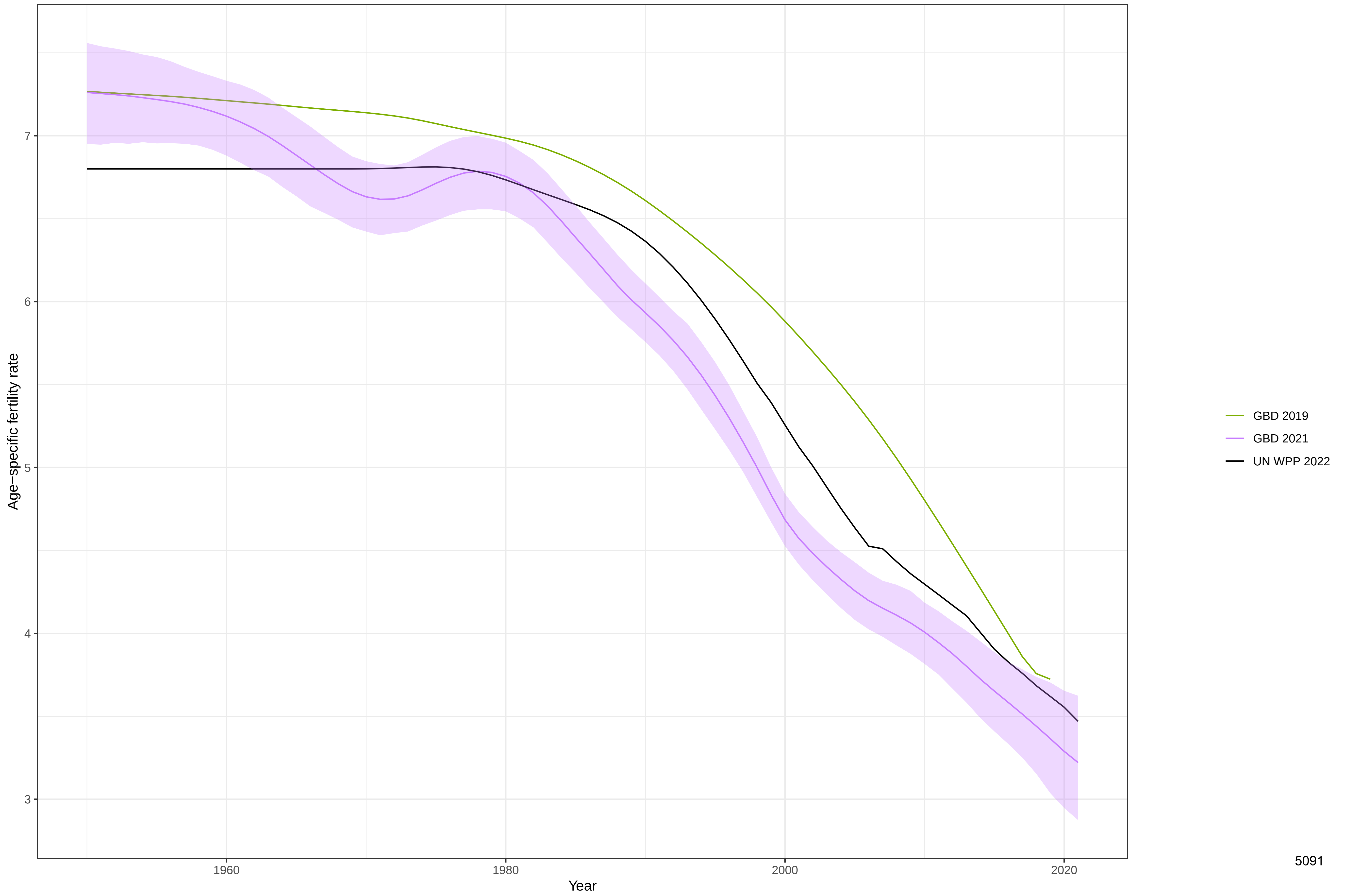

Age−specific fertility fitted trend lines, 1950−2021: Azad Jammu & Kashmir, Ages: 10−14
Data points enclosed with a diamond are reference sources

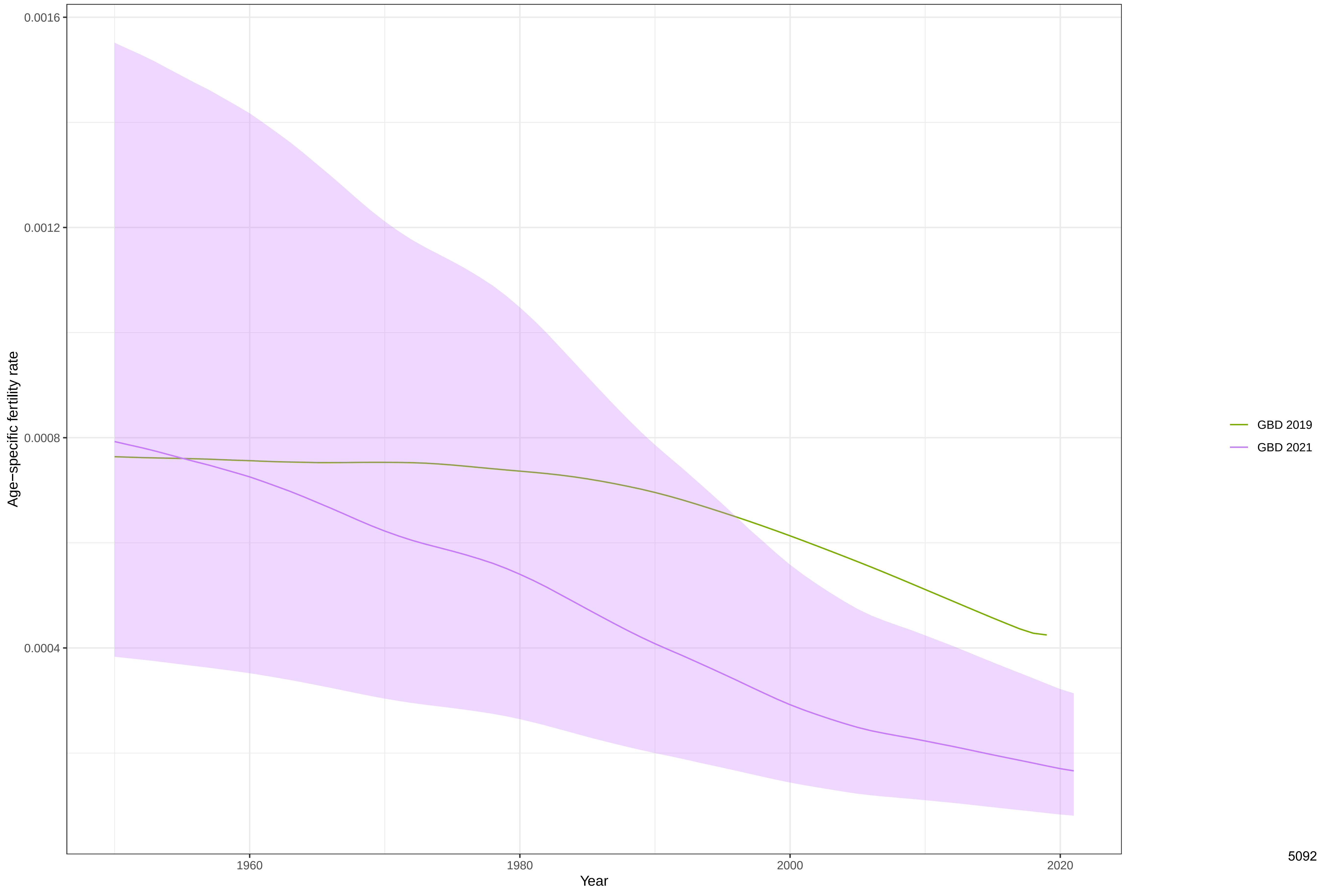

5092

Age−specific fertility fitted trend lines, 1950−2021: Azad Jammu & Kashmir, Ages: 15−19
Data points enclosed with a diamond are reference sources

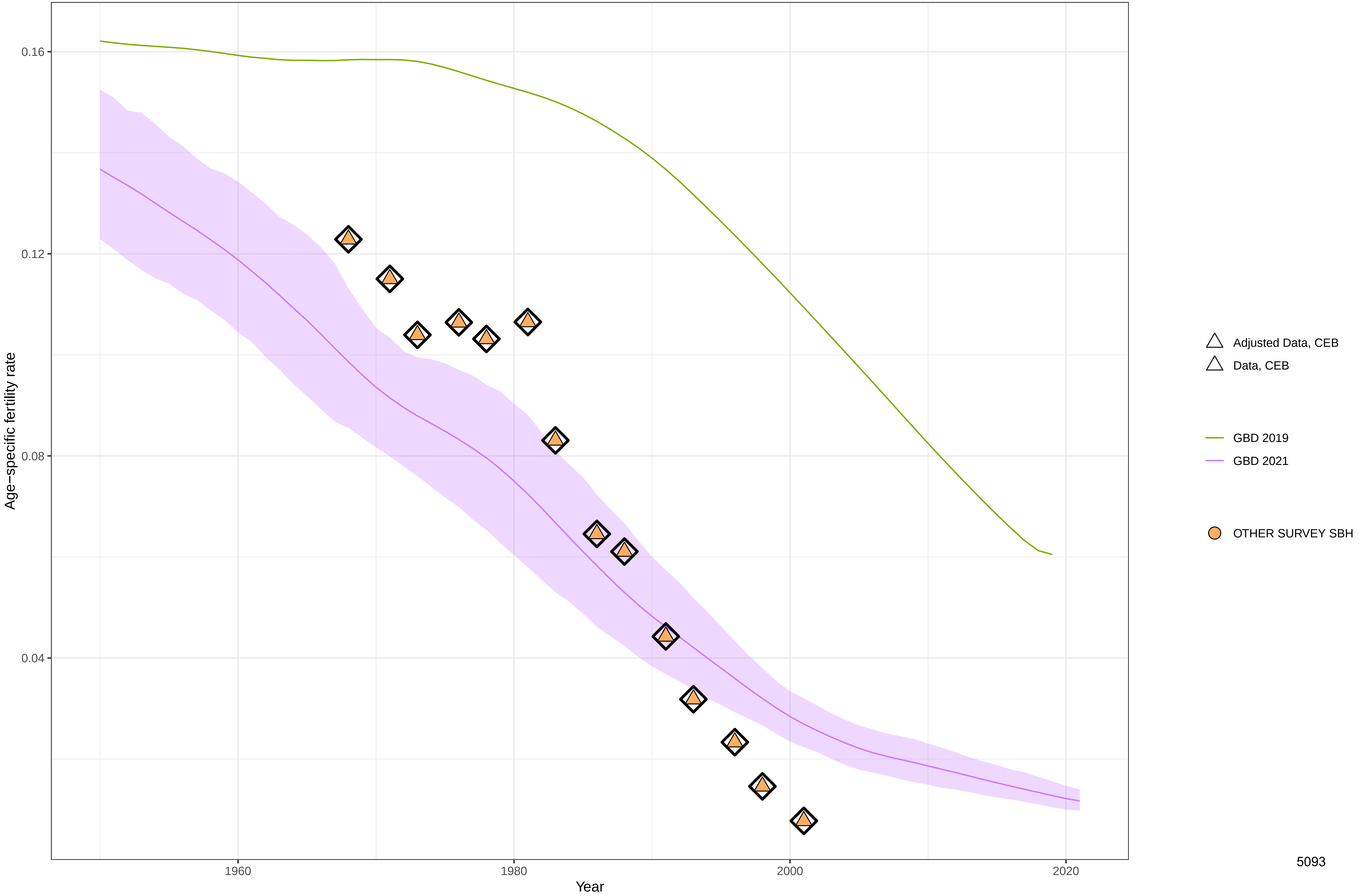

Age−specific fertility fitted trend lines, 1950−2021: Azad Jammu & Kashmir, Ages: 20−24
Data points enclosed with a diamond are reference sources

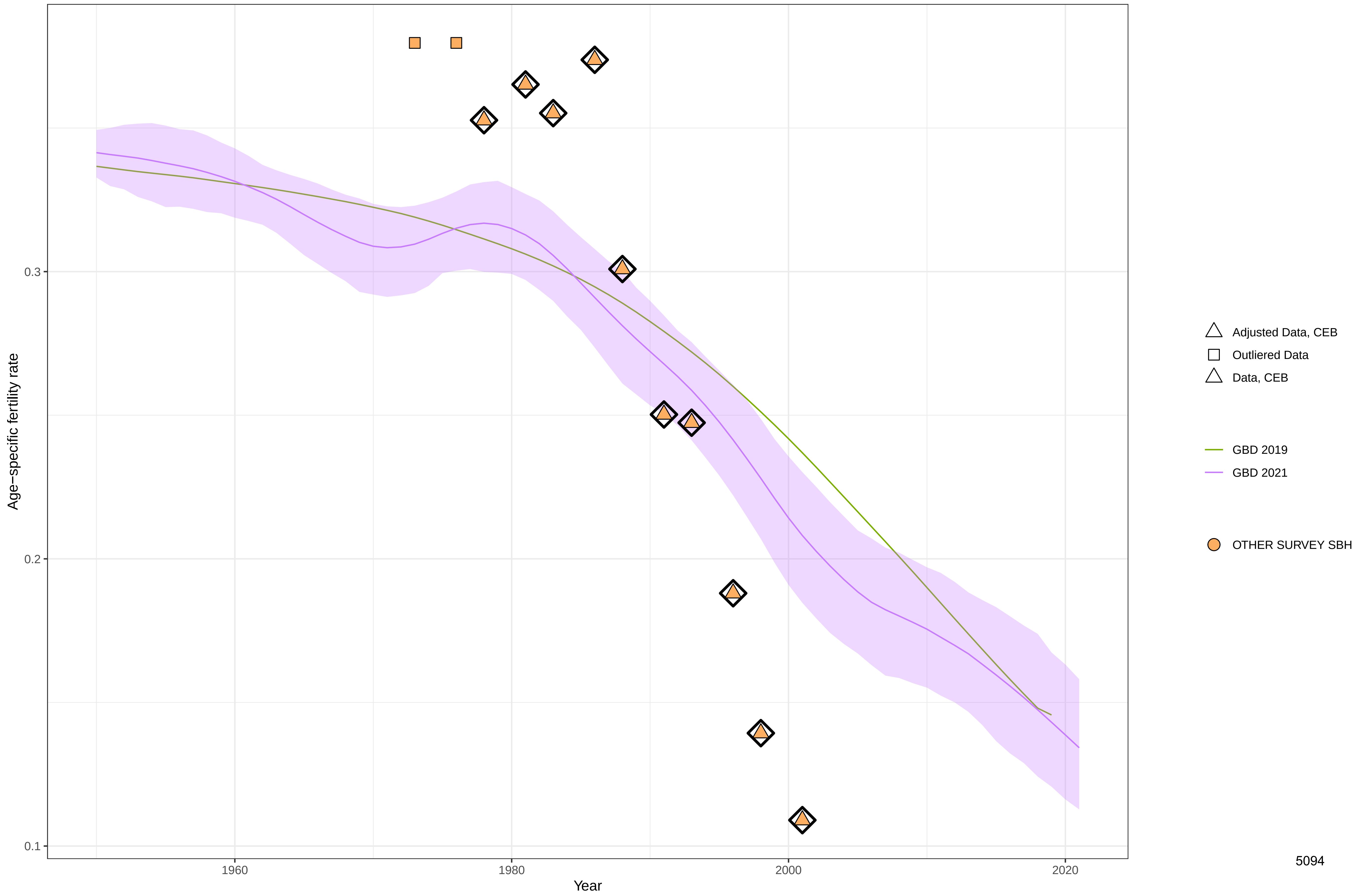

Age−specific fertility fitted trend lines, 1950−2021: Azad Jammu & Kashmir, Ages: 25−29
Data points enclosed with a diamond are reference sources

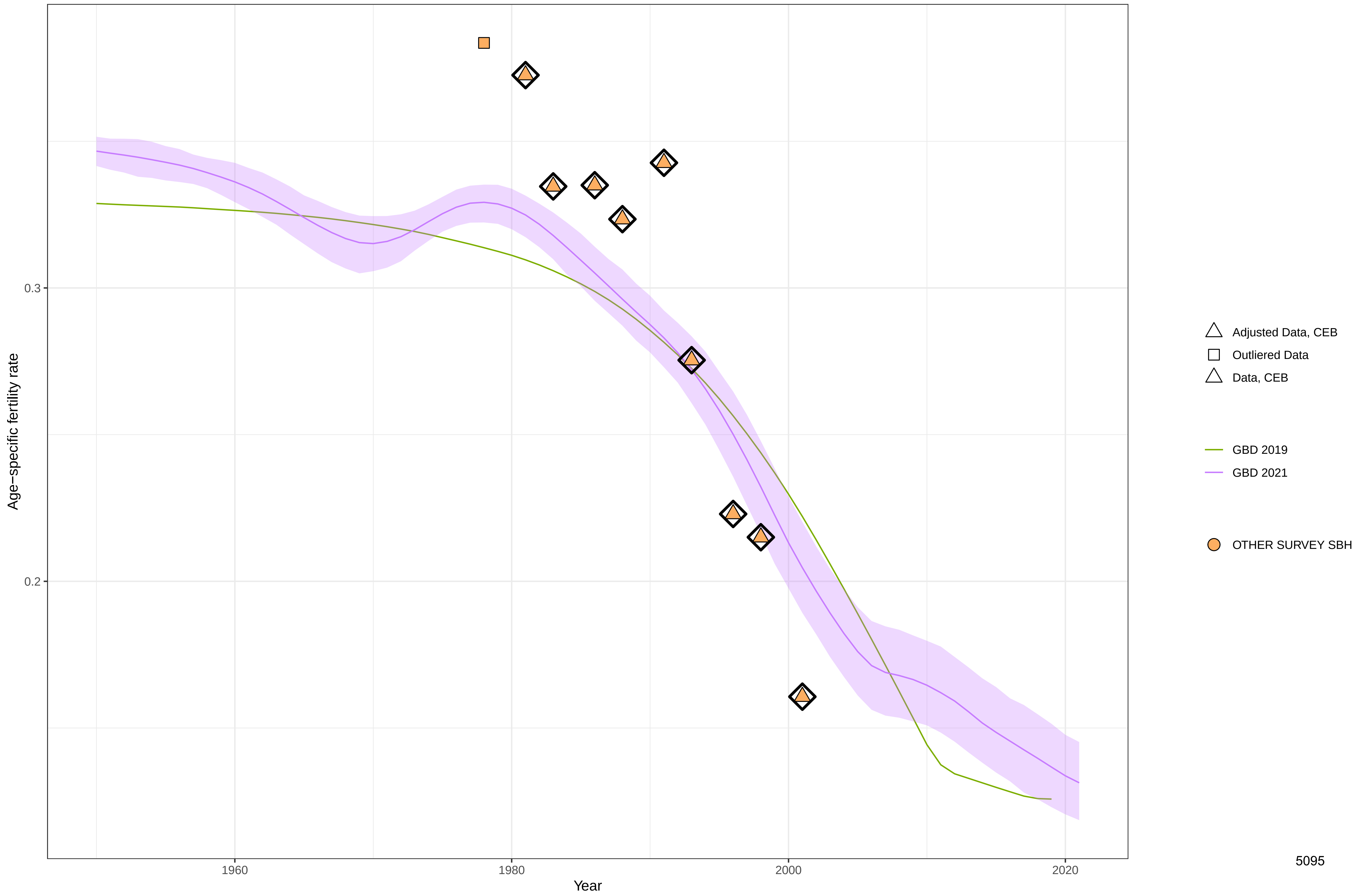

Age−specific fertility fitted trend lines, 1950−2021: Azad Jammu & Kashmir, Ages: 30−34
Data points enclosed with a diamond are reference sources

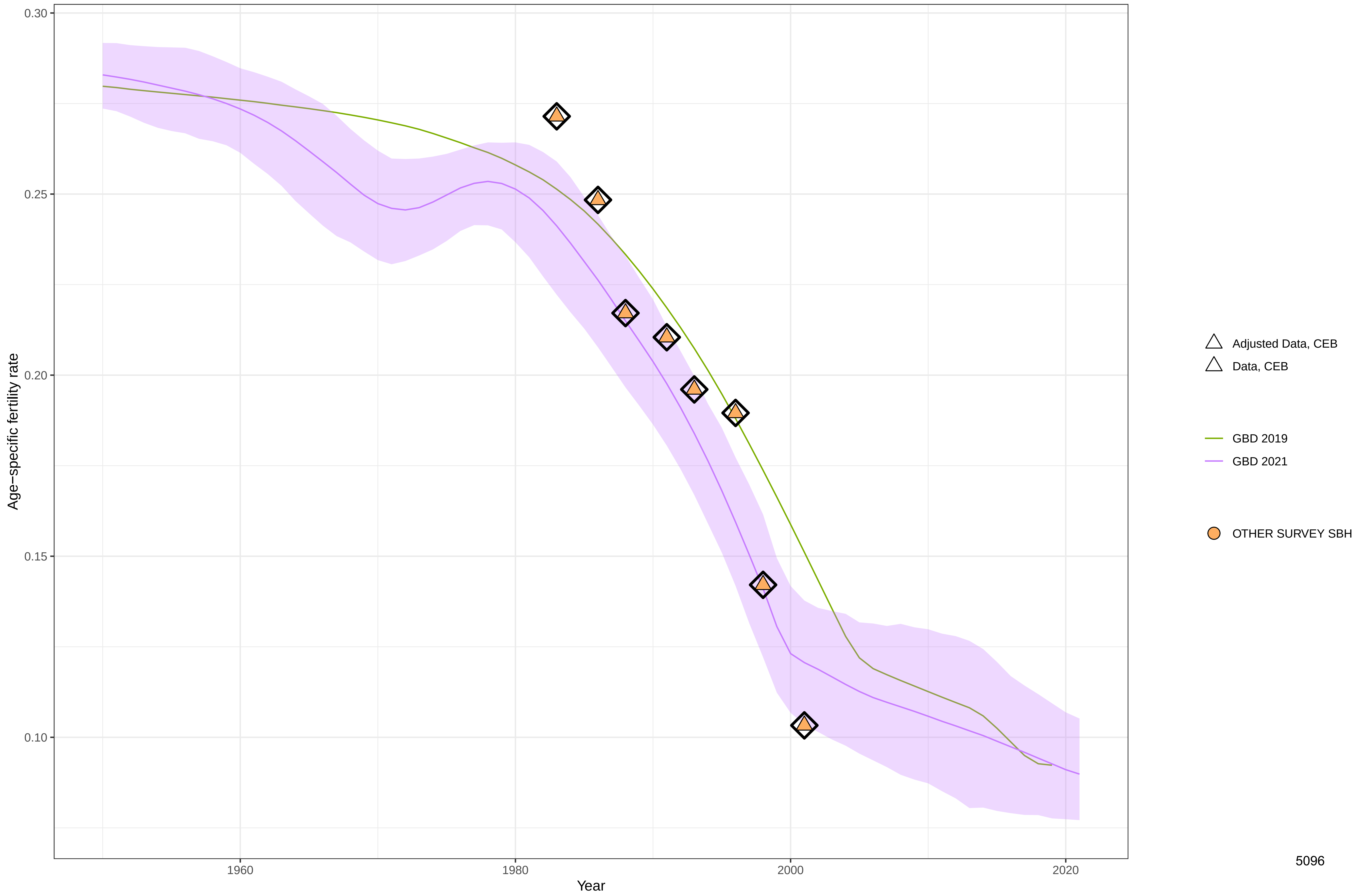

Age−specific fertility fitted trend lines, 1950−2021: Azad Jammu & Kashmir, Ages: 35−39
Data points enclosed with a diamond are reference sources

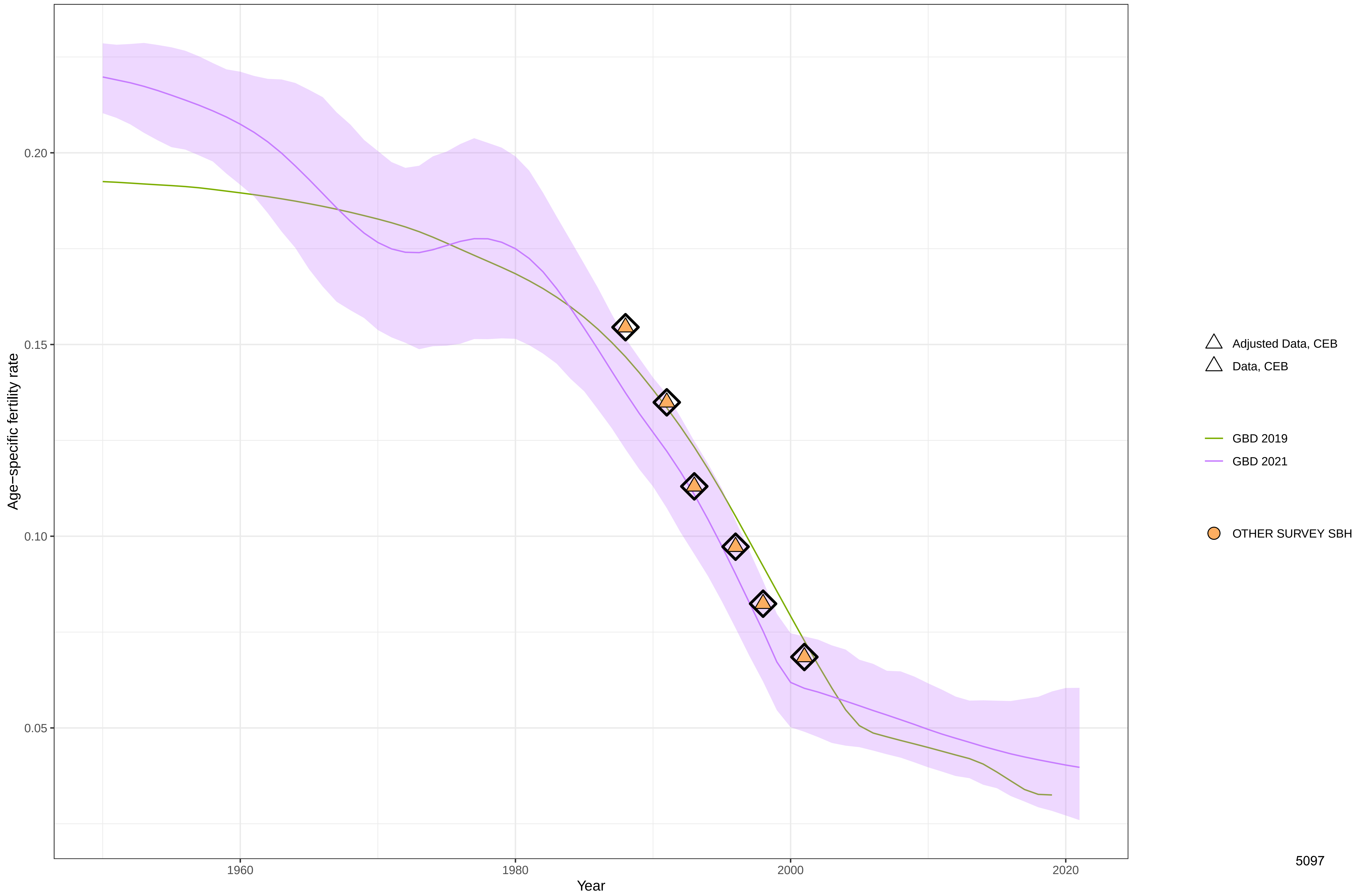

Age−specific fertility fitted trend lines, 1950−2021: Azad Jammu & Kashmir, Ages: 40−44
Data points enclosed with a diamond are reference sources

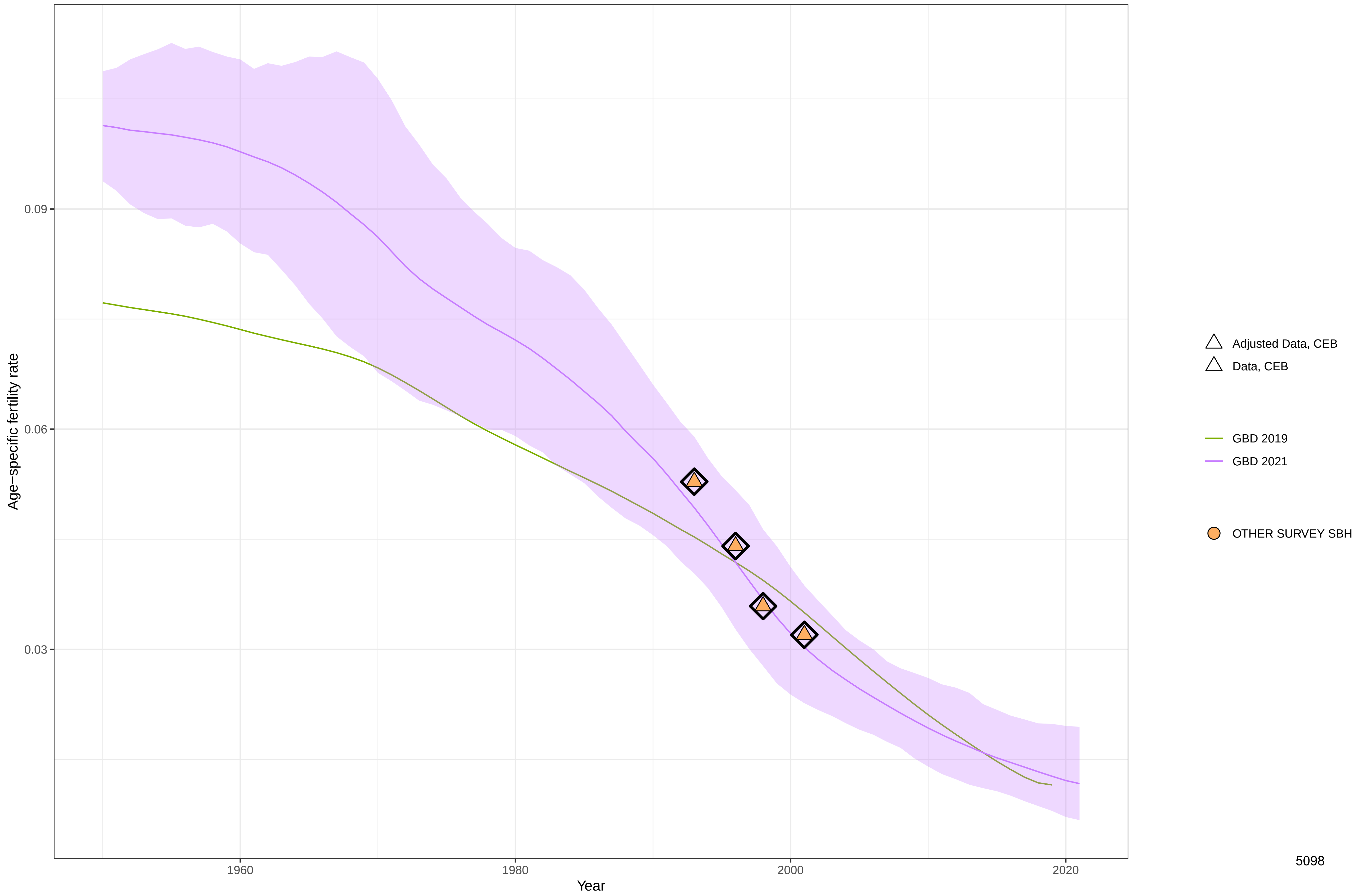

Age−specific fertility fitted trend lines, 1950−2021: Azad Jammu & Kashmir, Ages: 45−49
Data points enclosed with a diamond are reference sources

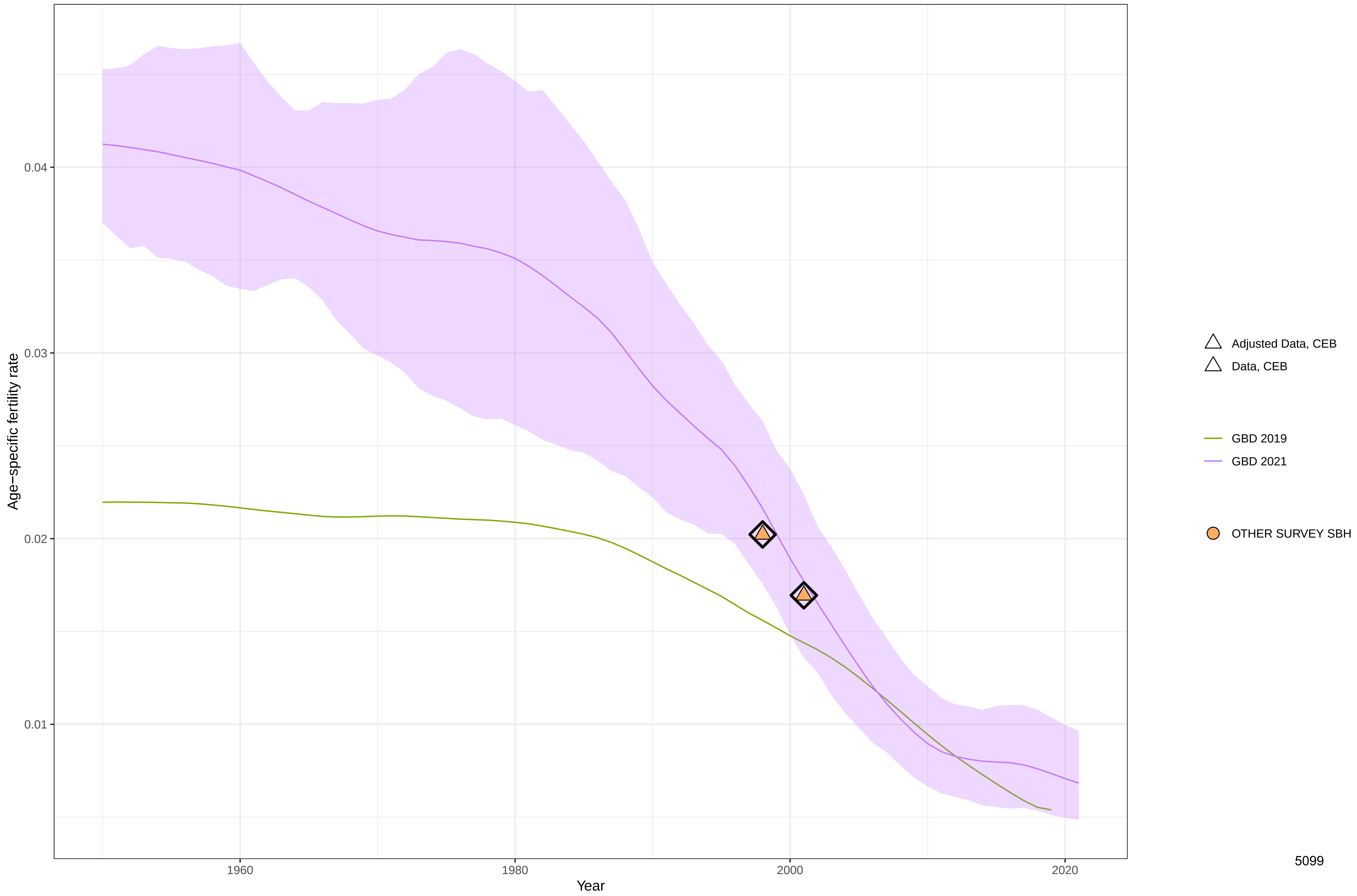

Age−specific fertility fitted trend lines, 1950−2021: Azad Jammu & Kashmir, Ages: 50−54
Data points enclosed with a diamond are reference sources

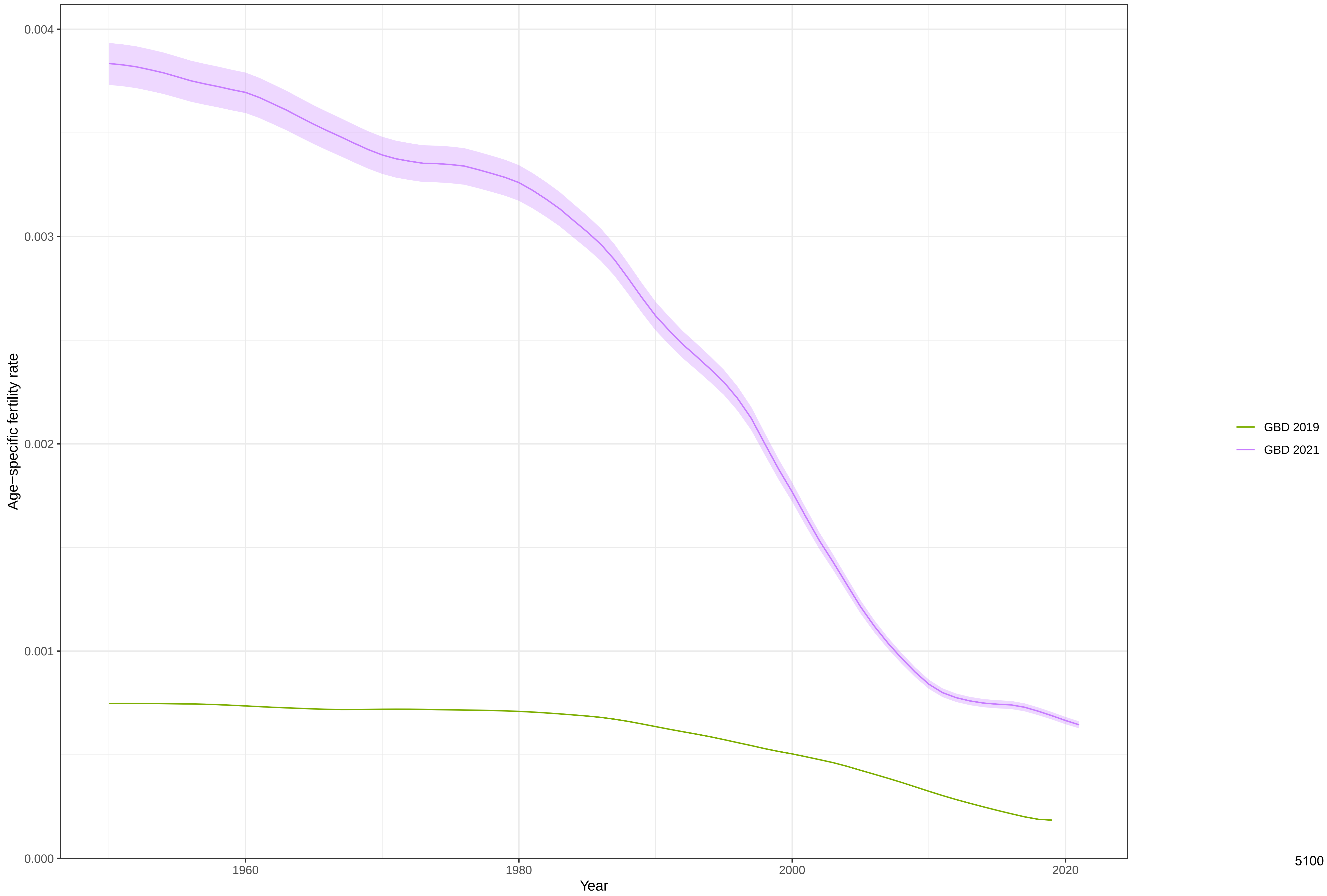

Total fertility rate fitted trend lines, 1950−2021: Azad Jammu & Kashmir TFR
Data points enclosed with a diamond are reference sources

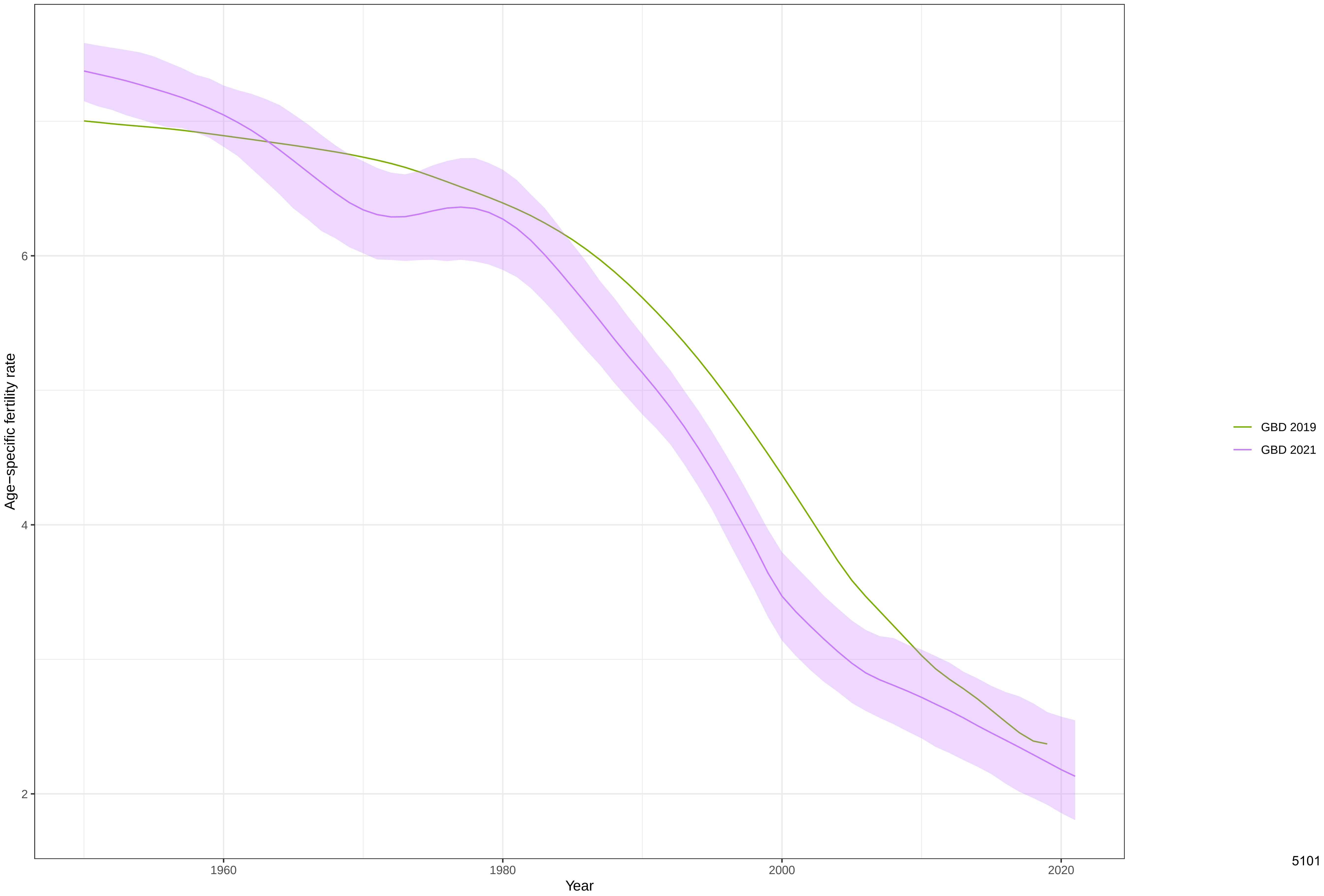

Age−specific fertility fitted trend lines, 1950−2021: Balochistan, Ages: 10−14
Data points enclosed with a diamond are reference sources

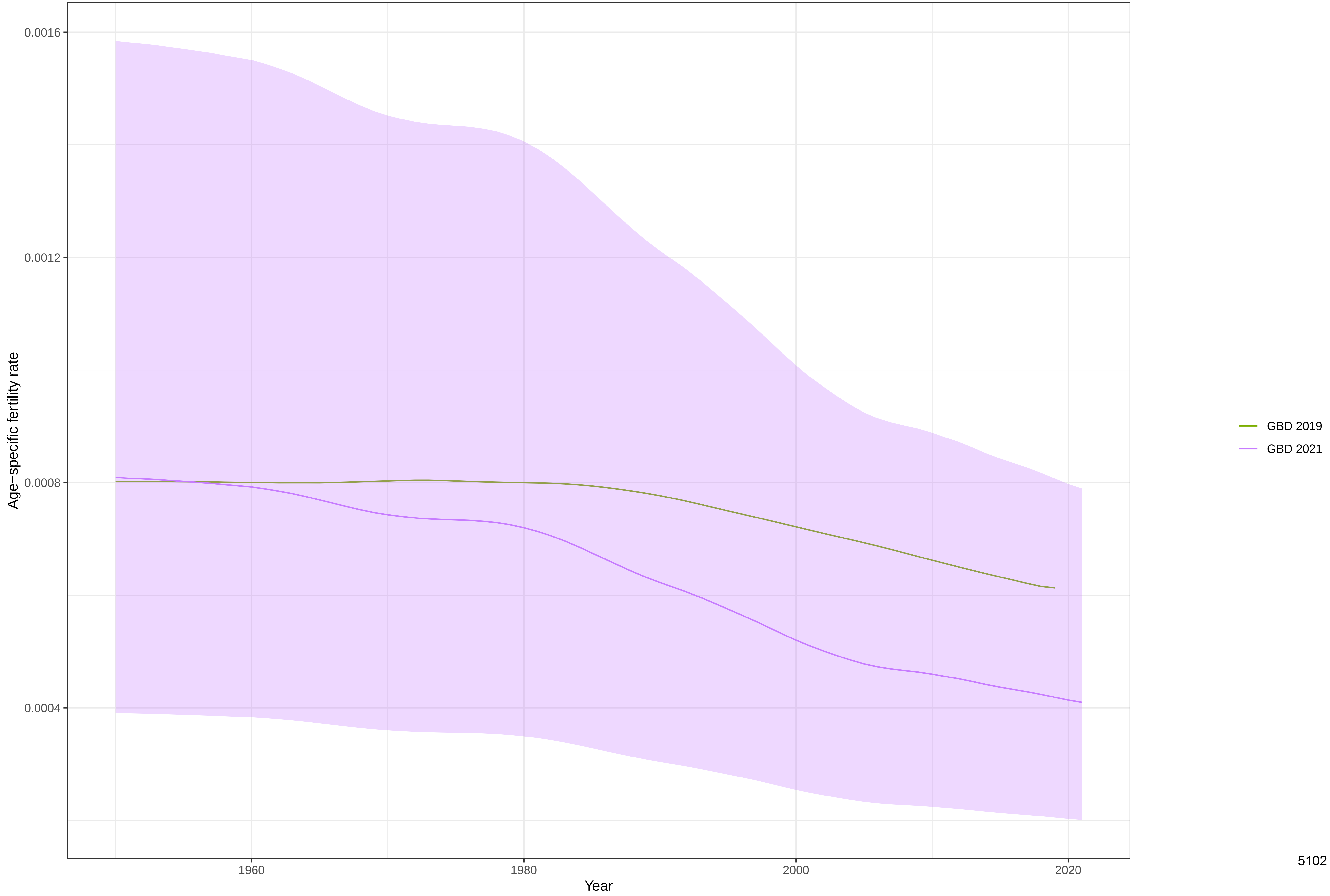

Age−specific fertility fitted trend lines, 1950−2021: Balochistan, Ages: 15−19
Data points enclosed with a diamond are reference sources

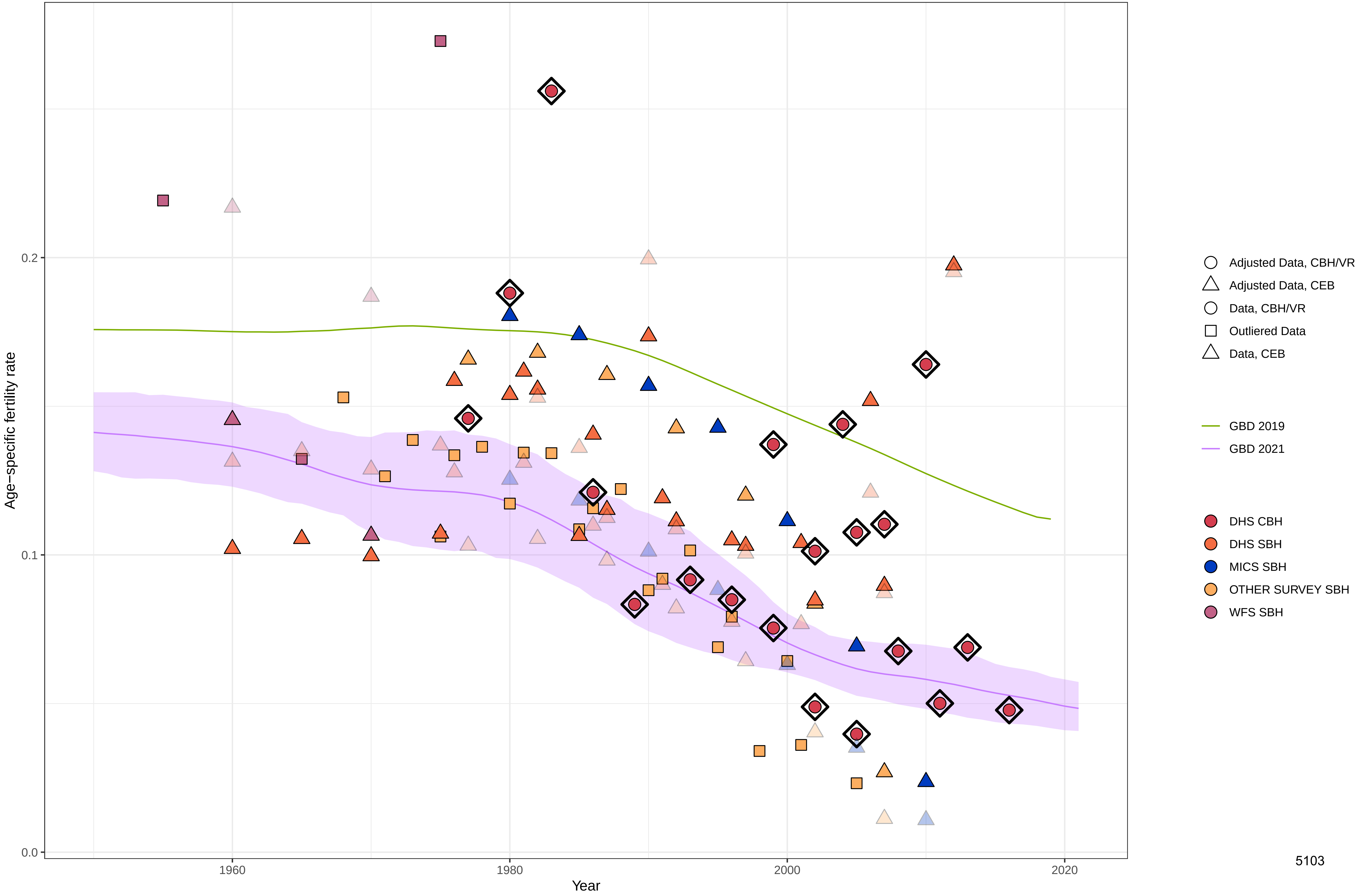

Age−specific fertility fitted trend lines, 1950−2021: Balochistan, Ages: 20−24
Data points enclosed with a diamond are reference sources

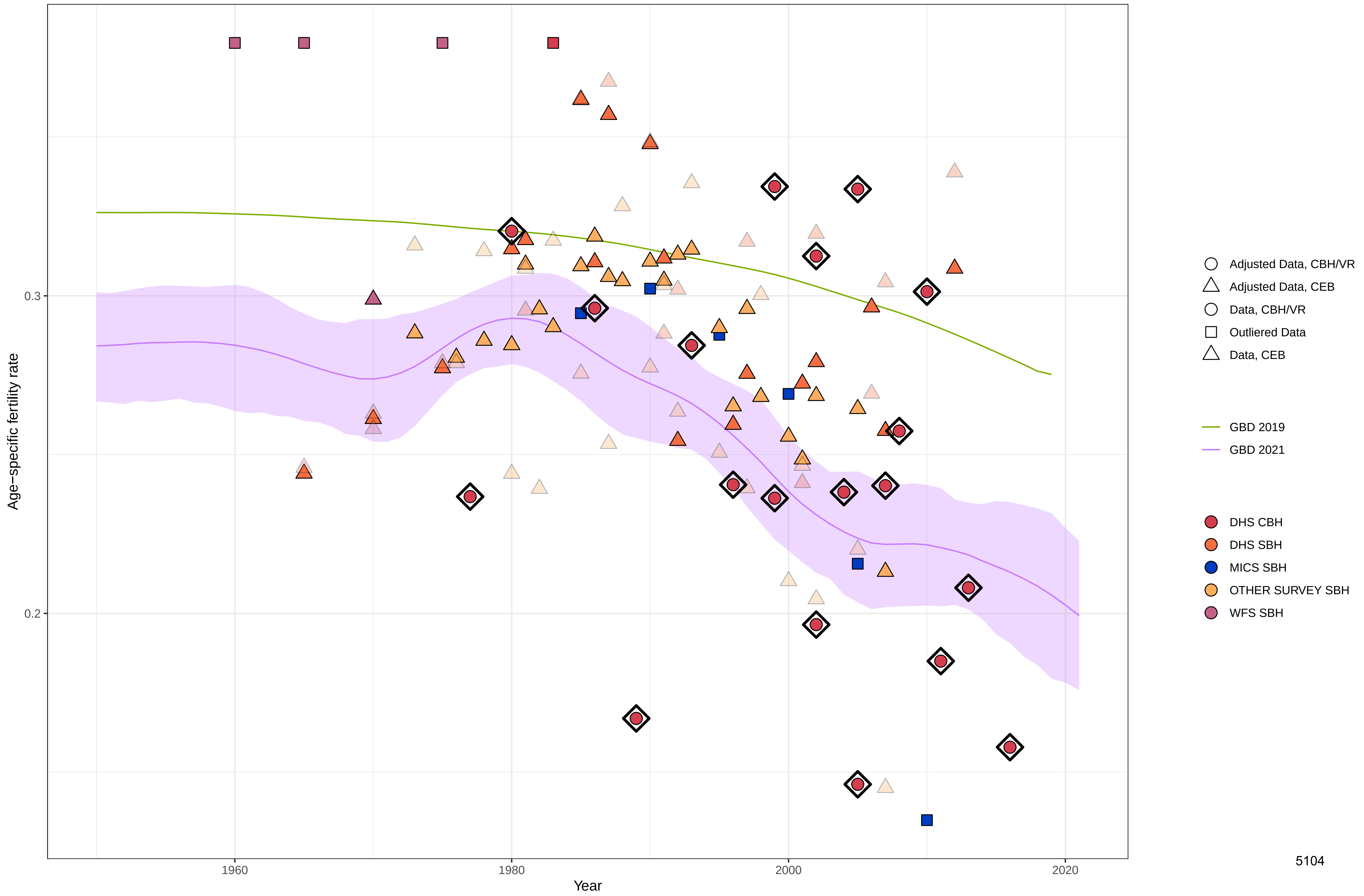

Age−specific fertility fitted trend lines, 1950−2021: Balochistan, Ages: 25−29
Data points enclosed with a diamond are reference sources

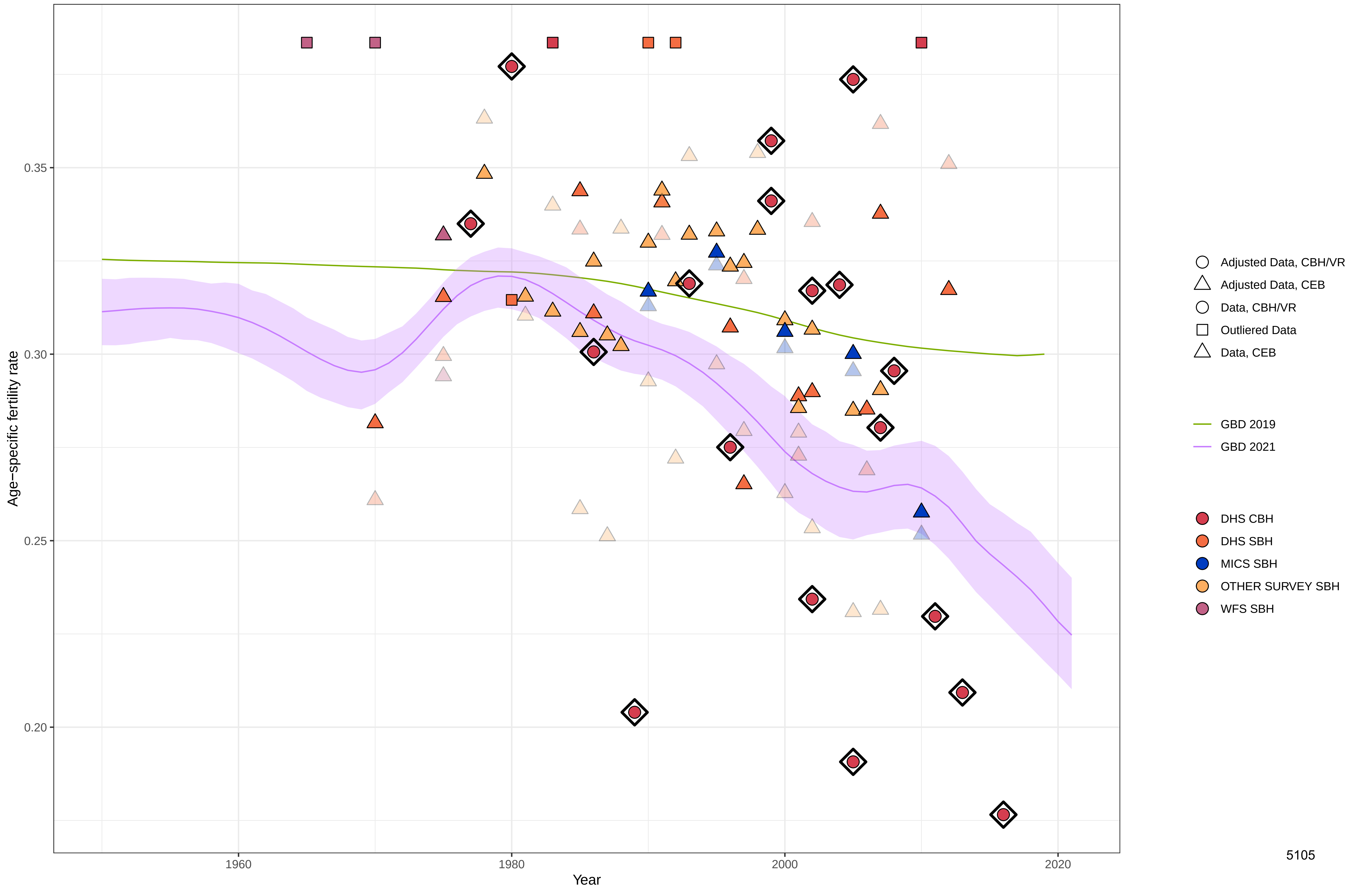

Age−specific fertility fitted trend lines, 1950−2021: Balochistan, Ages: 30−34
Data points enclosed with a diamond are reference sources

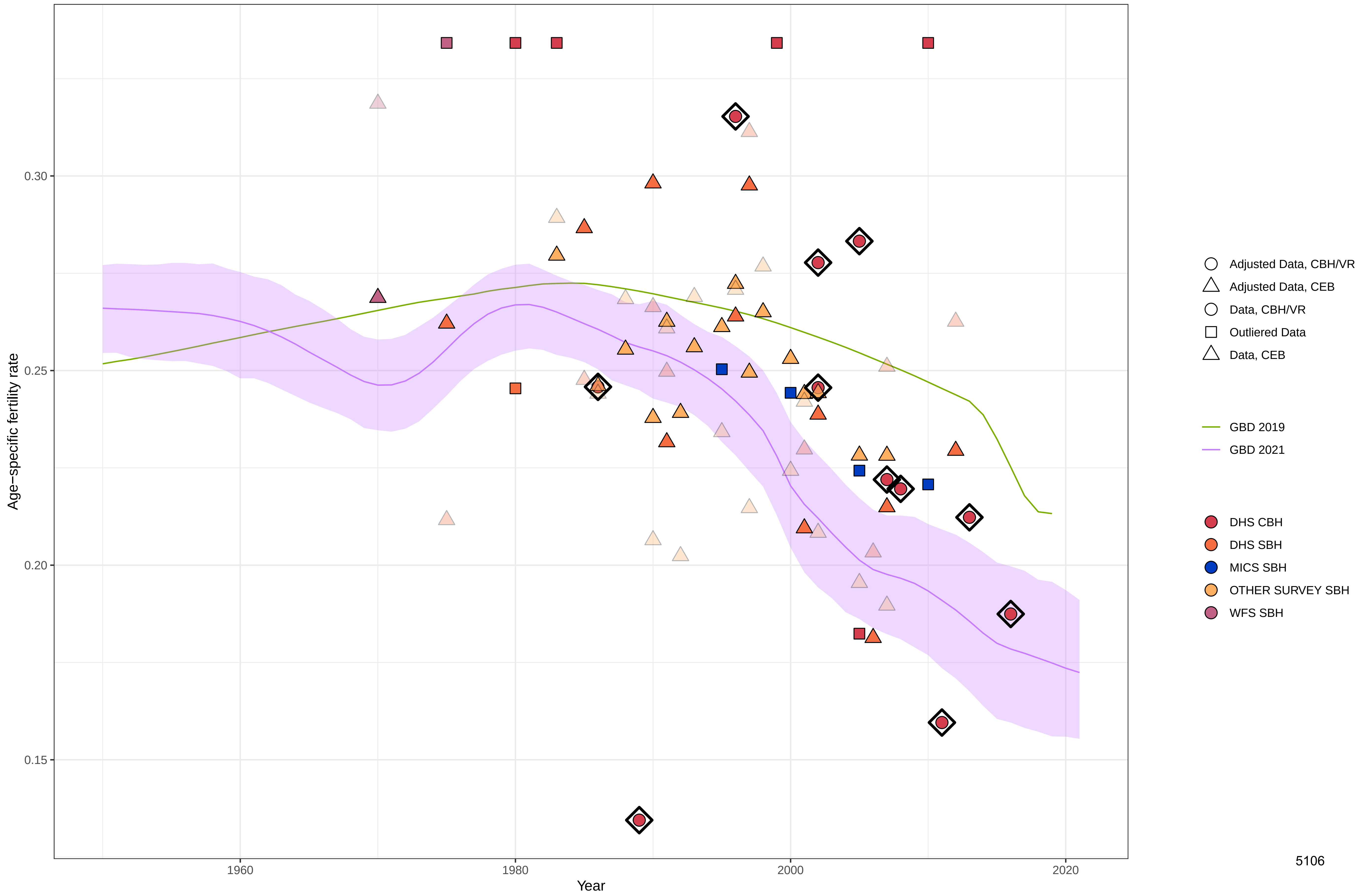

Age−specific fertility fitted trend lines, 1950−2021: Balochistan, Ages: 35−39
Data points enclosed with a diamond are reference sources

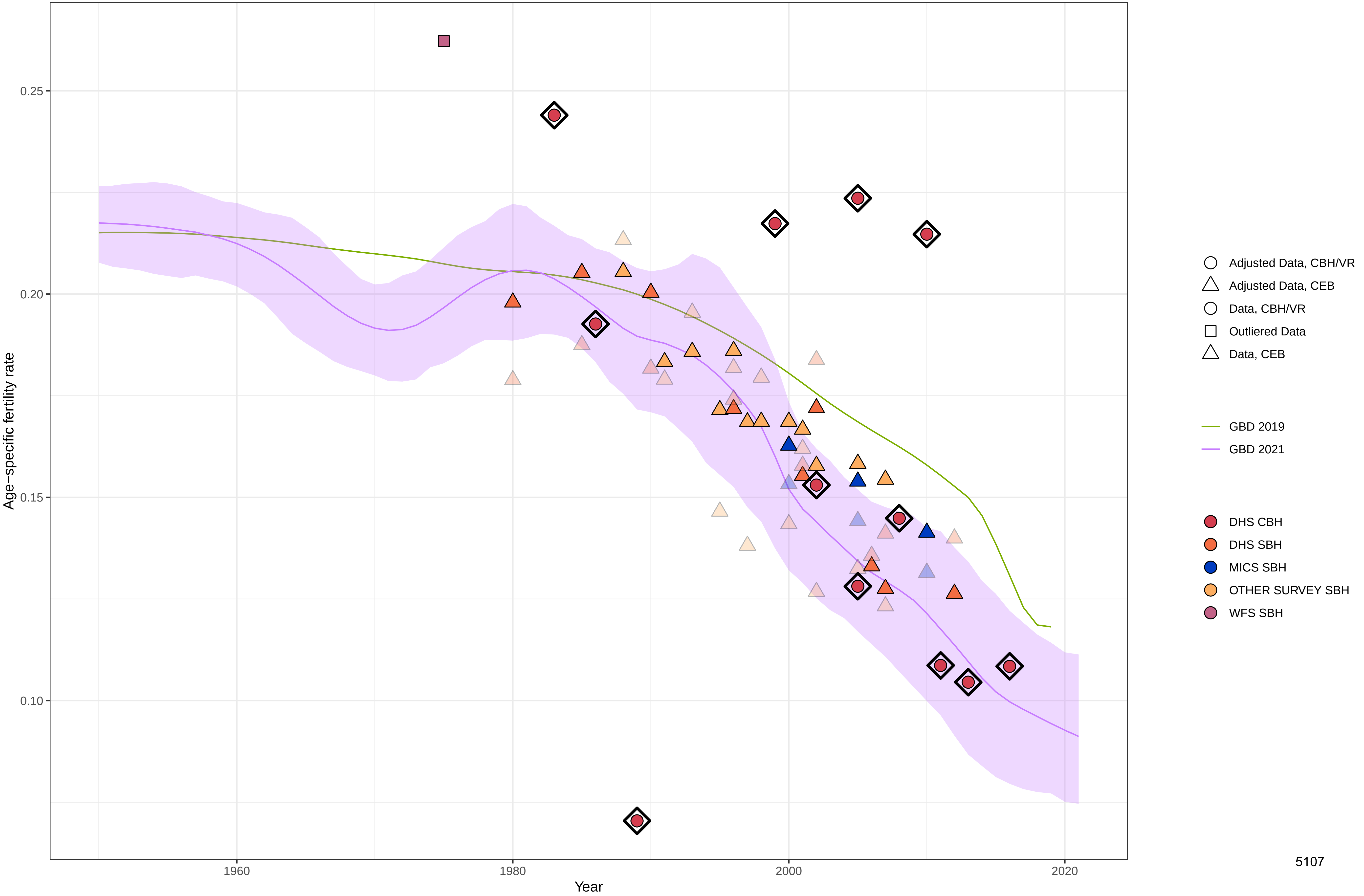

5107

Age−specific fertility fitted trend lines, 1950−2021: Balochistan, Ages: 40−44
Data points enclosed with a diamond are reference sources

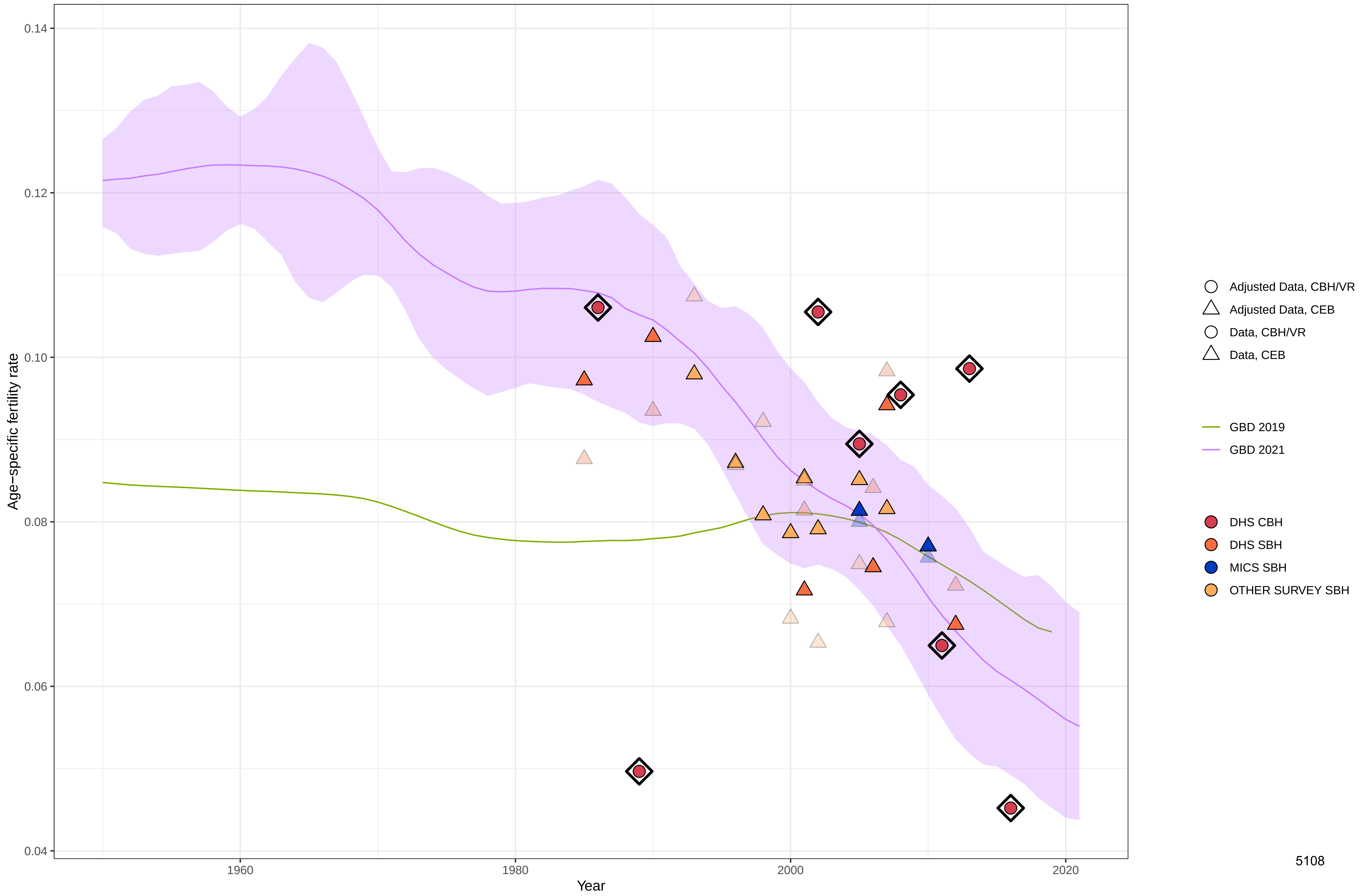

Age−specific fertility fitted trend lines, 1950−2021: Balochistan, Ages: 45−49
Data points enclosed with a diamond are reference sources

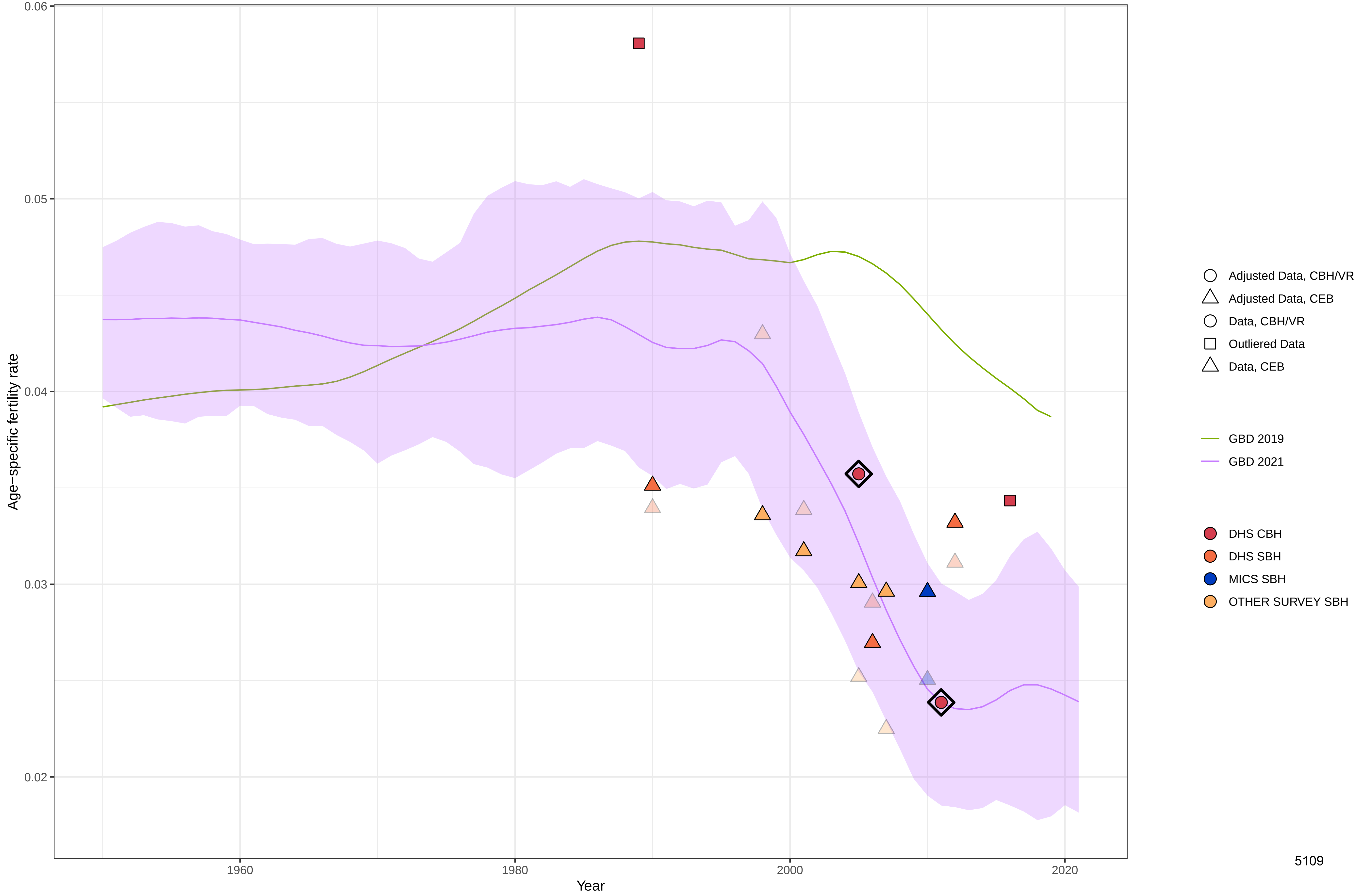

Age−specific fertility fitted trend lines, 1950−2021: Balochistan, Ages: 50−54
Data points enclosed with a diamond are reference sources

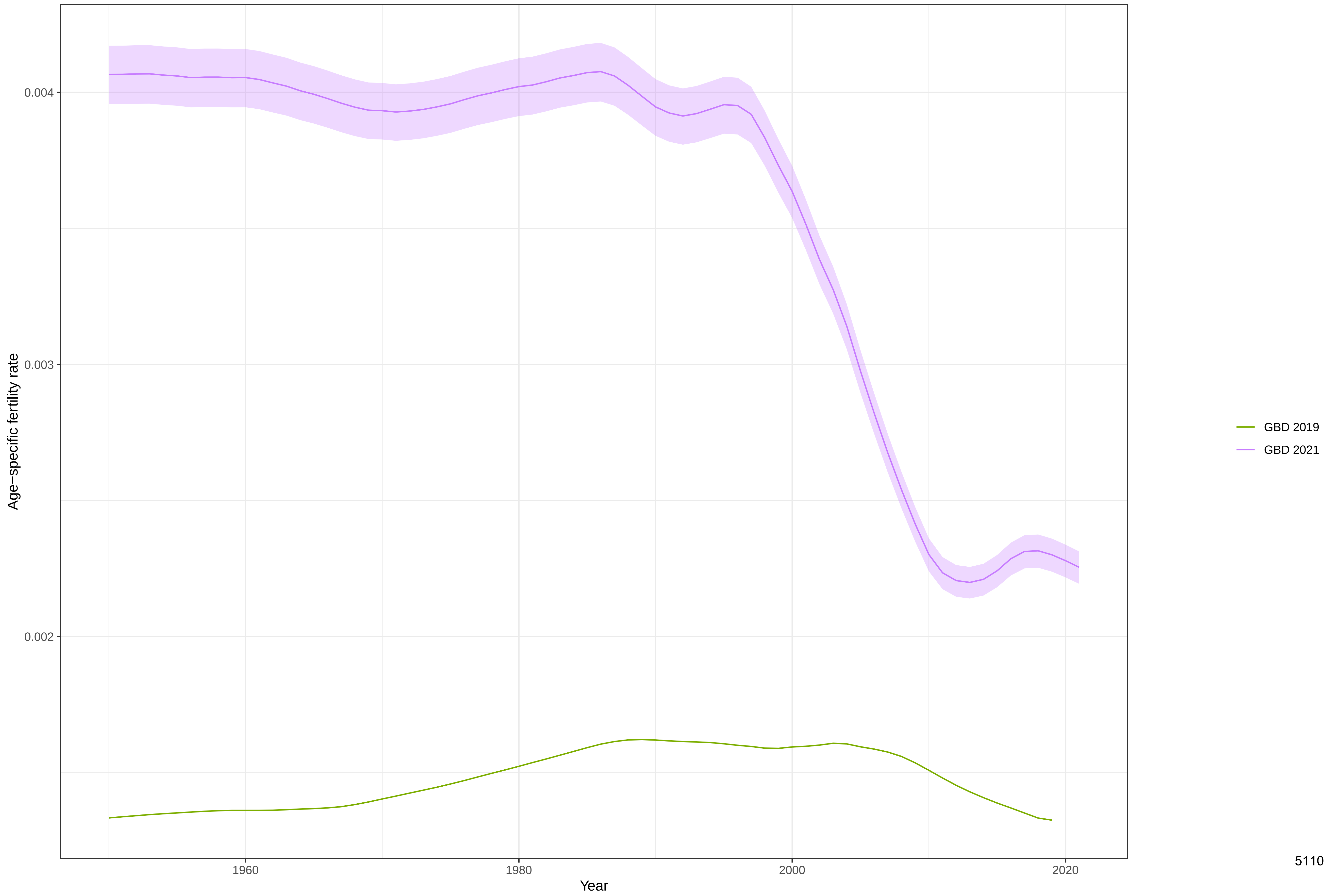

Total fertility rate fitted trend lines, 1950−2021: Balochistan TFR
Data points enclosed with a diamond are reference sources

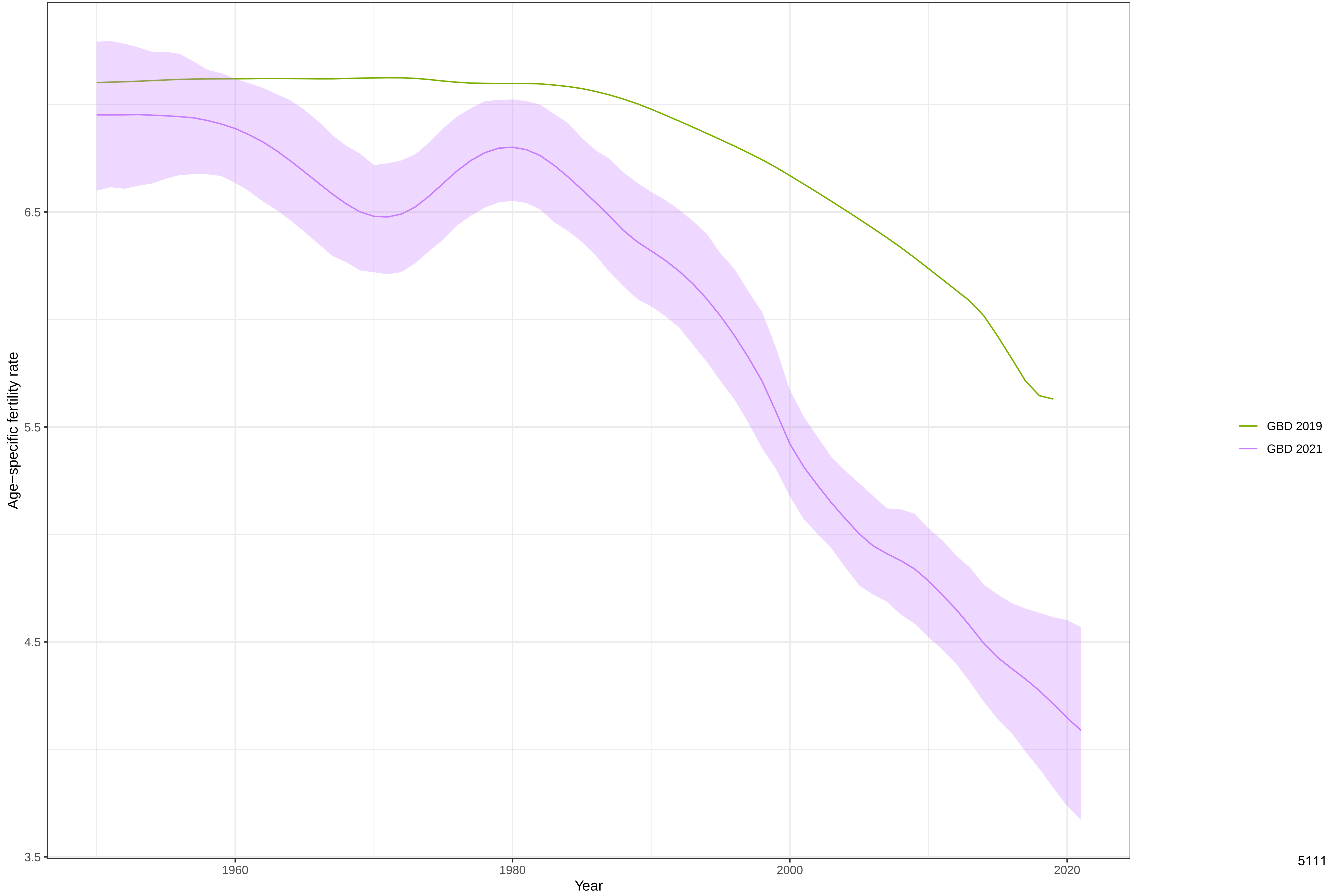

Age−specific fertility fitted trend lines, 1950−2021: Gilgit−Baltistan, Ages: 10−14
Data points enclosed with a diamond are reference sources

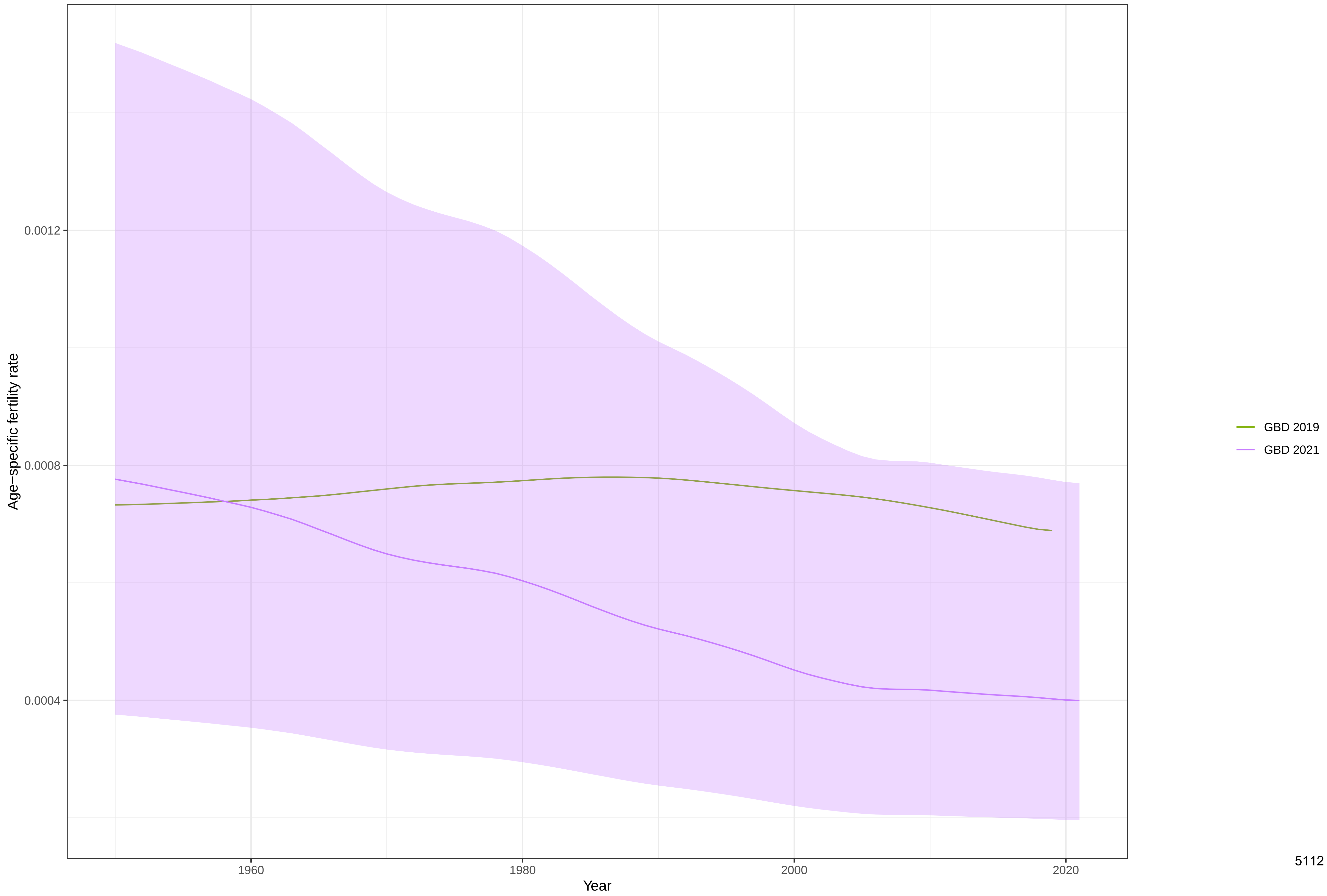

Age−specific fertility fitted trend lines, 1950−2021: Gilgit−Baltistan, Ages: 15−19
Data points enclosed with a diamond are reference sources

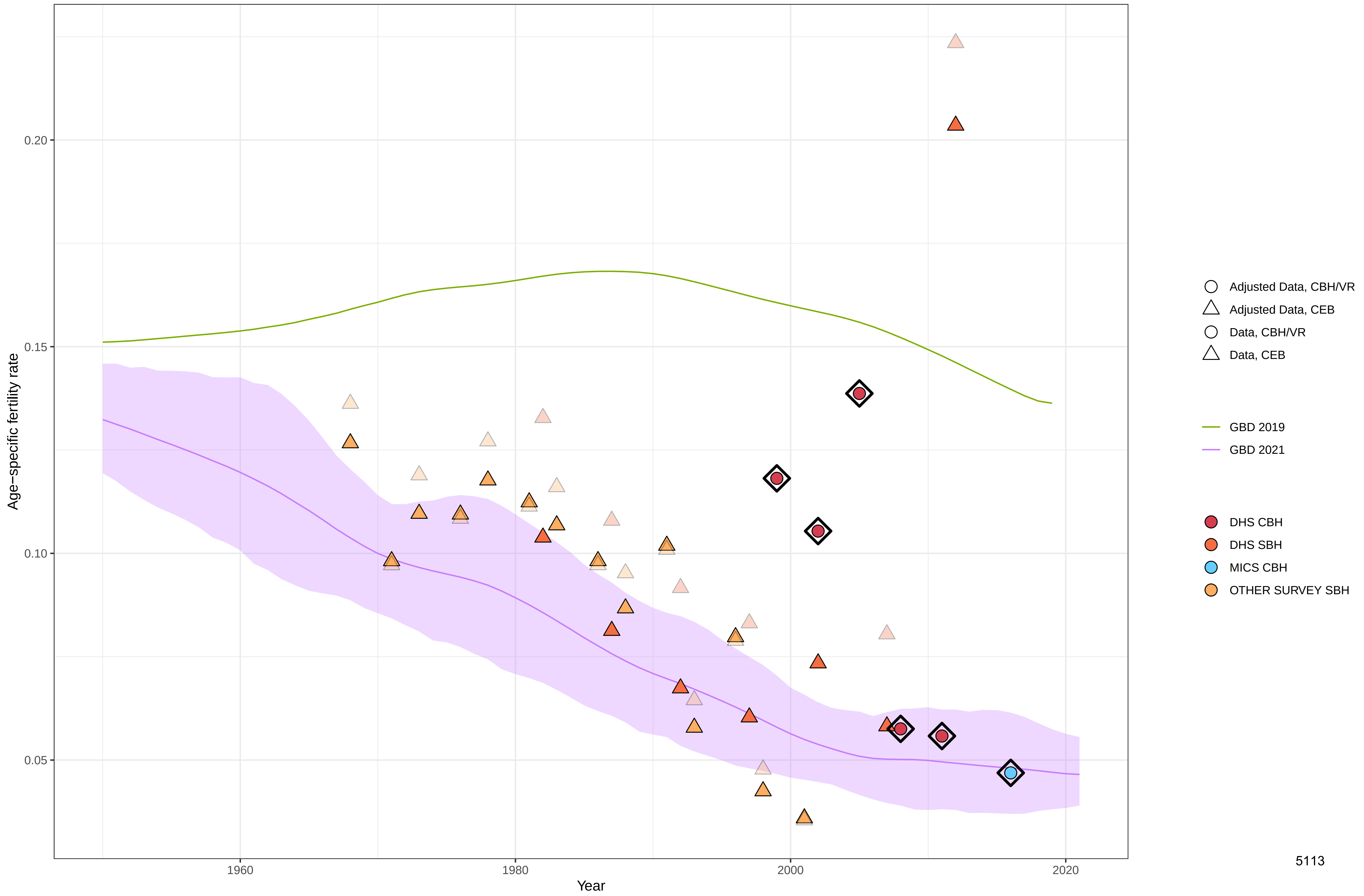

Age−specific fertility fitted trend lines, 1950−2021: Gilgit−Baltistan, Ages: 20−24
Data points enclosed with a diamond are reference sources

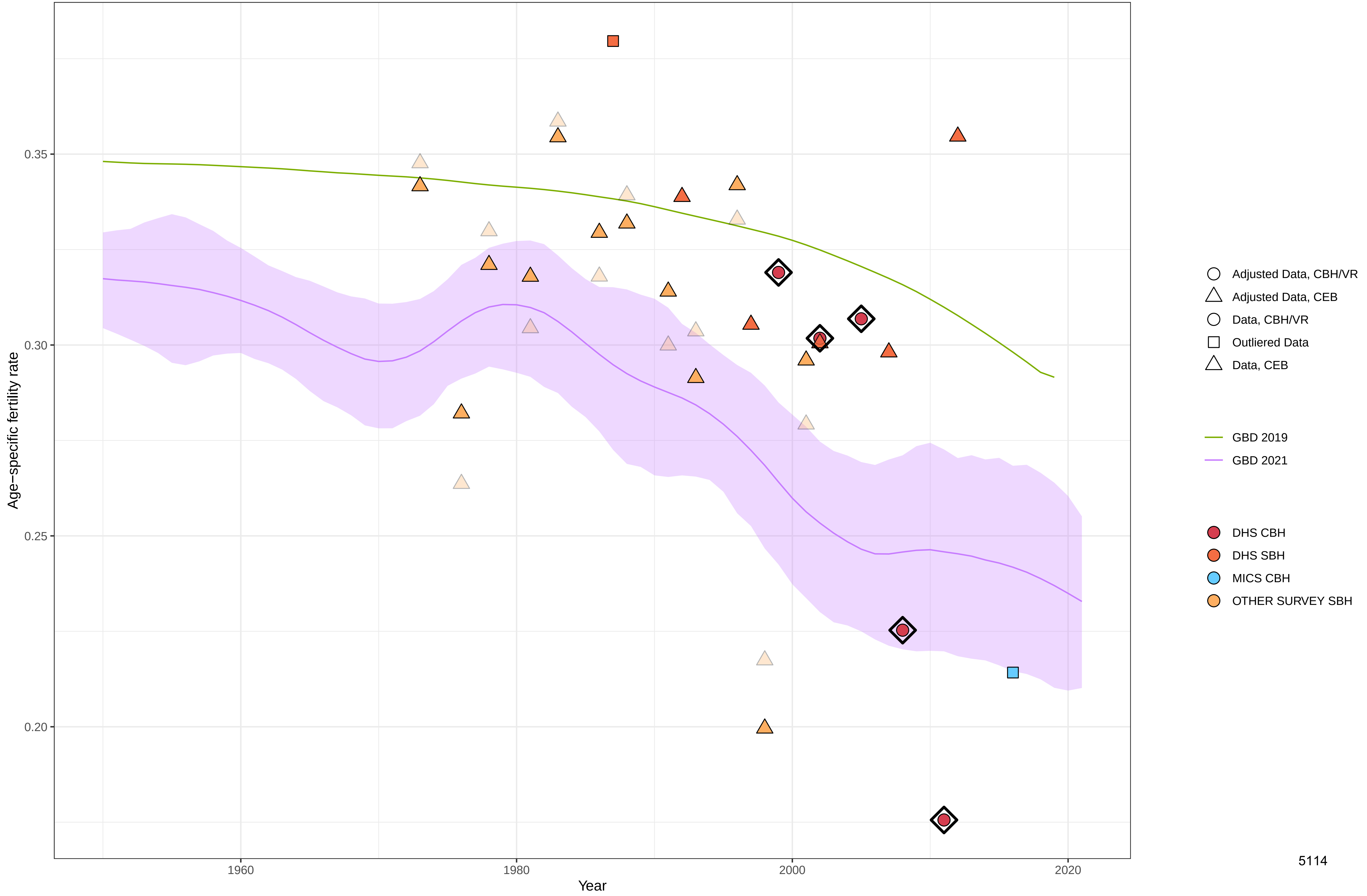

Age−specific fertility fitted trend lines, 1950−2021: Gilgit−Baltistan, Ages: 25−29
Data points enclosed with a diamond are reference sources

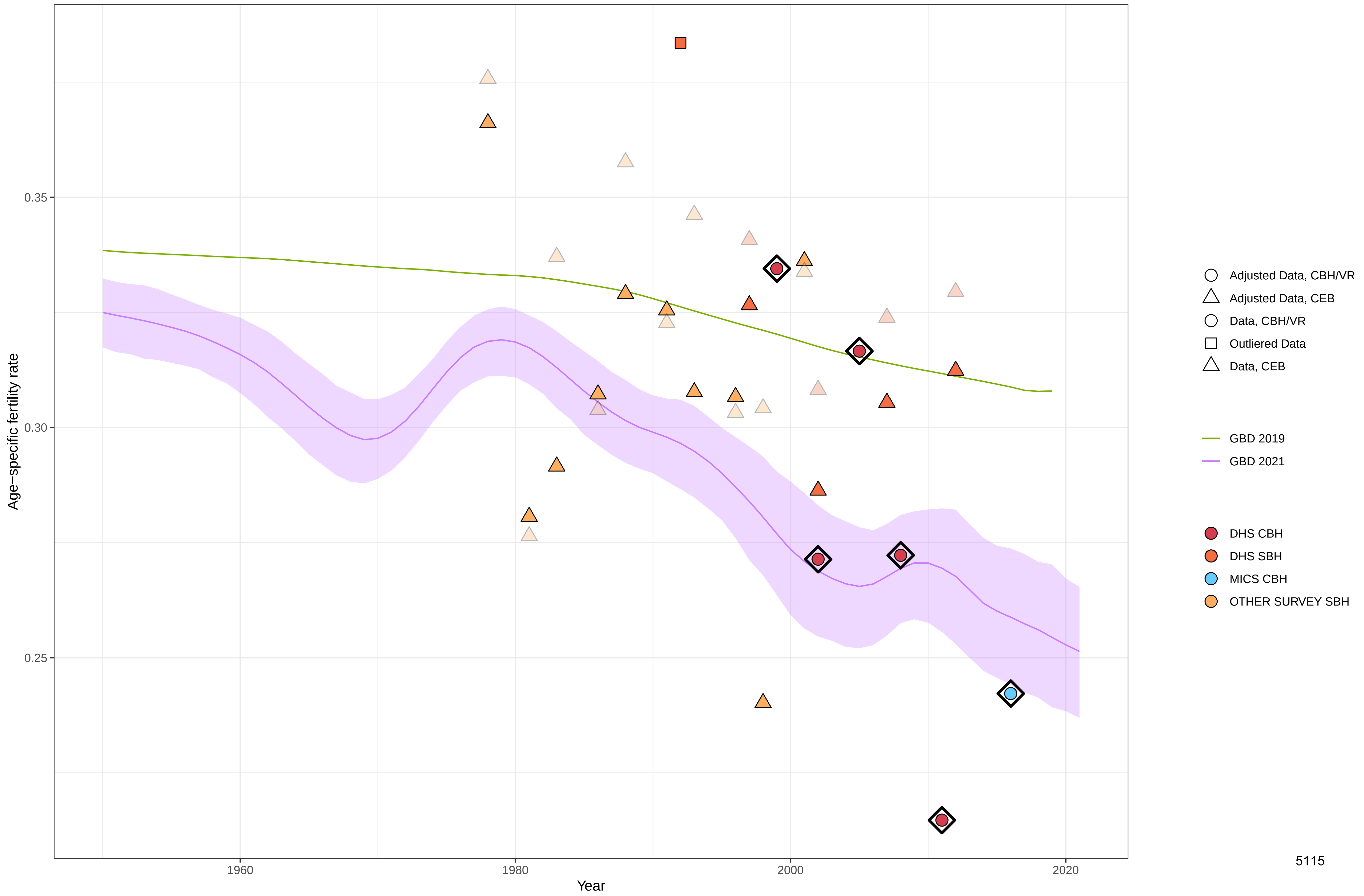

Age−specific fertility fitted trend lines, 1950−2021: Gilgit−Baltistan, Ages: 30−34
Data points enclosed with a diamond are reference sources

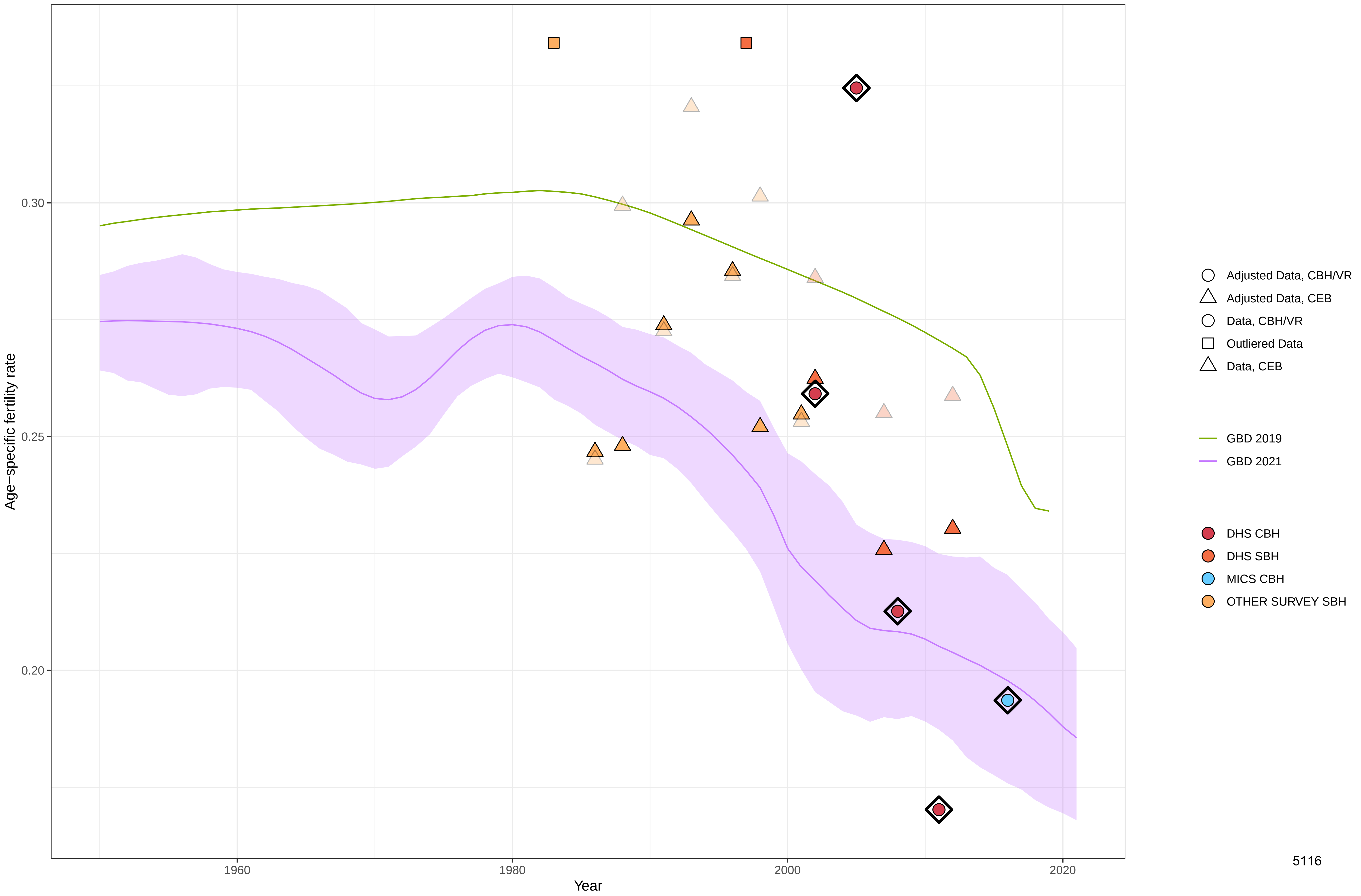

Age−specific fertility fitted trend lines, 1950−2021: Gilgit−Baltistan, Ages: 35−39
Data points enclosed with a diamond are reference sources

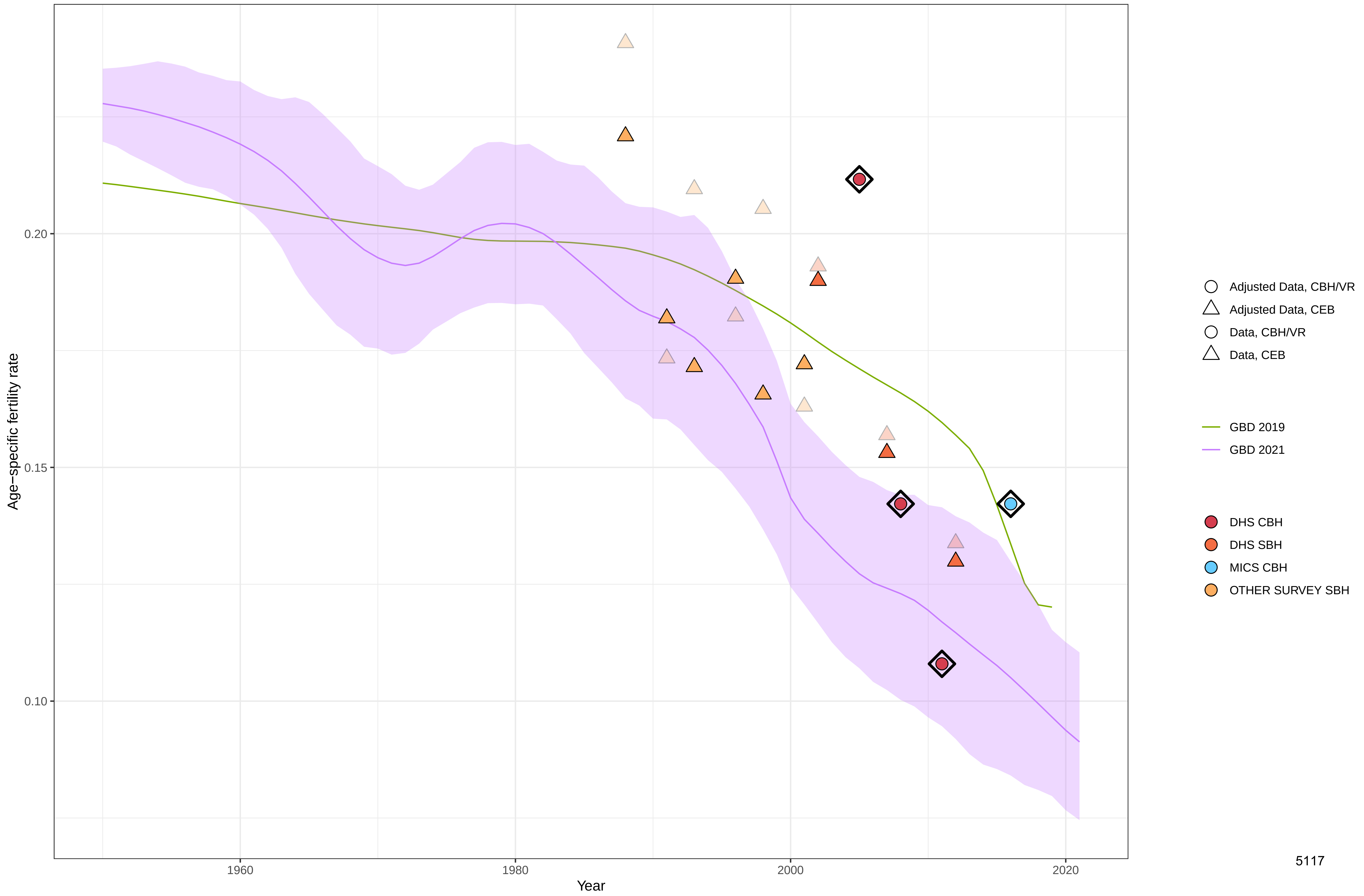

Age−specific fertility fitted trend lines, 1950−2021: Gilgit−Baltistan, Ages: 40−44
Data points enclosed with a diamond are reference sources

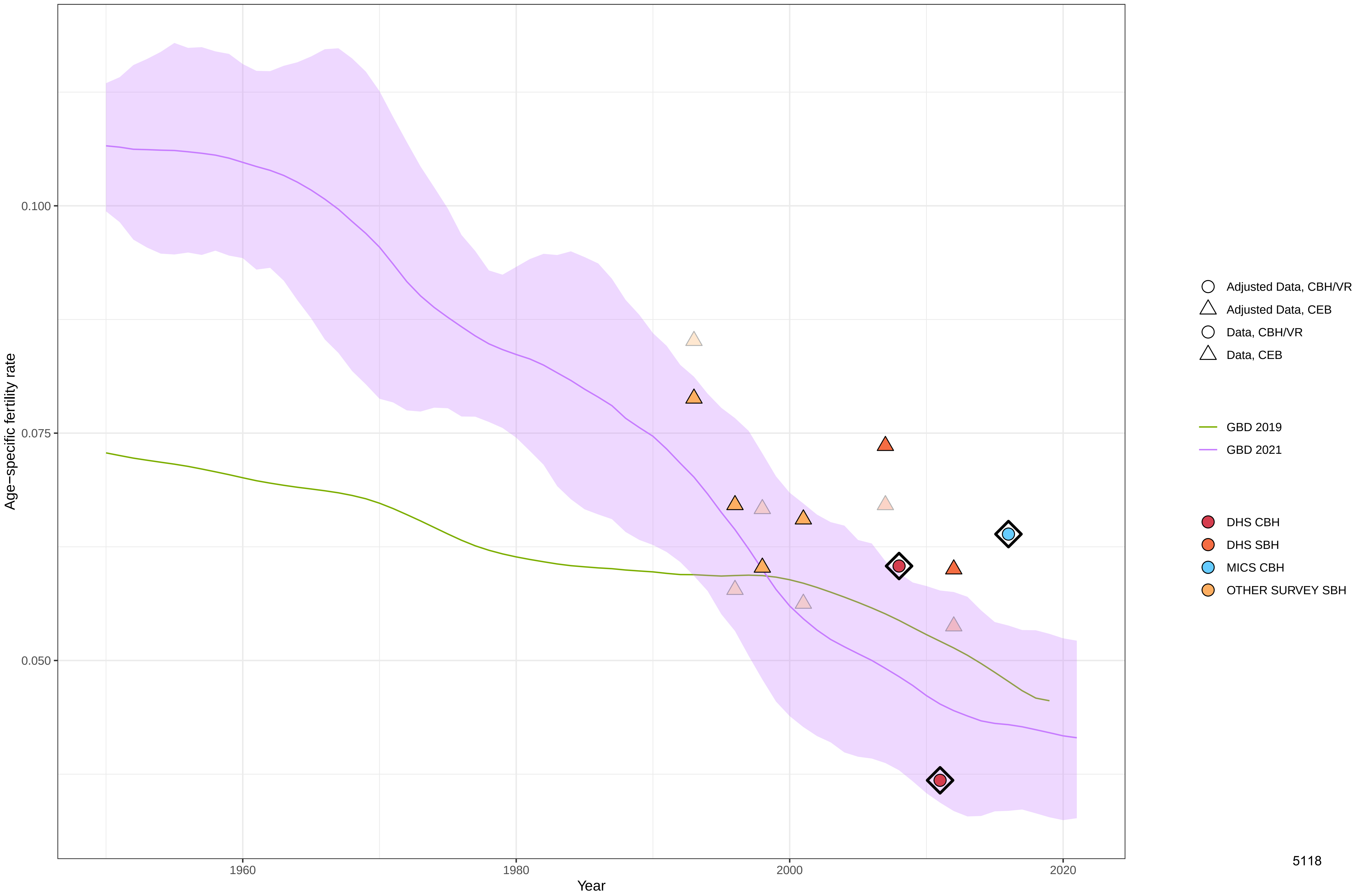

Age−specific fertility fitted trend lines, 1950−2021: Gilgit−Baltistan, Ages: 45−49
Data points enclosed with a diamond are reference sources

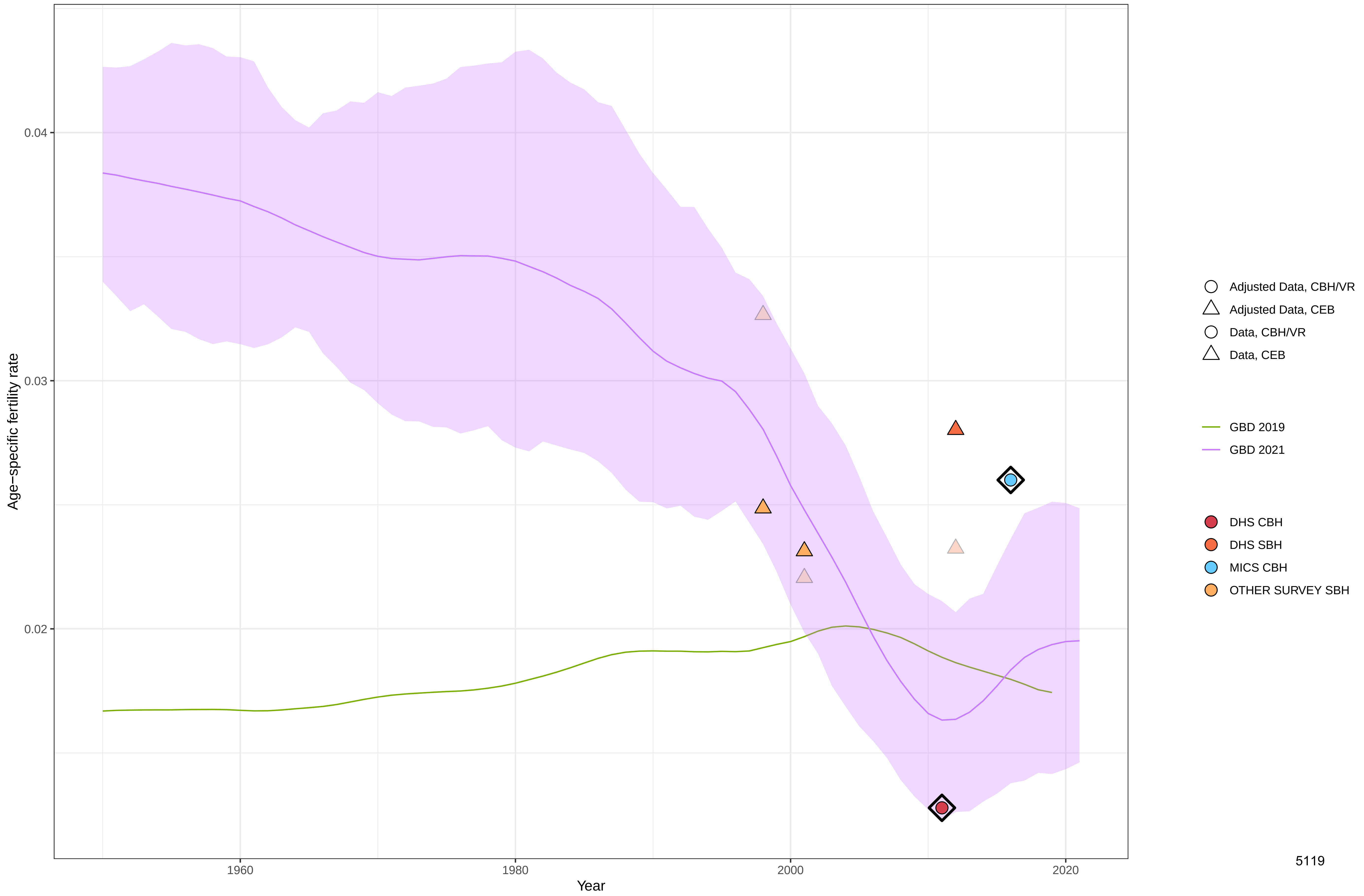

Age−specific fertility fitted trend lines, 1950−2021: Gilgit−Baltistan, Ages: 50−54
Data points enclosed with a diamond are reference sources

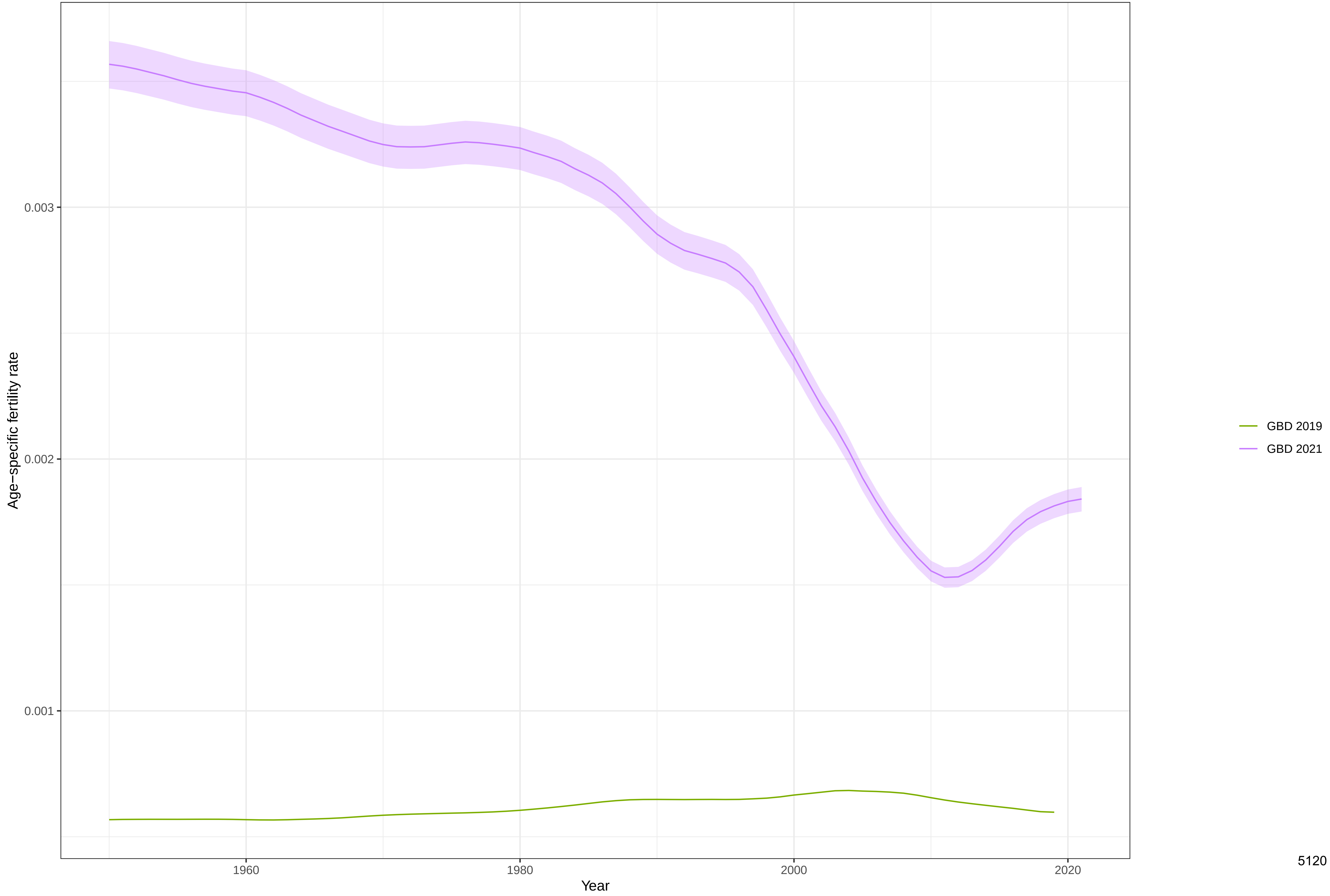

Total fertility rate fitted trend lines, 1950−2021: Gilgit−Baltistan TFR
Data points enclosed with a diamond are reference sources

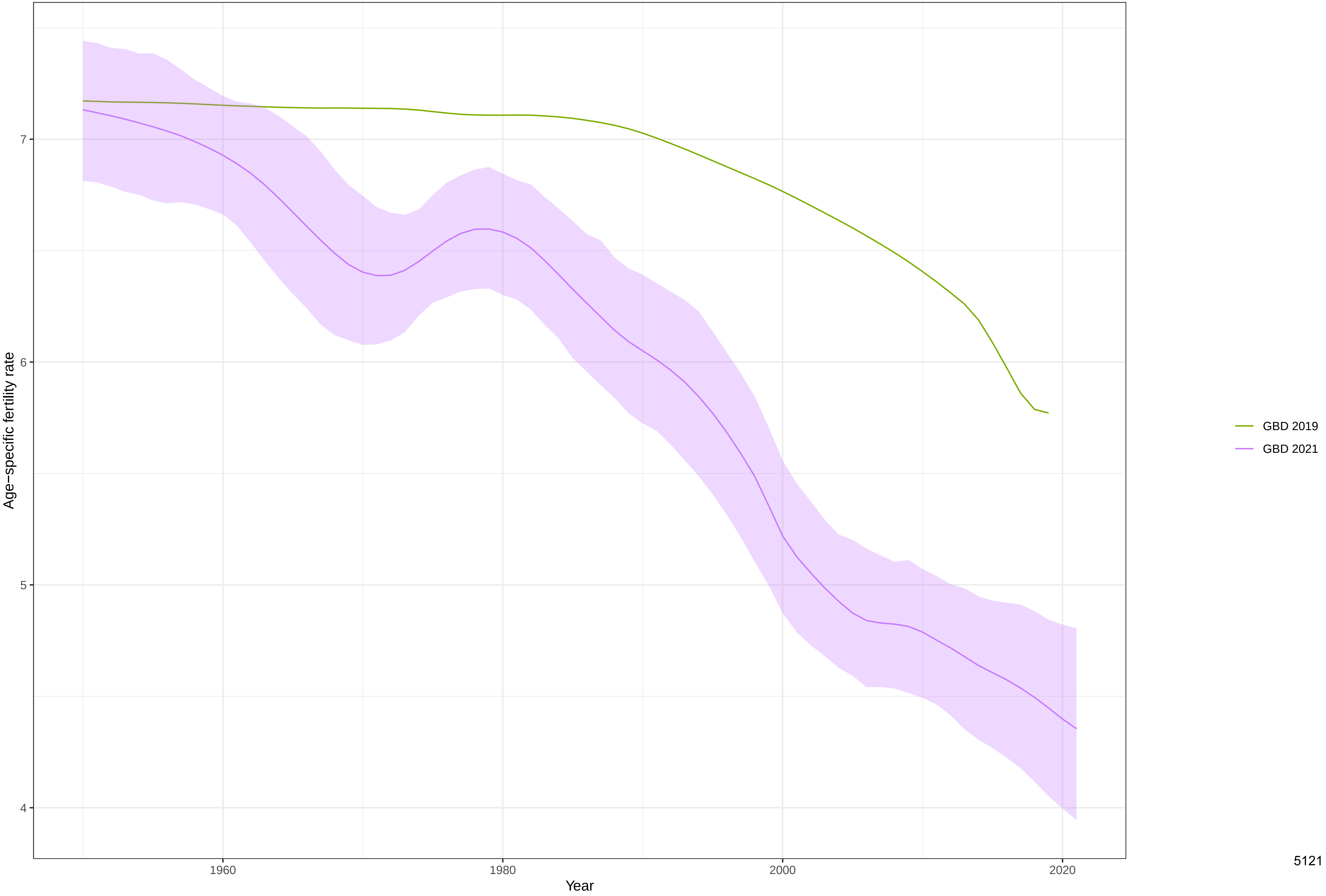

Age−specific fertility fitted trend lines, 1950−2021: Islamabad Capital Territory, Ages: 10−14
Data points enclosed with a diamond are reference sources

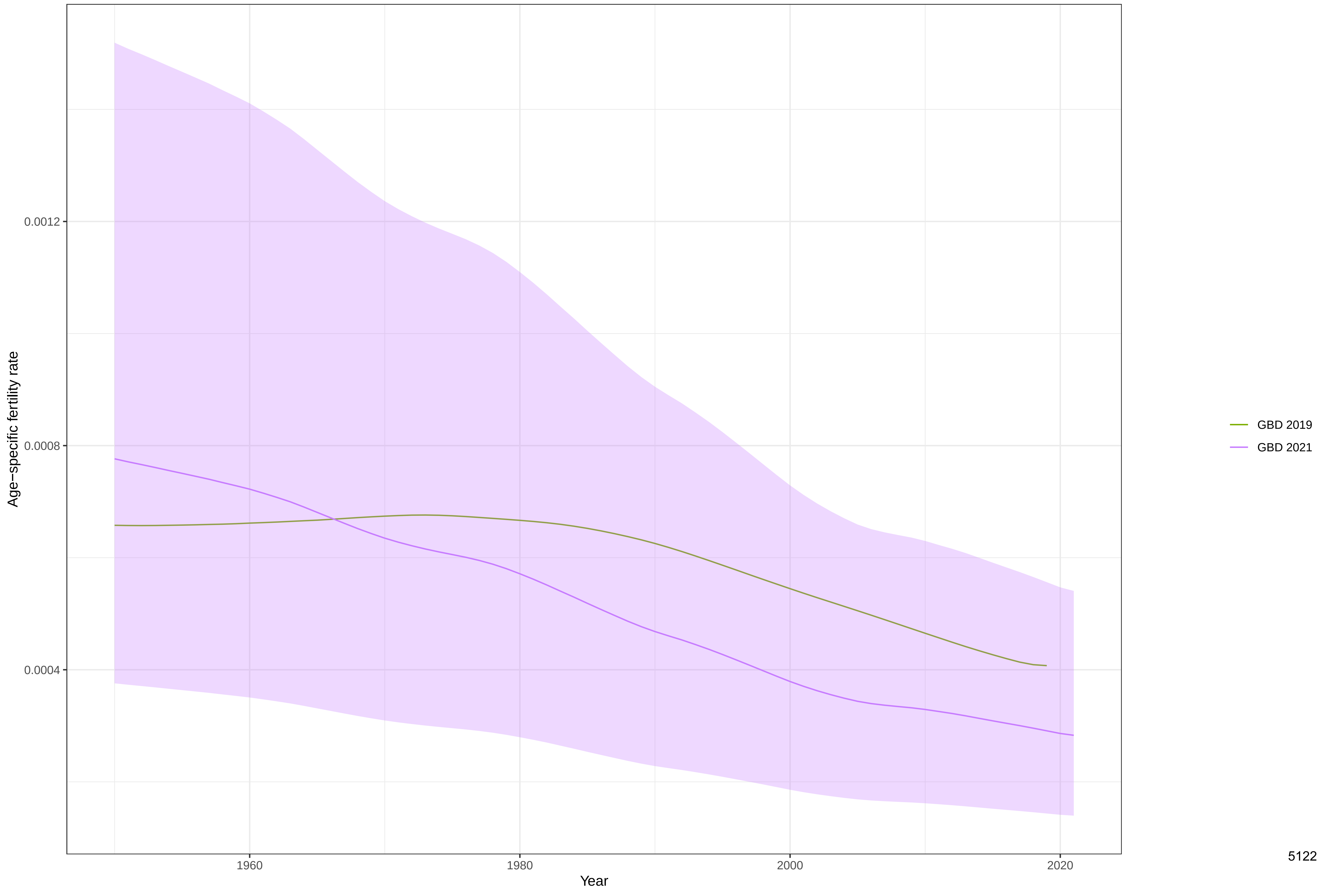

Age−specific fertility fitted trend lines, 1950−2021: Islamabad Capital Territory, Ages: 15−19
Data points enclosed with a diamond are reference sources

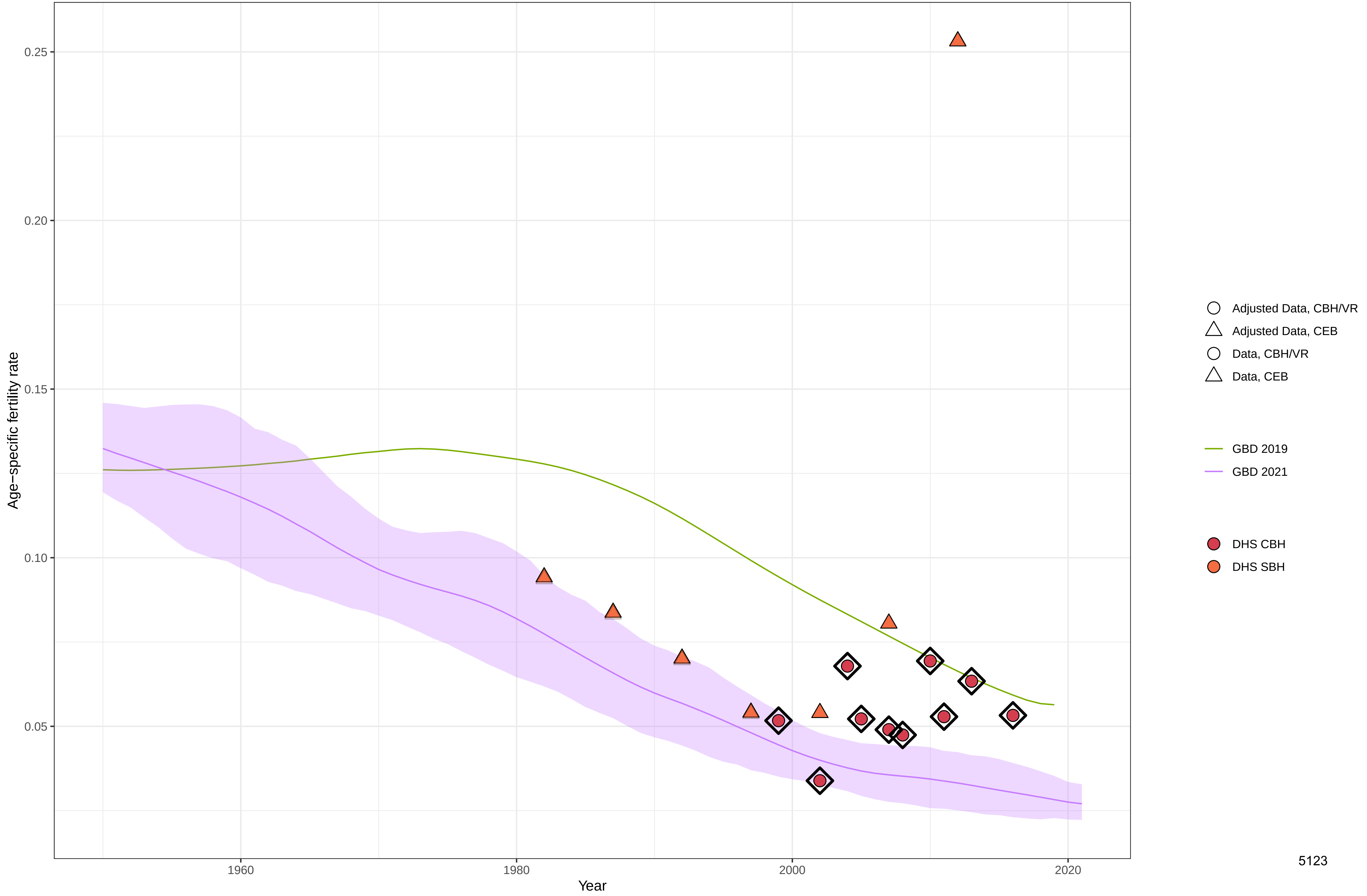

Age−specific fertility fitted trend lines, 1950−2021: Islamabad Capital Territory, Ages: 20−24
Data points enclosed with a diamond are reference sources

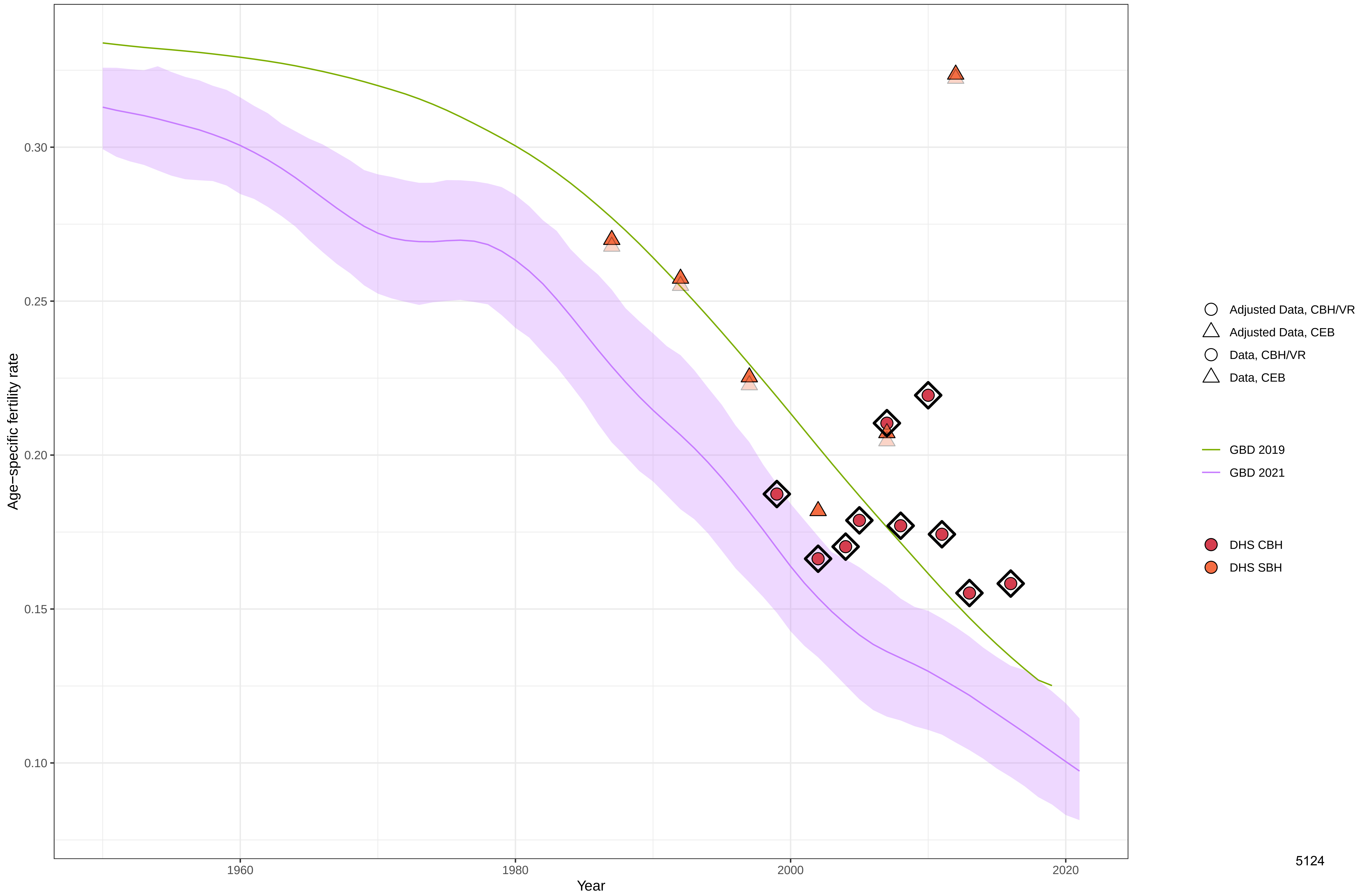

Age−specific fertility fitted trend lines, 1950−2021: Islamabad Capital Territory, Ages: 25−29
Data points enclosed with a diamond are reference sources

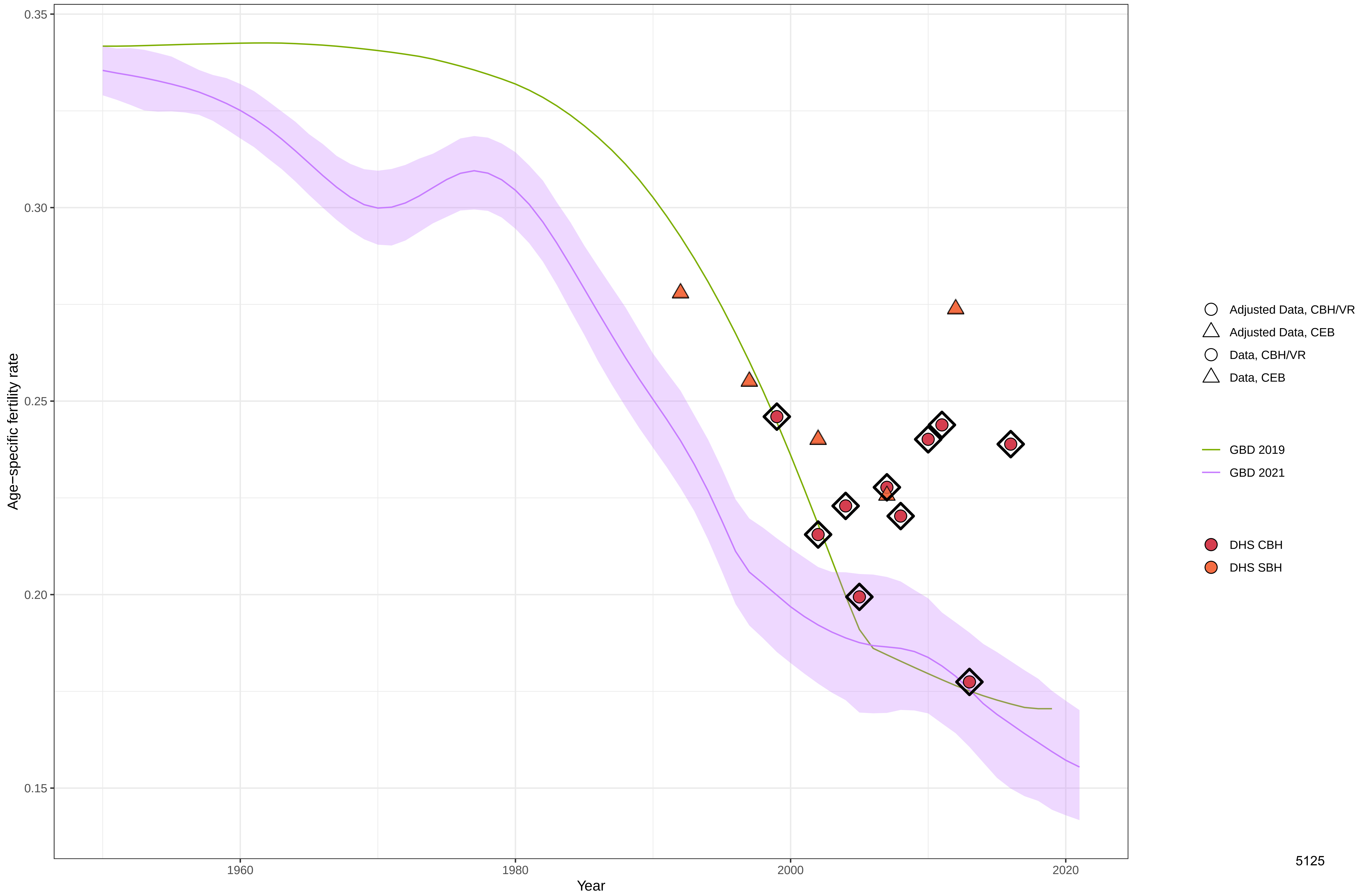

Age−specific fertility fitted trend lines, 1950−2021: Islamabad Capital Territory, Ages: 30−34
Data points enclosed with a diamond are reference sources

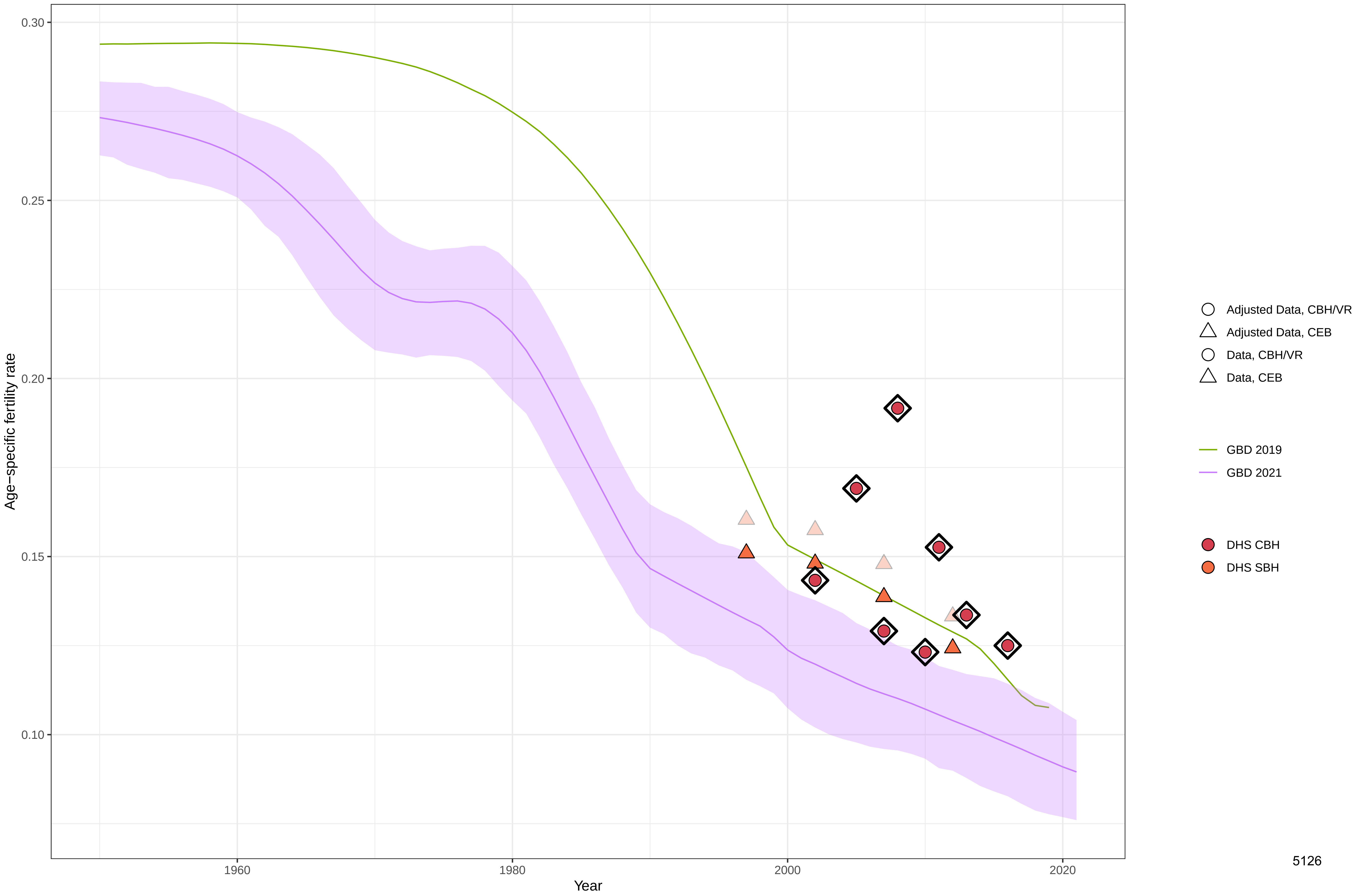

Age−specific fertility fitted trend lines, 1950−2021: Islamabad Capital Territory, Ages: 35−39
Data points enclosed with a diamond are reference sources

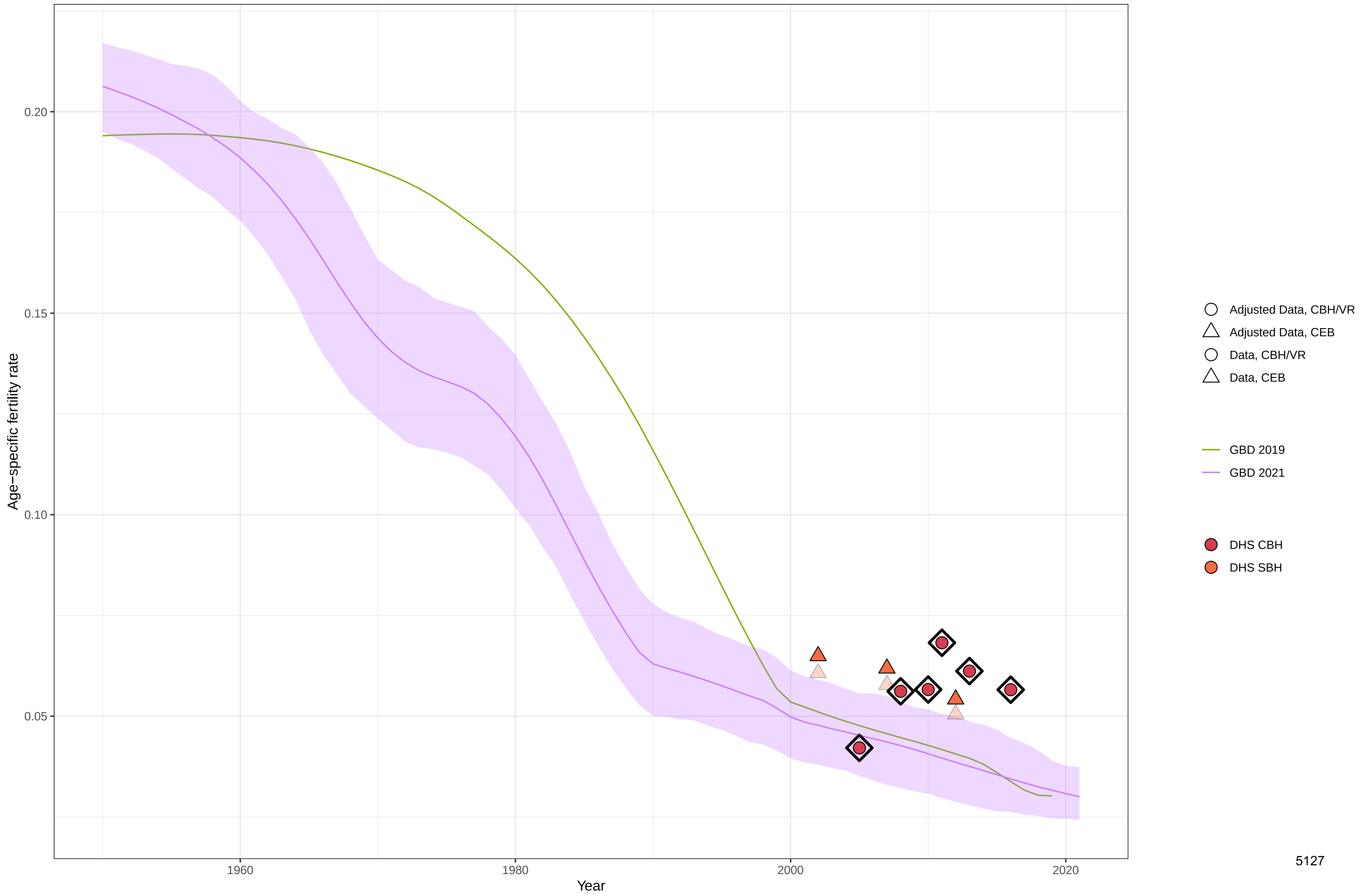

Age−specific fertility fitted trend lines, 1950−2021: Islamabad Capital Territory, Ages: 40−44
Data points enclosed with a diamond are reference sources

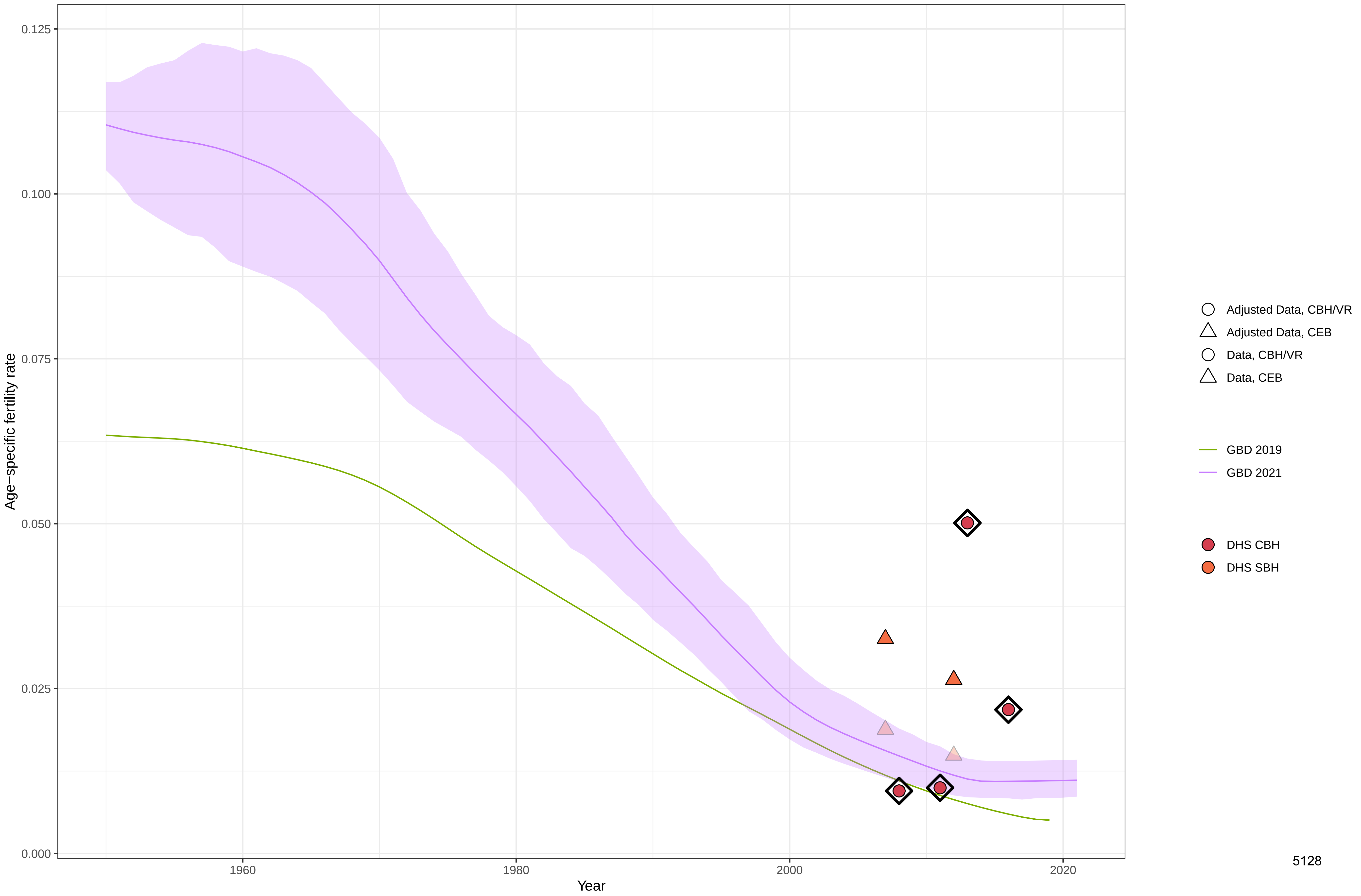

Age−specific fertility fitted trend lines, 1950−2021: Islamabad Capital Territory, Ages: 45−49
Data points enclosed with a diamond are reference sources

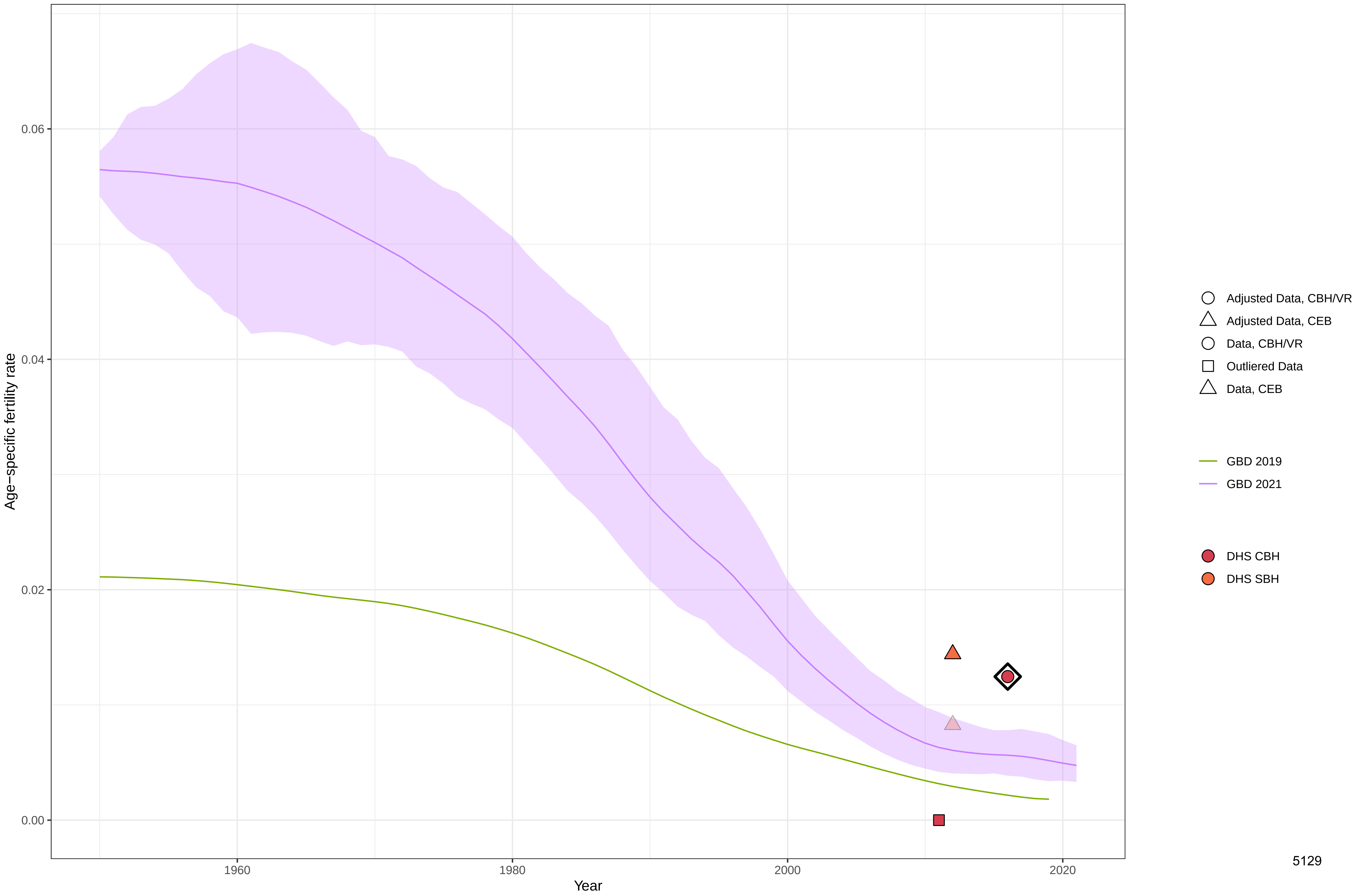

Age−specific fertility fitted trend lines, 1950−2021: Islamabad Capital Territory, Ages: 50−54
Data points enclosed with a diamond are reference sources

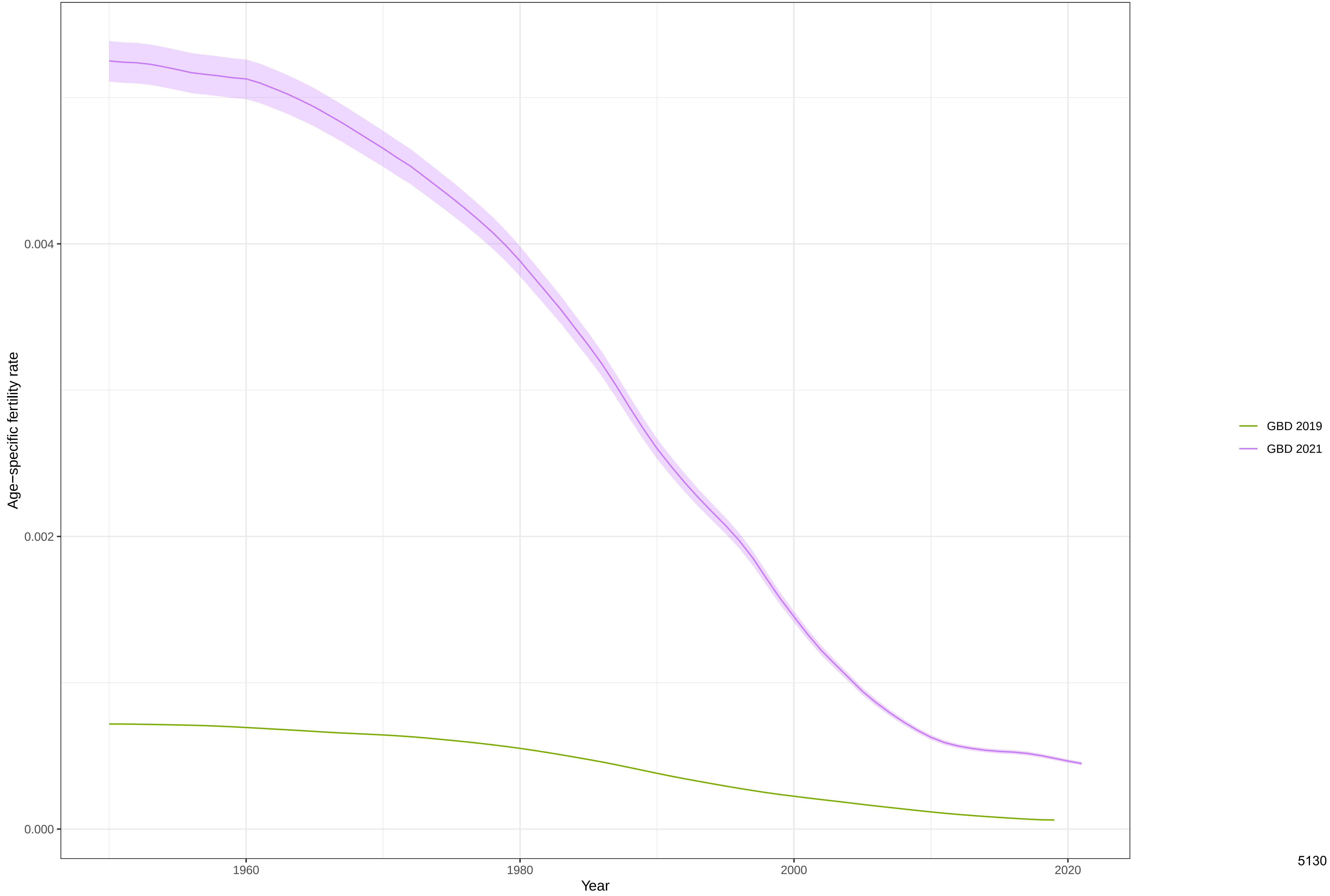

Total fertility rate fitted trend lines, 1950−2021: Islamabad Capital Territory TFR
Data points enclosed with a diamond are reference sources

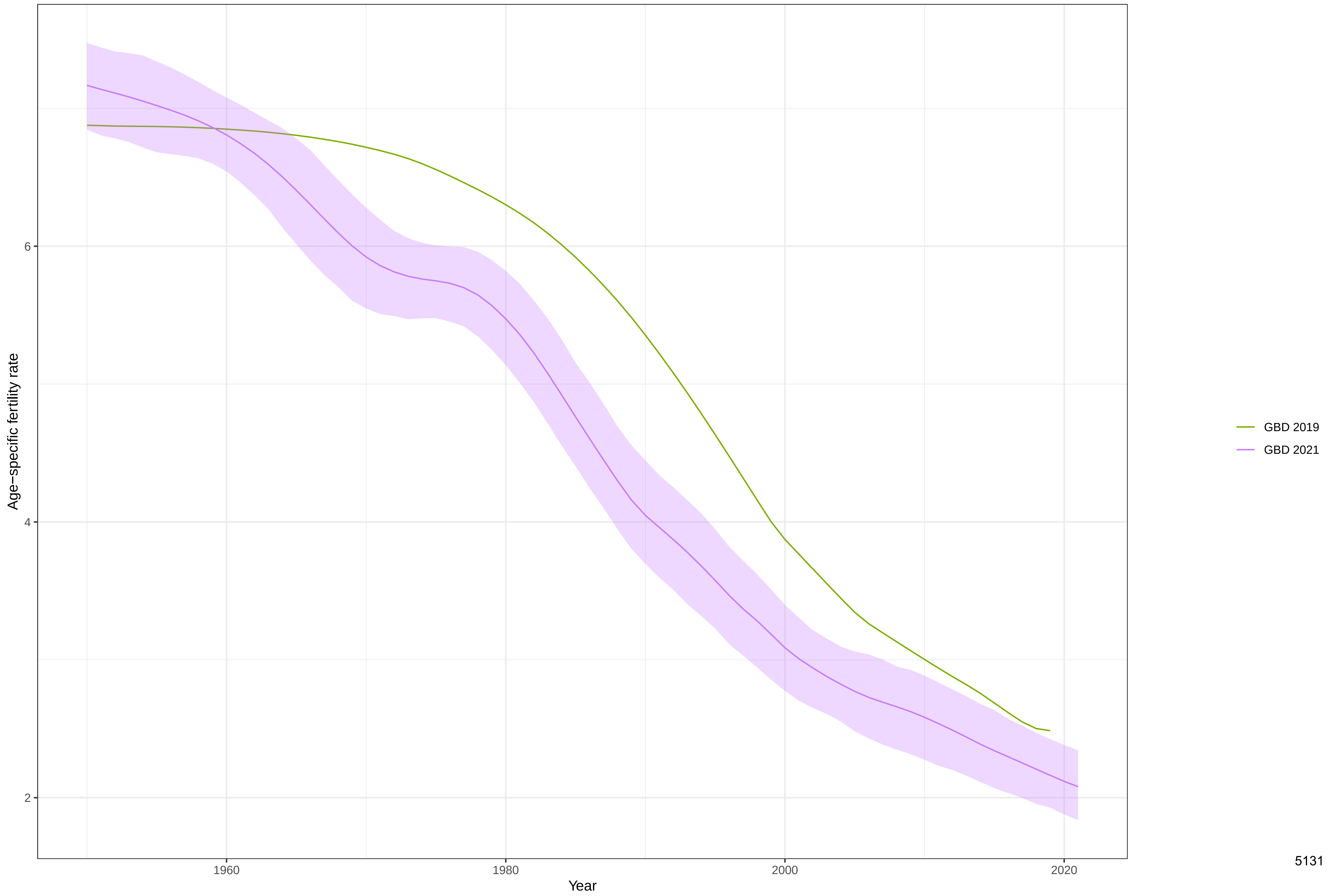

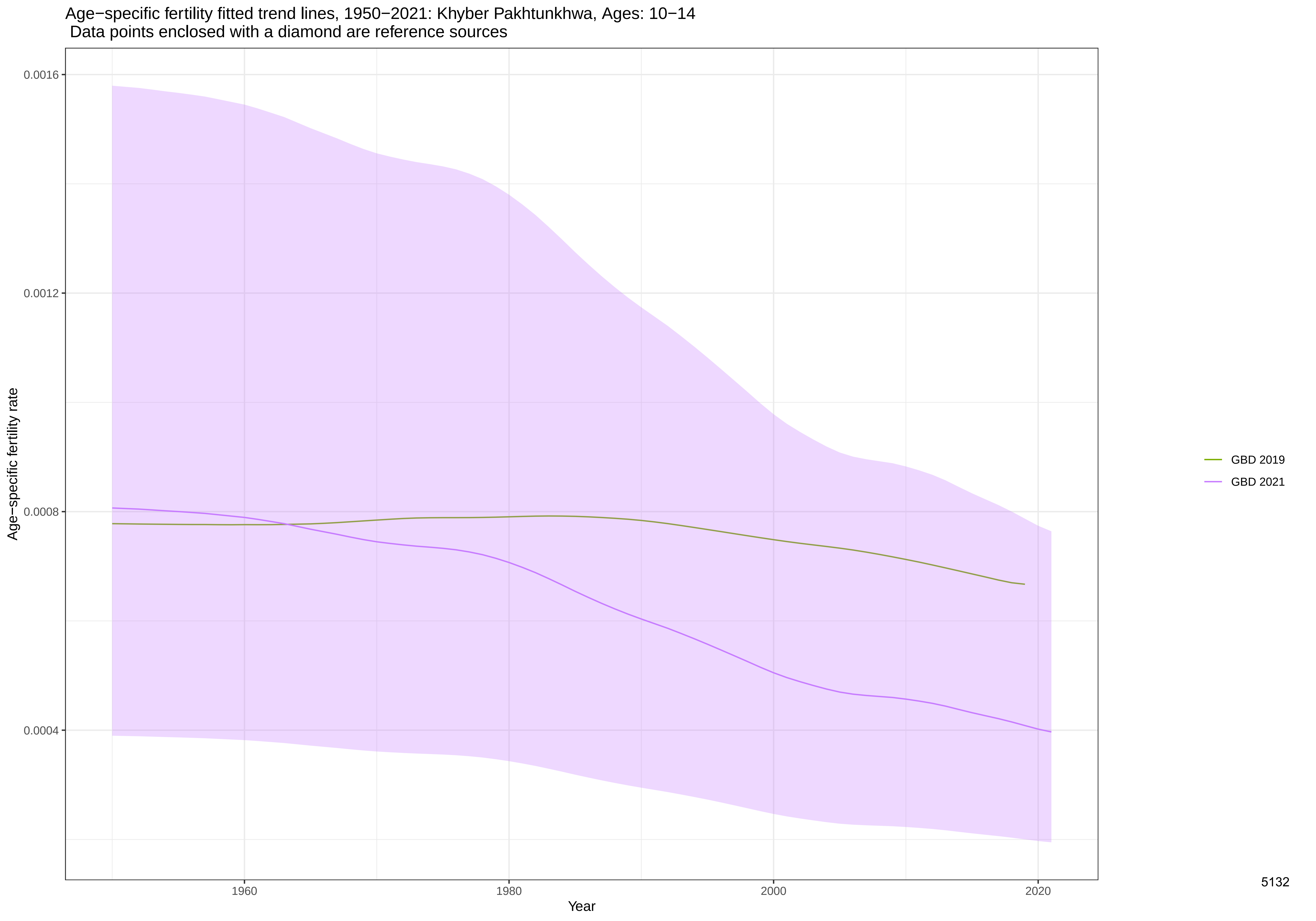

Age−specific fertility fitted trend lines, 1950−2021: Khyber Pakhtunkhwa, Ages: 10−14
Data points enclosed with a diamond are reference sources
Age−specific fertility rate
0.0016
0.0012
0.0008
0.0004
1960
1980
2000
2020
Year
GBD 2019
GBD 2021

Age−specific fertility fitted trend lines, 1950−2021: Khyber Pakhtunkhwa, Ages: 15−19
Data points enclosed with a diamond are reference sources

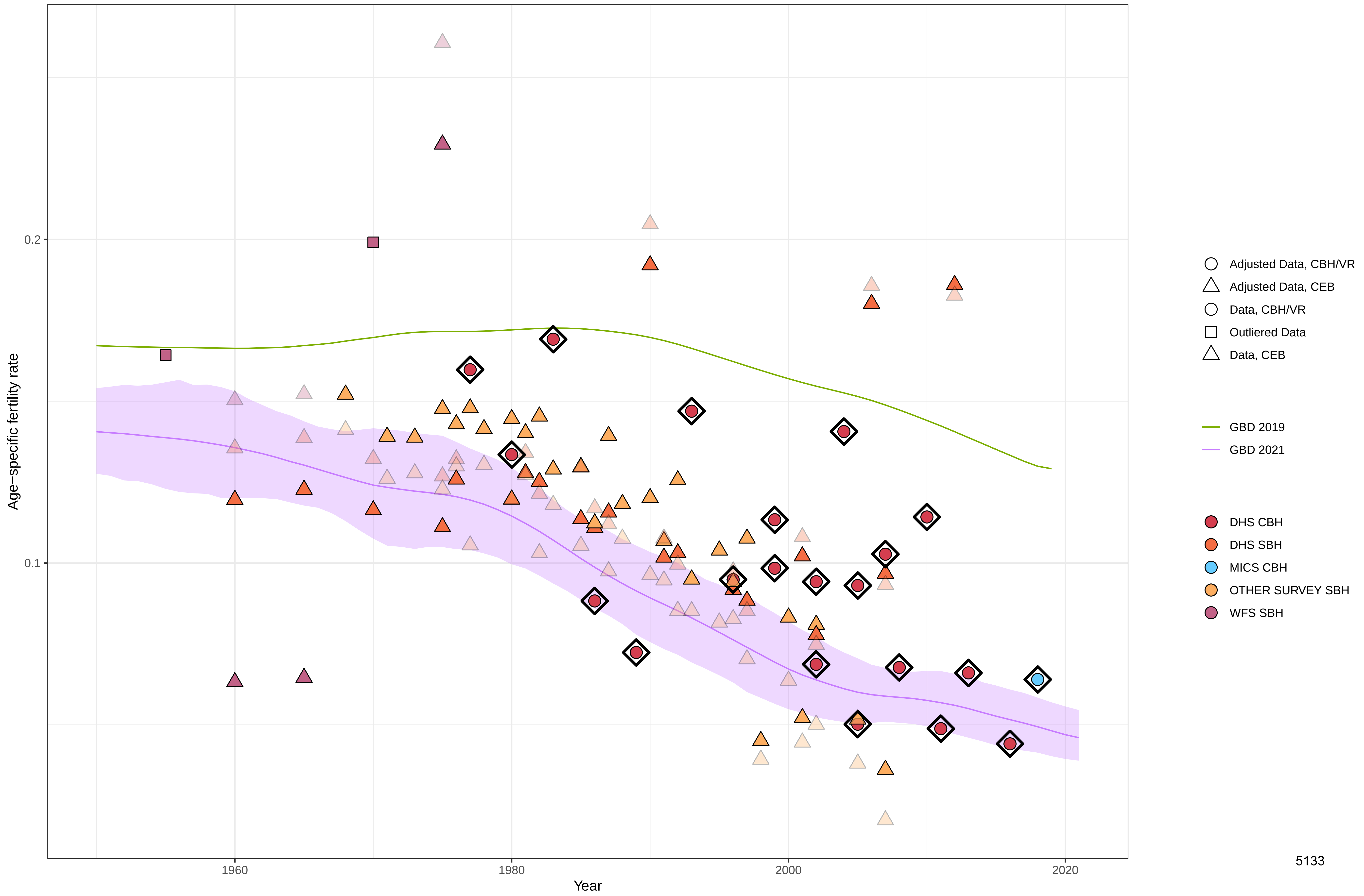

Age−specific fertility fitted trend lines, 1950−2021: Khyber Pakhtunkhwa, Ages: 20−24
Data points enclosed with a diamond are reference sources

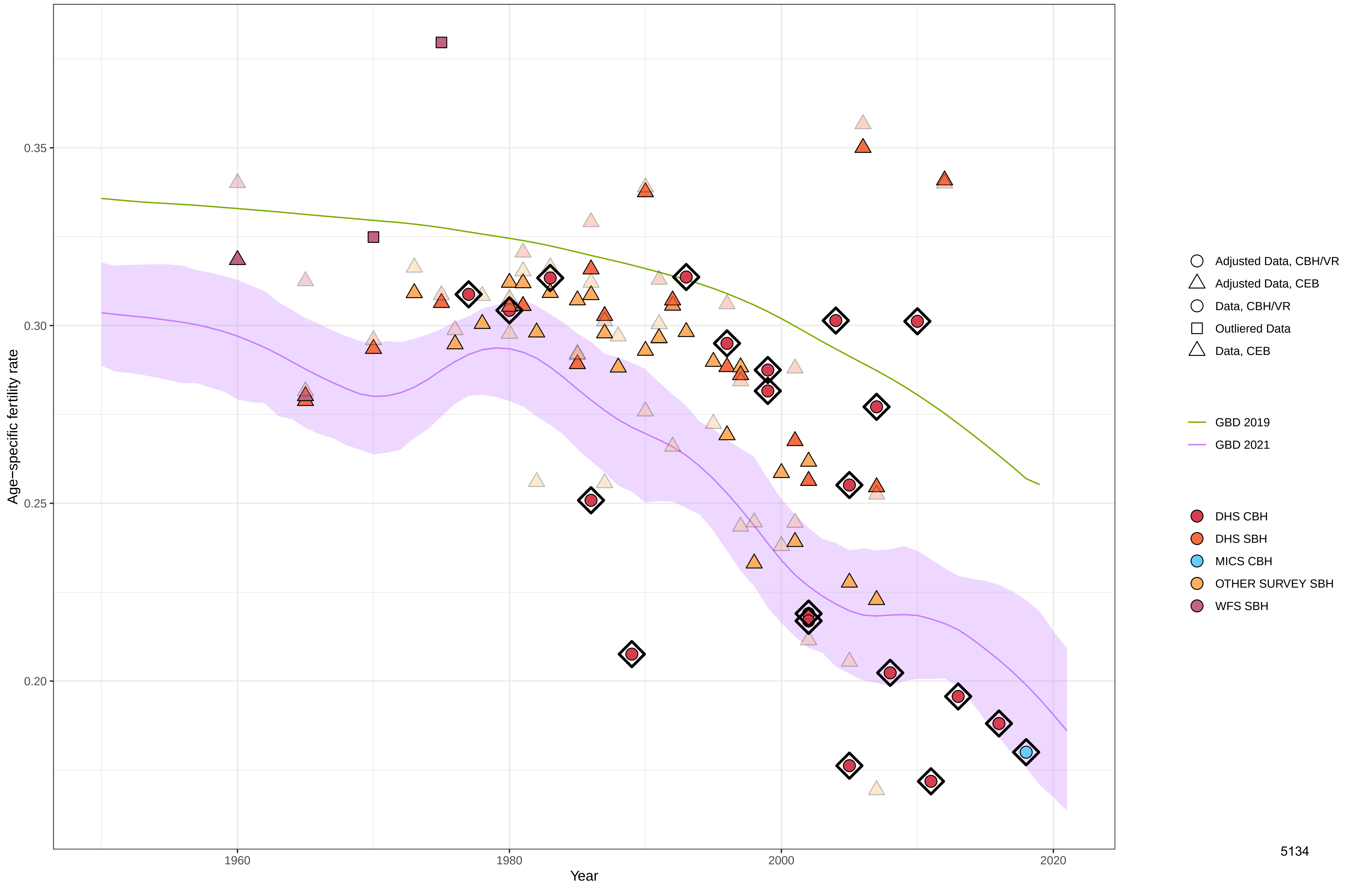

Age−specific fertility fitted trend lines, 1950−2021: Khyber Pakhtunkhwa, Ages: 25−29
Data points enclosed with a diamond are reference sources

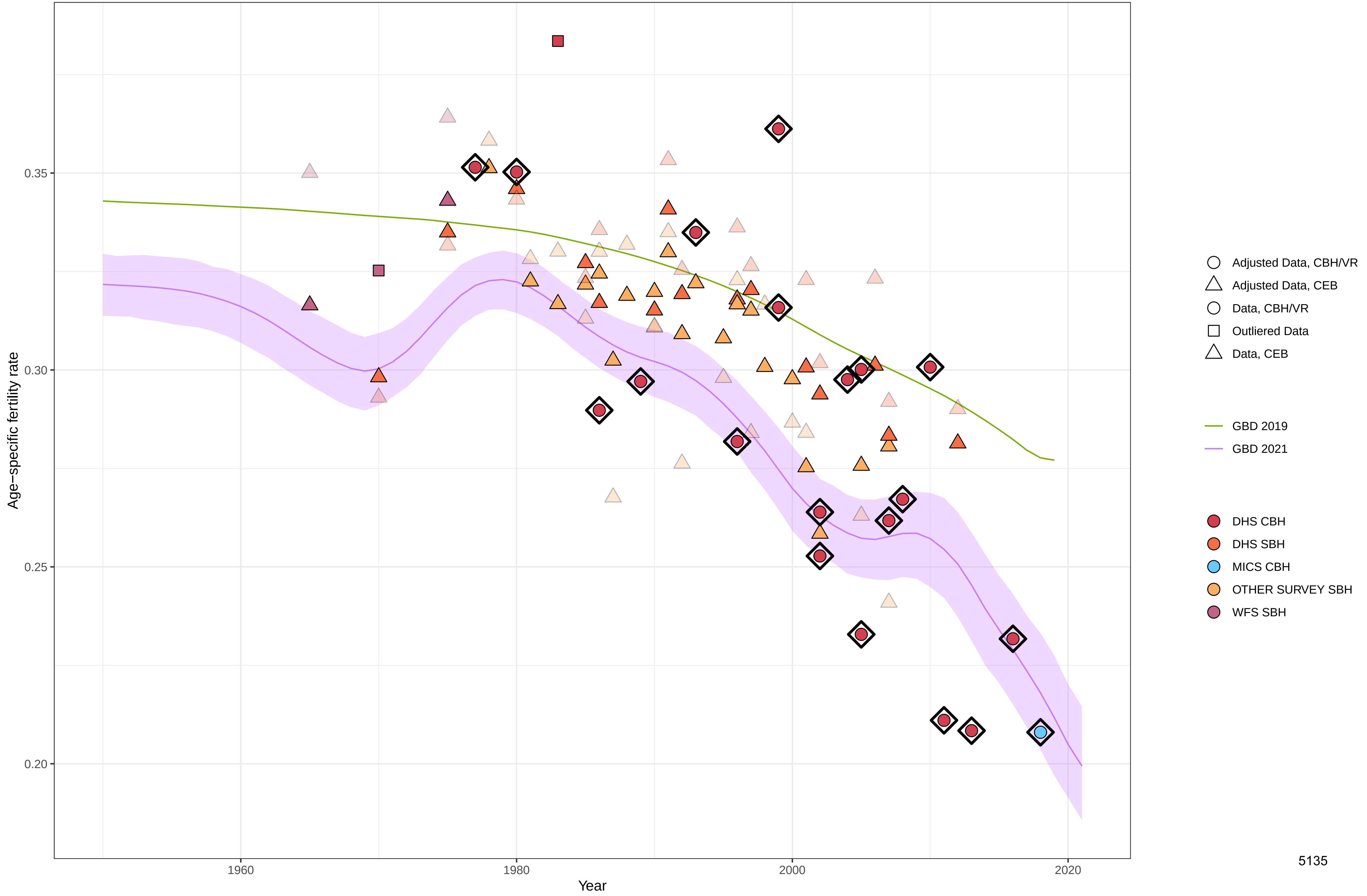

Age−specific fertility fitted trend lines, 1950−2021: Khyber Pakhtunkhwa, Ages: 30−34
Data points enclosed with a diamond are reference sources

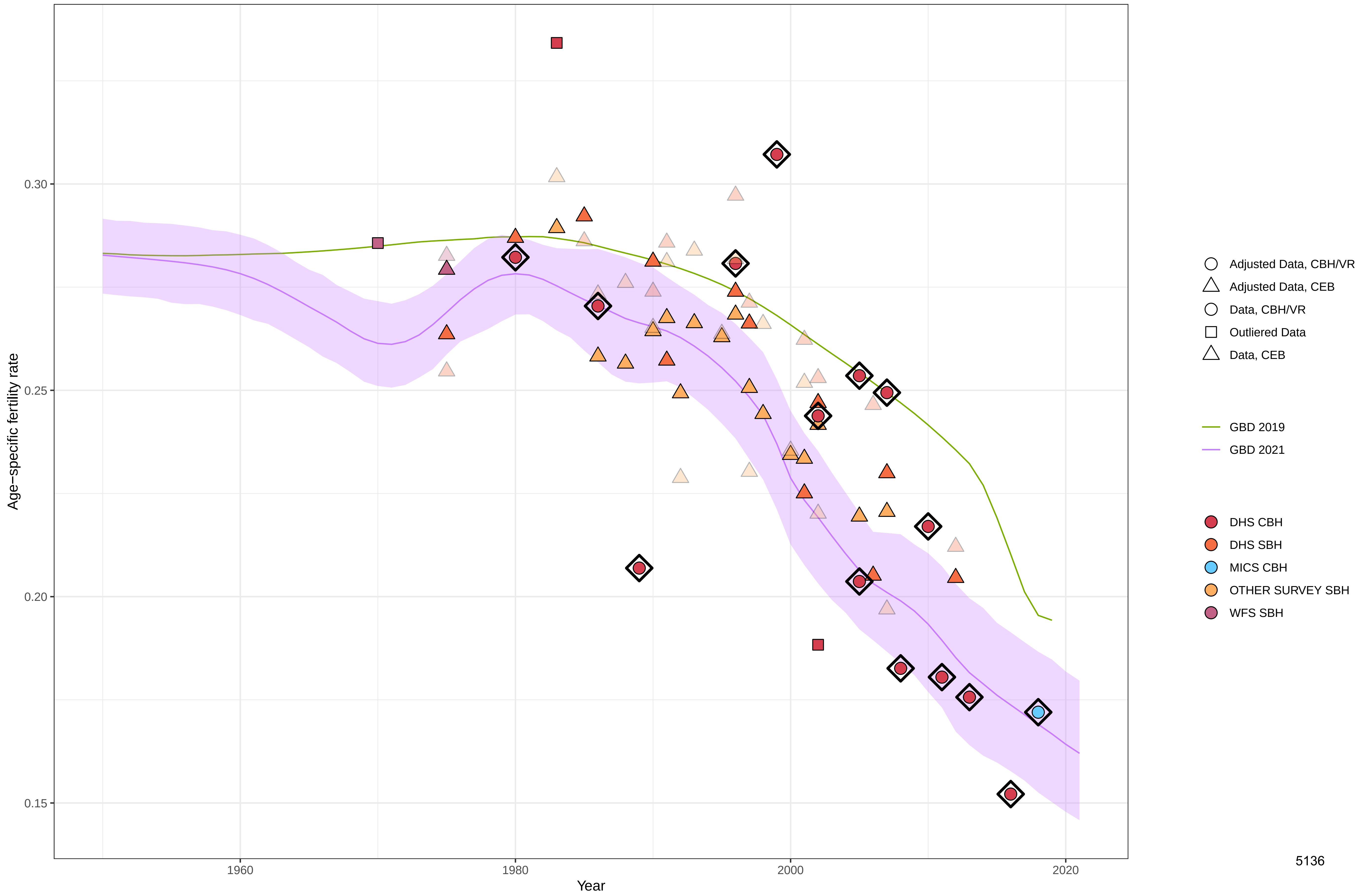

Age−specific fertility fitted trend lines, 1950−2021: Khyber Pakhtunkhwa, Ages: 35−39
Data points enclosed with a diamond are reference sources

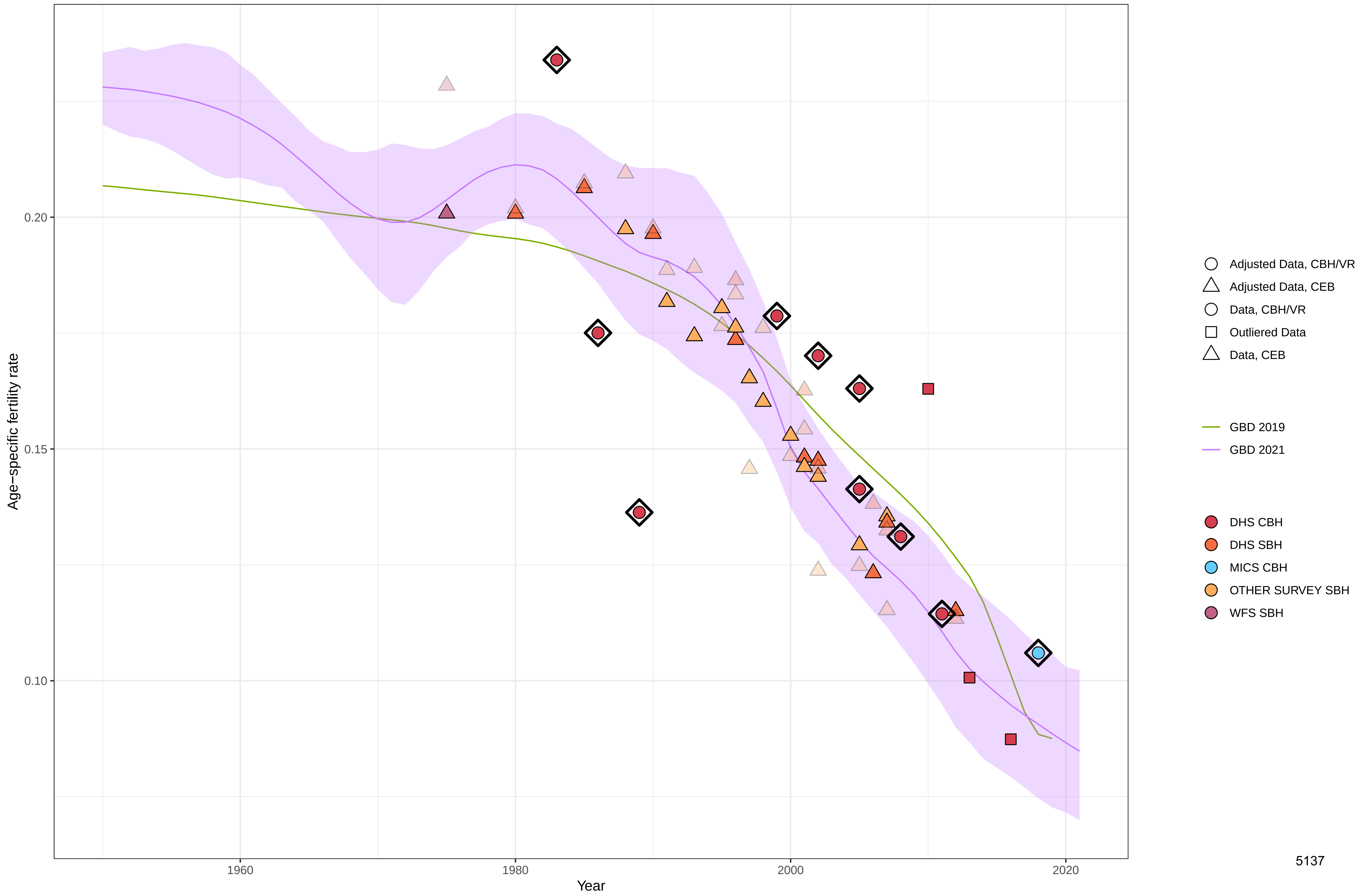

Age−specific fertility fitted trend lines, 1950−2021: Khyber Pakhtunkhwa, Ages: 40−44
Data points enclosed with a diamond are reference sources

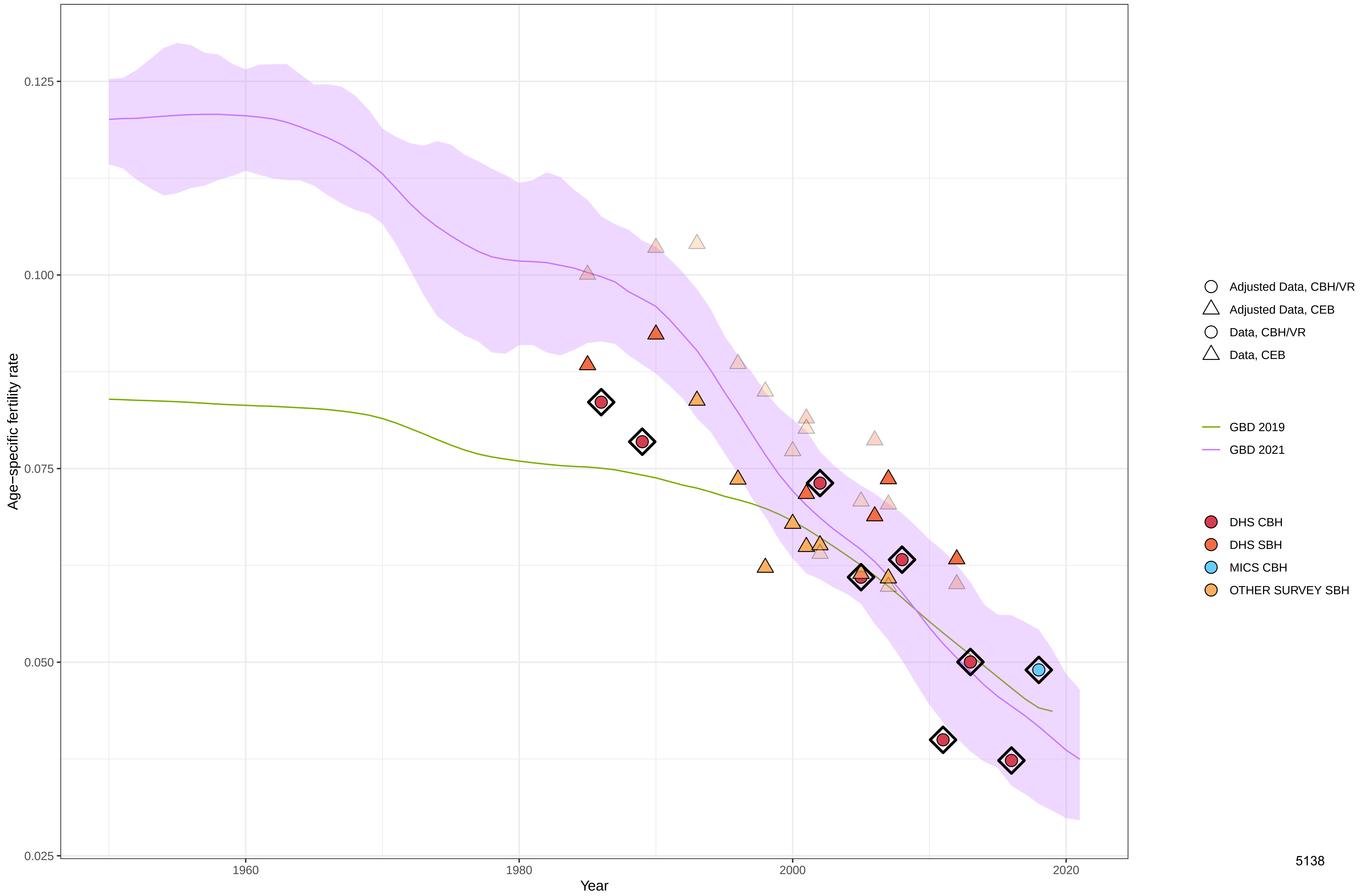

Age−specific fertility fitted trend lines, 1950−2021: Khyber Pakhtunkhwa, Ages: 45−49
Data points enclosed with a diamond are reference sources

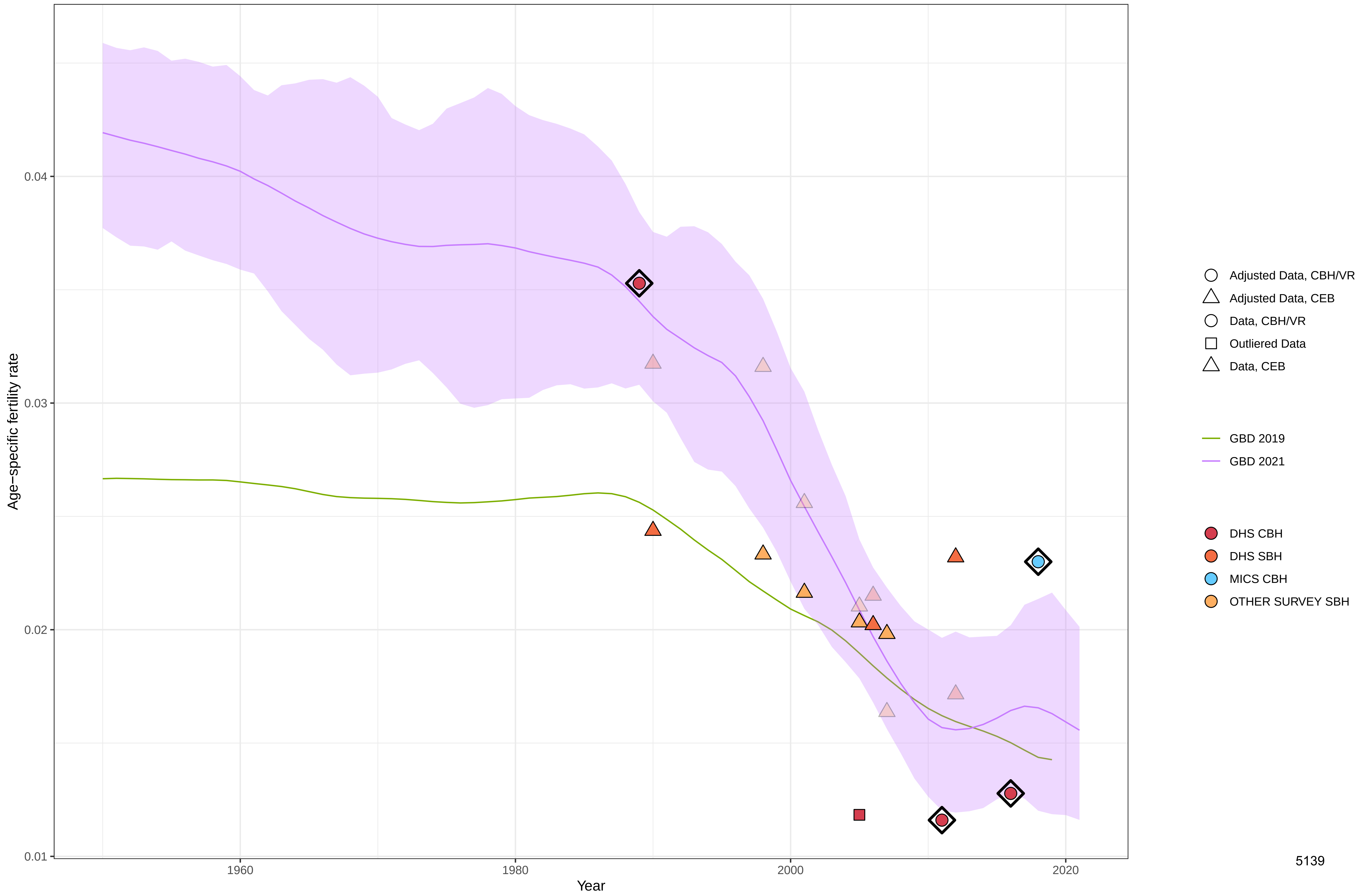

Age−specific fertility fitted trend lines, 1950−2021: Khyber Pakhtunkhwa, Ages: 50−54
Data points enclosed with a diamond are reference sources

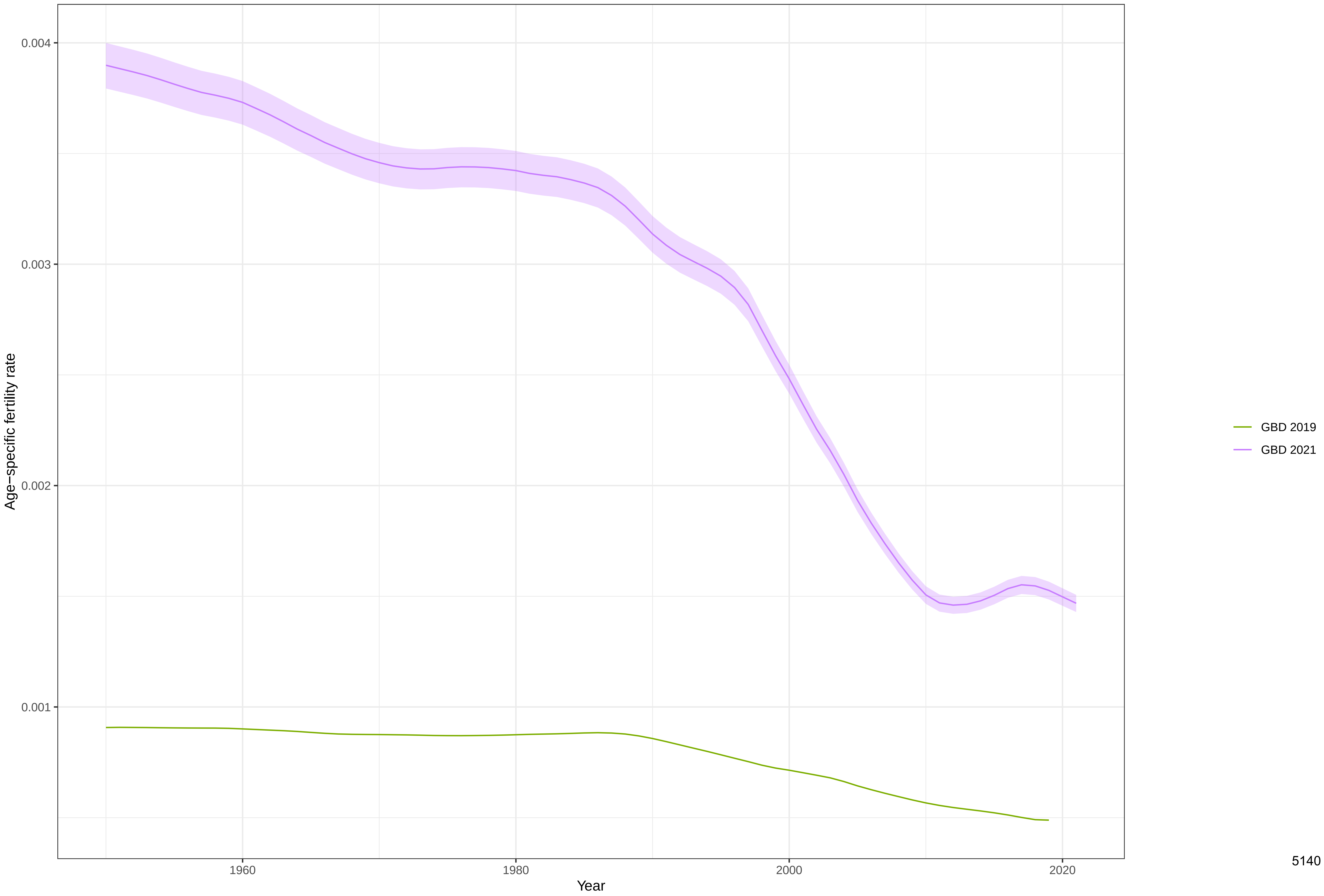

Total fertility rate fitted trend lines, 1950−2021: Khyber Pakhtunkhwa TFR
Data points enclosed with a diamond are reference sources

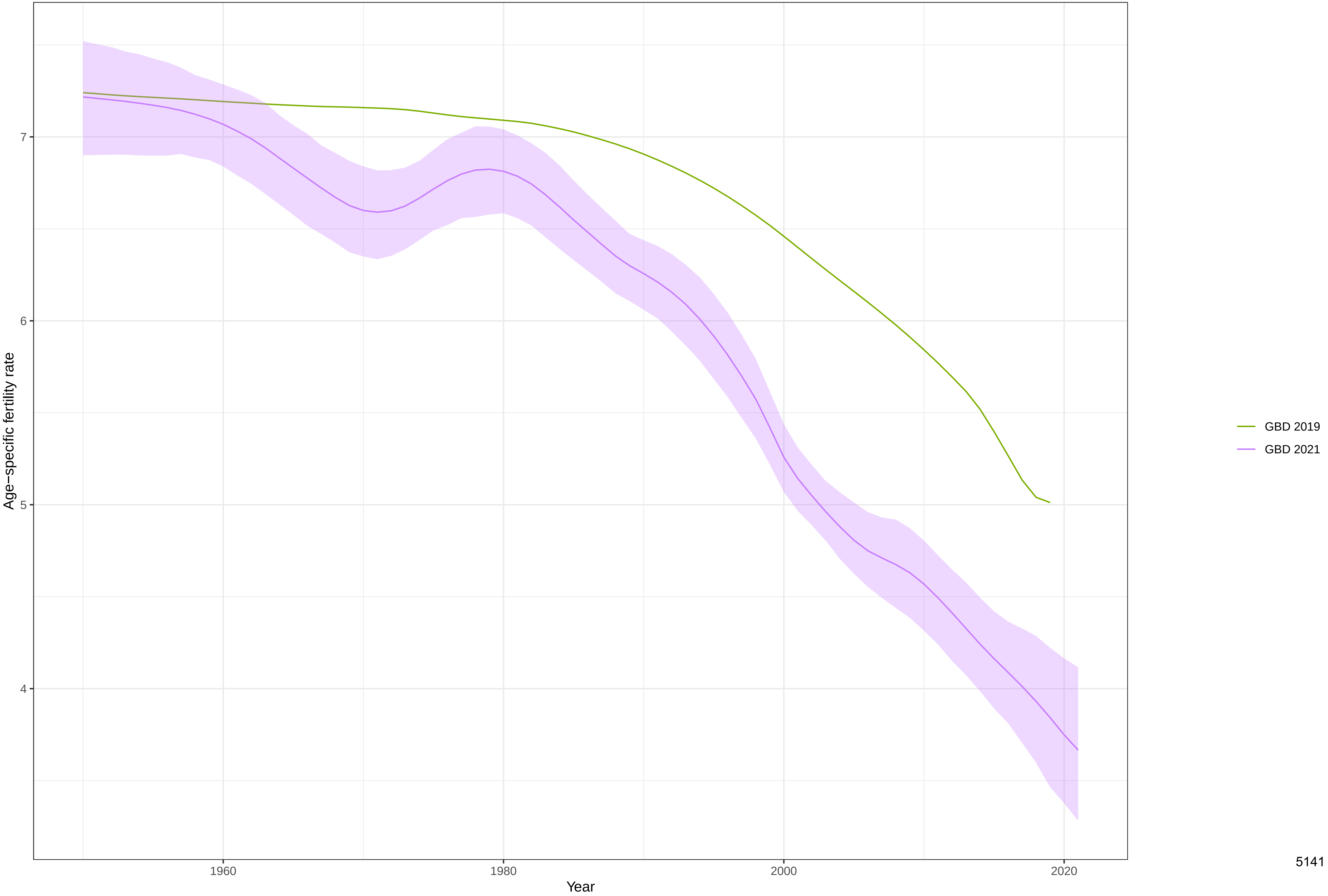

Age−specific fertility fitted trend lines, 1950−2021: Punjab, Ages: 10−14
Data points enclosed with a diamond are reference sources

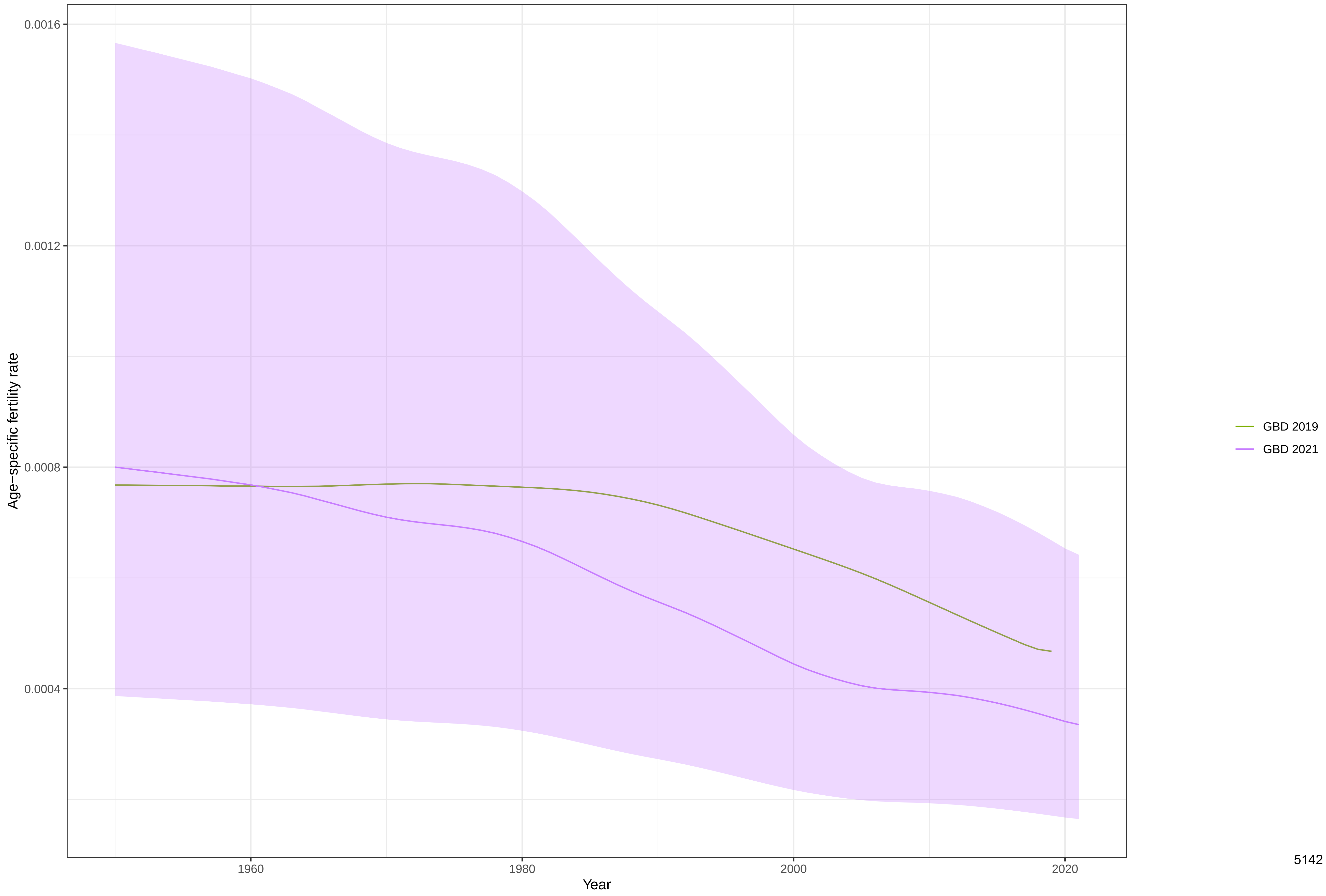

Age−specific fertility fitted trend lines, 1950−2021: Punjab, Ages: 15−19
Data points enclosed with a diamond are reference sources

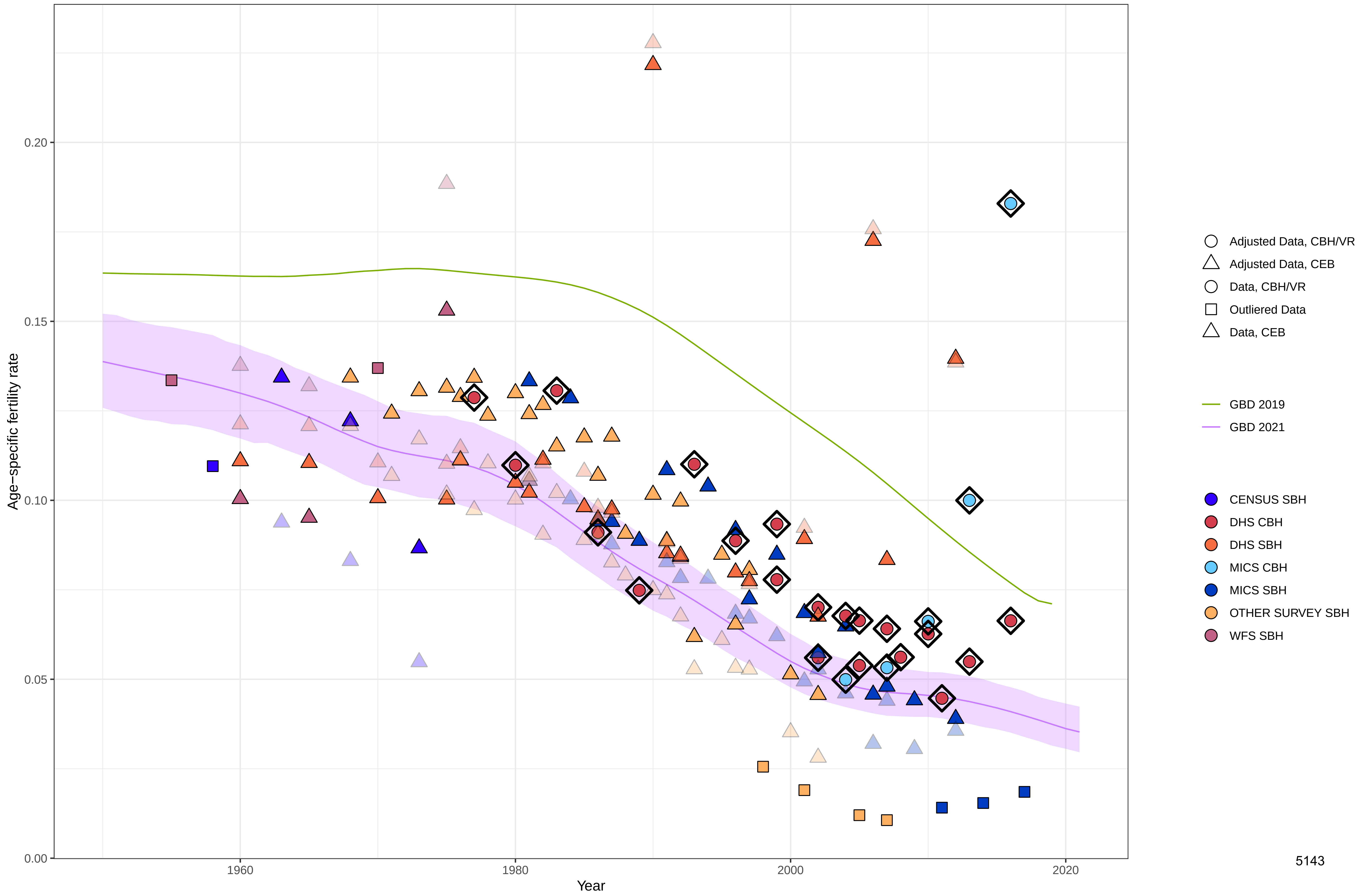

Age−specific fertility fitted trend lines, 1950−2021: Punjab, Ages: 20−24
Data points enclosed with a diamond are reference sources

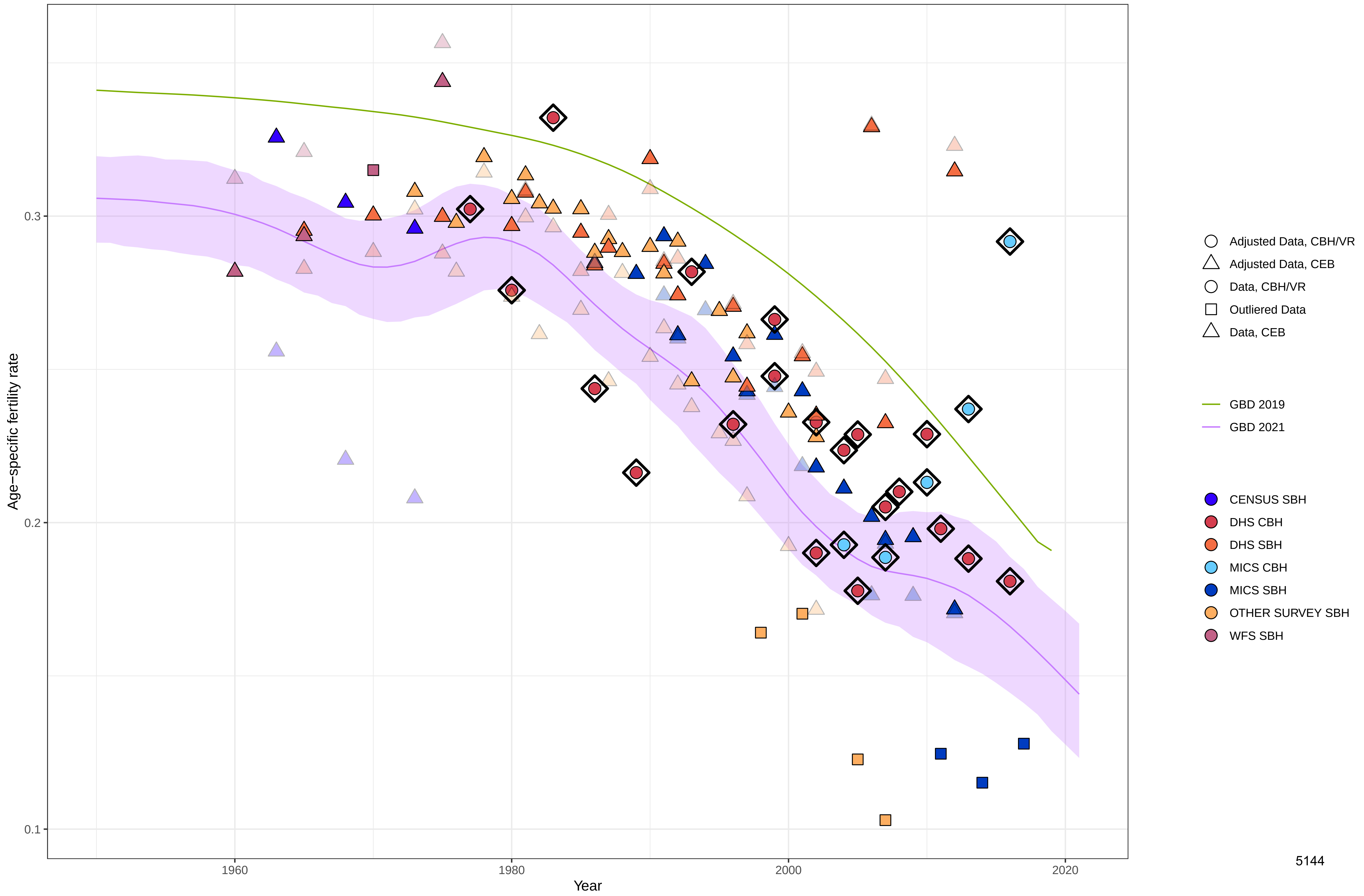

Age−specific fertility fitted trend lines, 1950−2021: Punjab, Ages: 25−29
Data points enclosed with a diamond are reference sources

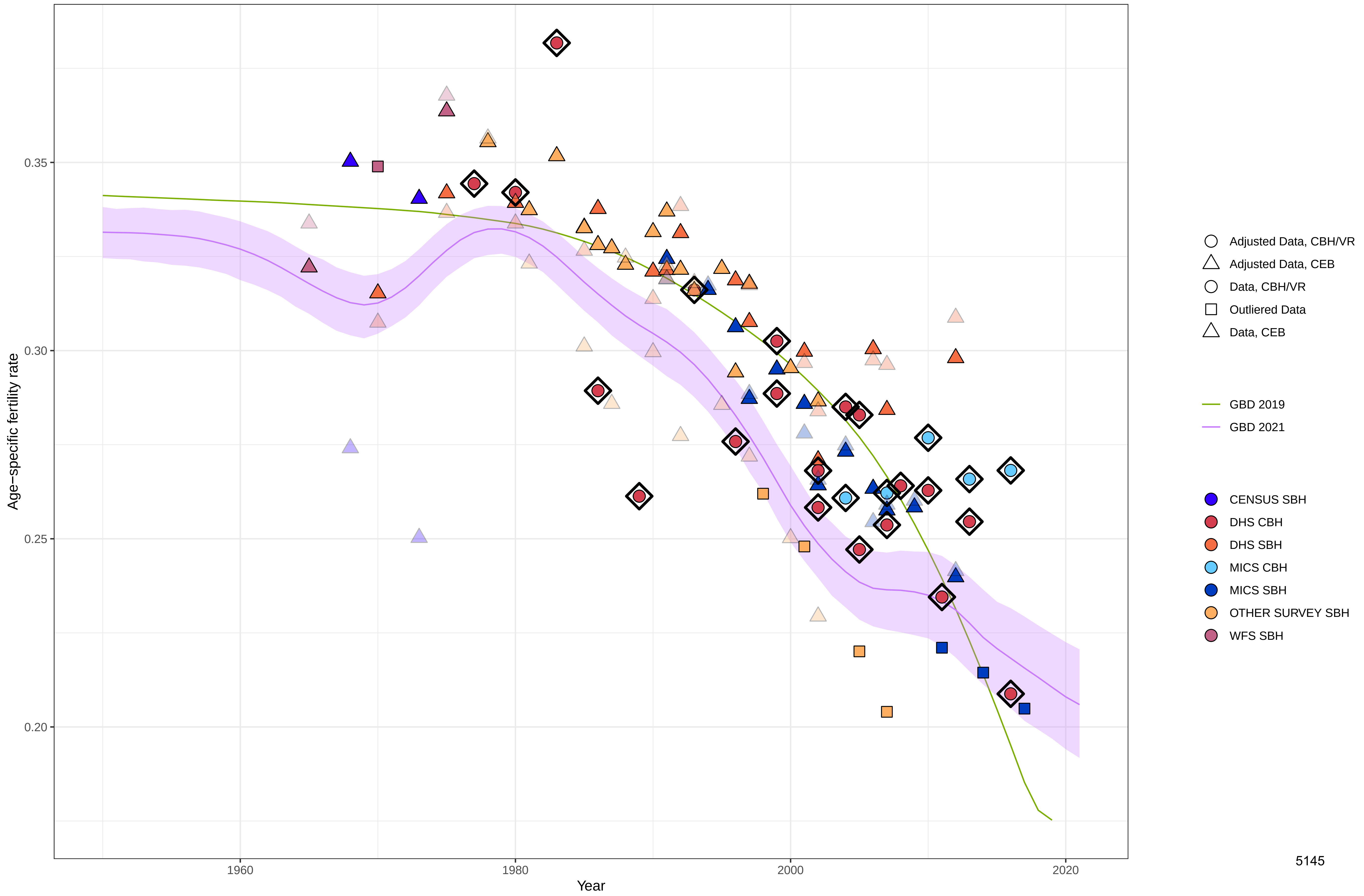

Age−specific fertility fitted trend lines, 1950−2021: Punjab, Ages: 30−34
Data points enclosed with a diamond are reference sources

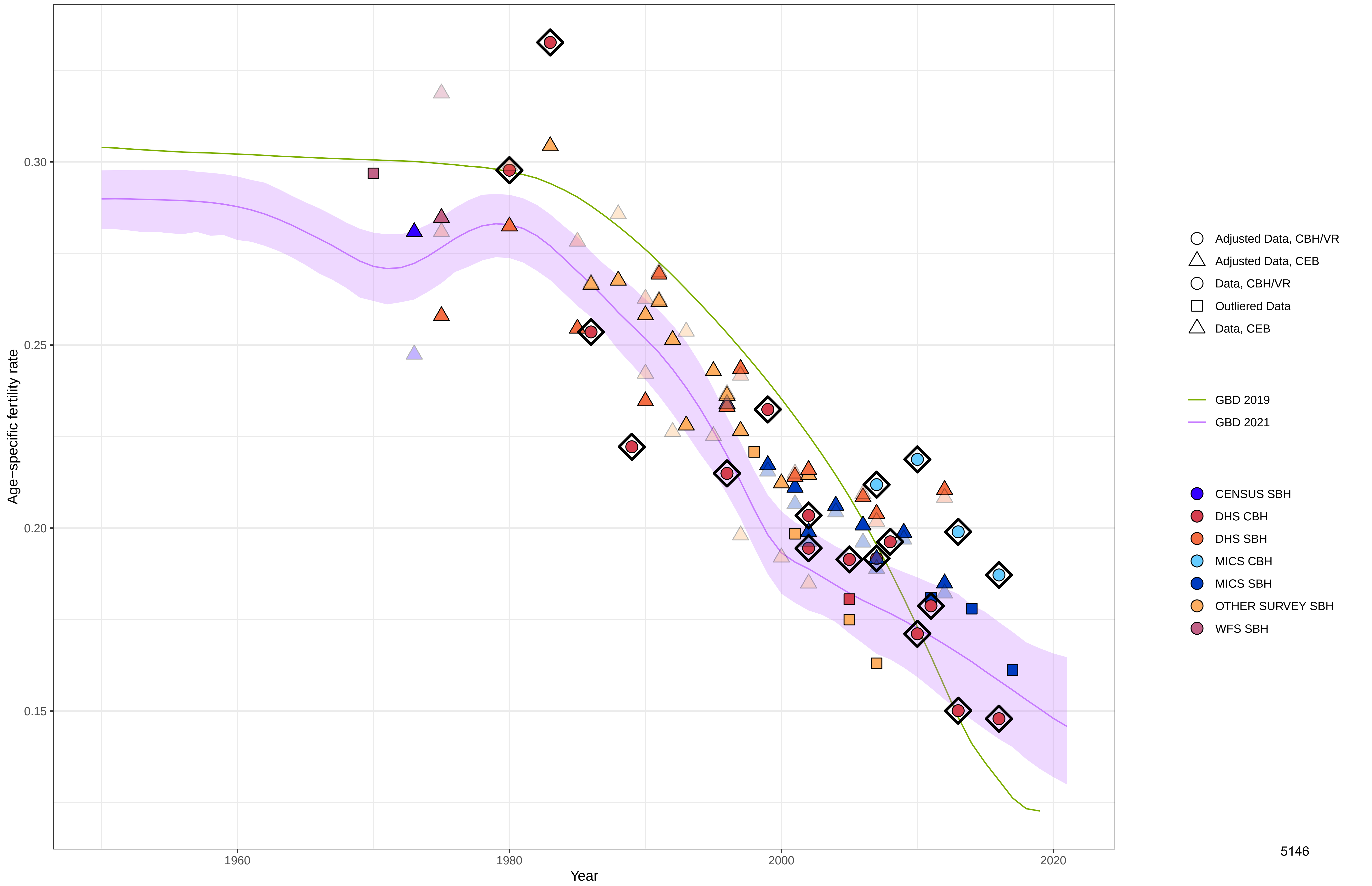

Age−specific fertility fitted trend lines, 1950−2021: Punjab, Ages: 35−39
Data points enclosed with a diamond are reference sources

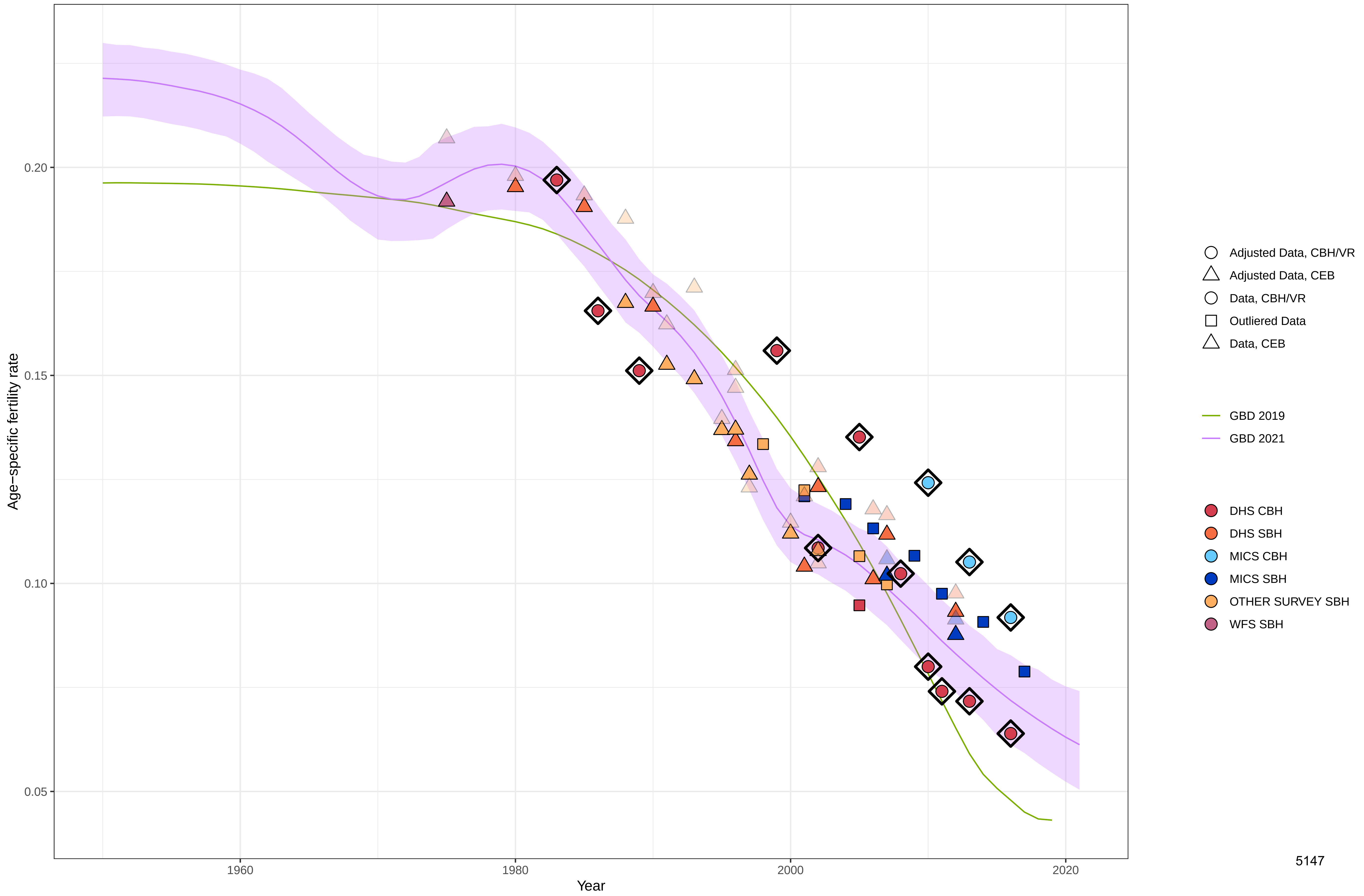

Age−specific fertility fitted trend lines, 1950−2021: Punjab, Ages: 40−44
Data points enclosed with a diamond are reference sources

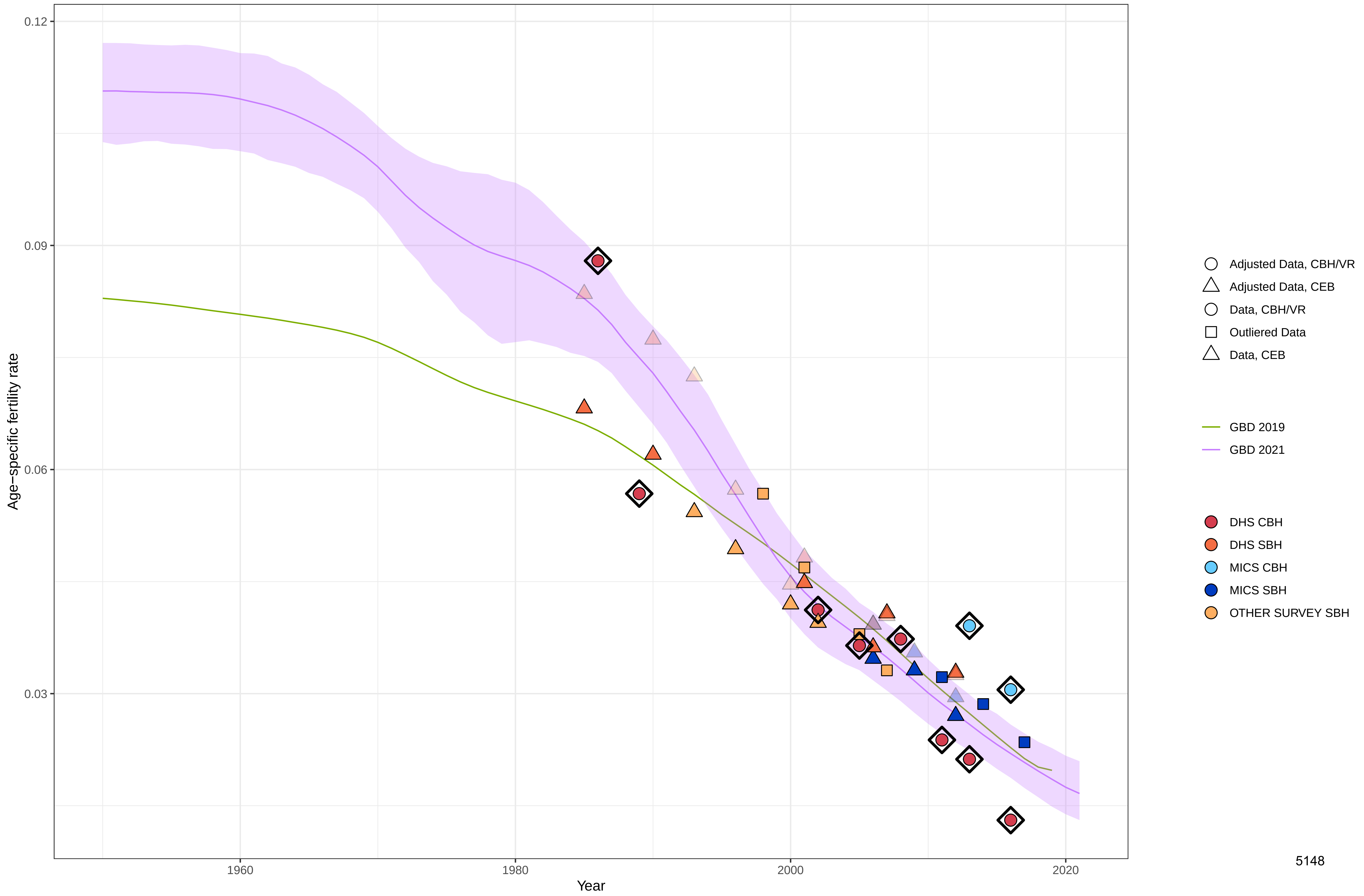

Age−specific fertility fitted trend lines, 1950−2021: Punjab, Ages: 45−49
Data points enclosed with a diamond are reference sources

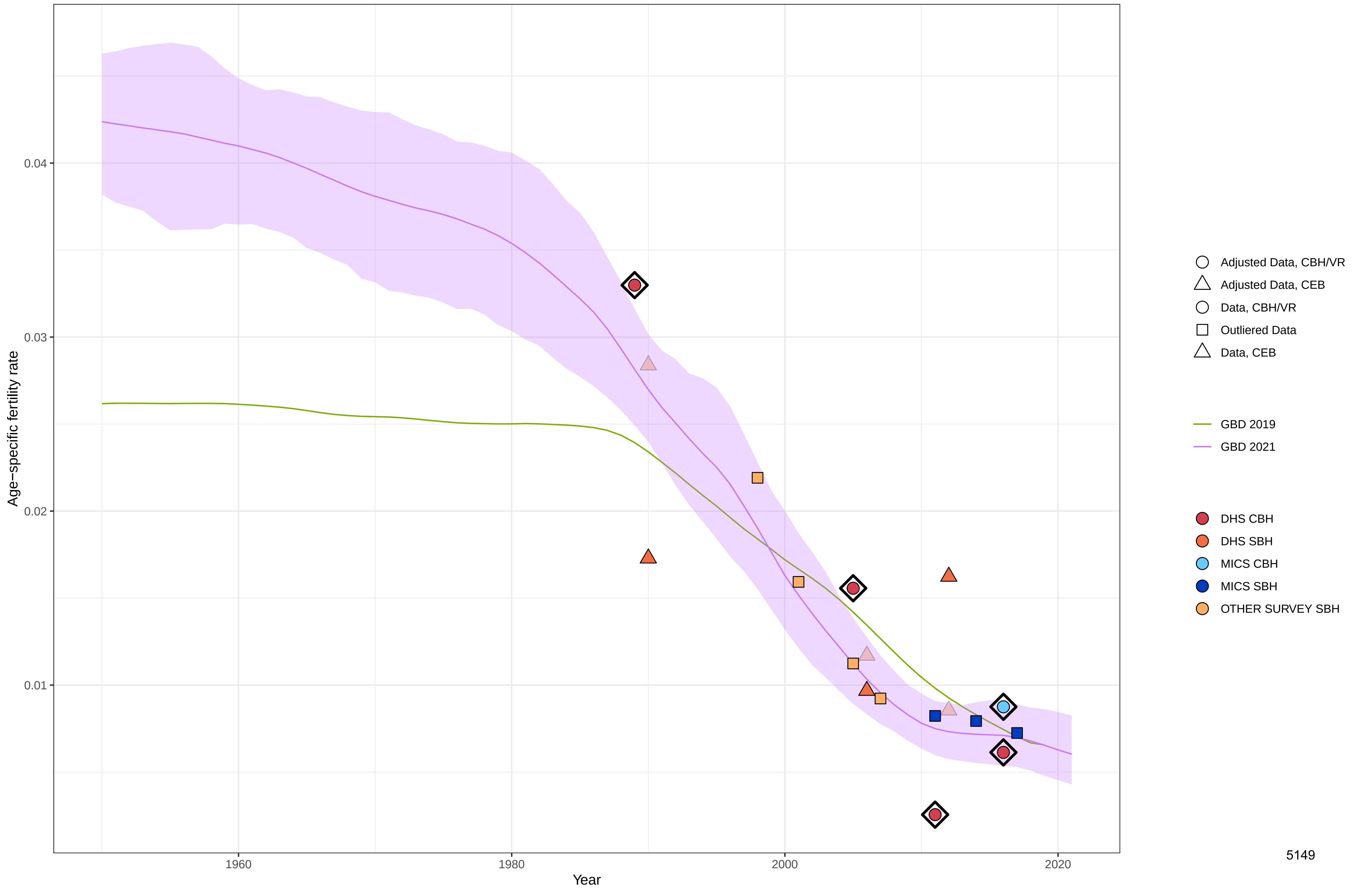

Age−specific fertility fitted trend lines, 1950−2021: Punjab, Ages: 50−54
Data points enclosed with a diamond are reference sources

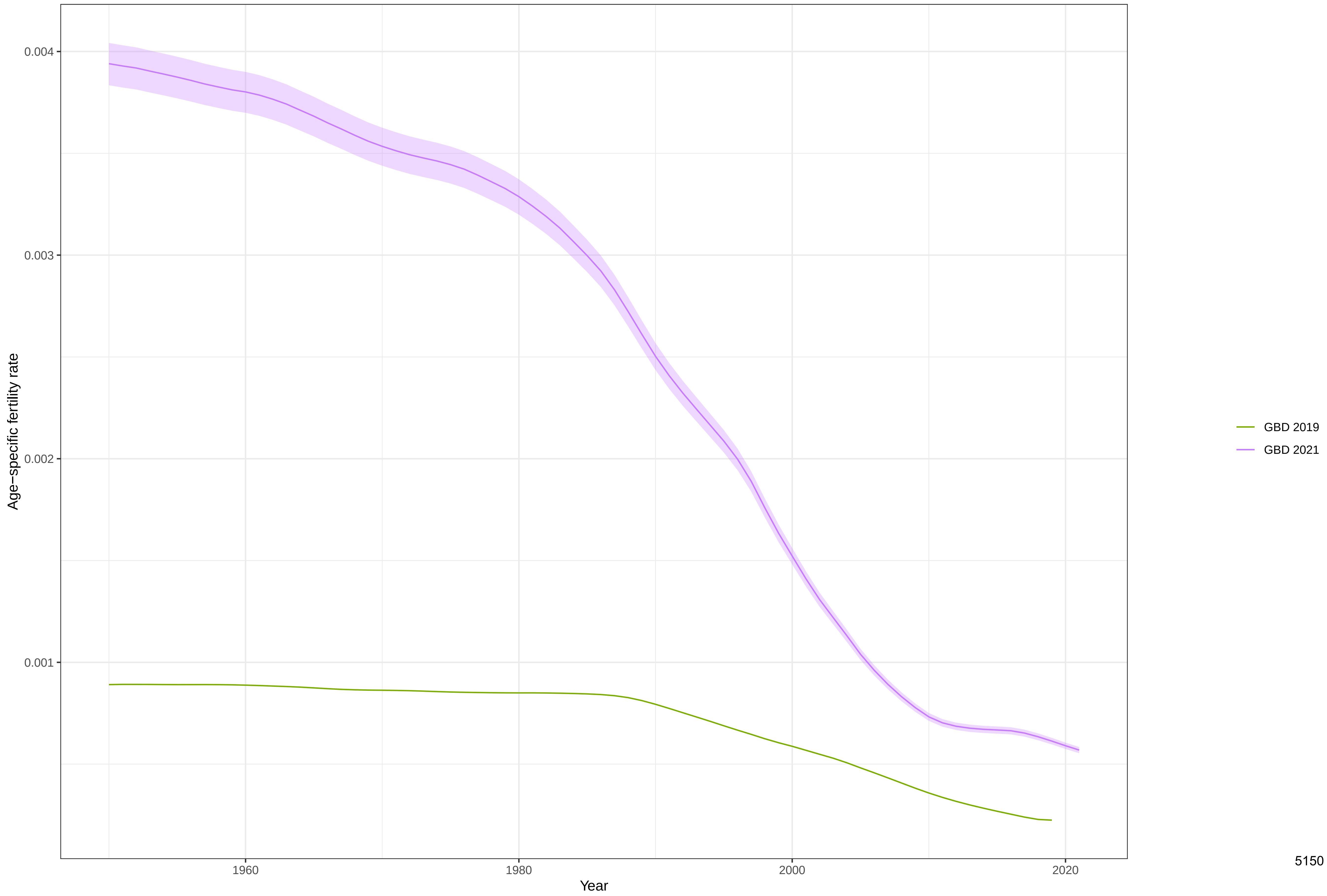

Total fertility rate fitted trend lines, 1950−2021: Punjab TFR
Data points enclosed with a diamond are reference sources

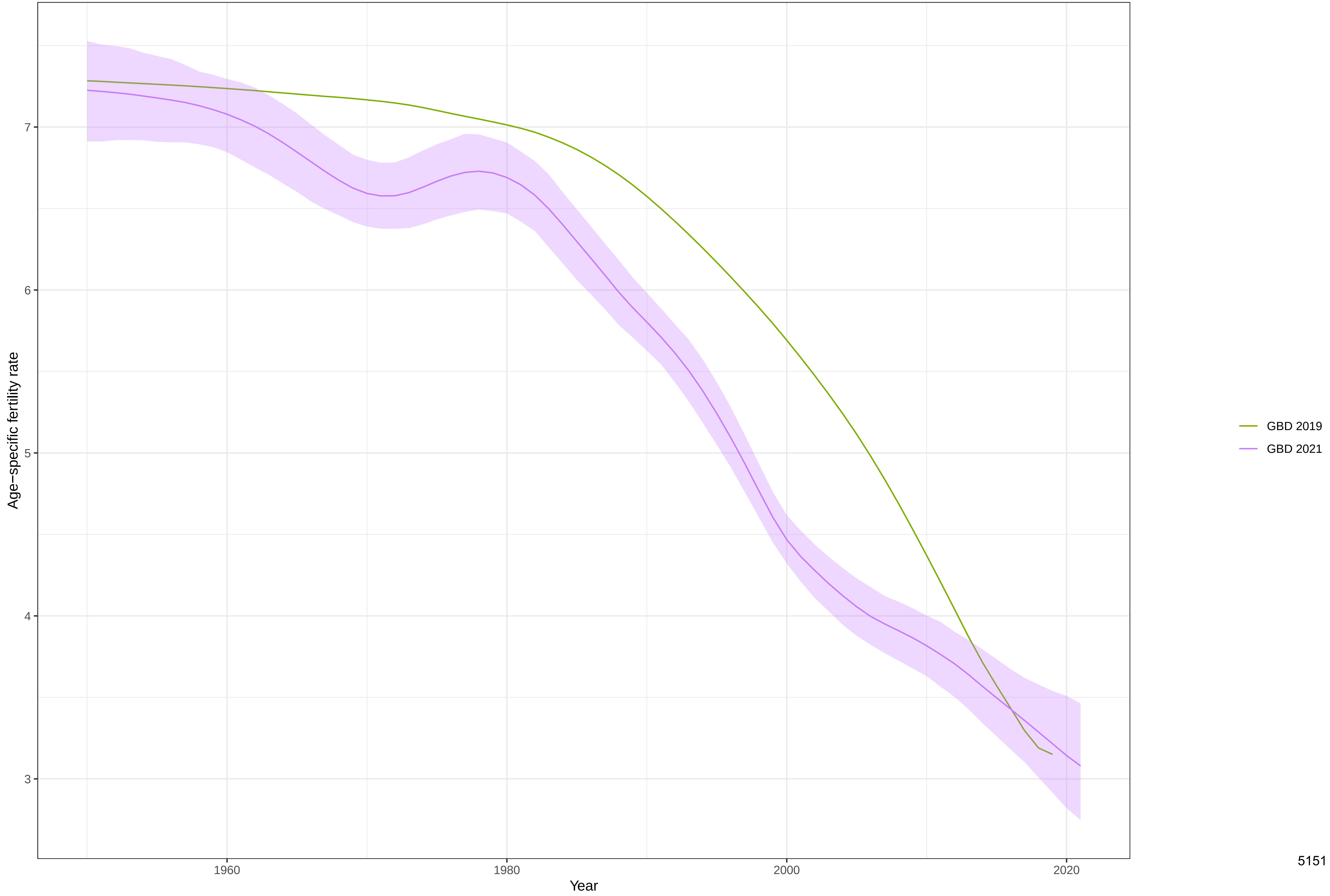

Age−specific fertility fitted trend lines, 1950−2021: Sindh, Ages: 10−14
Data points enclosed with a diamond are reference sources

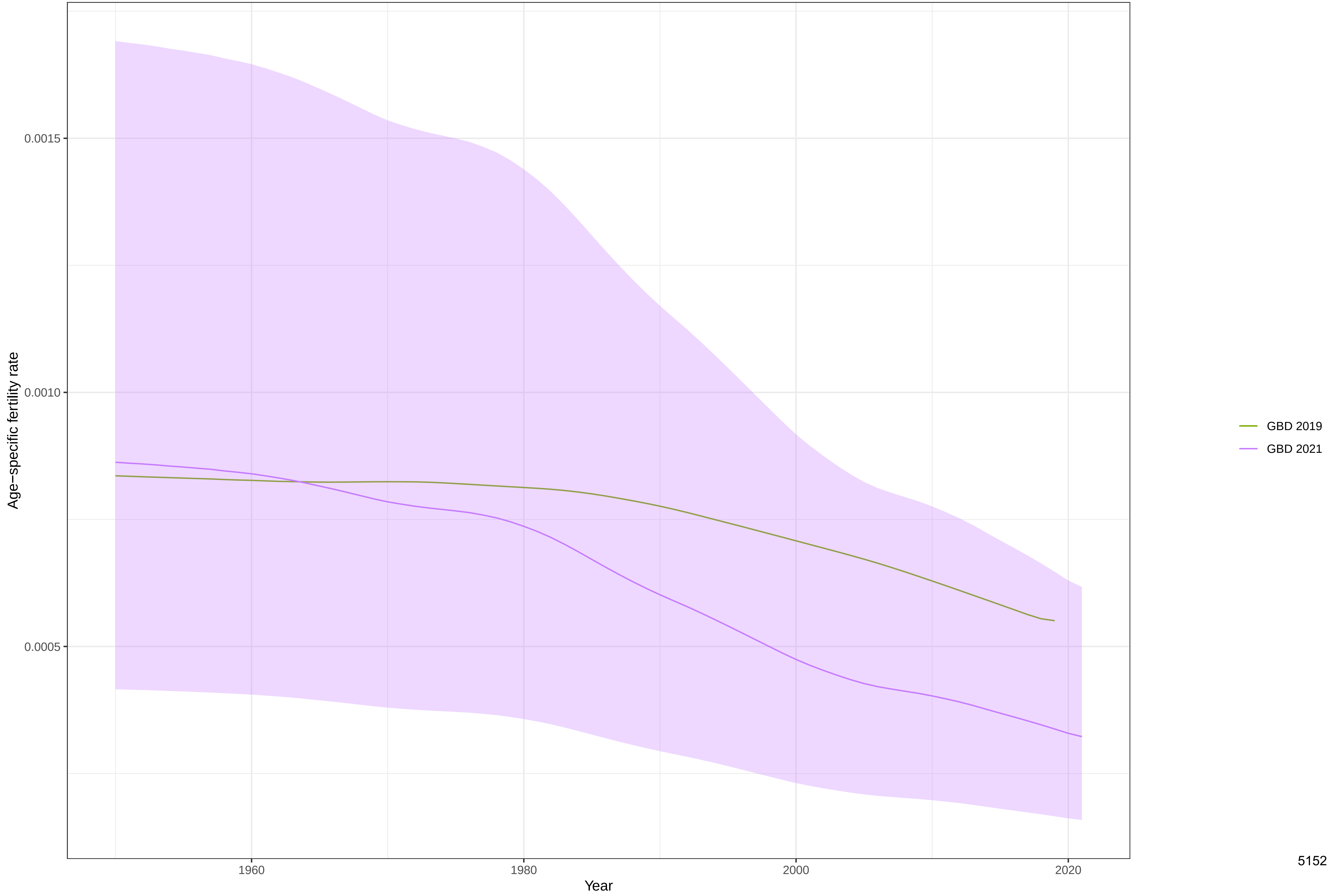

Age−specific fertility fitted trend lines, 1950−2021: Sindh, Ages: 15−19
Data points enclosed with a diamond are reference sources

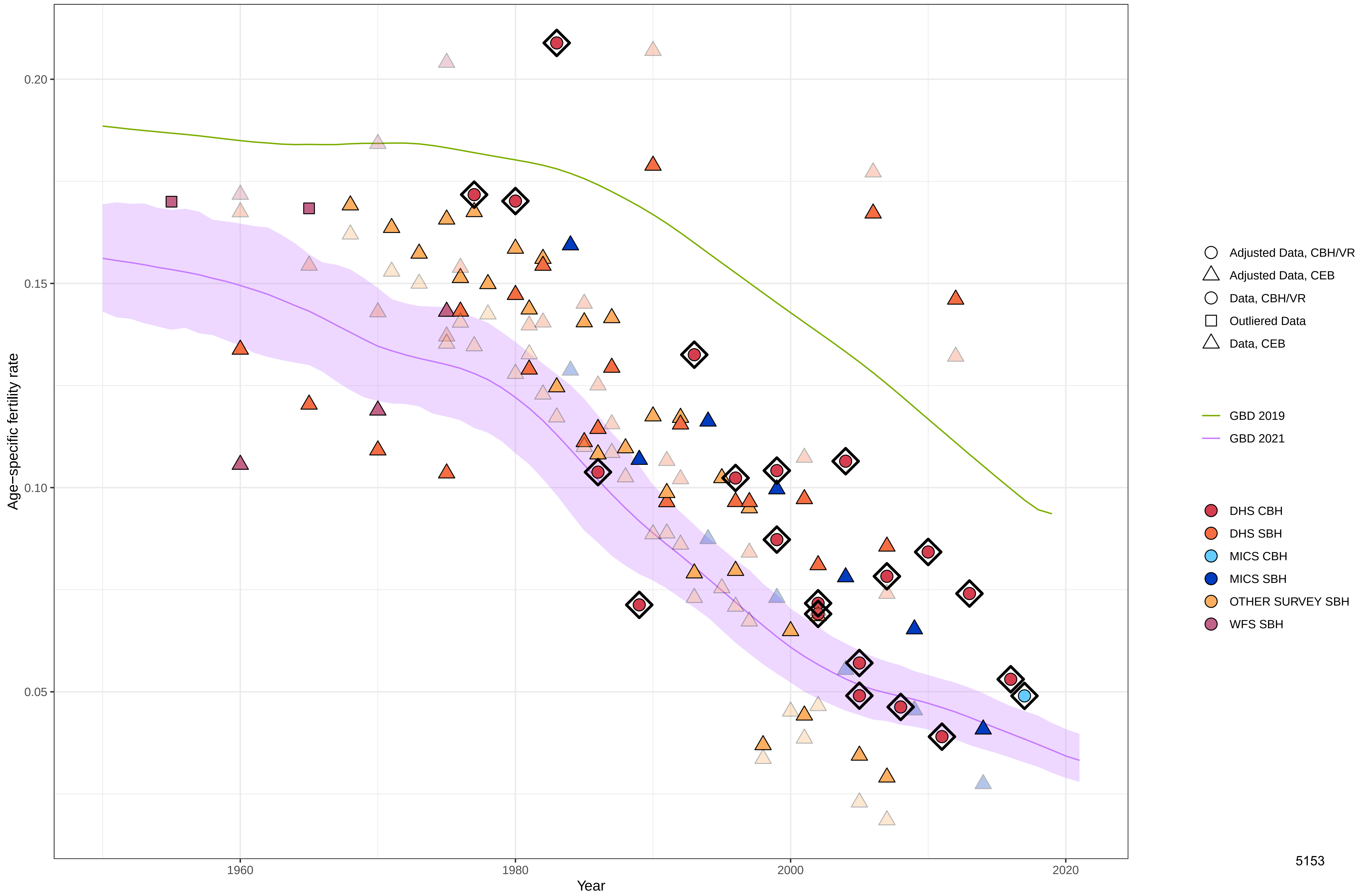

Age−specific fertility fitted trend lines, 1950−2021: Sindh, Ages: 20−24
Data points enclosed with a diamond are reference sources

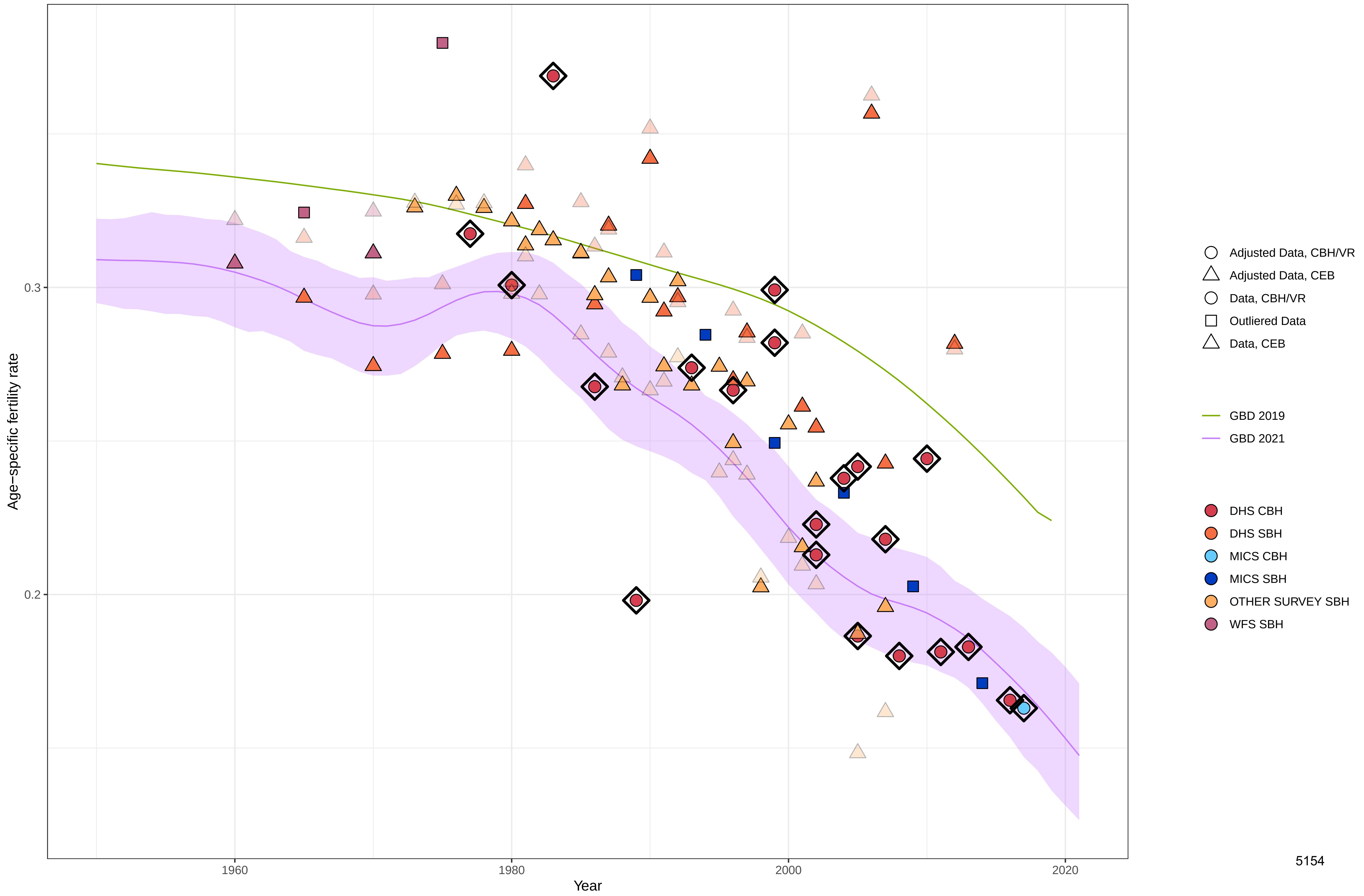

Age−specific fertility fitted trend lines, 1950−2021: Sindh, Ages: 25−29
Data points enclosed with a diamond are reference sources

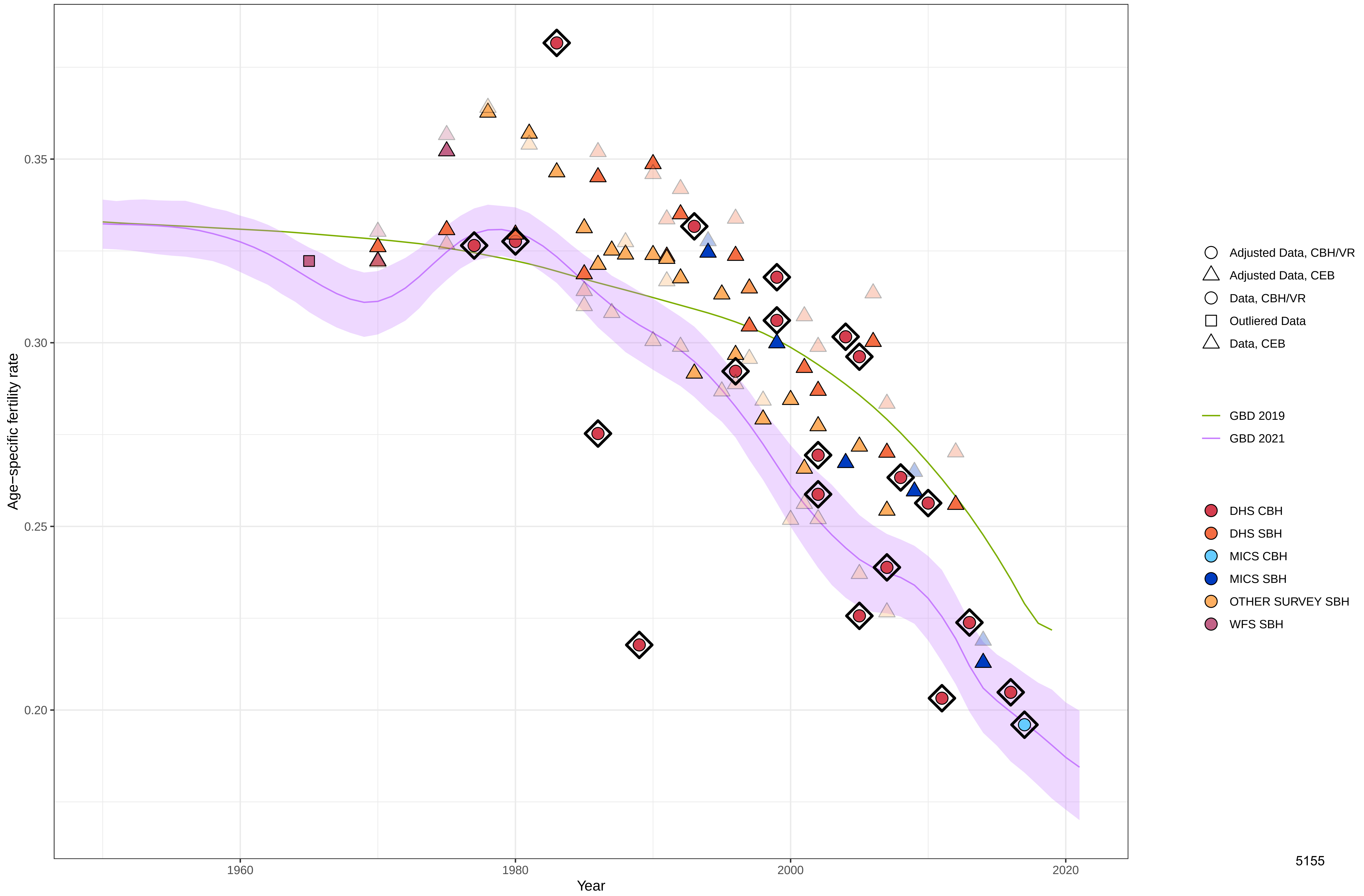

Age−specific fertility fitted trend lines, 1950−2021: Sindh, Ages: 30−34
Data points enclosed with a diamond are reference sources

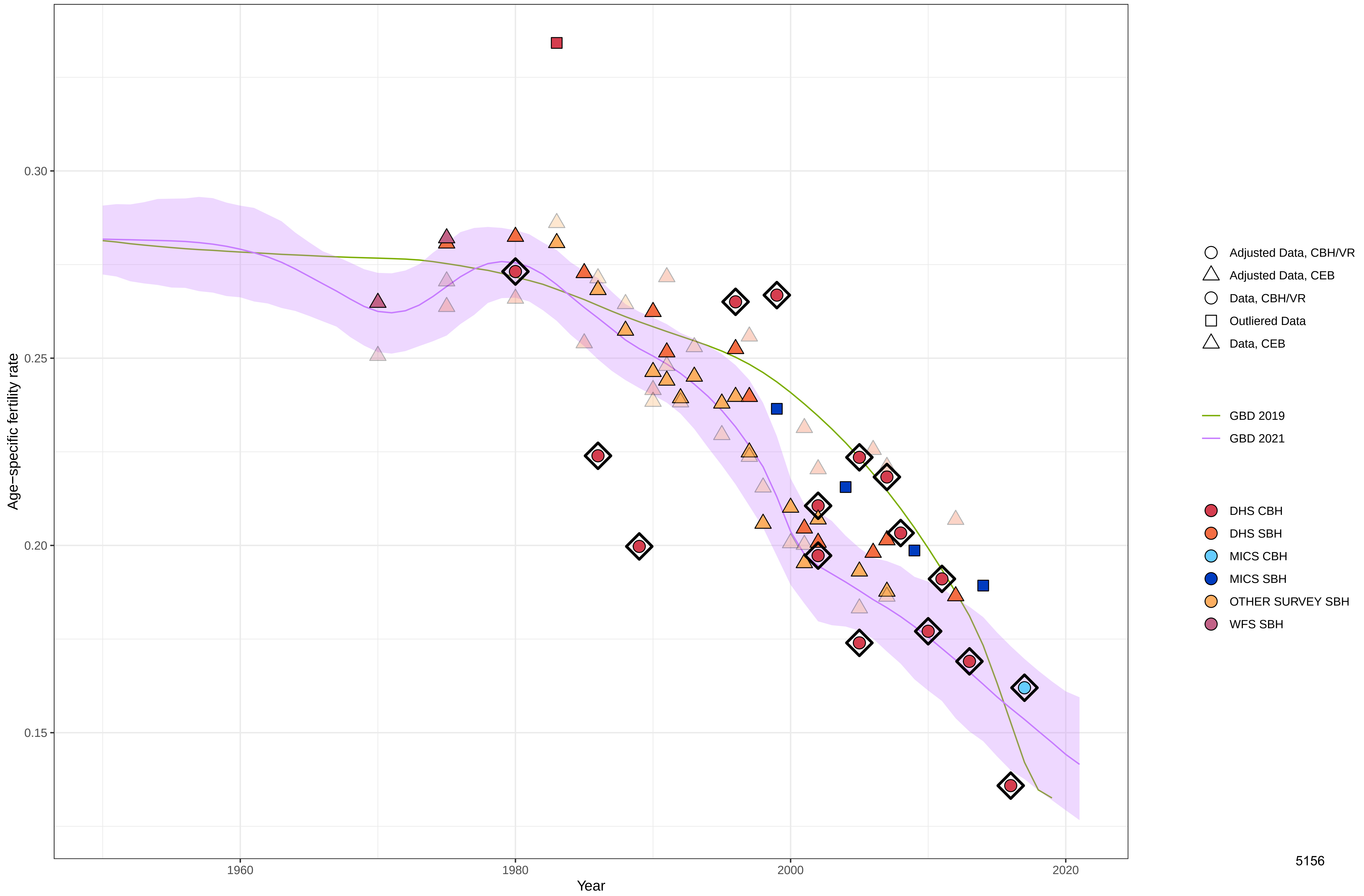

Age−specific fertility fitted trend lines, 1950−2021: Sindh, Ages: 35−39
Data points enclosed with a diamond are reference sources

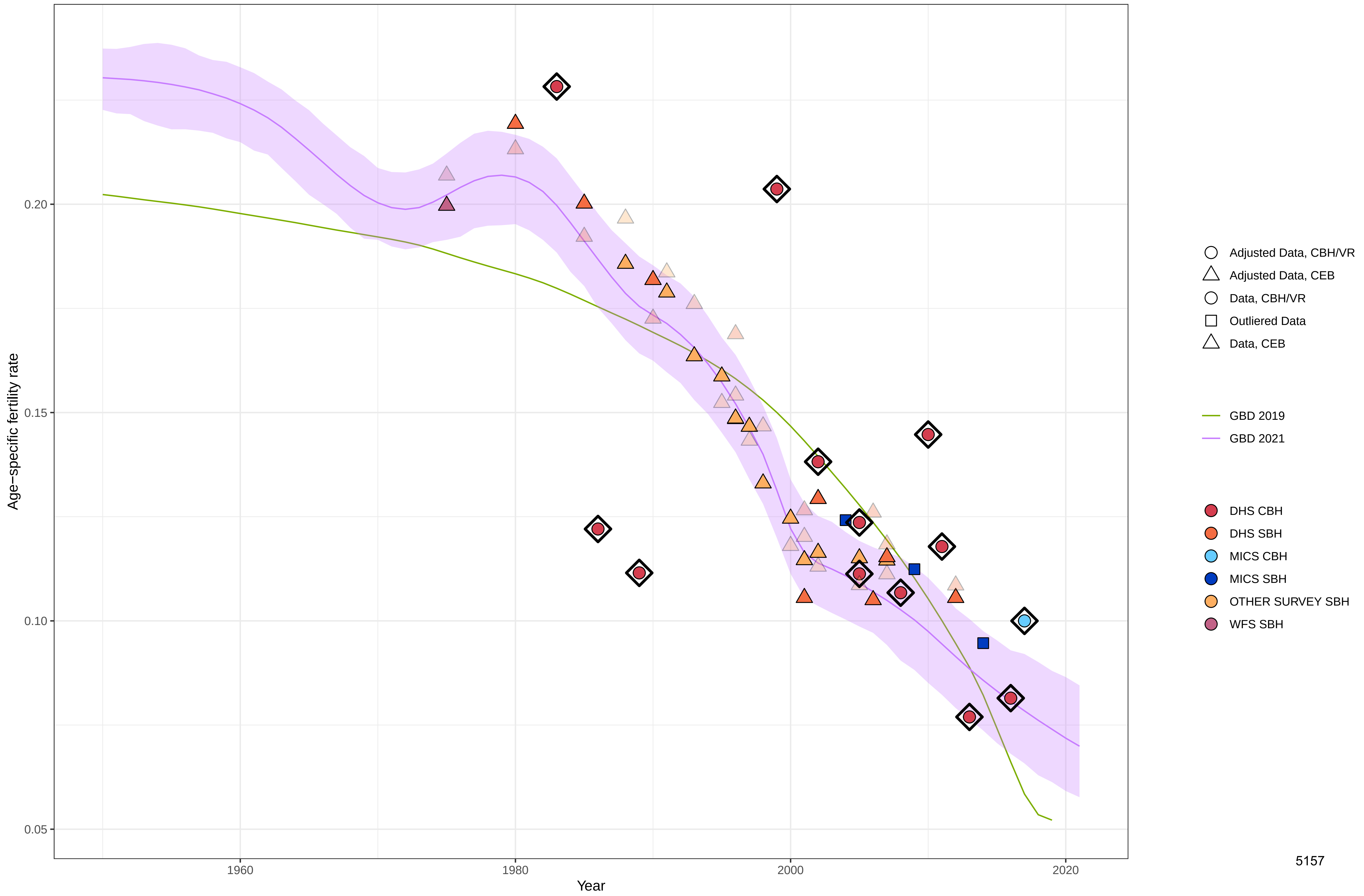

Age−specific fertility fitted trend lines, 1950−2021: Sindh, Ages: 40−44
Data points enclosed with a diamond are reference sources

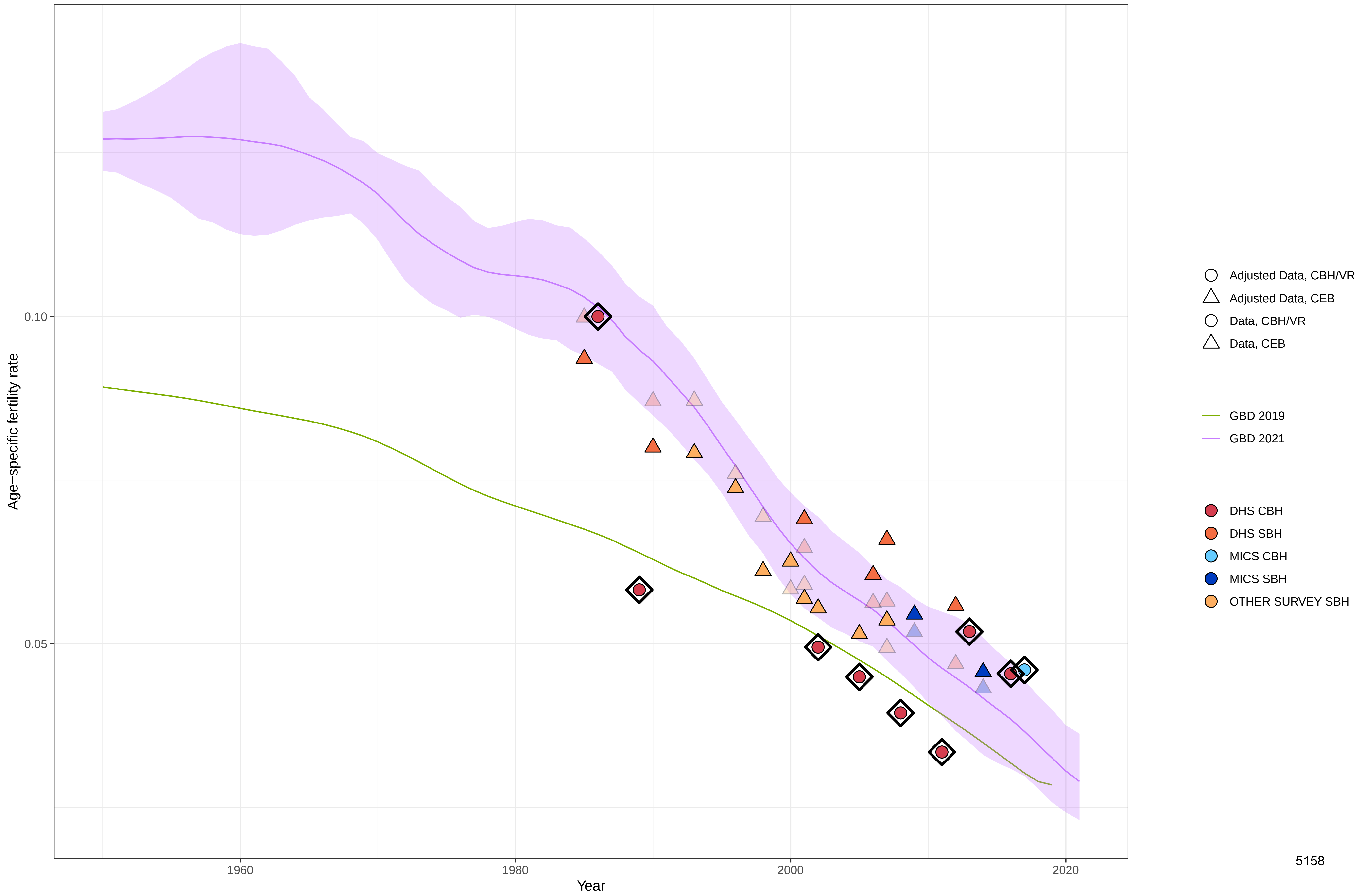

Age−specific fertility fitted trend lines, 1950−2021: Sindh, Ages: 45−49
Data points enclosed with a diamond are reference sources

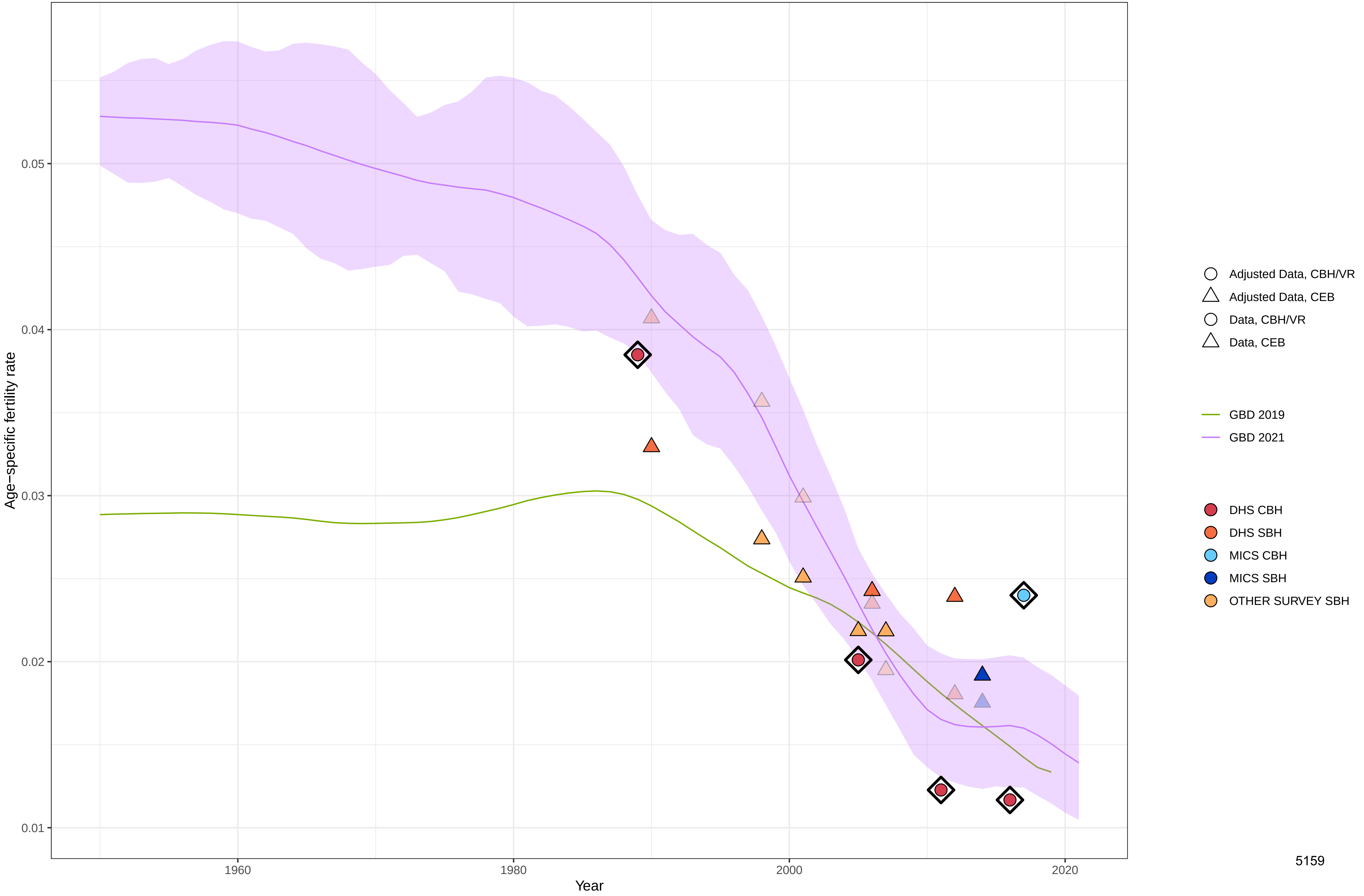

Age−specific fertility fitted trend lines, 1950−2021: Sindh, Ages: 50−54
Data points enclosed with a diamond are reference sources

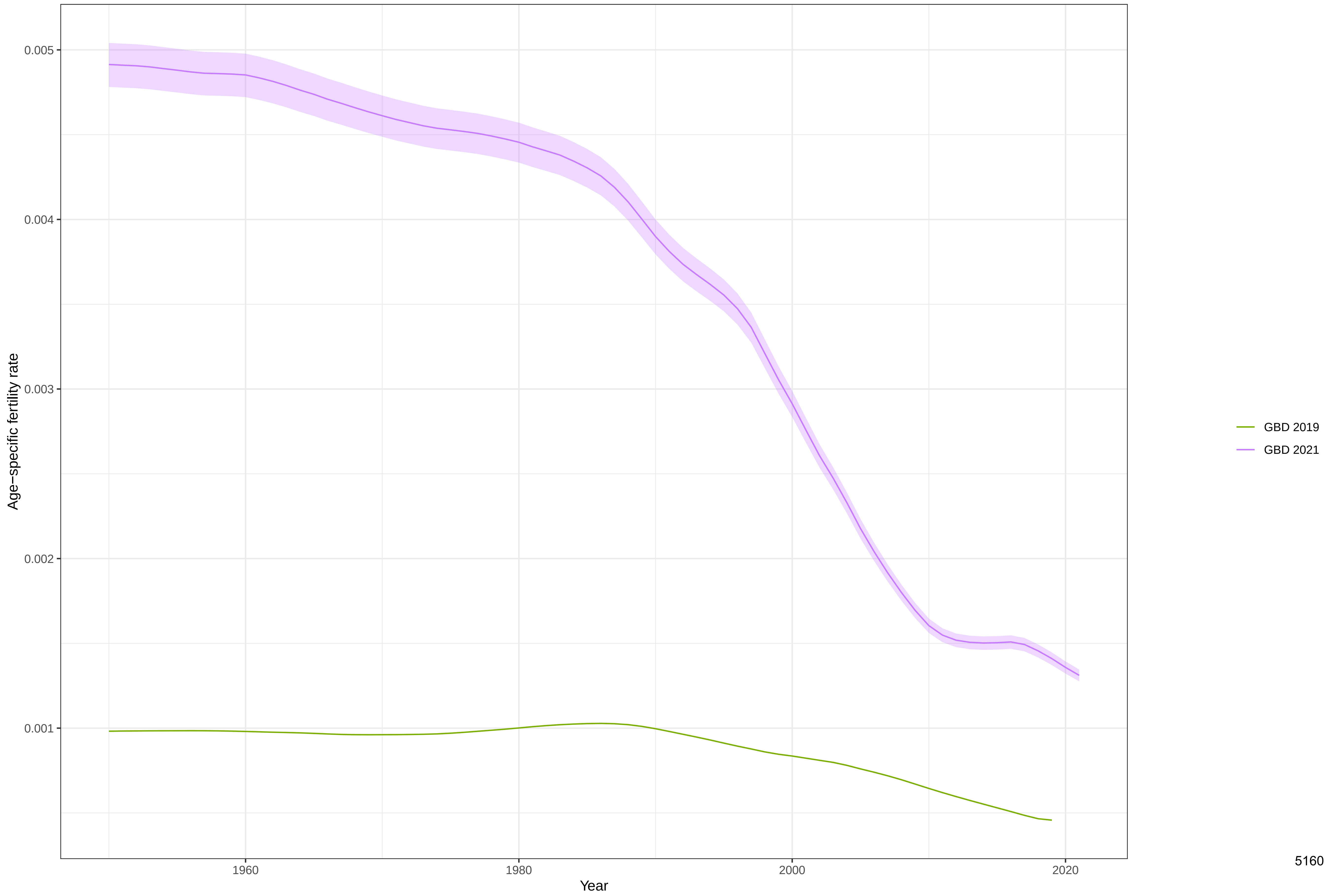

Total fertility rate fitted trend lines, 1950−2021: Sindh TFR
Data points enclosed with a diamond are reference sources

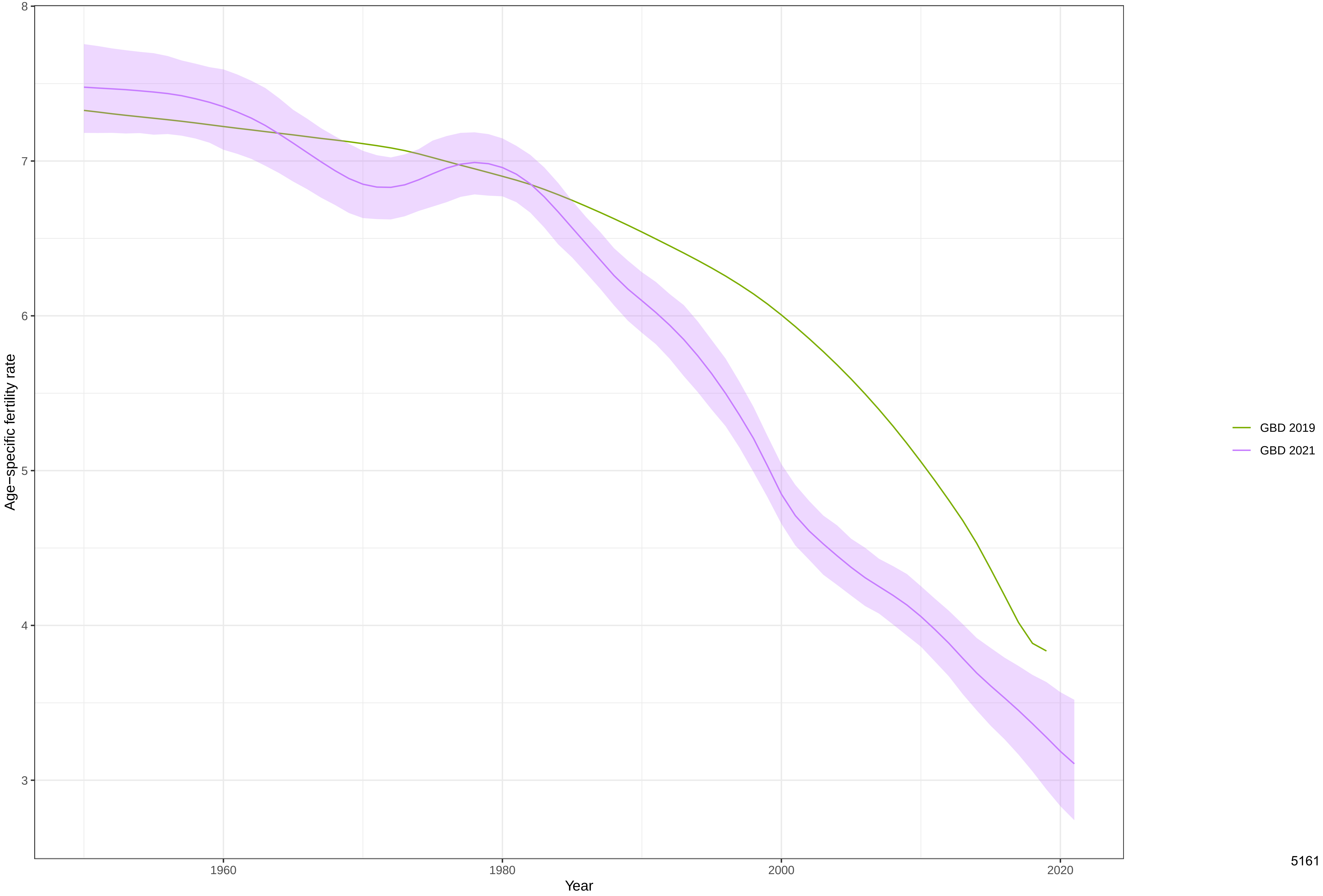

Age−specific fertility fitted trend lines, 1950−2021: North Korea, Ages: 10−14
Data points enclosed with a diamond are reference sources

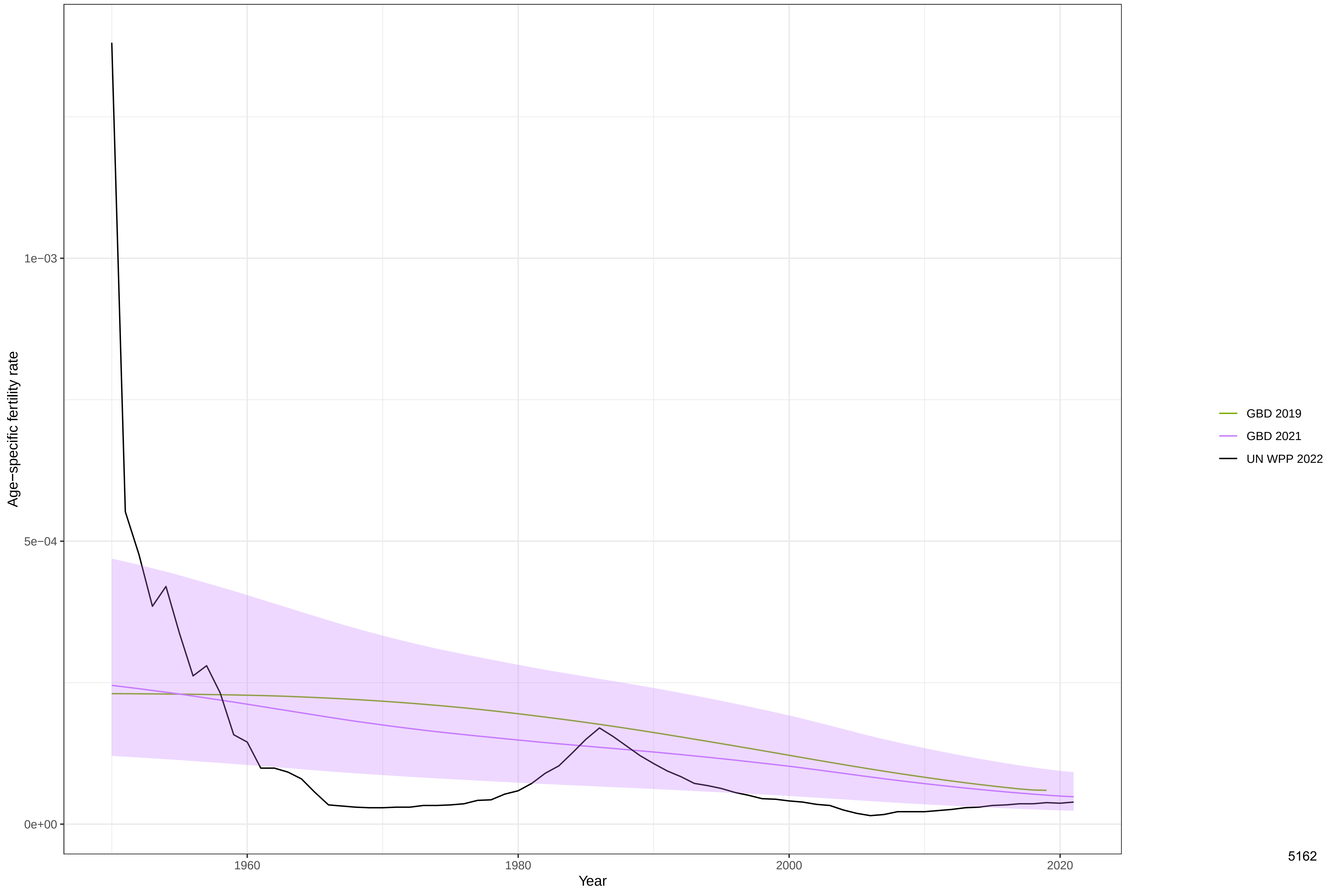

Age−specific fertility fitted trend lines, 1950−2021: North Korea, Ages: 15−19
Data points enclosed with a diamond are reference sources

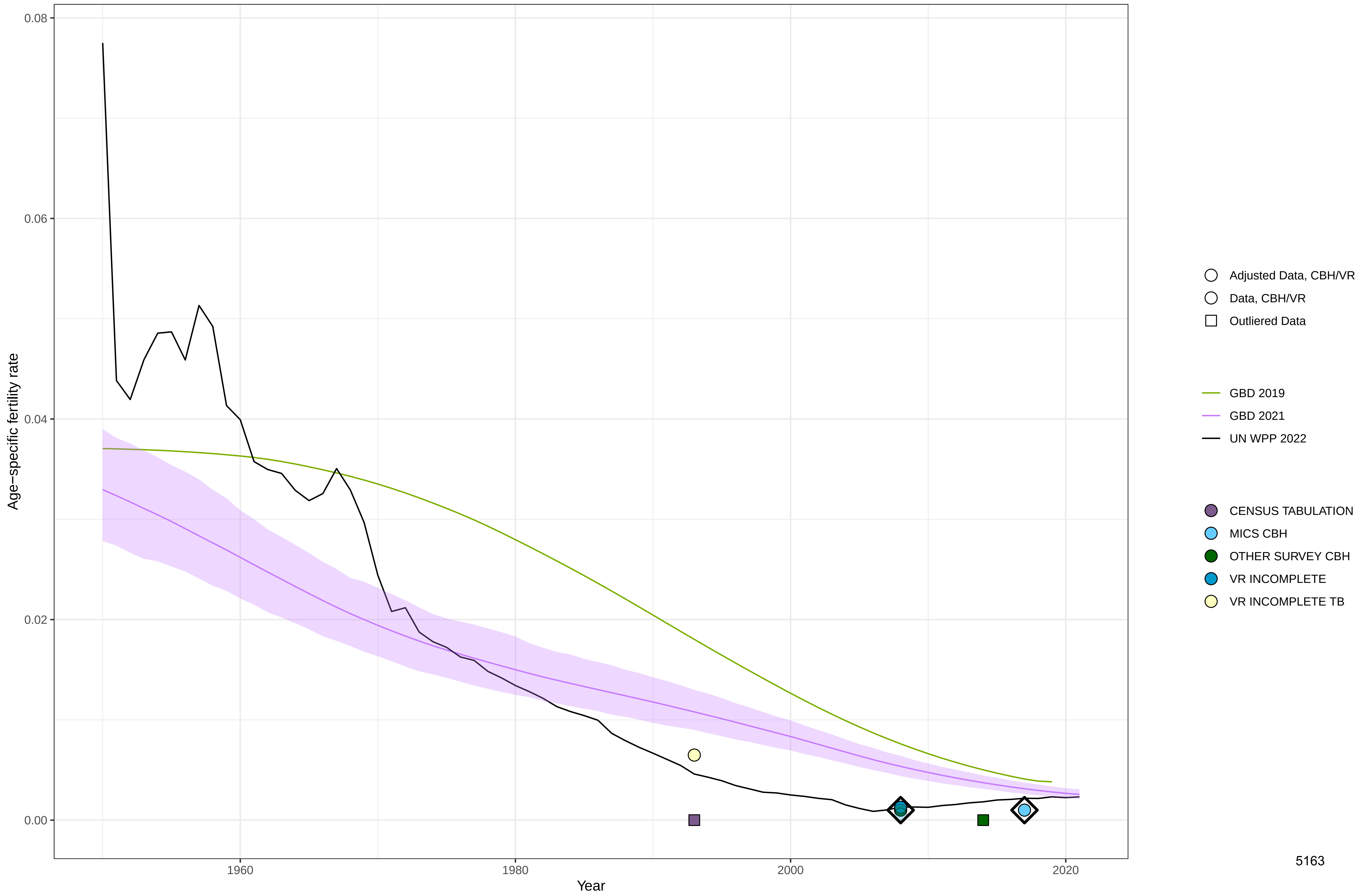

Age−specific fertility fitted trend lines, 1950−2021: North Korea, Ages: 20−24
Data points enclosed with a diamond are reference sources

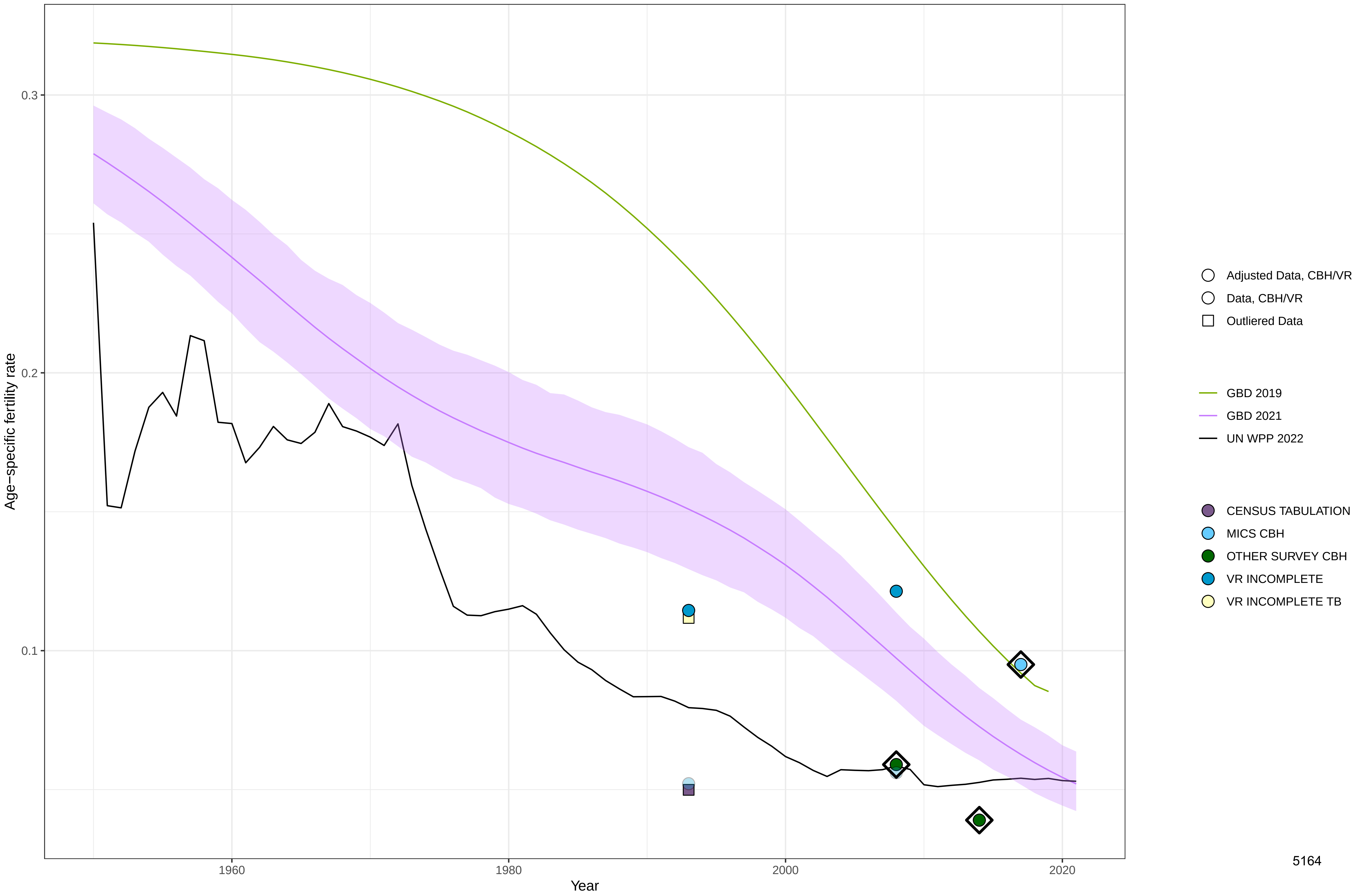

Age−specific fertility fitted trend lines, 1950−2021: North Korea, Ages: 25−29
Data points enclosed with a diamond are reference sources

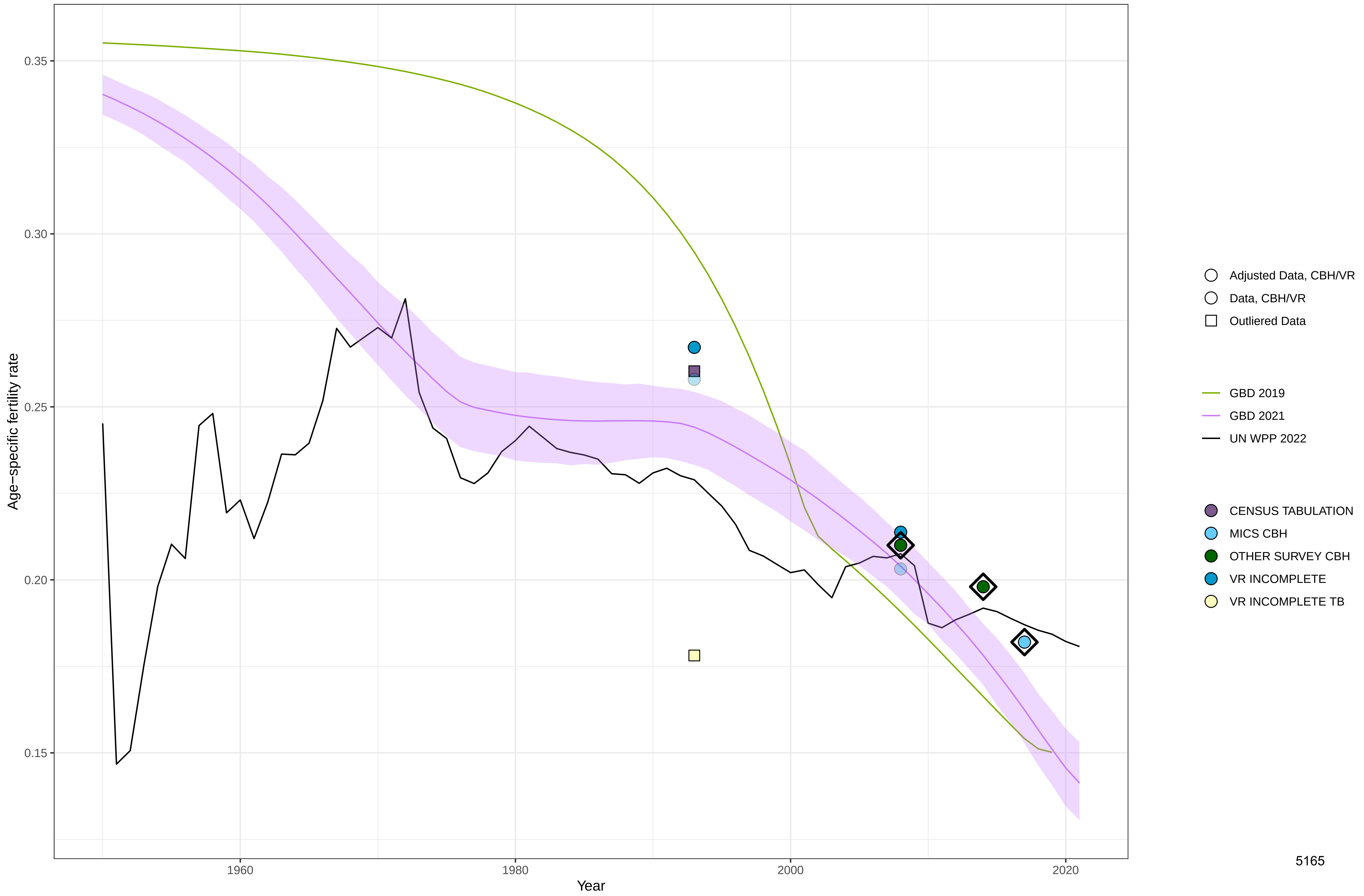

Age−specific fertility fitted trend lines, 1950−2021: North Korea, Ages: 30−34
Data points enclosed with a diamond are reference sources

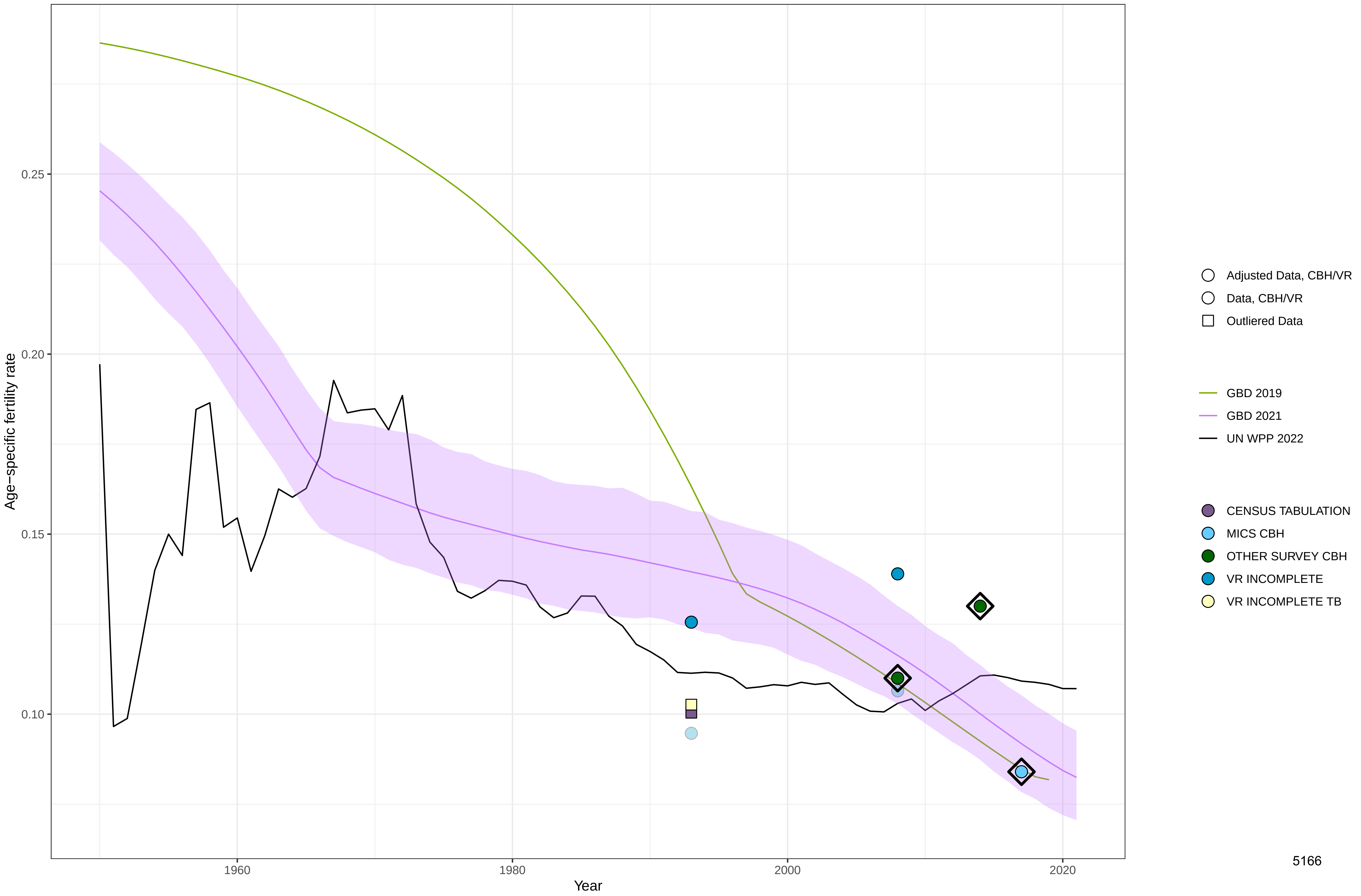

Age−specific fertility fitted trend lines, 1950−2021: North Korea, Ages: 35−39
Data points enclosed with a diamond are reference sources

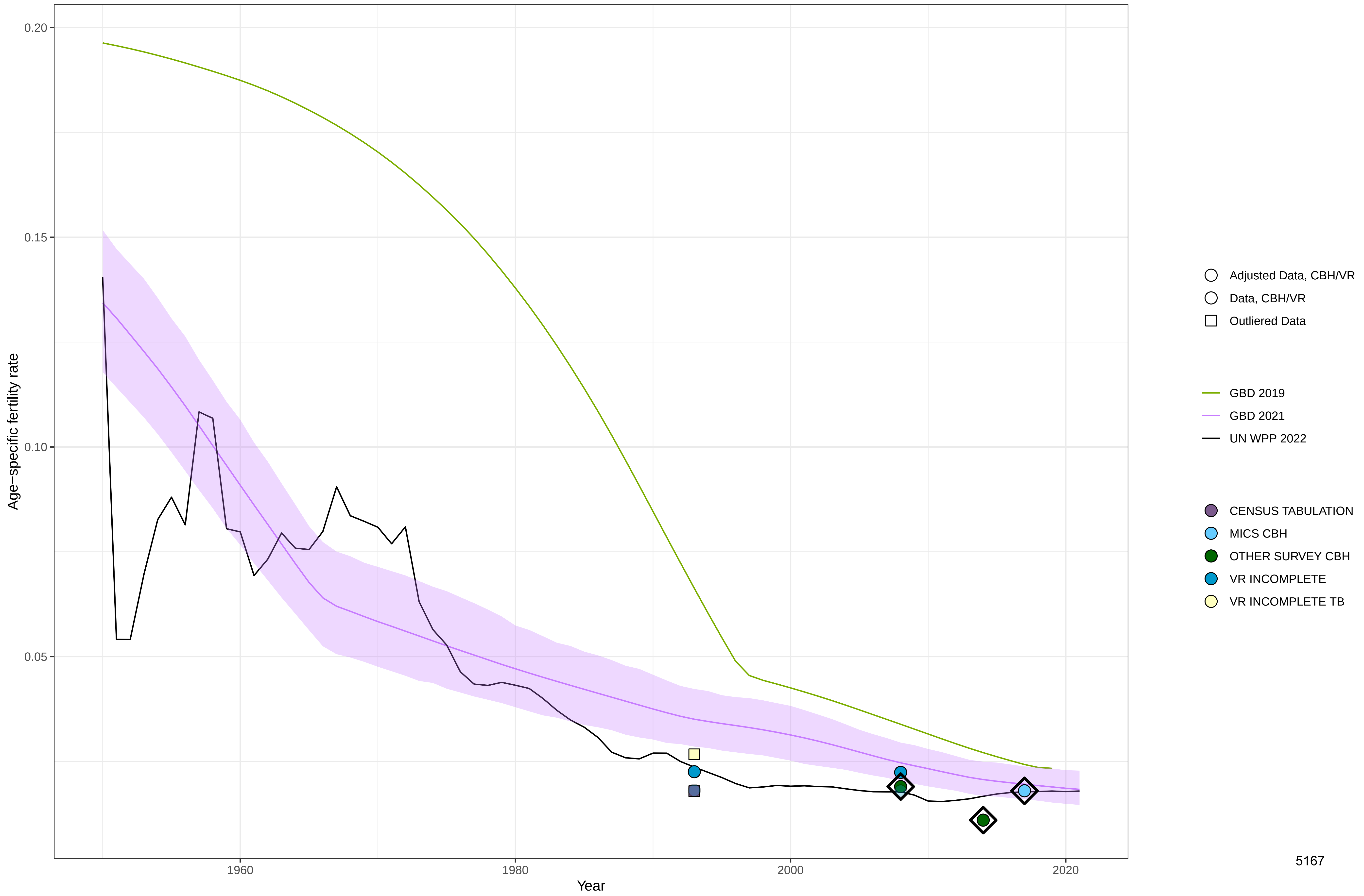

Age−specific fertility fitted trend lines, 1950−2021: North Korea, Ages: 40−44
Data points enclosed with a diamond are reference sources

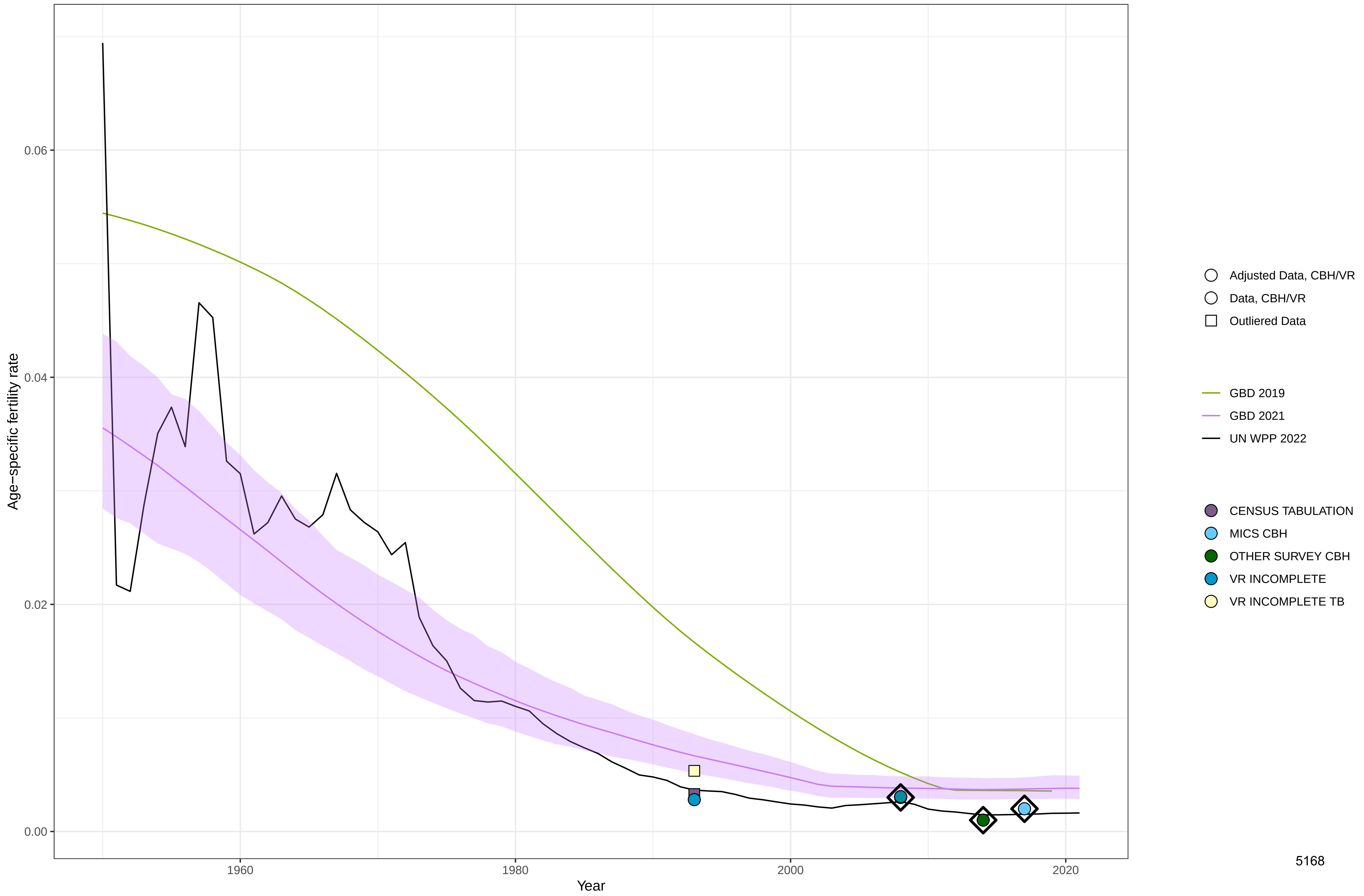

Age−specific fertility fitted trend lines, 1950−2021: North Korea, Ages: 45−49
Data points enclosed with a diamond are reference sources

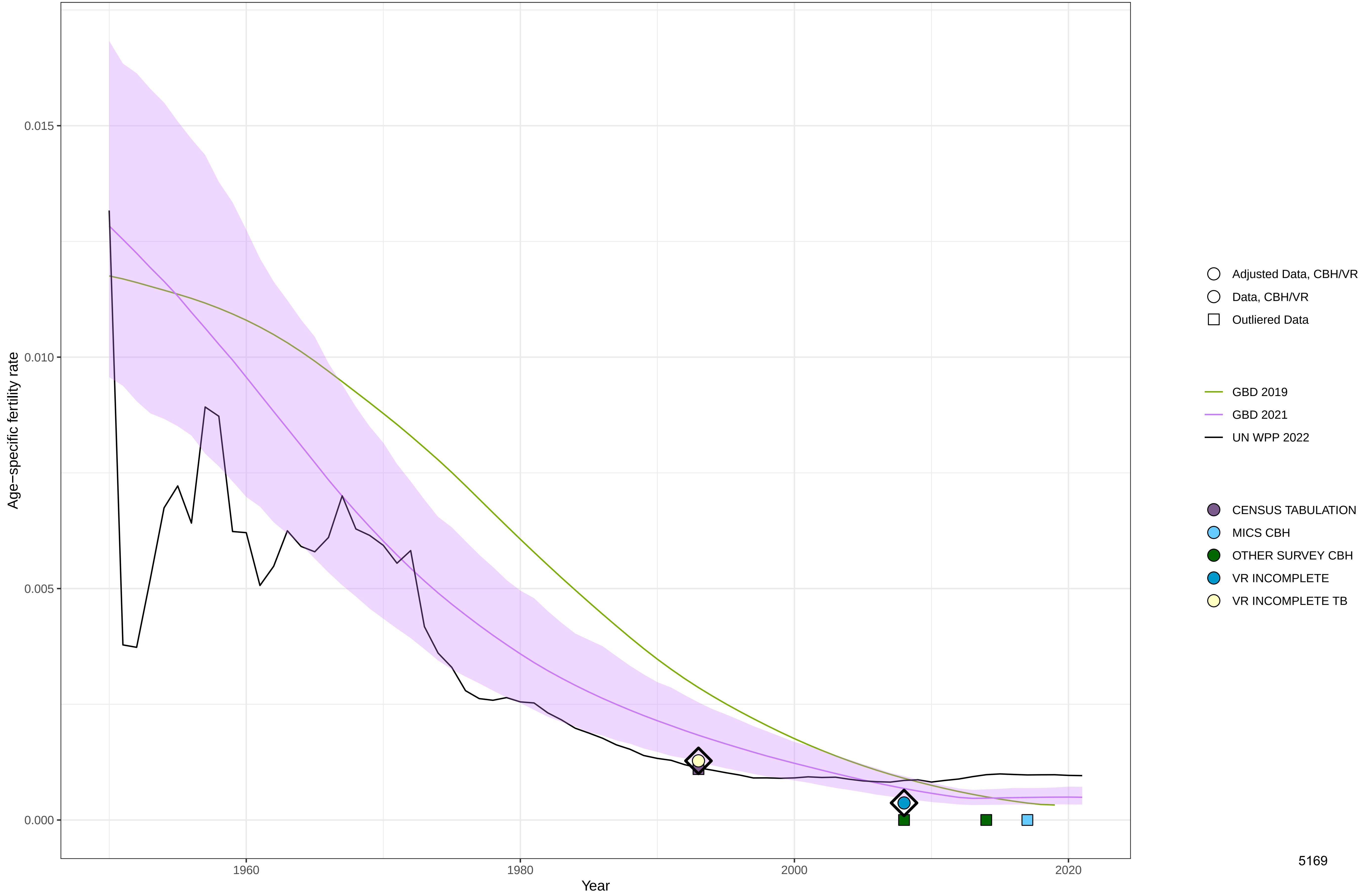

Age−specific fertility fitted trend lines, 1950−2021: North Korea, Ages: 50−54
Data points enclosed with a diamond are reference sources

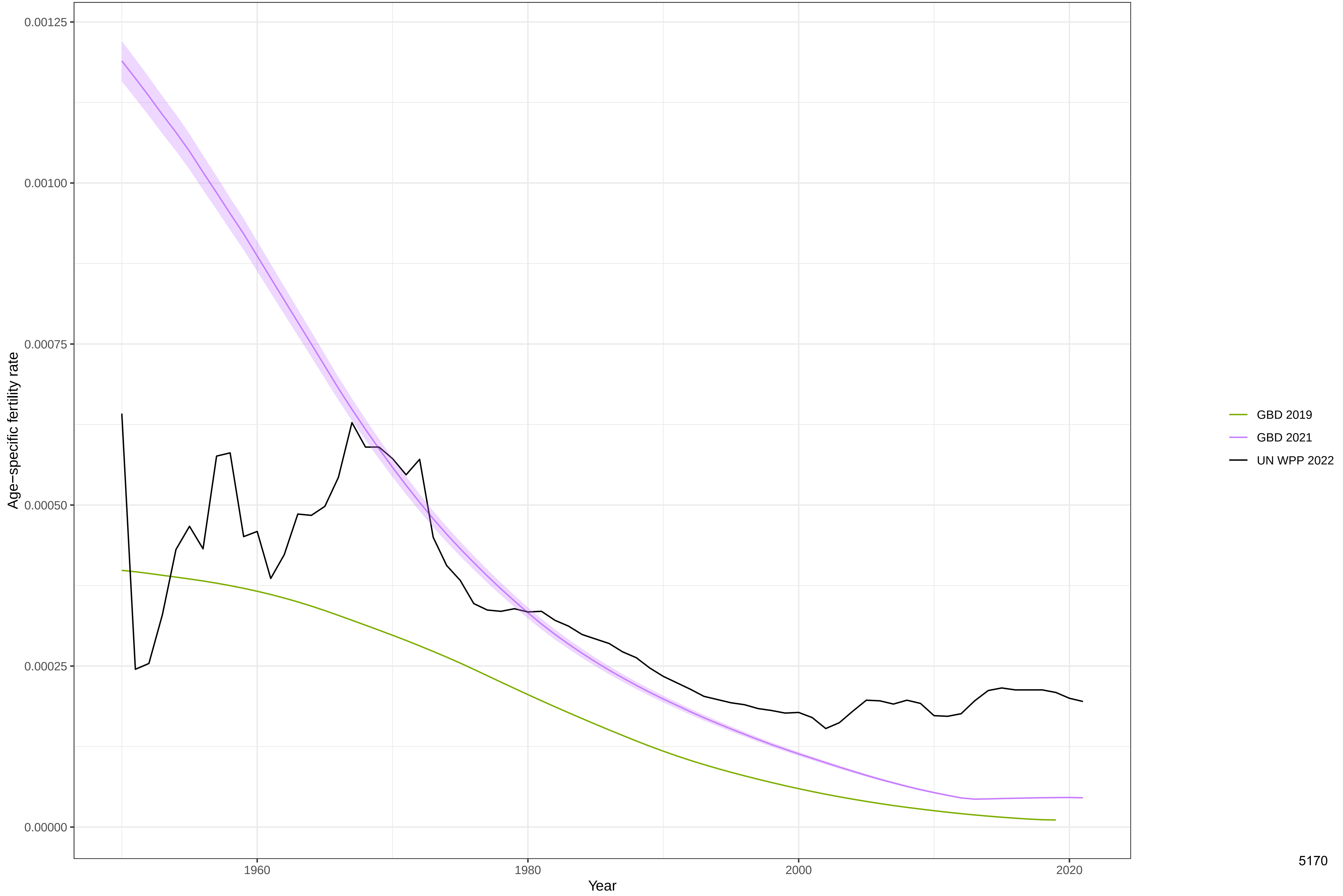

Total fertility rate fitted trend lines, 1950−2021: North Korea TFR
Data points enclosed with a diamond are reference sources

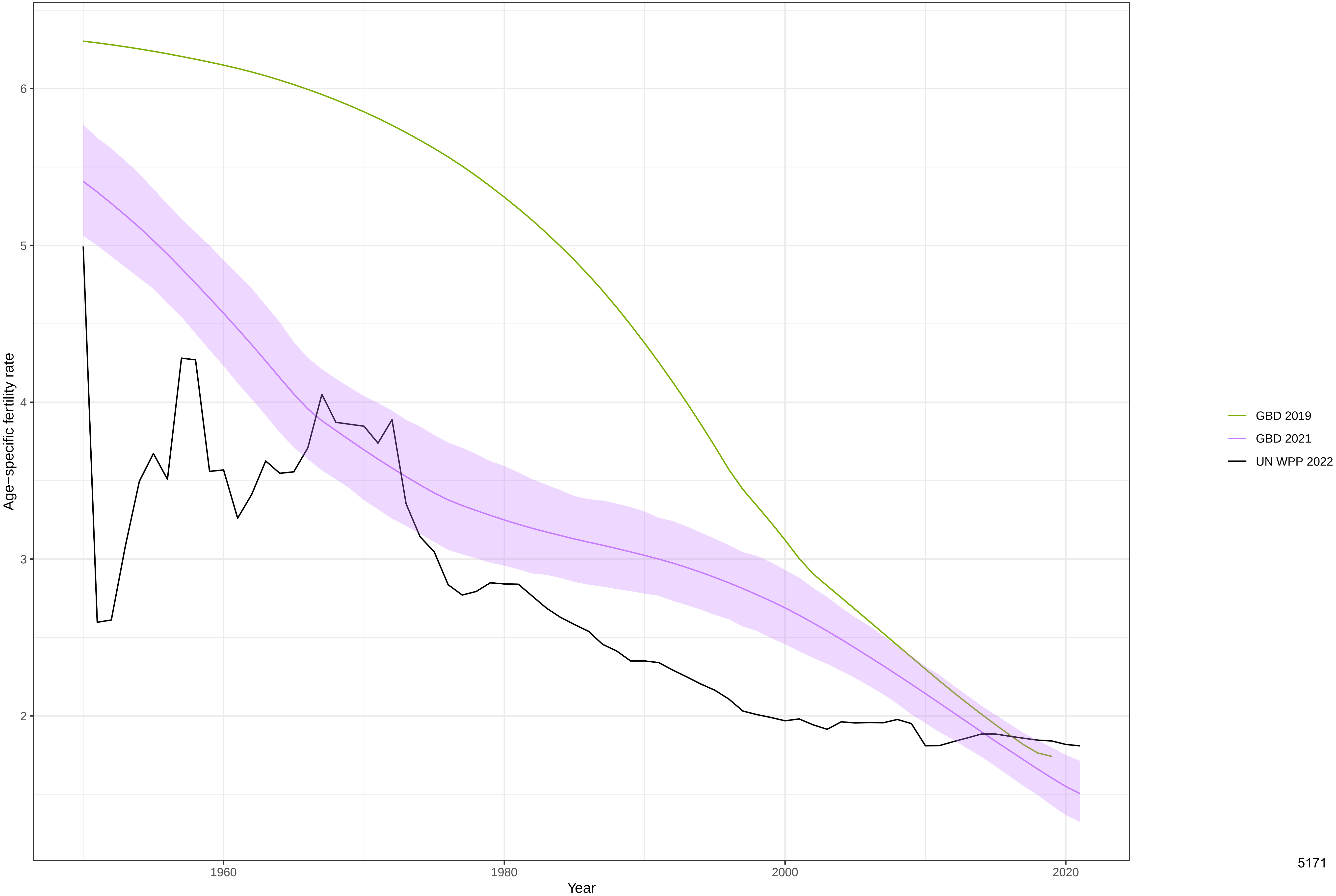

Age−specific fertility fitted trend lines, 1950−2021: Taiwan (province of China), Ages: 10−14
Data points enclosed with a diamond are reference sources

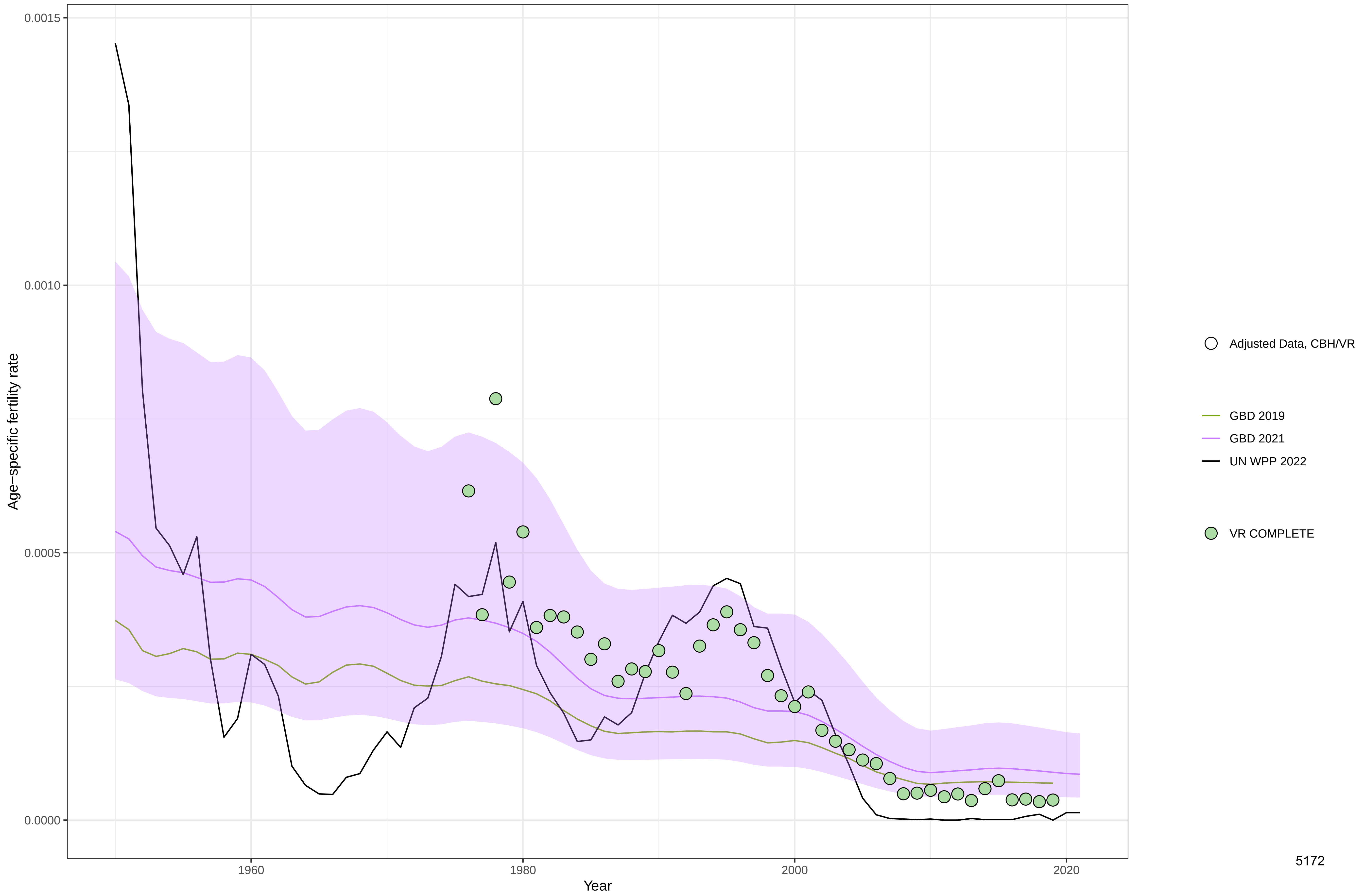

Age−specific fertility fitted trend lines, 1950−2021: Taiwan (province of China), Ages: 15−19
Data points enclosed with a diamond are reference sources

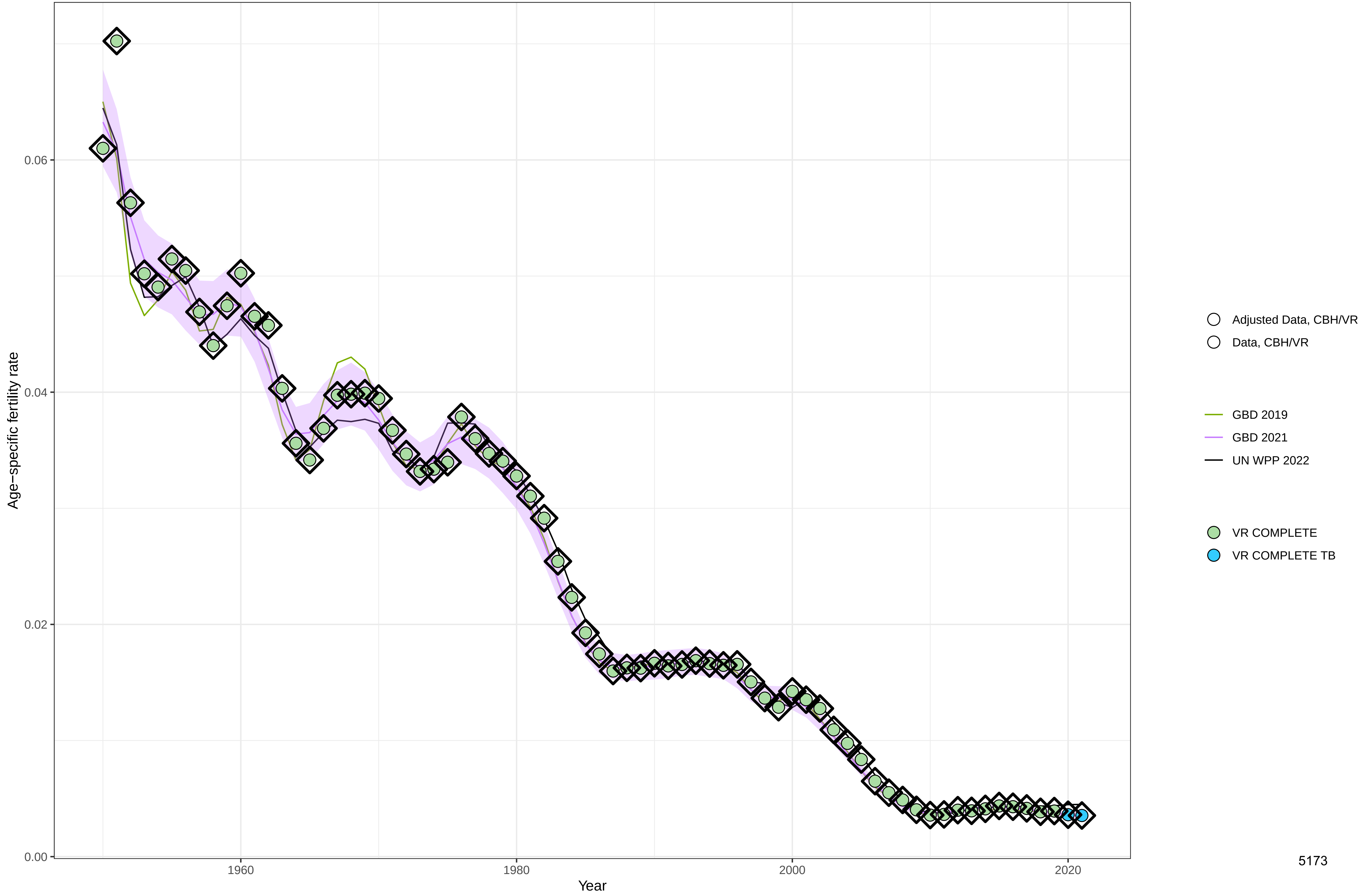

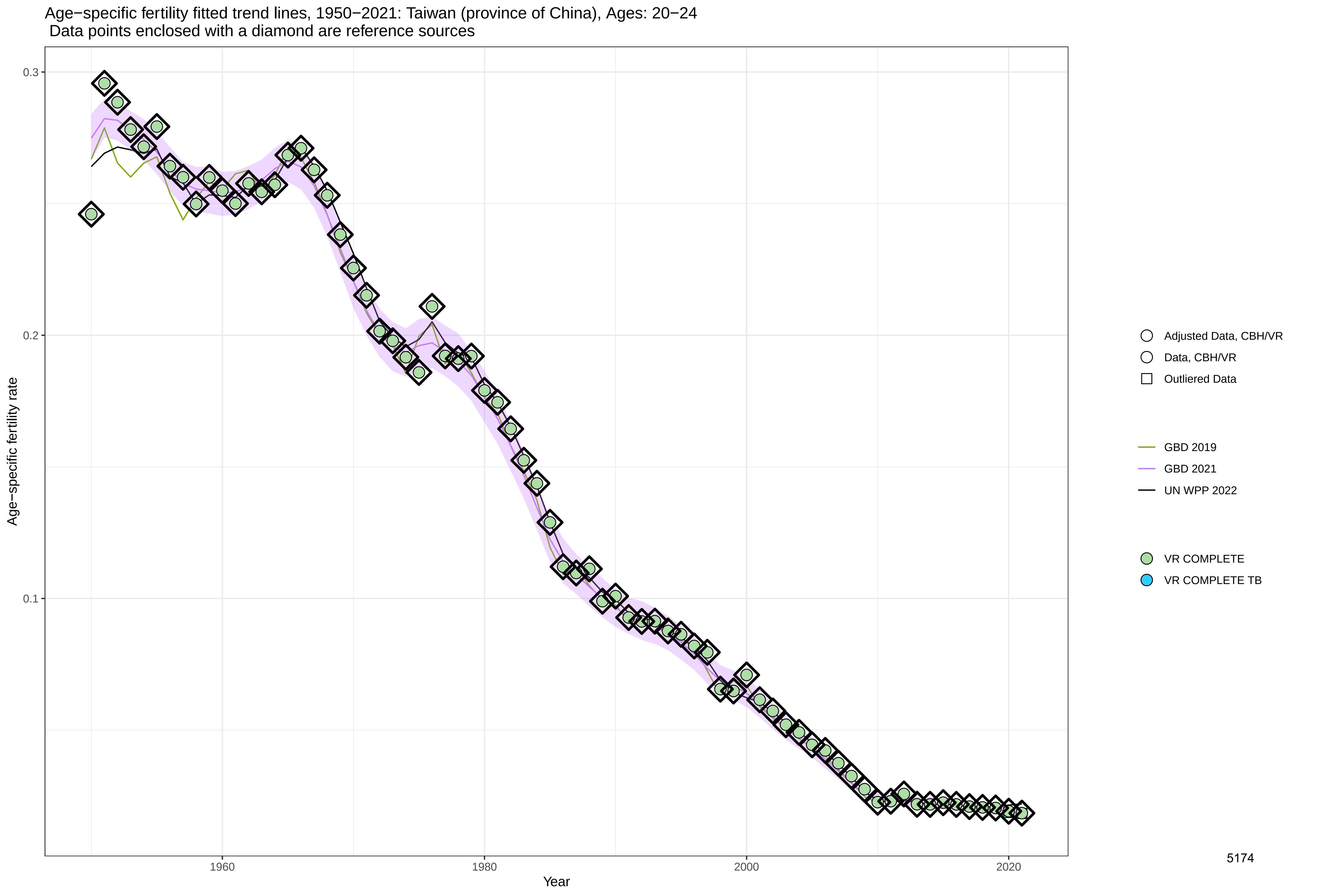
Age−specific fertility fitted trend lines, 1950−2021: Taiwan (province of China), Ages: 20−24
Data points enclosed with a diamond are reference sources
0.3
0.2
0.1
Age−specific fertility rate
1960
1980
2000
2020
Year
Adjusted Data, CBH/VR
Data, CBH/VR
Outliered Data
GBD 2019
GBD 2021
UN WPP 2022
VR COMPLETE
VR COMPLETE TB
5174

Age−specific fertility fitted trend lines, 1950−2021: Taiwan (province of China), Ages: 25−29
Data points enclosed with a diamond are reference sources

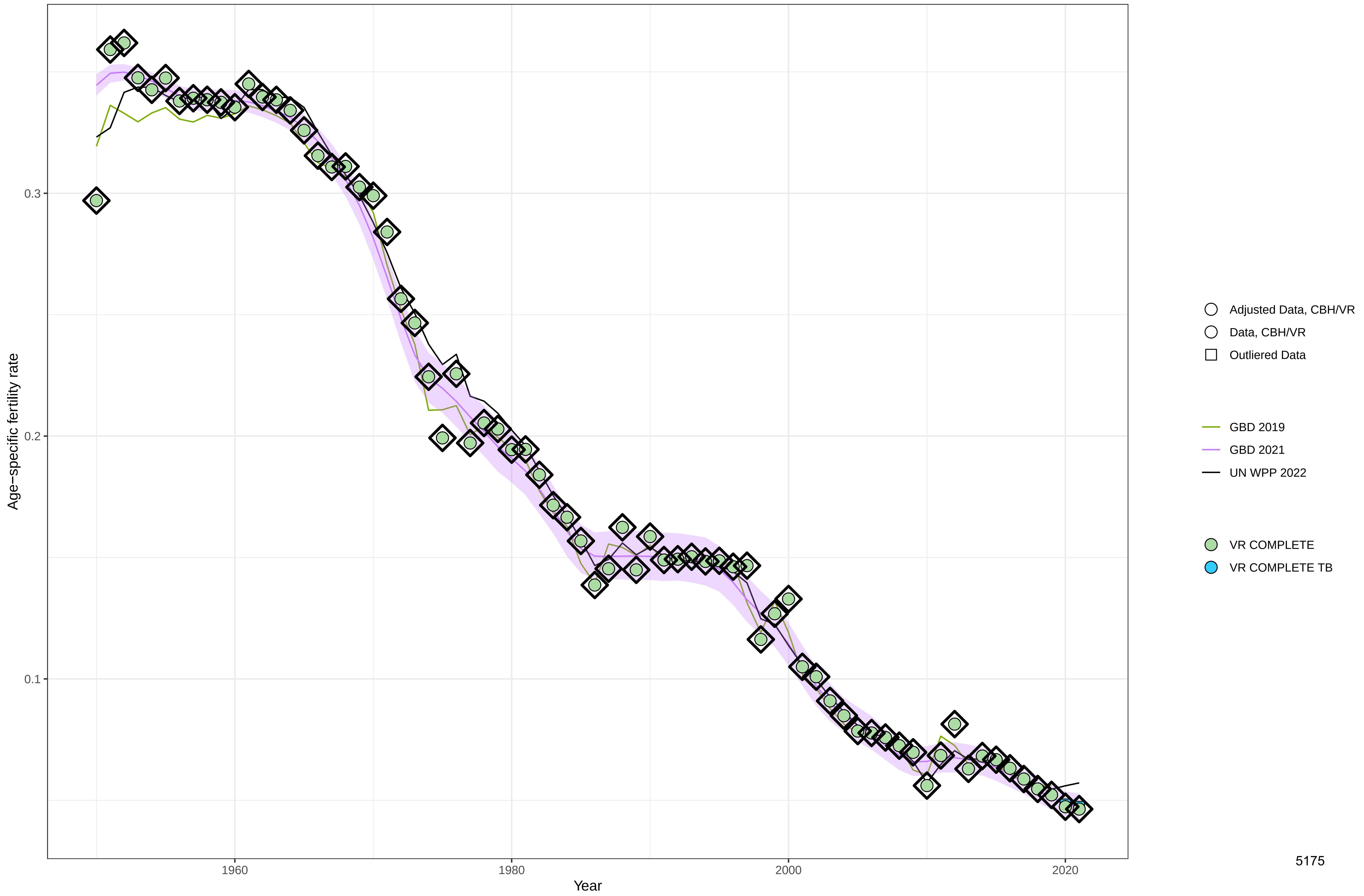

Age−specific fertility fitted trend lines, 1950−2021: Taiwan (province of China), Ages: 30−34
Data points enclosed with a diamond are reference sources

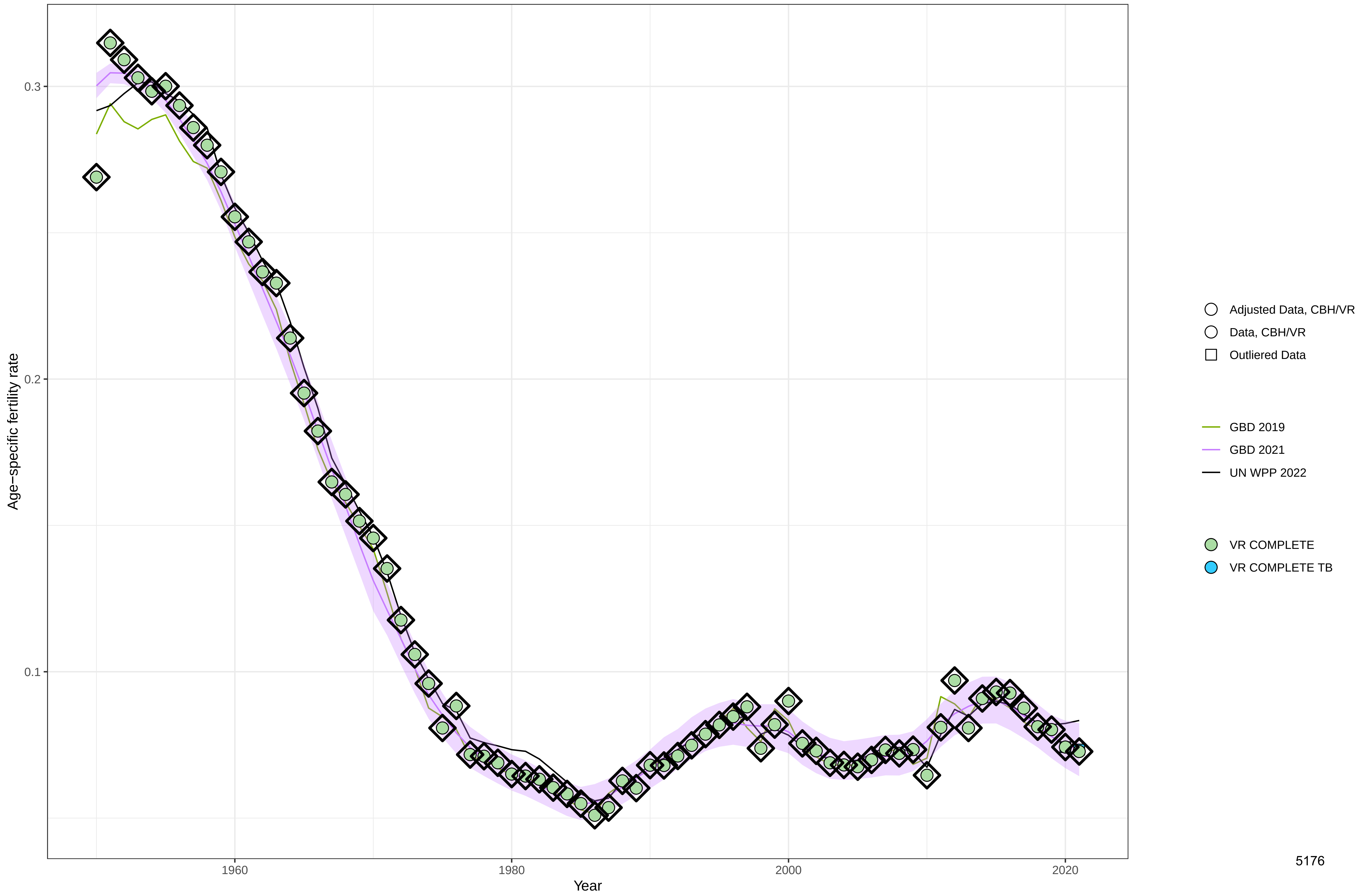

Age−specific fertility fitted trend lines, 1950−2021: Taiwan (province of China), Ages: 35−39
Data points enclosed with a diamond are reference sources

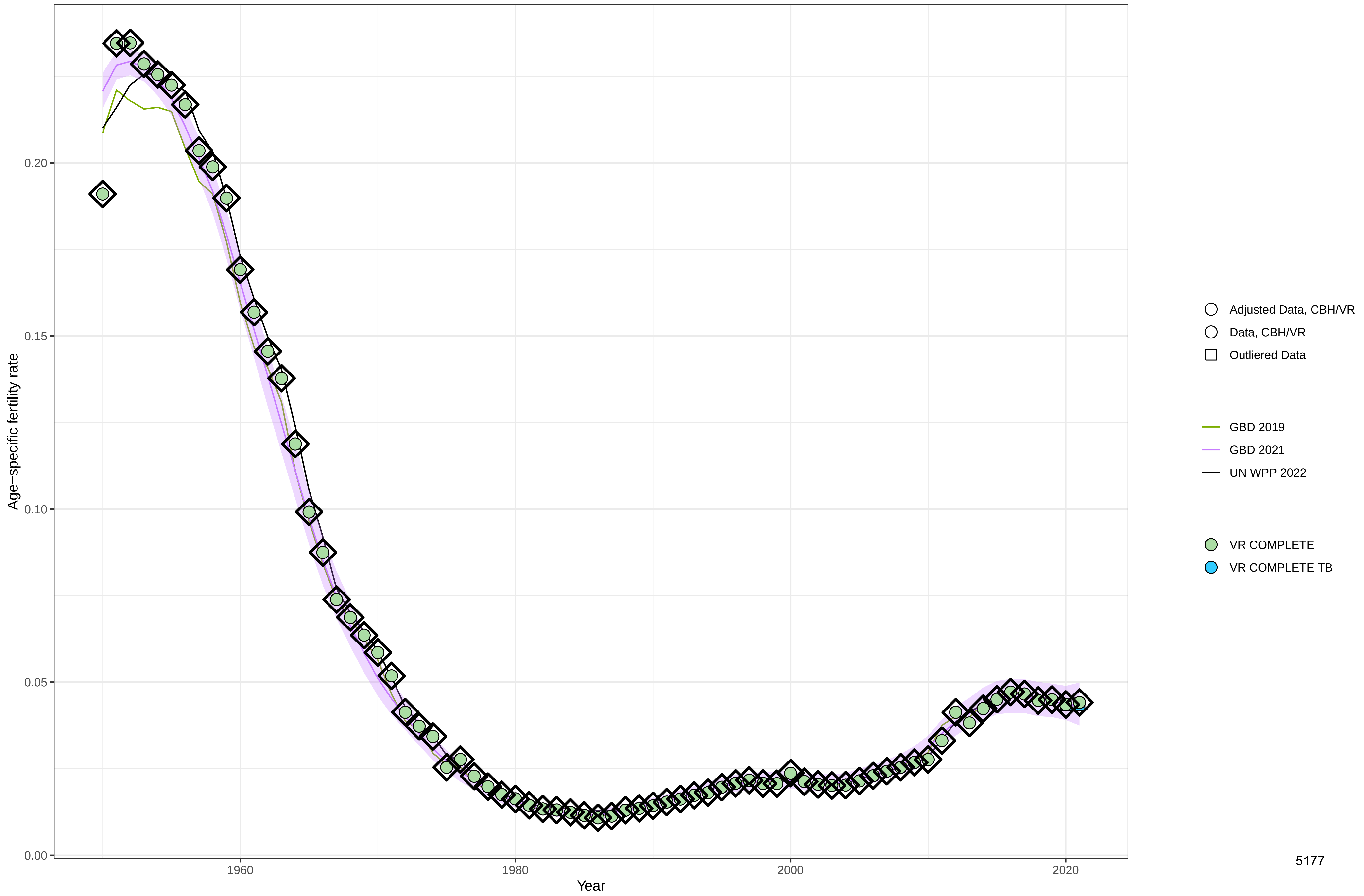

Age−specific fertility fitted trend lines, 1950−2021: Taiwan (province of China), Ages: 40−44
Data points enclosed with a diamond are reference sources

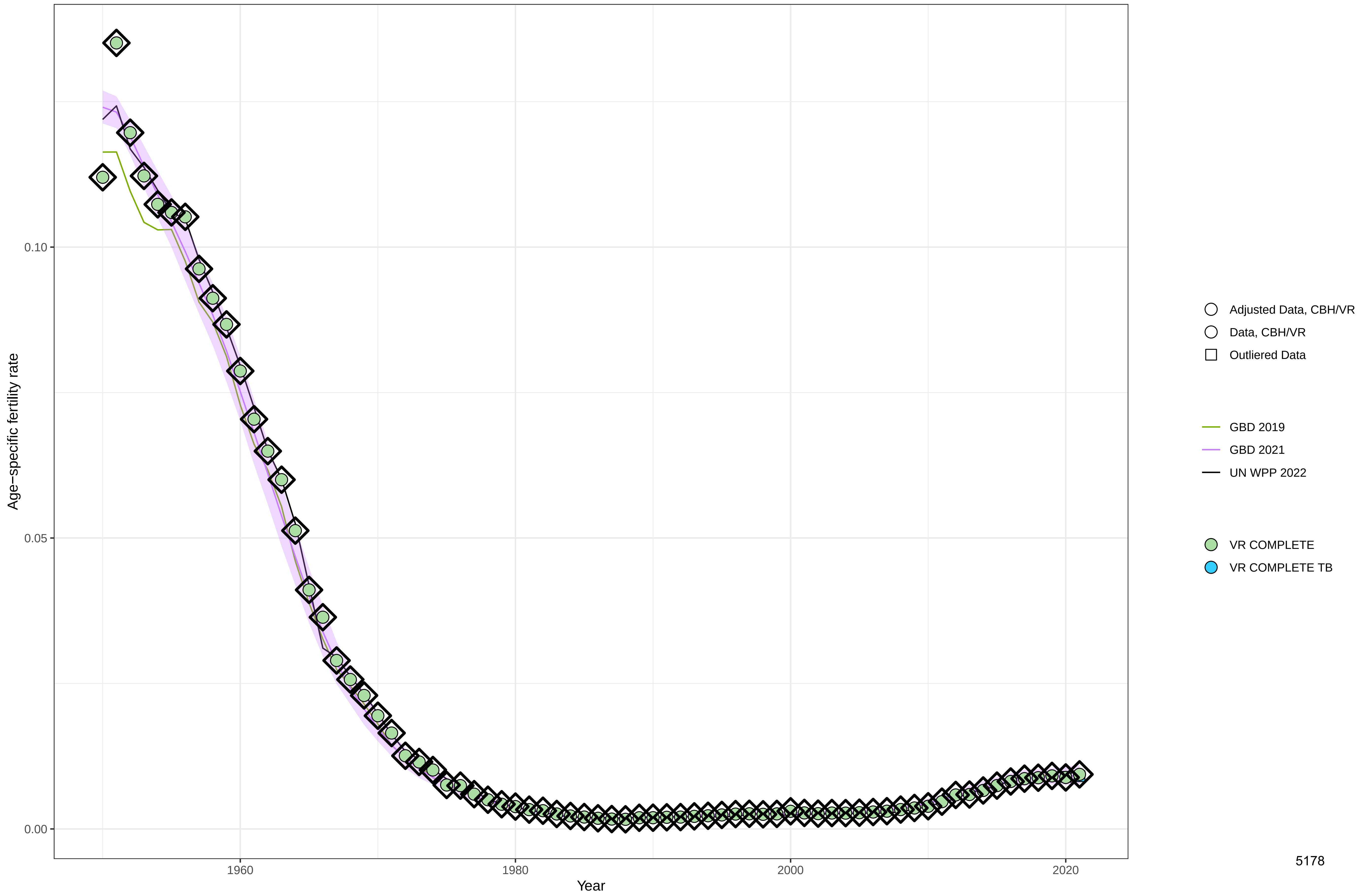

Age−specific fertility fitted trend lines, 1950−2021: Taiwan (province of China), Ages: 45−49
Data points enclosed with a diamond are reference sources

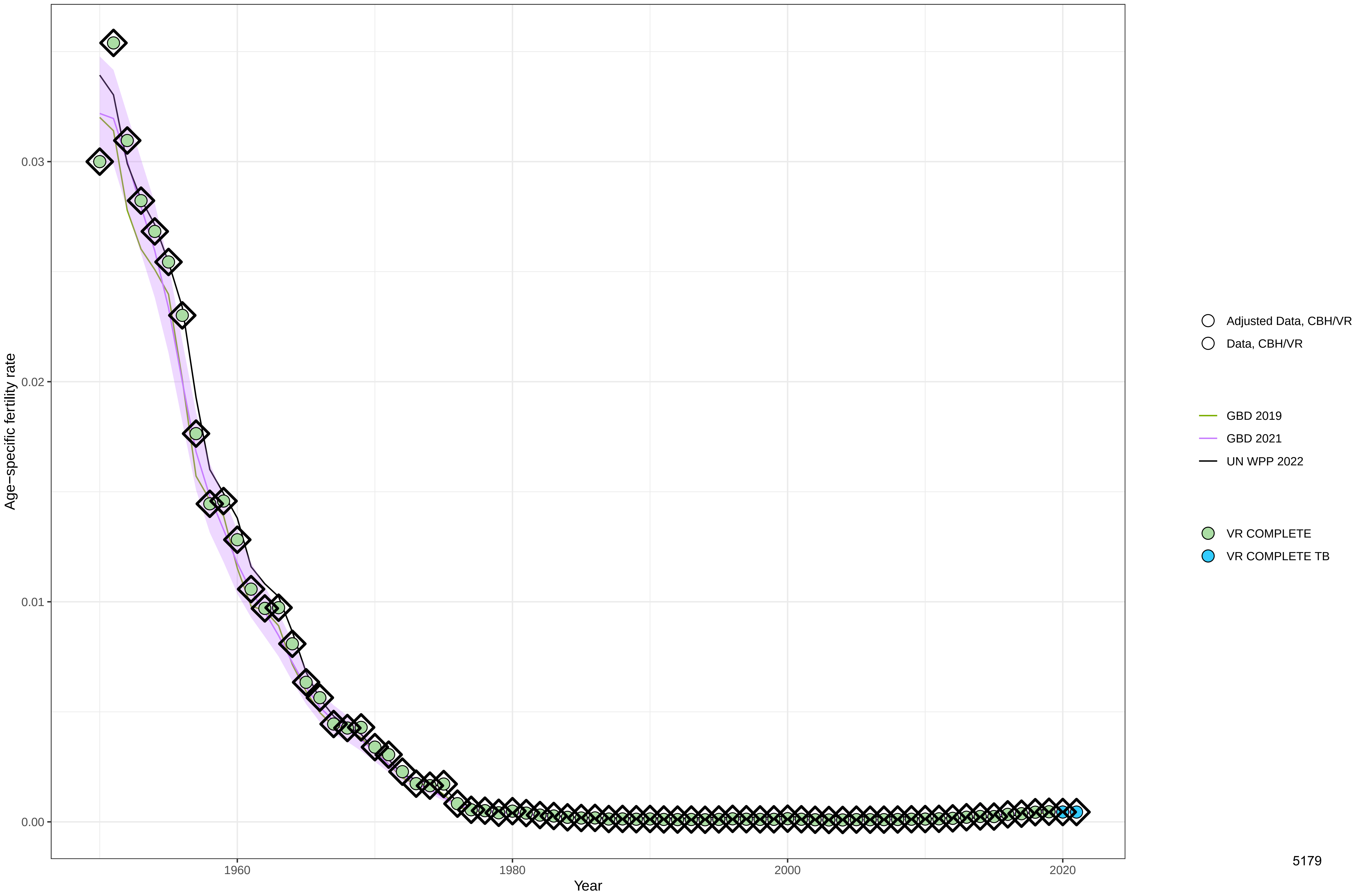

Age−specific fertility fitted trend lines, 1950−2021: Taiwan (province of China), Ages: 50−54
Data points enclosed with a diamond are reference sources

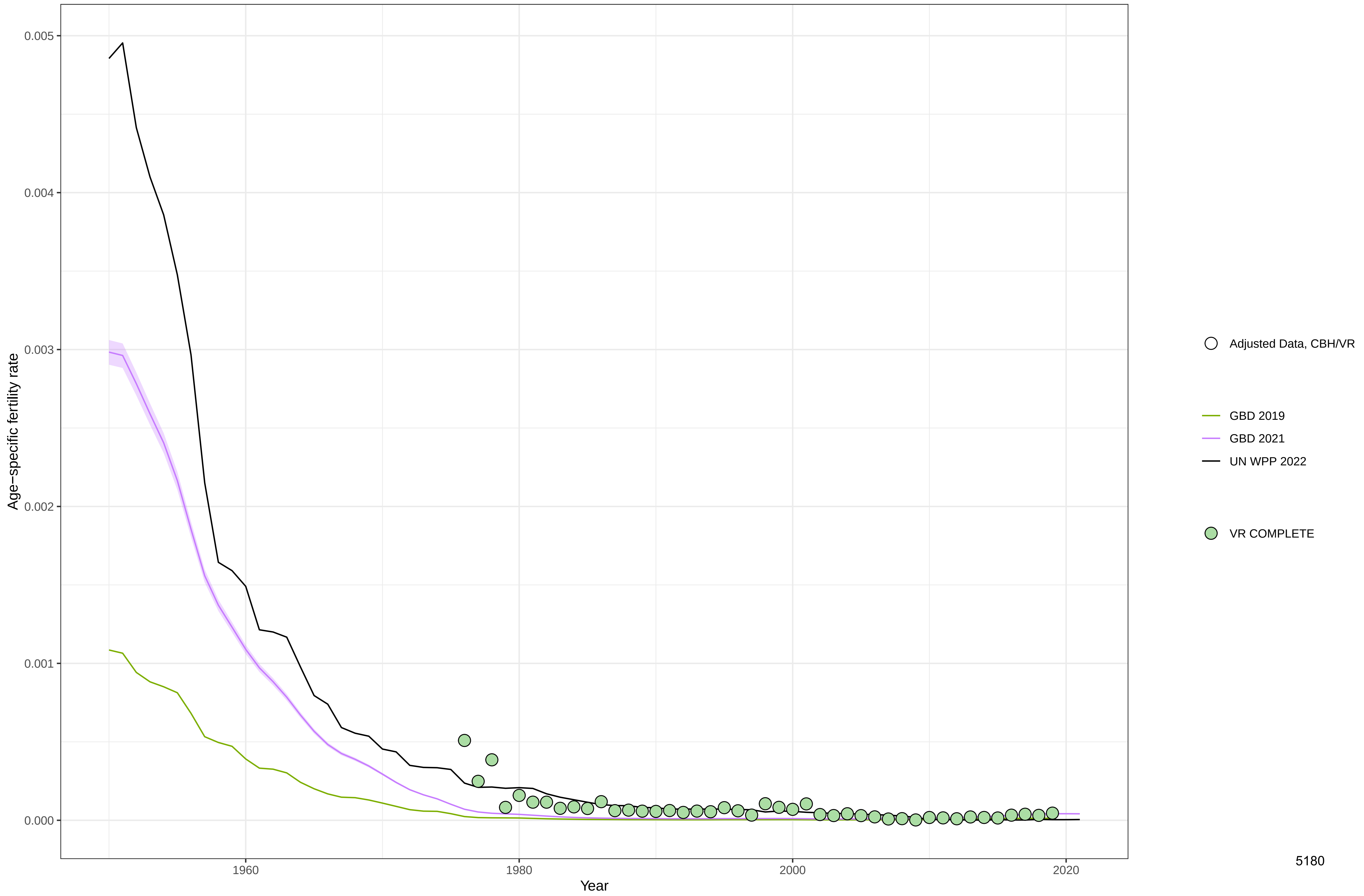

Total fertility rate fitted trend lines, 1950−2021: Taiwan (province of China) TFR
Data points enclosed with a diamond are reference sources

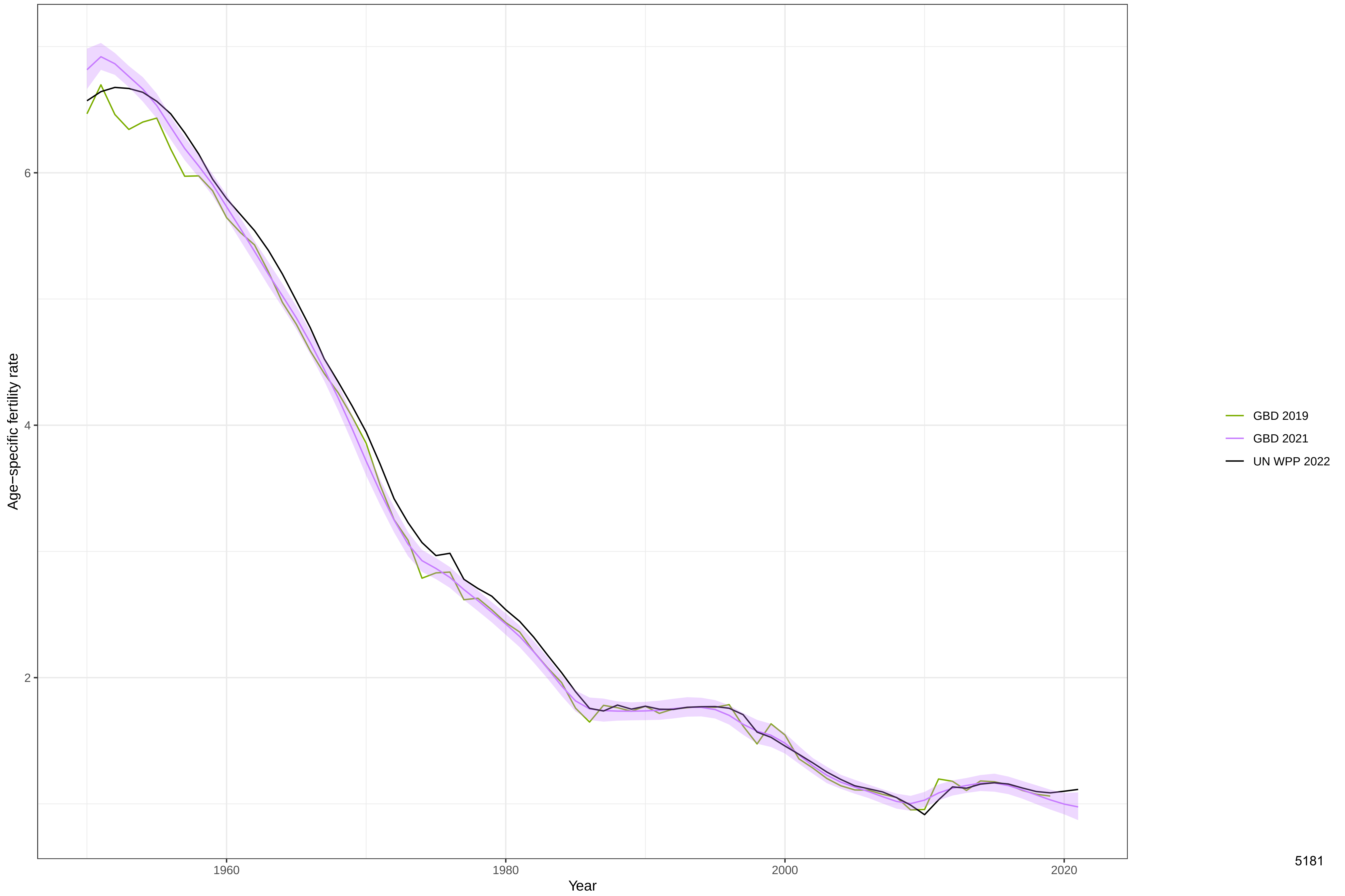

Age−specific fertility fitted trend lines, 1950−2021: American Samoa, Ages: 10−14
Data points enclosed with a diamond are reference sources

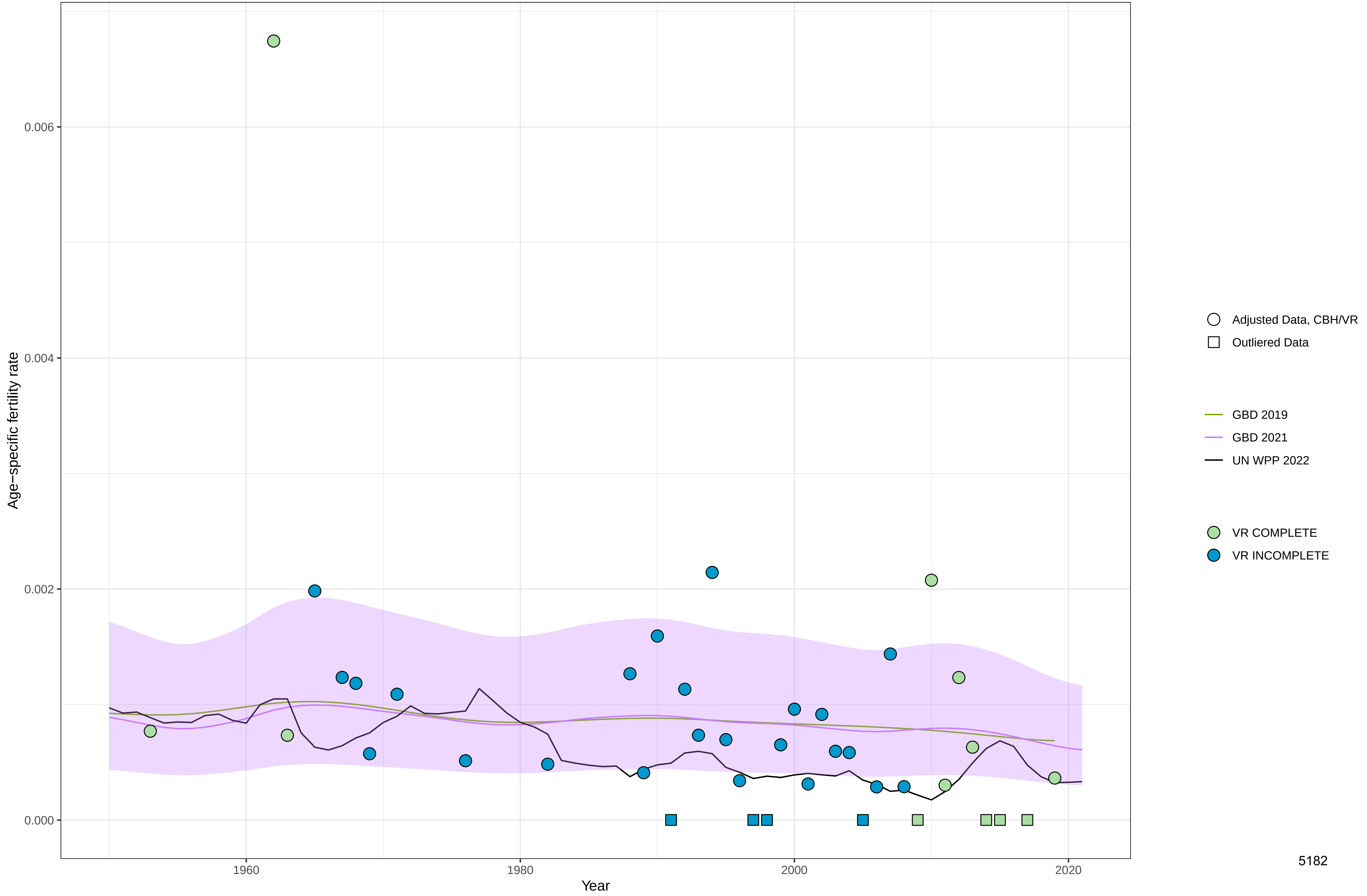

Age−specific fertility fitted trend lines, 1950−2021: American Samoa, Ages: 15−19
Data points enclosed with a diamond are reference sources

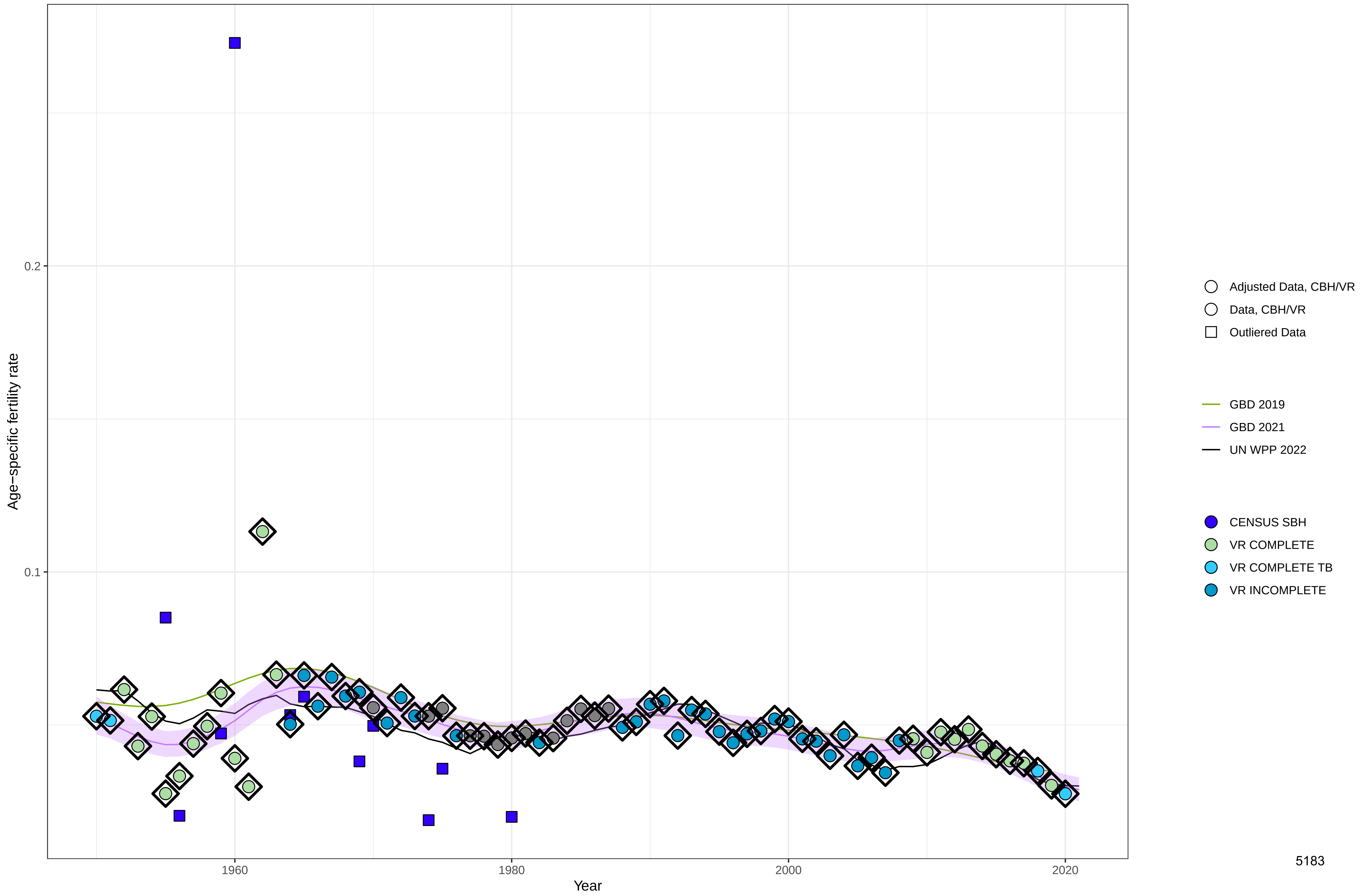

Age−specific fertility fitted trend lines, 1950−2021: American Samoa, Ages: 20−24
Data points enclosed with a diamond are reference sources

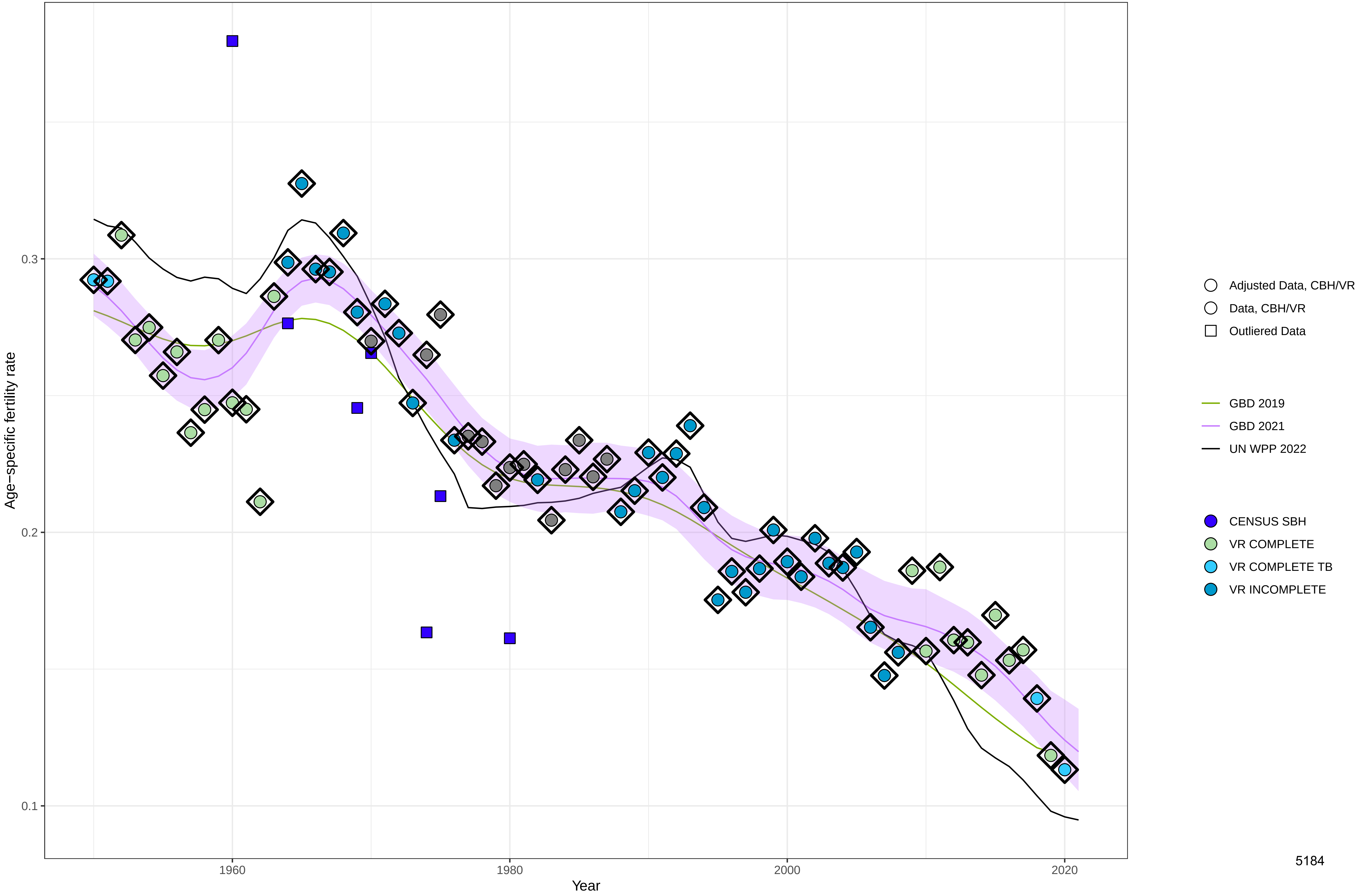

Age−specific fertility fitted trend lines, 1950−2021: American Samoa, Ages: 25−29
Data points enclosed with a diamond are reference sources

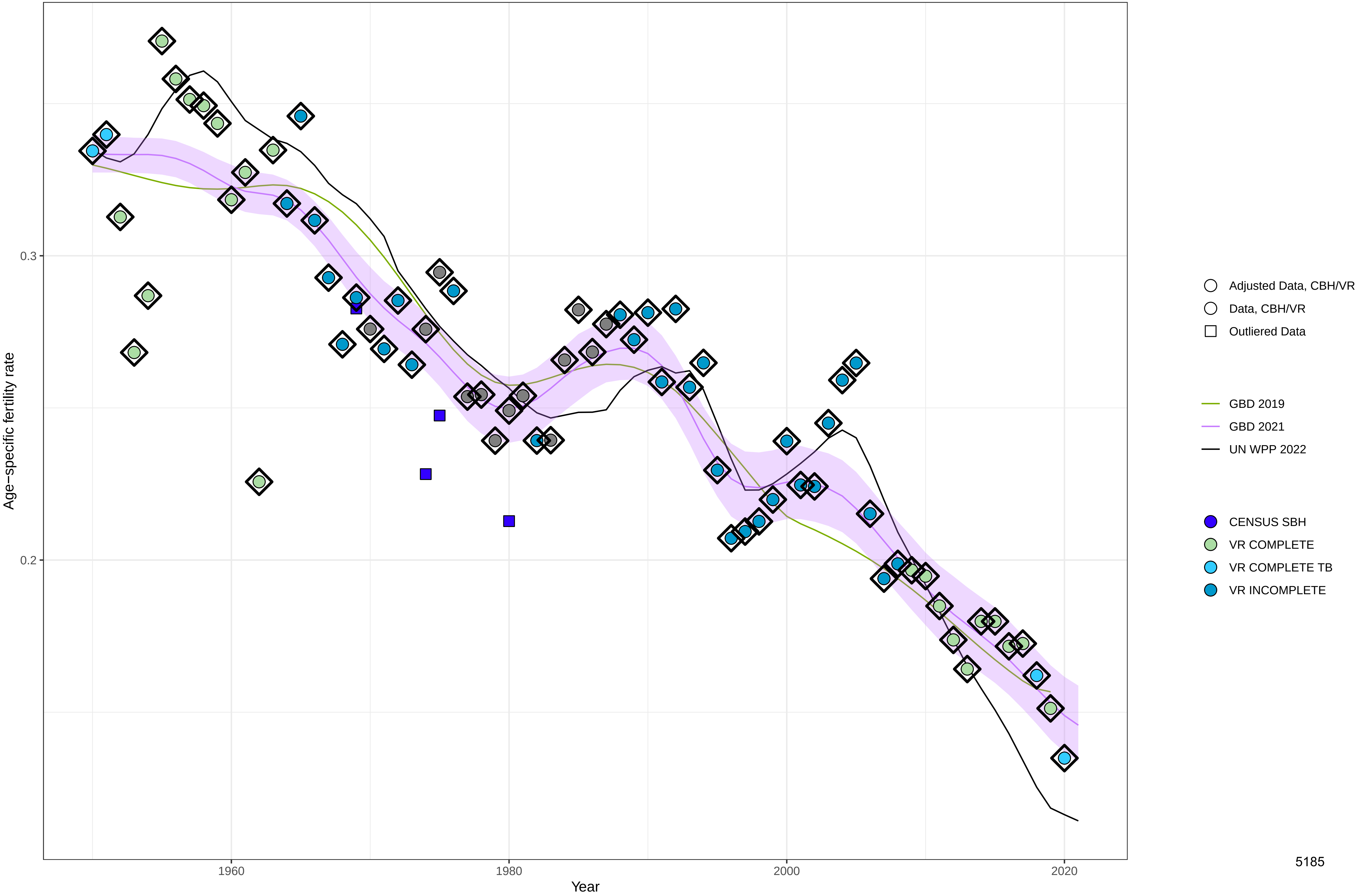

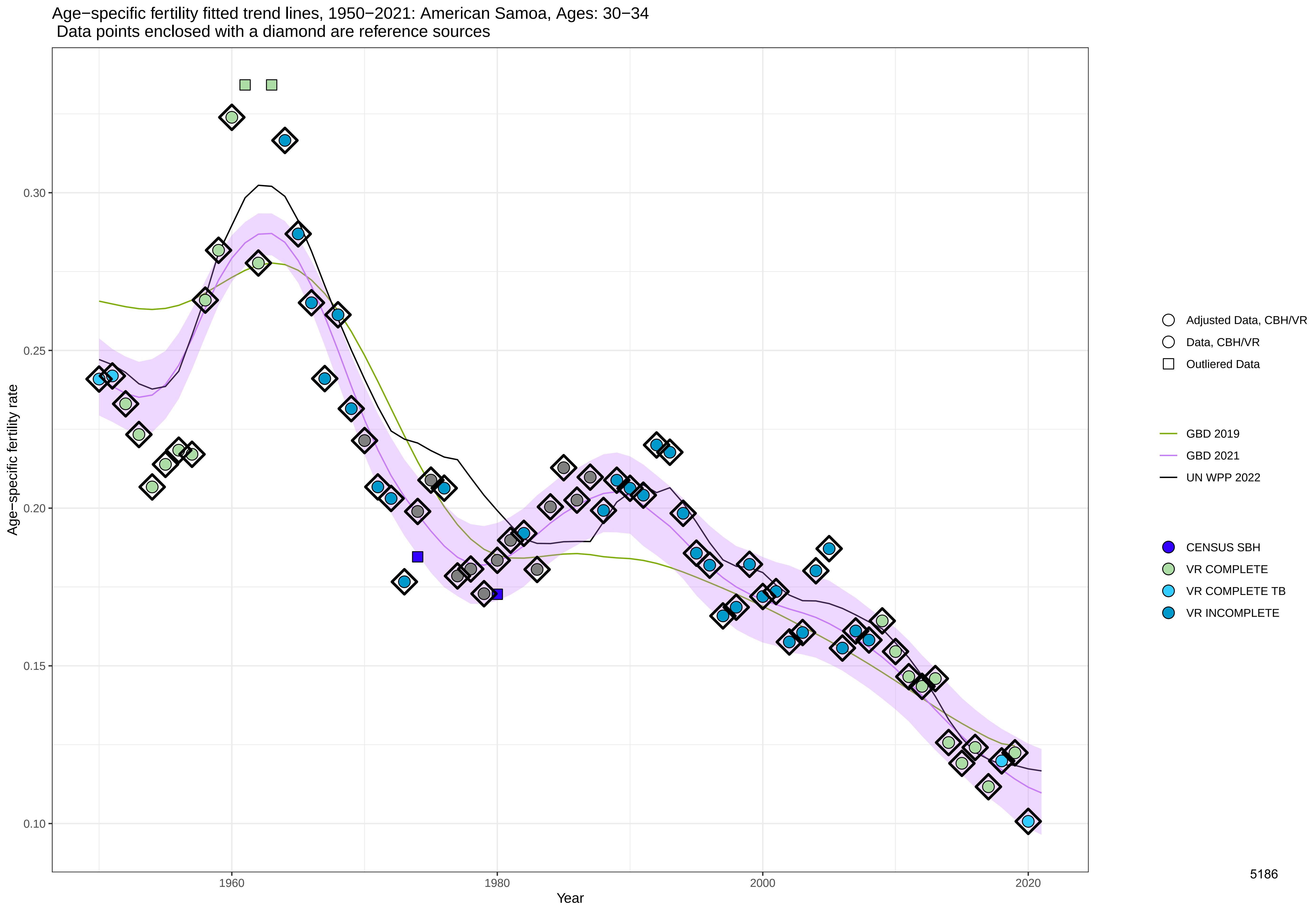

Age−specific fertility fitted trend lines, 1950−2021: American Samoa, Ages: 30−34
Data points enclosed with a diamond are reference sources
Age−specific fertility rate
0.30
0.25
0.20
0.15
0.10
1960
1980
2000
2020
Year
Adjusted Data, CBH/VR
Data, CBH/VR
Outliered Data
GBD 2019
GBD 2021
UN WPP 2022
CENSUS SBH
VR COMPLETE
VR COMPLETE TB
VR INCOMPLETE

Age−specific fertility fitted trend lines, 1950−2021: American Samoa, Ages: 35−39
Data points enclosed with a diamond are reference sources

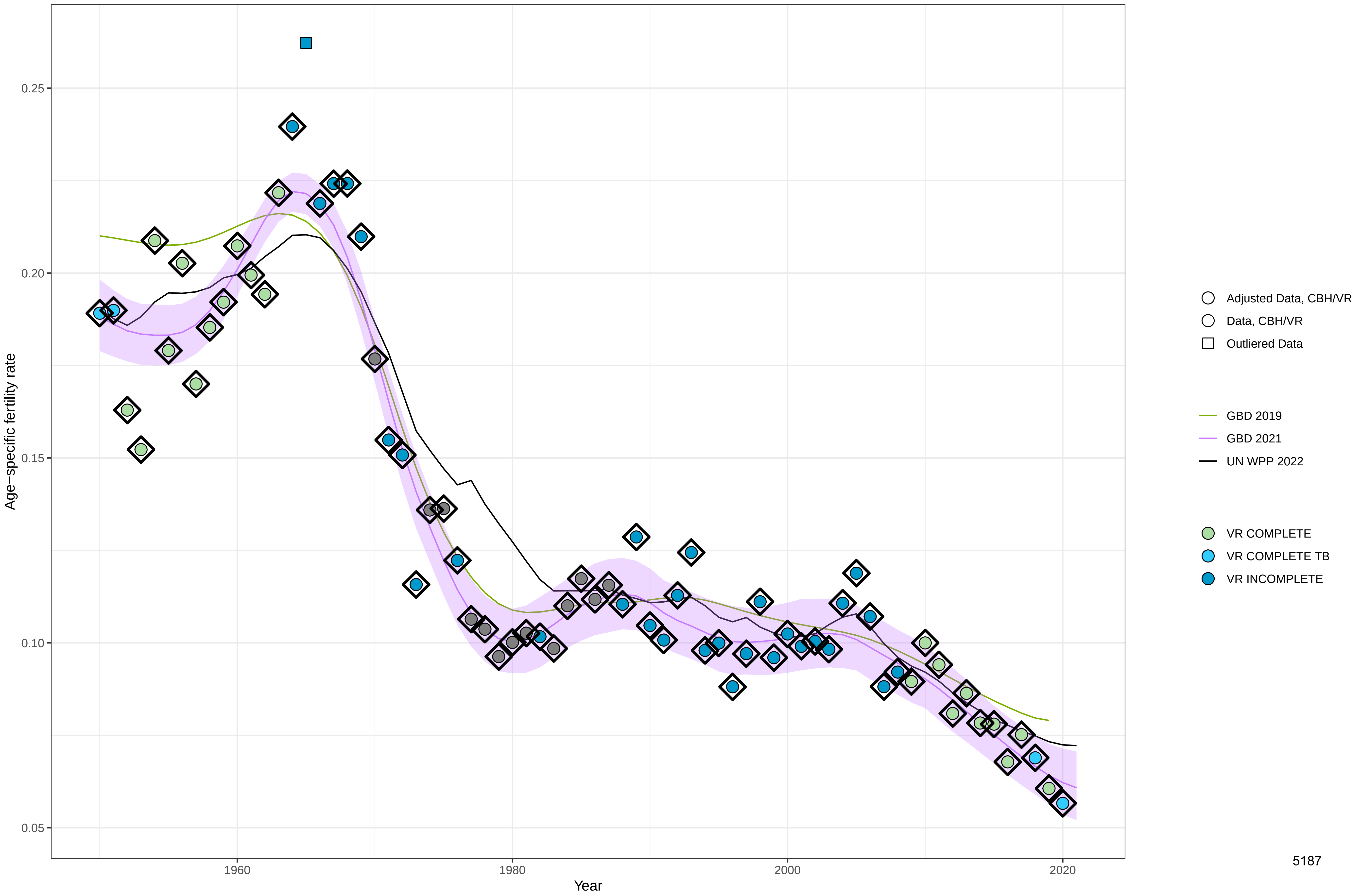

Age−specific fertility fitted trend lines, 1950−2021: American Samoa, Ages: 40−44
Data points enclosed with a diamond are reference sources

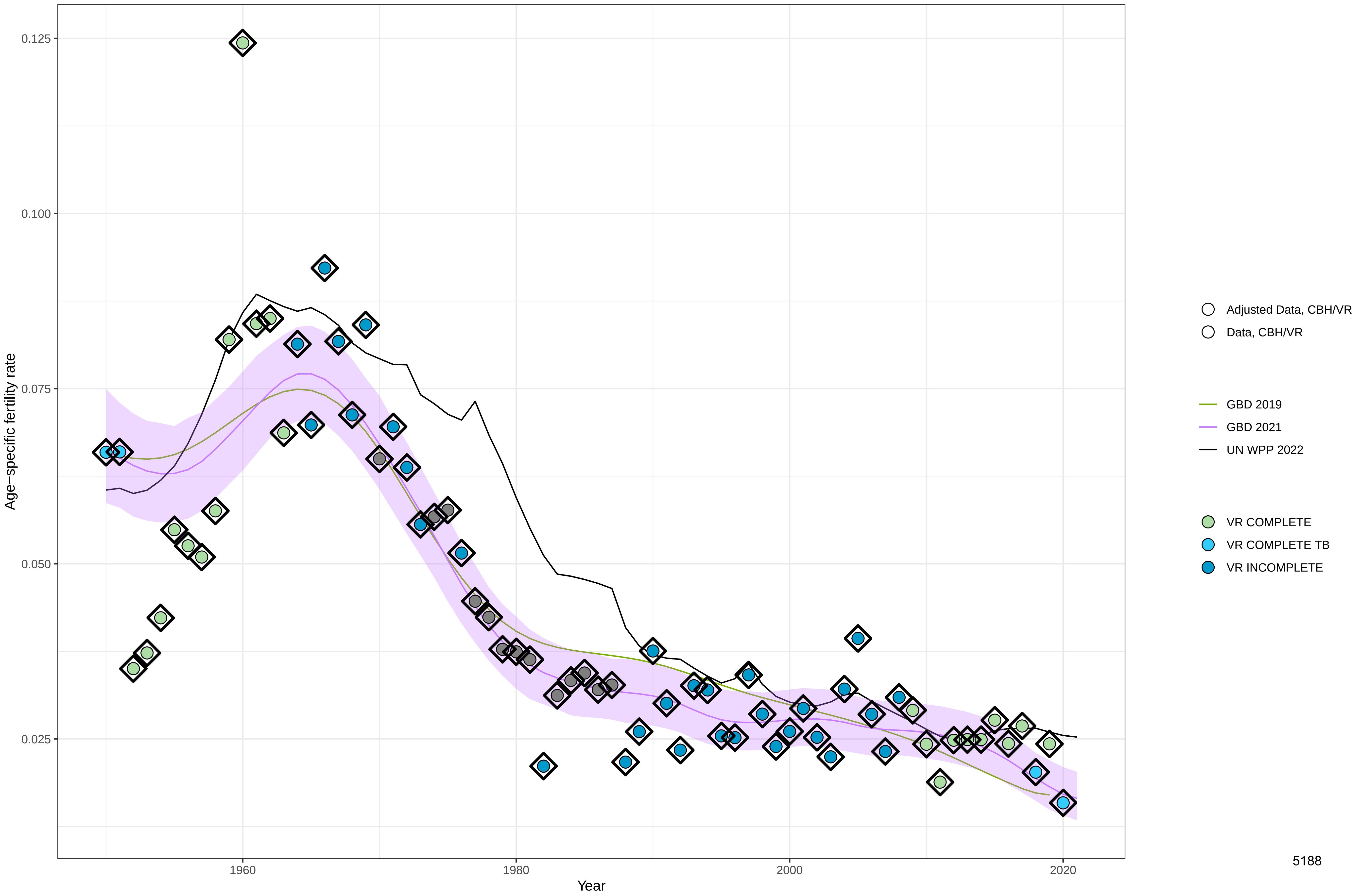

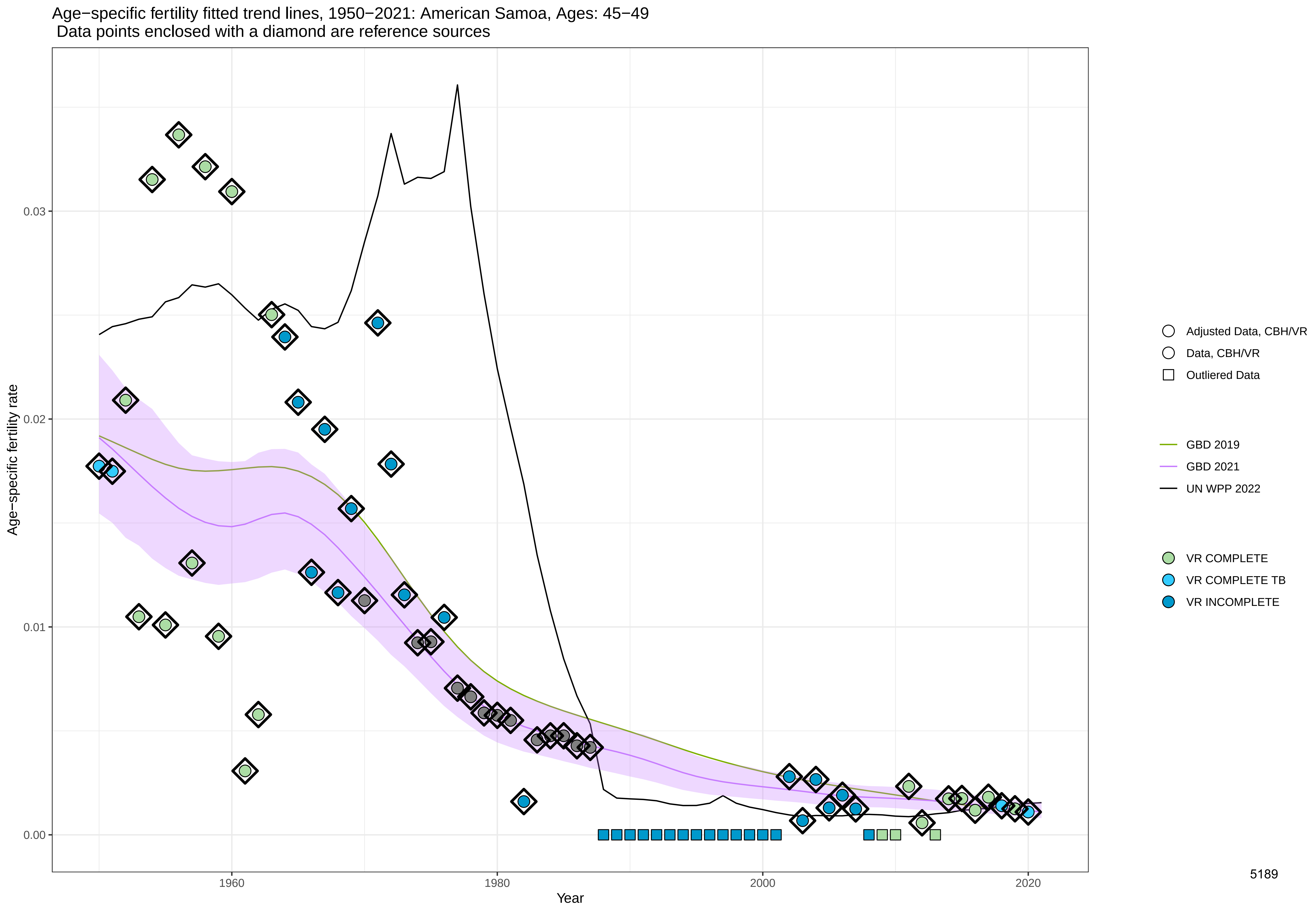

Age−specific fertility fitted trend lines, 1950−2021: American Samoa, Ages: 45−49
Data points enclosed with a diamond are reference sources
Age−specific fertility rate
0.00
0.01
0.02
0.03
Year
1960
1980
2000
2020
Adjusted Data, CBH/VR
Data, CBH/VR
Outliered Data
GBD 2019
GBD 2021
UN WPP 2022
VR COMPLETE
VR COMPLETE TB
VR INCOMPLETE

Age−specific fertility fitted trend lines, 1950−2021: American Samoa, Ages: 50−54
Data points enclosed with a diamond are reference sources

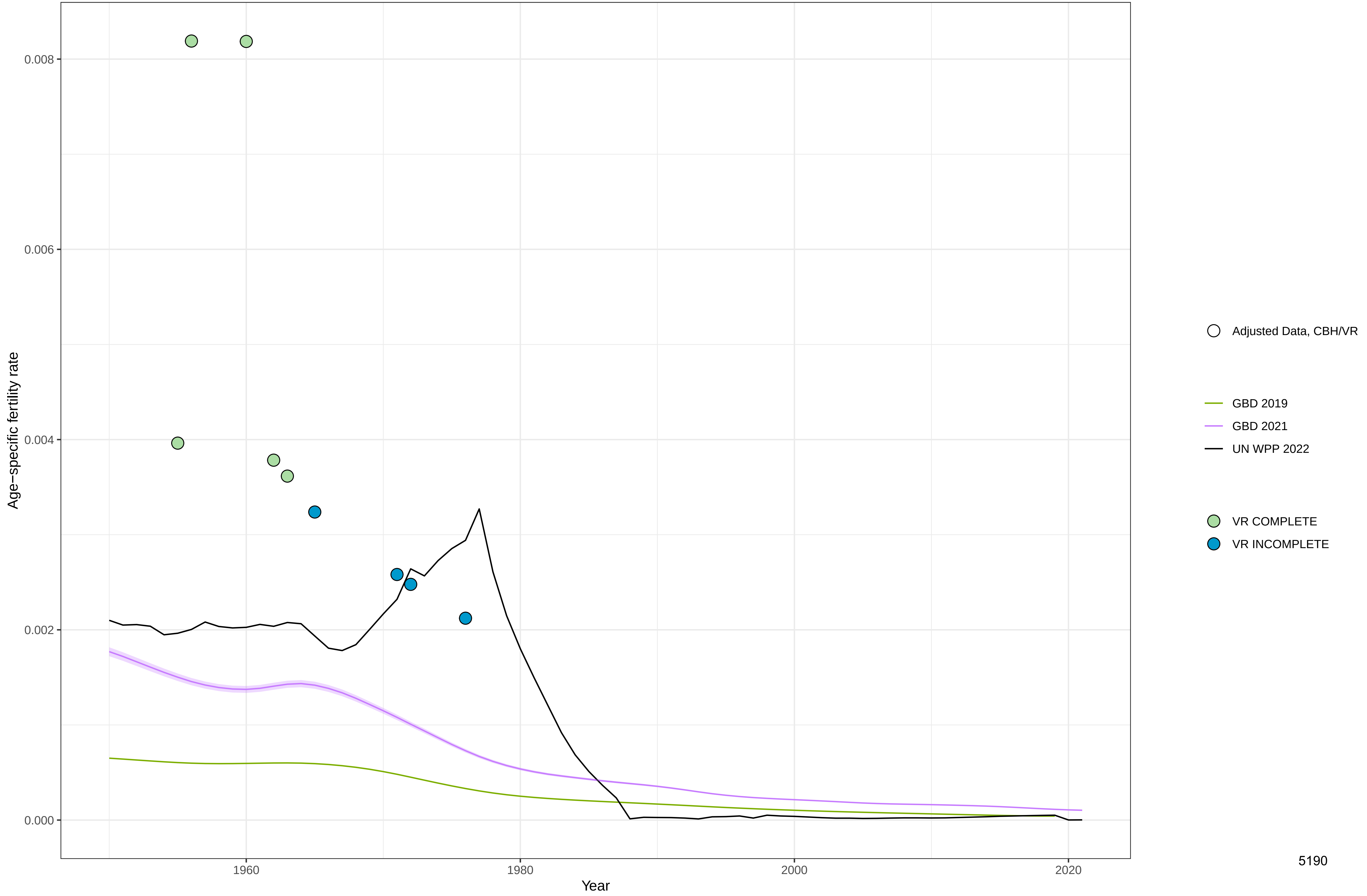

Total fertility rate fitted trend lines, 1950−2021: American Samoa TFR
Data points enclosed with a diamond are reference sources

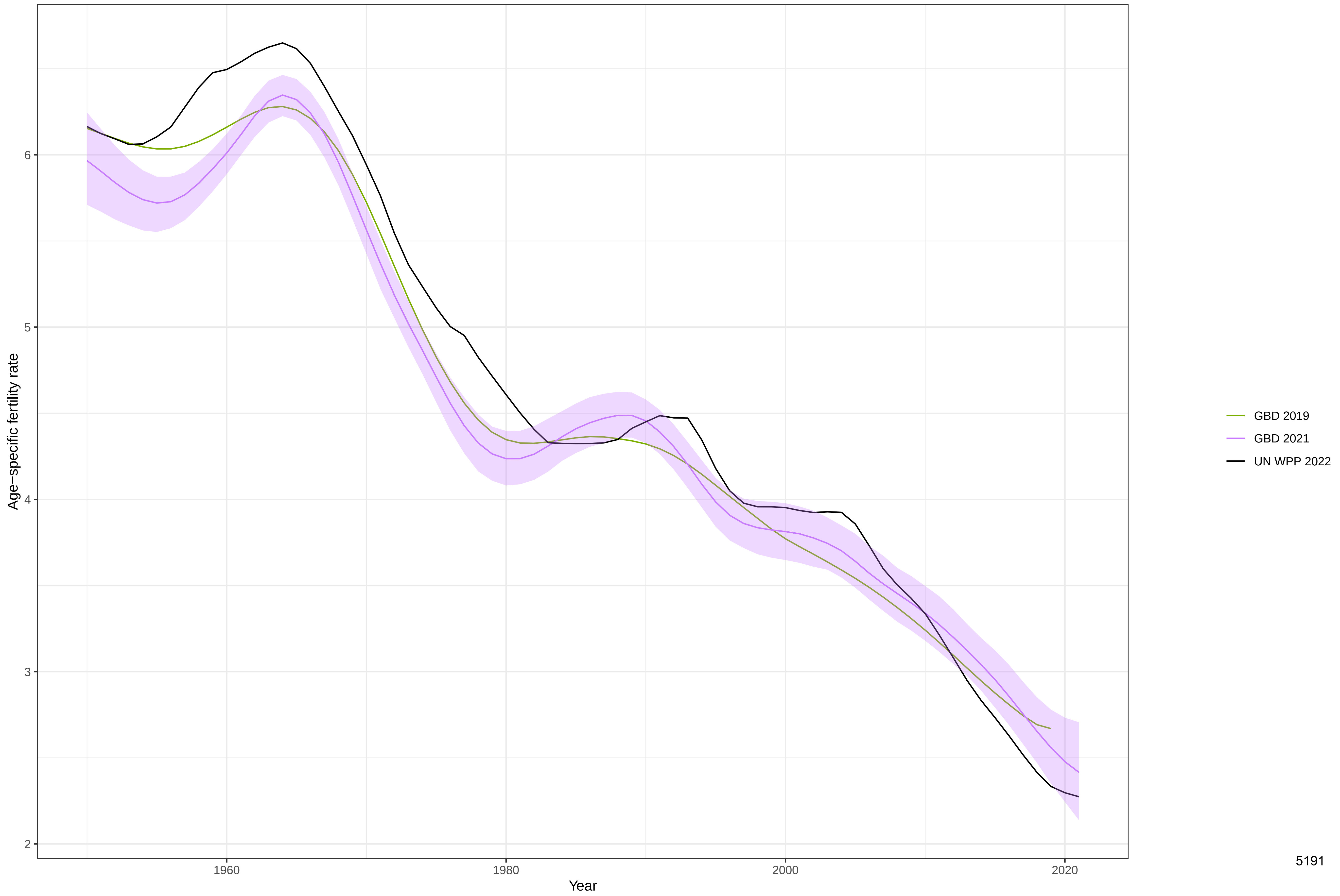

Age−specific fertility fitted trend lines, 1950−2021: Cook Islands, Ages: 10−14
Data points enclosed with a diamond are reference sources

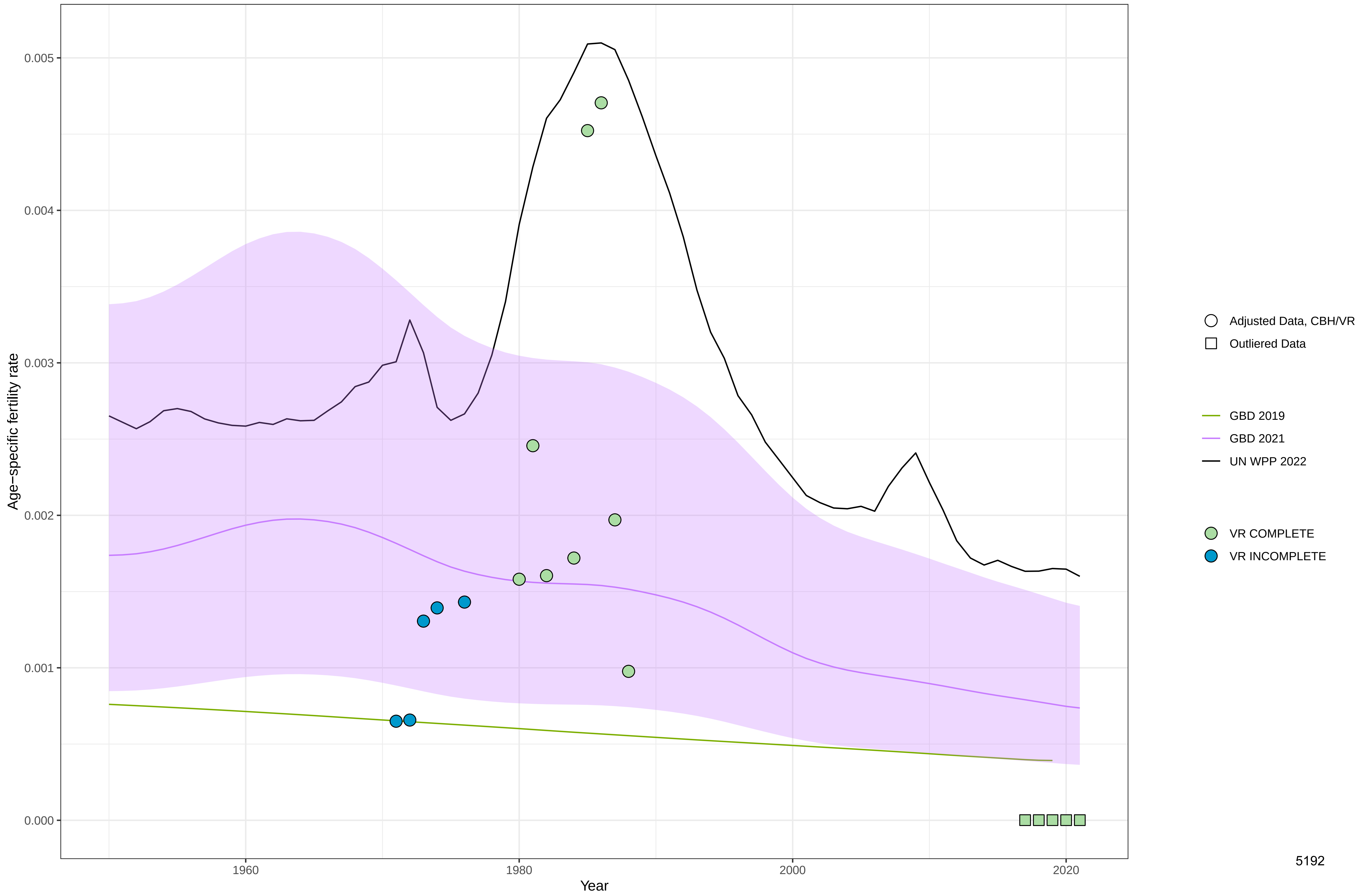

Age−specific fertility fitted trend lines, 1950−2021: Cook Islands, Ages: 15−19
Data points enclosed with a diamond are reference sources

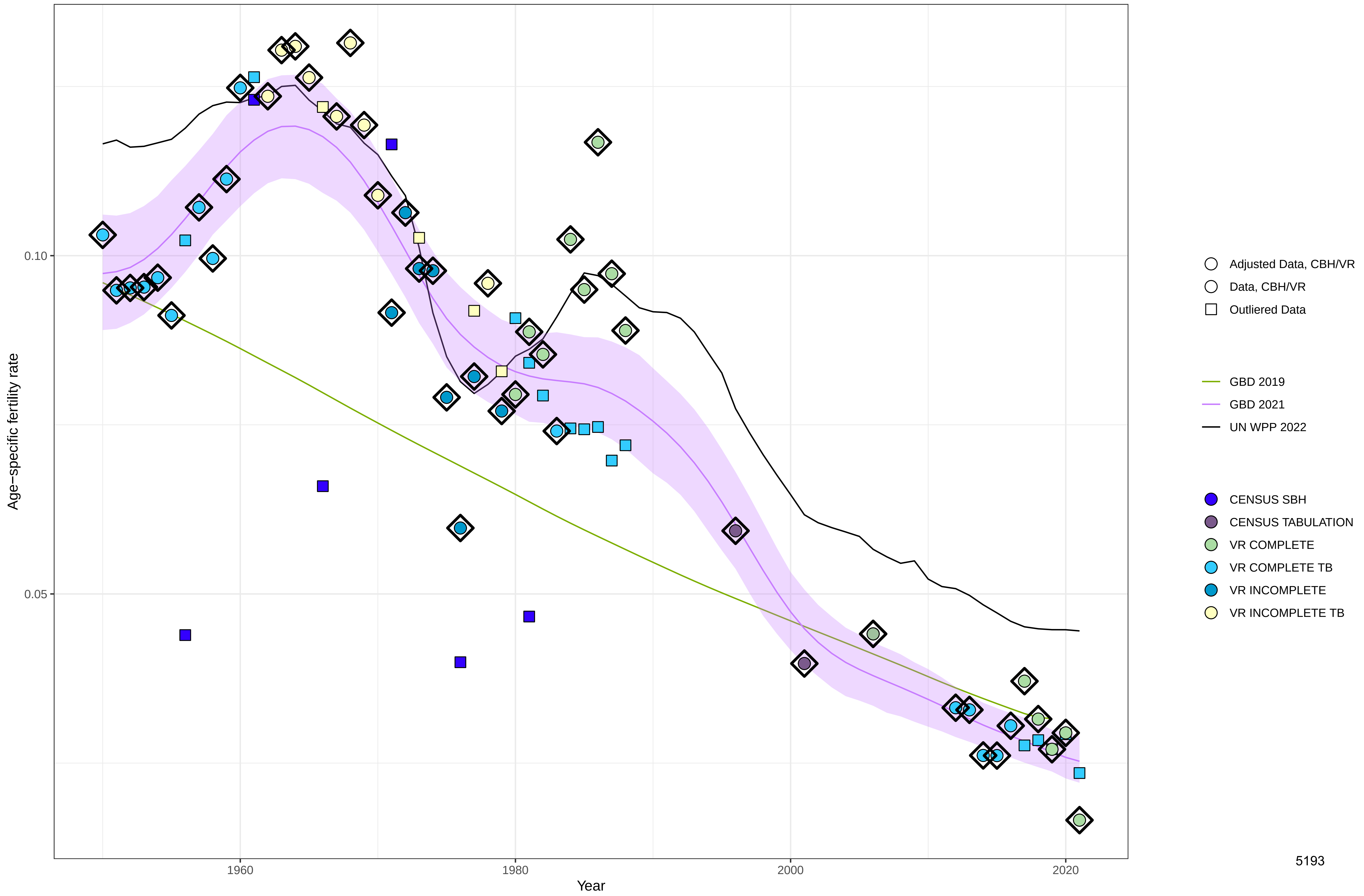

Age−specific fertility fitted trend lines, 1950−2021: Cook Islands, Ages: 20−24
Data points enclosed with a diamond are reference sources

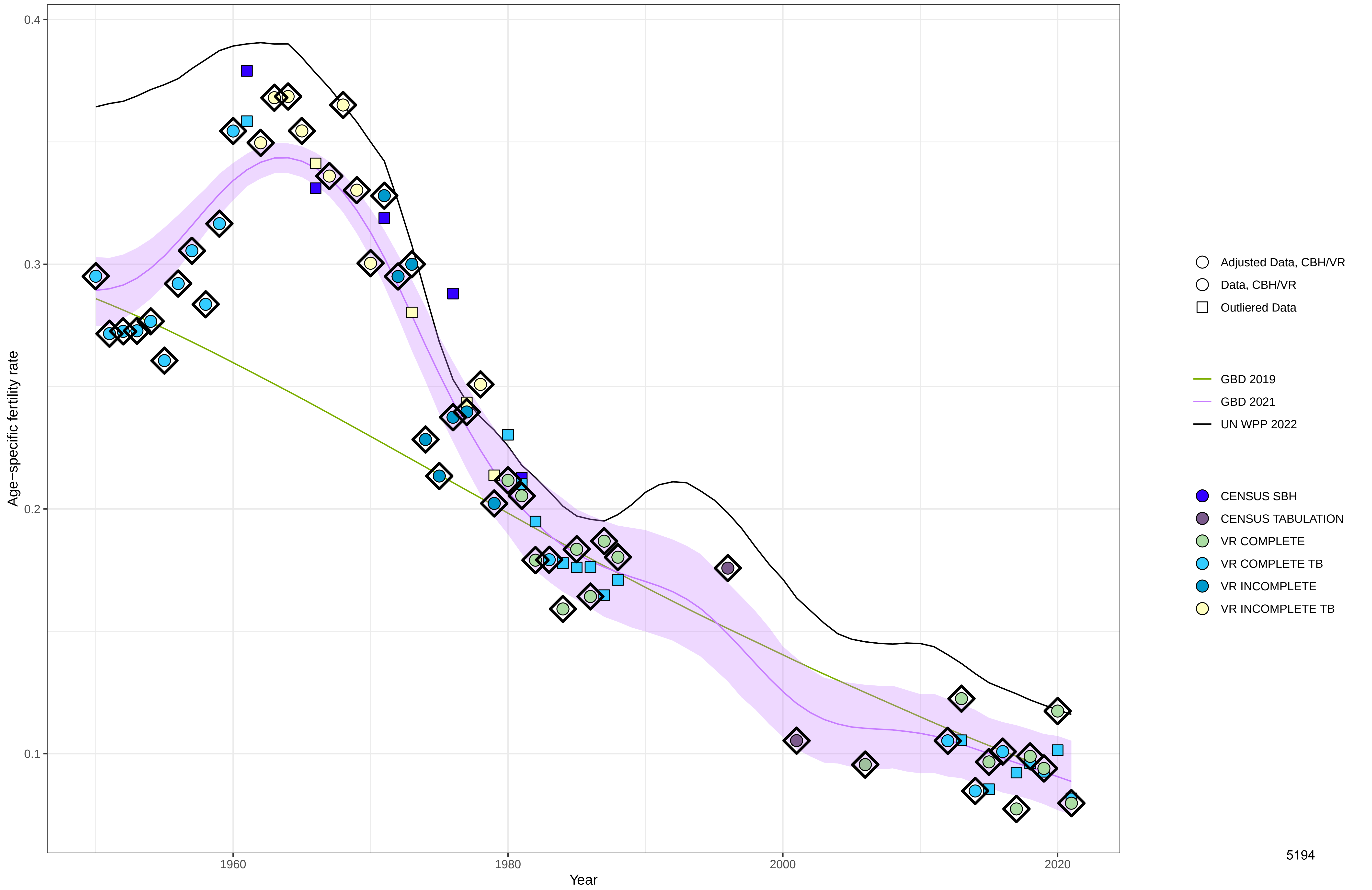

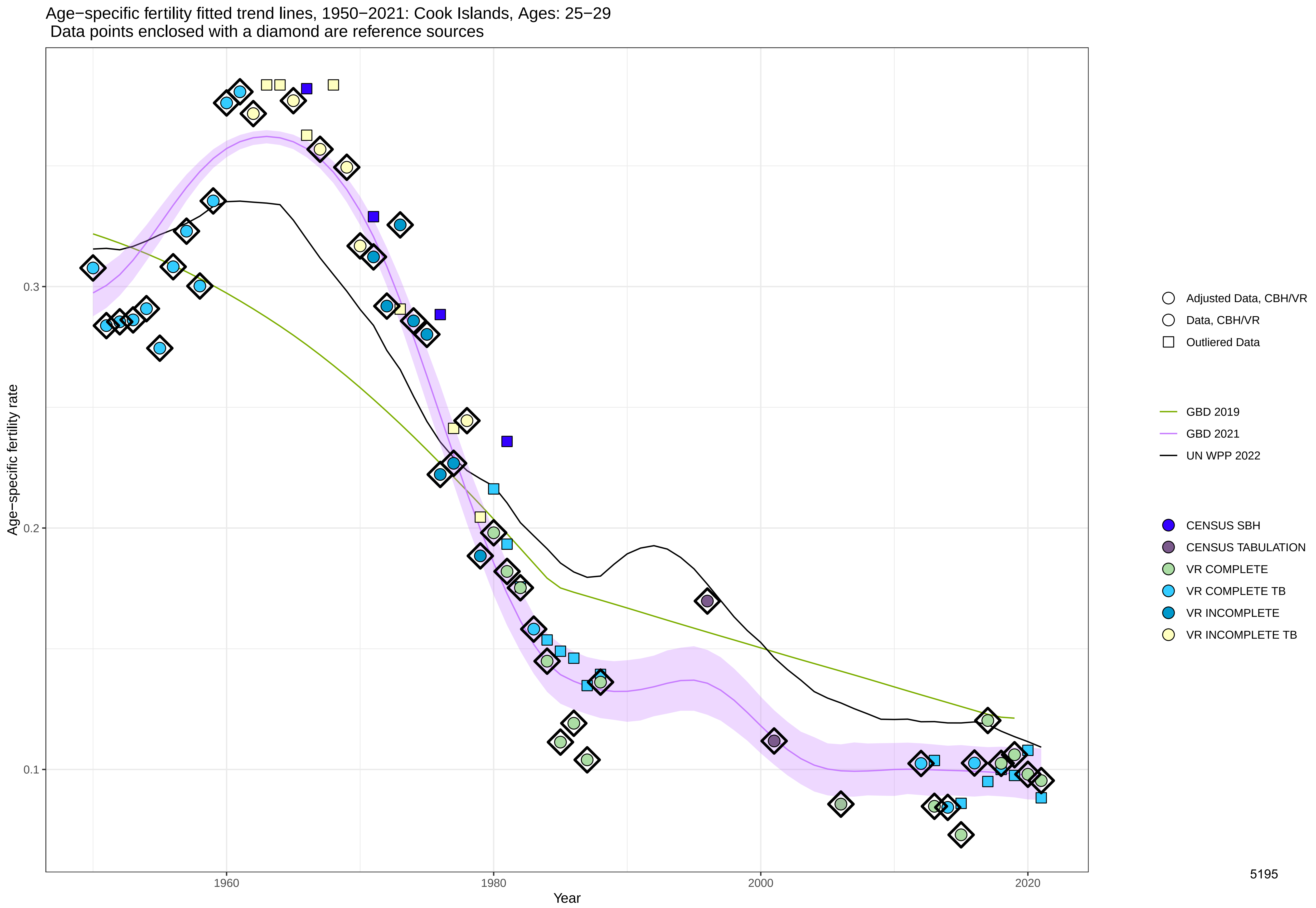

Age−specific fertility fitted trend lines, 1950−2021: Cook Islands, Ages: 25−29
Data points enclosed with a diamond are reference sources
Age−specific fertility rate
Year
0.1
0.2
0.3
1960
1980
2000
2020
Adjusted Data, CBH/VR
Data, CBH/VR
Outliered Data
GBD 2019
GBD 2021
UN WPP 2022
CENSUS SBH
CENSUS TABULATION
VR COMPLETE
VR COMPLETE TB
VR INCOMPLETE
VR INCOMPLETE TB

Age−specific fertility fitted trend lines, 1950−2021: Cook Islands, Ages: 30−34
Data points enclosed with a diamond are reference sources

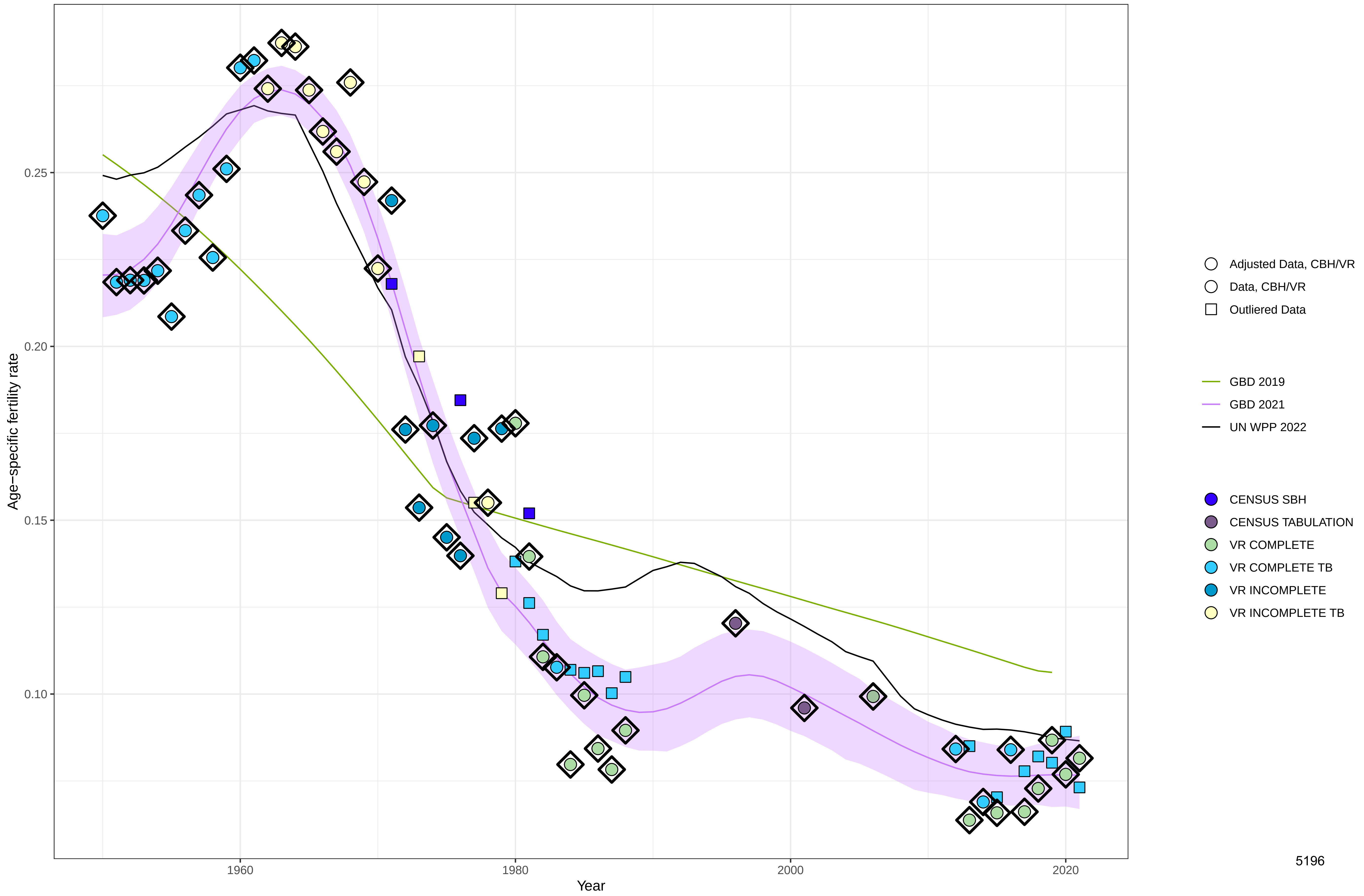

5196

Age−specific fertility fitted trend lines, 1950−2021: Cook Islands, Ages: 35−39
Data points enclosed with a diamond are reference sources

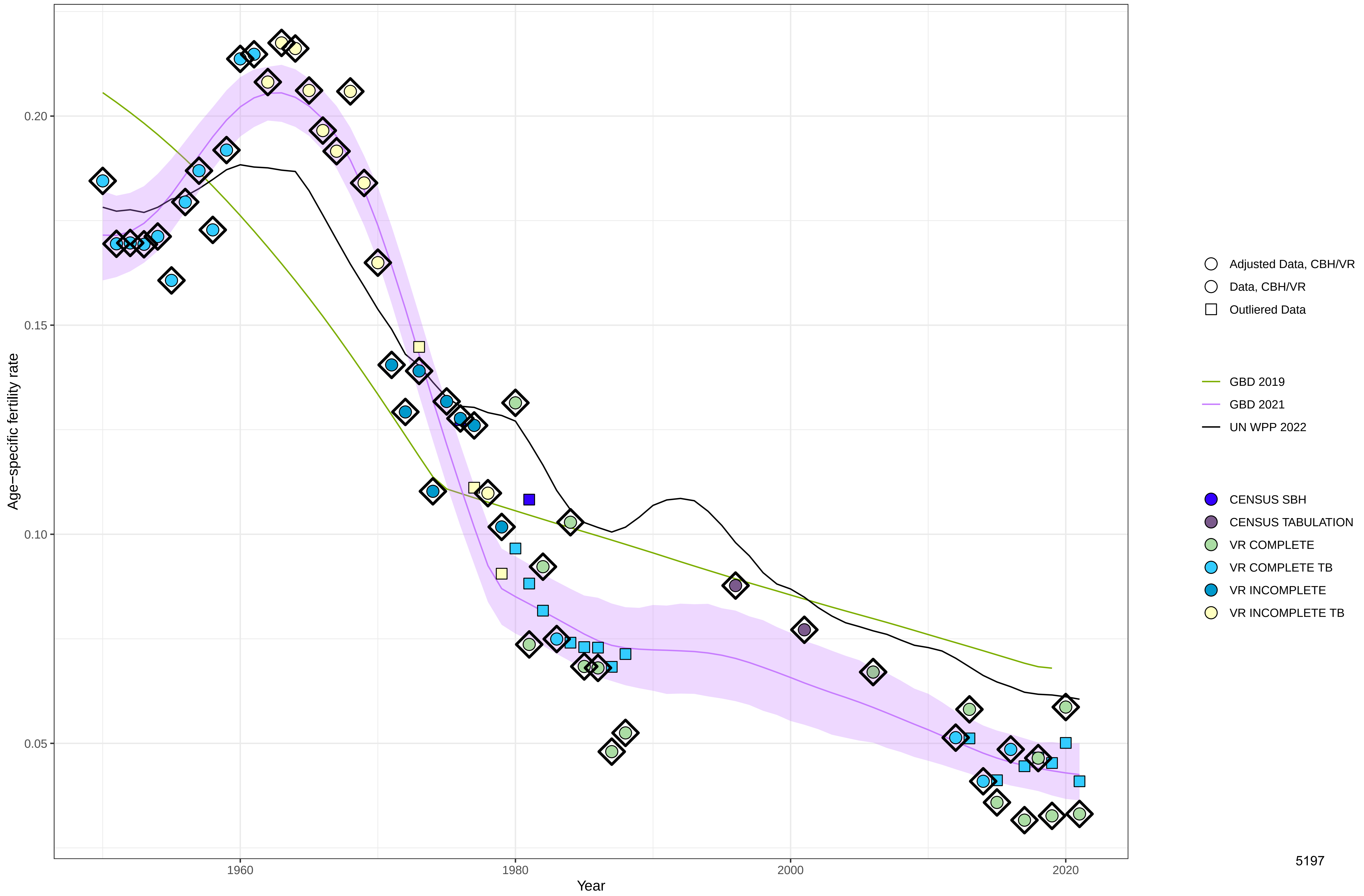

Age−specific fertility fitted trend lines, 1950−2021: Cook Islands, Ages: 40−44
Data points enclosed with a diamond are reference sources

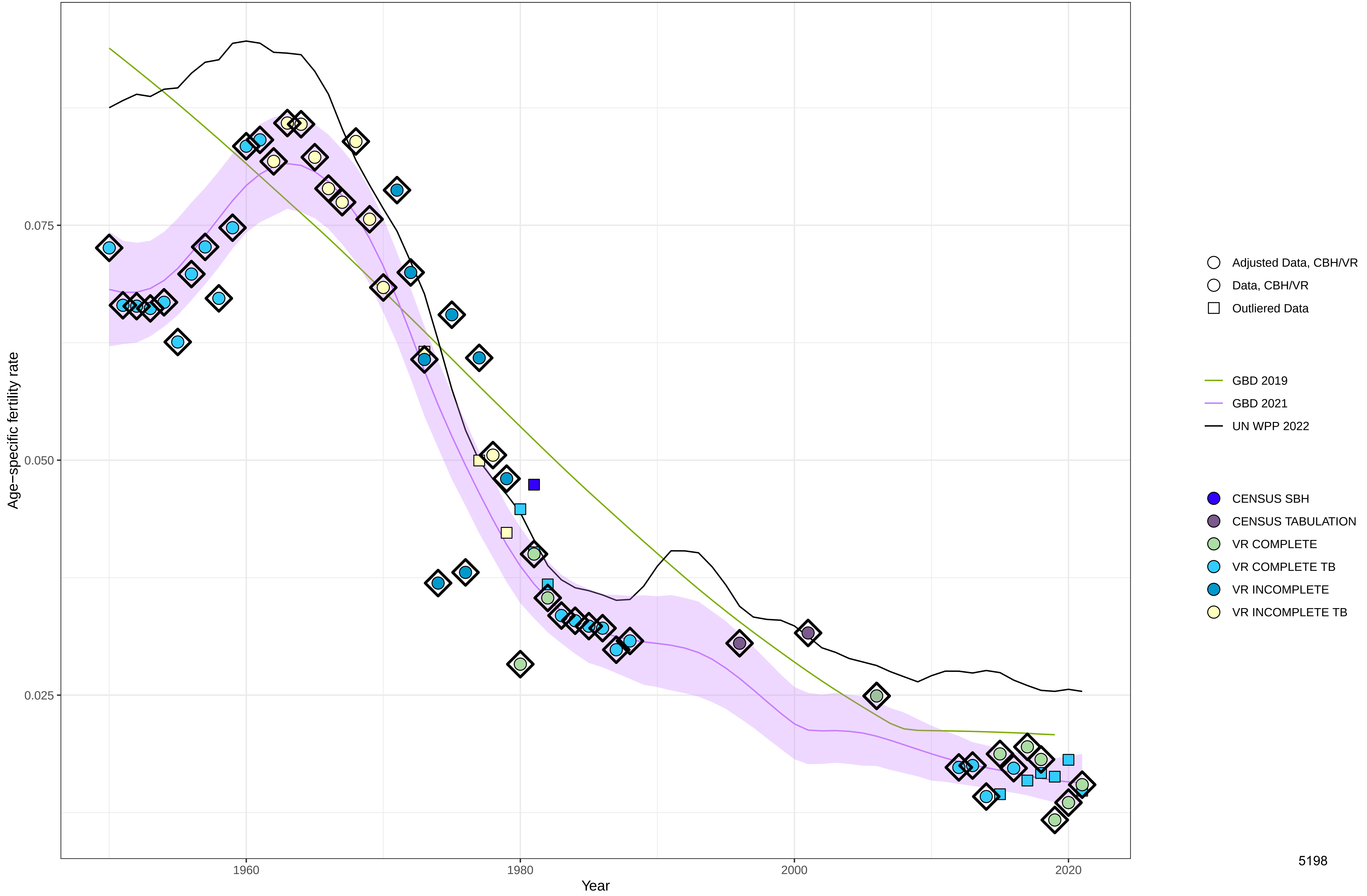

Age−specific fertility fitted trend lines, 1950−2021: Cook Islands, Ages: 45−49
Data points enclosed with a diamond are reference sources

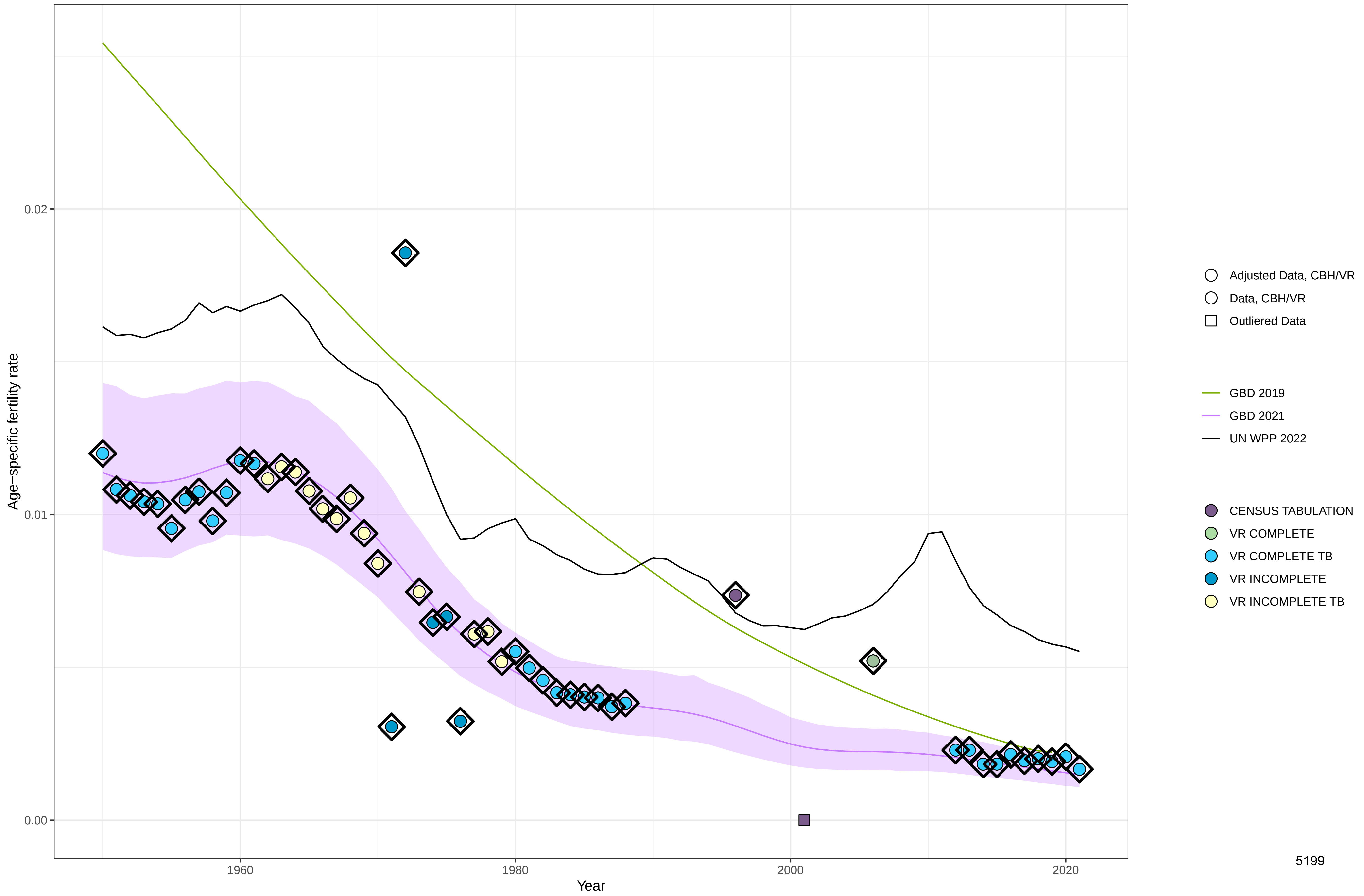

Age−specific fertility fitted trend lines, 1950−2021: Cook Islands, Ages: 50−54
Data points enclosed with a diamond are reference sources

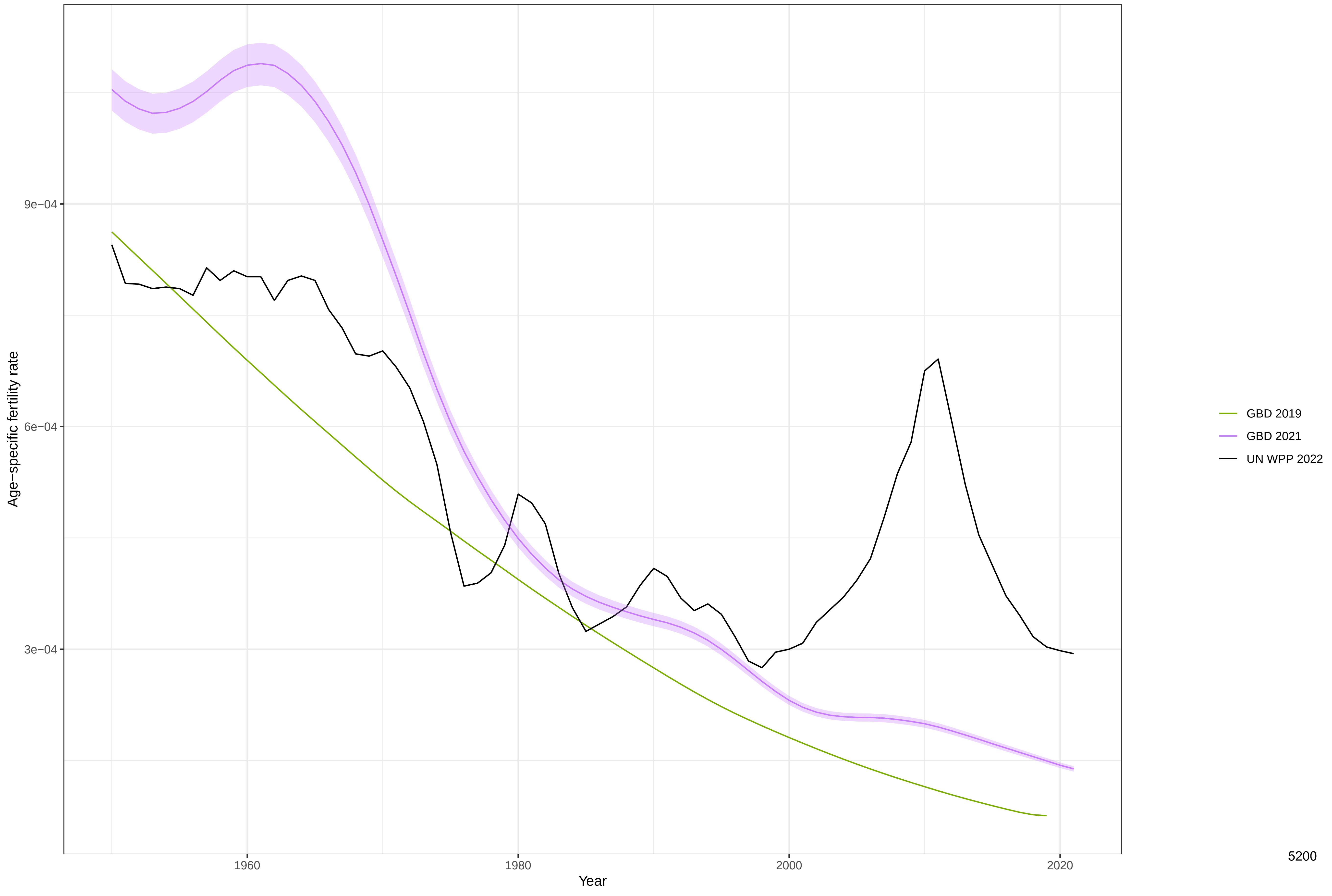

Total fertility rate fitted trend lines, 1950−2021: Cook Islands TFR
Data points enclosed with a diamond are reference sources

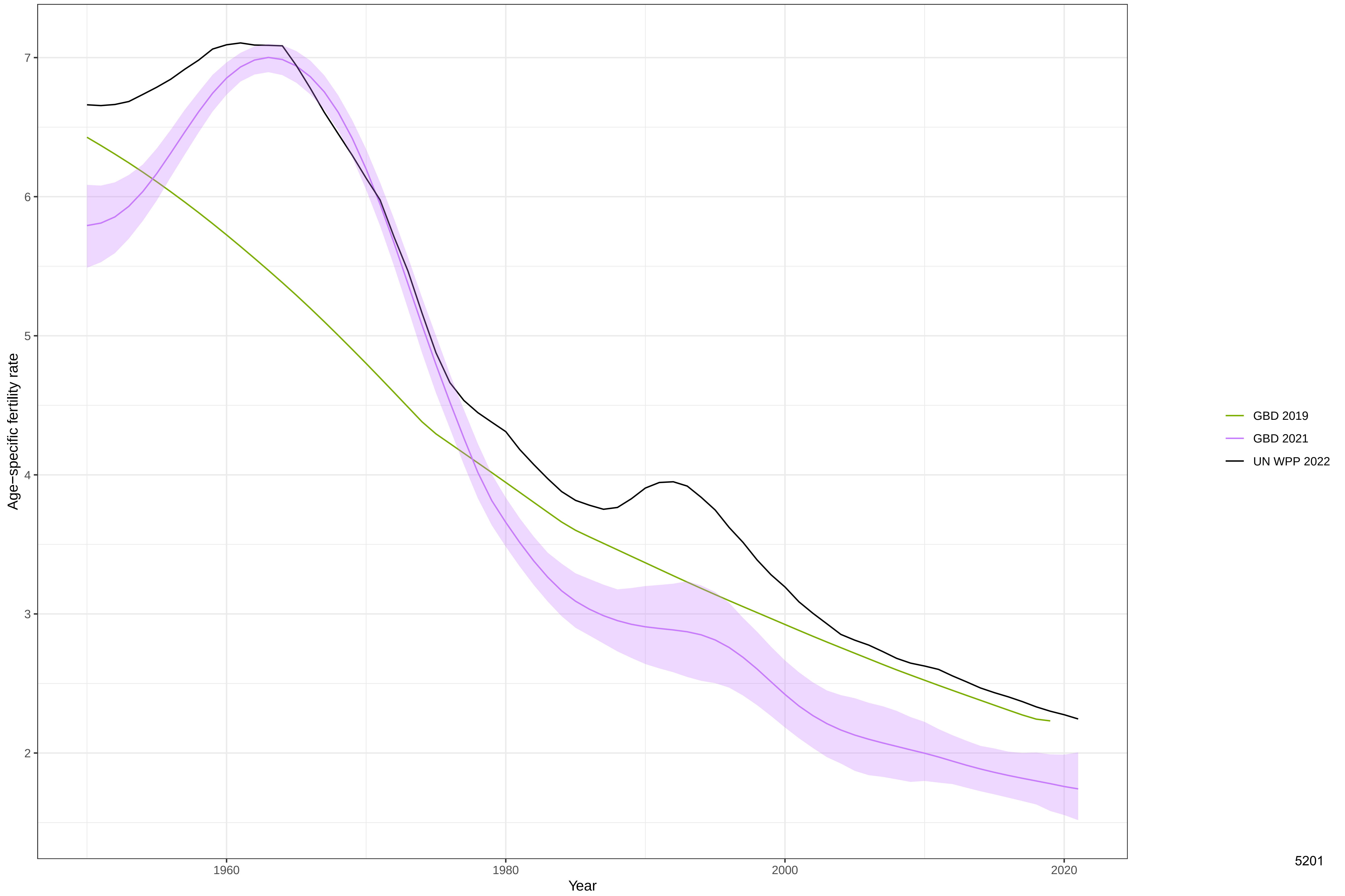

Age−specific fertility fitted trend lines, 1950−2021: Fiji, Ages: 10−14
Data points enclosed with a diamond are reference sources

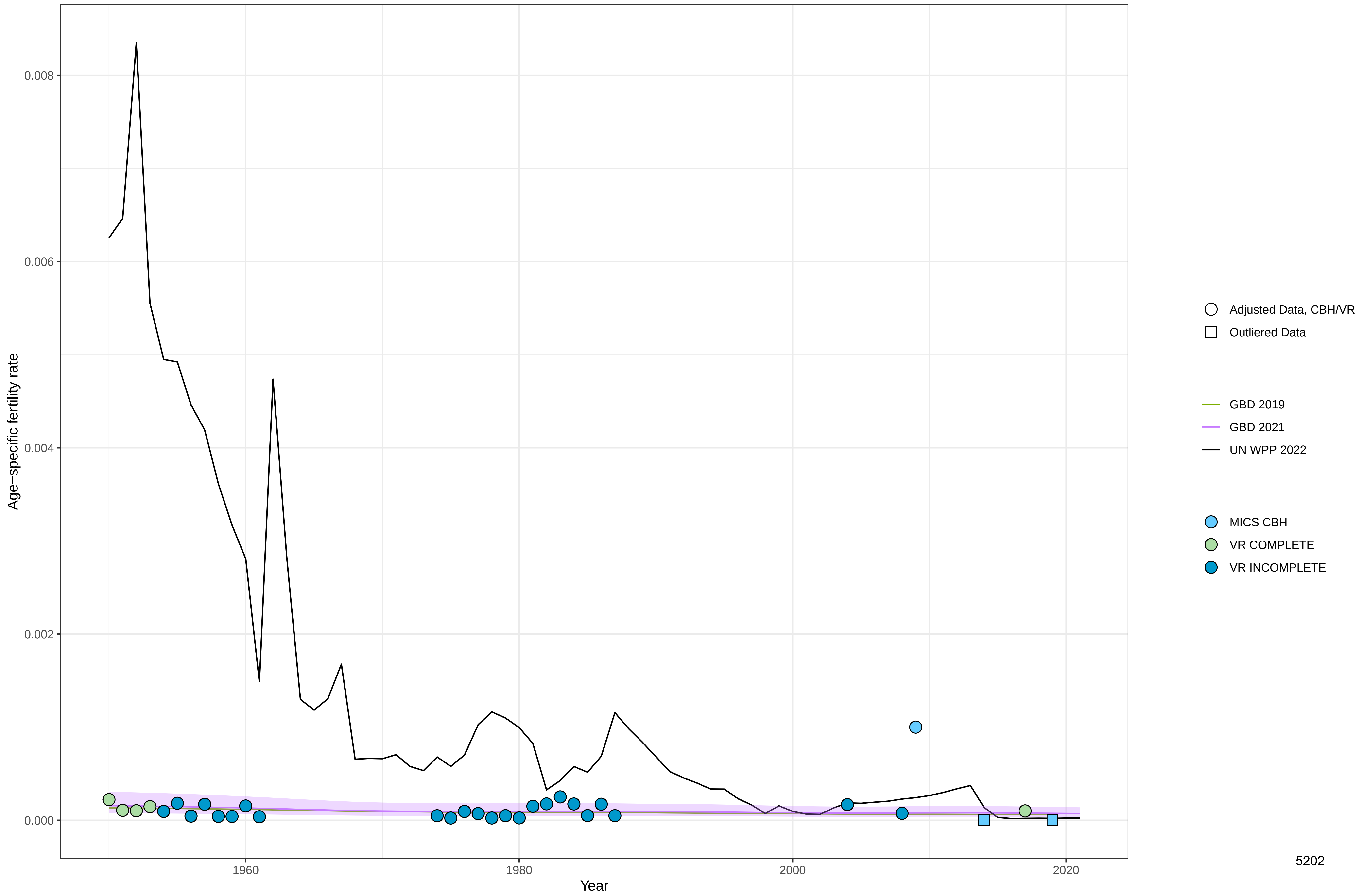

Age−specific fertility fitted trend lines, 1950−2021: Fiji, Ages: 15−19
Data points enclosed with a diamond are reference sources

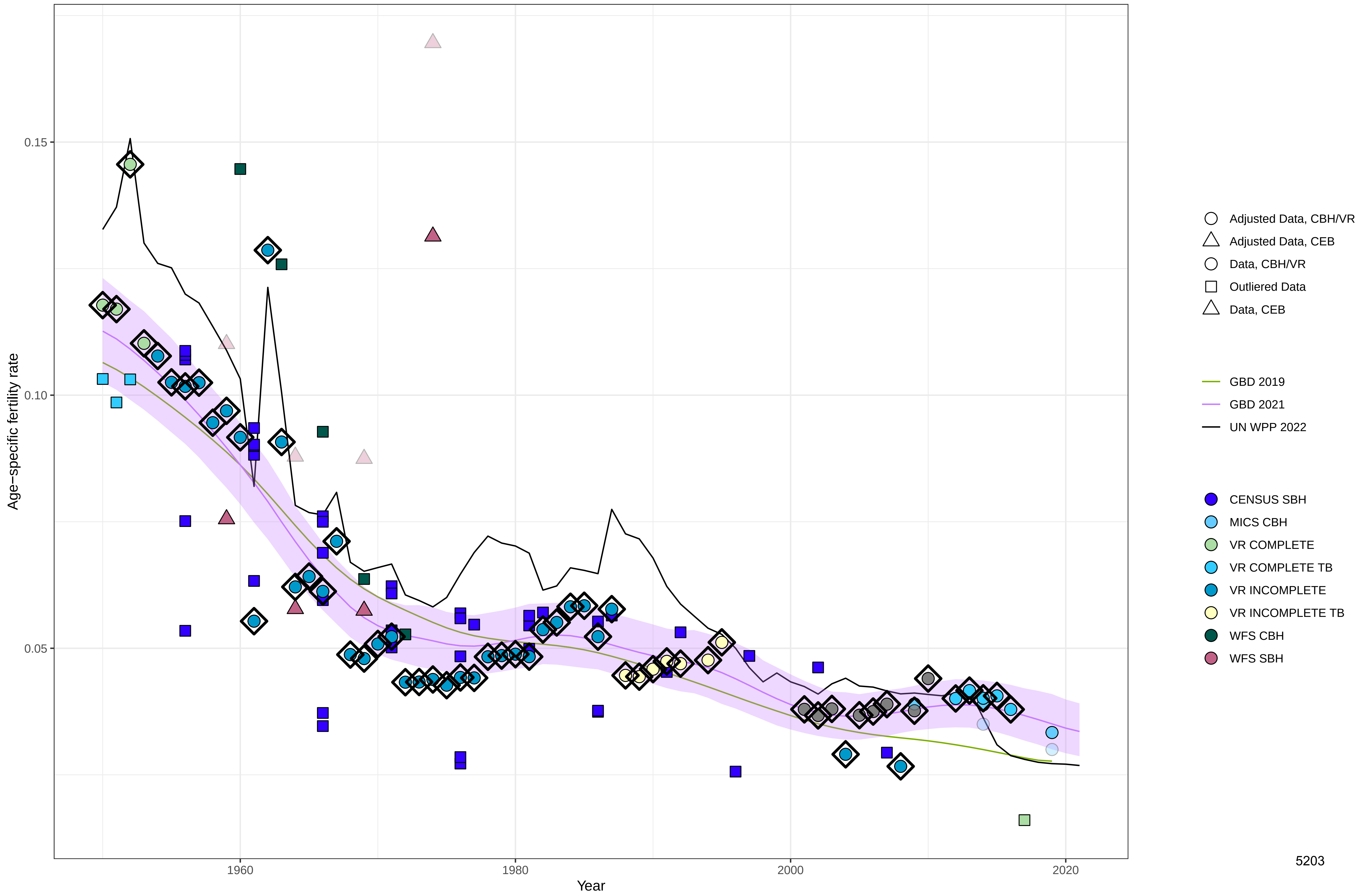

Age−specific fertility fitted trend lines, 1950−2021: Fiji, Ages: 20−24
Data points enclosed with a diamond are reference sources

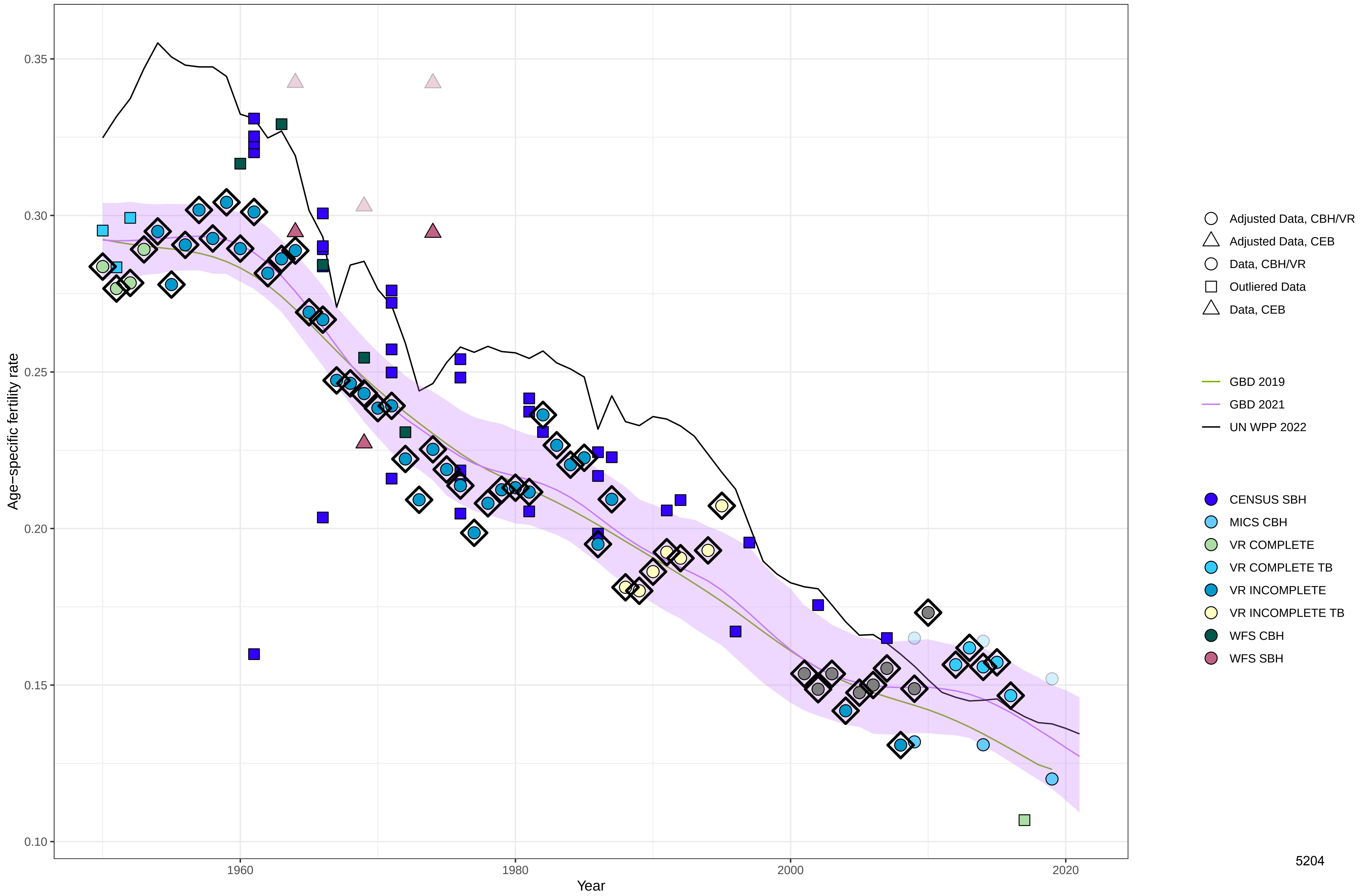

Age−specific fertility fitted trend lines, 1950−2021: Fiji, Ages: 25−29
Data points enclosed with a diamond are reference sources

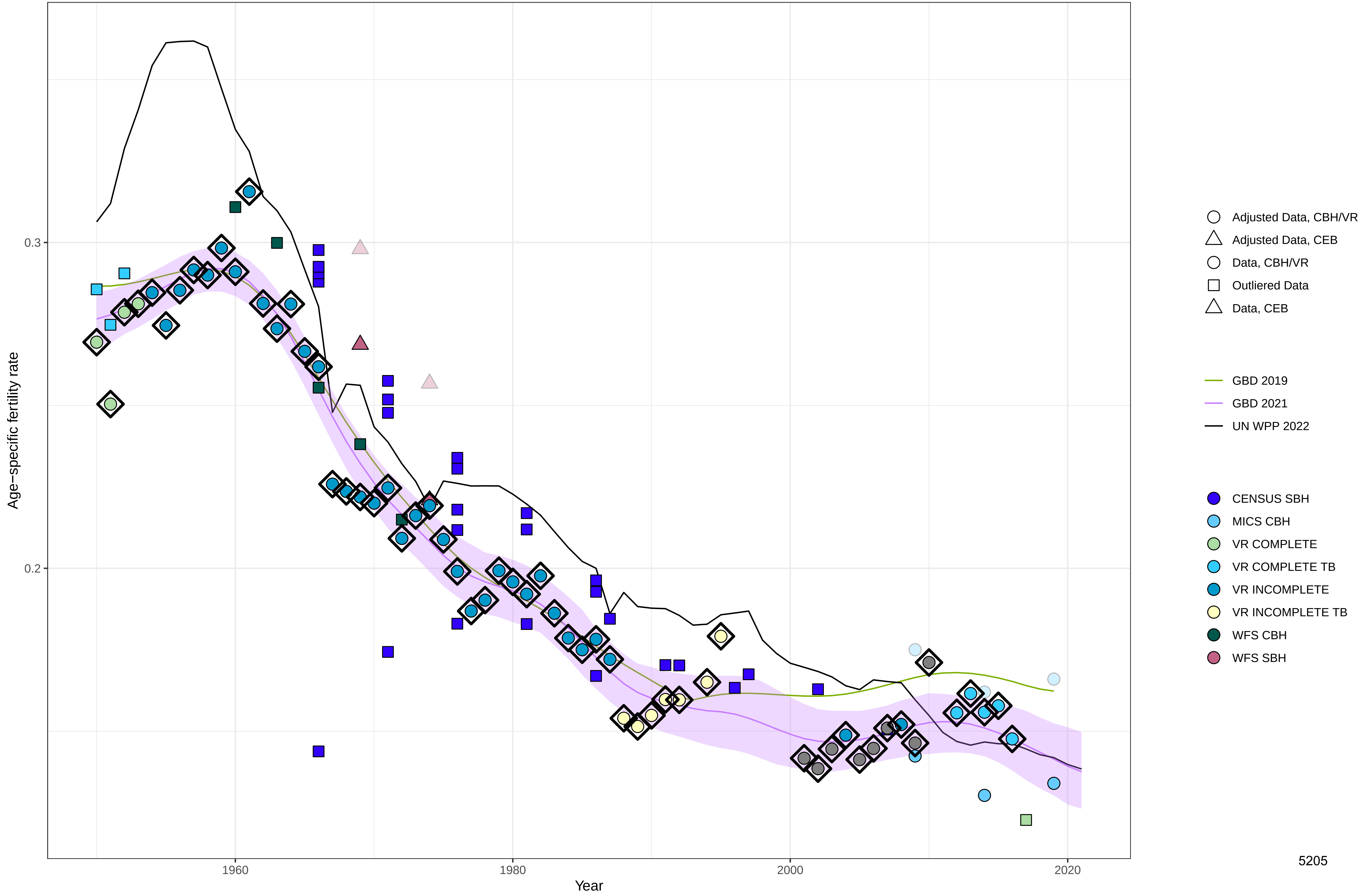

Age−specific fertility fitted trend lines, 1950−2021: Fiji, Ages: 30−34
Data points enclosed with a diamond are reference sources

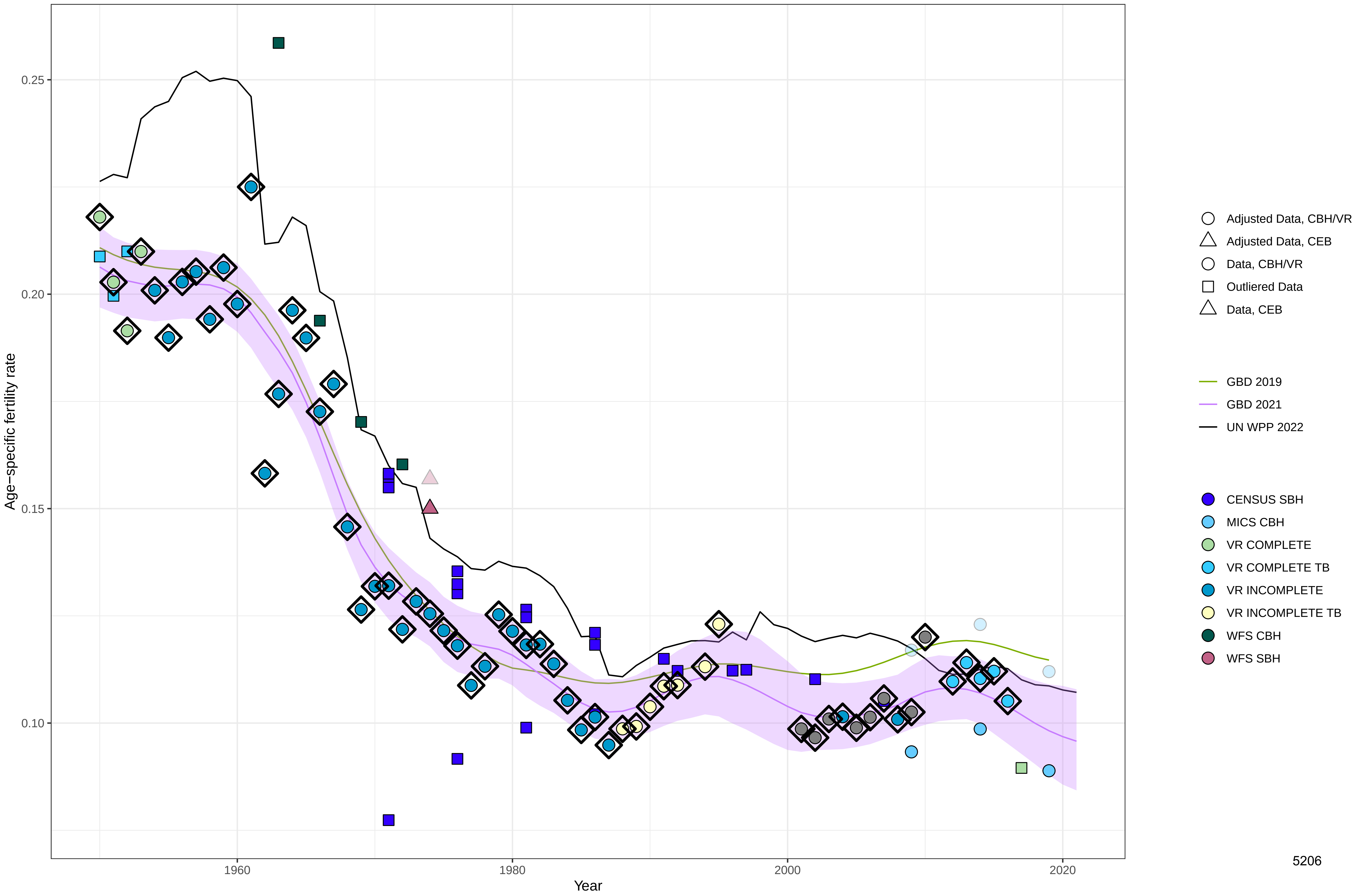

Age−specific fertility fitted trend lines, 1950−2021: Fiji, Ages: 35−39
Data points enclosed with a diamond are reference sources

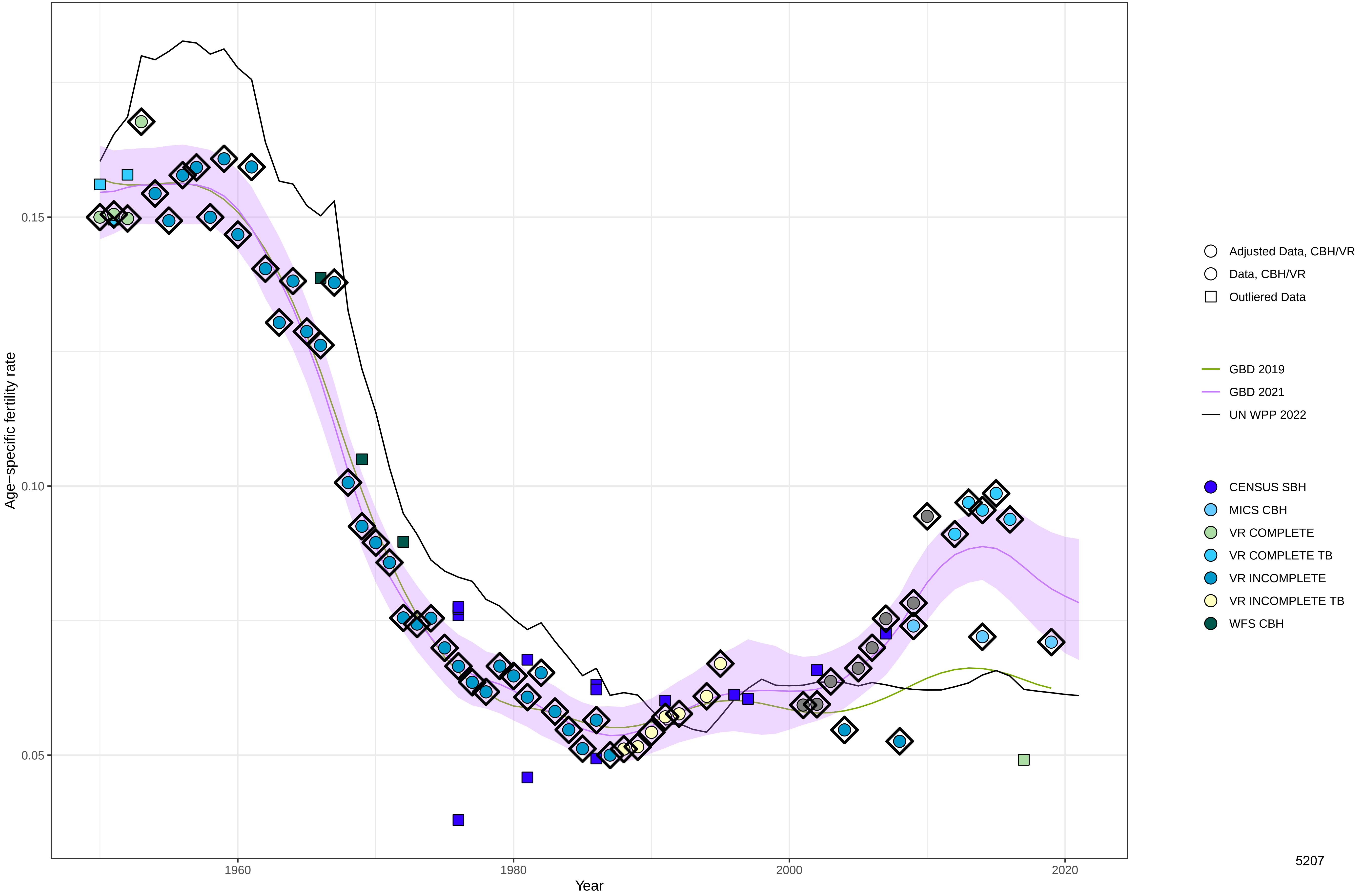

Age−specific fertility fitted trend lines, 1950−2021: Fiji, Ages: 40−44
Data points enclosed with a diamond are reference sources

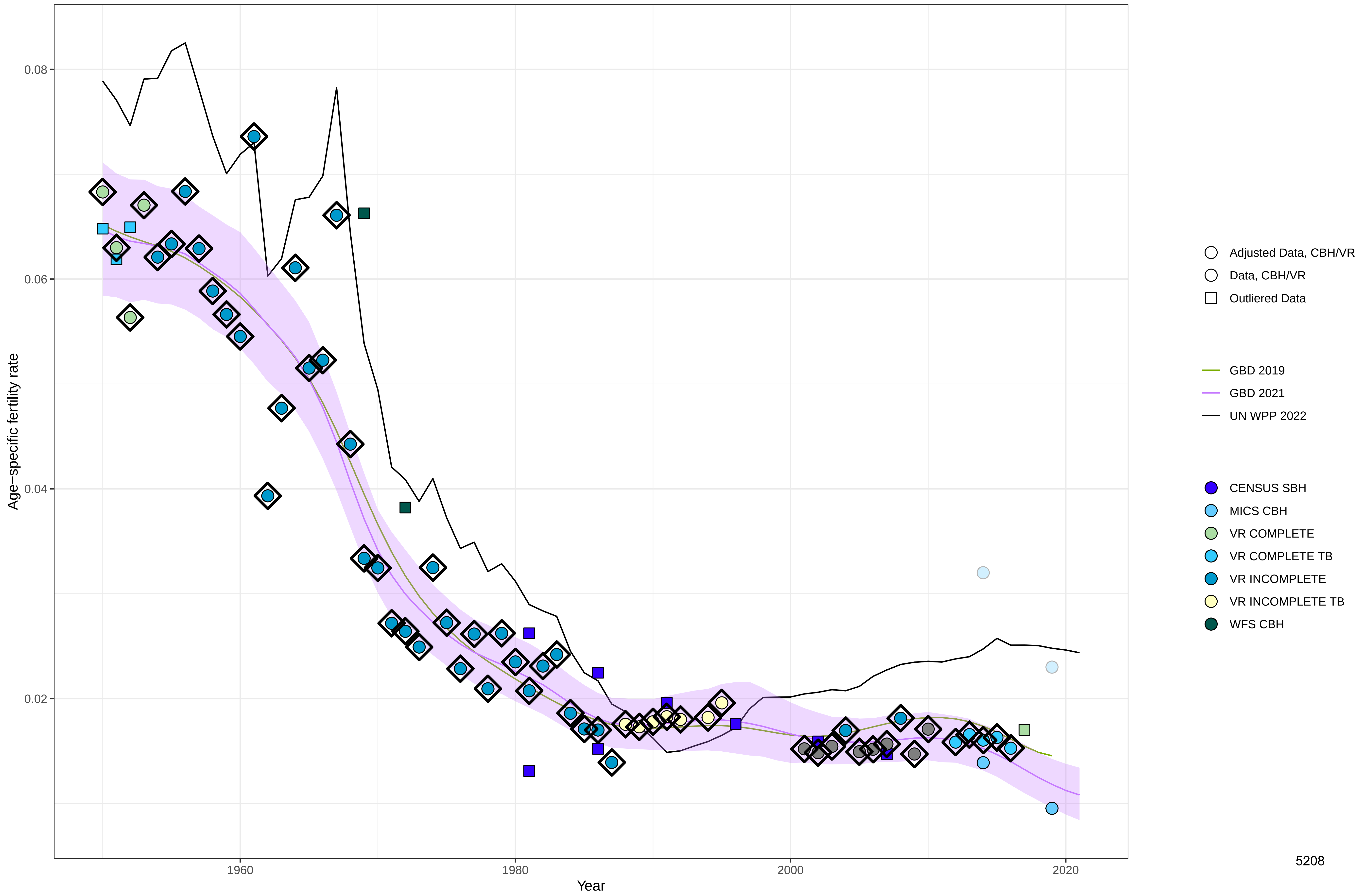

Age−specific fertility fitted trend lines, 1950−2021: Fiji, Ages: 45−49
Data points enclosed with a diamond are reference sources

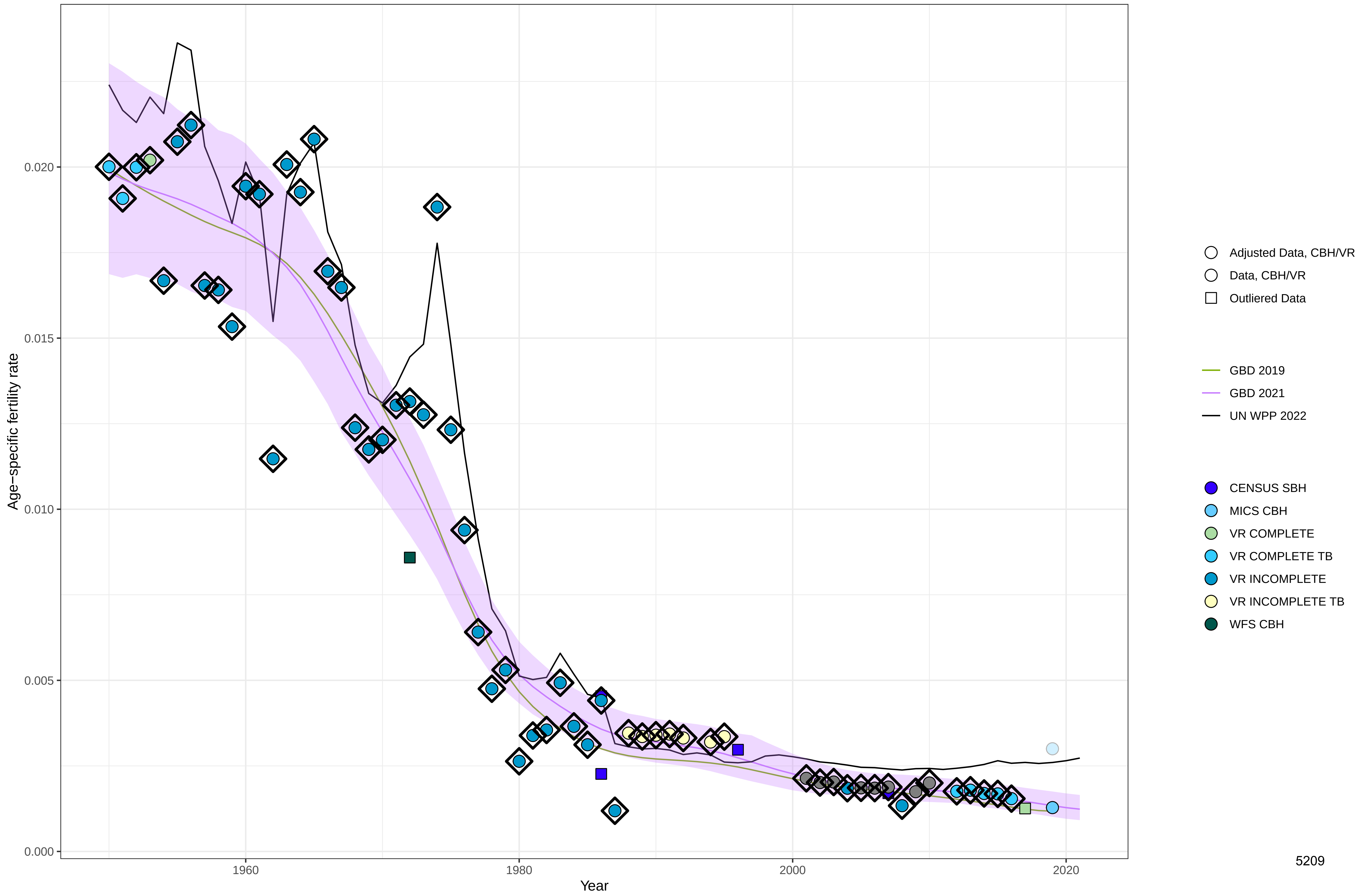

Age−specific fertility fitted trend lines, 1950−2021: Fiji, Ages: 50−54
Data points enclosed with a diamond are reference sources

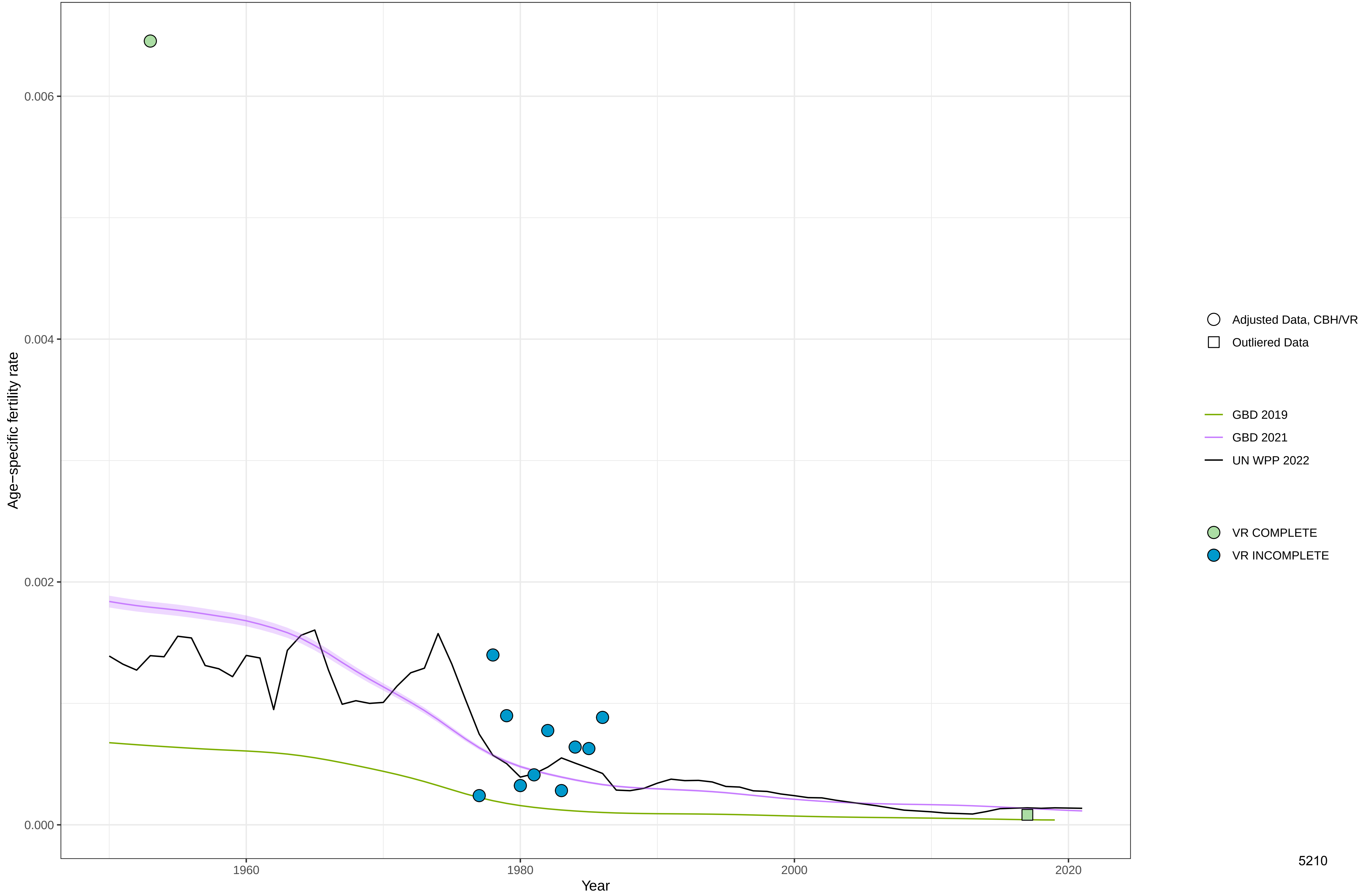

Total fertility rate fitted trend lines, 1950−2021: Fiji TFR
Data points enclosed with a diamond are reference sources

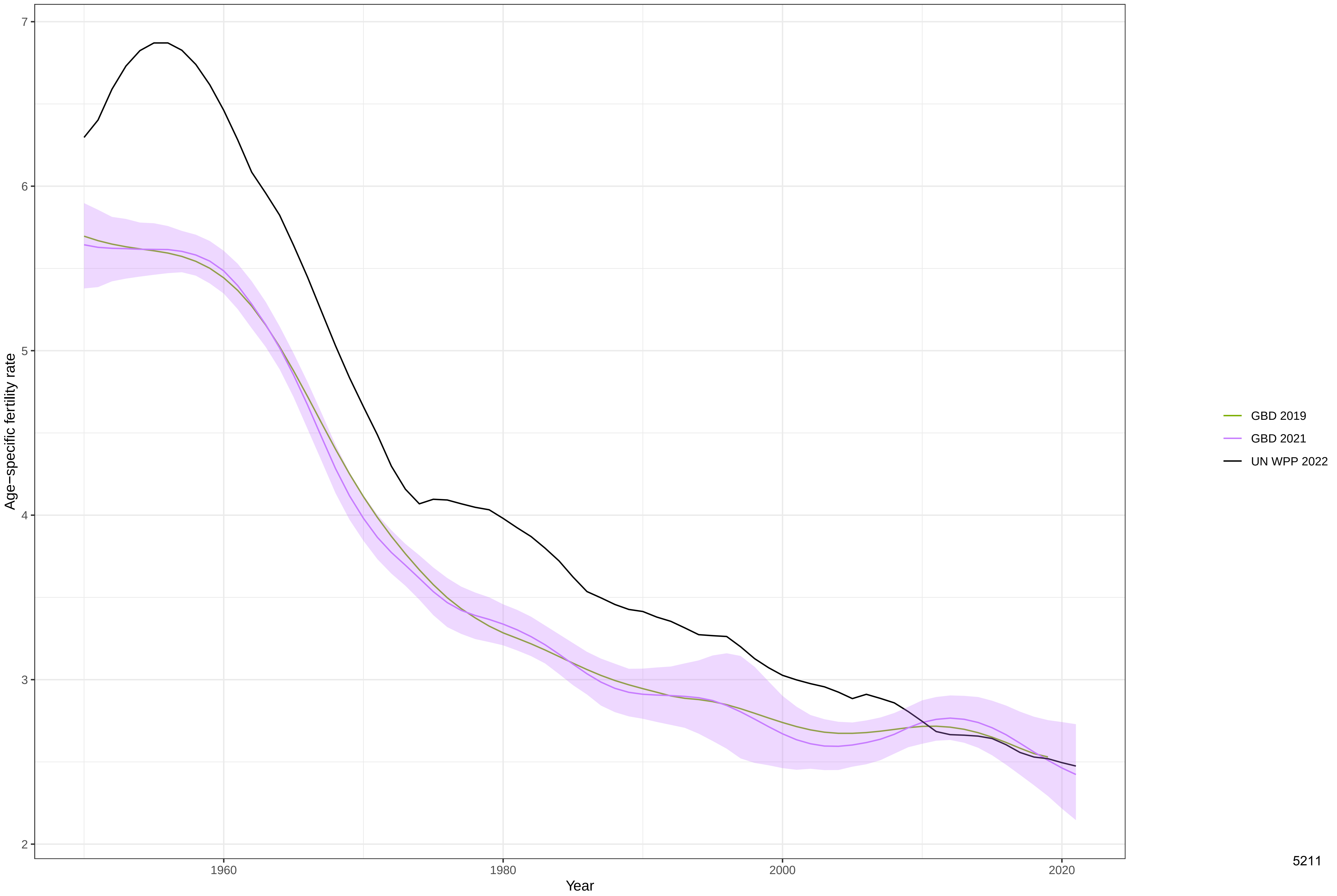

Age−specific fertility fitted trend lines, 1950−2021: Guam, Ages: 10−14
Data points enclosed with a diamond are reference sources

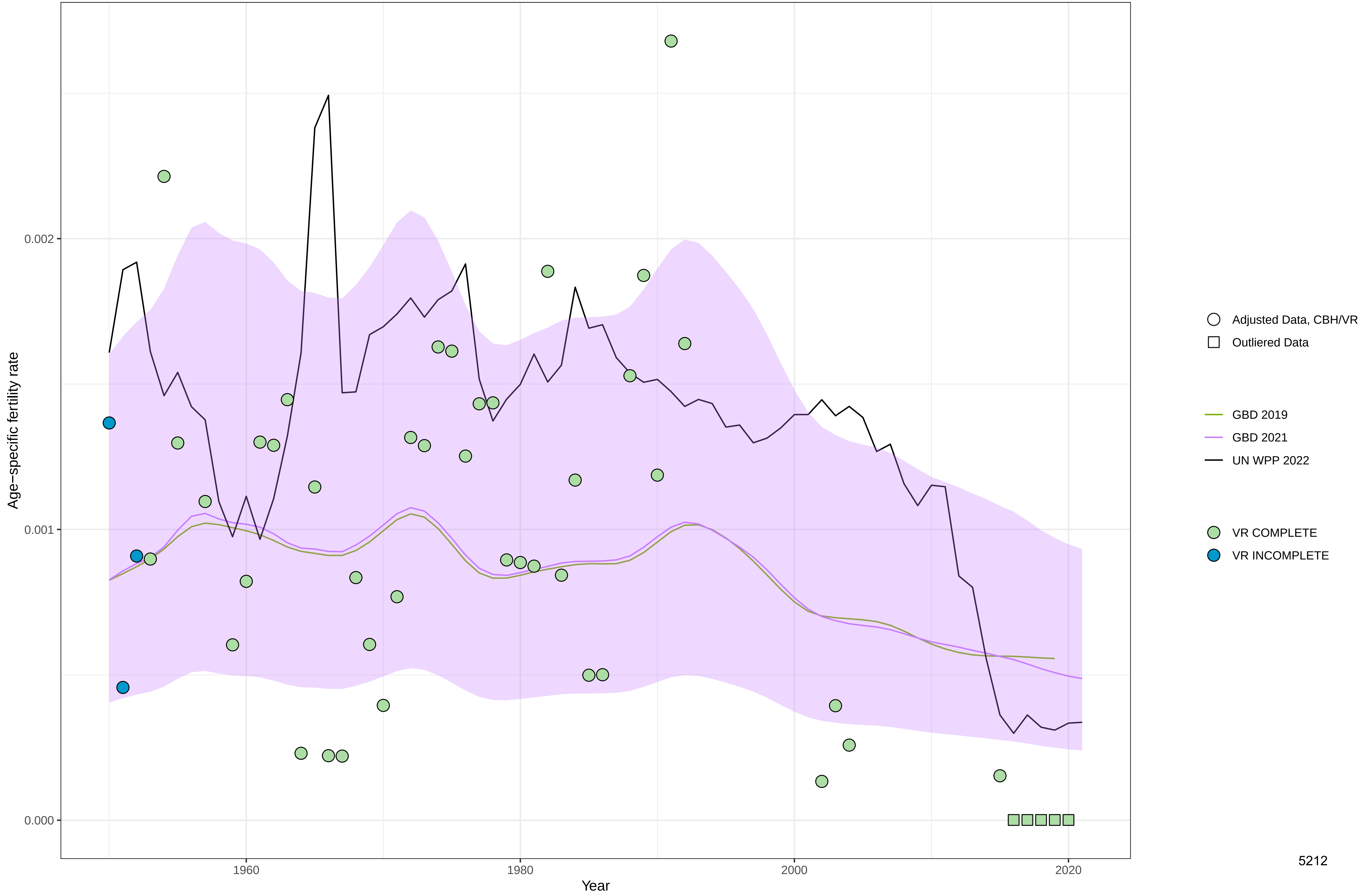

Age−specific fertility fitted trend lines, 1950−2021: Guam, Ages: 15−19
Data points enclosed with a diamond are reference sources

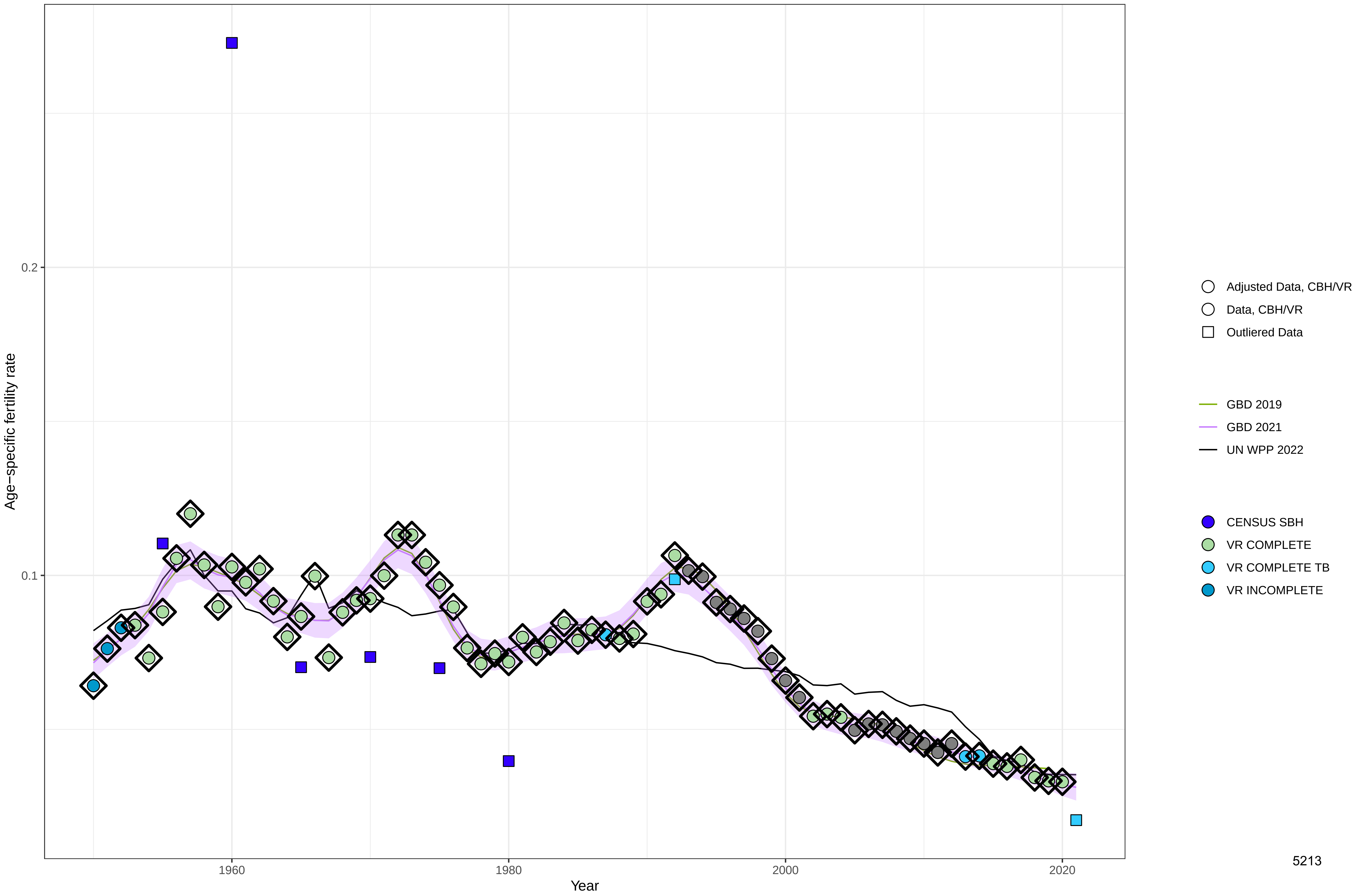

Age−specific fertility fitted trend lines, 1950−2021: Guam, Ages: 20−24
Data points enclosed with a diamond are reference sources

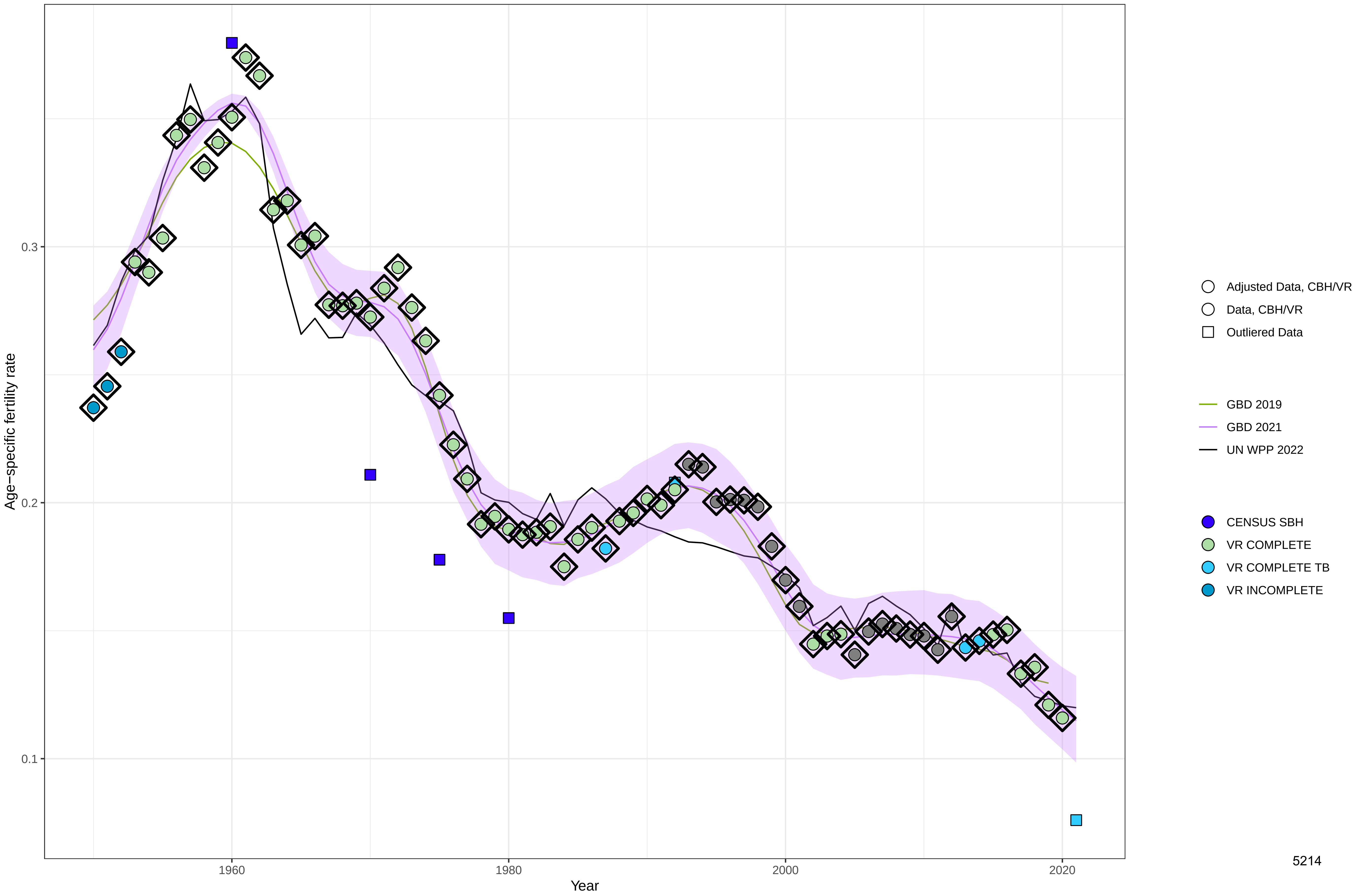

Age−specific fertility fitted trend lines, 1950−2021: Guam, Ages: 25−29
Data points enclosed with a diamond are reference sources

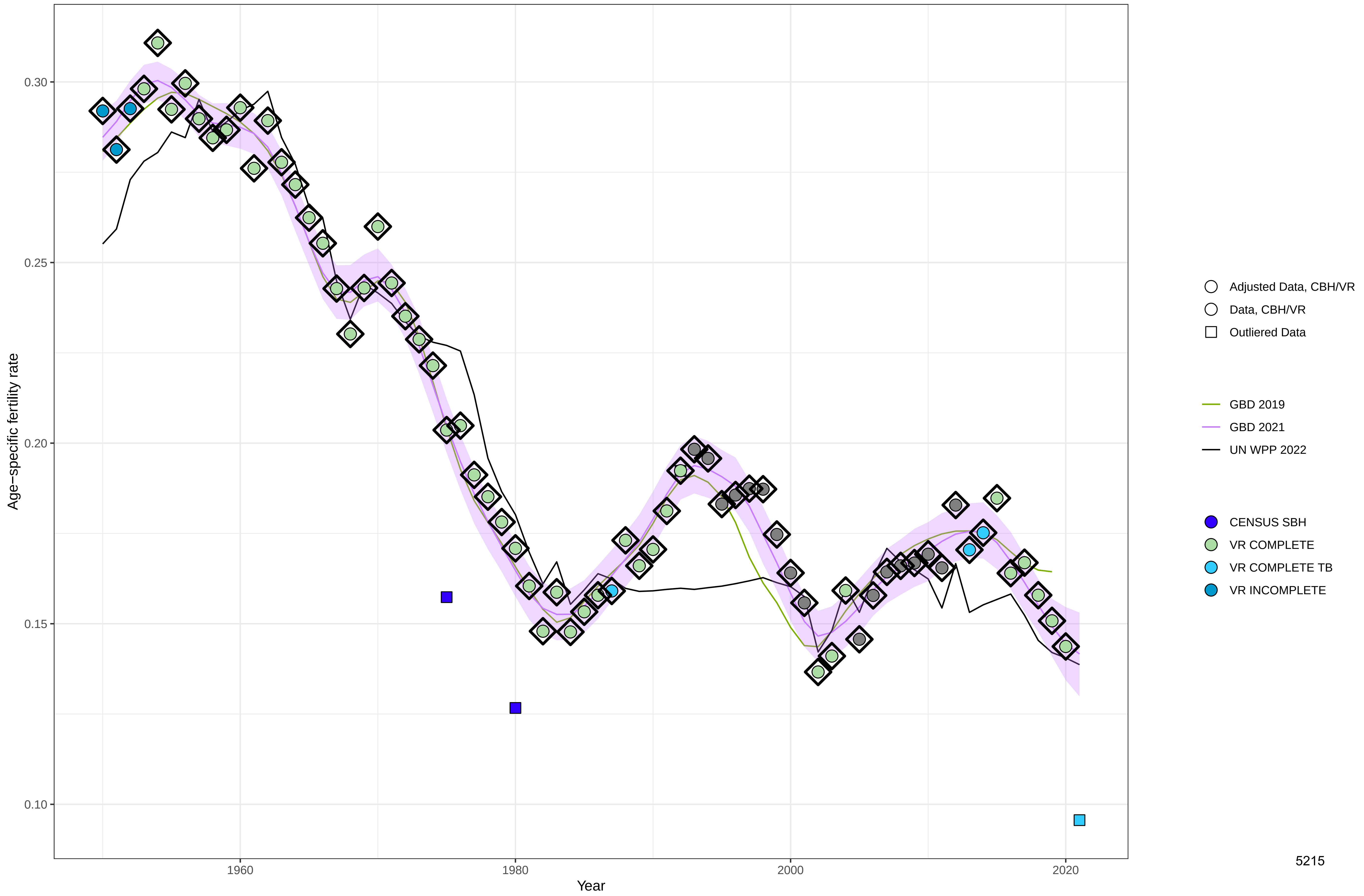

Age−specific fertility fitted trend lines, 1950−2021: Guam, Ages: 30−34
Data points enclosed with a diamond are reference sources

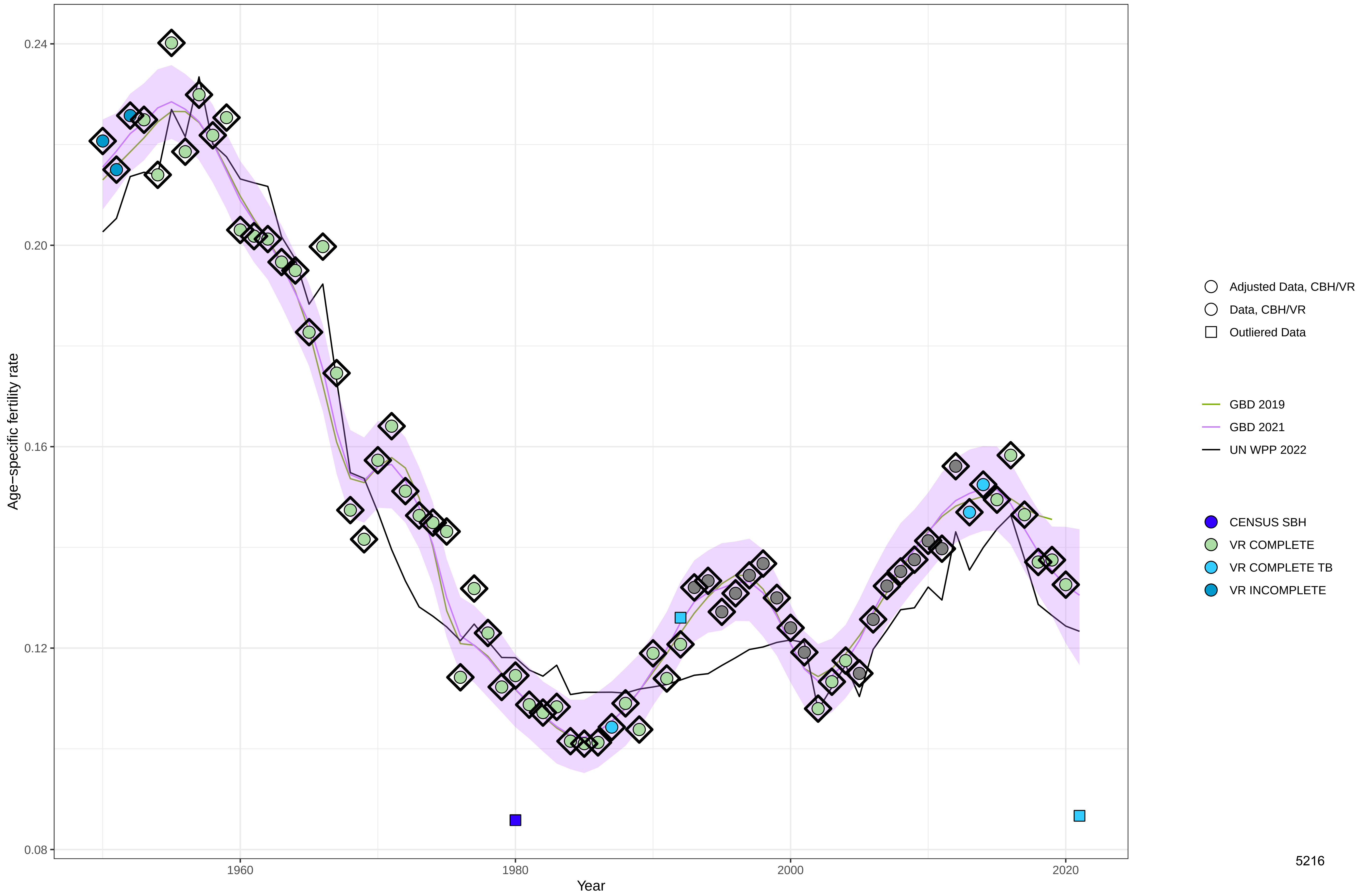

Age−specific fertility fitted trend lines, 1950−2021: Guam, Ages: 35−39
Data points enclosed with a diamond are reference sources

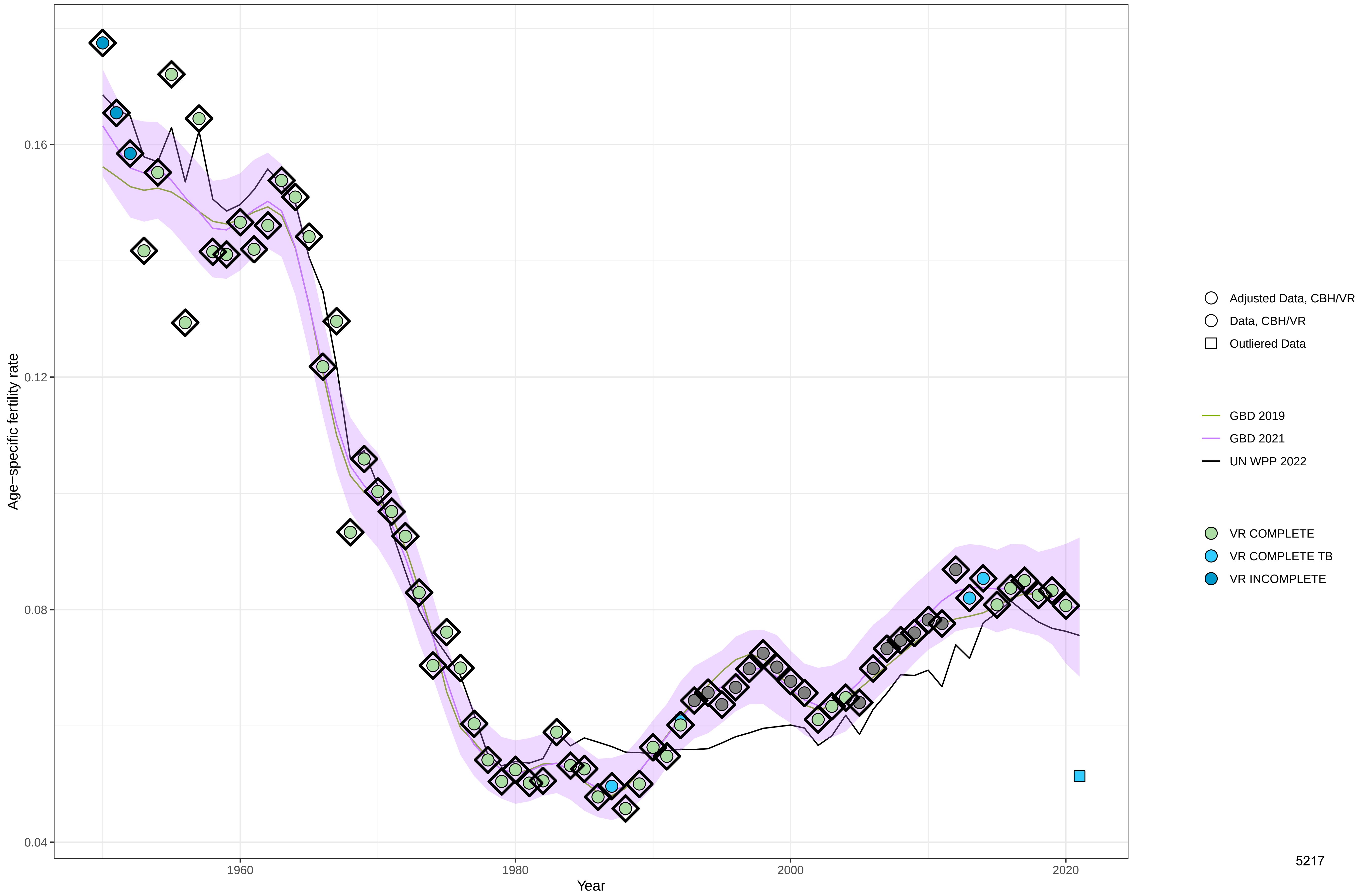

Age−specific fertility fitted trend lines, 1950−2021: Guam, Ages: 40−44
Data points enclosed with a diamond are reference sources

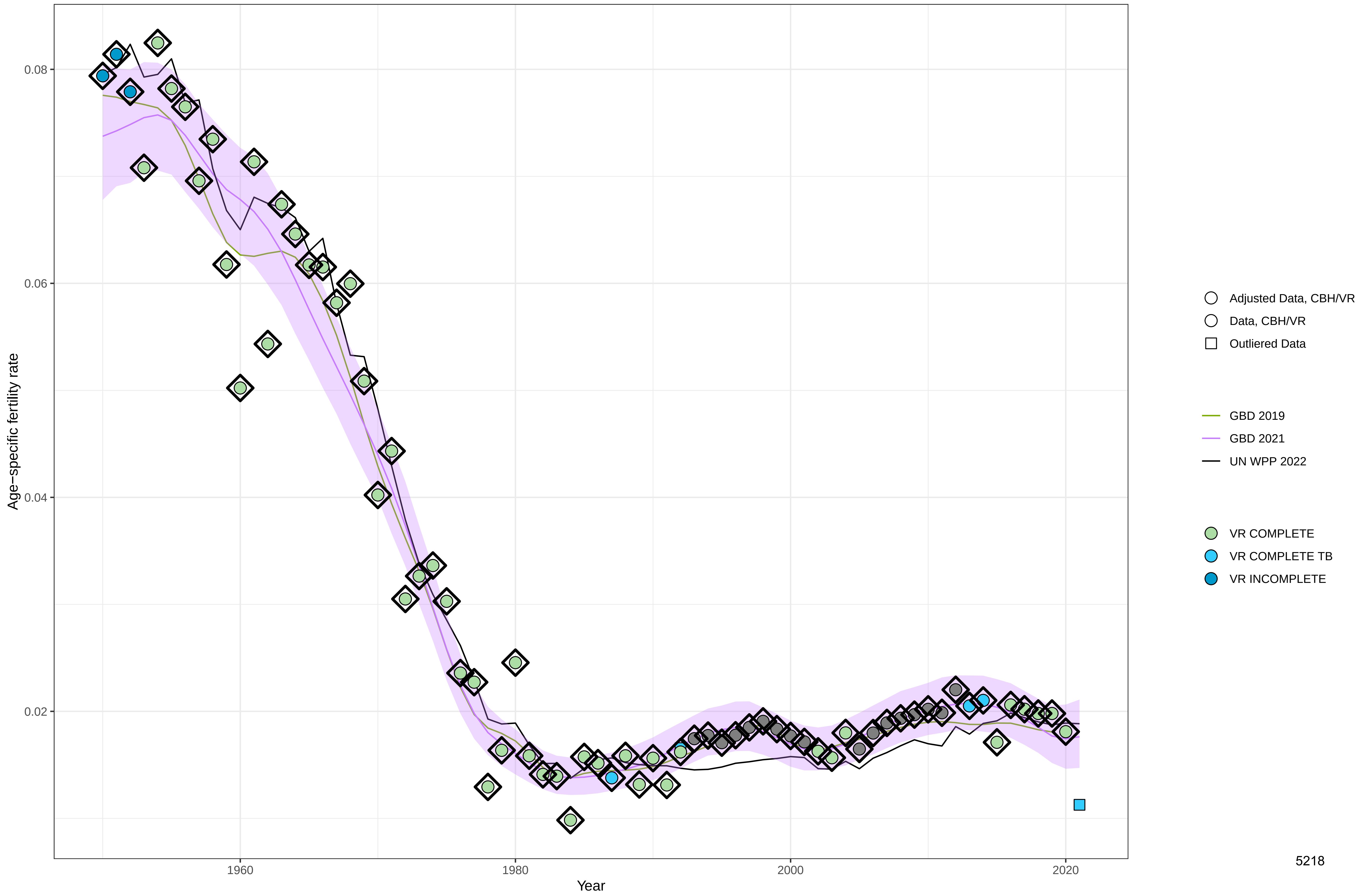

Age−specific fertility fitted trend lines, 1950−2021: Guam, Ages: 45−49
Data points enclosed with a diamond are reference sources

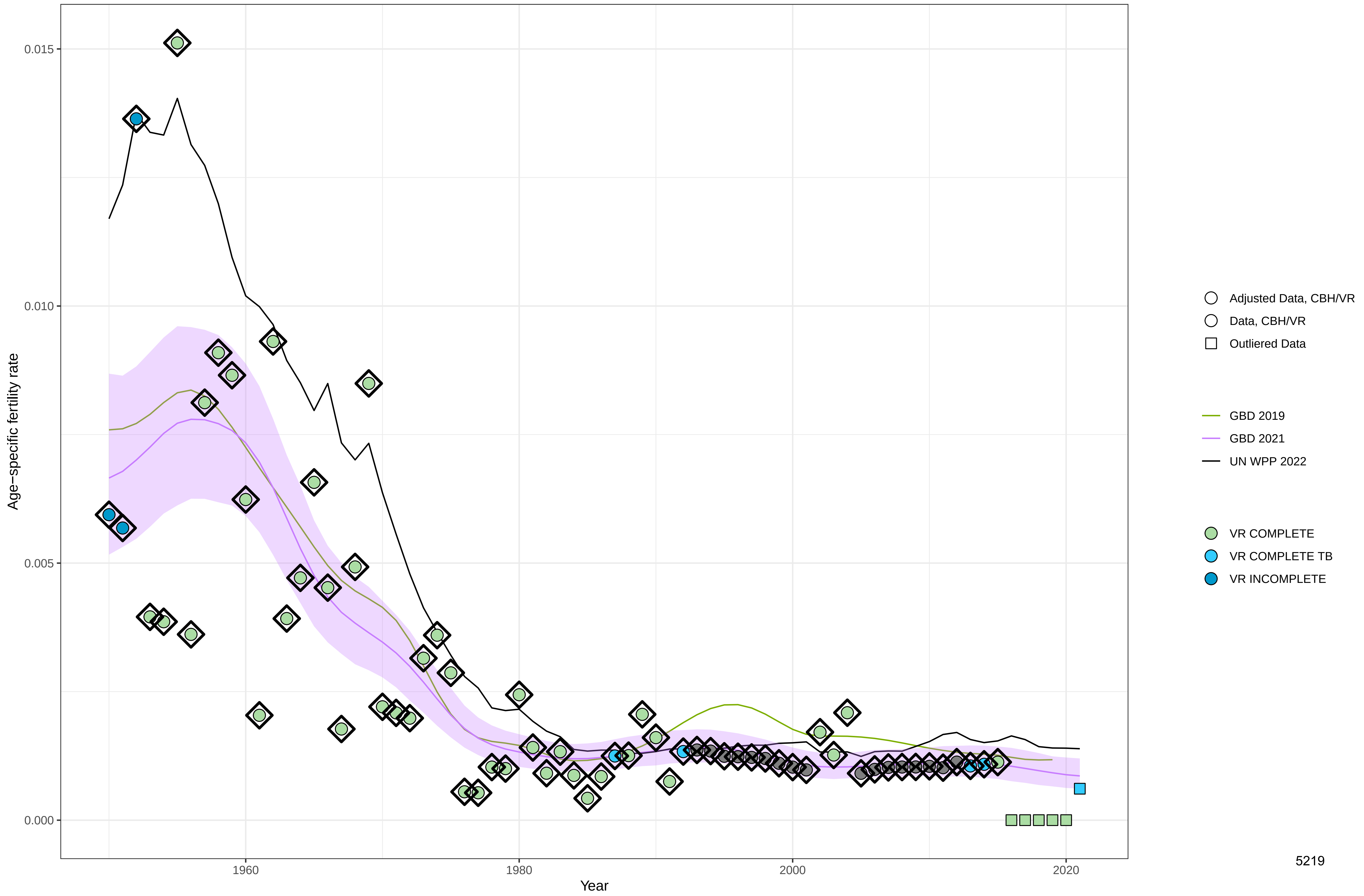

Age−specific fertility fitted trend lines, 1950−2021: Guam, Ages: 50−54
Data points enclosed with a diamond are reference sources

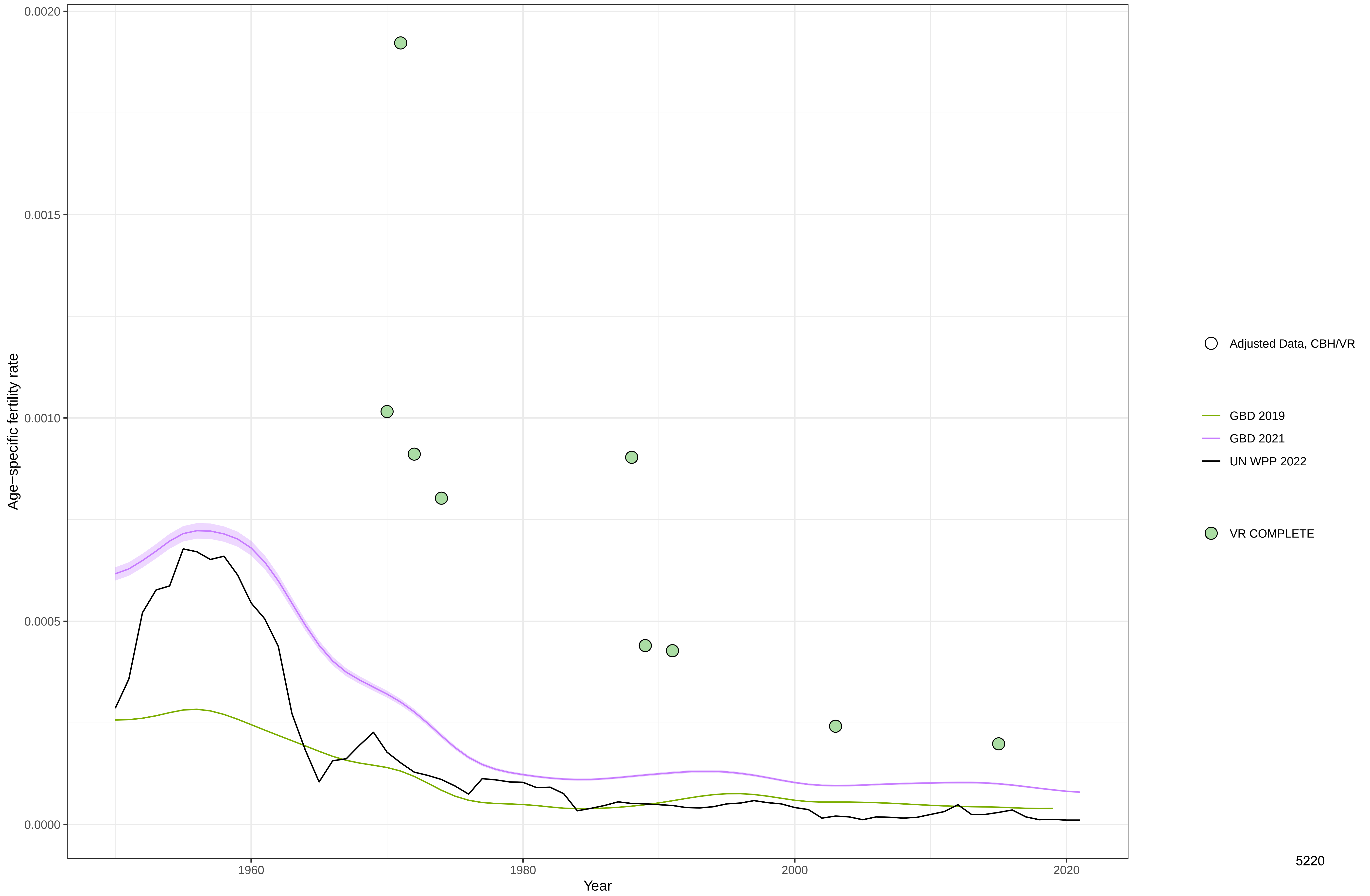

Total fertility rate fitted trend lines, 1950−2021: Guam TFR
Data points enclosed with a diamond are reference sources

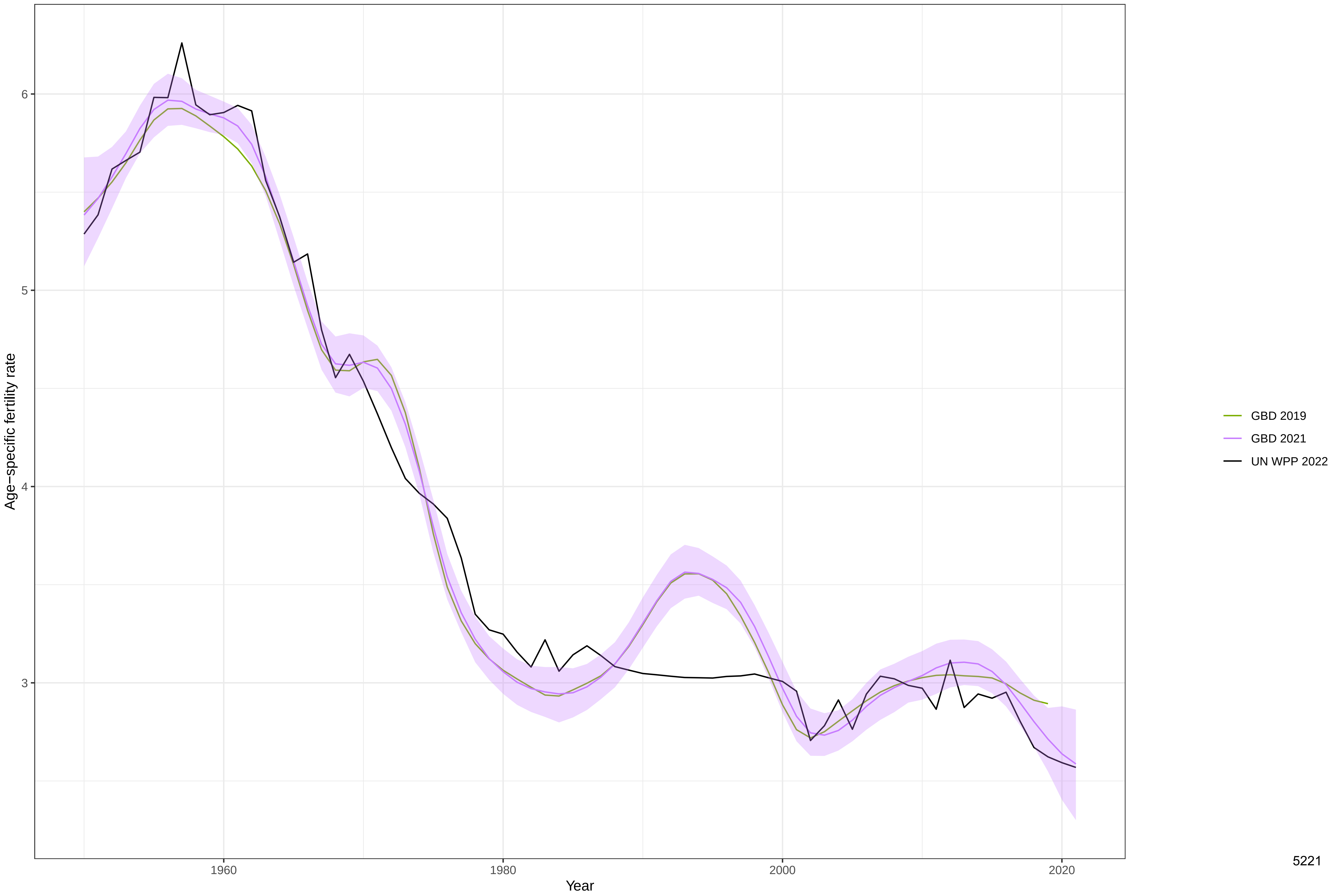

Age−specific fertility fitted trend lines, 1950−2021: Kiribati, Ages: 10−14
Data points enclosed with a diamond are reference sources

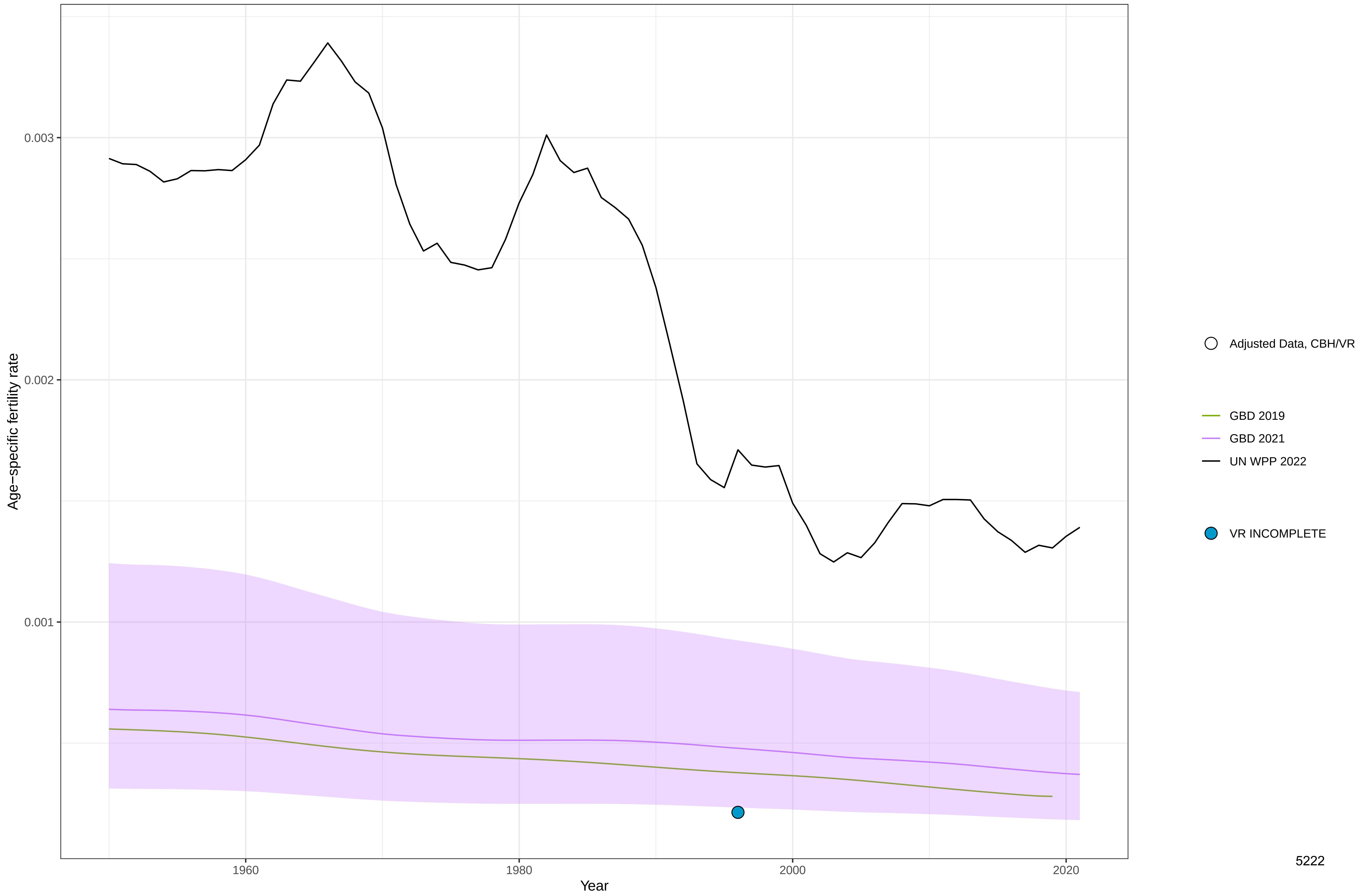

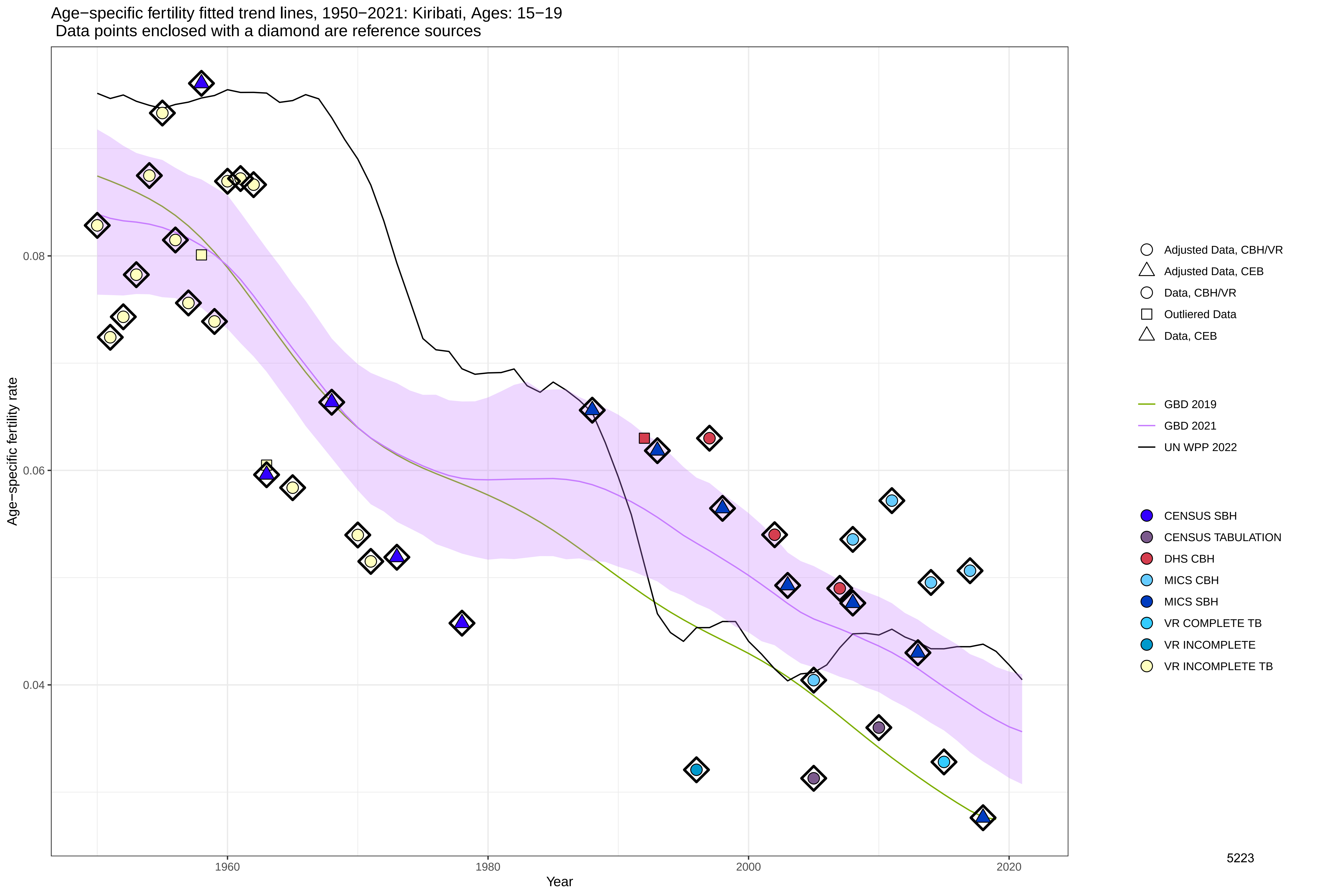
Age−specific fertility fitted trend lines, 1950−2021: Kiribati, Ages: 15−19
Data points enclosed with a diamond are reference sources
Age−specific fertility rate
0.08
0.06
0.04
1960
1980
2000
2020
Year
Adjusted Data, CBH/VR
Adjusted Data, CEB
Data, CBH/VR
Outliered Data
Data, CEB
GBD 2019
GBD 2021
UN WPP 2022
CENSUS SBH
CENSUS TABULATION
DHS CBH
MICS CBH
MICS SBH
VR COMPLETE TB
VR INCOMPLETE
VR INCOMPLETE TB
5223

Age−specific fertility fitted trend lines, 1950−2021: Kiribati, Ages: 20−24
Data points enclosed with a diamond are reference sources

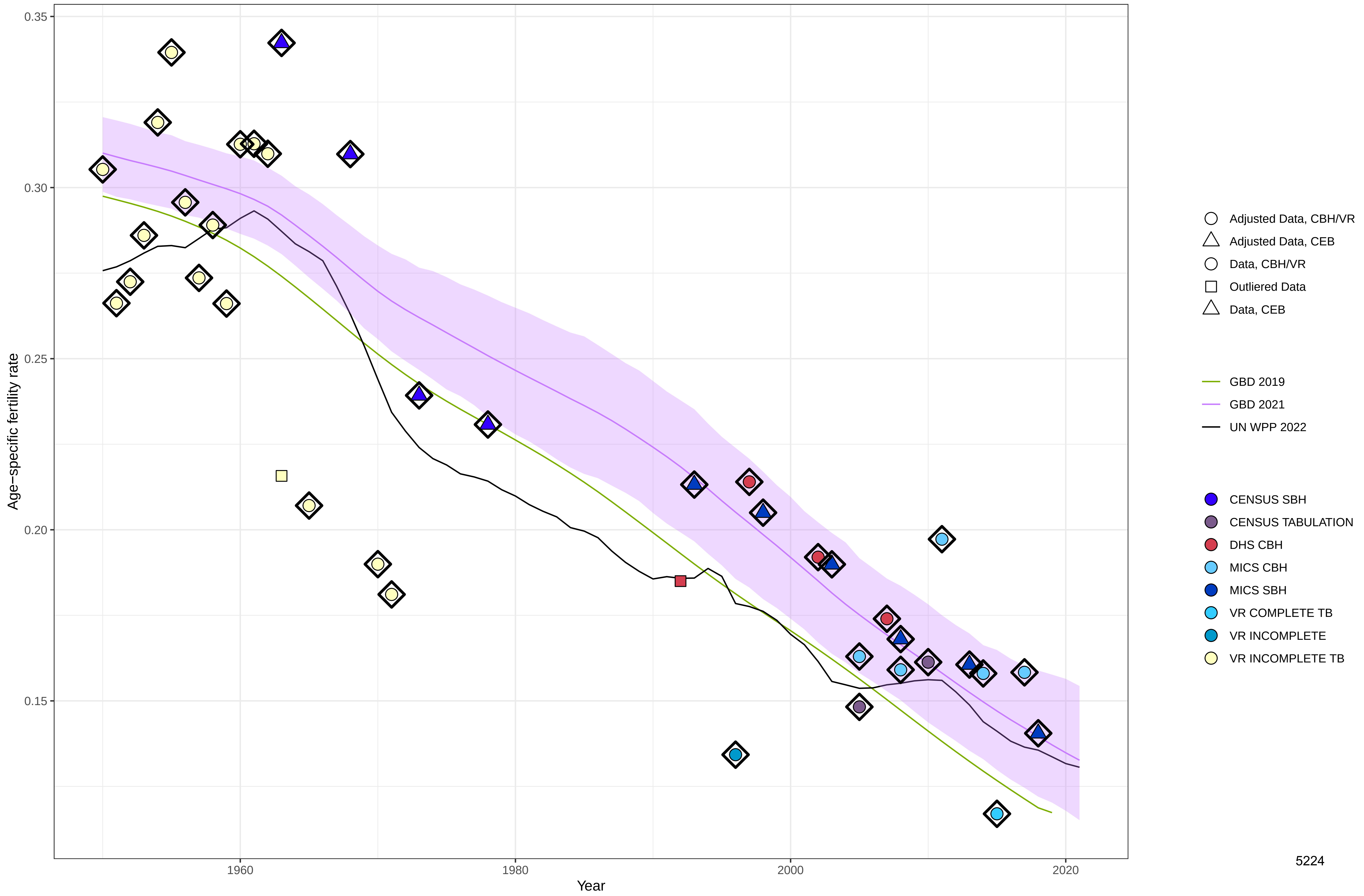

Age−specific fertility fitted trend lines, 1950−2021: Kiribati, Ages: 25−29
Data points enclosed with a diamond are reference sources

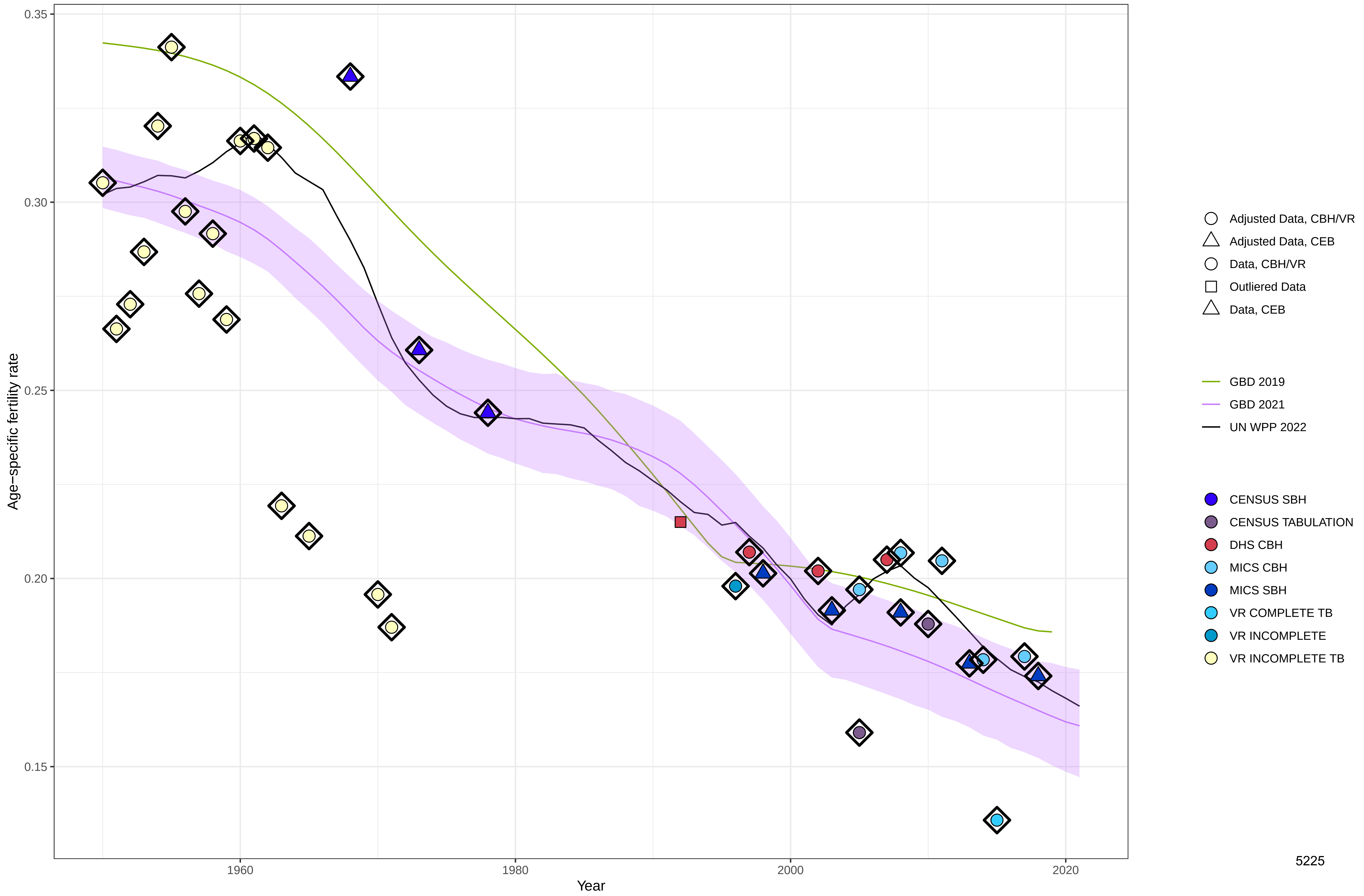

Age−specific fertility fitted trend lines, 1950−2021: Kiribati, Ages: 30−34
Data points enclosed with a diamond are reference sources

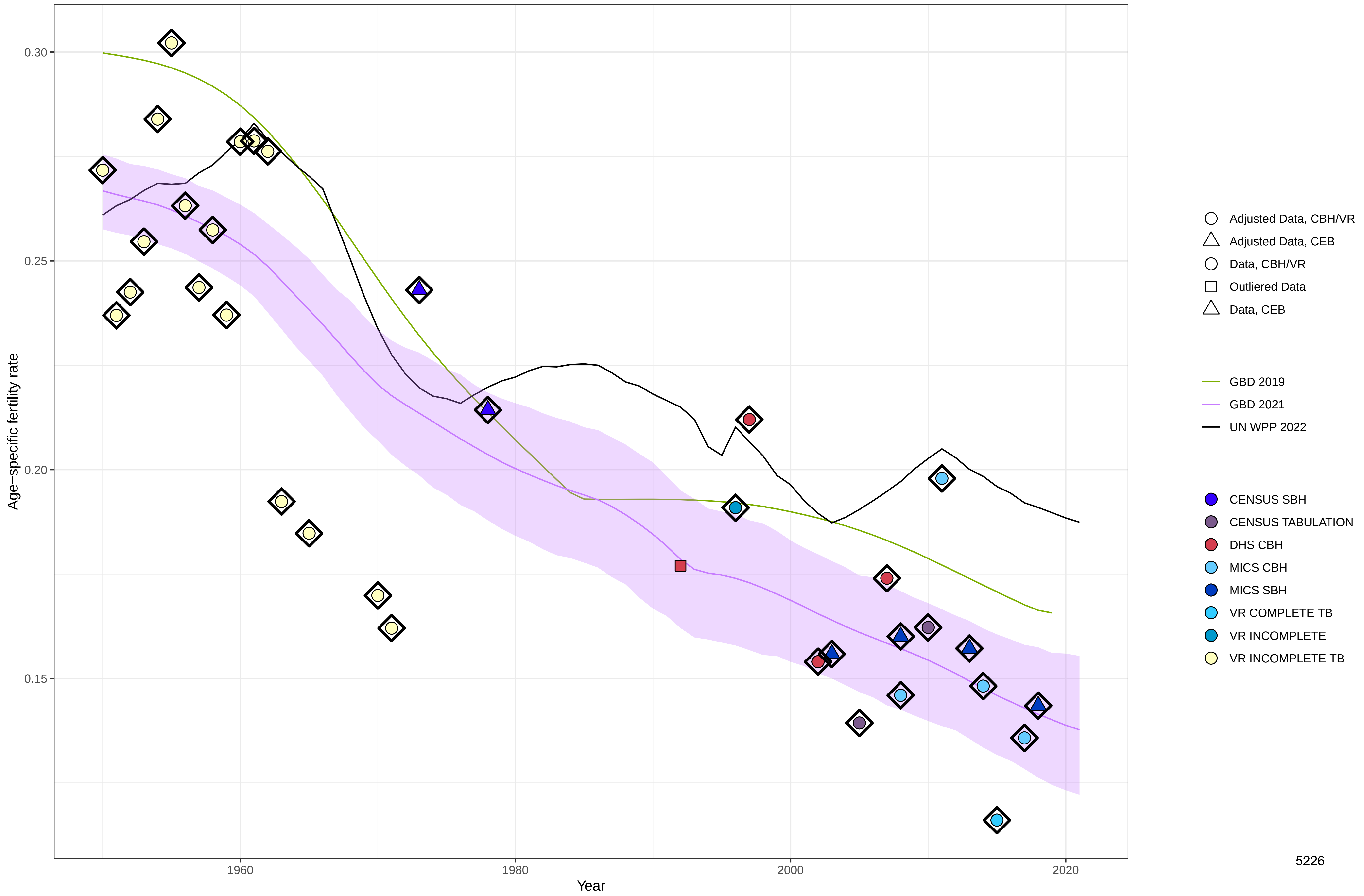

Age−specific fertility fitted trend lines, 1950−2021: Kiribati, Ages: 35−39
Data points enclosed with a diamond are reference sources

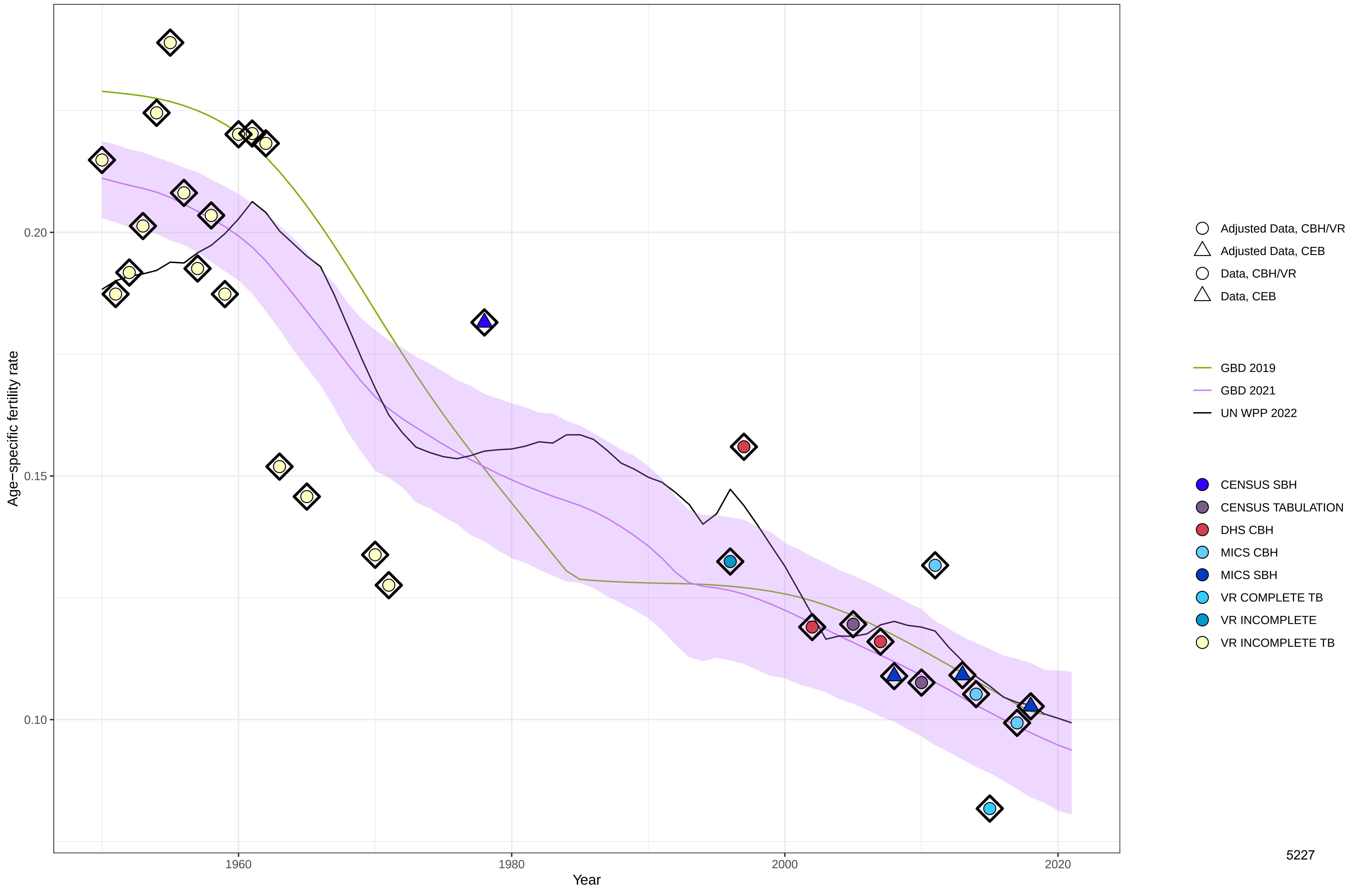

Age−specific fertility fitted trend lines, 1950−2021: Kiribati, Ages: 40−44
Data points enclosed with a diamond are reference sources

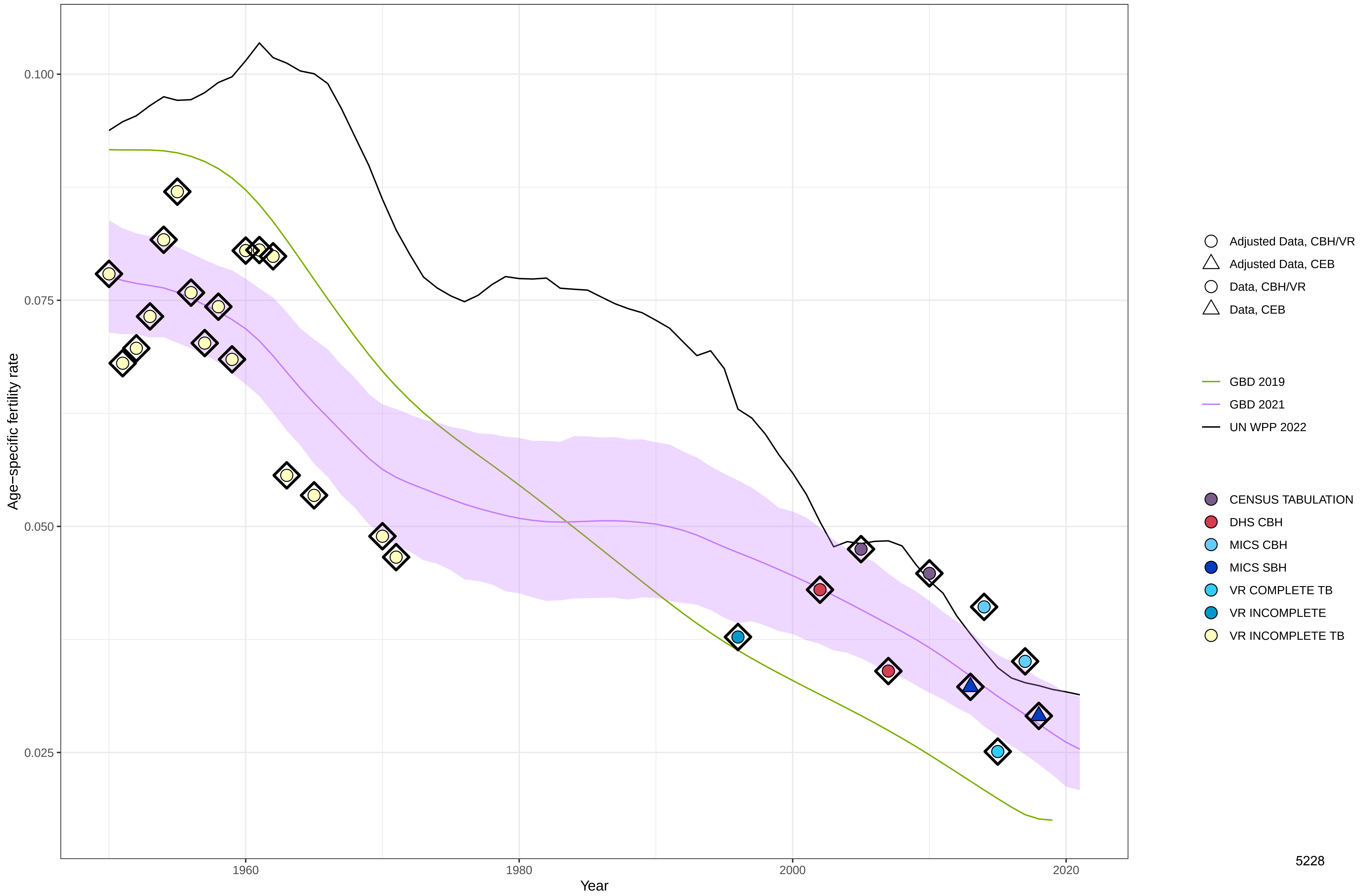

Age−specific fertility fitted trend lines, 1950−2021: Kiribati, Ages: 45−49
Data points enclosed with a diamond are reference sources

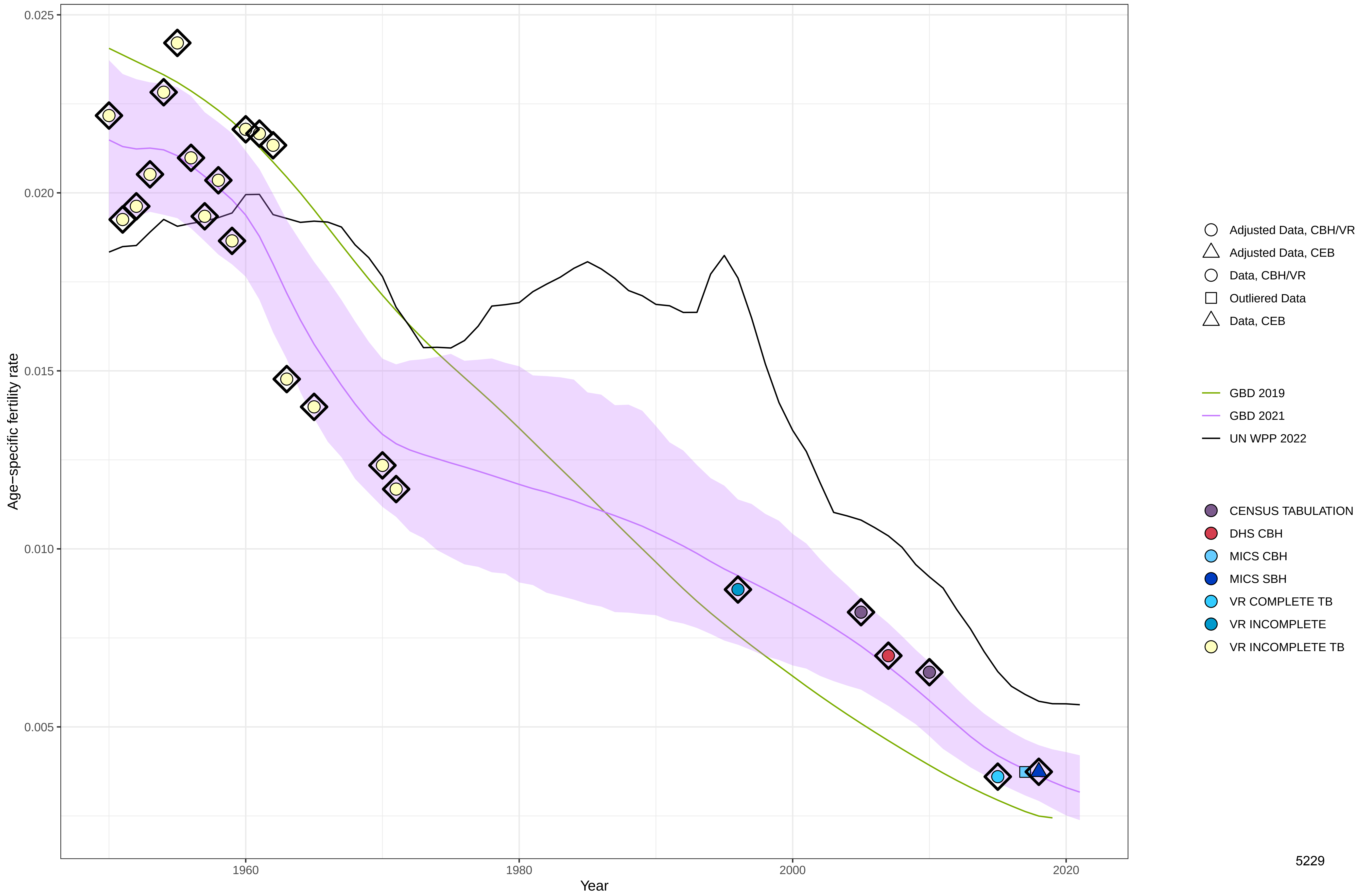

Age−specific fertility fitted trend lines, 1950−2021: Kiribati, Ages: 50−54
Data points enclosed with a diamond are reference sources

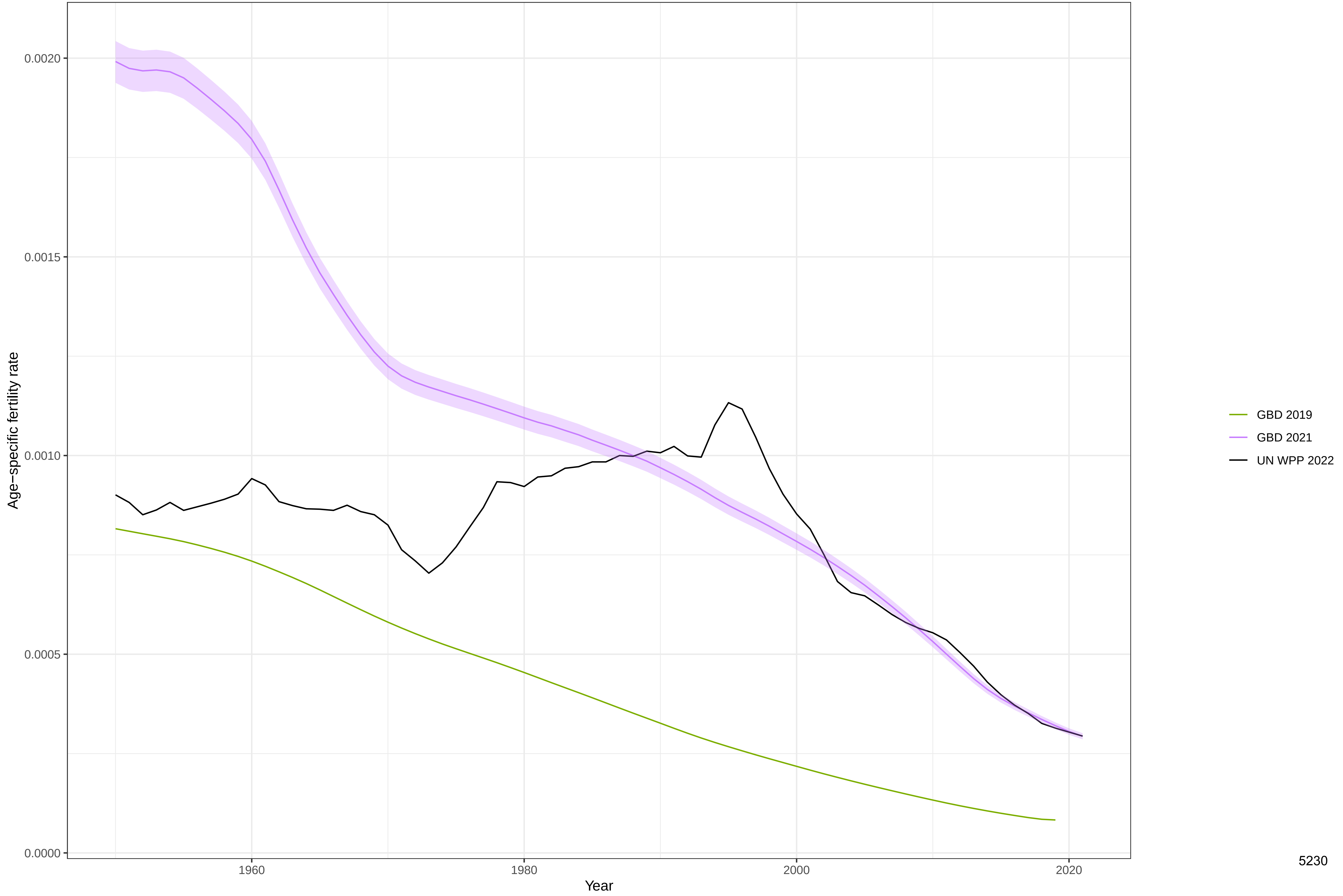

Total fertility rate fitted trend lines, 1950−2021: Kiribati TFR
Data points enclosed with a diamond are reference sources

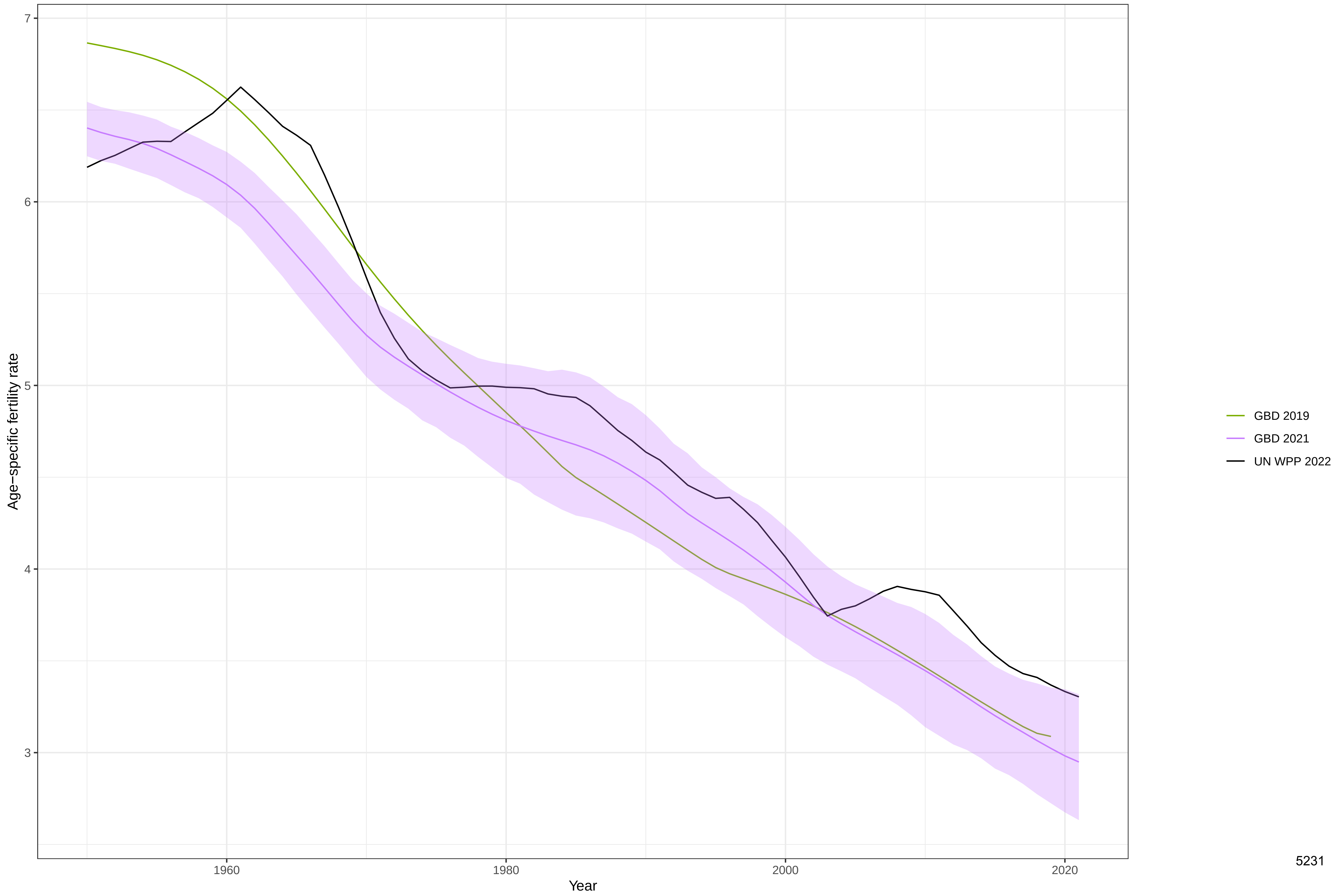

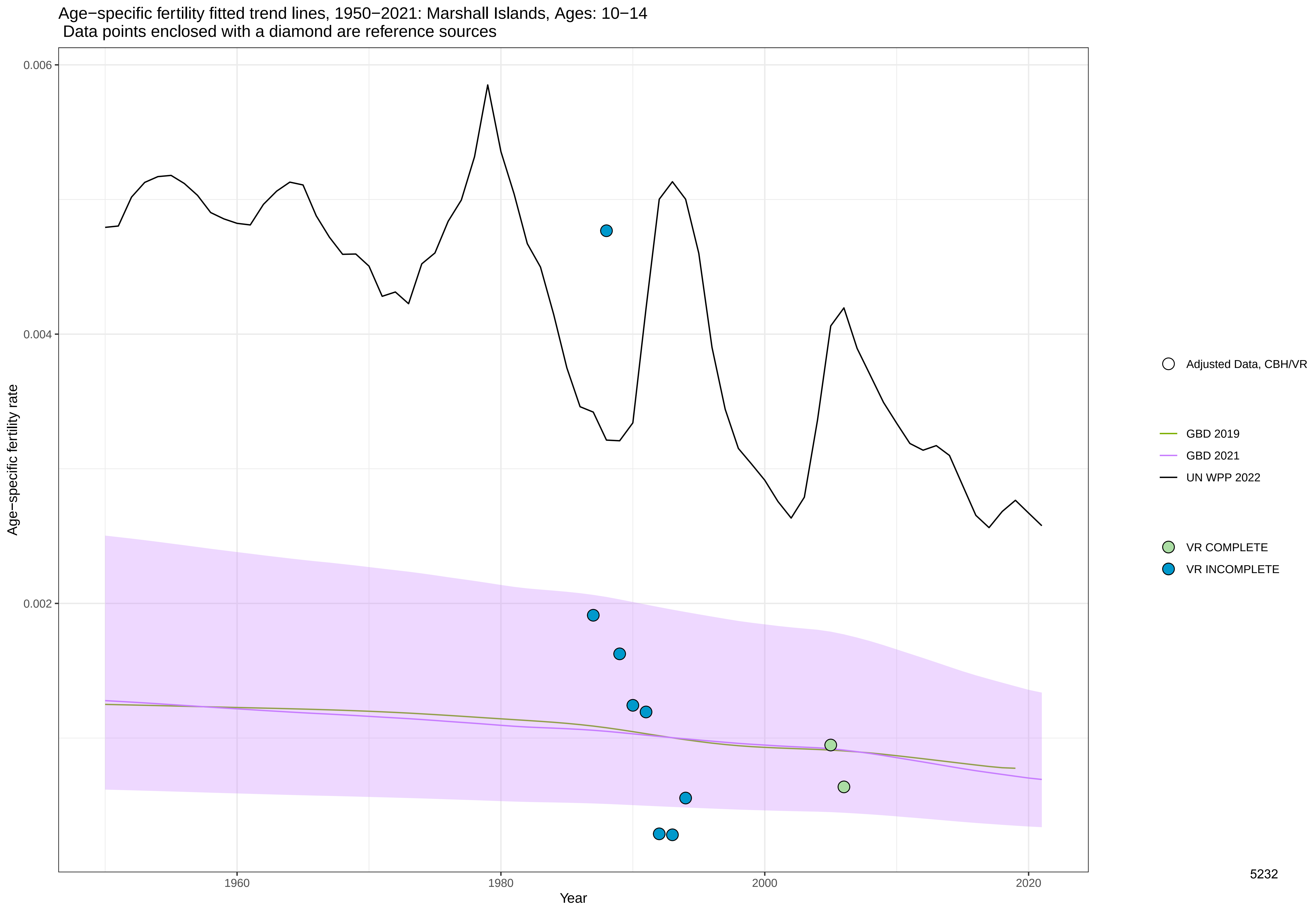
Age−specific fertility fitted trend lines, 1950−2021: Marshall Islands, Ages: 10−14
Data points enclosed with a diamond are reference sources
0.006
0.004
0.002
Age−specific fertility rate
1960
1980
2000
2020
Year
Adjusted Data, CBH/VR
GBD 2019
GBD 2021
UN WPP 2022
VR COMPLETE
VR INCOMPLETE

Age−specific fertility fitted trend lines, 1950−2021: Marshall Islands, Ages: 15−19
Data points enclosed with a diamond are reference sources

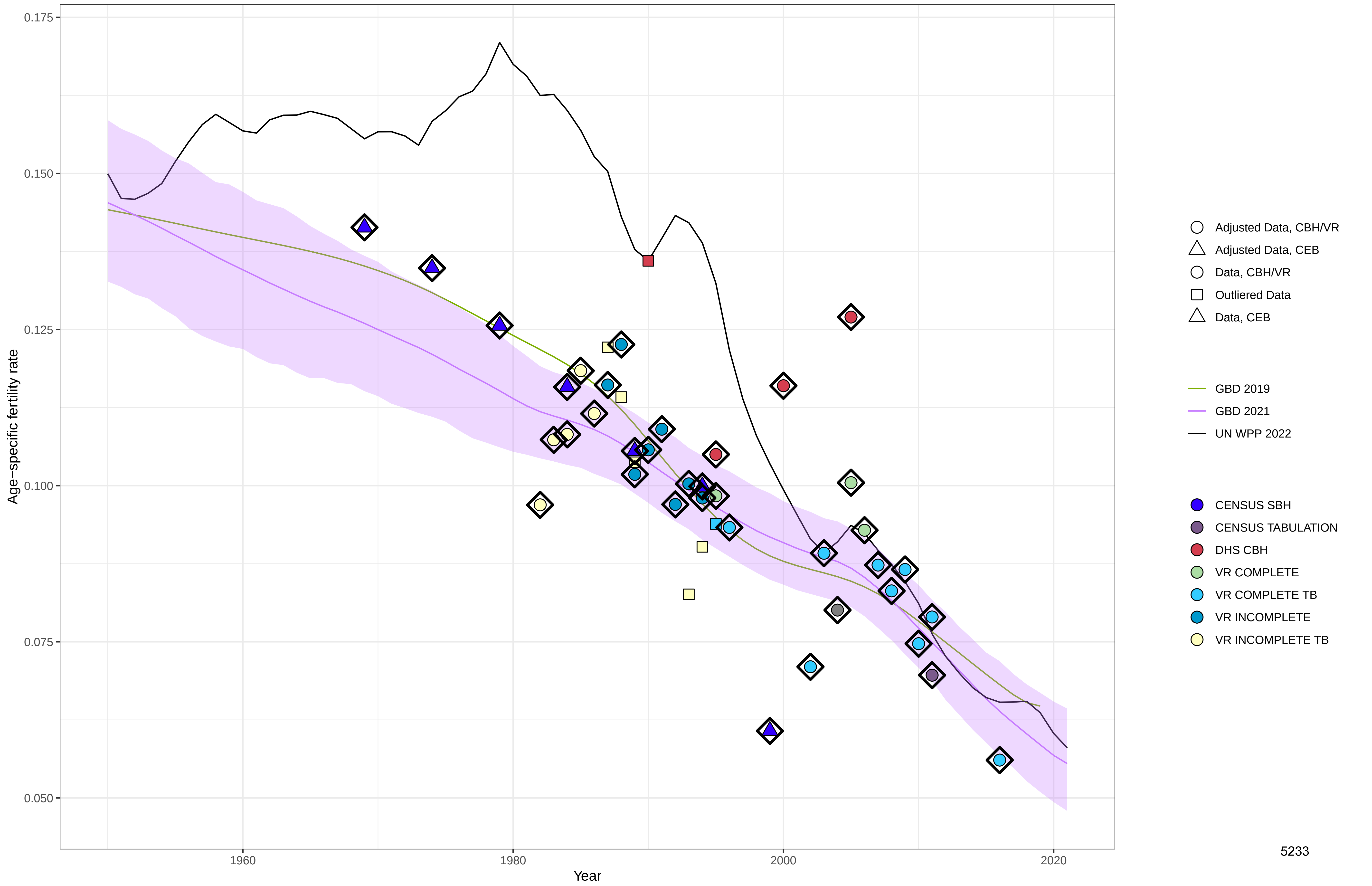

Age−specific fertility fitted trend lines, 1950−2021: Marshall Islands, Ages: 20−24
Data points enclosed with a diamond are reference sources

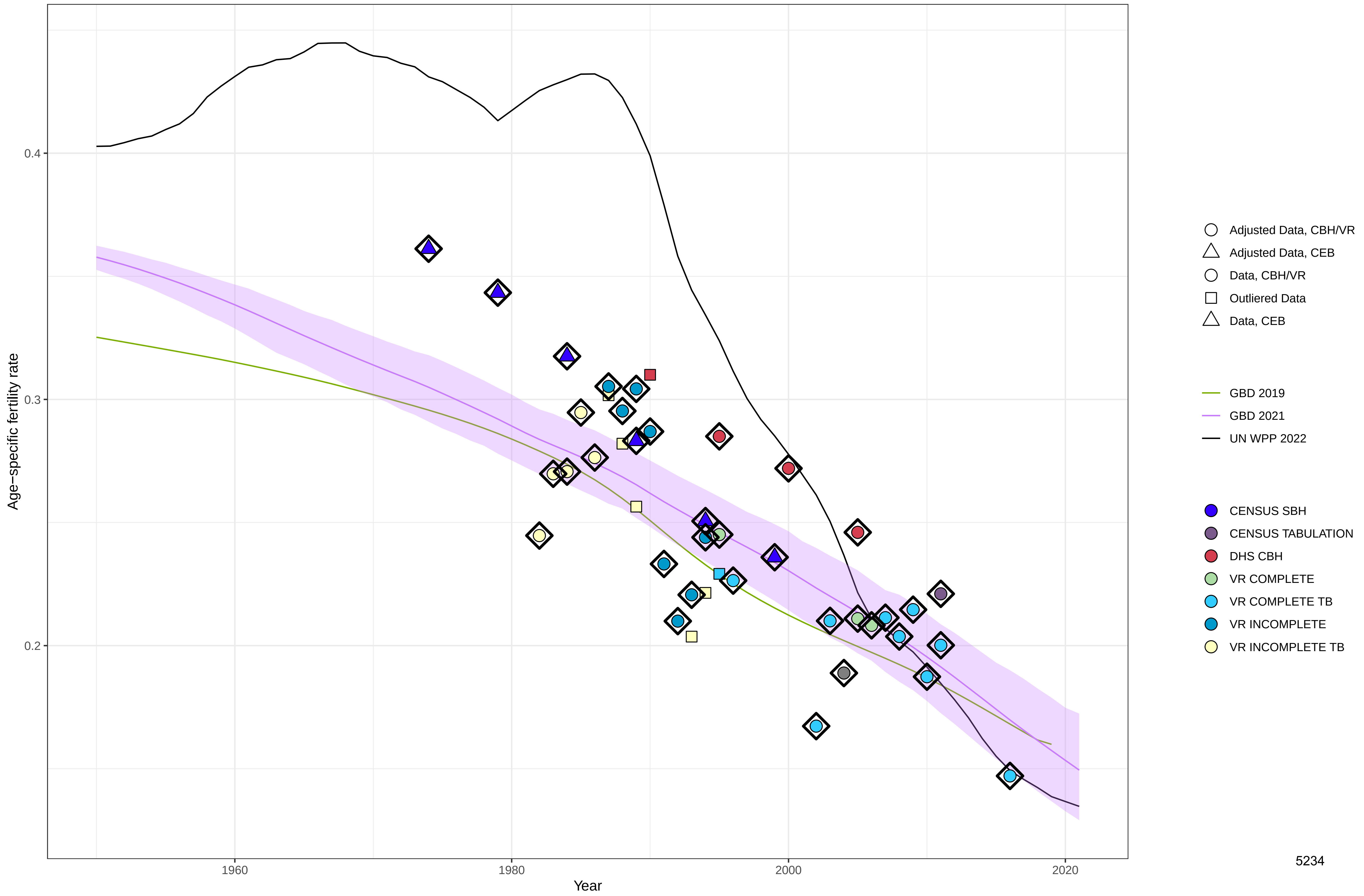

Age−specific fertility fitted trend lines, 1950−2021: Marshall Islands, Ages: 25−29
Data points enclosed with a diamond are reference sources

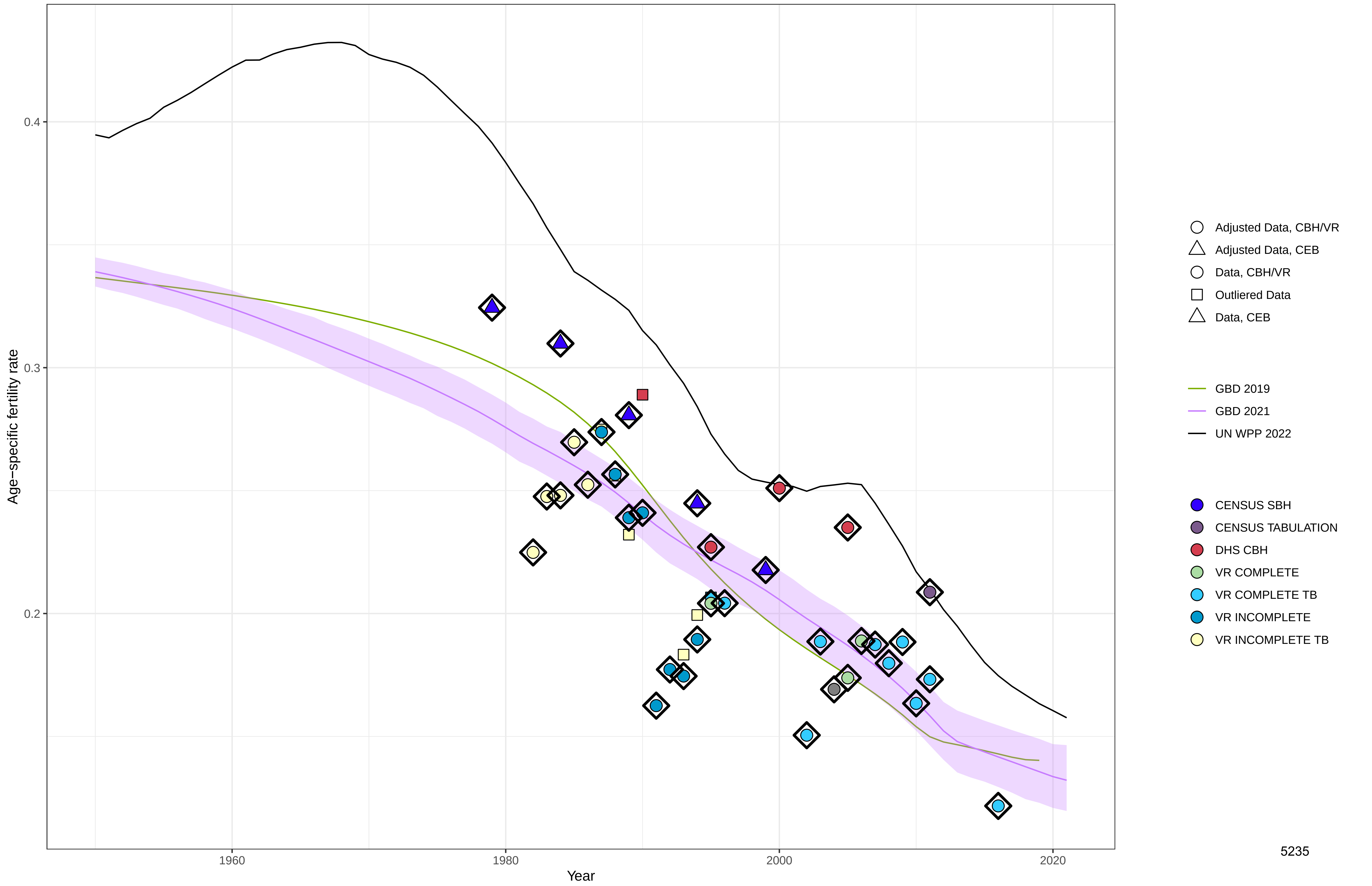

Age−specific fertility fitted trend lines, 1950−2021: Marshall Islands, Ages: 30−34
Data points enclosed with a diamond are reference sources

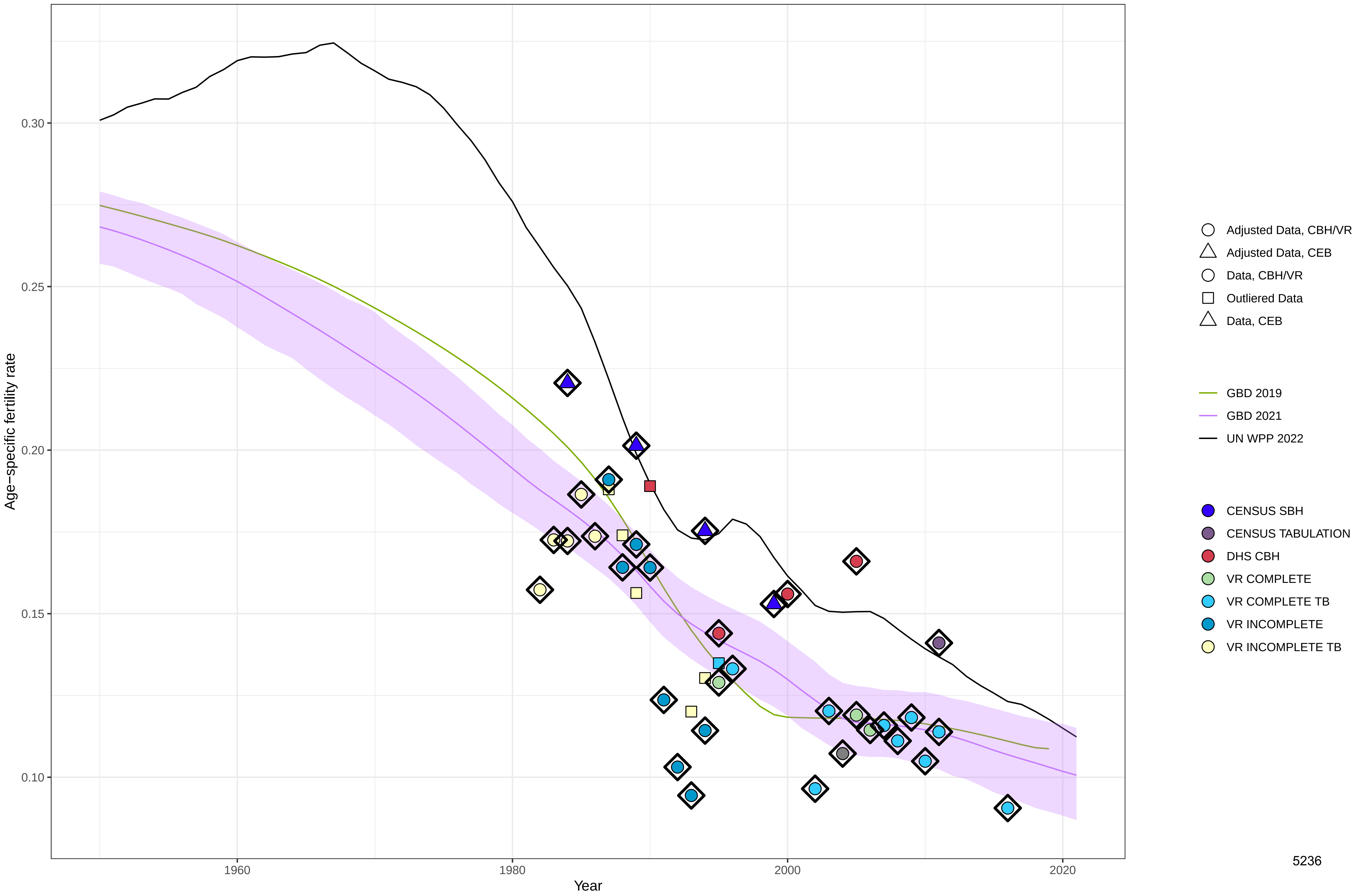

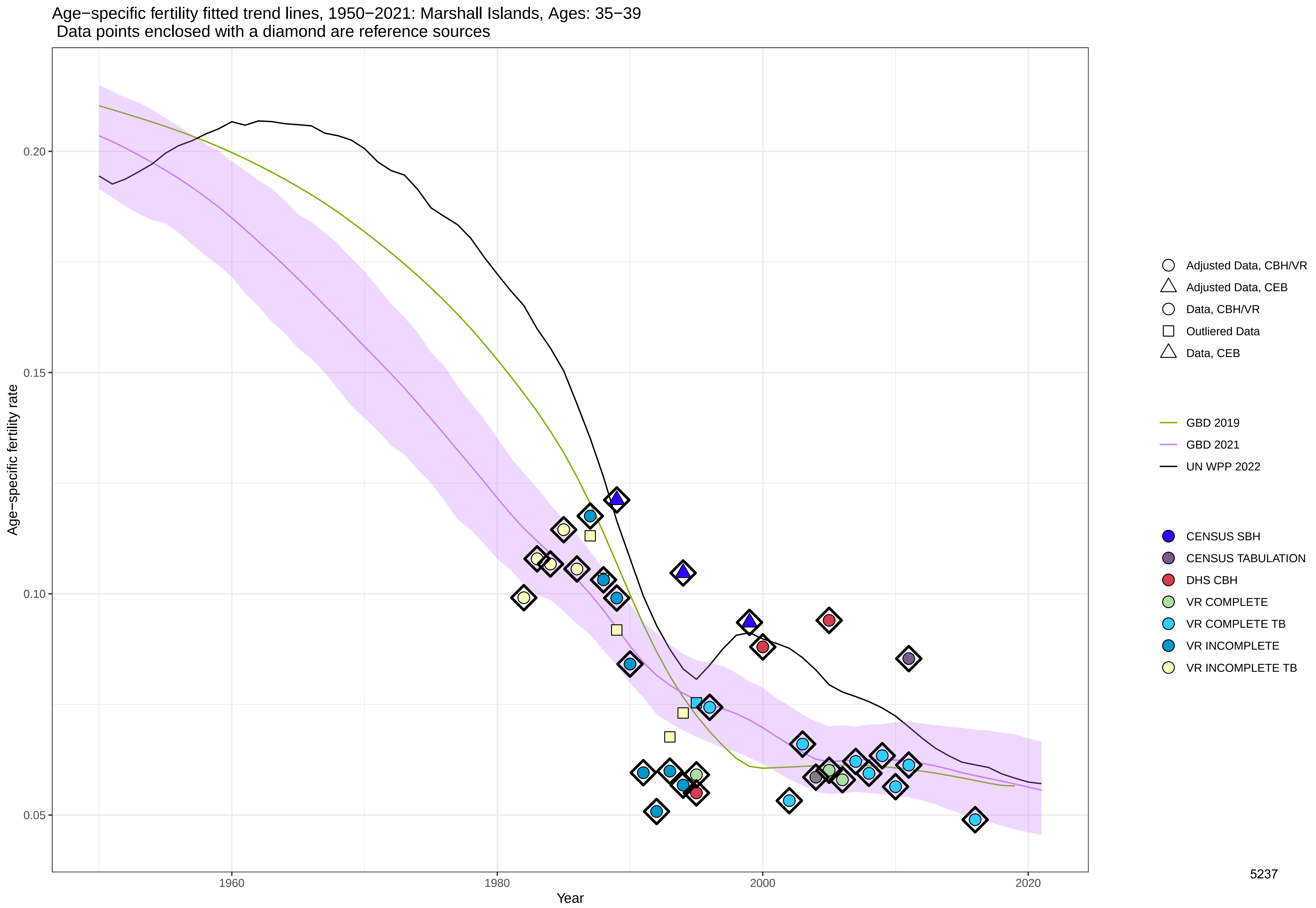

Age−specific fertility fitted trend lines, 1950−2021: Marshall Islands, Ages: 35−39
Data points enclosed with a diamond are reference sources
Age−specific fertility rate
0.05
0.10
0.15
0.20
1960
1980
2000
2020
Year
Adjusted Data, CBH/VR
Adjusted Data, CEB
Data, CBH/VR
Outliered Data
Data, CEB
GBD 2019
GBD 2021
UN WPP 2022
CENSUS SBH
CENSUS TABULATION
DHS CBH
VR COMPLETE
VR COMPLETE TB
VR INCOMPLETE
VR INCOMPLETE TB

5237

Age−specific fertility fitted trend lines, 1950−2021: Marshall Islands, Ages: 40−44
Data points enclosed with a diamond are reference sources

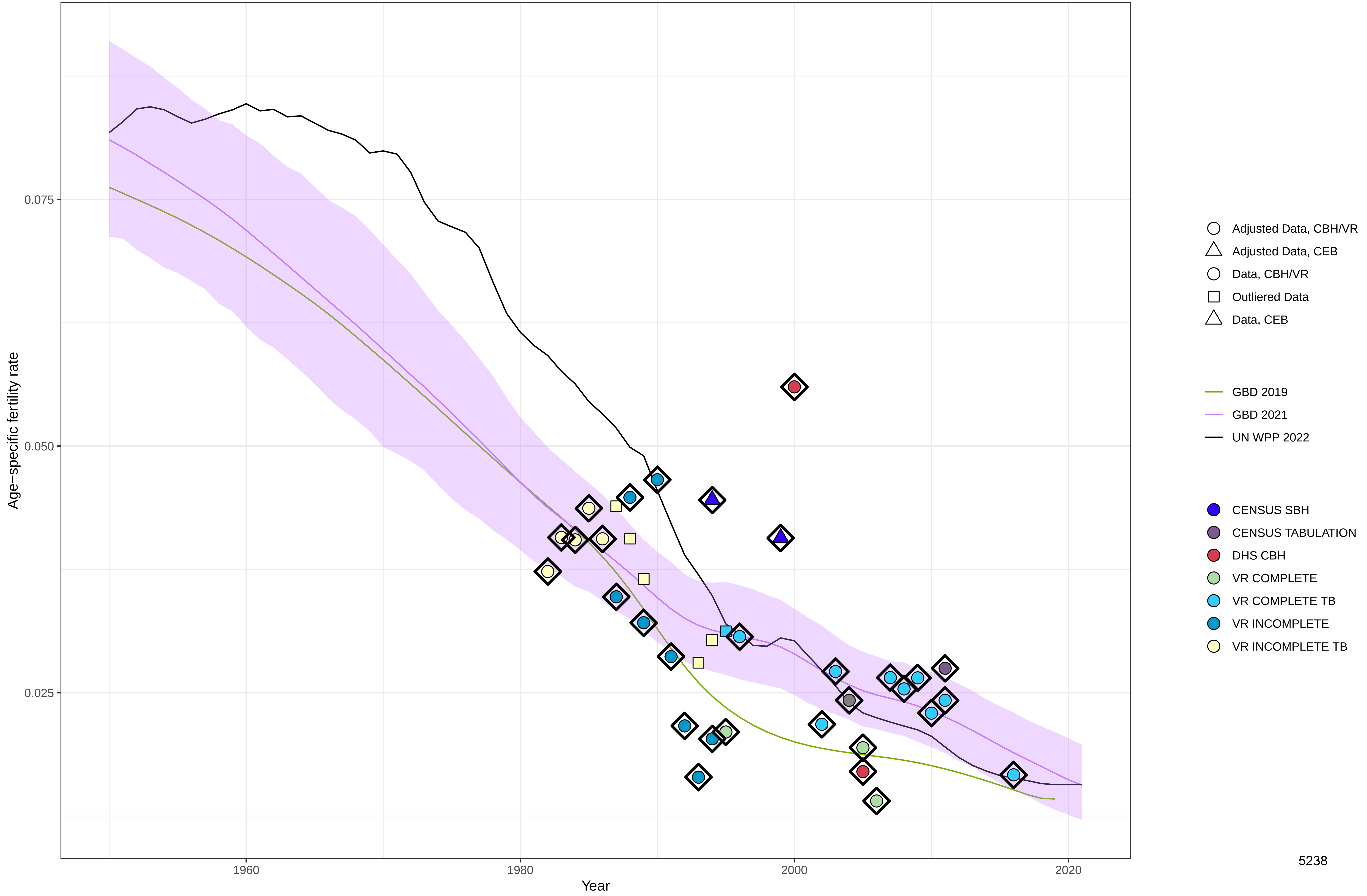

Age−specific fertility fitted trend lines, 1950−2021: Marshall Islands, Ages: 45−49
Data points enclosed with a diamond are reference sources

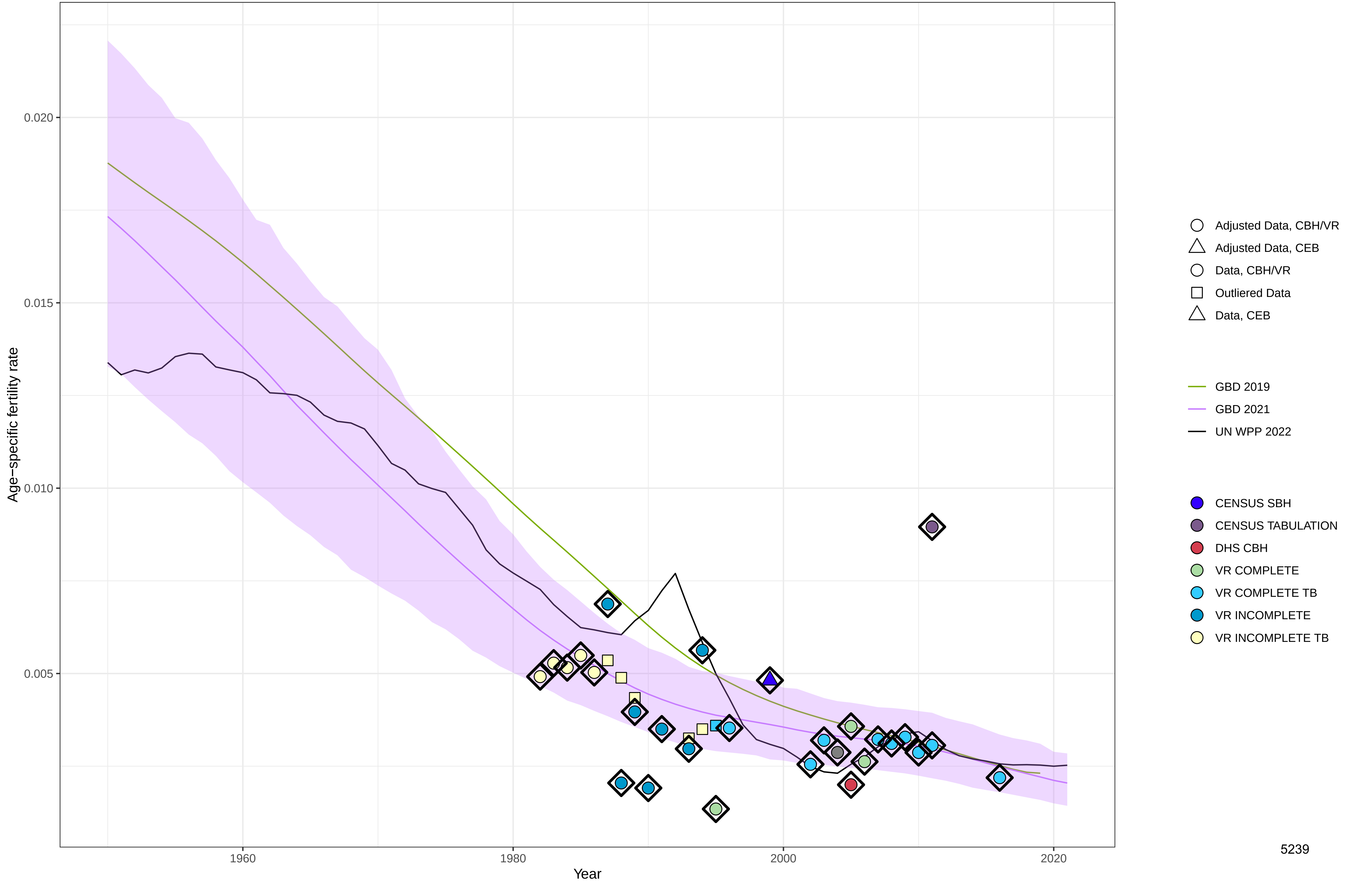

Age−specific fertility fitted trend lines, 1950−2021: Marshall Islands, Ages: 50−54
Data points enclosed with a diamond are reference sources

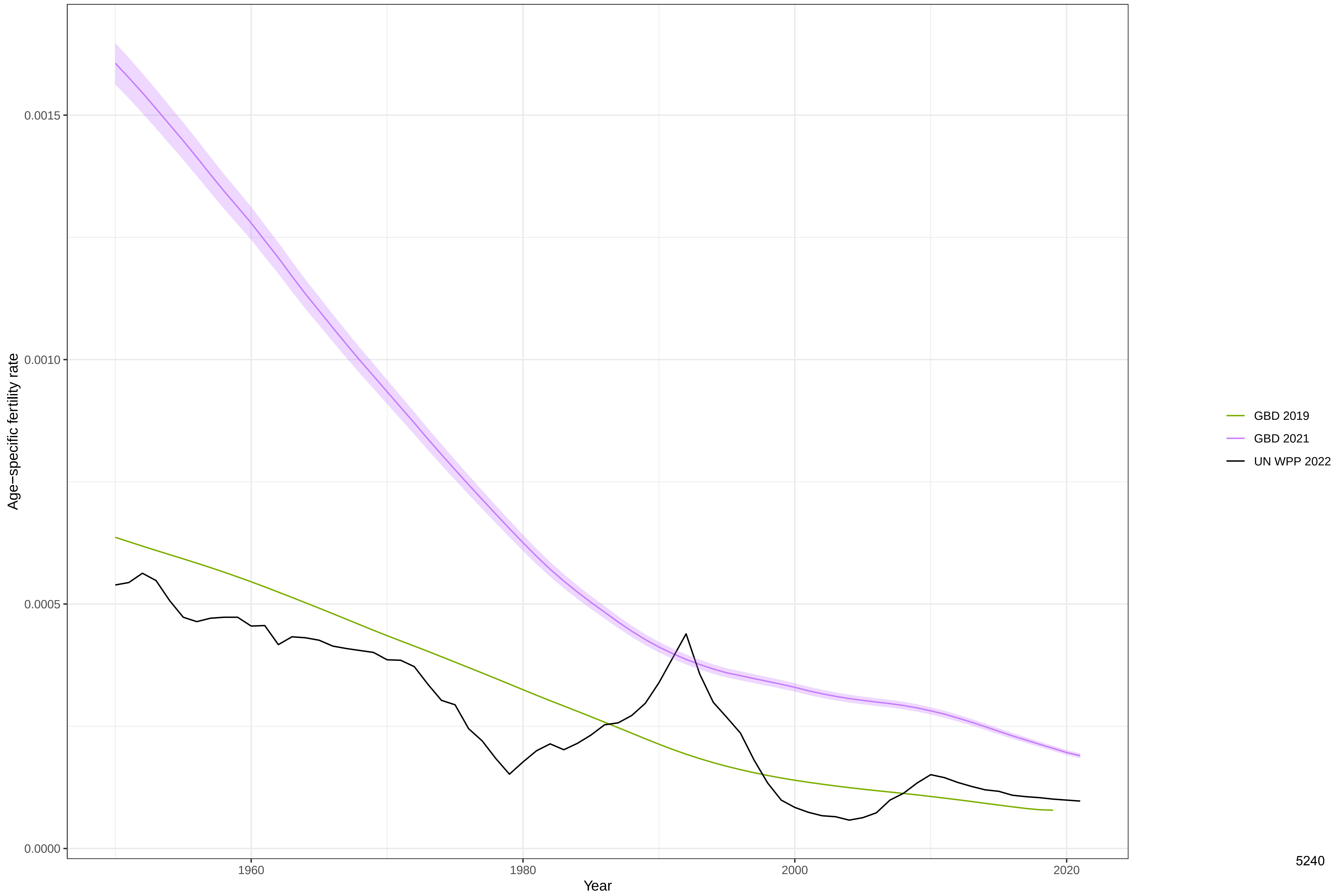

Total fertility rate fitted trend lines, 1950−2021: Marshall Islands TFR
Data points enclosed with a diamond are reference sources

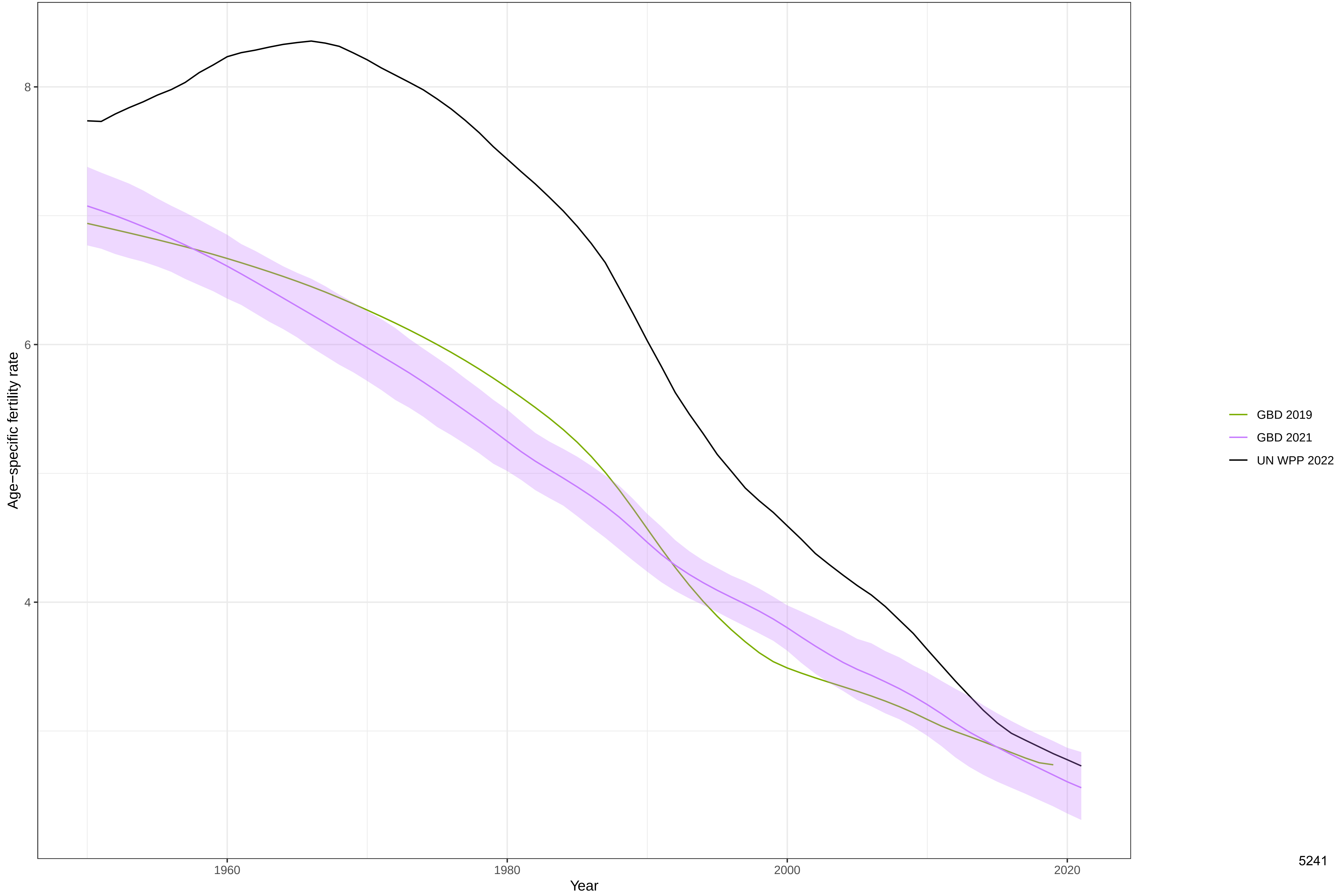

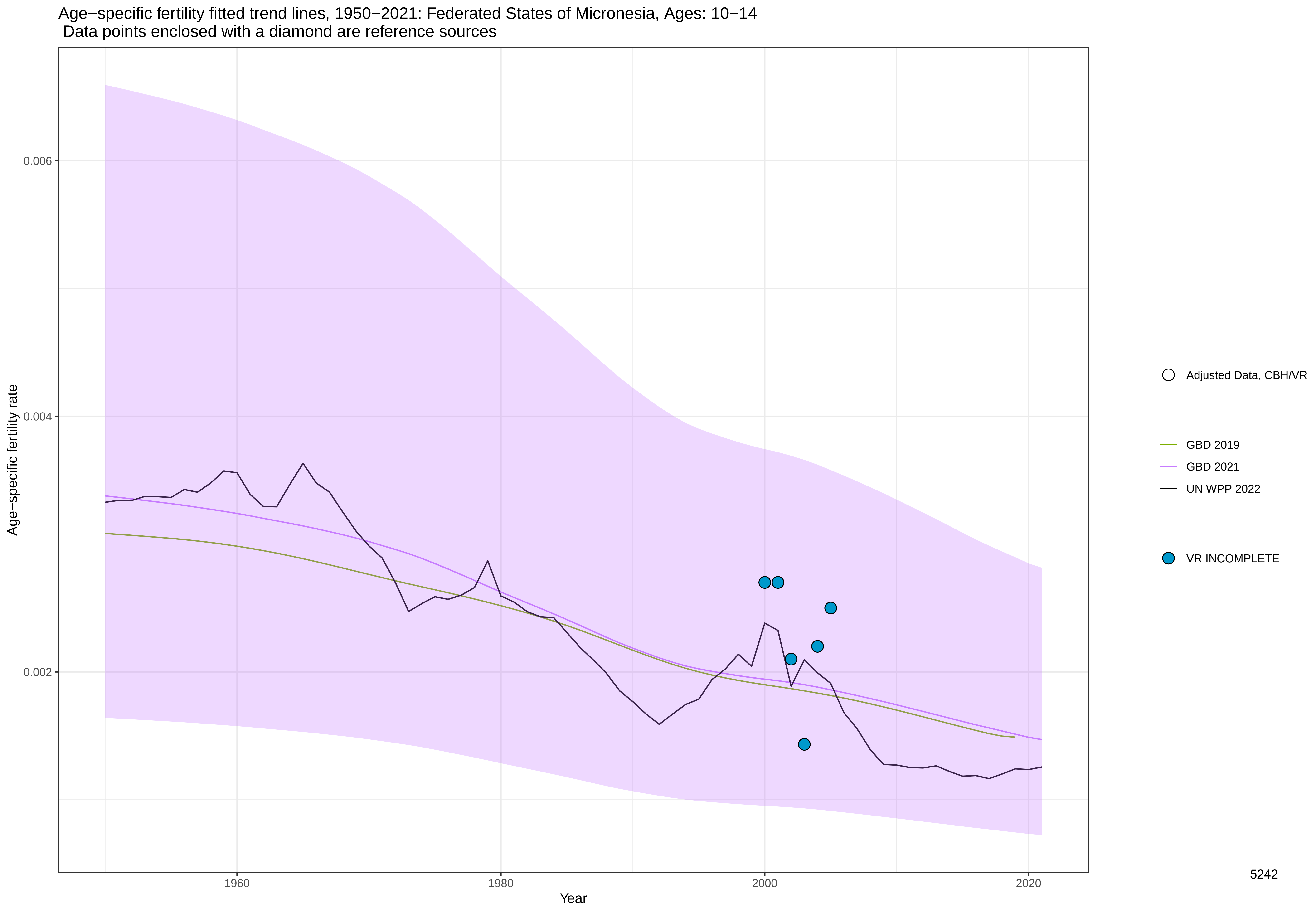
Age−specific fertility fitted trend lines, 1950−2021: Federated States of Micronesia, Ages: 10−14
Data points enclosed with a diamond are reference sources
Age−specific fertility rate
0.006
0.004
0.002
1960
1980
2000
2020
Year
Adjusted Data, CBH/VR
GBD 2019
GBD 2021
UN WPP 2022
VR INCOMPLETE

Age−specific fertility fitted trend lines, 1950−2021: Federated States of Micronesia, Ages: 15−19
Data points enclosed with a diamond are reference sources

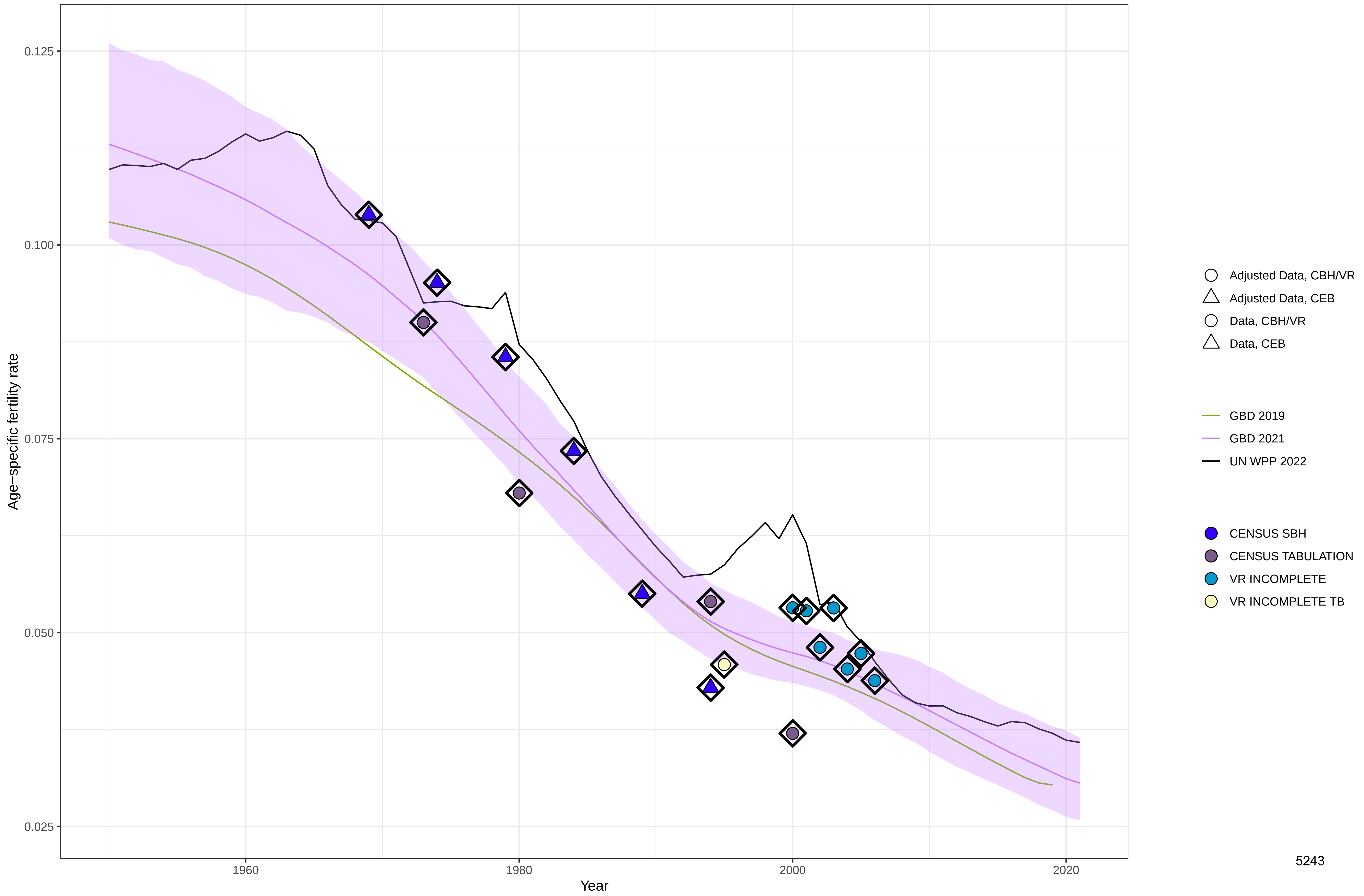

Age−specific fertility fitted trend lines, 1950−2021: Federated States of Micronesia, Ages: 20−24
Data points enclosed with a diamond are reference sources

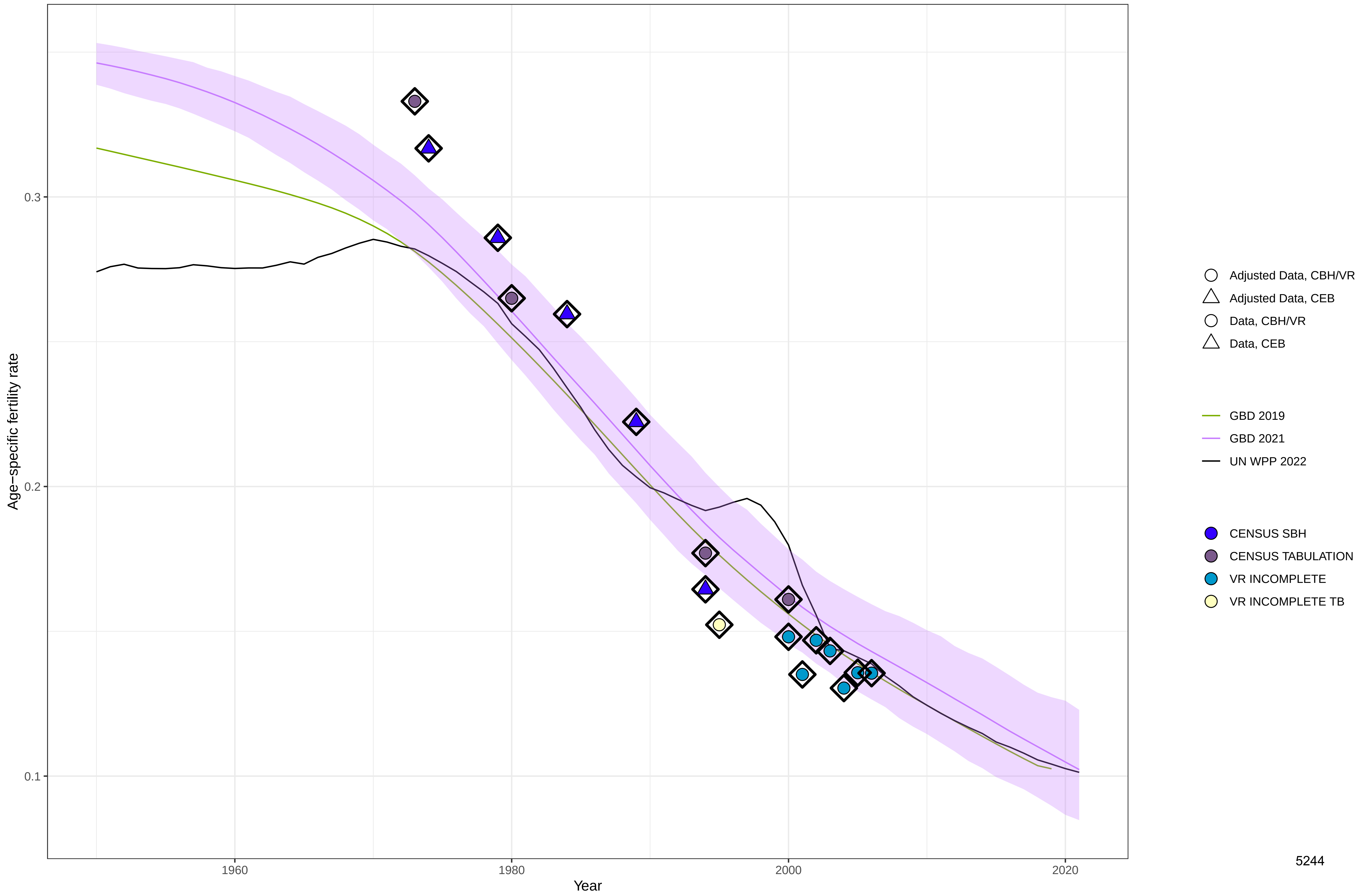

Age−specific fertility fitted trend lines, 1950−2021: Federated States of Micronesia, Ages: 25−29
Data points enclosed with a diamond are reference sources

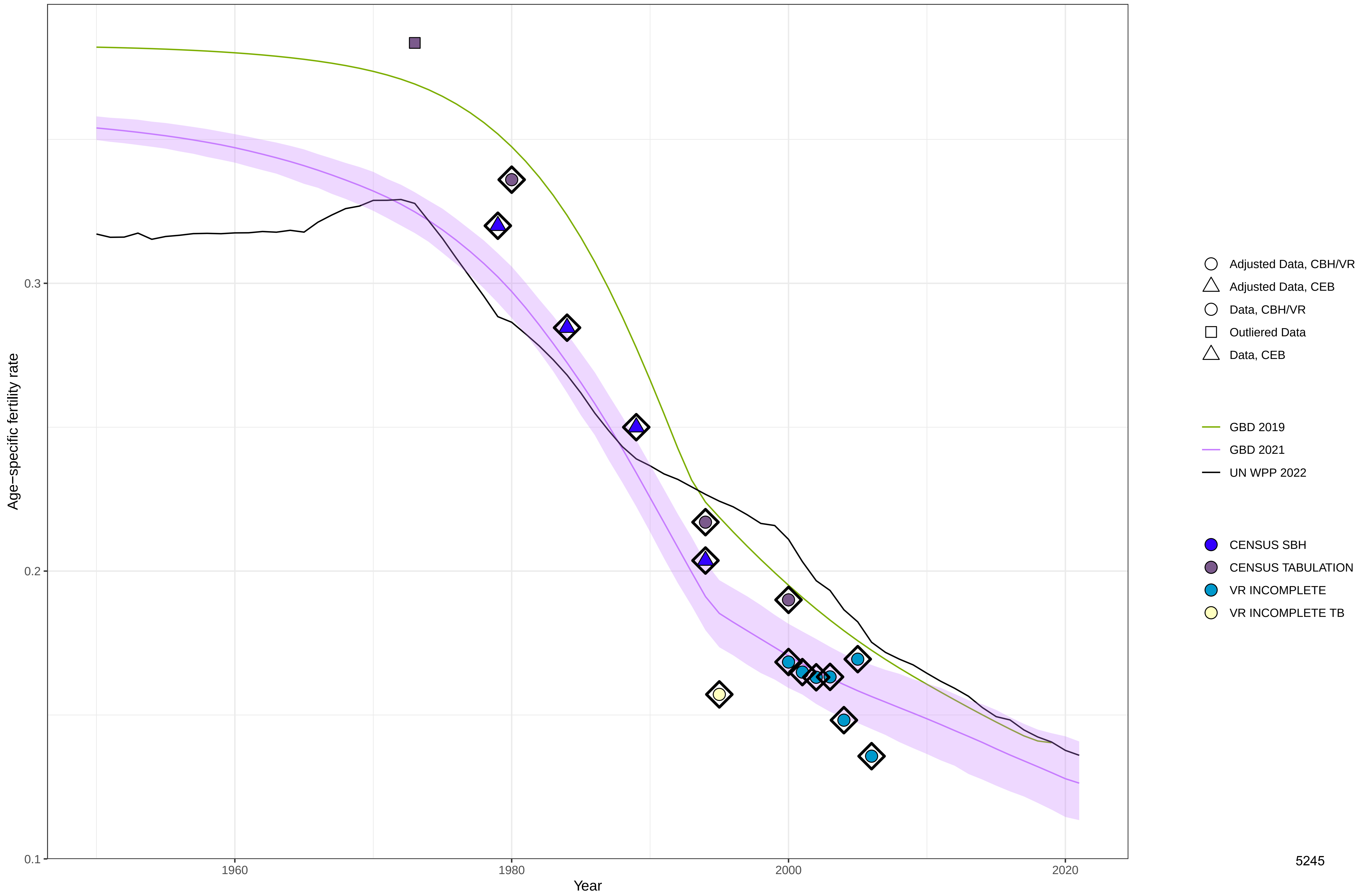

Age−specific fertility fitted trend lines, 1950−2021: Federated States of Micronesia, Ages: 30−34
Data points enclosed with a diamond are reference sources

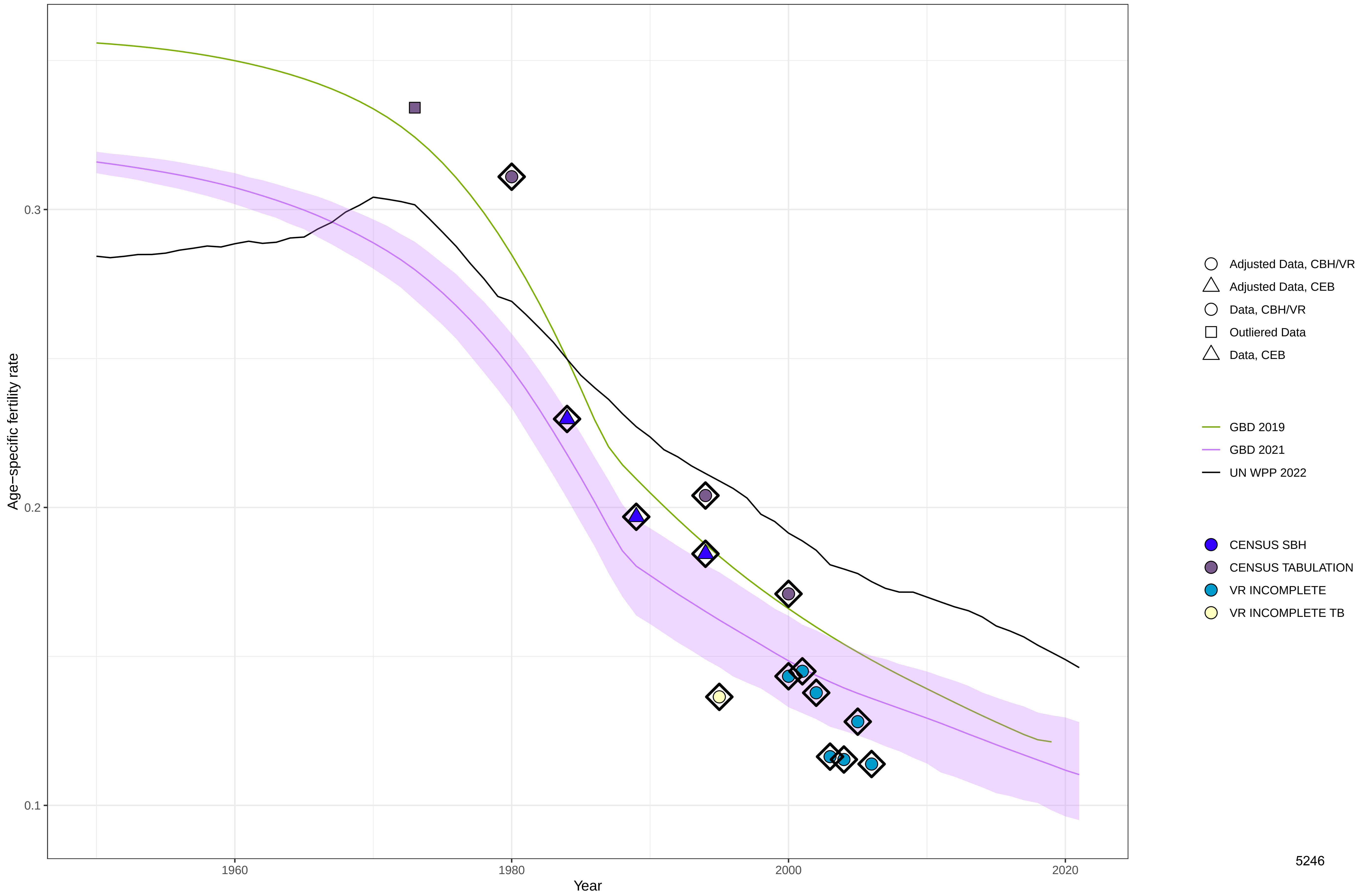

Age−specific fertility fitted trend lines, 1950−2021: Federated States of Micronesia, Ages: 35−39
Data points enclosed with a diamond are reference sources

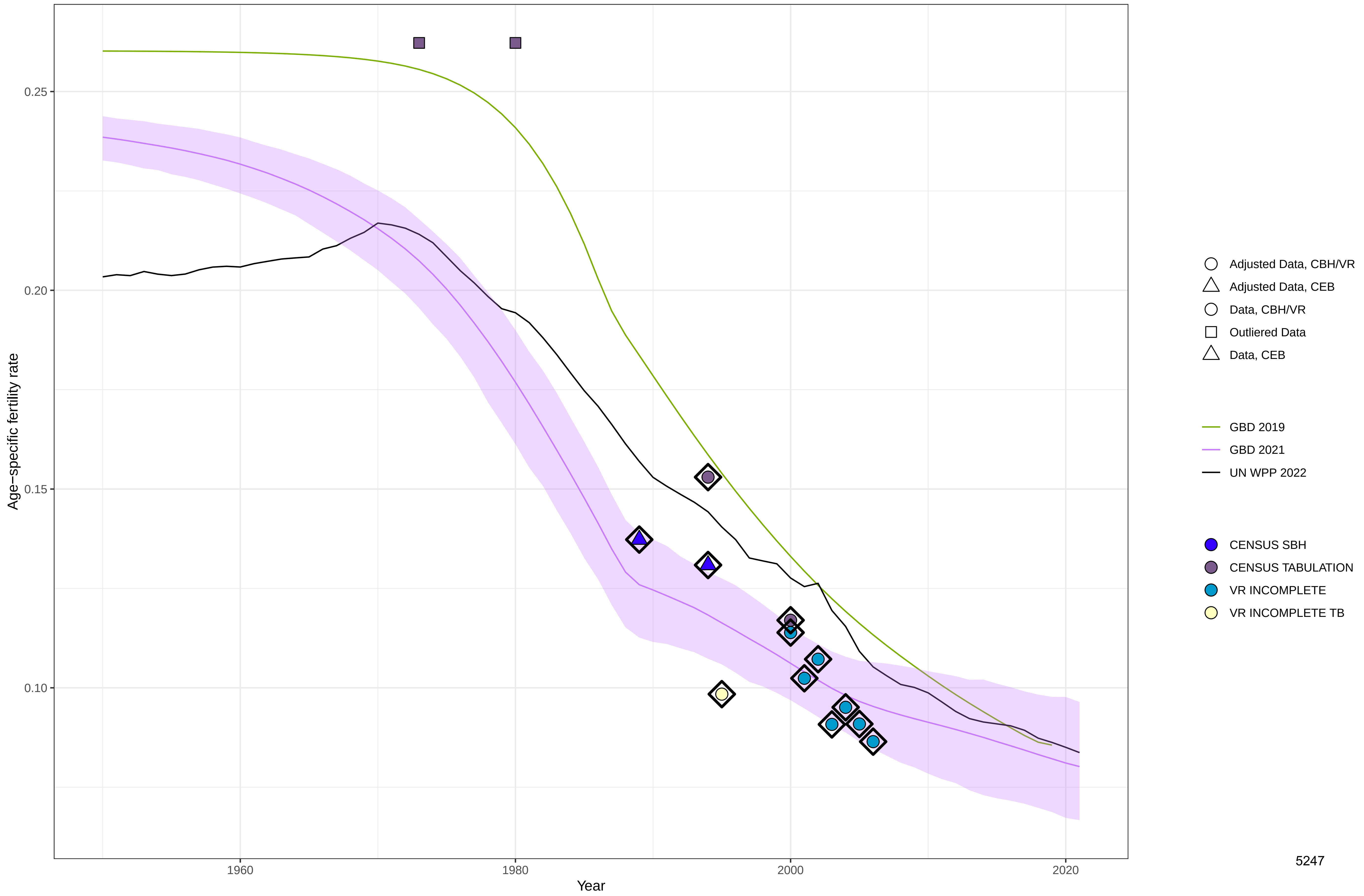

Age−specific fertility fitted trend lines, 1950−2021: Federated States of Micronesia, Ages: 40−44
Data points enclosed with a diamond are reference sources

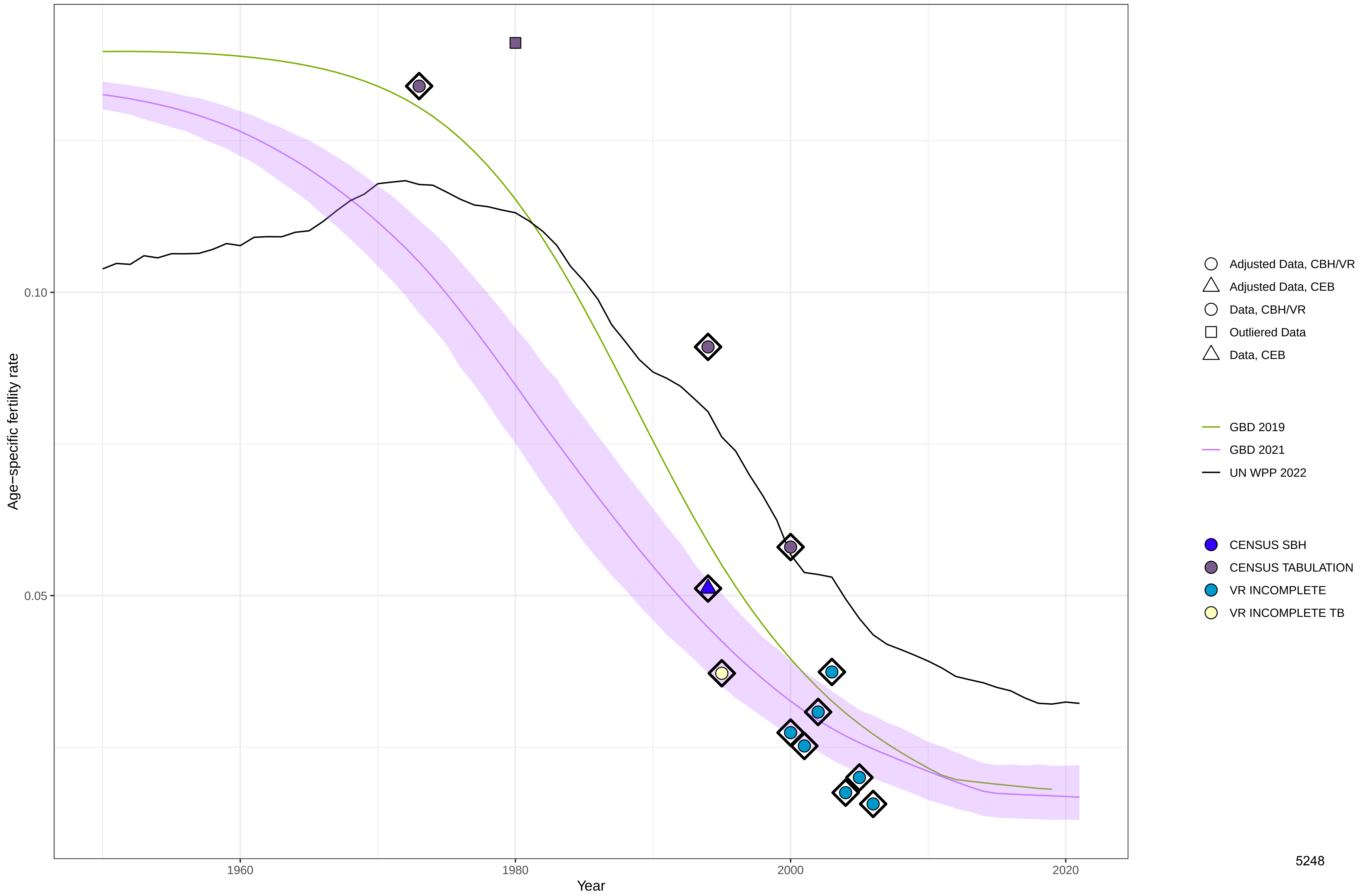

Age−specific fertility fitted trend lines, 1950−2021: Federated States of Micronesia, Ages: 45−49
Data points enclosed with a diamond are reference sources

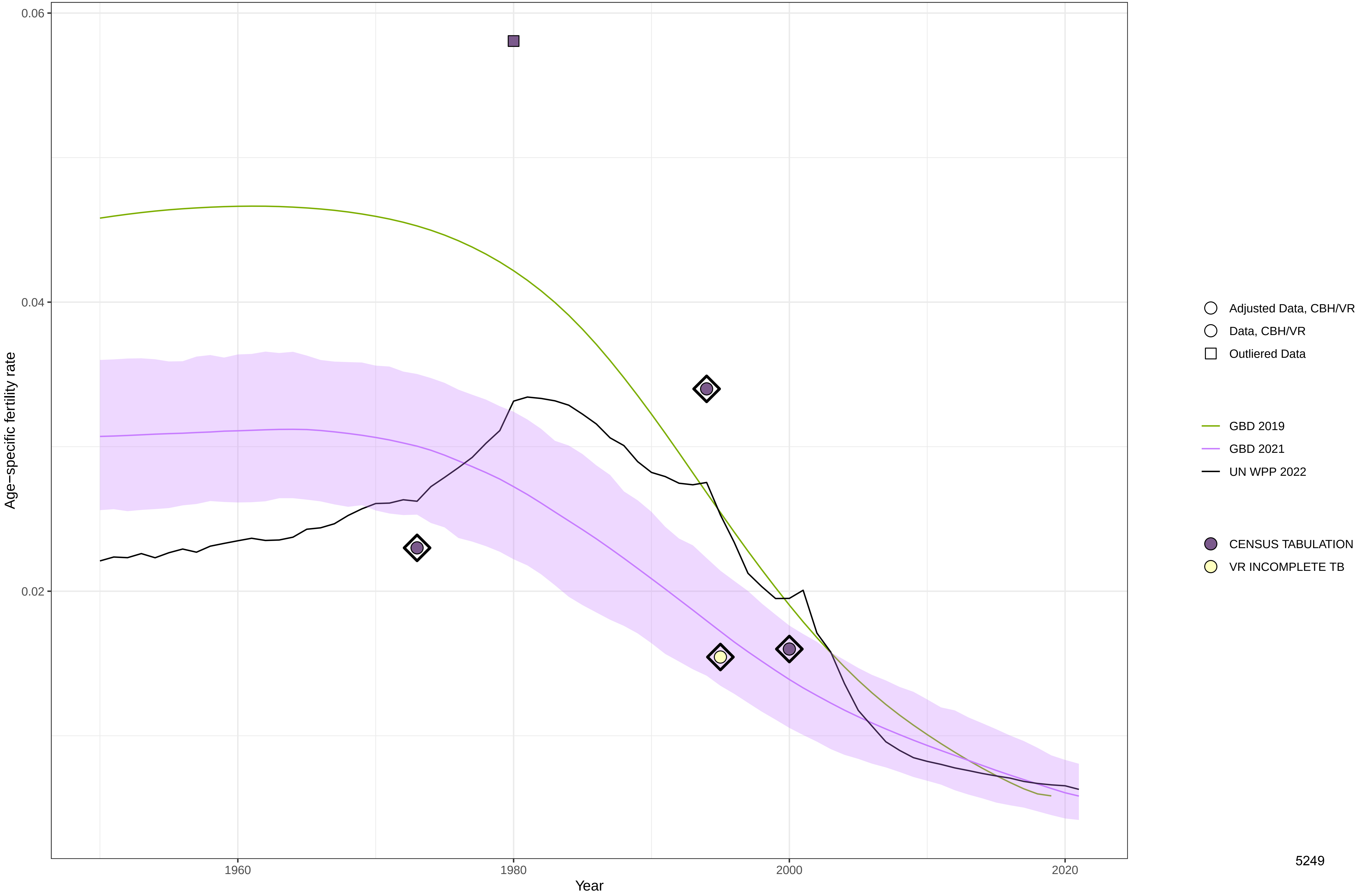

Age−specific fertility fitted trend lines, 1950−2021: Federated States of Micronesia, Ages: 50−54
Data points enclosed with a diamond are reference sources

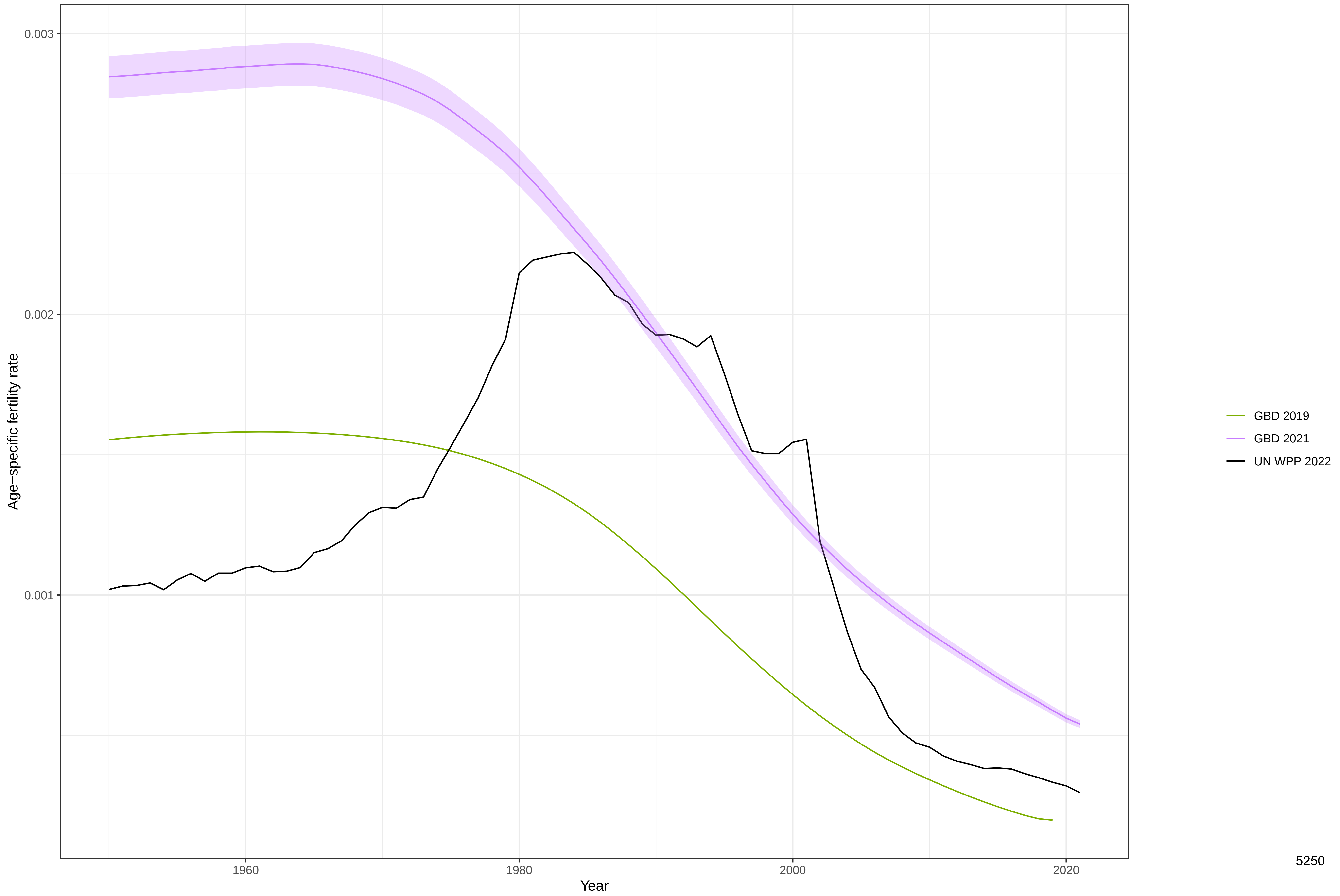

Total fertility rate fitted trend lines, 1950−2021: Federated States of Micronesia TFR
Data points enclosed with a diamond are reference sources

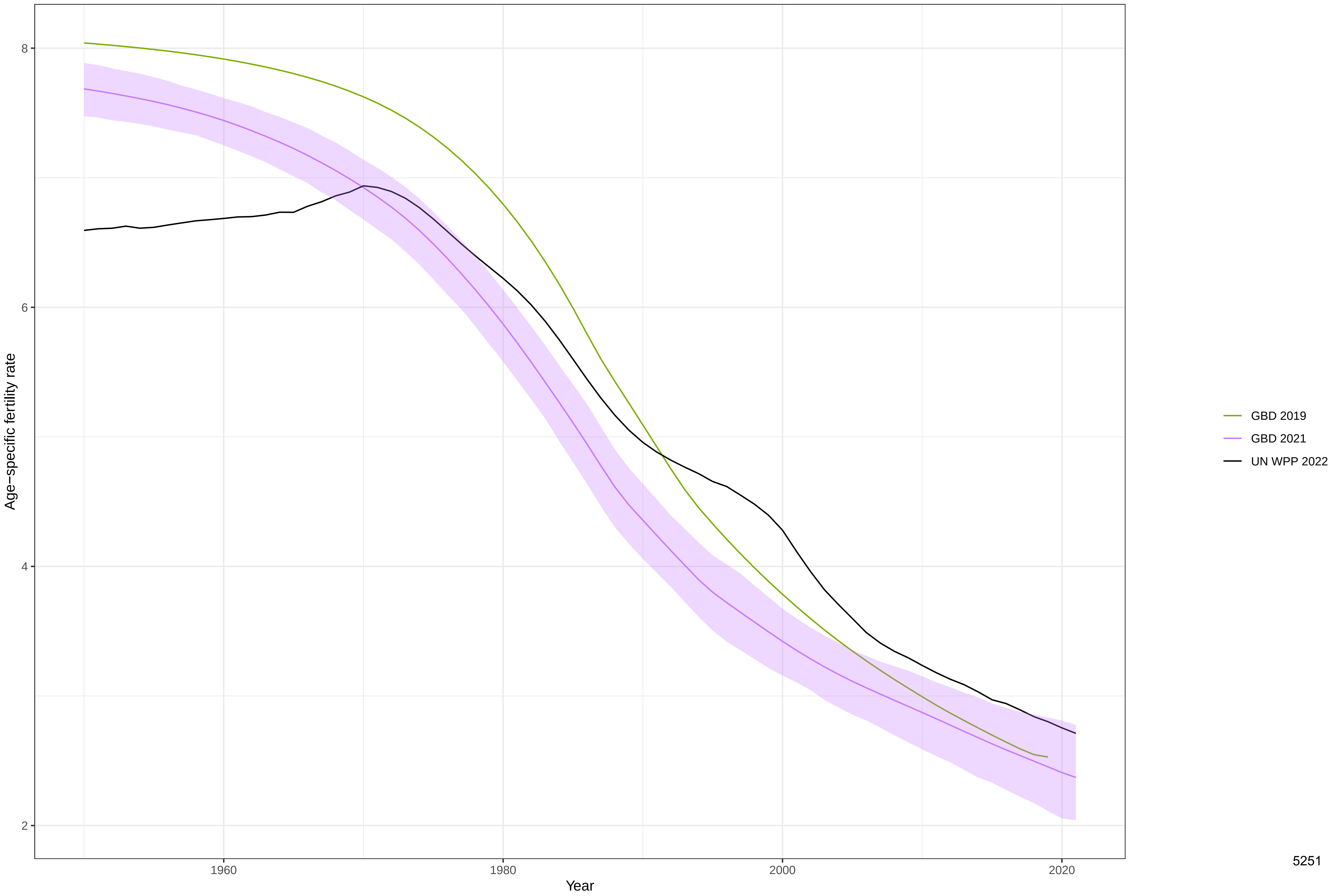

Age−specific fertility fitted trend lines, 1950−2021: Nauru, Ages: 10−14
Data points enclosed with a diamond are reference sources

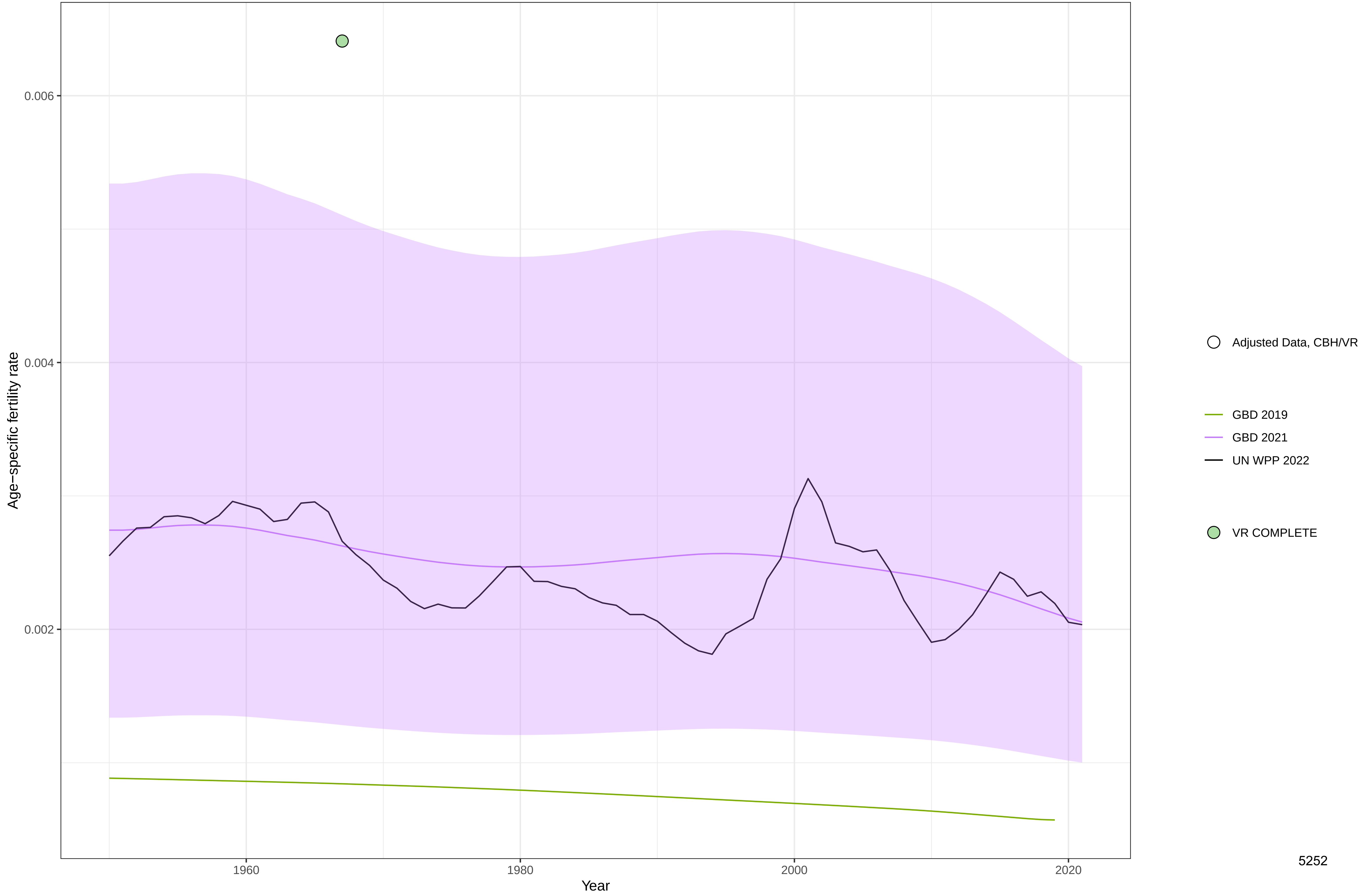

Age−specific fertility fitted trend lines, 1950−2021: Nauru, Ages: 15−19
Data points enclosed with a diamond are reference sources

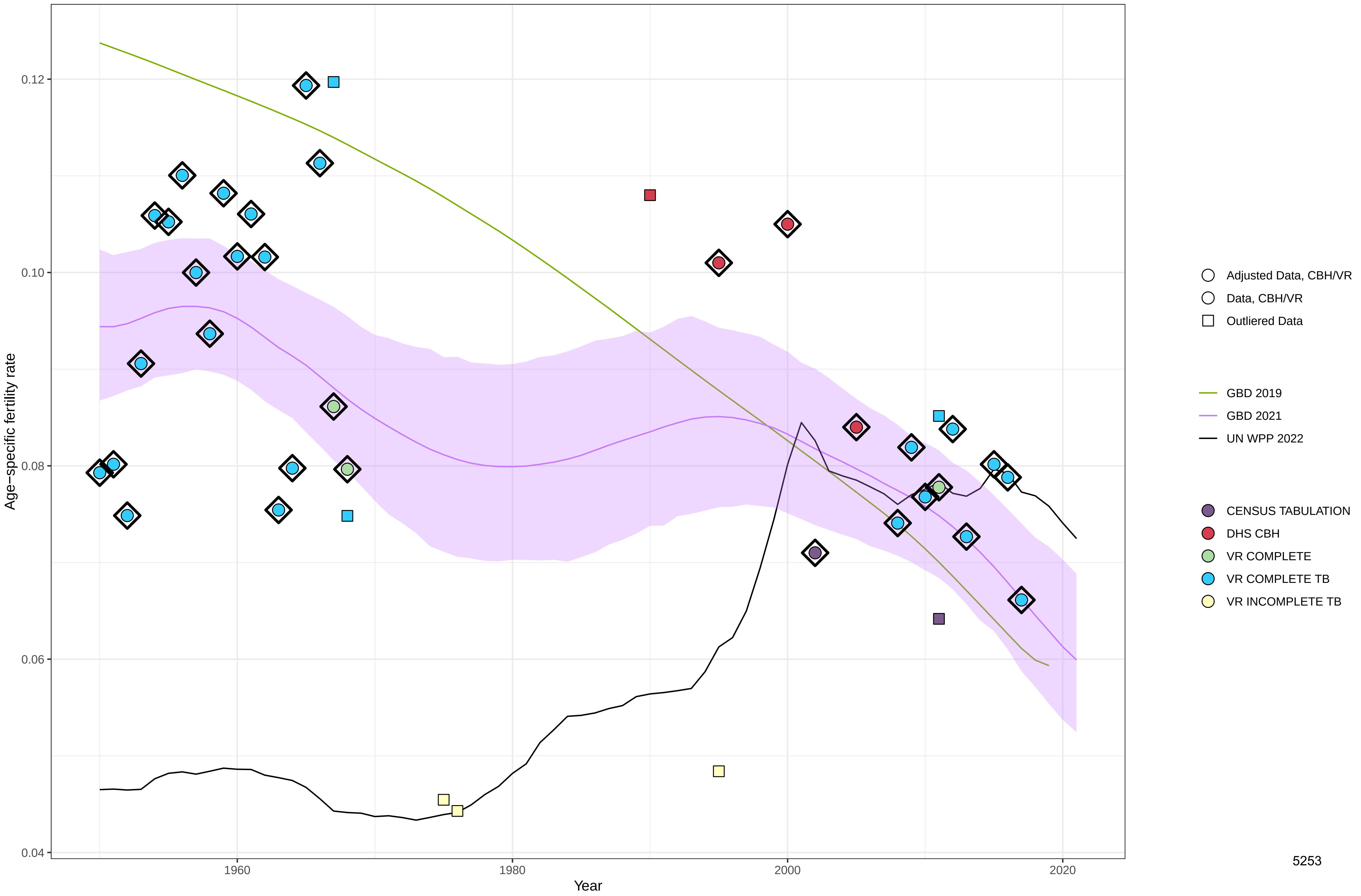

Age−specific fertility fitted trend lines, 1950−2021: Nauru, Ages: 20−24
Data points enclosed with a diamond are reference sources

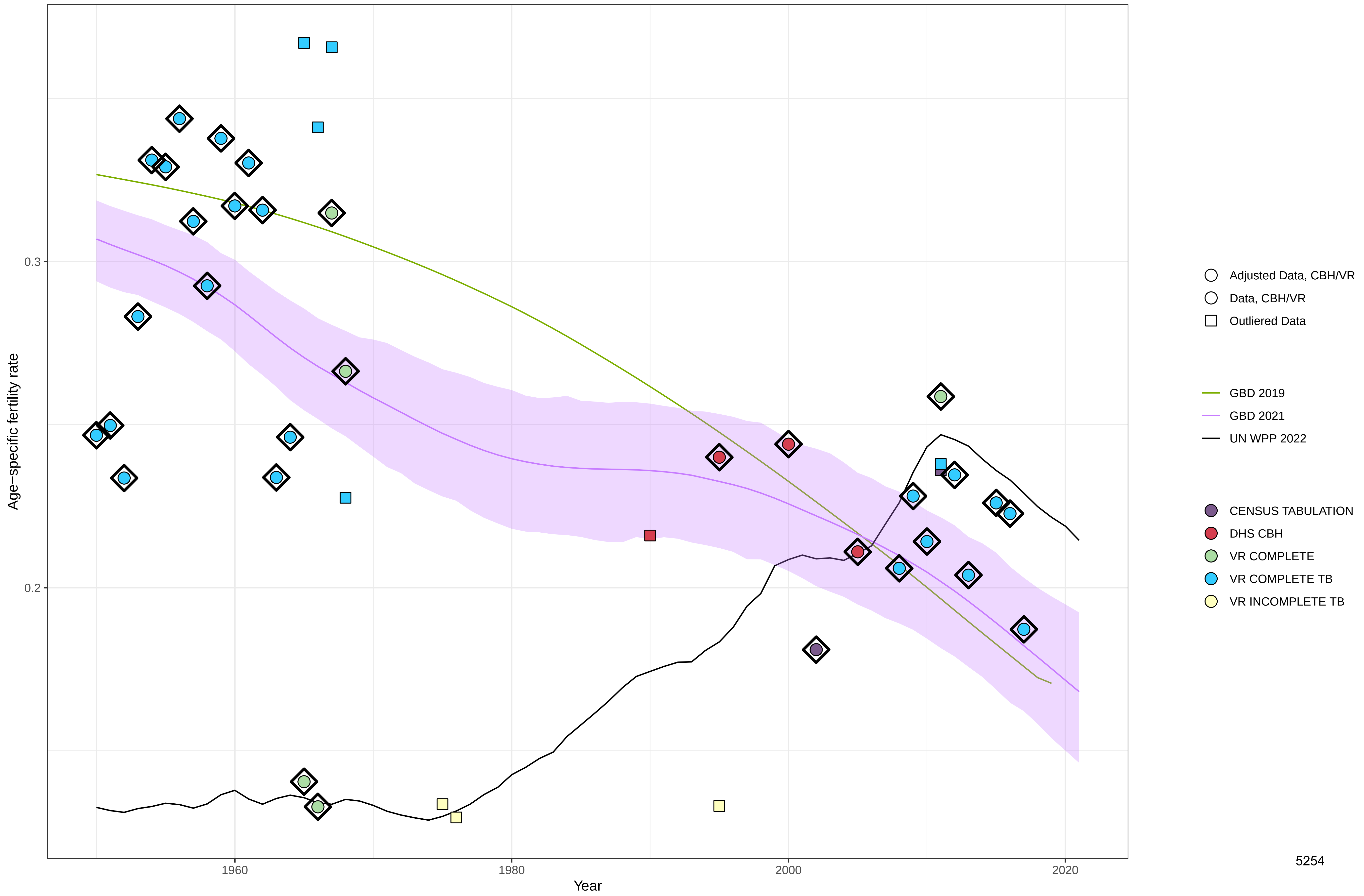

Age−specific fertility fitted trend lines, 1950−2021: Nauru, Ages: 25−29
 Data points enclosed with a diamond are reference sources

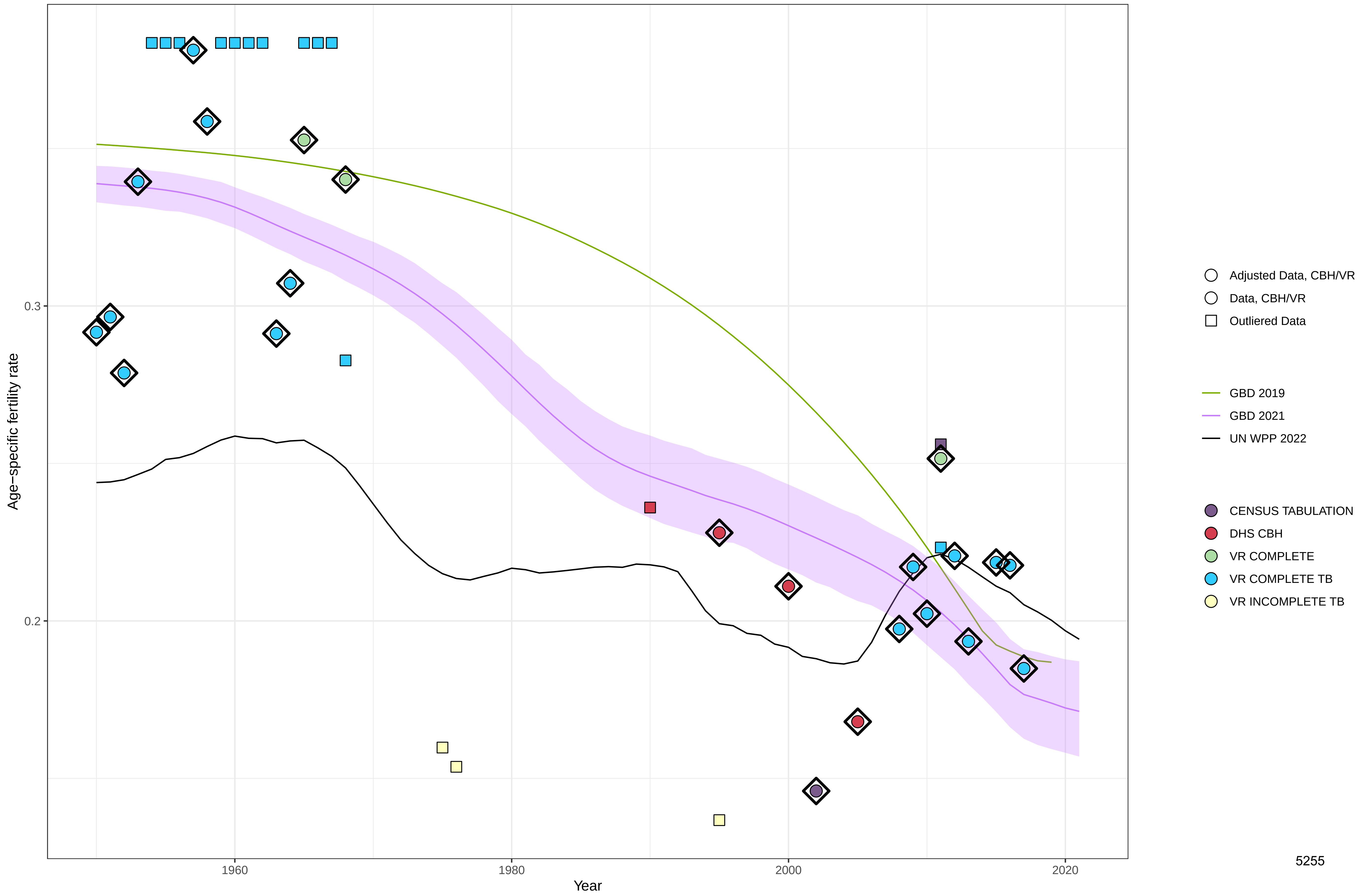

Age−specific fertility fitted trend lines, 1950−2021: Nauru, Ages: 30−34
Data points enclosed with a diamond are reference sources

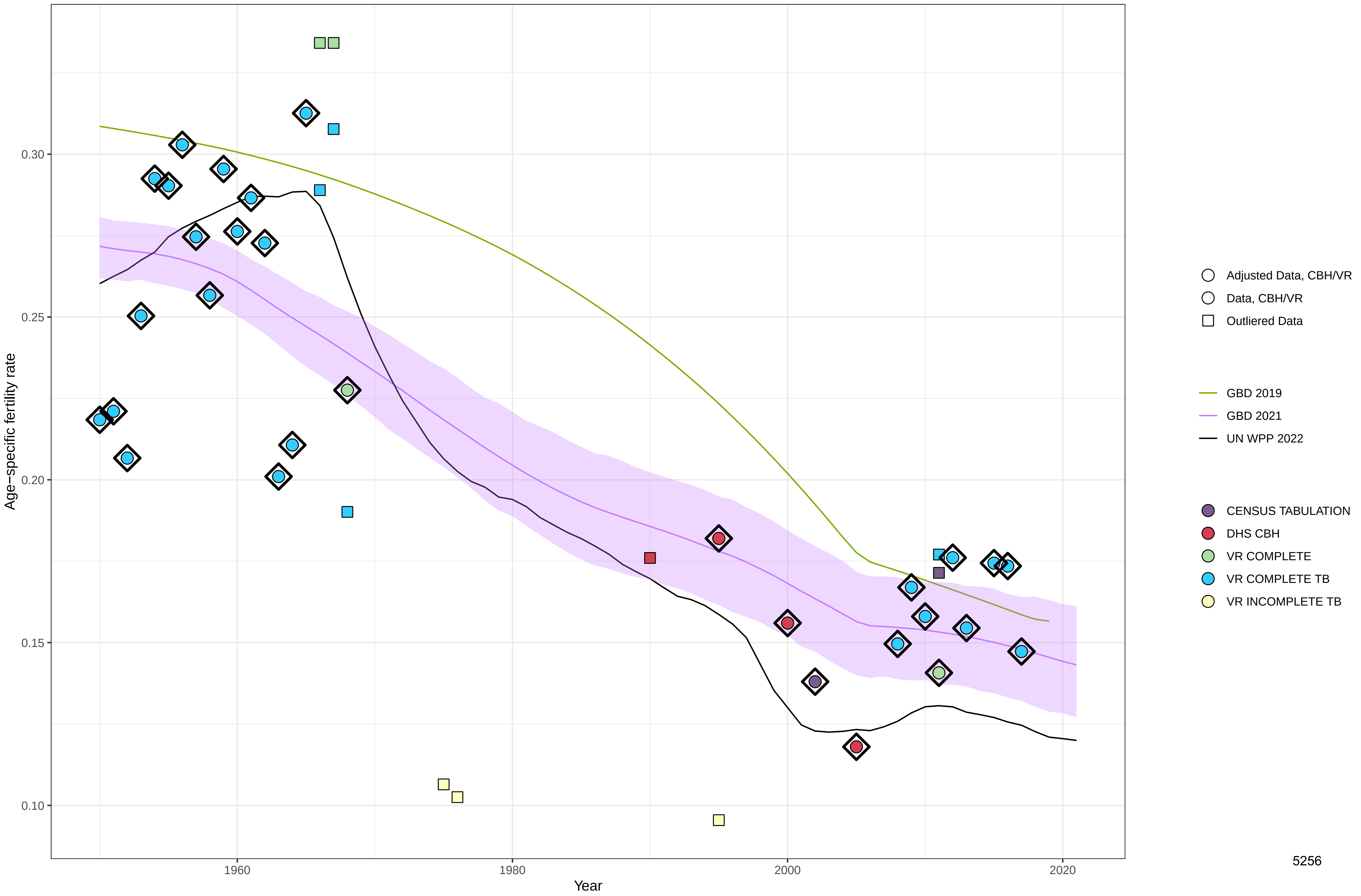

Age−specific fertility fitted trend lines, 1950−2021: Nauru, Ages: 35−39
Data points enclosed with a diamond are reference sources

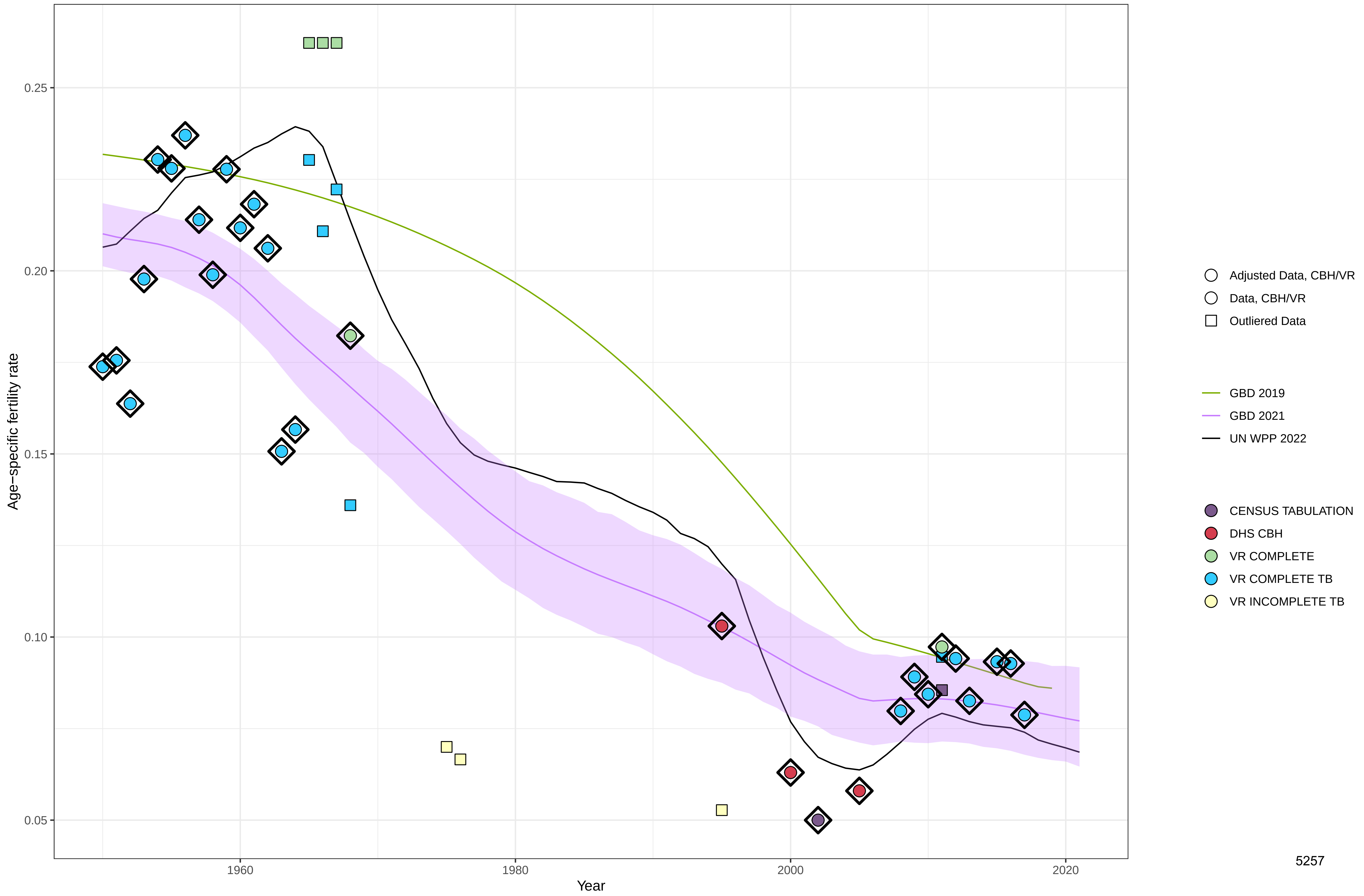

Age−specific fertility fitted trend lines, 1950−2021: Nauru, Ages: 40−44
Data points enclosed with a diamond are reference sources

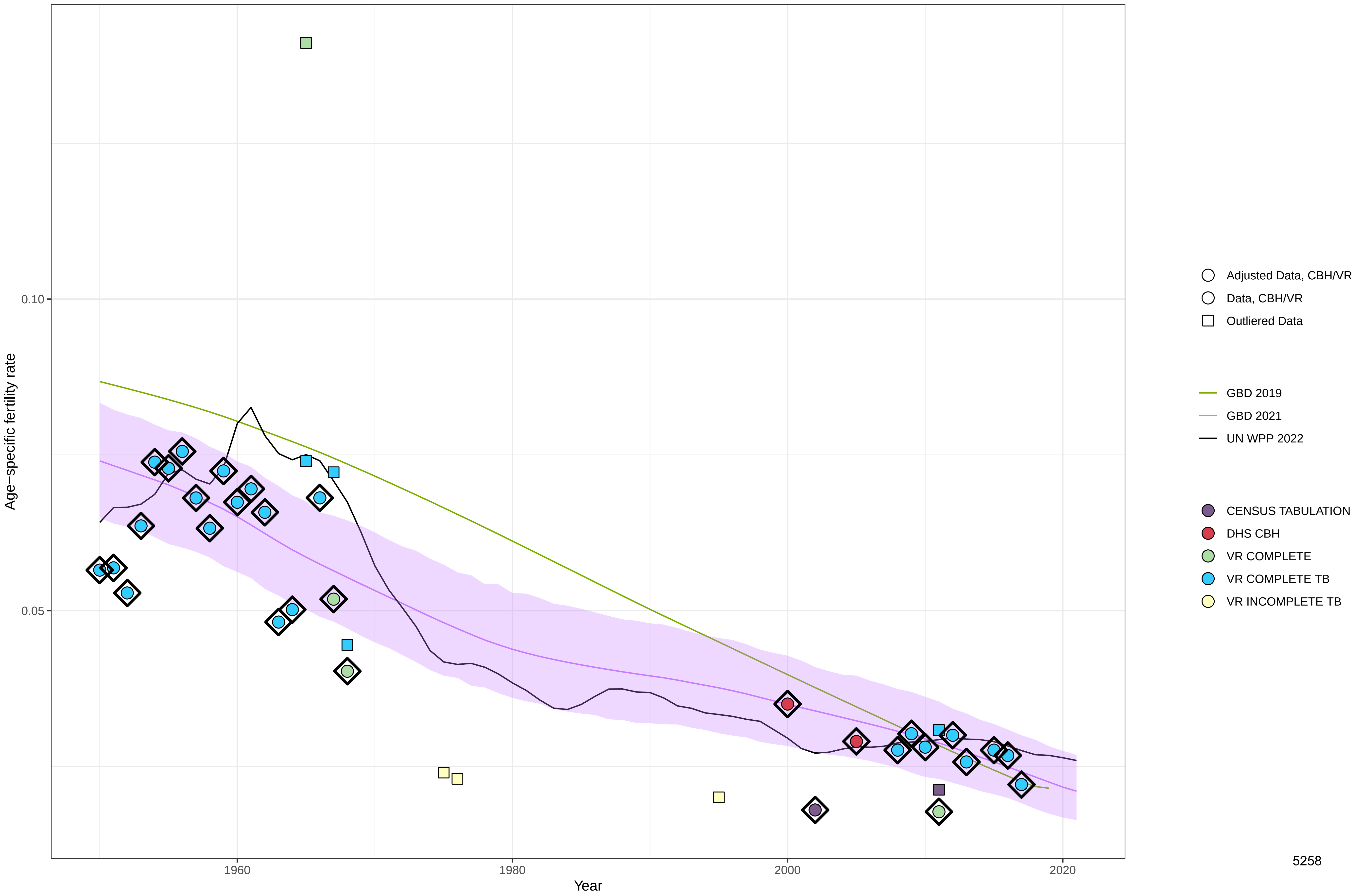

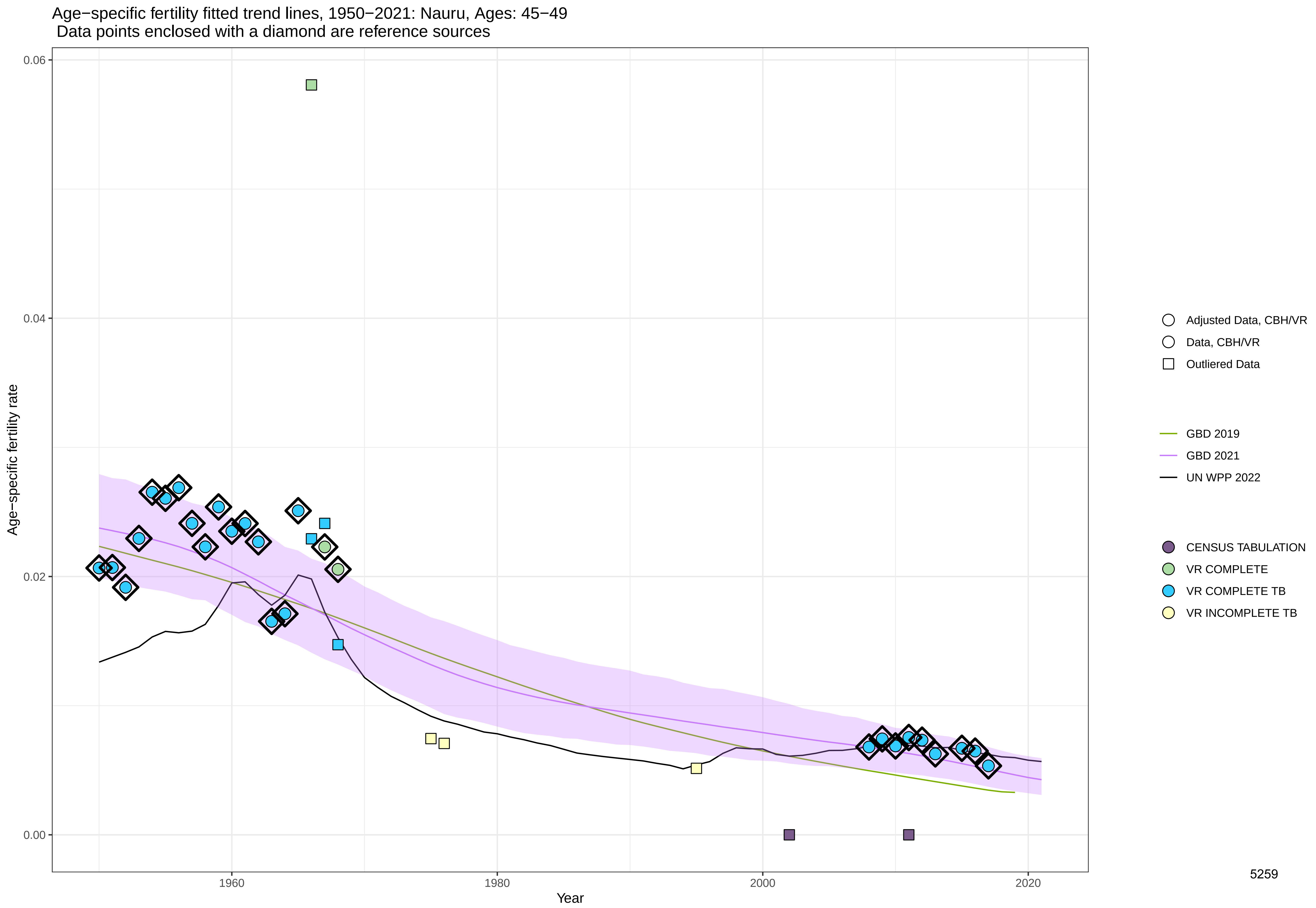

Age−specific fertility fitted trend lines, 1950−2021: Nauru, Ages: 45−49
Data points enclosed with a diamond are reference sources
Age−specific fertility rate
Year
0.00
0.02
0.04
0.06
1960
1980
2000
2020
Adjusted Data, CBH/VR
Data, CBH/VR
Outliered Data
GBD 2019
GBD 2021
UN WPP 2022
CENSUS TABULATION
VR COMPLETE
VR COMPLETE TB
VR INCOMPLETE TB

Age−specific fertility fitted trend lines, 1950−2021: Nauru, Ages: 50−54
Data points enclosed with a diamond are reference sources

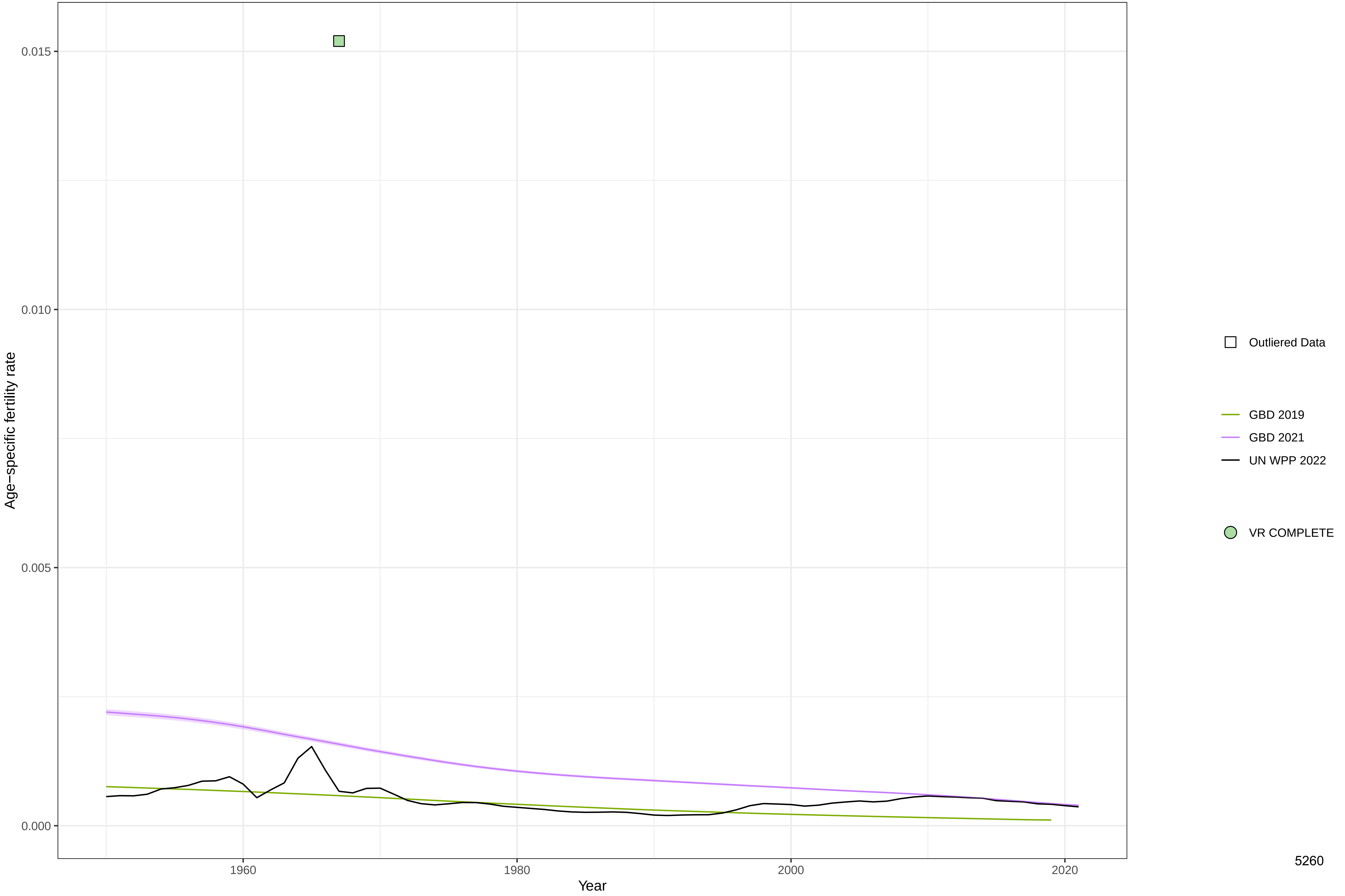

Total fertility rate fitted trend lines, 1950−2021: Nauru TFR
Data points enclosed with a diamond are reference sources

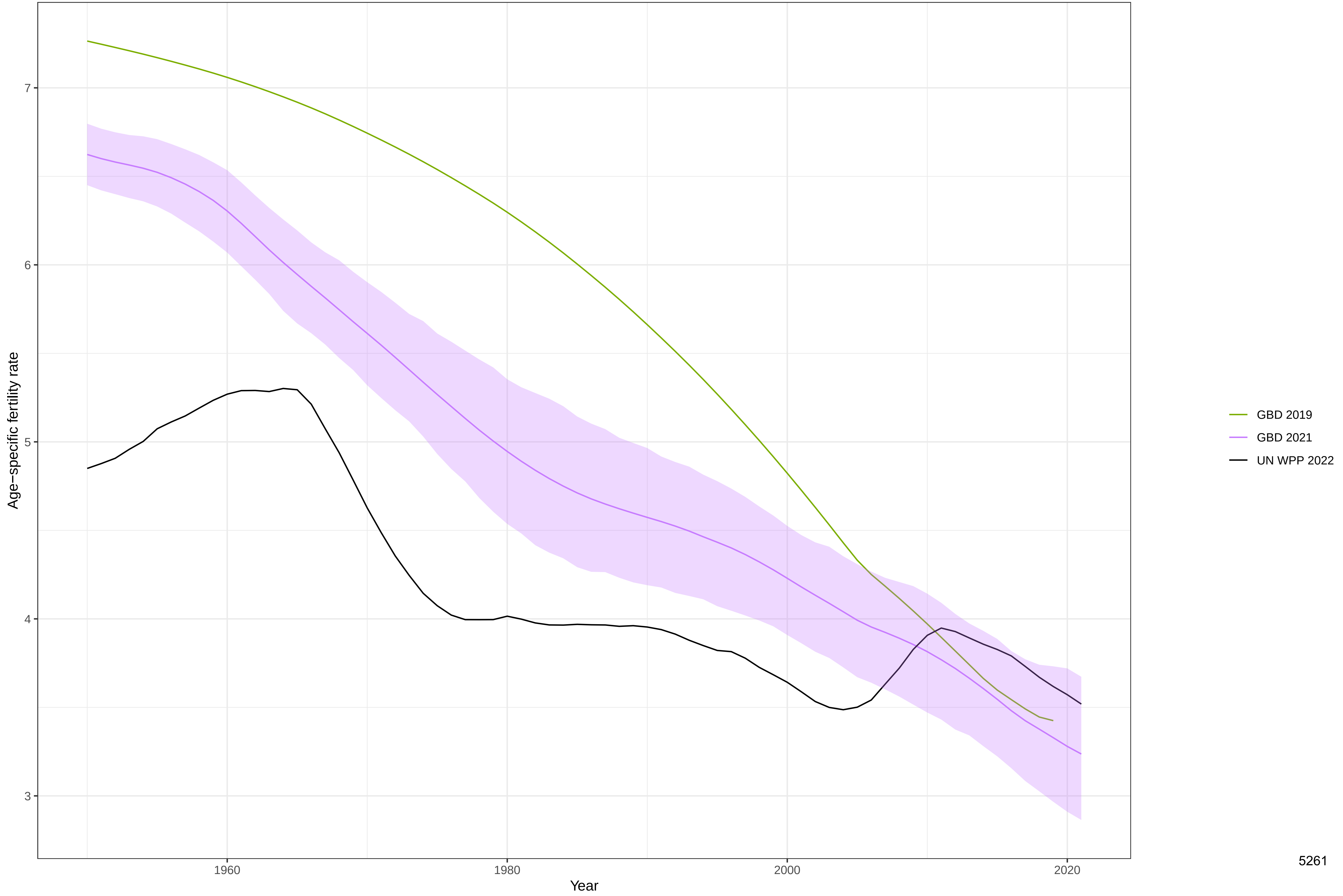

Age−specific fertility fitted trend lines, 1950−2021: Niue, Ages: 10−14
Data points enclosed with a diamond are reference sources

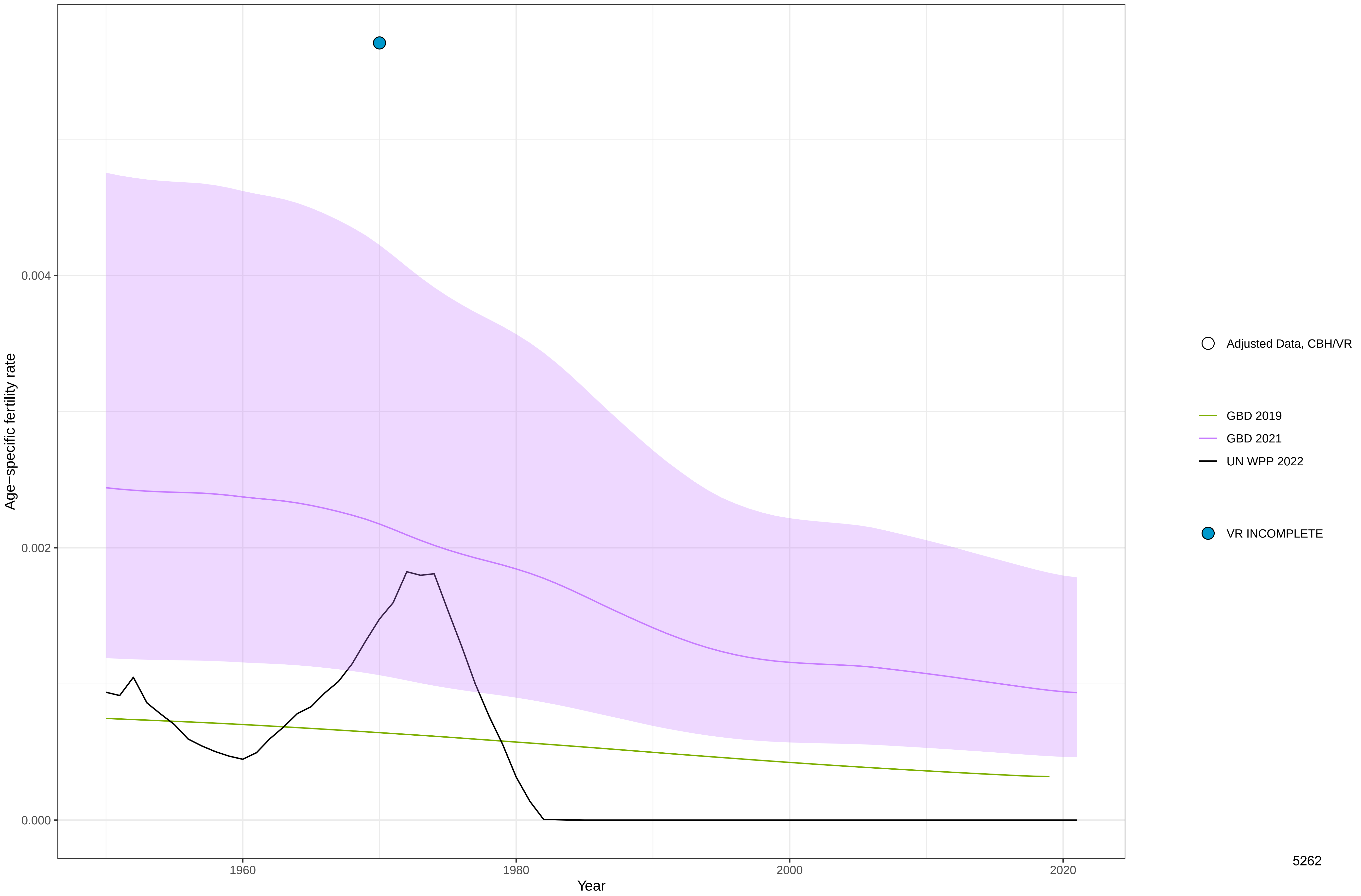

Age−specific fertility fitted trend lines, 1950−2021: Niue, Ages: 15−19
Data points enclosed with a diamond are reference sources

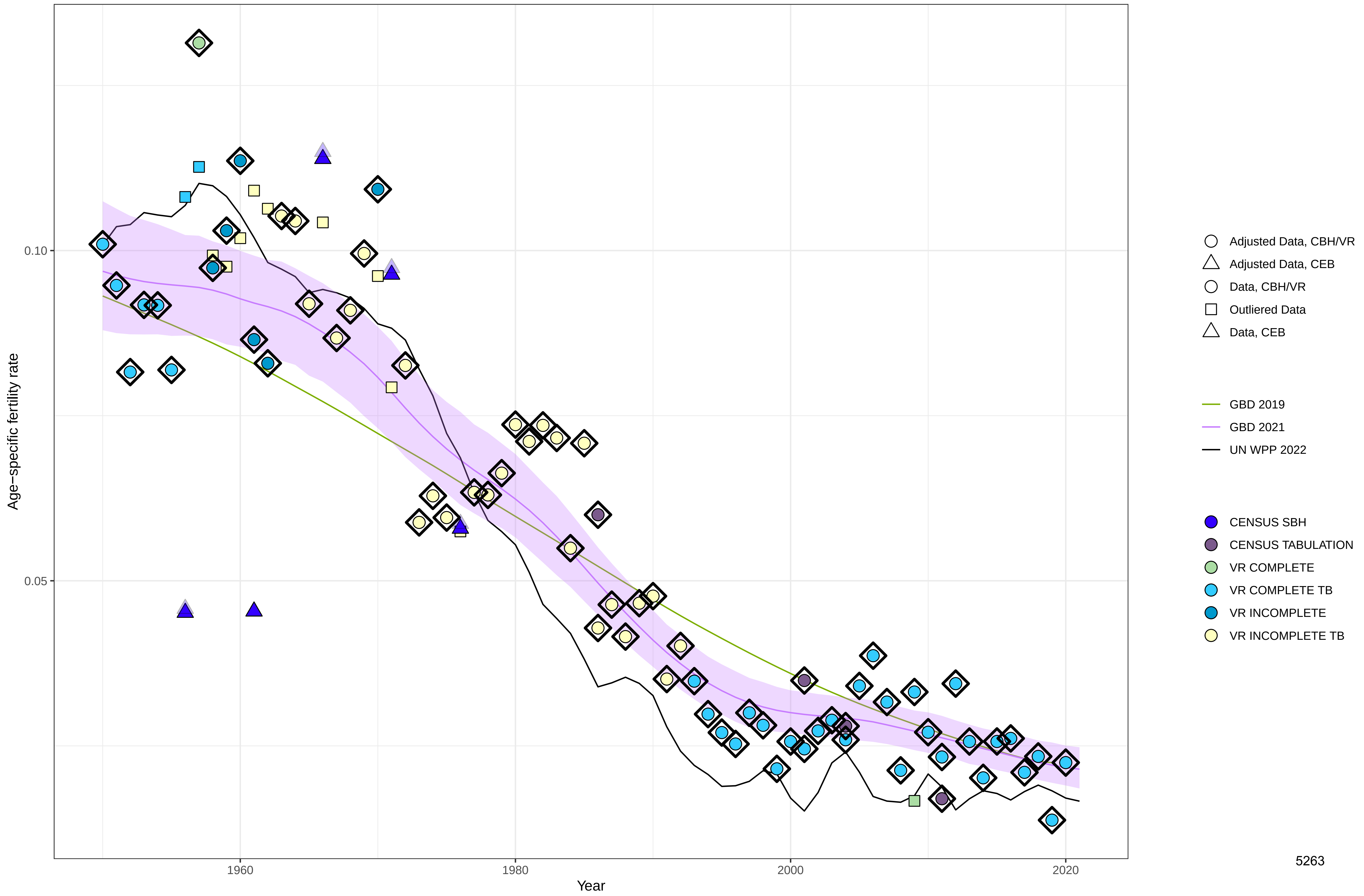

Age−specific fertility fitted trend lines, 1950−2021: Niue, Ages: 20−24
Data points enclosed with a diamond are reference sources

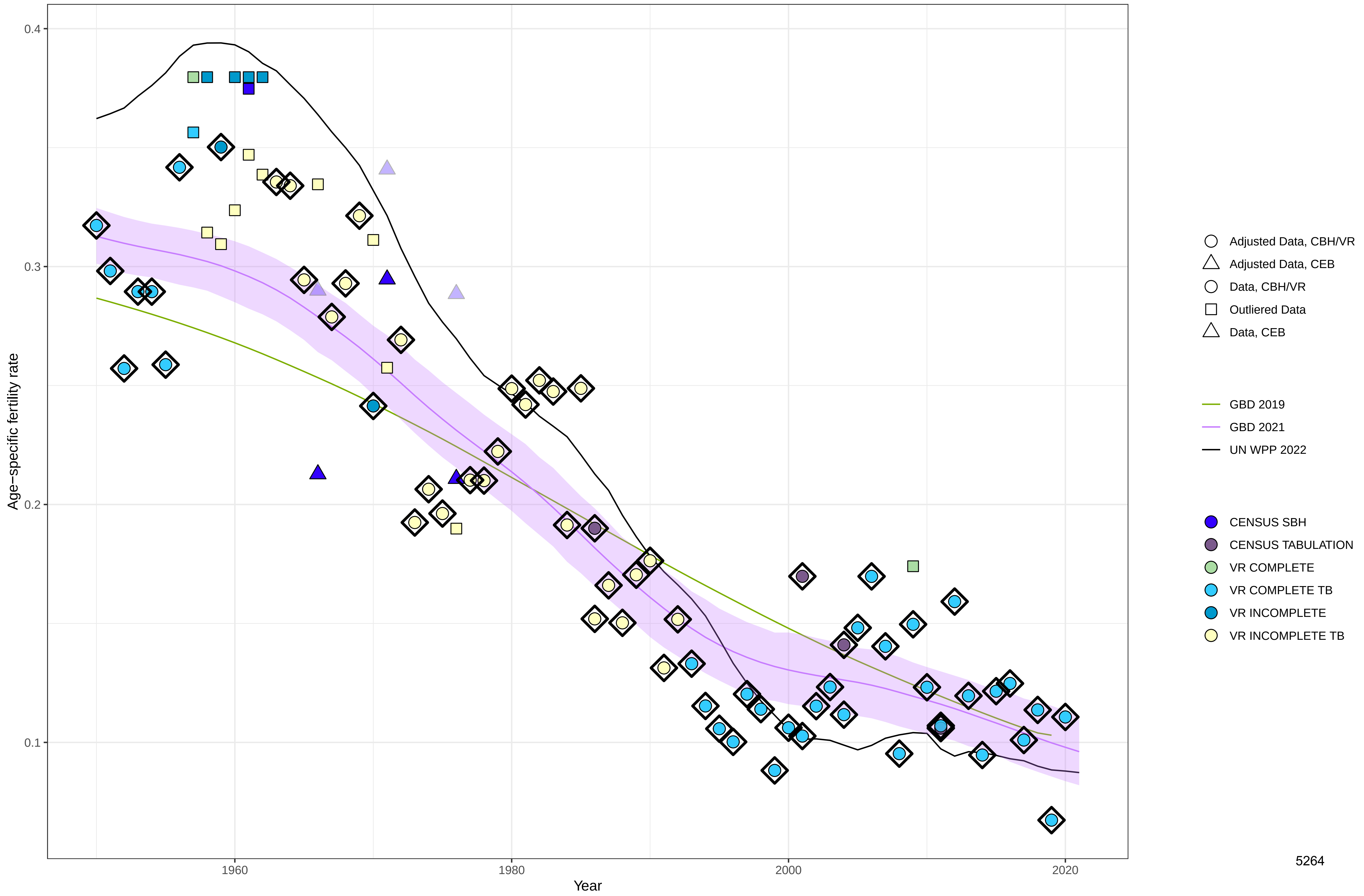

Age−specific fertility fitted trend lines, 1950−2021: Niue, Ages: 25−29
Data points enclosed with a diamond are reference sources

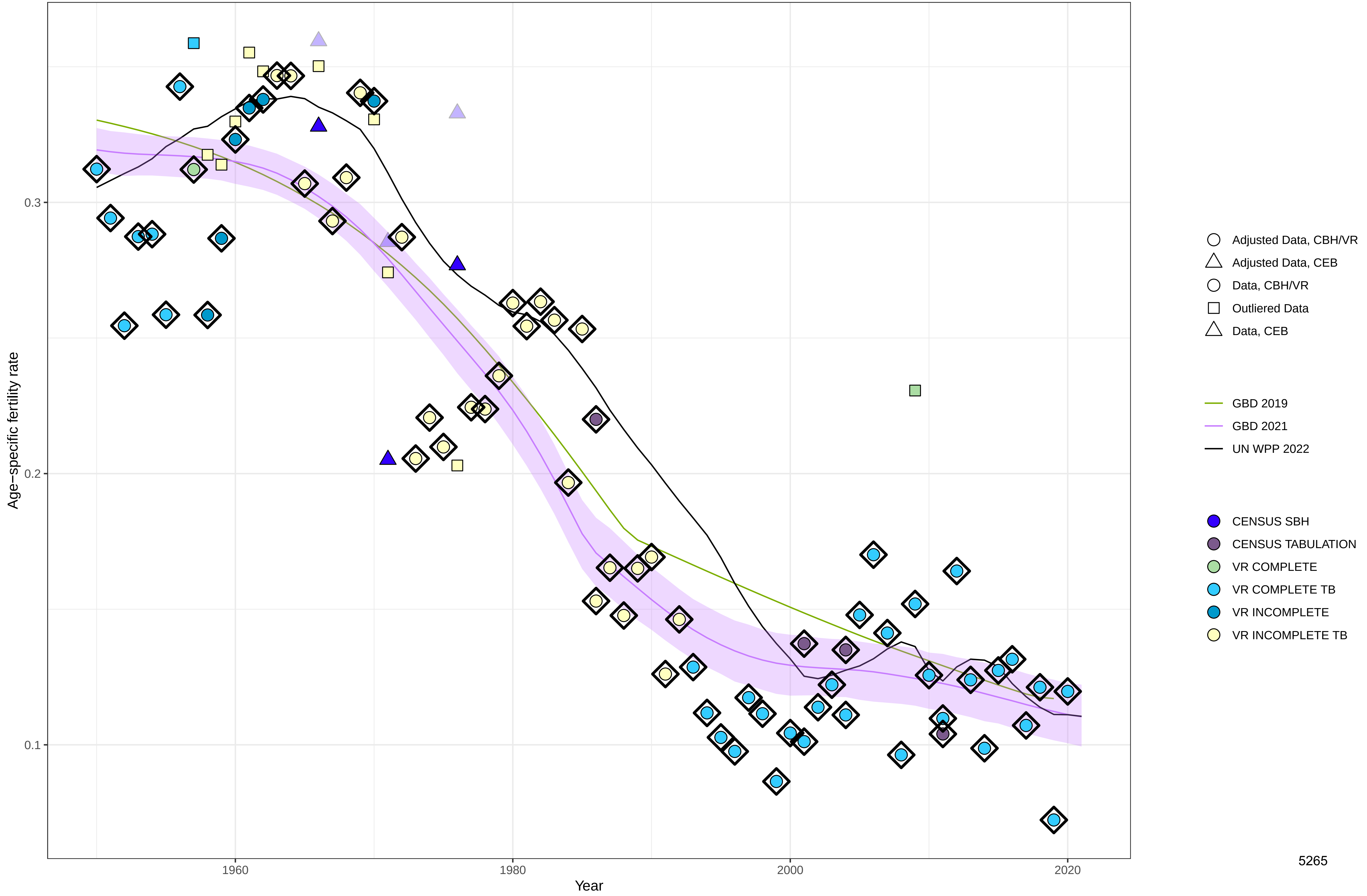

Age−specific fertility fitted trend lines, 1950−2021: Niue, Ages: 30−34
Data points enclosed with a diamond are reference sources

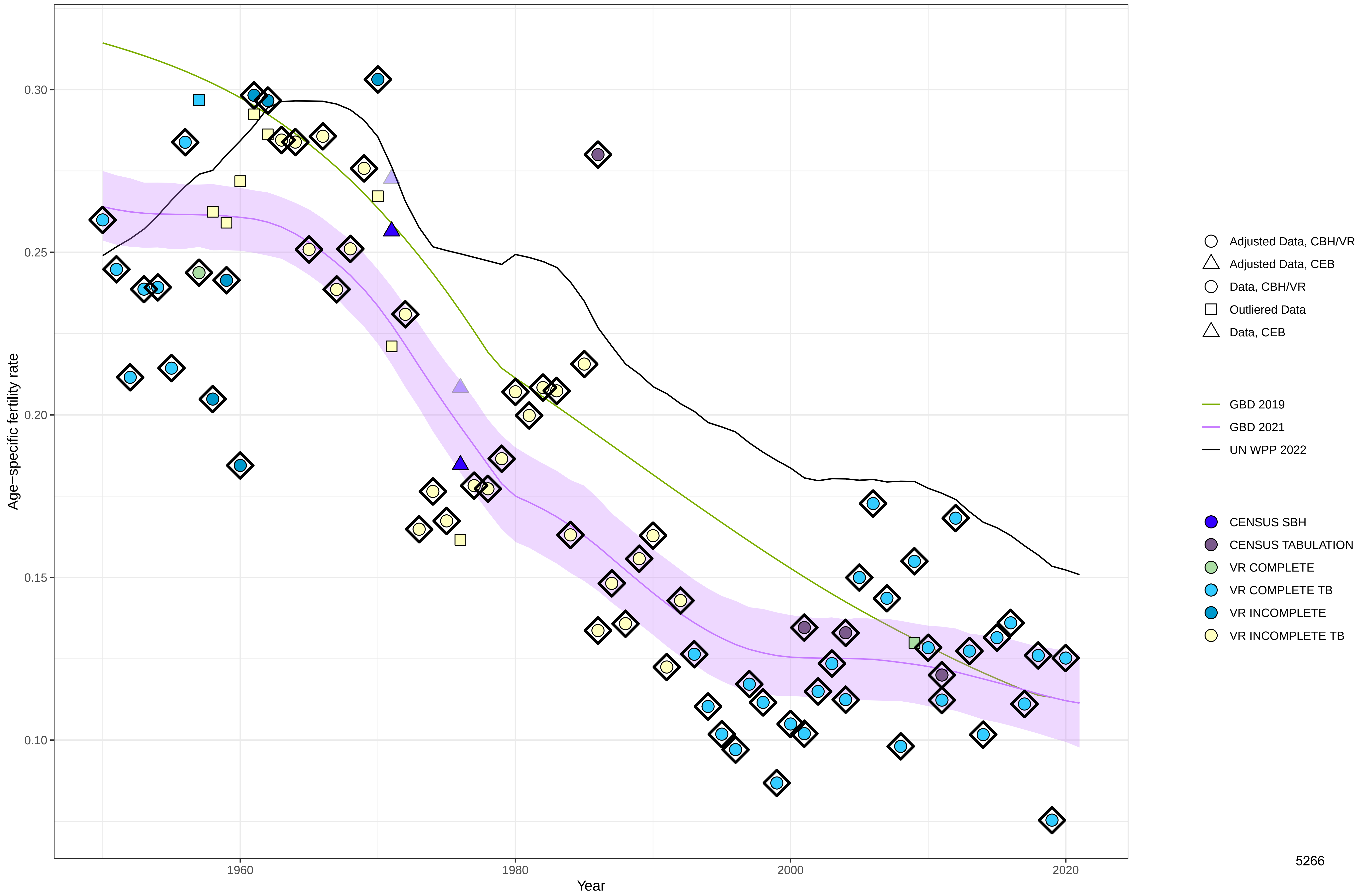

Age−specific fertility fitted trend lines, 1950−2021: Niue, Ages: 35−39
Data points enclosed with a diamond are reference sources

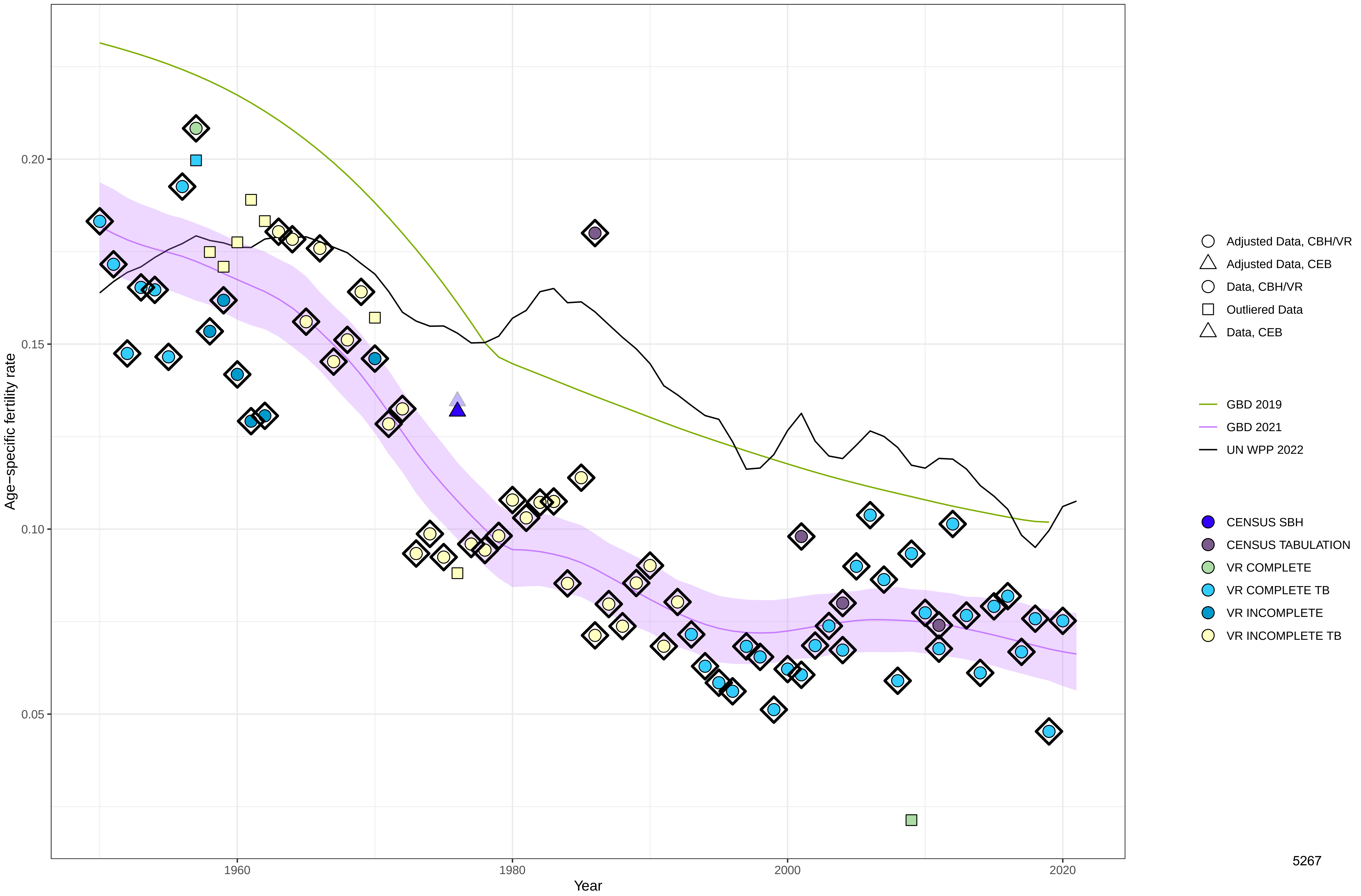

Age−specific fertility fitted trend lines, 1950−2021: Niue, Ages: 40−44
Data points enclosed with a diamond are reference sources

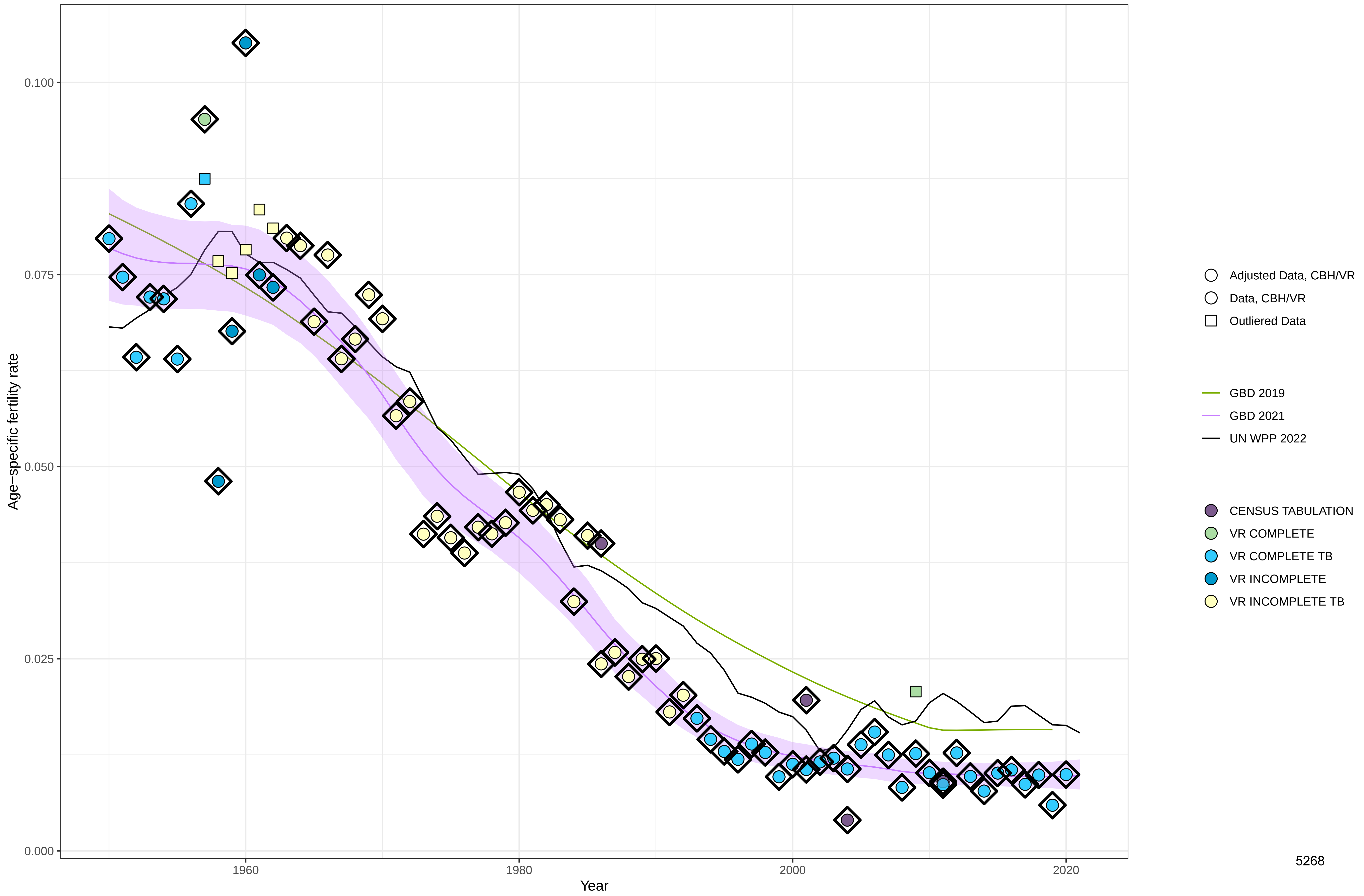

Age−specific fertility fitted trend lines, 1950−2021: Niue, Ages: 45−49
Data points enclosed with a diamond are reference sources

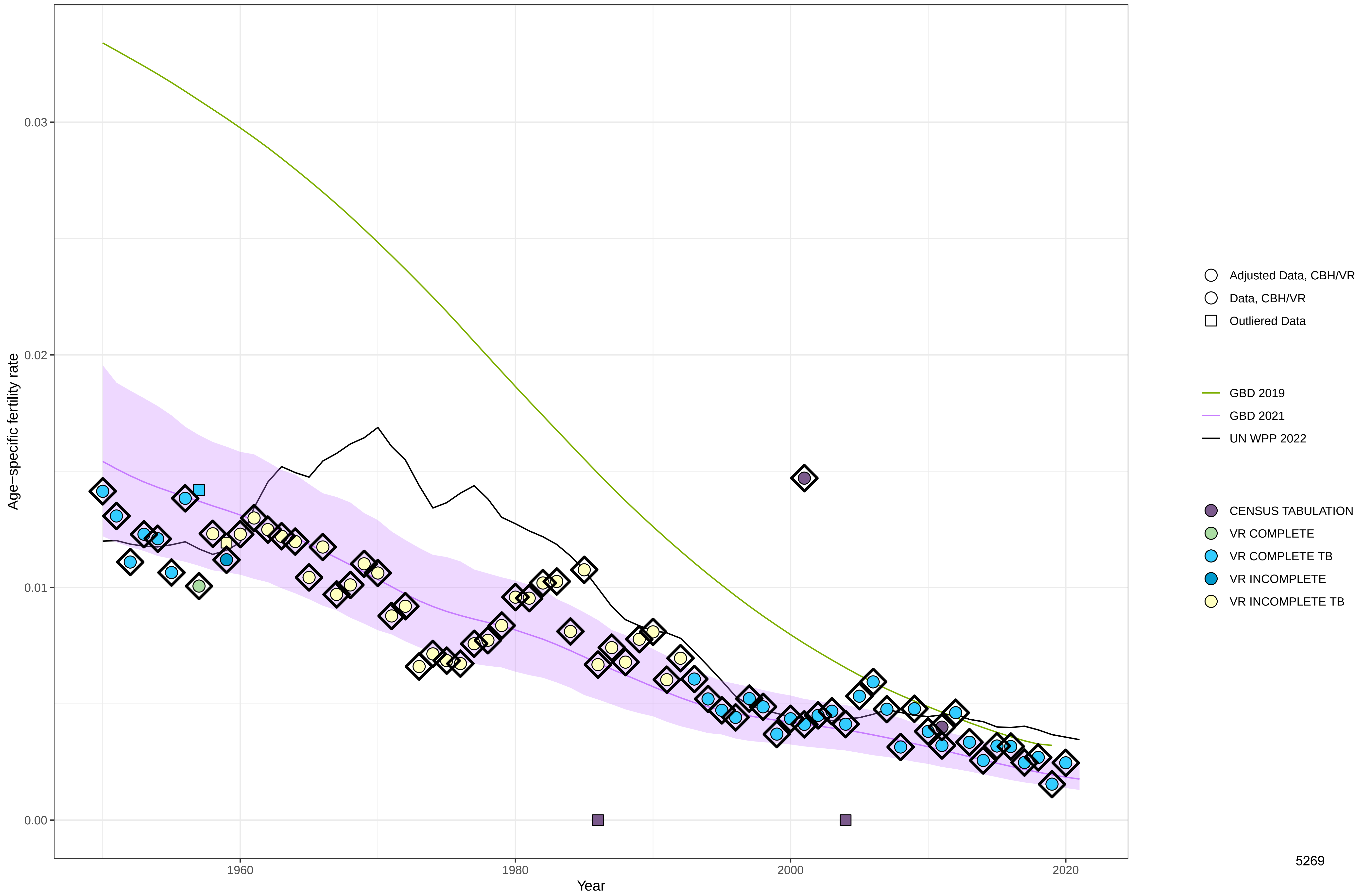

Age−specific fertility fitted trend lines, 1950−2021: Niue, Ages: 50−54
Data points enclosed with a diamond are reference sources

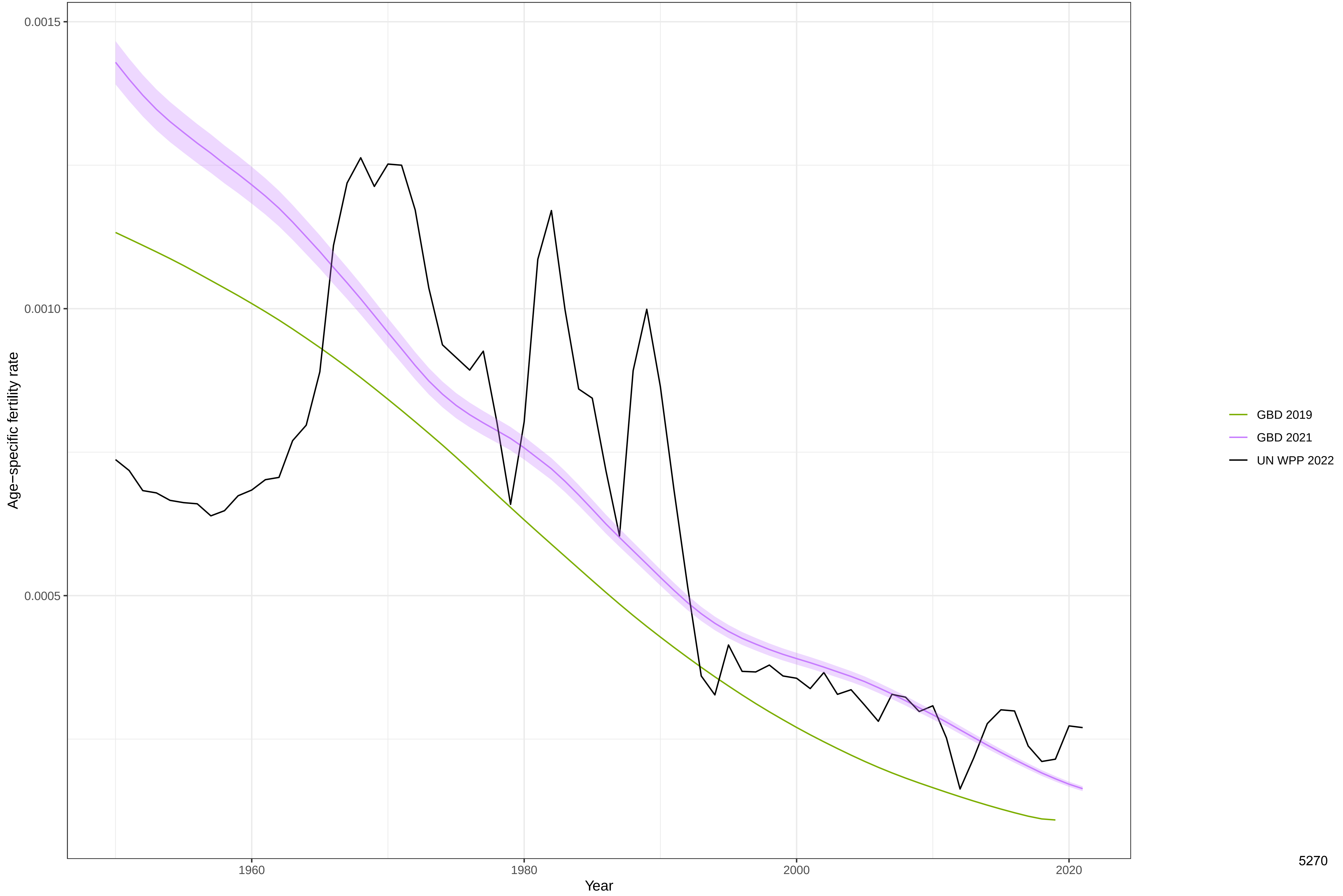

Total fertility rate fitted trend lines, 1950−2021: Niue TFR
Data points enclosed with a diamond are reference sources

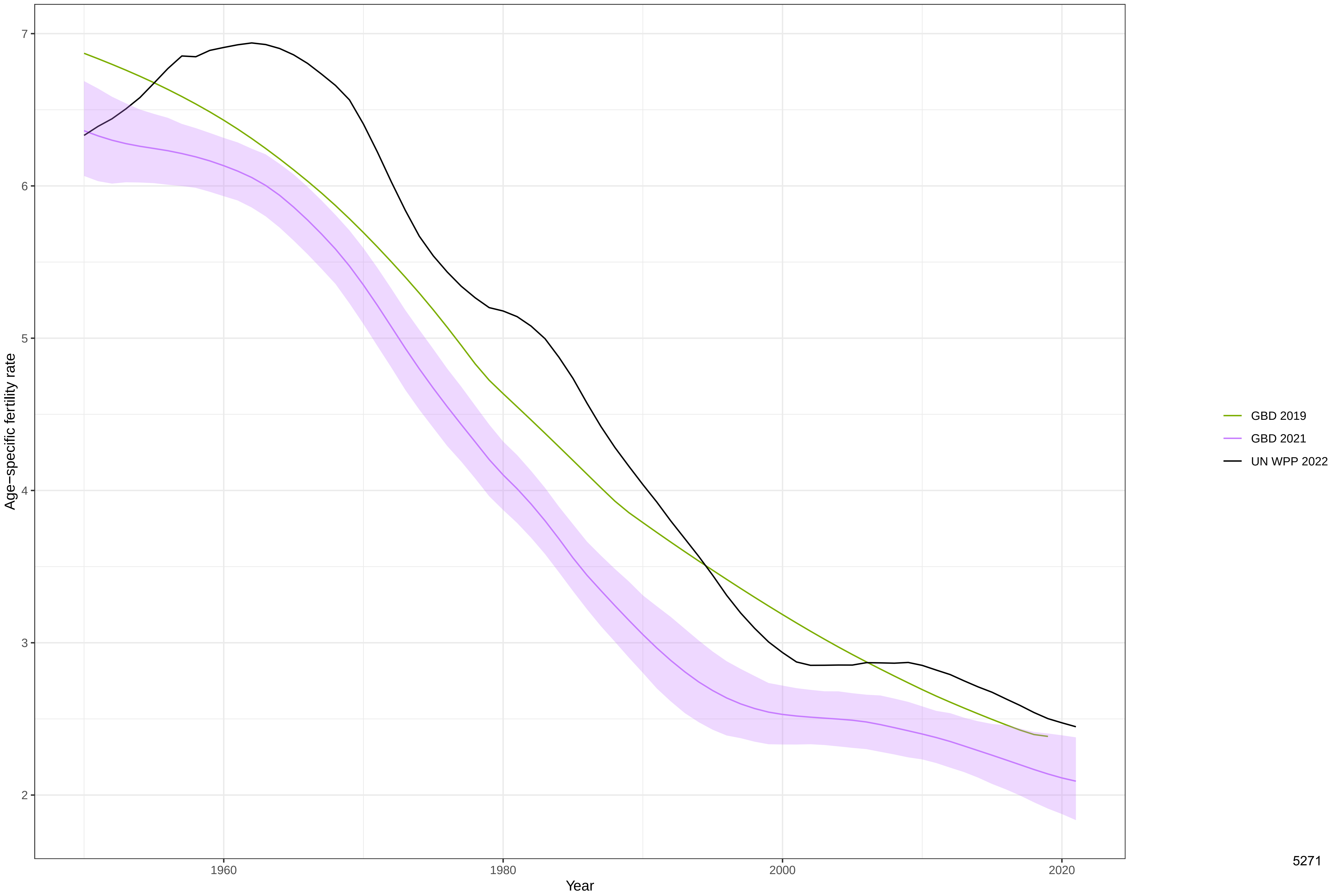

Age−specific fertility fitted trend lines, 1950−2021: Northern Mariana Islands, Ages: 10−14
Data points enclosed with a diamond are reference sources

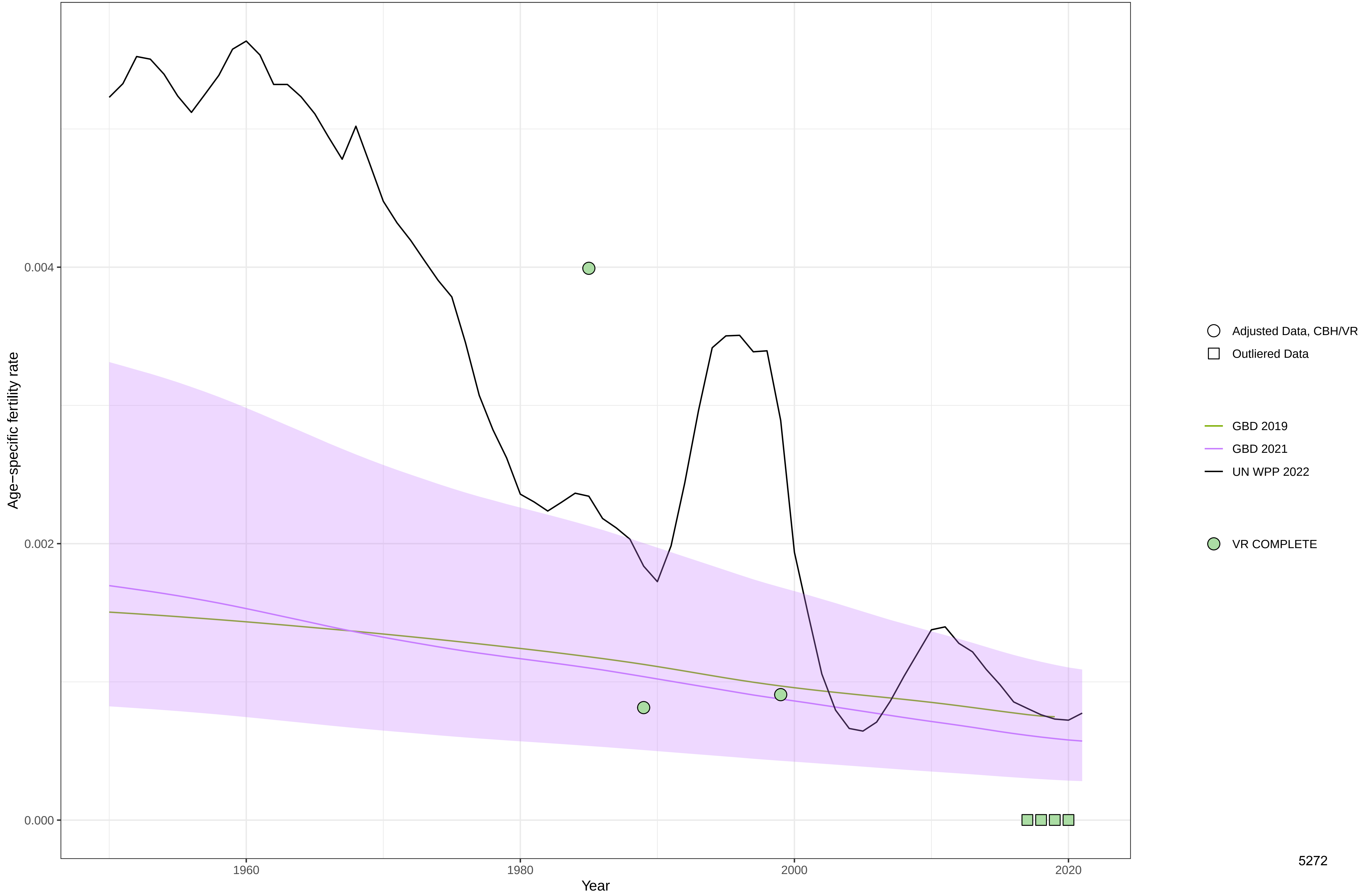

Age−specific fertility fitted trend lines, 1950−2021: Northern Mariana Islands, Ages: 15−19
Data points enclosed with a diamond are reference sources

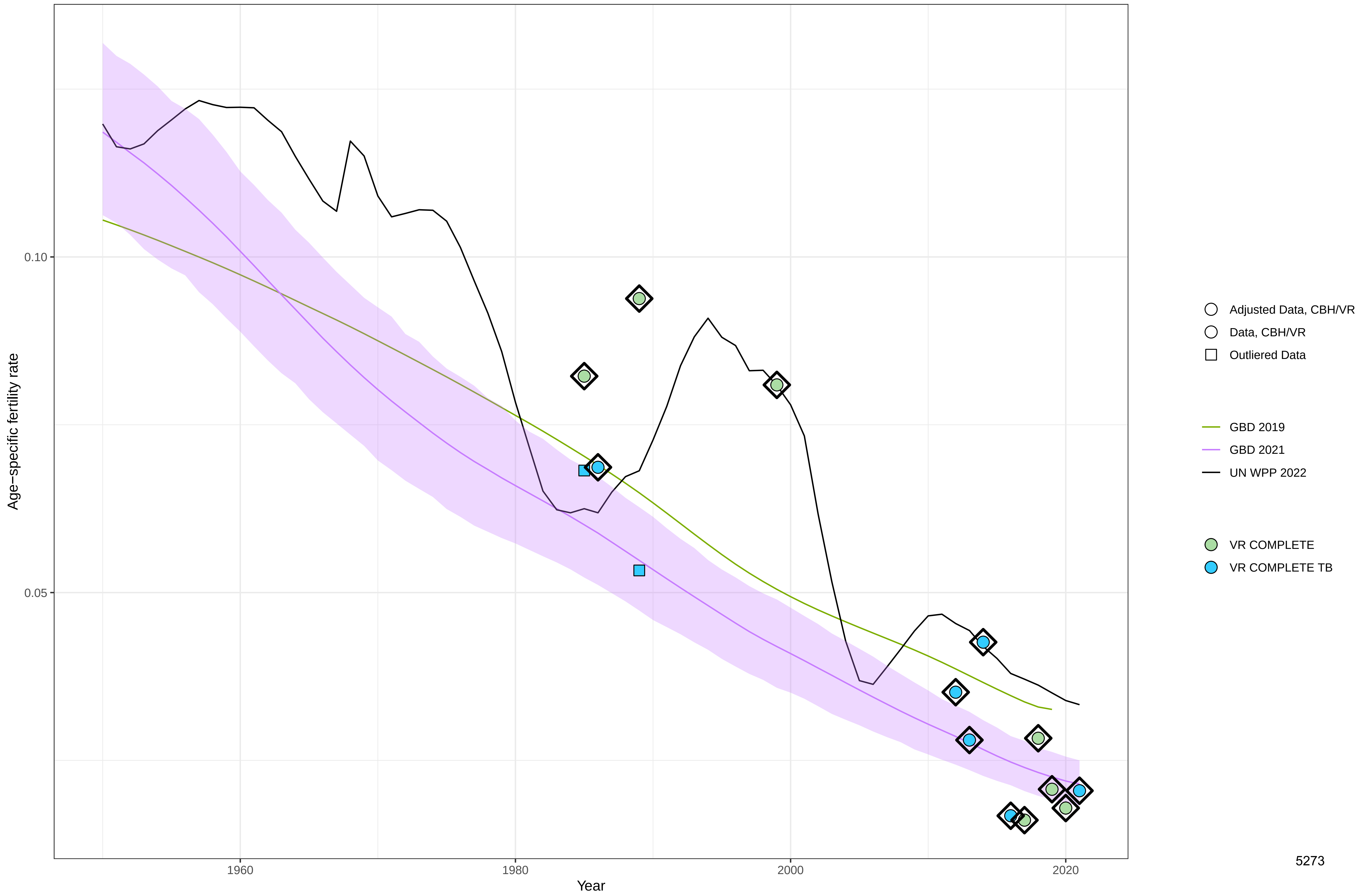

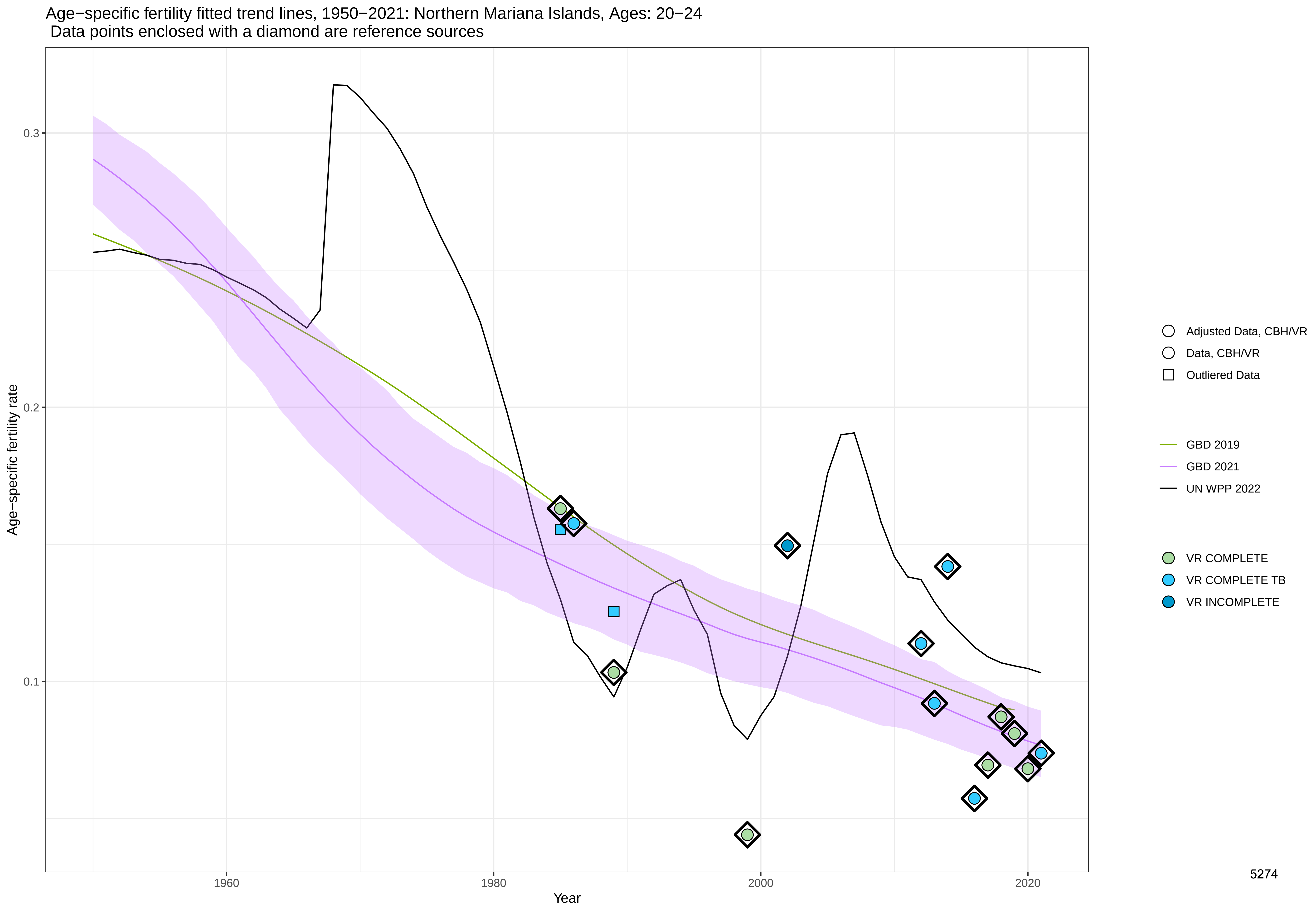
Age−specific fertility fitted trend lines, 1950−2021: Northern Mariana Islands, Ages: 20−24
Data points enclosed with a diamond are reference sources
Age−specific fertility rate
Year
0.3
0.2
0.1
1960
1980
2000
2020
Adjusted Data, CBH/VR
Data, CBH/VR
Outliered Data
GBD 2019
GBD 2021
UN WPP 2022
VR COMPLETE
VR COMPLETE TB
VR INCOMPLETE

Age−specific fertility fitted trend lines, 1950−2021: Northern Mariana Islands, Ages: 25−29
Data points enclosed with a diamond are reference sources

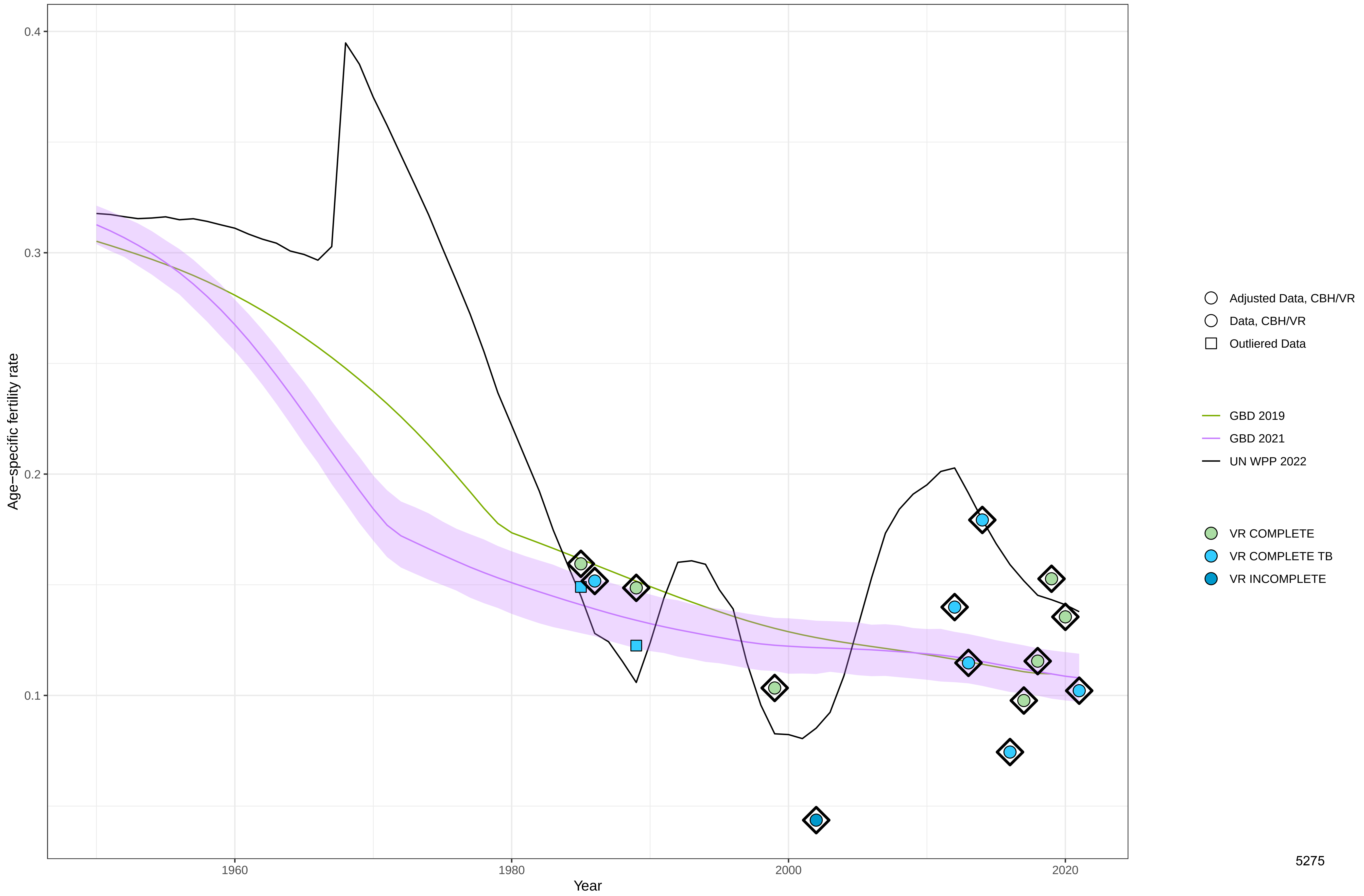

Age−specific fertility fitted trend lines, 1950−2021: Northern Mariana Islands, Ages: 30−34
Data points enclosed with a diamond are reference sources

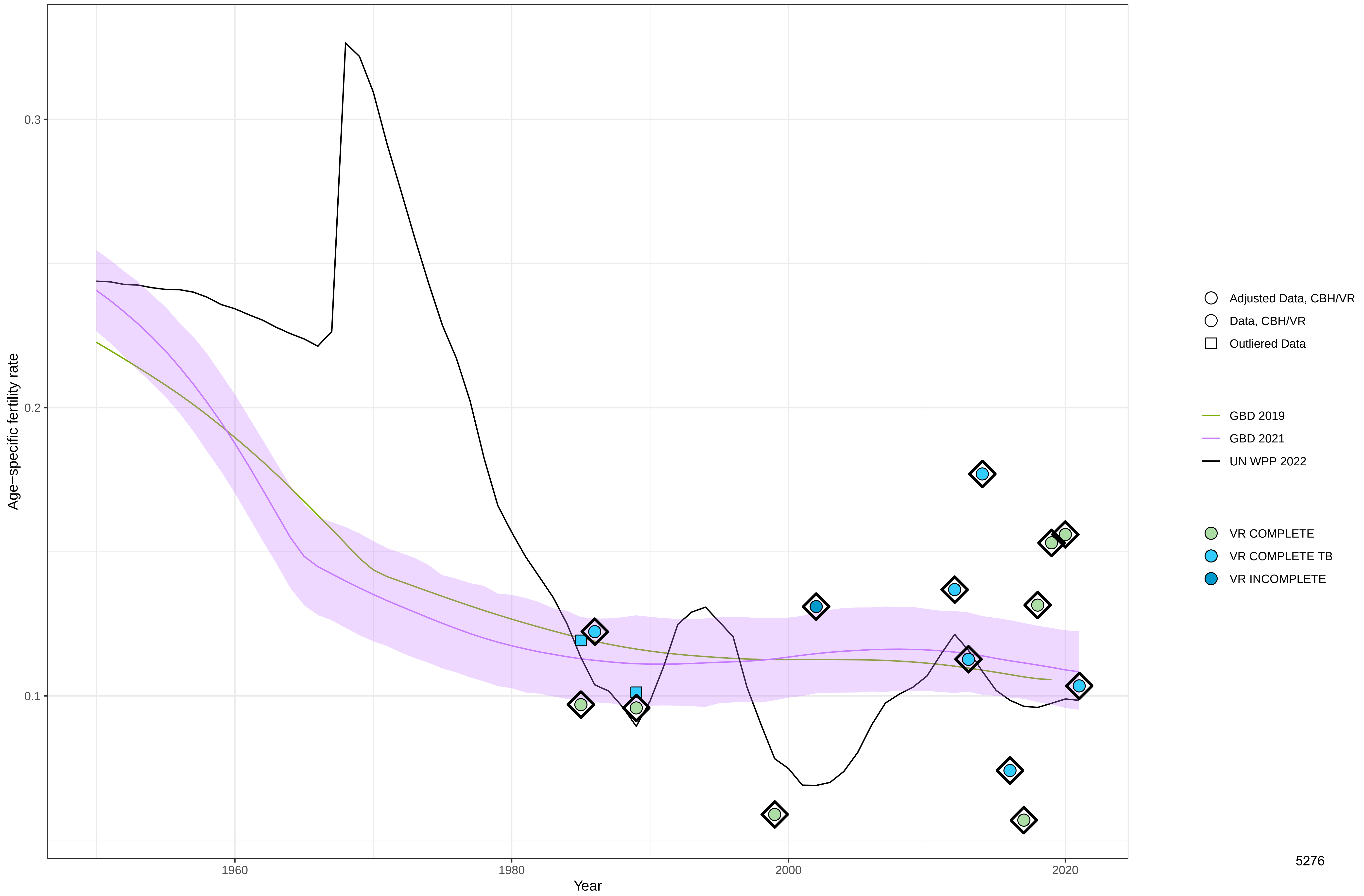

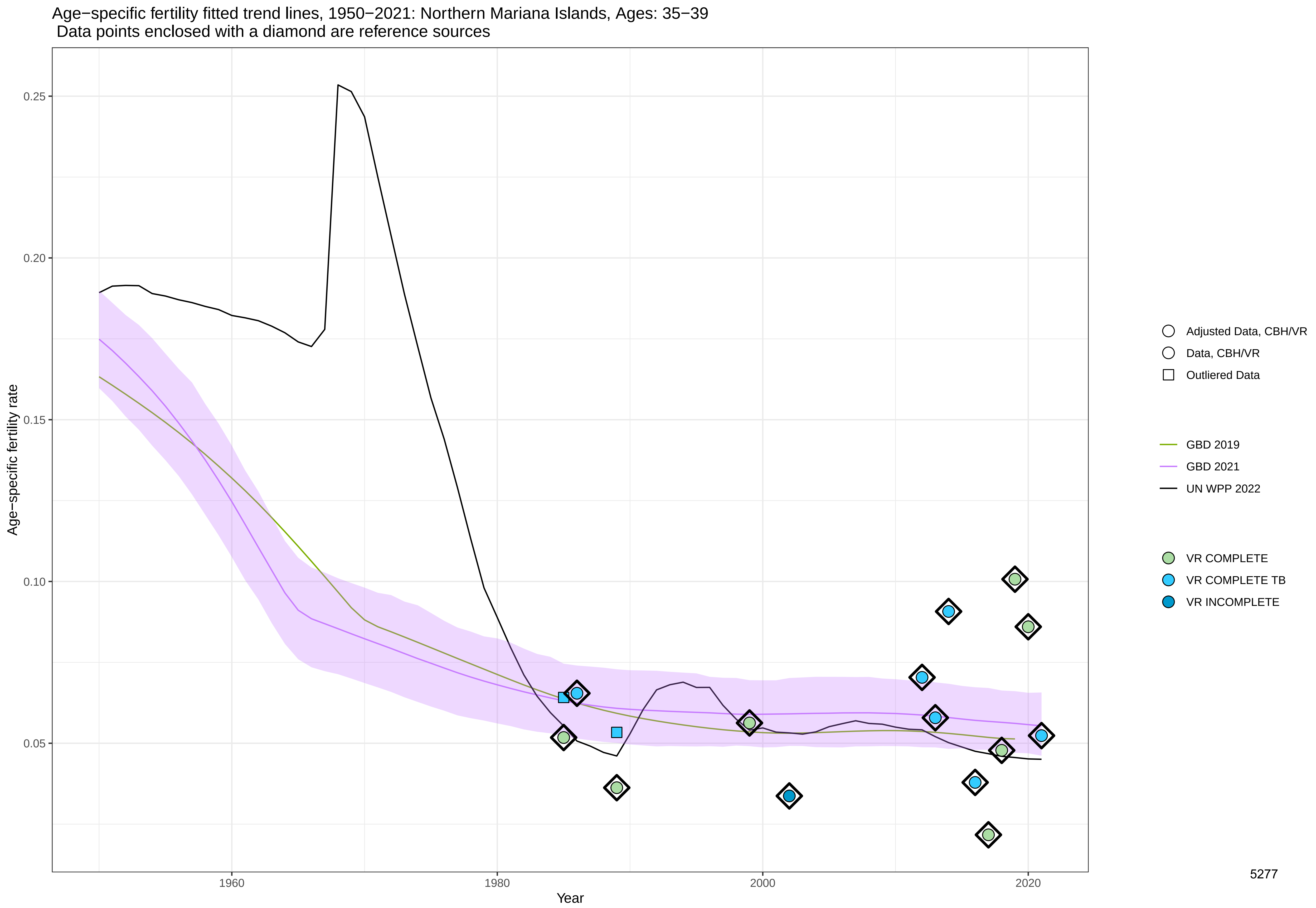

Age−specific fertility fitted trend lines, 1950−2021: Northern Mariana Islands, Ages: 35−39
Data points enclosed with a diamond are reference sources
Age−specific fertility rate
0.25
0.20
0.15
0.10
0.05
1960
1980
2000
2020
Year
Adjusted Data, CBH/VR
Data, CBH/VR
Outliered Data
GBD 2019
GBD 2021
UN WPP 2022
VR COMPLETE
VR COMPLETE TB
VR INCOMPLETE

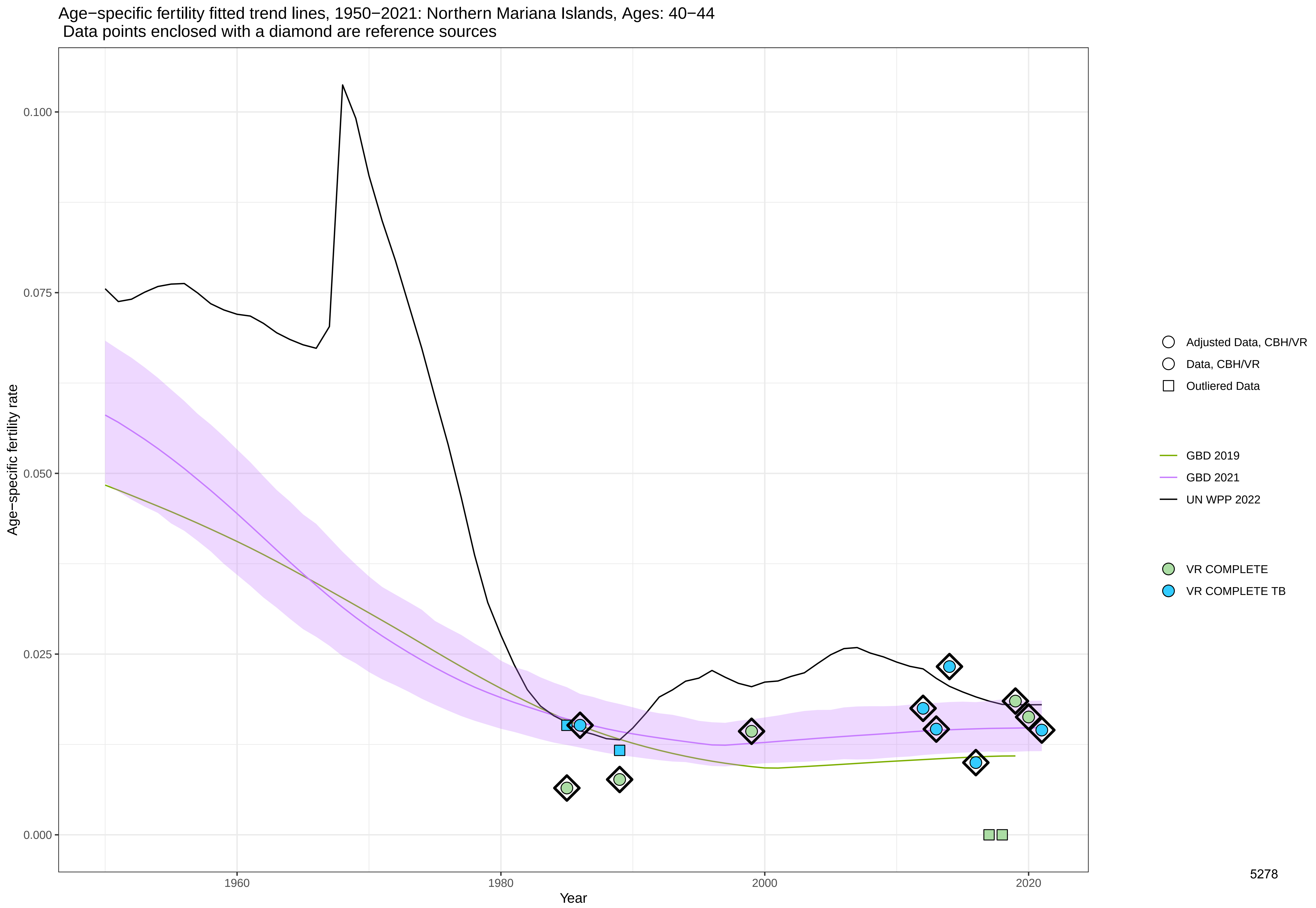

Age−specific fertility fitted trend lines, 1950−2021: Northern Mariana Islands, Ages: 40−44
Data points enclosed with a diamond are reference sources
Age−specific fertility rate
0.100
0.075
0.050
0.025
0.000
1960
1980
2000
2020
Year
Adjusted Data, CBH/VR
Data, CBH/VR
Outliered Data
GBD 2019
GBD 2021
UN WPP 2022
VR COMPLETE
VR COMPLETE TB

5278

Age−specific fertility fitted trend lines, 1950−2021: Northern Mariana Islands, Ages: 45−49
Data points enclosed with a diamond are reference sources

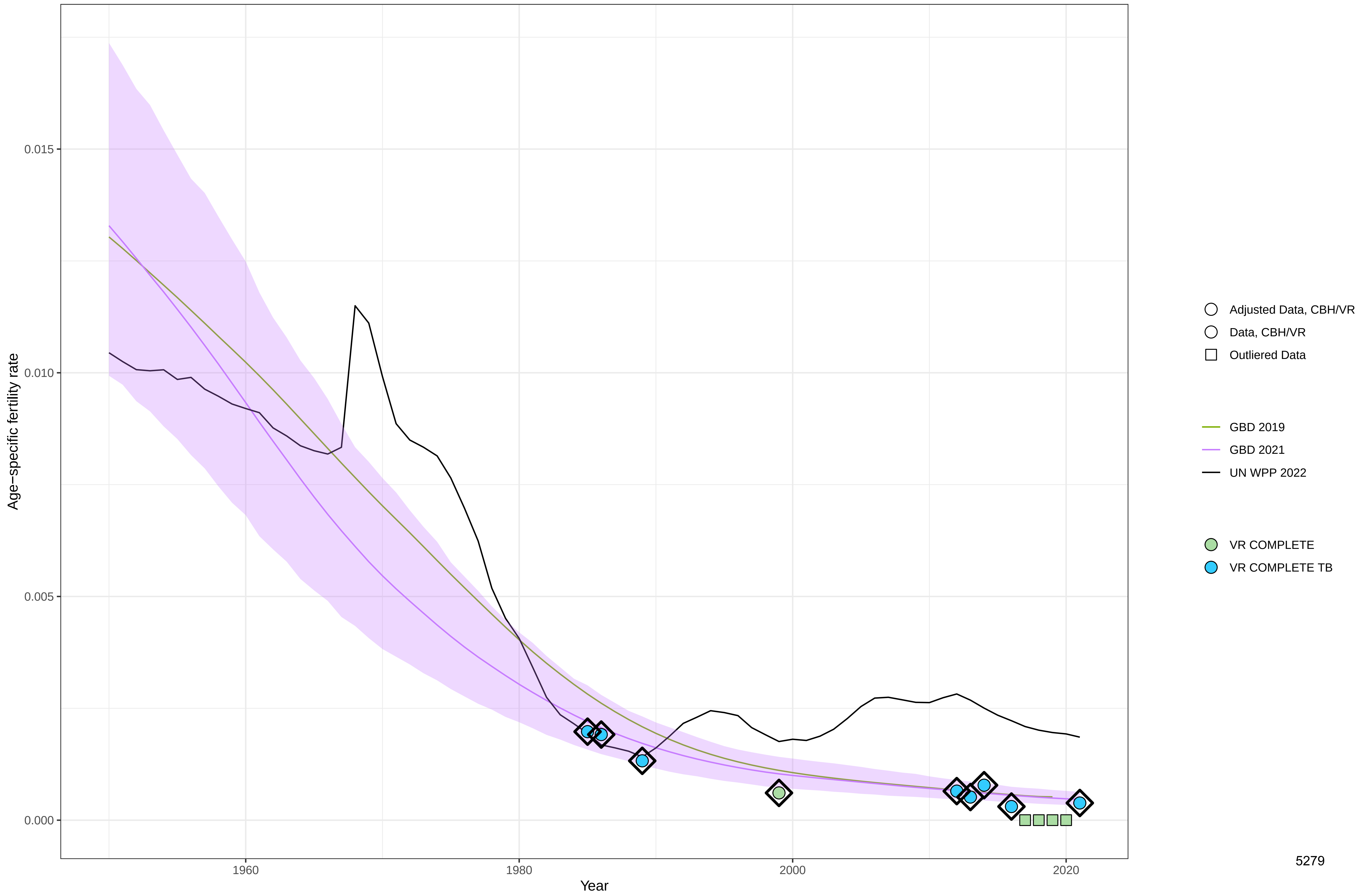

Age−specific fertility fitted trend lines, 1950−2021: Northern Mariana Islands, Ages: 50−54
Data points enclosed with a diamond are reference sources

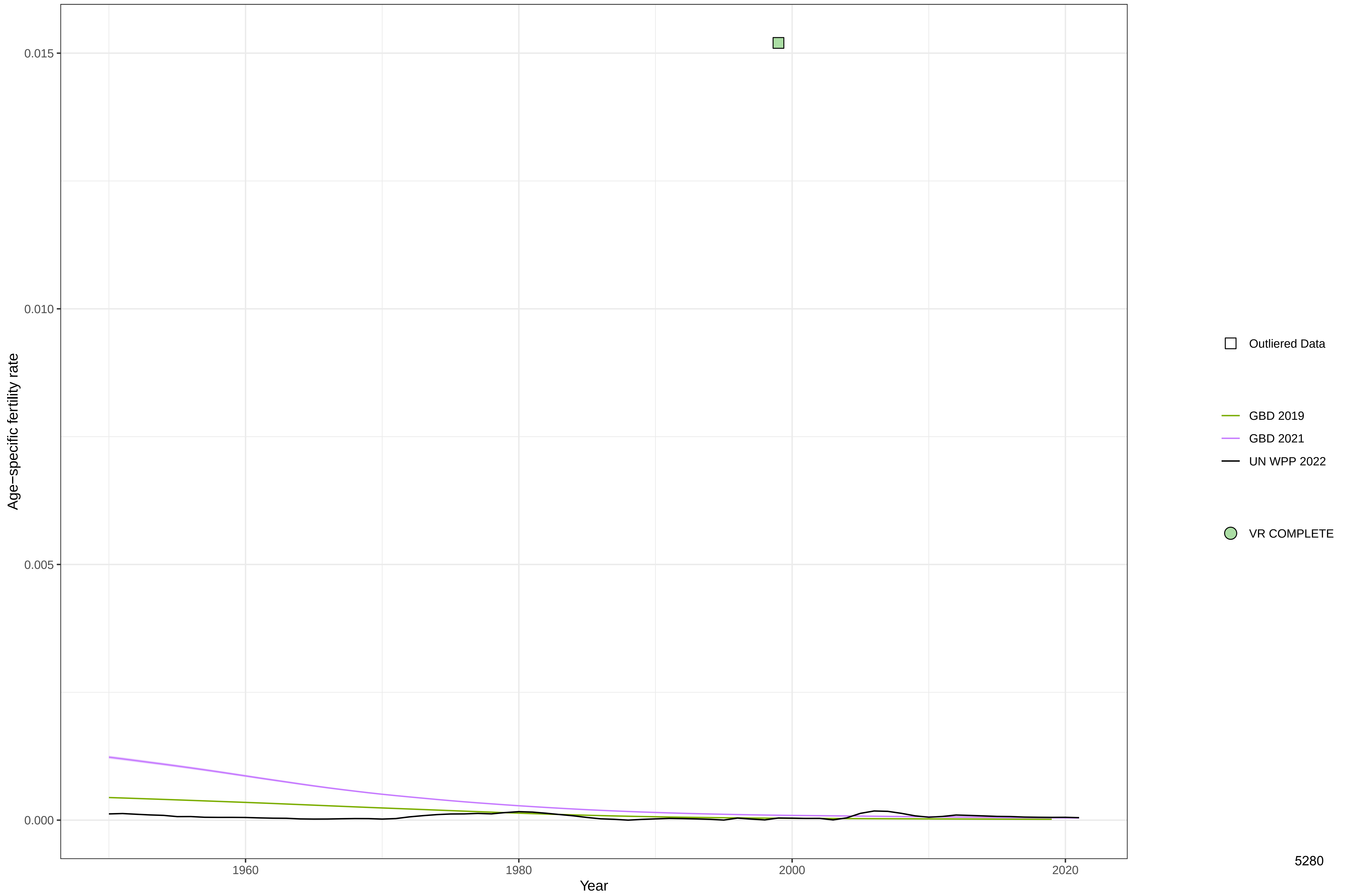

Total fertility rate fitted trend lines, 1950−2021: Northern Mariana Islands TFR
Data points enclosed with a diamond are reference sources

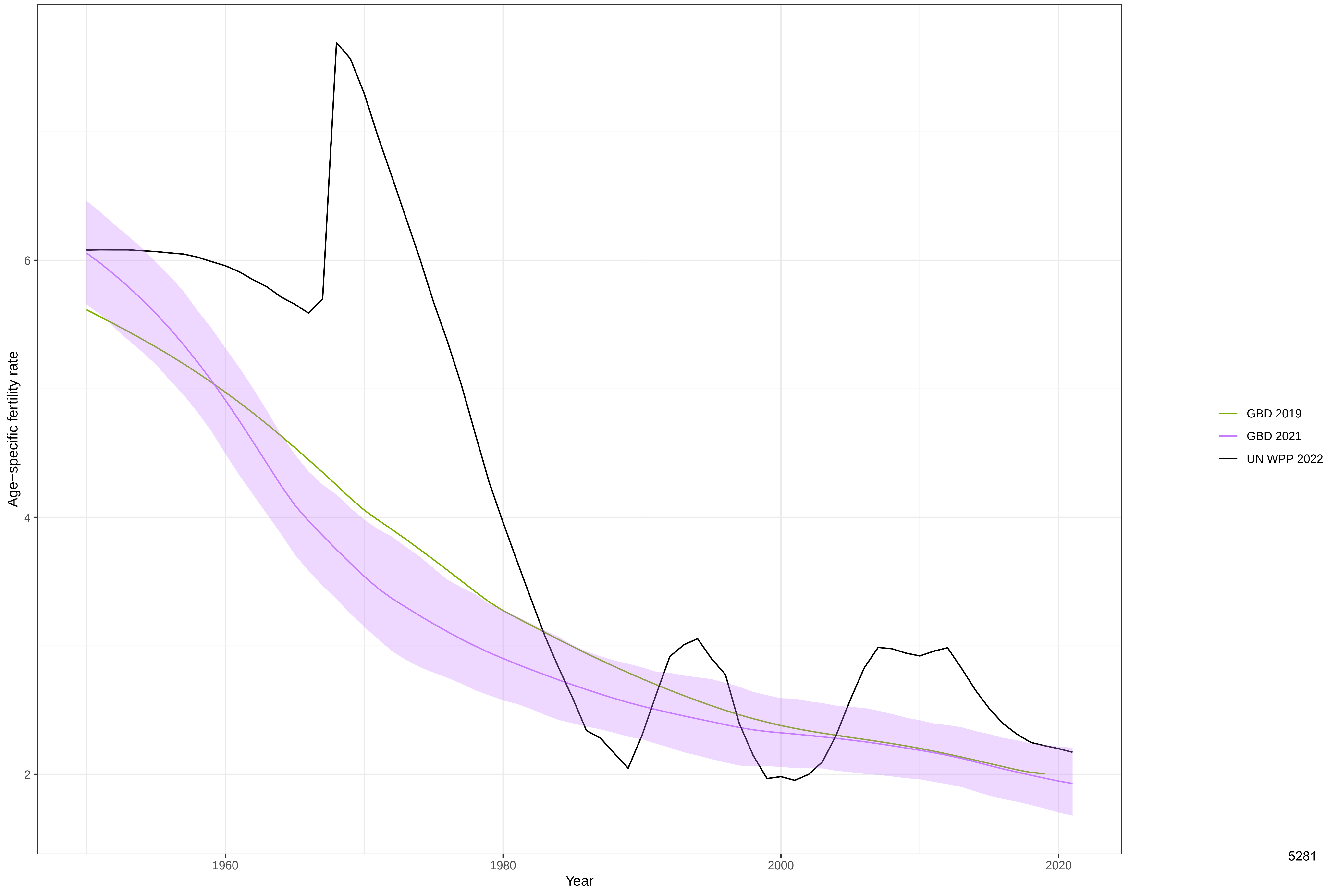

Age−specific fertility fitted trend lines, 1950−2021: Palau, Ages: 10−14
Data points enclosed with a diamond are reference sources

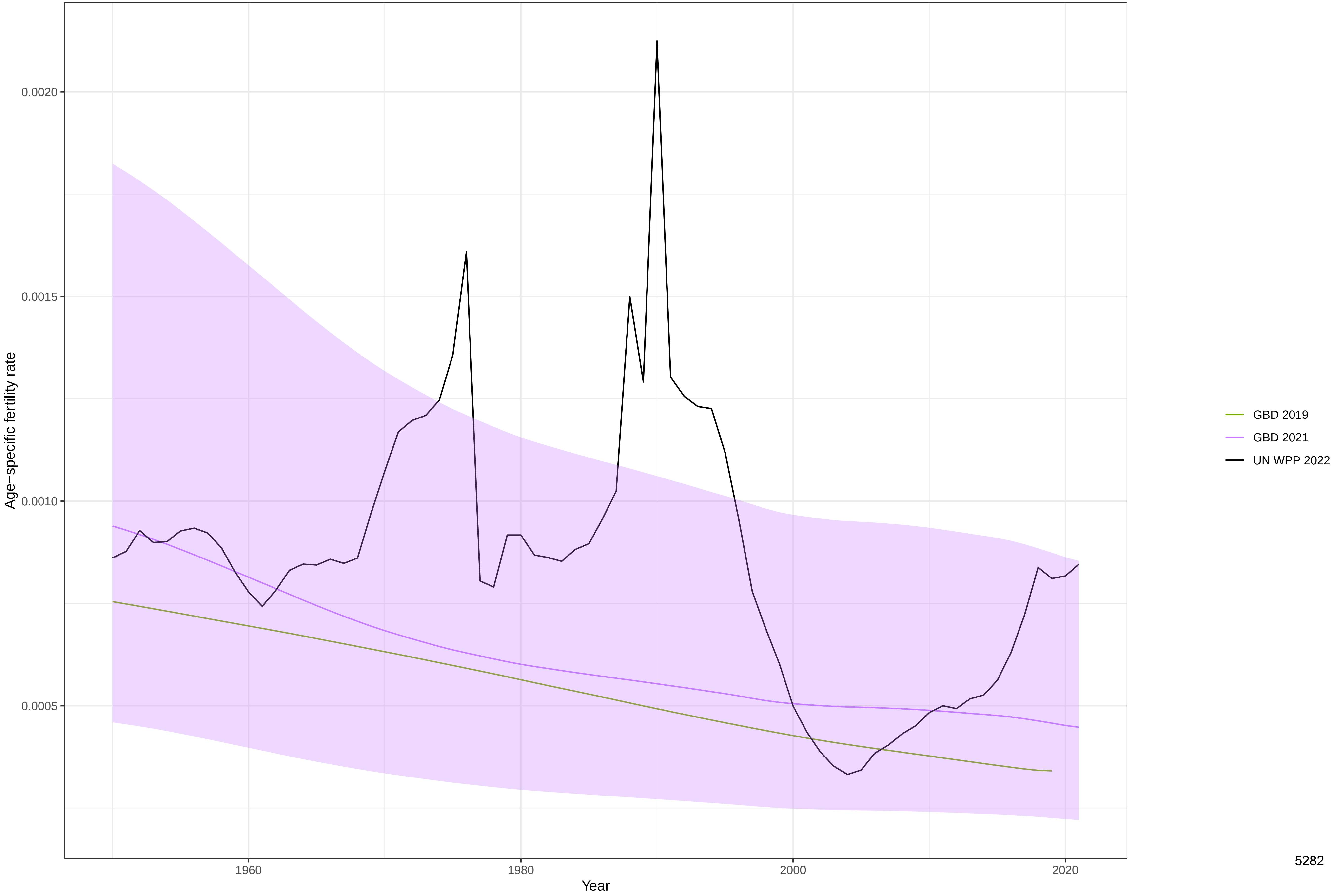

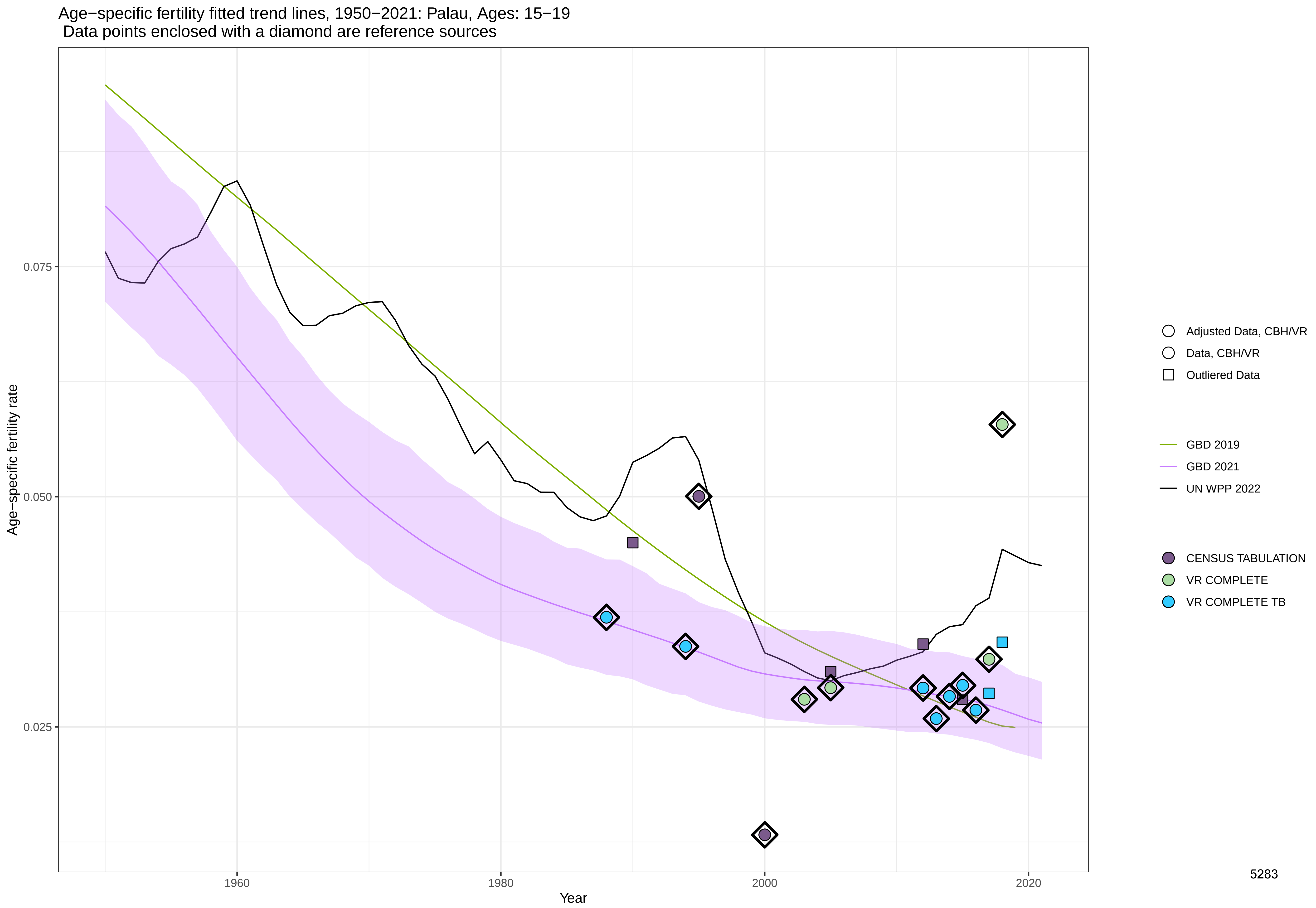

Age−specific fertility fitted trend lines, 1950−2021: Palau, Ages: 15−19
Data points enclosed with a diamond are reference sources
Age−specific fertility rate
0.075
0.050
0.025
1960
1980
2000
2020
Year
Adjusted Data, CBH/VR
Data, CBH/VR
Outliered Data
GBD 2019
GBD 2021
UN WPP 2022
CENSUS TABULATION
VR COMPLETE
VR COMPLETE TB

Age−specific fertility fitted trend lines, 1950−2021: Palau, Ages: 20−24
Data points enclosed with a diamond are reference sources

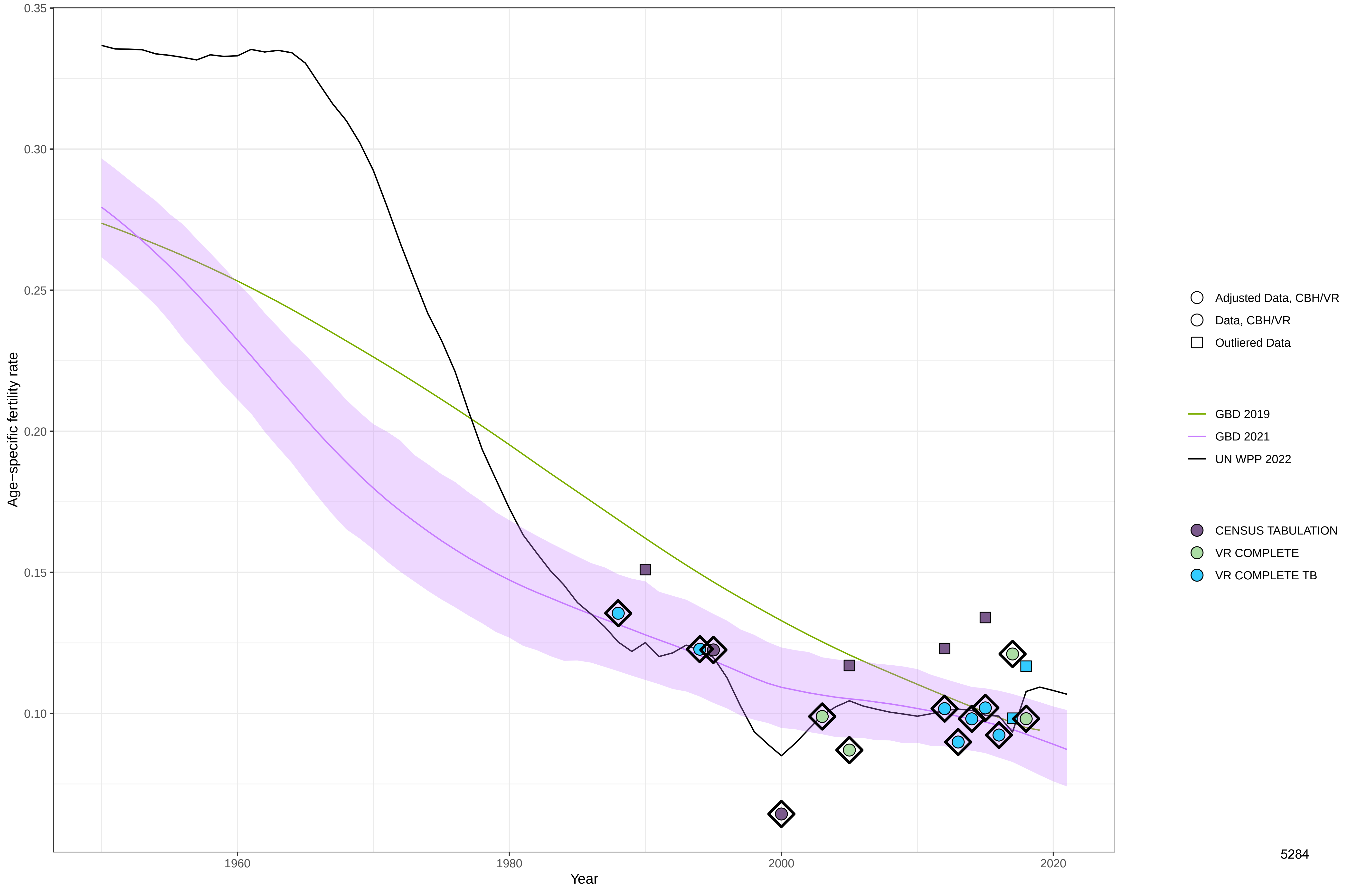

Age−specific fertility fitted trend lines, 1950−2021: Palau, Ages: 25−29
Data points enclosed with a diamond are reference sources

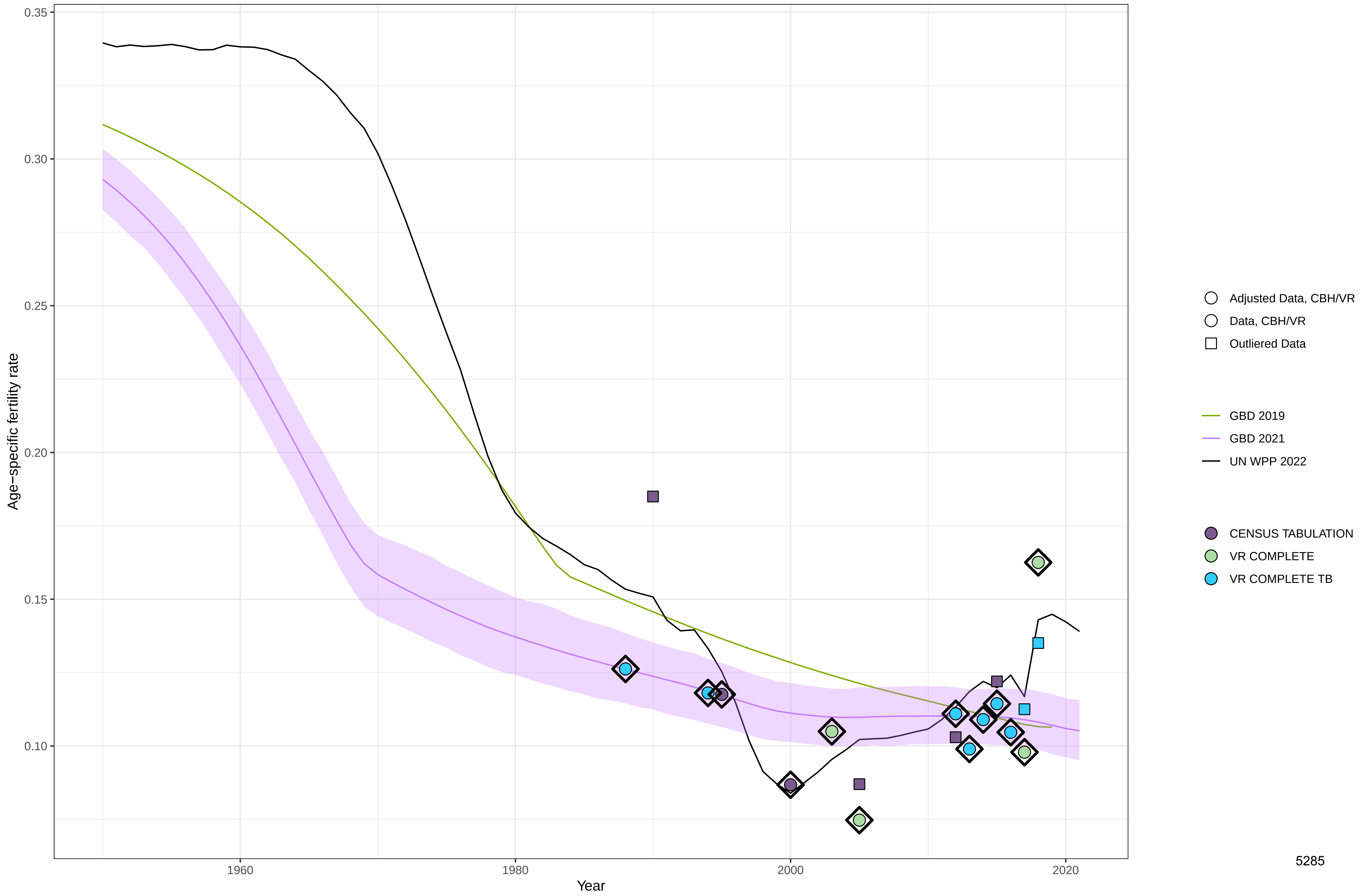

Age−specific fertility fitted trend lines, 1950−2021: Palau, Ages: 30−34
Data points enclosed with a diamond are reference sources

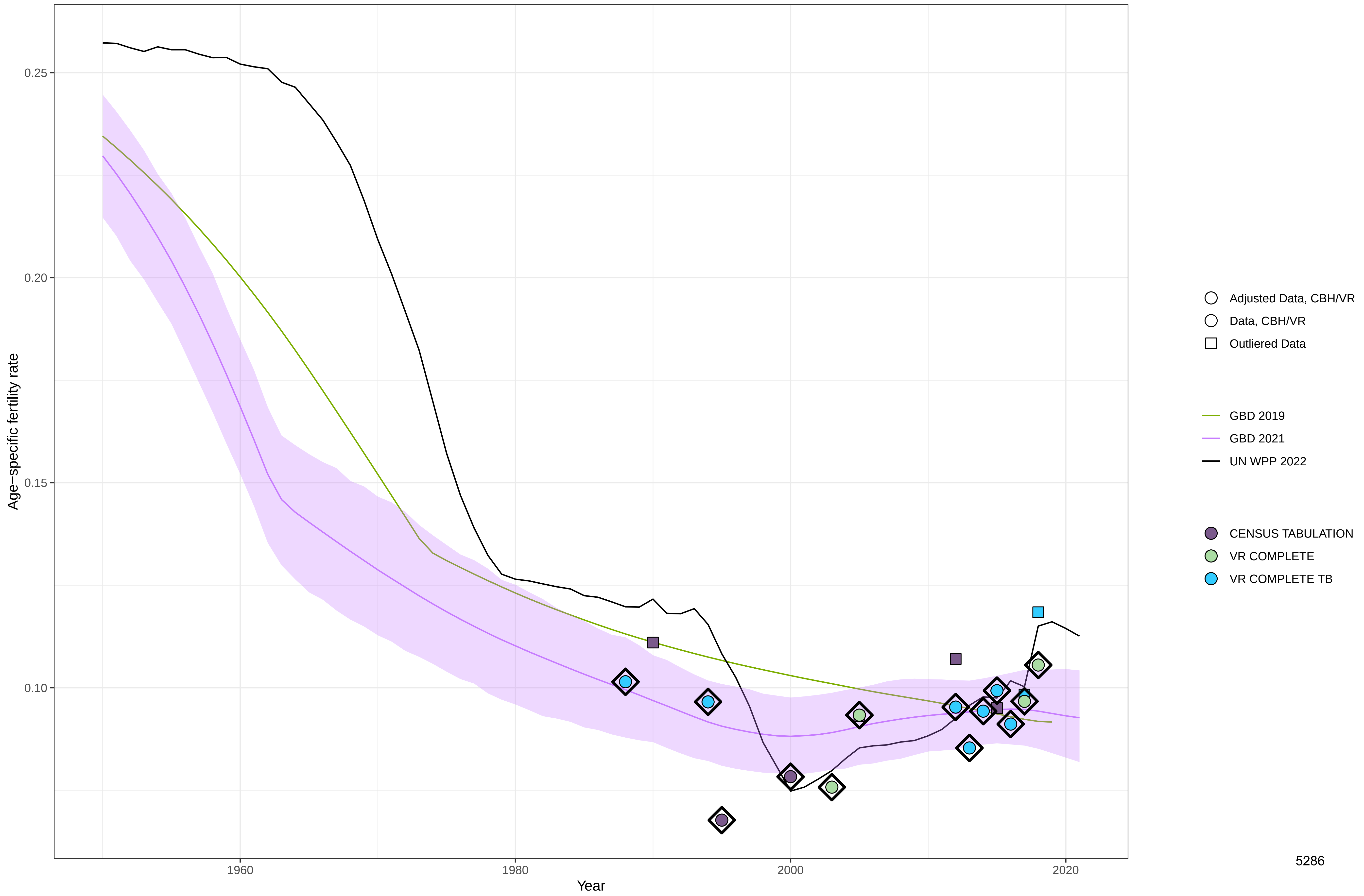

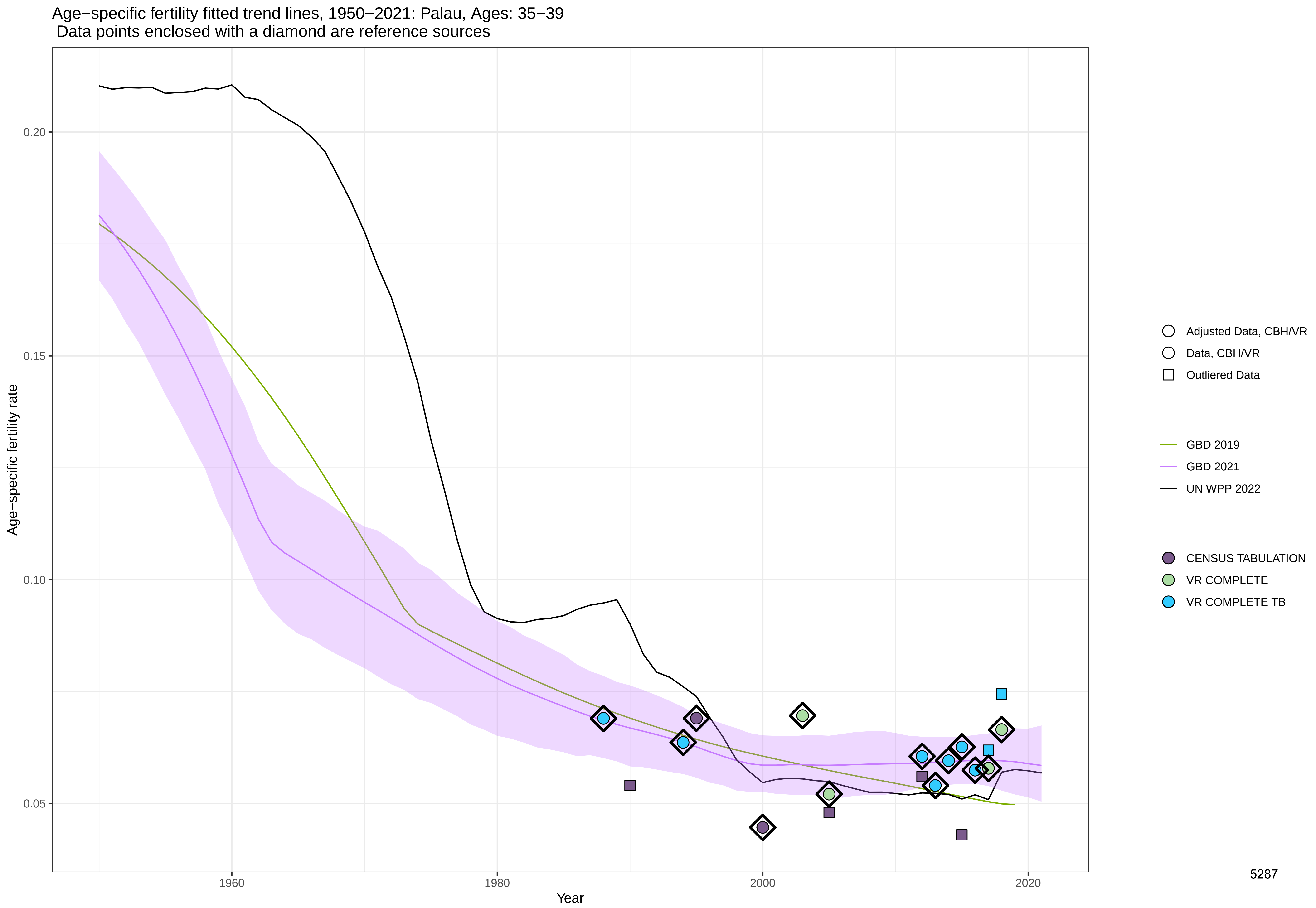
Age−specific fertility fitted trend lines, 1950−2021: Palau, Ages: 35−39
Data points enclosed with a diamond are reference sources
Age−specific fertility rate
0.20
0.15
0.10
0.05
1960
1980
2000
2020
Year
Adjusted Data, CBH/VR
Data, CBH/VR
Outliered Data
GBD 2019
GBD 2021
UN WPP 2022
CENSUS TABULATION
VR COMPLETE
VR COMPLETE TB

Age−specific fertility fitted trend lines, 1950−2021: Palau, Ages: 40−44
Data points enclosed with a diamond are reference sources

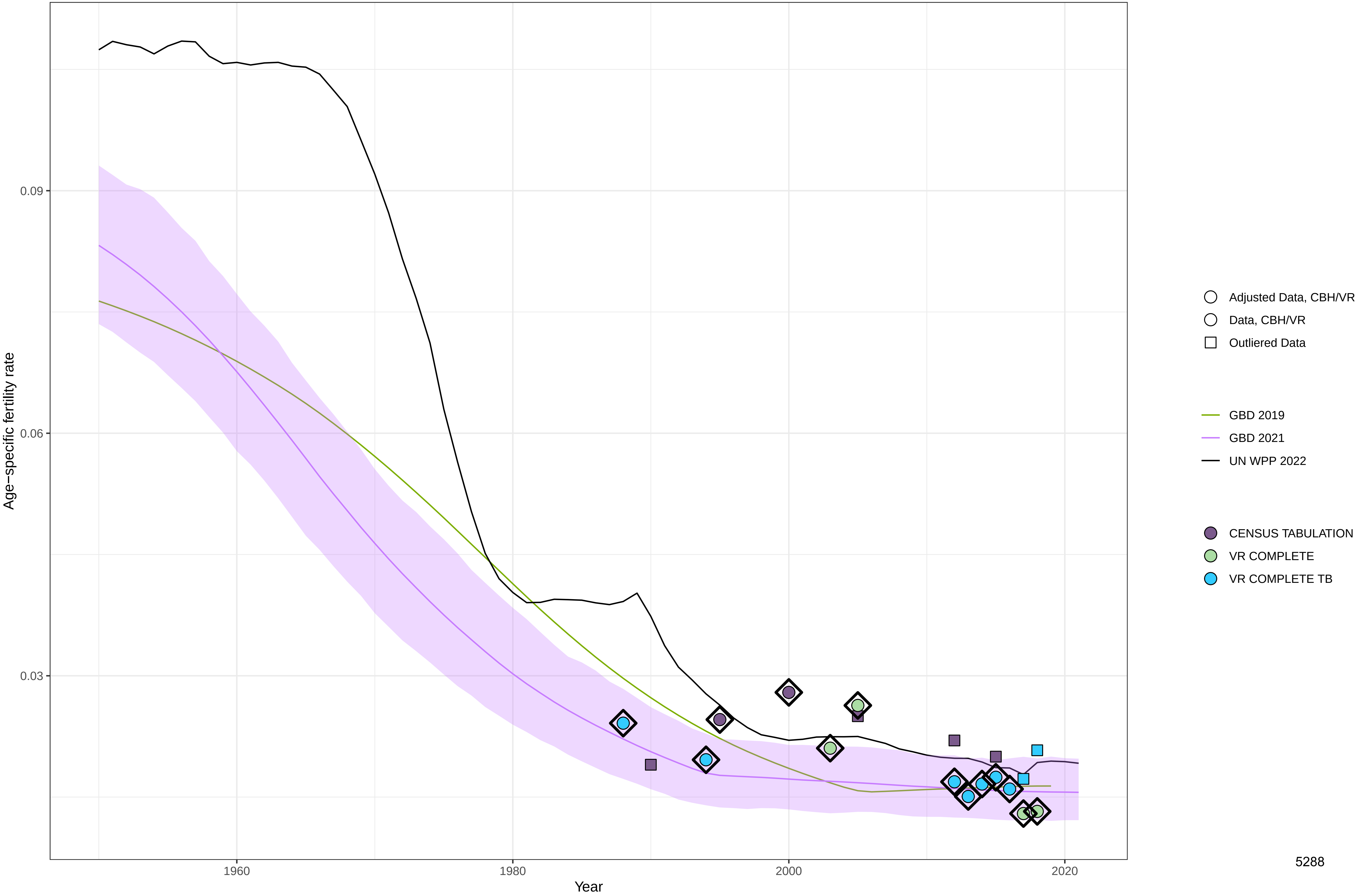

Age−specific fertility fitted trend lines, 1950−2021: Palau, Ages: 45−49
Data points enclosed with a diamond are reference sources

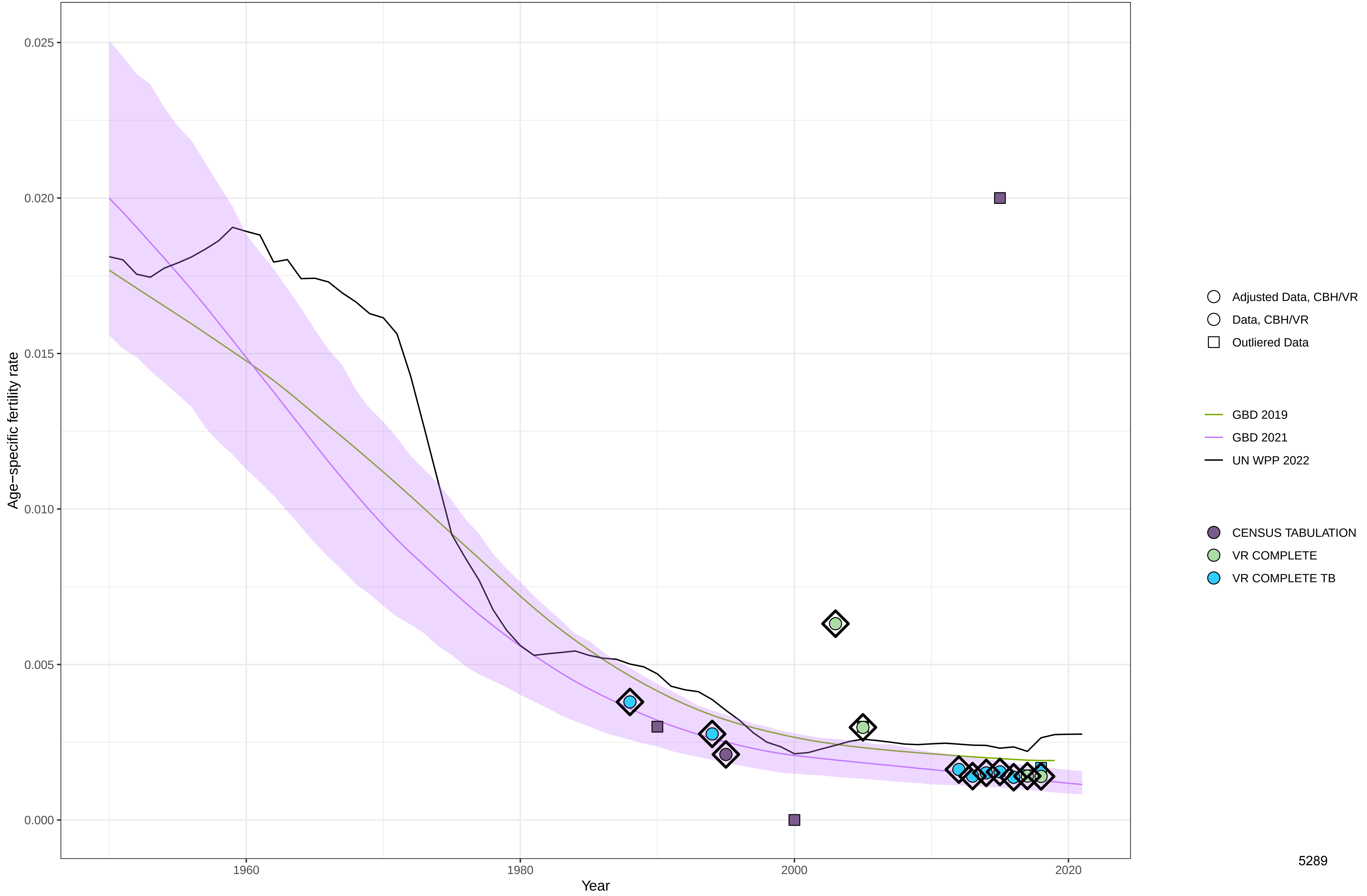

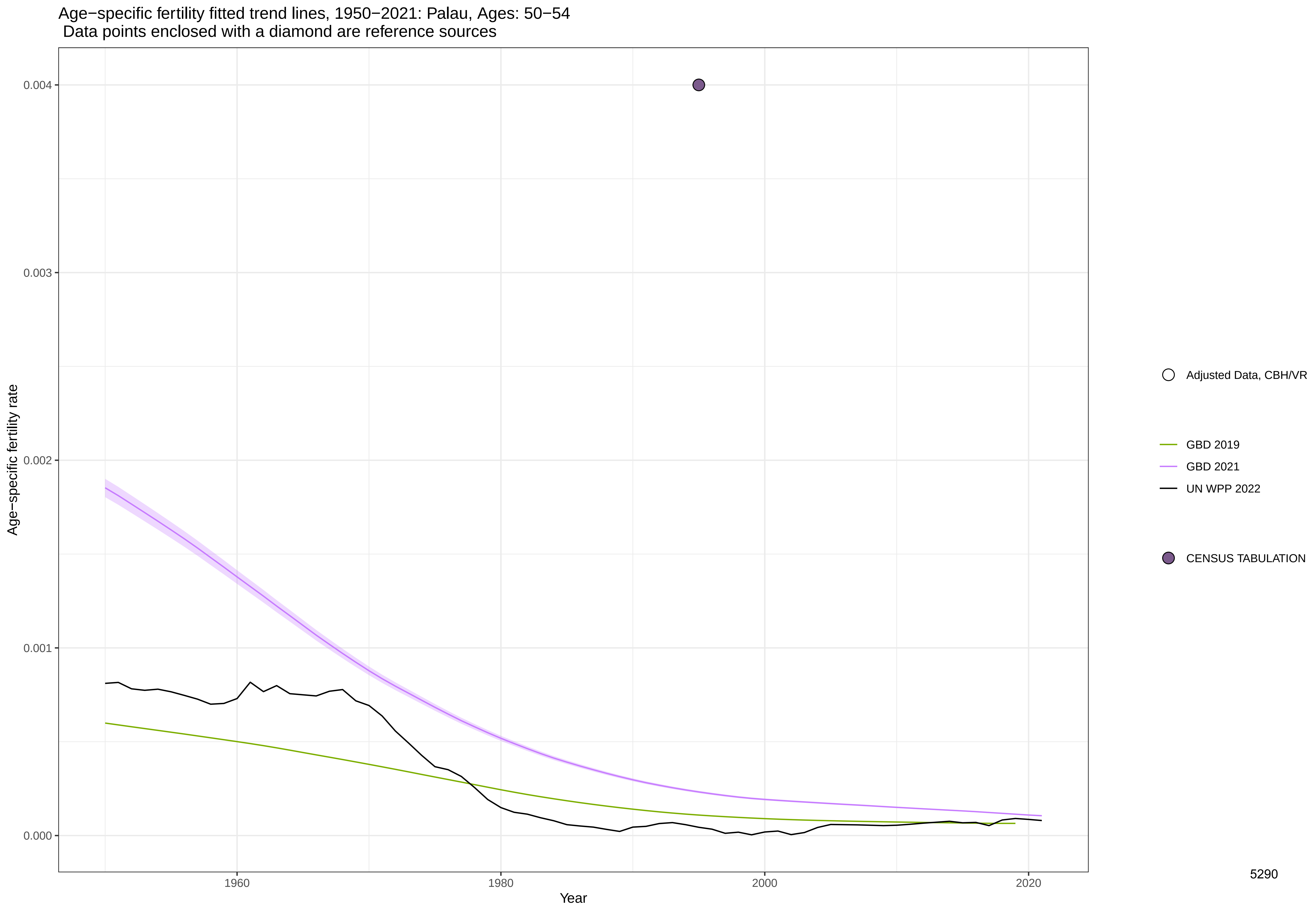
Age−specific fertility fitted trend lines, 1950−2021: Palau, Ages: 50−54
Data points enclosed with a diamond are reference sources
0.004
0.003
0.002
0.001
0.000
Age−specific fertility rate
1960
1980
2000
2020
Year
Adjusted Data, CBH/VR
GBD 2019
GBD 2021
UN WPP 2022
CENSUS TABULATION

Total fertility rate fitted trend lines, 1950−2021: Palau TFR
Data points enclosed with a diamond are reference sources

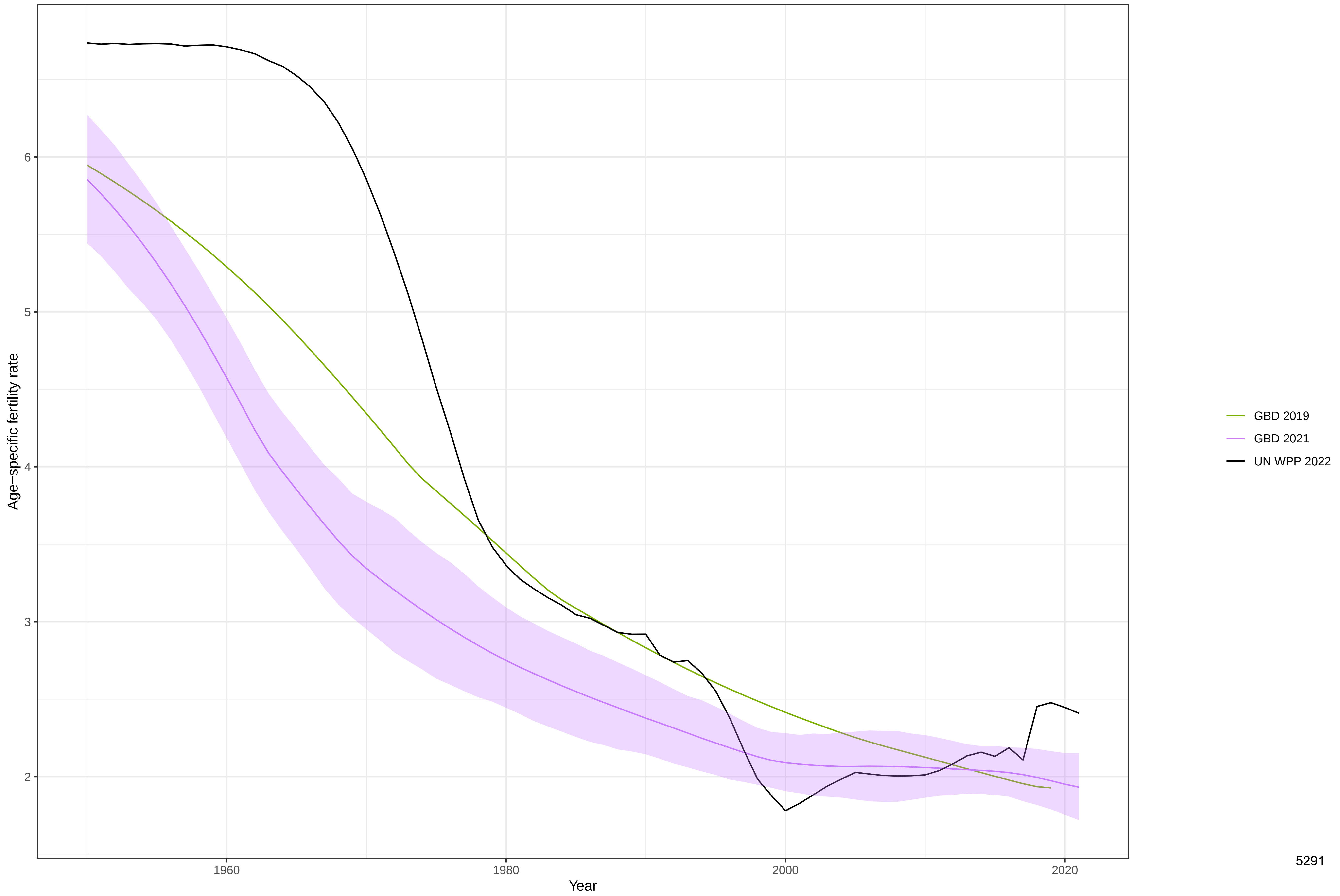

Age−specific fertility fitted trend lines, 1950−2021: Papua New Guinea, Ages: 10−14
Data points enclosed with a diamond are reference sources

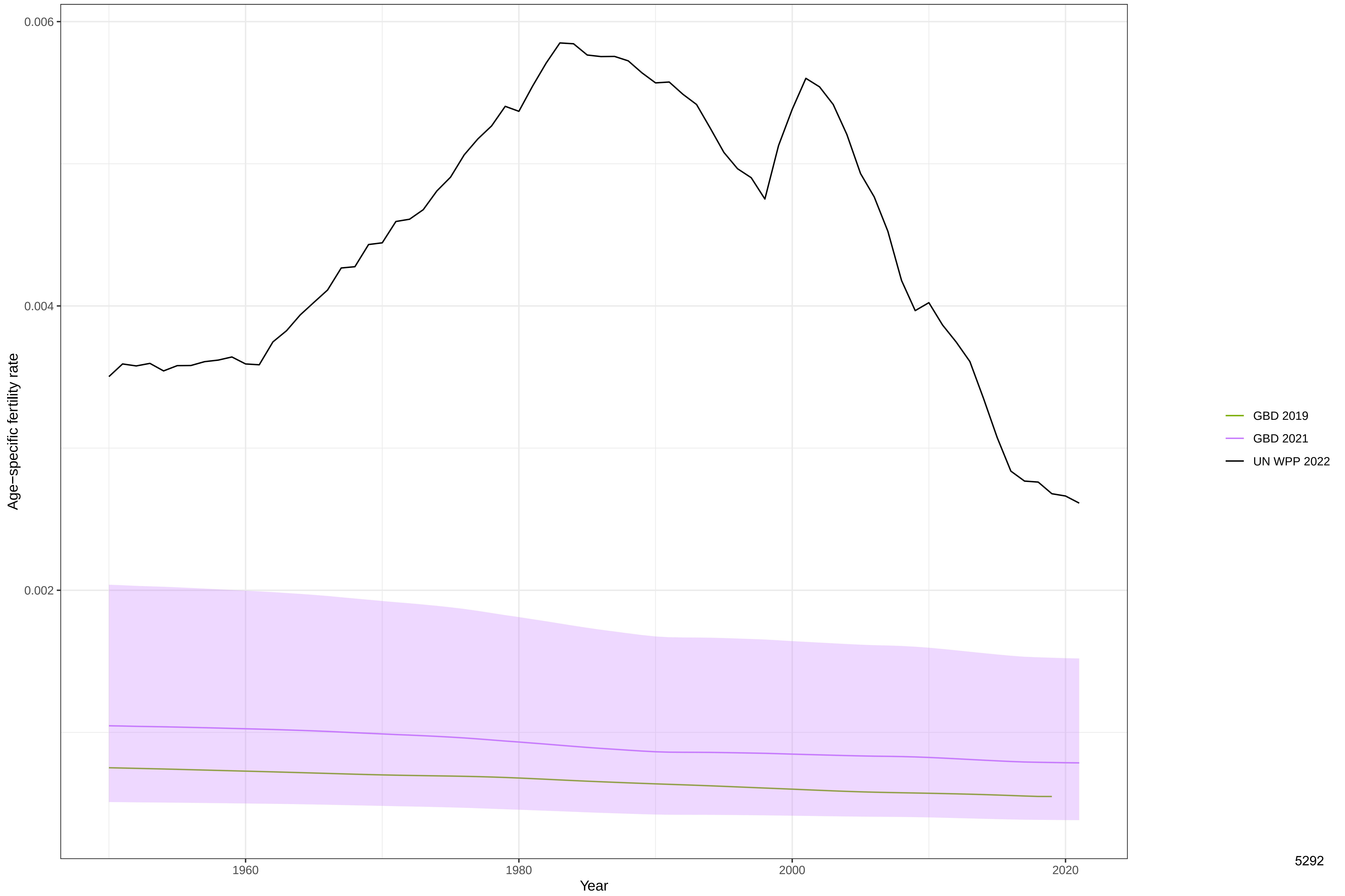

Age−specific fertility fitted trend lines, 1950−2021: Papua New Guinea, Ages: 15−19
Data points enclosed with a diamond are reference sources

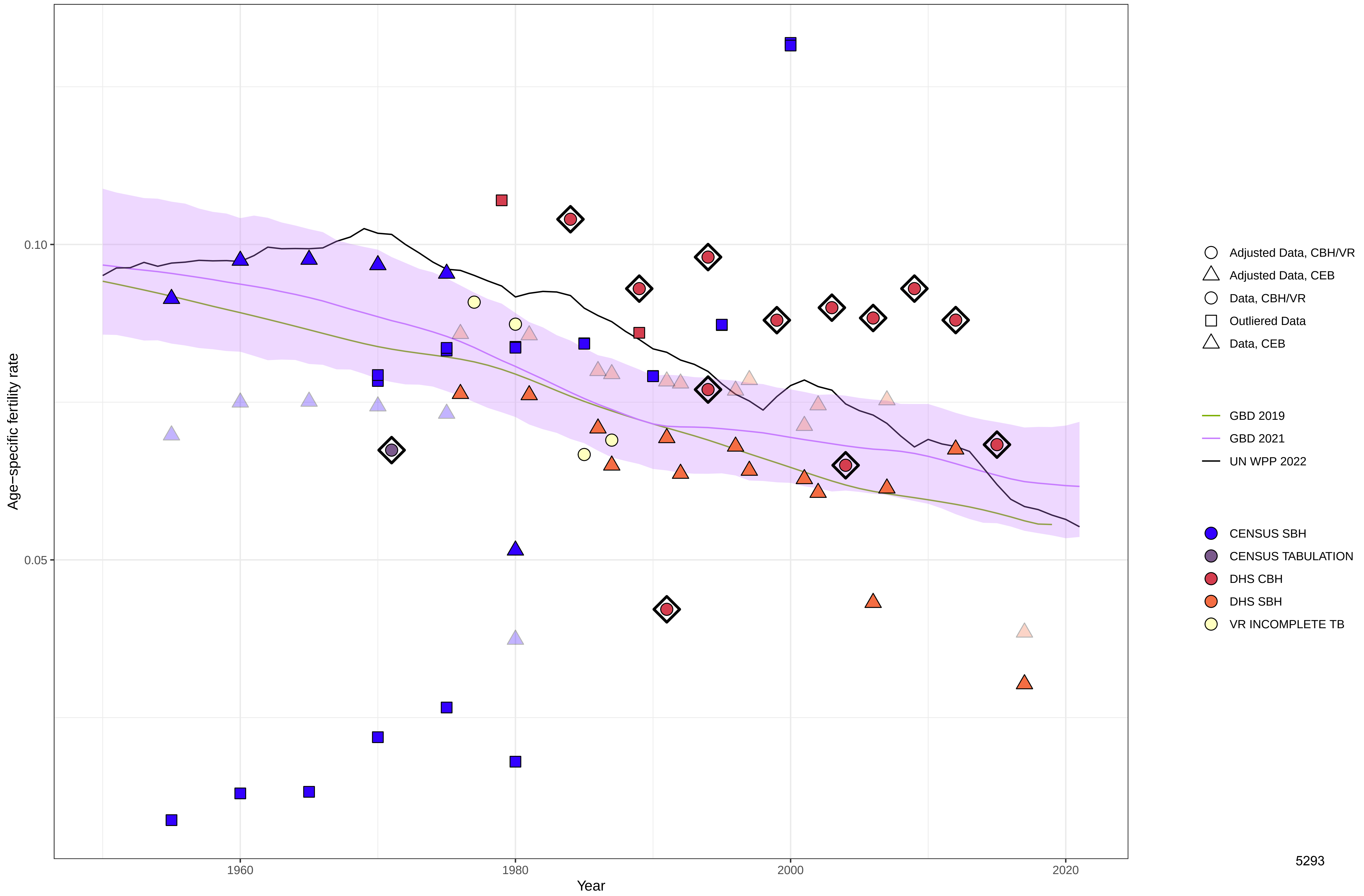

Age−specific fertility fitted trend lines, 1950−2021: Papua New Guinea, Ages: 20−24
Data points enclosed with a diamond are reference sources

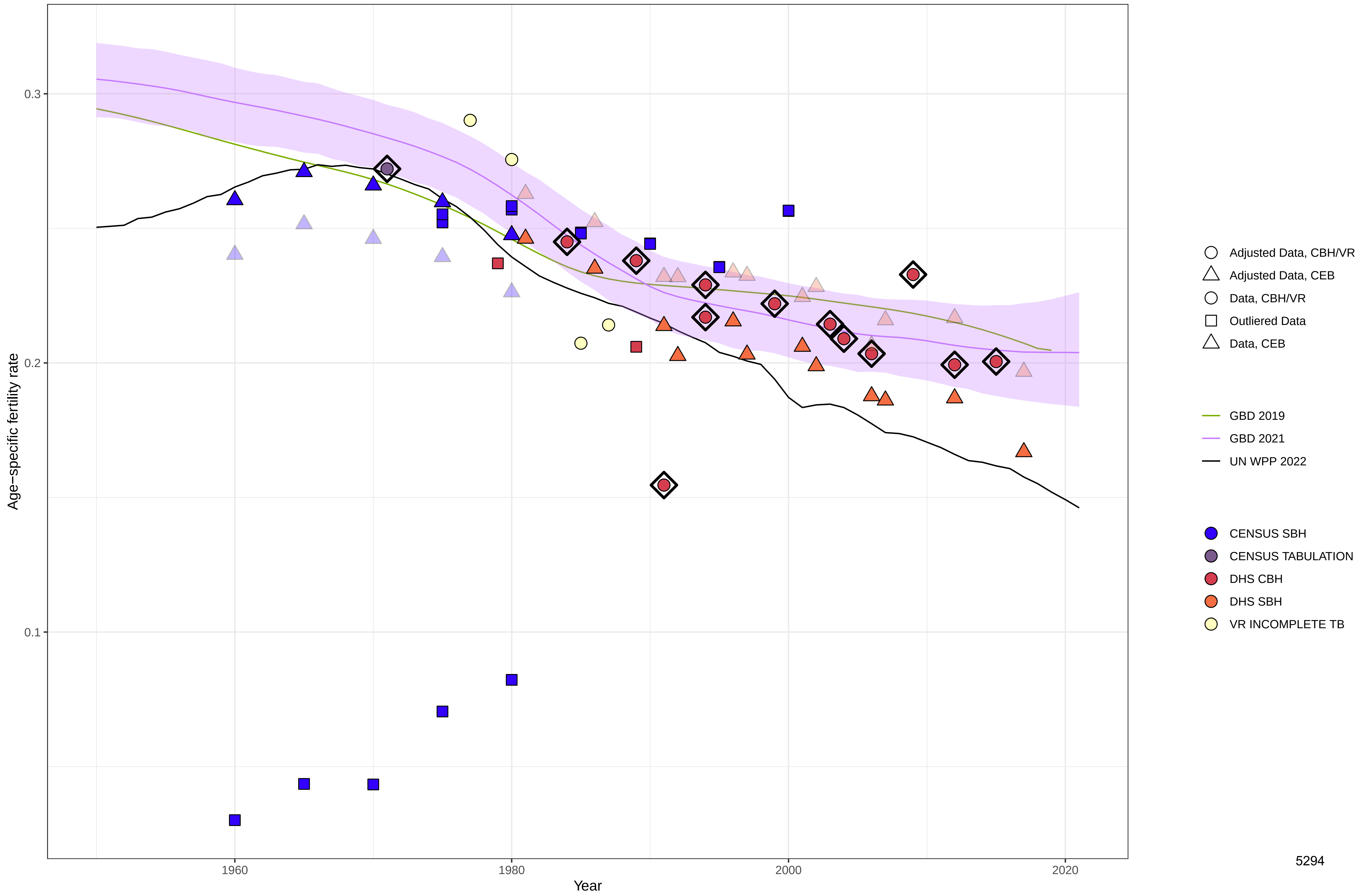

Age−specific fertility fitted trend lines, 1950−2021: Papua New Guinea, Ages: 25−29
Data points enclosed with a diamond are reference sources

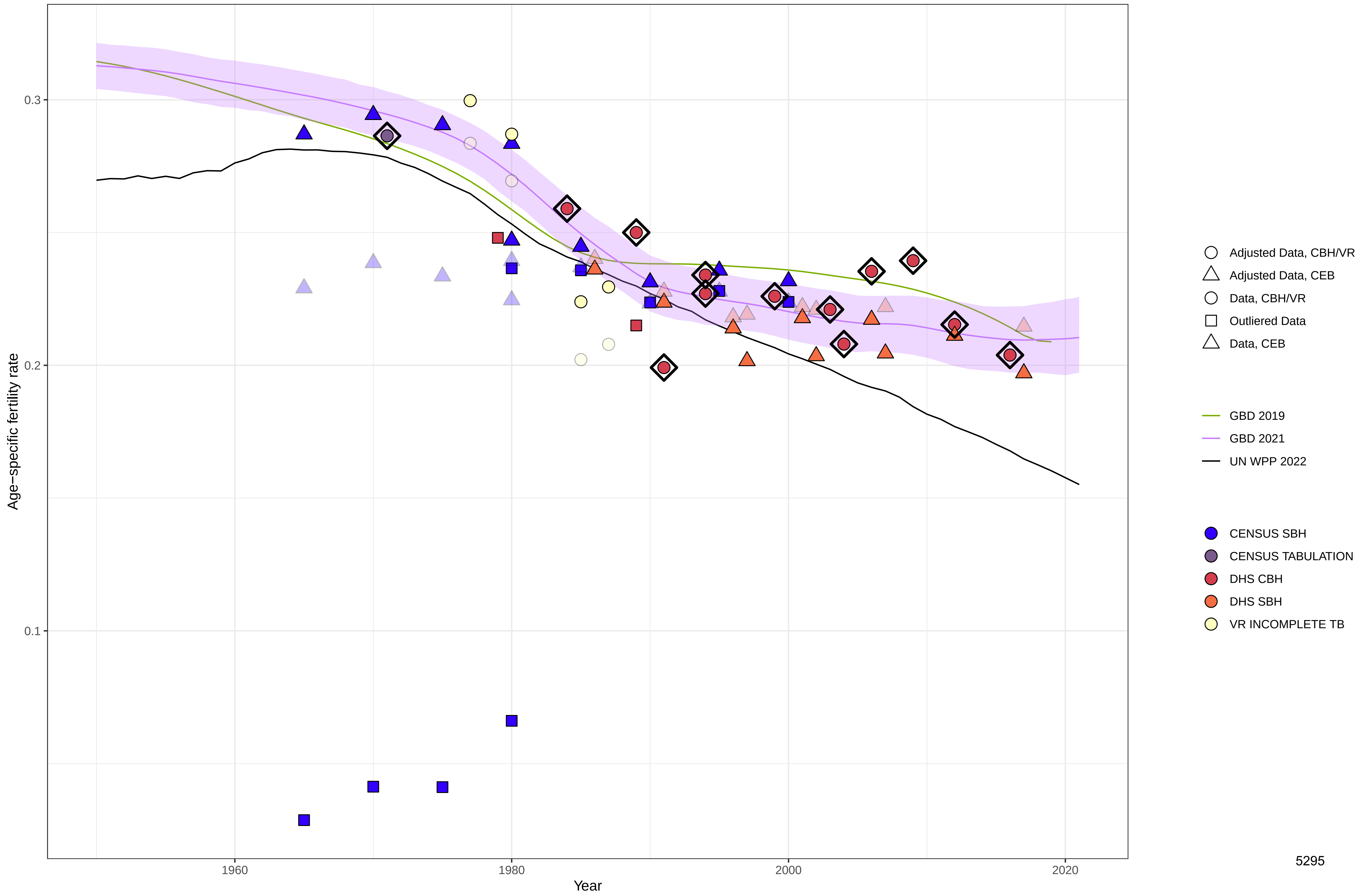

Age−specific fertility fitted trend lines, 1950−2021: Papua New Guinea, Ages: 30−34
Data points enclosed with a diamond are reference sources

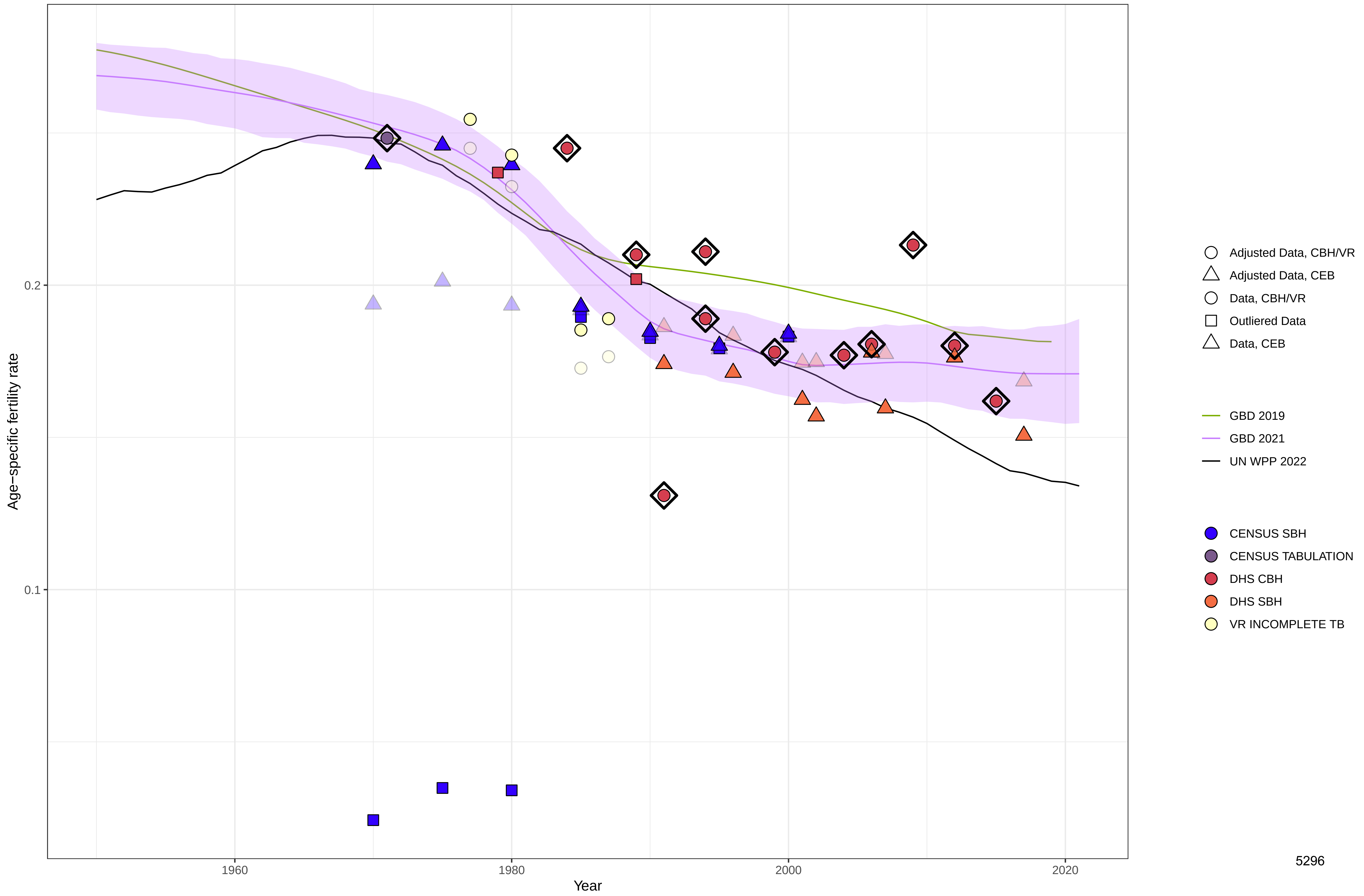

Age−specific fertility fitted trend lines, 1950−2021: Papua New Guinea, Ages: 35−39
Data points enclosed with a diamond are reference sources

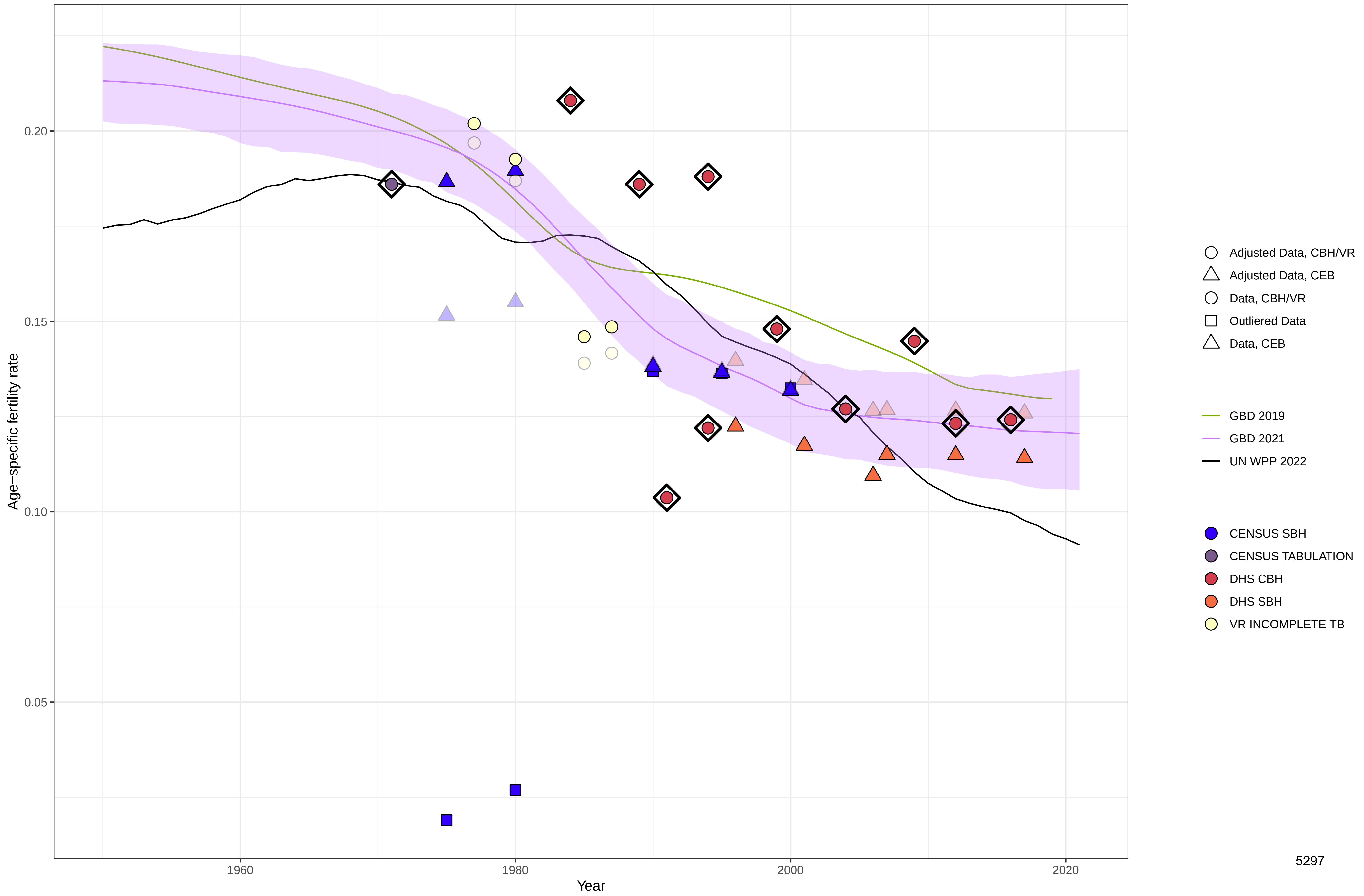

Age−specific fertility fitted trend lines, 1950−2021: Papua New Guinea, Ages: 40−44
Data points enclosed with a diamond are reference sources

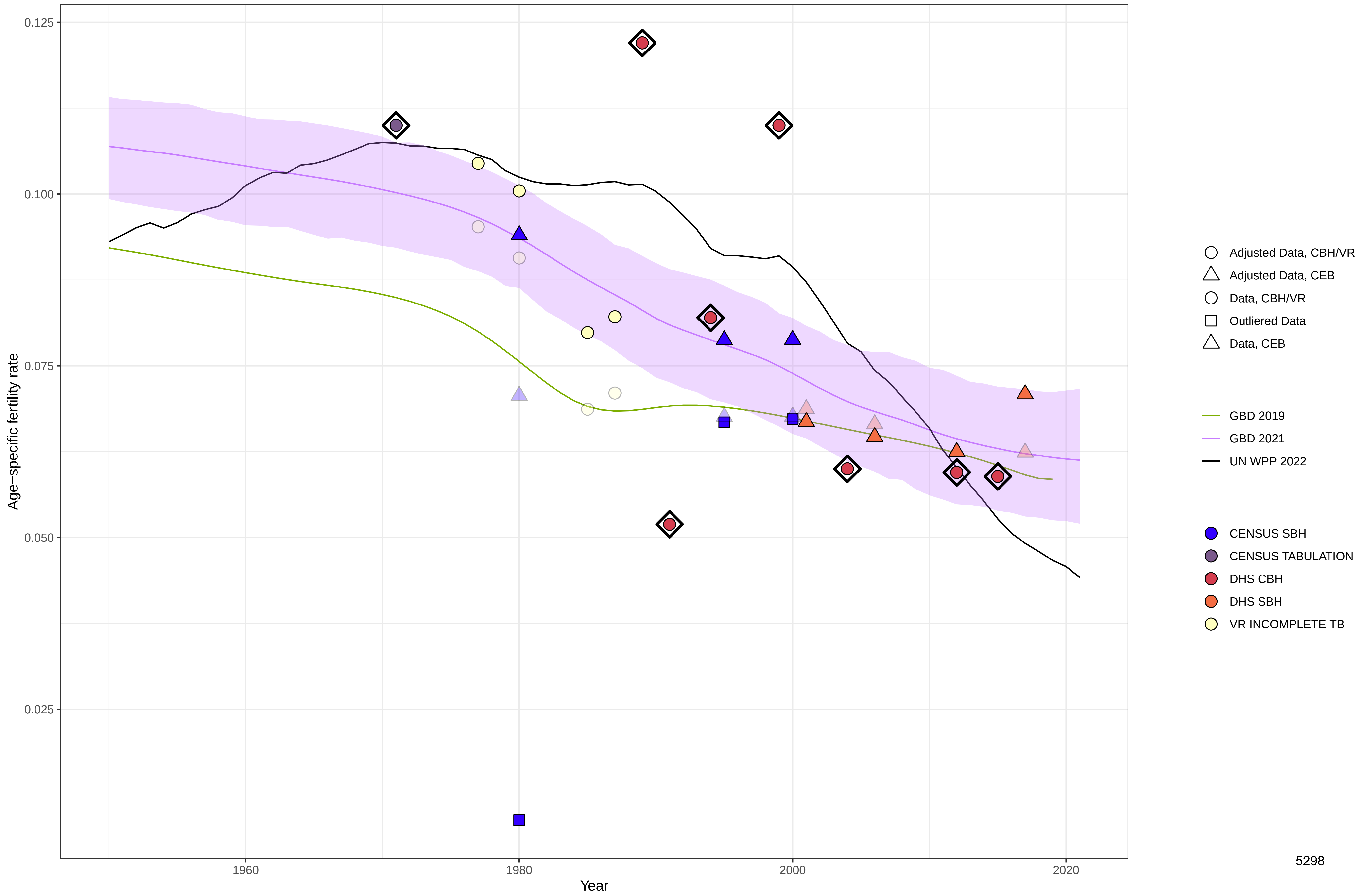

Age−specific fertility fitted trend lines, 1950−2021: Papua New Guinea, Ages: 45−49
Data points enclosed with a diamond are reference sources

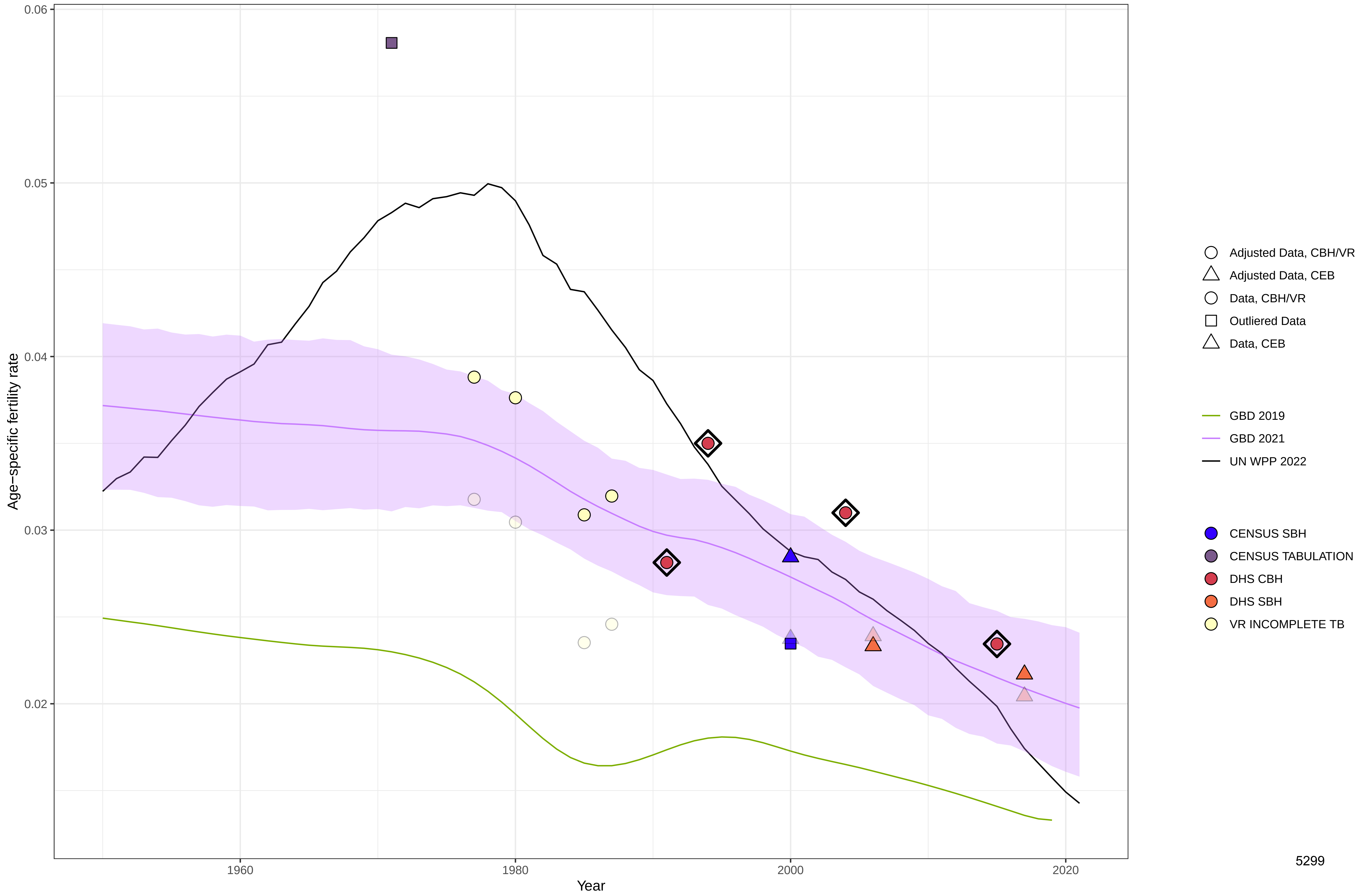

Age−specific fertility fitted trend lines, 1950−2021: Papua New Guinea, Ages: 50−54
Data points enclosed with a diamond are reference sources

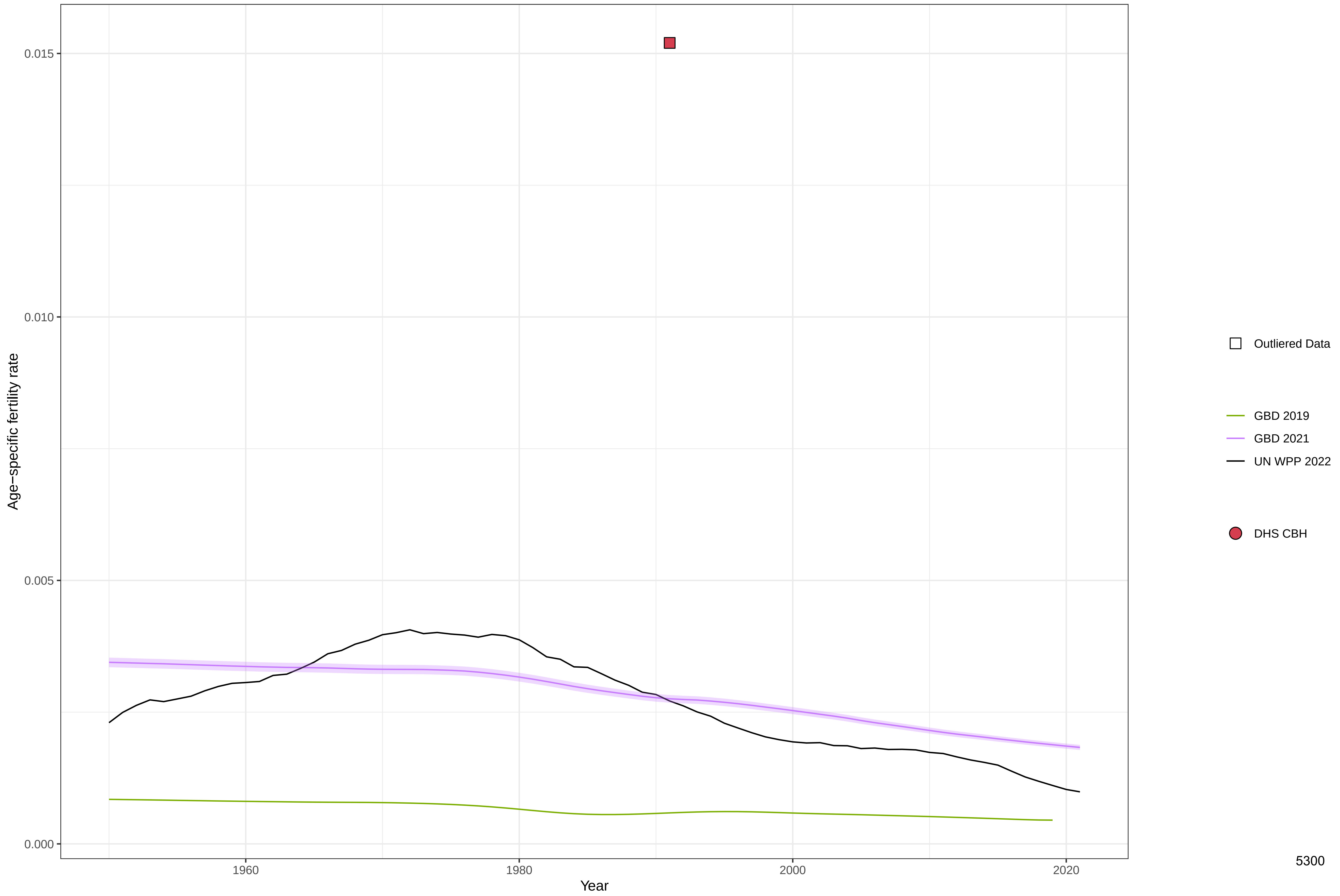

Total fertility rate fitted trend lines, 1950−2021: Papua New Guinea TFR
Data points enclosed with a diamond are reference sources

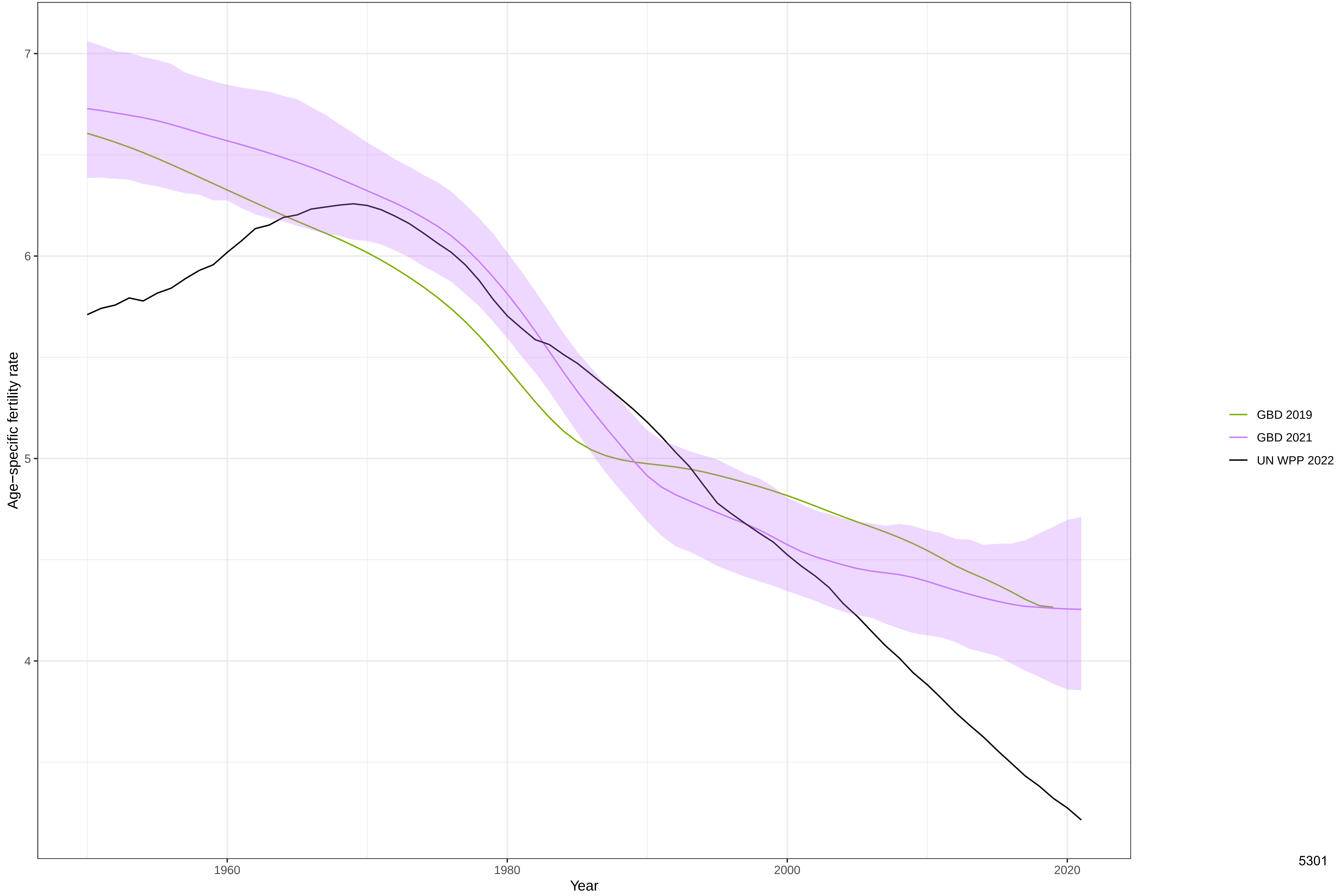

Age−specific fertility fitted trend lines, 1950−2021: Samoa, Ages: 10−14
Data points enclosed with a diamond are reference sources

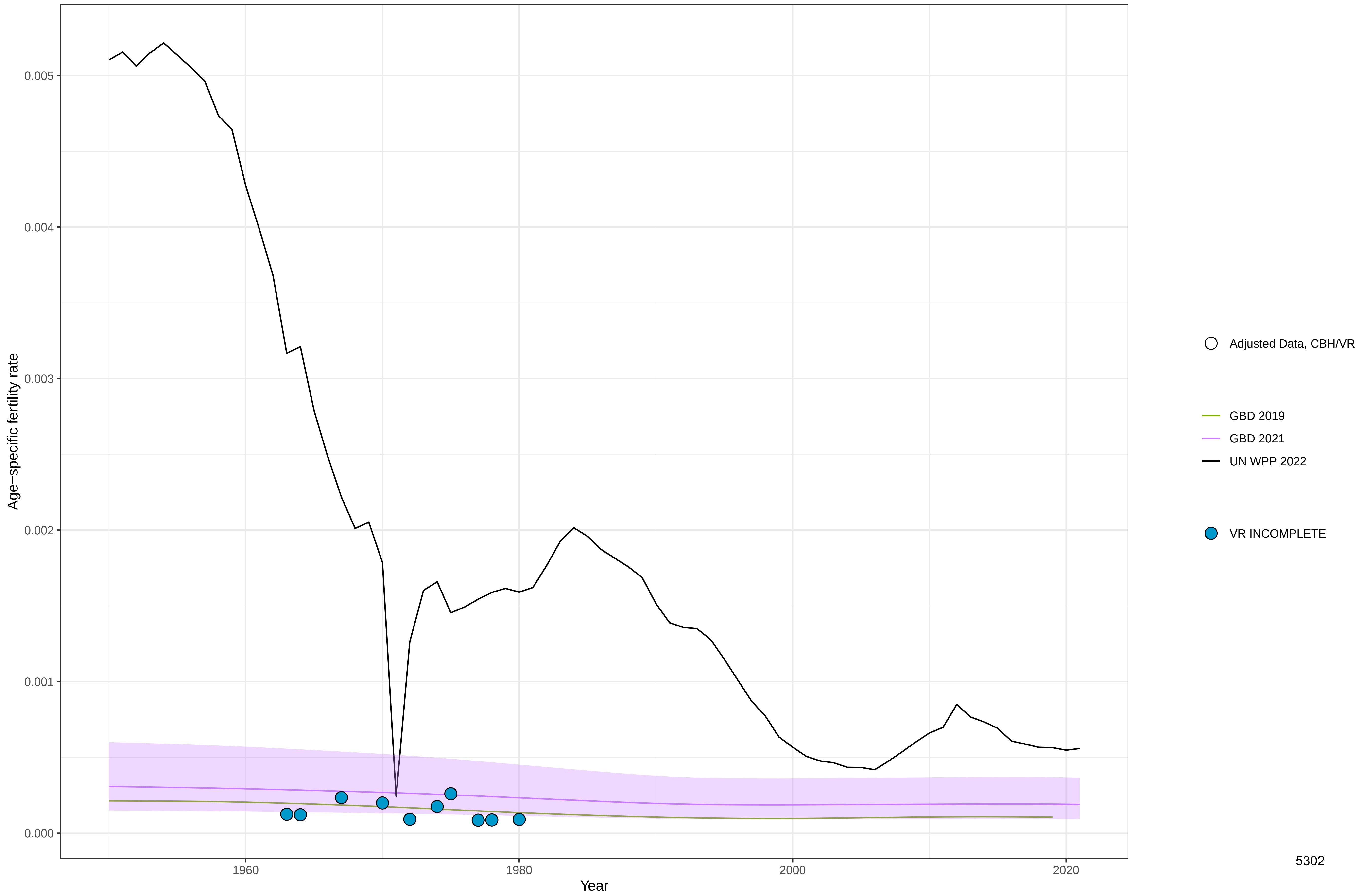

Age−specific fertility fitted trend lines, 1950−2021: Samoa, Ages: 15−19
Data points enclosed with a diamond are reference sources

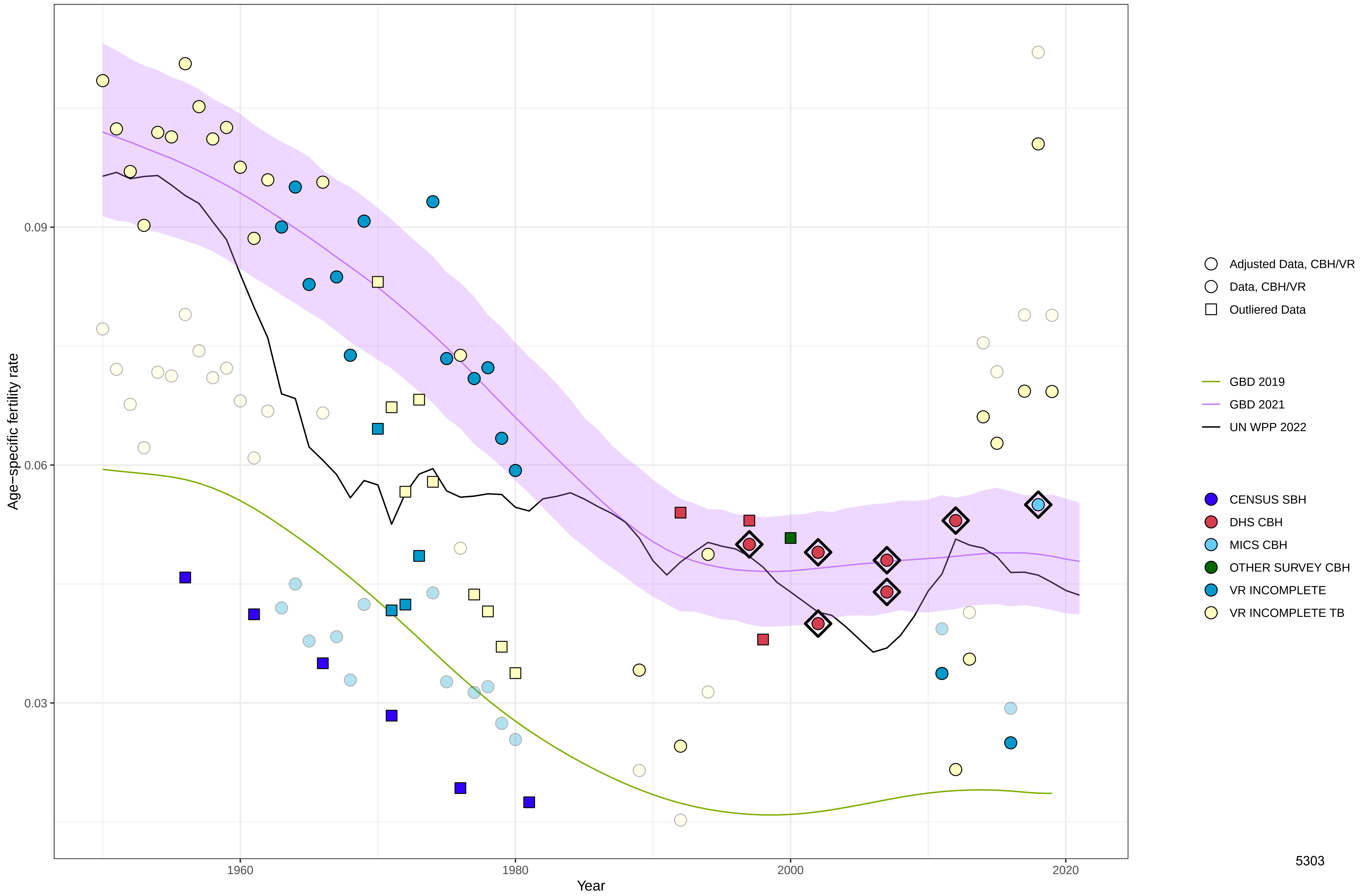

Age−specific fertility fitted trend lines, 1950−2021: Samoa, Ages: 20−24
Data points enclosed with a diamond are reference sources

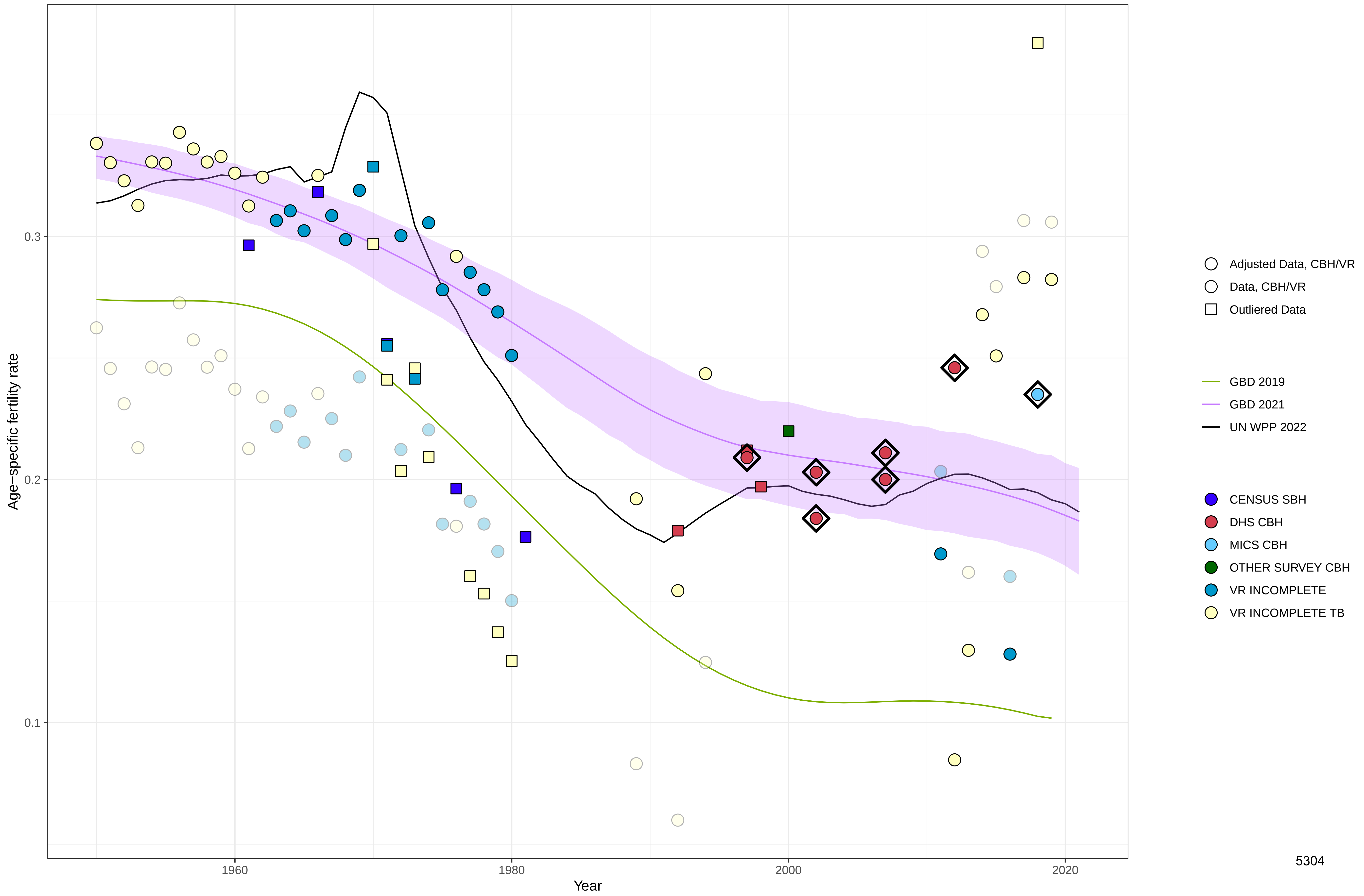

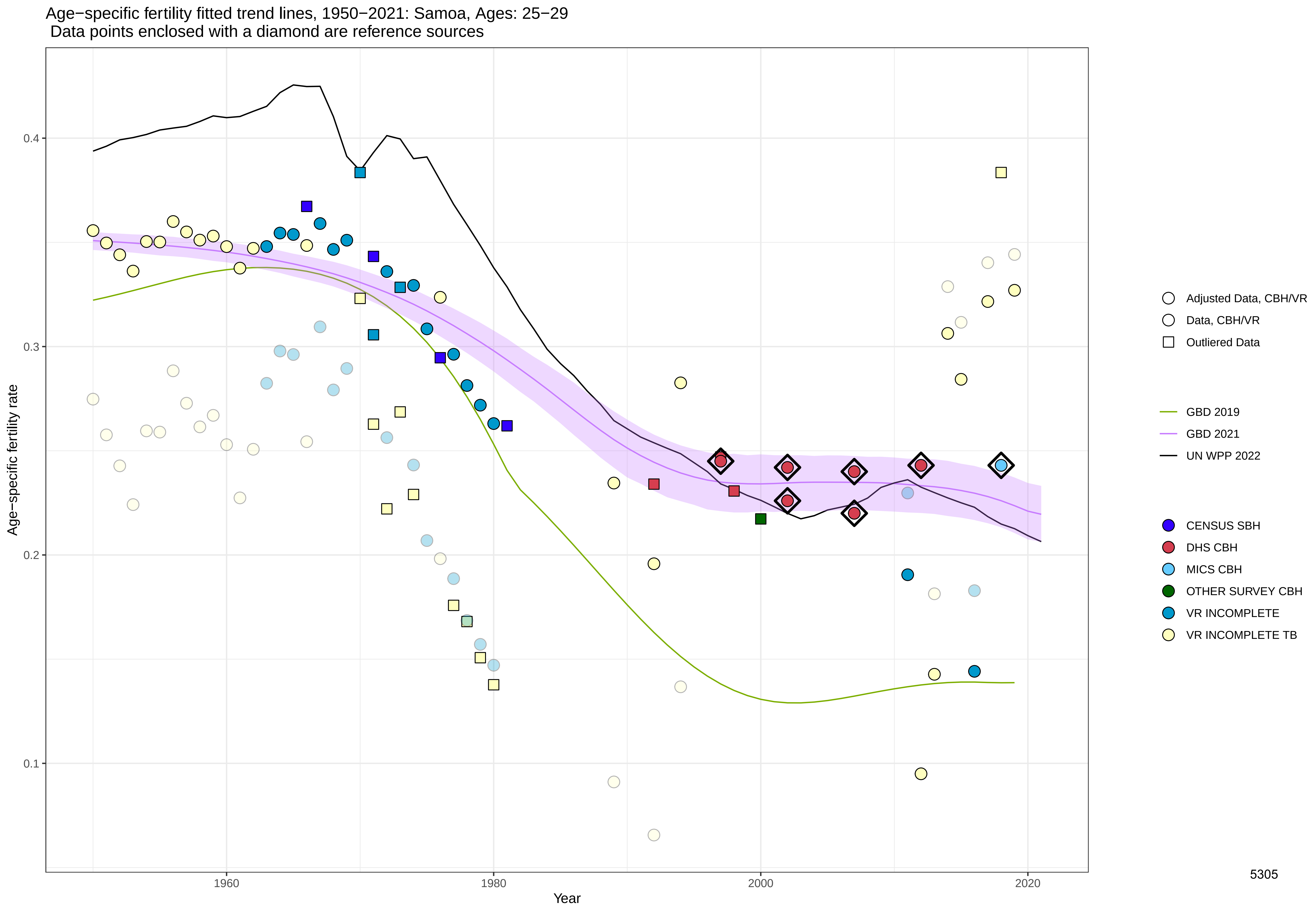

Age−specific fertility fitted trend lines, 1950−2021: Samoa, Ages: 25−29
Data points enclosed with a diamond are reference sources
Age−specific fertility rate
Year
0.1
0.2
0.3
0.4
1960
1980
2000
2020
Adjusted Data, CBH/VR
Data, CBH/VR
Outliered Data
GBD 2019
GBD 2021
UN WPP 2022
CENSUS SBH
DHS CBH
MICS CBH
OTHER SURVEY CBH
VR INCOMPLETE
VR INCOMPLETE TB

Age−specific fertility fitted trend lines, 1950−2021: Samoa, Ages: 30−34
Data points enclosed with a diamond are reference sources

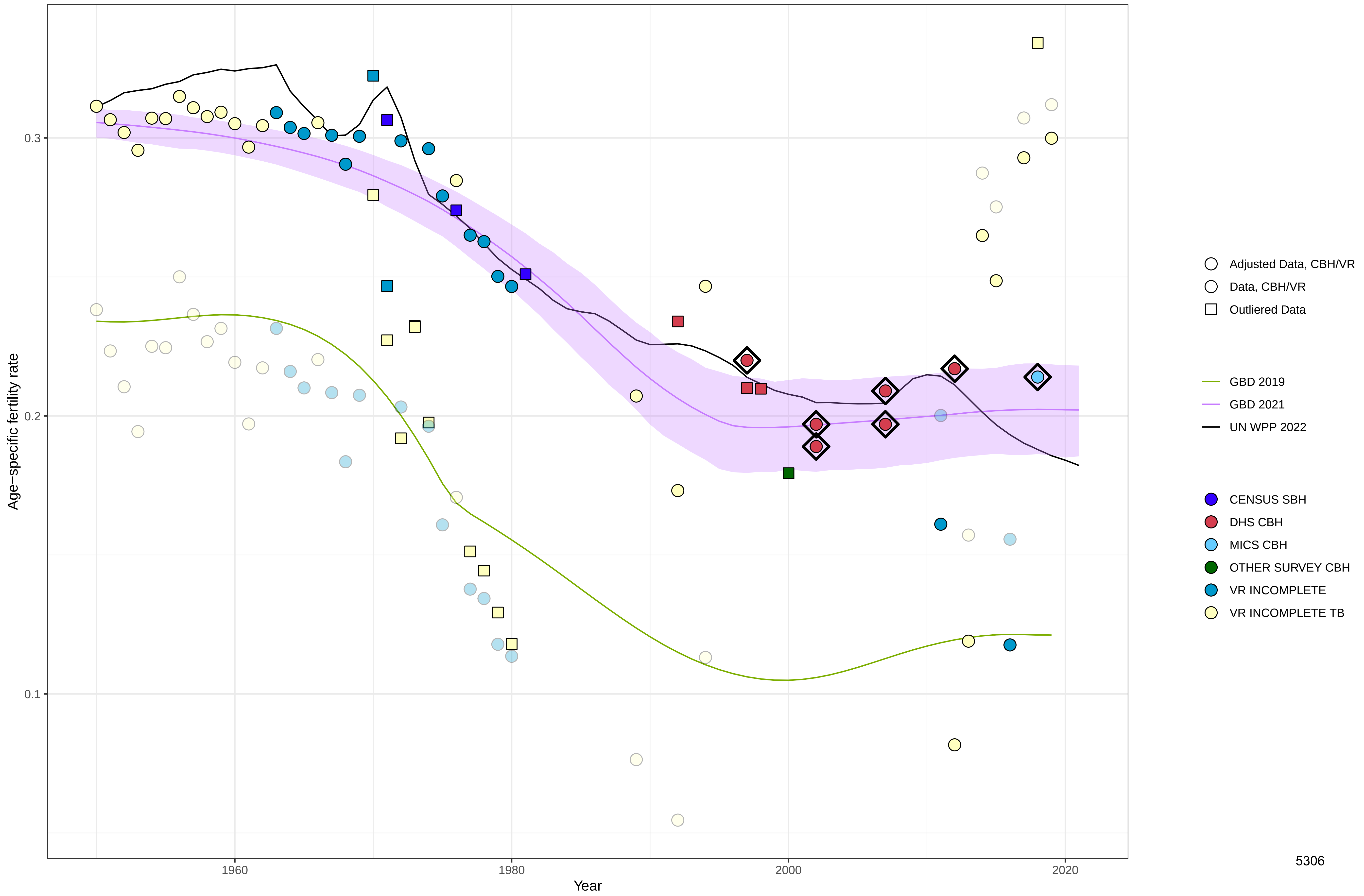

Age−specific fertility fitted trend lines, 1950−2021: Samoa, Ages: 35−39
Data points enclosed with a diamond are reference sources

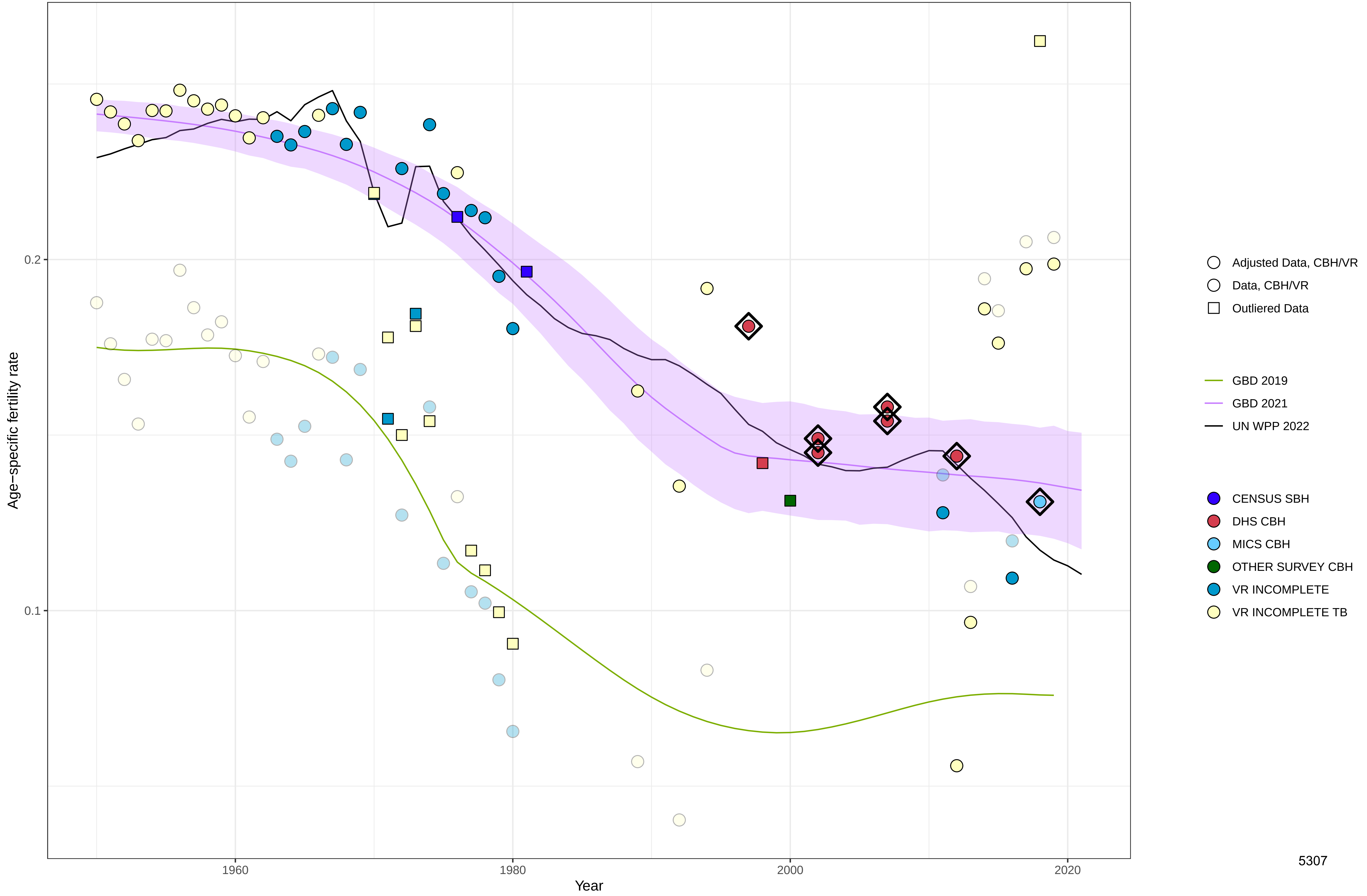

Age−specific fertility fitted trend lines, 1950−2021: Samoa, Ages: 40−44
Data points enclosed with a diamond are reference sources

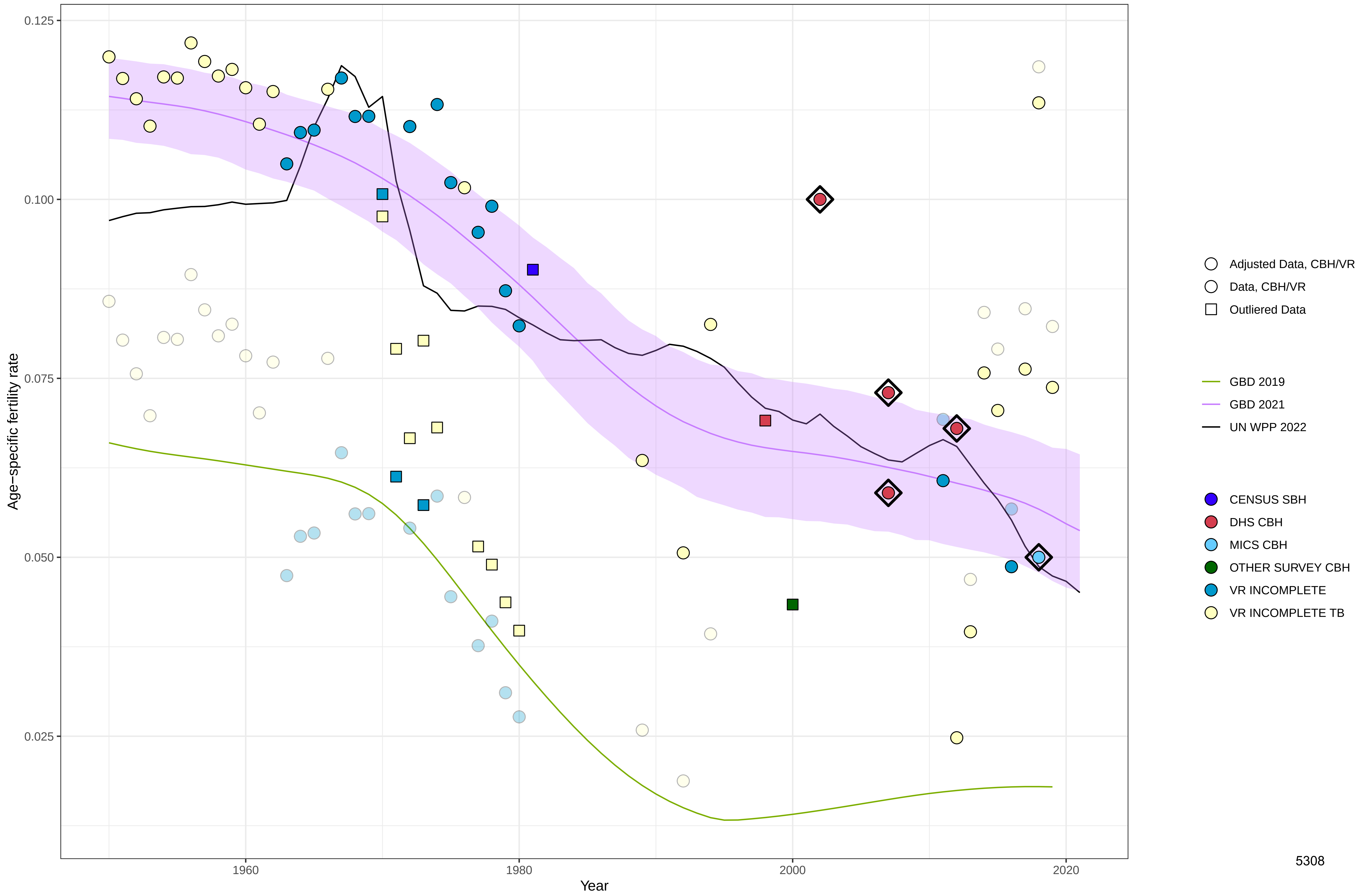

Age−specific fertility fitted trend lines, 1950−2021: Samoa, Ages: 45−49
Data points enclosed with a diamond are reference sources

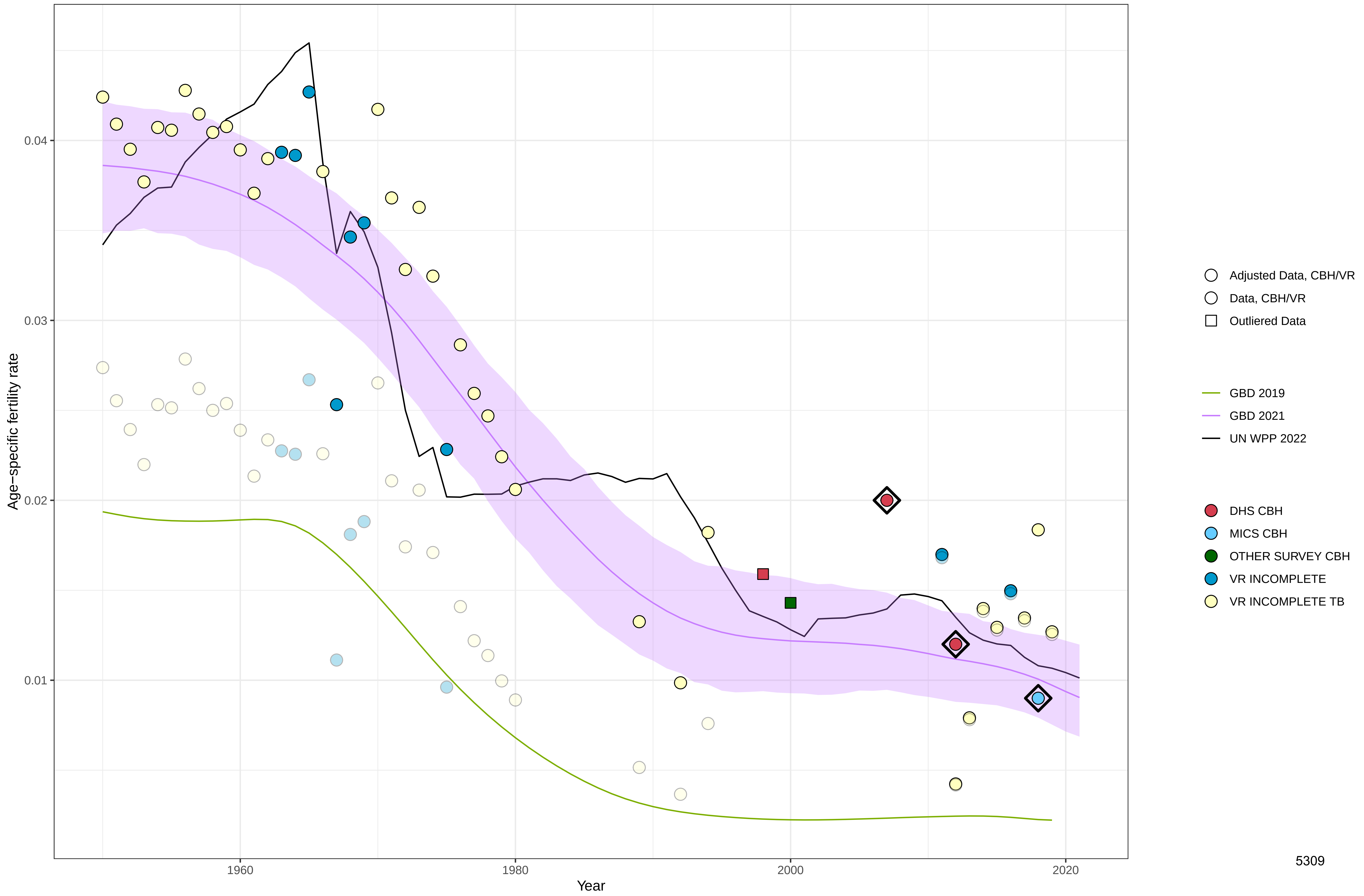

Age−specific fertility fitted trend lines, 1950−2021: Samoa, Ages: 50−54
Data points enclosed with a diamond are reference sources

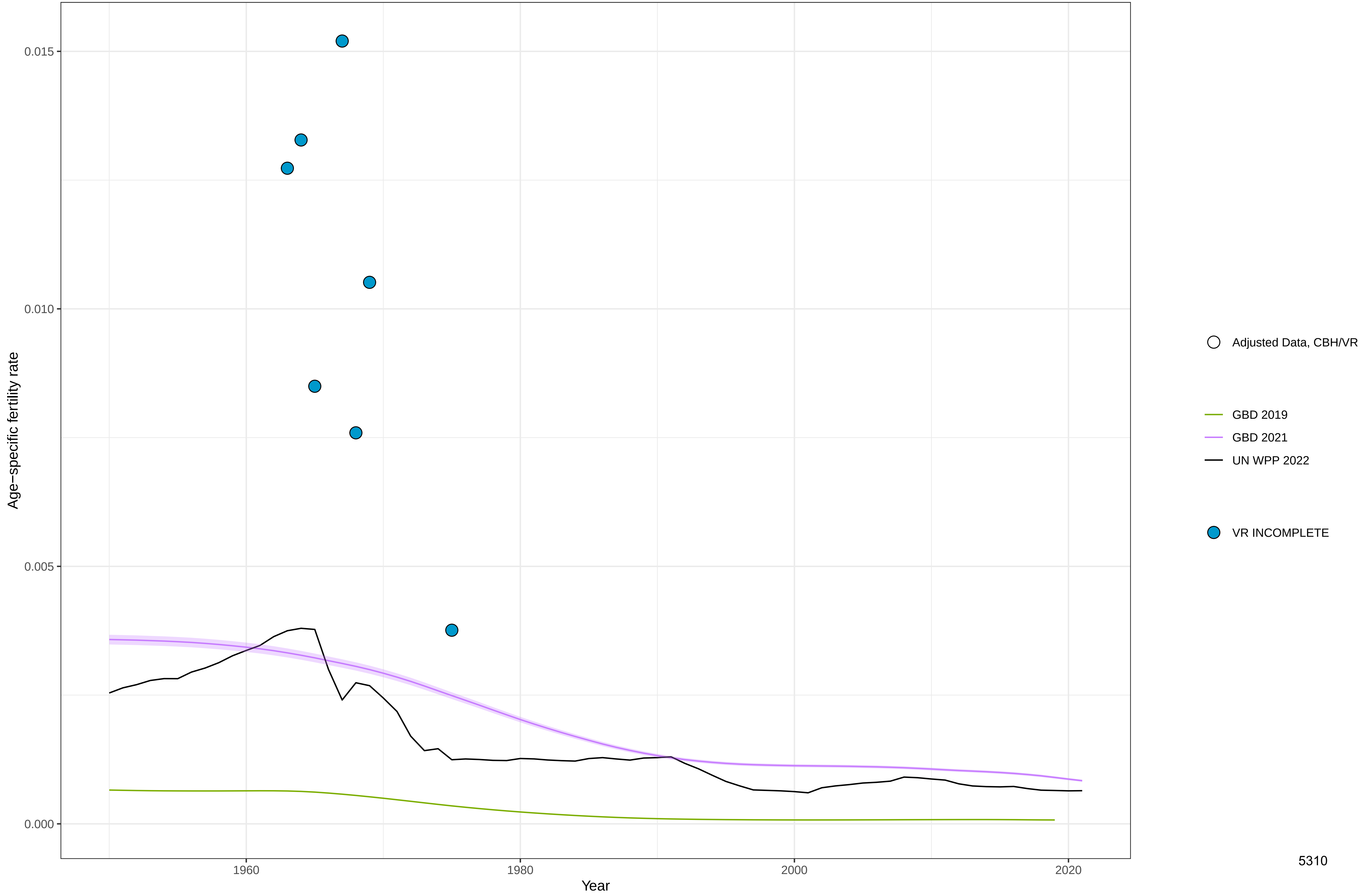

Total fertility rate fitted trend lines, 1950−2021: Samoa TFR
Data points enclosed with a diamond are reference sources

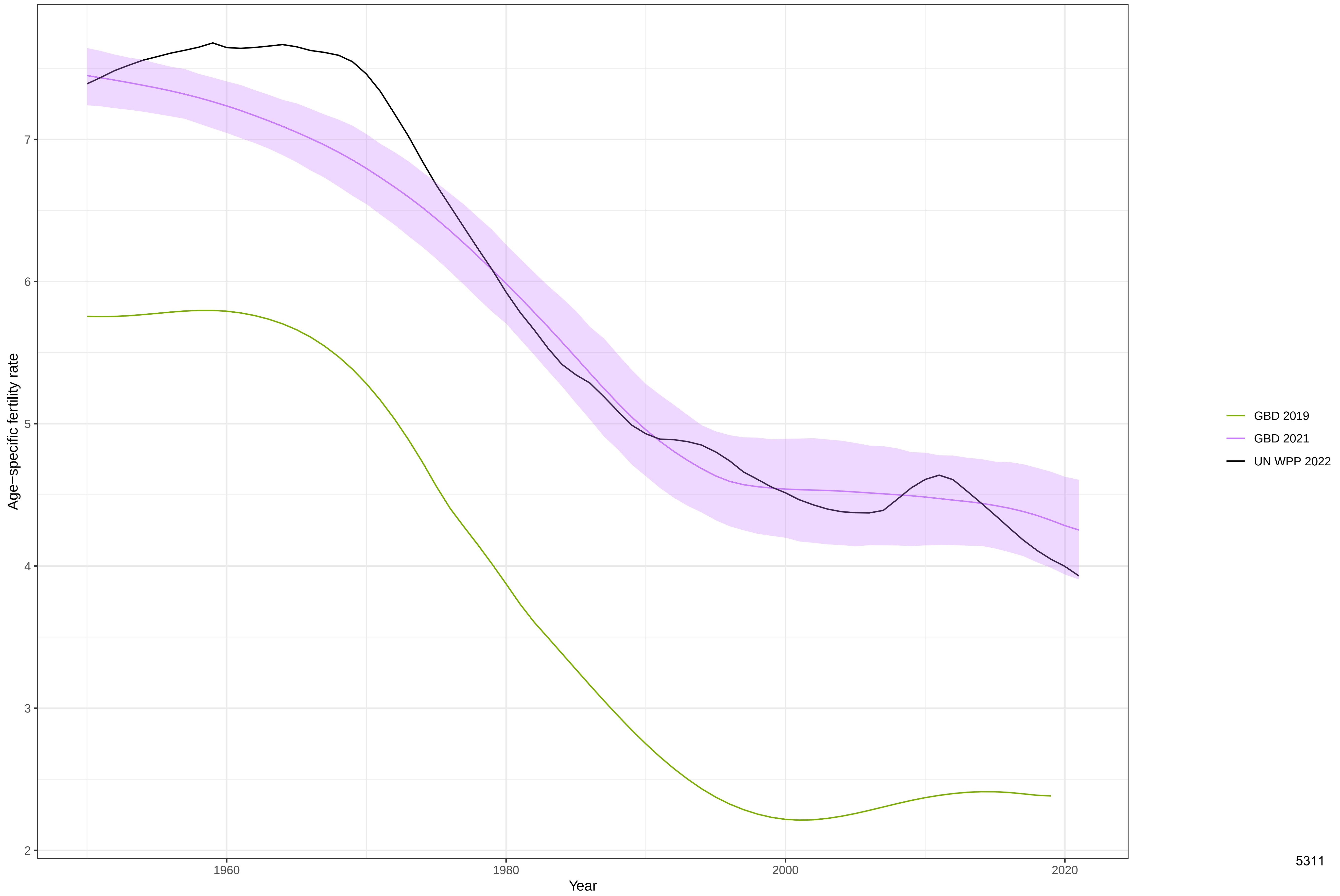

Age−specific fertility fitted trend lines, 1950−2021: Solomon Islands, Ages: 10−14
Data points enclosed with a diamond are reference sources

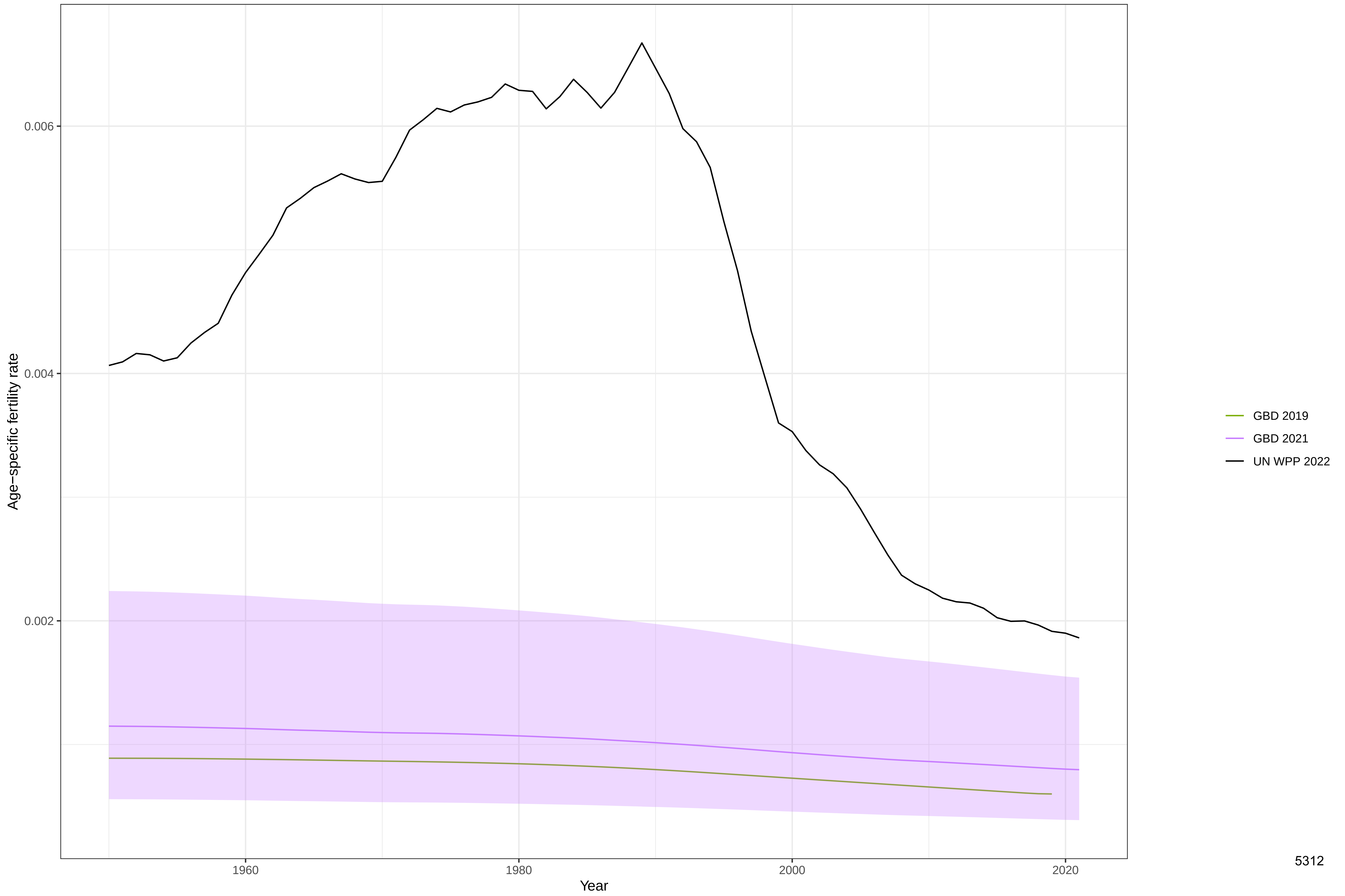

Age−specific fertility fitted trend lines, 1950−2021: Solomon Islands, Ages: 15−19
Data points enclosed with a diamond are reference sources

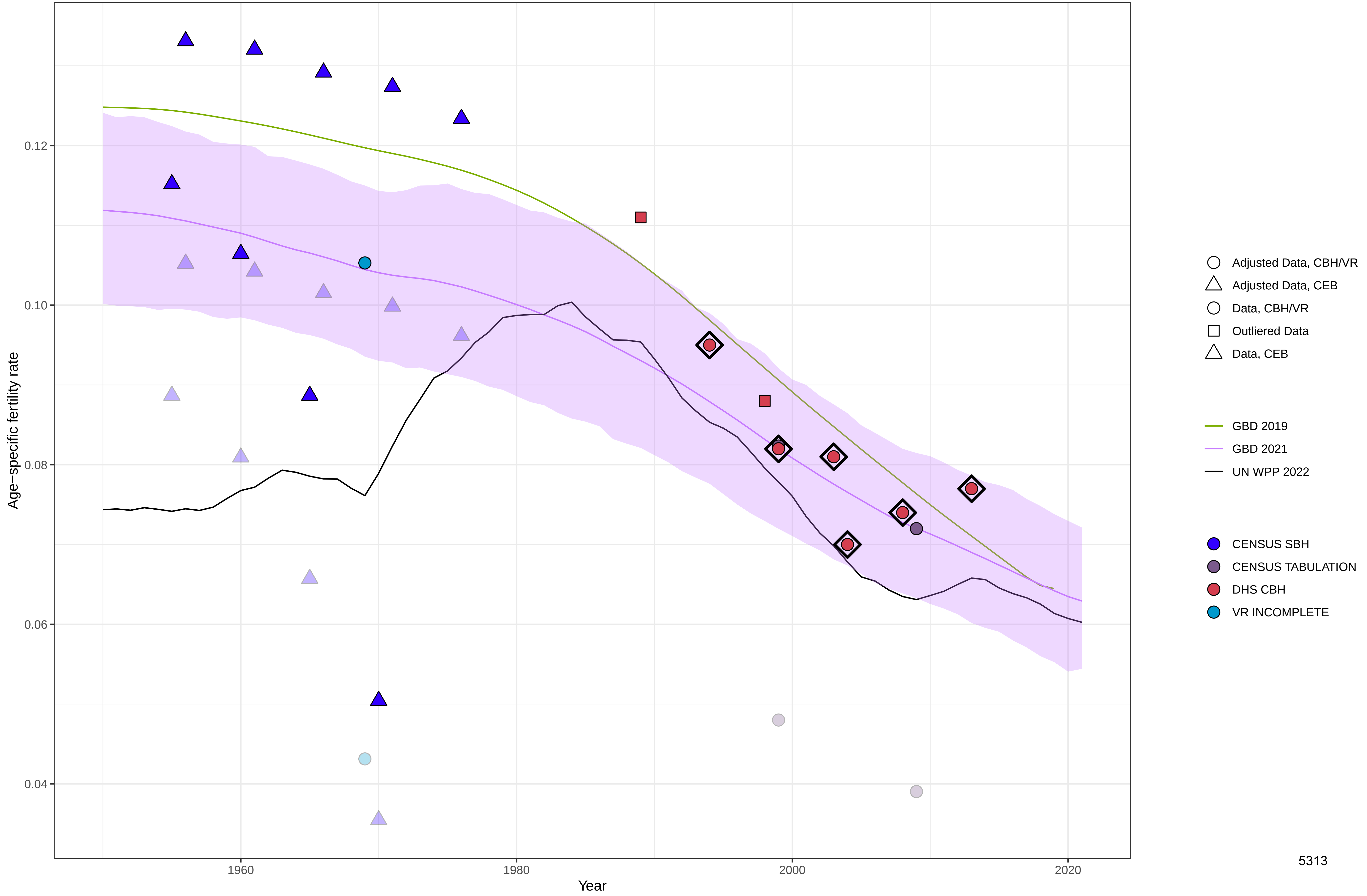

Age−specific fertility fitted trend lines, 1950−2021: Solomon Islands, Ages: 20−24
Data points enclosed with a diamond are reference sources

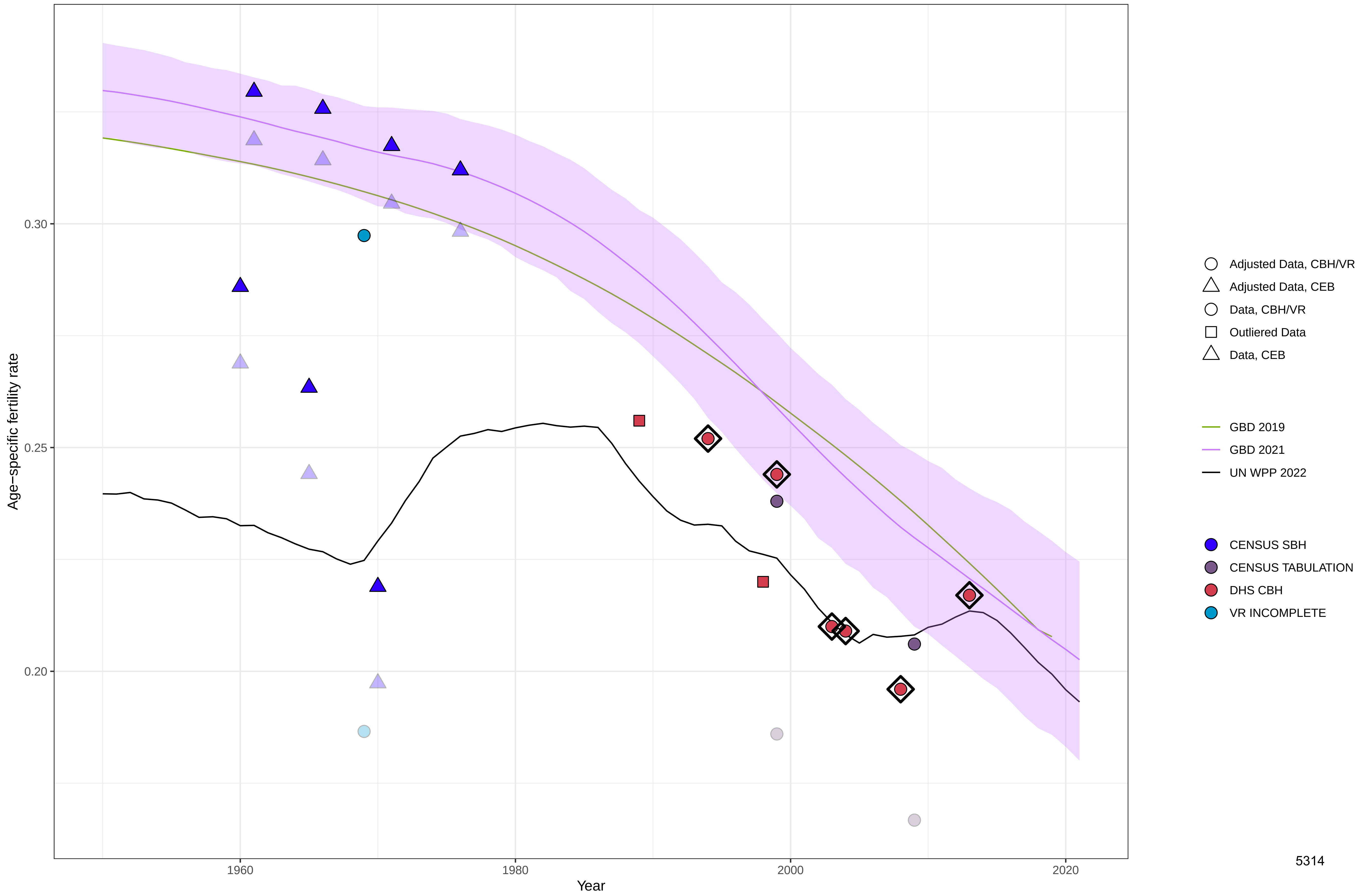

Age−specific fertility fitted trend lines, 1950−2021: Solomon Islands, Ages: 25−29
Data points enclosed with a diamond are reference sources

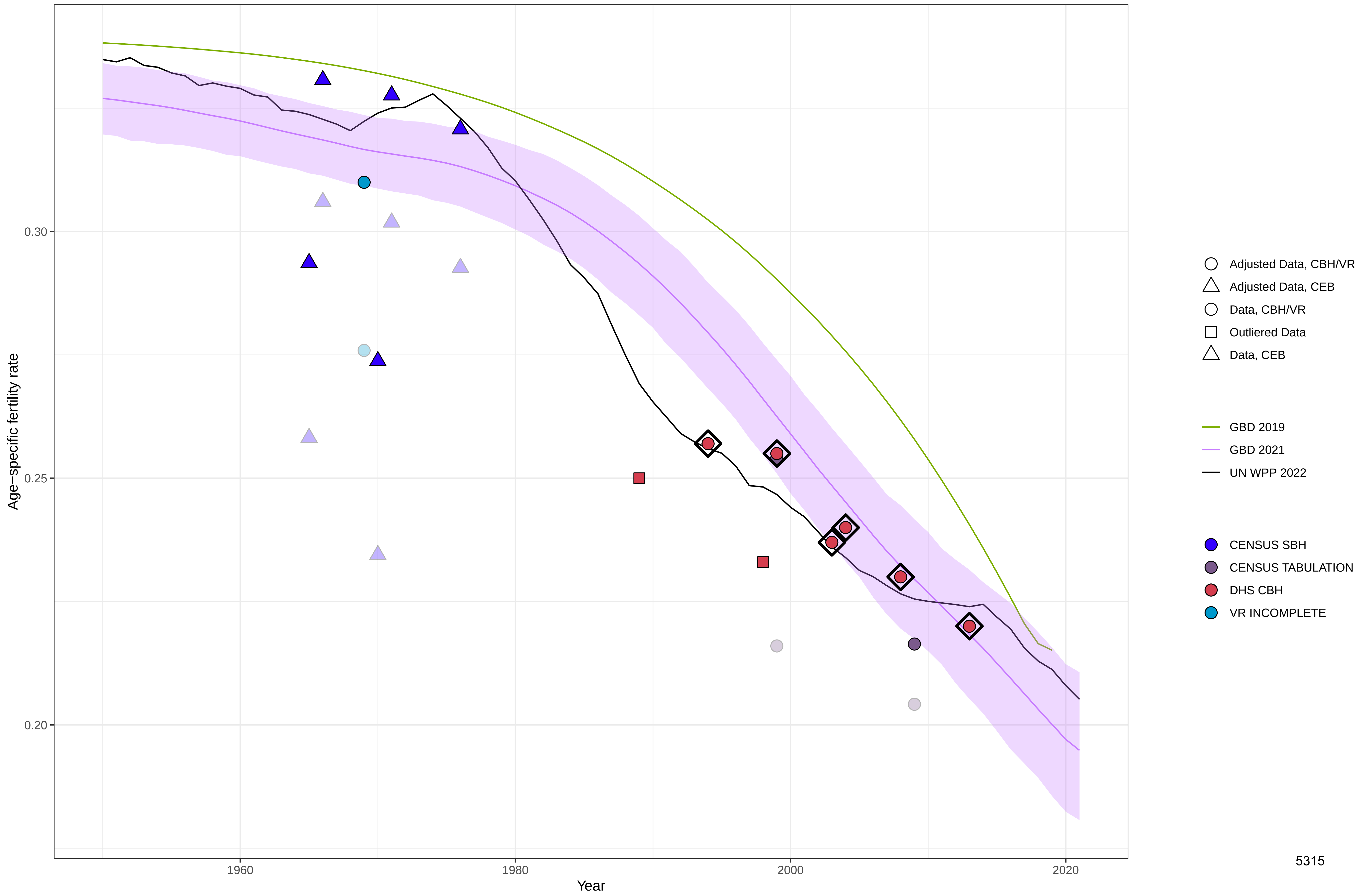

Age−specific fertility fitted trend lines, 1950−2021: Solomon Islands, Ages: 30−34
Data points enclosed with a diamond are reference sources

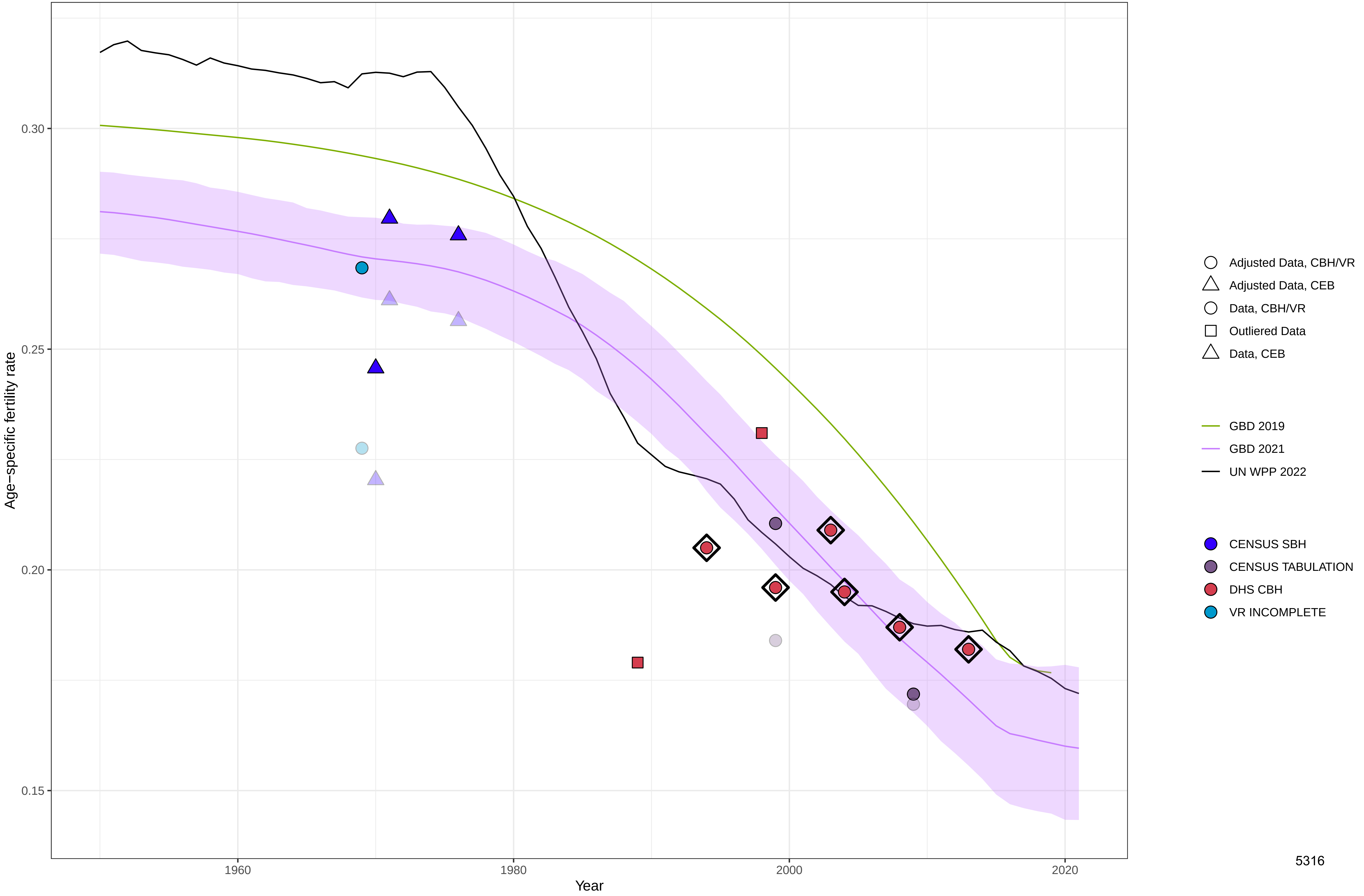

Age−specific fertility fitted trend lines, 1950−2021: Solomon Islands, Ages: 35−39
Data points enclosed with a diamond are reference sources

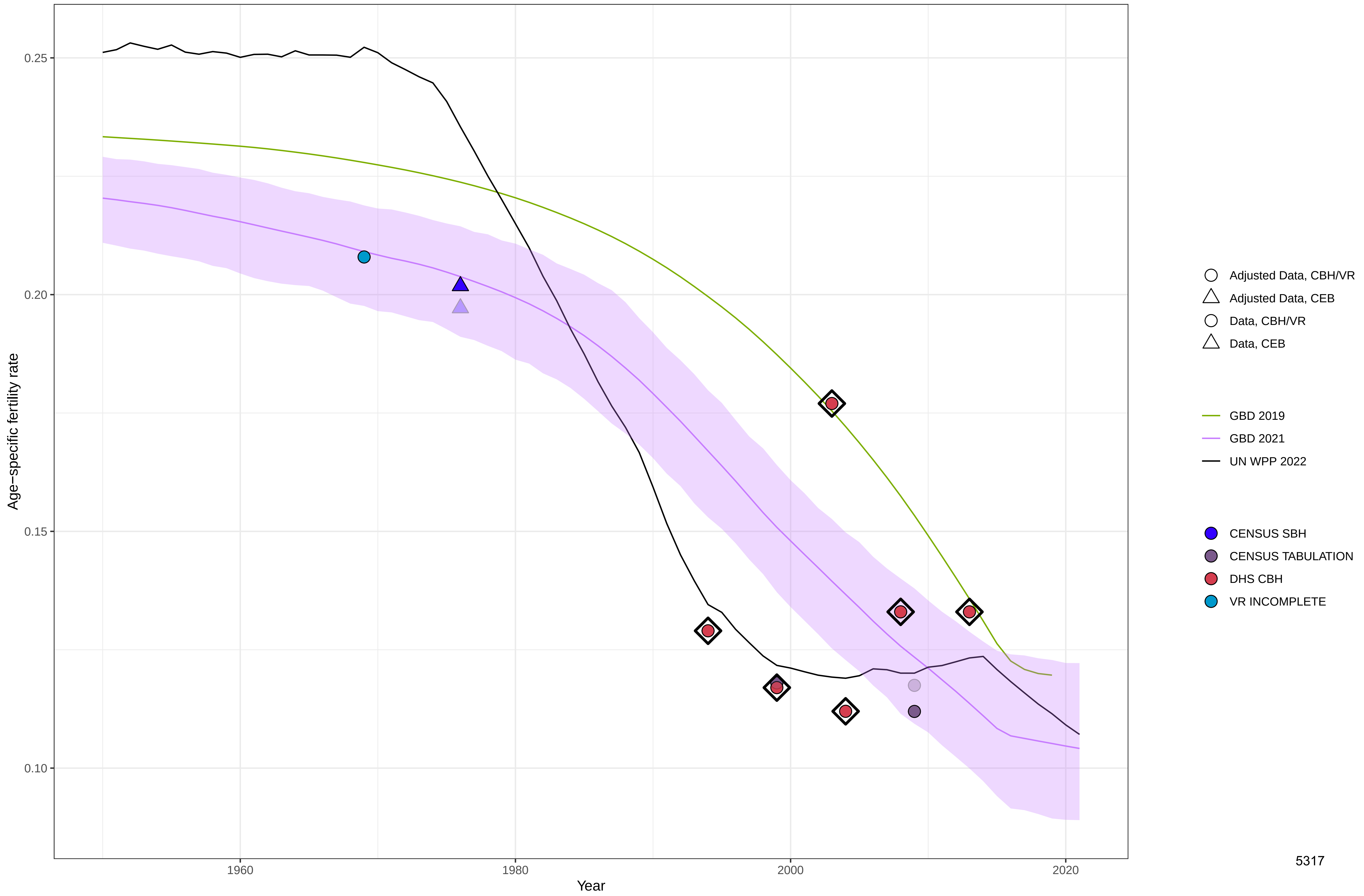

Age−specific fertility fitted trend lines, 1950−2021: Solomon Islands, Ages: 40−44
Data points enclosed with a diamond are reference sources

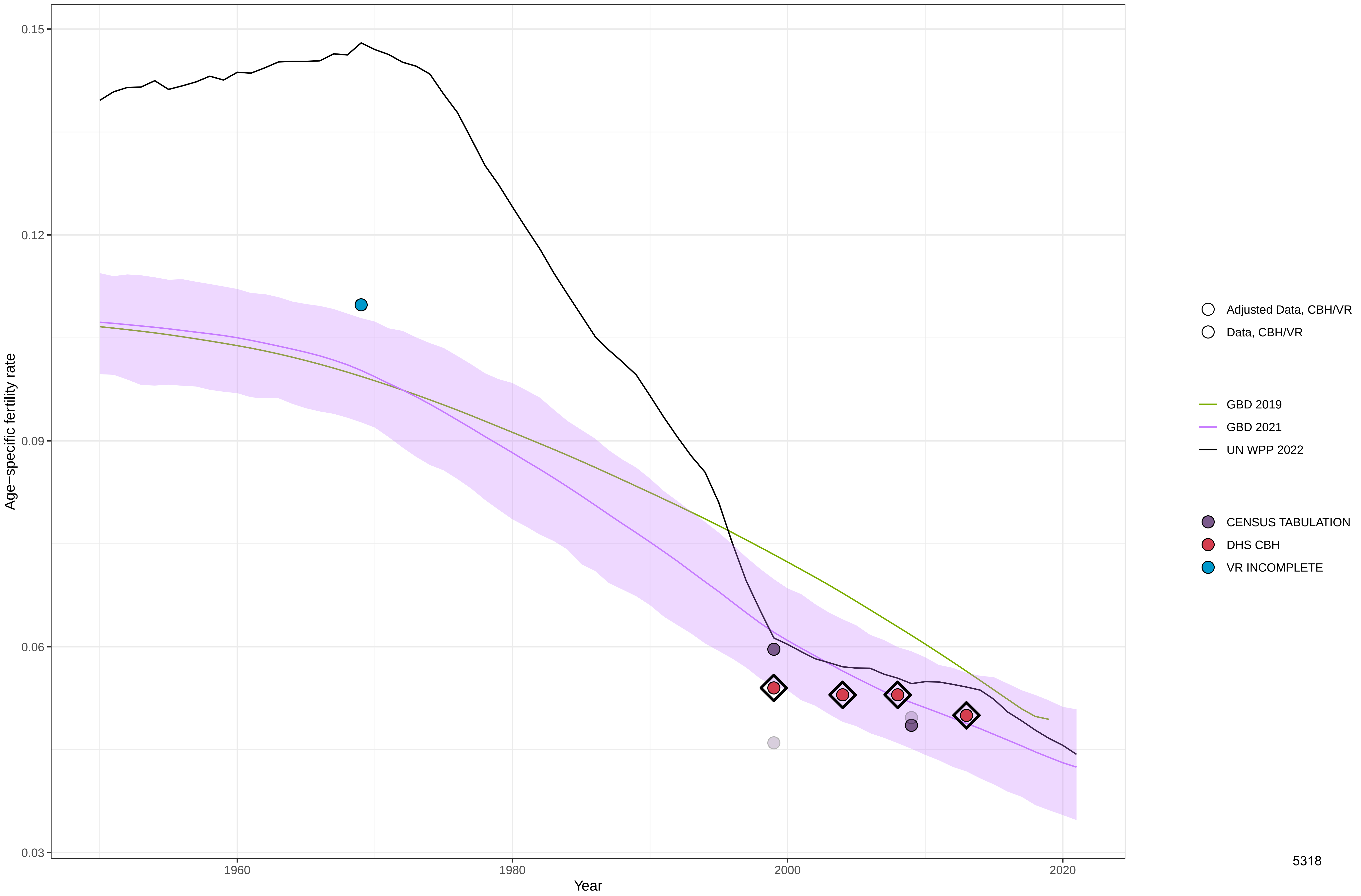

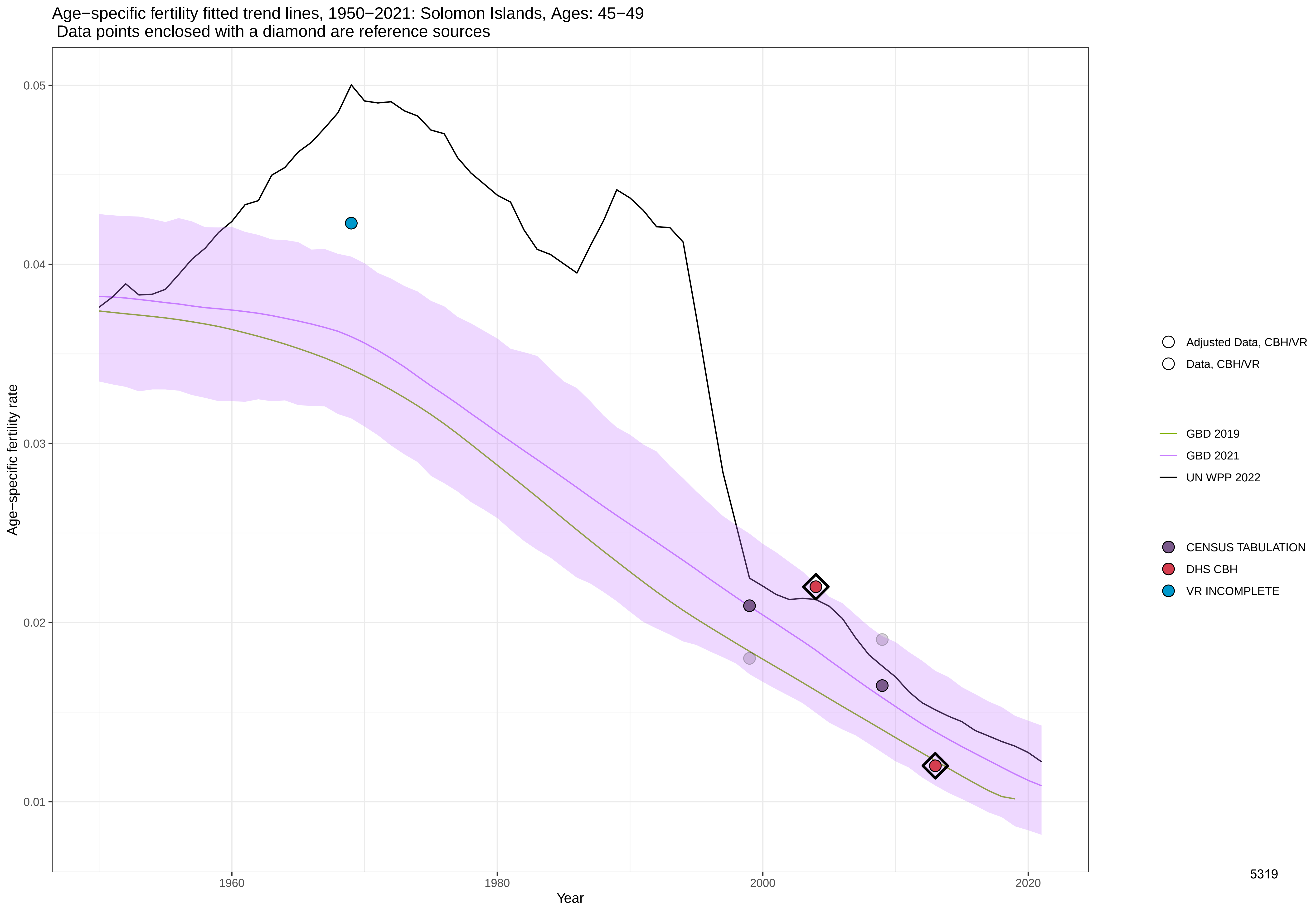

Age−specific fertility fitted trend lines, 1950−2021: Solomon Islands, Ages: 45−49
Data points enclosed with a diamond are reference sources
Age−specific fertility rate
Year
0.05
0.04
0.03
0.02
0.01
1960
1980
2000
2020
Adjusted Data, CBH/VR
Data, CBH/VR
GBD 2019
GBD 2021
UN WPP 2022
CENSUS TABULATION
DHS CBH
VR INCOMPLETE

Age−specific fertility fitted trend lines, 1950−2021: Solomon Islands, Ages: 50−54
Data points enclosed with a diamond are reference sources

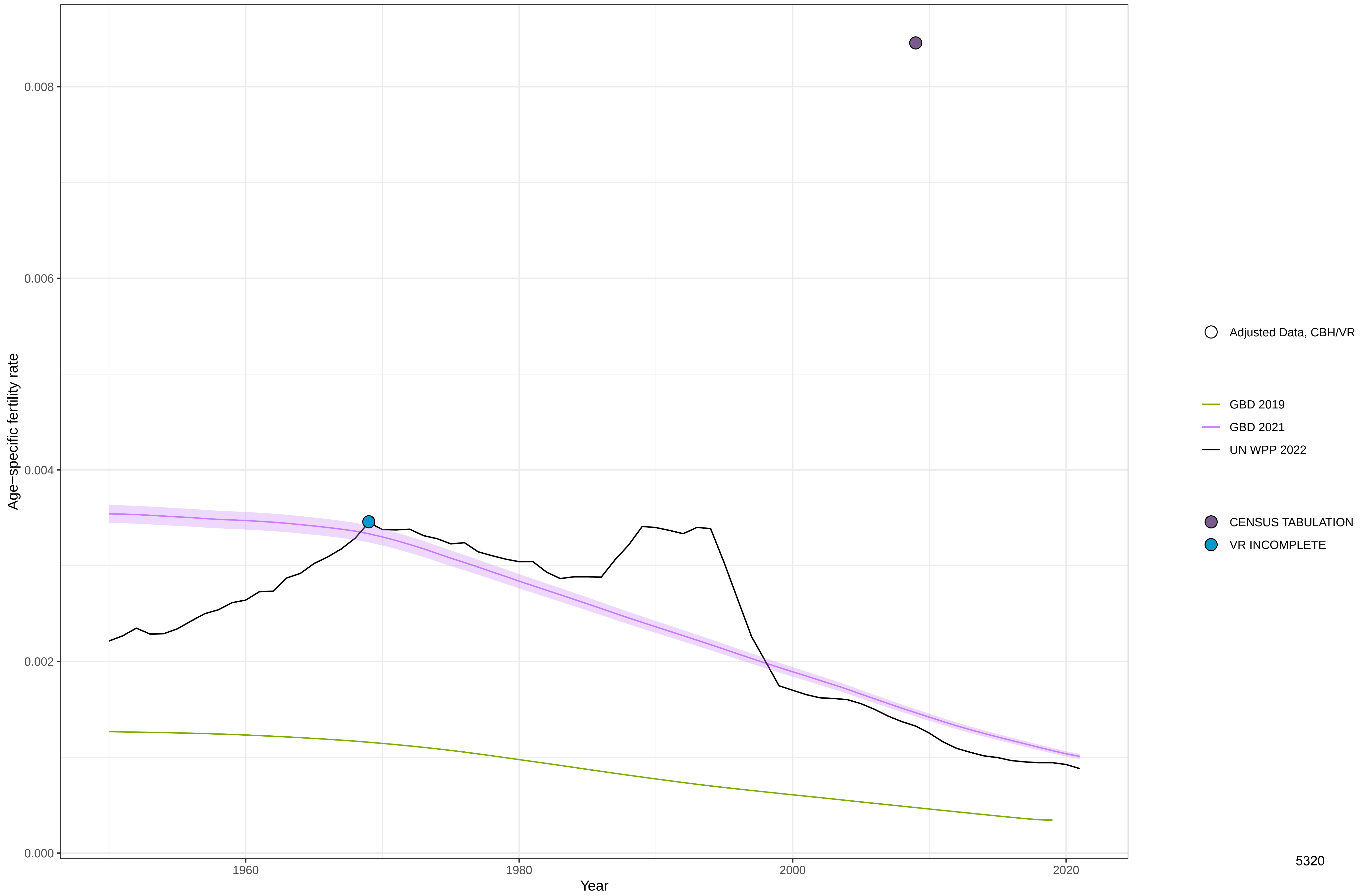

Total fertility rate fitted trend lines, 1950−2021: Solomon Islands TFR
Data points enclosed with a diamond are reference sources

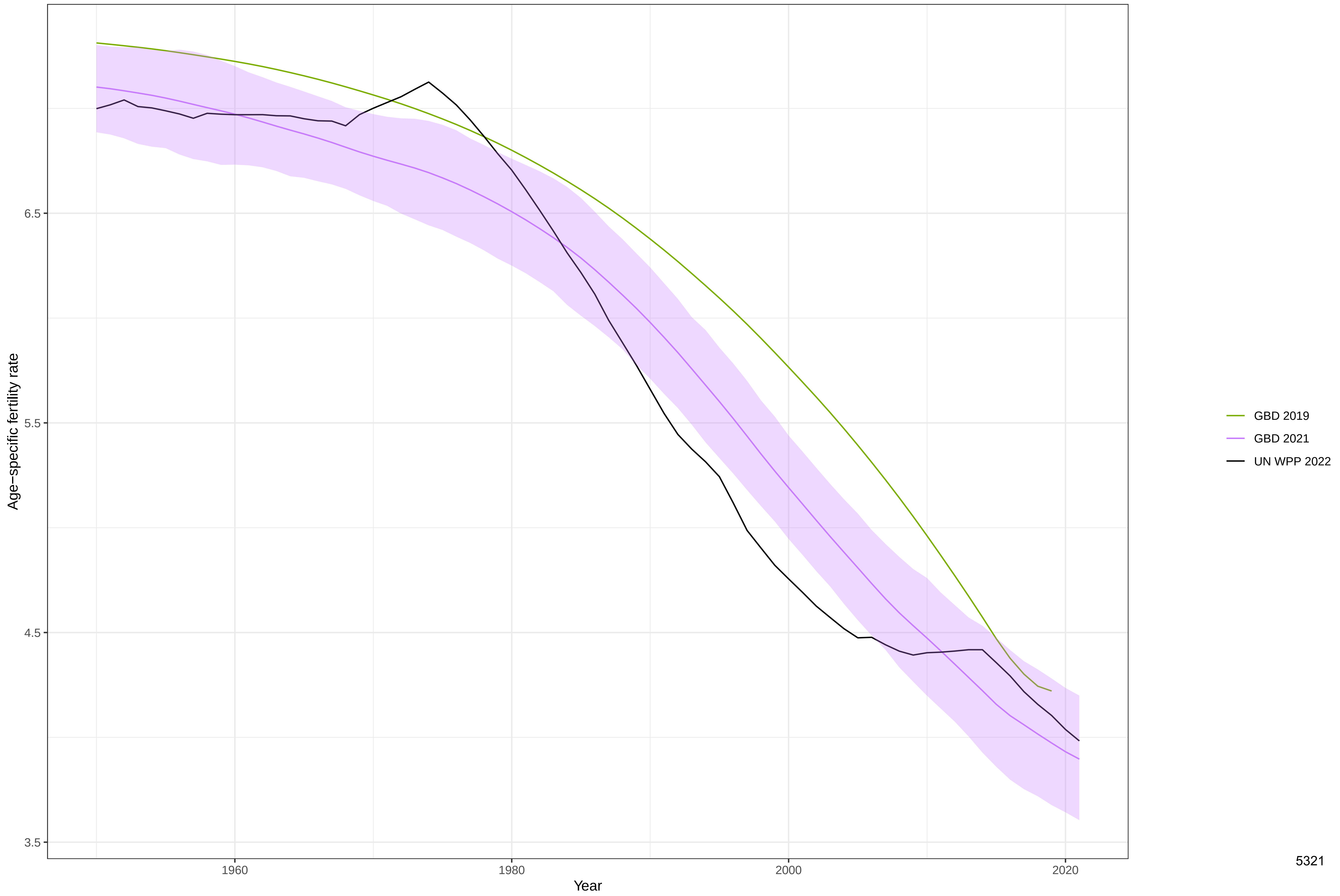

Age−specific fertility fitted trend lines, 1950−2021: Tokelau, Ages: 10−14
Data points enclosed with a diamond are reference sources

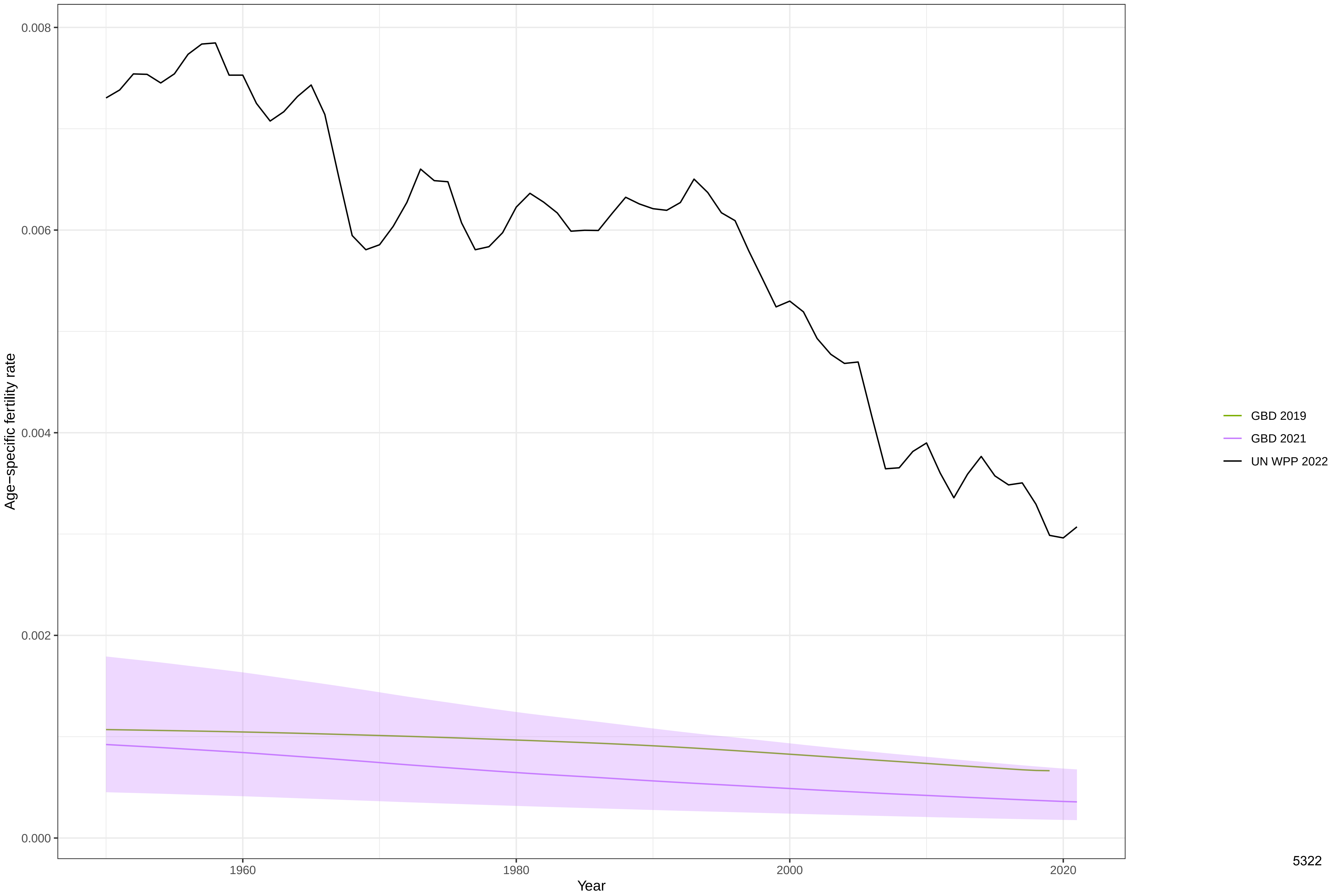

Age−specific fertility fitted trend lines, 1950−2021: Tokelau, Ages: 15−19
Data points enclosed with a diamond are reference sources

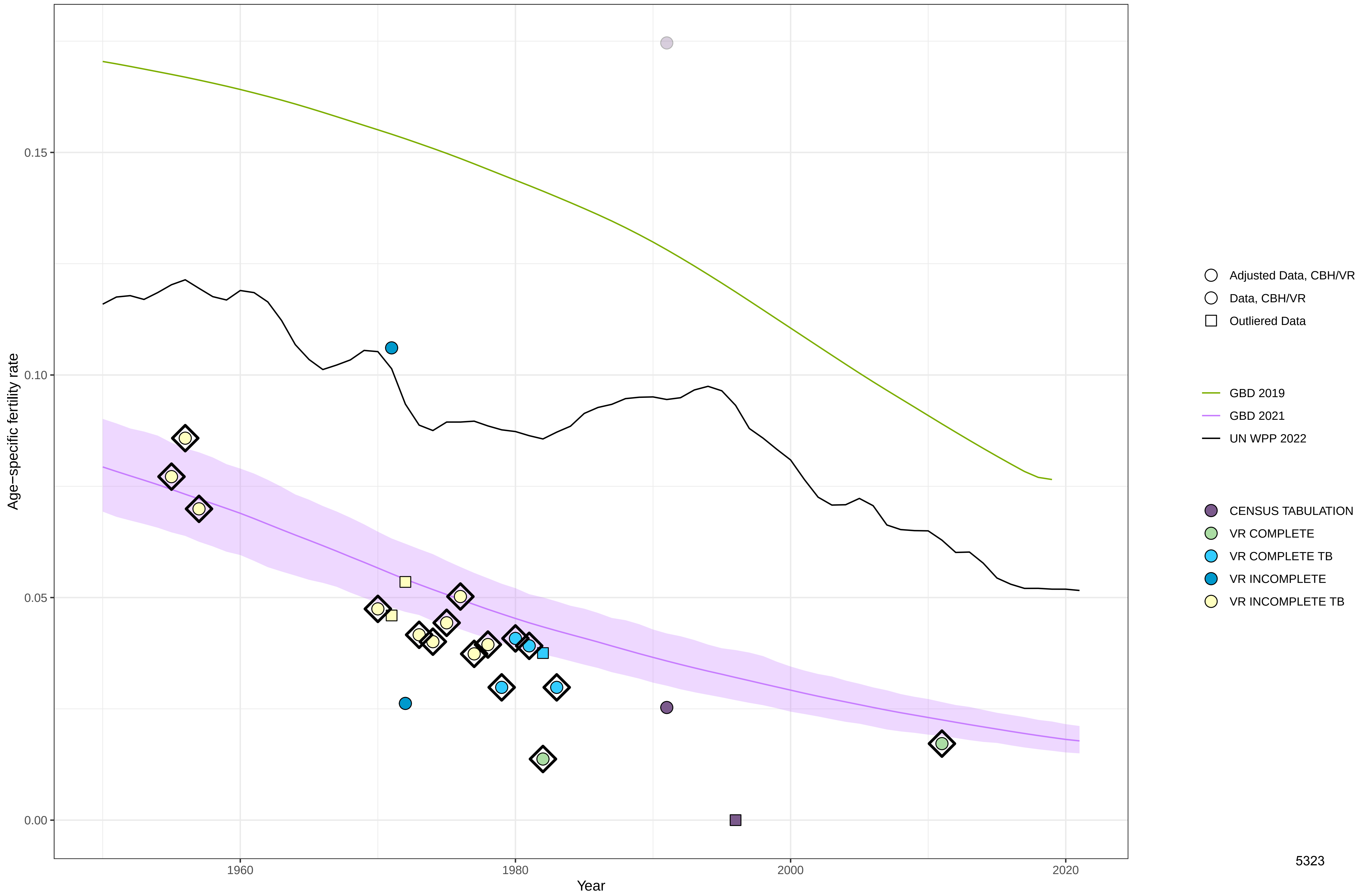

Age−specific fertility fitted trend lines, 1950−2021: Tokelau, Ages: 20−24
Data points enclosed with a diamond are reference sources

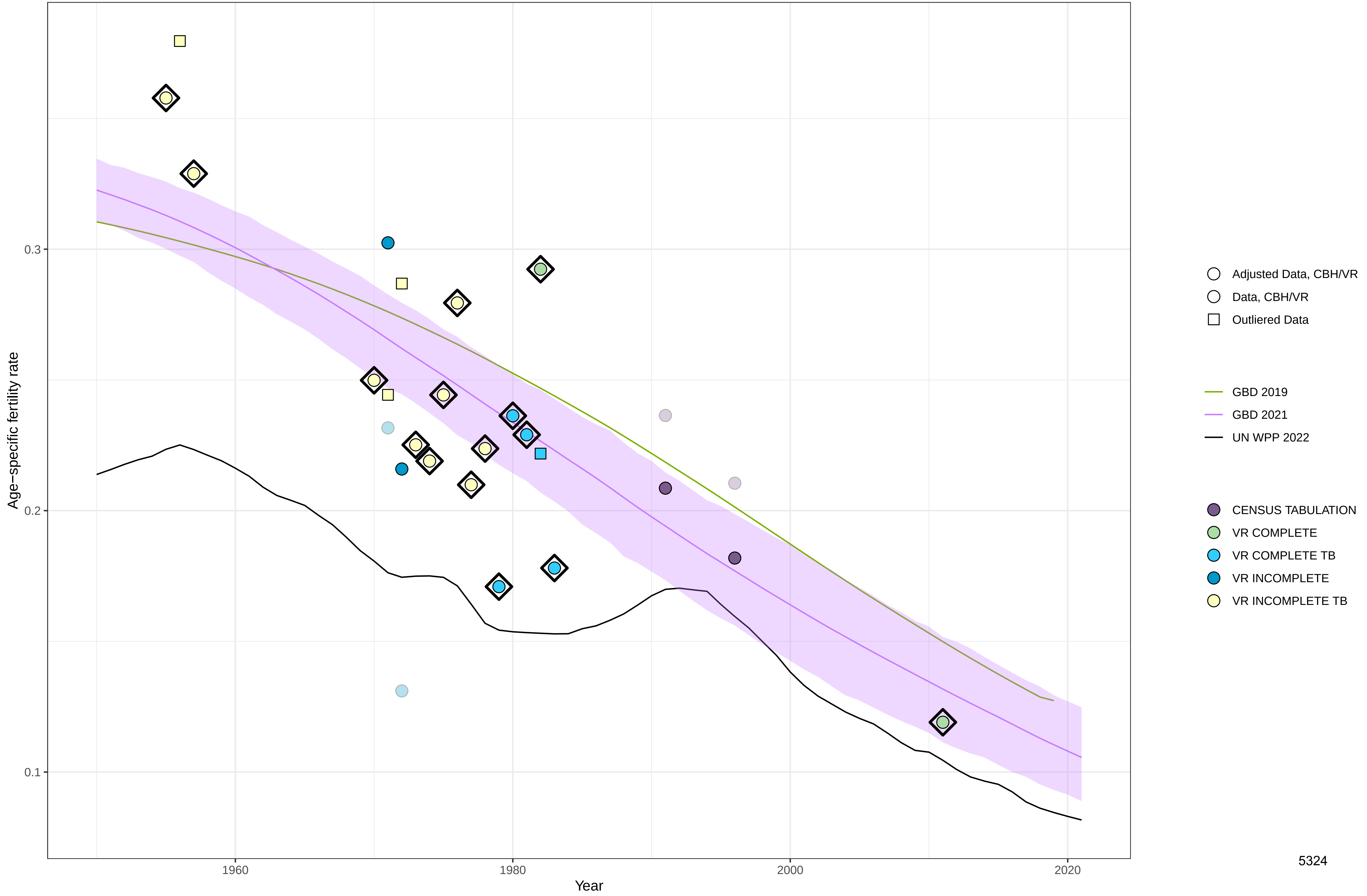

Age−specific fertility fitted trend lines, 1950−2021: Tokelau, Ages: 25−29
Data points enclosed with a diamond are reference sources

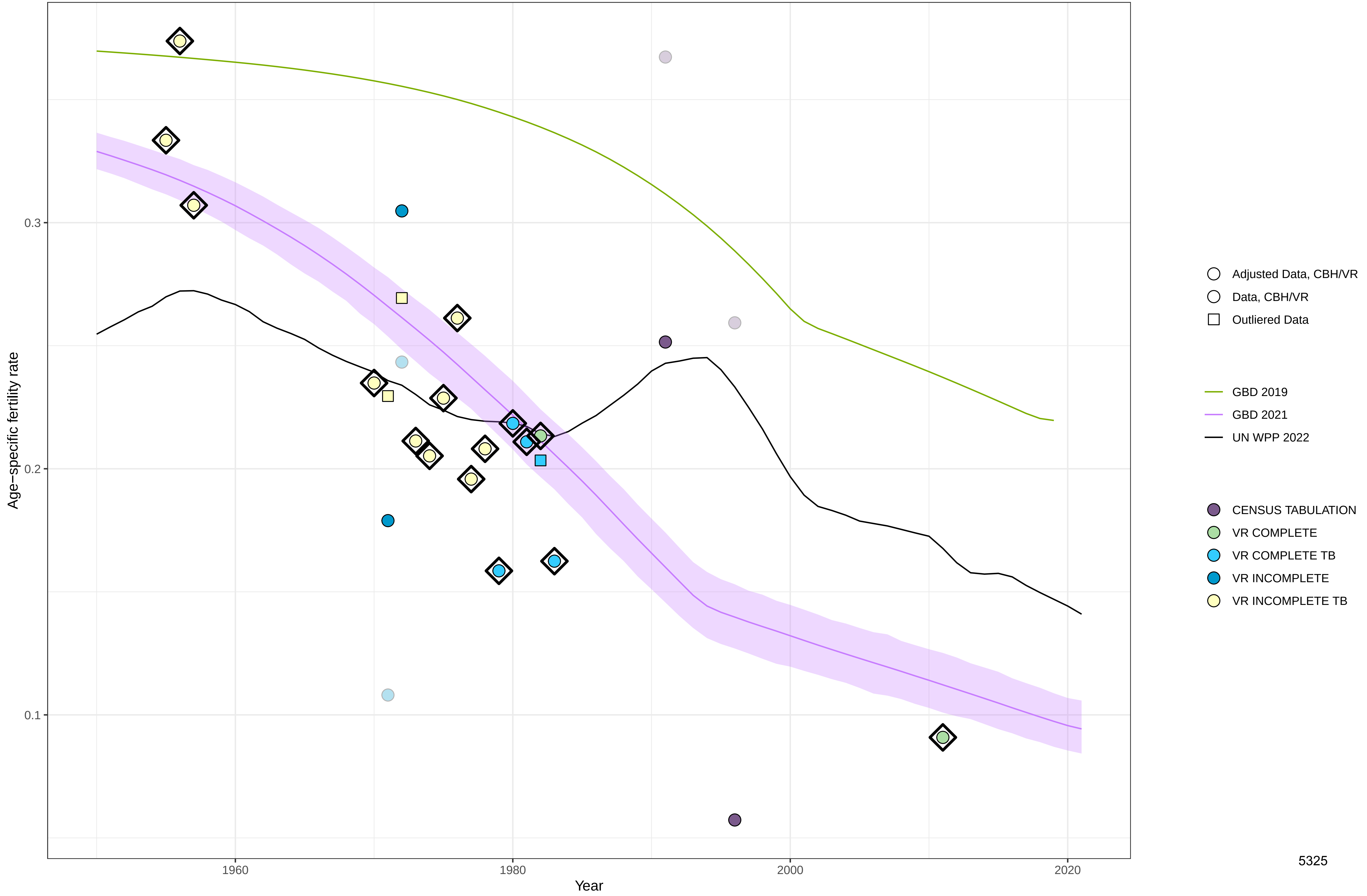

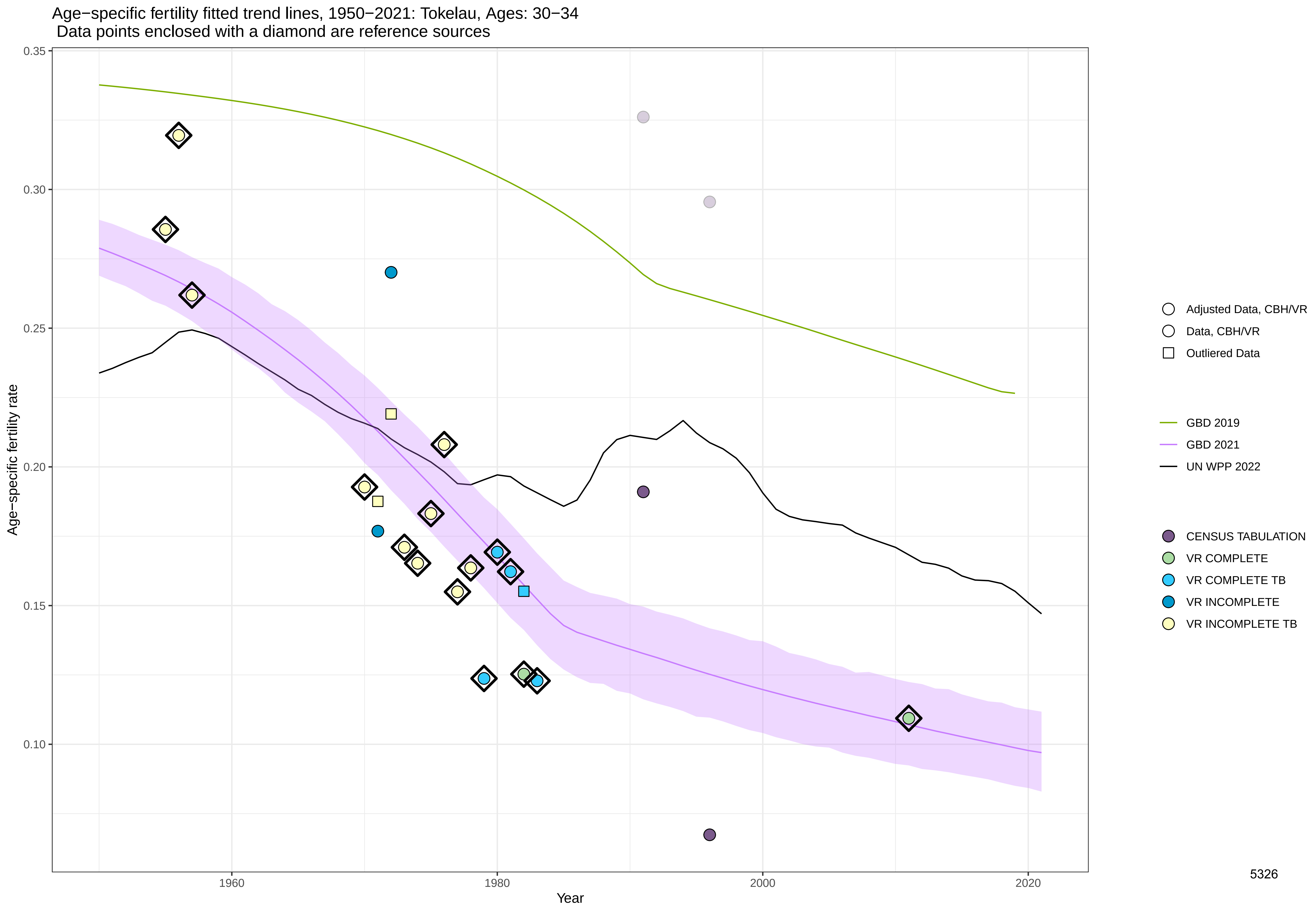

Age−specific fertility fitted trend lines, 1950−2021: Tokelau, Ages: 30−34
Data points enclosed with a diamond are reference sources
0.35
0.30
0.25
0.20
0.15
0.10
Age−specific fertility rate
1960
1980
2000
2020
Year
Adjusted Data, CBH/VR
Data, CBH/VR
Outliered Data
GBD 2019
GBD 2021
UN WPP 2022
CENSUS TABULATION
VR COMPLETE
VR COMPLETE TB
VR INCOMPLETE
VR INCOMPLETE TB

Age−specific fertility fitted trend lines, 1950−2021: Tokelau, Ages: 35−39
Data points enclosed with a diamond are reference sources

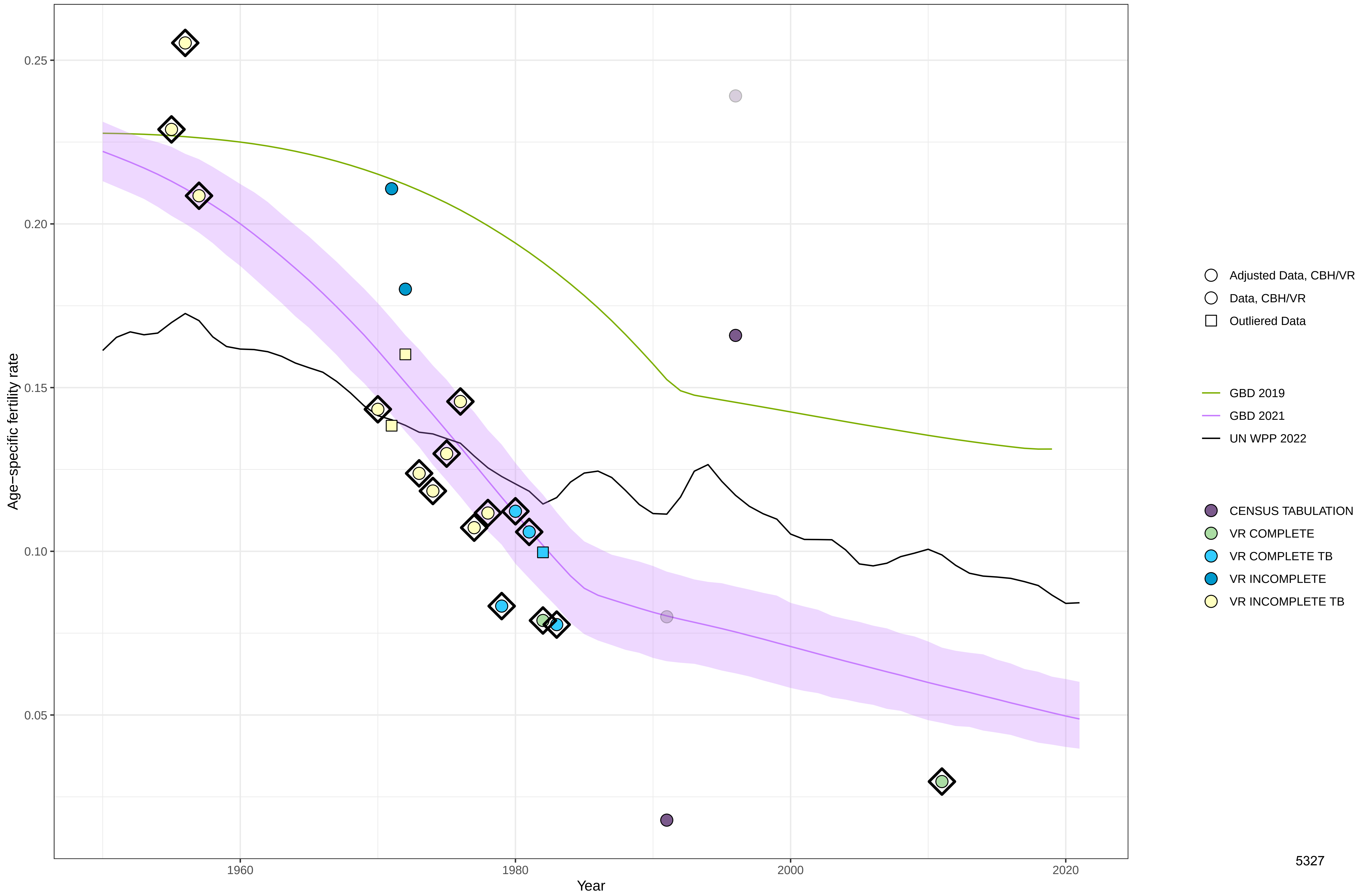

Age−specific fertility fitted trend lines, 1950−2021: Tokelau, Ages: 40−44
Data points enclosed with a diamond are reference sources

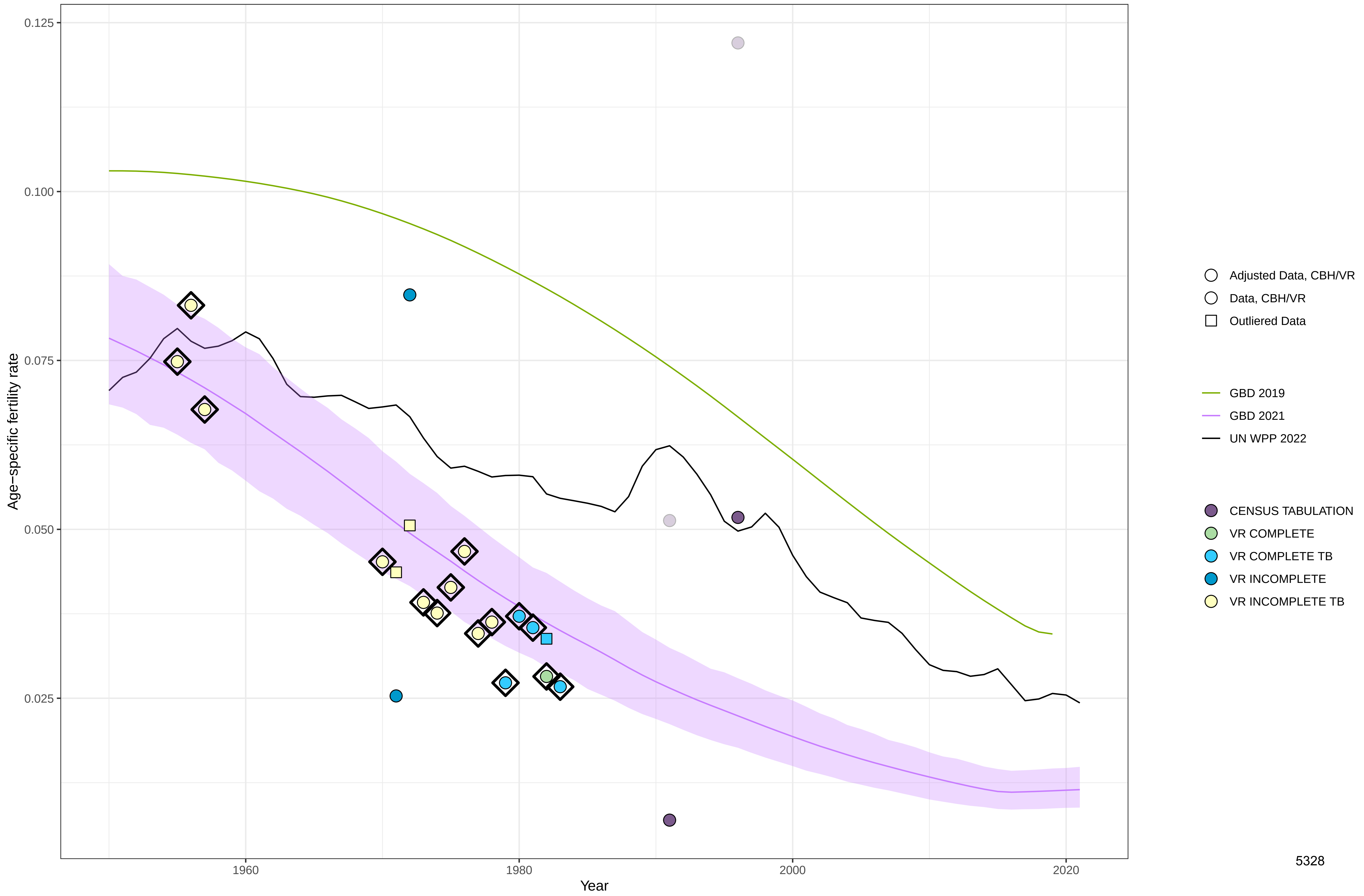

Age−specific fertility fitted trend lines, 1950−2021: Tokelau, Ages: 45−49
Data points enclosed with a diamond are reference sources

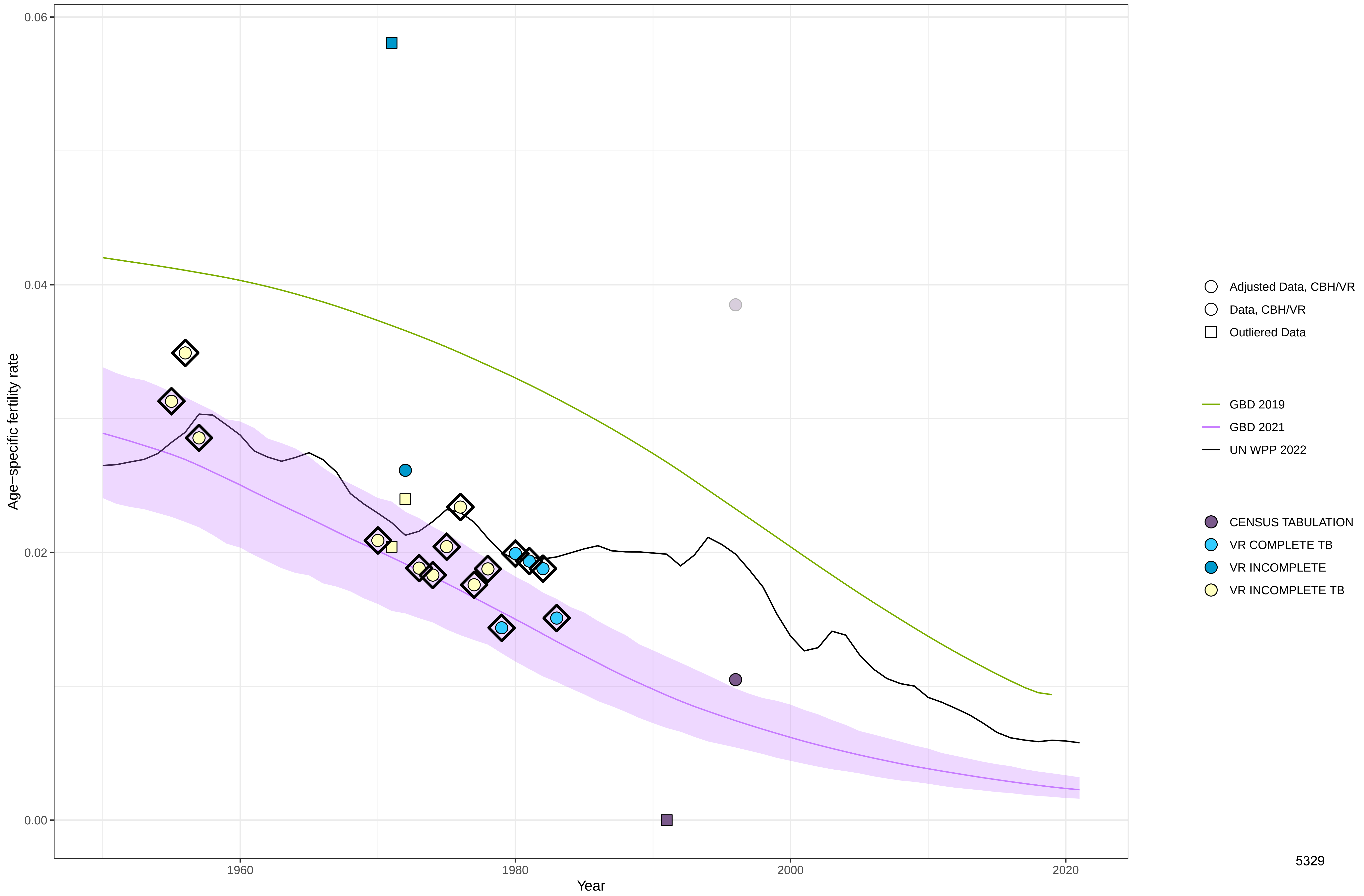

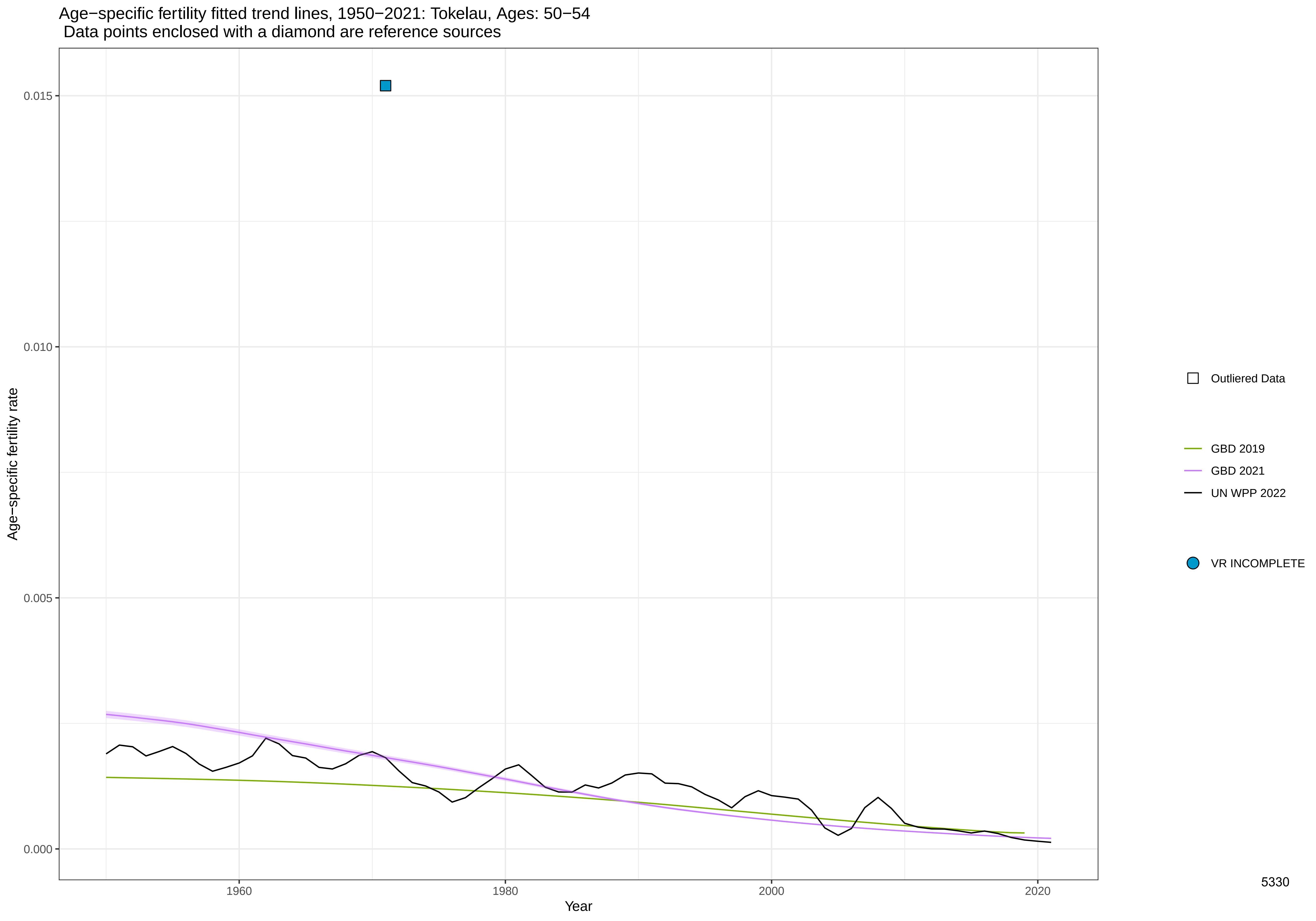

Age−specific fertility fitted trend lines, 1950−2021: Tokelau, Ages: 50−54
Data points enclosed with a diamond are reference sources
0.015
0.010
0.005
0.000
Age−specific fertility rate
1960
1980
2000
2020
Year
Outliered Data
GBD 2019
GBD 2021
UN WPP 2022
VR INCOMPLETE

Total fertility rate fitted trend lines, 1950−2021: Tokelau TFR
Data points enclosed with a diamond are reference sources

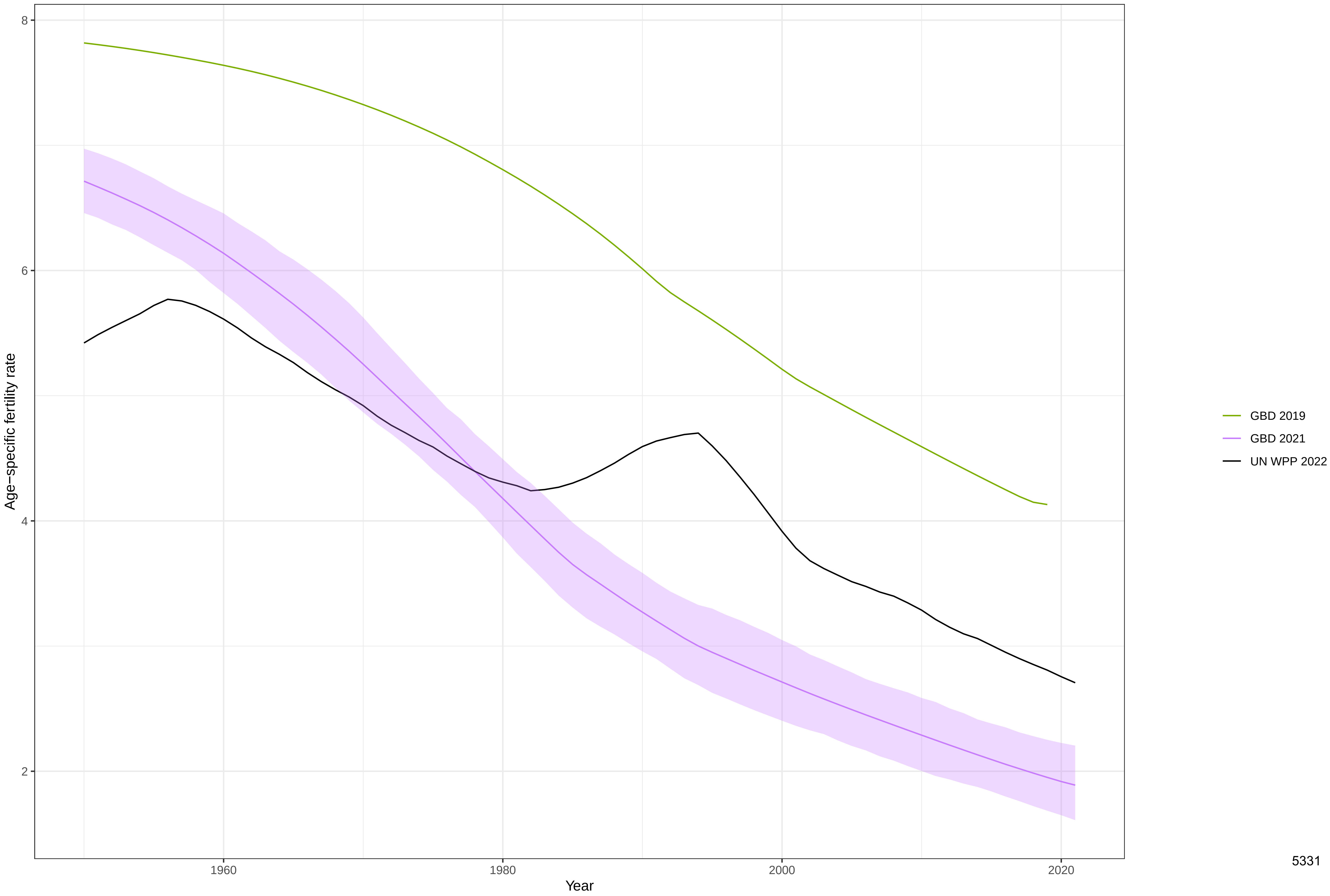

Age−specific fertility fitted trend lines, 1950−2021: Tonga, Ages: 10−14
Data points enclosed with a diamond are reference sources

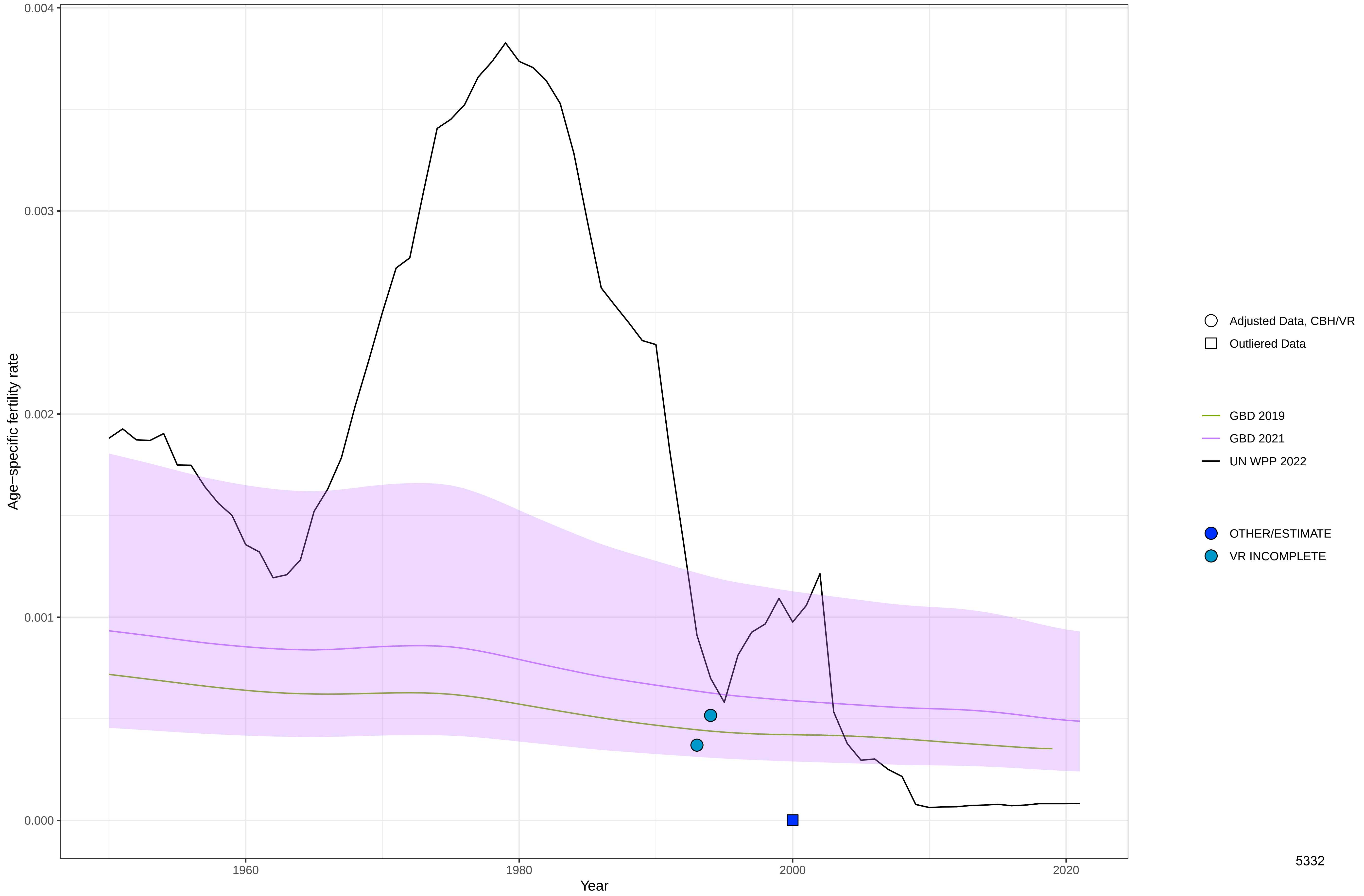

Age−specific fertility fitted trend lines, 1950−2021: Tonga, Ages: 15−19
Data points enclosed with a diamond are reference sources

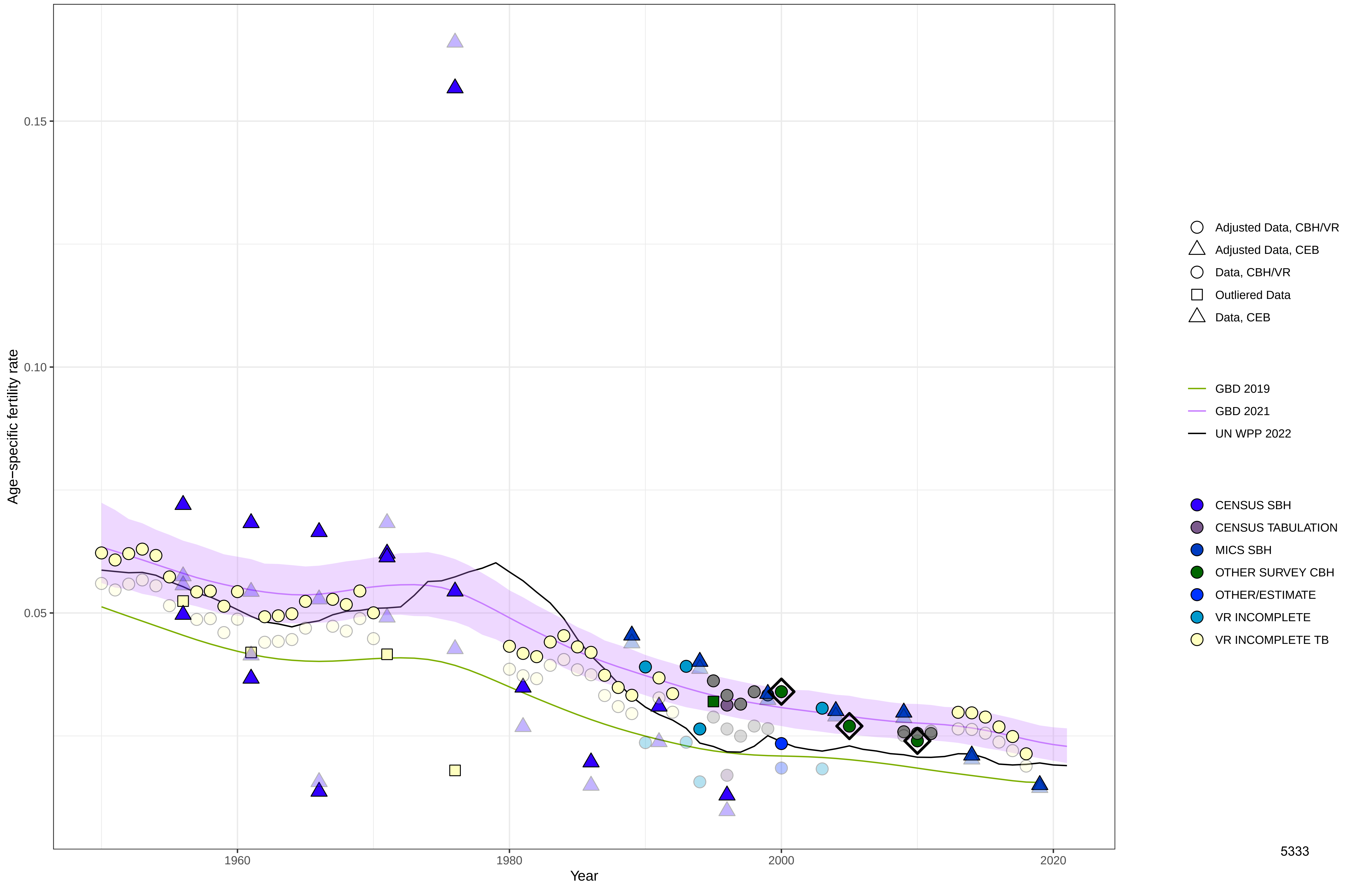

Age−specific fertility fitted trend lines, 1950−2021: Tonga, Ages: 20−24
Data points enclosed with a diamond are reference sources

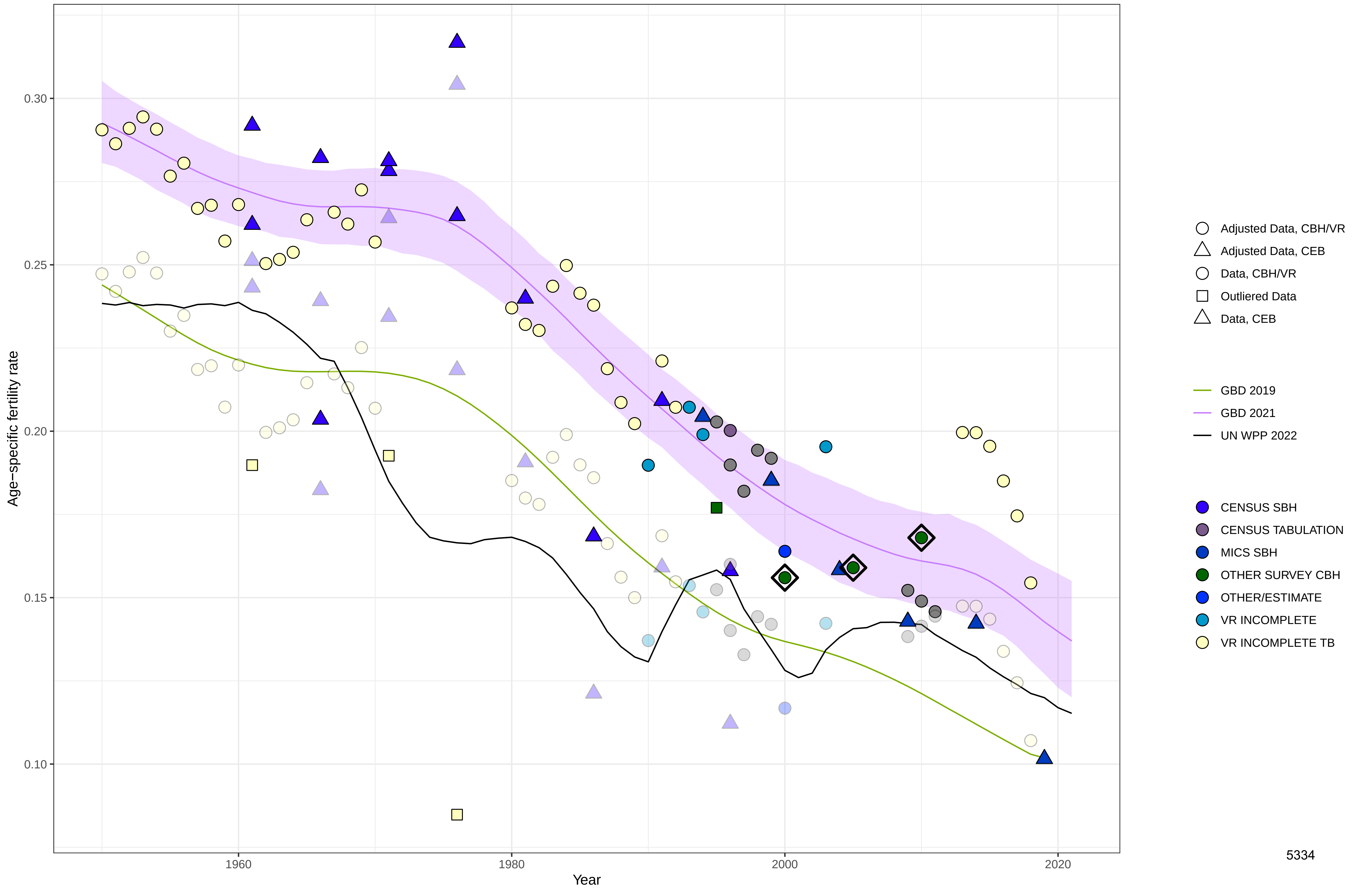

Age−specific fertility fitted trend lines, 1950−2021: Tonga, Ages: 25−29
Data points enclosed with a diamond are reference sources

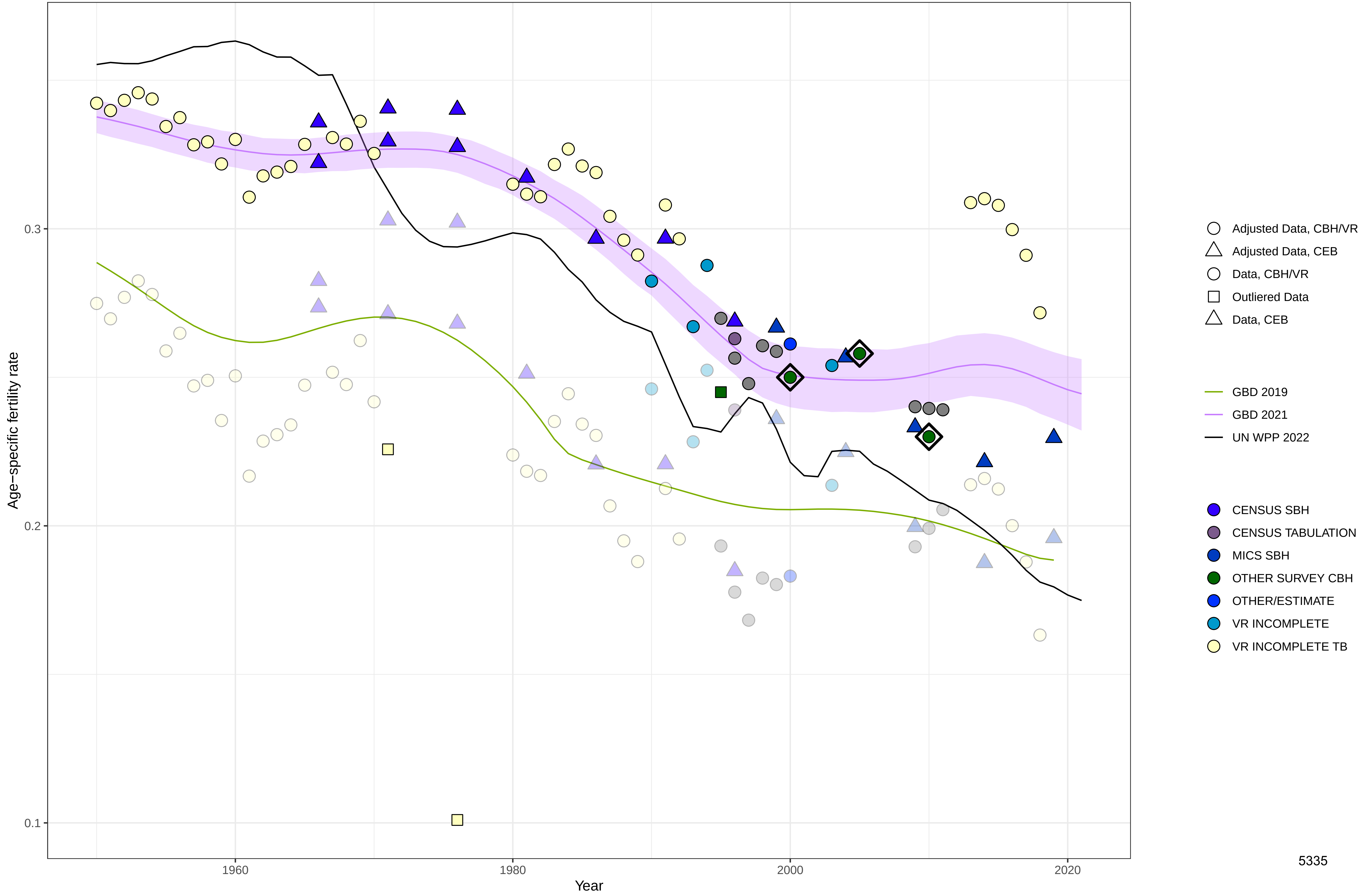

Age−specific fertility fitted trend lines, 1950−2021: Tonga, Ages: 30−34
Data points enclosed with a diamond are reference sources

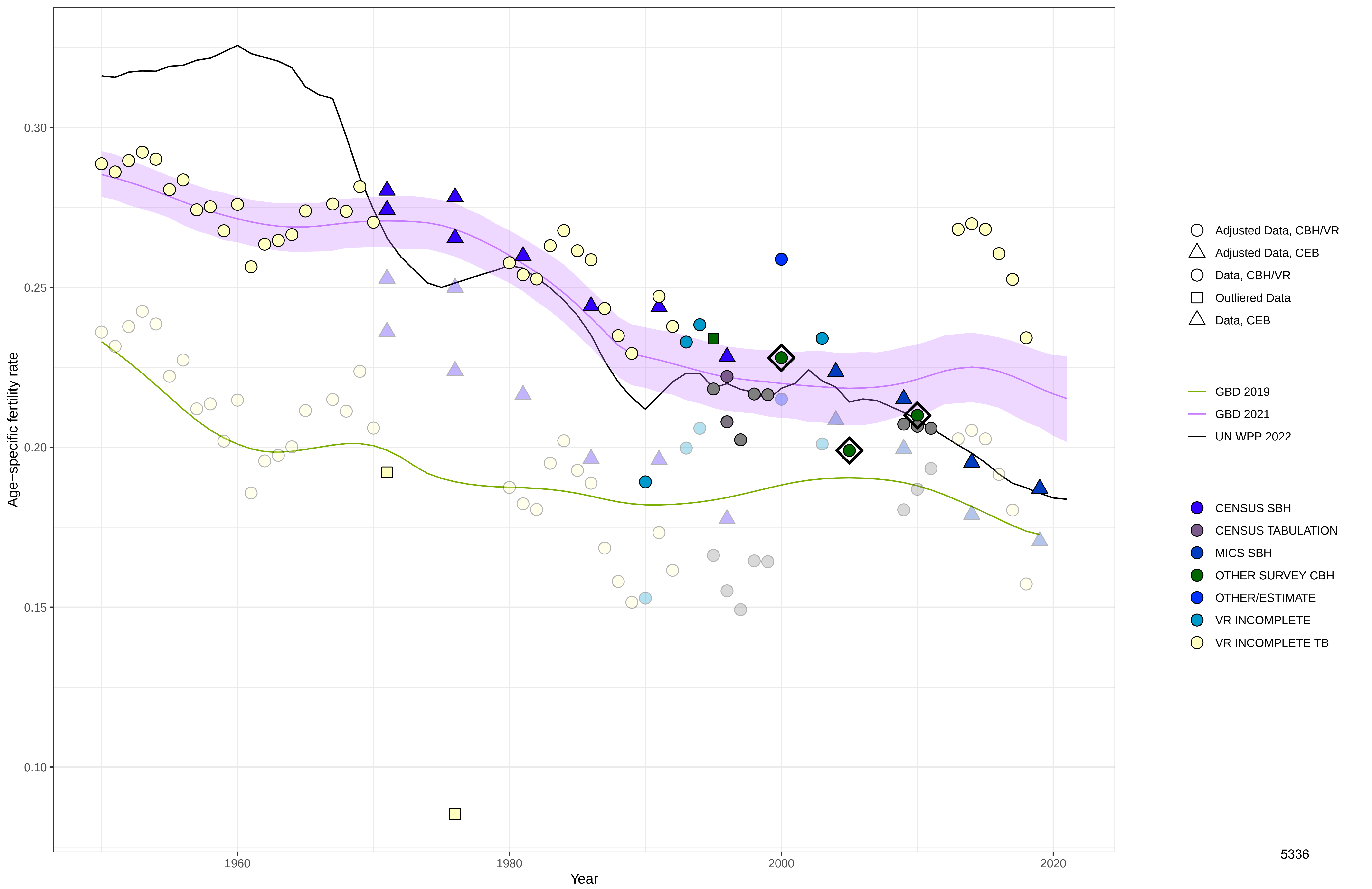

Age−specific fertility fitted trend lines, 1950−2021: Tonga, Ages: 35−39
Data points enclosed with a diamond are reference sources

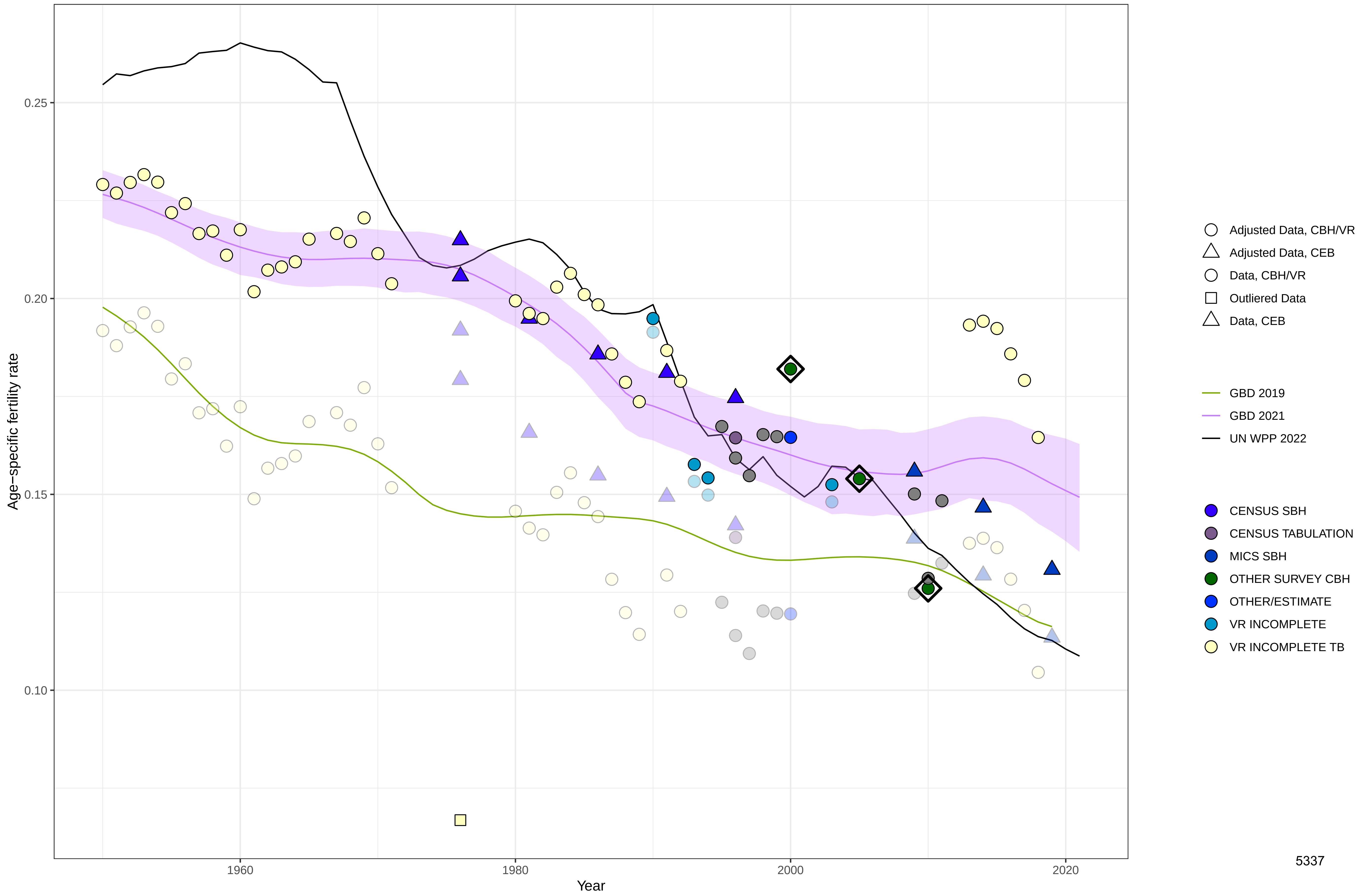

Age−specific fertility fitted trend lines, 1950−2021: Tonga, Ages: 40−44
Data points enclosed with a diamond are reference sources

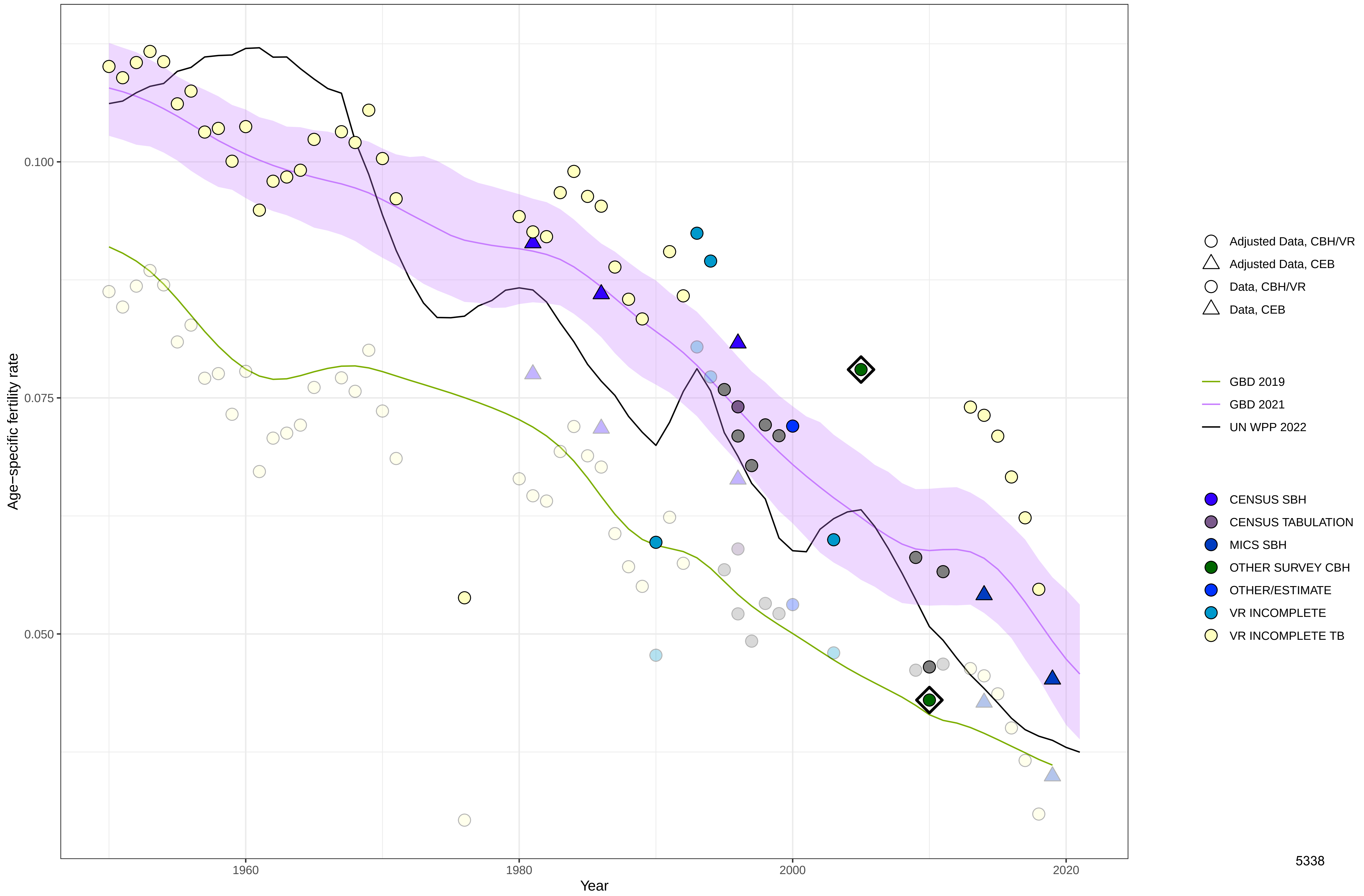

Age−specific fertility fitted trend lines, 1950−2021: Tonga, Ages: 45−49
Data points enclosed with a diamond are reference sources

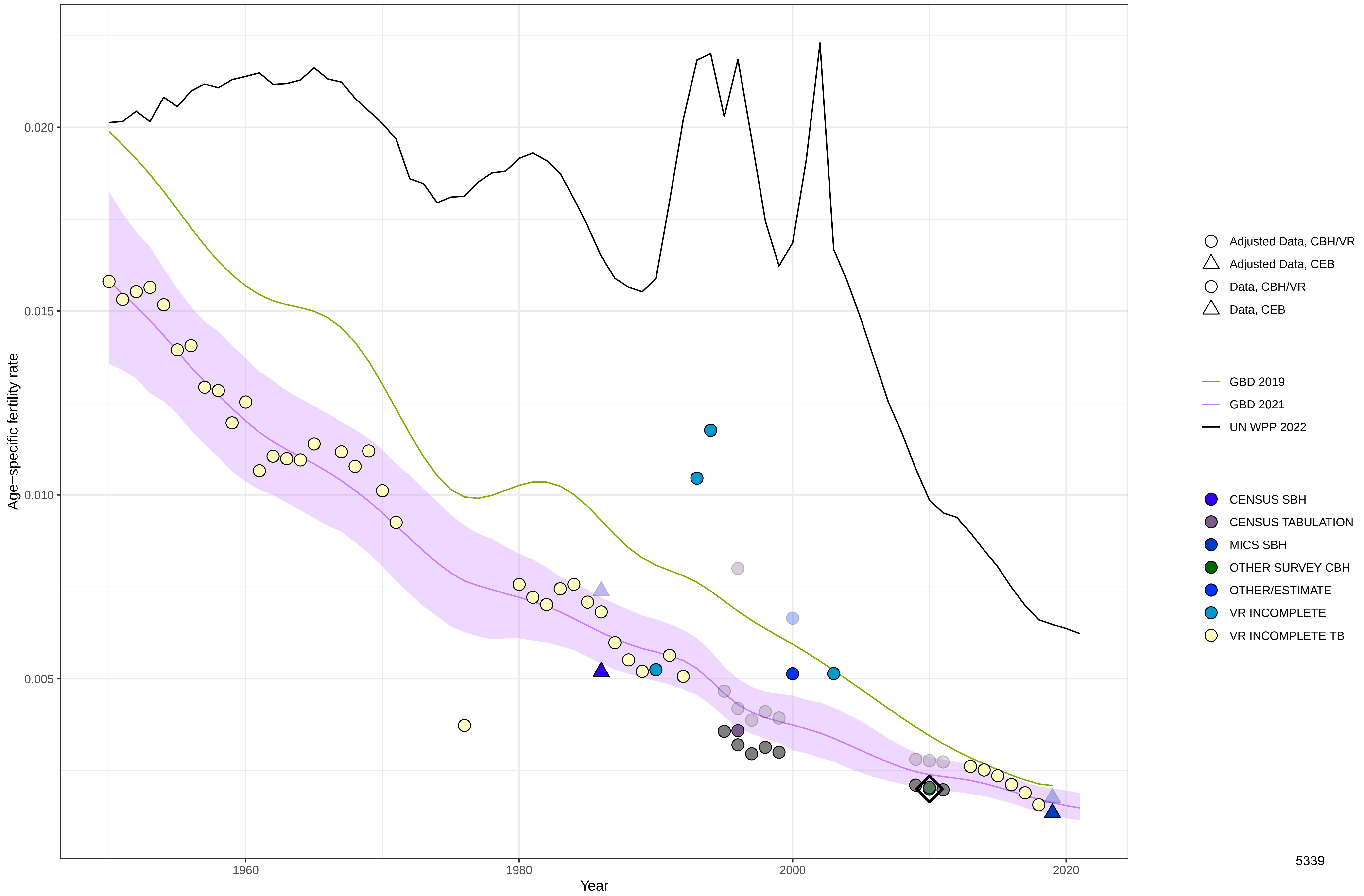

Age−specific fertility fitted trend lines, 1950−2021: Tonga, Ages: 50−54
Data points enclosed with a diamond are reference sources

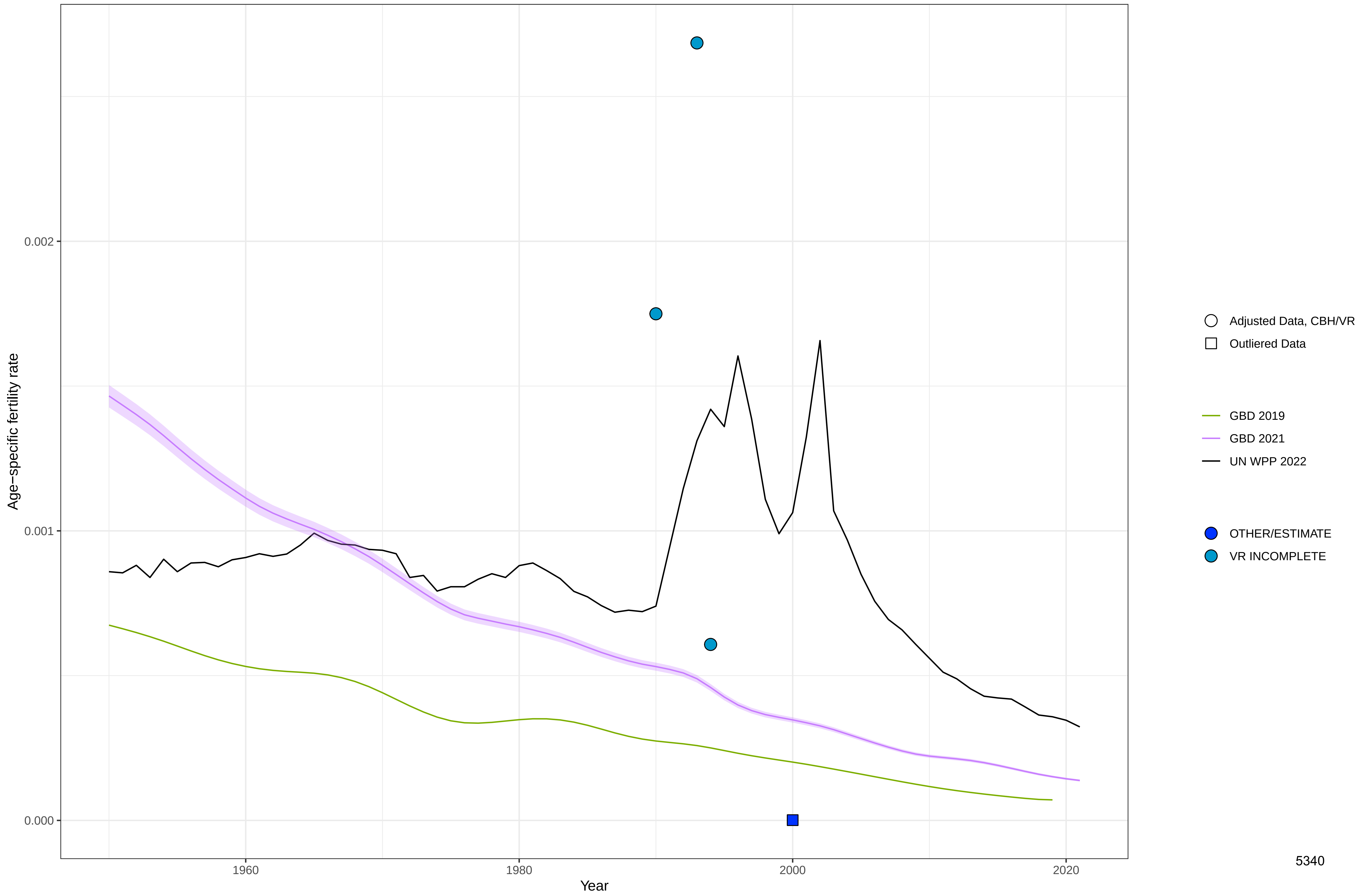

Total fertility rate fitted trend lines, 1950−2021: Tonga TFR
Data points enclosed with a diamond are reference sources

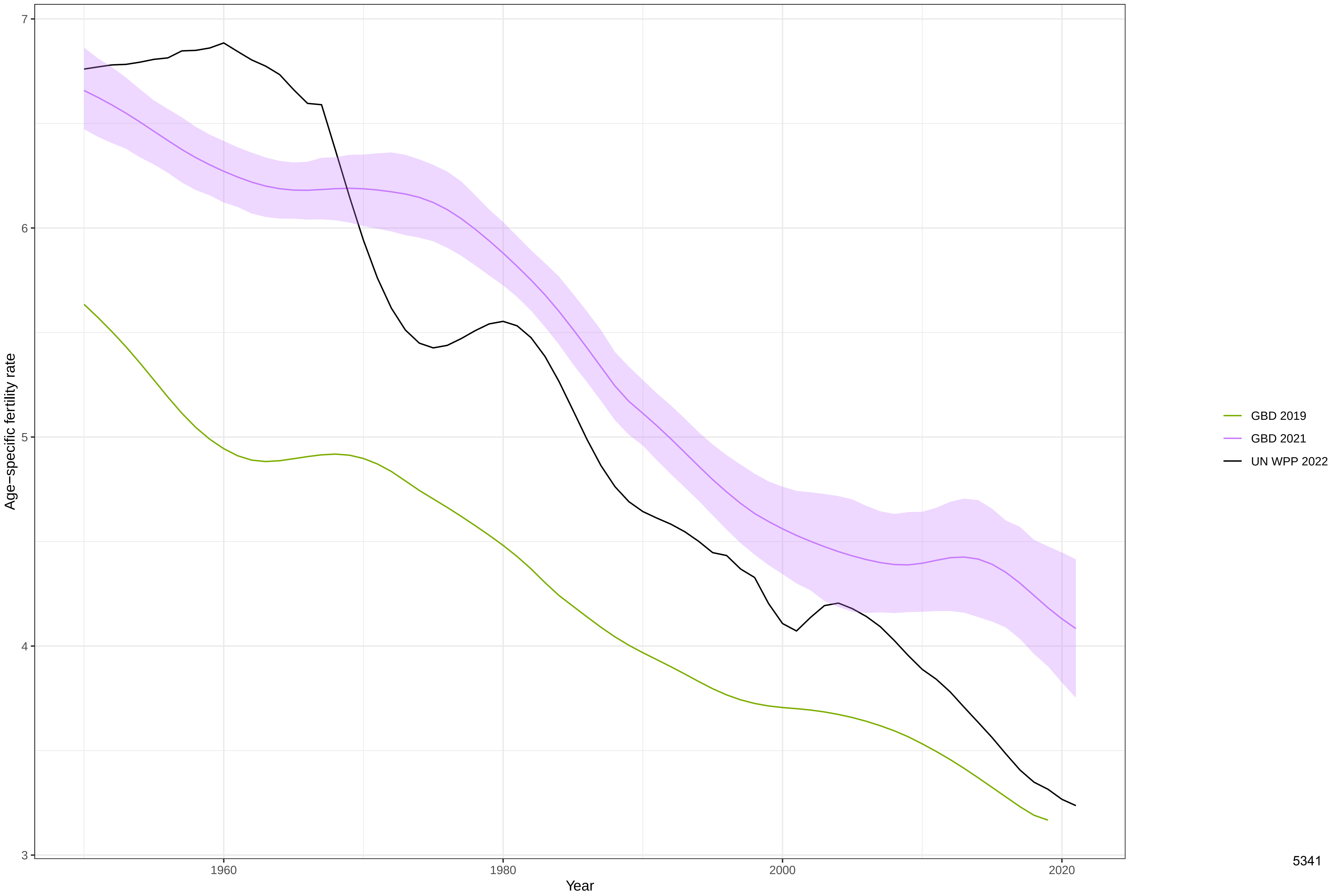

Age−specific fertility fitted trend lines, 1950−2021: Tuvalu, Ages: 10−14
Data points enclosed with a diamond are reference sources

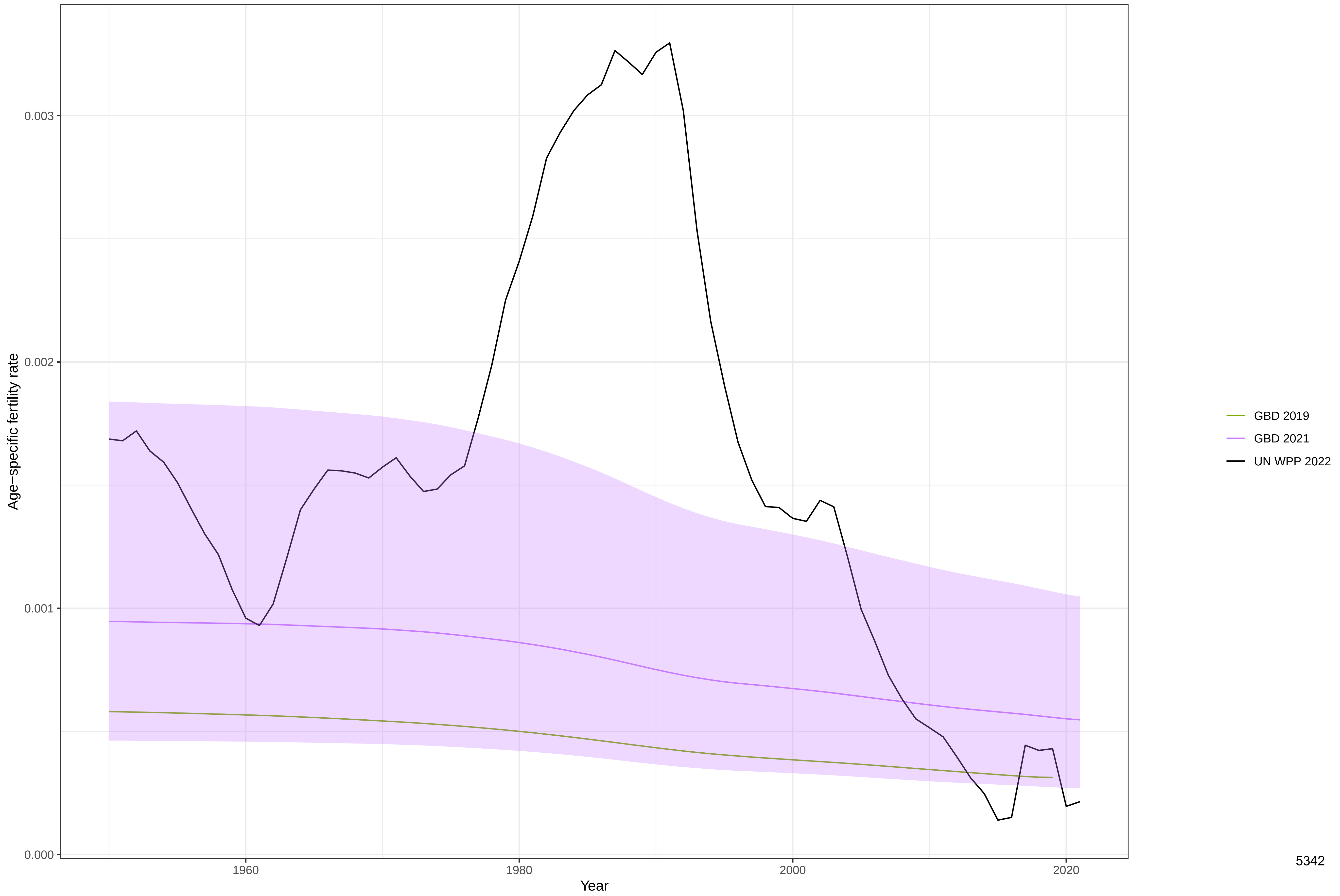

Age−specific fertility fitted trend lines, 1950−2021: Tuvalu, Ages: 15−19
Data points enclosed with a diamond are reference sources

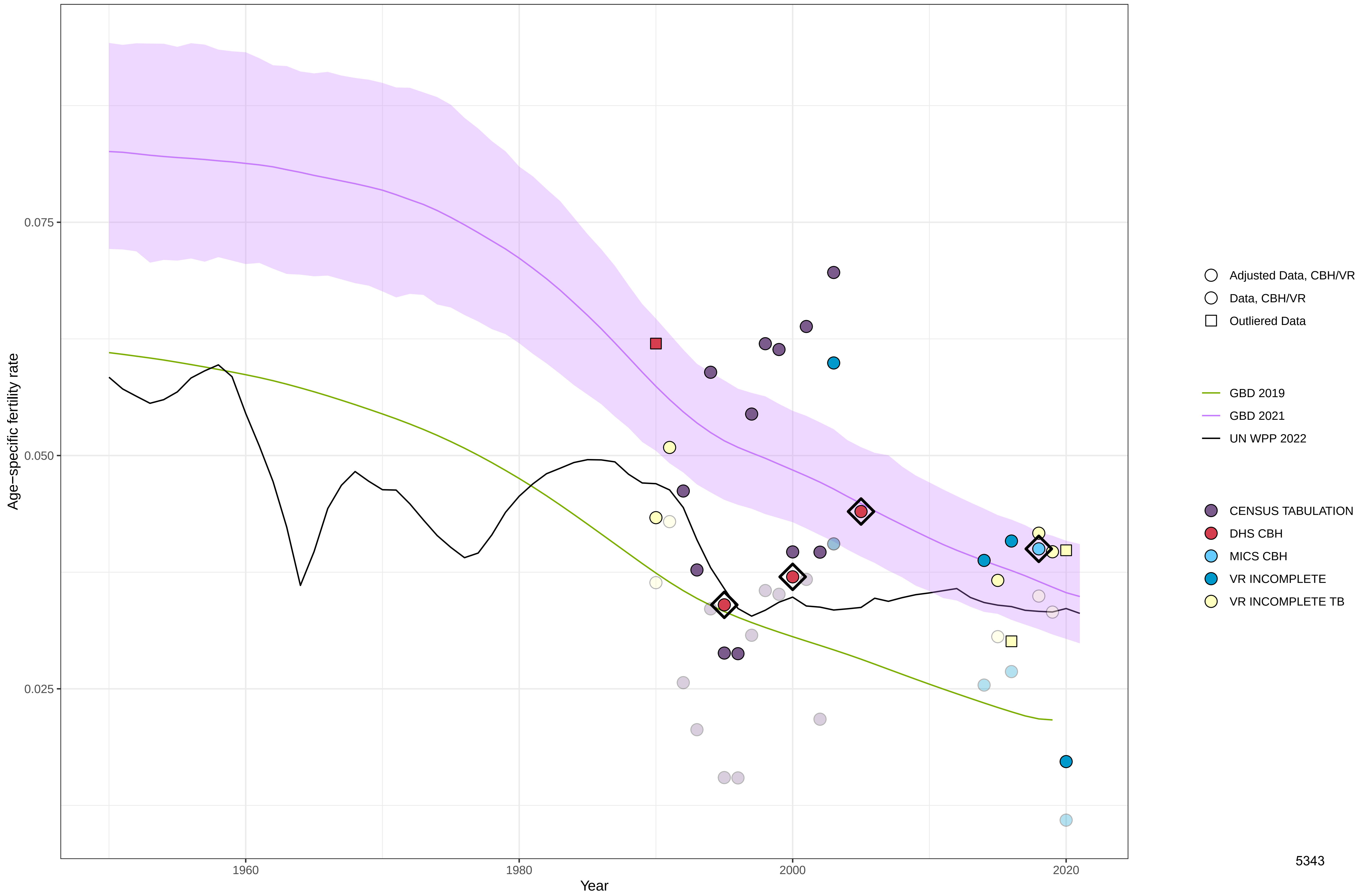

Age−specific fertility fitted trend lines, 1950−2021: Tuvalu, Ages: 20−24
Data points enclosed with a diamond are reference sources

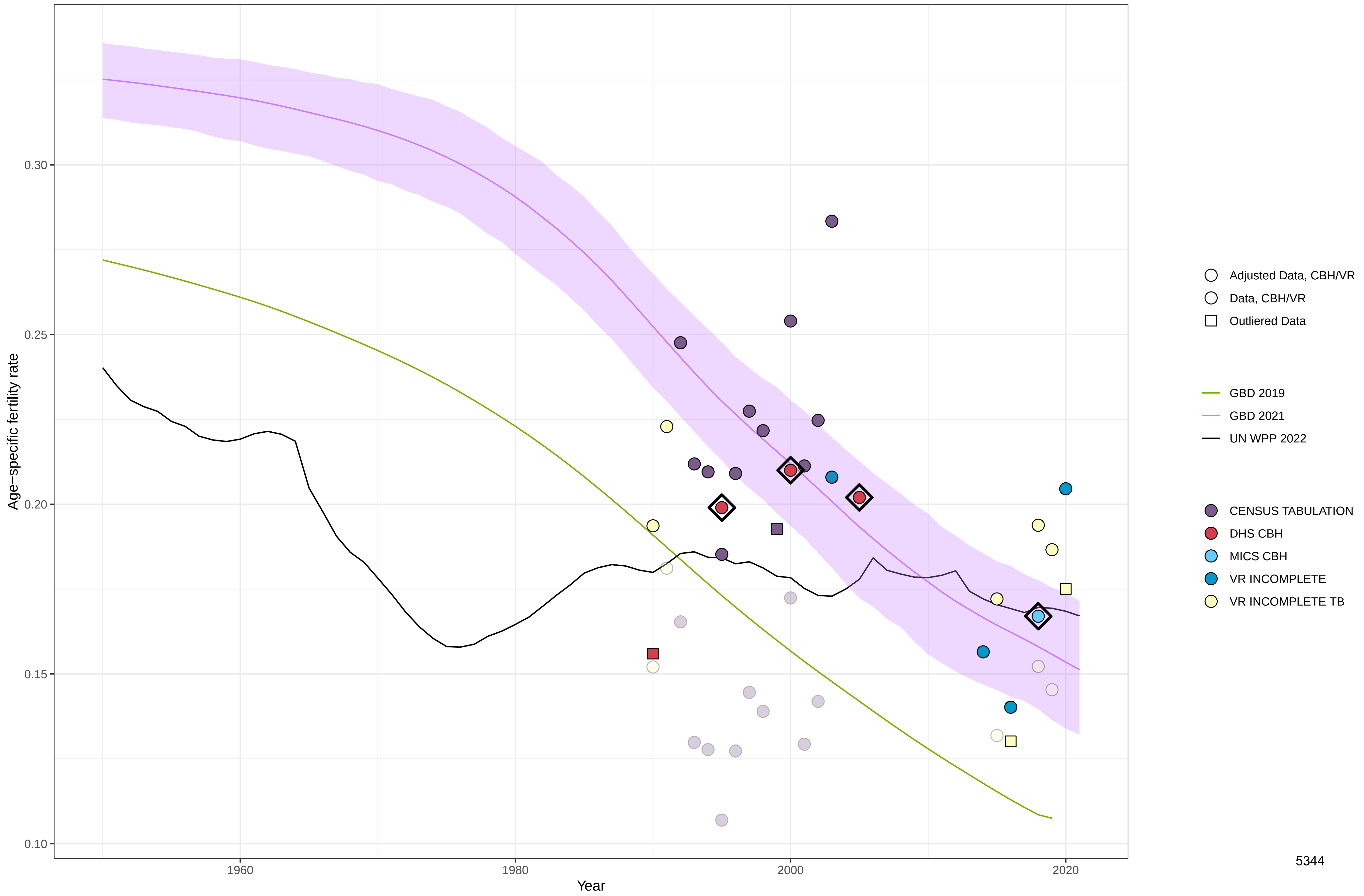

Age−specific fertility fitted trend lines, 1950−2021: Tuvalu, Ages: 25−29
Data points enclosed with a diamond are reference sources

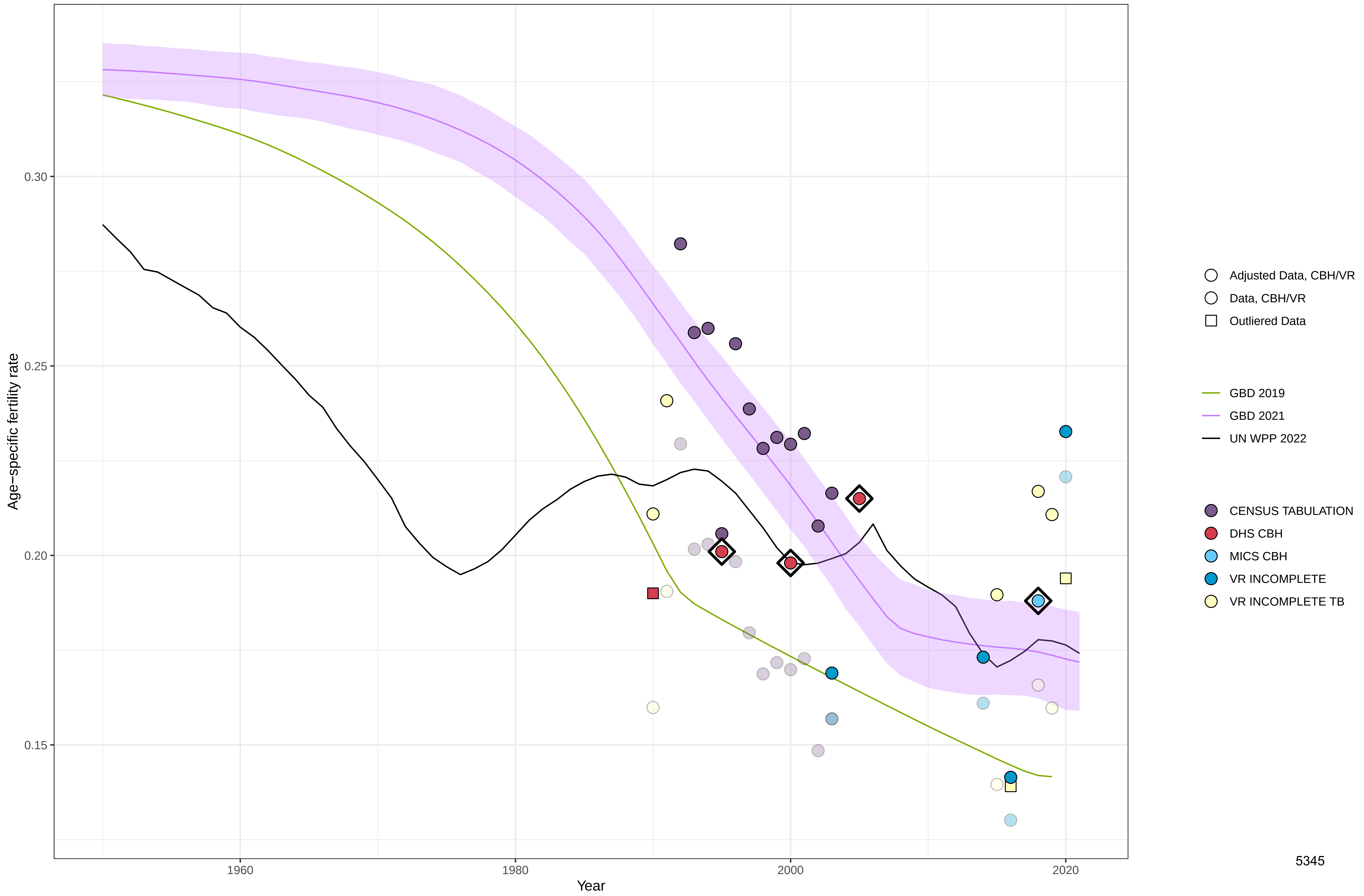

Age−specific fertility fitted trend lines, 1950−2021: Tuvalu, Ages: 30−34
Data points enclosed with a diamond are reference sources

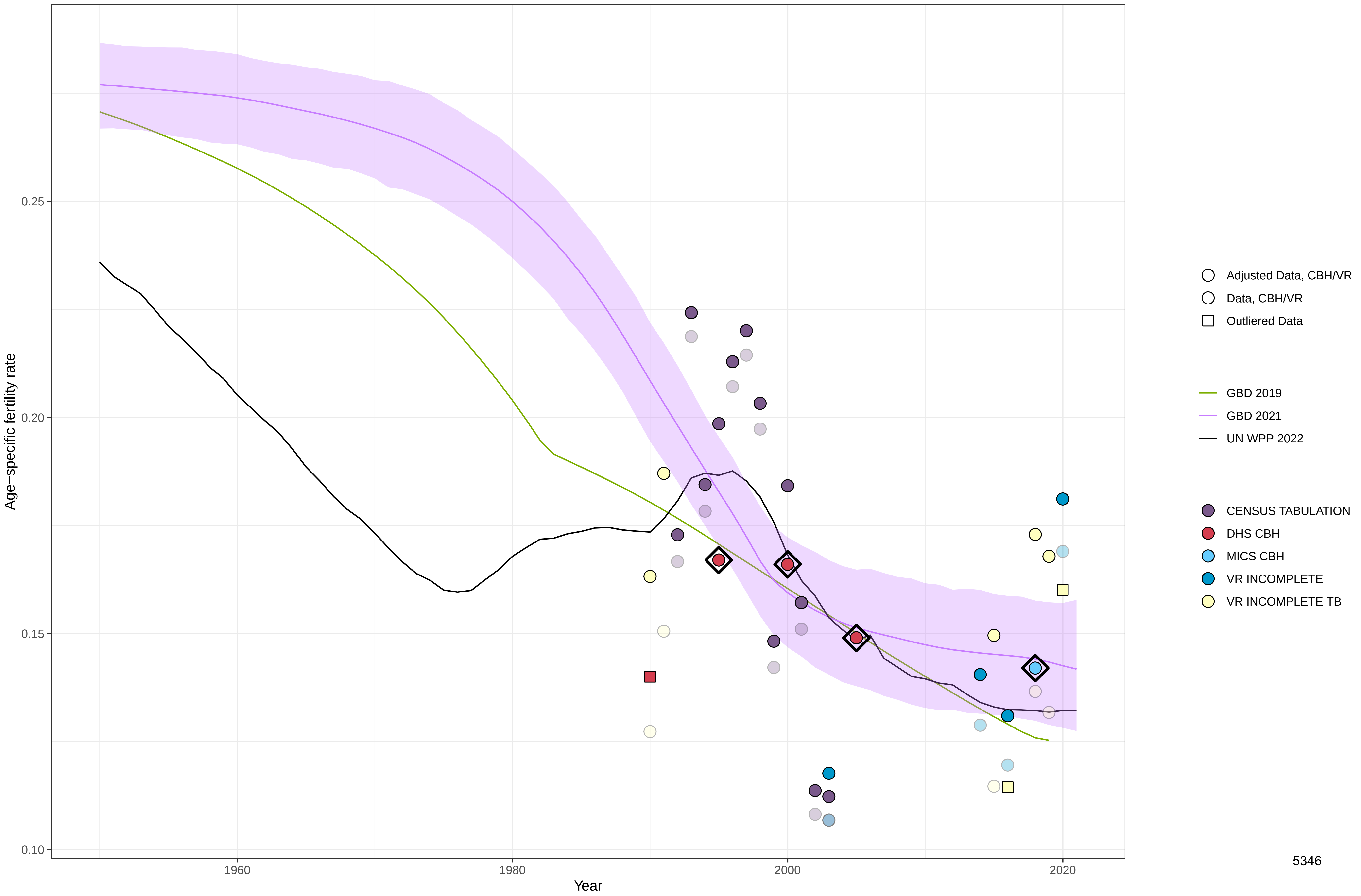

Age−specific fertility fitted trend lines, 1950−2021: Tuvalu, Ages: 35−39
Data points enclosed with a diamond are reference sources

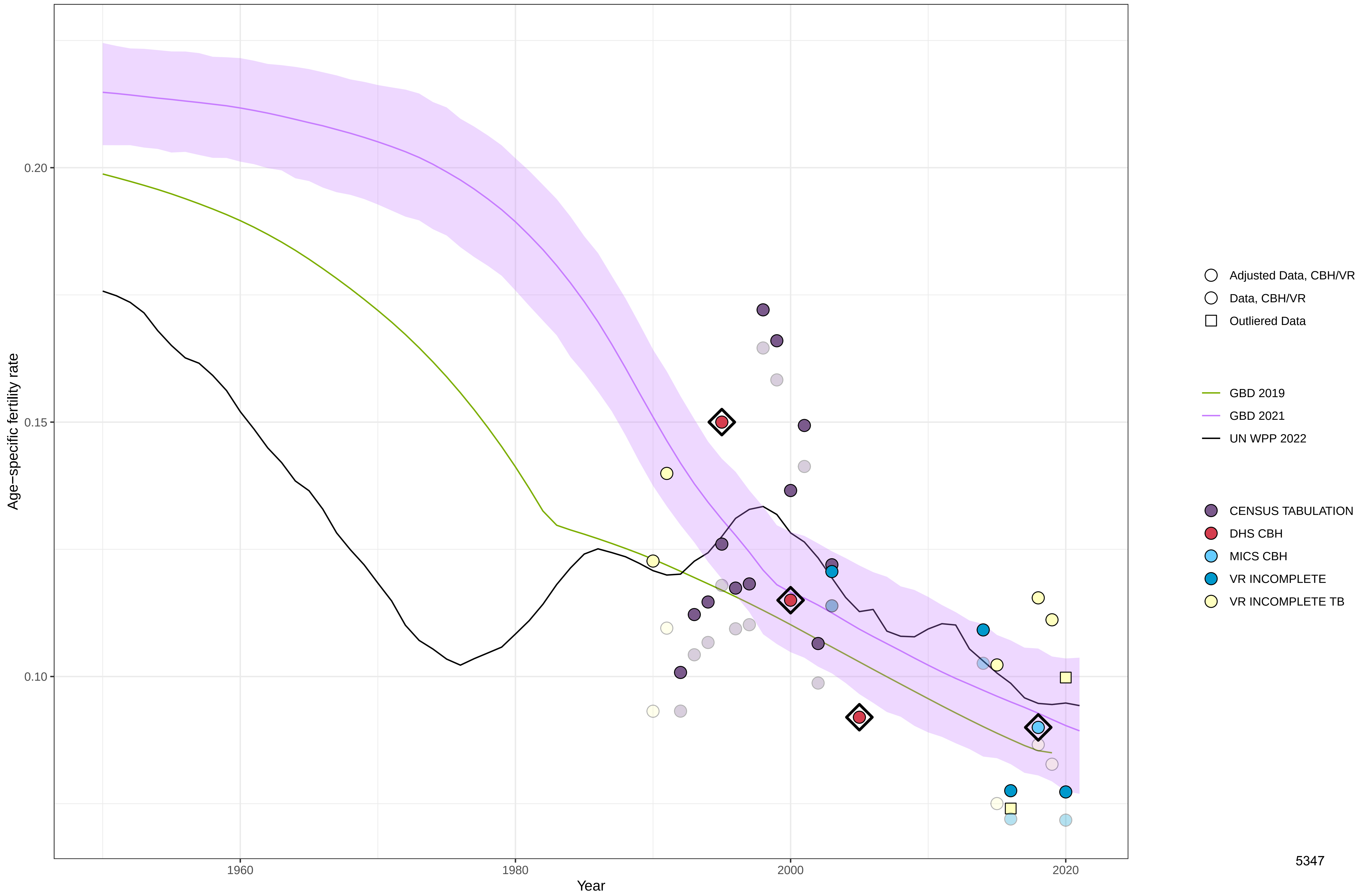

Age−specific fertility fitted trend lines, 1950−2021: Tuvalu, Ages: 40−44
Data points enclosed with a diamond are reference sources

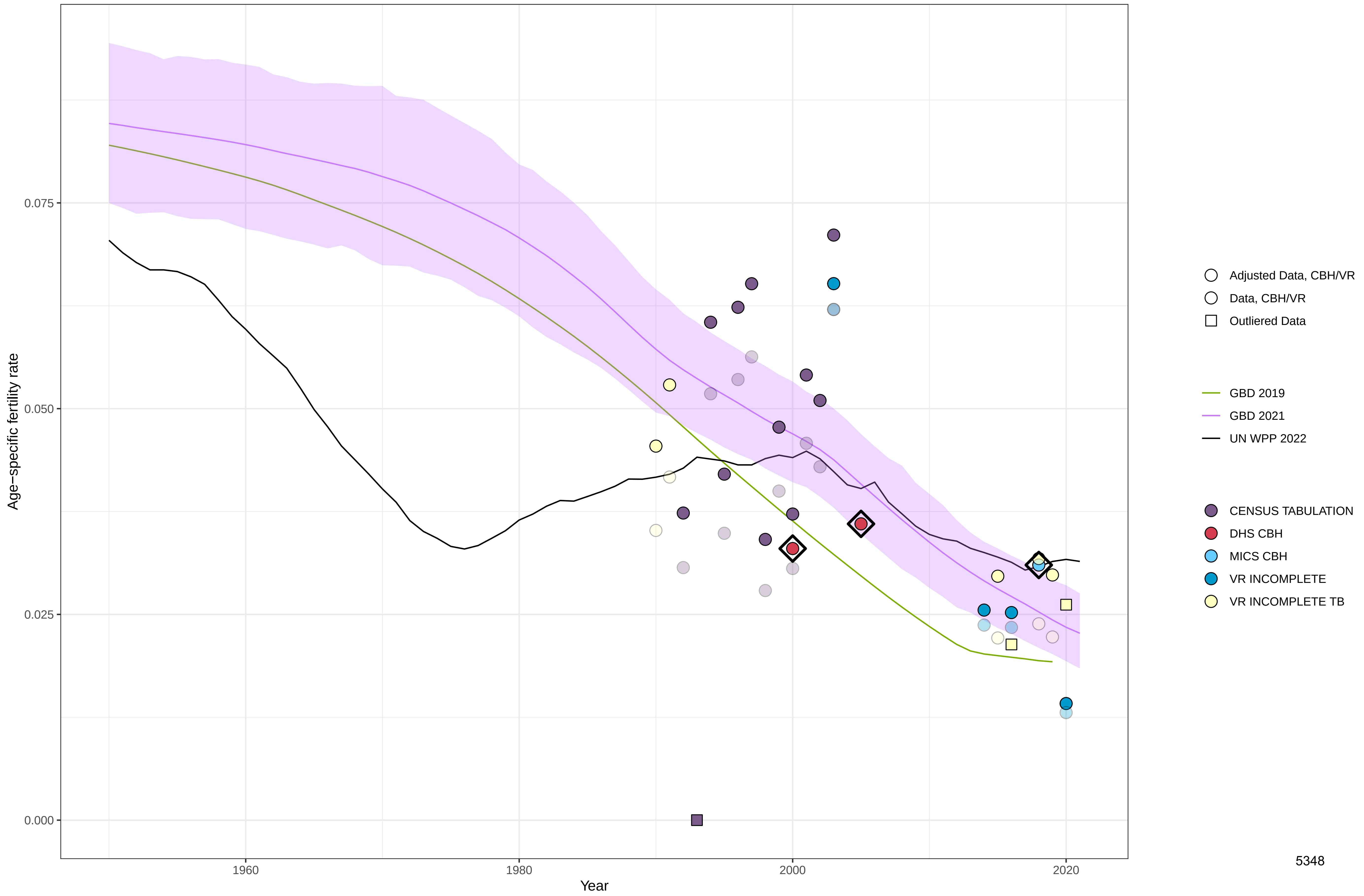

Age−specific fertility fitted trend lines, 1950−2021: Tuvalu, Ages: 45−49
Data points enclosed with a diamond are reference sources

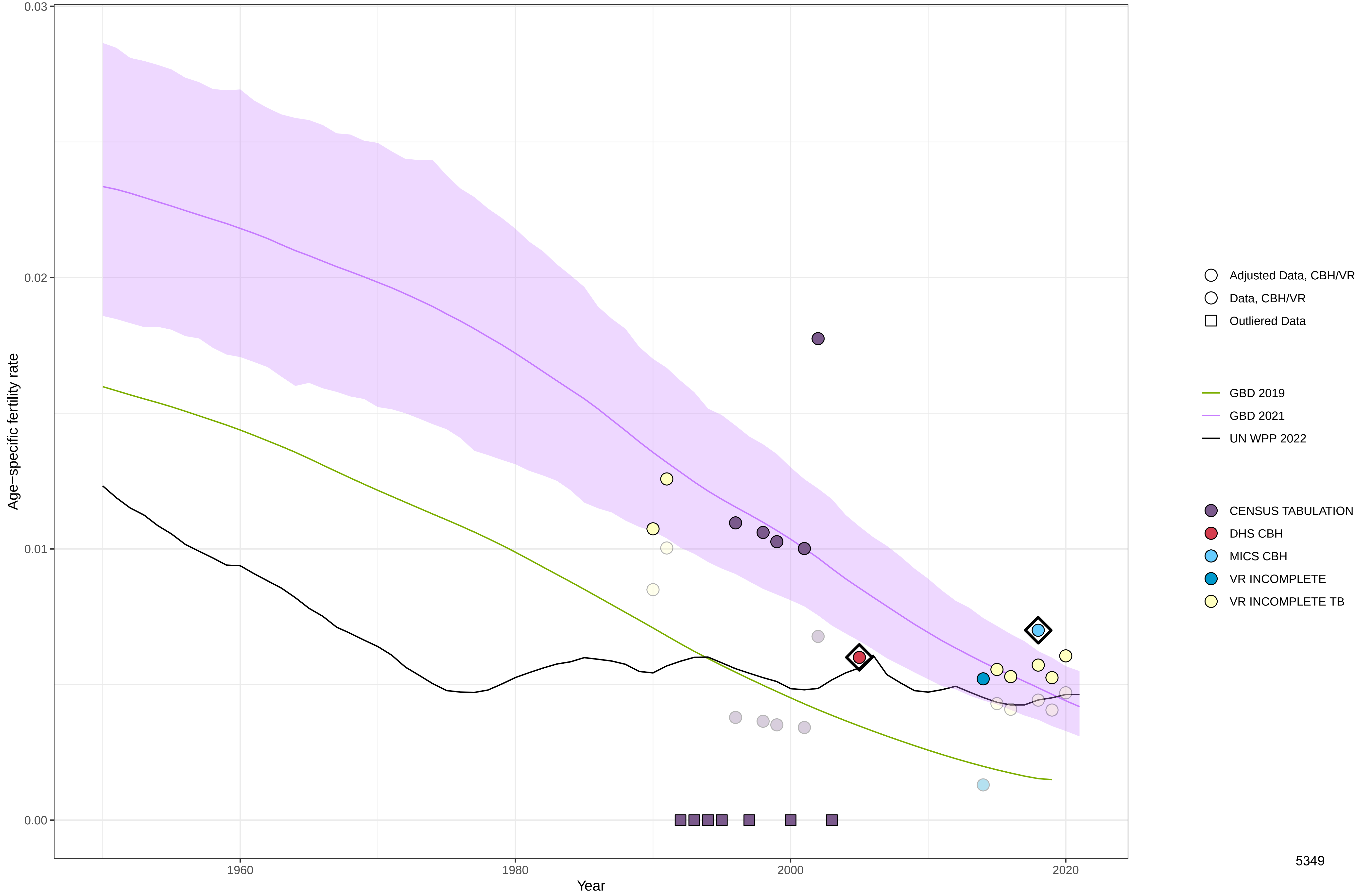

Age−specific fertility fitted trend lines, 1950−2021: Tuvalu, Ages: 50−54
Data points enclosed with a diamond are reference sources

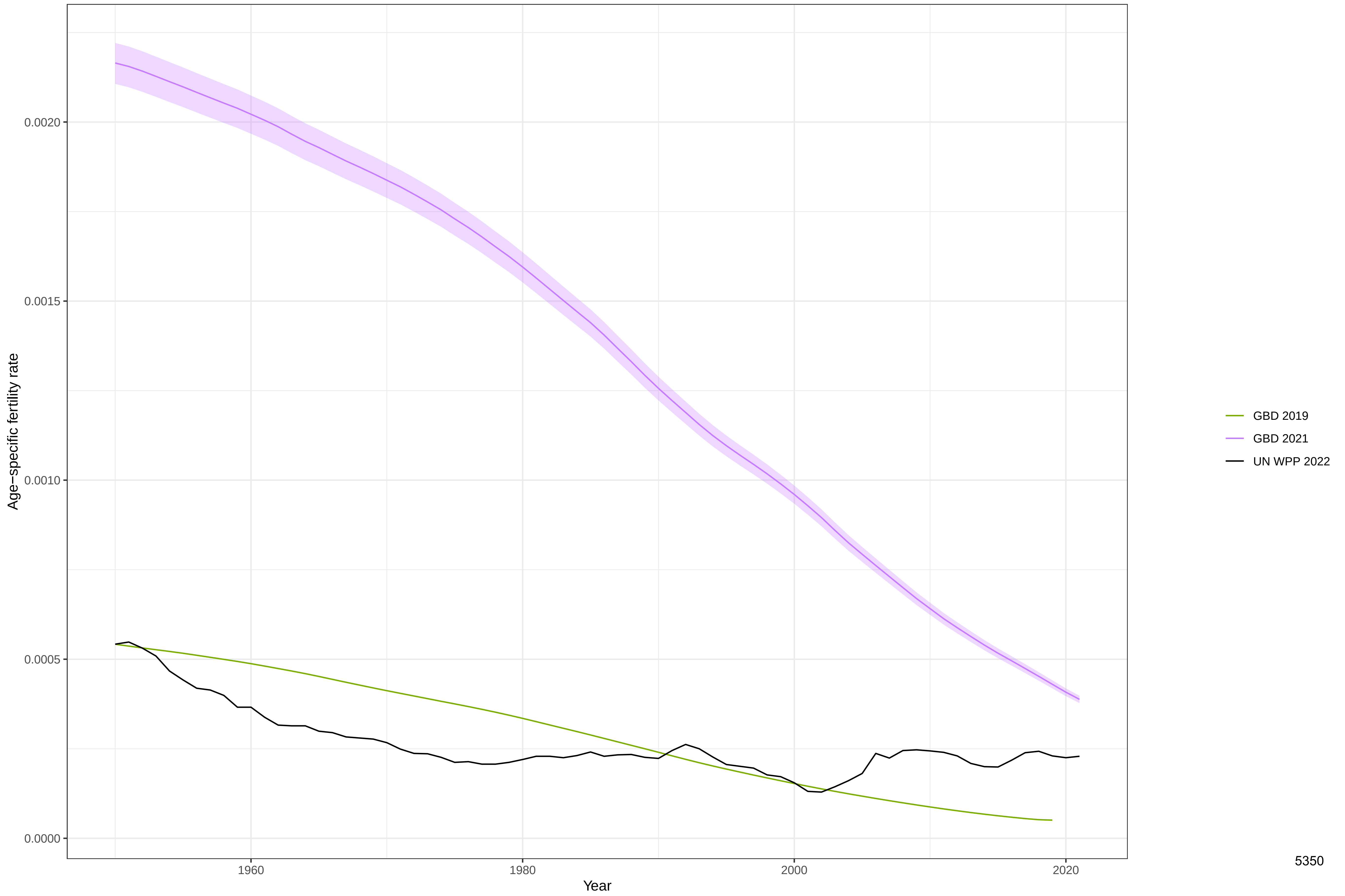

Total fertility rate fitted trend lines, 1950−2021: Tuvalu TFR
Data points enclosed with a diamond are reference sources

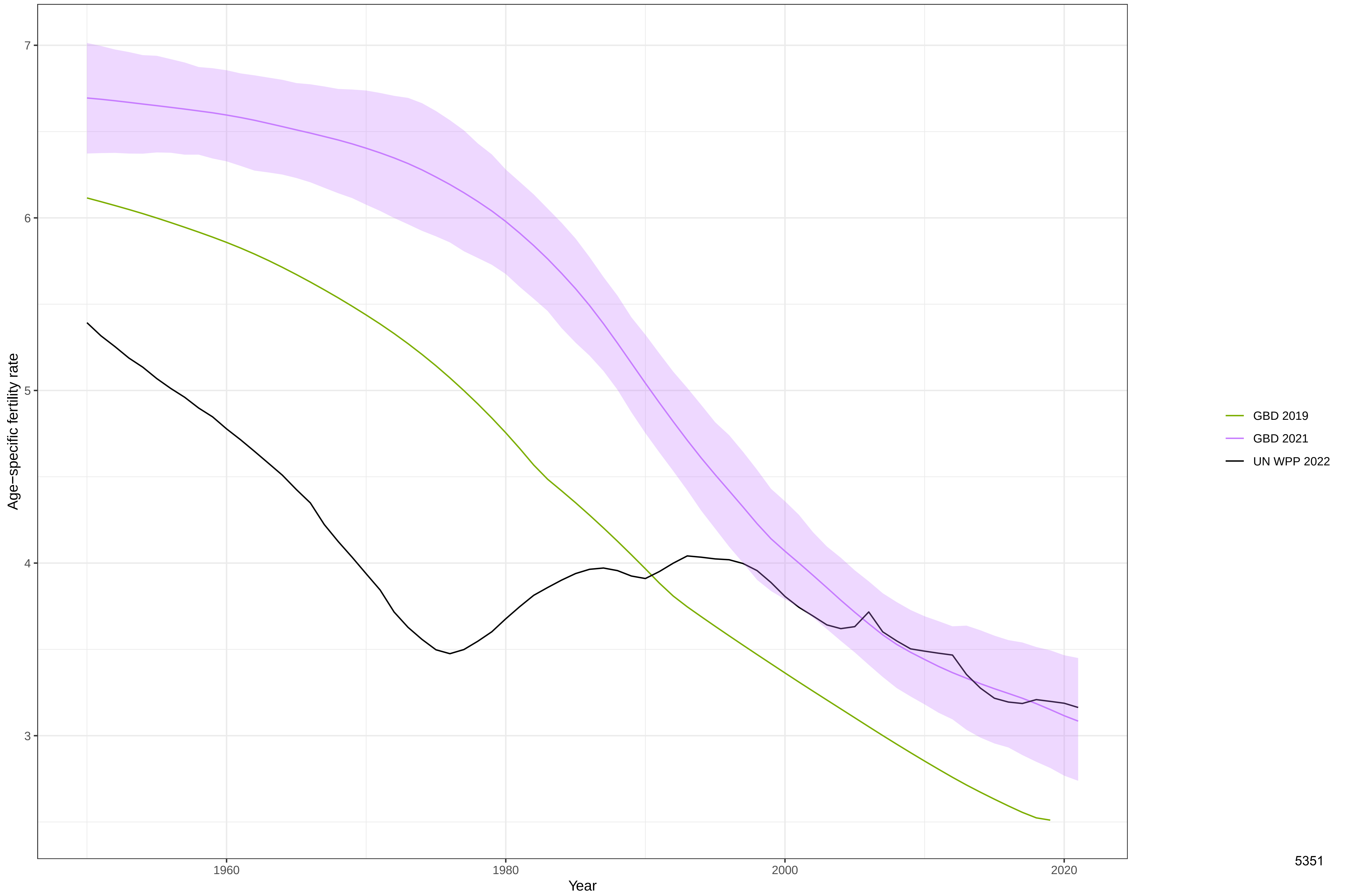

Age−specific fertility fitted trend lines, 1950−2021: Vanuatu, Ages: 10−14
Data points enclosed with a diamond are reference sources

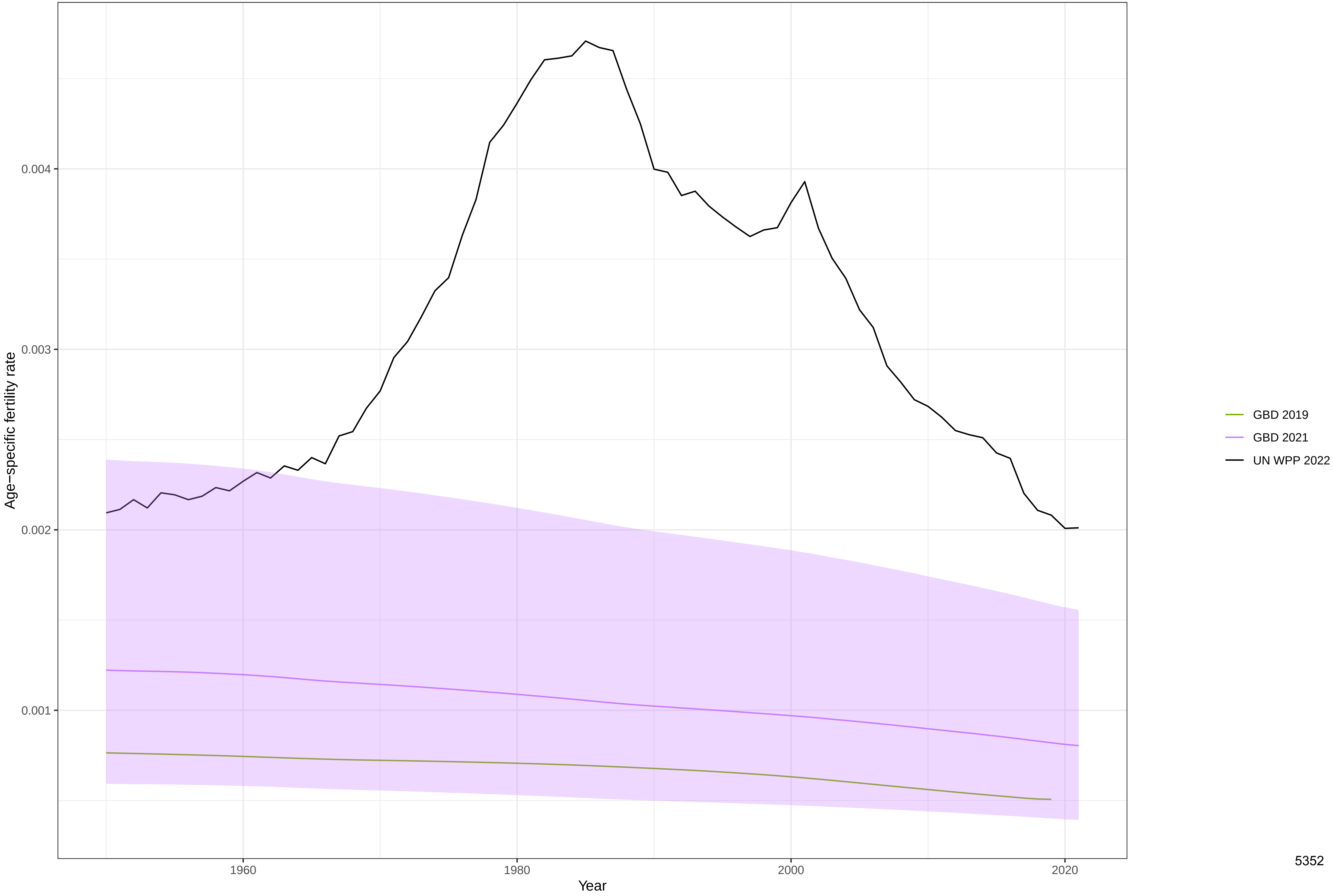

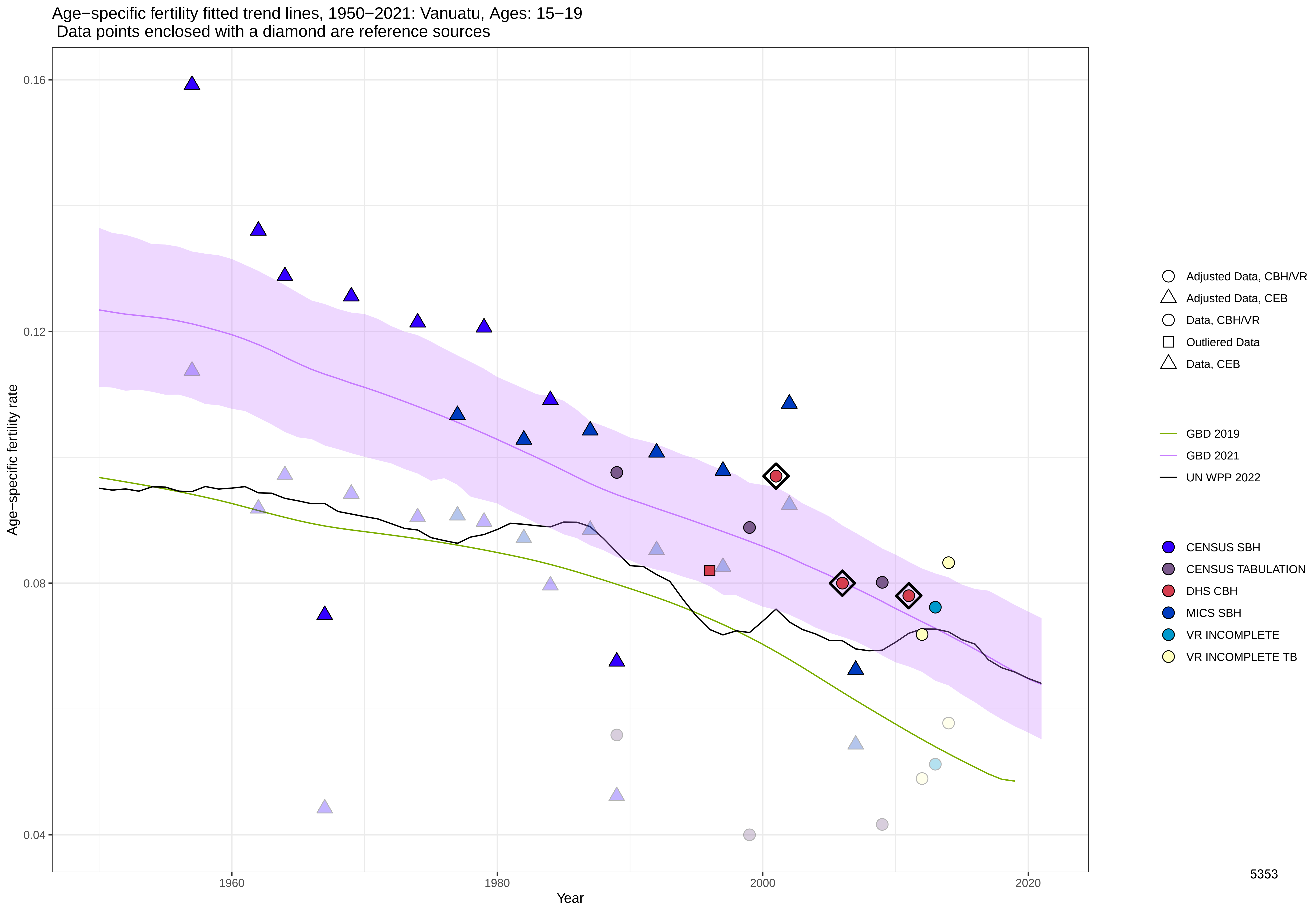
Age−specific fertility fitted trend lines, 1950−2021: Vanuatu, Ages: 15−19
Data points enclosed with a diamond are reference sources
Age−specific fertility rate
0.16
0.12
0.08
0.04
1960
1980
2000
2020
Year
Adjusted Data, CBH/VR
Adjusted Data, CEB
Data, CBH/VR
Outliered Data
Data, CEB
GBD 2019
GBD 2021
UN WPP 2022
CENSUS SBH
CENSUS TABULATION
DHS CBH
MICS SBH
VR INCOMPLETE
VR INCOMPLETE TB

Age−specific fertility fitted trend lines, 1950−2021: Vanuatu, Ages: 20−24
Data points enclosed with a diamond are reference sources

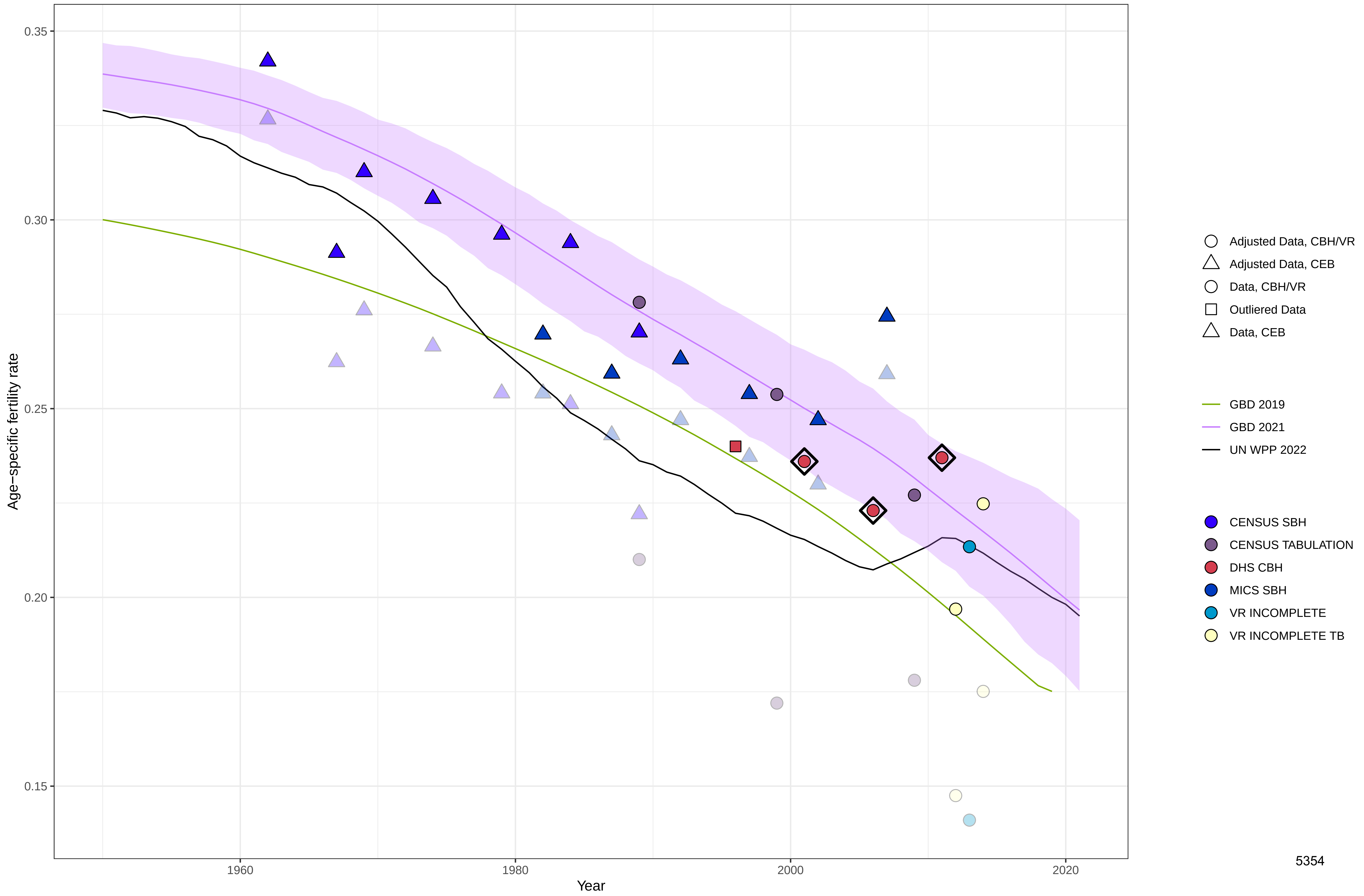

Age−specific fertility fitted trend lines, 1950−2021: Vanuatu, Ages: 25−29
Data points enclosed with a diamond are reference sources

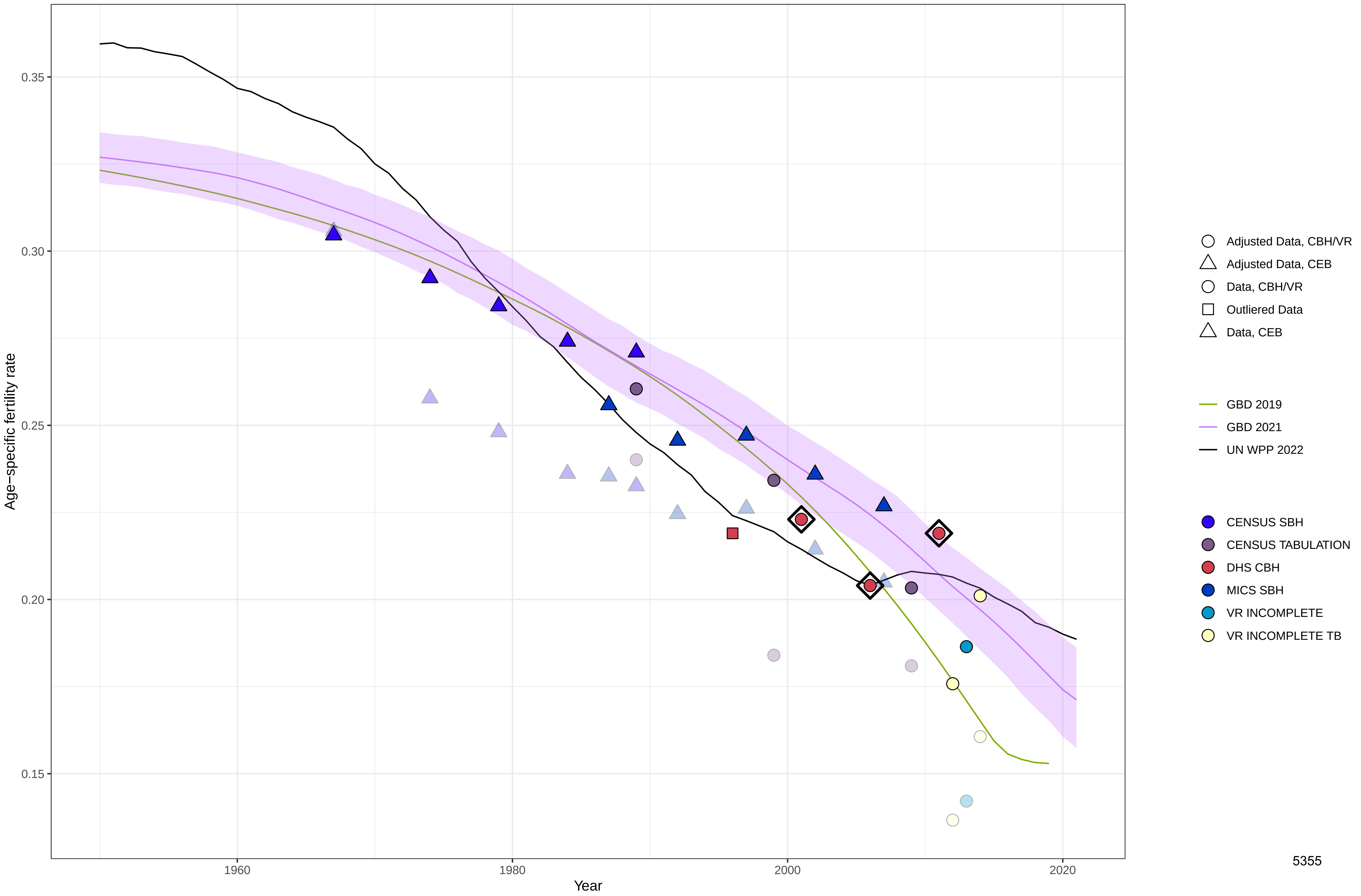

Age−specific fertility fitted trend lines, 1950−2021: Vanuatu, Ages: 30−34
Data points enclosed with a diamond are reference sources

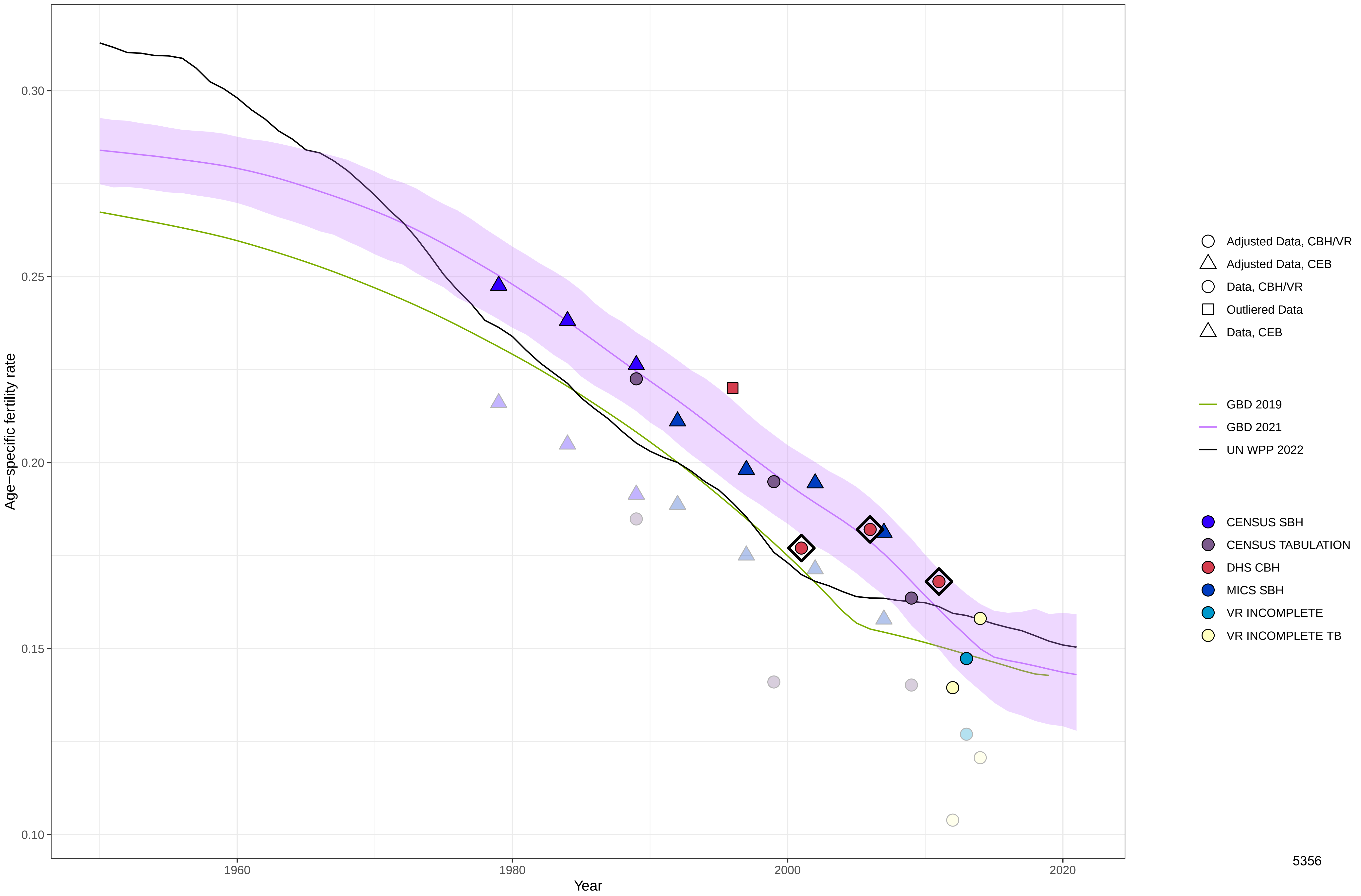

Age−specific fertility fitted trend lines, 1950−2021: Vanuatu, Ages: 35−39
Data points enclosed with a diamond are reference sources

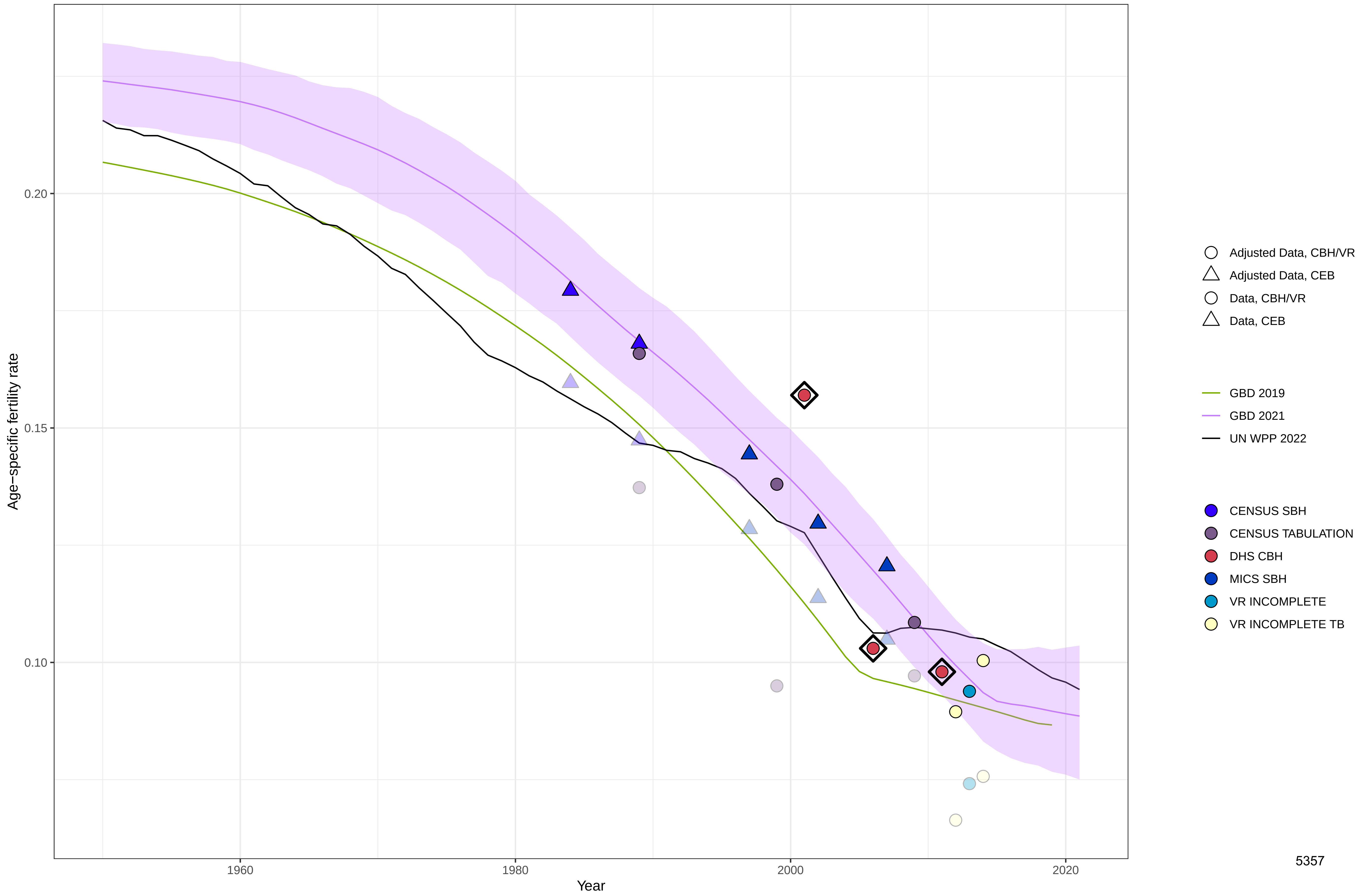

Age−specific fertility fitted trend lines, 1950−2021: Vanuatu, Ages: 40−44
Data points enclosed with a diamond are reference sources

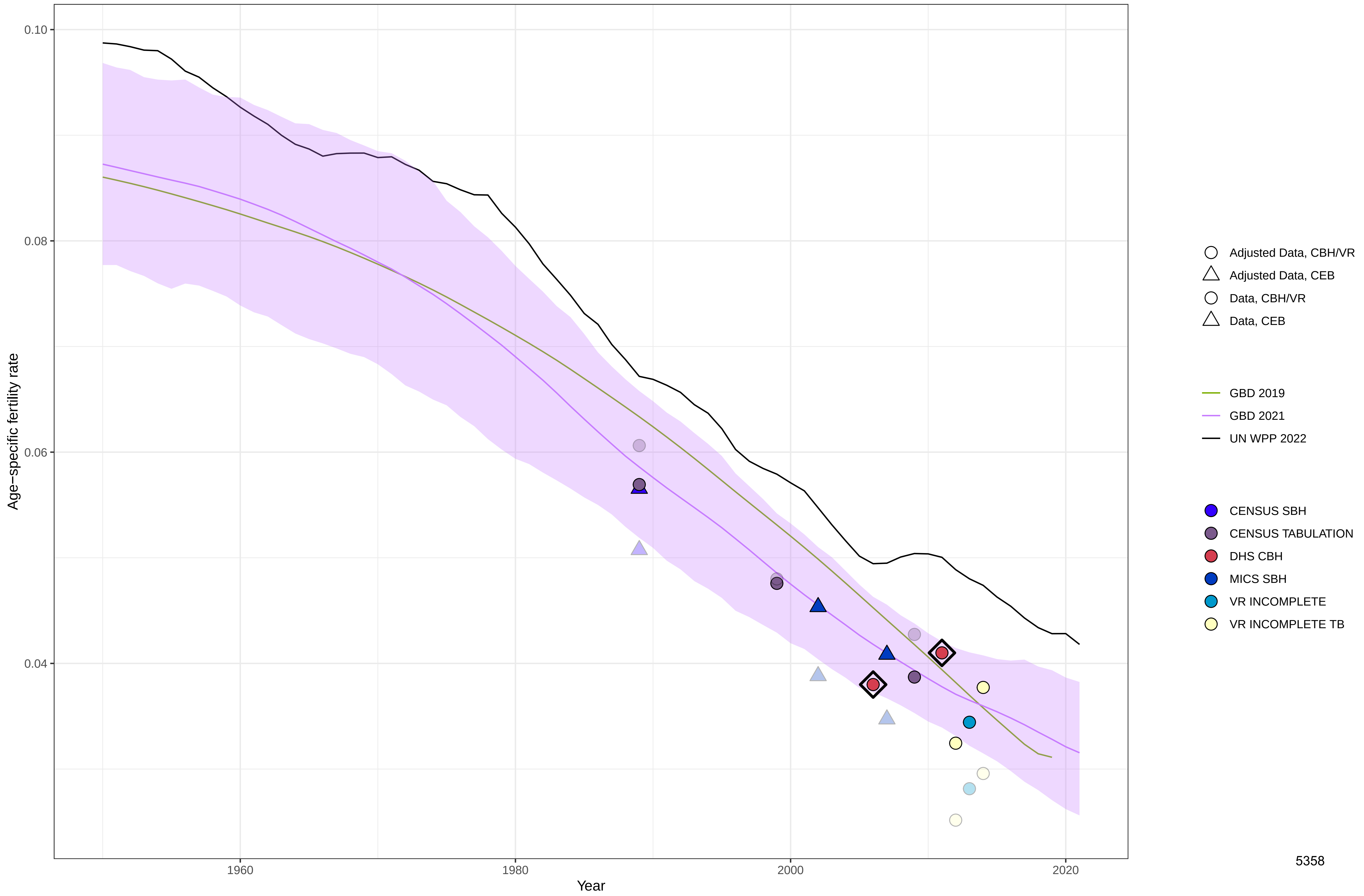

Age−specific fertility fitted trend lines, 1950−2021: Vanuatu, Ages: 45−49
Data points enclosed with a diamond are reference sources

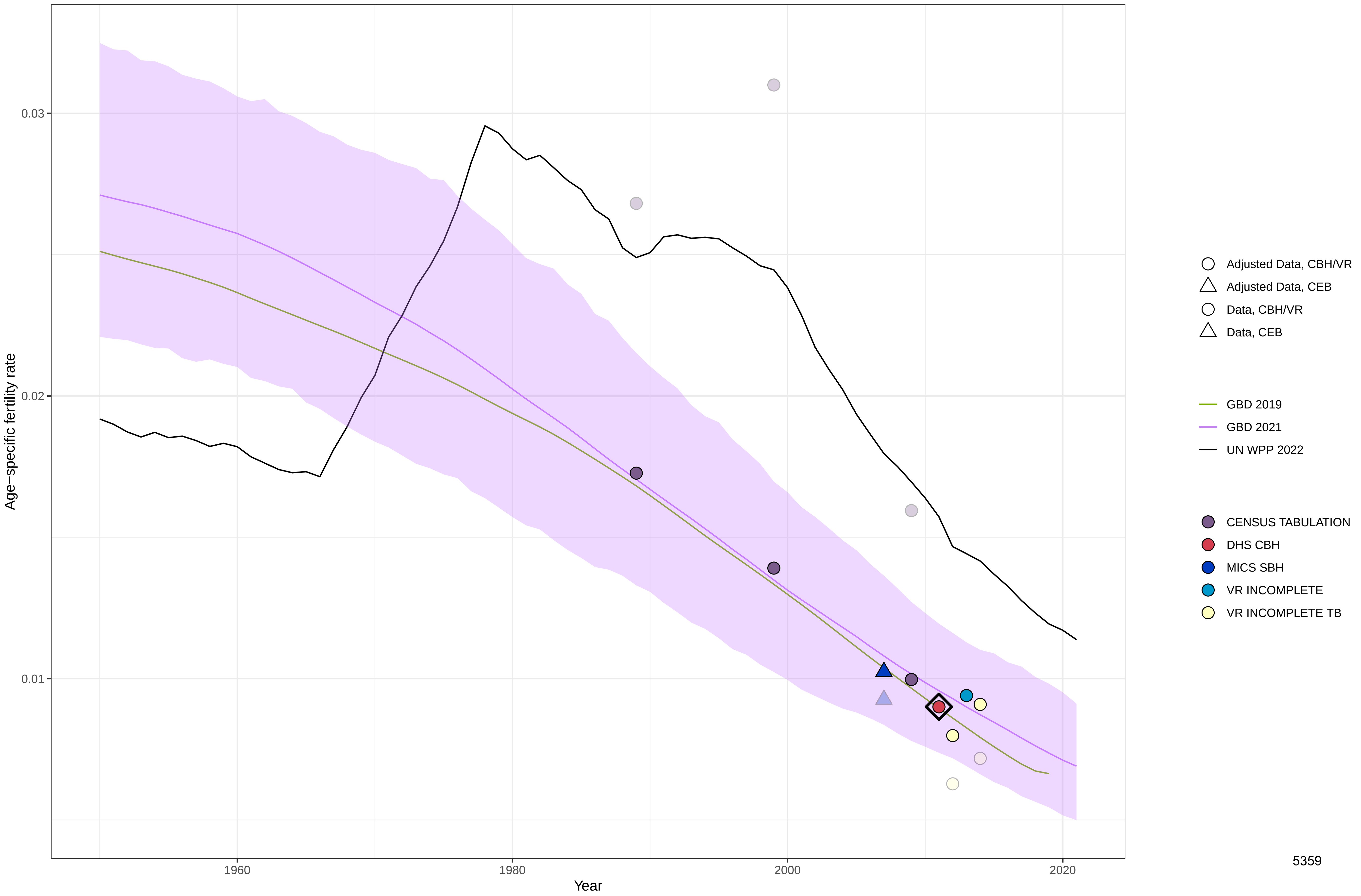

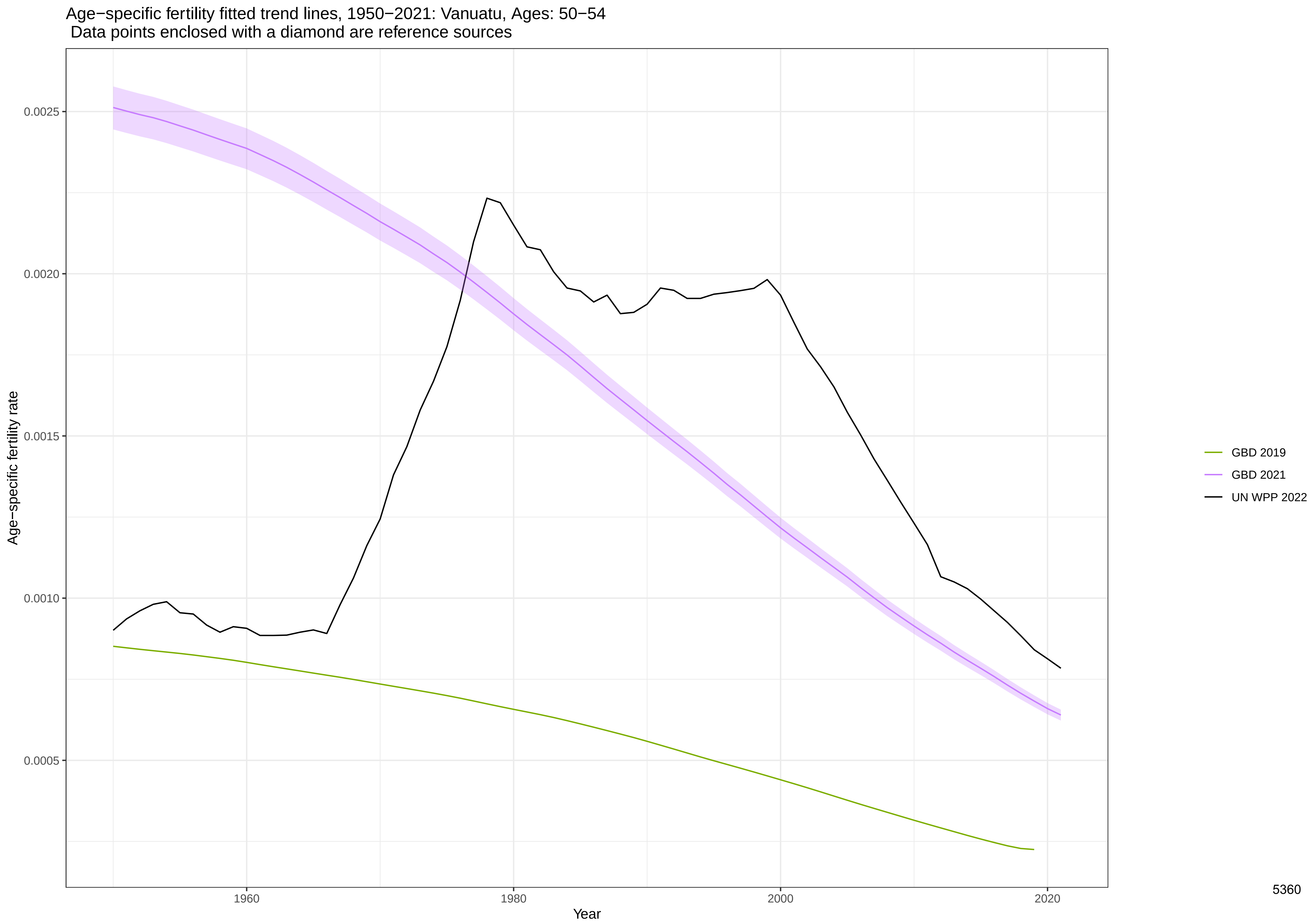

Age−specific fertility fitted trend lines, 1950−2021: Vanuatu, Ages: 50−54
Data points enclosed with a diamond are reference sources
0.0025
0.0020
0.0015
0.0010
0.0005
Age−specific fertility rate
1960
1980
2000
2020
Year
GBD 2019
GBD 2021
UN WPP 2022

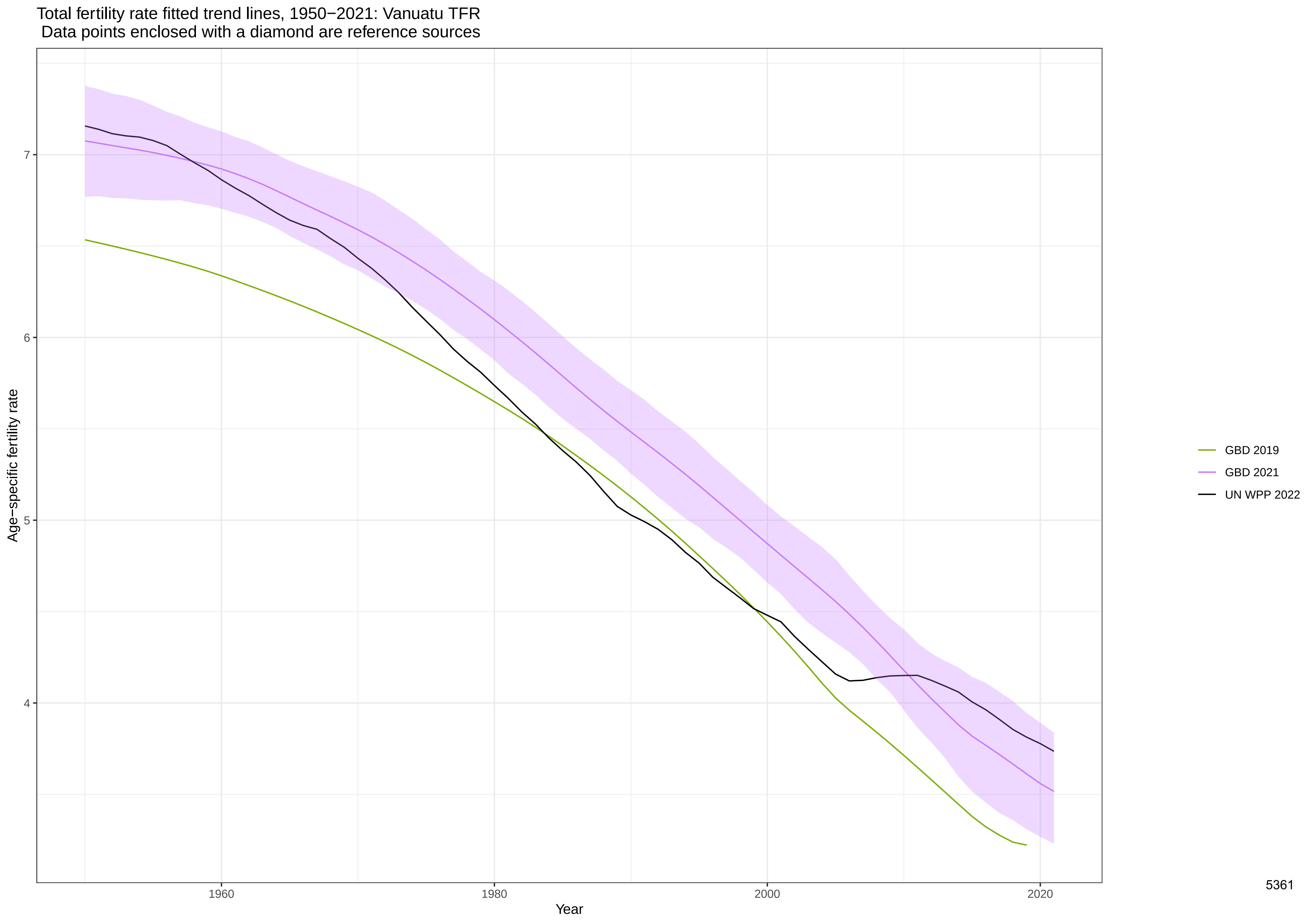

Total fertility rate fitted trend lines, 1950−2021: Vanuatu TFR
Data points enclosed with a diamond are reference sources
Age−specific fertility rate
7
6
5
4
1960
1980
2000
2020
Year
GBD 2019
GBD 2021
UN WPP 2022

Age−specific fertility fitted trend lines, 1950−2021: Cambodia, Ages: 10−14
Data points enclosed with a diamond are reference sources

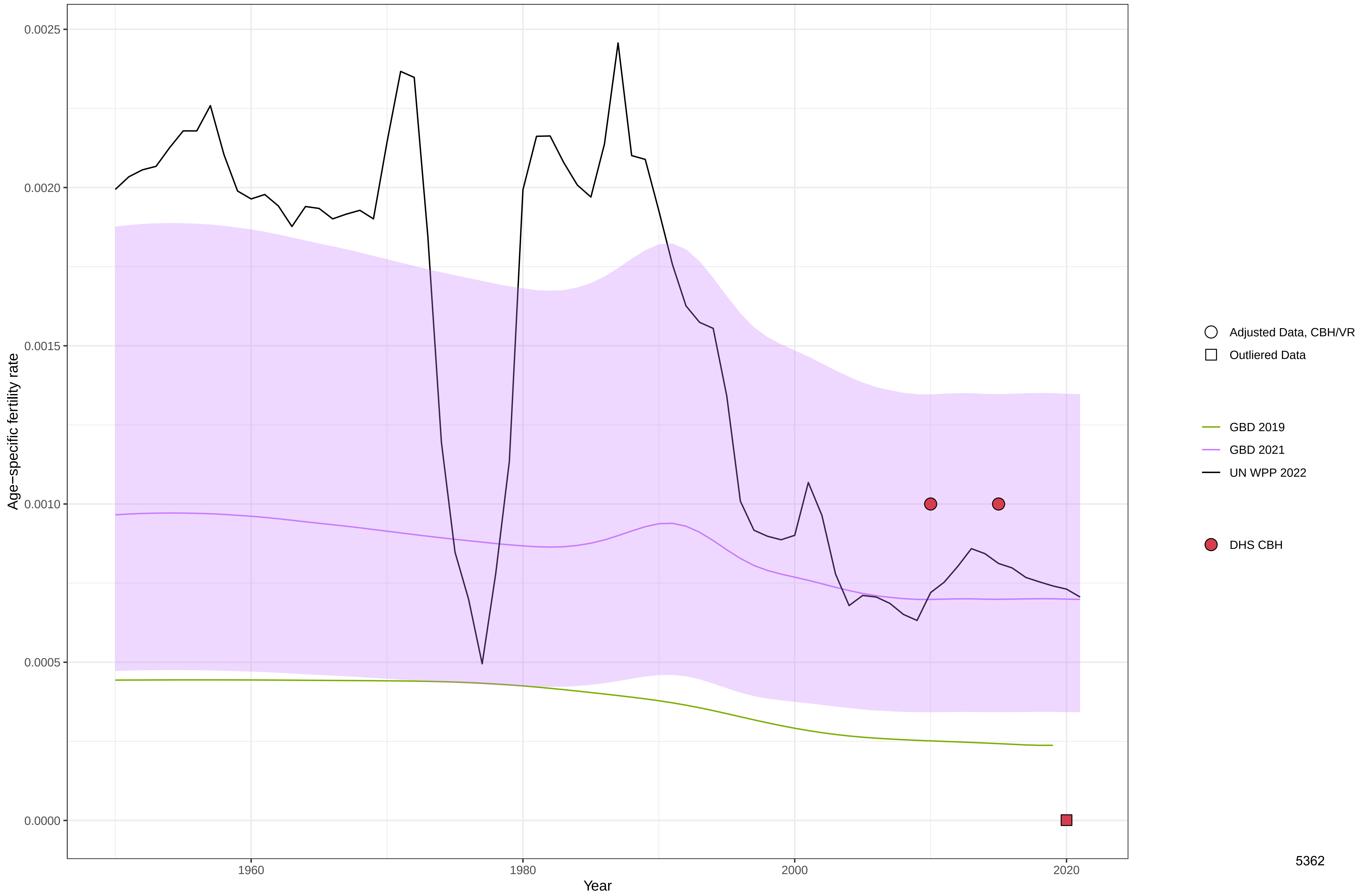

Age−specific fertility fitted trend lines, 1950−2021: Cambodia, Ages: 15−19
Data points enclosed with a diamond are reference sources

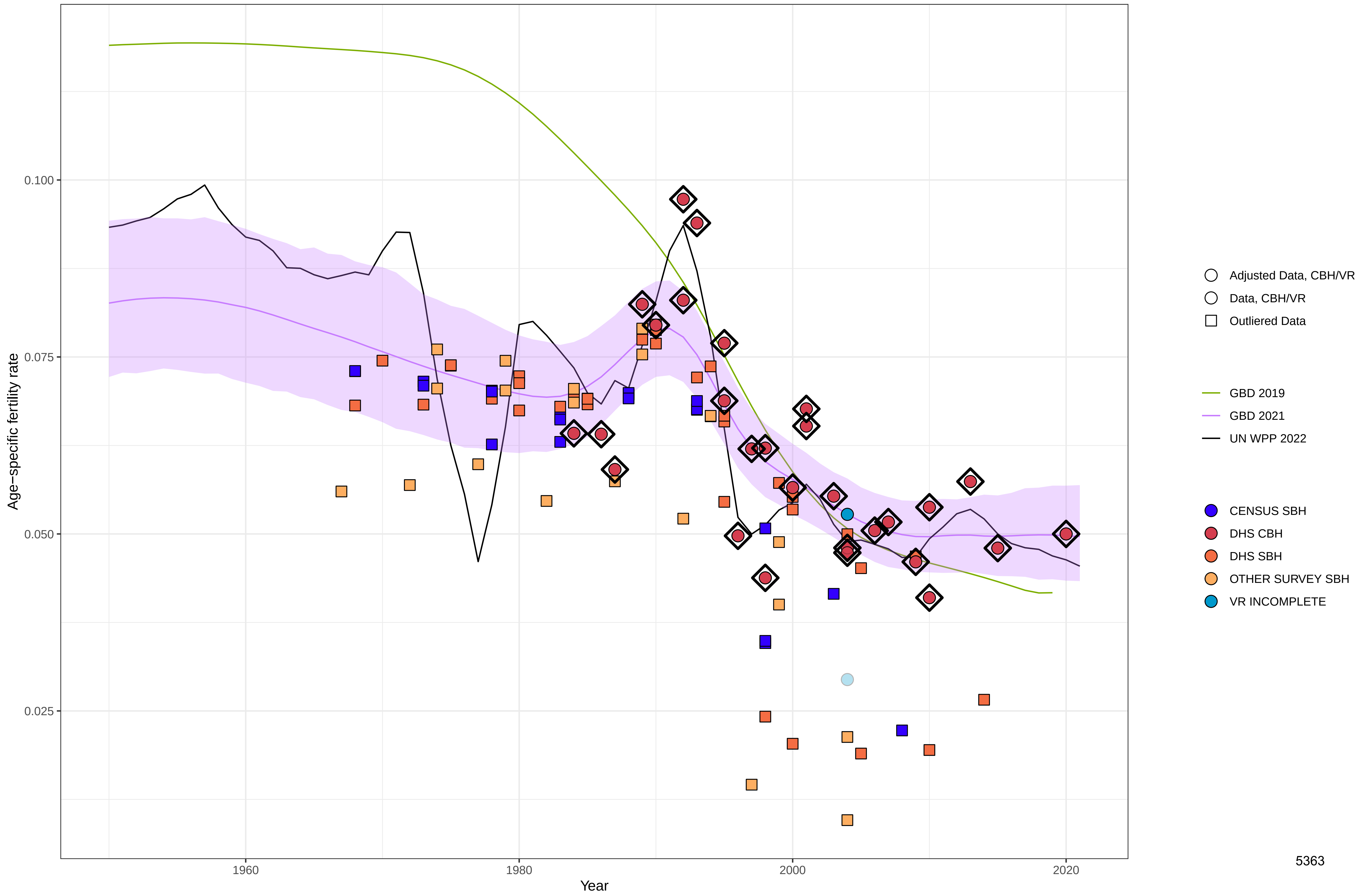

Age−specific fertility fitted trend lines, 1950−2021: Cambodia, Ages: 20−24
Data points enclosed with a diamond are reference sources

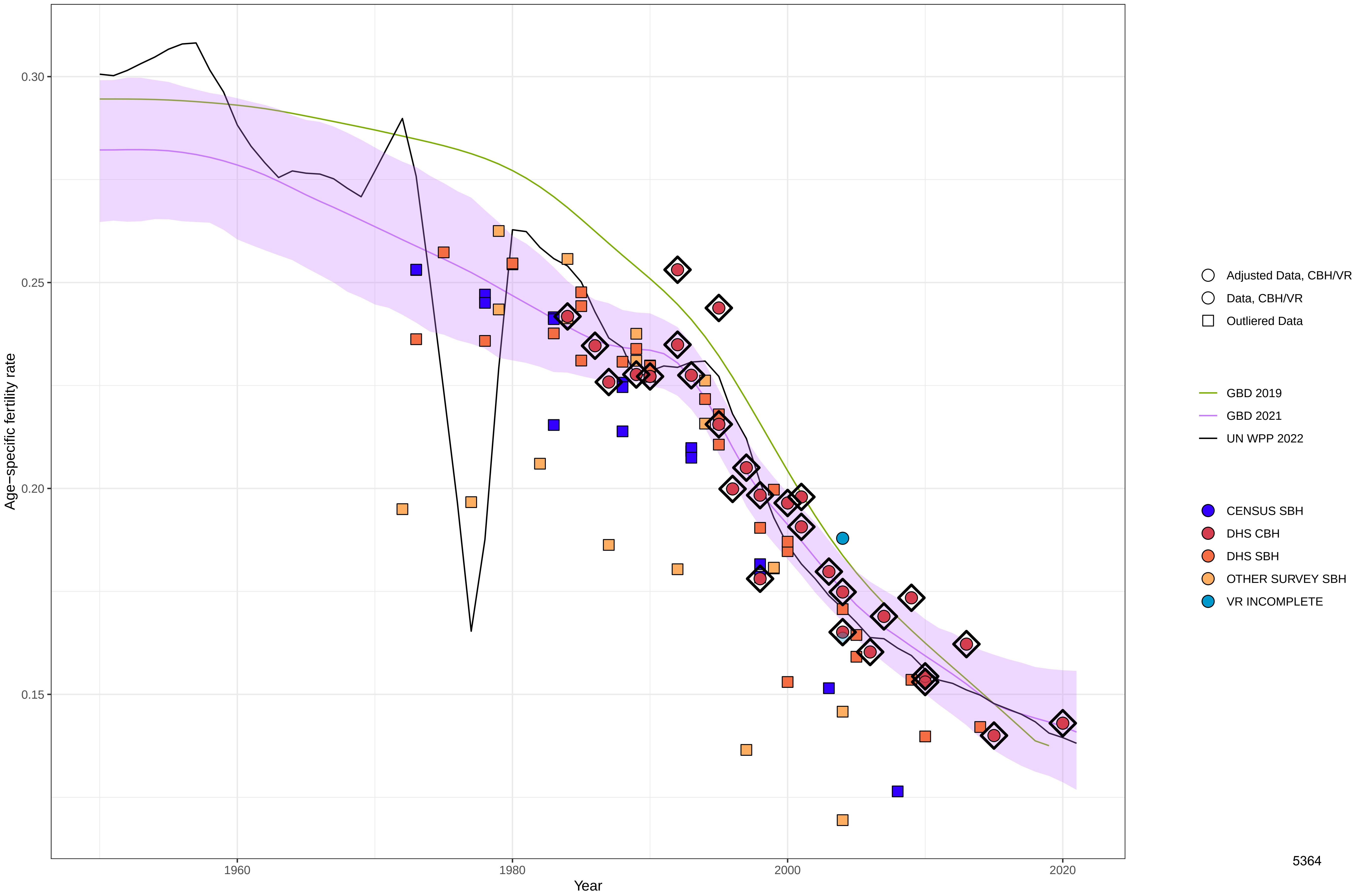

Age−specific fertility fitted trend lines, 1950−2021: Cambodia, Ages: 25−29
Data points enclosed with a diamond are reference sources

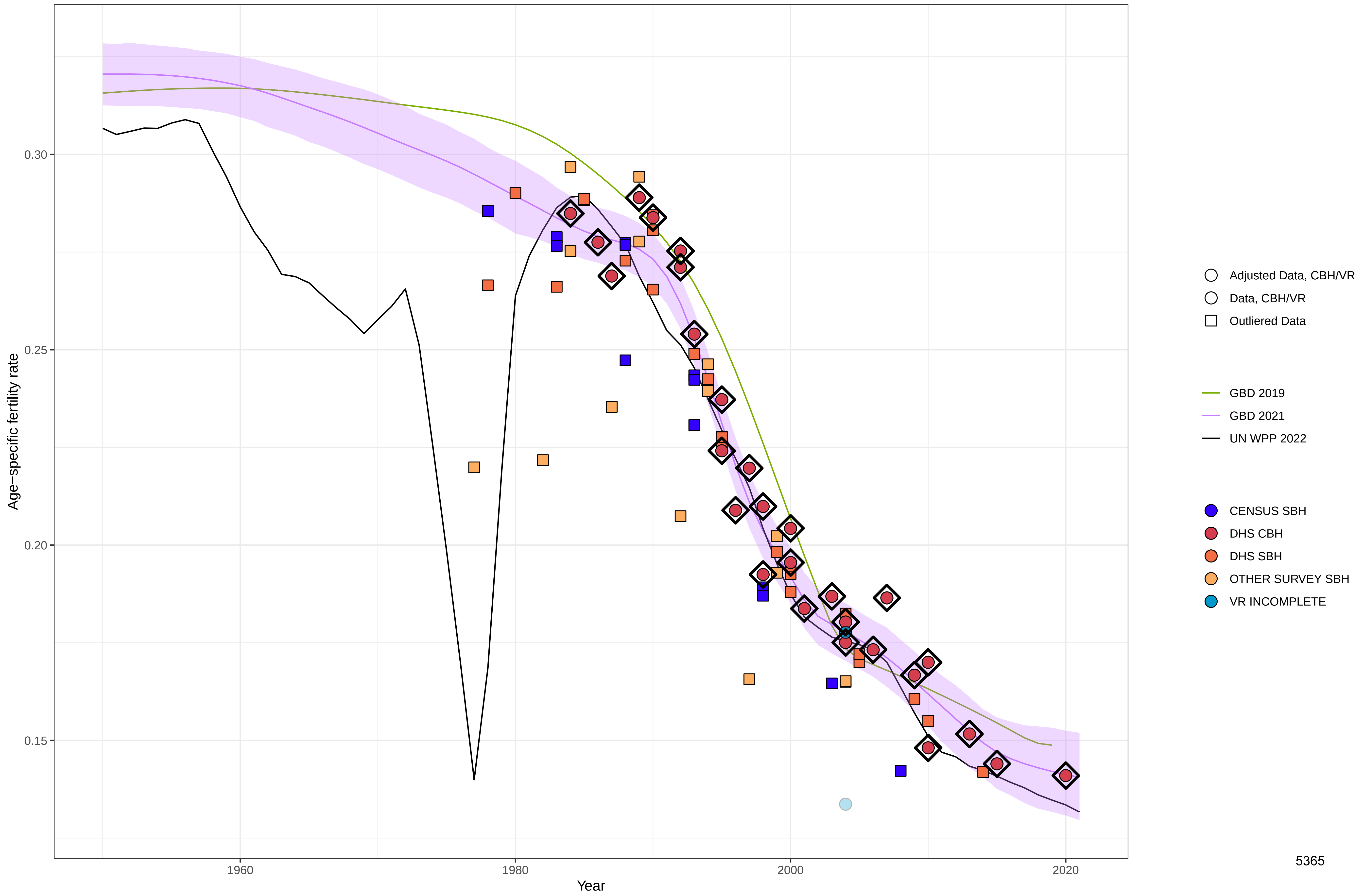

Age−specific fertility fitted trend lines, 1950−2021: Cambodia, Ages: 30−34
Data points enclosed with a diamond are reference sources

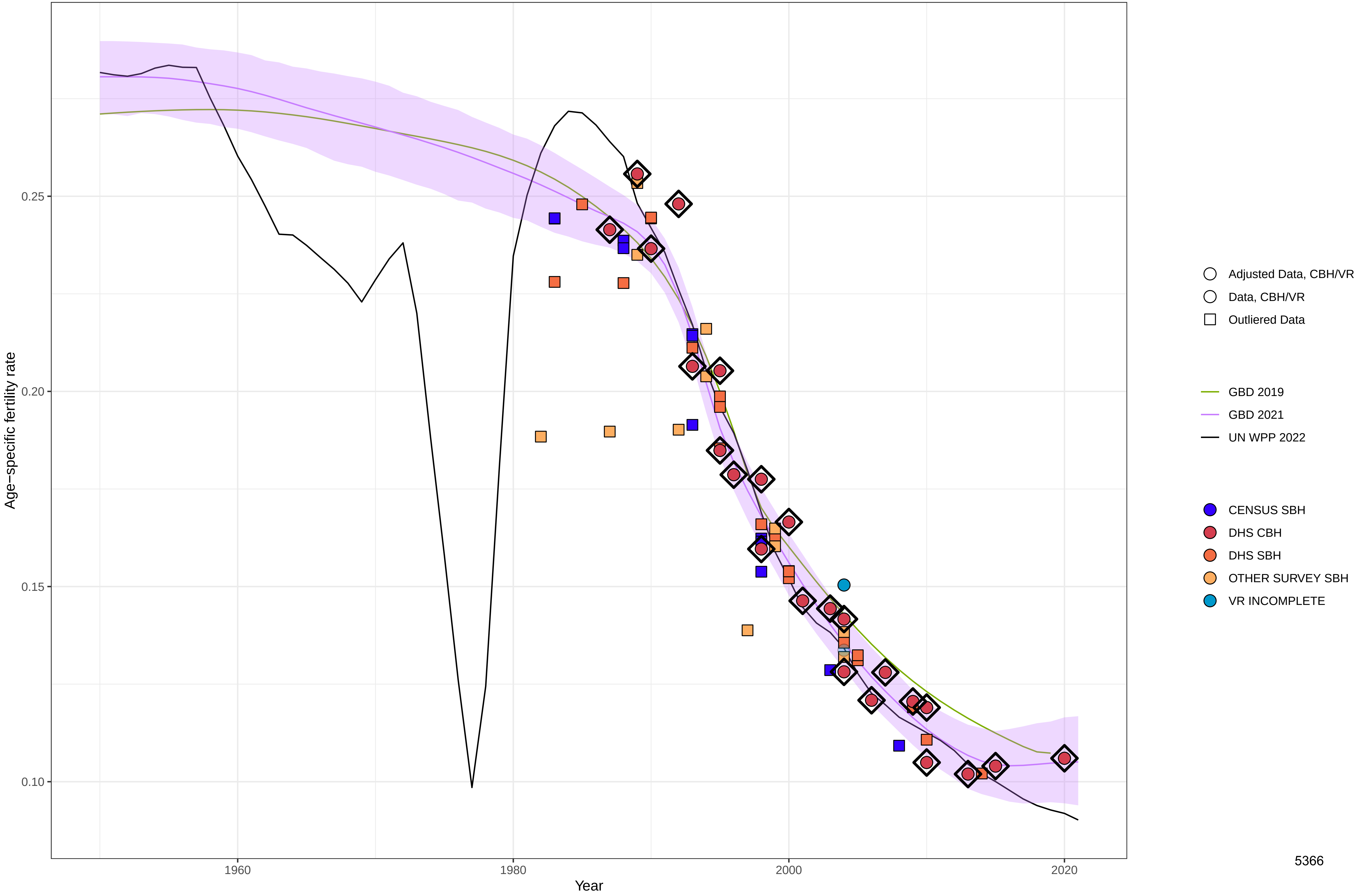

Age−specific fertility fitted trend lines, 1950−2021: Cambodia, Ages: 35−39
Data points enclosed with a diamond are reference sources

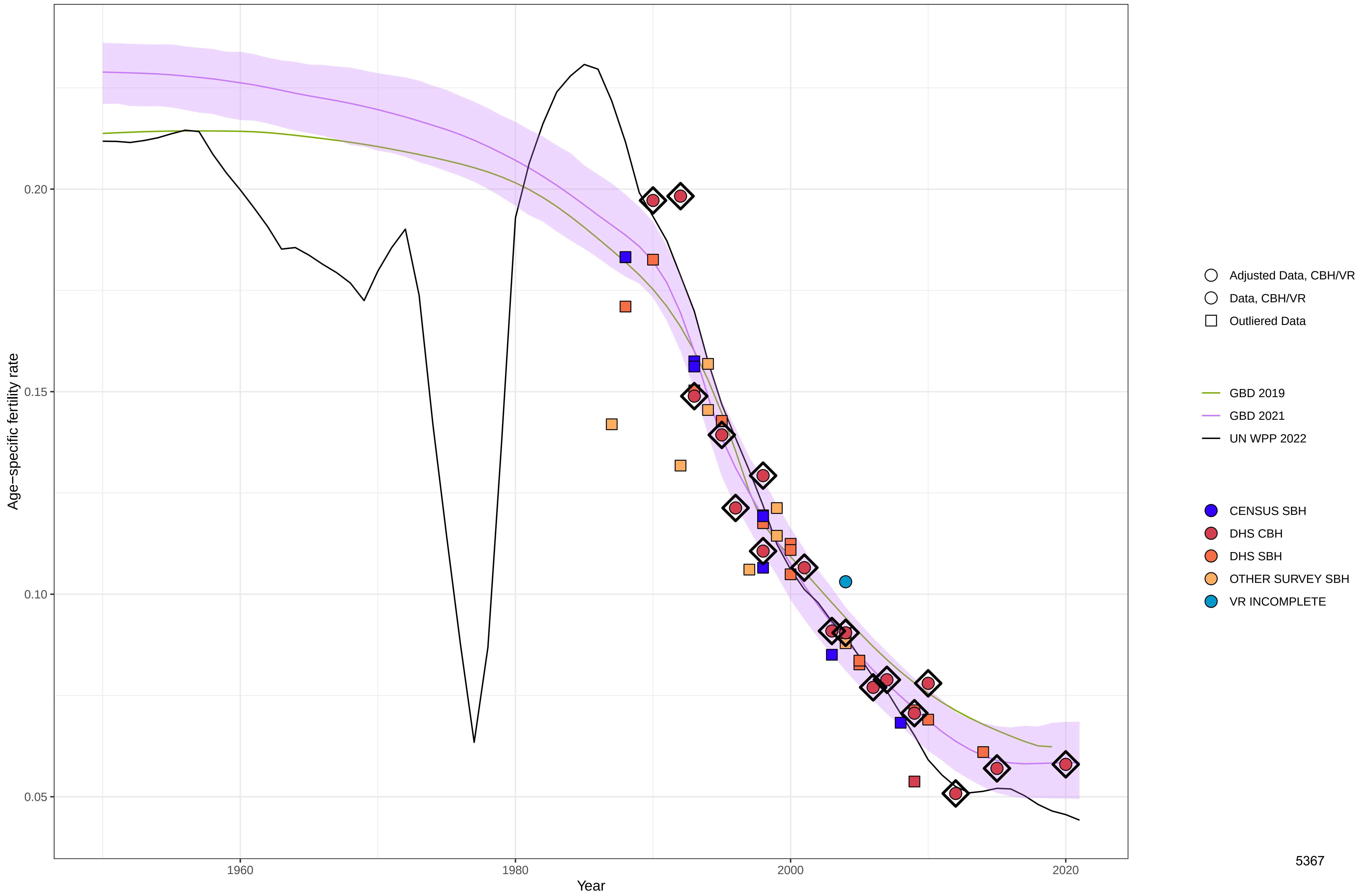

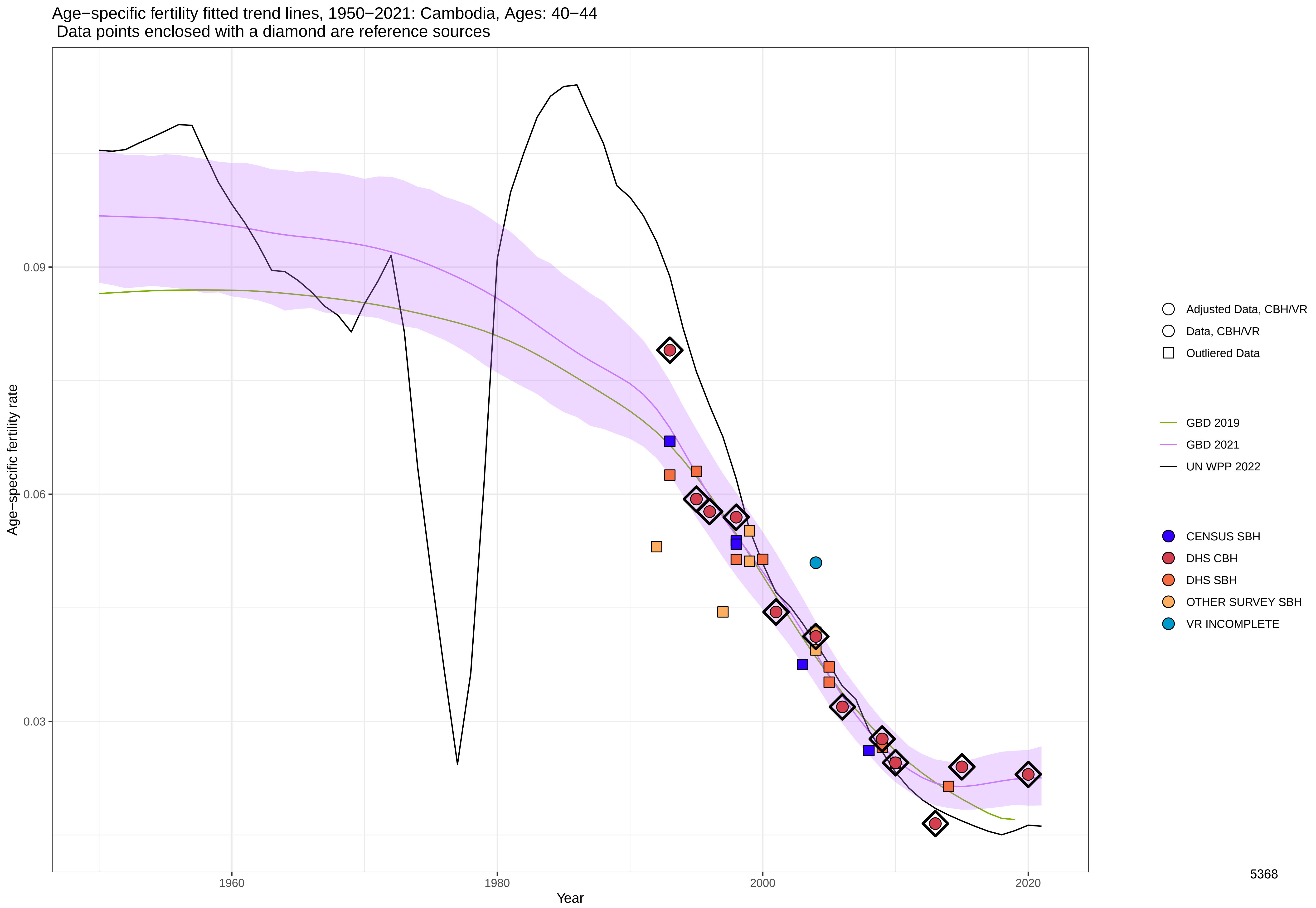

Age−specific fertility fitted trend lines, 1950−2021: Cambodia, Ages: 40−44
Data points enclosed with a diamond are reference sources
Age−specific fertility rate
0.09
0.06
0.03
1960
1980
2000
2020
Year
Adjusted Data, CBH/VR
Data, CBH/VR
Outliered Data
GBD 2019
GBD 2021
UN WPP 2022
CENSUS SBH
DHS CBH
DHS SBH
OTHER SURVEY SBH
VR INCOMPLETE

Age−specific fertility fitted trend lines, 1950−2021: Cambodia, Ages: 45−49
Data points enclosed with a diamond are reference sources

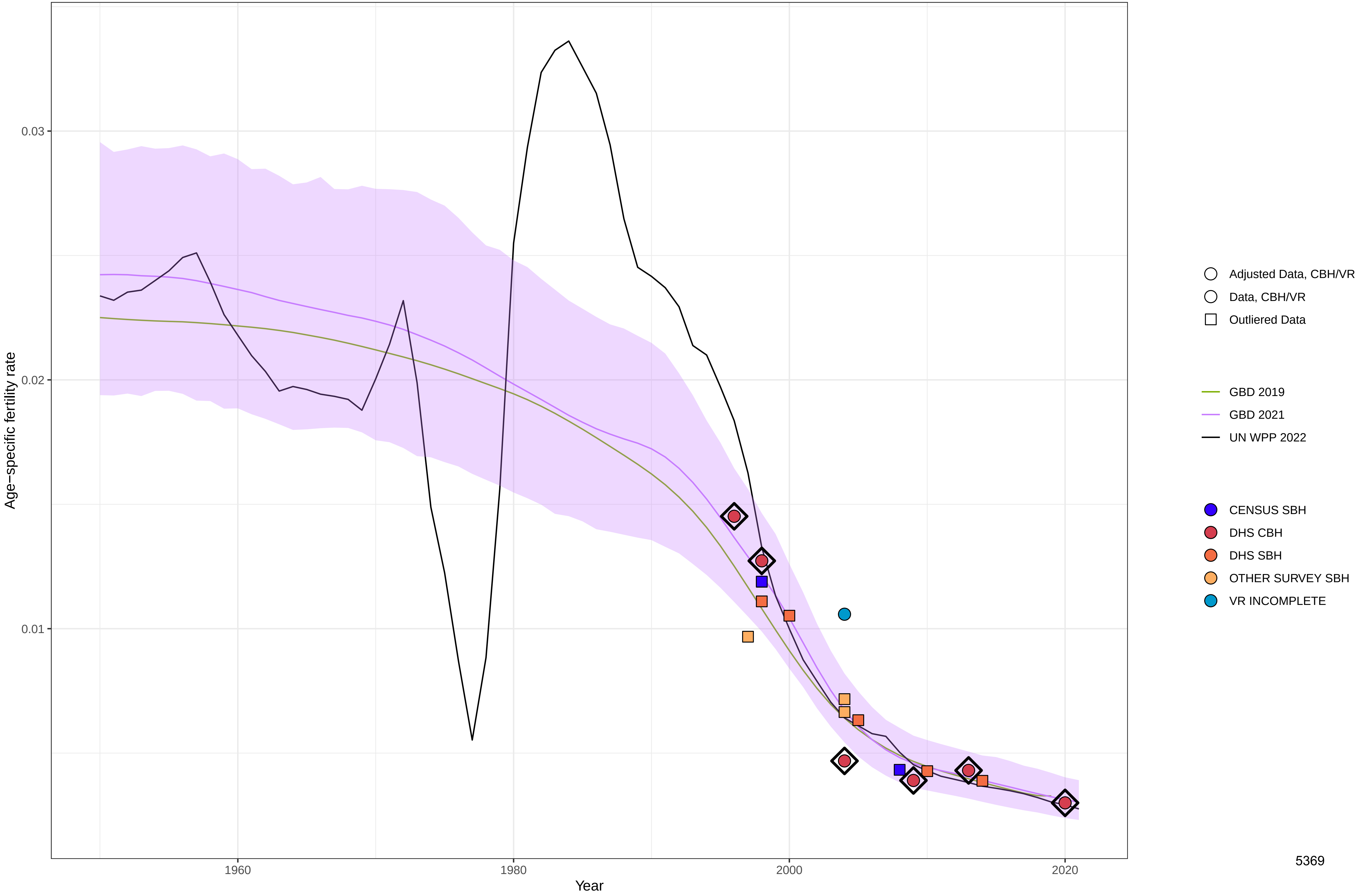

Age−specific fertility fitted trend lines, 1950−2021: Cambodia, Ages: 50−54
Data points enclosed with a diamond are reference sources

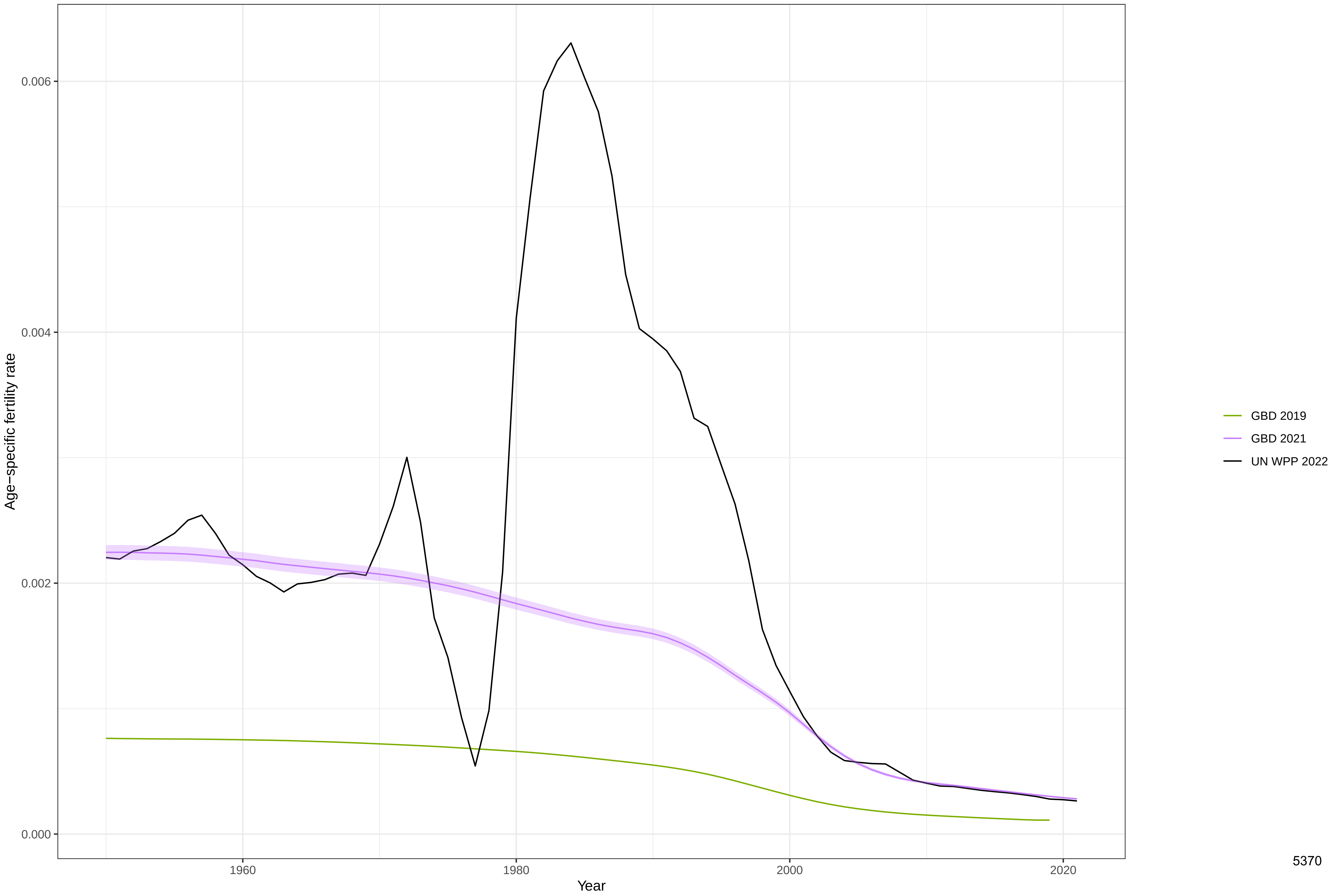

Total fertility rate fitted trend lines, 1950−2021: Cambodia TFR
Data points enclosed with a diamond are reference sources

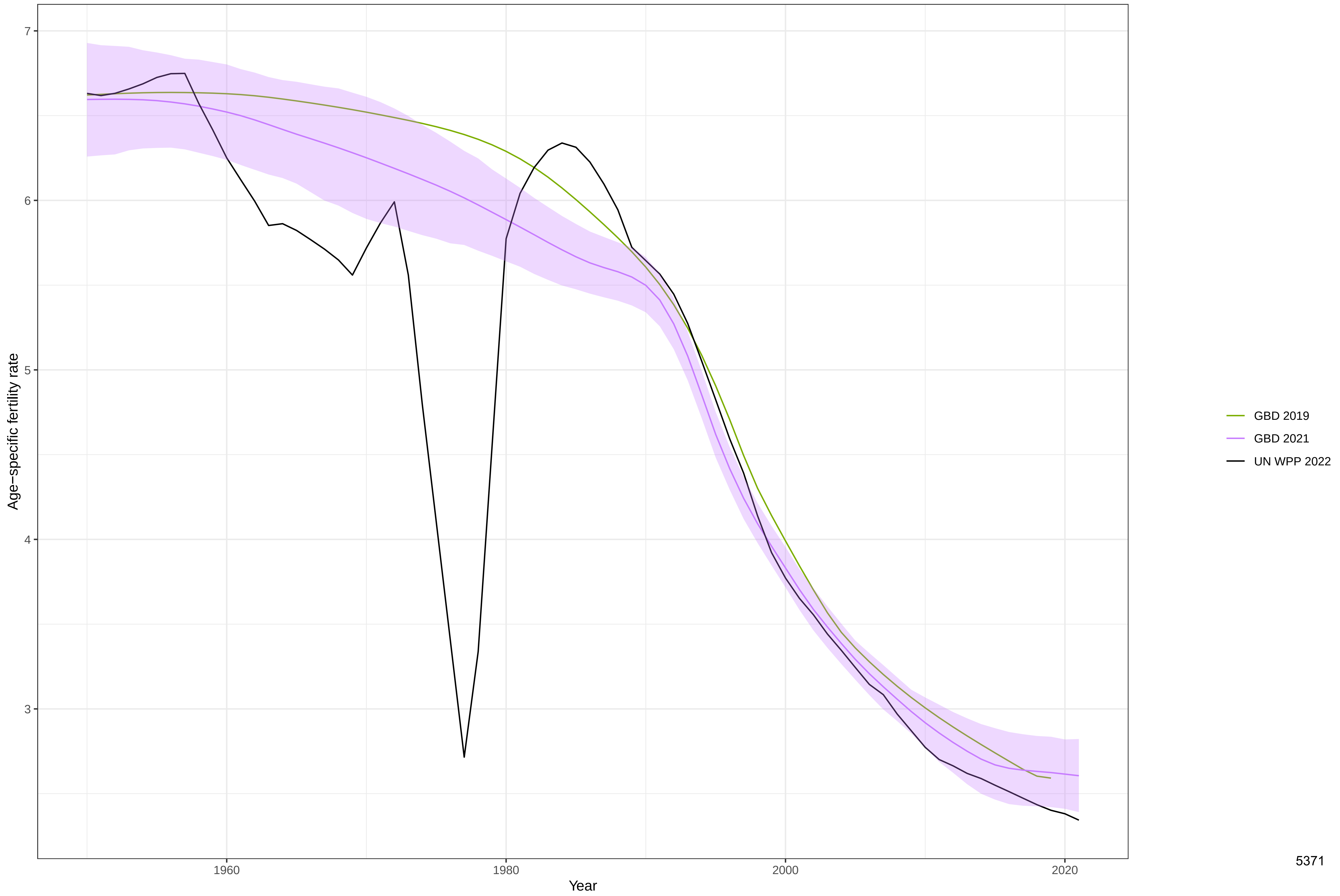

Age−specific fertility fitted trend lines, 1950−2021: Indonesia, Ages: 10−14
Data points enclosed with a diamond are reference sources

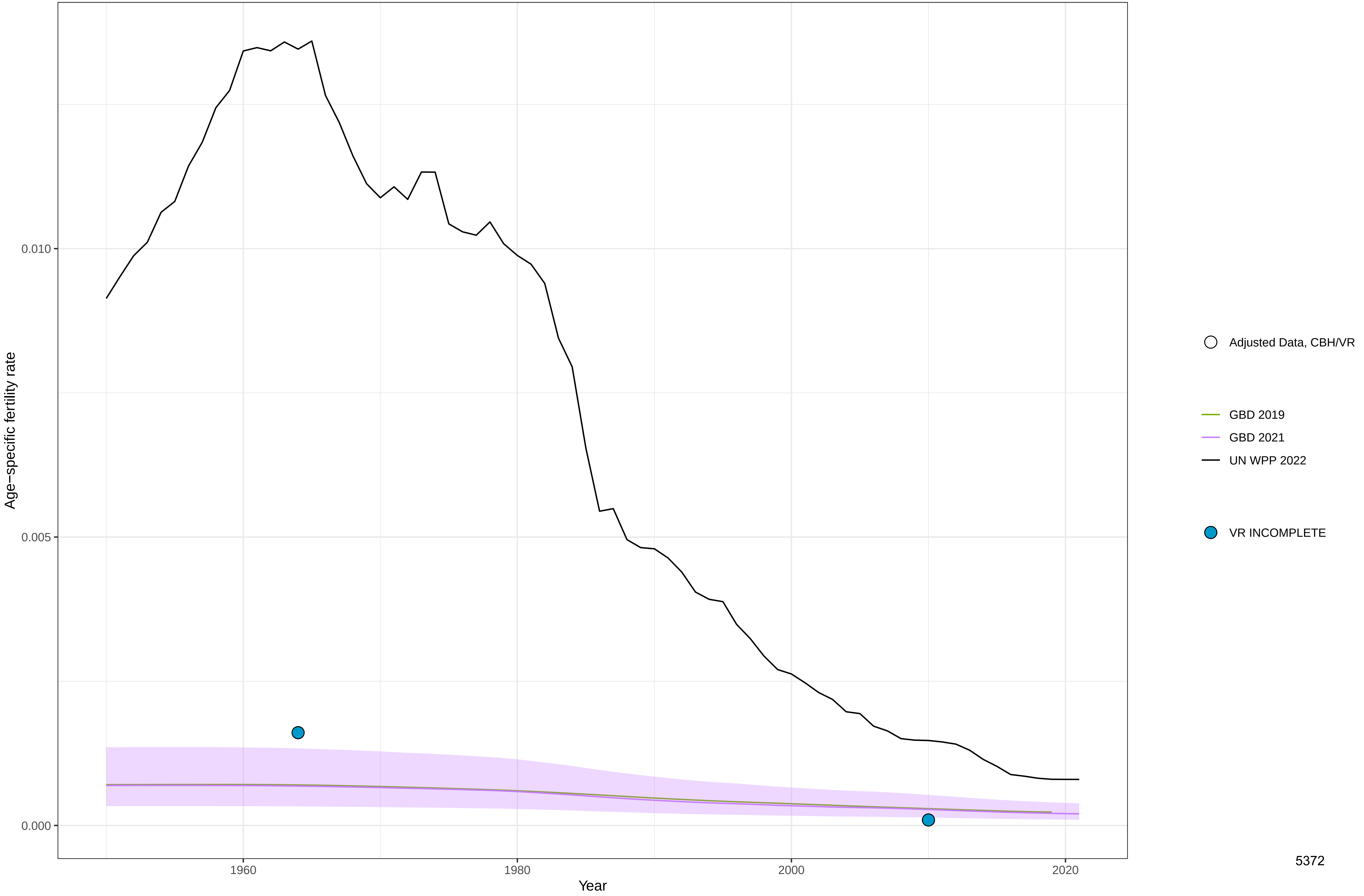

Age−specific fertility fitted trend lines, 1950−2021: Indonesia, Ages: 15−19
Data points enclosed with a diamond are reference sources

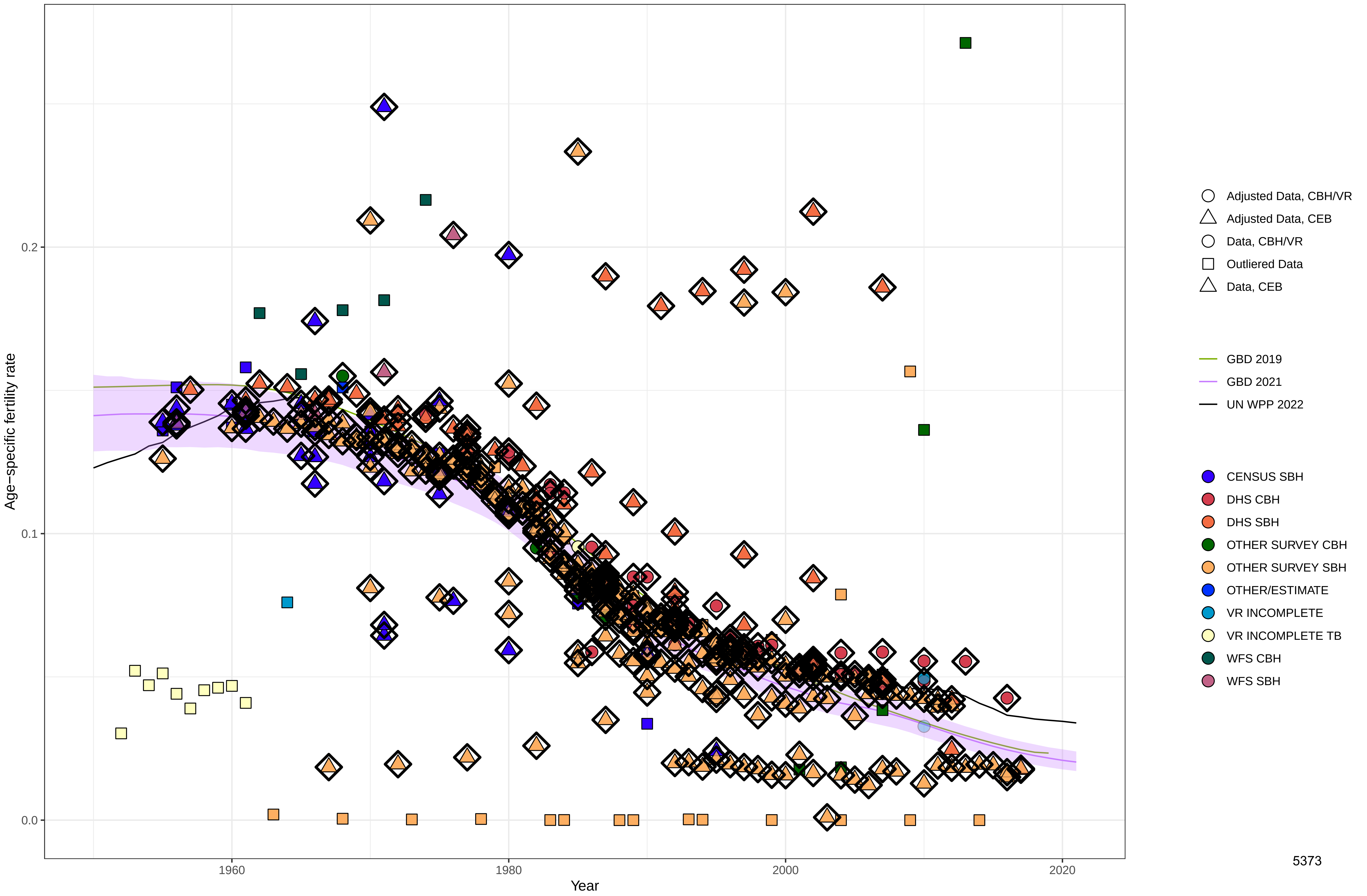

Age−specific fertility fitted trend lines, 1950−2021: Indonesia, Ages: 20−24
Data points enclosed with a diamond are reference sources

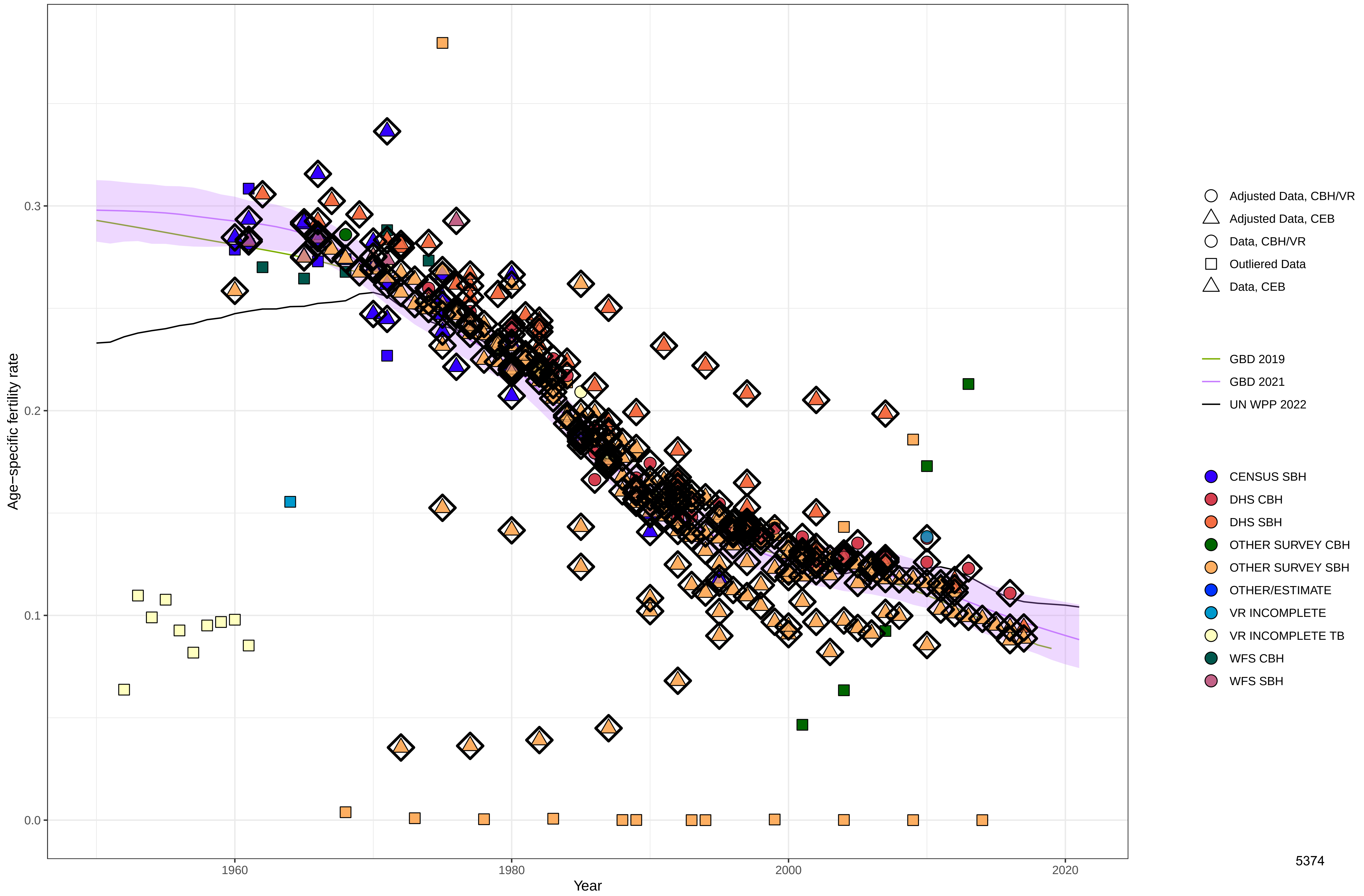

Age−specific fertility fitted trend lines, 1950−2021: Indonesia, Ages: 25−29
Data points enclosed with a diamond are reference sources

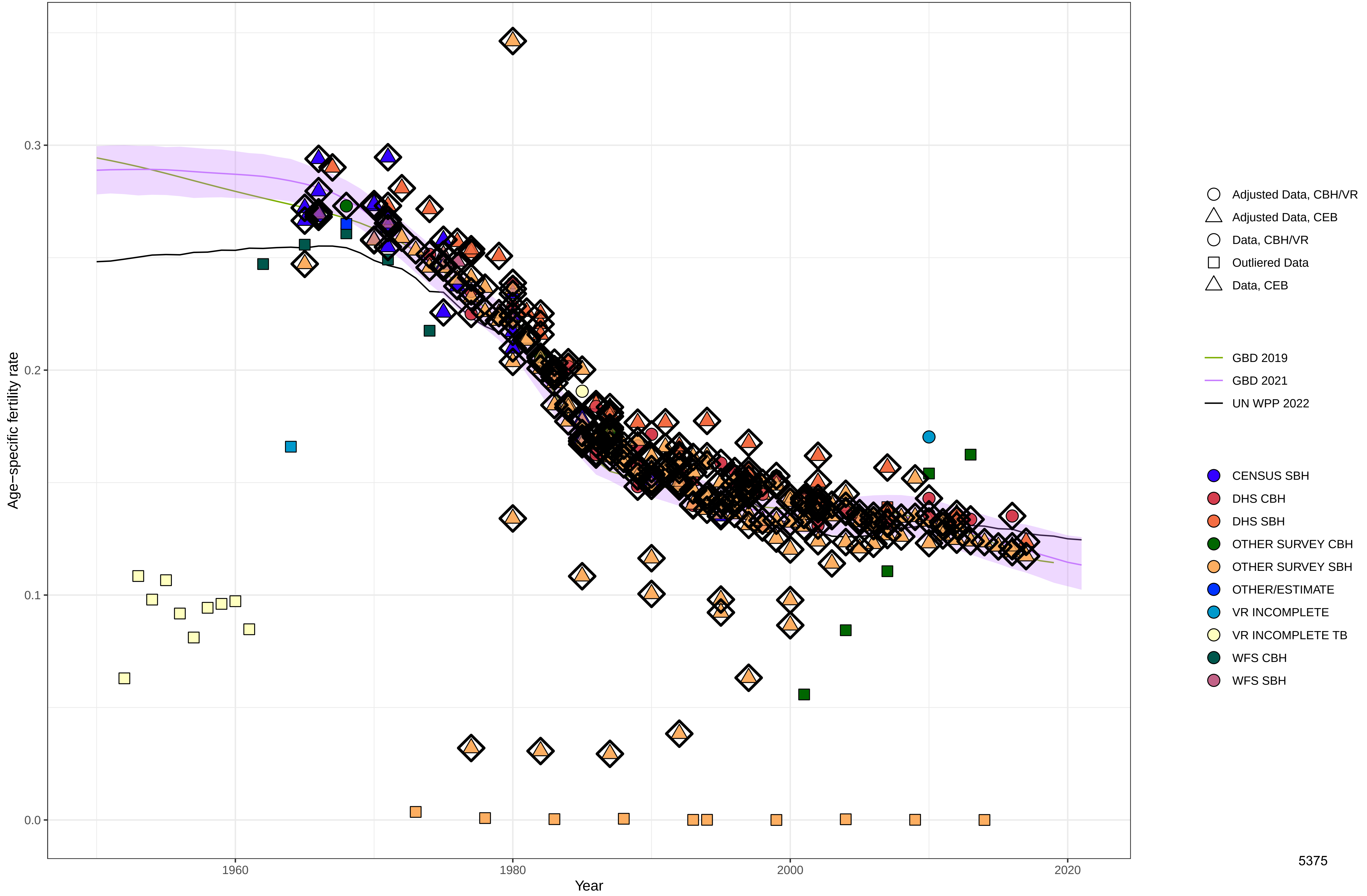

Age−specific fertility fitted trend lines, 1950−2021: Indonesia, Ages: 30−34
Data points enclosed with a diamond are reference sources

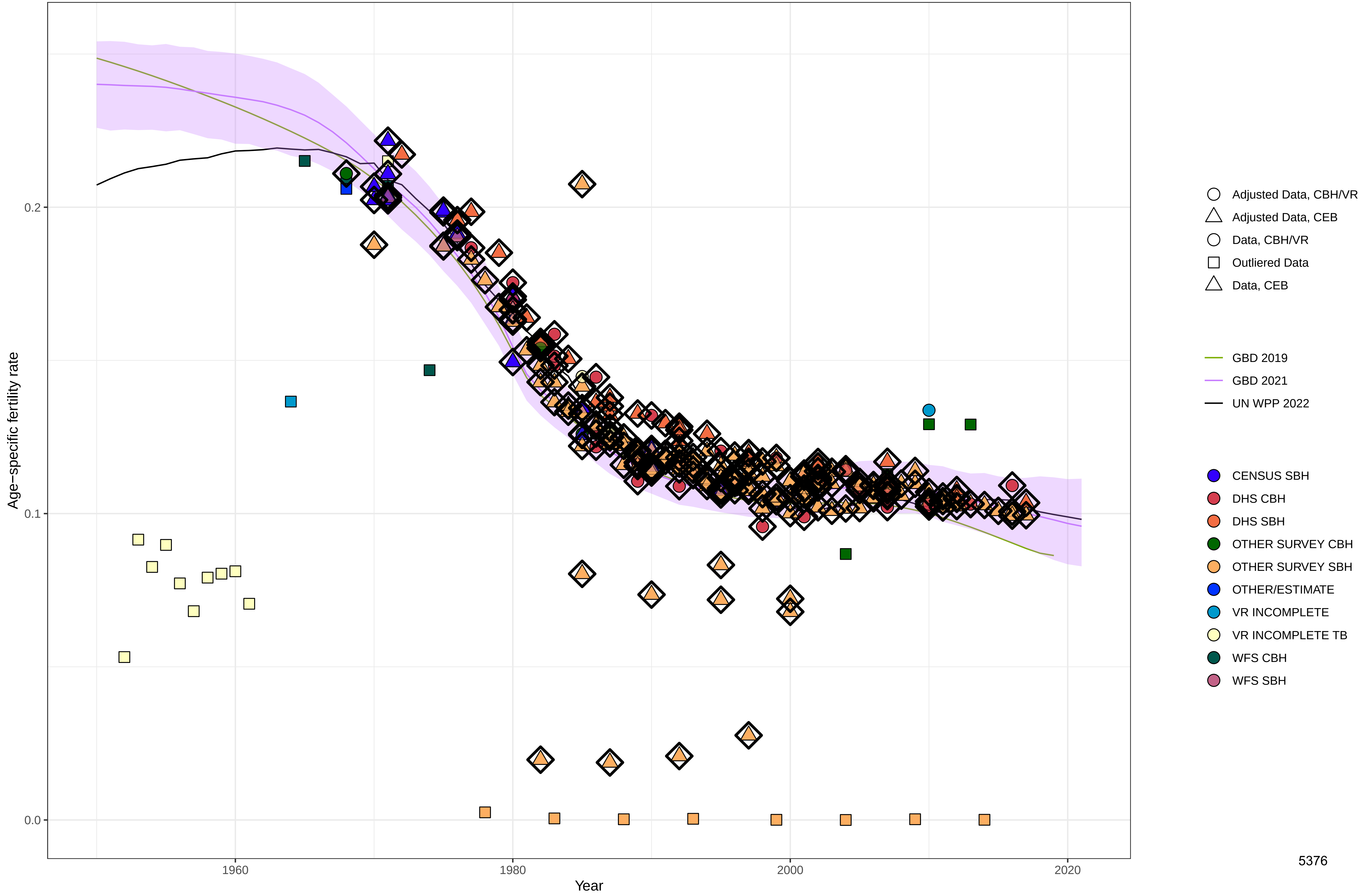

Age−specific fertility fitted trend lines, 1950−2021: Indonesia, Ages: 35−39
Data points enclosed with a diamond are reference sources

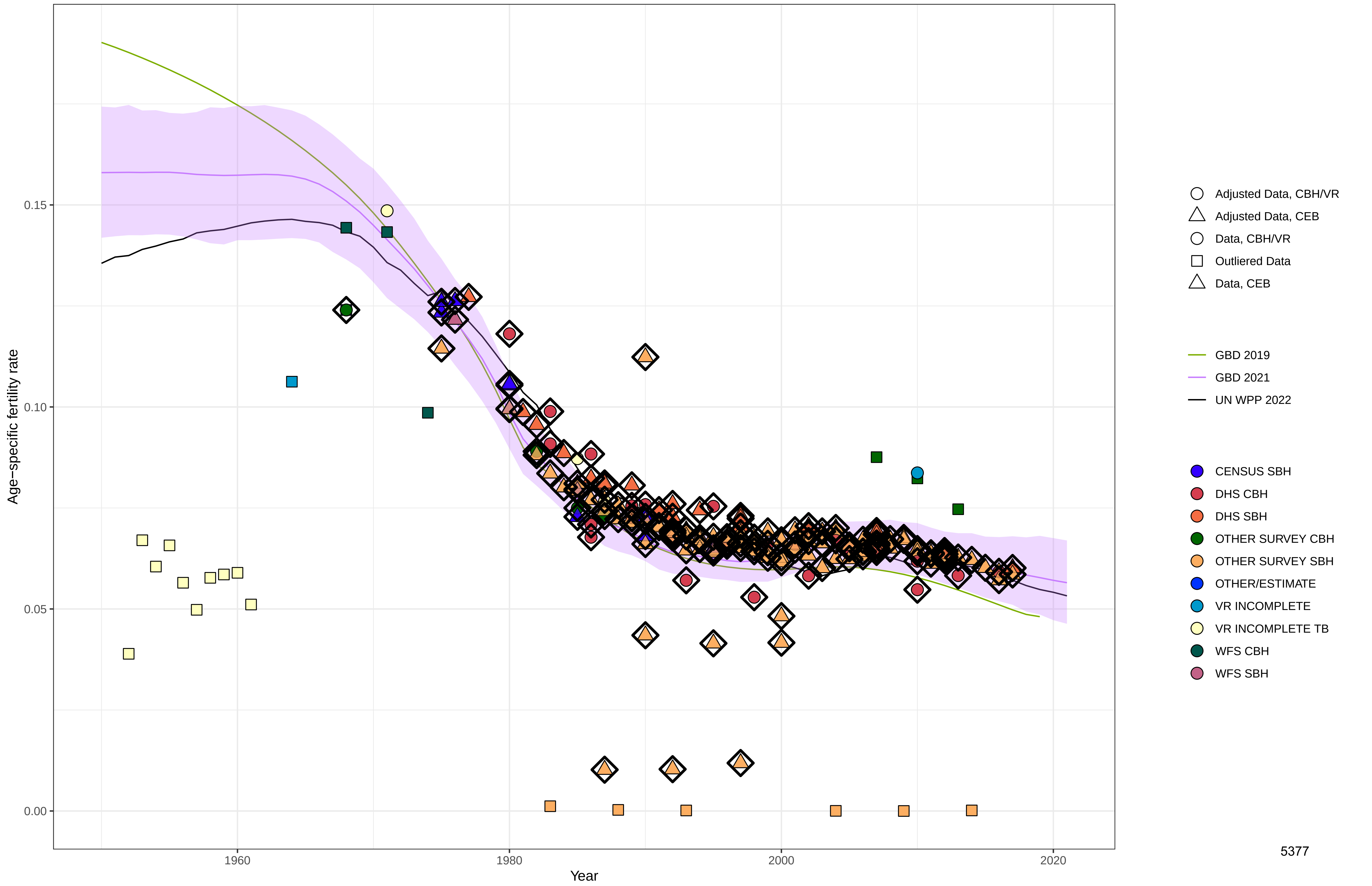

Age−specific fertility fitted trend lines, 1950−2021: Indonesia, Ages: 40−44
Data points enclosed with a diamond are reference sources

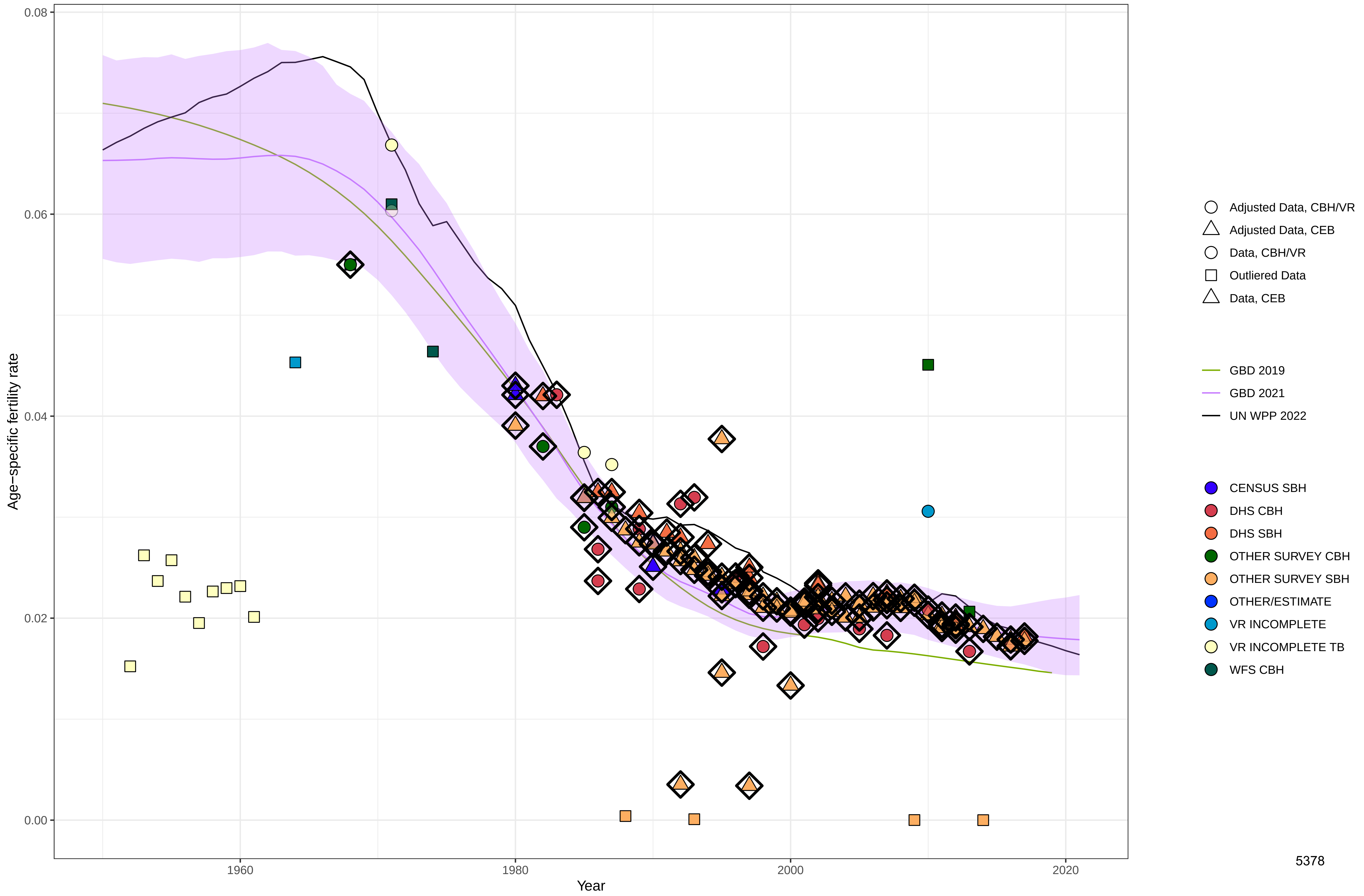

Age−specific fertility fitted trend lines, 1950−2021: Indonesia, Ages: 45−49
Data points enclosed with a diamond are reference sources

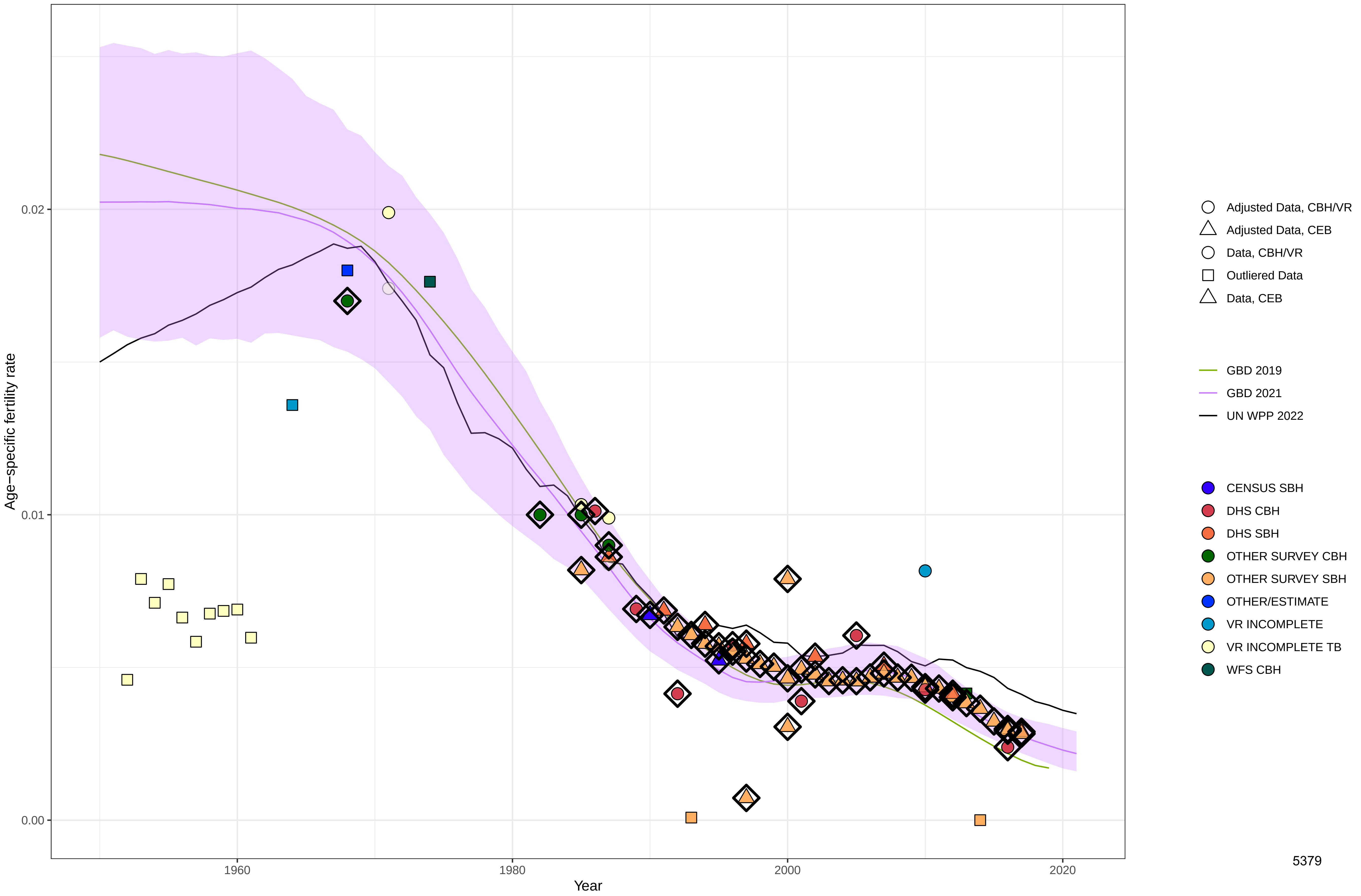

Age−specific fertility fitted trend lines, 1950−2021: Indonesia, Ages: 50−54
Data points enclosed with a diamond are reference sources

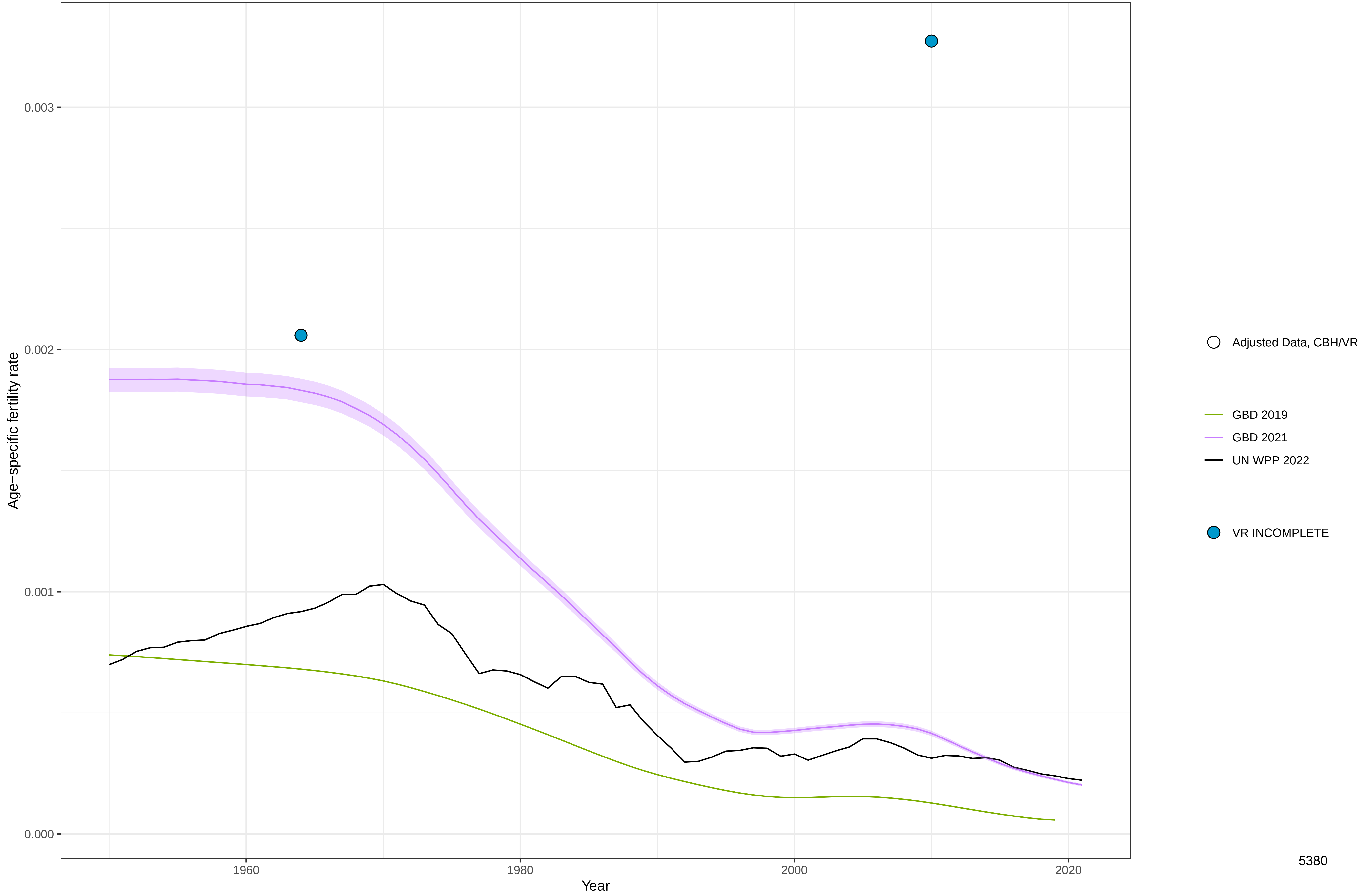

Total fertility rate fitted trend lines, 1950−2021: Indonesia TFR
Data points enclosed with a diamond are reference sources

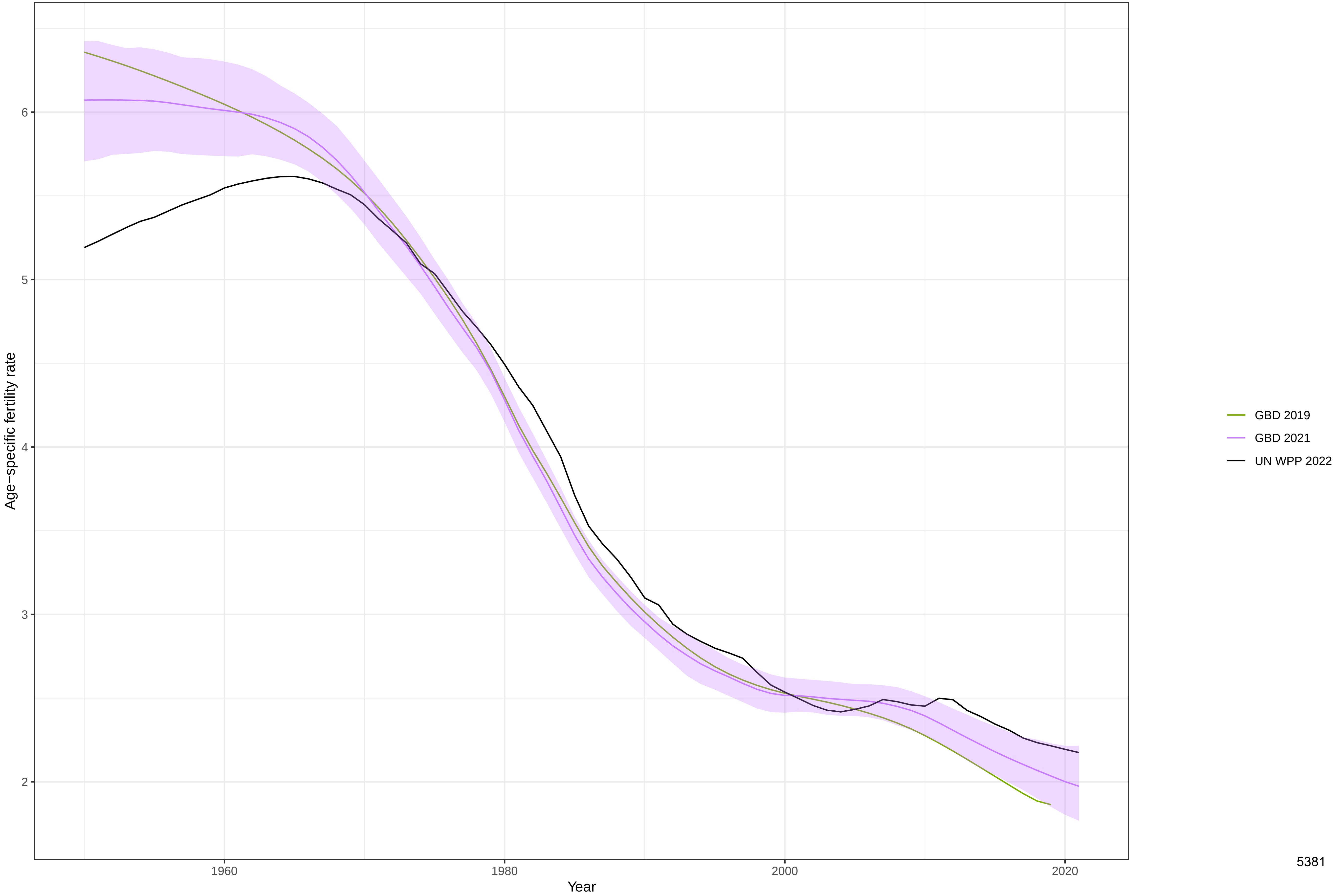

Age−specific fertility fitted trend lines, 1950−2021: Aceh, Ages: 10−14
Data points enclosed with a diamond are reference sources

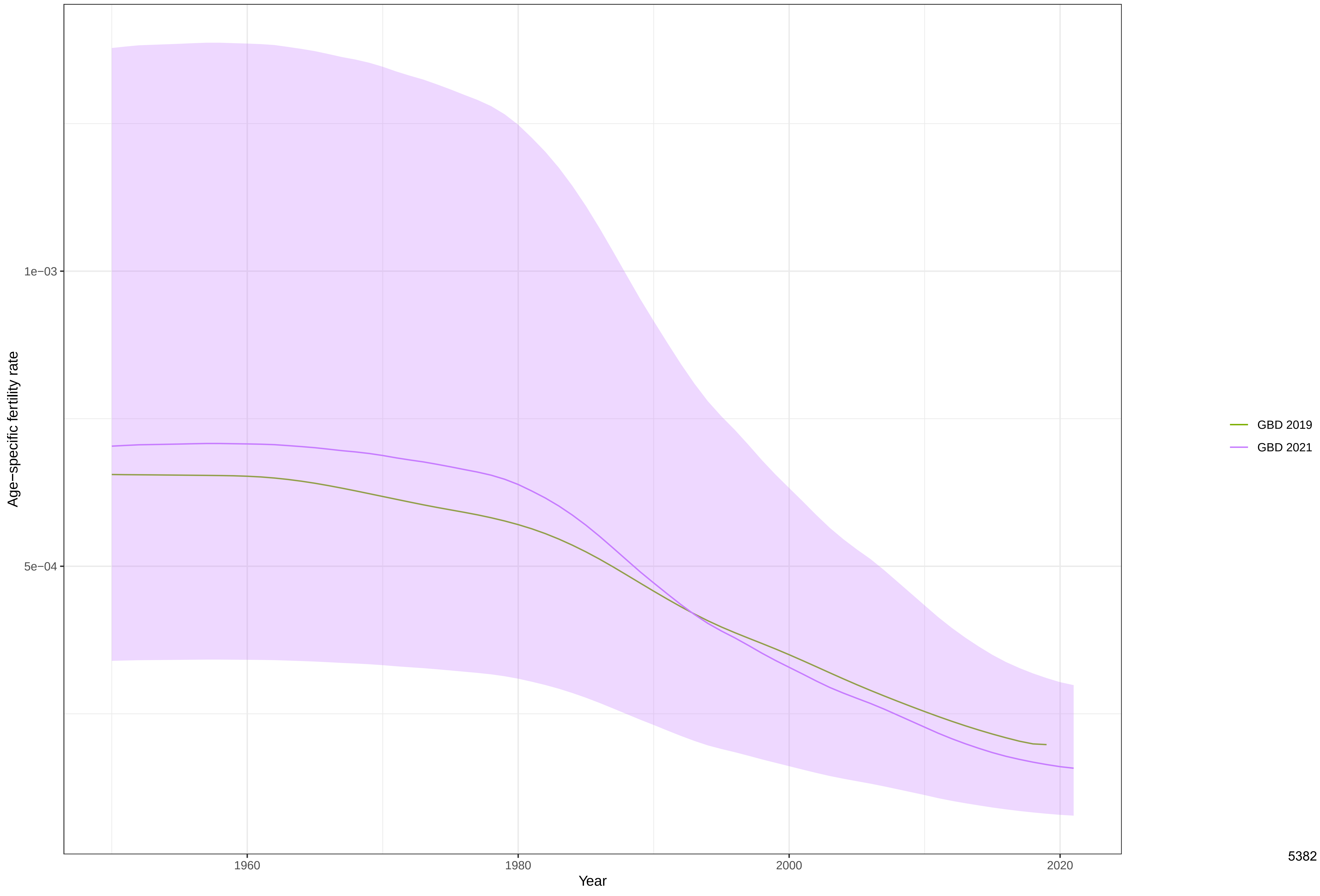

Age−specific fertility fitted trend lines, 1950−2021: Aceh, Ages: 15−19
Data points enclosed with a diamond are reference sources

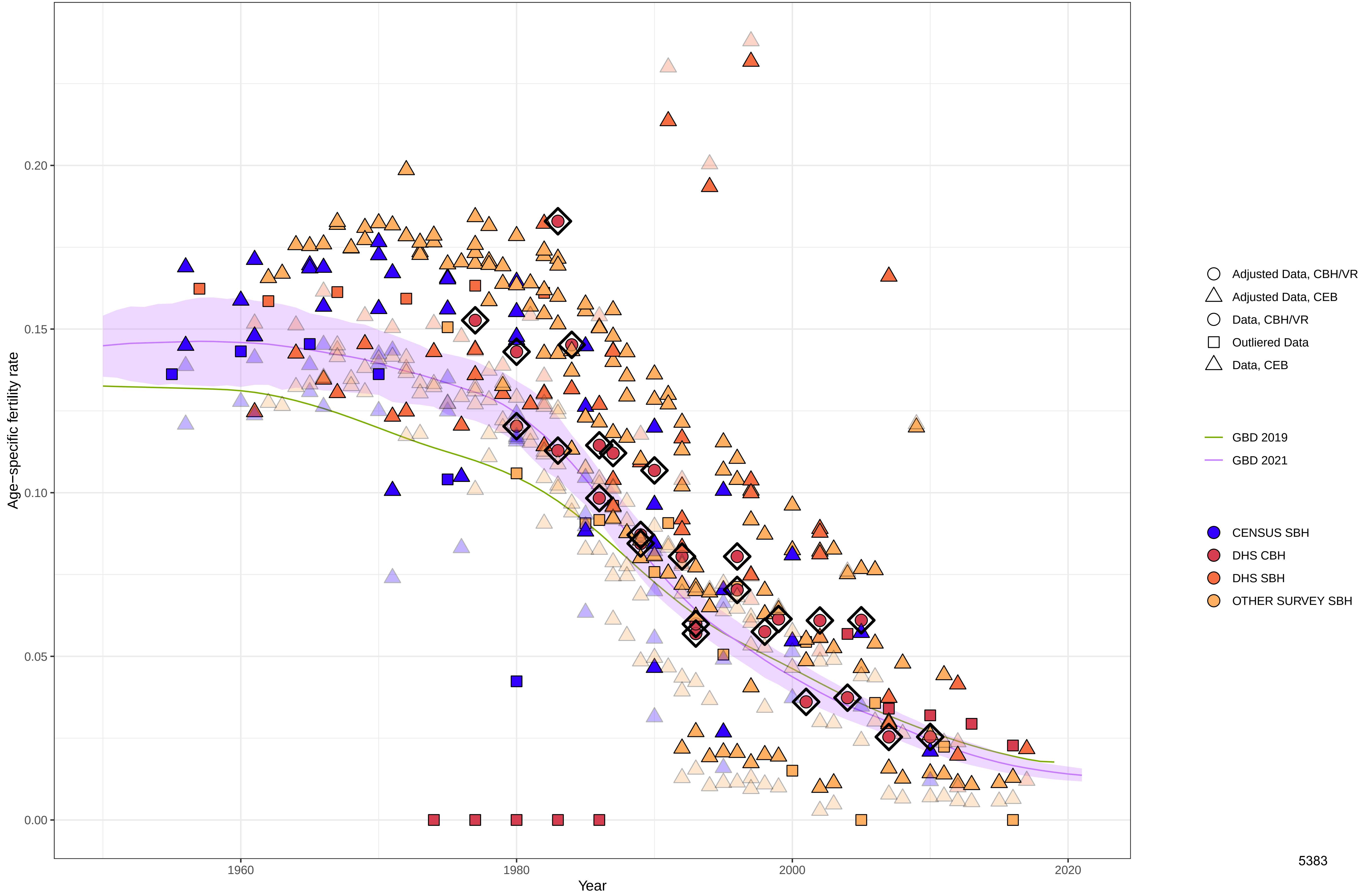

Age−specific fertility fitted trend lines, 1950−2021: Aceh, Ages: 20−24
Data points enclosed with a diamond are reference sources

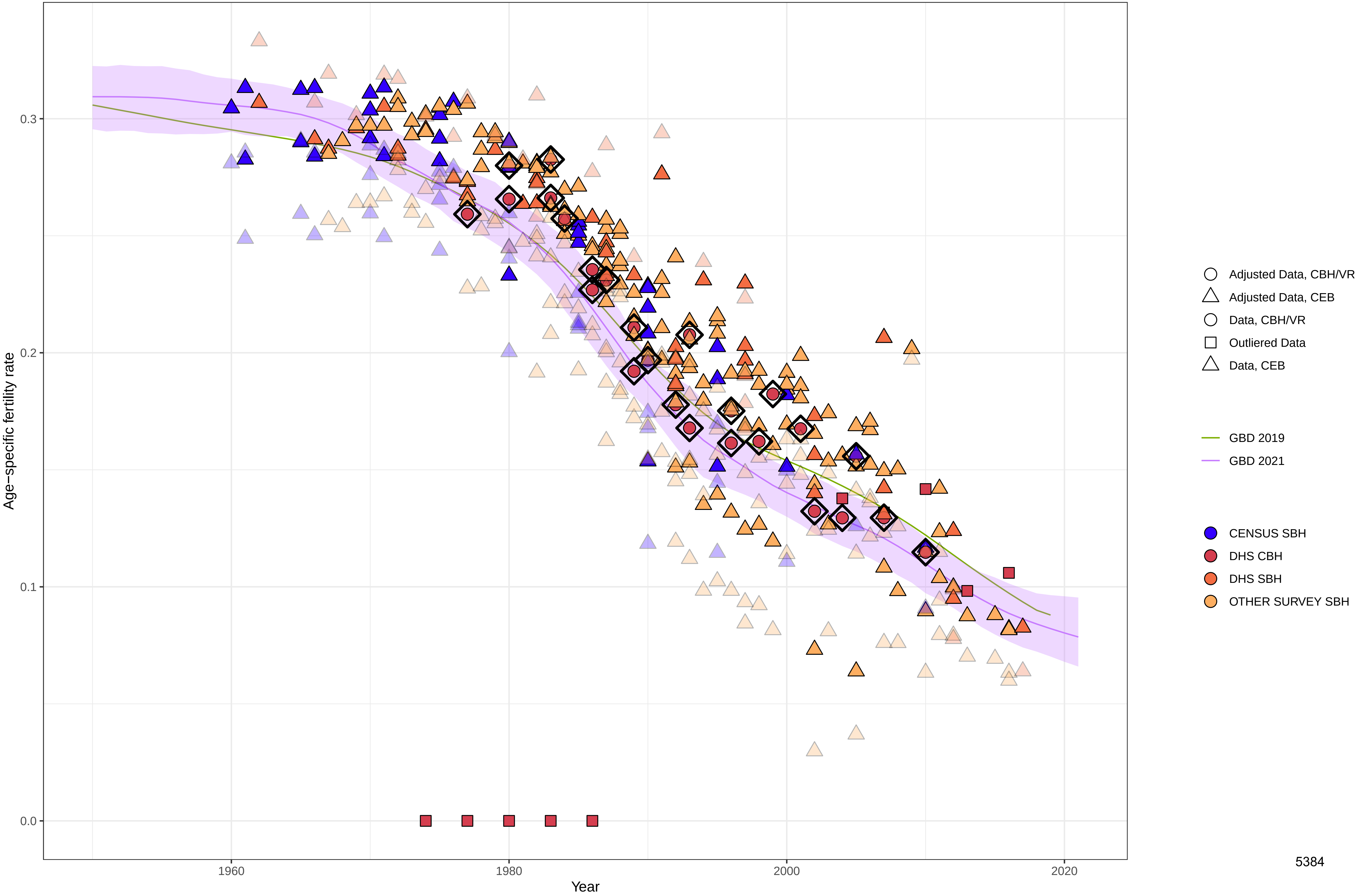

Age−specific fertility fitted trend lines, 1950−2021: Aceh, Ages: 25−29
Data points enclosed with a diamond are reference sources

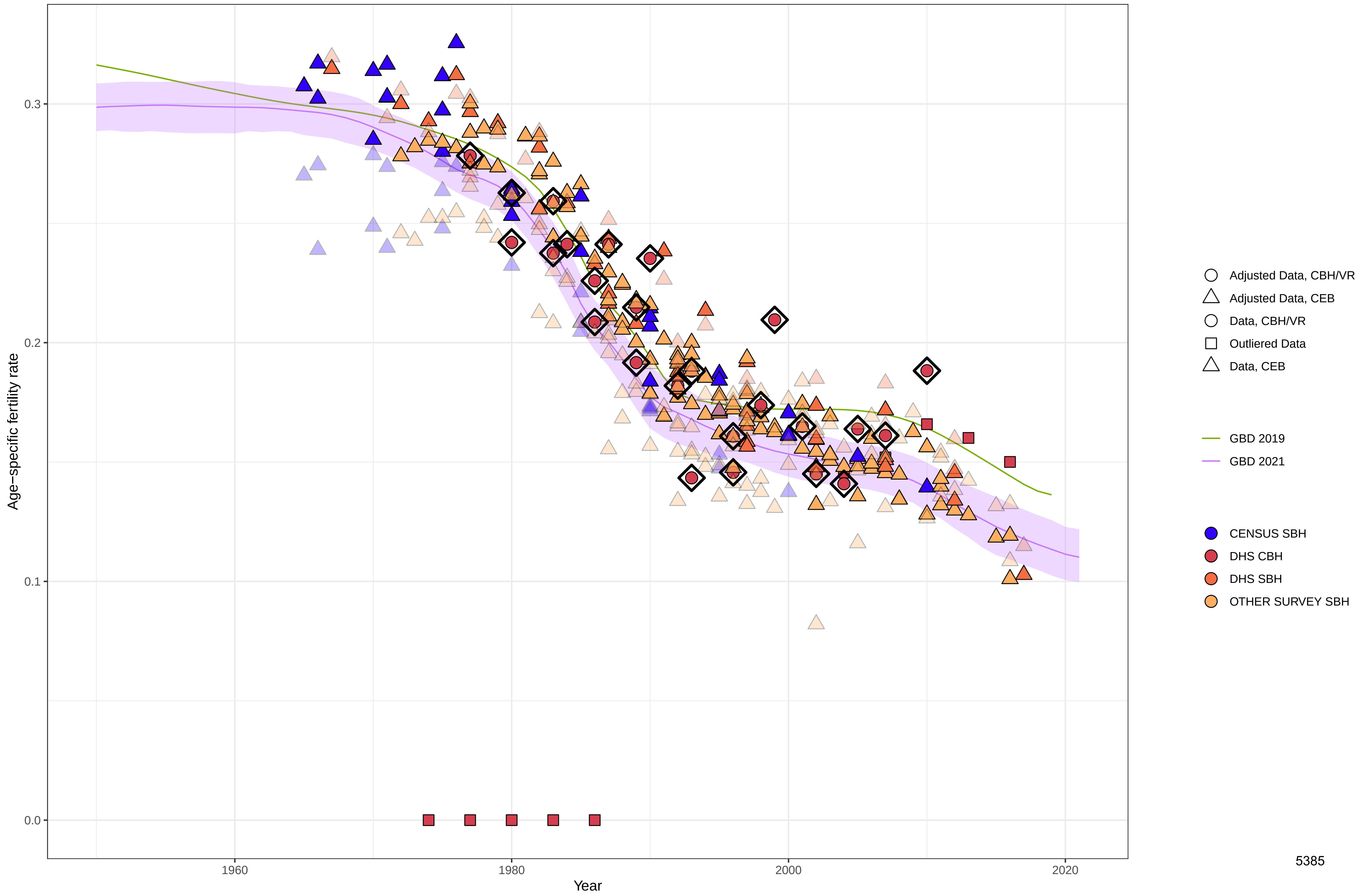

Age−specific fertility fitted trend lines, 1950−2021: Aceh, Ages: 30−34
Data points enclosed with a diamond are reference sources

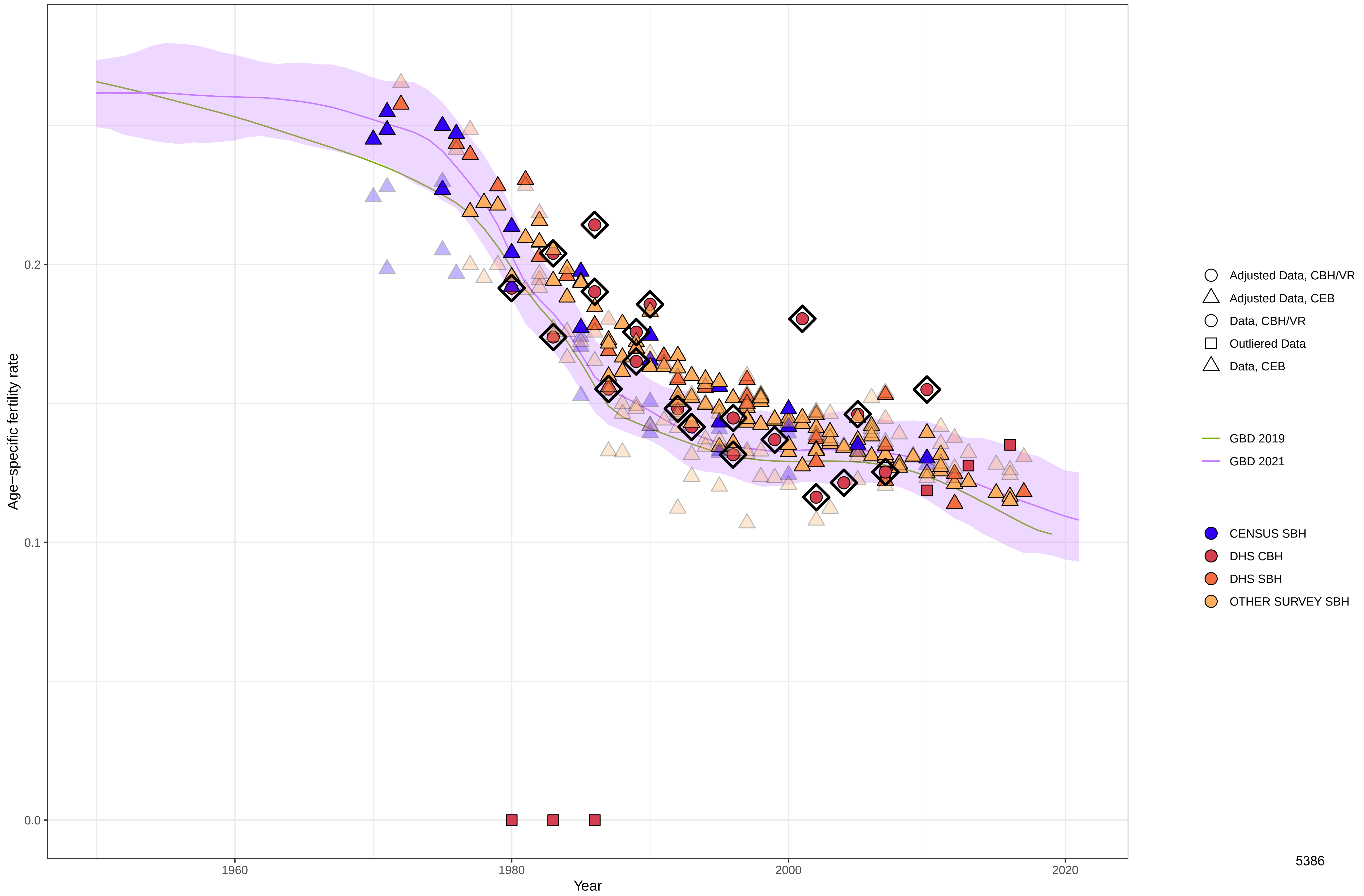

Age−specific fertility fitted trend lines, 1950−2021: Aceh, Ages: 35−39
Data points enclosed with a diamond are reference sources

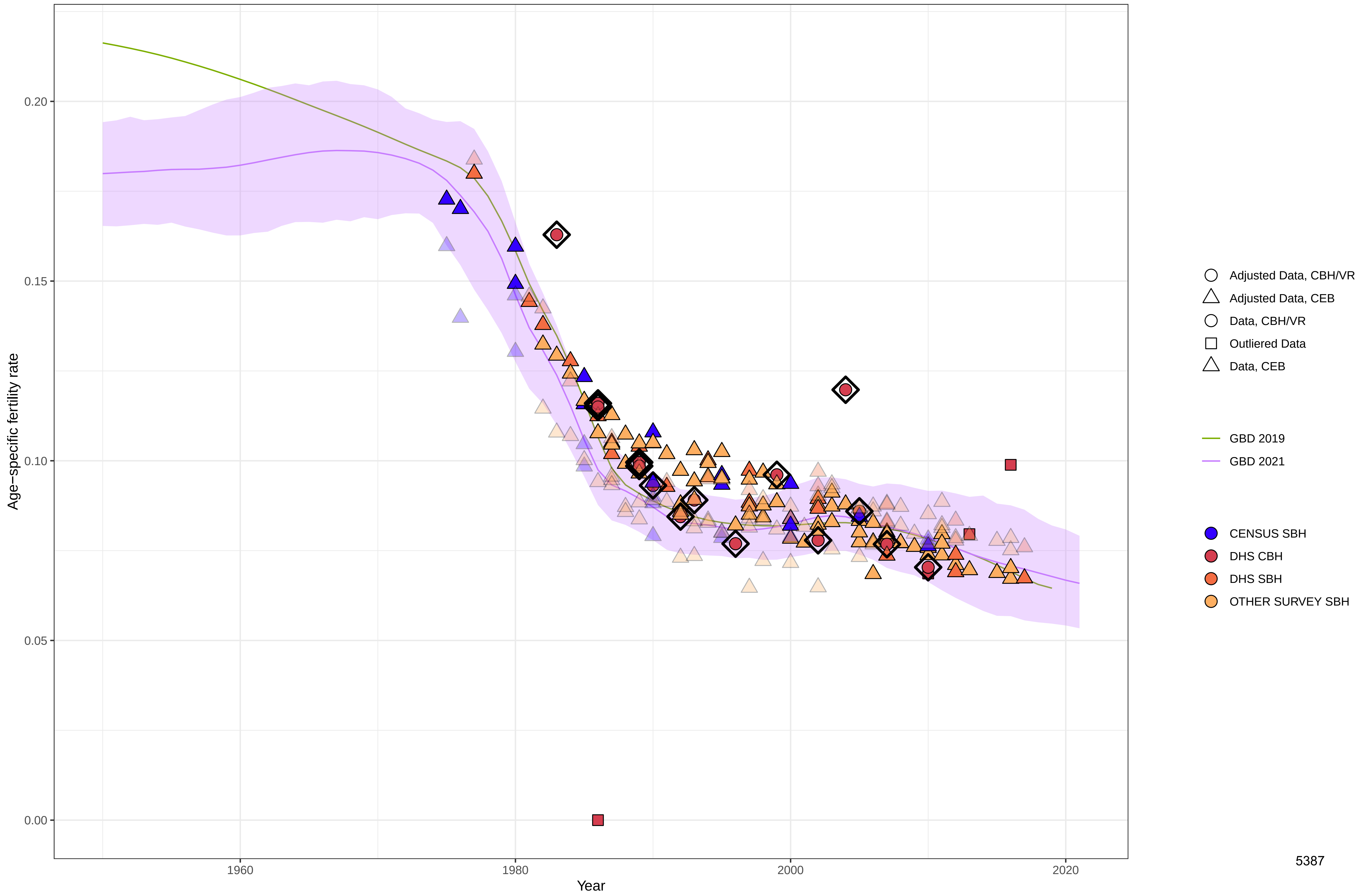

Age−specific fertility fitted trend lines, 1950−2021: Aceh, Ages: 40−44
Data points enclosed with a diamond are reference sources

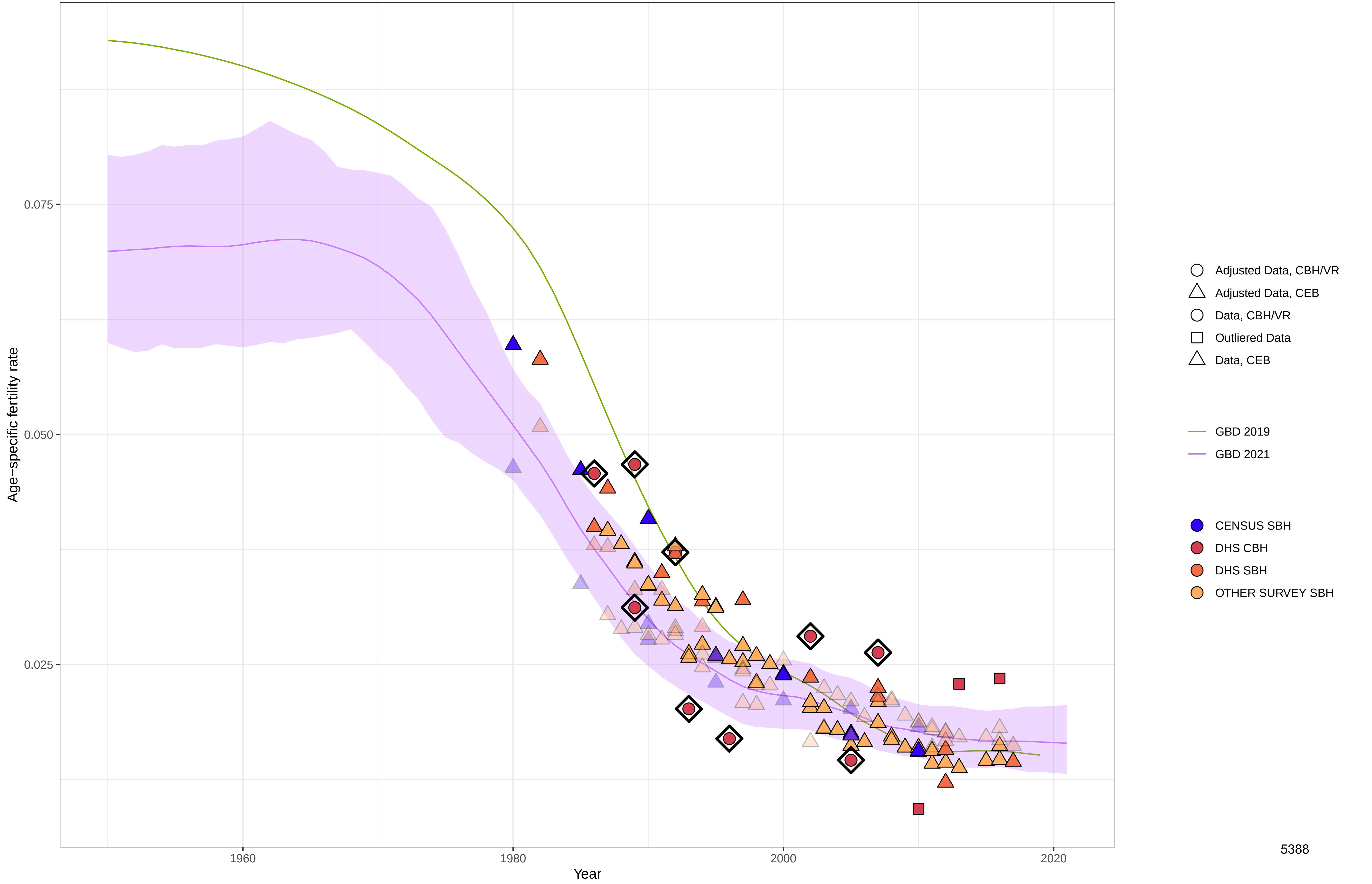

Age−specific fertility fitted trend lines, 1950−2021: Aceh, Ages: 45−49
Data points enclosed with a diamond are reference sources

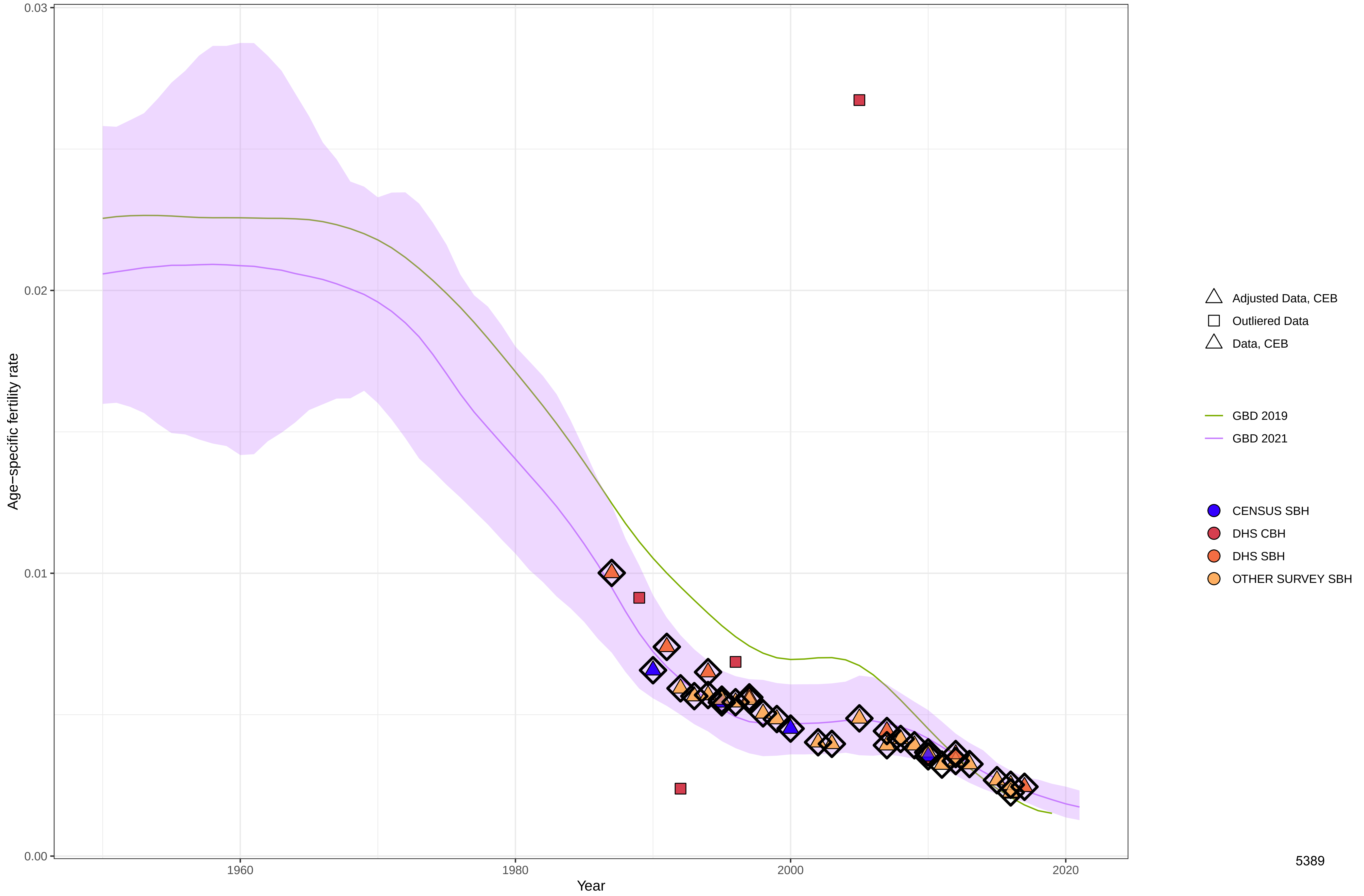

Age−specific fertility fitted trend lines, 1950−2021: Aceh, Ages: 50−54
Data points enclosed with a diamond are reference sources

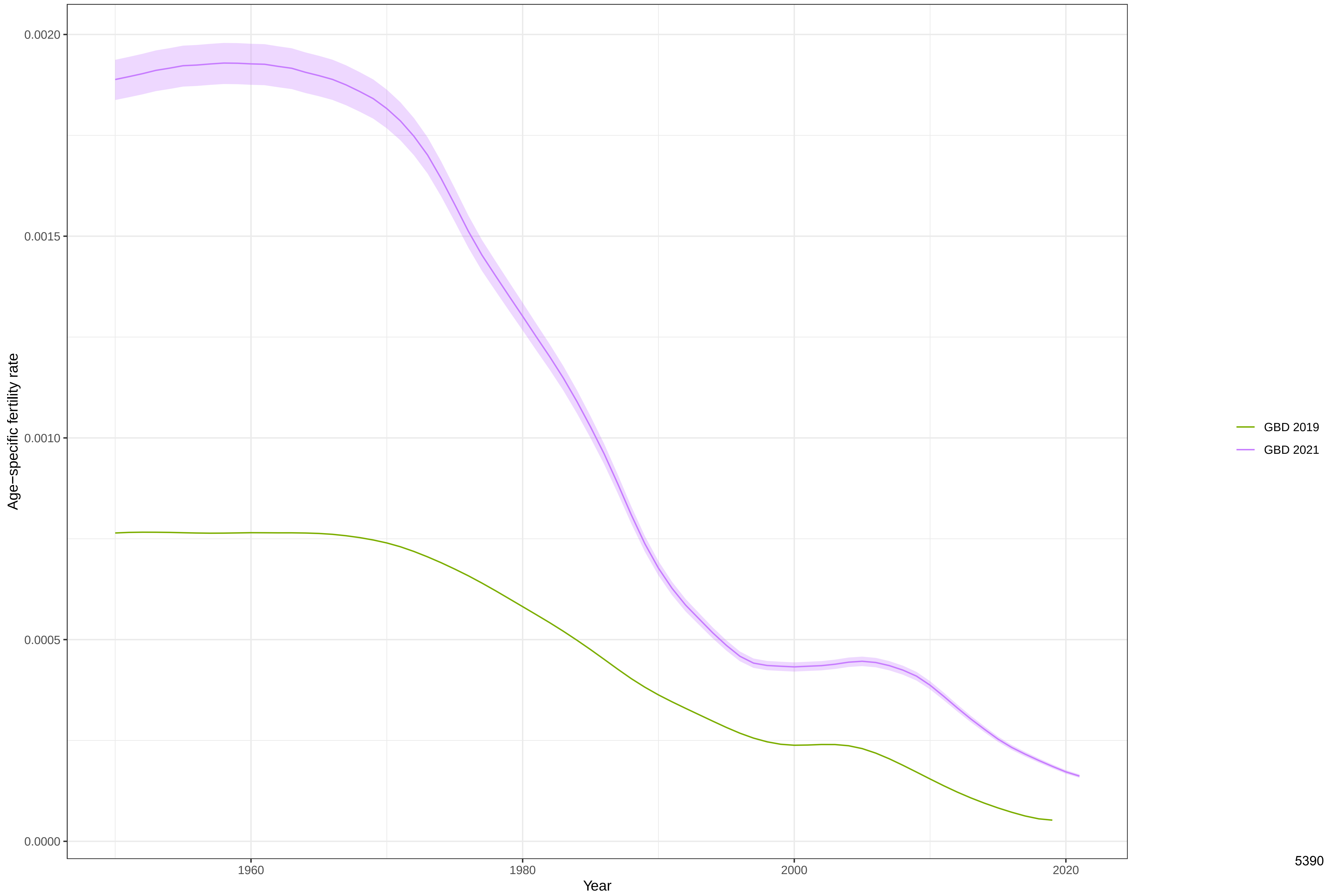

Total fertility rate fitted trend lines, 1950−2021: Aceh TFR
Data points enclosed with a diamond are reference sources

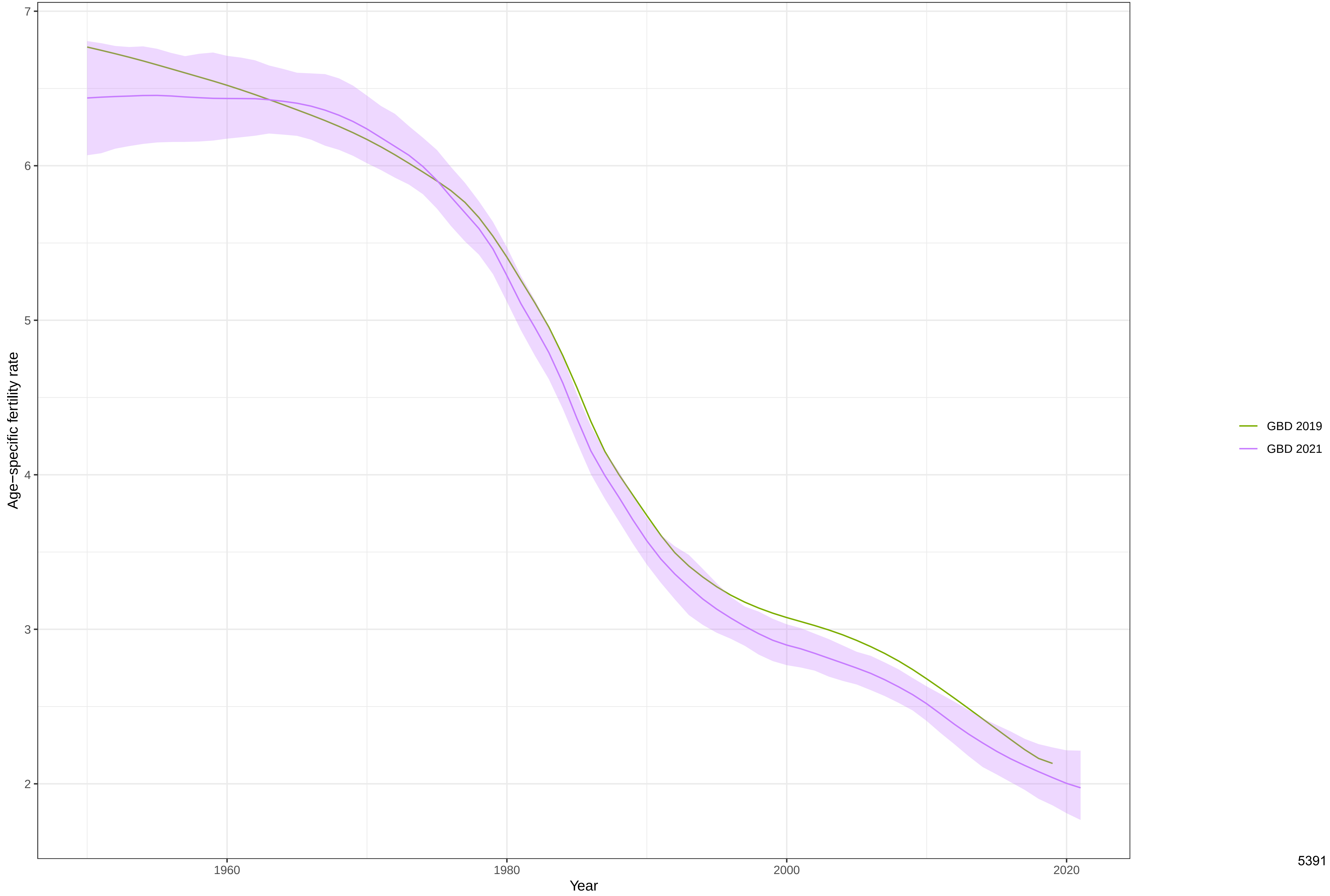

Age−specific fertility fitted trend lines, 1950−2021: Bali, Ages: 10−14
Data points enclosed with a diamond are reference sources

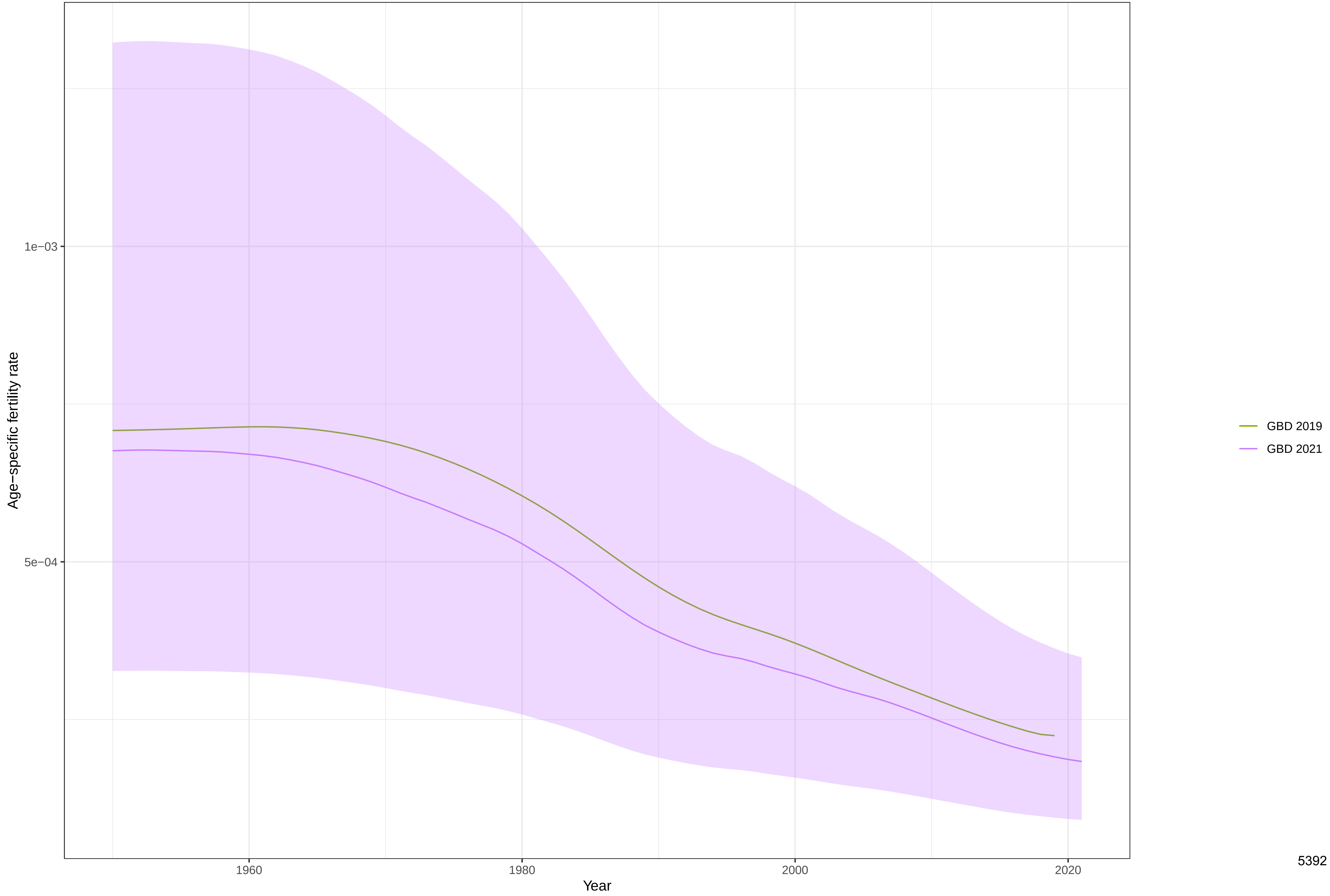

Age−specific fertility fitted trend lines, 1950−2021: Bali, Ages: 15−19
Data points enclosed with a diamond are reference sources

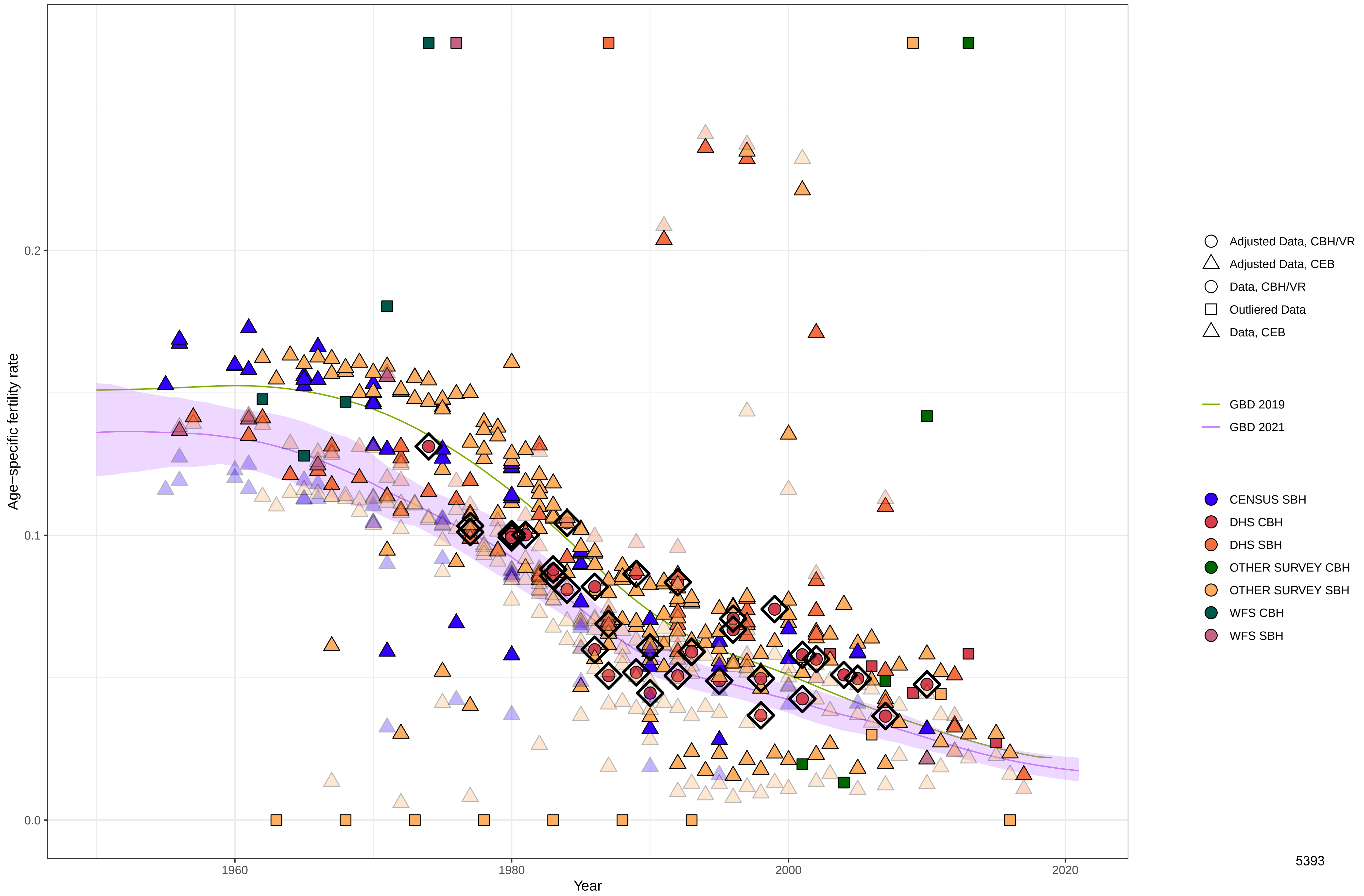

Age−specific fertility fitted trend lines, 1950−2021: Bali, Ages: 20−24
Data points enclosed with a diamond are reference sources

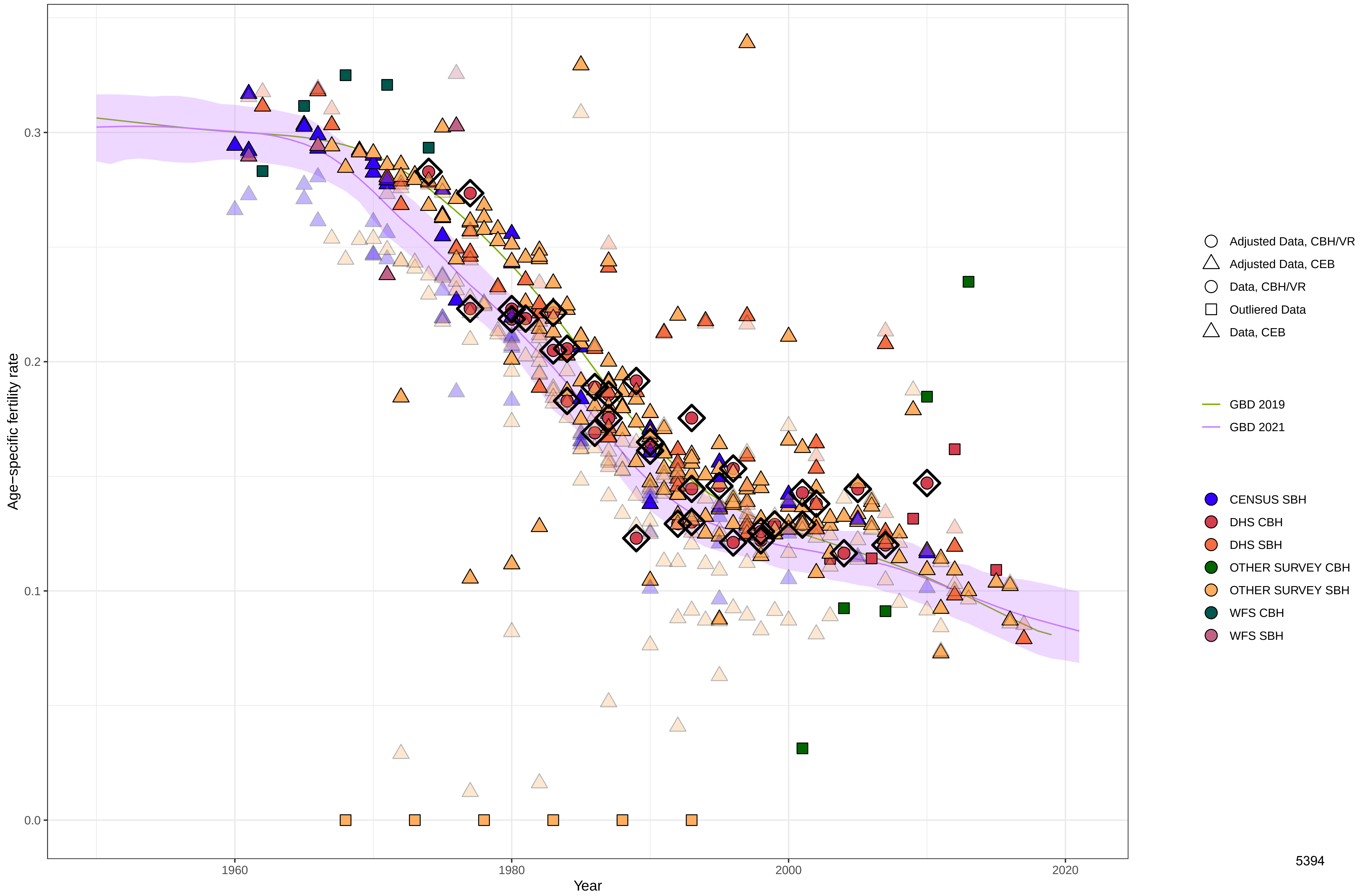

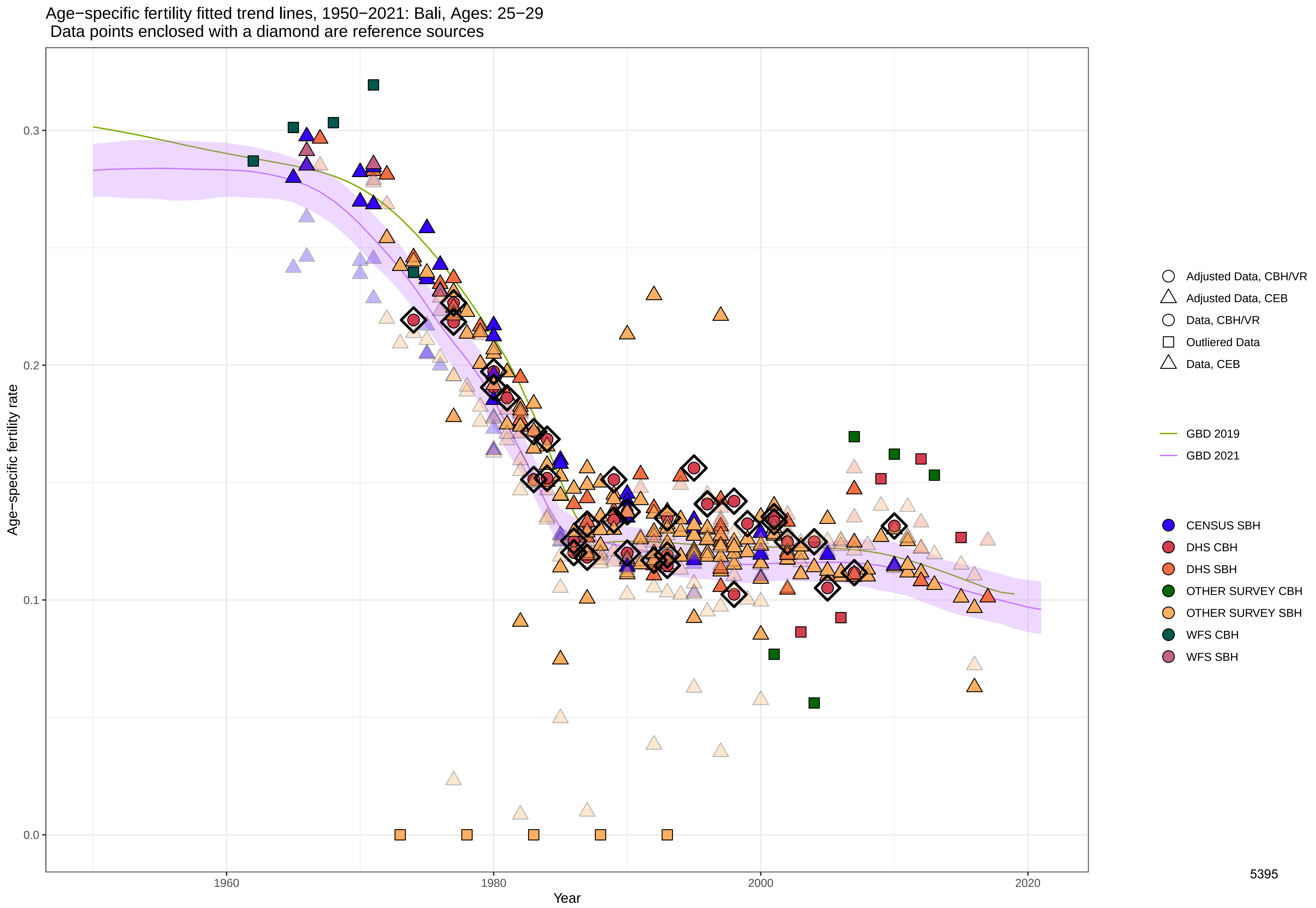

Age−specific fertility fitted trend lines, 1950−2021: Bali, Ages: 25−29
Data points enclosed with a diamond are reference sources
Age−specific fertility rate
Year
0.0
0.1
0.2
0.3
1960
1980
2000
2020
Adjusted Data, CBH/VR
Adjusted Data, CEB
Data, CBH/VR
Outliered Data
Data, CEB
GBD 2019
GBD 2021
CENSUS SBH
DHS CBH
DHS SBH
OTHER SURVEY CBH
OTHER SURVEY SBH
WFS CBH
WFS SBH

Age−specific fertility fitted trend lines, 1950−2021: Bali, Ages: 30−34
Data points enclosed with a diamond are reference sources

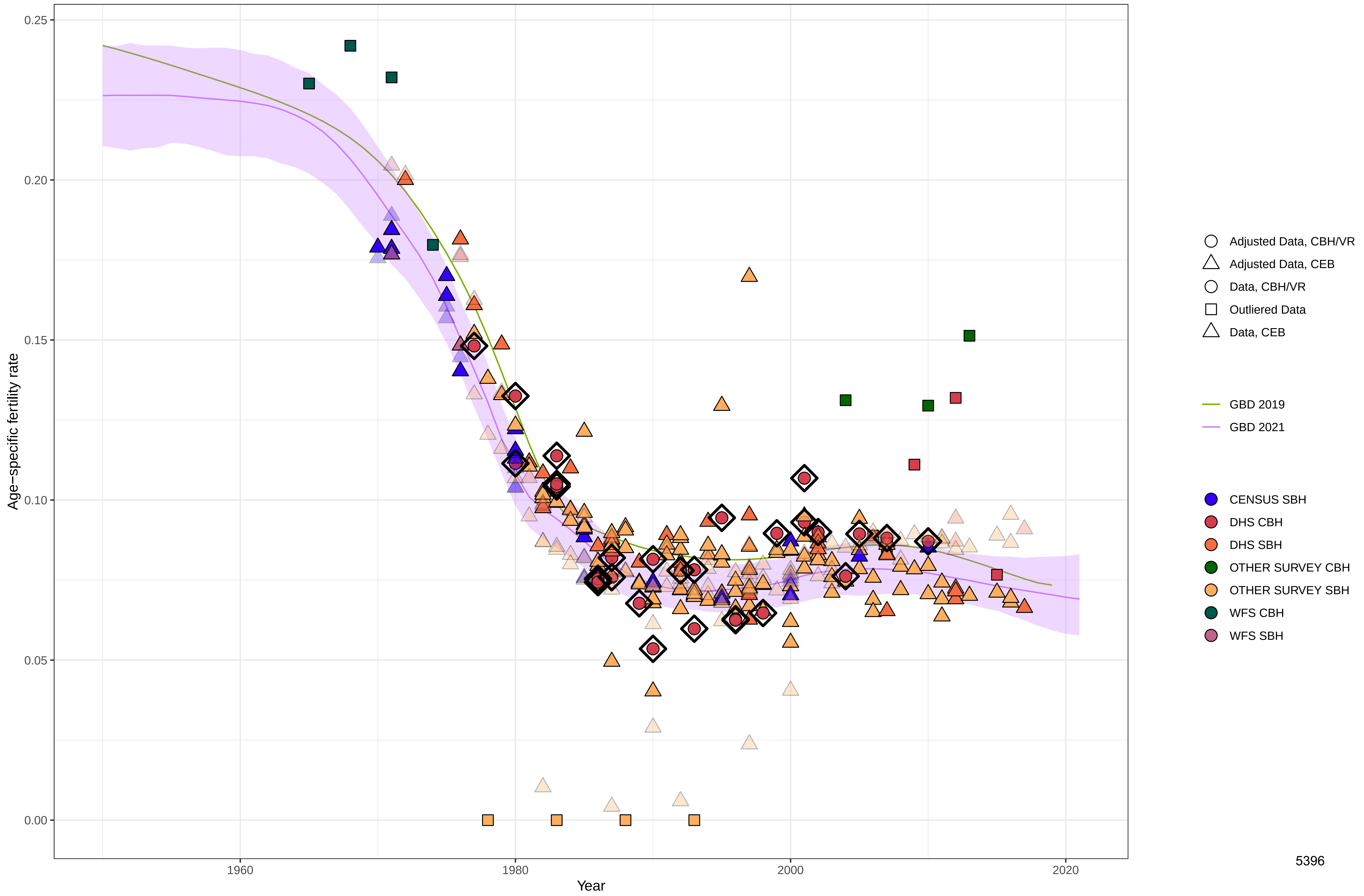

Age−specific fertility fitted trend lines, 1950−2021: Bali, Ages: 35−39
Data points enclosed with a diamond are reference sources

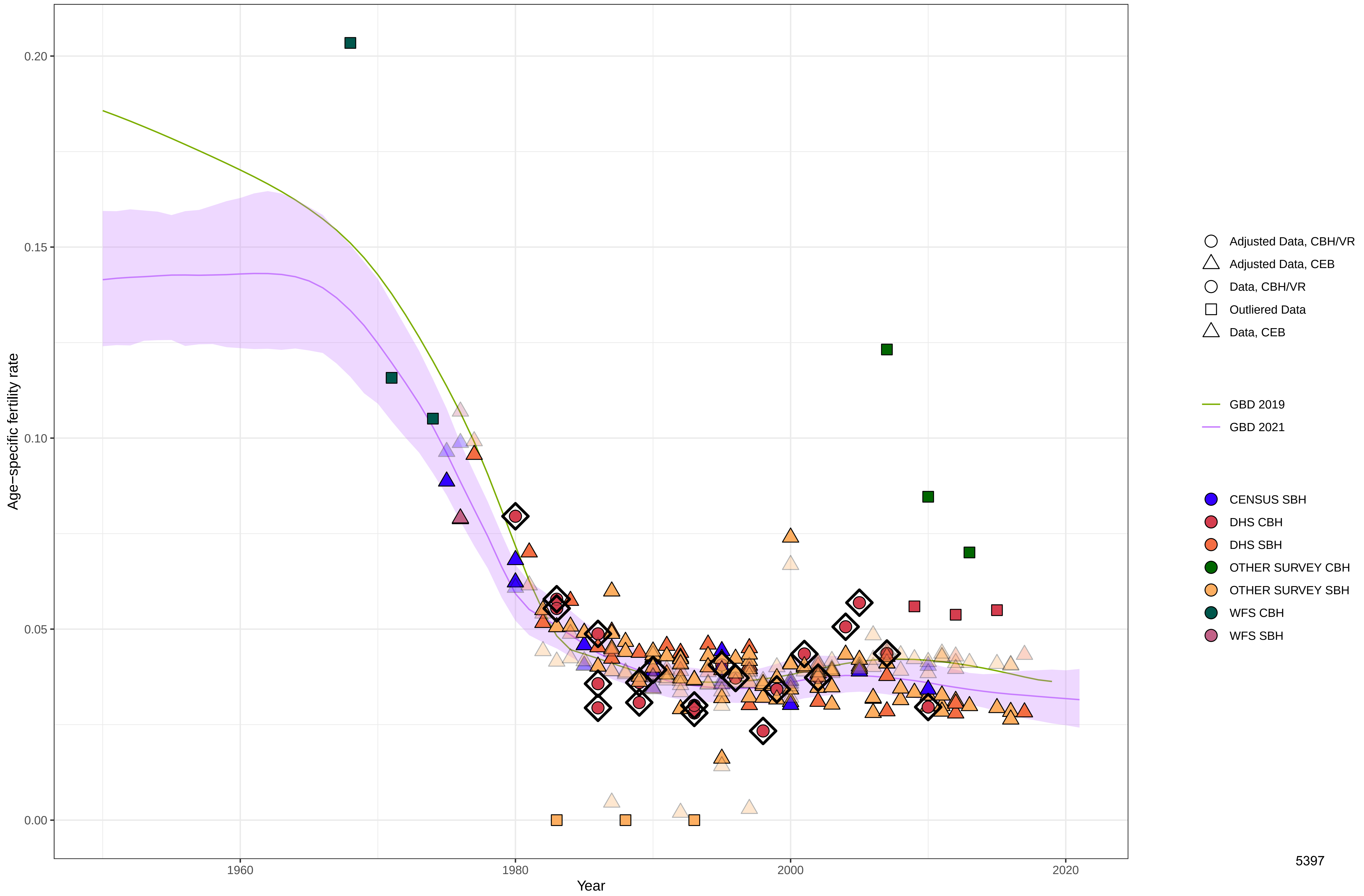

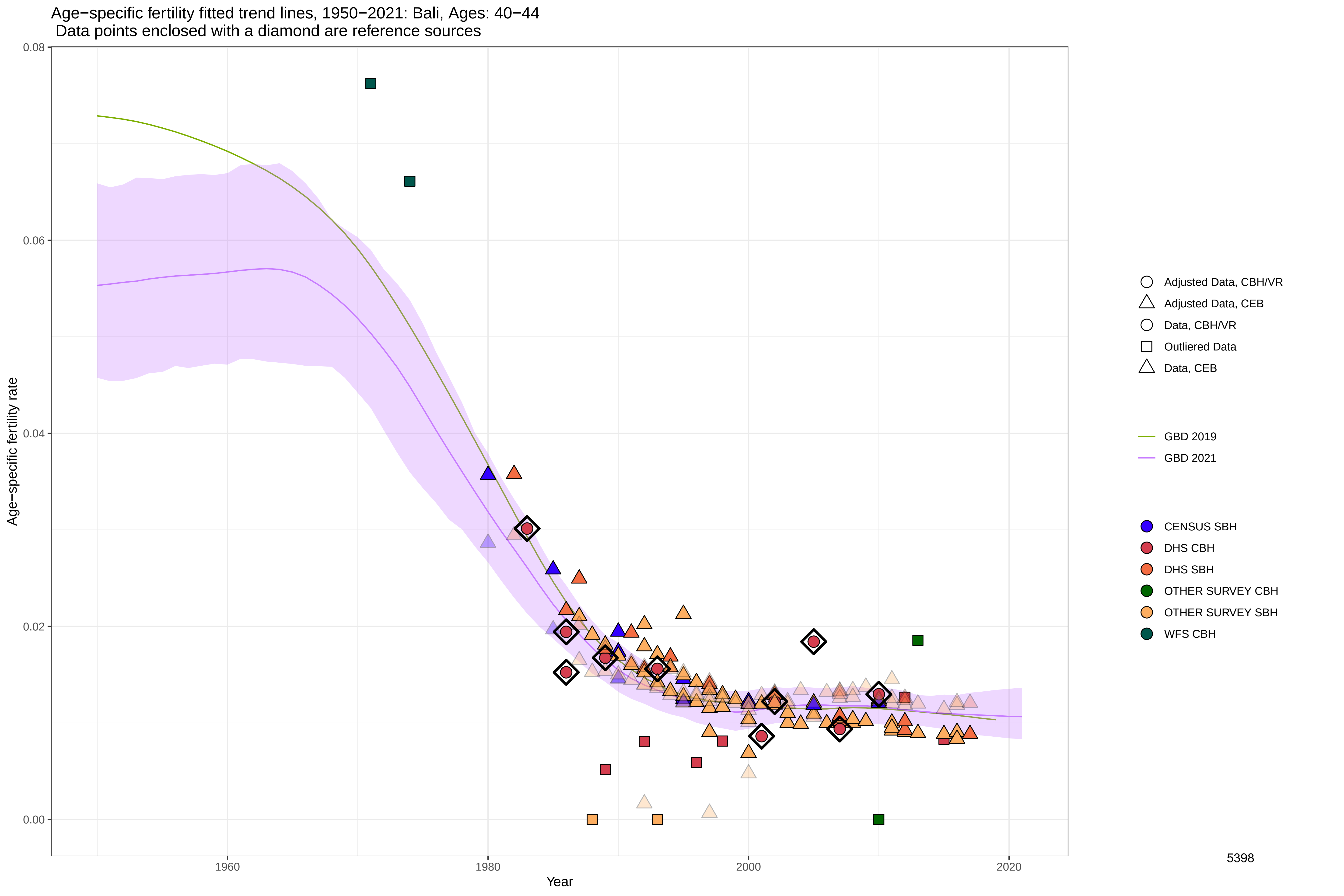

Age−specific fertility fitted trend lines, 1950−2021: Bali, Ages: 40−44
Data points enclosed with a diamond are reference sources
0.08
0.06
0.04
0.02
0.00
Age−specific fertility rate
1960
1980
2000
2020
Year
Adjusted Data, CBH/VR
Adjusted Data, CEB
Data, CBH/VR
Outliered Data
Data, CEB
GBD 2019
GBD 2021
CENSUS SBH
DHS CBH
DHS SBH
OTHER SURVEY CBH
OTHER SURVEY SBH
WFS CBH
5398

Age−specific fertility fitted trend lines, 1950−2021: Bali, Ages: 45−49
Data points enclosed with a diamond are reference sources

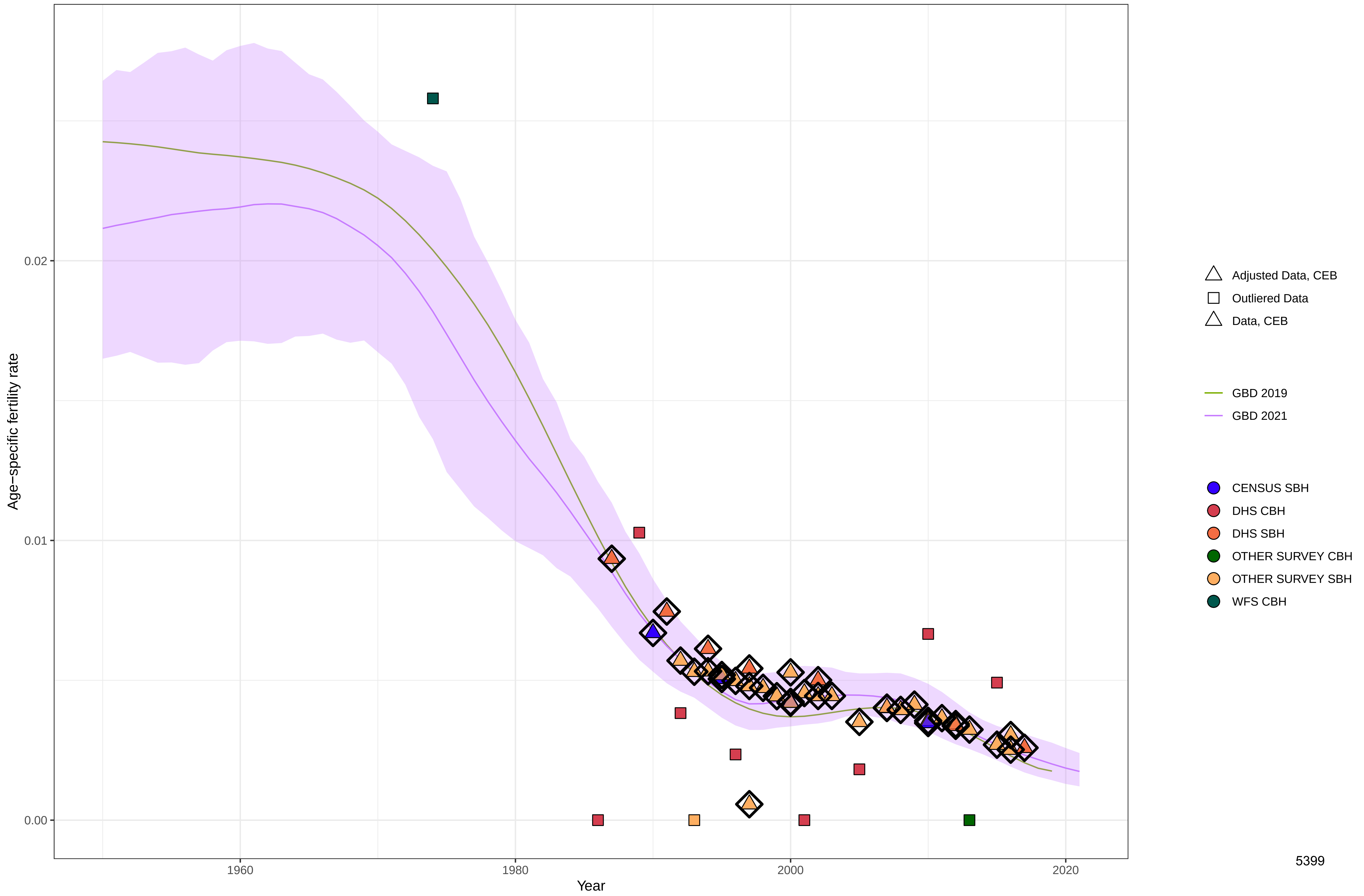

Age−specific fertility fitted trend lines, 1950−2021: Bali, Ages: 50−54
Data points enclosed with a diamond are reference sources

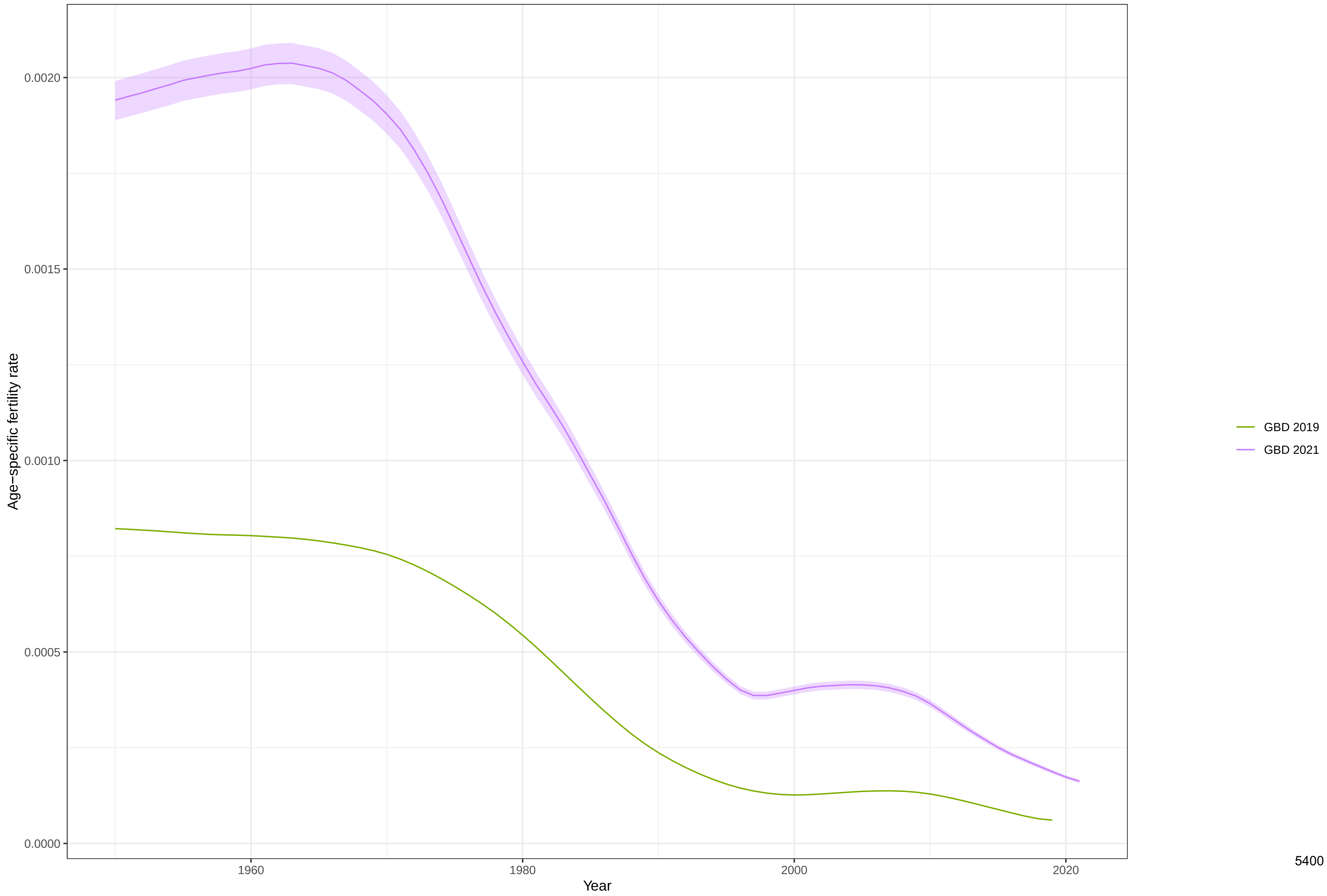

Total fertility rate fitted trend lines, 1950−2021: Bali TFR
Data points enclosed with a diamond are reference sources

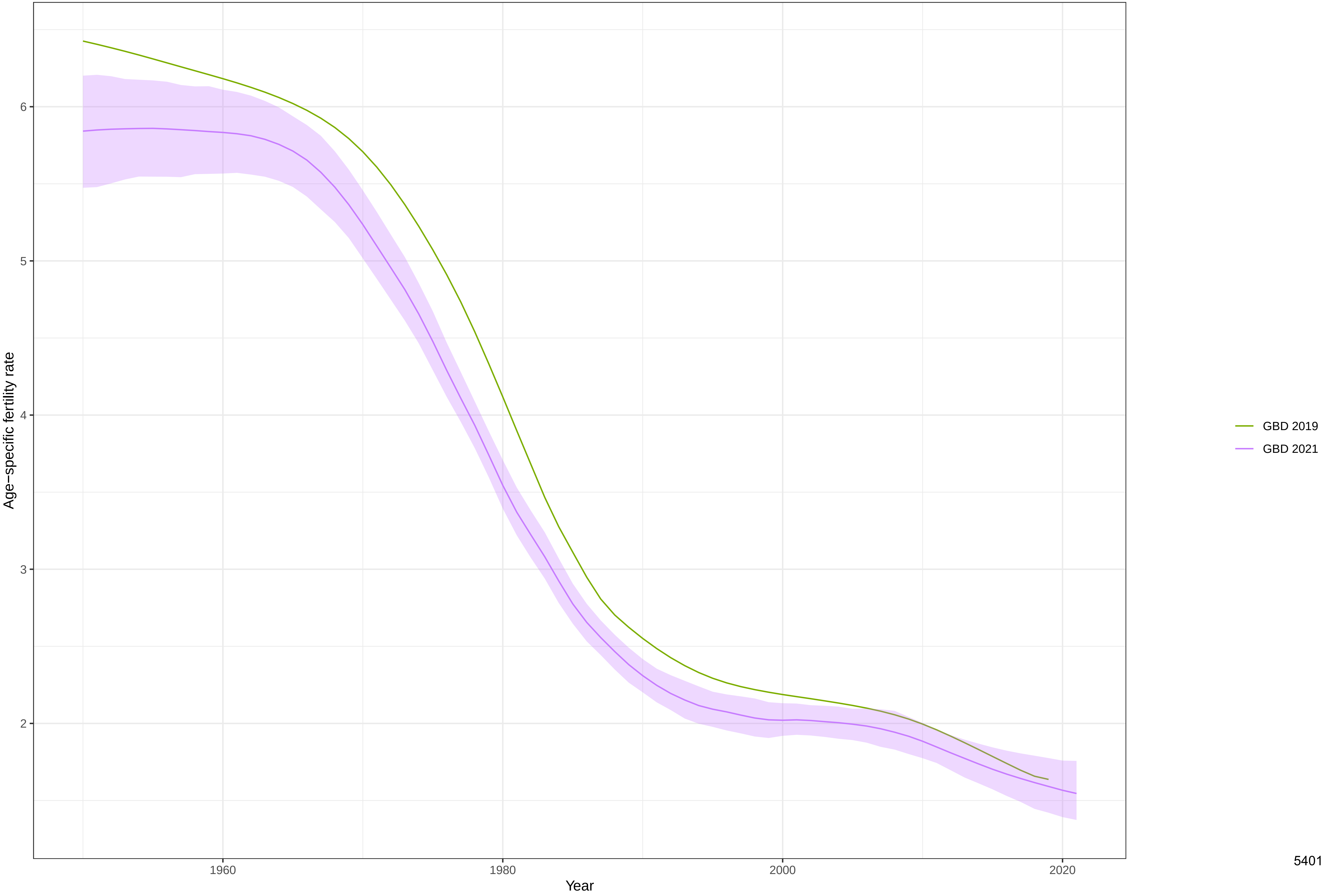

Age−specific fertility fitted trend lines, 1950−2021: Bangka−Belitung Islands, Ages: 10−14
Data points enclosed with a diamond are reference sources

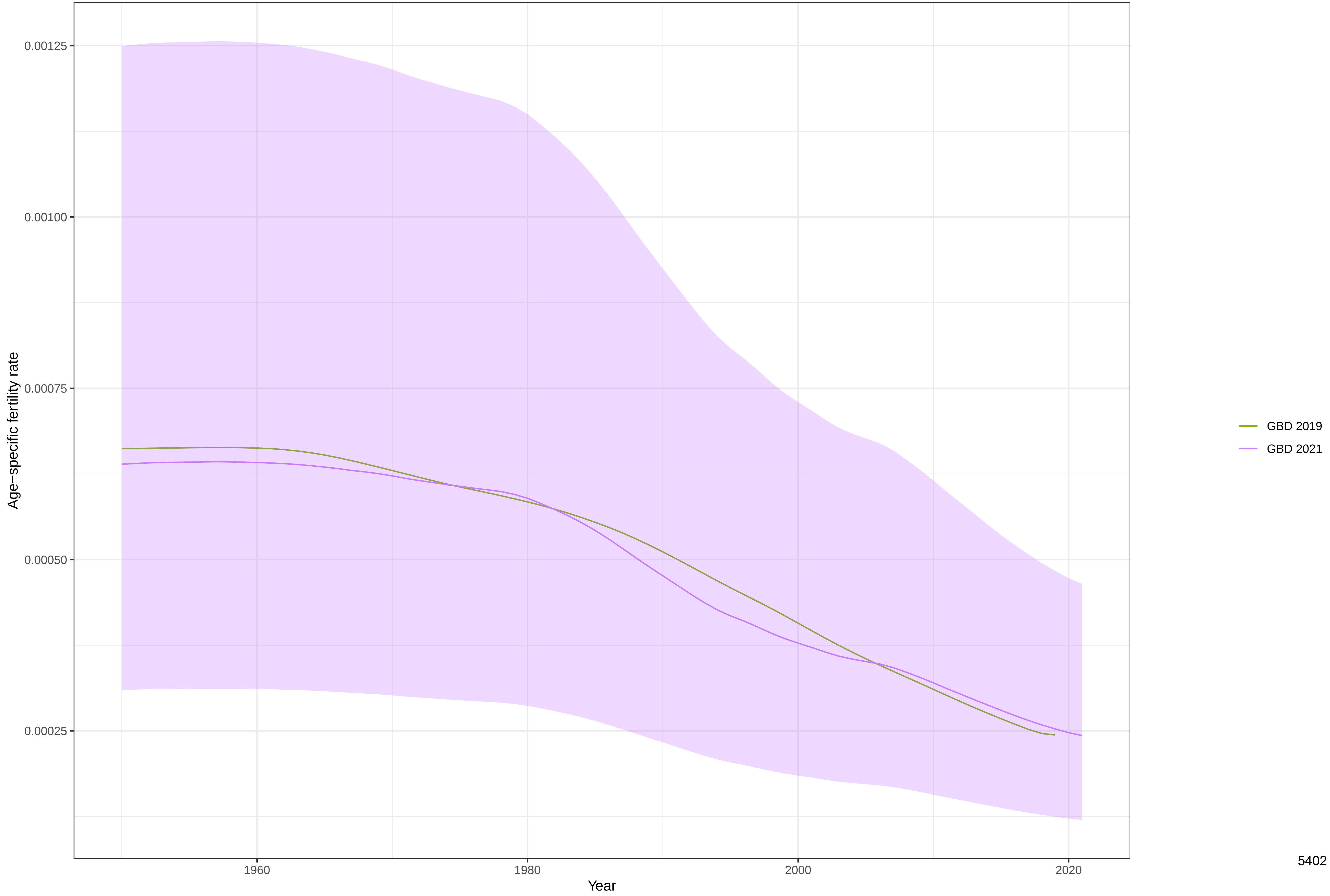

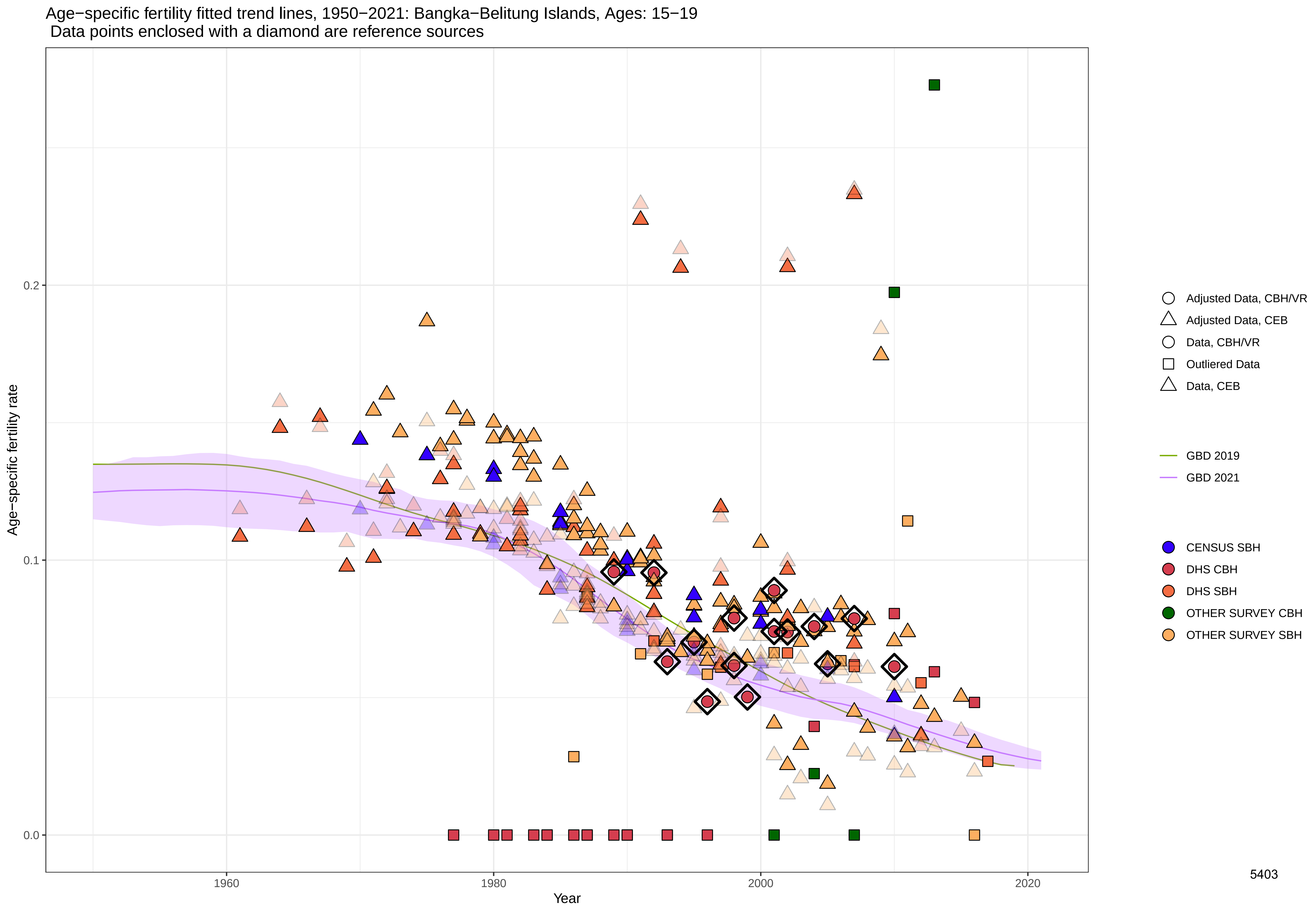

Age−specific fertility fitted trend lines, 1950−2021: Bangka−Belitung Islands, Ages: 15−19
Data points enclosed with a diamond are reference sources
Age−specific fertility rate
Year
0.0
0.1
0.2
1960
1980
2000
2020
Adjusted Data, CBH/VR
Adjusted Data, CEB
Data, CBH/VR
Outliered Data
Data, CEB
GBD 2019
GBD 2021
CENSUS SBH
DHS CBH
DHS SBH
OTHER SURVEY CBH
OTHER SURVEY SBH

Age−specific fertility fitted trend lines, 1950−2021: Bangka−Belitung Islands, Ages: 20−24
Data points enclosed with a diamond are reference sources

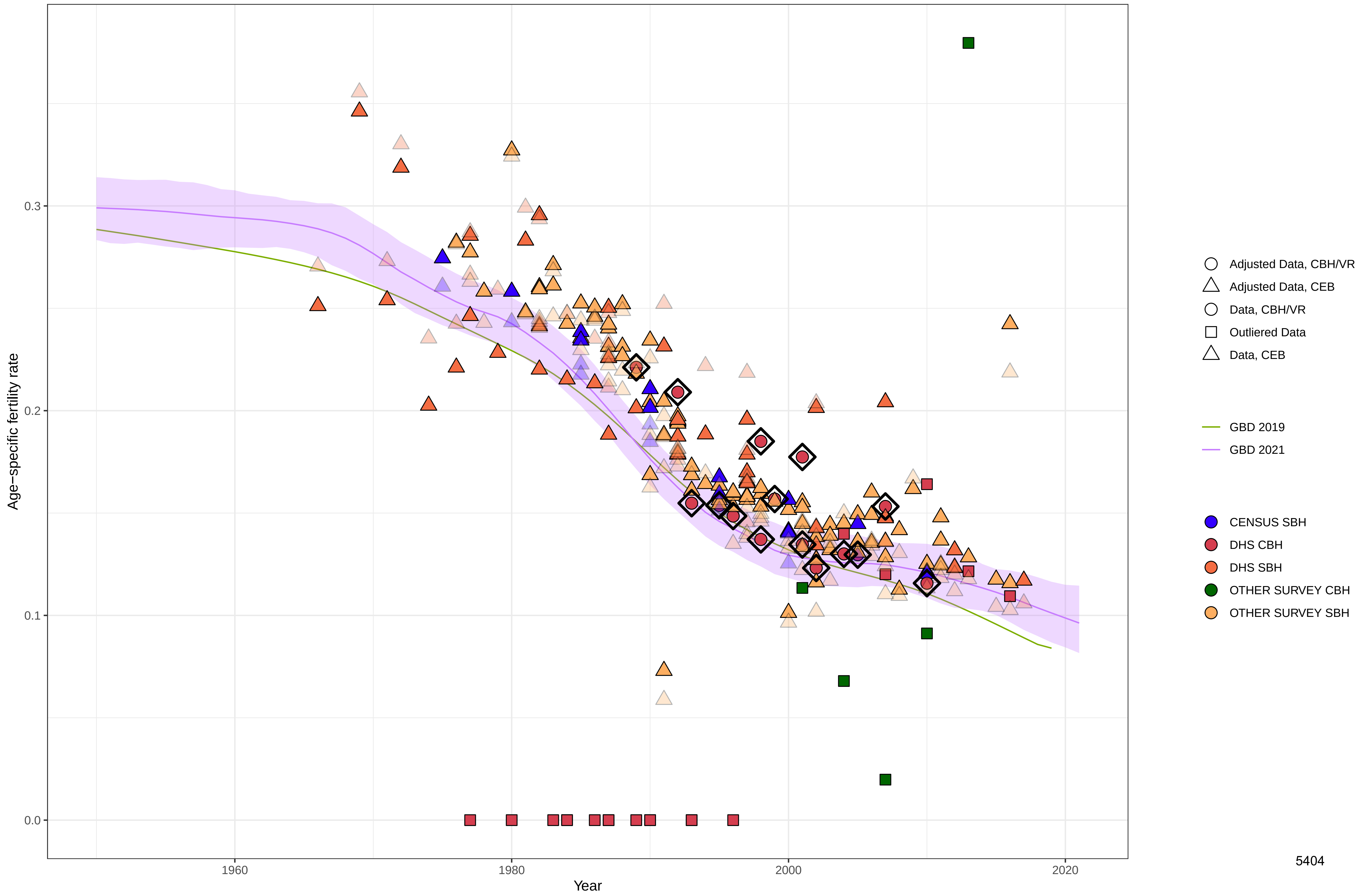

Age−specific fertility fitted trend lines, 1950−2021: Bangka−Belitung Islands, Ages: 25−29
Data points enclosed with a diamond are reference sources

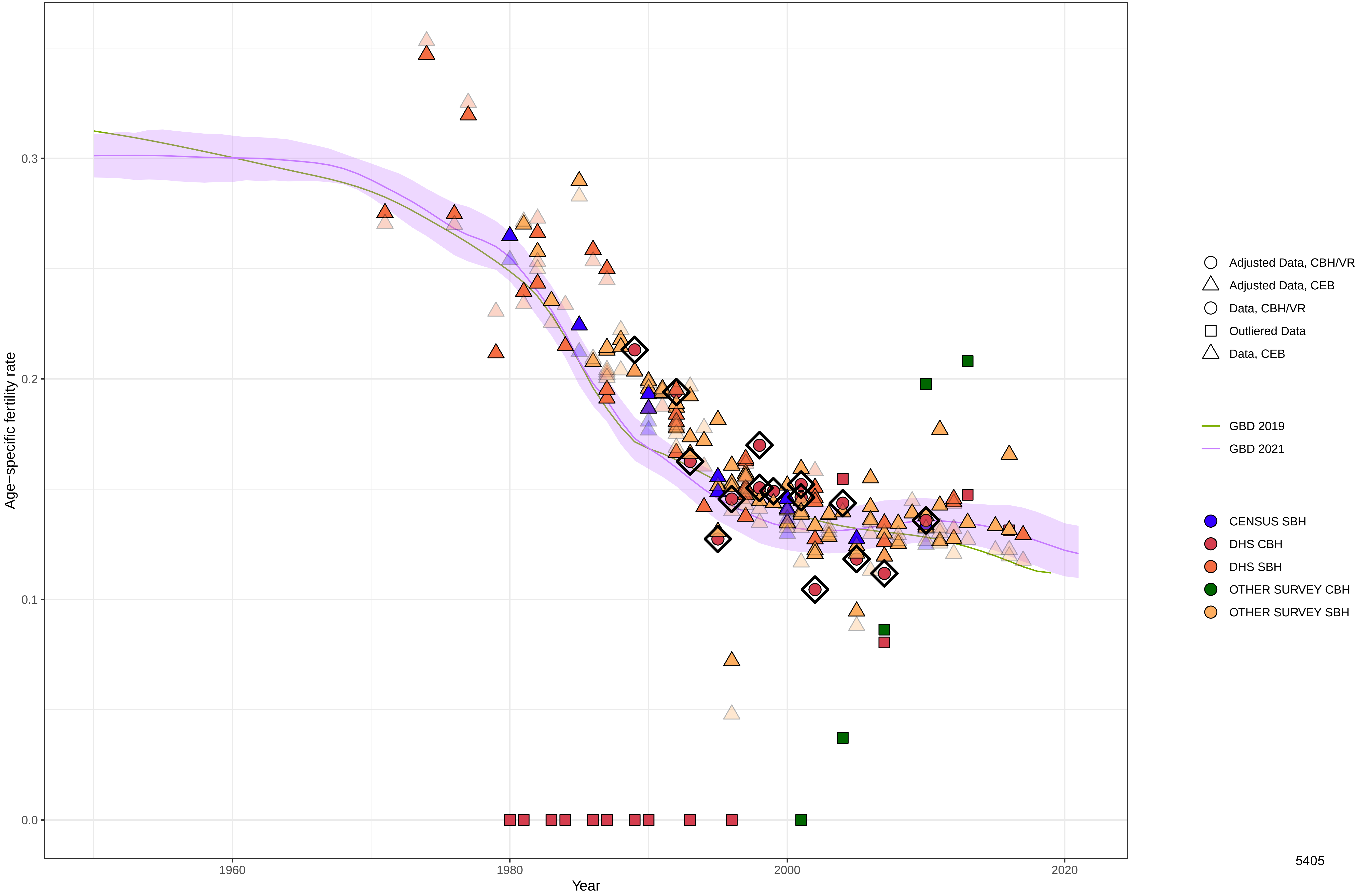

Age−specific fertility fitted trend lines, 1950−2021: Bangka−Belitung Islands, Ages: 30−34
Data points enclosed with a diamond are reference sources

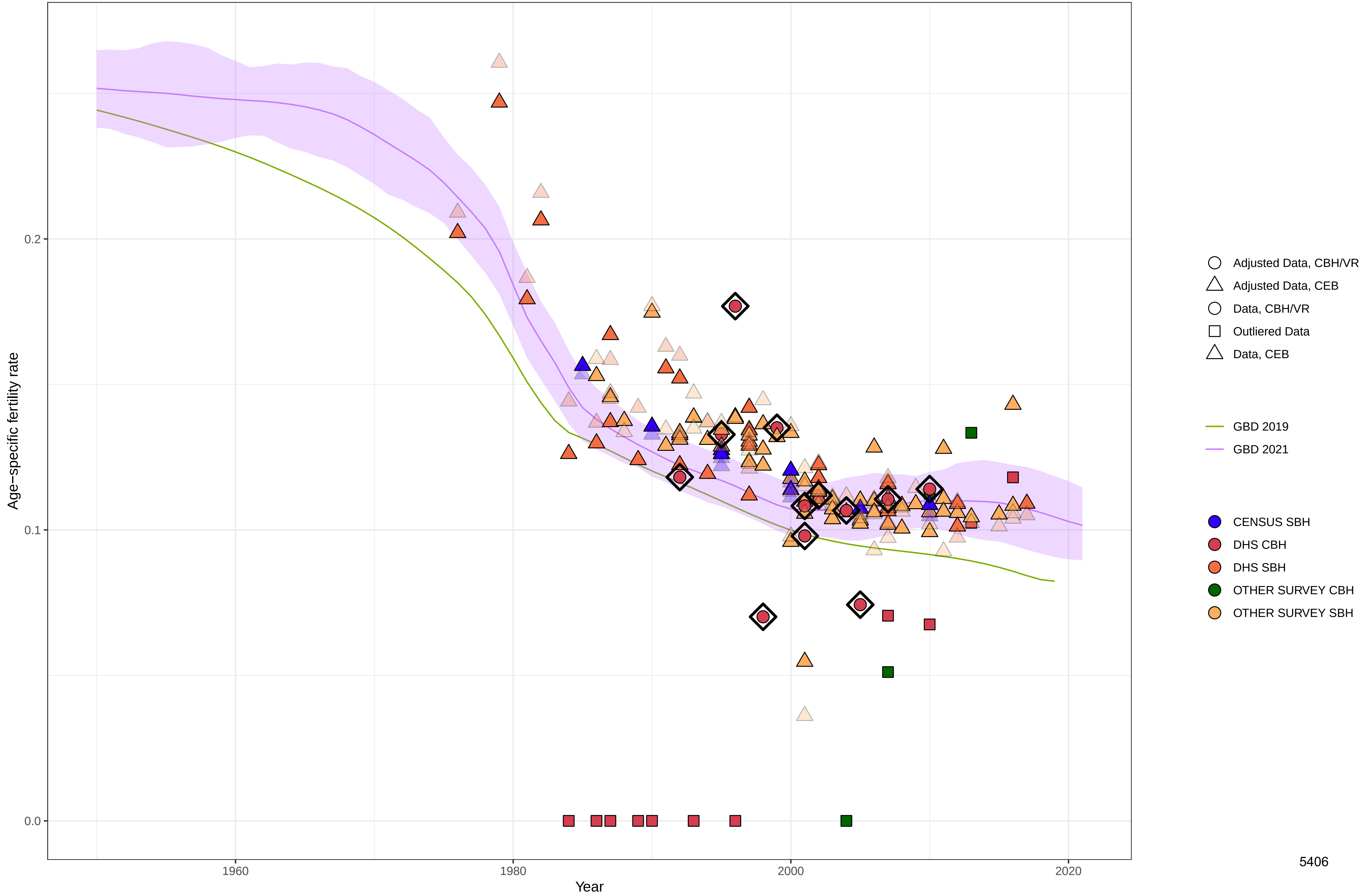

Age−specific fertility fitted trend lines, 1950−2021: Bangka−Belitung Islands, Ages: 35−39
Data points enclosed with a diamond are reference sources

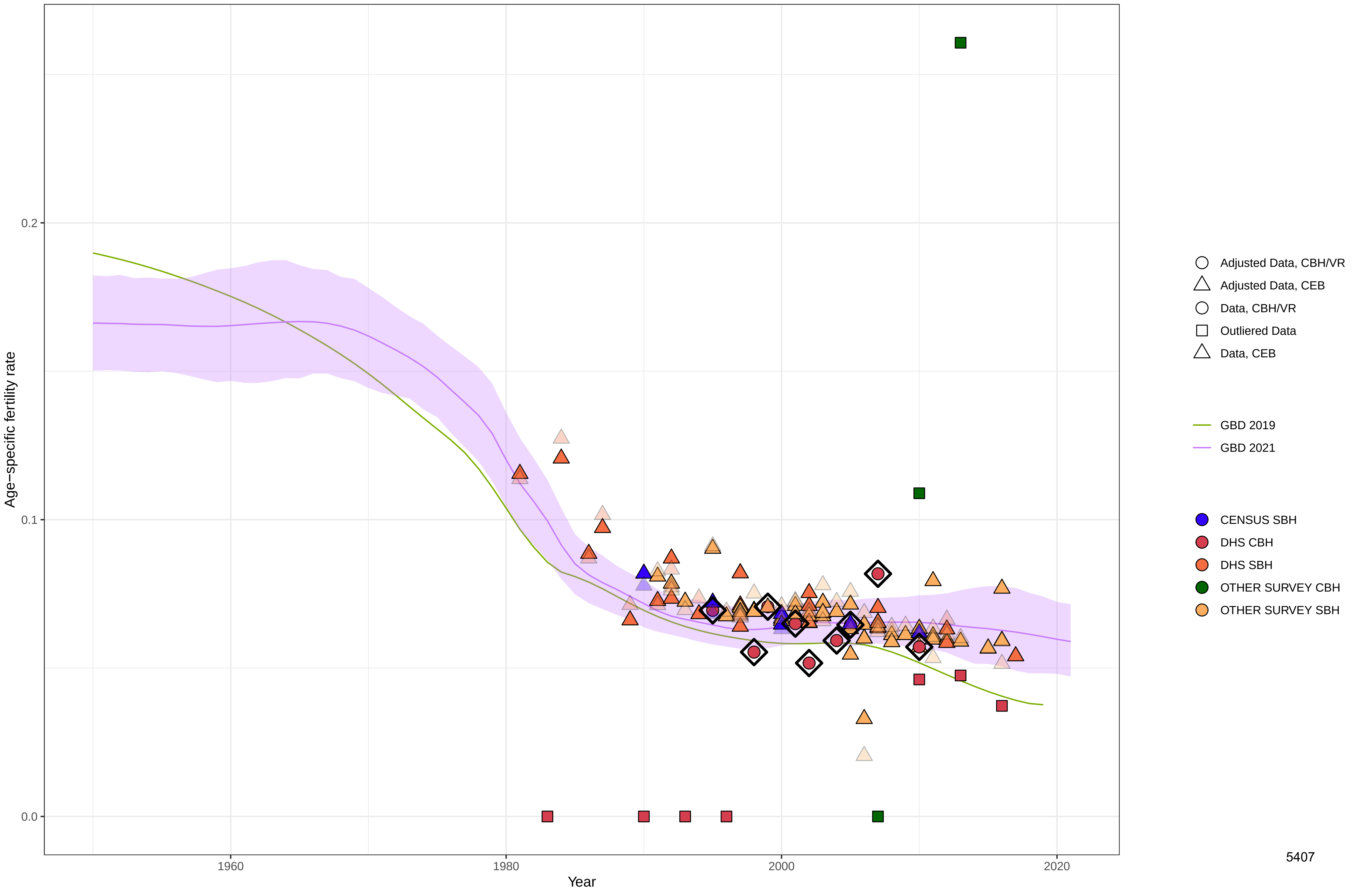

5407

Age−specific fertility fitted trend lines, 1950−2021: Bangka−Belitung Islands, Ages: 40−44
Data points enclosed with a diamond are reference sources

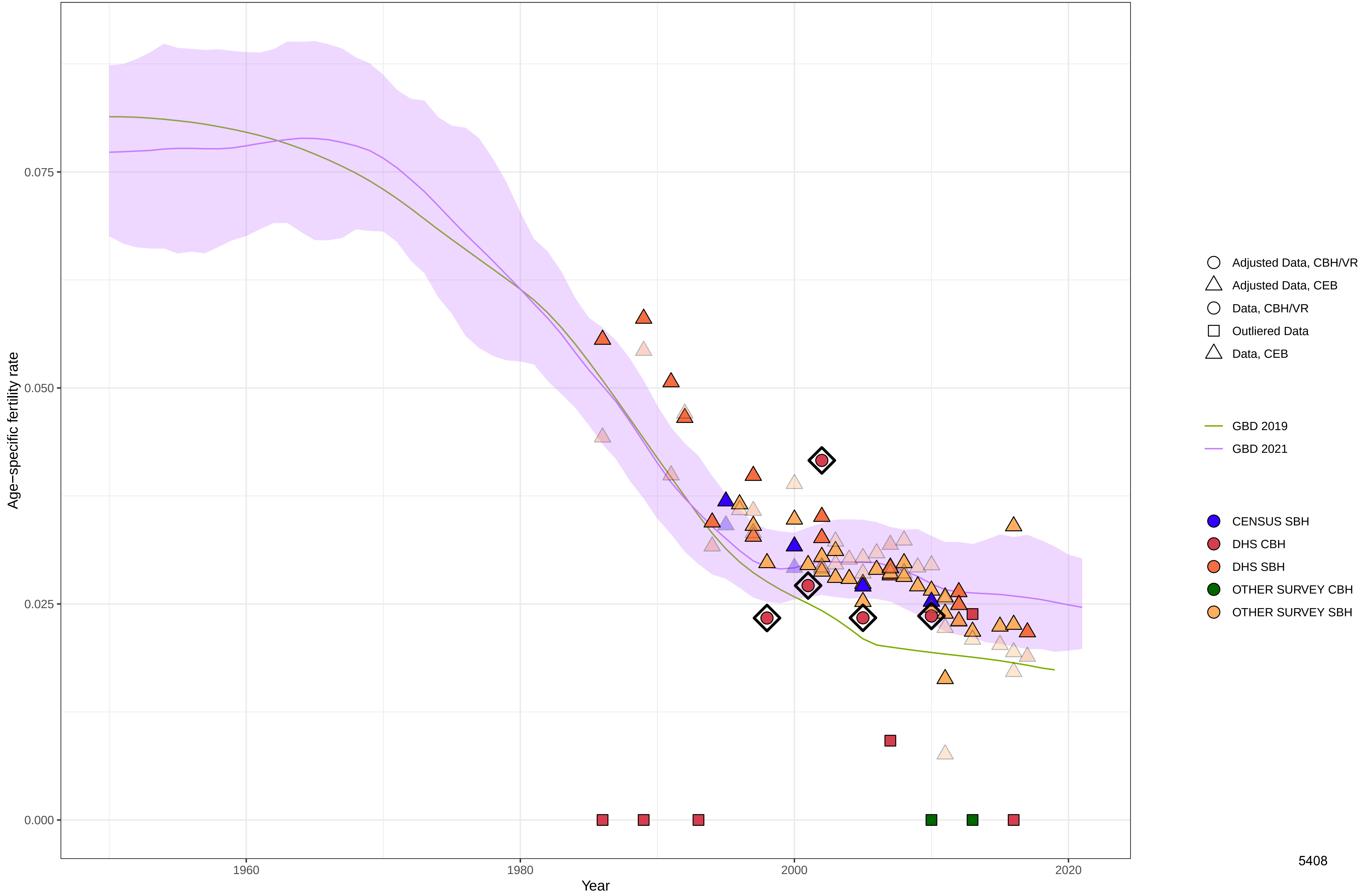

Age−specific fertility fitted trend lines, 1950−2021: Bangka−Belitung Islands, Ages: 45−49
Data points enclosed with a diamond are reference sources

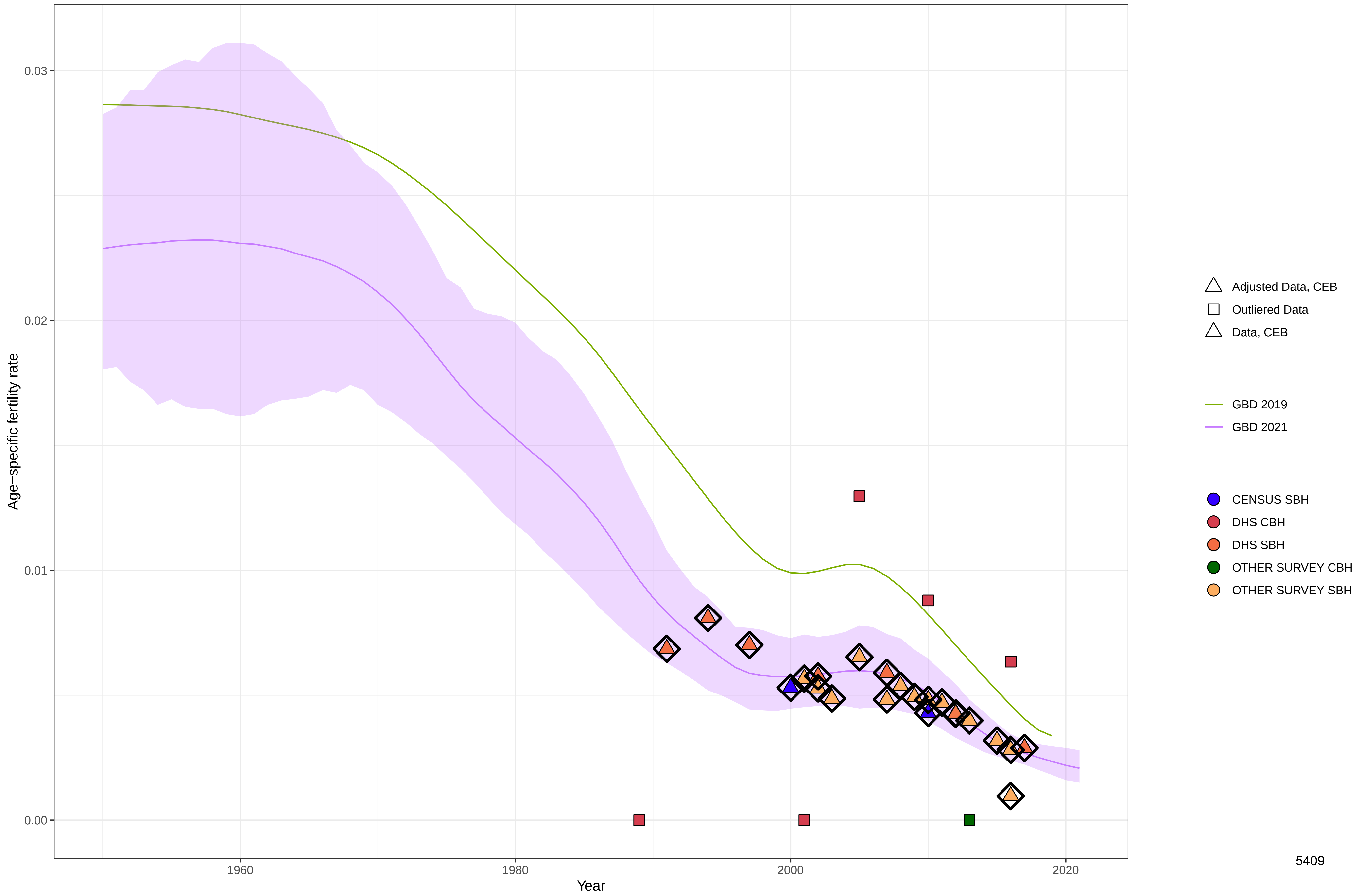

Age−specific fertility fitted trend lines, 1950−2021: Bangka−Belitung Islands, Ages: 50−54
Data points enclosed with a diamond are reference sources

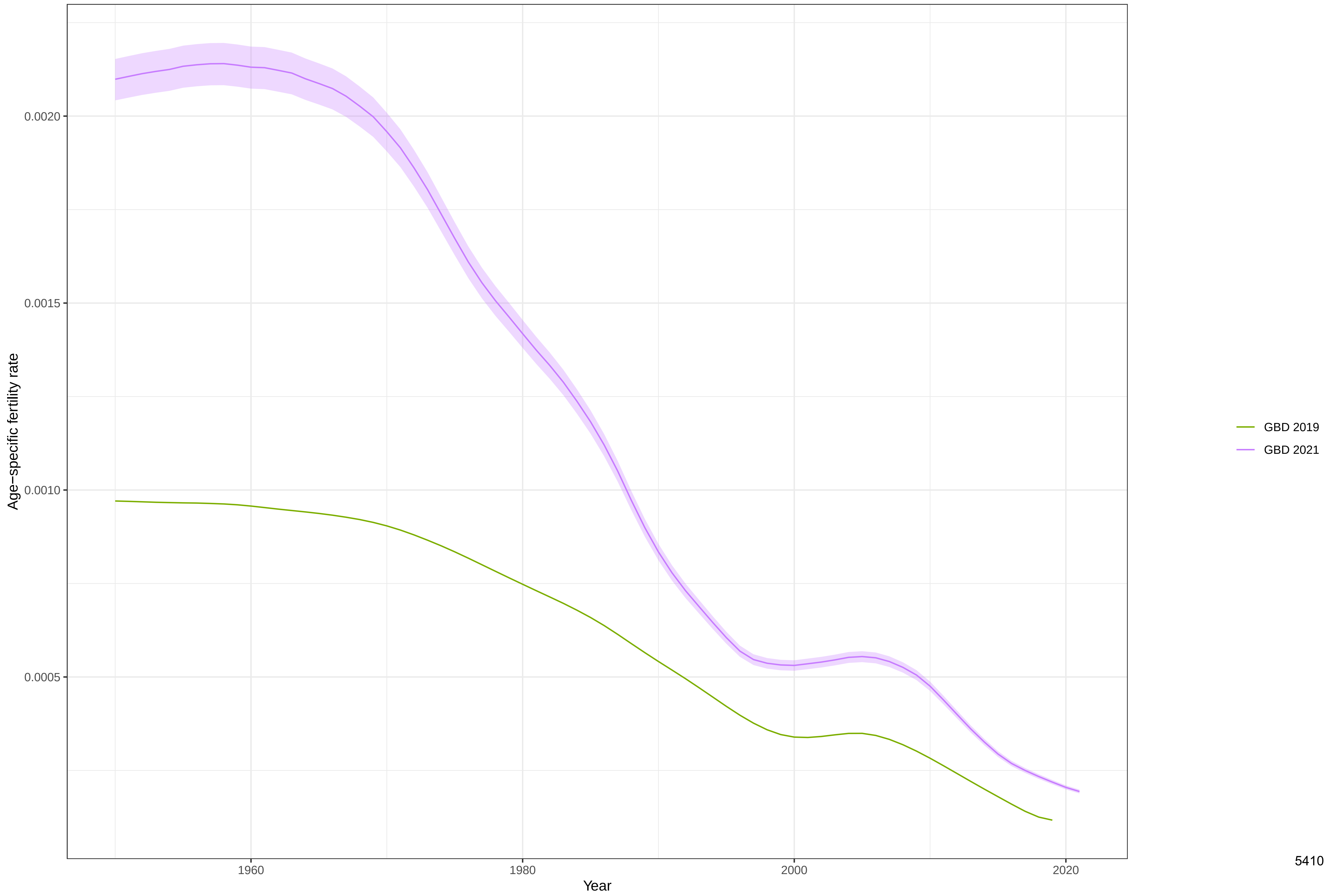

Total fertility rate fitted trend lines, 1950−2021: Bangka−Belitung Islands TFR
Data points enclosed with a diamond are reference sources

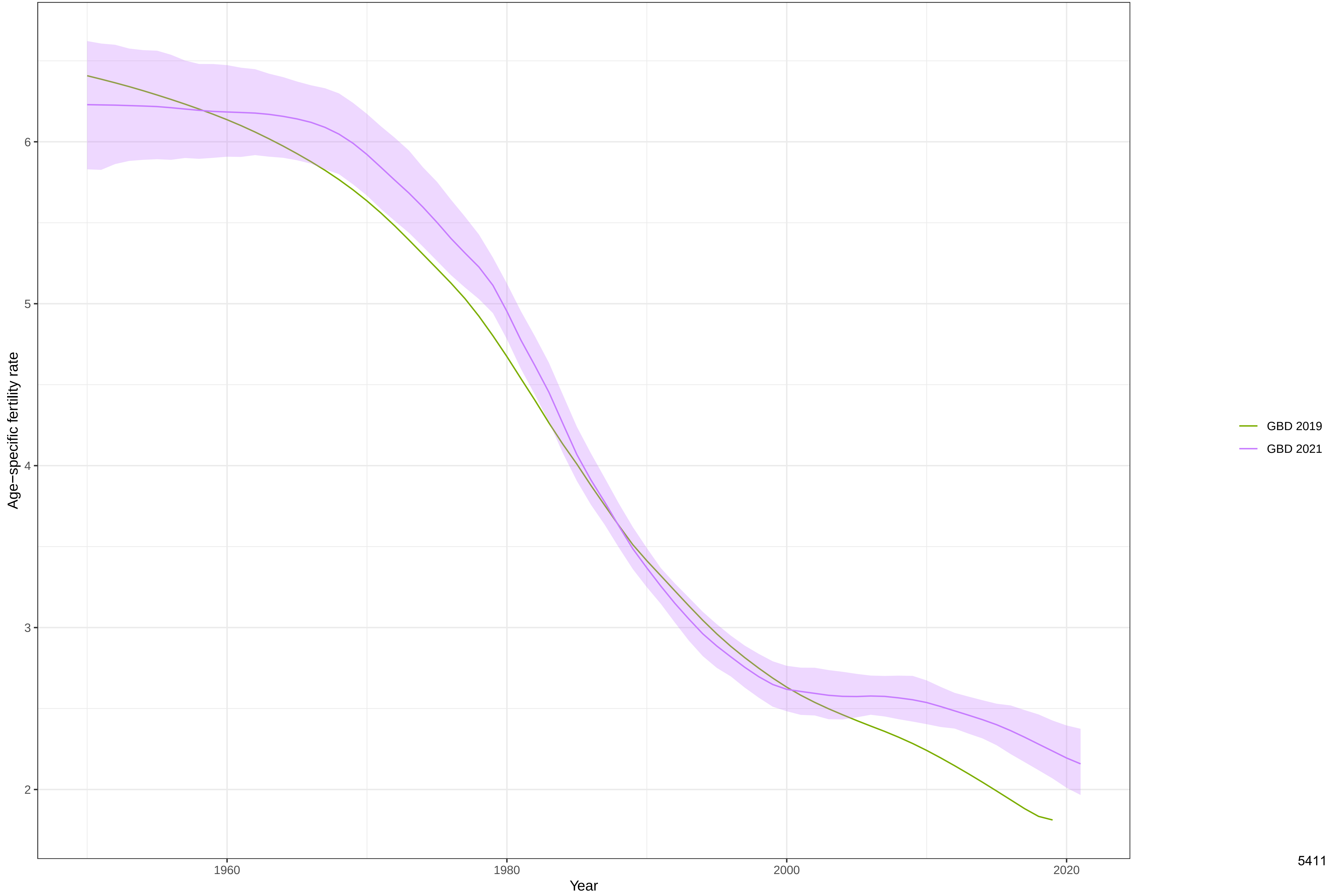

Age−specific fertility fitted trend lines, 1950−2021: Banten, Ages: 10−14
Data points enclosed with a diamond are reference sources

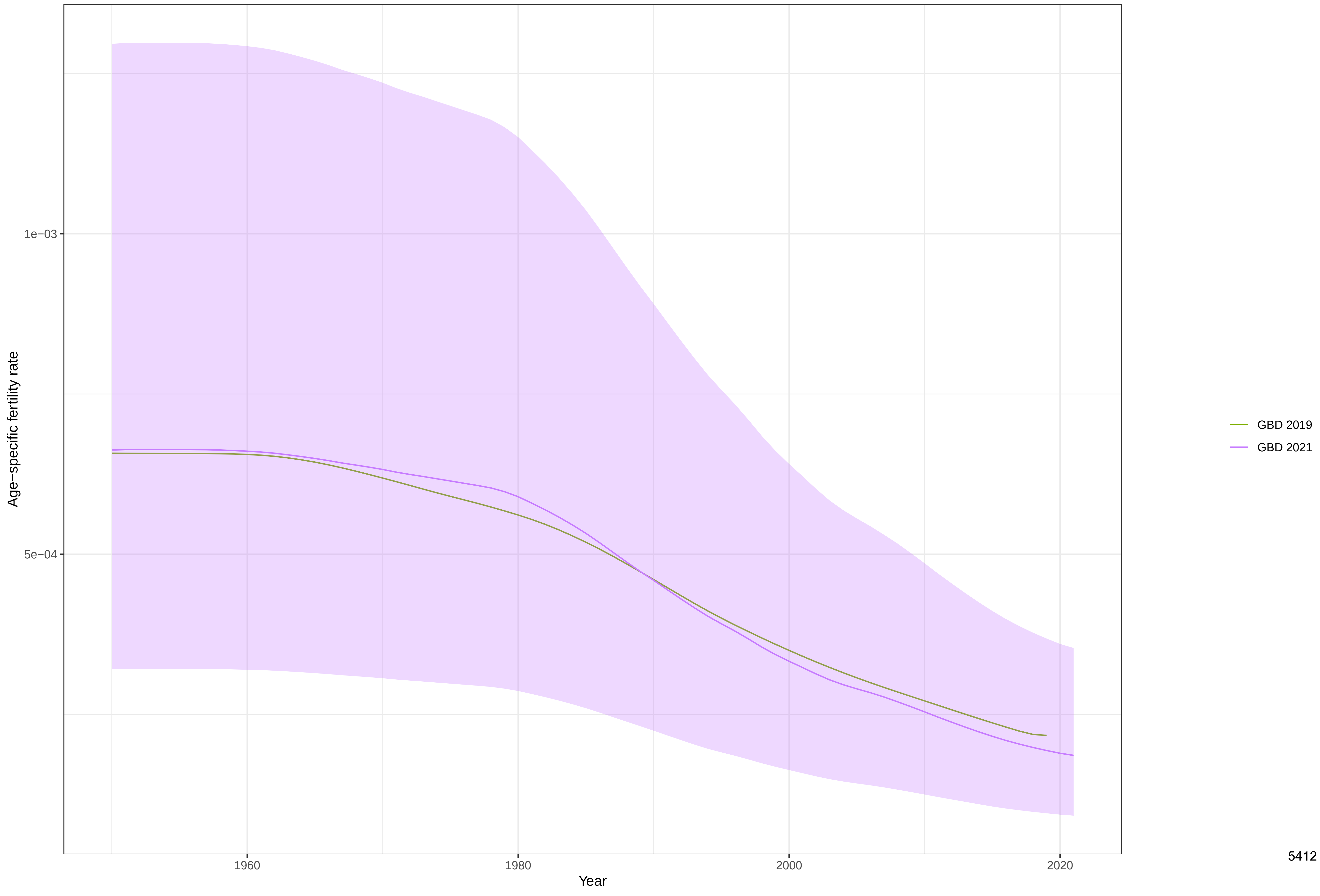

Age−specific fertility fitted trend lines, 1950−2021: Banten, Ages: 15−19
Data points enclosed with a diamond are reference sources

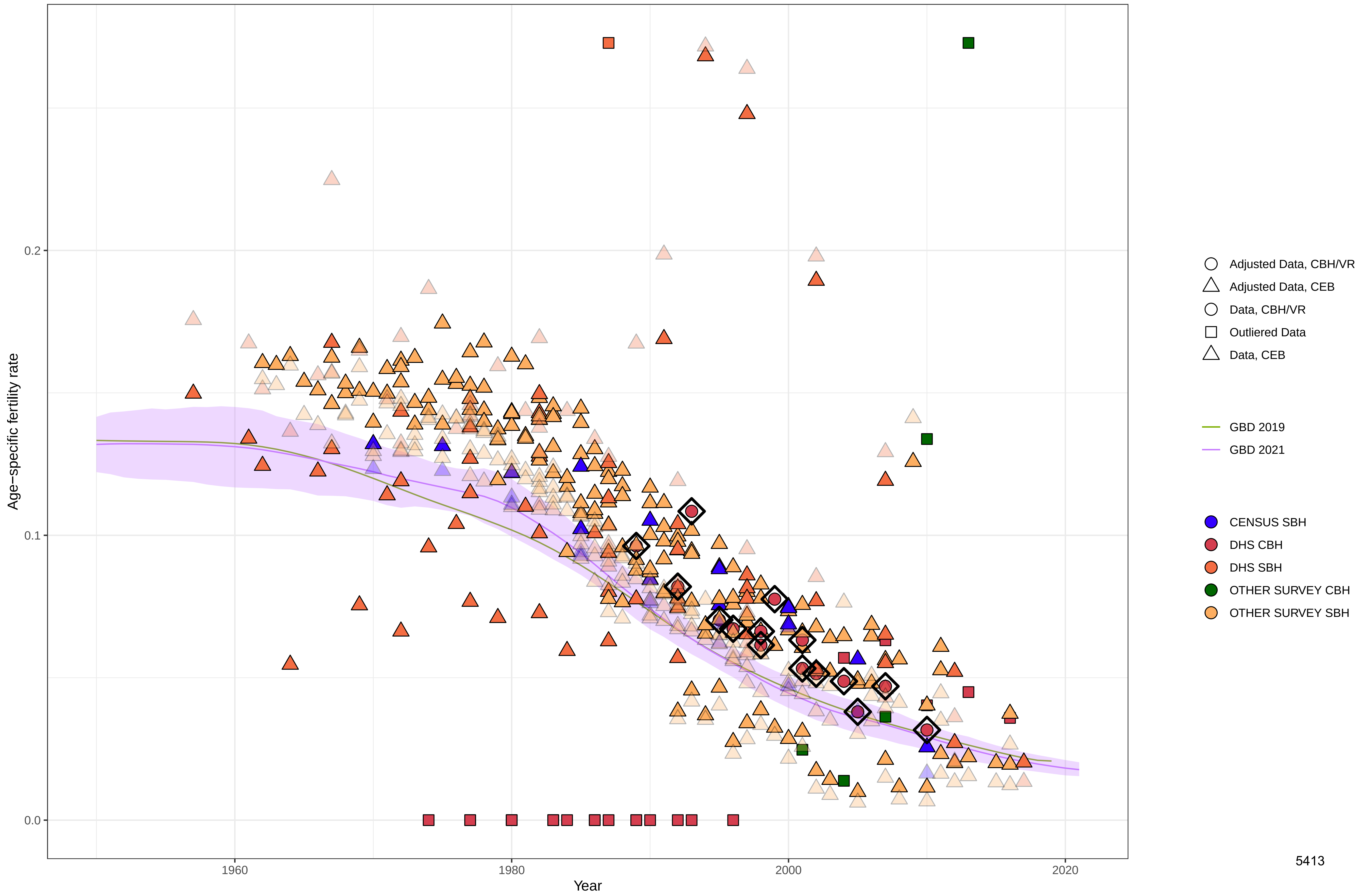

Age−specific fertility fitted trend lines, 1950−2021: Banten, Ages: 20−24
Data points enclosed with a diamond are reference sources

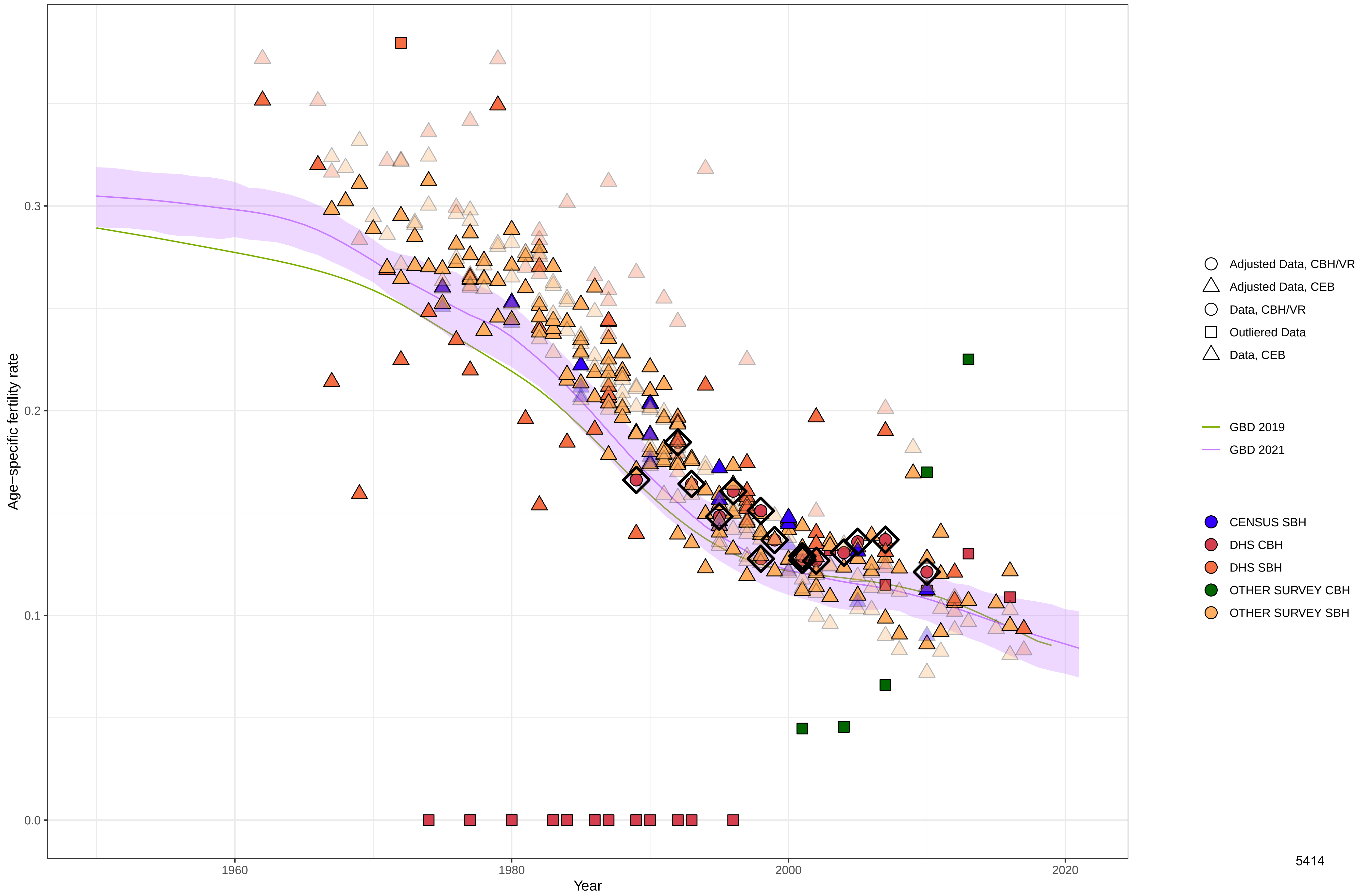

Age−specific fertility fitted trend lines, 1950−2021: Banten, Ages: 25−29
Data points enclosed with a diamond are reference sources

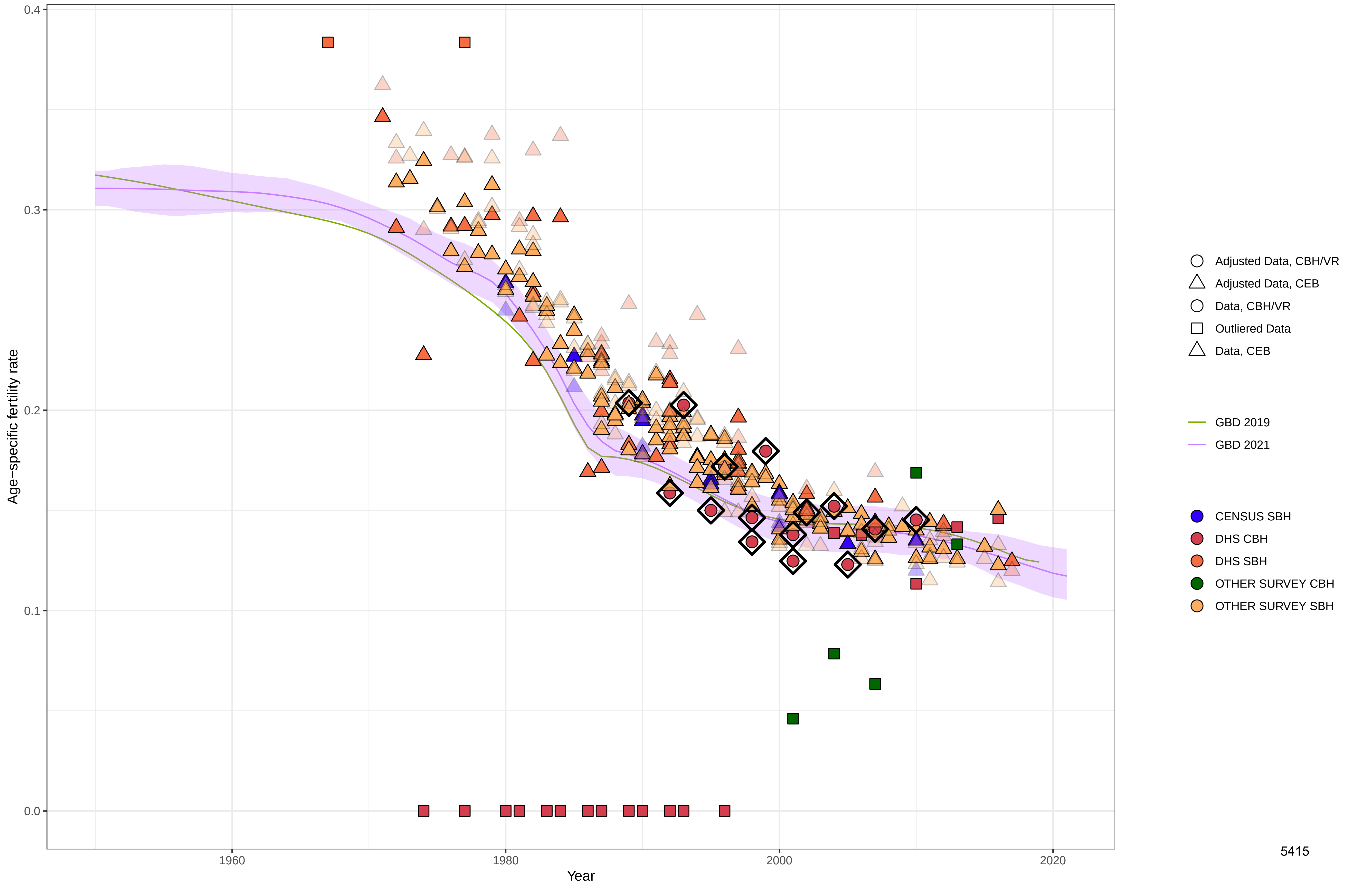

Age−specific fertility fitted trend lines, 1950−2021: Banten, Ages: 30−34
Data points enclosed with a diamond are reference sources

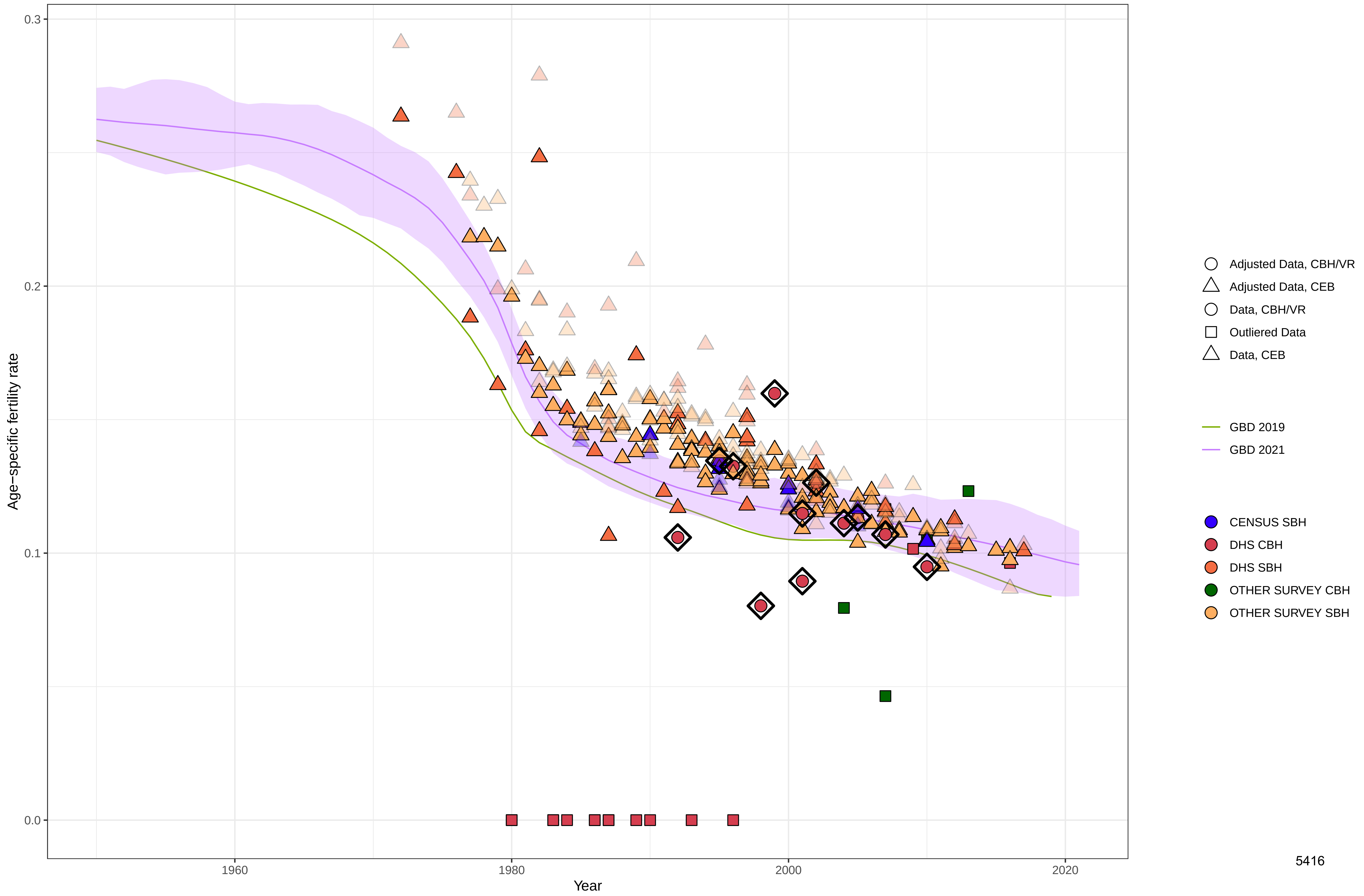

Age−specific fertility fitted trend lines, 1950−2021: Banten, Ages: 35−39
Data points enclosed with a diamond are reference sources

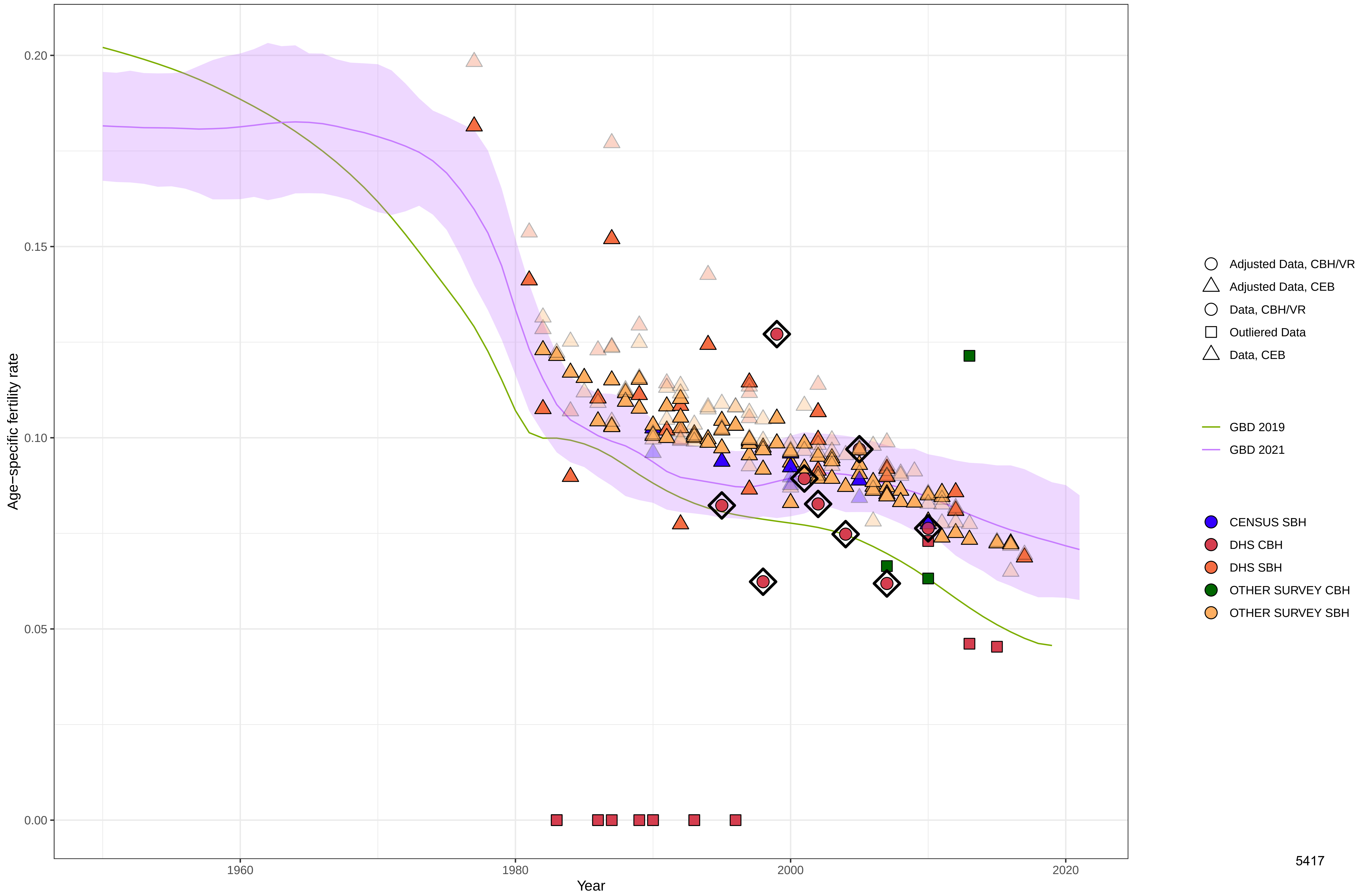

Age−specific fertility fitted trend lines, 1950−2021: Banten, Ages: 40−44
Data points enclosed with a diamond are reference sources

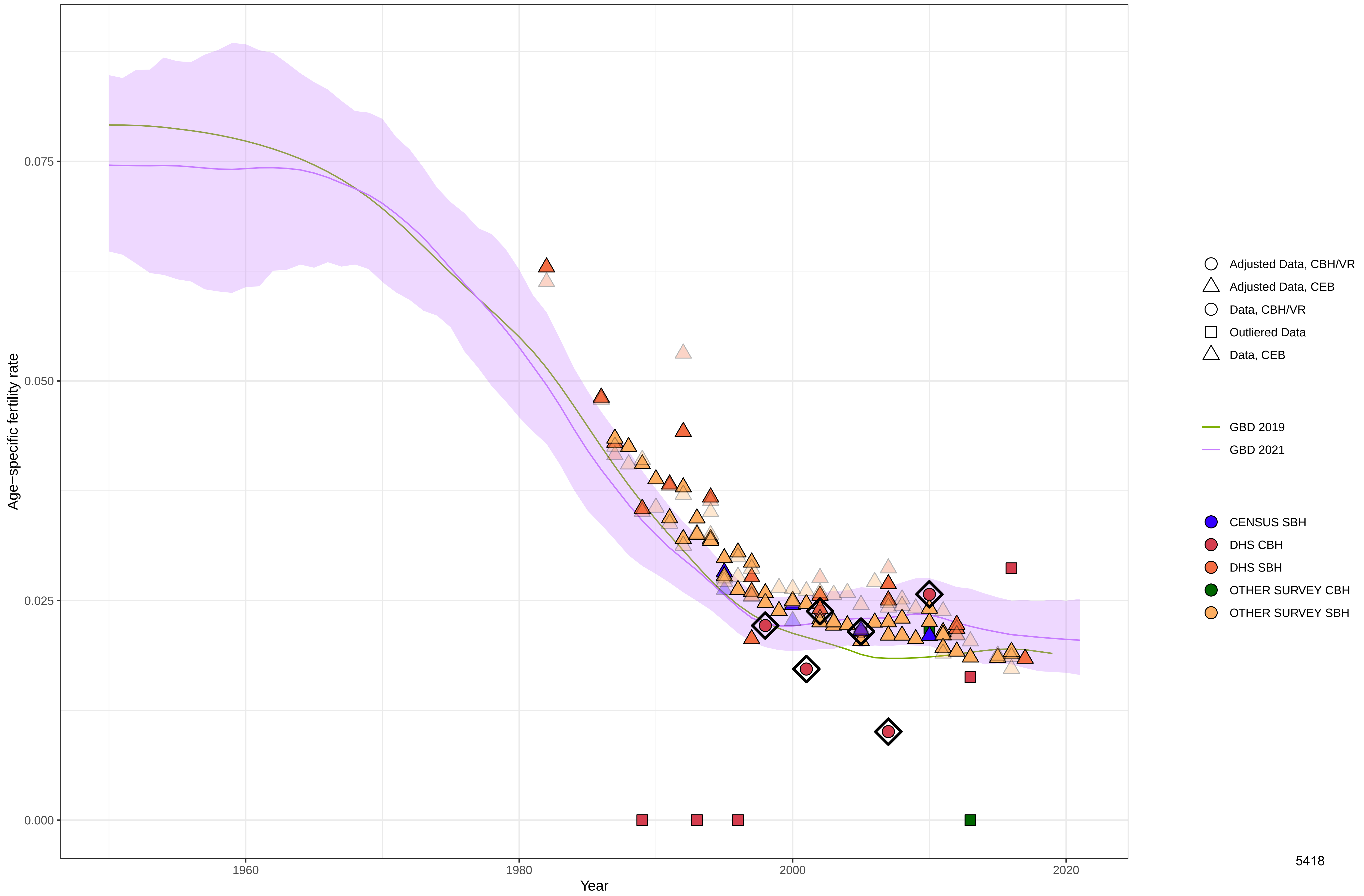

Age−specific fertility fitted trend lines, 1950−2021: Banten, Ages: 45−49
Data points enclosed with a diamond are reference sources

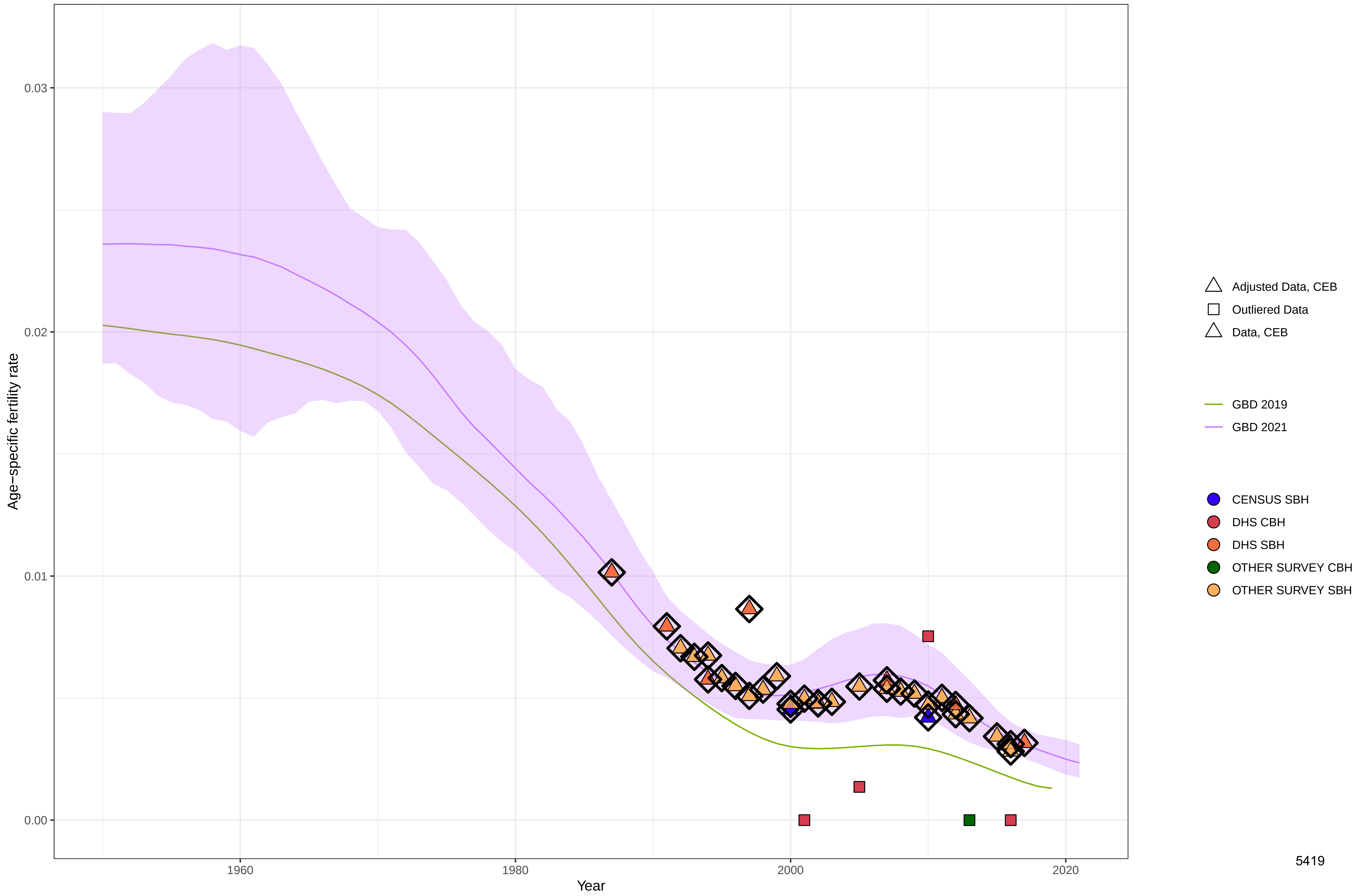

Age−specific fertility fitted trend lines, 1950−2021: Banten, Ages: 50−54
Data points enclosed with a diamond are reference sources

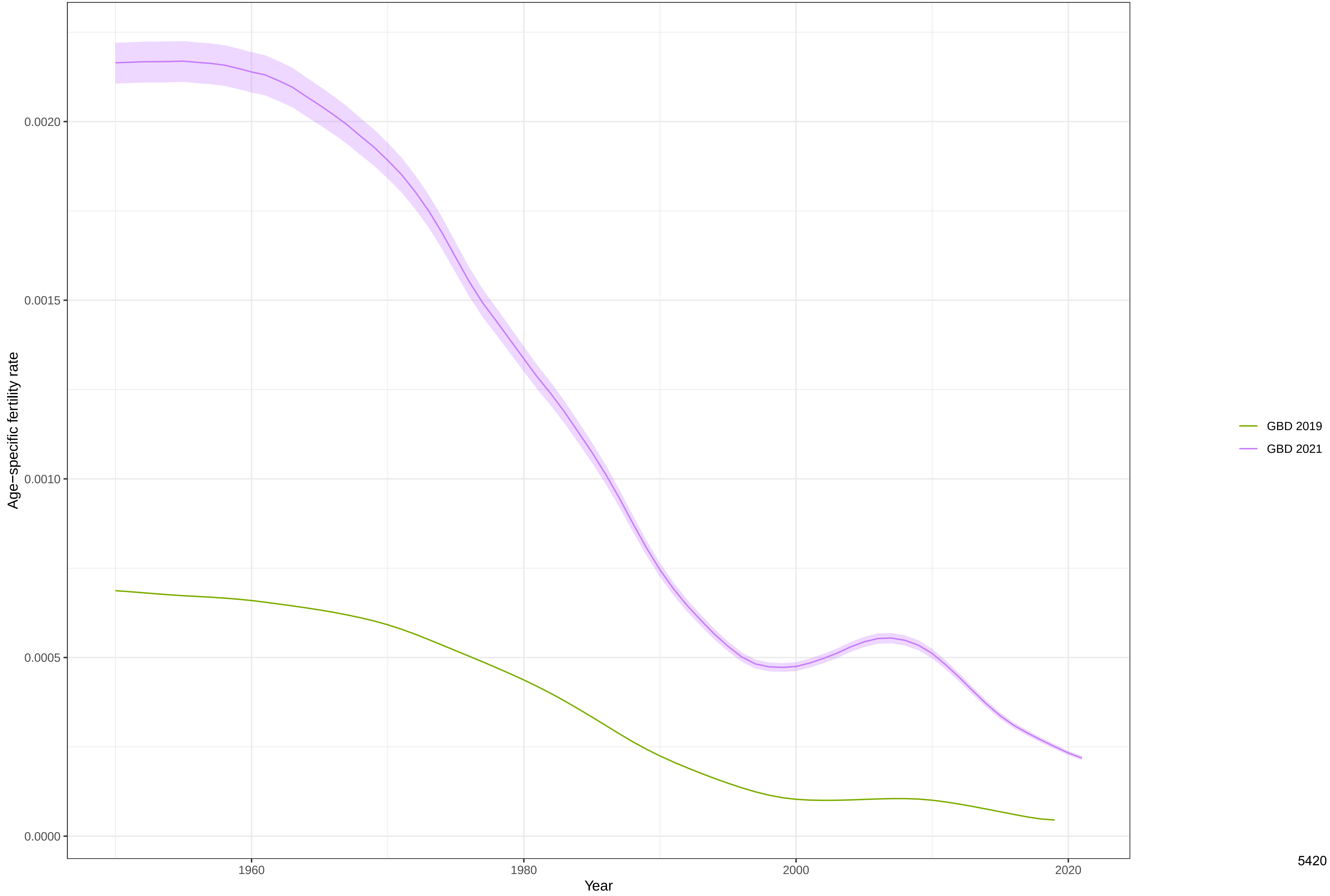

Total fertility rate fitted trend lines, 1950−2021: Banten TFR
Data points enclosed with a diamond are reference sources

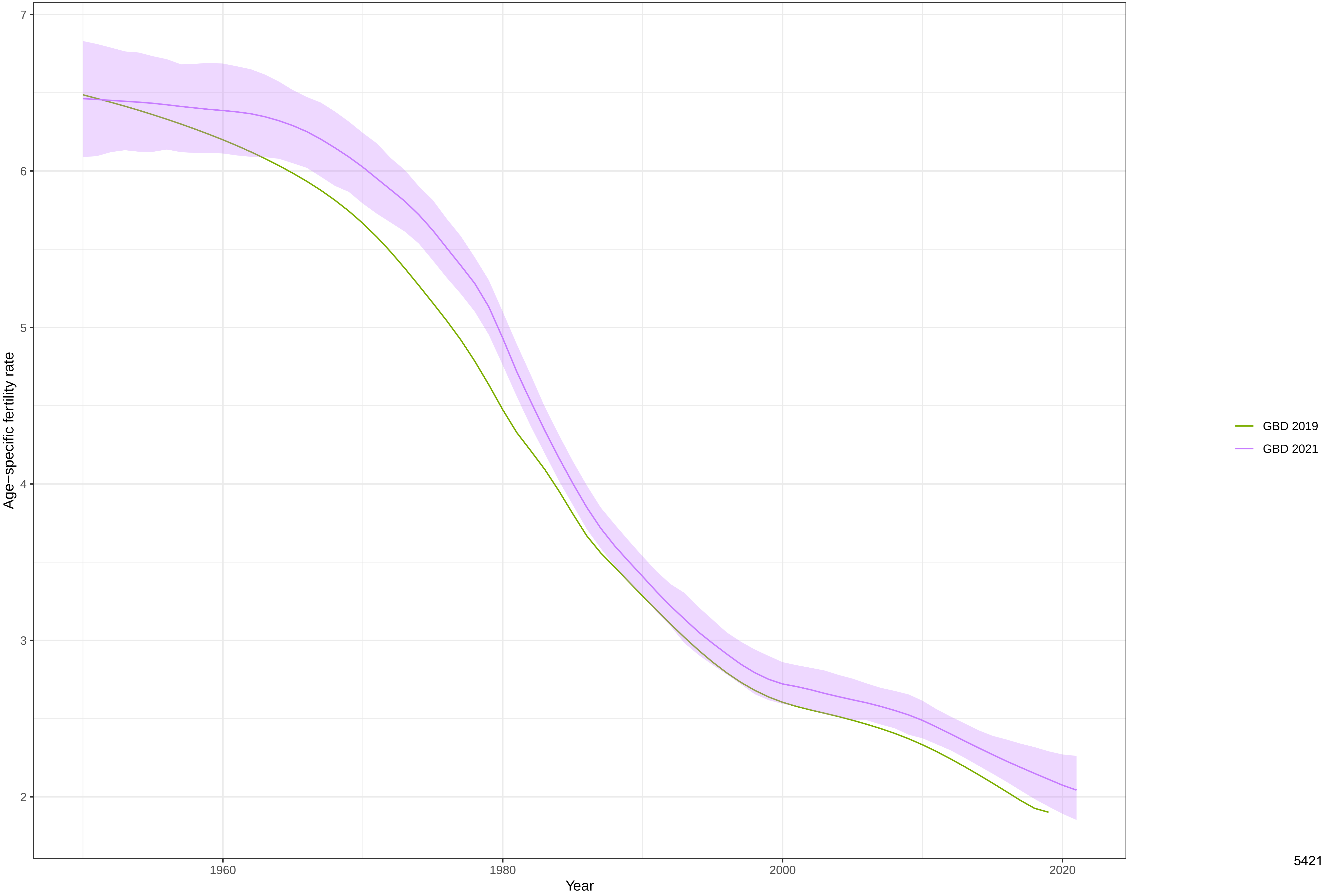

Age−specific fertility fitted trend lines, 1950−2021: Bengkulu, Ages: 10−14
Data points enclosed with a diamond are reference sources

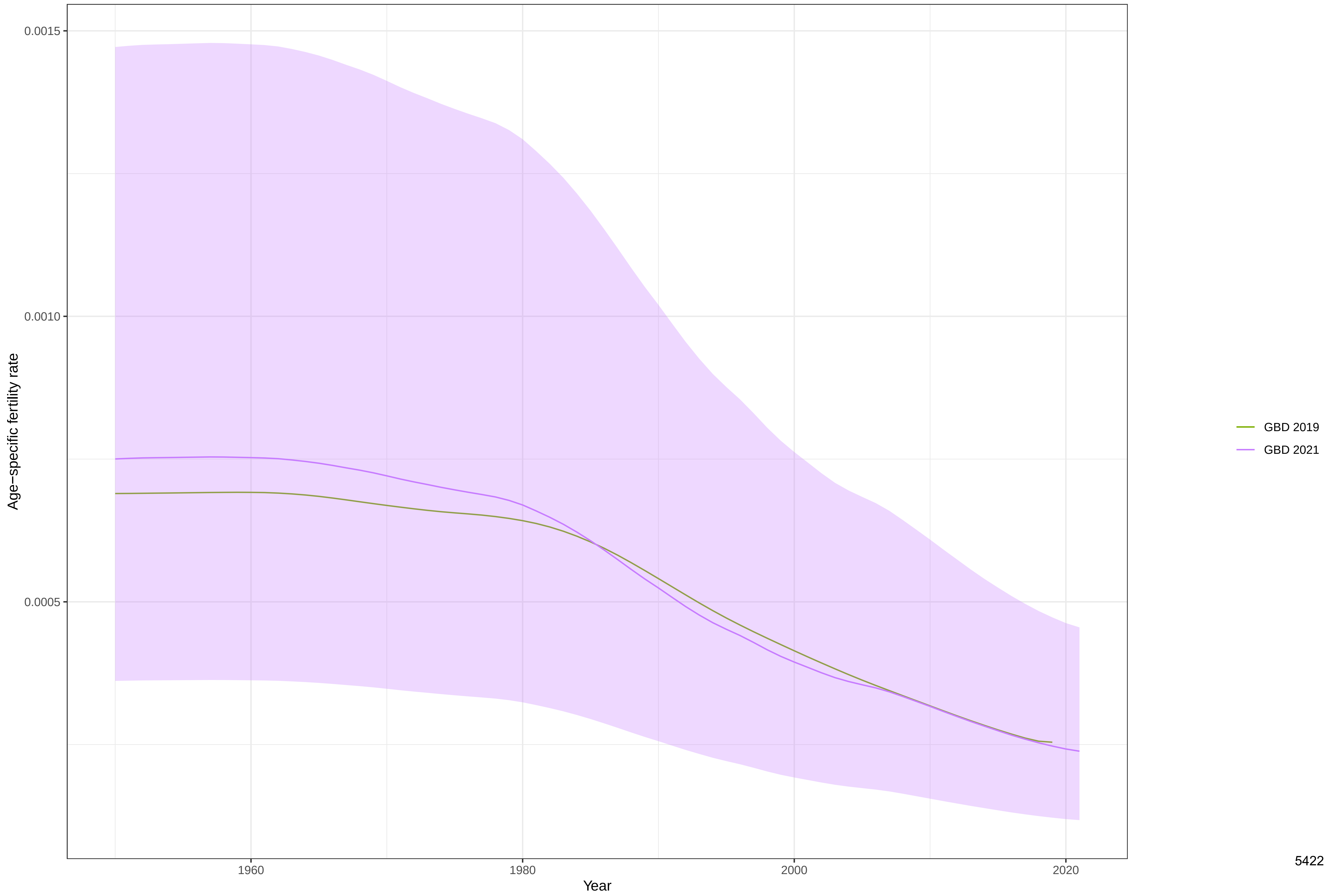

Age−specific fertility fitted trend lines, 1950−2021: Bengkulu, Ages: 15−19
Data points enclosed with a diamond are reference sources

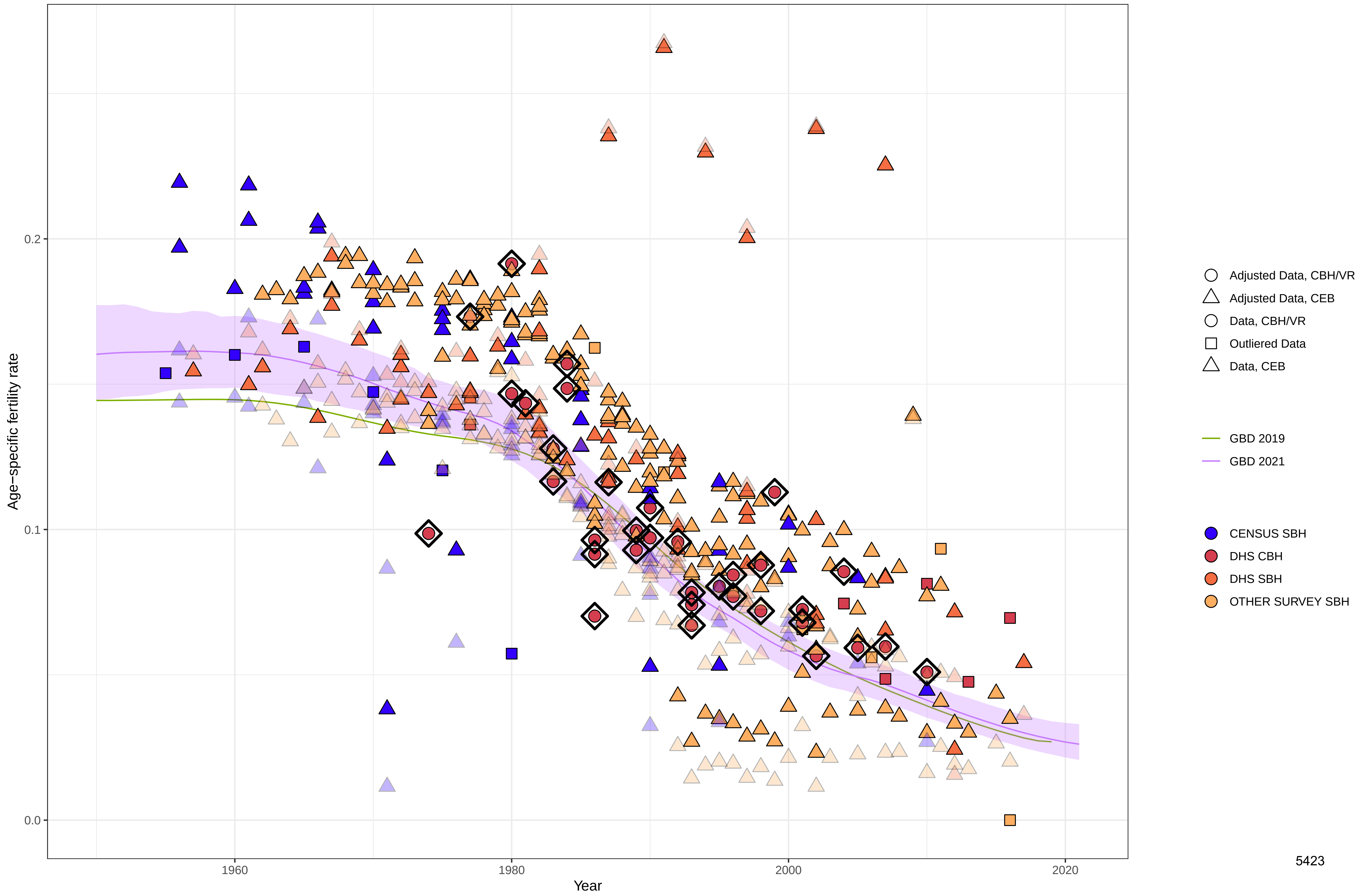

Age−specific fertility fitted trend lines, 1950−2021: Bengkulu, Ages: 20−24
Data points enclosed with a diamond are reference sources

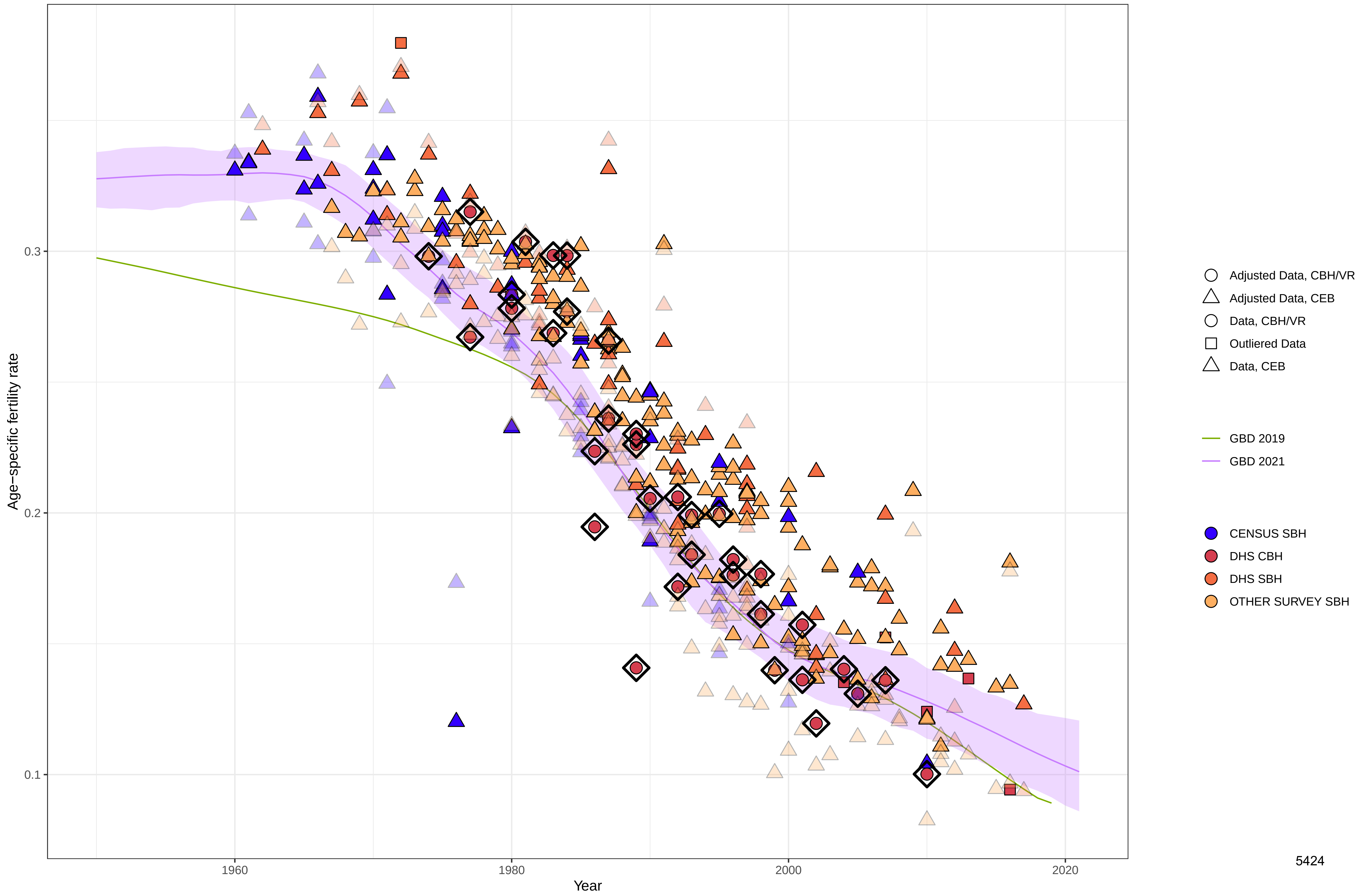

Age−specific fertility fitted trend lines, 1950−2021: Bengkulu, Ages: 25−29
Data points enclosed with a diamond are reference sources

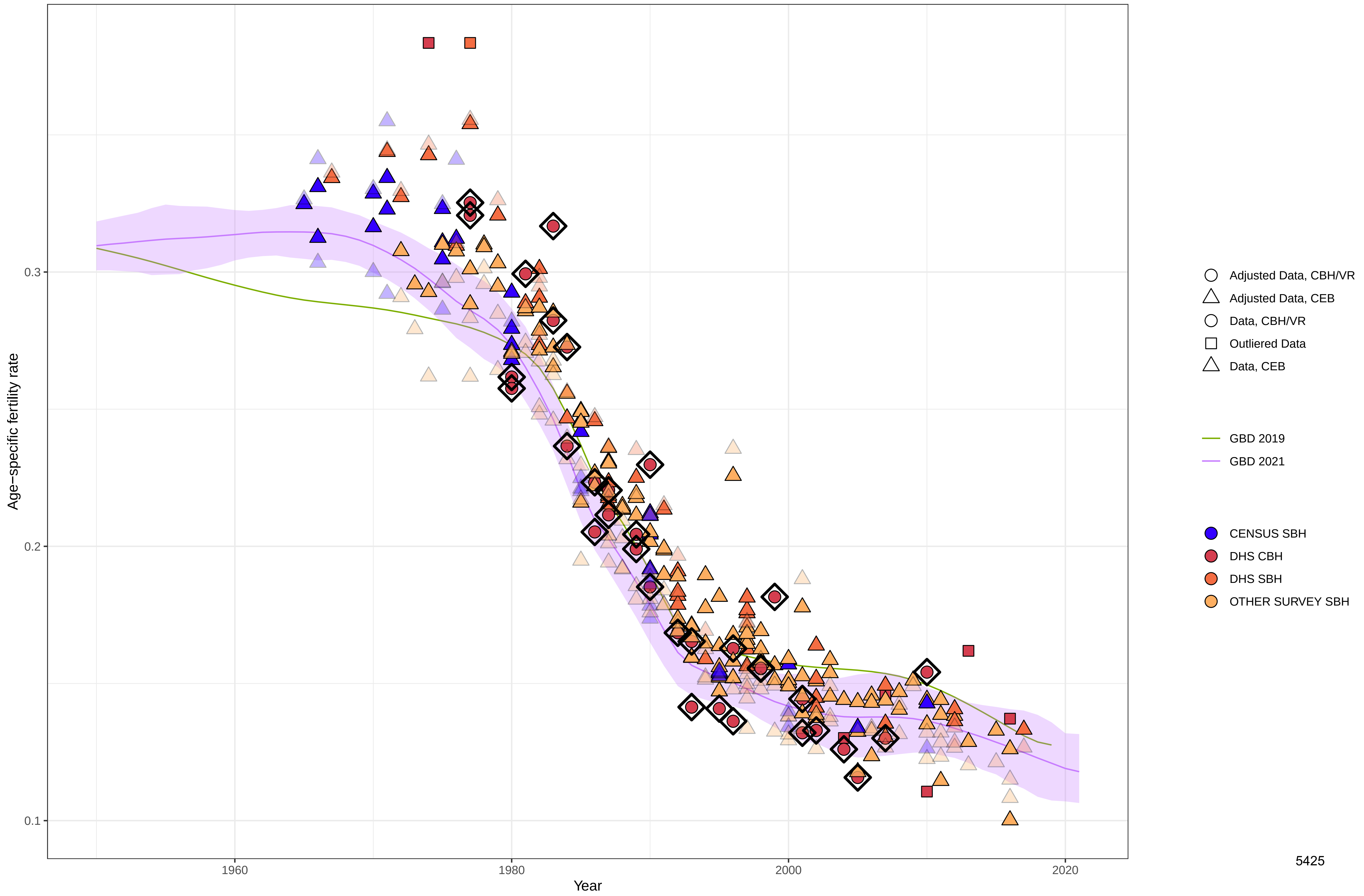

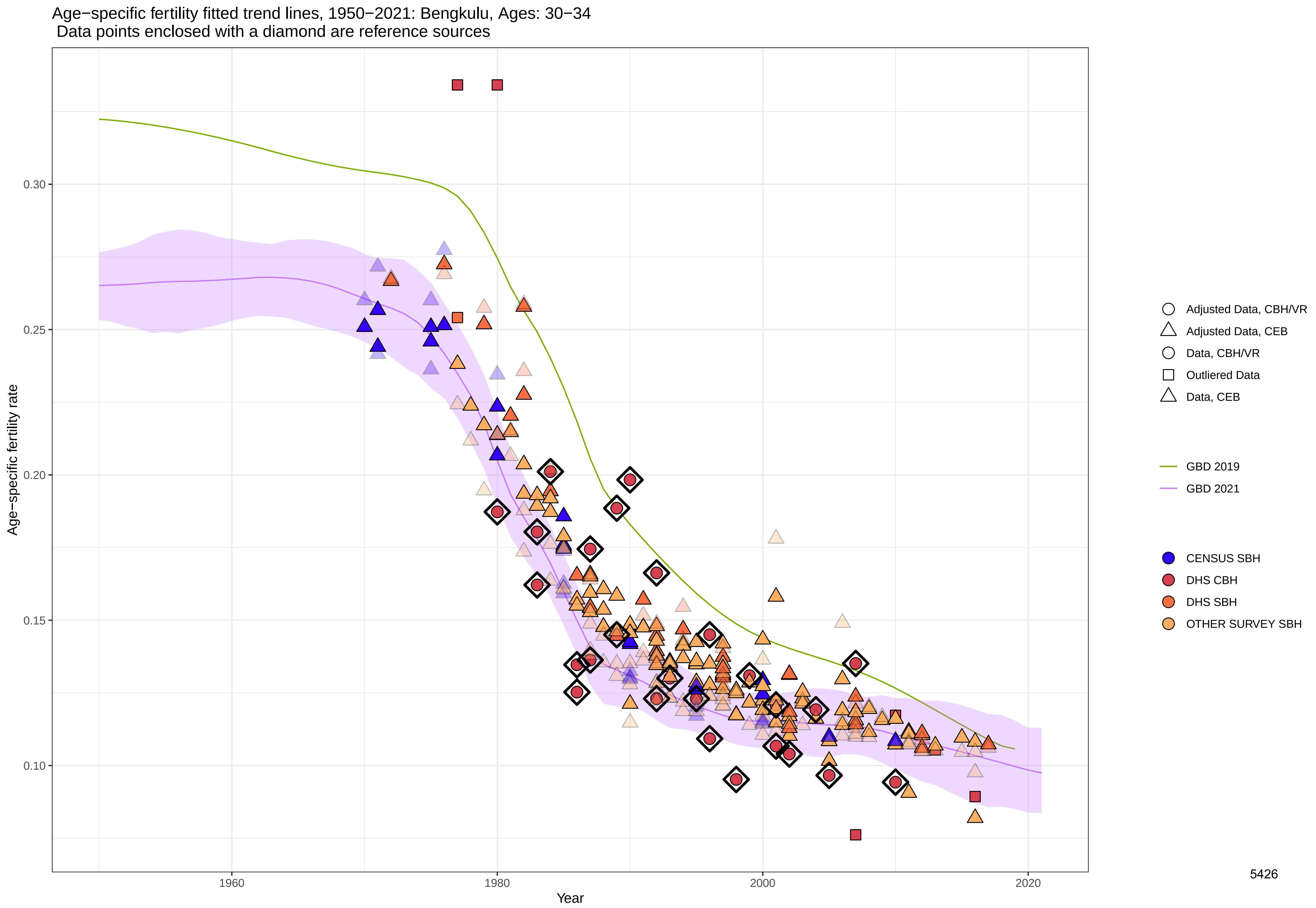

Age−specific fertility fitted trend lines, 1950−2021: Bengkulu, Ages: 30−34
Data points enclosed with a diamond are reference sources
Age−specific fertility rate
0.30
0.25
0.20
0.15
0.10
1960
1980
2000
2020
Year
Adjusted Data, CBH/VR
Adjusted Data, CEB
Data, CBH/VR
Outliered Data
Data, CEB
GBD 2019
GBD 2021
CENSUS SBH
DHS CBH
DHS SBH
OTHER SURVEY SBH

Age−specific fertility fitted trend lines, 1950−2021: Bengkulu, Ages: 35−39
Data points enclosed with a diamond are reference sources

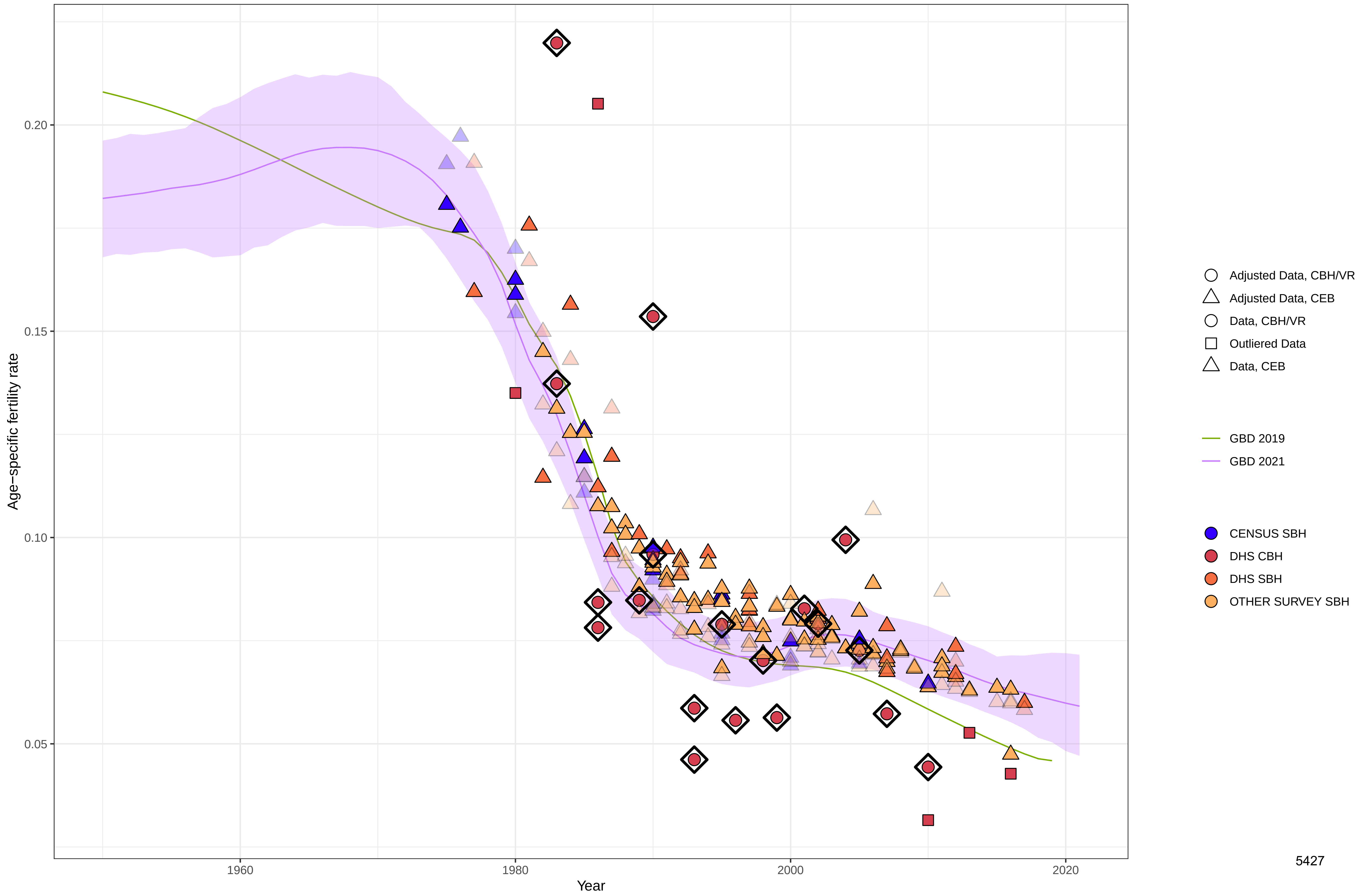

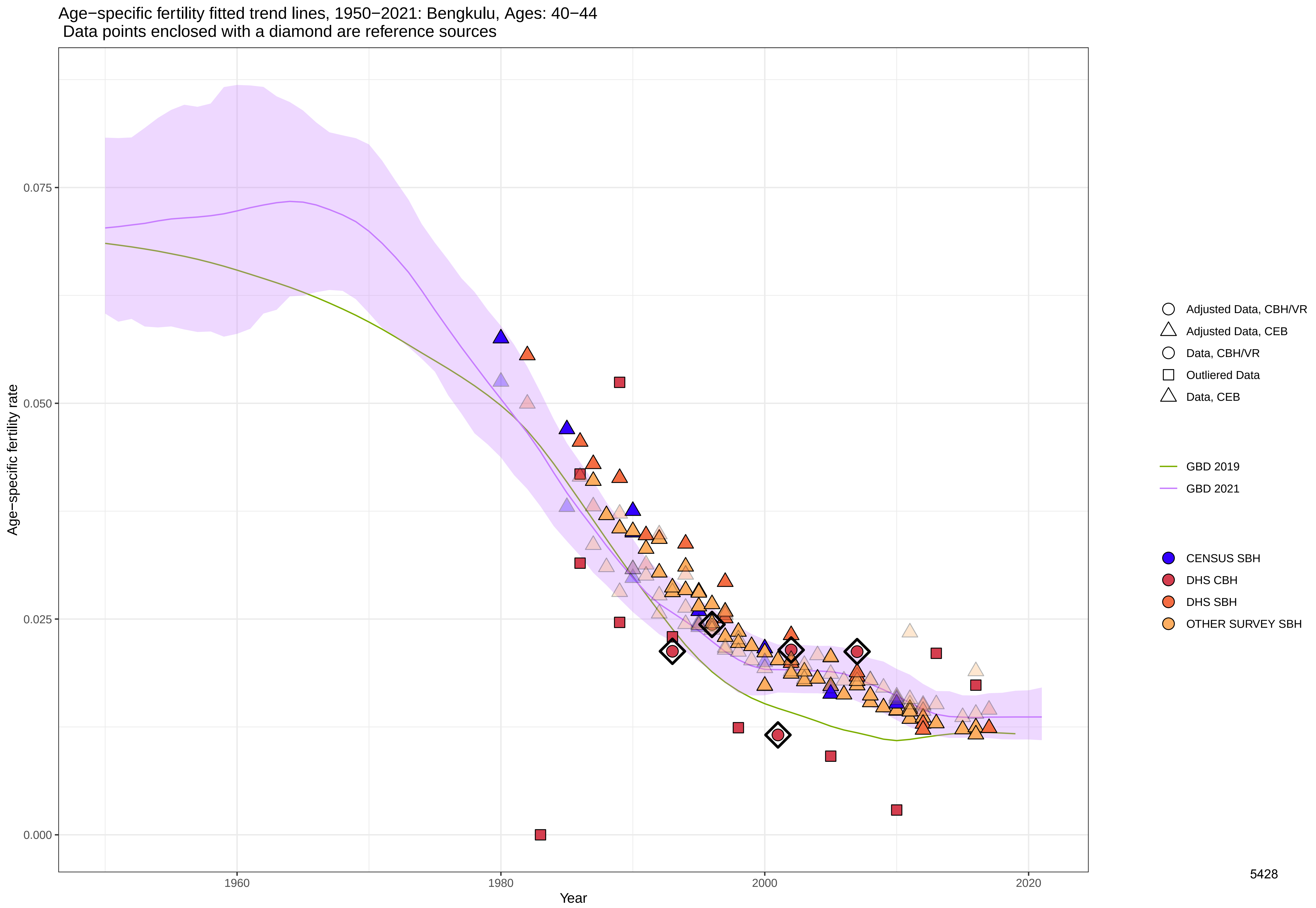

Age−specific fertility fitted trend lines, 1950−2021: Bengkulu, Ages: 40−44
Data points enclosed with a diamond are reference sources
Age−specific fertility rate
Year
0.000
0.025
0.050
0.075
1960
1980
2000
2020
Adjusted Data, CBH/VR
Adjusted Data, CEB
Data, CBH/VR
Outliered Data
Data, CEB
GBD 2019
GBD 2021
CENSUS SBH
DHS CBH
DHS SBH
OTHER SURVEY SBH

Age−specific fertility fitted trend lines, 1950−2021: Bengkulu, Ages: 45−49
Data points enclosed with a diamond are reference sources

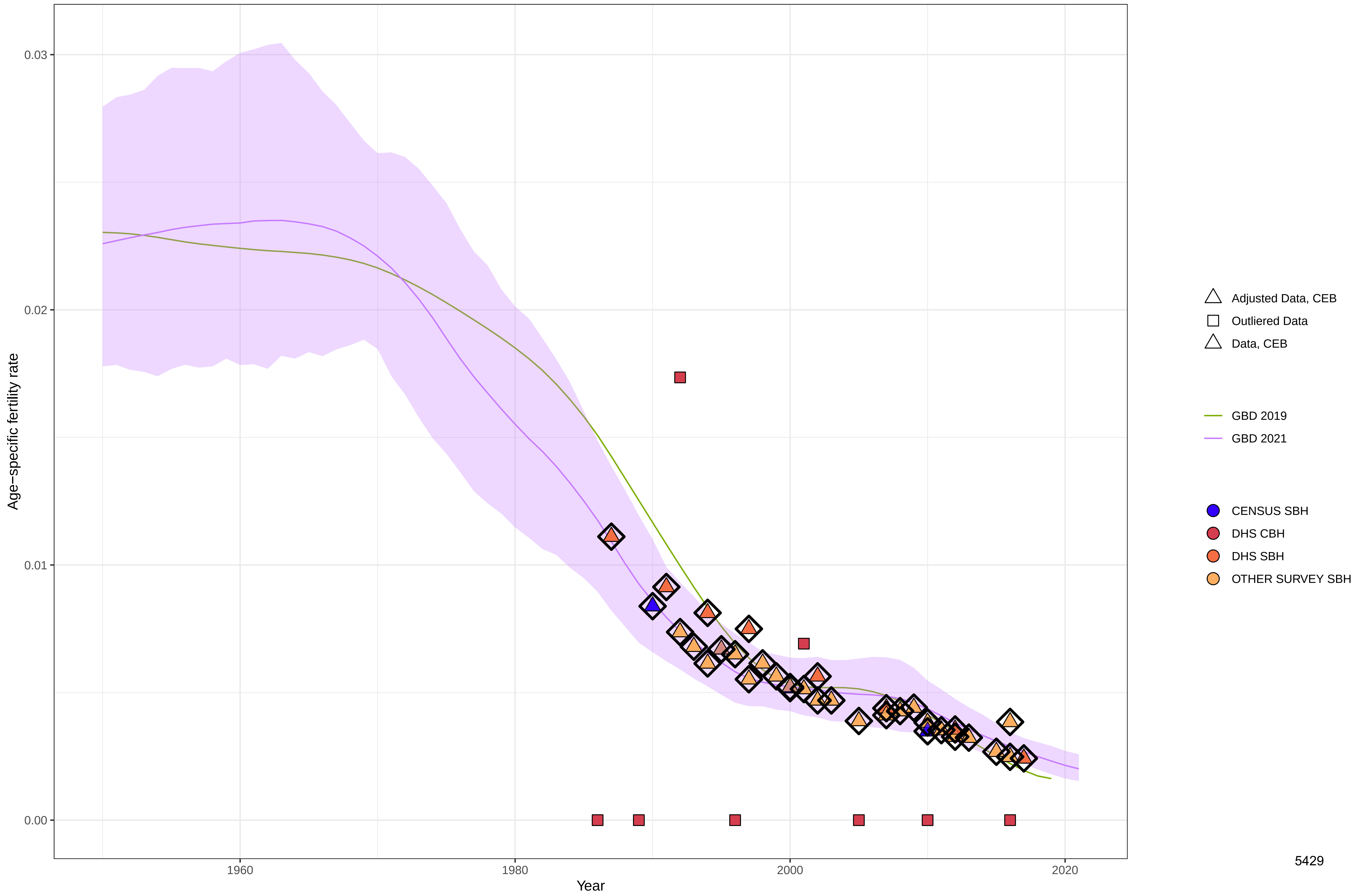

Age−specific fertility fitted trend lines, 1950−2021: Bengkulu, Ages: 50−54
Data points enclosed with a diamond are reference sources

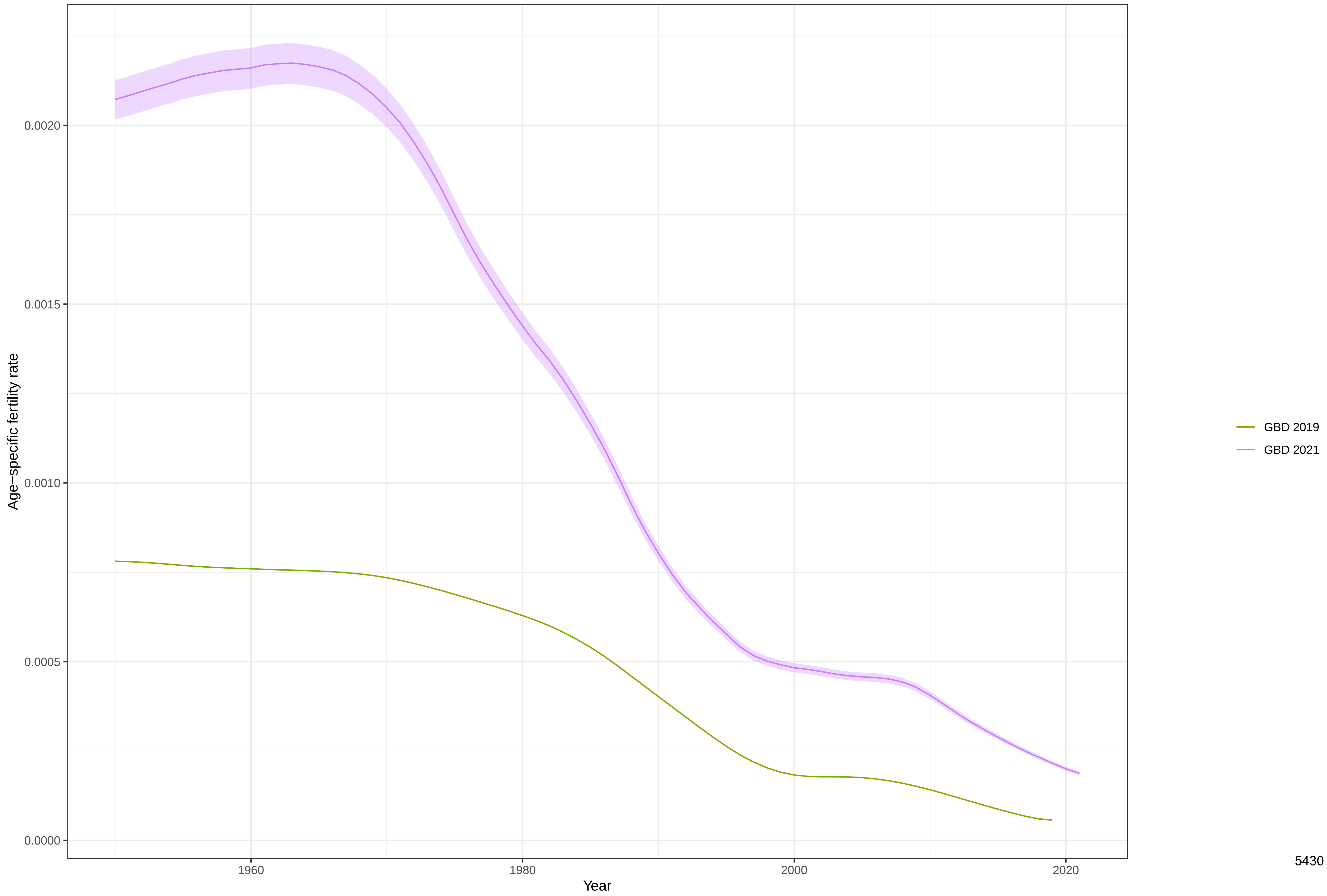

Total fertility rate fitted trend lines, 1950−2021: Bengkulu TFR
Data points enclosed with a diamond are reference sources

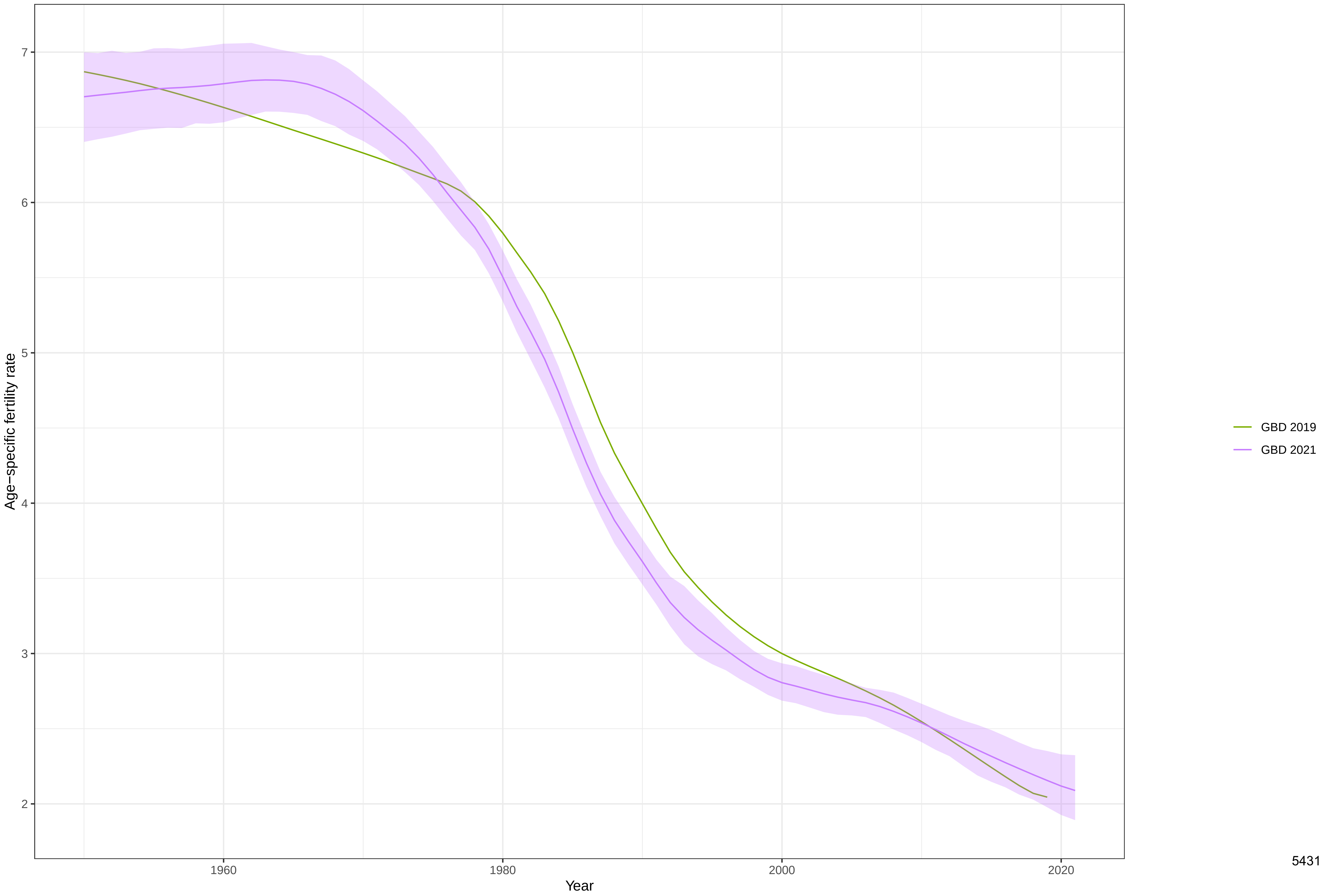

Age−specific fertility fitted trend lines, 1950−2021: Gorontalo, Ages: 10−14
Data points enclosed with a diamond are reference sources

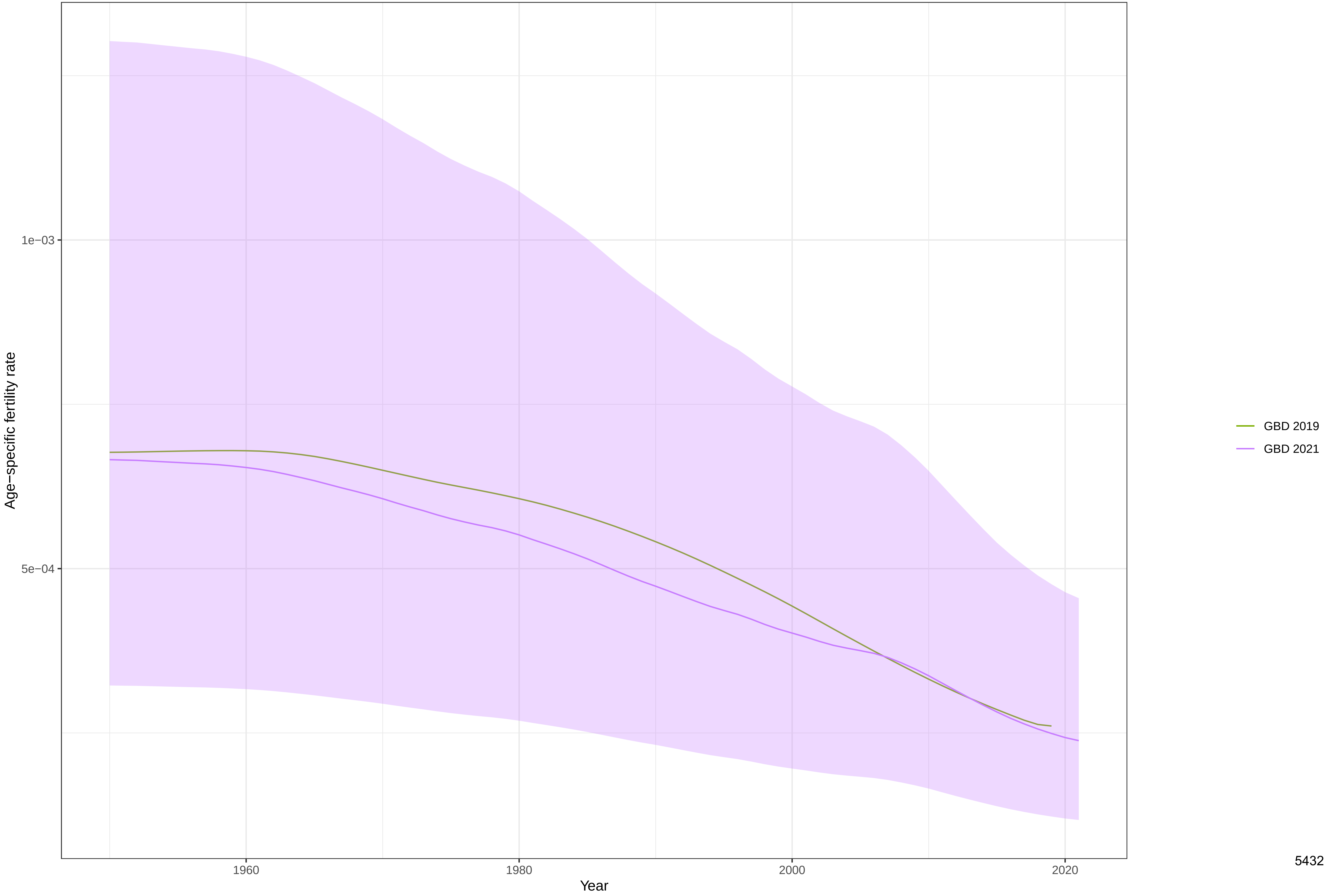

Age−specific fertility fitted trend lines, 1950−2021: Gorontalo, Ages: 15−19
Data points enclosed with a diamond are reference sources

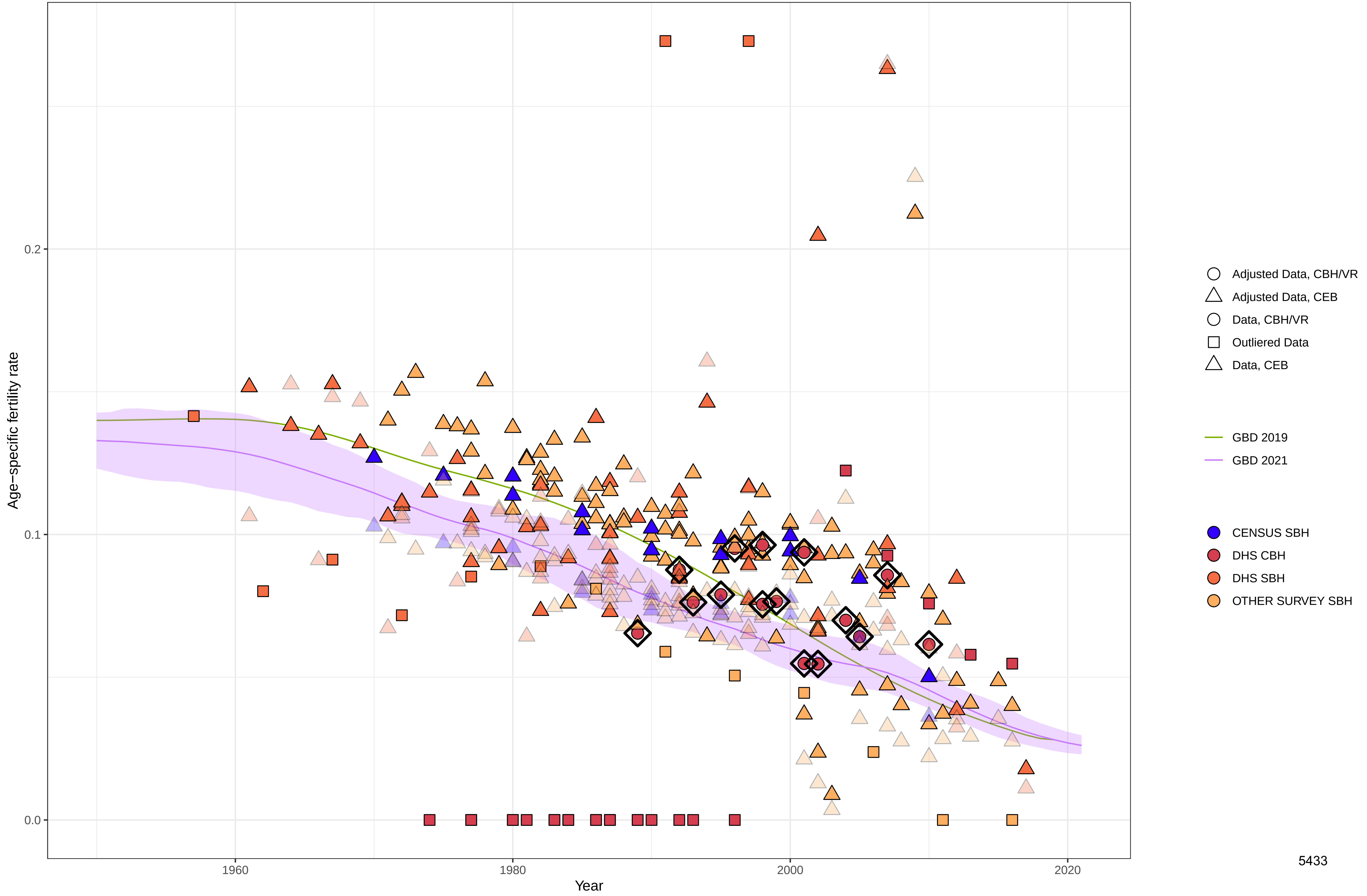

Age−specific fertility fitted trend lines, 1950−2021: Gorontalo, Ages: 20−24
Data points enclosed with a diamond are reference sources

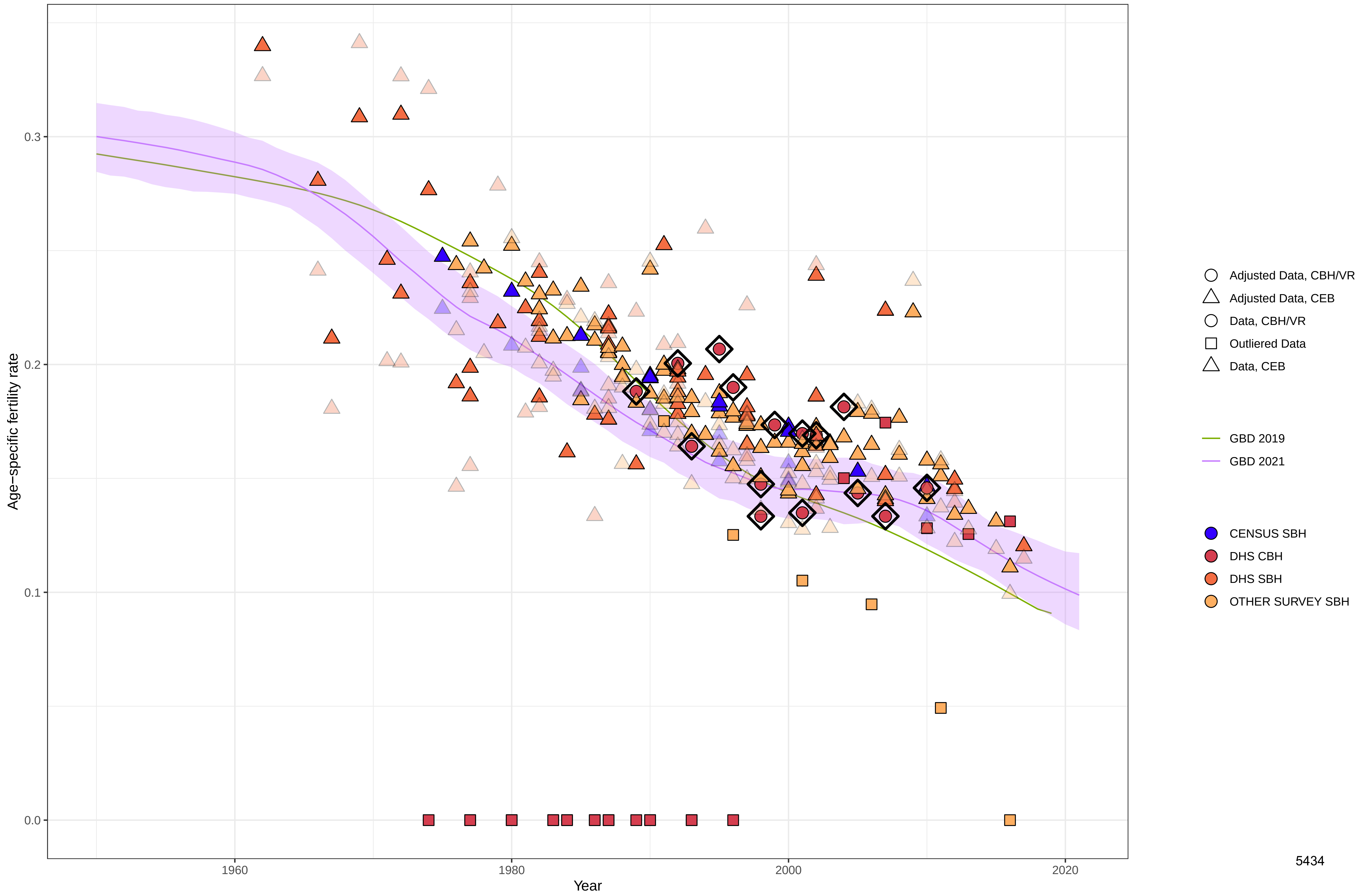

Age−specific fertility fitted trend lines, 1950−2021: Gorontalo, Ages: 25−29
Data points enclosed with a diamond are reference sources

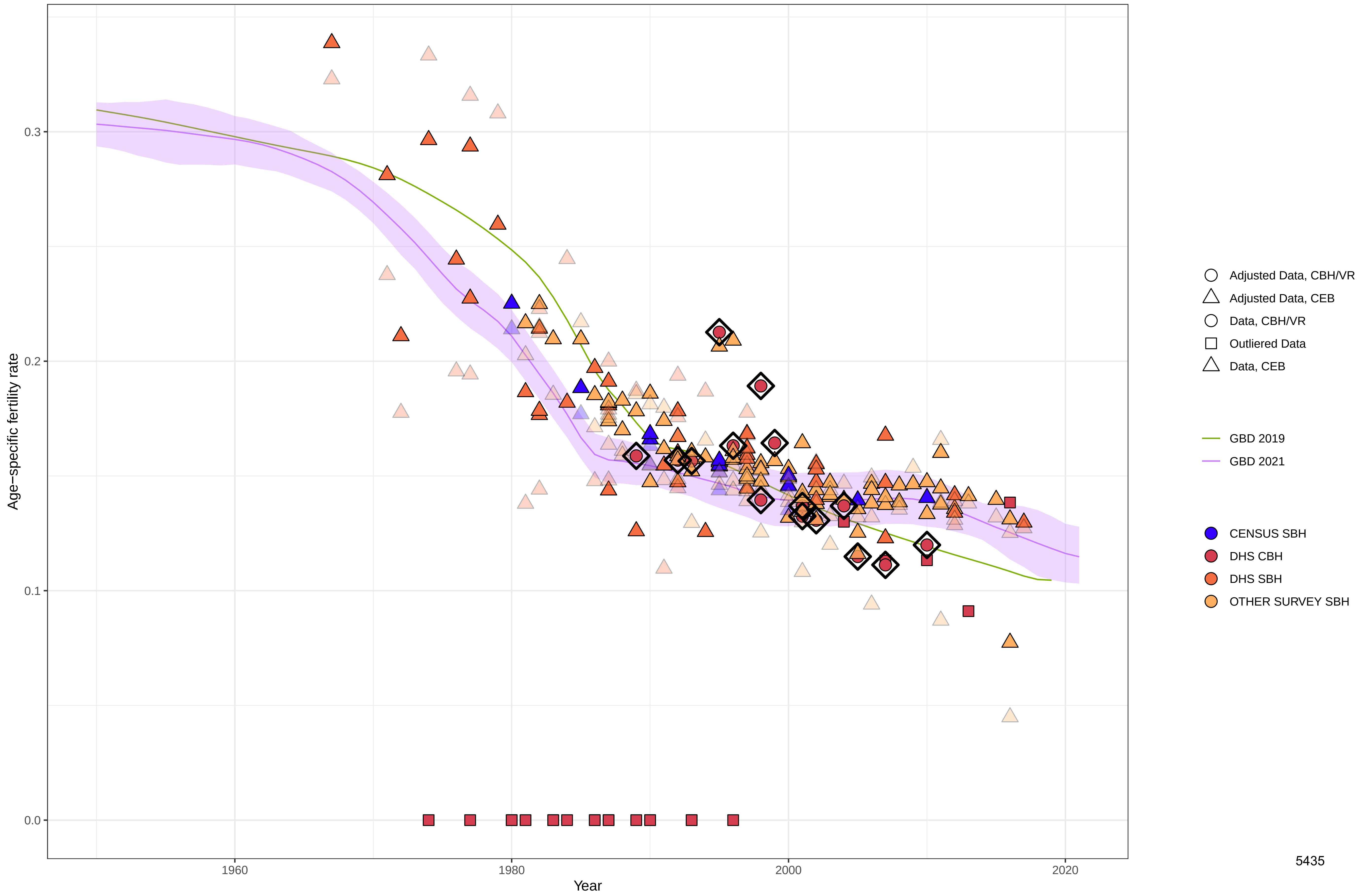

Age−specific fertility fitted trend lines, 1950−2021: Gorontalo, Ages: 30−34
Data points enclosed with a diamond are reference sources

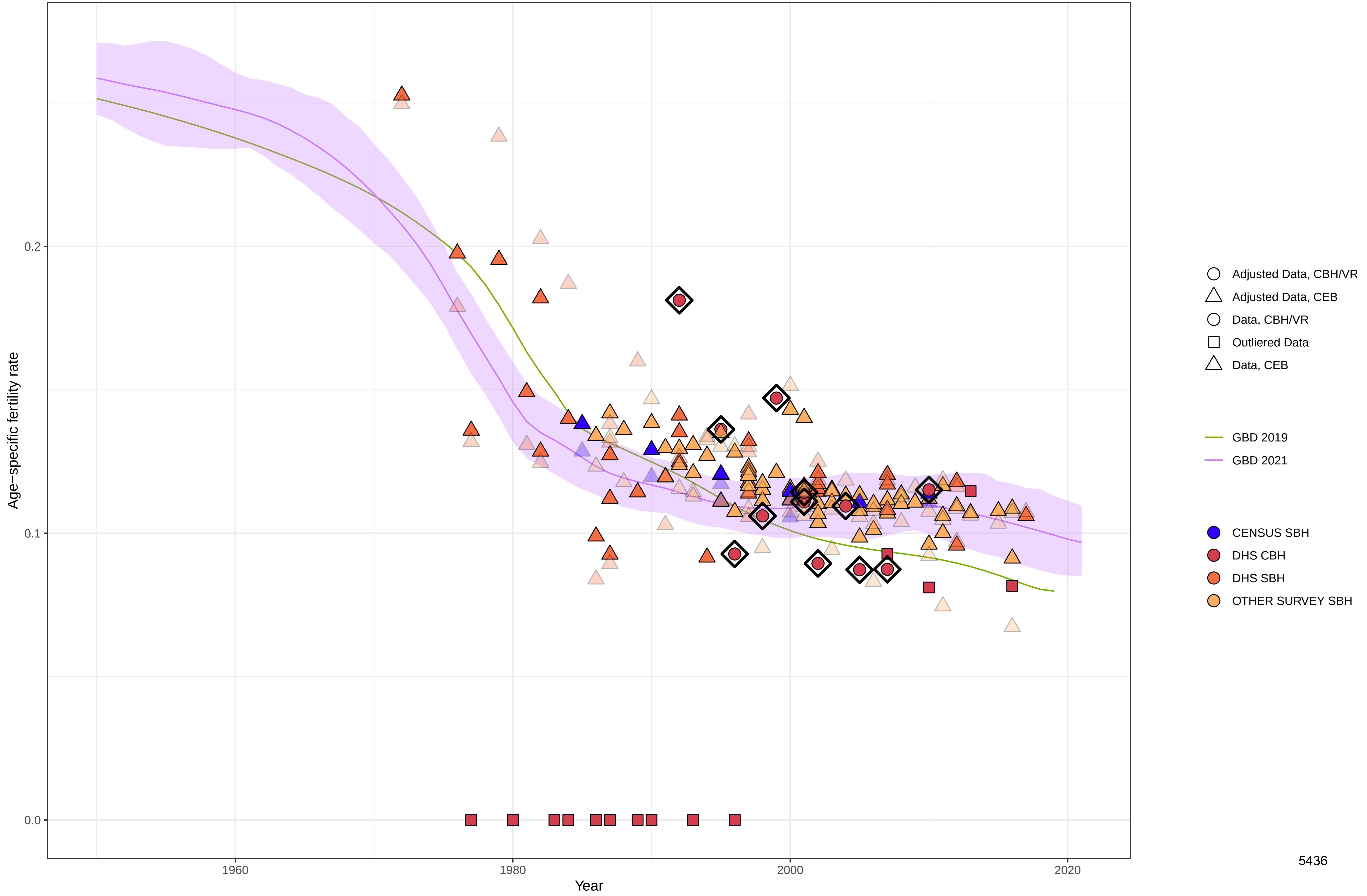

Age−specific fertility fitted trend lines, 1950−2021: Gorontalo, Ages: 35−39
Data points enclosed with a diamond are reference sources

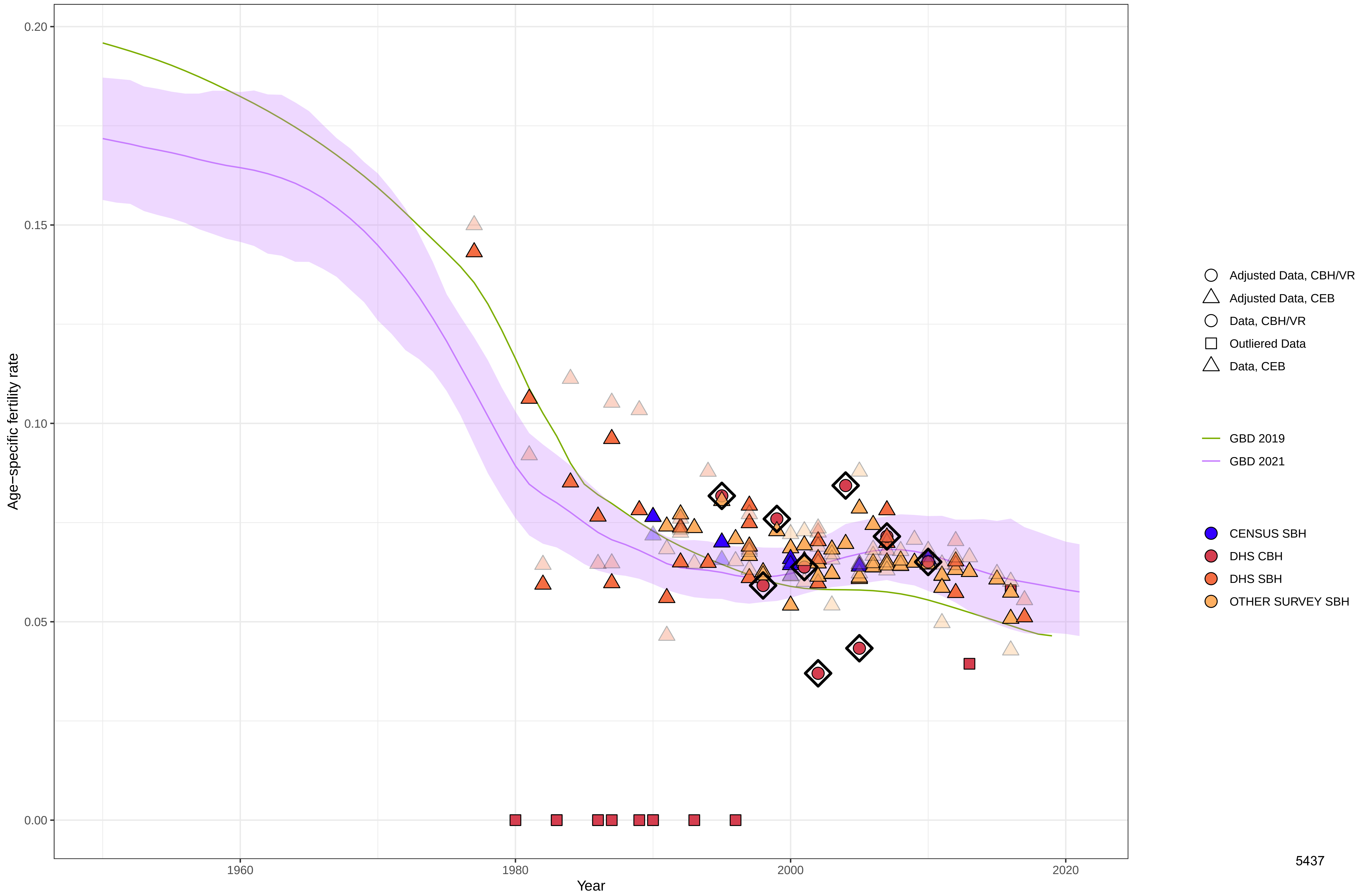

Age−specific fertility fitted trend lines, 1950−2021: Gorontalo, Ages: 40−44
Data points enclosed with a diamond are reference sources

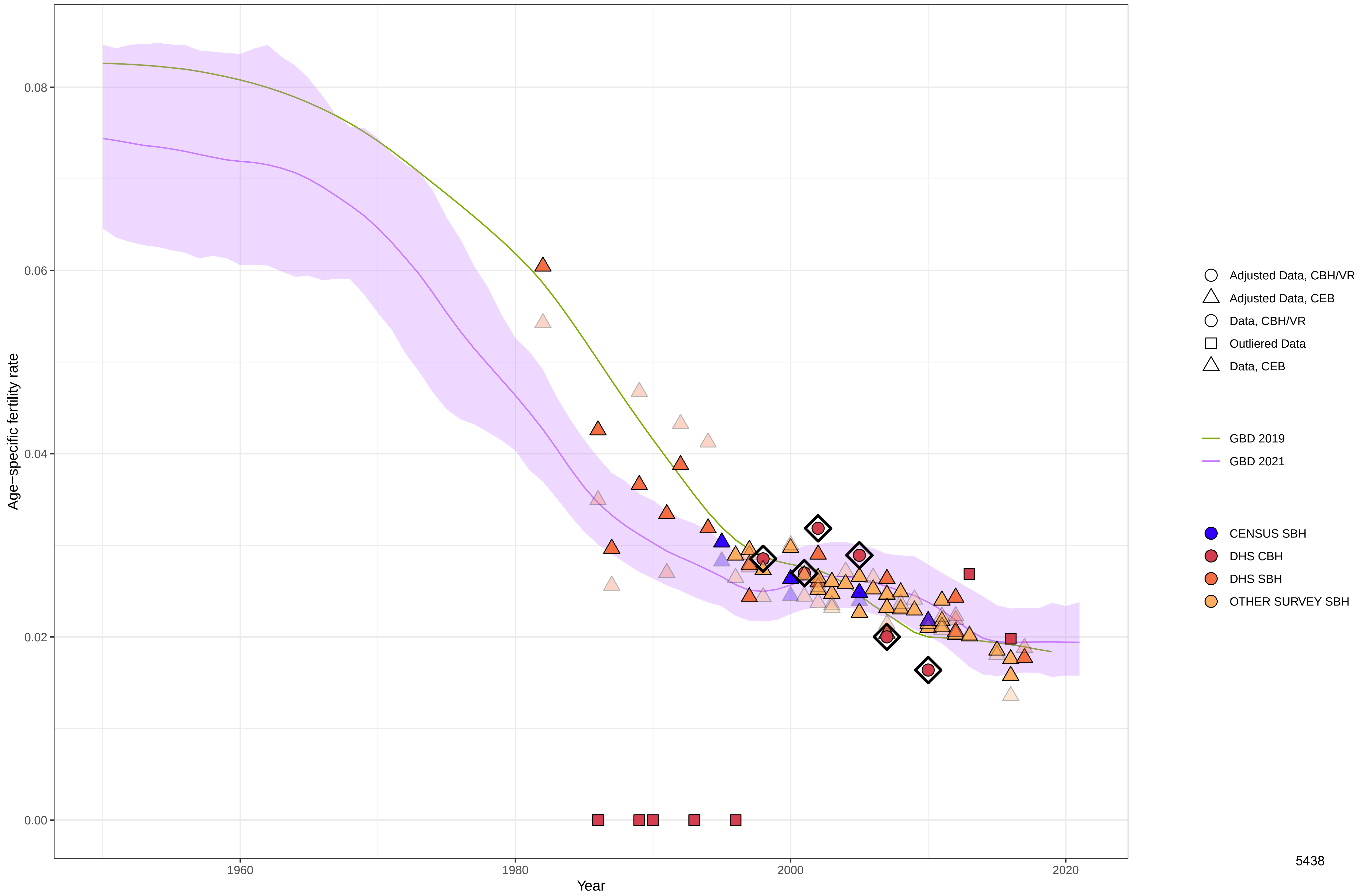

Age−specific fertility fitted trend lines, 1950−2021: Gorontalo, Ages: 45−49
Data points enclosed with a diamond are reference sources

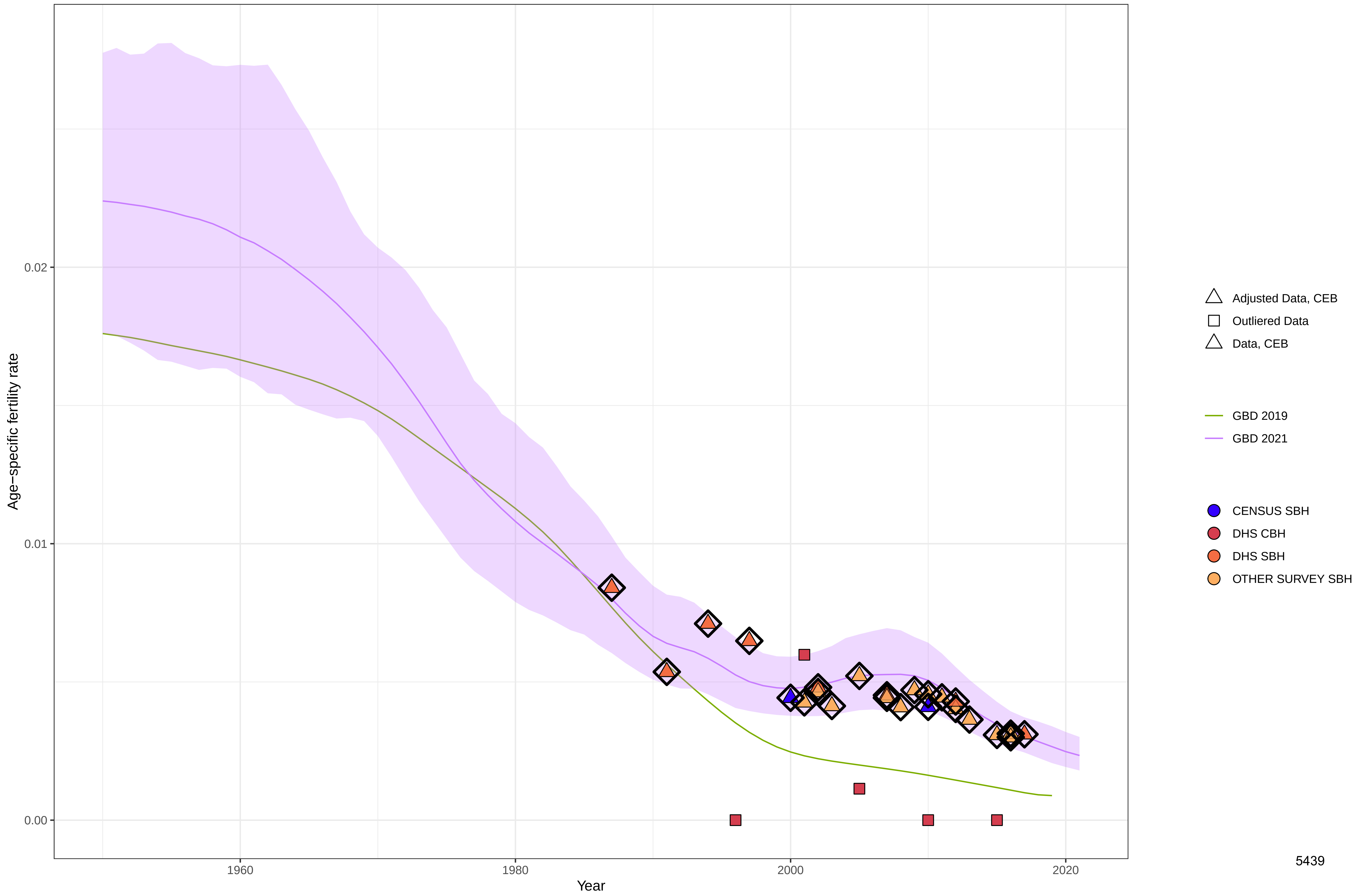

Age−specific fertility fitted trend lines, 1950−2021: Gorontalo, Ages: 50−54
Data points enclosed with a diamond are reference sources

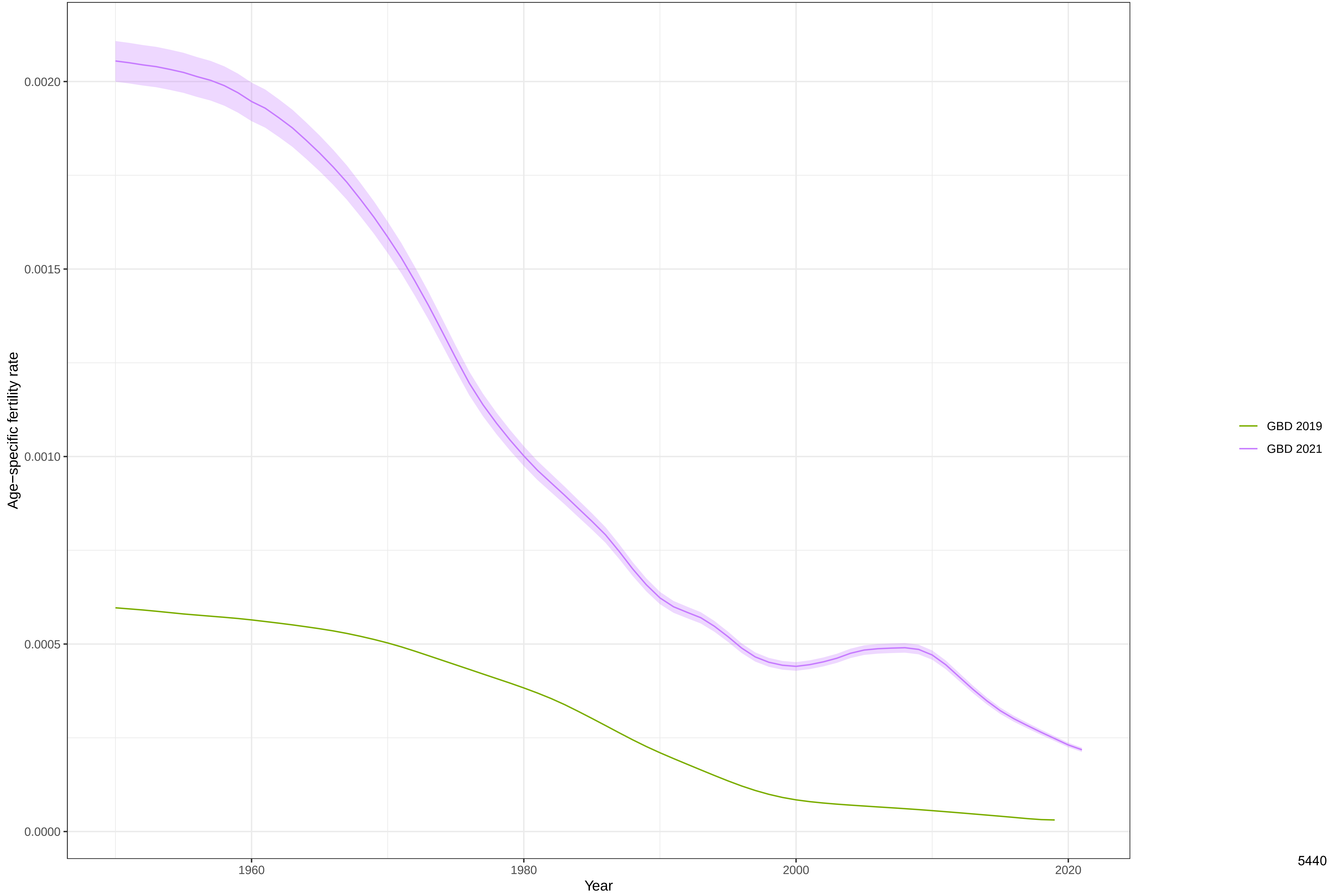

Total fertility rate fitted trend lines, 1950−2021: Gorontalo TFR
Data points enclosed with a diamond are reference sources

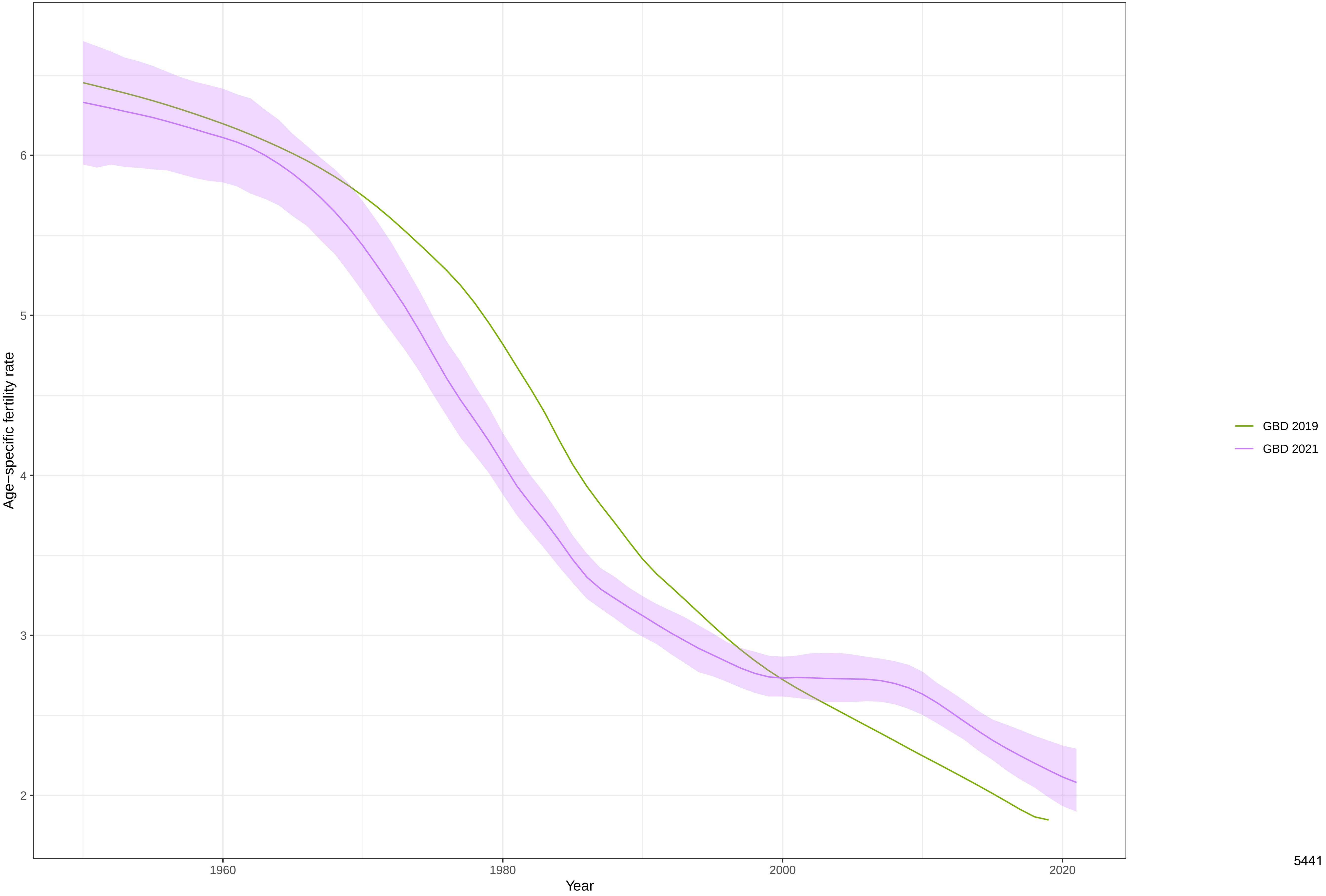

Age−specific fertility fitted trend lines, 1950−2021: Jakarta, Ages: 10−14
Data points enclosed with a diamond are reference sources

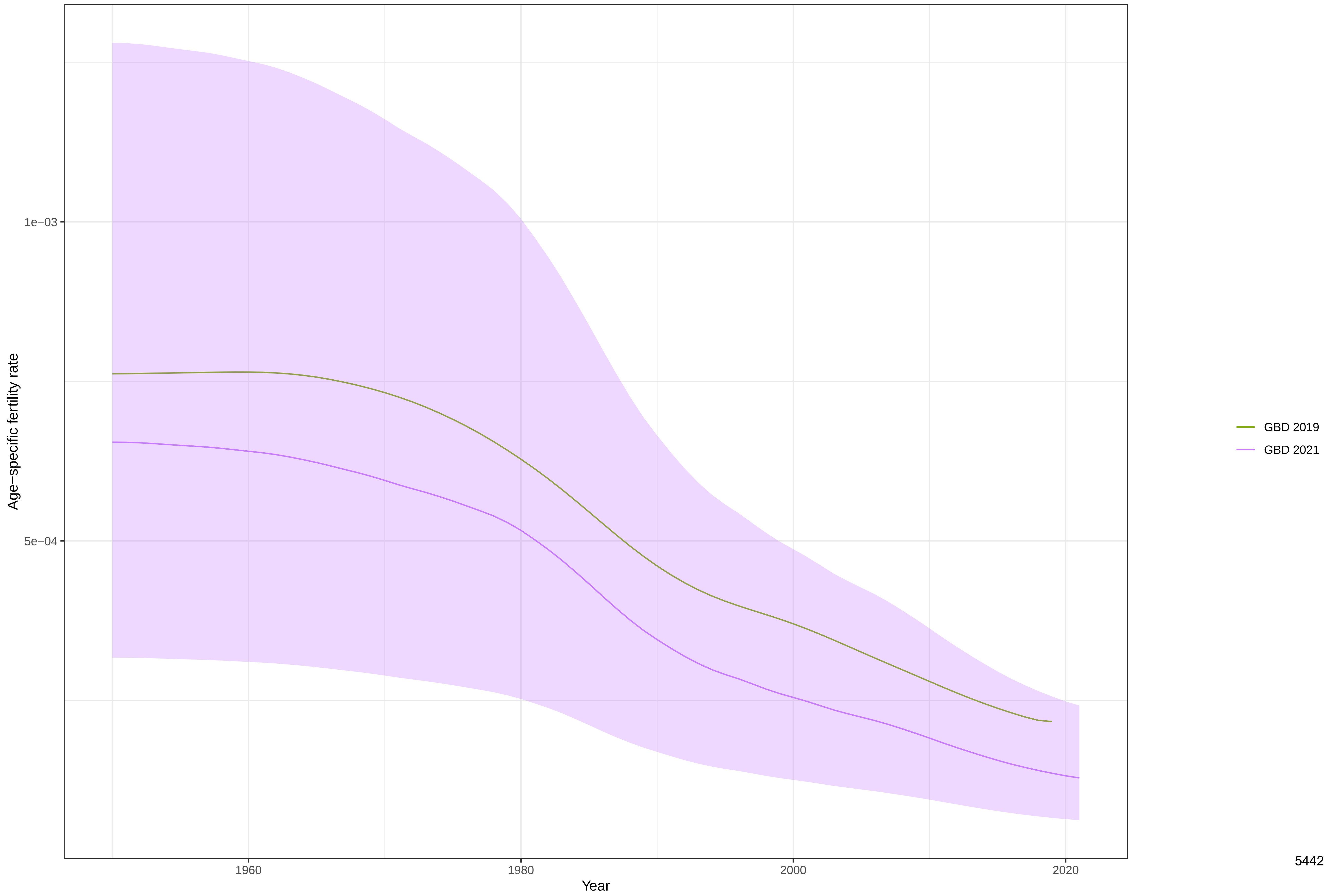

Age−specific fertility fitted trend lines, 1950−2021: Jakarta, Ages: 15−19
Data points enclosed with a diamond are reference sources

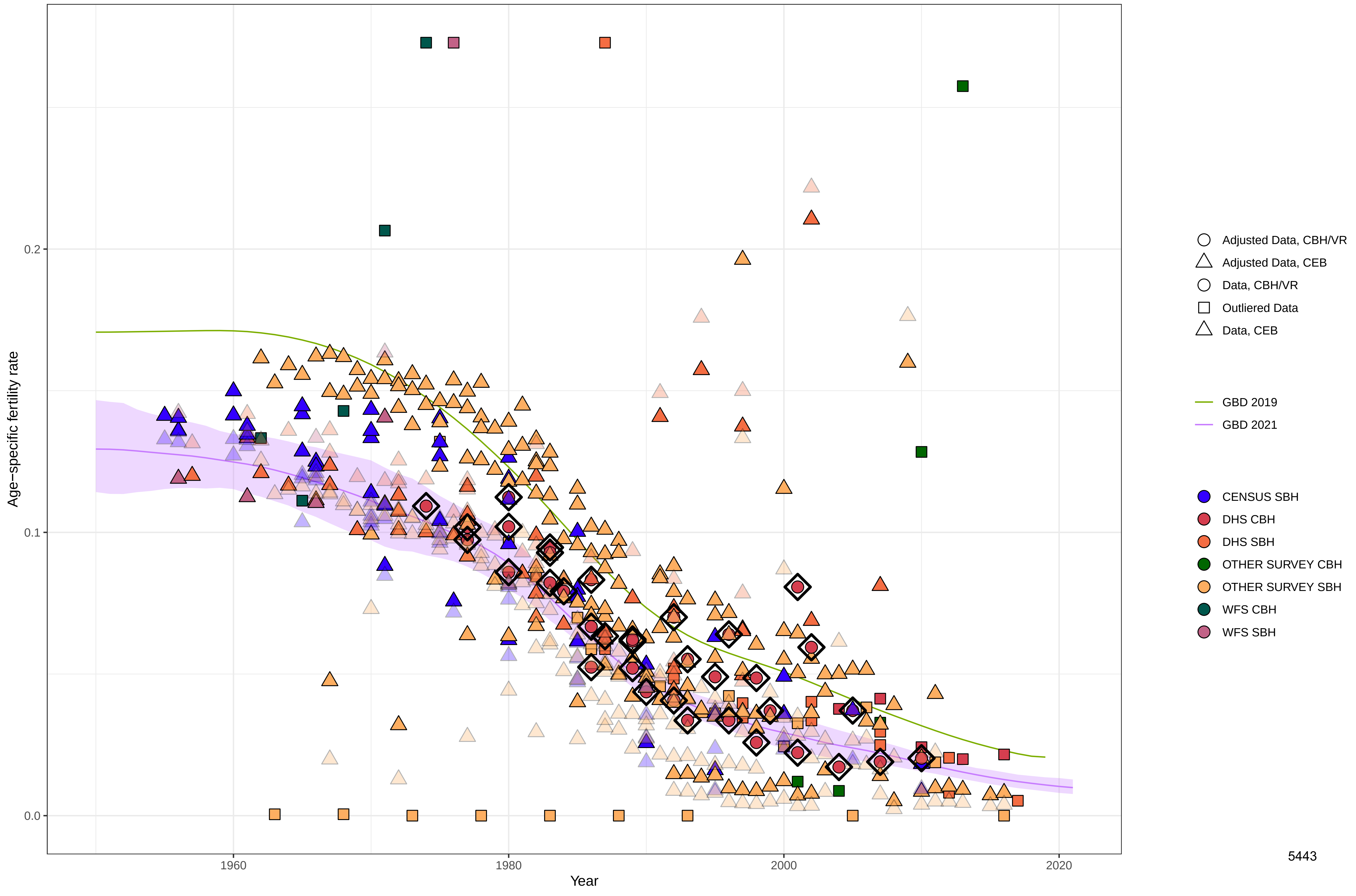

Age−specific fertility fitted trend lines, 1950−2021: Jakarta, Ages: 20−24
Data points enclosed with a diamond are reference sources

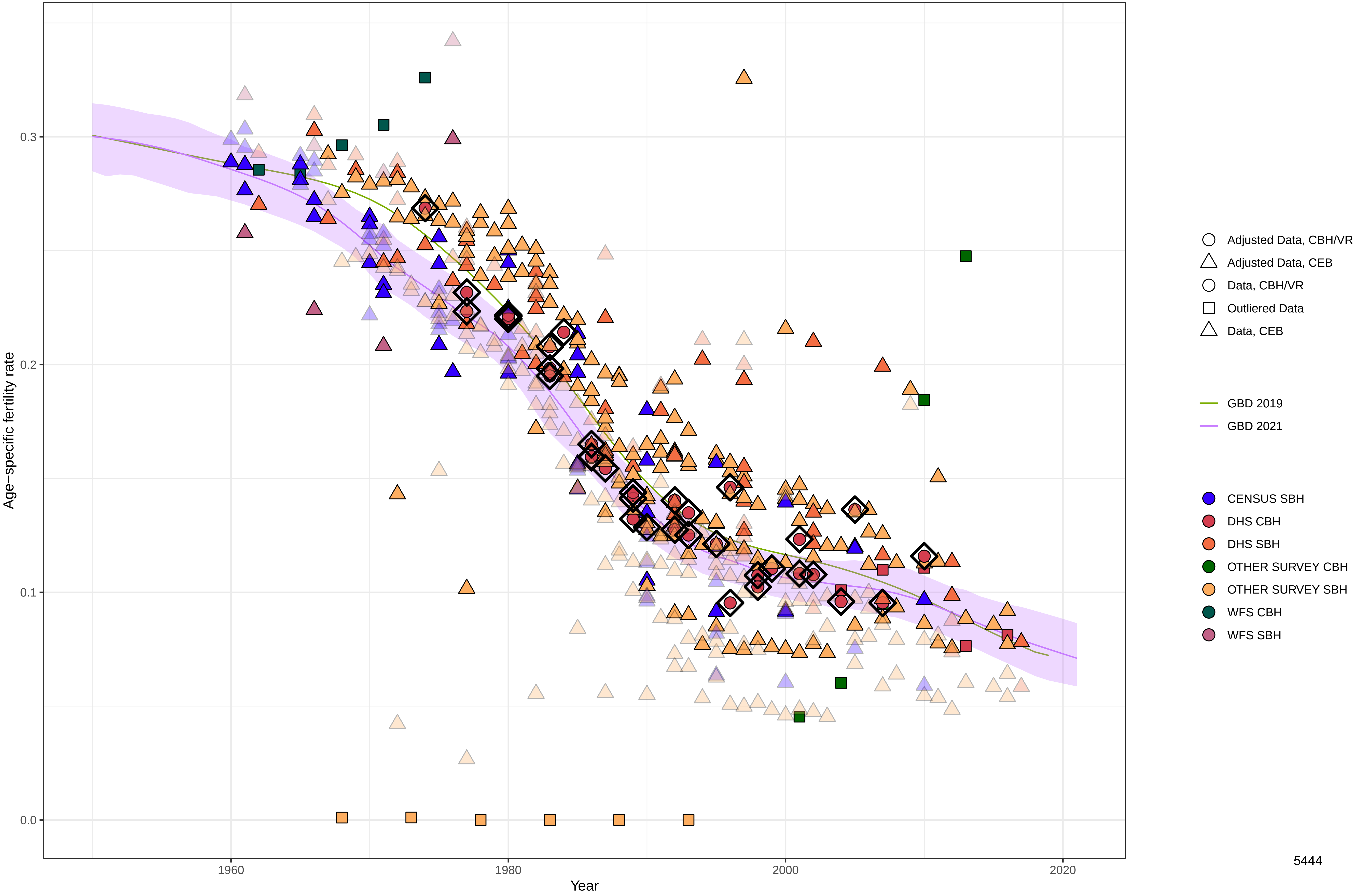

Age−specific fertility fitted trend lines, 1950−2021: Jakarta, Ages: 25−29
Data points enclosed with a diamond are reference sources

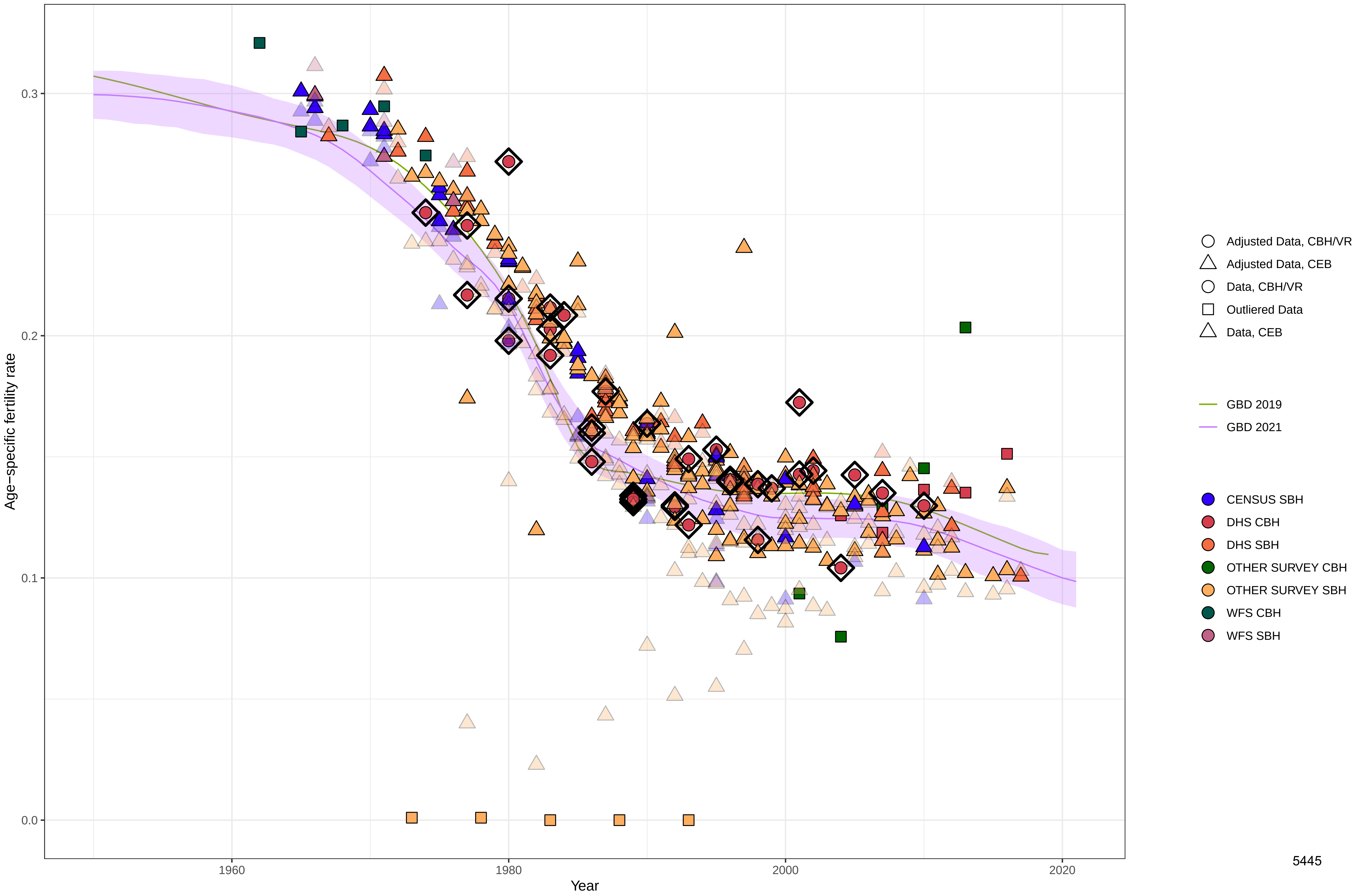

Age−specific fertility fitted trend lines, 1950−2021: Jakarta, Ages: 30−34
Data points enclosed with a diamond are reference sources

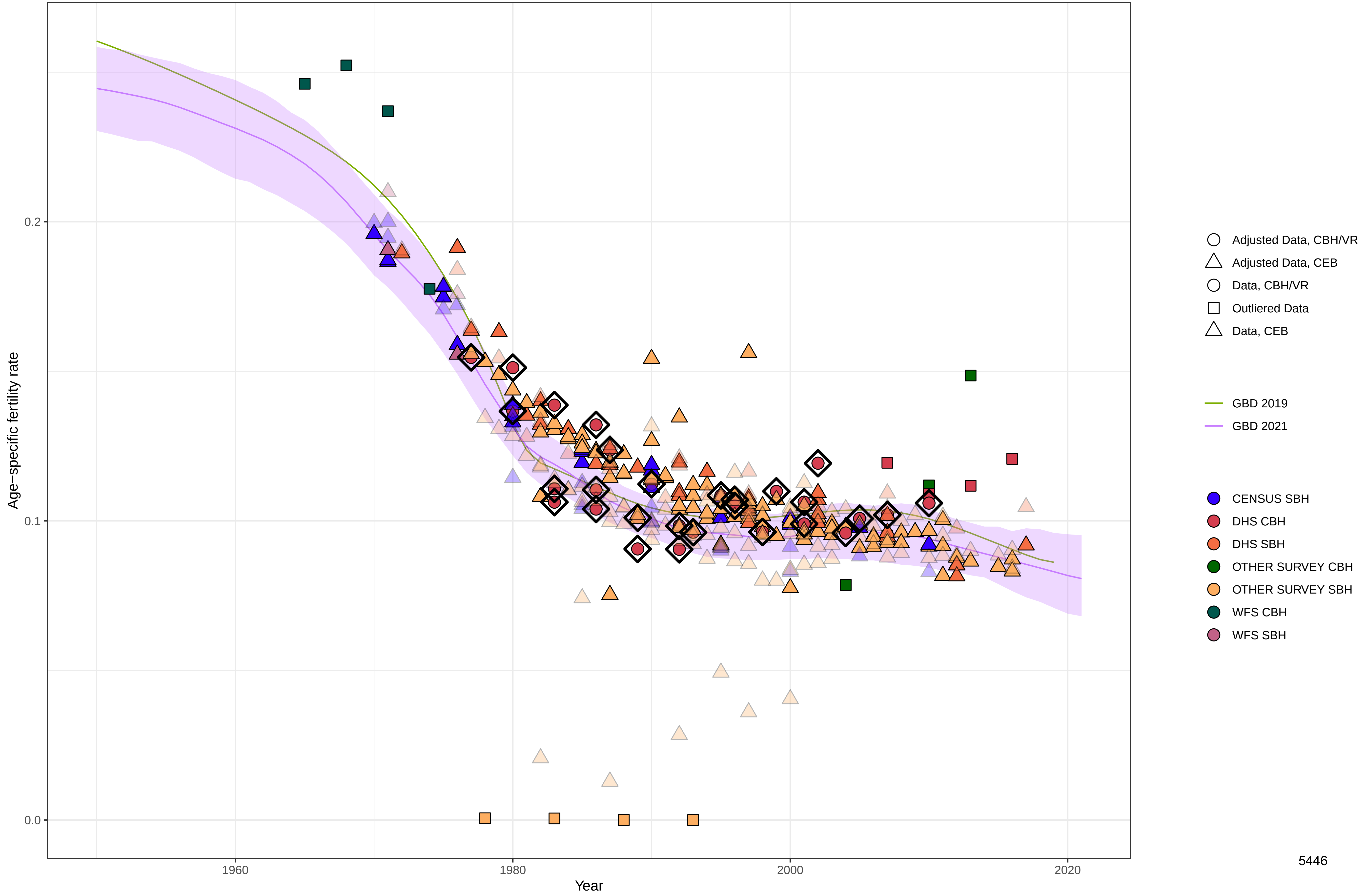

Age−specific fertility fitted trend lines, 1950−2021: Jakarta, Ages: 35−39
Data points enclosed with a diamond are reference sources

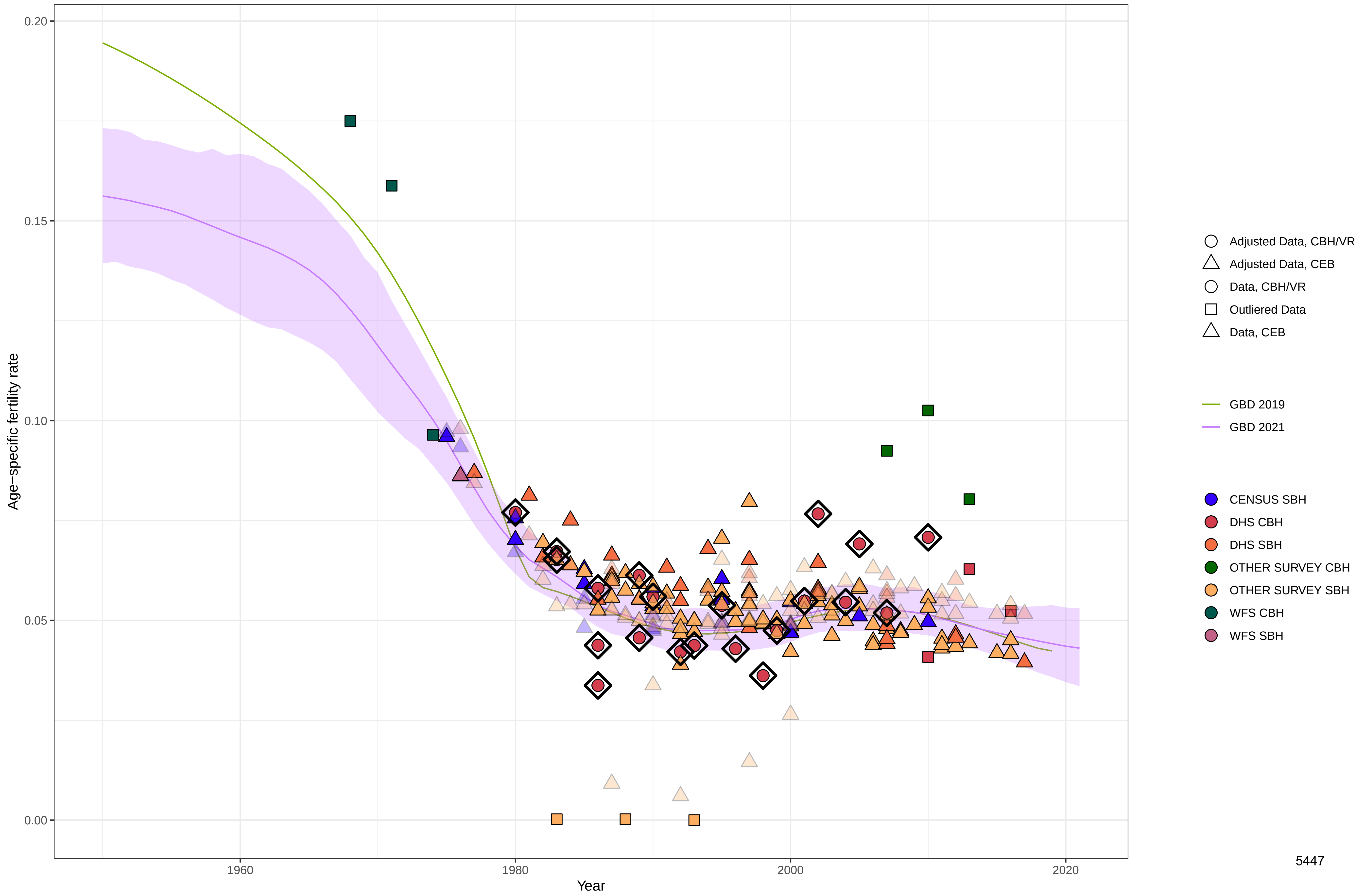

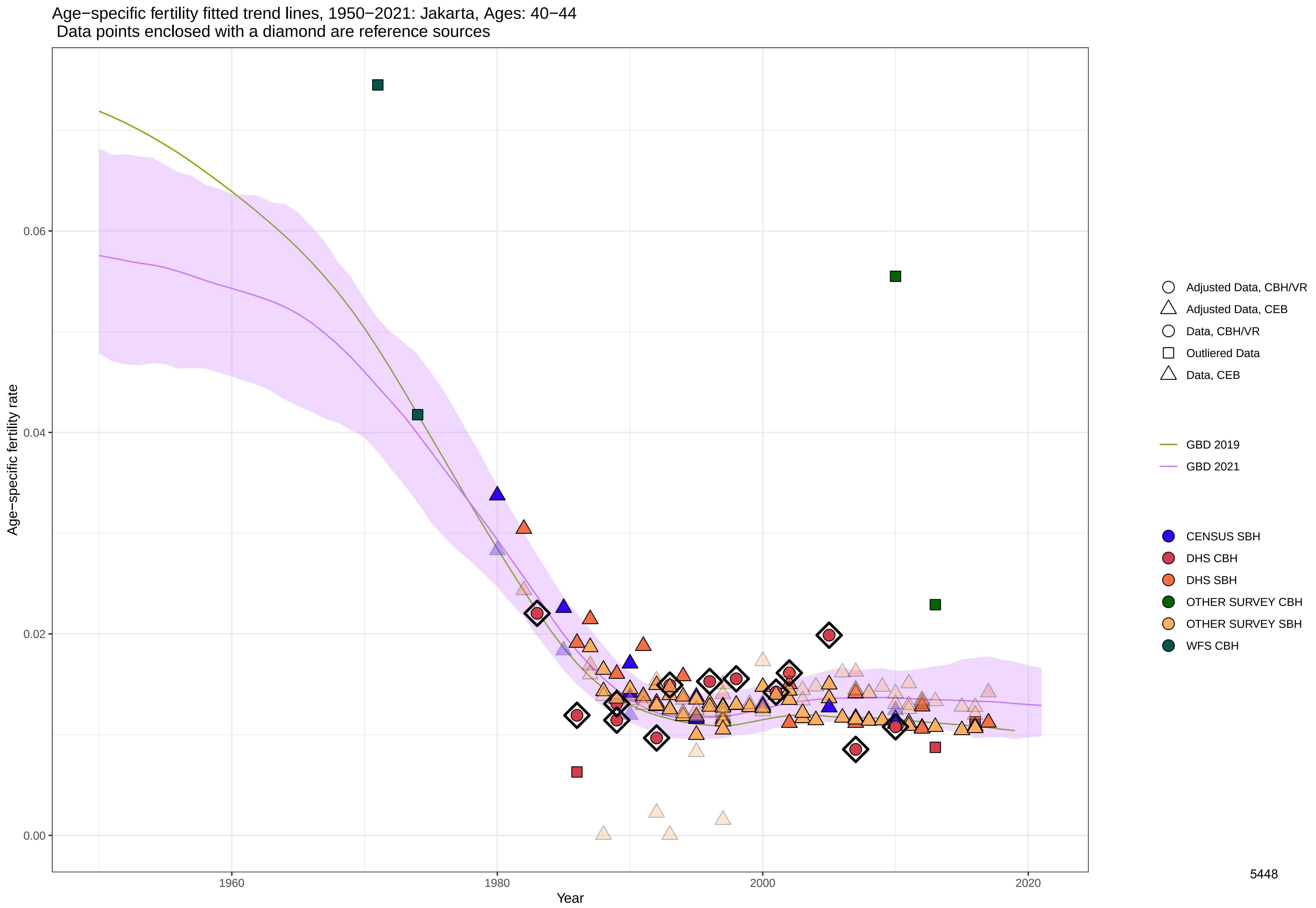
Age−specific fertility fitted trend lines, 1950−2021: Jakarta, Ages: 40−44
Data points enclosed with a diamond are reference sources
Age−specific fertility rate
Year
0.00
0.02
0.04
0.06
1960
1980
2000
2020
Adjusted Data, CBH/VR
Adjusted Data, CEB
Data, CBH/VR
Outliered Data
Data, CEB
GBD 2019
GBD 2021
CENSUS SBH
DHS CBH
DHS SBH
OTHER SURVEY CBH
OTHER SURVEY SBH
WFS CBH

Age−specific fertility fitted trend lines, 1950−2021: Jakarta, Ages: 45−49
Data points enclosed with a diamond are reference sources

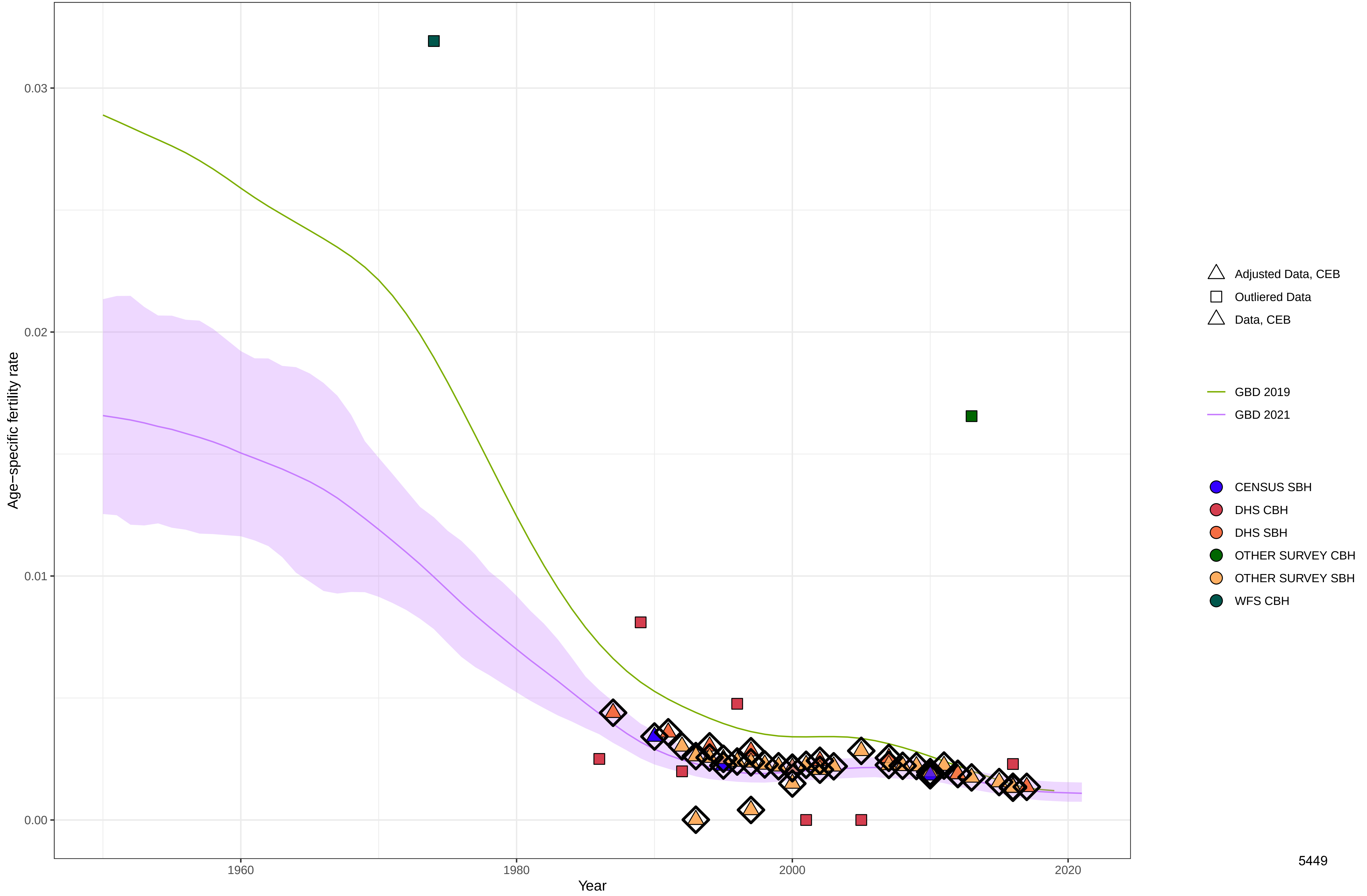

Age−specific fertility fitted trend lines, 1950−2021: Jakarta, Ages: 50−54
Data points enclosed with a diamond are reference sources

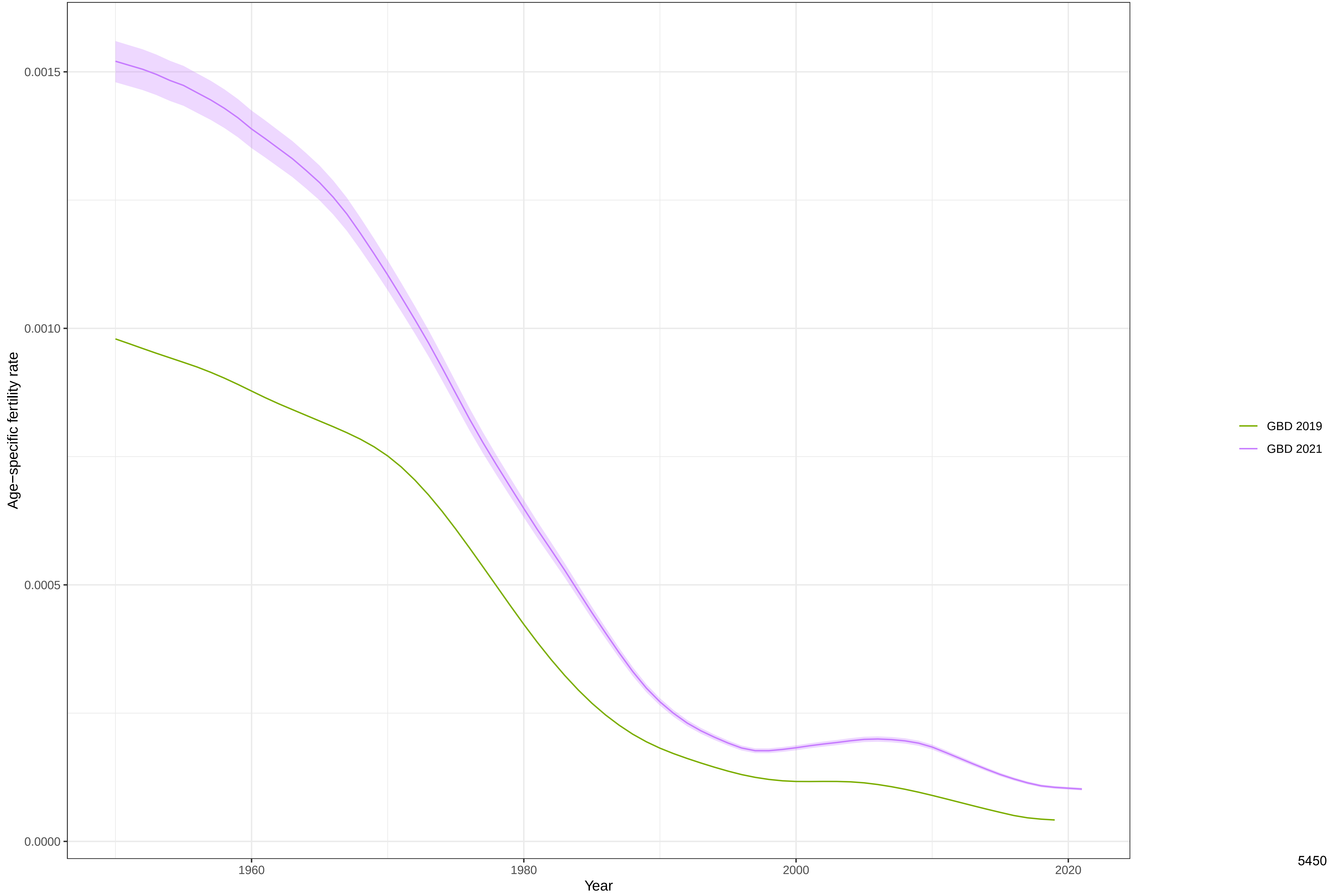

Total fertility rate fitted trend lines, 1950−2021: Jakarta TFR
Data points enclosed with a diamond are reference sources

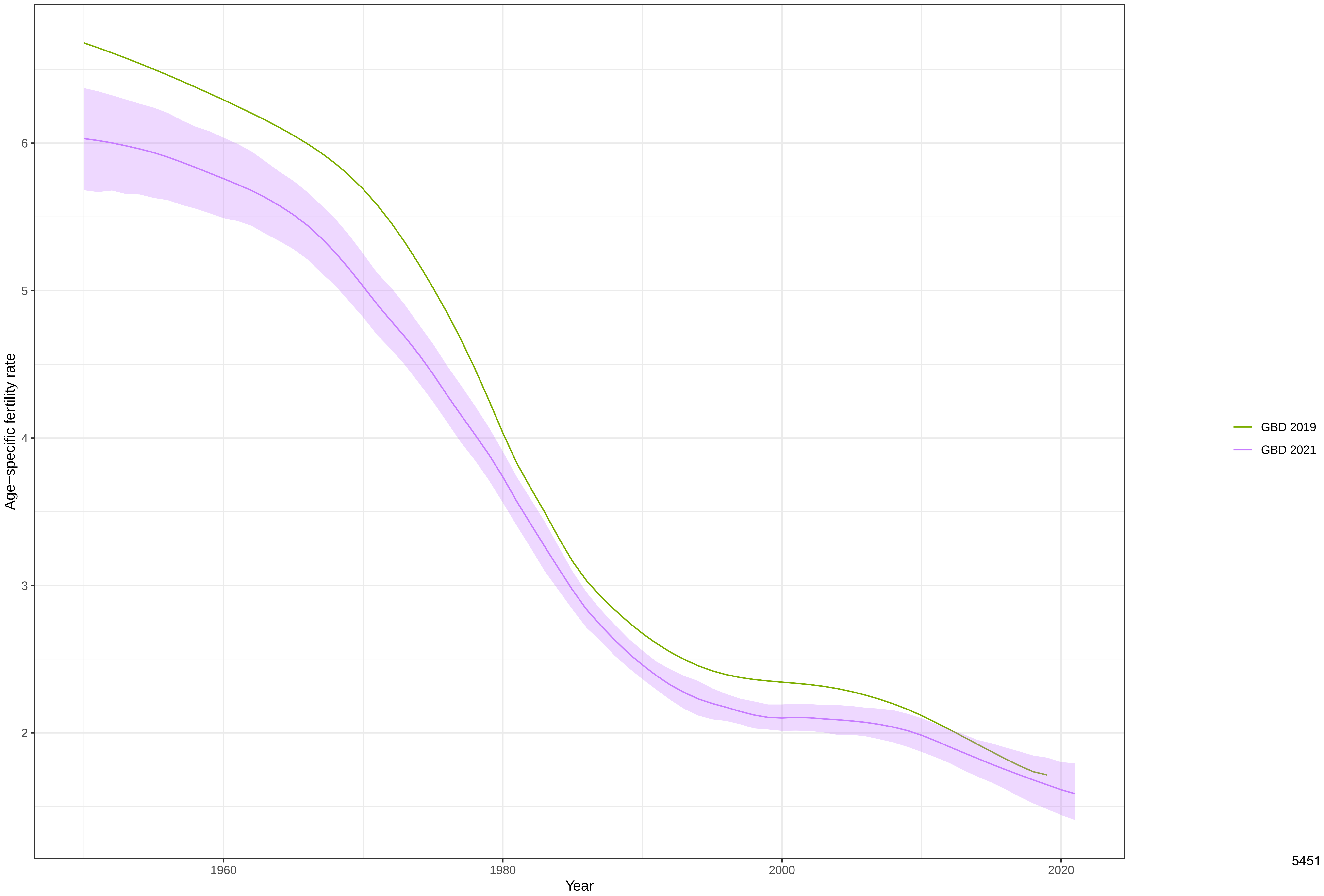

5451

Age−specific fertility fitted trend lines, 1950−2021: Jambi, Ages: 10−14
Data points enclosed with a diamond are reference sources

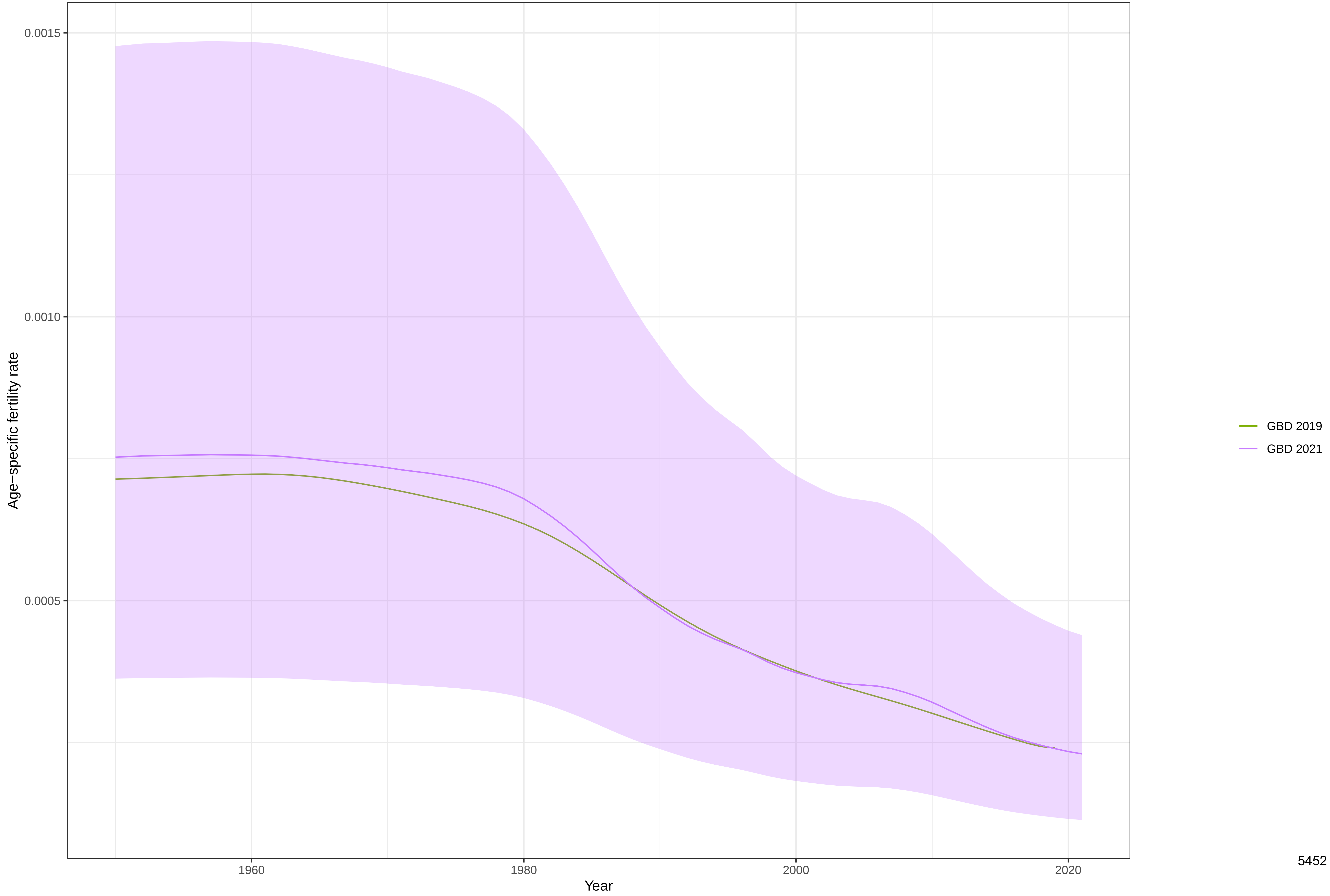

Age−specific fertility fitted trend lines, 1950−2021: Jambi, Ages: 15−19
Data points enclosed with a diamond are reference sources

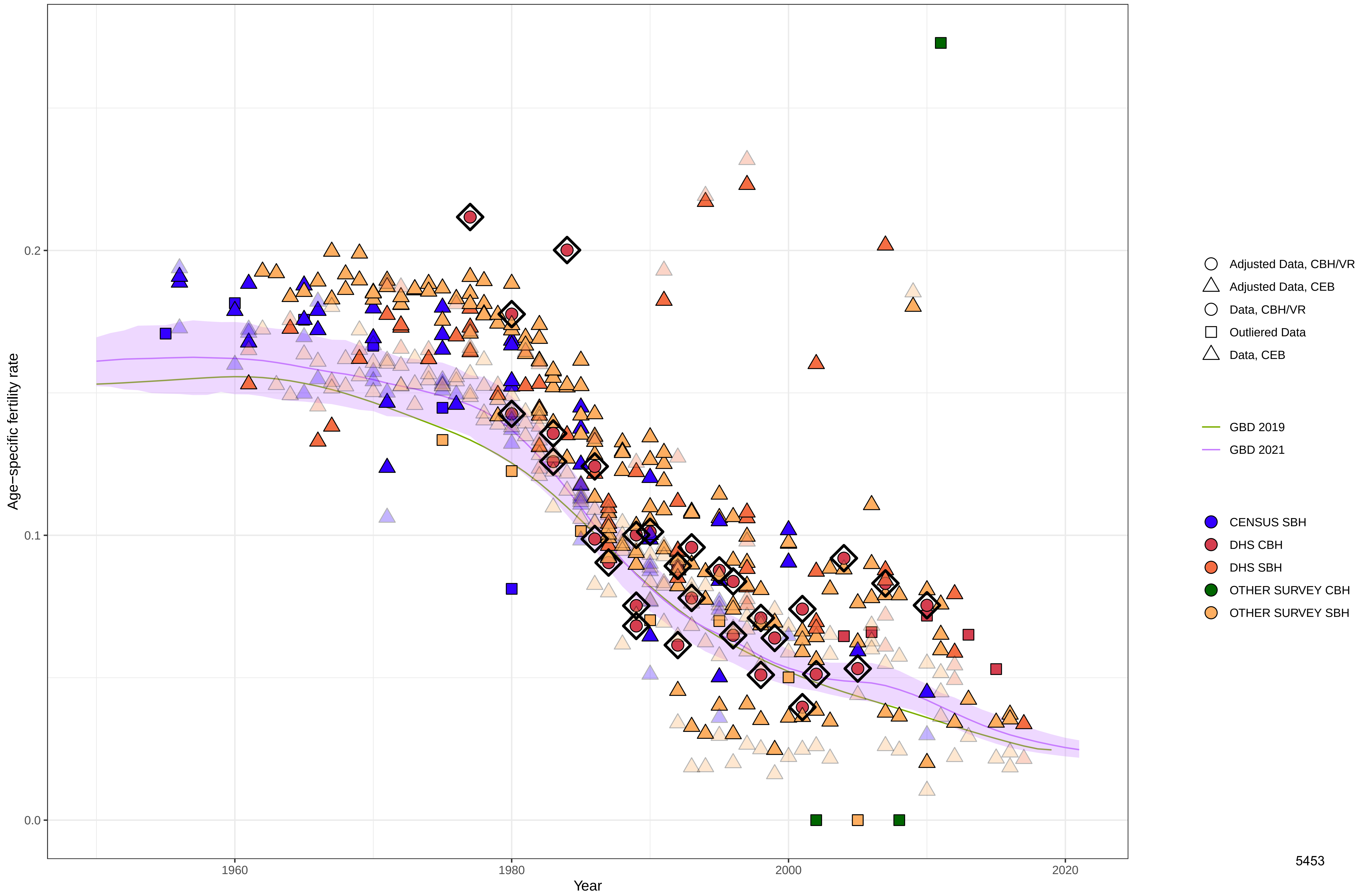

Age−specific fertility fitted trend lines, 1950−2021: Jambi, Ages: 20−24
Data points enclosed with a diamond are reference sources

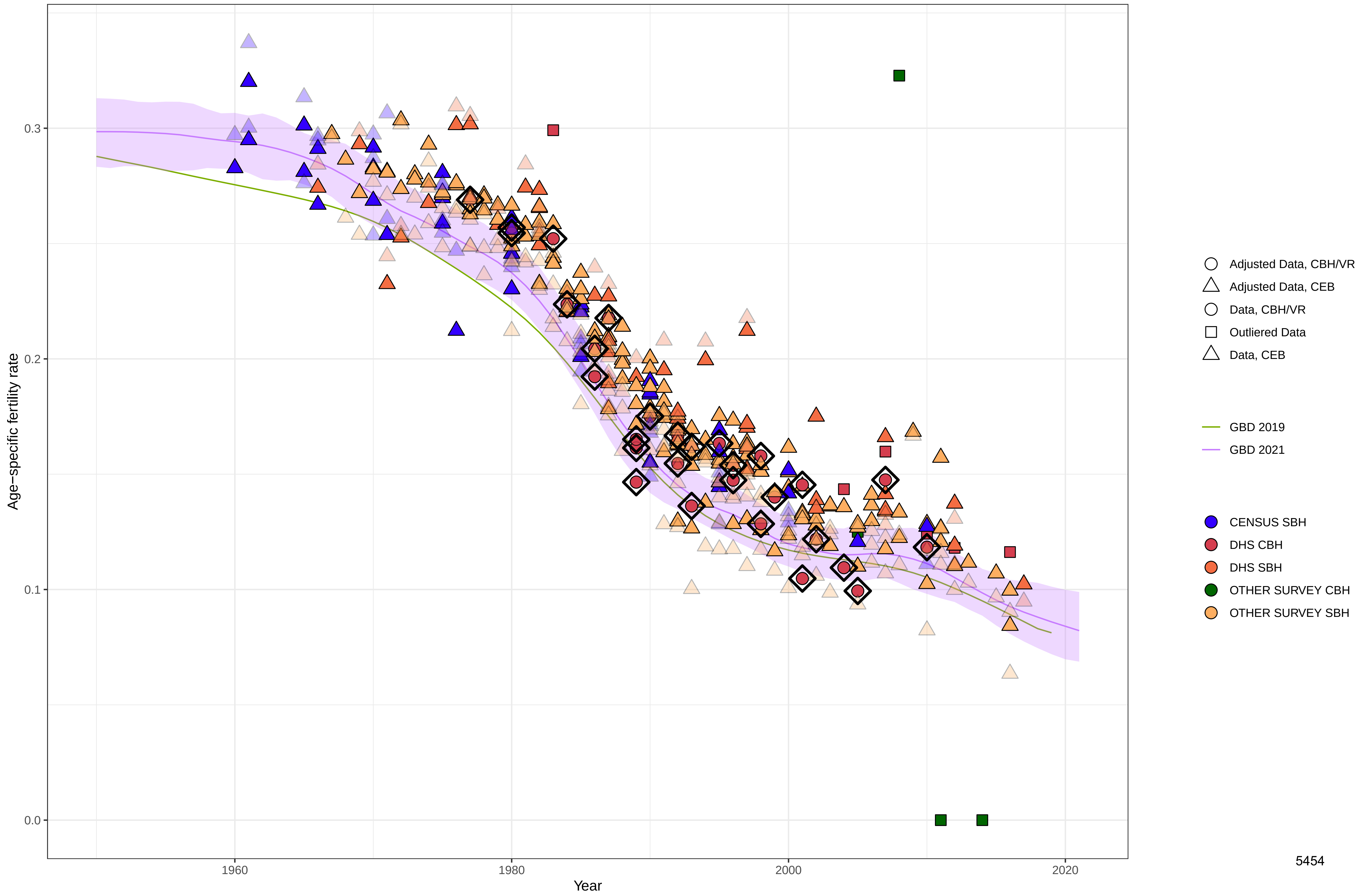

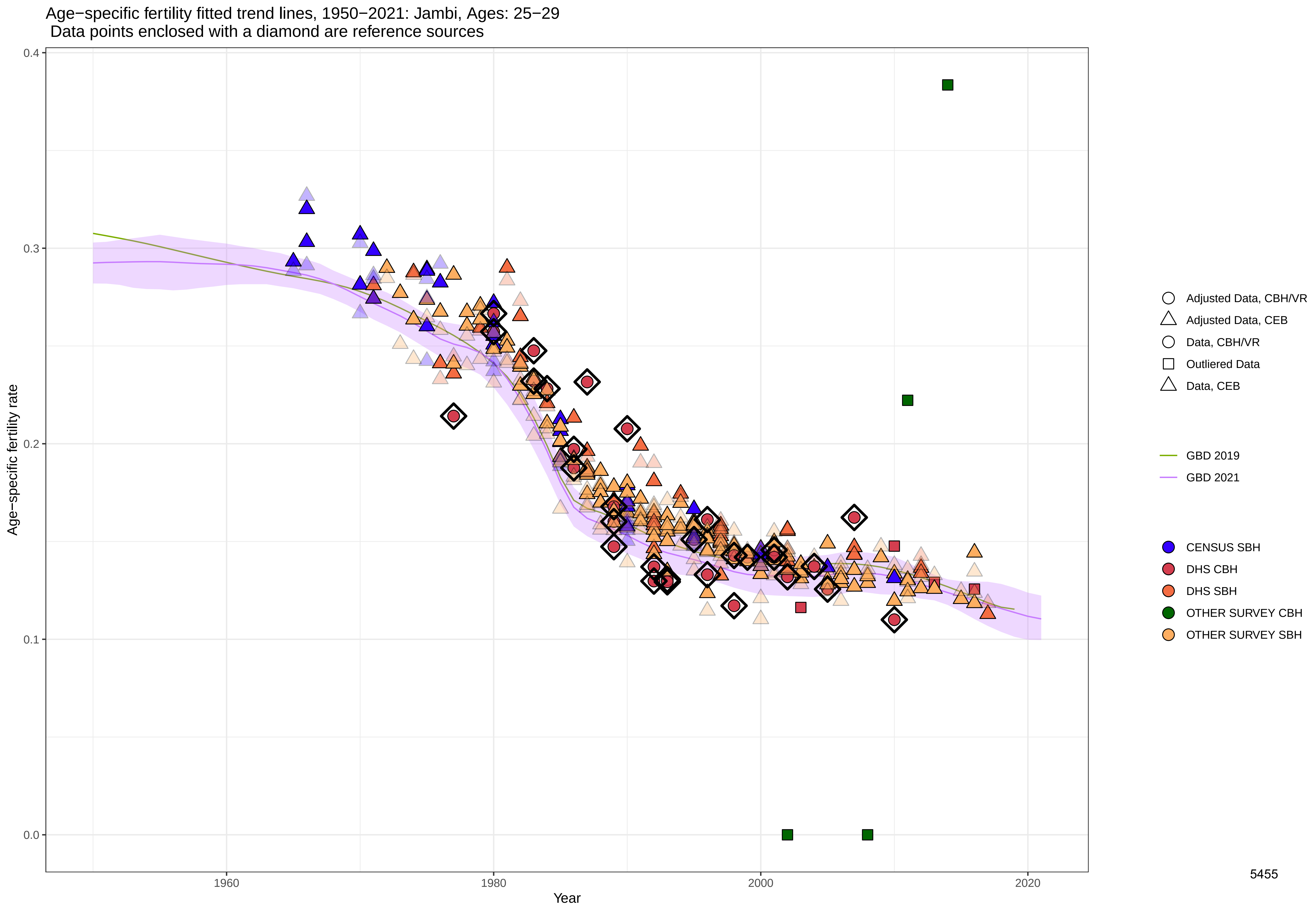
Age−specific fertility fitted trend lines, 1950−2021: Jambi, Ages: 25−29
Data points enclosed with a diamond are reference sources
Age−specific fertility rate
Year
0.0
0.1
0.2
0.3
0.4
1960
1980
2000
2020
Adjusted Data, CBH/VR
Adjusted Data, CEB
Data, CBH/VR
Outliered Data
Data, CEB
GBD 2019
GBD 2021
CENSUS SBH
DHS CBH
DHS SBH
OTHER SURVEY CBH
OTHER SURVEY SBH

Age−specific fertility fitted trend lines, 1950−2021: Jambi, Ages: 30−34
Data points enclosed with a diamond are reference sources

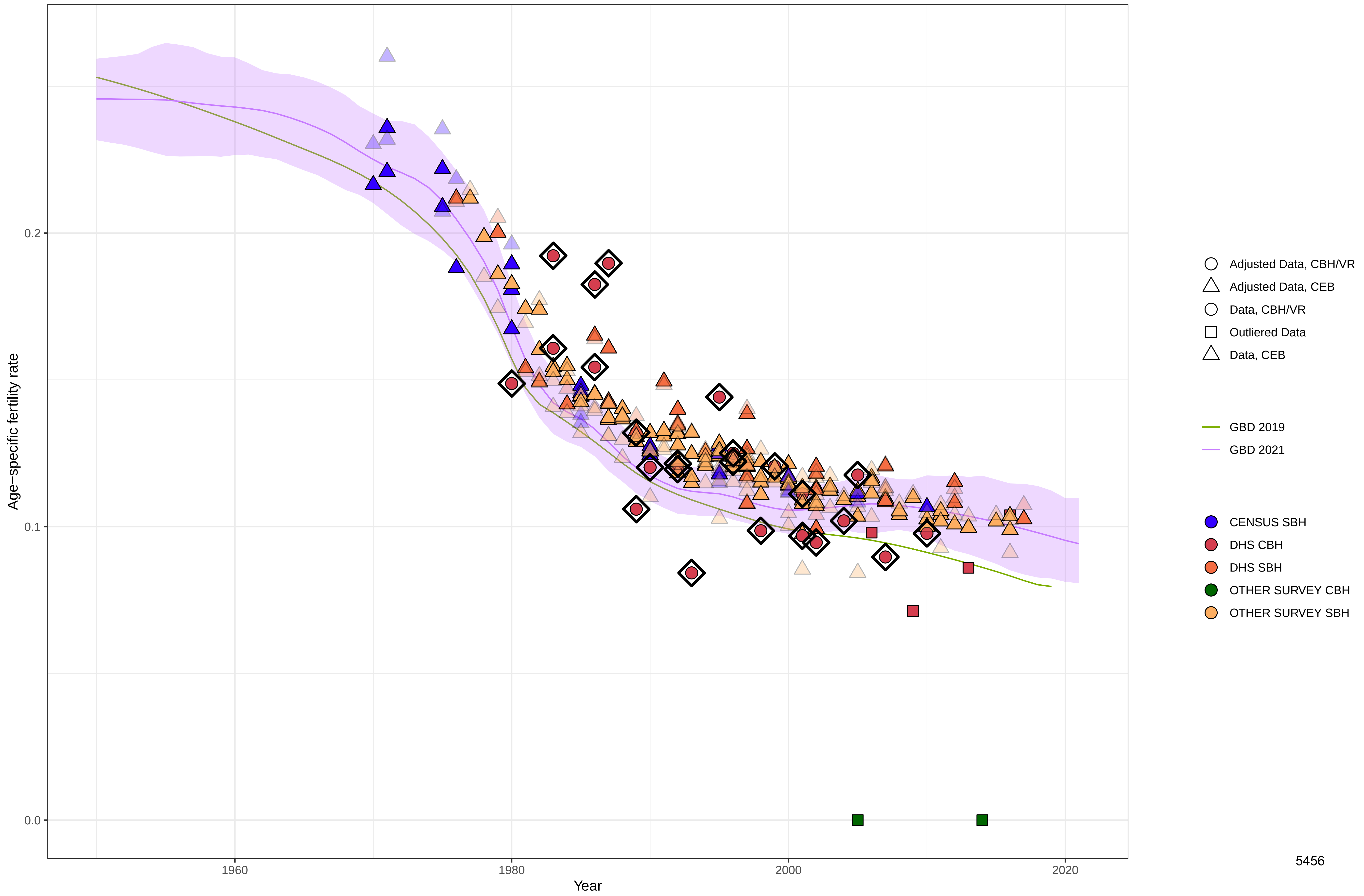

Age−specific fertility fitted trend lines, 1950−2021: Jambi, Ages: 35−39
Data points enclosed with a diamond are reference sources

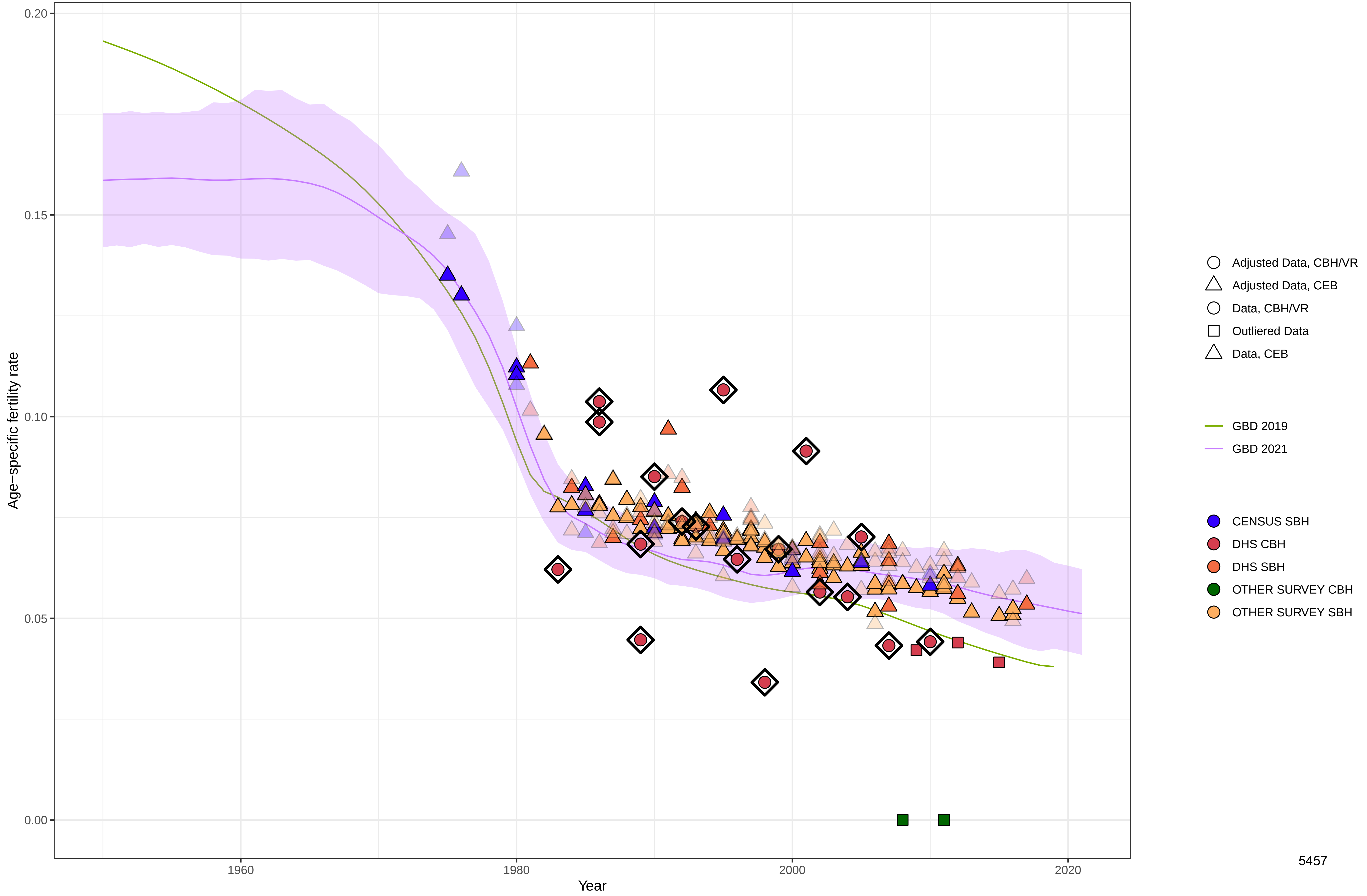

5457

Age−specific fertility fitted trend lines, 1950−2021: Jambi, Ages: 40−44
Data points enclosed with a diamond are reference sources

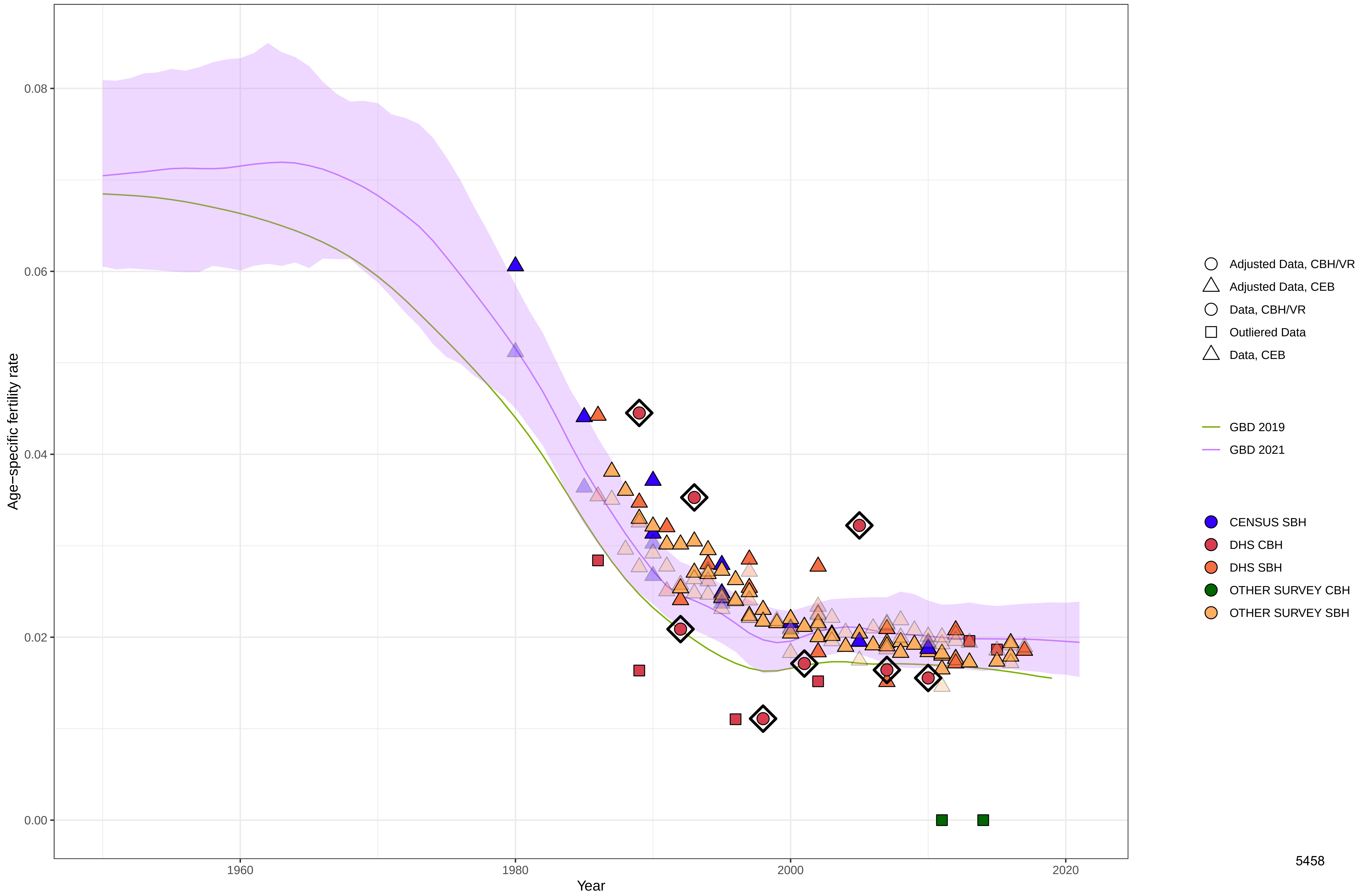

Age−specific fertility fitted trend lines, 1950−2021: Jambi, Ages: 45−49
Data points enclosed with a diamond are reference sources

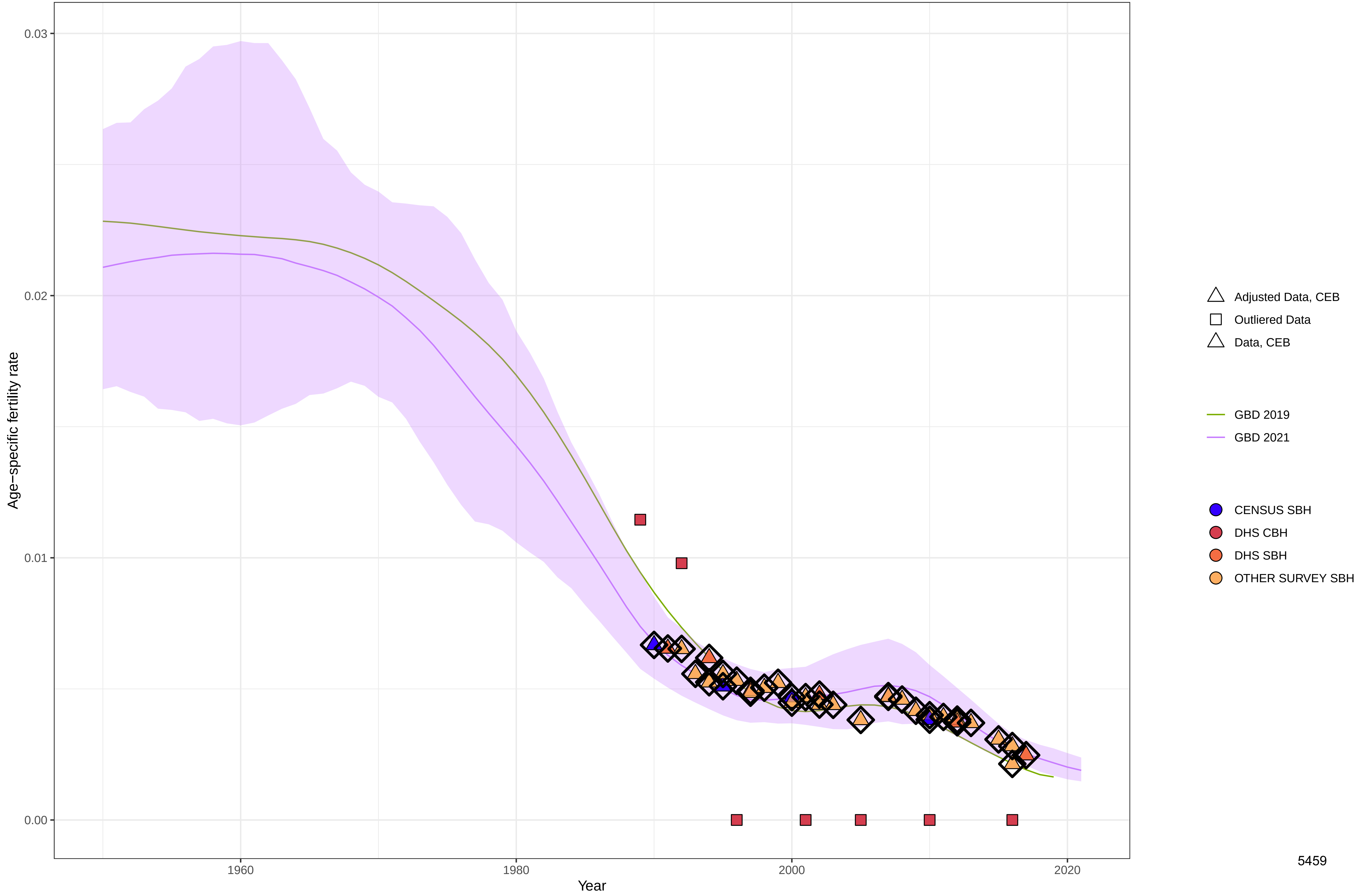

Age−specific fertility fitted trend lines, 1950−2021: Jambi, Ages: 50−54
Data points enclosed with a diamond are reference sources

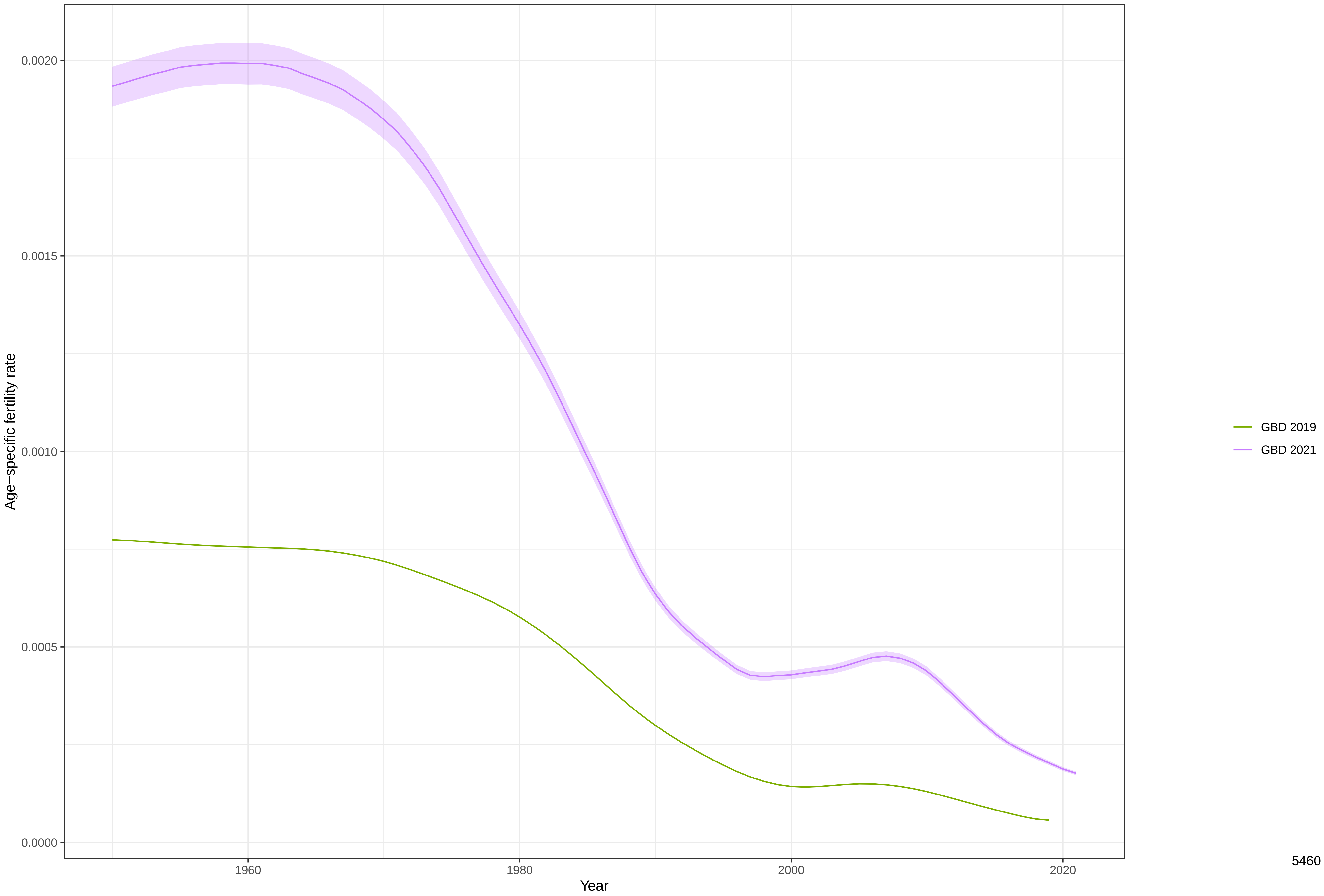

Total fertility rate fitted trend lines, 1950−2021: Jambi TFR
Data points enclosed with a diamond are reference sources

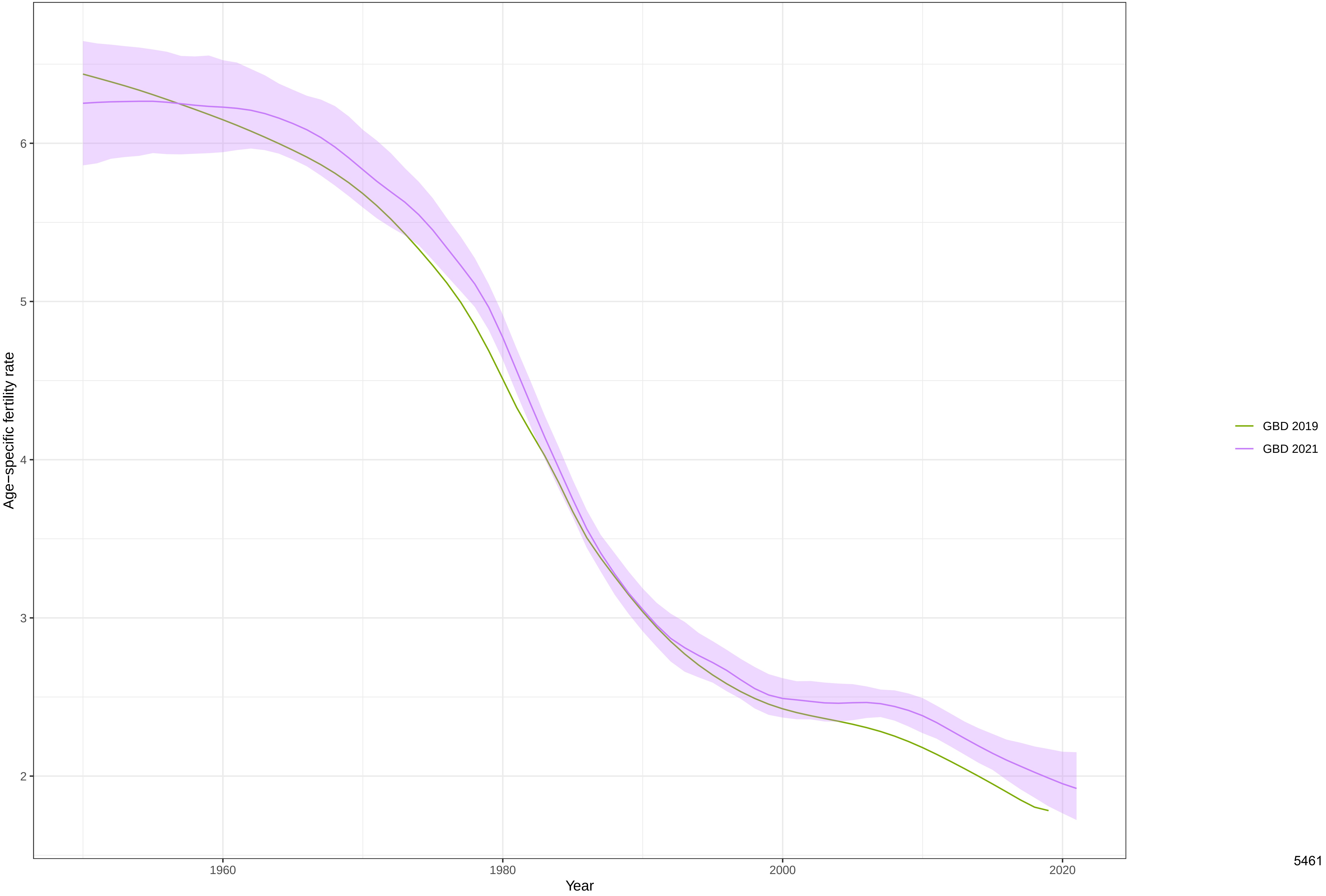

Age−specific fertility fitted trend lines, 1950−2021: West Java, Ages: 10−14
Data points enclosed with a diamond are reference sources

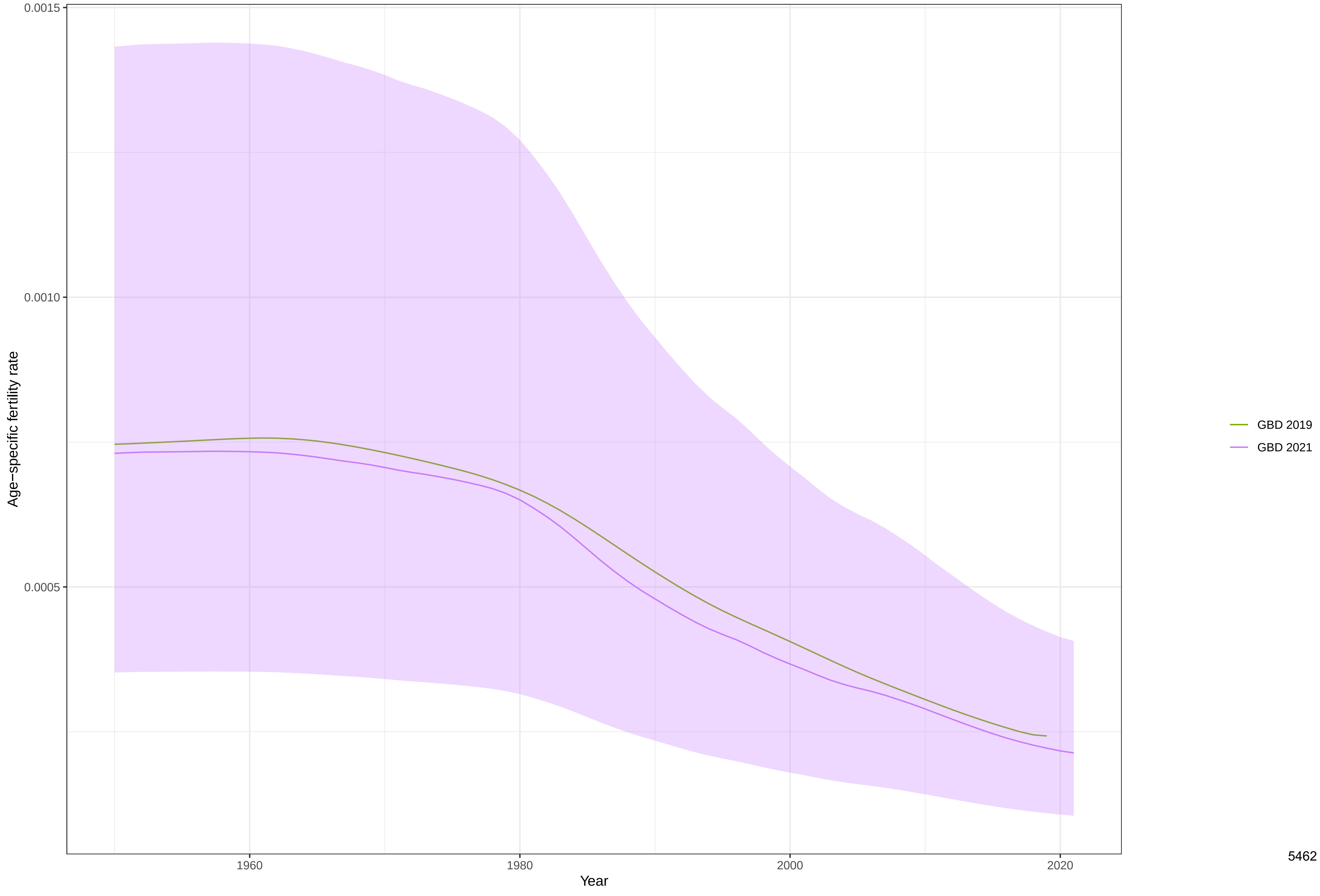

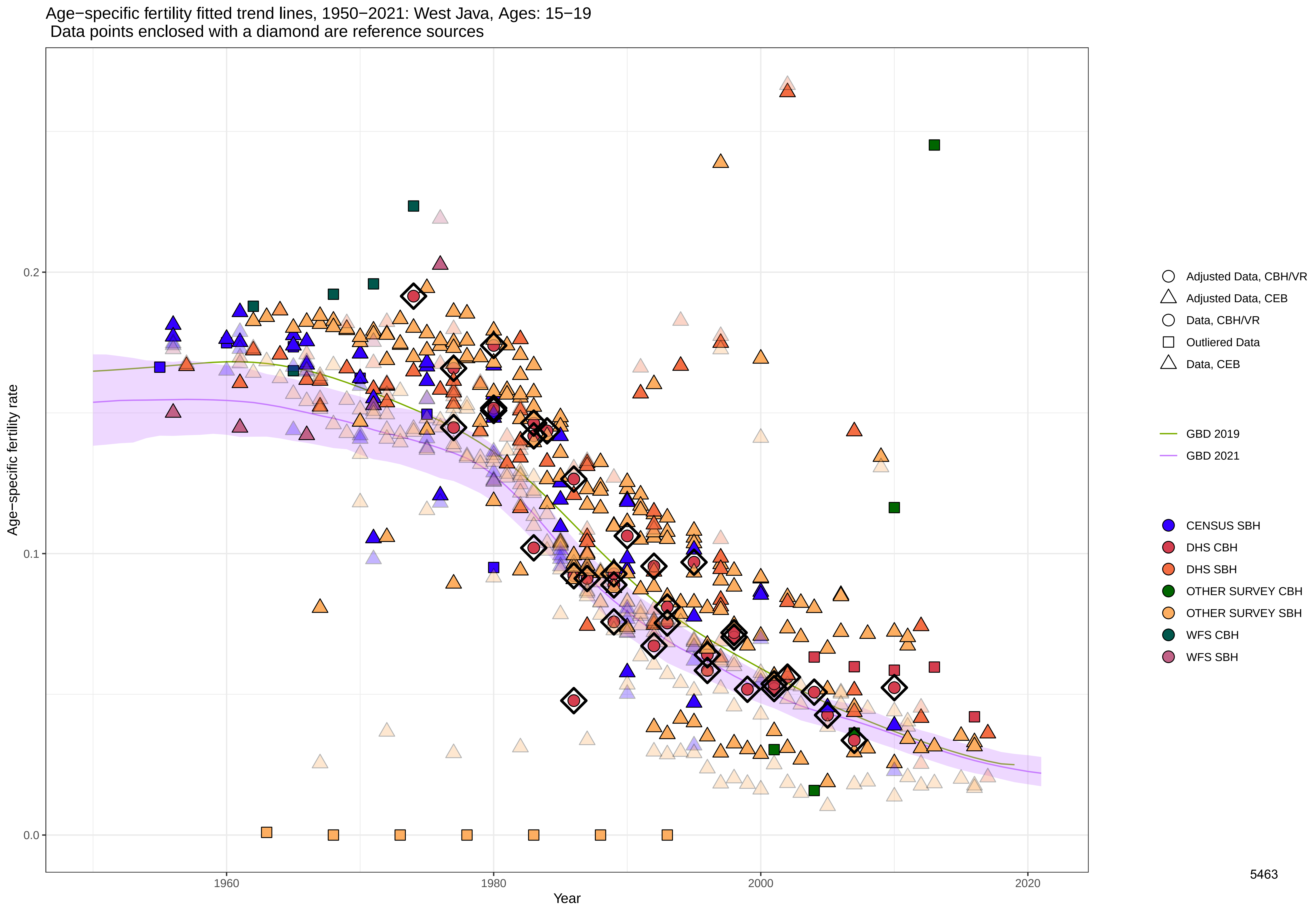

Age−specific fertility fitted trend lines, 1950−2021: West Java, Ages: 15−19
Data points enclosed with a diamond are reference sources
Age−specific fertility rate
0.0
0.1
0.2
Year
1960
1980
2000
2020
Adjusted Data, CBH/VR
Adjusted Data, CEB
Data, CBH/VR
Outliered Data
Data, CEB
GBD 2019
GBD 2021
CENSUS SBH
DHS CBH
DHS SBH
OTHER SURVEY CBH
OTHER SURVEY SBH
WFS CBH
WFS SBH

Age−specific fertility fitted trend lines, 1950−2021: West Java, Ages: 20−24
Data points enclosed with a diamond are reference sources

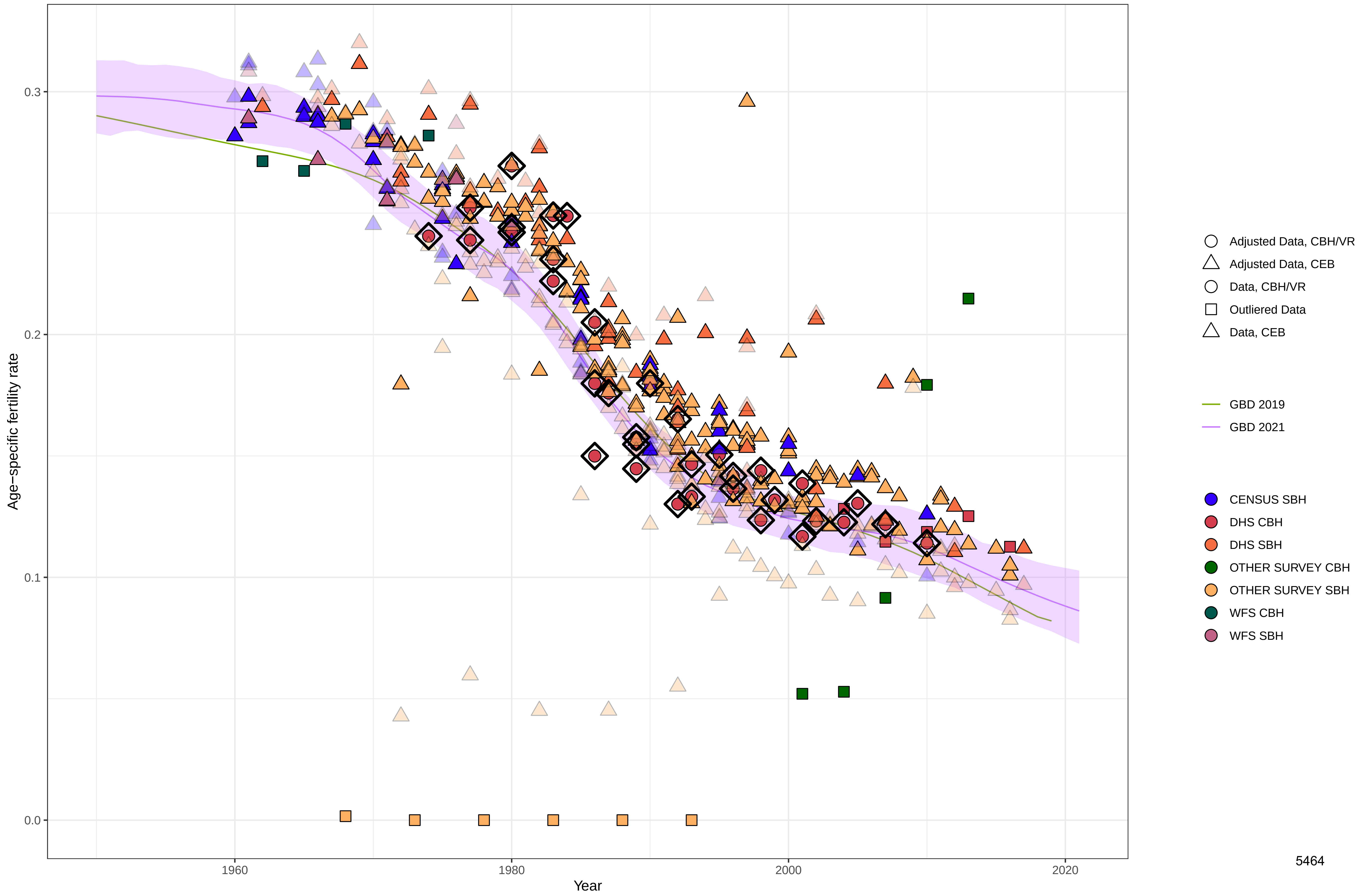

Age−specific fertility fitted trend lines, 1950−2021: West Java, Ages: 25−29
Data points enclosed with a diamond are reference sources

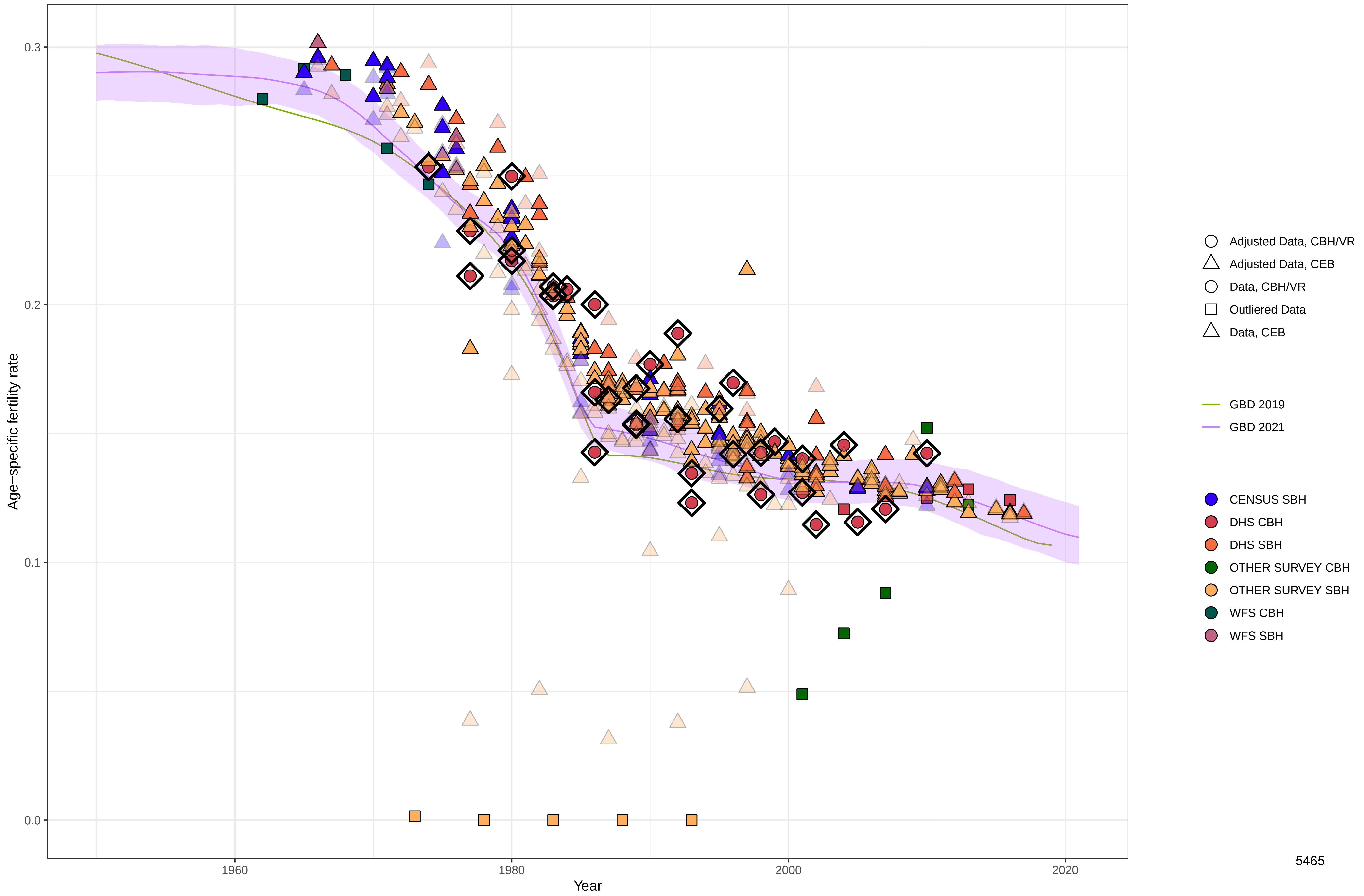

Age−specific fertility fitted trend lines, 1950−2021: West Java, Ages: 30−34
Data points enclosed with a diamond are reference sources

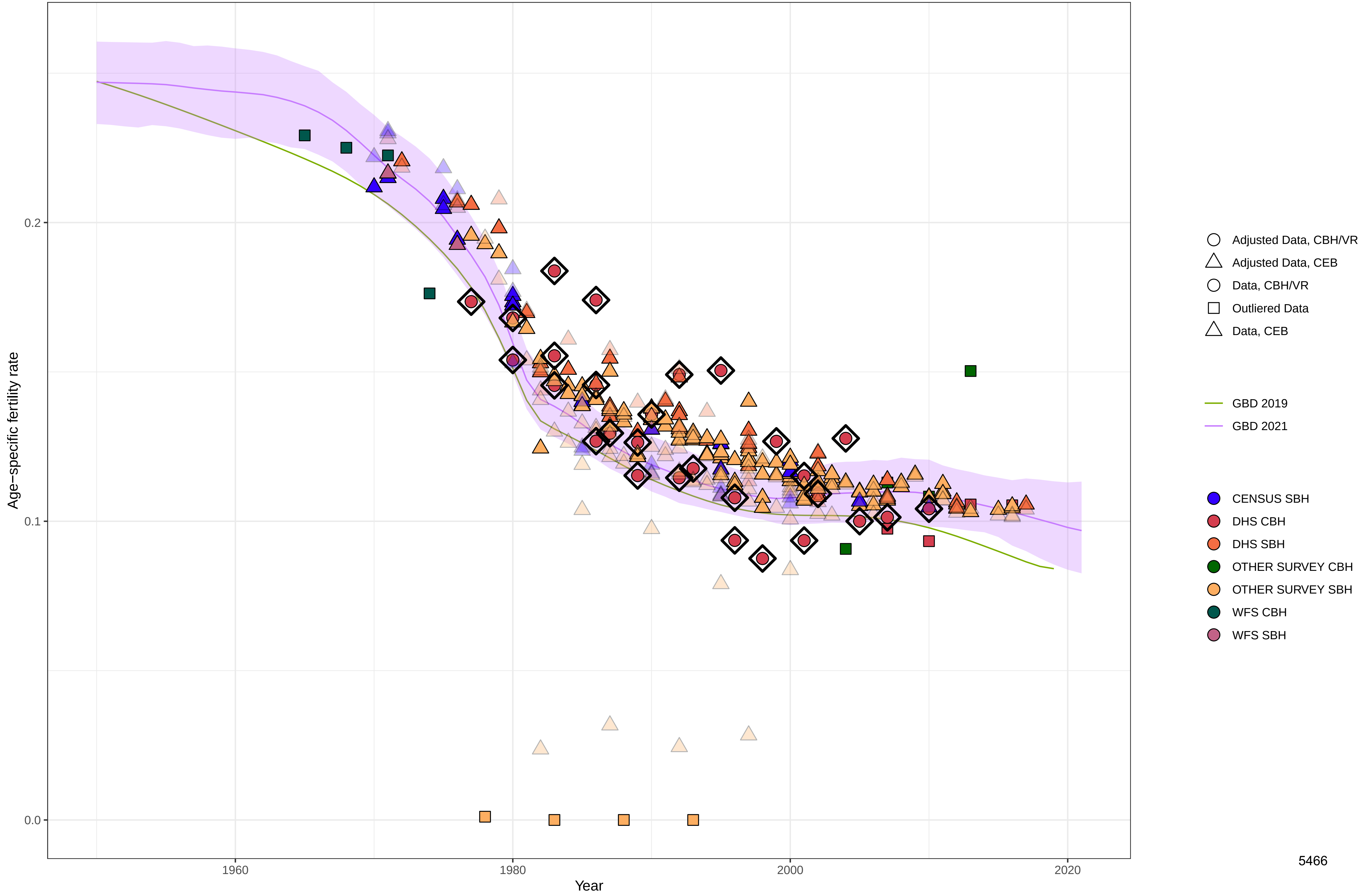

Age−specific fertility fitted trend lines, 1950−2021: West Java, Ages: 35−39
Data points enclosed with a diamond are reference sources

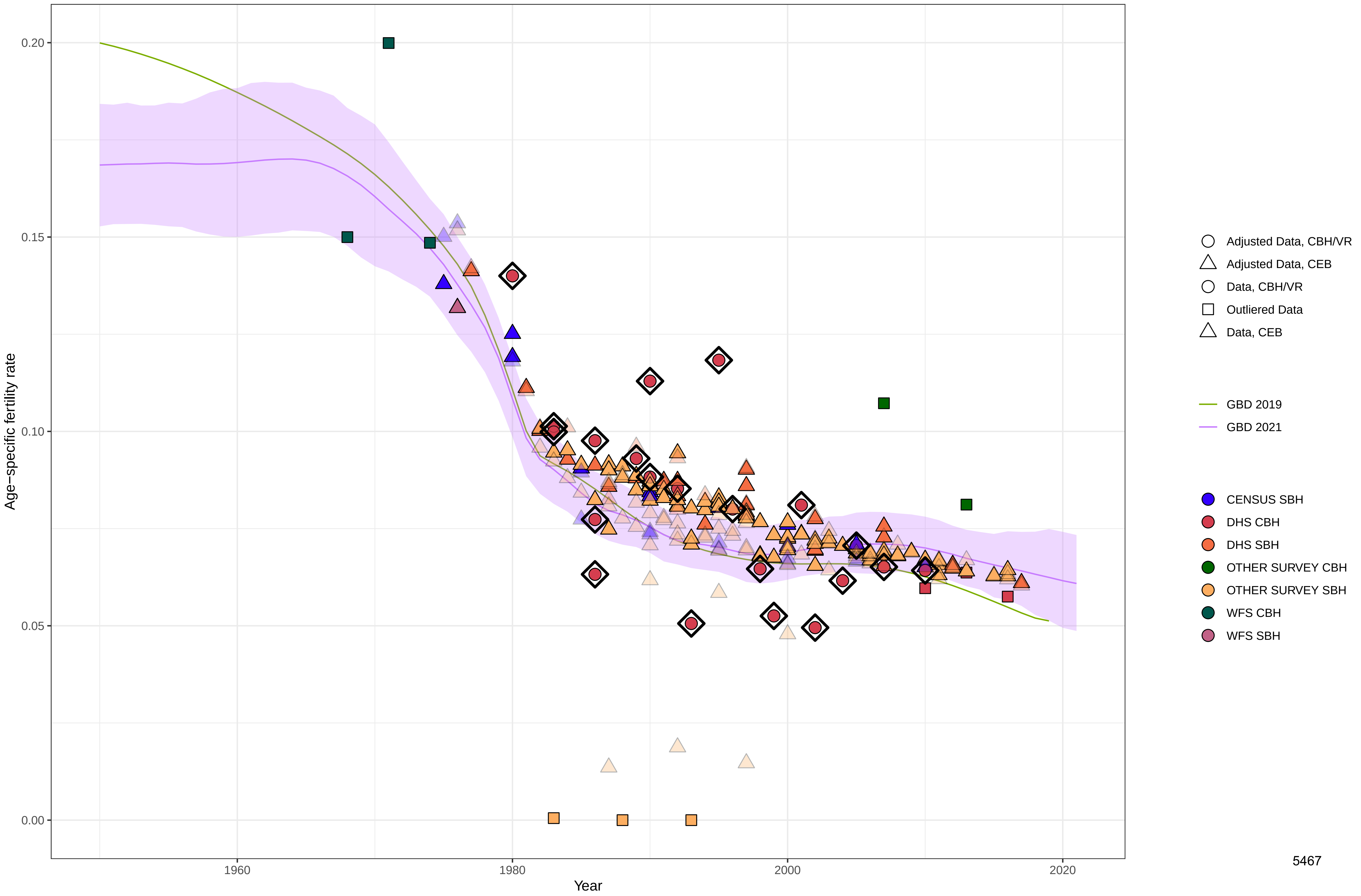

Age−specific fertility fitted trend lines, 1950−2021: West Java, Ages: 40−44
Data points enclosed with a diamond are reference sources

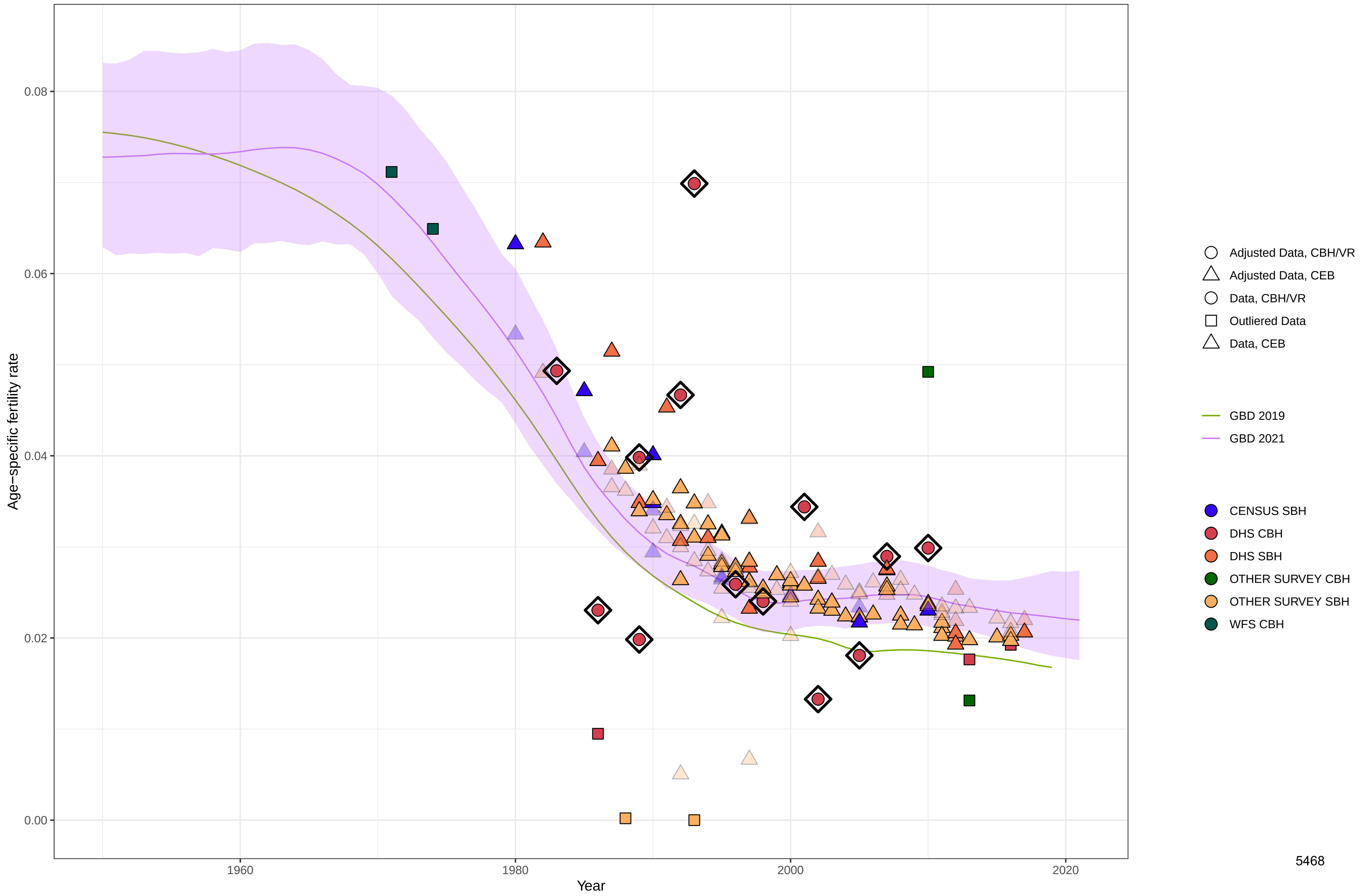

Age−specific fertility fitted trend lines, 1950−2021: West Java, Ages: 45−49
Data points enclosed with a diamond are reference sources

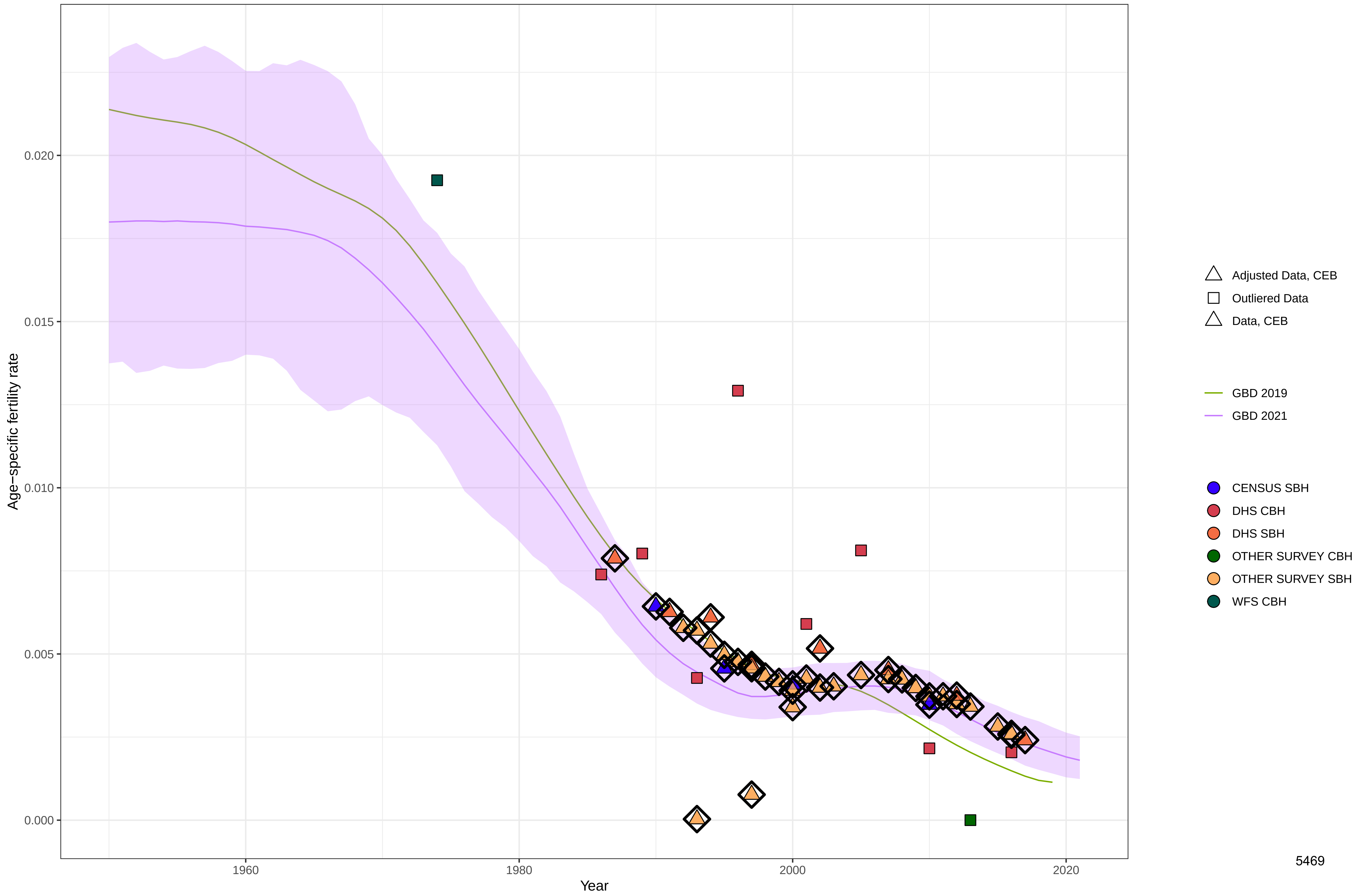

Age−specific fertility fitted trend lines, 1950−2021: West Java, Ages: 50−54
Data points enclosed with a diamond are reference sources

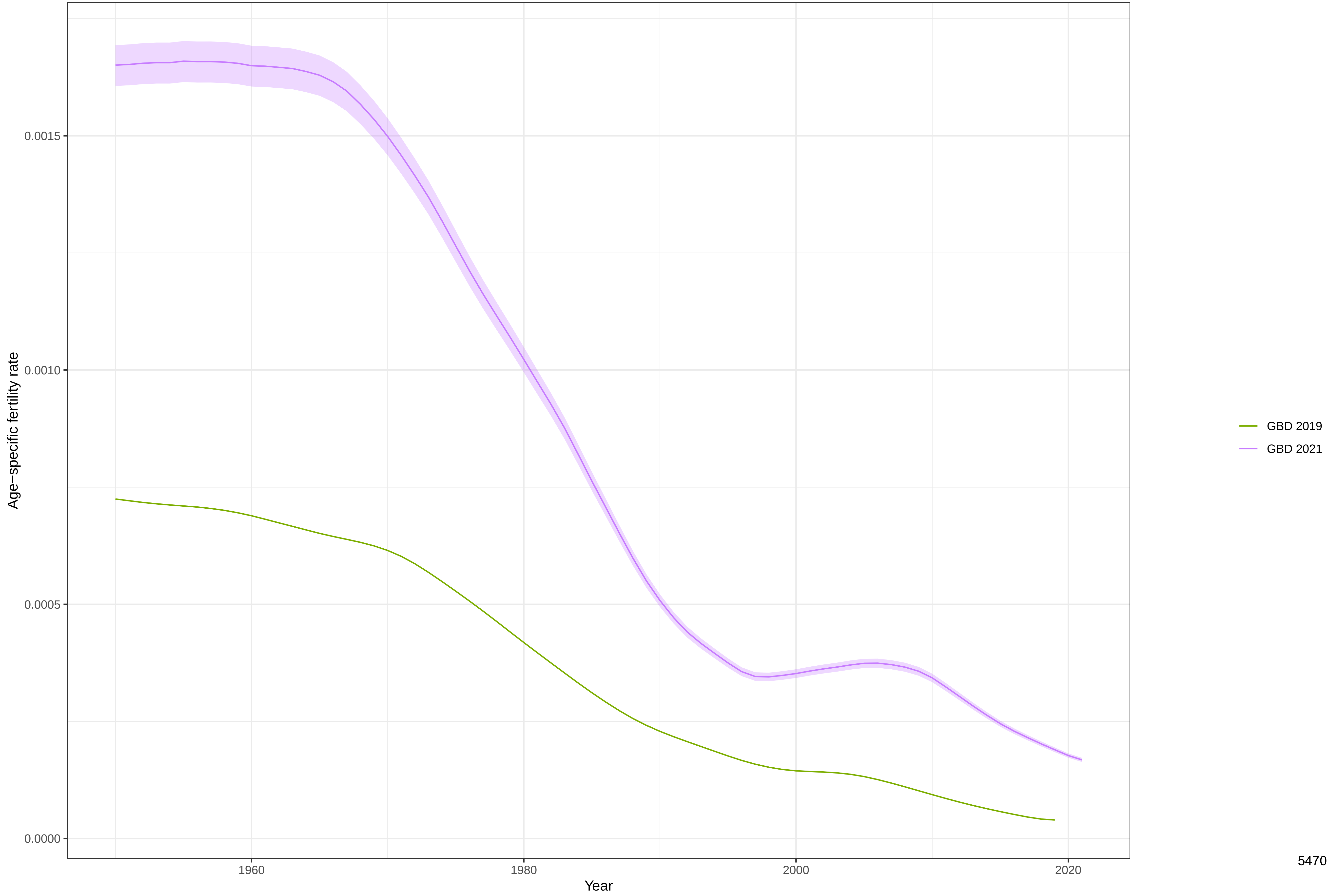

Total fertility rate fitted trend lines, 1950−2021: West Java TFR
Data points enclosed with a diamond are reference sources

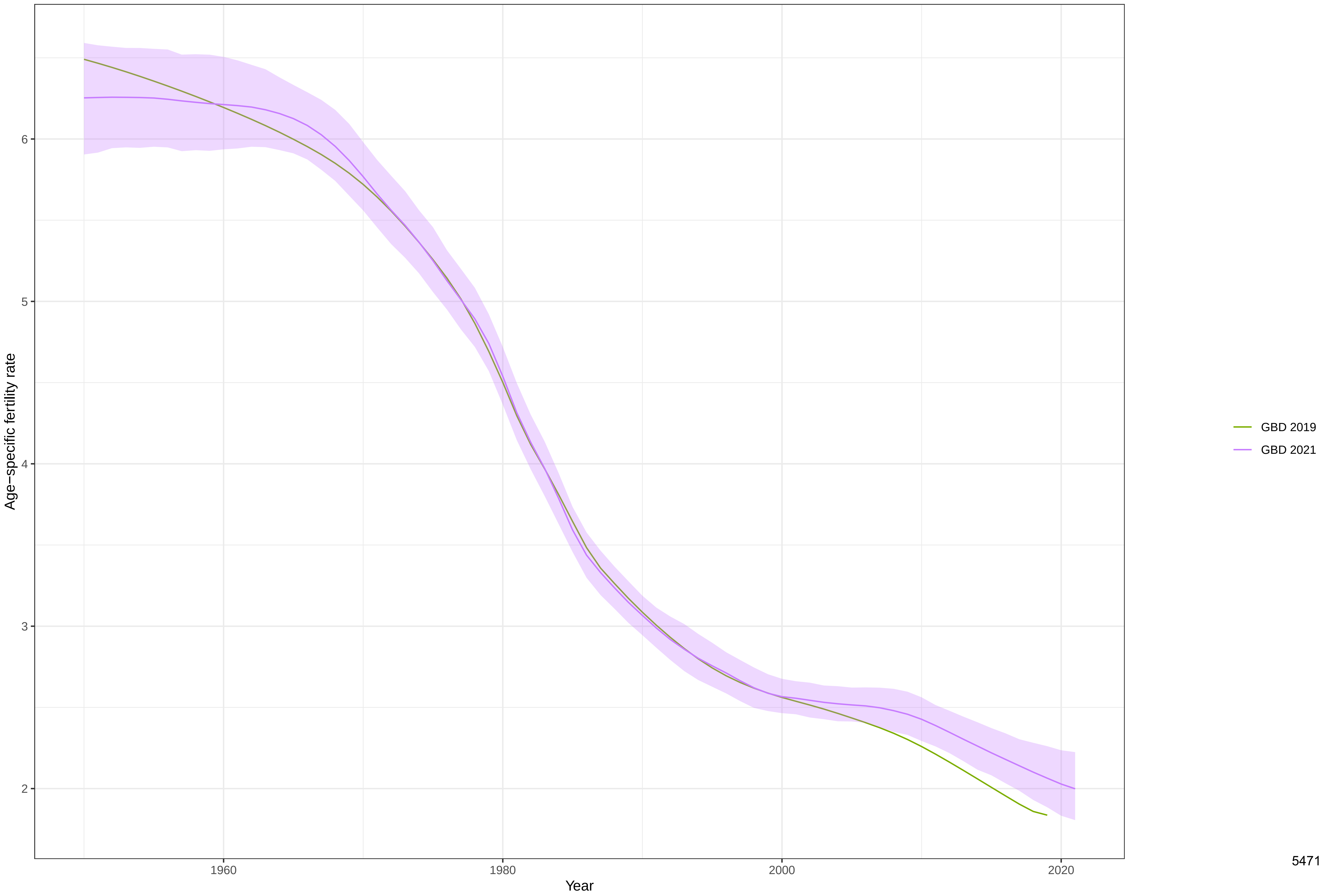

Age−specific fertility fitted trend lines, 1950−2021: Central Java, Ages: 10−14
Data points enclosed with a diamond are reference sources

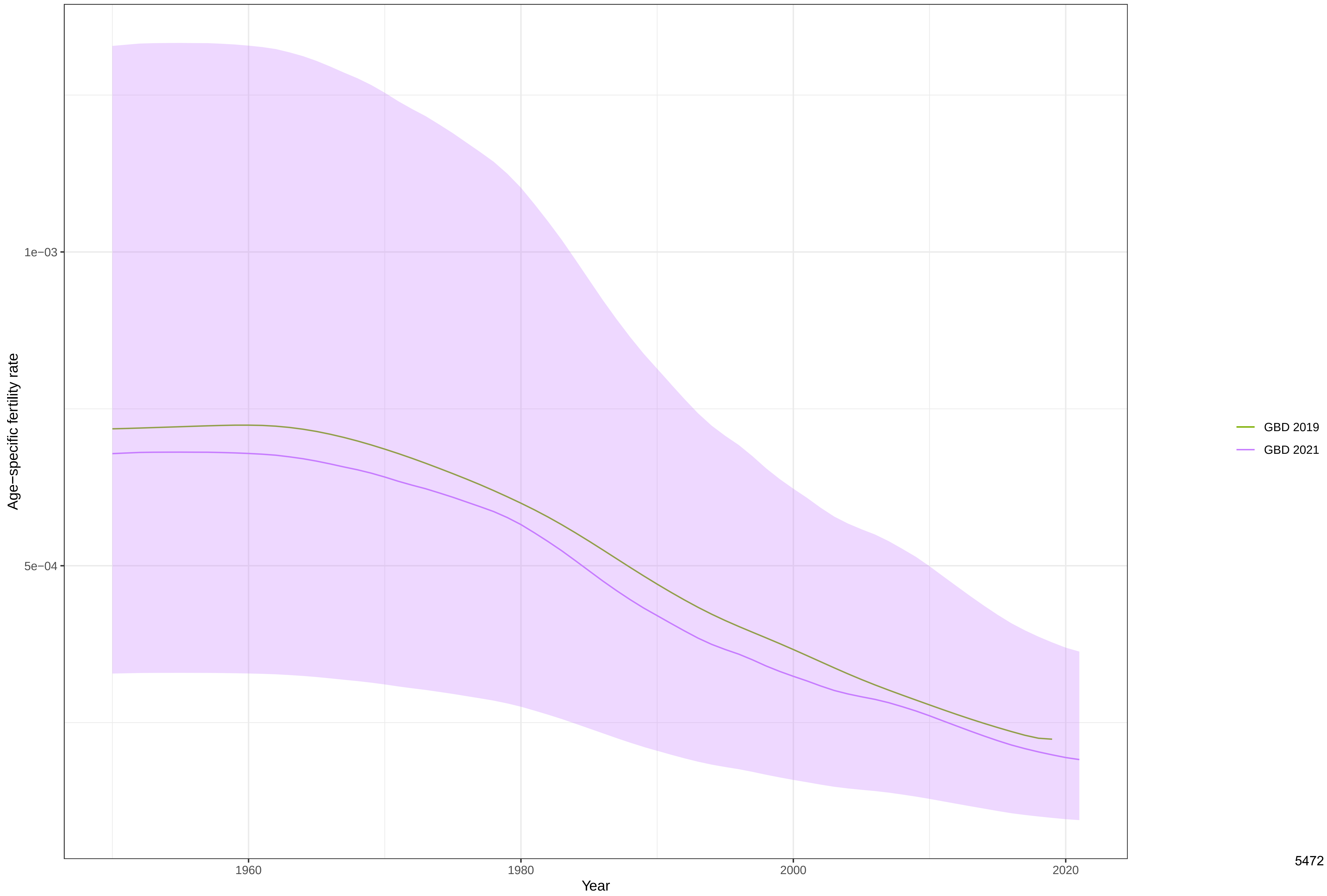

Age−specific fertility fitted trend lines, 1950−2021: Central Java, Ages: 15−19
Data points enclosed with a diamond are reference sources

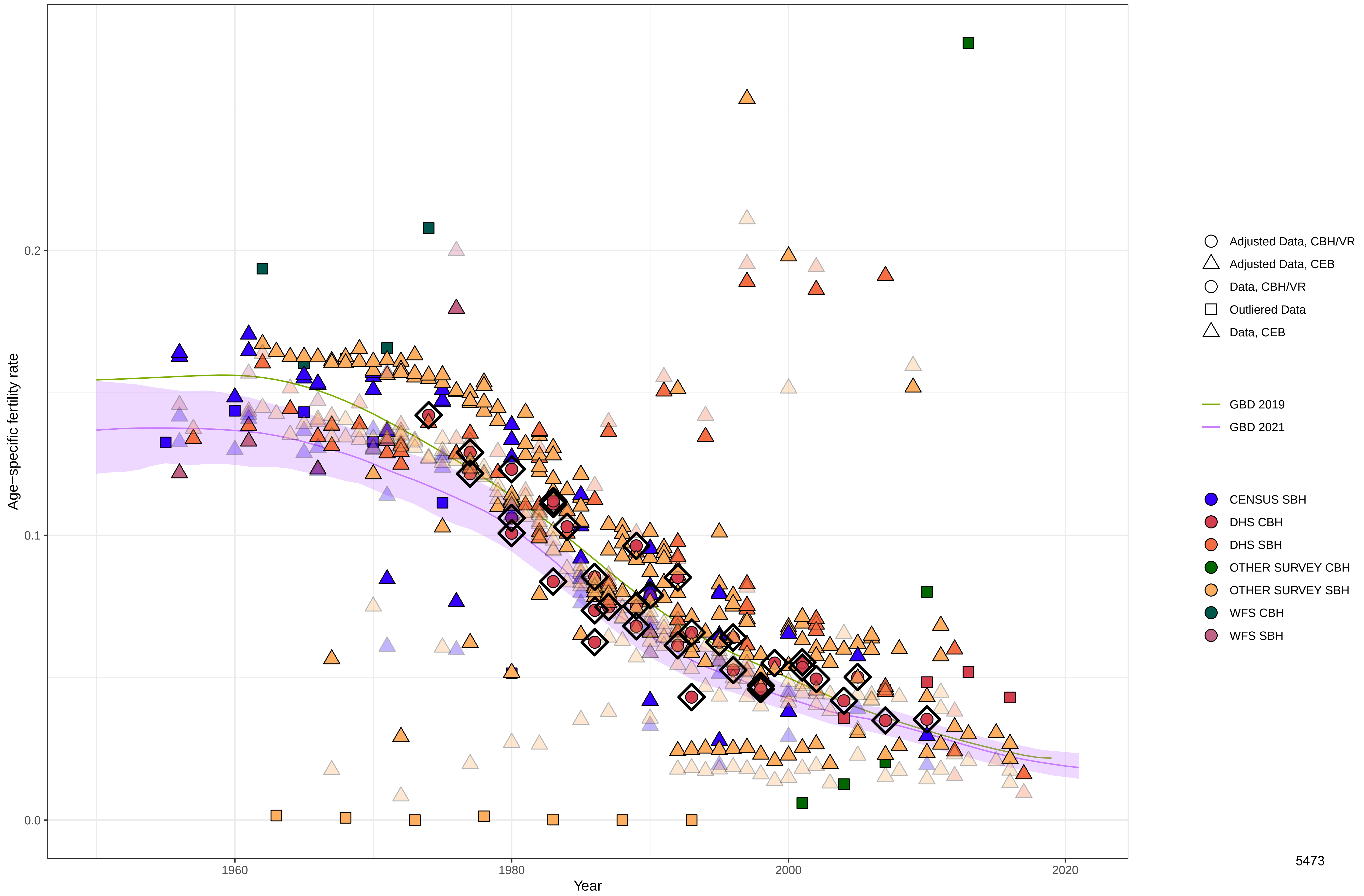

Age−specific fertility fitted trend lines, 1950−2021: Central Java, Ages: 20−24
Data points enclosed with a diamond are reference sources

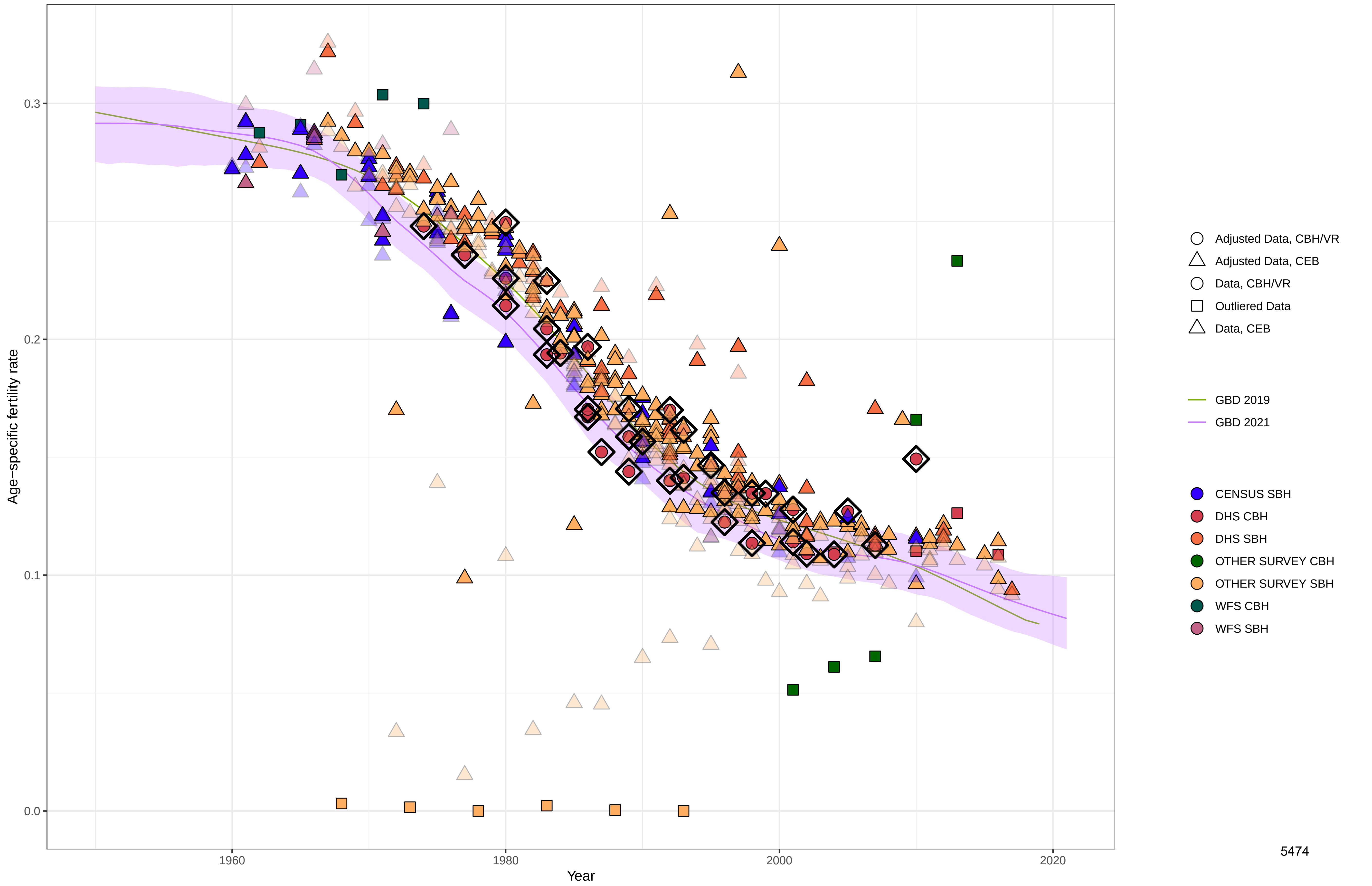

Age−specific fertility fitted trend lines, 1950−2021: Central Java, Ages: 25−29
Data points enclosed with a diamond are reference sources

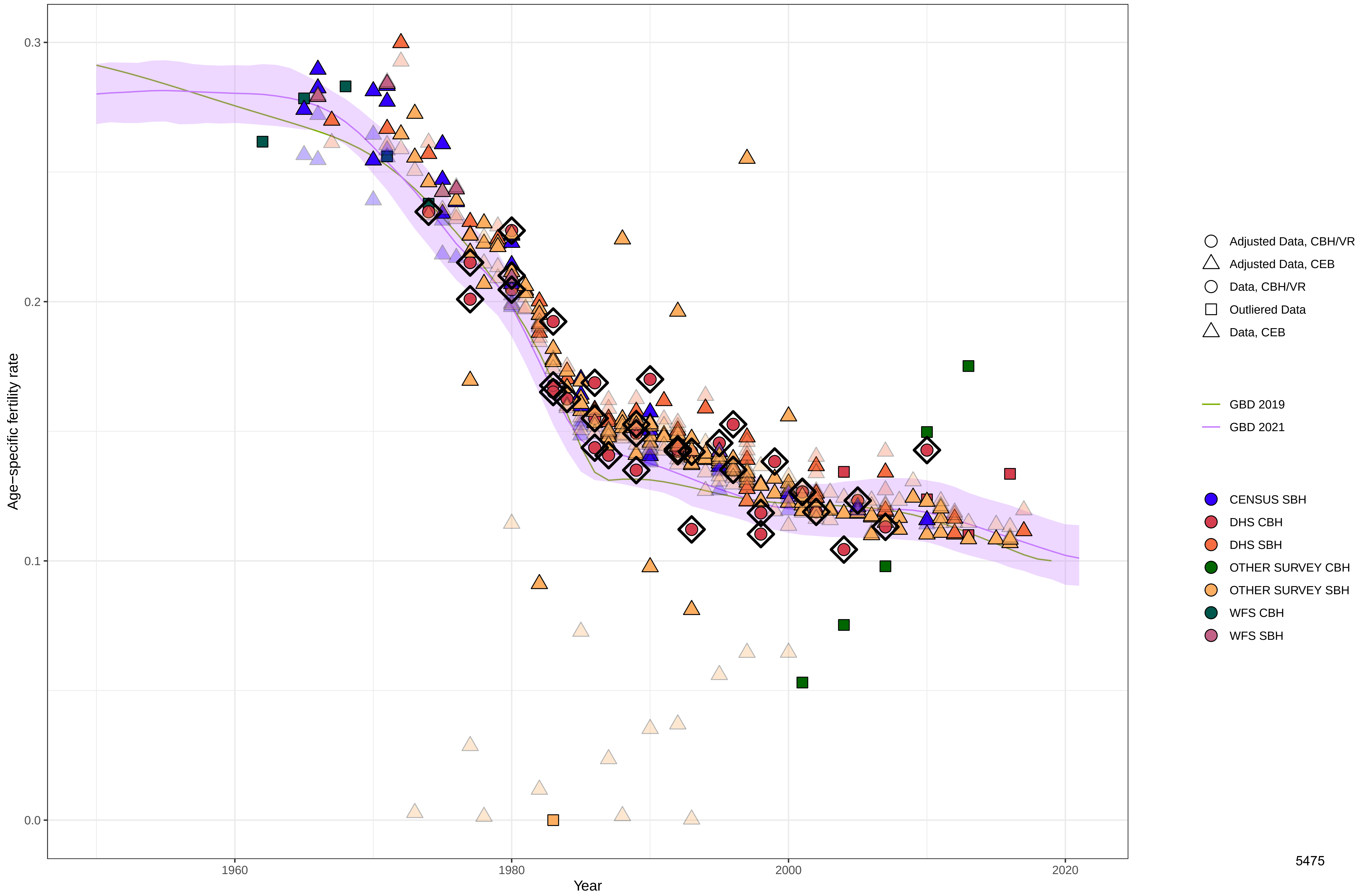

Age−specific fertility fitted trend lines, 1950−2021: Central Java, Ages: 30−34
Data points enclosed with a diamond are reference sources

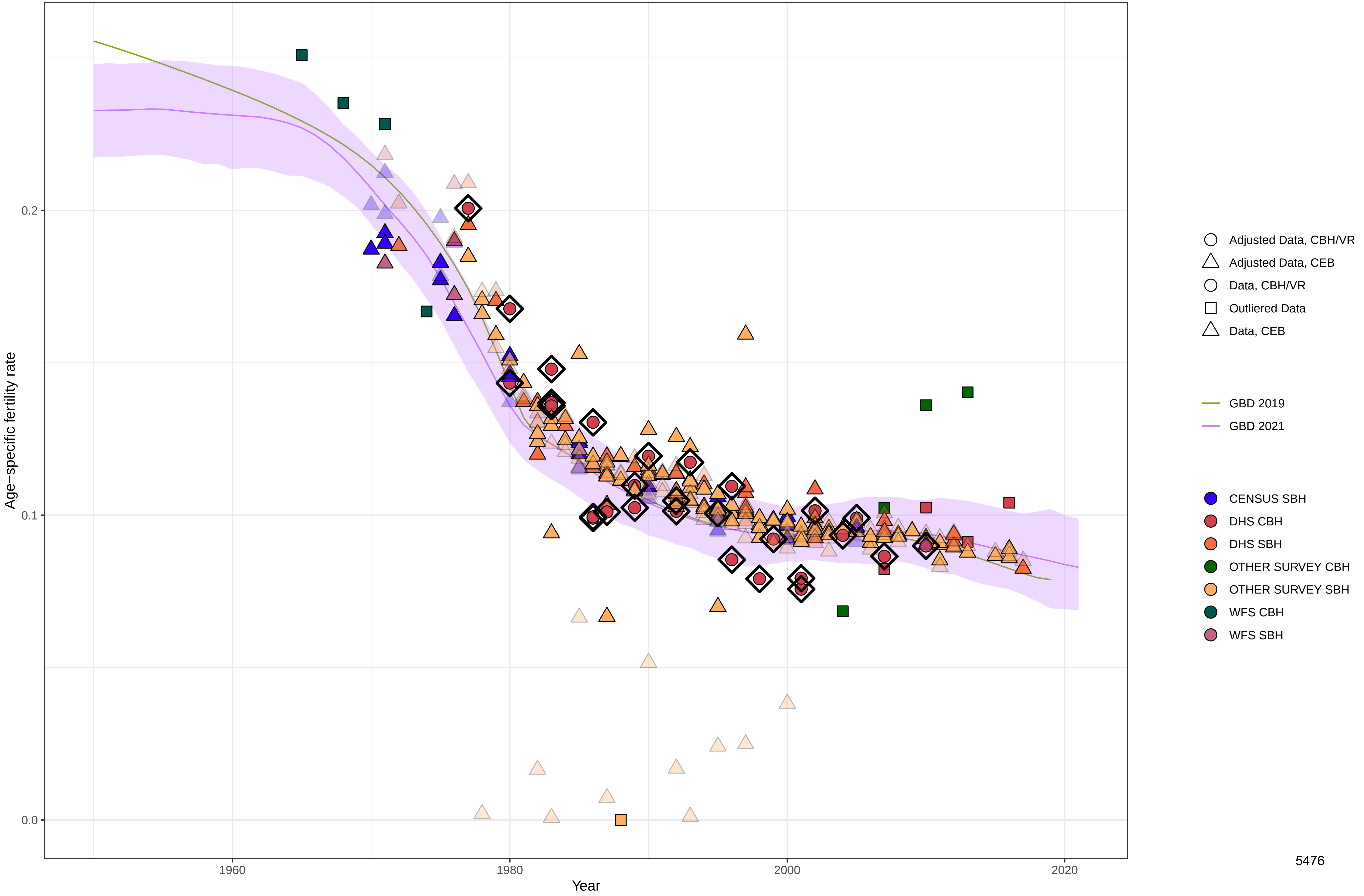

Age−specific fertility fitted trend lines, 1950−2021: Central Java, Ages: 35−39
Data points enclosed with a diamond are reference sources

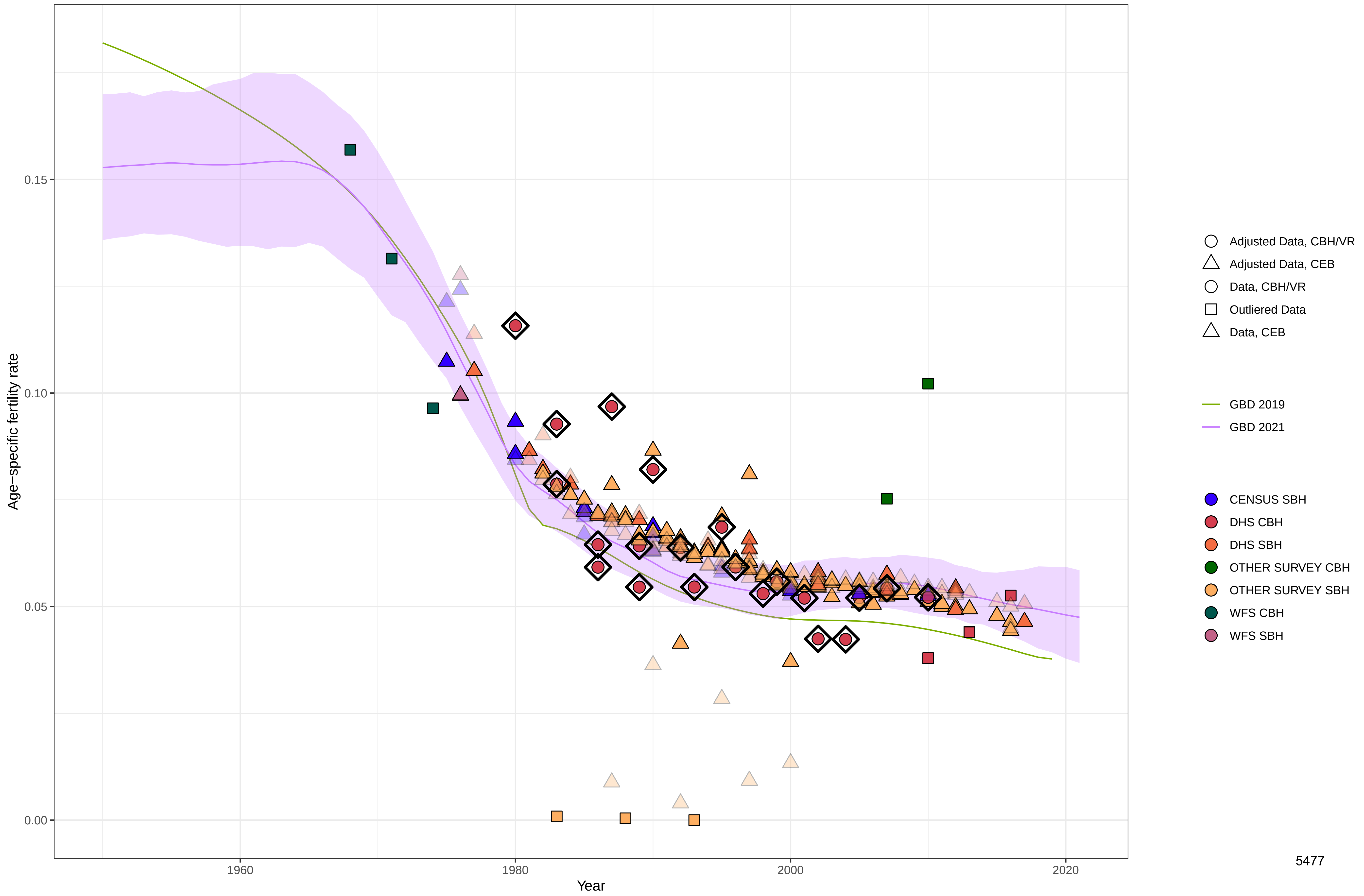

Age−specific fertility fitted trend lines, 1950−2021: Central Java, Ages: 40−44
Data points enclosed with a diamond are reference sources

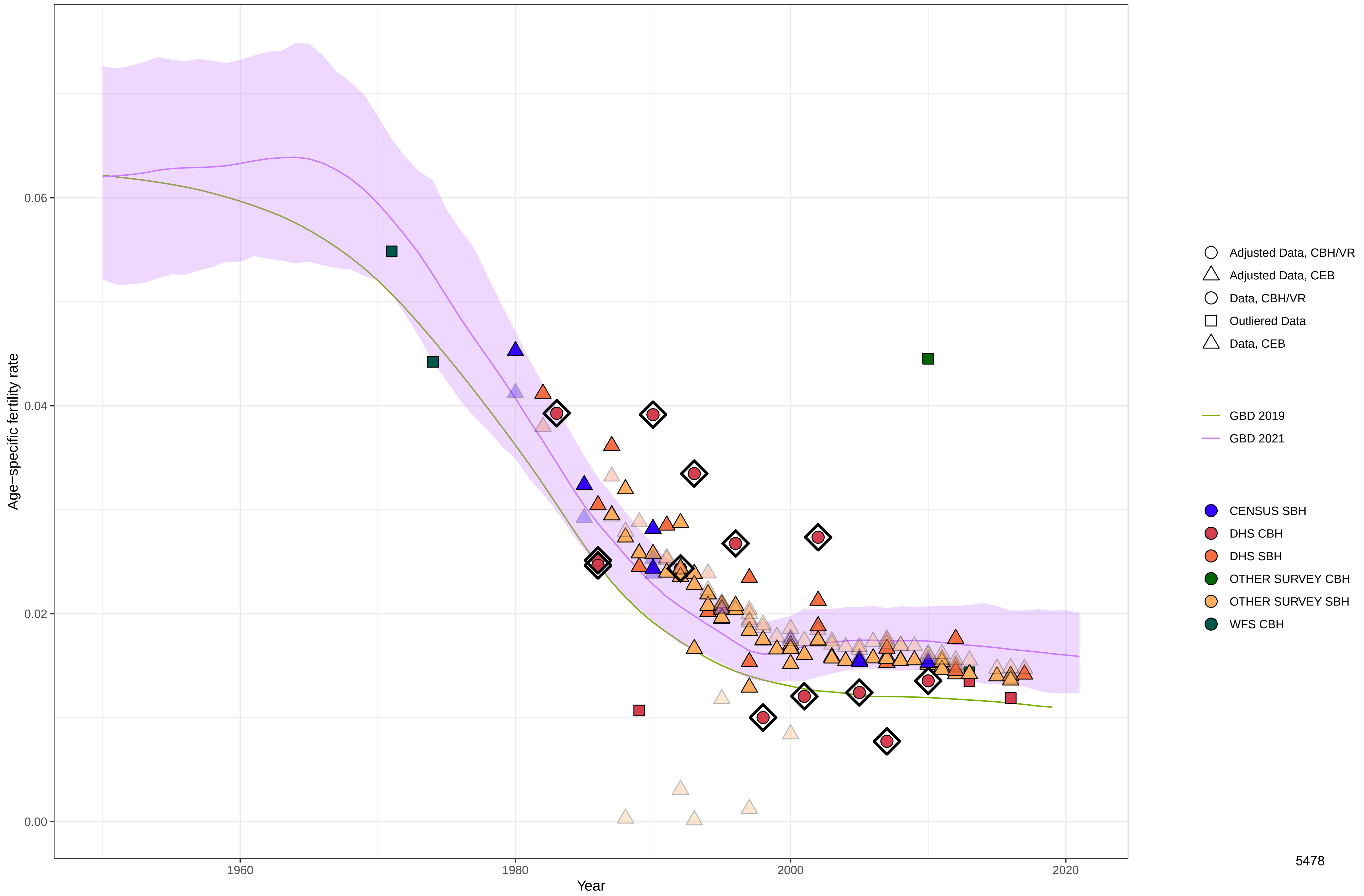

Age−specific fertility fitted trend lines, 1950−2021: Central Java, Ages: 45−49
Data points enclosed with a diamond are reference sources

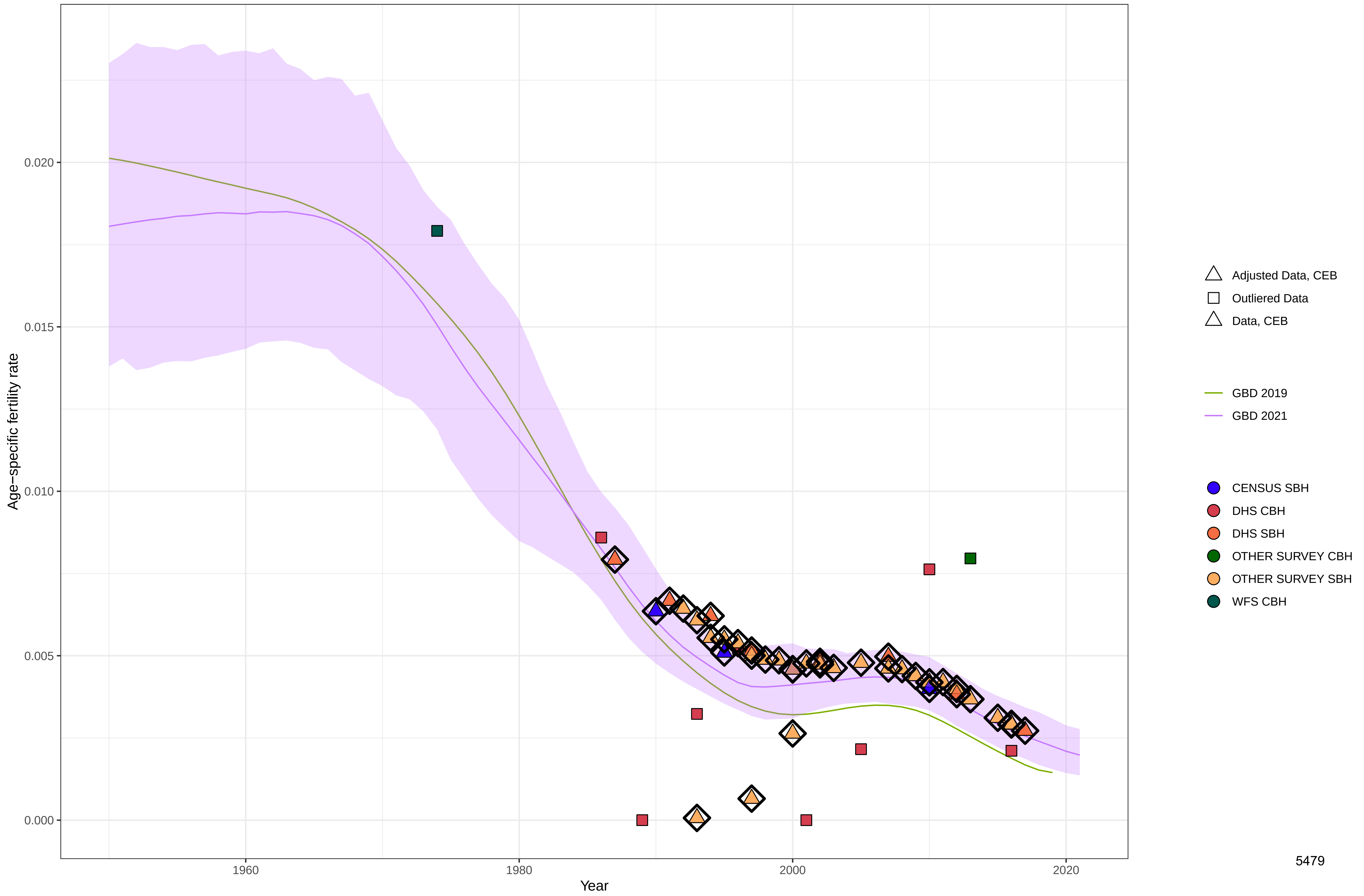

Age−specific fertility fitted trend lines, 1950−2021: Central Java, Ages: 50−54
Data points enclosed with a diamond are reference sources

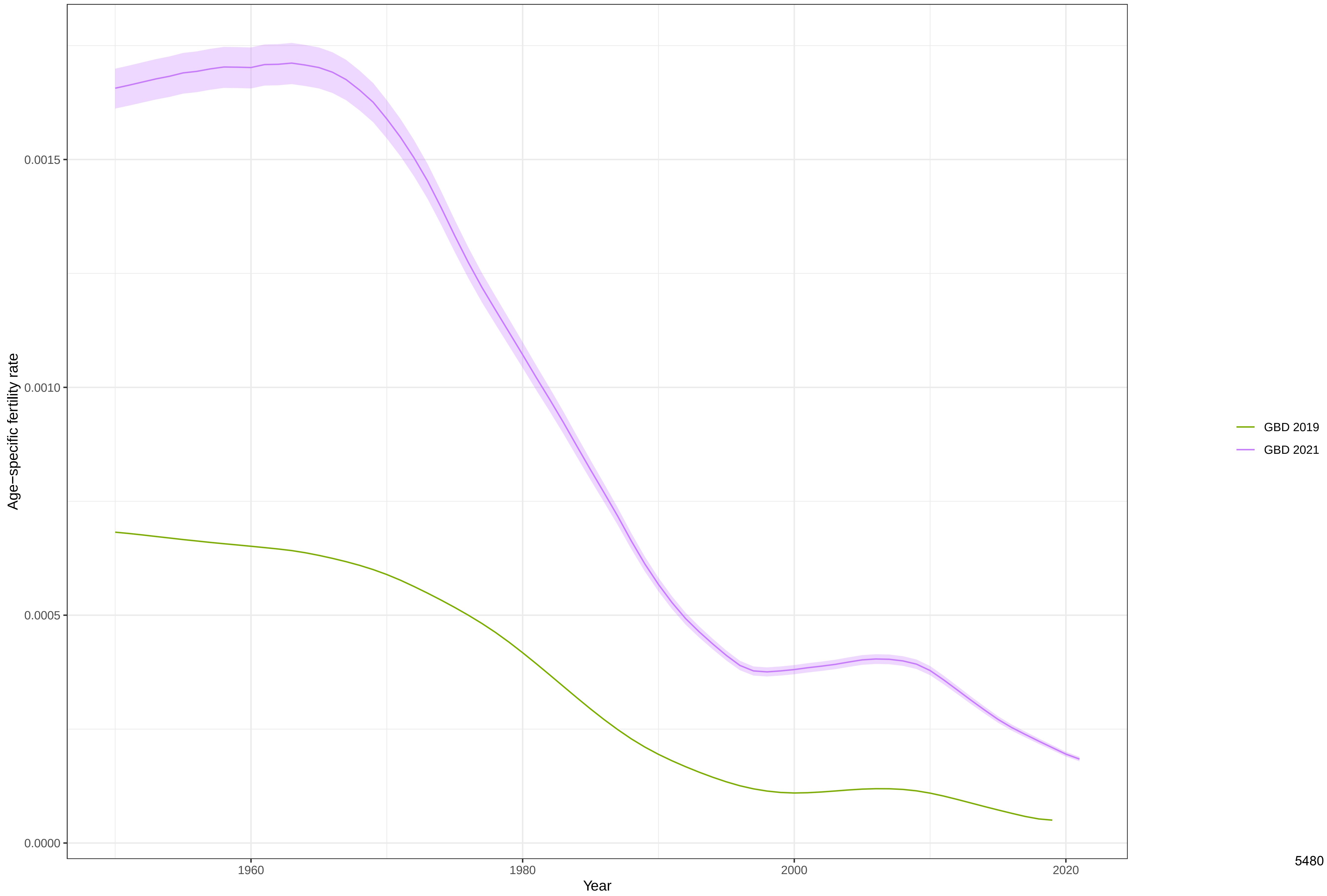

Total fertility rate fitted trend lines, 1950−2021: Central Java TFR
Data points enclosed with a diamond are reference sources

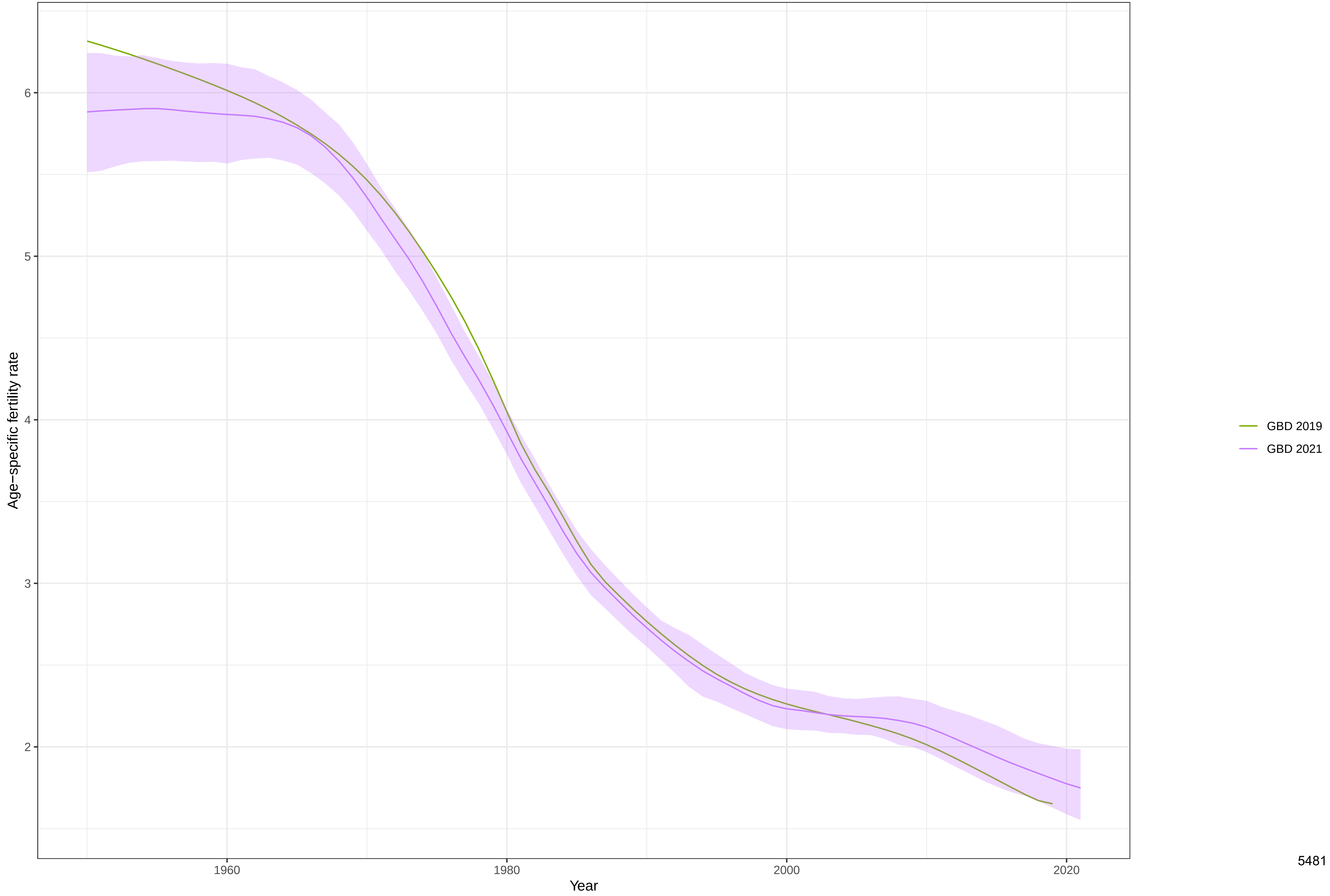

Age−specific fertility fitted trend lines, 1950−2021: East Java, Ages: 10−14
Data points enclosed with a diamond are reference sources

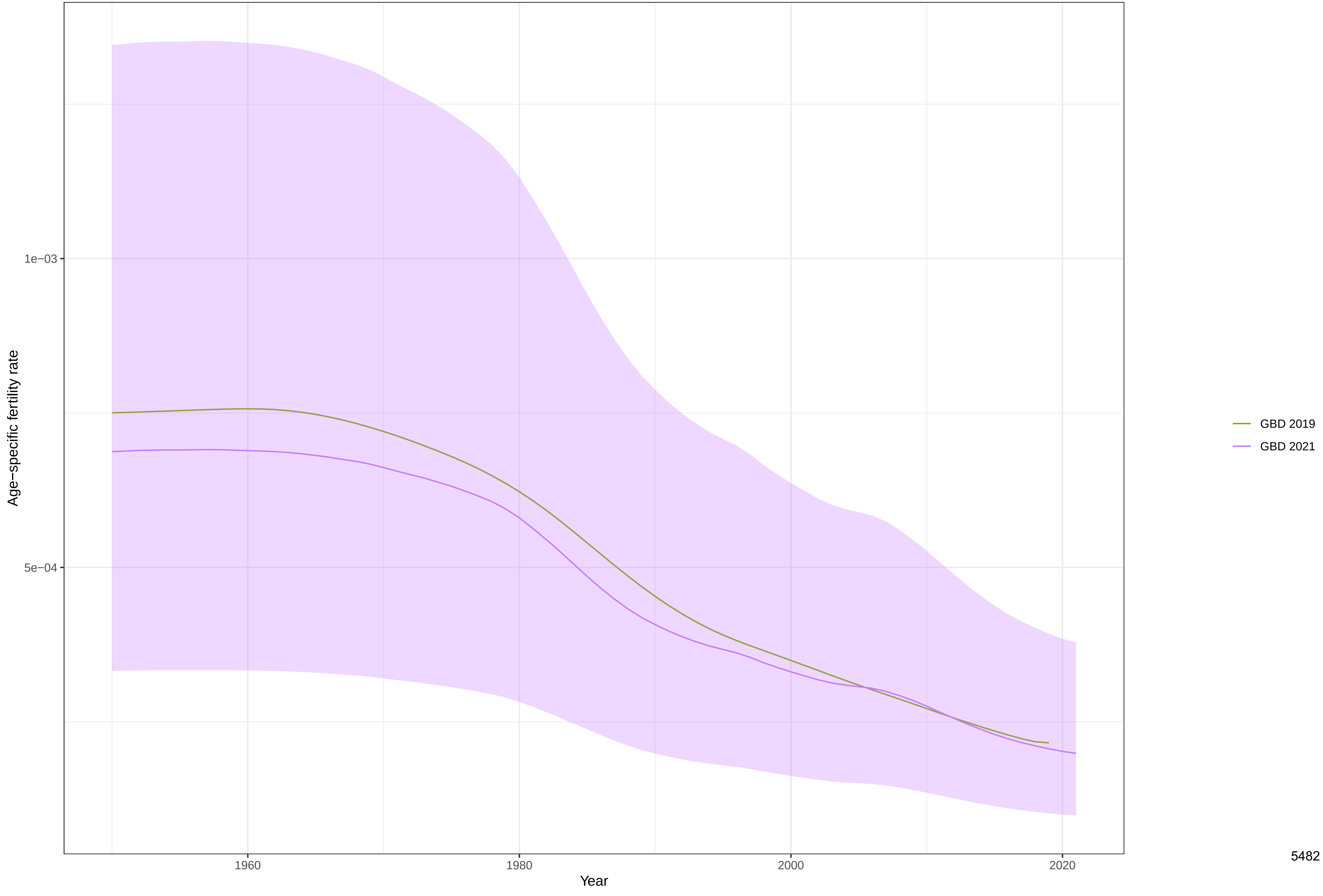

Age−specific fertility fitted trend lines, 1950−2021: East Java, Ages: 15−19
Data points enclosed with a diamond are reference sources

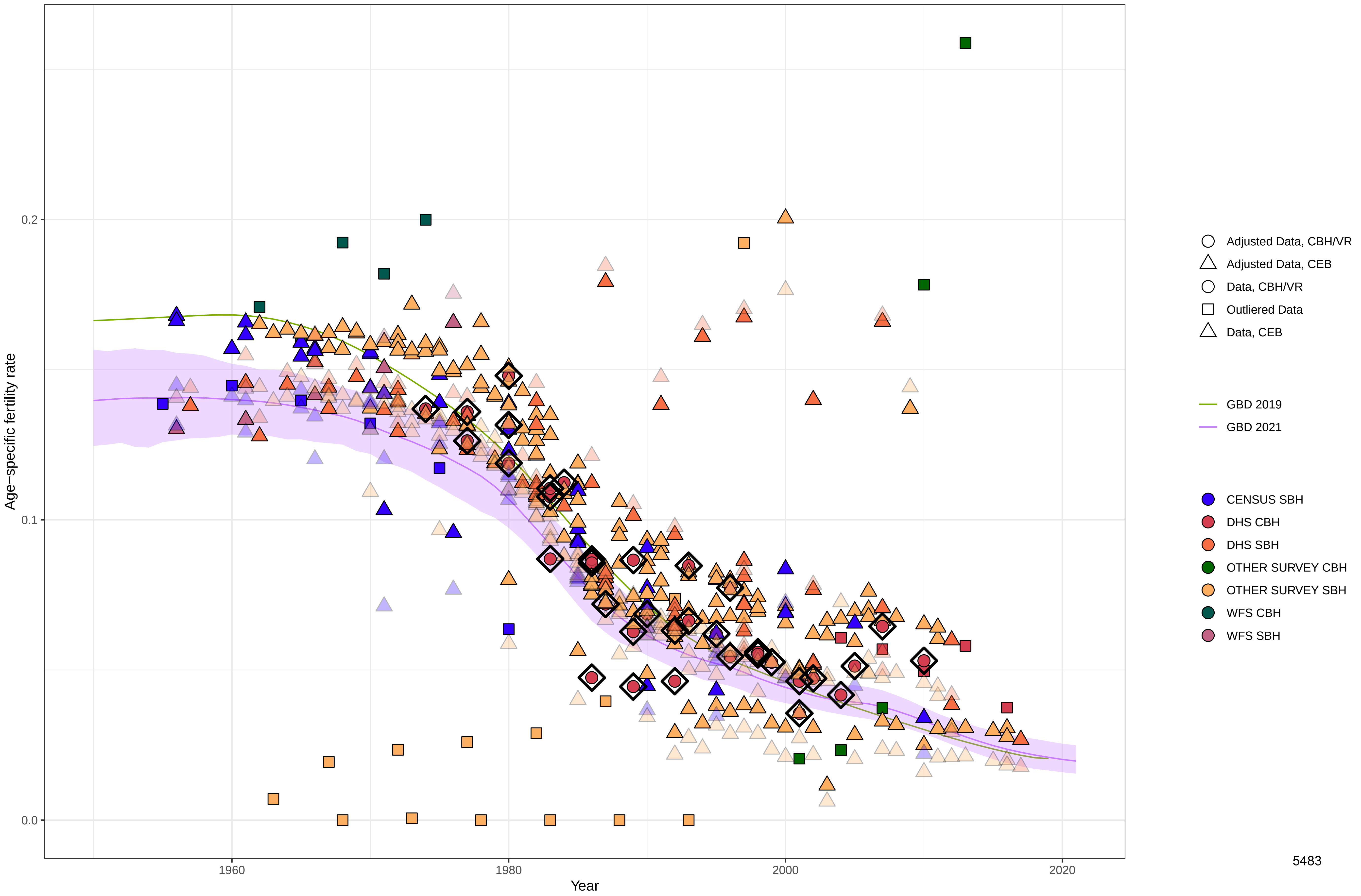

Age−specific fertility fitted trend lines, 1950−2021: East Java, Ages: 20−24
Data points enclosed with a diamond are reference sources

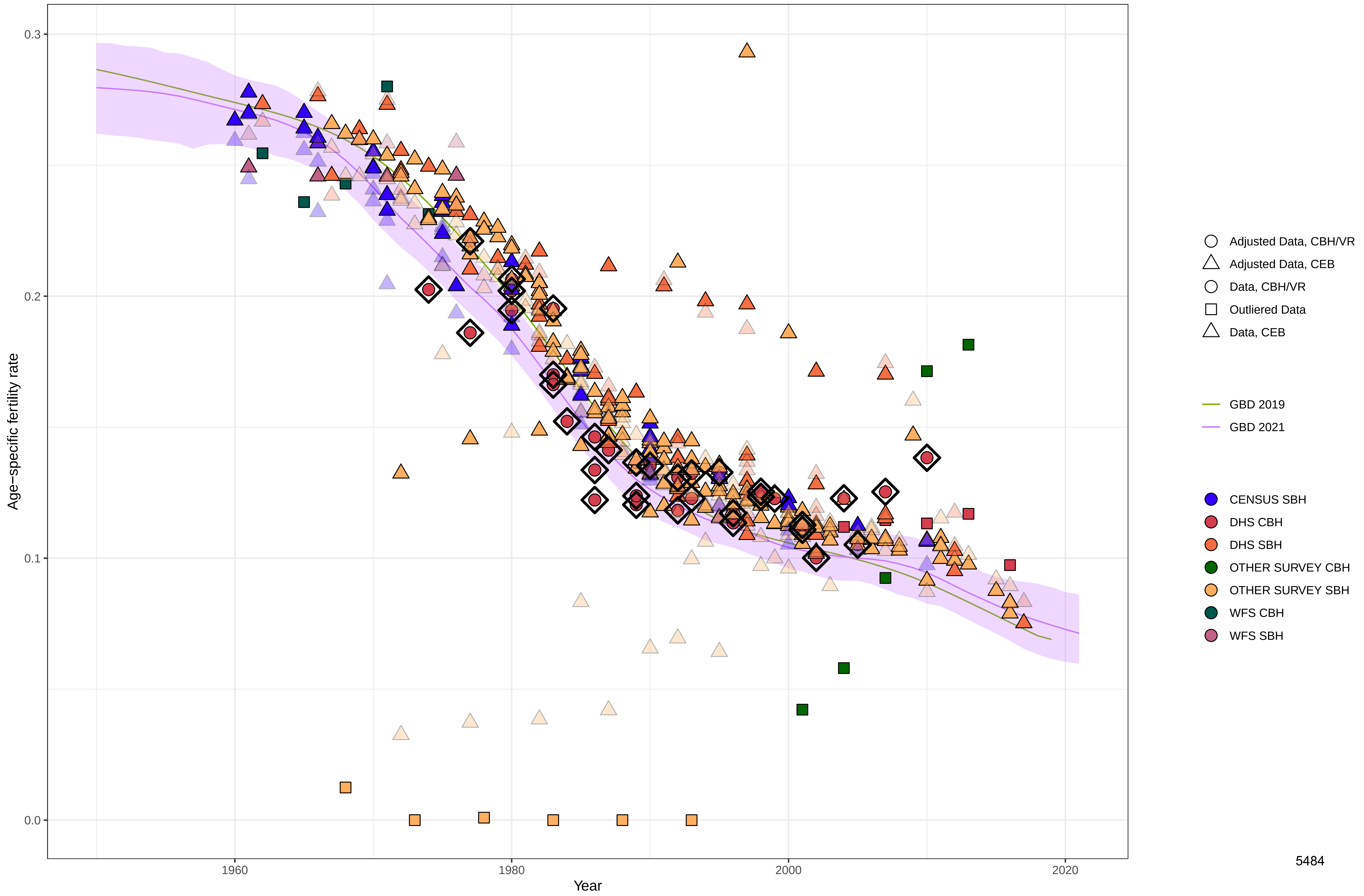

Age−specific fertility fitted trend lines, 1950−2021: East Java, Ages: 25−29
Data points enclosed with a diamond are reference sources

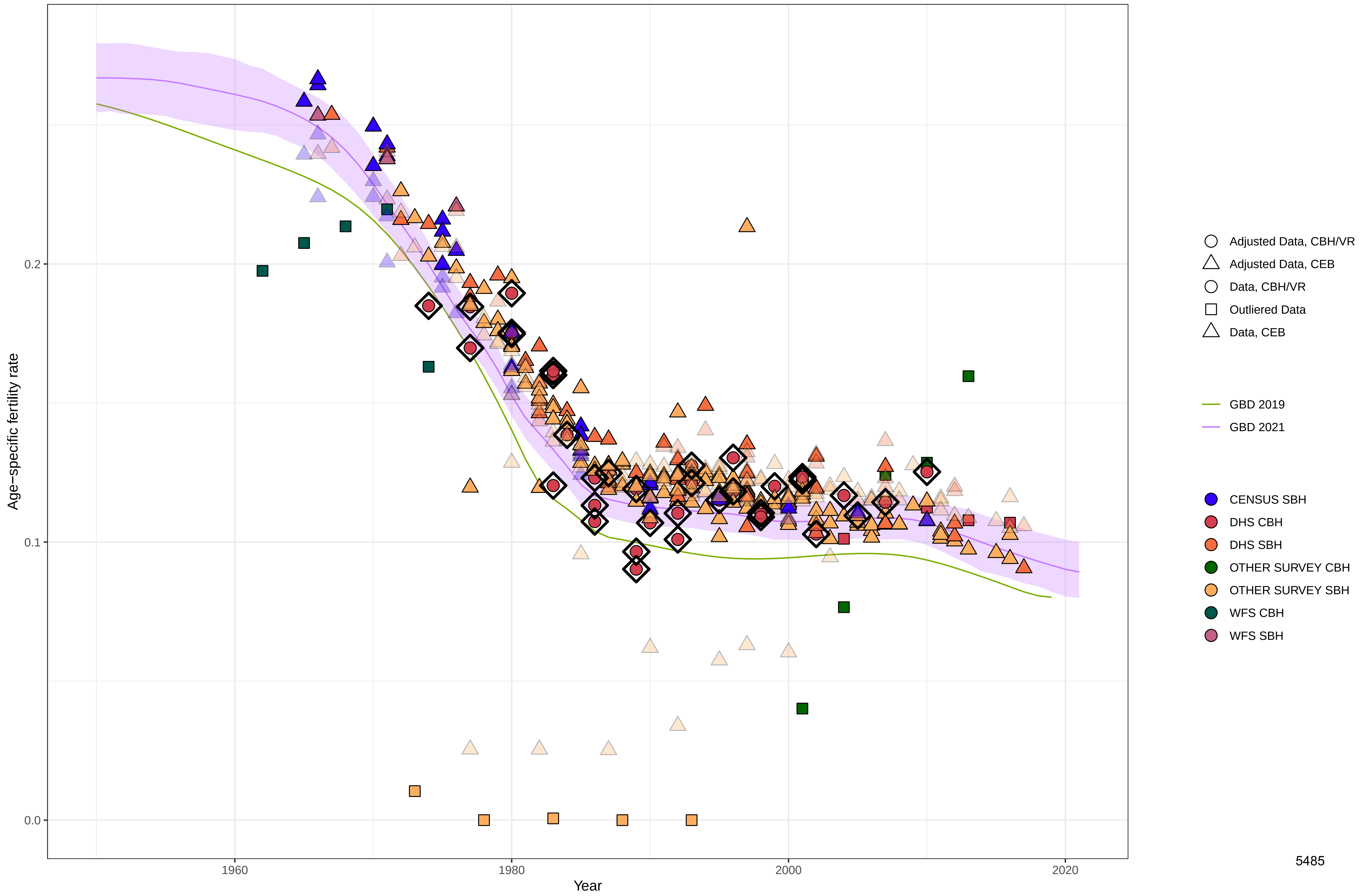

Age−specific fertility fitted trend lines, 1950−2021: East Java, Ages: 30−34
Data points enclosed with a diamond are reference sources

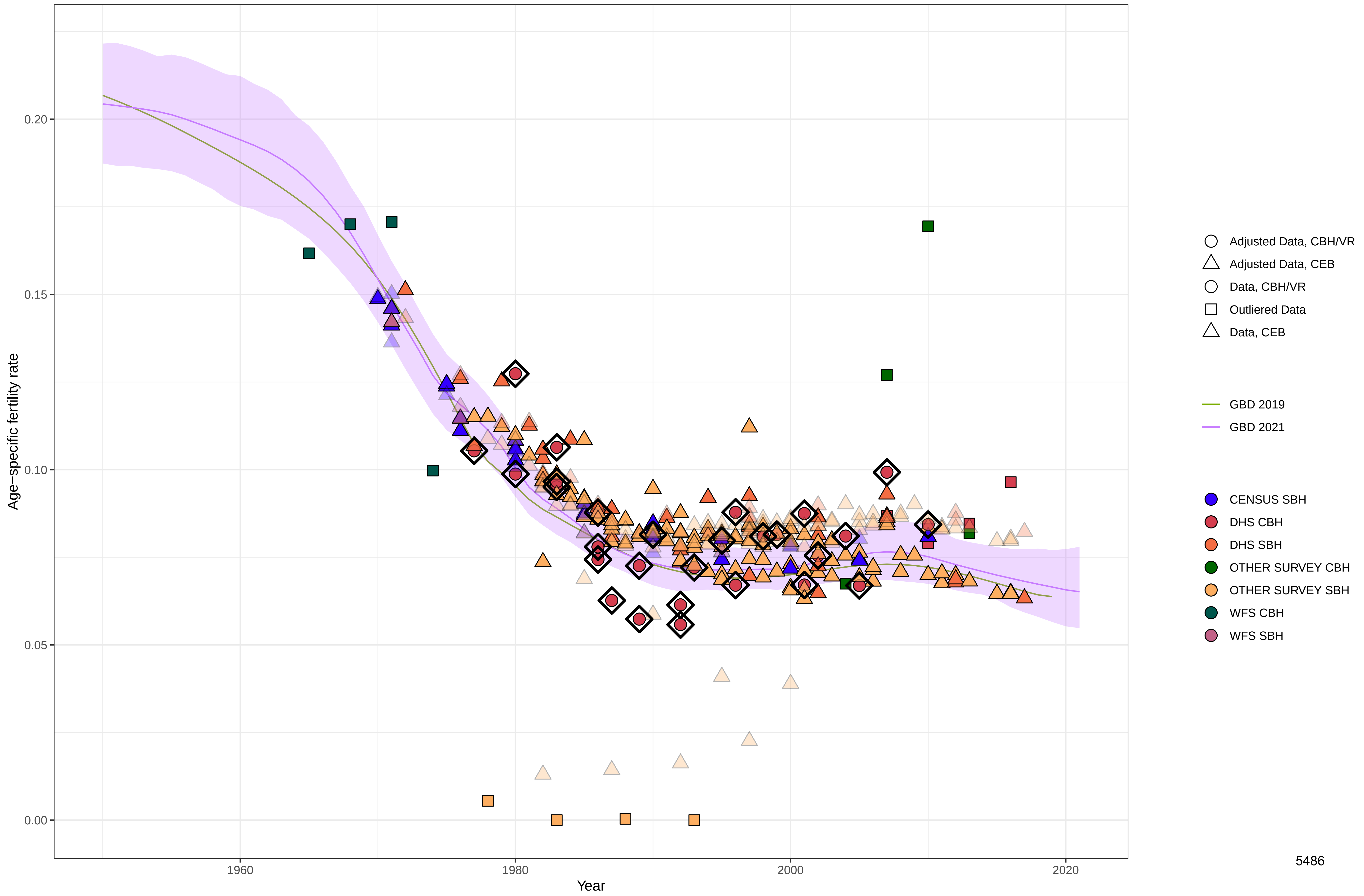

Age−specific fertility fitted trend lines, 1950−2021: East Java, Ages: 35−39
Data points enclosed with a diamond are reference sources

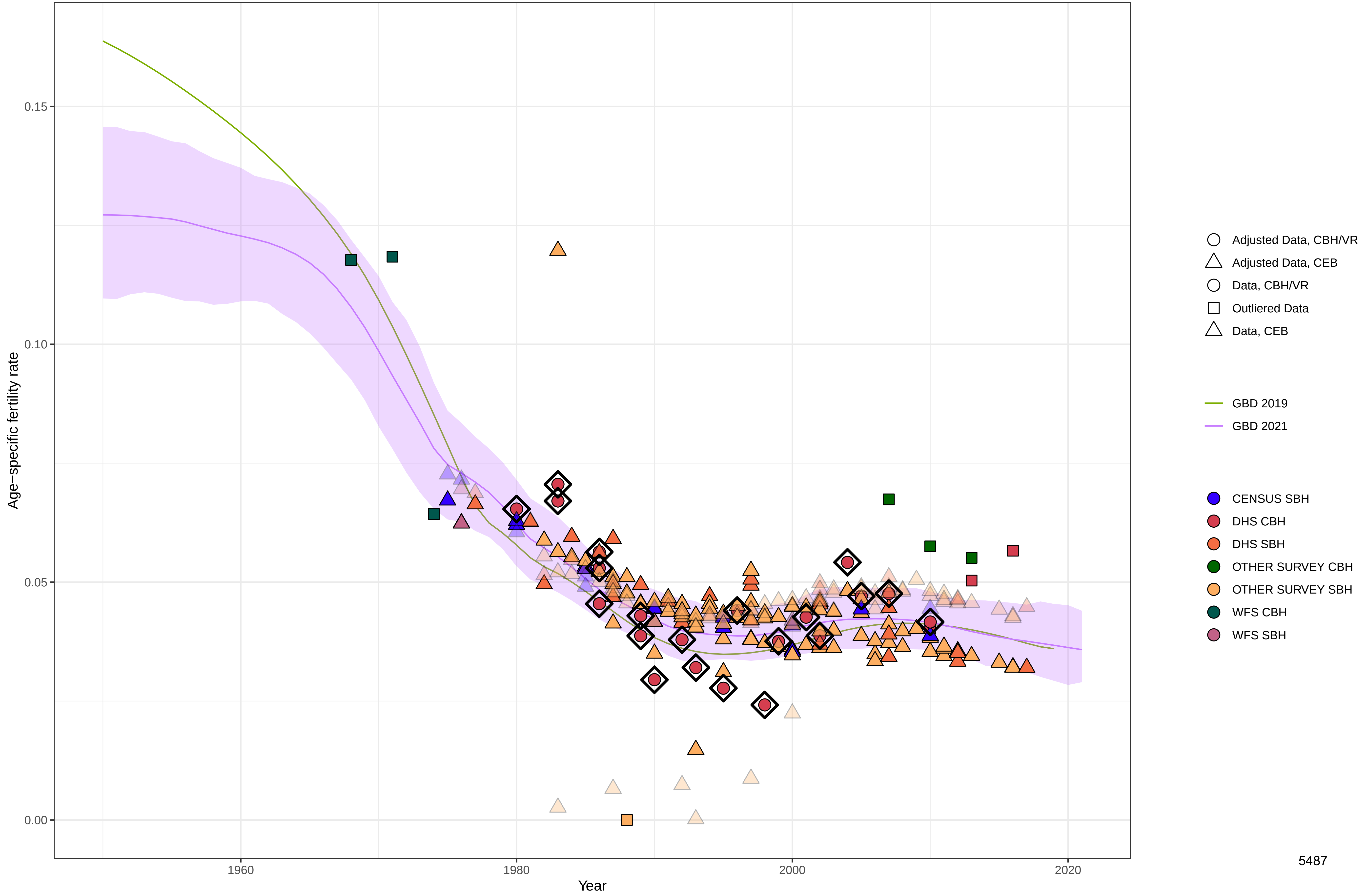

Age−specific fertility fitted trend lines, 1950−2021: East Java, Ages: 40−44
Data points enclosed with a diamond are reference sources

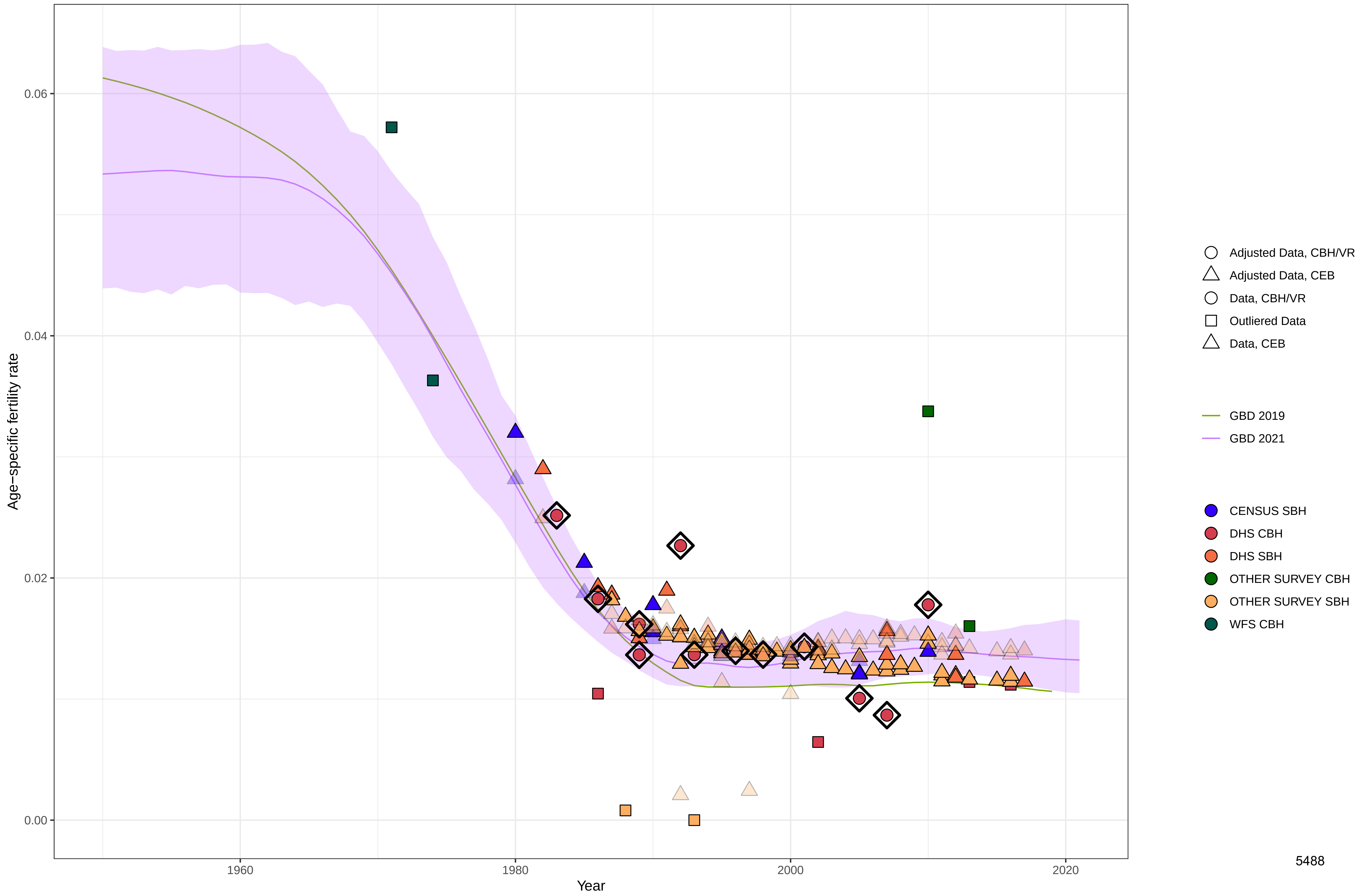

Age−specific fertility fitted trend lines, 1950−2021: East Java, Ages: 45−49
Data points enclosed with a diamond are reference sources

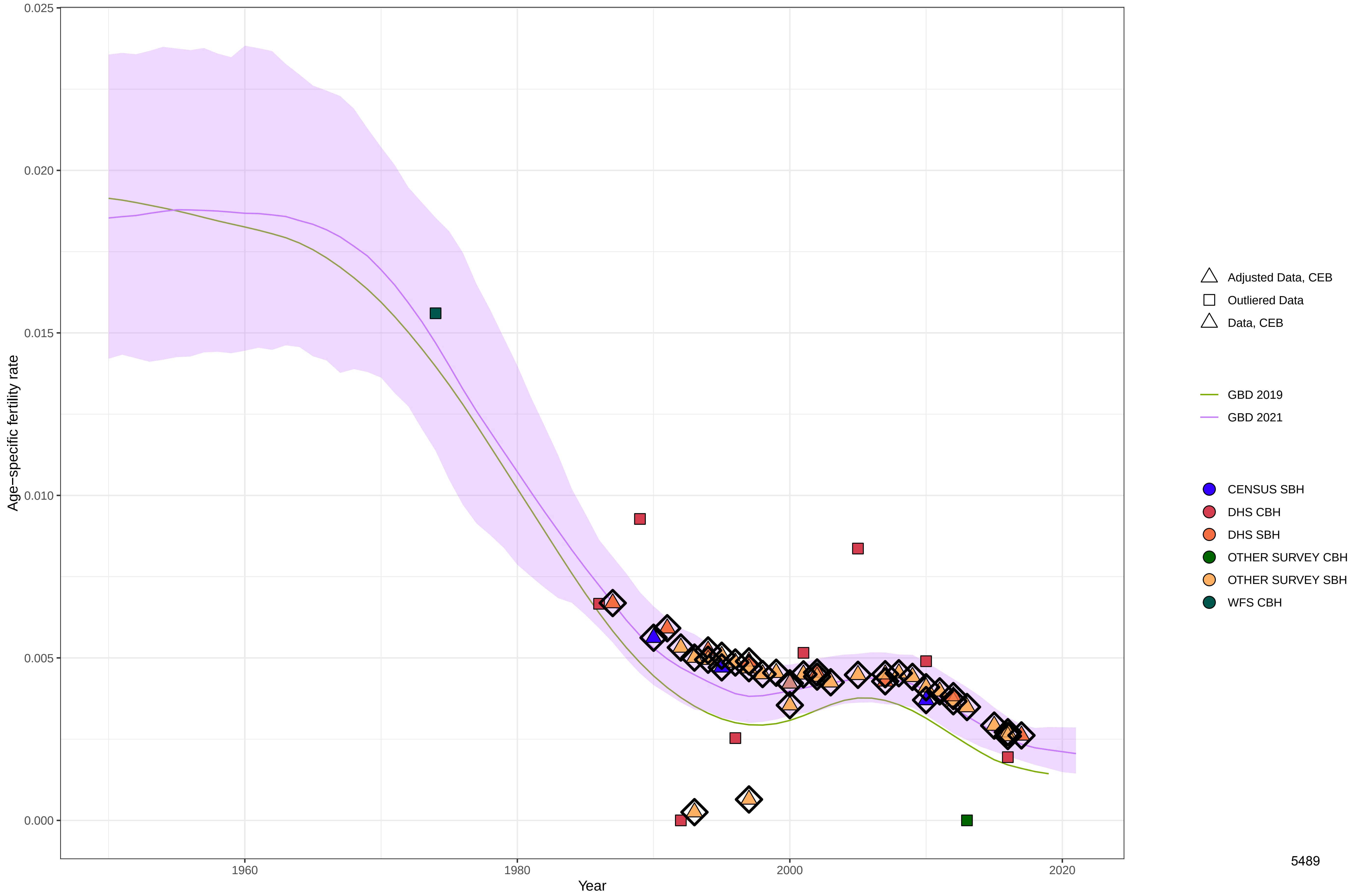

Age−specific fertility fitted trend lines, 1950−2021: East Java, Ages: 50−54
Data points enclosed with a diamond are reference sources

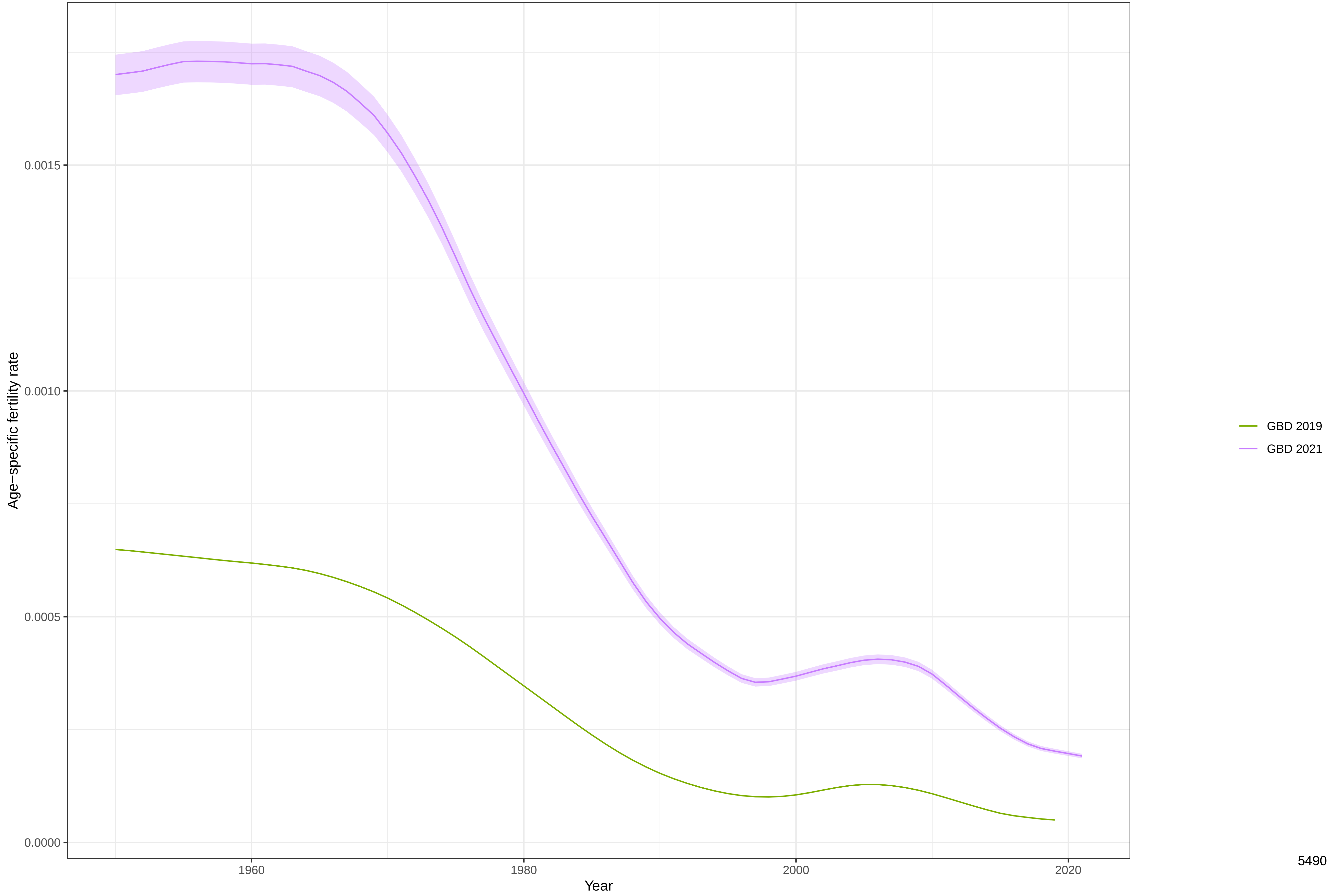

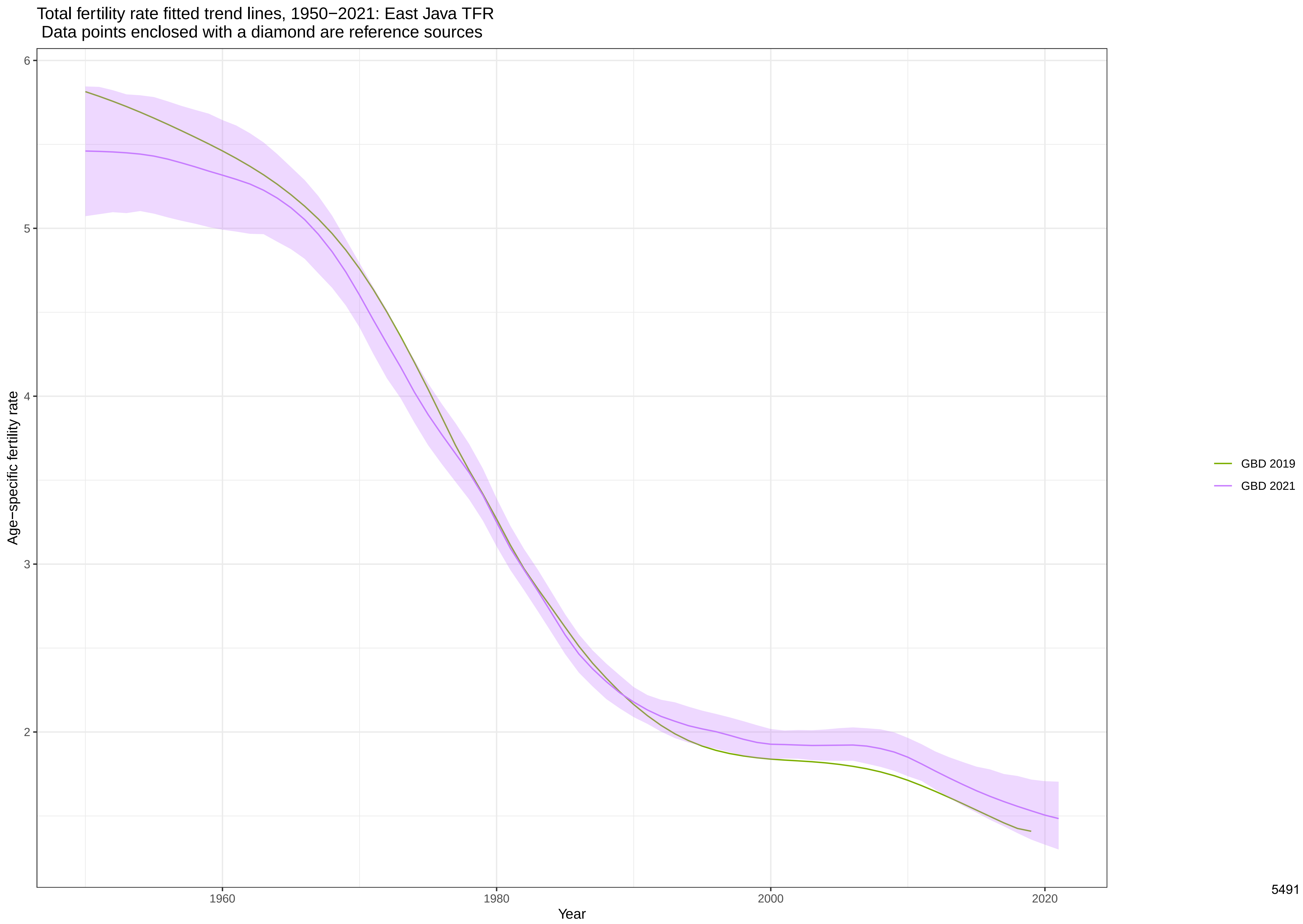

Total fertility rate fitted trend lines, 1950−2021: East Java TFR
Data points enclosed with a diamond are reference sources
Age−specific fertility rate
Year
6
5
4
3
2
1960
1980
2000
2020
GBD 2019
GBD 2021

Age−specific fertility fitted trend lines, 1950−2021: West Kalimantan, Ages: 10−14
Data points enclosed with a diamond are reference sources

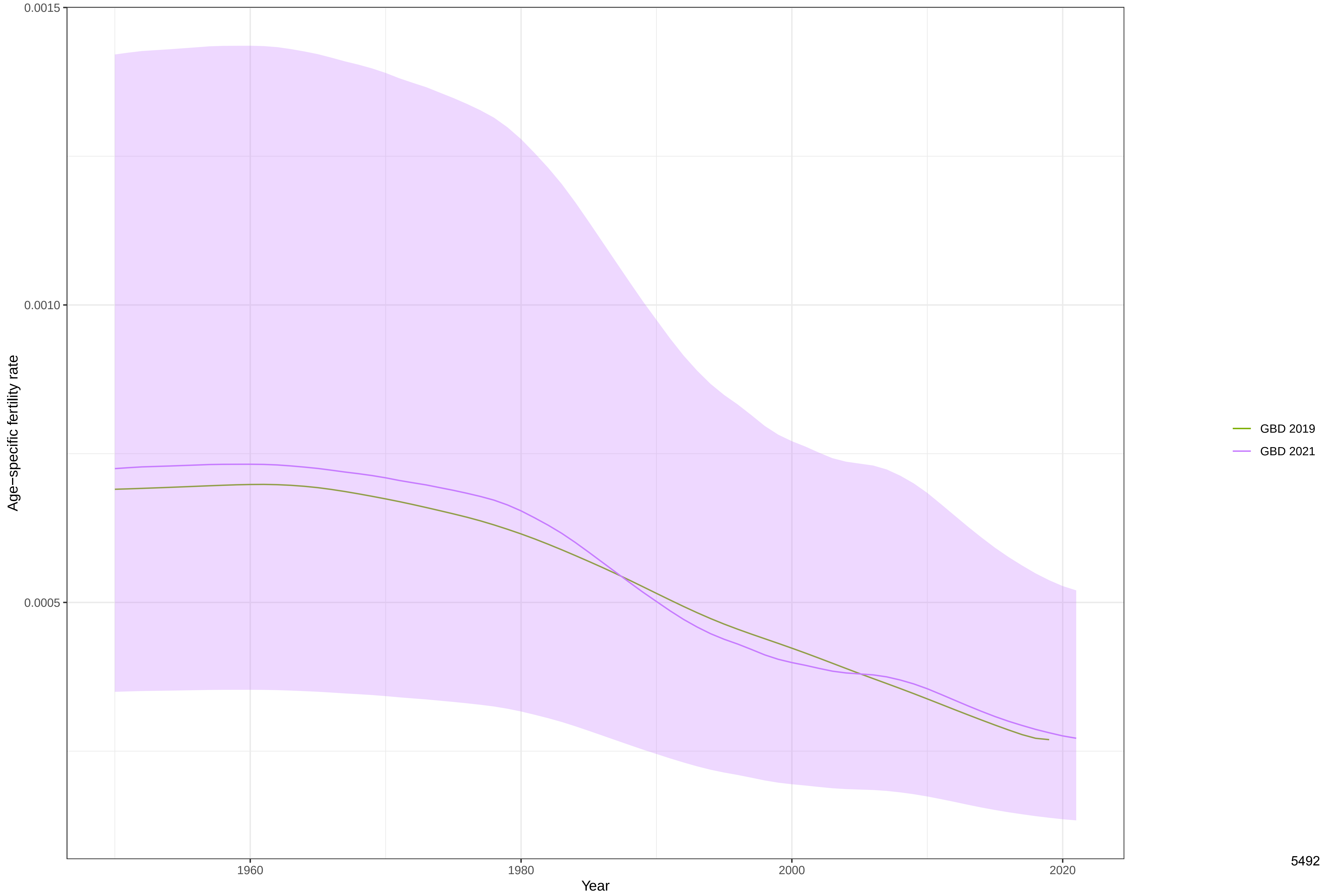

Age−specific fertility fitted trend lines, 1950−2021: West Kalimantan, Ages: 15−19
Data points enclosed with a diamond are reference sources

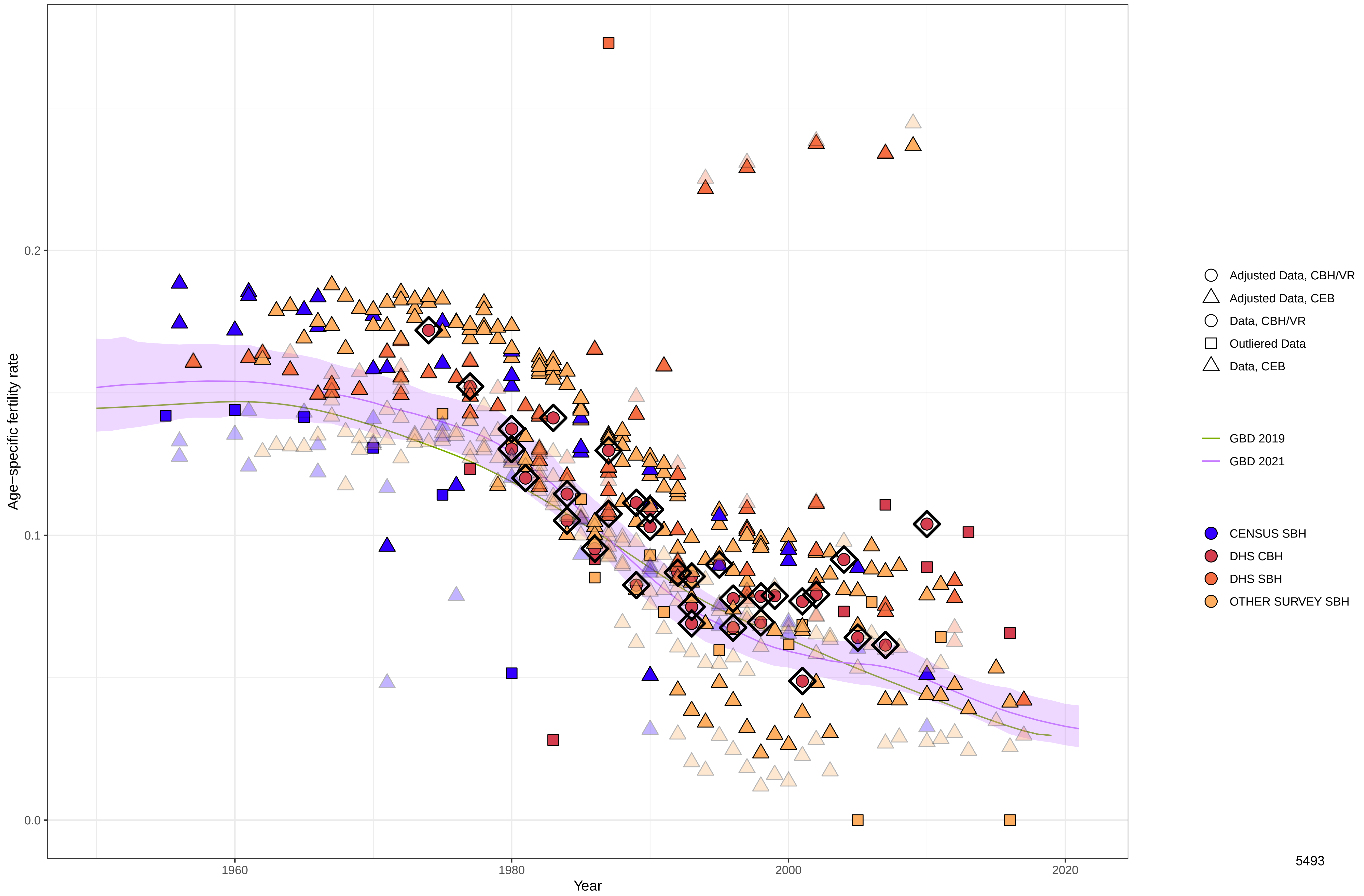

Age−specific fertility fitted trend lines, 1950−2021: West Kalimantan, Ages: 20−24
Data points enclosed with a diamond are reference sources

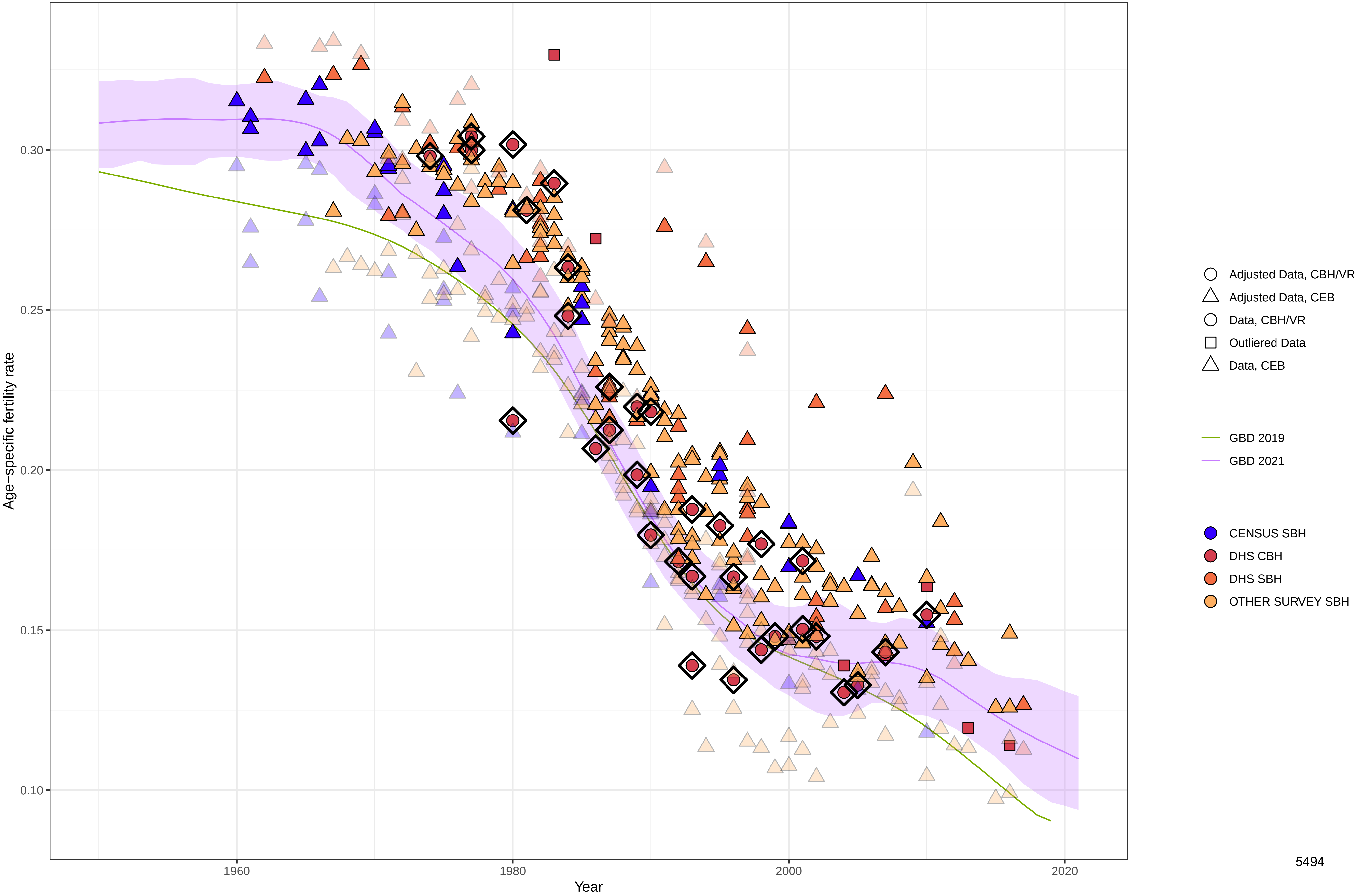

Age−specific fertility fitted trend lines, 1950−2021: West Kalimantan, Ages: 25−29
Data points enclosed with a diamond are reference sources

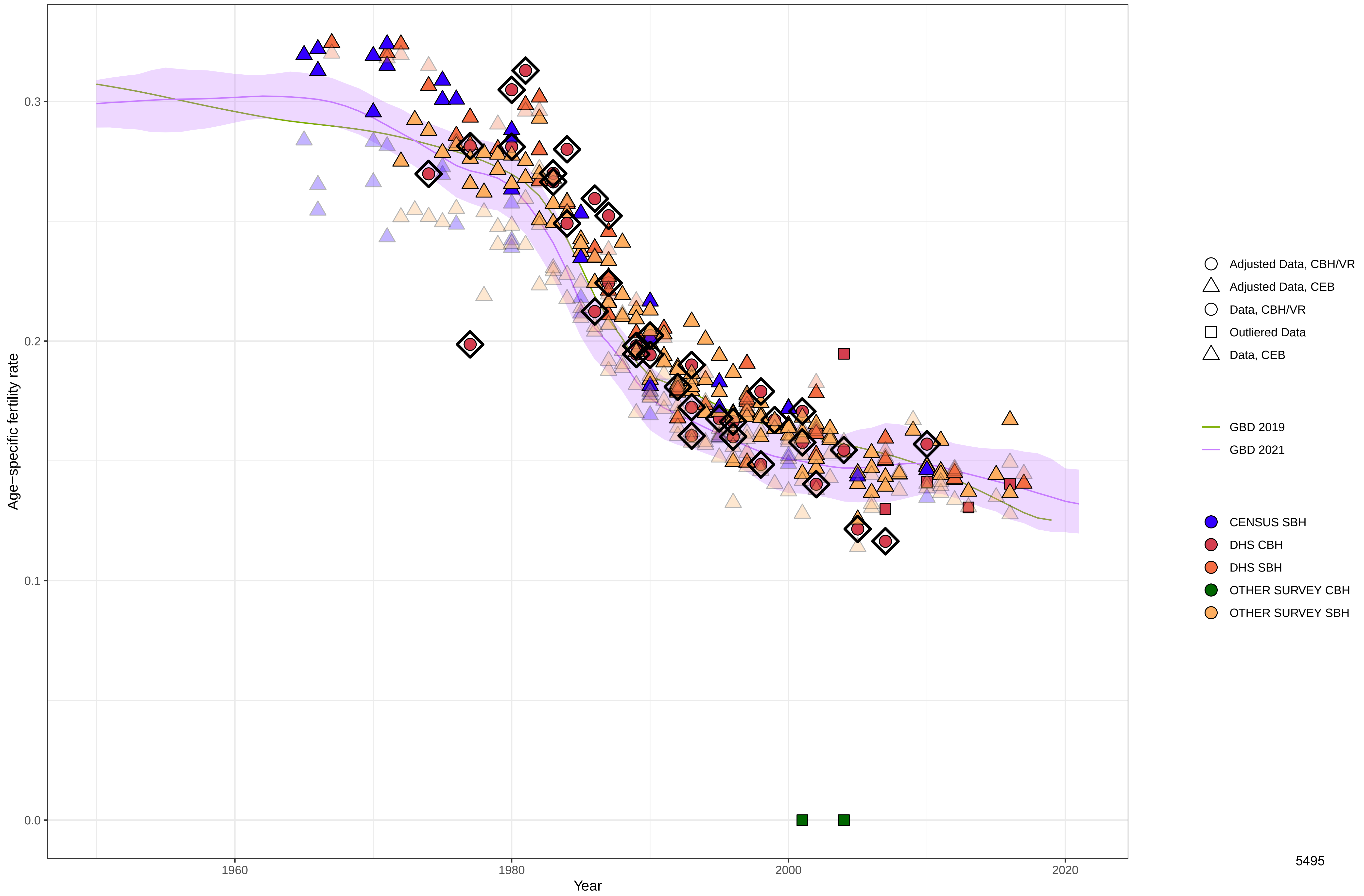

Age−specific fertility fitted trend lines, 1950−2021: West Kalimantan, Ages: 30−34
Data points enclosed with a diamond are reference sources

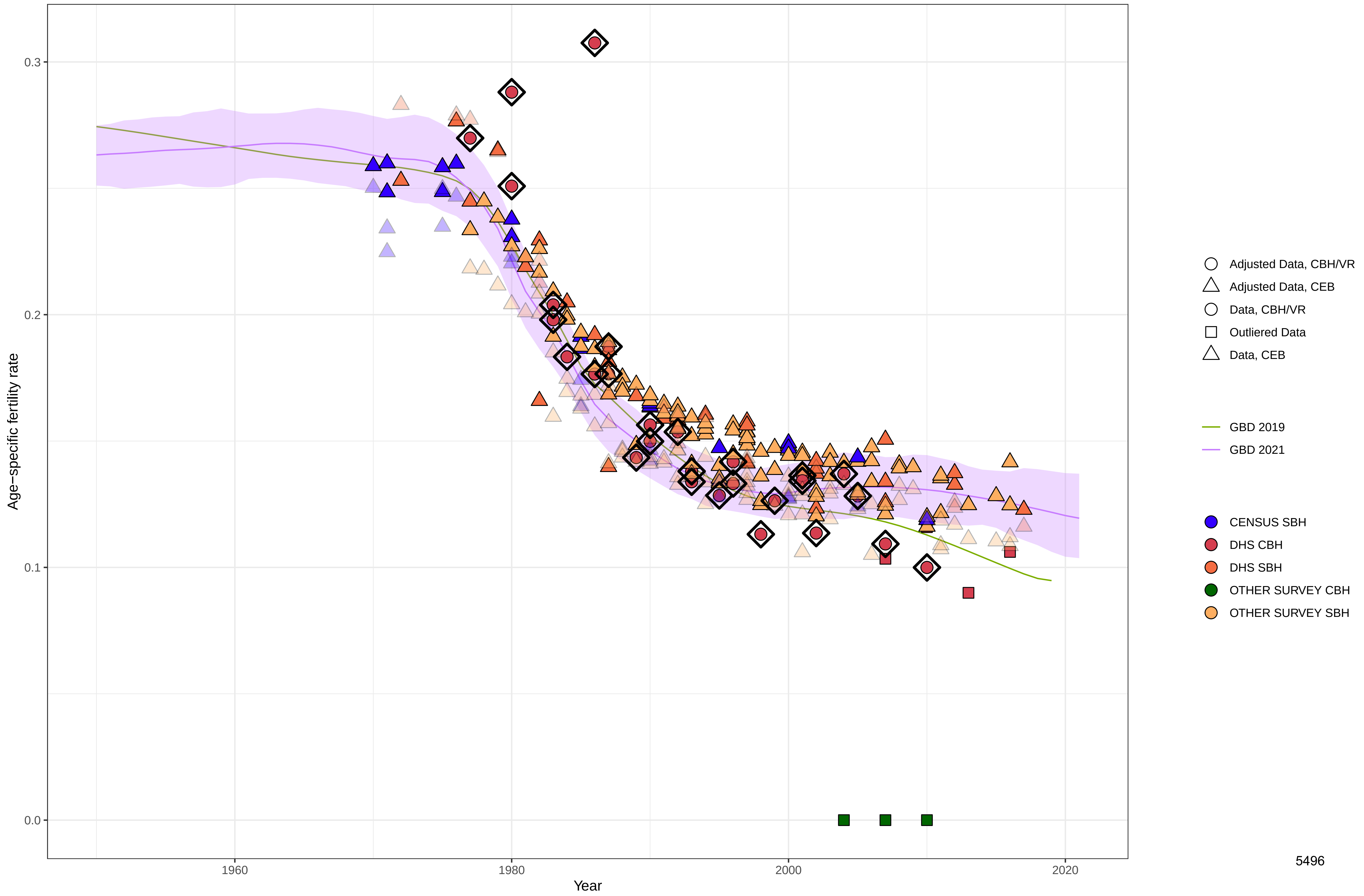

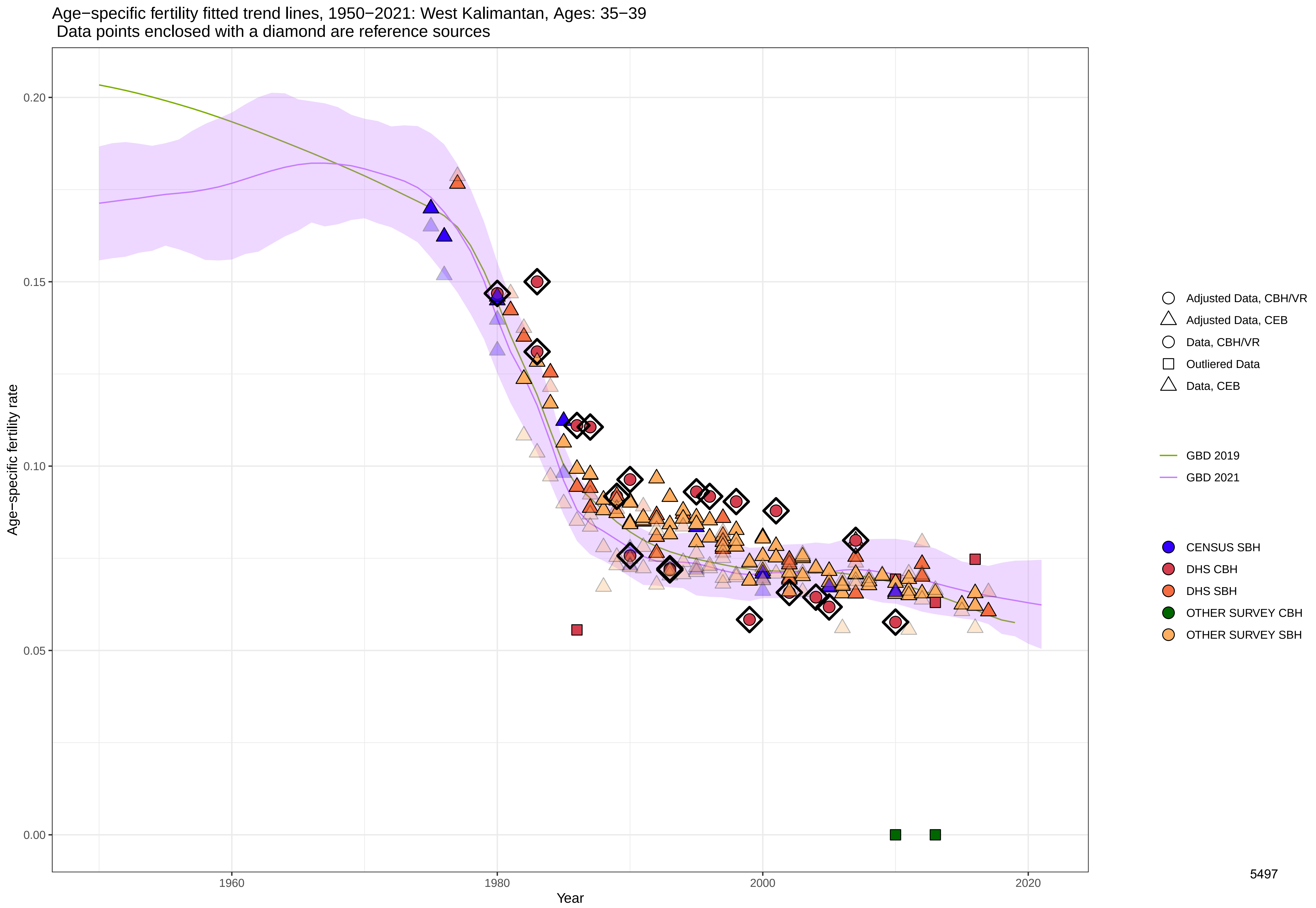

Age−specific fertility fitted trend lines, 1950−2021: West Kalimantan, Ages: 35−39
Data points enclosed with a diamond are reference sources
Age−specific fertility rate
Year
0.00
0.05
0.10
0.15
0.20
1960
1980
2000
2020
Adjusted Data, CBH/VR
Adjusted Data, CEB
Data, CBH/VR
Outliered Data
Data, CEB
GBD 2019
GBD 2021
CENSUS SBH
DHS CBH
DHS SBH
OTHER SURVEY CBH
OTHER SURVEY SBH

Age−specific fertility fitted trend lines, 1950−2021: West Kalimantan, Ages: 40−44
Data points enclosed with a diamond are reference sources

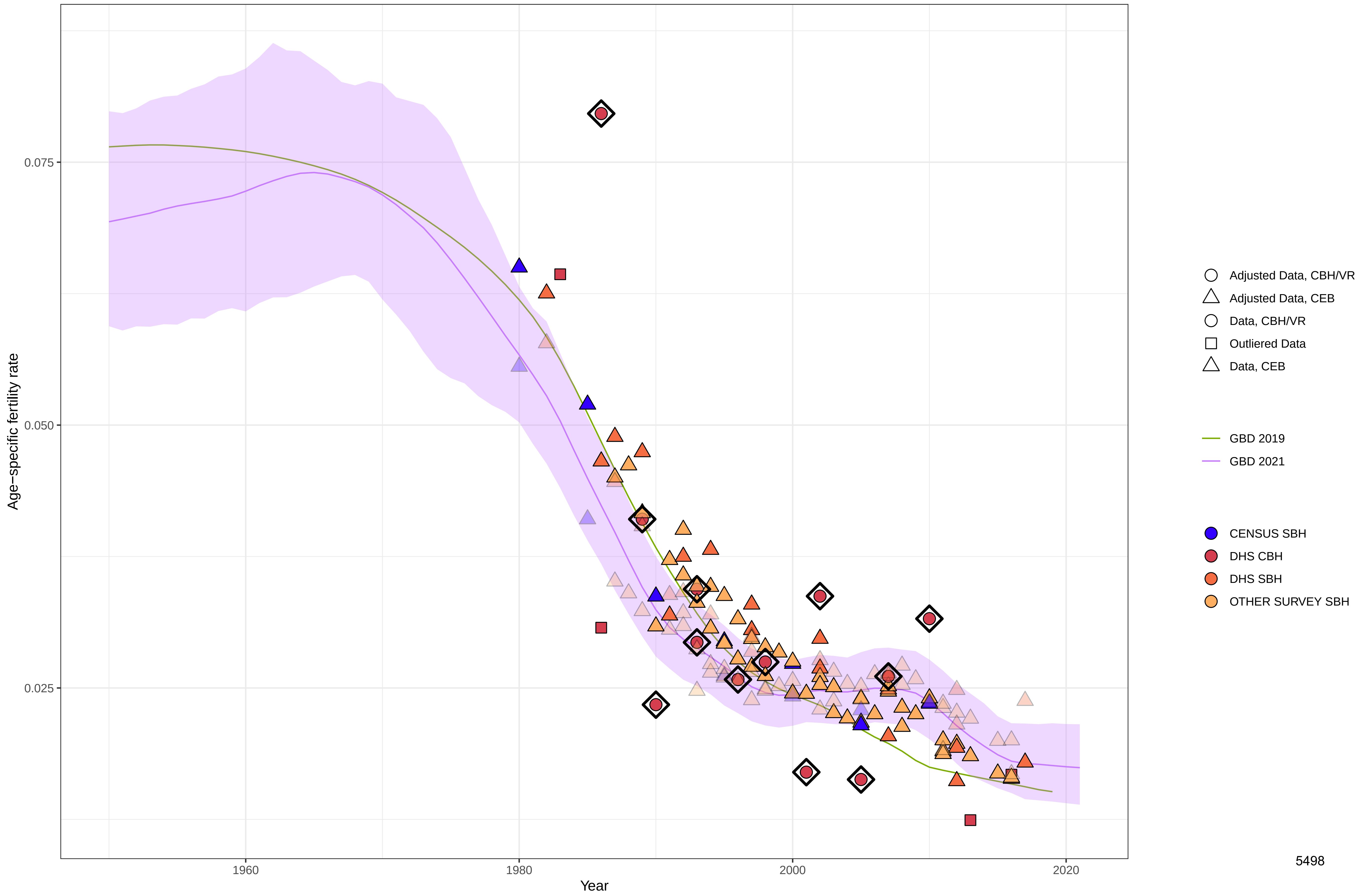

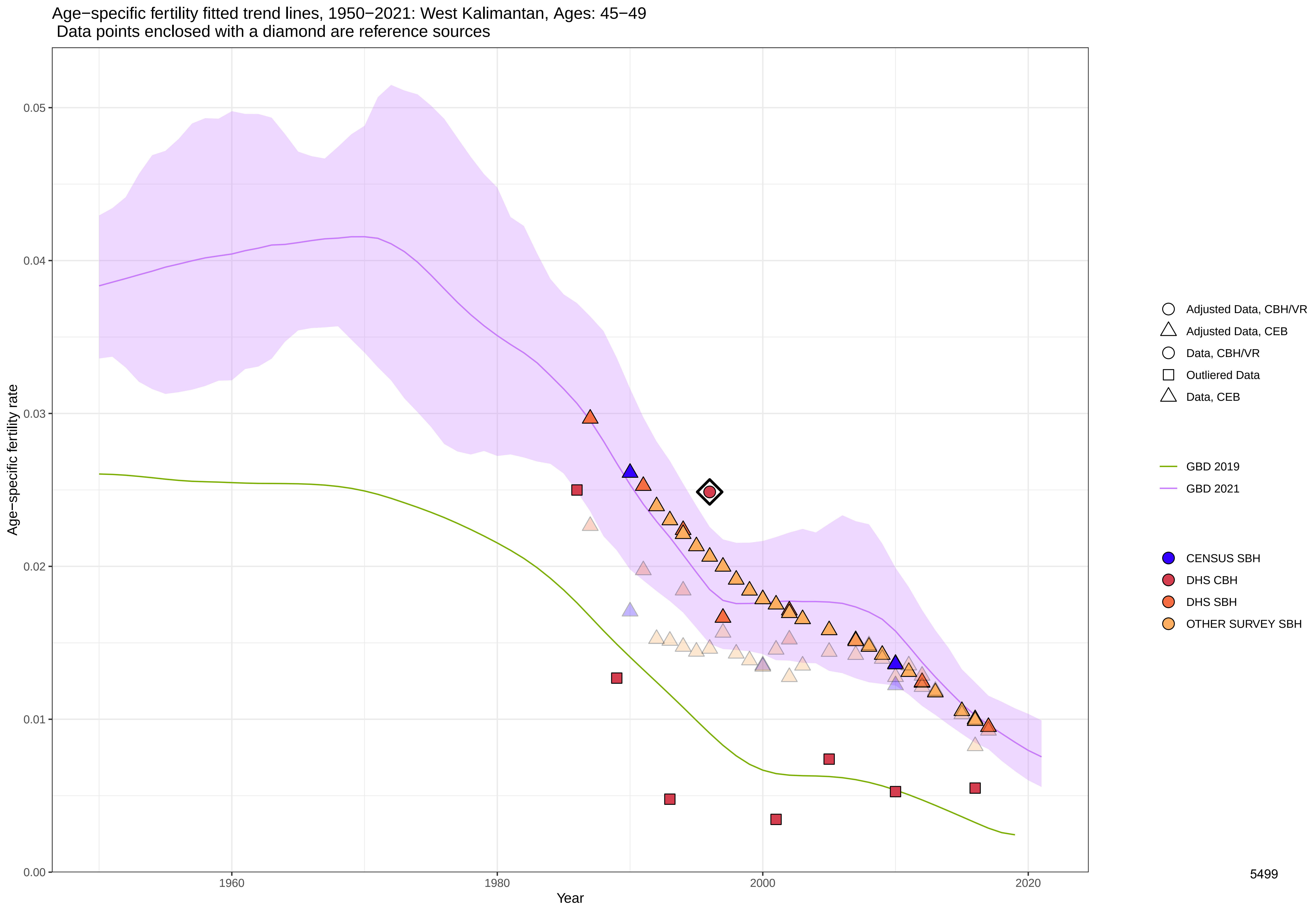

Age−specific fertility fitted trend lines, 1950−2021: West Kalimantan, Ages: 45−49
Data points enclosed with a diamond are reference sources
Age−specific fertility rate
Year
0.00
0.01
0.02
0.03
0.04
0.05
1960
1980
2000
2020
Adjusted Data, CBH/VR
Adjusted Data, CEB
Data, CBH/VR
Outliered Data
Data, CEB
GBD 2019
GBD 2021
CENSUS SBH
DHS CBH
DHS SBH
OTHER SURVEY SBH

Age−specific fertility fitted trend lines, 1950−2021: West Kalimantan, Ages: 50−54
Data points enclosed with a diamond are reference sources

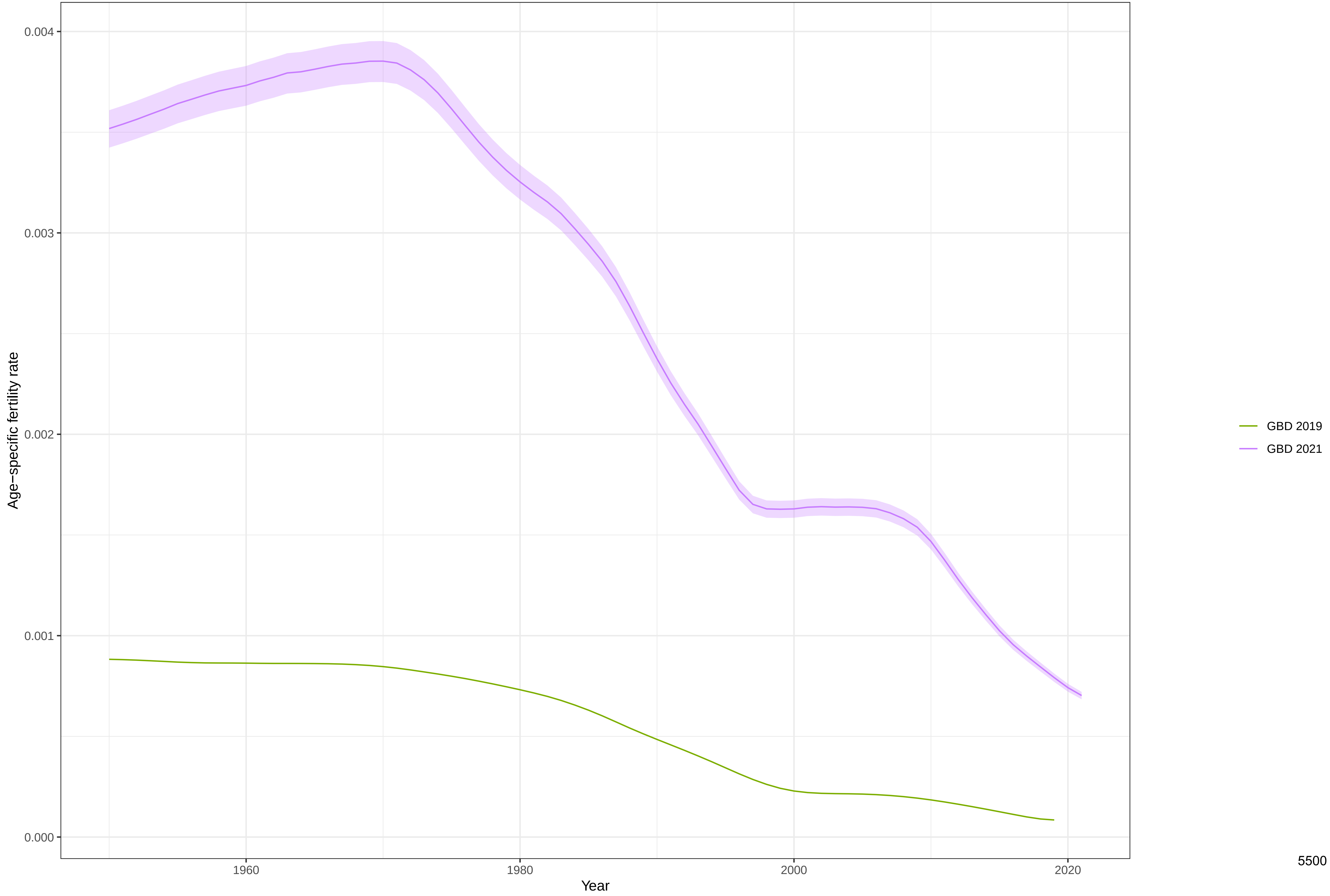

Total fertility rate fitted trend lines, 1950−2021: West Kalimantan TFR
Data points enclosed with a diamond are reference sources

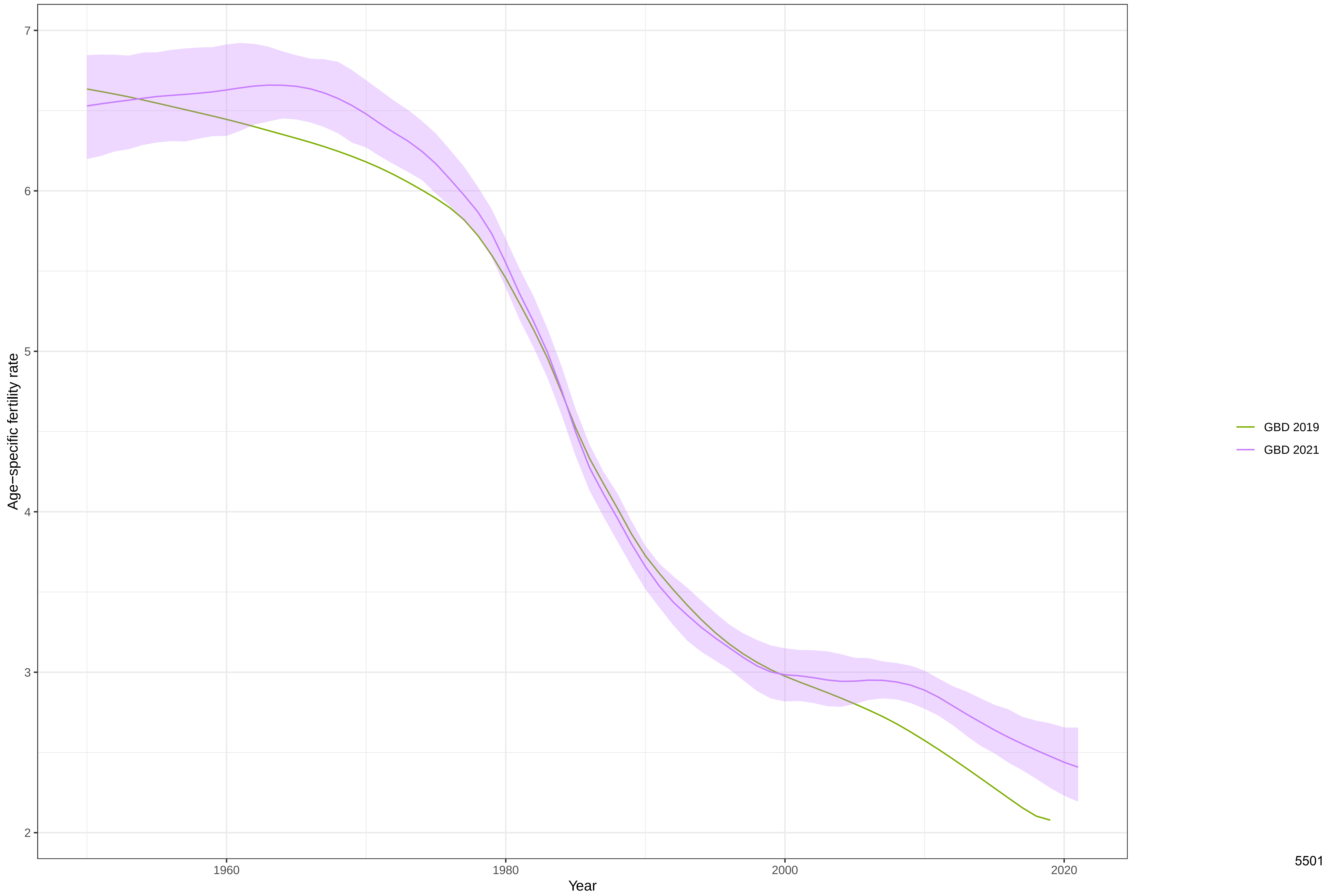

Age−specific fertility fitted trend lines, 1950−2021: South Kalimantan, Ages: 10−14
Data points enclosed with a diamond are reference sources

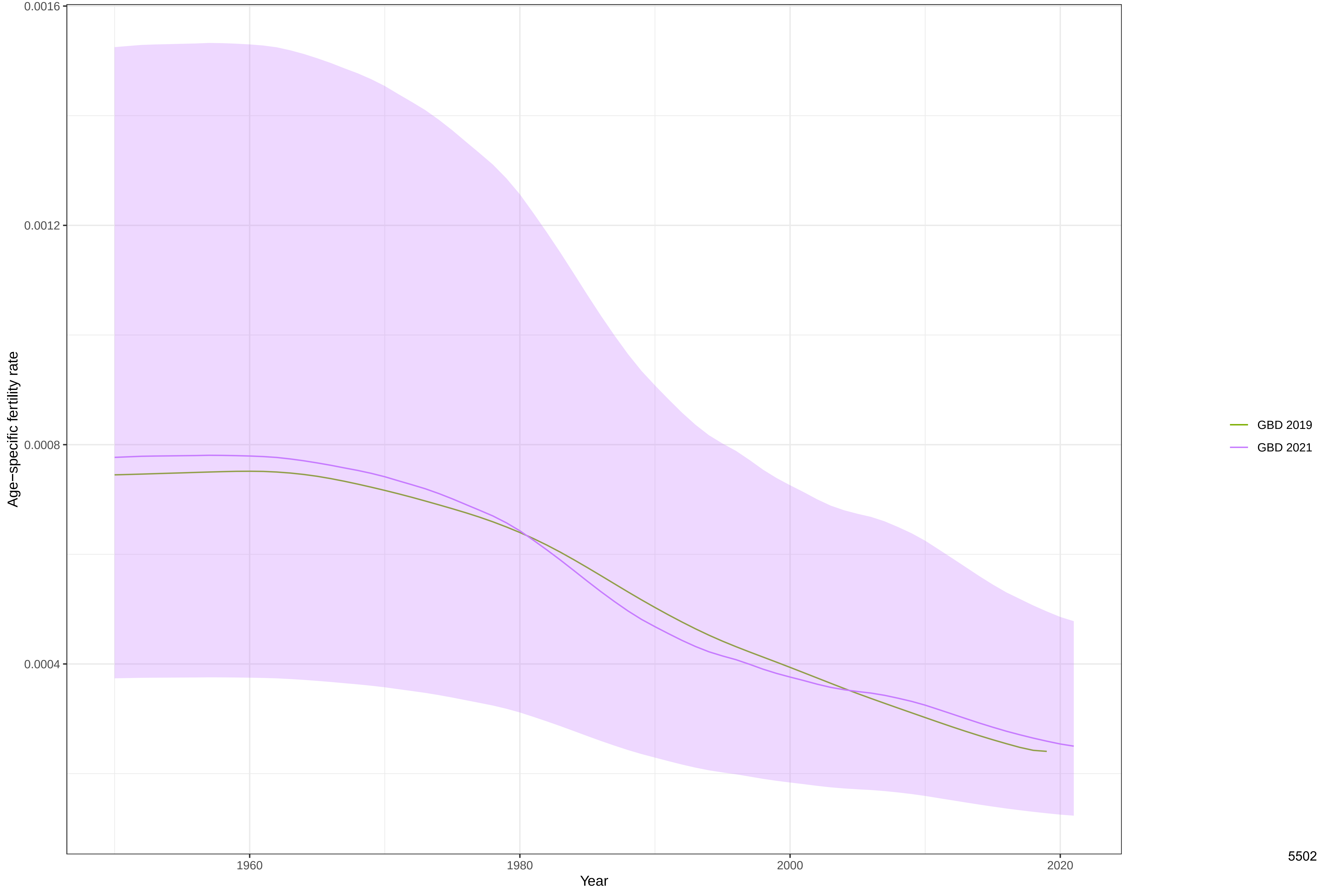

Age−specific fertility fitted trend lines, 1950−2021: South Kalimantan, Ages: 15−19
Data points enclosed with a diamond are reference sources

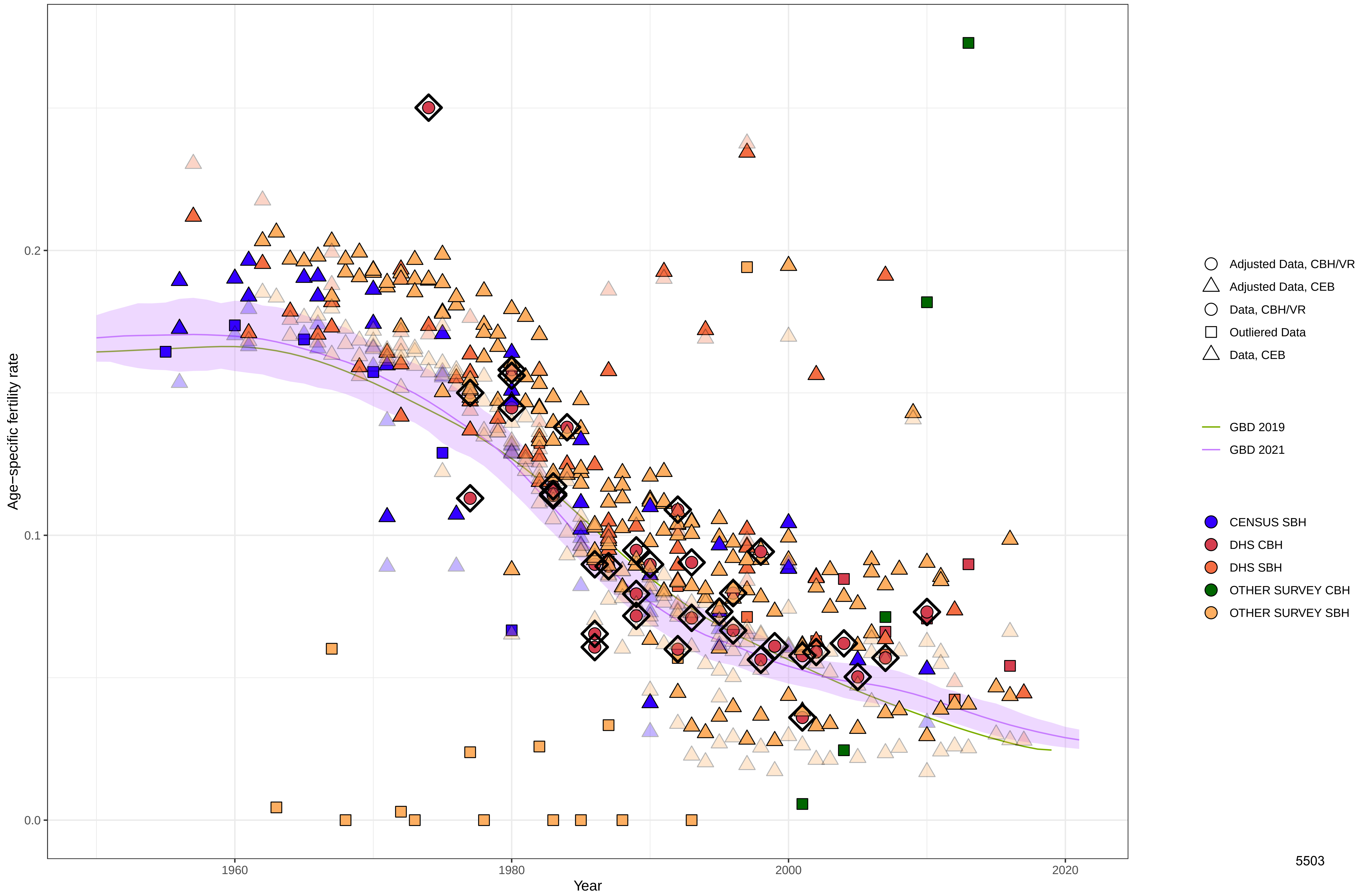

Age−specific fertility fitted trend lines, 1950−2021: South Kalimantan, Ages: 20−24
Data points enclosed with a diamond are reference sources

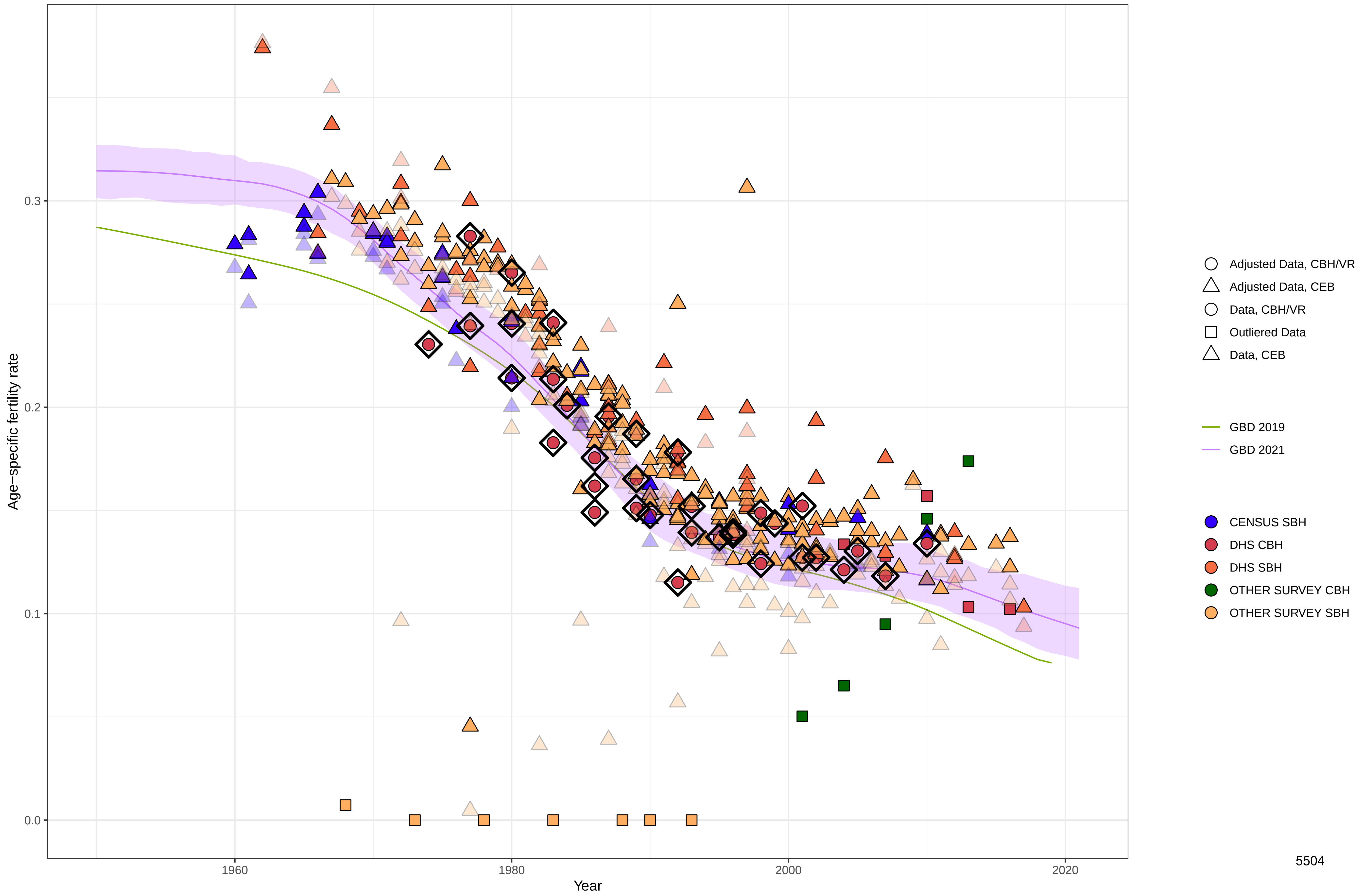

Age−specific fertility fitted trend lines, 1950−2021: South Kalimantan, Ages: 25−29
Data points enclosed with a diamond are reference sources

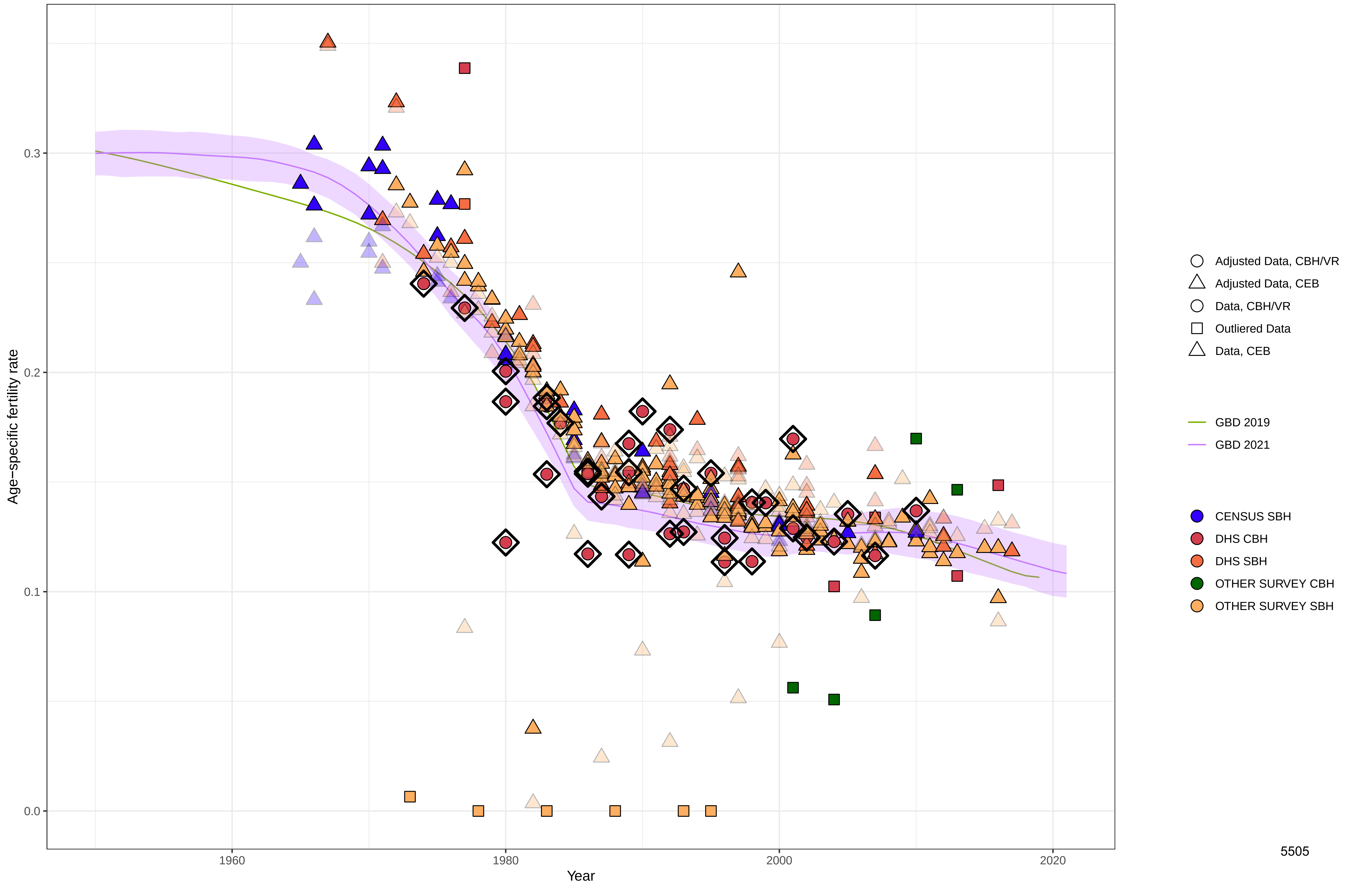

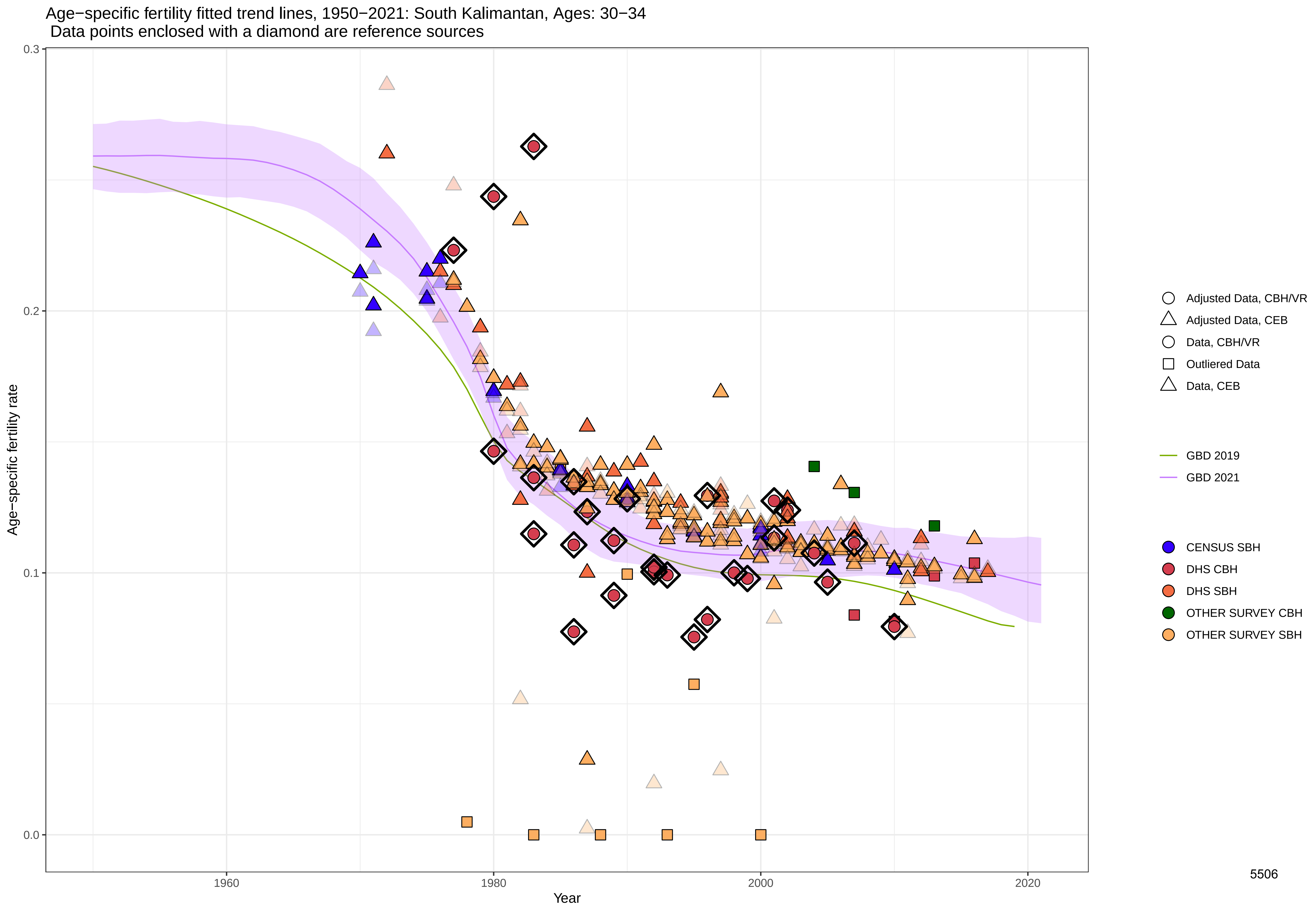
Age−specific fertility fitted trend lines, 1950−2021: South Kalimantan, Ages: 30−34
Data points enclosed with a diamond are reference sources
Age−specific fertility rate
Year
0.0
0.1
0.2
0.3
1960
1980
2000
2020
Adjusted Data, CBH/VR
Adjusted Data, CEB
Data, CBH/VR
Outliered Data
Data, CEB
GBD 2019
GBD 2021
CENSUS SBH
DHS CBH
DHS SBH
OTHER SURVEY CBH
OTHER SURVEY SBH

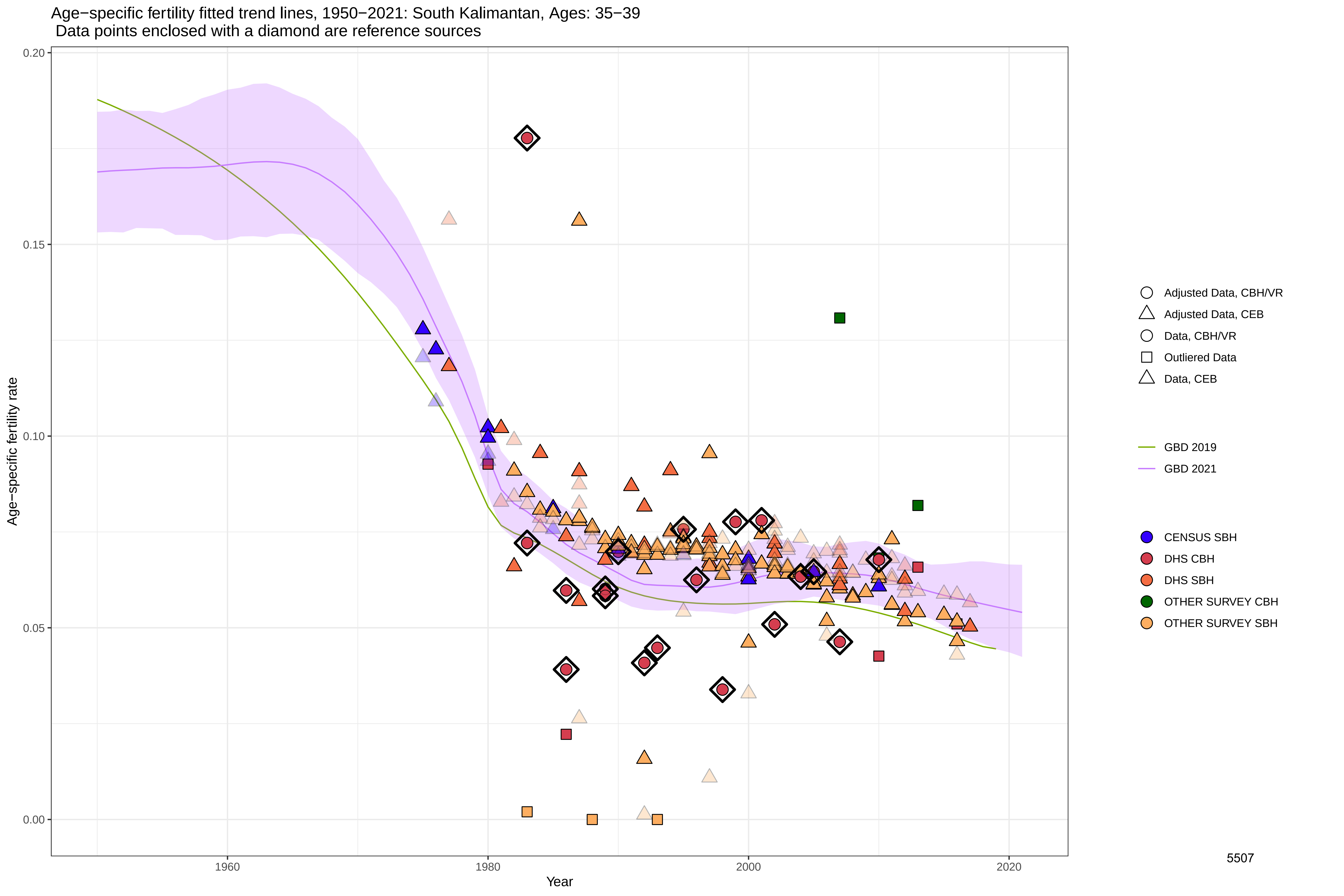

Age−specific fertility fitted trend lines, 1950−2021: South Kalimantan, Ages: 35−39
Data points enclosed with a diamond are reference sources
Age−specific fertility rate
Year
0.00
0.05
0.10
0.15
0.20
1960
1980
2000
2020
Adjusted Data, CBH/VR
Adjusted Data, CEB
Data, CBH/VR
Outliered Data
Data, CEB
GBD 2019
GBD 2021
CENSUS SBH
DHS CBH
DHS SBH
OTHER SURVEY CBH
OTHER SURVEY SBH
5507

Age−specific fertility fitted trend lines, 1950−2021: South Kalimantan, Ages: 40−44
Data points enclosed with a diamond are reference sources

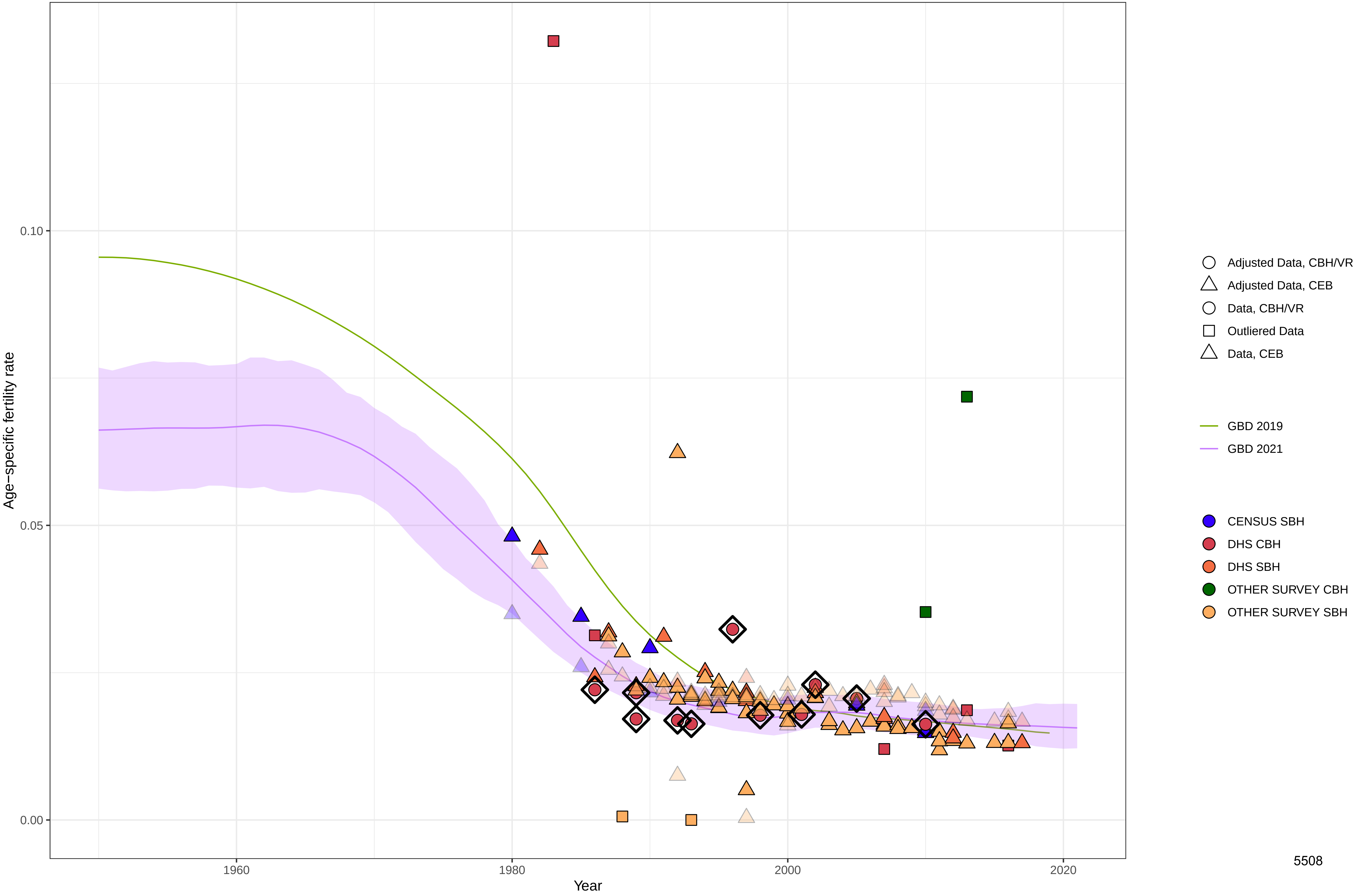

5508

Age−specific fertility fitted trend lines, 1950−2021: South Kalimantan, Ages: 45−49
Data points enclosed with a diamond are reference sources

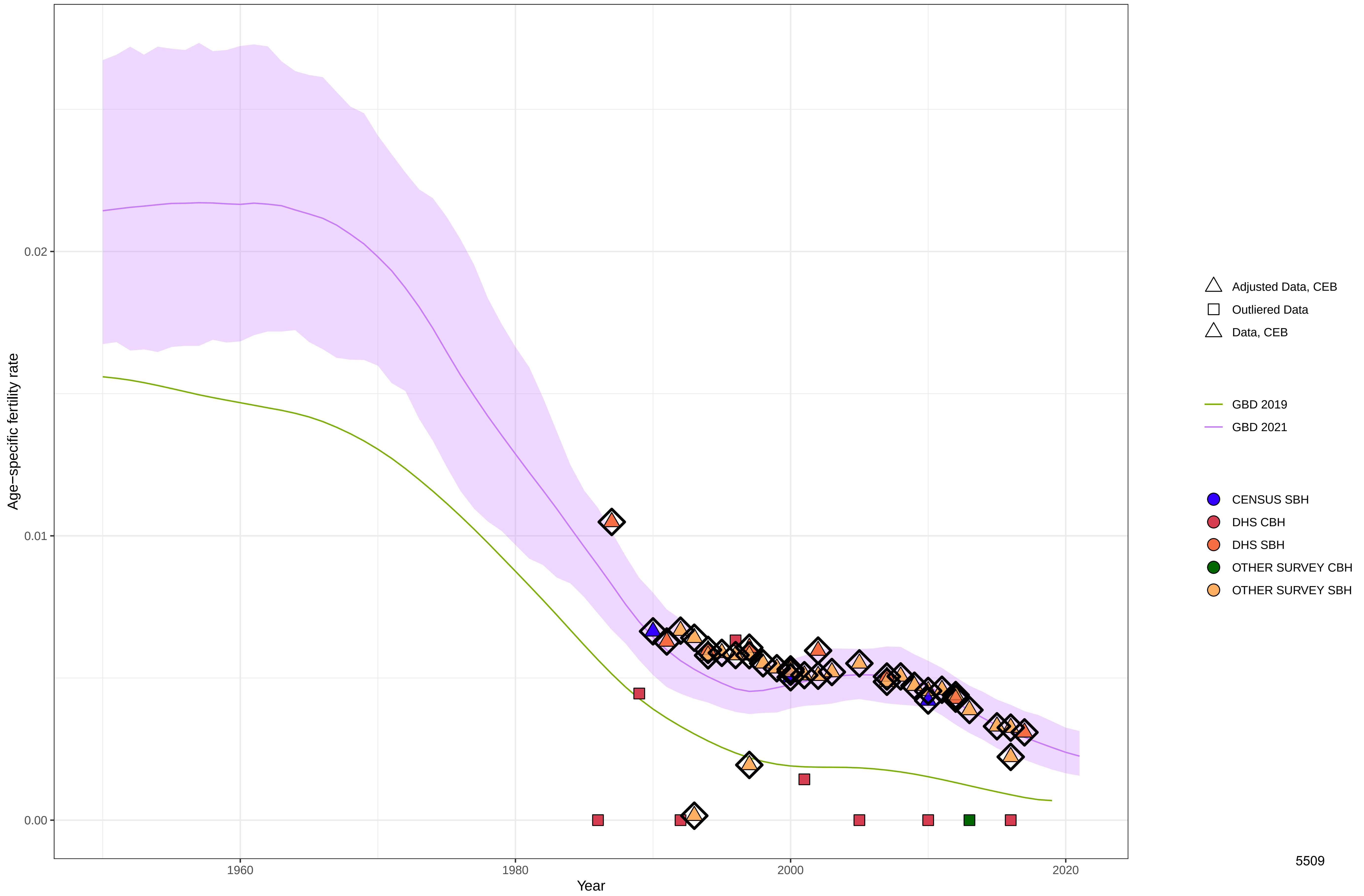

Age−specific fertility fitted trend lines, 1950−2021: South Kalimantan, Ages: 50−54
Data points enclosed with a diamond are reference sources

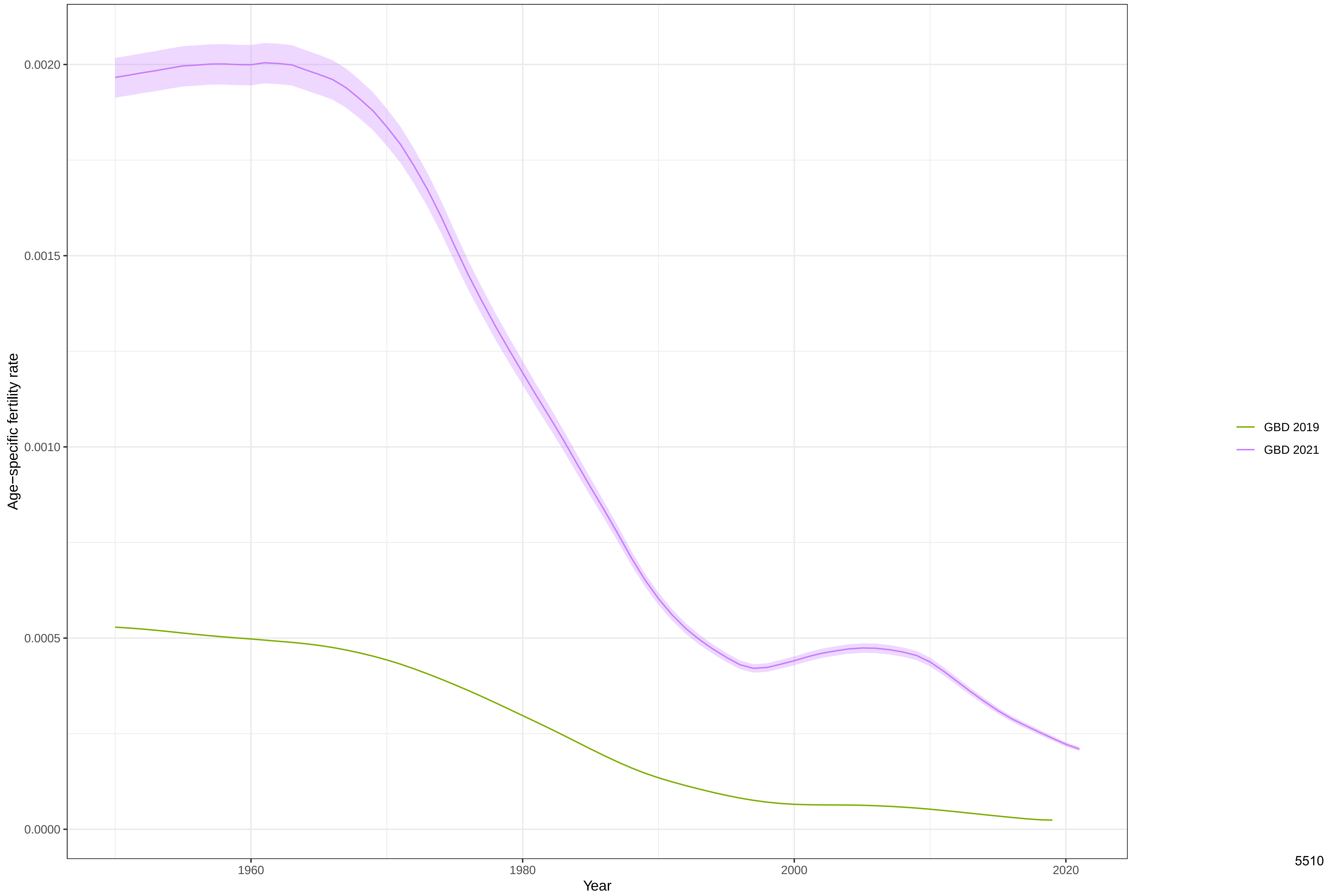

Total fertility rate fitted trend lines, 1950−2021: South Kalimantan TFR
Data points enclosed with a diamond are reference sources

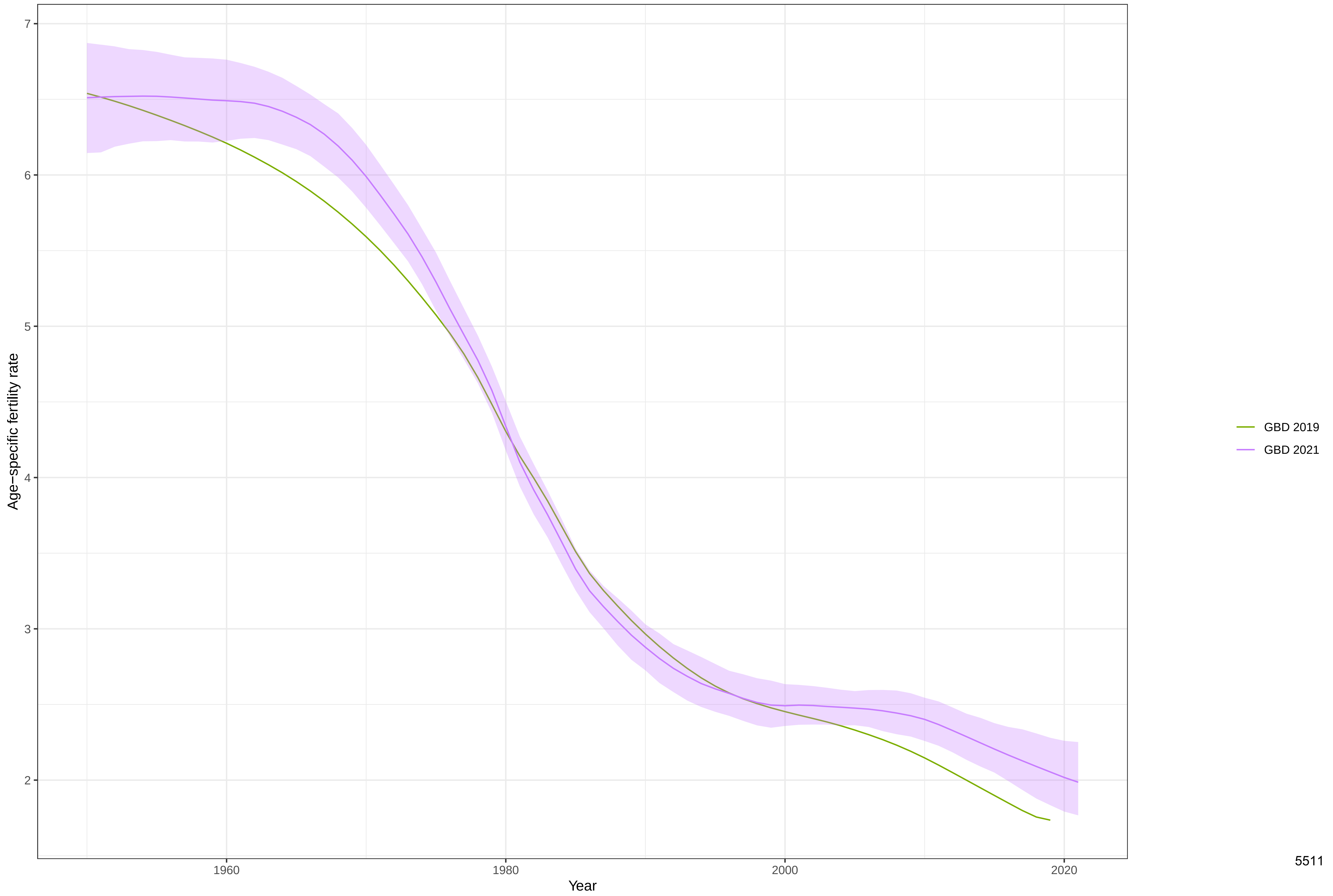

Age−specific fertility fitted trend lines, 1950−2021: Central Kalimantan, Ages: 10−14
Data points enclosed with a diamond are reference sources

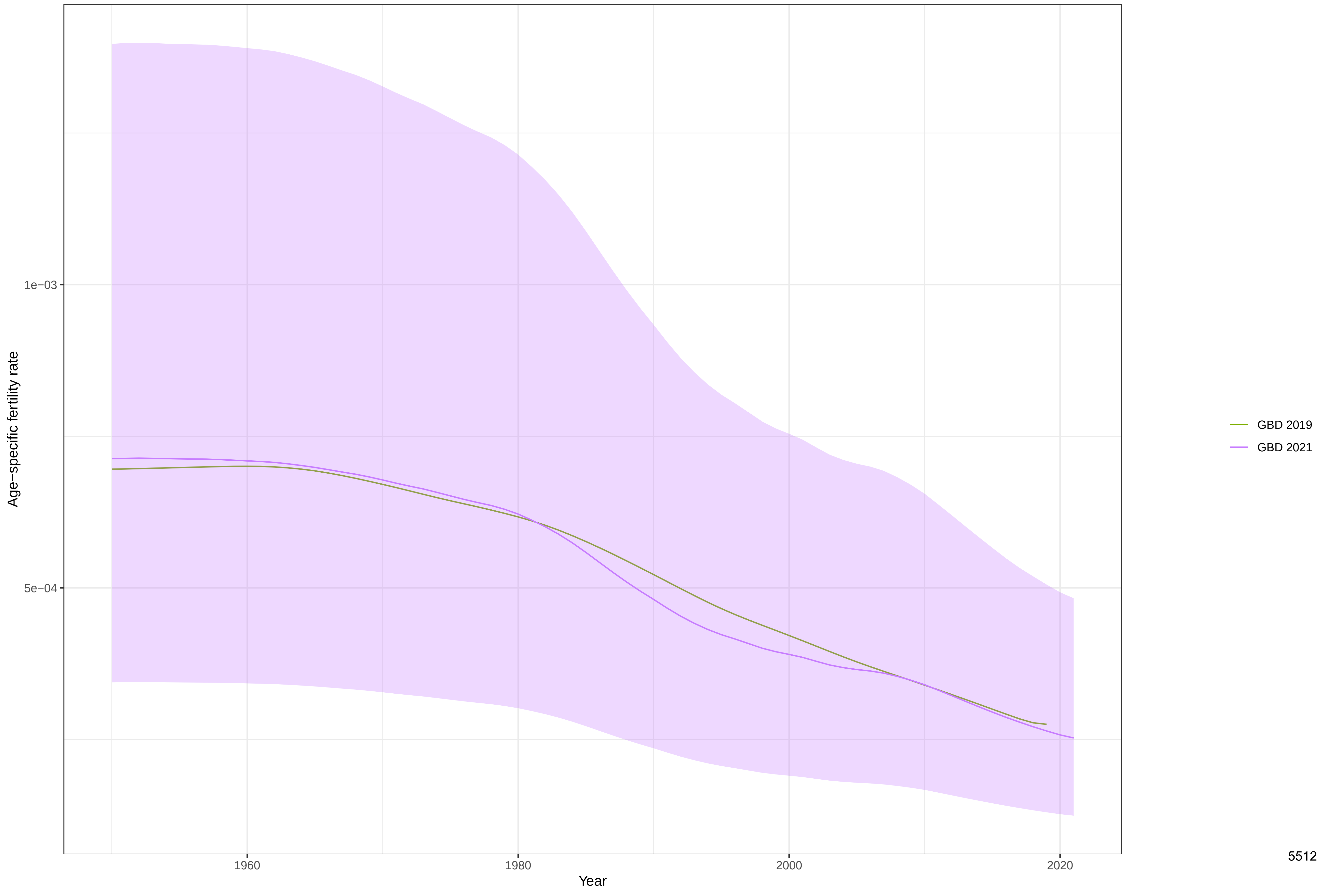

Age−specific fertility fitted trend lines, 1950−2021: Central Kalimantan, Ages: 15−19
Data points enclosed with a diamond are reference sources

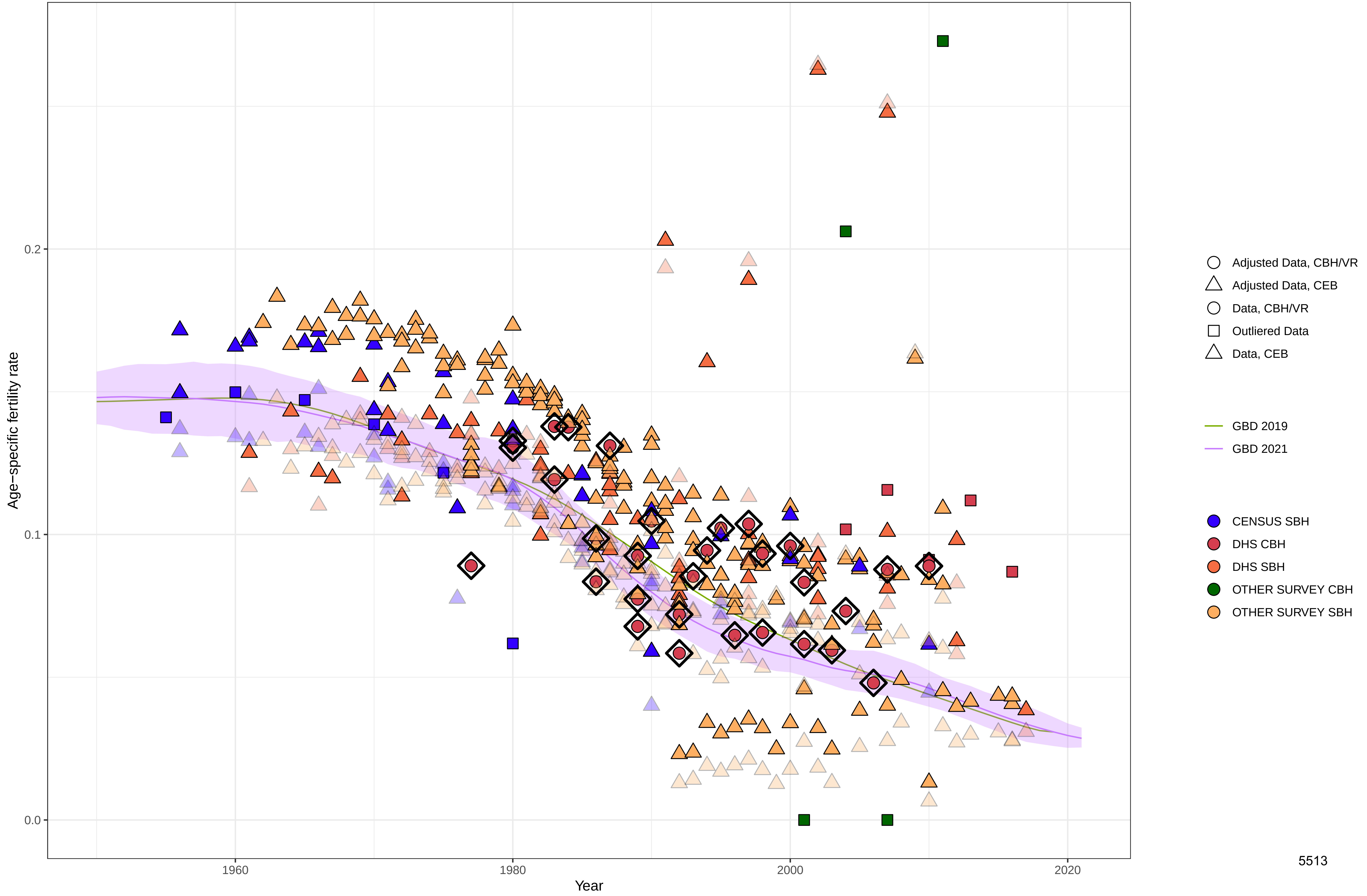

Age−specific fertility fitted trend lines, 1950−2021: Central Kalimantan, Ages: 20−24
Data points enclosed with a diamond are reference sources

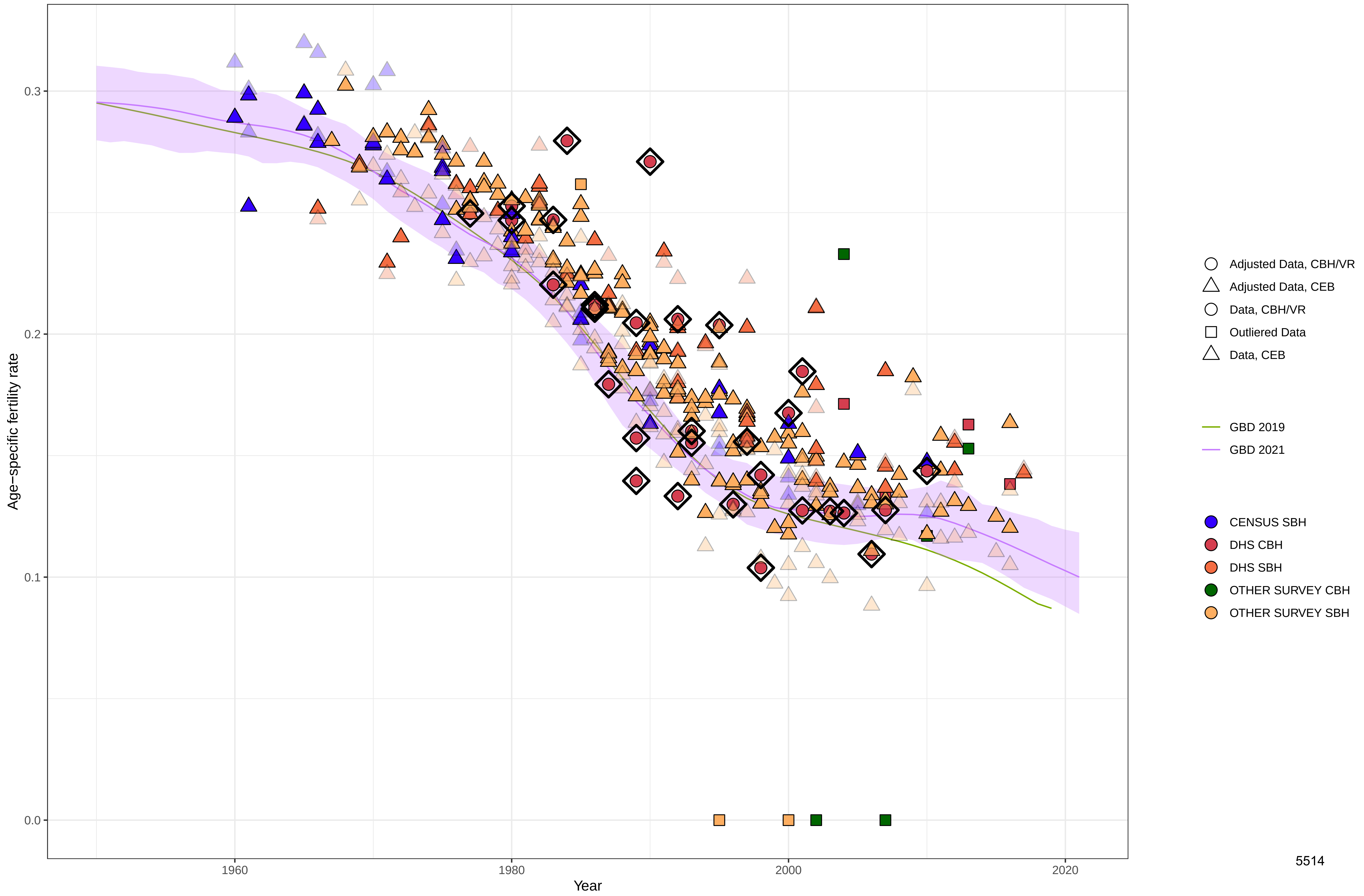

Age−specific fertility fitted trend lines, 1950−2021: Central Kalimantan, Ages: 25−29
Data points enclosed with a diamond are reference sources

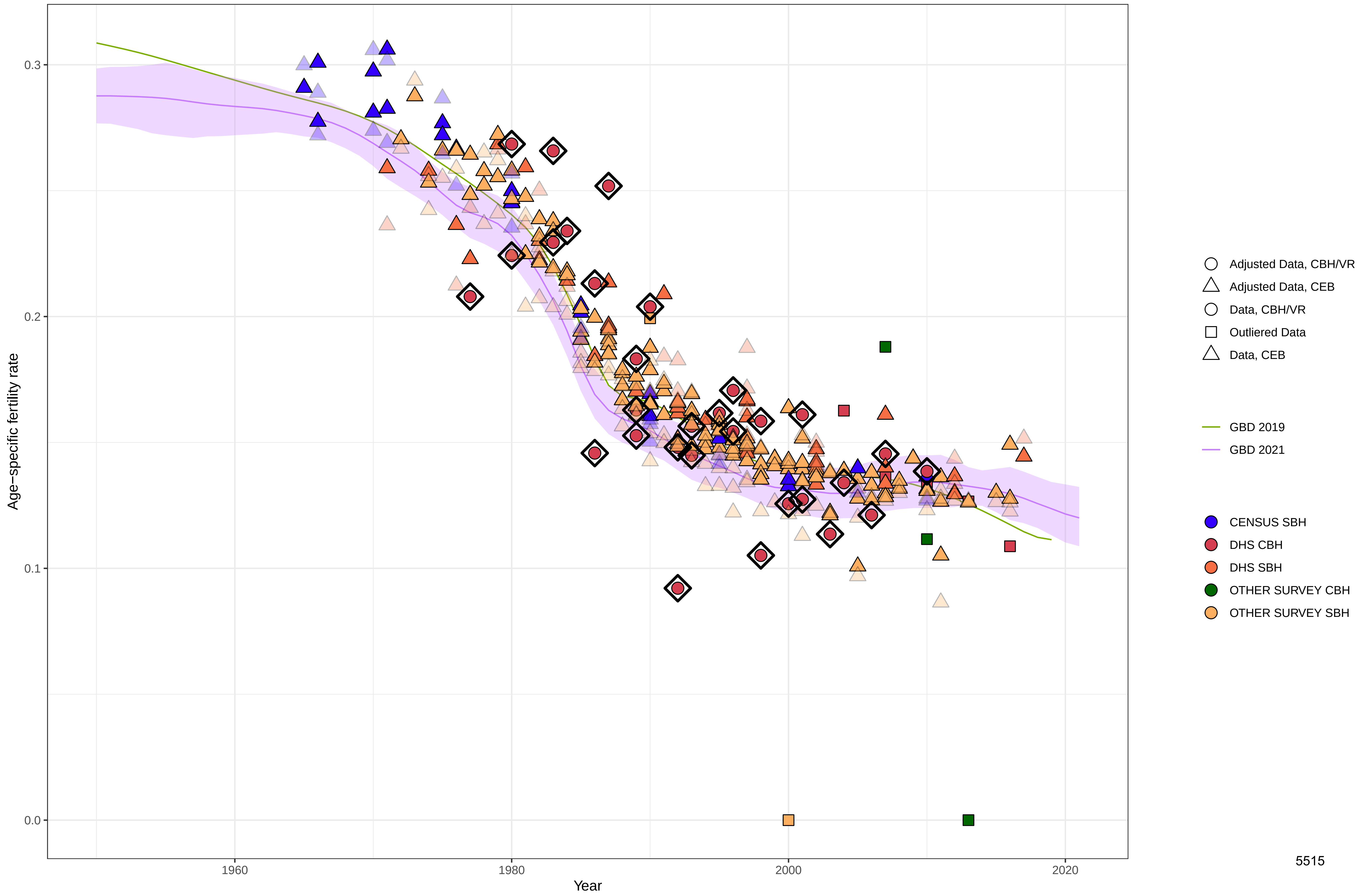

Age−specific fertility fitted trend lines, 1950−2021: Central Kalimantan, Ages: 30−34
Data points enclosed with a diamond are reference sources

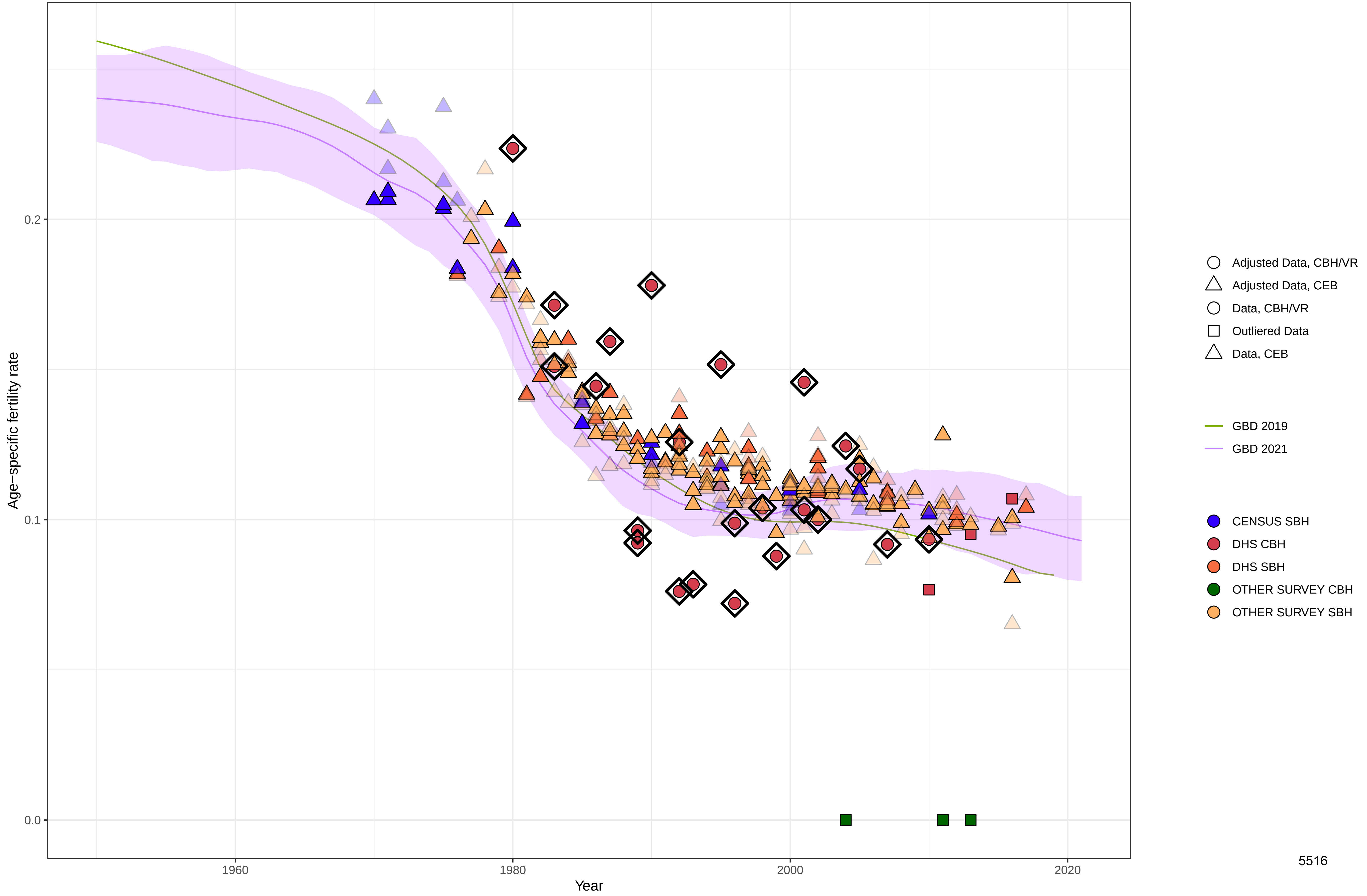

Age−specific fertility fitted trend lines, 1950−2021: Central Kalimantan, Ages: 35−39
Data points enclosed with a diamond are reference sources

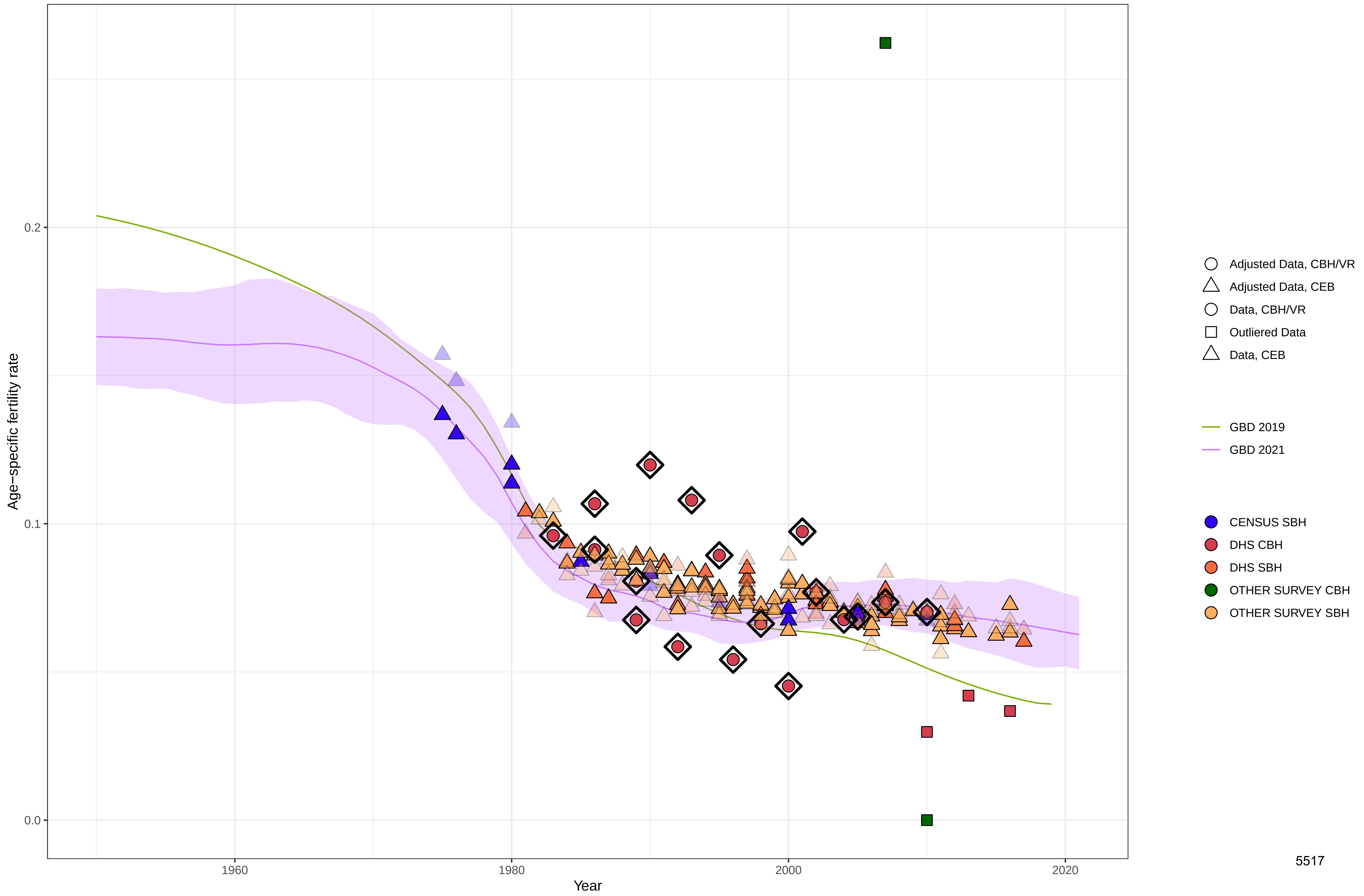

Age−specific fertility fitted trend lines, 1950−2021: Central Kalimantan, Ages: 40−44
Data points enclosed with a diamond are reference sources

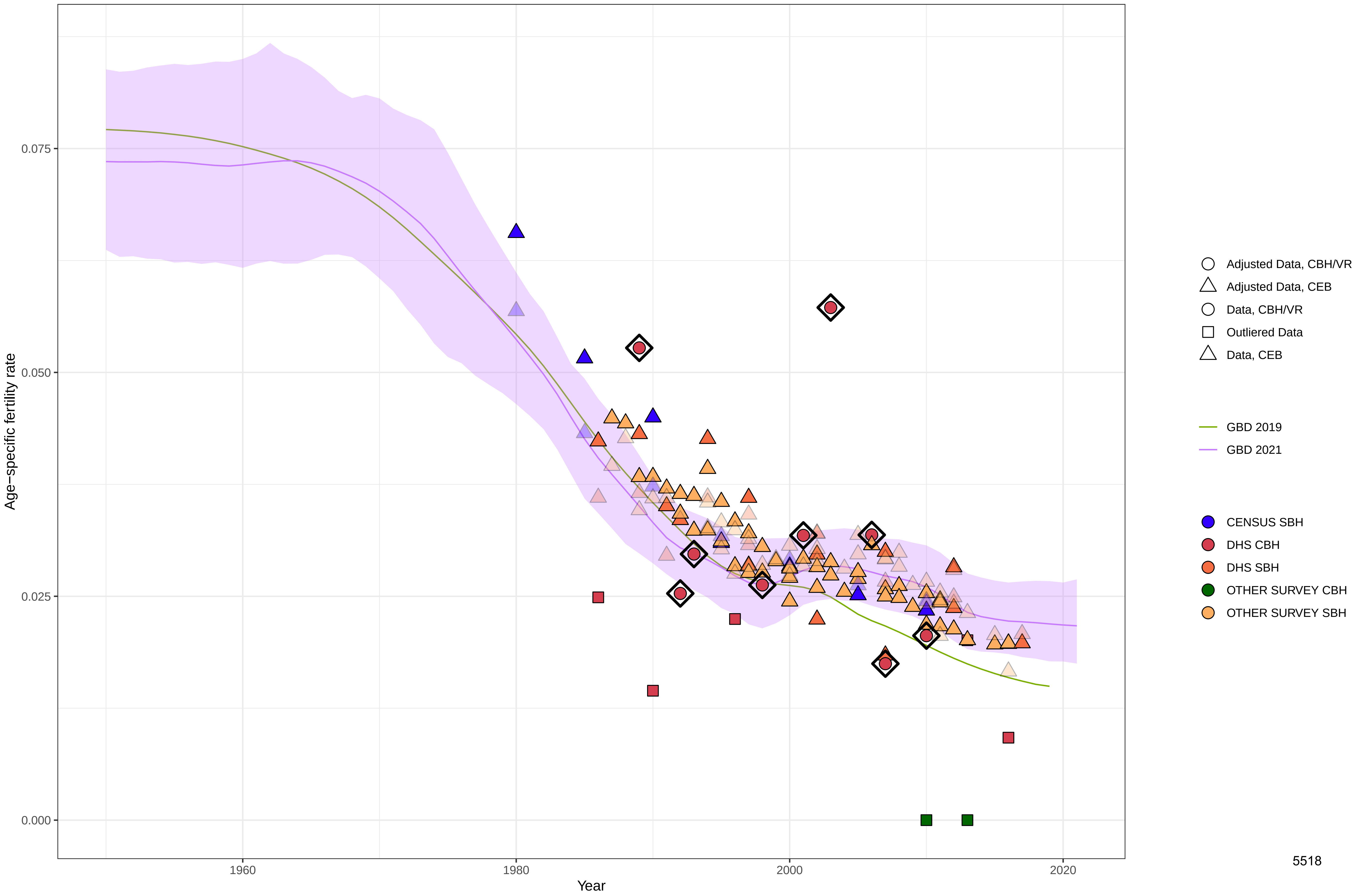

Age−specific fertility fitted trend lines, 1950−2021: Central Kalimantan, Ages: 45−49
Data points enclosed with a diamond are reference sources

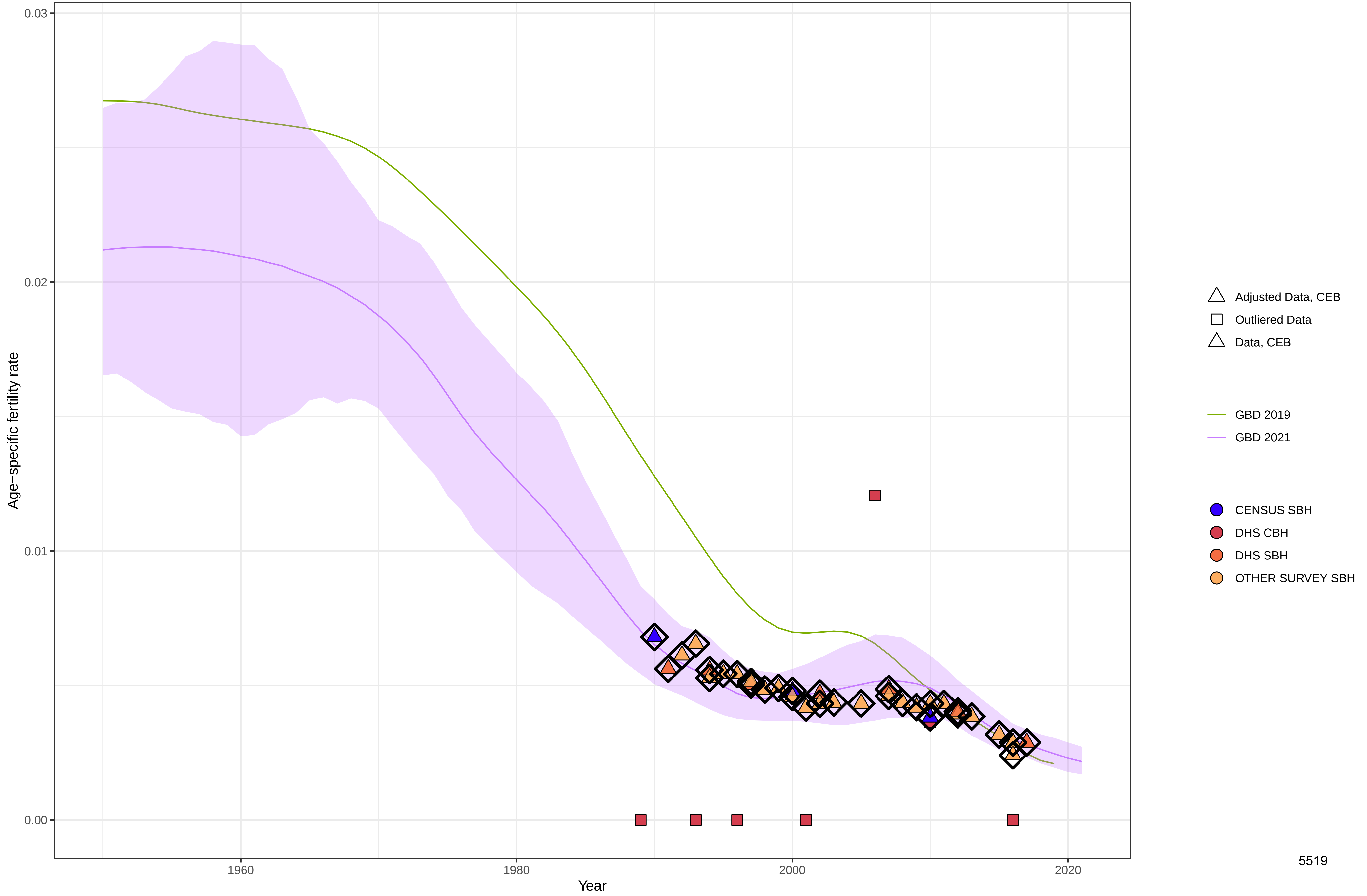

Age−specific fertility fitted trend lines, 1950−2021: Central Kalimantan, Ages: 50−54
Data points enclosed with a diamond are reference sources

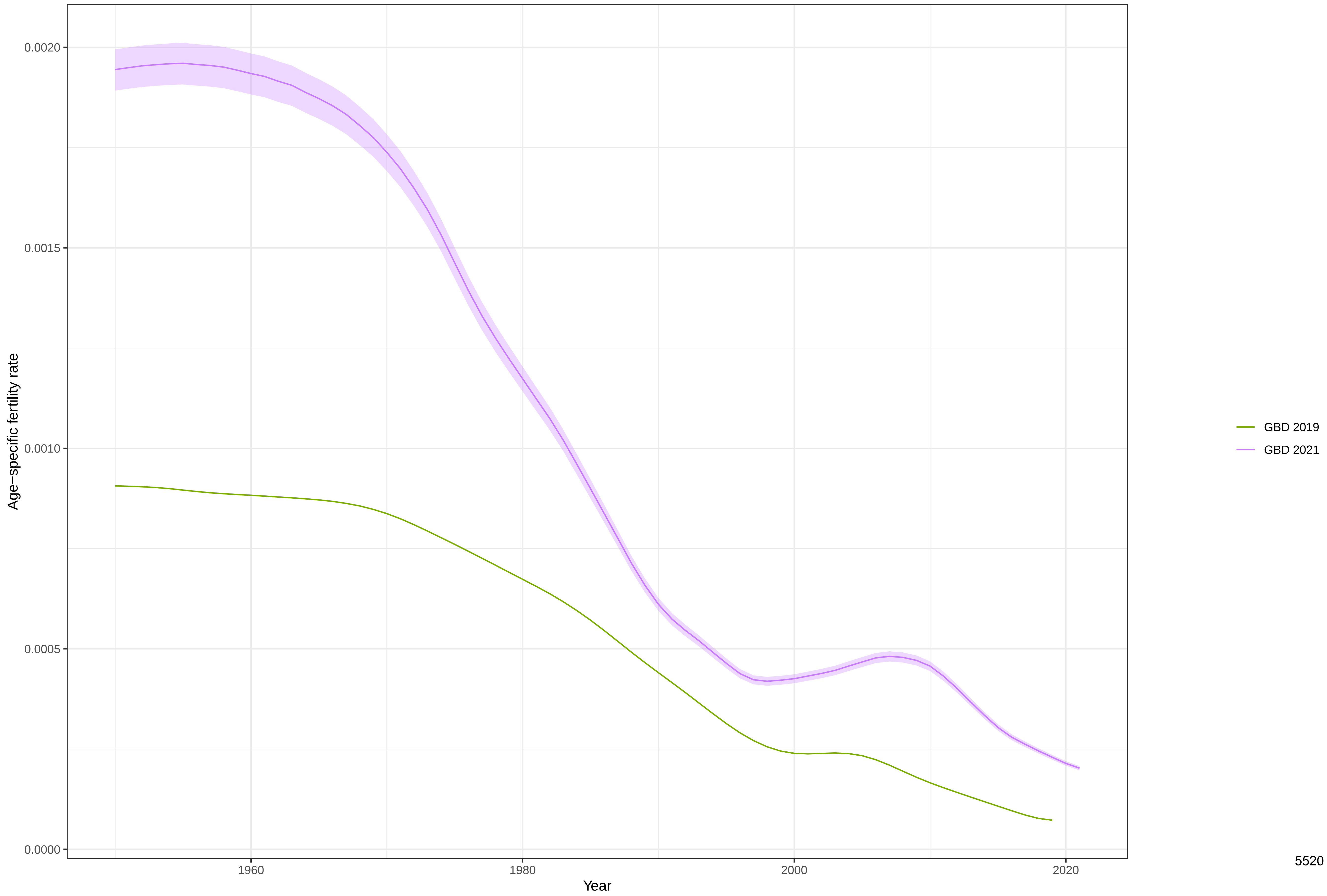

Total fertility rate fitted trend lines, 1950−2021: Central Kalimantan TFR
Data points enclosed with a diamond are reference sources

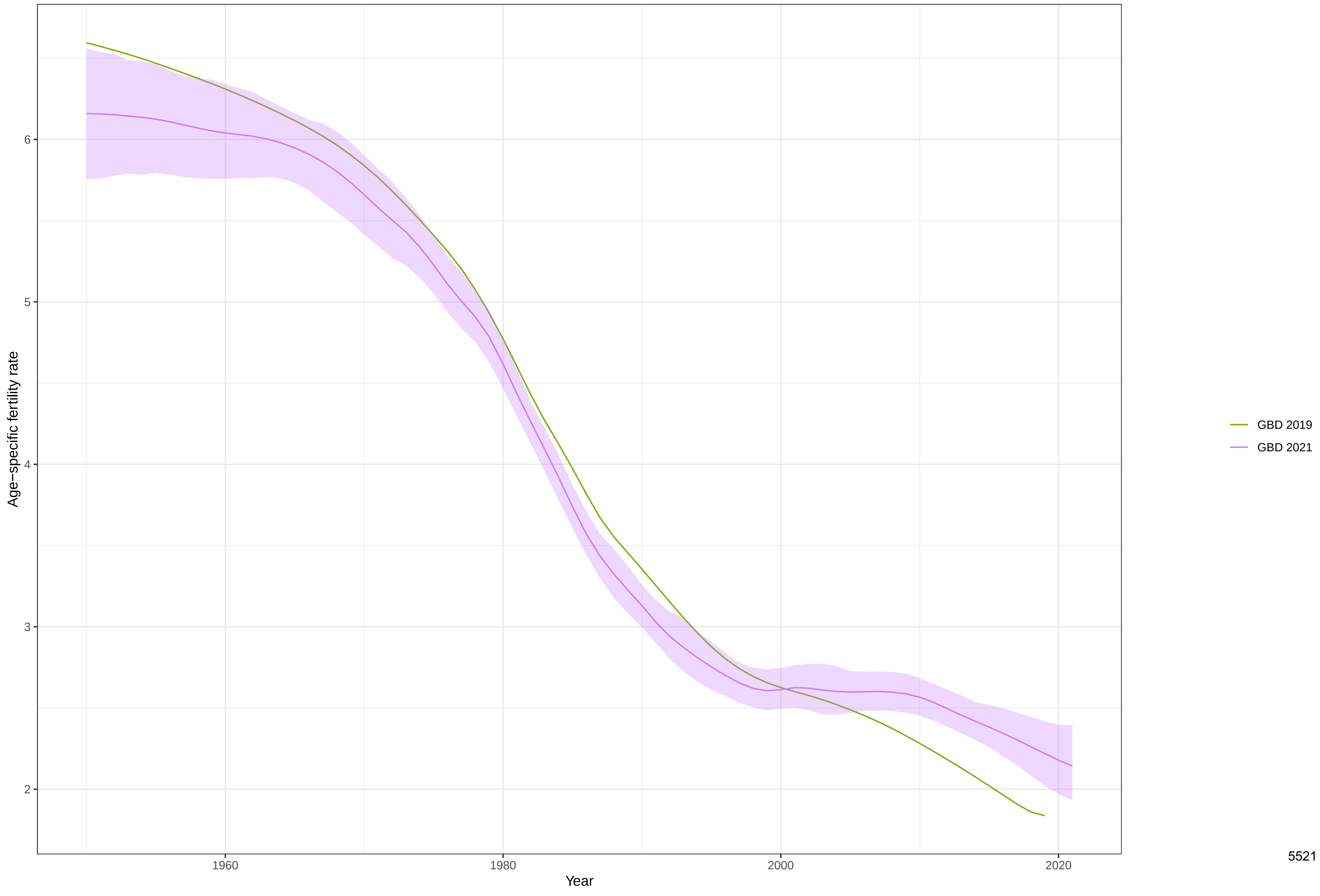

Age−specific fertility fitted trend lines, 1950−2021: East Kalimantan, Ages: 10−14
Data points enclosed with a diamond are reference sources

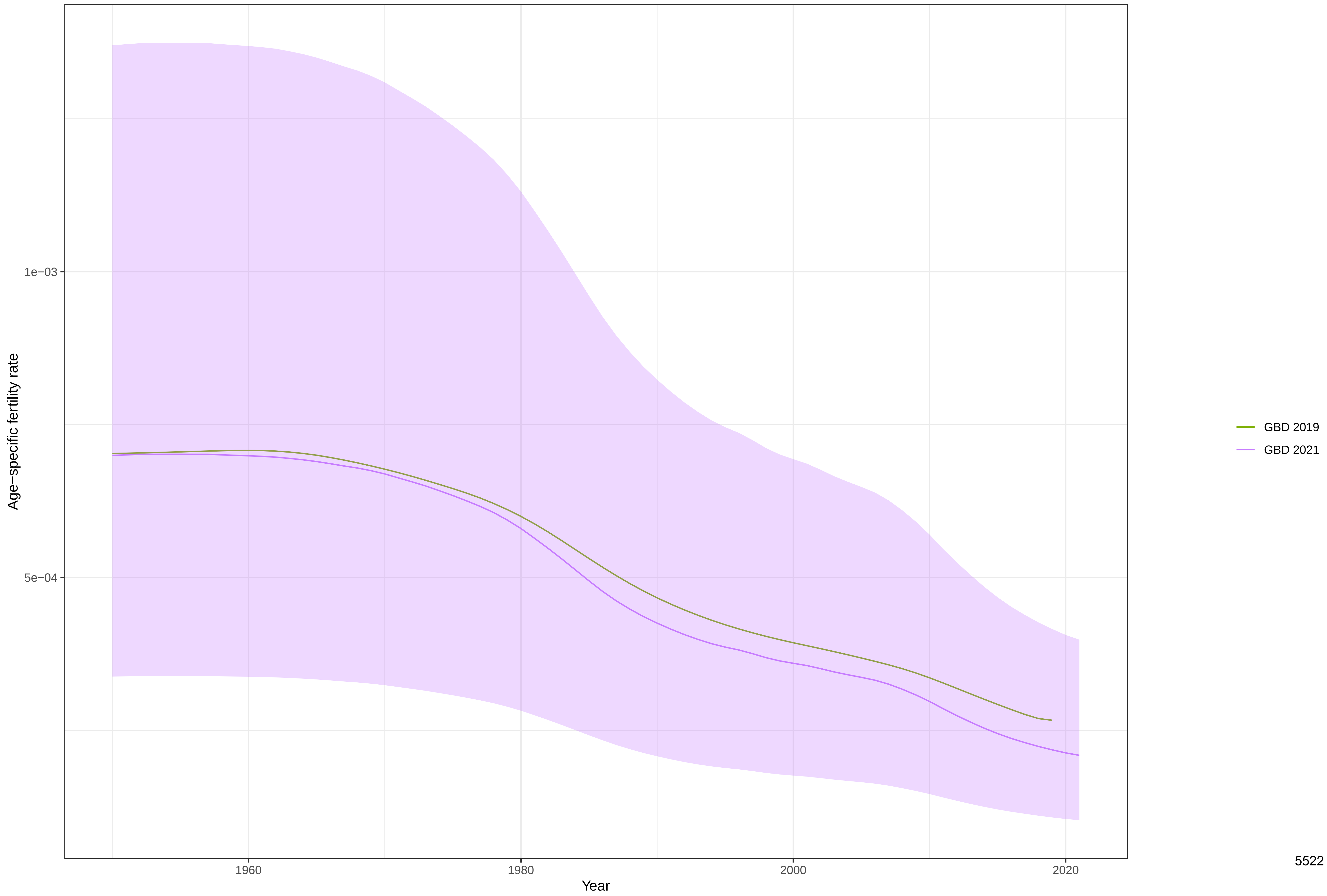

Age−specific fertility fitted trend lines, 1950−2021: East Kalimantan, Ages: 15−19
Data points enclosed with a diamond are reference sources

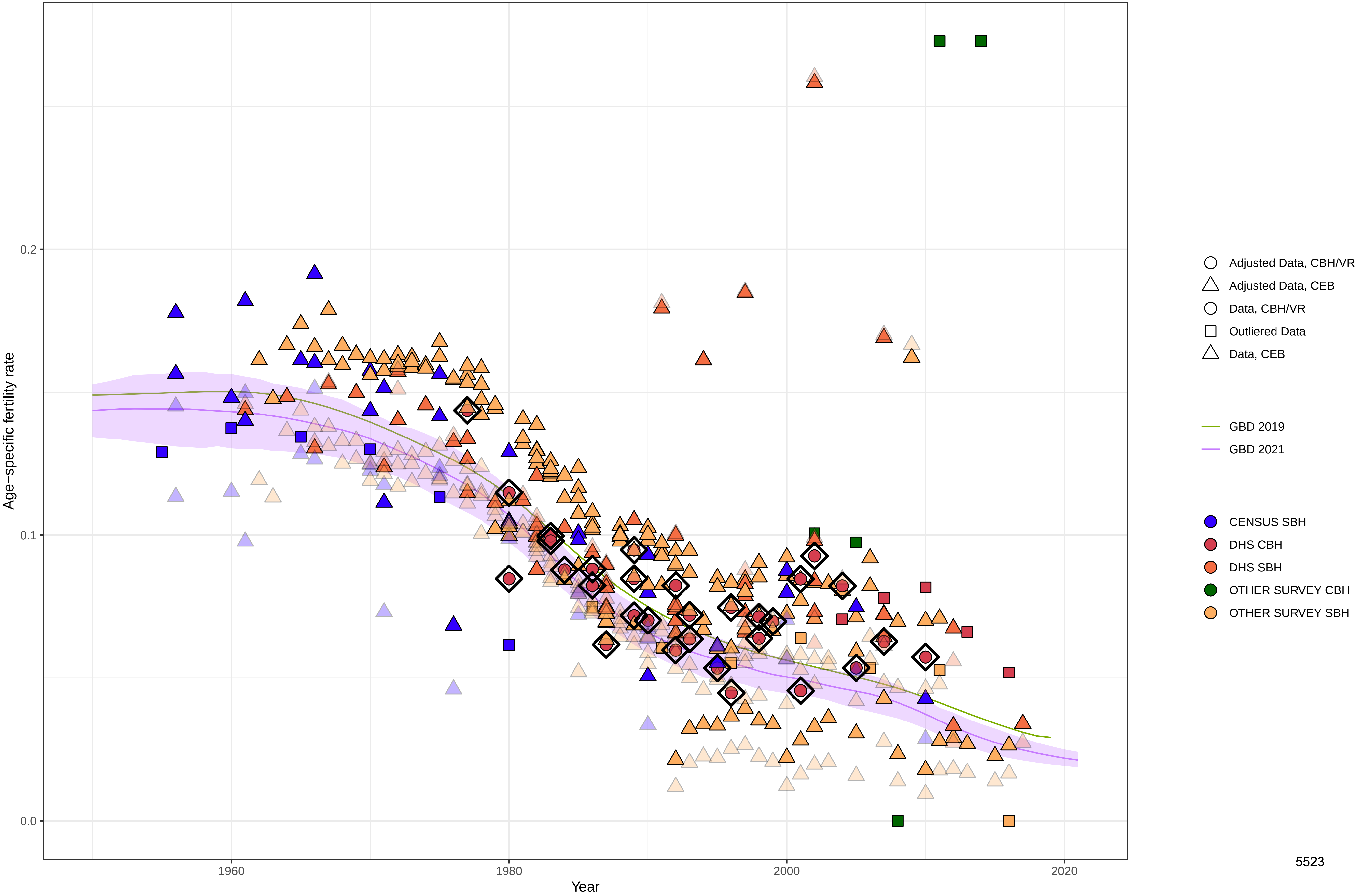

Age−specific fertility fitted trend lines, 1950−2021: East Kalimantan, Ages: 20−24
Data points enclosed with a diamond are reference sources

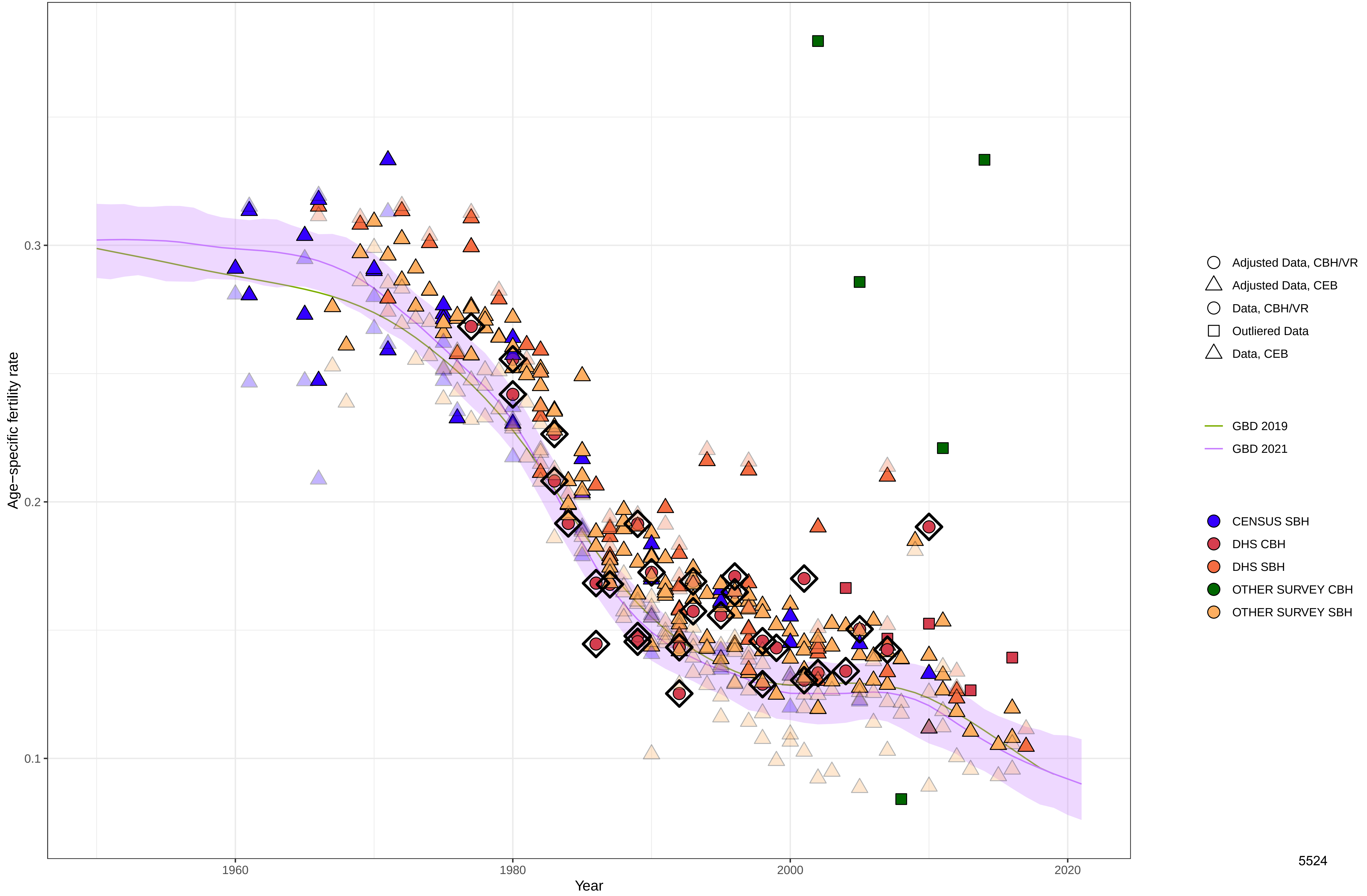

Age−specific fertility fitted trend lines, 1950−2021: East Kalimantan, Ages: 25−29
Data points enclosed with a diamond are reference sources

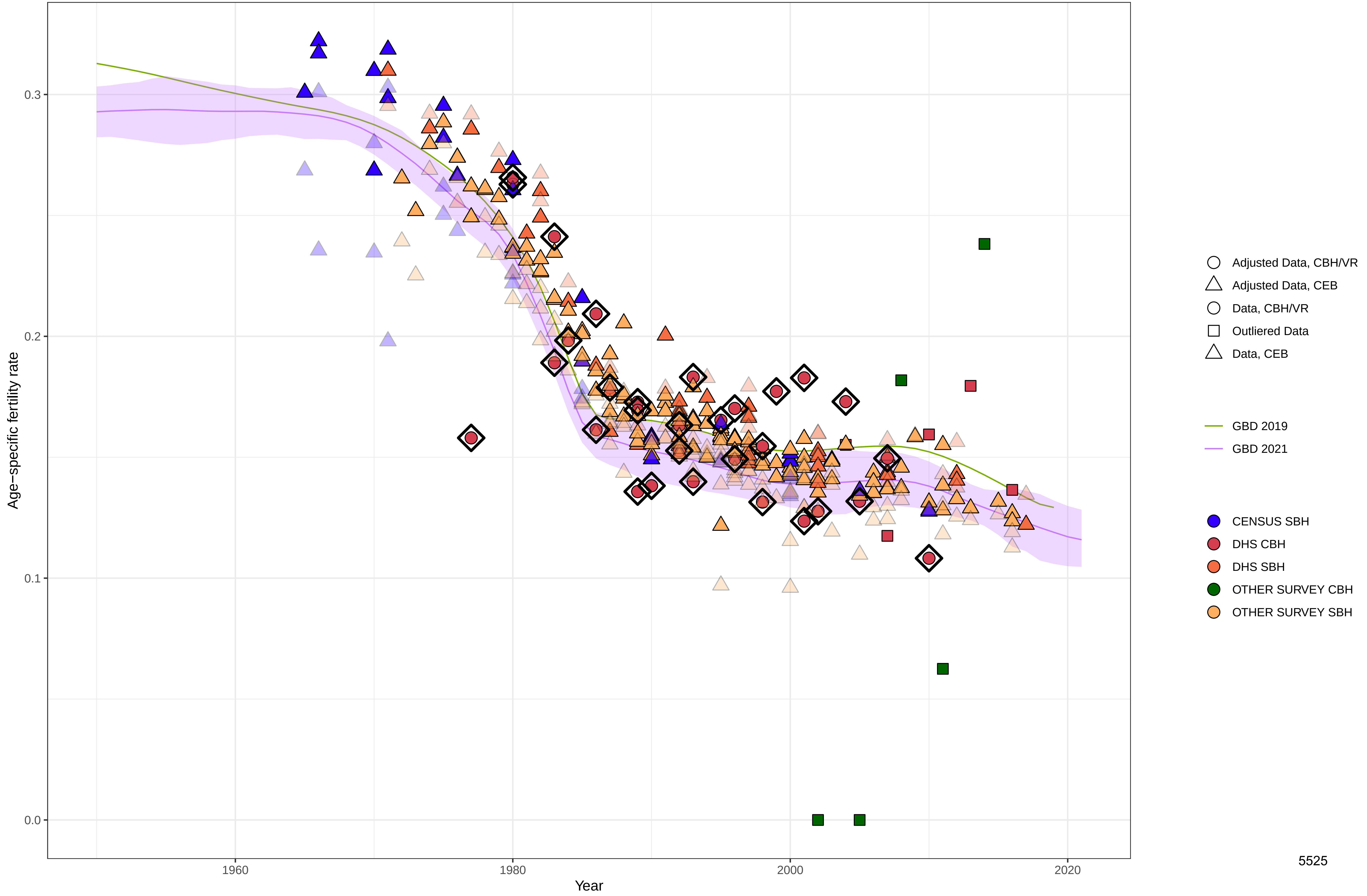

Age−specific fertility fitted trend lines, 1950−2021: East Kalimantan, Ages: 30−34
Data points enclosed with a diamond are reference sources

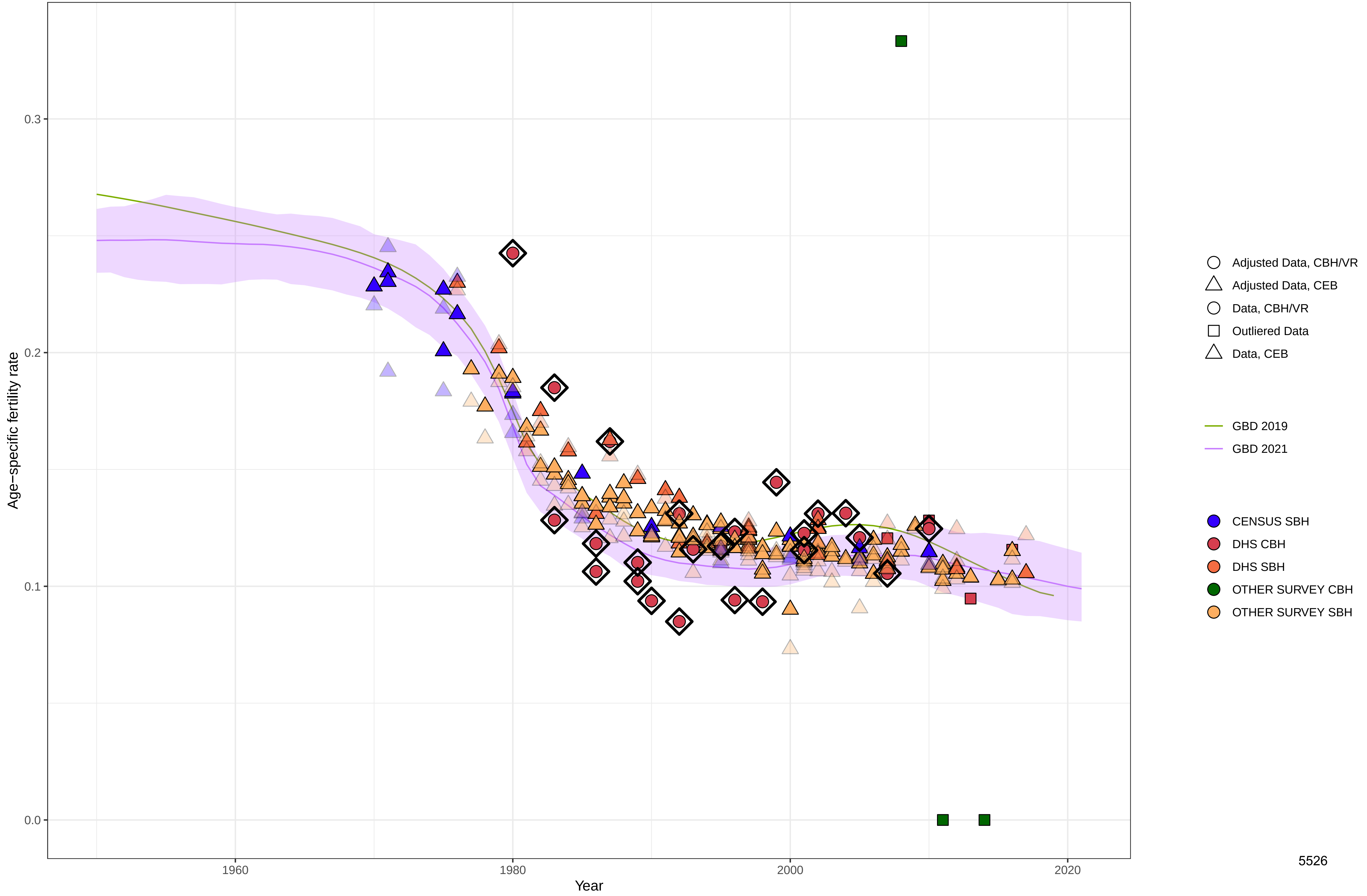

Age−specific fertility fitted trend lines, 1950−2021: East Kalimantan, Ages: 35−39
Data points enclosed with a diamond are reference sources

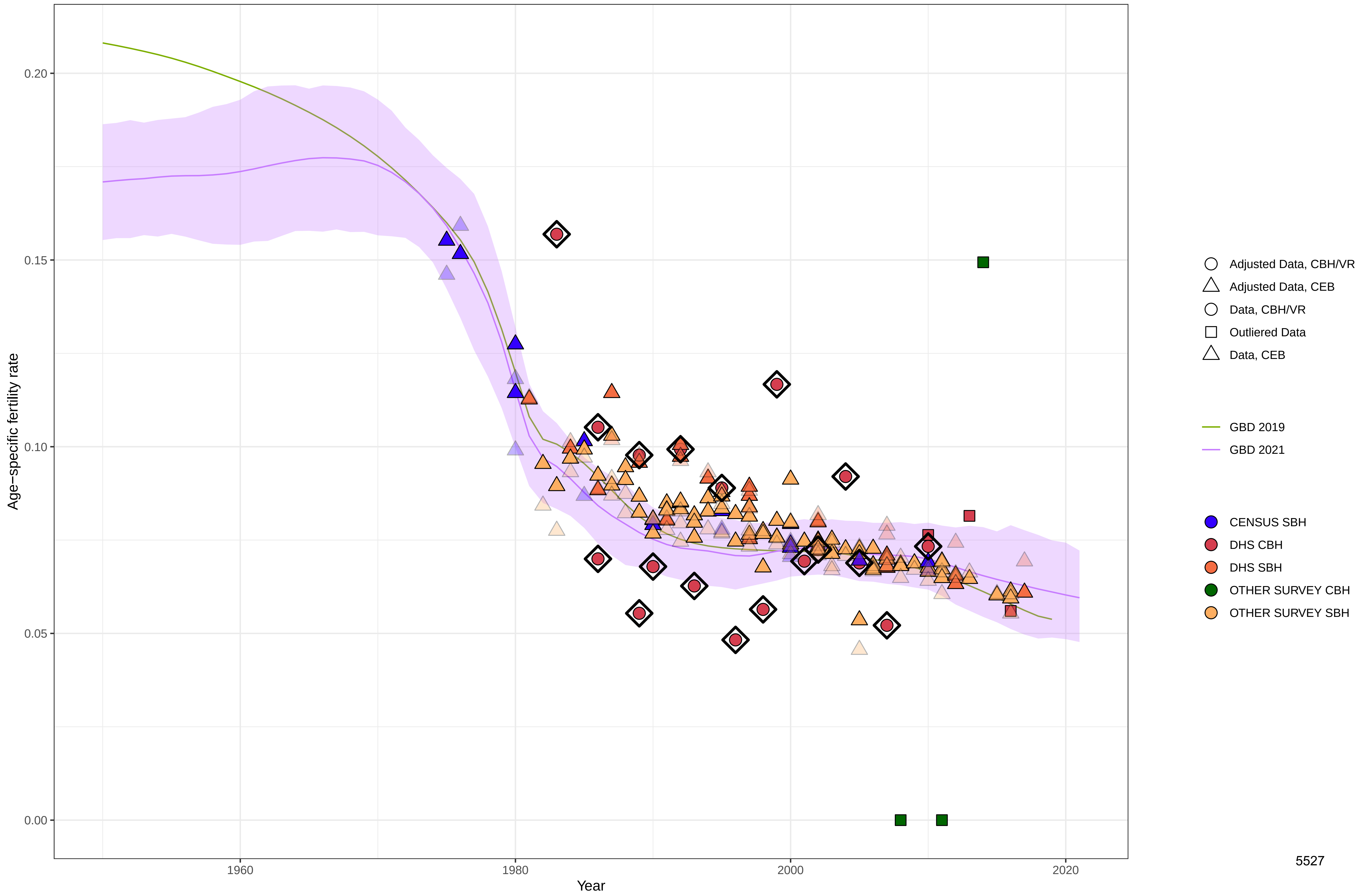

Age−specific fertility fitted trend lines, 1950−2021: East Kalimantan, Ages: 40−44
Data points enclosed with a diamond are reference sources

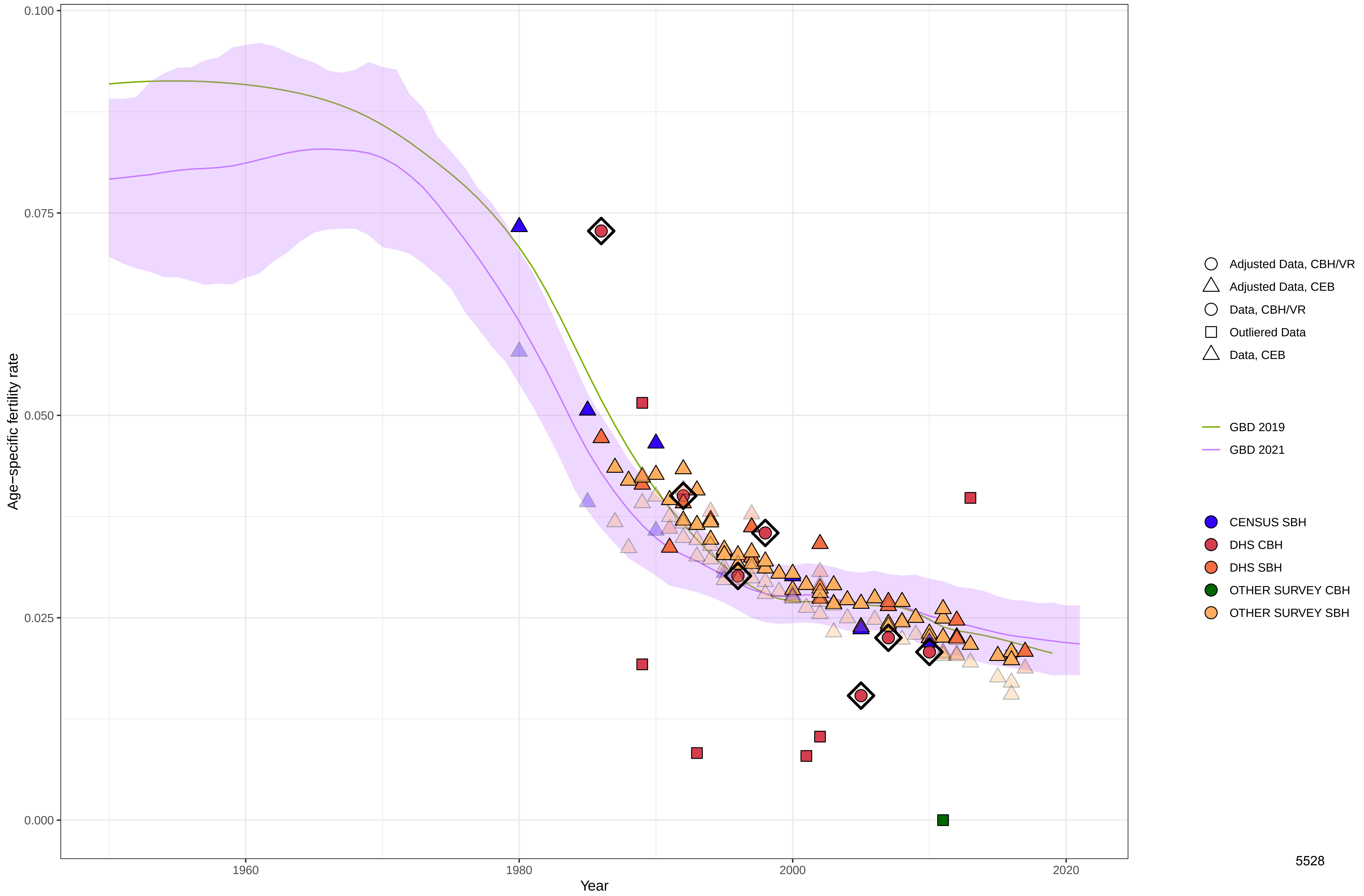

Age−specific fertility fitted trend lines, 1950−2021: East Kalimantan, Ages: 45−49
Data points enclosed with a diamond are reference sources

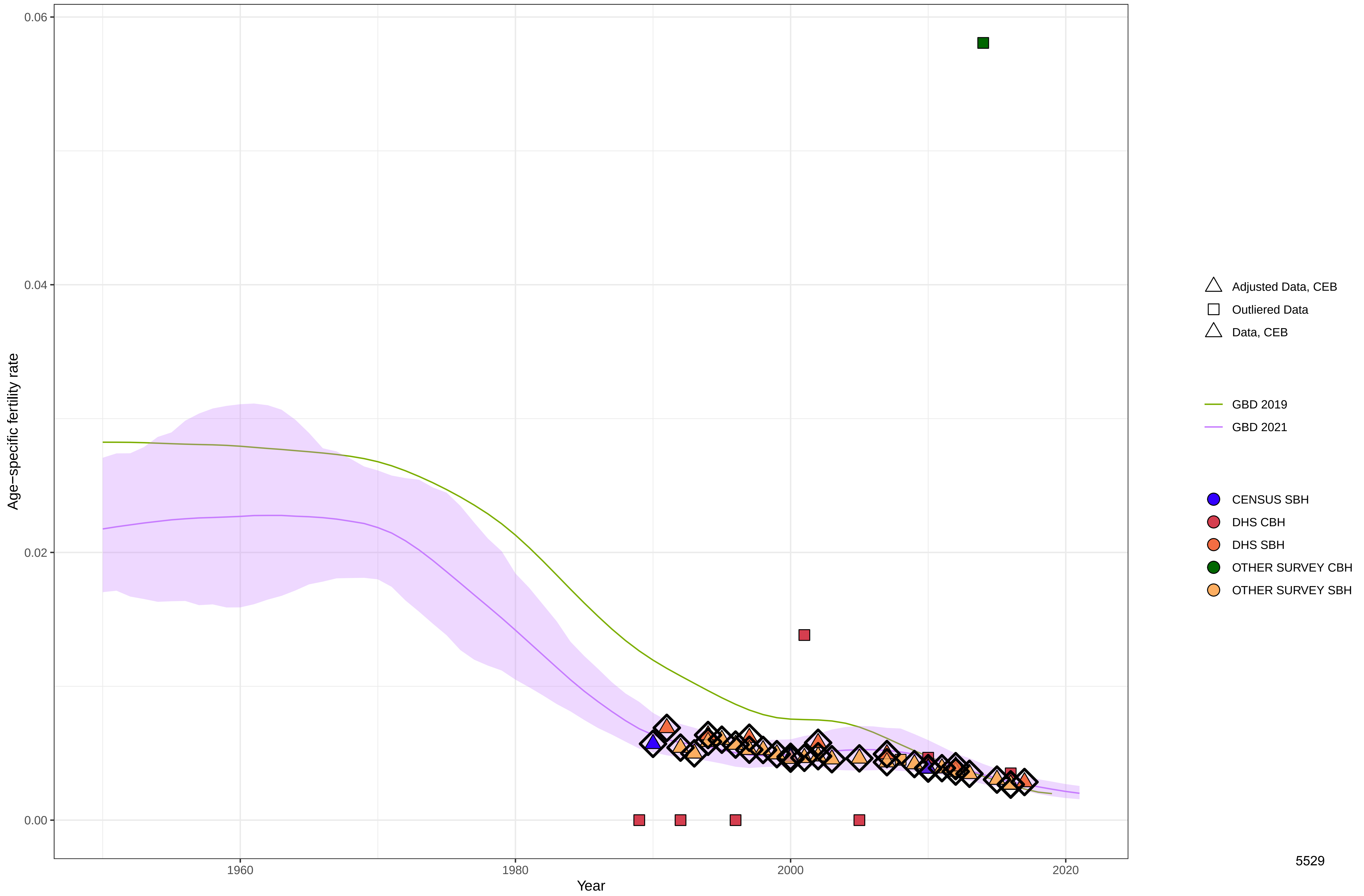

Age−specific fertility fitted trend lines, 1950−2021: East Kalimantan, Ages: 50−54
Data points enclosed with a diamond are reference sources

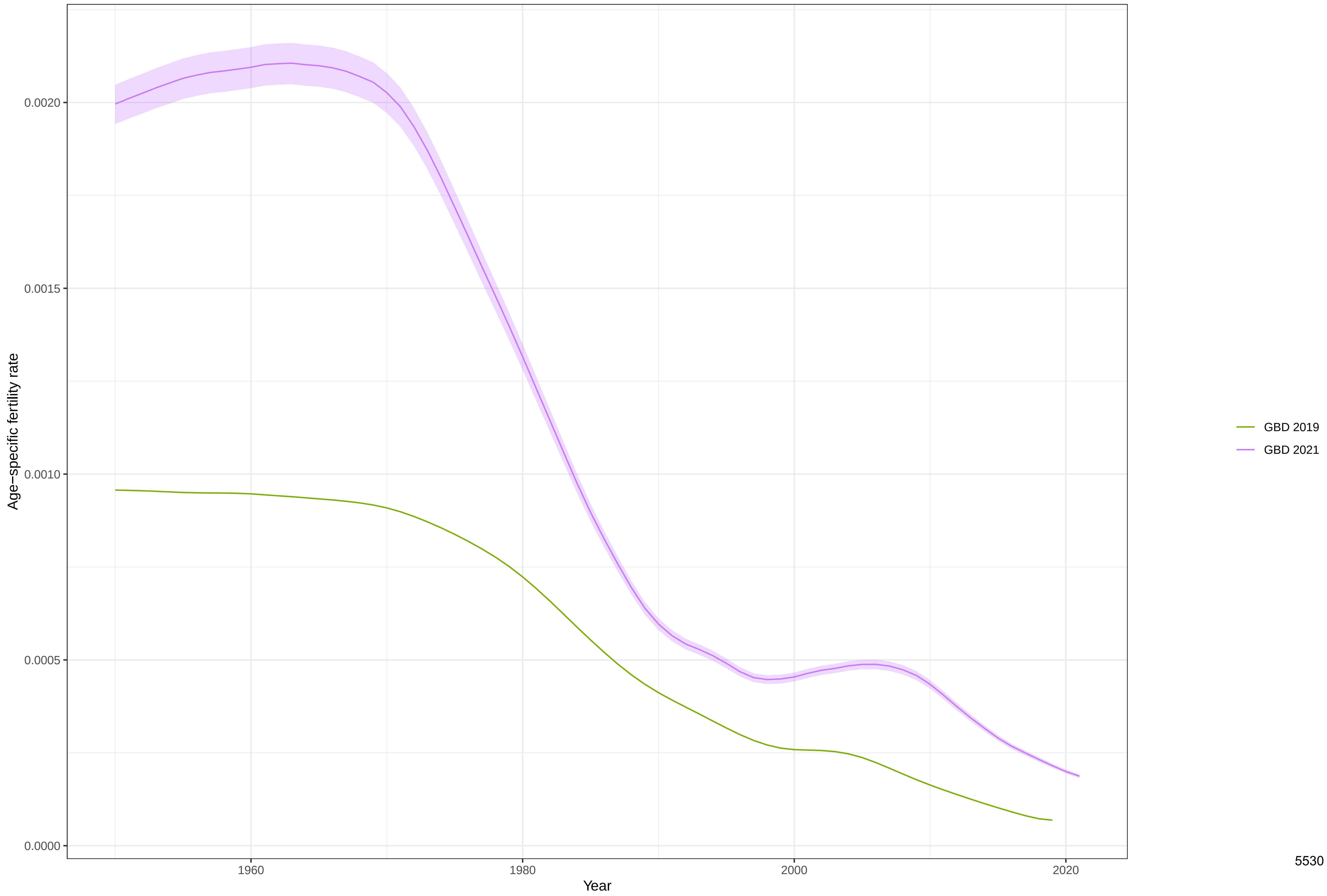

Total fertility rate fitted trend lines, 1950−2021: East Kalimantan TFR
Data points enclosed with a diamond are reference sources

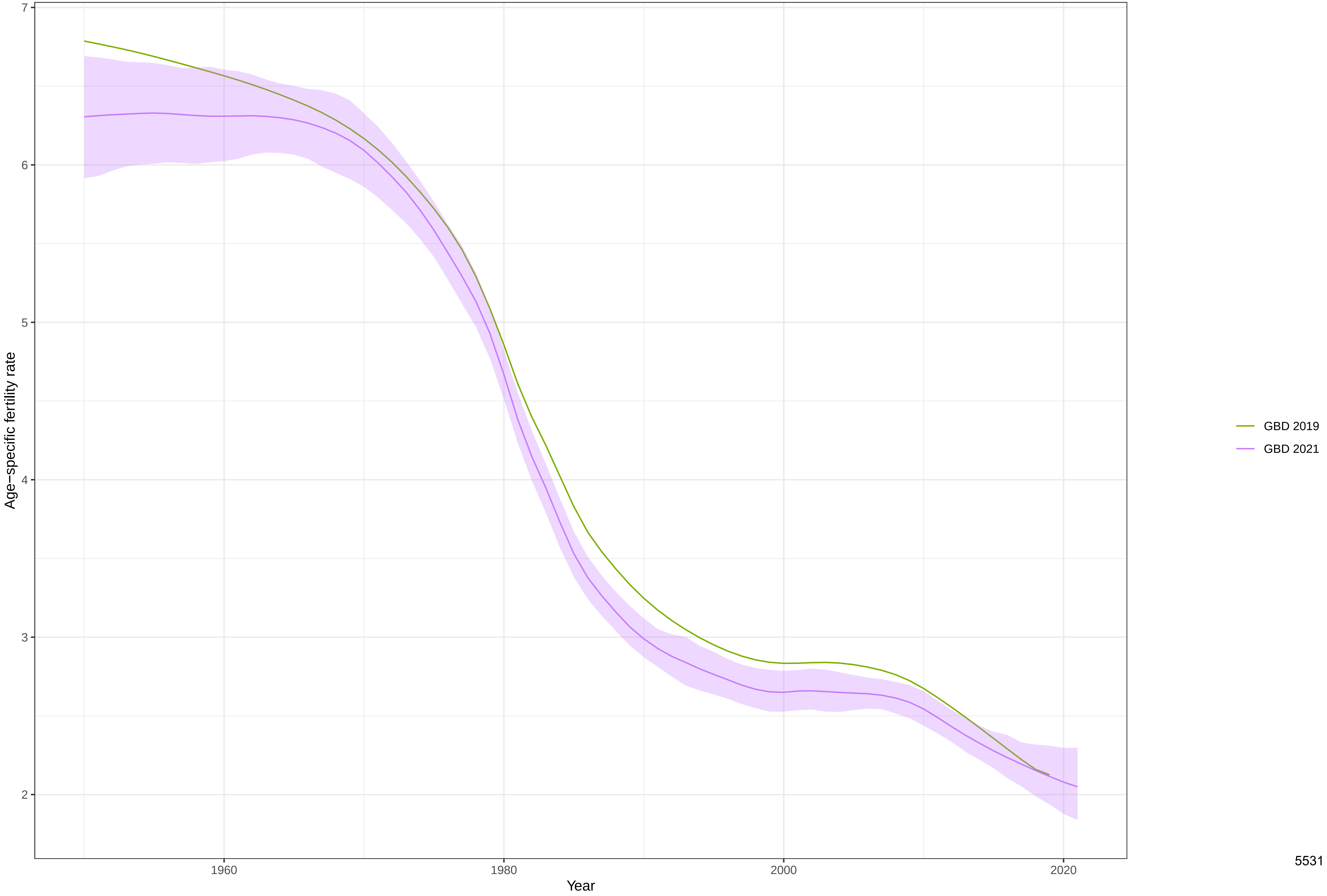

Age−specific fertility fitted trend lines, 1950−2021: North Kalimantan, Ages: 10−14
Data points enclosed with a diamond are reference sources

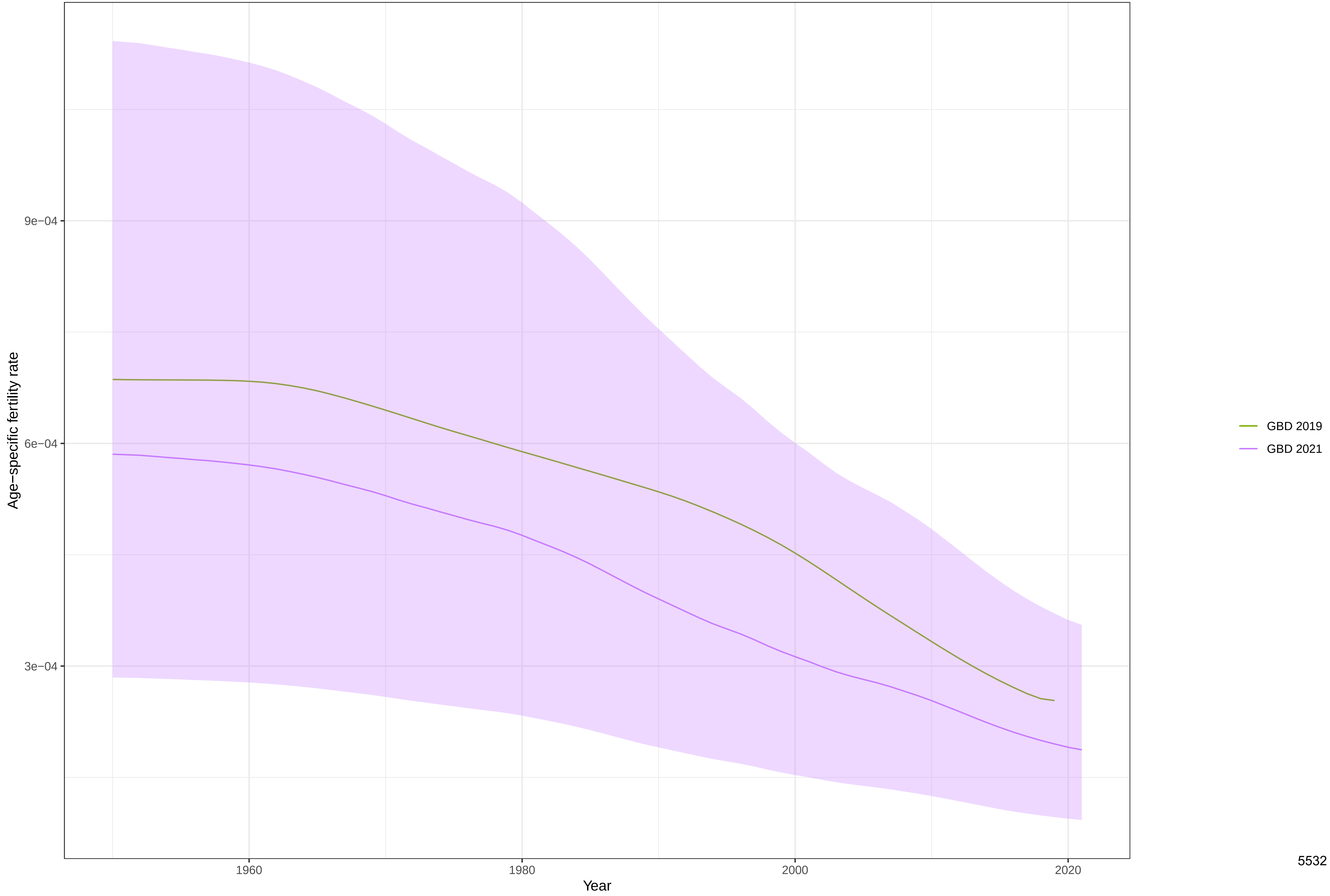

Age−specific fertility fitted trend lines, 1950−2021: North Kalimantan, Ages: 15−19
Data points enclosed with a diamond are reference sources

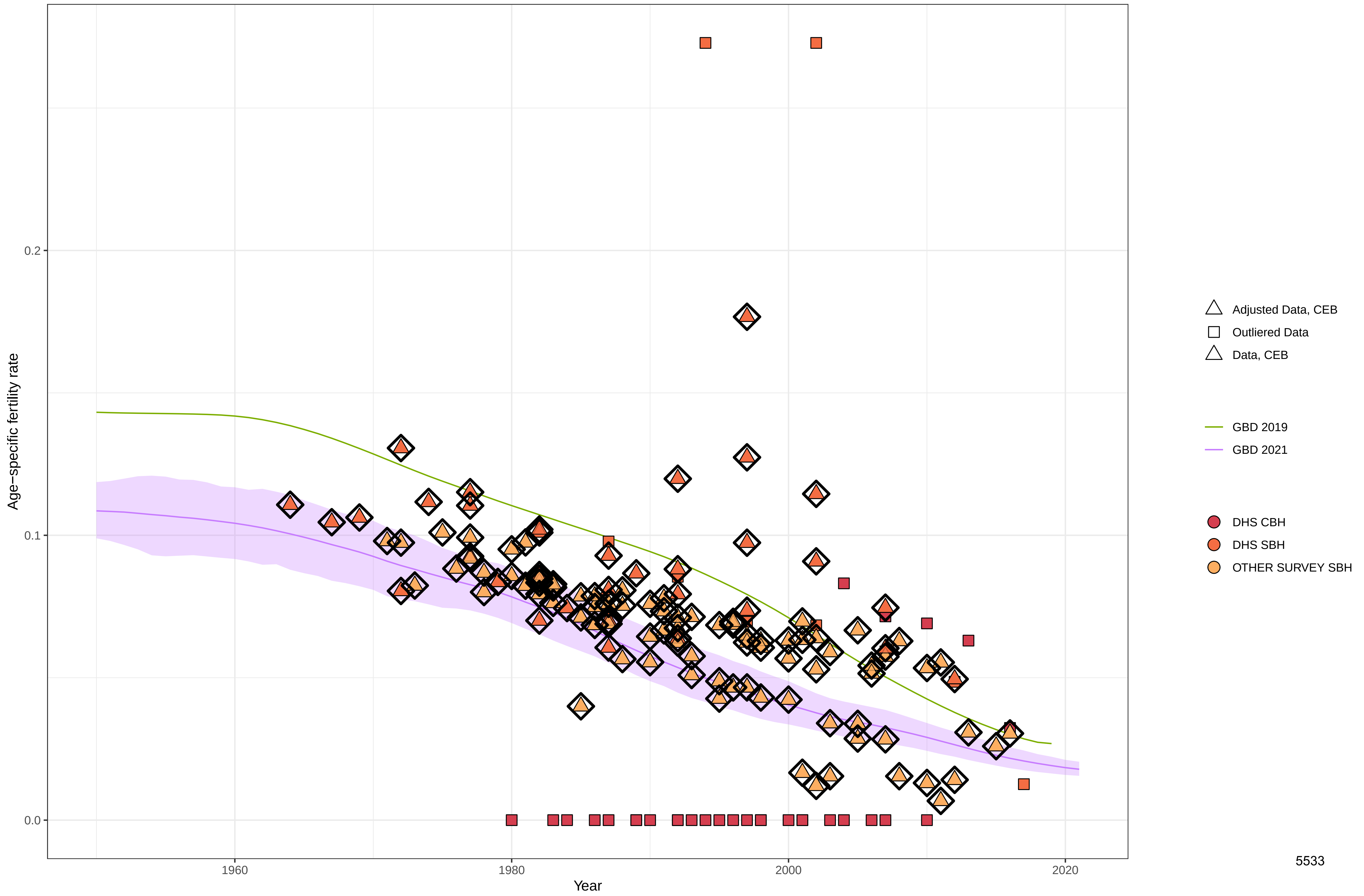

Age−specific fertility fitted trend lines, 1950−2021: North Kalimantan, Ages: 20−24
Data points enclosed with a diamond are reference sources

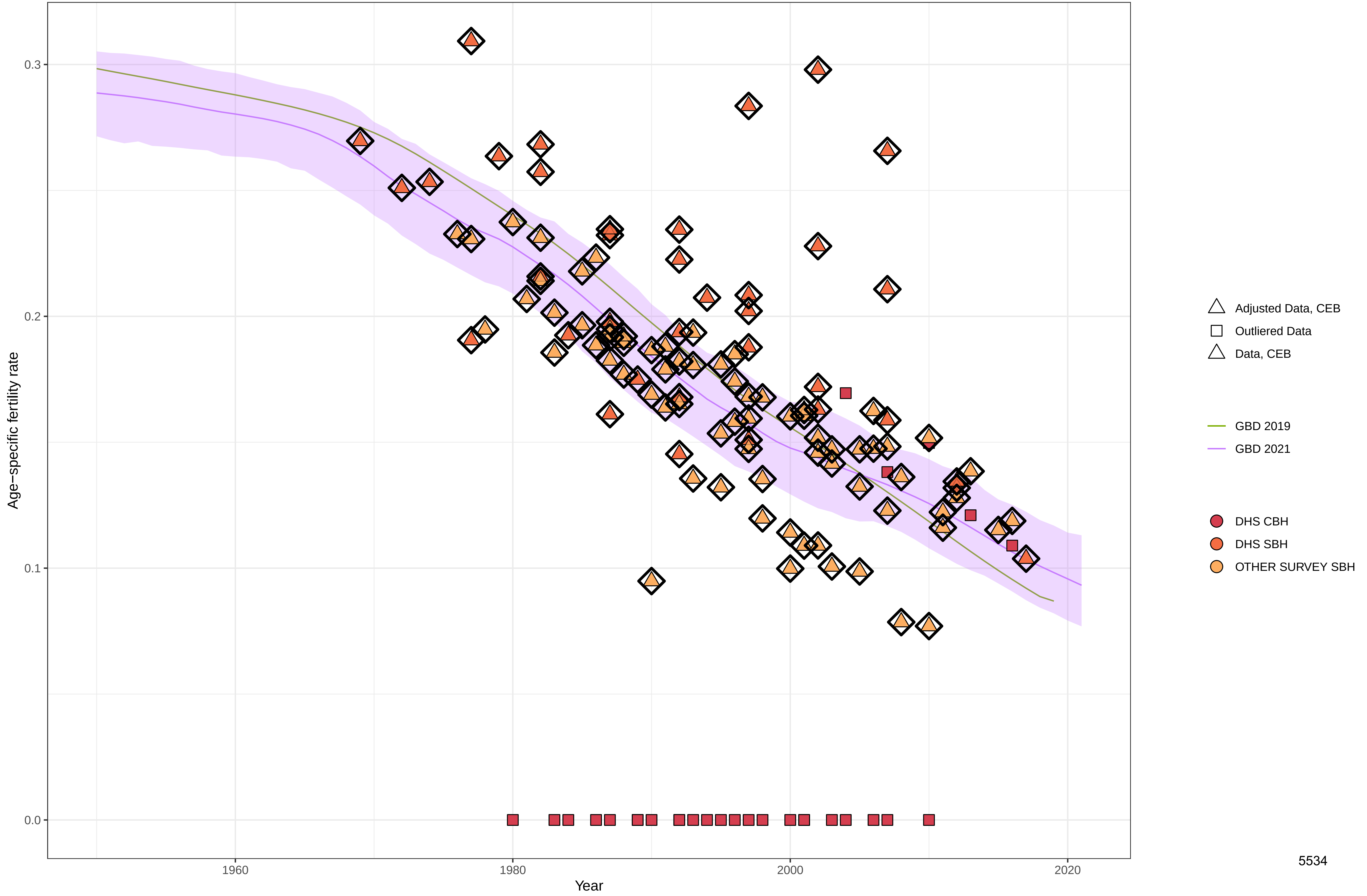

Age−specific fertility fitted trend lines, 1950−2021: North Kalimantan, Ages: 25−29
Data points enclosed with a diamond are reference sources

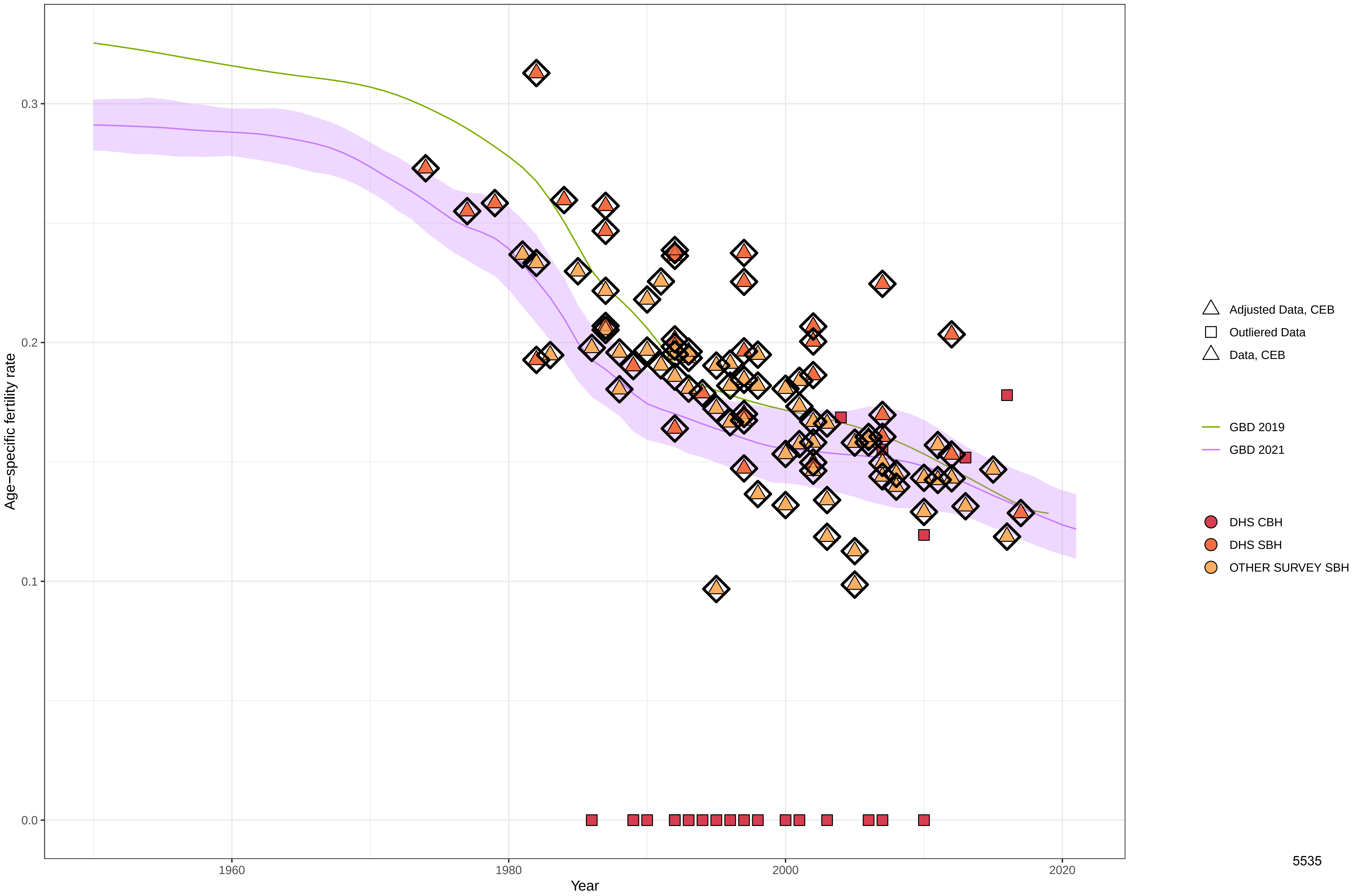

Age−specific fertility fitted trend lines, 1950−2021: North Kalimantan, Ages: 30−34
Data points enclosed with a diamond are reference sources

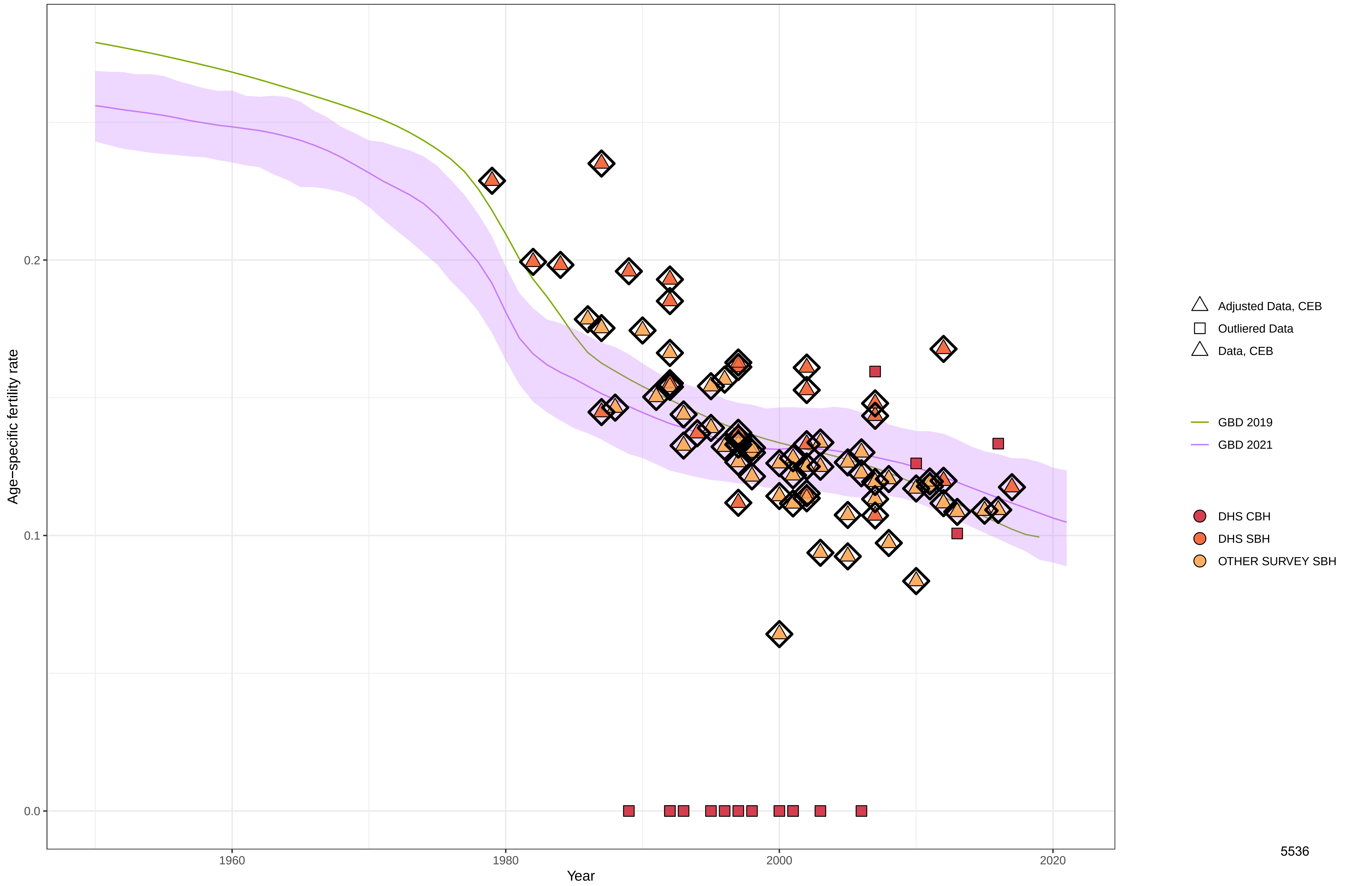

Age−specific fertility fitted trend lines, 1950−2021: North Kalimantan, Ages: 35−39
Data points enclosed with a diamond are reference sources

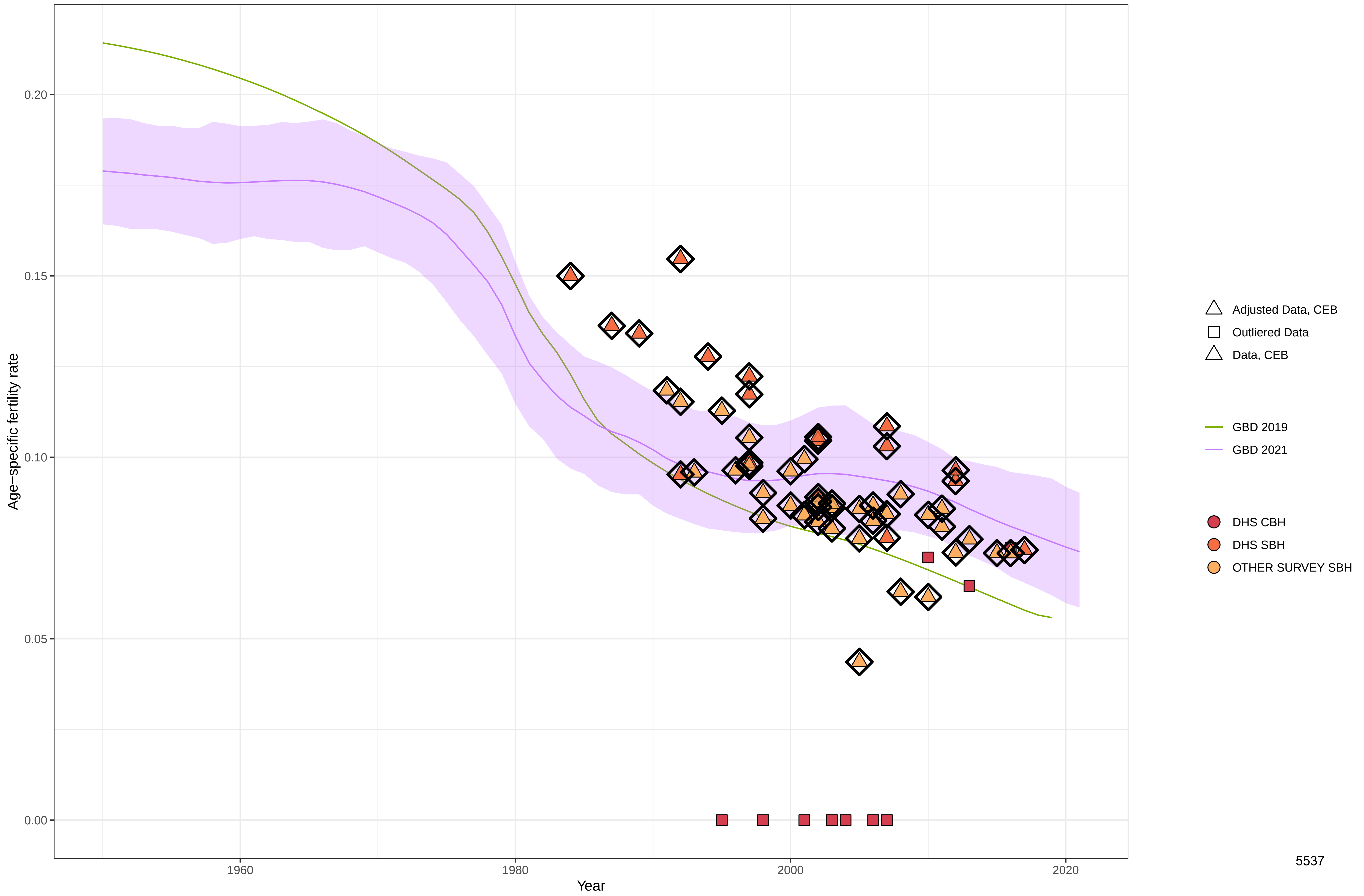

Age−specific fertility fitted trend lines, 1950−2021: North Kalimantan, Ages: 40−44
Data points enclosed with a diamond are reference sources

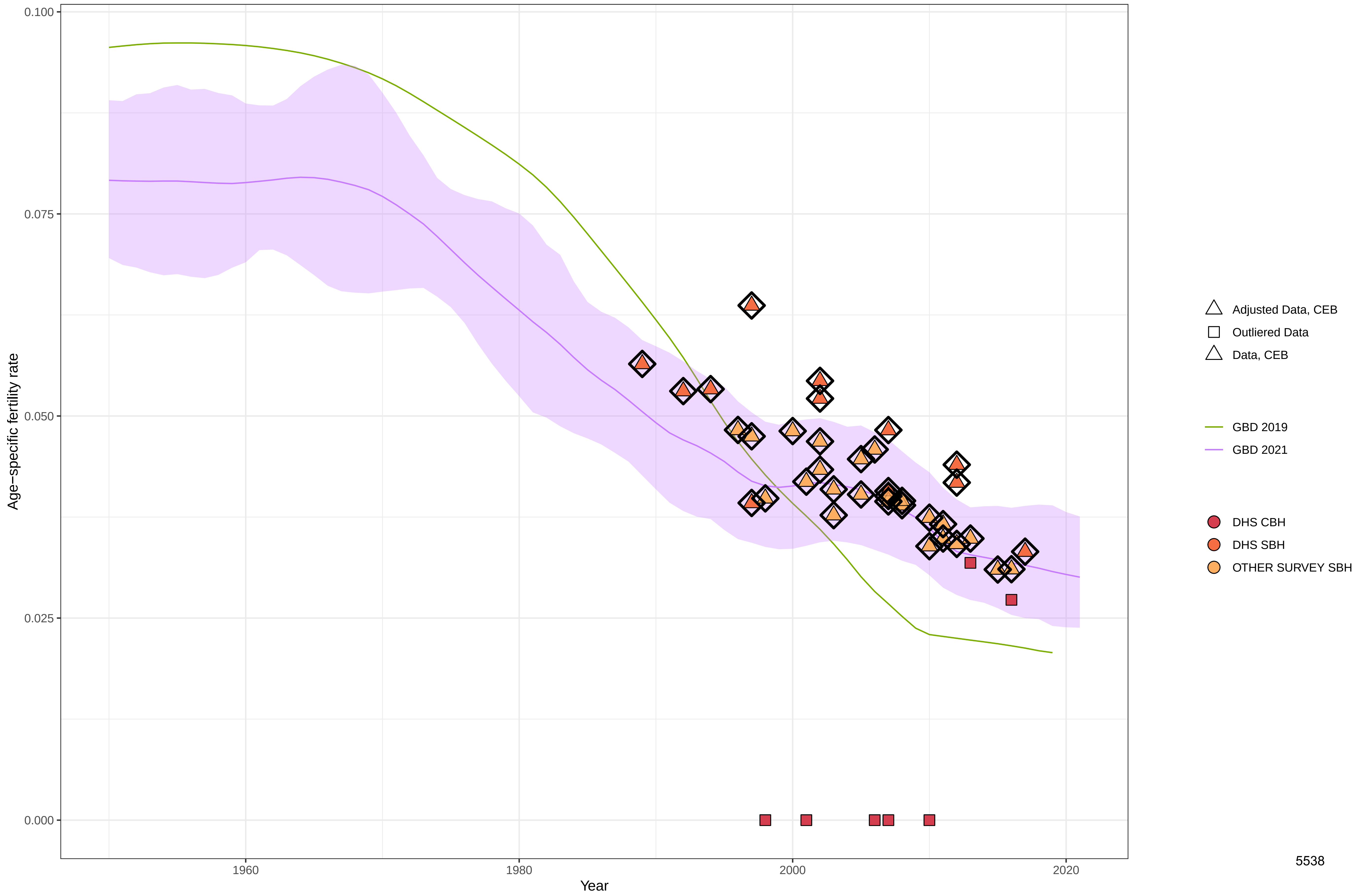

Age−specific fertility fitted trend lines, 1950−2021: North Kalimantan, Ages: 45−49
Data points enclosed with a diamond are reference sources

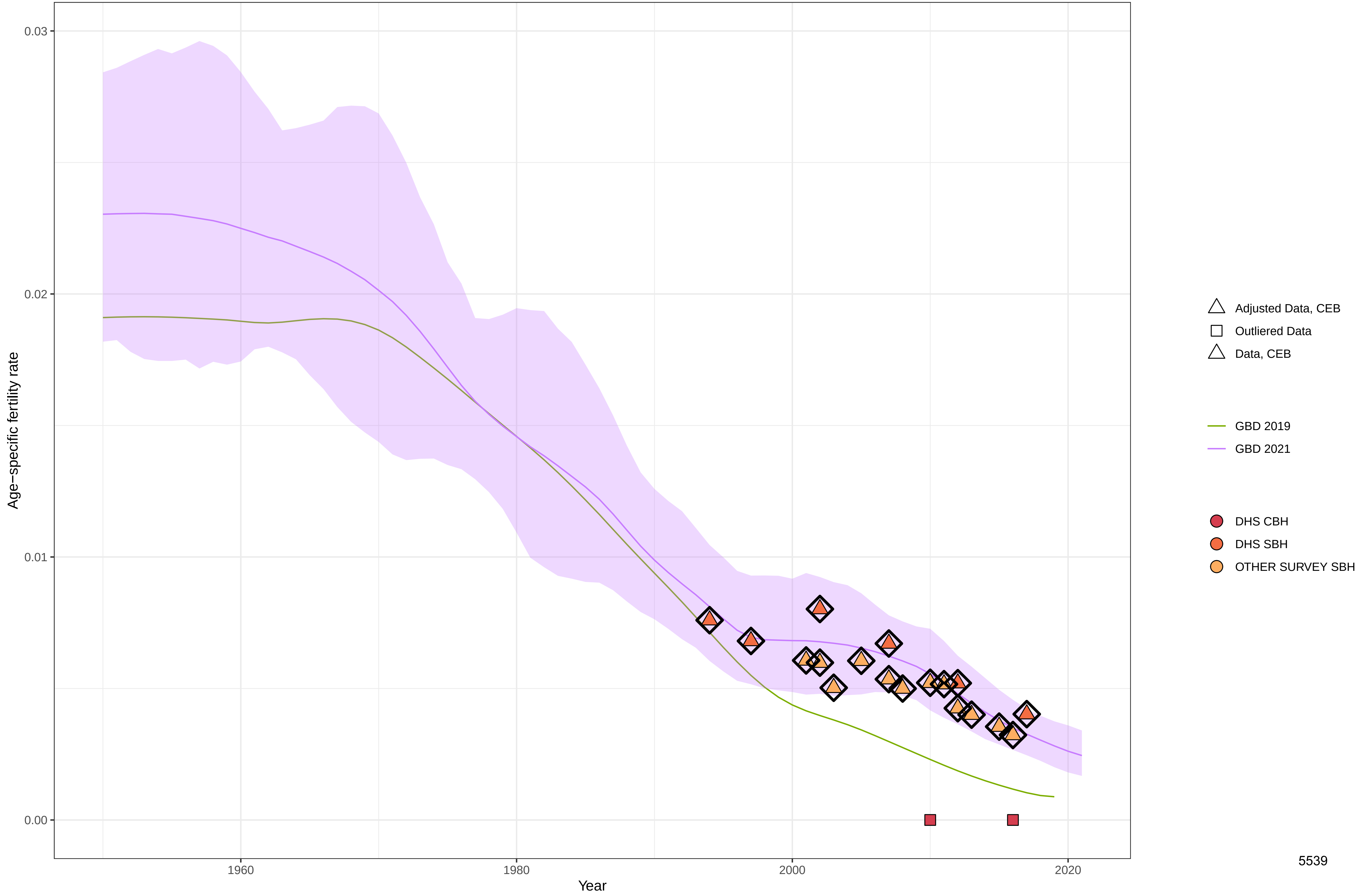

Age−specific fertility fitted trend lines, 1950−2021: North Kalimantan, Ages: 50−54
Data points enclosed with a diamond are reference sources

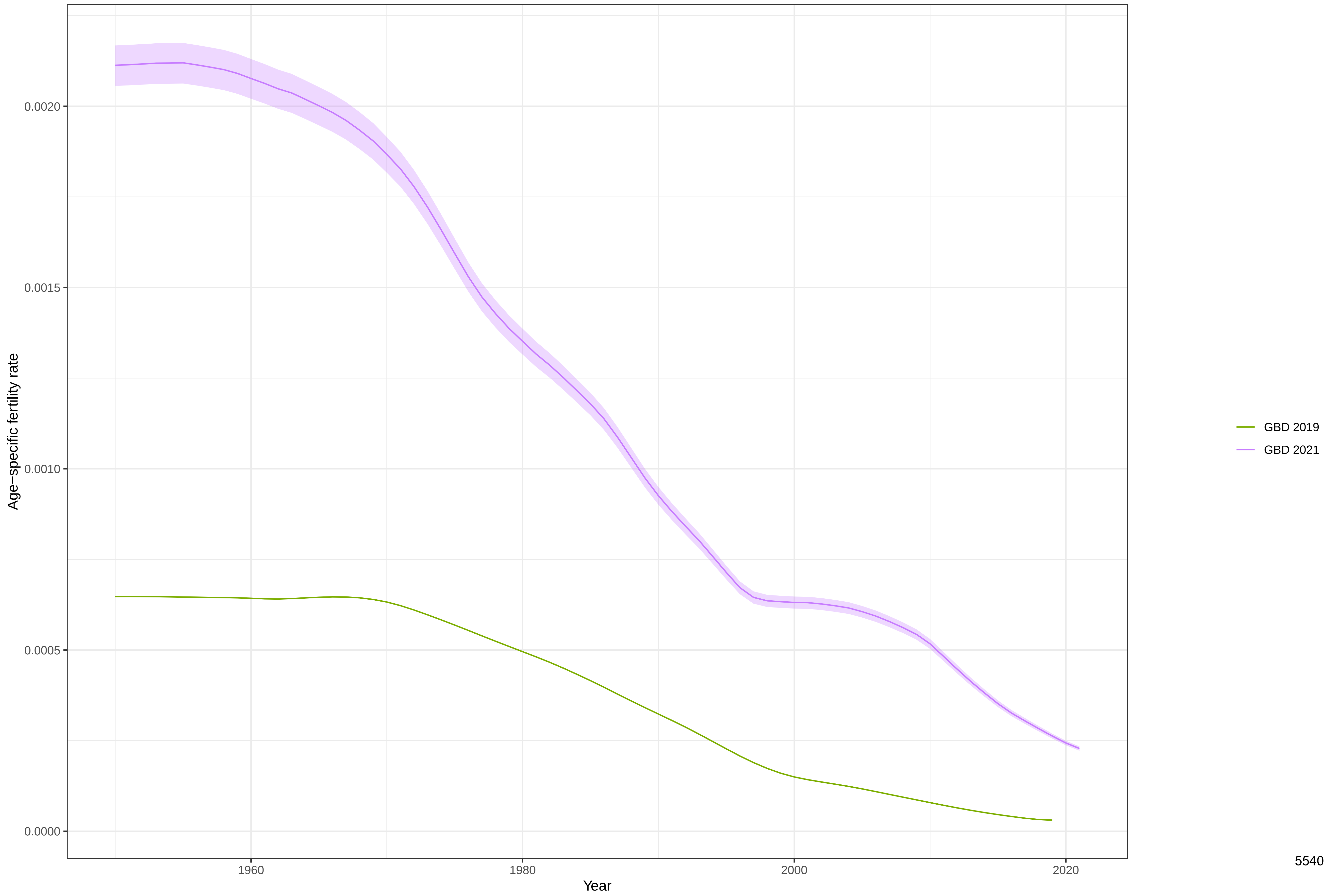

Total fertility rate fitted trend lines, 1950−2021: North Kalimantan TFR
Data points enclosed with a diamond are reference sources

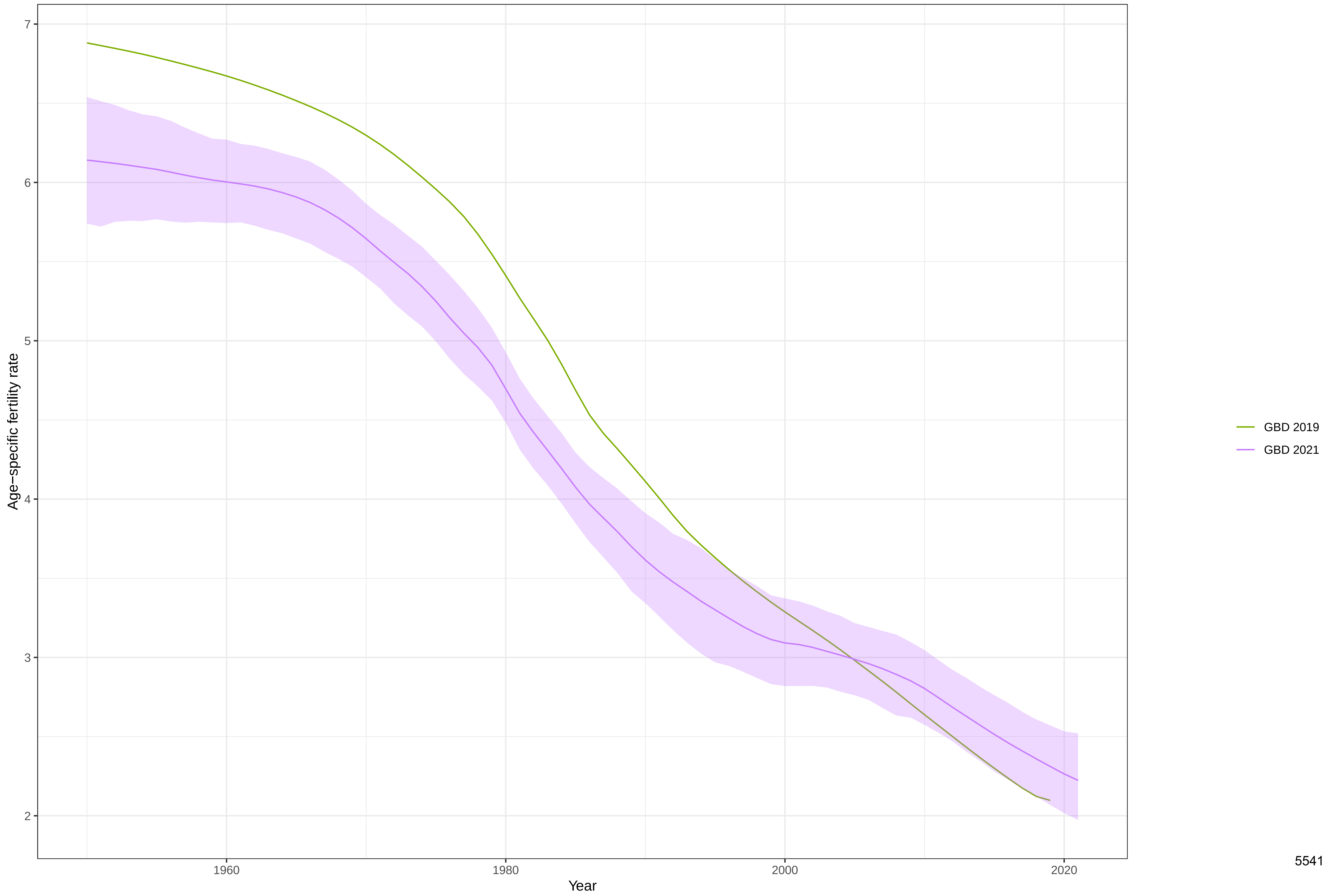

Age−specific fertility fitted trend lines, 1950−2021: Riau Islands, Ages: 10−14
Data points enclosed with a diamond are reference sources

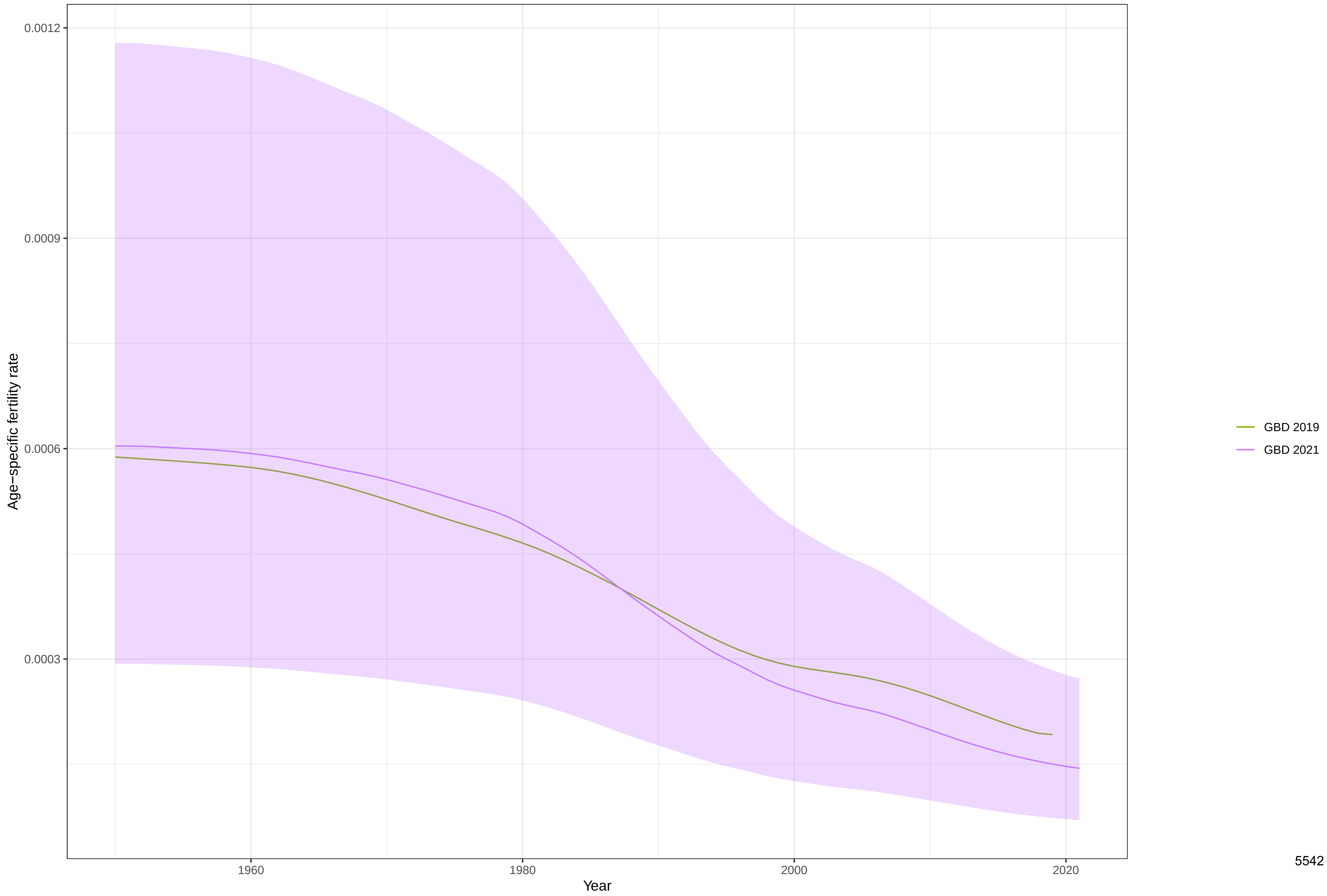

Age−specific fertility fitted trend lines, 1950−2021: Riau Islands, Ages: 15−19
Data points enclosed with a diamond are reference sources

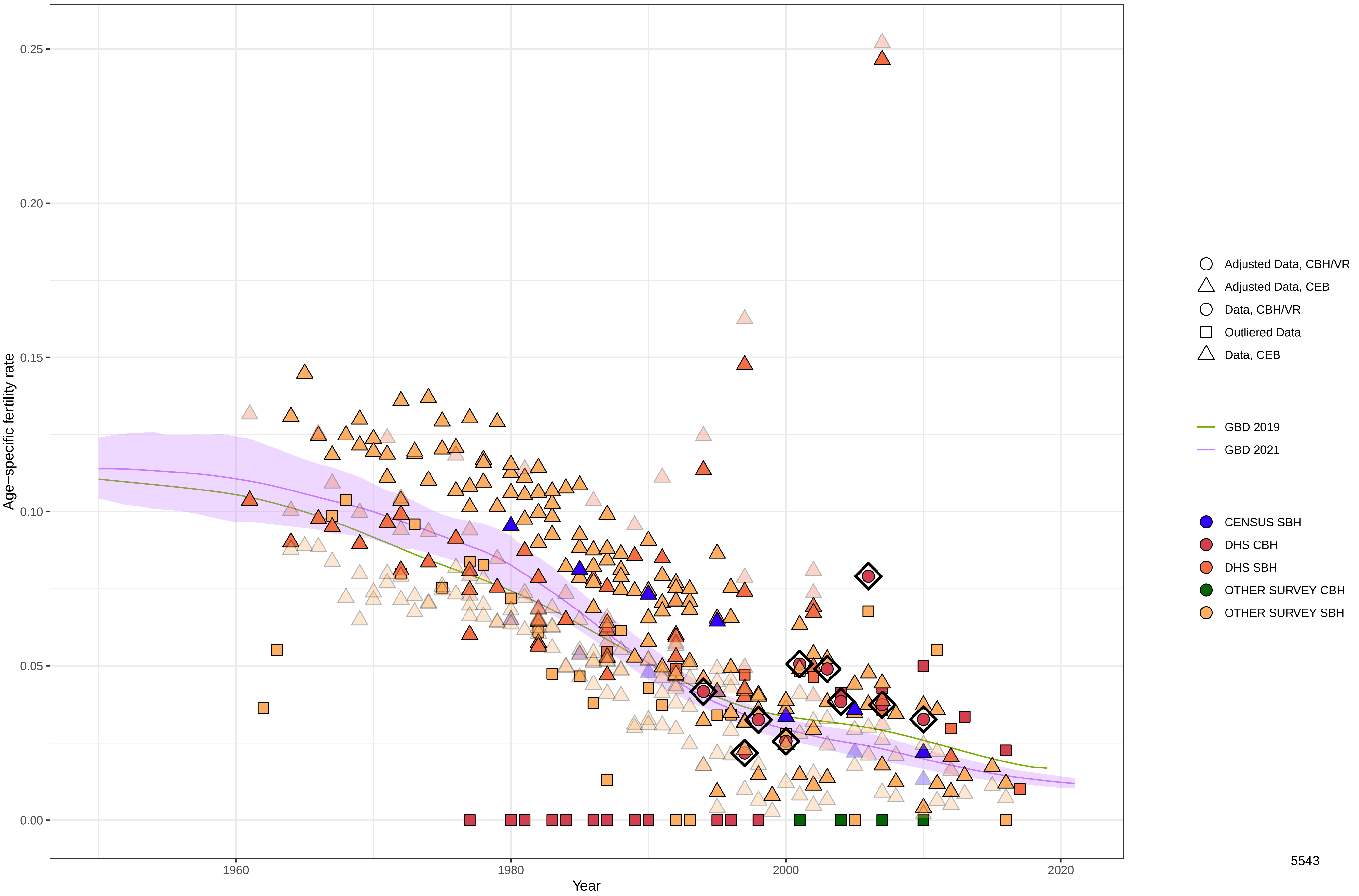

Age−specific fertility fitted trend lines, 1950−2021: Riau Islands, Ages: 20−24
Data points enclosed with a diamond are reference sources

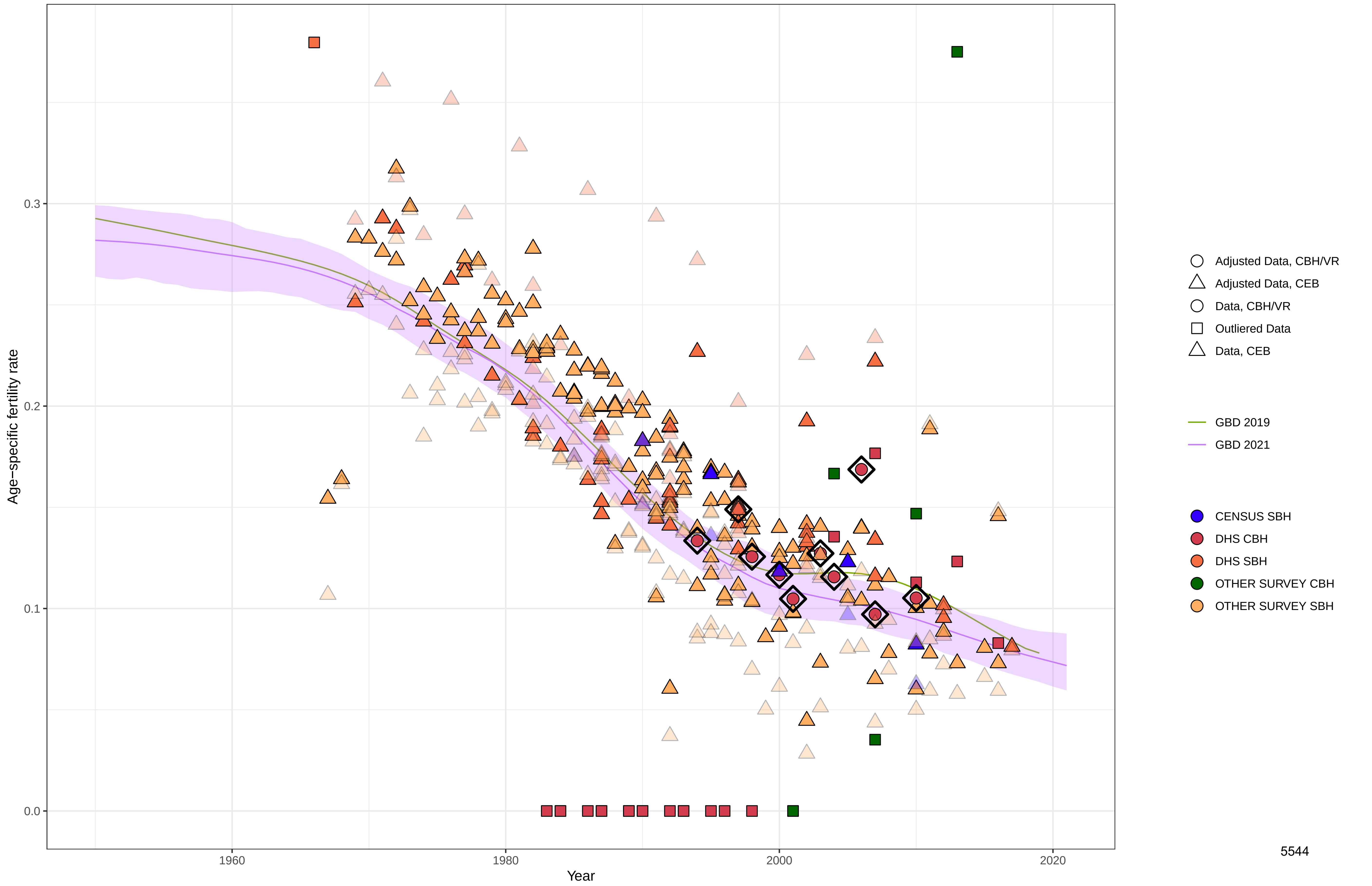

5544

Age−specific fertility fitted trend lines, 1950−2021: Riau Islands, Ages: 25−29
Data points enclosed with a diamond are reference sources

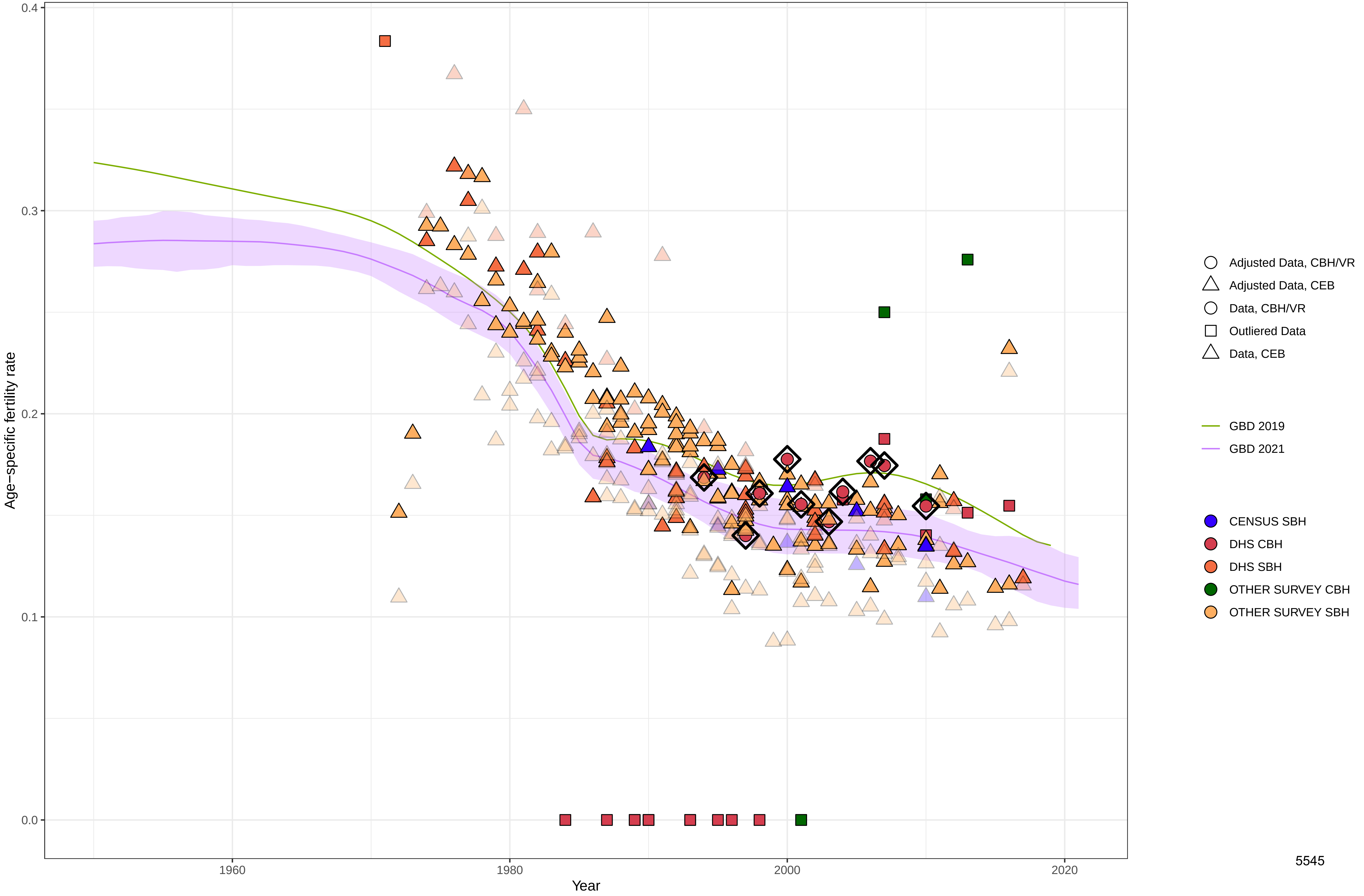

Age−specific fertility fitted trend lines, 1950−2021: Riau Islands, Ages: 30−34
Data points enclosed with a diamond are reference sources

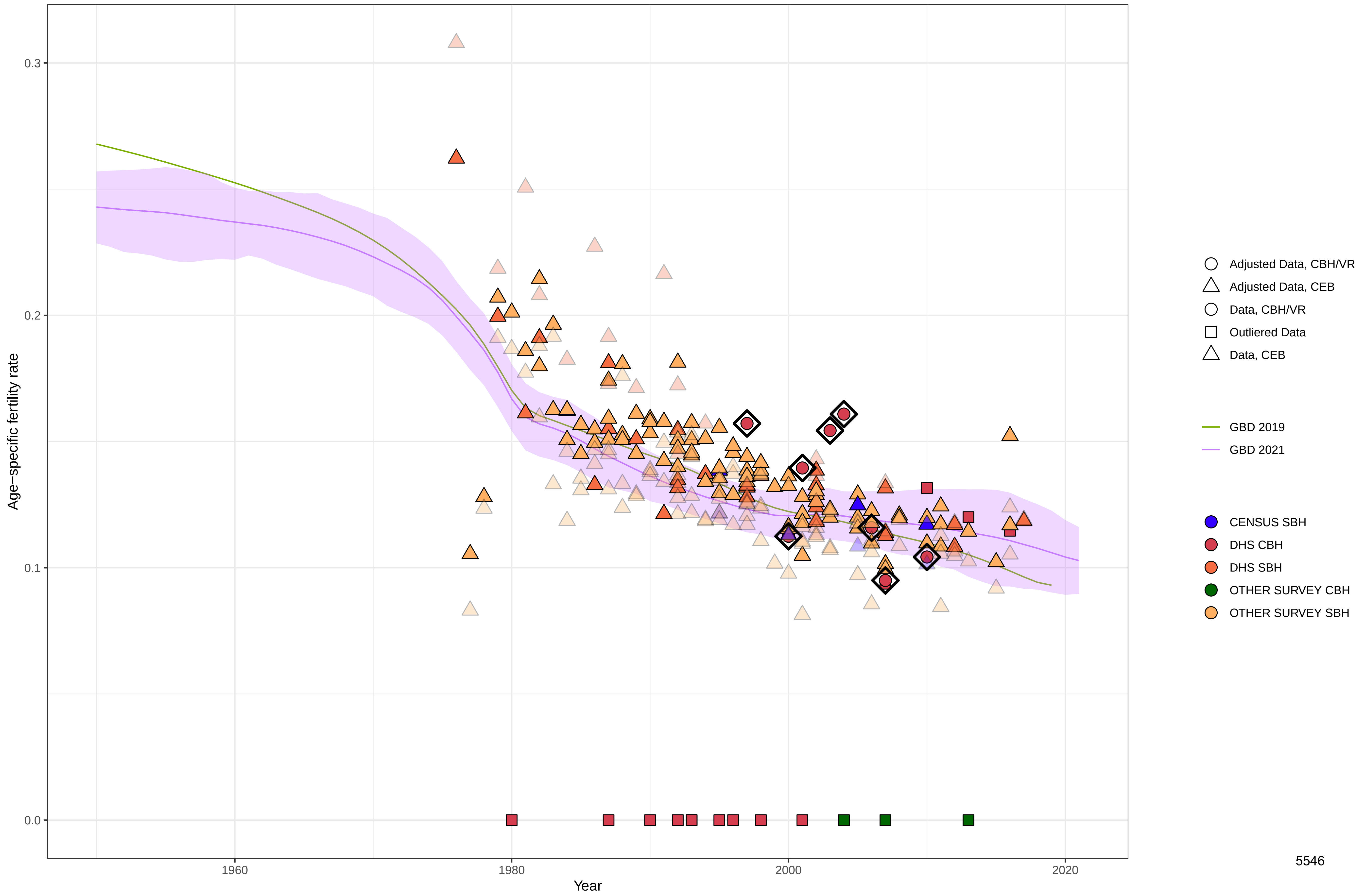

Age−specific fertility fitted trend lines, 1950−2021: Riau Islands, Ages: 35−39
Data points enclosed with a diamond are reference sources

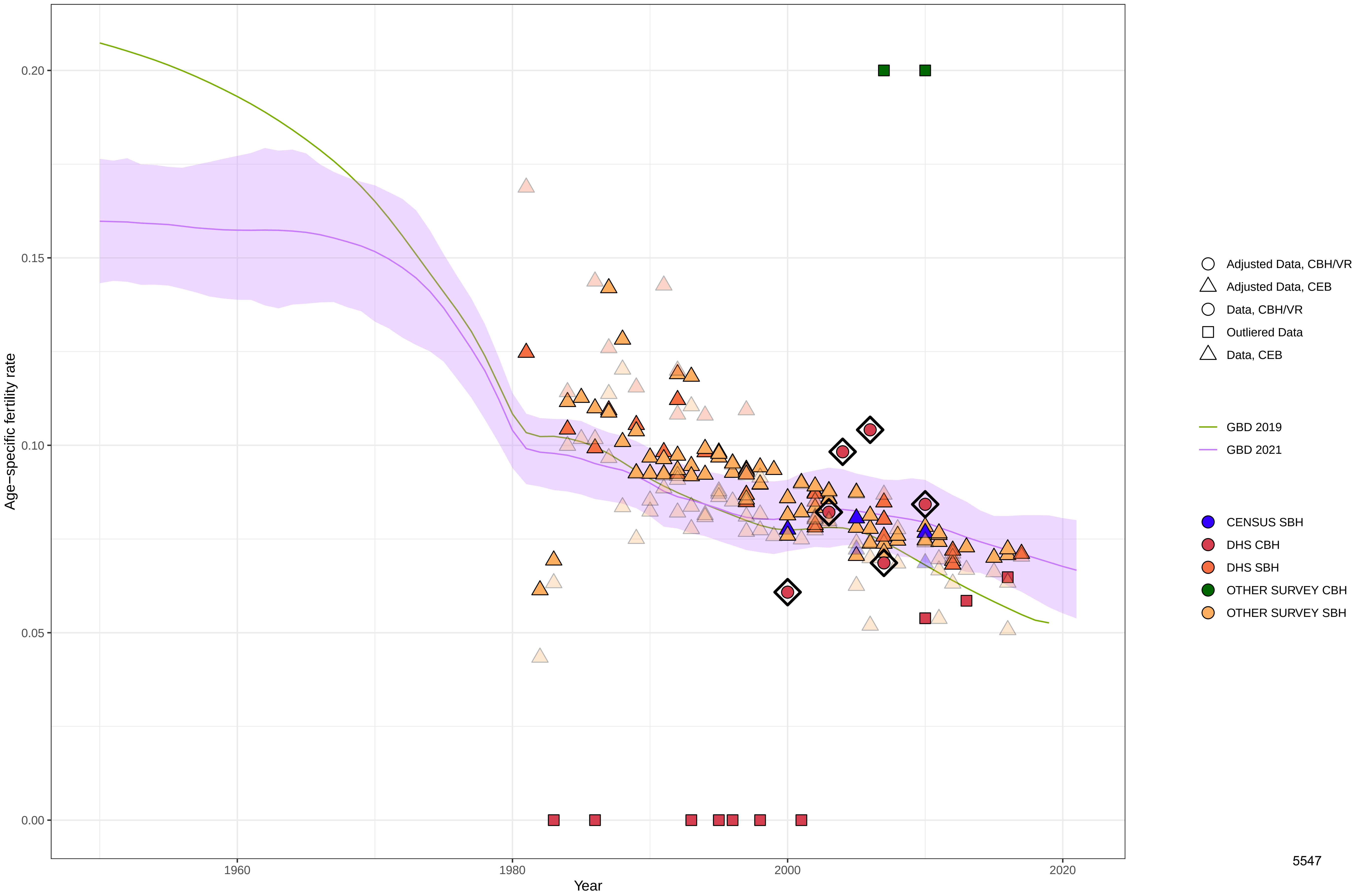

Age−specific fertility fitted trend lines, 1950−2021: Riau Islands, Ages: 40−44
Data points enclosed with a diamond are reference sources

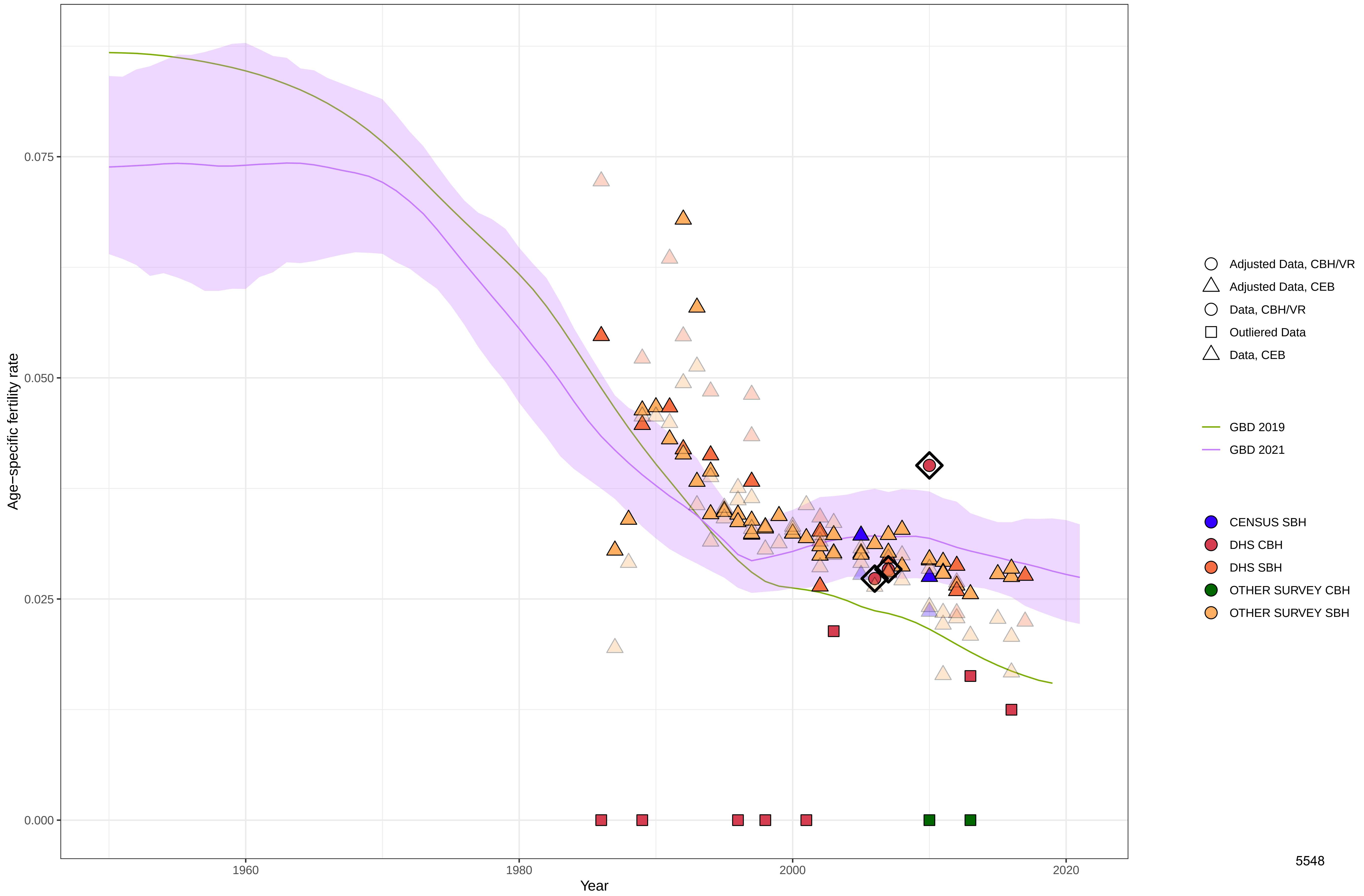

Age−specific fertility fitted trend lines, 1950−2021: Riau Islands, Ages: 45−49
Data points enclosed with a diamond are reference sources

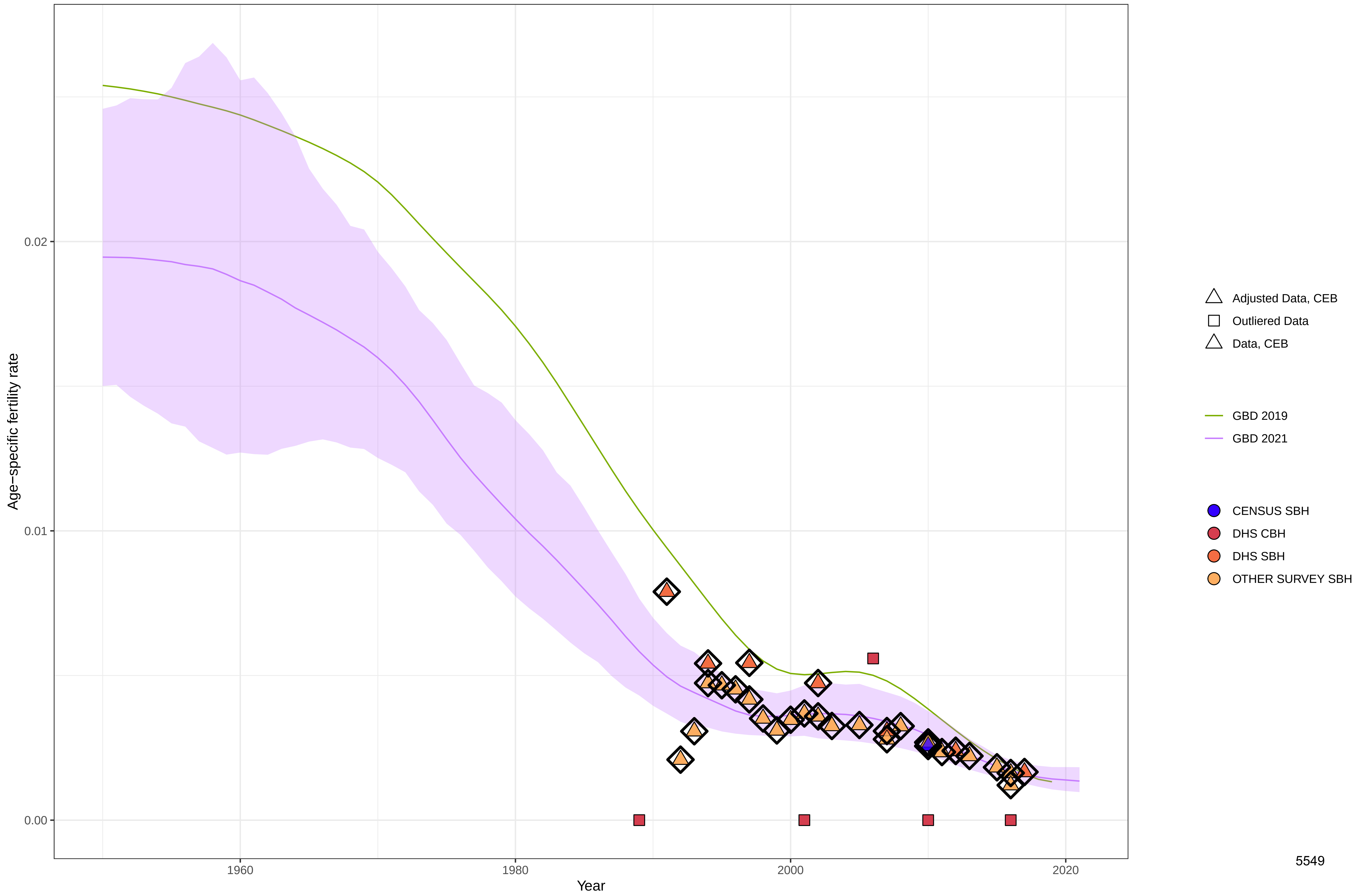

Age−specific fertility fitted trend lines, 1950−2021: Riau Islands, Ages: 50−54
Data points enclosed with a diamond are reference sources

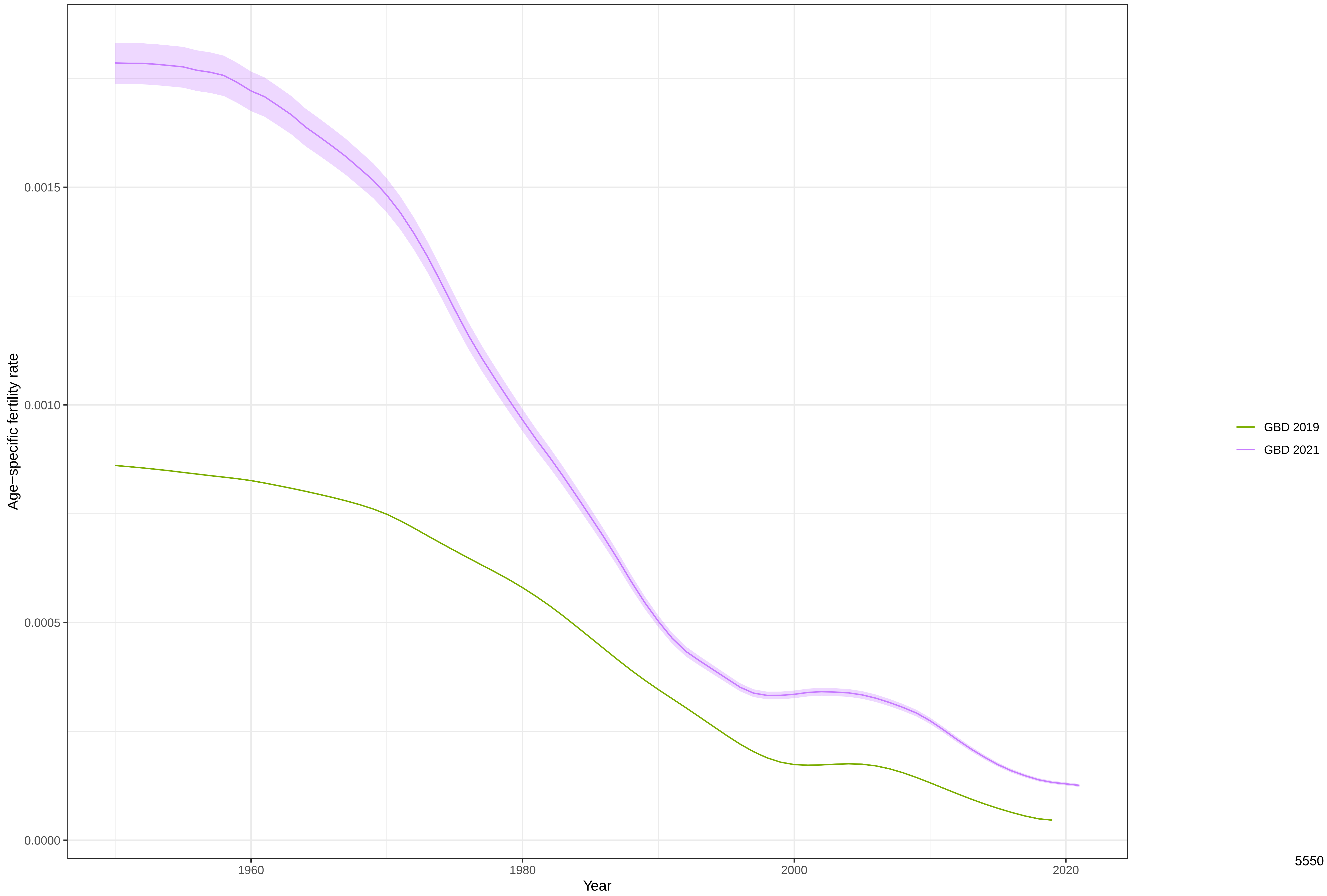

Total fertility rate fitted trend lines, 1950−2021: Riau Islands TFR
Data points enclosed with a diamond are reference sources

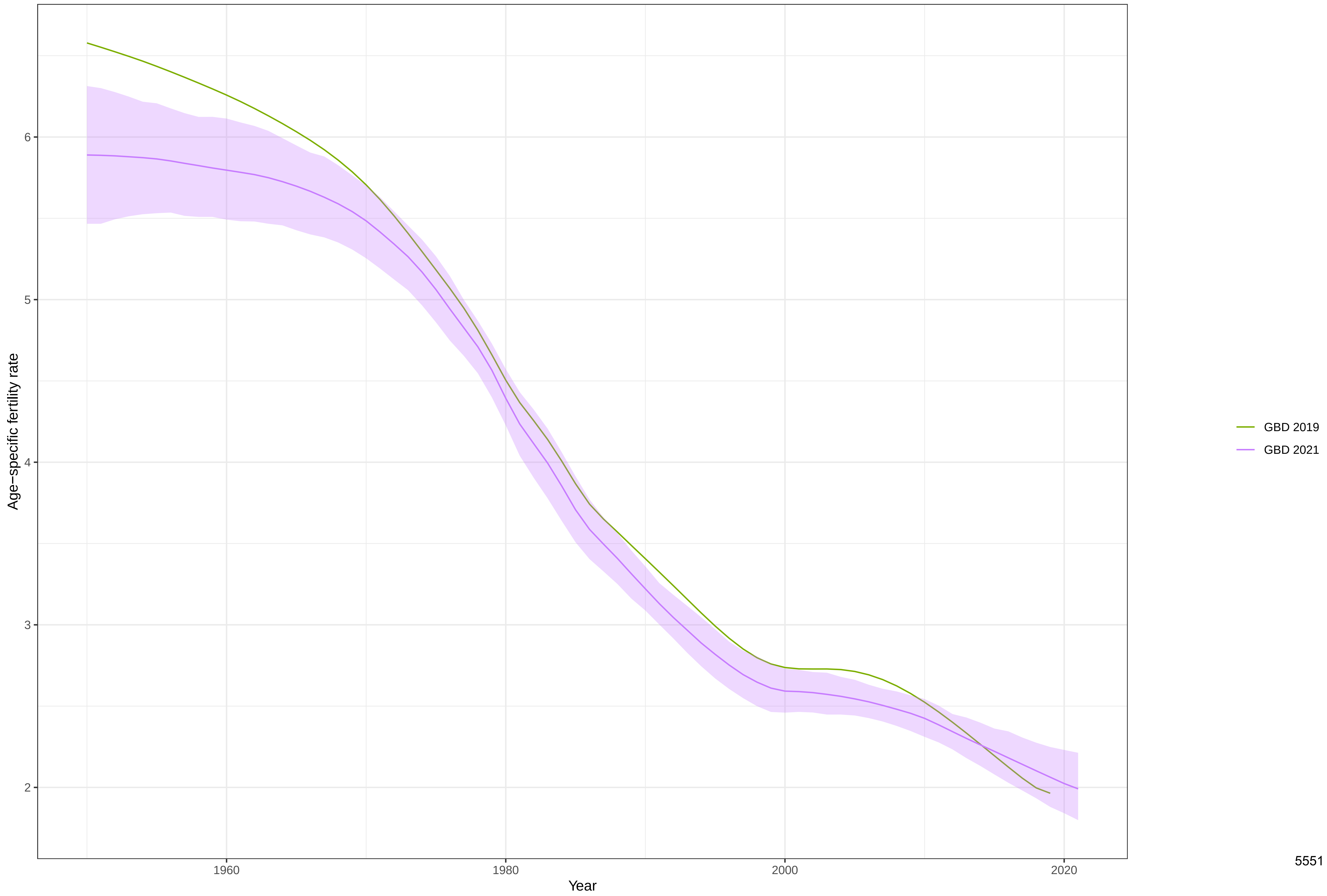

Age−specific fertility fitted trend lines, 1950−2021: Lampung, Ages: 10−14
Data points enclosed with a diamond are reference sources

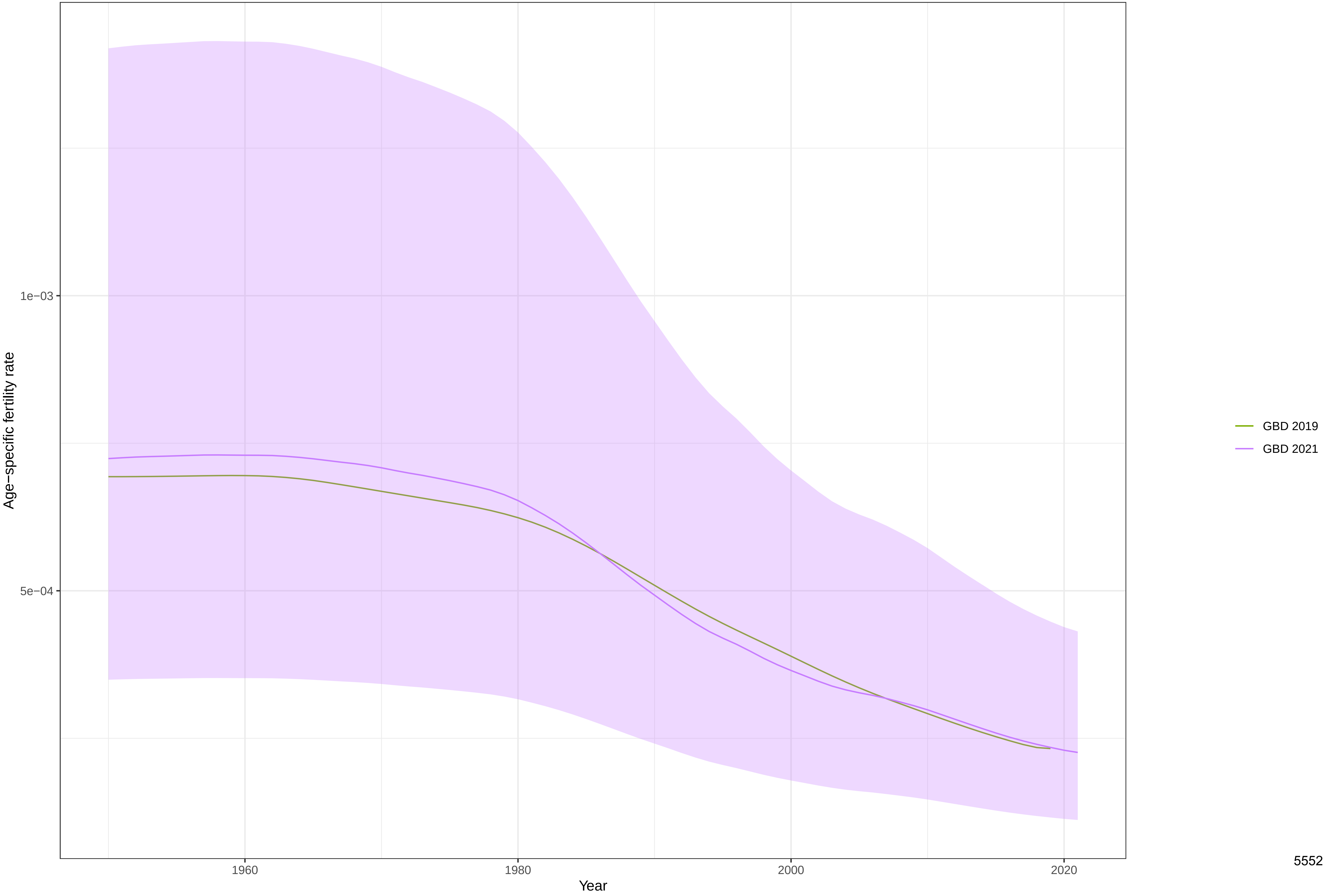

Age−specific fertility fitted trend lines, 1950−2021: Lampung, Ages: 15−19
Data points enclosed with a diamond are reference sources

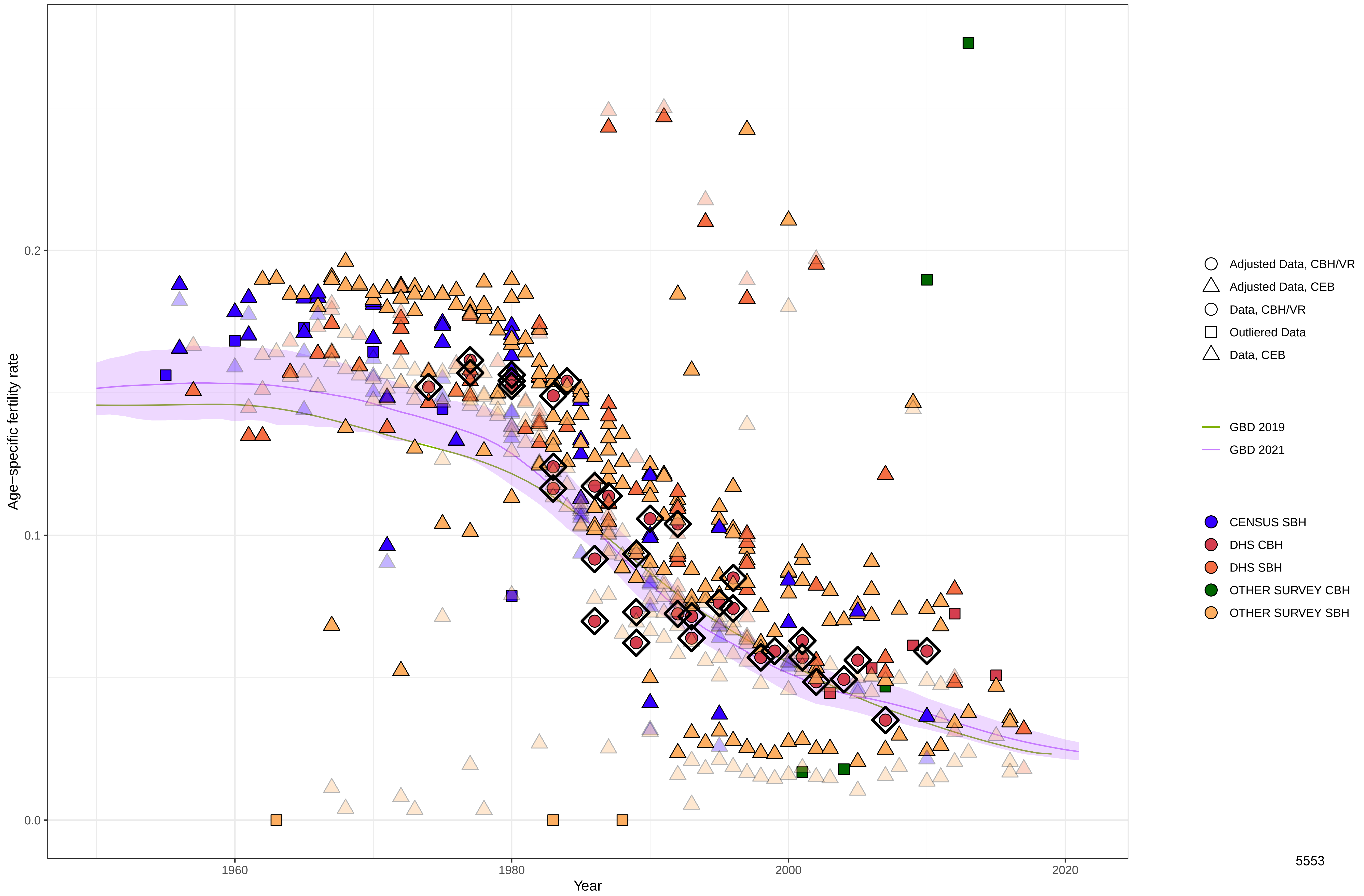

Age−specific fertility fitted trend lines, 1950−2021: Lampung, Ages: 20−24
Data points enclosed with a diamond are reference sources

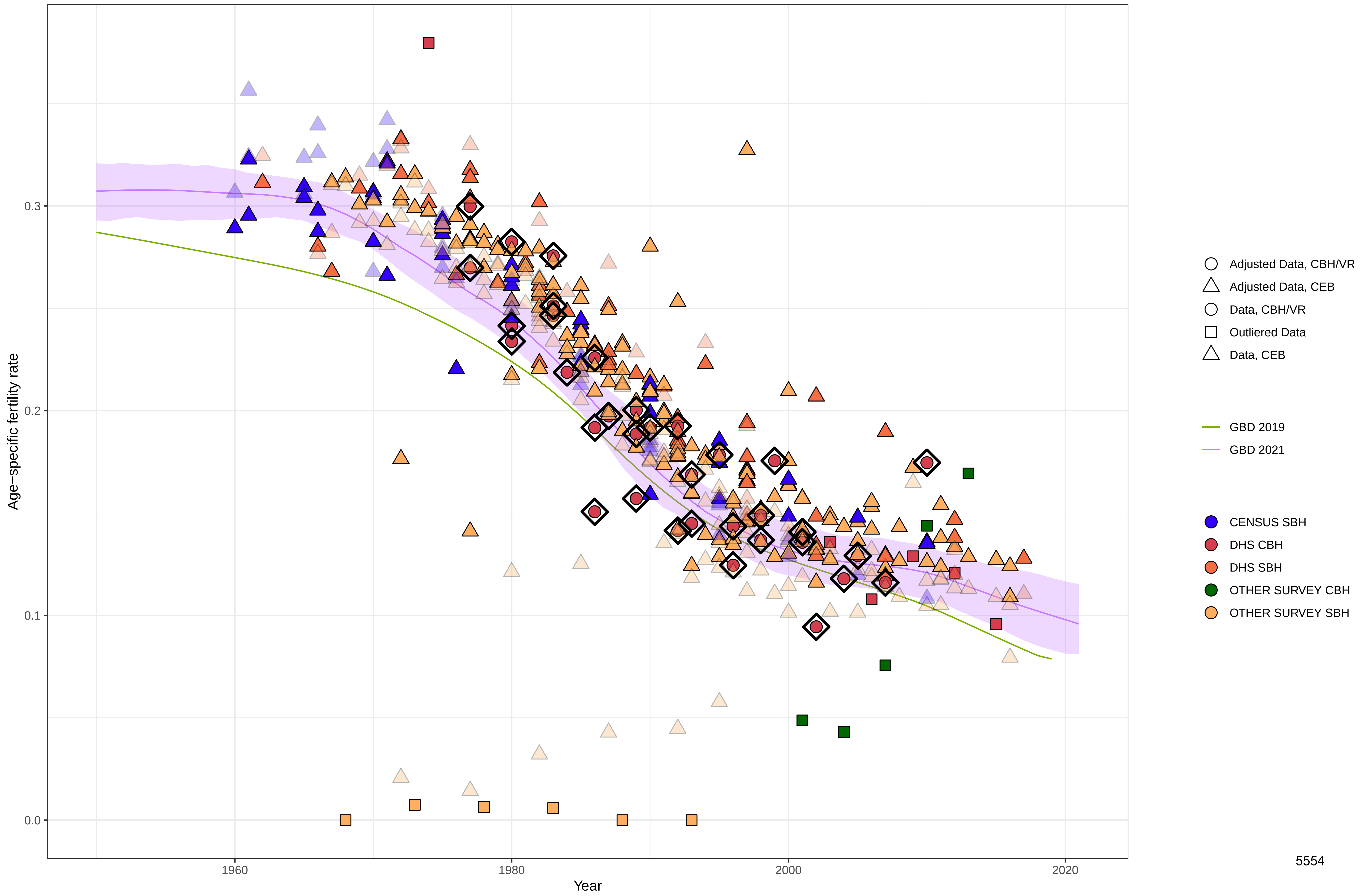

Age−specific fertility fitted trend lines, 1950−2021: Lampung, Ages: 25−29
Data points enclosed with a diamond are reference sources

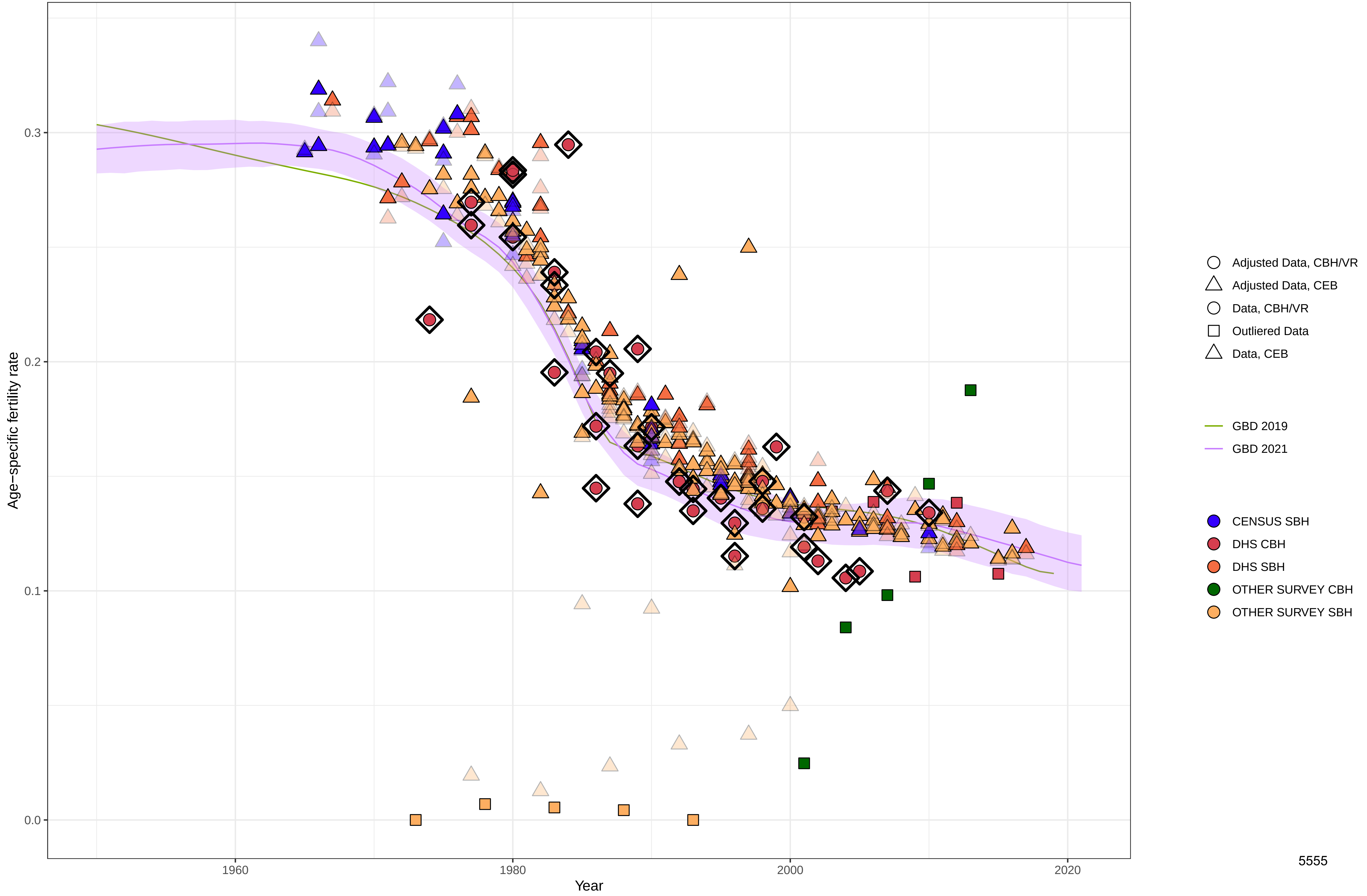

Age−specific fertility fitted trend lines, 1950−2021: Lampung, Ages: 30−34
Data points enclosed with a diamond are reference sources

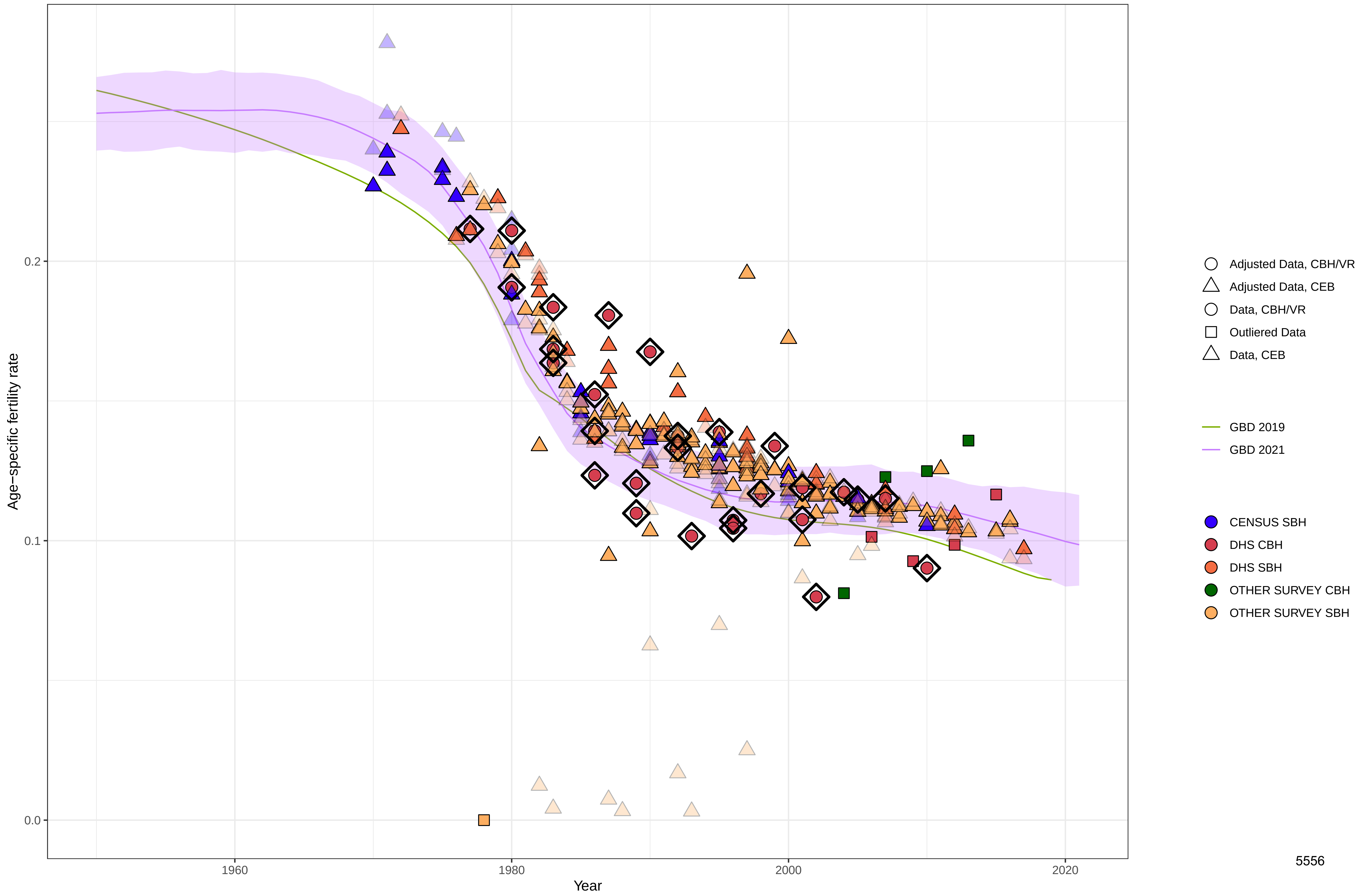

Age−specific fertility fitted trend lines, 1950−2021: Lampung, Ages: 35−39
Data points enclosed with a diamond are reference sources

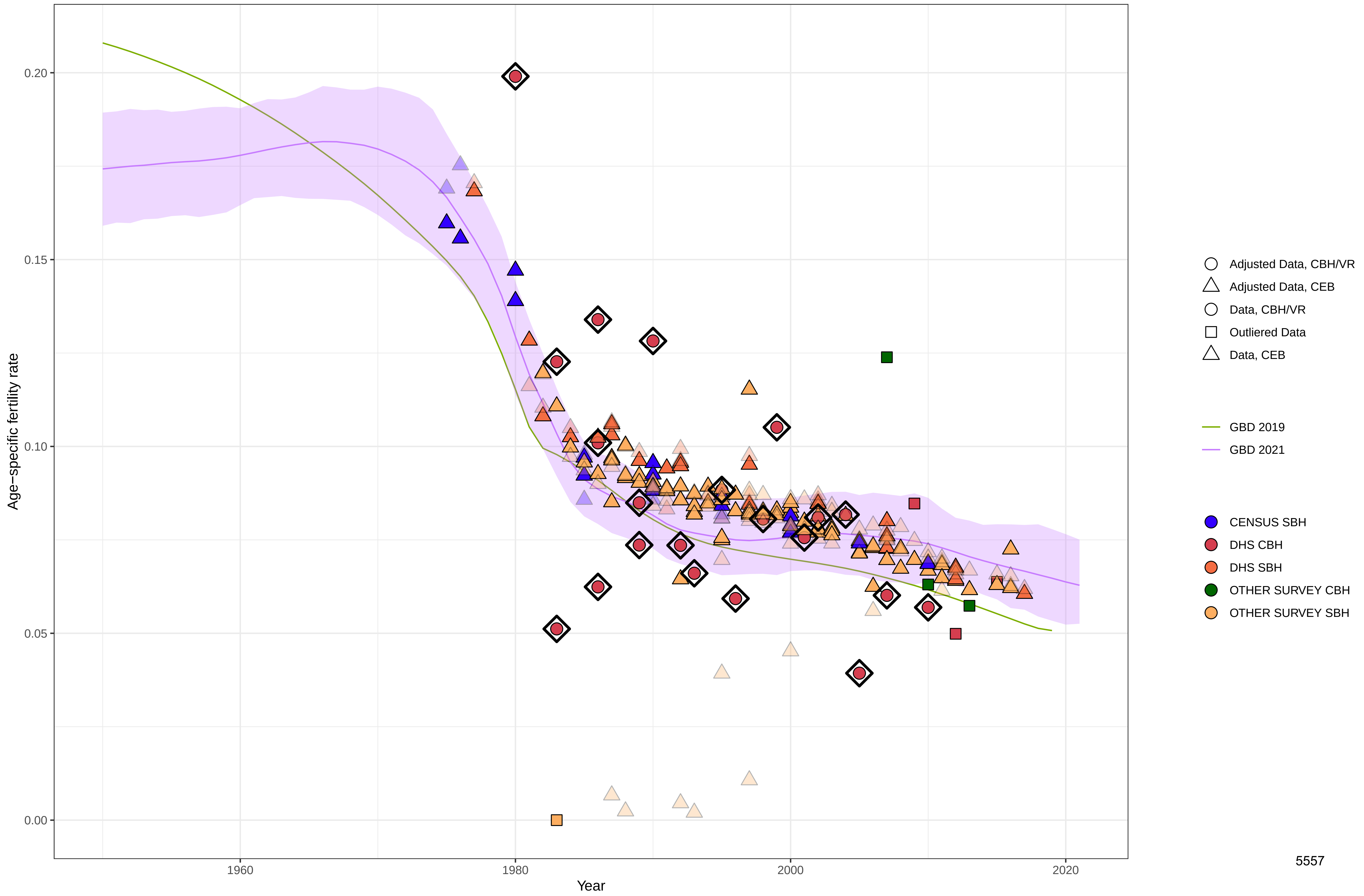

Age−specific fertility fitted trend lines, 1950−2021: Lampung, Ages: 40−44
Data points enclosed with a diamond are reference sources

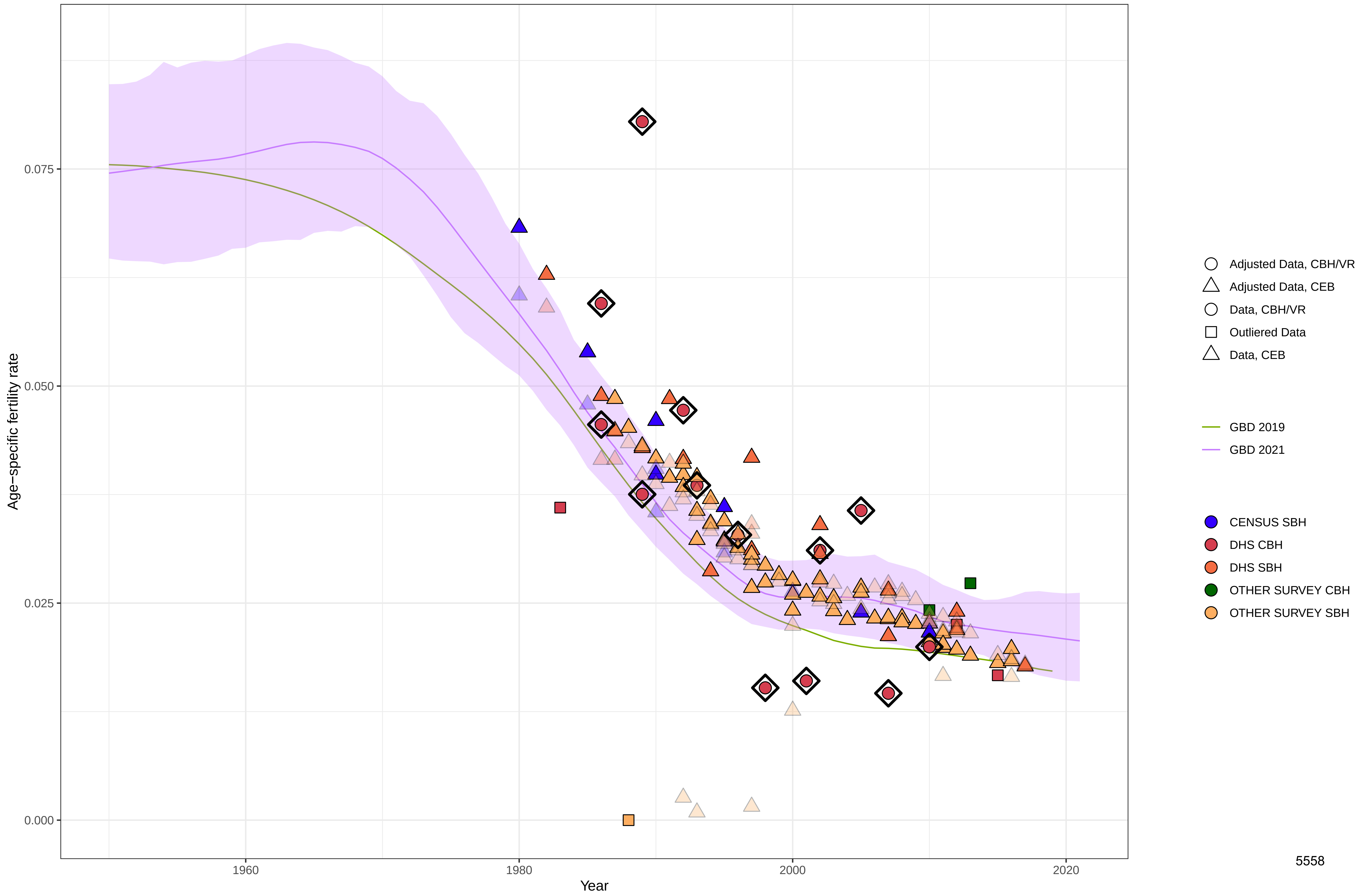

Age−specific fertility fitted trend lines, 1950−2021: Lampung, Ages: 45−49
Data points enclosed with a diamond are reference sources

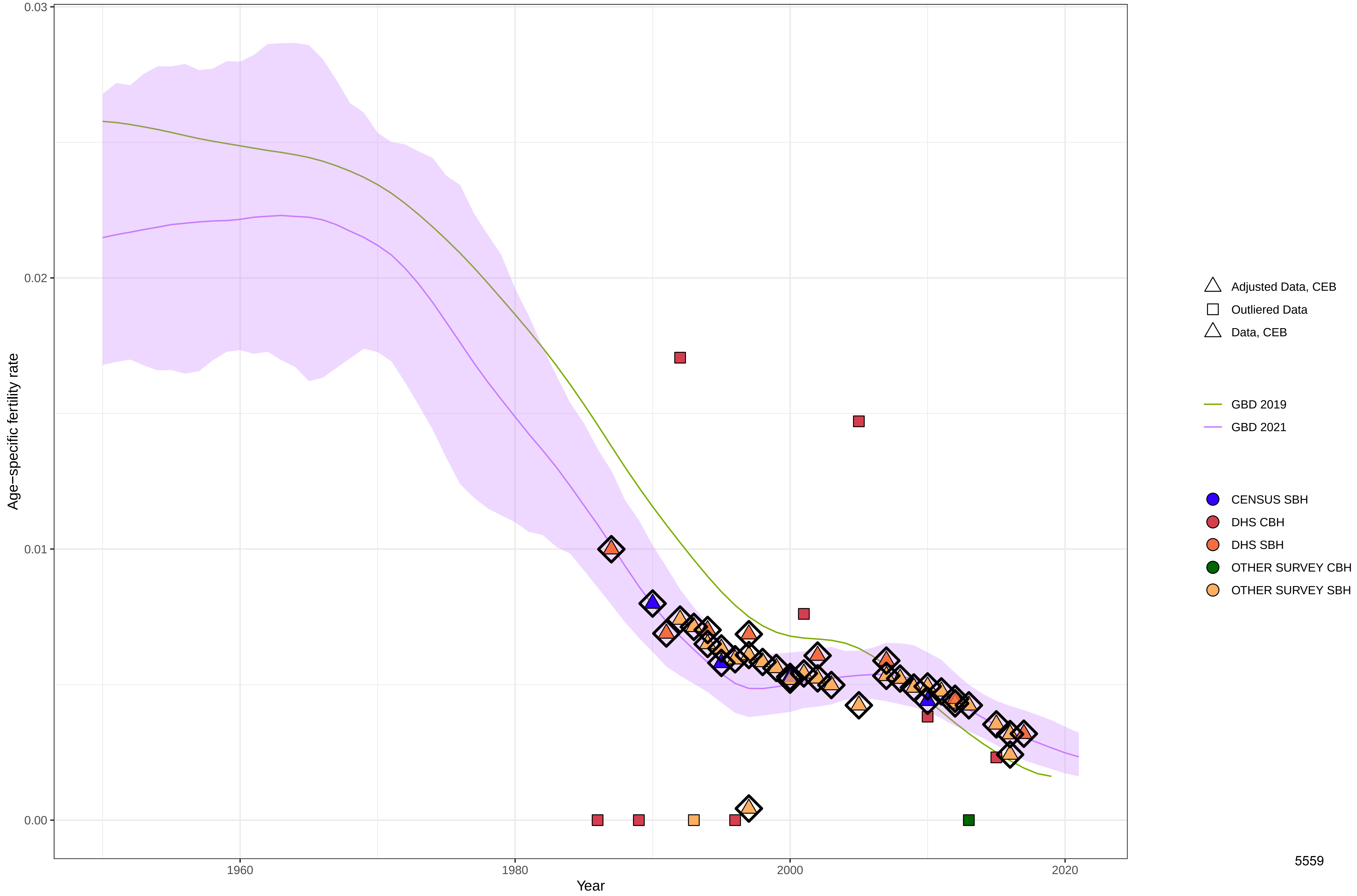

5559

Age−specific fertility fitted trend lines, 1950−2021: Lampung, Ages: 50−54
Data points enclosed with a diamond are reference sources

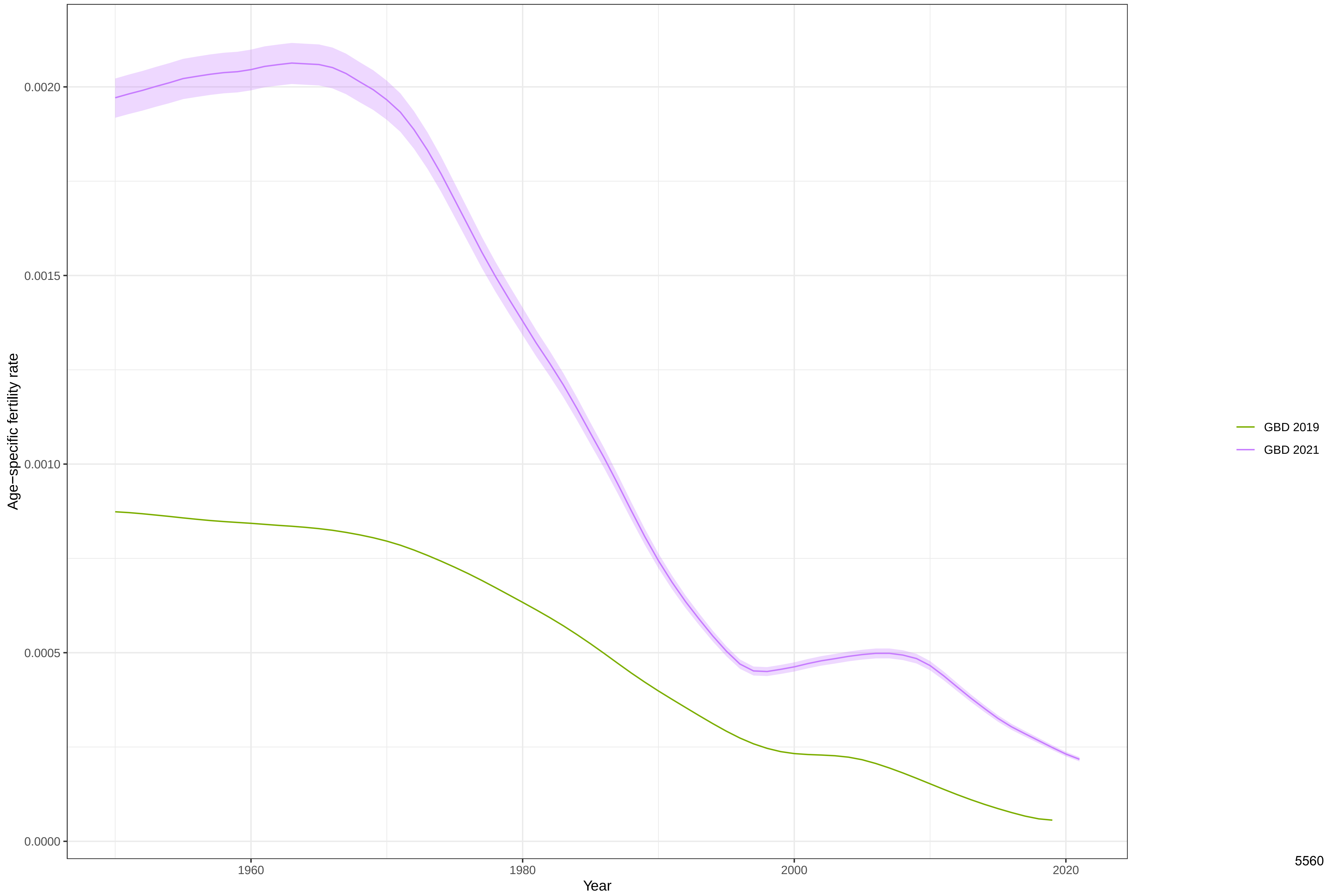

Total fertility rate fitted trend lines, 1950−2021: Lampung TFR
Data points enclosed with a diamond are reference sources

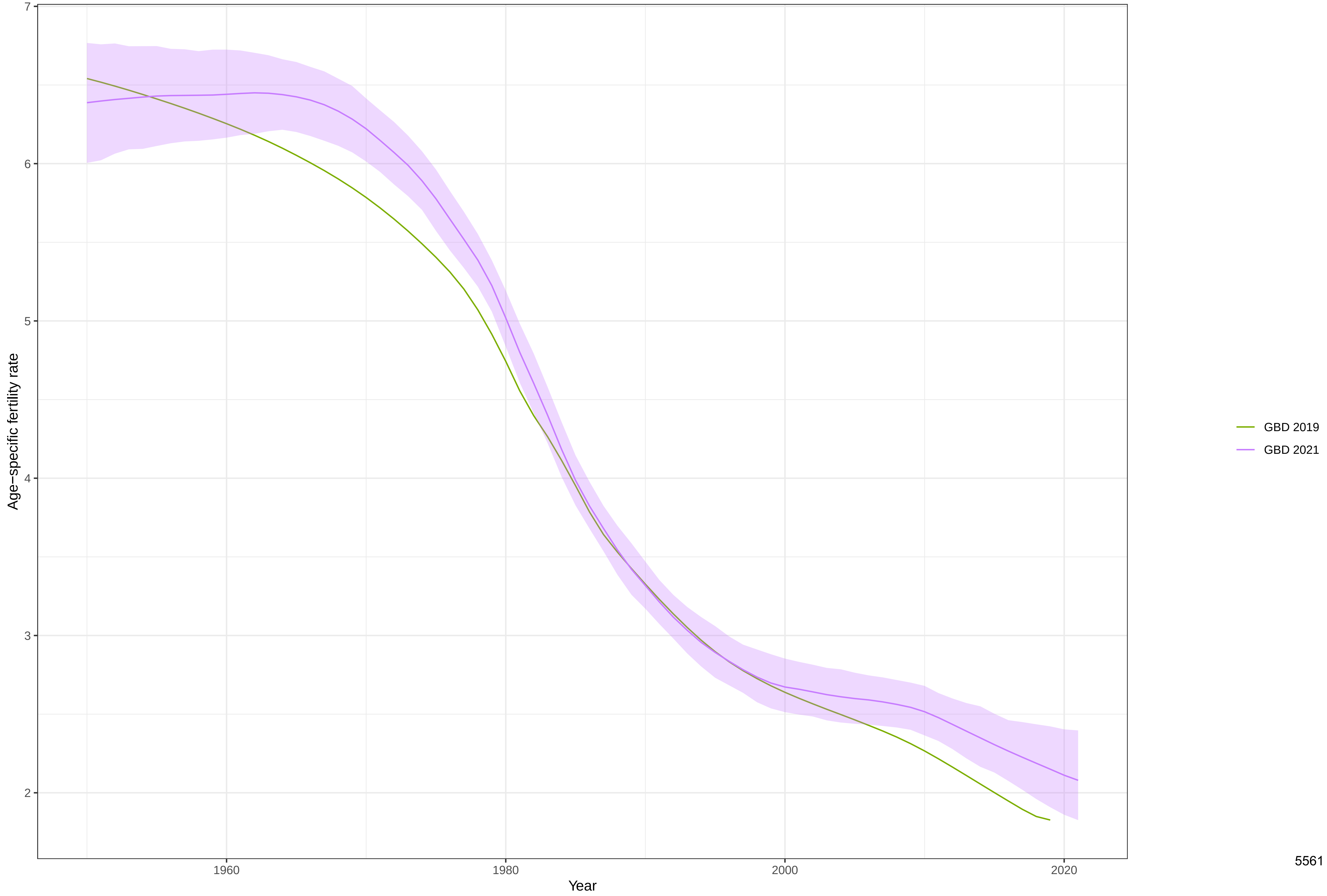

Age−specific fertility fitted trend lines, 1950−2021: Maluku, Ages: 10−14
Data points enclosed with a diamond are reference sources

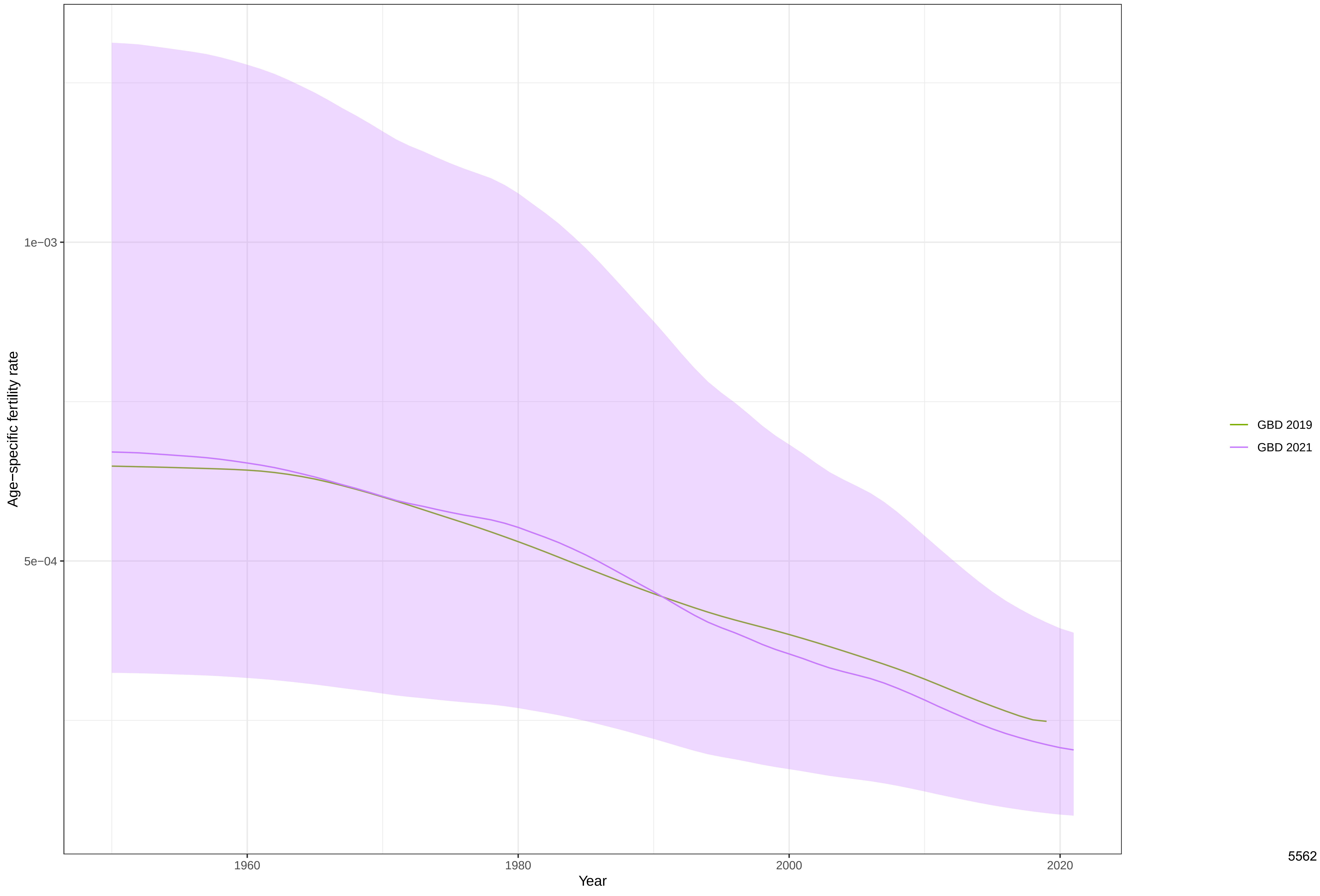

Age−specific fertility fitted trend lines, 1950−2021: Maluku, Ages: 15−19
Data points enclosed with a diamond are reference sources

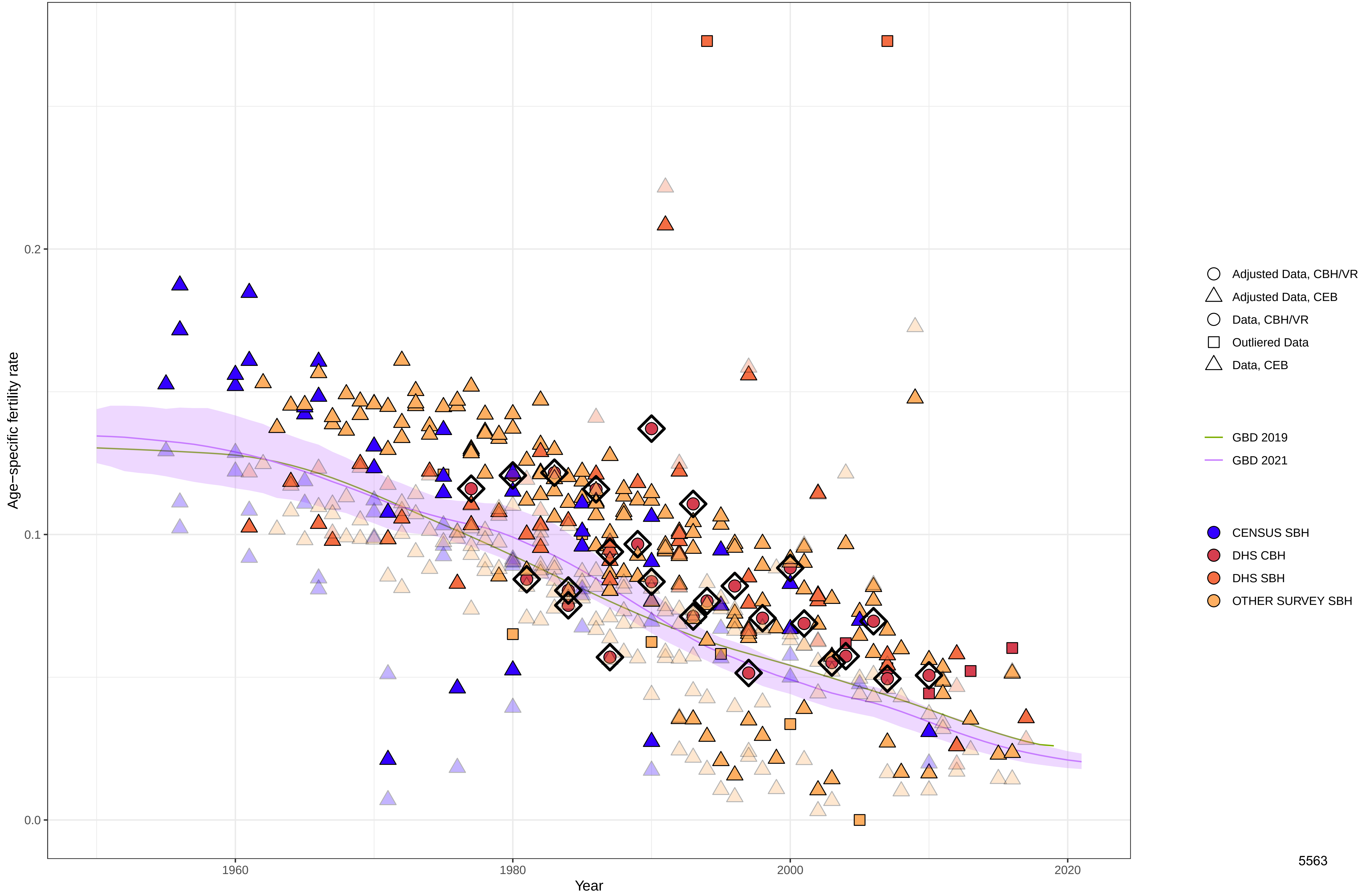

Age−specific fertility fitted trend lines, 1950−2021: Maluku, Ages: 20−24
Data points enclosed with a diamond are reference sources

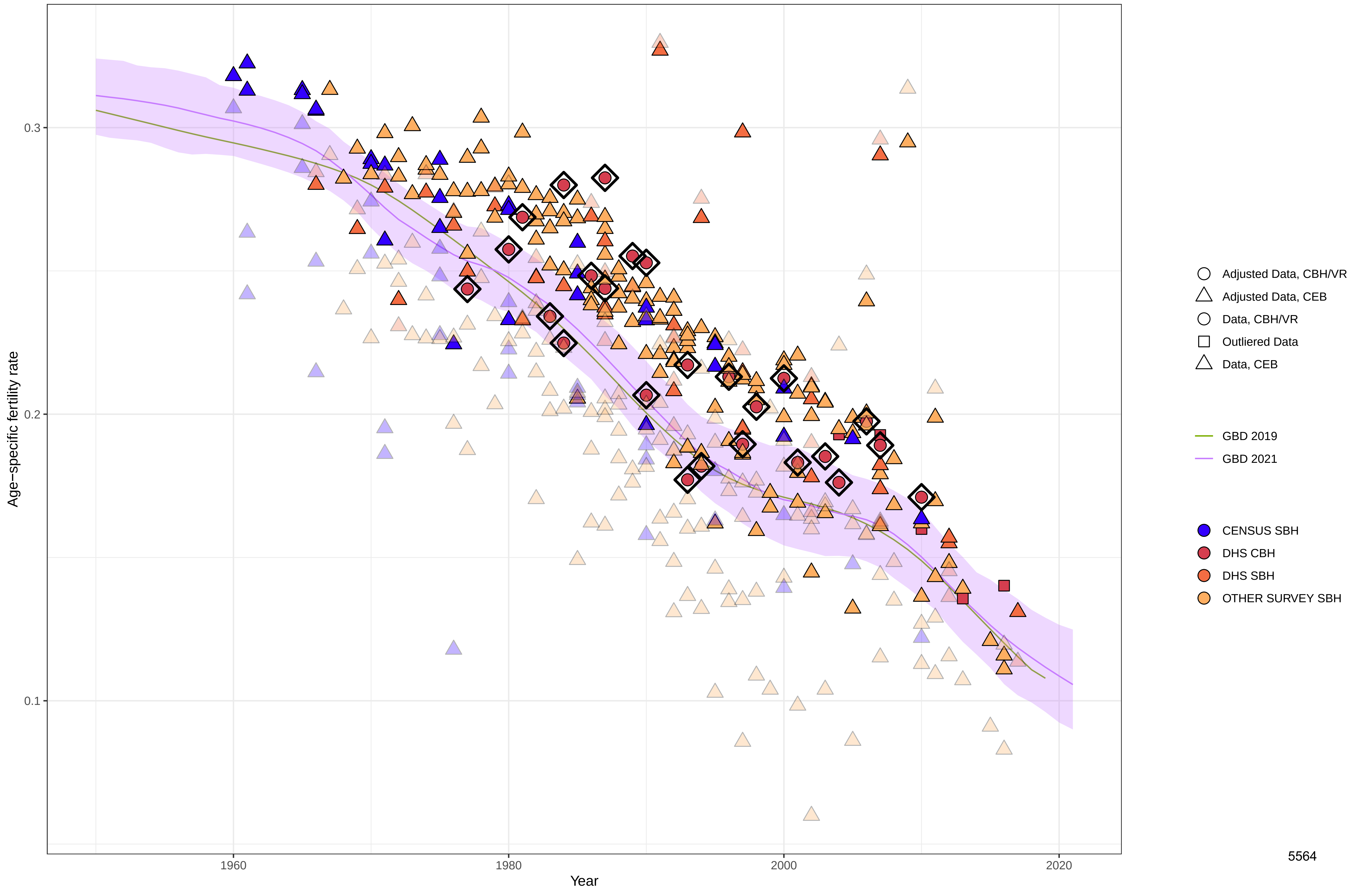

Age−specific fertility fitted trend lines, 1950−2021: Maluku, Ages: 25−29
Data points enclosed with a diamond are reference sources

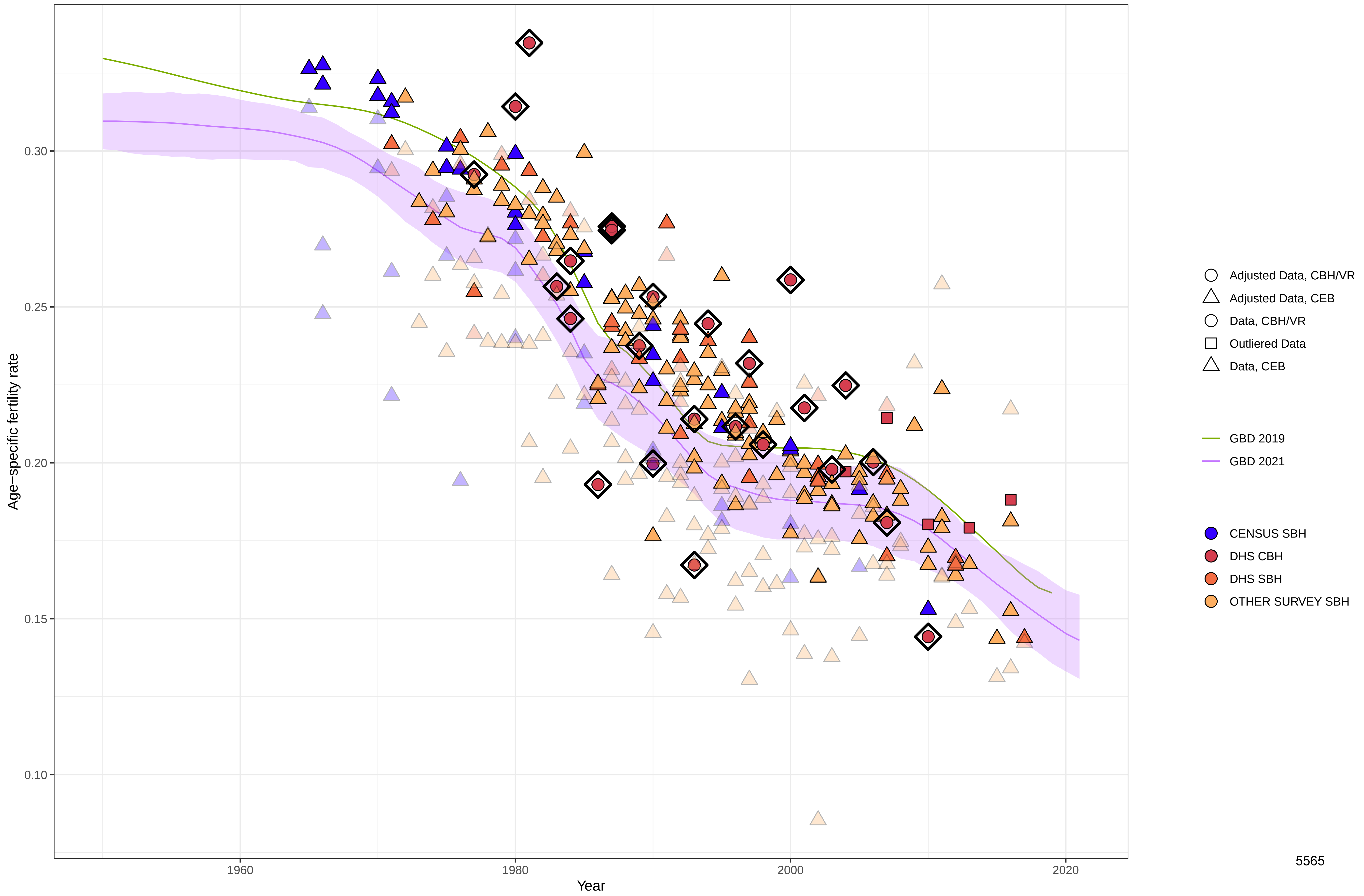

Age−specific fertility fitted trend lines, 1950−2021: Maluku, Ages: 30−34
Data points enclosed with a diamond are reference sources

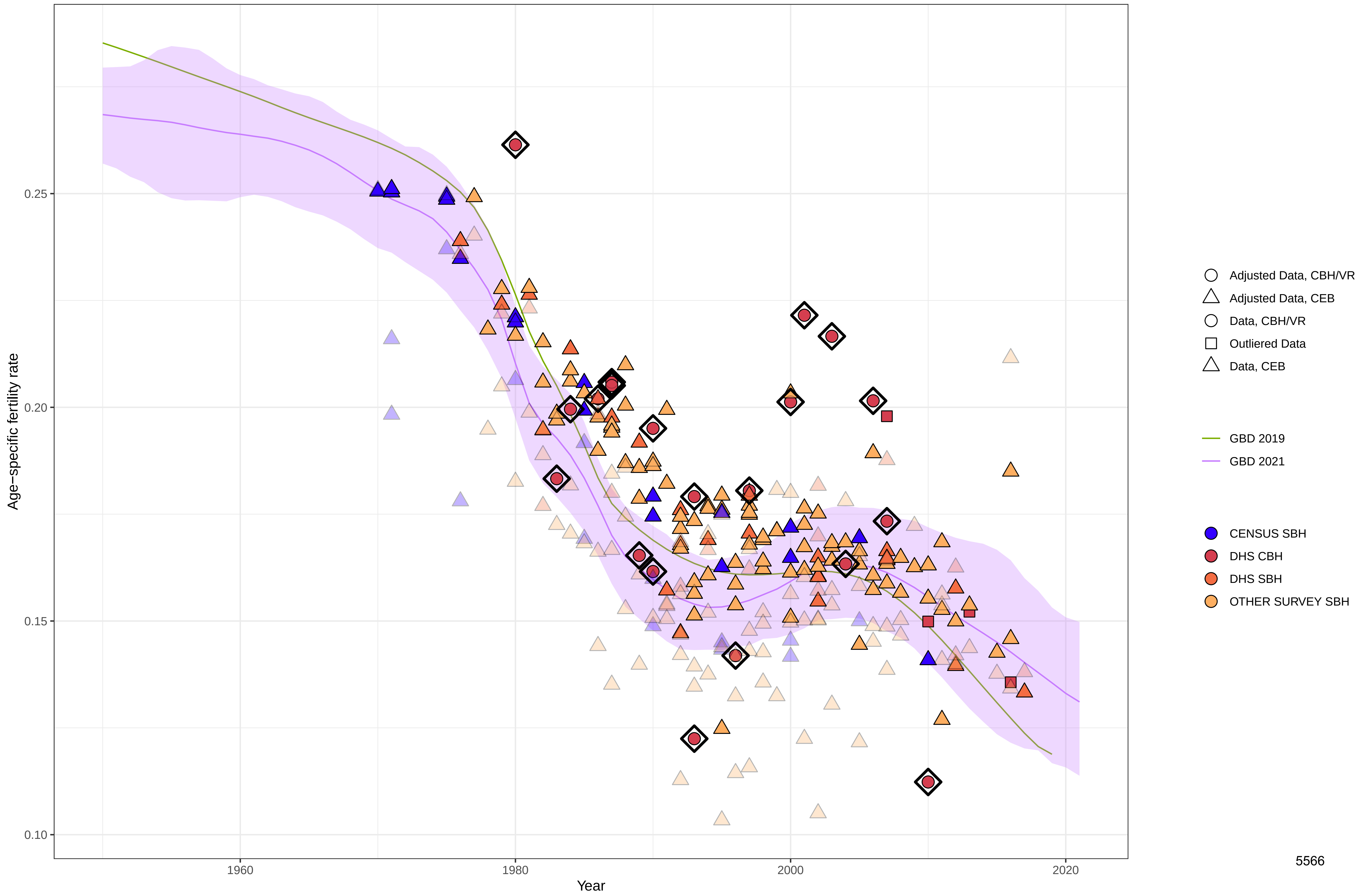

Age−specific fertility fitted trend lines, 1950−2021: Maluku, Ages: 35−39
Data points enclosed with a diamond are reference sources

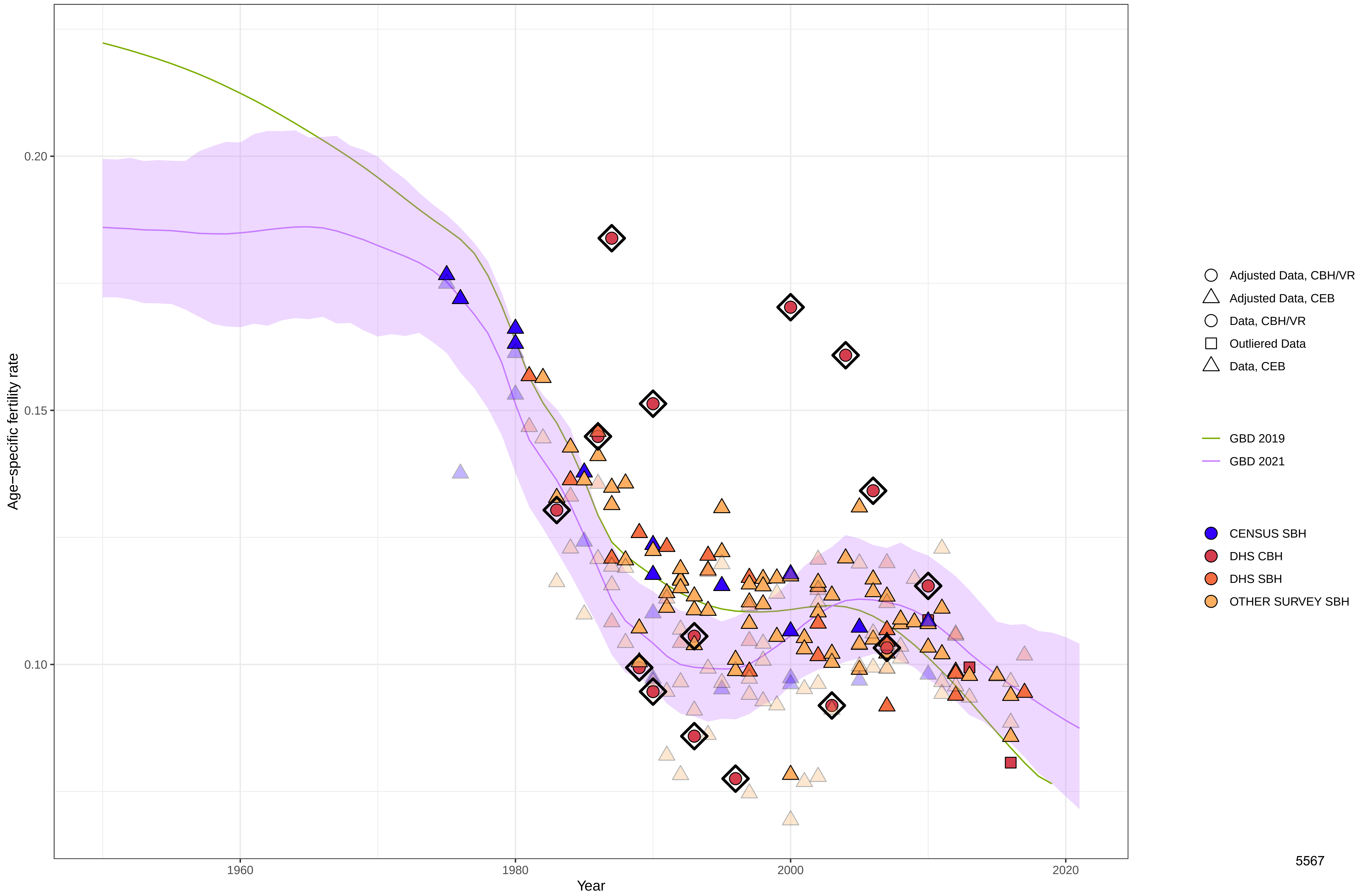

Age−specific fertility fitted trend lines, 1950−2021: Maluku, Ages: 40−44
Data points enclosed with a diamond are reference sources

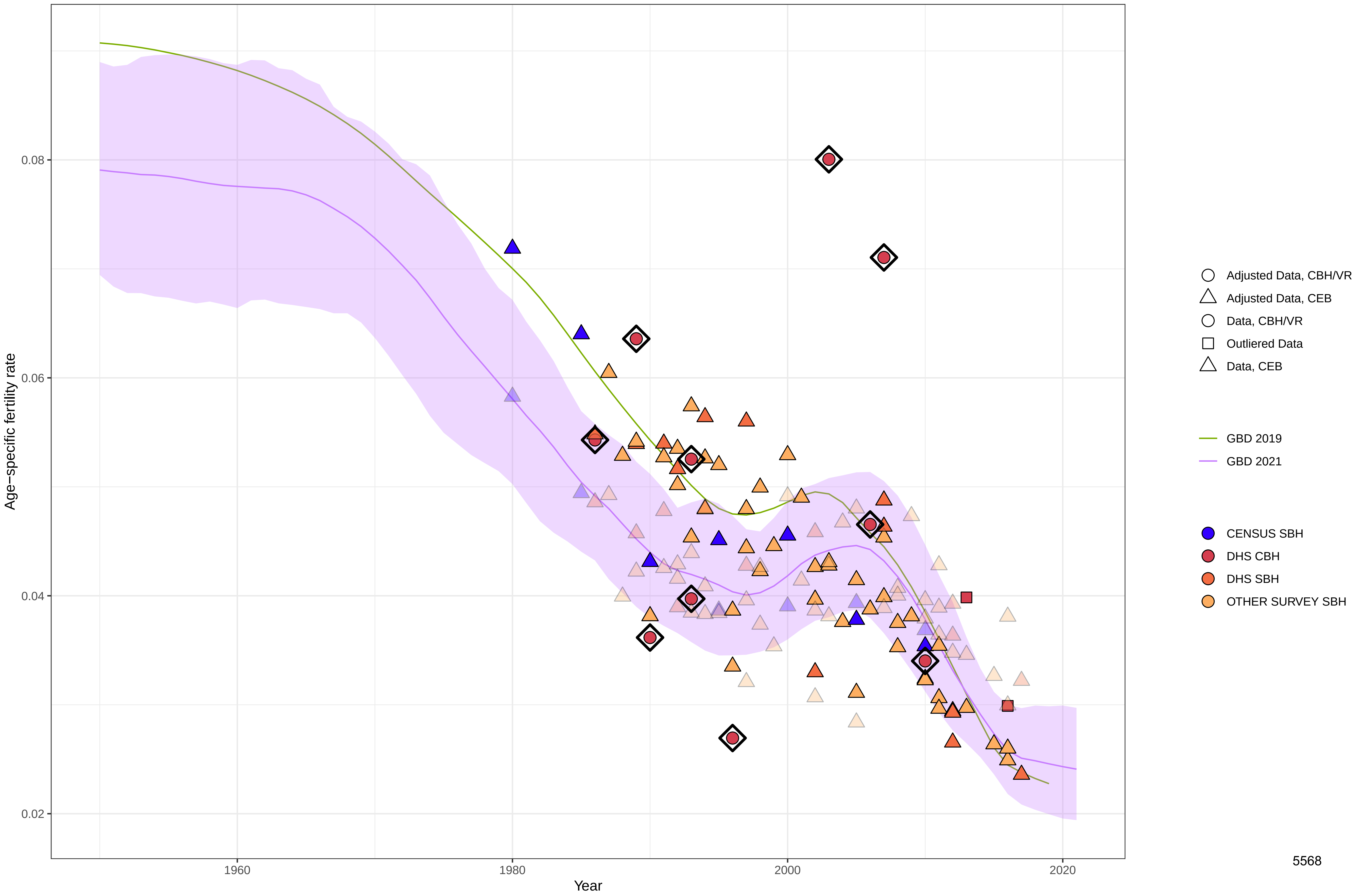

Age−specific fertility fitted trend lines, 1950−2021: Maluku, Ages: 45−49
Data points enclosed with a diamond are reference sources

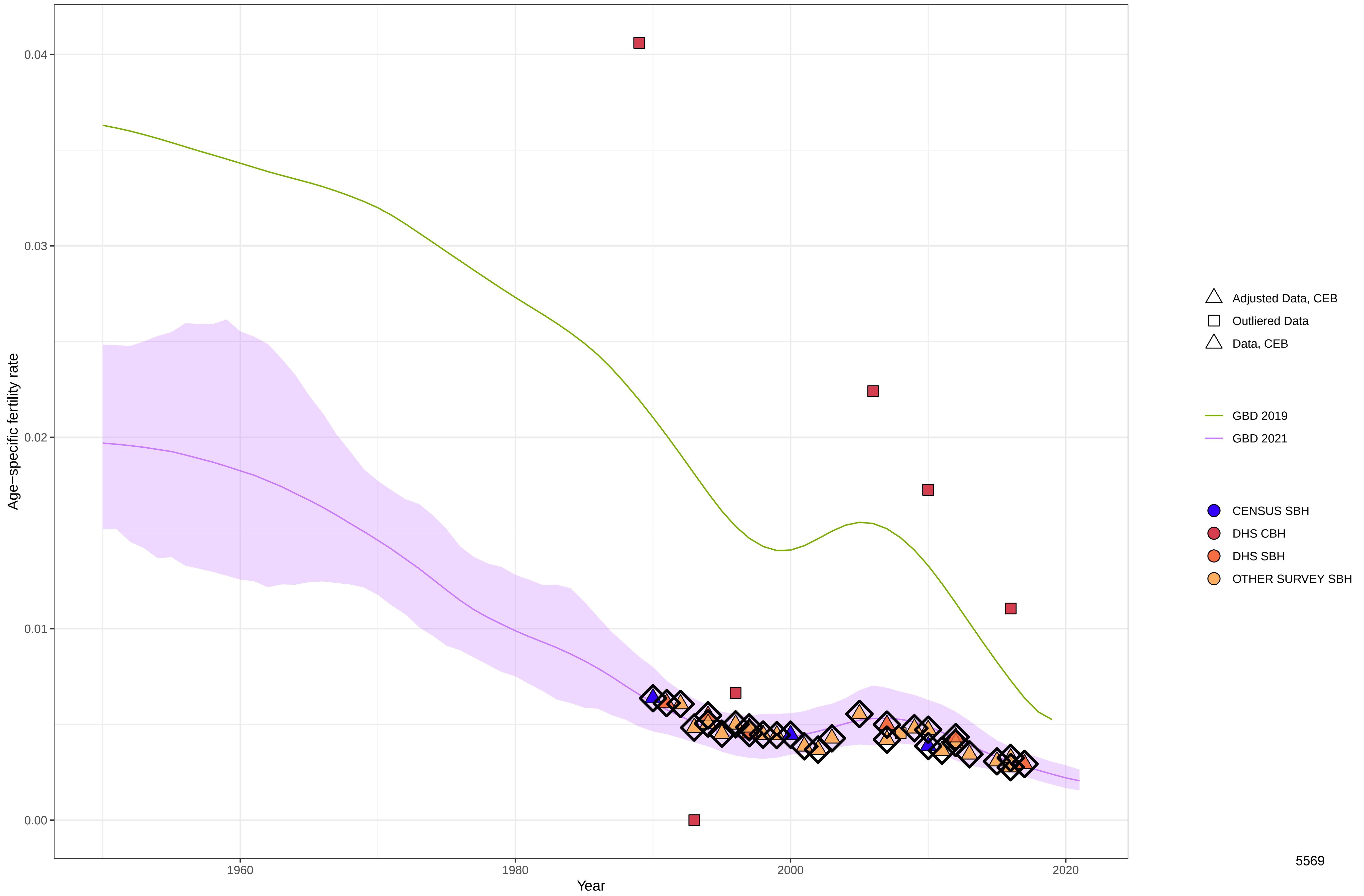

Age−specific fertility fitted trend lines, 1950−2021: Maluku, Ages: 50−54
Data points enclosed with a diamond are reference sources

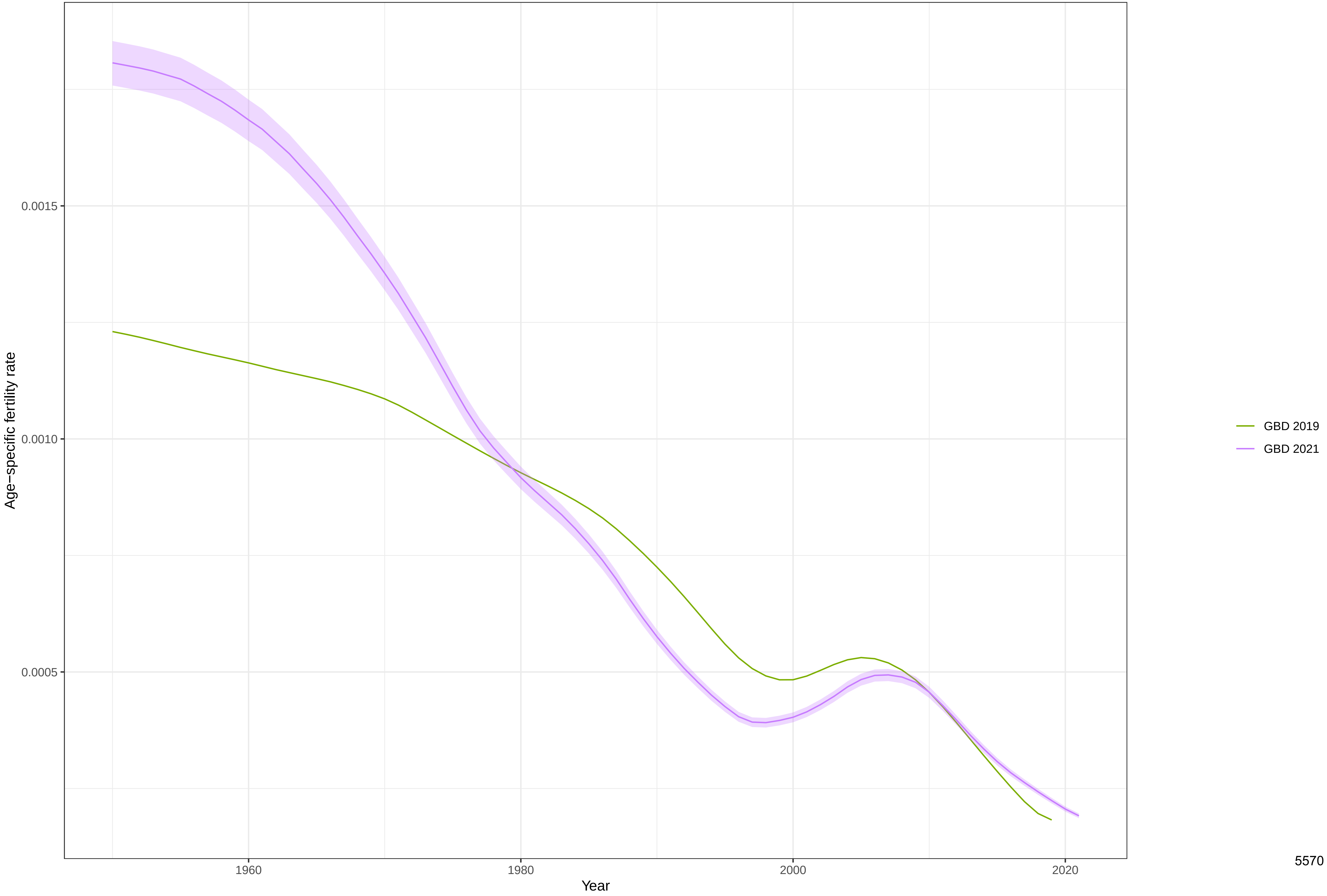

Total fertility rate fitted trend lines, 1950−2021: Maluku TFR
Data points enclosed with a diamond are reference sources

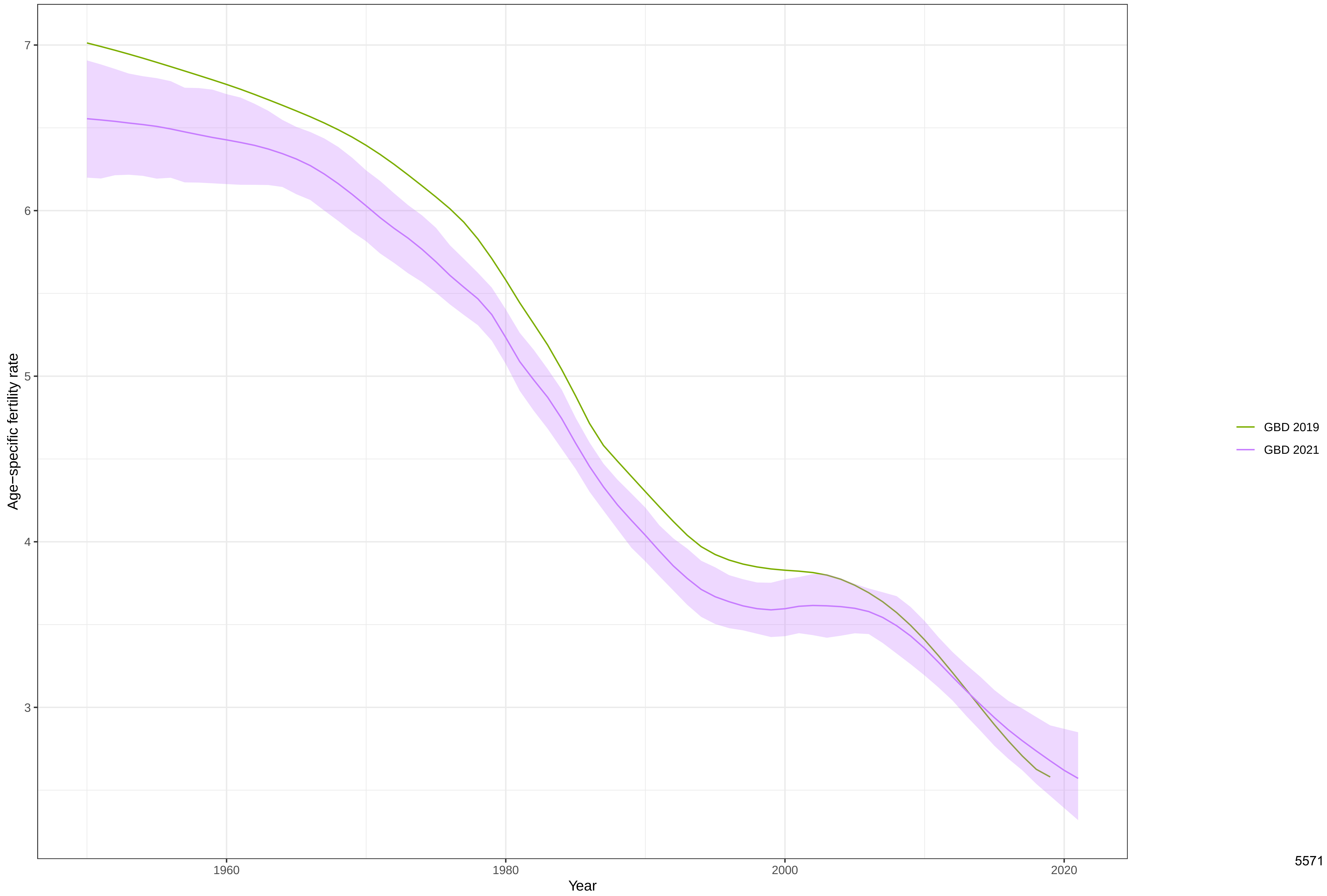

Age−specific fertility fitted trend lines, 1950−2021: North Maluku, Ages: 10−14
Data points enclosed with a diamond are reference sources

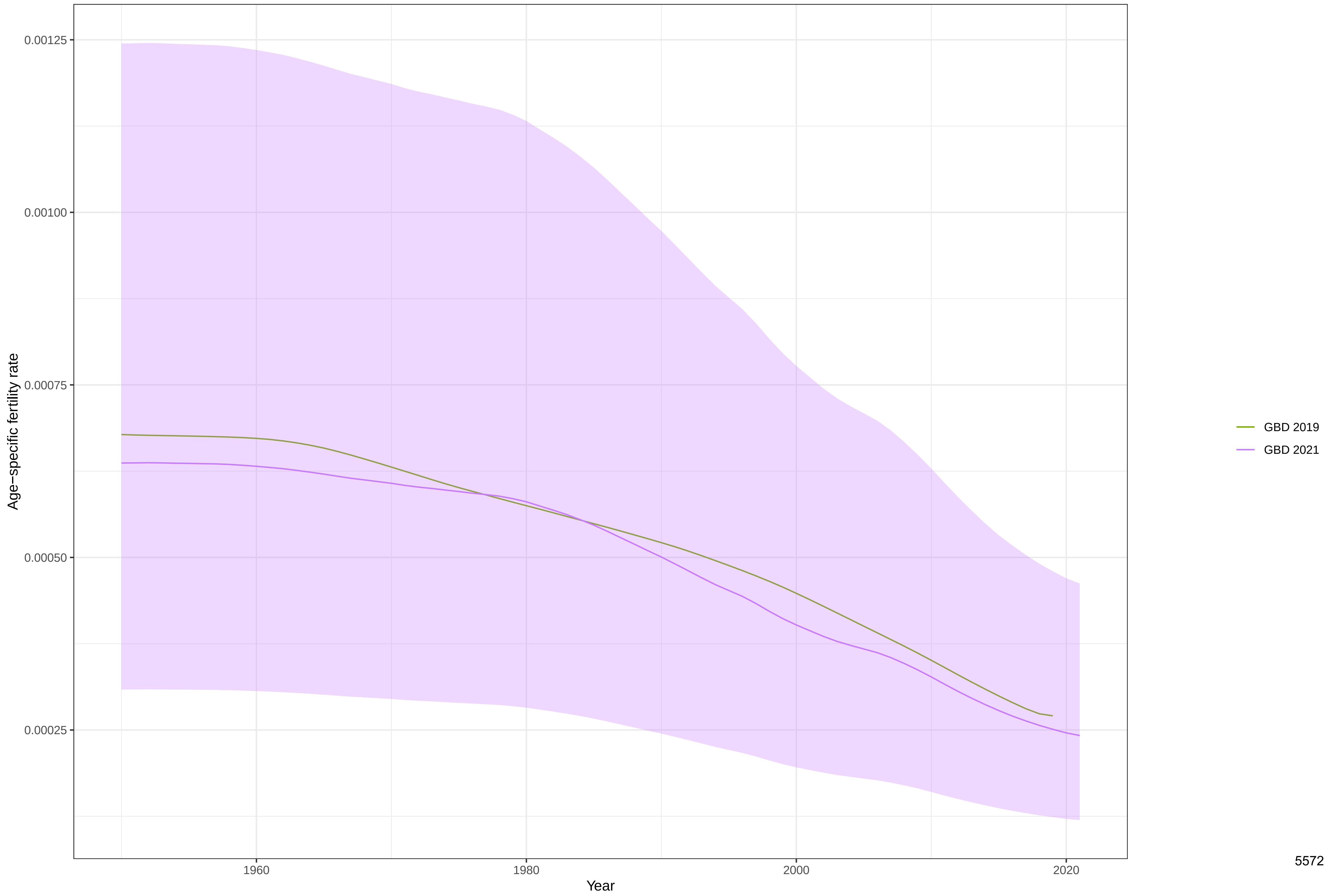

Age−specific fertility fitted trend lines, 1950−2021: North Maluku, Ages: 15−19
Data points enclosed with a diamond are reference sources

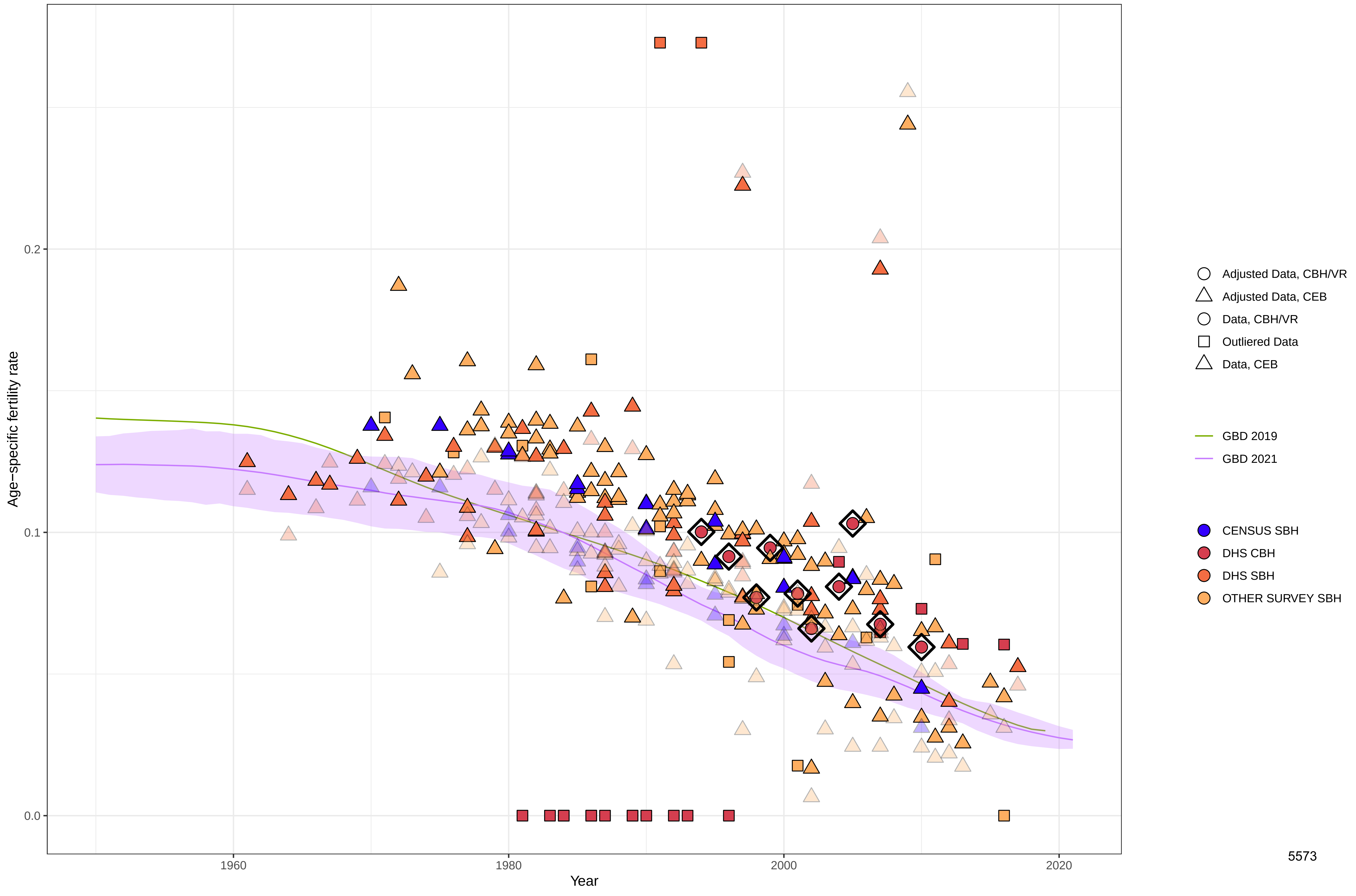

Age−specific fertility fitted trend lines, 1950−2021: North Maluku, Ages: 20−24
Data points enclosed with a diamond are reference sources

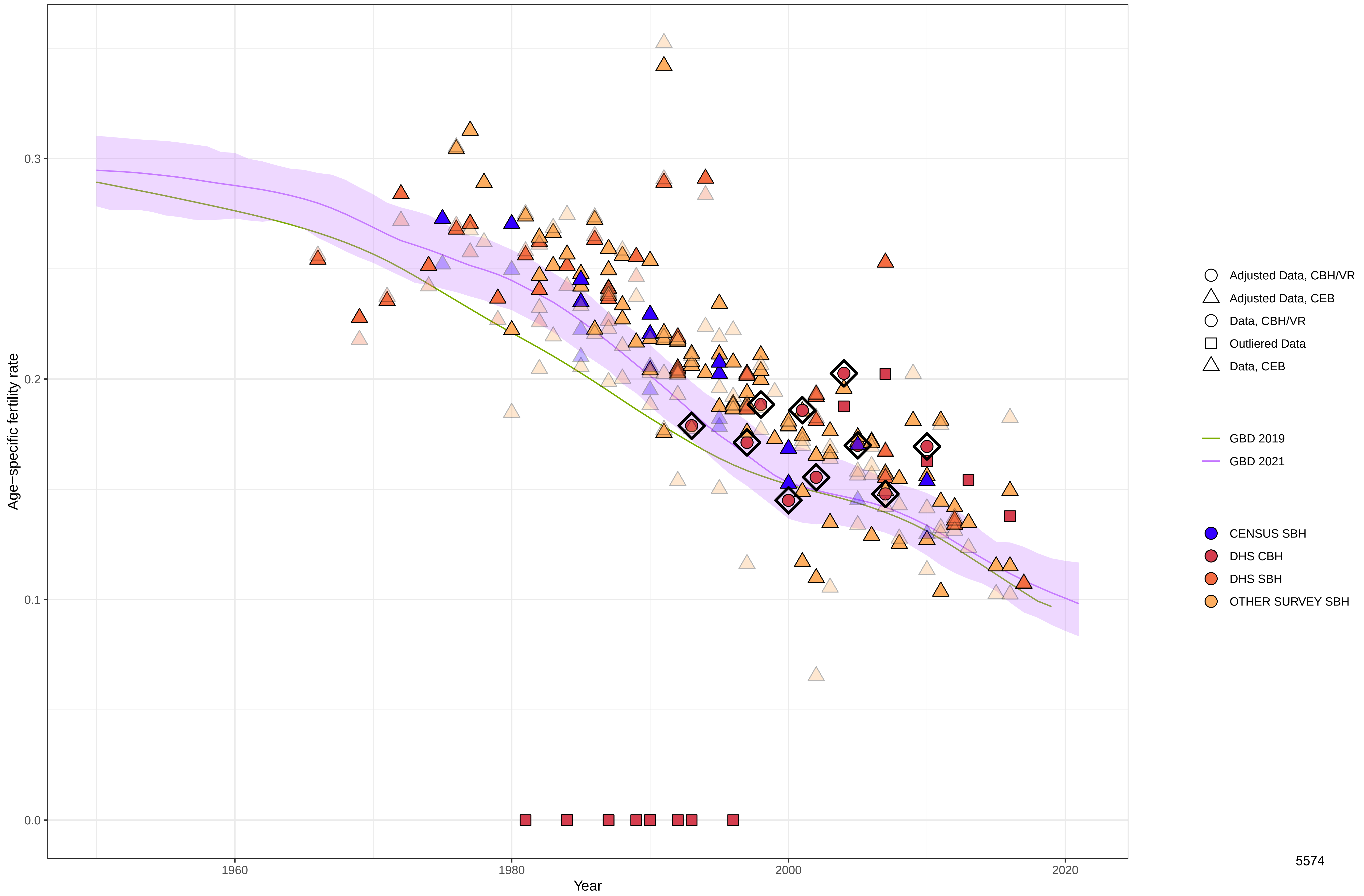

Age−specific fertility fitted trend lines, 1950−2021: North Maluku, Ages: 25−29
Data points enclosed with a diamond are reference sources

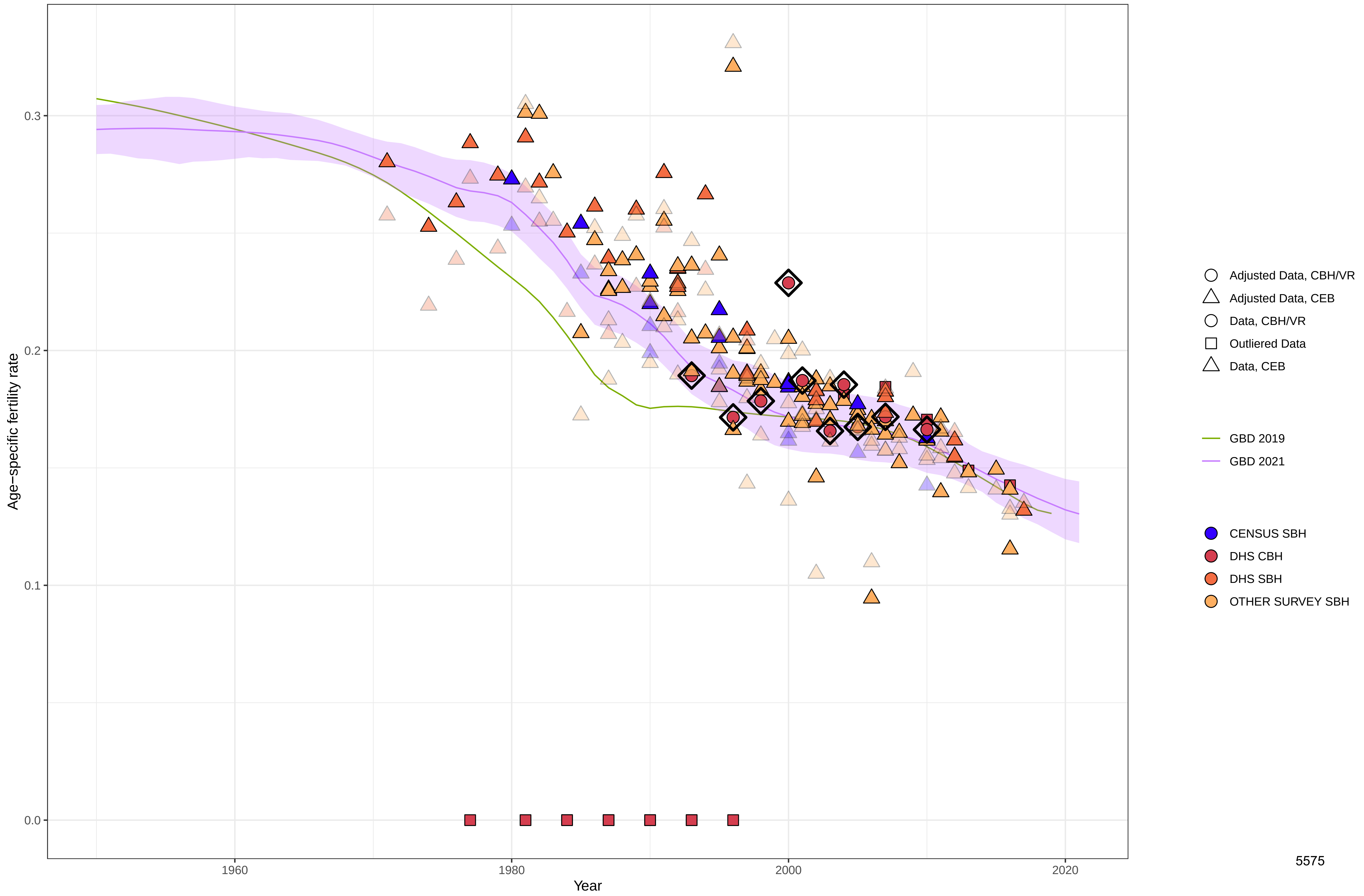

Age−specific fertility fitted trend lines, 1950−2021: North Maluku, Ages: 30−34
Data points enclosed with a diamond are reference sources

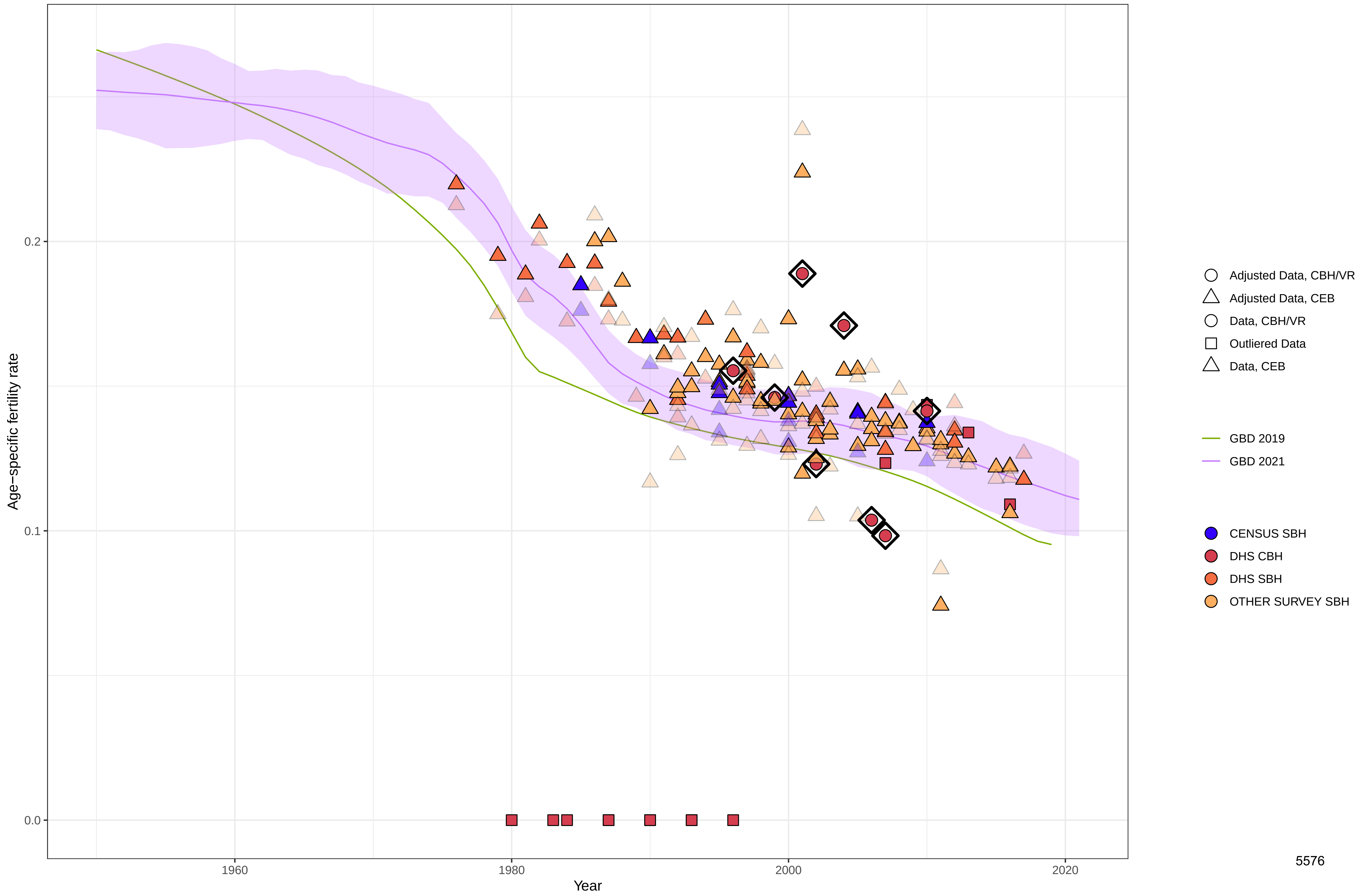

Age−specific fertility fitted trend lines, 1950−2021: North Maluku, Ages: 35−39
Data points enclosed with a diamond are reference sources

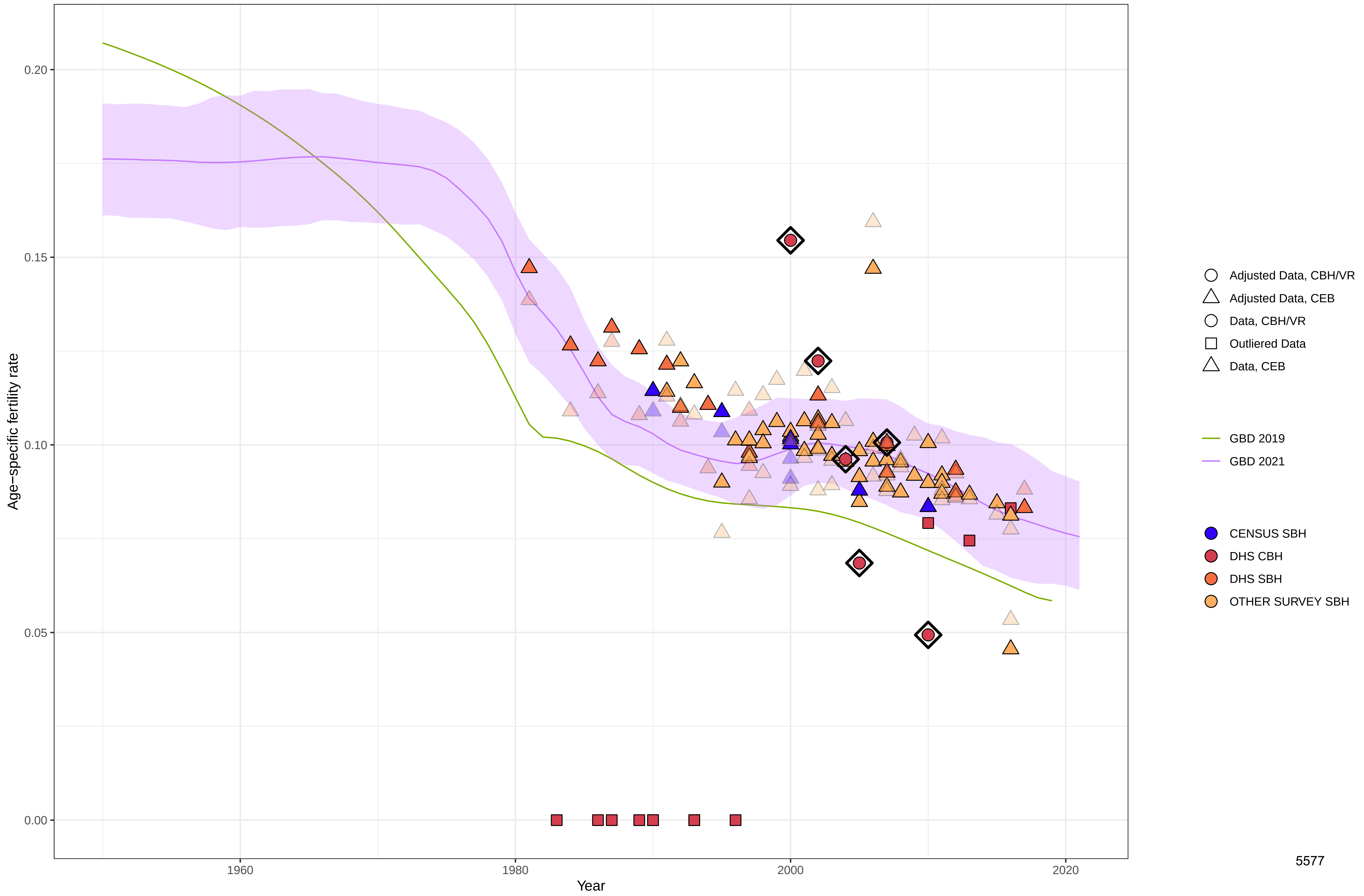

Age−specific fertility fitted trend lines, 1950−2021: North Maluku, Ages: 40−44
Data points enclosed with a diamond are reference sources

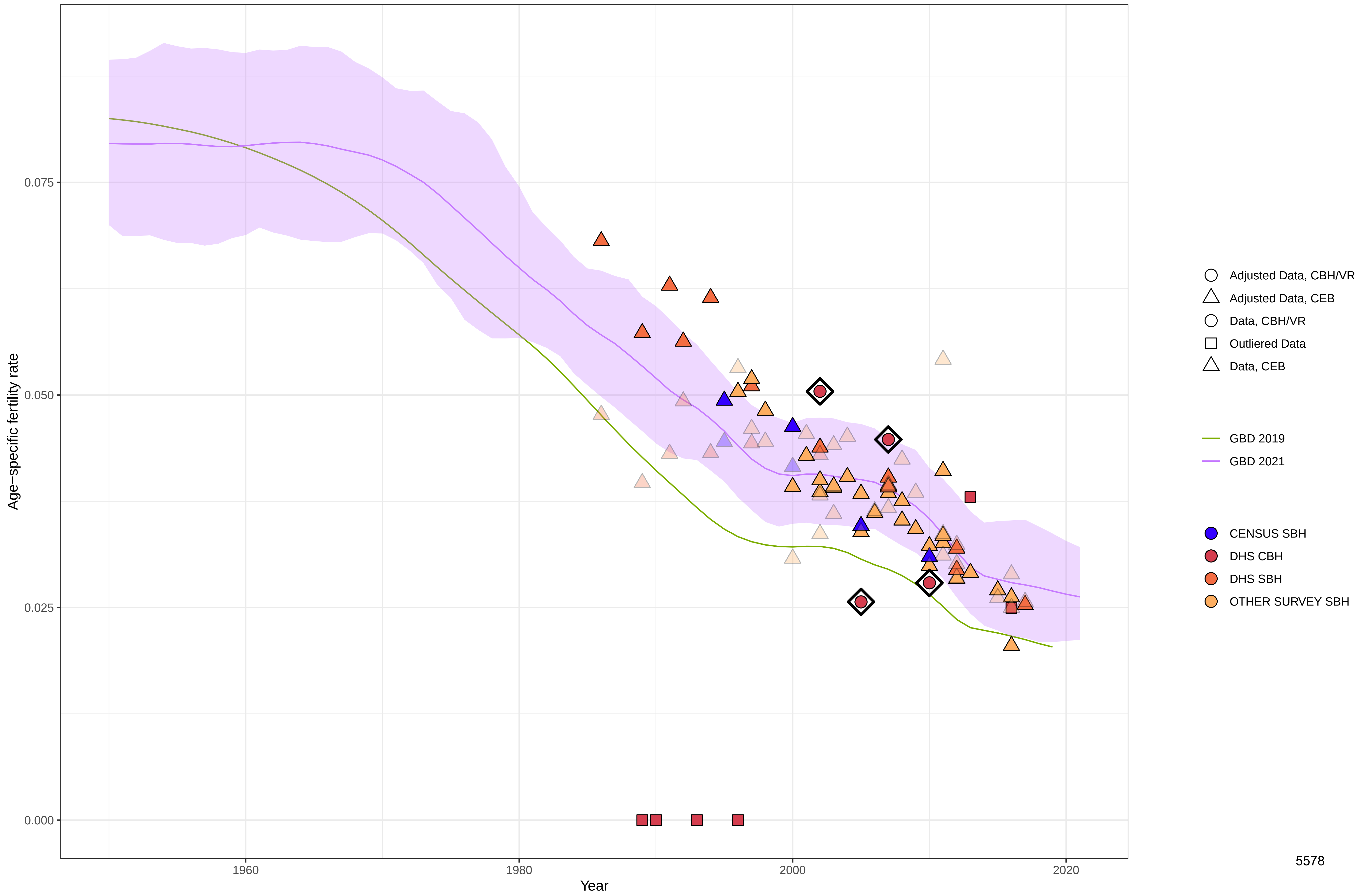

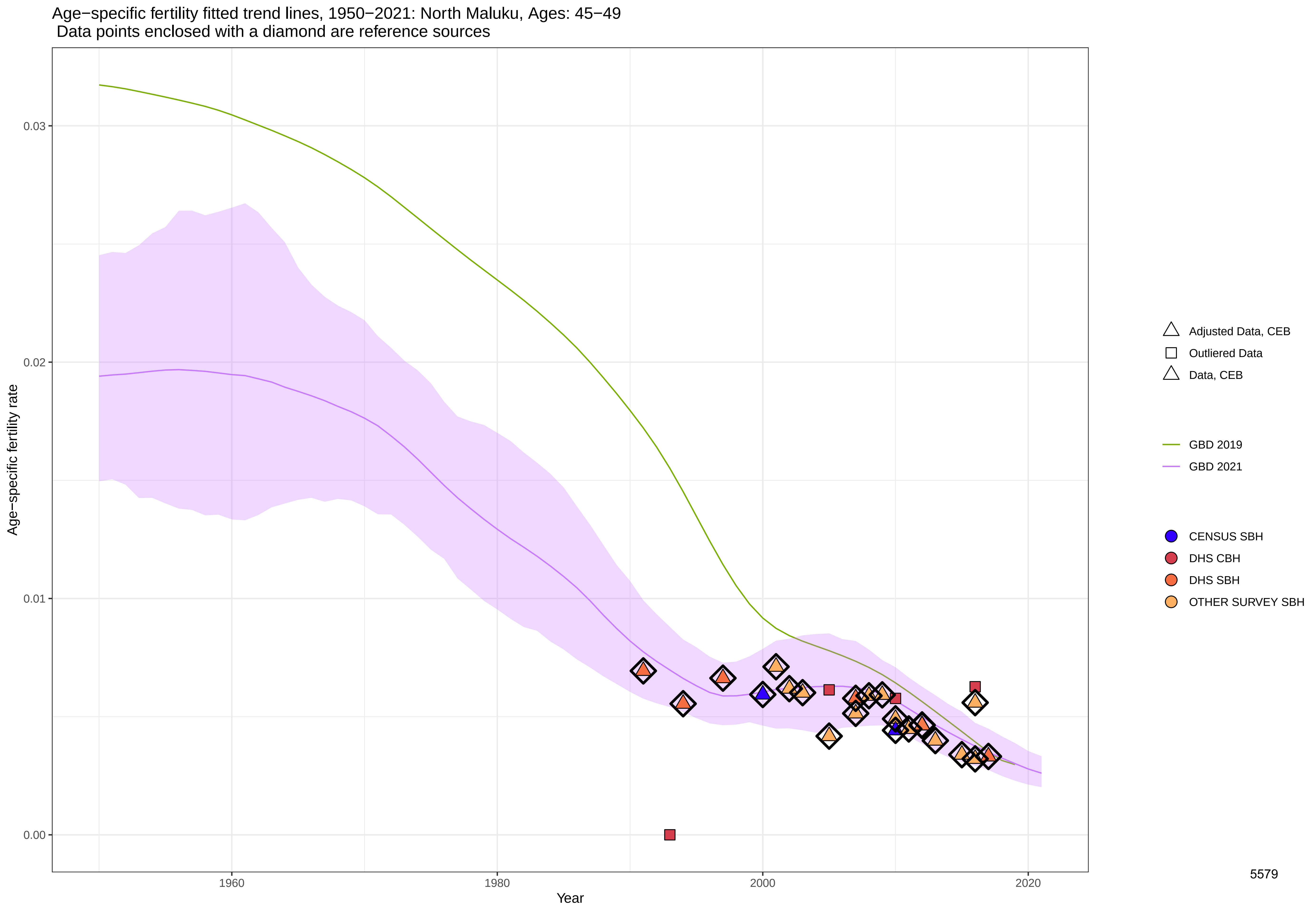

Age−specific fertility fitted trend lines, 1950−2021: North Maluku, Ages: 45−49
Data points enclosed with a diamond are reference sources
Age−specific fertility rate
0.00
0.01
0.02
0.03
1960
1980
2000
2020
Year
Adjusted Data, CEB
Outliered Data
Data, CEB
GBD 2019
GBD 2021
CENSUS SBH
DHS CBH
DHS SBH
OTHER SURVEY SBH

Age−specific fertility fitted trend lines, 1950−2021: North Maluku, Ages: 50−54
Data points enclosed with a diamond are reference sources

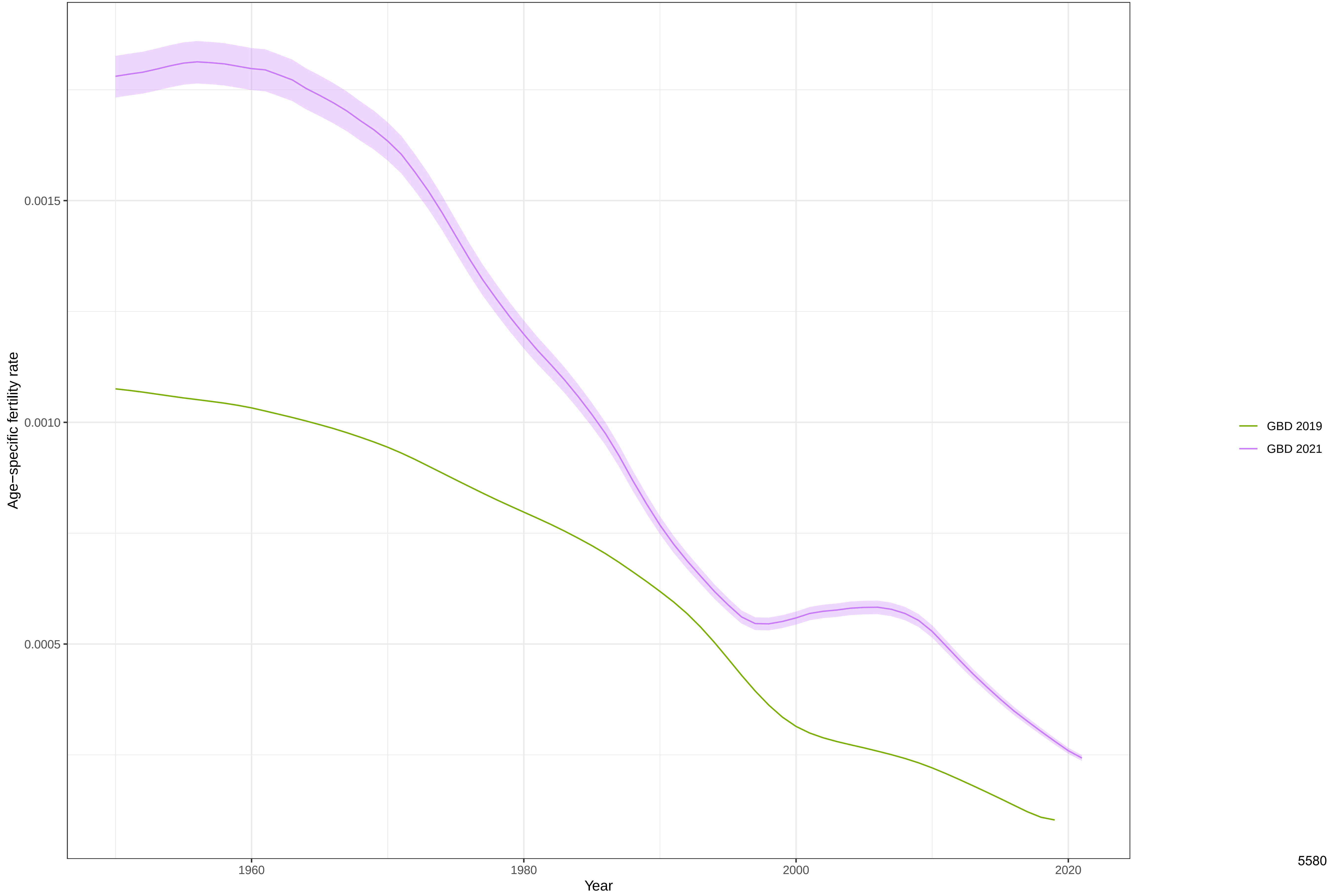

Total fertility rate fitted trend lines, 1950−2021: North Maluku TFR
Data points enclosed with a diamond are reference sources

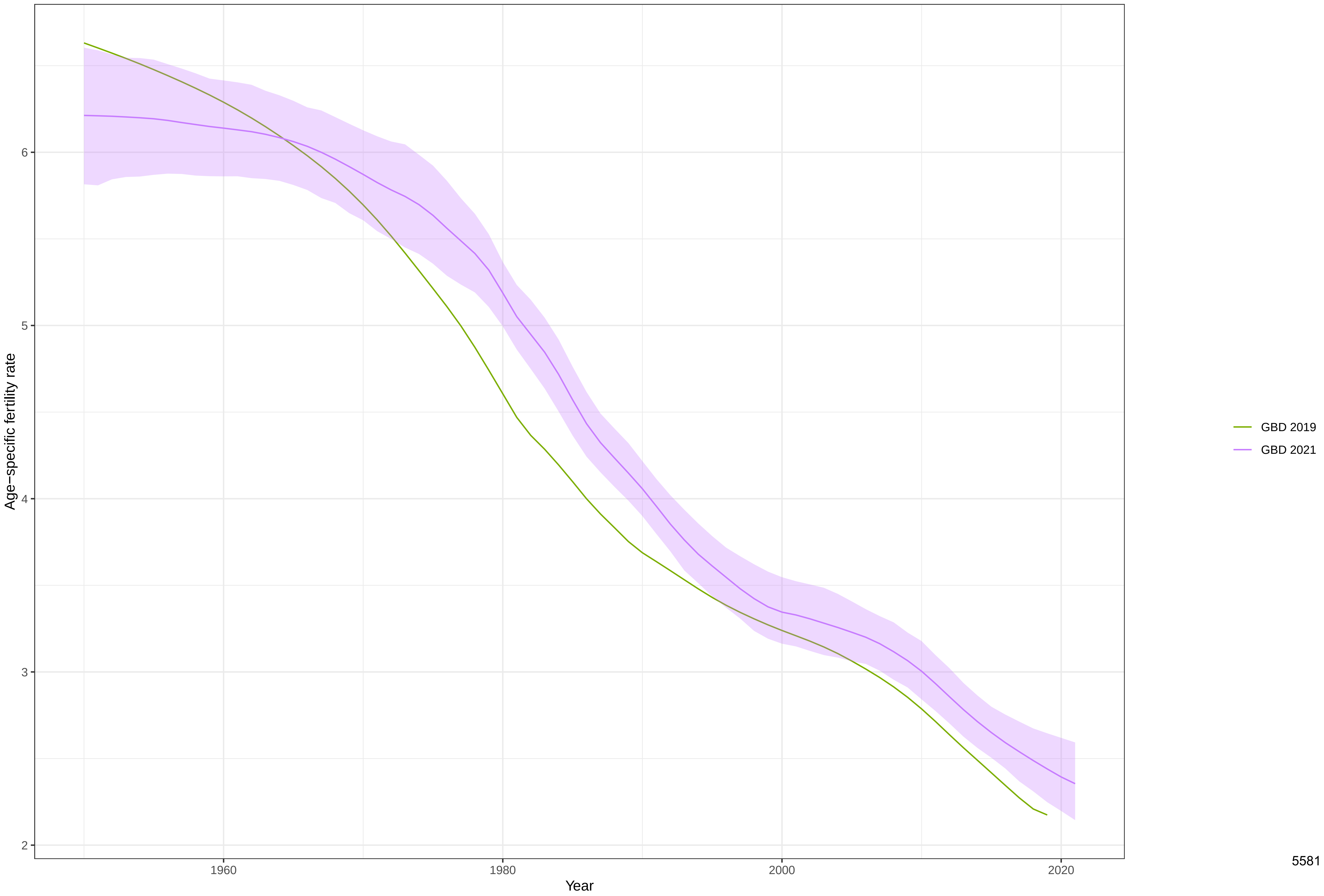

Age−specific fertility fitted trend lines, 1950−2021: West Nusa Tenggara, Ages: 10−14
Data points enclosed with a diamond are reference sources

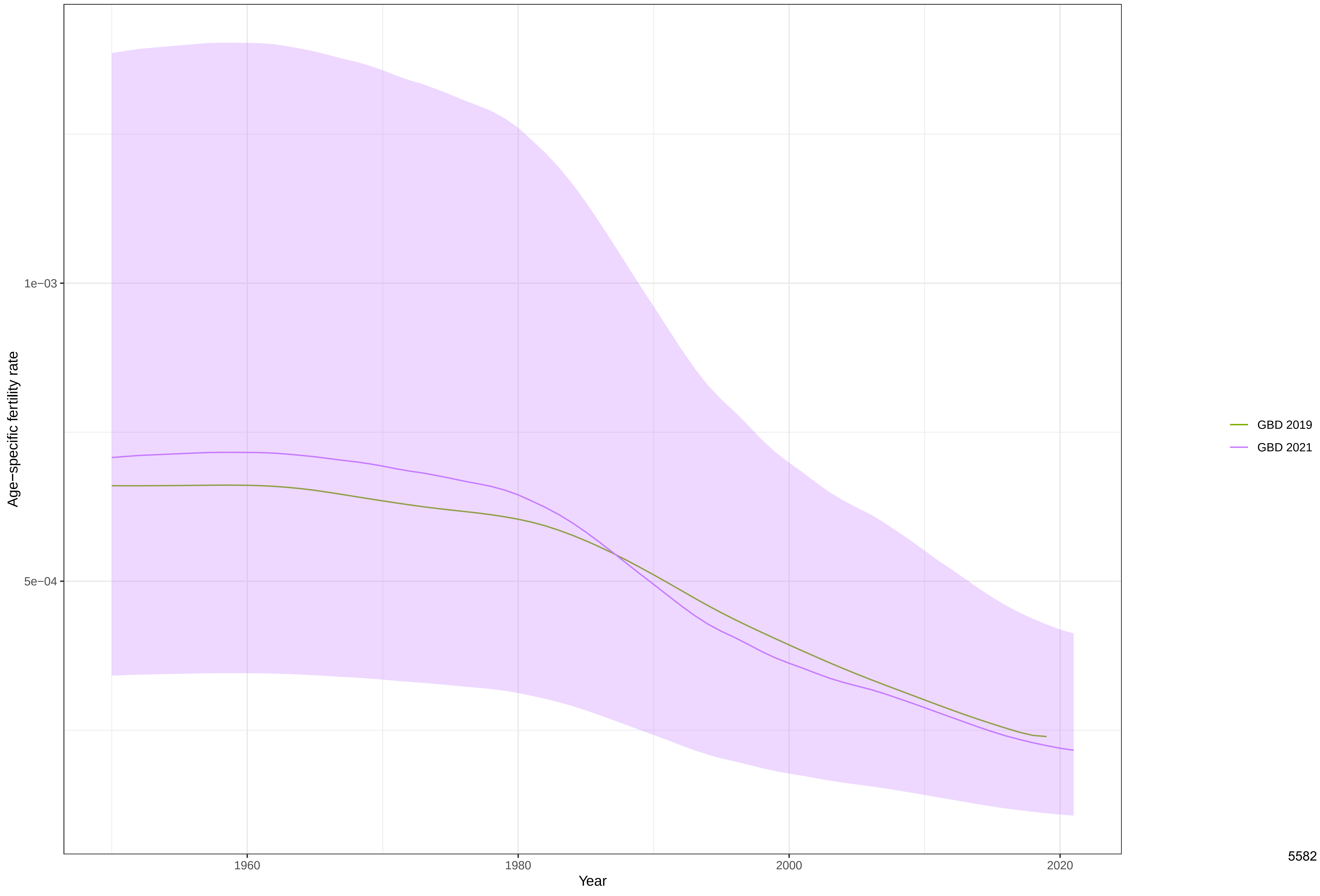

Age−specific fertility fitted trend lines, 1950−2021: West Nusa Tenggara, Ages: 15−19
Data points enclosed with a diamond are reference sources

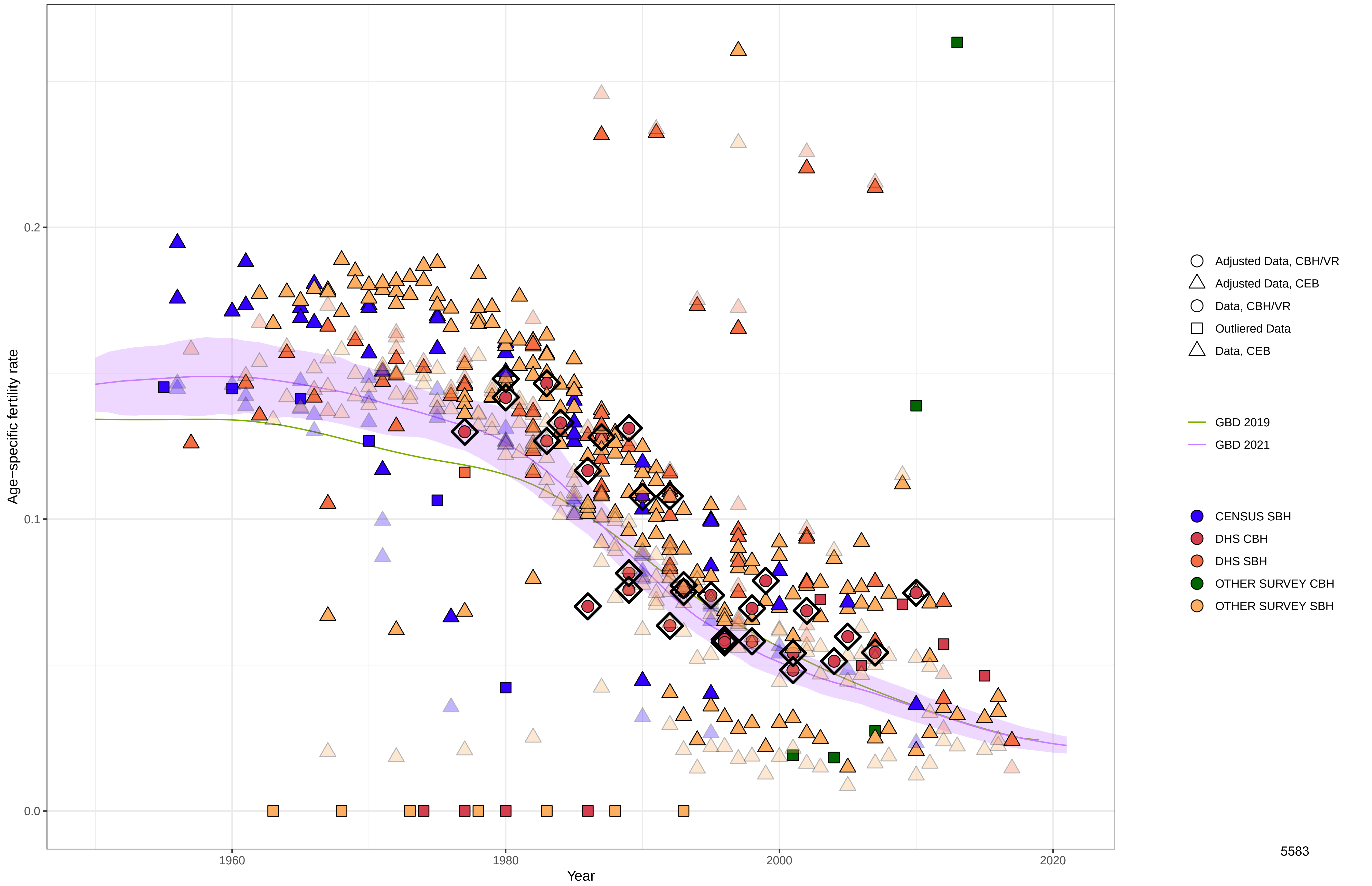

5583

Age−specific fertility fitted trend lines, 1950−2021: West Nusa Tenggara, Ages: 20−24
Data points enclosed with a diamond are reference sources

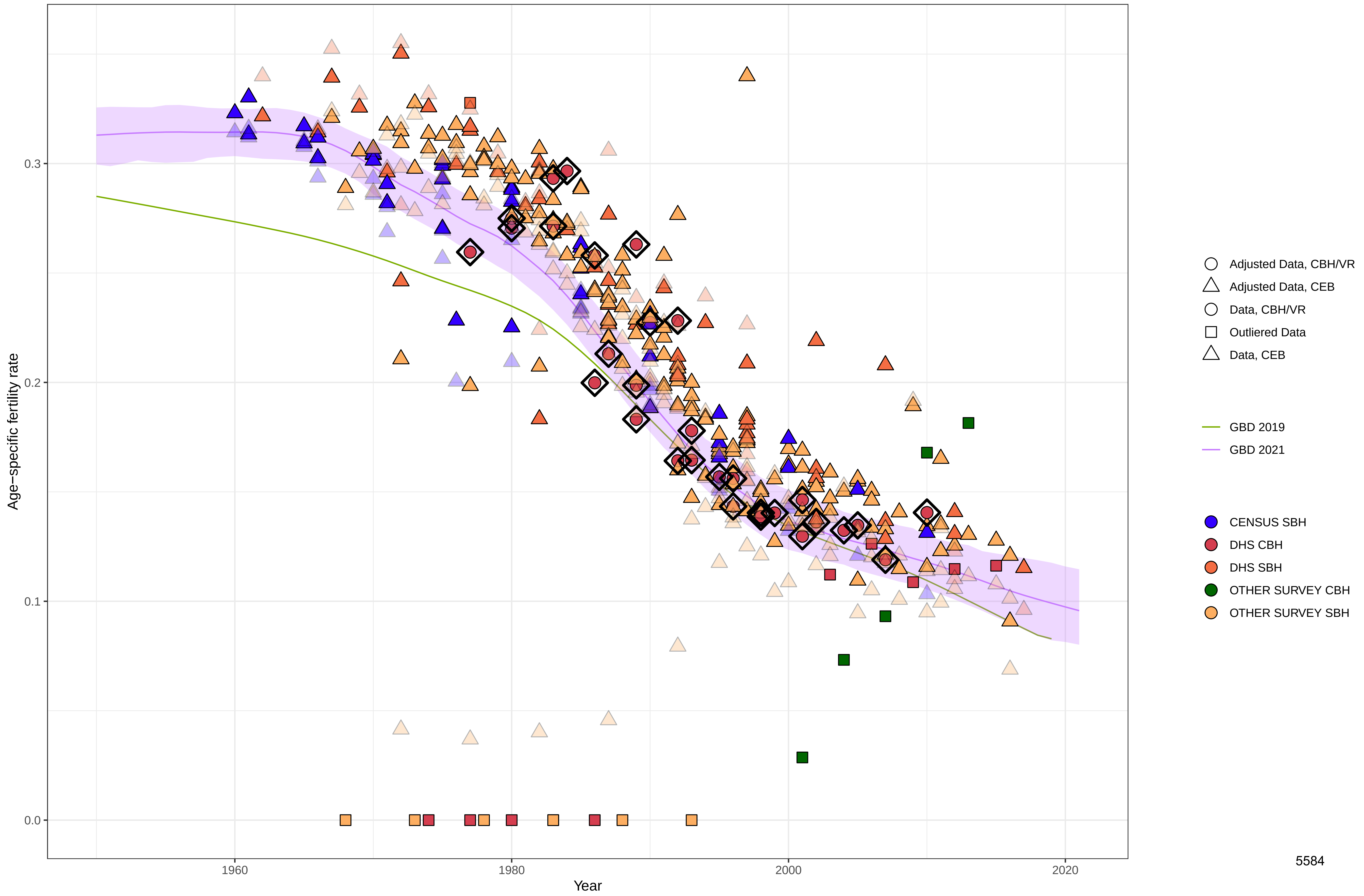

Age−specific fertility fitted trend lines, 1950−2021: West Nusa Tenggara, Ages: 25−29
Data points enclosed with a diamond are reference sources

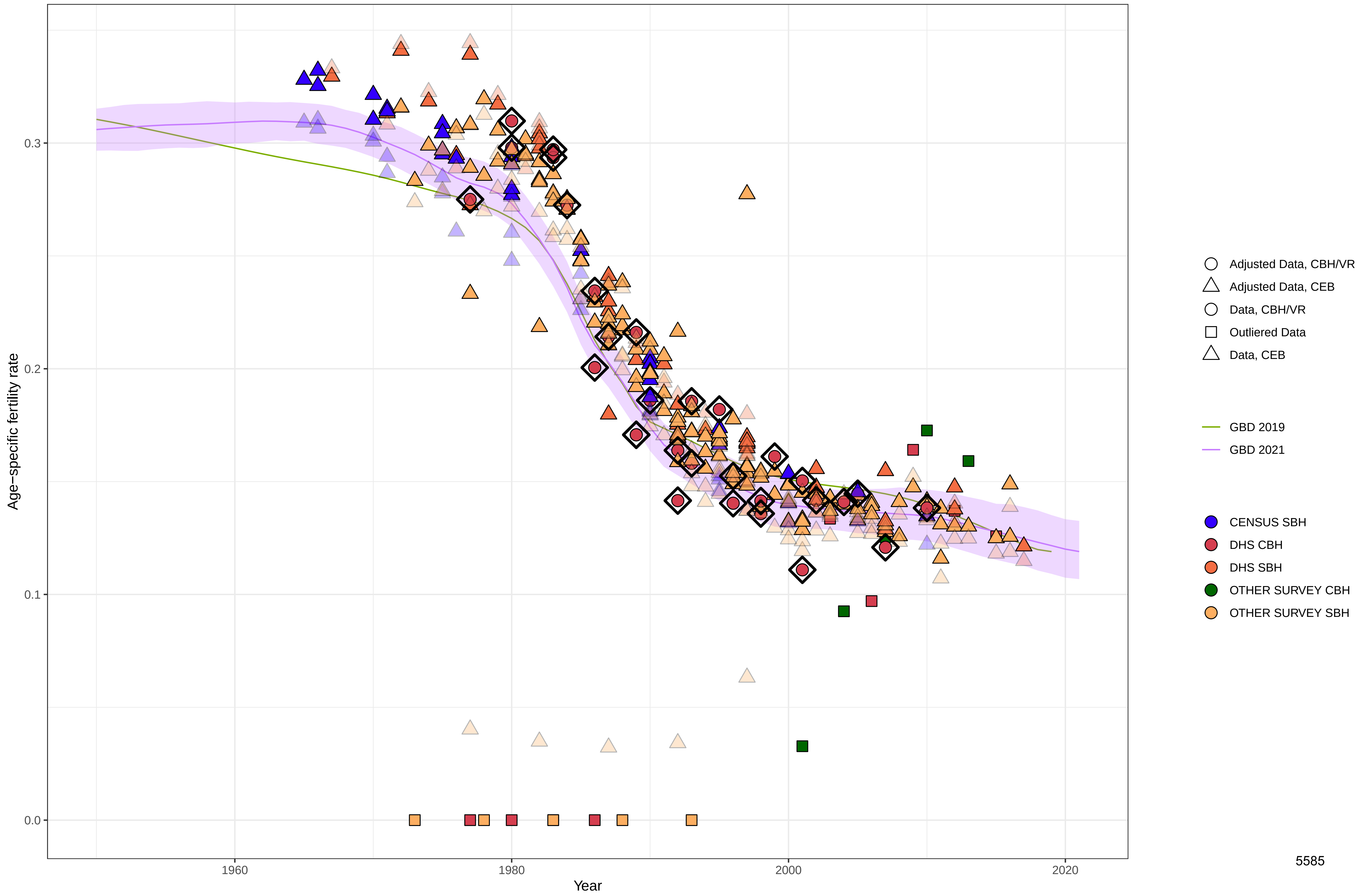

Age−specific fertility fitted trend lines, 1950−2021: West Nusa Tenggara, Ages: 30−34
Data points enclosed with a diamond are reference sources

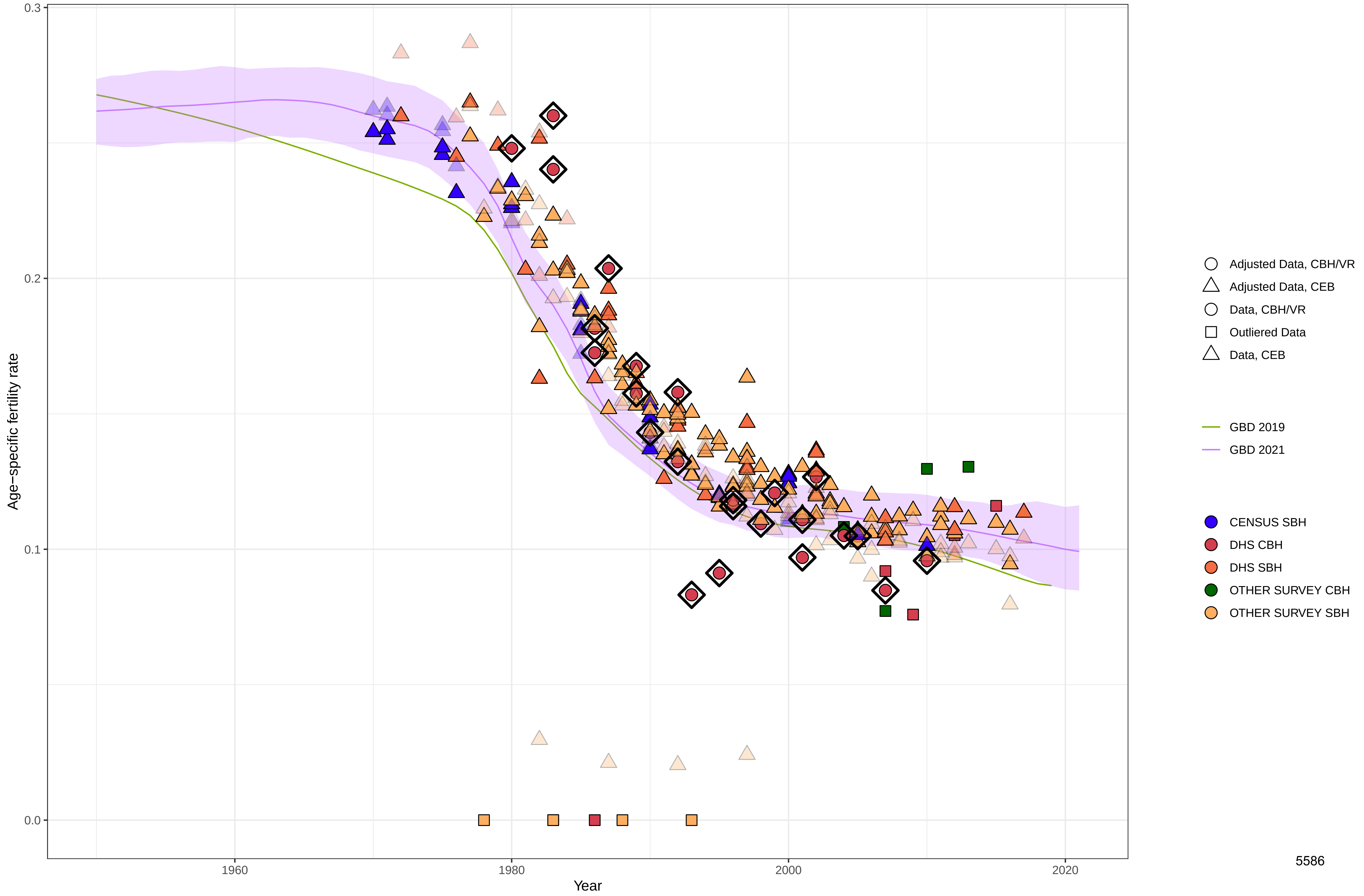

Age−specific fertility fitted trend lines, 1950−2021: West Nusa Tenggara, Ages: 35−39
Data points enclosed with a diamond are reference sources

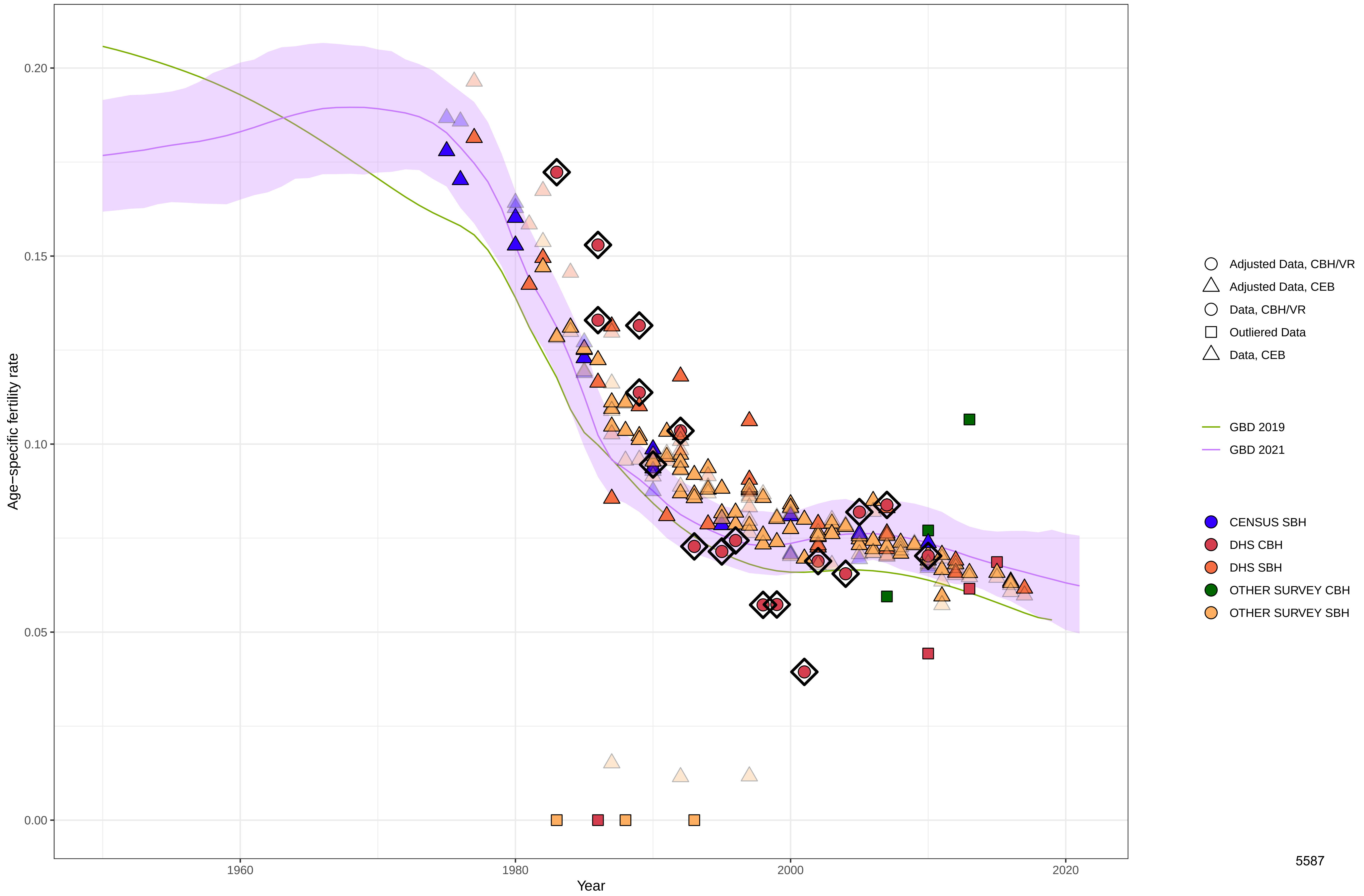

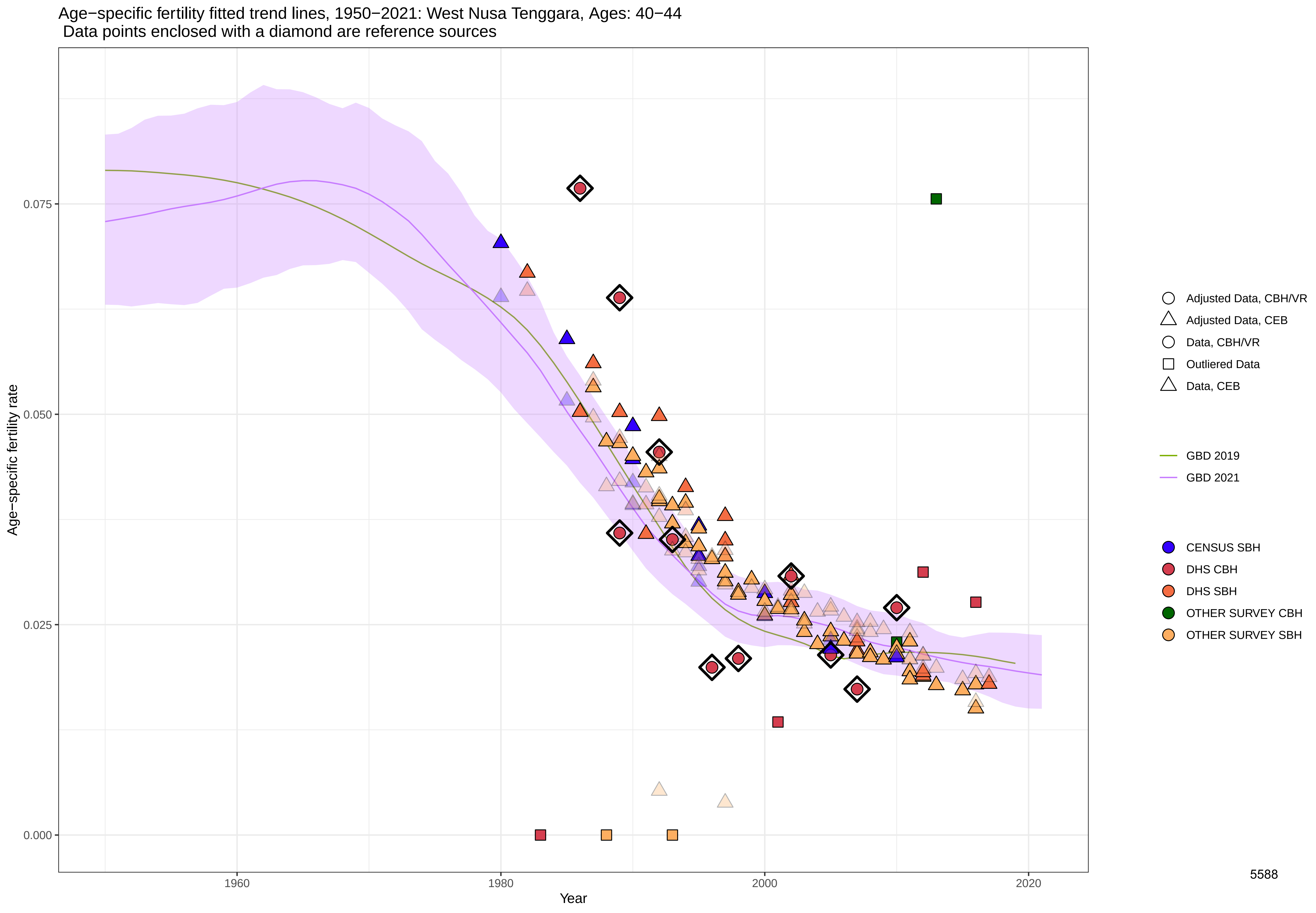

Age−specific fertility fitted trend lines, 1950−2021: West Nusa Tenggara, Ages: 40−44
Data points enclosed with a diamond are reference sources
Age−specific fertility rate
0.000
0.025
0.050
0.075
1960
1980
2000
2020
Year
Adjusted Data, CBH/VR
Adjusted Data, CEB
Data, CBH/VR
Outliered Data
Data, CEB
GBD 2019
GBD 2021
CENSUS SBH
DHS CBH
DHS SBH
OTHER SURVEY CBH
OTHER SURVEY SBH

Age−specific fertility fitted trend lines, 1950−2021: West Nusa Tenggara, Ages: 45−49
Data points enclosed with a diamond are reference sources

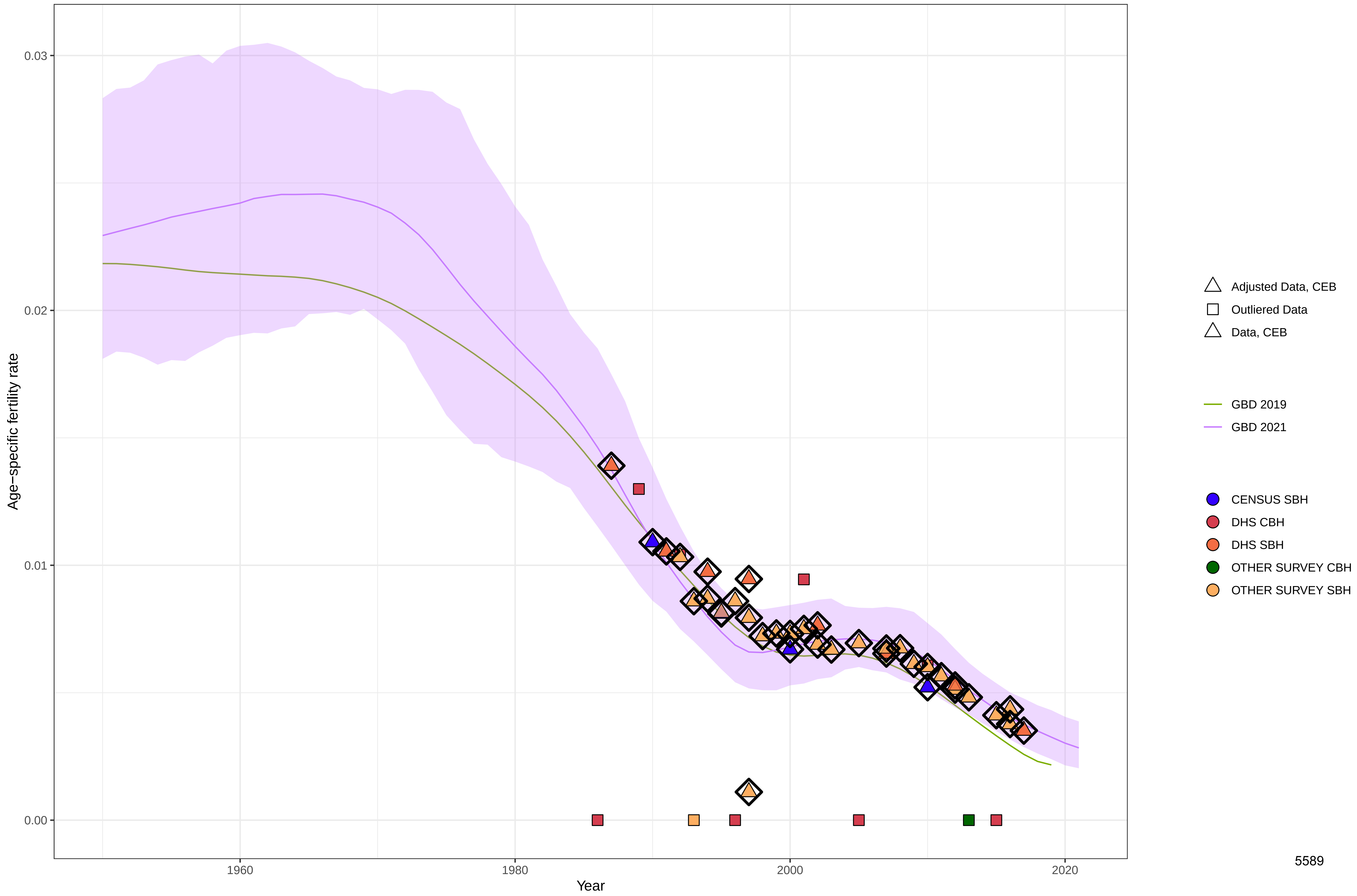

Age−specific fertility fitted trend lines, 1950−2021: West Nusa Tenggara, Ages: 50−54
Data points enclosed with a diamond are reference sources

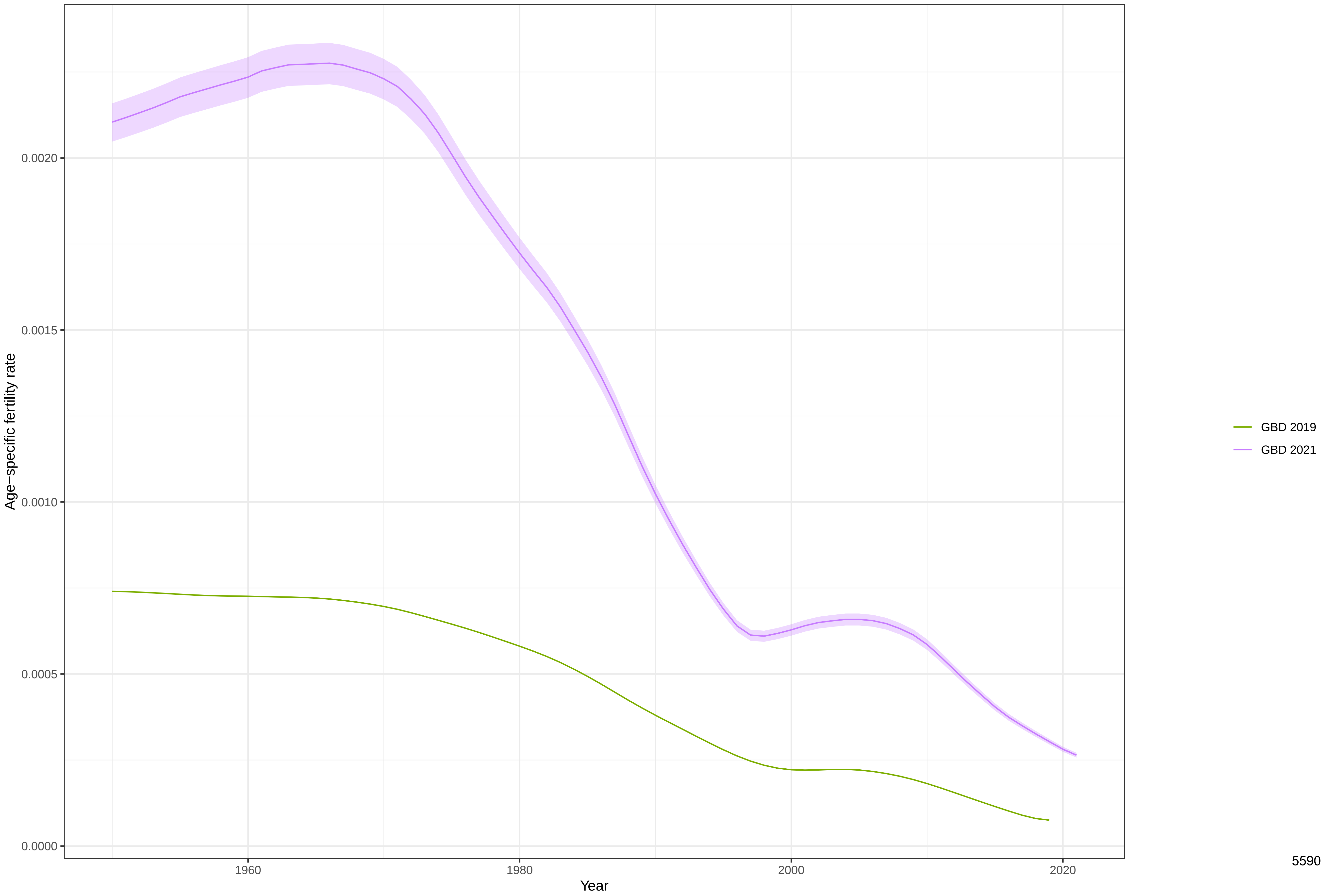

Total fertility rate fitted trend lines, 1950−2021: West Nusa Tenggara TFR
Data points enclosed with a diamond are reference sources

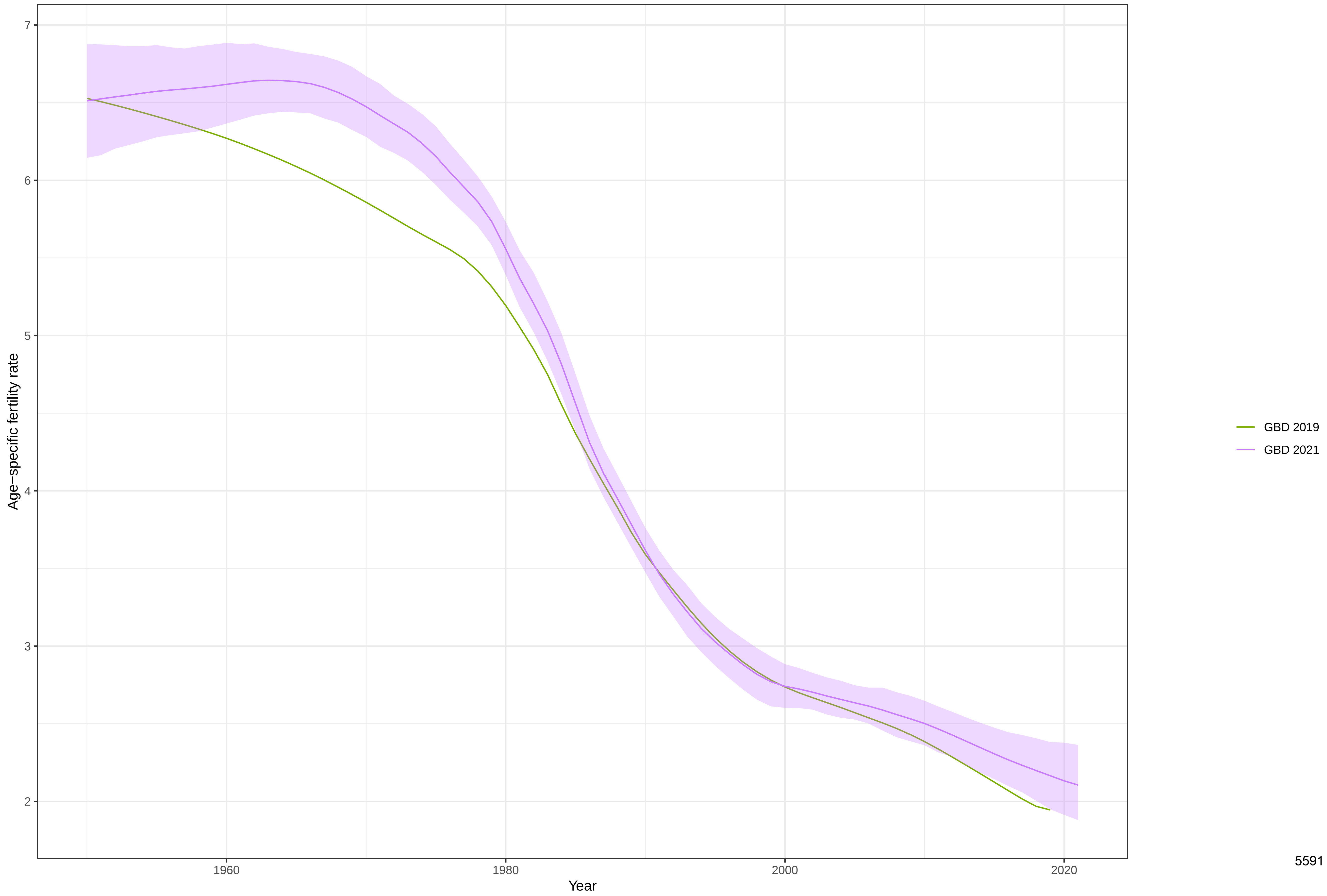

Age−specific fertility fitted trend lines, 1950−2021: East Nusa Tenggara, Ages: 10−14
Data points enclosed with a diamond are reference sources

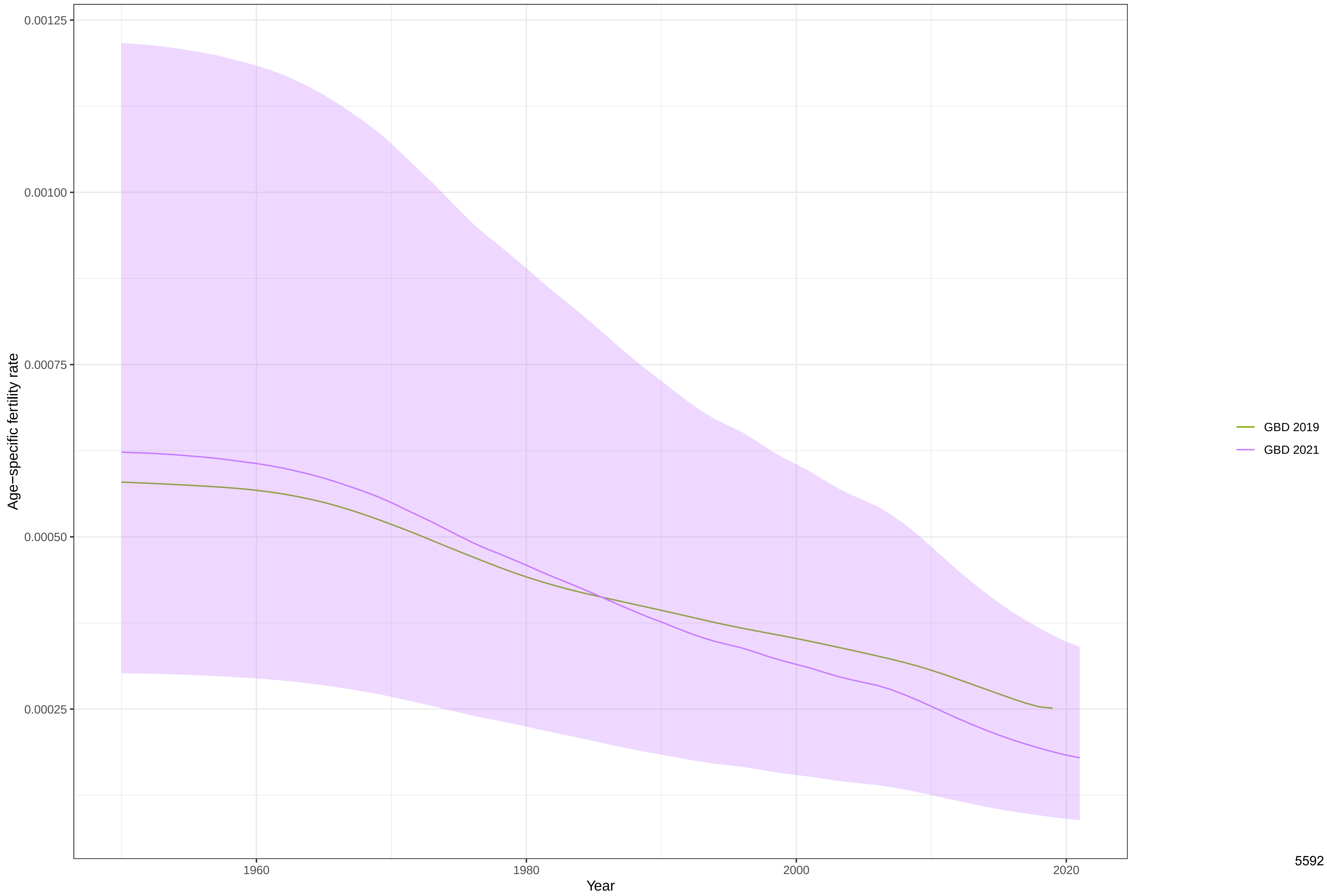

Age−specific fertility fitted trend lines, 1950−2021: East Nusa Tenggara, Ages: 15−19
Data points enclosed with a diamond are reference sources

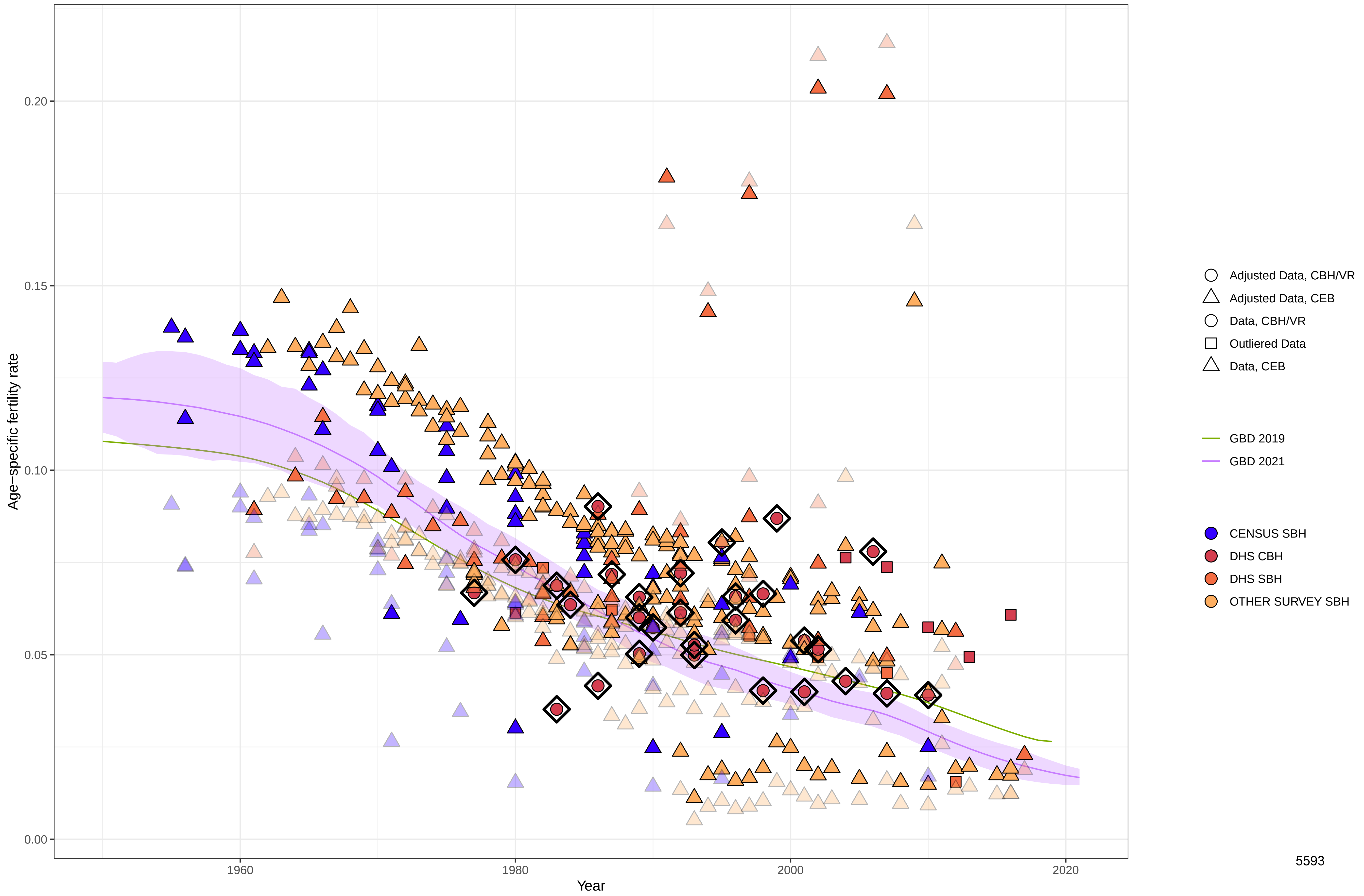

Age−specific fertility fitted trend lines, 1950−2021: East Nusa Tenggara, Ages: 20−24
Data points enclosed with a diamond are reference sources

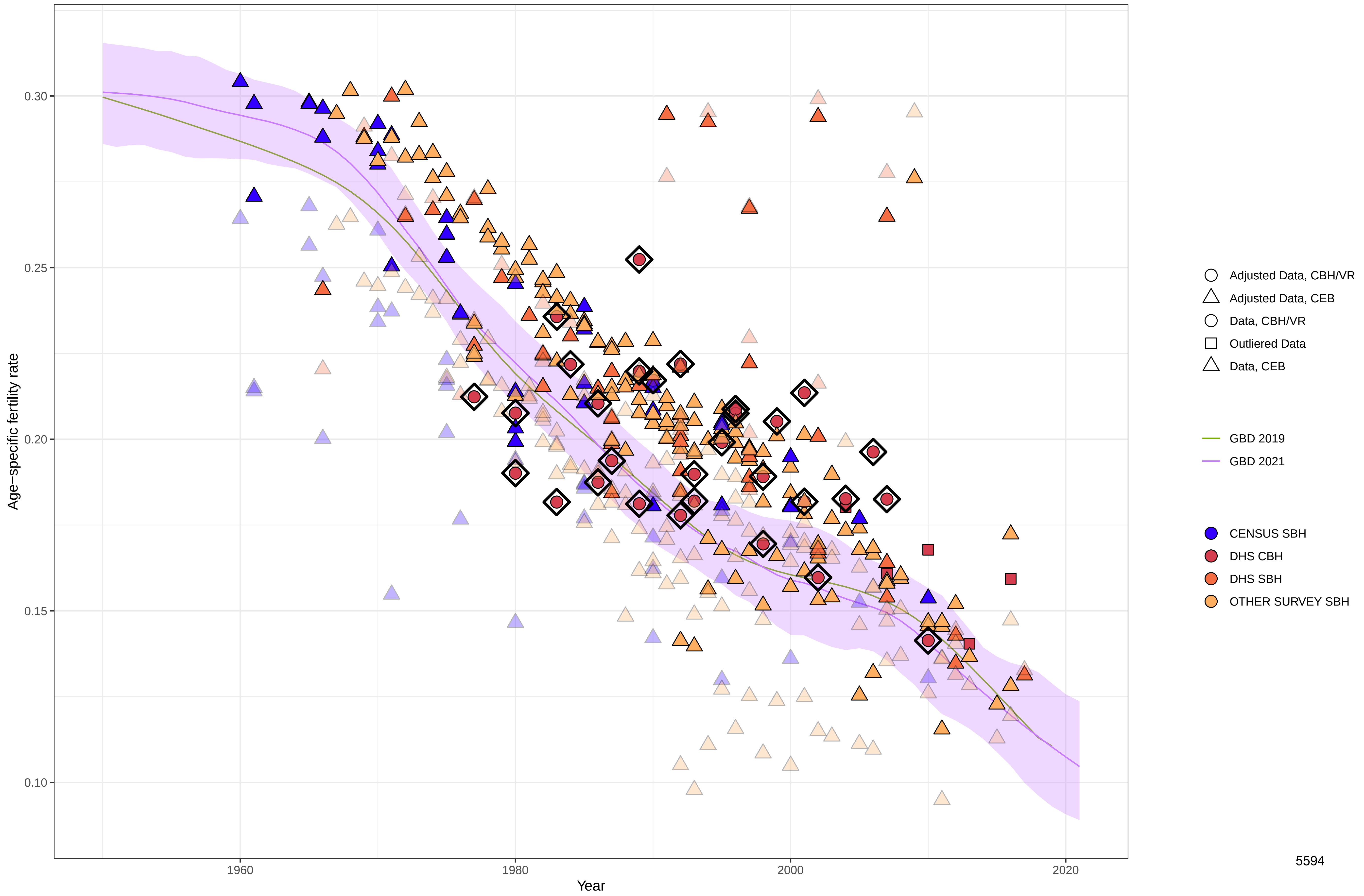

Age−specific fertility fitted trend lines, 1950−2021: East Nusa Tenggara, Ages: 25−29
Data points enclosed with a diamond are reference sources

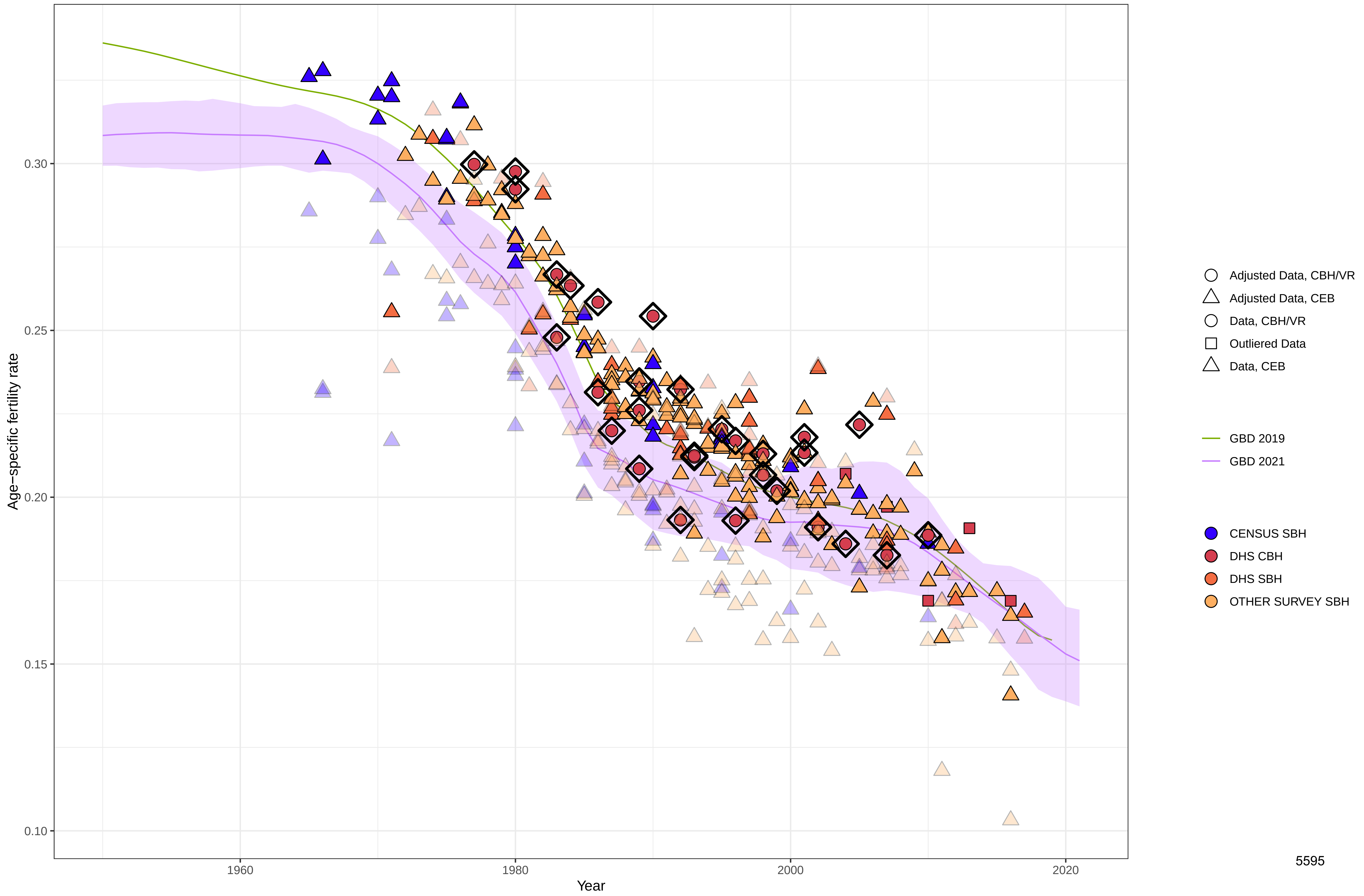

Age−specific fertility fitted trend lines, 1950−2021: East Nusa Tenggara, Ages: 30−34
Data points enclosed with a diamond are reference sources

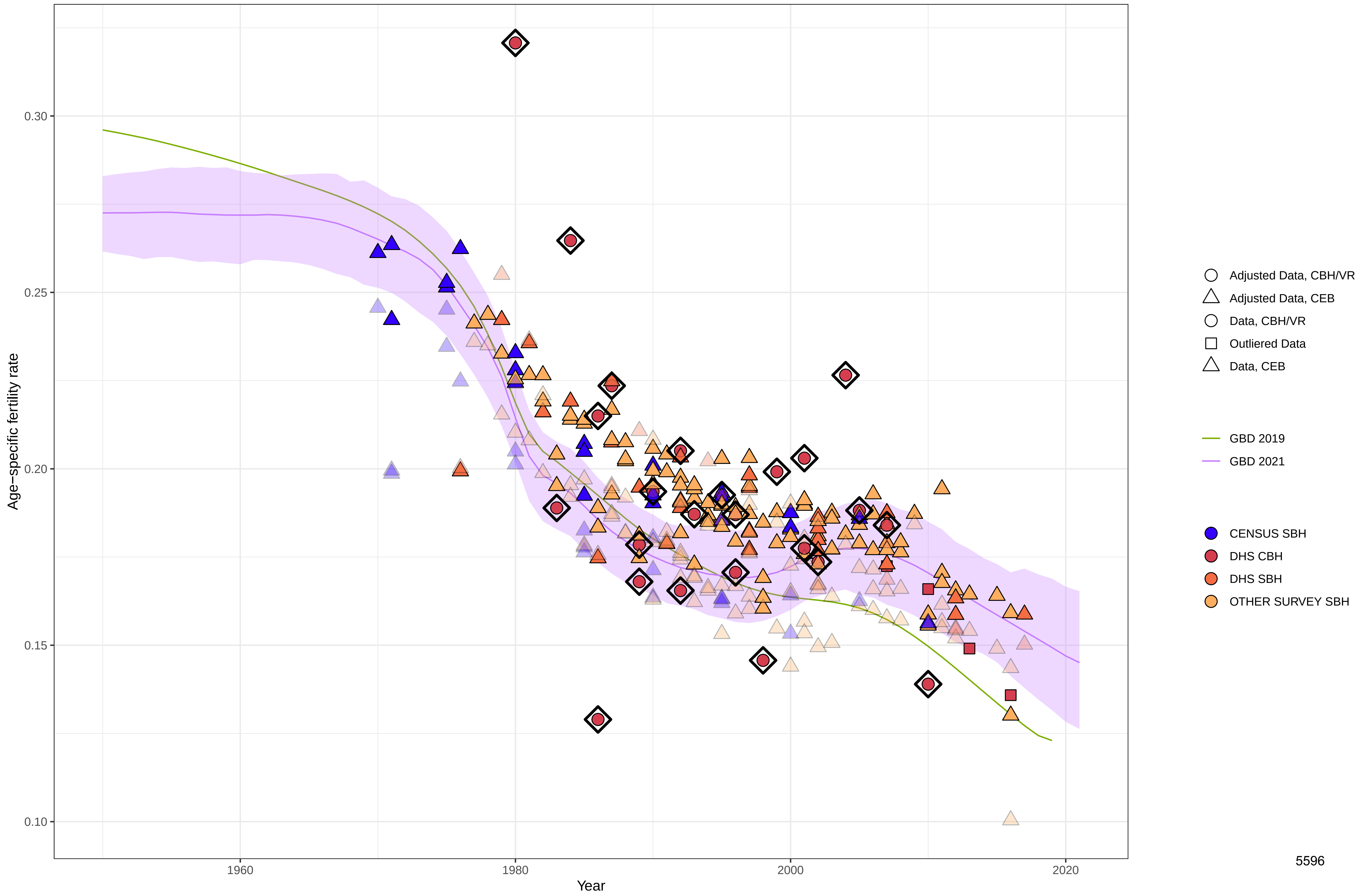

Age−specific fertility fitted trend lines, 1950−2021: East Nusa Tenggara, Ages: 35−39
Data points enclosed with a diamond are reference sources

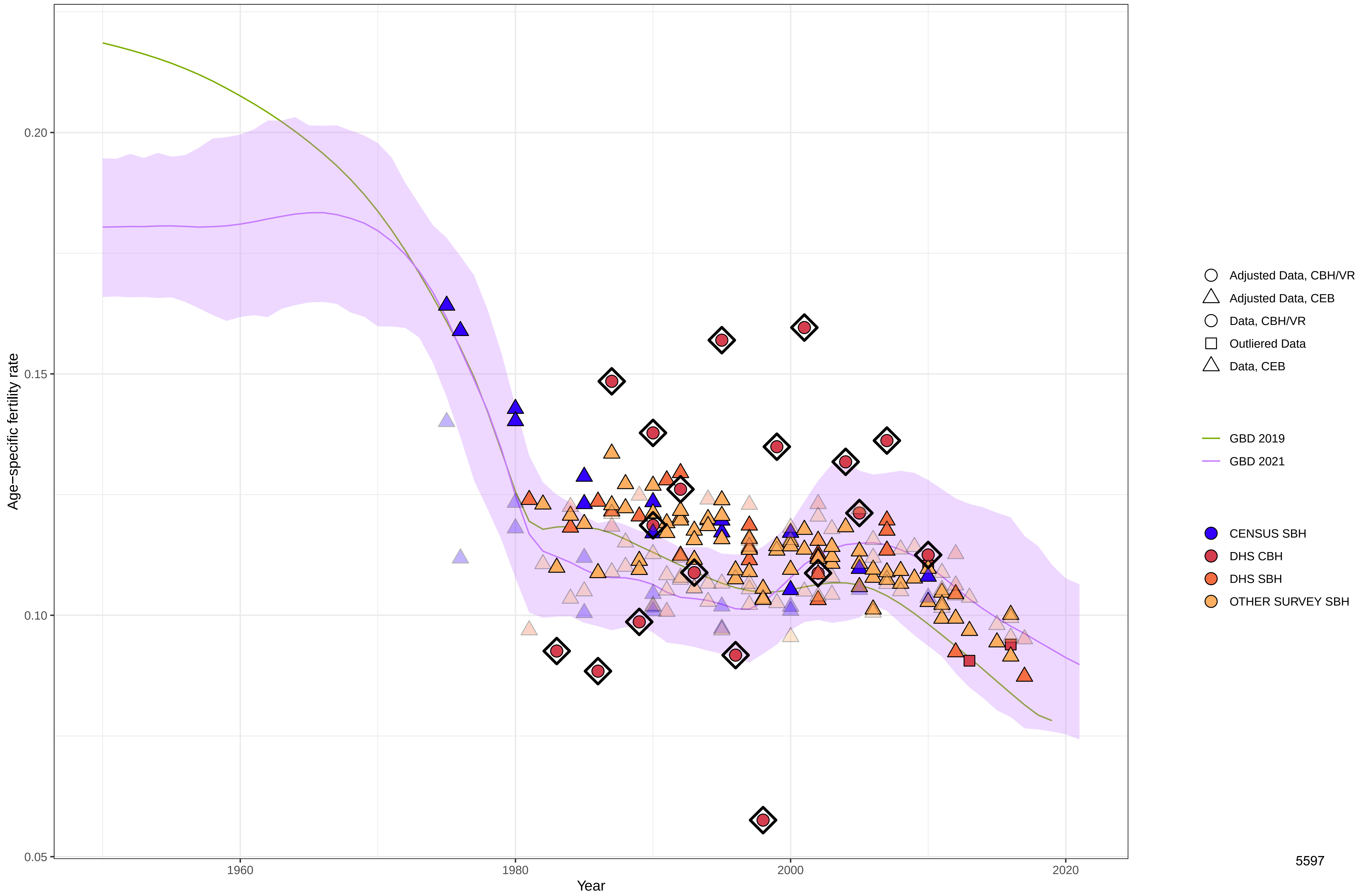

Age−specific fertility fitted trend lines, 1950−2021: East Nusa Tenggara, Ages: 40−44
Data points enclosed with a diamond are reference sources

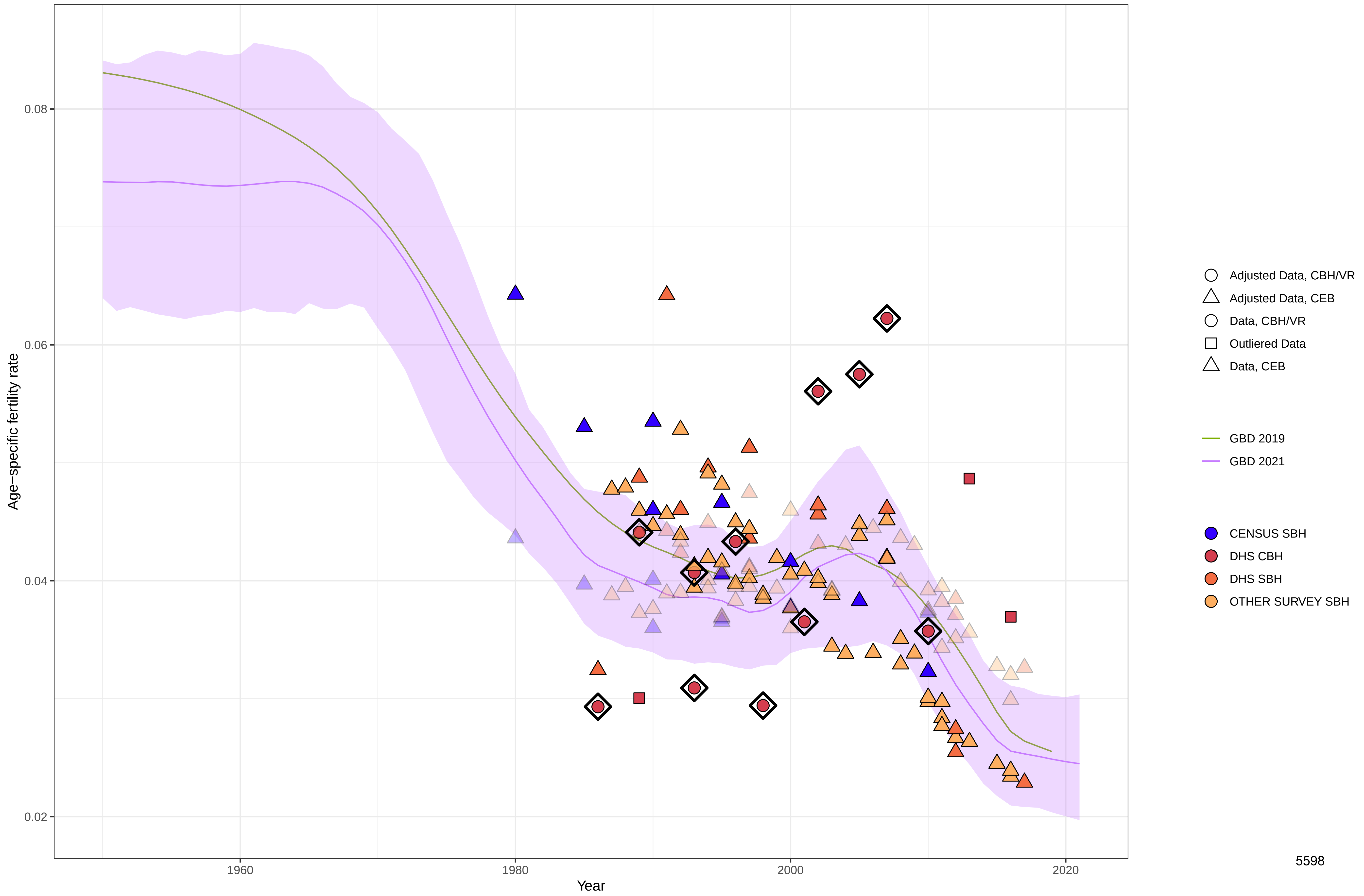

Age−specific fertility fitted trend lines, 1950−2021: East Nusa Tenggara, Ages: 45−49
Data points enclosed with a diamond are reference sources

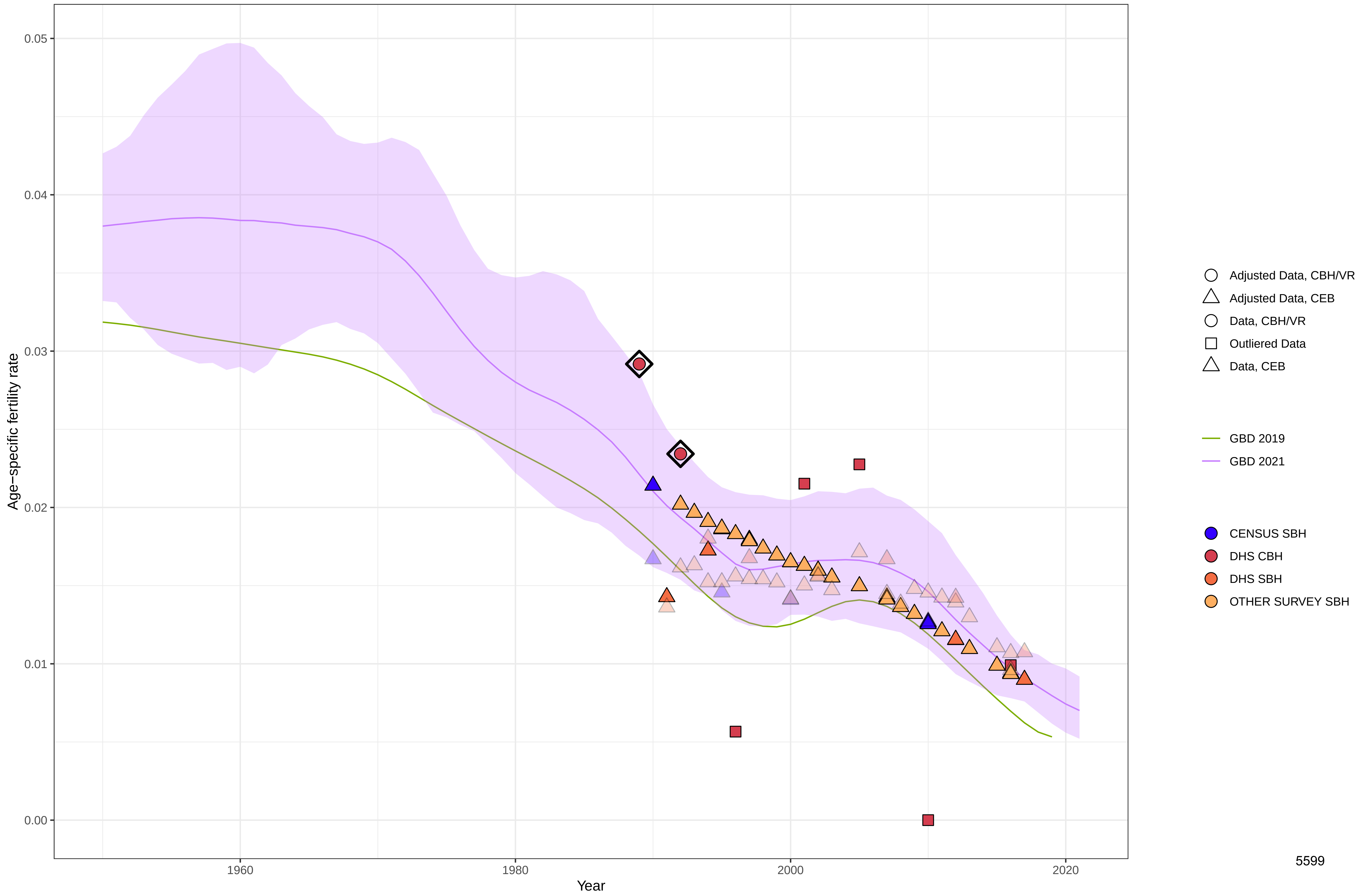

Age−specific fertility fitted trend lines, 1950−2021: East Nusa Tenggara, Ages: 50−54
Data points enclosed with a diamond are reference sources

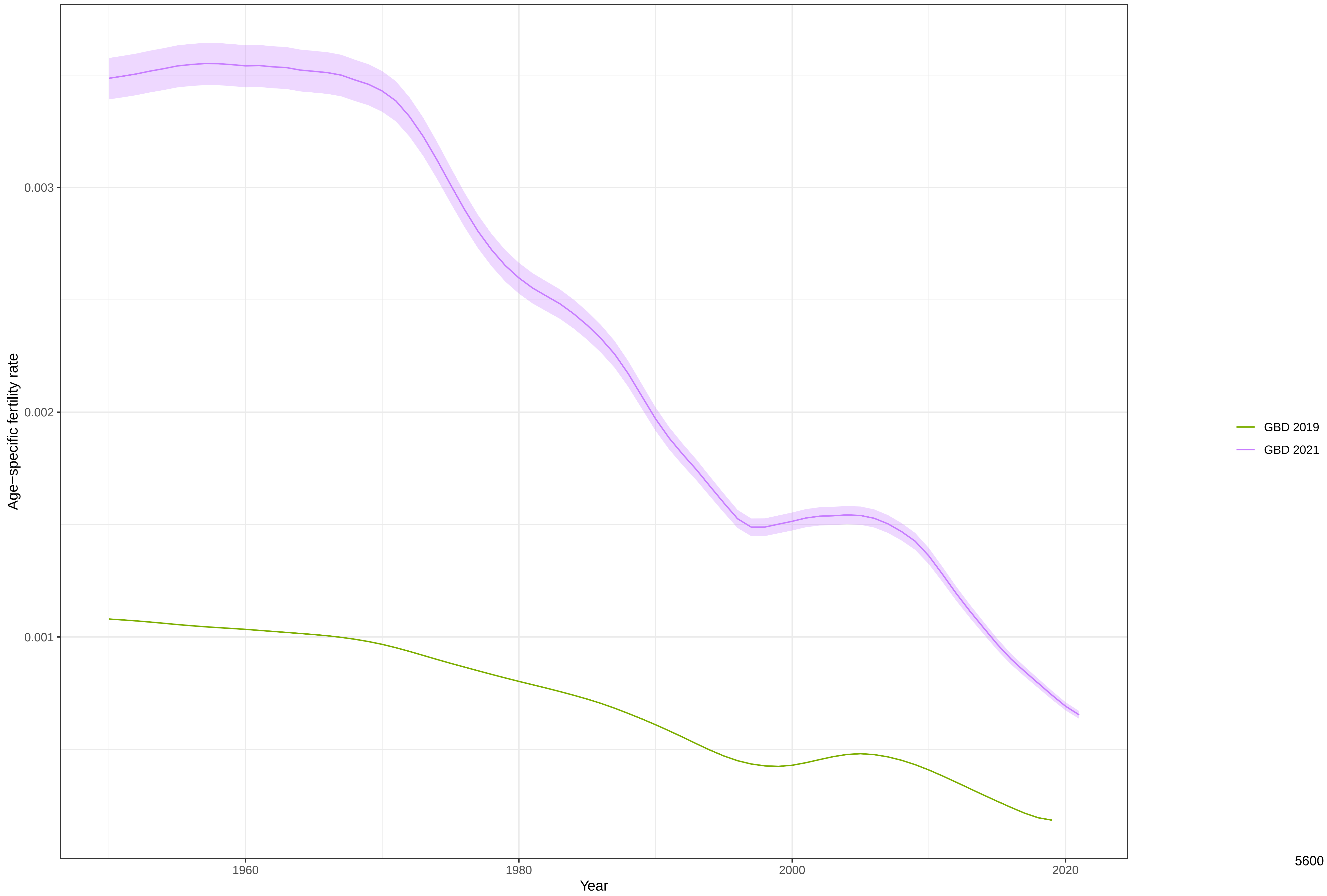

Total fertility rate fitted trend lines, 1950−2021: East Nusa Tenggara TFR
Data points enclosed with a diamond are reference sources

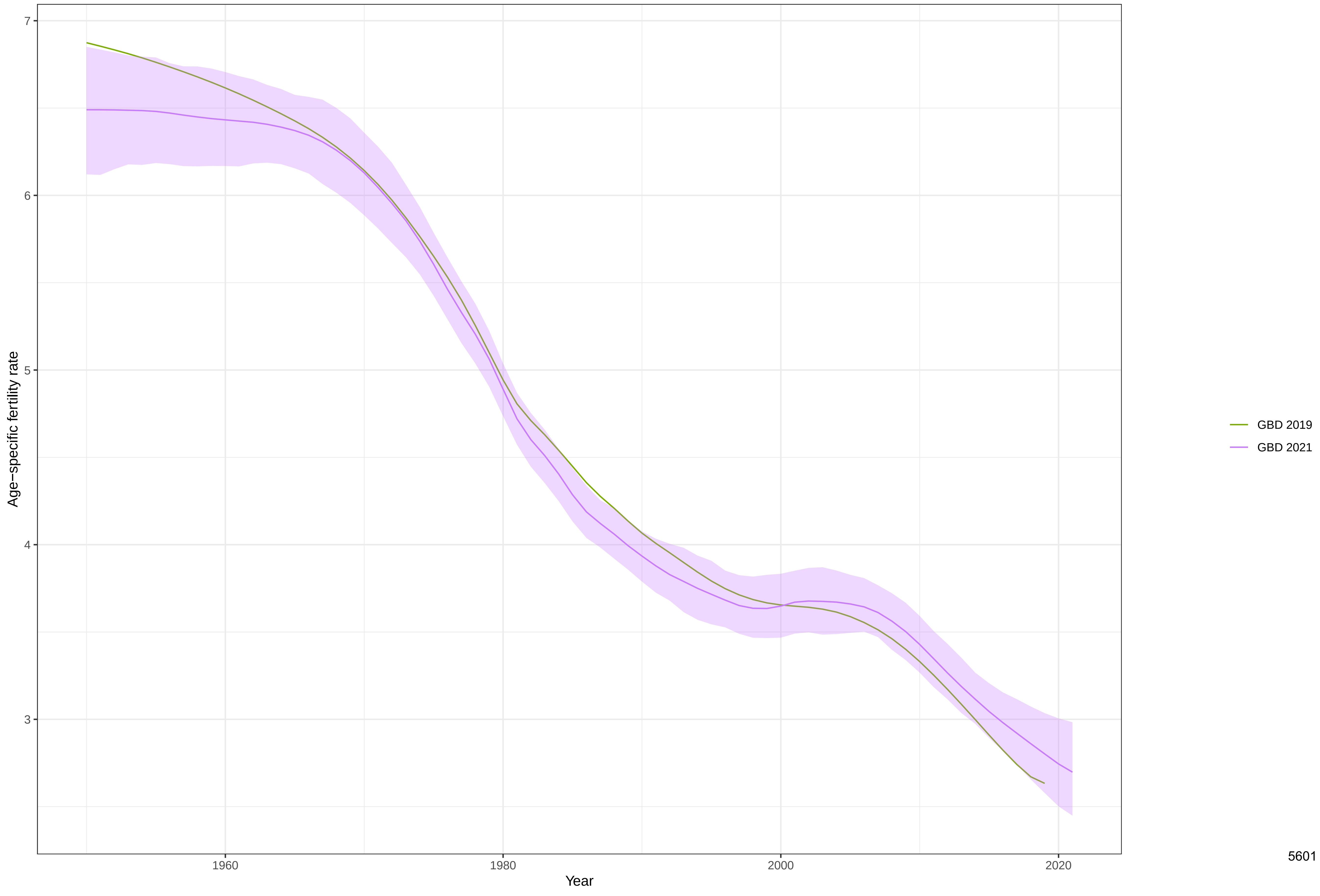

Age−specific fertility fitted trend lines, 1950−2021: Papua, Ages: 10−14
 Data points enclosed with a diamond are reference sources

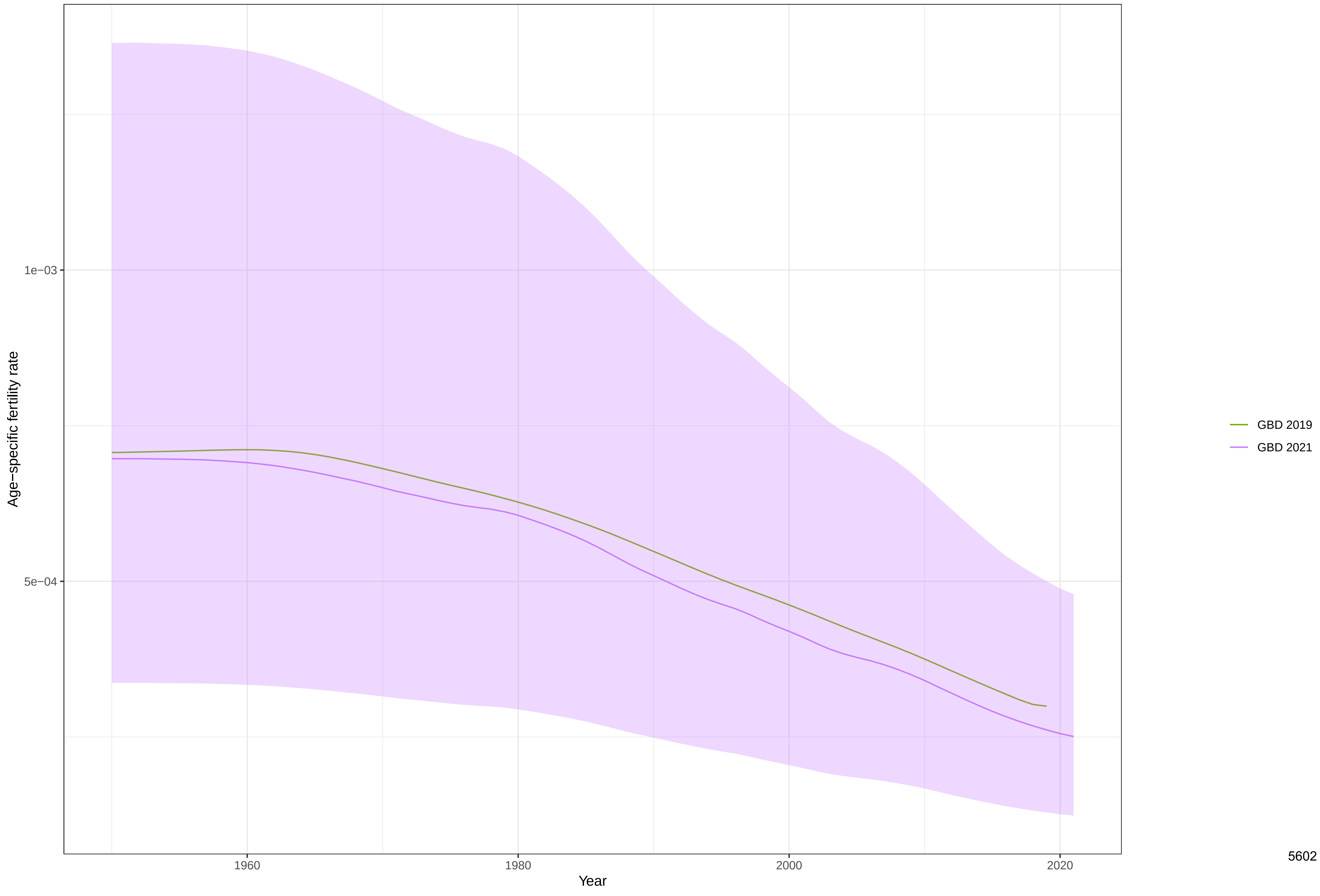

Age−specific fertility fitted trend lines, 1950−2021: Papua, Ages: 15−19
Data points enclosed with a diamond are reference sources

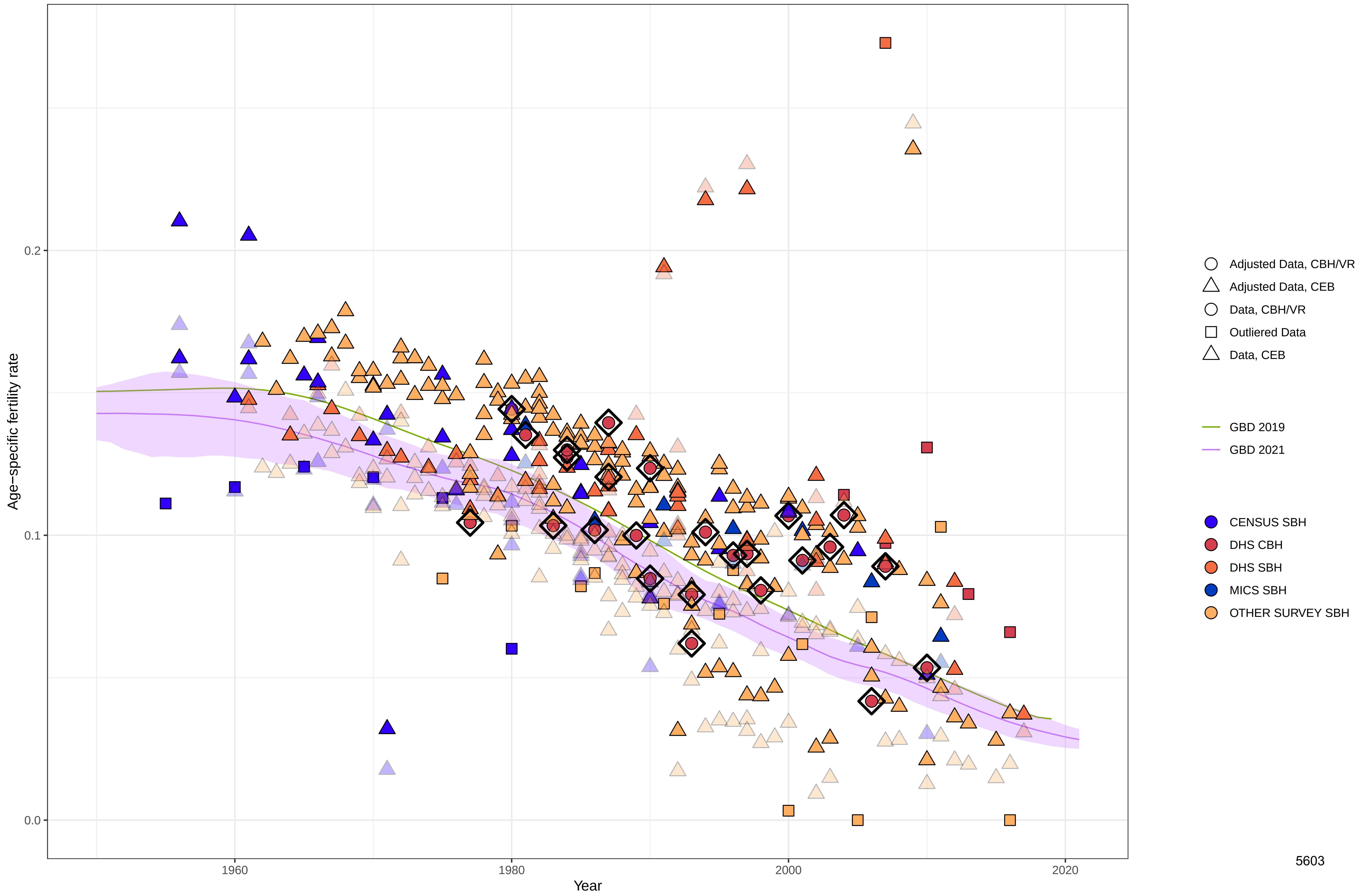

Age−specific fertility fitted trend lines, 1950−2021: Papua, Ages: 20−24
Data points enclosed with a diamond are reference sources

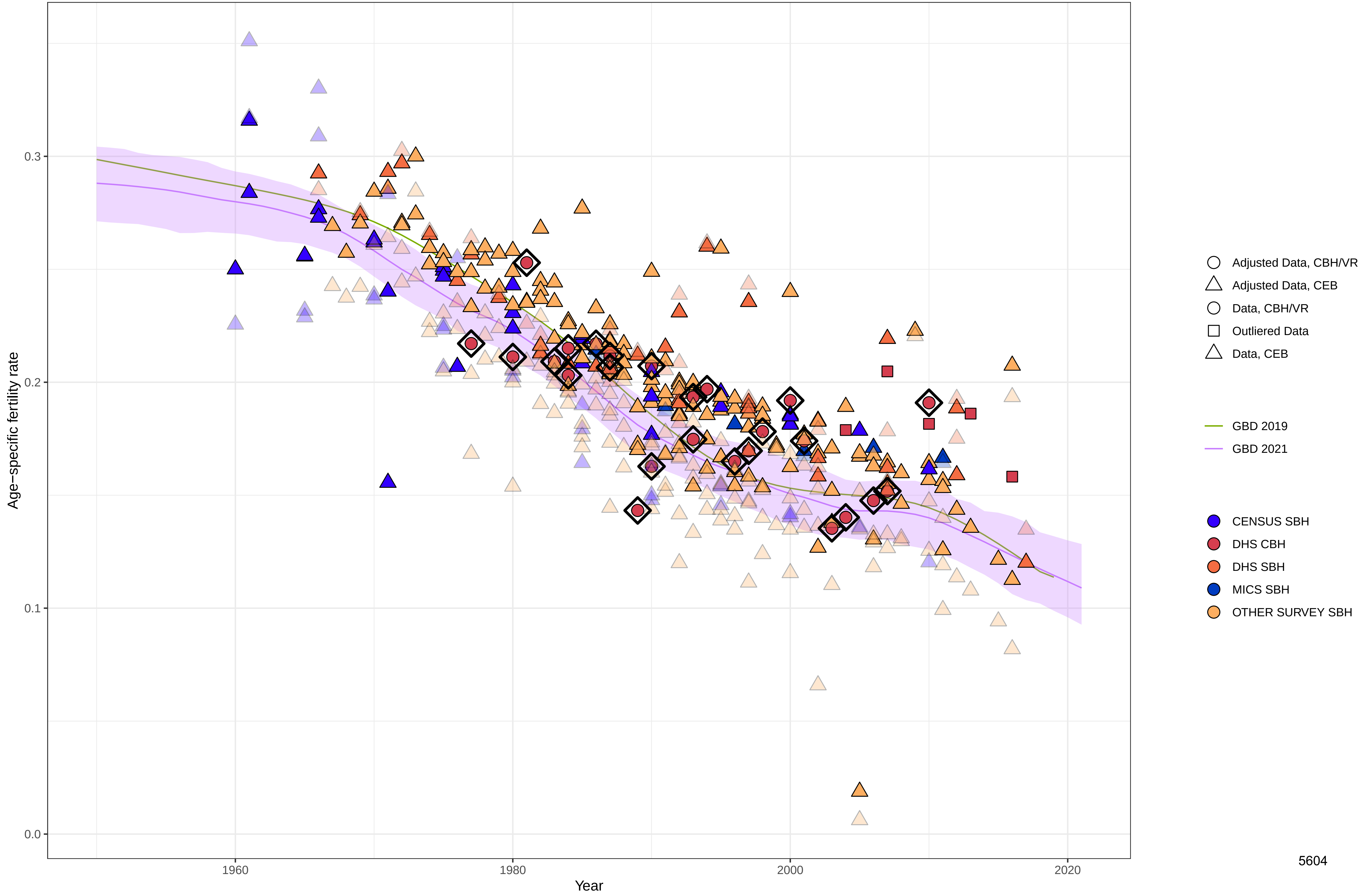

Age−specific fertility fitted trend lines, 1950−2021: Papua, Ages: 25−29
Data points enclosed with a diamond are reference sources

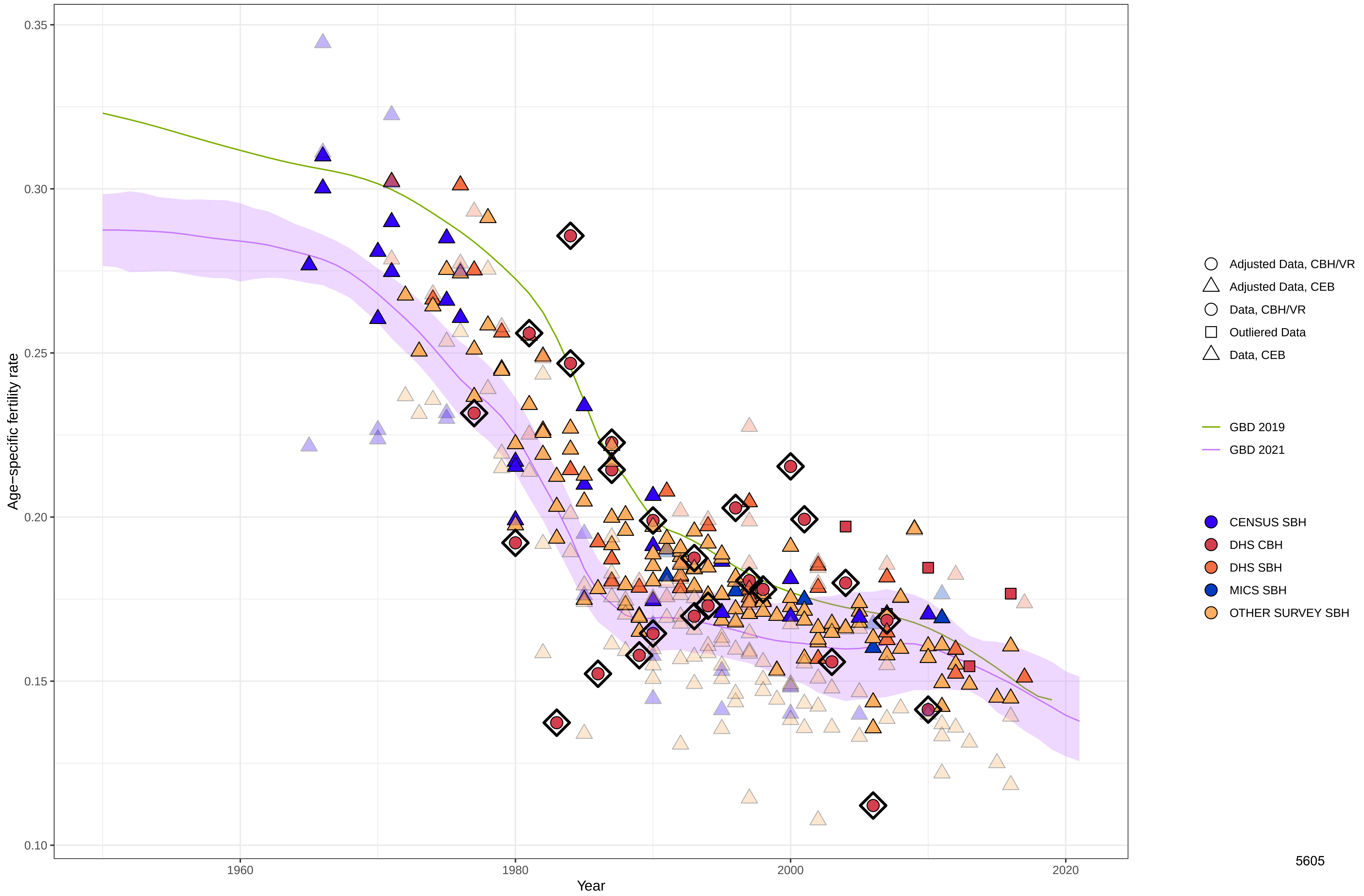

Age−specific fertility fitted trend lines, 1950−2021: Papua, Ages: 30−34
Data points enclosed with a diamond are reference sources

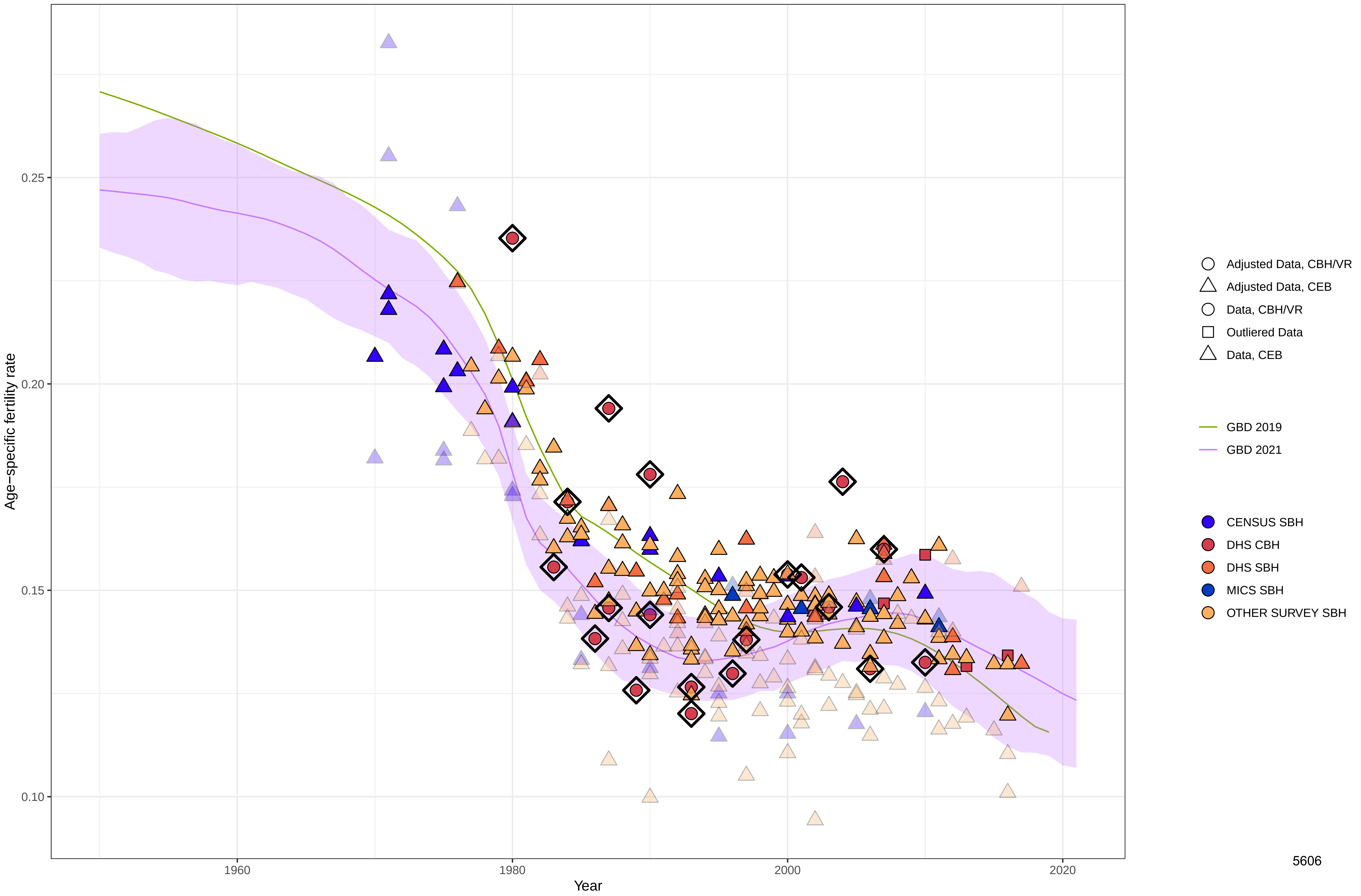

Age−specific fertility fitted trend lines, 1950−2021: Papua, Ages: 35−39
Data points enclosed with a diamond are reference sources

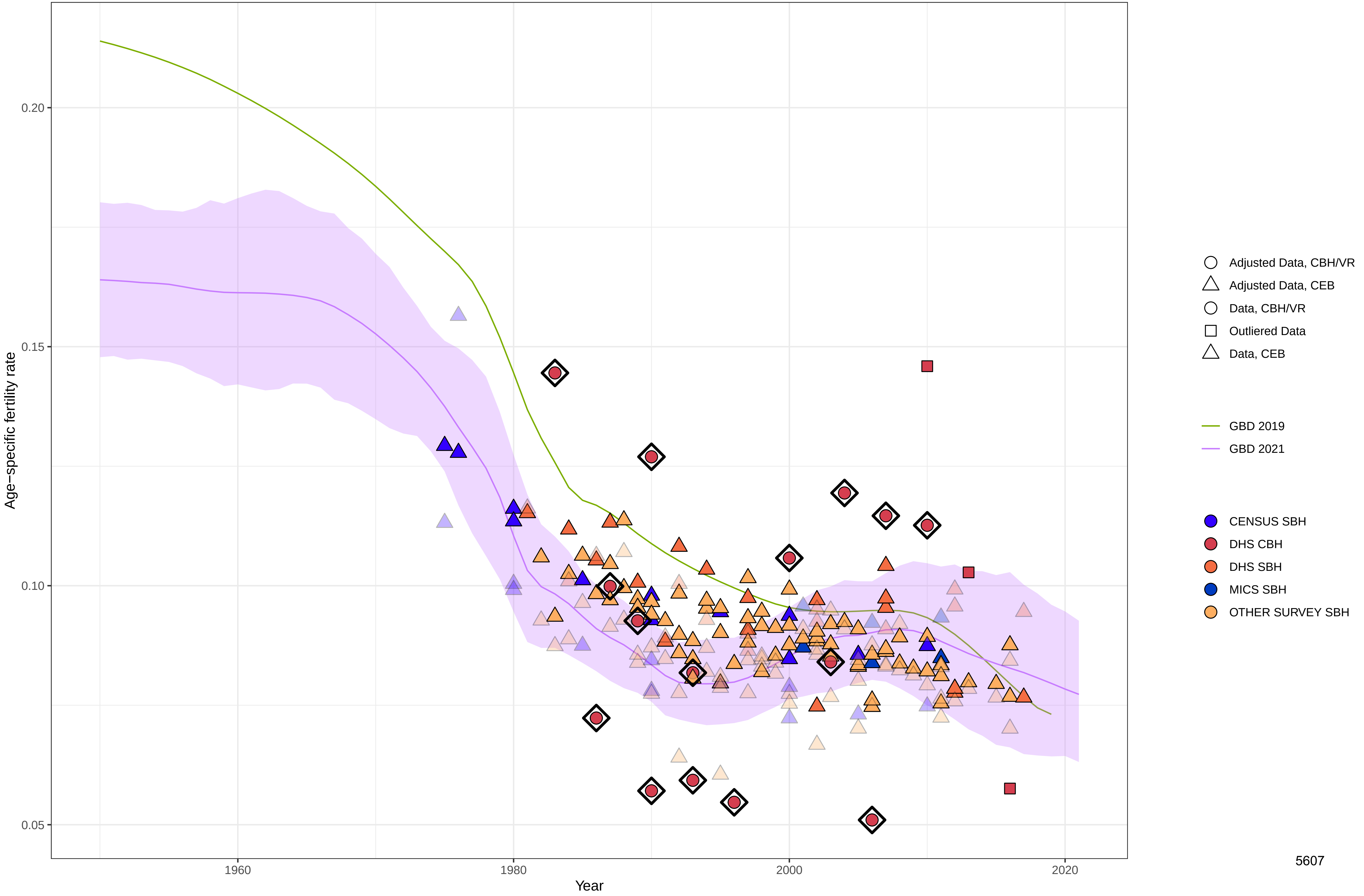

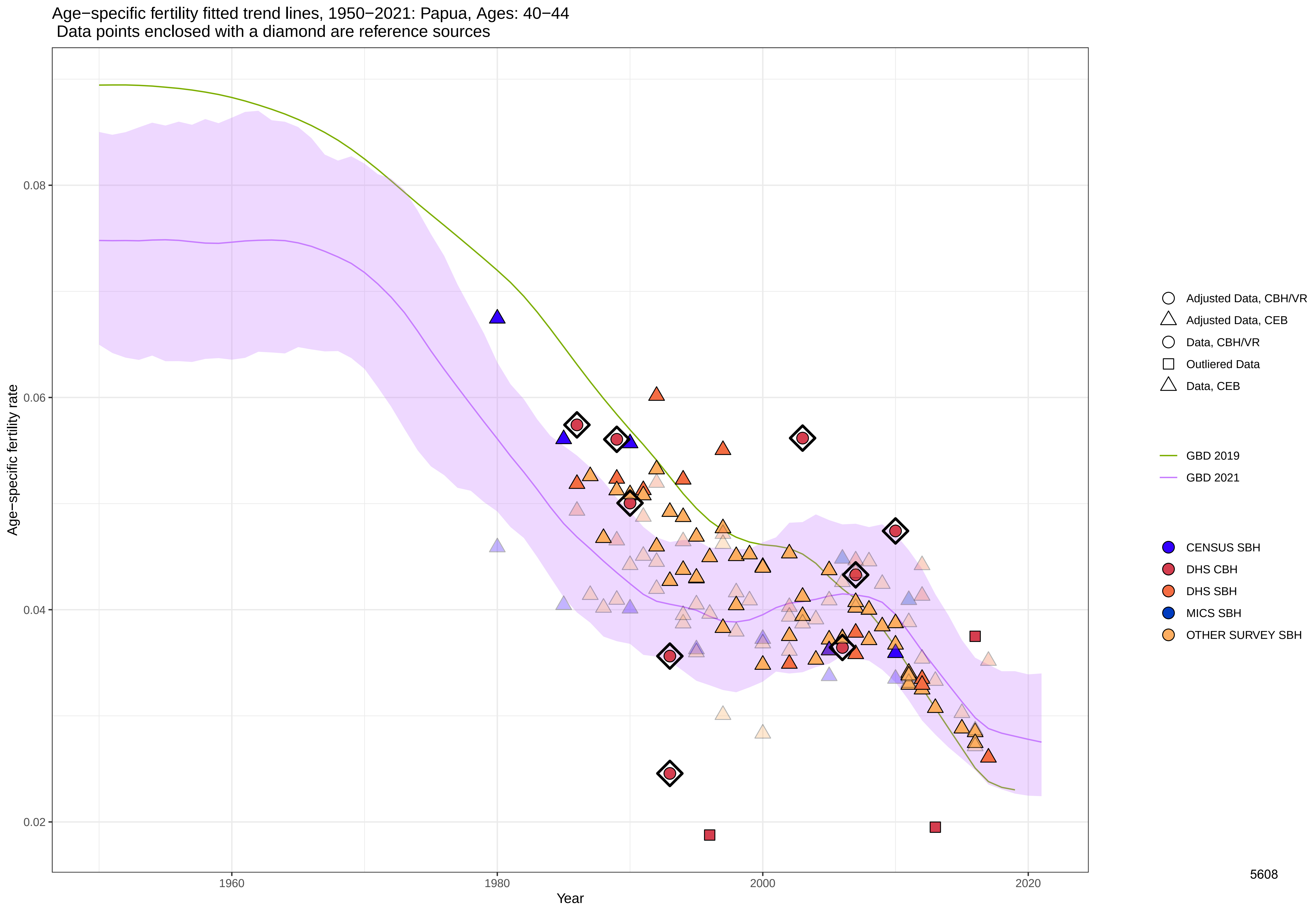

Age−specific fertility fitted trend lines, 1950−2021: Papua, Ages: 40−44
Data points enclosed with a diamond are reference sources
Age−specific fertility rate
0.08
0.06
0.04
0.02
1960
1980
2000
2020
Year
Adjusted Data, CBH/VR
Adjusted Data, CEB
Data, CBH/VR
Outliered Data
Data, CEB
GBD 2019
GBD 2021
CENSUS SBH
DHS CBH
DHS SBH
MICS SBH
OTHER SURVEY SBH

5608

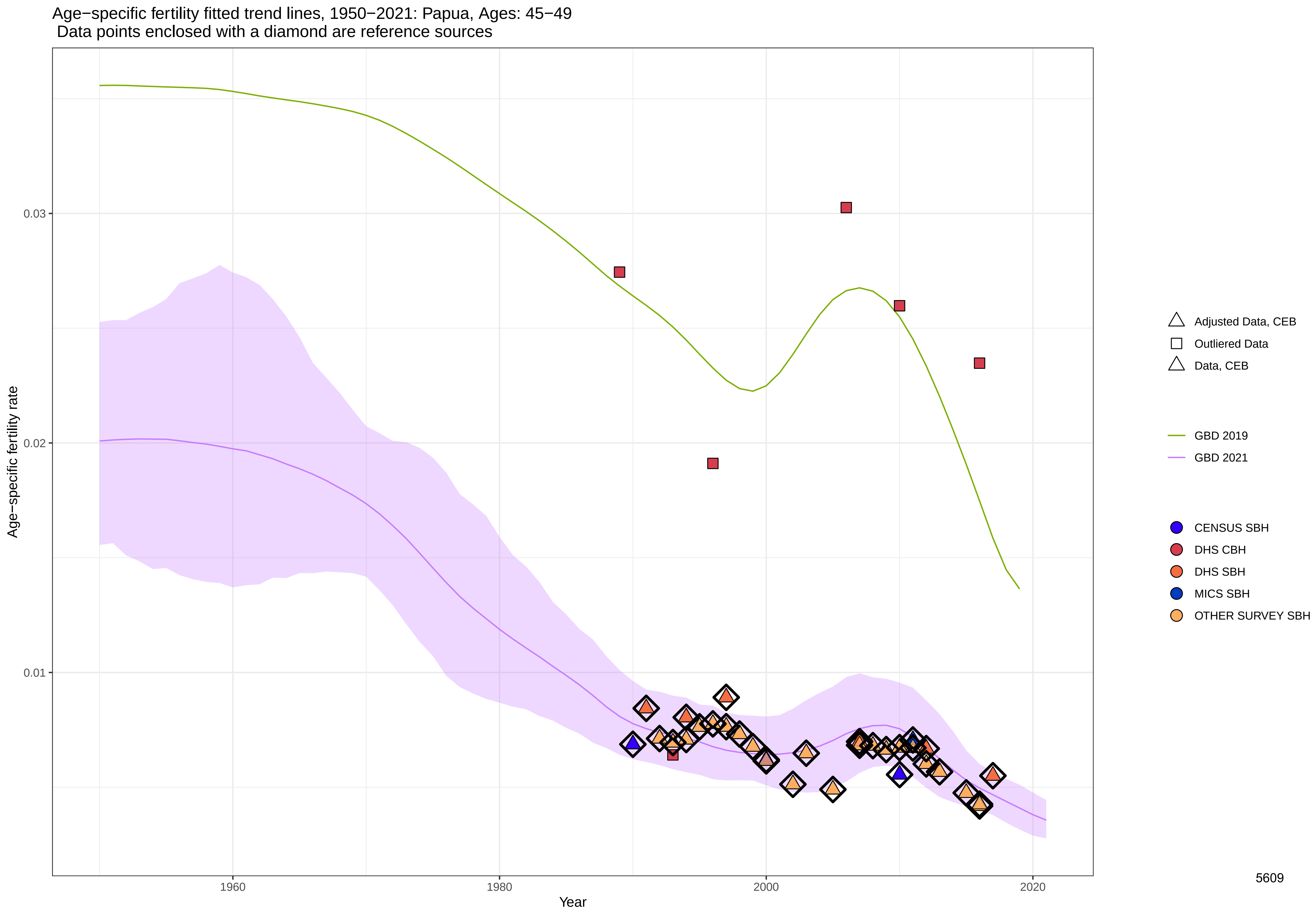
Age−specific fertility fitted trend lines, 1950−2021: Papua, Ages: 45−49
Data points enclosed with a diamond are reference sources
Age−specific fertility rate
0.03
0.02
0.01
1960
1980
2000
2020
Year
Adjusted Data, CEB
Outliered Data
Data, CEB
GBD 2019
GBD 2021
CENSUS SBH
DHS CBH
DHS SBH
MICS SBH
OTHER SURVEY SBH

Age−specific fertility fitted trend lines, 1950−2021: Papua, Ages: 50−54
Data points enclosed with a diamond are reference sources

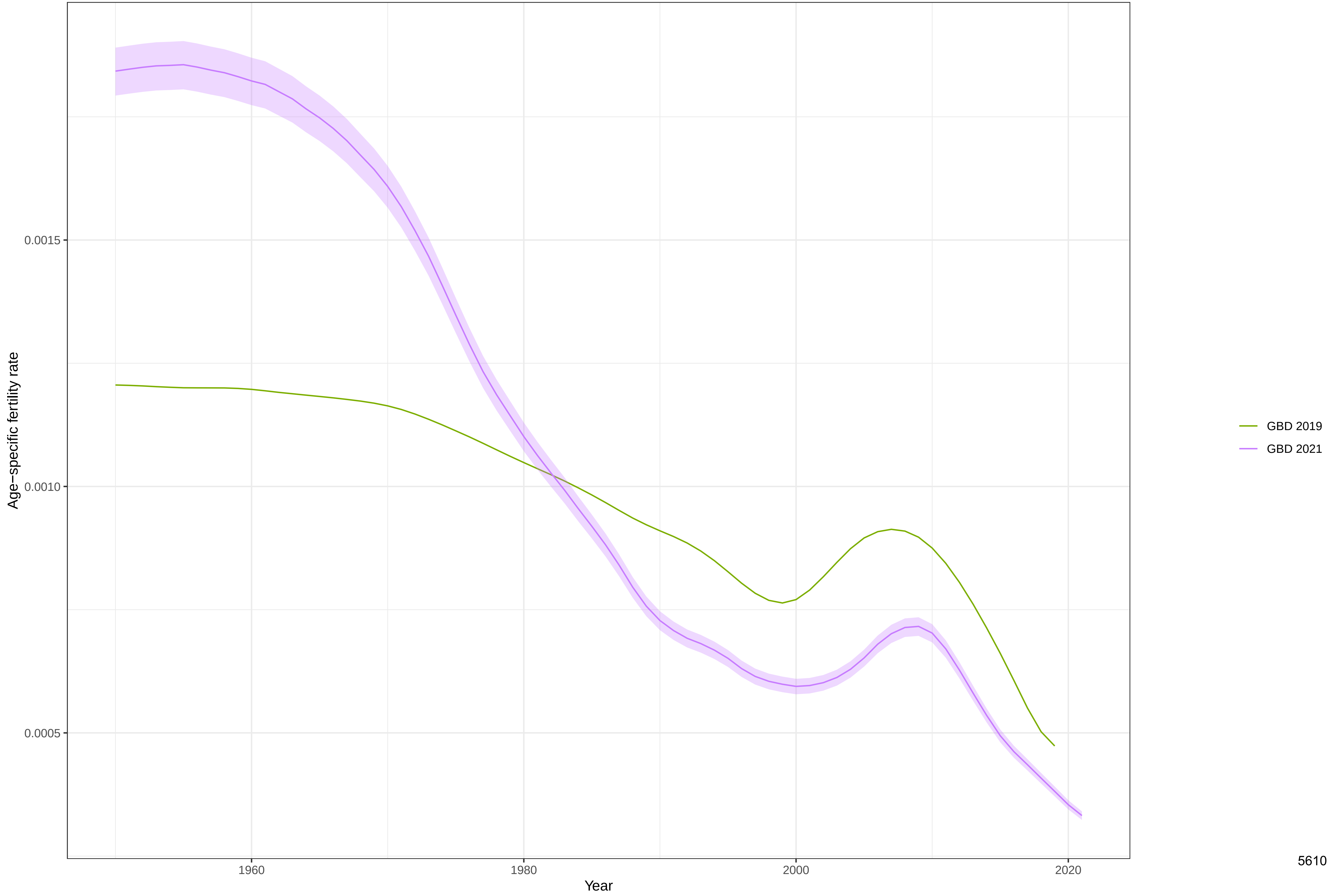

Total fertility rate fitted trend lines, 1950−2021: Papua TFR
Data points enclosed with a diamond are reference sources

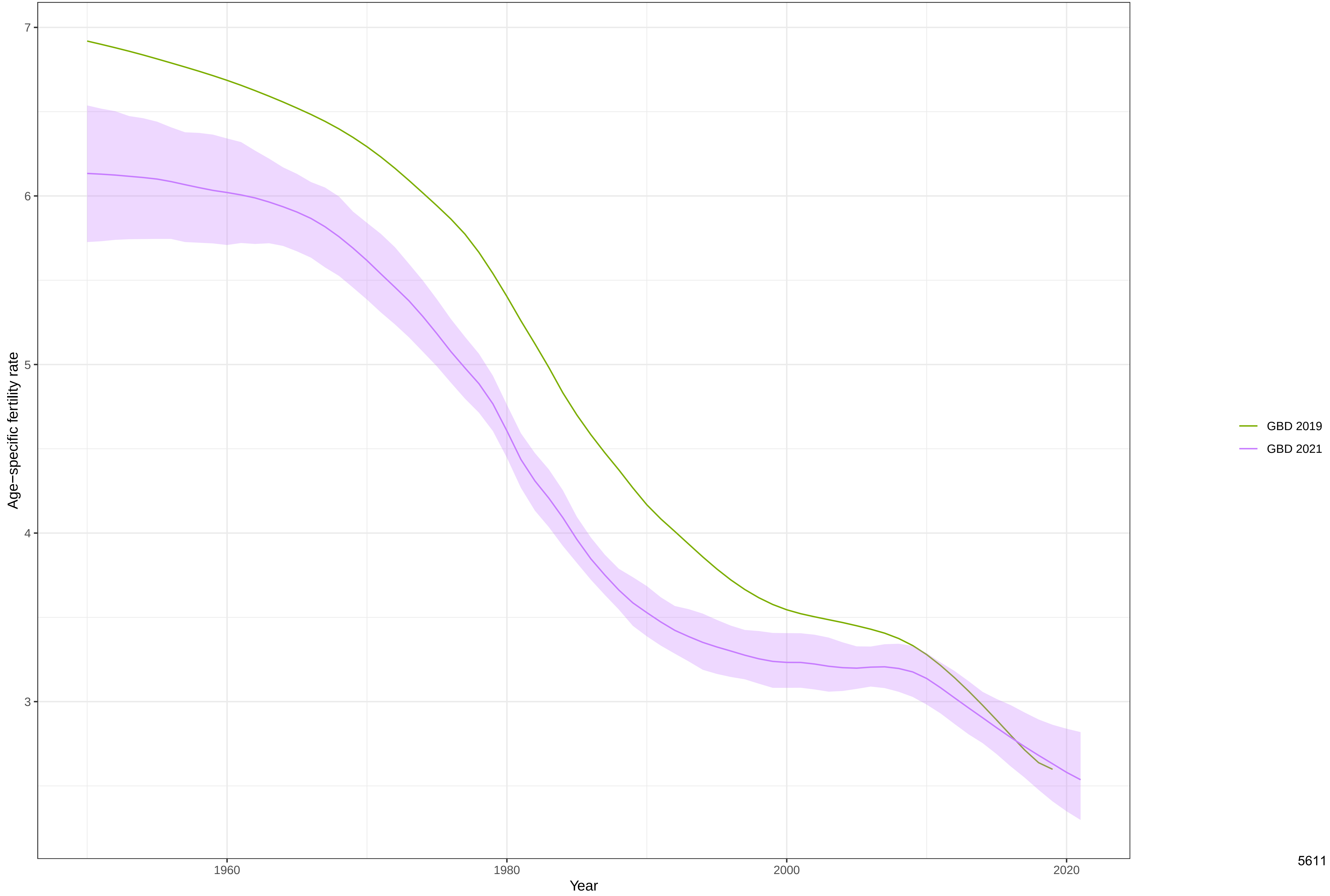

Age−specific fertility fitted trend lines, 1950−2021: West Papua, Ages: 10−14
Data points enclosed with a diamond are reference sources

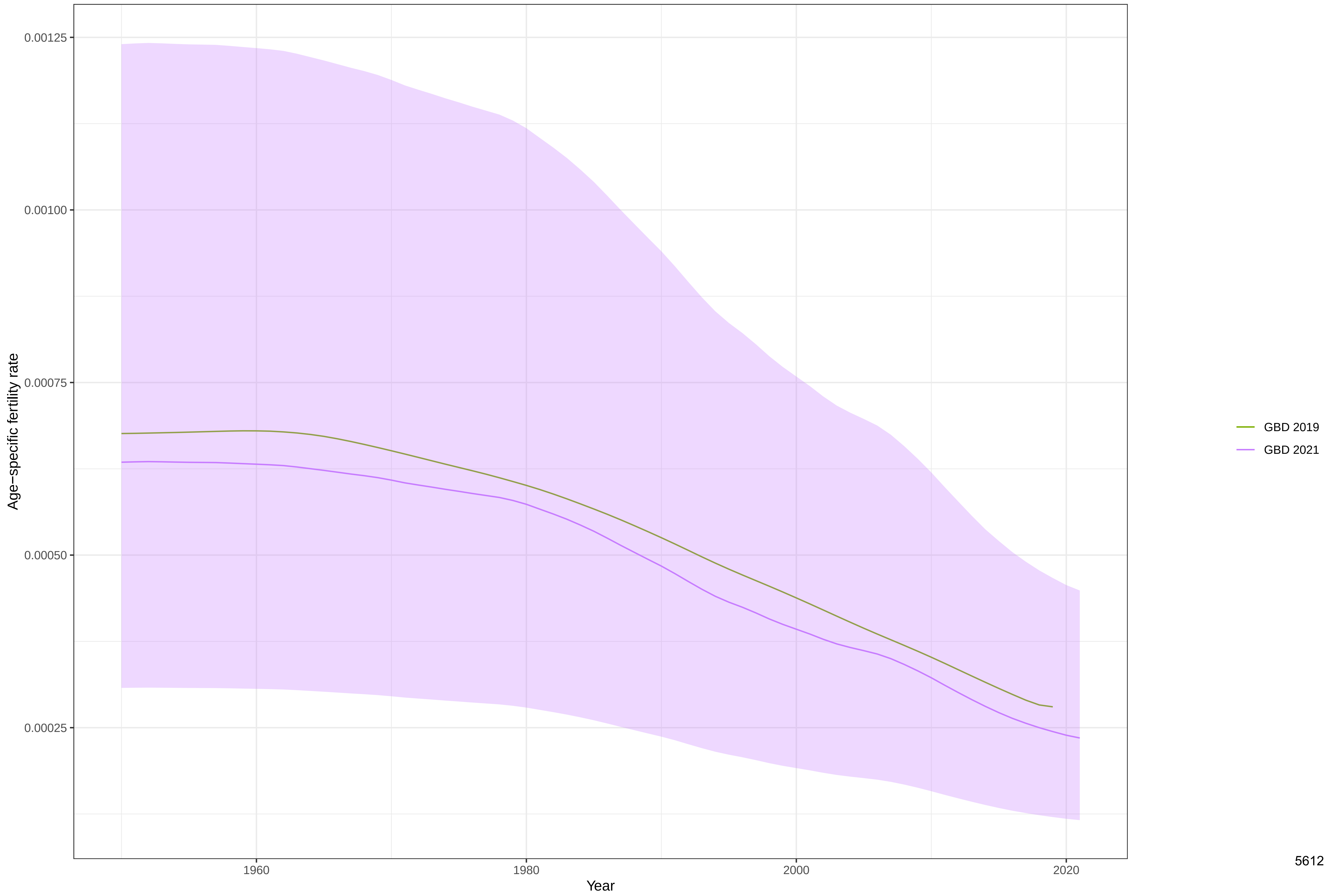

Age−specific fertility fitted trend lines, 1950−2021: West Papua, Ages: 15−19
Data points enclosed with a diamond are reference sources

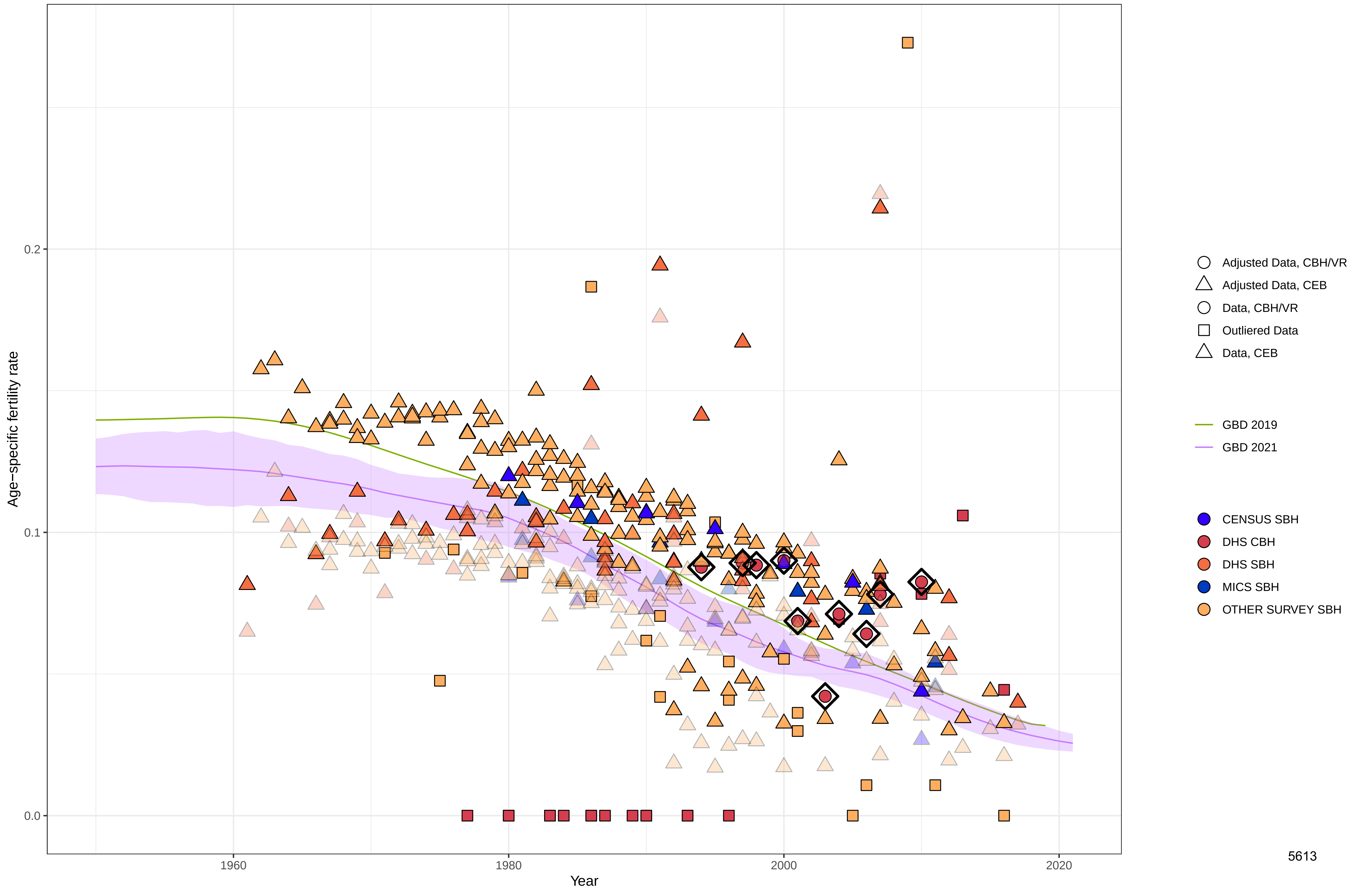

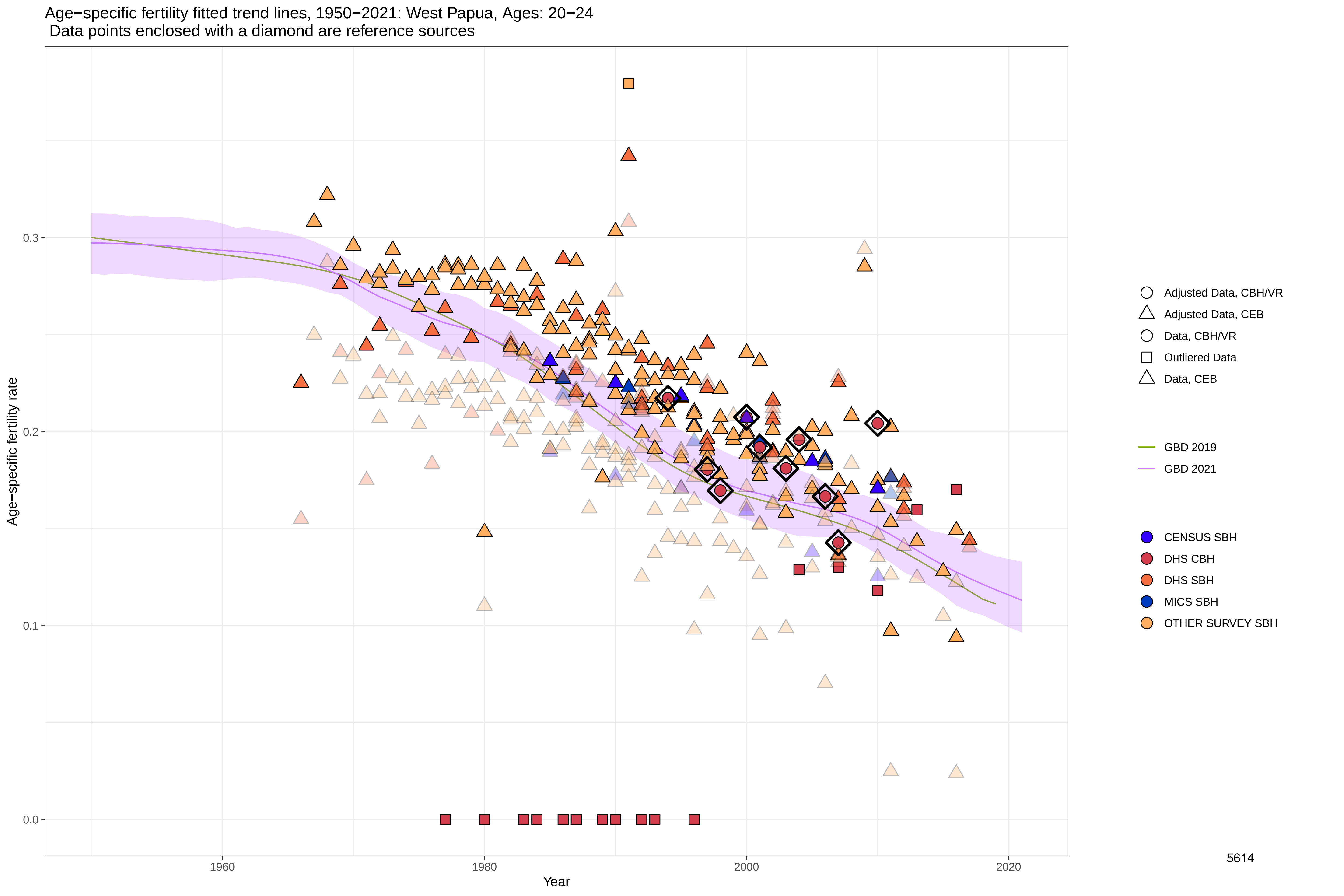

Age−specific fertility fitted trend lines, 1950−2021: West Papua, Ages: 20−24
Data points enclosed with a diamond are reference sources
Age−specific fertility rate
Year
0.0
0.1
0.2
0.3
1960
1980
2000
2020
Adjusted Data, CBH/VR
Adjusted Data, CEB
Data, CBH/VR
Outliered Data
Data, CEB
GBD 2019
GBD 2021
CENSUS SBH
DHS CBH
DHS SBH
MICS SBH
OTHER SURVEY SBH
5614

Age−specific fertility fitted trend lines, 1950−2021: West Papua, Ages: 25−29
Data points enclosed with a diamond are reference sources

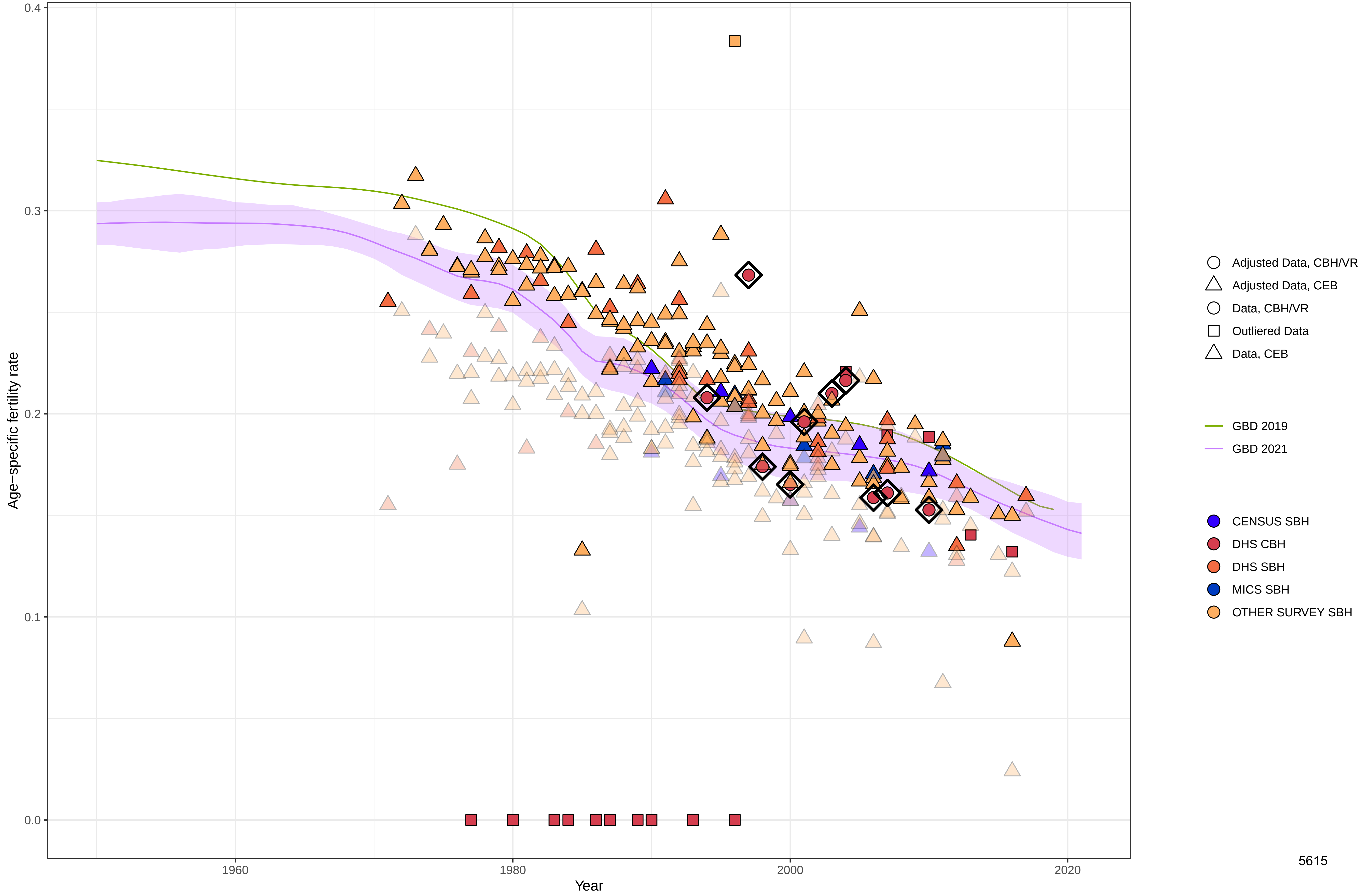

Age−specific fertility fitted trend lines, 1950−2021: West Papua, Ages: 30−34
Data points enclosed with a diamond are reference sources

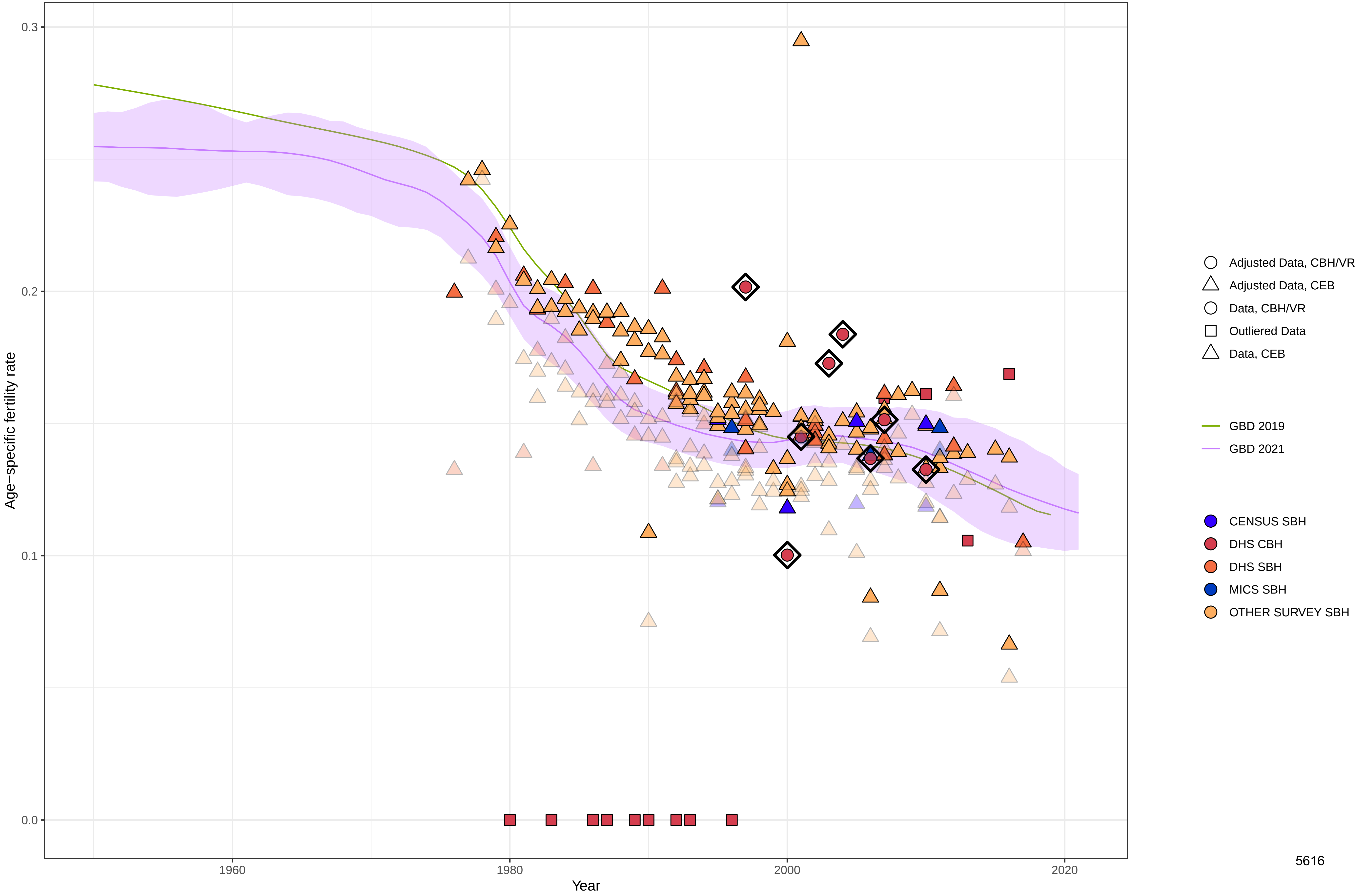

Age−specific fertility fitted trend lines, 1950−2021: West Papua, Ages: 35−39
Data points enclosed with a diamond are reference sources

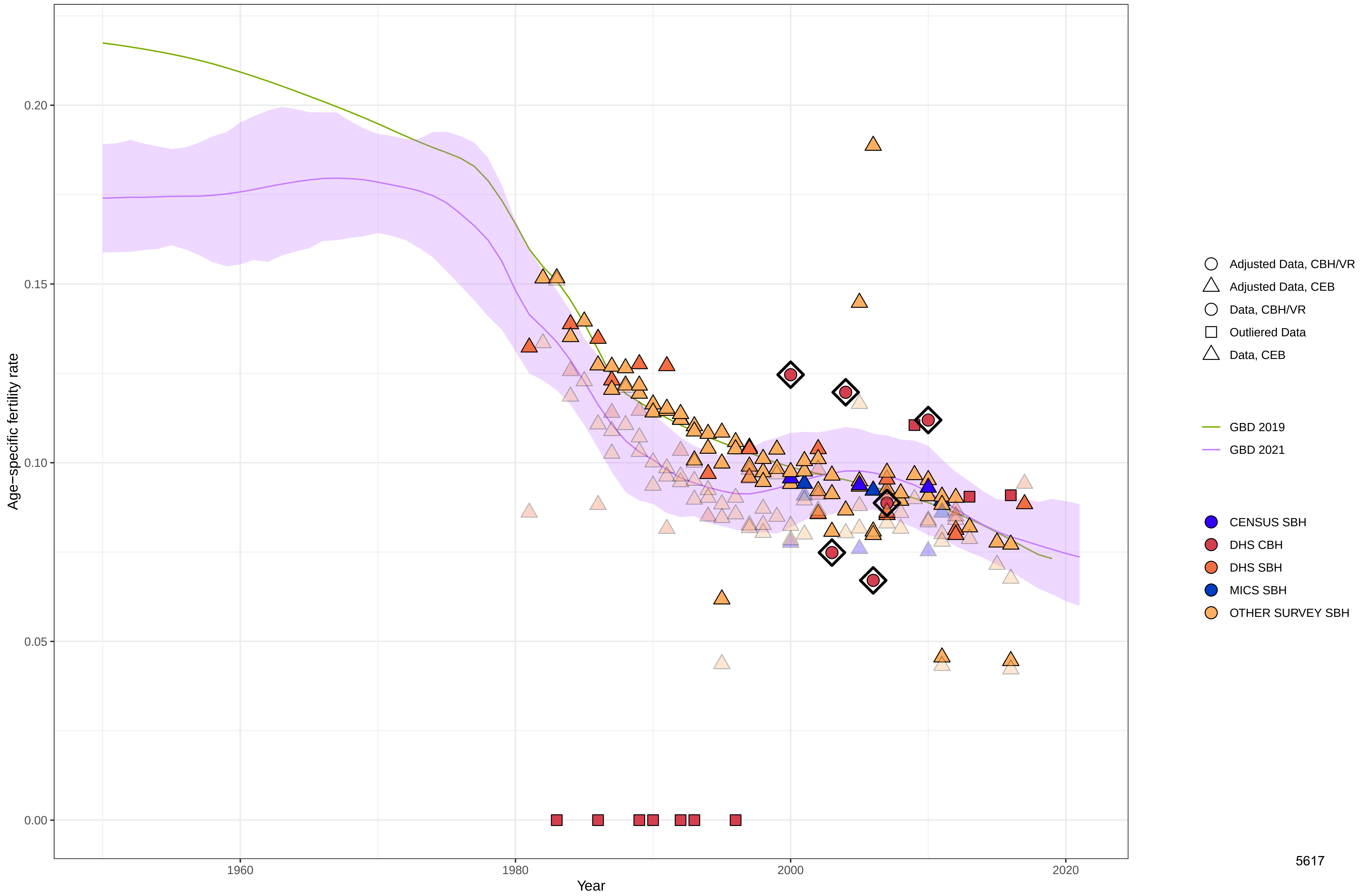

Age−specific fertility fitted trend lines, 1950−2021: West Papua, Ages: 40−44
Data points enclosed with a diamond are reference sources

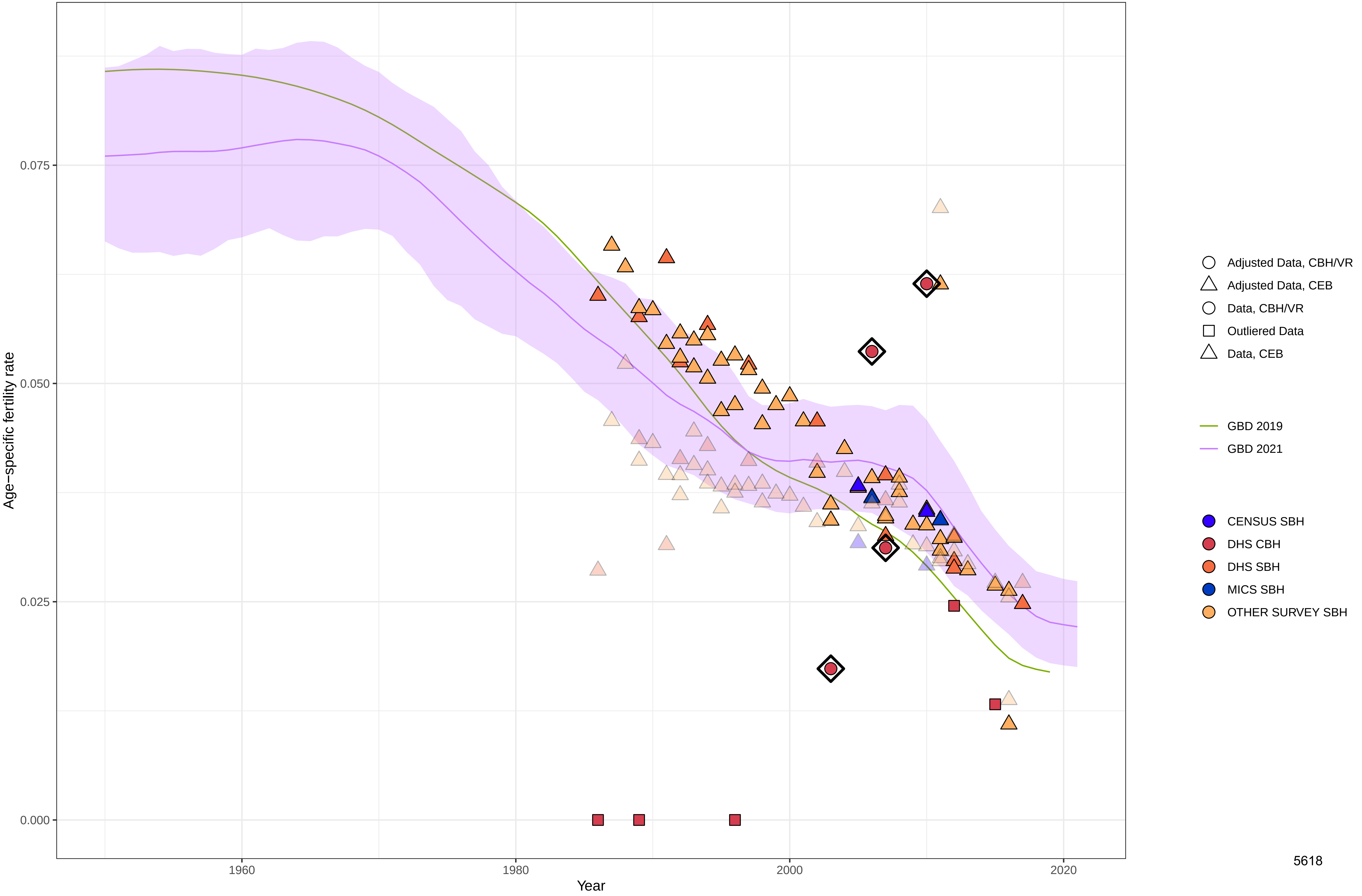

Age−specific fertility fitted trend lines, 1950−2021: West Papua, Ages: 45−49
Data points enclosed with a diamond are reference sources

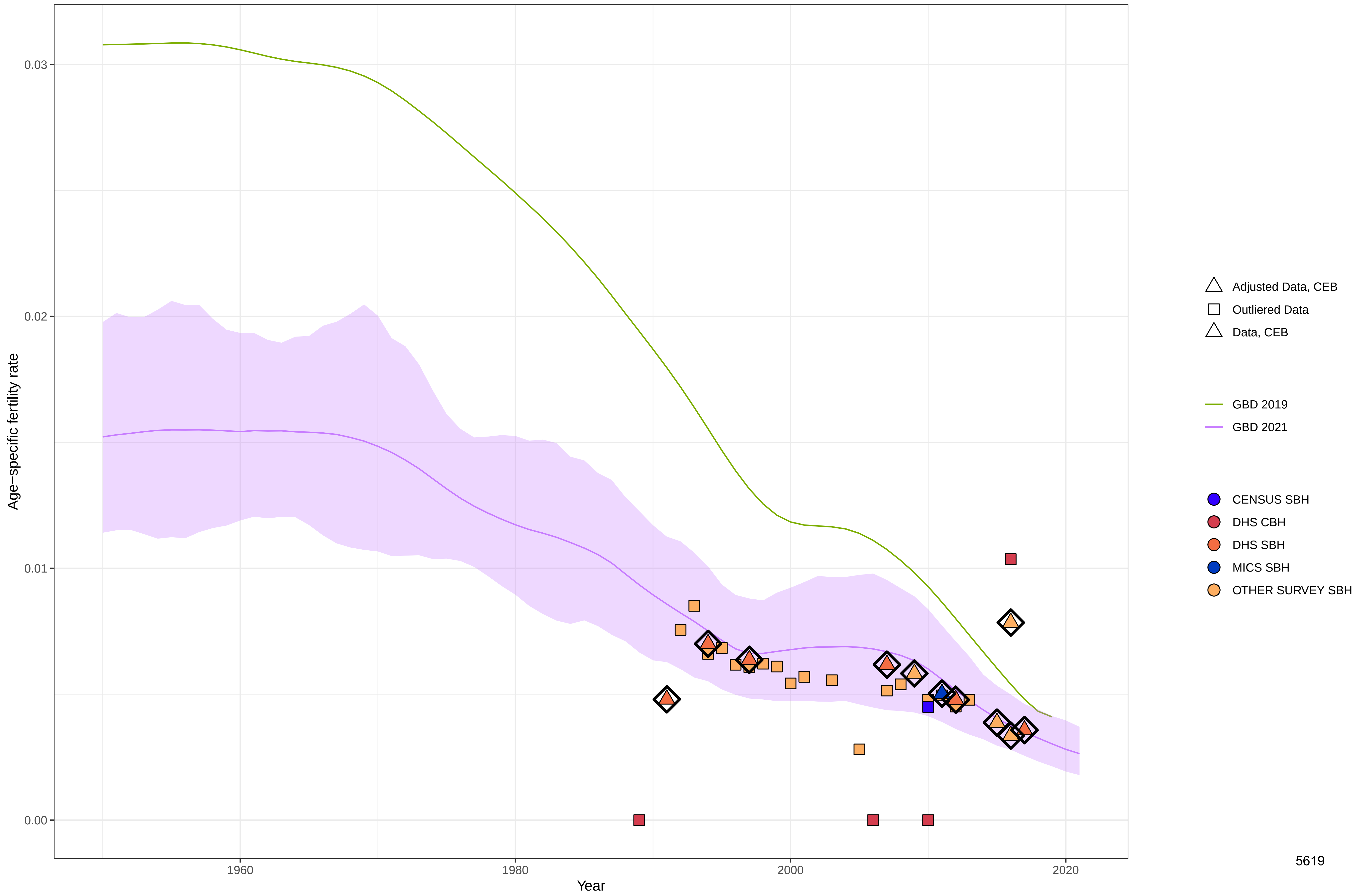

Age−specific fertility fitted trend lines, 1950−2021: West Papua, Ages: 50−54
Data points enclosed with a diamond are reference sources

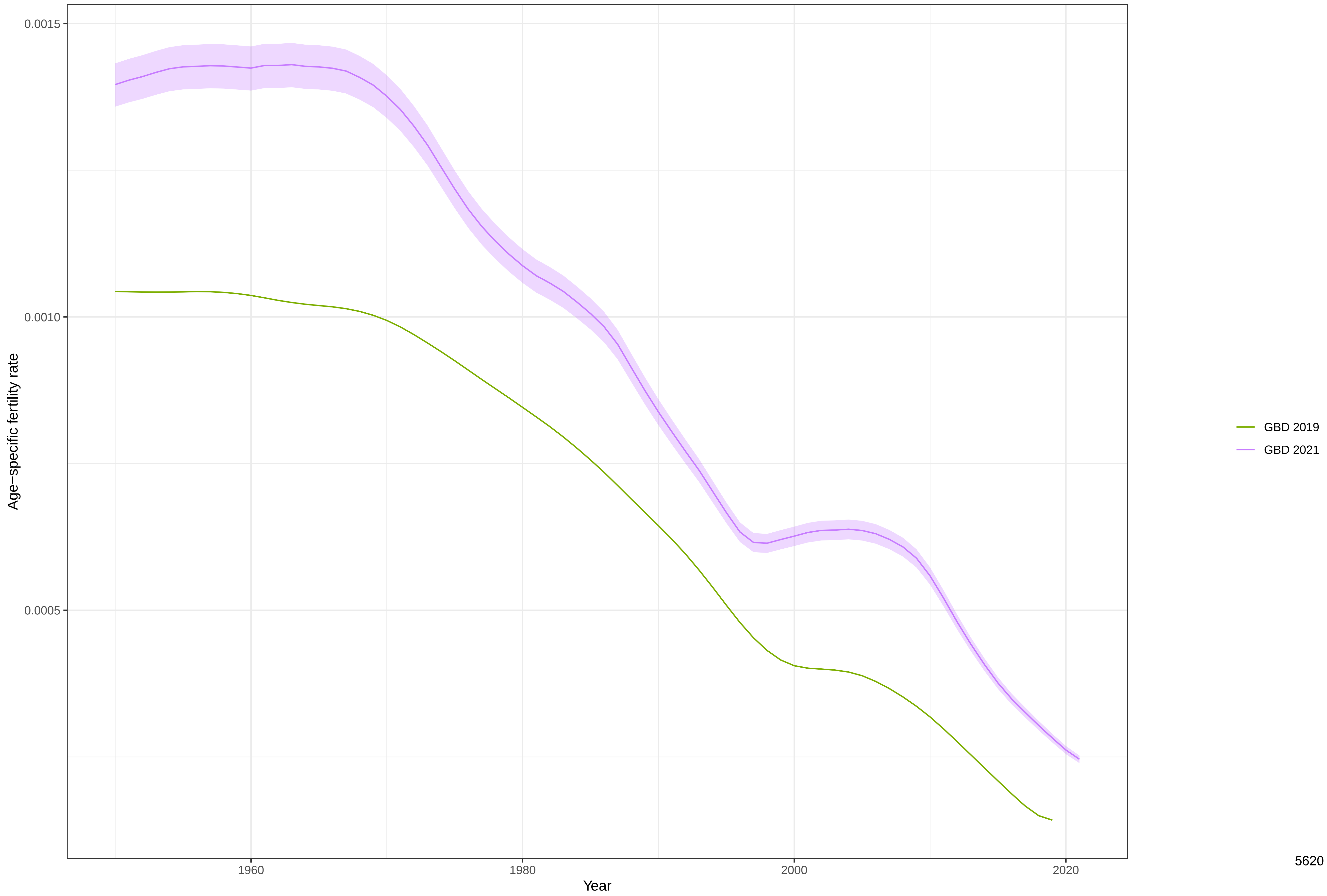

Total fertility rate fitted trend lines, 1950−2021: West Papua TFR
Data points enclosed with a diamond are reference sources

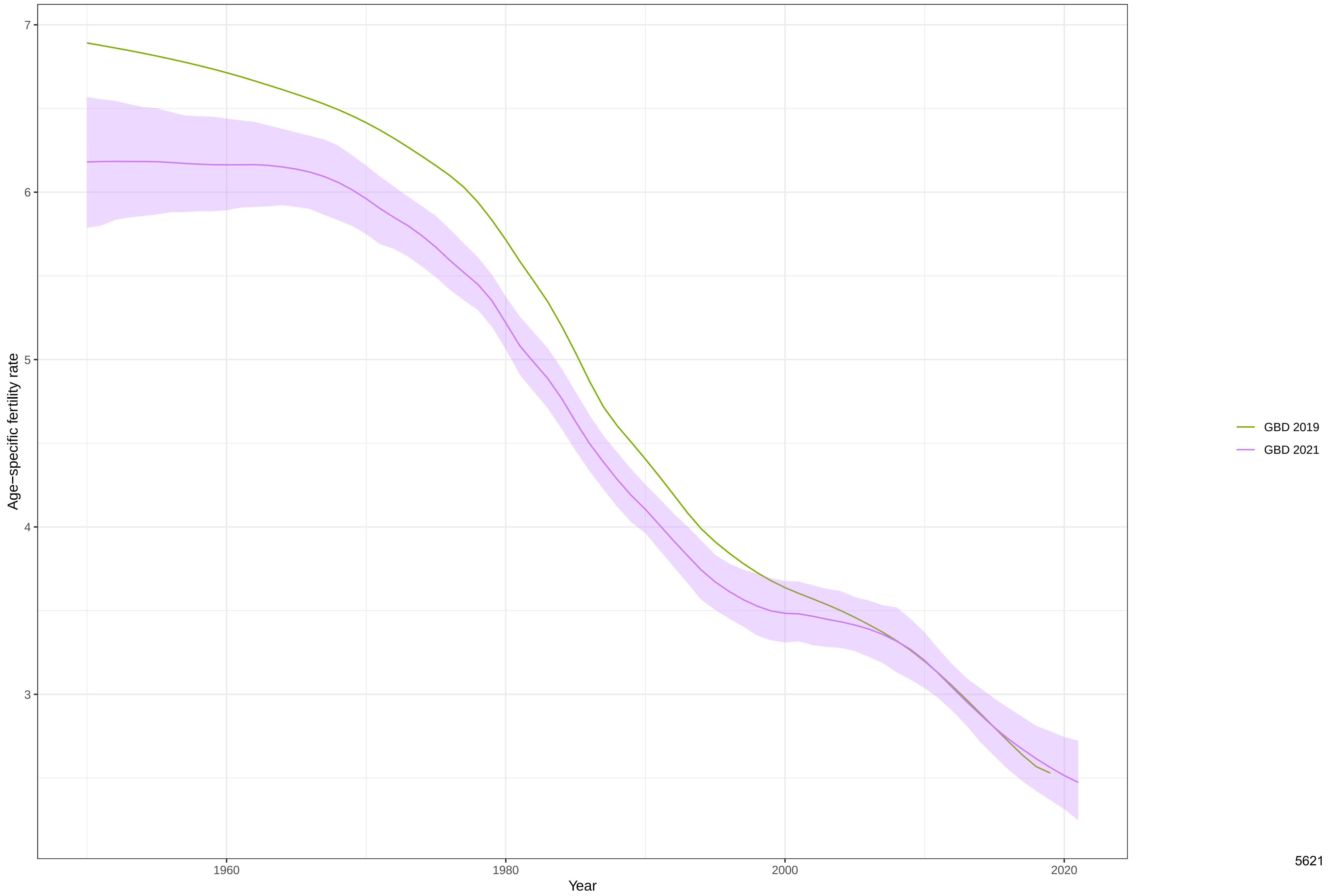

Age−specific fertility fitted trend lines, 1950−2021: Riau, Ages: 10−14
Data points enclosed with a diamond are reference sources

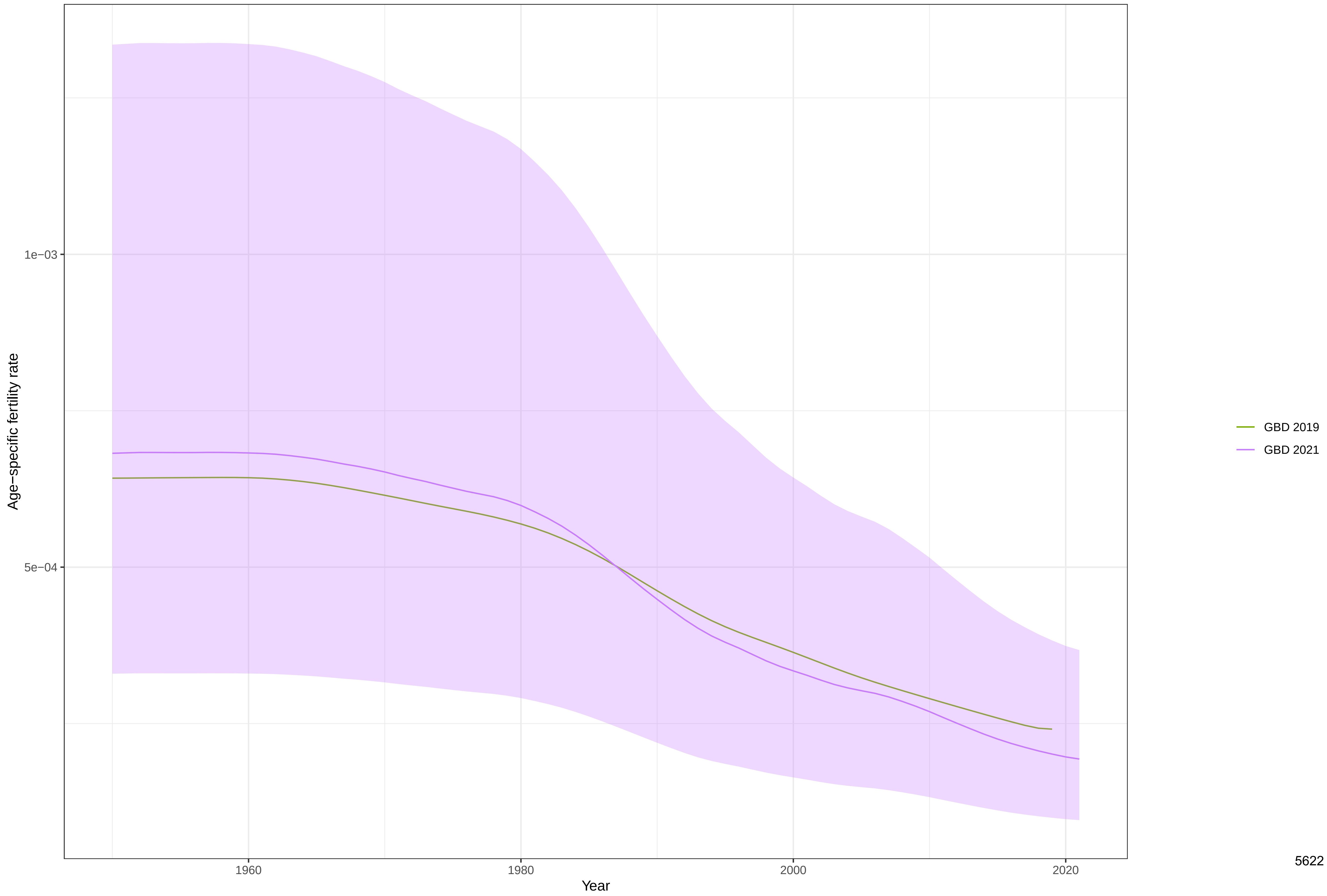

Age−specific fertility fitted trend lines, 1950−2021: Riau, Ages: 15−19
Data points enclosed with a diamond are reference sources

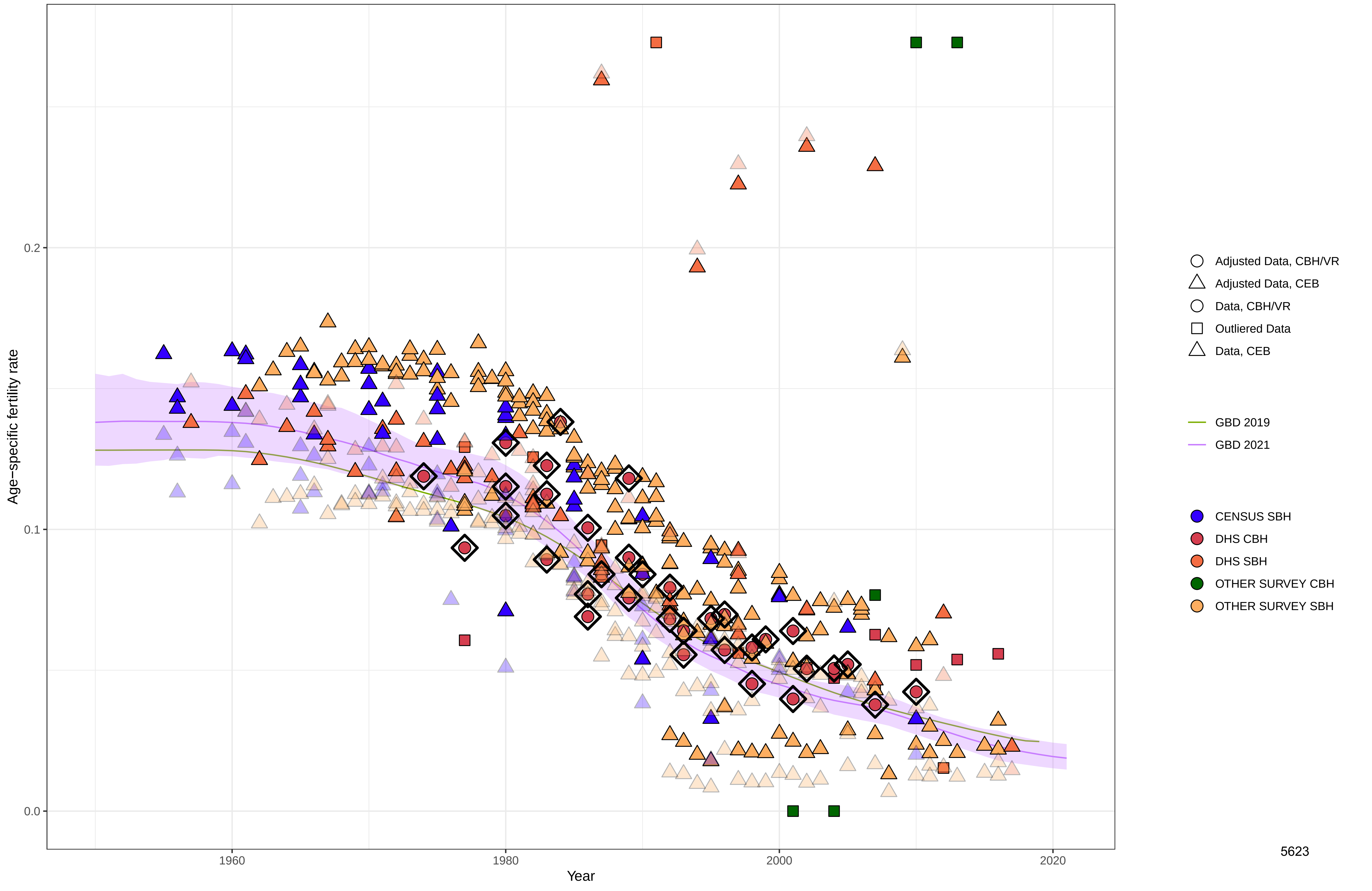

Age−specific fertility fitted trend lines, 1950−2021: Riau, Ages: 20−24
Data points enclosed with a diamond are reference sources

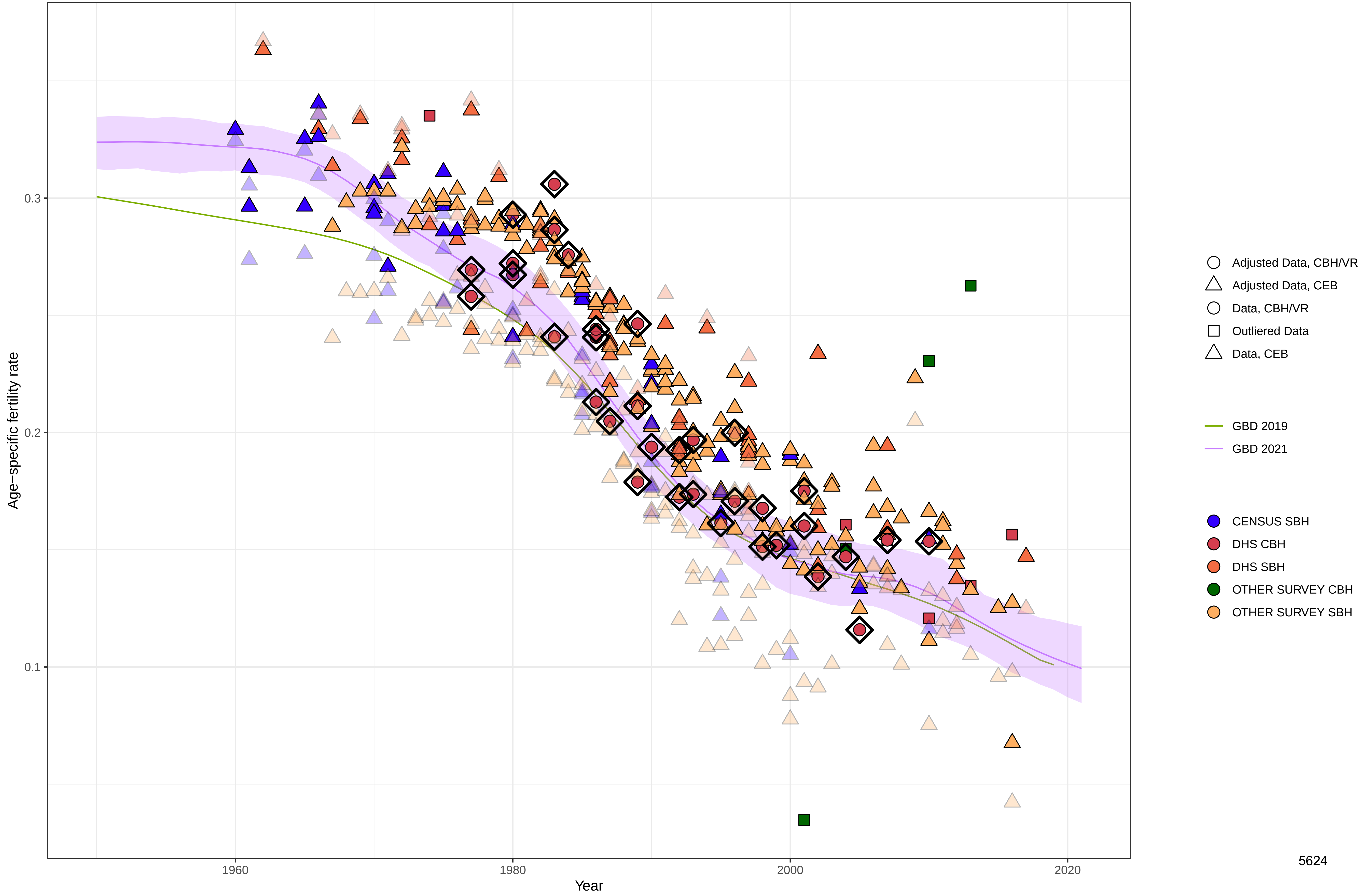

Age−specific fertility fitted trend lines, 1950−2021: Riau, Ages: 25−29
Data points enclosed with a diamond are reference sources

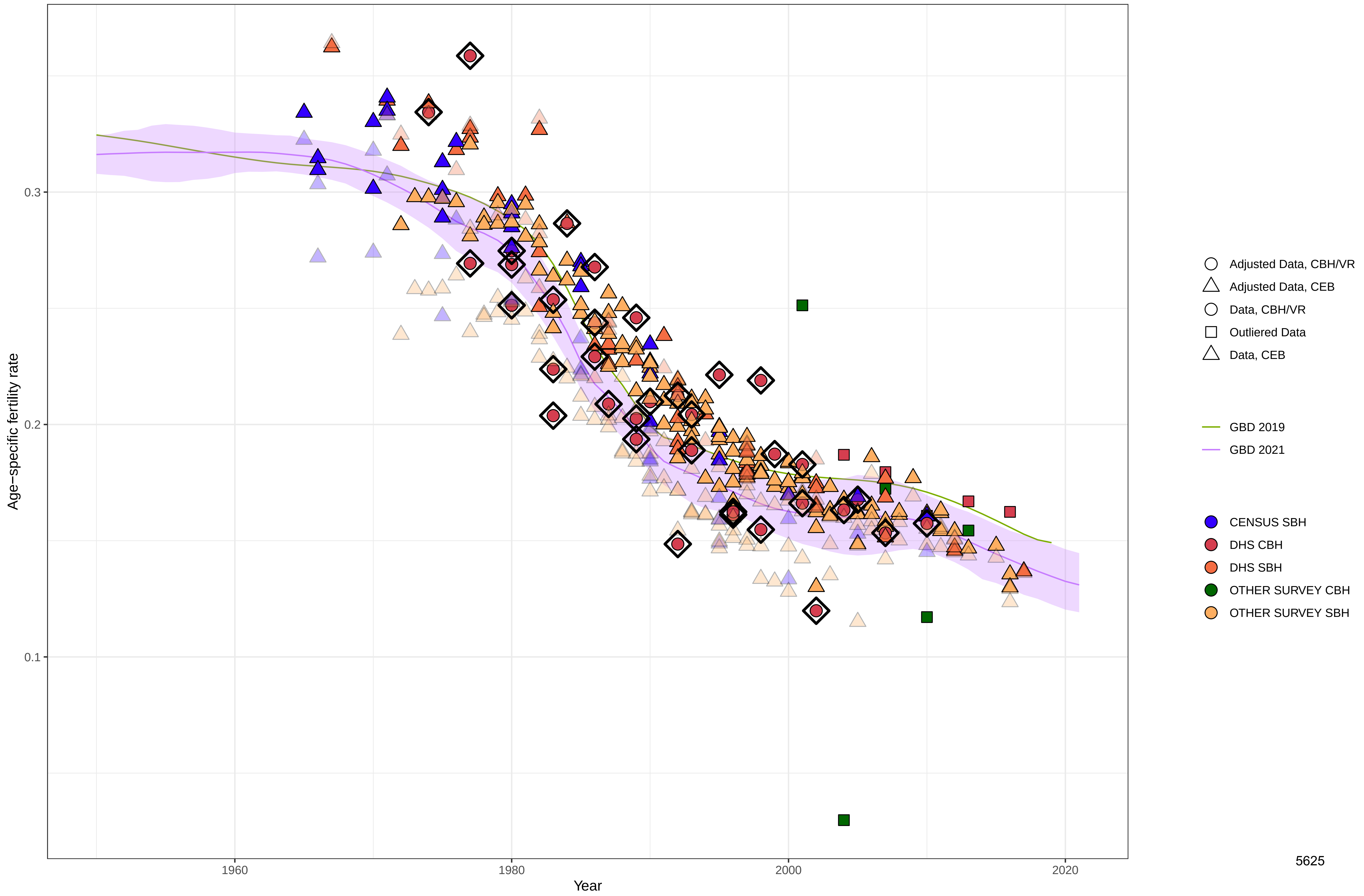

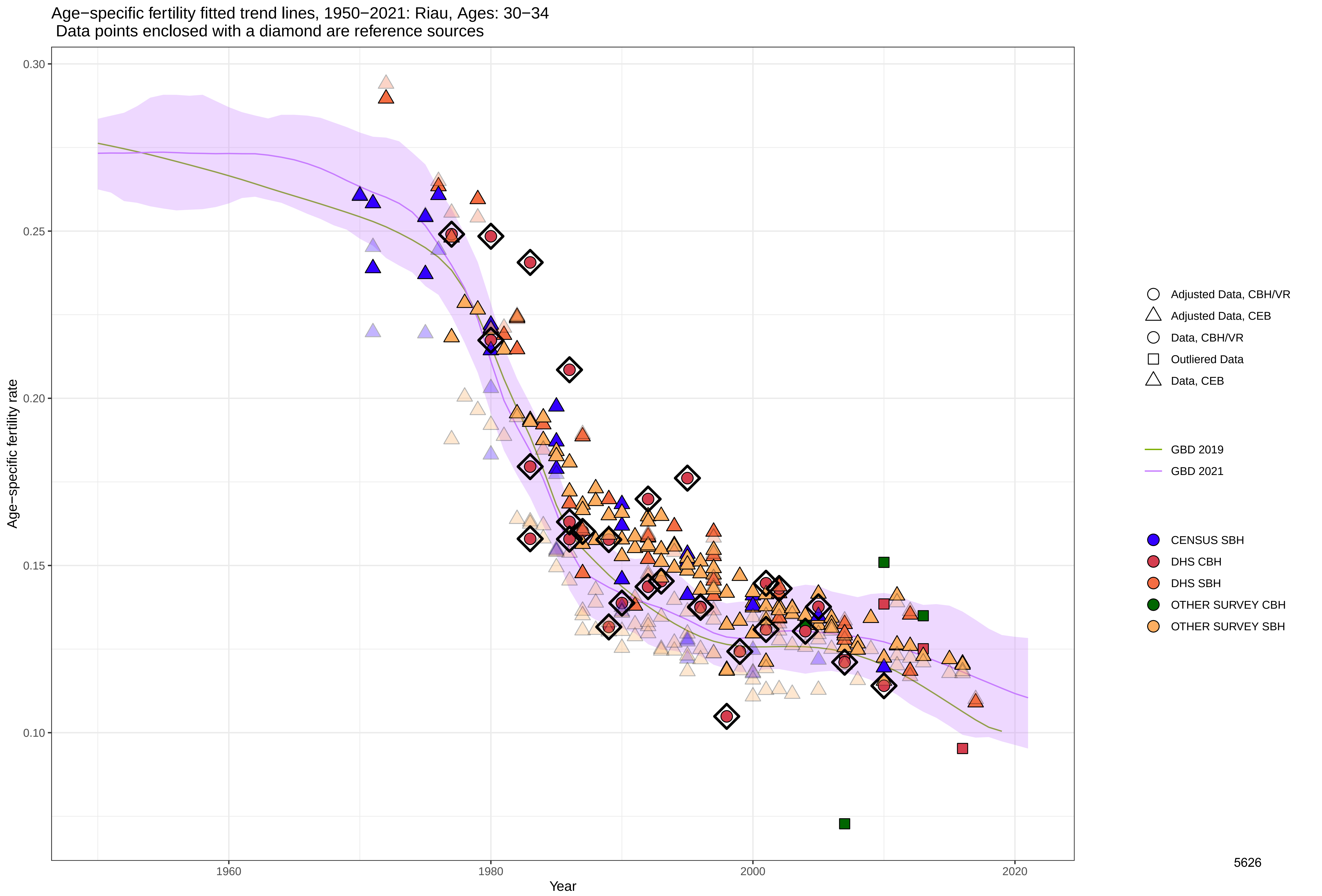

Age−specific fertility fitted trend lines, 1950−2021: Riau, Ages: 30−34
Data points enclosed with a diamond are reference sources
Age−specific fertility rate
Year
0.30
0.25
0.20
0.15
0.10
1960
1980
2000
2020
Adjusted Data, CBH/VR
Adjusted Data, CEB
Data, CBH/VR
Outliered Data
Data, CEB
GBD 2019
GBD 2021
CENSUS SBH
DHS CBH
DHS SBH
OTHER SURVEY CBH
OTHER SURVEY SBH

5626

Age−specific fertility fitted trend lines, 1950−2021: Riau, Ages: 35−39
Data points enclosed with a diamond are reference sources

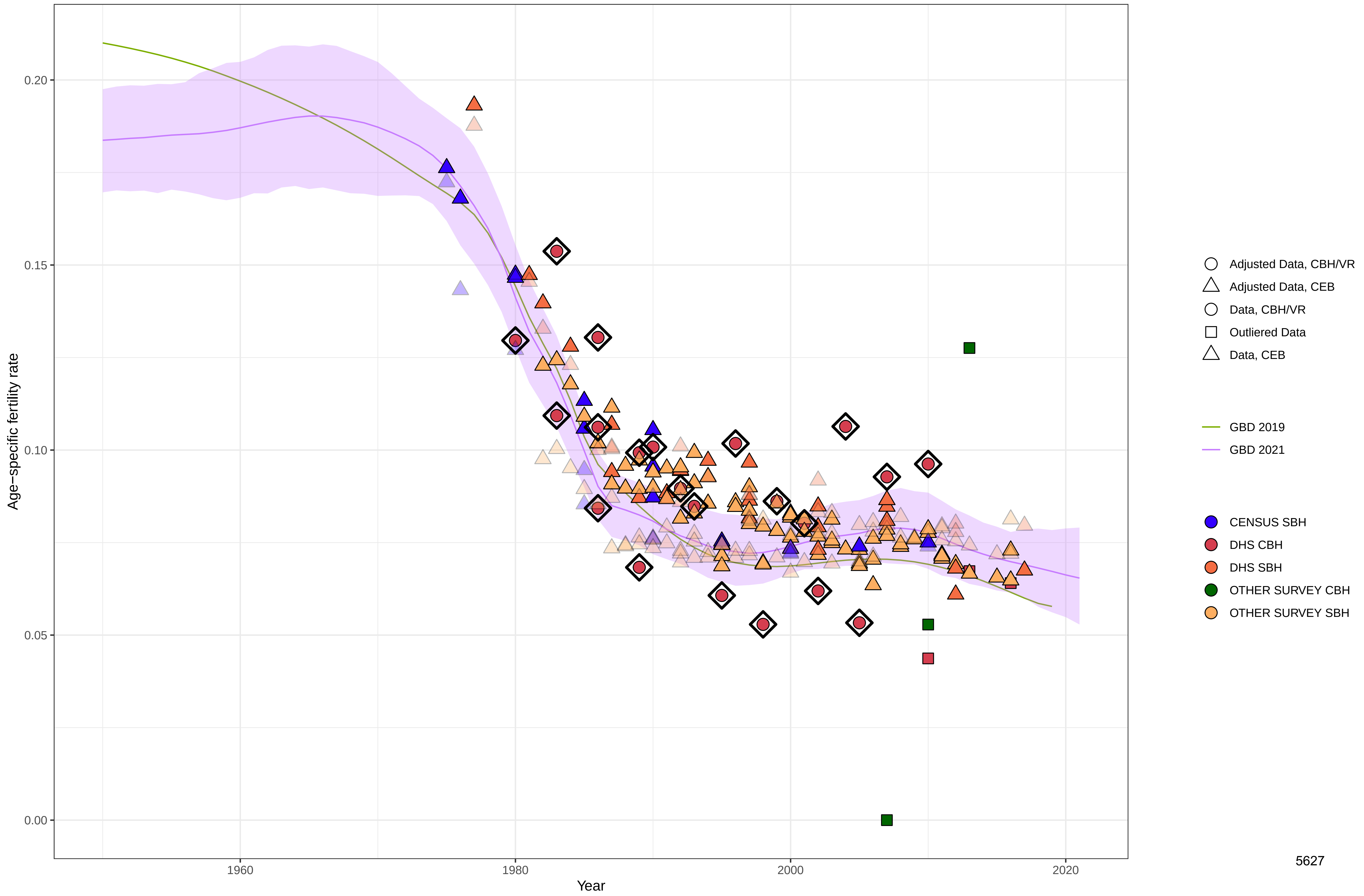

Age−specific fertility fitted trend lines, 1950−2021: Riau, Ages: 40−44
Data points enclosed with a diamond are reference sources

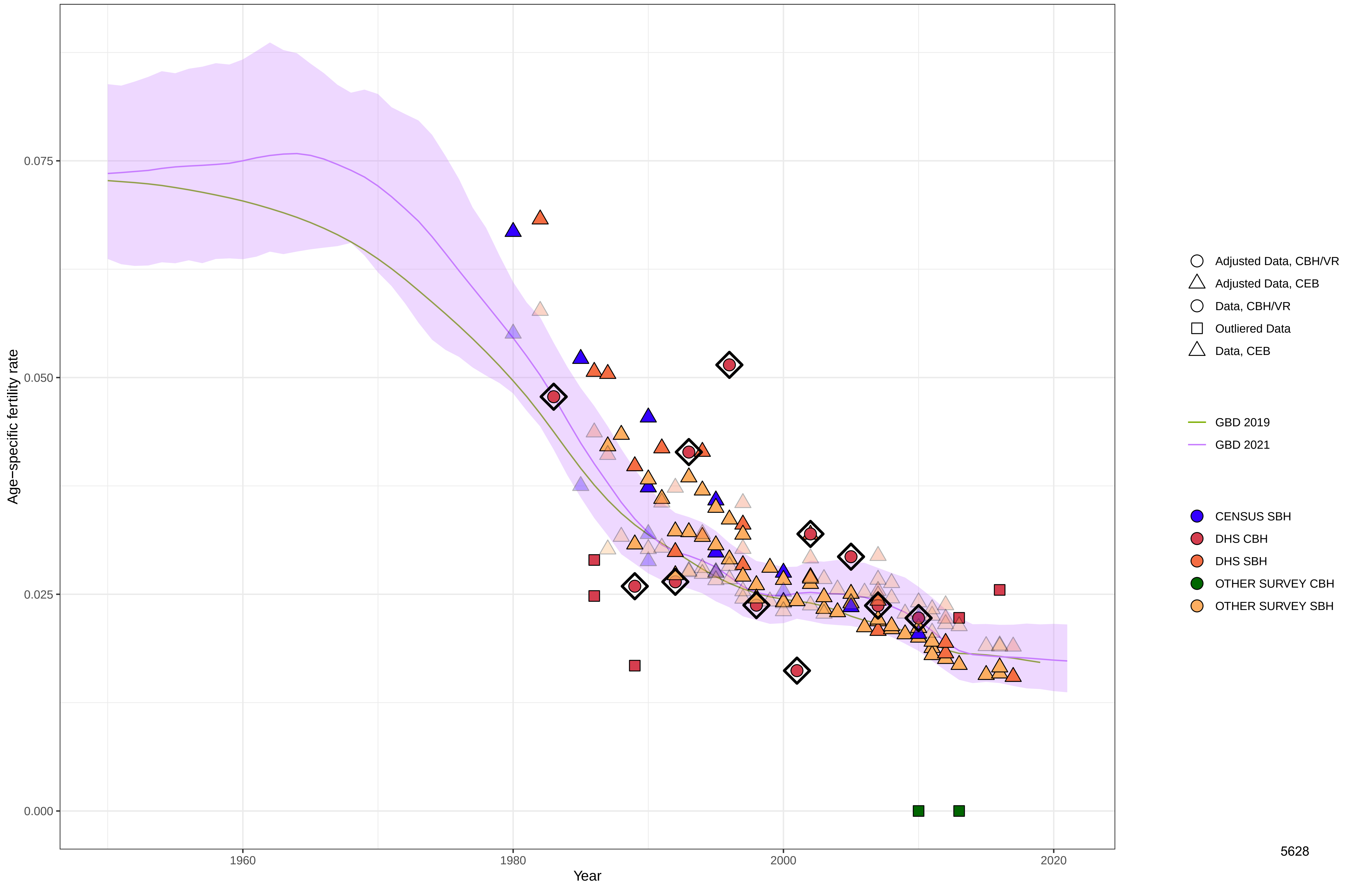

Age−specific fertility fitted trend lines, 1950−2021: Riau, Ages: 45−49
Data points enclosed with a diamond are reference sources

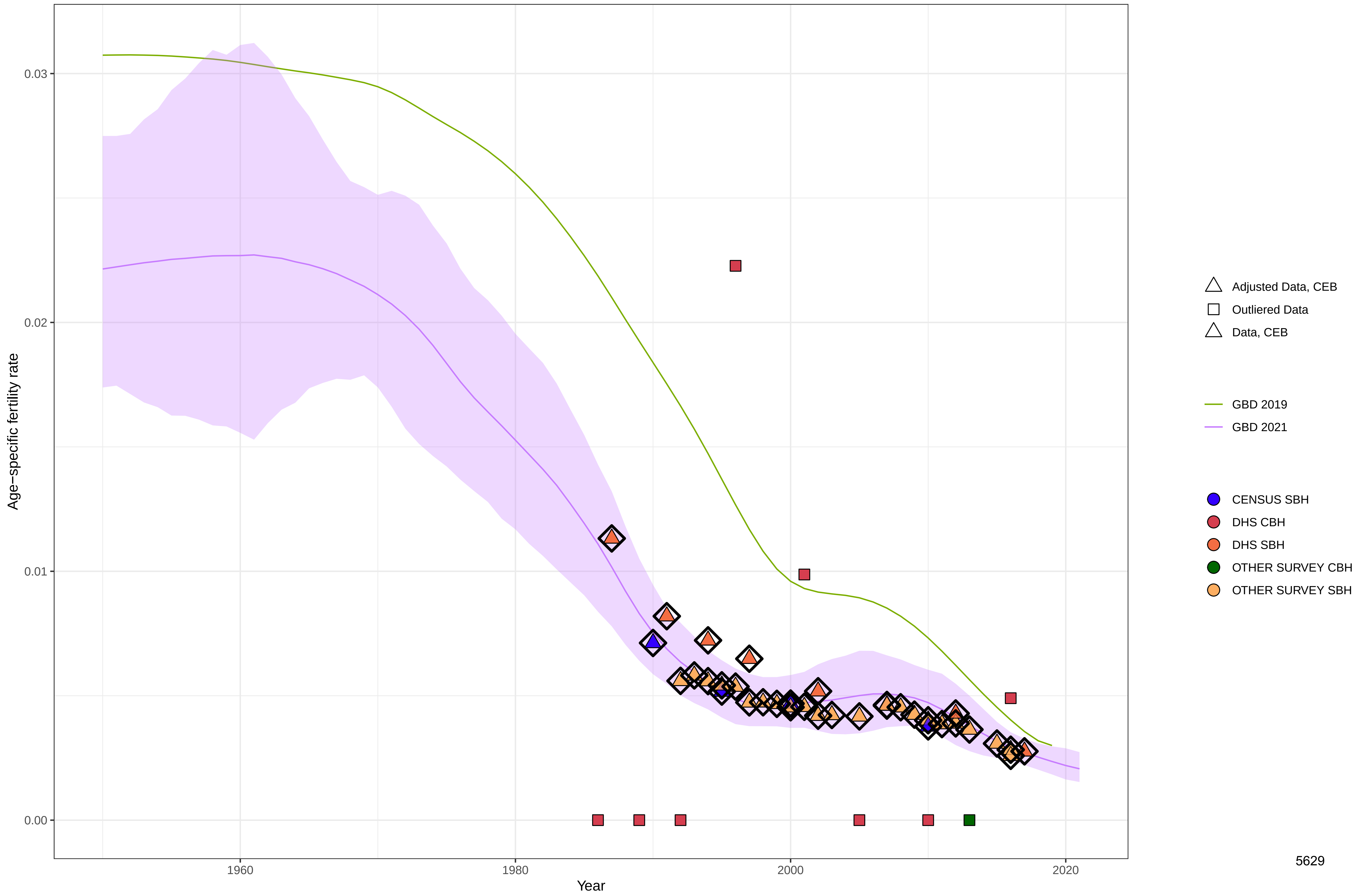

5629

Age−specific fertility fitted trend lines, 1950−2021: Riau, Ages: 50−54
Data points enclosed with a diamond are reference sources

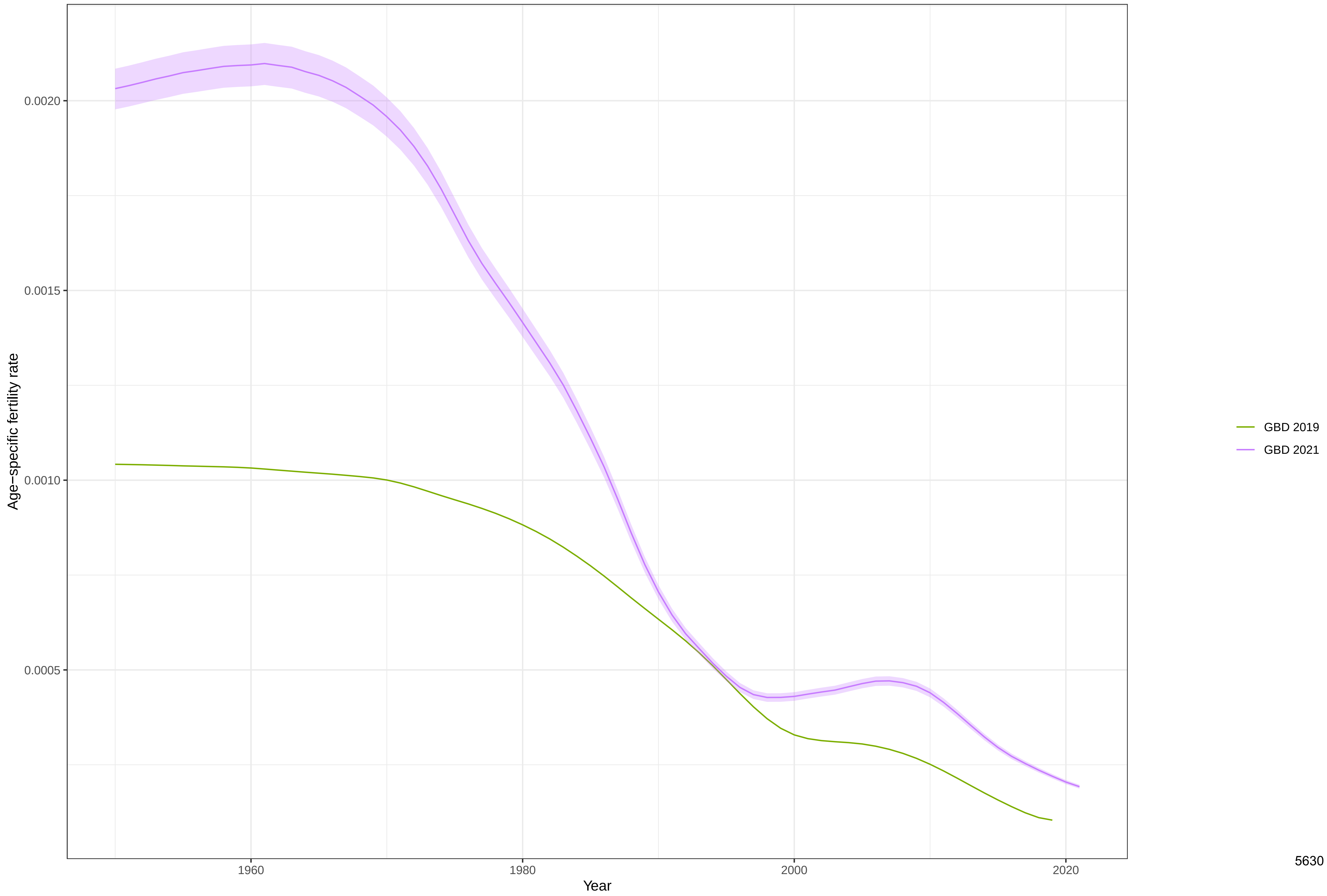

Total fertility rate fitted trend lines, 1950−2021: Riau TFR
Data points enclosed with a diamond are reference sources

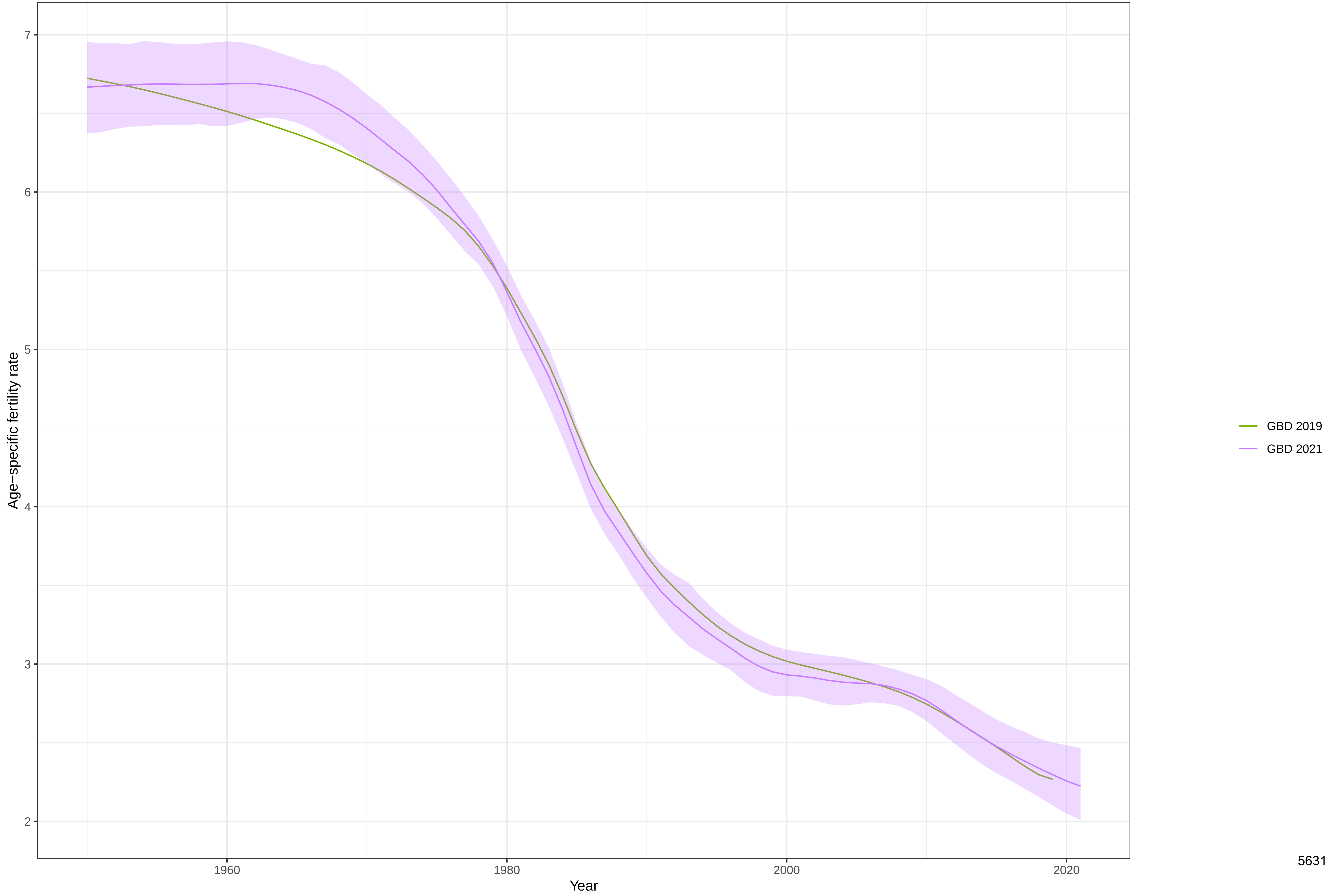

Age−specific fertility fitted trend lines, 1950−2021: West Sulawesi, Ages: 10−14
Data points enclosed with a diamond are reference sources

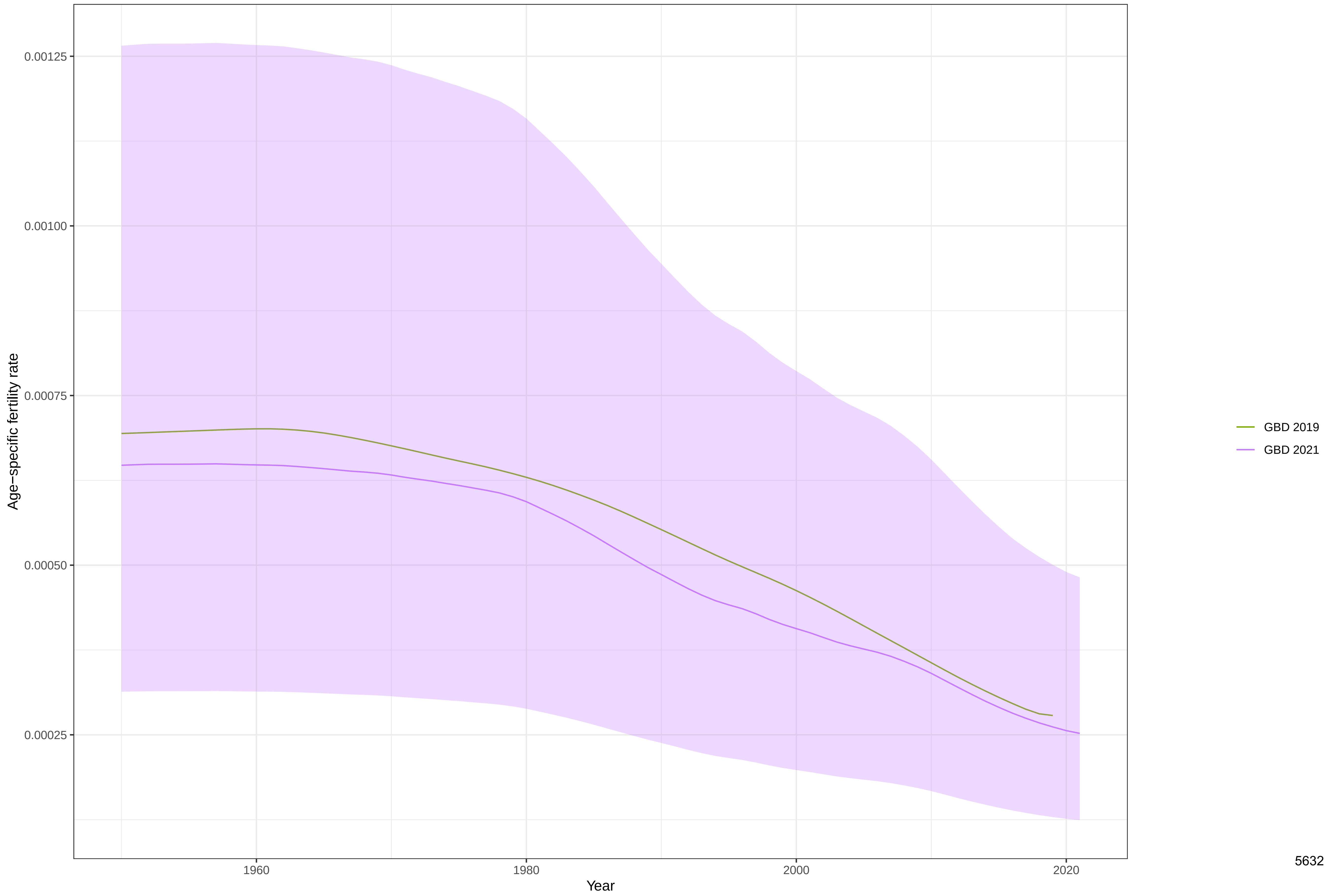

Age−specific fertility fitted trend lines, 1950−2021: West Sulawesi, Ages: 15−19
Data points enclosed with a diamond are reference sources

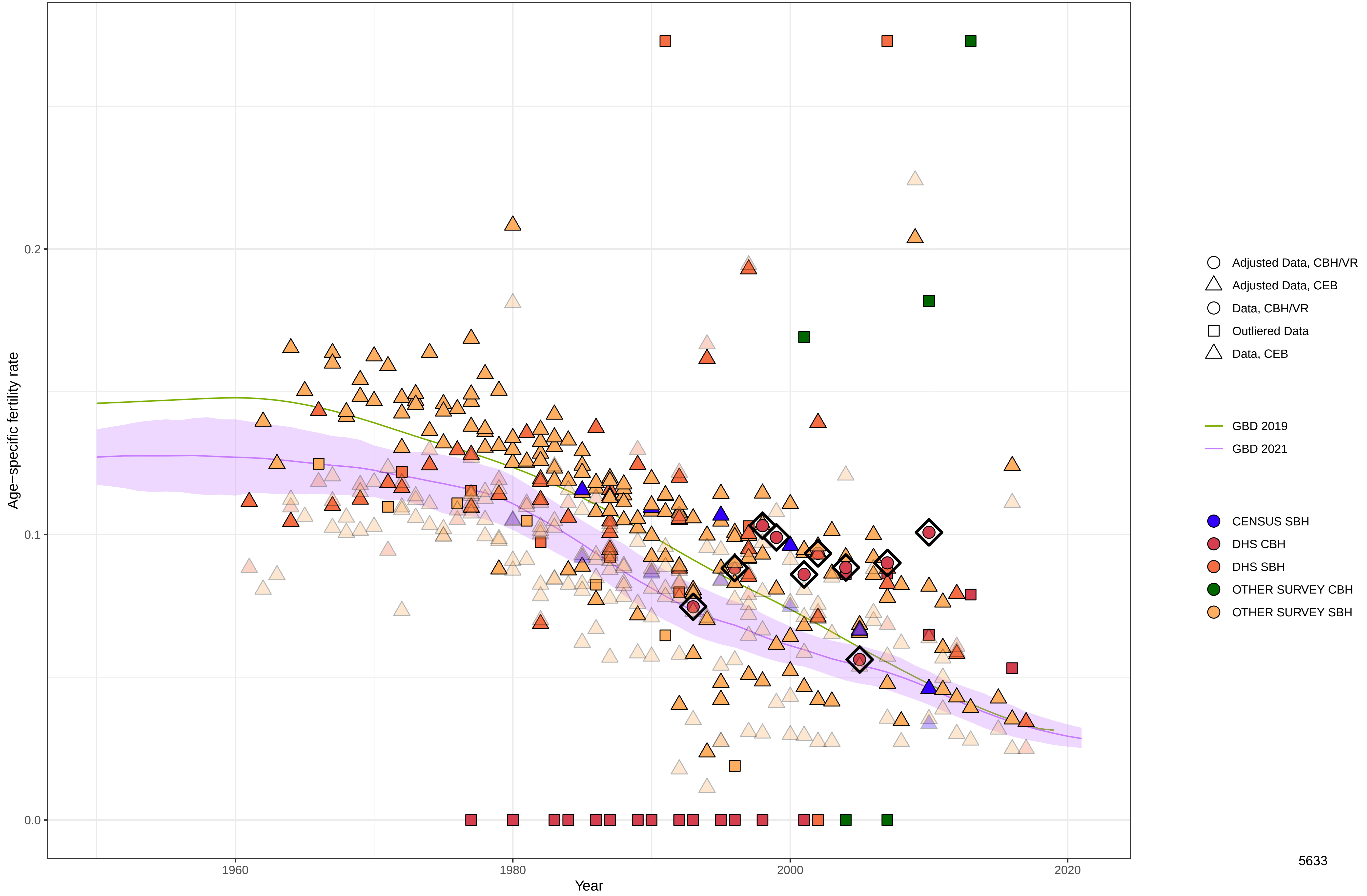

Age−specific fertility fitted trend lines, 1950−2021: West Sulawesi, Ages: 20−24
Data points enclosed with a diamond are reference sources

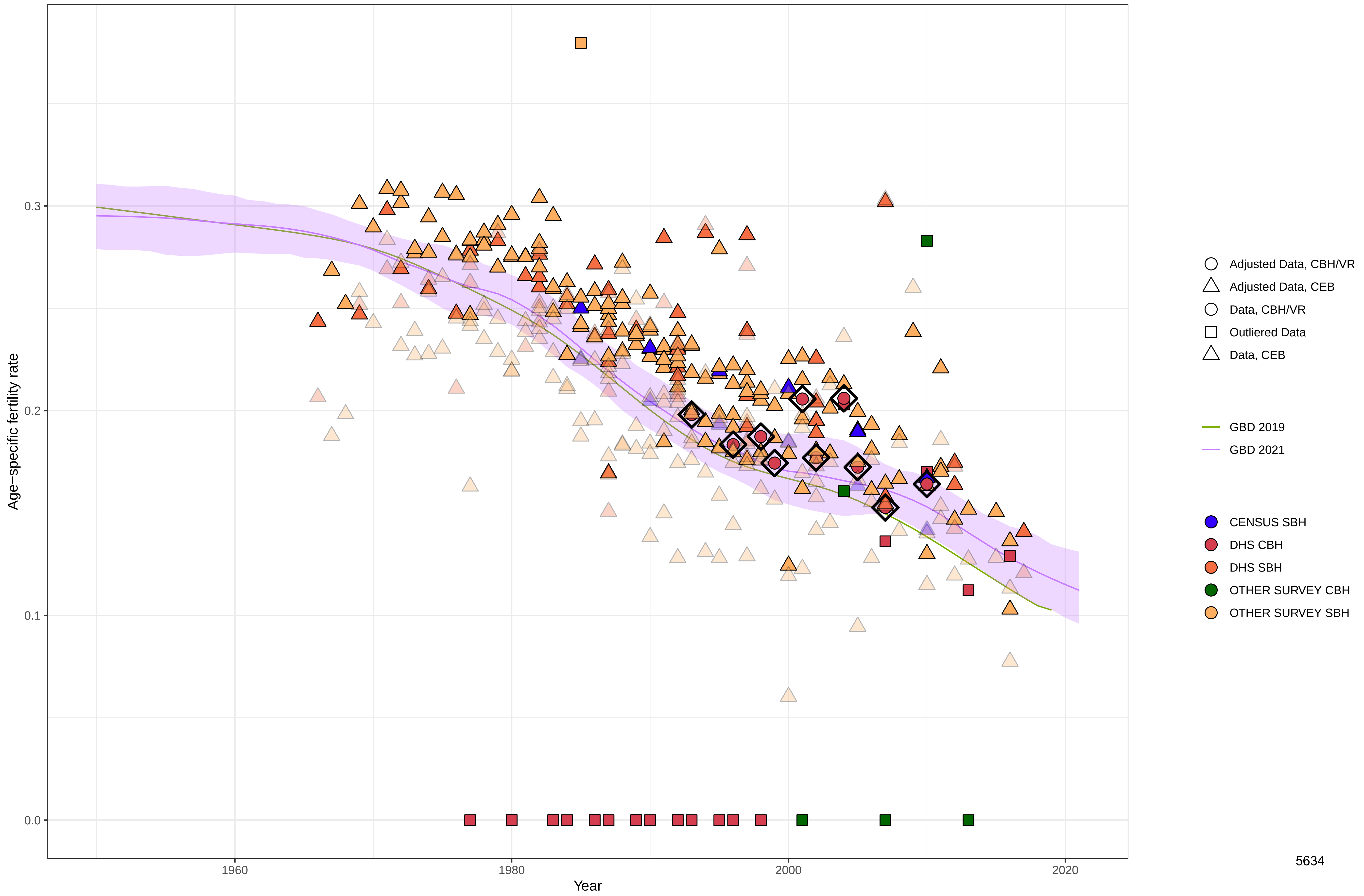

Age−specific fertility fitted trend lines, 1950−2021: West Sulawesi, Ages: 25−29
Data points enclosed with a diamond are reference sources

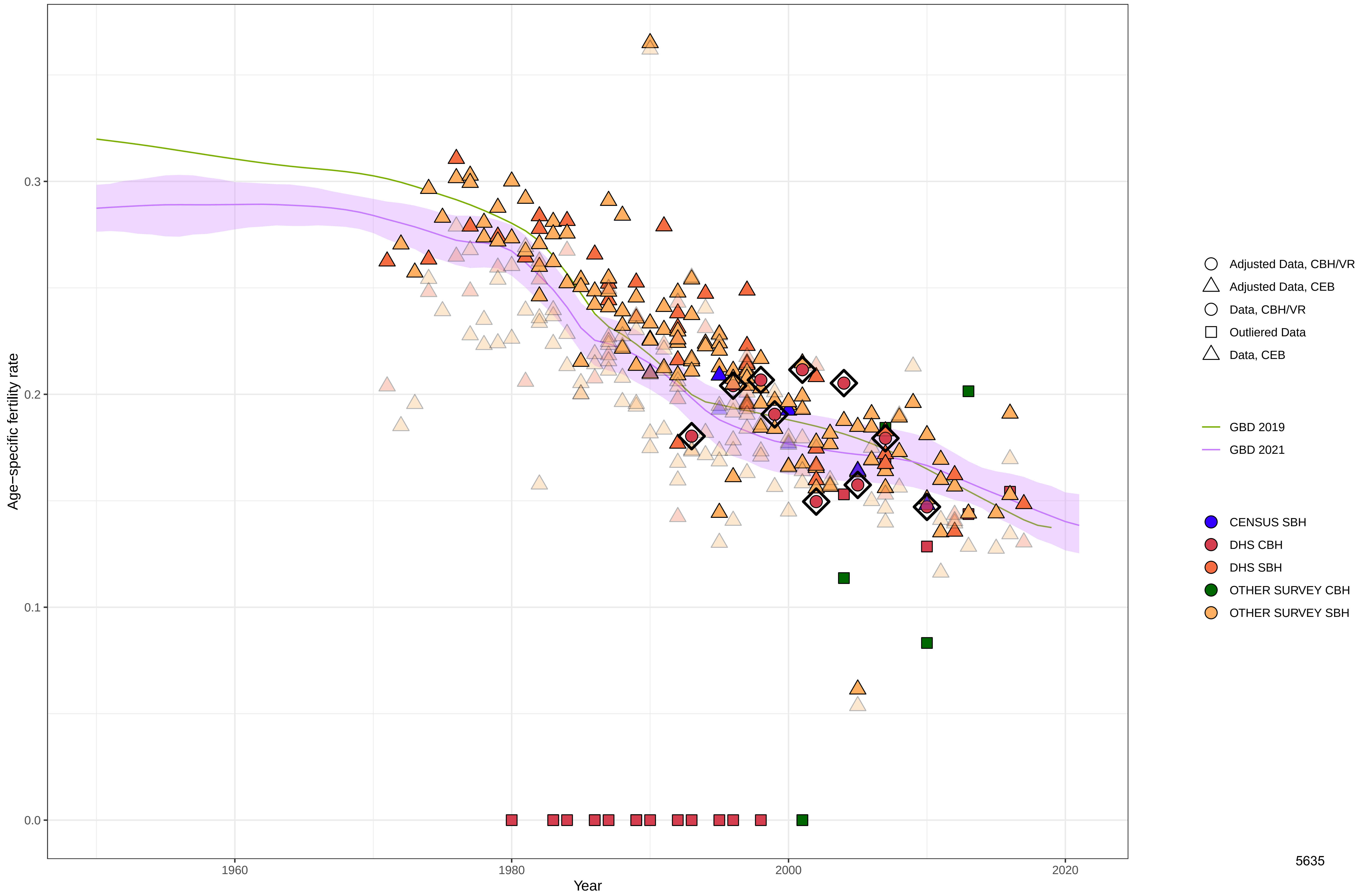

Age−specific fertility fitted trend lines, 1950−2021: West Sulawesi, Ages: 30−34
Data points enclosed with a diamond are reference sources

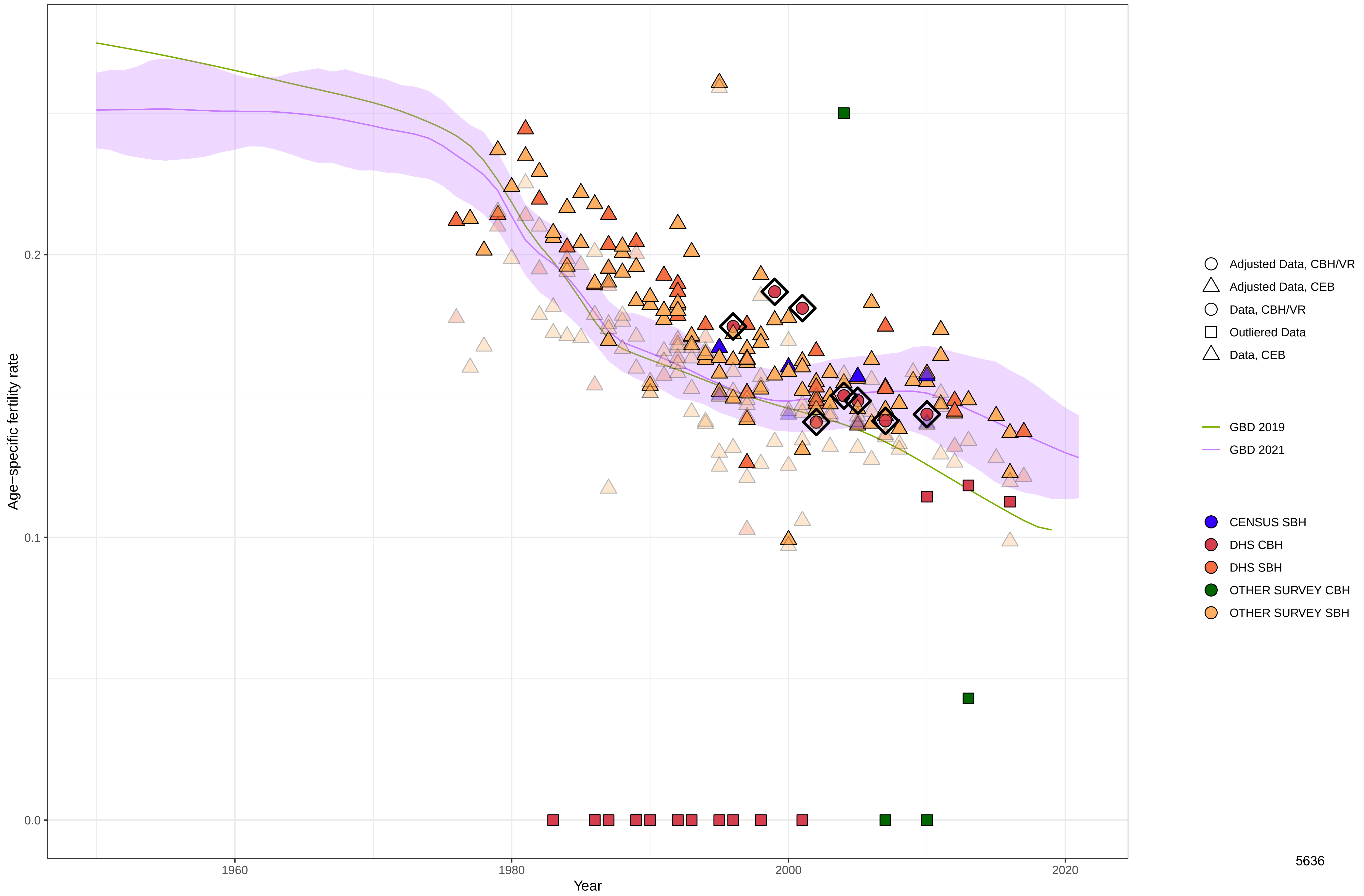

Age−specific fertility fitted trend lines, 1950−2021: West Sulawesi, Ages: 35−39
Data points enclosed with a diamond are reference sources

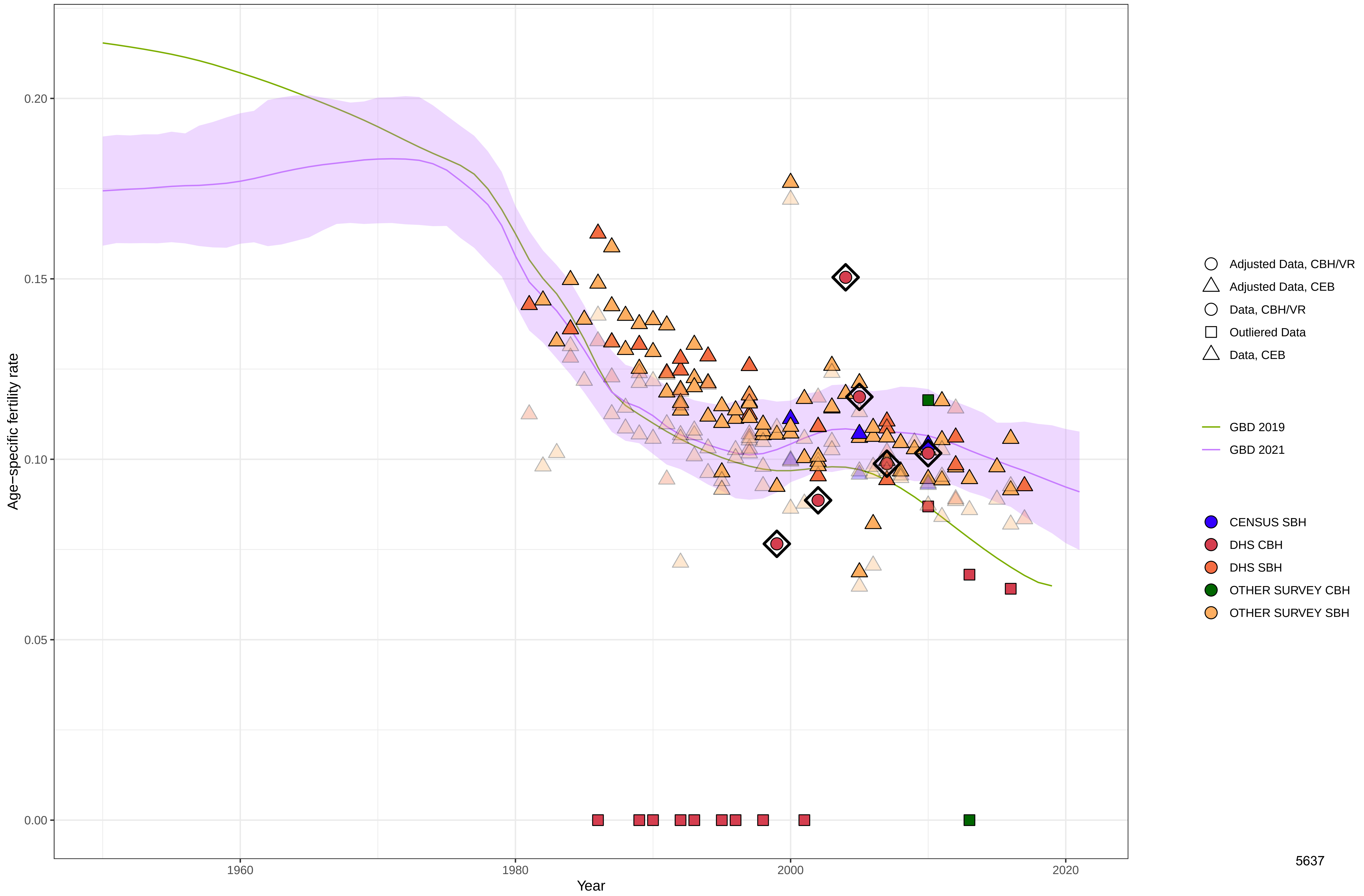

Age−specific fertility fitted trend lines, 1950−2021: West Sulawesi, Ages: 40−44
Data points enclosed with a diamond are reference sources

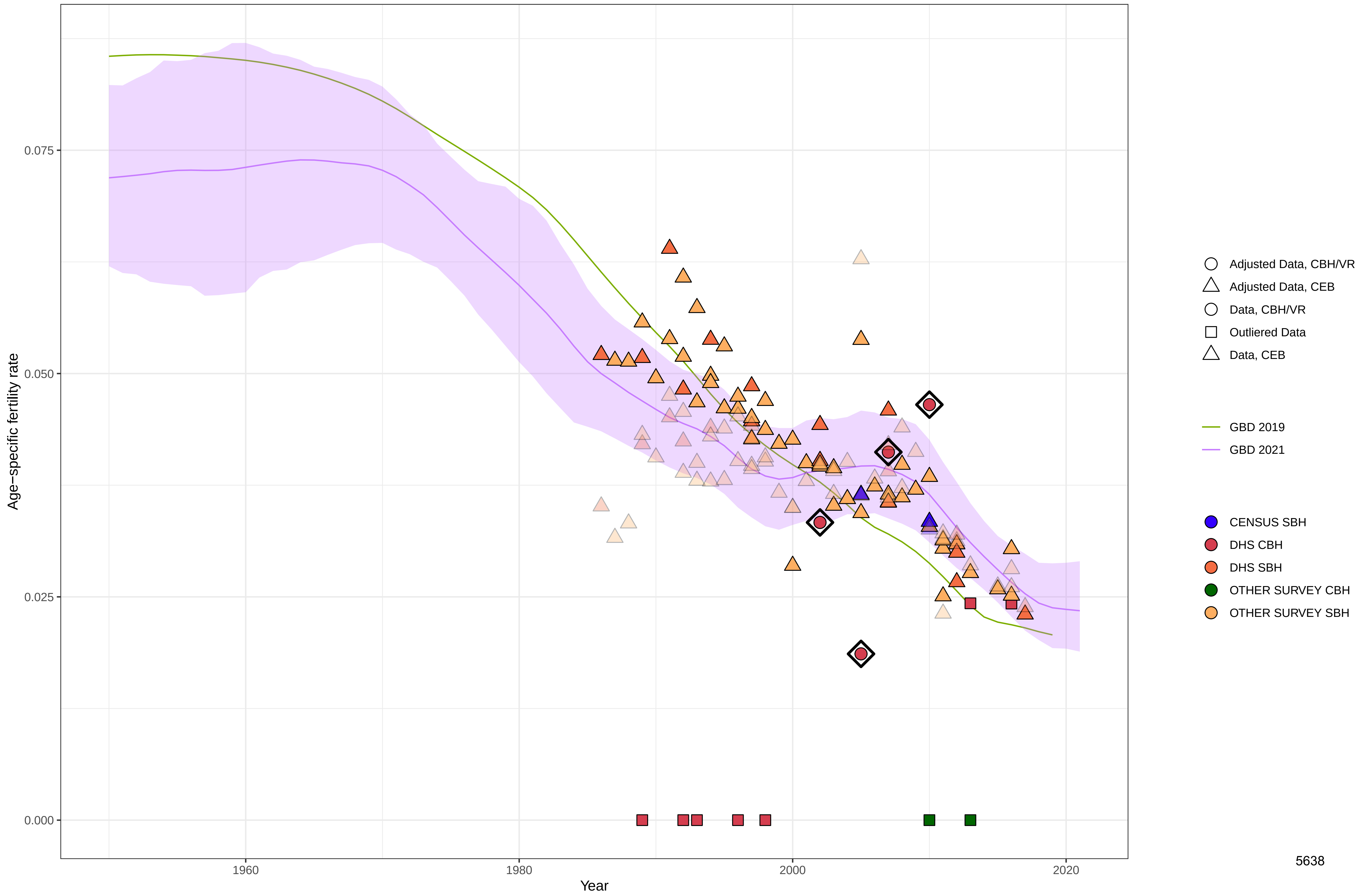

Age−specific fertility fitted trend lines, 1950−2021: West Sulawesi, Ages: 45−49
Data points enclosed with a diamond are reference sources

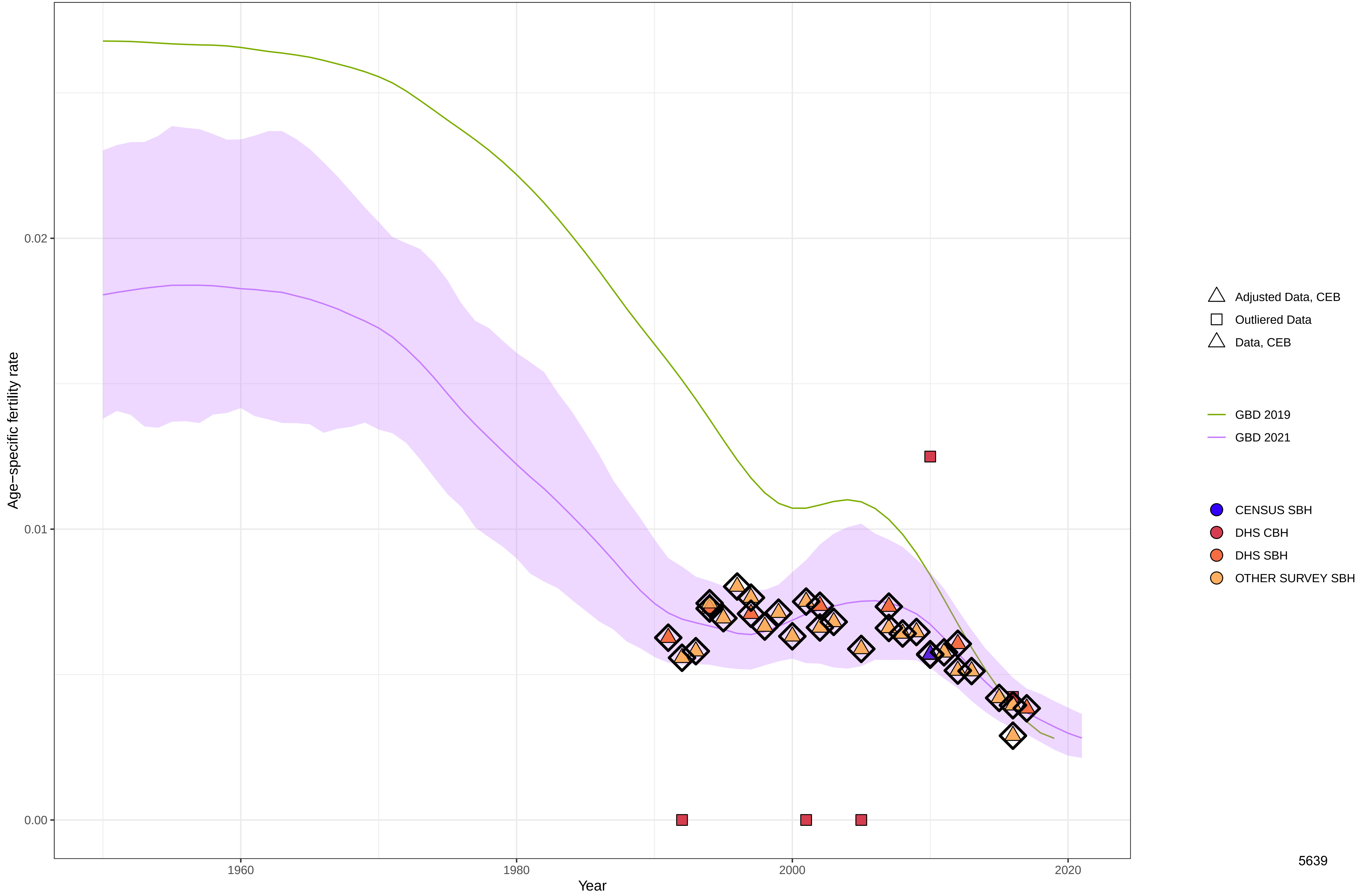

Age−specific fertility fitted trend lines, 1950−2021: West Sulawesi, Ages: 50−54
Data points enclosed with a diamond are reference sources

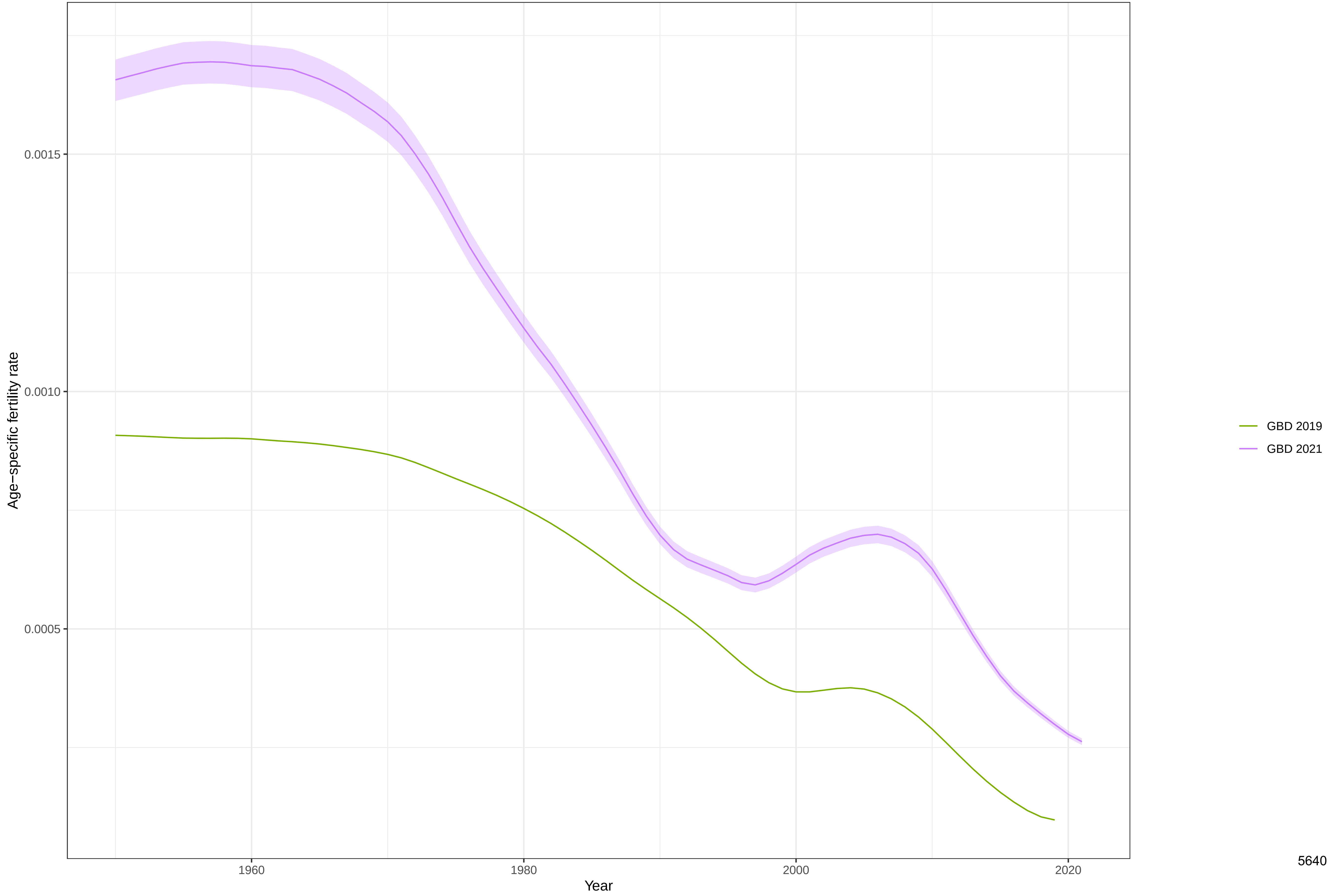

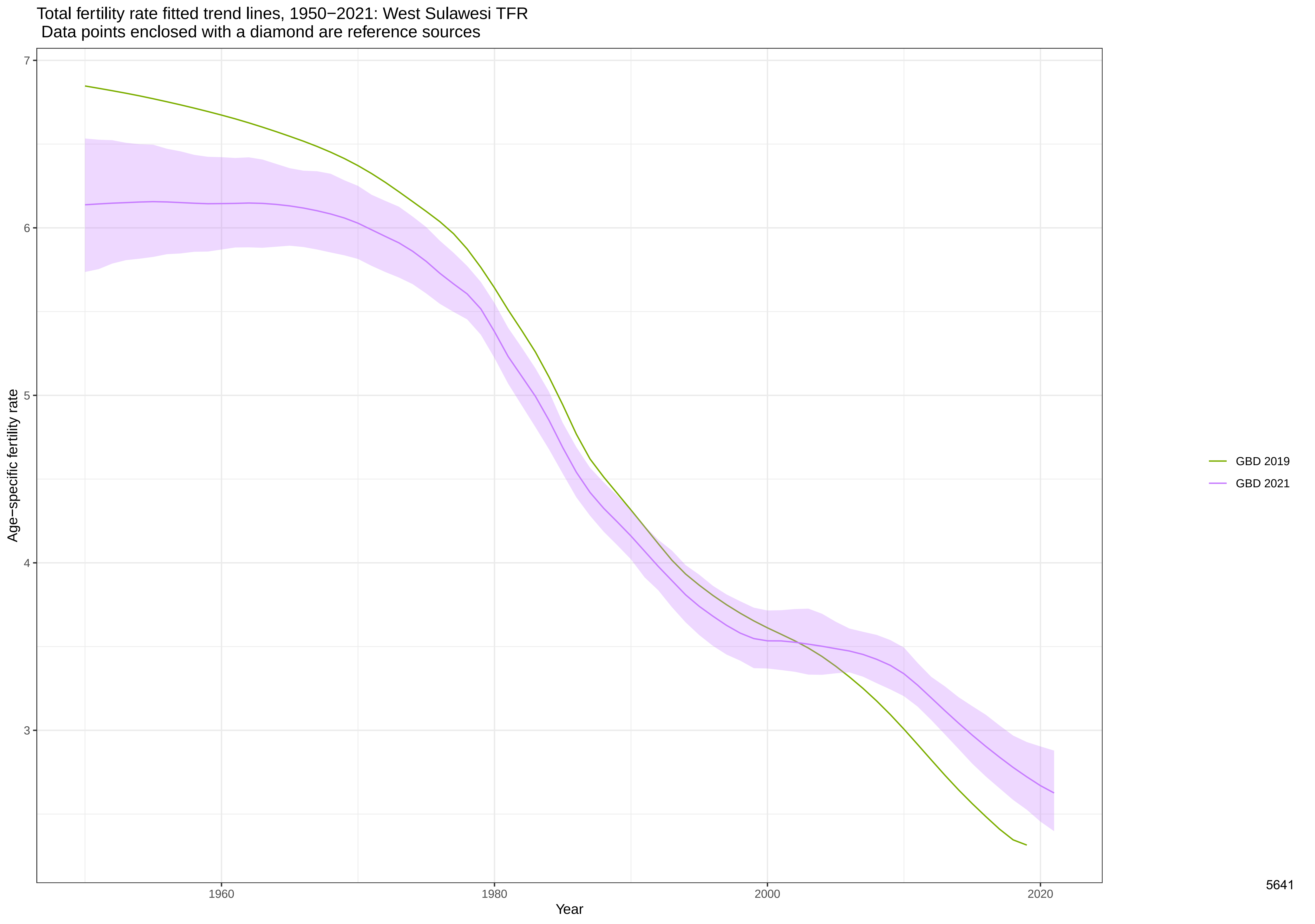

Total fertility rate fitted trend lines, 1950−2021: West Sulawesi TFR
Data points enclosed with a diamond are reference sources
Age−specific fertility rate
Year
7
6
5
4
3
1960
1980
2000
2020
GBD 2019
GBD 2021

Age−specific fertility fitted trend lines, 1950−2021: South Sulawesi, Ages: 10−14
Data points enclosed with a diamond are reference sources

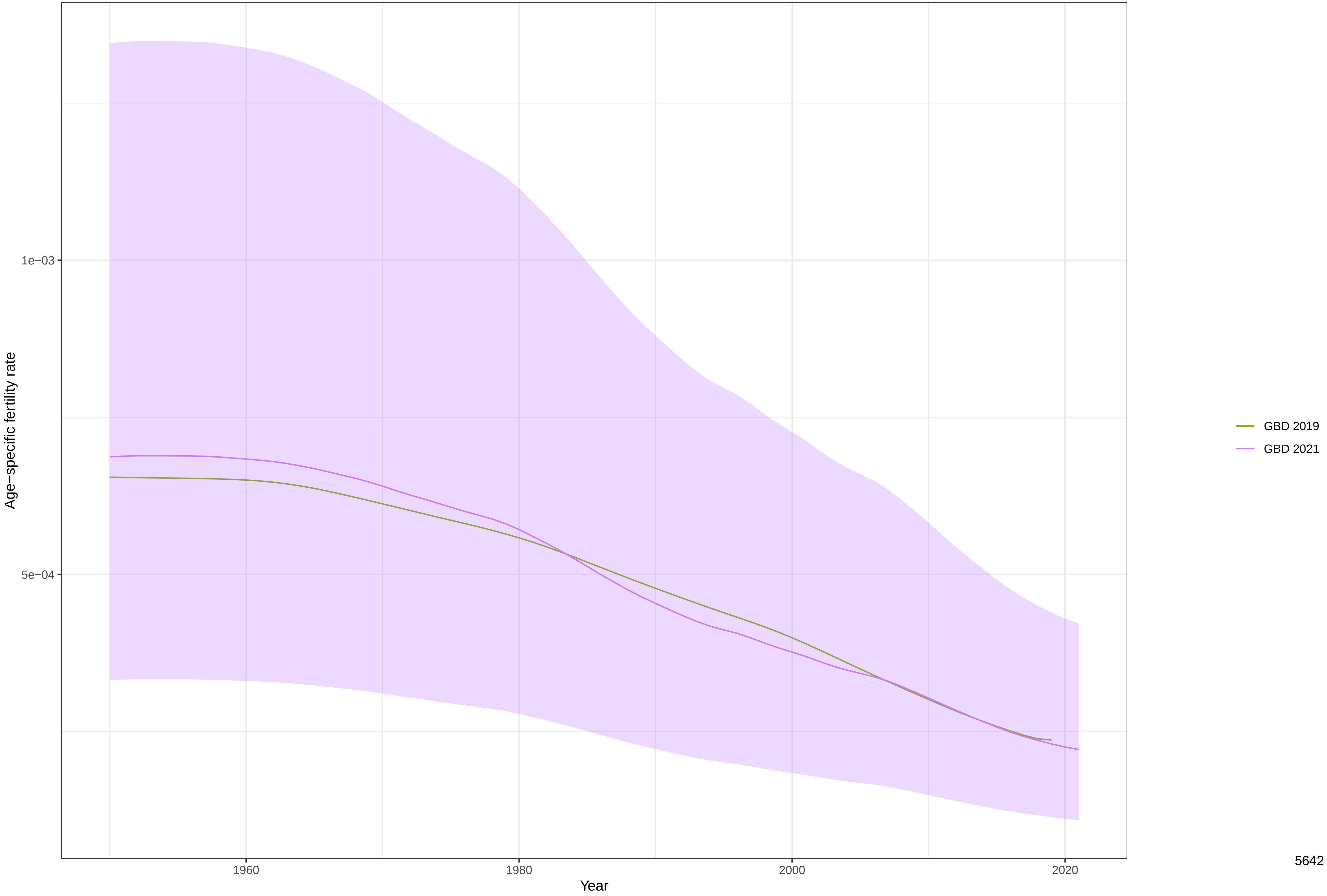

Age−specific fertility fitted trend lines, 1950−2021: South Sulawesi, Ages: 15−19
Data points enclosed with a diamond are reference sources

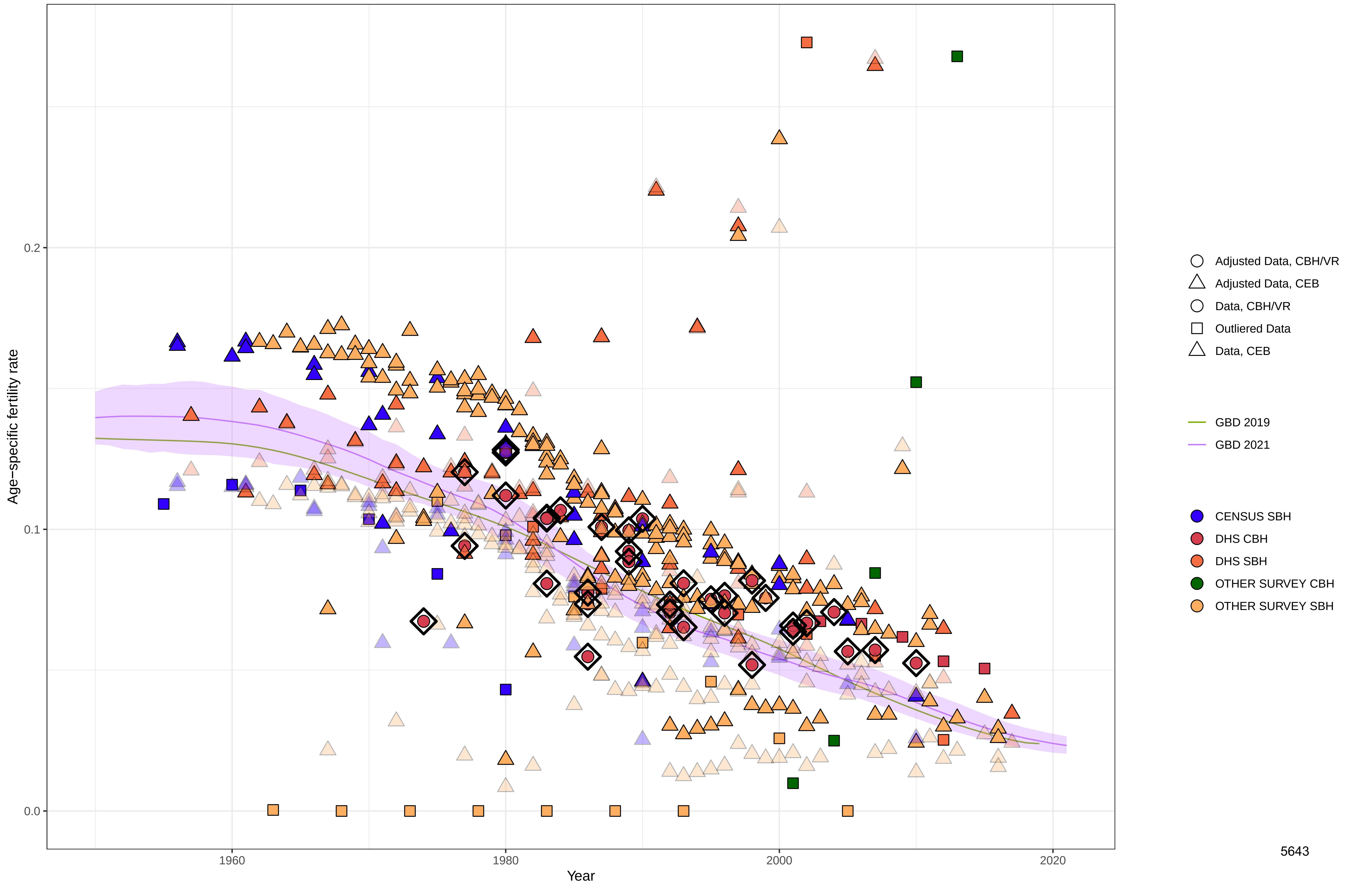

Age−specific fertility fitted trend lines, 1950−2021: South Sulawesi, Ages: 20−24
Data points enclosed with a diamond are reference sources

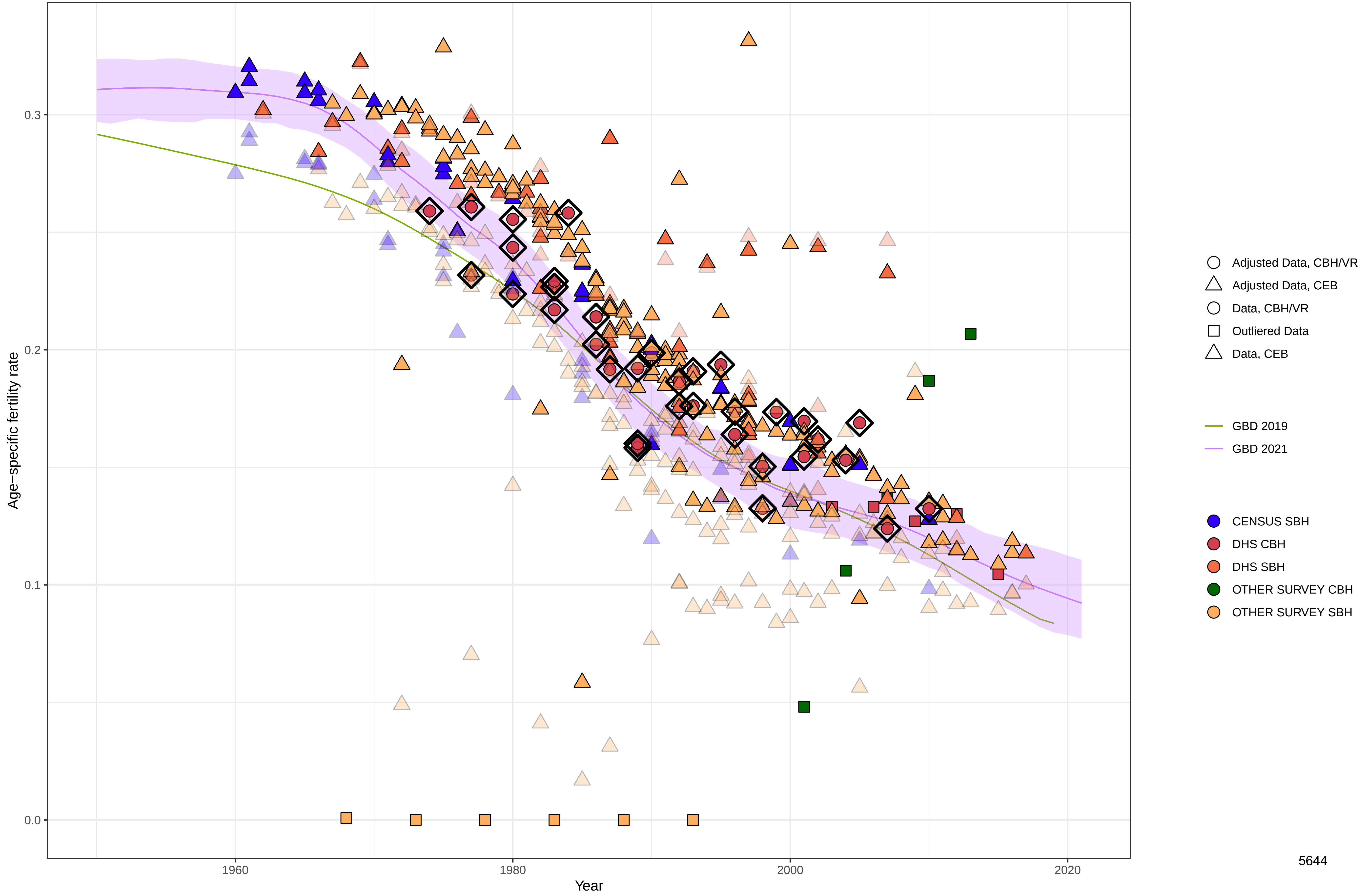

Age−specific fertility fitted trend lines, 1950−2021: South Sulawesi, Ages: 25−29
Data points enclosed with a diamond are reference sources

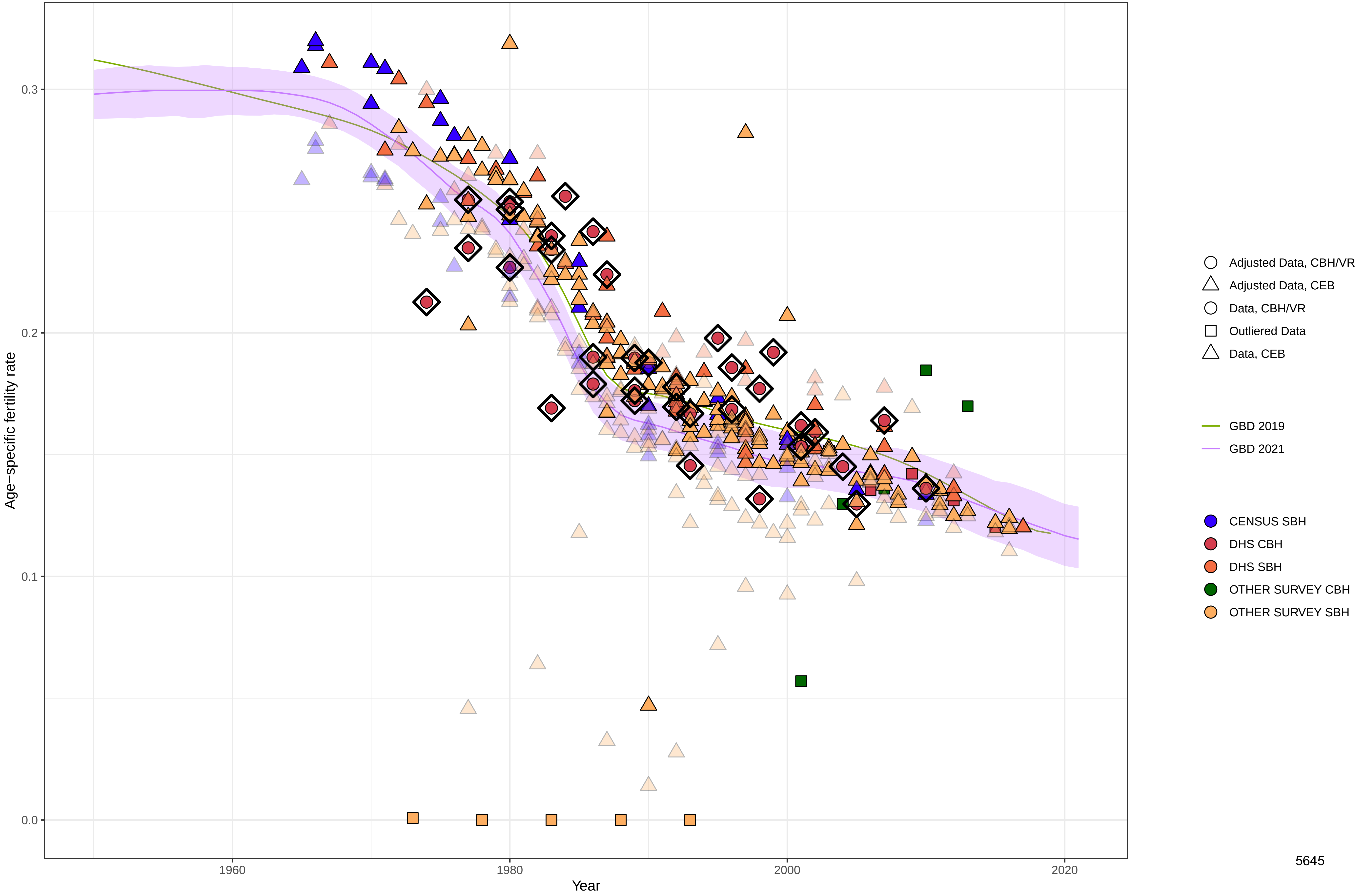

Age−specific fertility fitted trend lines, 1950−2021: South Sulawesi, Ages: 30−34
Data points enclosed with a diamond are reference sources

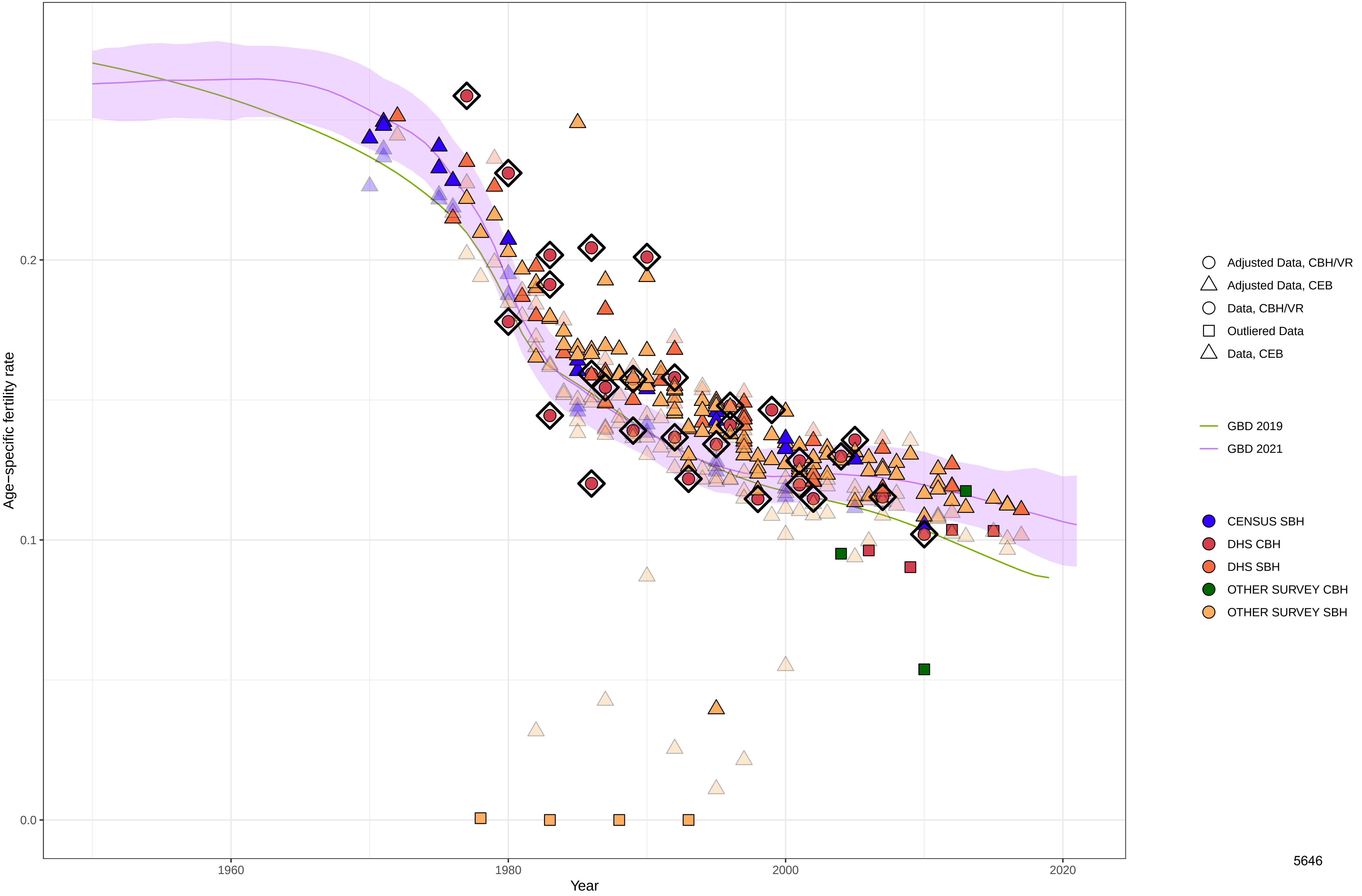

Age−specific fertility fitted trend lines, 1950−2021: South Sulawesi, Ages: 35−39
Data points enclosed with a diamond are reference sources

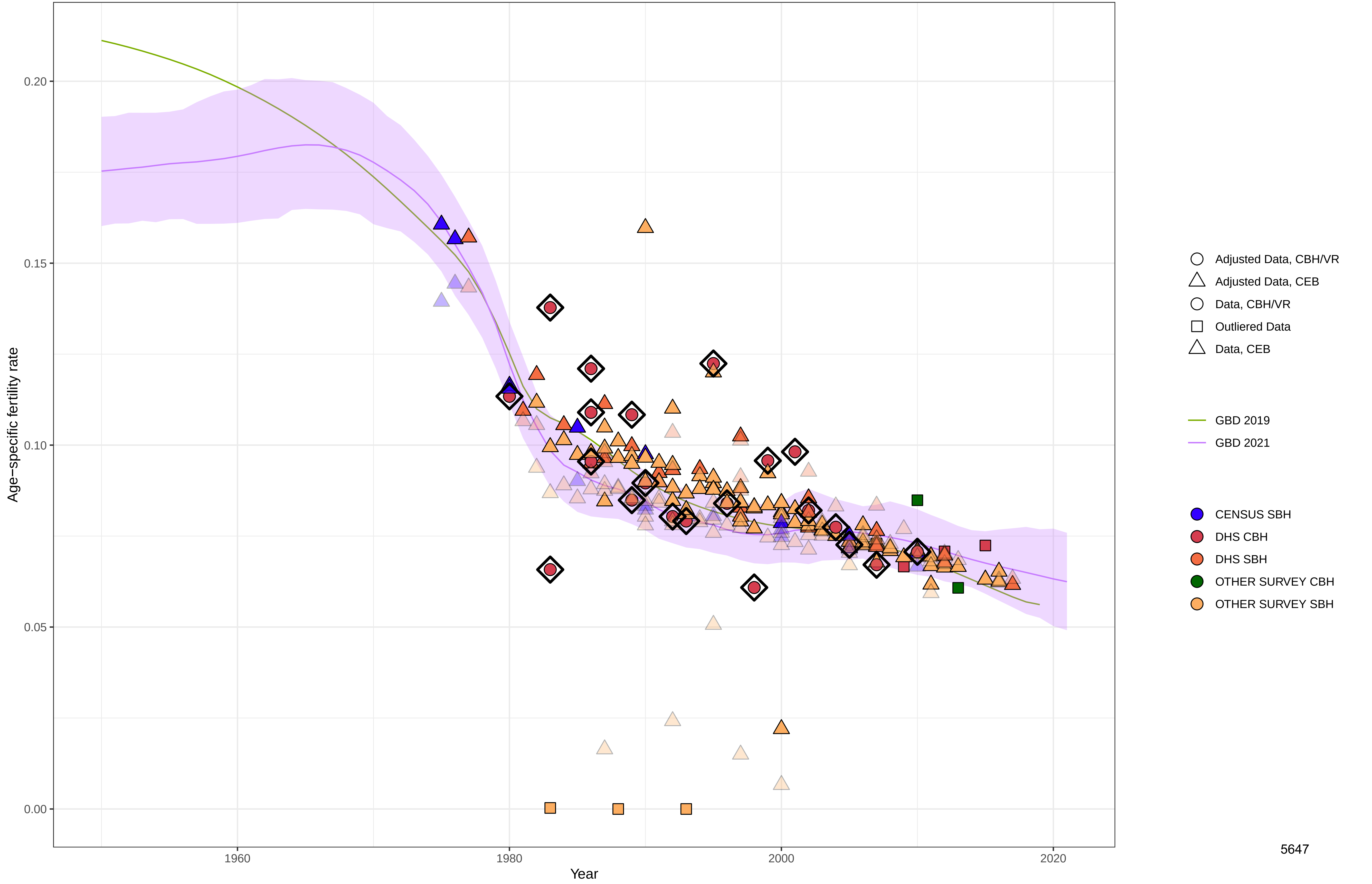

Age−specific fertility fitted trend lines, 1950−2021: South Sulawesi, Ages: 40−44
Data points enclosed with a diamond are reference sources

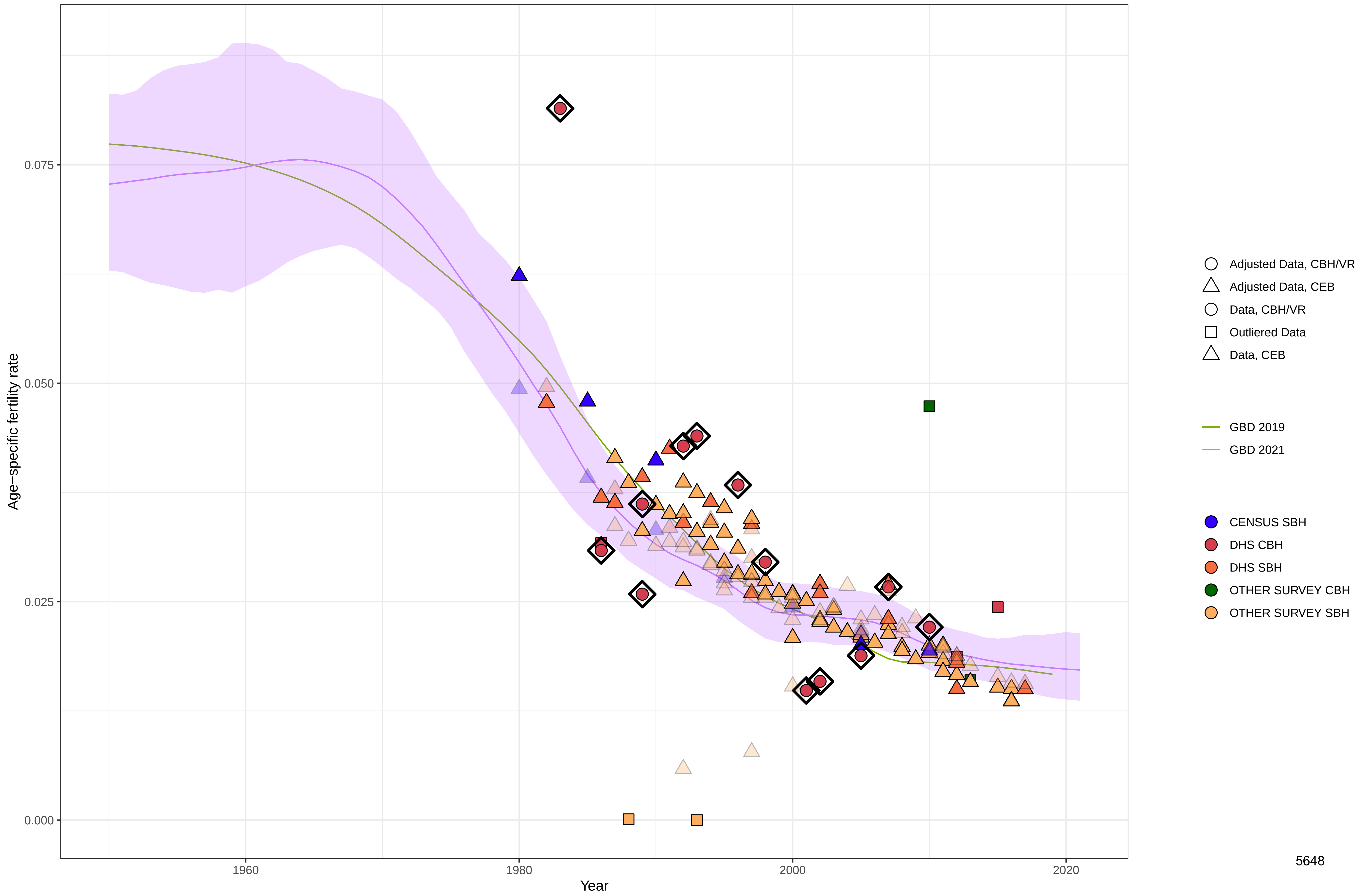

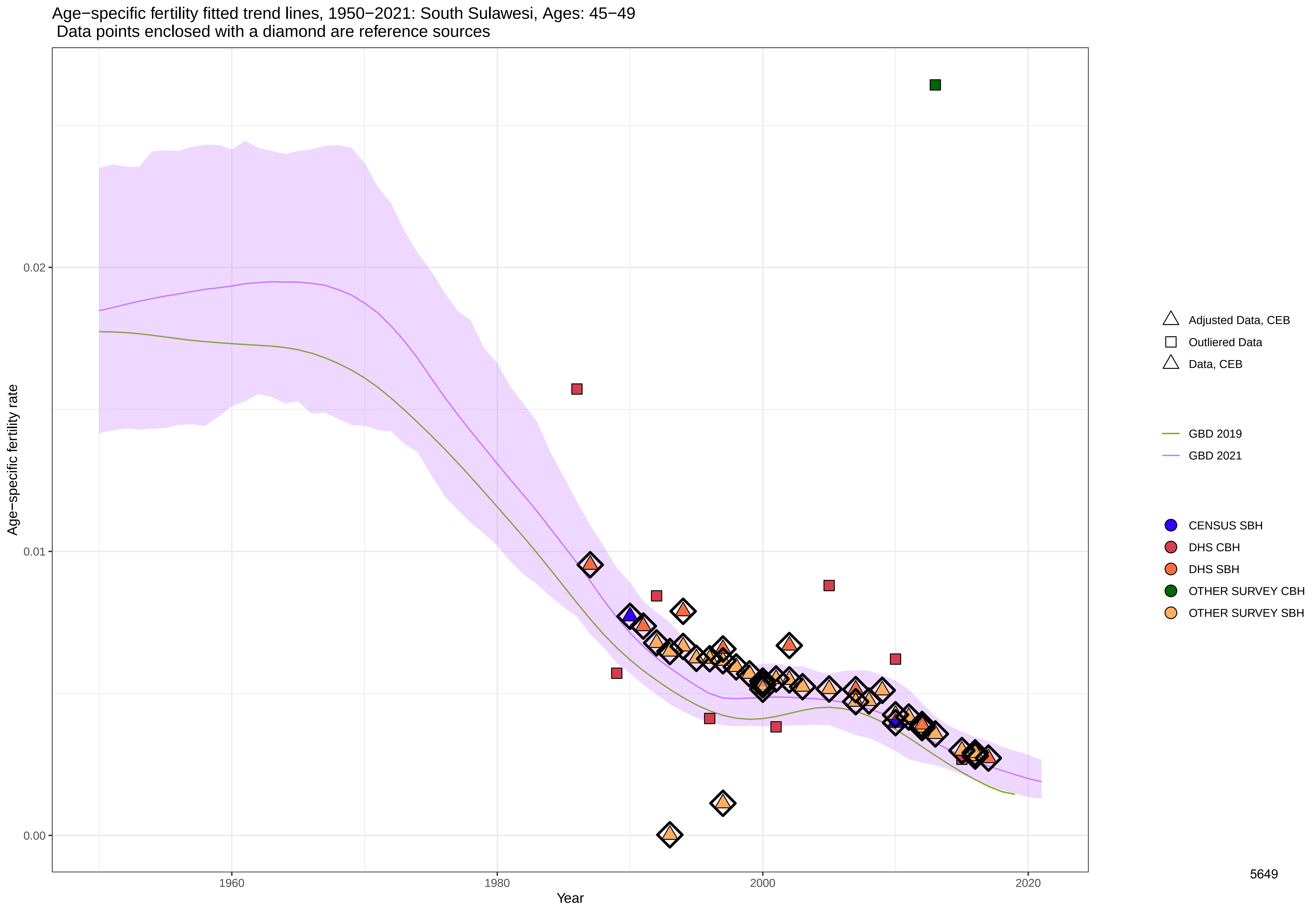

Age−specific fertility fitted trend lines, 1950−2021: South Sulawesi, Ages: 45−49
Data points enclosed with a diamond are reference sources
Age−specific fertility rate
0.00
0.01
0.02
1960
1980
2000
2020
Year
Adjusted Data, CEB
Outliered Data
Data, CEB
GBD 2019
GBD 2021
CENSUS SBH
DHS CBH
DHS SBH
OTHER SURVEY CBH
OTHER SURVEY SBH

Age−specific fertility fitted trend lines, 1950−2021: South Sulawesi, Ages: 50−54
Data points enclosed with a diamond are reference sources

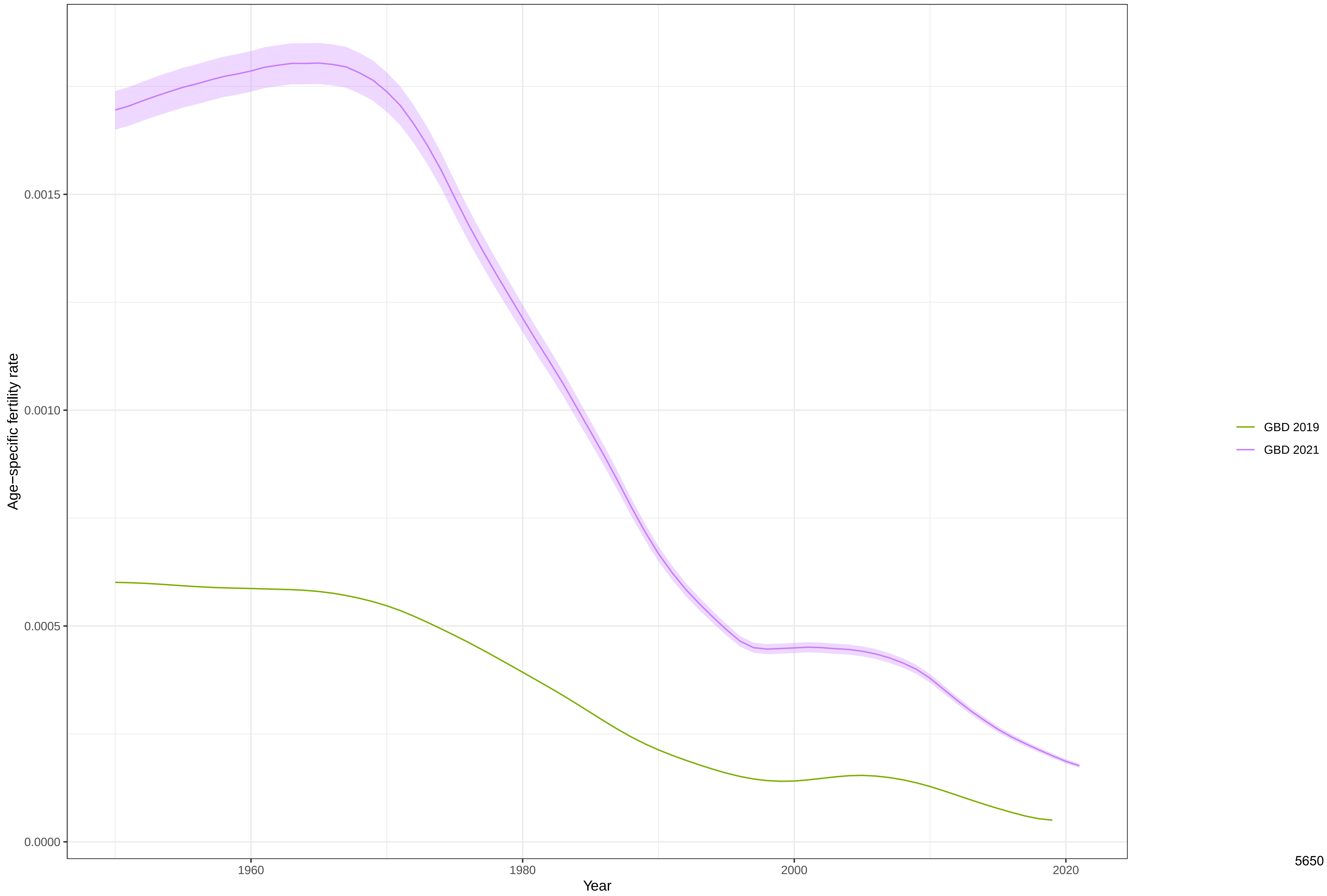

Total fertility rate fitted trend lines, 1950−2021: South Sulawesi TFR
Data points enclosed with a diamond are reference sources

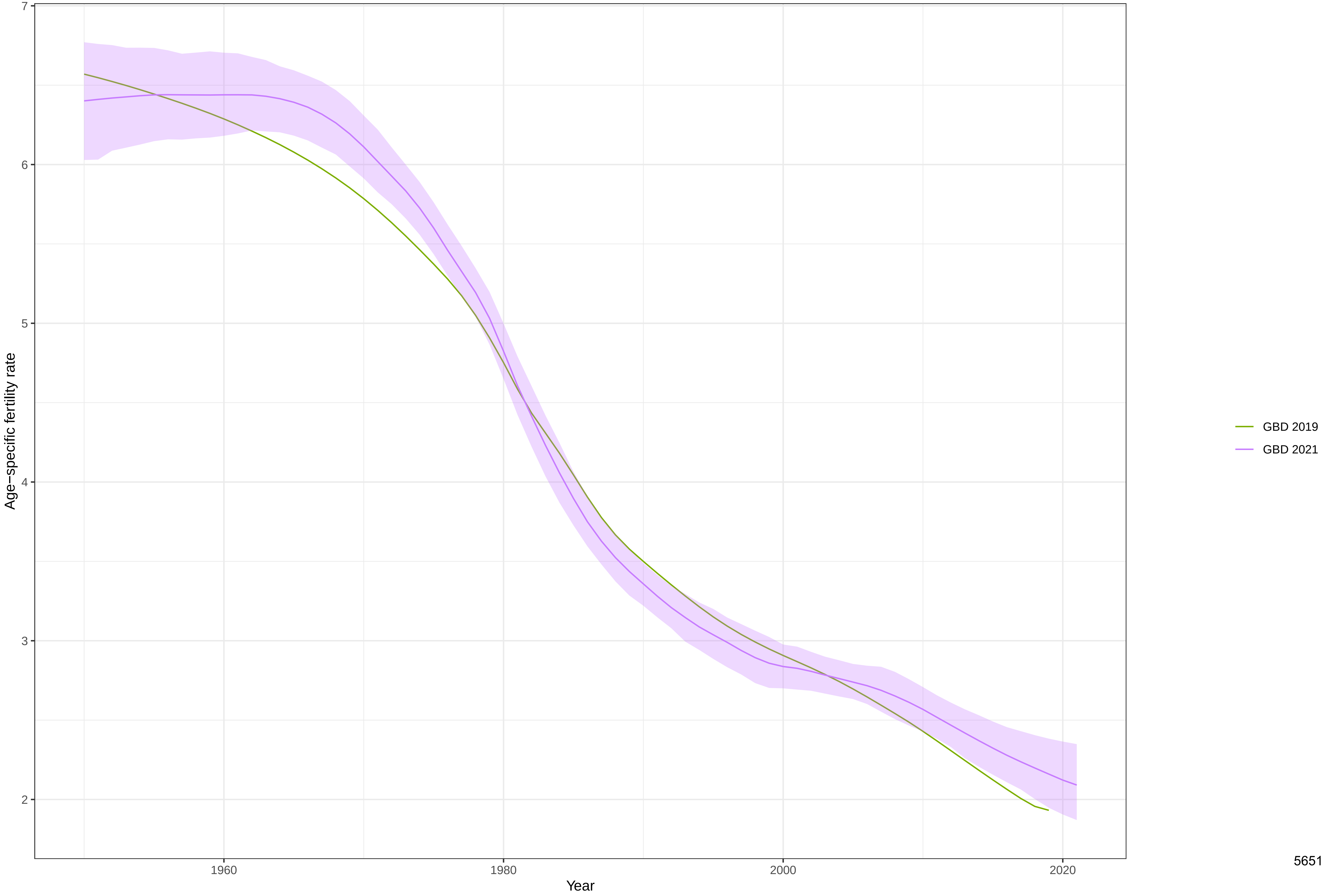

Age−specific fertility fitted trend lines, 1950−2021: Central Sulawesi, Ages: 10−14
Data points enclosed with a diamond are reference sources

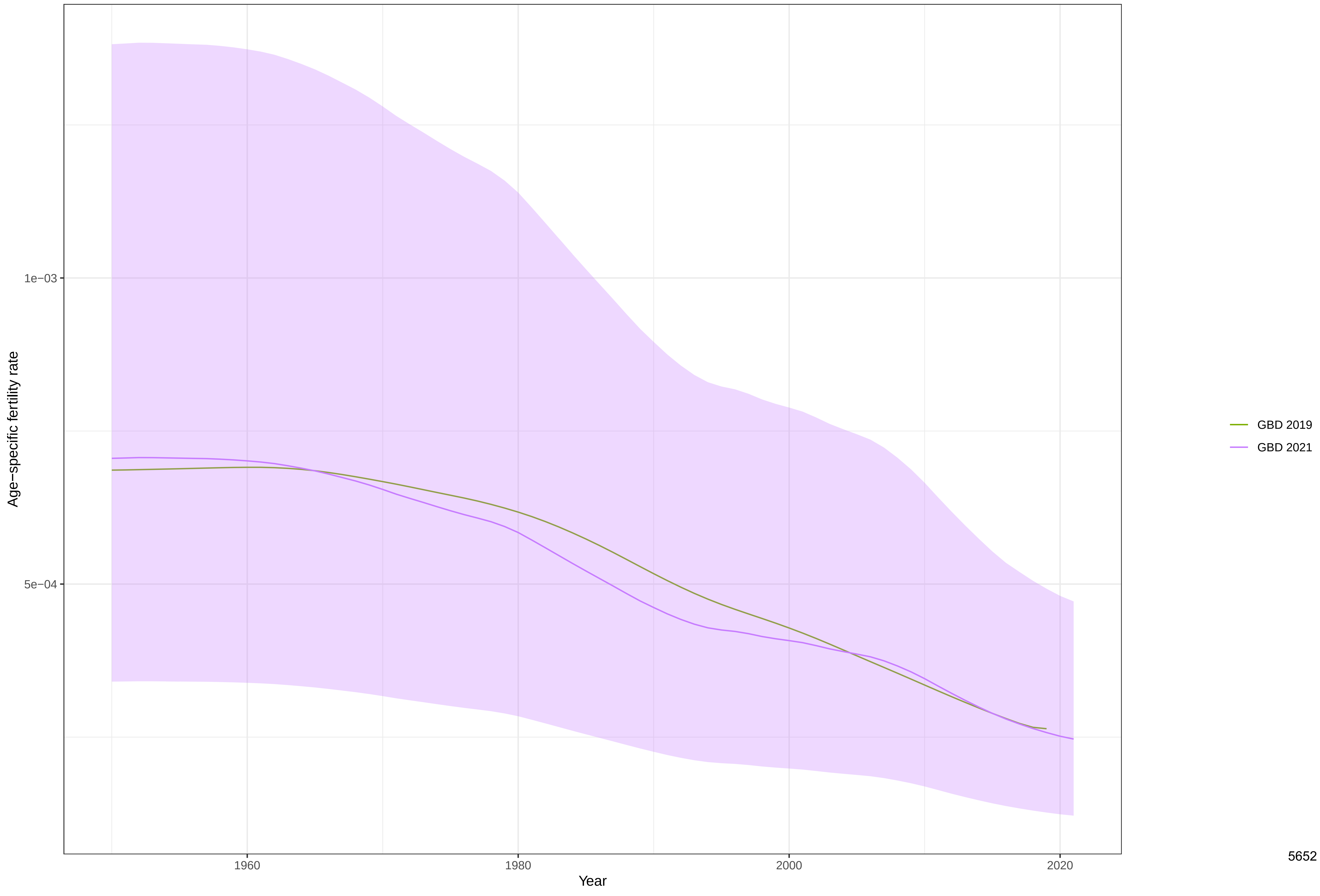

Age−specific fertility fitted trend lines, 1950−2021: Central Sulawesi, Ages: 15−19
Data points enclosed with a diamond are reference sources

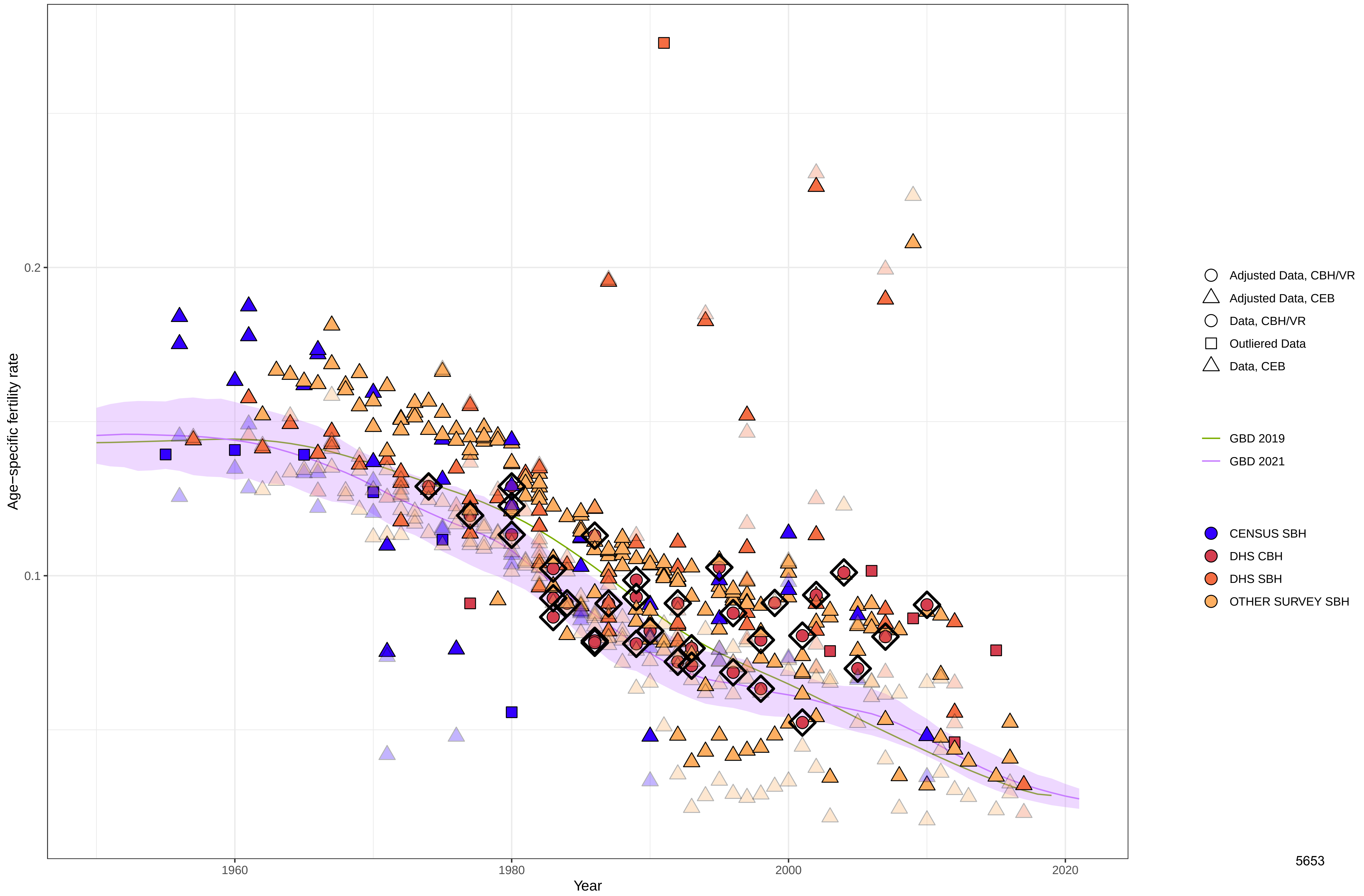

Age−specific fertility fitted trend lines, 1950−2021: Central Sulawesi, Ages: 20−24
Data points enclosed with a diamond are reference sources

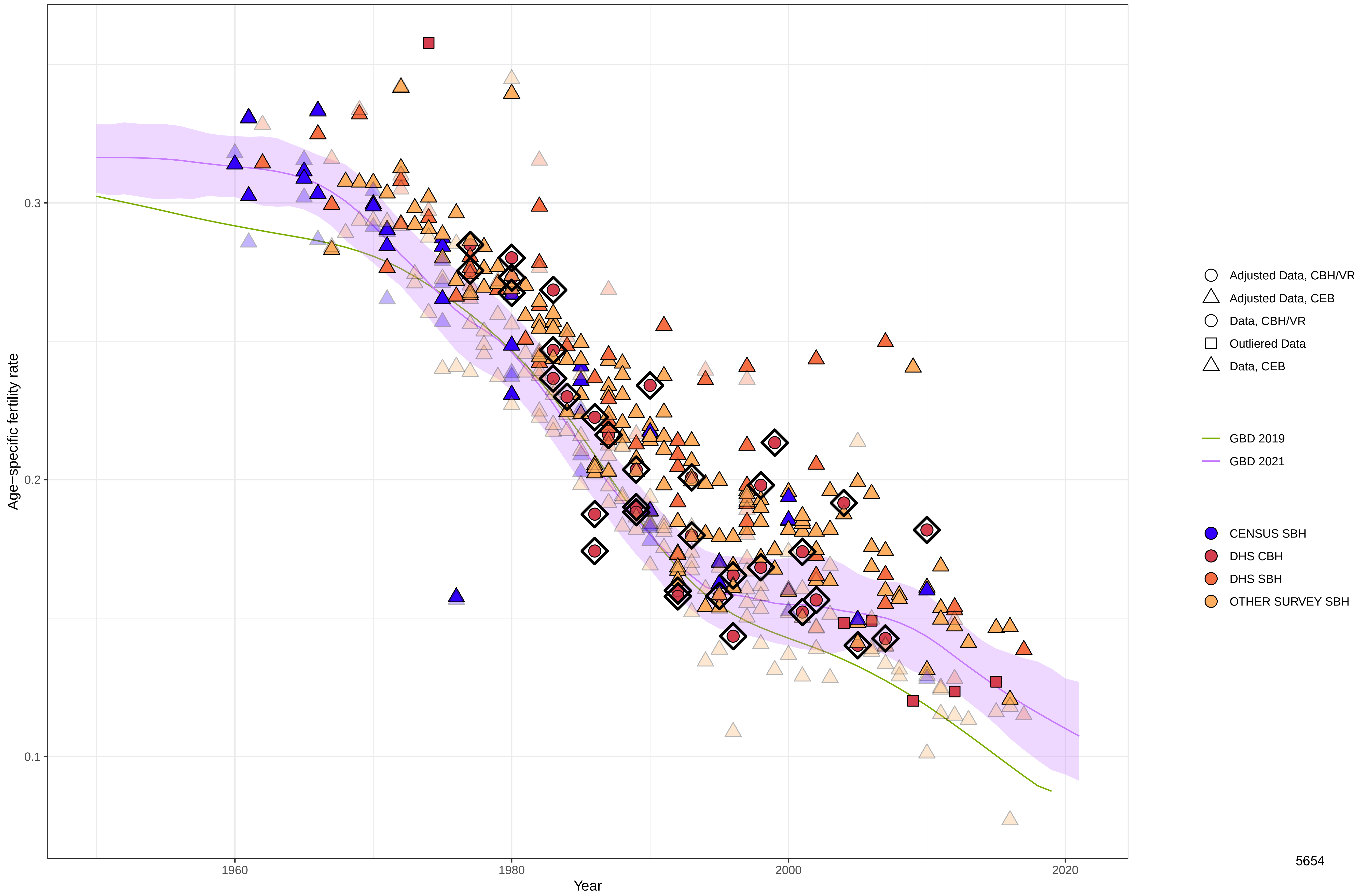

Age−specific fertility fitted trend lines, 1950−2021: Central Sulawesi, Ages: 25−29
Data points enclosed with a diamond are reference sources

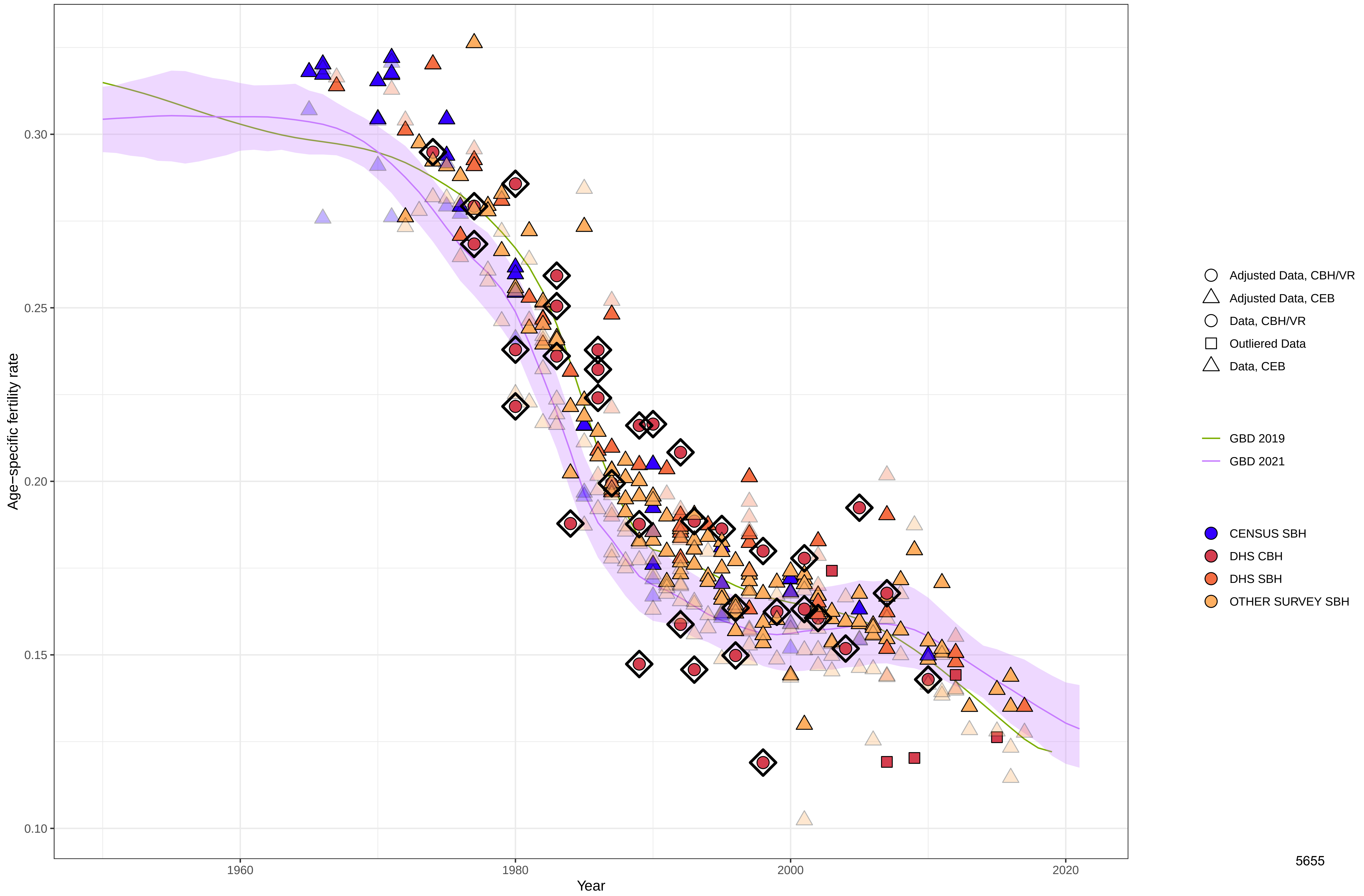

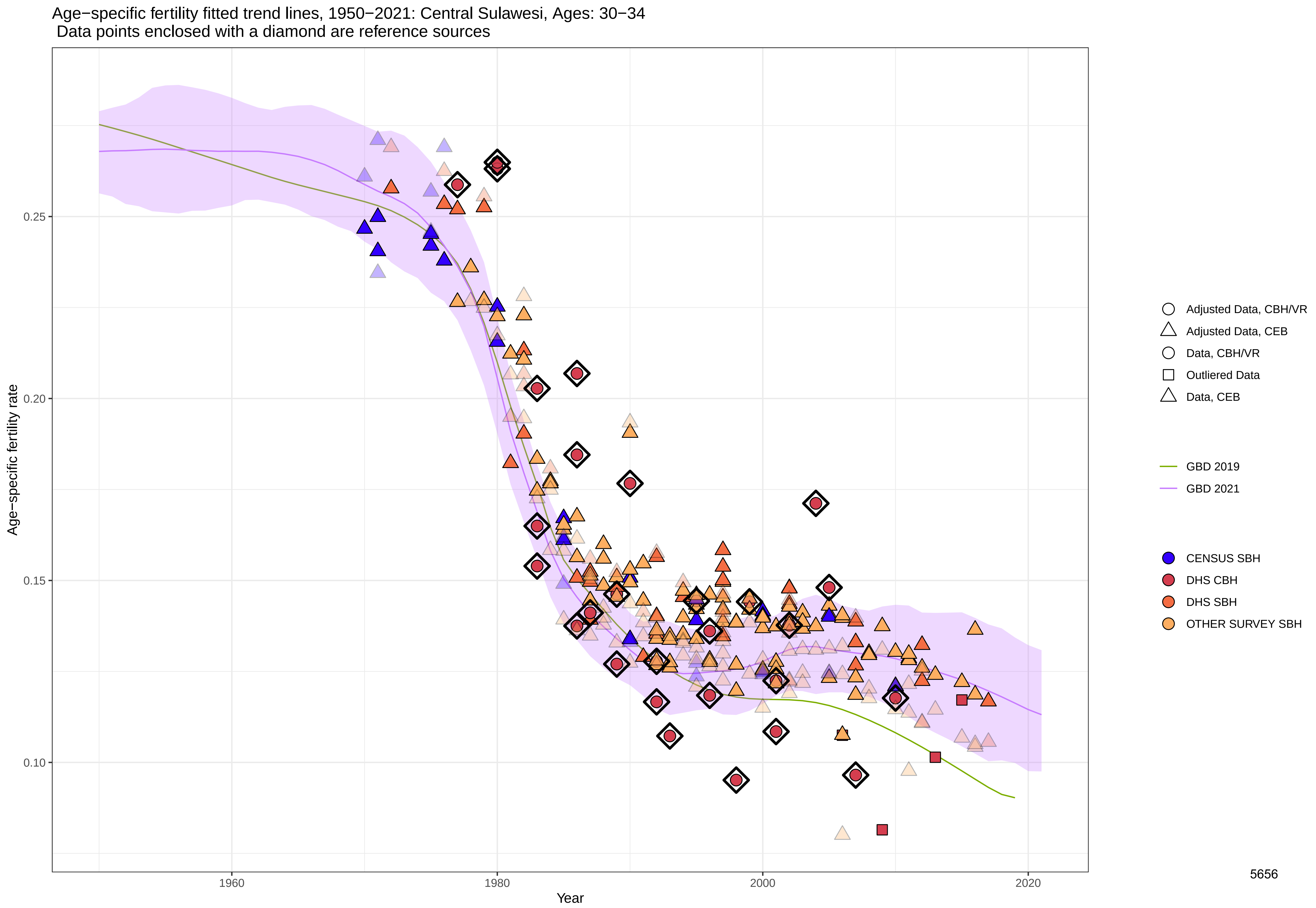

Age−specific fertility fitted trend lines, 1950−2021: Central Sulawesi, Ages: 30−34
Data points enclosed with a diamond are reference sources
Age−specific fertility rate
0.10
0.15
0.20
0.25
Year
1960
1980
2000
2020
Adjusted Data, CBH/VR
Adjusted Data, CEB
Data, CBH/VR
Outliered Data
Data, CEB
GBD 2019
GBD 2021
CENSUS SBH
DHS CBH
DHS SBH
OTHER SURVEY SBH

Age−specific fertility fitted trend lines, 1950−2021: Central Sulawesi, Ages: 35−39
Data points enclosed with a diamond are reference sources

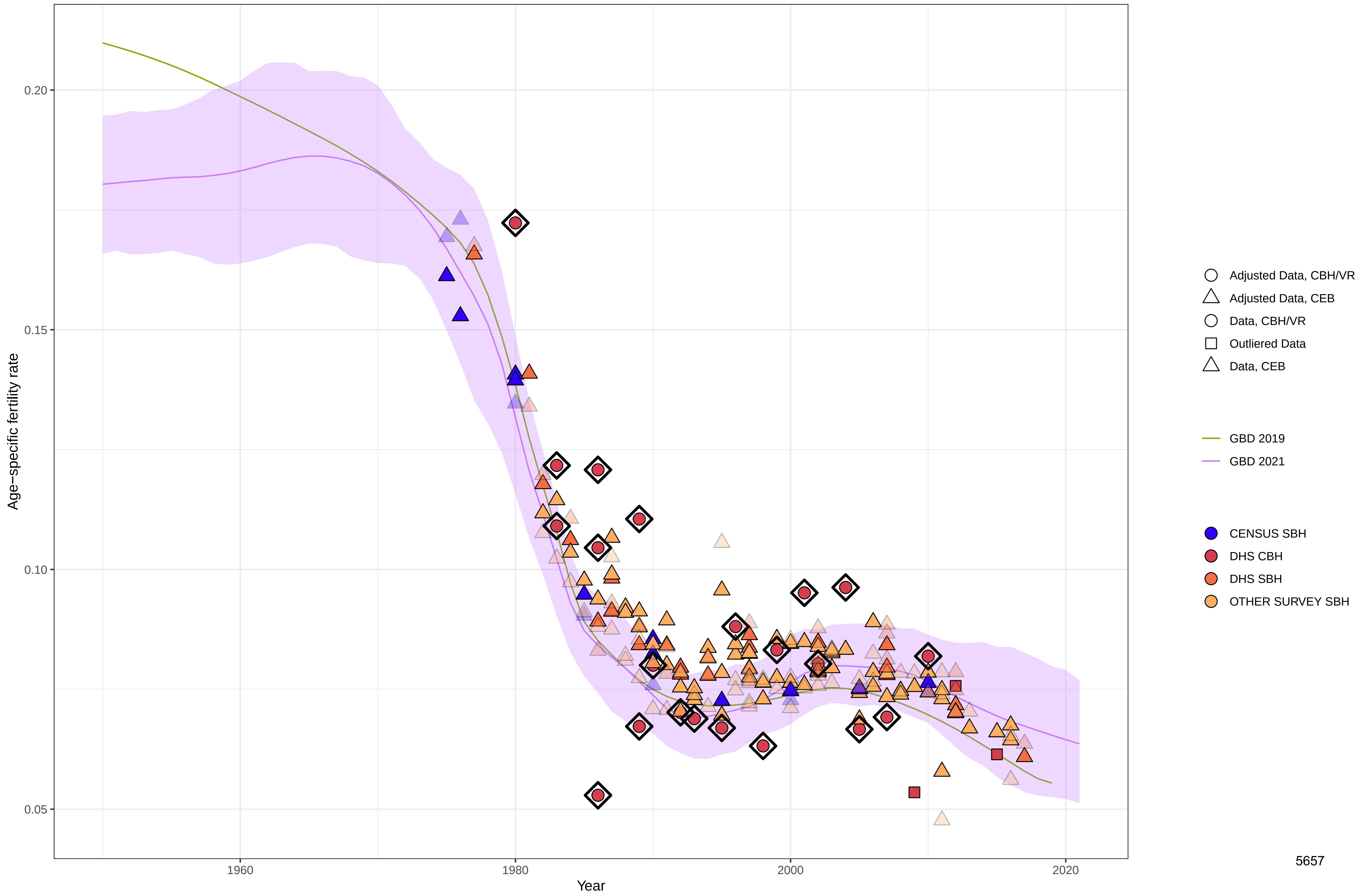

Age−specific fertility fitted trend lines, 1950−2021: Central Sulawesi, Ages: 40−44
Data points enclosed with a diamond are reference sources

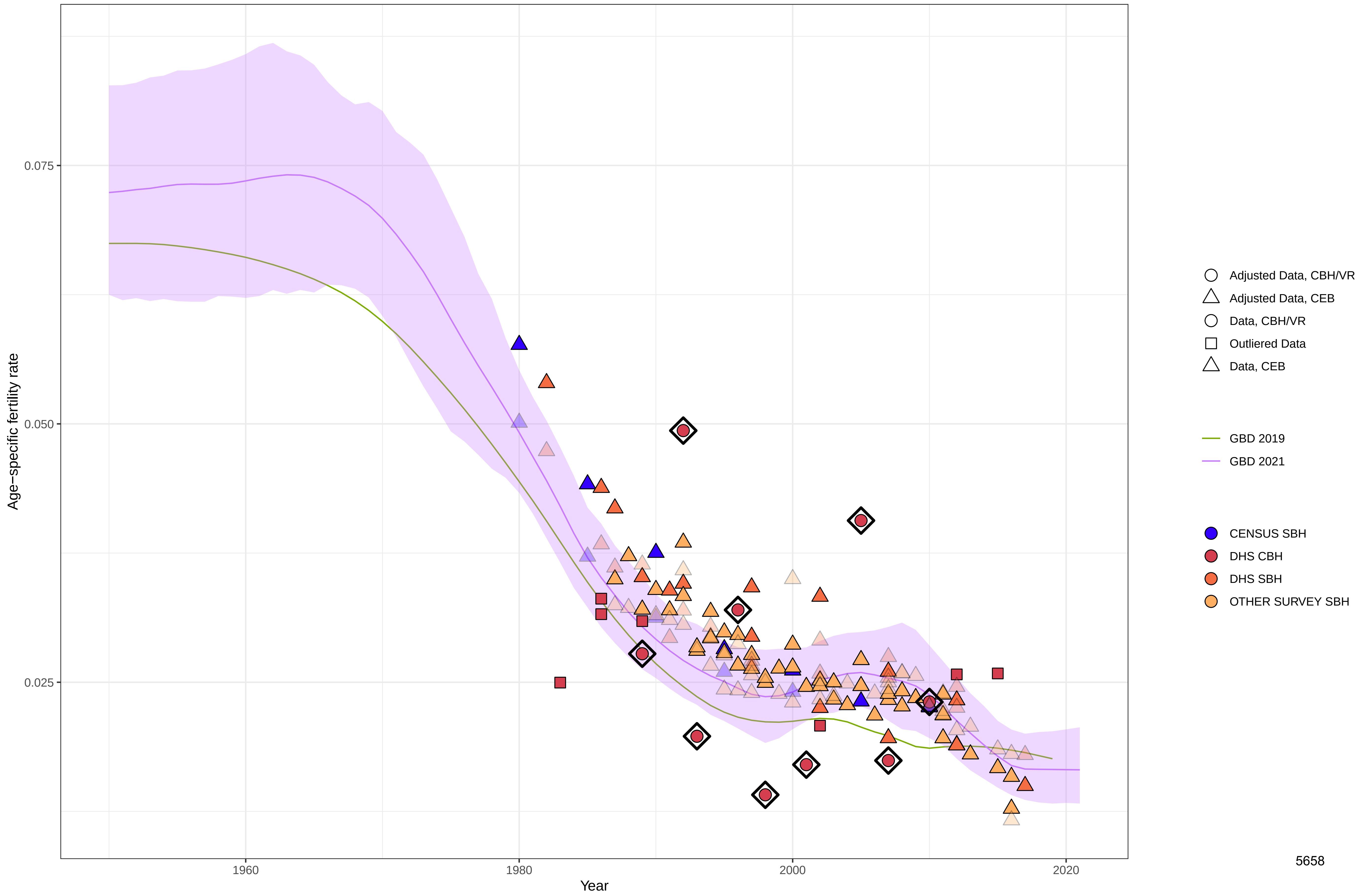

Age−specific fertility fitted trend lines, 1950−2021: Central Sulawesi, Ages: 45−49
Data points enclosed with a diamond are reference sources

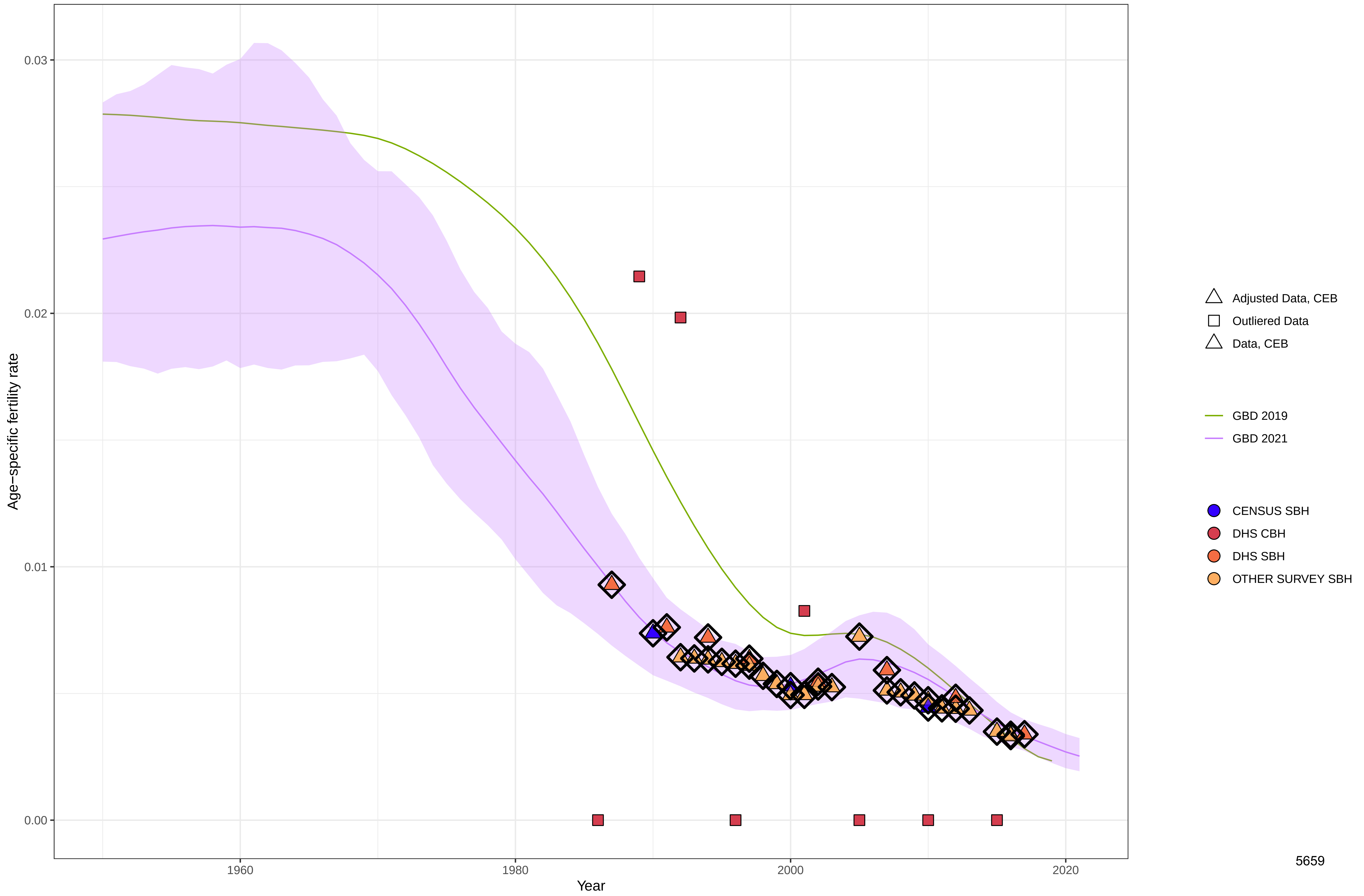

Age−specific fertility fitted trend lines, 1950−2021: Central Sulawesi, Ages: 50−54
Data points enclosed with a diamond are reference sources

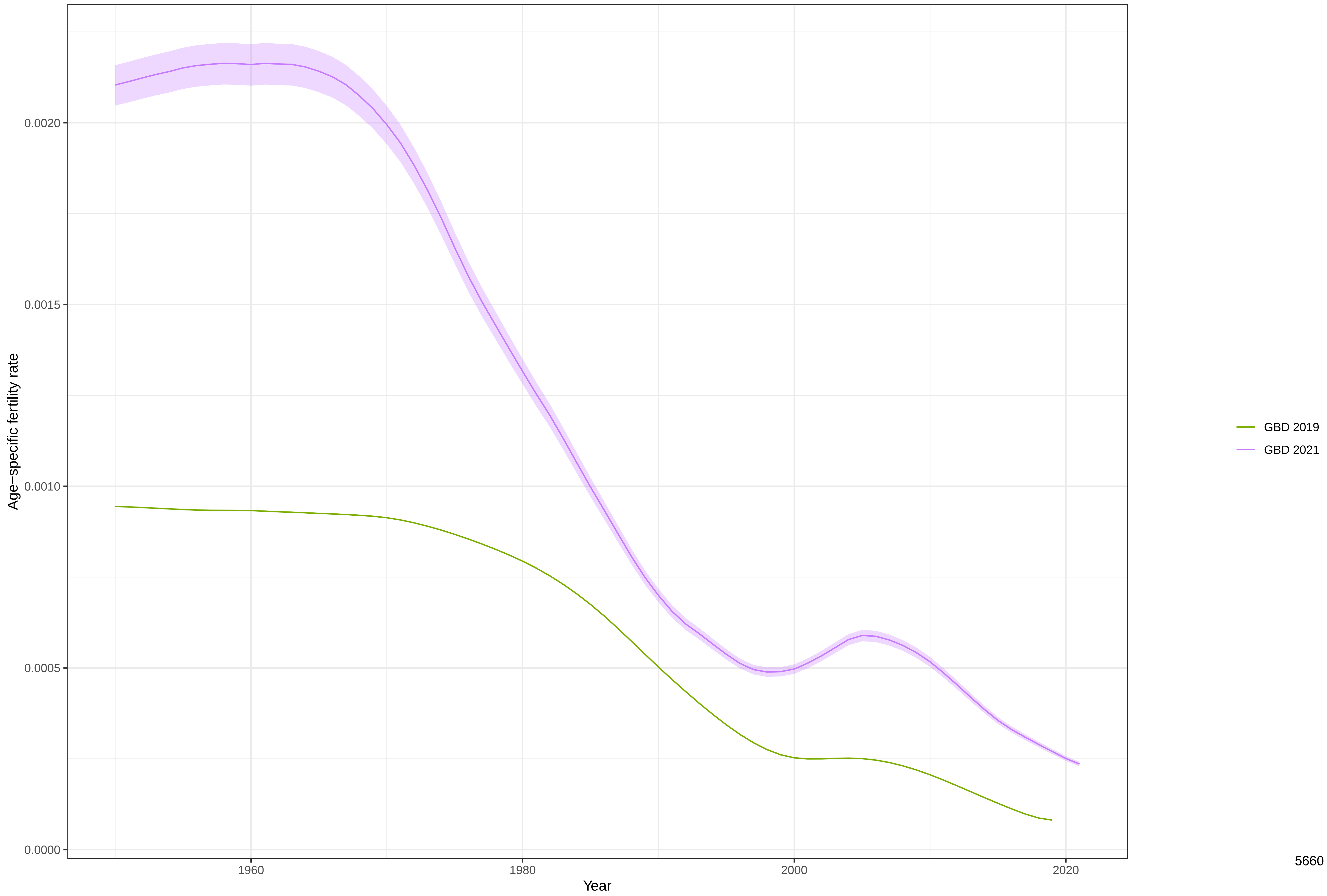

Total fertility rate fitted trend lines, 1950−2021: Central Sulawesi TFR
Data points enclosed with a diamond are reference sources

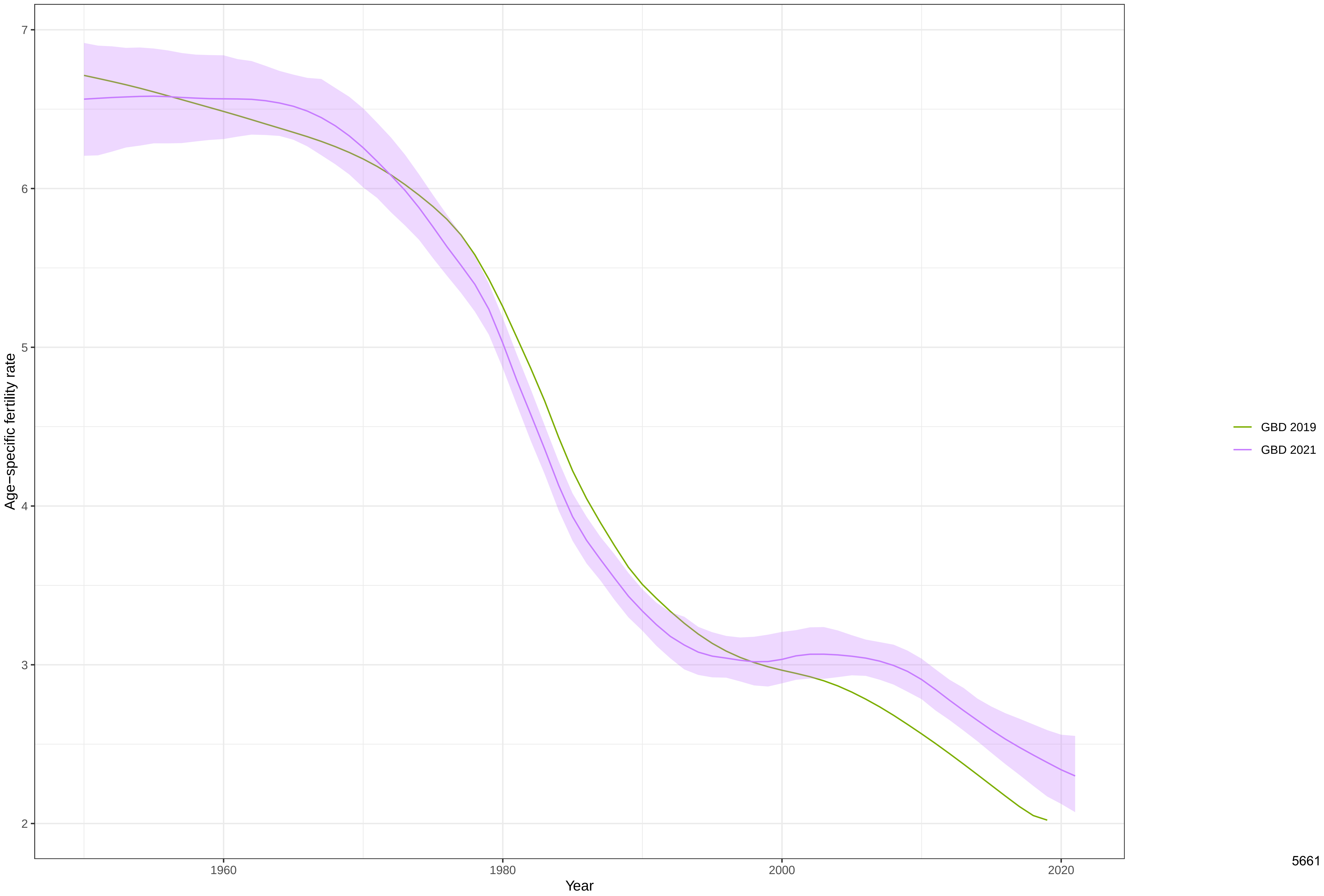

Age−specific fertility fitted trend lines, 1950−2021: Southeast Sulawesi, Ages: 10−14
Data points enclosed with a diamond are reference sources

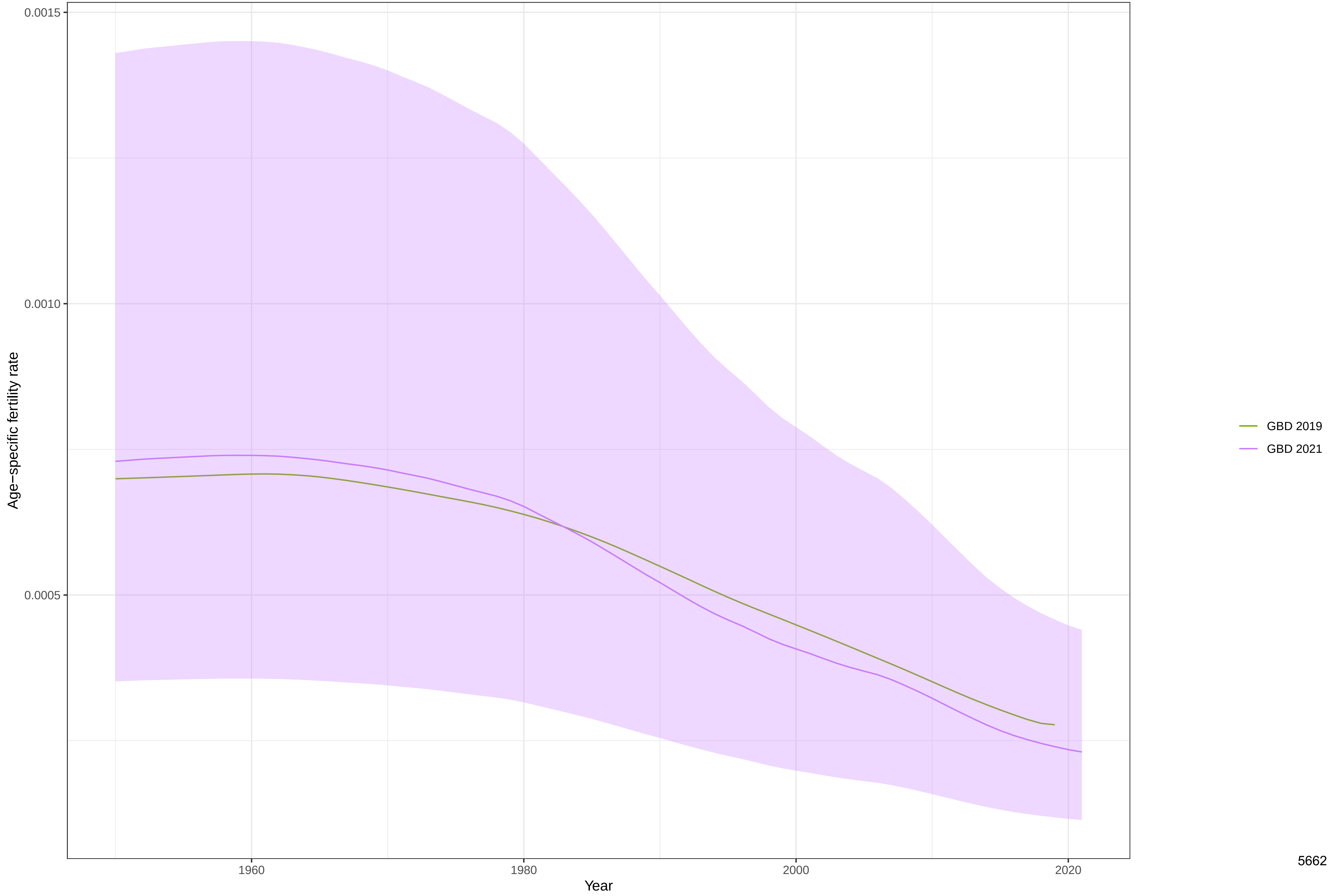

5662

Age−specific fertility fitted trend lines, 1950−2021: Southeast Sulawesi, Ages: 15−19
Data points enclosed with a diamond are reference sources

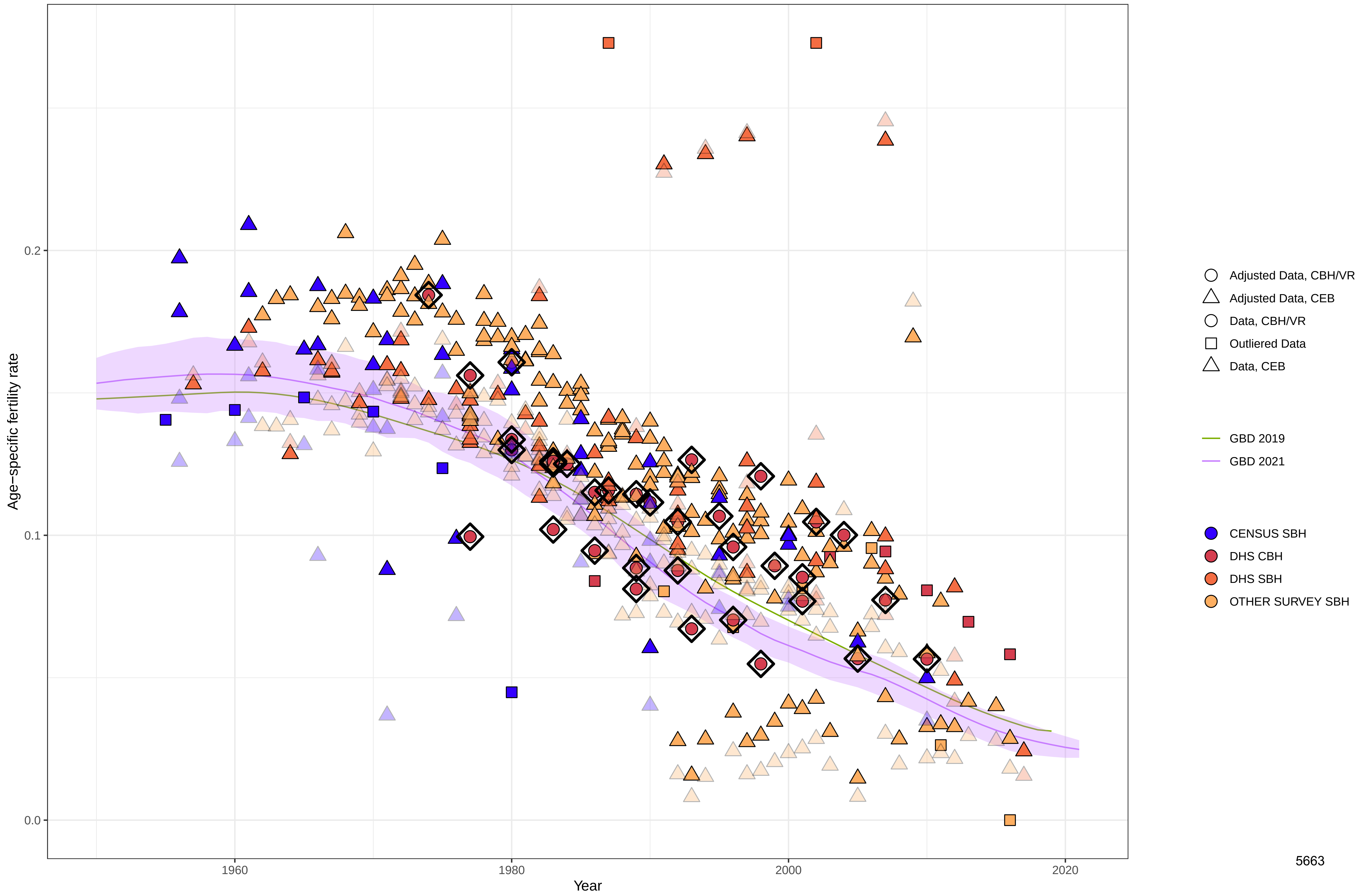

Age−specific fertility fitted trend lines, 1950−2021: Southeast Sulawesi, Ages: 20−24
Data points enclosed with a diamond are reference sources

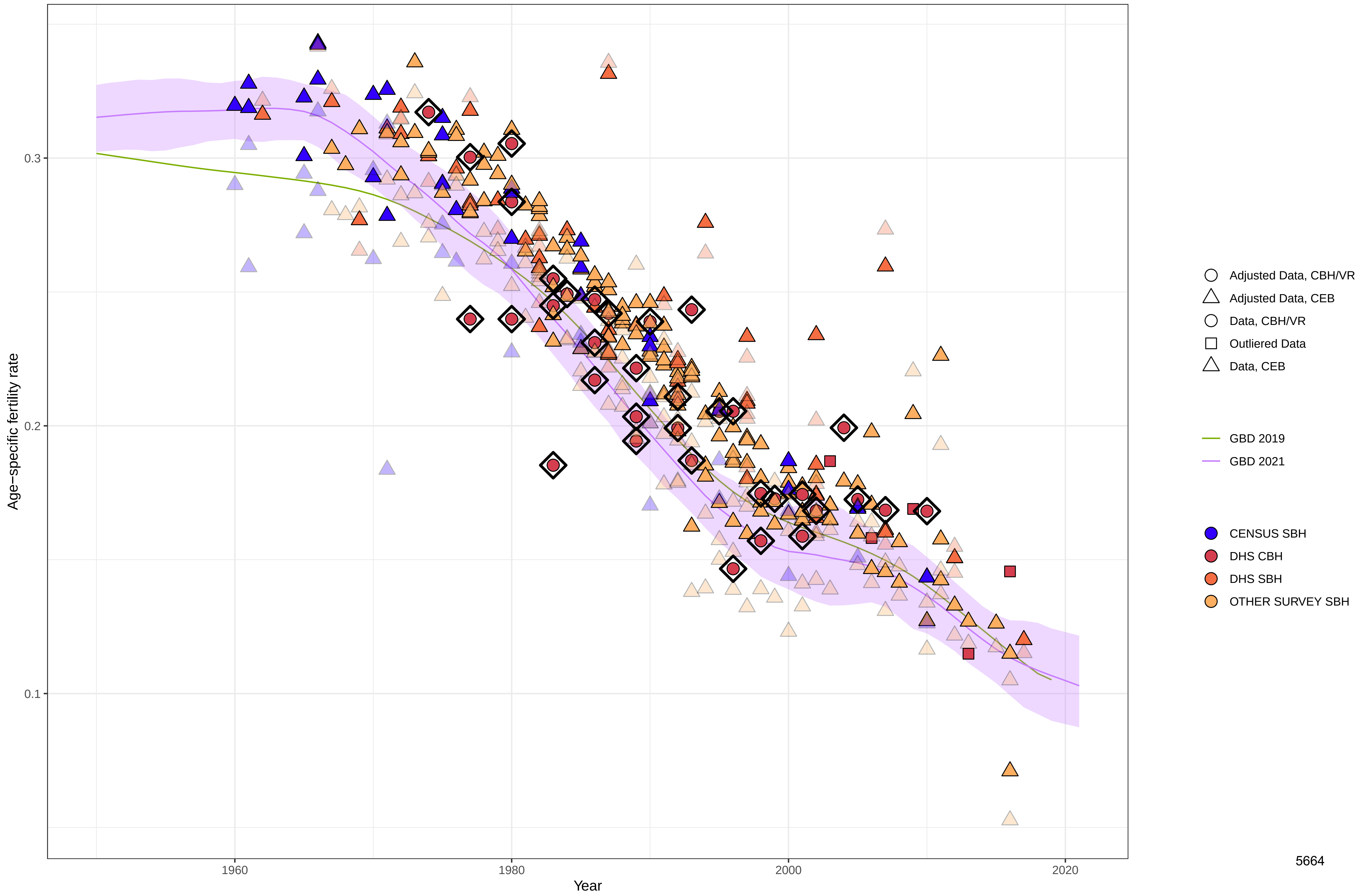

Age−specific fertility fitted trend lines, 1950−2021: Southeast Sulawesi, Ages: 25−29
Data points enclosed with a diamond are reference sources

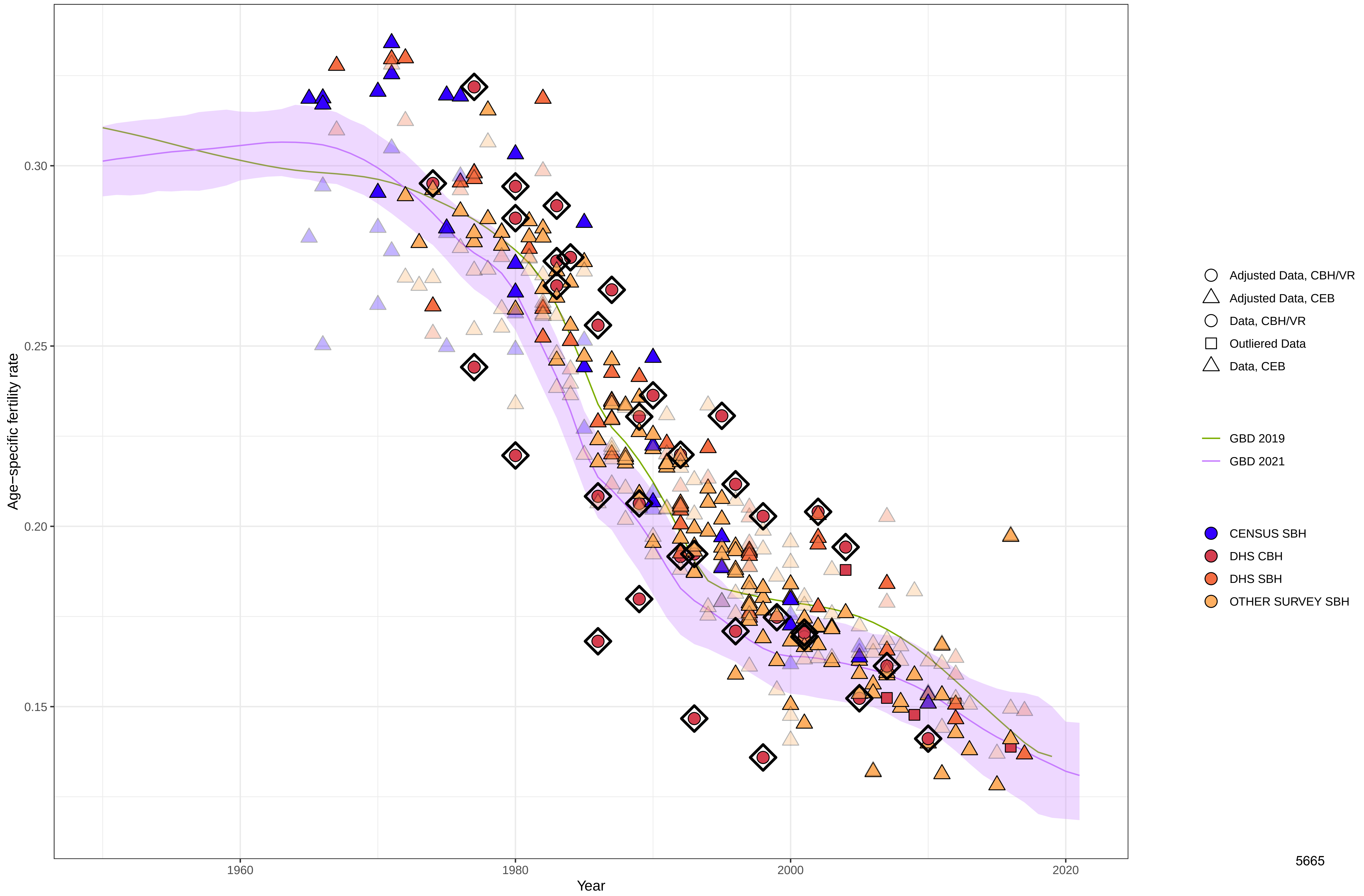

Age−specific fertility fitted trend lines, 1950−2021: Southeast Sulawesi, Ages: 30−34
Data points enclosed with a diamond are reference sources

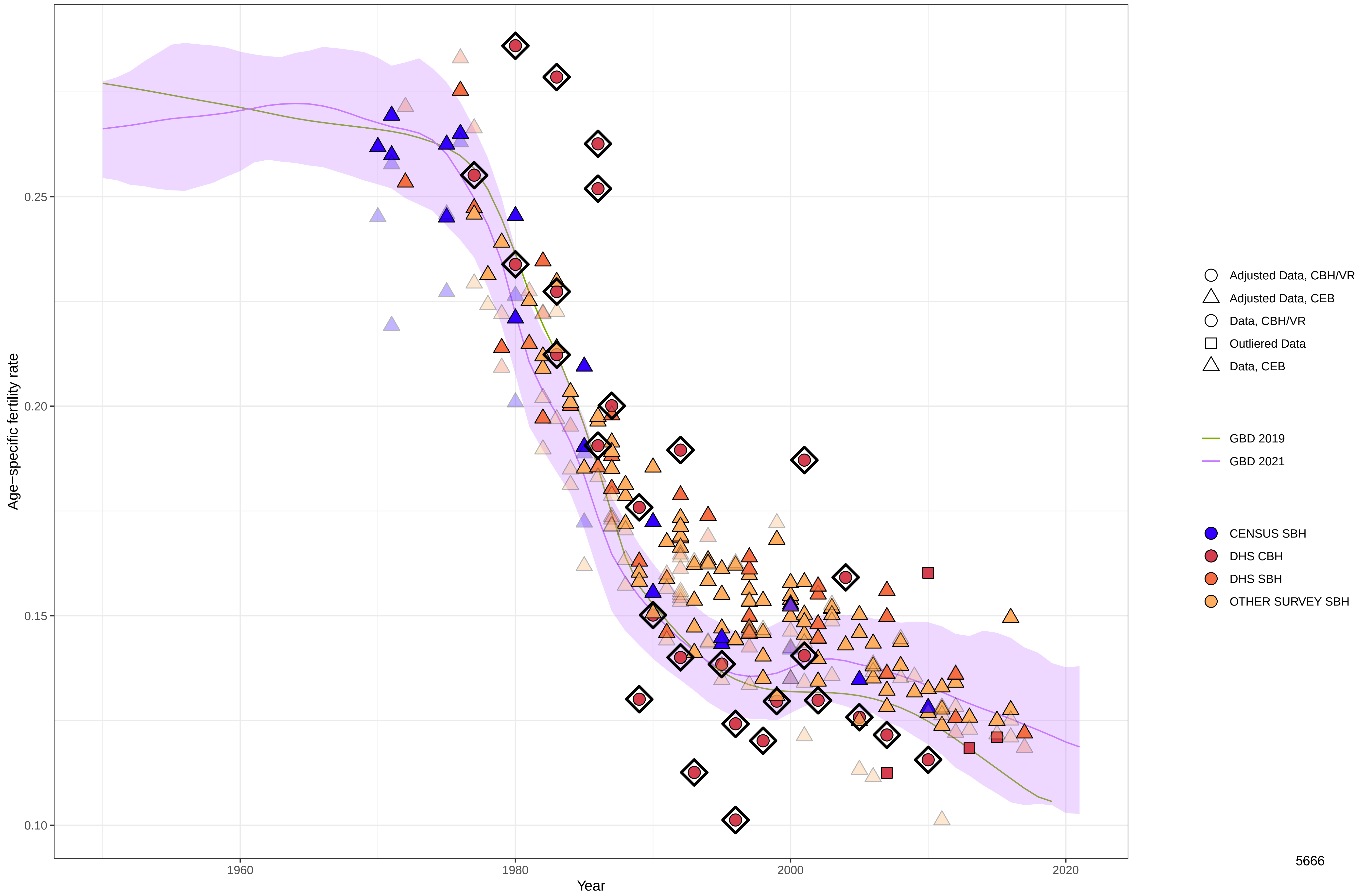

Age−specific fertility fitted trend lines, 1950−2021: Southeast Sulawesi, Ages: 35−39
Data points enclosed with a diamond are reference sources

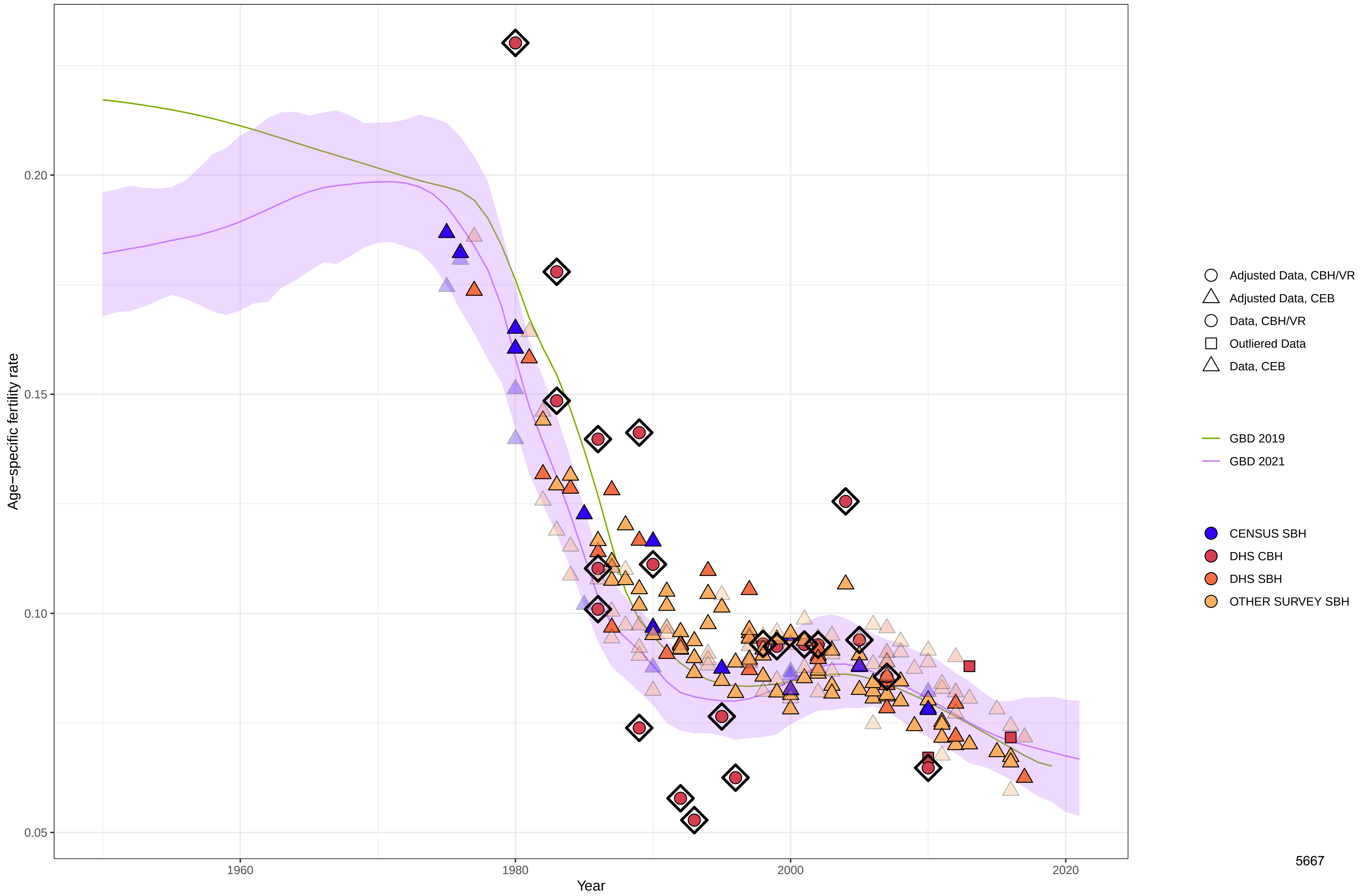

Age−specific fertility fitted trend lines, 1950−2021: Southeast Sulawesi, Ages: 40−44
Data points enclosed with a diamond are reference sources

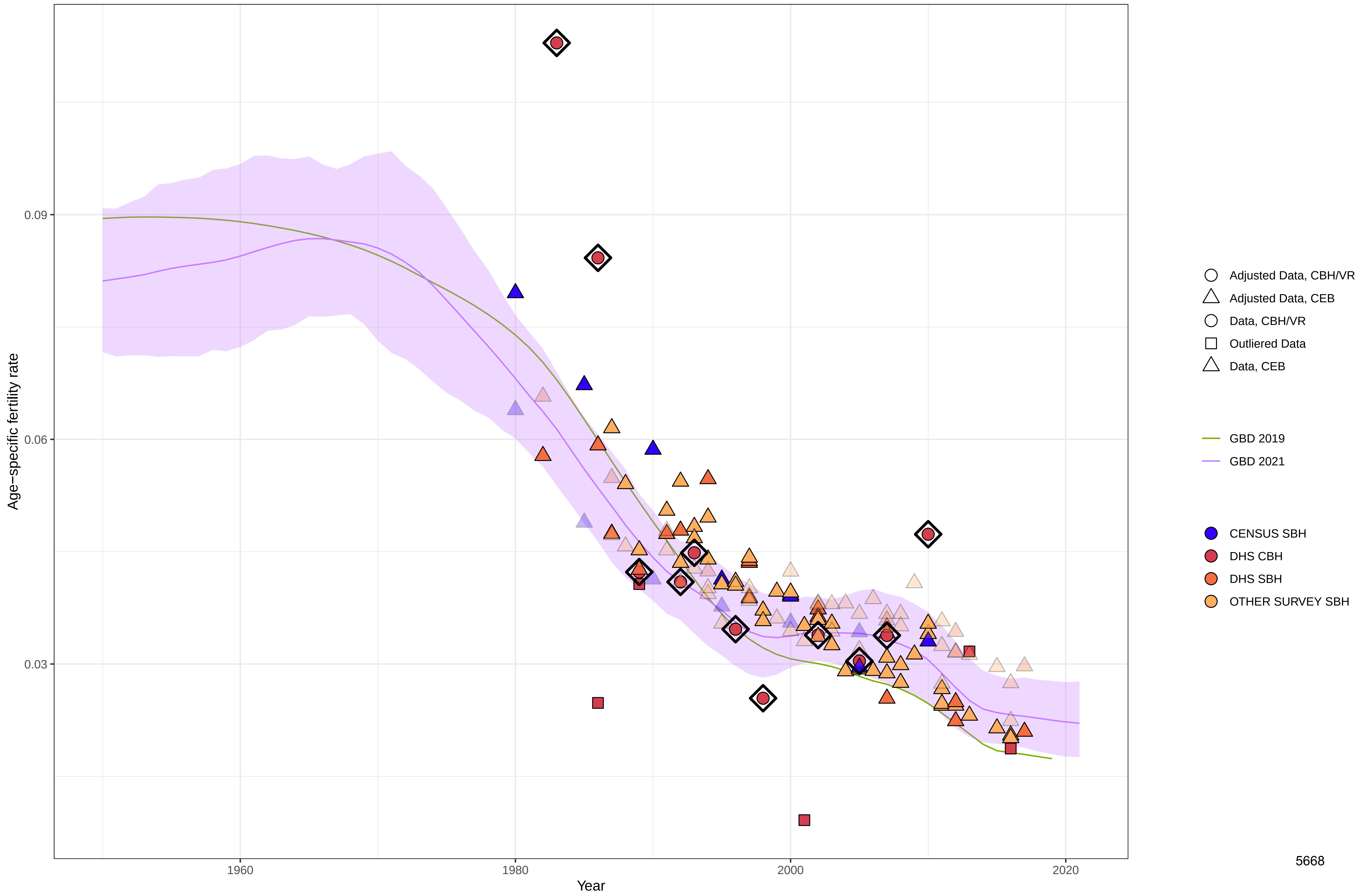

5668

Age−specific fertility fitted trend lines, 1950−2021: Southeast Sulawesi, Ages: 45−49
Data points enclosed with a diamond are reference sources

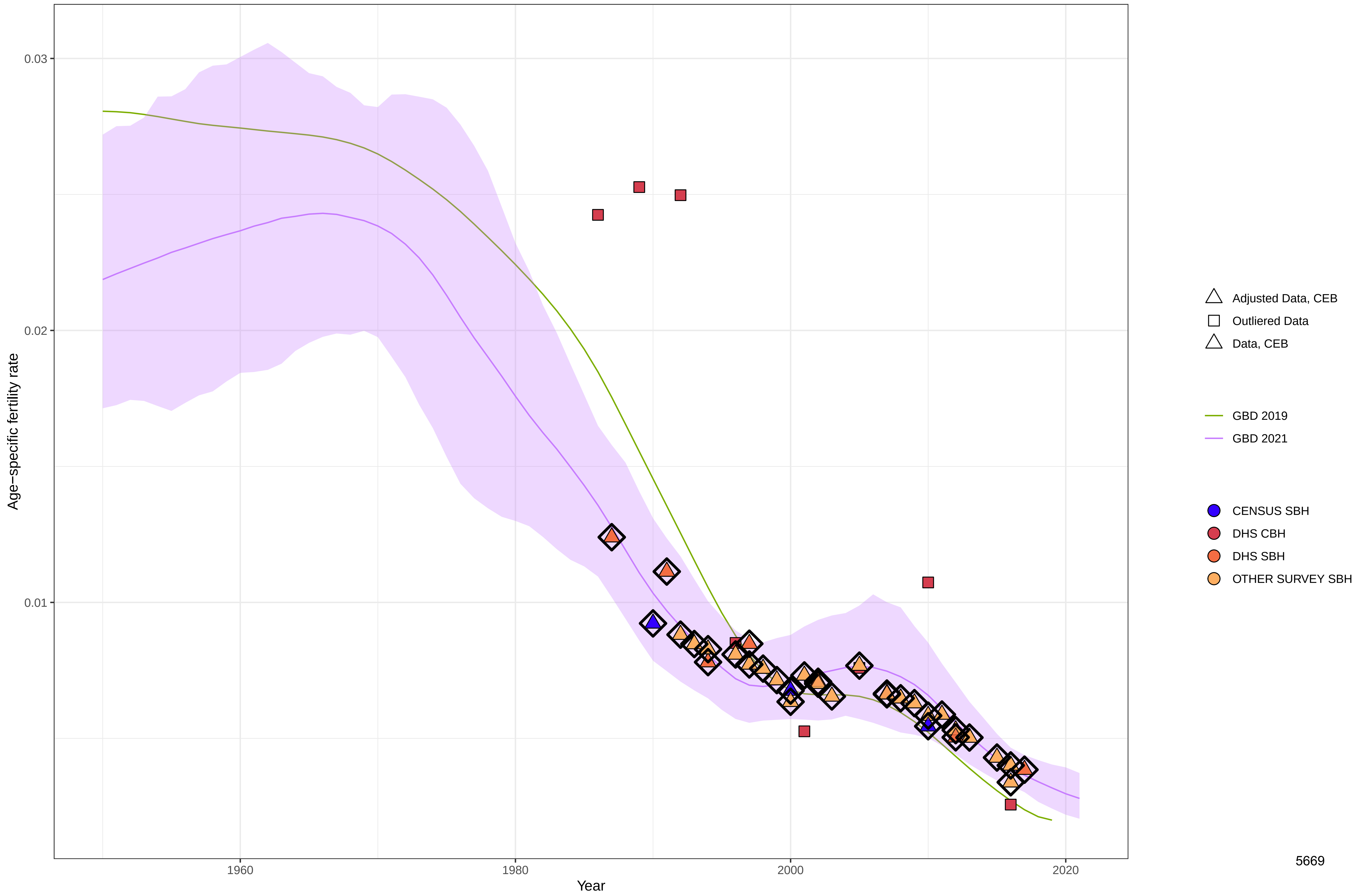

Age−specific fertility fitted trend lines, 1950−2021: Southeast Sulawesi, Ages: 50−54
Data points enclosed with a diamond are reference sources

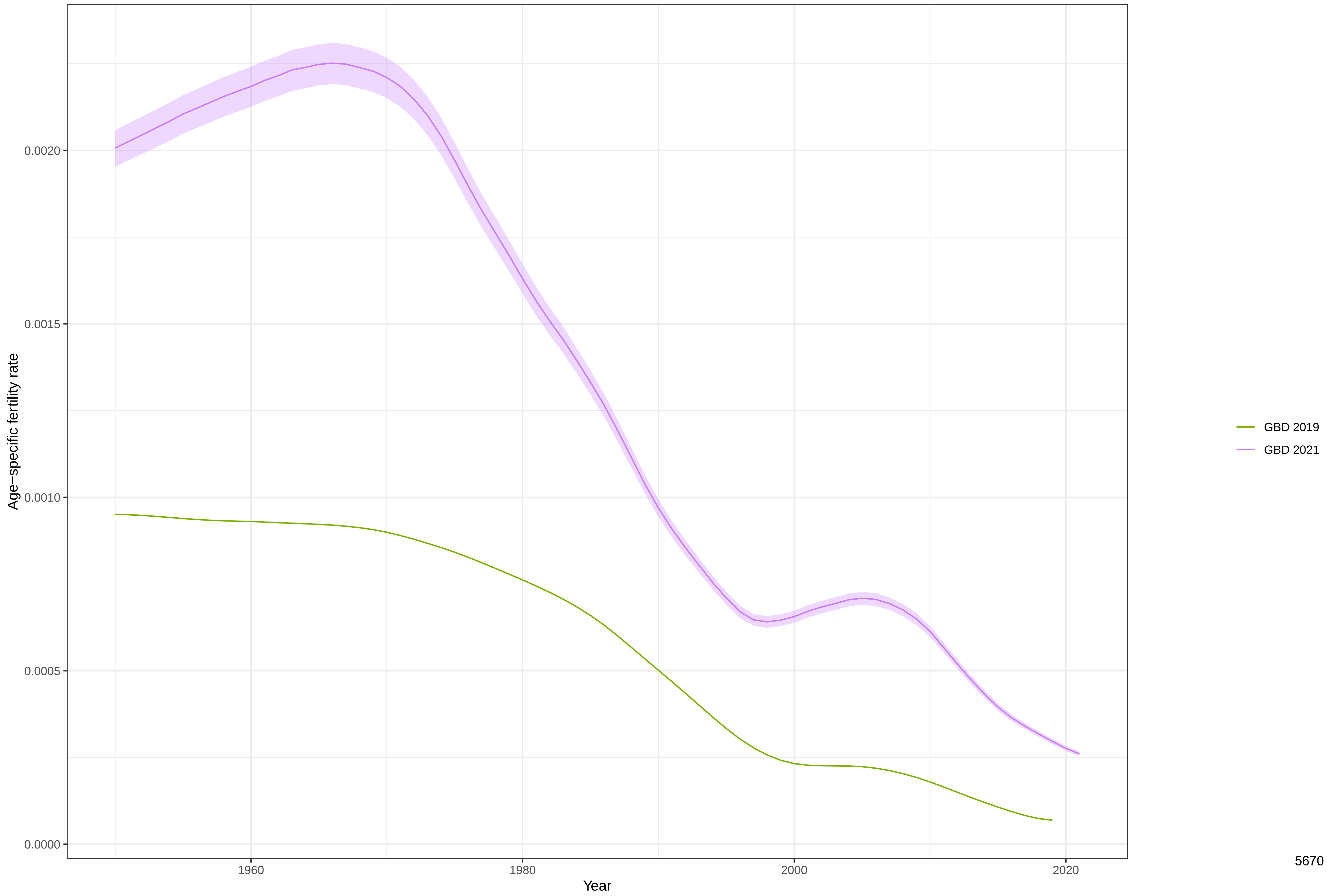

Total fertility rate fitted trend lines, 1950−2021: Southeast Sulawesi TFR
Data points enclosed with a diamond are reference sources

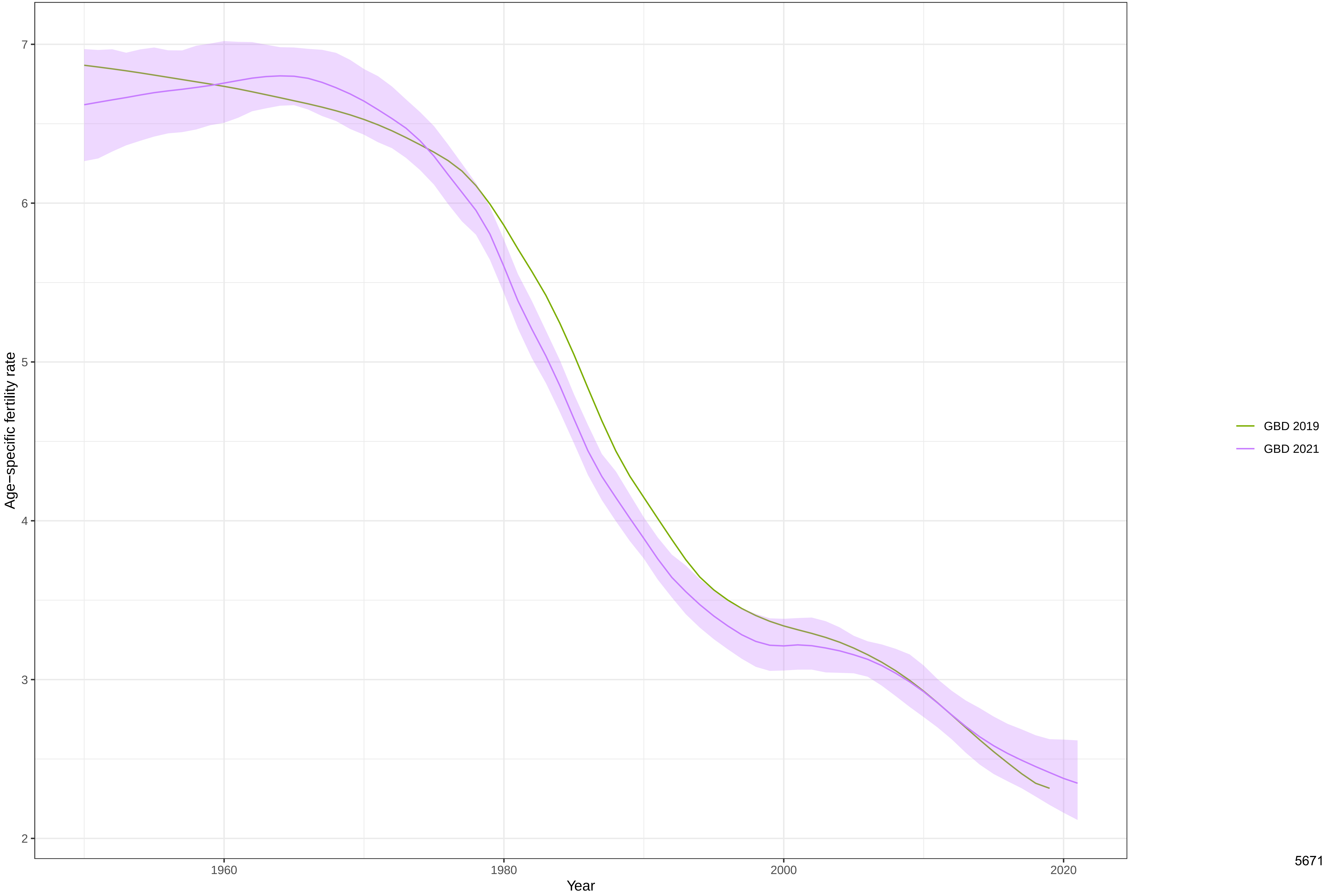

Age−specific fertility fitted trend lines, 1950−2021: North Sulawesi, Ages: 10−14
Data points enclosed with a diamond are reference sources

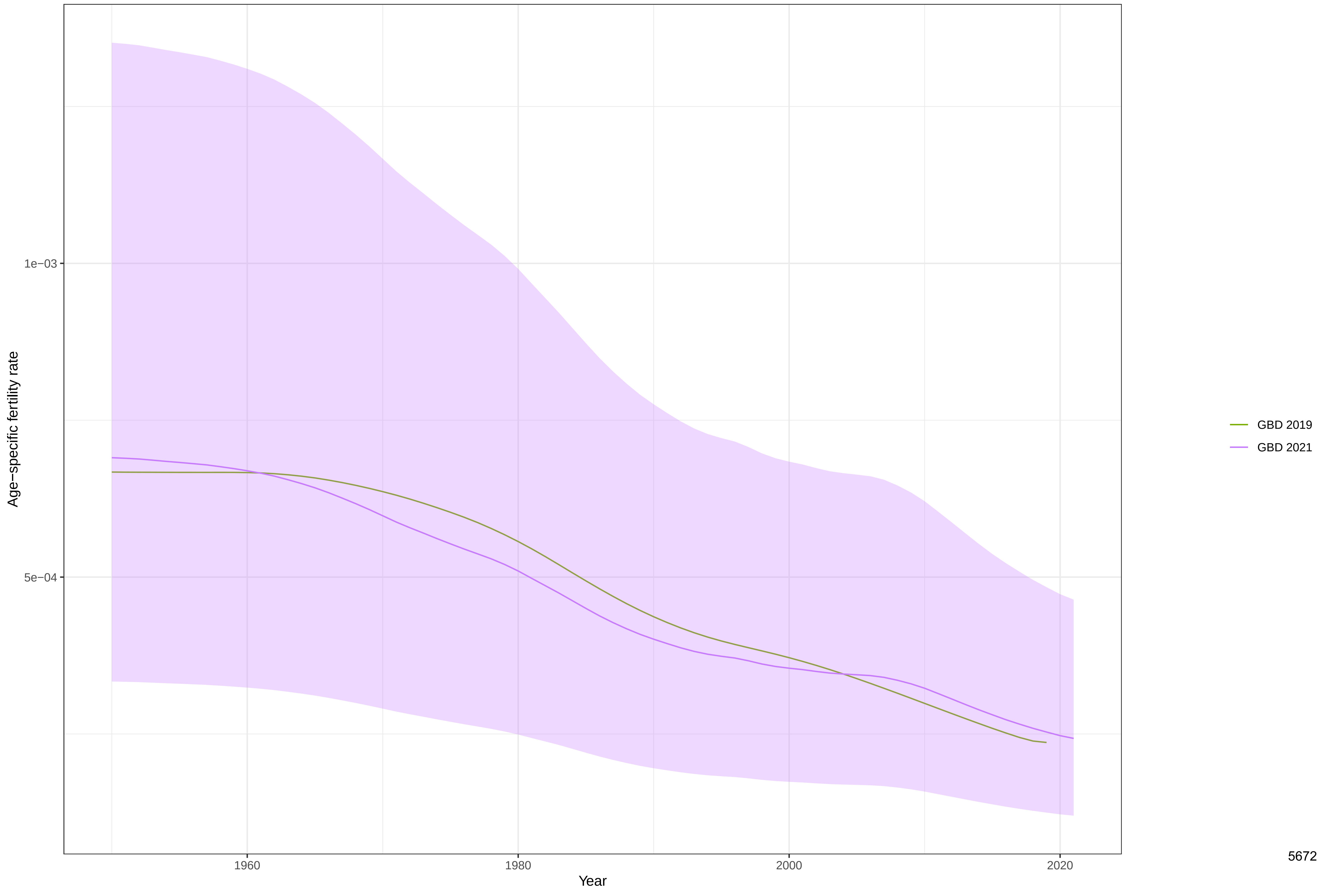

Age−specific fertility fitted trend lines, 1950−2021: North Sulawesi, Ages: 15−19
Data points enclosed with a diamond are reference sources

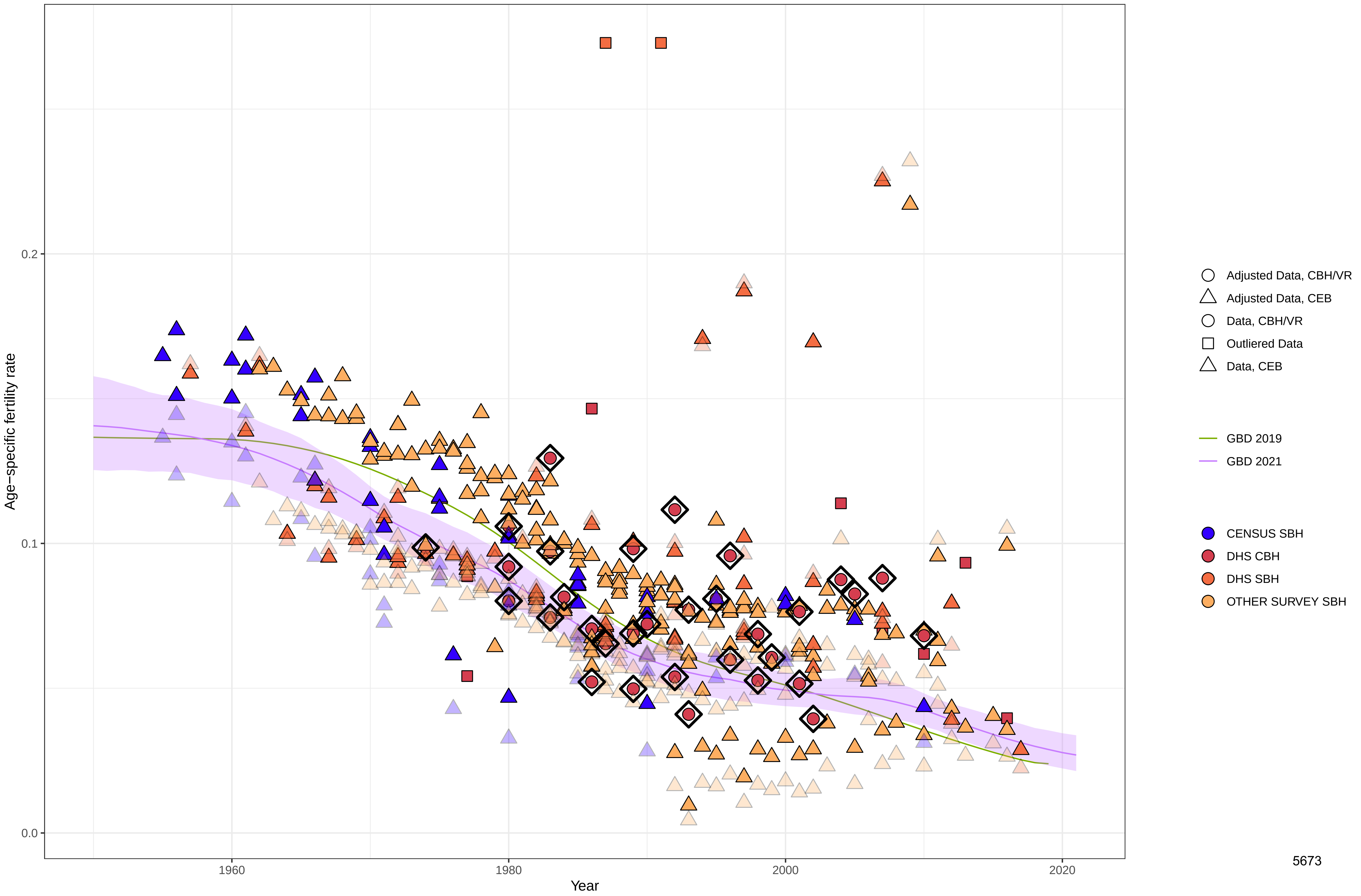

Age−specific fertility fitted trend lines, 1950−2021: North Sulawesi, Ages: 20−24
Data points enclosed with a diamond are reference sources

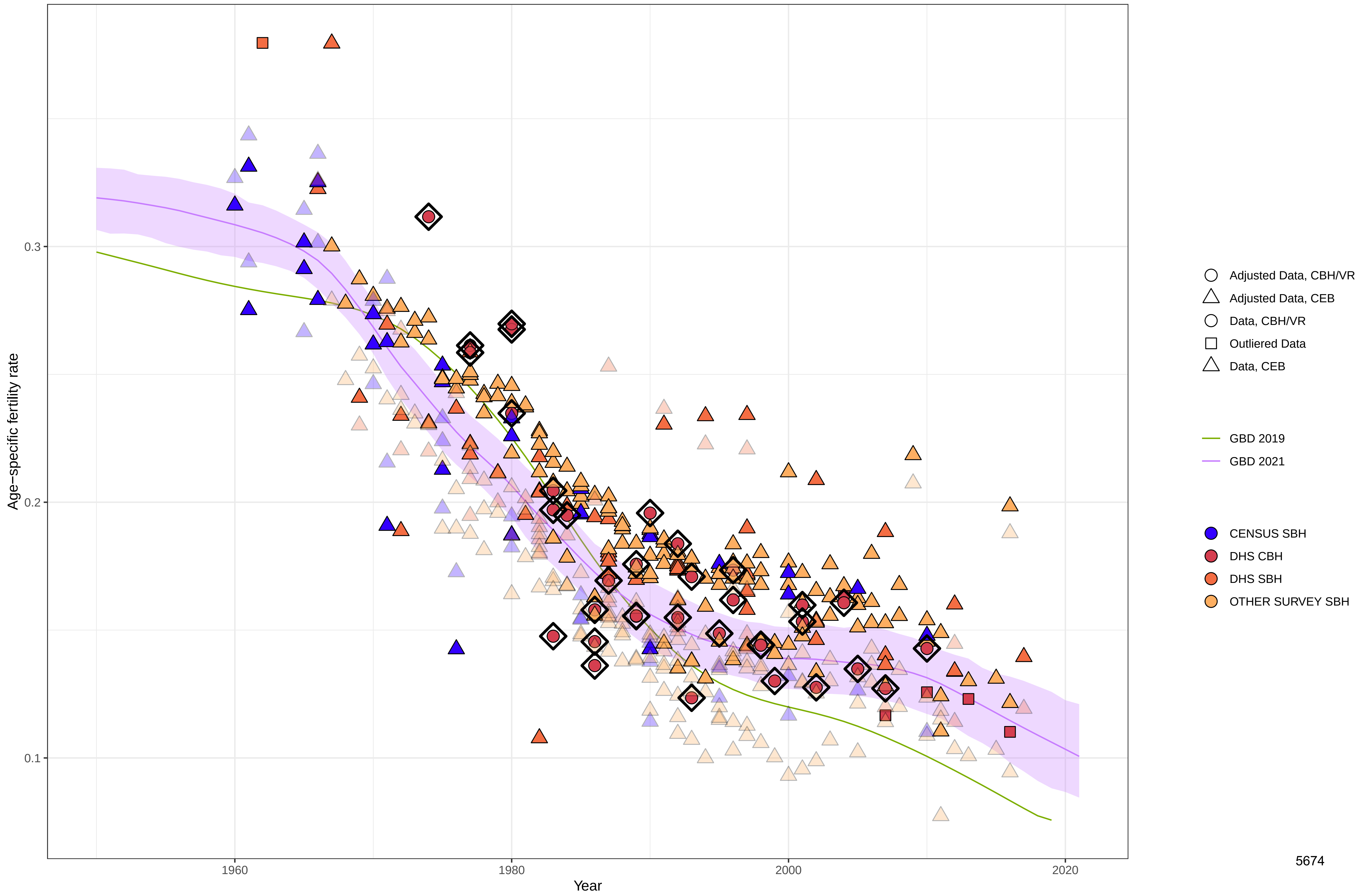

Age−specific fertility fitted trend lines, 1950−2021: North Sulawesi, Ages: 25−29
Data points enclosed with a diamond are reference sources

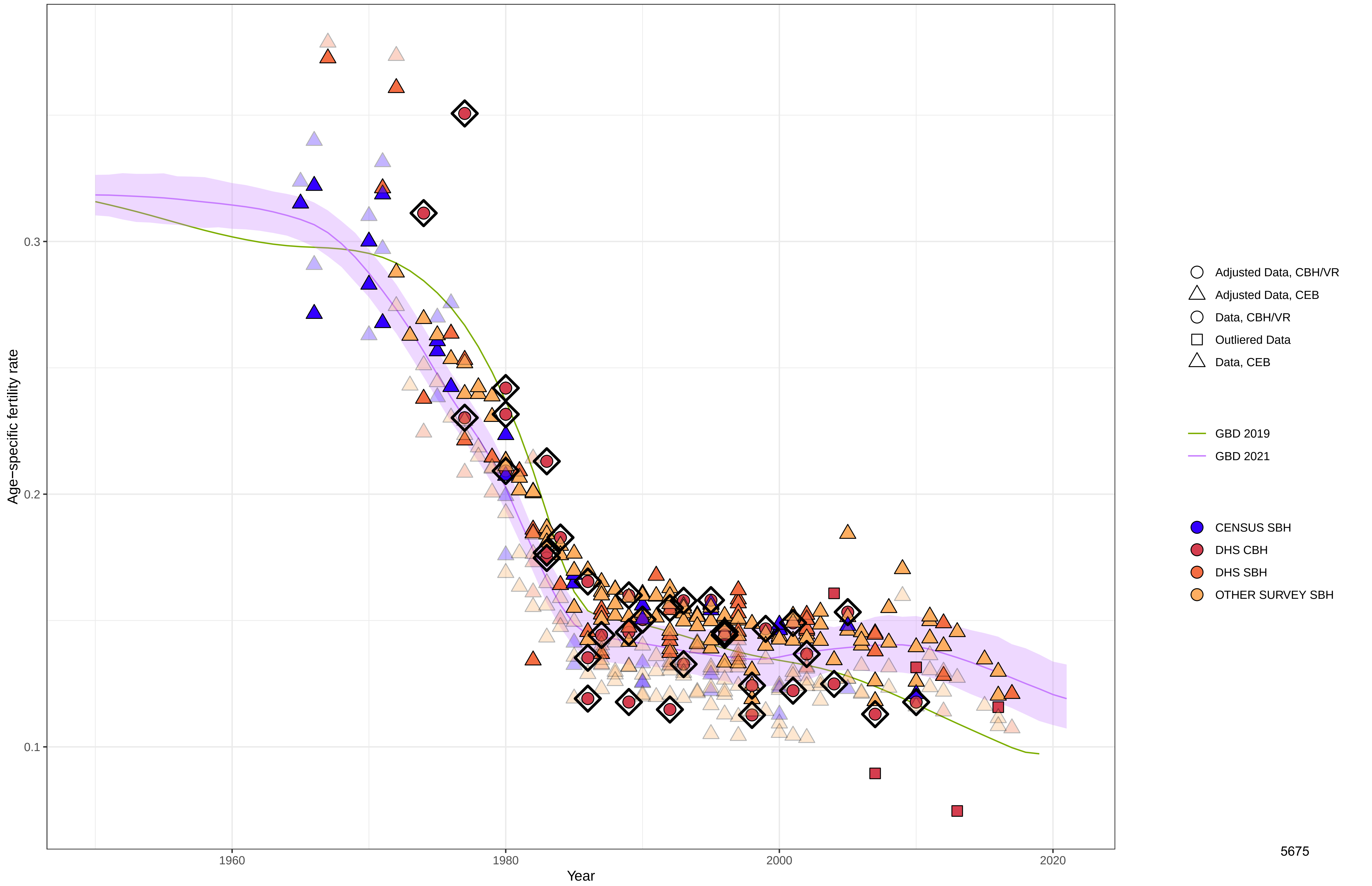

5675

Age−specific fertility fitted trend lines, 1950−2021: North Sulawesi, Ages: 30−34
Data points enclosed with a diamond are reference sources

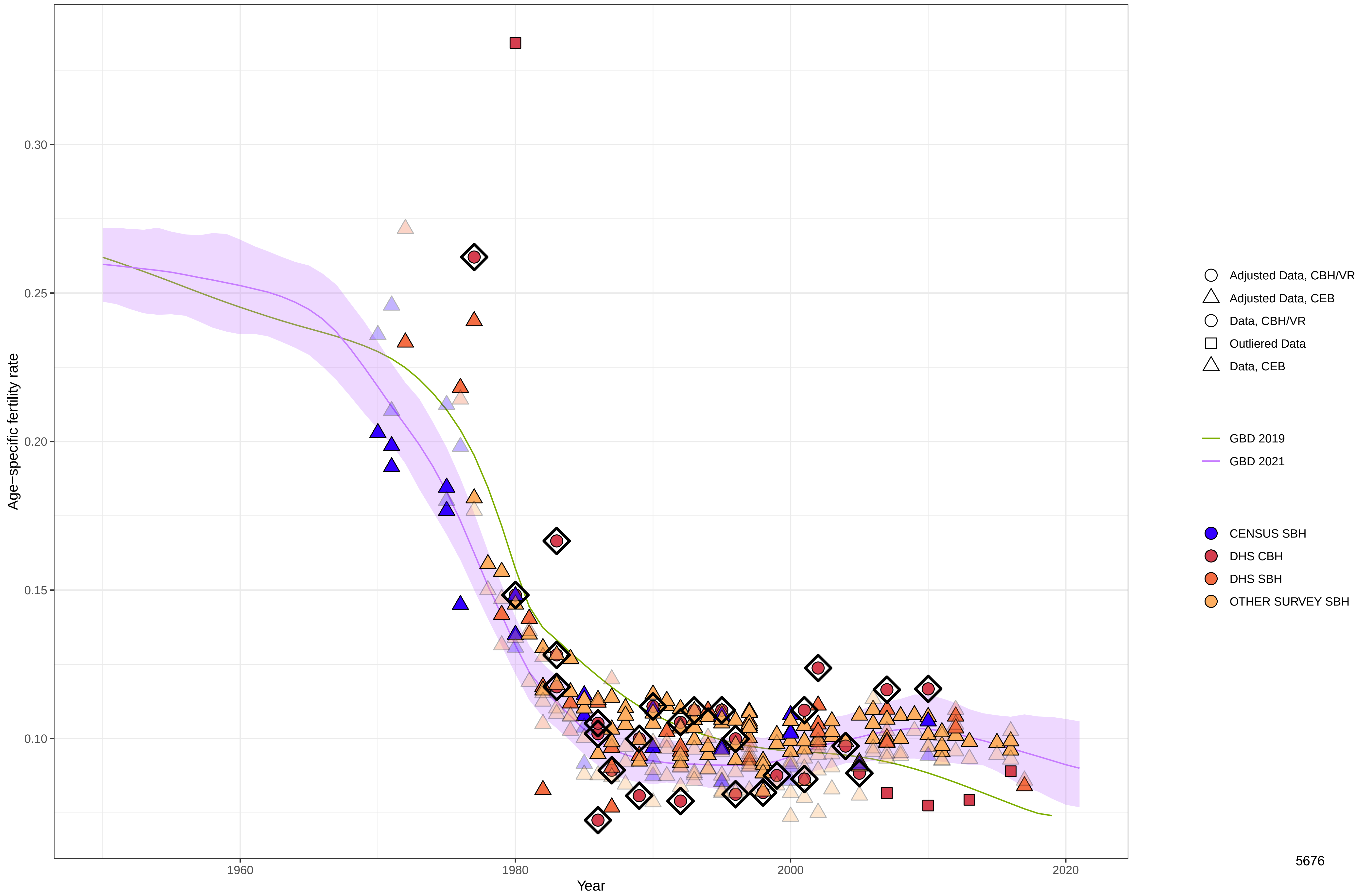

Age−specific fertility fitted trend lines, 1950−2021: North Sulawesi, Ages: 35−39
Data points enclosed with a diamond are reference sources

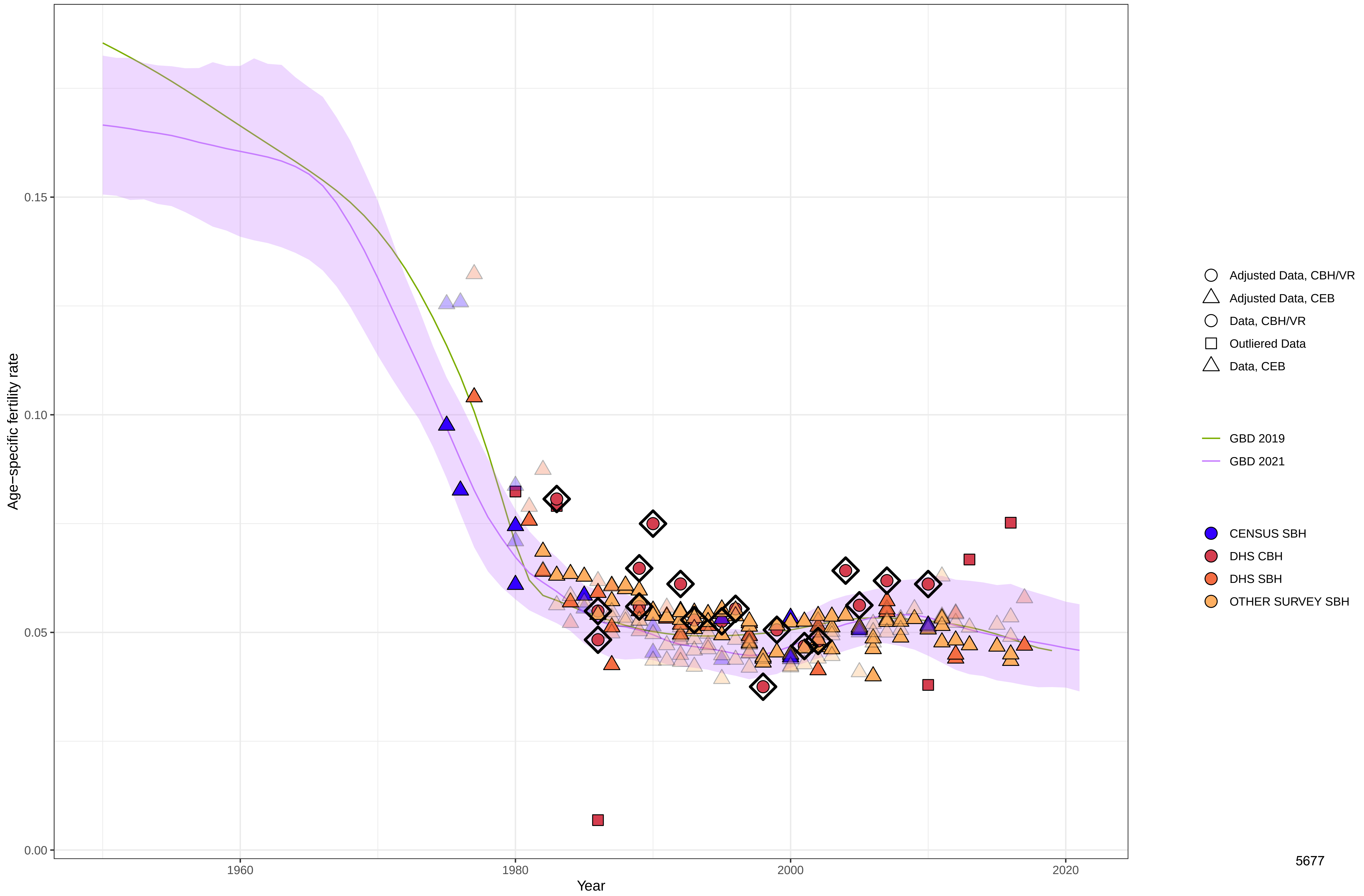

Age−specific fertility fitted trend lines, 1950−2021: North Sulawesi, Ages: 40−44
Data points enclosed with a diamond are reference sources

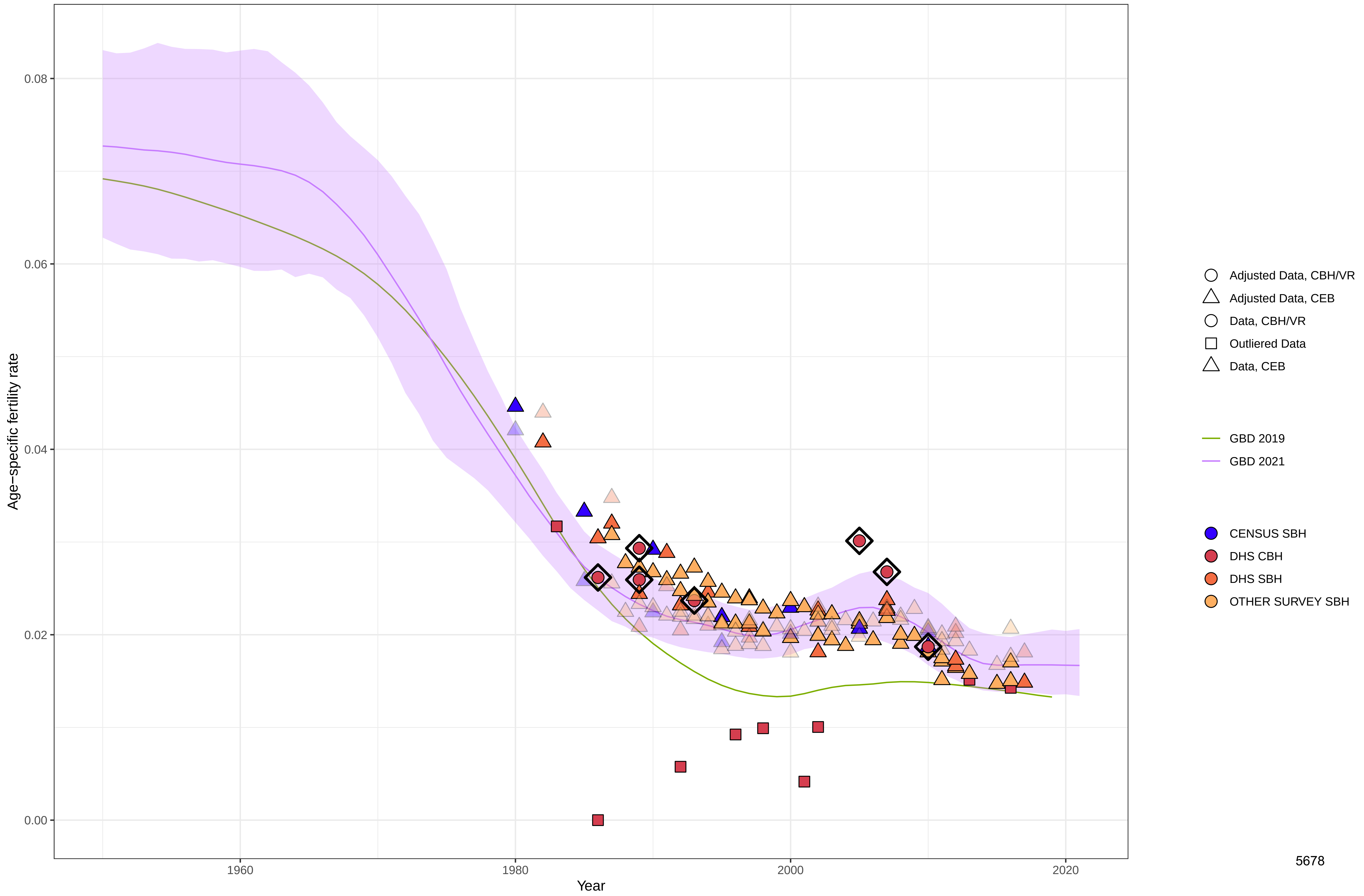

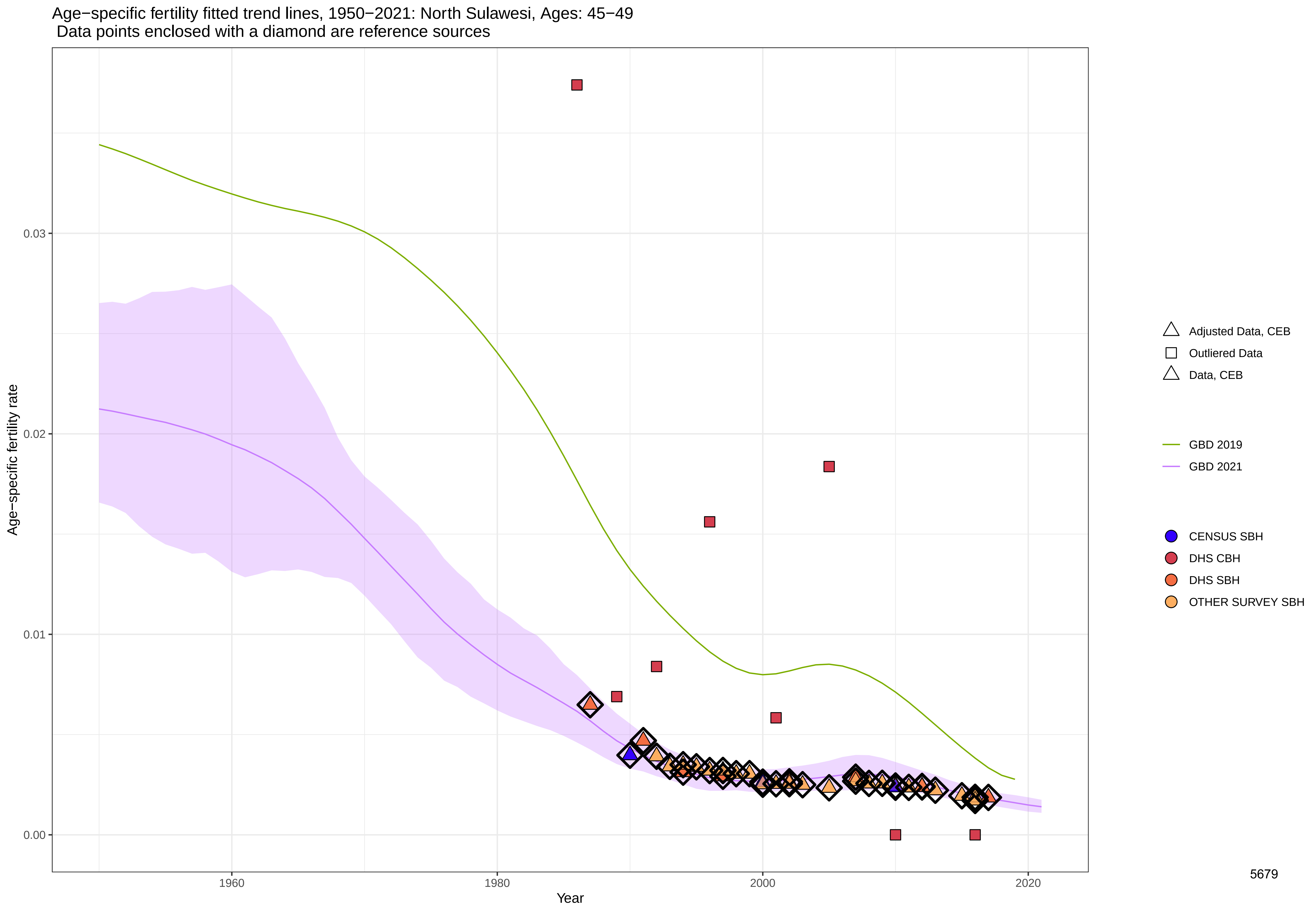

Age−specific fertility fitted trend lines, 1950−2021: North Sulawesi, Ages: 45−49
Data points enclosed with a diamond are reference sources
Age−specific fertility rate
0.00
0.01
0.02
0.03
1960
1980
2000
2020
Year
Adjusted Data, CEB
Outliered Data
Data, CEB
GBD 2019
GBD 2021
CENSUS SBH
DHS CBH
DHS SBH
OTHER SURVEY SBH

Age−specific fertility fitted trend lines, 1950−2021: North Sulawesi, Ages: 50−54
Data points enclosed with a diamond are reference sources

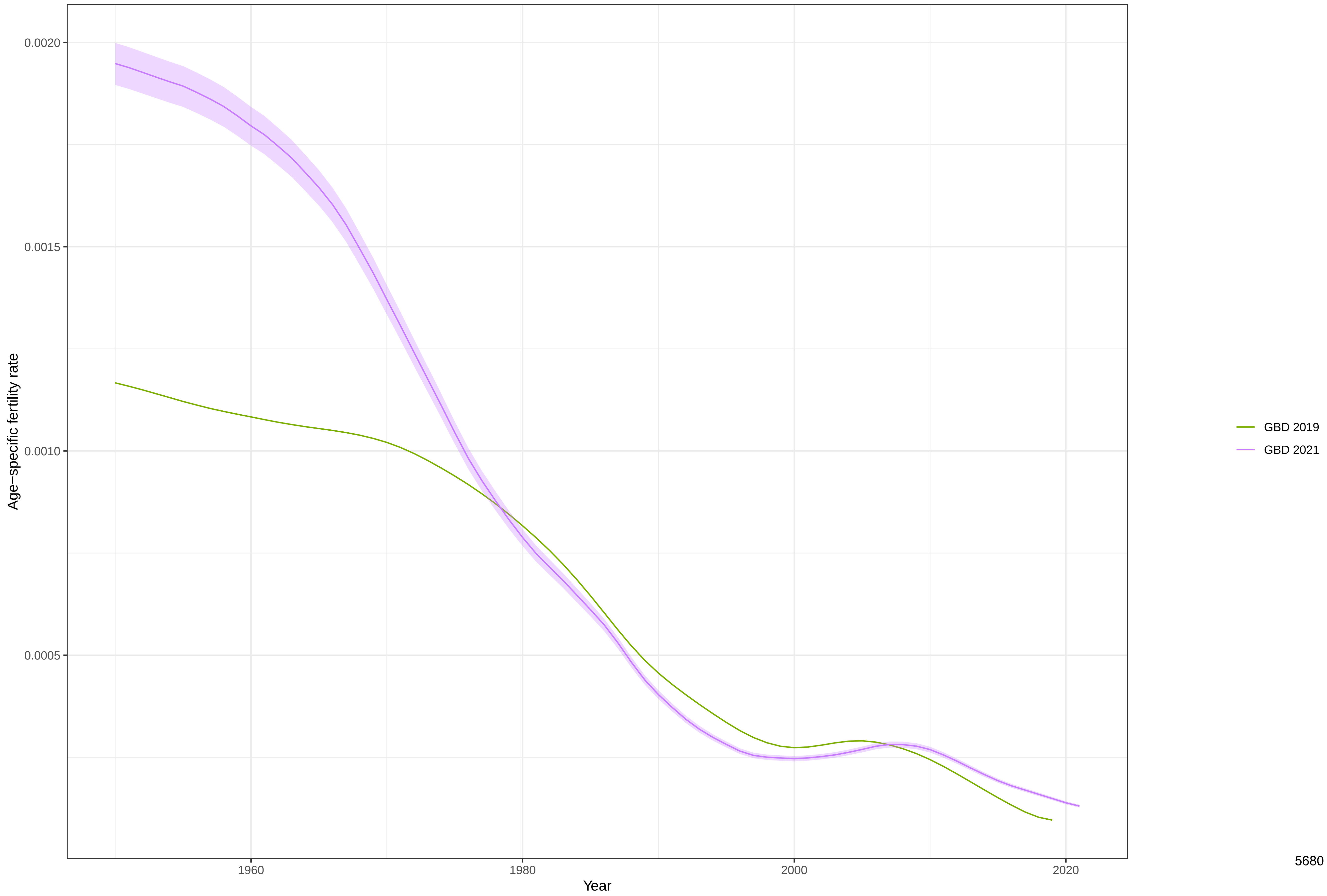

Total fertility rate fitted trend lines, 1950−2021: North Sulawesi TFR
Data points enclosed with a diamond are reference sources

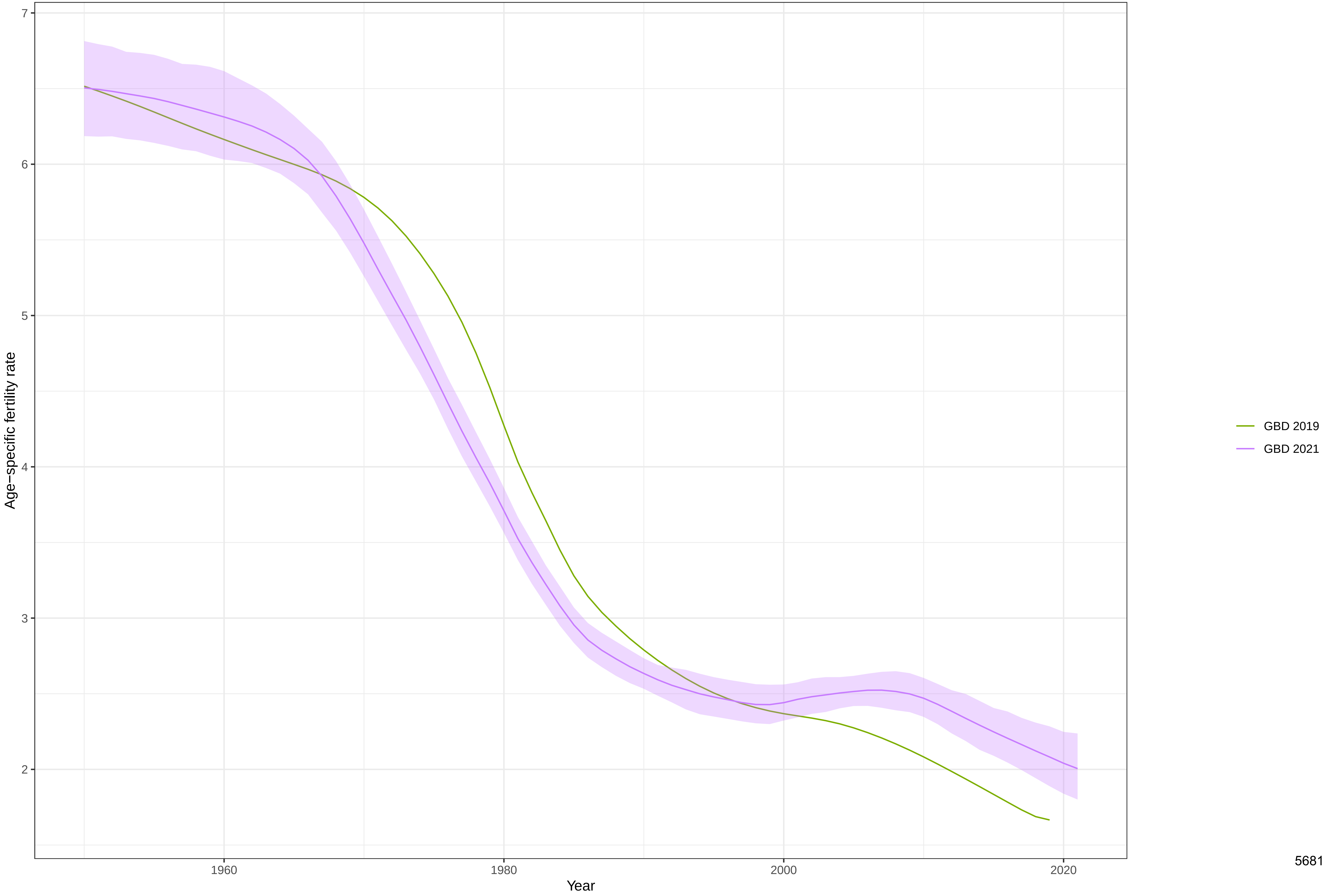

Age−specific fertility fitted trend lines, 1950−2021: West Sumatra, Ages: 10−14
Data points enclosed with a diamond are reference sources

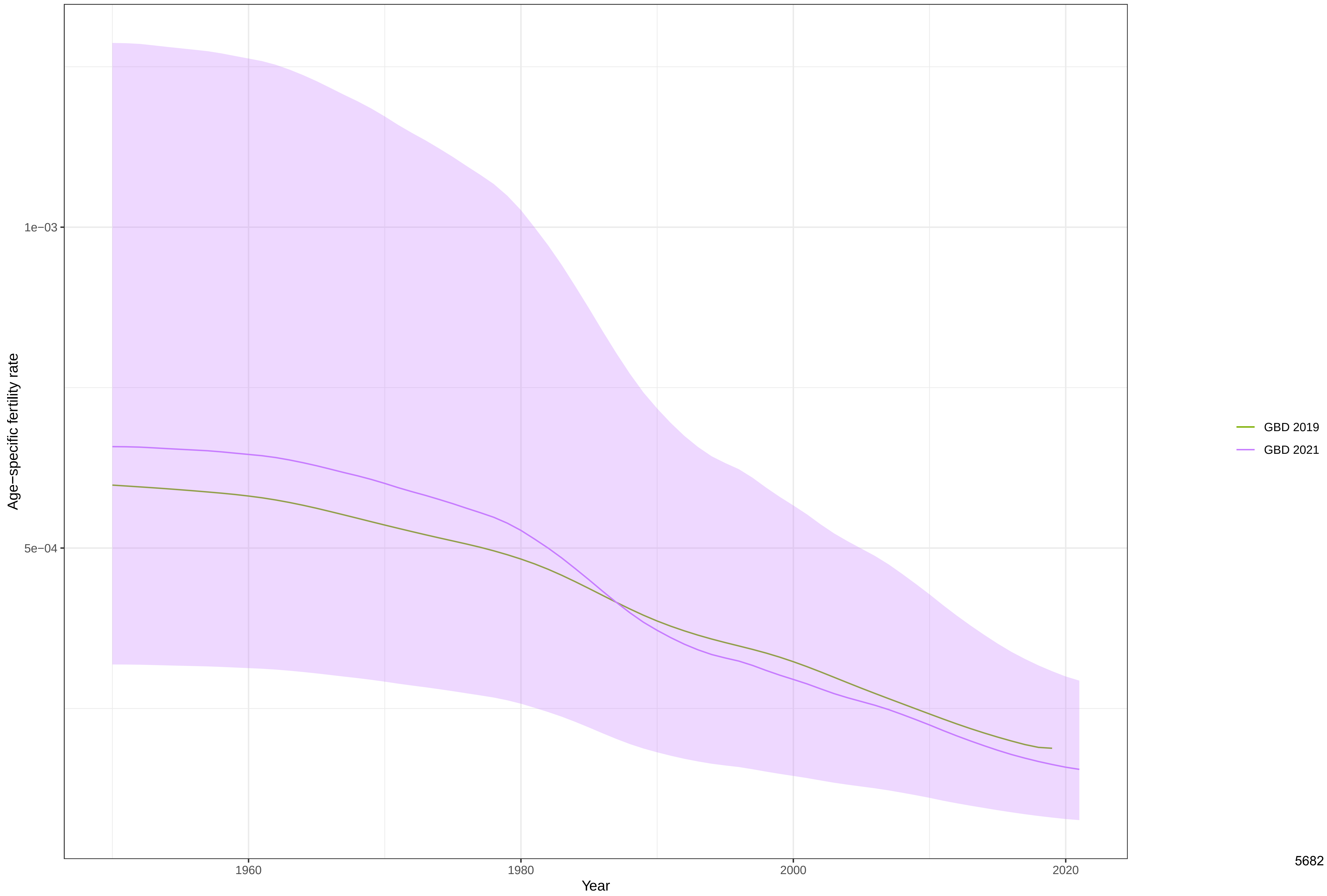

Age−specific fertility fitted trend lines, 1950−2021: West Sumatra, Ages: 15−19
Data points enclosed with a diamond are reference sources

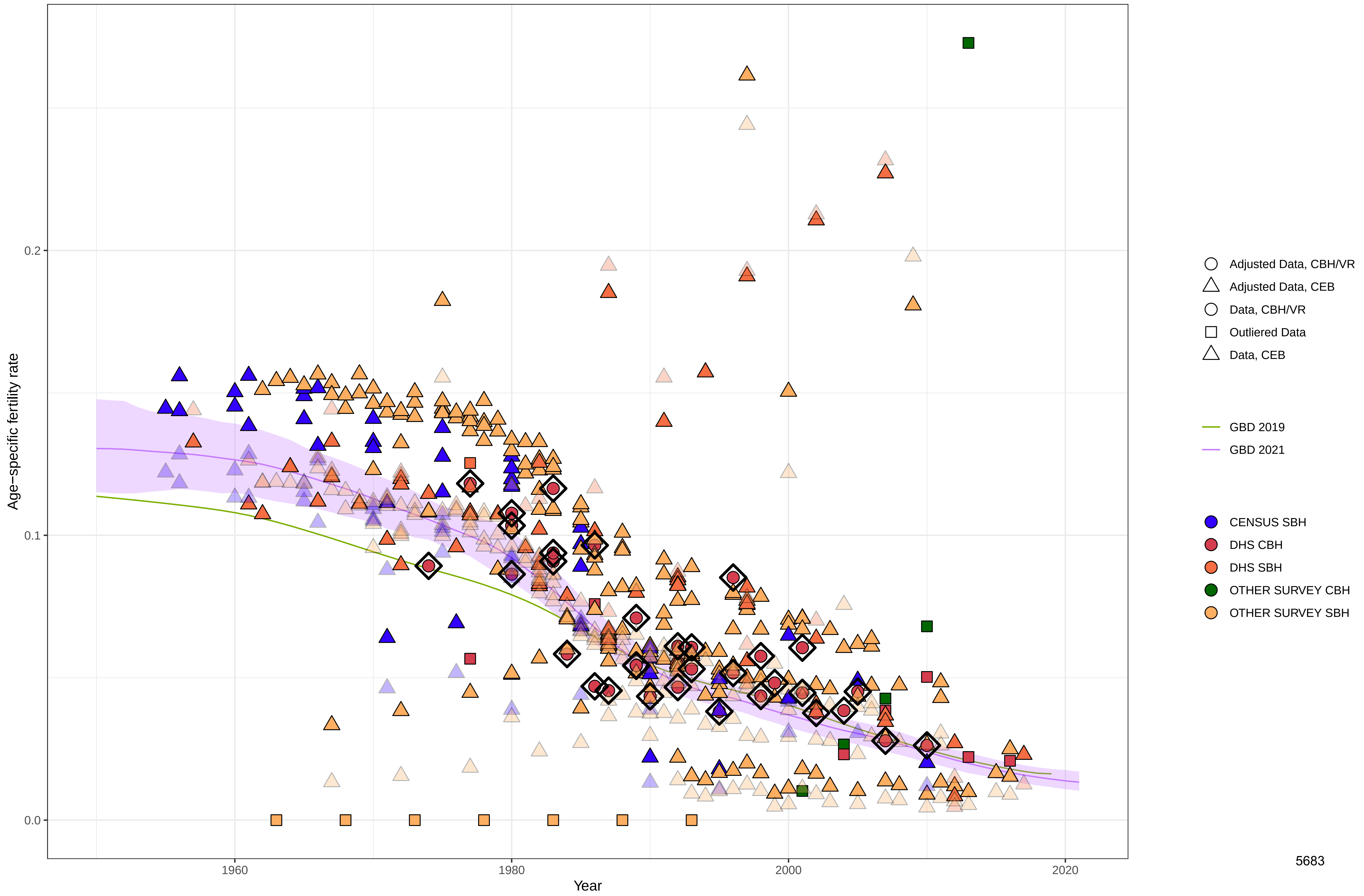

Age−specific fertility fitted trend lines, 1950−2021: West Sumatra, Ages: 20−24
Data points enclosed with a diamond are reference sources

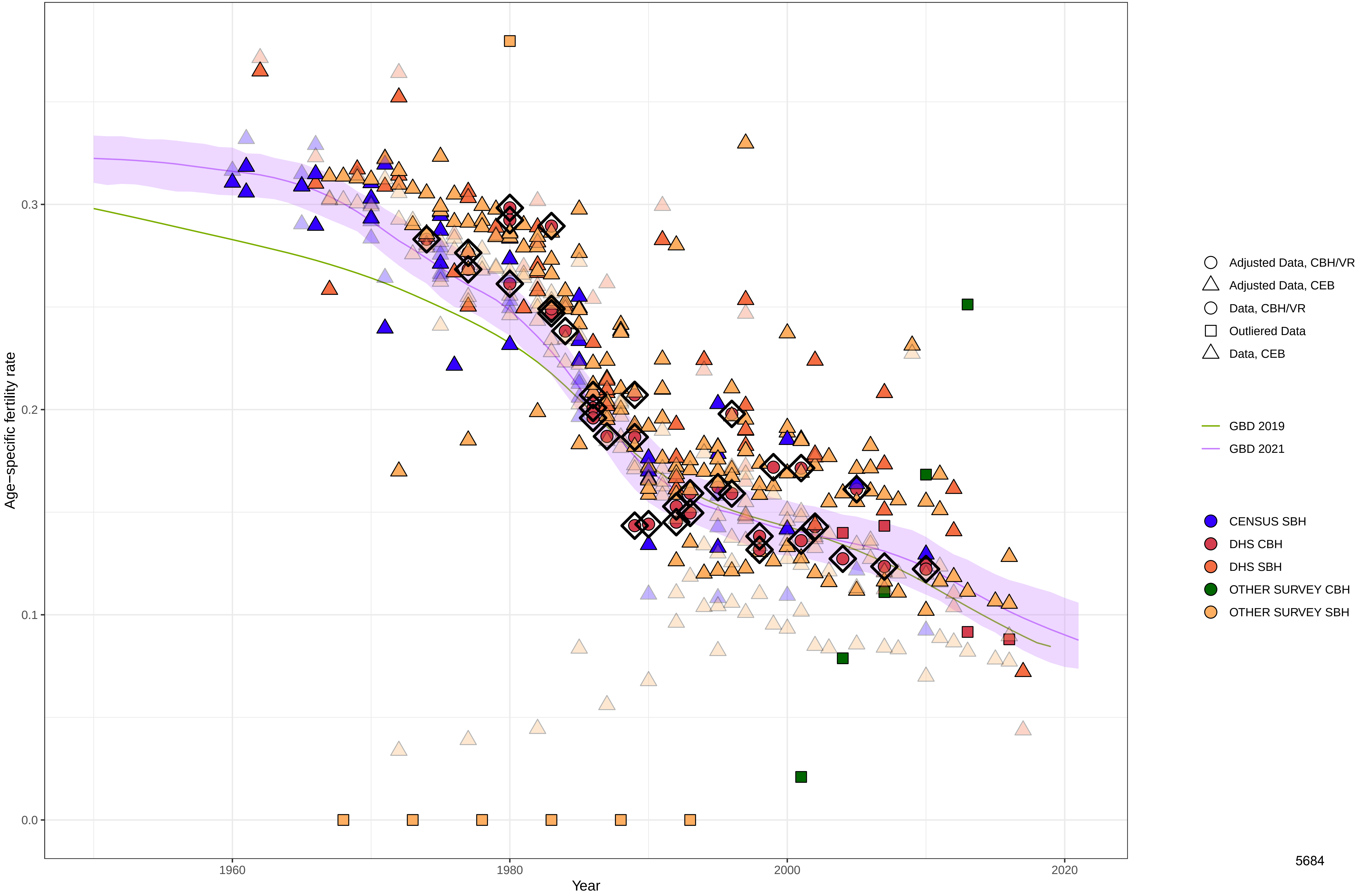

Age−specific fertility fitted trend lines, 1950−2021: West Sumatra, Ages: 25−29
Data points enclosed with a diamond are reference sources

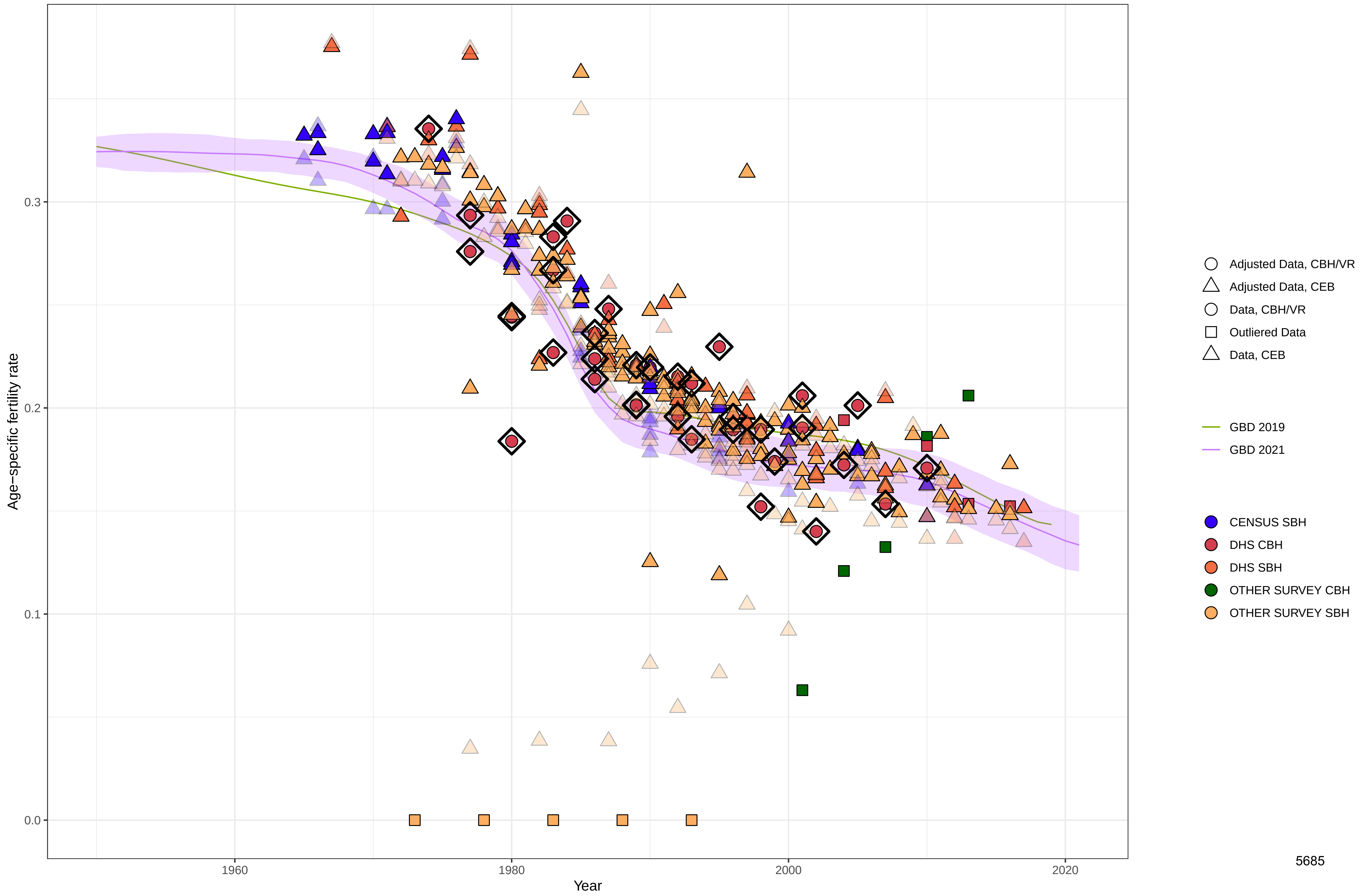

Age−specific fertility fitted trend lines, 1950−2021: West Sumatra, Ages: 30−34
Data points enclosed with a diamond are reference sources

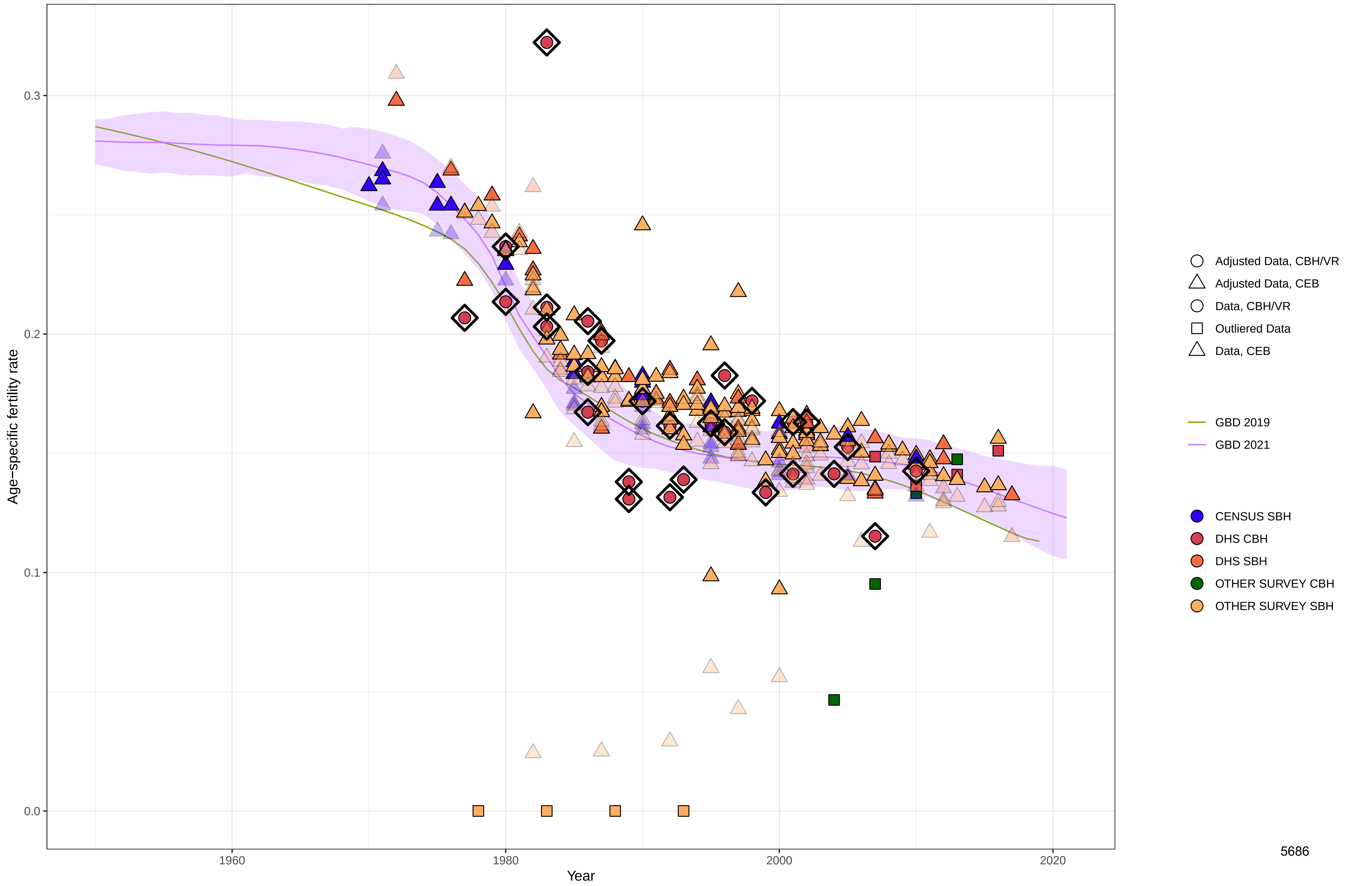

5686

Age−specific fertility fitted trend lines, 1950−2021: West Sumatra, Ages: 35−39
Data points enclosed with a diamond are reference sources

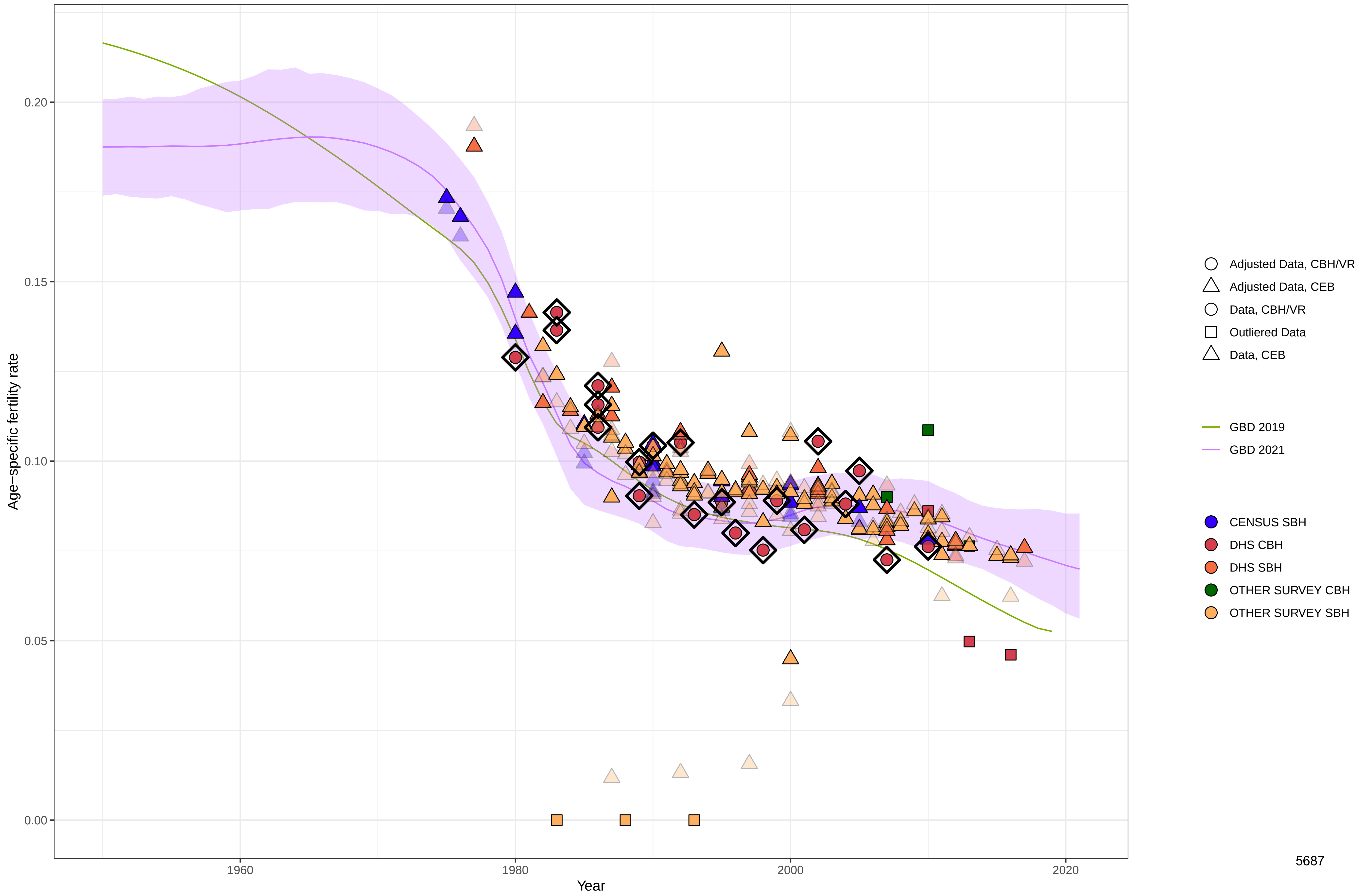

Age−specific fertility fitted trend lines, 1950−2021: West Sumatra, Ages: 40−44
Data points enclosed with a diamond are reference sources

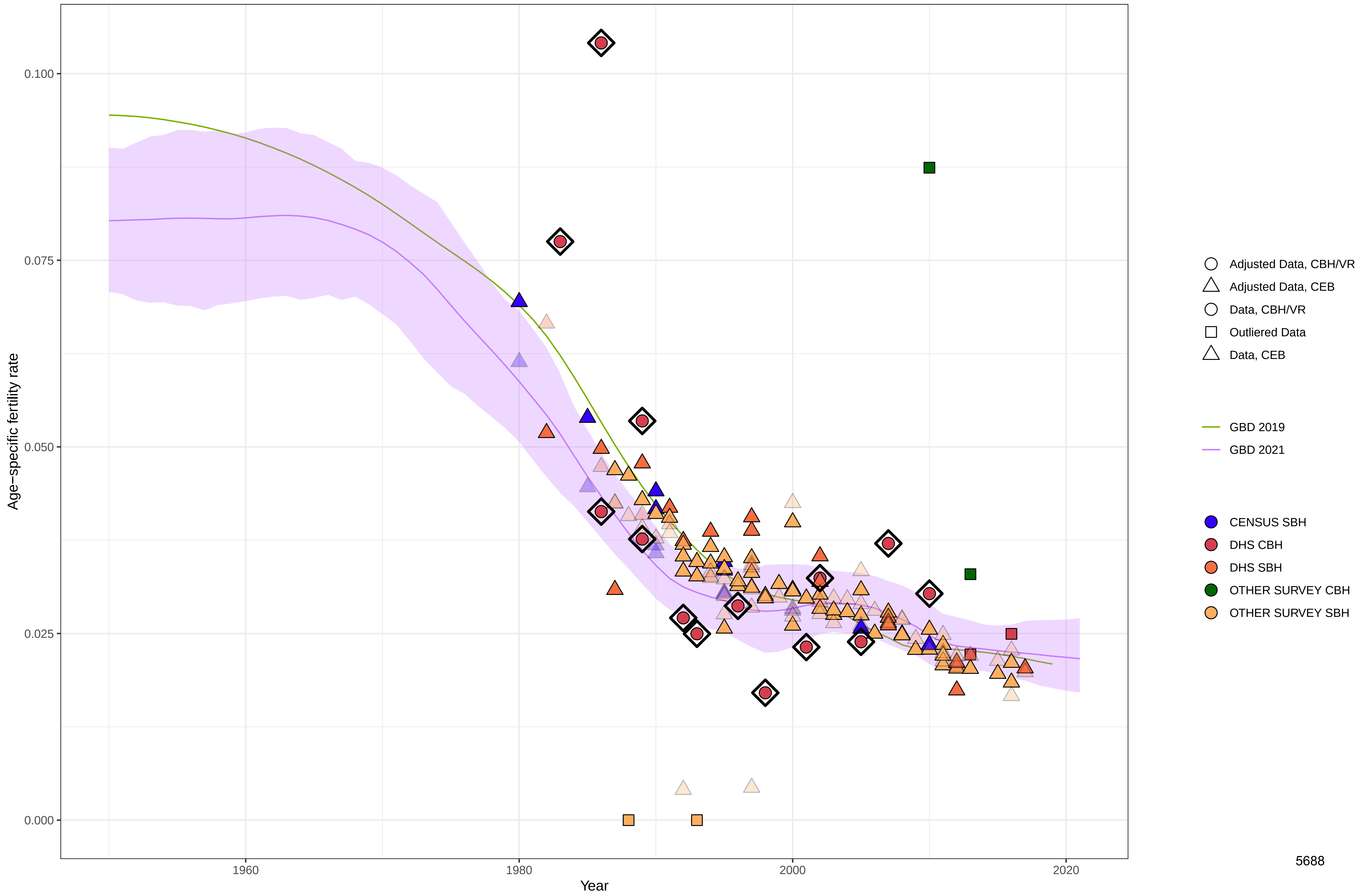

Age−specific fertility fitted trend lines, 1950−2021: West Sumatra, Ages: 45−49
Data points enclosed with a diamond are reference sources

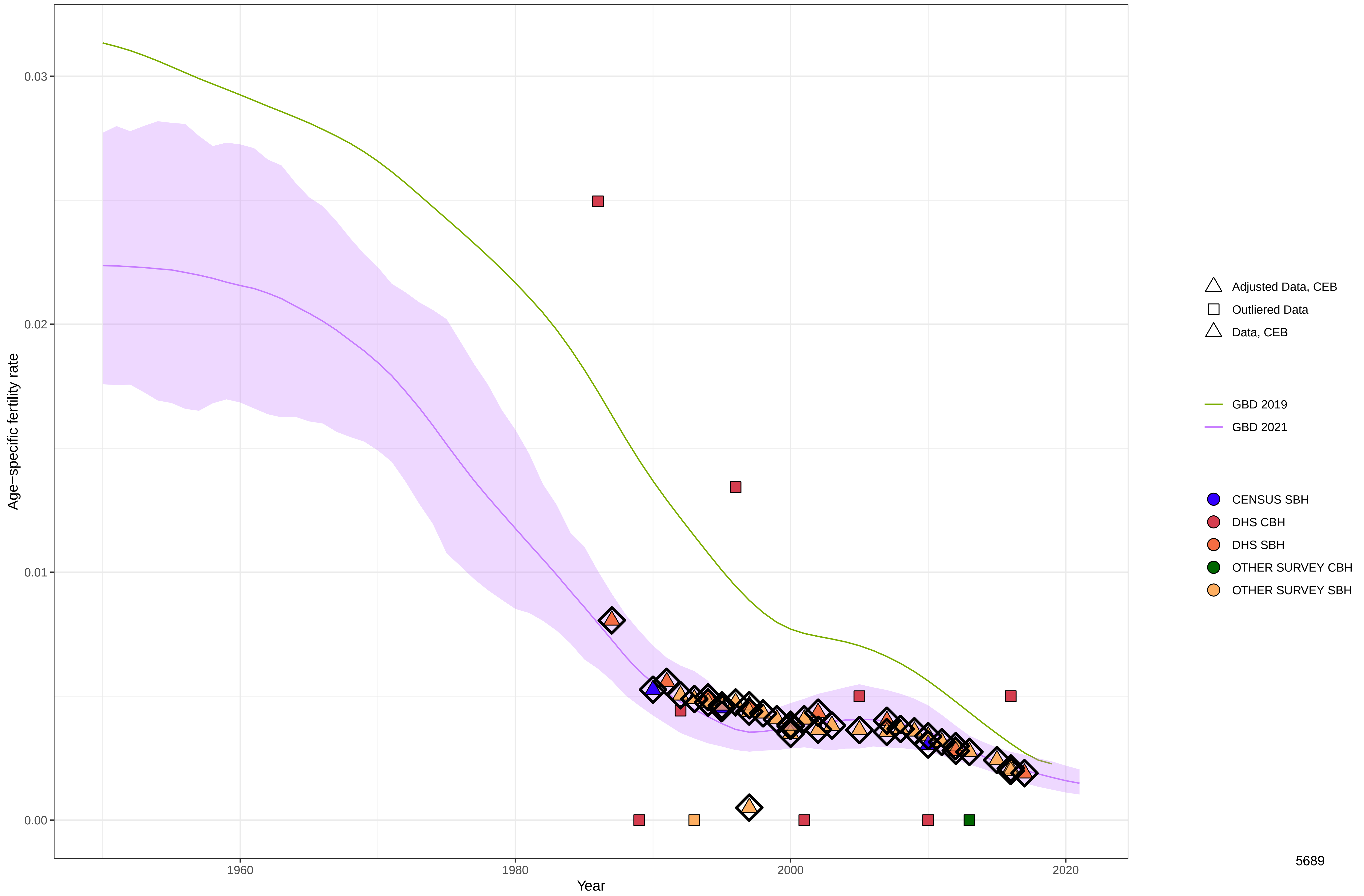

Age−specific fertility fitted trend lines, 1950−2021: West Sumatra, Ages: 50−54
Data points enclosed with a diamond are reference sources

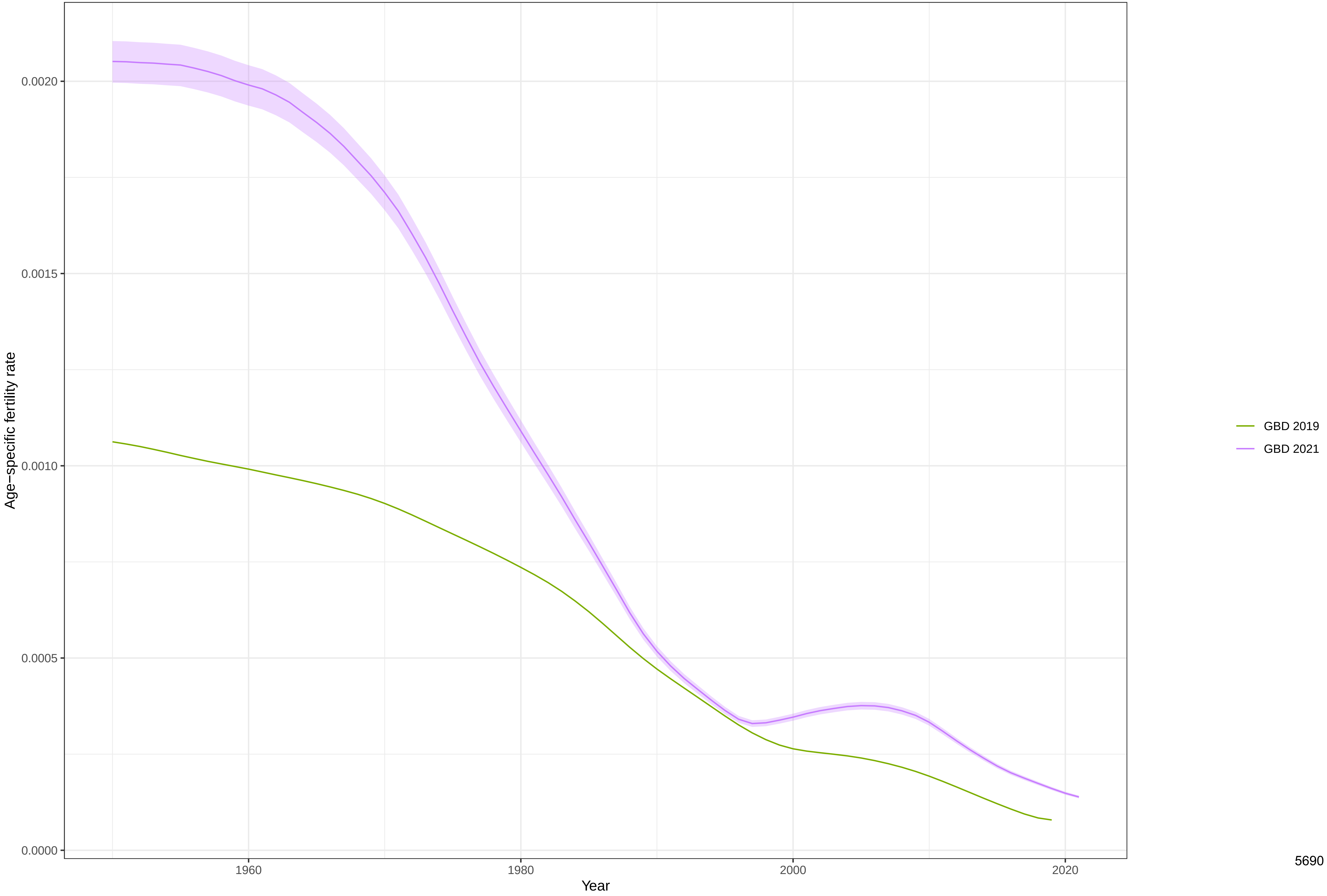

5690

Total fertility rate fitted trend lines, 1950−2021: West Sumatra TFR
Data points enclosed with a diamond are reference sources

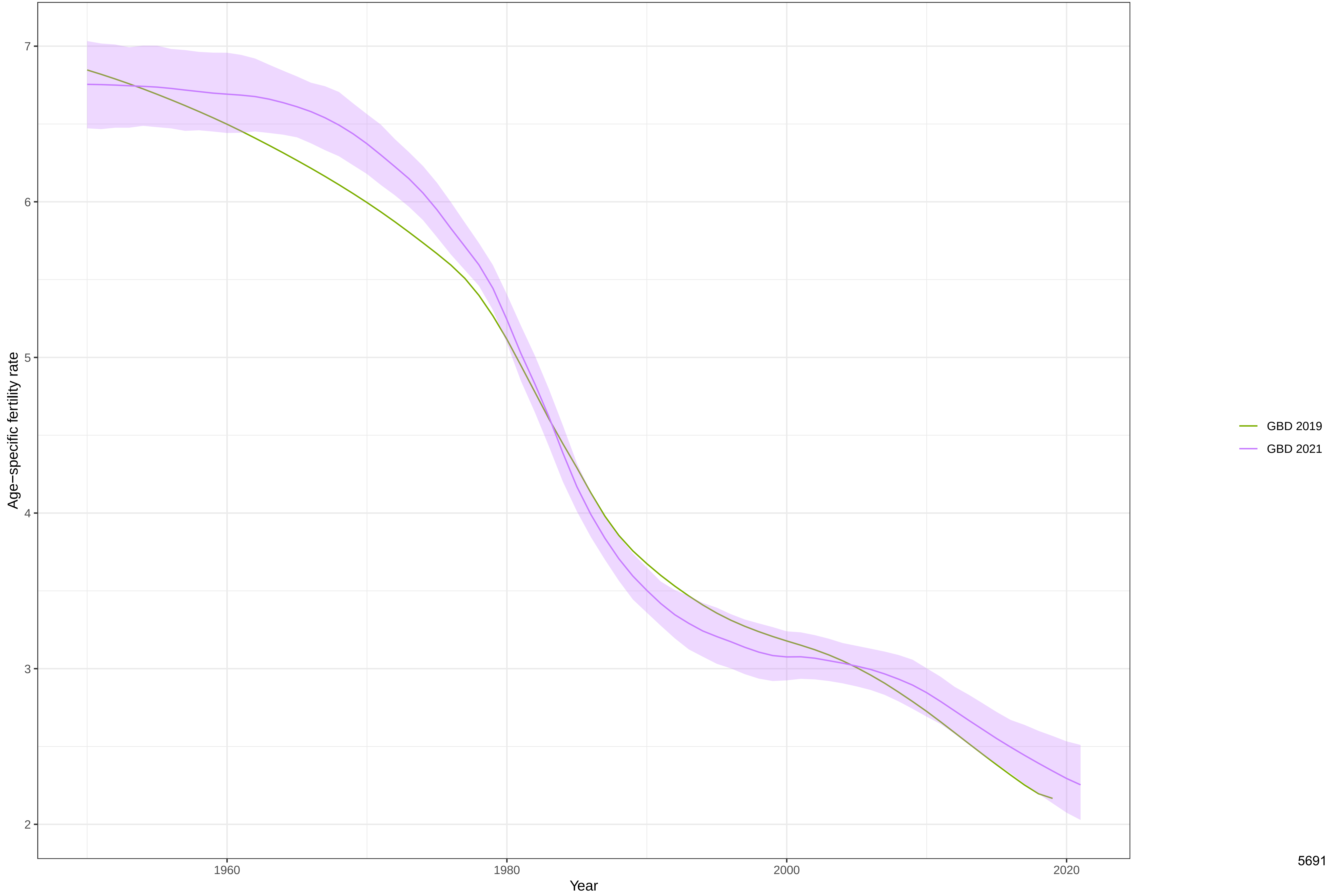

5691

Age−specific fertility fitted trend lines, 1950−2021: South Sumatra, Ages: 10−14
 Data points enclosed with a diamond are reference sources

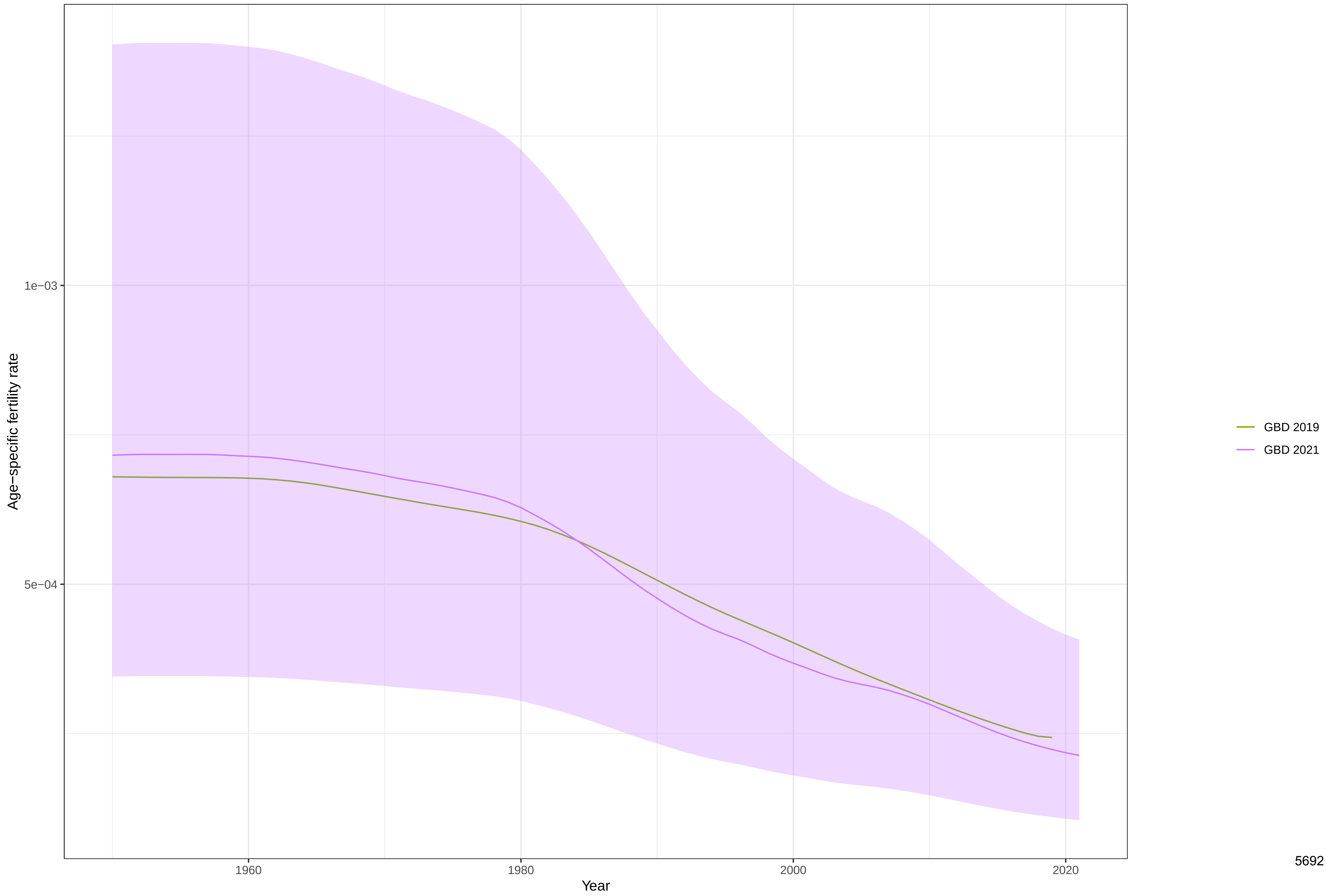

Age−specific fertility fitted trend lines, 1950−2021: South Sumatra, Ages: 15−19
Data points enclosed with a diamond are reference sources

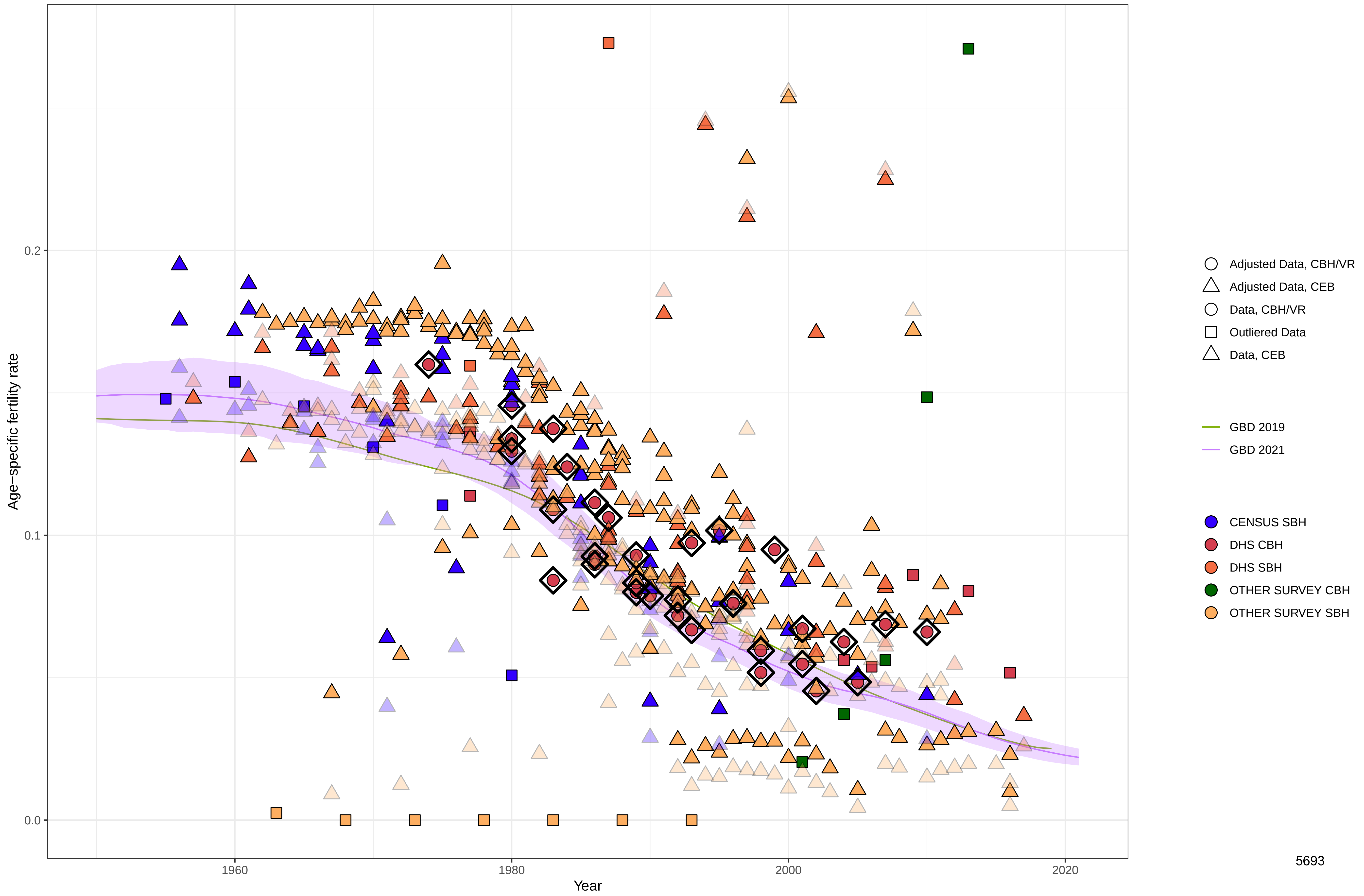

Age−specific fertility fitted trend lines, 1950−2021: South Sumatra, Ages: 20−24
Data points enclosed with a diamond are reference sources

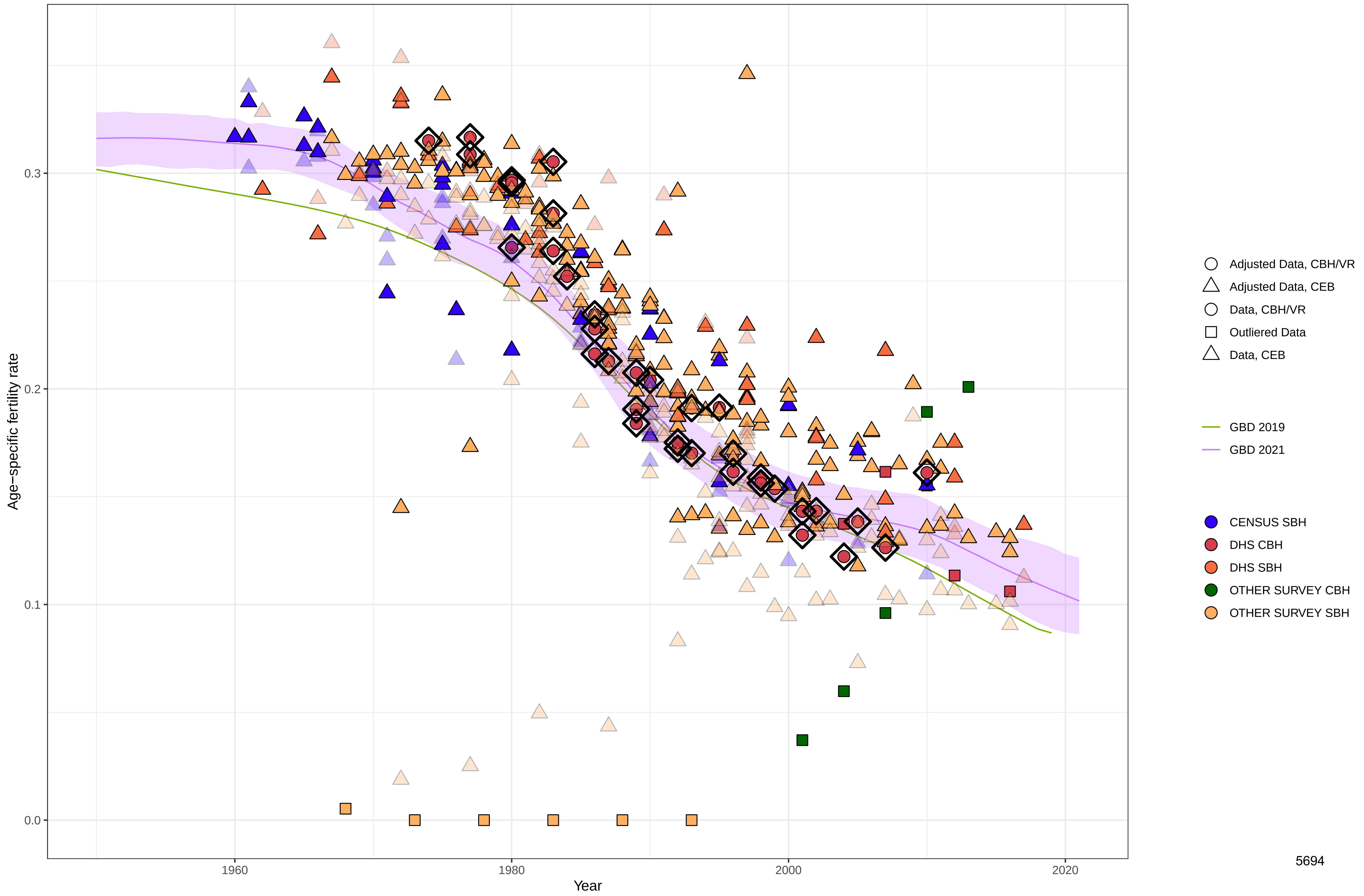

Age−specific fertility fitted trend lines, 1950−2021: South Sumatra, Ages: 25−29
Data points enclosed with a diamond are reference sources

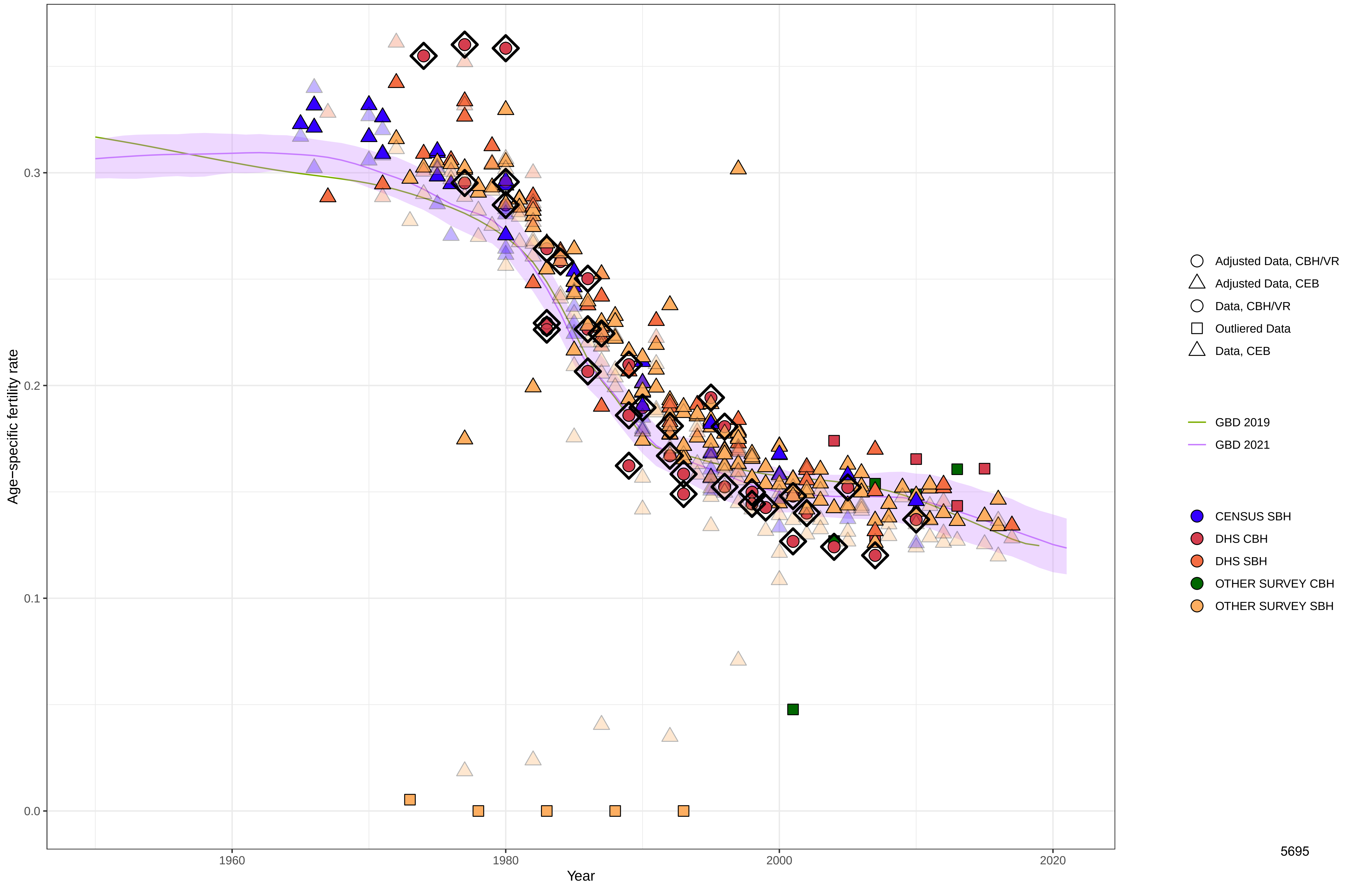

Age−specific fertility fitted trend lines, 1950−2021: South Sumatra, Ages: 30−34
Data points enclosed with a diamond are reference sources

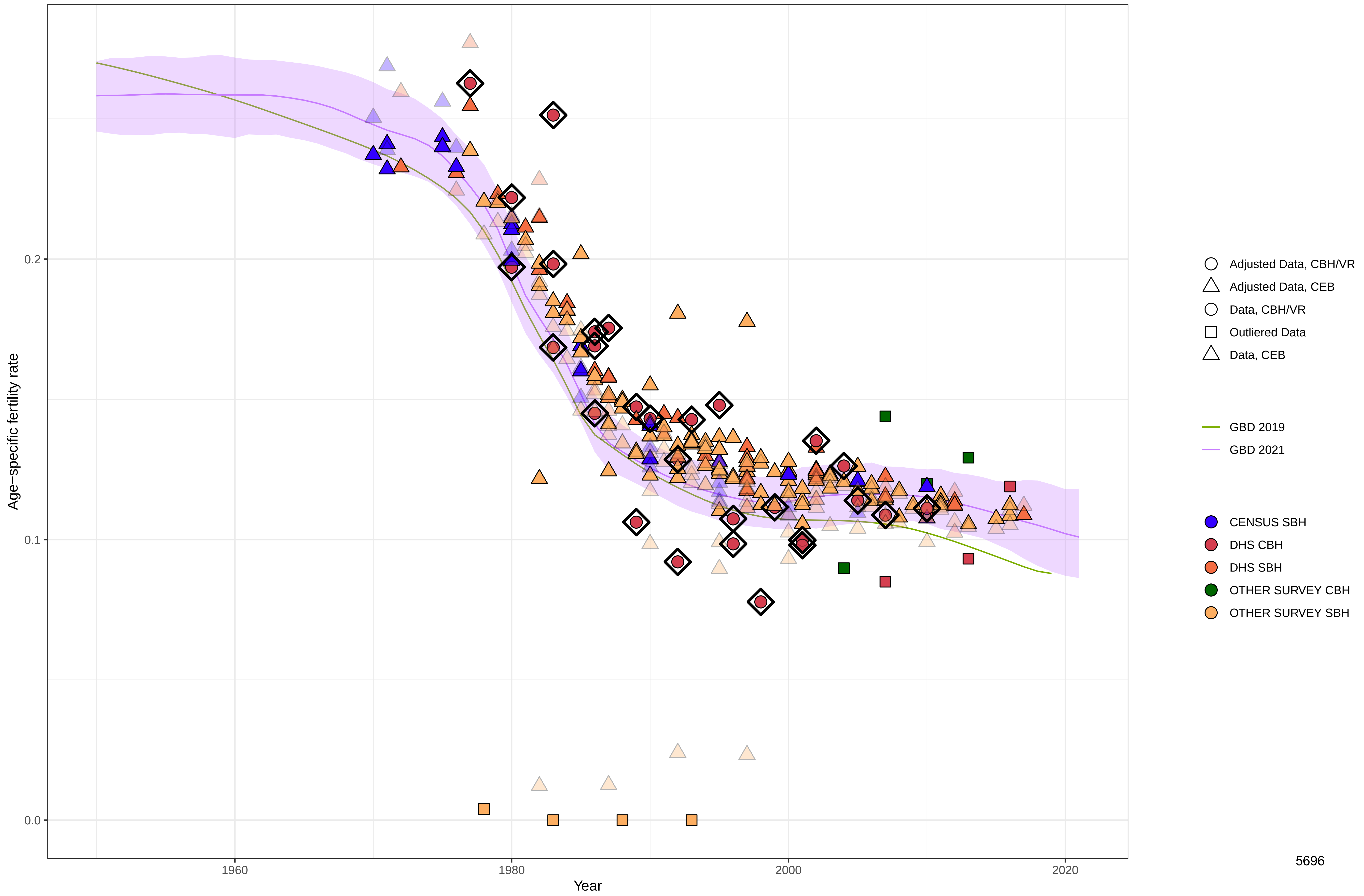

Age−specific fertility fitted trend lines, 1950−2021: South Sumatra, Ages: 35−39
Data points enclosed with a diamond are reference sources

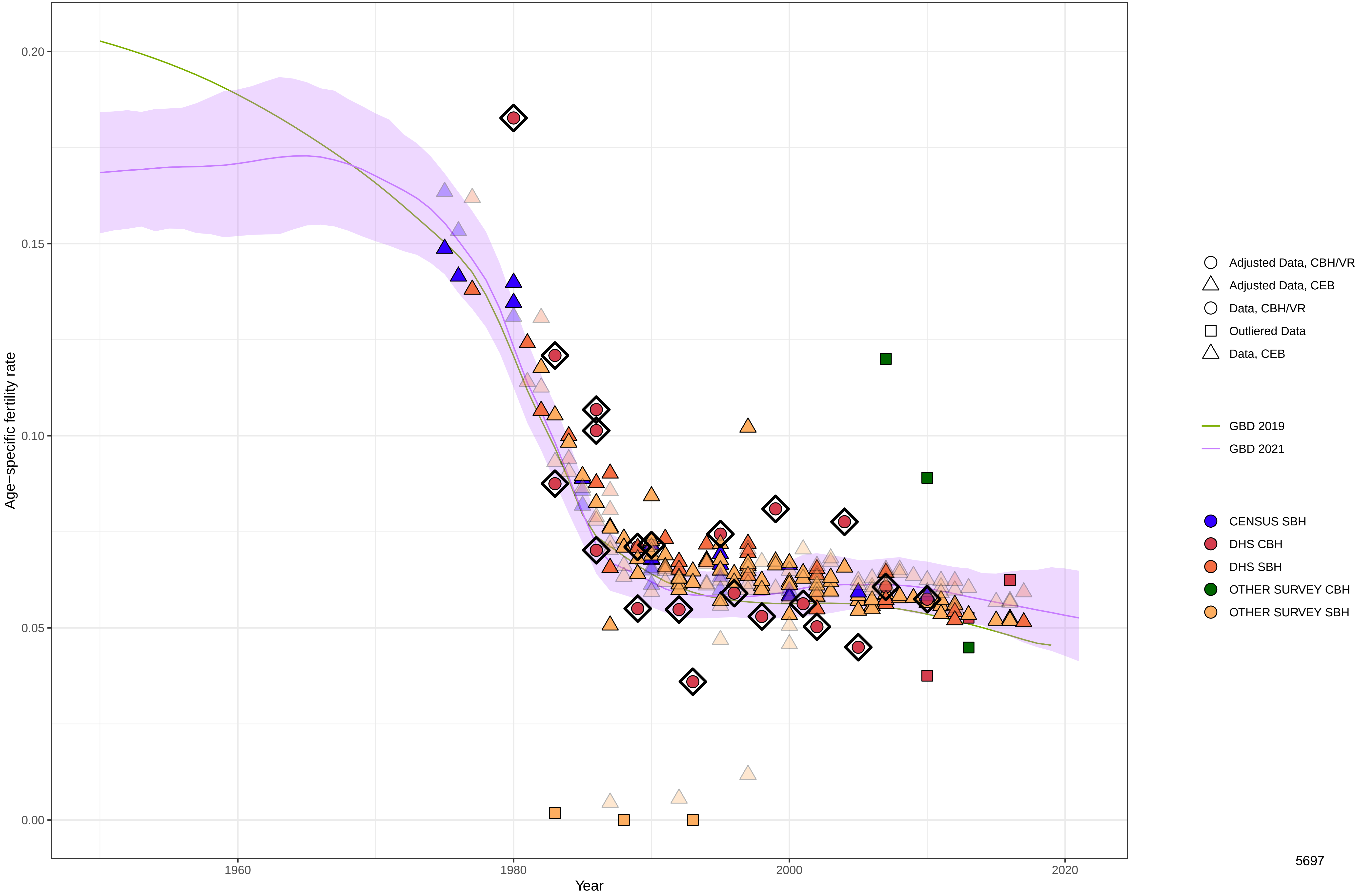

Age−specific fertility fitted trend lines, 1950−2021: South Sumatra, Ages: 40−44
Data points enclosed with a diamond are reference sources

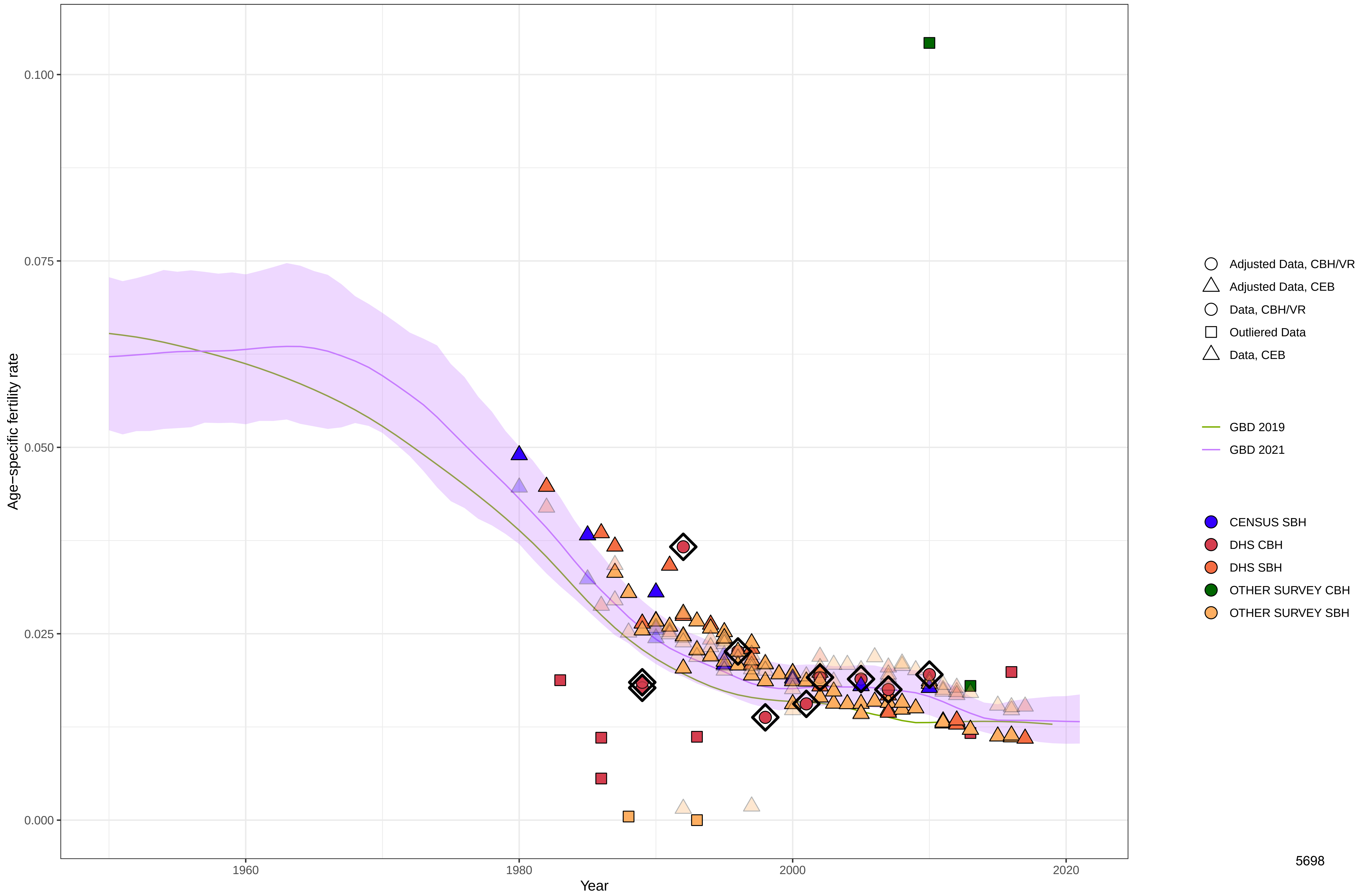

Age−specific fertility fitted trend lines, 1950−2021: South Sumatra, Ages: 45−49
Data points enclosed with a diamond are reference sources

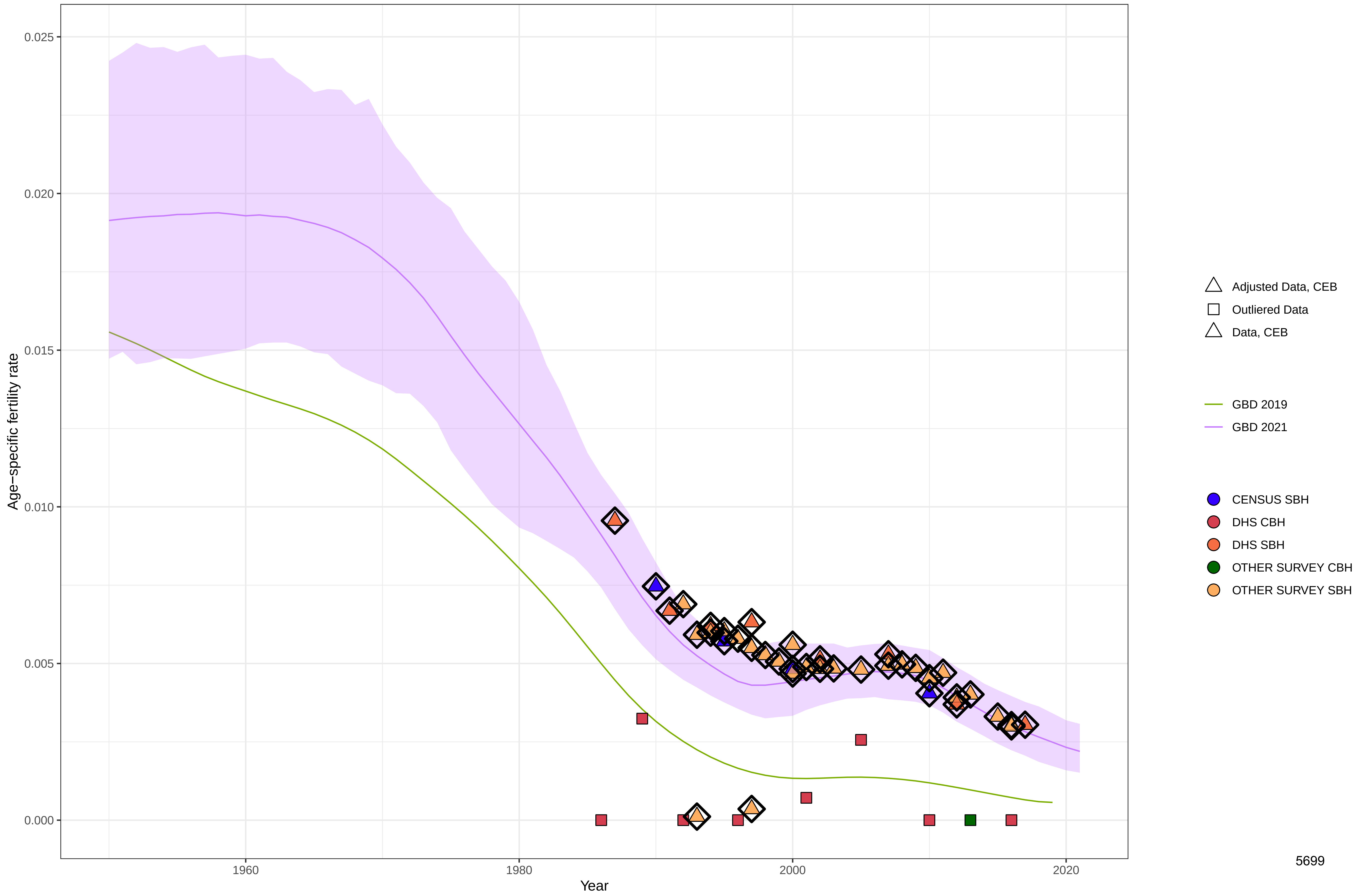

Age−specific fertility fitted trend lines, 1950−2021: South Sumatra, Ages: 50−54
Data points enclosed with a diamond are reference sources

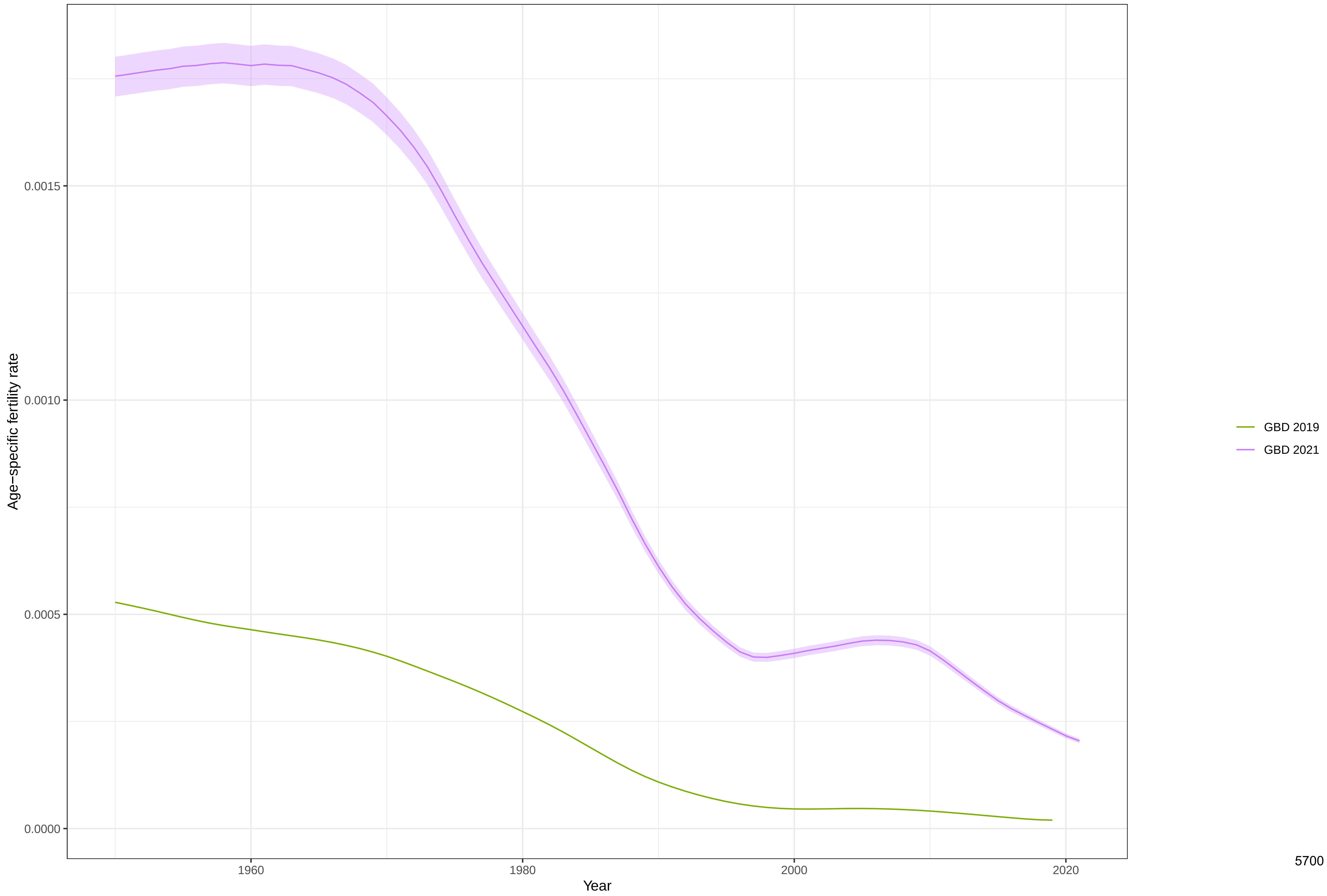

Total fertility rate fitted trend lines, 1950−2021: South Sumatra TFR
Data points enclosed with a diamond are reference sources

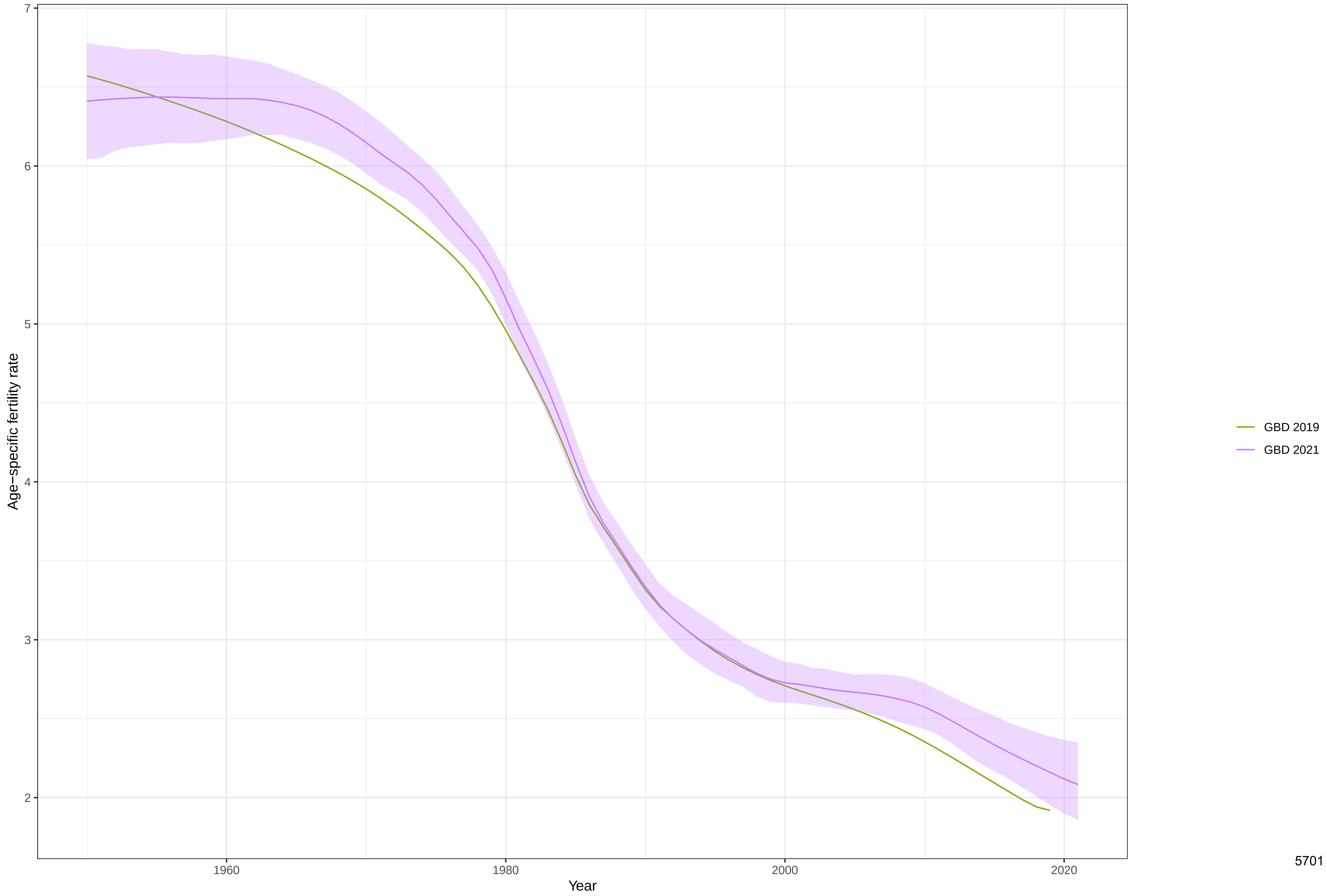

Age−specific fertility fitted trend lines, 1950−2021: North Sumatra, Ages: 10−14
Data points enclosed with a diamond are reference sources

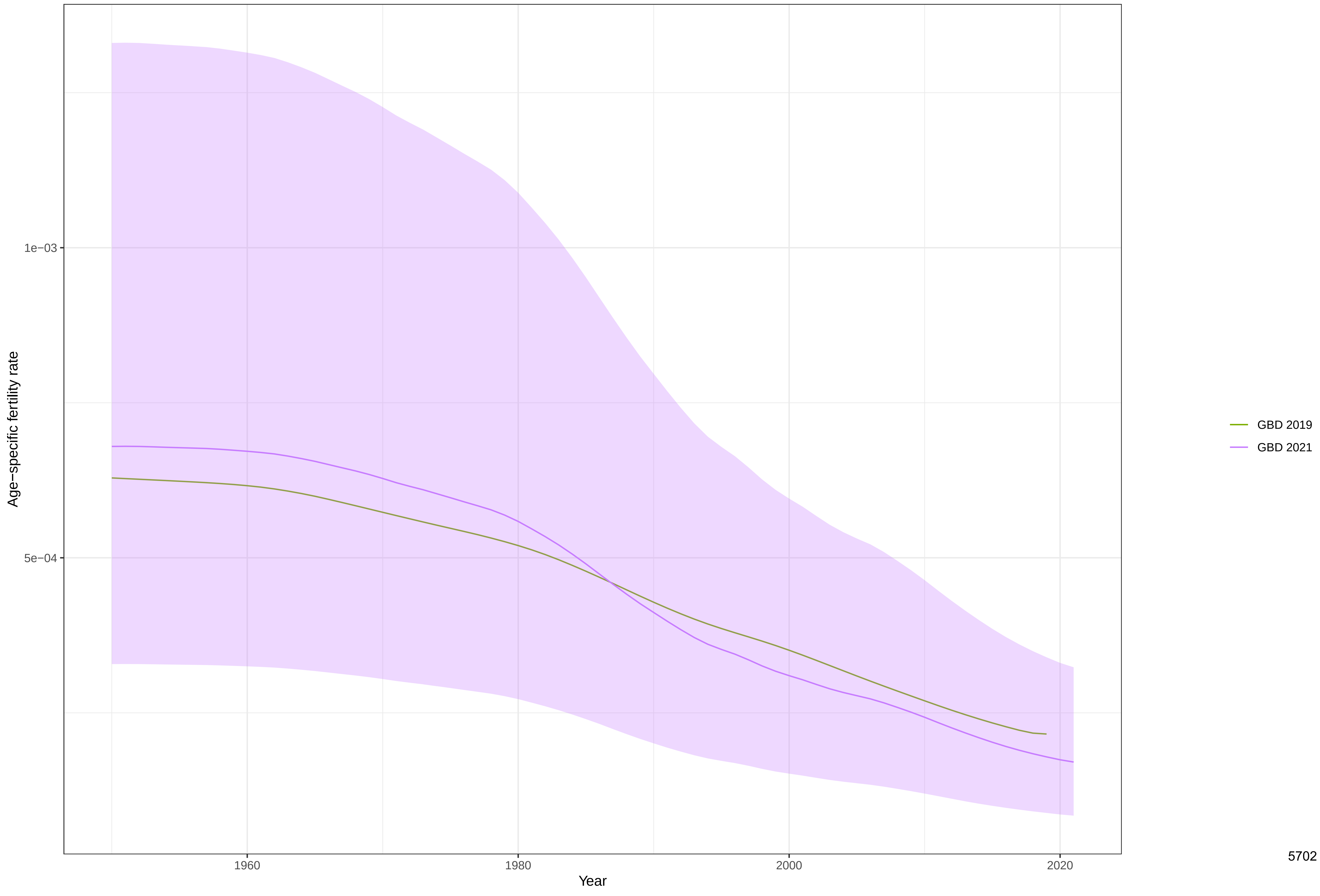

Age−specific fertility fitted trend lines, 1950−2021: North Sumatra, Ages: 15−19
Data points enclosed with a diamond are reference sources

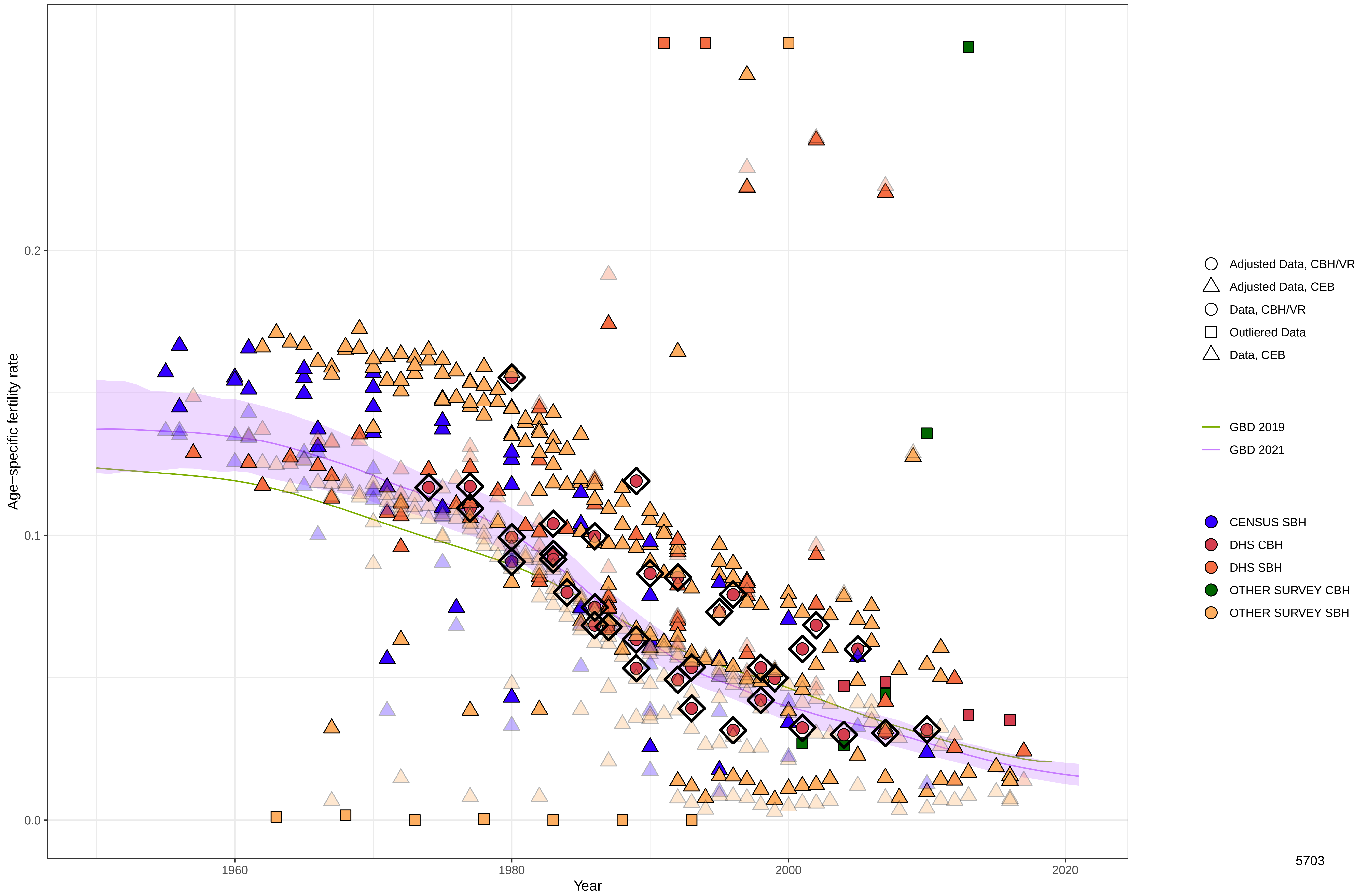

Age−specific fertility fitted trend lines, 1950−2021: North Sumatra, Ages: 20−24
Data points enclosed with a diamond are reference sources

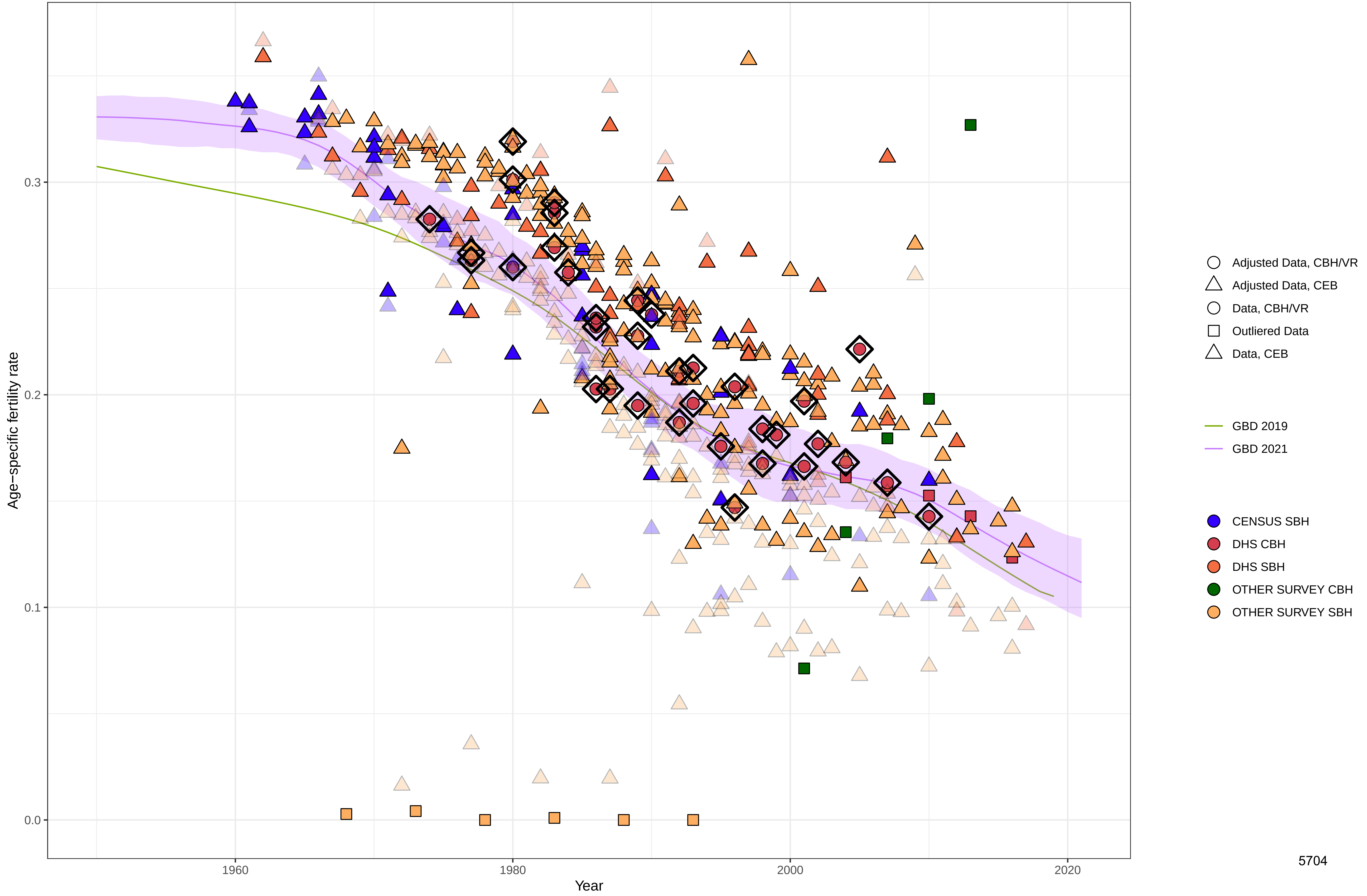

Age−specific fertility fitted trend lines, 1950−2021: North Sumatra, Ages: 25−29
Data points enclosed with a diamond are reference sources

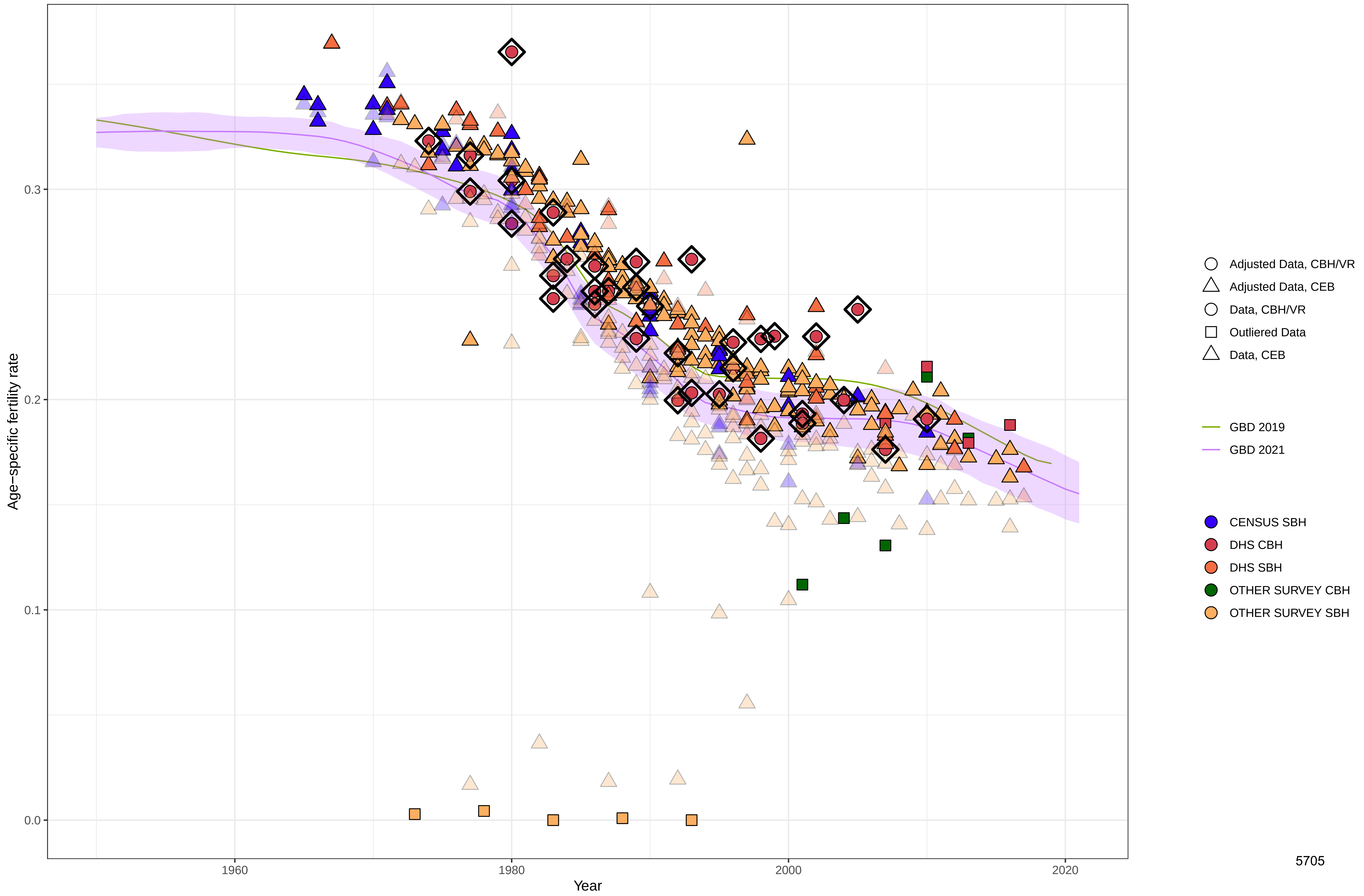

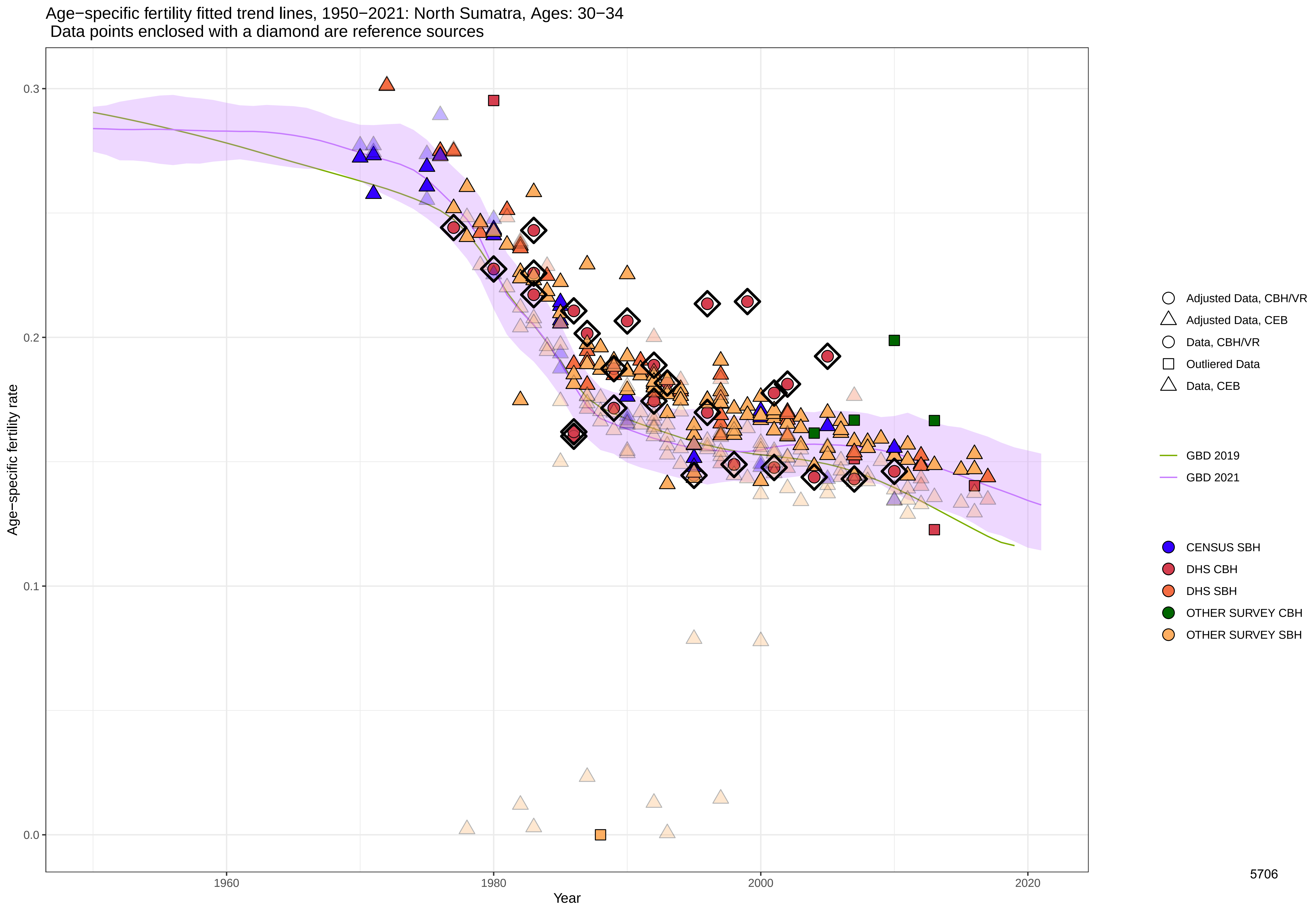
Age−specific fertility fitted trend lines, 1950−2021: North Sumatra, Ages: 30−34
Data points enclosed with a diamond are reference sources
Age−specific fertility rate
Year
0.0
0.1
0.2
0.3
1960
1980
2000
2020
Adjusted Data, CBH/VR
Adjusted Data, CEB
Data, CBH/VR
Outliered Data
Data, CEB
GBD 2019
GBD 2021
CENSUS SBH
DHS CBH
DHS SBH
OTHER SURVEY CBH
OTHER SURVEY SBH

Age−specific fertility fitted trend lines, 1950−2021: North Sumatra, Ages: 35−39
Data points enclosed with a diamond are reference sources

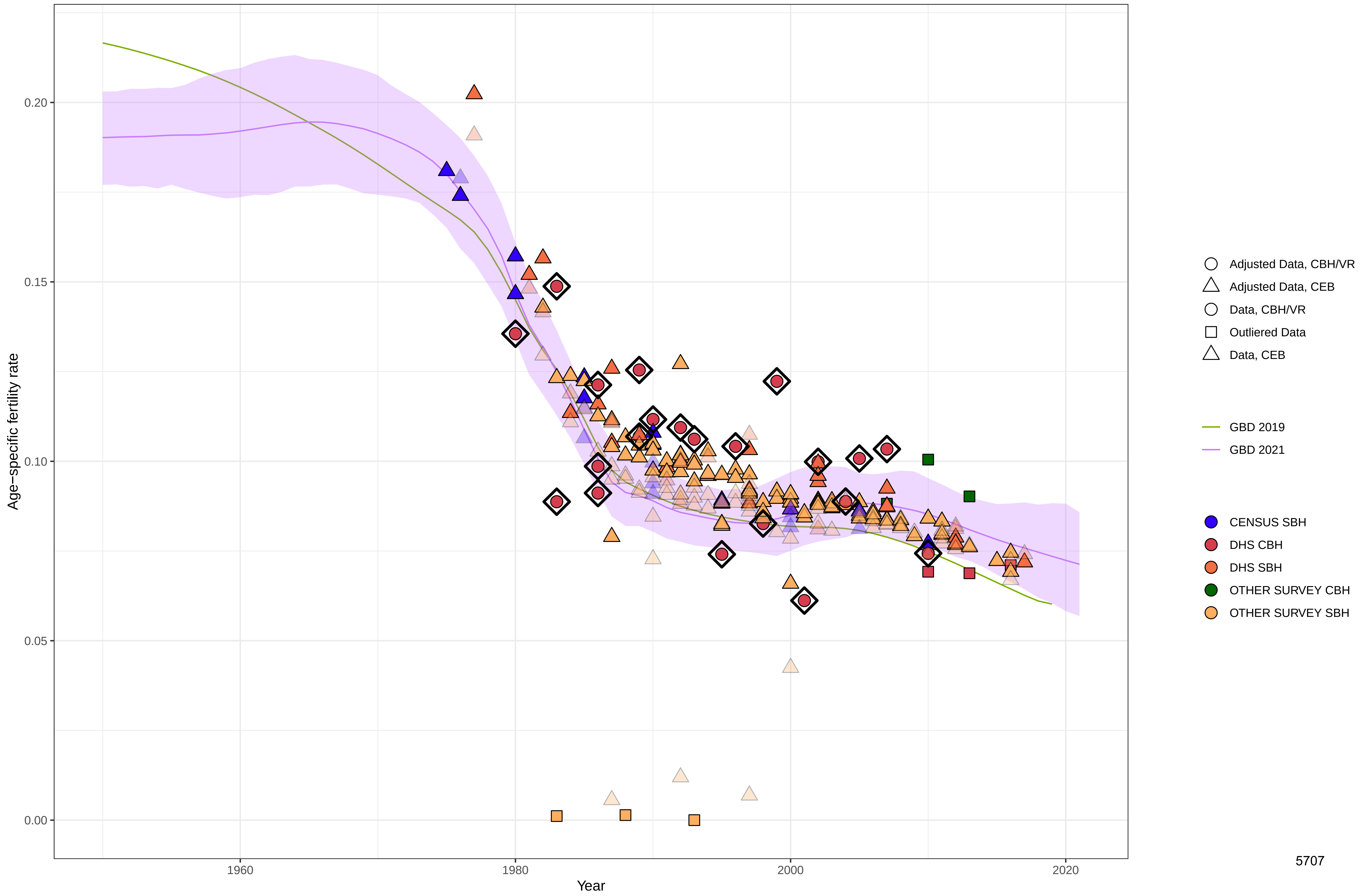

Age−specific fertility fitted trend lines, 1950−2021: North Sumatra, Ages: 40−44
Data points enclosed with a diamond are reference sources

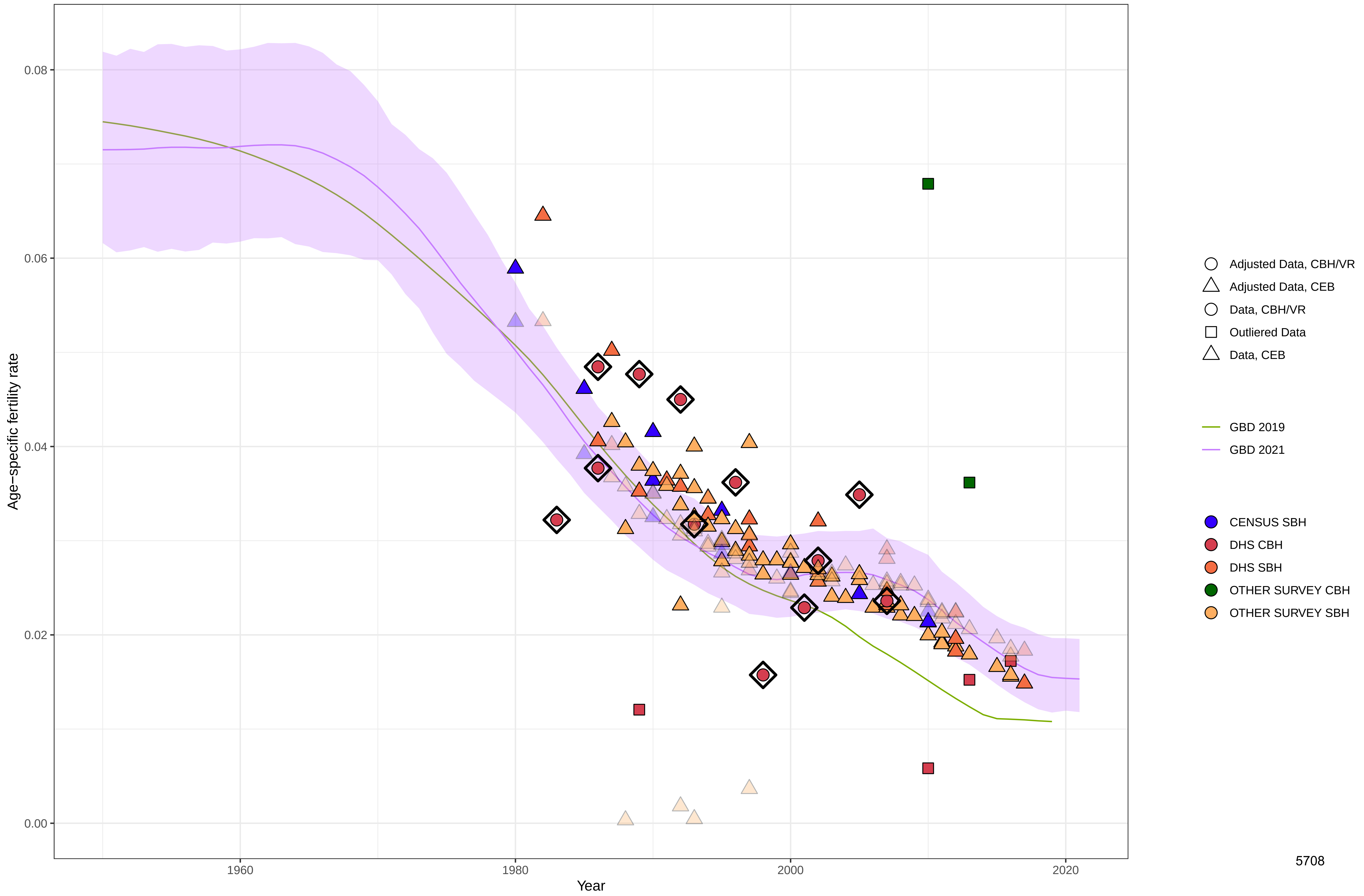

Age−specific fertility fitted trend lines, 1950−2021: North Sumatra, Ages: 45−49
Data points enclosed with a diamond are reference sources

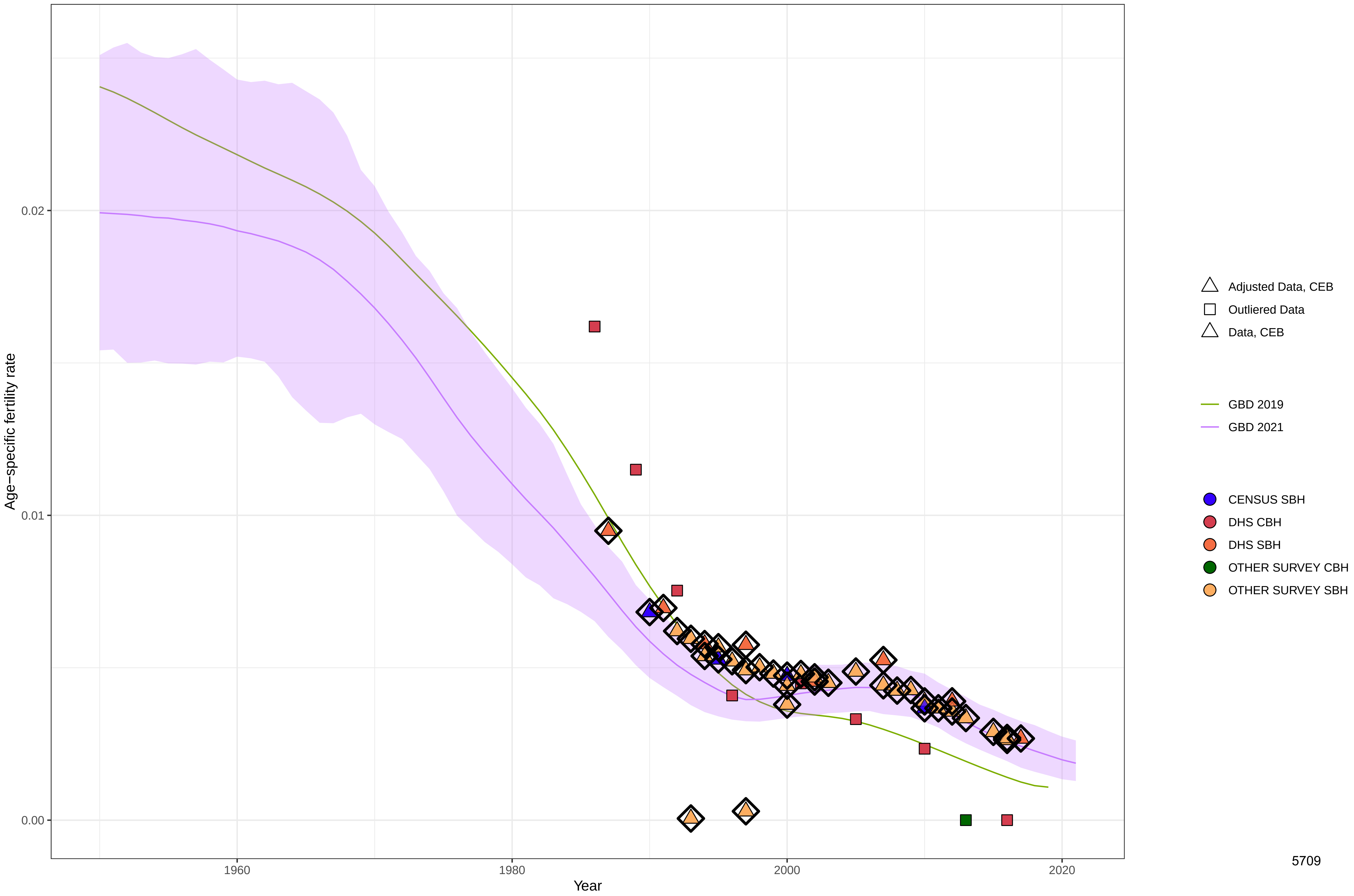

Age−specific fertility fitted trend lines, 1950−2021: North Sumatra, Ages: 50−54
Data points enclosed with a diamond are reference sources

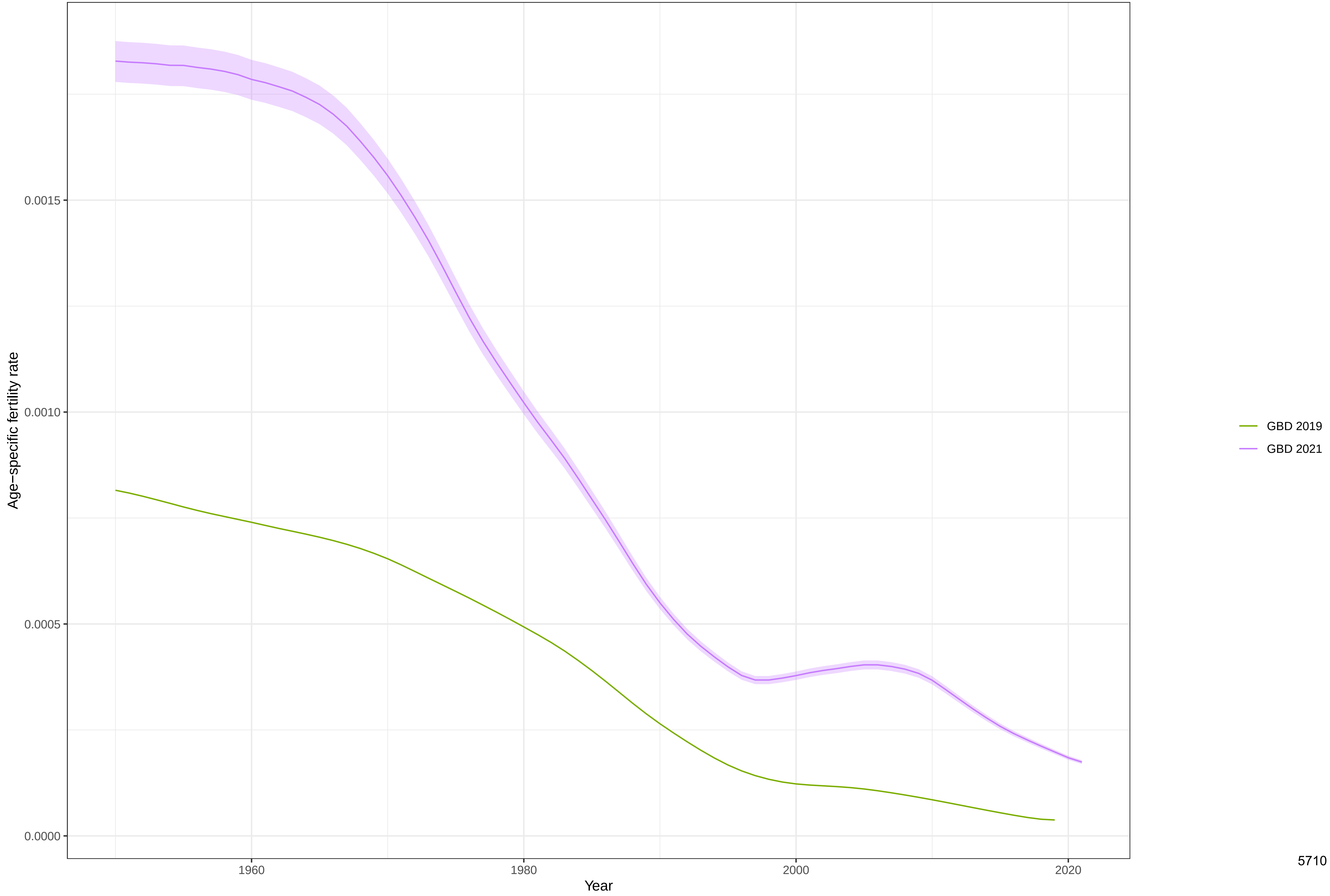

Total fertility rate fitted trend lines, 1950−2021: North Sumatra TFR
Data points enclosed with a diamond are reference sources

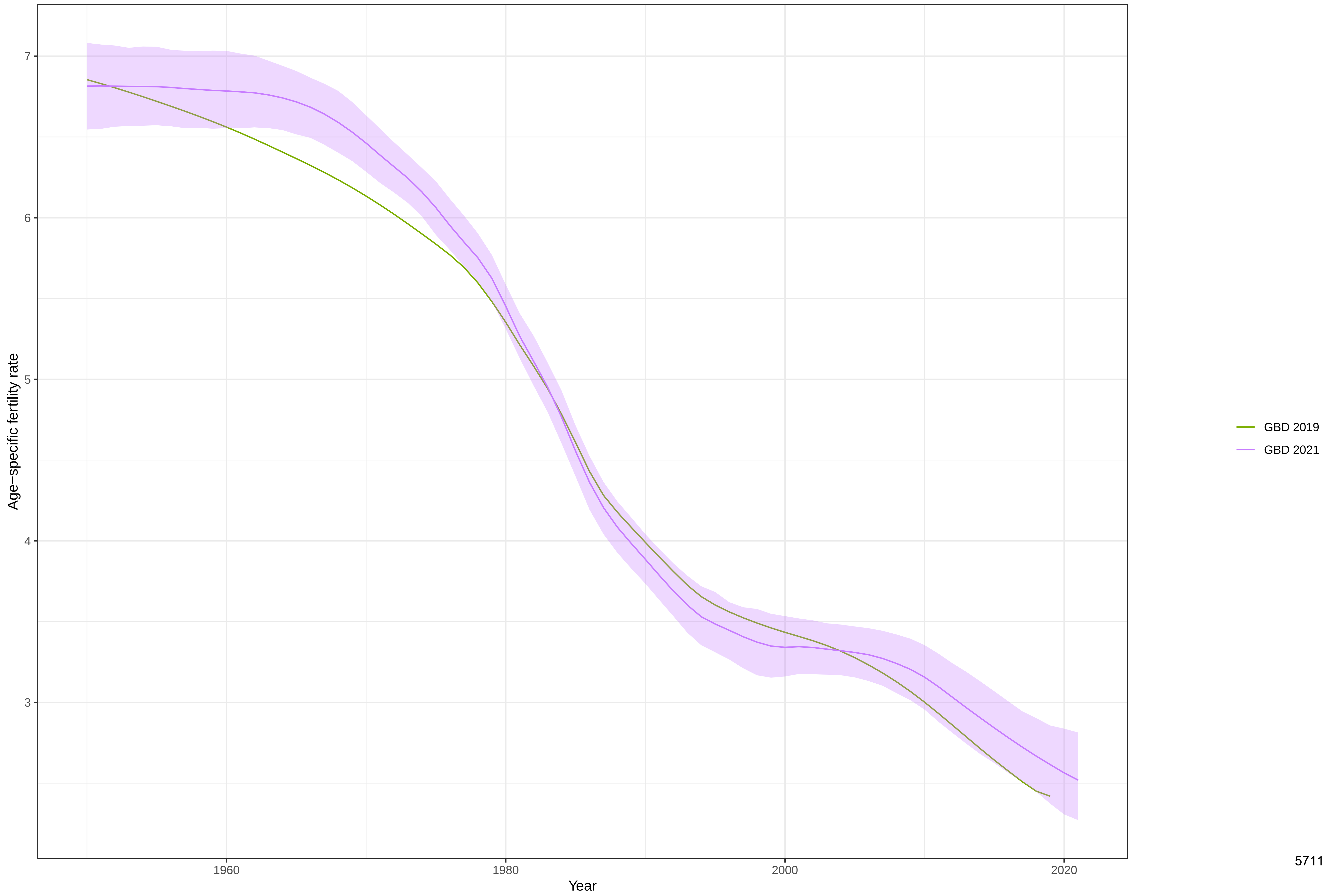

5711

Age−specific fertility fitted trend lines, 1950−2021: Yogyakarta, Ages: 10−14
Data points enclosed with a diamond are reference sources

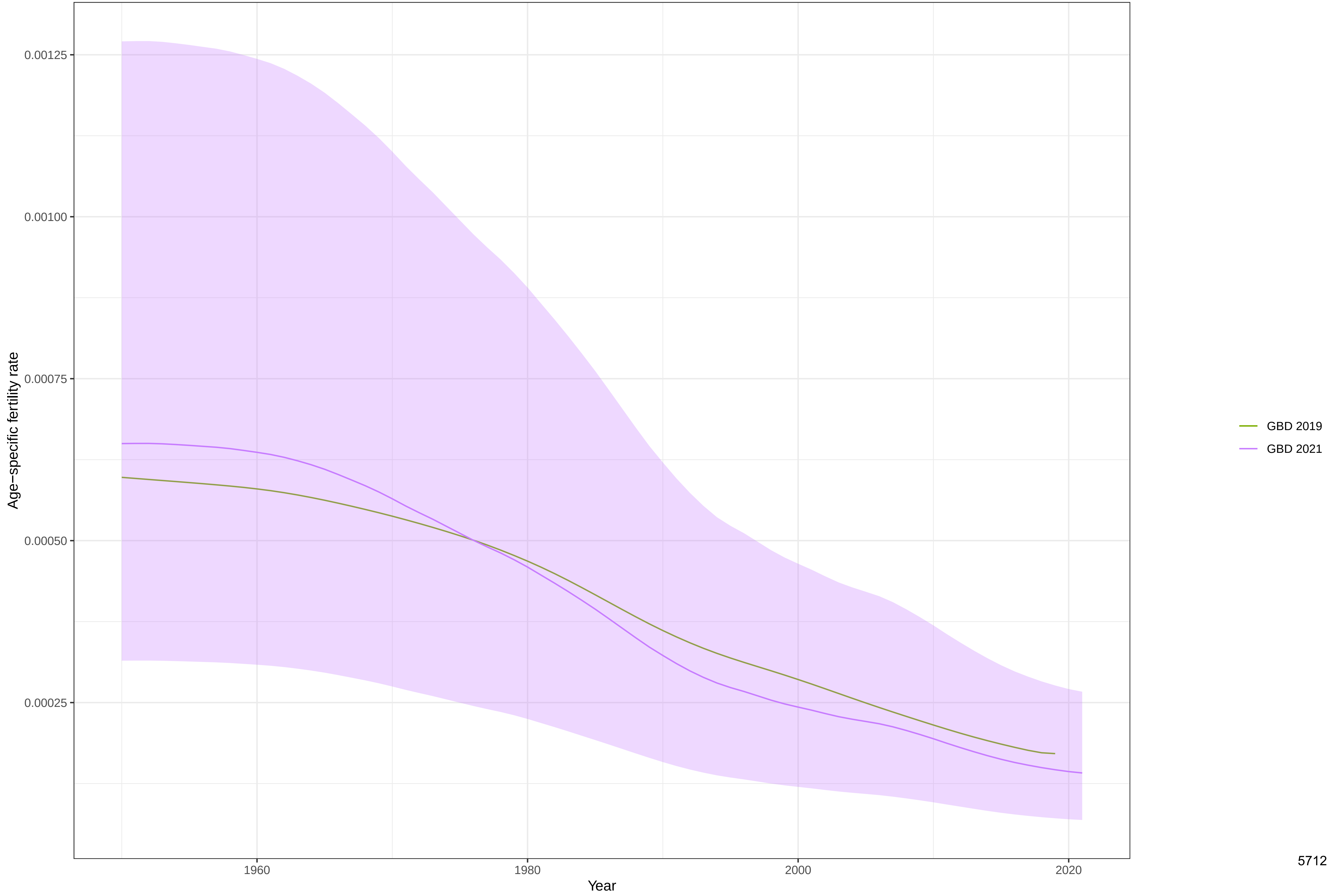

5712

Age−specific fertility fitted trend lines, 1950−2021: Yogyakarta, Ages: 15−19
Data points enclosed with a diamond are reference sources

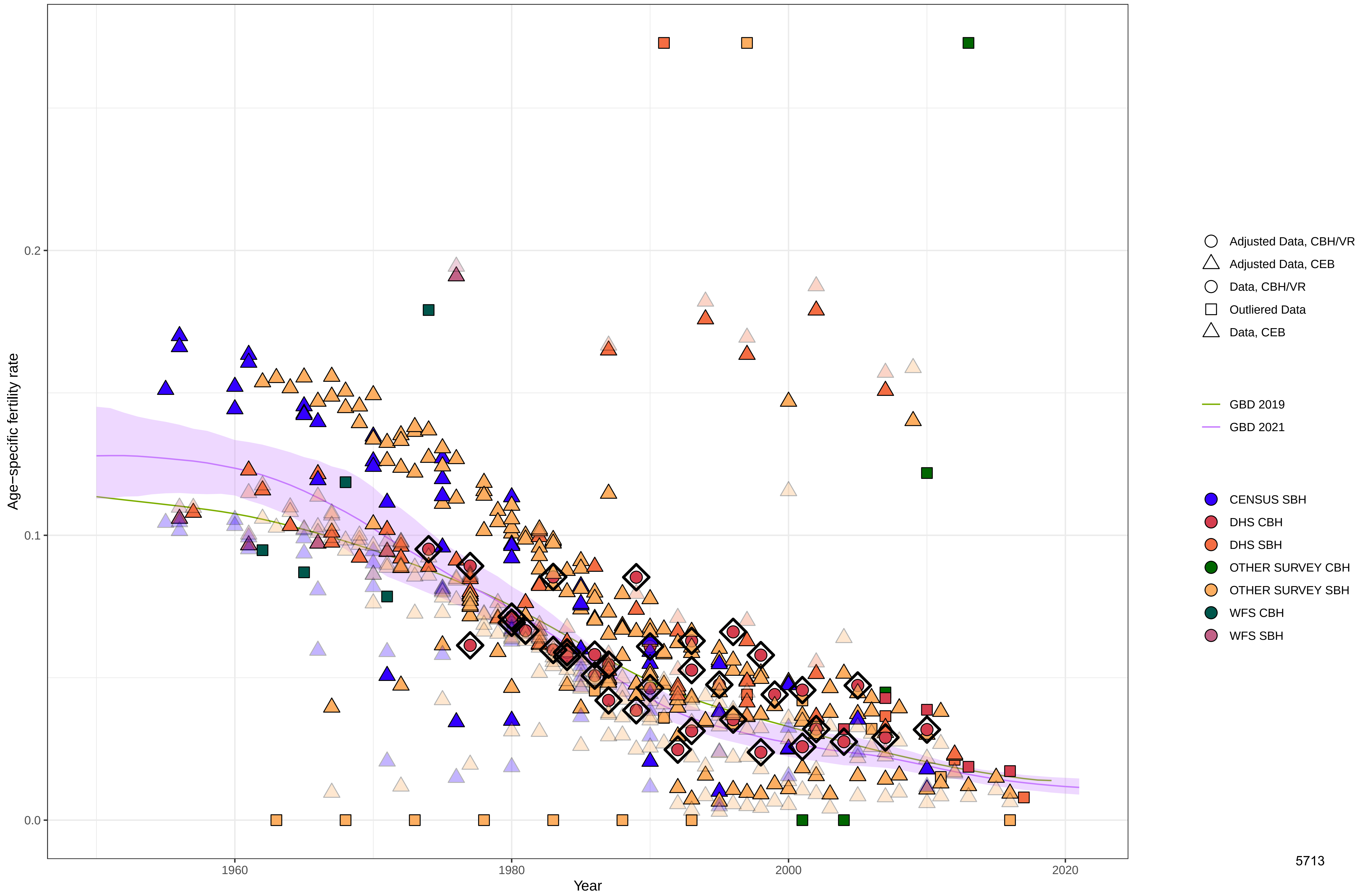

5713

Age−specific fertility fitted trend lines, 1950−2021: Yogyakarta, Ages: 20−24
Data points enclosed with a diamond are reference sources

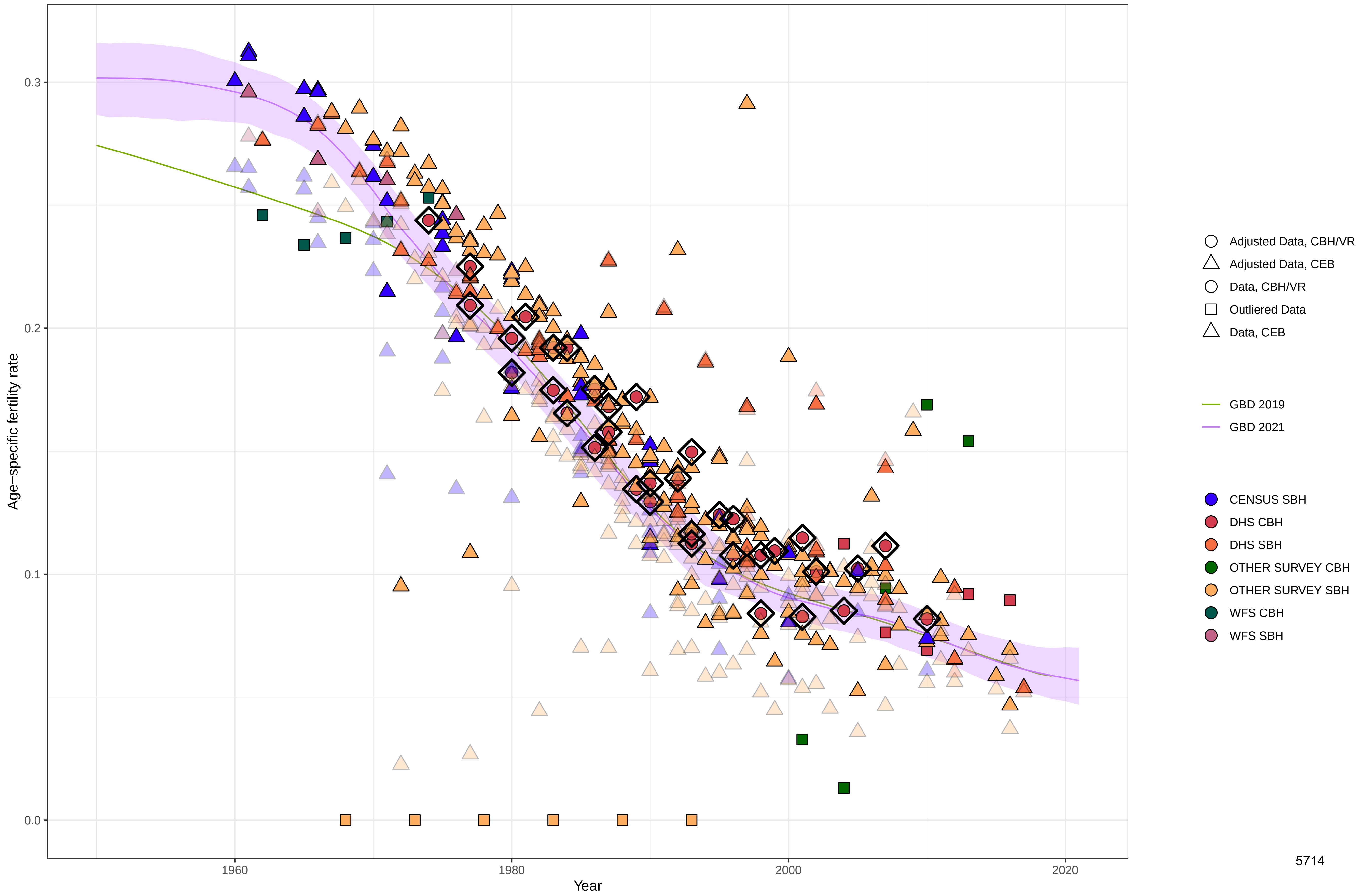

Age−specific fertility fitted trend lines, 1950−2021: Yogyakarta, Ages: 25−29
Data points enclosed with a diamond are reference sources

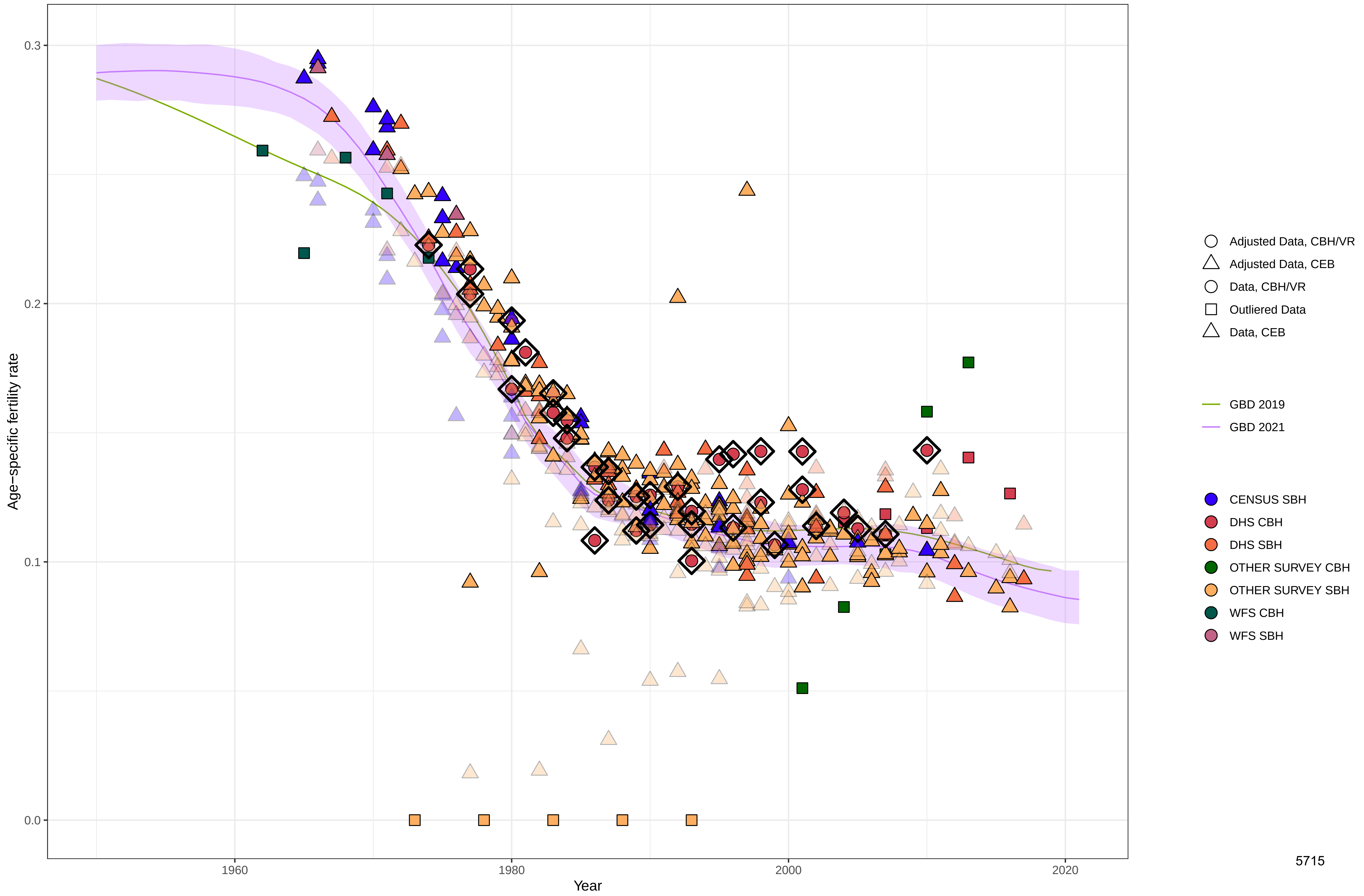

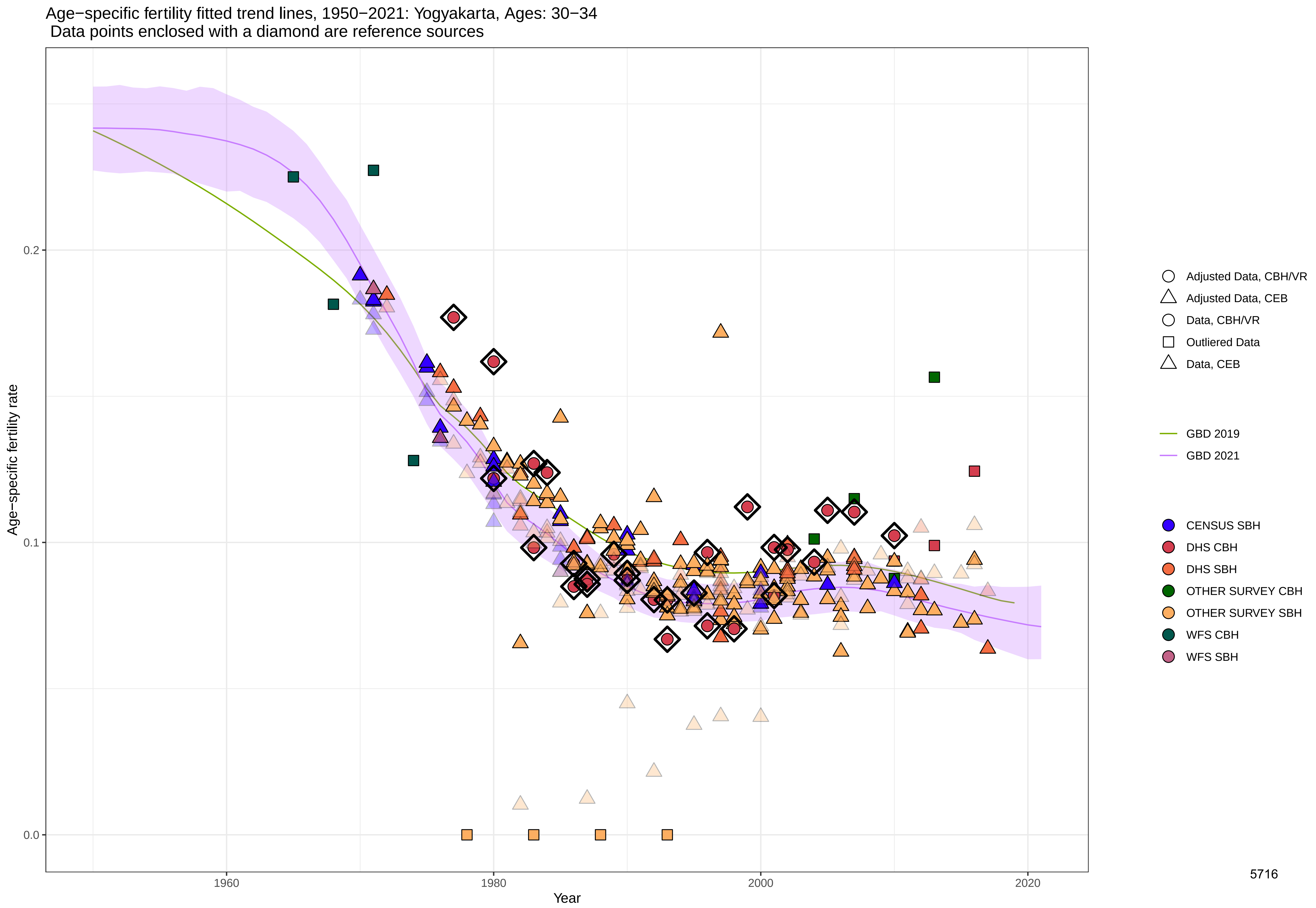

Age−specific fertility fitted trend lines, 1950−2021: Yogyakarta, Ages: 30−34
Data points enclosed with a diamond are reference sources
Age−specific fertility rate
Year
0.0
0.1
0.2
1960
1980
2000
2020
Adjusted Data, CBH/VR
Adjusted Data, CEB
Data, CBH/VR
Outliered Data
Data, CEB
GBD 2019
GBD 2021
CENSUS SBH
DHS CBH
DHS SBH
OTHER SURVEY CBH
OTHER SURVEY SBH
WFS CBH
WFS SBH

Age−specific fertility fitted trend lines, 1950−2021: Yogyakarta, Ages: 35−39
Data points enclosed with a diamond are reference sources

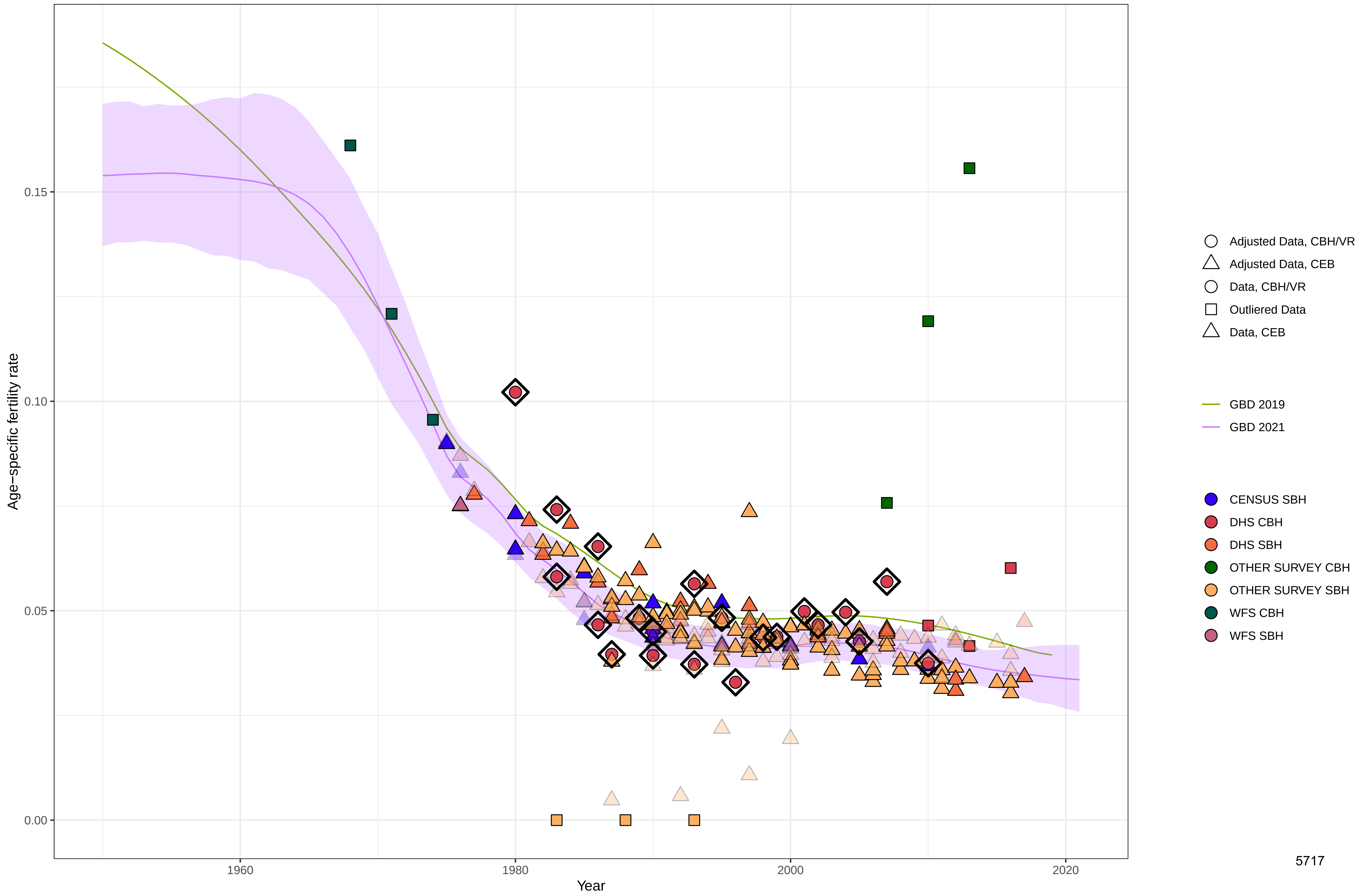

Age−specific fertility fitted trend lines, 1950−2021: Yogyakarta, Ages: 40−44
Data points enclosed with a diamond are reference sources

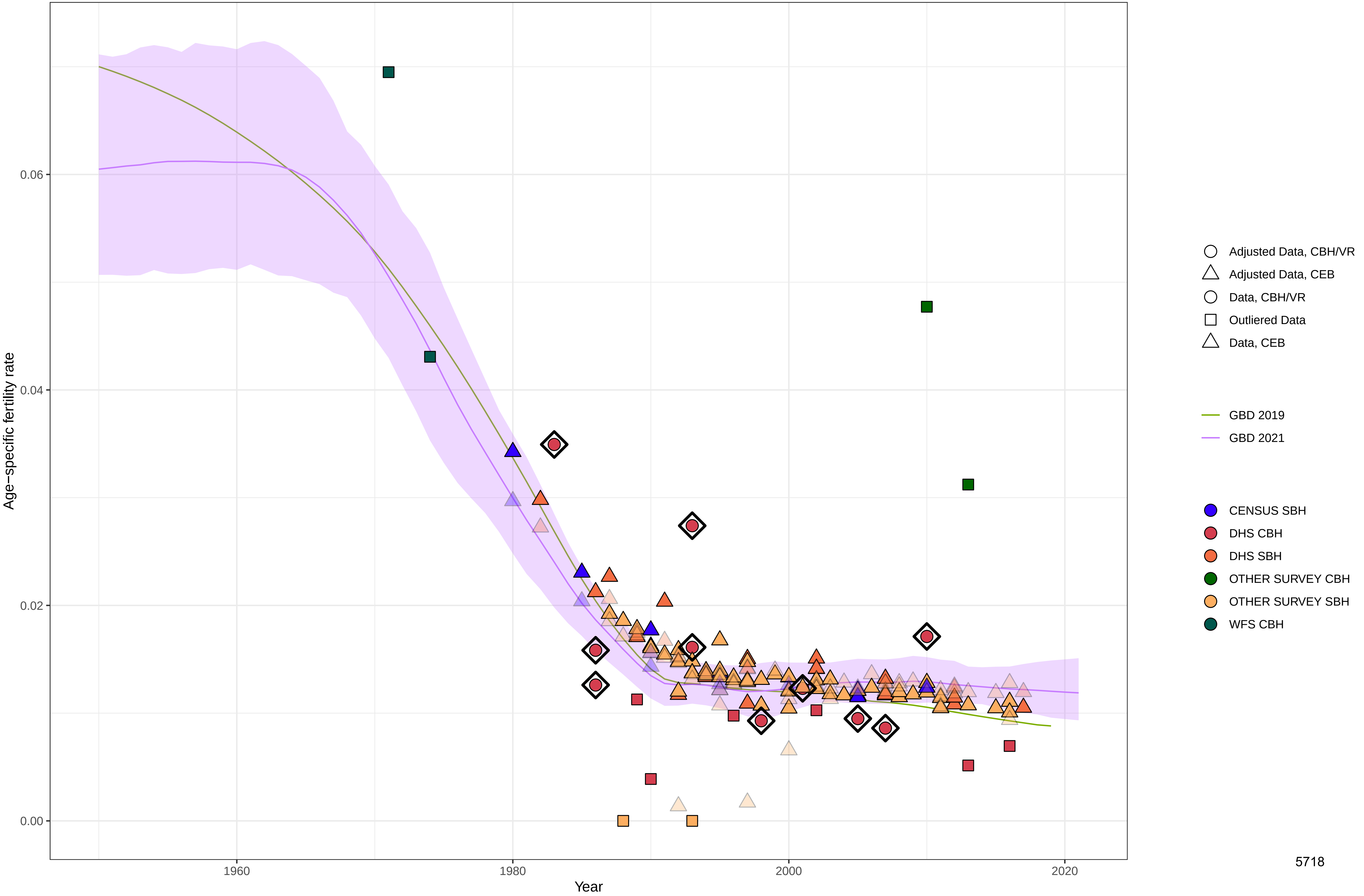

Age−specific fertility fitted trend lines, 1950−2021: Yogyakarta, Ages: 45−49
Data points enclosed with a diamond are reference sources

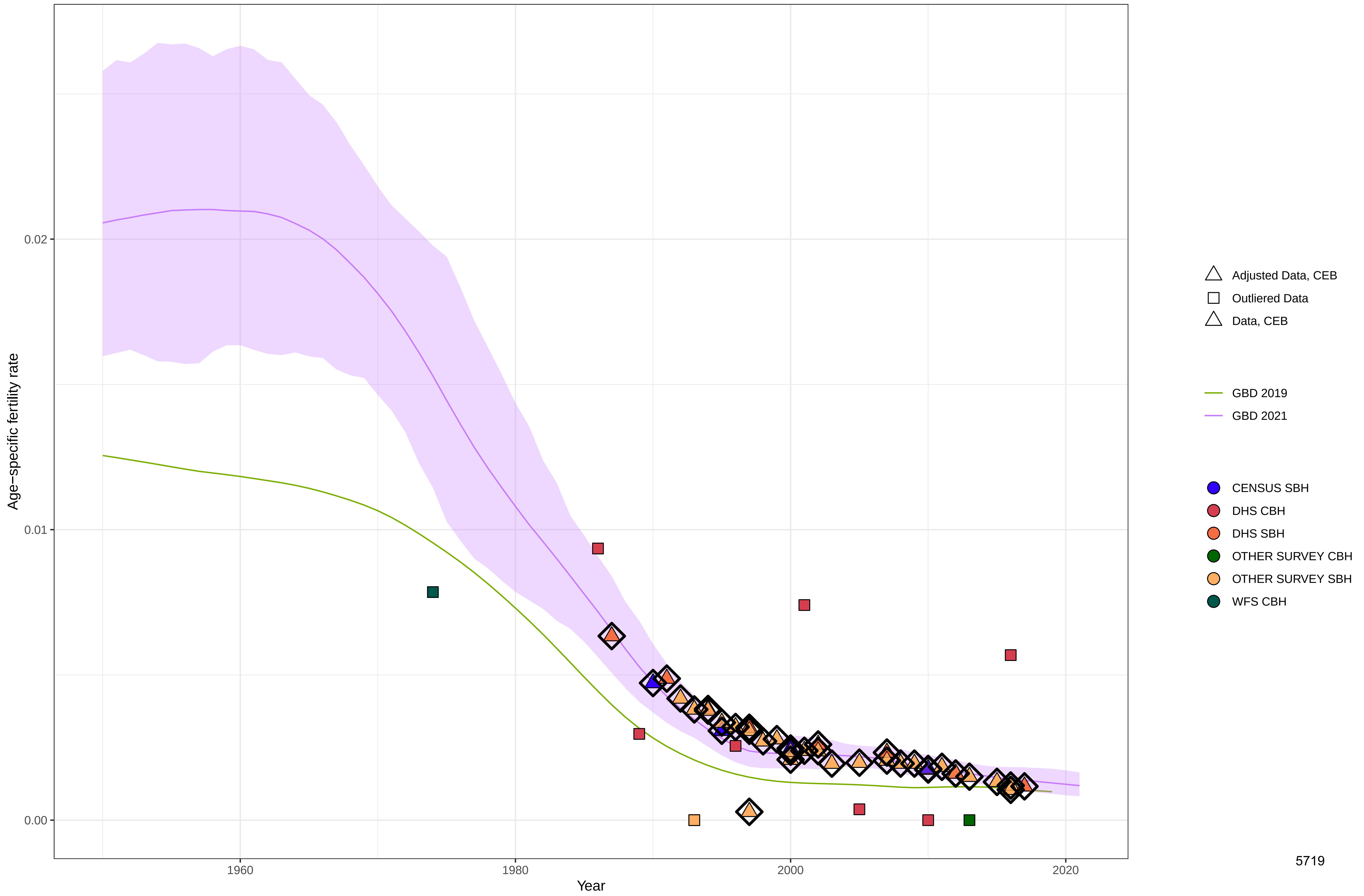

5719

Age−specific fertility fitted trend lines, 1950−2021: Yogyakarta, Ages: 50−54
Data points enclosed with a diamond are reference sources

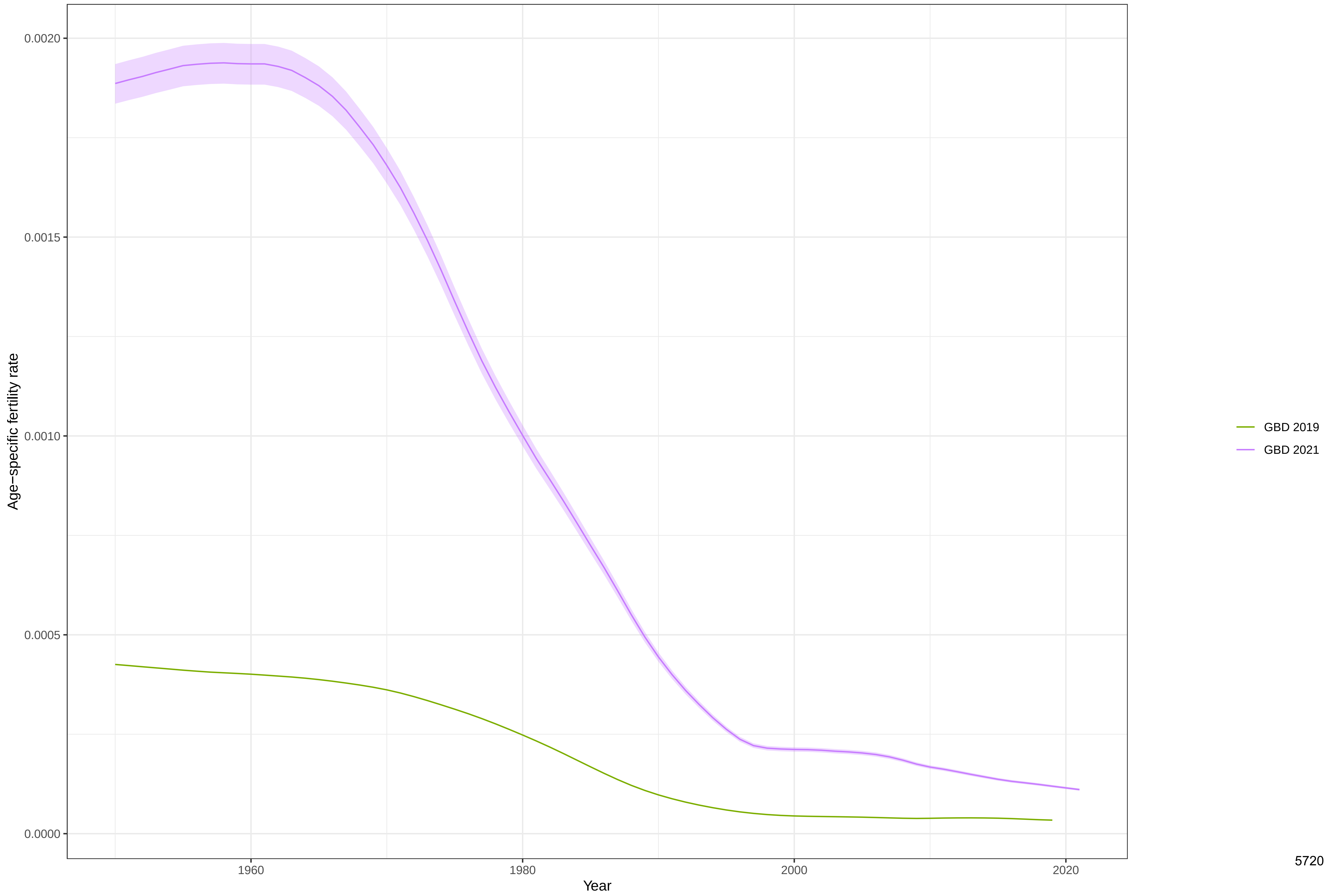

Total fertility rate fitted trend lines, 1950−2021: Yogyakarta TFR
Data points enclosed with a diamond are reference sources

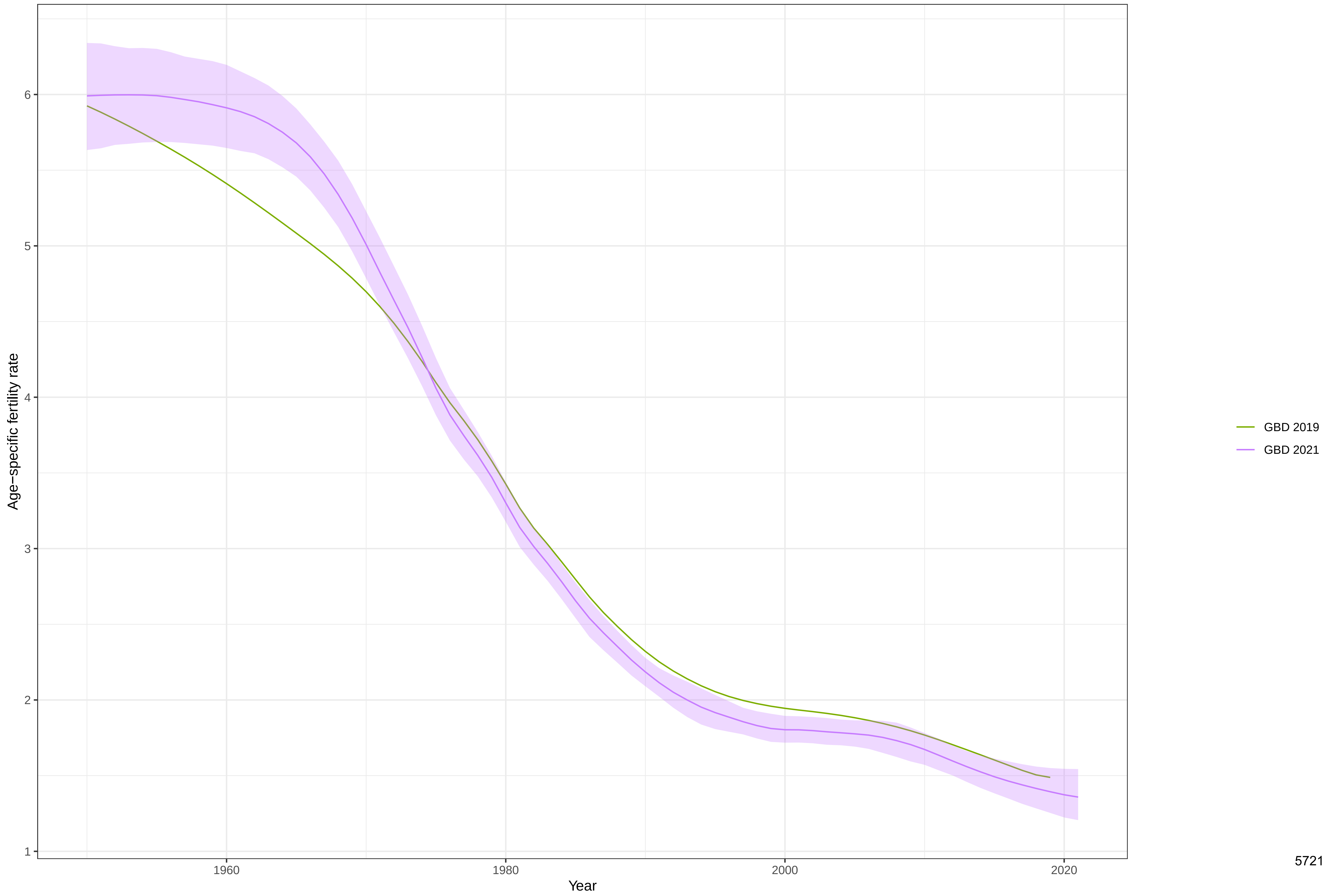

Age−specific fertility fitted trend lines, 1950−2021: Laos, Ages: 10−14
Data points enclosed with a diamond are reference sources

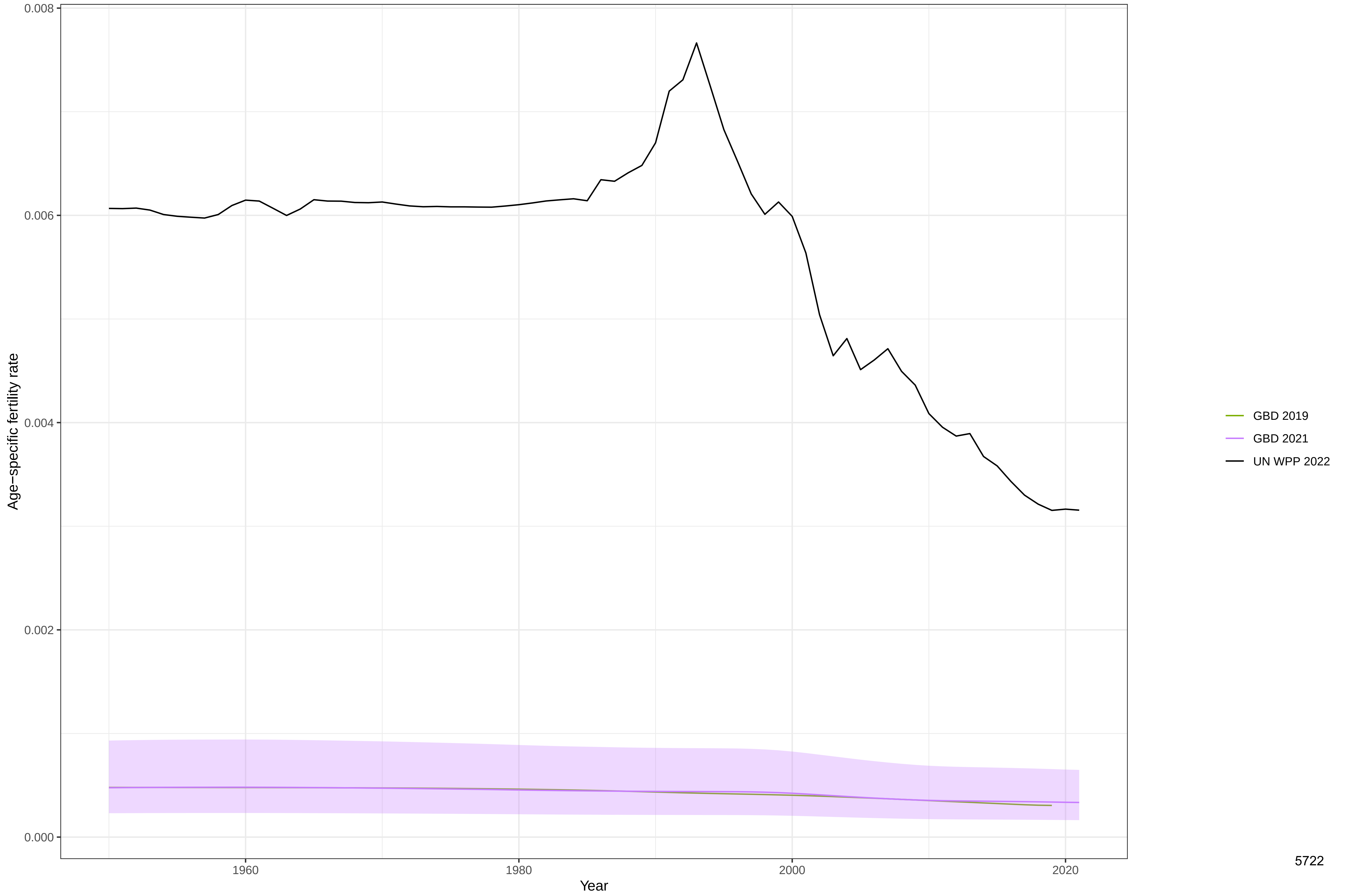

Age−specific fertility fitted trend lines, 1950−2021: Laos, Ages: 15−19
Data points enclosed with a diamond are reference sources

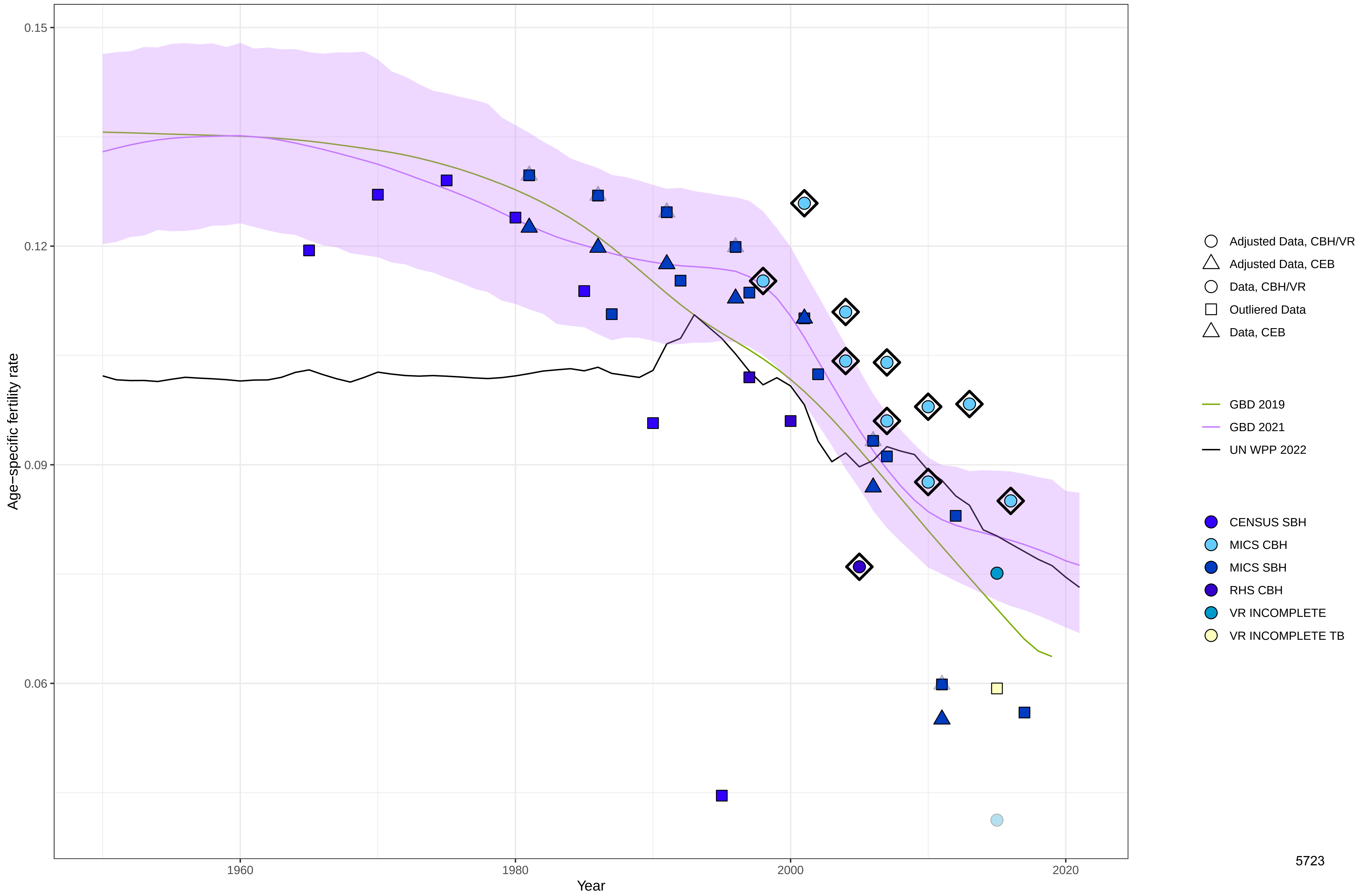

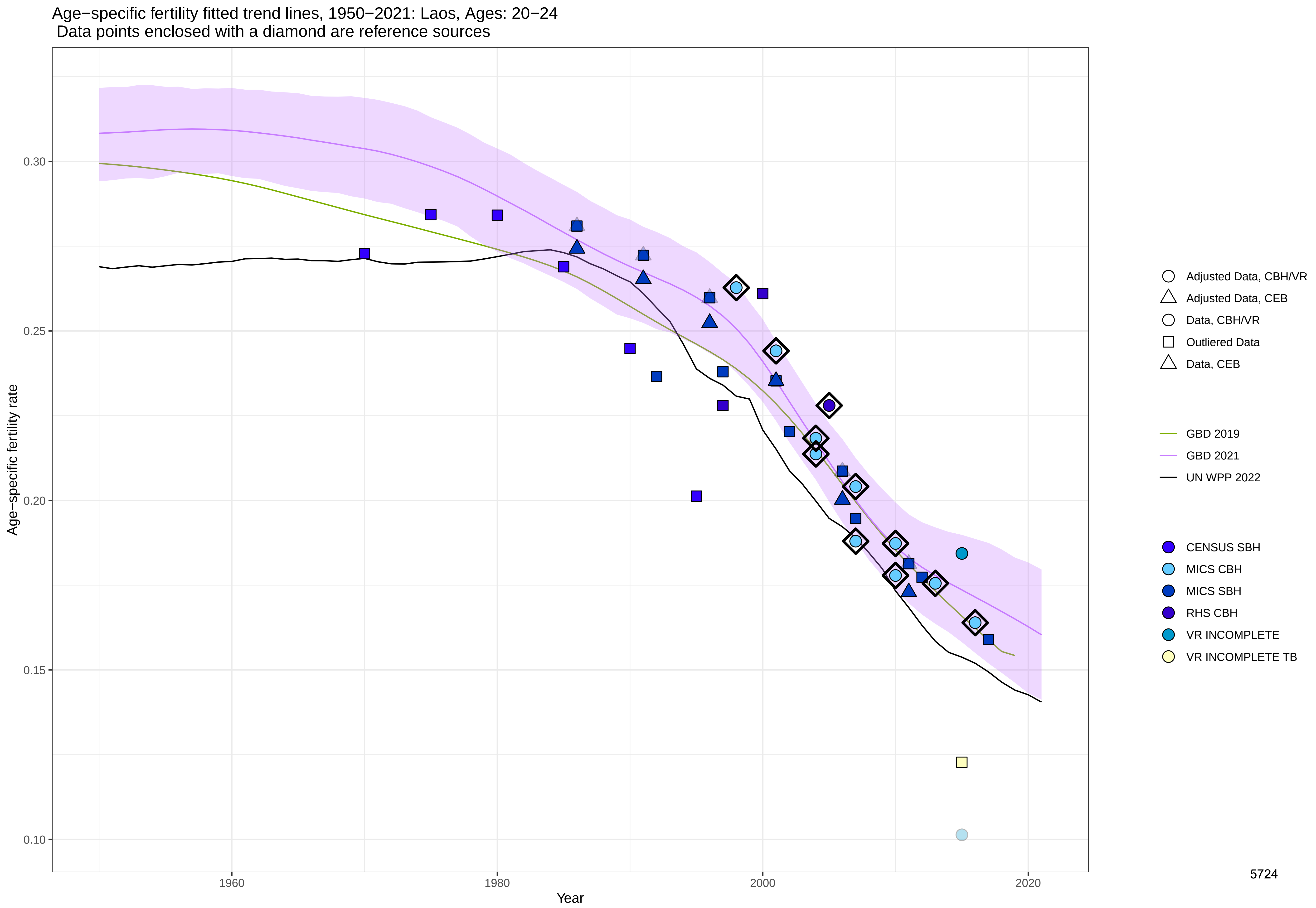

Age−specific fertility fitted trend lines, 1950−2021: Laos, Ages: 20−24
Data points enclosed with a diamond are reference sources
Age−specific fertility rate
Year
0.10
0.15
0.20
0.25
0.30
1960
1980
2000
2020
Adjusted Data, CBH/VR
Adjusted Data, CEB
Data, CBH/VR
Outliered Data
Data, CEB
GBD 2019
GBD 2021
UN WPP 2022
CENSUS SBH
MICS CBH
MICS SBH
RHS CBH
VR INCOMPLETE
VR INCOMPLETE TB

Age−specific fertility fitted trend lines, 1950−2021: Laos, Ages: 25−29
Data points enclosed with a diamond are reference sources

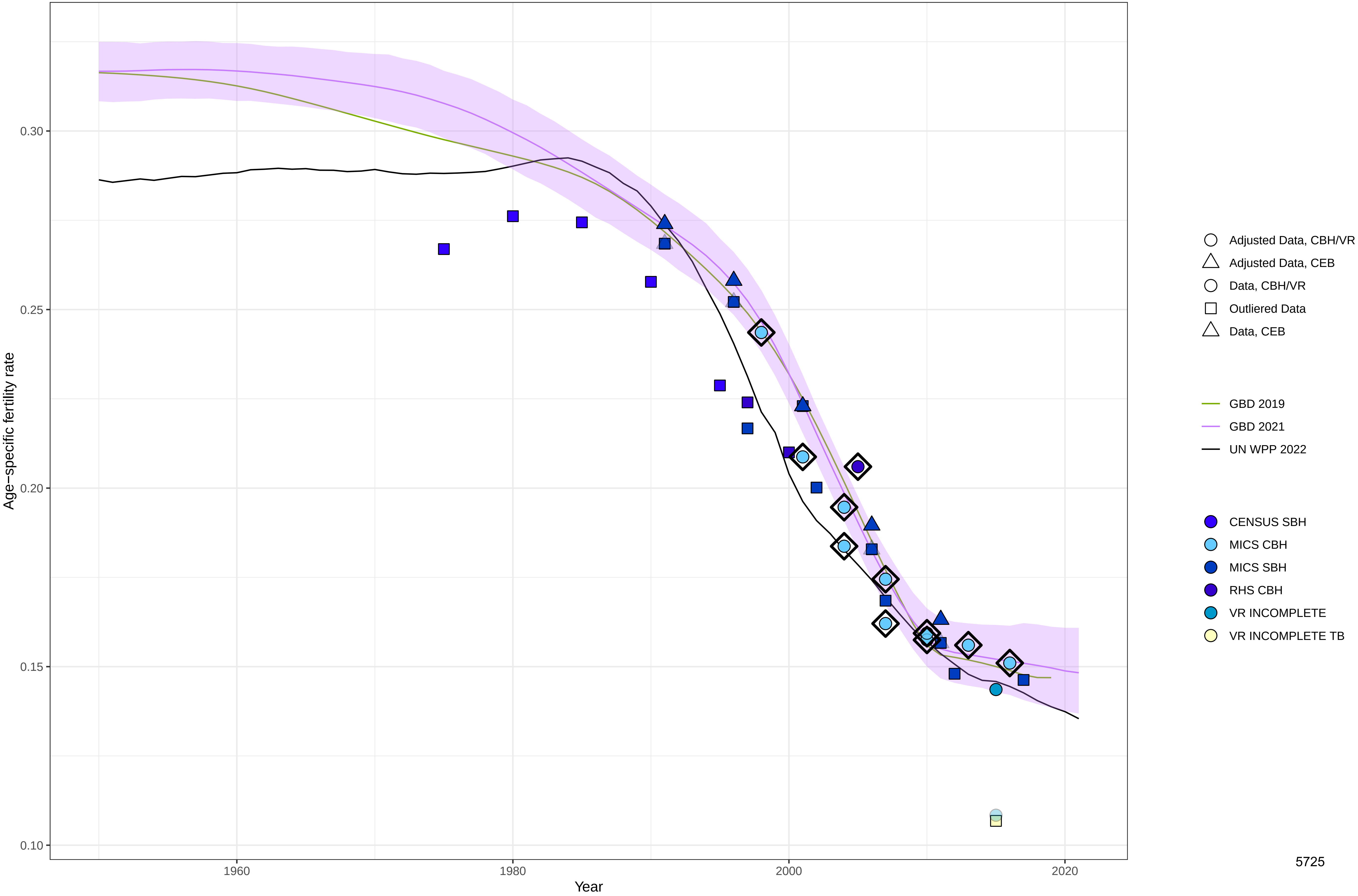

Age−specific fertility fitted trend lines, 1950−2021: Laos, Ages: 30−34
Data points enclosed with a diamond are reference sources

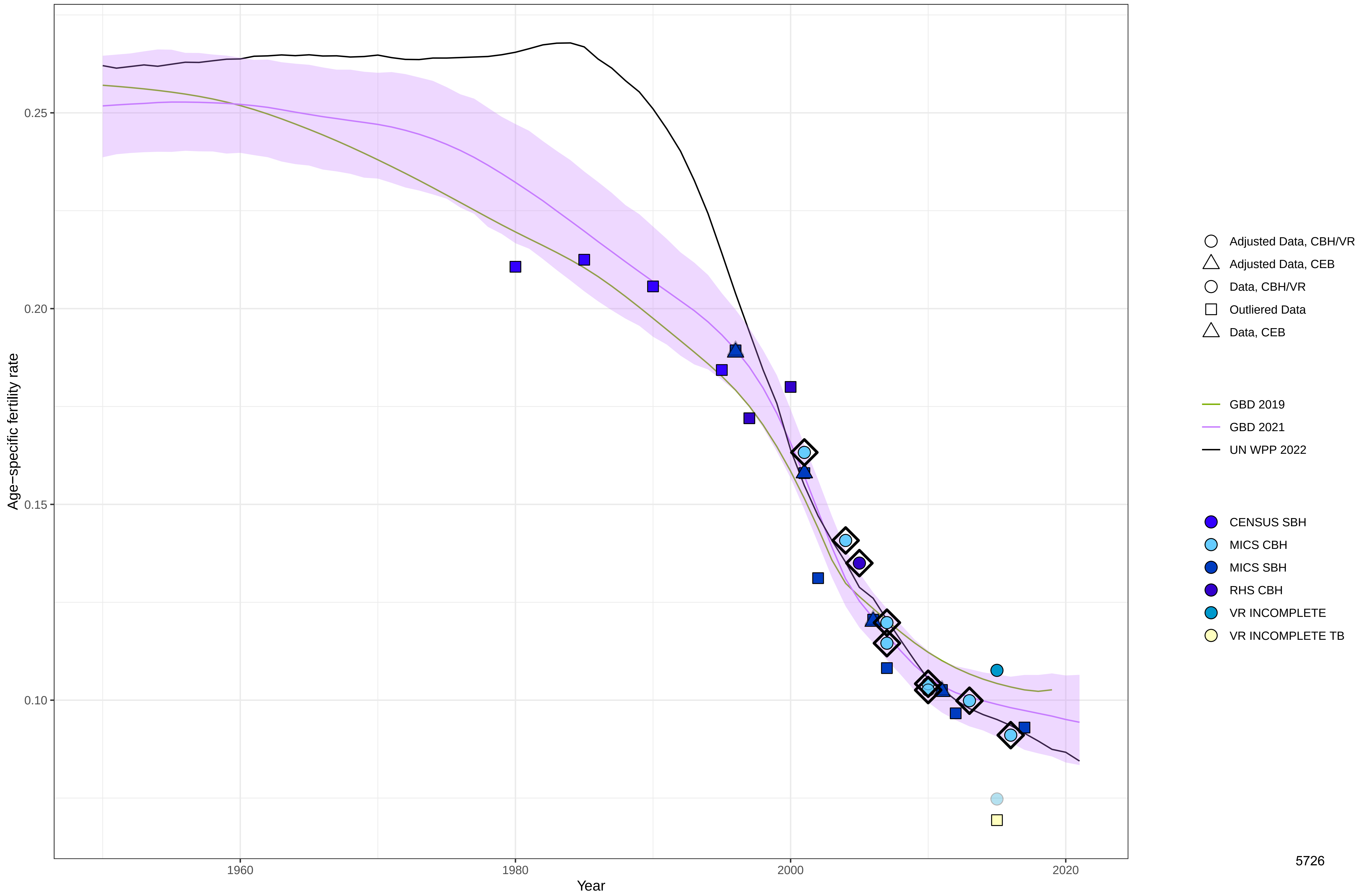

Age−specific fertility fitted trend lines, 1950−2021: Laos, Ages: 35−39
Data points enclosed with a diamond are reference sources

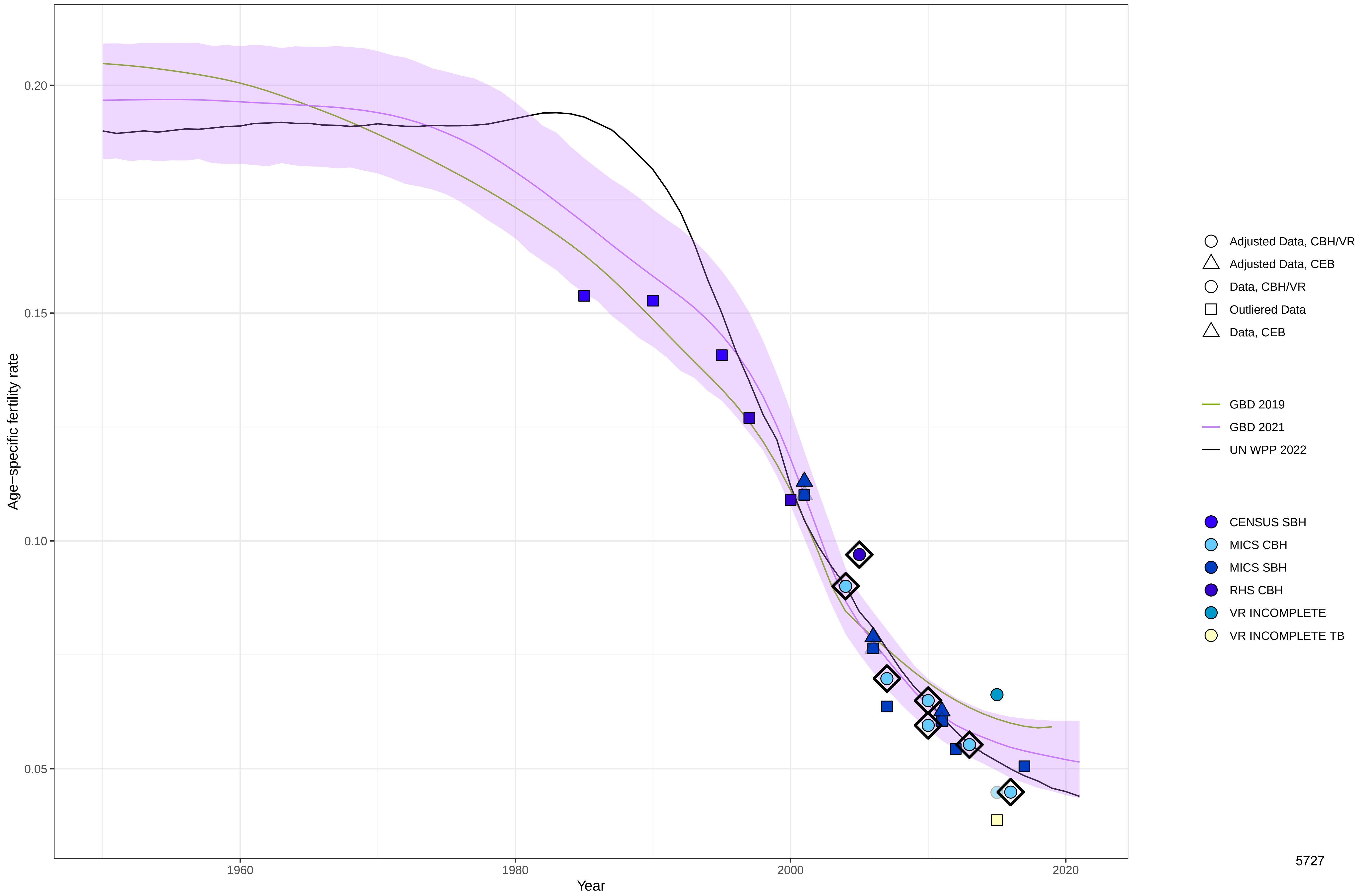

Age−specific fertility fitted trend lines, 1950−2021: Laos, Ages: 40−44
Data points enclosed with a diamond are reference sources

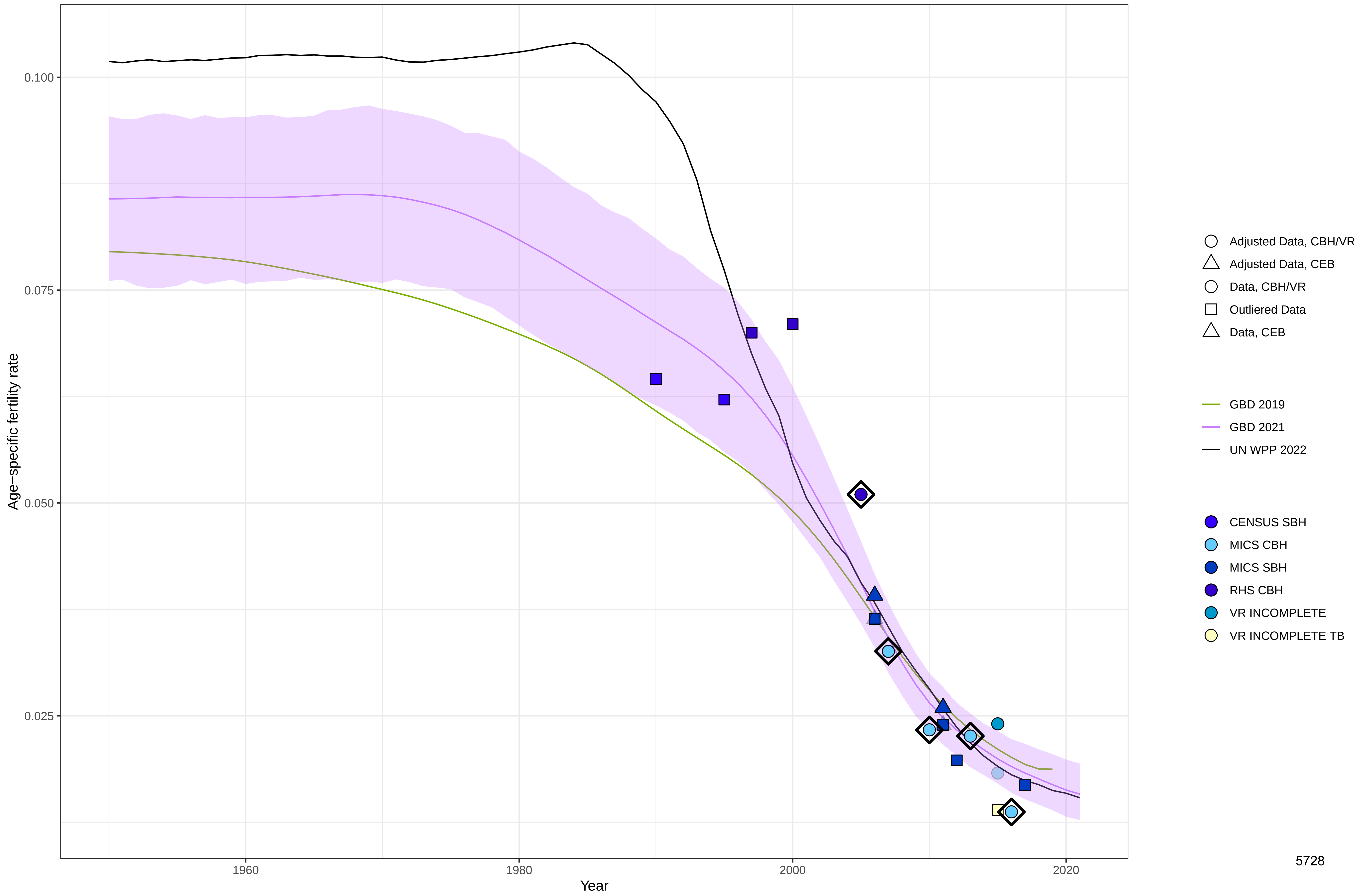

Age−specific fertility fitted trend lines, 1950−2021: Laos, Ages: 45−49
Data points enclosed with a diamond are reference sources

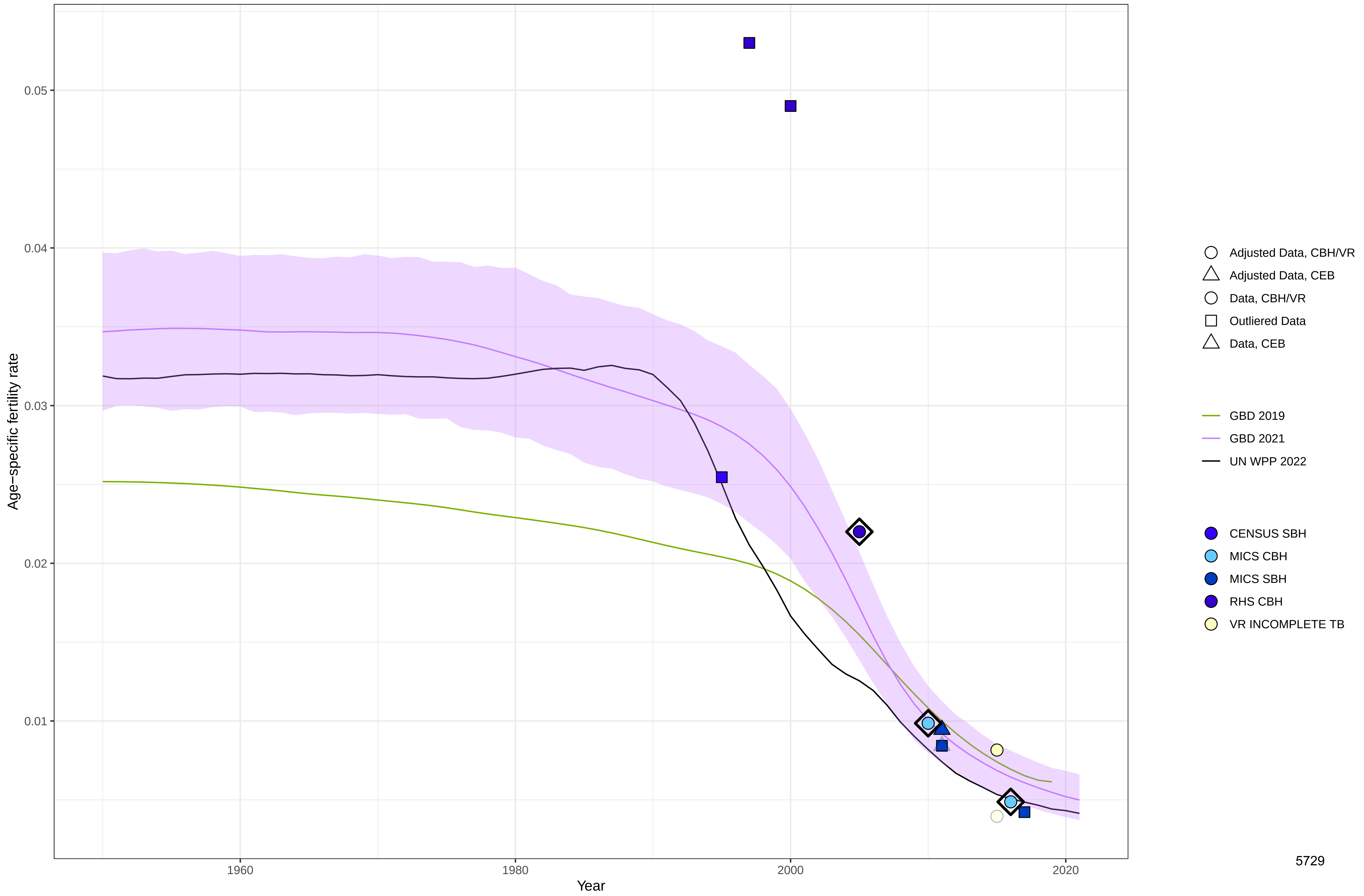

Age−specific fertility fitted trend lines, 1950−2021: Laos, Ages: 50−54
Data points enclosed with a diamond are reference sources

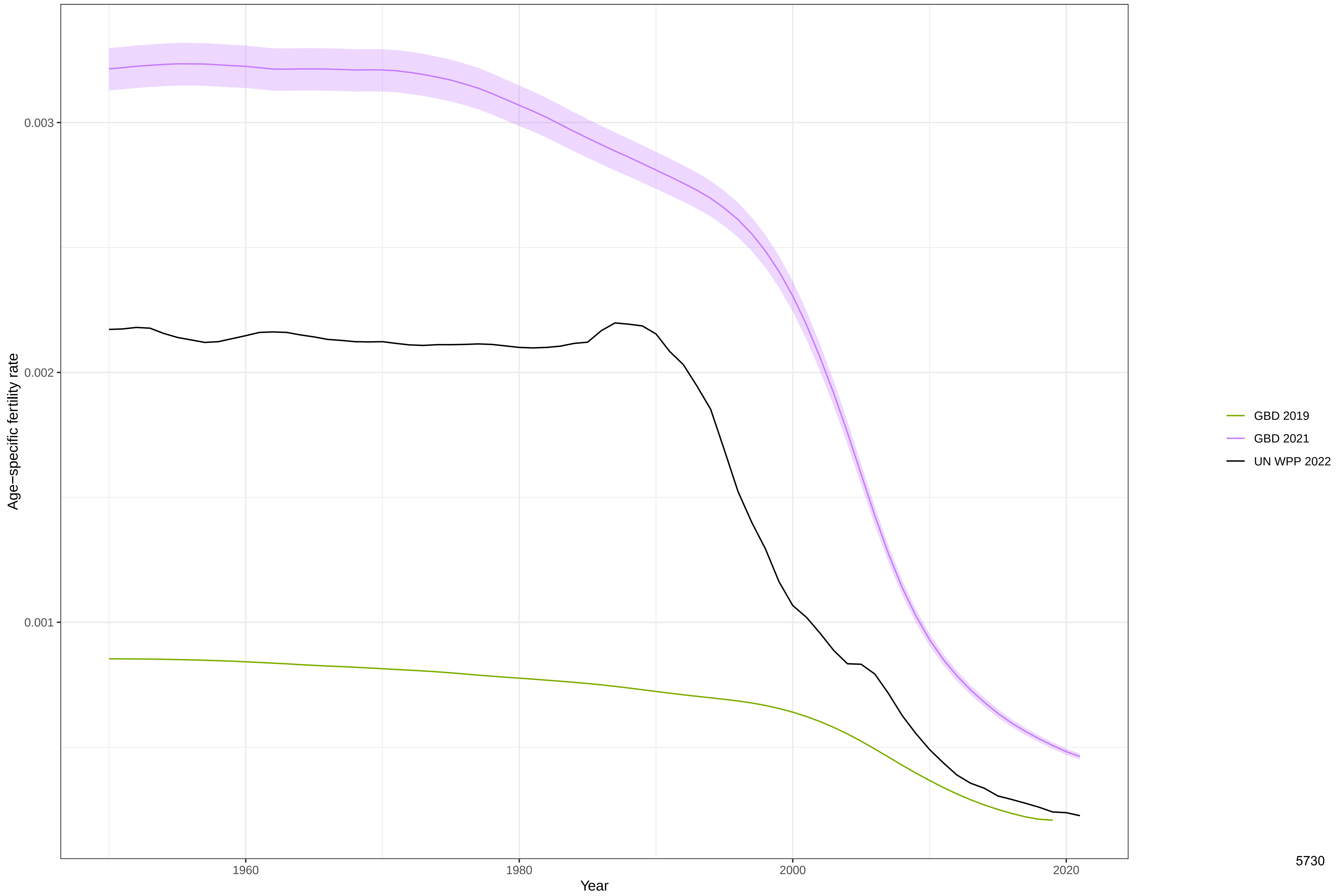

Total fertility rate fitted trend lines, 1950−2021: Laos TFR
Data points enclosed with a diamond are reference sources

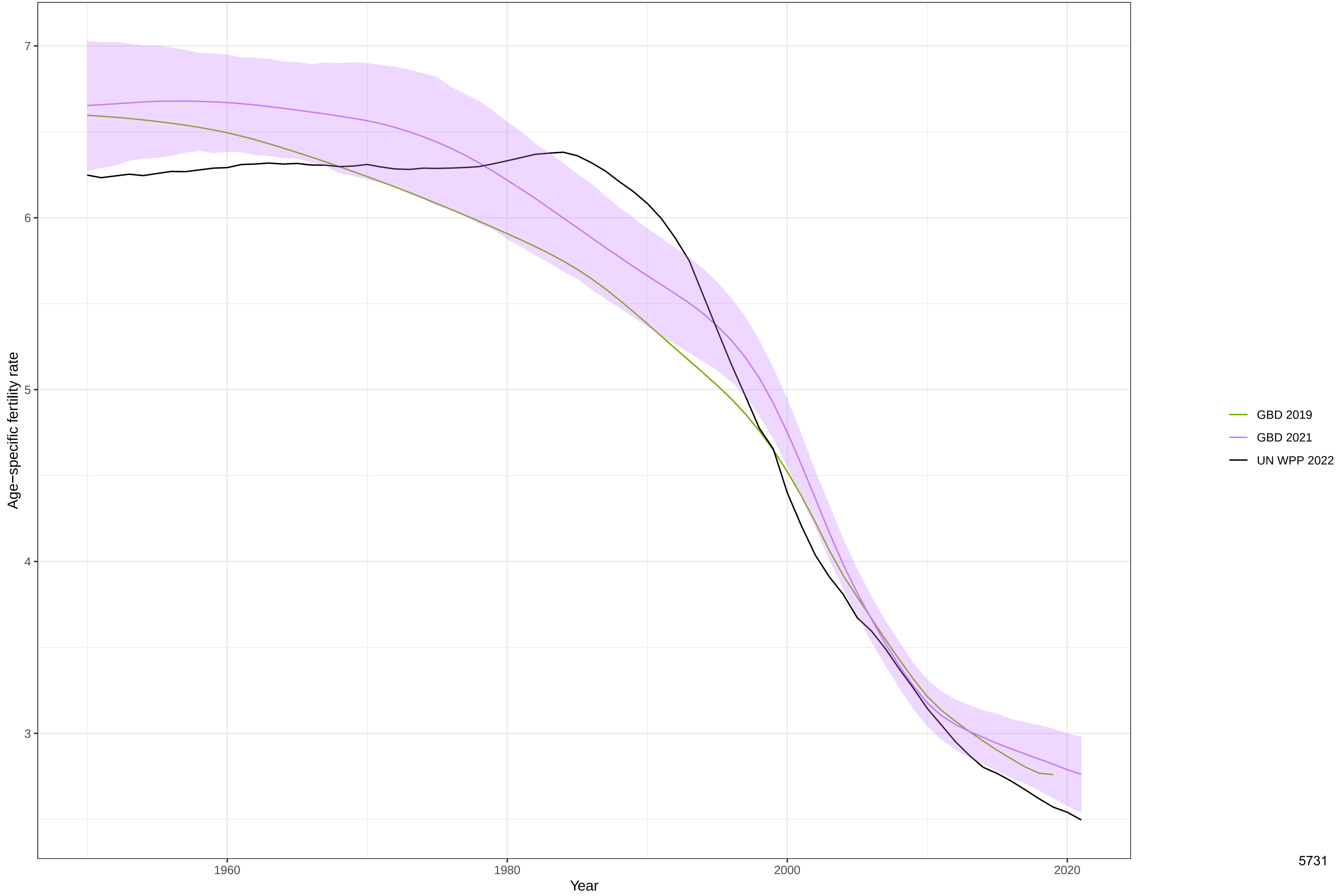

Age−specific fertility fitted trend lines, 1950−2021: Malaysia, Ages: 10−14
Data points enclosed with a diamond are reference sources

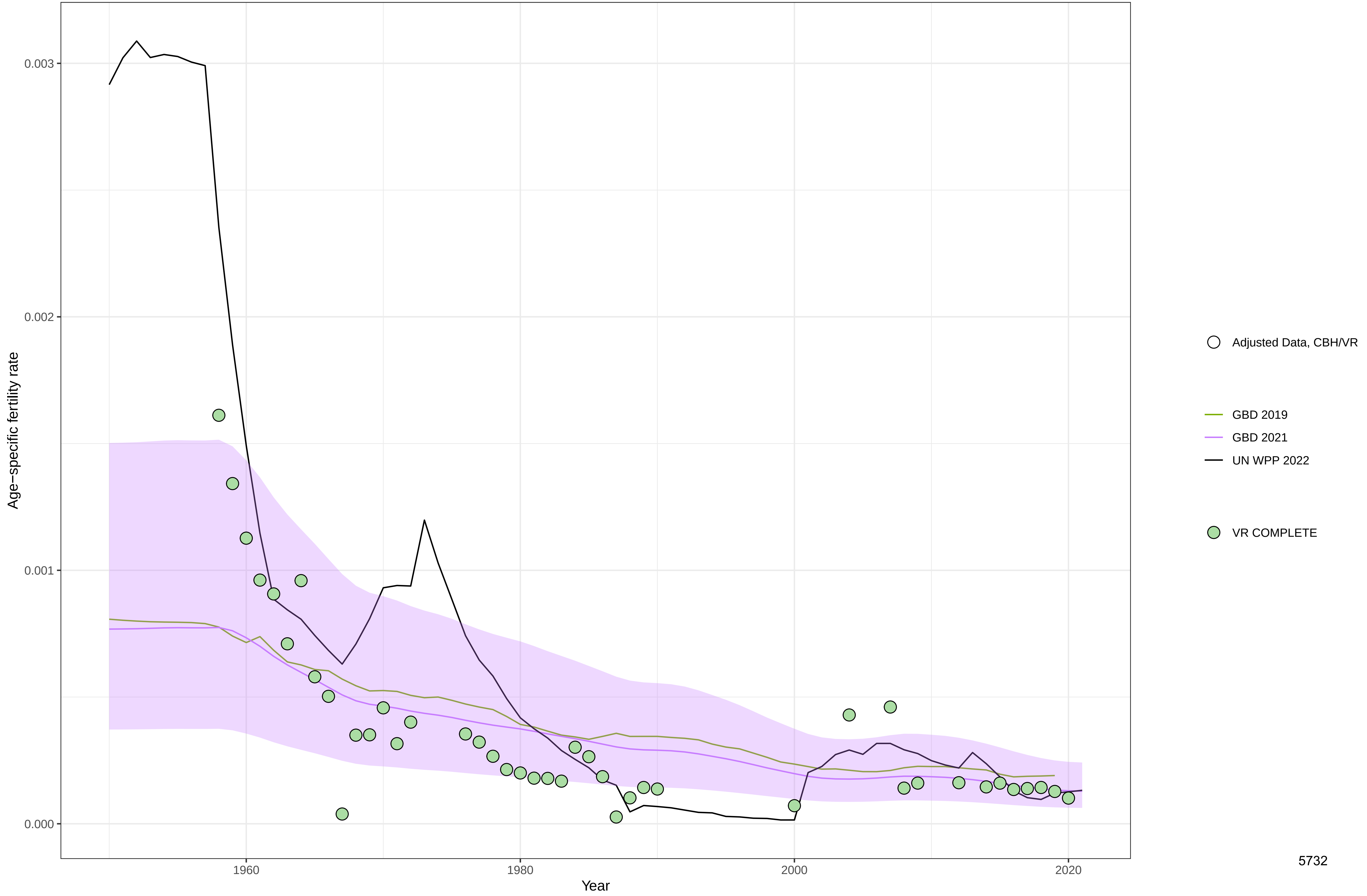

Age−specific fertility fitted trend lines, 1950−2021: Malaysia, Ages: 15−19
Data points enclosed with a diamond are reference sources

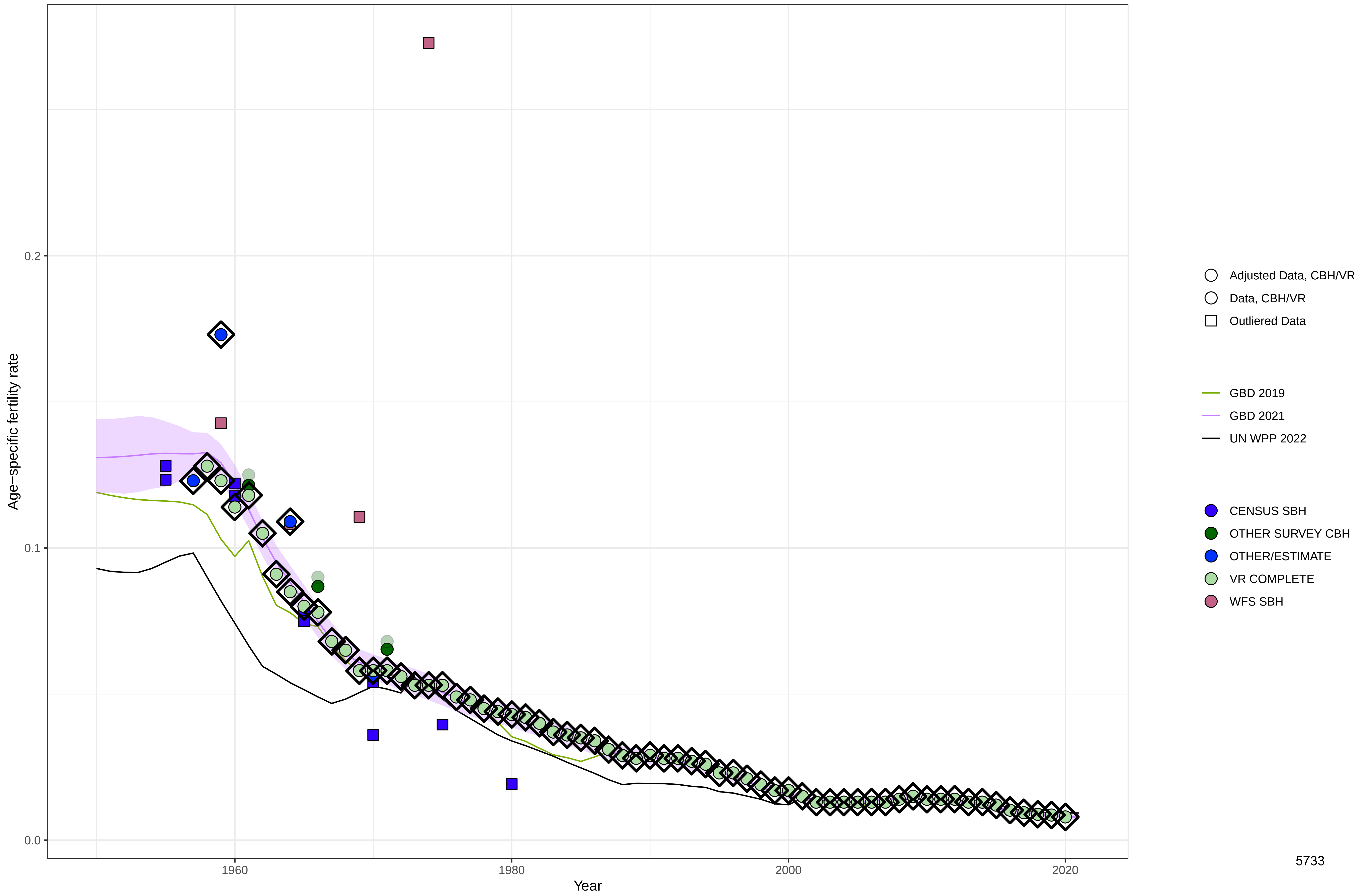

5733

Age−specific fertility fitted trend lines, 1950−2021: Malaysia, Ages: 20−24
Data points enclosed with a diamond are reference sources

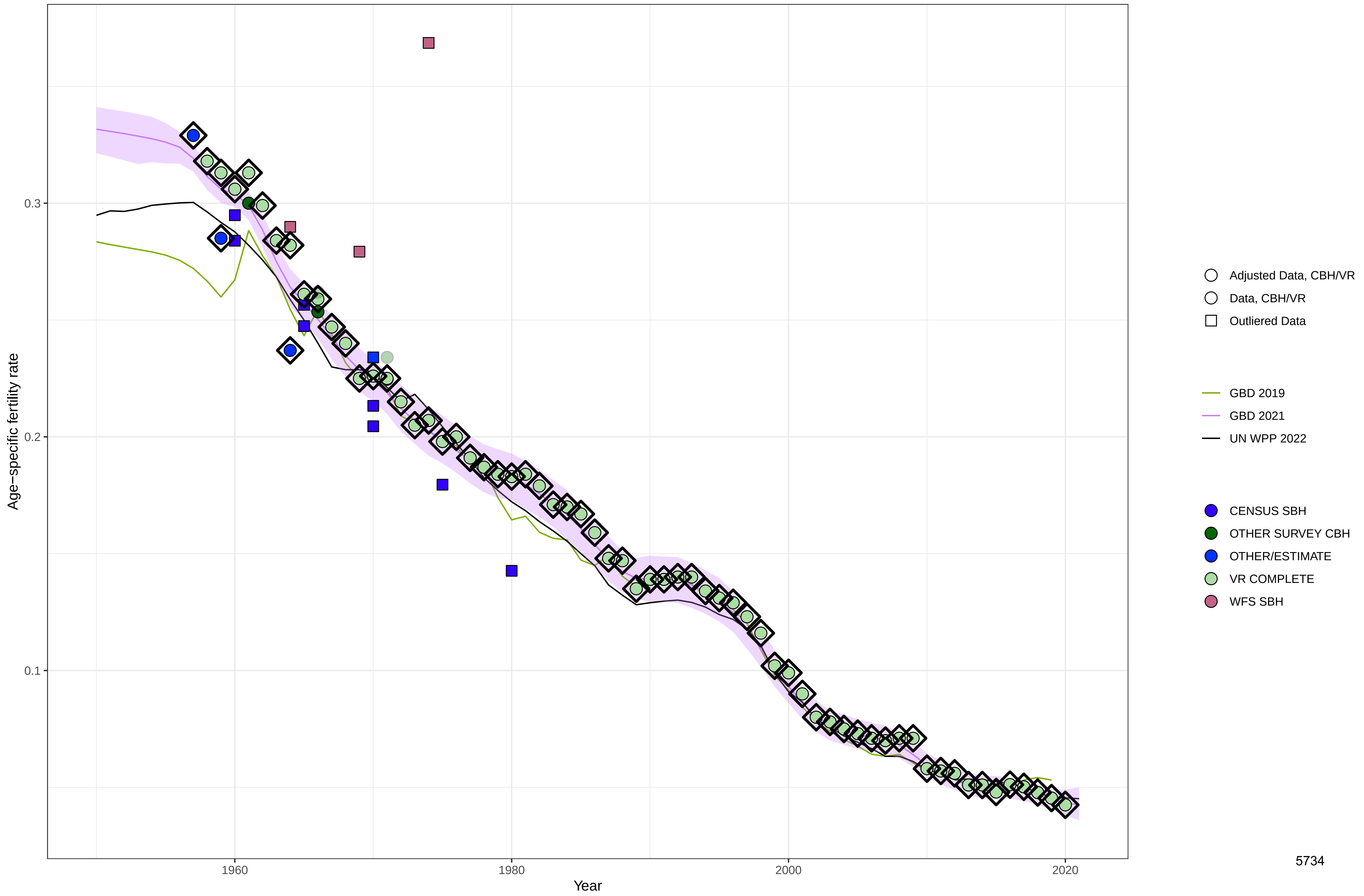

Age−specific fertility fitted trend lines, 1950−2021: Malaysia, Ages: 25−29
Data points enclosed with a diamond are reference sources

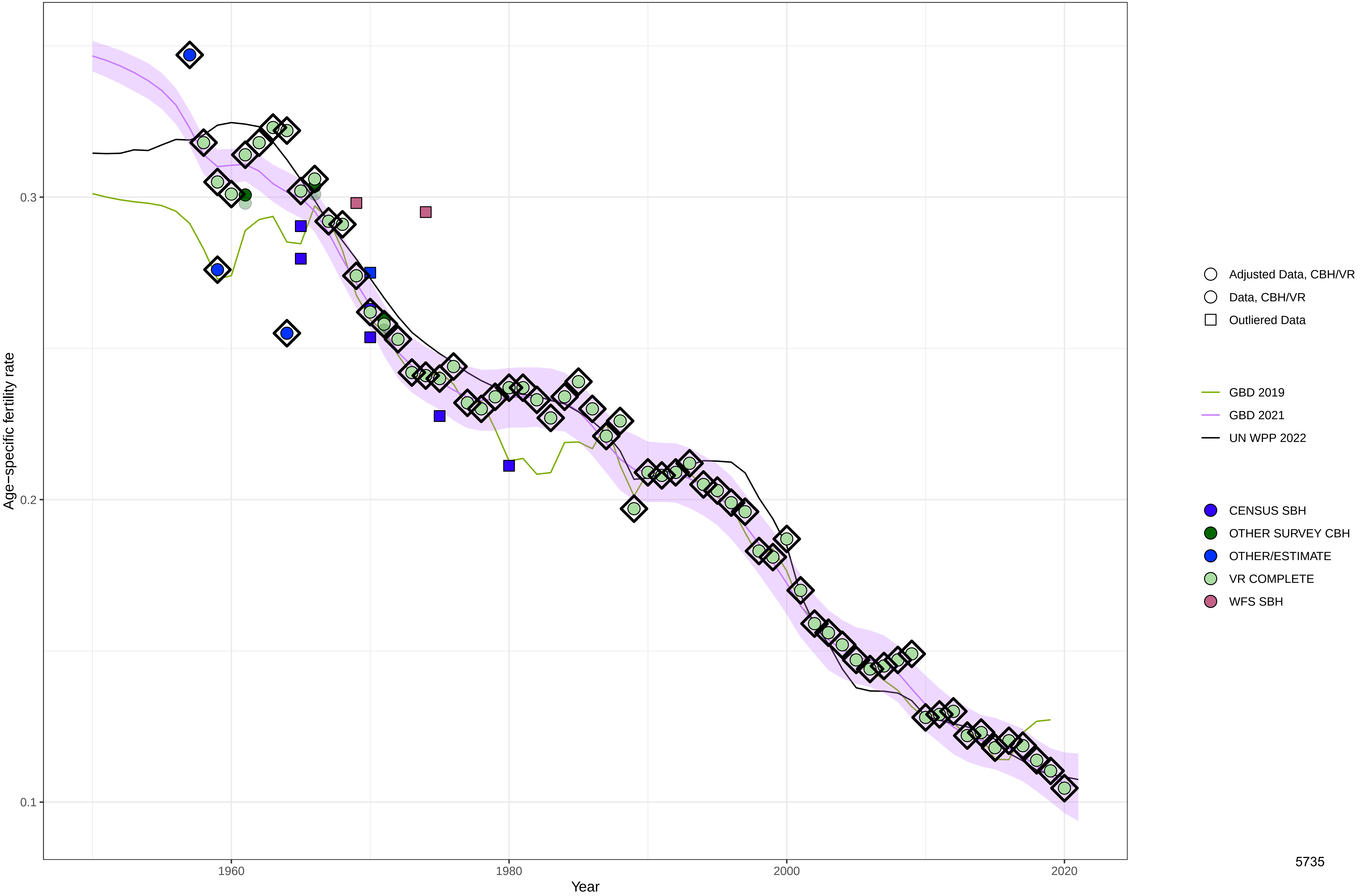

Age−specific fertility fitted trend lines, 1950−2021: Malaysia, Ages: 30−34
Data points enclosed with a diamond are reference sources

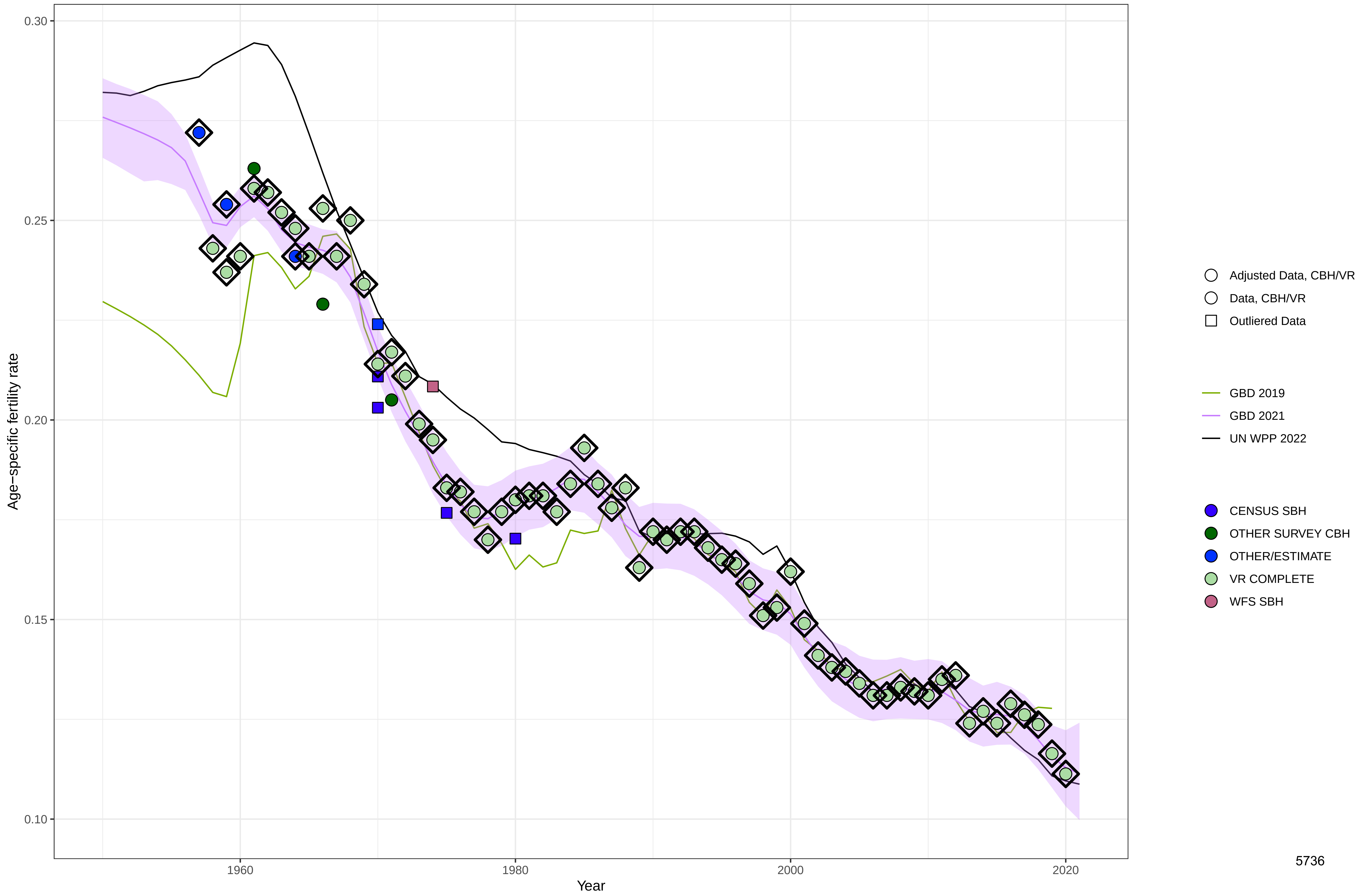

Age−specific fertility fitted trend lines, 1950−2021: Malaysia, Ages: 35−39
Data points enclosed with a diamond are reference sources

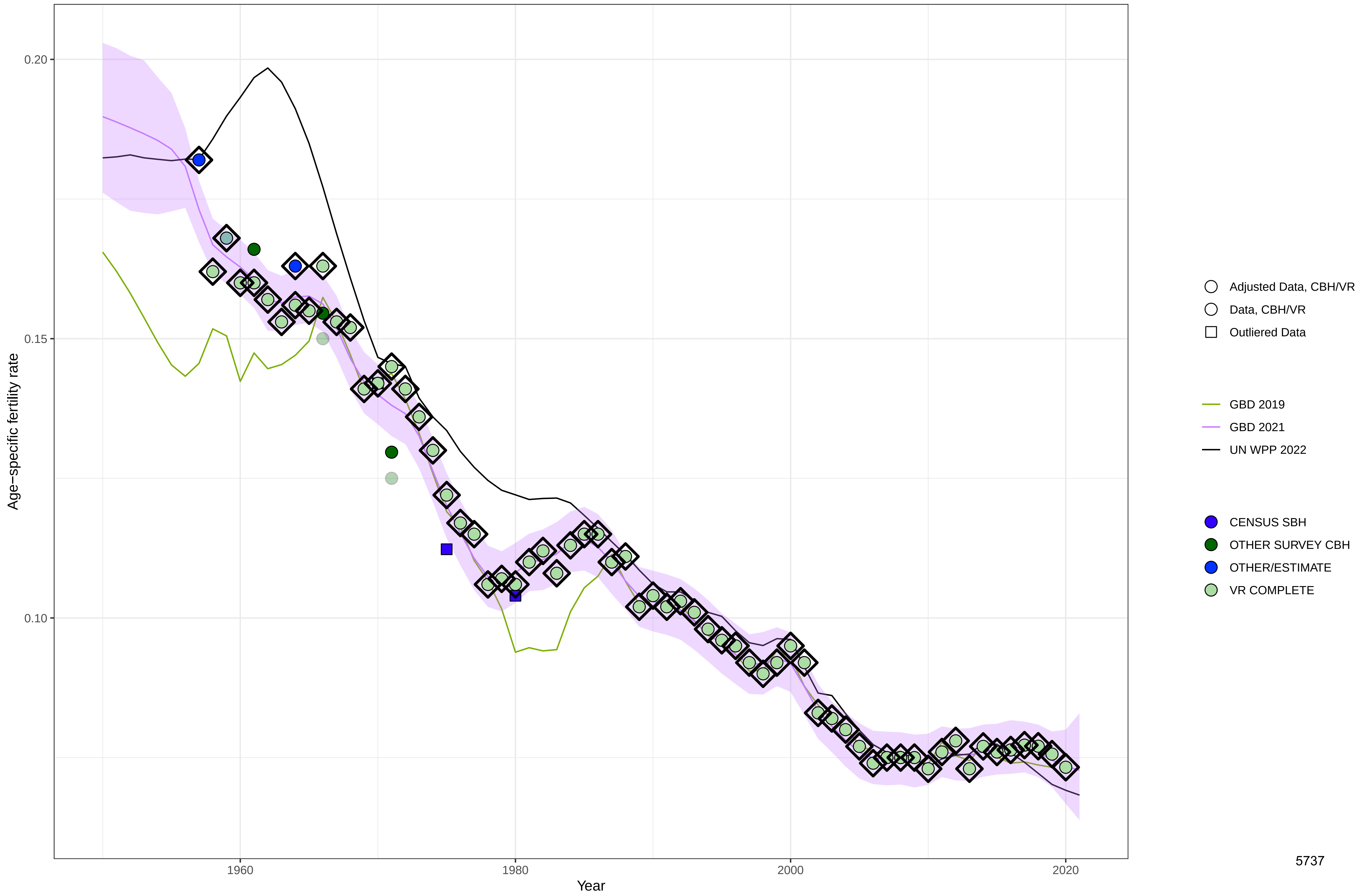

Age−specific fertility fitted trend lines, 1950−2021: Malaysia, Ages: 40−44
Data points enclosed with a diamond are reference sources

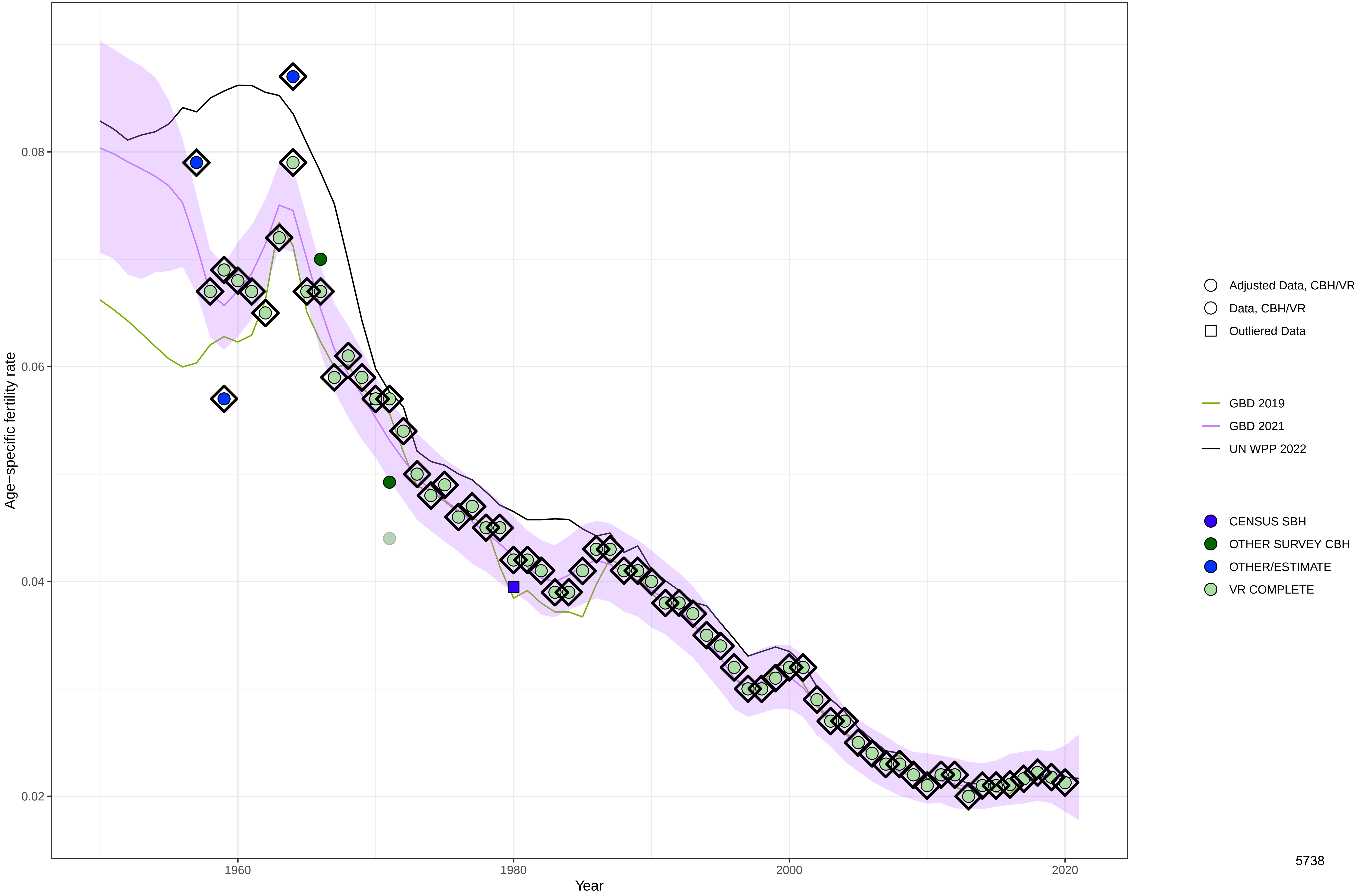

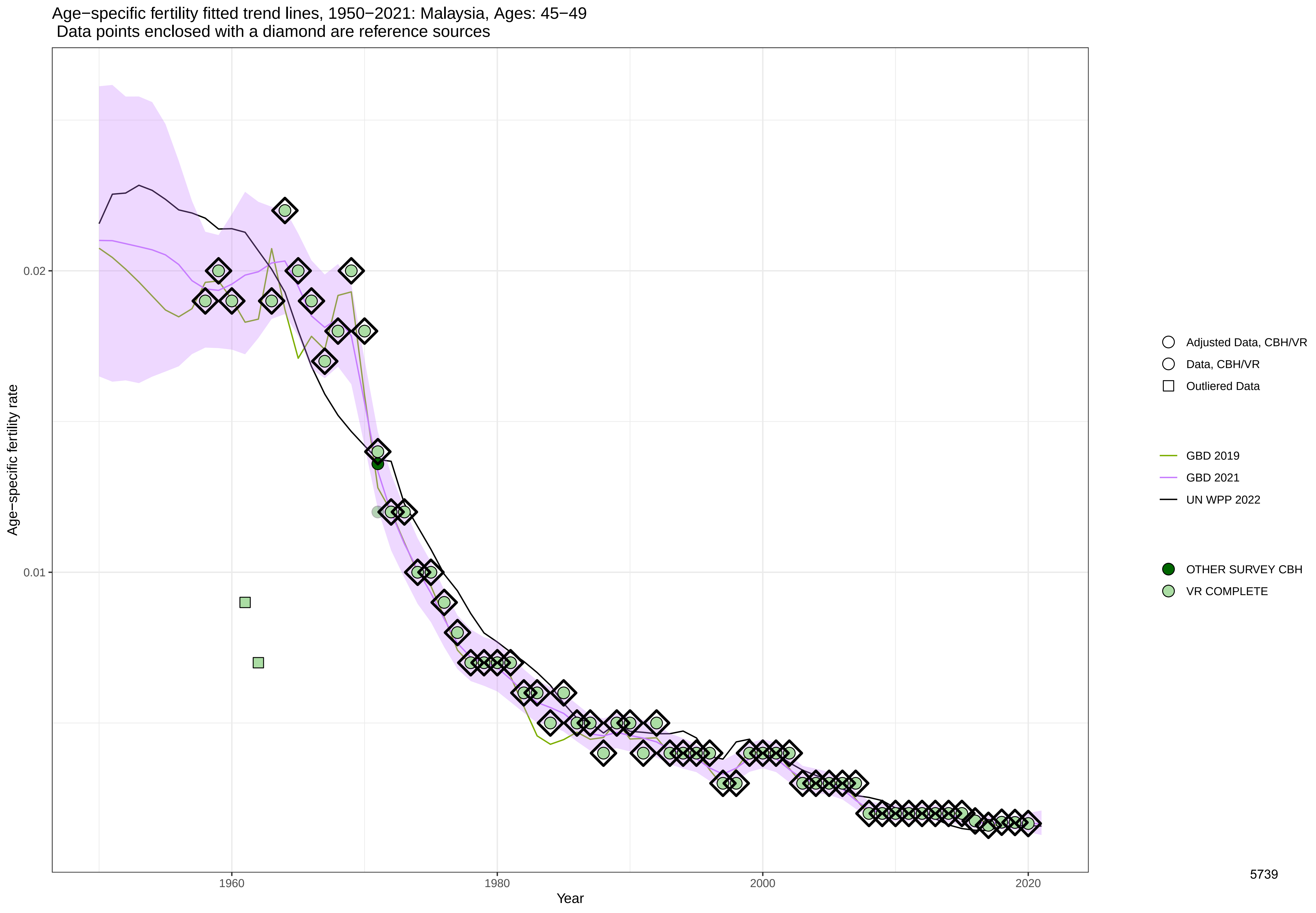
Age−specific fertility fitted trend lines, 1950−2021: Malaysia, Ages: 45−49
Data points enclosed with a diamond are reference sources
Age−specific fertility rate
0.02
0.01
1960
1980
2000
2020
Year
Adjusted Data, CBH/VR
Data, CBH/VR
Outliered Data
GBD 2019
GBD 2021
UN WPP 2022
OTHER SURVEY CBH
VR COMPLETE

Age−specific fertility fitted trend lines, 1950−2021: Malaysia, Ages: 50−54
Data points enclosed with a diamond are reference sources

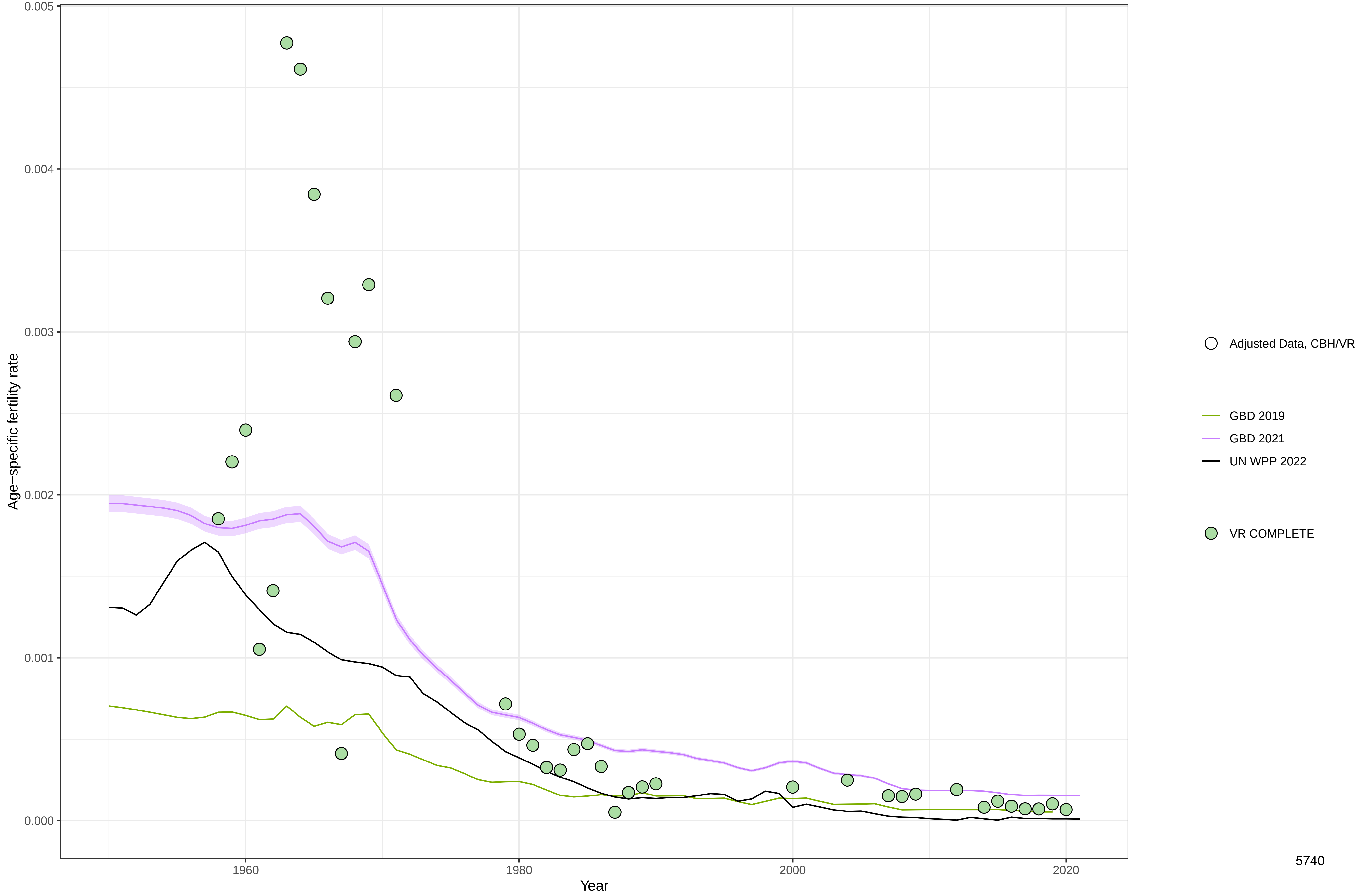

Total fertility rate fitted trend lines, 1950−2021: Malaysia TFR
Data points enclosed with a diamond are reference sources

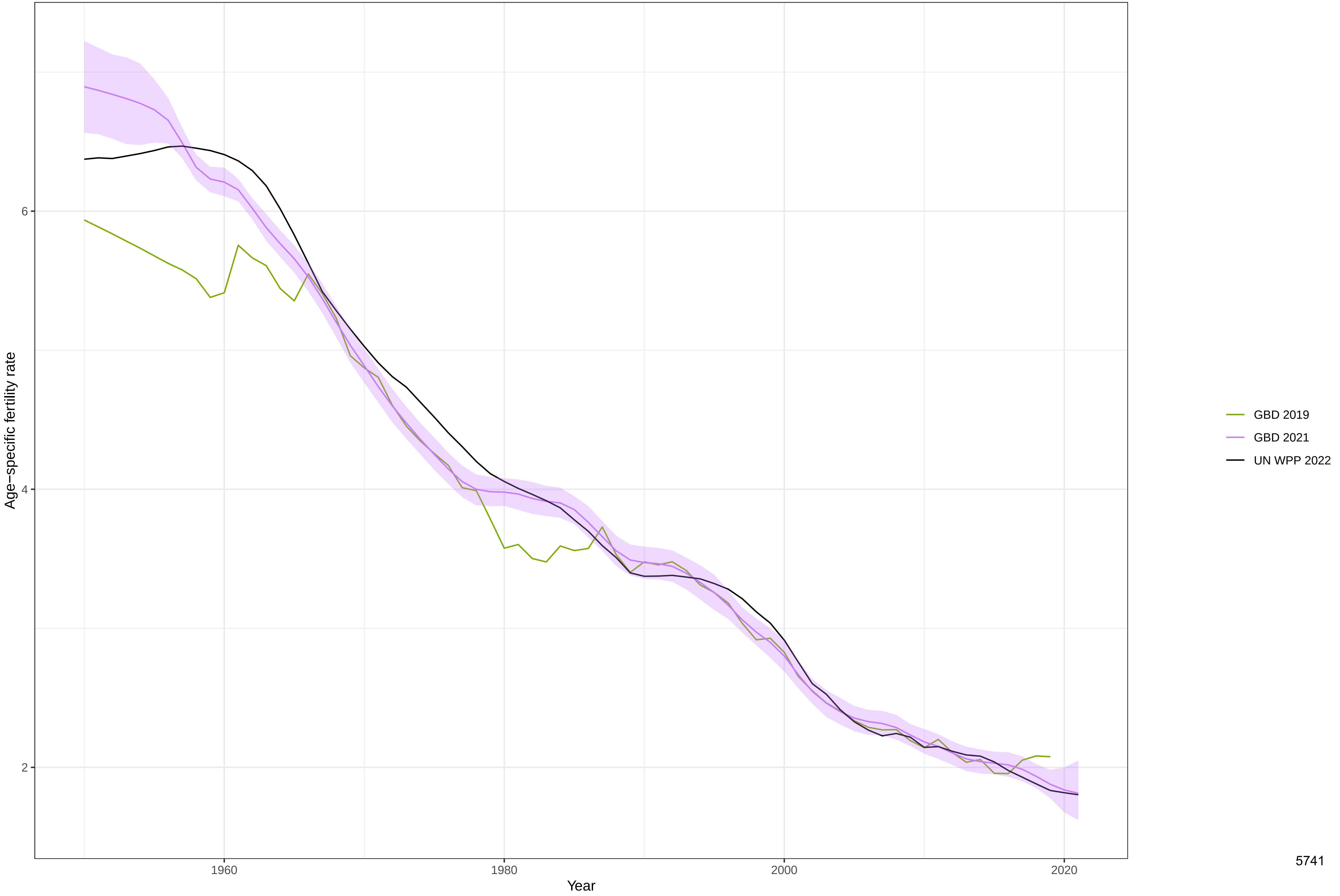

Age−specific fertility fitted trend lines, 1950−2021: Maldives, Ages: 10−14
Data points enclosed with a diamond are reference sources

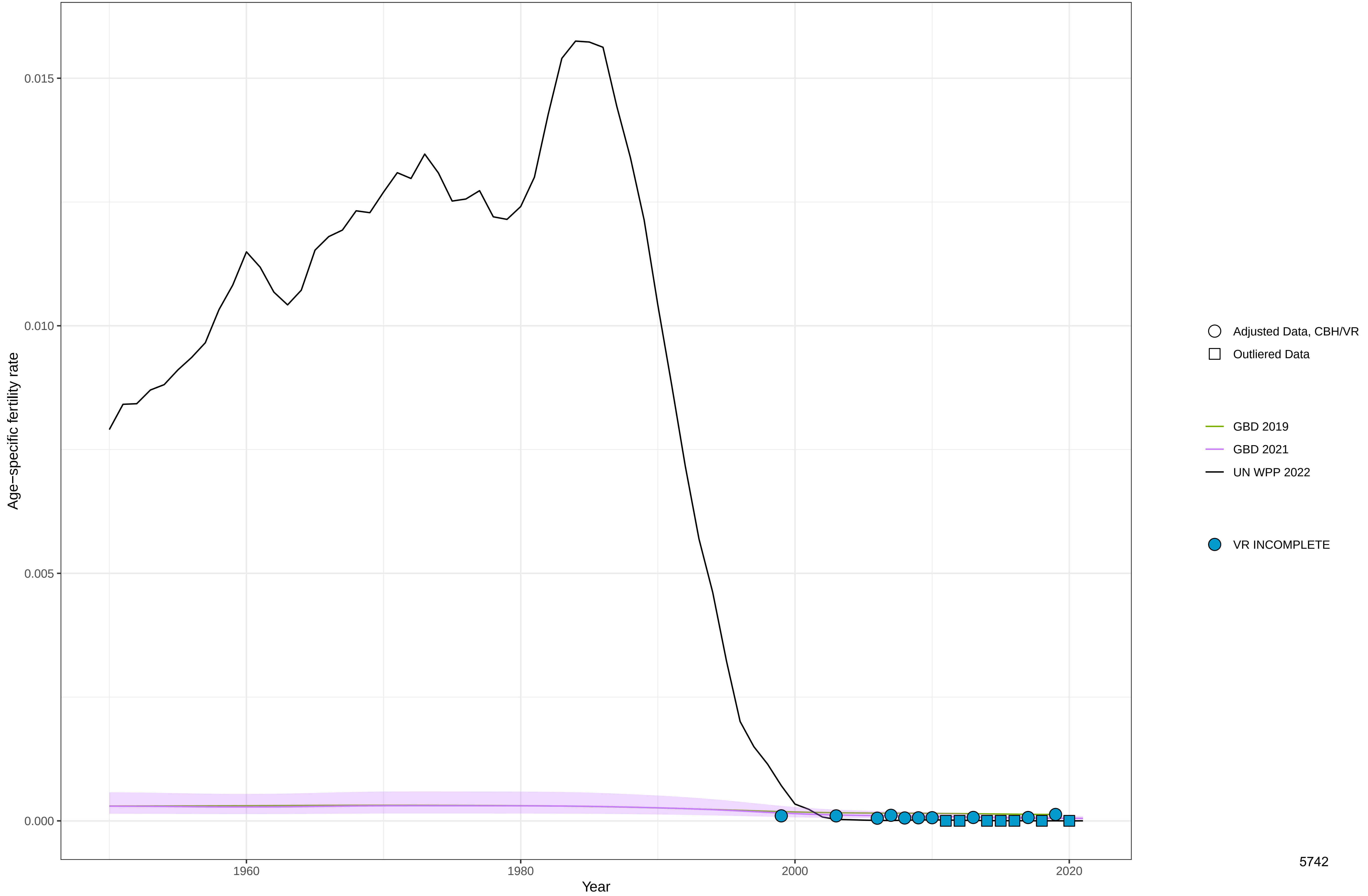

Age−specific fertility fitted trend lines, 1950−2021: Maldives, Ages: 15−19
Data points enclosed with a diamond are reference sources

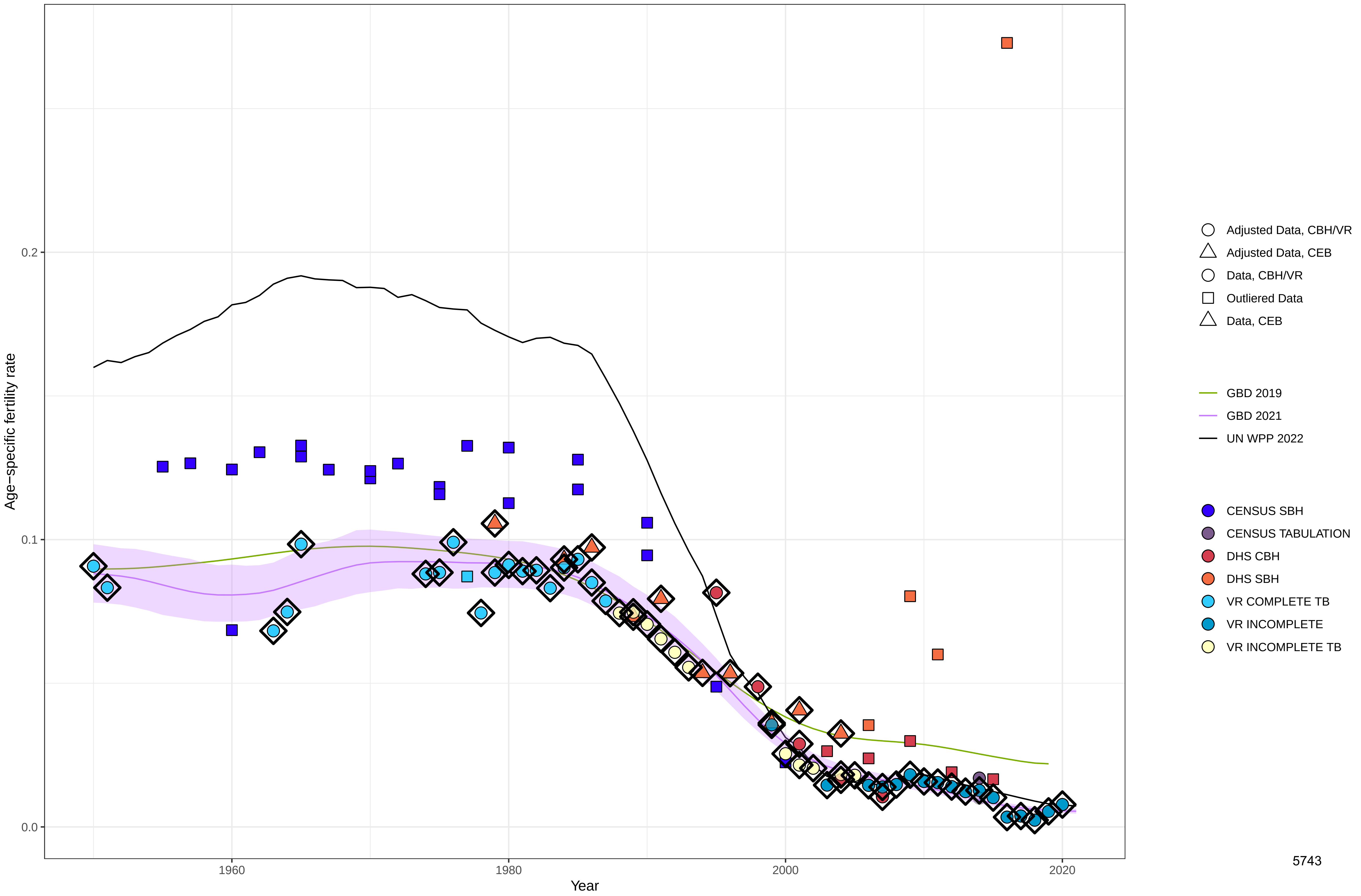

Age−specific fertility fitted trend lines, 1950−2021: Maldives, Ages: 20−24
Data points enclosed with a diamond are reference sources

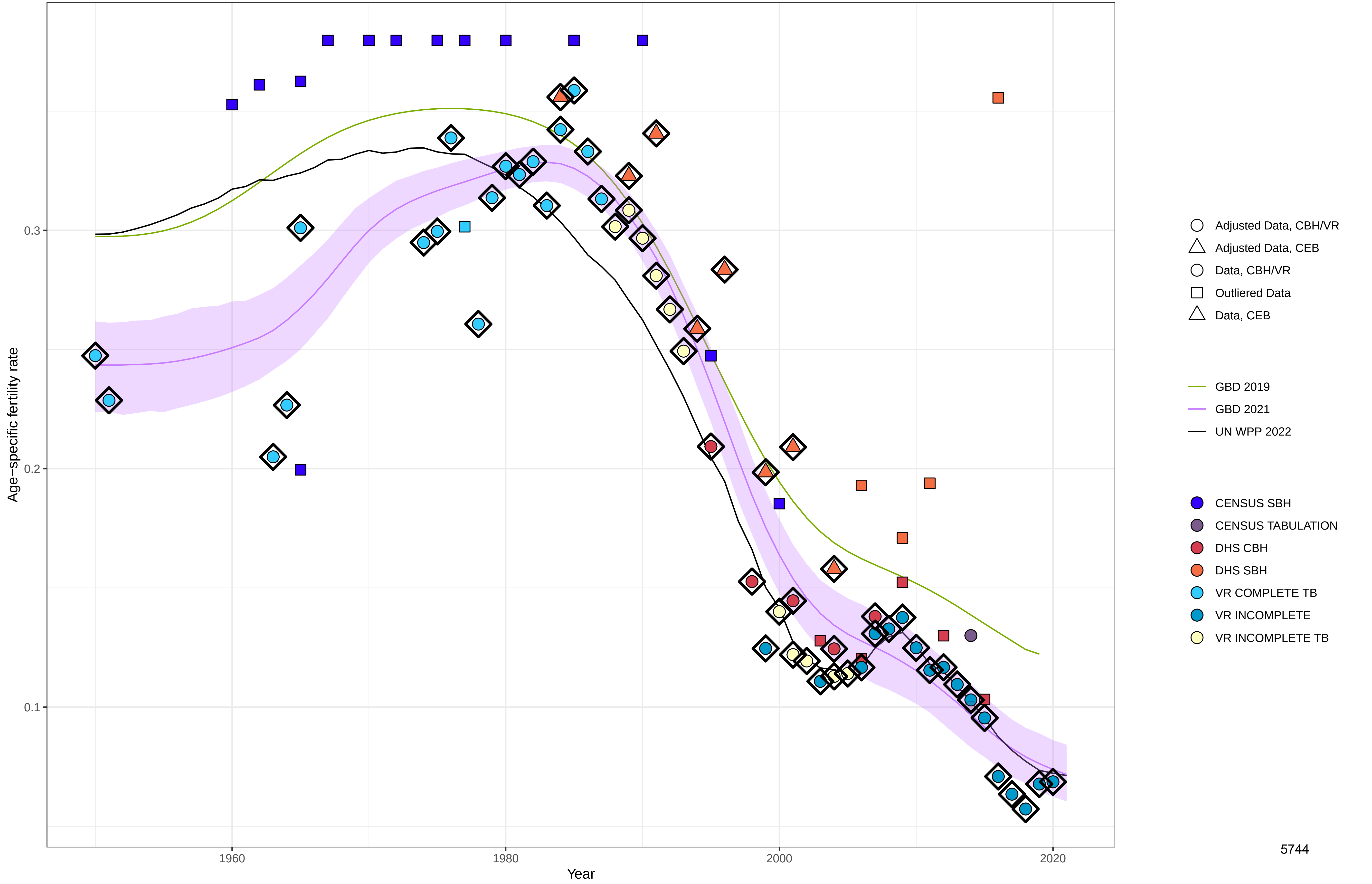

Age−specific fertility fitted trend lines, 1950−2021: Maldives, Ages: 25−29
Data points enclosed with a diamond are reference sources

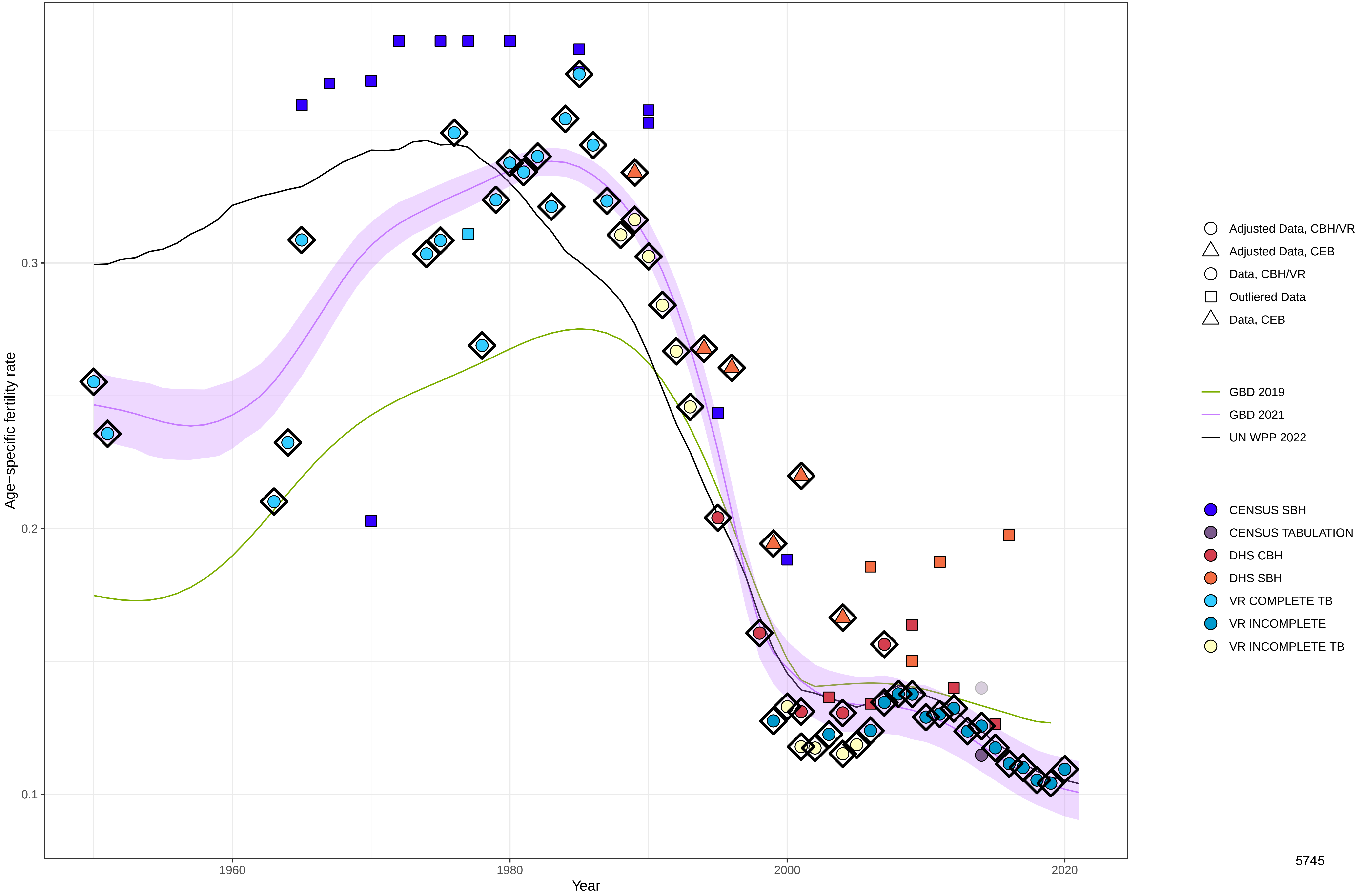

Age−specific fertility fitted trend lines, 1950−2021: Maldives, Ages: 30−34
Data points enclosed with a diamond are reference sources

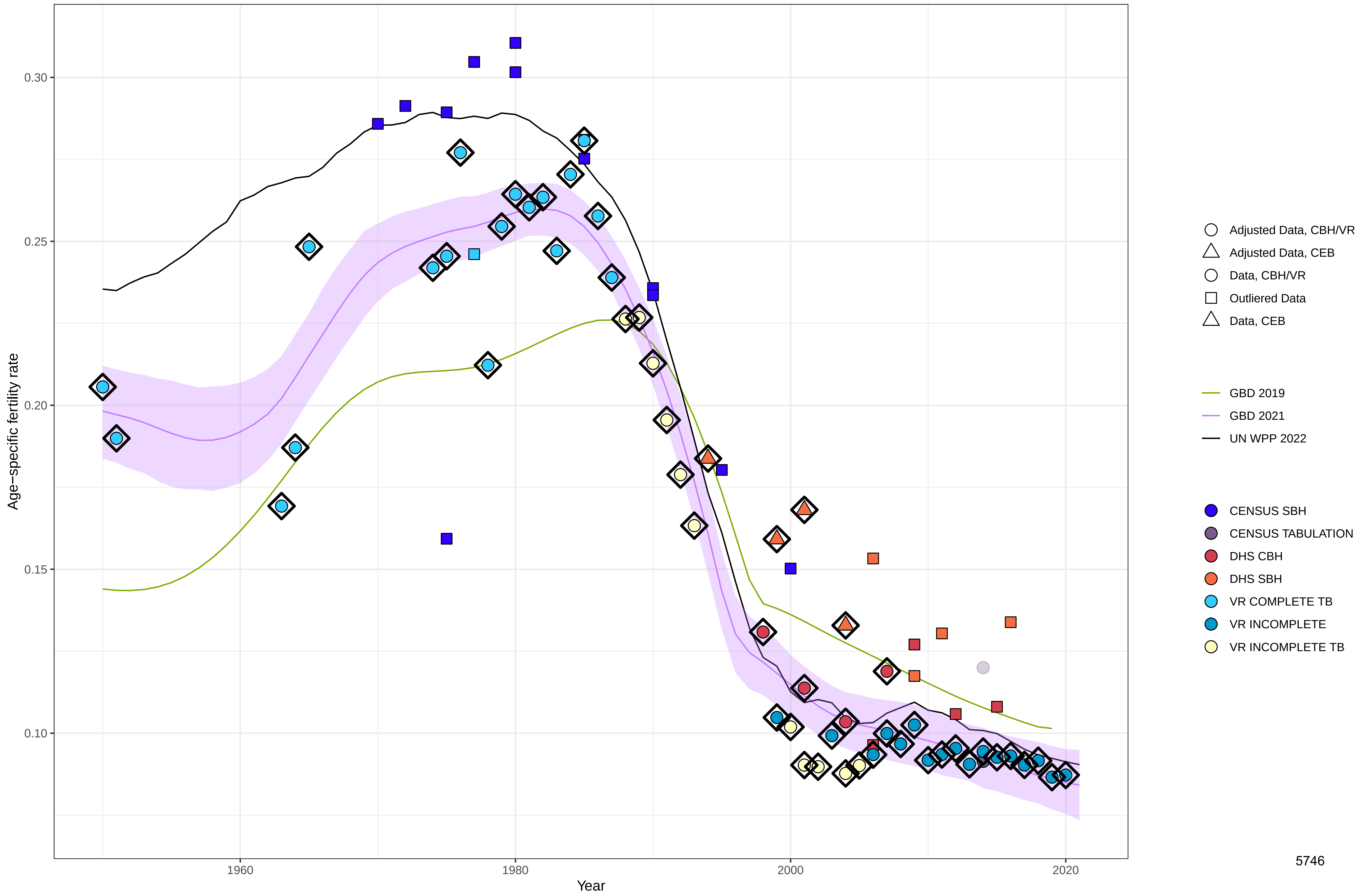

Age−specific fertility fitted trend lines, 1950−2021: Maldives, Ages: 35−39
Data points enclosed with a diamond are reference sources

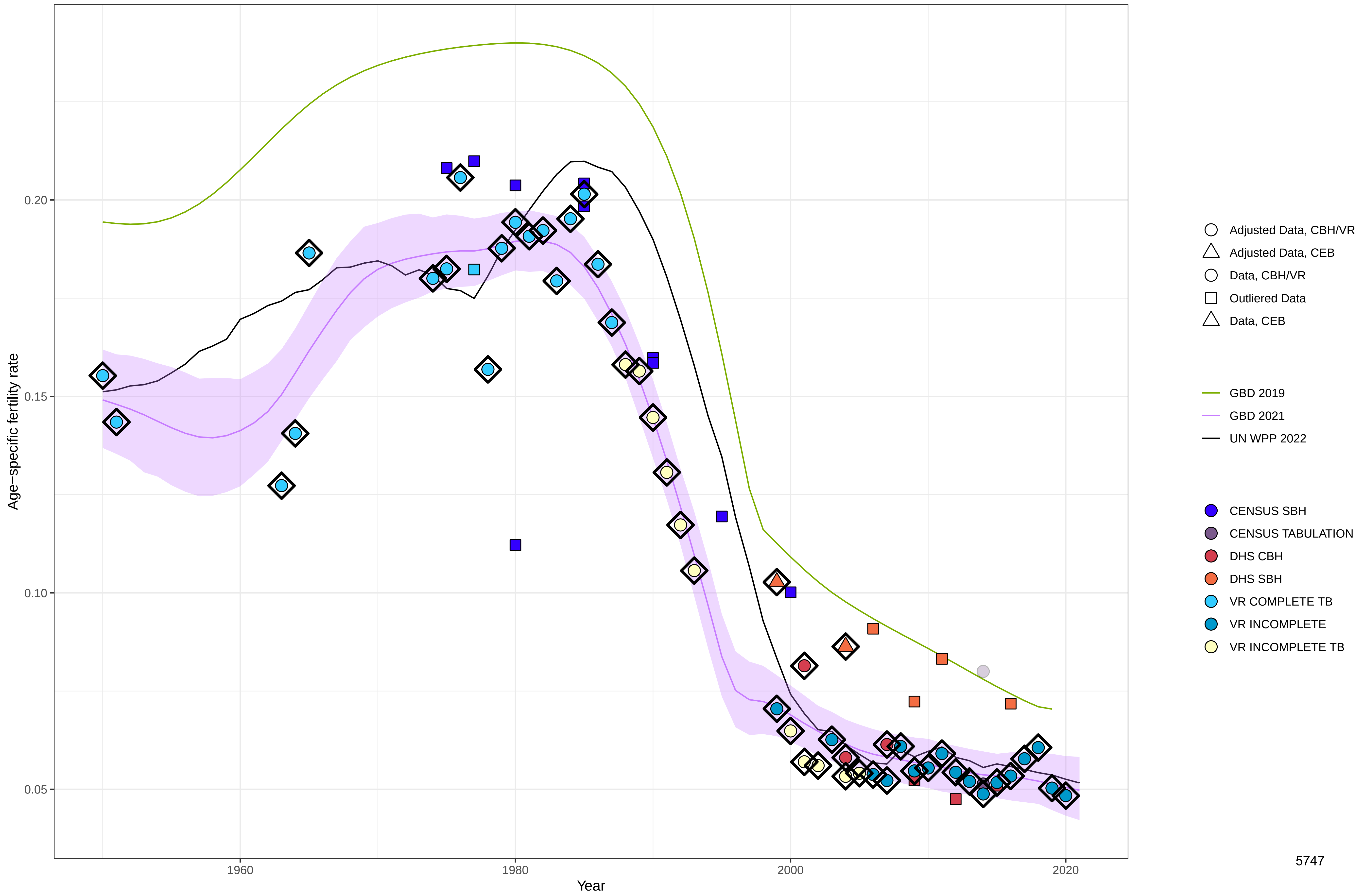

5747

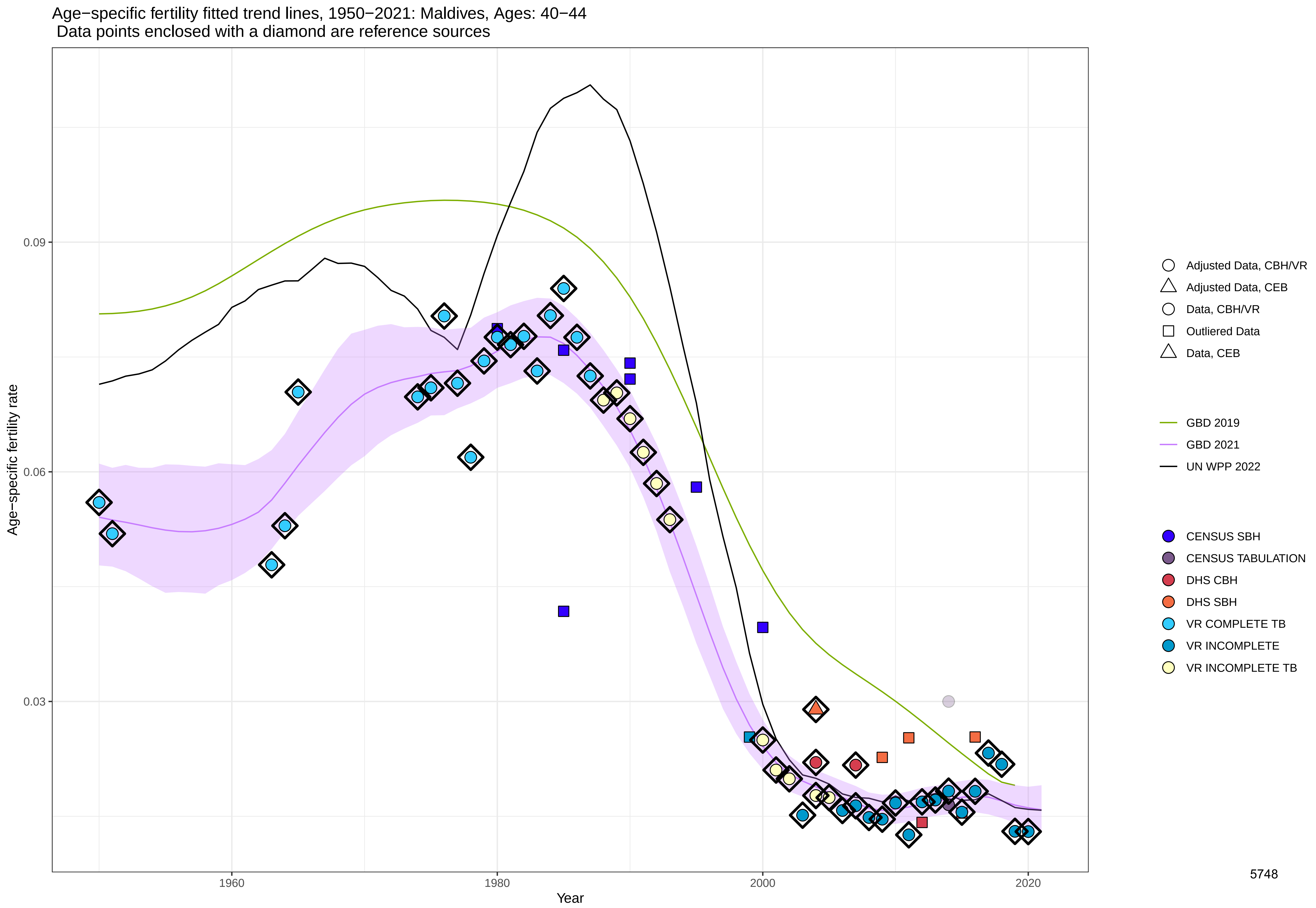

Age−specific fertility fitted trend lines, 1950−2021: Maldives, Ages: 40−44
Data points enclosed with a diamond are reference sources
Age−specific fertility rate
0.09
0.06
0.03
1960
1980
2000
2020
Year
Adjusted Data, CBH/VR
Adjusted Data, CEB
Data, CBH/VR
Outliered Data
Data, CEB
GBD 2019
GBD 2021
UN WPP 2022
CENSUS SBH
CENSUS TABULATION
DHS CBH
DHS SBH
VR COMPLETE TB
VR INCOMPLETE
VR INCOMPLETE TB

Age−specific fertility fitted trend lines, 1950−2021: Maldives, Ages: 45−49
Data points enclosed with a diamond are reference sources

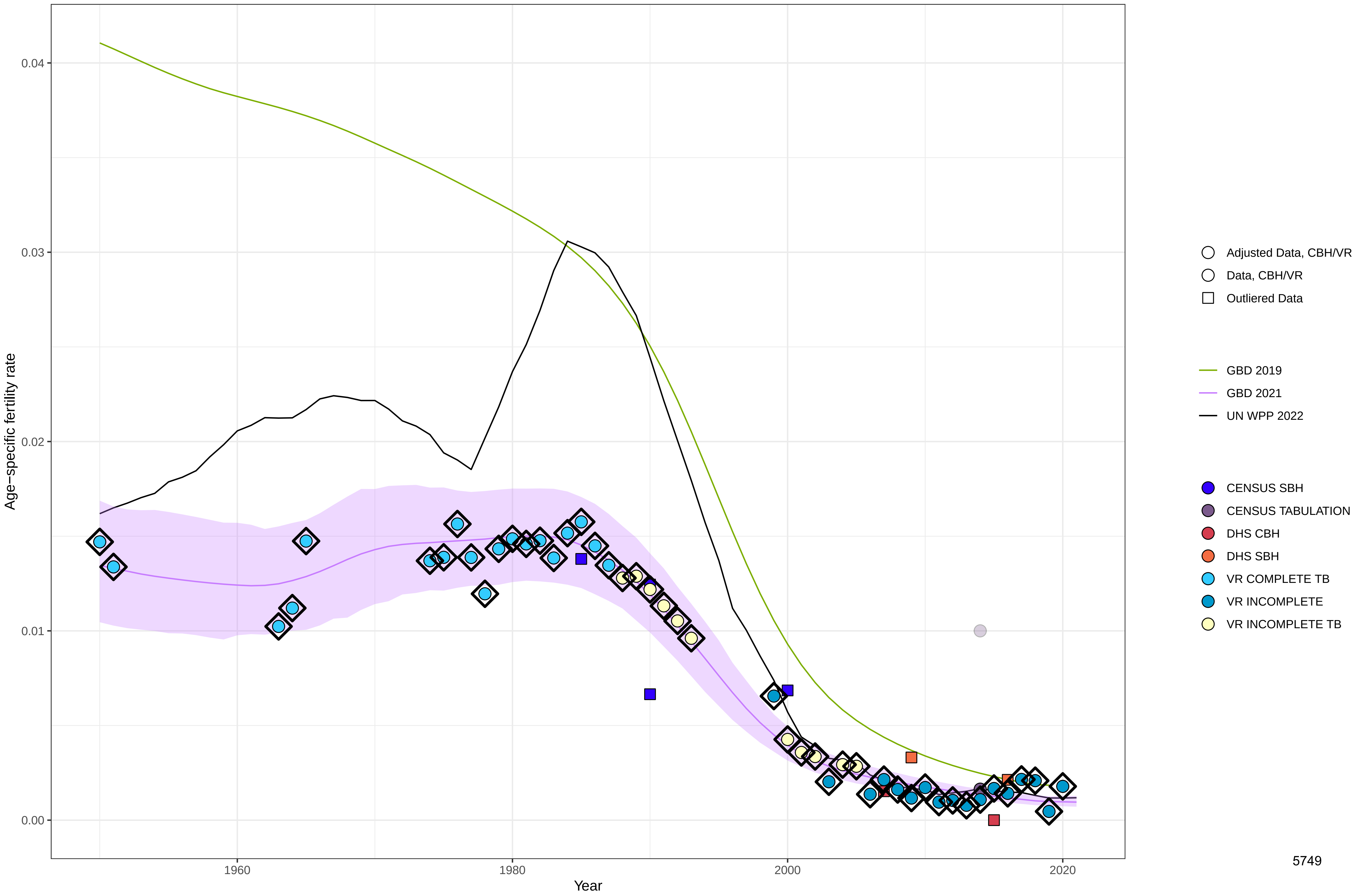

Age−specific fertility fitted trend lines, 1950−2021: Maldives, Ages: 50−54
Data points enclosed with a diamond are reference sources

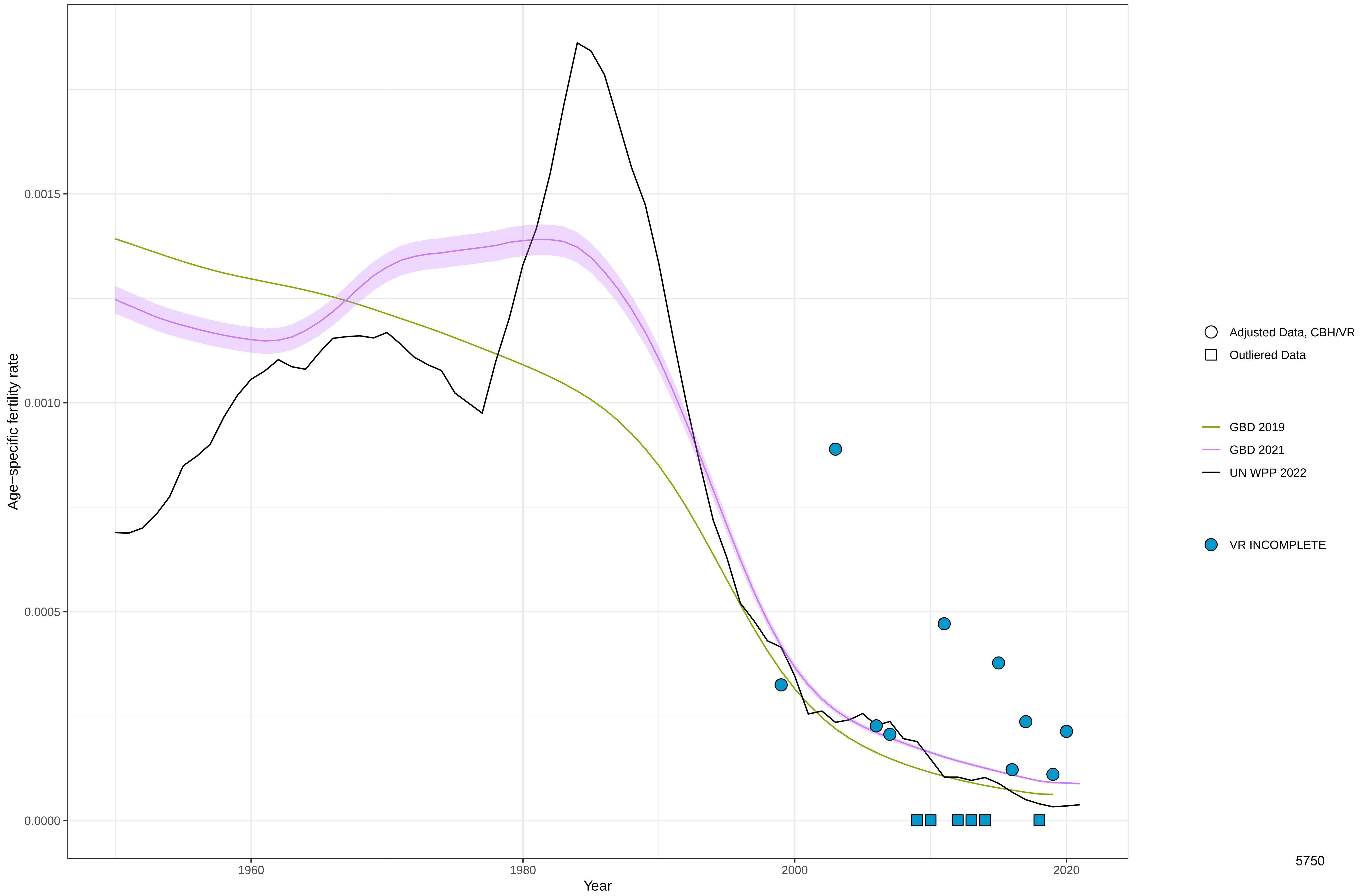

Total fertility rate fitted trend lines, 1950−2021: Maldives TFR
Data points enclosed with a diamond are reference sources

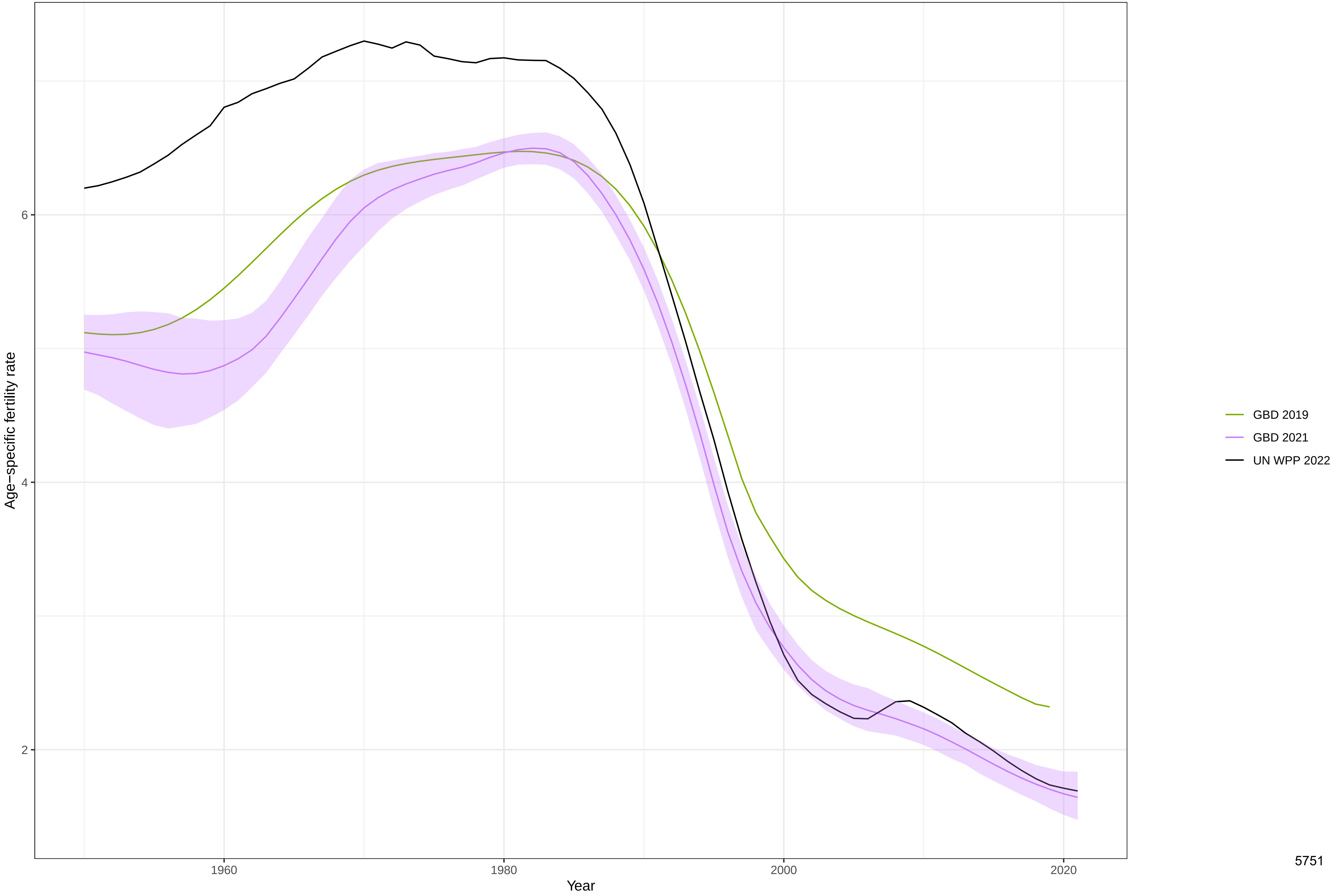

Age−specific fertility fitted trend lines, 1950−2021: Mauritius, Ages: 10−14
Data points enclosed with a diamond are reference sources

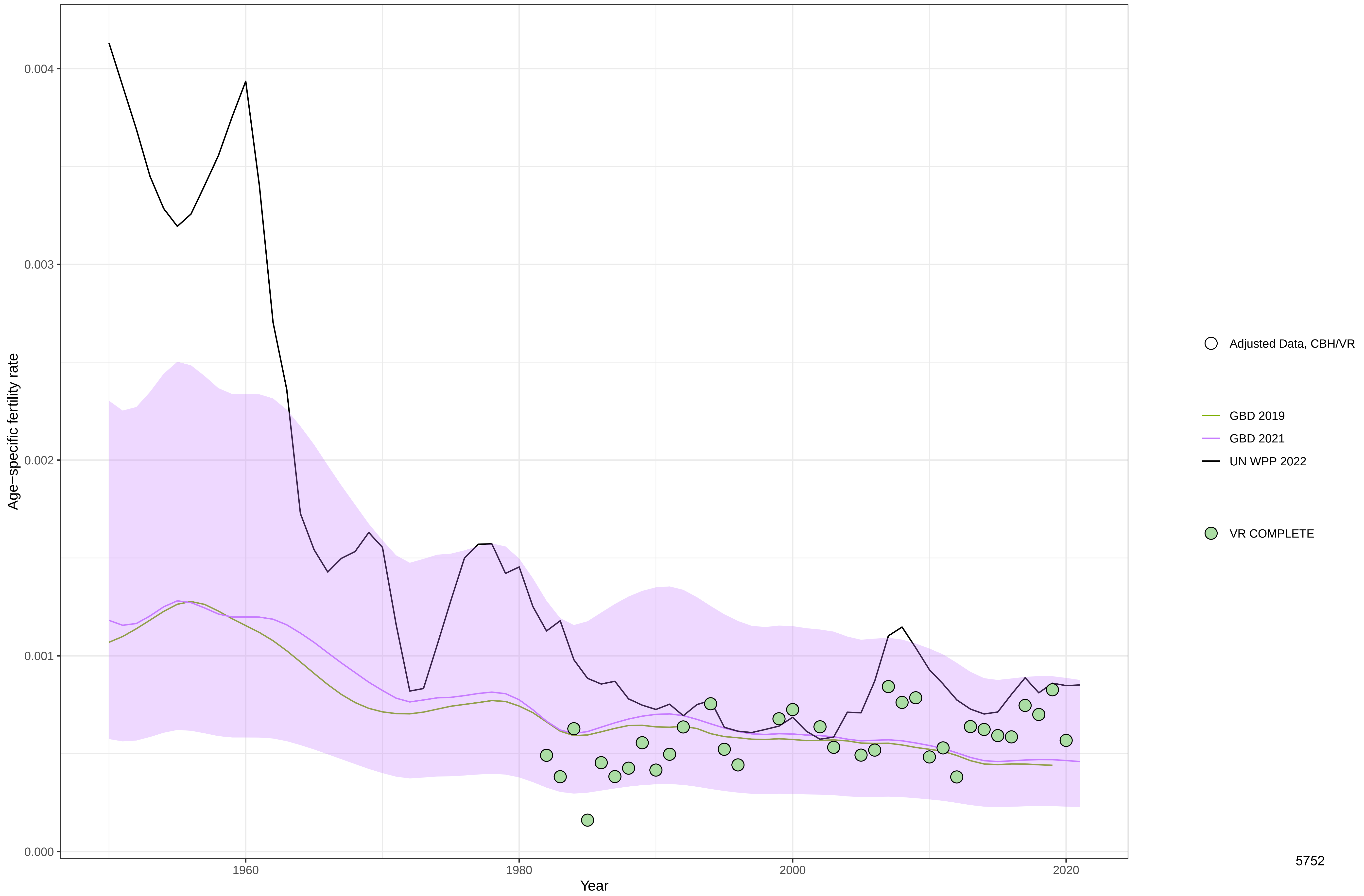

Age−specific fertility fitted trend lines, 1950−2021: Mauritius, Ages: 15−19
Data points enclosed with a diamond are reference sources

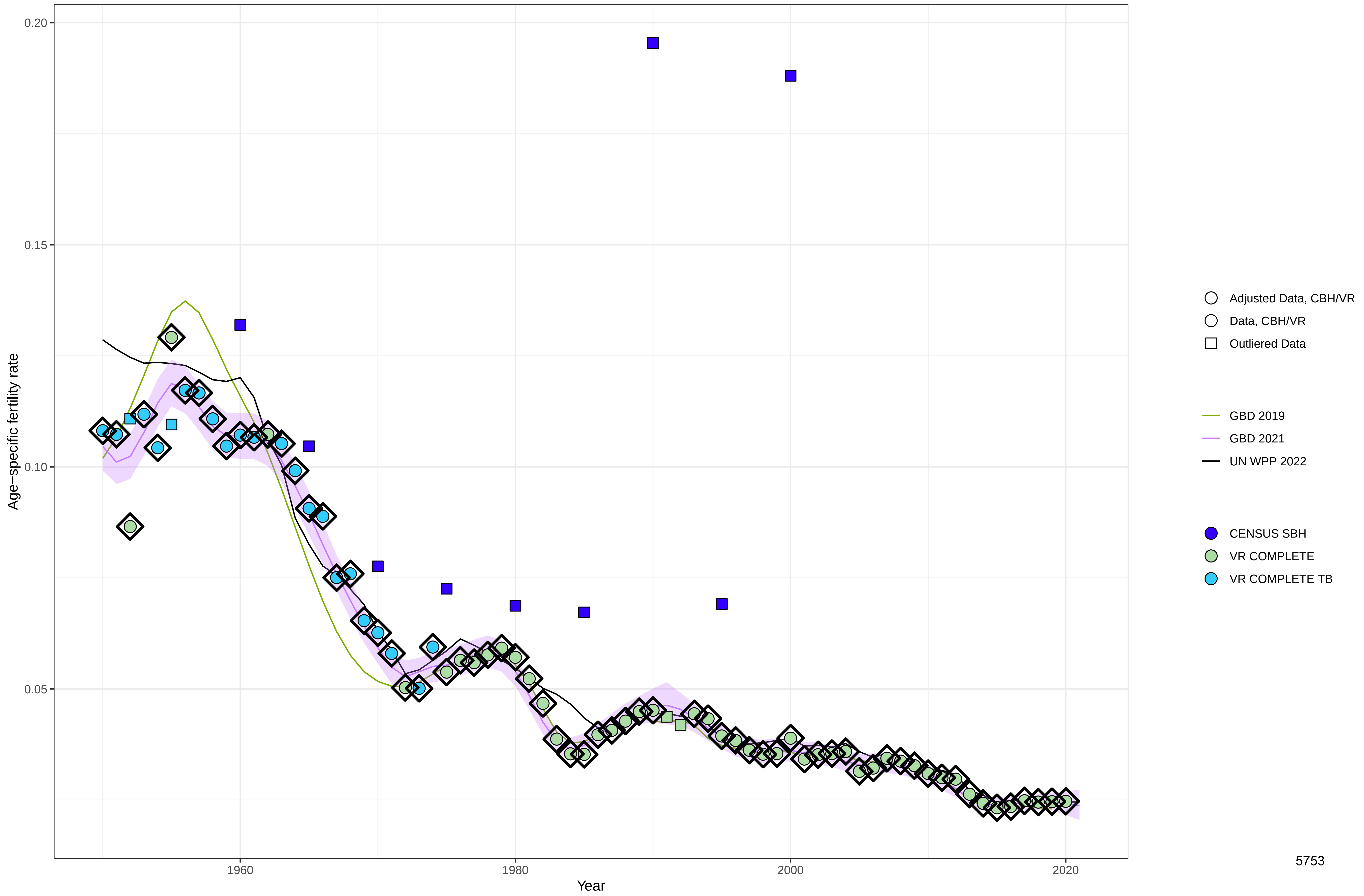

Age−specific fertility fitted trend lines, 1950−2021: Mauritius, Ages: 20−24
Data points enclosed with a diamond are reference sources

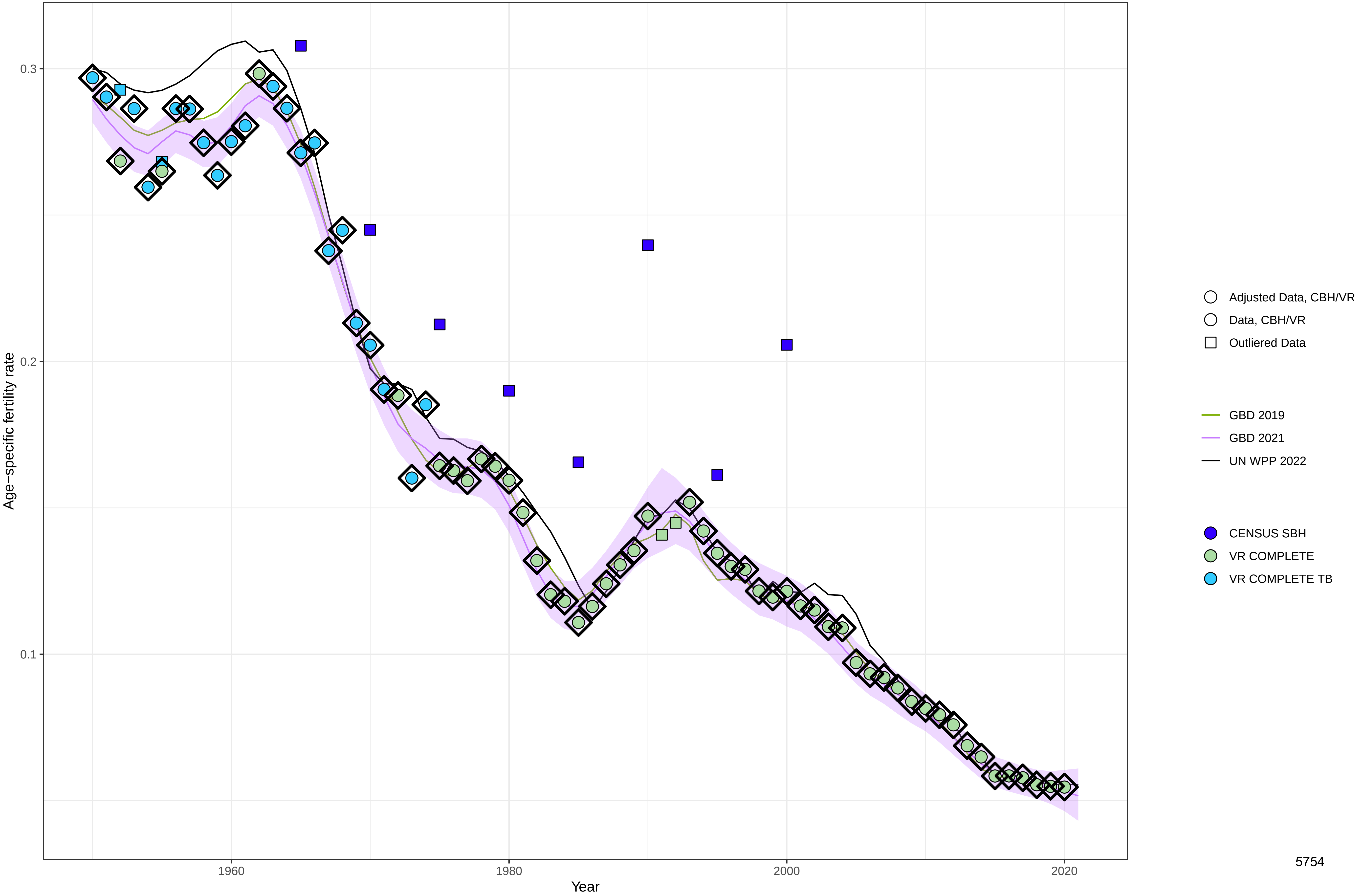

Age−specific fertility fitted trend lines, 1950−2021: Mauritius, Ages: 25−29
Data points enclosed with a diamond are reference sources

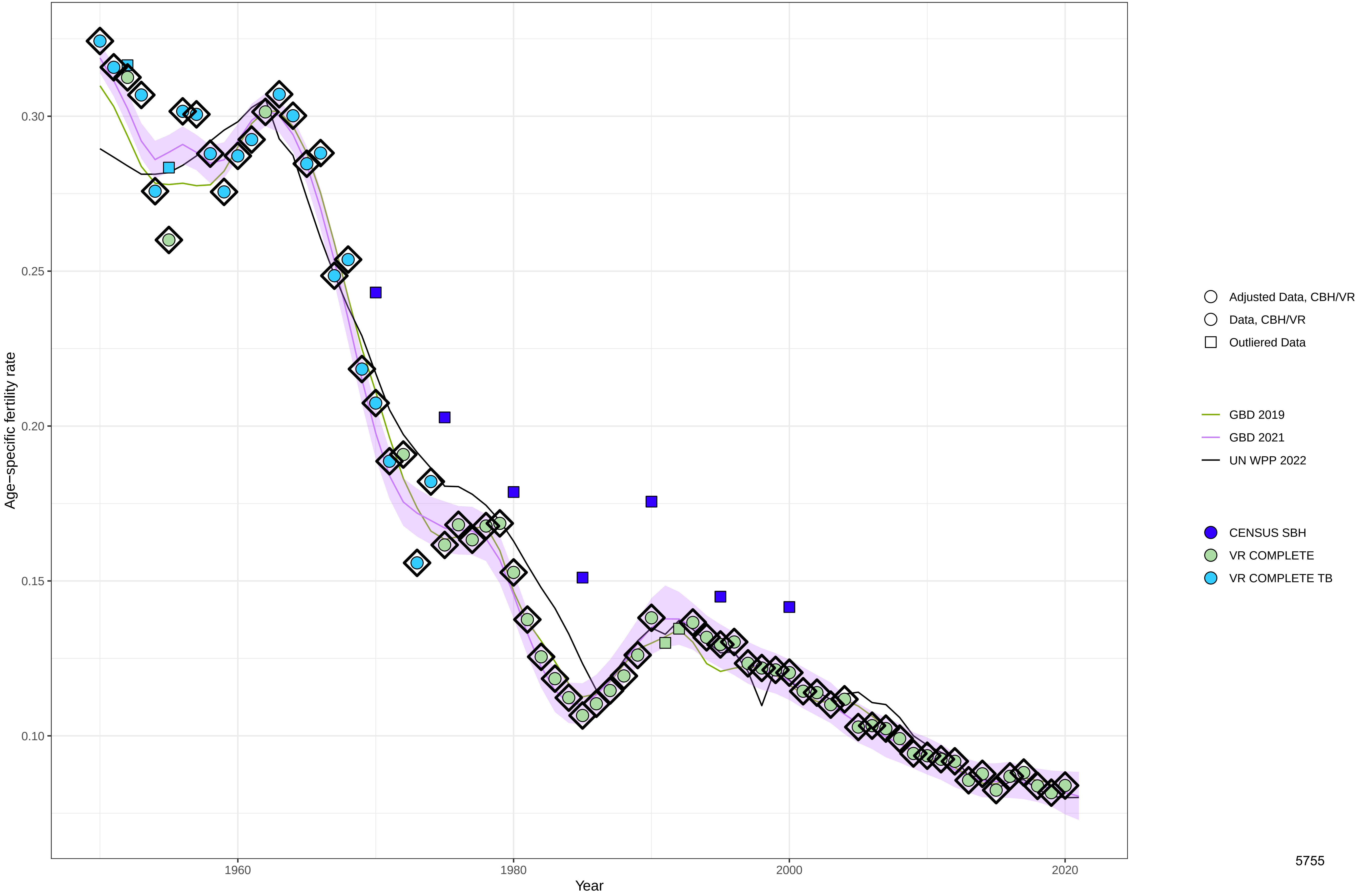

Age−specific fertility fitted trend lines, 1950−2021: Mauritius, Ages: 30−34
Data points enclosed with a diamond are reference sources

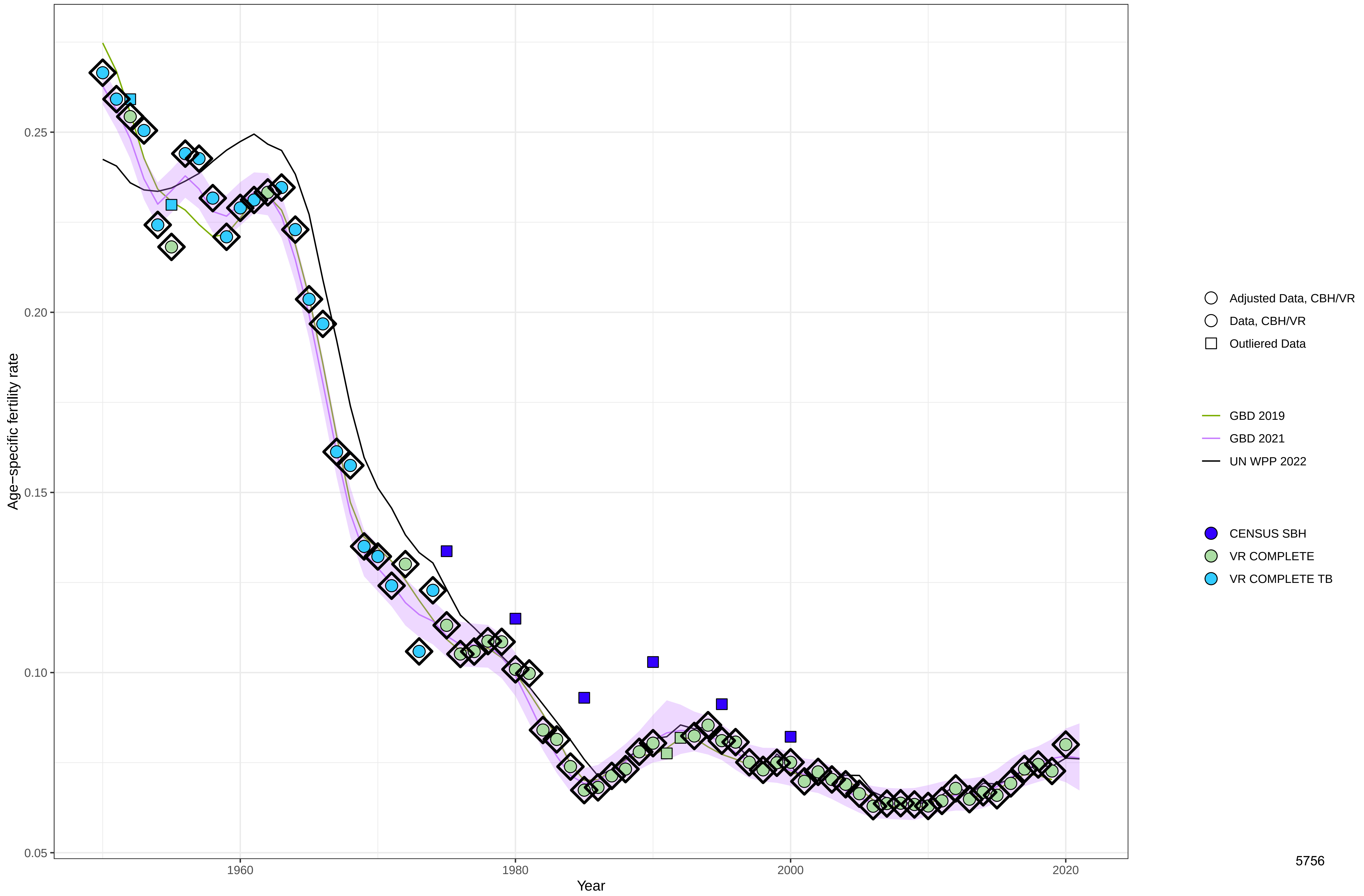

Age−specific fertility fitted trend lines, 1950−2021: Mauritius, Ages: 35−39
Data points enclosed with a diamond are reference sources

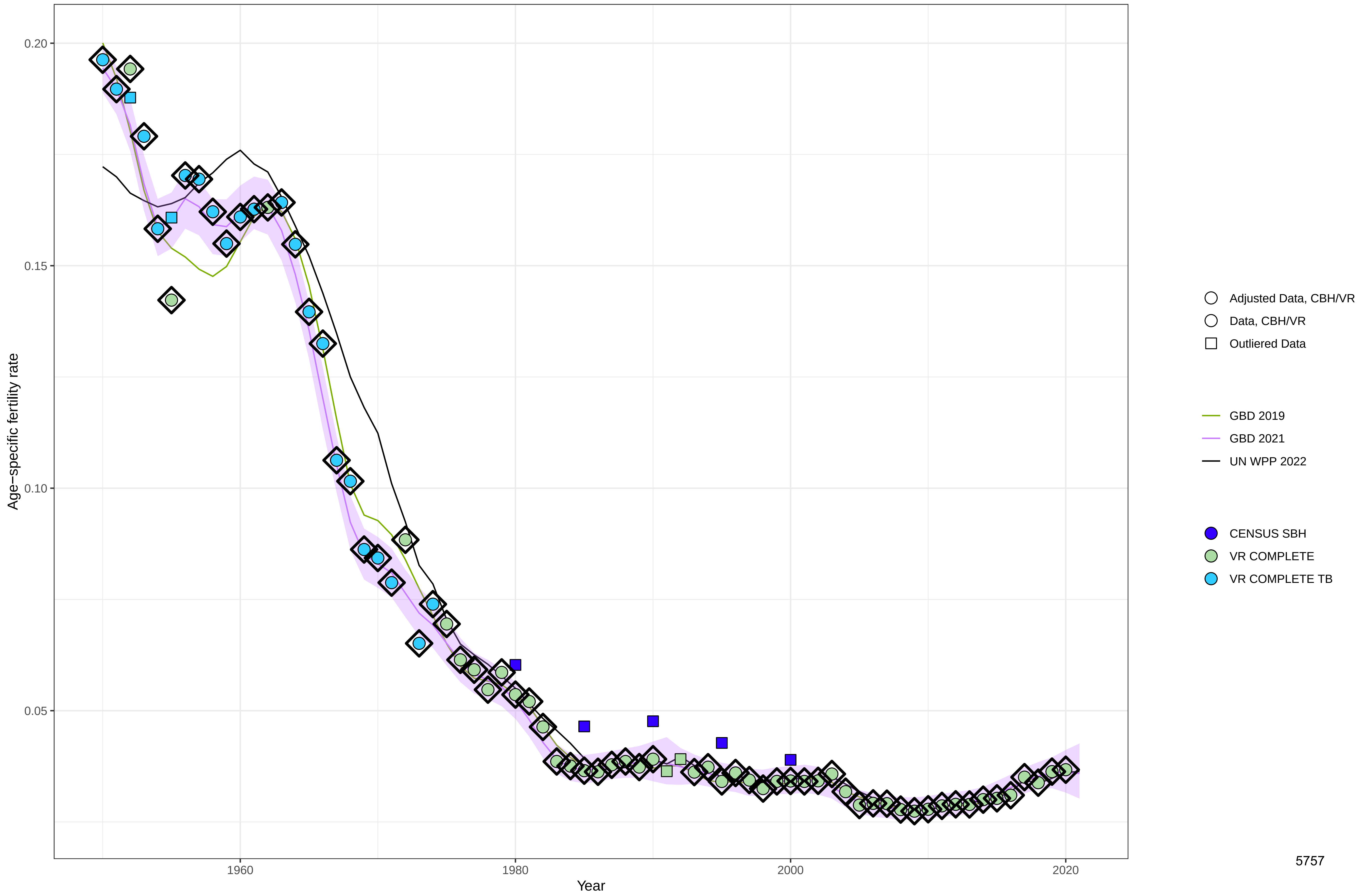

Age−specific fertility fitted trend lines, 1950−2021: Mauritius, Ages: 40−44
Data points enclosed with a diamond are reference sources

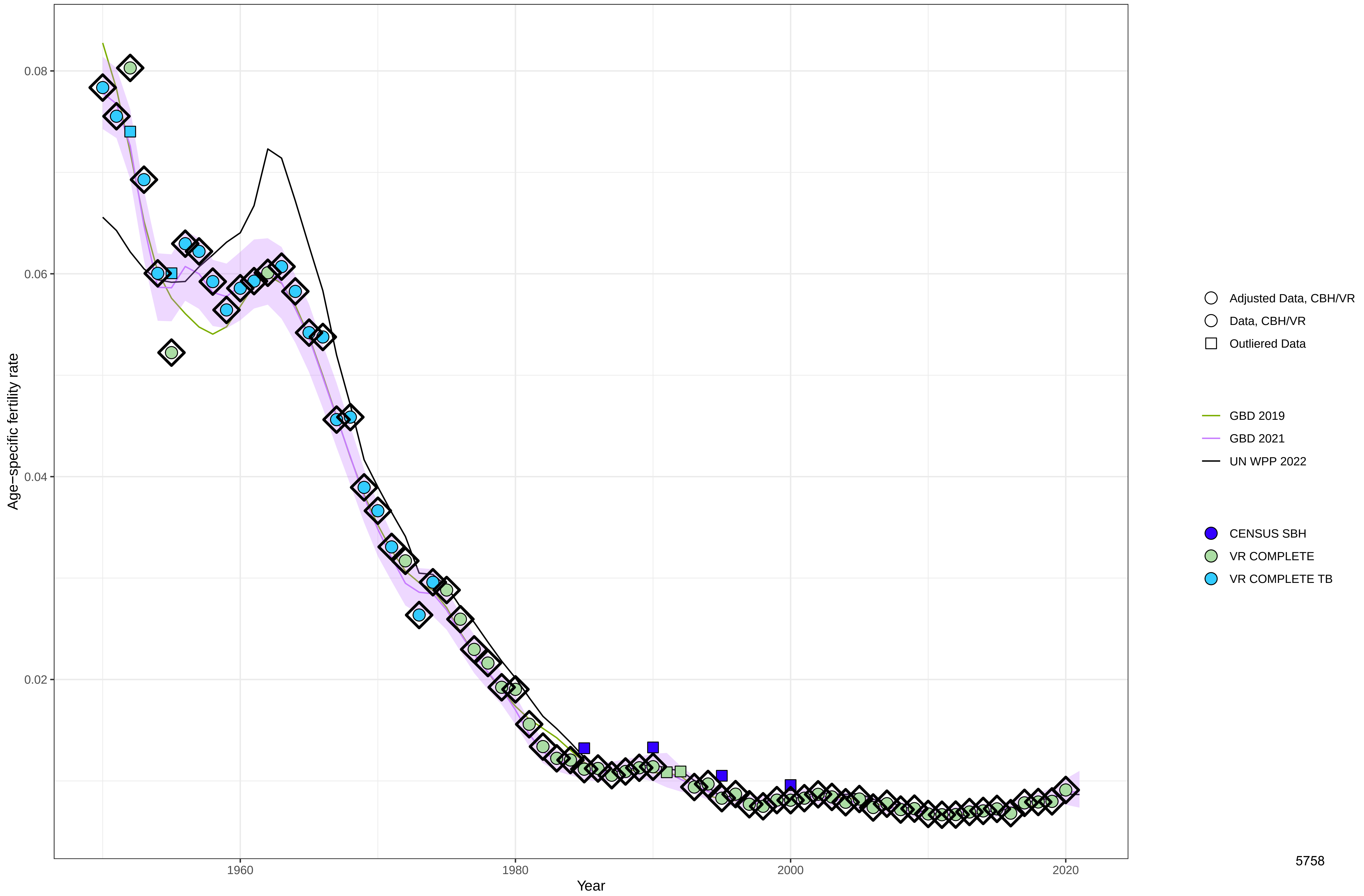

Age−specific fertility fitted trend lines, 1950−2021: Mauritius, Ages: 45−49
Data points enclosed with a diamond are reference sources

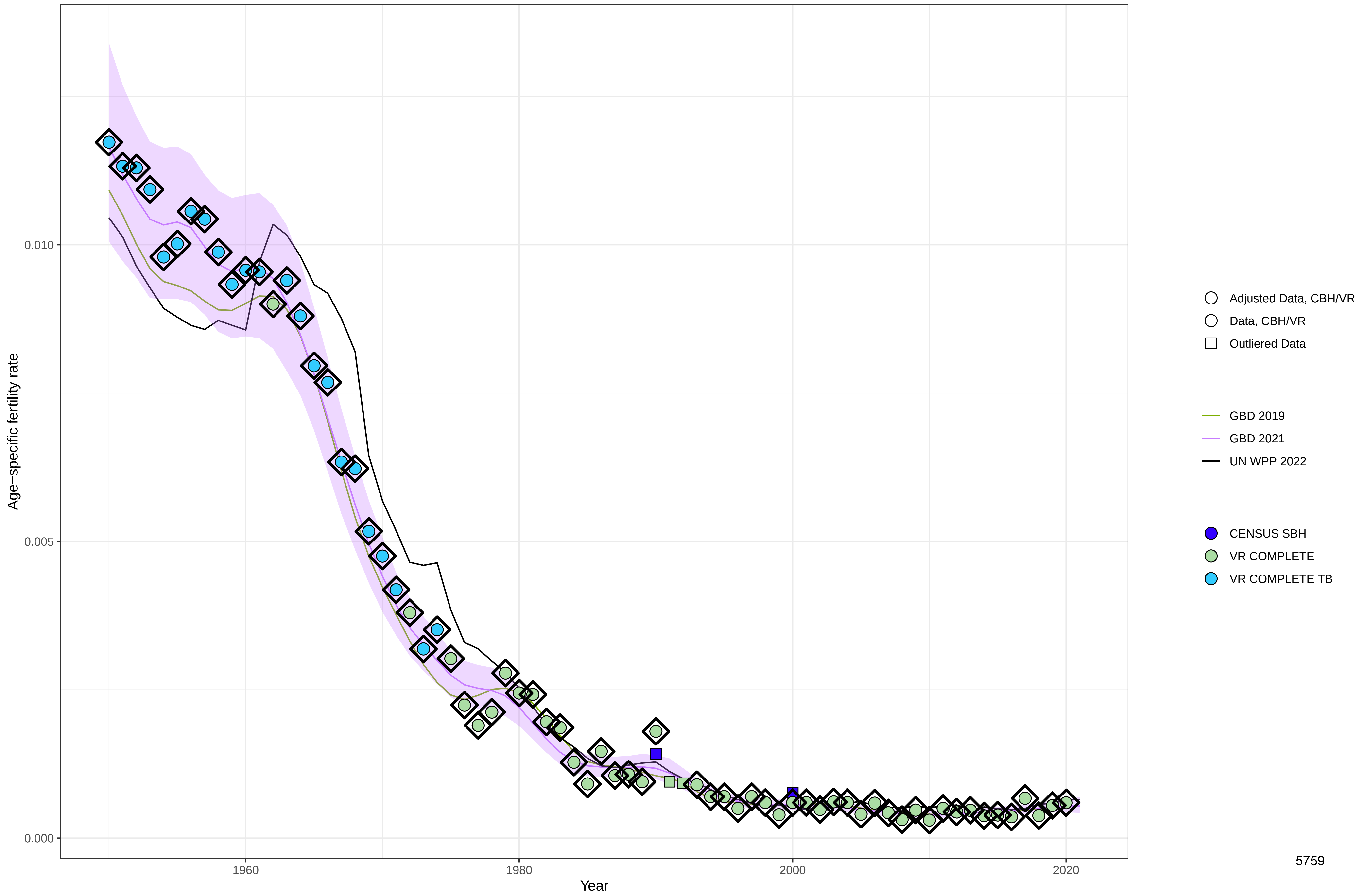

Age−specific fertility fitted trend lines, 1950−2021: Mauritius, Ages: 50−54
Data points enclosed with a diamond are reference sources

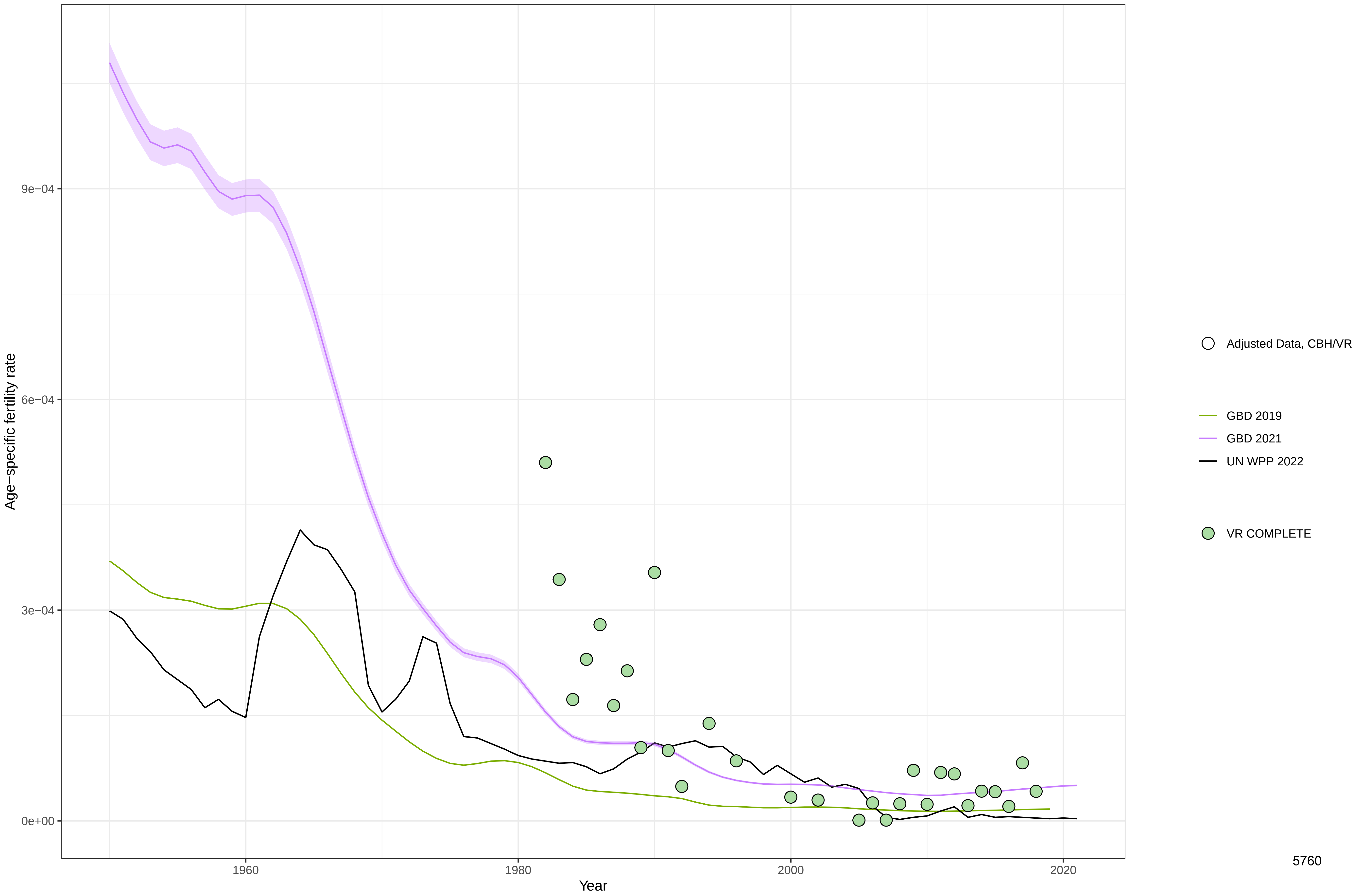

Total fertility rate fitted trend lines, 1950−2021: Mauritius TFR
Data points enclosed with a diamond are reference sources

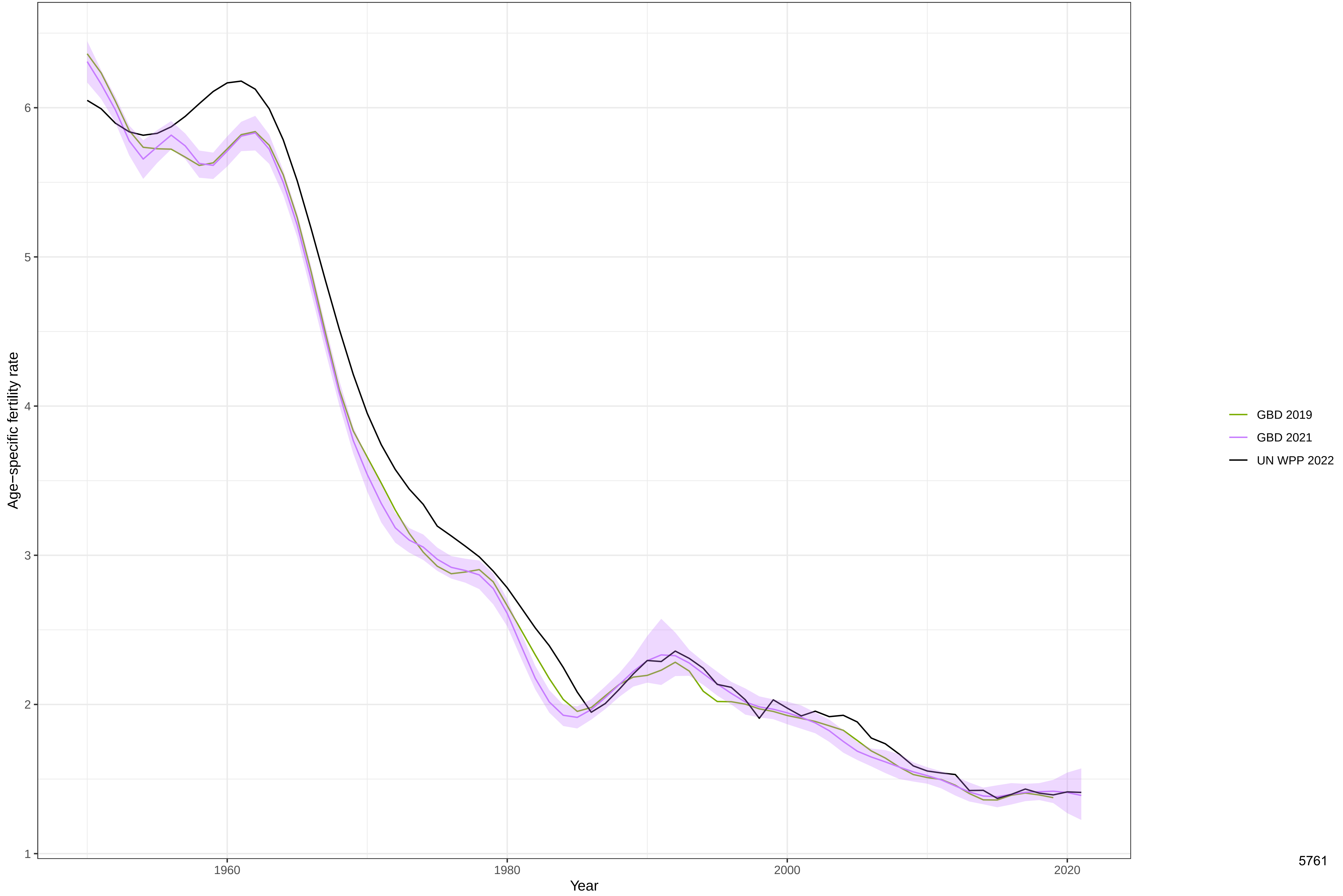

Age−specific fertility fitted trend lines, 1950−2021: Myanmar, Ages: 10−14
Data points enclosed with a diamond are reference sources

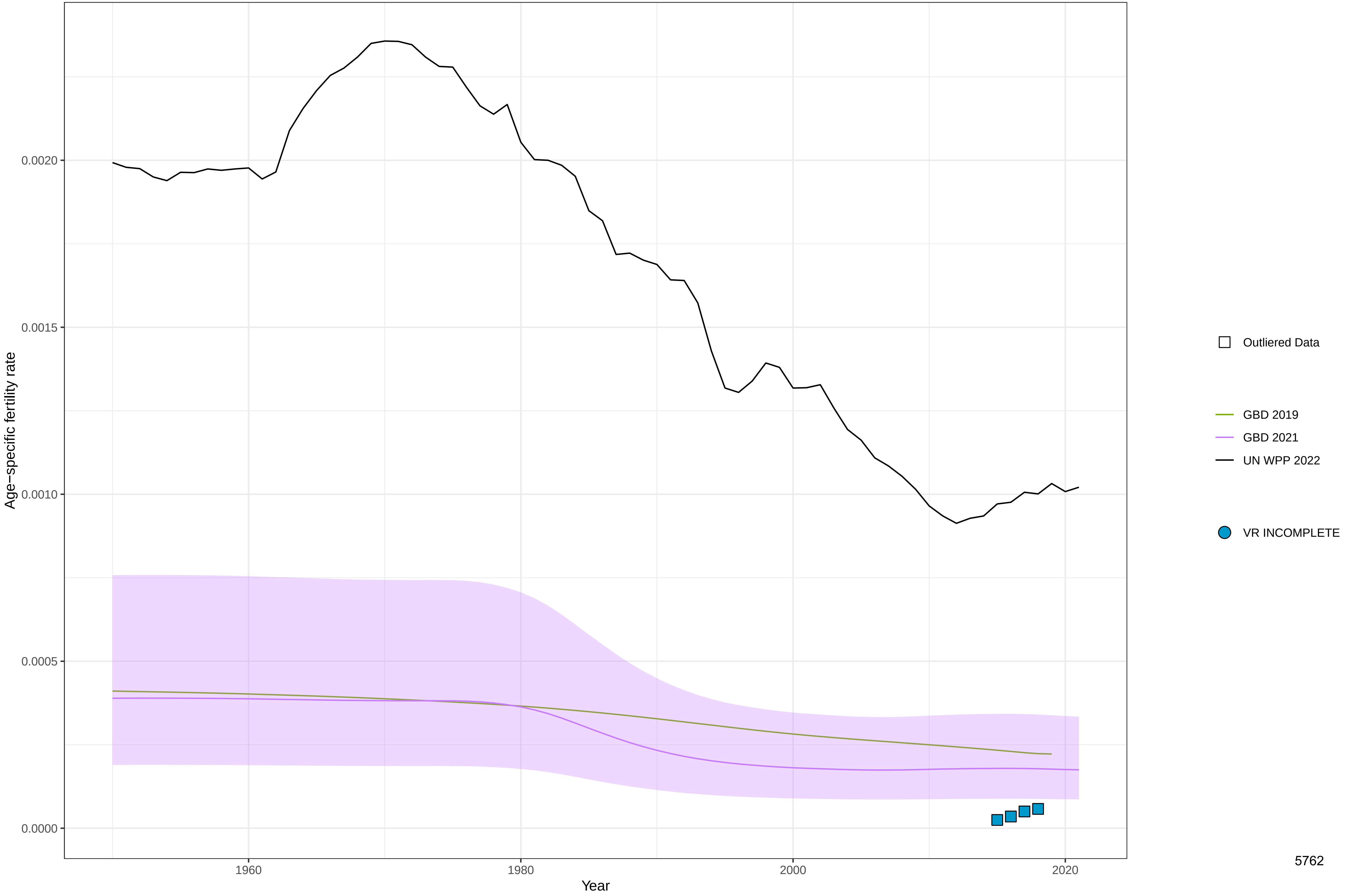

Age−specific fertility fitted trend lines, 1950−2021: Myanmar, Ages: 15−19
Data points enclosed with a diamond are reference sources

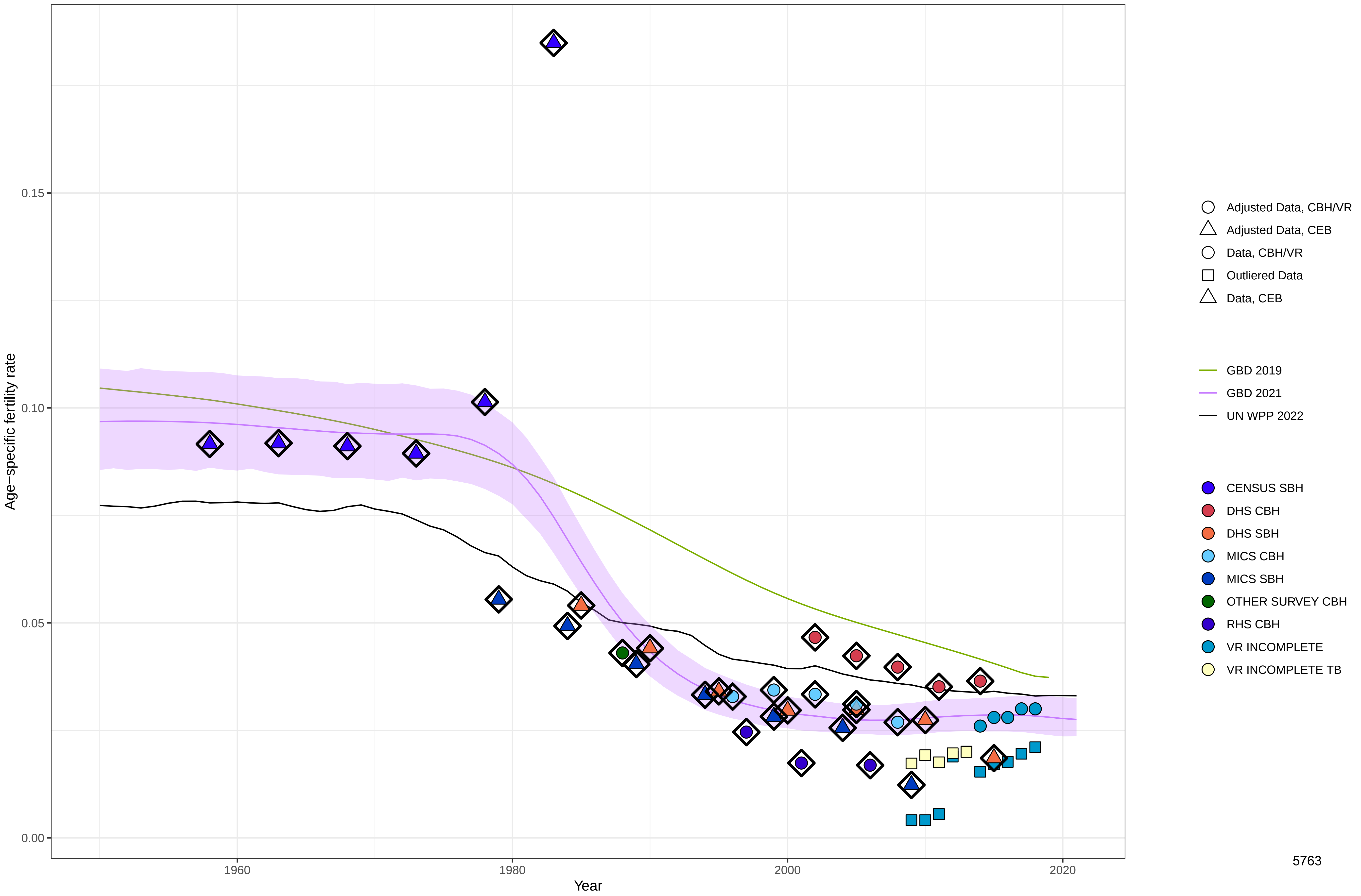

Age−specific fertility fitted trend lines, 1950−2021: Myanmar, Ages: 20−24
Data points enclosed with a diamond are reference sources

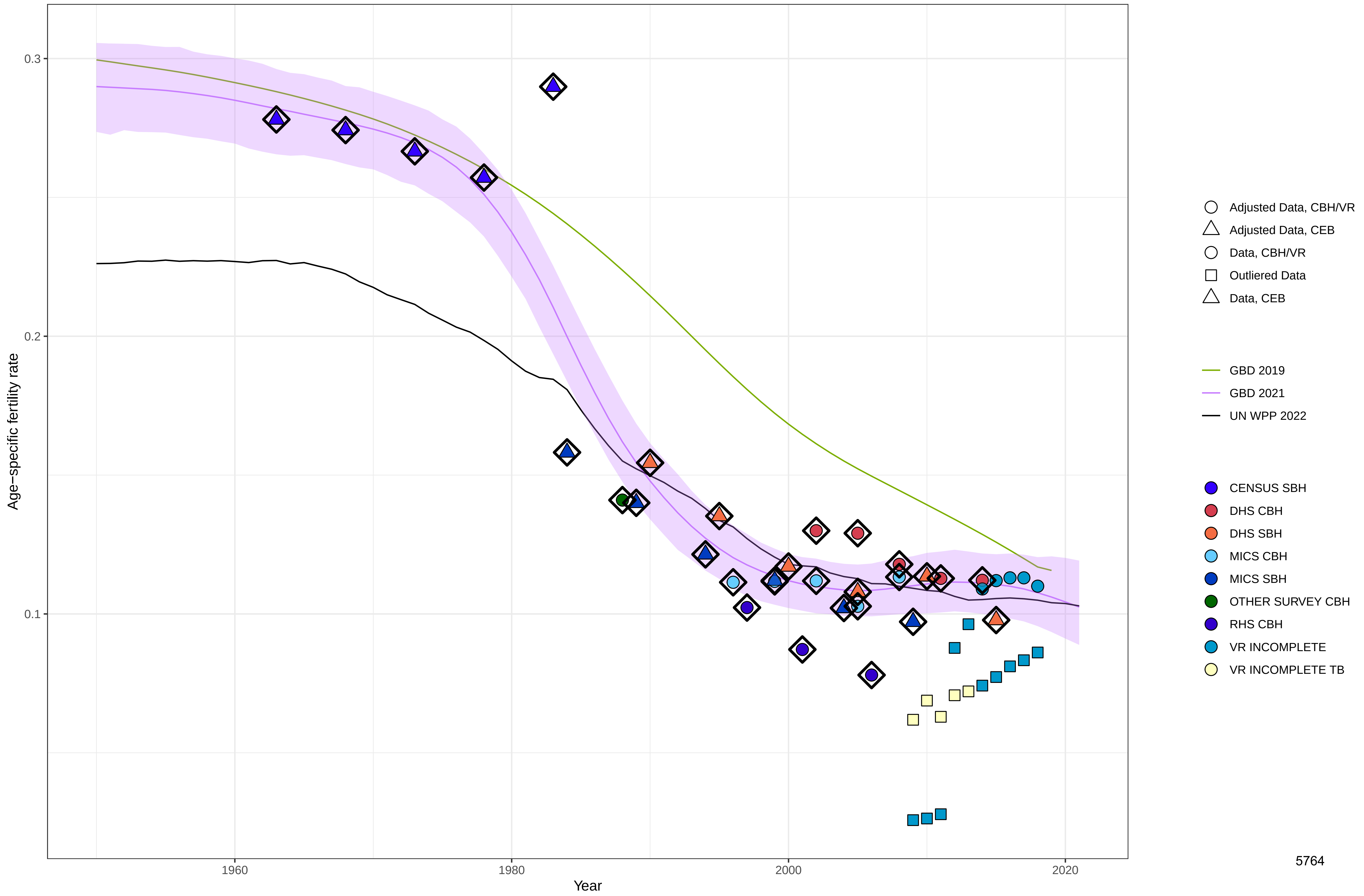

Age−specific fertility fitted trend lines, 1950−2021: Myanmar, Ages: 25−29
Data points enclosed with a diamond are reference sources

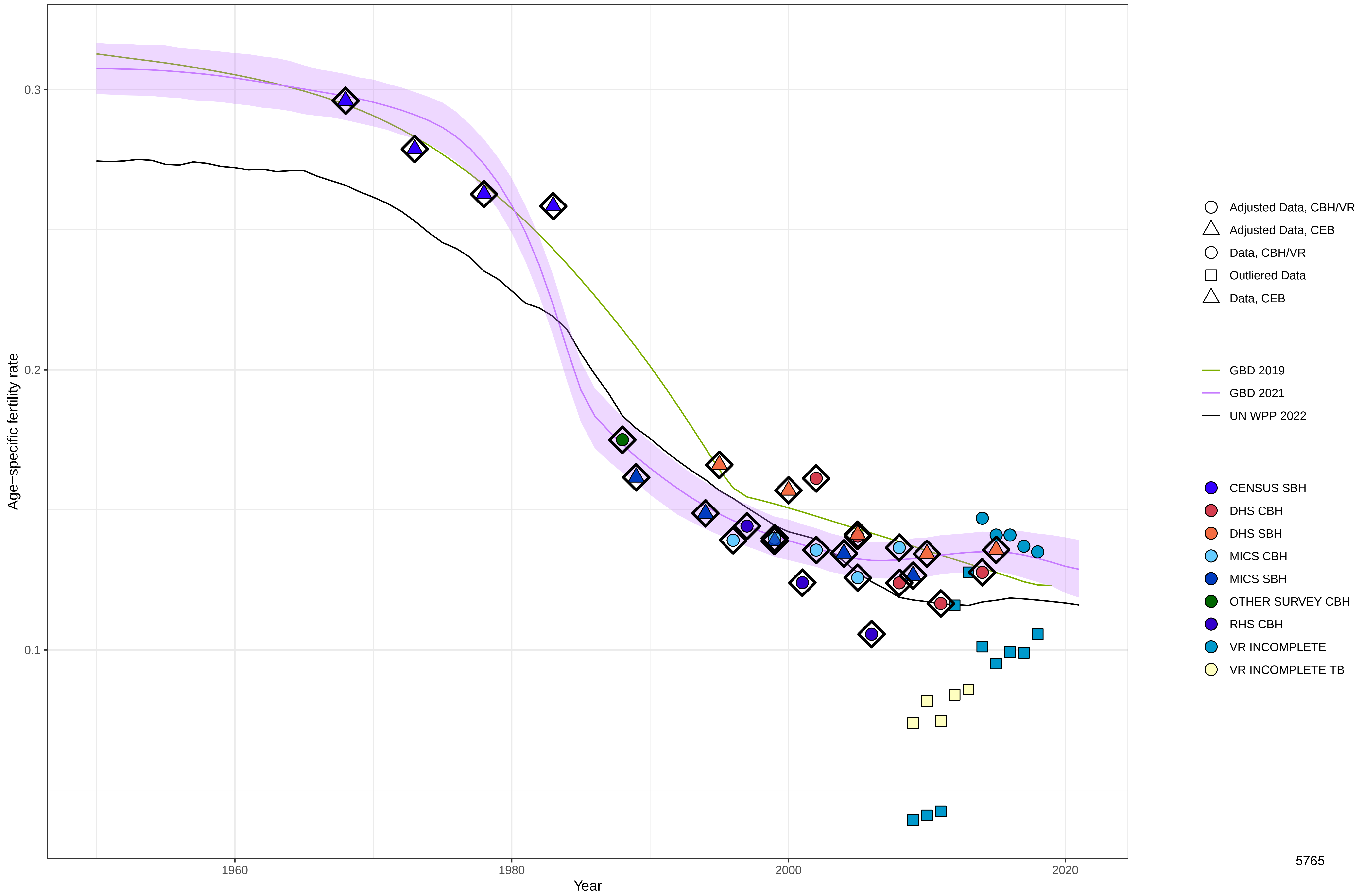

Age−specific fertility fitted trend lines, 1950−2021: Myanmar, Ages: 30−34
Data points enclosed with a diamond are reference sources

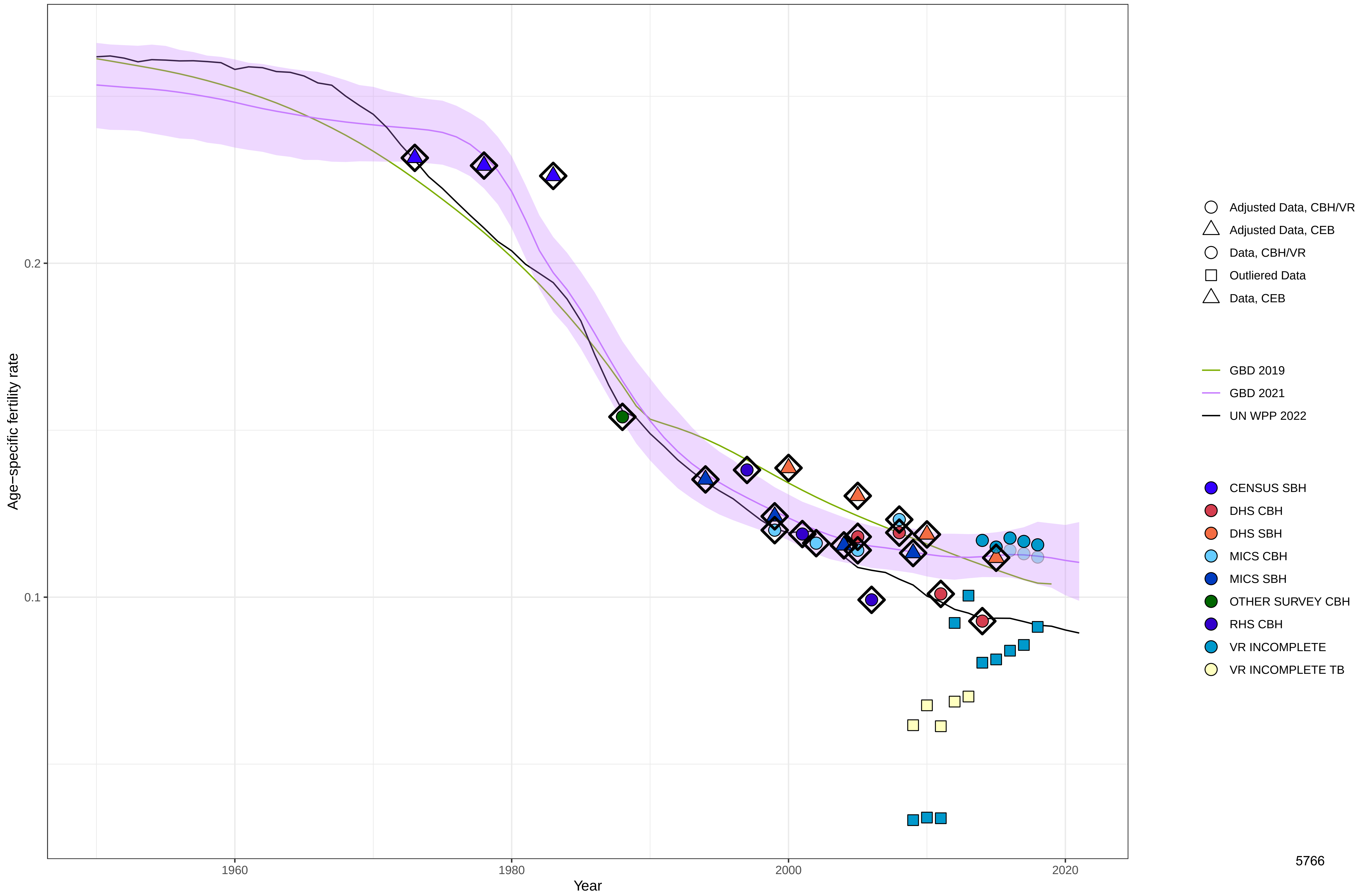

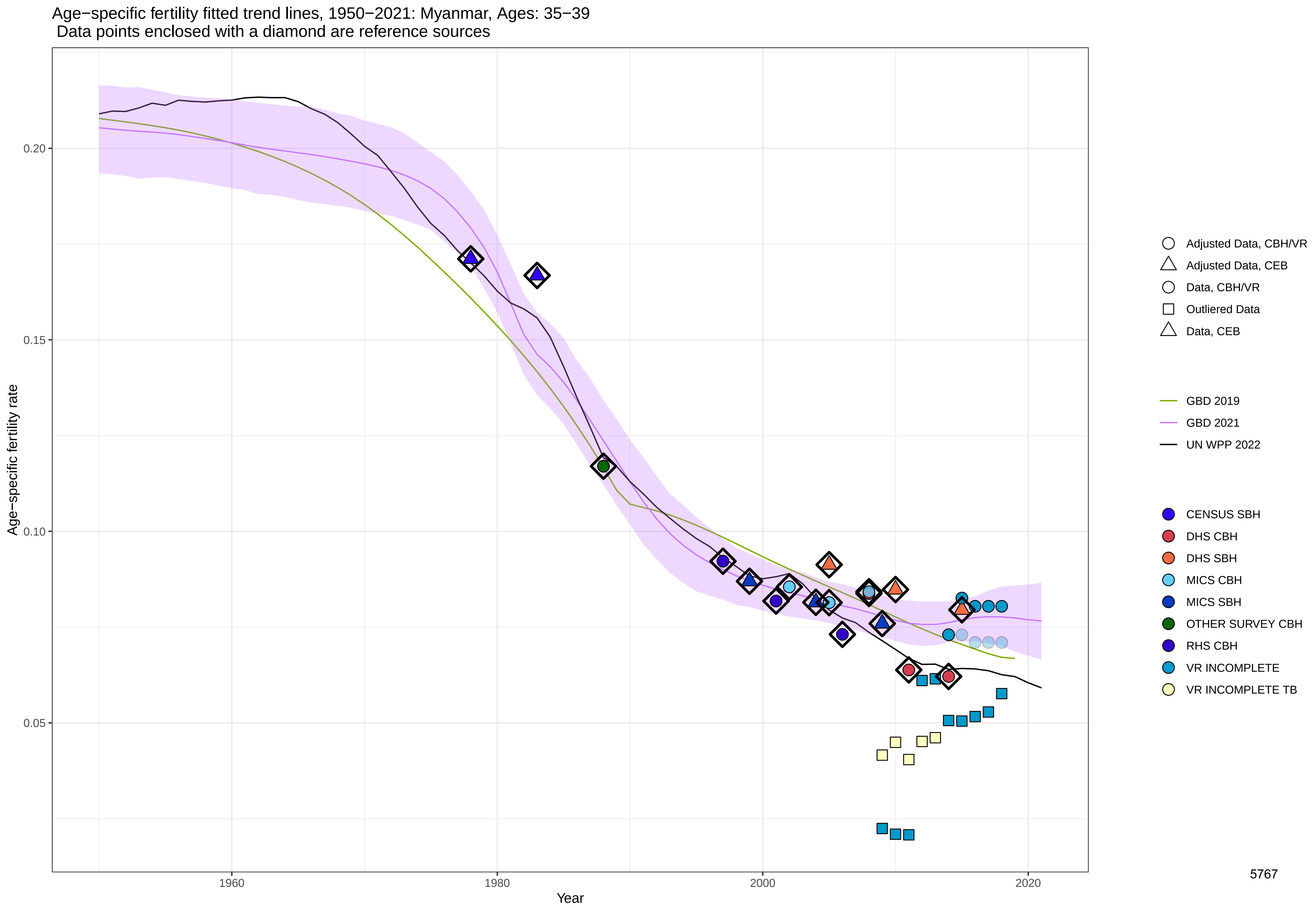

Age−specific fertility fitted trend lines, 1950−2021: Myanmar, Ages: 35−39
Data points enclosed with a diamond are reference sources
Age−specific fertility rate
0.20
0.15
0.10
0.05
1960
1980
2000
2020
Year
Adjusted Data, CBH/VR
Adjusted Data, CEB
Data, CBH/VR
Outliered Data
Data, CEB
GBD 2019
GBD 2021
UN WPP 2022
CENSUS SBH
DHS CBH
DHS SBH
MICS CBH
MICS SBH
OTHER SURVEY CBH
RHS CBH
VR INCOMPLETE
VR INCOMPLETE TB

Age−specific fertility fitted trend lines, 1950−2021: Myanmar, Ages: 40−44
Data points enclosed with a diamond are reference sources

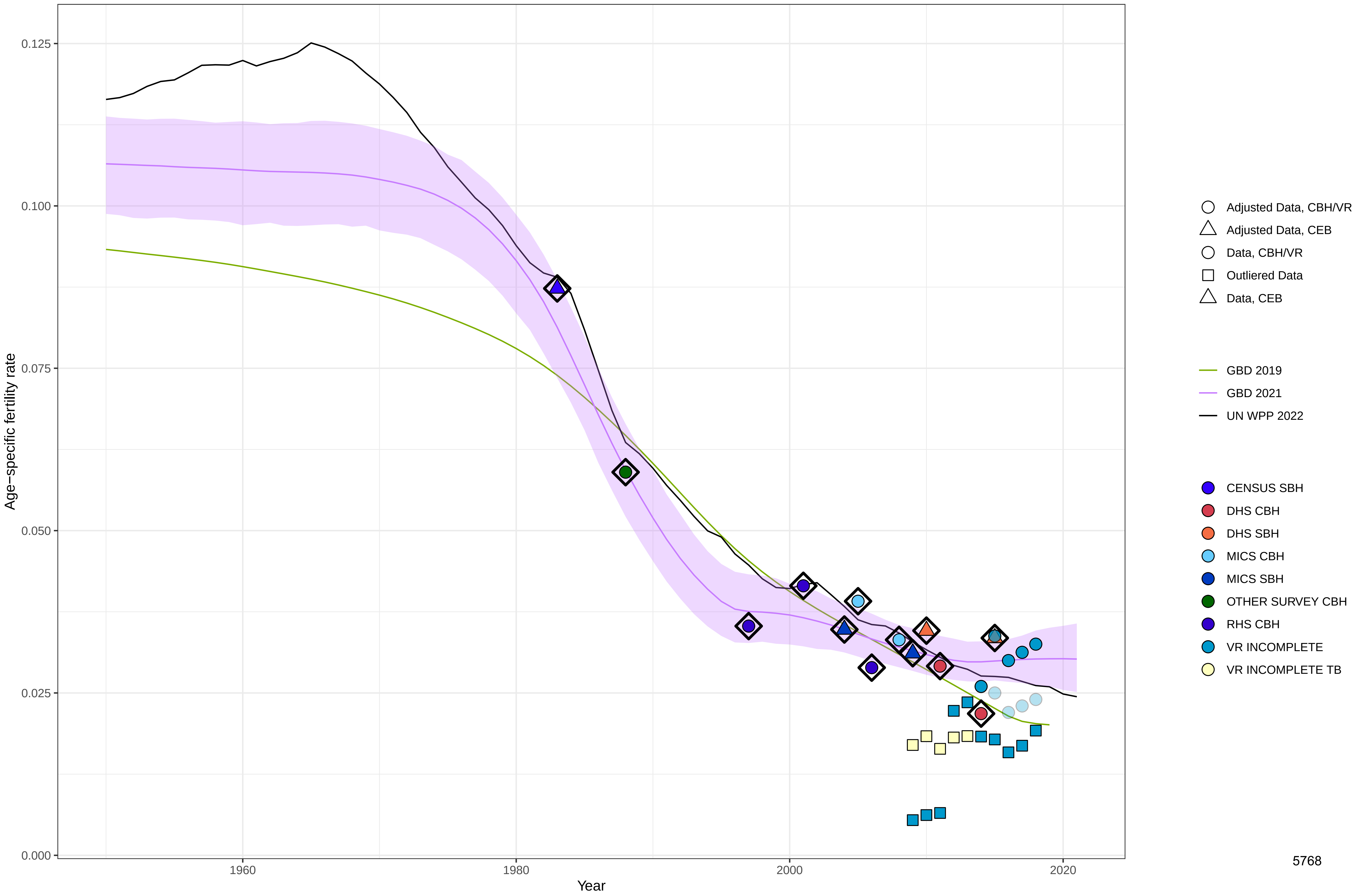

Age−specific fertility fitted trend lines, 1950−2021: Myanmar, Ages: 45−49
Data points enclosed with a diamond are reference sources

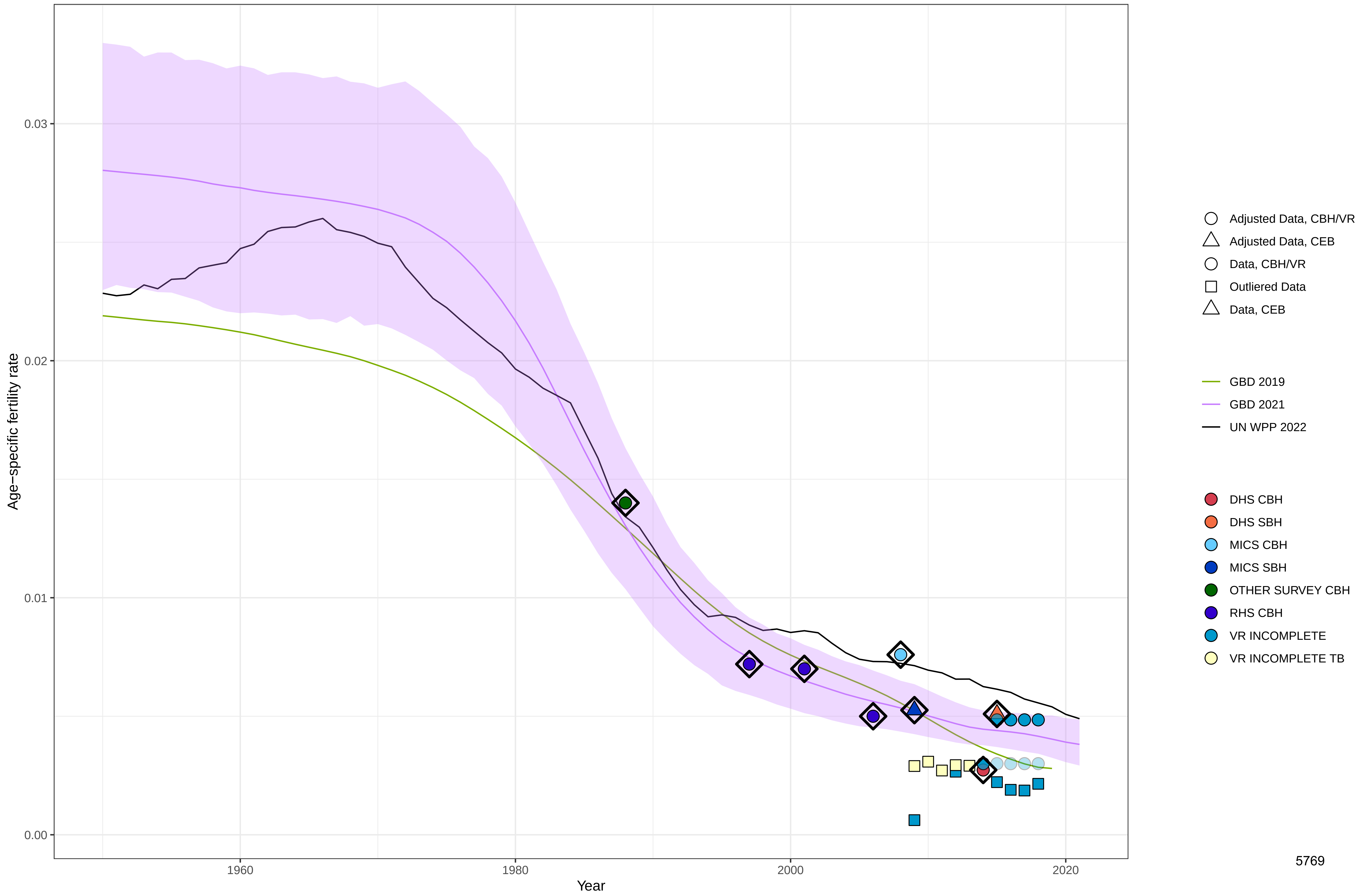

Age−specific fertility fitted trend lines, 1950−2021: Myanmar, Ages: 50−54
Data points enclosed with a diamond are reference sources

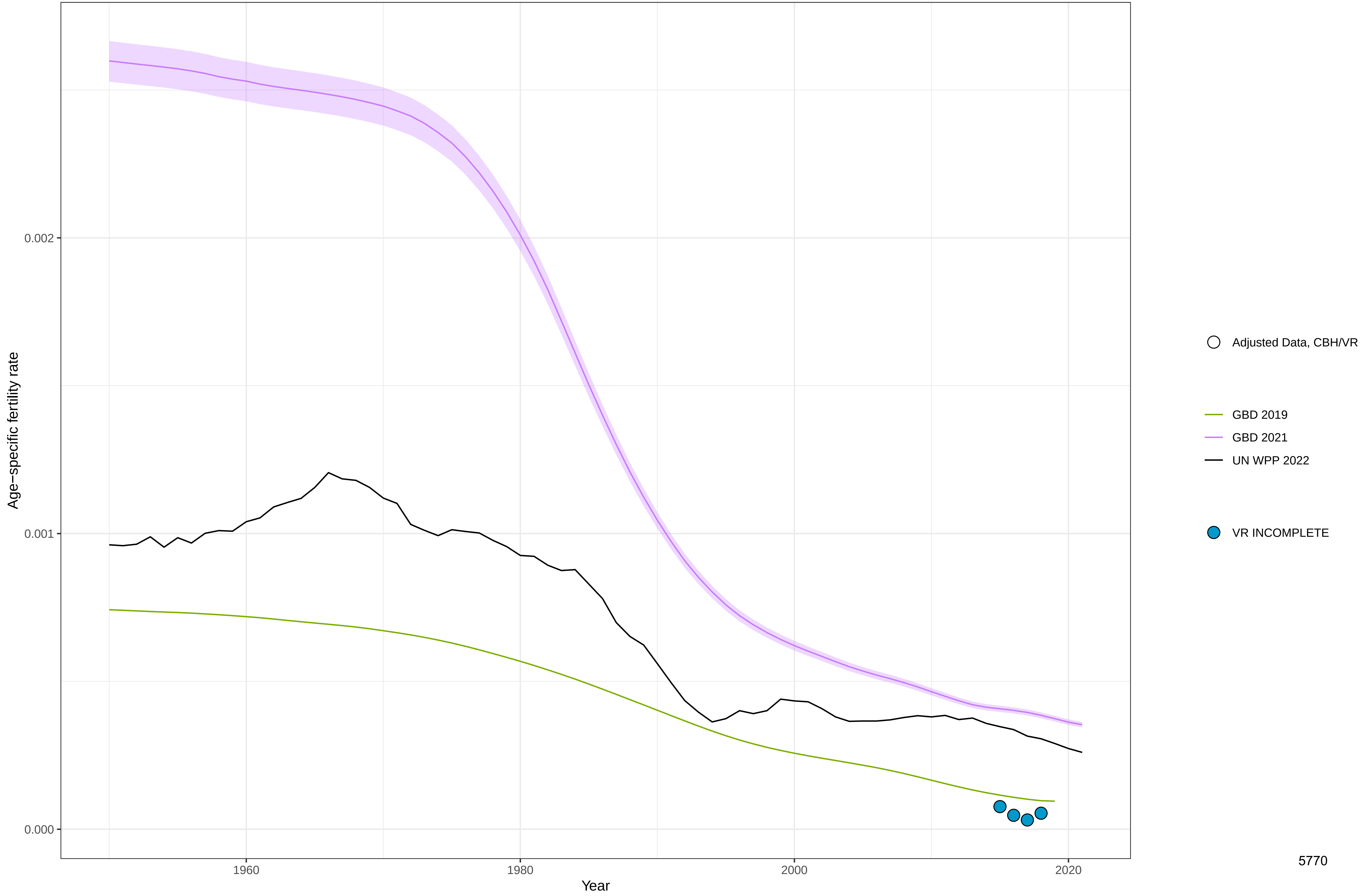

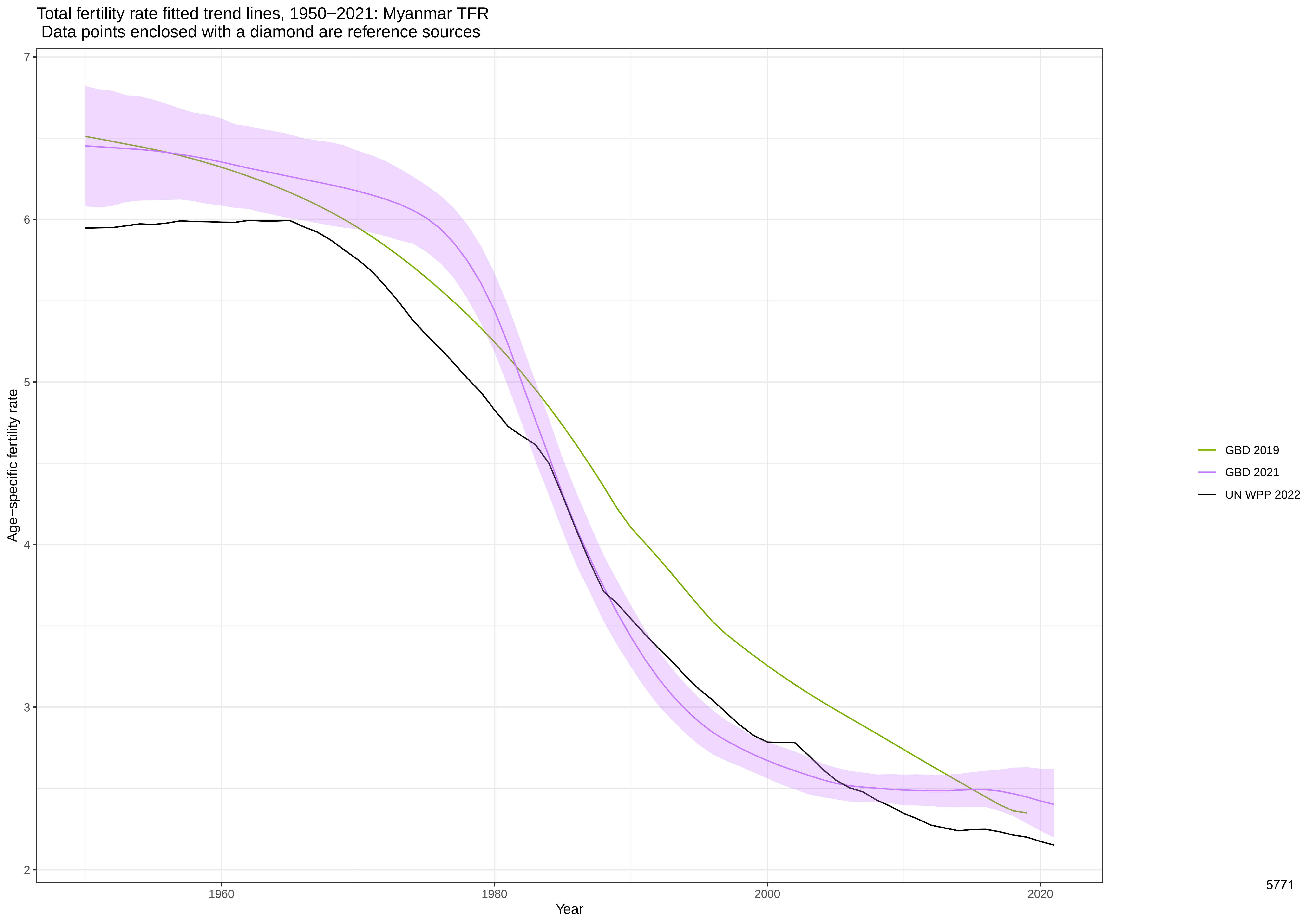
Total fertility rate fitted trend lines, 1950−2021: Myanmar TFR
Data points enclosed with a diamond are reference sources
Age−specific fertility rate
Year
7
6
5
4
3
2
1960
1980
2000
2020
GBD 2019
GBD 2021
UN WPP 2022

Age−specific fertility fitted trend lines, 1950−2021: Philippines, Ages: 10−14
Data points enclosed with a diamond are reference sources

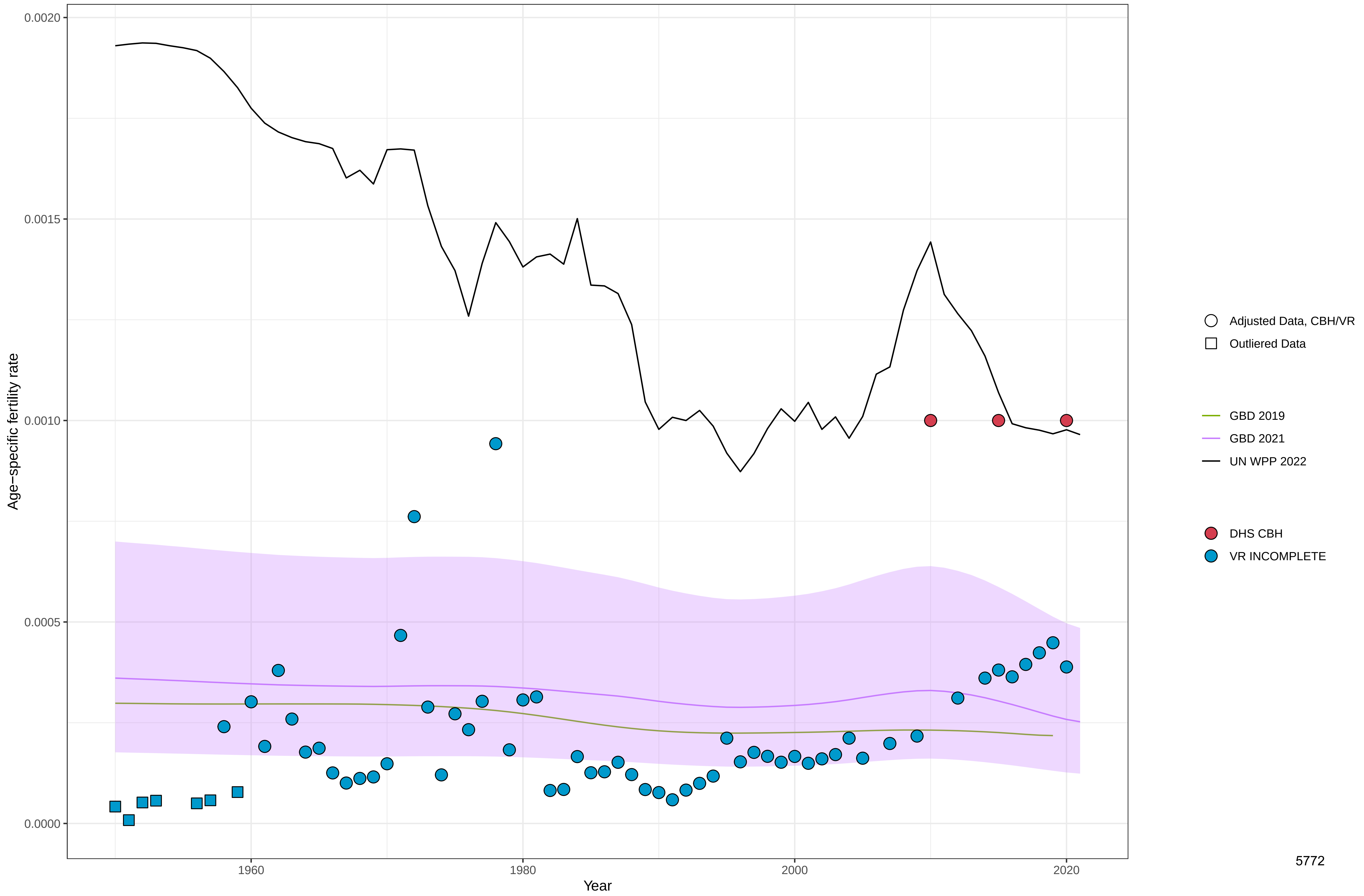

Age−specific fertility fitted trend lines, 1950−2021: Philippines, Ages: 15−19
Data points enclosed with a diamond are reference sources

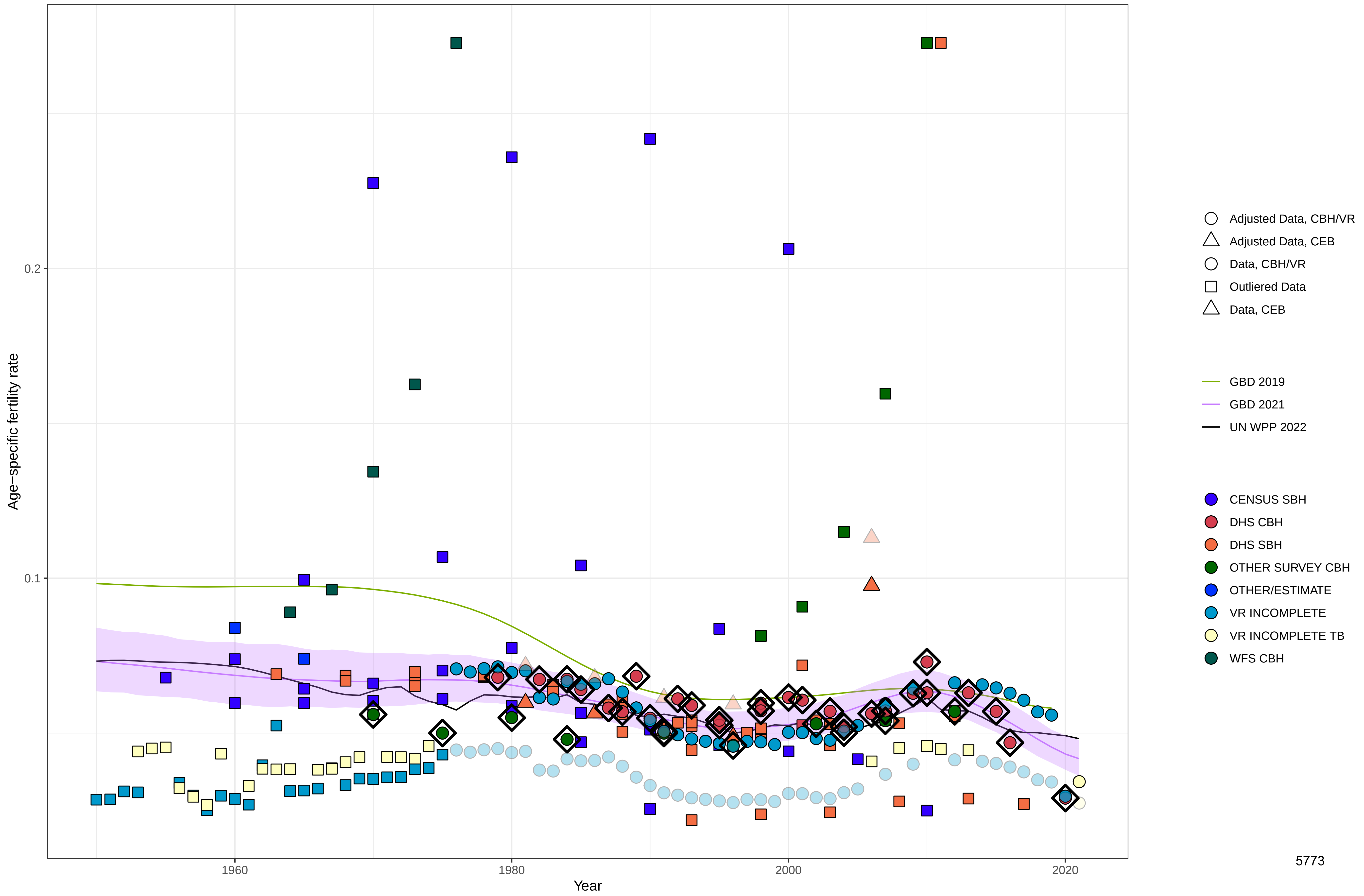

Age−specific fertility fitted trend lines, 1950−2021: Philippines, Ages: 20−24
Data points enclosed with a diamond are reference sources

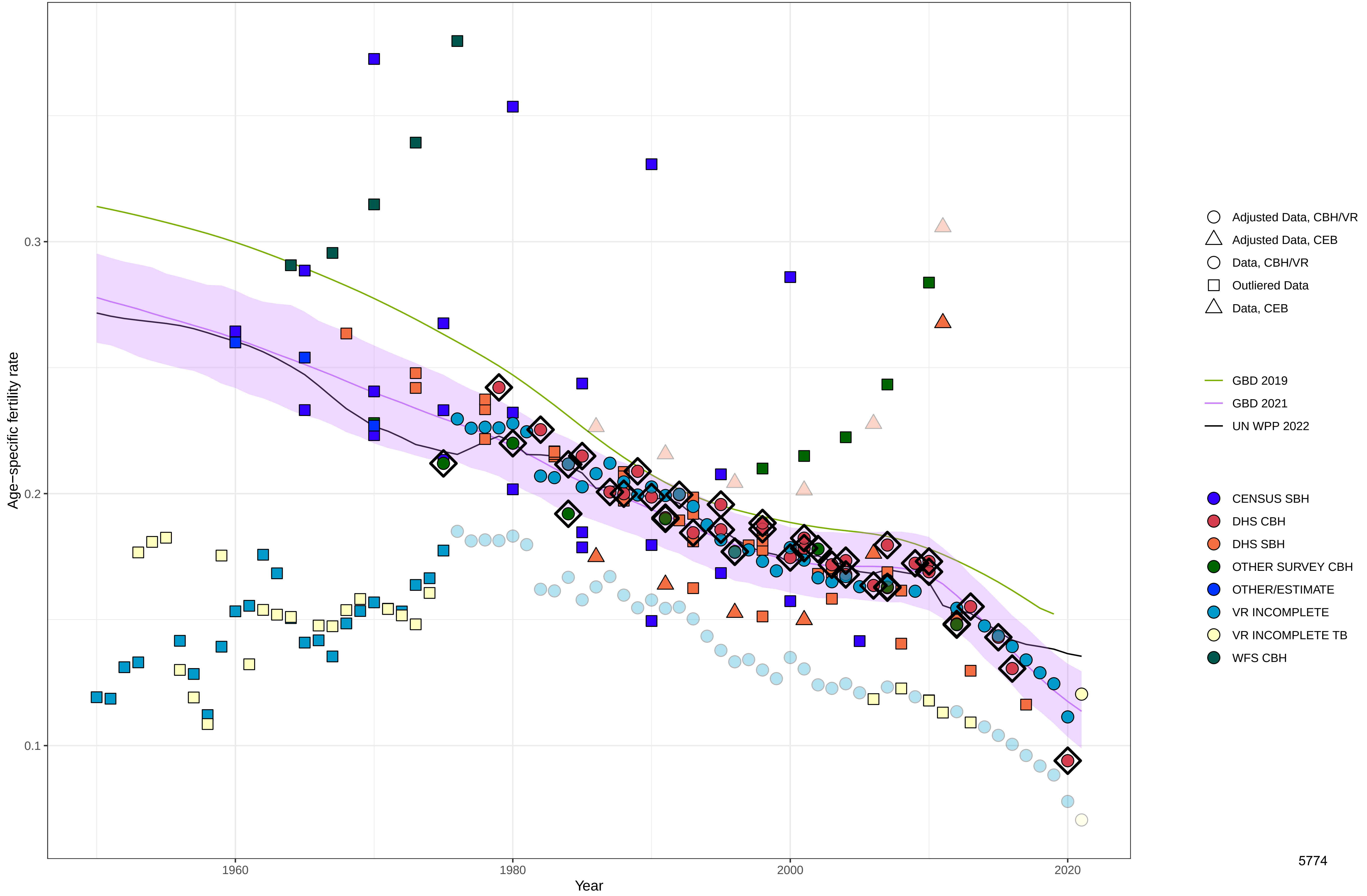

Age−specific fertility fitted trend lines, 1950−2021: Philippines, Ages: 25−29
Data points enclosed with a diamond are reference sources

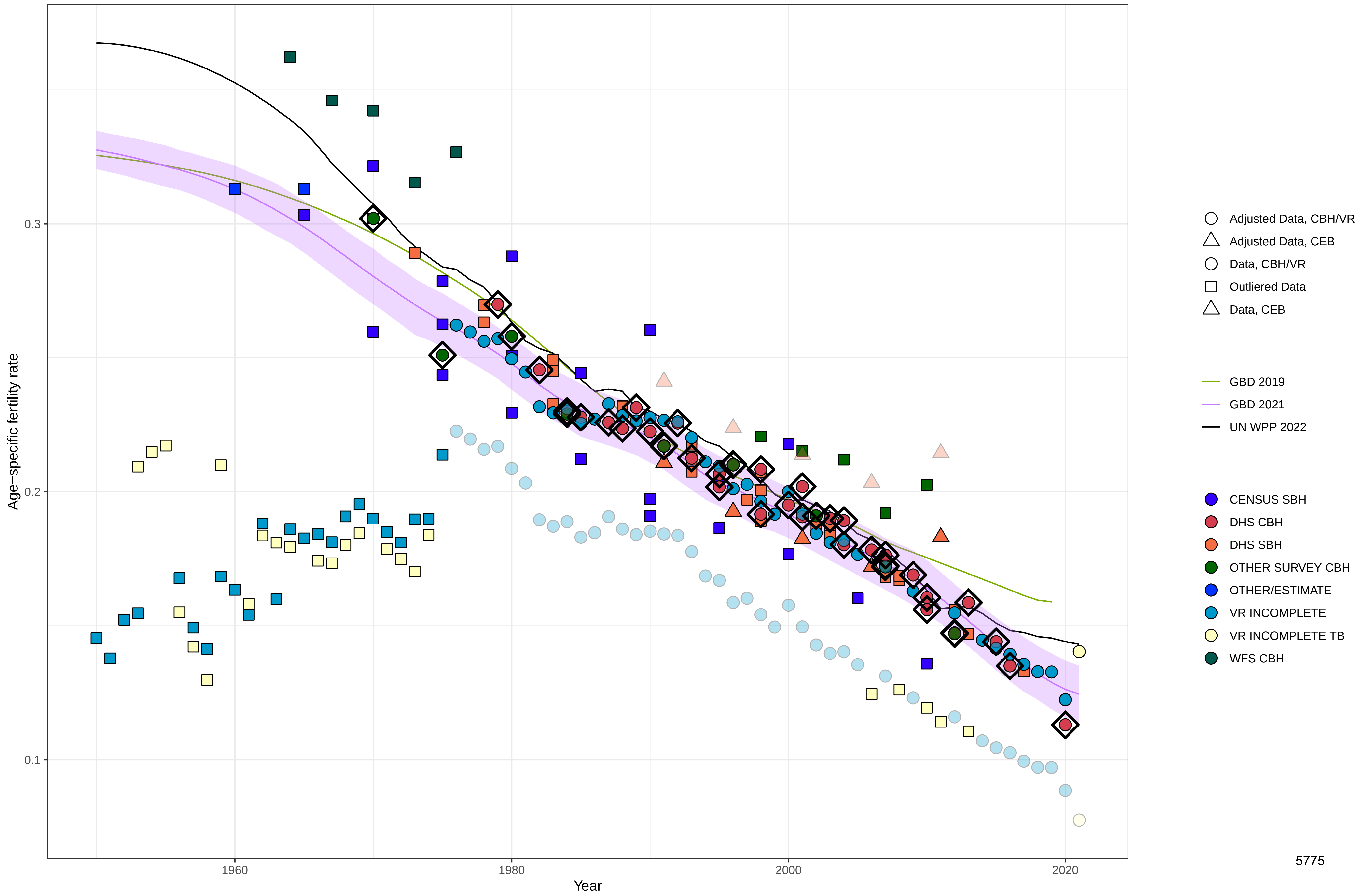

Age−specific fertility fitted trend lines, 1950−2021: Philippines, Ages: 30−34
Data points enclosed with a diamond are reference sources

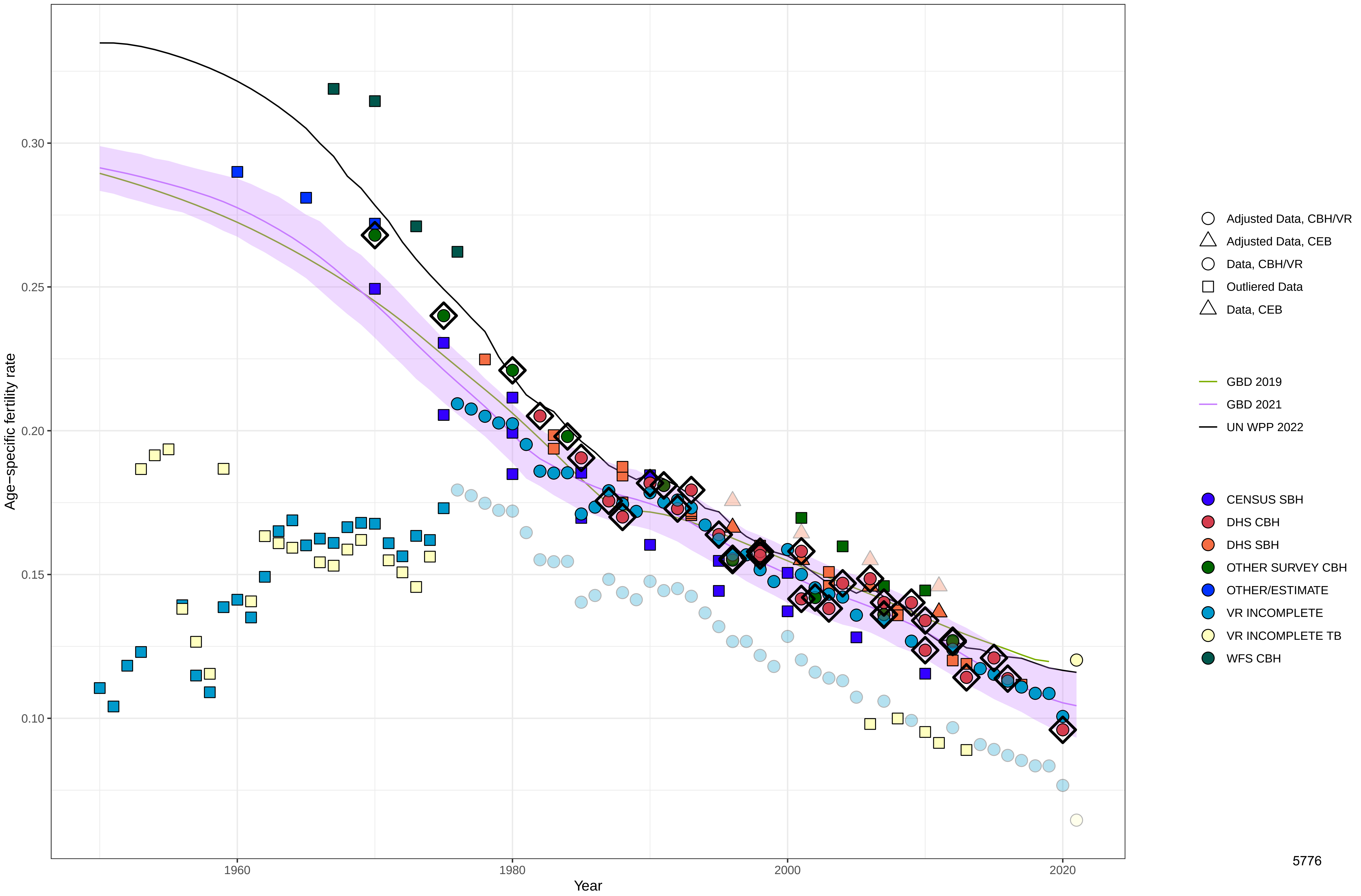

Age−specific fertility fitted trend lines, 1950−2021: Philippines, Ages: 35−39
Data points enclosed with a diamond are reference sources

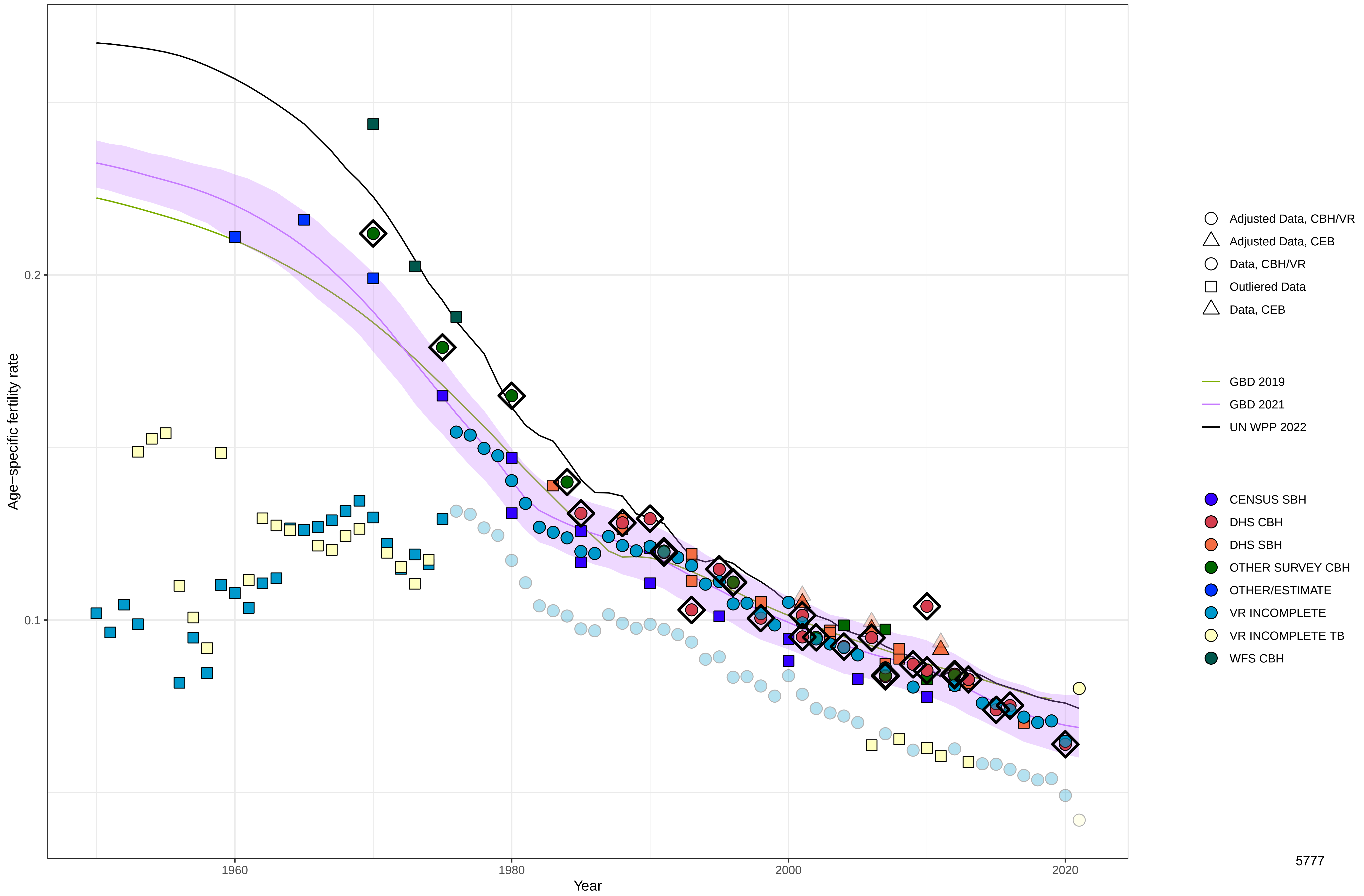

Age−specific fertility fitted trend lines, 1950−2021: Philippines, Ages: 40−44
Data points enclosed with a diamond are reference sources

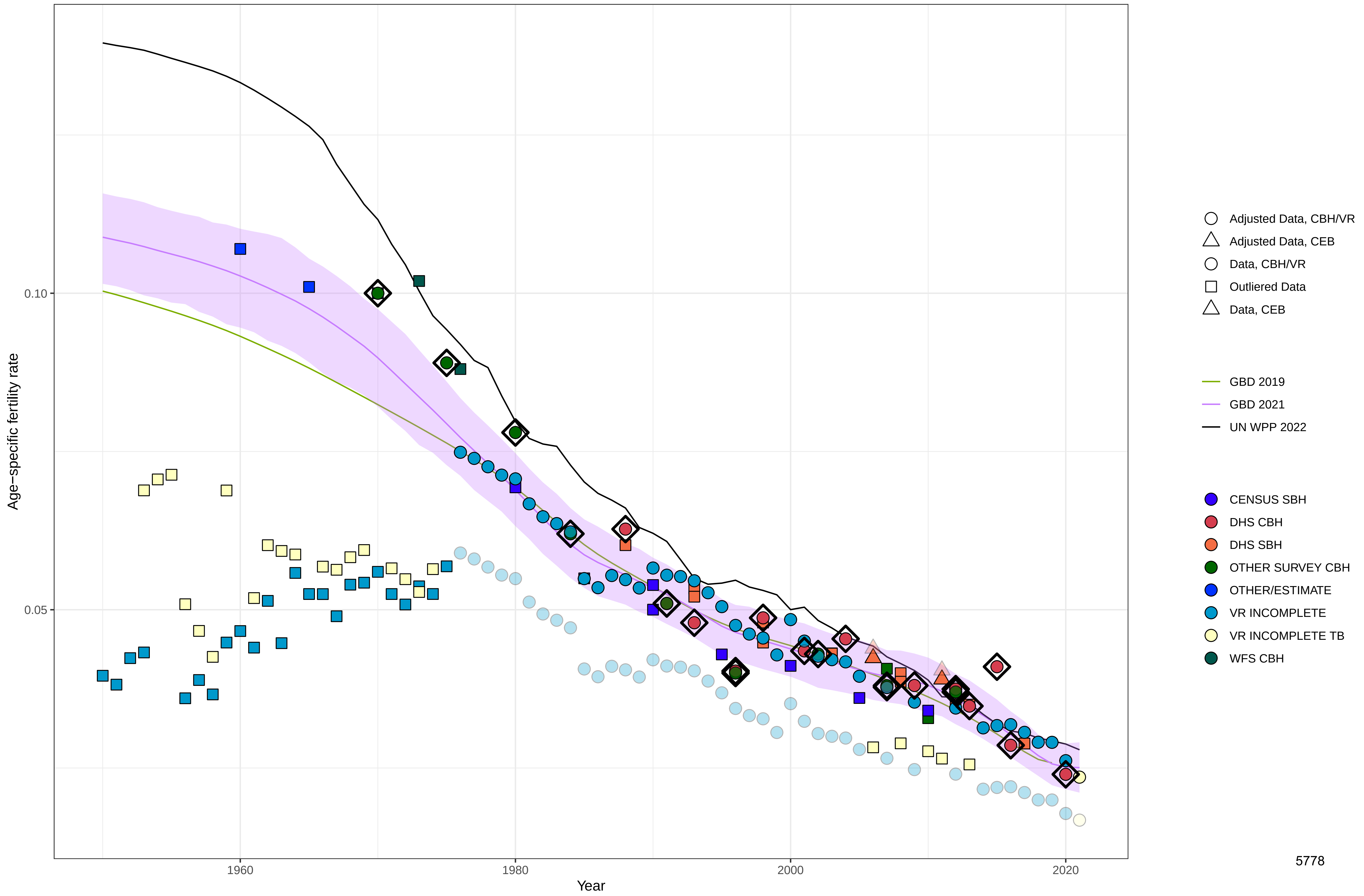

Age−specific fertility fitted trend lines, 1950−2021: Philippines, Ages: 45−49
Data points enclosed with a diamond are reference sources

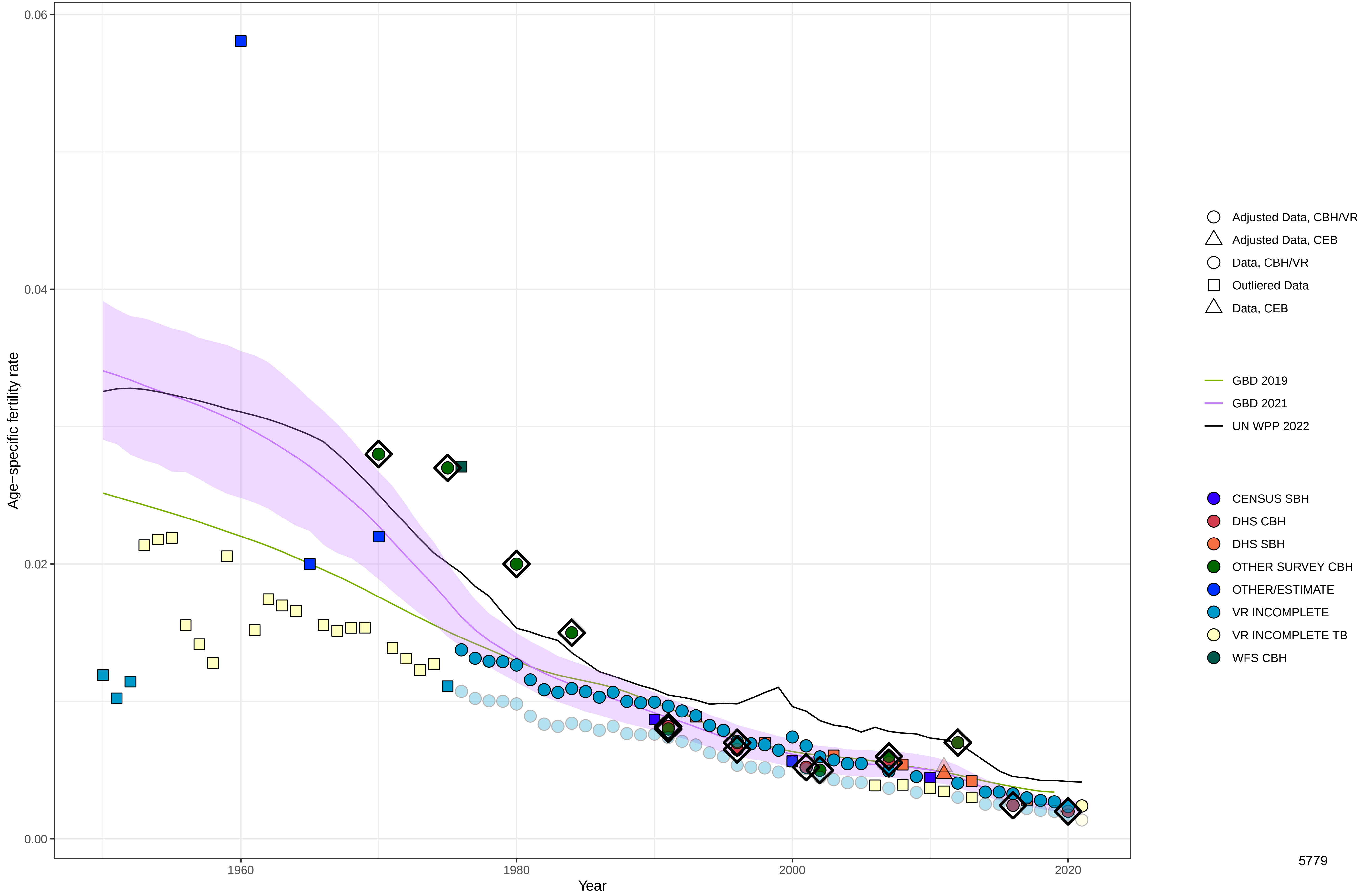

Age−specific fertility fitted trend lines, 1950−2021: Philippines, Ages: 50−54
Data points enclosed with a diamond are reference sources

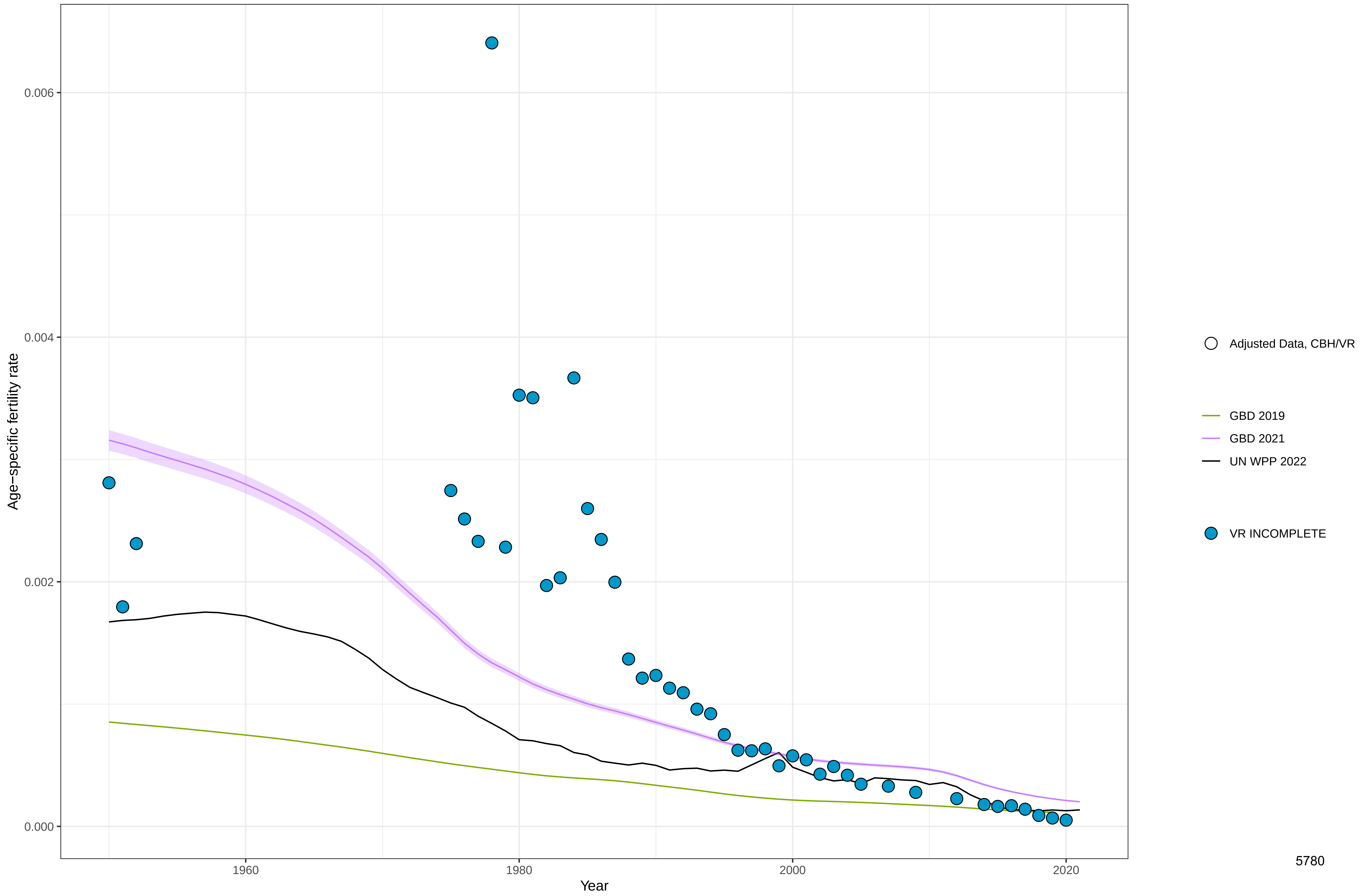

Total fertility rate fitted trend lines, 1950−2021: Philippines TFR
Data points enclosed with a diamond are reference sources

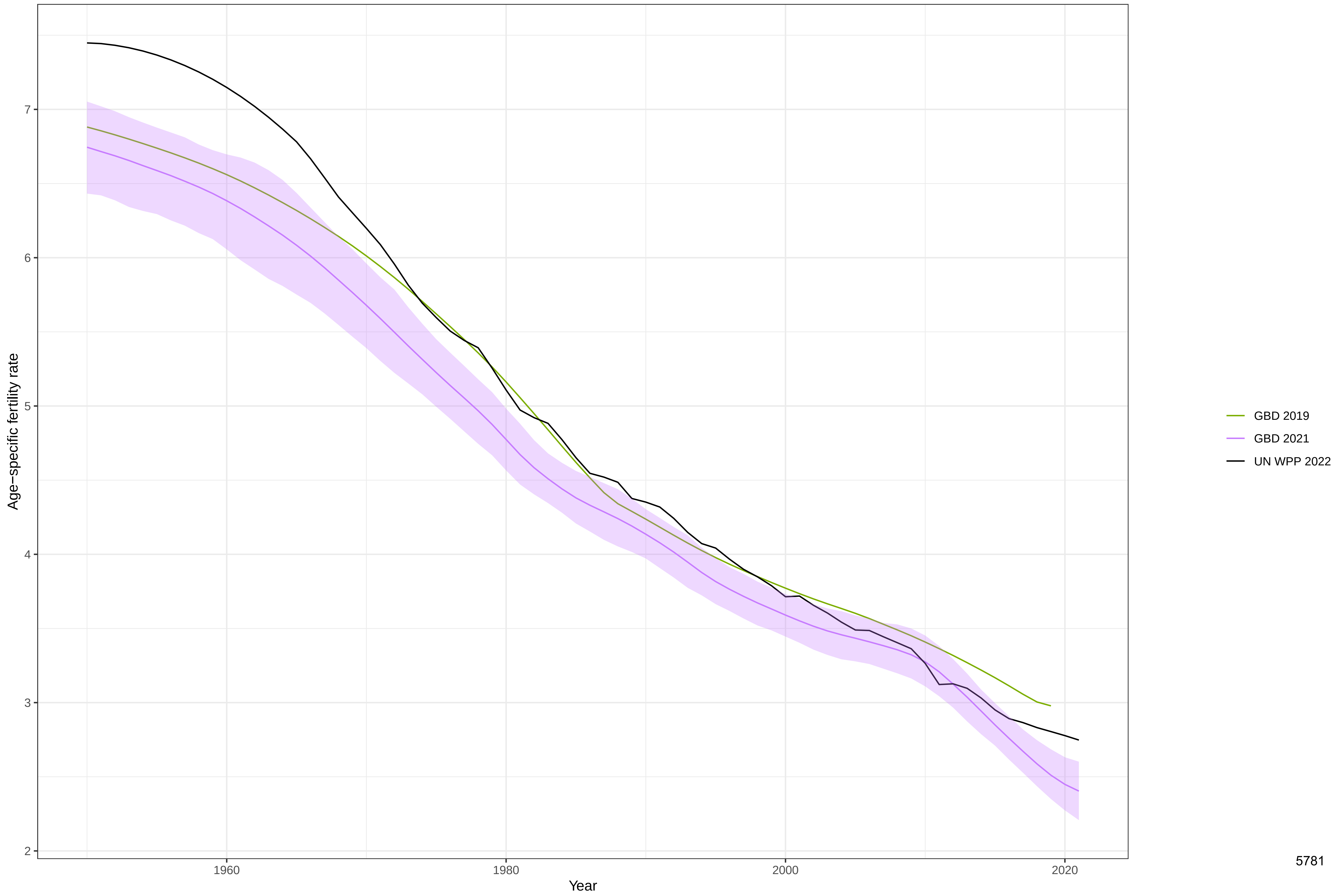

Age−specific fertility fitted trend lines, 1950−2021: Abra, Ages: 10−14
Data points enclosed with a diamond are reference sources

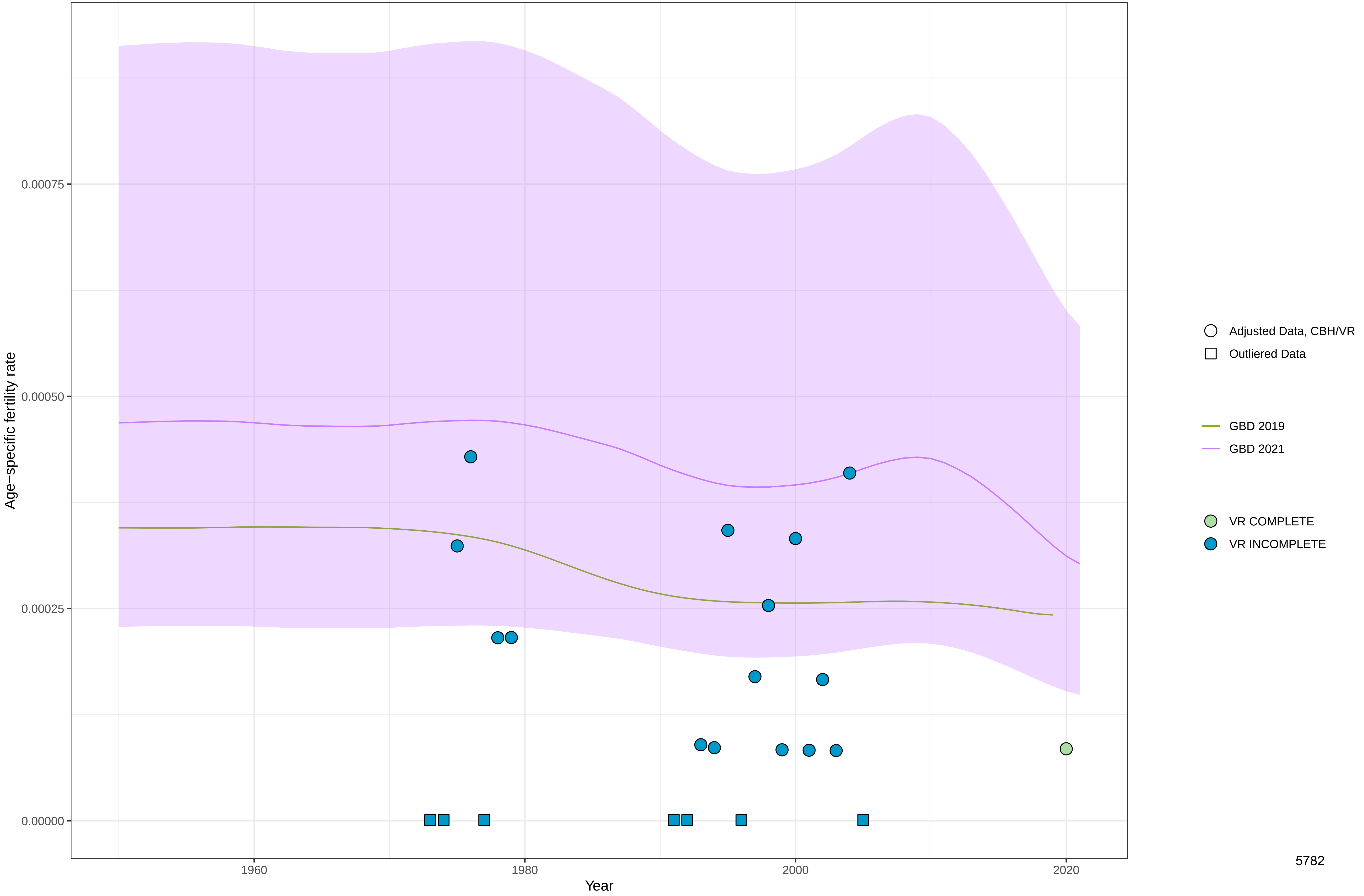

Age−specific fertility fitted trend lines, 1950−2021: Abra, Ages: 15−19
Data points enclosed with a diamond are reference sources

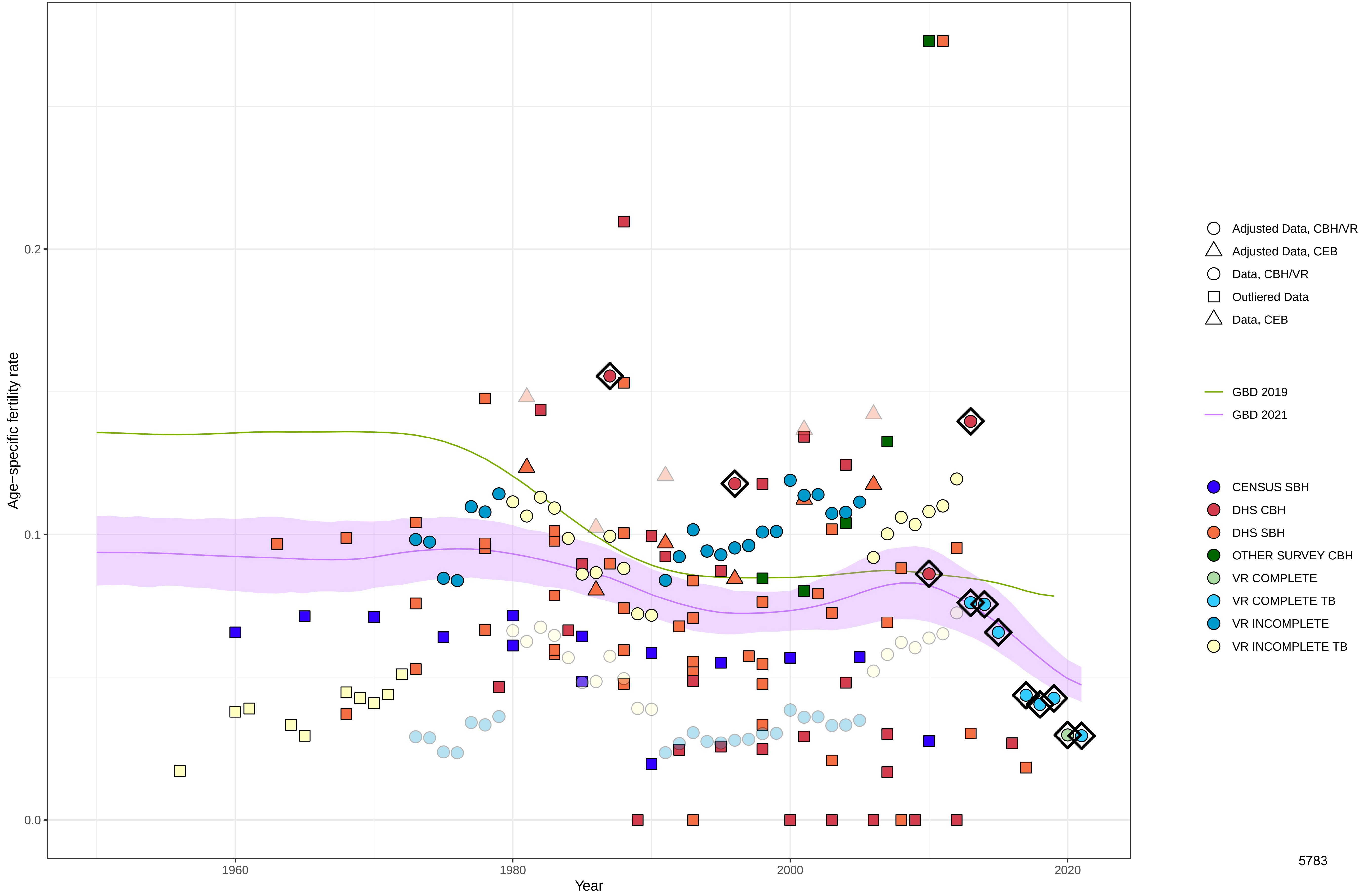

Age−specific fertility fitted trend lines, 1950−2021: Abra, Ages: 20−24
Data points enclosed with a diamond are reference sources

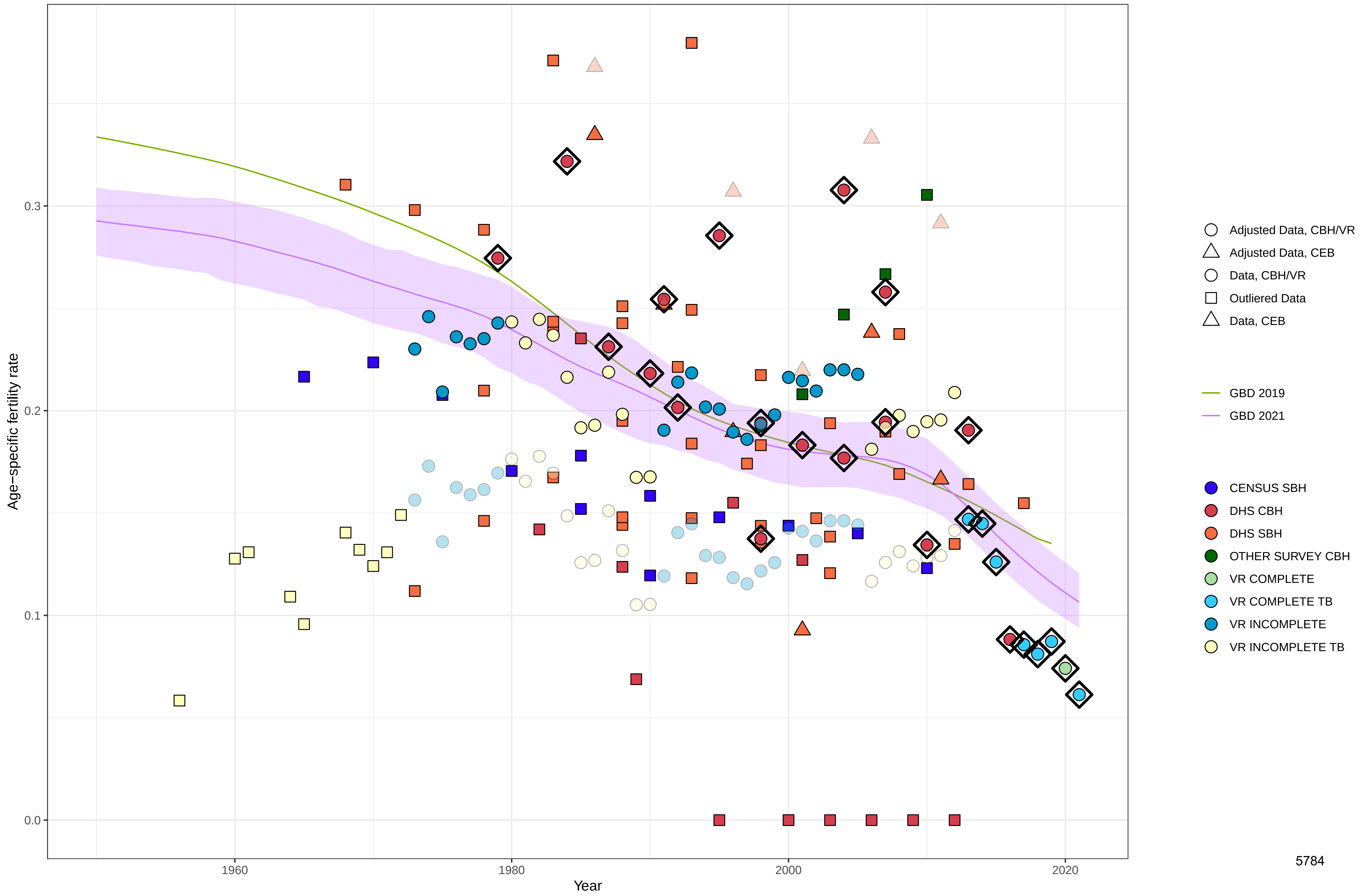

Age−specific fertility fitted trend lines, 1950−2021: Abra, Ages: 25−29
Data points enclosed with a diamond are reference sources

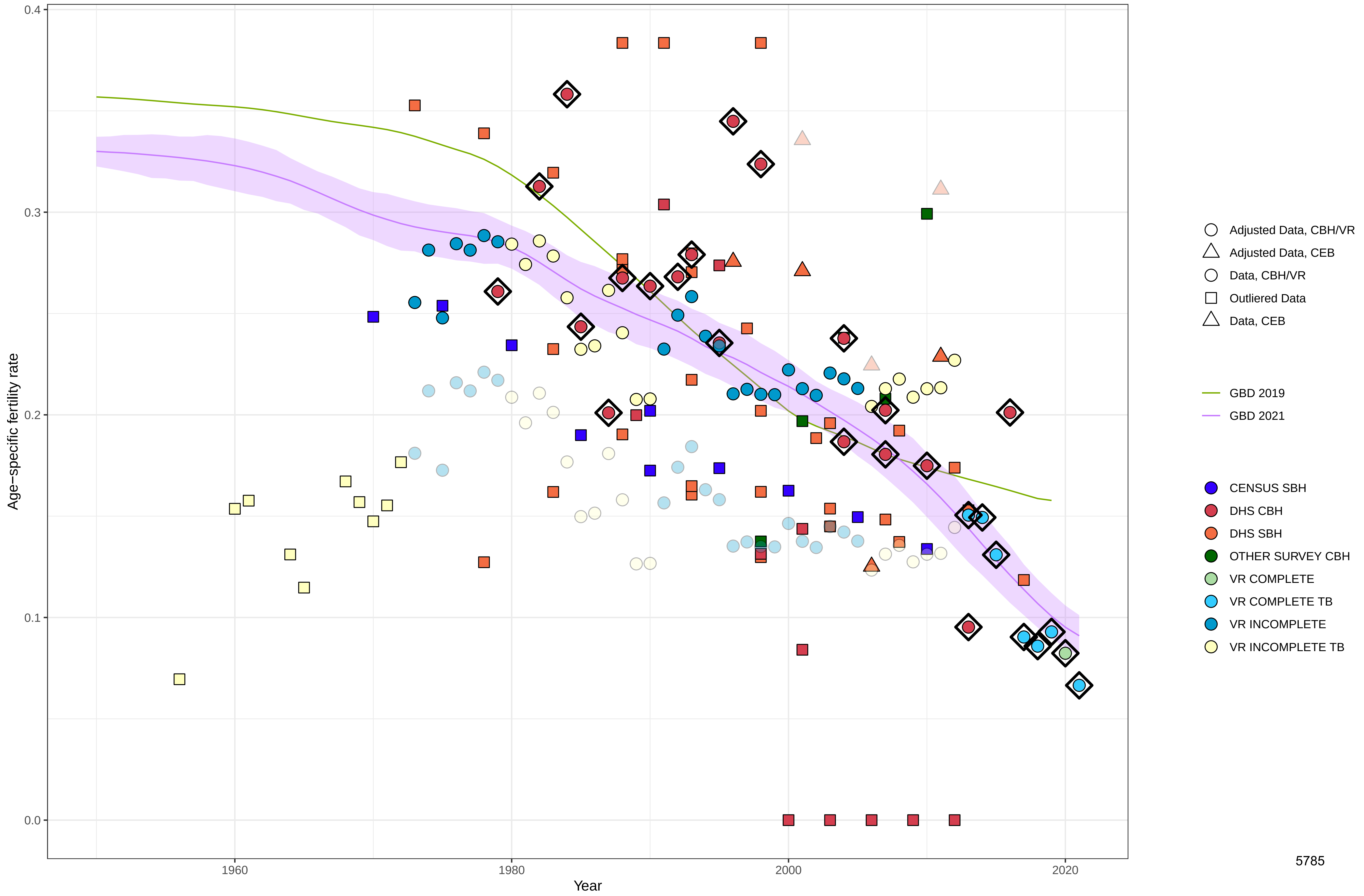

Age−specific fertility fitted trend lines, 1950−2021: Abra, Ages: 30−34
Data points enclosed with a diamond are reference sources

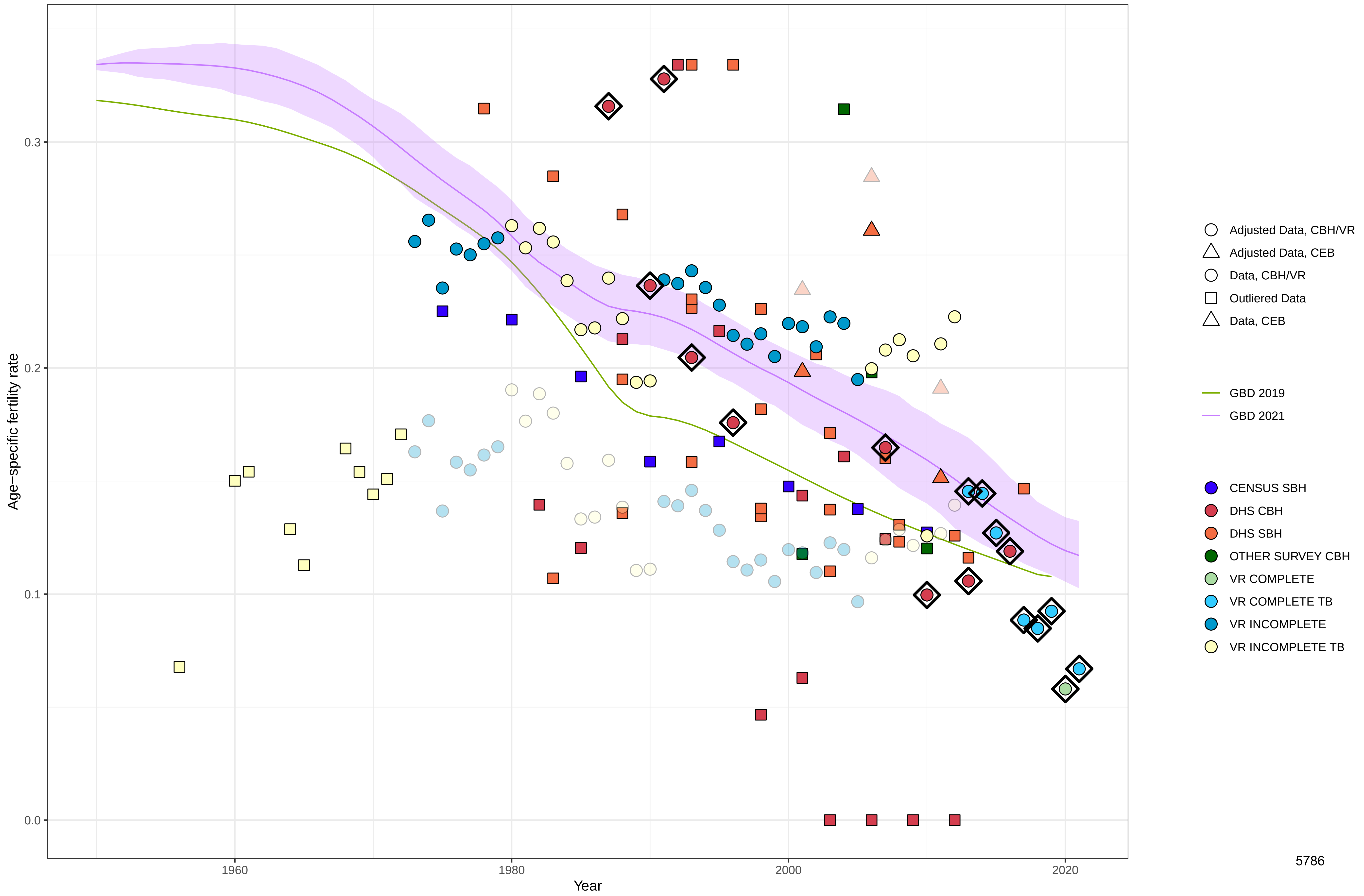

Age−specific fertility fitted trend lines, 1950−2021: Abra, Ages: 35−39
Data points enclosed with a diamond are reference sources

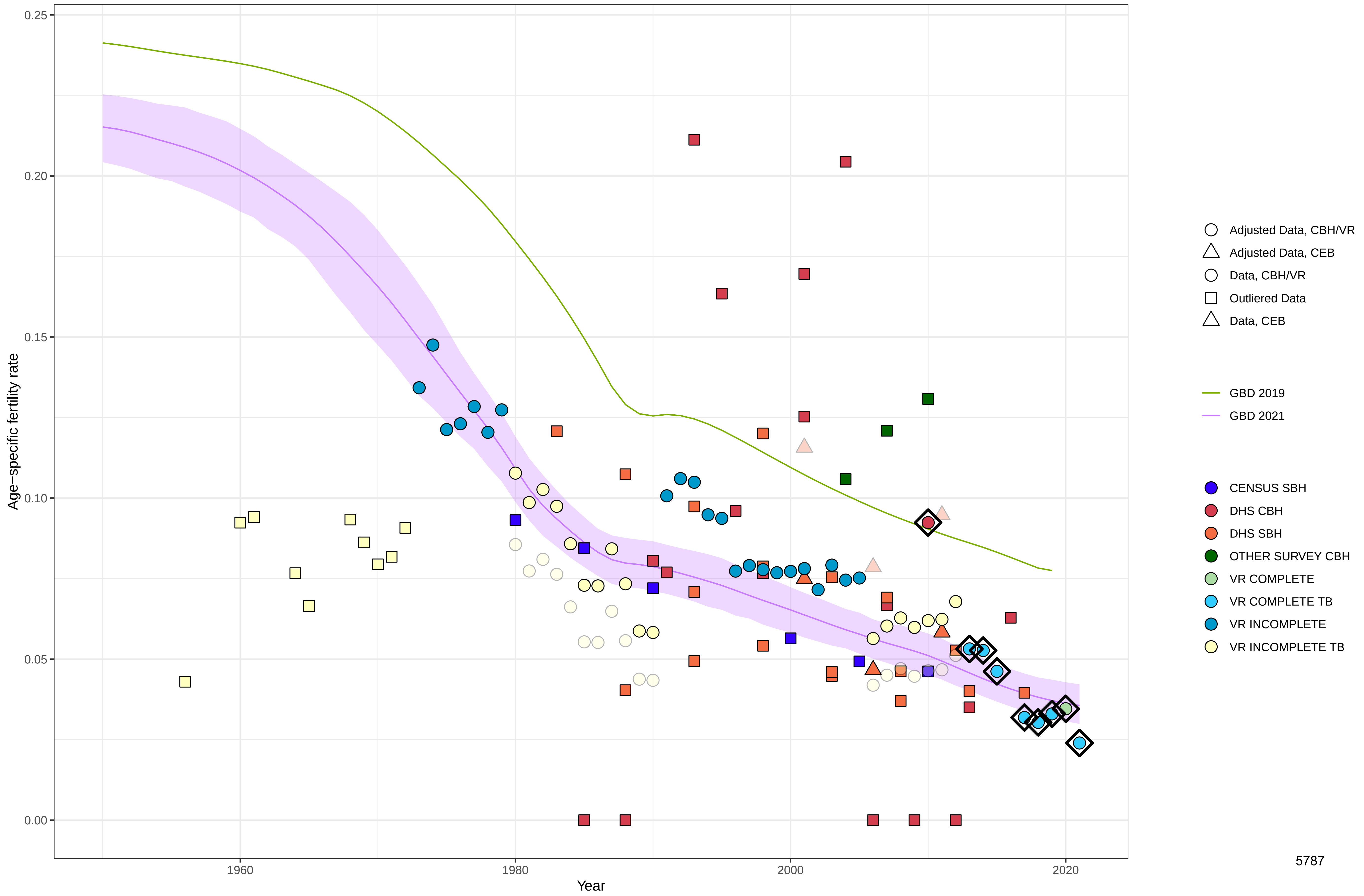

Age−specific fertility fitted trend lines, 1950−2021: Abra, Ages: 40−44
Data points enclosed with a diamond are reference sources

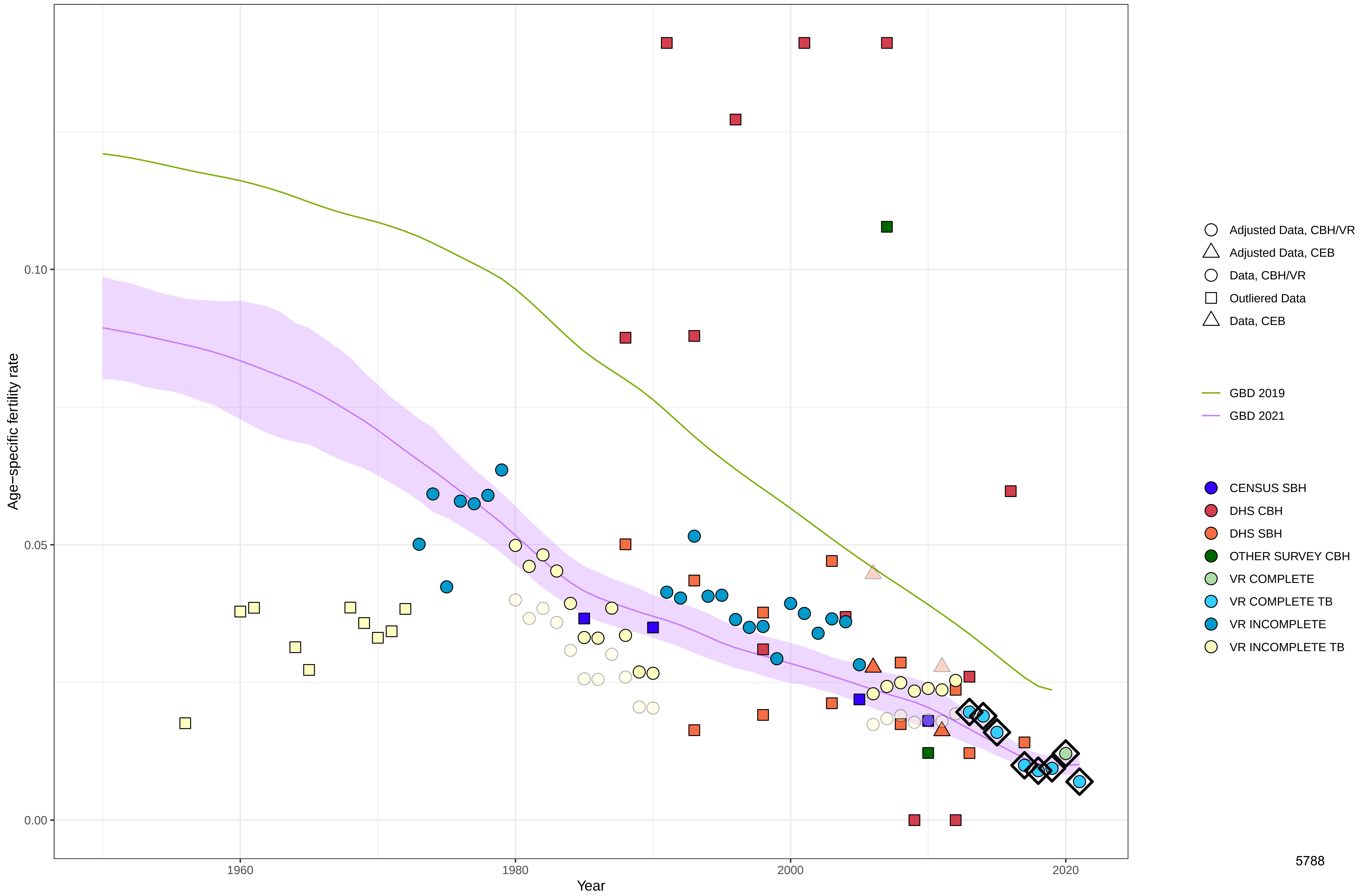

Age−specific fertility fitted trend lines, 1950−2021: Abra, Ages: 45−49
Data points enclosed with a diamond are reference sources

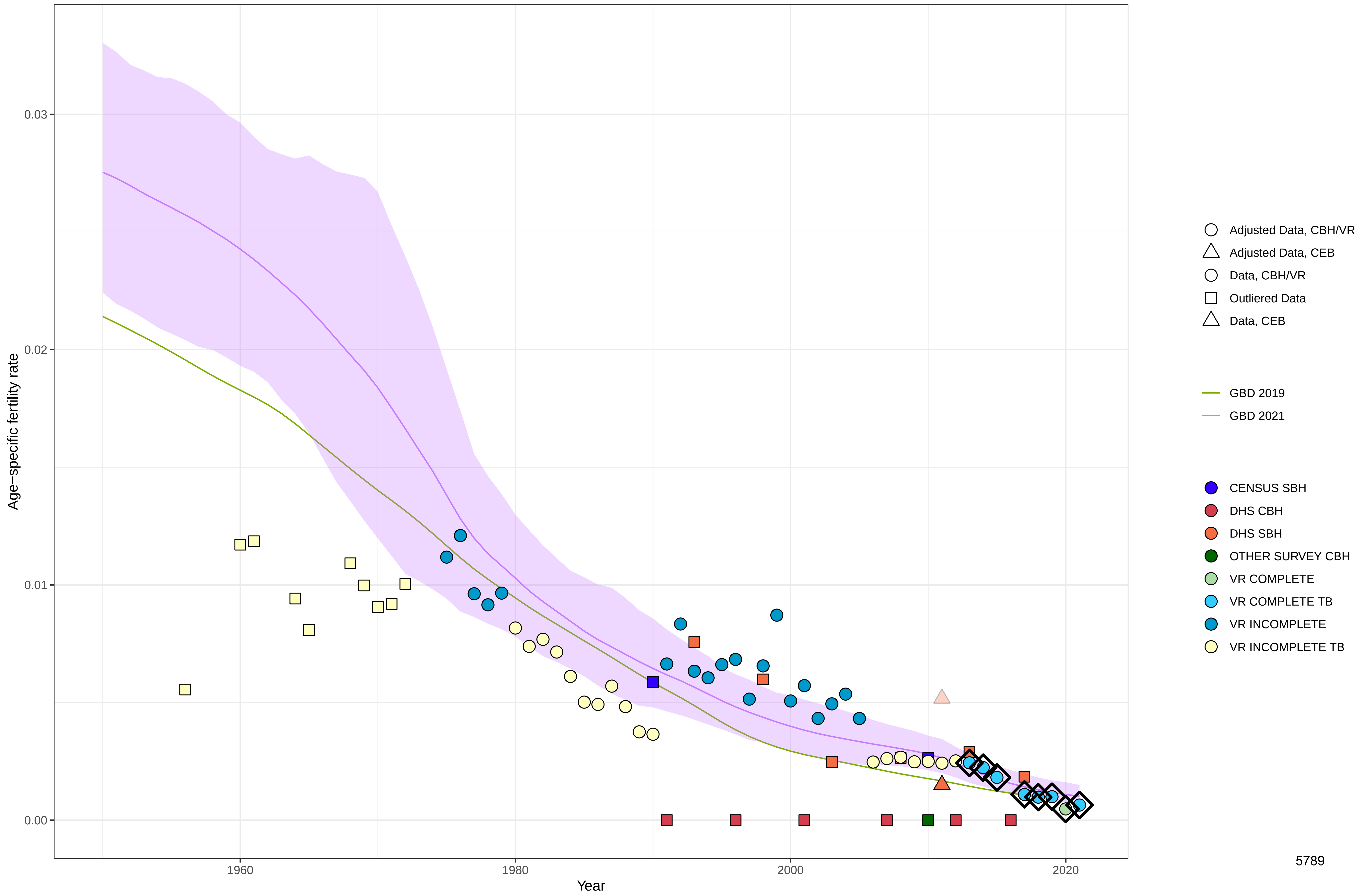

Age−specific fertility fitted trend lines, 1950−2021: Abra, Ages: 50−54
Data points enclosed with a diamond are reference sources

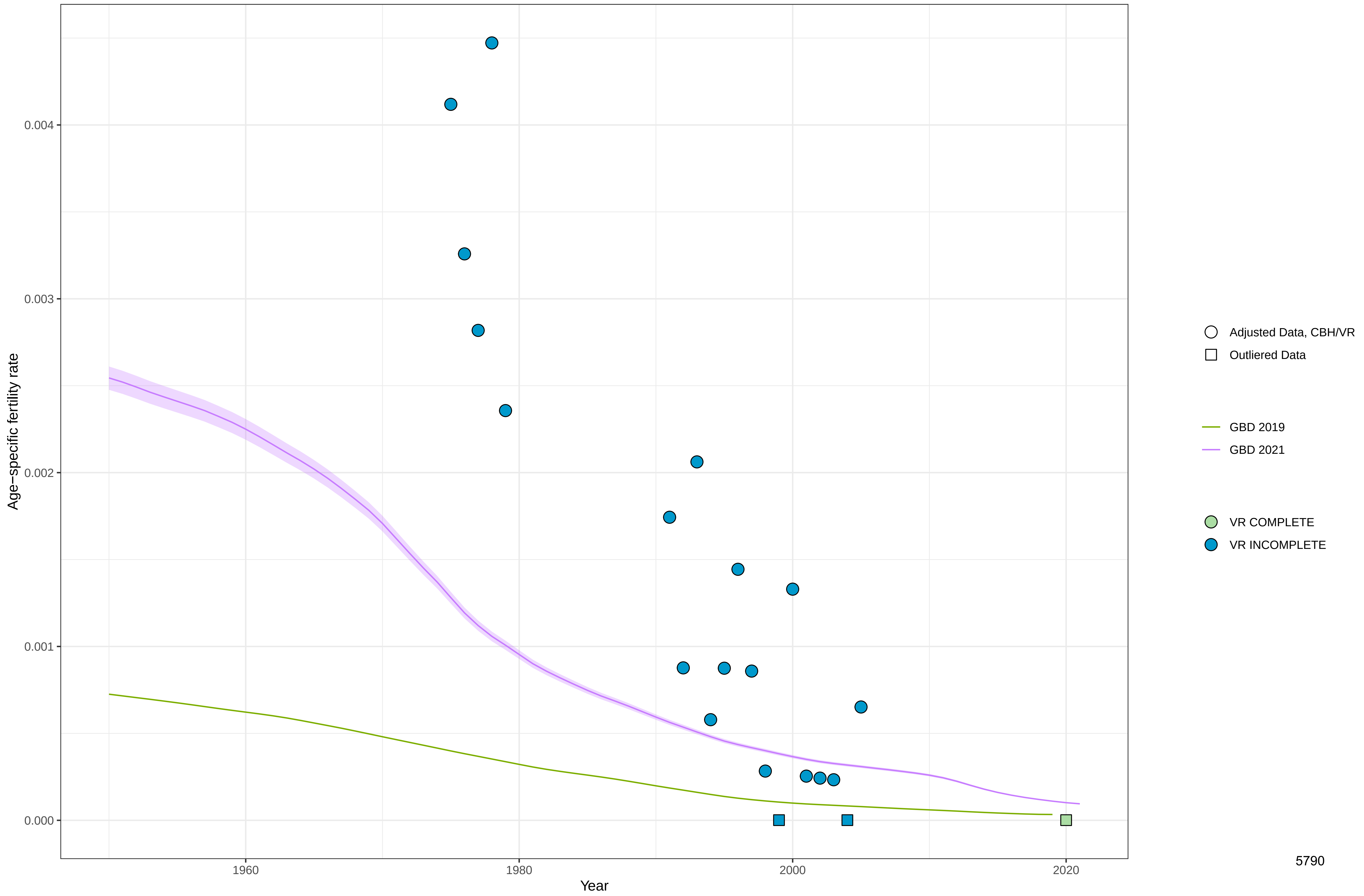

Total fertility rate fitted trend lines, 1950−2021: Abra TFR
Data points enclosed with a diamond are reference sources

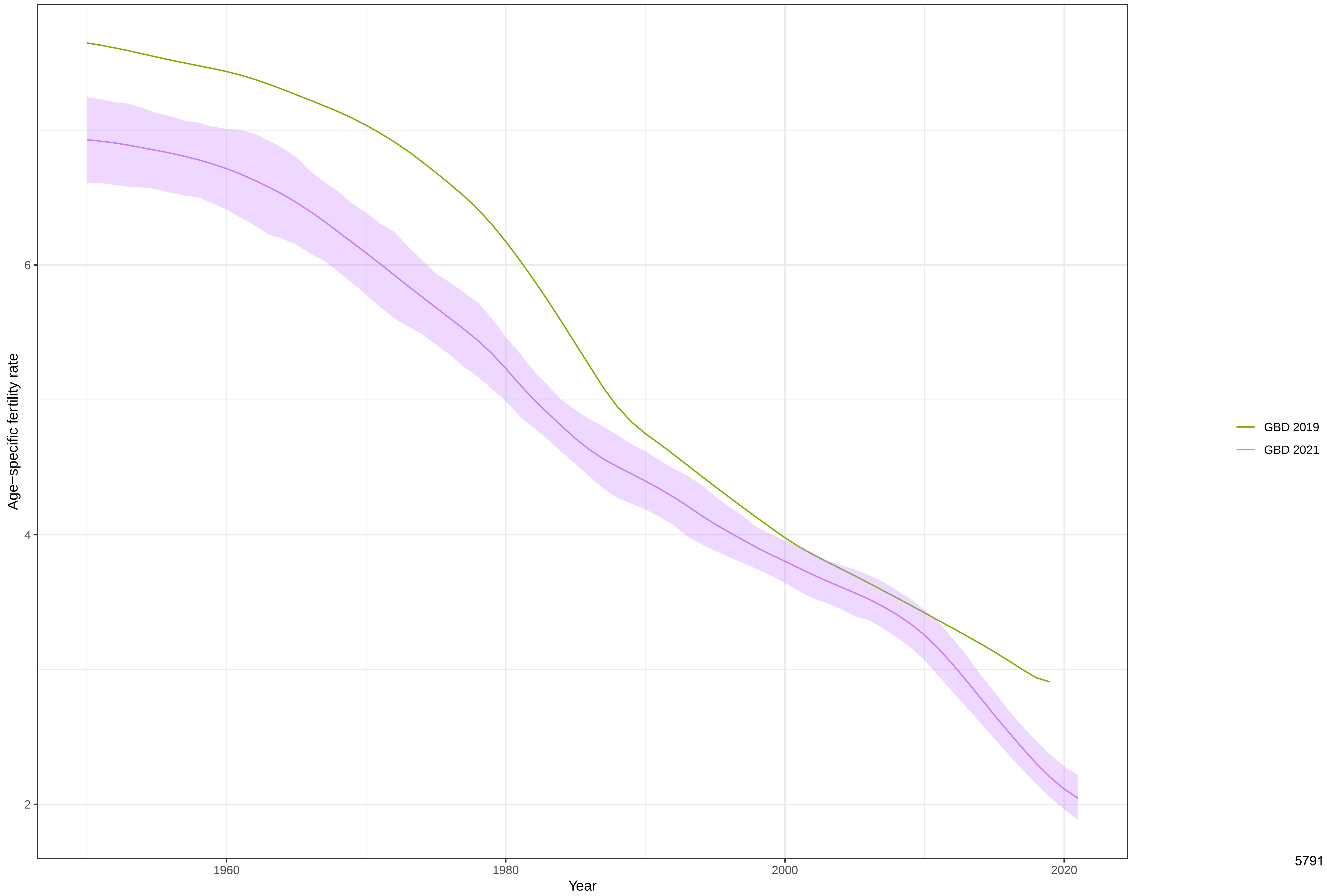

5791

Age−specific fertility fitted trend lines, 1950−2021: Agusan Del Norte, Ages: 10−14
Data points enclosed with a diamond are reference sources

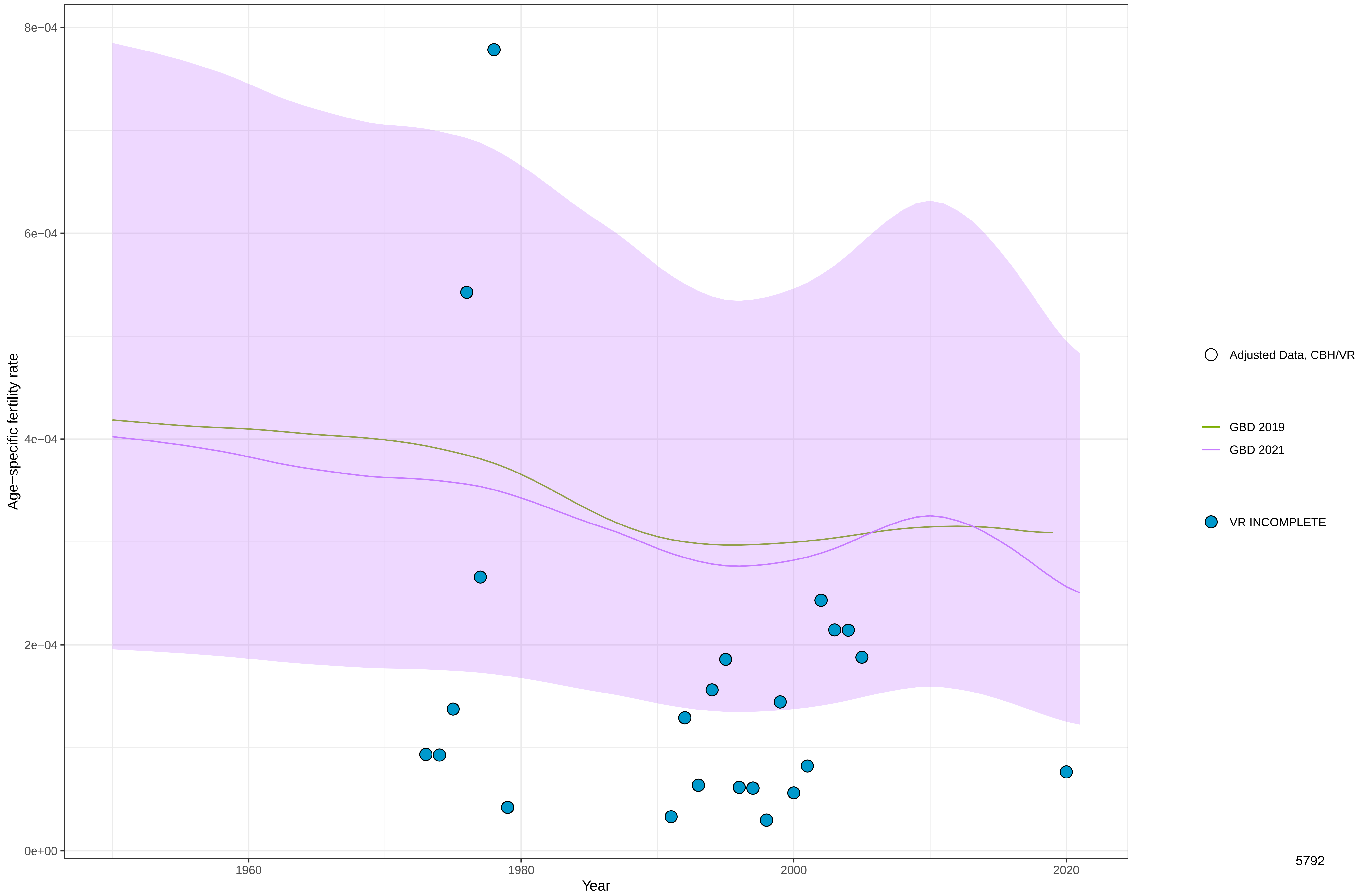

Age−specific fertility fitted trend lines, 1950−2021: Agusan Del Norte, Ages: 15−19
Data points enclosed with a diamond are reference sources

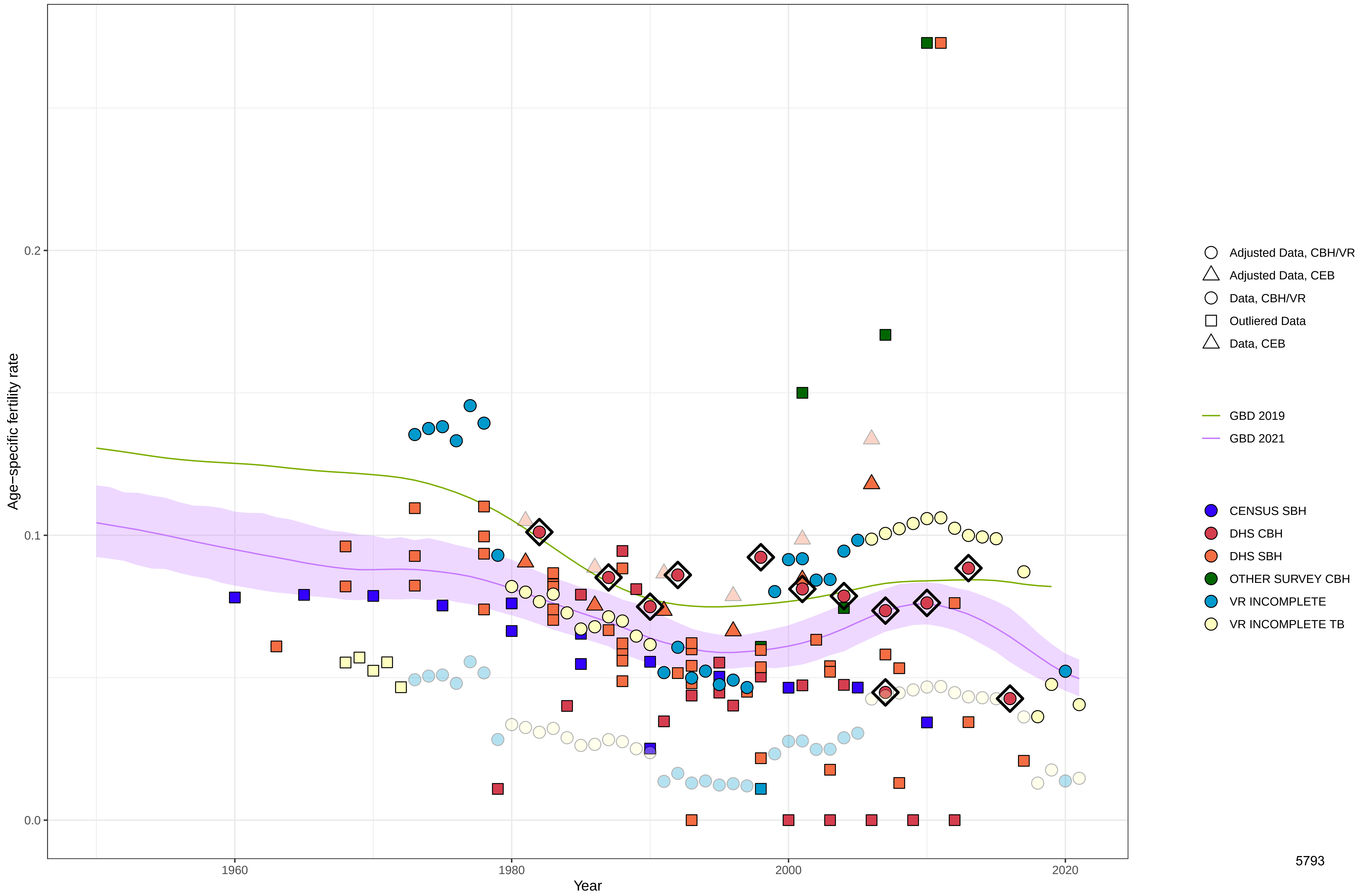

Age−specific fertility fitted trend lines, 1950−2021: Agusan Del Norte, Ages: 20−24
Data points enclosed with a diamond are reference sources

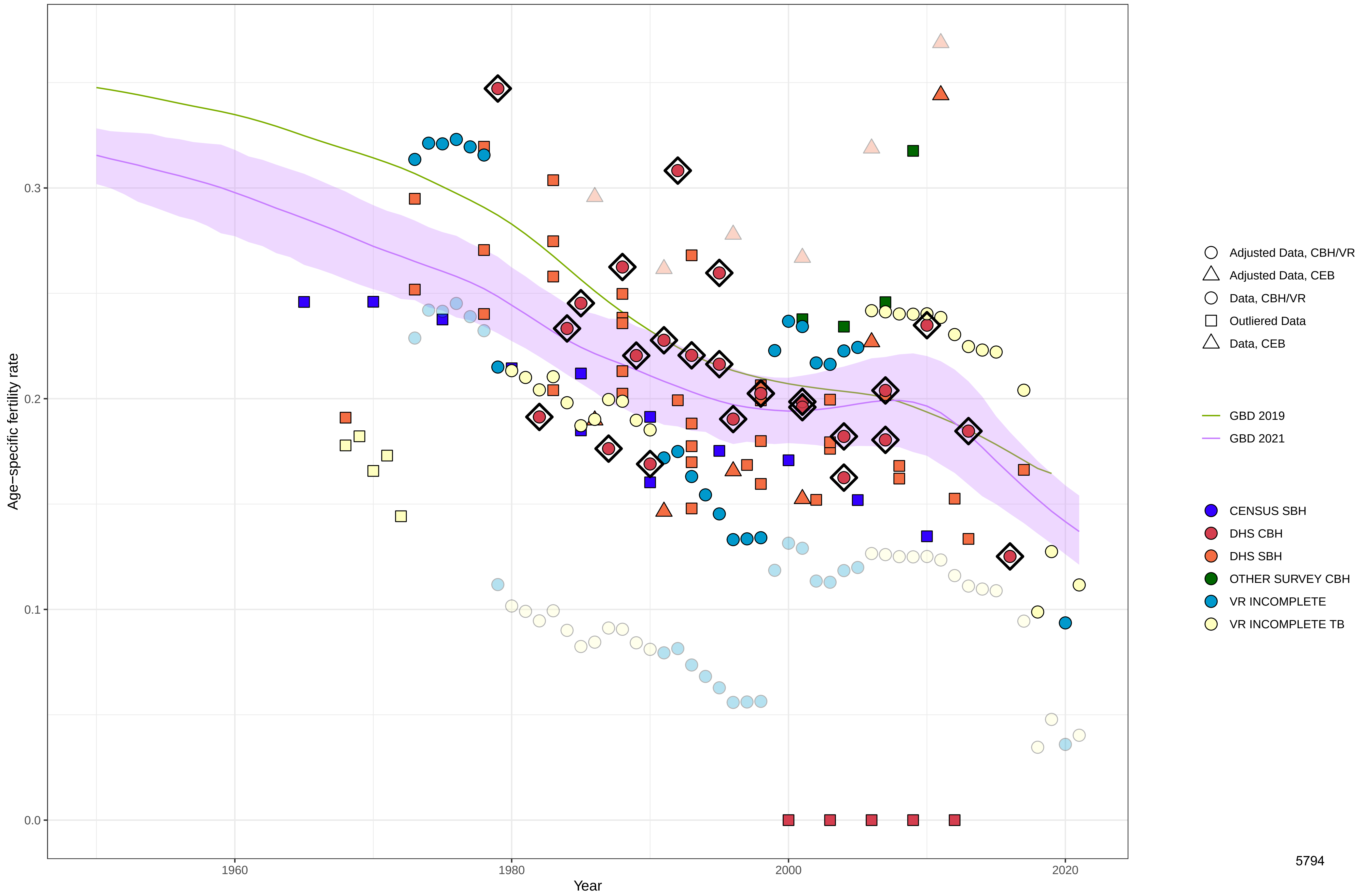

Age−specific fertility fitted trend lines, 1950−2021: Agusan Del Norte, Ages: 25−29
Data points enclosed with a diamond are reference sources

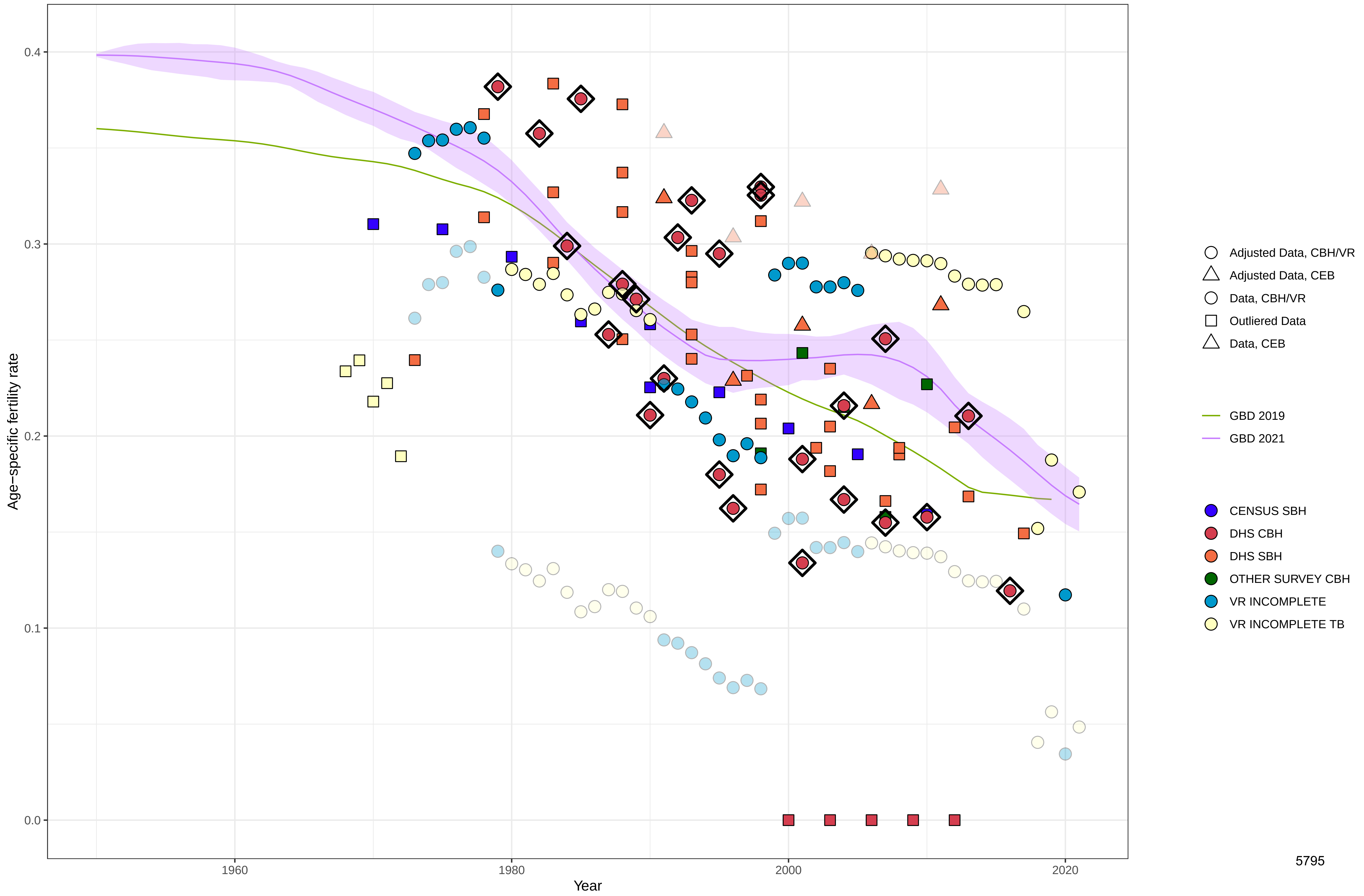

Age−specific fertility fitted trend lines, 1950−2021: Agusan Del Norte, Ages: 30−34
Data points enclosed with a diamond are reference sources

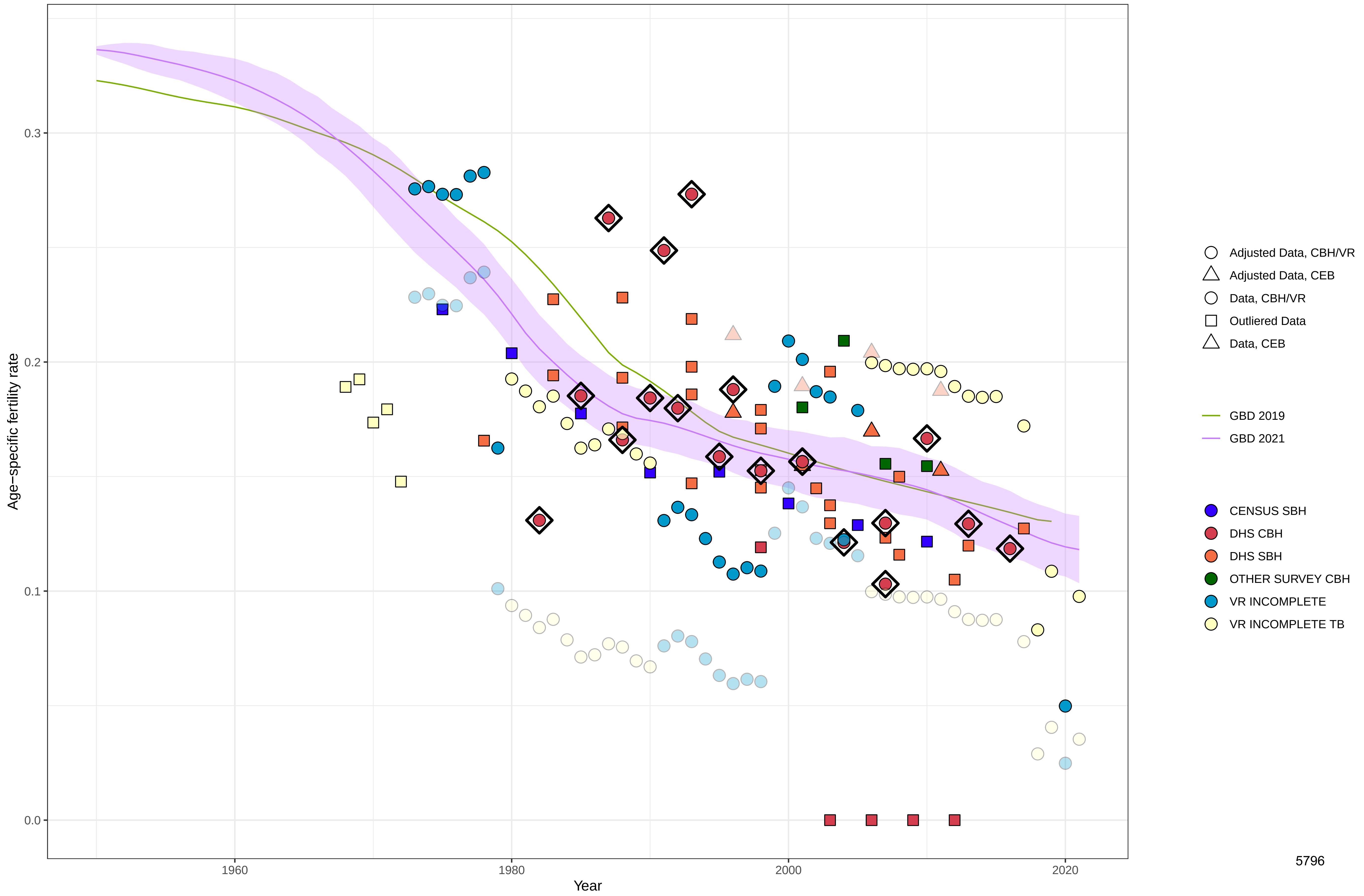

Age−specific fertility fitted trend lines, 1950−2021: Agusan Del Norte, Ages: 35−39
Data points enclosed with a diamond are reference sources

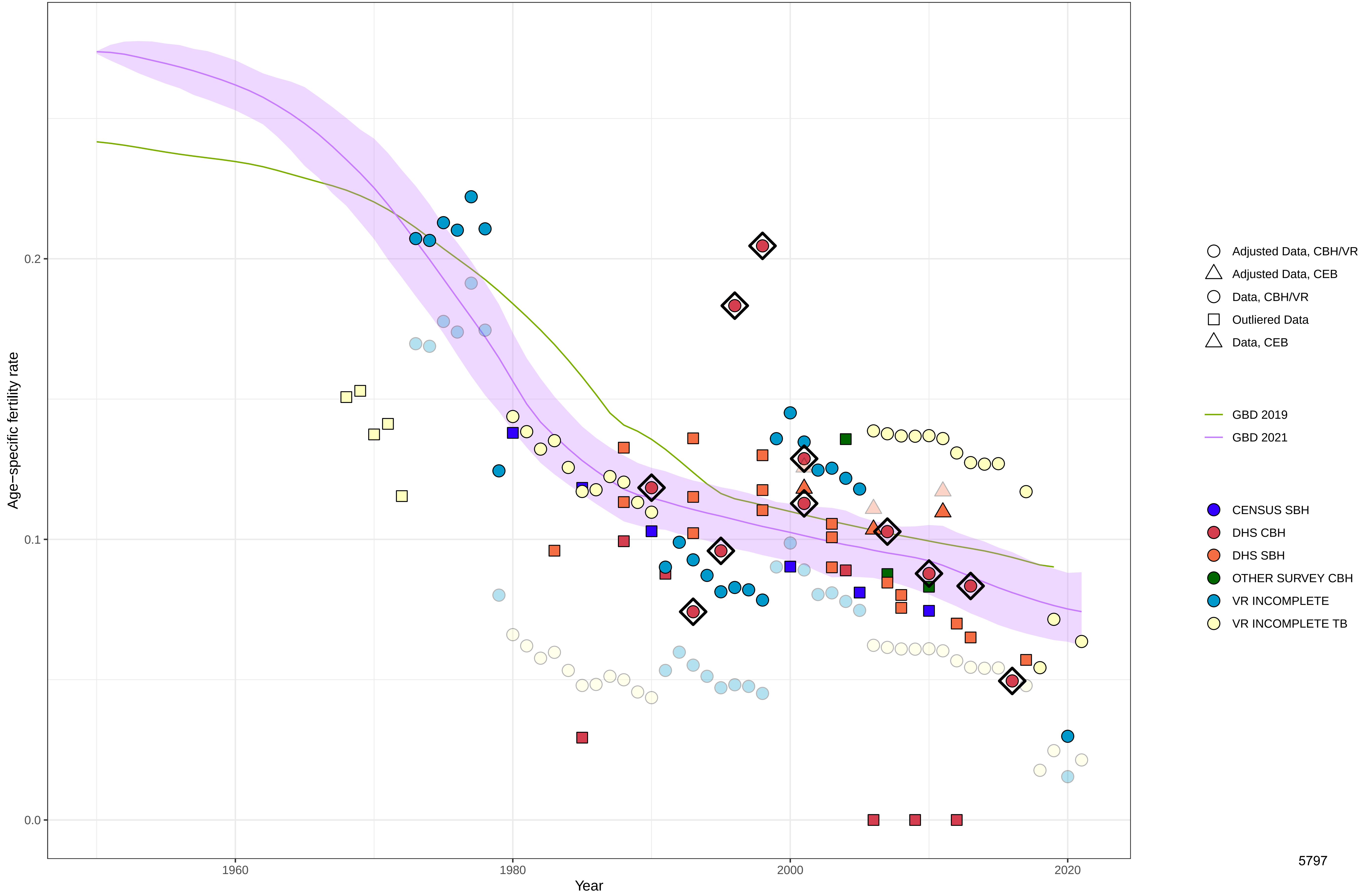

Age−specific fertility fitted trend lines, 1950−2021: Agusan Del Norte, Ages: 40−44
Data points enclosed with a diamond are reference sources

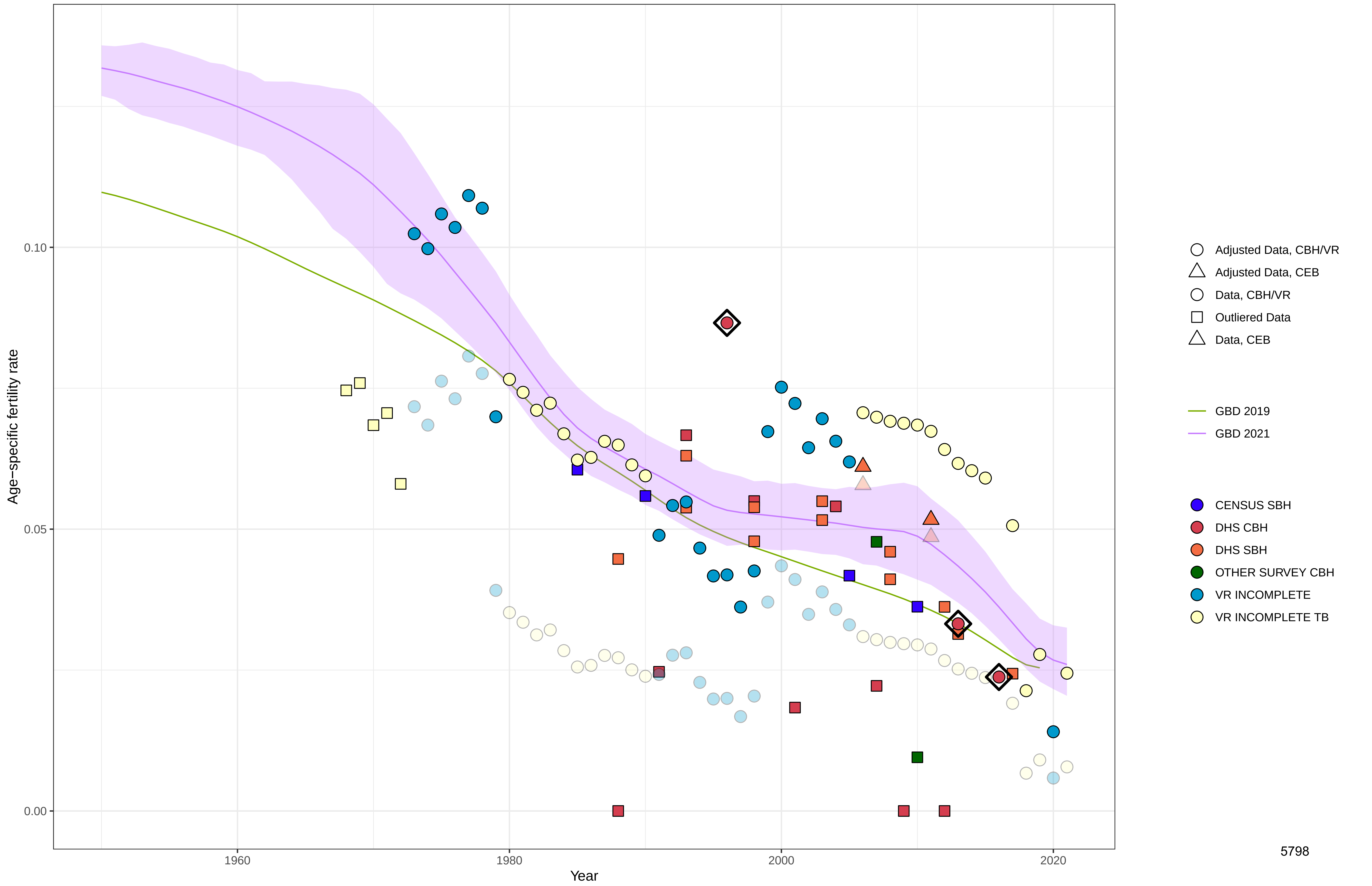

Age−specific fertility fitted trend lines, 1950−2021: Agusan Del Norte, Ages: 45−49
Data points enclosed with a diamond are reference sources

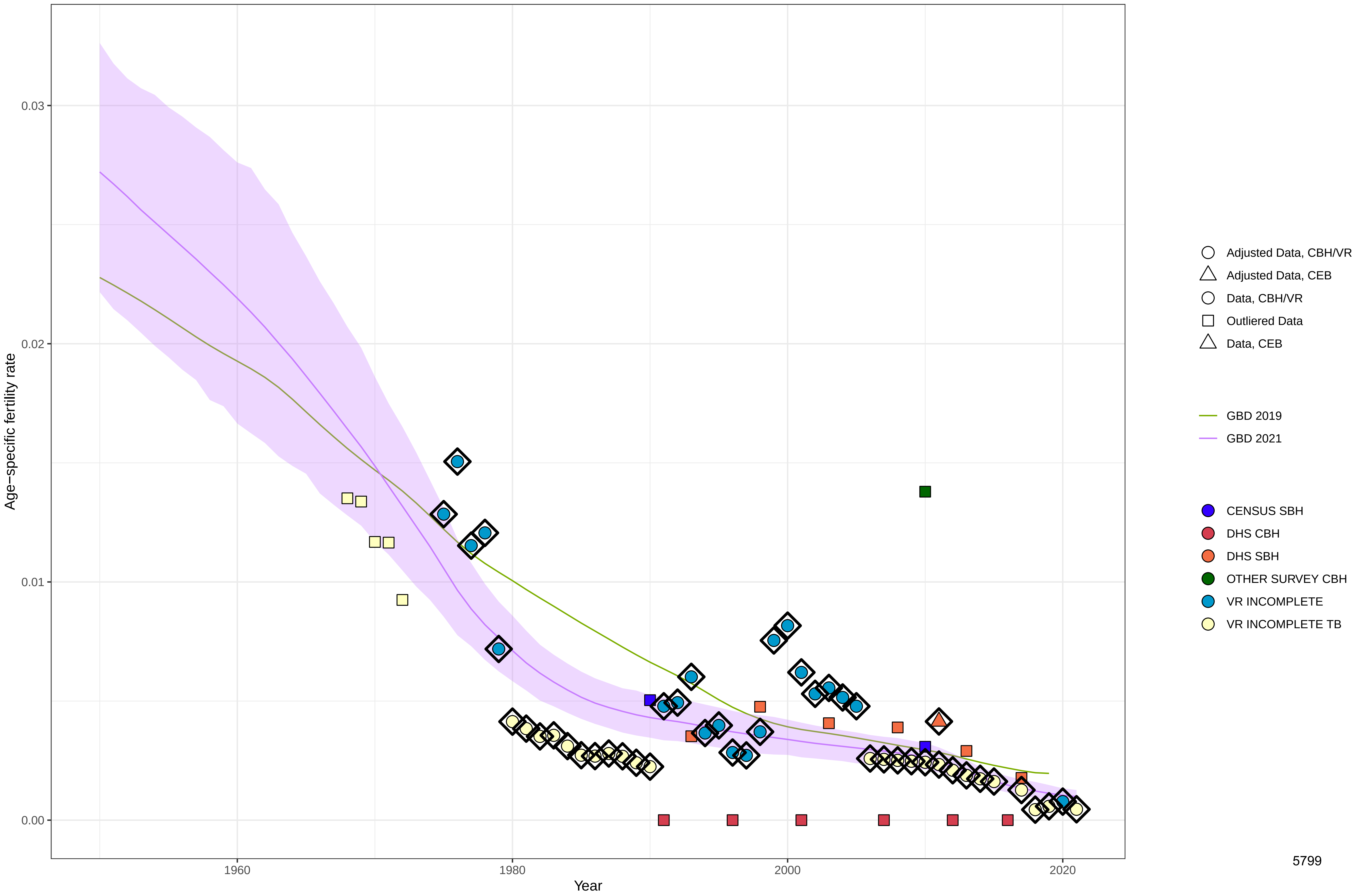

Age−specific fertility fitted trend lines, 1950−2021: Agusan Del Norte, Ages: 50−54
Data points enclosed with a diamond are reference sources

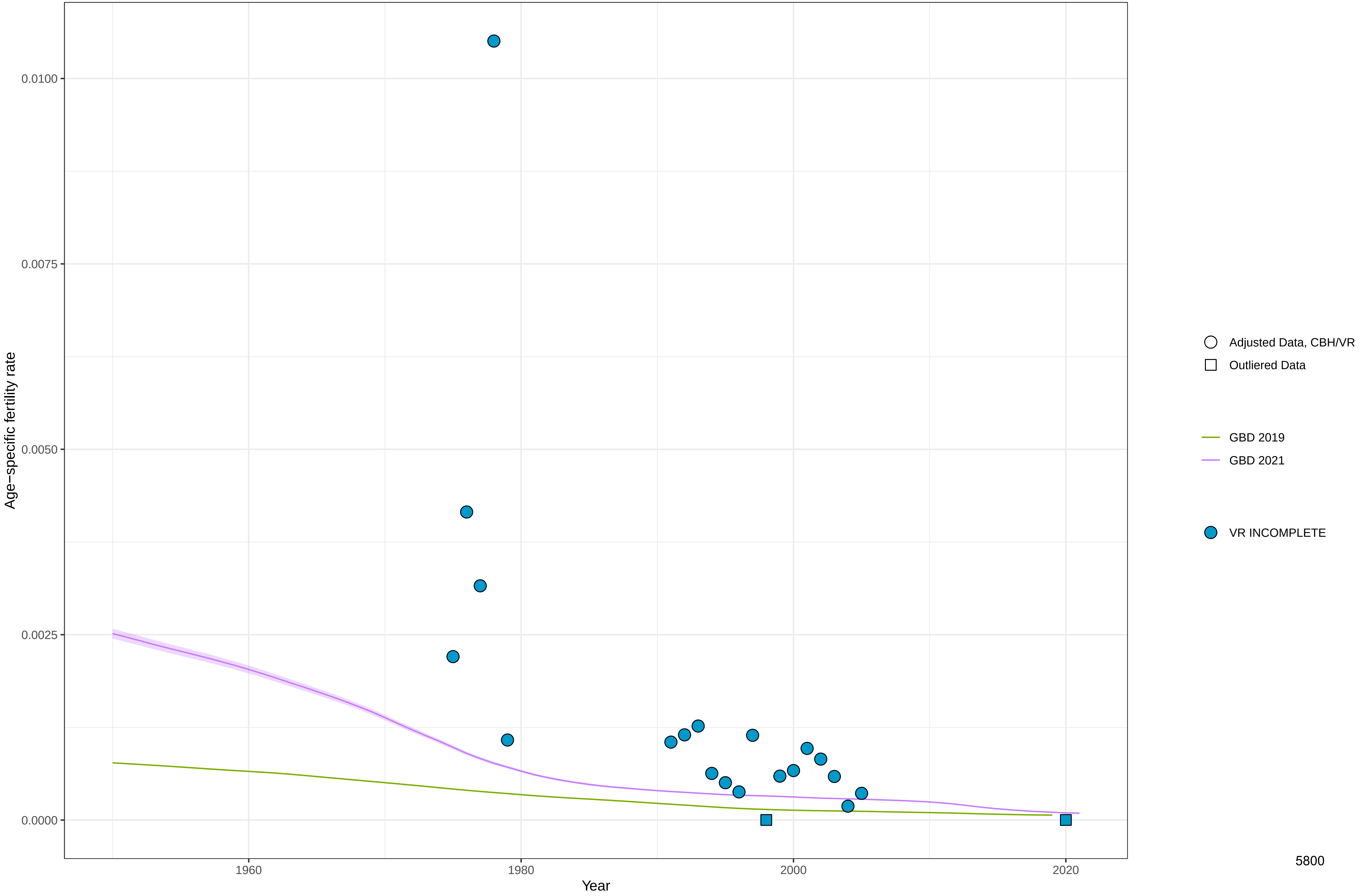

Total fertility rate fitted trend lines, 1950−2021: Agusan Del Norte TFR
Data points enclosed with a diamond are reference sources

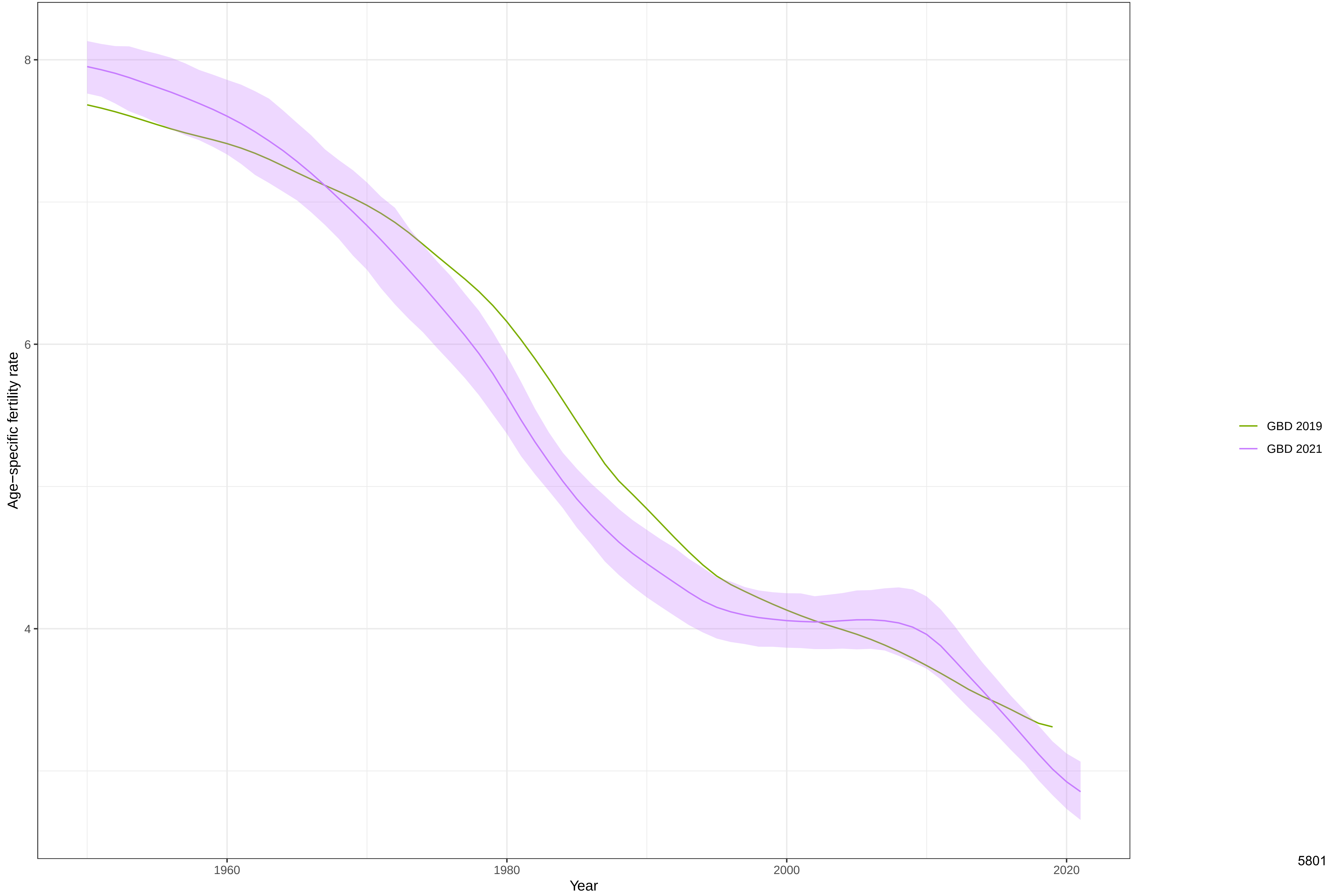

Age−specific fertility fitted trend lines, 1950−2021: Agusan Del Sur, Ages: 10−14
Data points enclosed with a diamond are reference sources

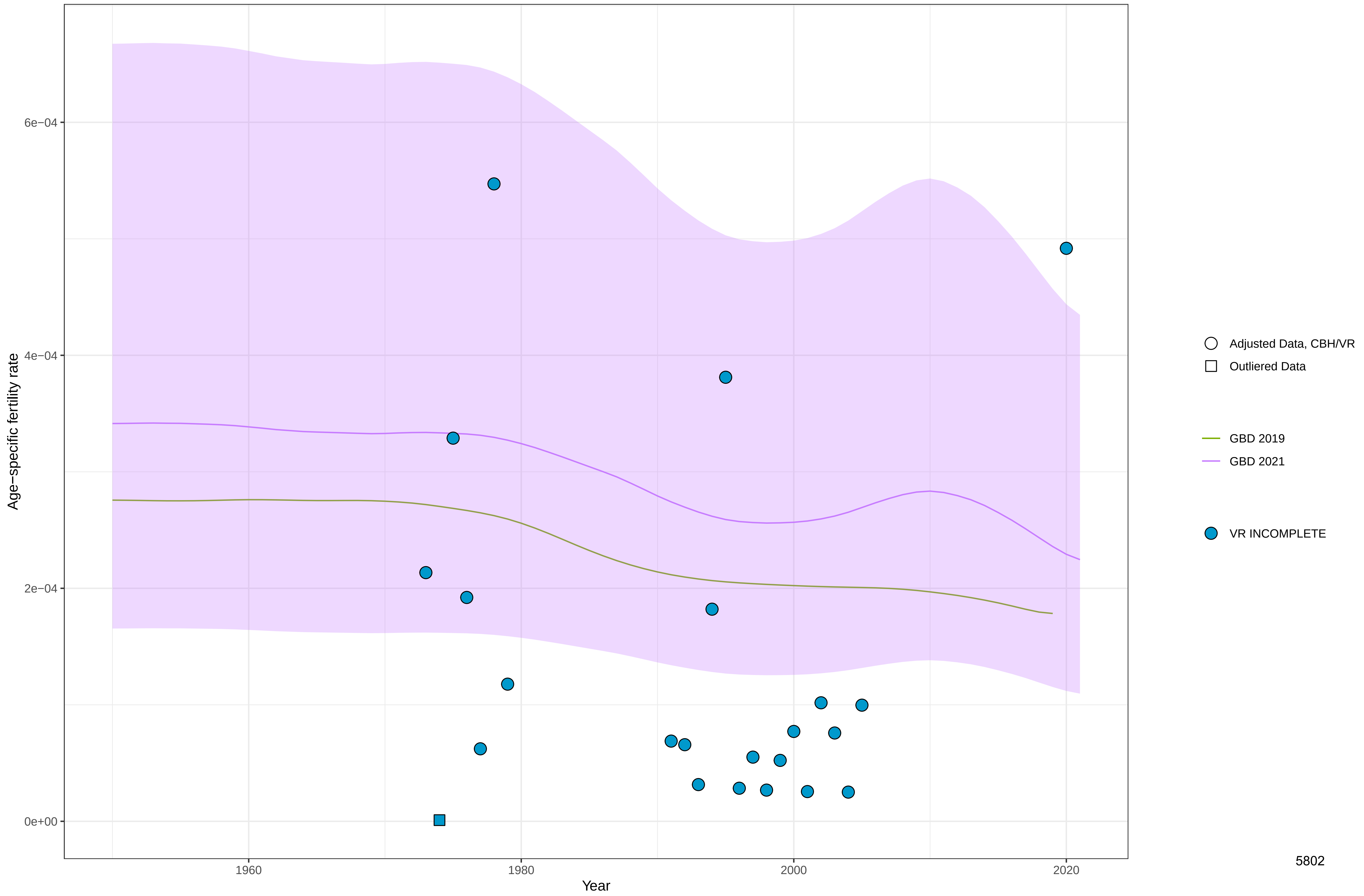

Age−specific fertility fitted trend lines, 1950−2021: Agusan Del Sur, Ages: 15−19
Data points enclosed with a diamond are reference sources

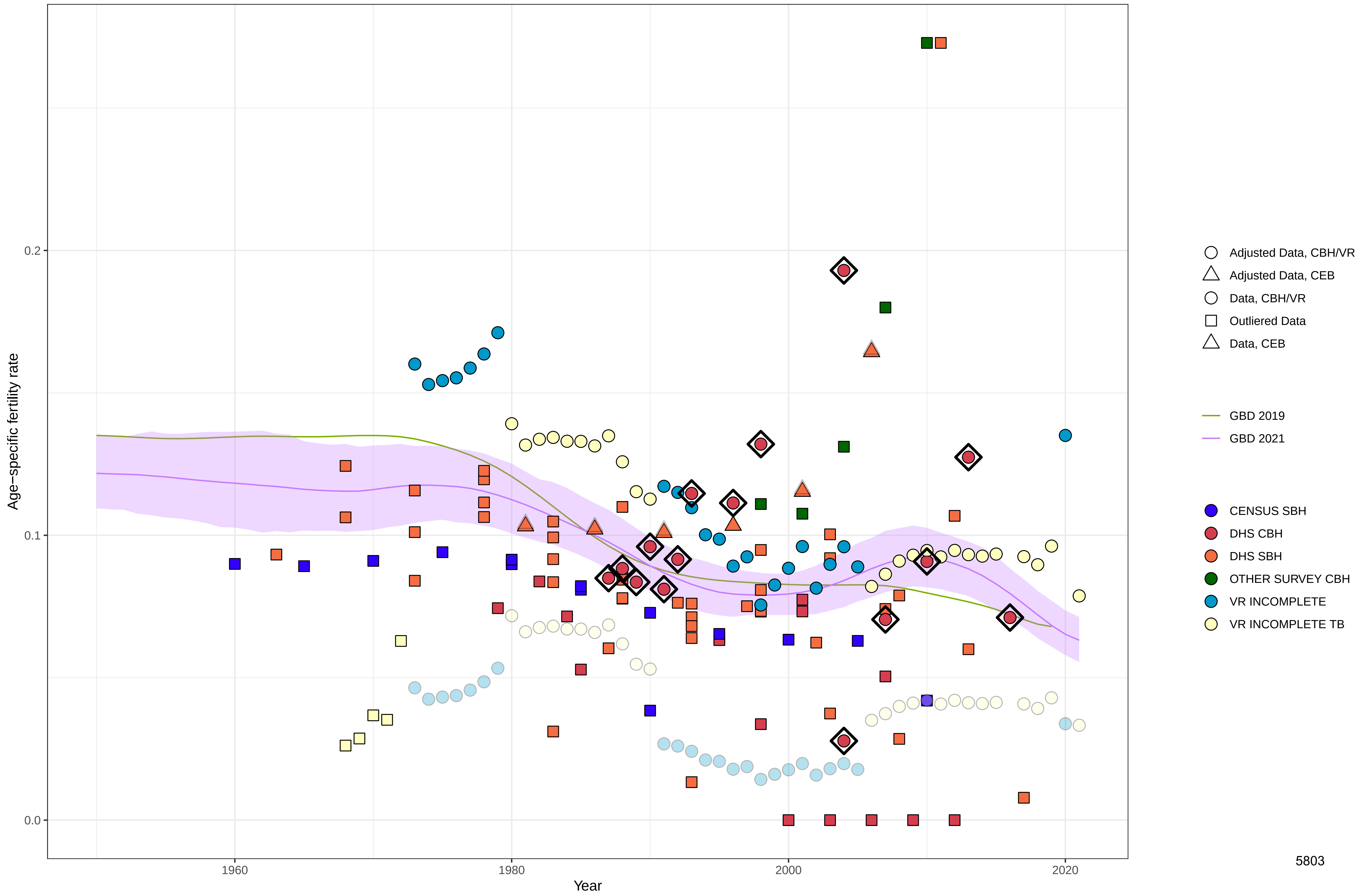

Age−specific fertility fitted trend lines, 1950−2021: Agusan Del Sur, Ages: 20−24
Data points enclosed with a diamond are reference sources

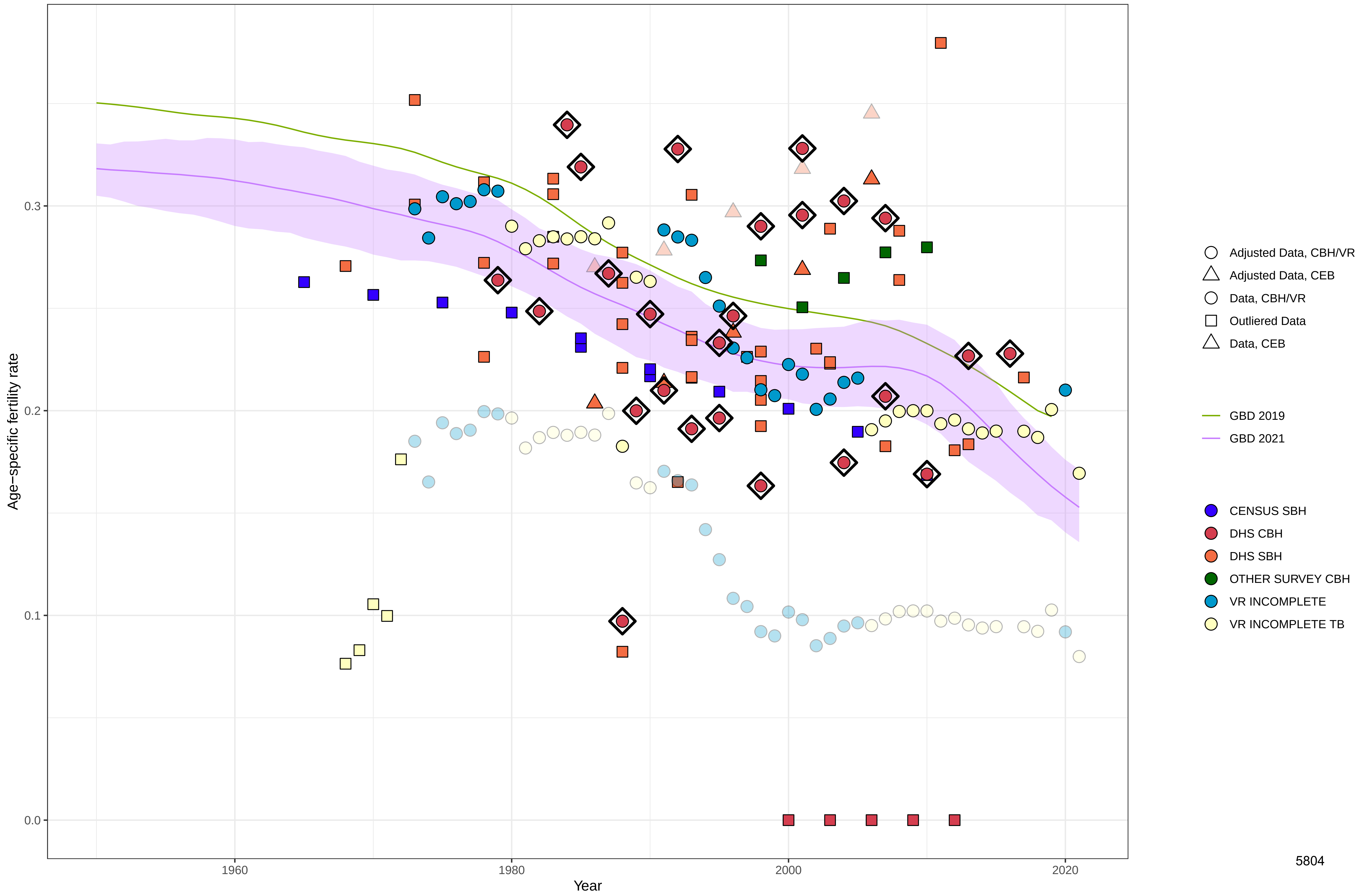

Age−specific fertility fitted trend lines, 1950−2021: Agusan Del Sur, Ages: 25−29
Data points enclosed with a diamond are reference sources

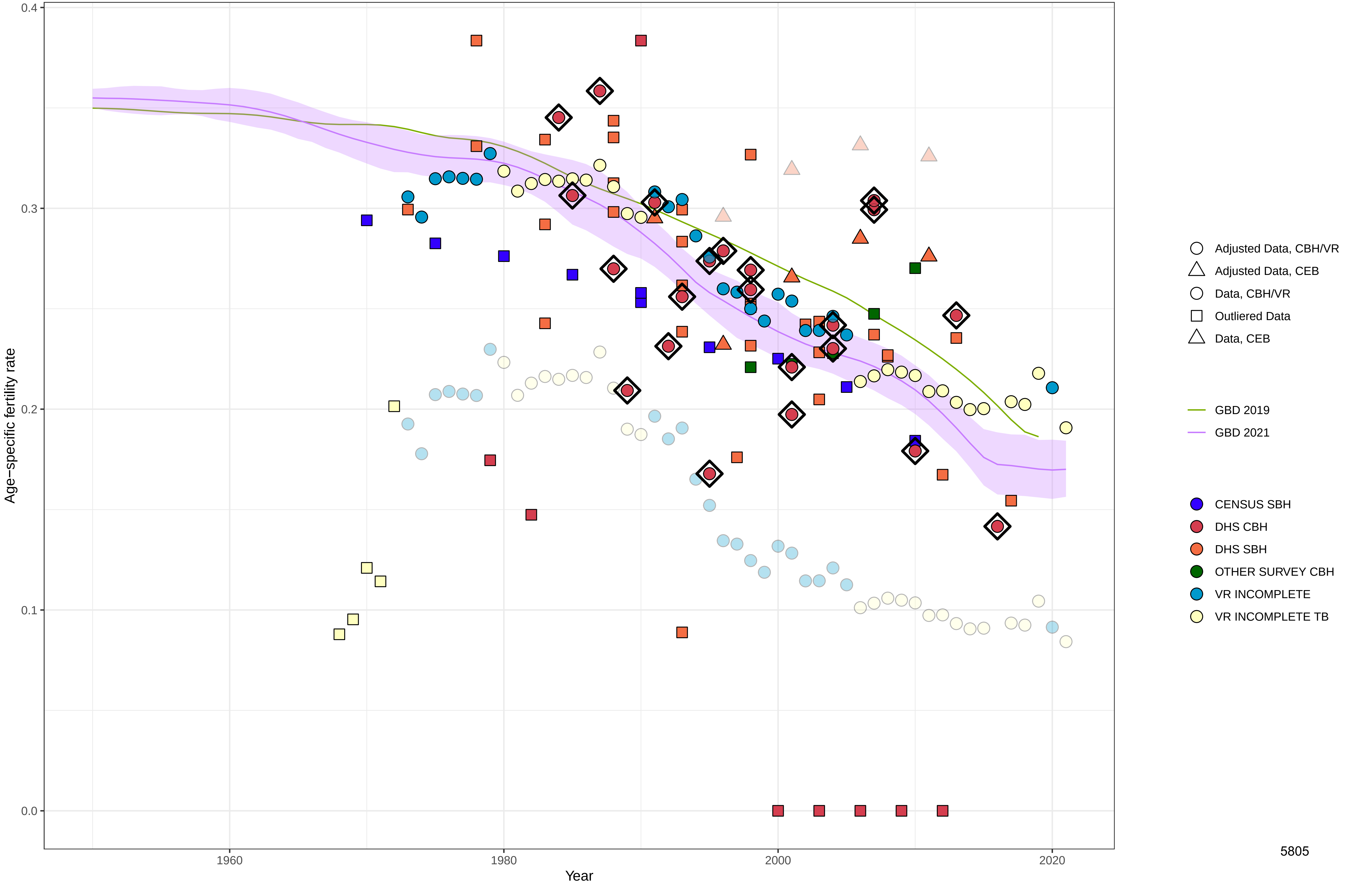

Age−specific fertility fitted trend lines, 1950−2021: Agusan Del Sur, Ages: 30−34
Data points enclosed with a diamond are reference sources

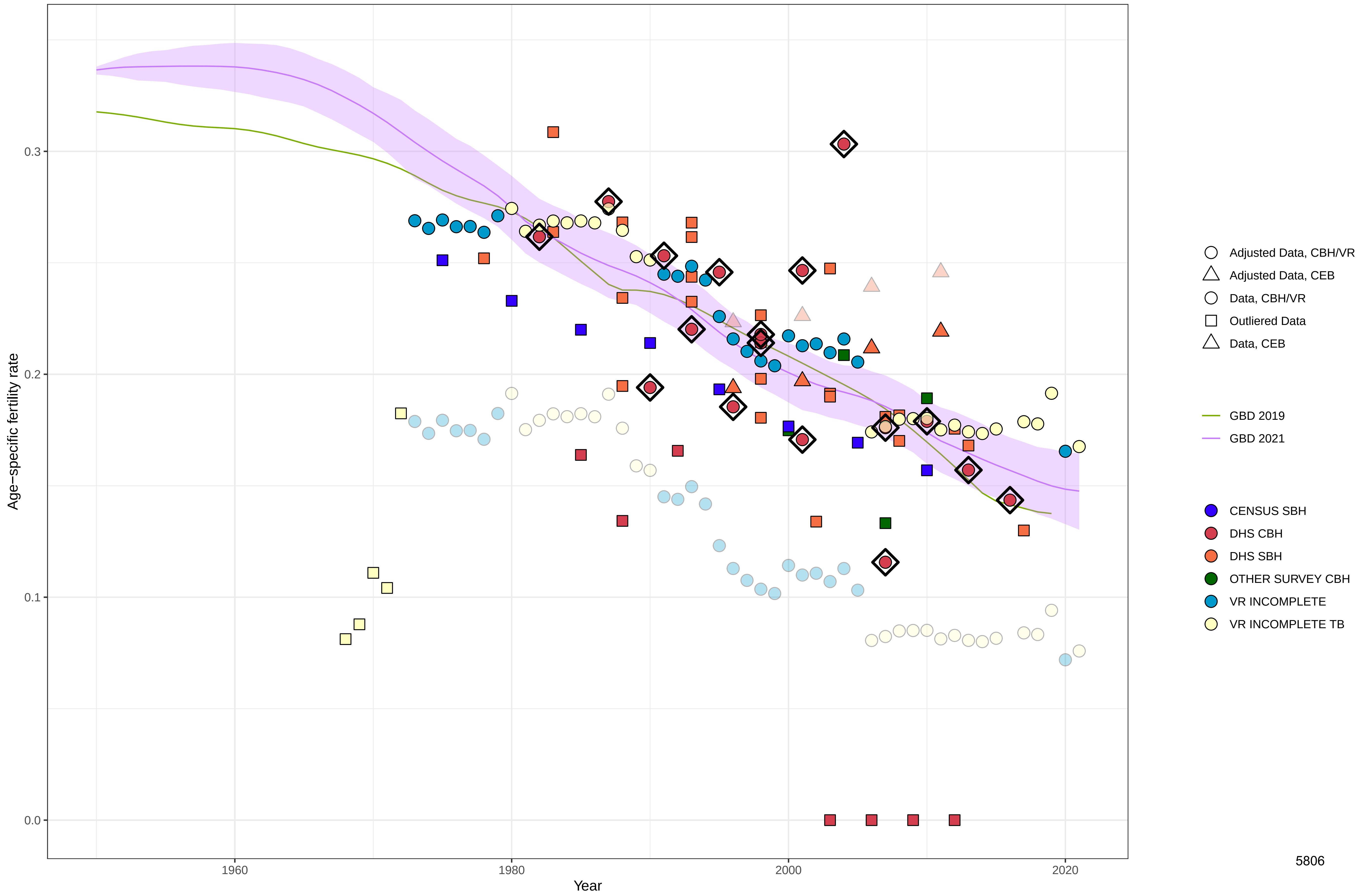

Age−specific fertility fitted trend lines, 1950−2021: Agusan Del Sur, Ages: 35−39
Data points enclosed with a diamond are reference sources

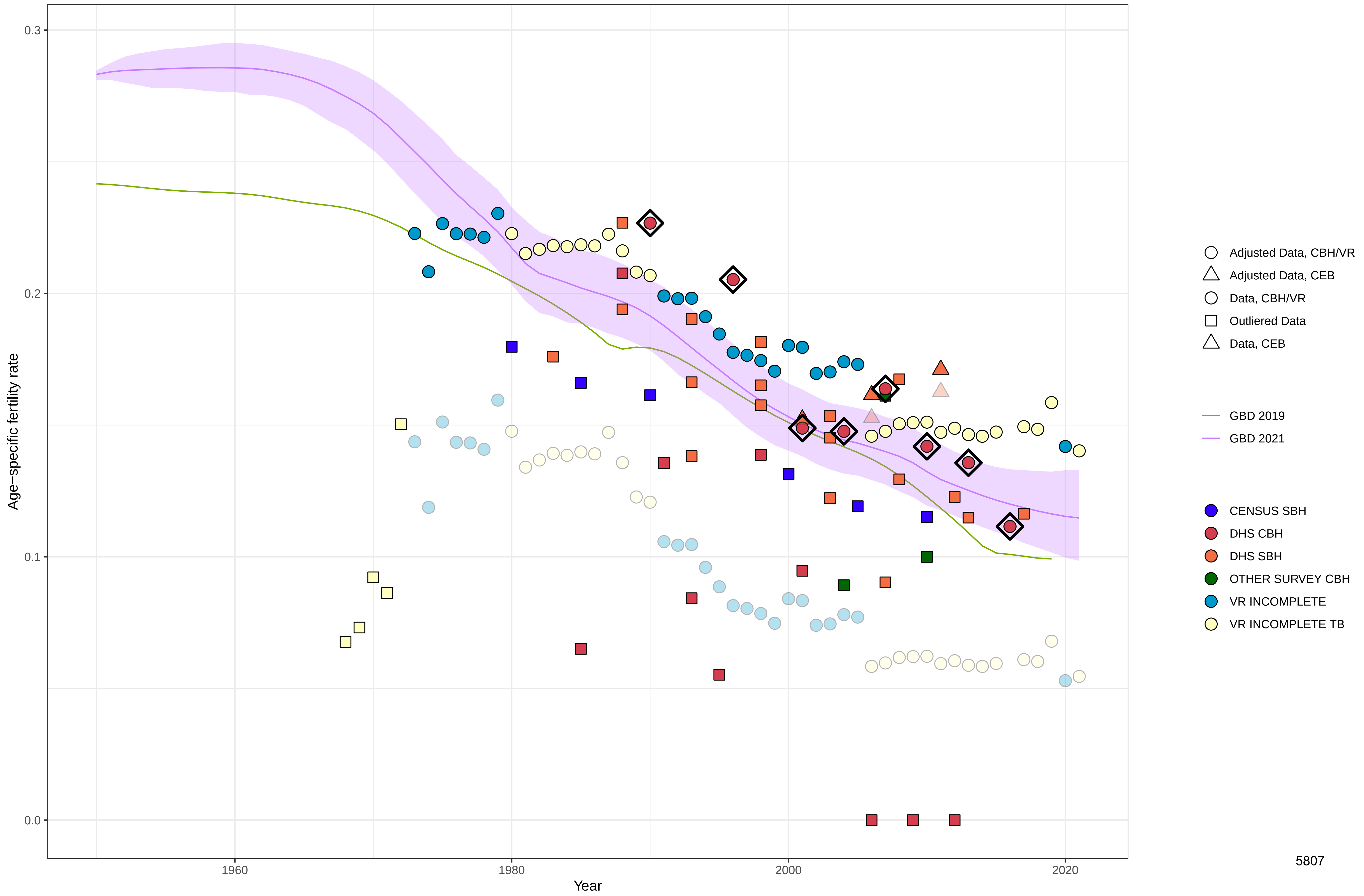

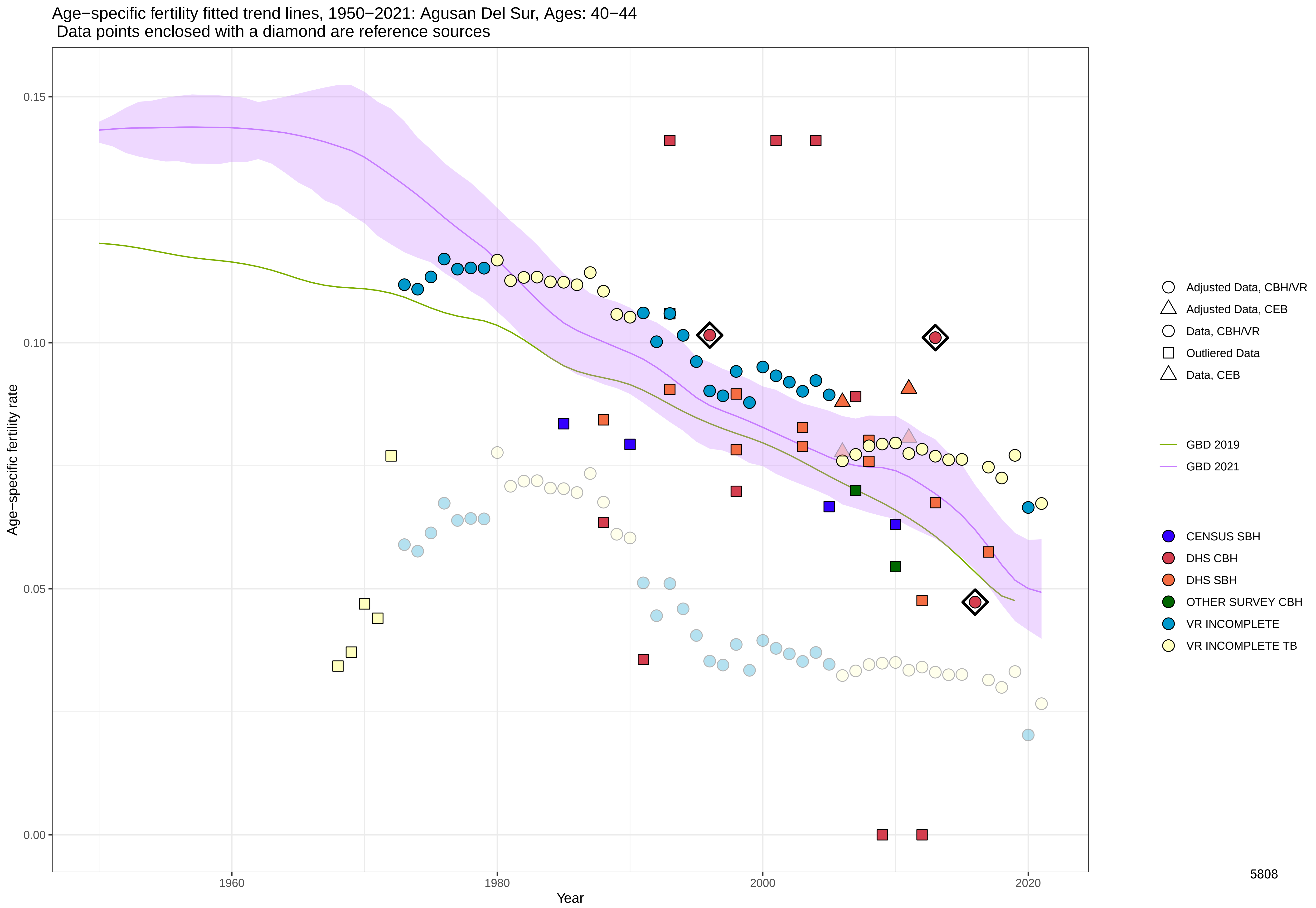

Age−specific fertility fitted trend lines, 1950−2021: Agusan Del Sur, Ages: 40−44
Data points enclosed with a diamond are reference sources
Age−specific fertility rate
Year
0.00
0.05
0.10
0.15
1960
1980
2000
2020
Adjusted Data, CBH/VR
Adjusted Data, CEB
Data, CBH/VR
Outliered Data
Data, CEB
GBD 2019
GBD 2021
CENSUS SBH
DHS CBH
DHS SBH
OTHER SURVEY CBH
VR INCOMPLETE
VR INCOMPLETE TB

Age−specific fertility fitted trend lines, 1950−2021: Agusan Del Sur, Ages: 45−49
Data points enclosed with a diamond are reference sources

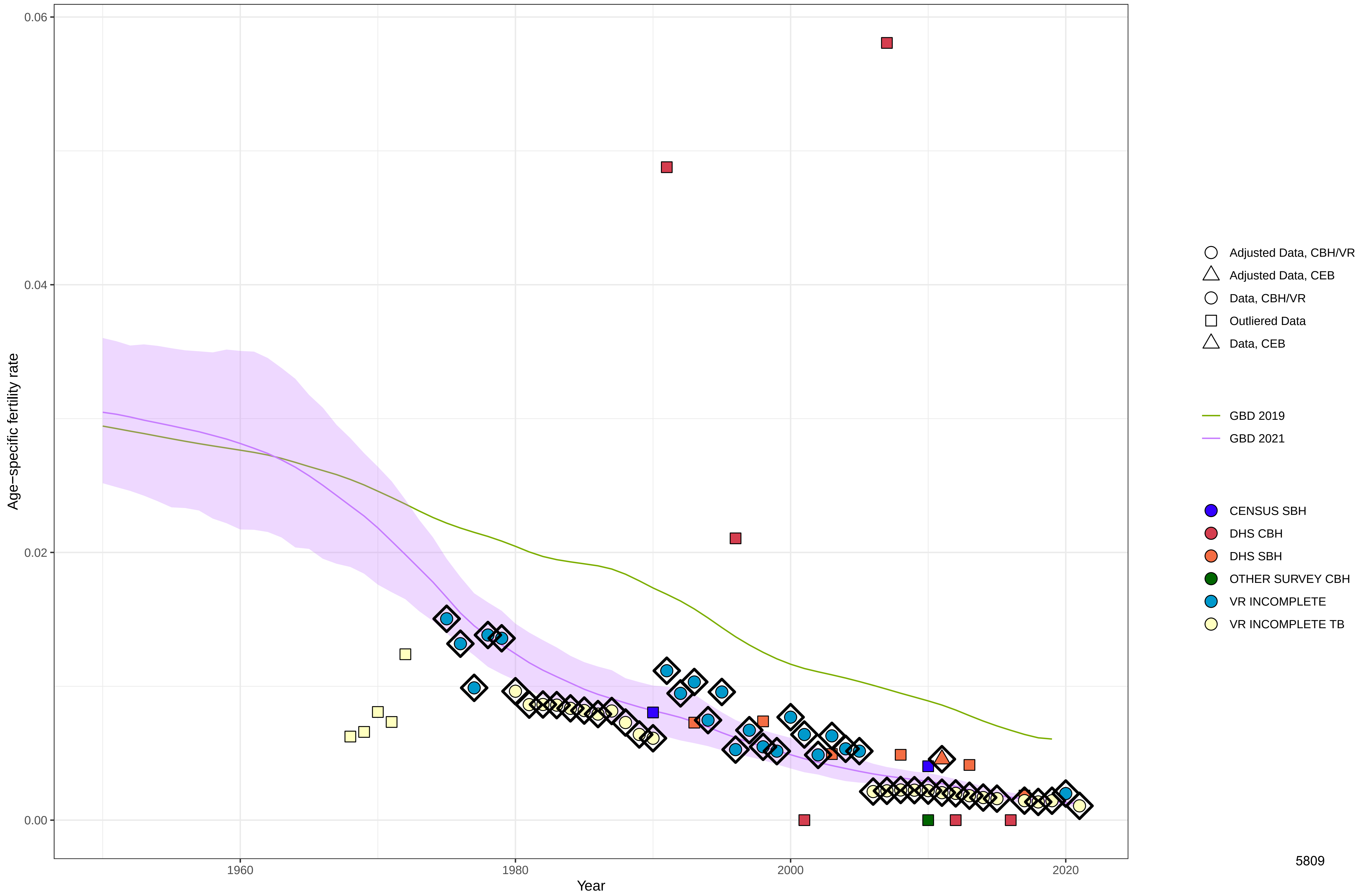

Age−specific fertility fitted trend lines, 1950−2021: Agusan Del Sur, Ages: 50−54
Data points enclosed with a diamond are reference sources

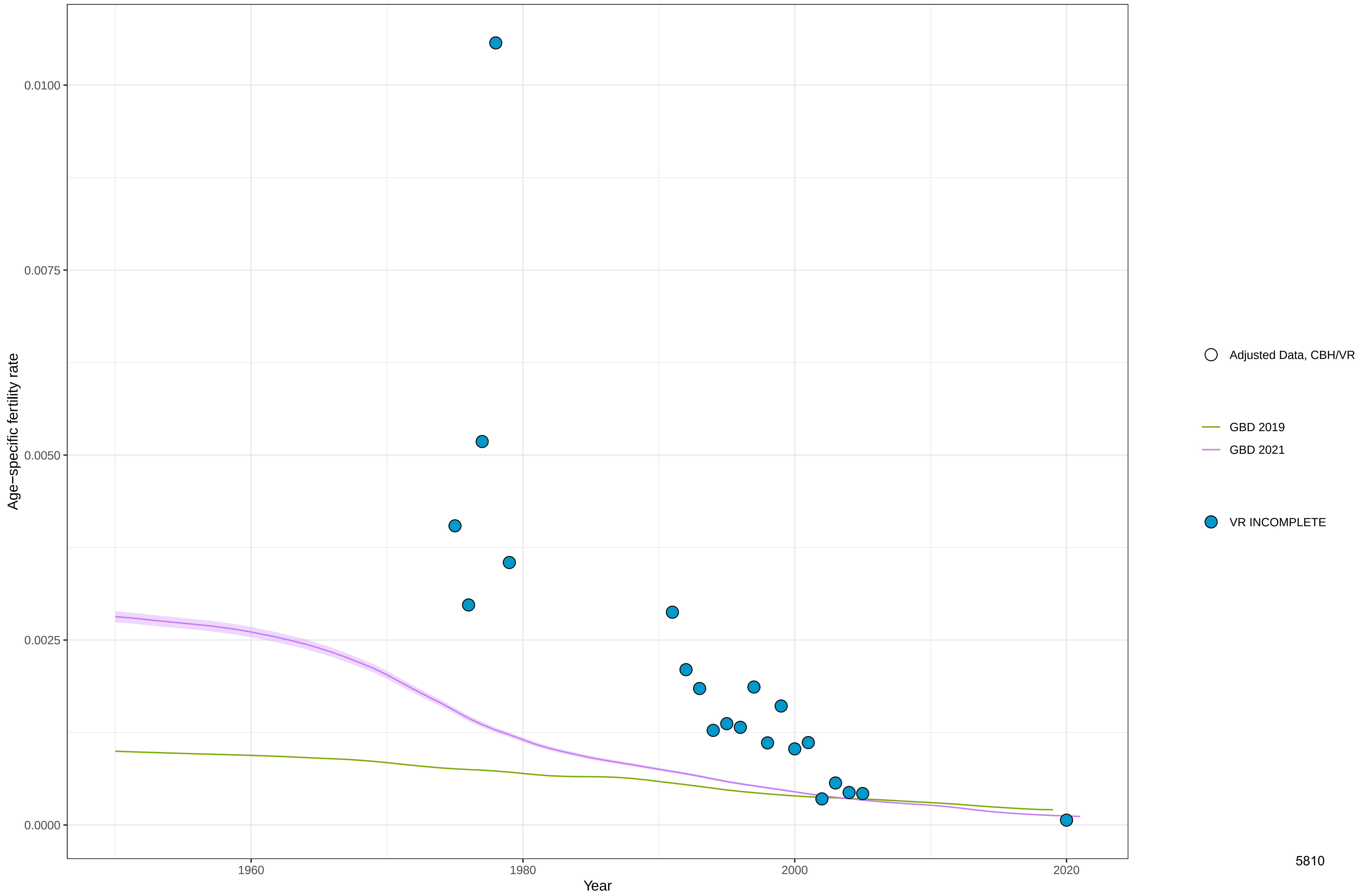

Total fertility rate fitted trend lines, 1950−2021: Agusan Del Sur TFR
Data points enclosed with a diamond are reference sources

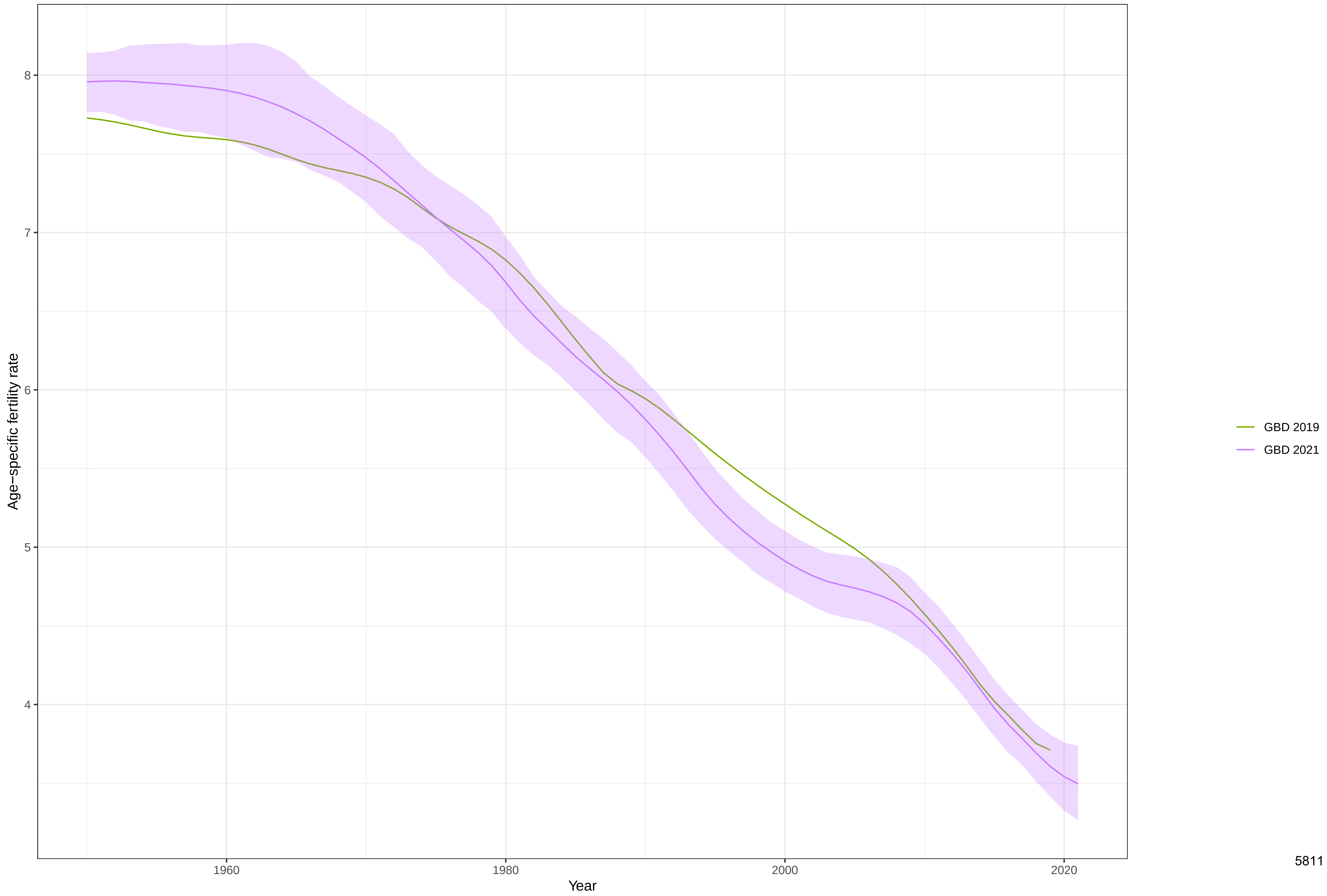

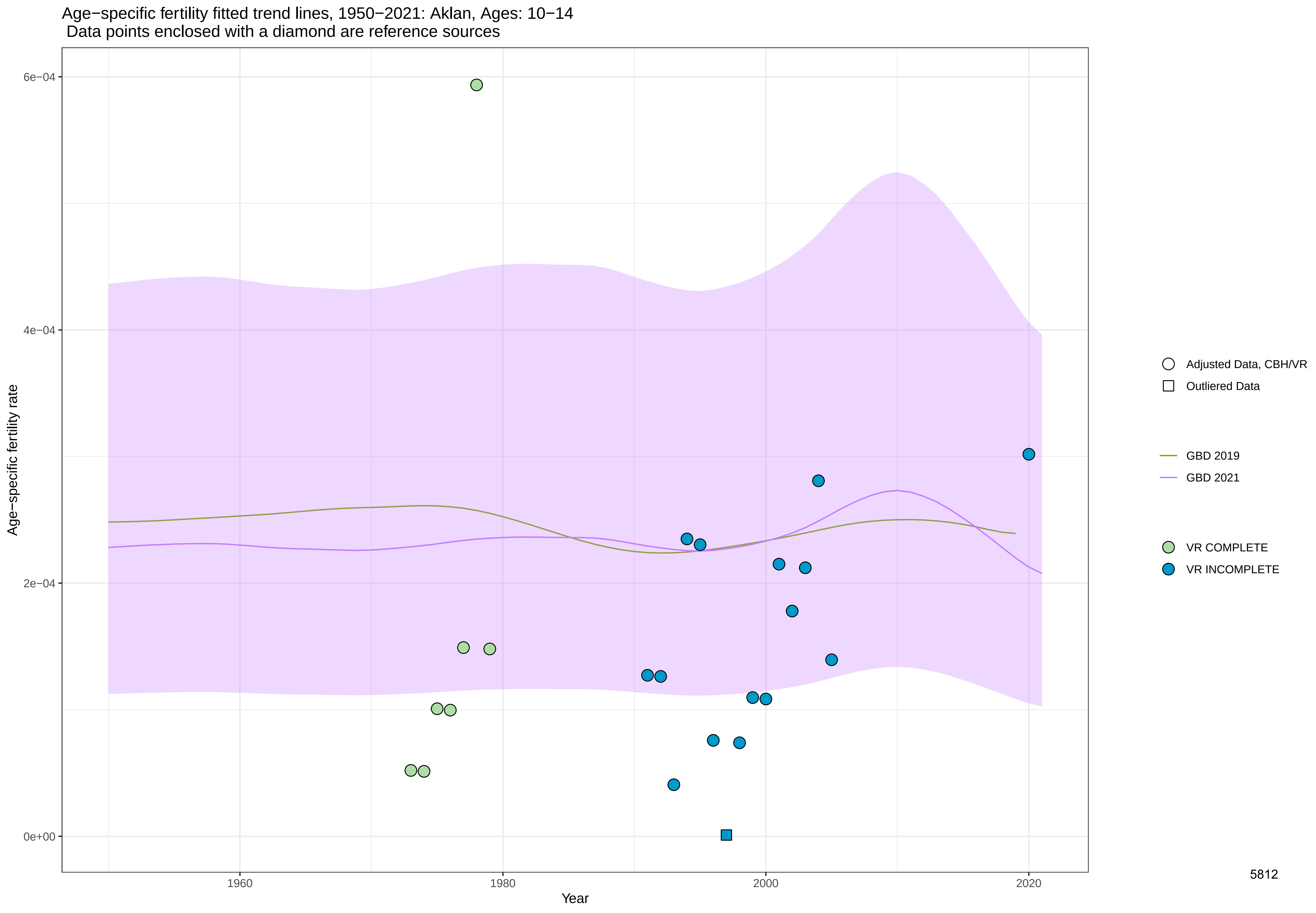

Age−specific fertility fitted trend lines, 1950−2021: Aklan, Ages: 10−14
Data points enclosed with a diamond are reference sources
Age−specific fertility rate
6e−04
4e−04
2e−04
0e+00
1960
1980
2000
2020
Year
Adjusted Data, CBH/VR
Outliered Data
GBD 2019
GBD 2021
VR COMPLETE
VR INCOMPLETE

Age−specific fertility fitted trend lines, 1950−2021: Aklan, Ages: 15−19
Data points enclosed with a diamond are reference sources

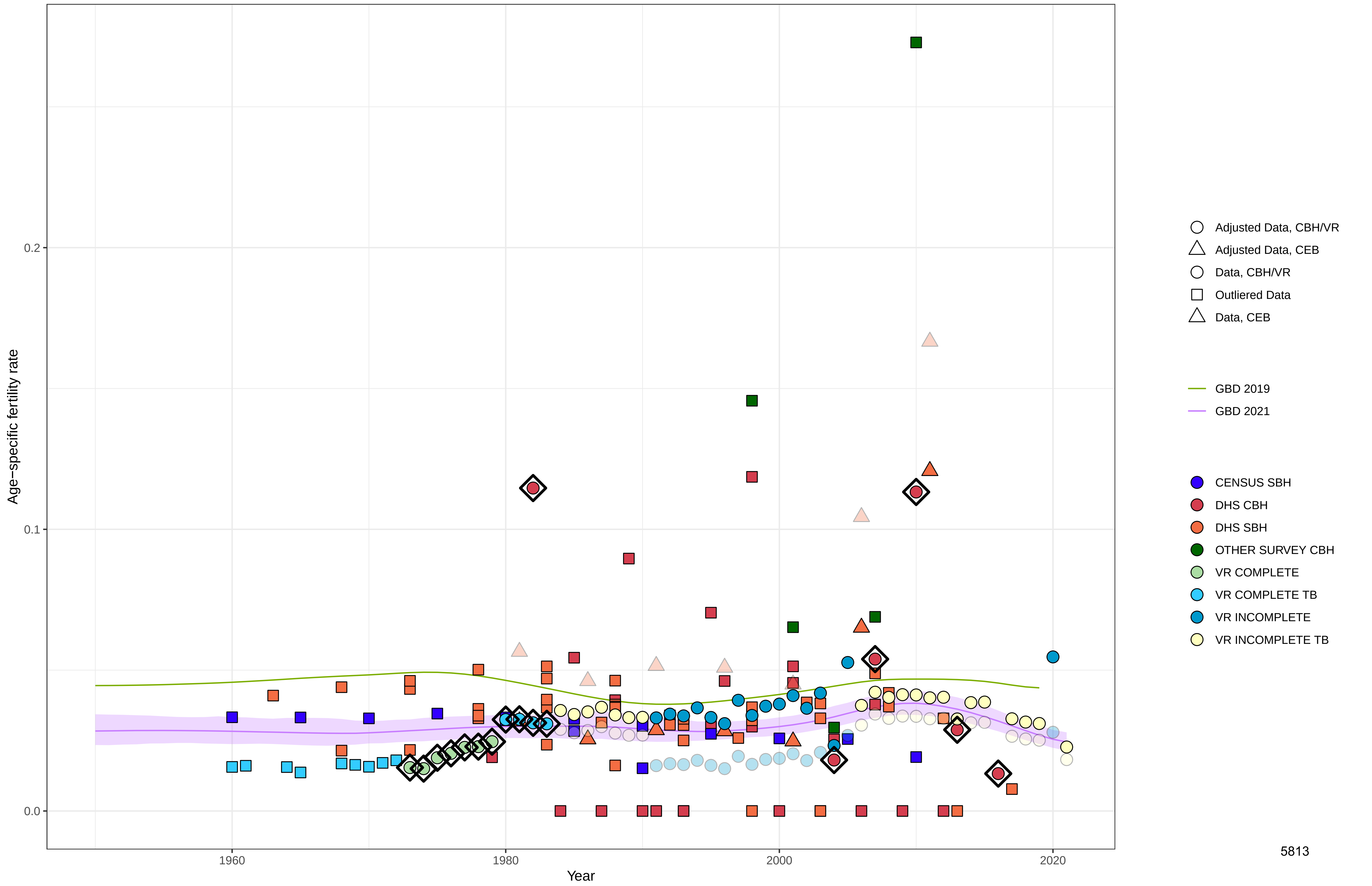

Age−specific fertility fitted trend lines, 1950−2021: Aklan, Ages: 20−24
Data points enclosed with a diamond are reference sources

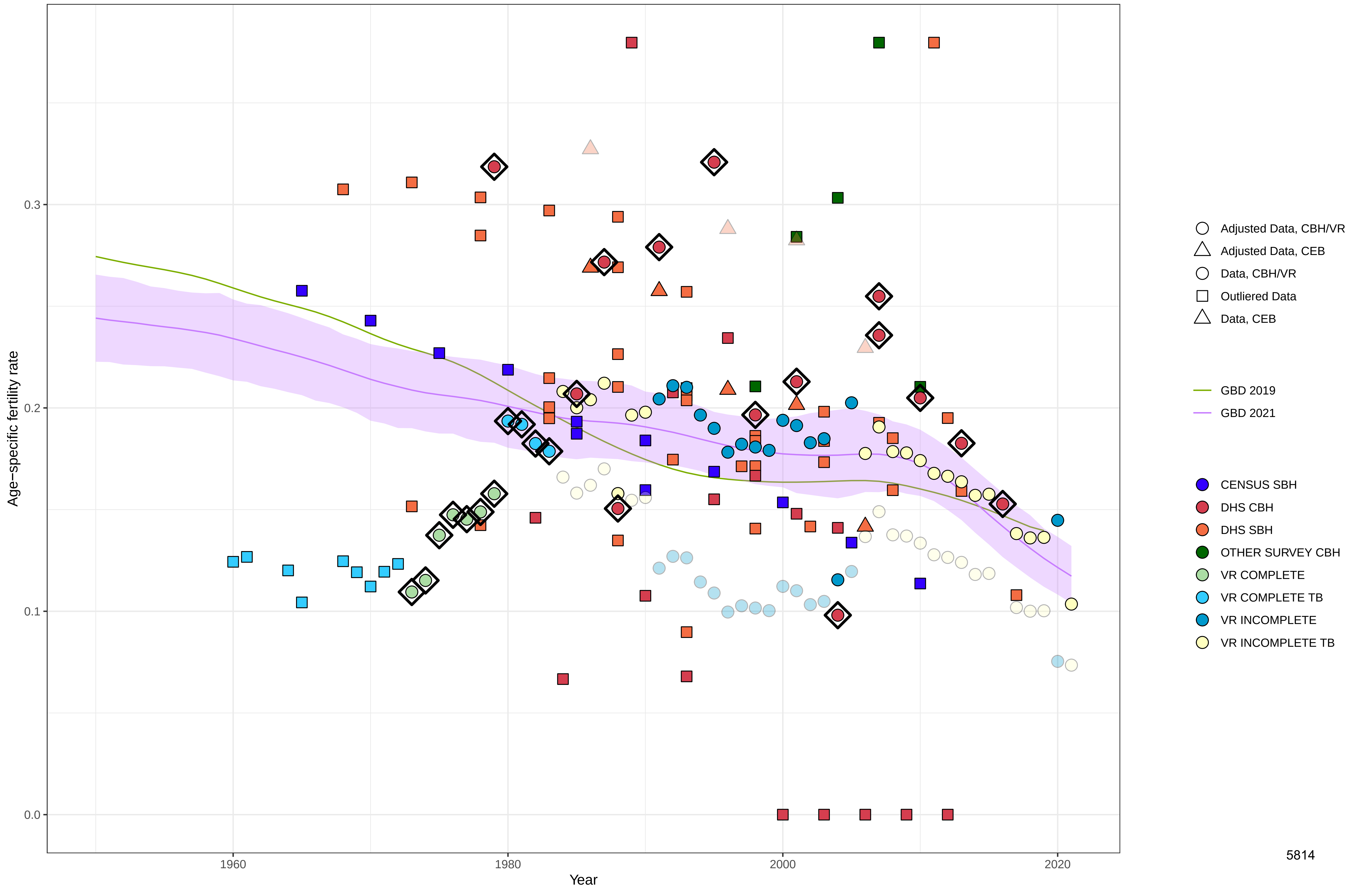

Age−specific fertility fitted trend lines, 1950−2021: Aklan, Ages: 25−29
Data points enclosed with a diamond are reference sources

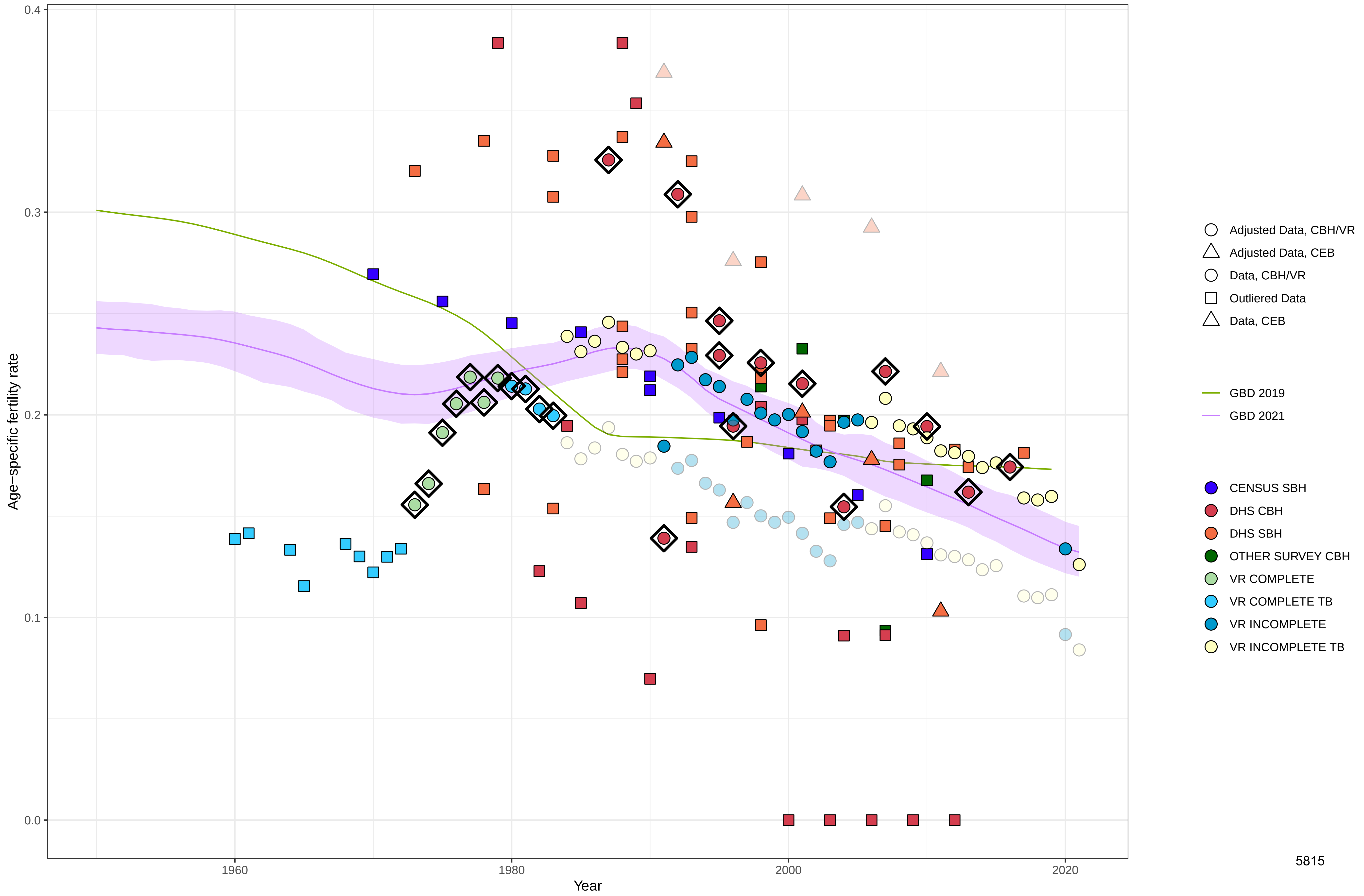

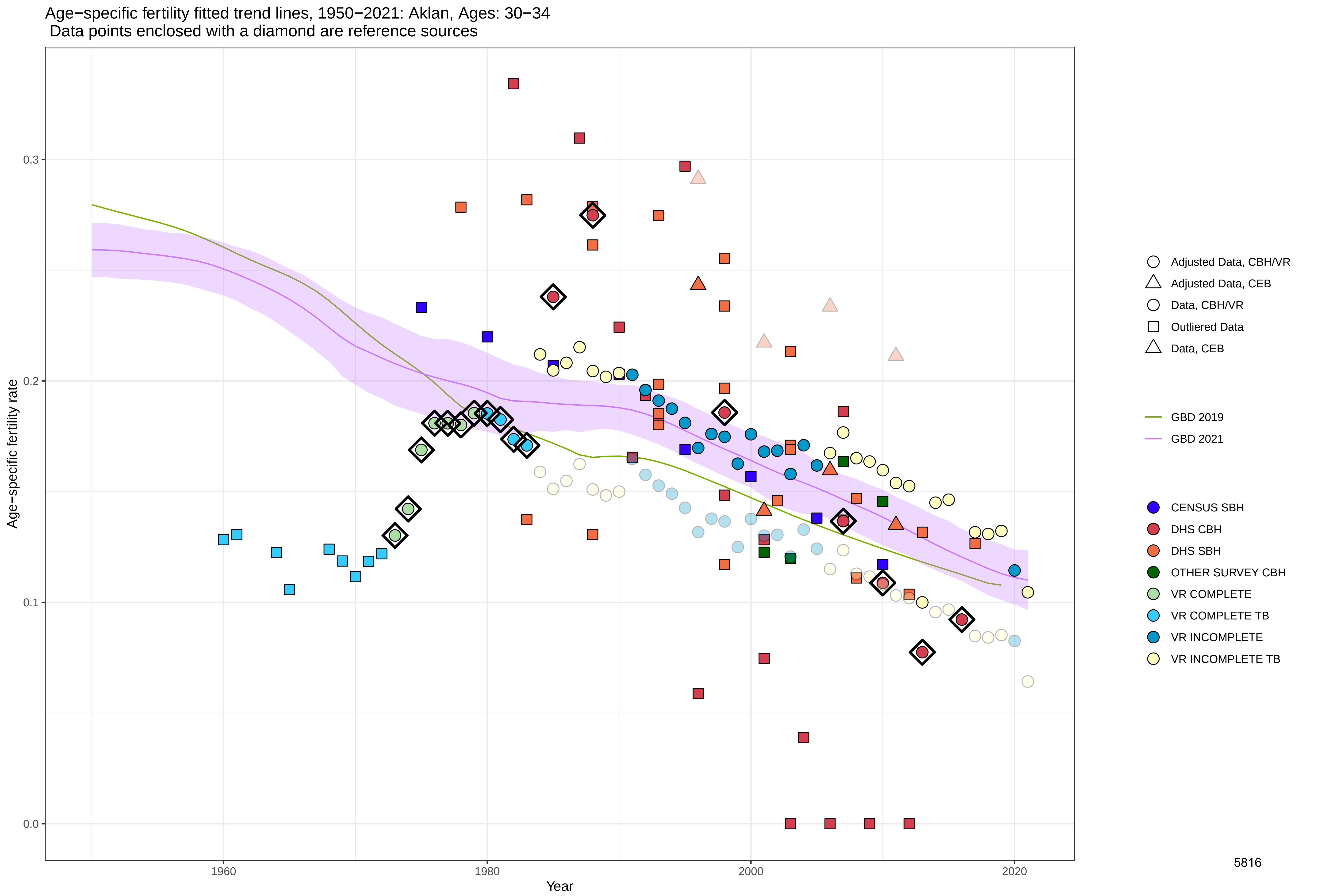

Age−specific fertility fitted trend lines, 1950−2021: Aklan, Ages: 30−34
Data points enclosed with a diamond are reference sources
Age−specific fertility rate
Year
0.0
0.1
0.2
0.3
1960
1980
2000
2020
Adjusted Data, CBH/VR
Adjusted Data, CEB
Data, CBH/VR
Outliered Data
Data, CEB
GBD 2019
GBD 2021
CENSUS SBH
DHS CBH
DHS SBH
OTHER SURVEY CBH
VR COMPLETE
VR COMPLETE TB
VR INCOMPLETE
VR INCOMPLETE TB

Age−specific fertility fitted trend lines, 1950−2021: Aklan, Ages: 35−39
Data points enclosed with a diamond are reference sources

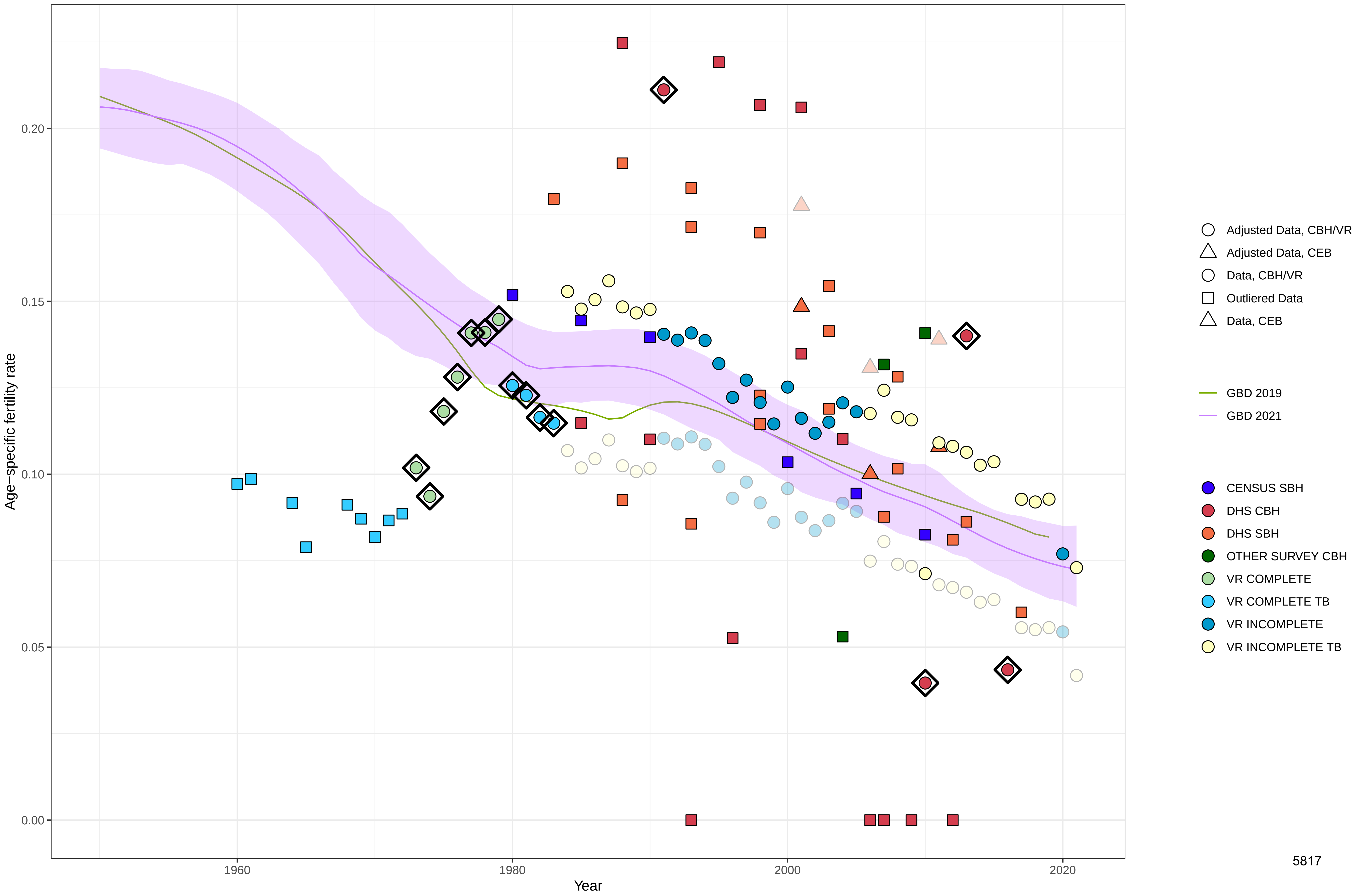

Age−specific fertility fitted trend lines, 1950−2021: Aklan, Ages: 40−44
Data points enclosed with a diamond are reference sources

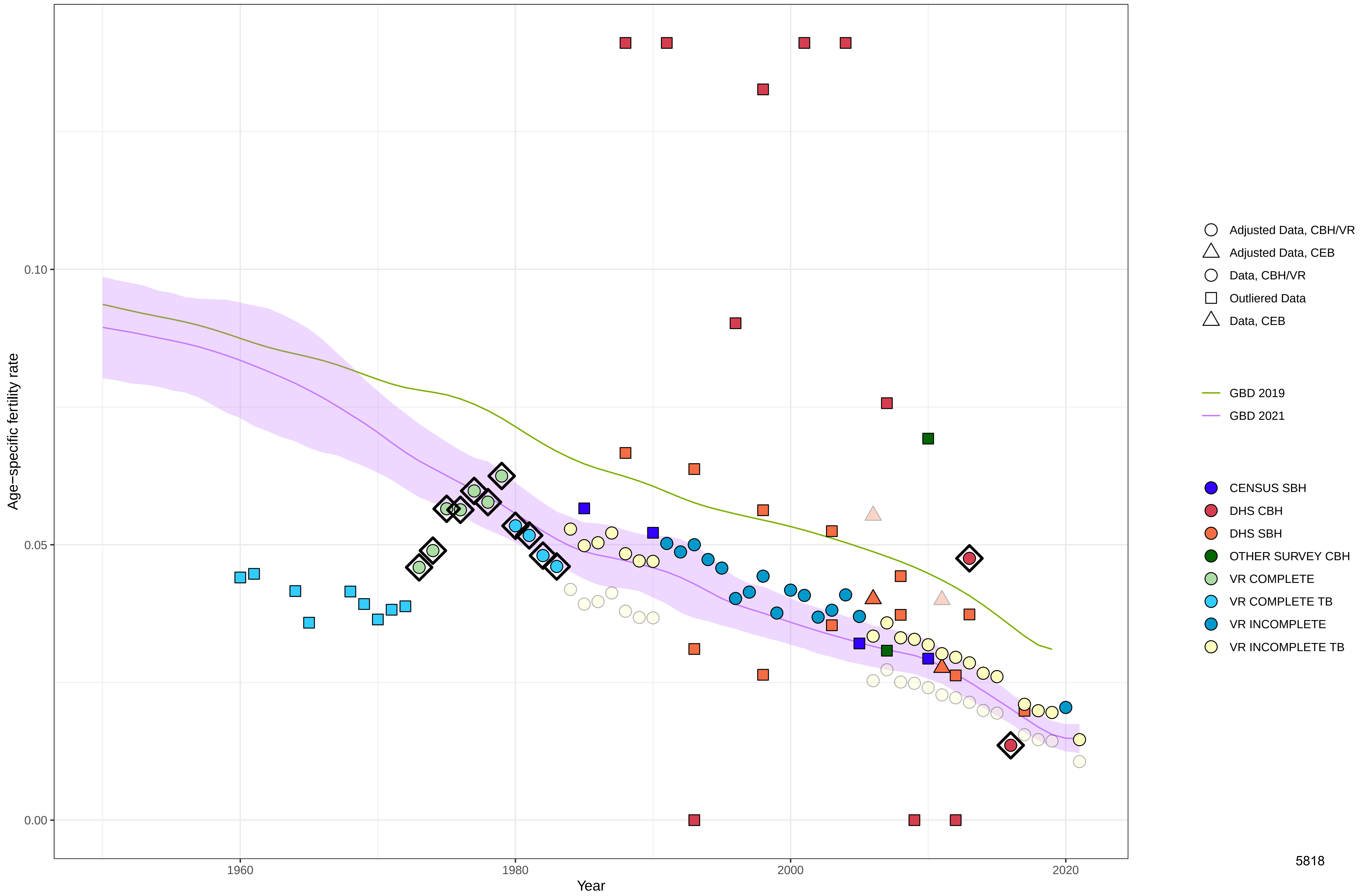

Age−specific fertility fitted trend lines, 1950−2021: Aklan, Ages: 45−49
Data points enclosed with a diamond are reference sources

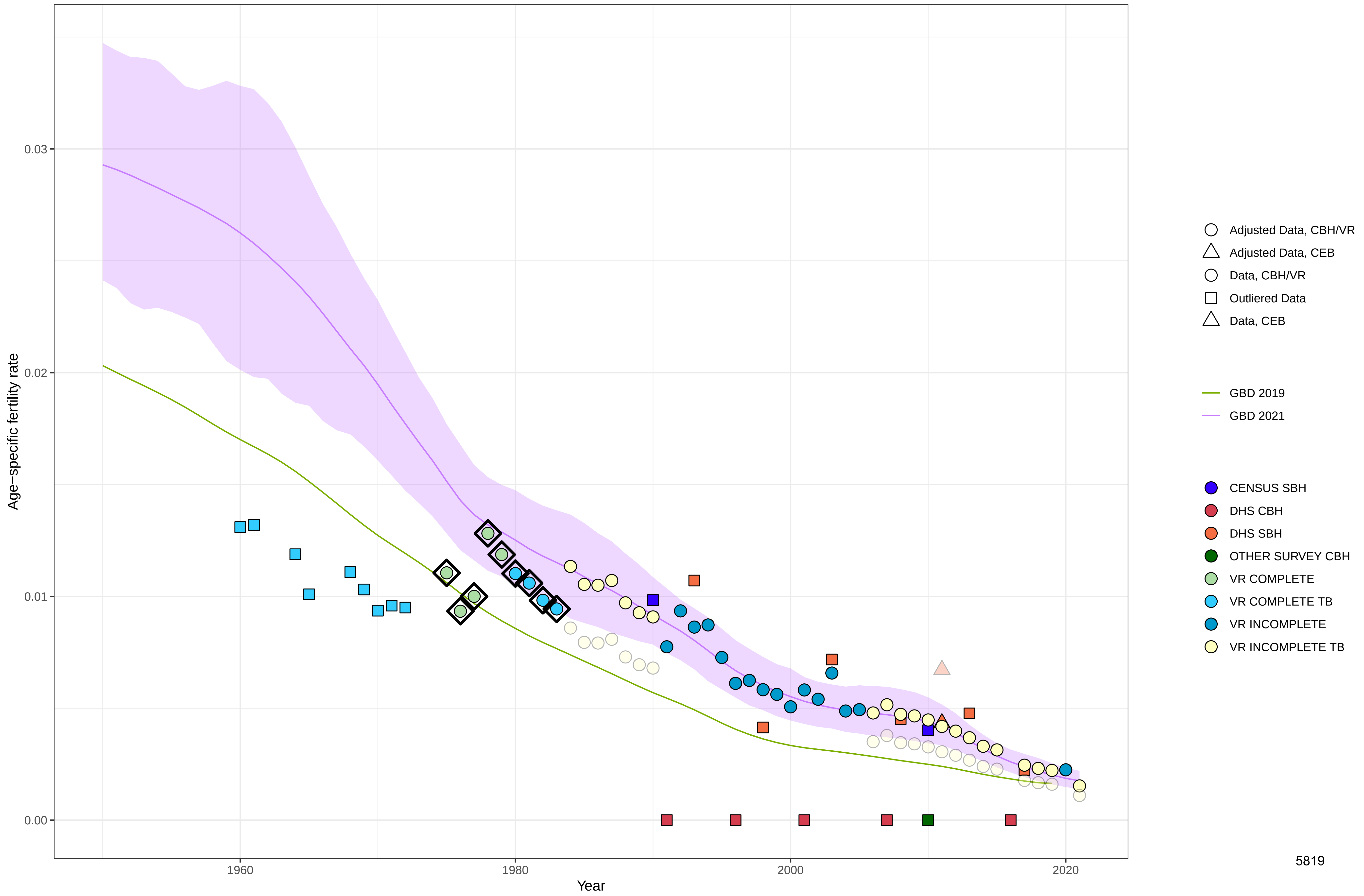

Age−specific fertility fitted trend lines, 1950−2021: Aklan, Ages: 50−54
Data points enclosed with a diamond are reference sources

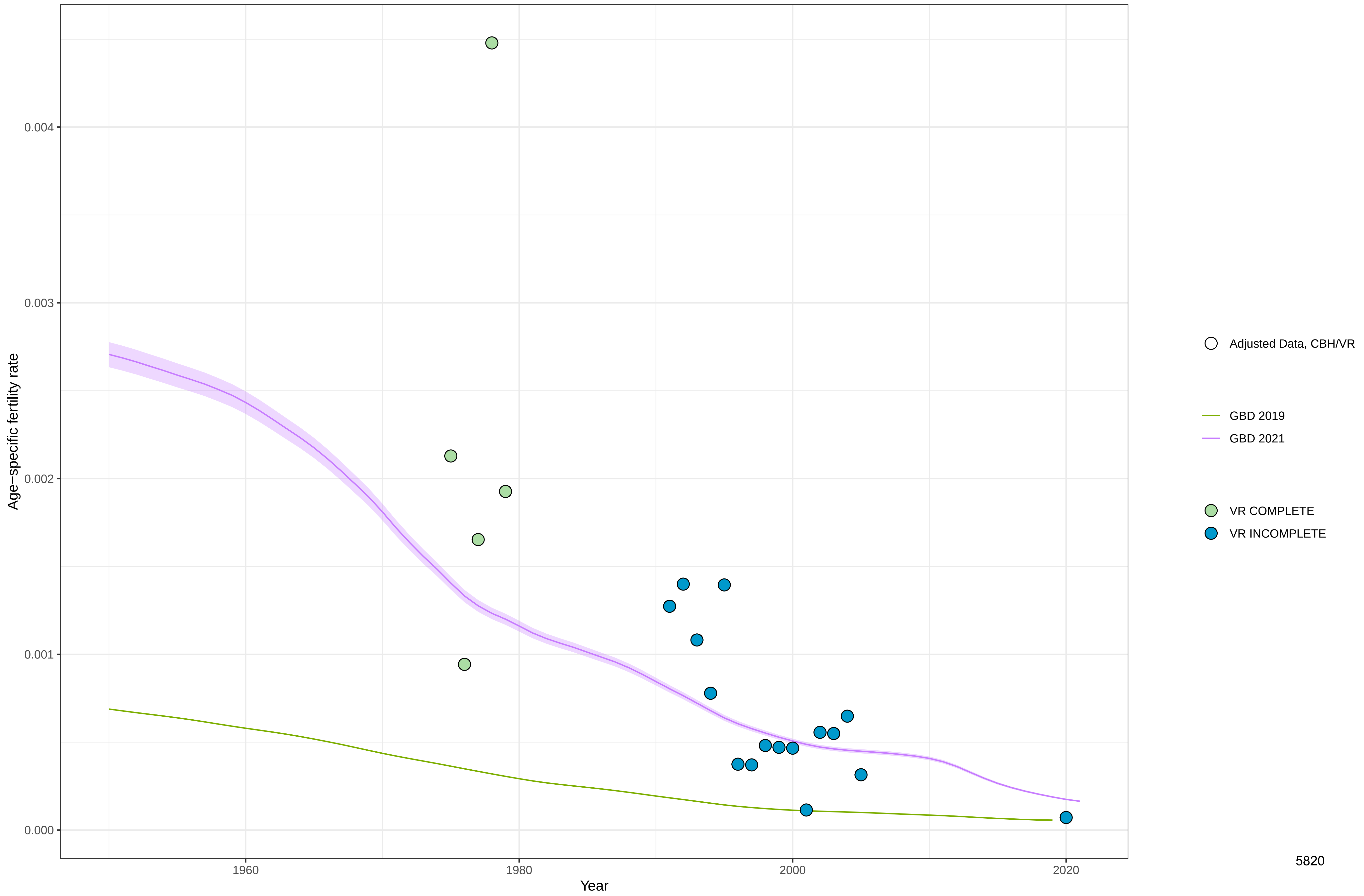

Total fertility rate fitted trend lines, 1950−2021: Aklan TFR
Data points enclosed with a diamond are reference sources

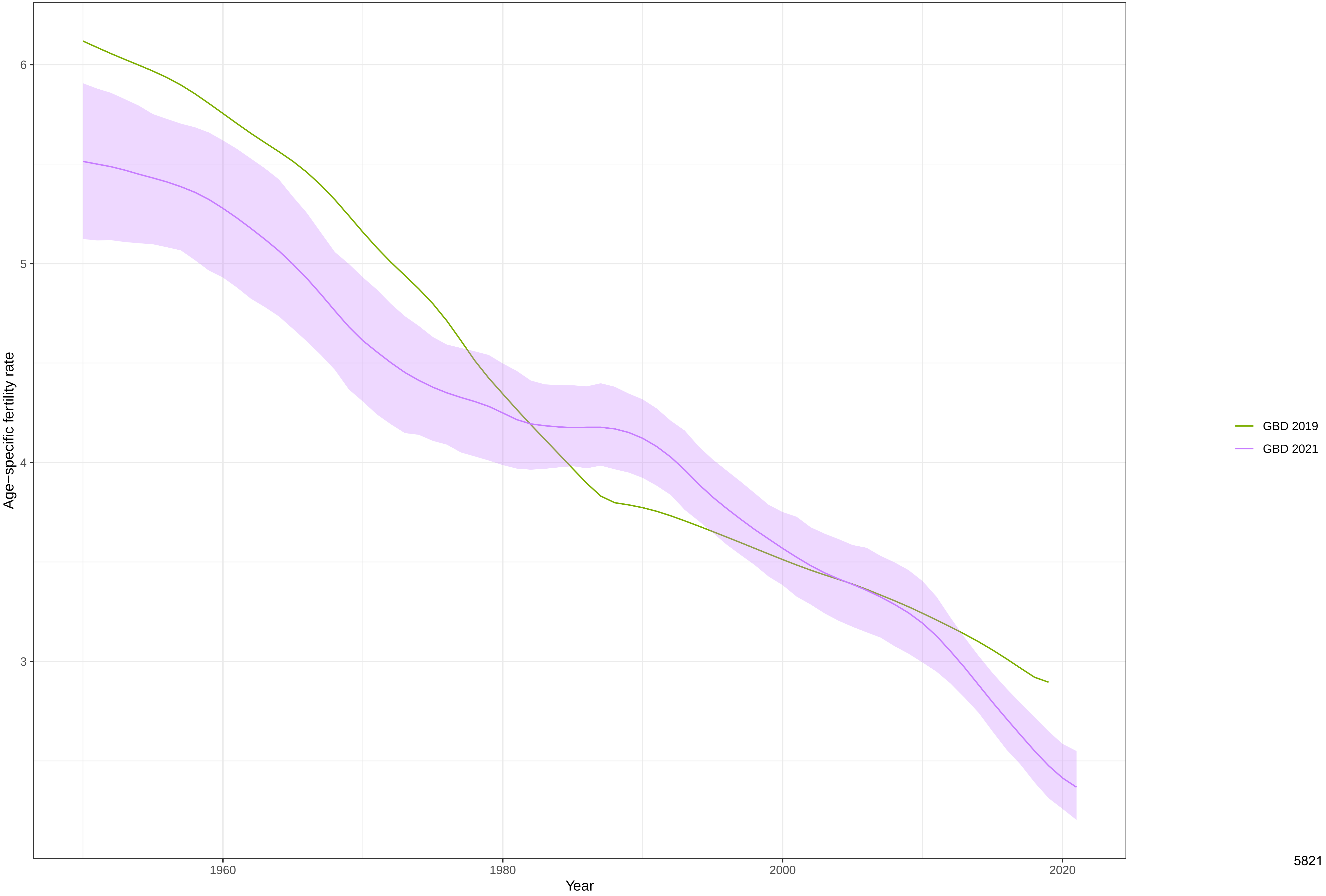

Age−specific fertility fitted trend lines, 1950−2021: Albay, Ages: 10−14
Data points enclosed with a diamond are reference sources

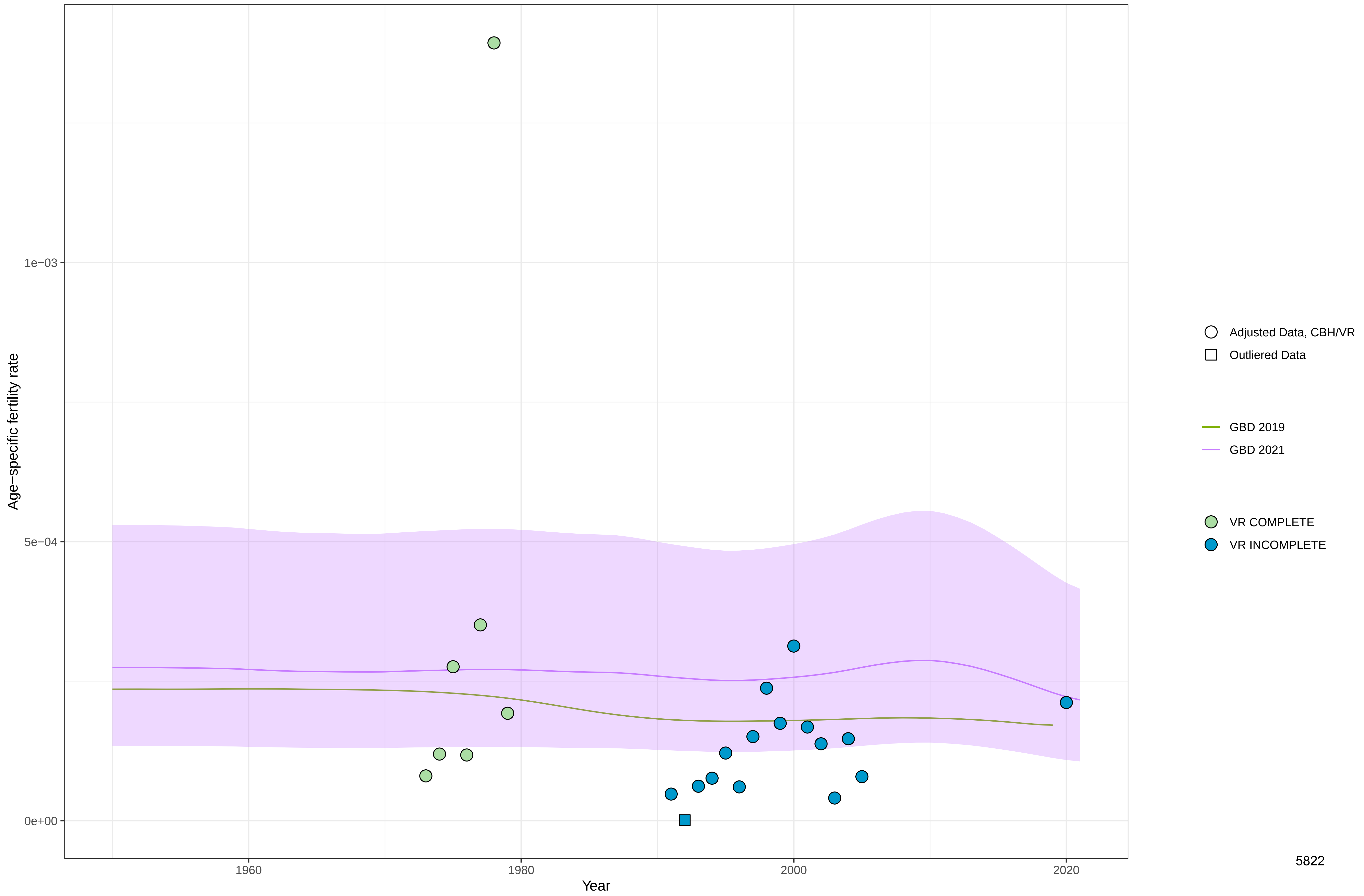

Age−specific fertility fitted trend lines, 1950−2021: Albay, Ages: 15−19
Data points enclosed with a diamond are reference sources

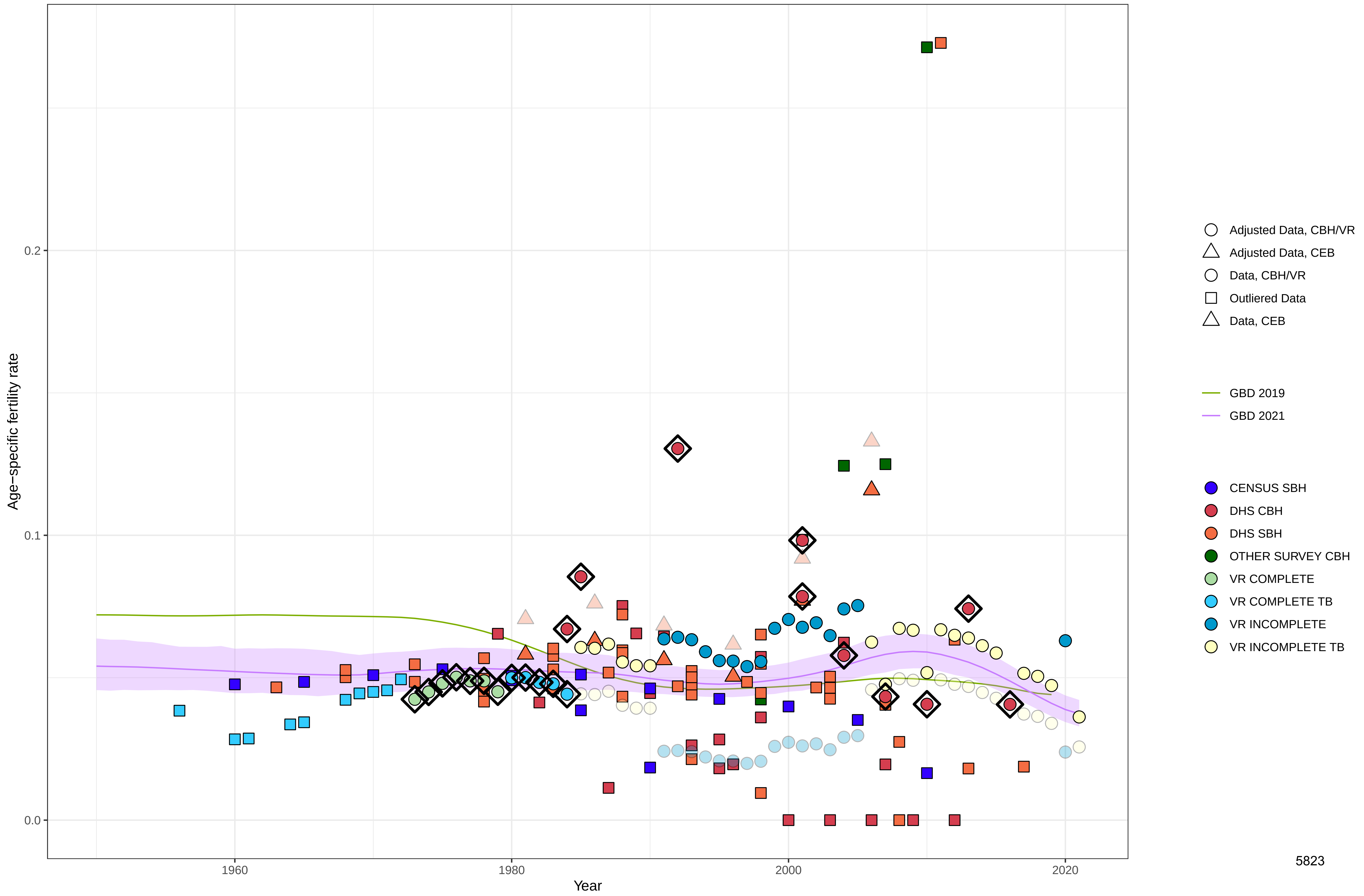

Age−specific fertility fitted trend lines, 1950−2021: Albay, Ages: 20−24
Data points enclosed with a diamond are reference sources

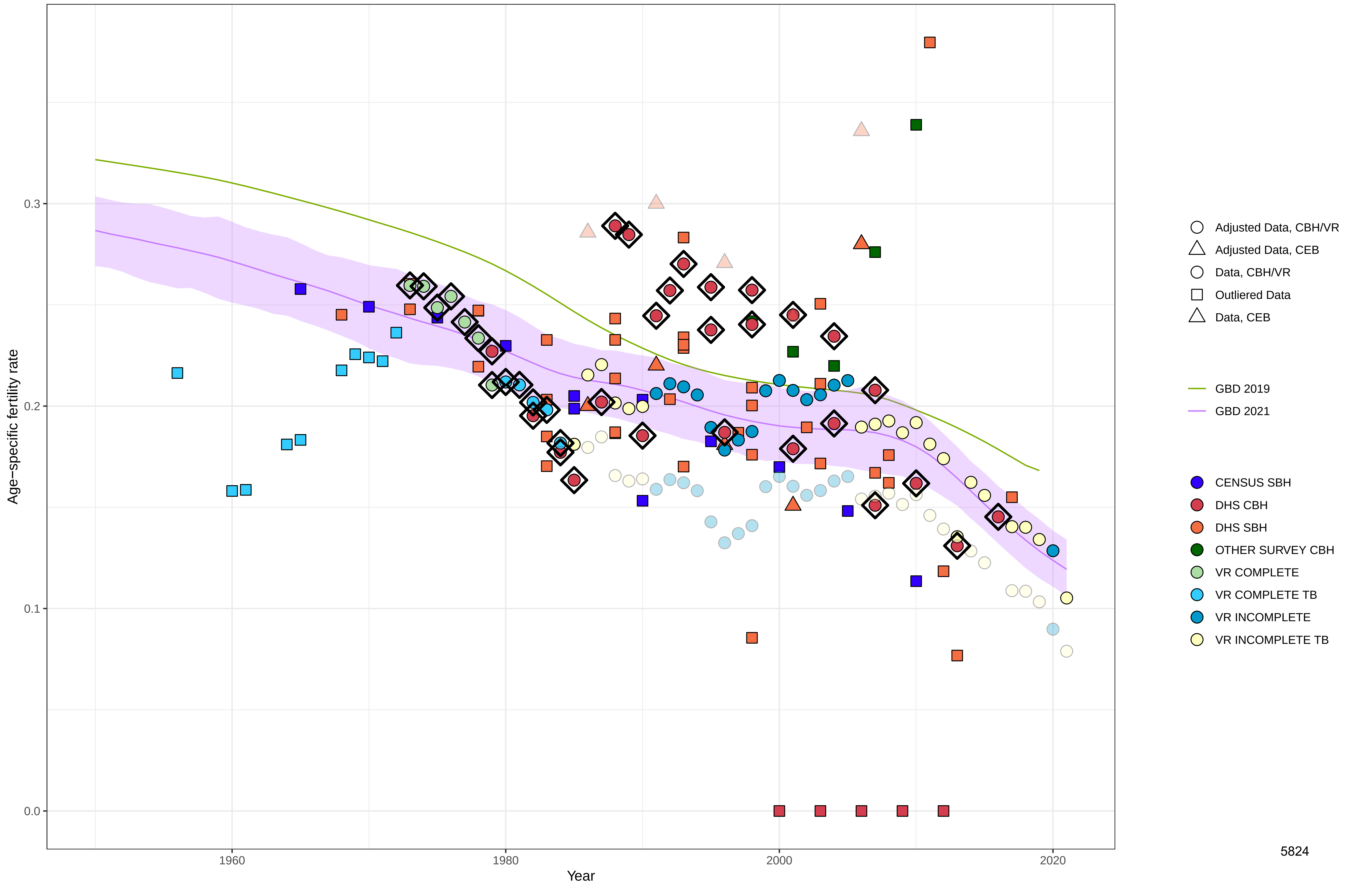

Age−specific fertility fitted trend lines, 1950−2021: Albay, Ages: 25−29
Data points enclosed with a diamond are reference sources

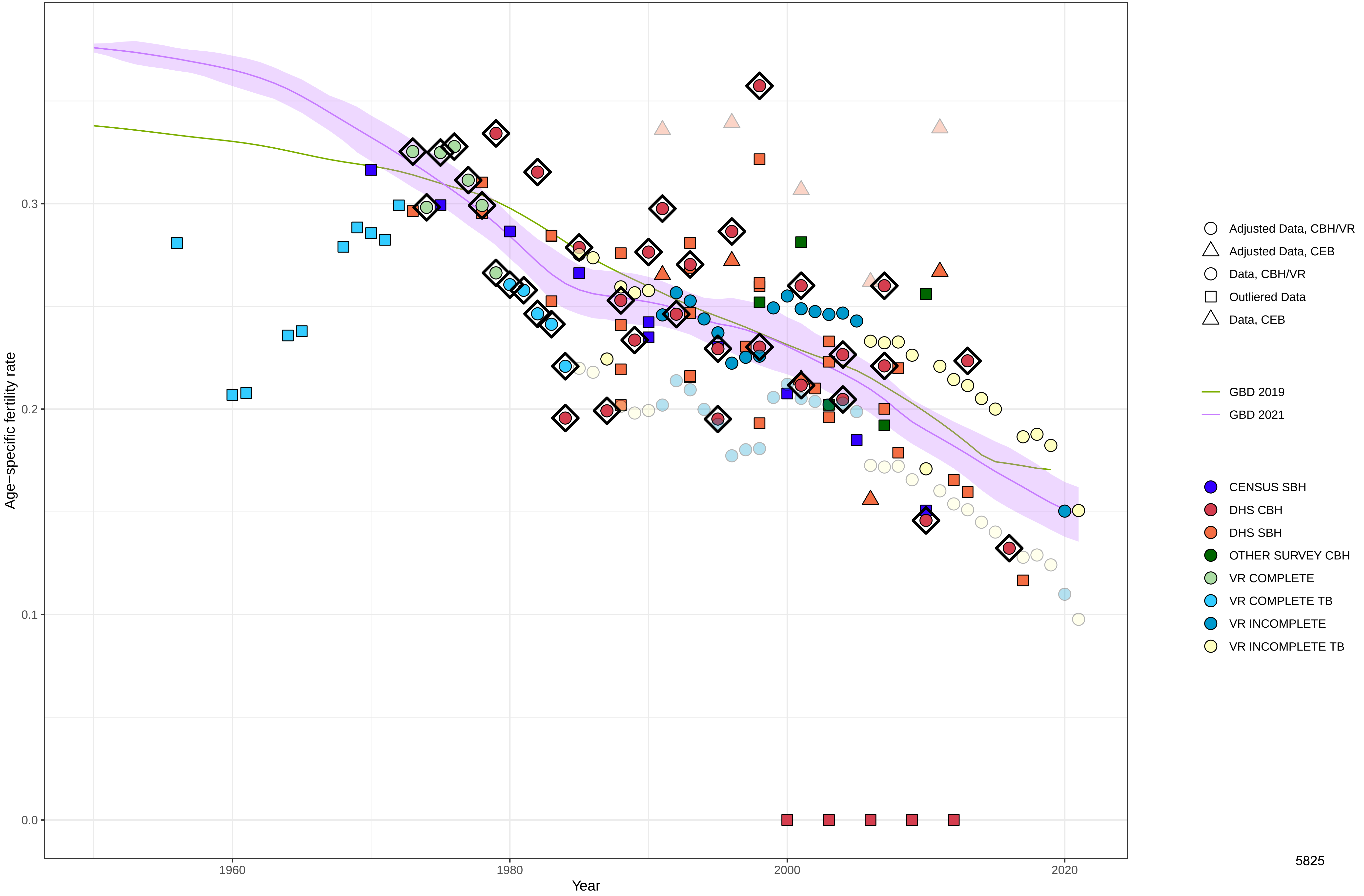

Age−specific fertility fitted trend lines, 1950−2021: Albay, Ages: 30−34
Data points enclosed with a diamond are reference sources

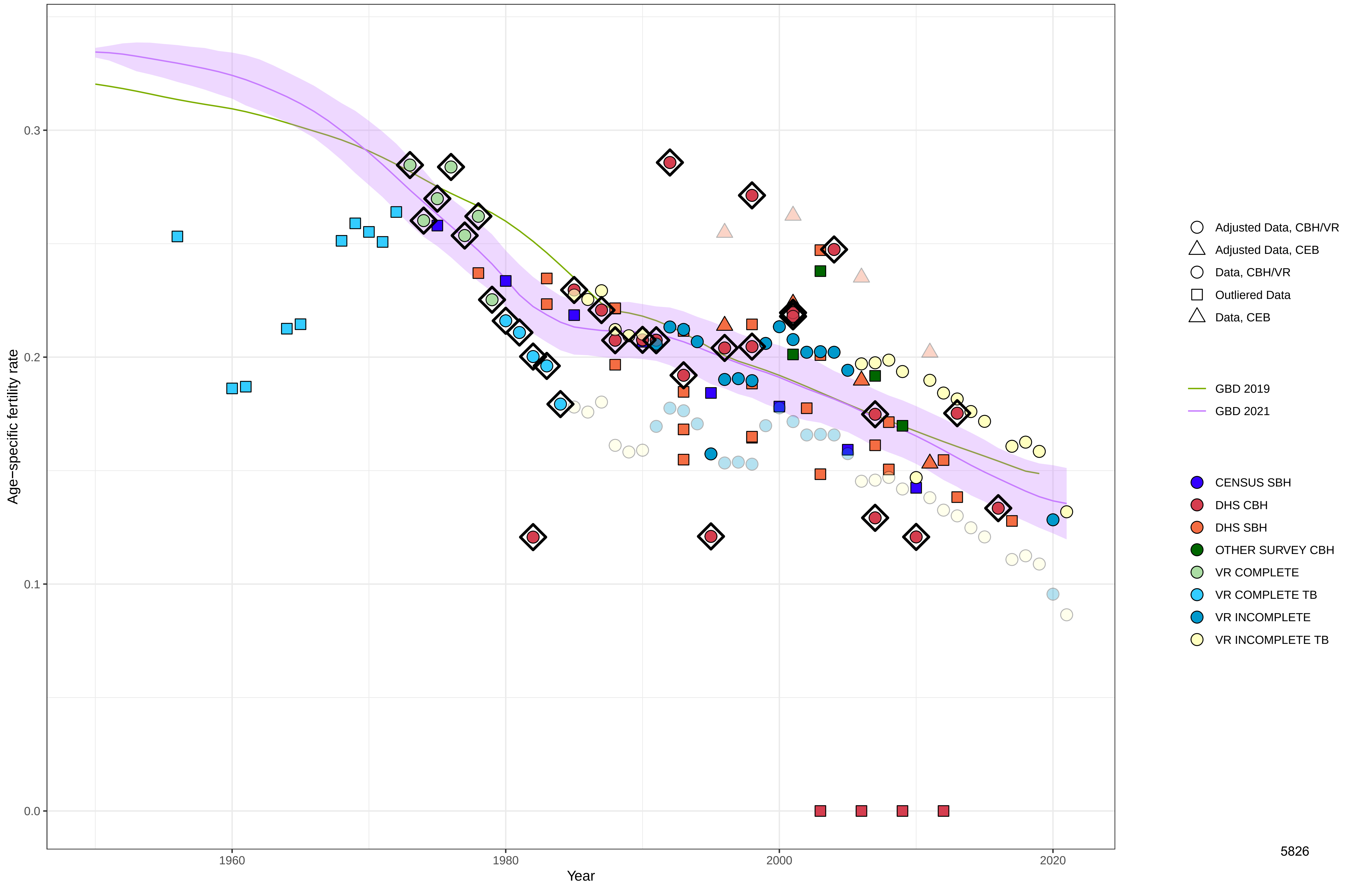

Age−specific fertility fitted trend lines, 1950−2021: Albay, Ages: 35−39
Data points enclosed with a diamond are reference sources

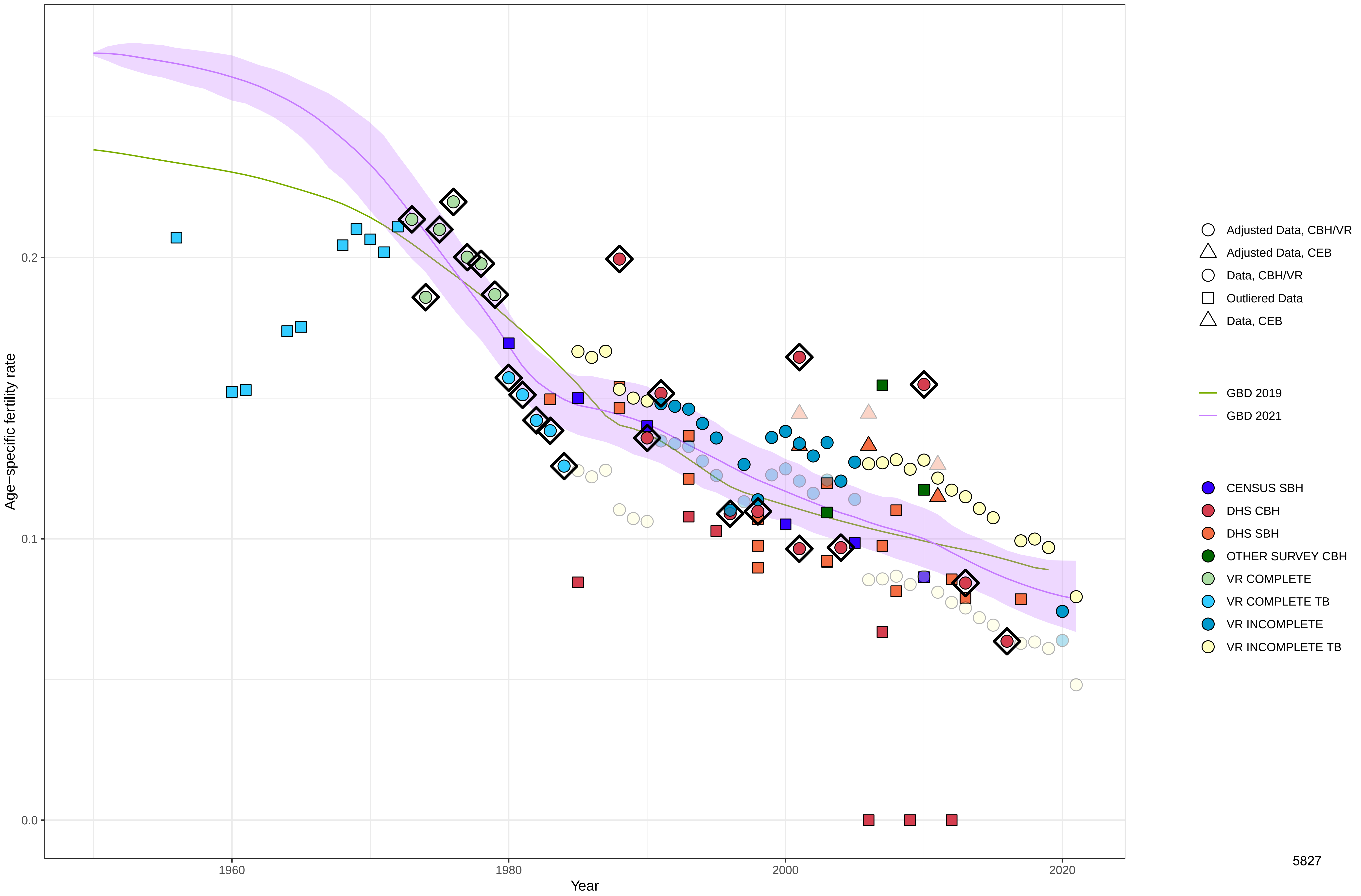

Age−specific fertility fitted trend lines, 1950−2021: Albay, Ages: 40−44
Data points enclosed with a diamond are reference sources

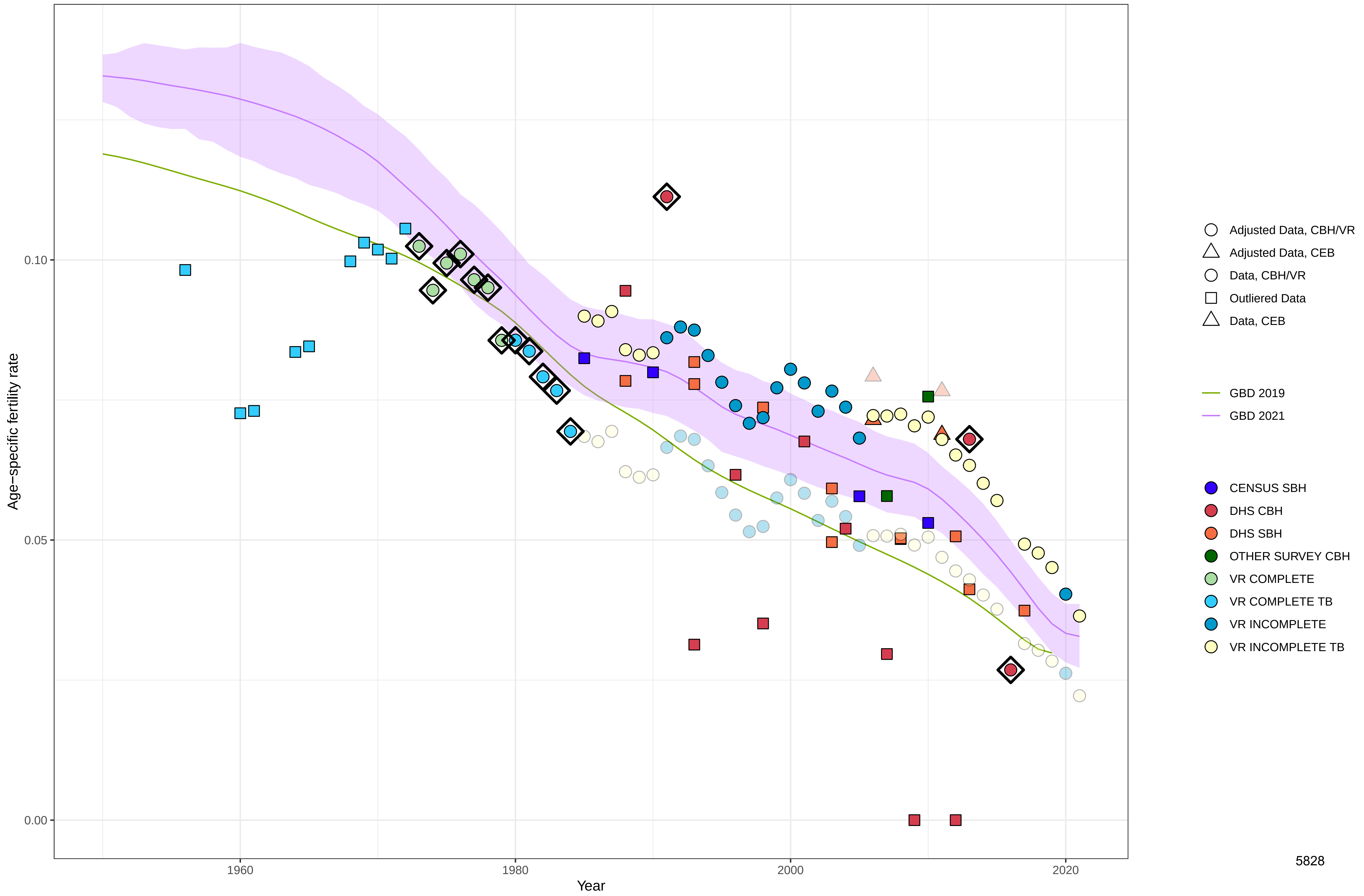

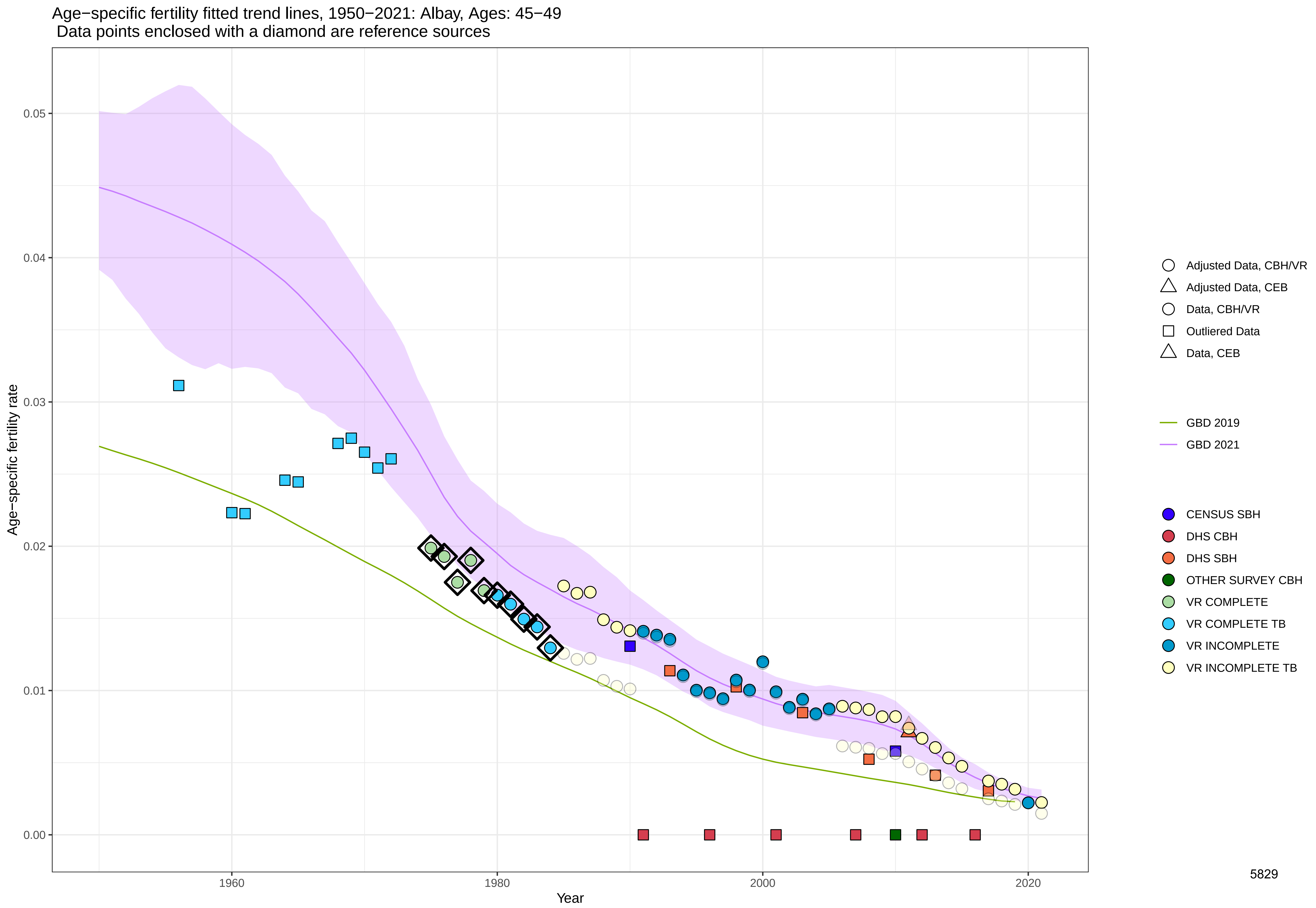

Age−specific fertility fitted trend lines, 1950−2021: Albay, Ages: 45−49
Data points enclosed with a diamond are reference sources
Age−specific fertility rate
0.00
0.01
0.02
0.03
0.04
0.05
1960
1980
2000
2020
Year
Adjusted Data, CBH/VR
Adjusted Data, CEB
Data, CBH/VR
Outliered Data
Data, CEB
GBD 2019
GBD 2021
CENSUS SBH
DHS CBH
DHS SBH
OTHER SURVEY CBH
VR COMPLETE
VR COMPLETE TB
VR INCOMPLETE
VR INCOMPLETE TB

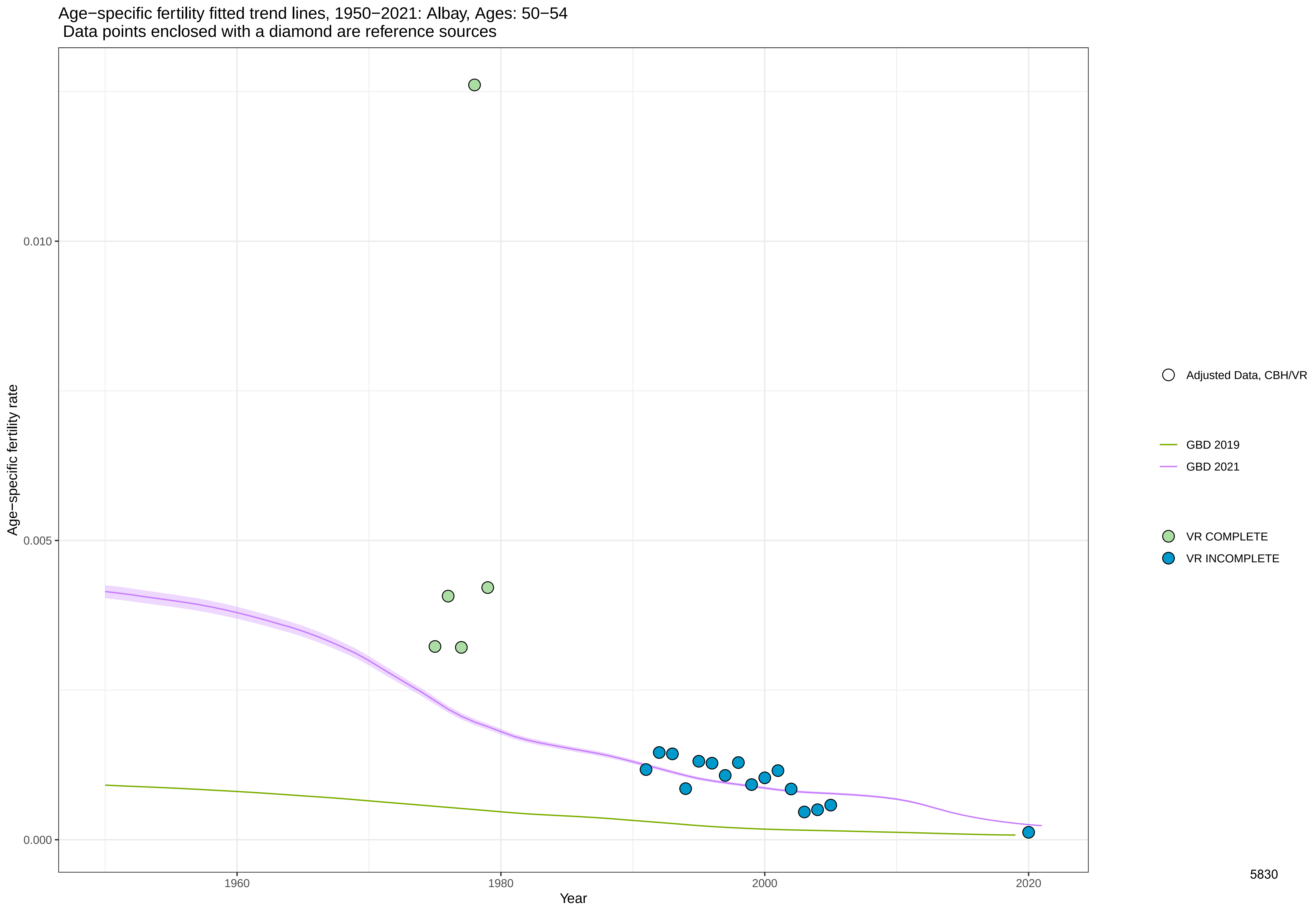

Age−specific fertility fitted trend lines, 1950−2021: Albay, Ages: 50−54
Data points enclosed with a diamond are reference sources
Age−specific fertility rate
0.010
0.005
0.000
1960
1980
2000
2020
Year
Adjusted Data, CBH/VR
GBD 2019
GBD 2021
VR COMPLETE
VR INCOMPLETE

Total fertility rate fitted trend lines, 1950−2021: Albay TFR
Data points enclosed with a diamond are reference sources

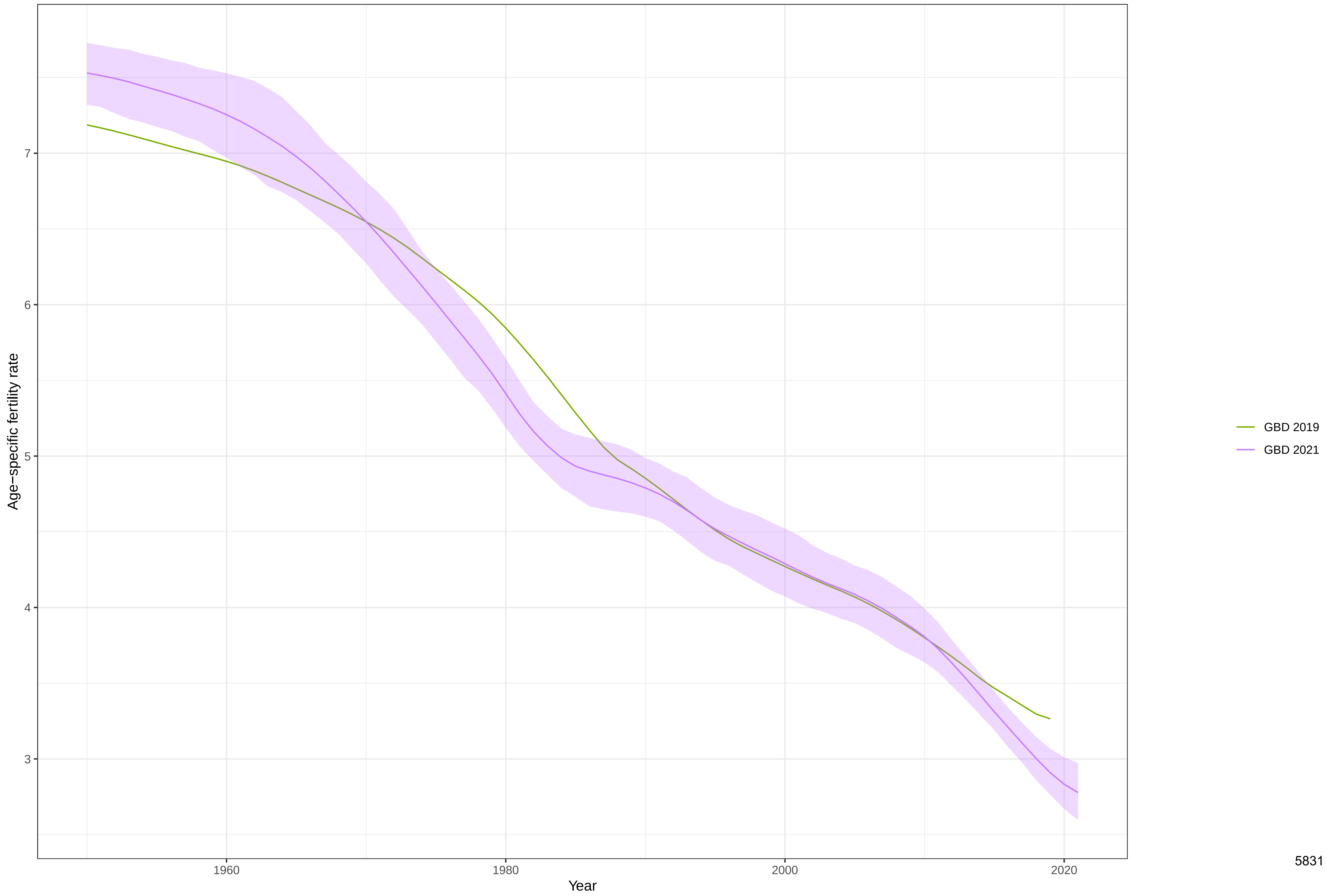

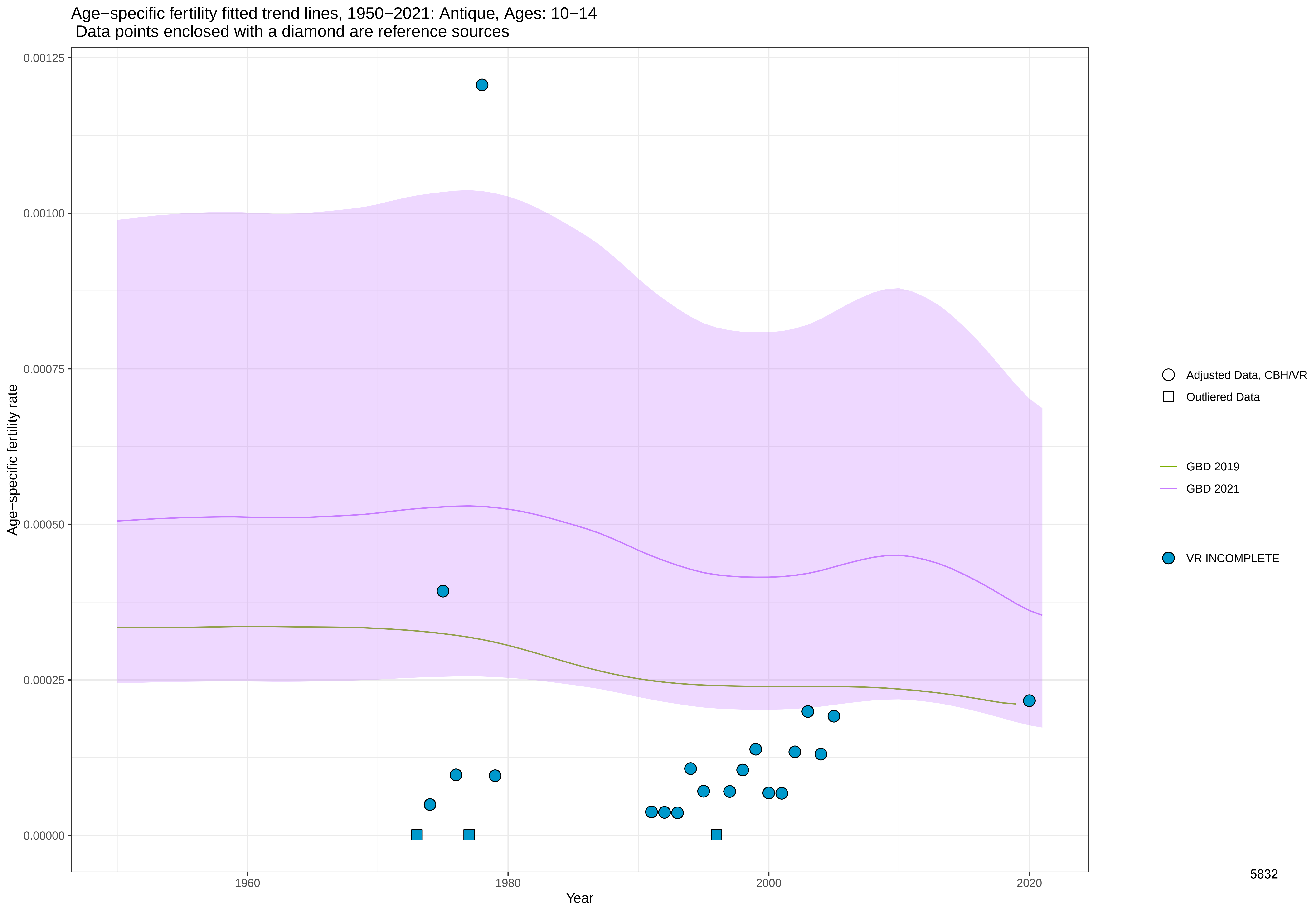

Age−specific fertility fitted trend lines, 1950−2021: Antique, Ages: 10−14
Data points enclosed with a diamond are reference sources
Age−specific fertility rate
0.00125
0.00100
0.00075
0.00050
0.00025
0.00000
1960
1980
2000
2020
Year
Adjusted Data, CBH/VR
Outliered Data
GBD 2019
GBD 2021
VR INCOMPLETE

5832

Age−specific fertility fitted trend lines, 1950−2021: Antique, Ages: 15−19
Data points enclosed with a diamond are reference sources

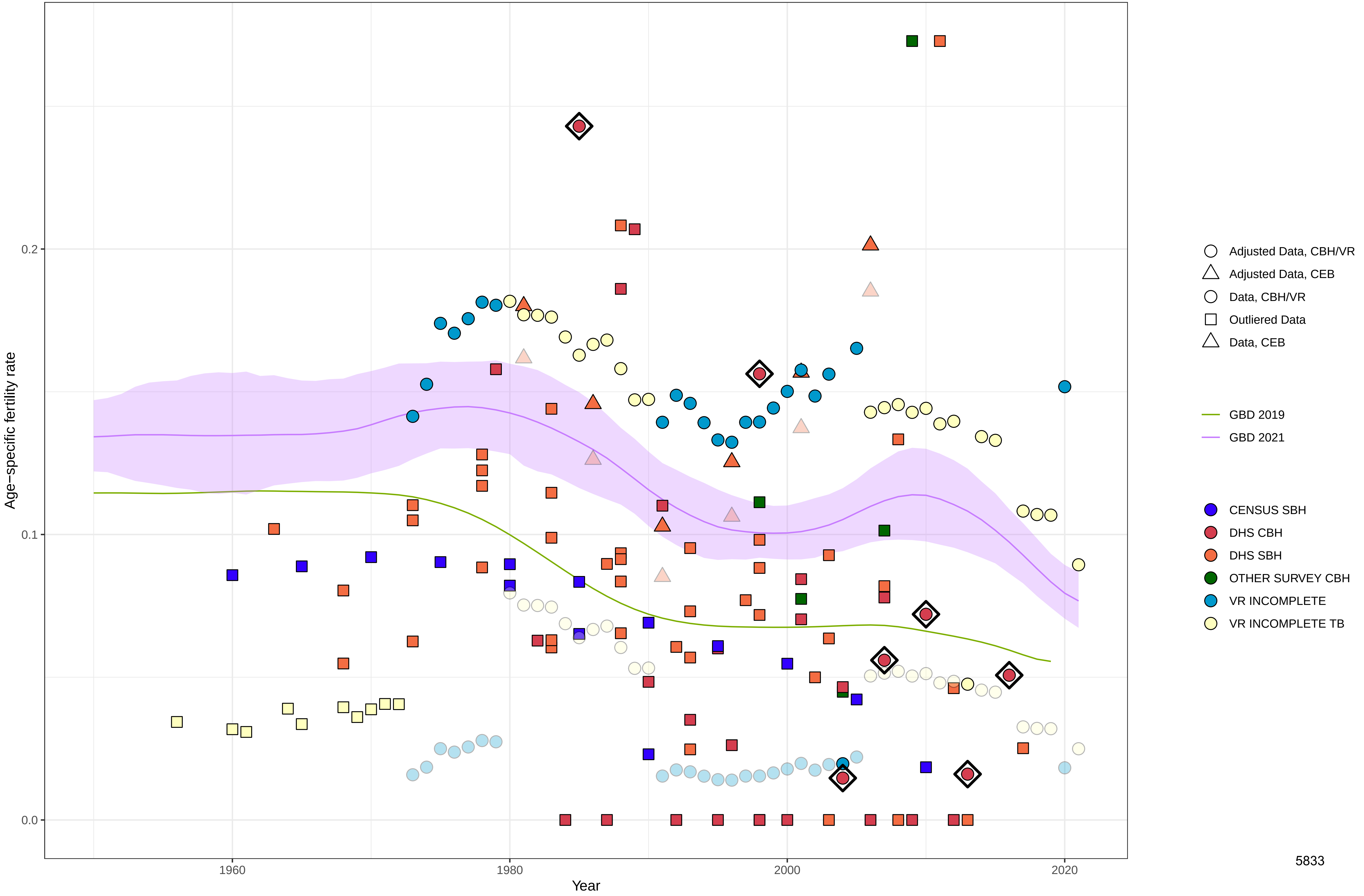

Age−specific fertility fitted trend lines, 1950−2021: Antique, Ages: 20−24
Data points enclosed with a diamond are reference sources

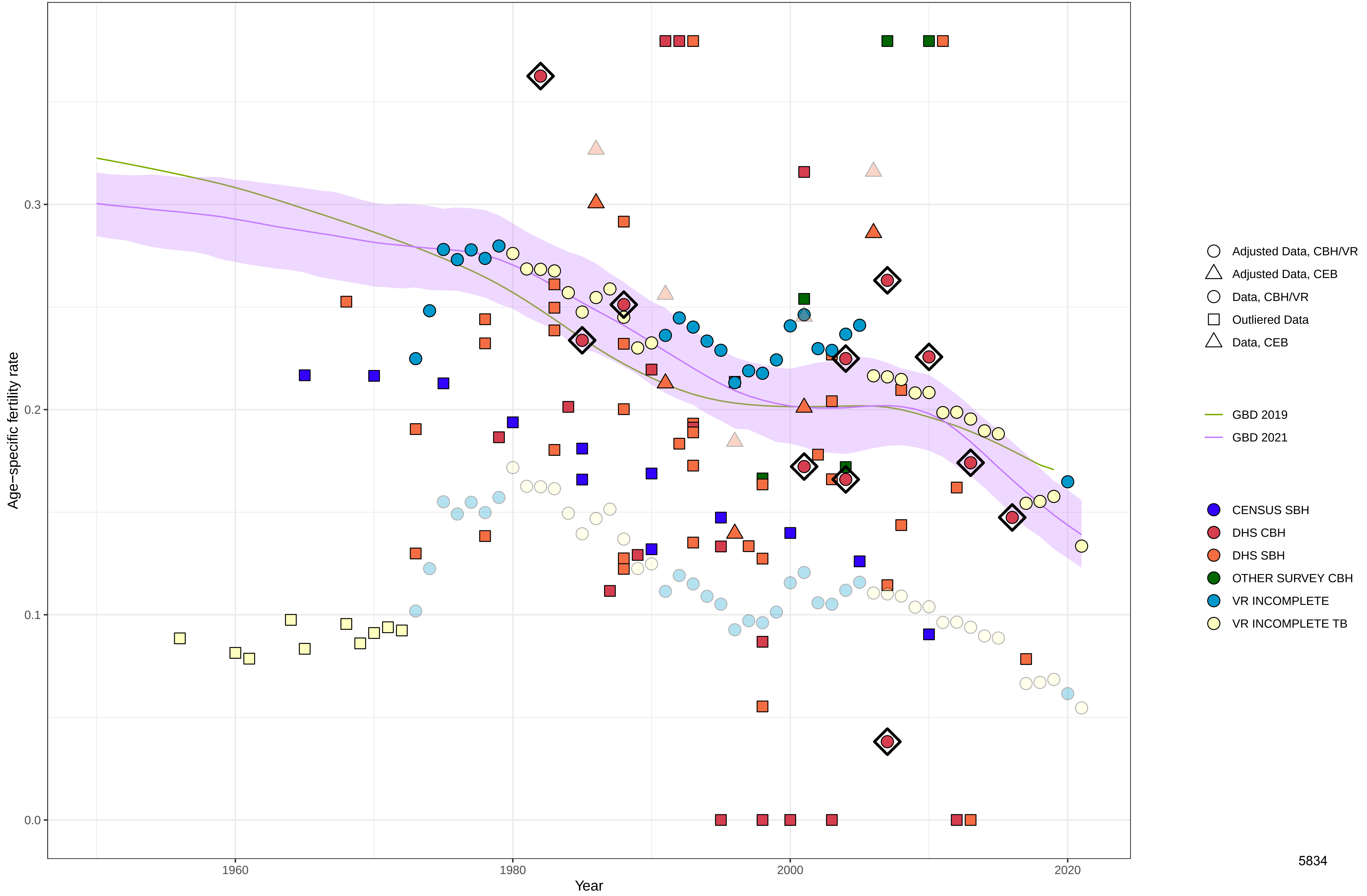

Age−specific fertility fitted trend lines, 1950−2021: Antique, Ages: 25−29
Data points enclosed with a diamond are reference sources

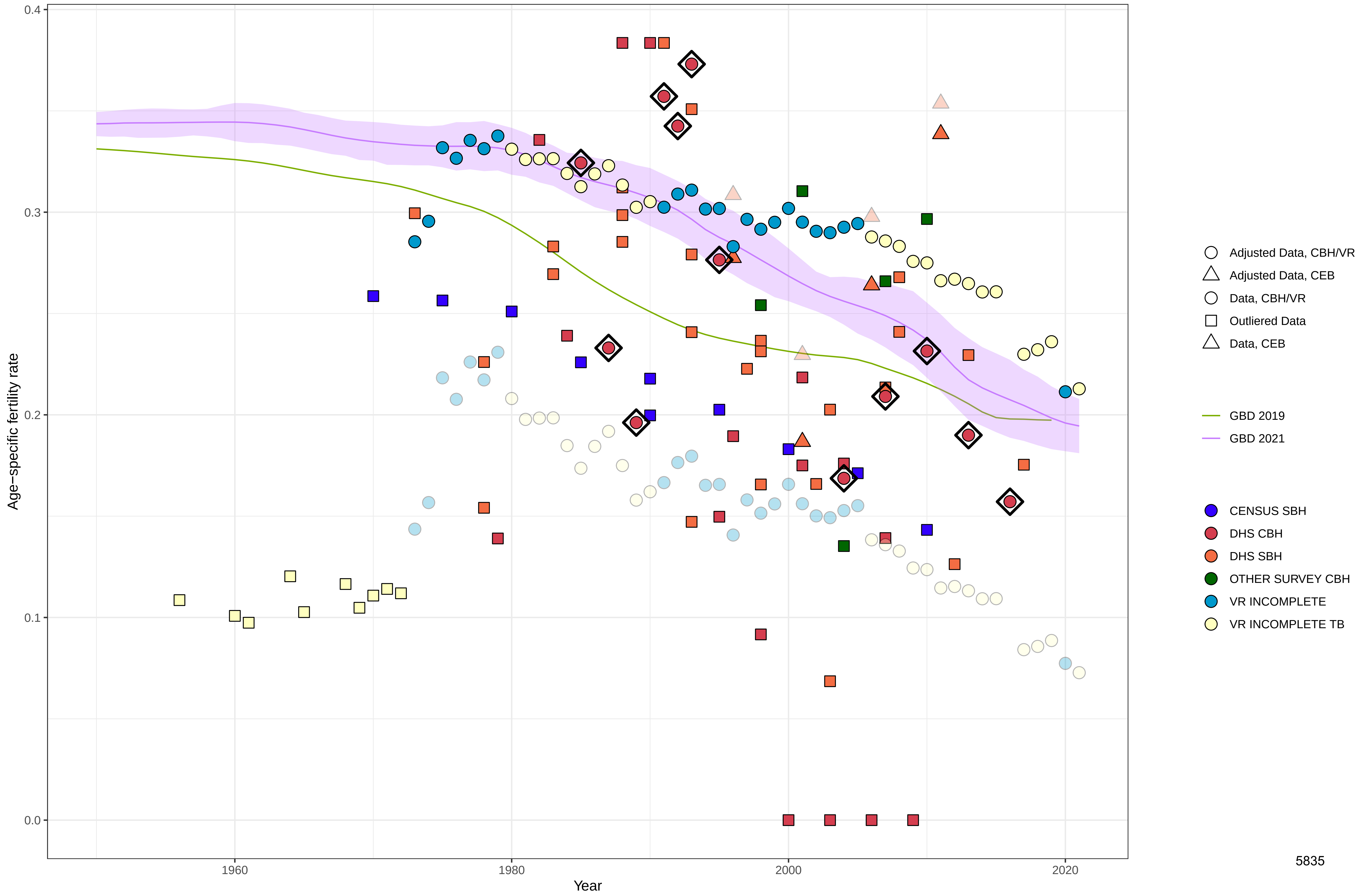

Age−specific fertility fitted trend lines, 1950−2021: Antique, Ages: 30−34
Data points enclosed with a diamond are reference sources

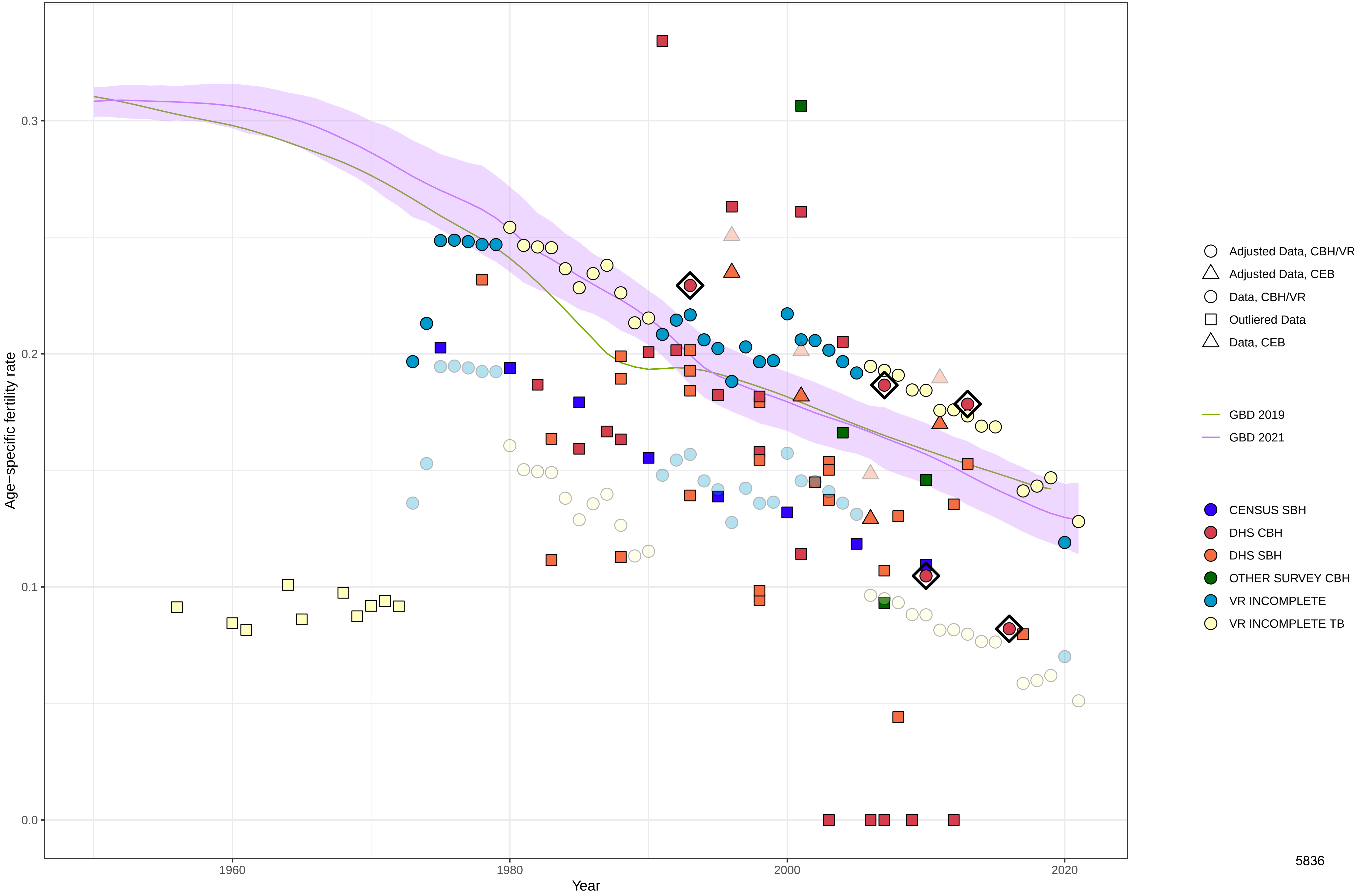

Age−specific fertility fitted trend lines, 1950−2021: Antique, Ages: 35−39
Data points enclosed with a diamond are reference sources

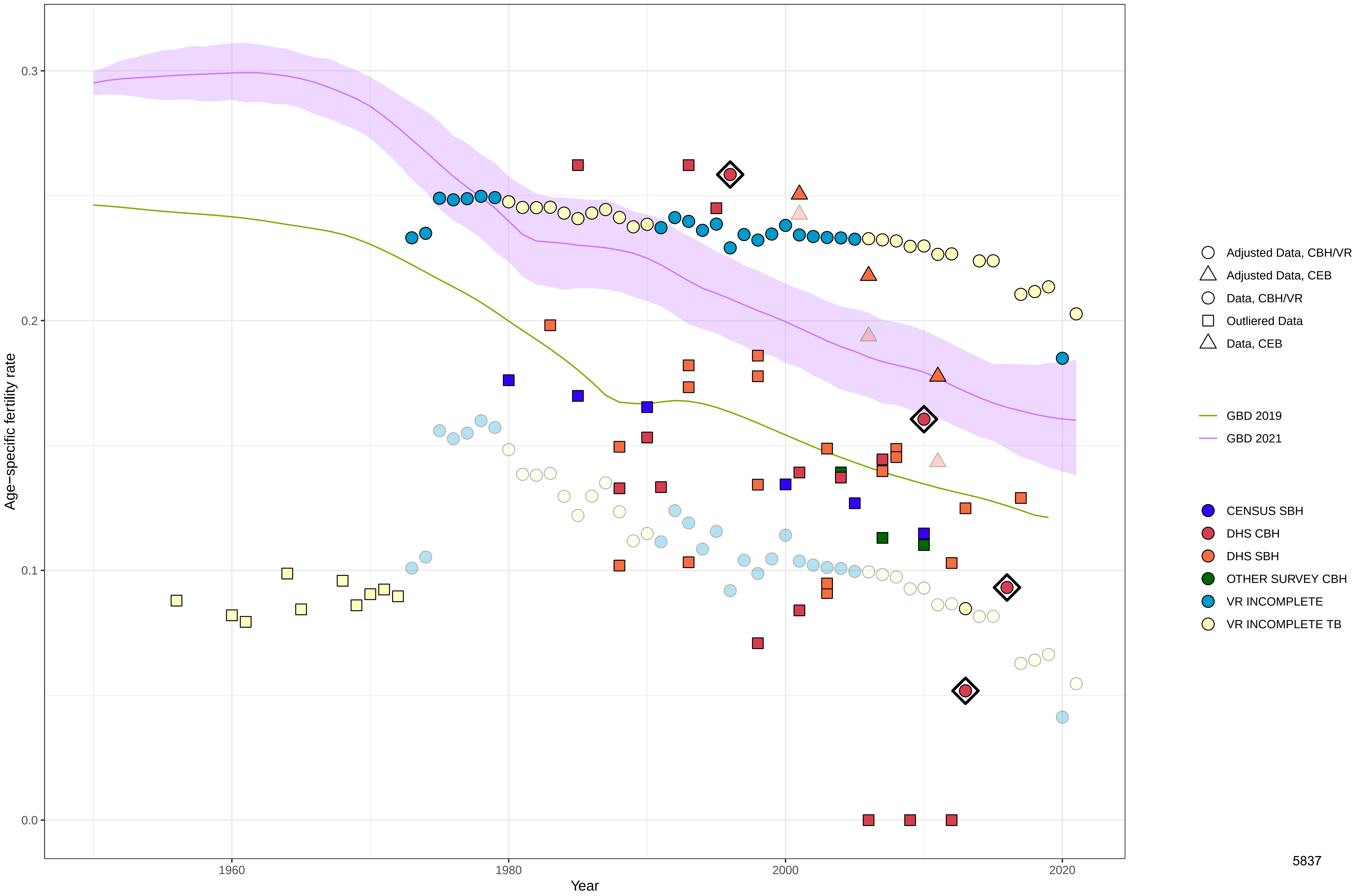

Age−specific fertility fitted trend lines, 1950−2021: Antique, Ages: 40−44
Data points enclosed with a diamond are reference sources

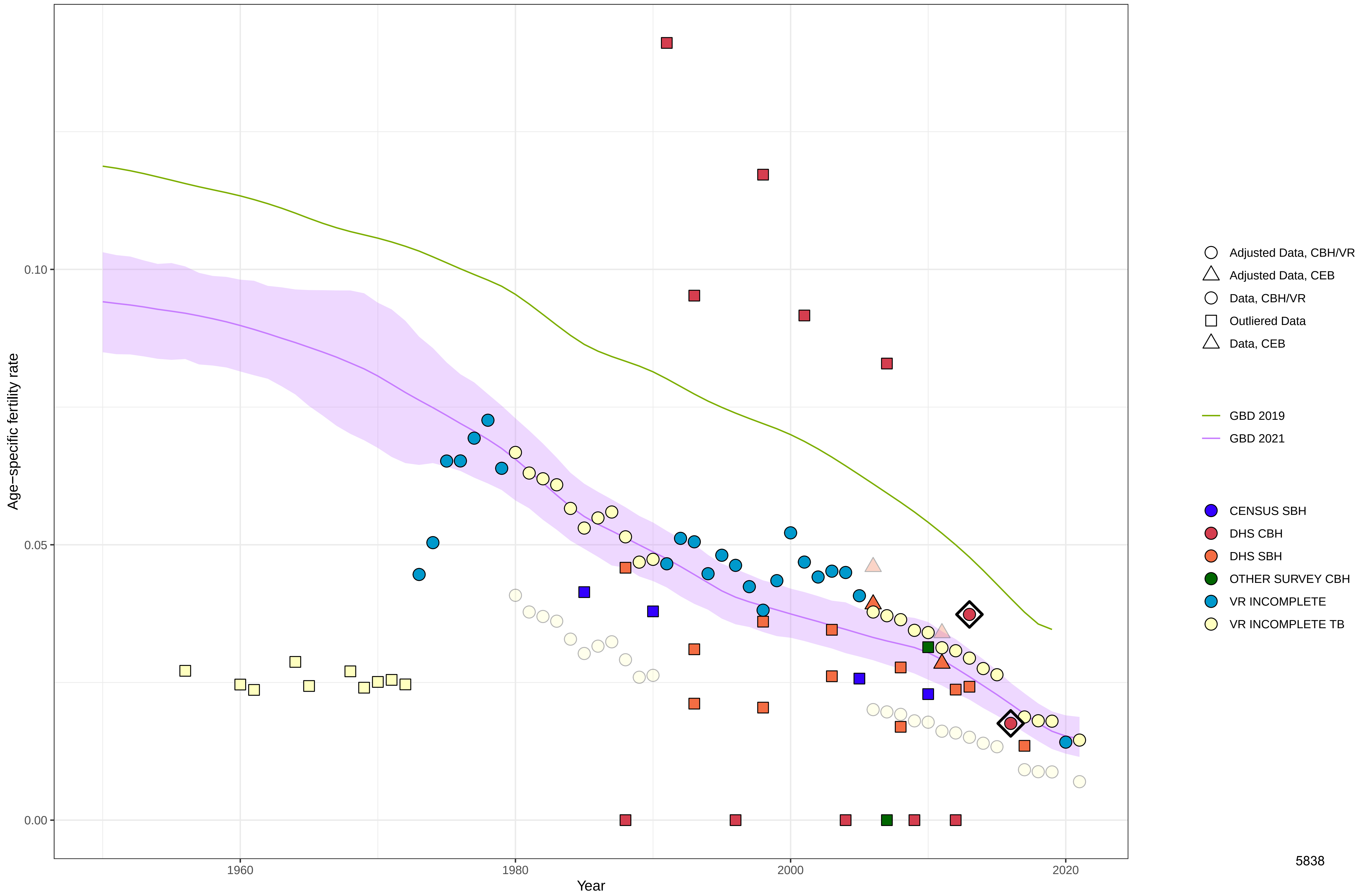

Age−specific fertility fitted trend lines, 1950−2021: Antique, Ages: 45−49
Data points enclosed with a diamond are reference sources

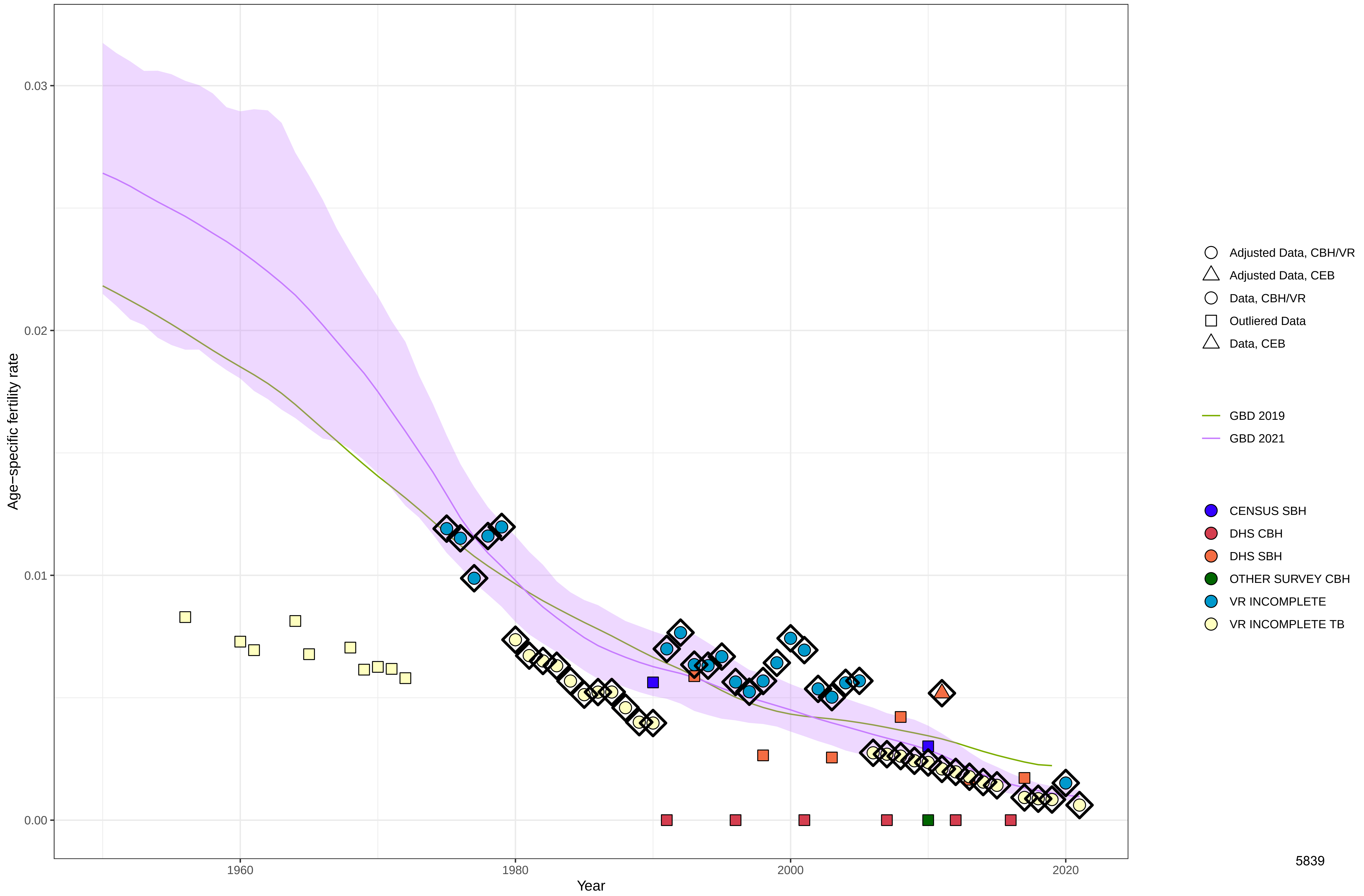

Age−specific fertility fitted trend lines, 1950−2021: Antique, Ages: 50−54
Data points enclosed with a diamond are reference sources

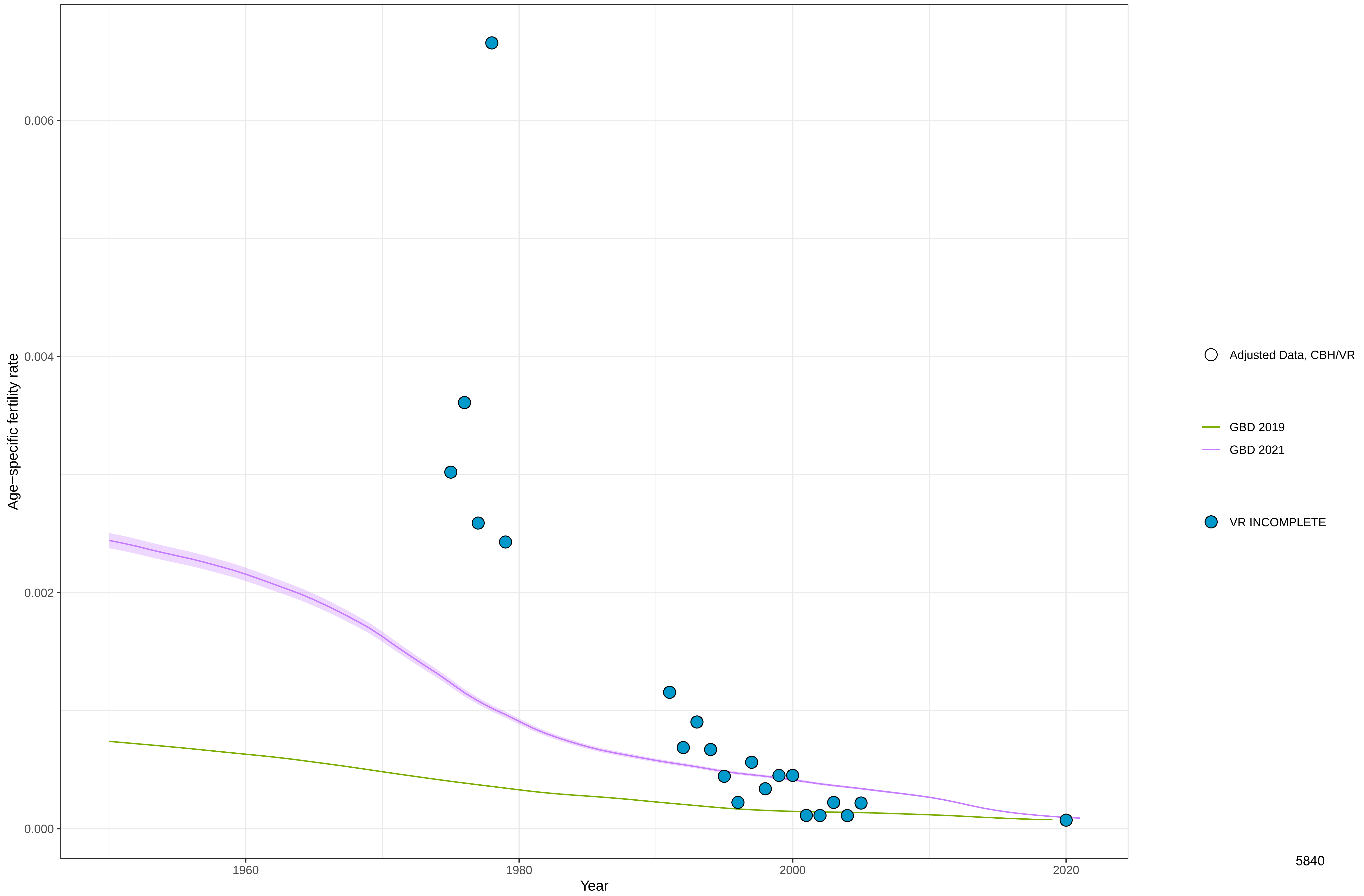

Total fertility rate fitted trend lines, 1950−2021: Antique TFR
Data points enclosed with a diamond are reference sources

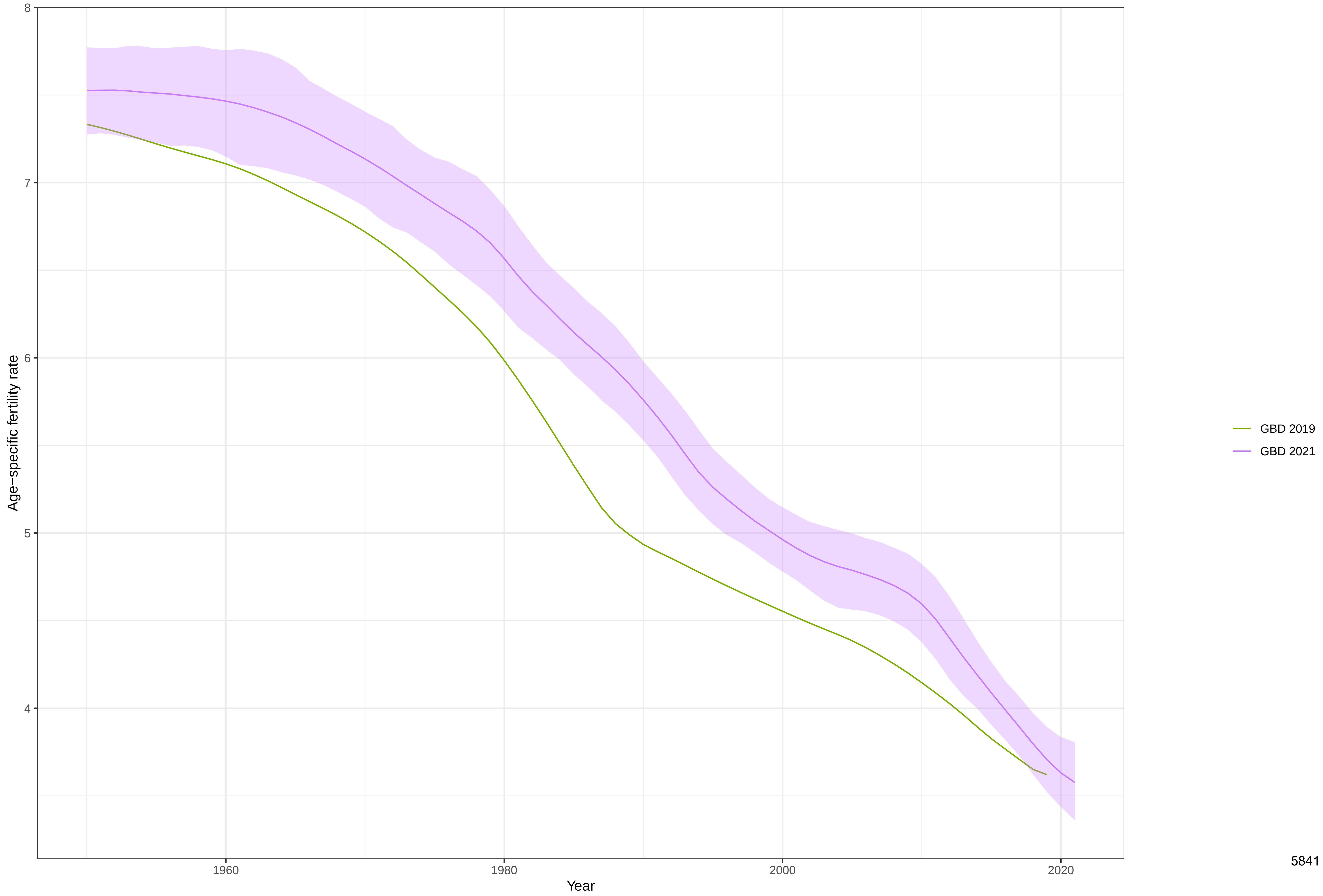

5841

Age−specific fertility fitted trend lines, 1950−2021: Apayao, Ages: 10−14
Data points enclosed with a diamond are reference sources

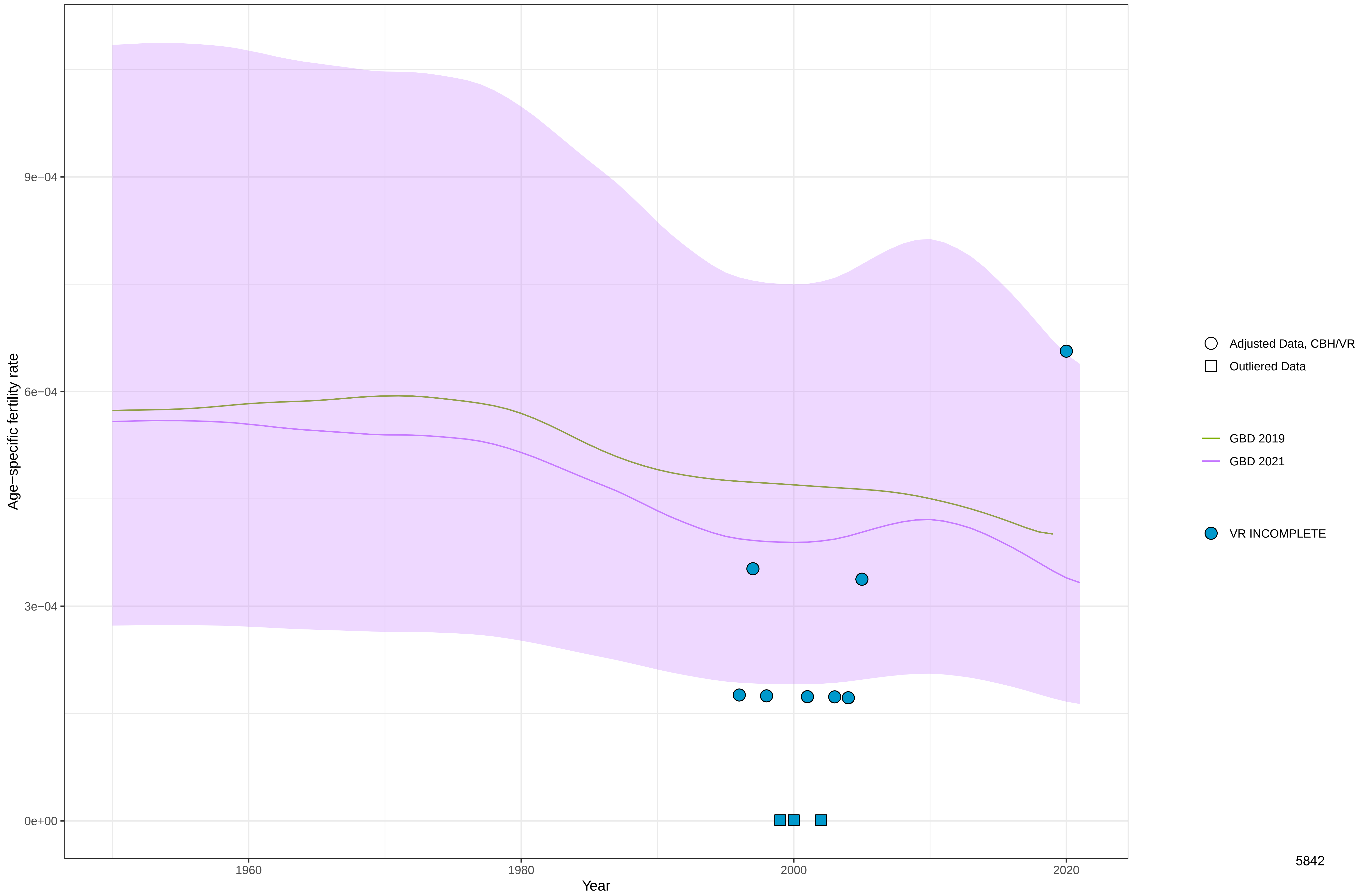

Age−specific fertility fitted trend lines, 1950−2021: Apayao, Ages: 15−19
Data points enclosed with a diamond are reference sources

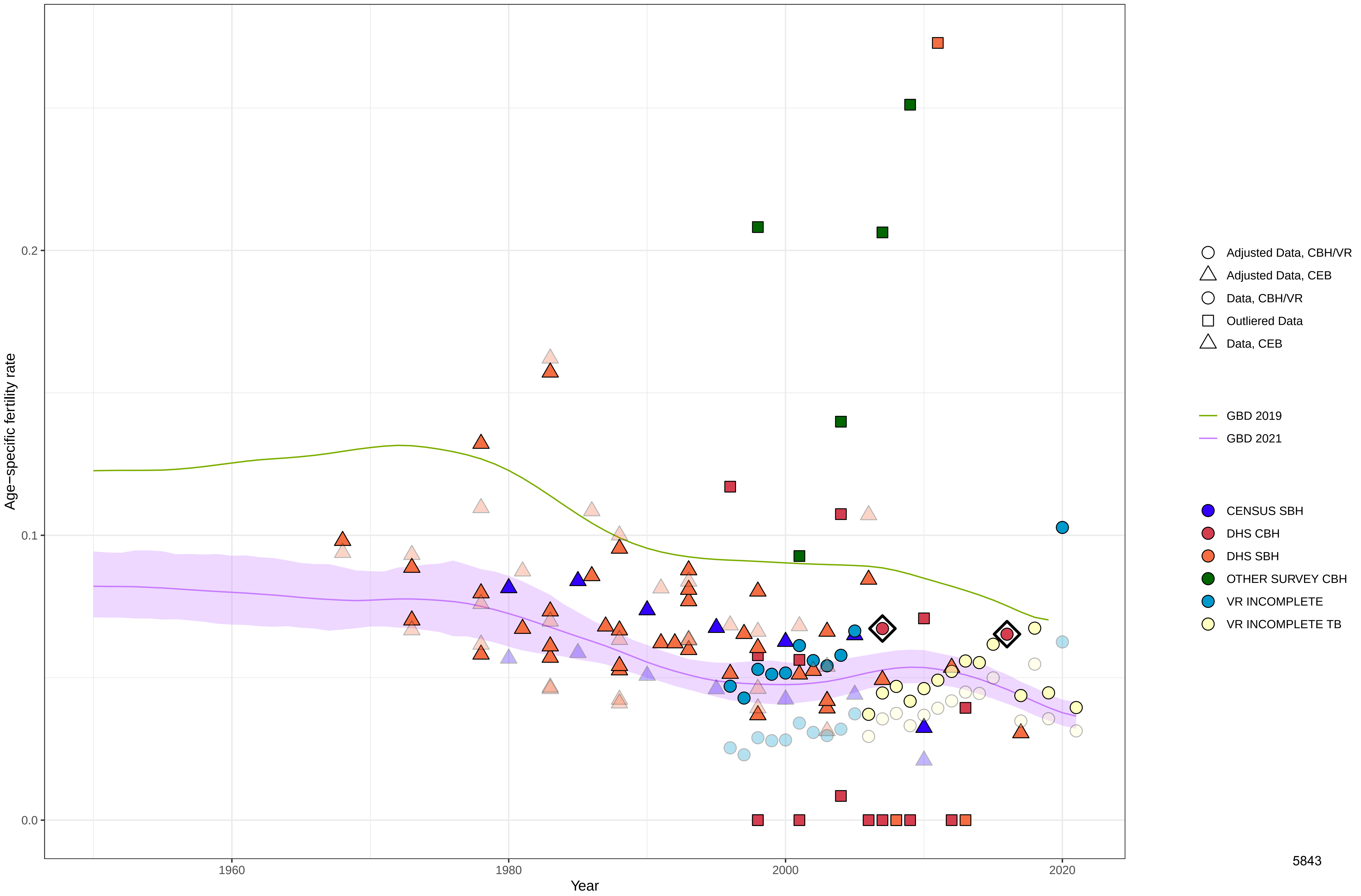

Age−specific fertility fitted trend lines, 1950−2021: Apayao, Ages: 20−24
Data points enclosed with a diamond are reference sources

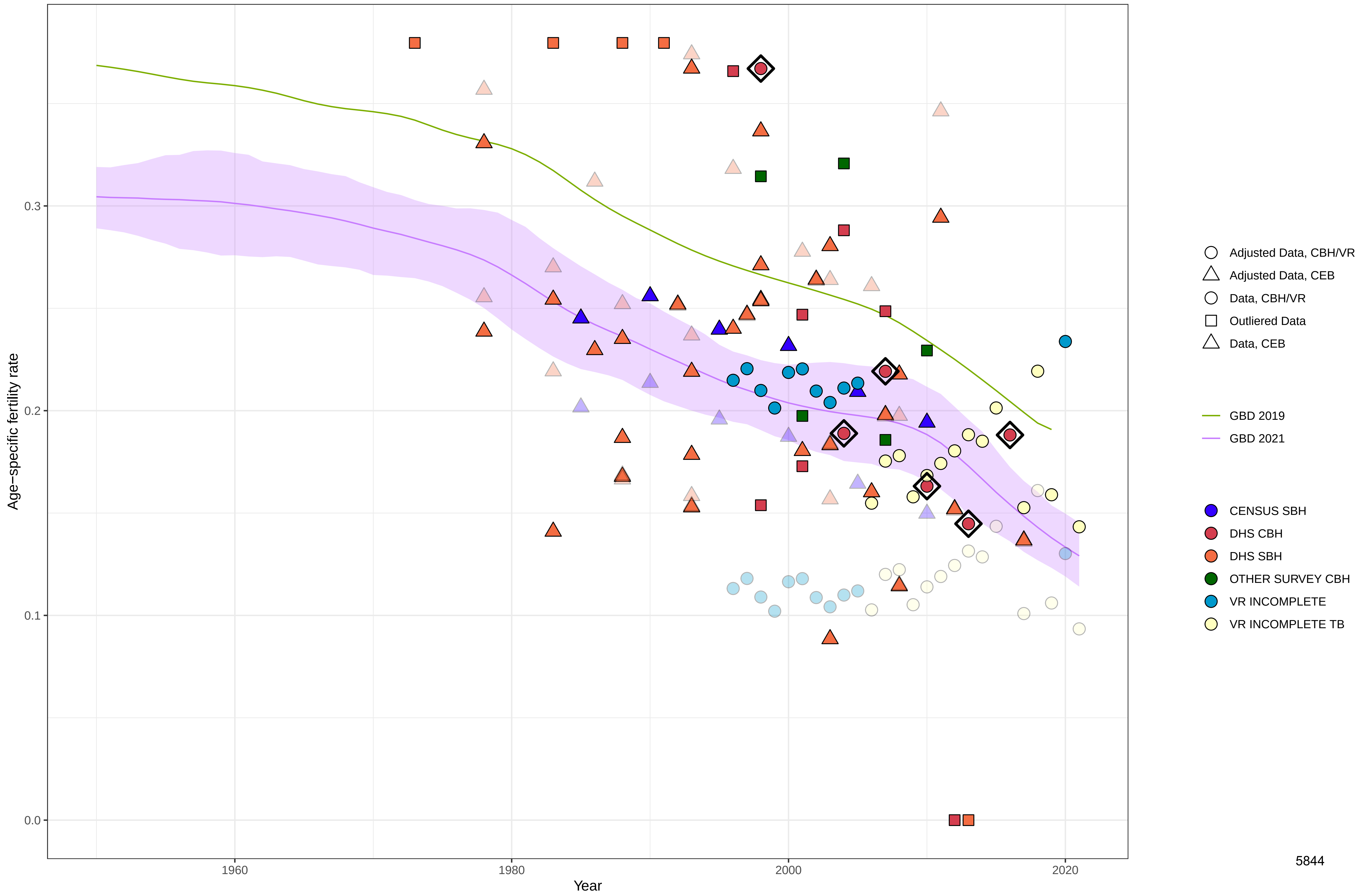

Age−specific fertility fitted trend lines, 1950−2021: Apayao, Ages: 25−29
Data points enclosed with a diamond are reference sources

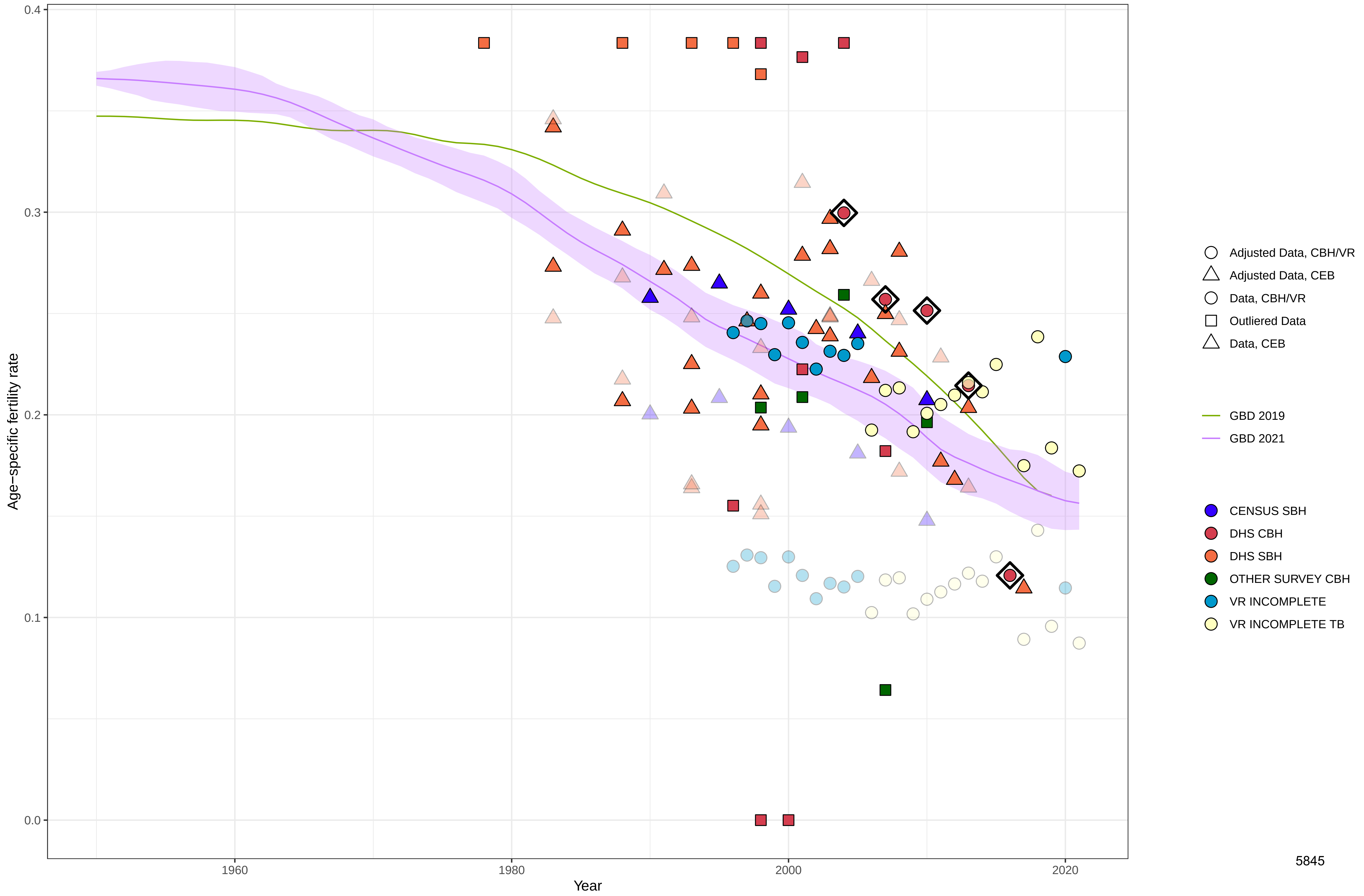

Age−specific fertility fitted trend lines, 1950−2021: Apayao, Ages: 30−34
Data points enclosed with a diamond are reference sources

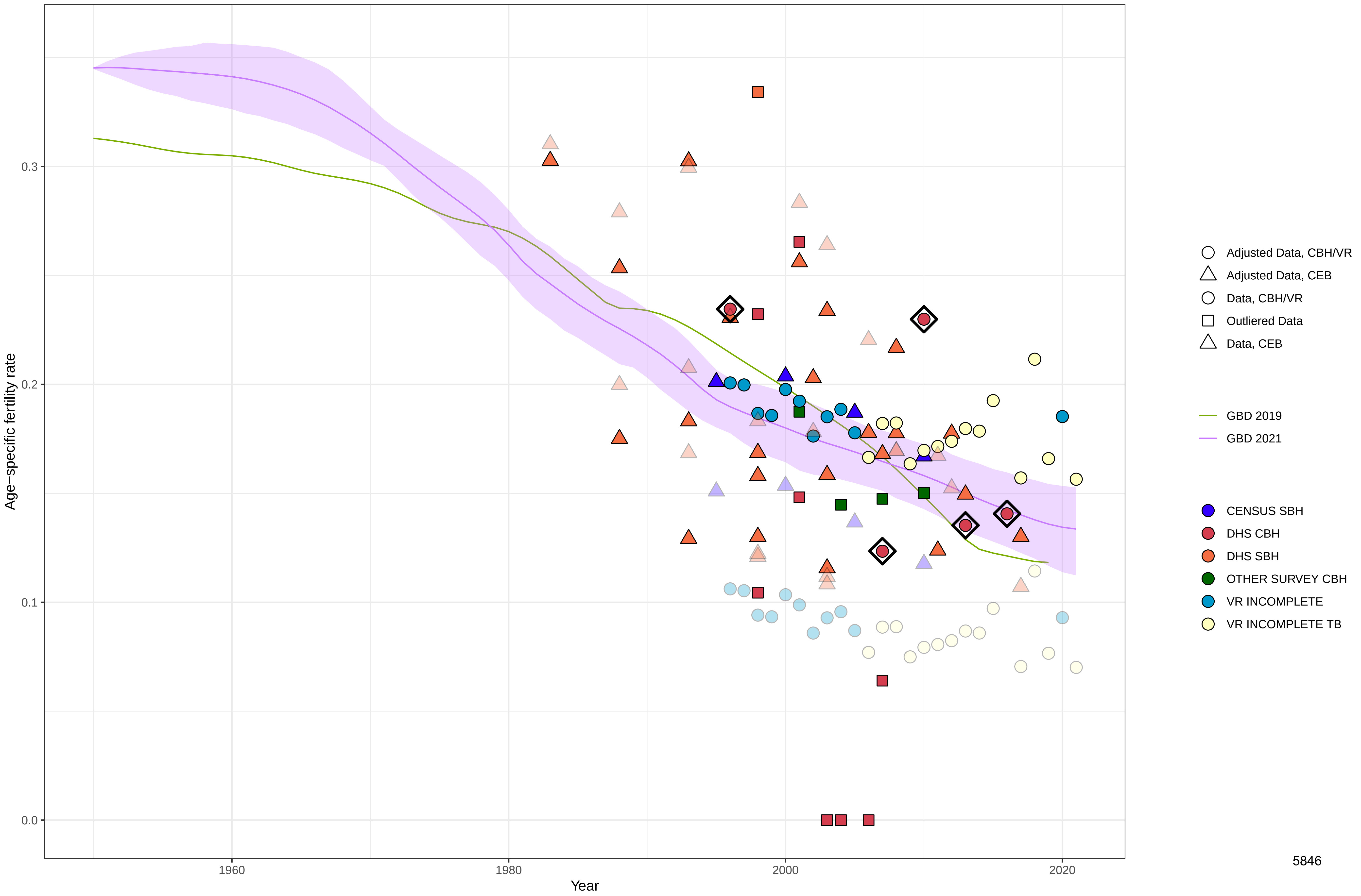

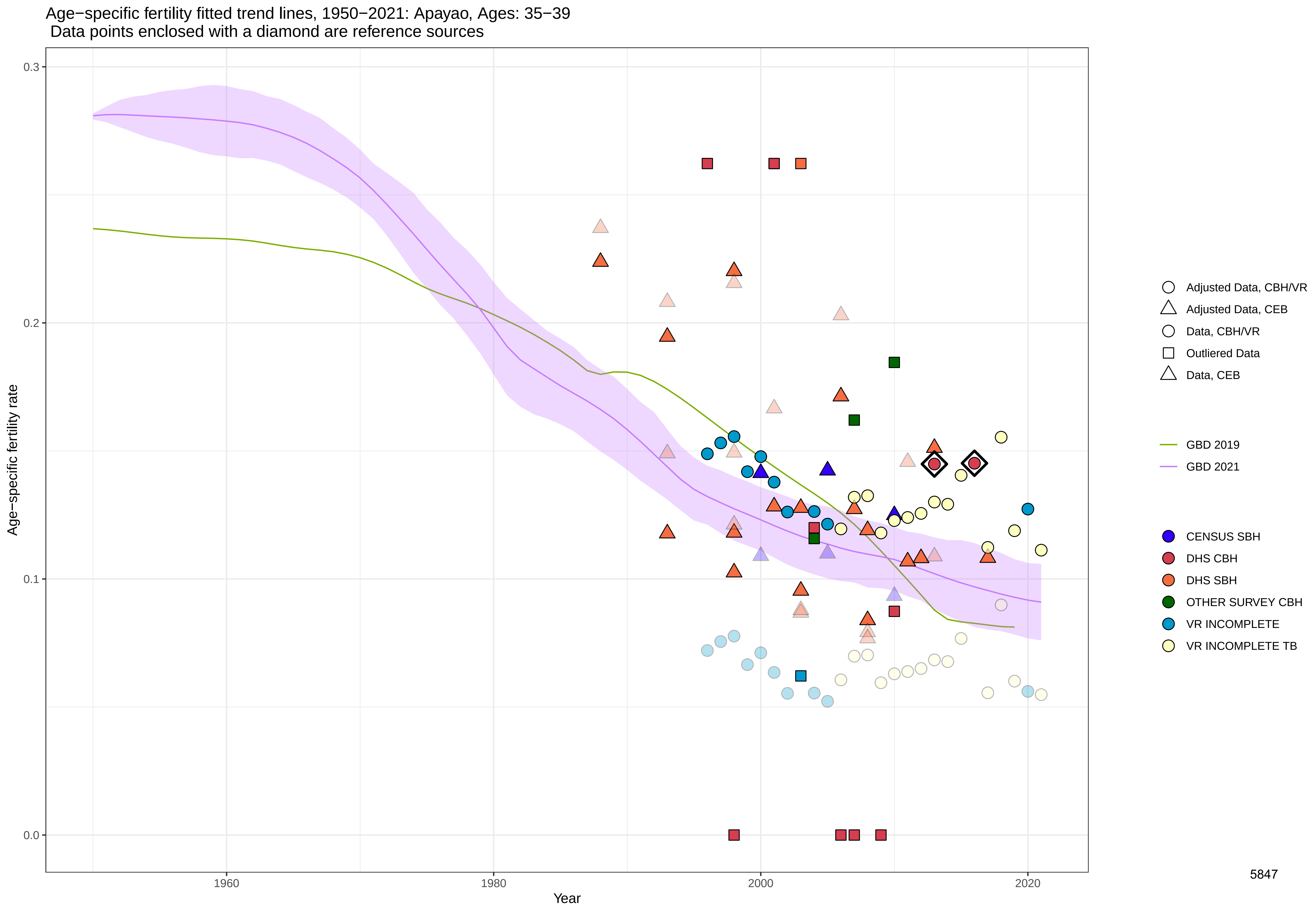

Age−specific fertility fitted trend lines, 1950−2021: Apayao, Ages: 35−39
Data points enclosed with a diamond are reference sources
Age−specific fertility rate
Year
0.0
0.1
0.2
0.3
1960
1980
2000
2020
Adjusted Data, CBH/VR
Adjusted Data, CEB
Data, CBH/VR
Outliered Data
Data, CEB
GBD 2019
GBD 2021
CENSUS SBH
DHS CBH
DHS SBH
OTHER SURVEY CBH
VR INCOMPLETE
VR INCOMPLETE TB

5847

Age−specific fertility fitted trend lines, 1950−2021: Apayao, Ages: 40−44
Data points enclosed with a diamond are reference sources

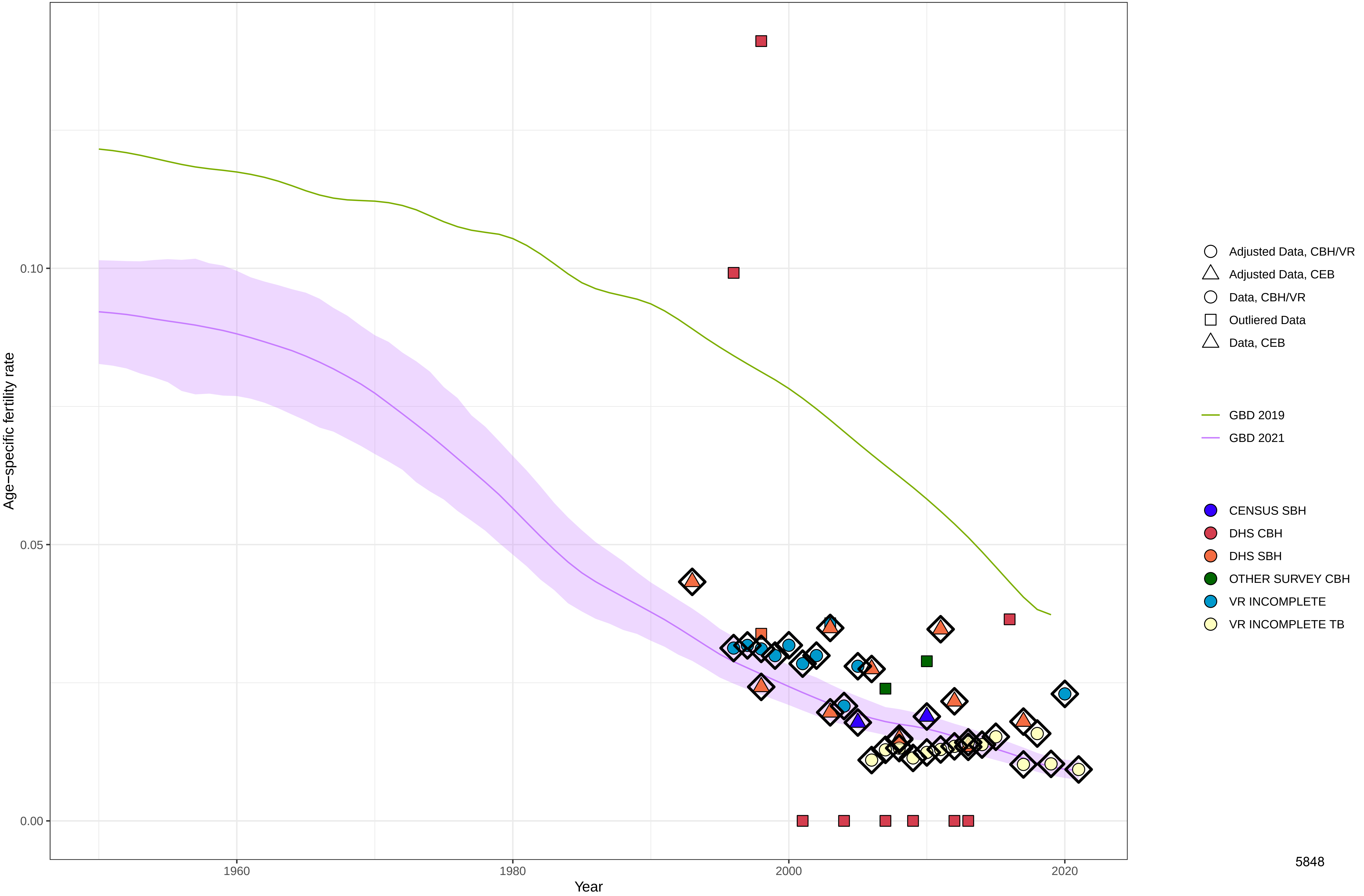

Age−specific fertility fitted trend lines, 1950−2021: Apayao, Ages: 45−49
Data points enclosed with a diamond are reference sources

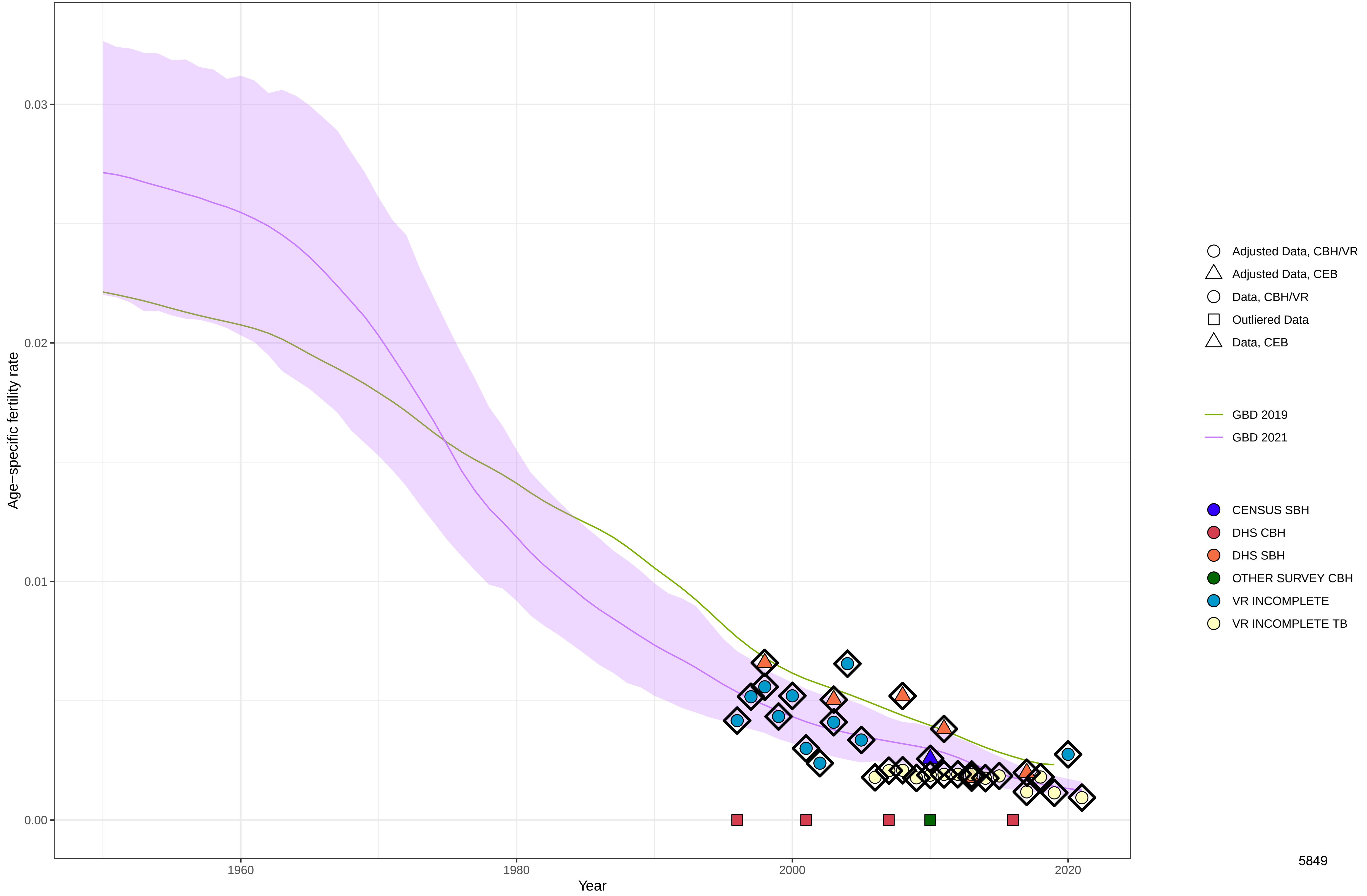

Age−specific fertility fitted trend lines, 1950−2021: Apayao, Ages: 50−54
Data points enclosed with a diamond are reference sources

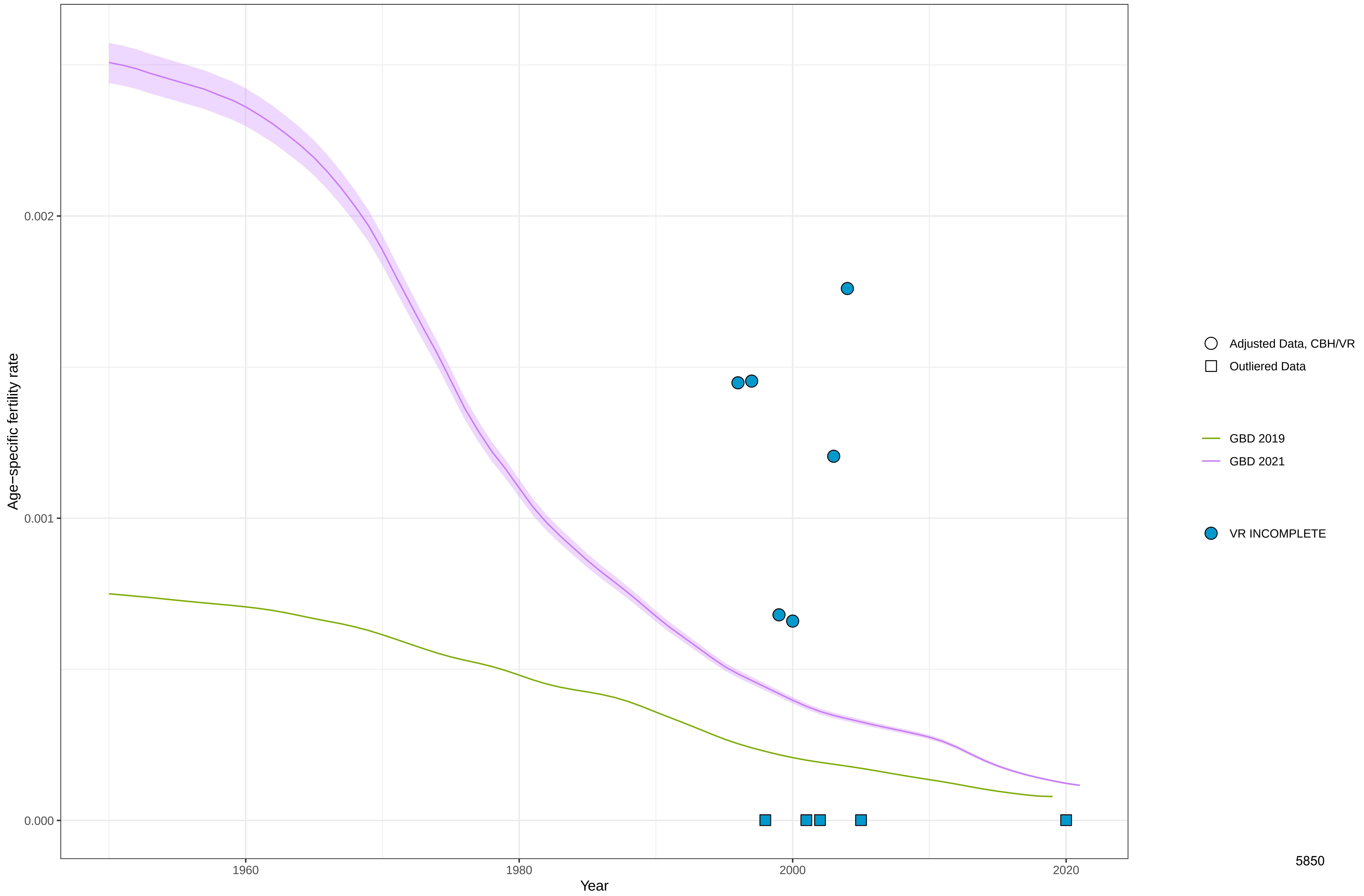

Total fertility rate fitted trend lines, 1950−2021: Apayao TFR
Data points enclosed with a diamond are reference sources

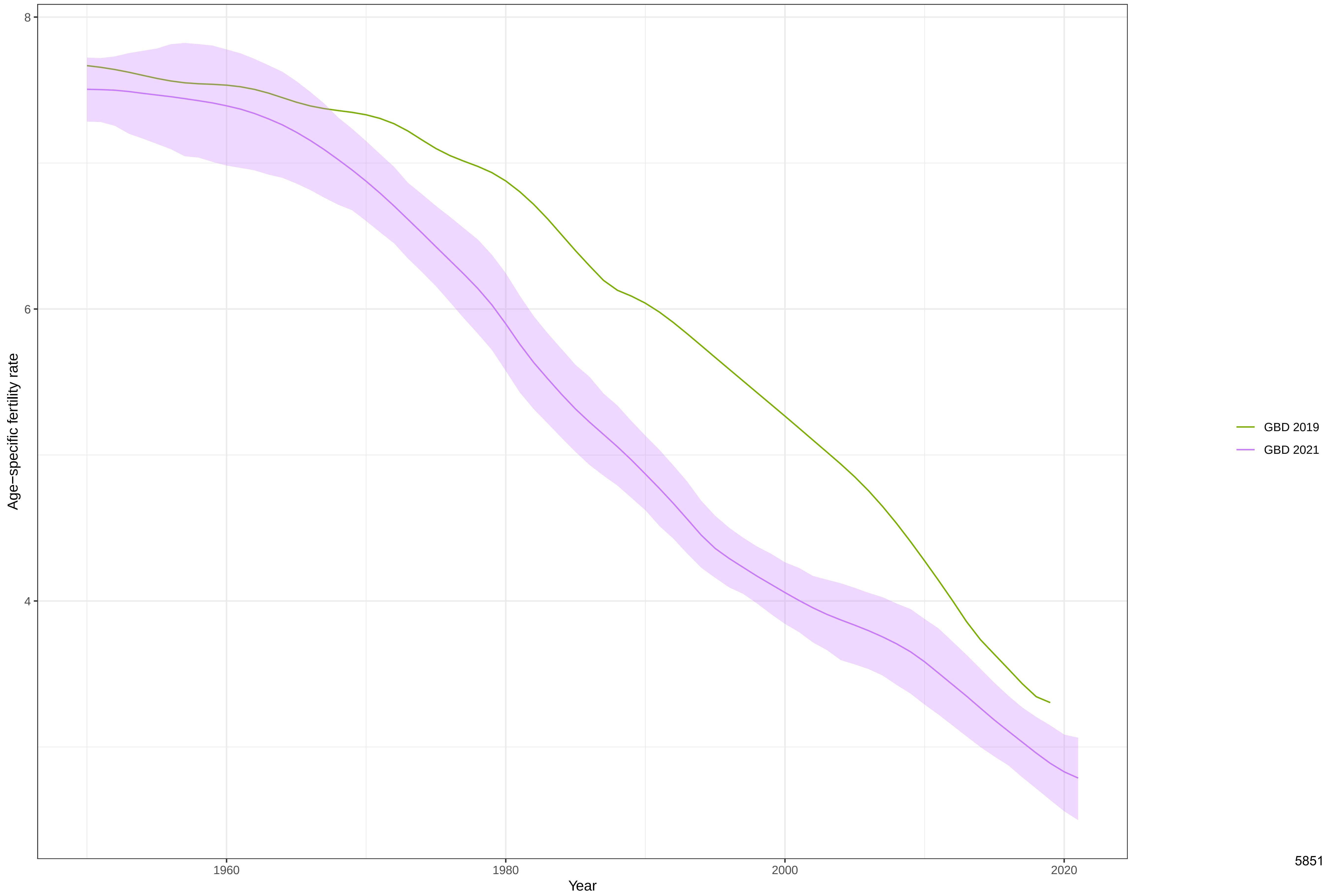

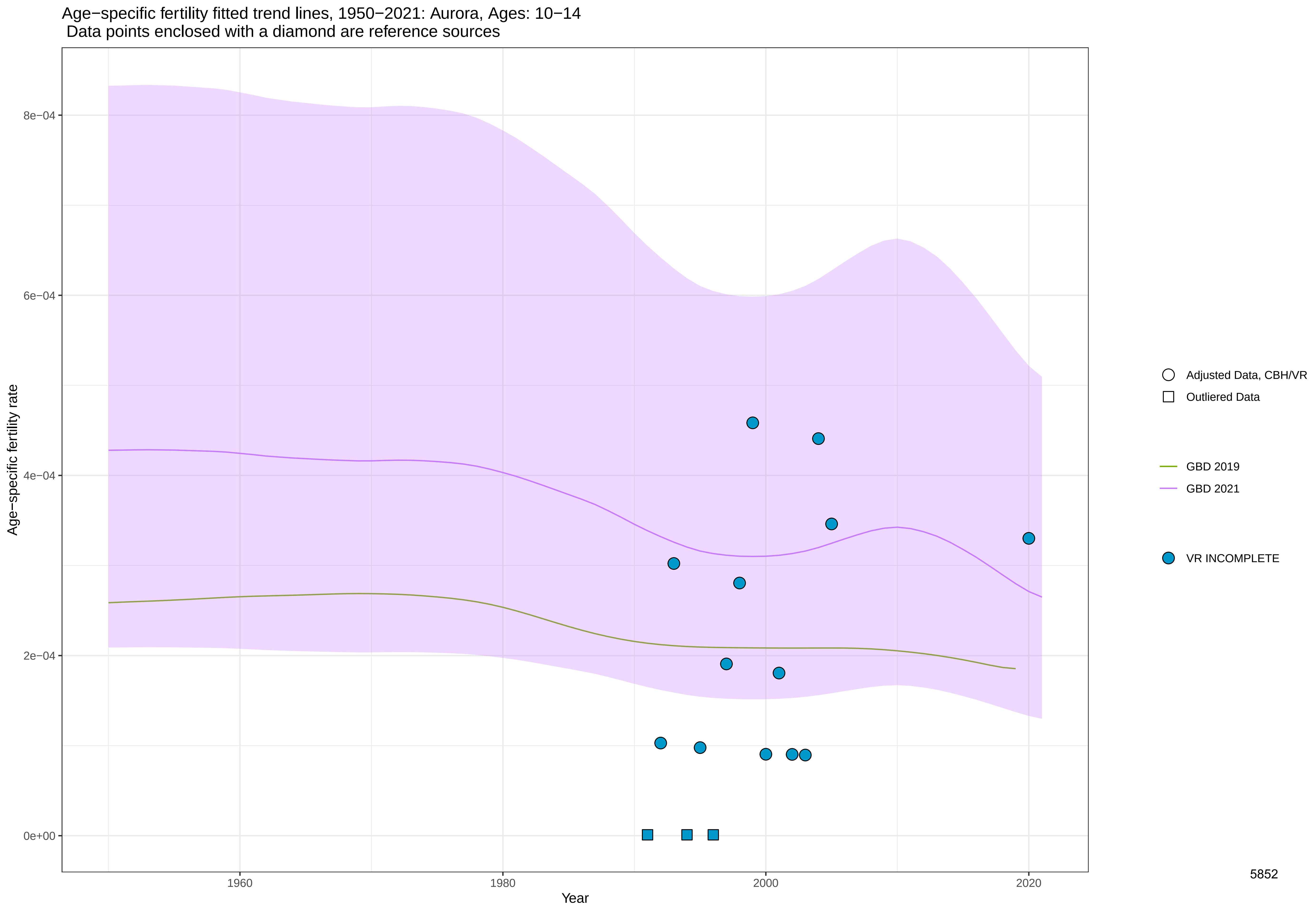
Age−specific fertility fitted trend lines, 1950−2021: Aurora, Ages: 10−14
Data points enclosed with a diamond are reference sources
Age−specific fertility rate
8e−04
6e−04
4e−04
2e−04
0e+00
1960
1980
2000
2020
Year
Adjusted Data, CBH/VR
Outliered Data
GBD 2019
GBD 2021
VR INCOMPLETE

Age−specific fertility fitted trend lines, 1950−2021: Aurora, Ages: 15−19
Data points enclosed with a diamond are reference sources

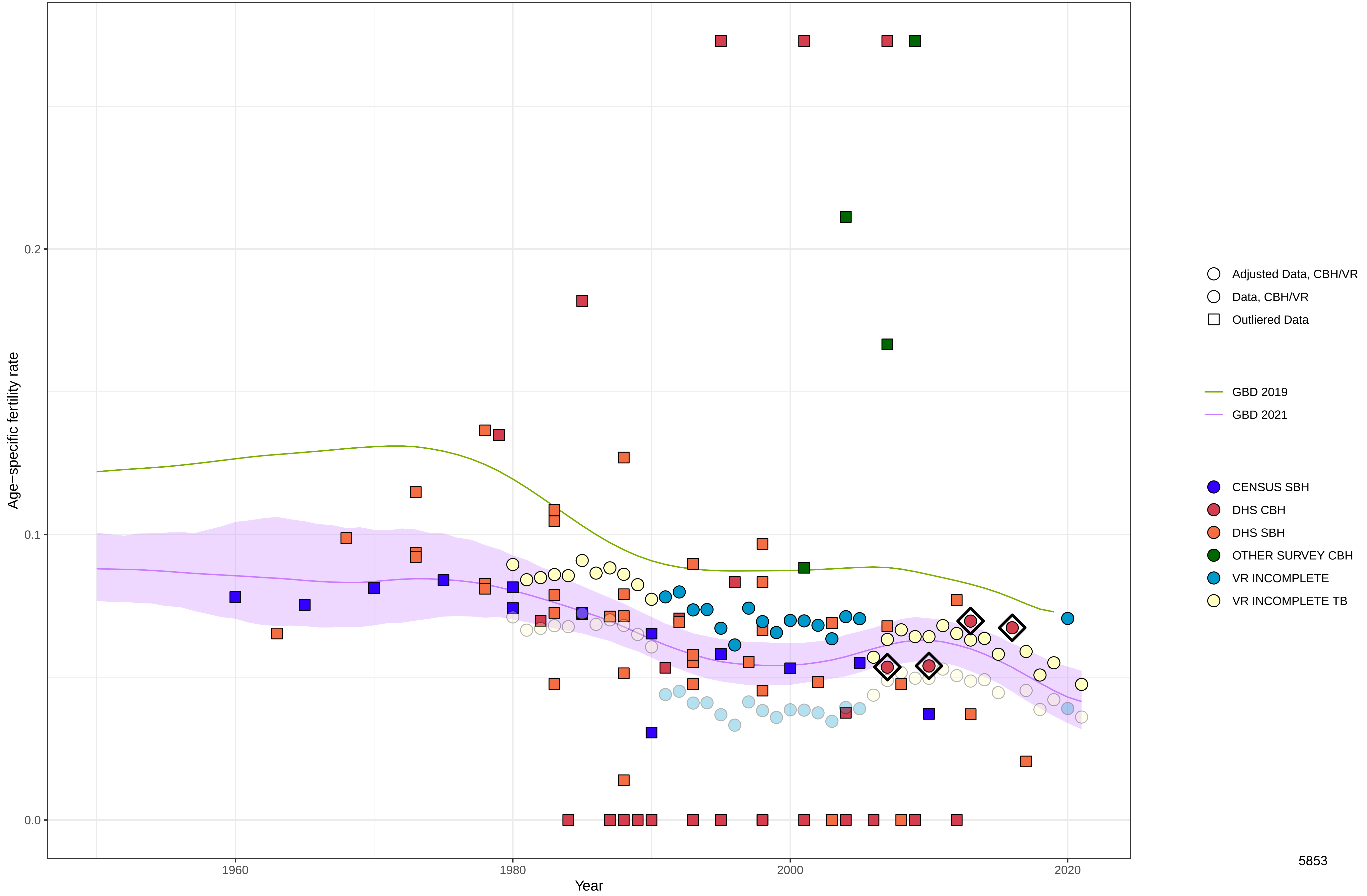

Age−specific fertility fitted trend lines, 1950−2021: Aurora, Ages: 20−24
Data points enclosed with a diamond are reference sources

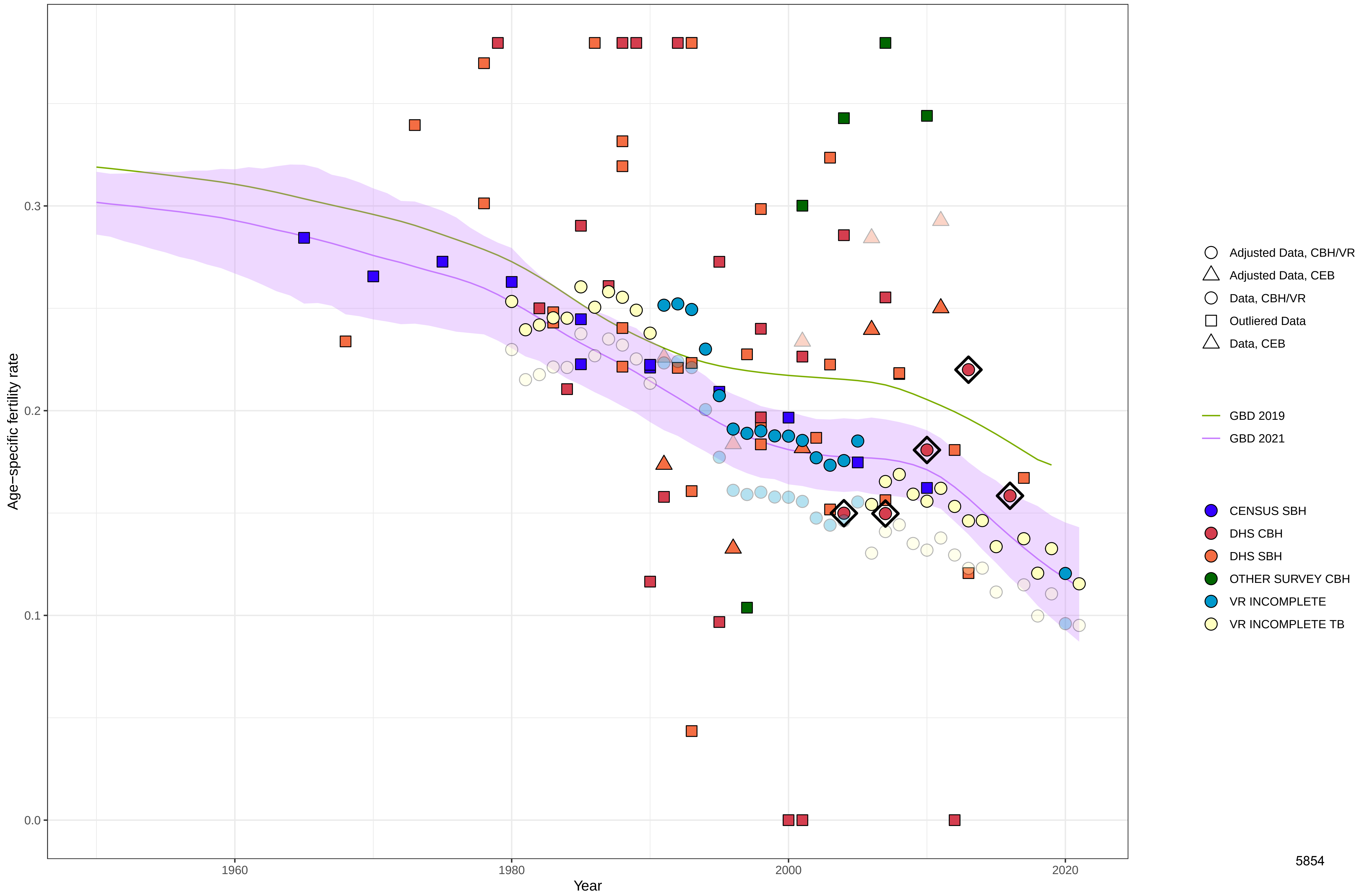

Age−specific fertility fitted trend lines, 1950−2021: Aurora, Ages: 25−29
Data points enclosed with a diamond are reference sources

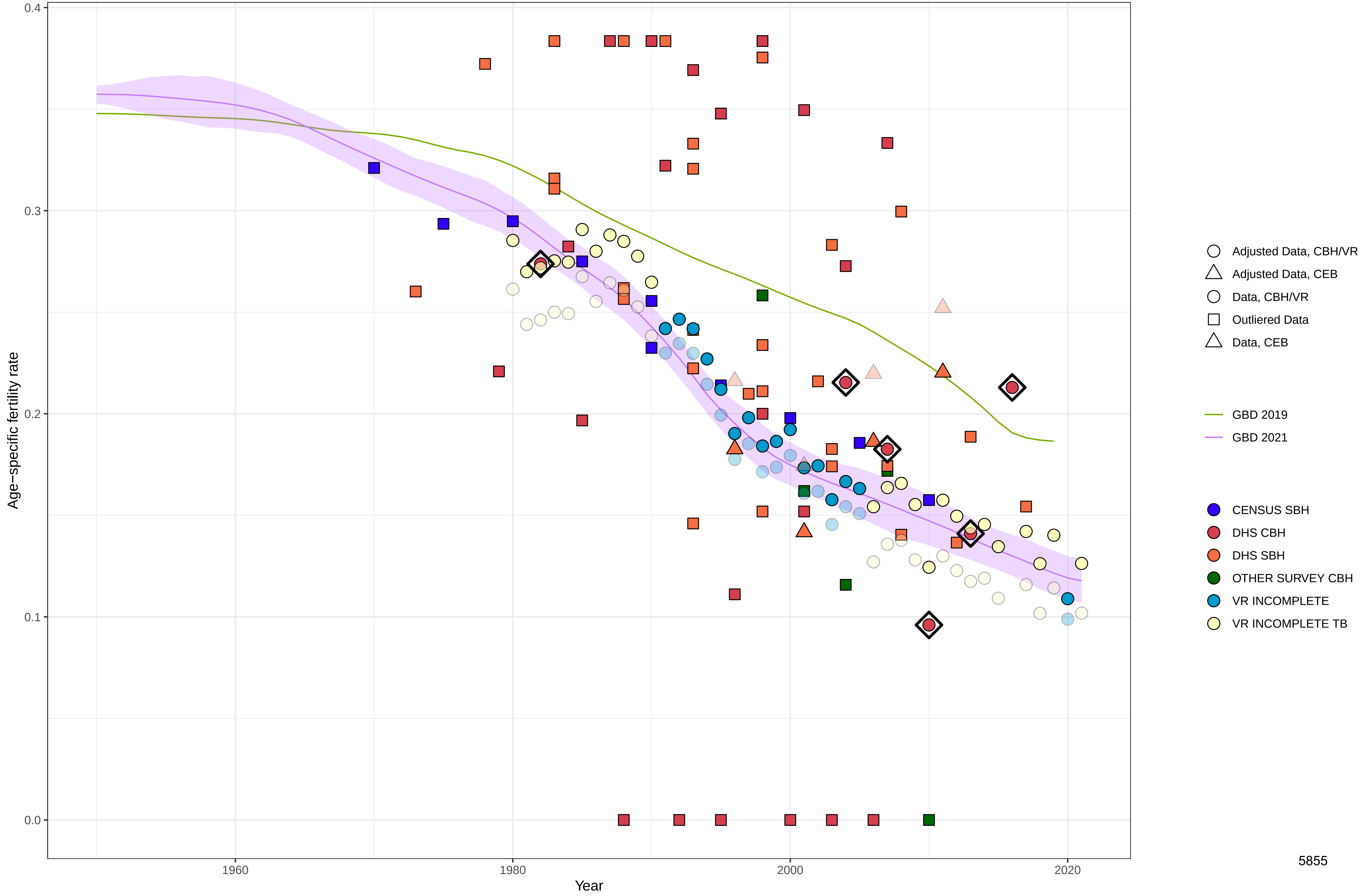

Age−specific fertility fitted trend lines, 1950−2021: Aurora, Ages: 30−34
Data points enclosed with a diamond are reference sources

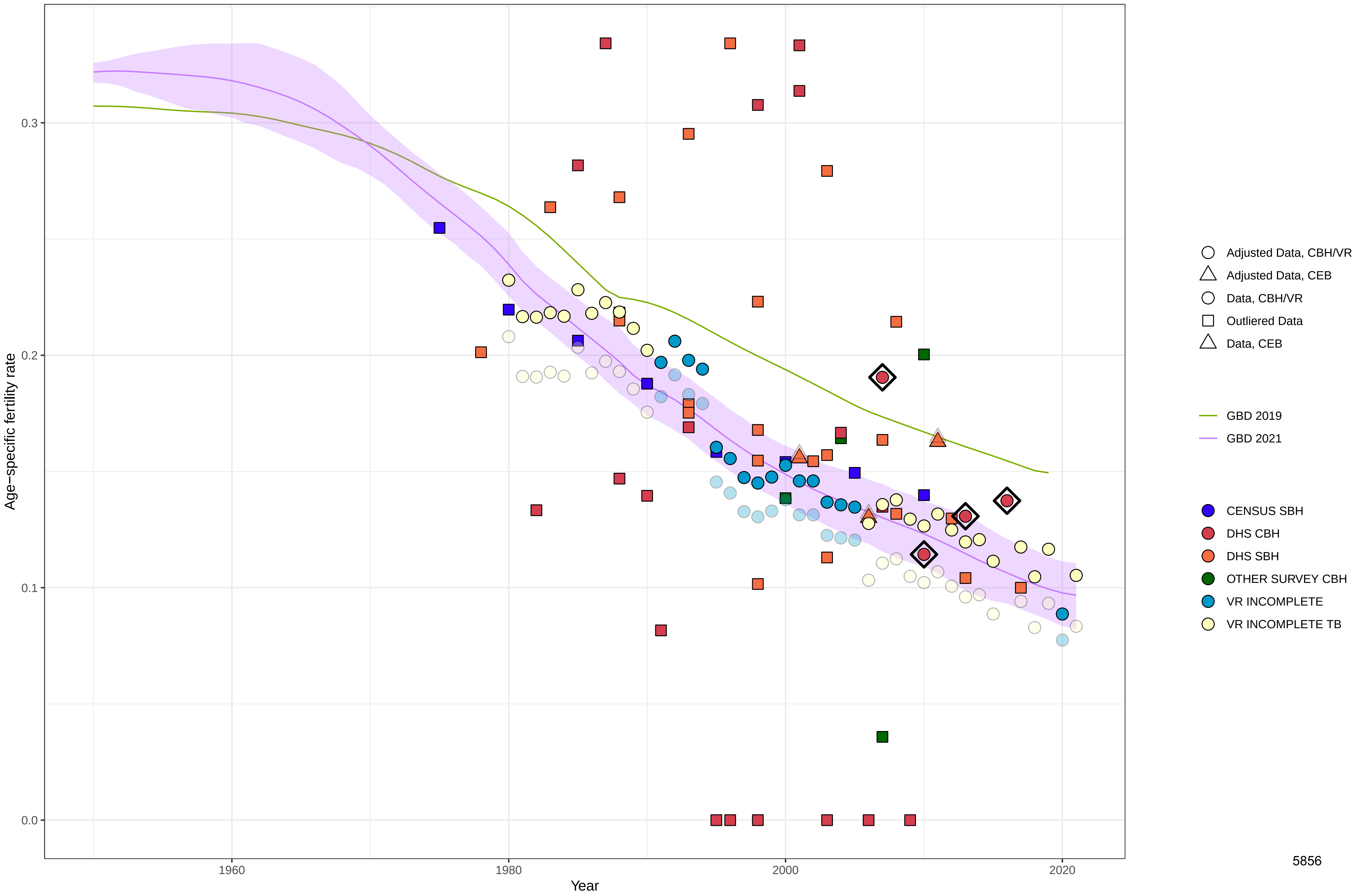

Age−specific fertility fitted trend lines, 1950−2021: Aurora, Ages: 35−39
Data points enclosed with a diamond are reference sources

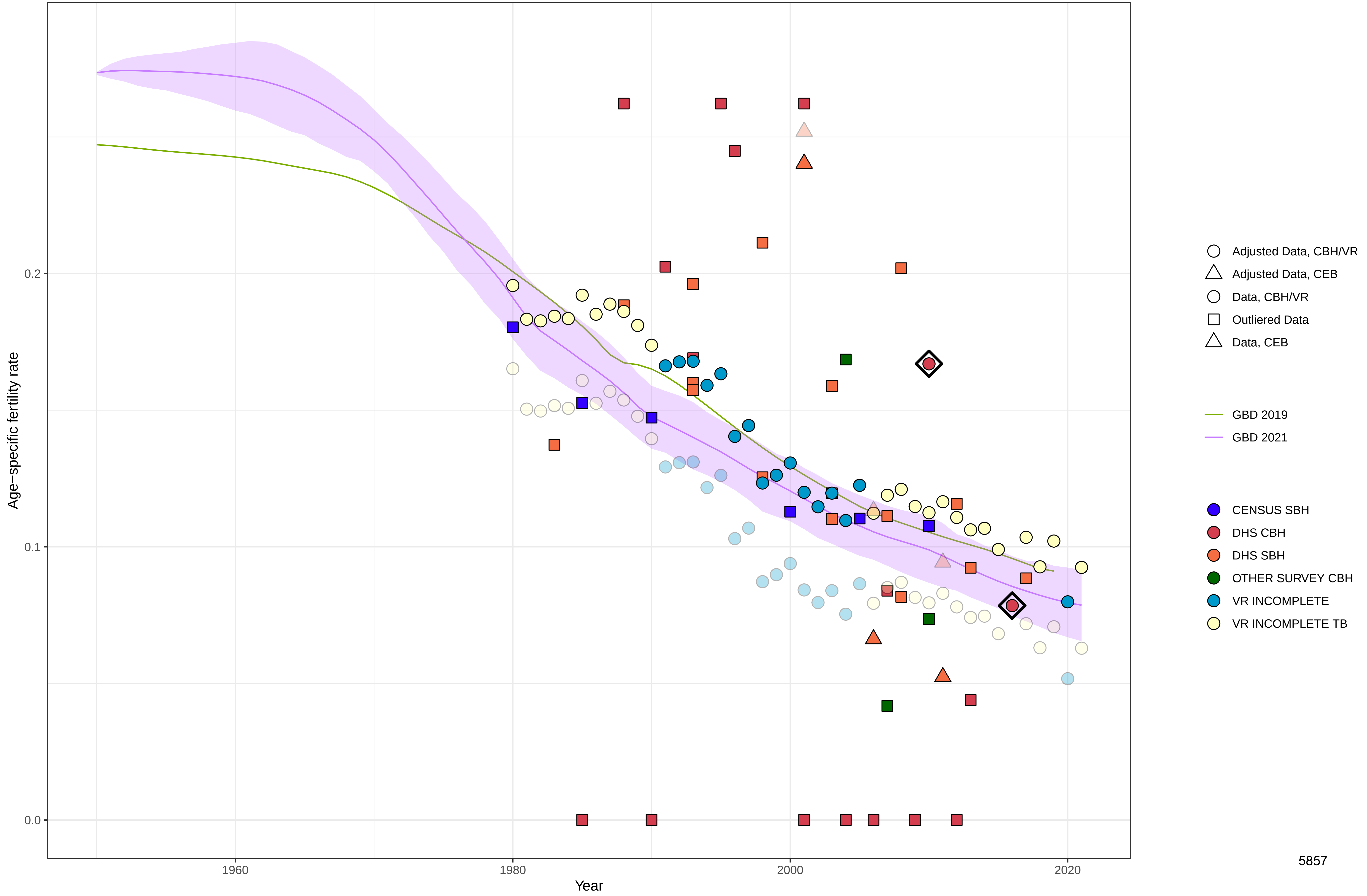

Age−specific fertility fitted trend lines, 1950−2021: Aurora, Ages: 40−44
Data points enclosed with a diamond are reference sources

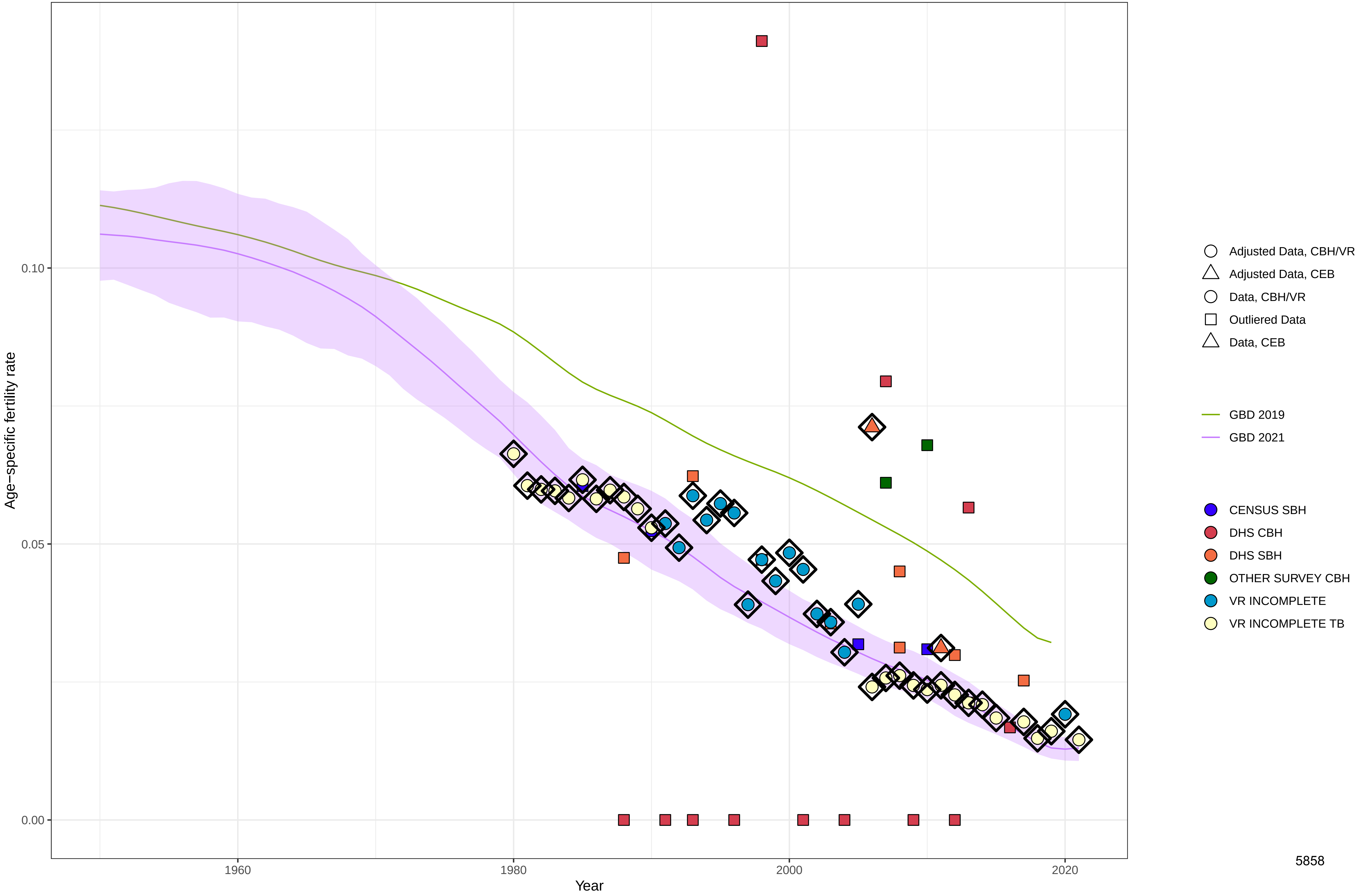

Age−specific fertility fitted trend lines, 1950−2021: Aurora, Ages: 45−49
Data points enclosed with a diamond are reference sources

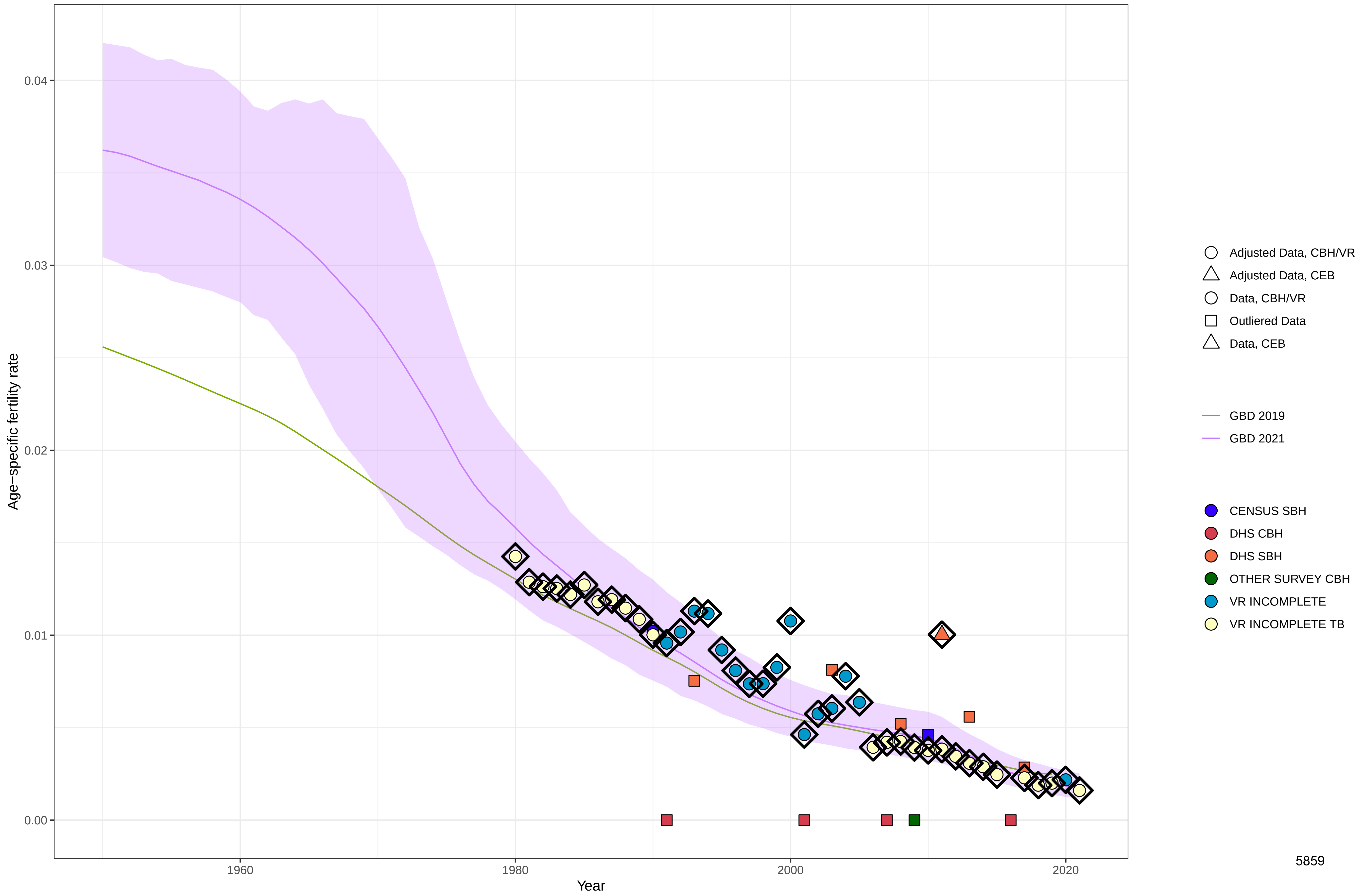

Age−specific fertility fitted trend lines, 1950−2021: Aurora, Ages: 50−54
Data points enclosed with a diamond are reference sources

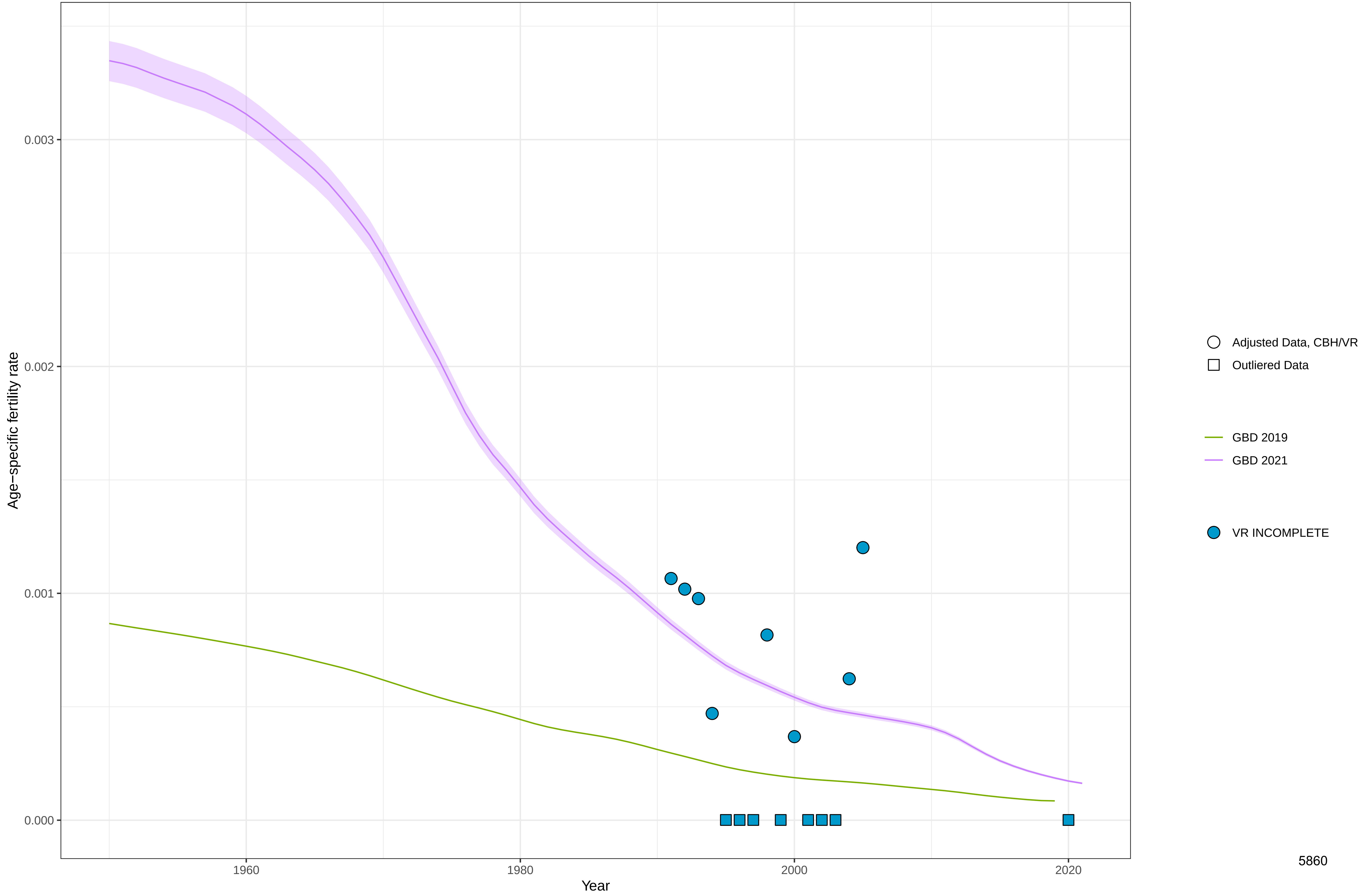

Total fertility rate fitted trend lines, 1950−2021: Aurora TFR
Data points enclosed with a diamond are reference sources

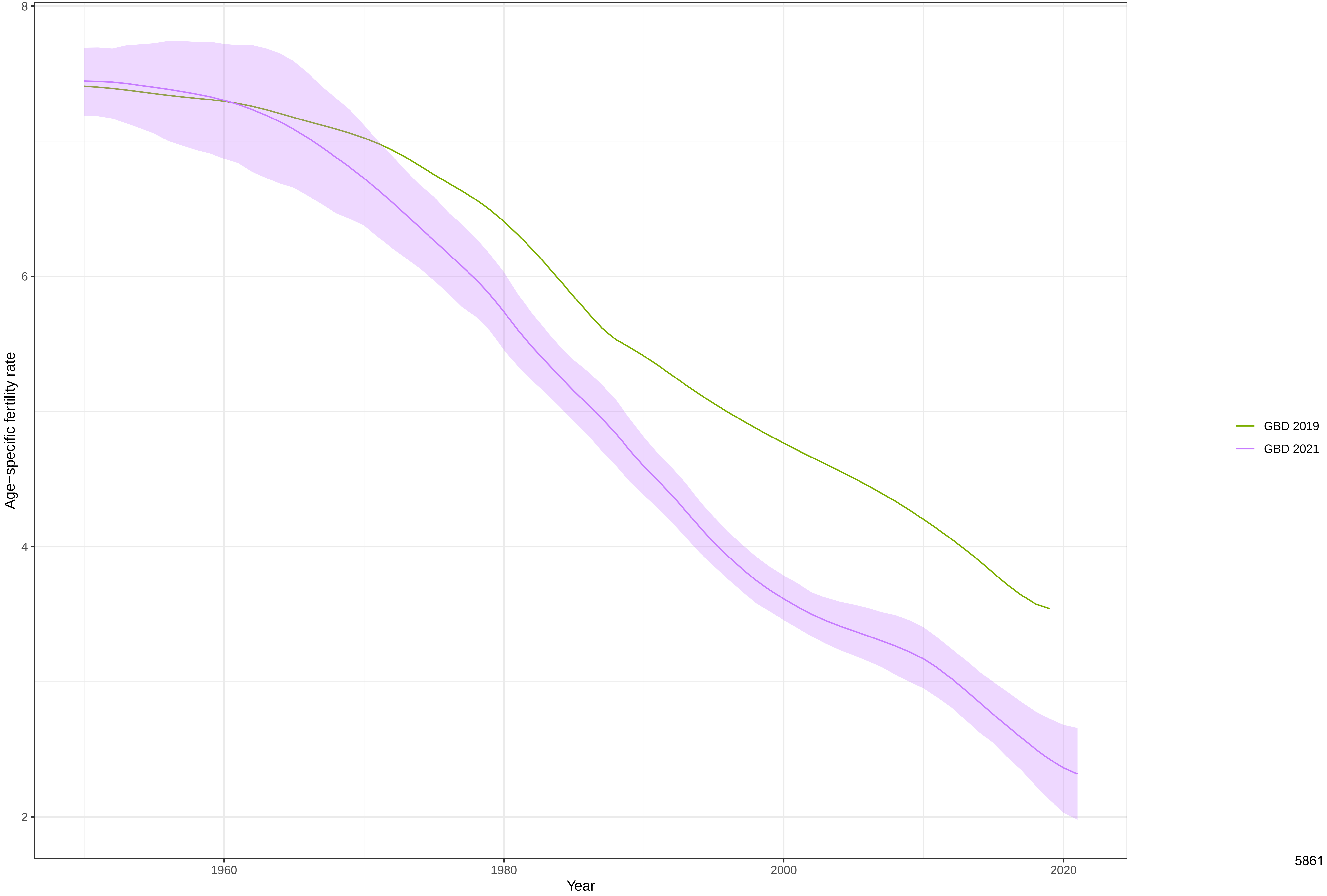

Age−specific fertility fitted trend lines, 1950−2021: Basilan, Ages: 10−14
Data points enclosed with a diamond are reference sources

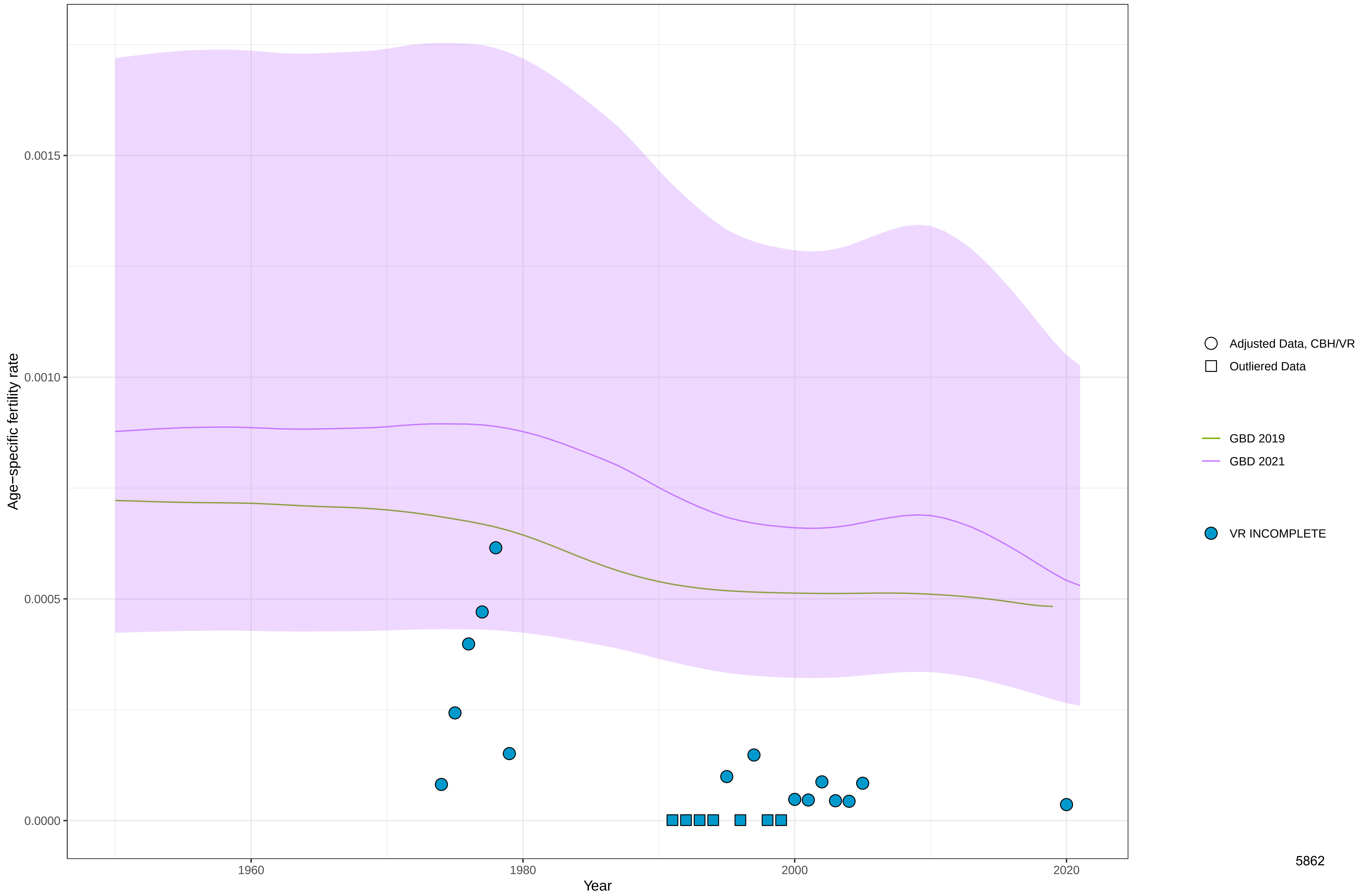

Age−specific fertility fitted trend lines, 1950−2021: Basilan, Ages: 15−19
Data points enclosed with a diamond are reference sources

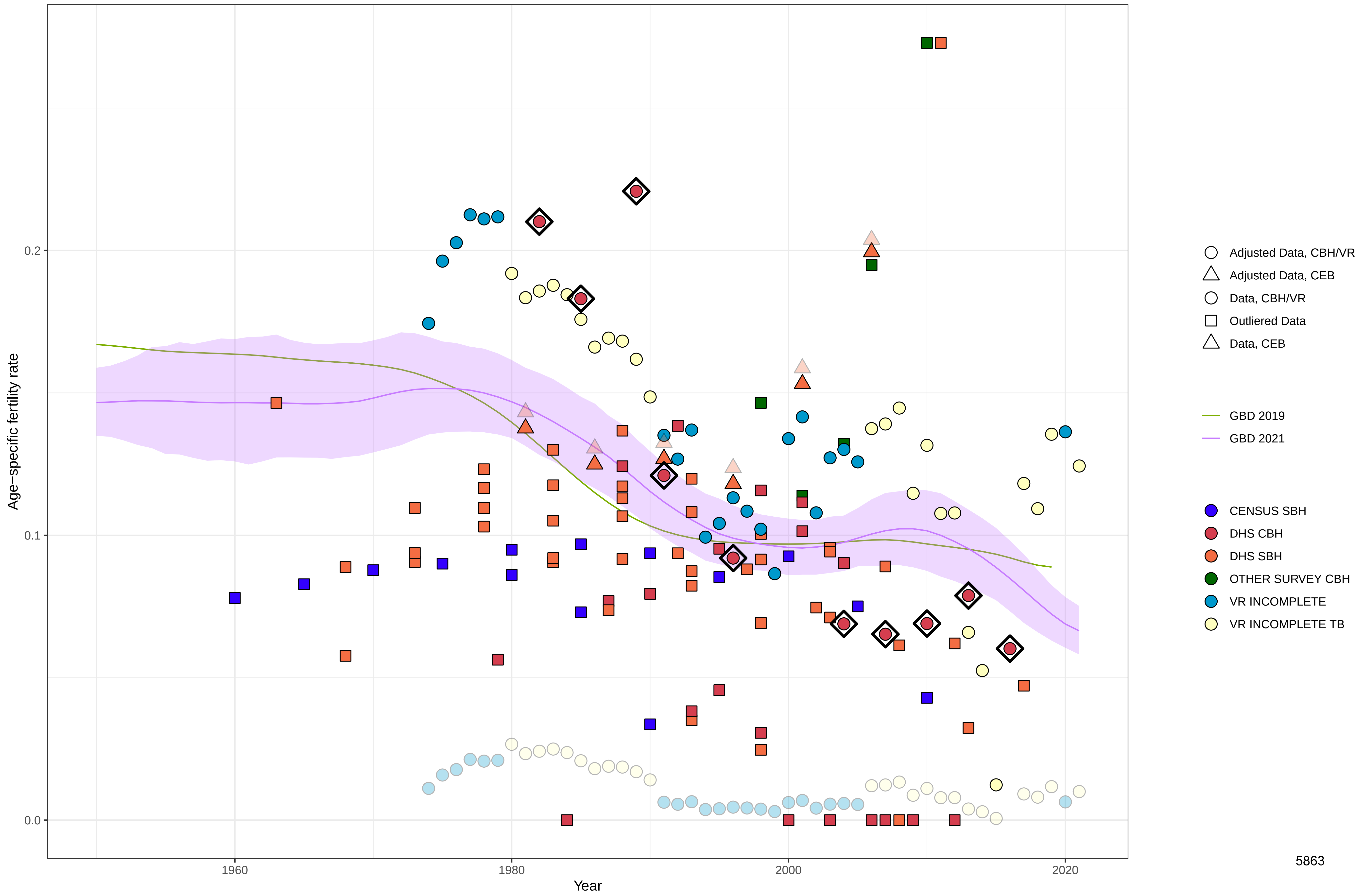

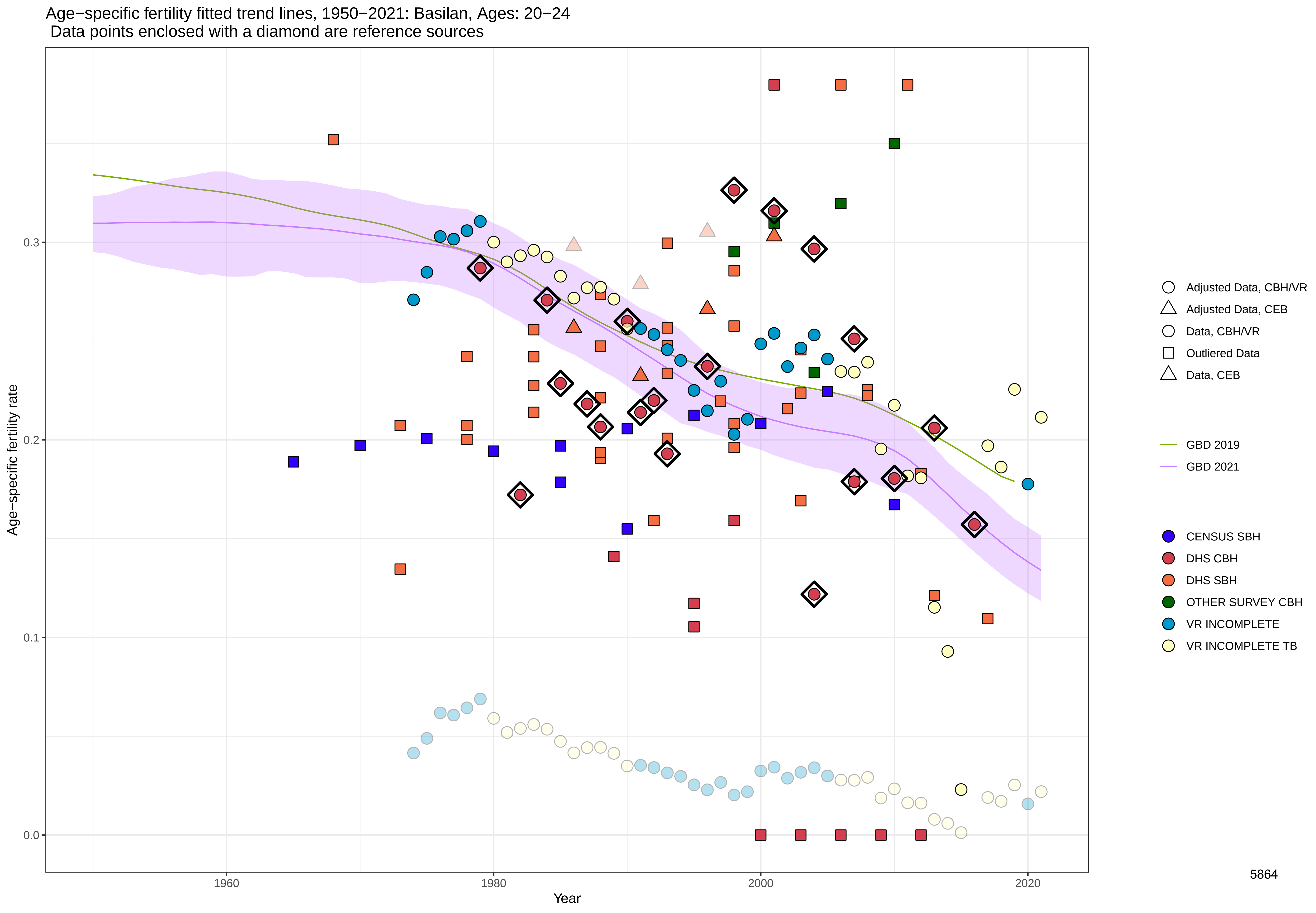

Age−specific fertility fitted trend lines, 1950−2021: Basilan, Ages: 20−24
Data points enclosed with a diamond are reference sources
Age−specific fertility rate
Year
0.0
0.1
0.2
0.3
1960
1980
2000
2020
Adjusted Data, CBH/VR
Adjusted Data, CEB
Data, CBH/VR
Outliered Data
Data, CEB
GBD 2019
GBD 2021
CENSUS SBH
DHS CBH
DHS SBH
OTHER SURVEY CBH
VR INCOMPLETE
VR INCOMPLETE TB

Age−specific fertility fitted trend lines, 1950−2021: Basilan, Ages: 25−29
Data points enclosed with a diamond are reference sources

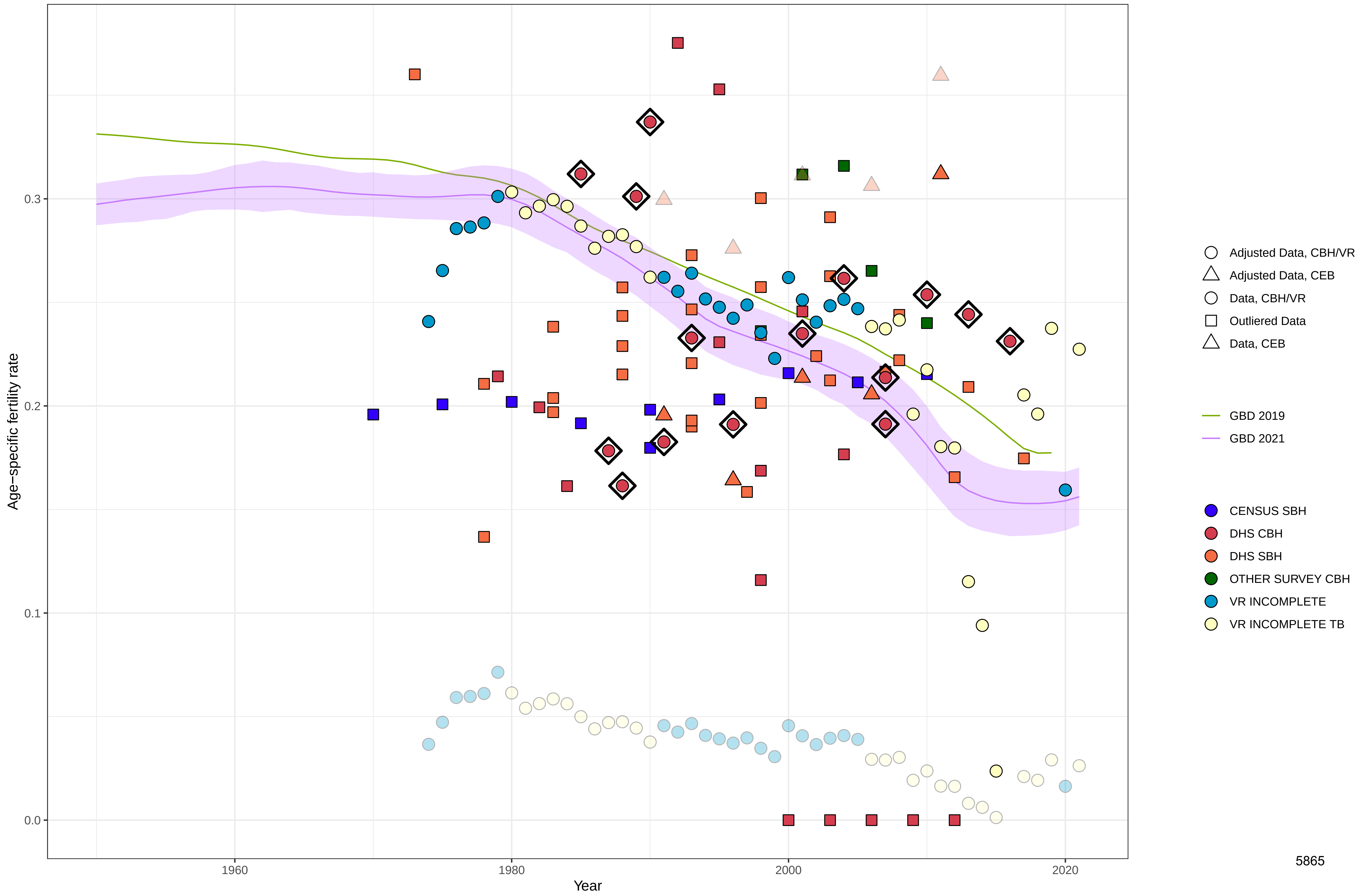

Age−specific fertility fitted trend lines, 1950−2021: Basilan, Ages: 30−34
Data points enclosed with a diamond are reference sources

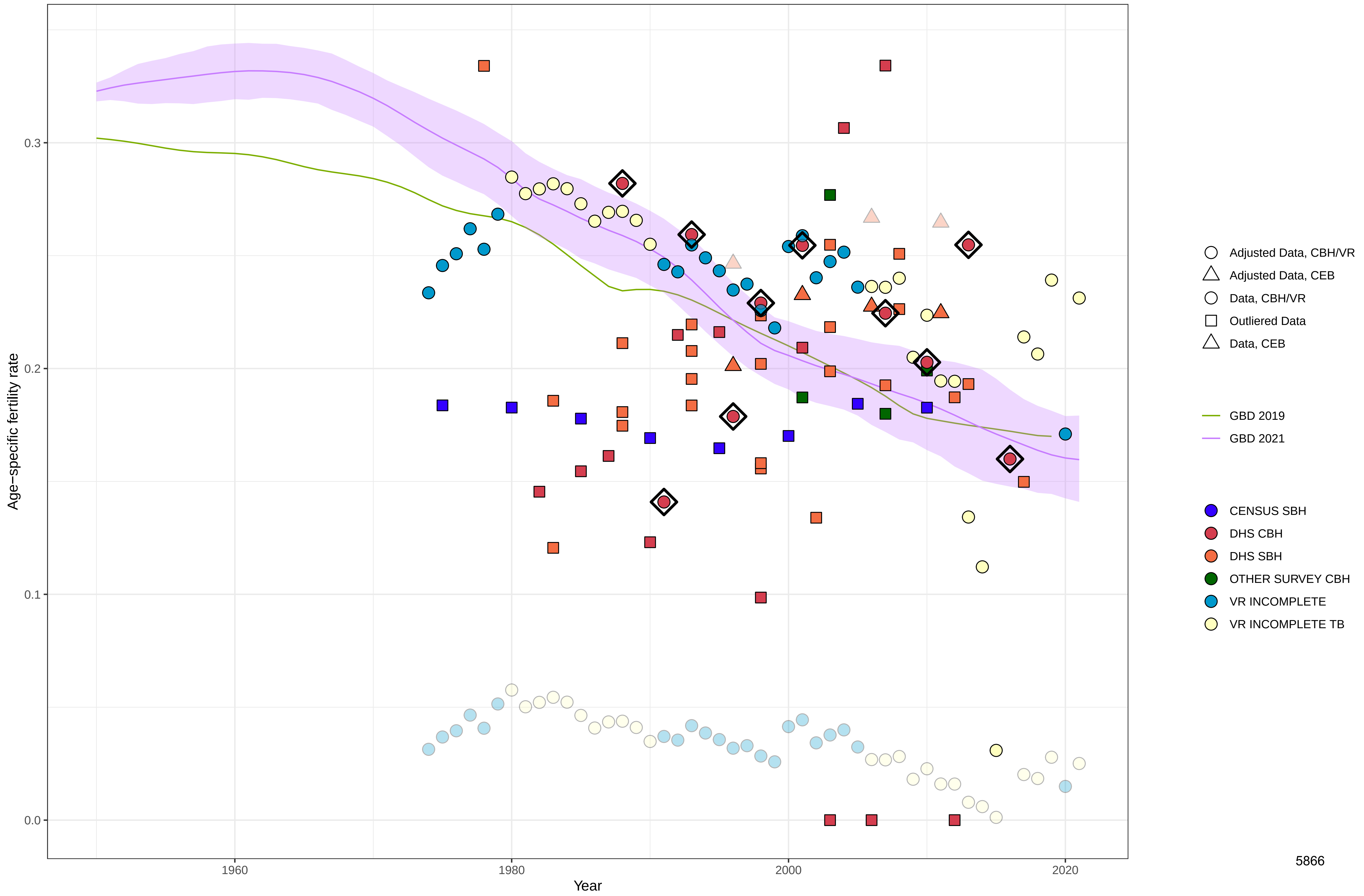

Age−specific fertility fitted trend lines, 1950−2021: Basilan, Ages: 35−39
Data points enclosed with a diamond are reference sources

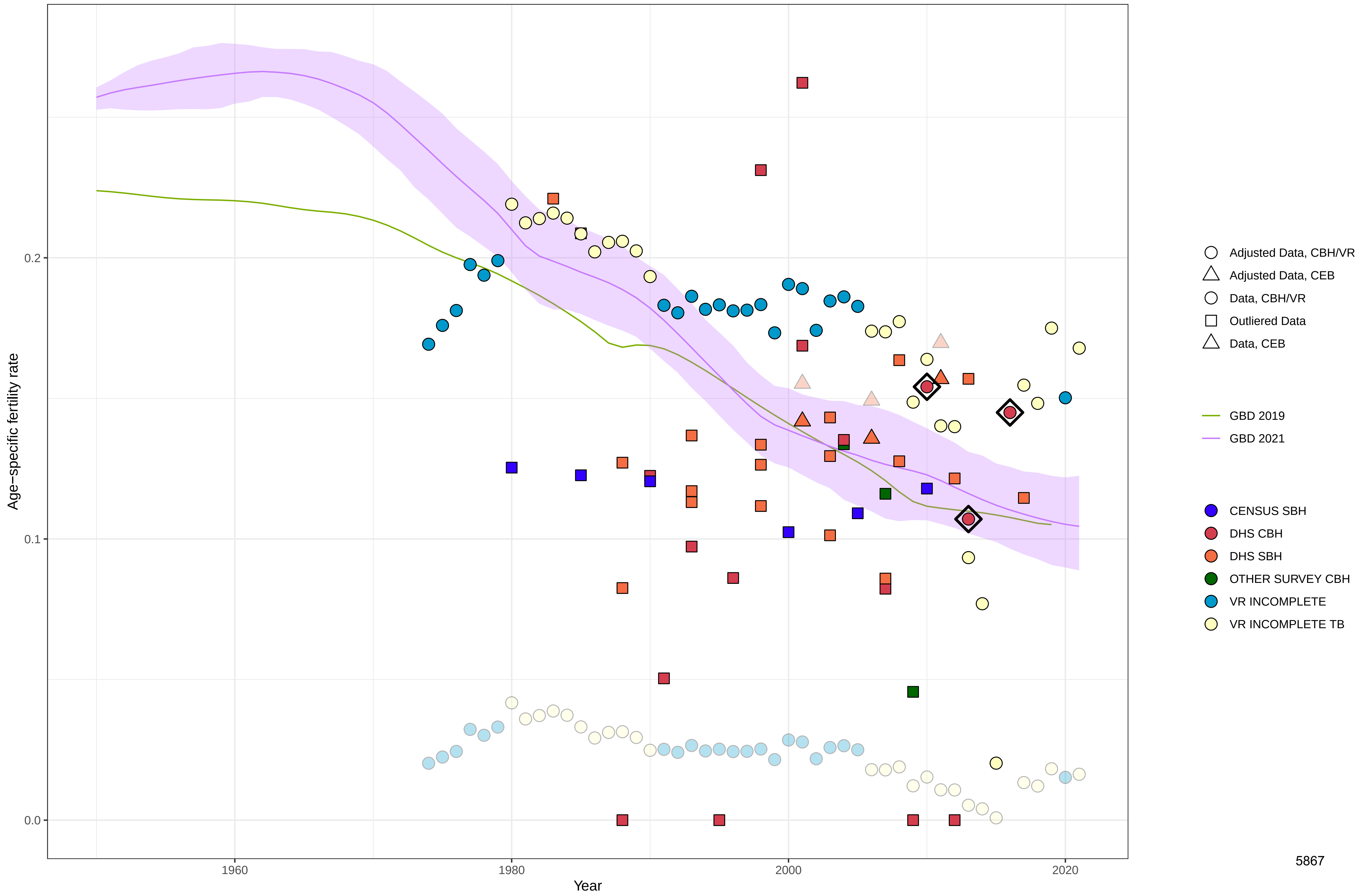

Age−specific fertility fitted trend lines, 1950−2021: Basilan, Ages: 40−44
Data points enclosed with a diamond are reference sources

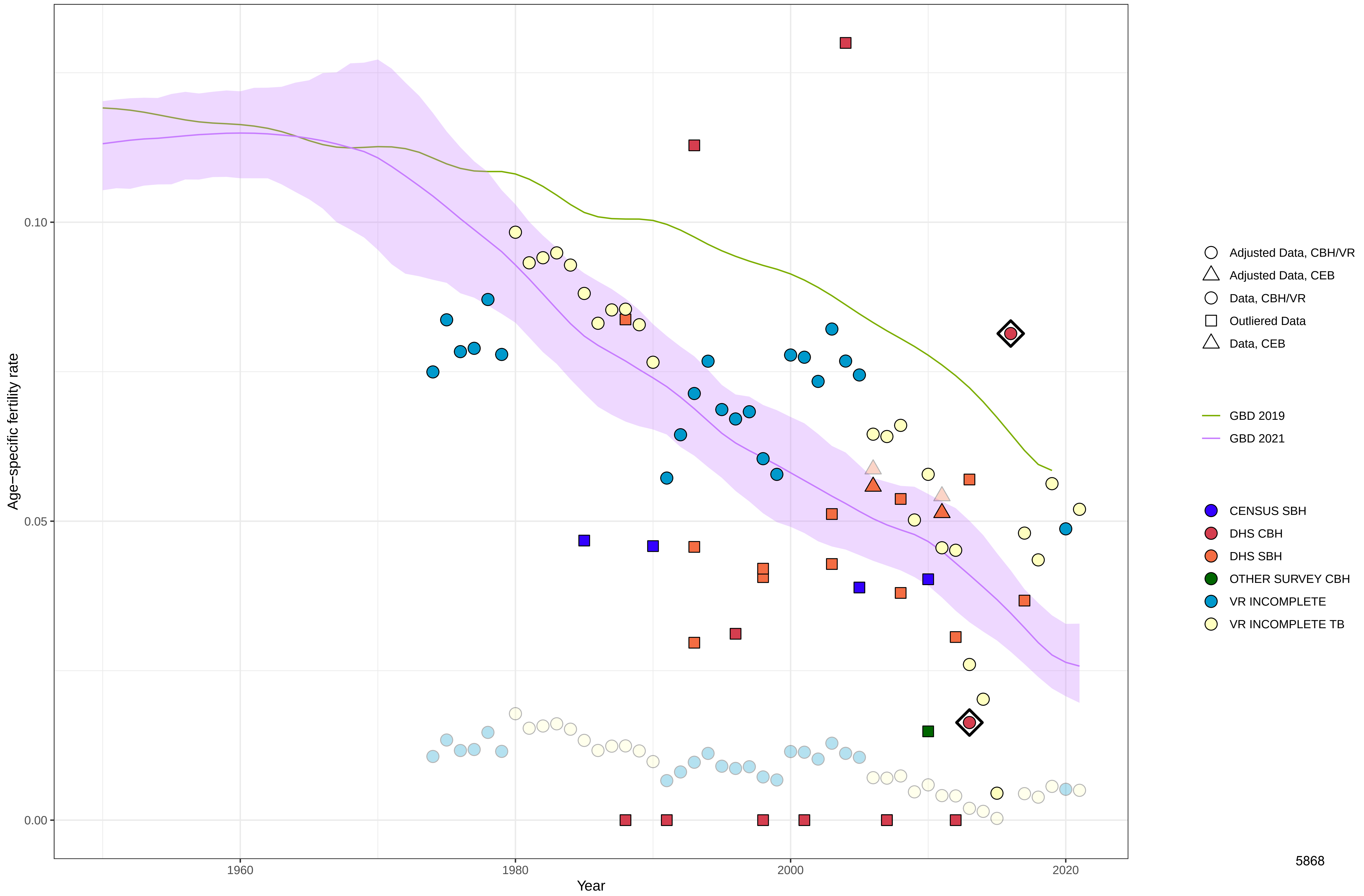

Age−specific fertility fitted trend lines, 1950−2021: Basilan, Ages: 45−49
Data points enclosed with a diamond are reference sources

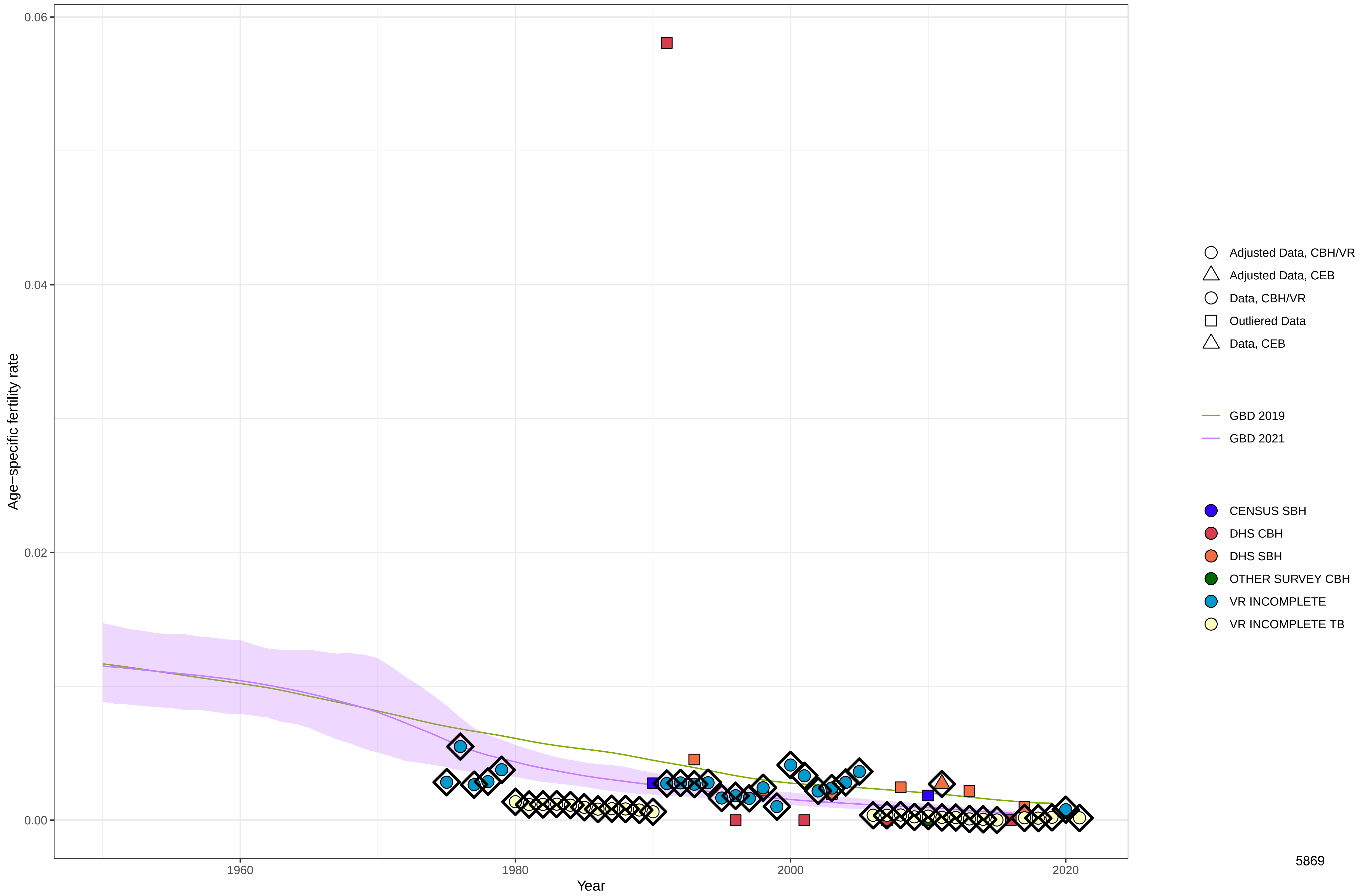

Age−specific fertility fitted trend lines, 1950−2021: Basilan, Ages: 50−54
Data points enclosed with a diamond are reference sources

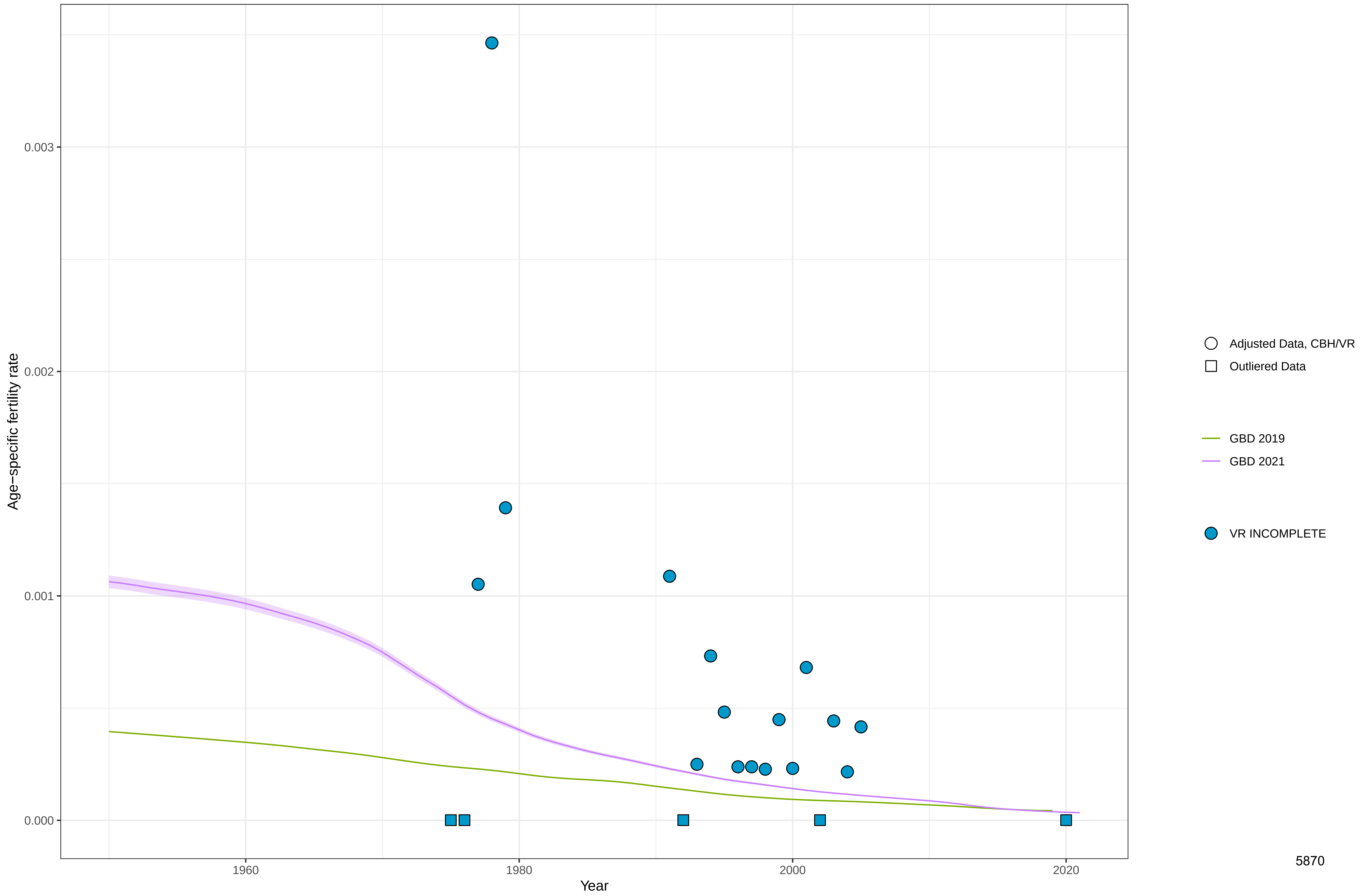

Total fertility rate fitted trend lines, 1950−2021: Basilan TFR
Data points enclosed with a diamond are reference sources

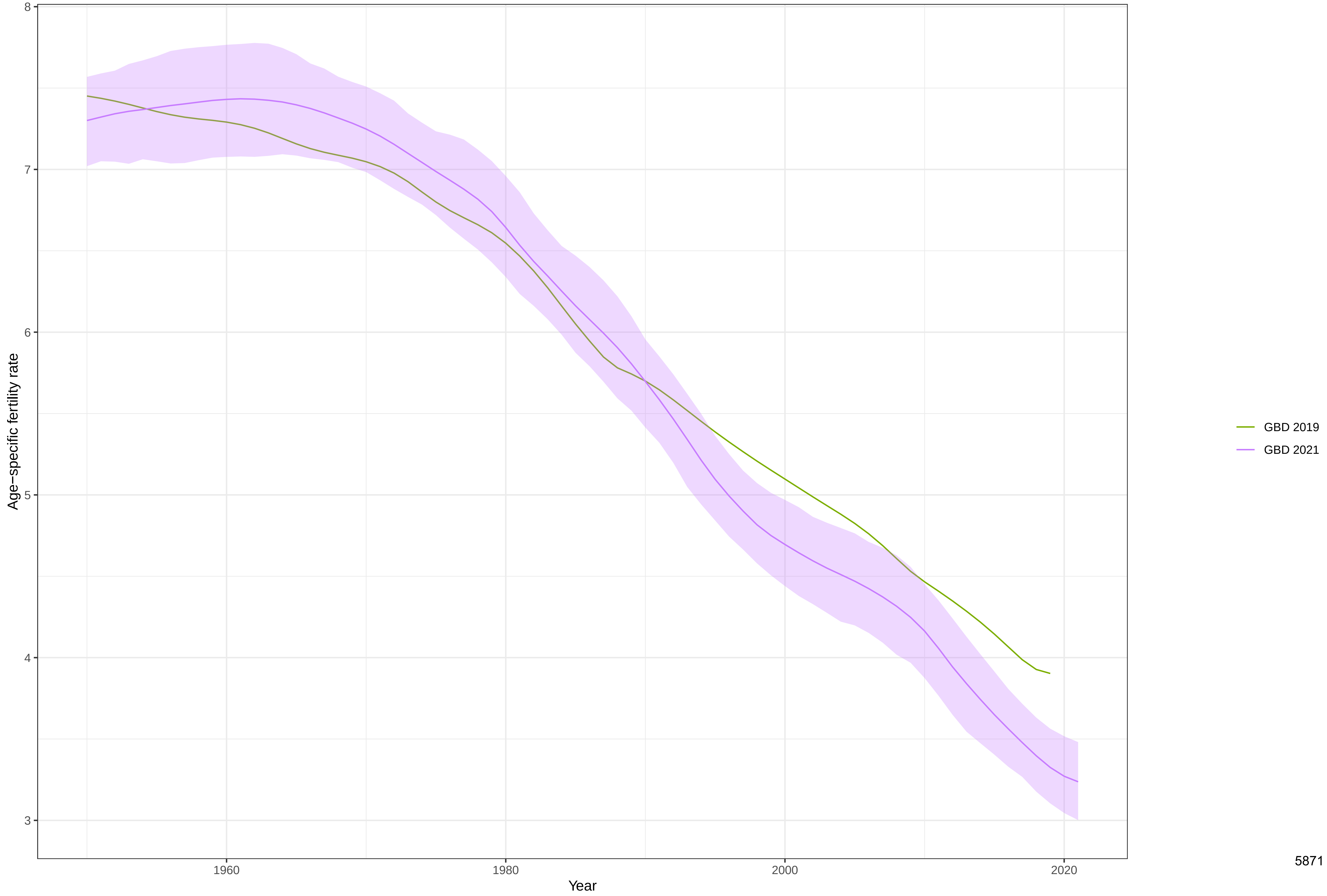

Age−specific fertility fitted trend lines, 1950−2021: Bataan, Ages: 10−14
Data points enclosed with a diamond are reference sources

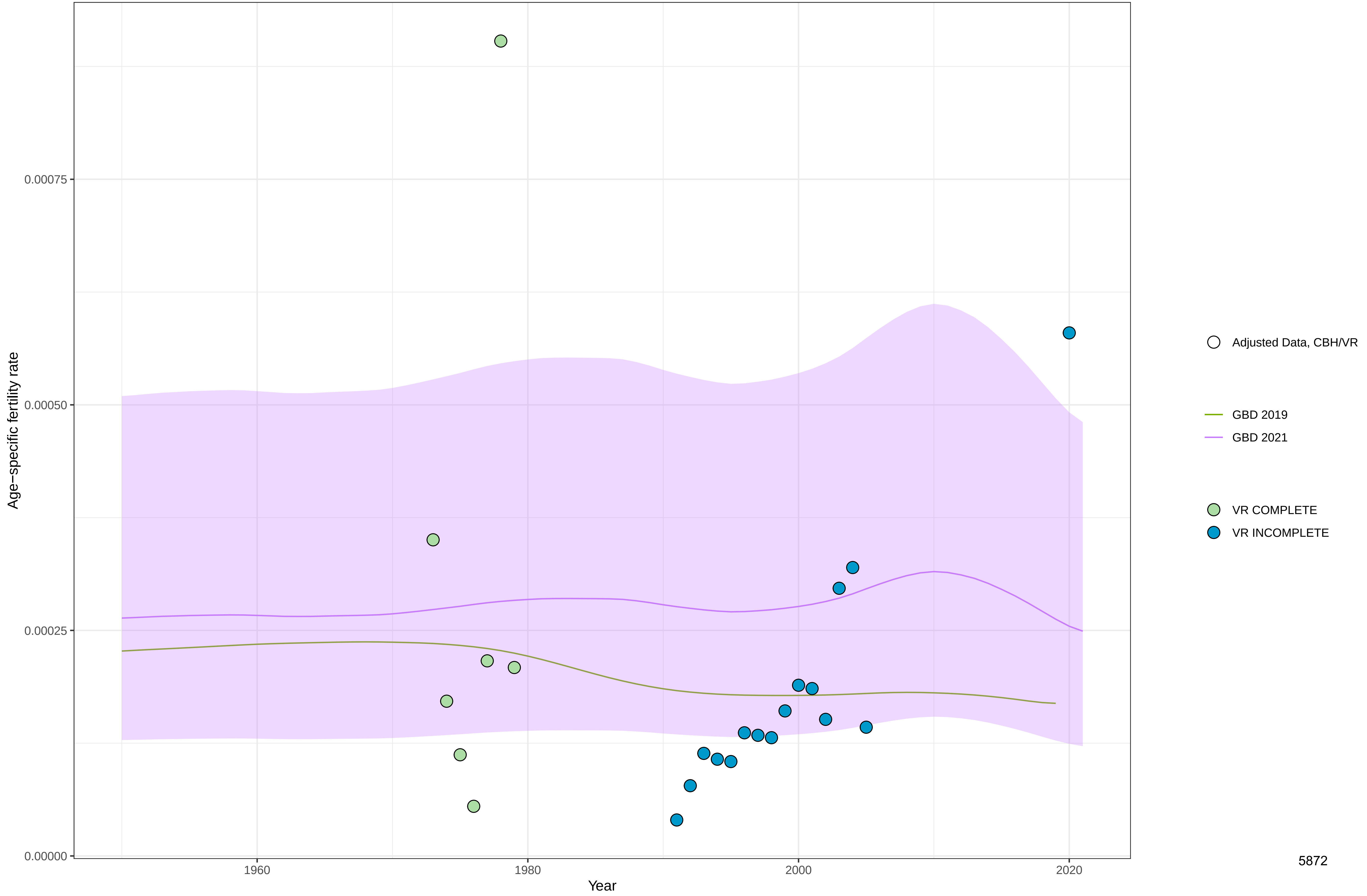

Age−specific fertility fitted trend lines, 1950−2021: Bataan, Ages: 15−19
Data points enclosed with a diamond are reference sources

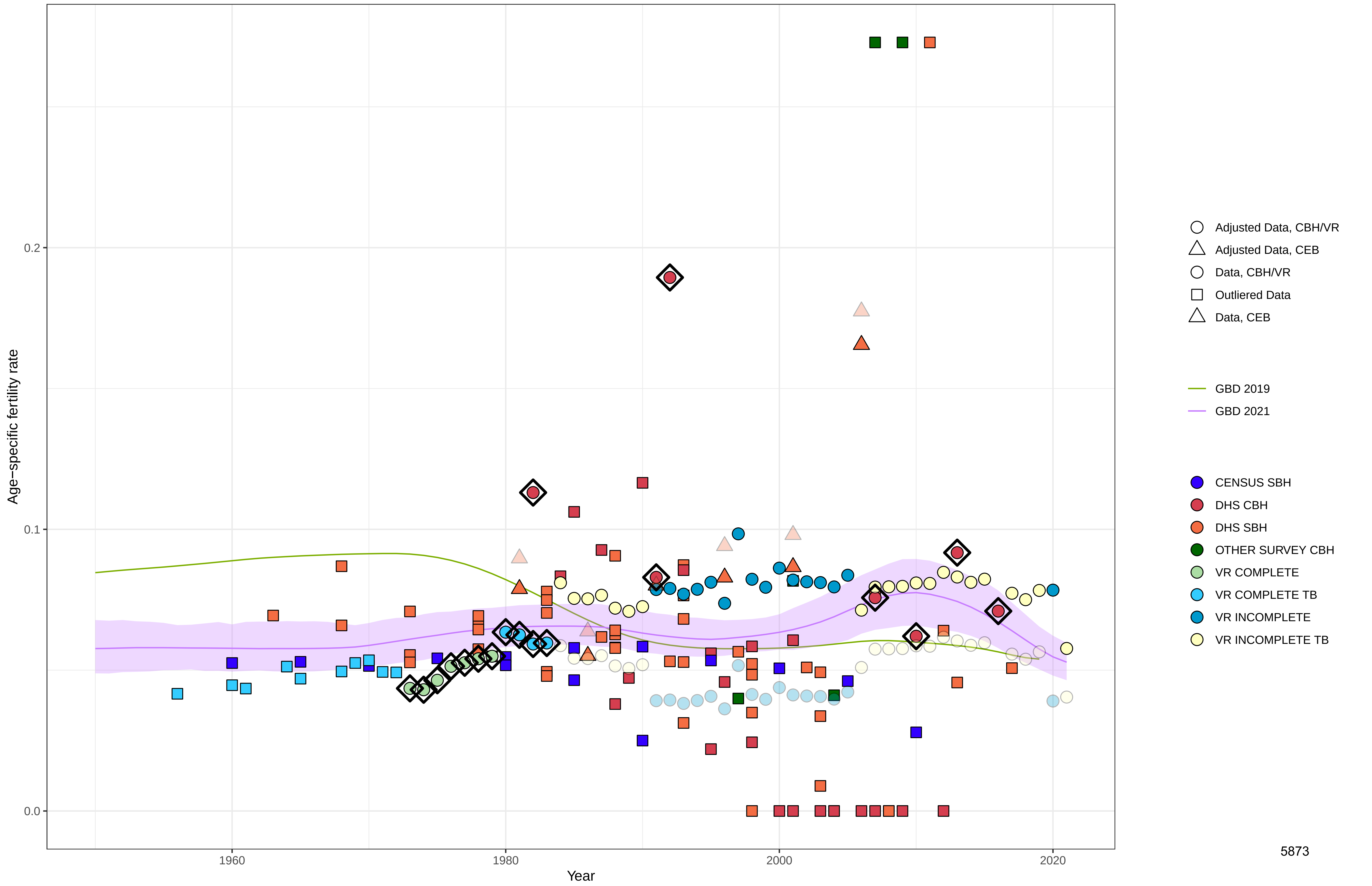

Age−specific fertility fitted trend lines, 1950−2021: Bataan, Ages: 20−24
Data points enclosed with a diamond are reference sources

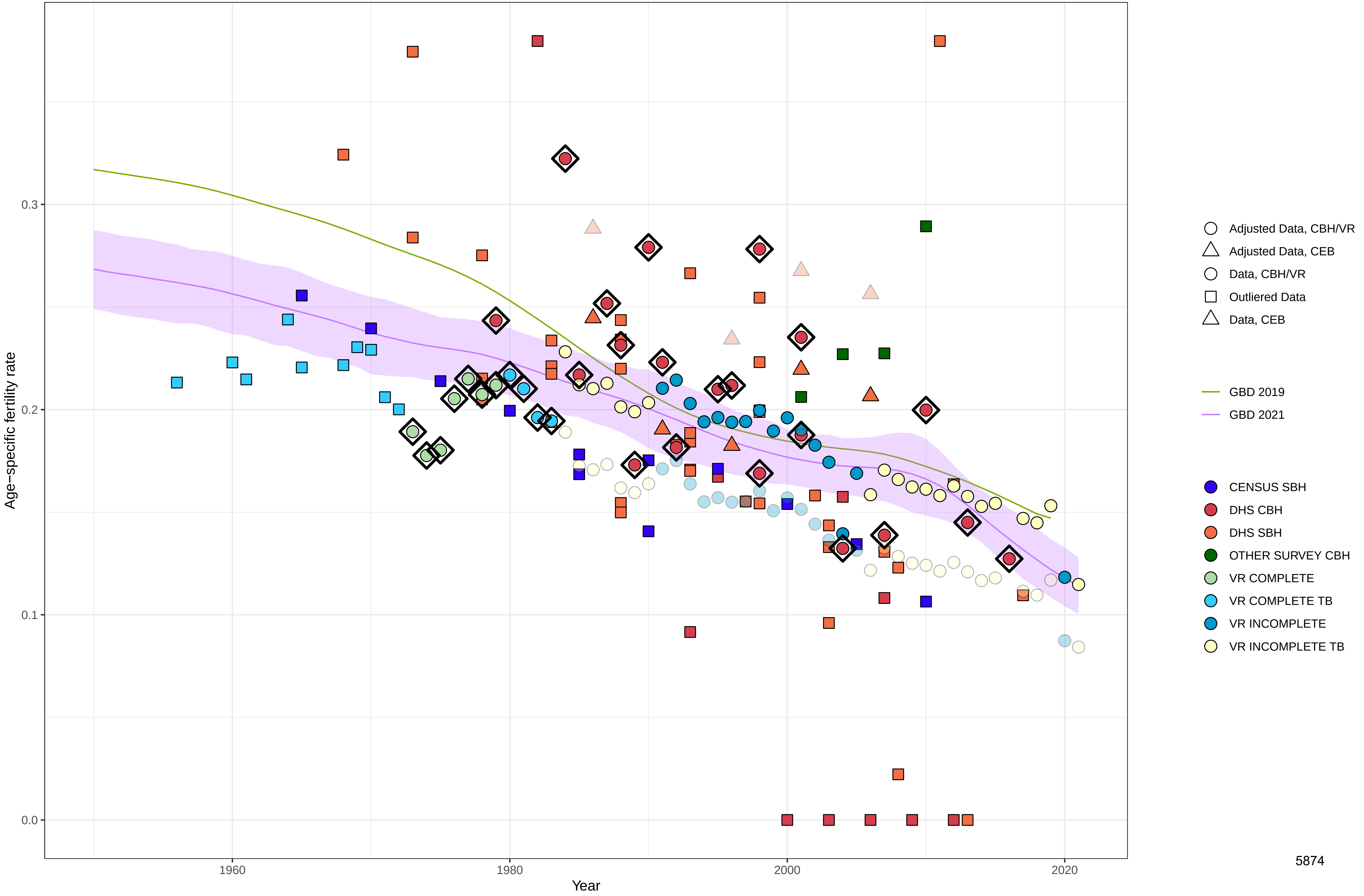

5874

Age−specific fertility fitted trend lines, 1950−2021: Bataan, Ages: 25−29
Data points enclosed with a diamond are reference sources

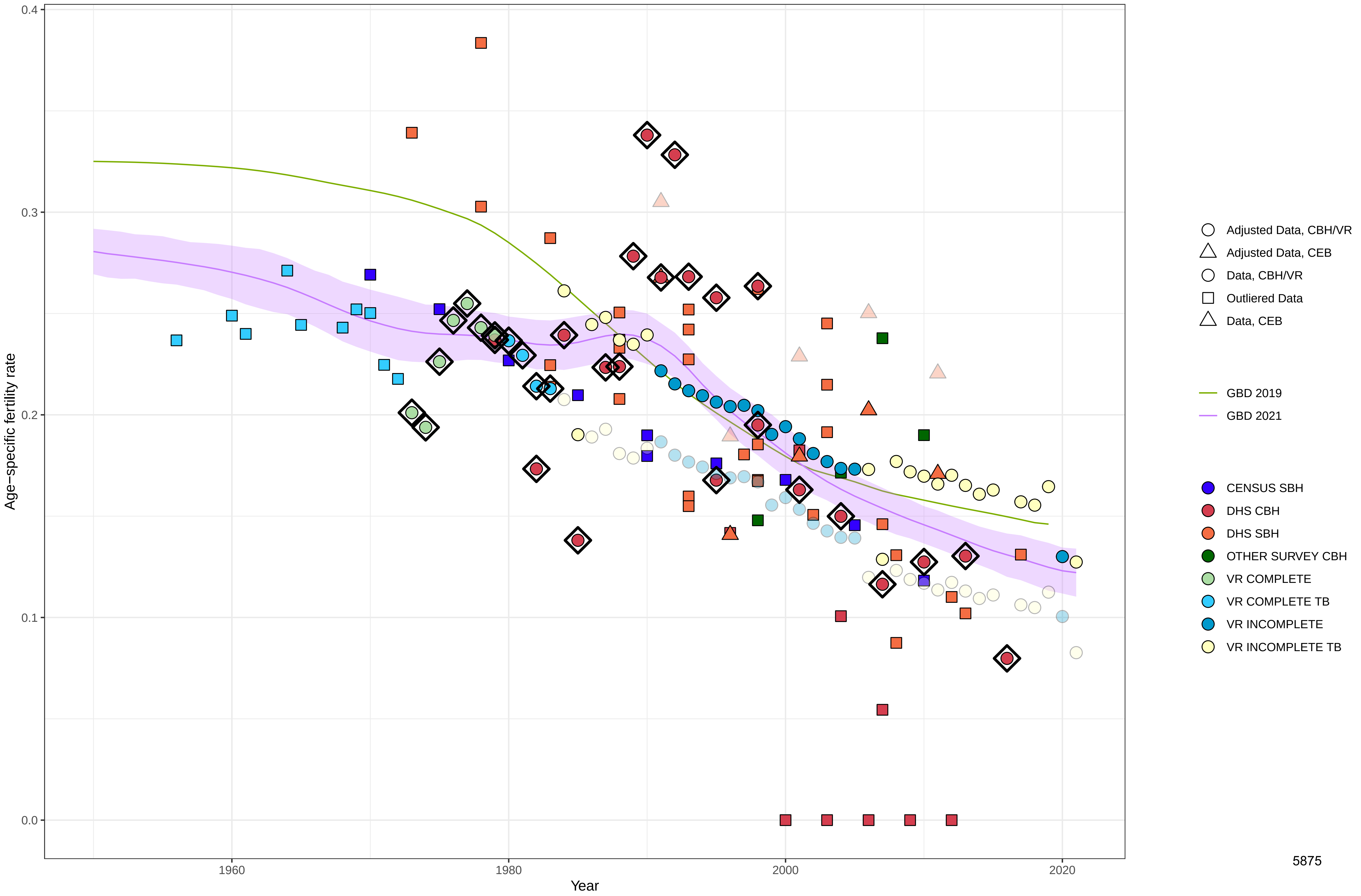

Age−specific fertility fitted trend lines, 1950−2021: Bataan, Ages: 30−34
Data points enclosed with a diamond are reference sources

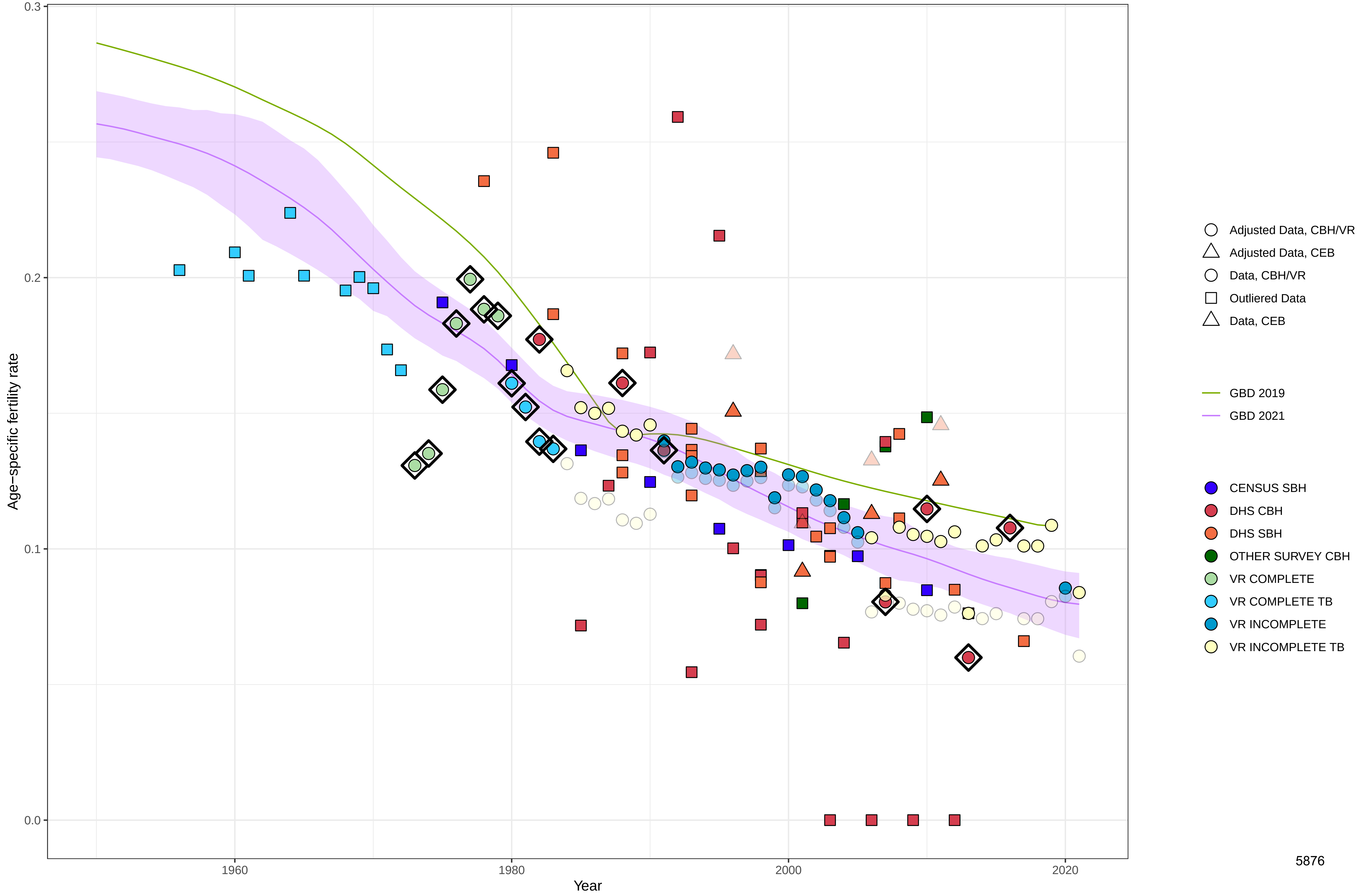

Age−specific fertility fitted trend lines, 1950−2021: Bataan, Ages: 35−39
Data points enclosed with a diamond are reference sources

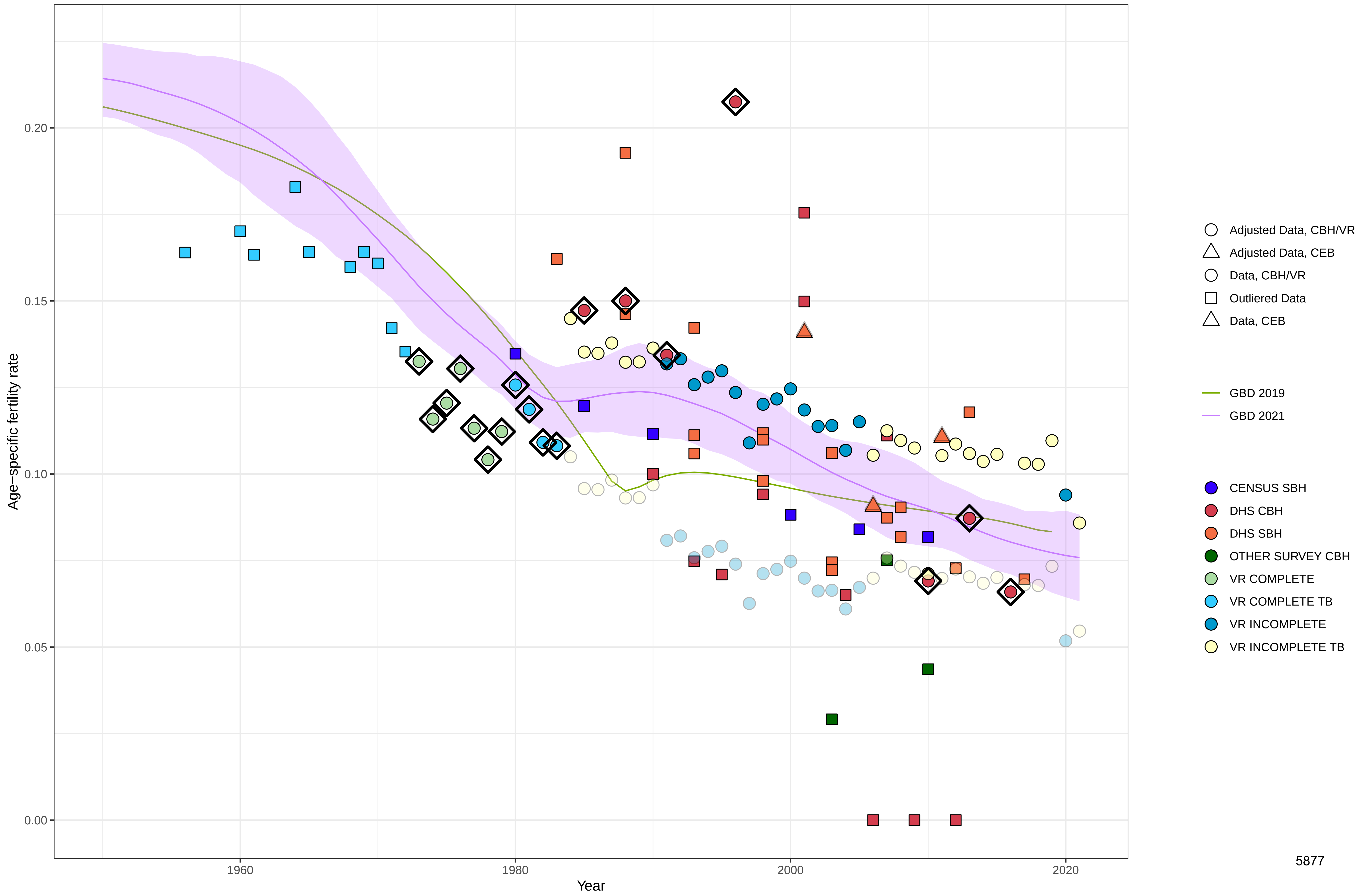

Age−specific fertility fitted trend lines, 1950−2021: Bataan, Ages: 40−44
Data points enclosed with a diamond are reference sources

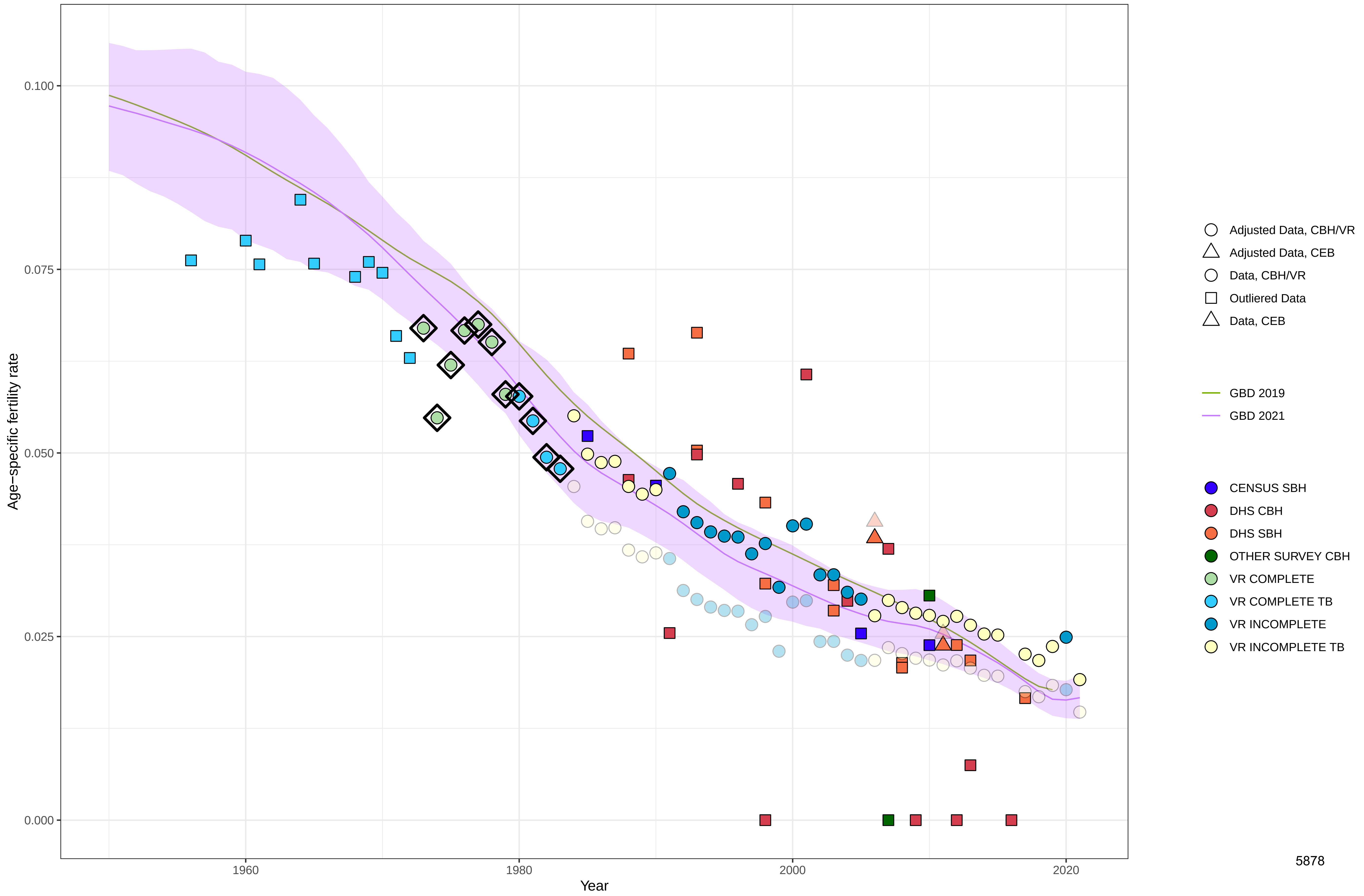

Age−specific fertility fitted trend lines, 1950−2021: Bataan, Ages: 45−49
Data points enclosed with a diamond are reference sources

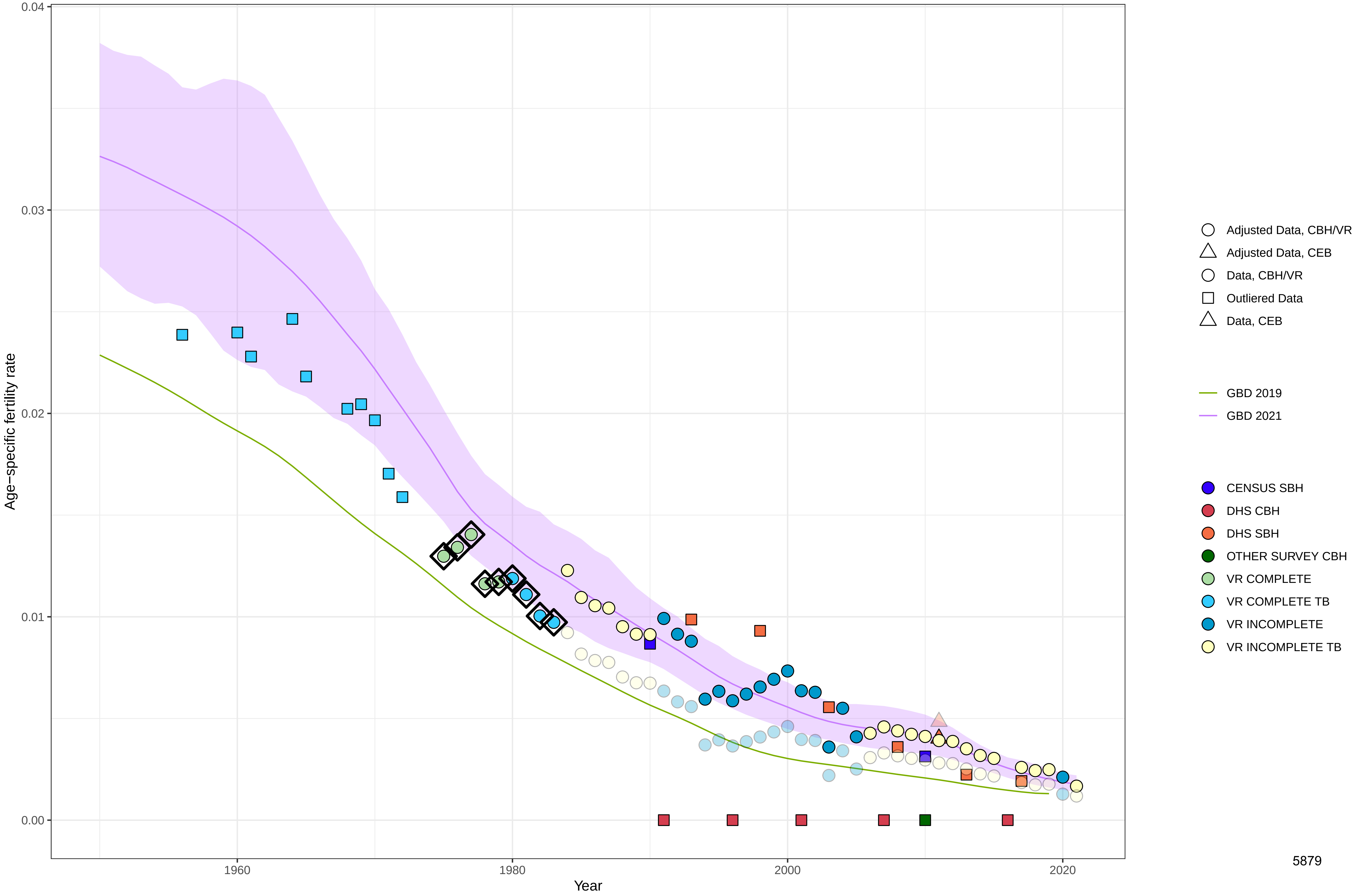

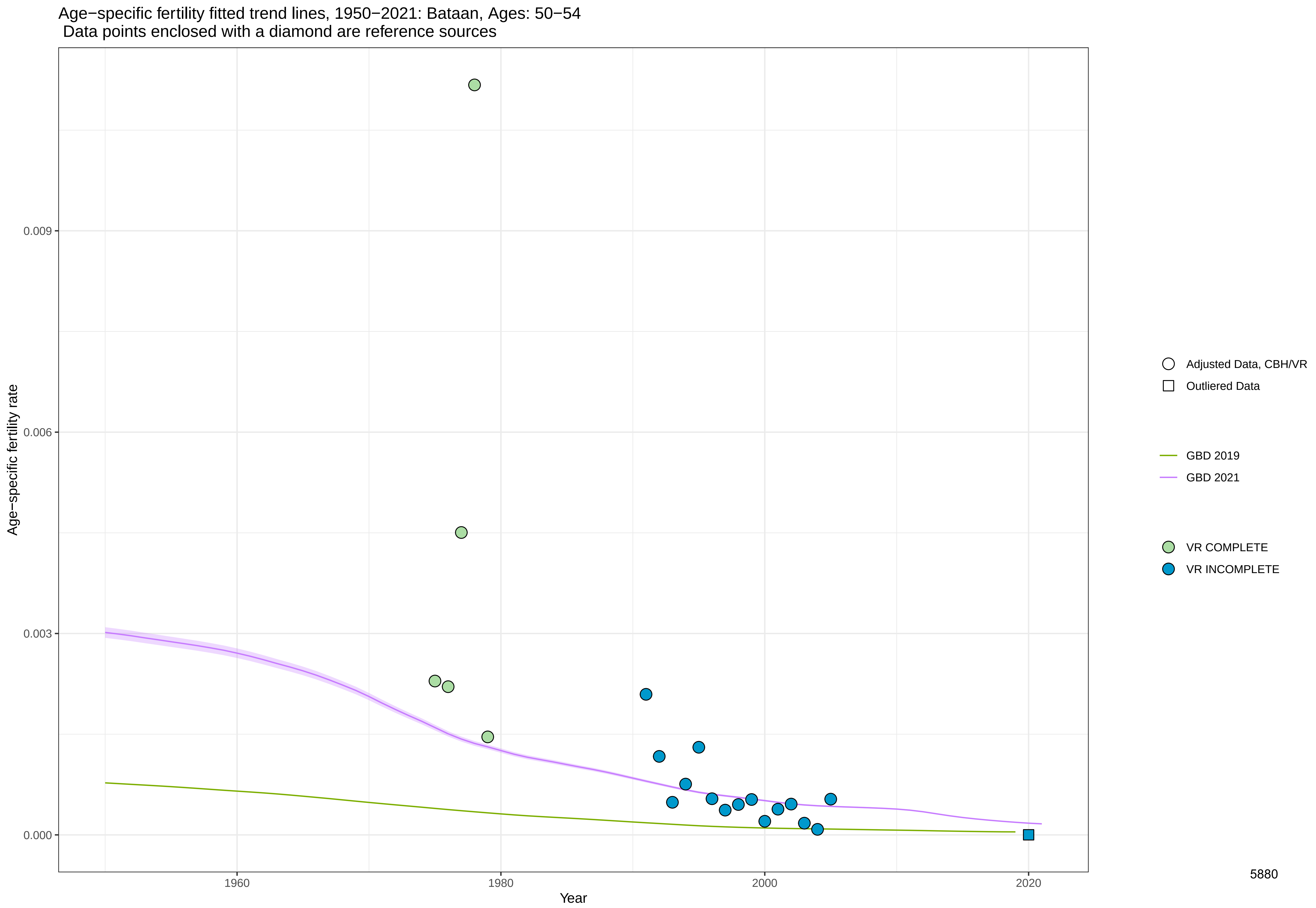

Age−specific fertility fitted trend lines, 1950−2021: Bataan, Ages: 50−54
Data points enclosed with a diamond are reference sources
Age−specific fertility rate
0.009
0.006
0.003
0.000
1960
1980
2000
2020
Year
Adjusted Data, CBH/VR
Outliered Data
GBD 2019
GBD 2021
VR COMPLETE
VR INCOMPLETE

Total fertility rate fitted trend lines, 1950−2021: Bataan TFR
Data points enclosed with a diamond are reference sources

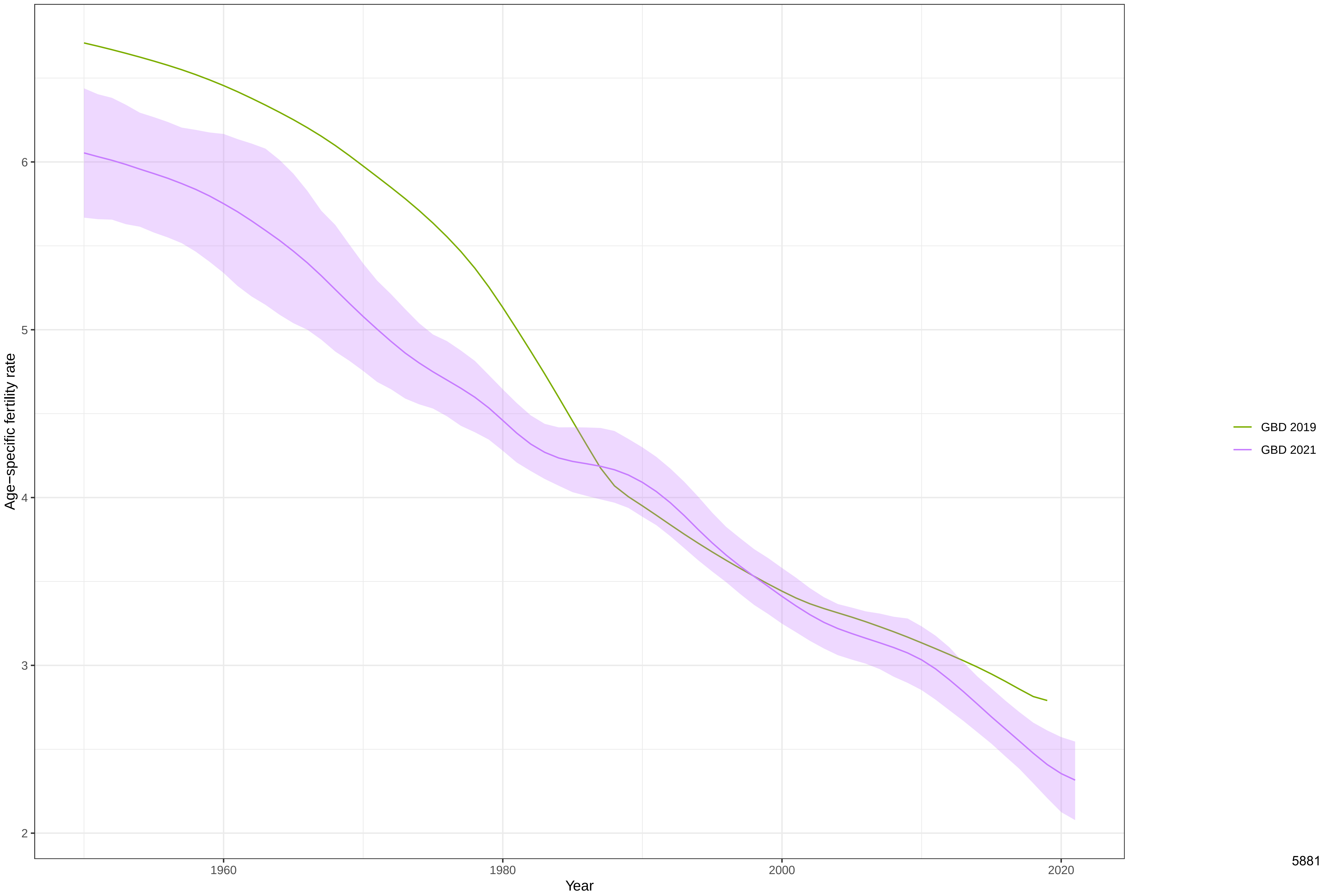

Age−specific fertility fitted trend lines, 1950−2021: Batanes, Ages: 10−14
Data points enclosed with a diamond are reference sources

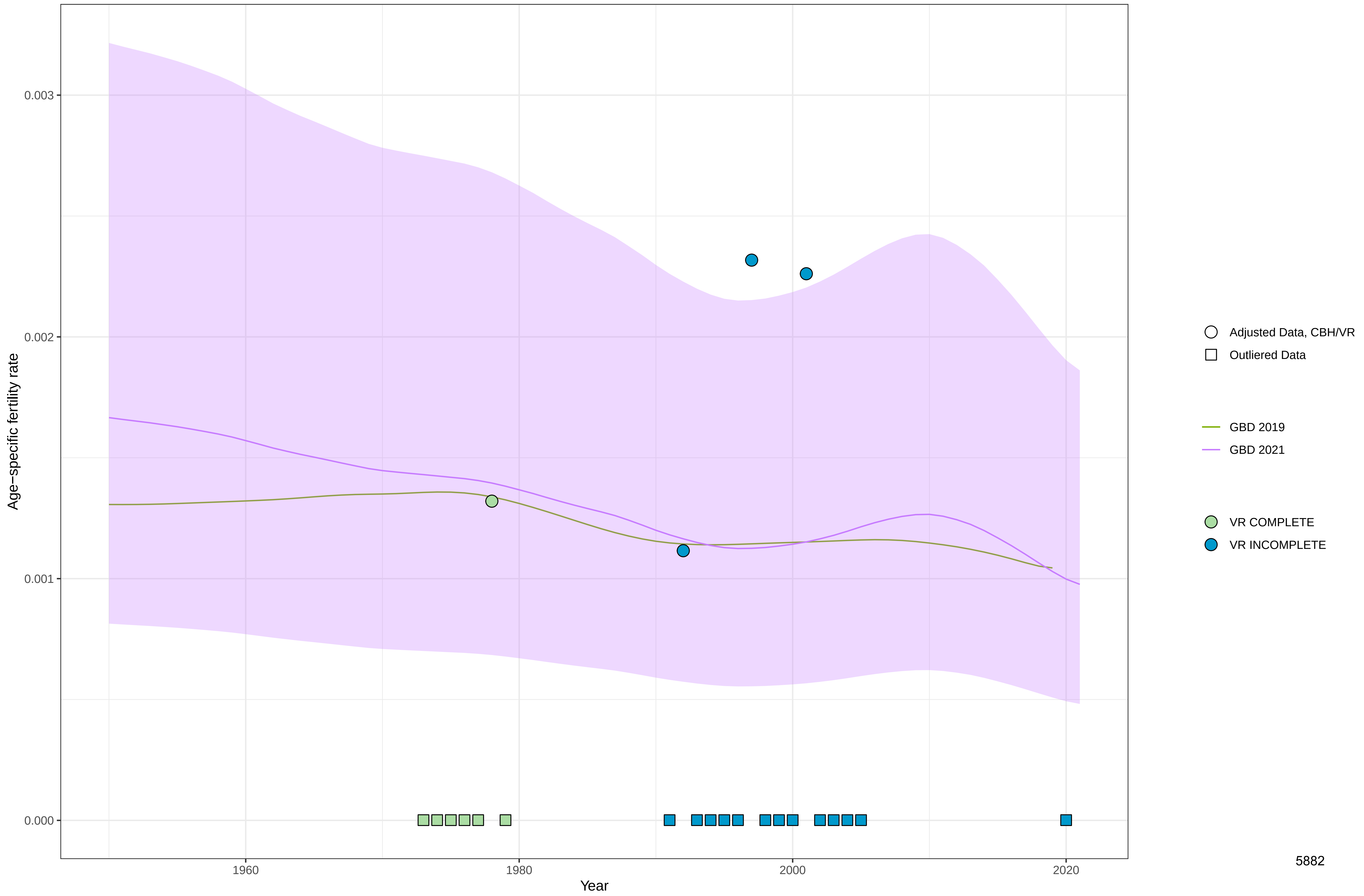

Age−specific fertility fitted trend lines, 1950−2021: Batanes, Ages: 15−19
Data points enclosed with a diamond are reference sources

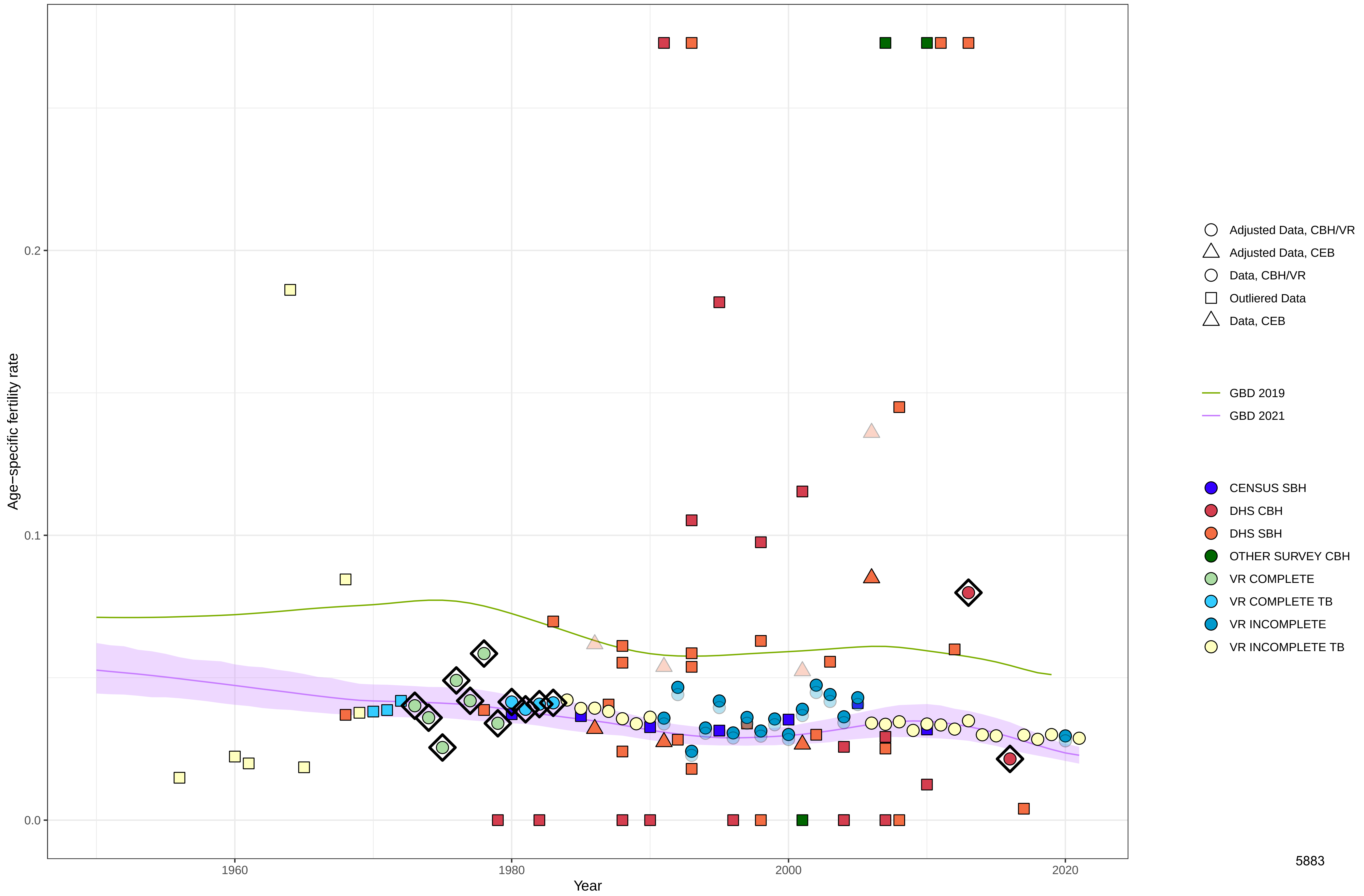

Age−specific fertility fitted trend lines, 1950−2021: Batanes, Ages: 20−24
Data points enclosed with a diamond are reference sources

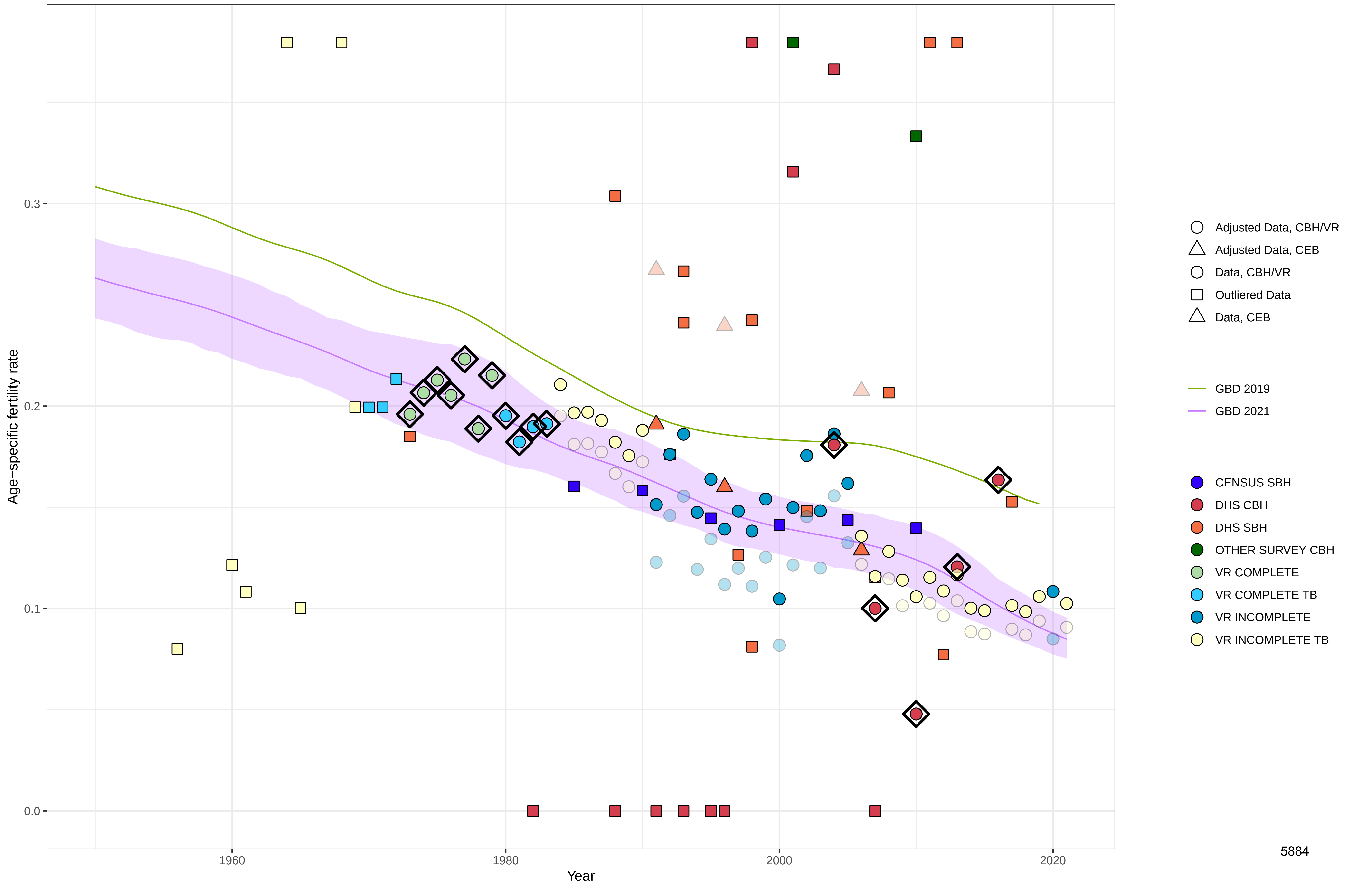

Age−specific fertility fitted trend lines, 1950−2021: Batanes, Ages: 25−29
Data points enclosed with a diamond are reference sources

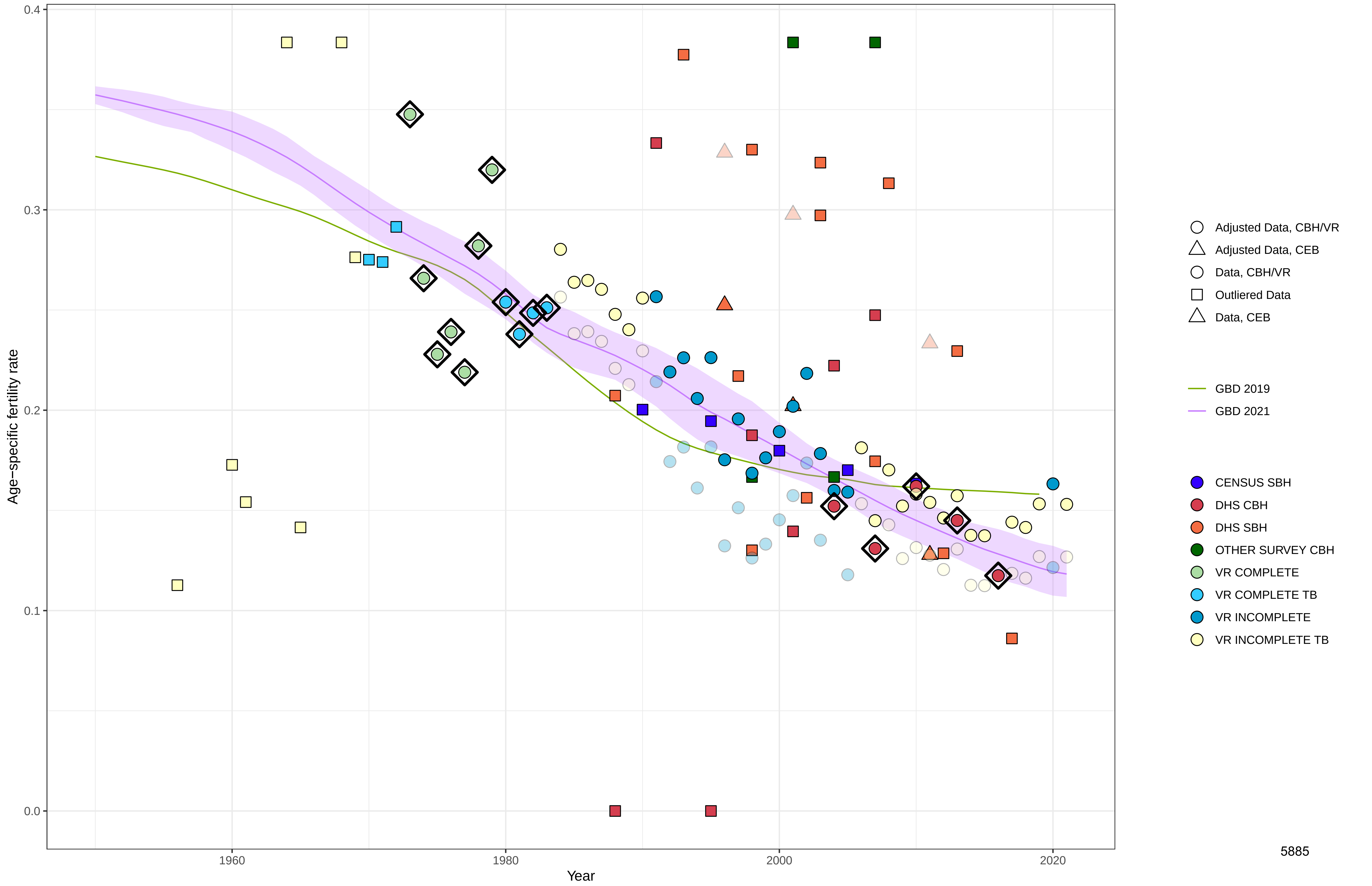

Age−specific fertility fitted trend lines, 1950−2021: Batanes, Ages: 30−34
Data points enclosed with a diamond are reference sources

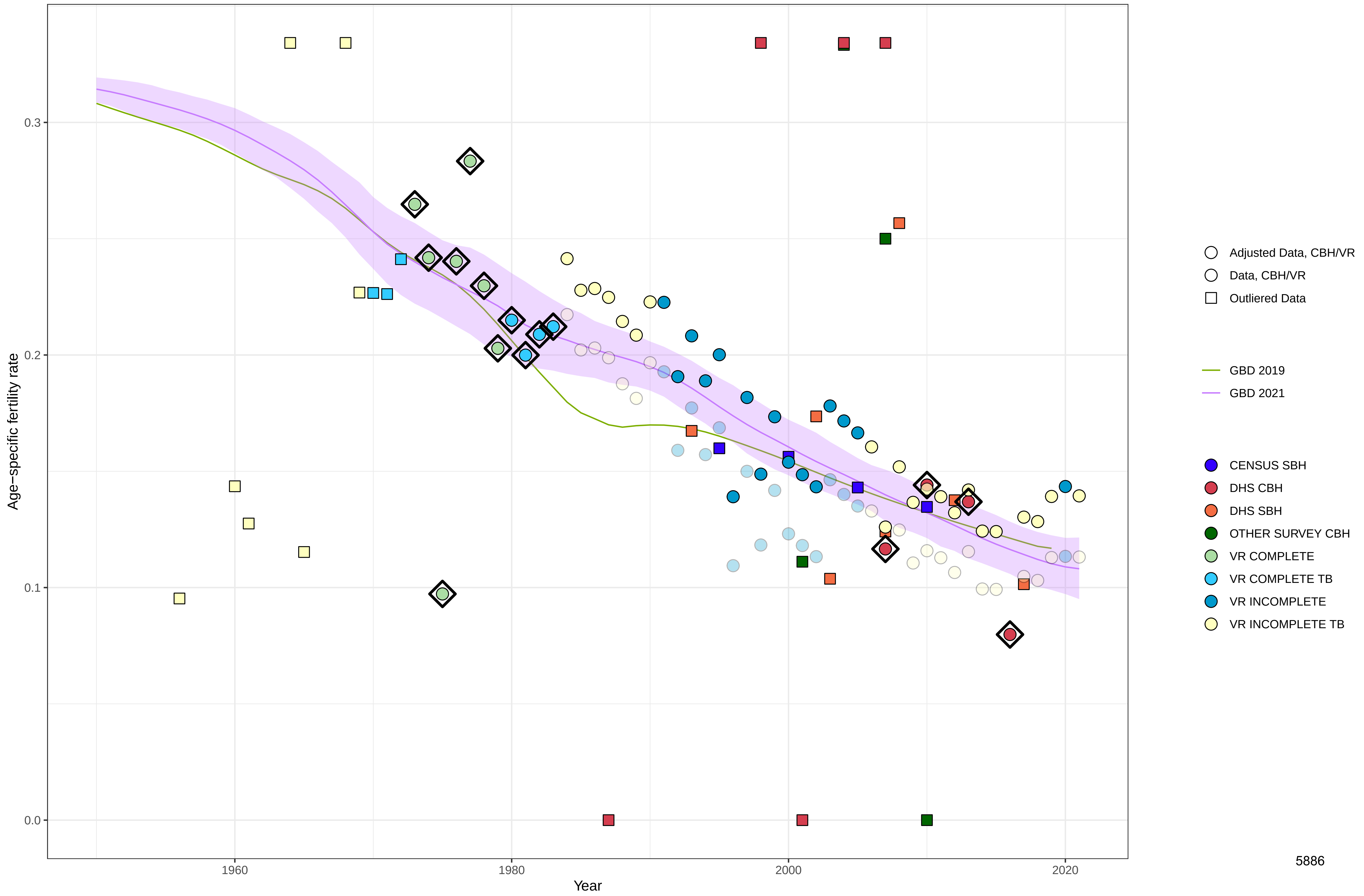

Age−specific fertility fitted trend lines, 1950−2021: Batanes, Ages: 35−39
Data points enclosed with a diamond are reference sources

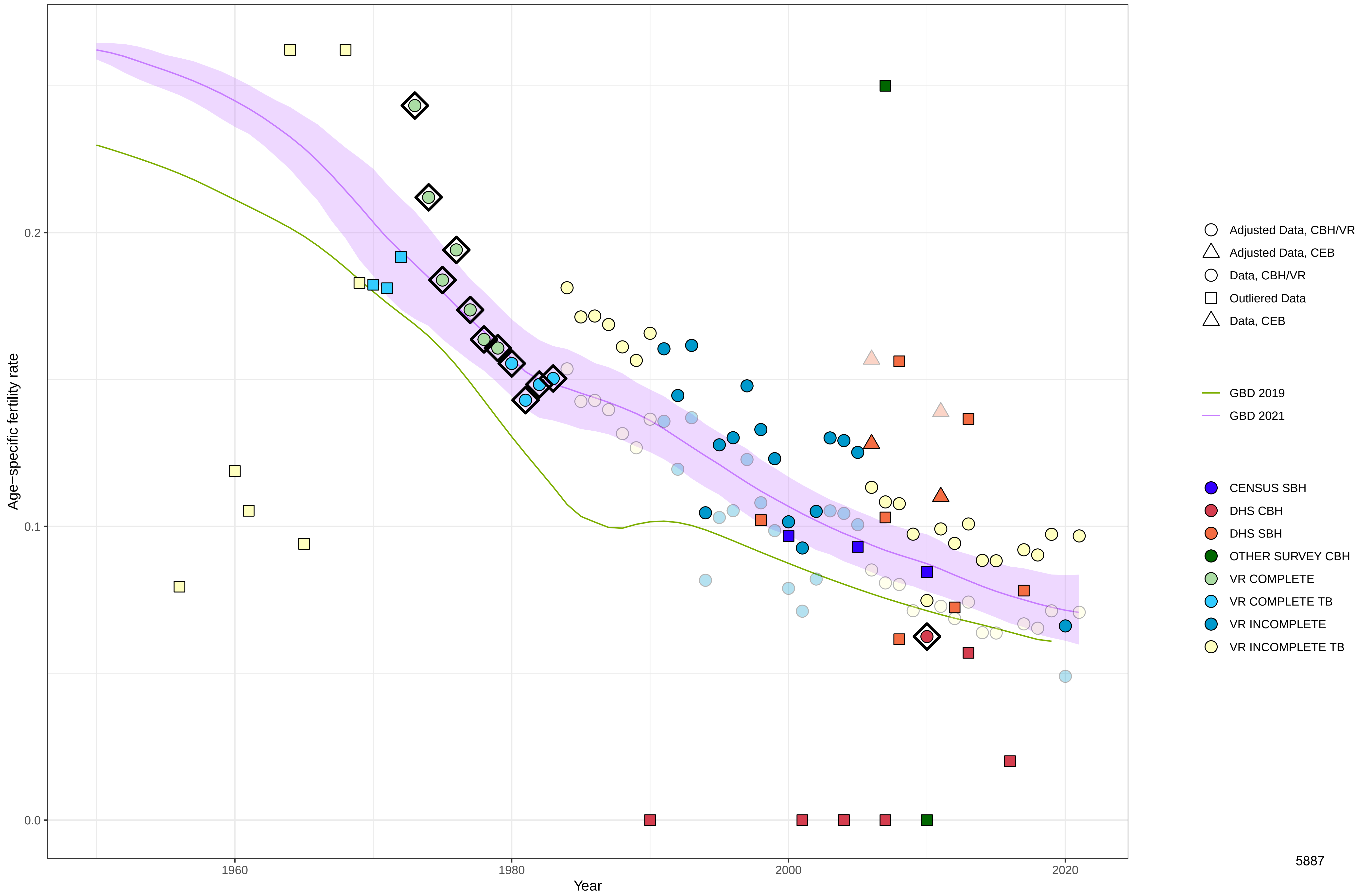

Age−specific fertility fitted trend lines, 1950−2021: Batanes, Ages: 40−44
Data points enclosed with a diamond are reference sources

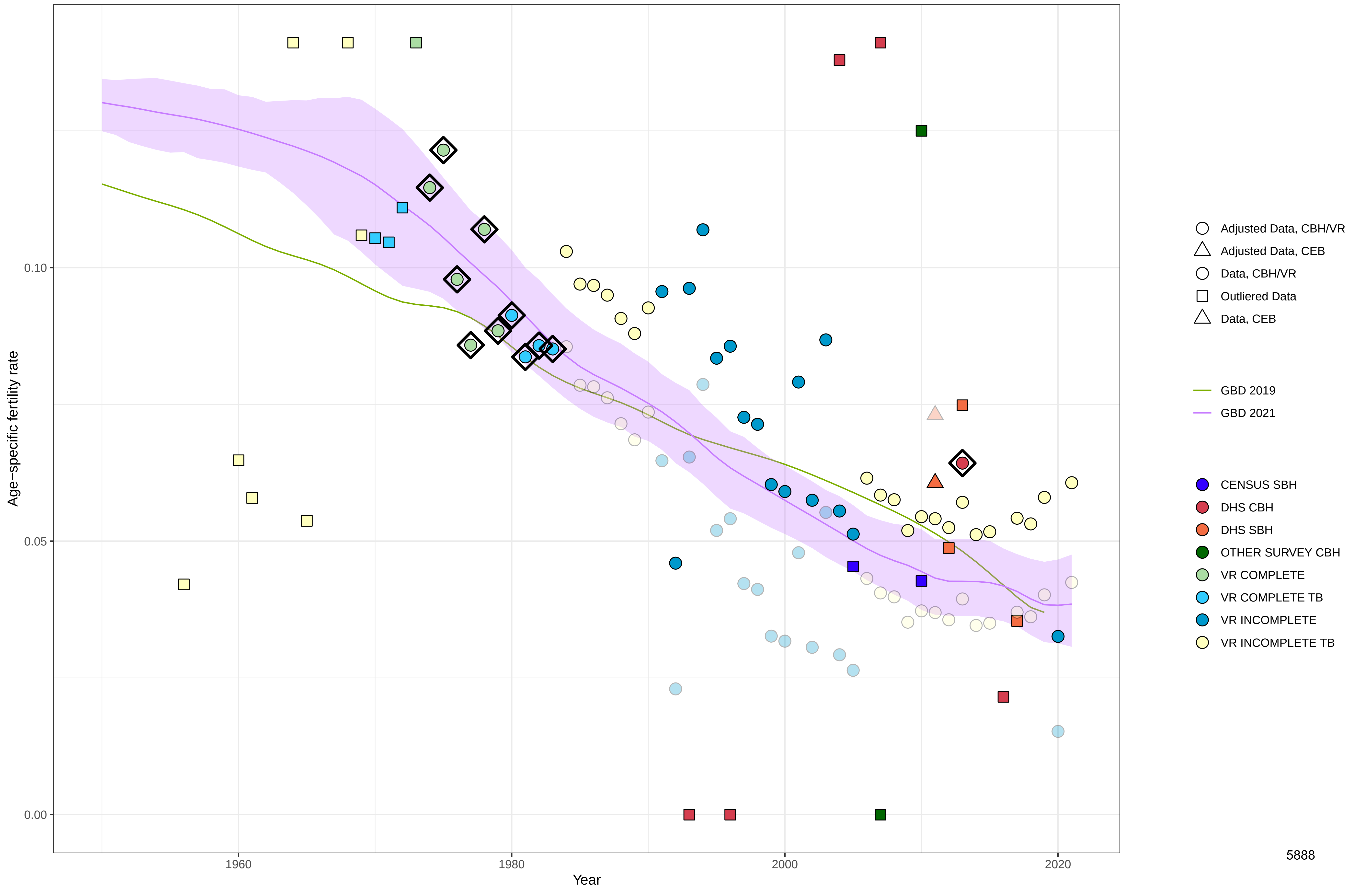

Age−specific fertility fitted trend lines, 1950−2021: Batanes, Ages: 45−49
Data points enclosed with a diamond are reference sources

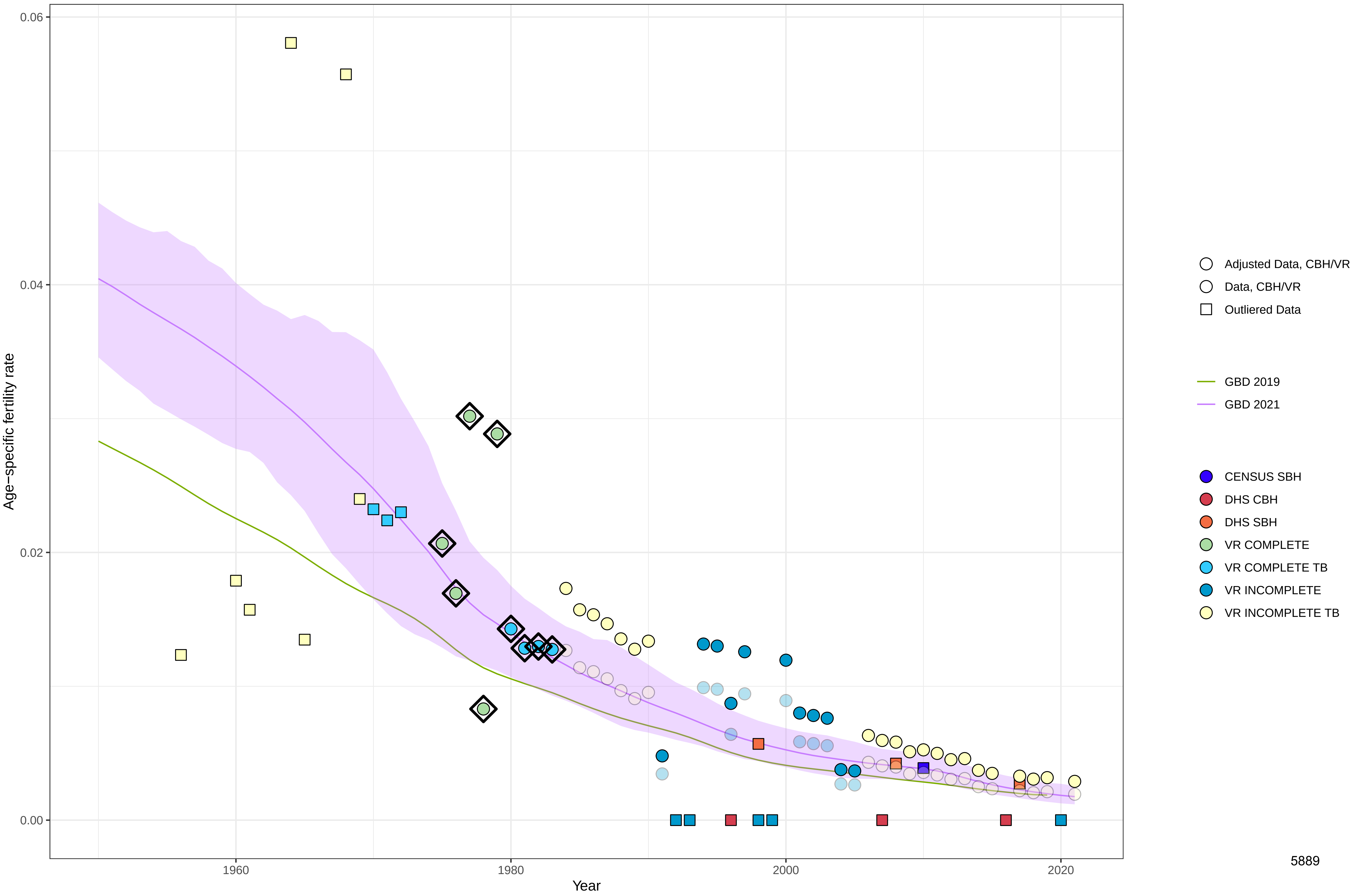

Age−specific fertility fitted trend lines, 1950−2021: Batanes, Ages: 50−54
Data points enclosed with a diamond are reference sources

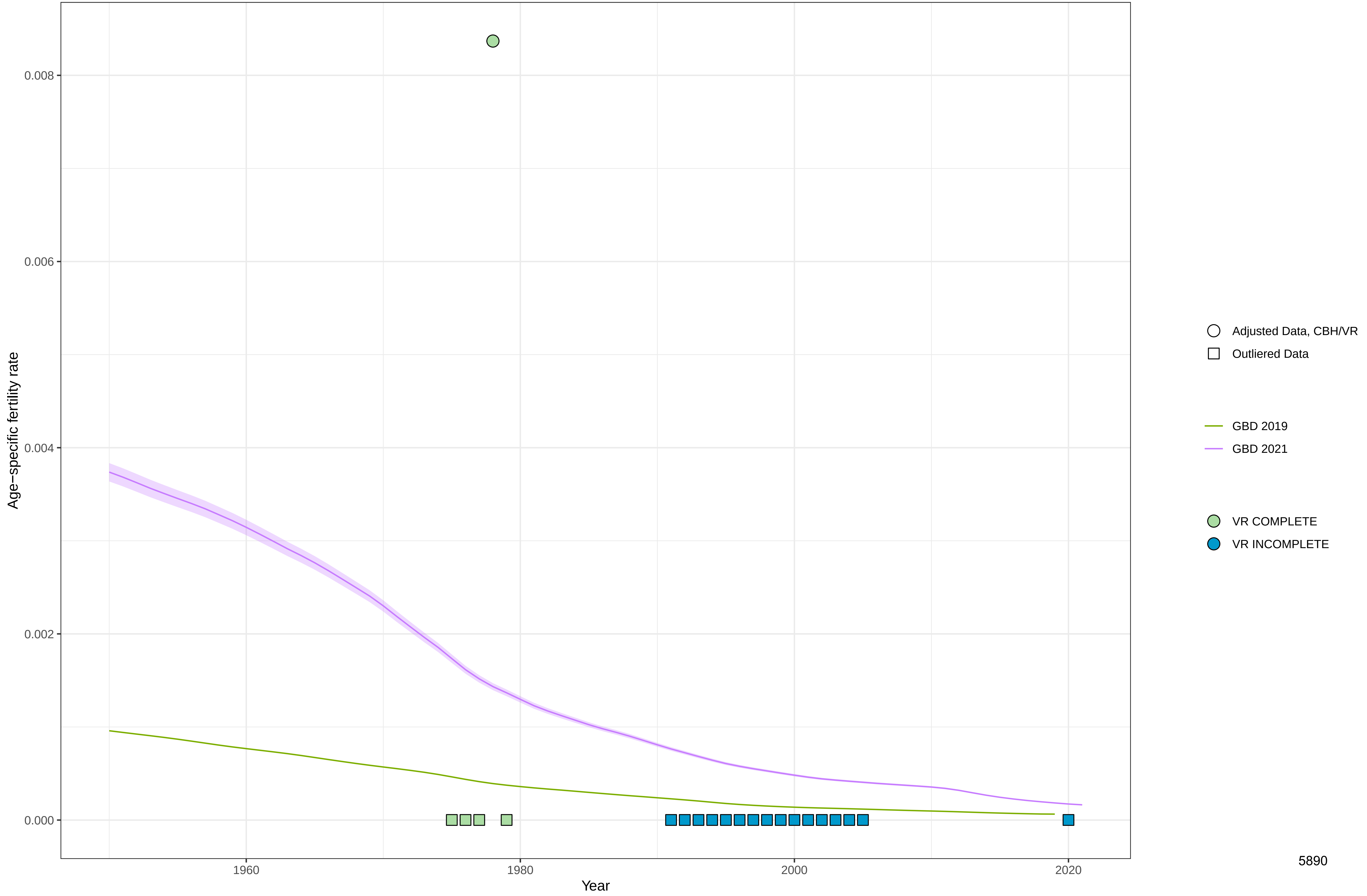

Total fertility rate fitted trend lines, 1950−2021: Batanes TFR
Data points enclosed with a diamond are reference sources

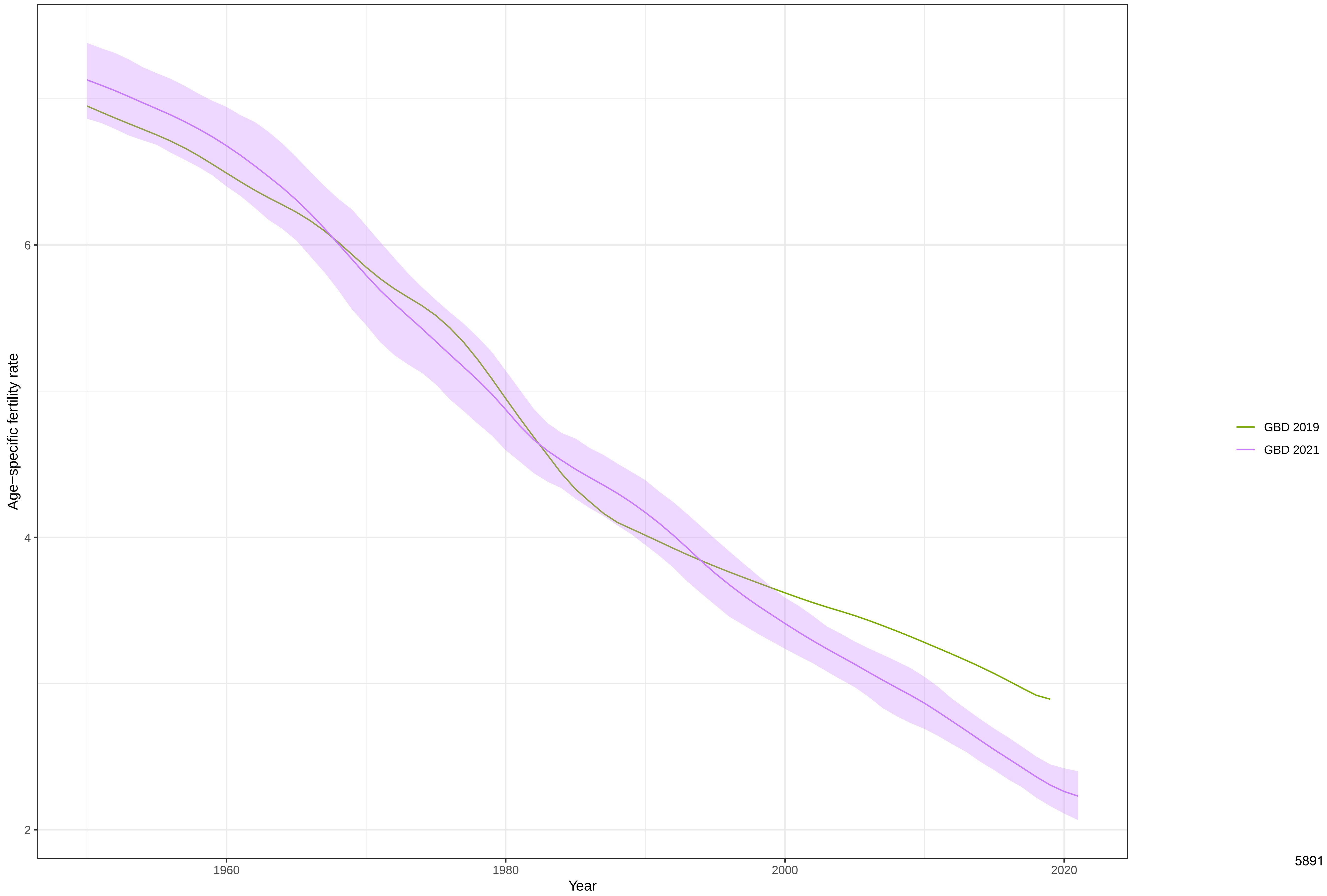

Age−specific fertility fitted trend lines, 1950−2021: Batangas, Ages: 10−14
Data points enclosed with a diamond are reference sources

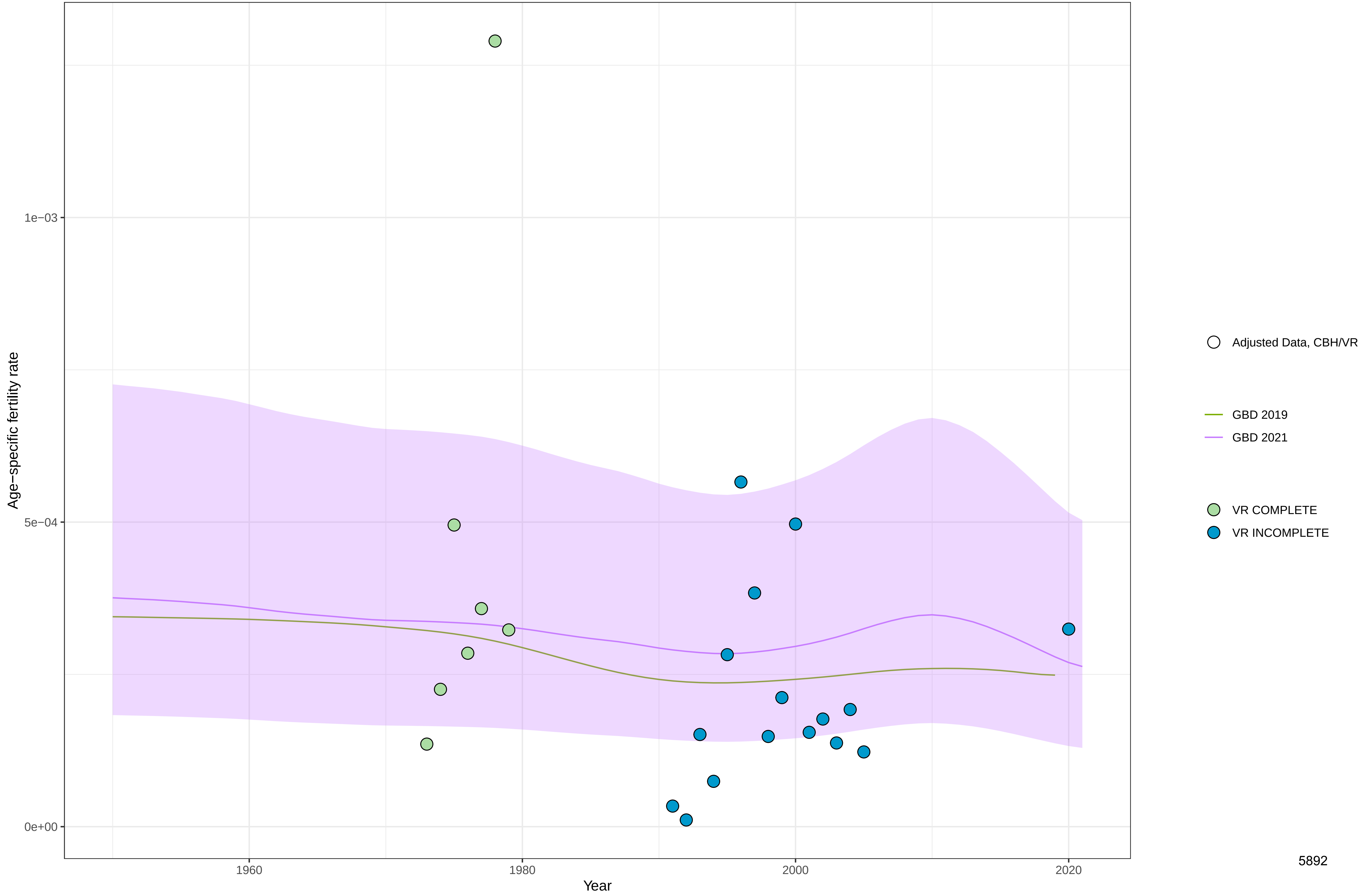

5892

Age−specific fertility fitted trend lines, 1950−2021: Batangas, Ages: 15−19
Data points enclosed with a diamond are reference sources

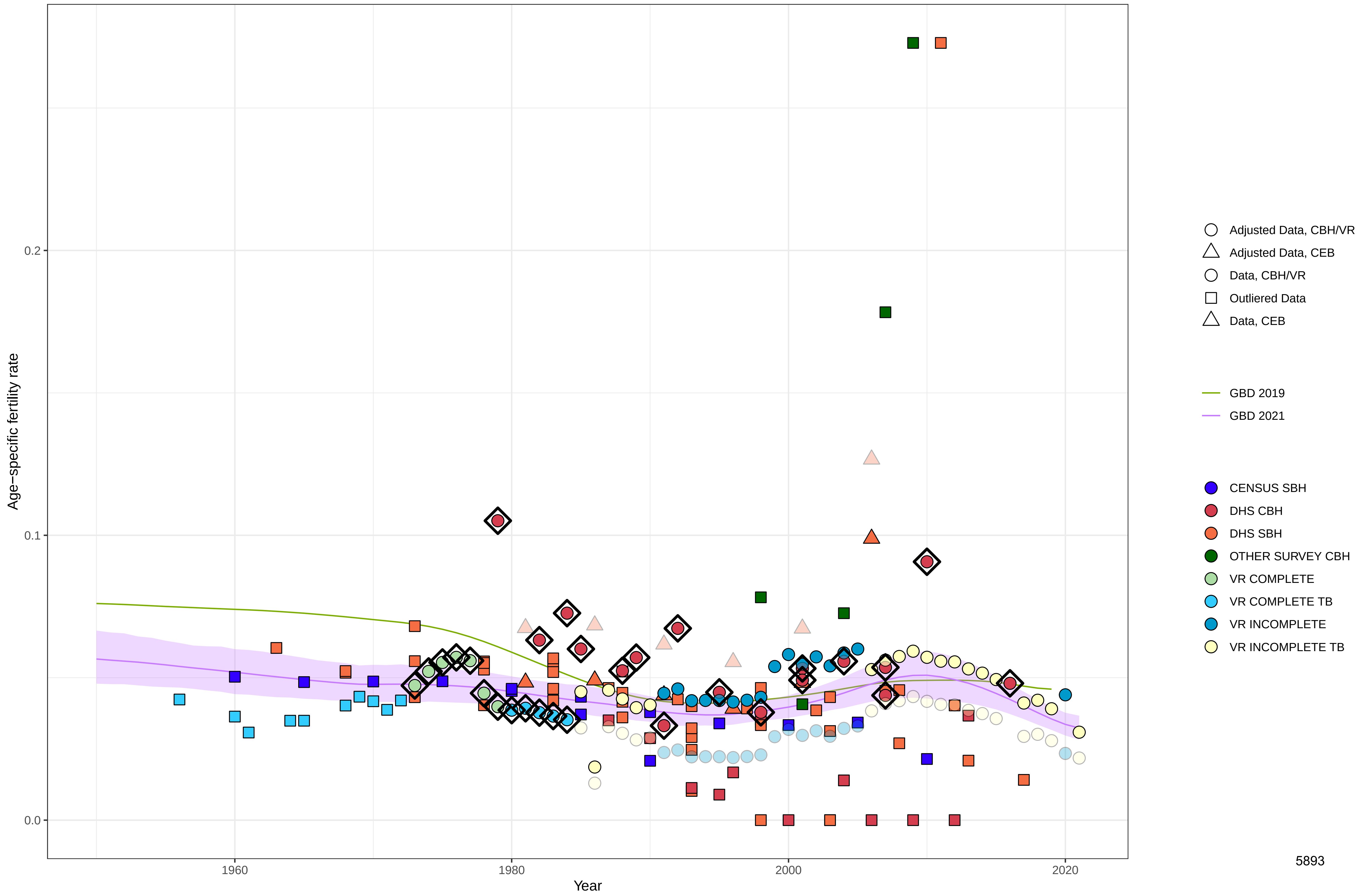

Age−specific fertility fitted trend lines, 1950−2021: Batangas, Ages: 20−24
Data points enclosed with a diamond are reference sources

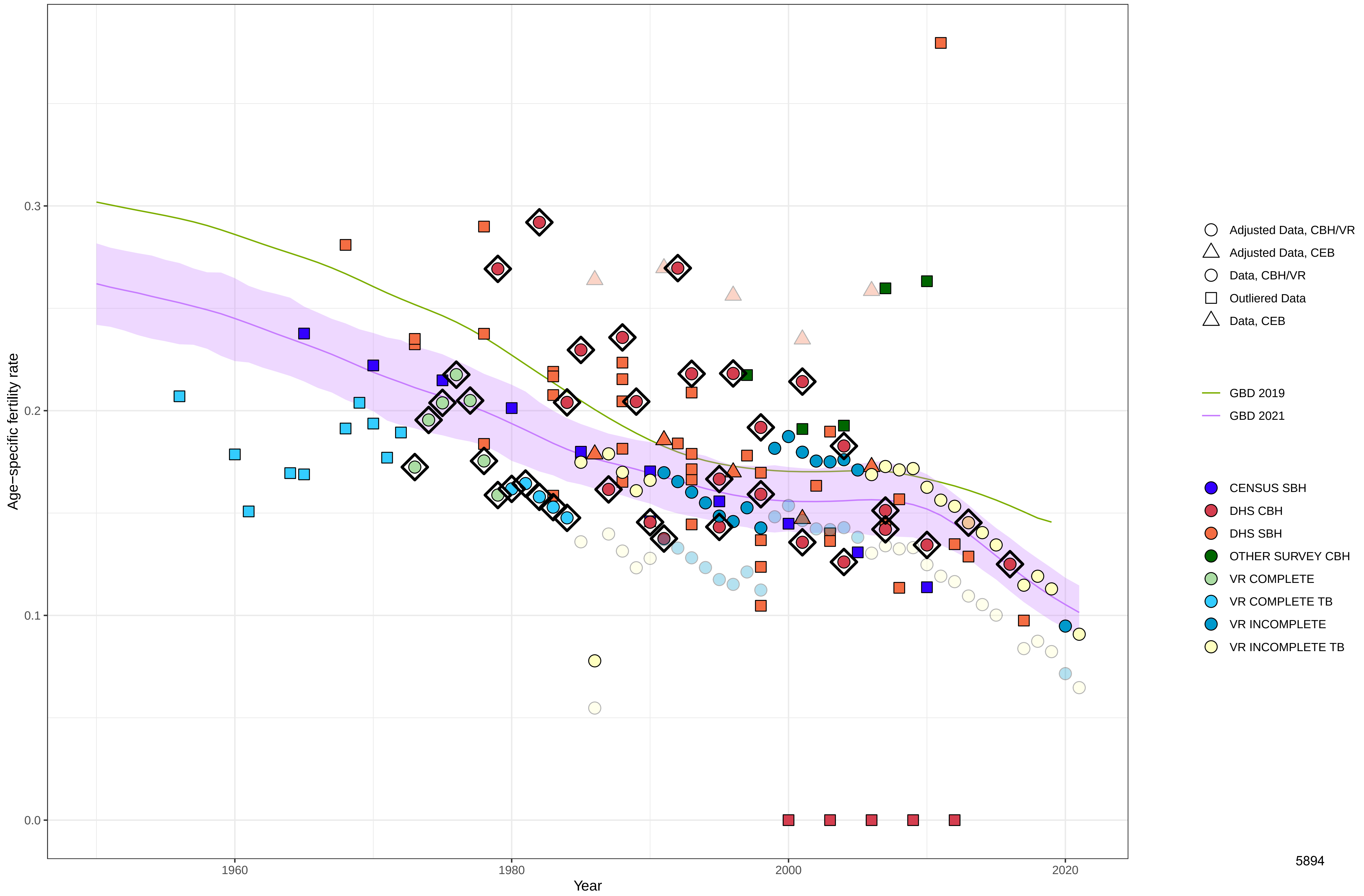

Age−specific fertility fitted trend lines, 1950−2021: Batangas, Ages: 25−29
Data points enclosed with a diamond are reference sources

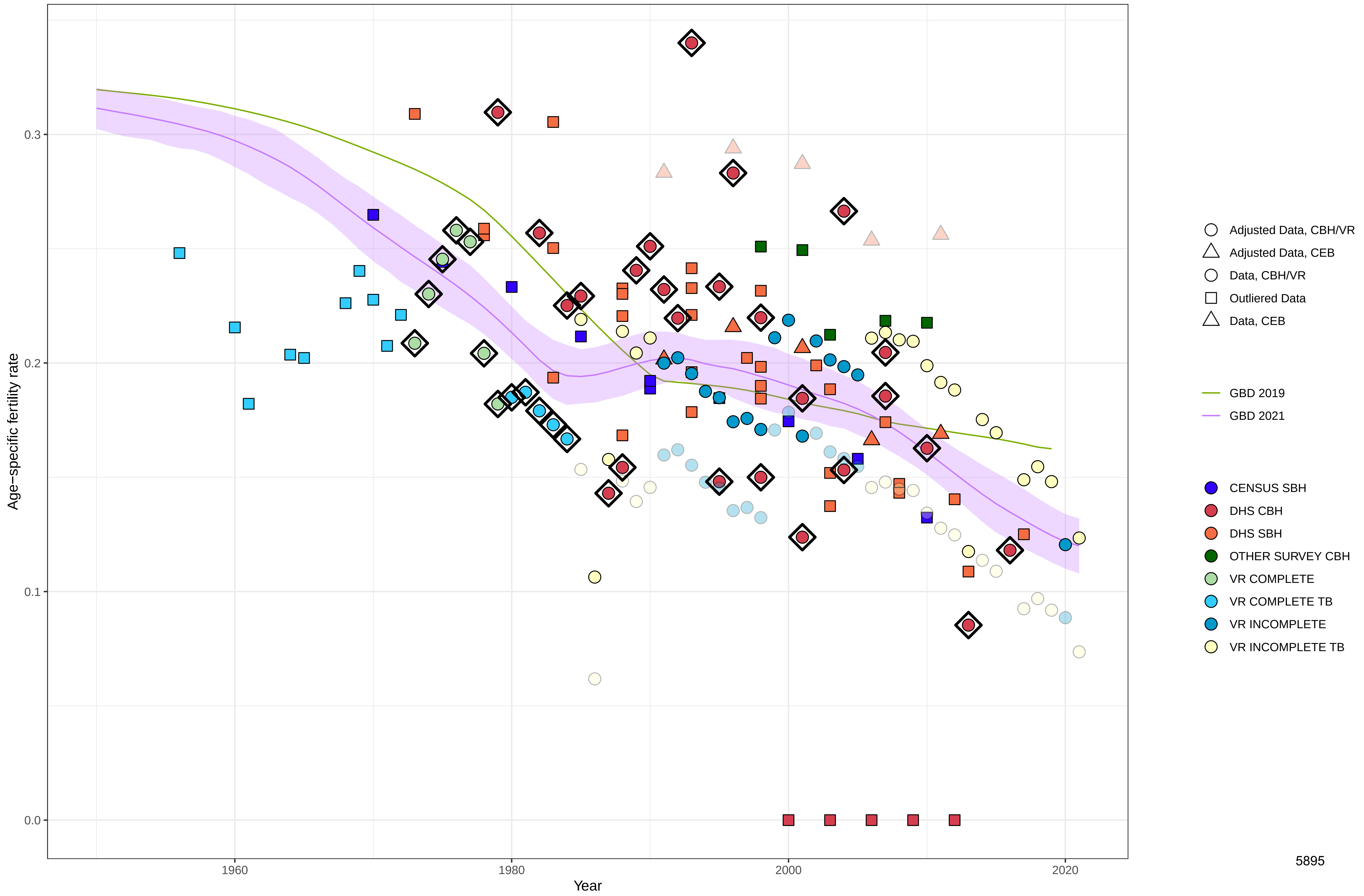

Age−specific fertility fitted trend lines, 1950−2021: Batangas, Ages: 30−34
Data points enclosed with a diamond are reference sources

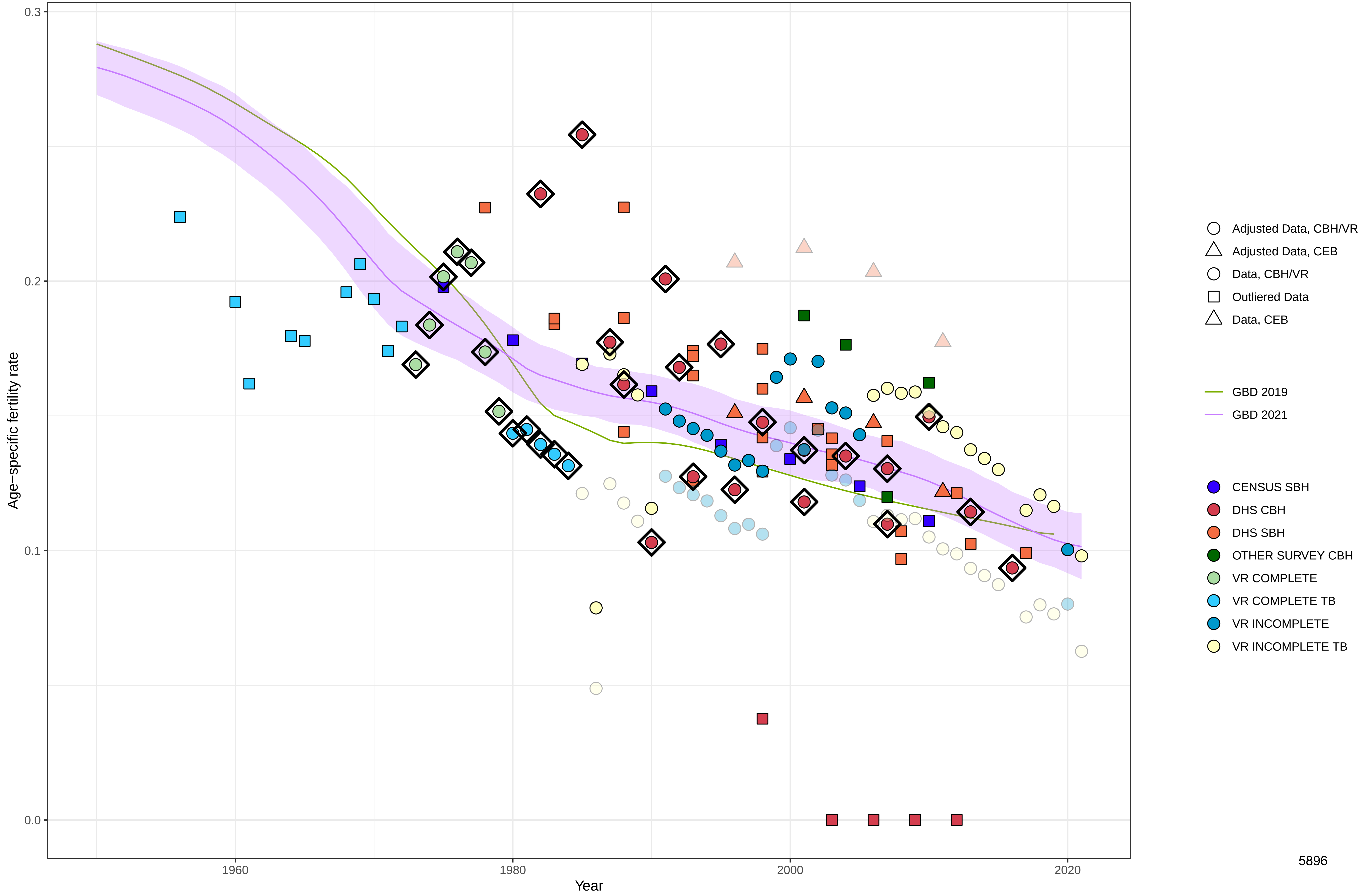

Age−specific fertility fitted trend lines, 1950−2021: Batangas, Ages: 35−39
Data points enclosed with a diamond are reference sources

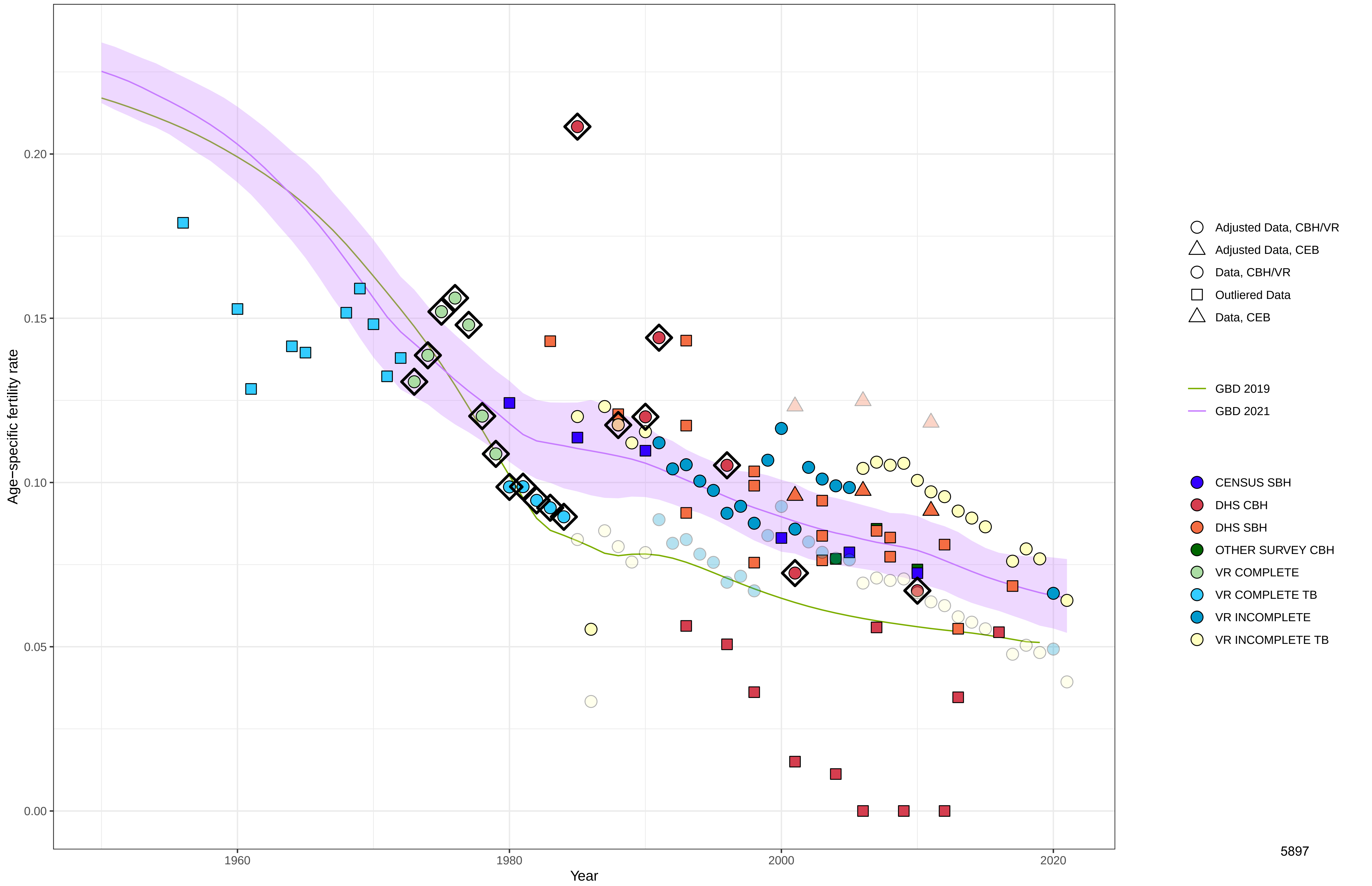

Age−specific fertility fitted trend lines, 1950−2021: Batangas, Ages: 40−44
Data points enclosed with a diamond are reference sources

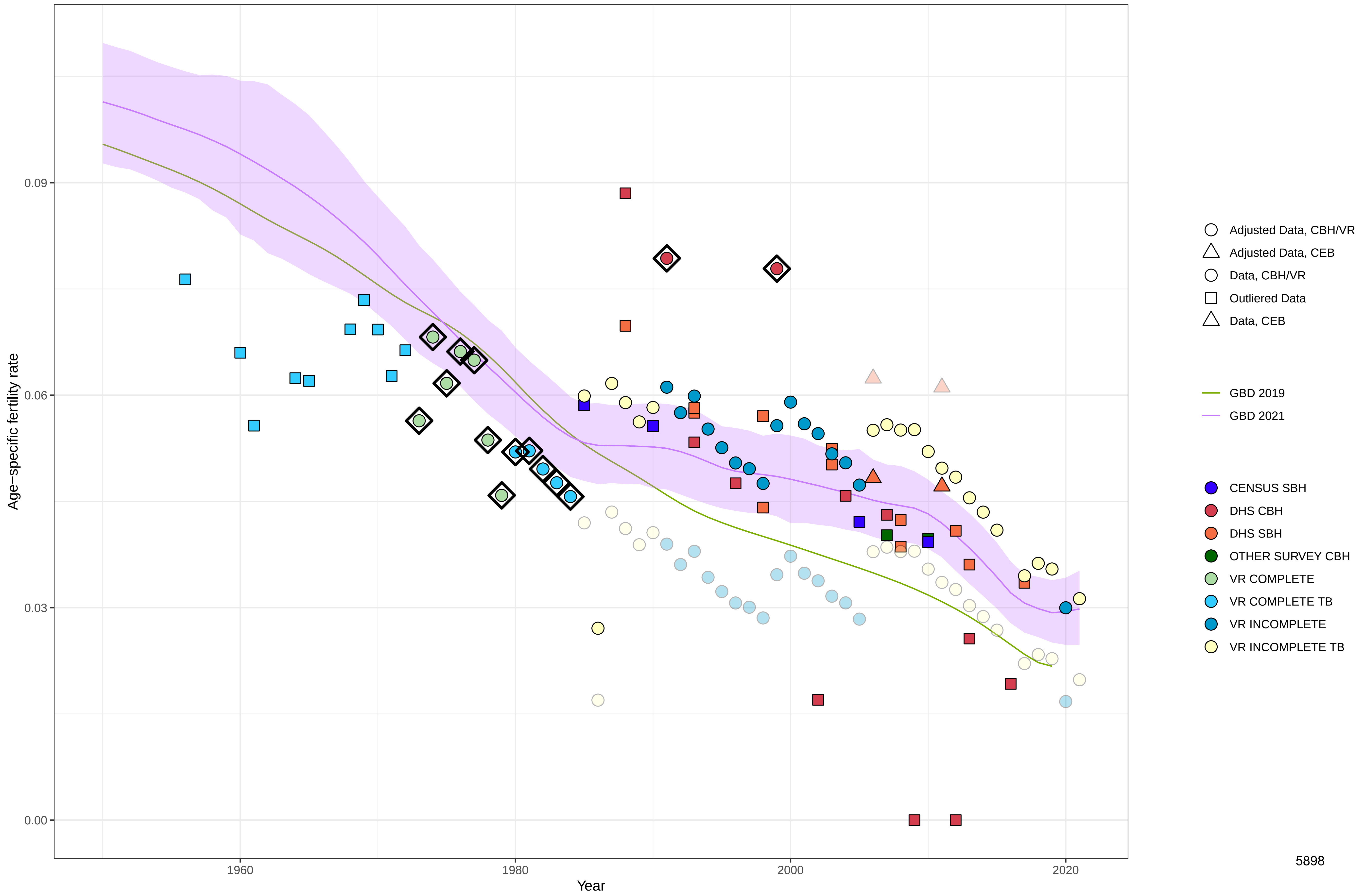

Age−specific fertility fitted trend lines, 1950−2021: Batangas, Ages: 45−49
Data points enclosed with a diamond are reference sources

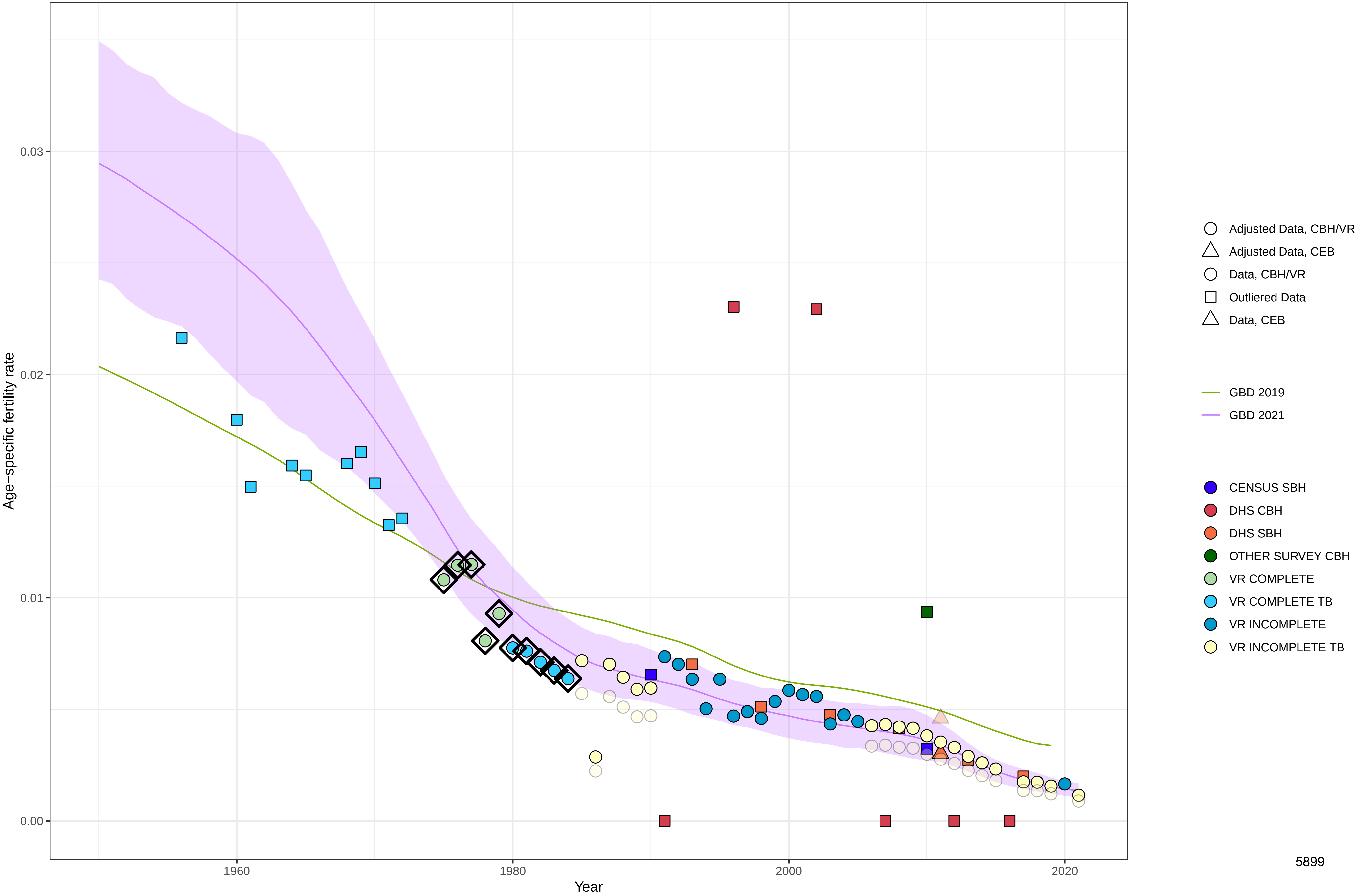

Age−specific fertility fitted trend lines, 1950−2021: Batangas, Ages: 50−54
Data points enclosed with a diamond are reference sources

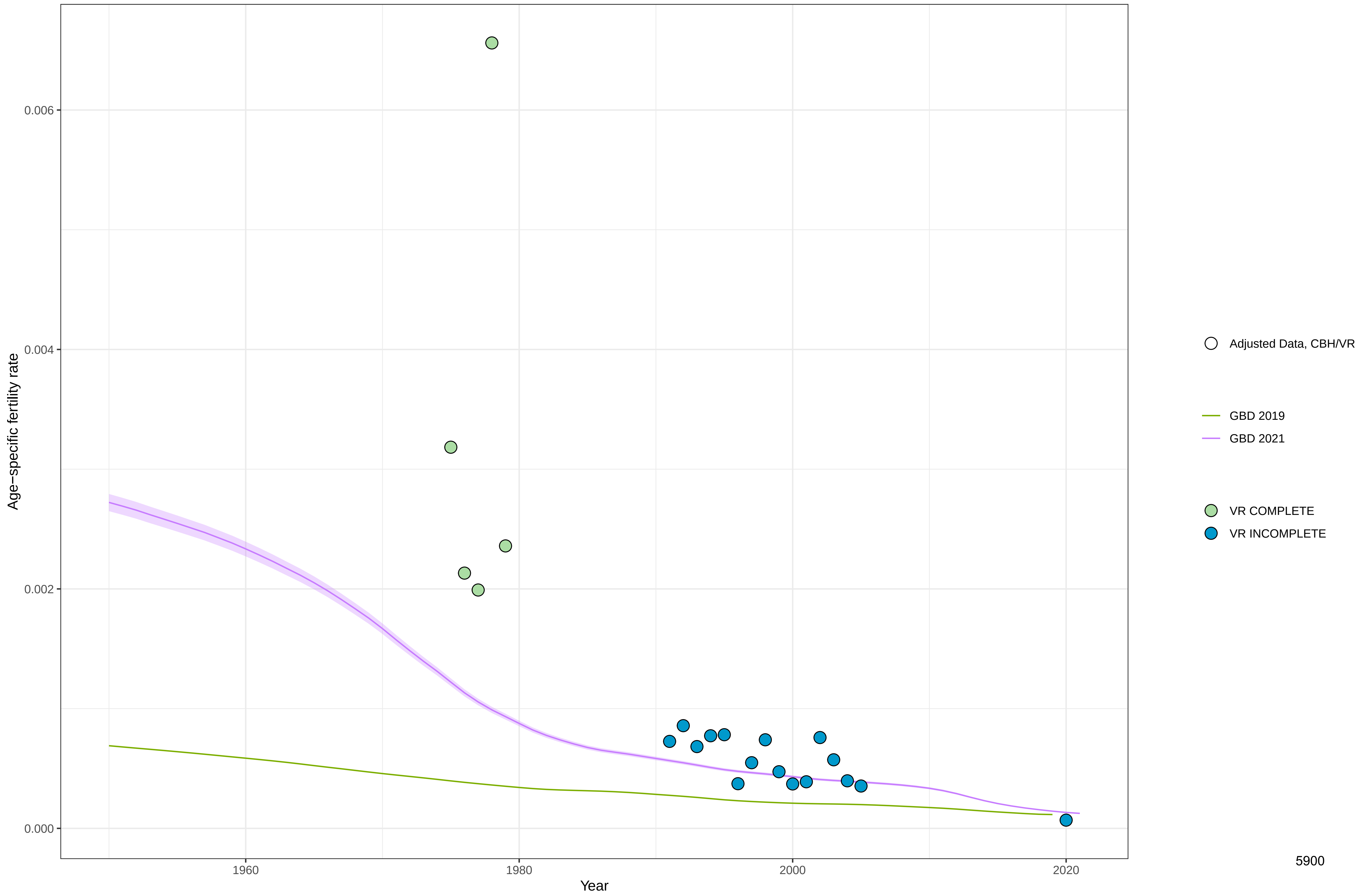

Total fertility rate fitted trend lines, 1950−2021: Batangas TFR
Data points enclosed with a diamond are reference sources

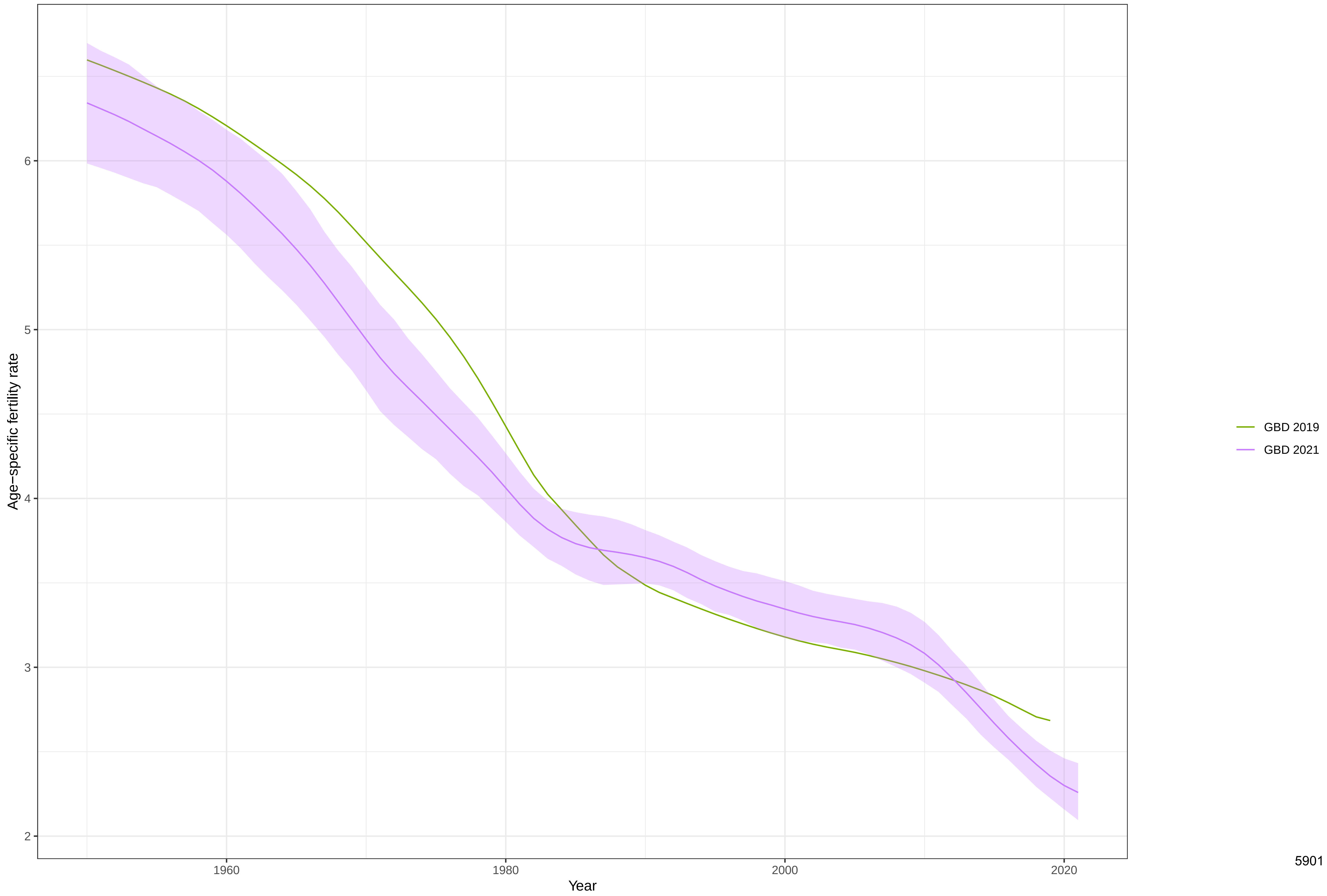

Age−specific fertility fitted trend lines, 1950−2021: Benguet, Ages: 10−14
Data points enclosed with a diamond are reference sources

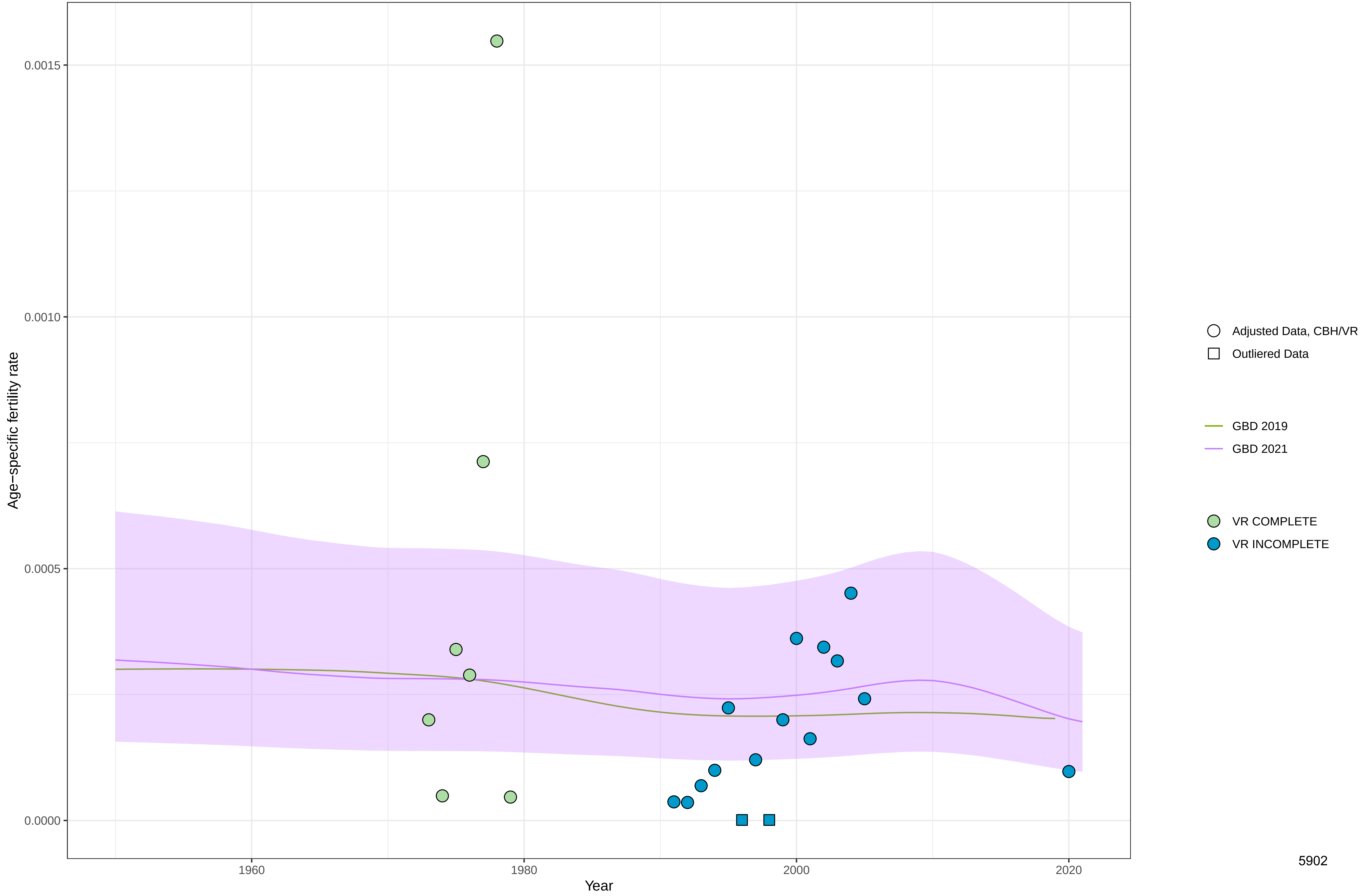

Age−specific fertility fitted trend lines, 1950−2021: Benguet, Ages: 15−19
Data points enclosed with a diamond are reference sources

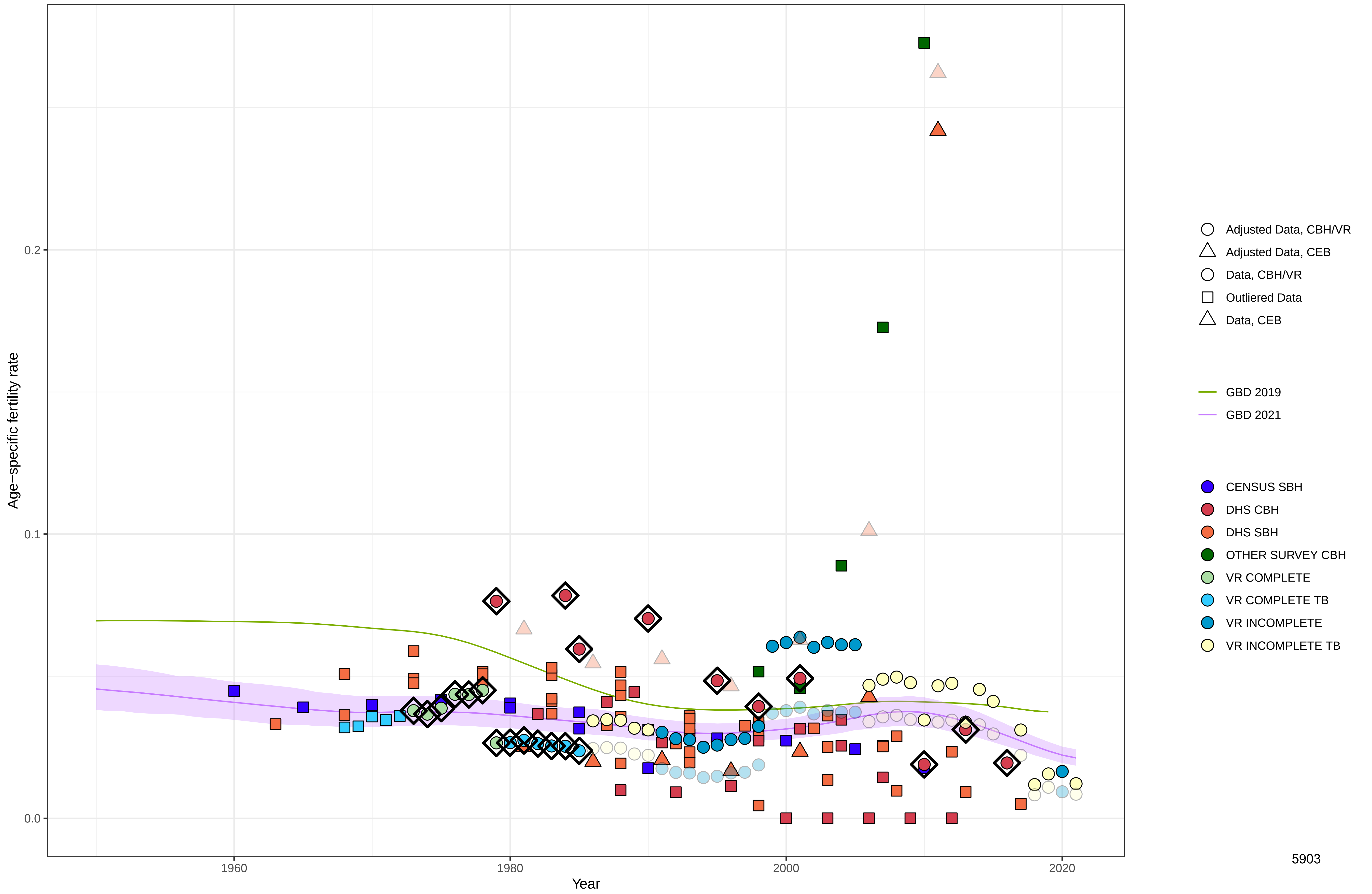

Age−specific fertility fitted trend lines, 1950−2021: Benguet, Ages: 20−24
Data points enclosed with a diamond are reference sources

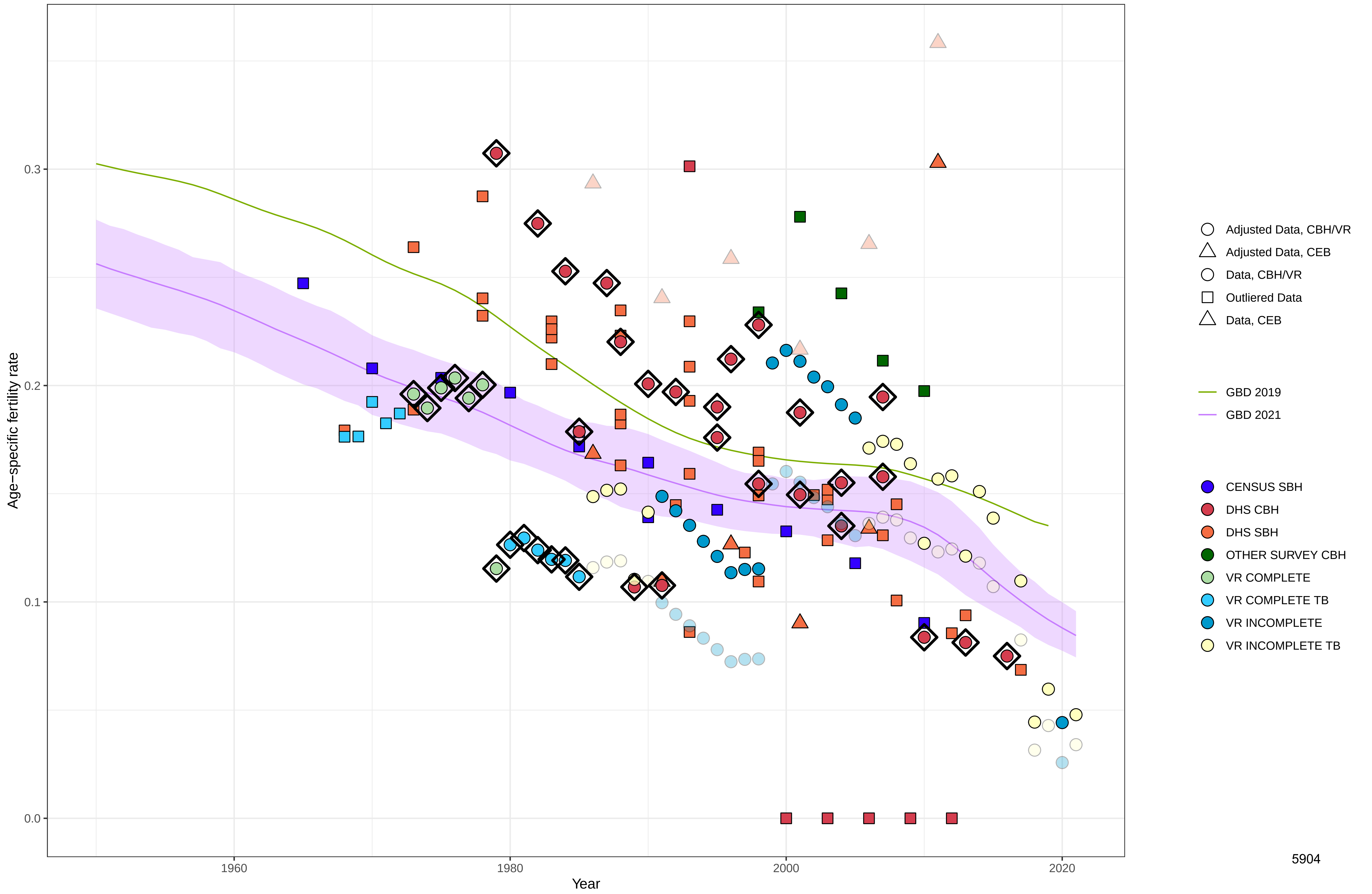

Age−specific fertility fitted trend lines, 1950−2021: Benguet, Ages: 25−29
Data points enclosed with a diamond are reference sources

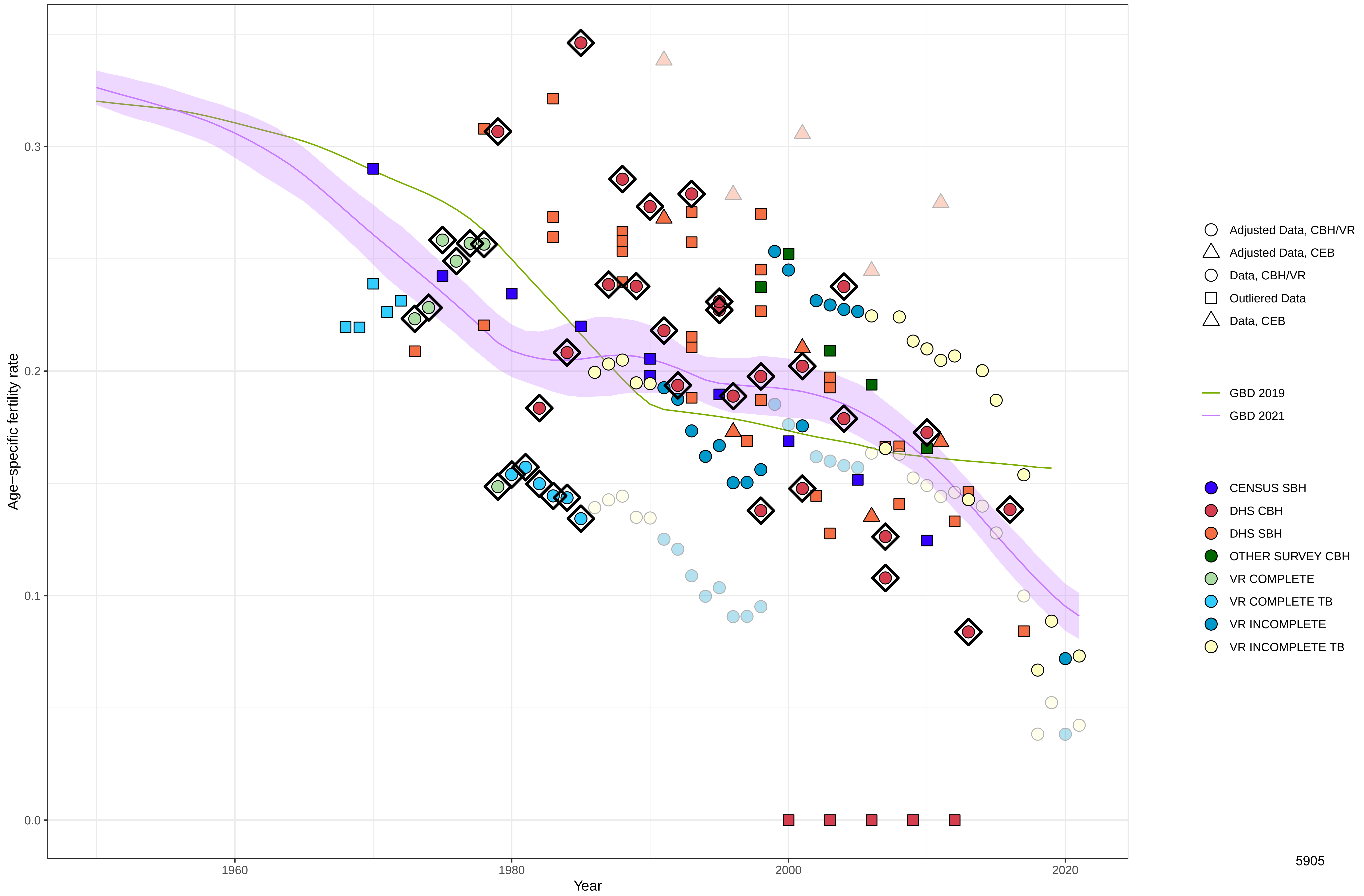

Age−specific fertility fitted trend lines, 1950−2021: Benguet, Ages: 30−34
Data points enclosed with a diamond are reference sources

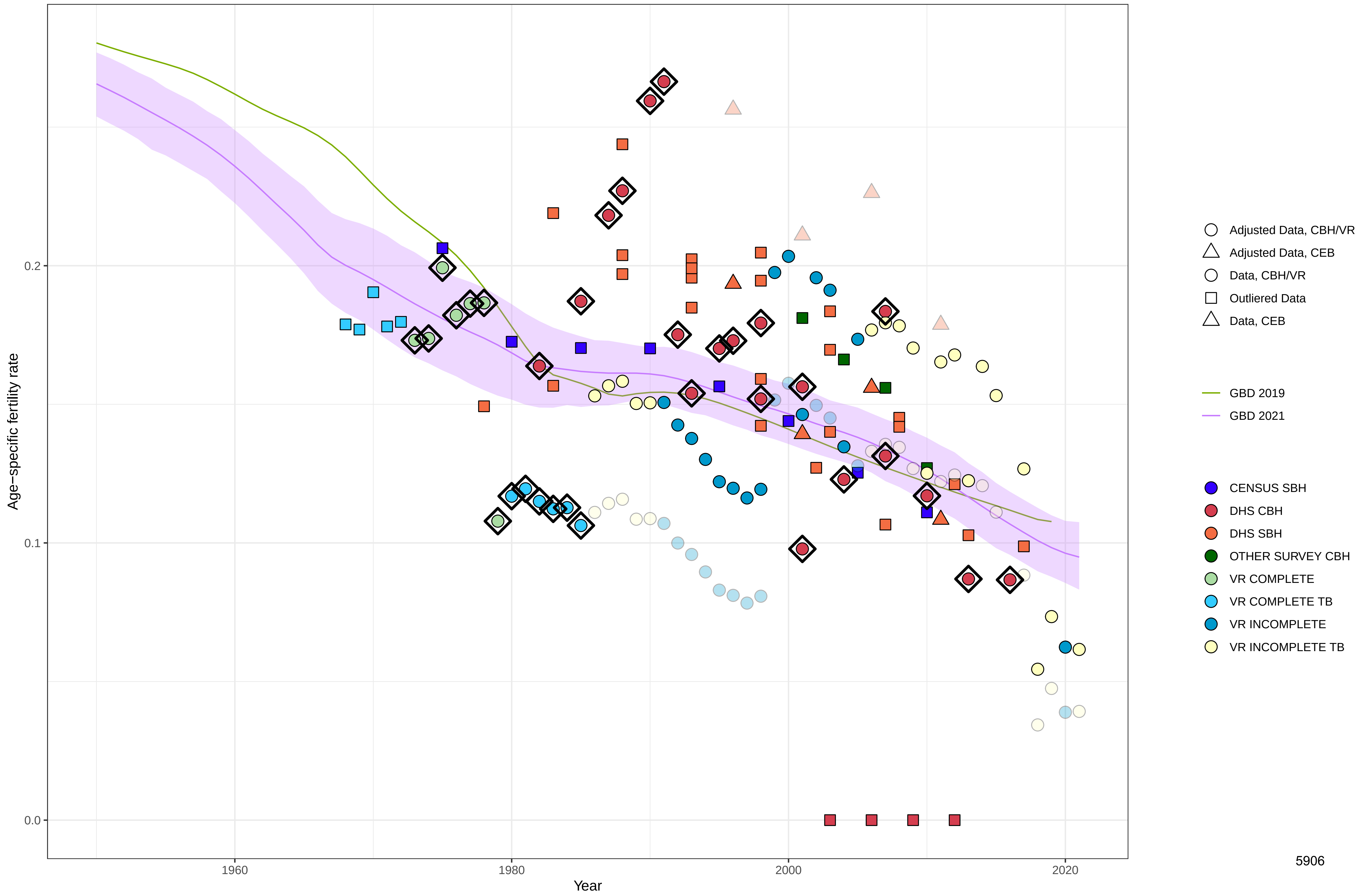

Age−specific fertility fitted trend lines, 1950−2021: Benguet, Ages: 35−39
Data points enclosed with a diamond are reference sources

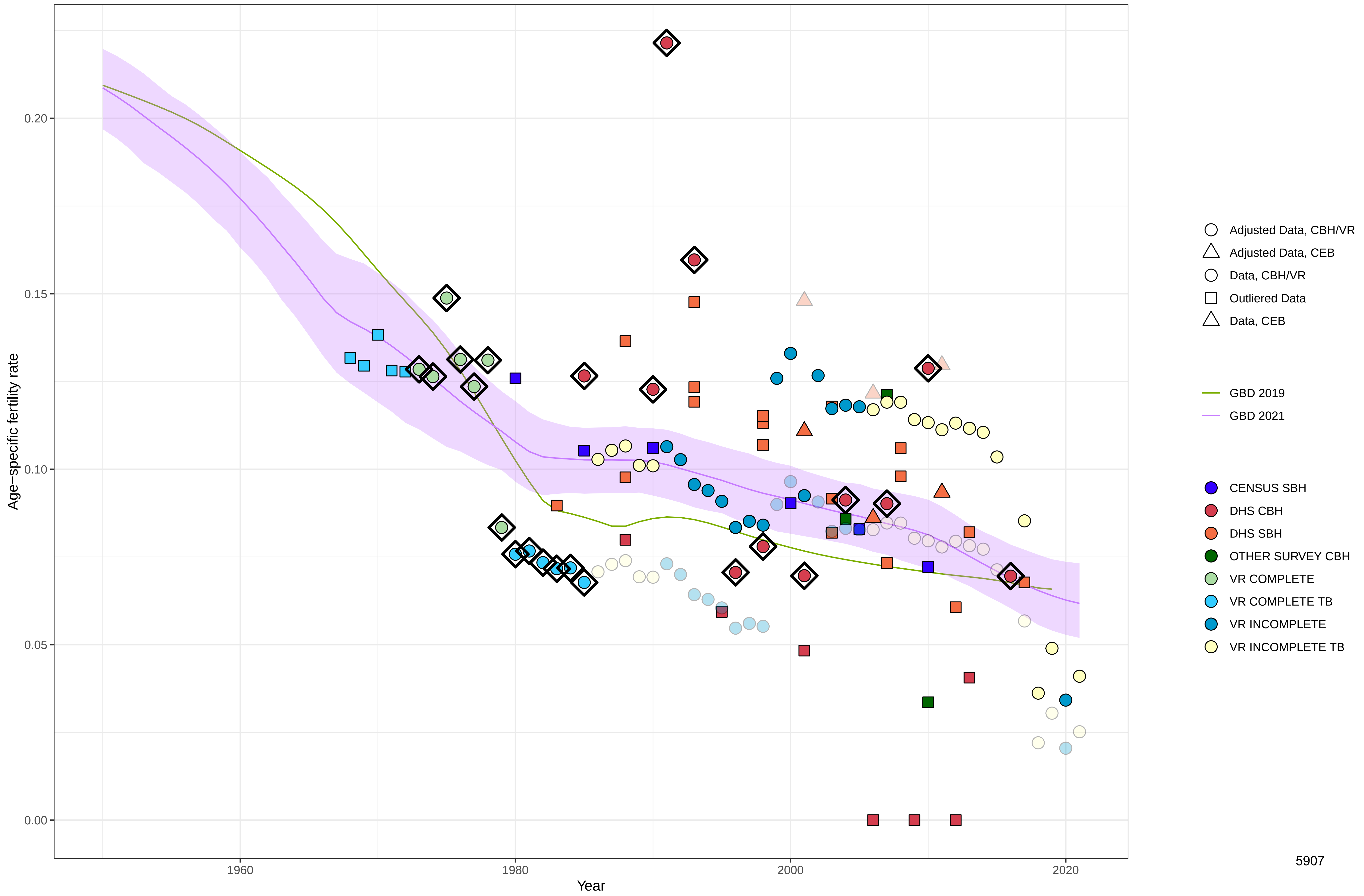

Age−specific fertility fitted trend lines, 1950−2021: Benguet, Ages: 40−44
Data points enclosed with a diamond are reference sources

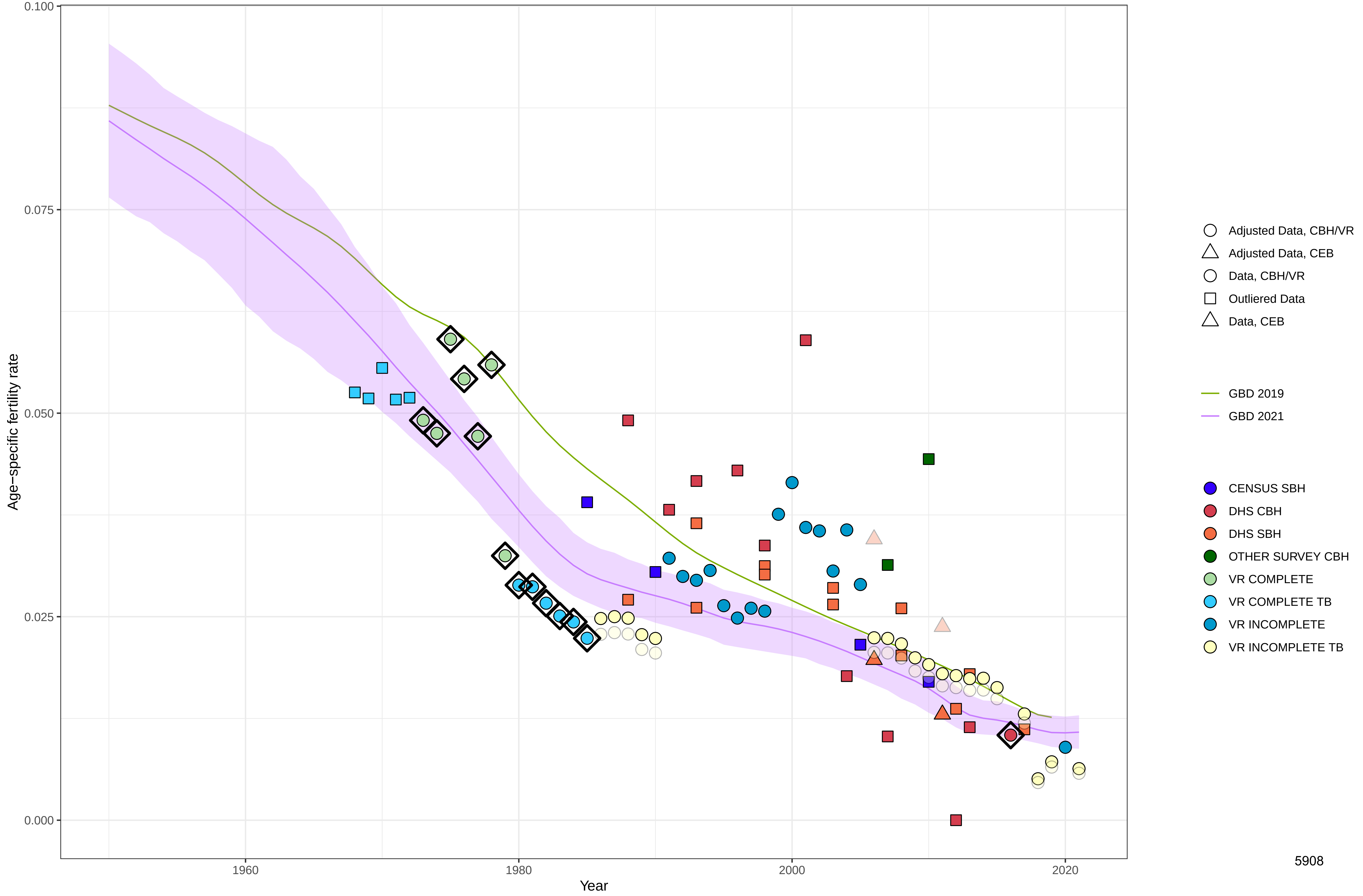

Age−specific fertility fitted trend lines, 1950−2021: Benguet, Ages: 45−49
Data points enclosed with a diamond are reference sources

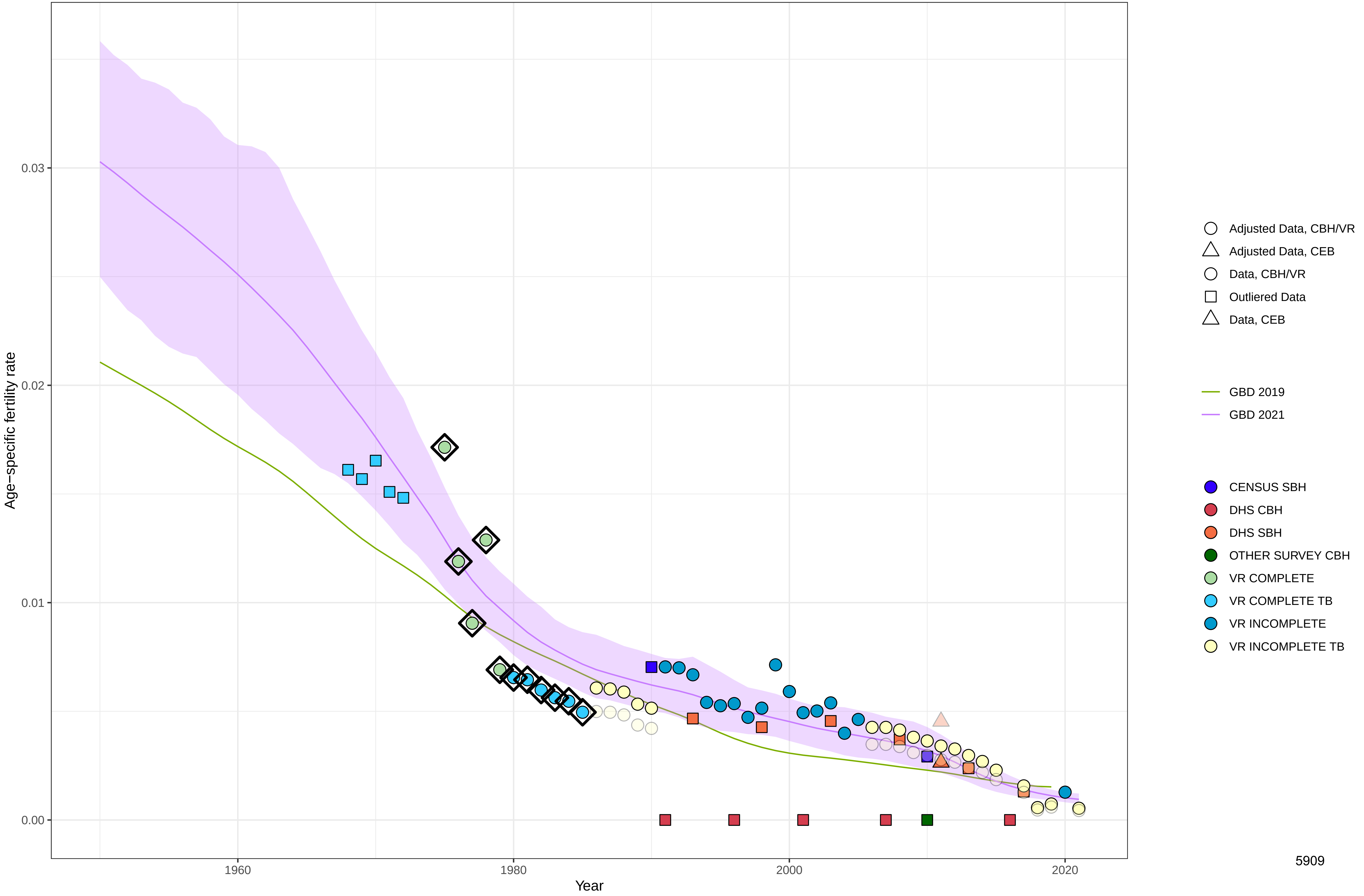

Age−specific fertility fitted trend lines, 1950−2021: Benguet, Ages: 50−54
Data points enclosed with a diamond are reference sources

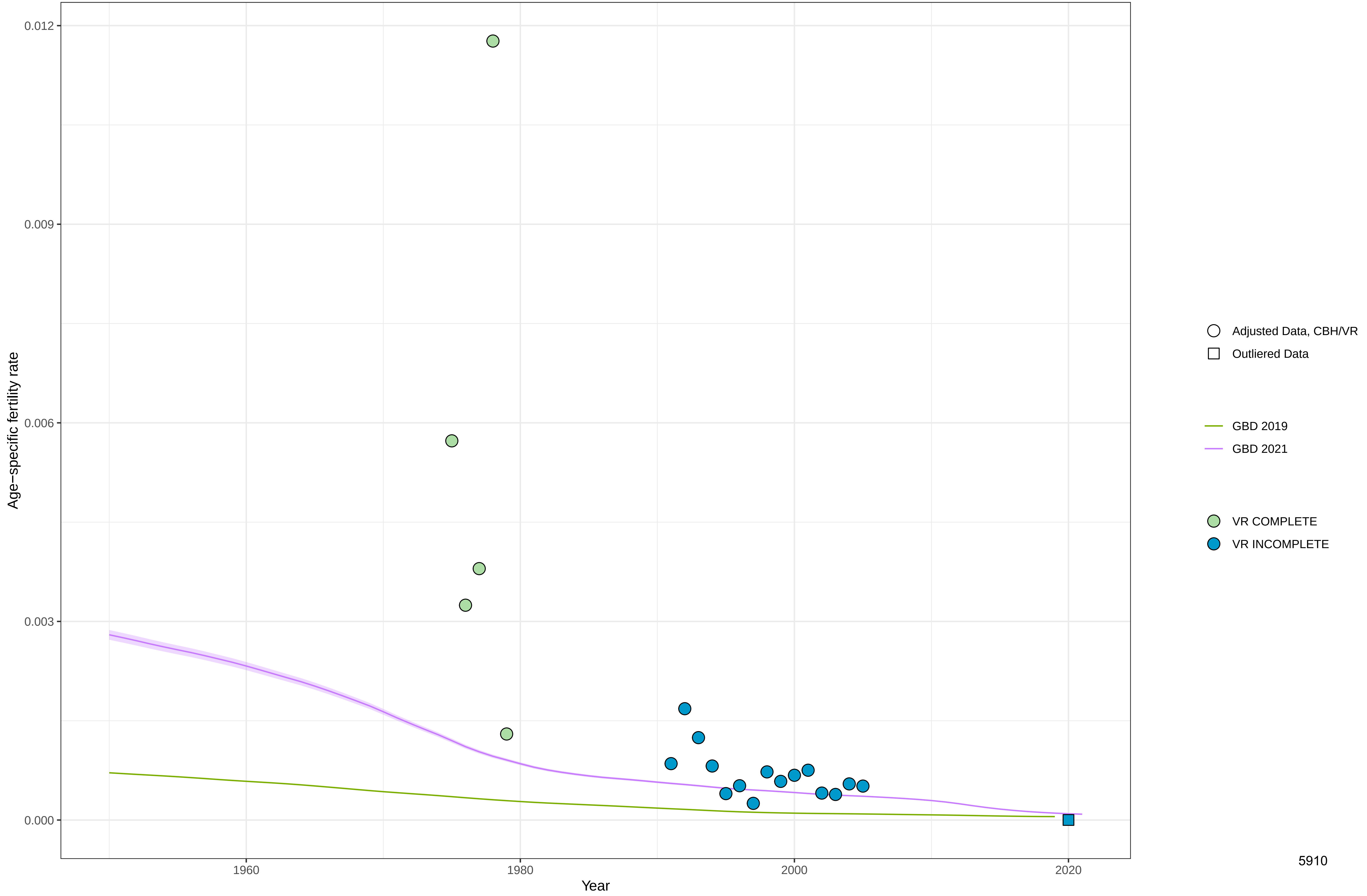

Total fertility rate fitted trend lines, 1950−2021: Benguet TFR
Data points enclosed with a diamond are reference sources

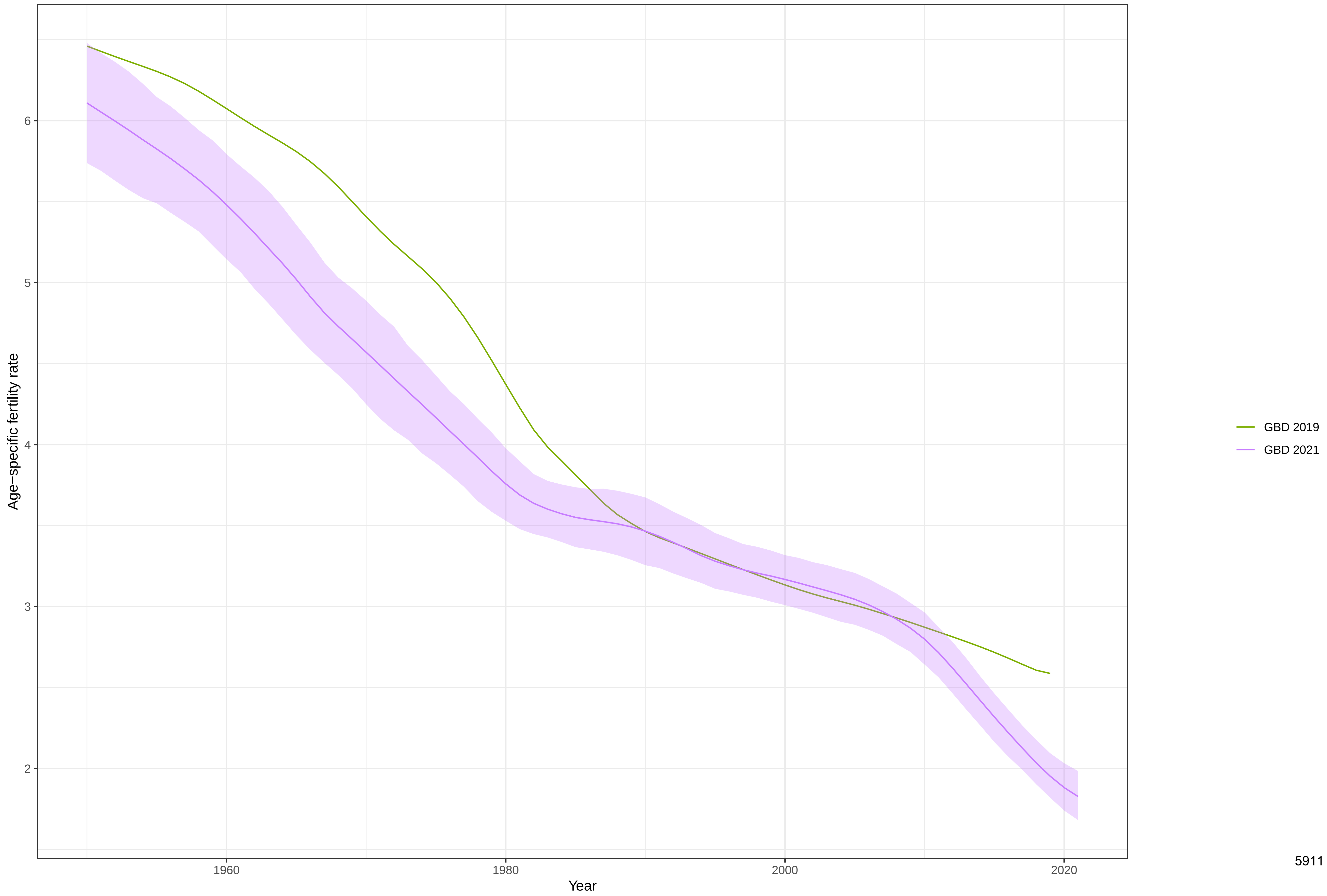

5911

Age−specific fertility fitted trend lines, 1950−2021: Biliran, Ages: 10−14
Data points enclosed with a diamond are reference sources

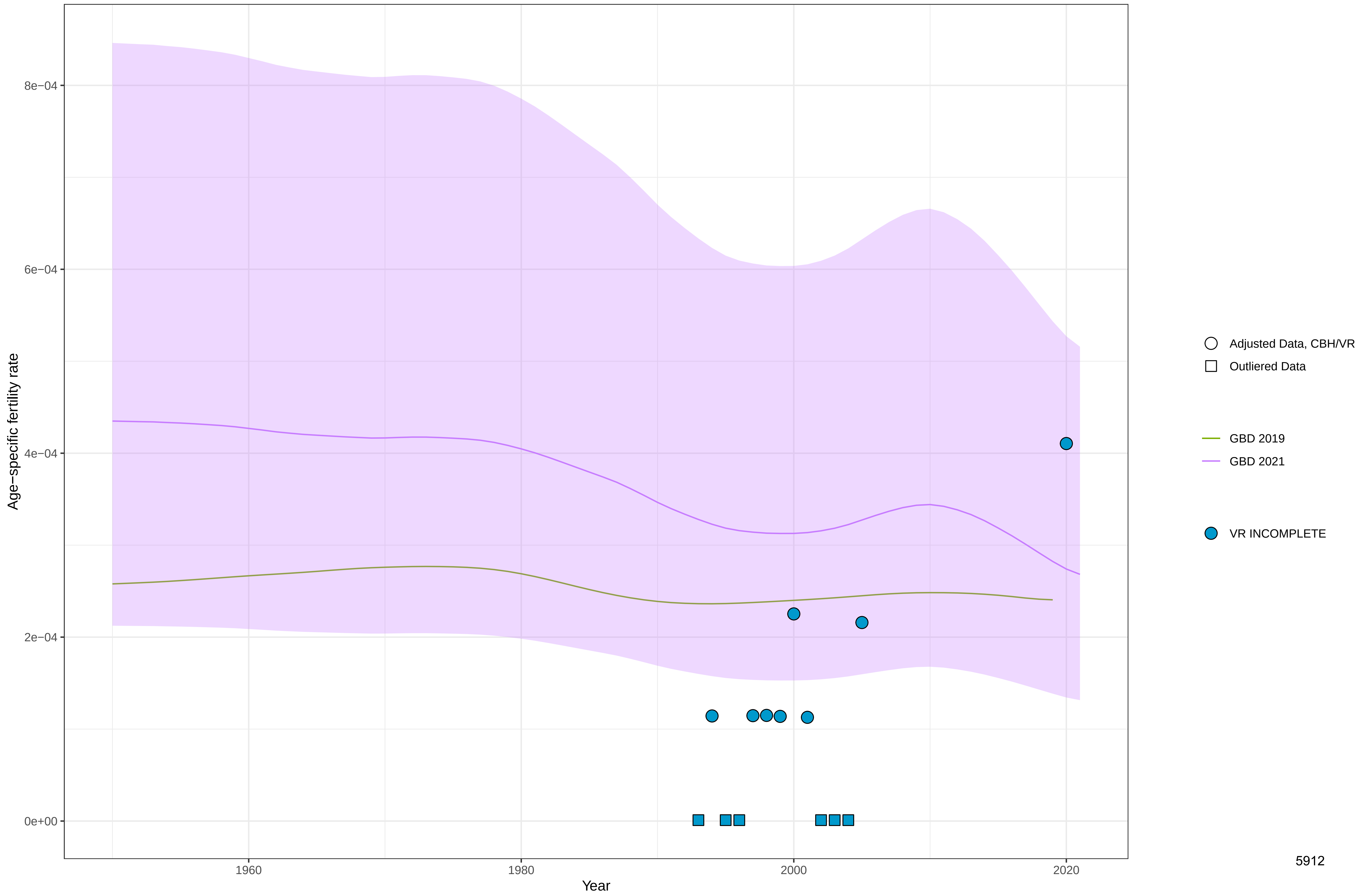

Age−specific fertility fitted trend lines, 1950−2021: Biliran, Ages: 15−19
Data points enclosed with a diamond are reference sources

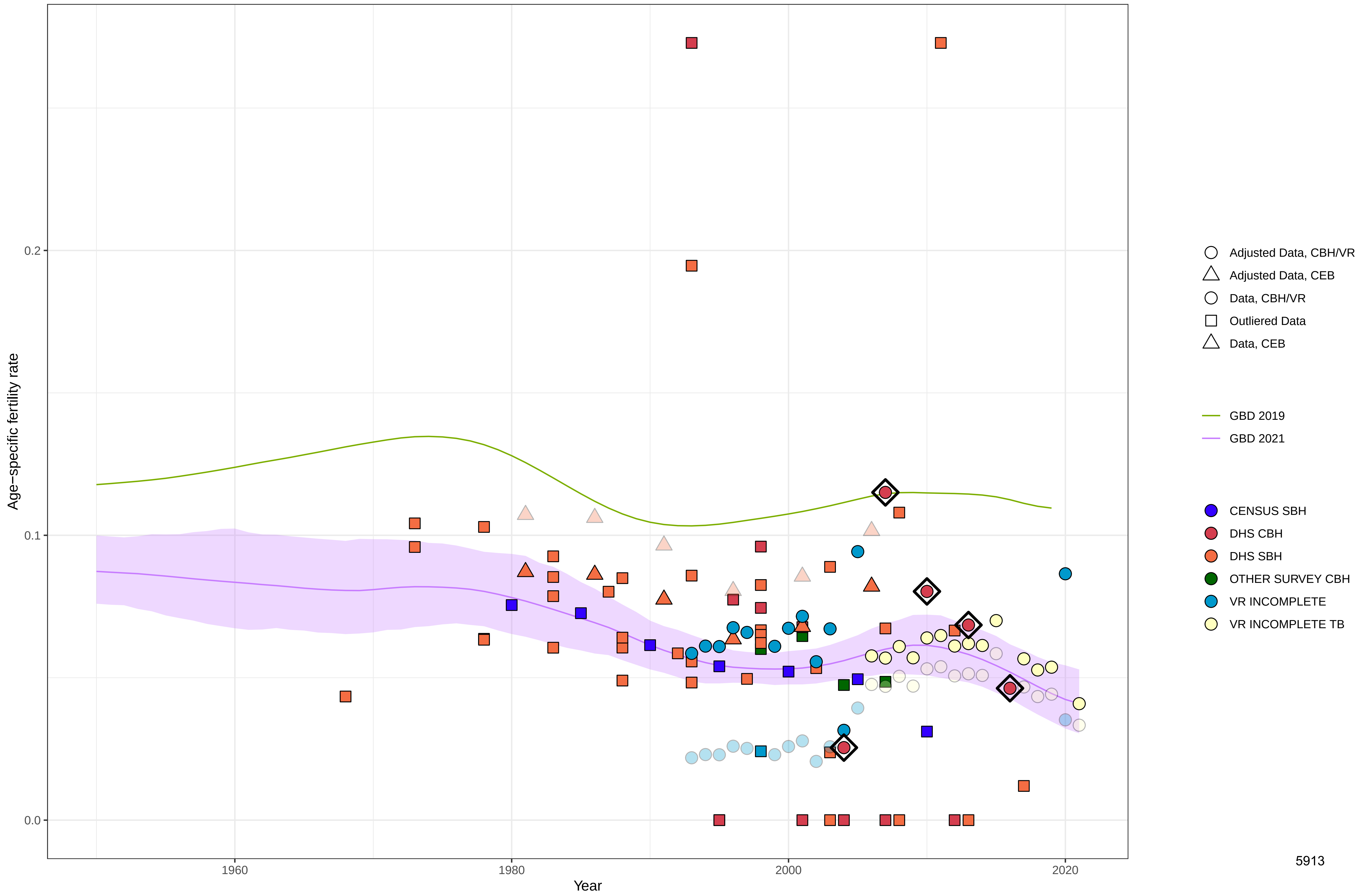

Age−specific fertility fitted trend lines, 1950−2021: Biliran, Ages: 20−24
Data points enclosed with a diamond are reference sources

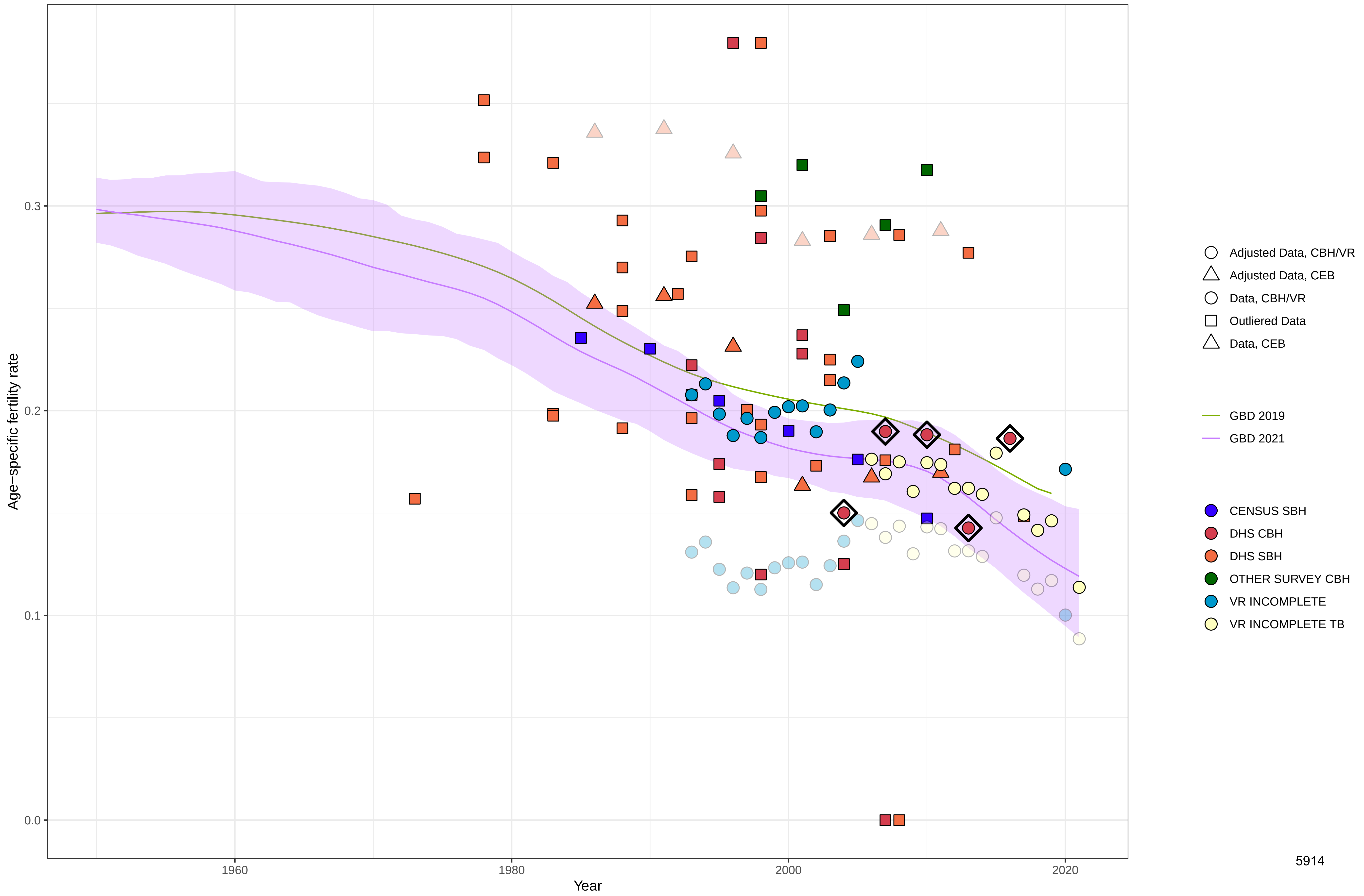

Age−specific fertility fitted trend lines, 1950−2021: Biliran, Ages: 25−29
Data points enclosed with a diamond are reference sources

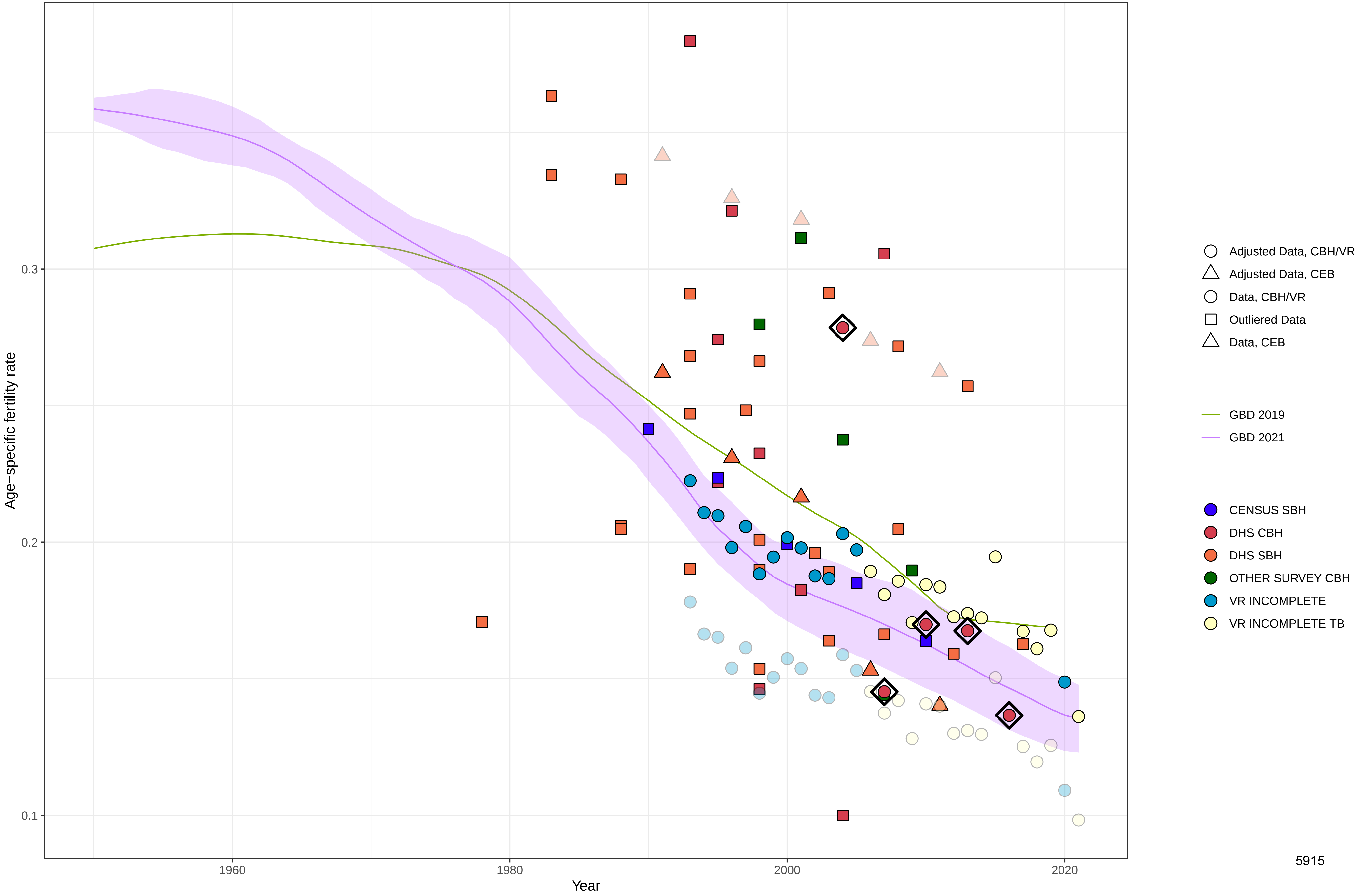

Age−specific fertility fitted trend lines, 1950−2021: Biliran, Ages: 30−34
Data points enclosed with a diamond are reference sources

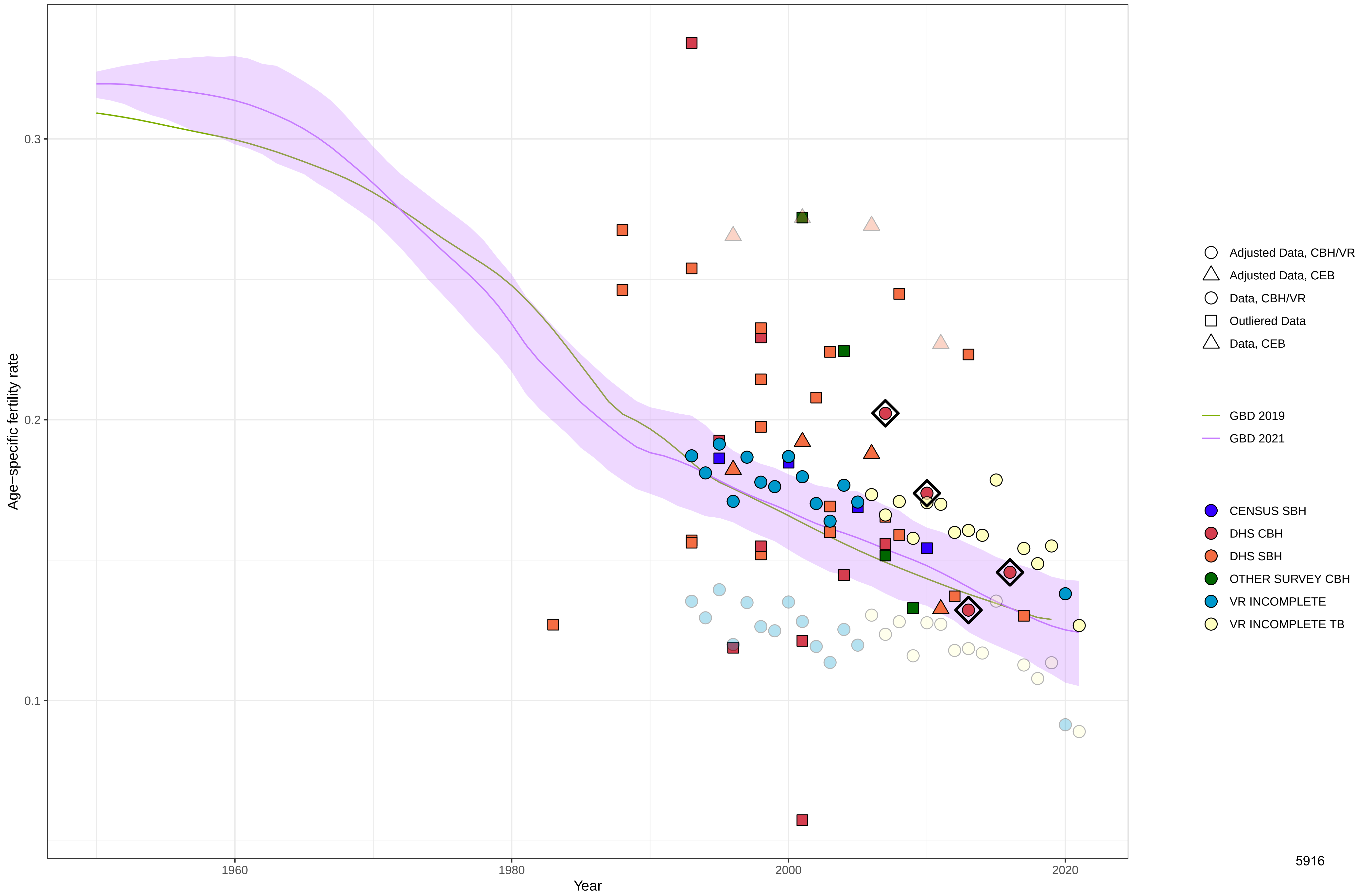

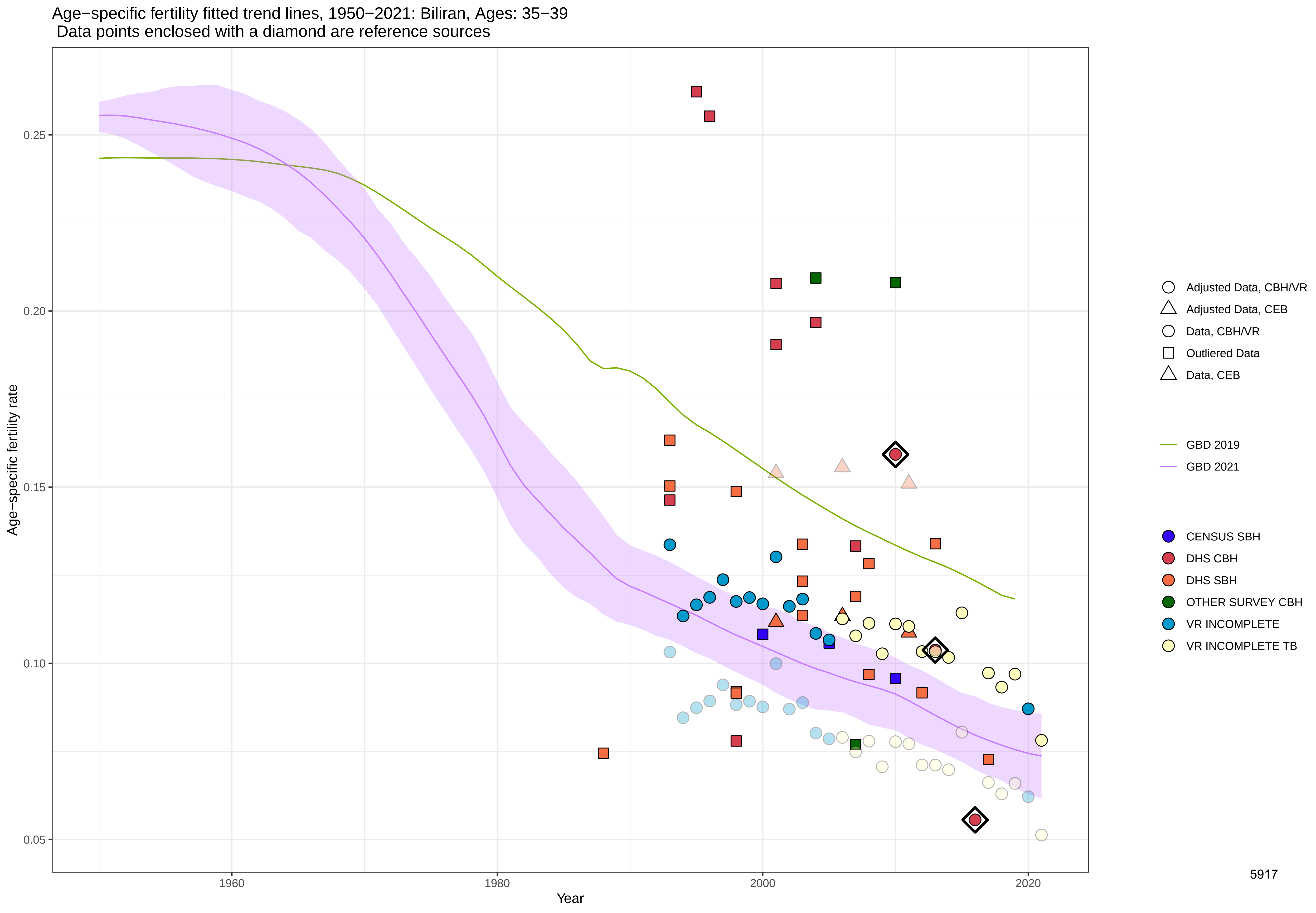

Age−specific fertility fitted trend lines, 1950−2021: Biliran, Ages: 35−39
Data points enclosed with a diamond are reference sources
Age−specific fertility rate
0.25
0.20
0.15
0.10
0.05
1960
1980
2000
2020
Year
Adjusted Data, CBH/VR
Adjusted Data, CEB
Data, CBH/VR
Outliered Data
Data, CEB
GBD 2019
GBD 2021
CENSUS SBH
DHS CBH
DHS SBH
OTHER SURVEY CBH
VR INCOMPLETE
VR INCOMPLETE TB

5917

Age−specific fertility fitted trend lines, 1950−2021: Biliran, Ages: 40−44
Data points enclosed with a diamond are reference sources

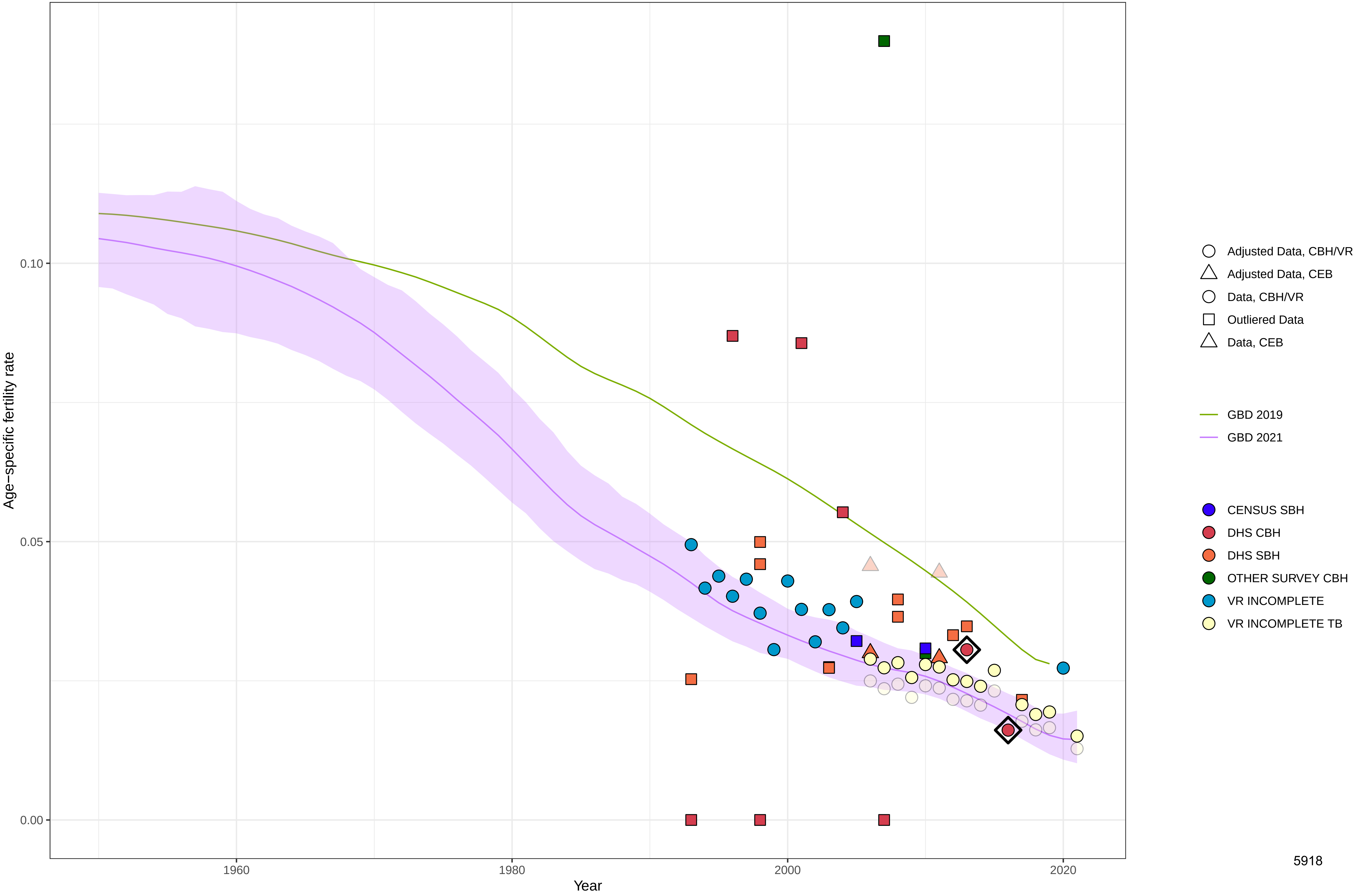

Age−specific fertility fitted trend lines, 1950−2021: Biliran, Ages: 45−49
Data points enclosed with a diamond are reference sources

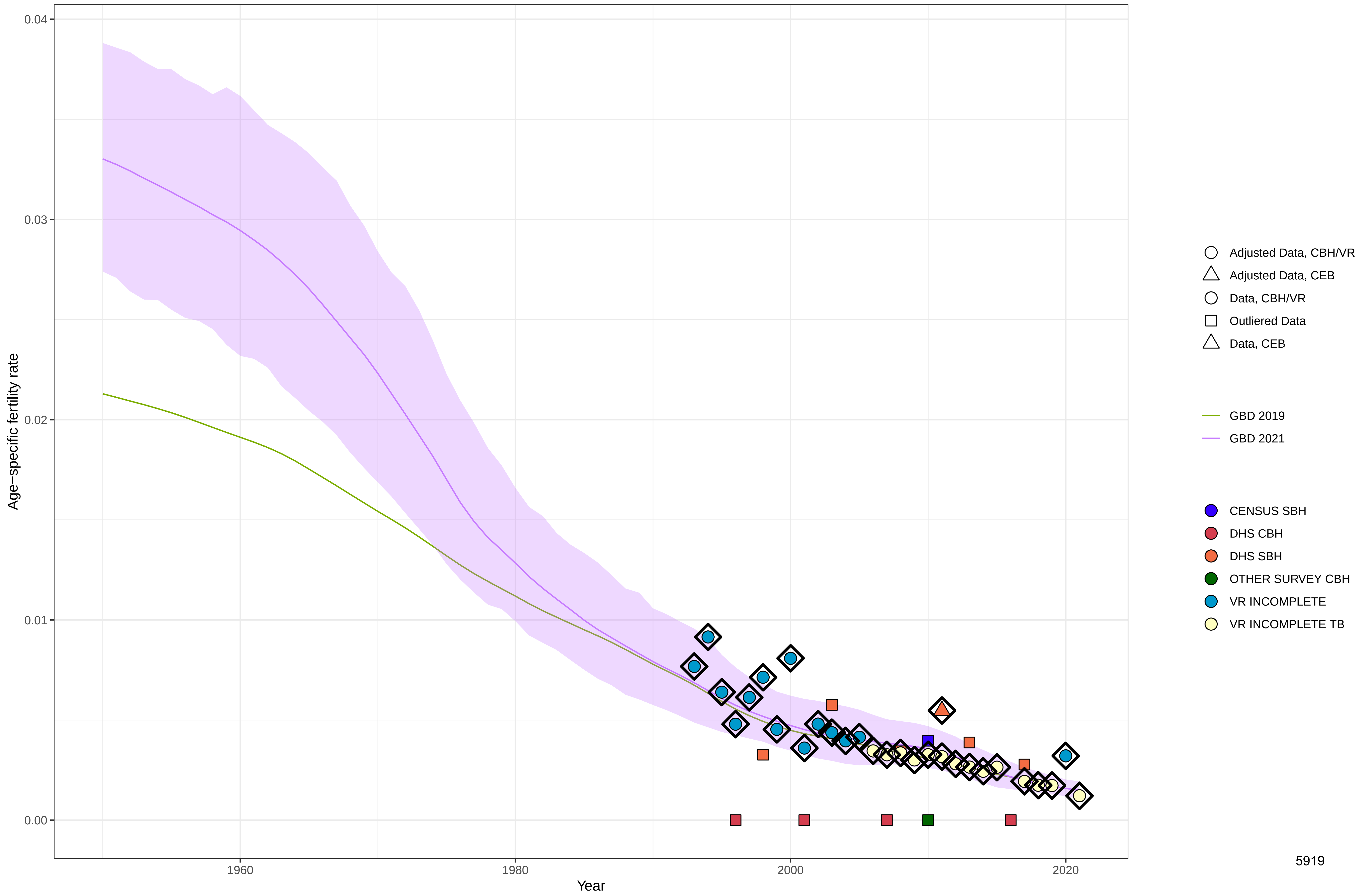

Age−specific fertility fitted trend lines, 1950−2021: Biliran, Ages: 50−54
Data points enclosed with a diamond are reference sources

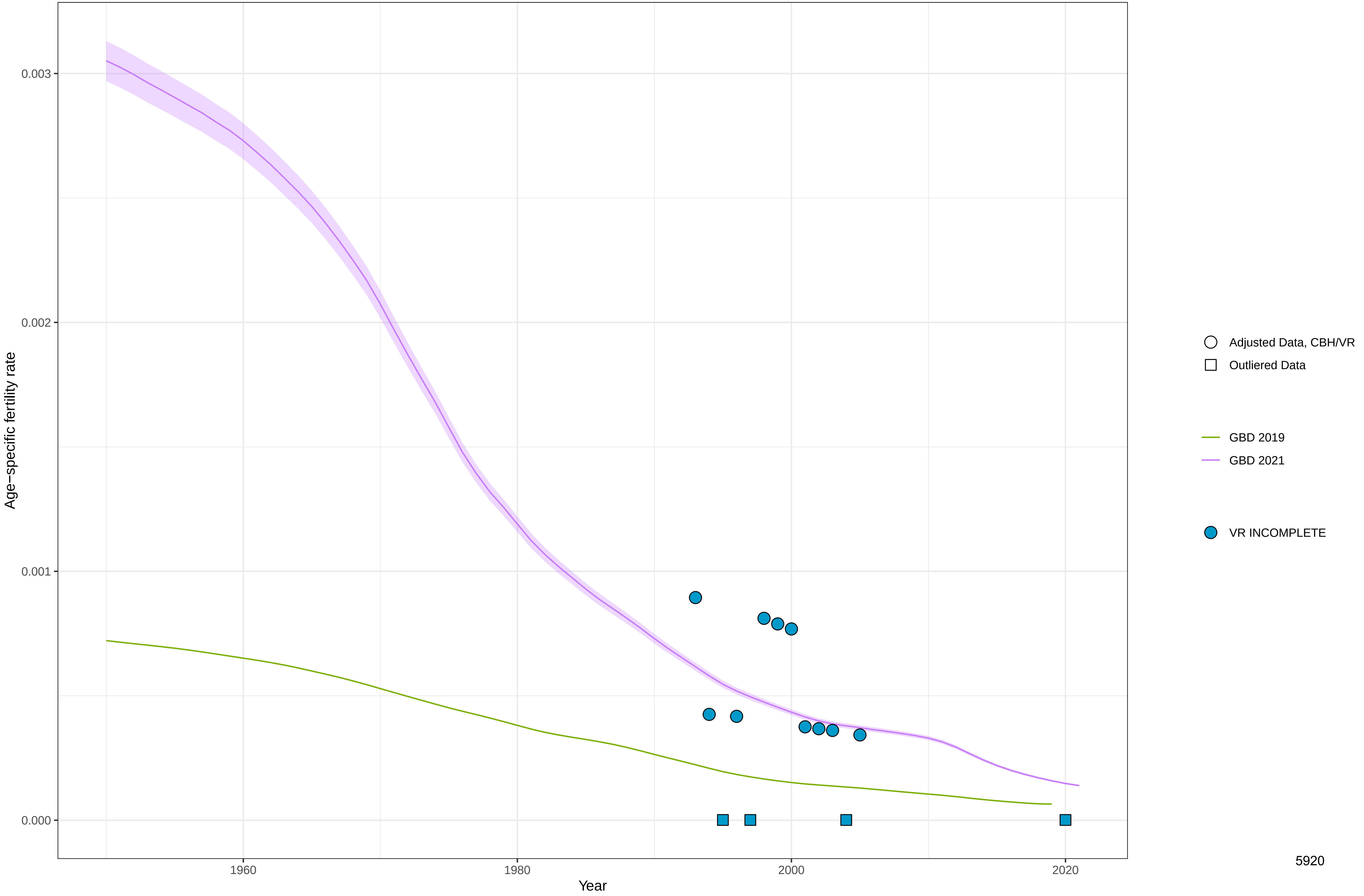

Total fertility rate fitted trend lines, 1950−2021: Biliran TFR
Data points enclosed with a diamond are reference sources

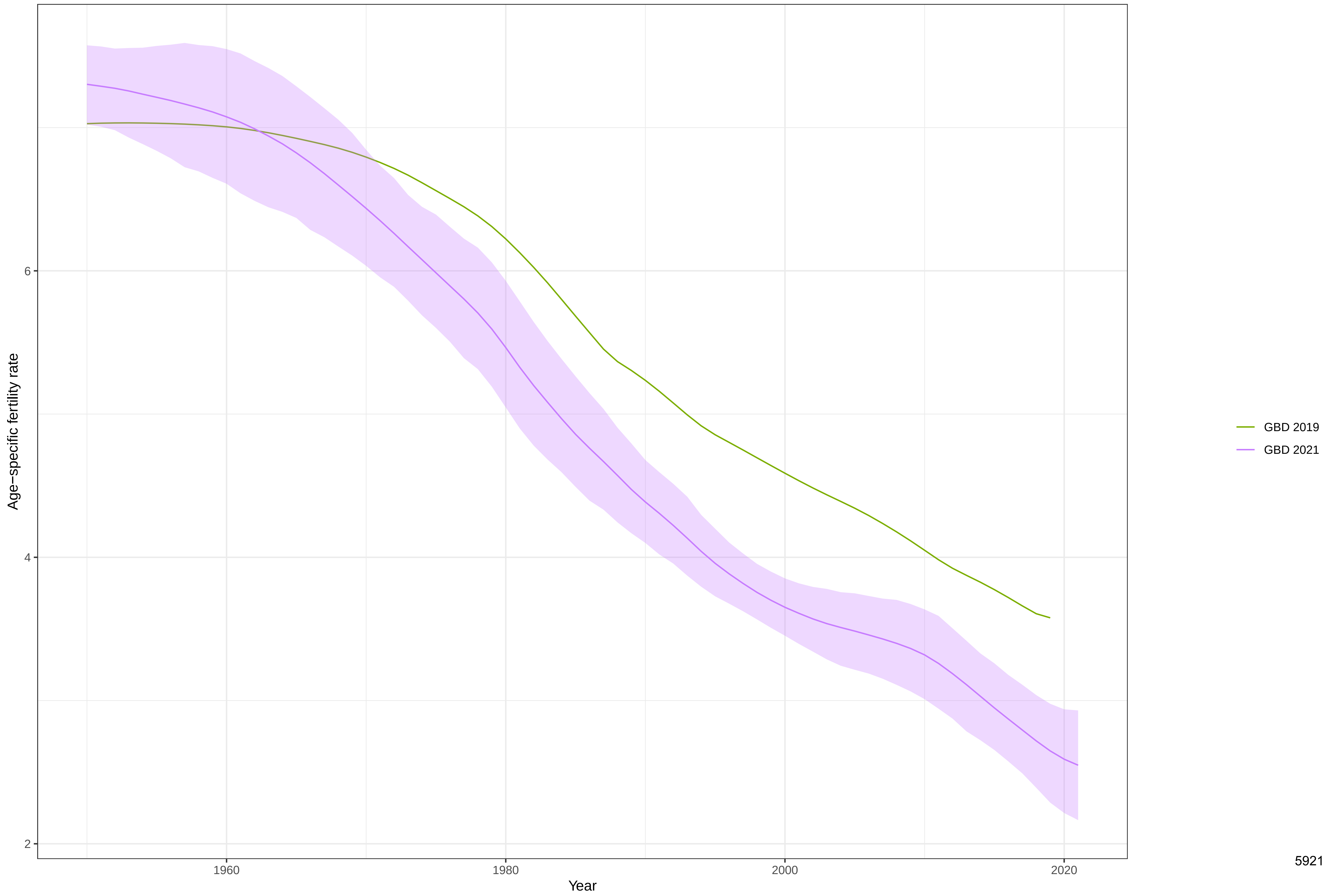

Age−specific fertility fitted trend lines, 1950−2021: Bohol, Ages: 10−14
Data points enclosed with a diamond are reference sources

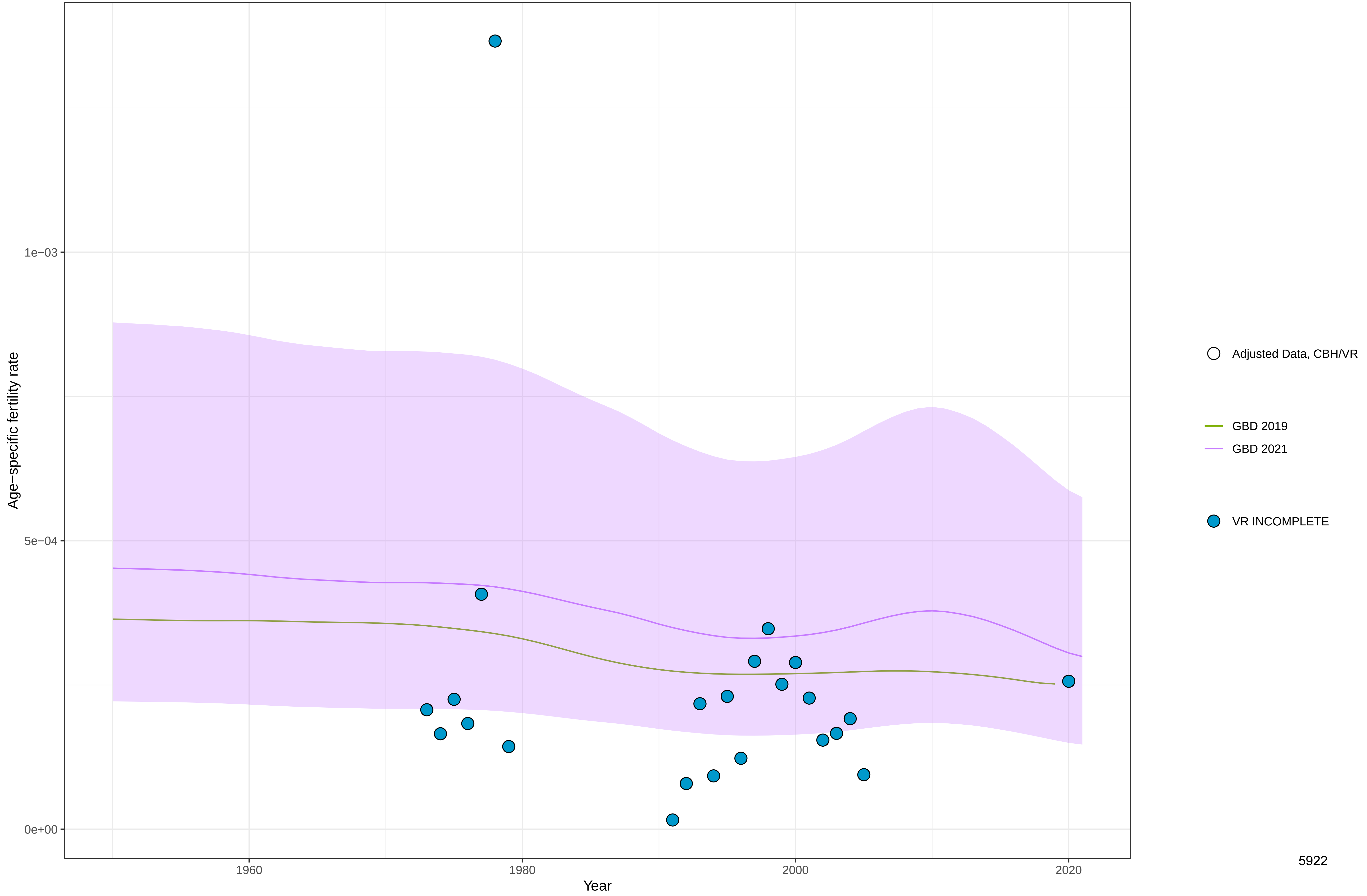

Age−specific fertility fitted trend lines, 1950−2021: Bohol, Ages: 15−19
Data points enclosed with a diamond are reference sources

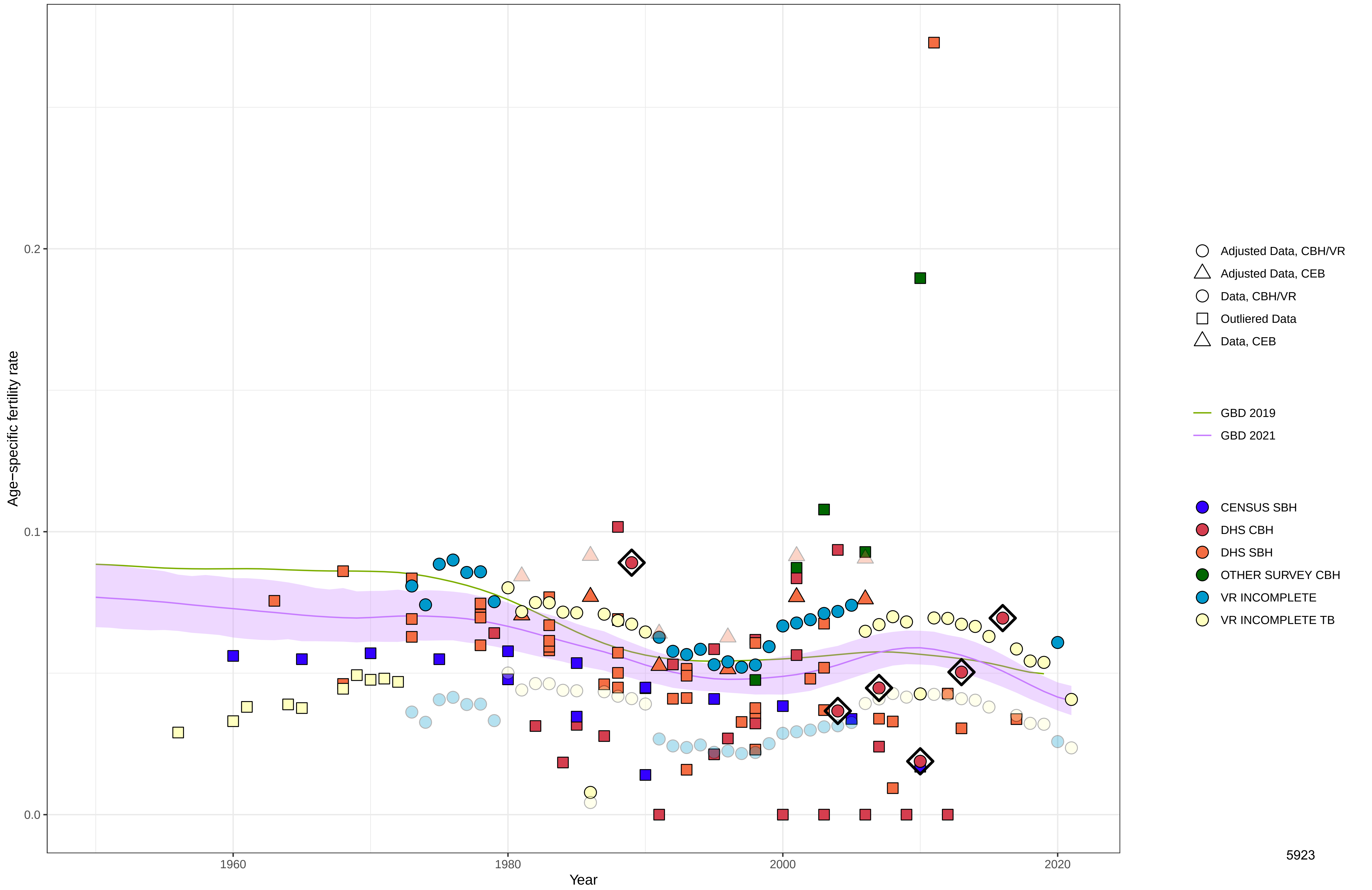

Age−specific fertility fitted trend lines, 1950−2021: Bohol, Ages: 20−24
Data points enclosed with a diamond are reference sources

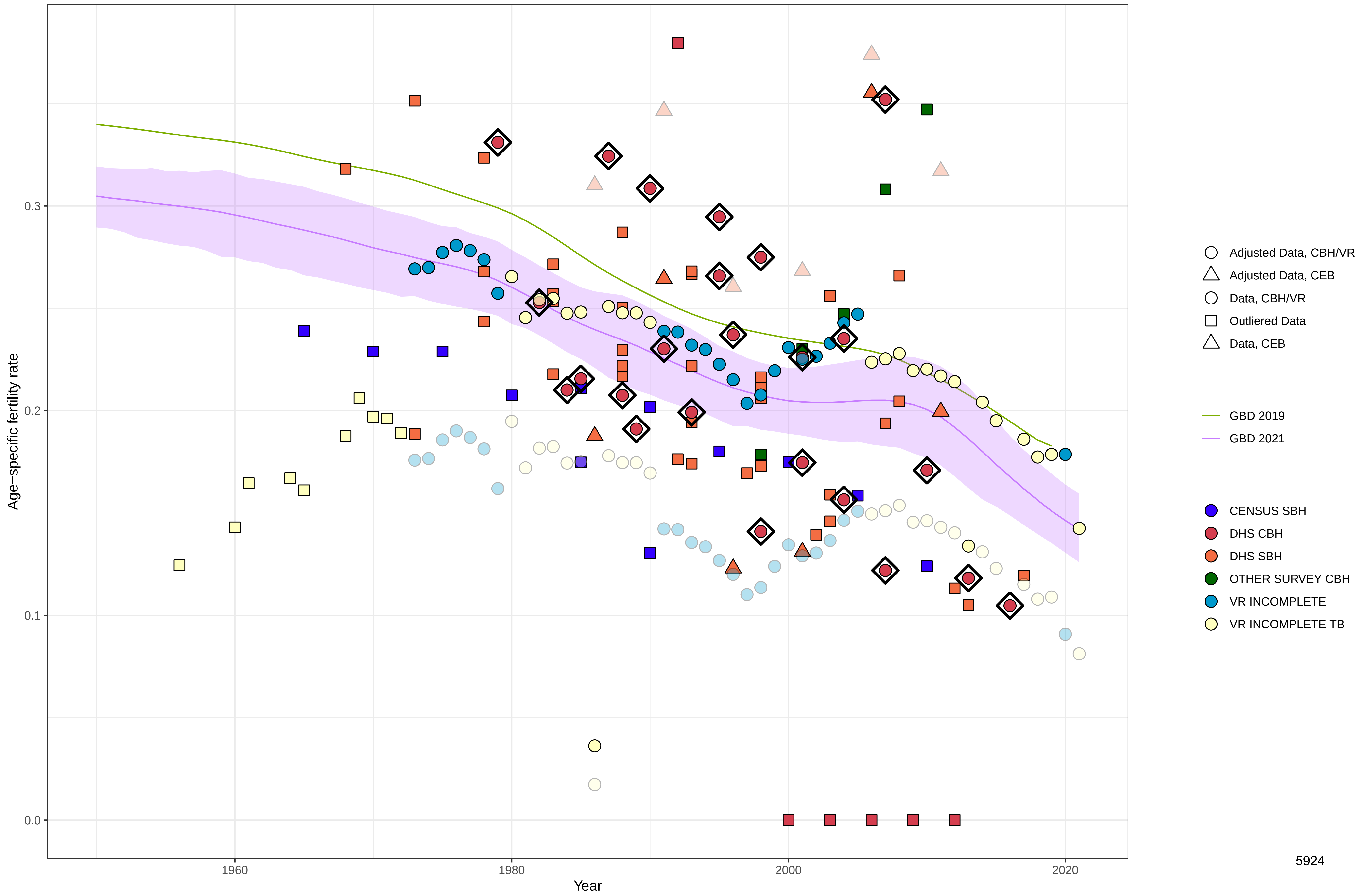

Age−specific fertility fitted trend lines, 1950−2021: Bohol, Ages: 25−29
Data points enclosed with a diamond are reference sources

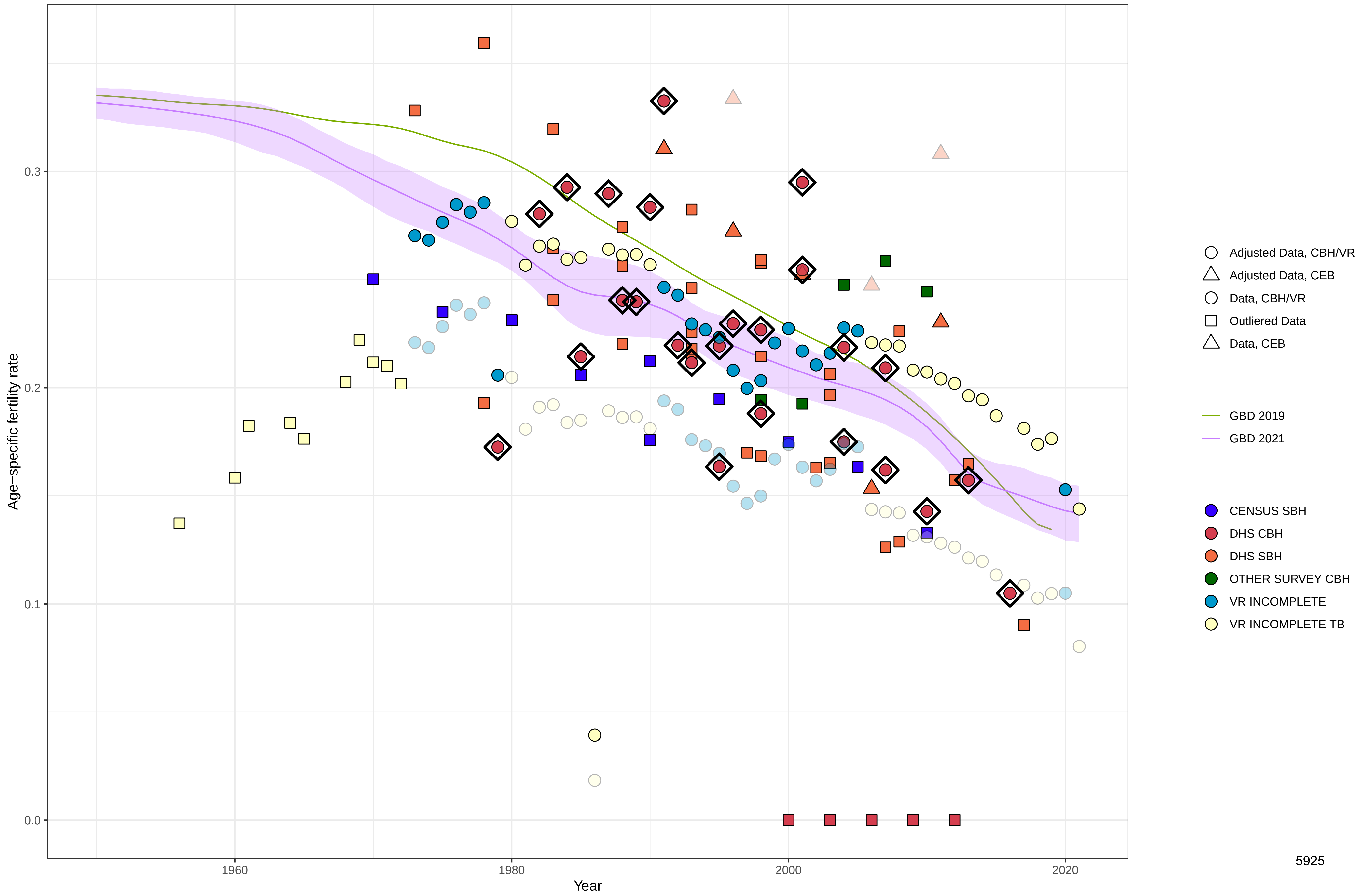

Age−specific fertility fitted trend lines, 1950−2021: Bohol, Ages: 30−34
Data points enclosed with a diamond are reference sources

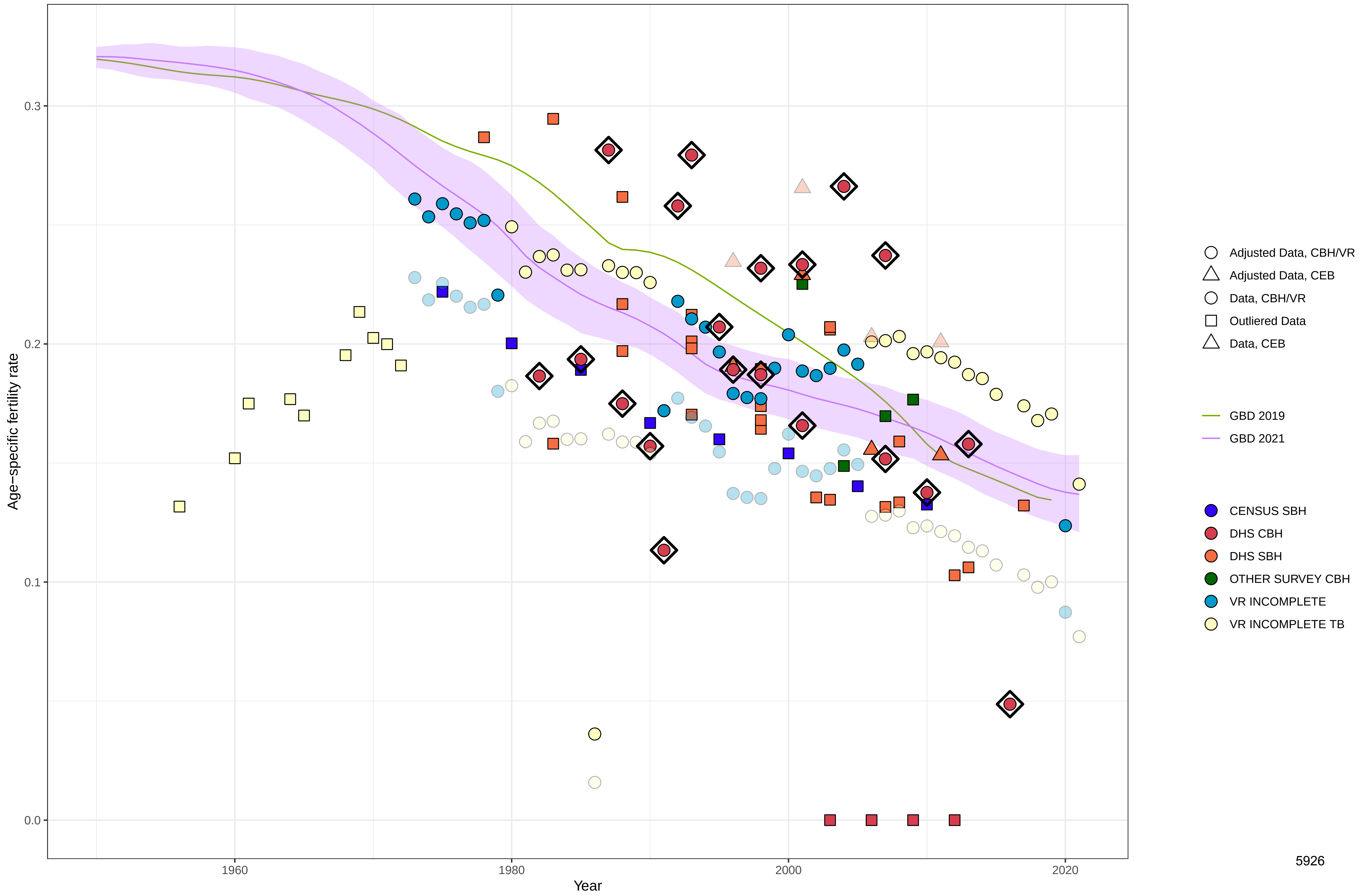

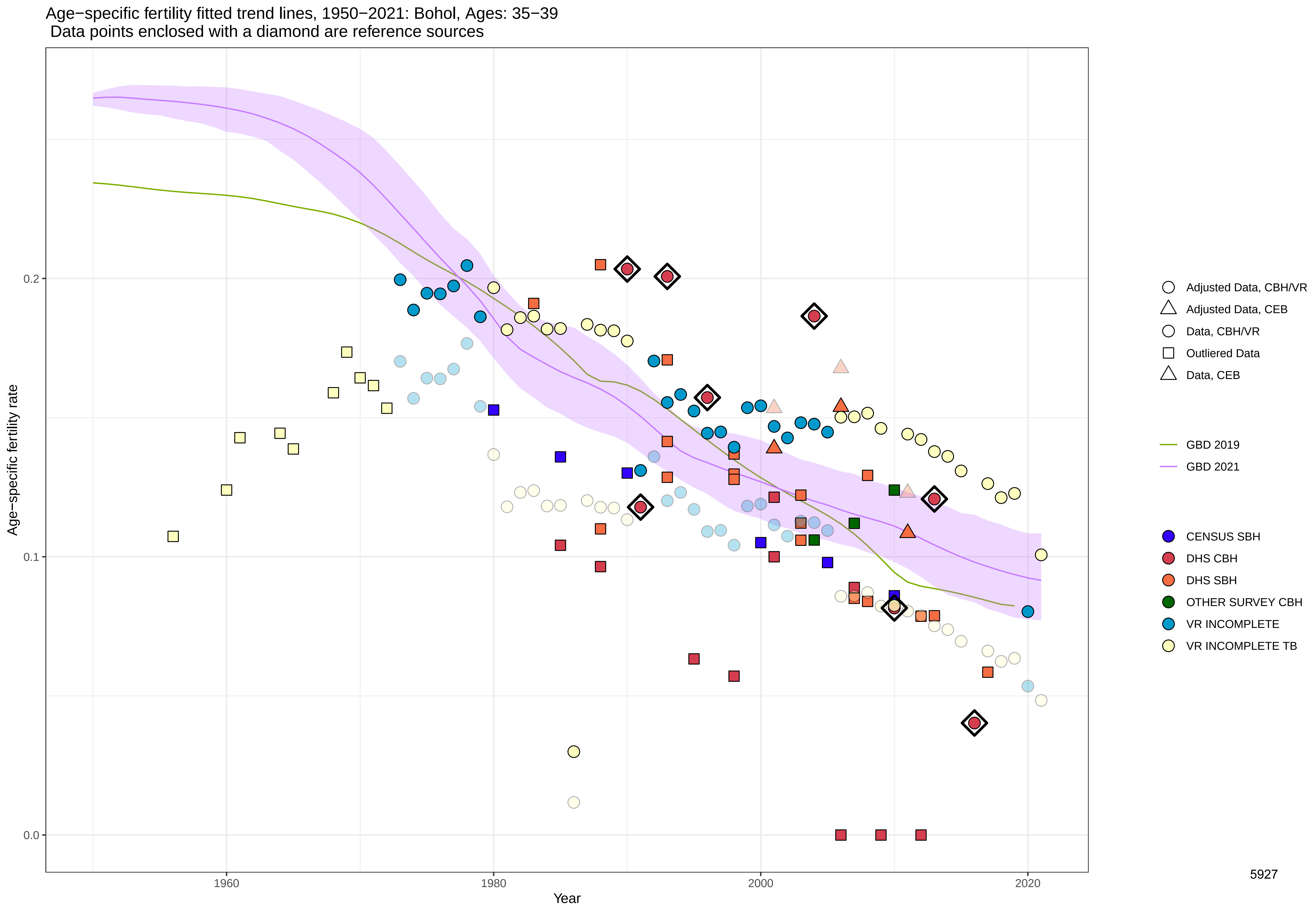

Age−specific fertility fitted trend lines, 1950−2021: Bohol, Ages: 35−39
Data points enclosed with a diamond are reference sources
Age−specific fertility rate
Year
0.0
0.1
0.2
1960
1980
2000
2020
Adjusted Data, CBH/VR
Adjusted Data, CEB
Data, CBH/VR
Outliered Data
Data, CEB
GBD 2019
GBD 2021
CENSUS SBH
DHS CBH
DHS SBH
OTHER SURVEY CBH
VR INCOMPLETE
VR INCOMPLETE TB

Age−specific fertility fitted trend lines, 1950−2021: Bohol, Ages: 40−44
Data points enclosed with a diamond are reference sources

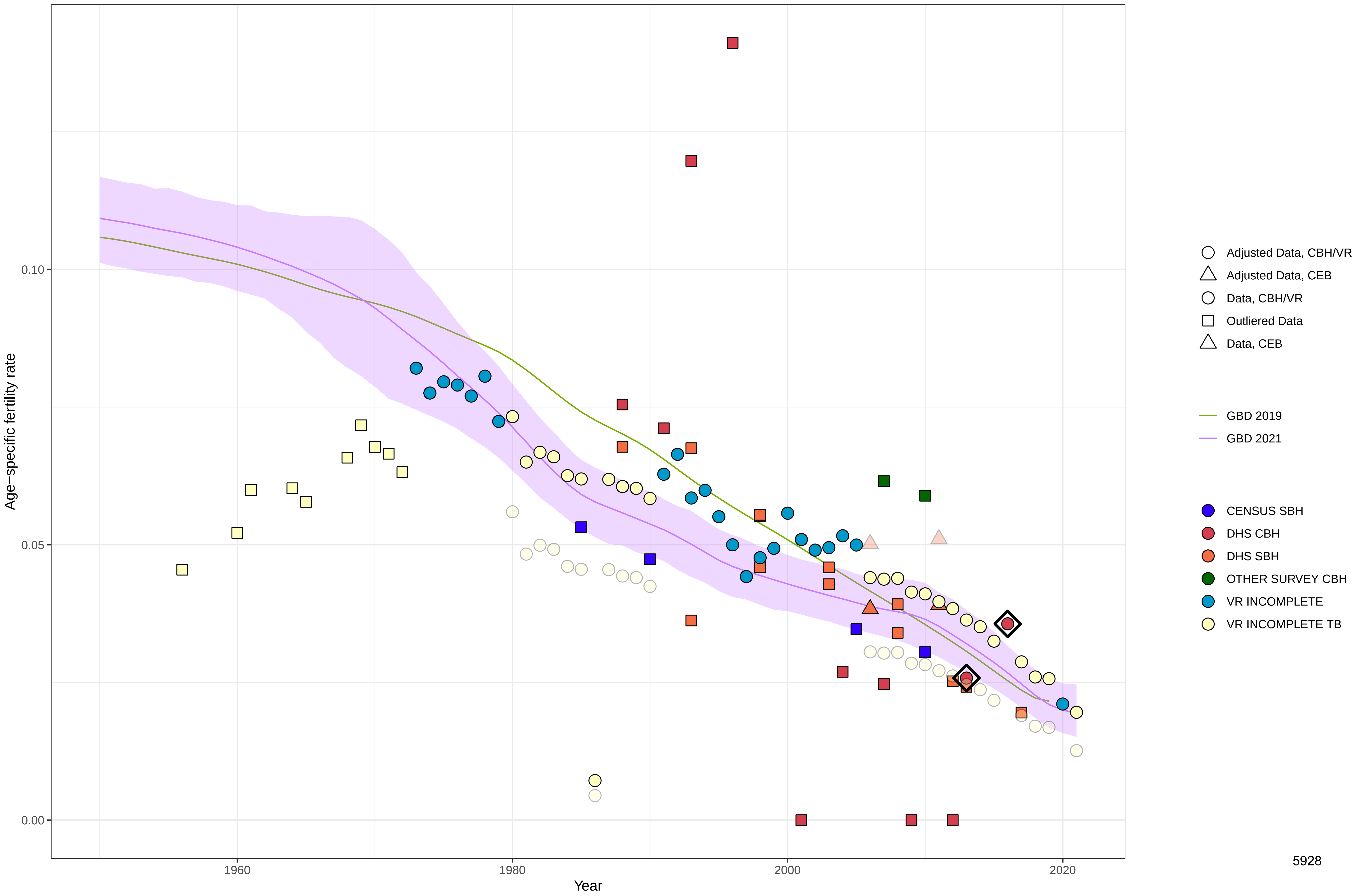

Age−specific fertility fitted trend lines, 1950−2021: Bohol, Ages: 45−49
Data points enclosed with a diamond are reference sources

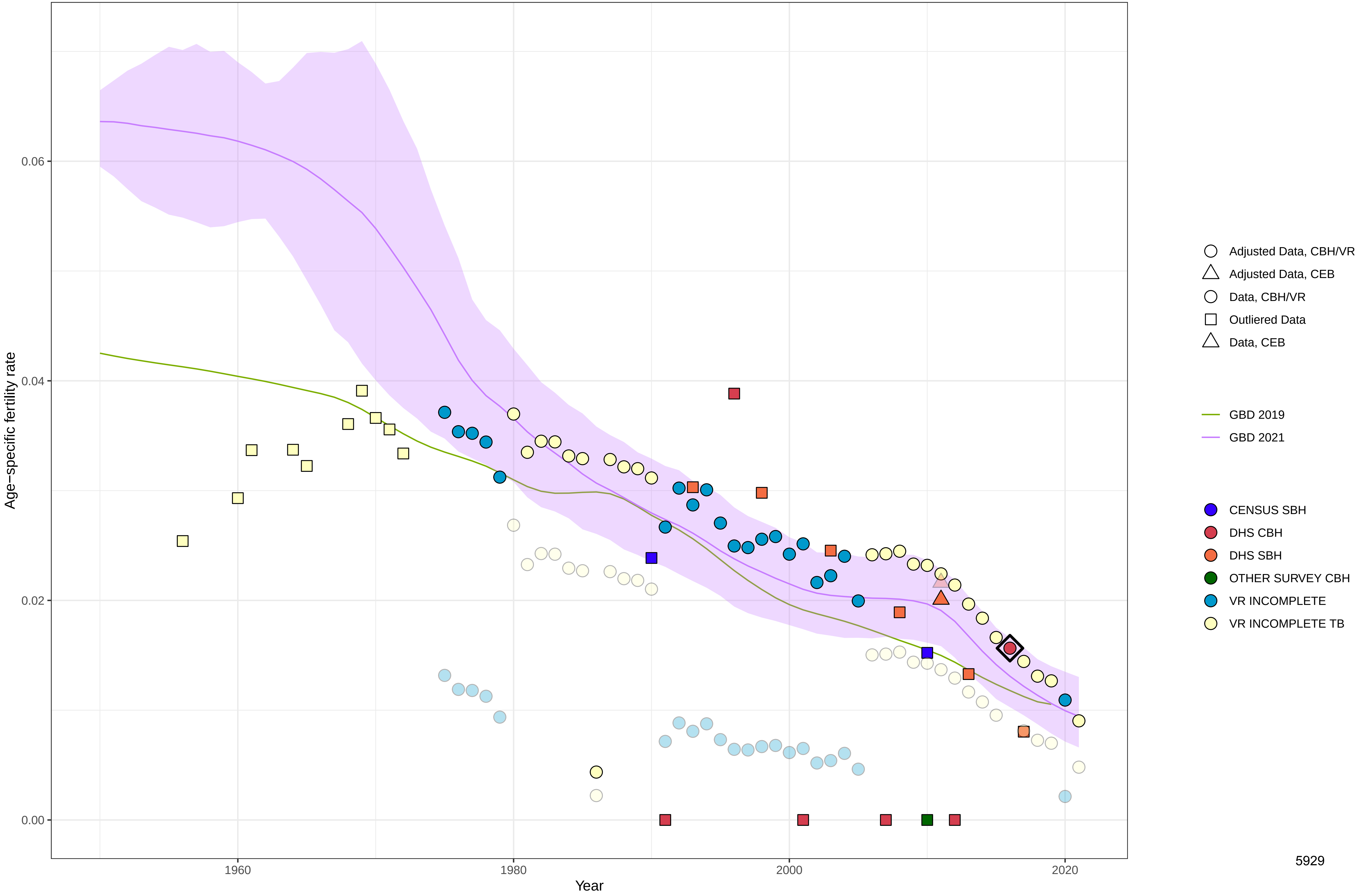

Age−specific fertility fitted trend lines, 1950−2021: Bohol, Ages: 50−54
Data points enclosed with a diamond are reference sources

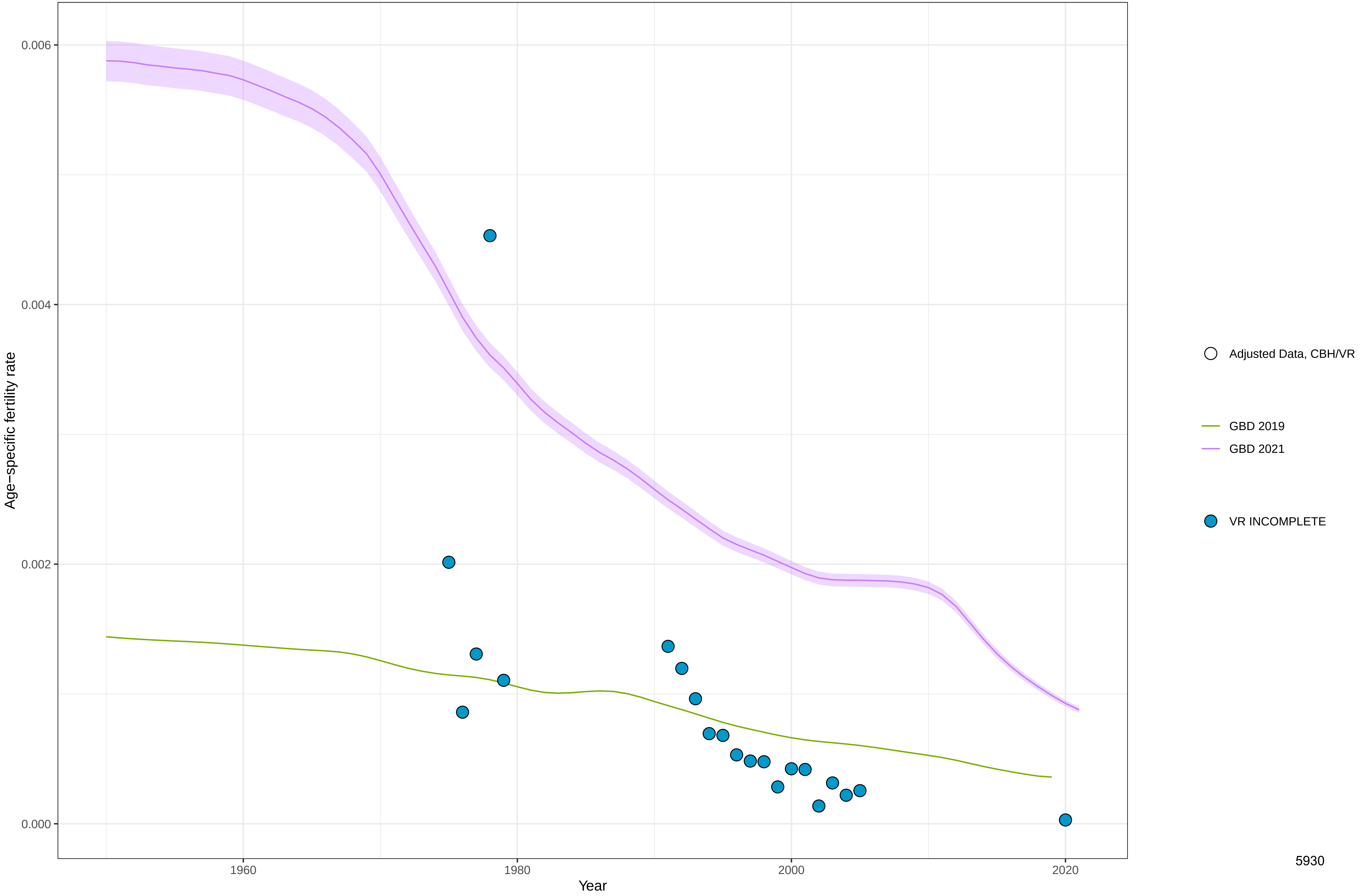

Total fertility rate fitted trend lines, 1950−2021: Bohol TFR
Data points enclosed with a diamond are reference sources

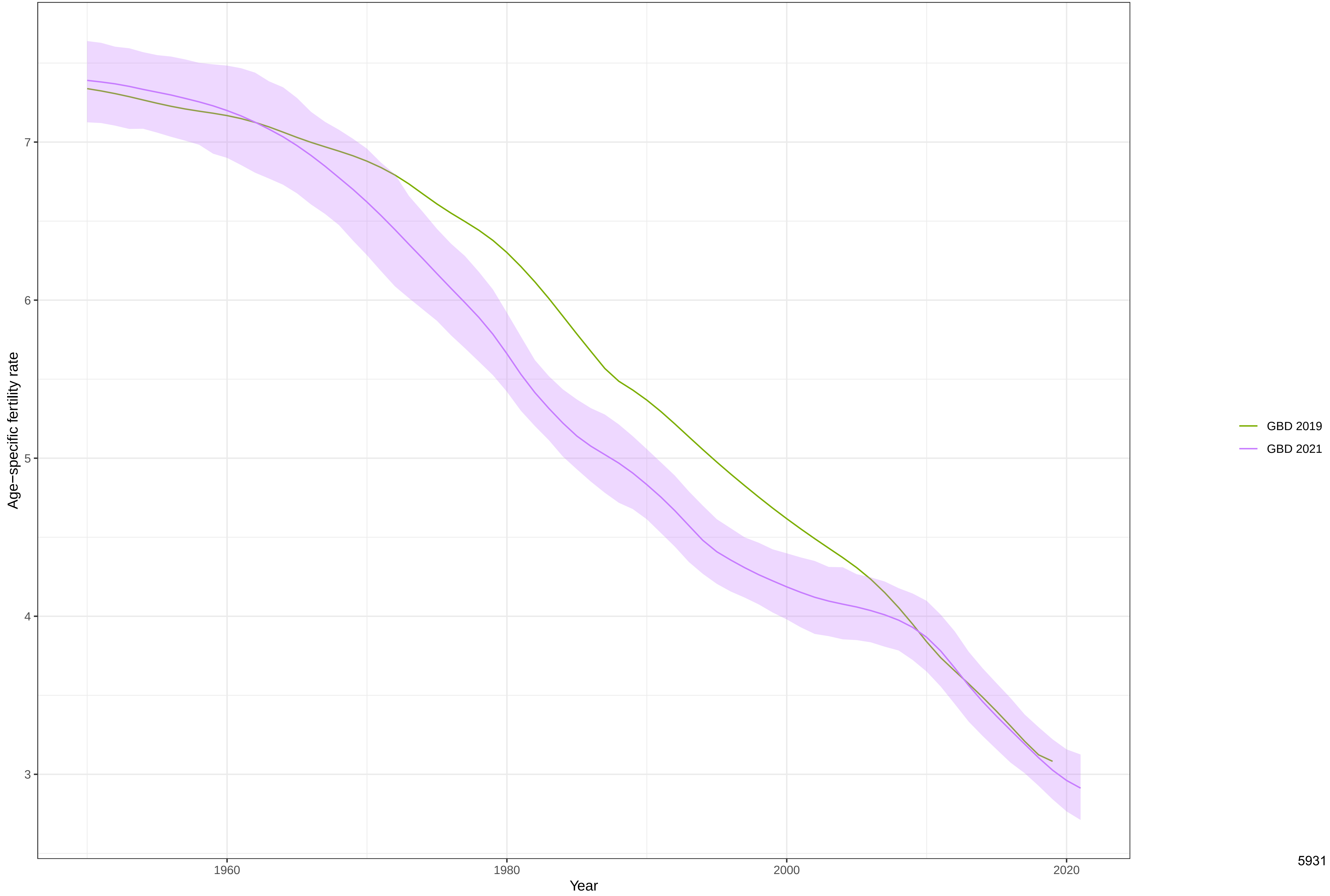

Age−specific fertility fitted trend lines, 1950−2021: Bukidnon, Ages: 10−14
Data points enclosed with a diamond are reference sources

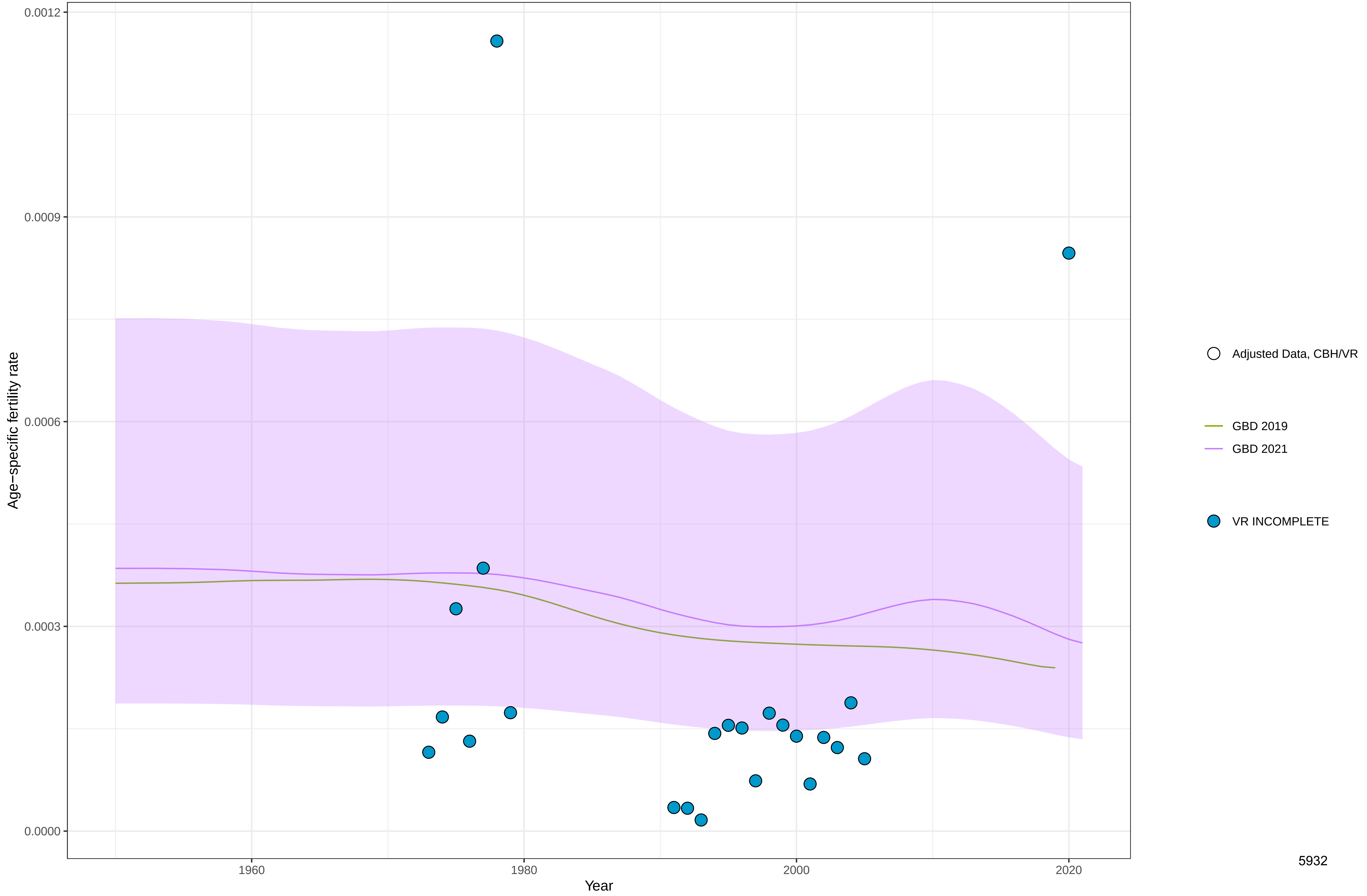

Age−specific fertility fitted trend lines, 1950−2021: Bukidnon, Ages: 15−19
Data points enclosed with a diamond are reference sources

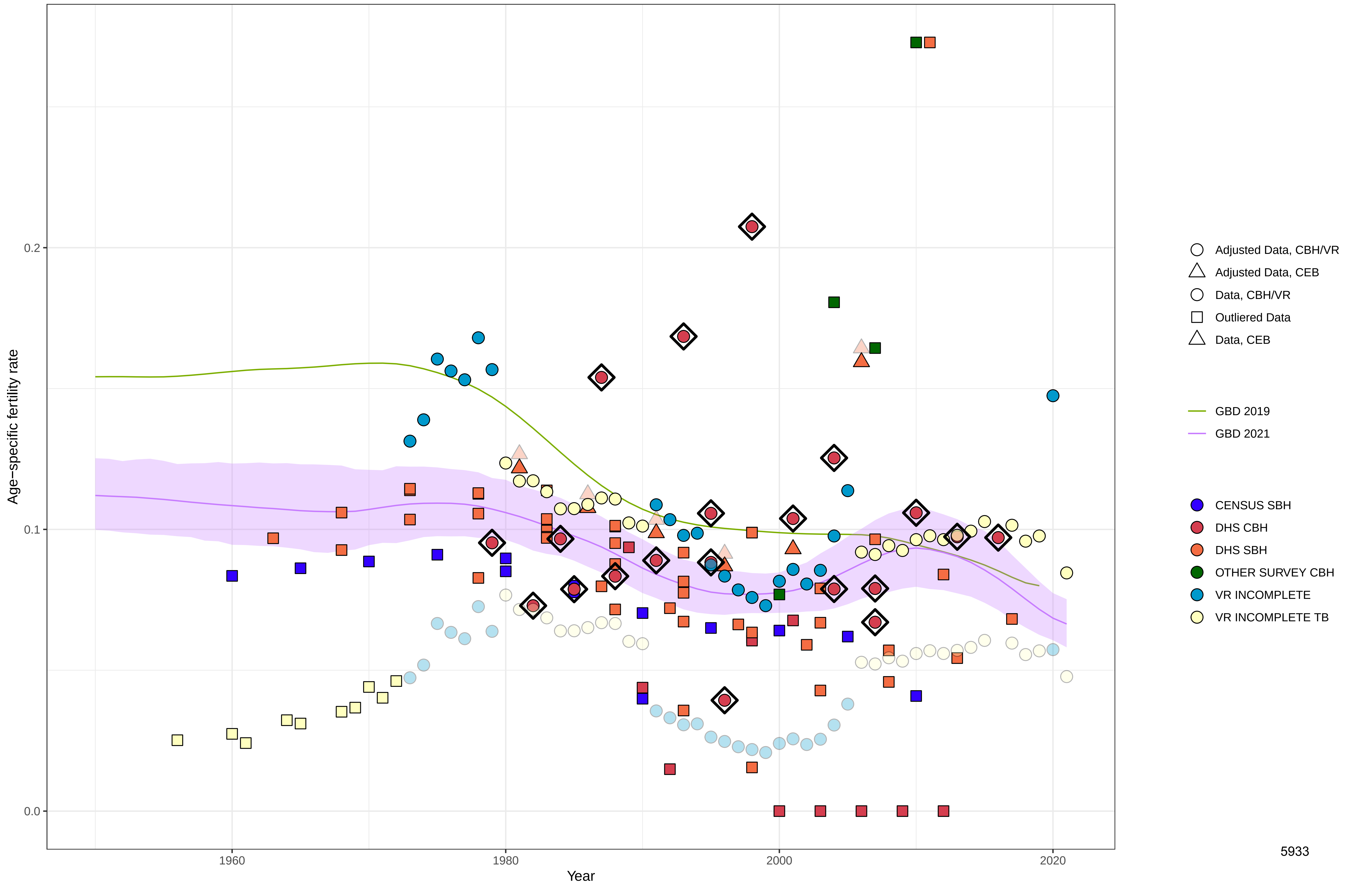

Age−specific fertility fitted trend lines, 1950−2021: Bukidnon, Ages: 20−24
Data points enclosed with a diamond are reference sources

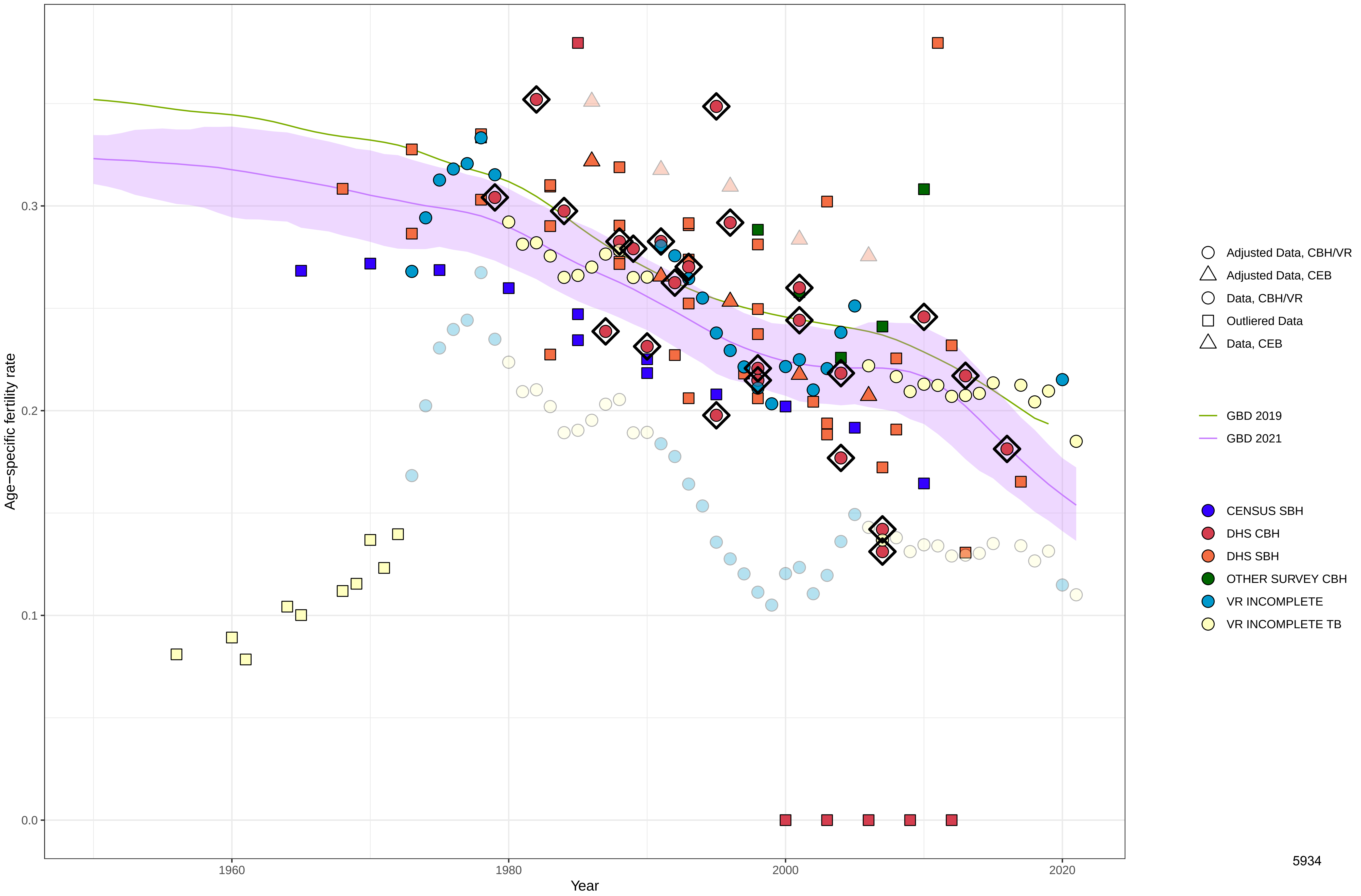

Age−specific fertility fitted trend lines, 1950−2021: Bukidnon, Ages: 25−29
Data points enclosed with a diamond are reference sources

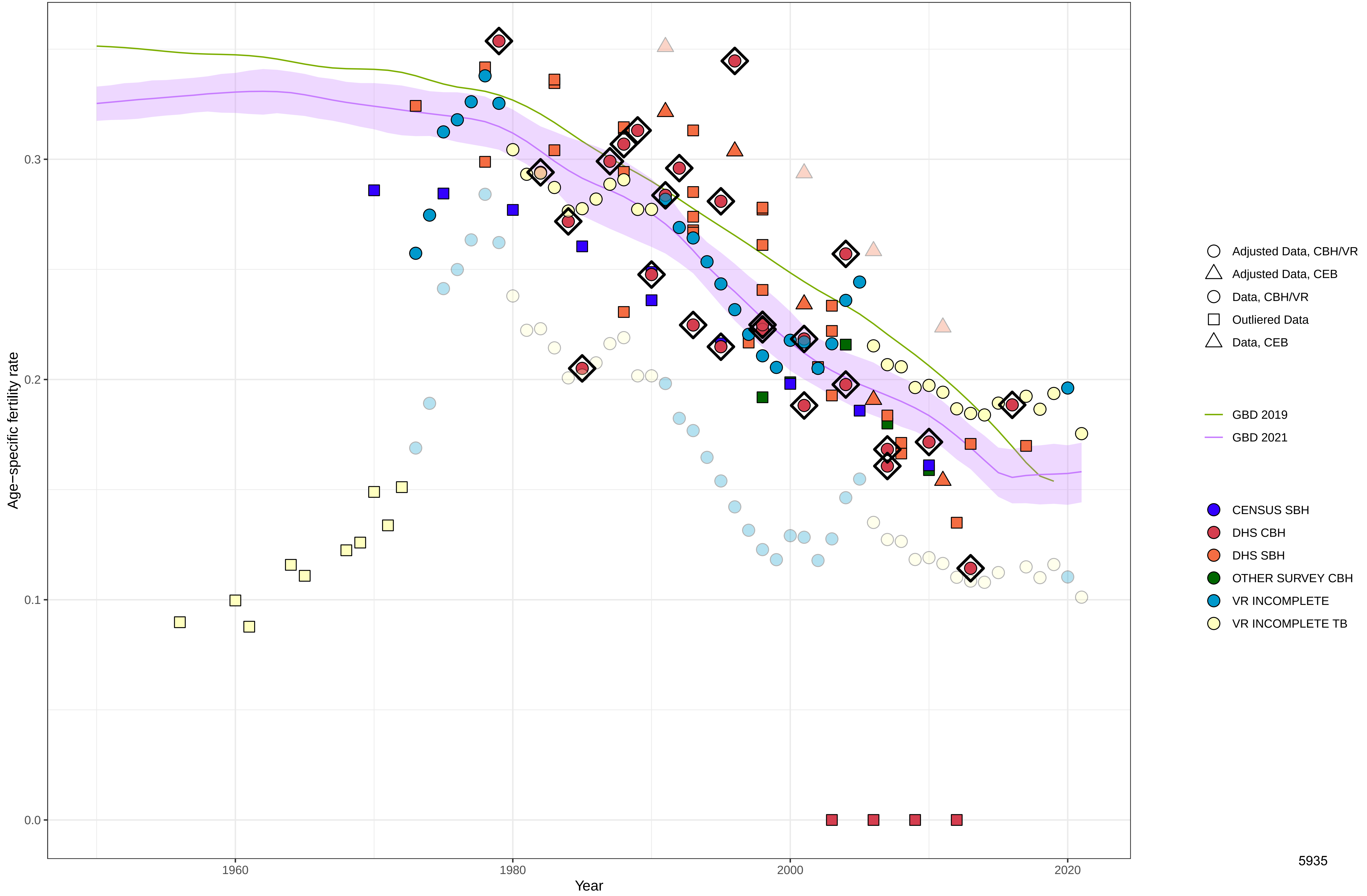

Age−specific fertility fitted trend lines, 1950−2021: Bukidnon, Ages: 30−34
Data points enclosed with a diamond are reference sources

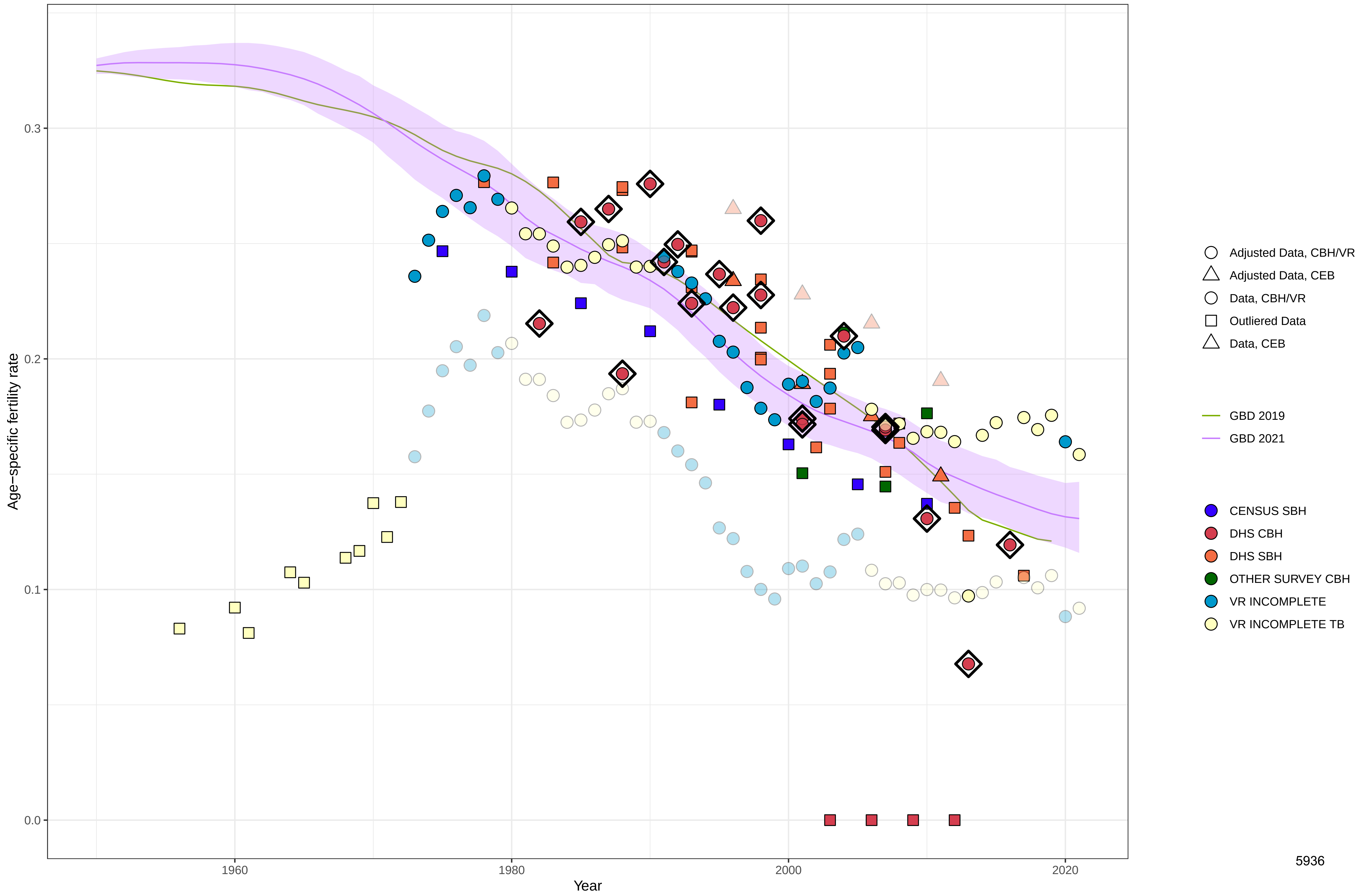

Age−specific fertility fitted trend lines, 1950−2021: Bukidnon, Ages: 35−39
Data points enclosed with a diamond are reference sources

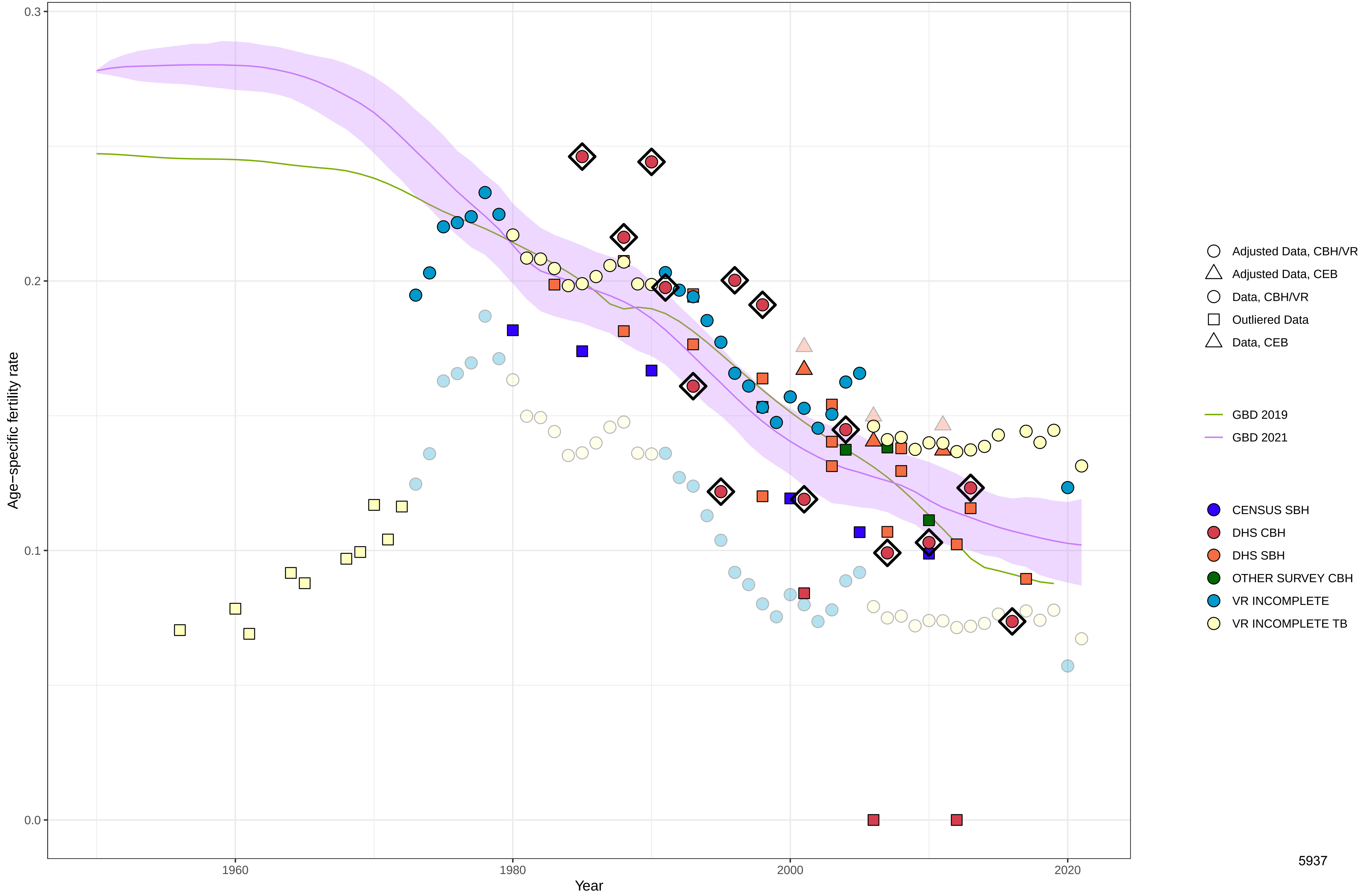

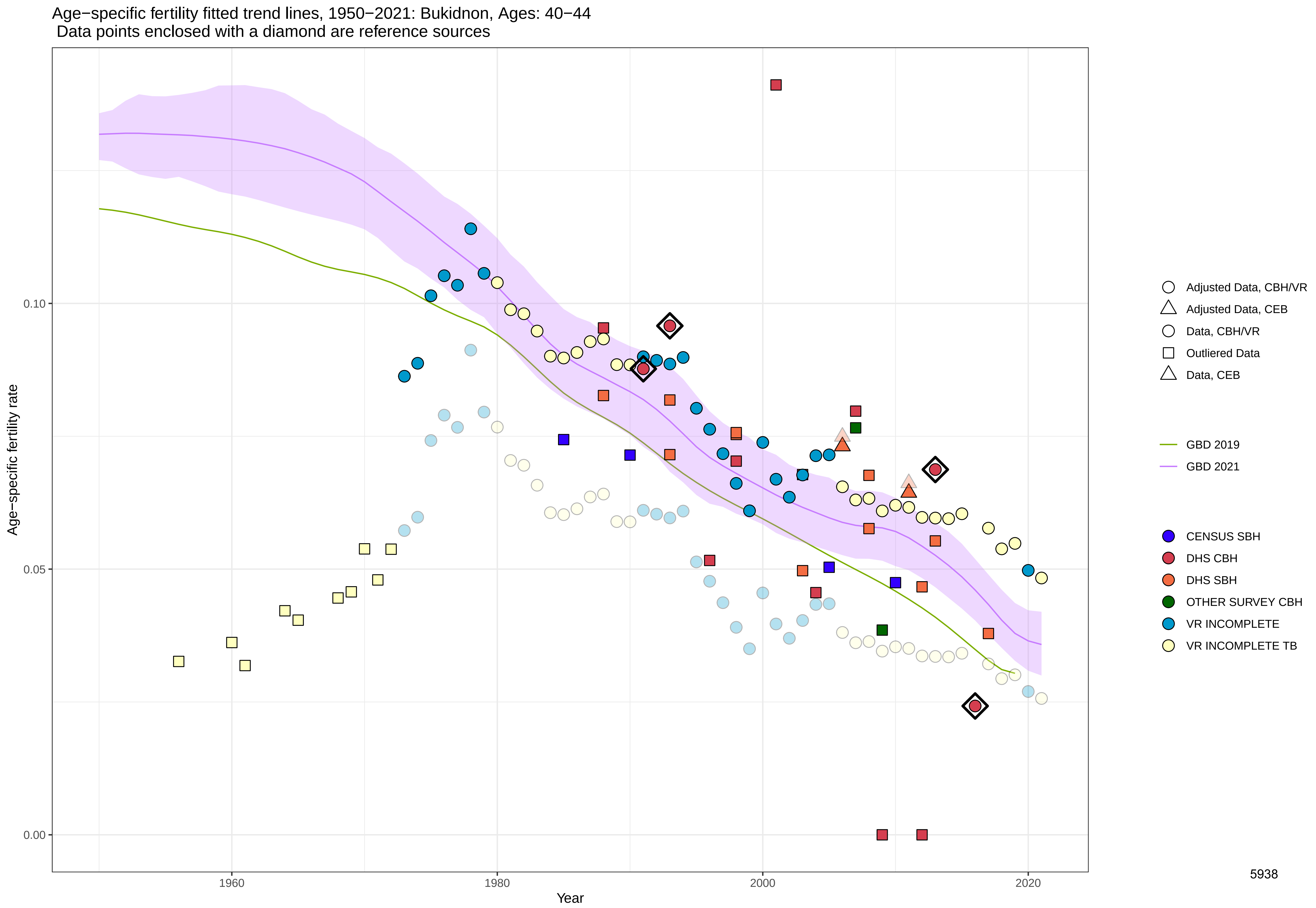

Age−specific fertility fitted trend lines, 1950−2021: Bukidnon, Ages: 40−44
Data points enclosed with a diamond are reference sources
Age−specific fertility rate
0.00
0.05
0.10
Year
1960
1980
2000
2020
Adjusted Data, CBH/VR
Adjusted Data, CEB
Data, CBH/VR
Outliered Data
Data, CEB
GBD 2019
GBD 2021
CENSUS SBH
DHS CBH
DHS SBH
OTHER SURVEY CBH
VR INCOMPLETE
VR INCOMPLETE TB

Age−specific fertility fitted trend lines, 1950−2021: Bukidnon, Ages: 45−49
Data points enclosed with a diamond are reference sources

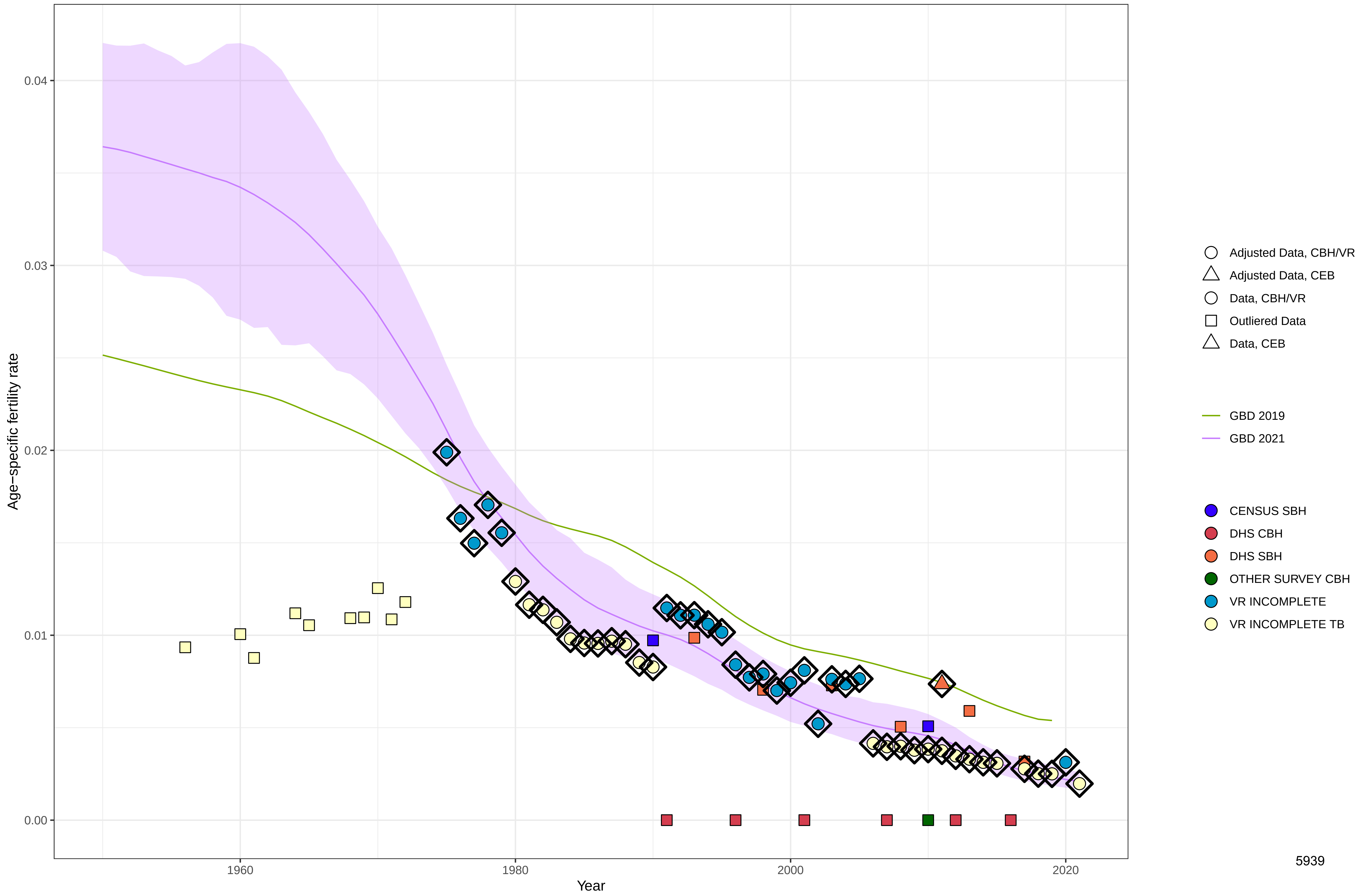

Age−specific fertility fitted trend lines, 1950−2021: Bukidnon, Ages: 50−54
Data points enclosed with a diamond are reference sources

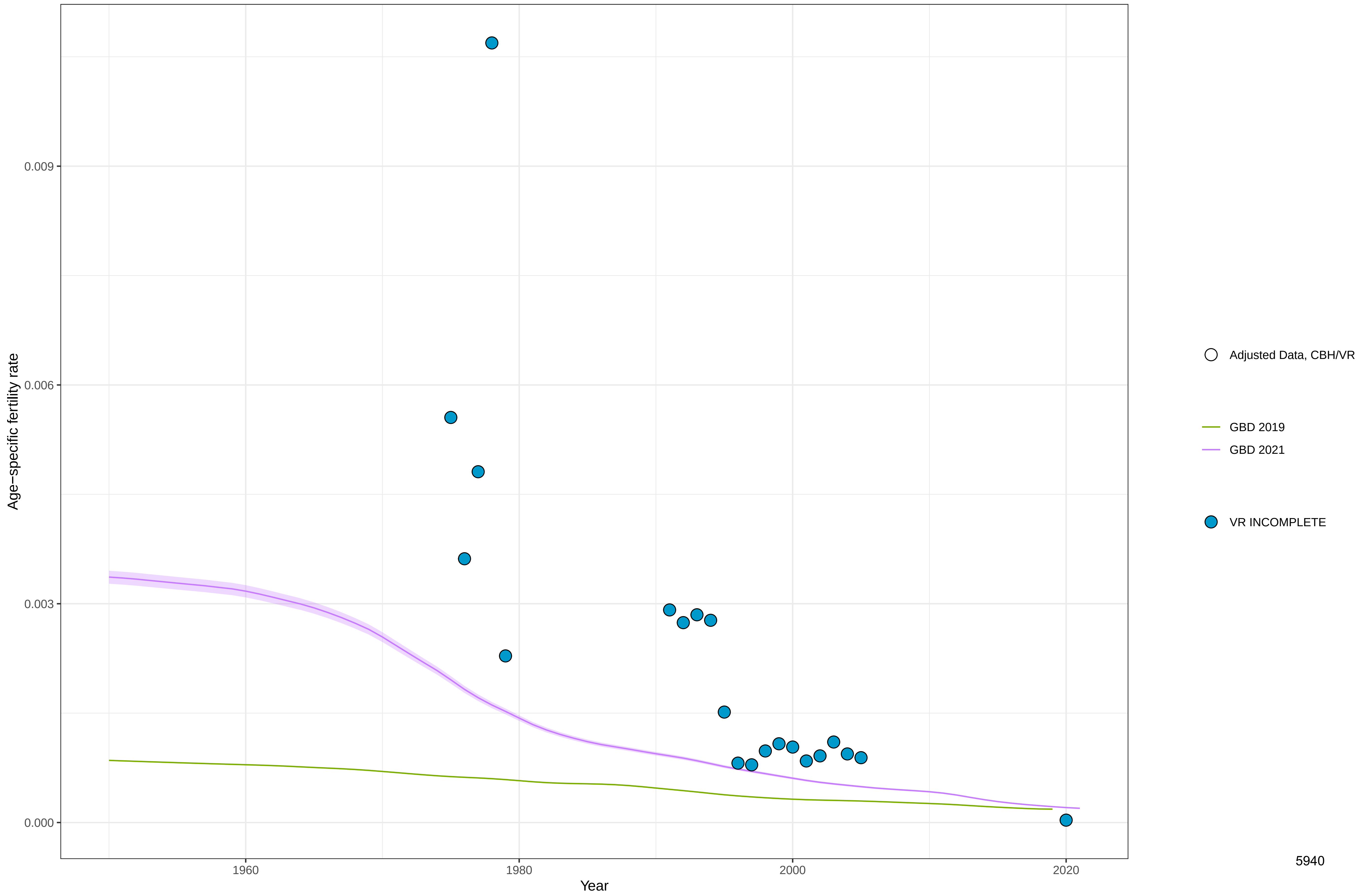

Total fertility rate fitted trend lines, 1950−2021: Bukidnon TFR
Data points enclosed with a diamond are reference sources

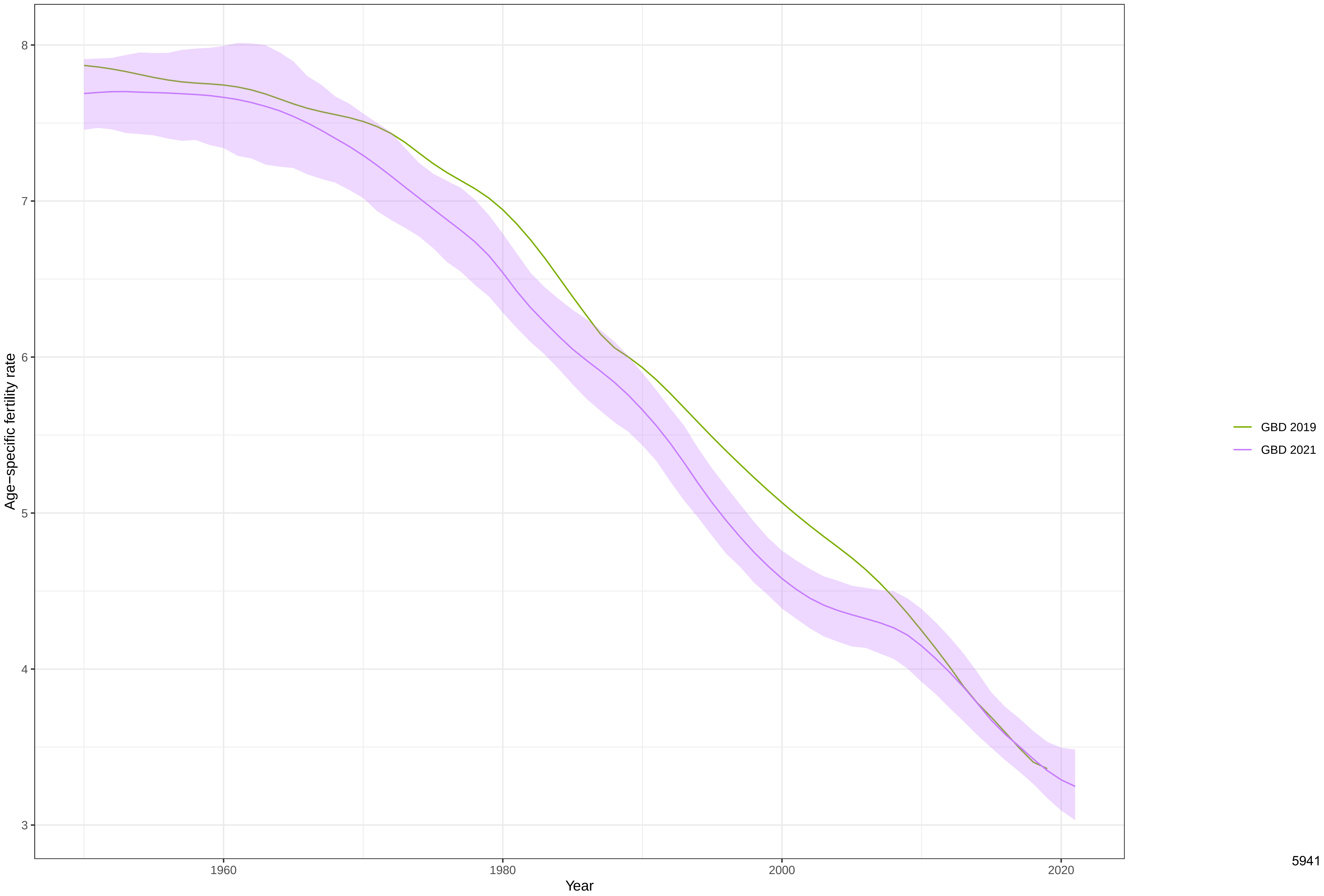

Age−specific fertility fitted trend lines, 1950−2021: Bulacan, Ages: 10−14
Data points enclosed with a diamond are reference sources

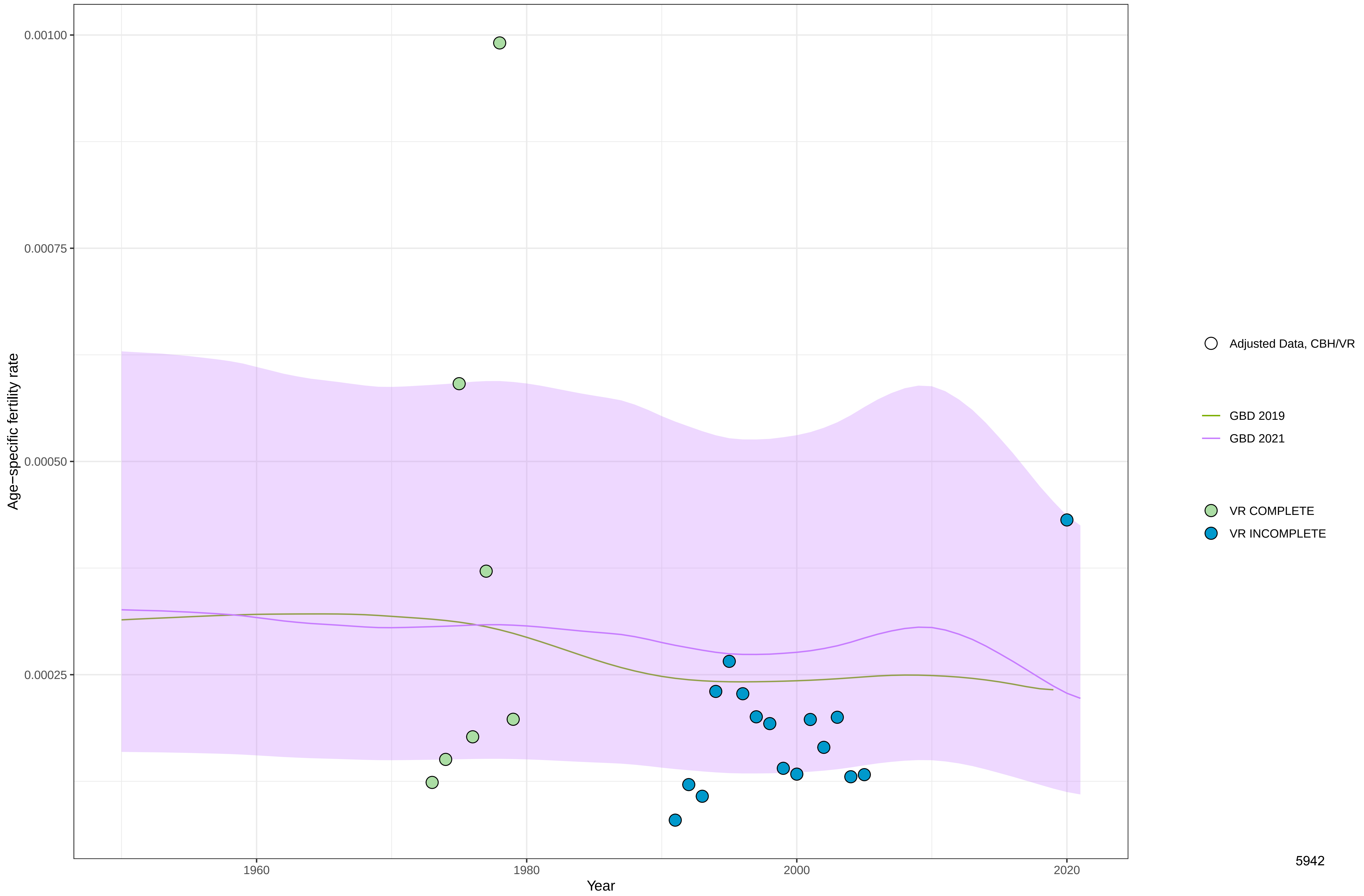

5942

Age−specific fertility fitted trend lines, 1950−2021: Bulacan, Ages: 15−19
Data points enclosed with a diamond are reference sources

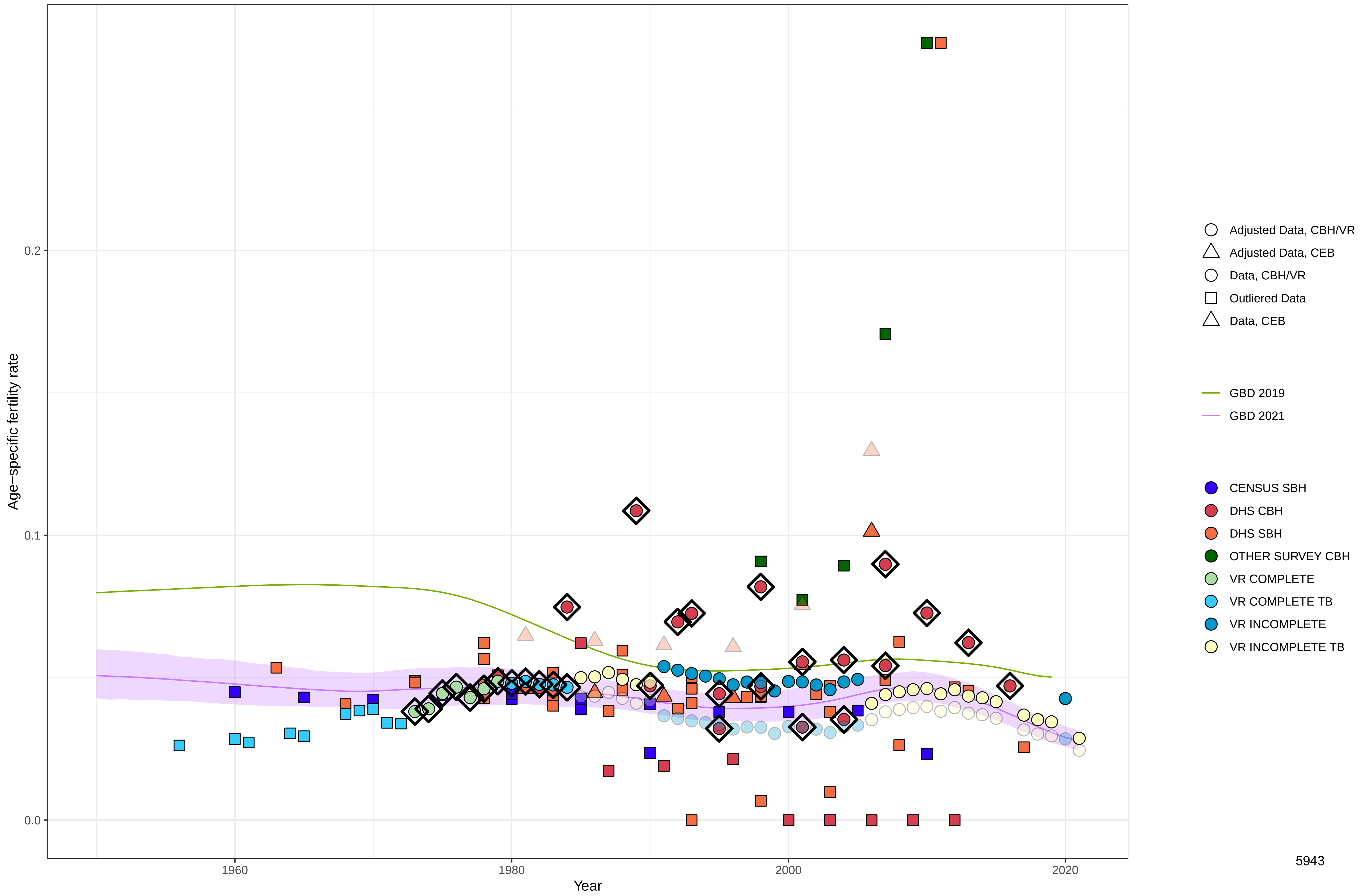

Age−specific fertility fitted trend lines, 1950−2021: Bulacan, Ages: 20−24
Data points enclosed with a diamond are reference sources

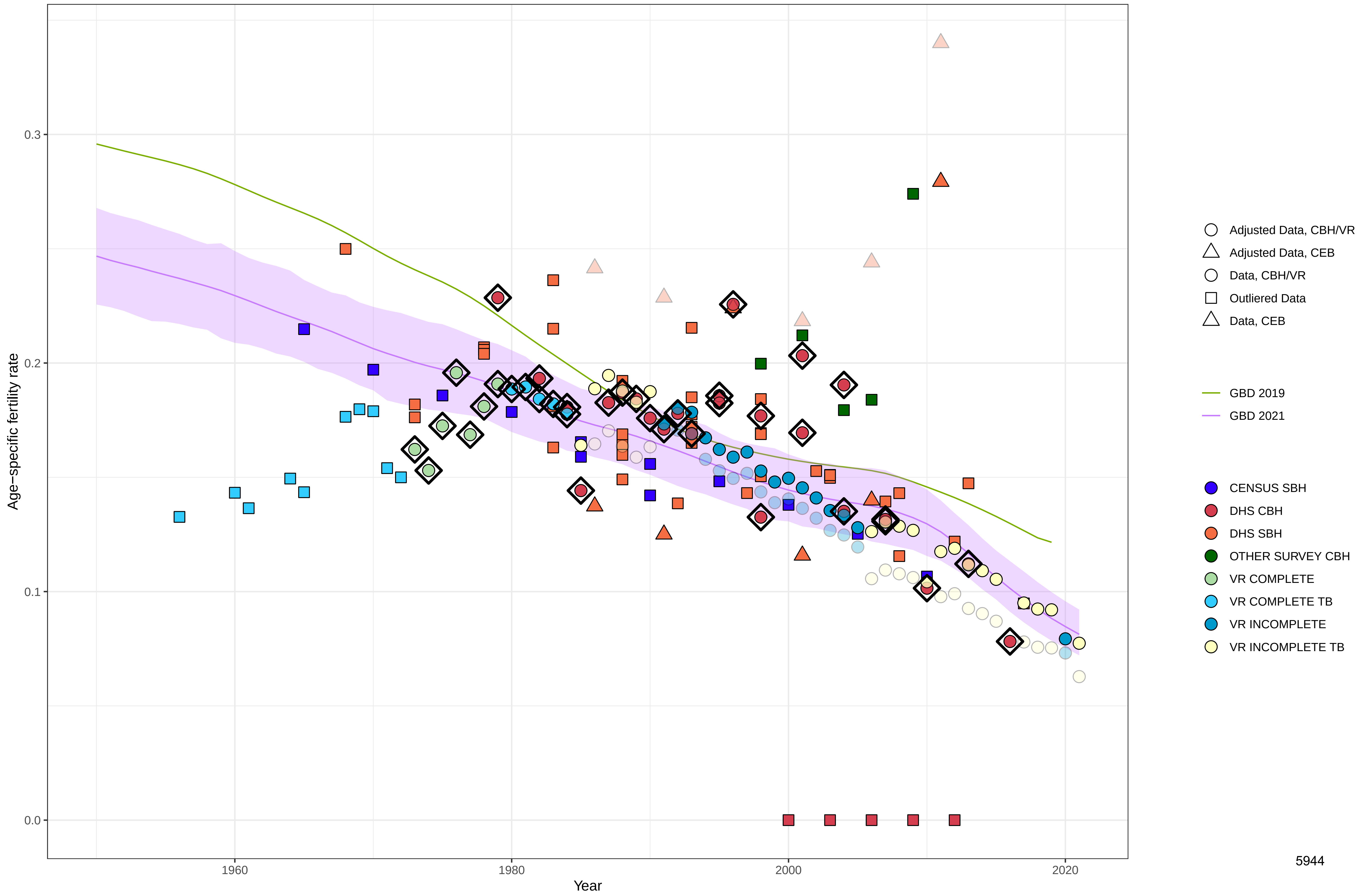

Age−specific fertility fitted trend lines, 1950−2021: Bulacan, Ages: 25−29
Data points enclosed with a diamond are reference sources

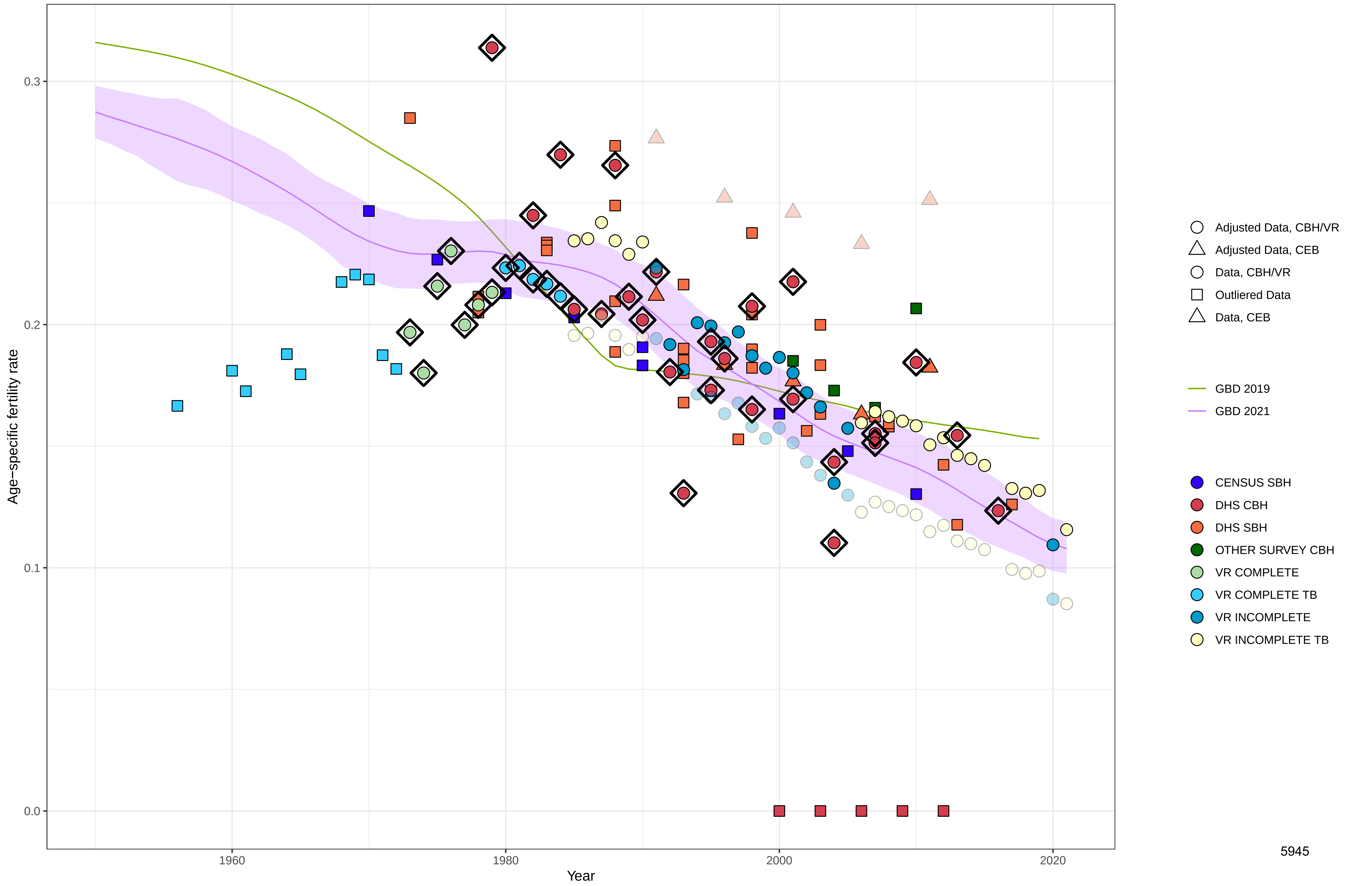

Age−specific fertility fitted trend lines, 1950−2021: Bulacan, Ages: 30−34
Data points enclosed with a diamond are reference sources

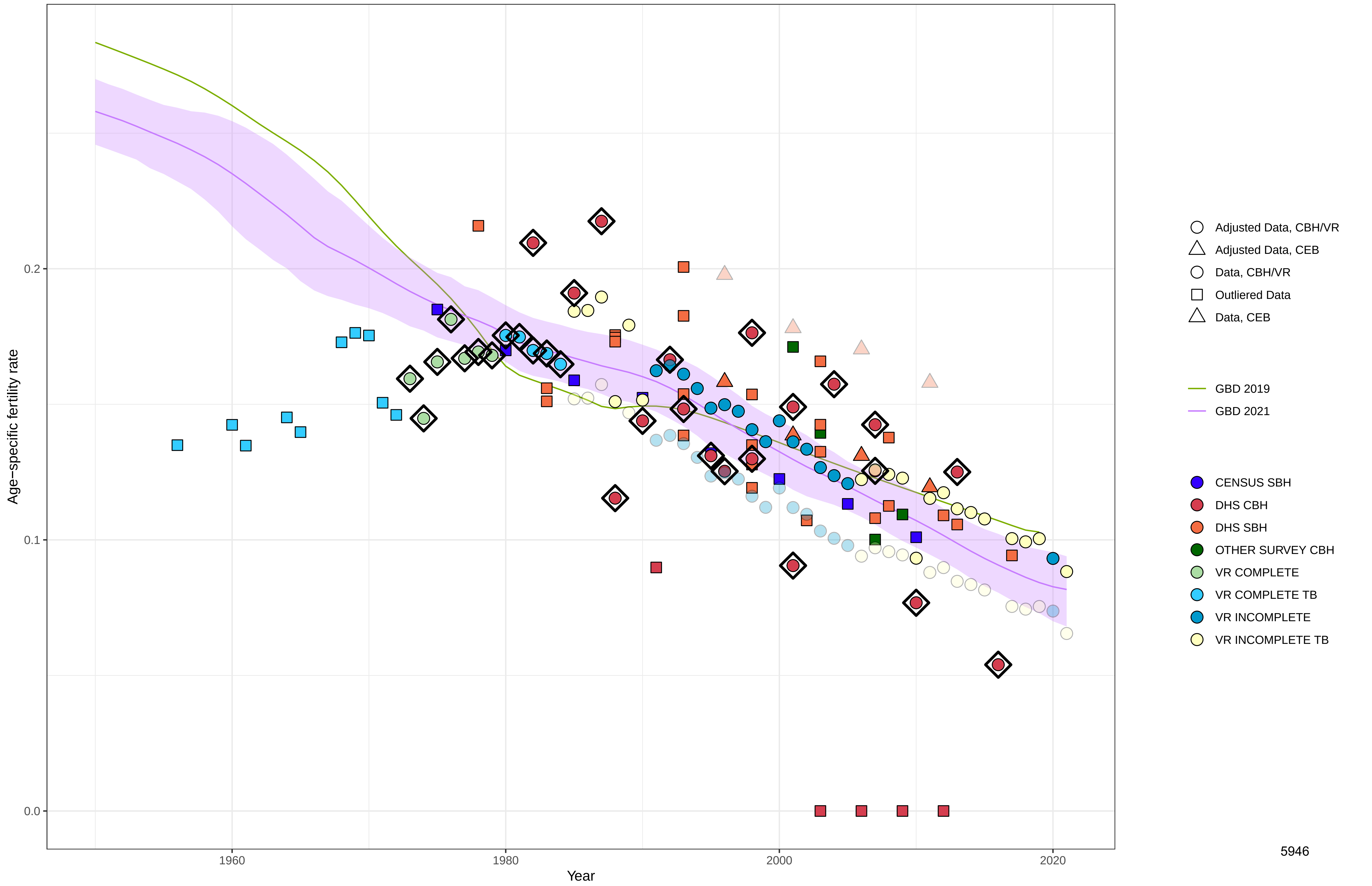

Age−specific fertility fitted trend lines, 1950−2021: Bulacan, Ages: 35−39
Data points enclosed with a diamond are reference sources

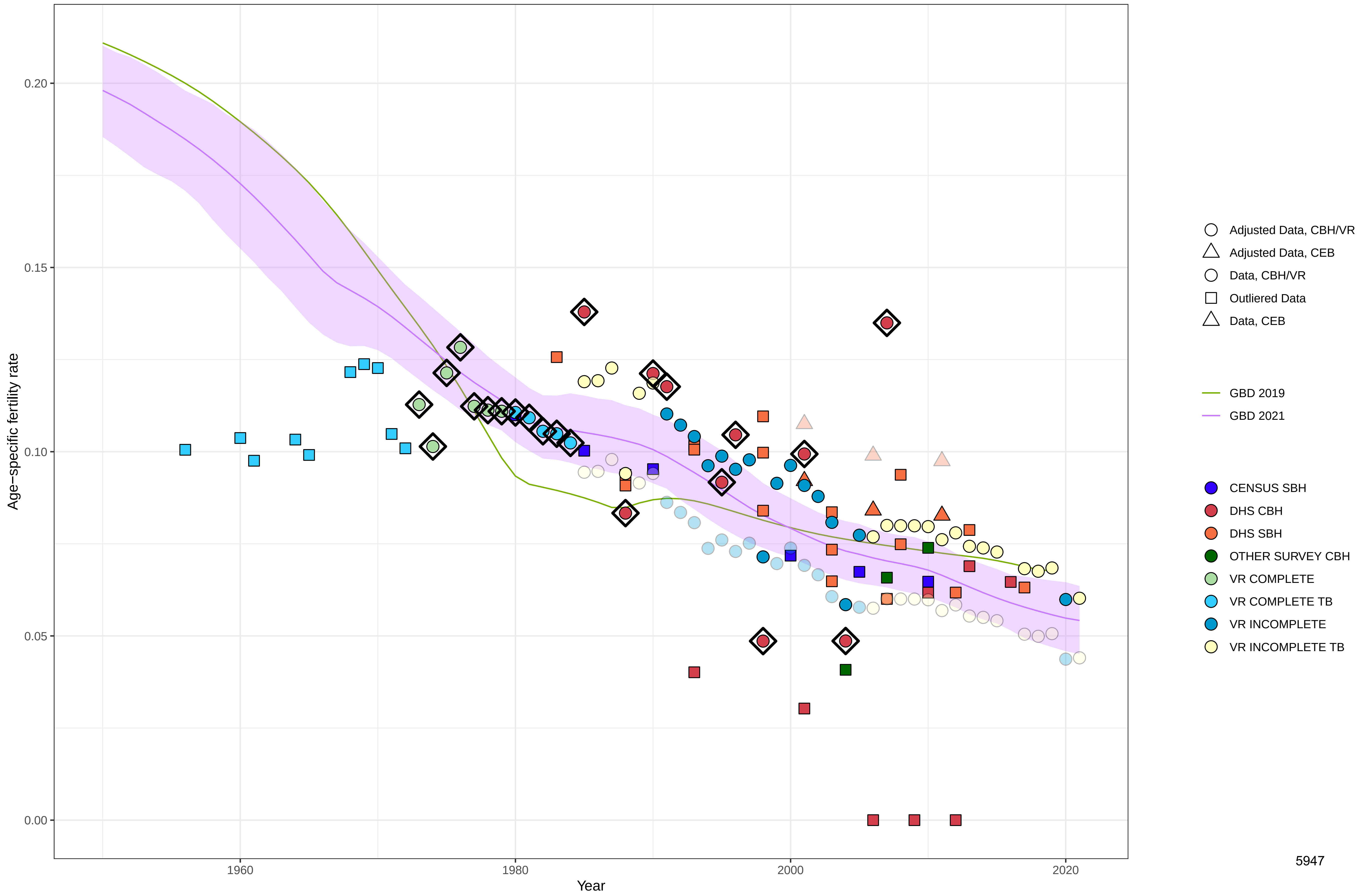

Age−specific fertility fitted trend lines, 1950−2021: Bulacan, Ages: 40−44
Data points enclosed with a diamond are reference sources

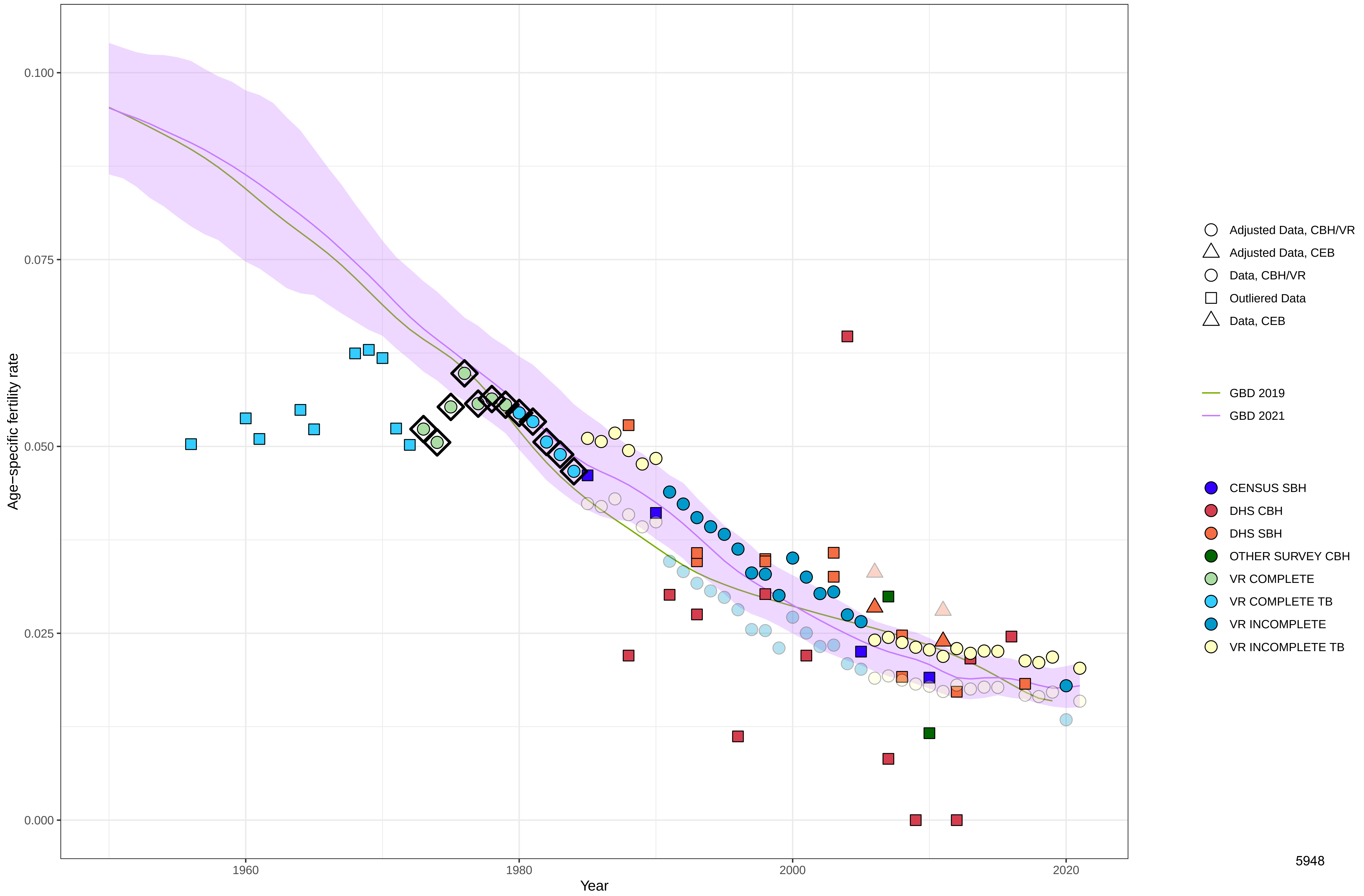

Age−specific fertility fitted trend lines, 1950−2021: Bulacan, Ages: 45−49
Data points enclosed with a diamond are reference sources

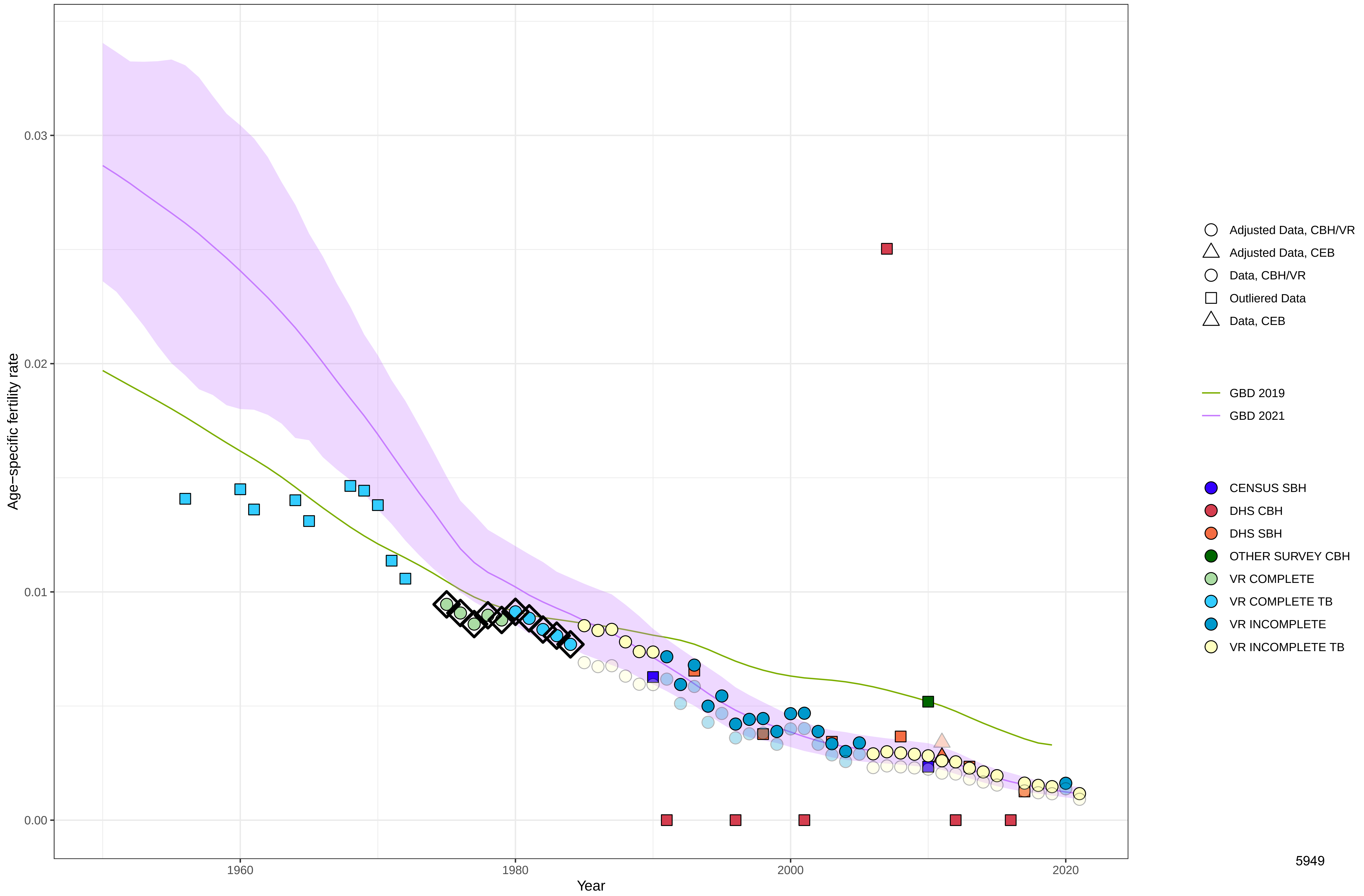

Age−specific fertility fitted trend lines, 1950−2021: Bulacan, Ages: 50−54
Data points enclosed with a diamond are reference sources

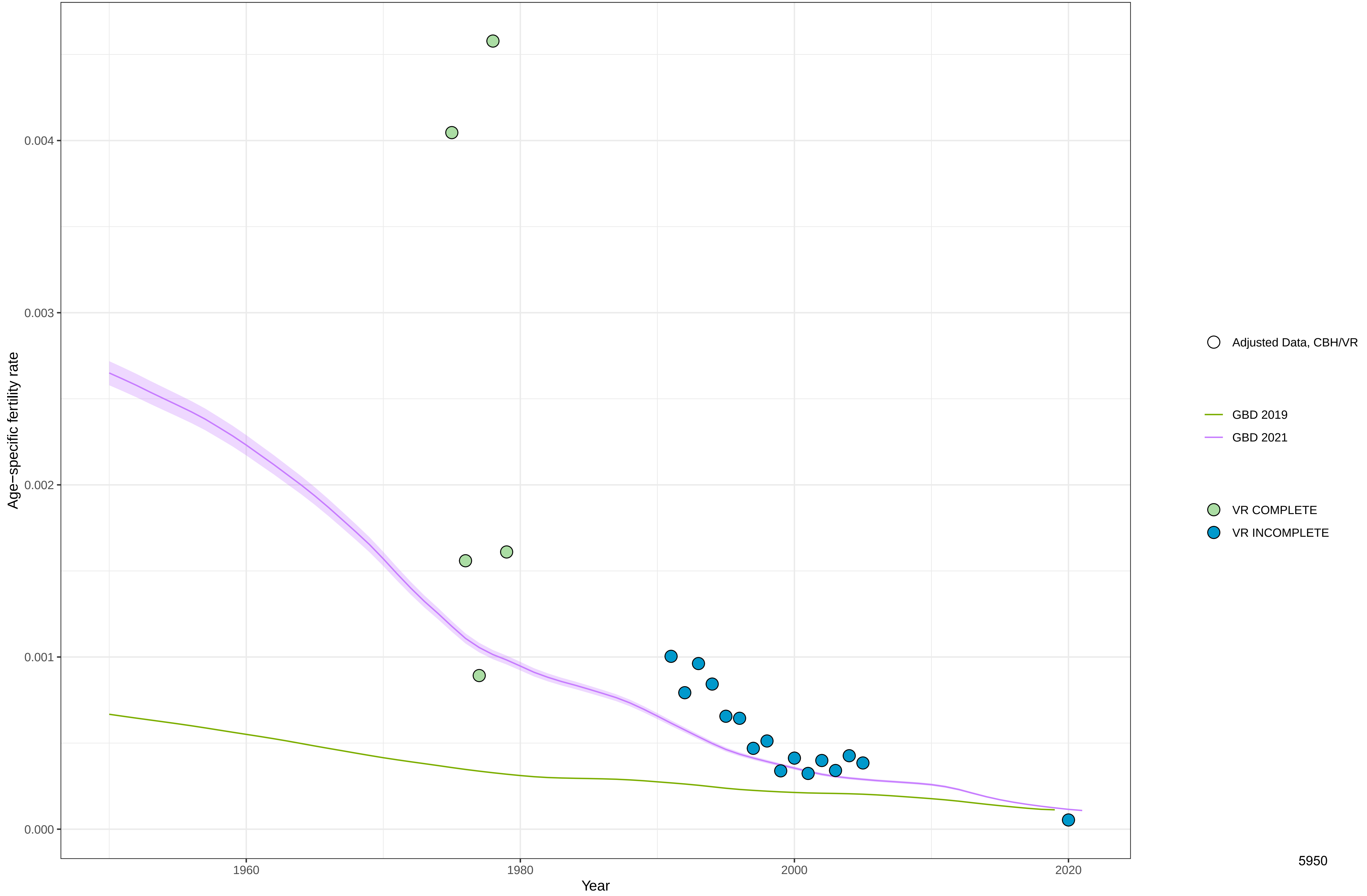

Total fertility rate fitted trend lines, 1950−2021: Bulacan TFR
Data points enclosed with a diamond are reference sources

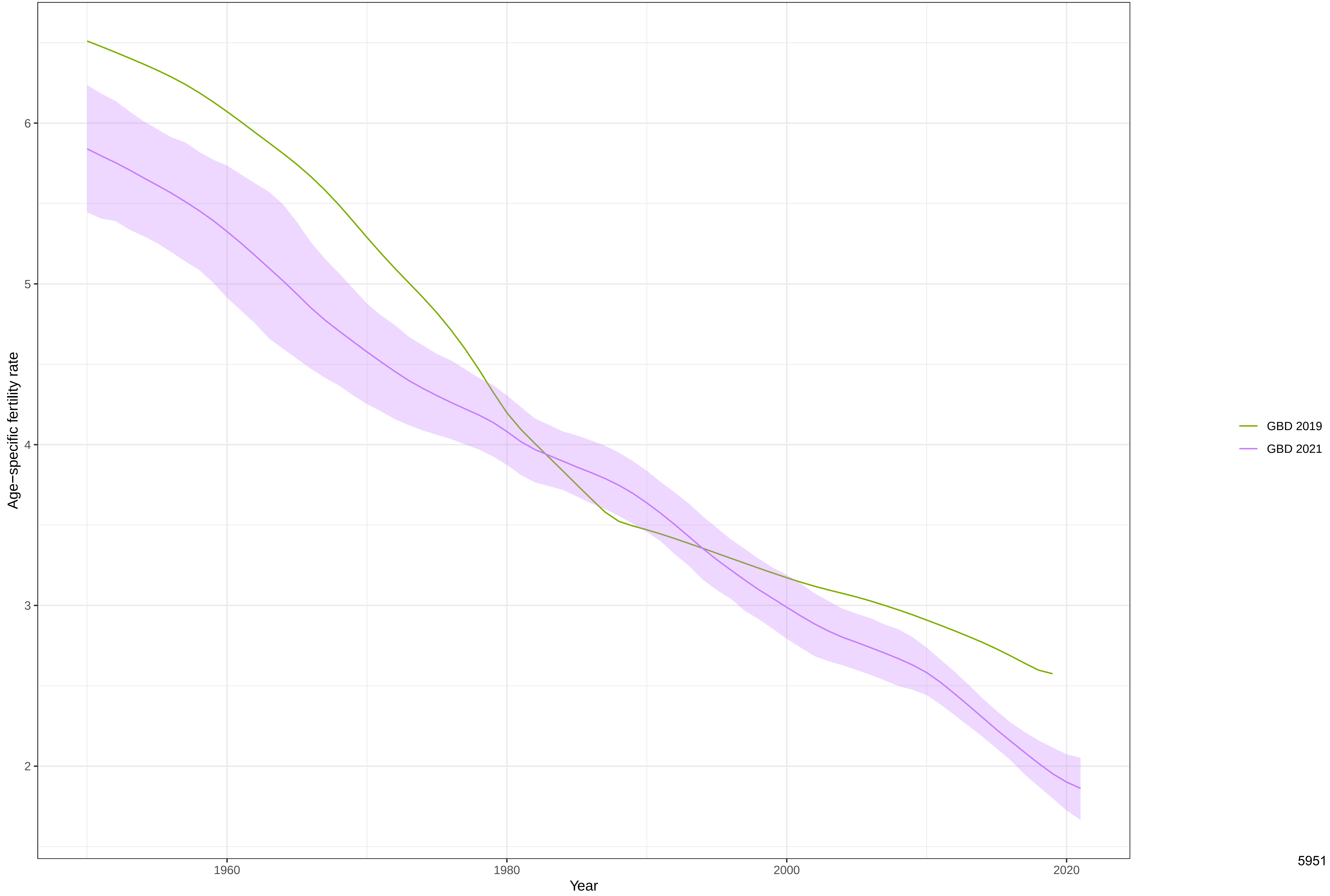

Age−specific fertility fitted trend lines, 1950−2021: Cagayan, Ages: 10−14
Data points enclosed with a diamond are reference sources

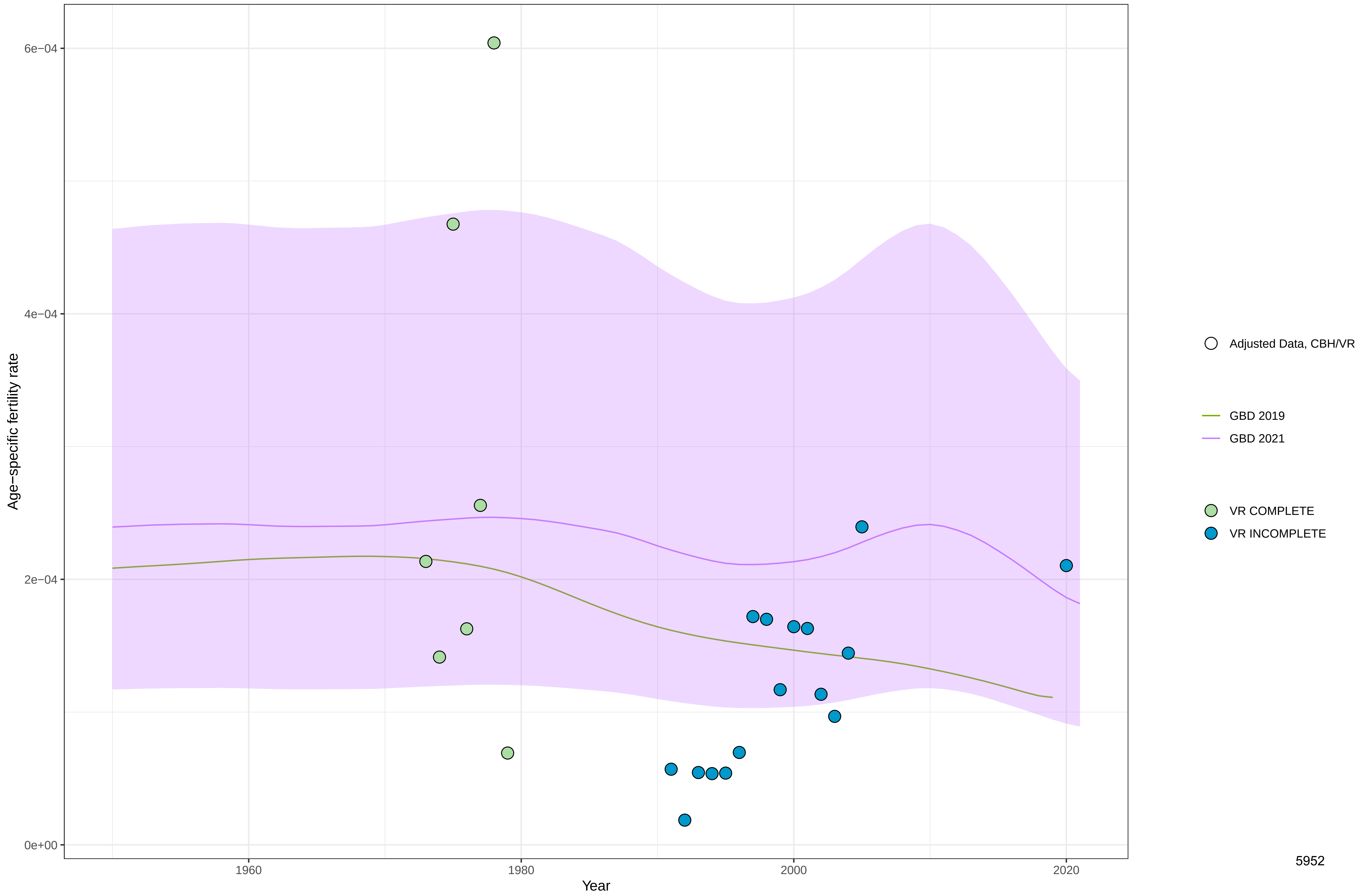

Age−specific fertility fitted trend lines, 1950−2021: Cagayan, Ages: 15−19
Data points enclosed with a diamond are reference sources

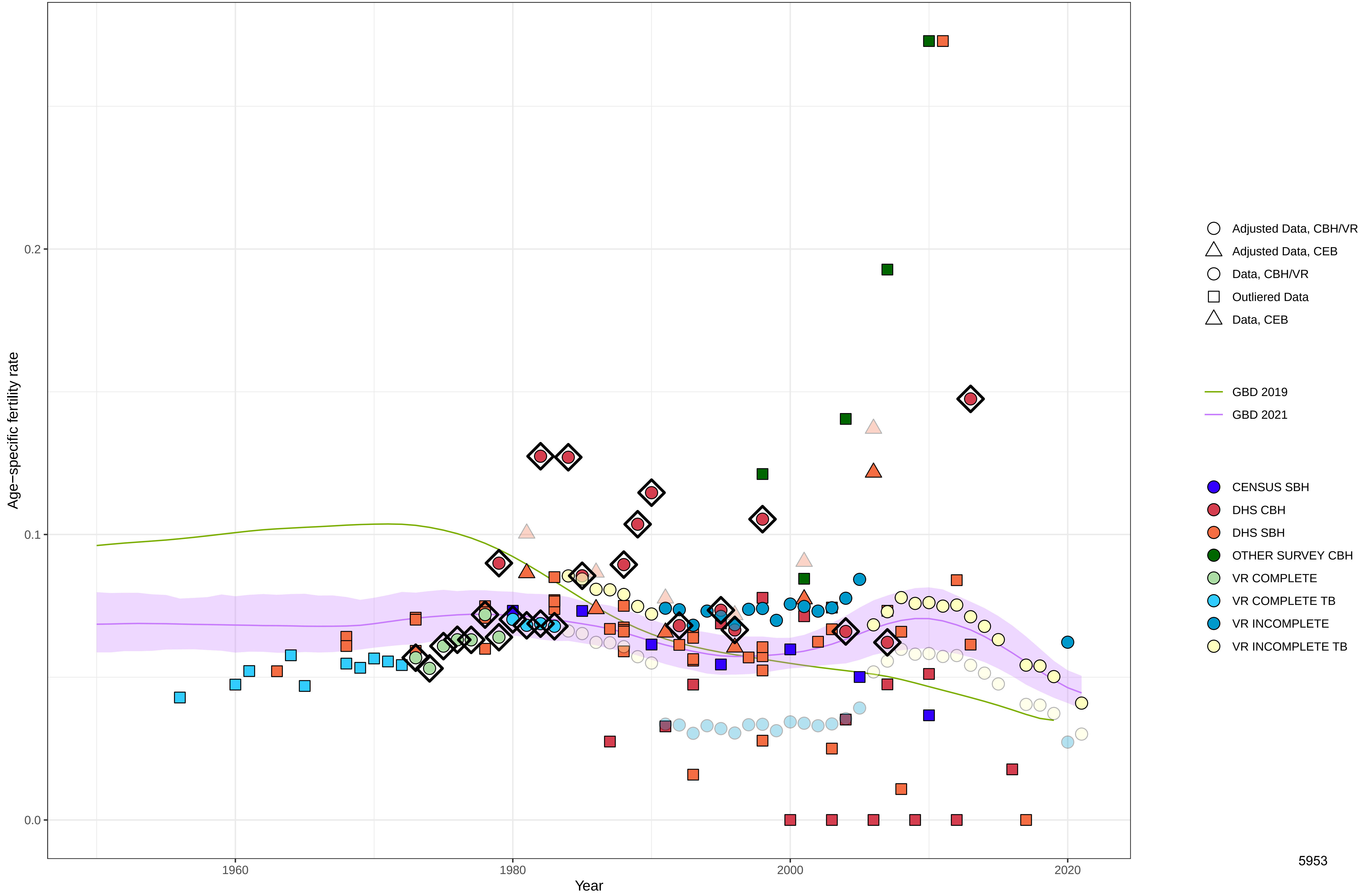

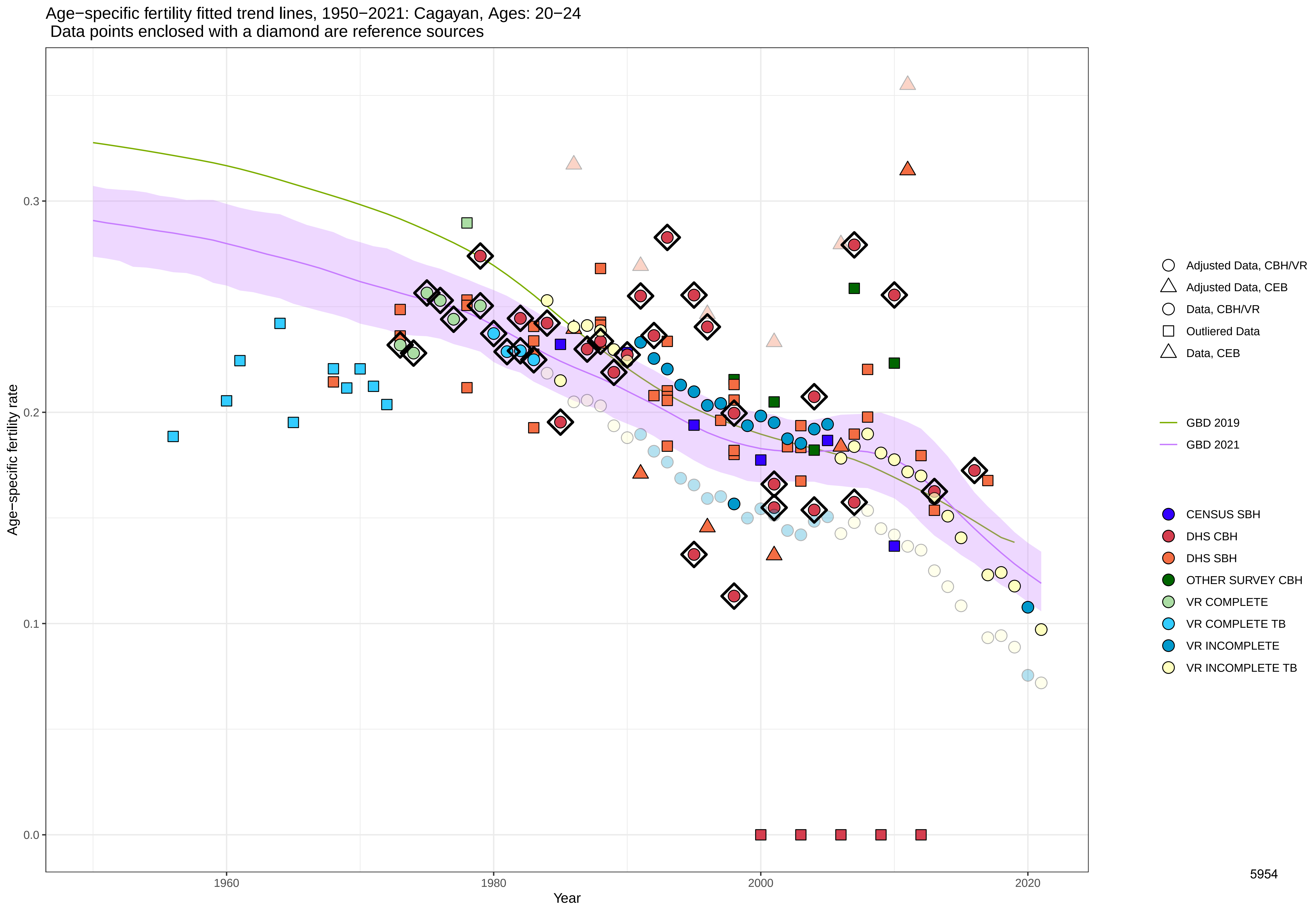

Age−specific fertility fitted trend lines, 1950−2021: Cagayan, Ages: 20−24
Data points enclosed with a diamond are reference sources
Age−specific fertility rate
Year
0.0
0.1
0.2
0.3
1960
1980
2000
2020
Adjusted Data, CBH/VR
Adjusted Data, CEB
Data, CBH/VR
Outliered Data
Data, CEB
GBD 2019
GBD 2021
CENSUS SBH
DHS CBH
DHS SBH
OTHER SURVEY CBH
VR COMPLETE
VR COMPLETE TB
VR INCOMPLETE
VR INCOMPLETE TB

Age−specific fertility fitted trend lines, 1950−2021: Cagayan, Ages: 25−29
Data points enclosed with a diamond are reference sources

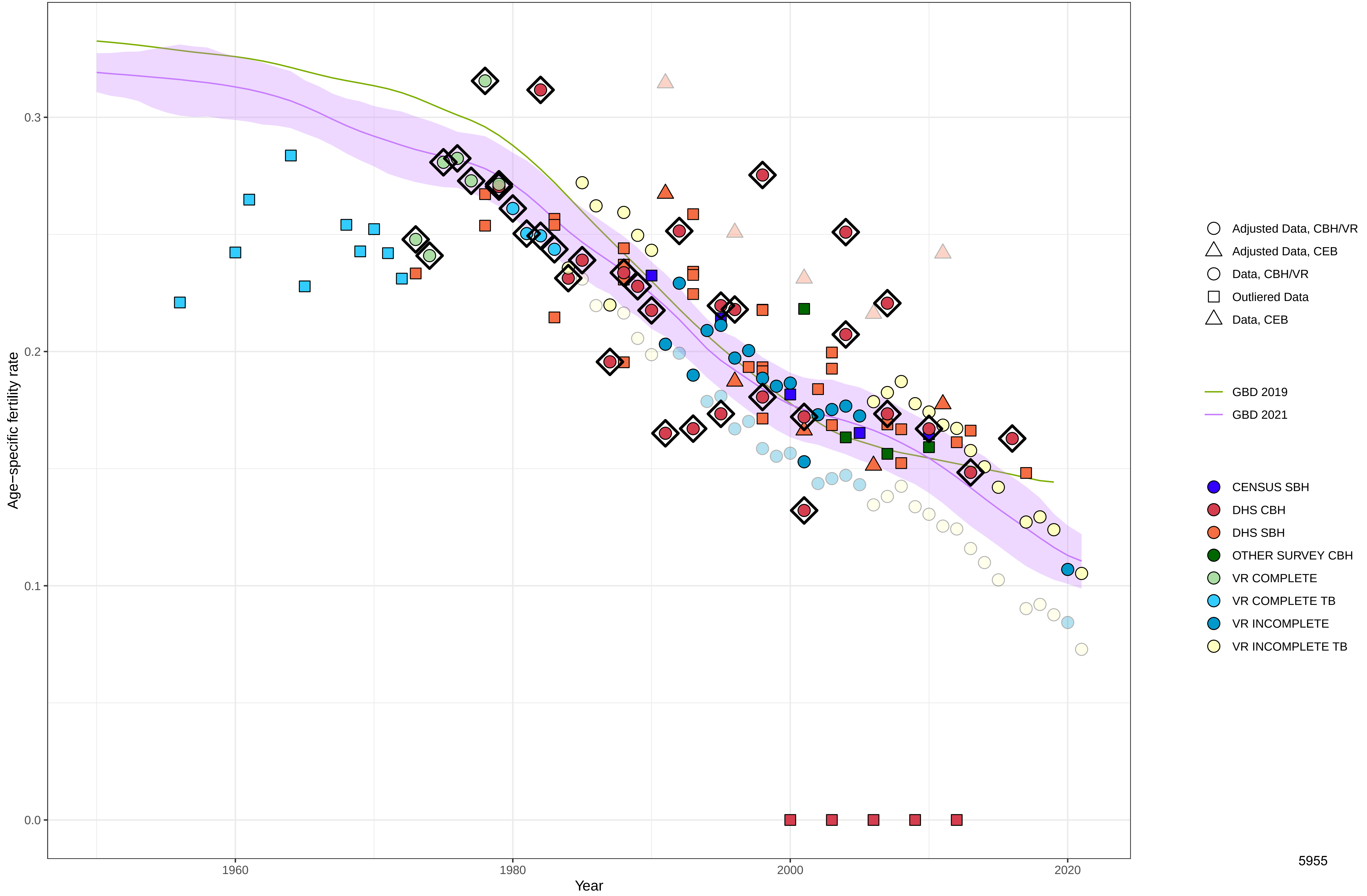

Age−specific fertility fitted trend lines, 1950−2021: Cagayan, Ages: 30−34
Data points enclosed with a diamond are reference sources

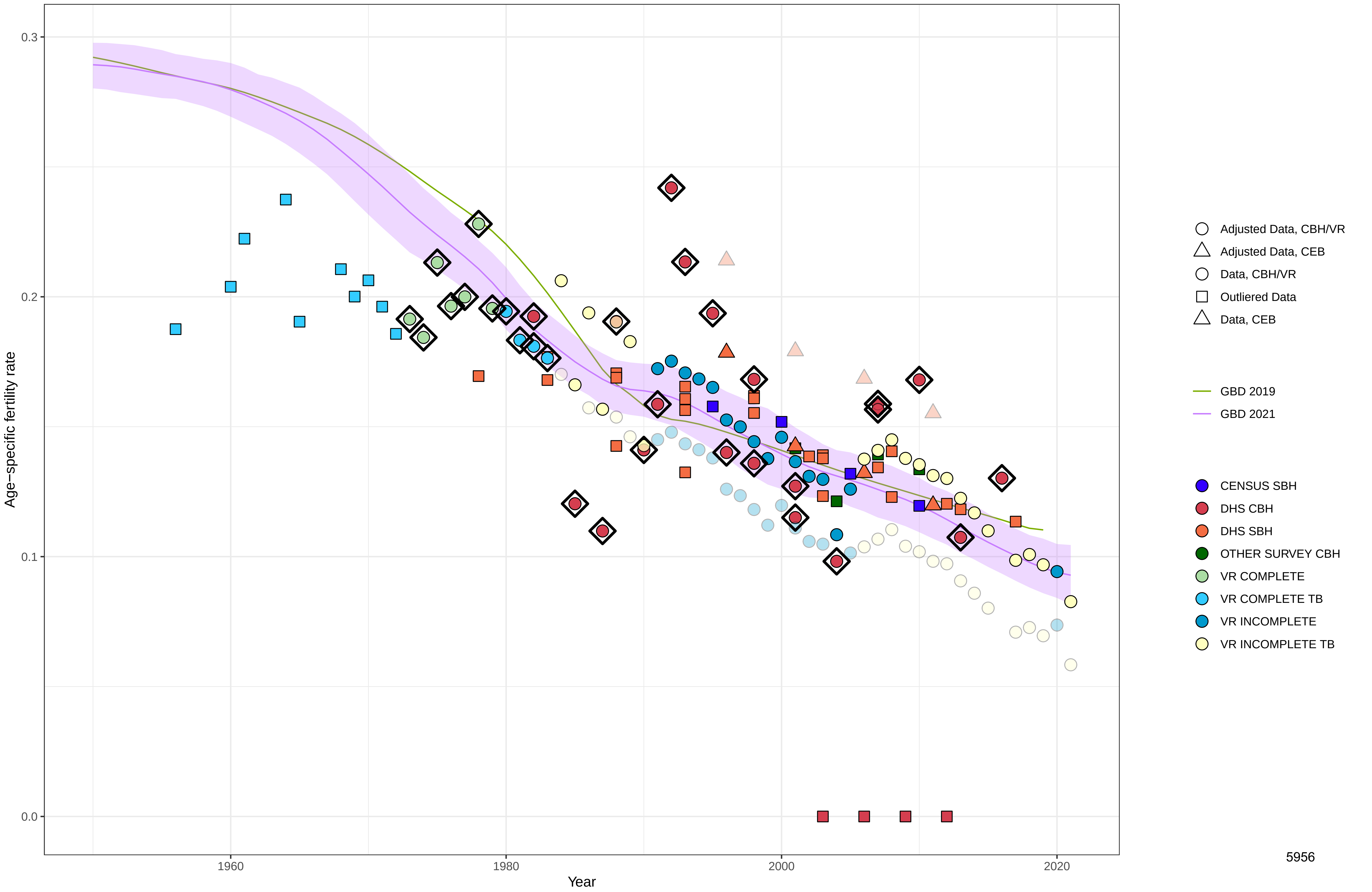

Age−specific fertility fitted trend lines, 1950−2021: Cagayan, Ages: 35−39
Data points enclosed with a diamond are reference sources

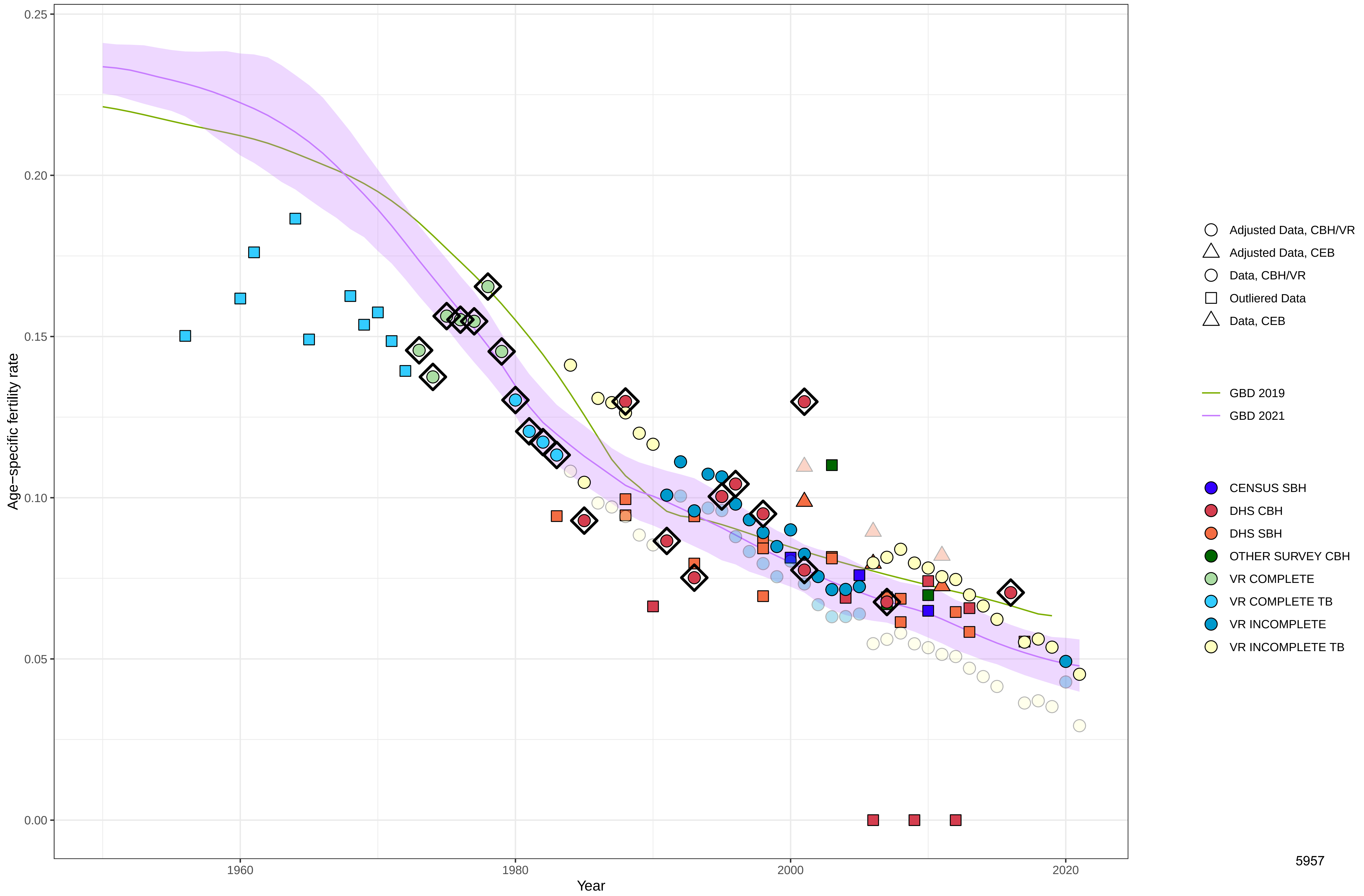

Age−specific fertility fitted trend lines, 1950−2021: Cagayan, Ages: 40−44
Data points enclosed with a diamond are reference sources

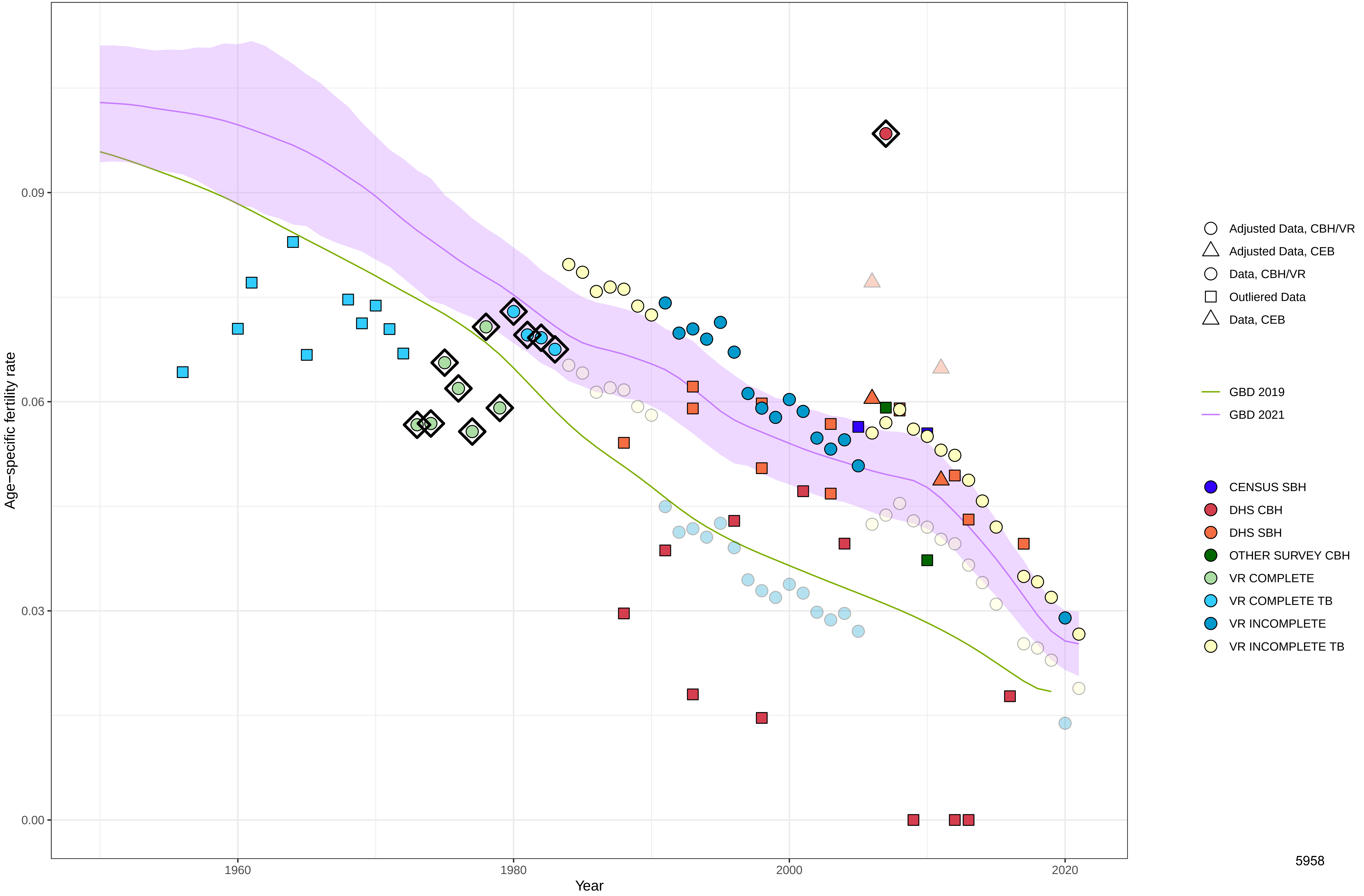

Age−specific fertility fitted trend lines, 1950−2021: Cagayan, Ages: 45−49
Data points enclosed with a diamond are reference sources

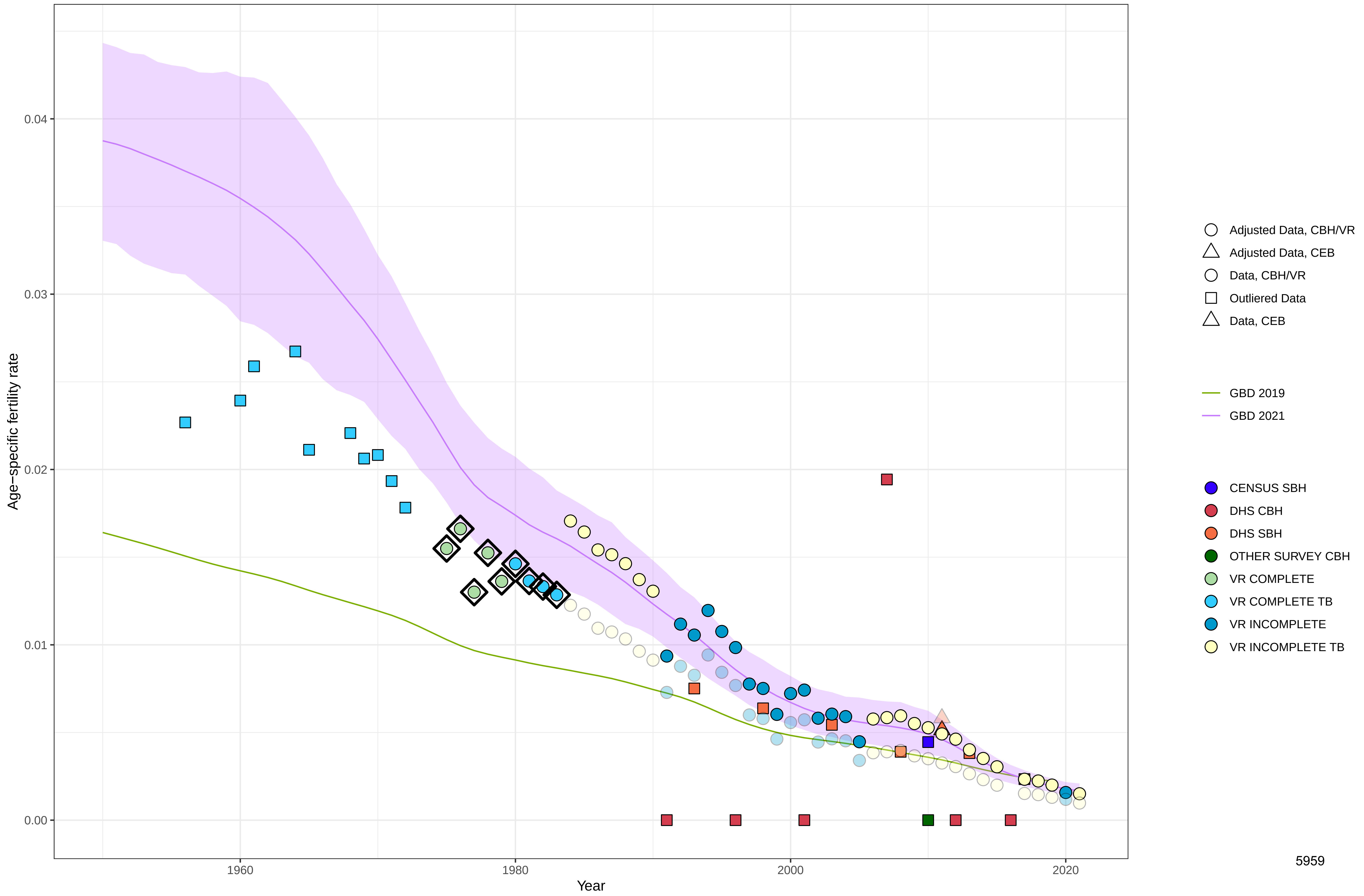

Age−specific fertility fitted trend lines, 1950−2021: Cagayan, Ages: 50−54
Data points enclosed with a diamond are reference sources

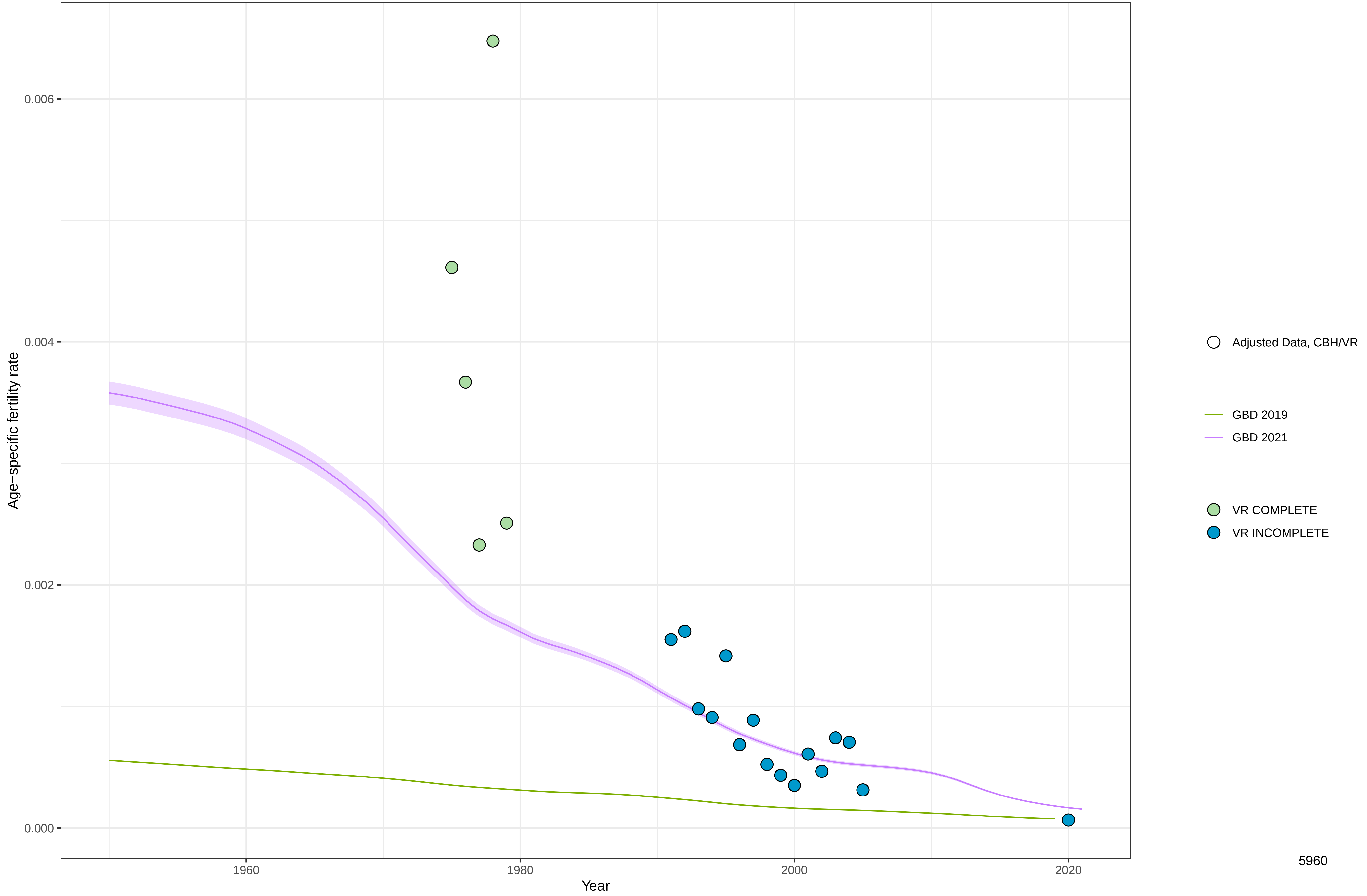

Total fertility rate fitted trend lines, 1950−2021: Cagayan TFR
Data points enclosed with a diamond are reference sources

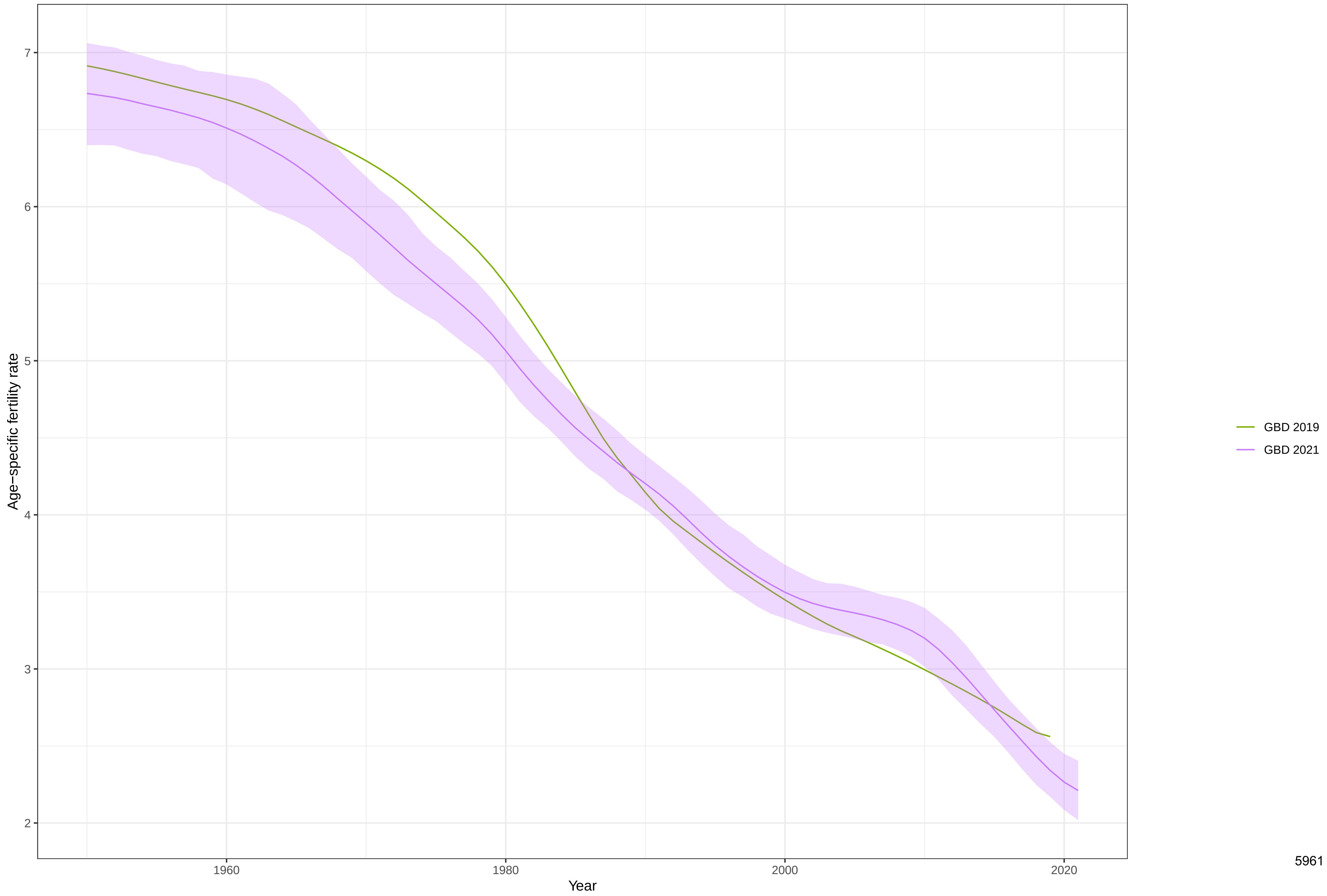

Age−specific fertility fitted trend lines, 1950−2021: Camarines Norte, Ages: 10−14
Data points enclosed with a diamond are reference sources

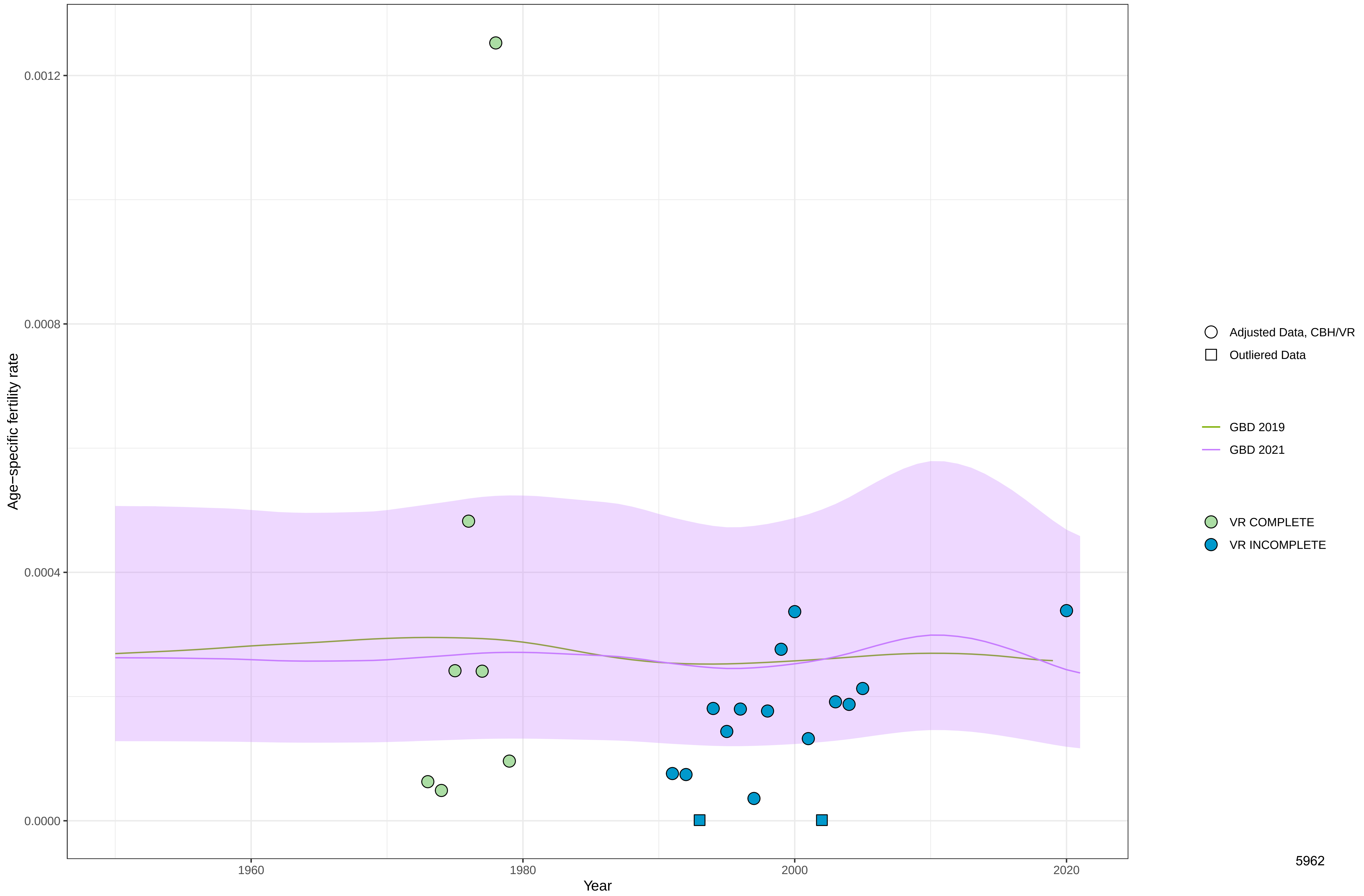

Age−specific fertility fitted trend lines, 1950−2021: Camarines Norte, Ages: 15−19
Data points enclosed with a diamond are reference sources

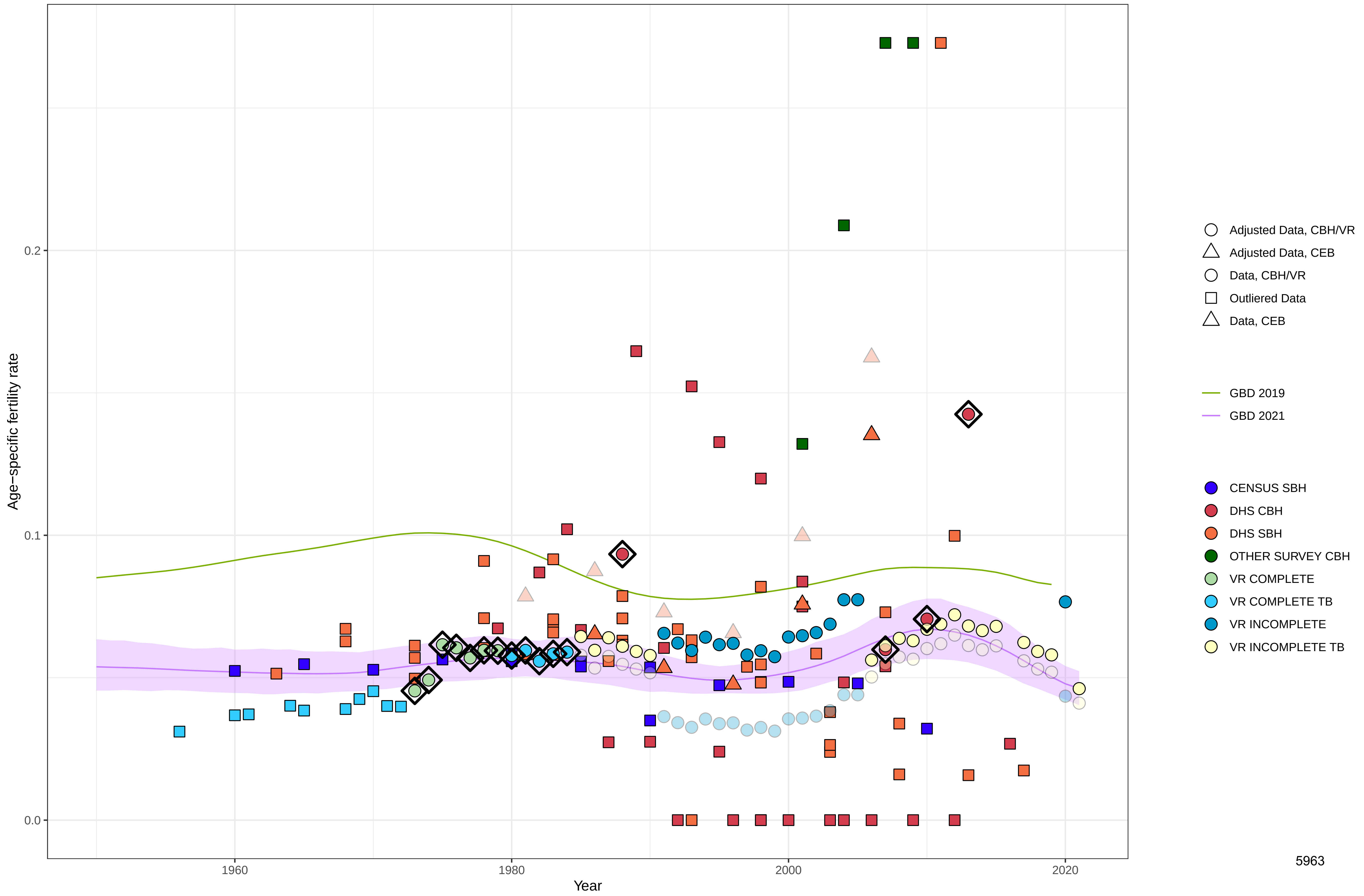

Age−specific fertility fitted trend lines, 1950−2021: Camarines Norte, Ages: 20−24
Data points enclosed with a diamond are reference sources

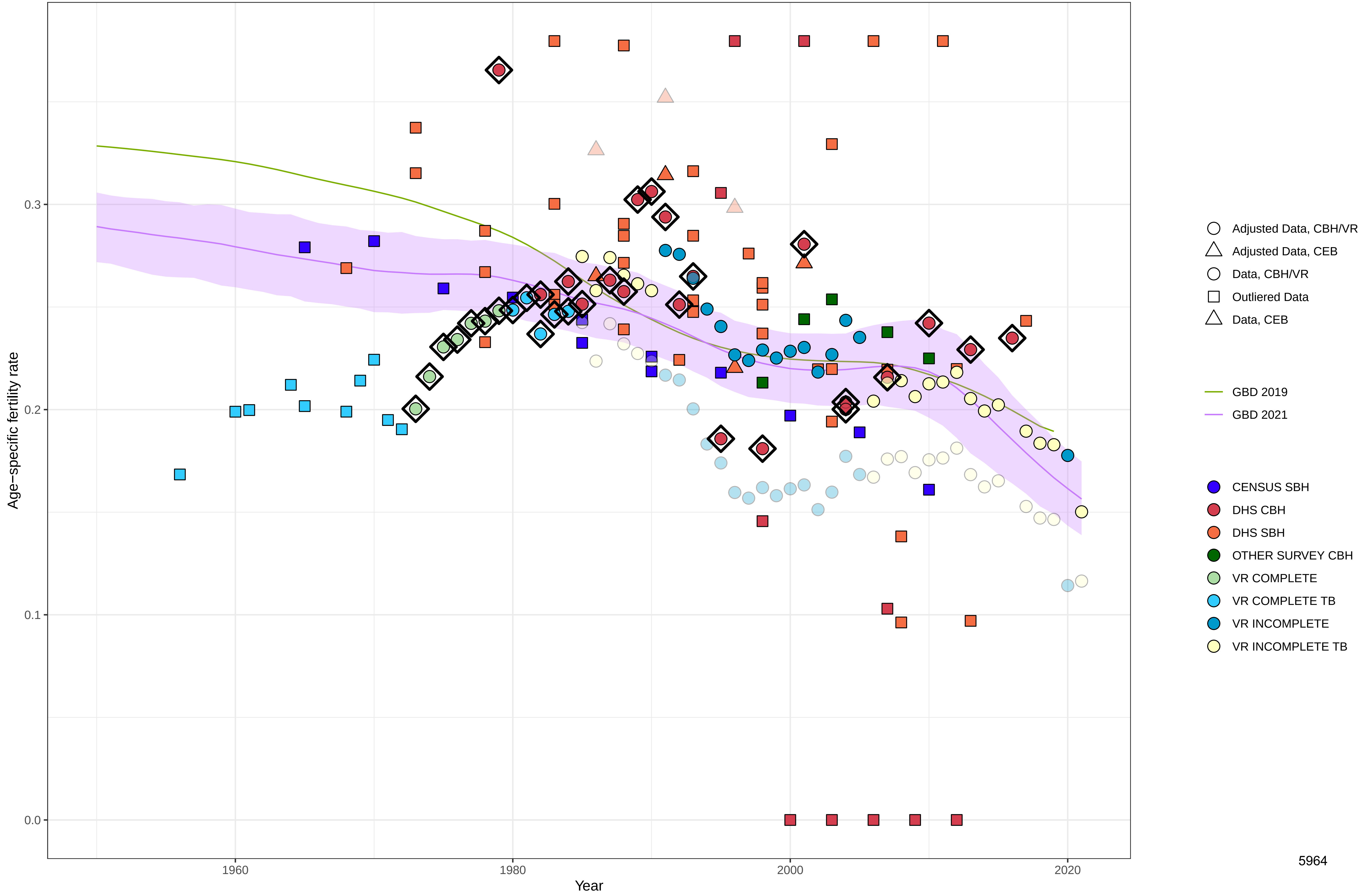

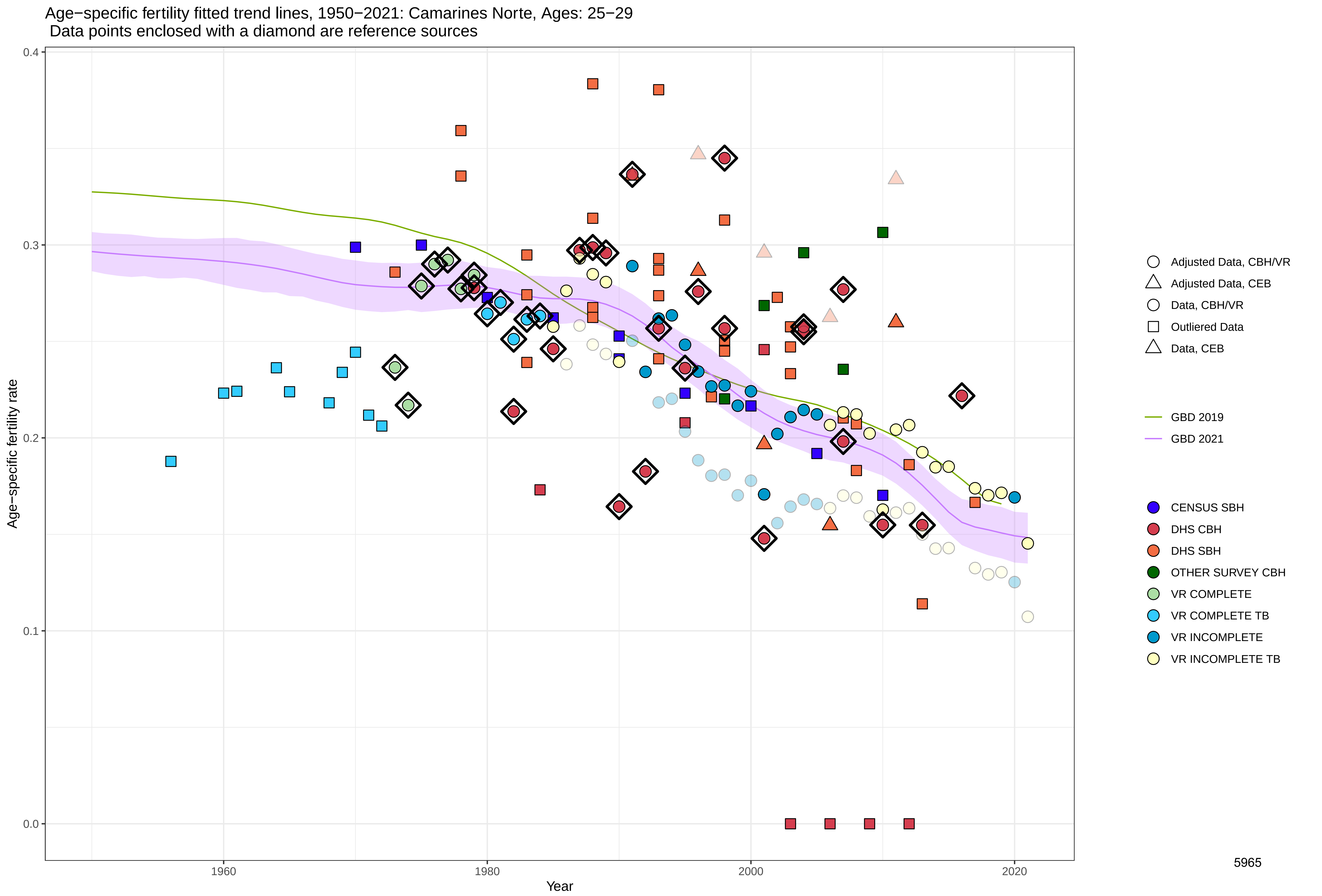
Age−specific fertility fitted trend lines, 1950−2021: Camarines Norte, Ages: 25−29
Data points enclosed with a diamond are reference sources
Age−specific fertility rate
Year
0.0
0.1
0.2
0.3
0.4
1960
1980
2000
2020
Adjusted Data, CBH/VR
Adjusted Data, CEB
Data, CBH/VR
Outliered Data
Data, CEB
GBD 2019
GBD 2021
CENSUS SBH
DHS CBH
DHS SBH
OTHER SURVEY CBH
VR COMPLETE
VR COMPLETE TB
VR INCOMPLETE
VR INCOMPLETE TB

Age−specific fertility fitted trend lines, 1950−2021: Camarines Norte, Ages: 30−34
Data points enclosed with a diamond are reference sources

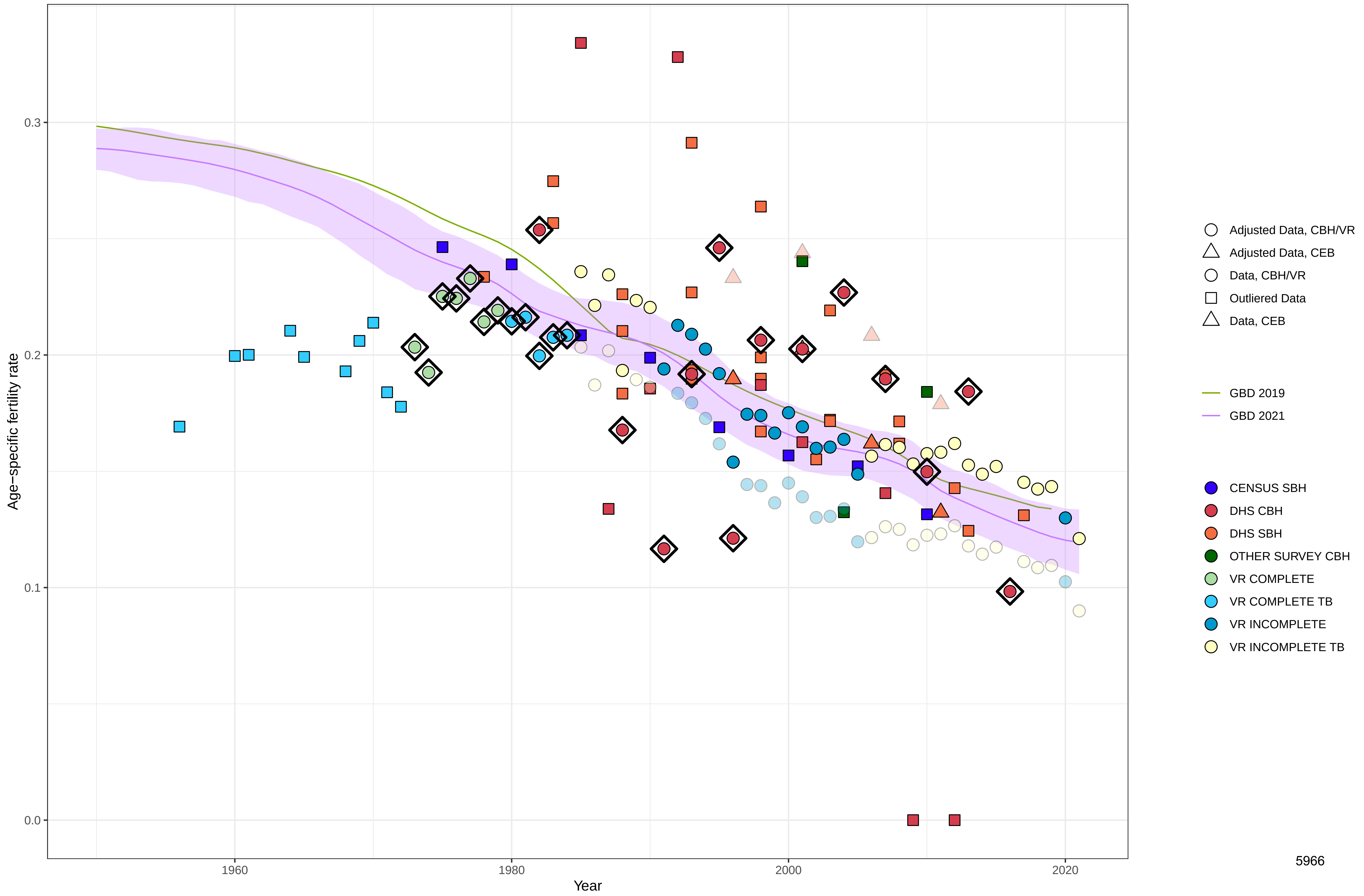

Age−specific fertility fitted trend lines, 1950−2021: Camarines Norte, Ages: 35−39
Data points enclosed with a diamond are reference sources

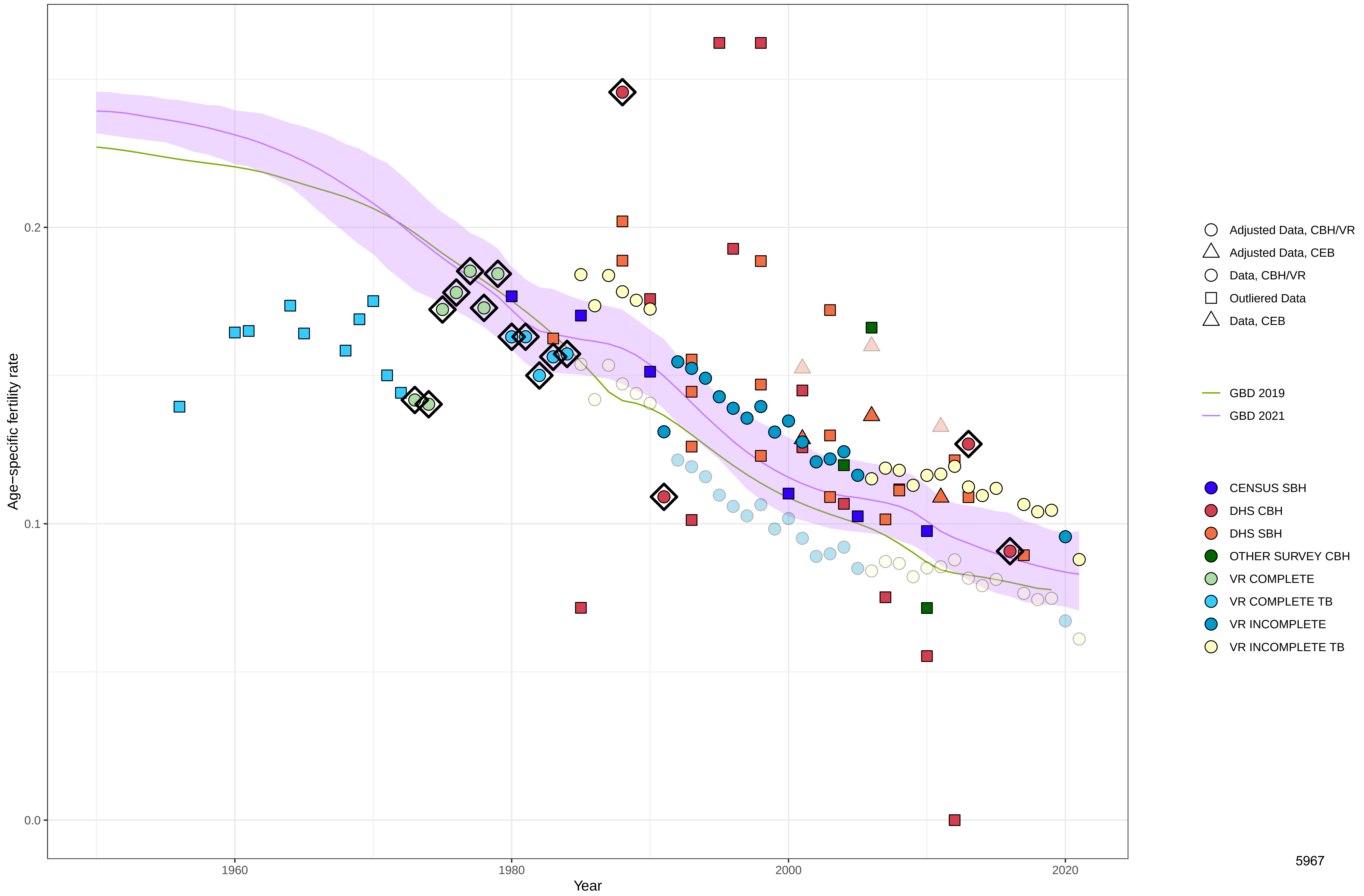

Age−specific fertility fitted trend lines, 1950−2021: Camarines Norte, Ages: 40−44
Data points enclosed with a diamond are reference sources

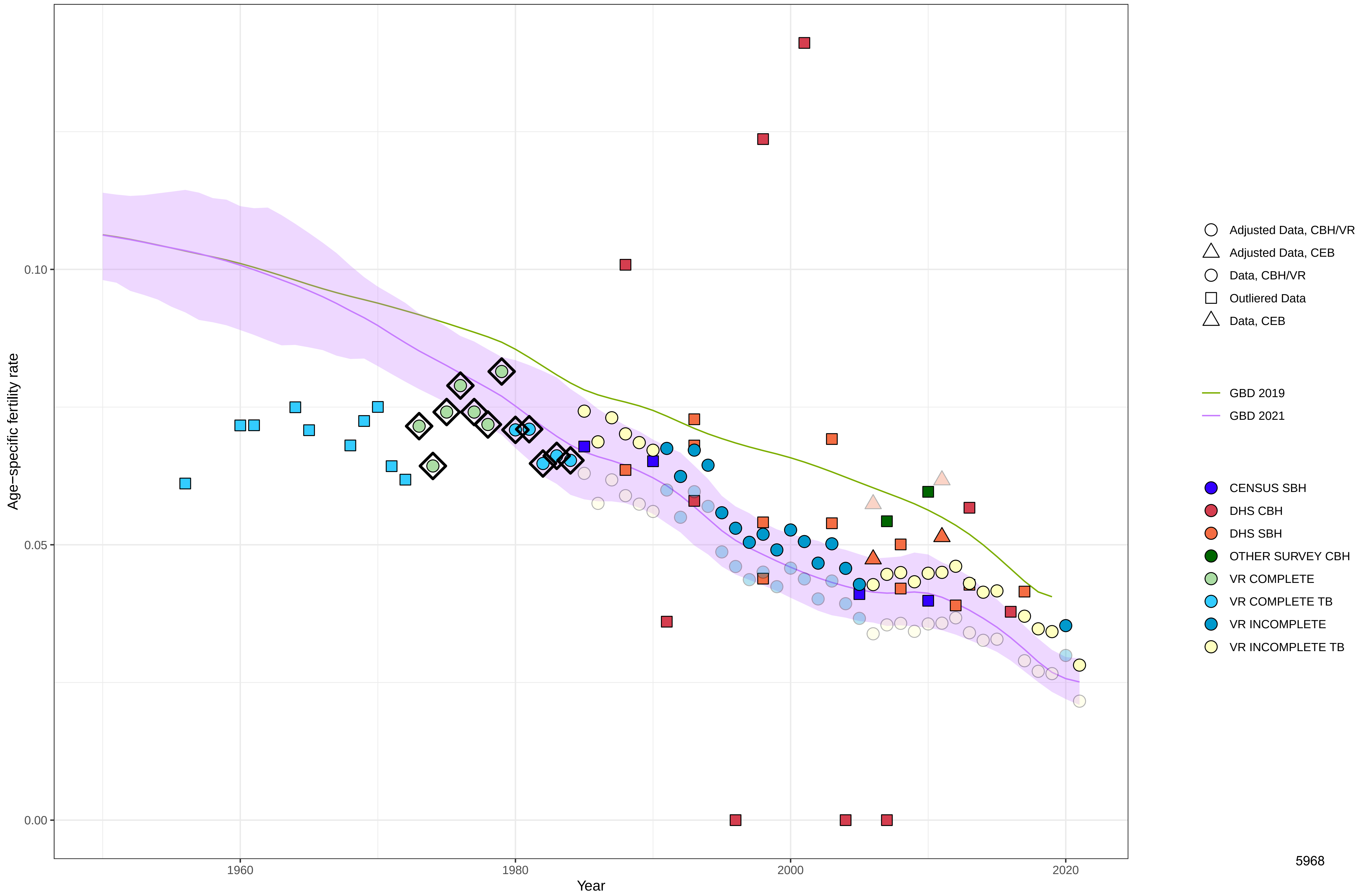

Age−specific fertility fitted trend lines, 1950−2021: Camarines Norte, Ages: 45−49
Data points enclosed with a diamond are reference sources

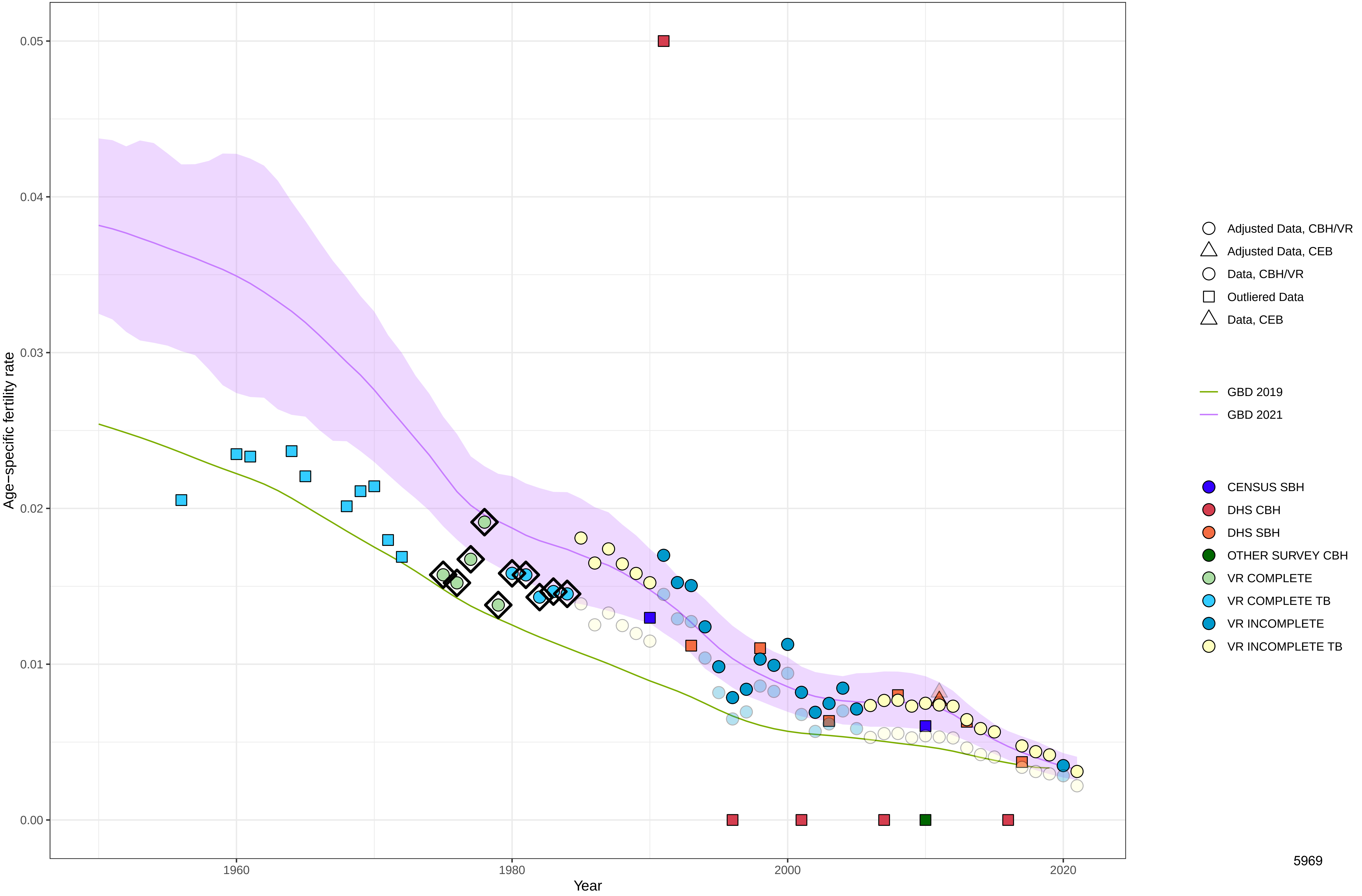

5969

Age−specific fertility fitted trend lines, 1950−2021: Camarines Norte, Ages: 50−54
Data points enclosed with a diamond are reference sources

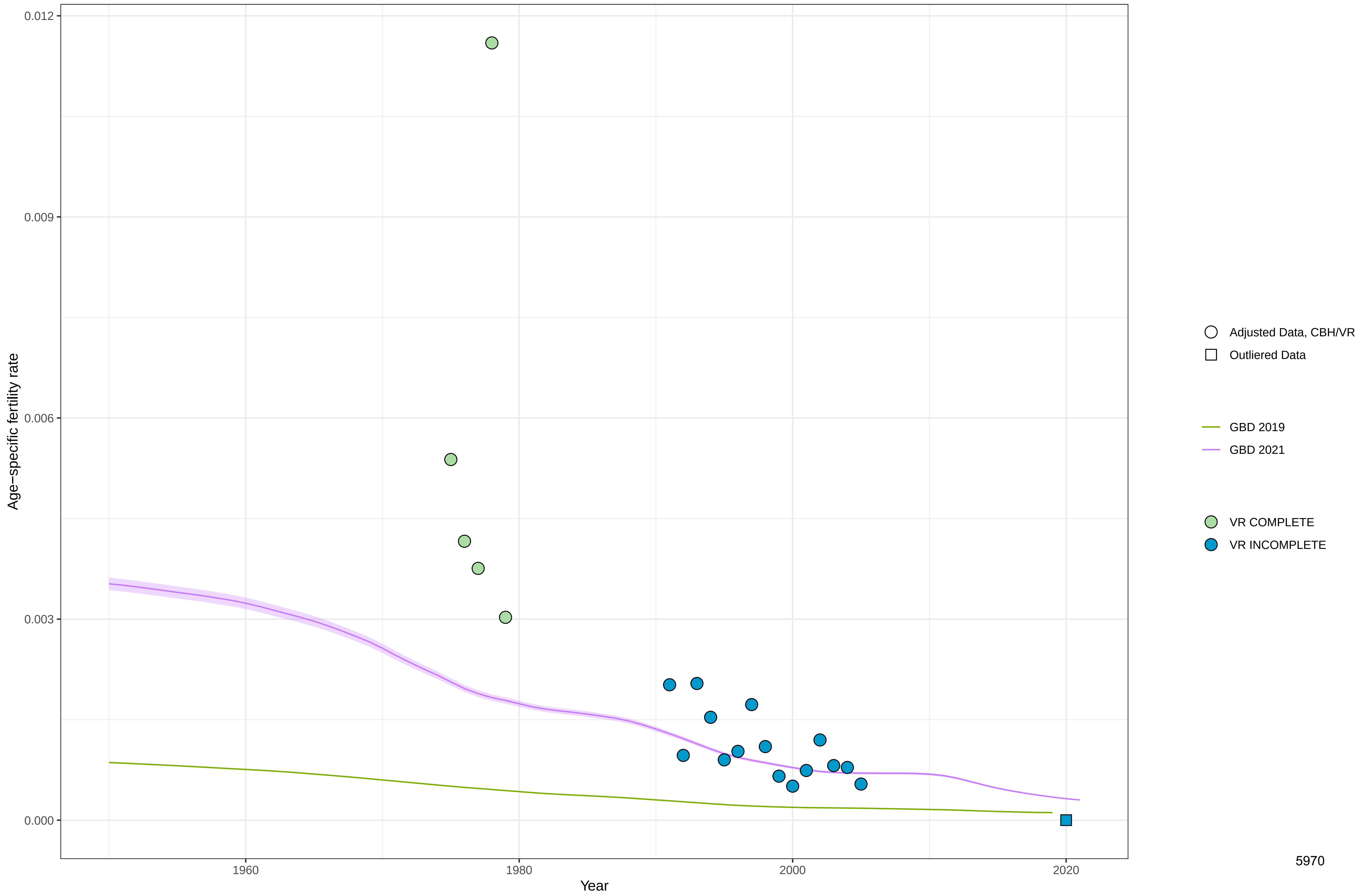

Total fertility rate fitted trend lines, 1950−2021: Camarines Norte TFR
Data points enclosed with a diamond are reference sources

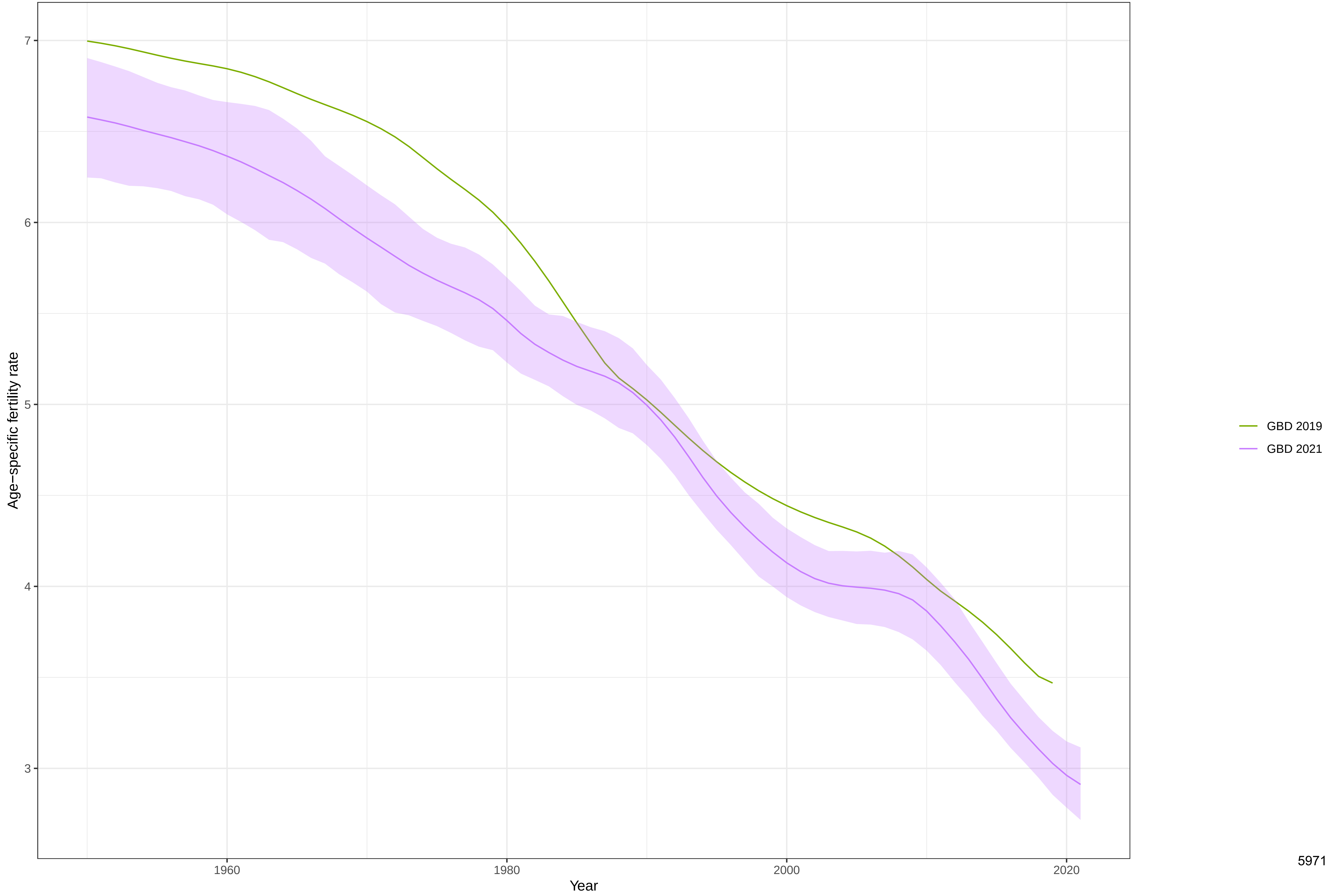

Age−specific fertility fitted trend lines, 1950−2021: Camarines Sur, Ages: 10−14
Data points enclosed with a diamond are reference sources

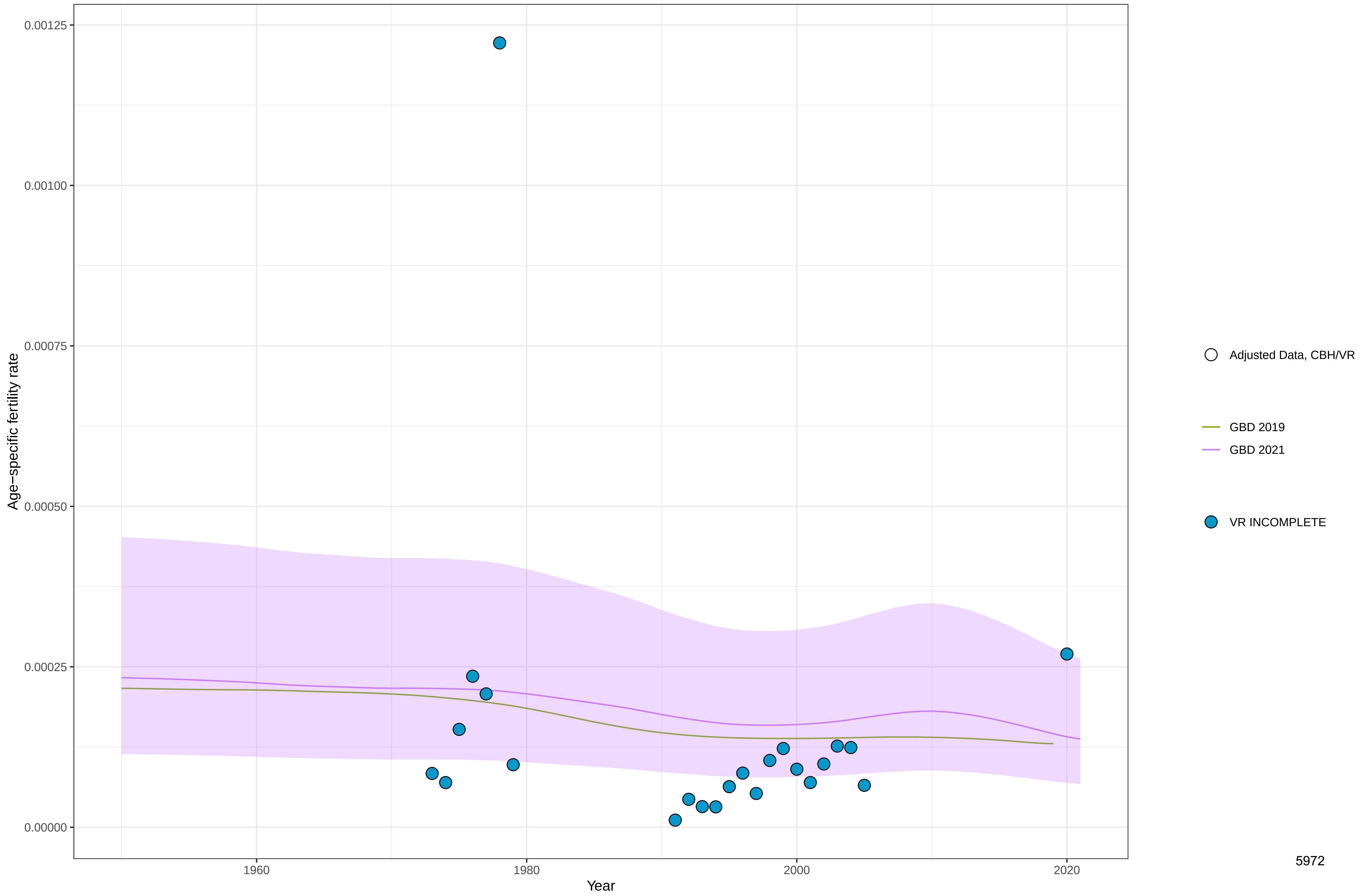

Age−specific fertility fitted trend lines, 1950−2021: Camarines Sur, Ages: 15−19
Data points enclosed with a diamond are reference sources

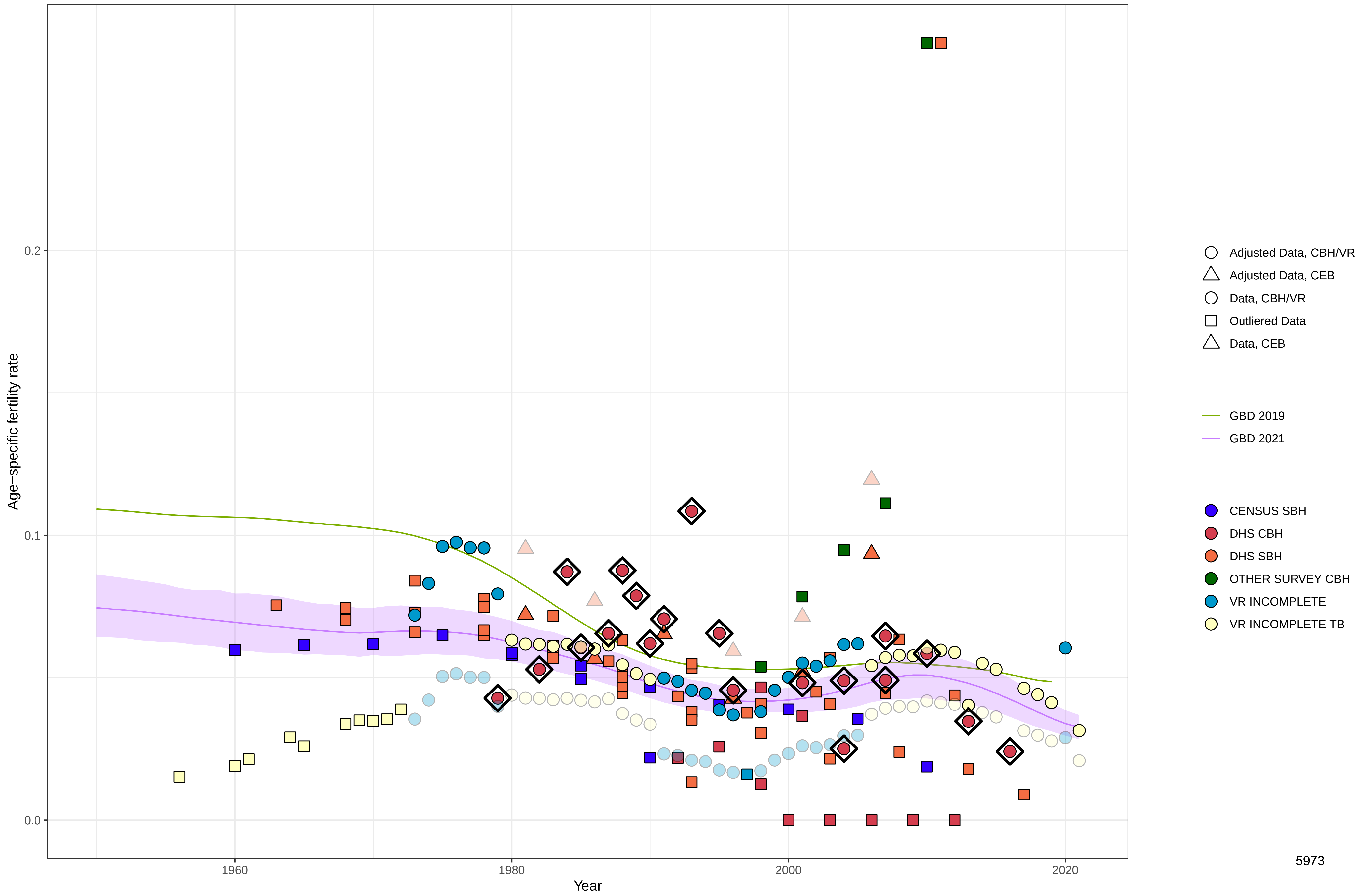

Age−specific fertility fitted trend lines, 1950−2021: Camarines Sur, Ages: 20−24
Data points enclosed with a diamond are reference sources

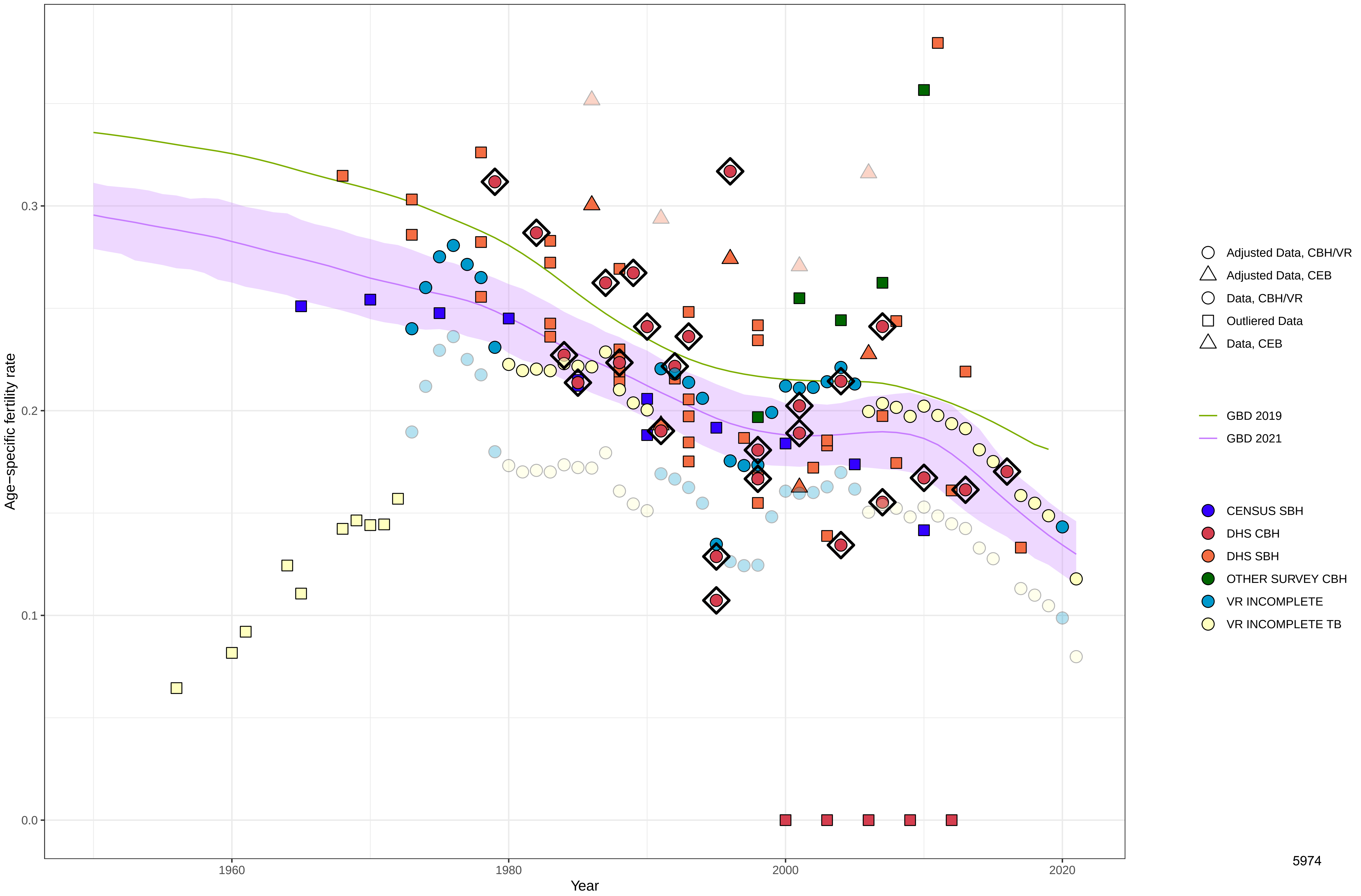

5974

Age−specific fertility fitted trend lines, 1950−2021: Camarines Sur, Ages: 25−29
Data points enclosed with a diamond are reference sources

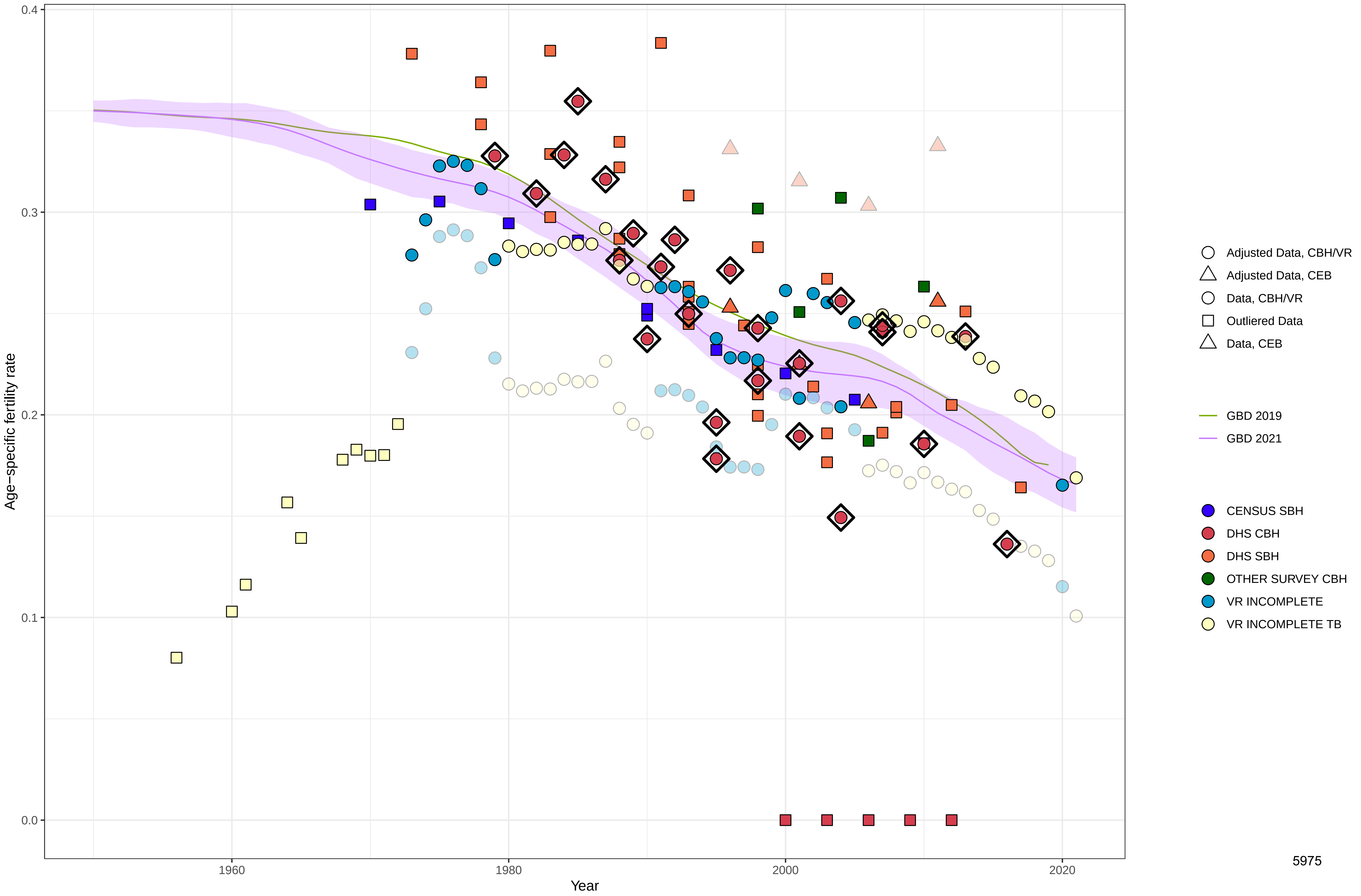

Age−specific fertility fitted trend lines, 1950−2021: Camarines Sur, Ages: 30−34
Data points enclosed with a diamond are reference sources

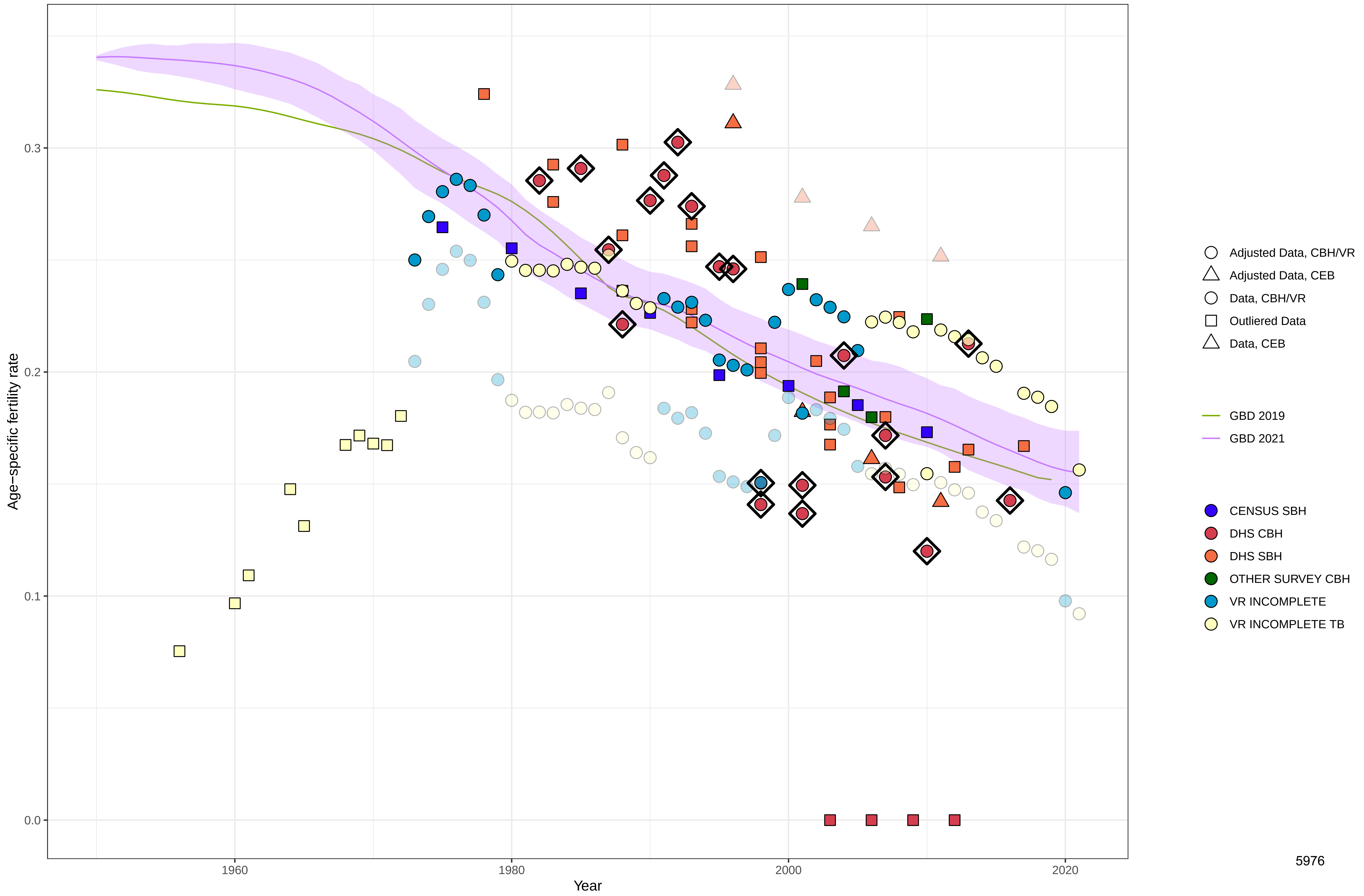

Age−specific fertility fitted trend lines, 1950−2021: Camarines Sur, Ages: 35−39
Data points enclosed with a diamond are reference sources

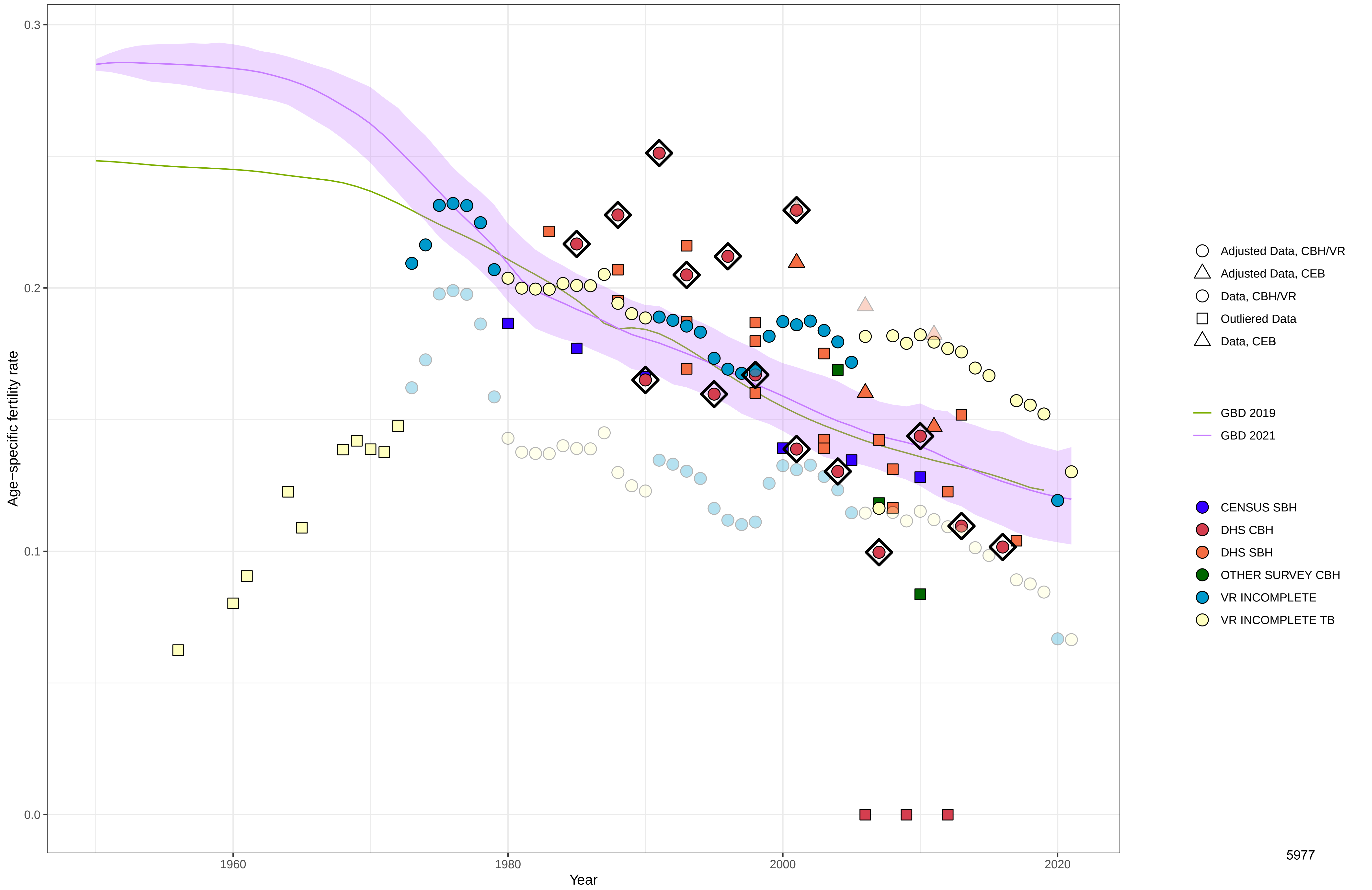

5977

Age−specific fertility fitted trend lines, 1950−2021: Camarines Sur, Ages: 40−44
Data points enclosed with a diamond are reference sources

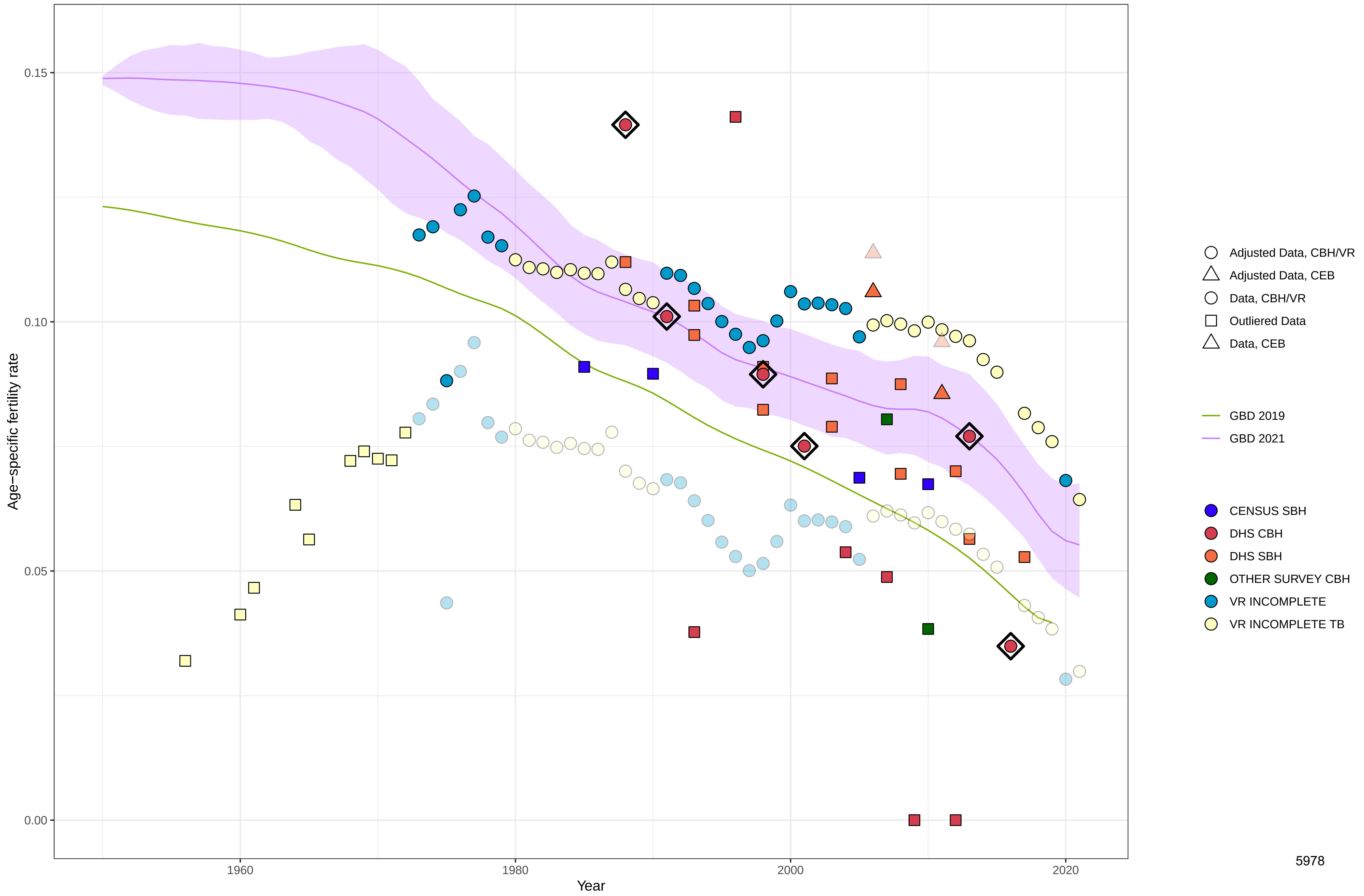

Age−specific fertility fitted trend lines, 1950−2021: Camarines Sur, Ages: 45−49
Data points enclosed with a diamond are reference sources

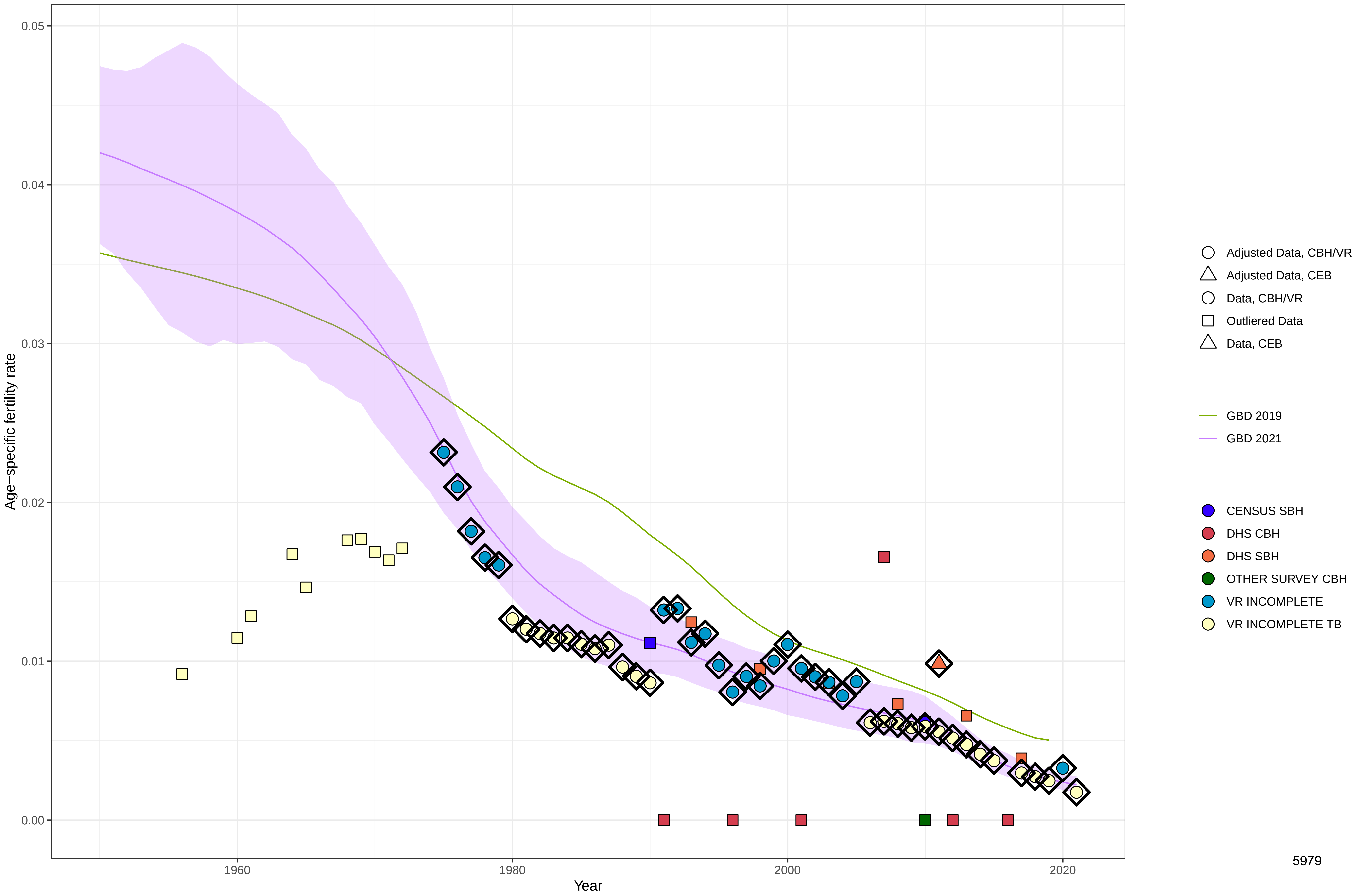

Age−specific fertility fitted trend lines, 1950−2021: Camarines Sur, Ages: 50−54
Data points enclosed with a diamond are reference sources

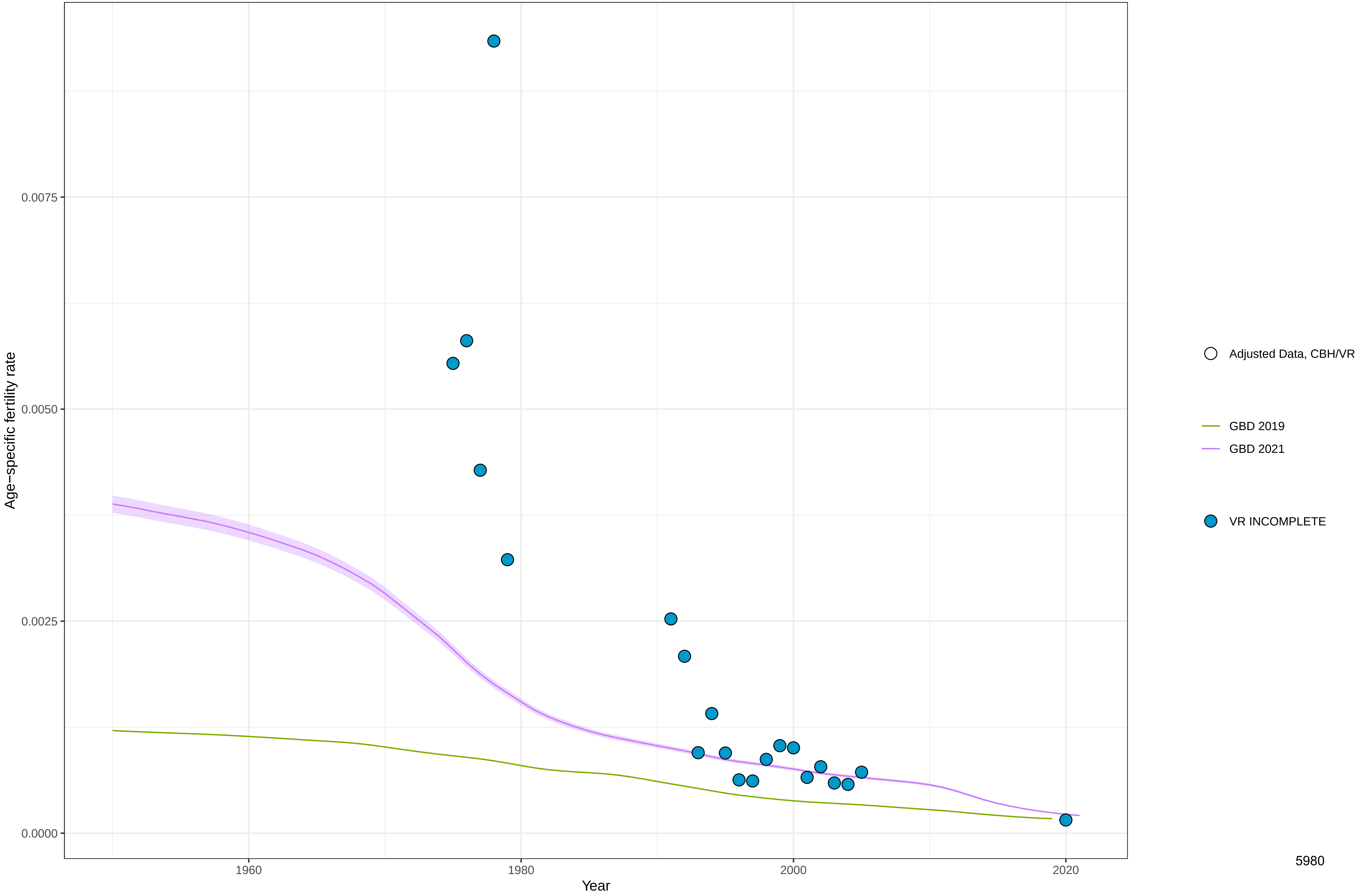

Total fertility rate fitted trend lines, 1950−2021: Camarines Sur TFR
Data points enclosed with a diamond are reference sources

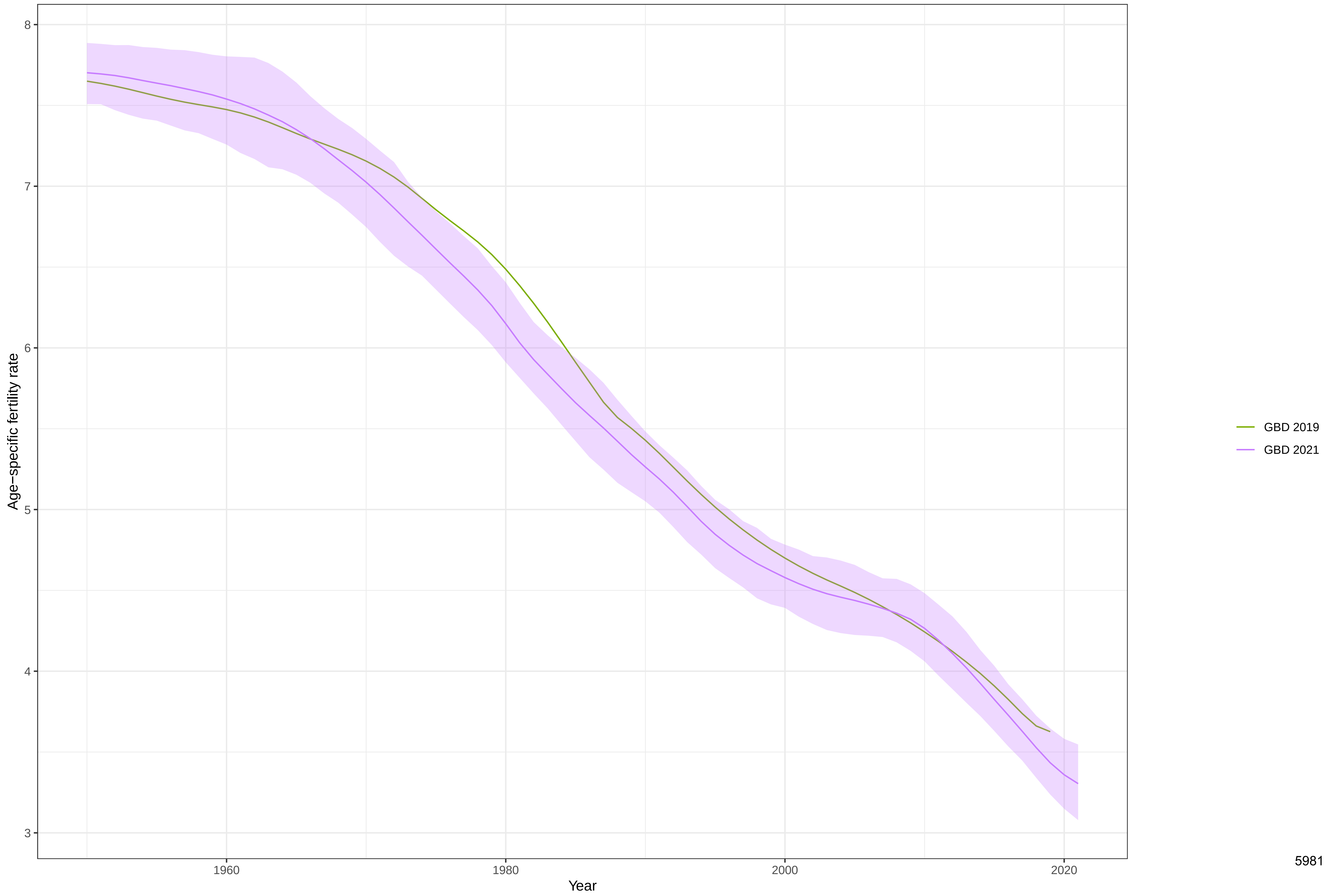

Age−specific fertility fitted trend lines, 1950−2021: Camiguin, Ages: 10−14
Data points enclosed with a diamond are reference sources

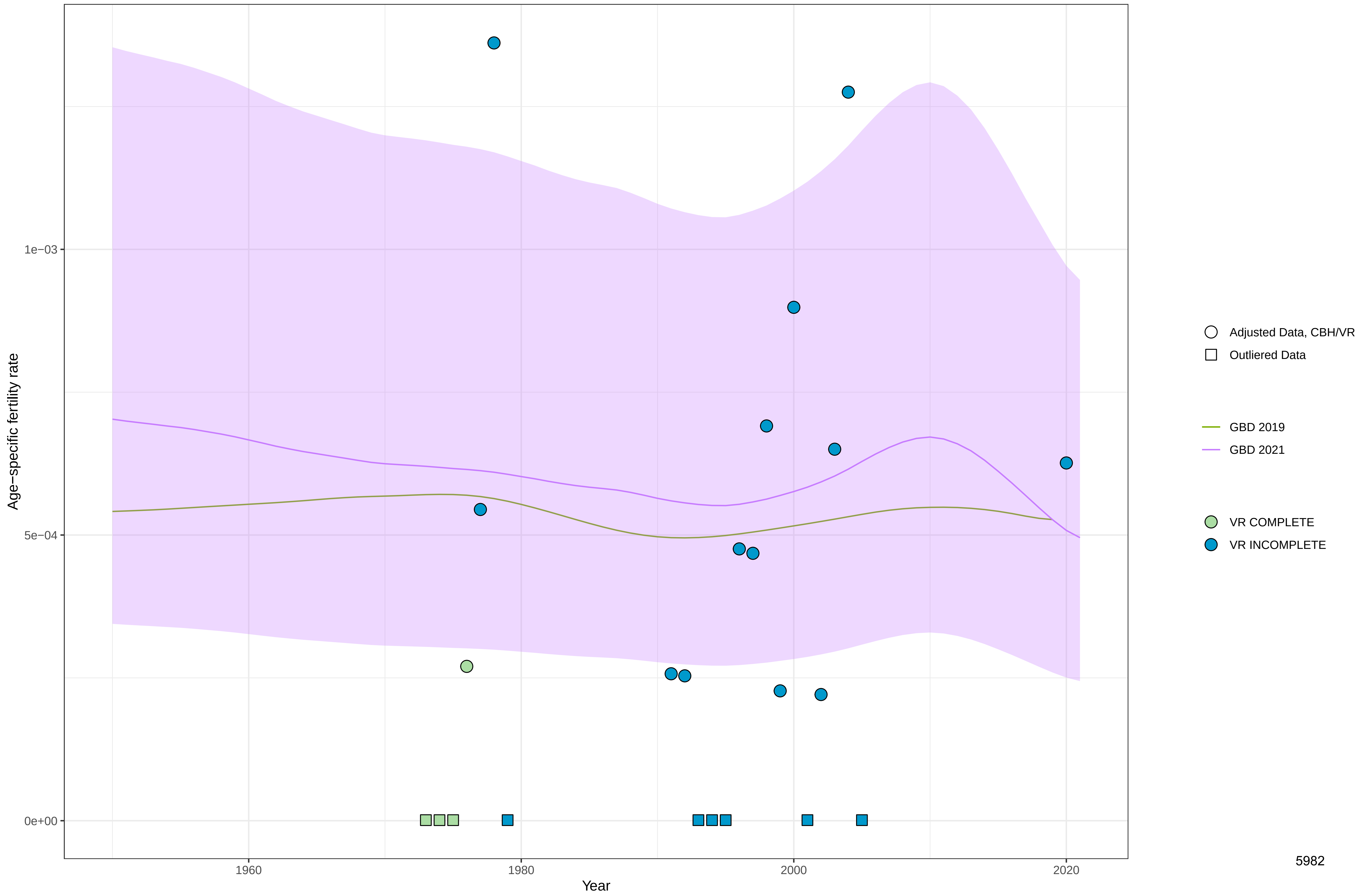

Age−specific fertility fitted trend lines, 1950−2021: Camiguin, Ages: 15−19
Data points enclosed with a diamond are reference sources

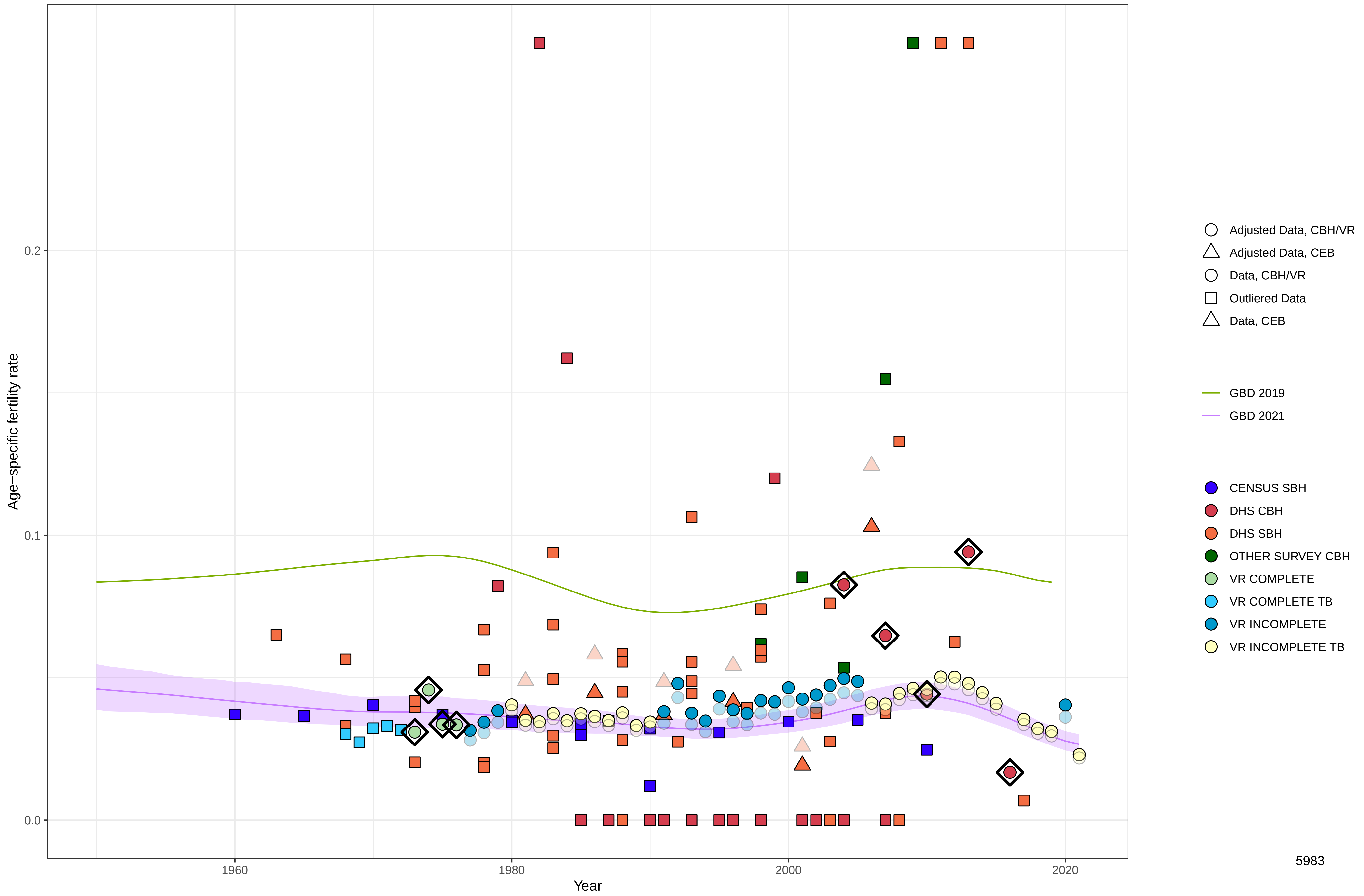

Age−specific fertility fitted trend lines, 1950−2021: Camiguin, Ages: 20−24
Data points enclosed with a diamond are reference sources

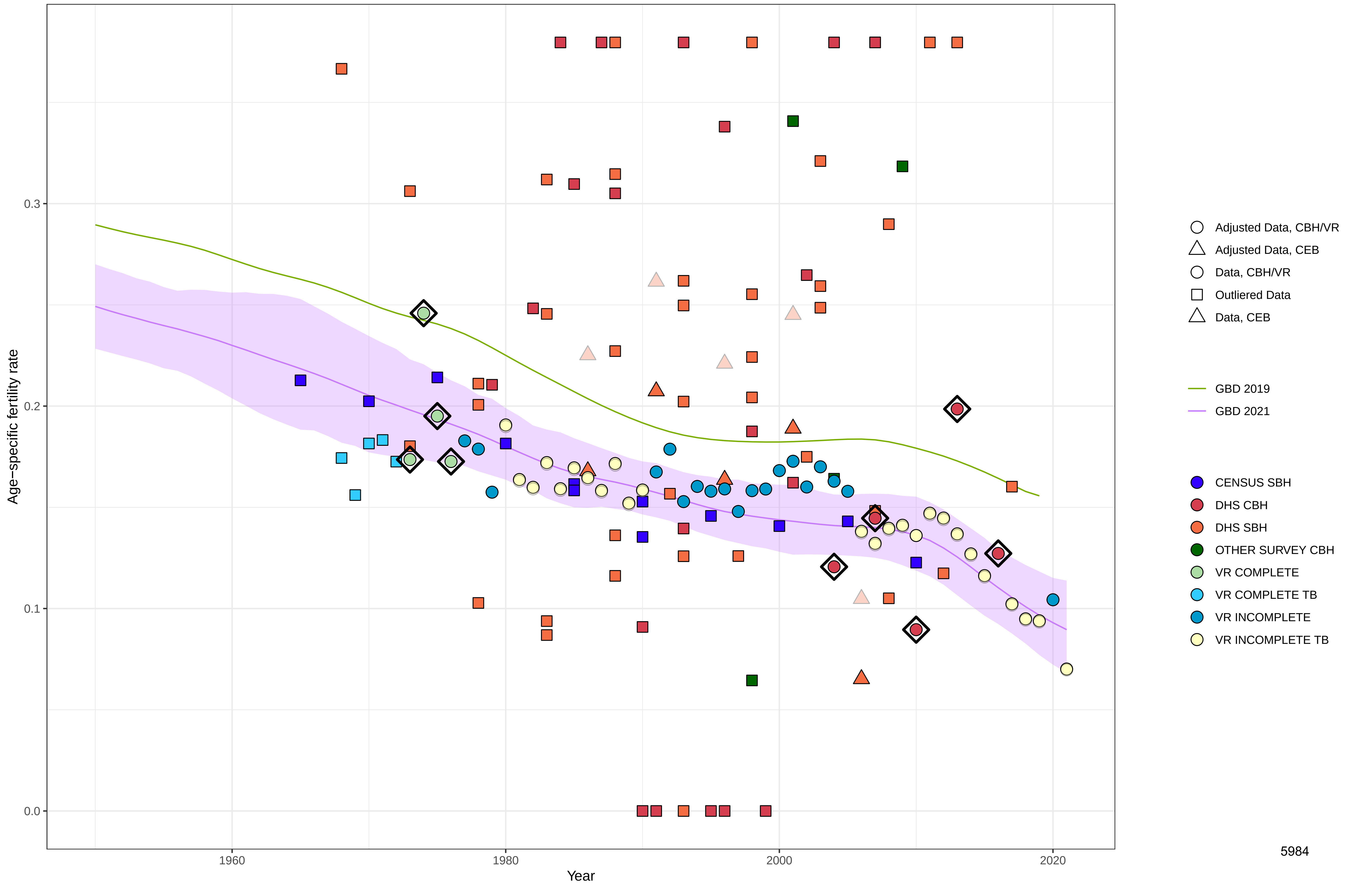

Age−specific fertility fitted trend lines, 1950−2021: Camiguin, Ages: 25−29
Data points enclosed with a diamond are reference sources

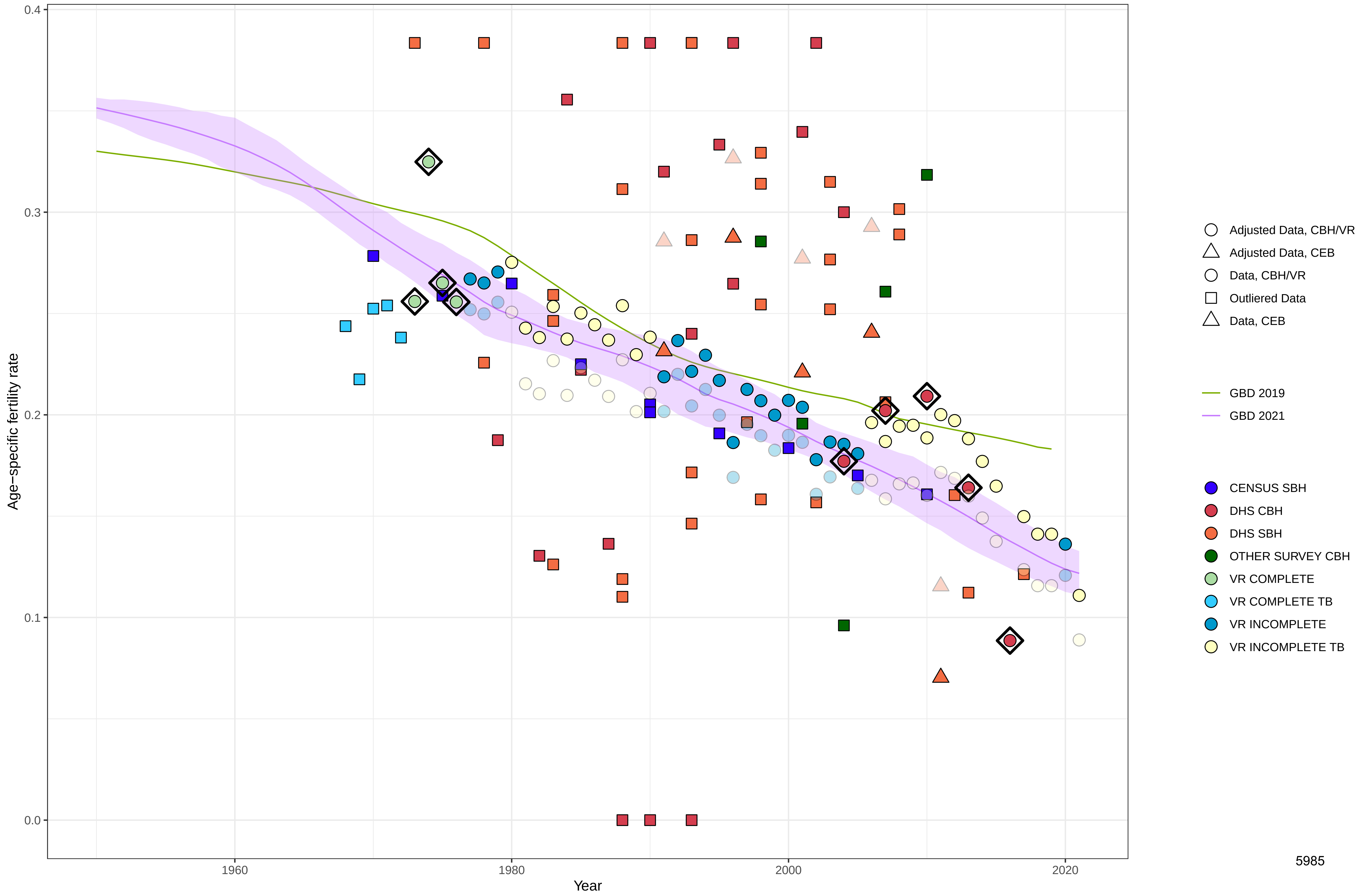

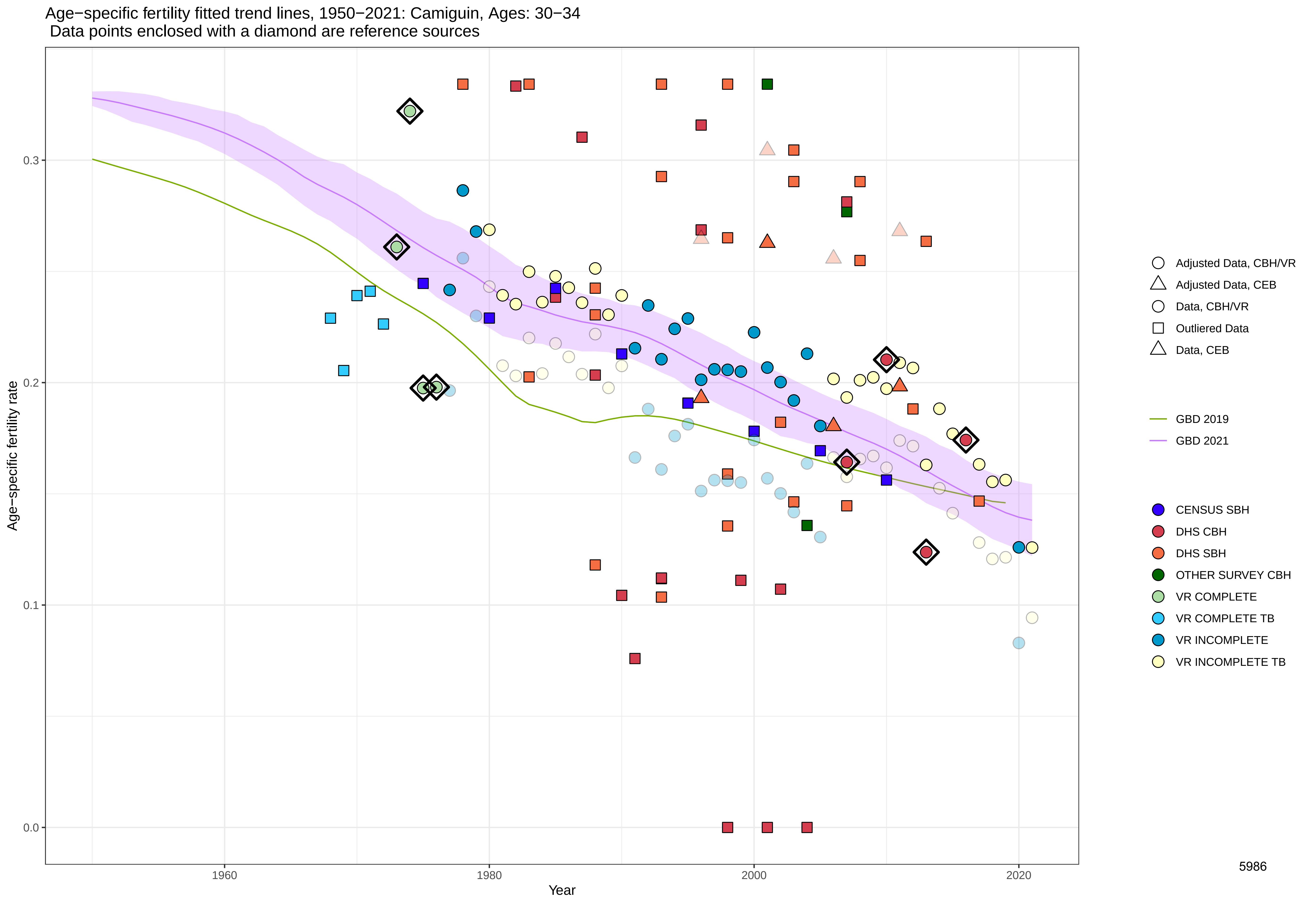
Age−specific fertility fitted trend lines, 1950−2021: Camiguin, Ages: 30−34
Data points enclosed with a diamond are reference sources
Age−specific fertility rate
Year
0.0
0.1
0.2
0.3
1960
1980
2000
2020
Adjusted Data, CBH/VR
Adjusted Data, CEB
Data, CBH/VR
Outliered Data
Data, CEB
GBD 2019
GBD 2021
CENSUS SBH
DHS CBH
DHS SBH
OTHER SURVEY CBH
VR COMPLETE
VR COMPLETE TB
VR INCOMPLETE
VR INCOMPLETE TB

Age−specific fertility fitted trend lines, 1950−2021: Camiguin, Ages: 35−39
Data points enclosed with a diamond are reference sources

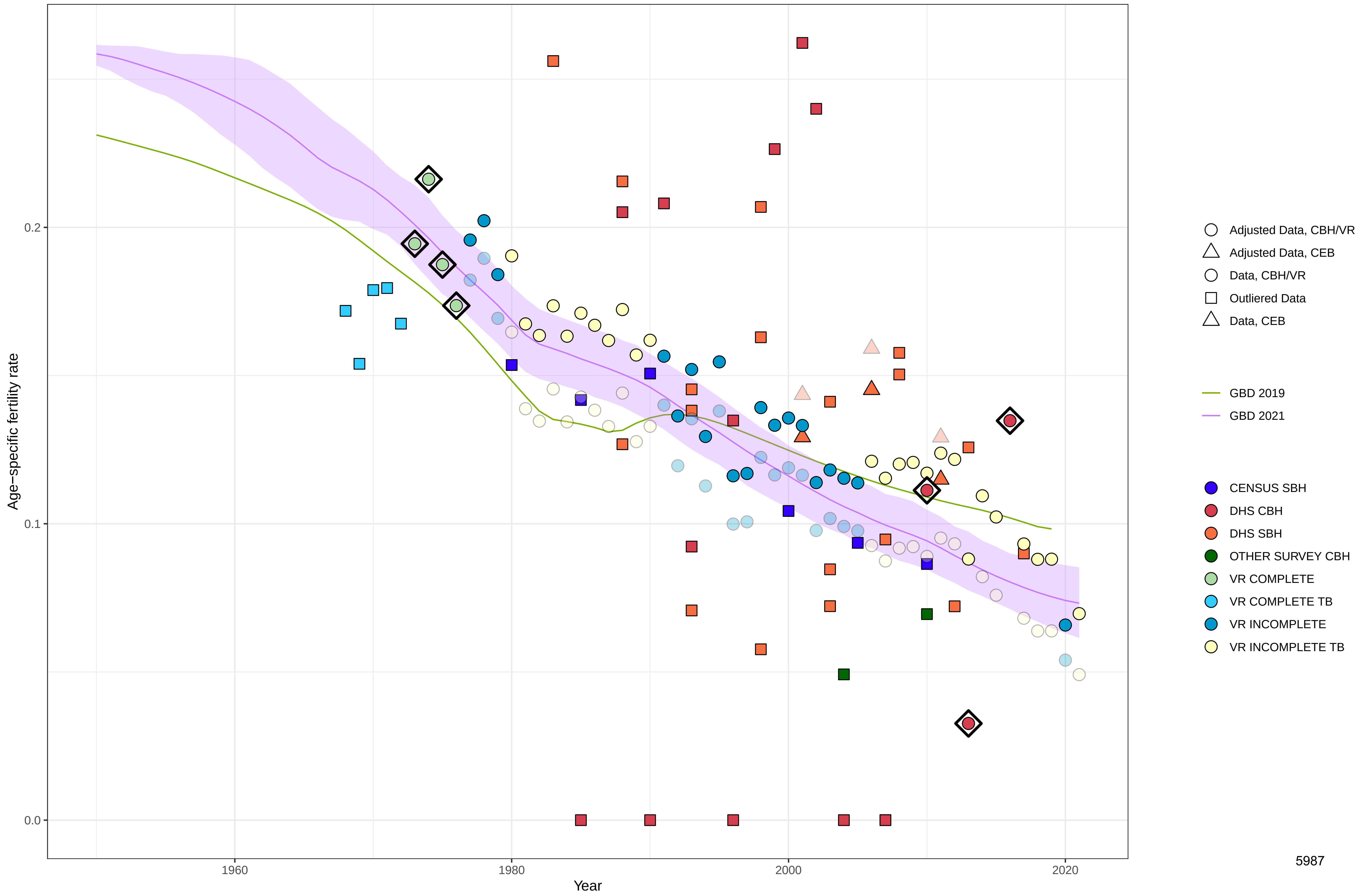

Age−specific fertility fitted trend lines, 1950−2021: Camiguin, Ages: 40−44
Data points enclosed with a diamond are reference sources

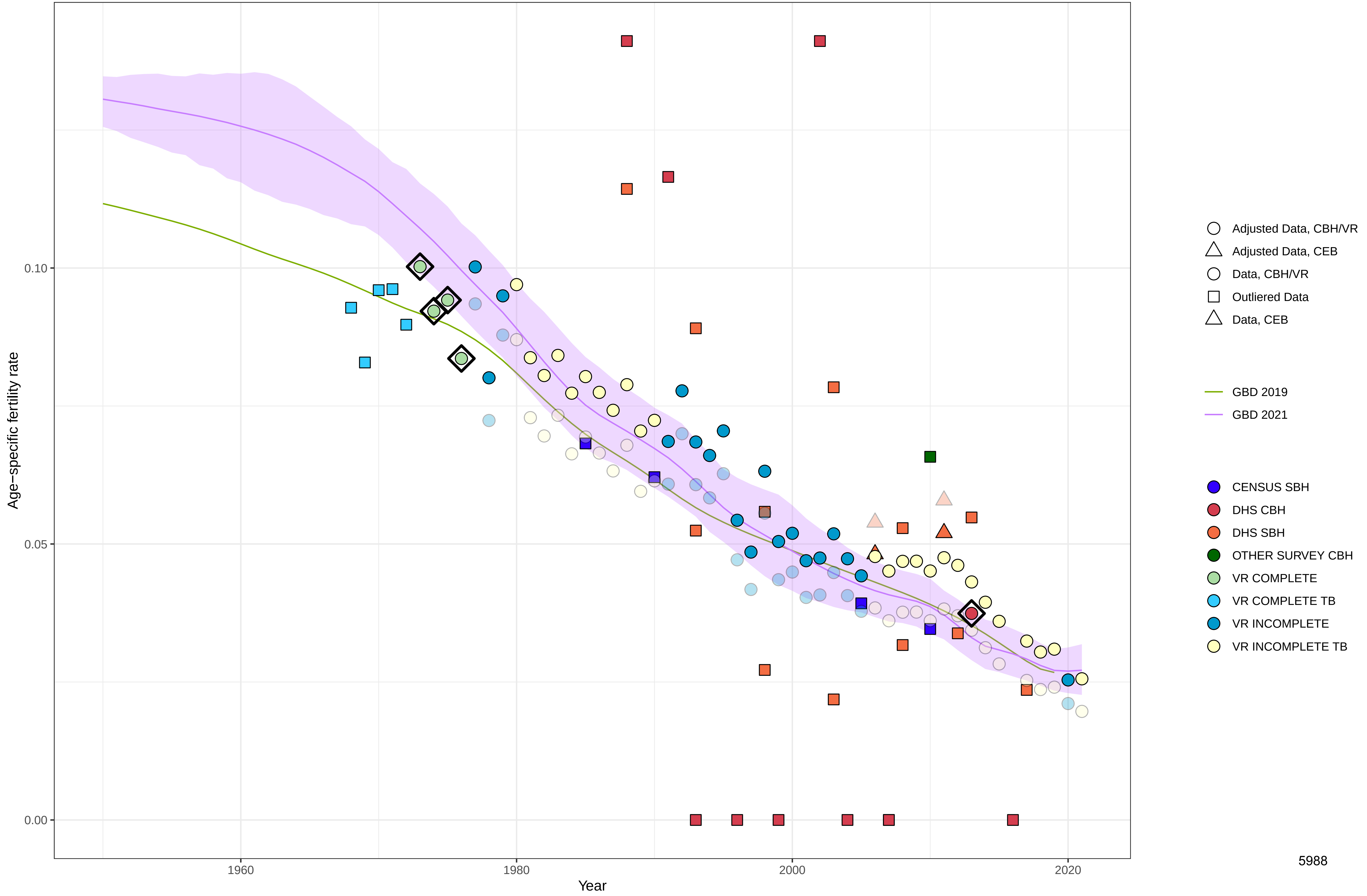

Age−specific fertility fitted trend lines, 1950−2021: Camiguin, Ages: 45−49
Data points enclosed with a diamond are reference sources

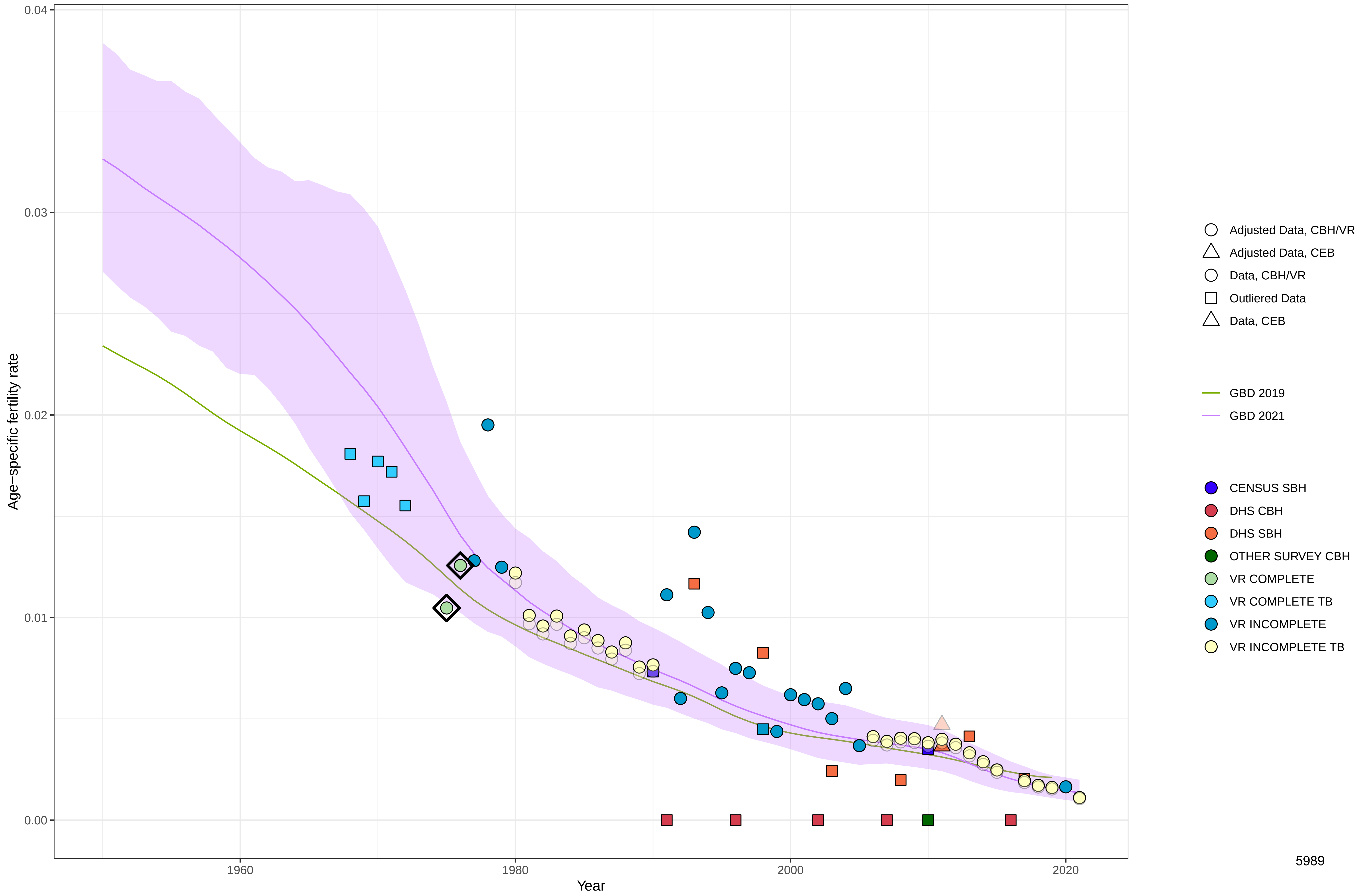

Age−specific fertility fitted trend lines, 1950−2021: Camiguin, Ages: 50−54
Data points enclosed with a diamond are reference sources

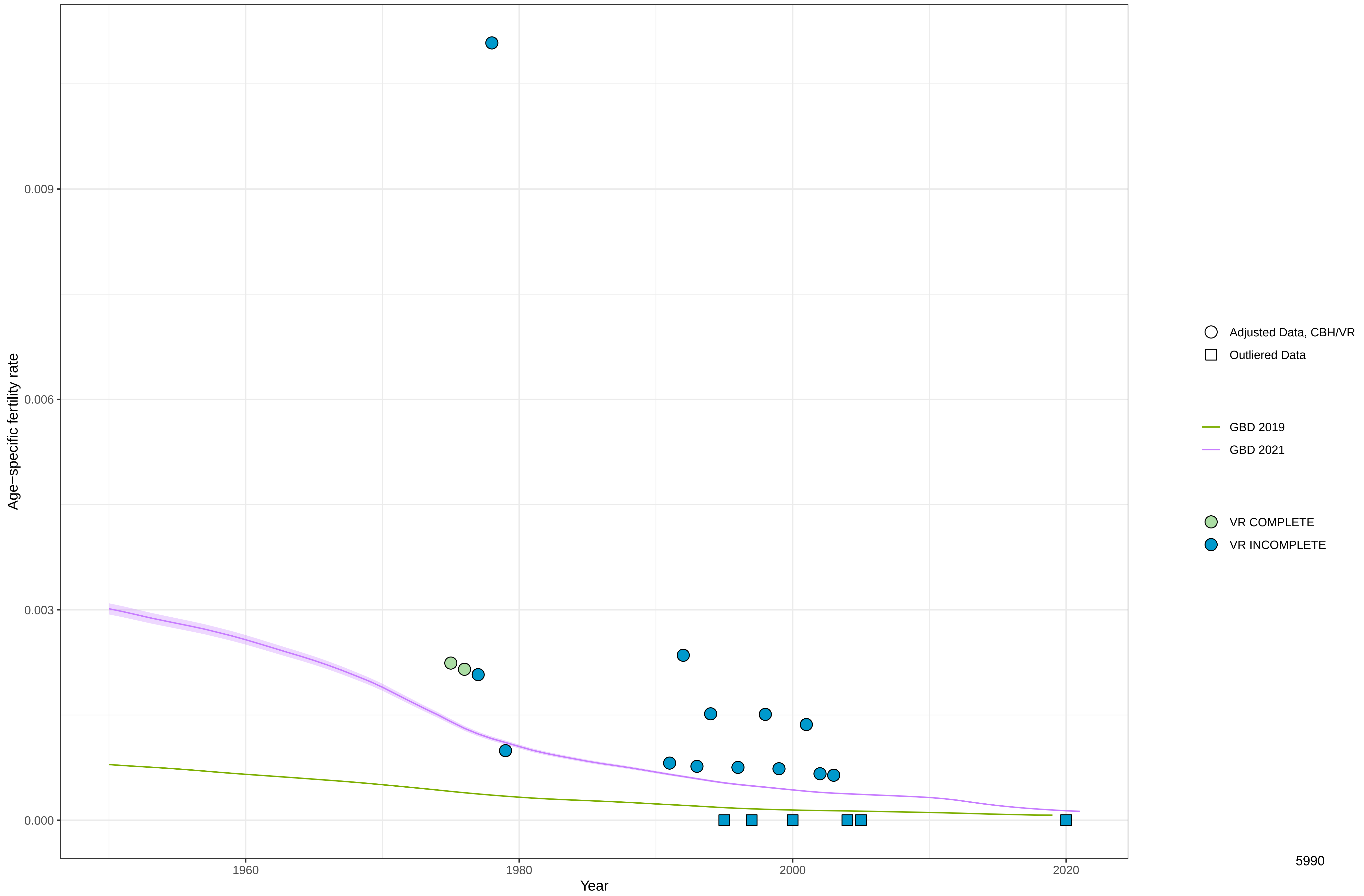

Total fertility rate fitted trend lines, 1950−2021: Camiguin TFR
Data points enclosed with a diamond are reference sources

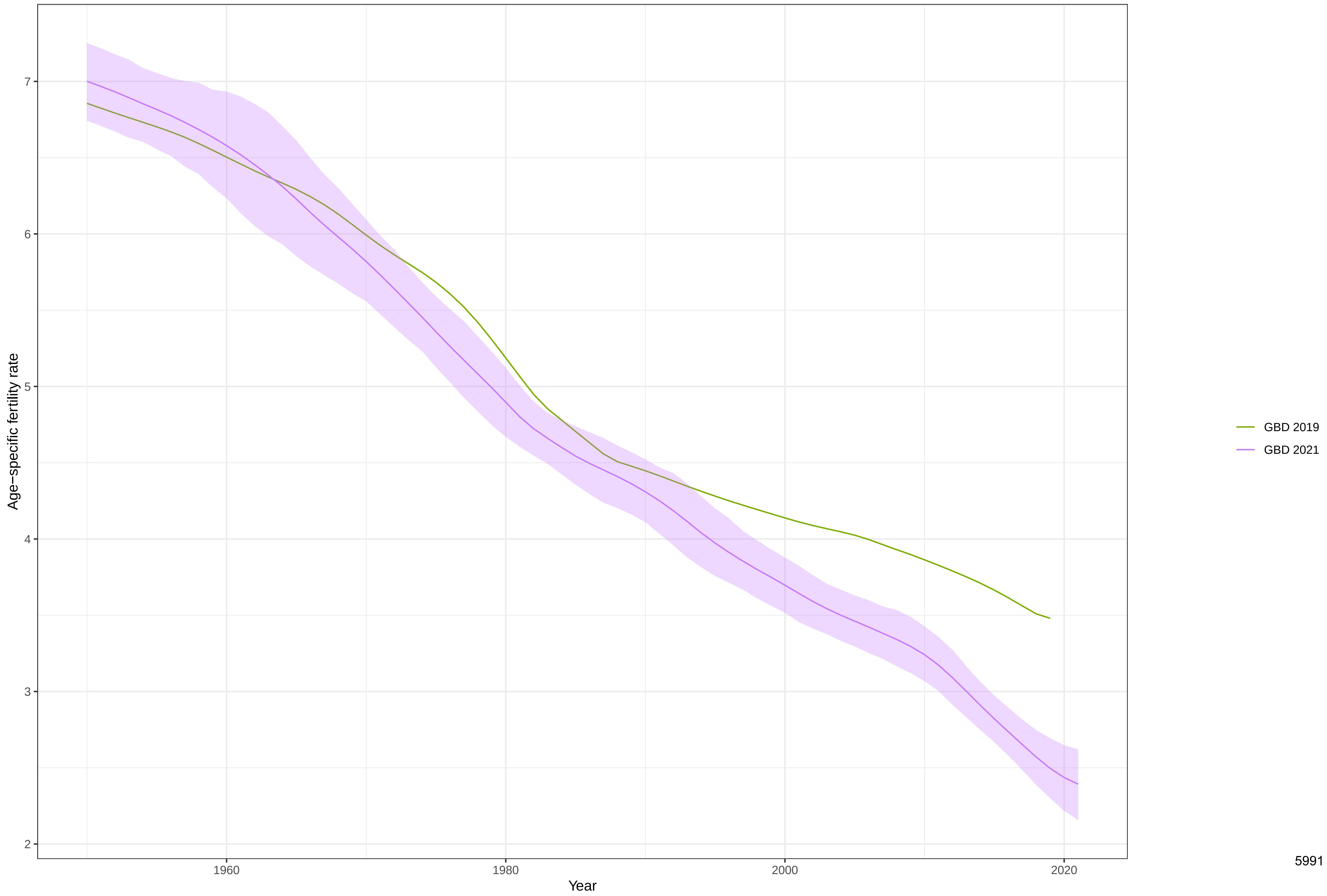

Age−specific fertility fitted trend lines, 1950−2021: Capiz, Ages: 10−14
 Data points enclosed with a diamond are reference sources

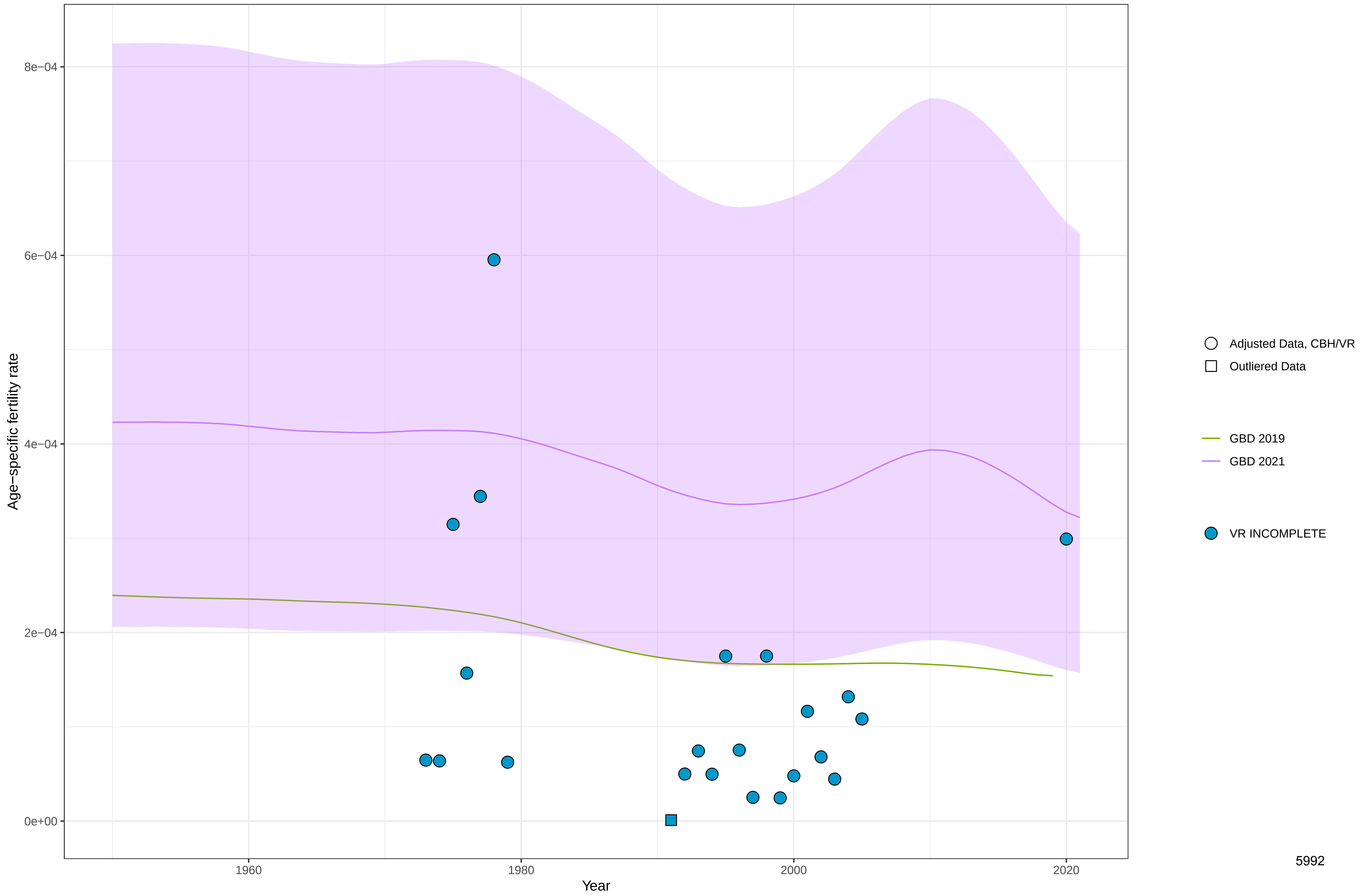

Age−specific fertility fitted trend lines, 1950−2021: Capiz, Ages: 15−19
Data points enclosed with a diamond are reference sources

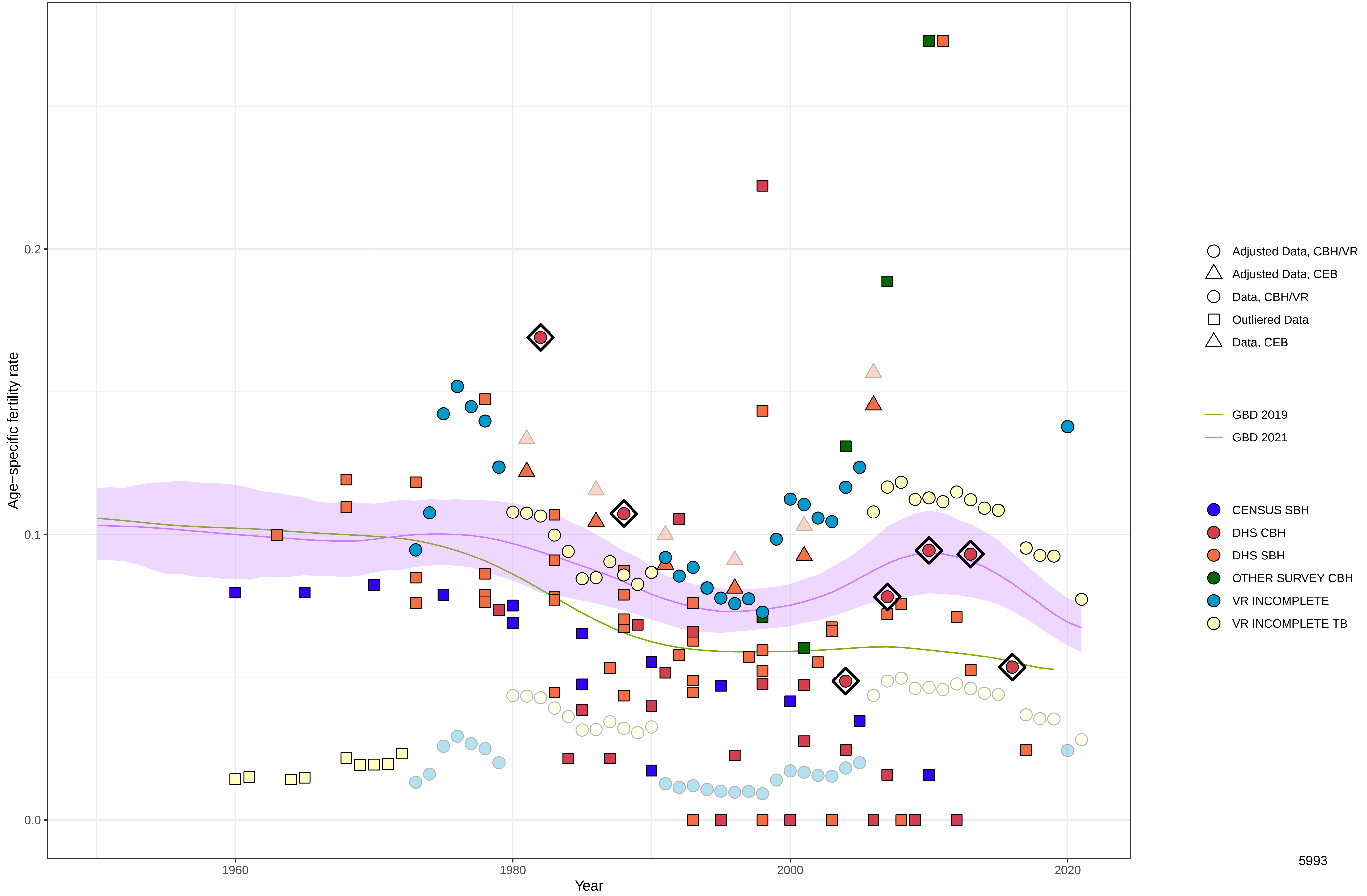

Age−specific fertility fitted trend lines, 1950−2021: Capiz, Ages: 20−24
Data points enclosed with a diamond are reference sources

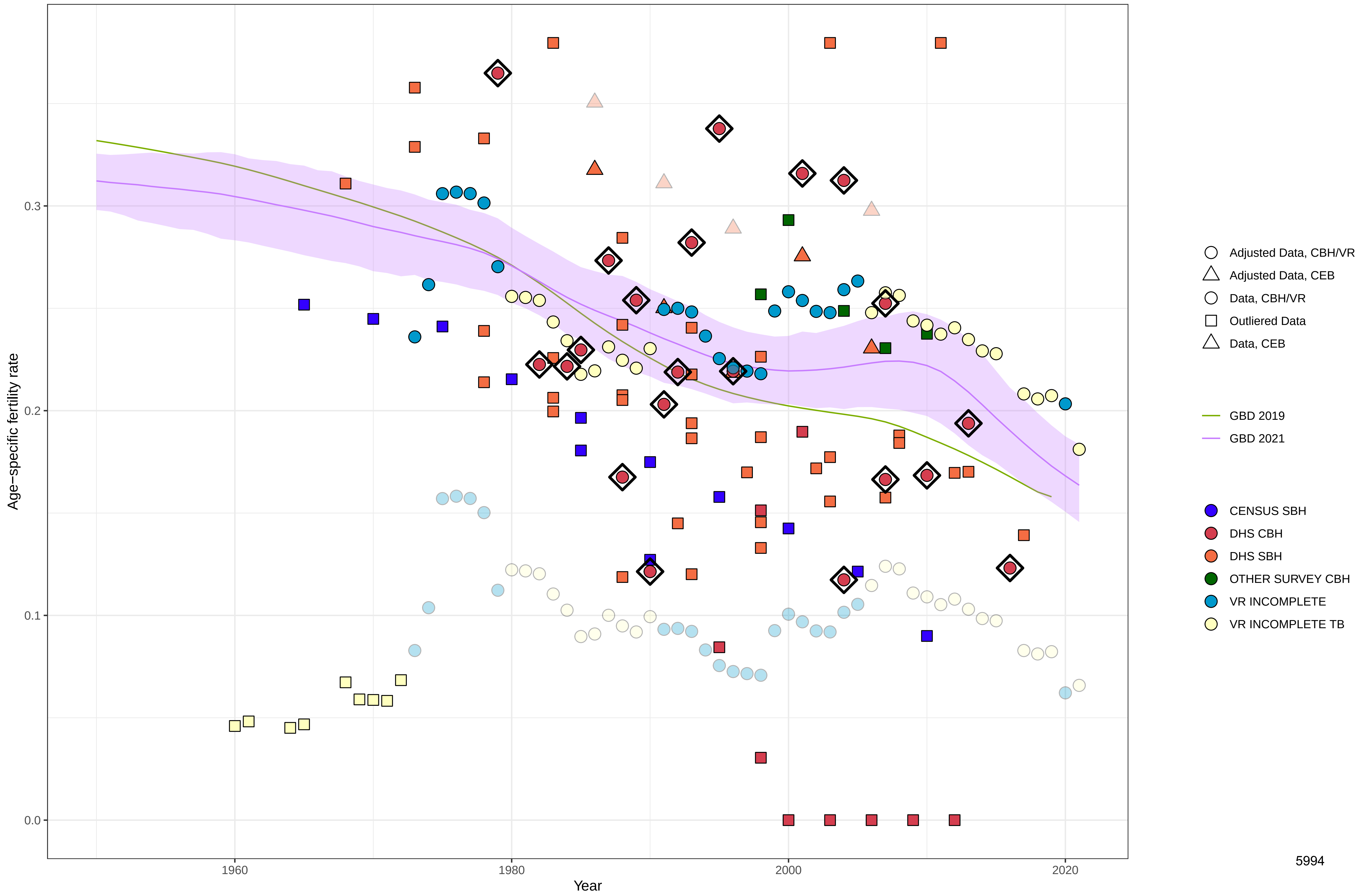

Age−specific fertility fitted trend lines, 1950−2021: Capiz, Ages: 25−29
Data points enclosed with a diamond are reference sources

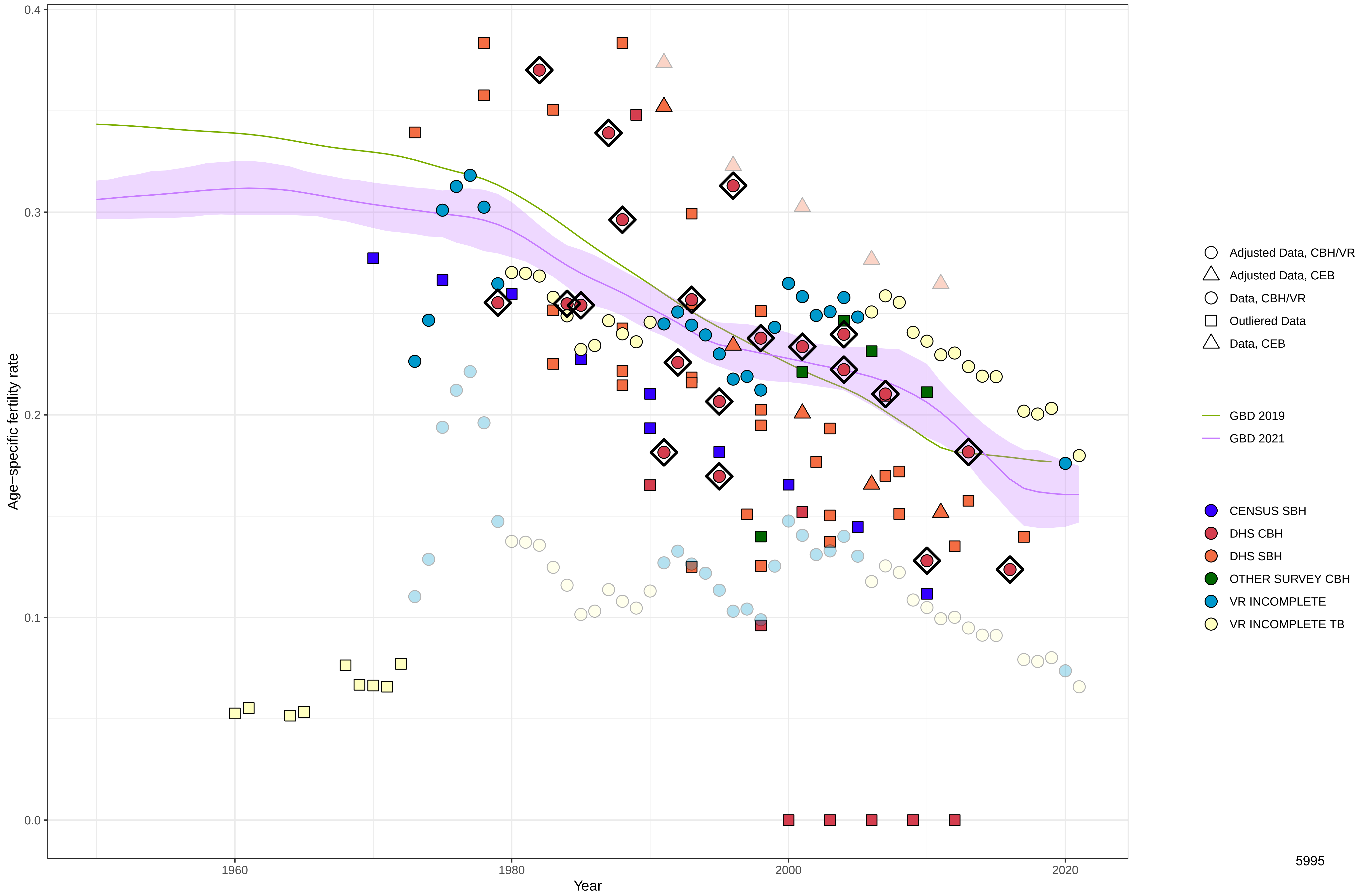

Age−specific fertility fitted trend lines, 1950−2021: Capiz, Ages: 30−34
Data points enclosed with a diamond are reference sources

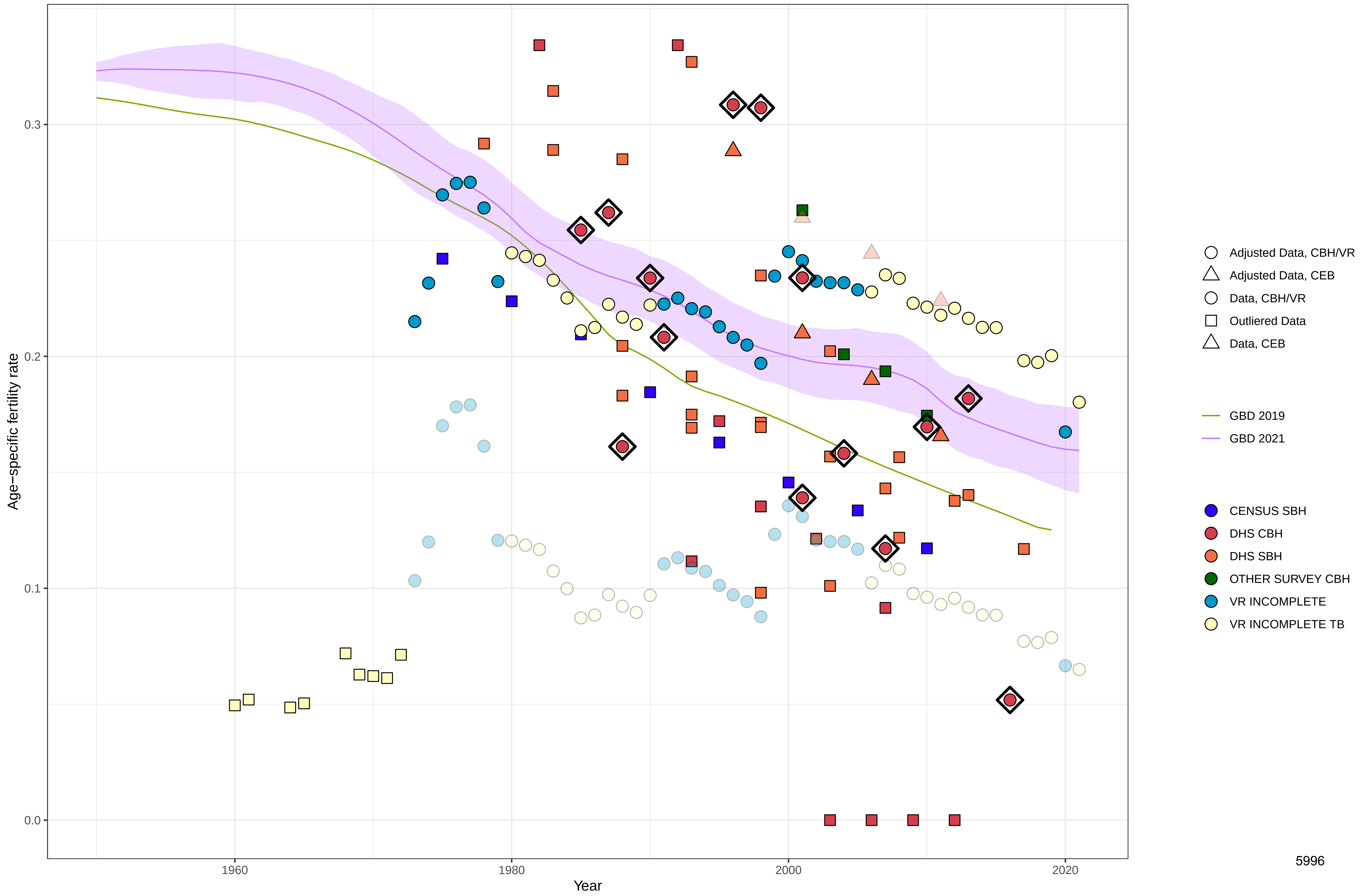

Age−specific fertility fitted trend lines, 1950−2021: Capiz, Ages: 35−39
Data points enclosed with a diamond are reference sources

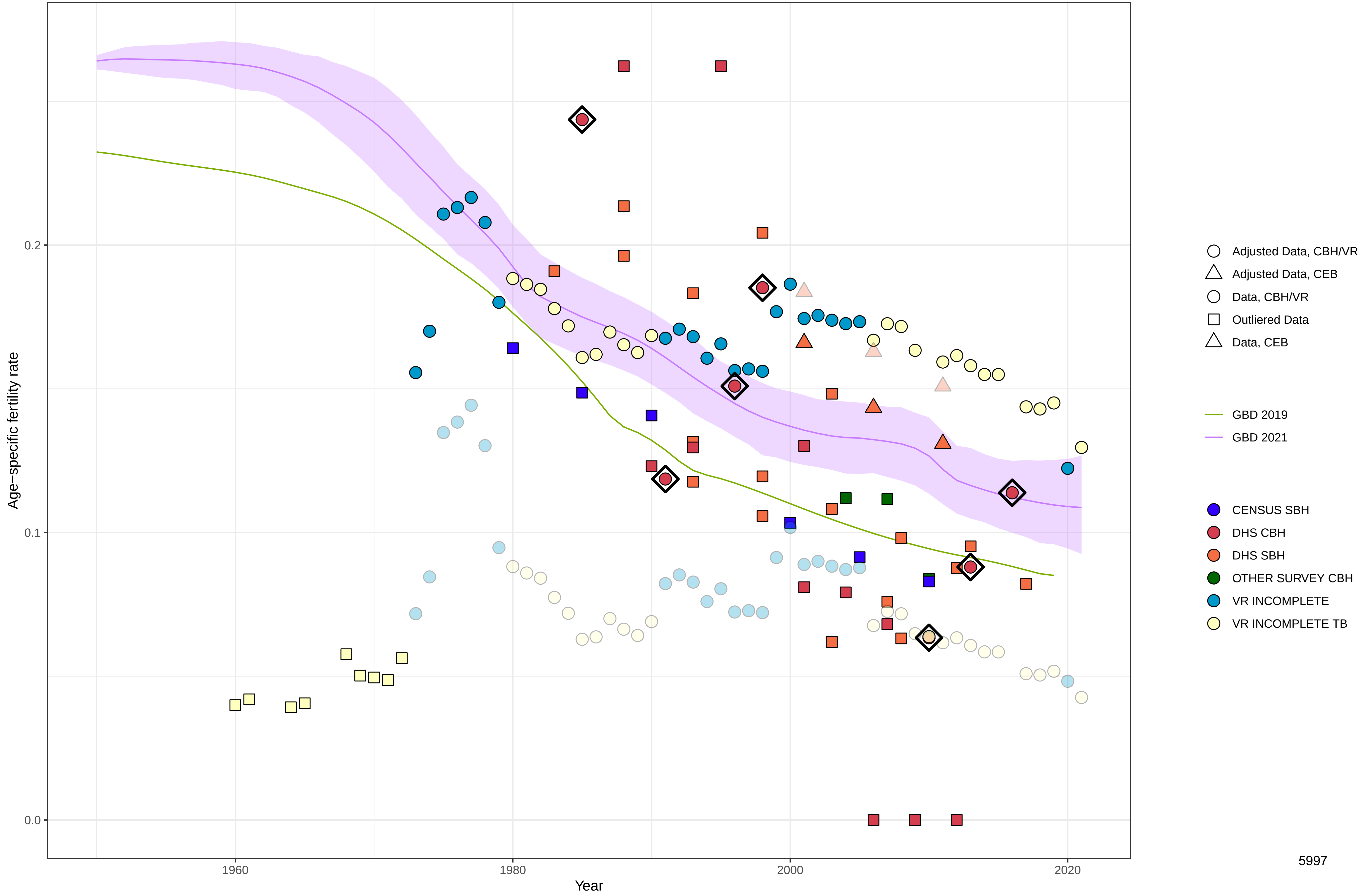

Age−specific fertility fitted trend lines, 1950−2021: Capiz, Ages: 40−44
Data points enclosed with a diamond are reference sources

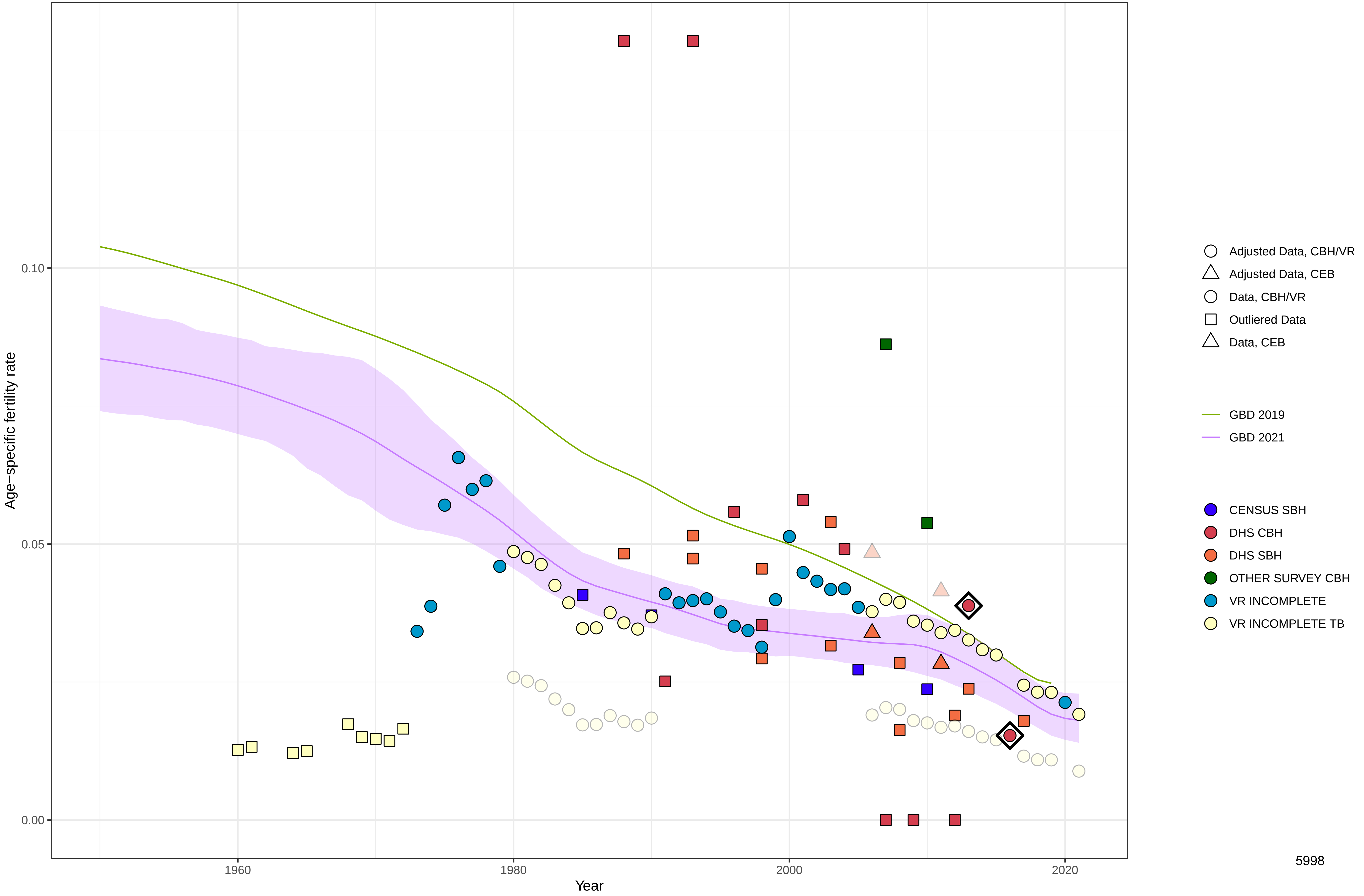

Age−specific fertility fitted trend lines, 1950−2021: Capiz, Ages: 45−49
Data points enclosed with a diamond are reference sources

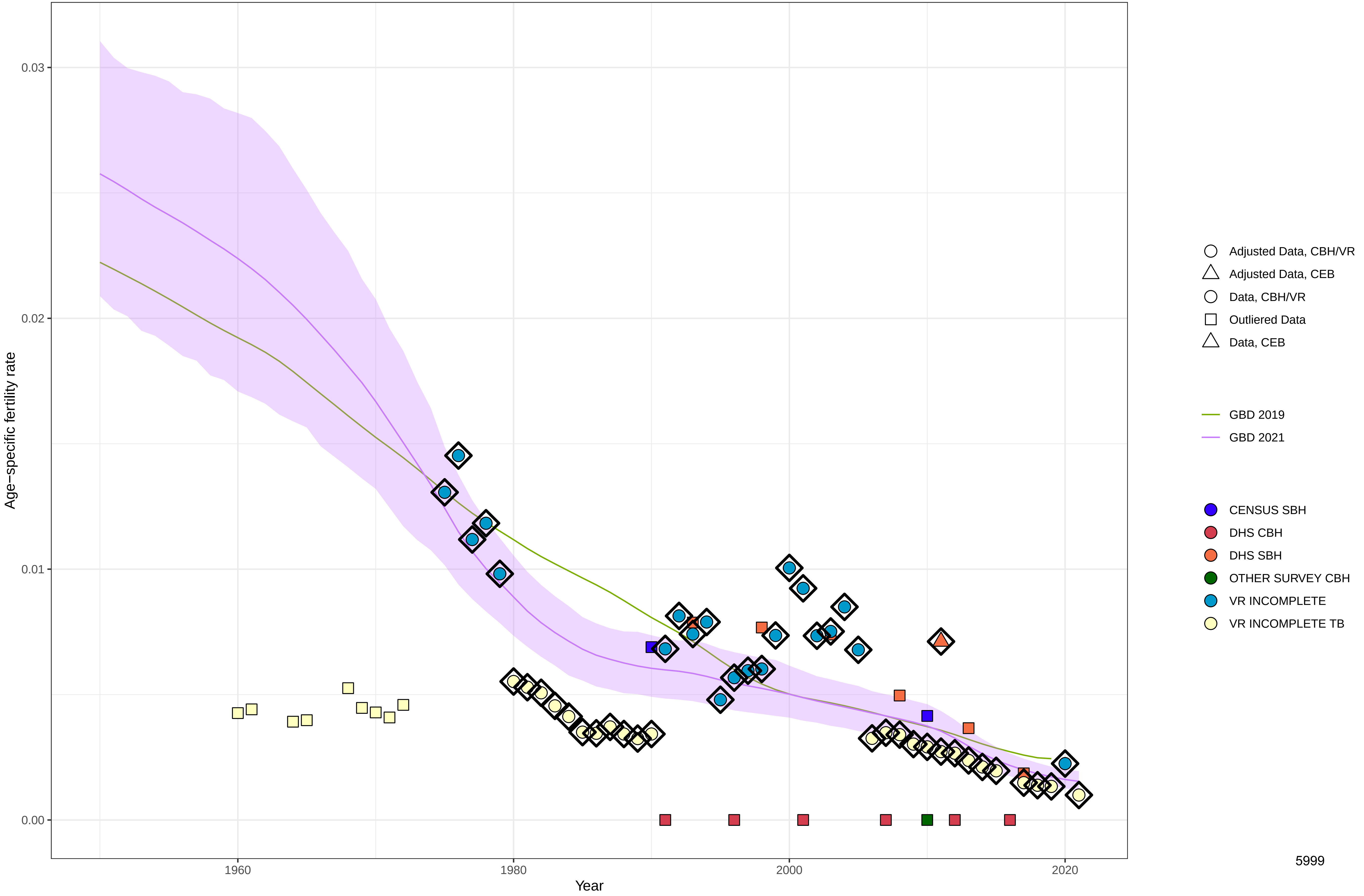

Age−specific fertility fitted trend lines, 1950−2021: Capiz, Ages: 50−54
Data points enclosed with a diamond are reference sources

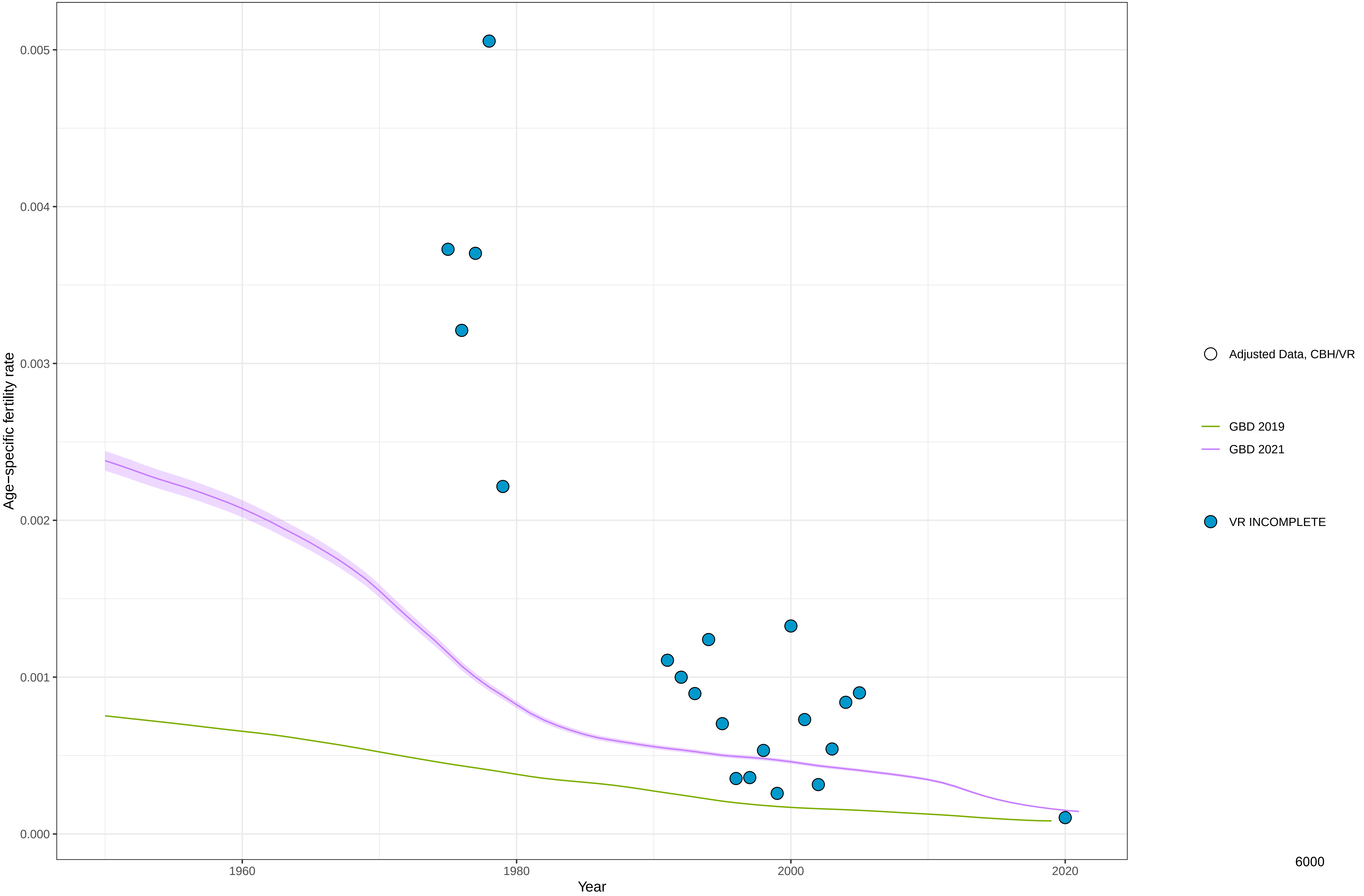

Total fertility rate fitted trend lines, 1950−2021: Capiz TFR
Data points enclosed with a diamond are reference sources

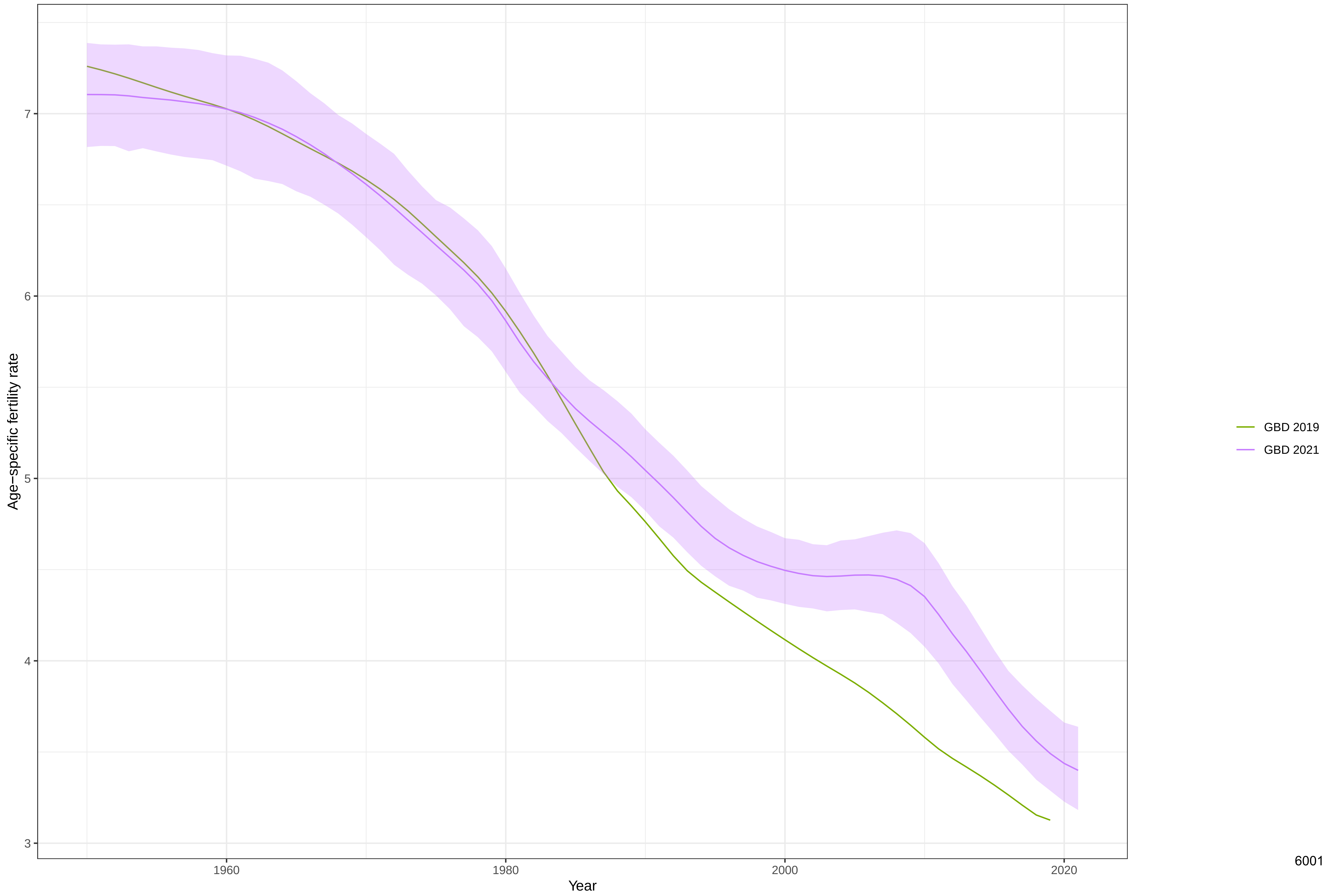

Age−specific fertility fitted trend lines, 1950−2021: Catanduanes, Ages: 10−14
Data points enclosed with a diamond are reference sources

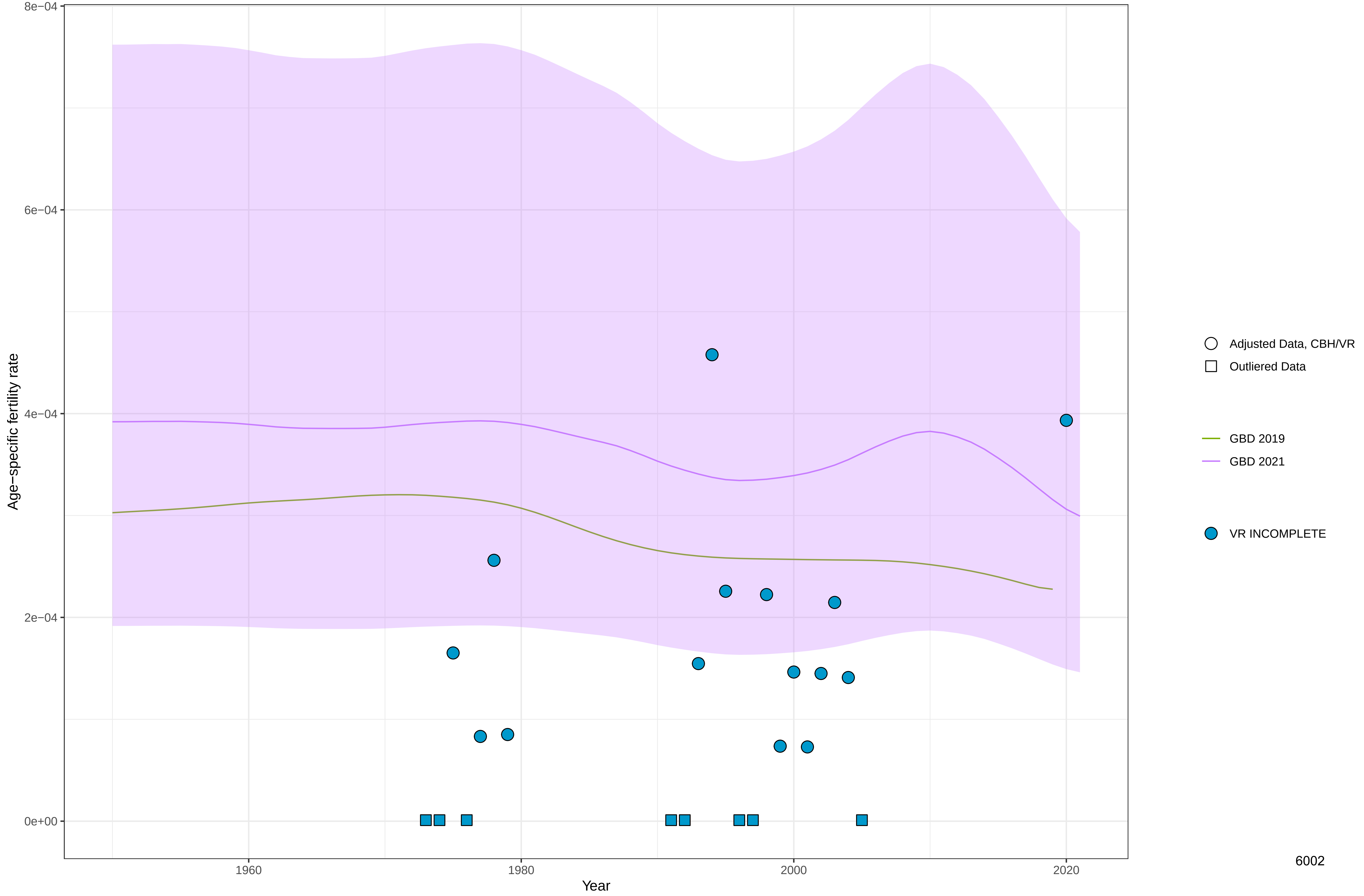

Age−specific fertility fitted trend lines, 1950−2021: Catanduanes, Ages: 15−19
Data points enclosed with a diamond are reference sources

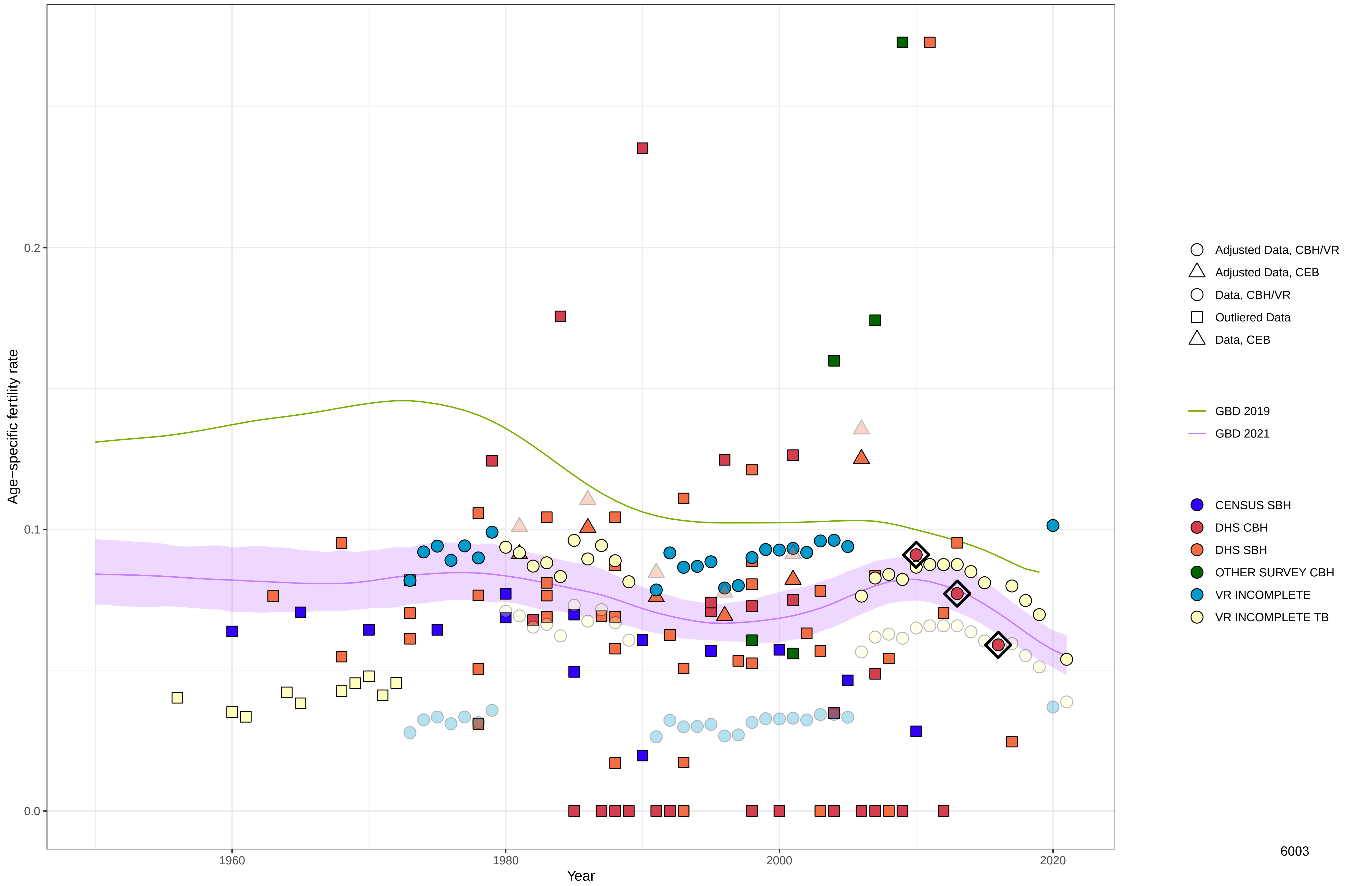

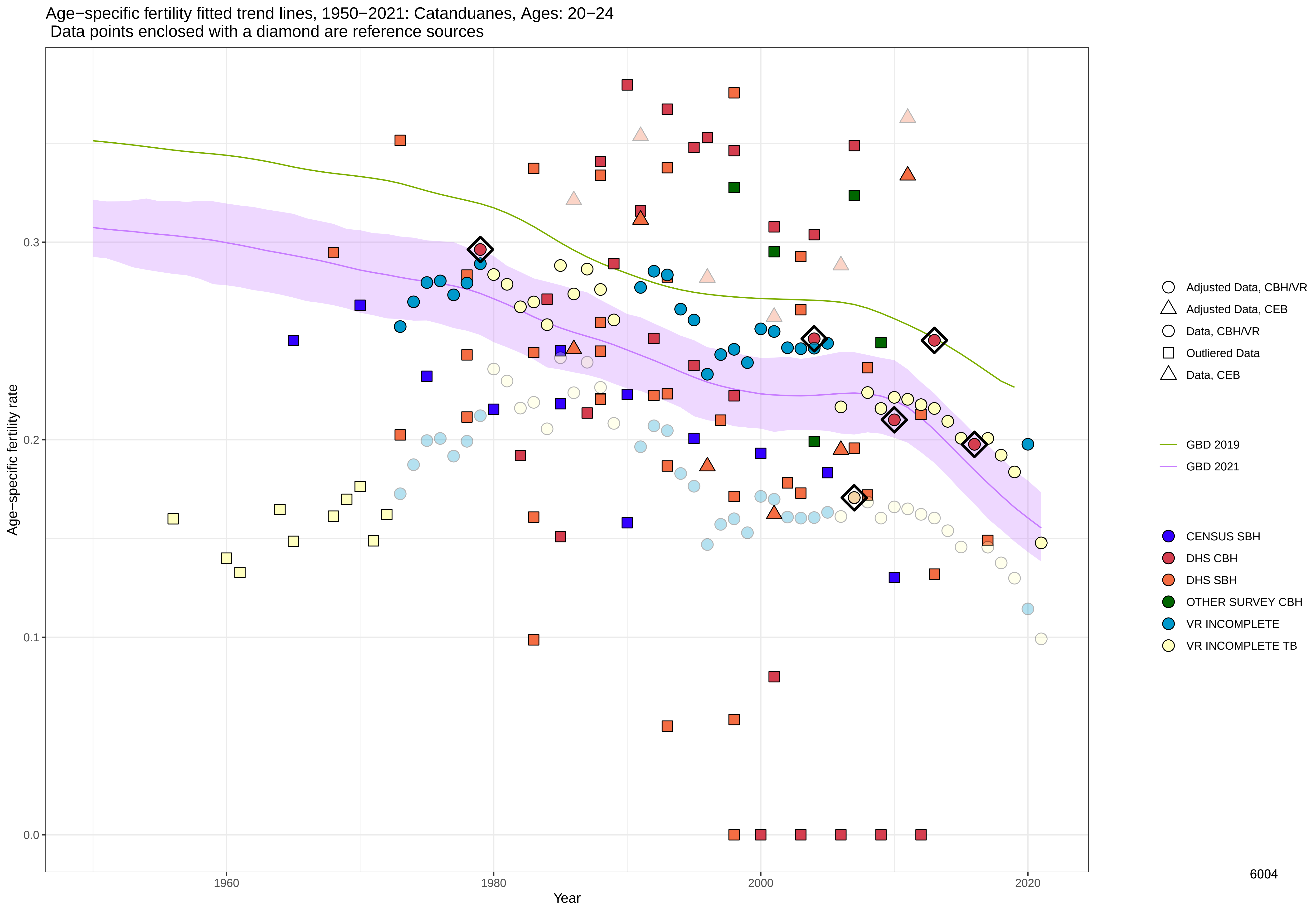

Age−specific fertility fitted trend lines, 1950−2021: Catanduanes, Ages: 20−24
Data points enclosed with a diamond are reference sources
Age−specific fertility rate
Year
0.0
0.1
0.2
0.3
1960
1980
2000
2020
Adjusted Data, CBH/VR
Adjusted Data, CEB
Data, CBH/VR
Outliered Data
Data, CEB
GBD 2019
GBD 2021
CENSUS SBH
DHS CBH
DHS SBH
OTHER SURVEY CBH
VR INCOMPLETE
VR INCOMPLETE TB

Age−specific fertility fitted trend lines, 1950−2021: Catanduanes, Ages: 25−29
Data points enclosed with a diamond are reference sources

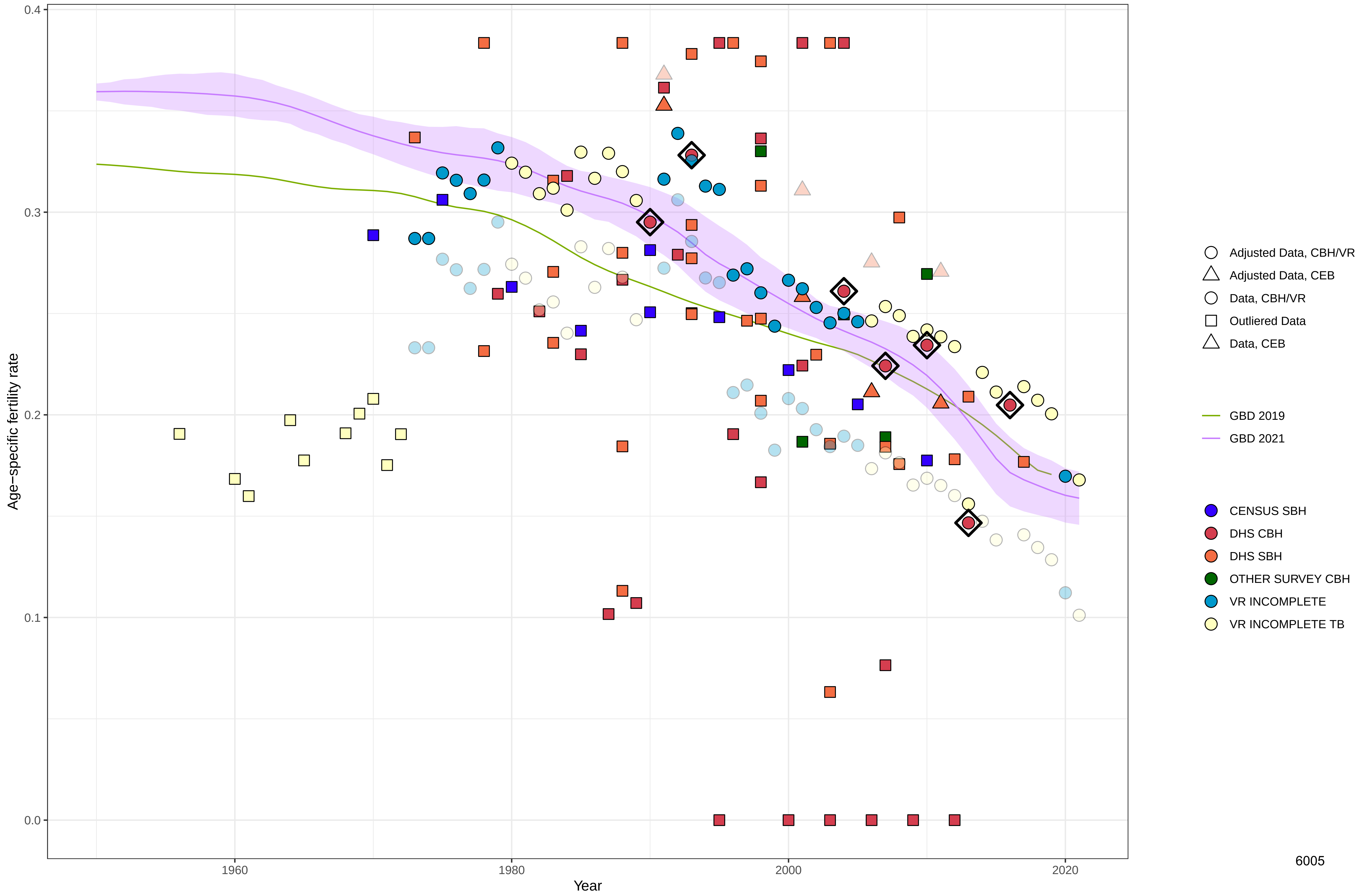

Age−specific fertility fitted trend lines, 1950−2021: Catanduanes, Ages: 30−34
Data points enclosed with a diamond are reference sources

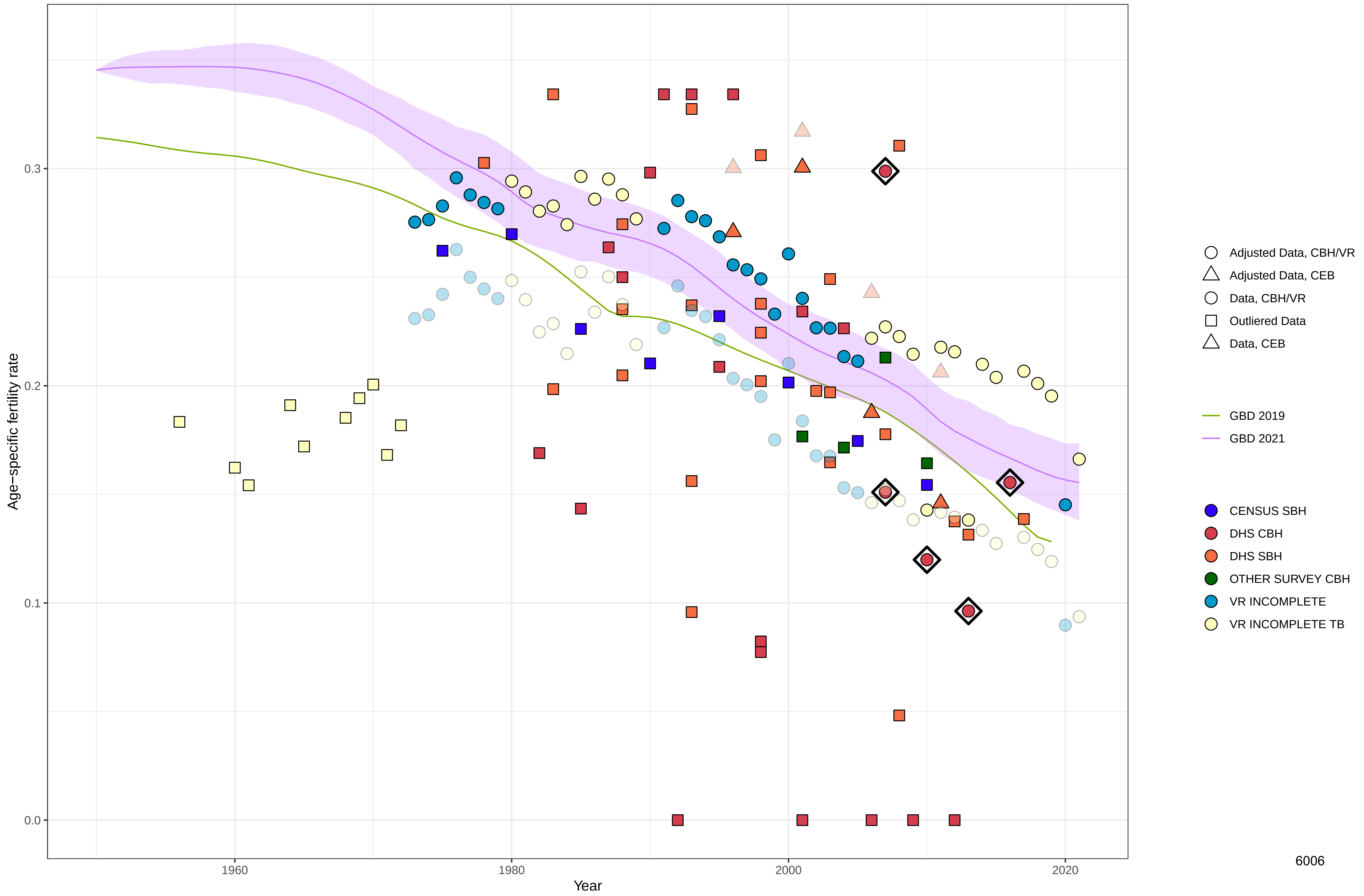

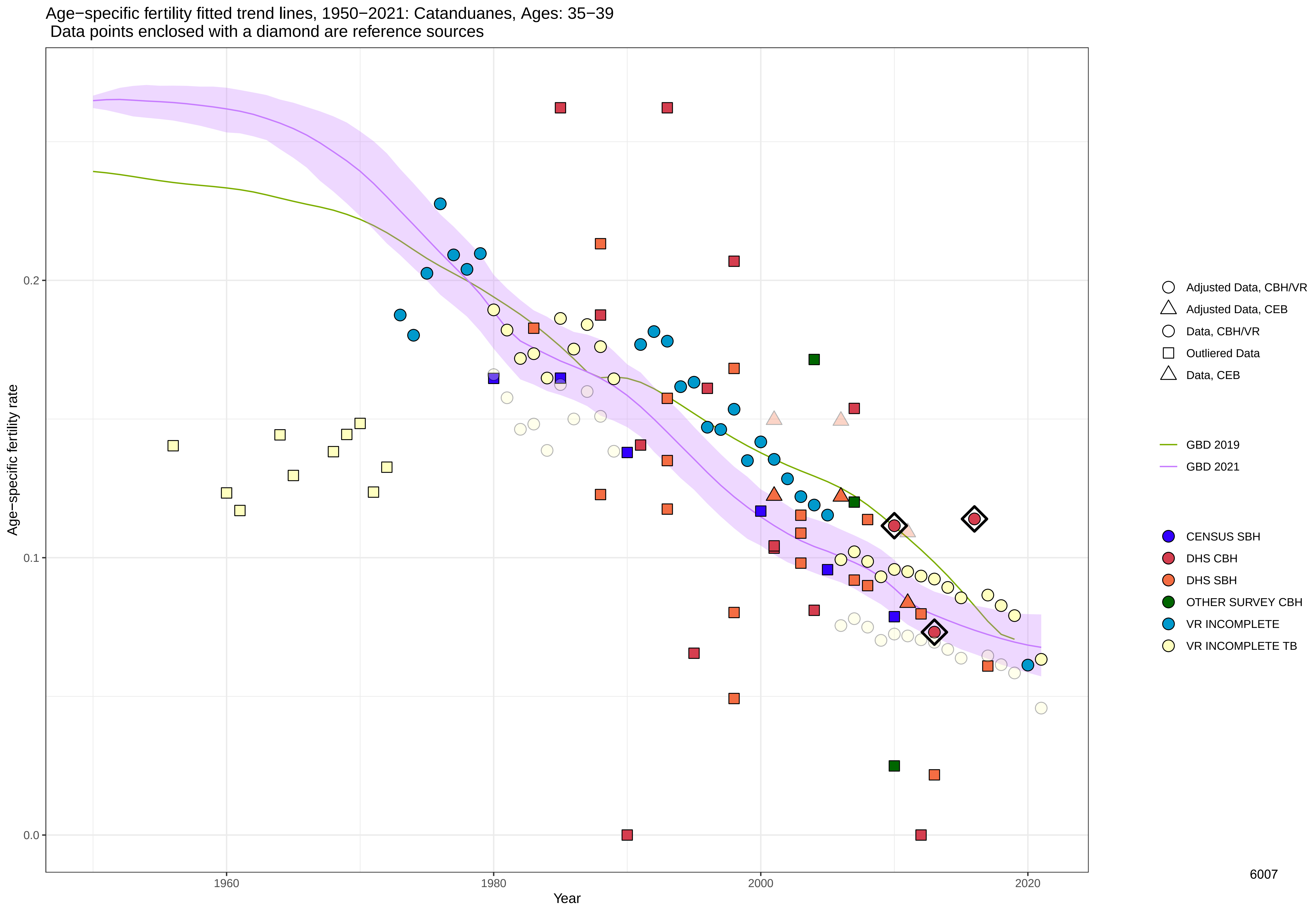

Age−specific fertility fitted trend lines, 1950−2021: Catanduanes, Ages: 35−39
Data points enclosed with a diamond are reference sources
Age−specific fertility rate
Year
0.0
0.1
0.2
1960
1980
2000
2020
Adjusted Data, CBH/VR
Adjusted Data, CEB
Data, CBH/VR
Outliered Data
Data, CEB
GBD 2019
GBD 2021
CENSUS SBH
DHS CBH
DHS SBH
OTHER SURVEY CBH
VR INCOMPLETE
VR INCOMPLETE TB

Age−specific fertility fitted trend lines, 1950−2021: Catanduanes, Ages: 40−44
Data points enclosed with a diamond are reference sources

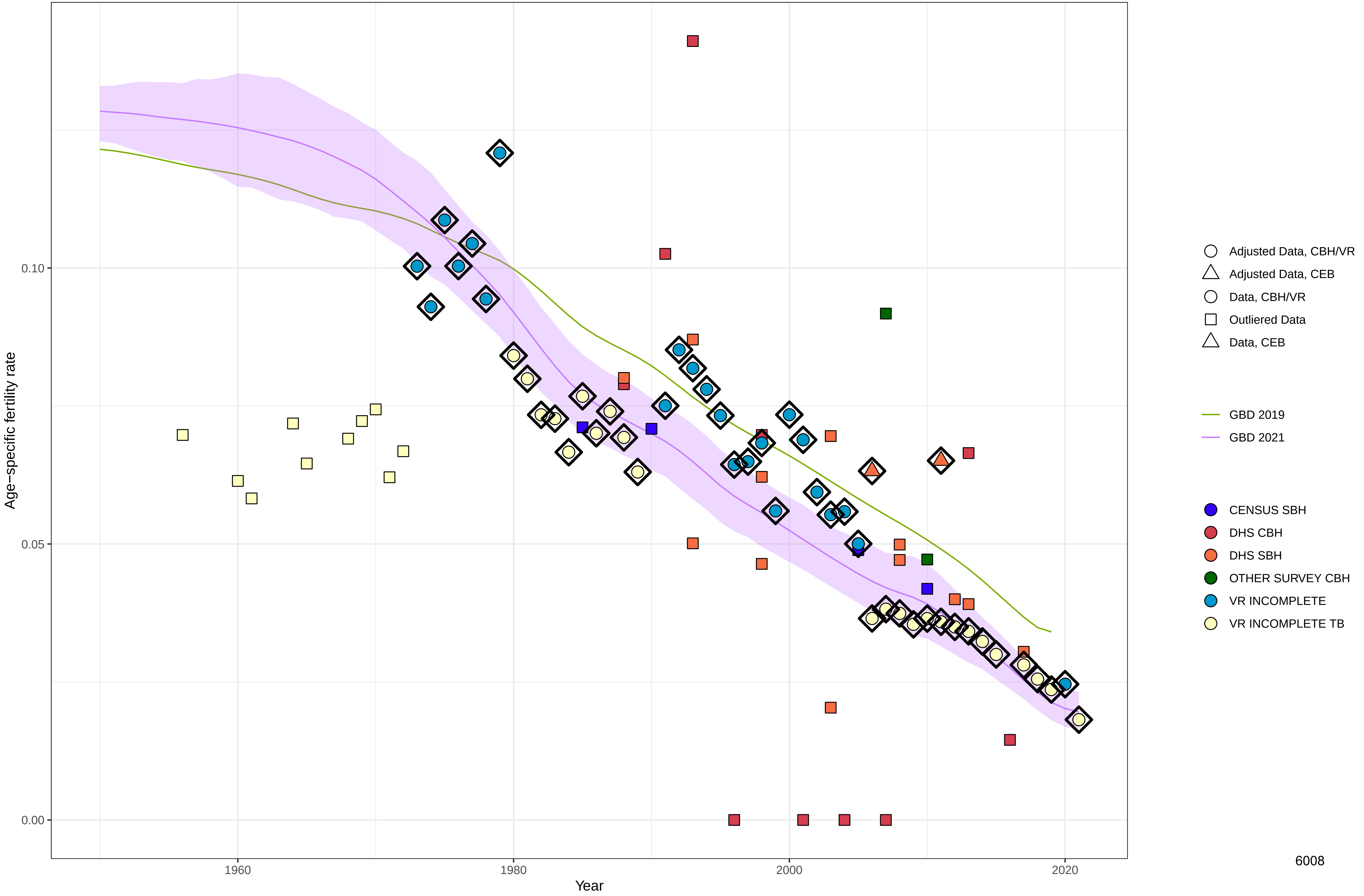

Age−specific fertility fitted trend lines, 1950−2021: Catanduanes, Ages: 45−49
Data points enclosed with a diamond are reference sources

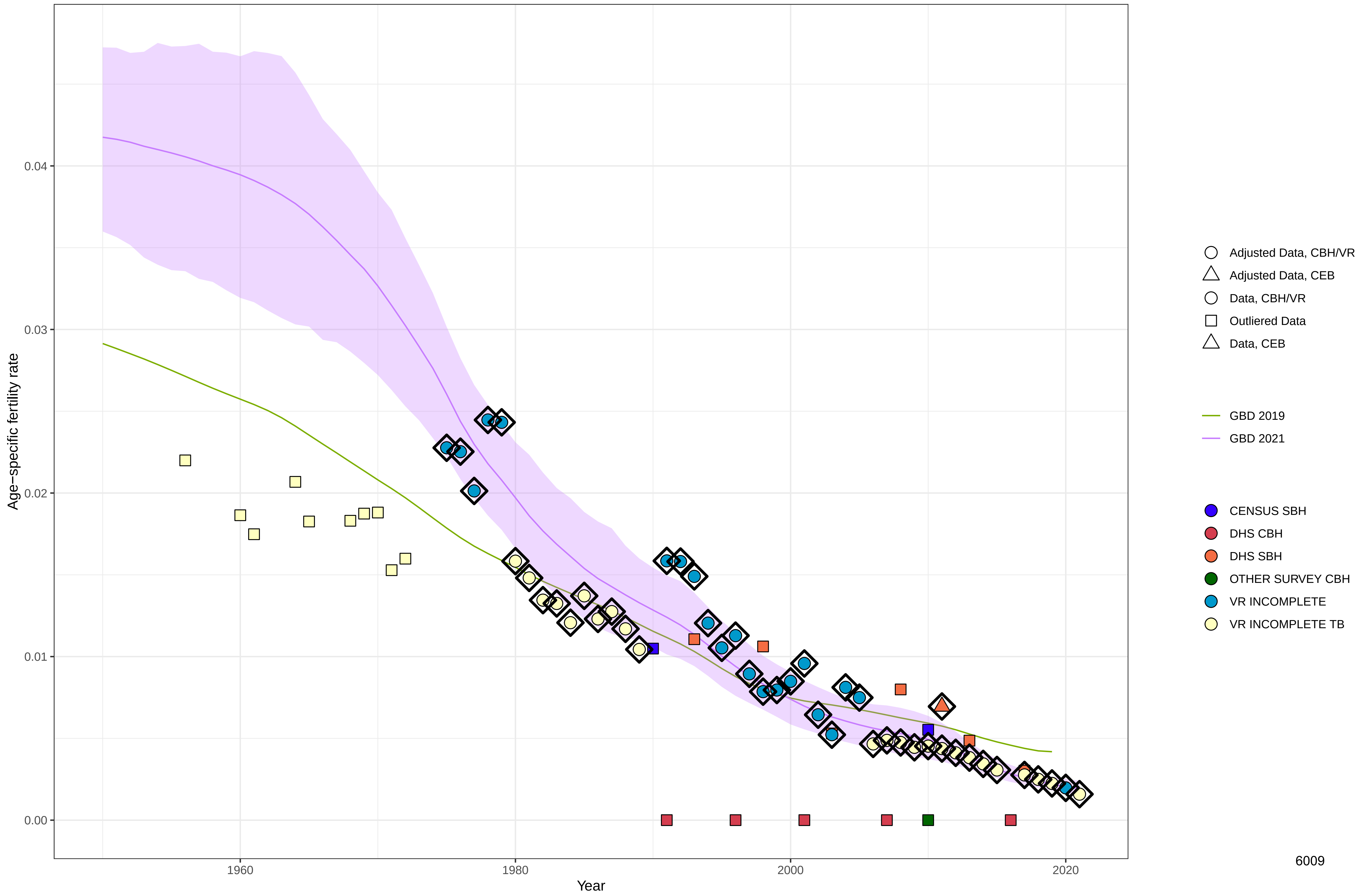

Age−specific fertility fitted trend lines, 1950−2021: Catanduanes, Ages: 50−54
 Data points enclosed with a diamond are reference sources

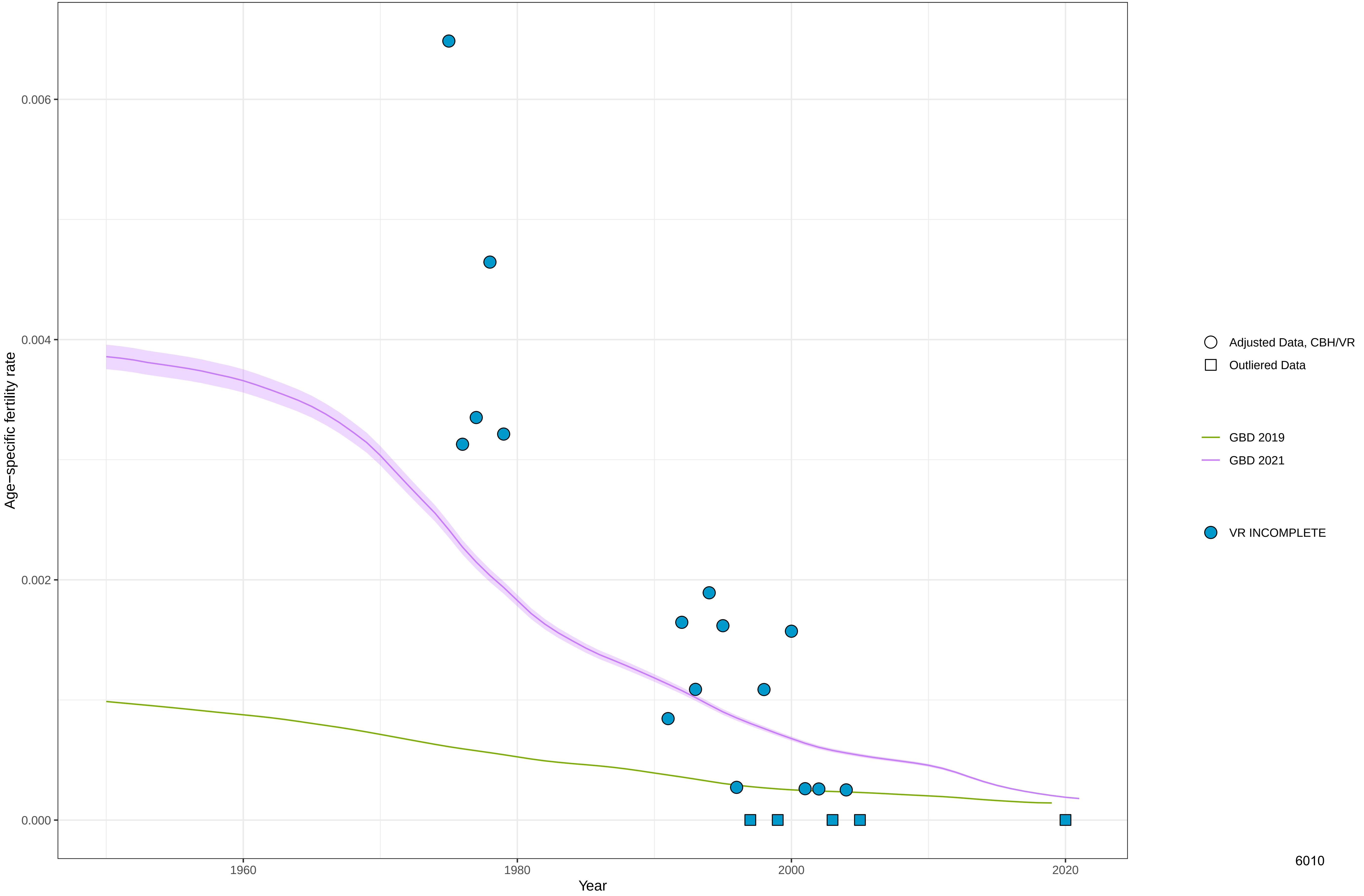

Total fertility rate fitted trend lines, 1950−2021: Catanduanes TFR
Data points enclosed with a diamond are reference sources

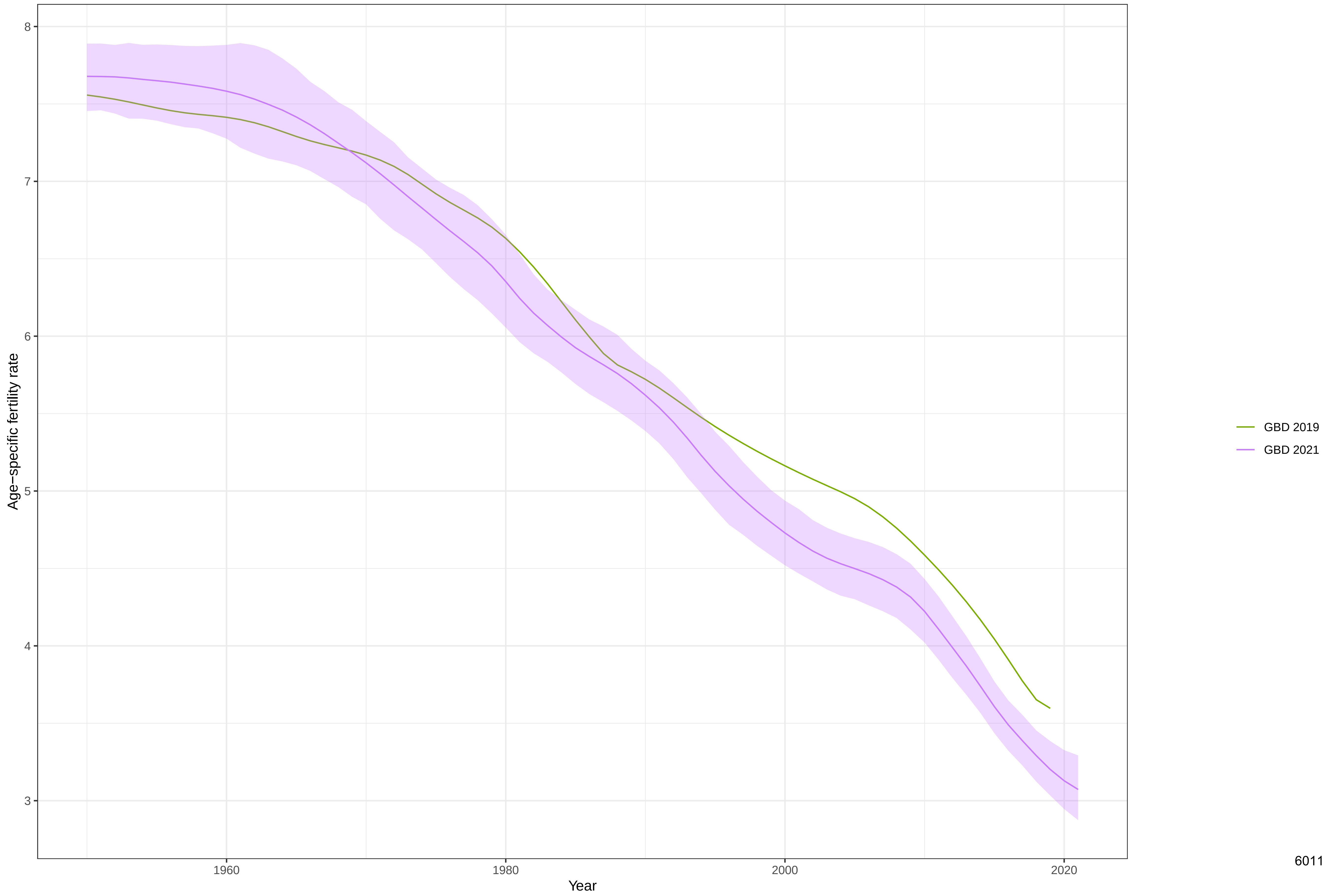

Age−specific fertility fitted trend lines, 1950−2021: Cavite, Ages: 10−14
Data points enclosed with a diamond are reference sources

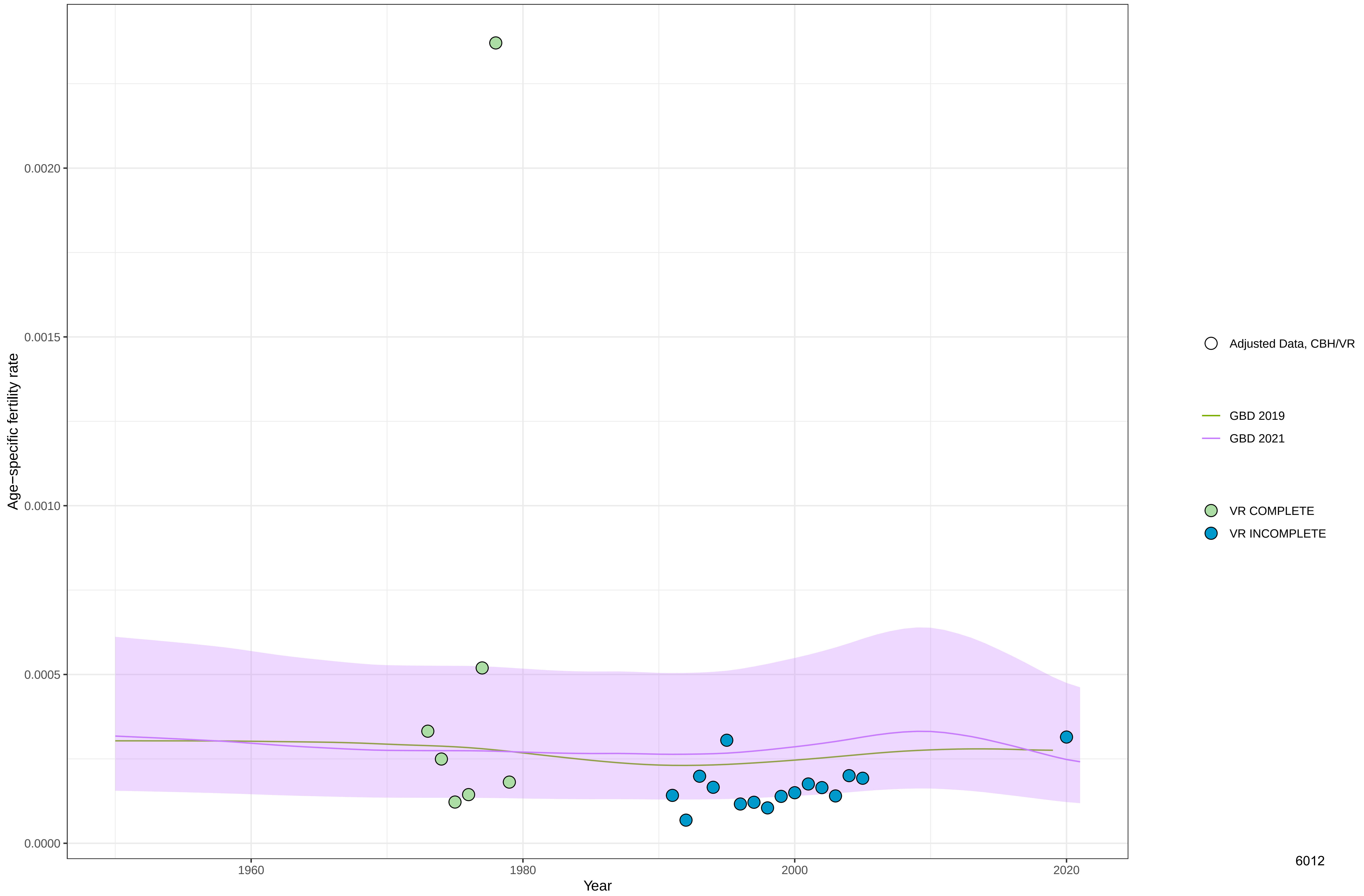

Age−specific fertility fitted trend lines, 1950−2021: Cavite, Ages: 15−19
Data points enclosed with a diamond are reference sources

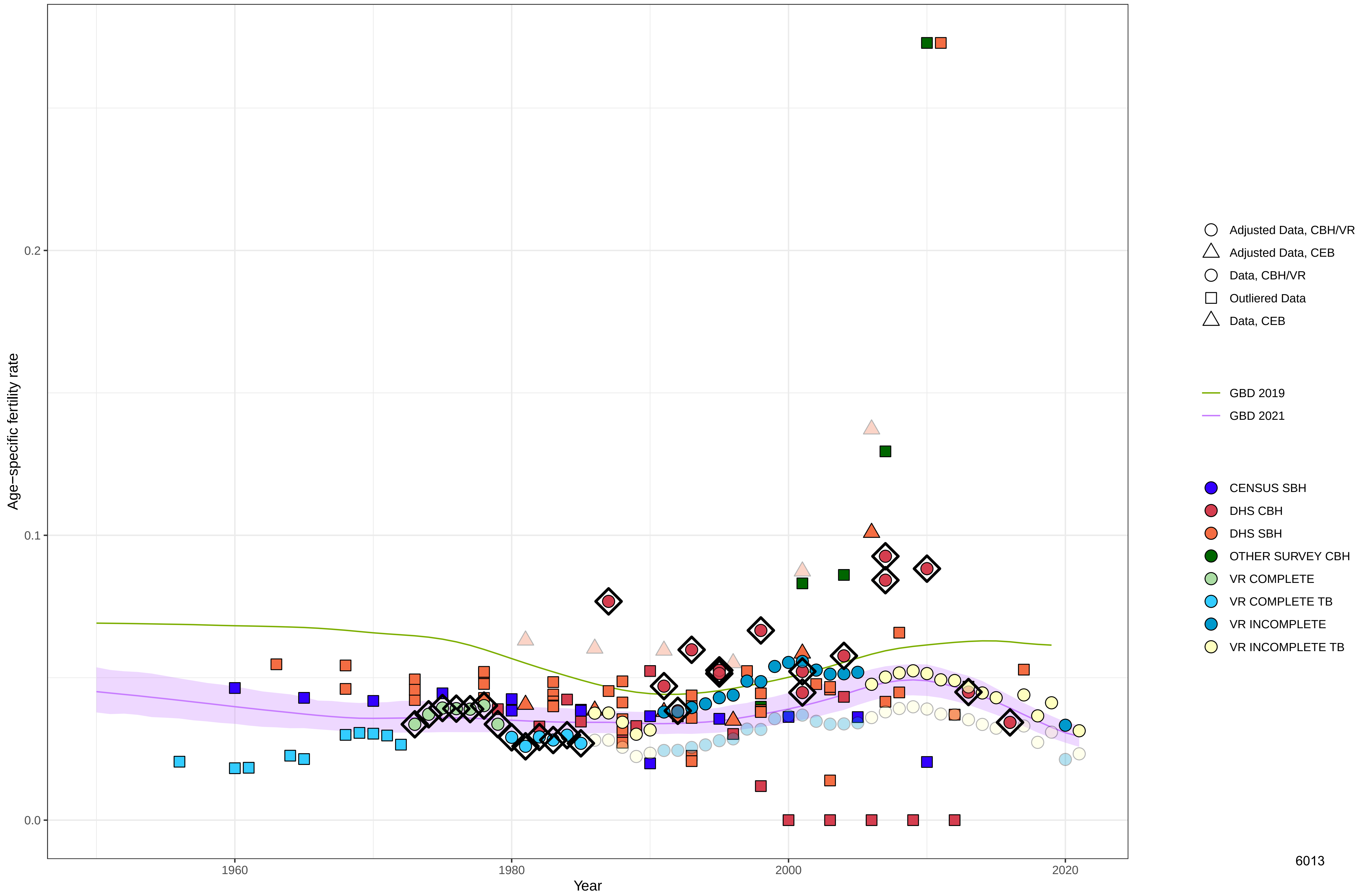

Age−specific fertility fitted trend lines, 1950−2021: Cavite, Ages: 20−24
Data points enclosed with a diamond are reference sources

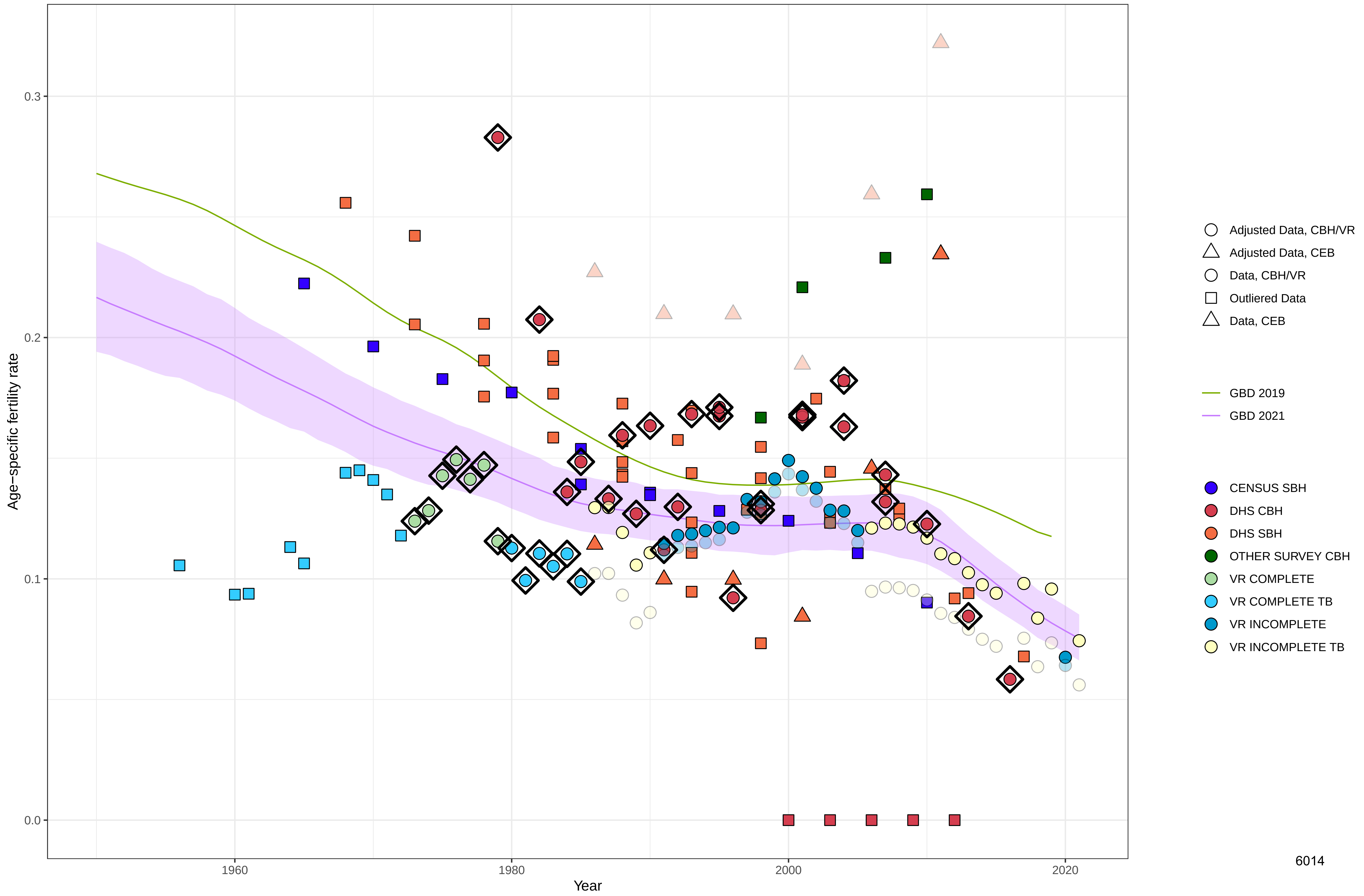

Age−specific fertility fitted trend lines, 1950−2021: Cavite, Ages: 25−29
Data points enclosed with a diamond are reference sources

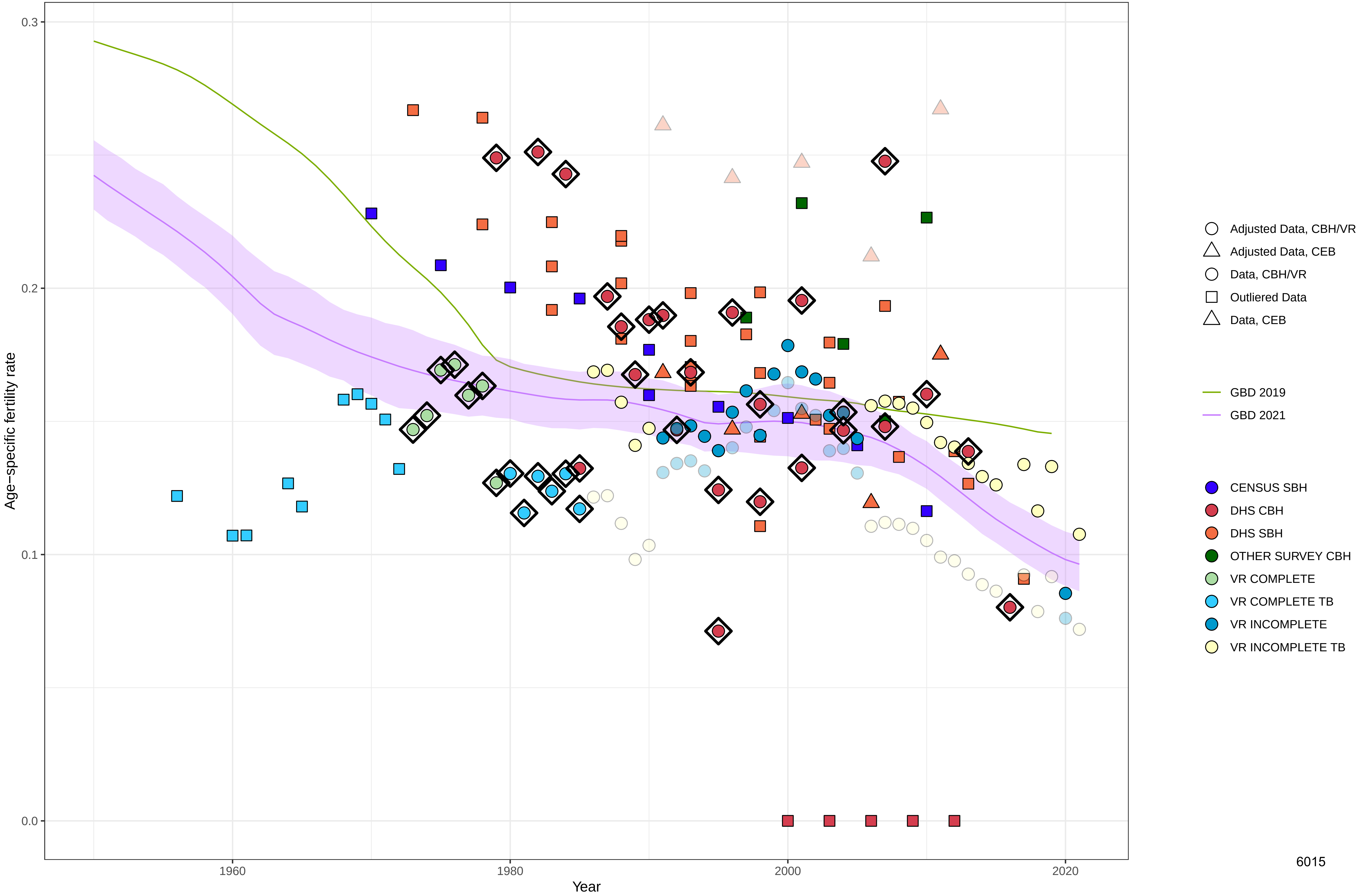

Age−specific fertility fitted trend lines, 1950−2021: Cavite, Ages: 30−34
Data points enclosed with a diamond are reference sources

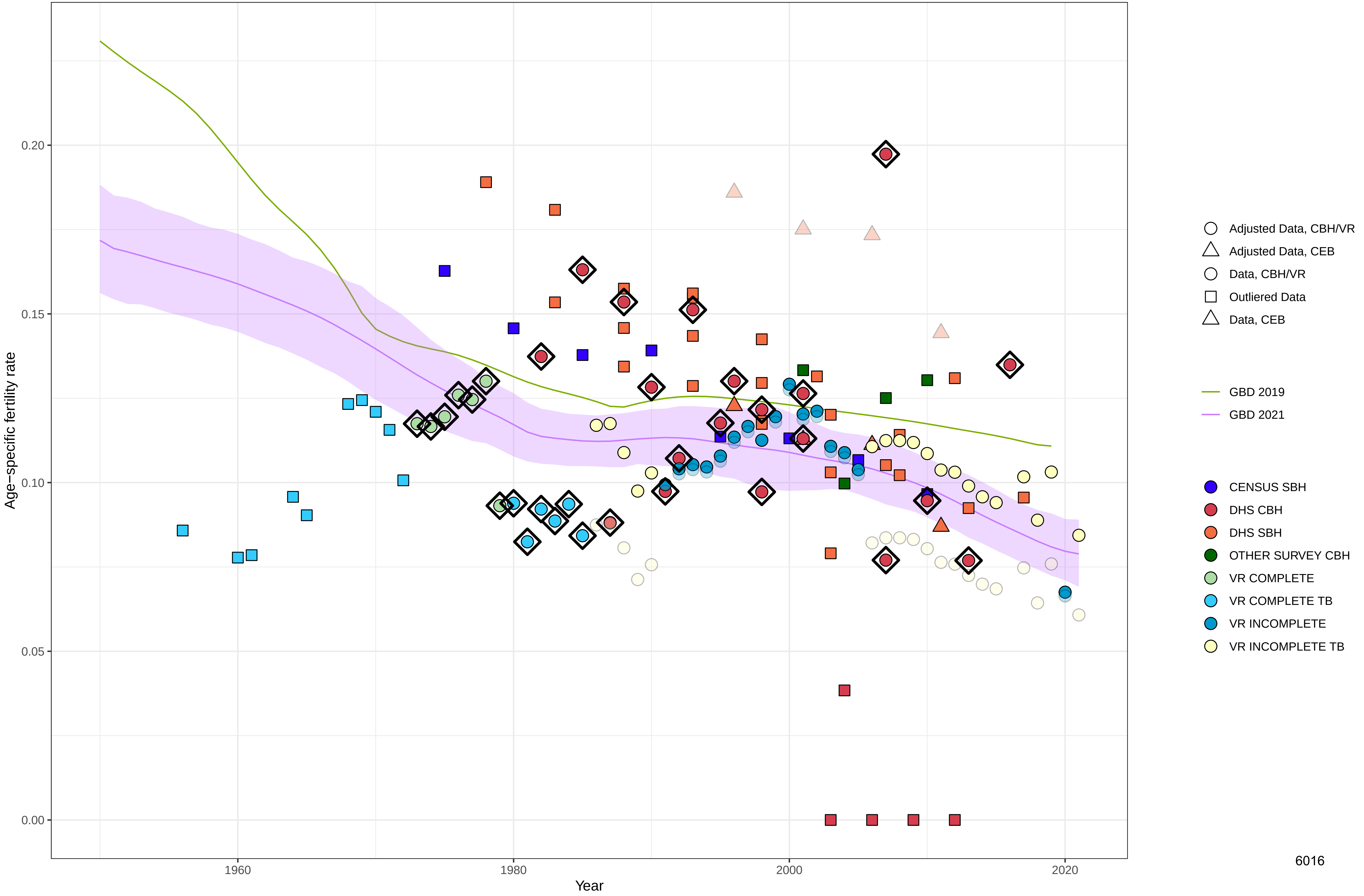

Age−specific fertility fitted trend lines, 1950−2021: Cavite, Ages: 35−39
Data points enclosed with a diamond are reference sources

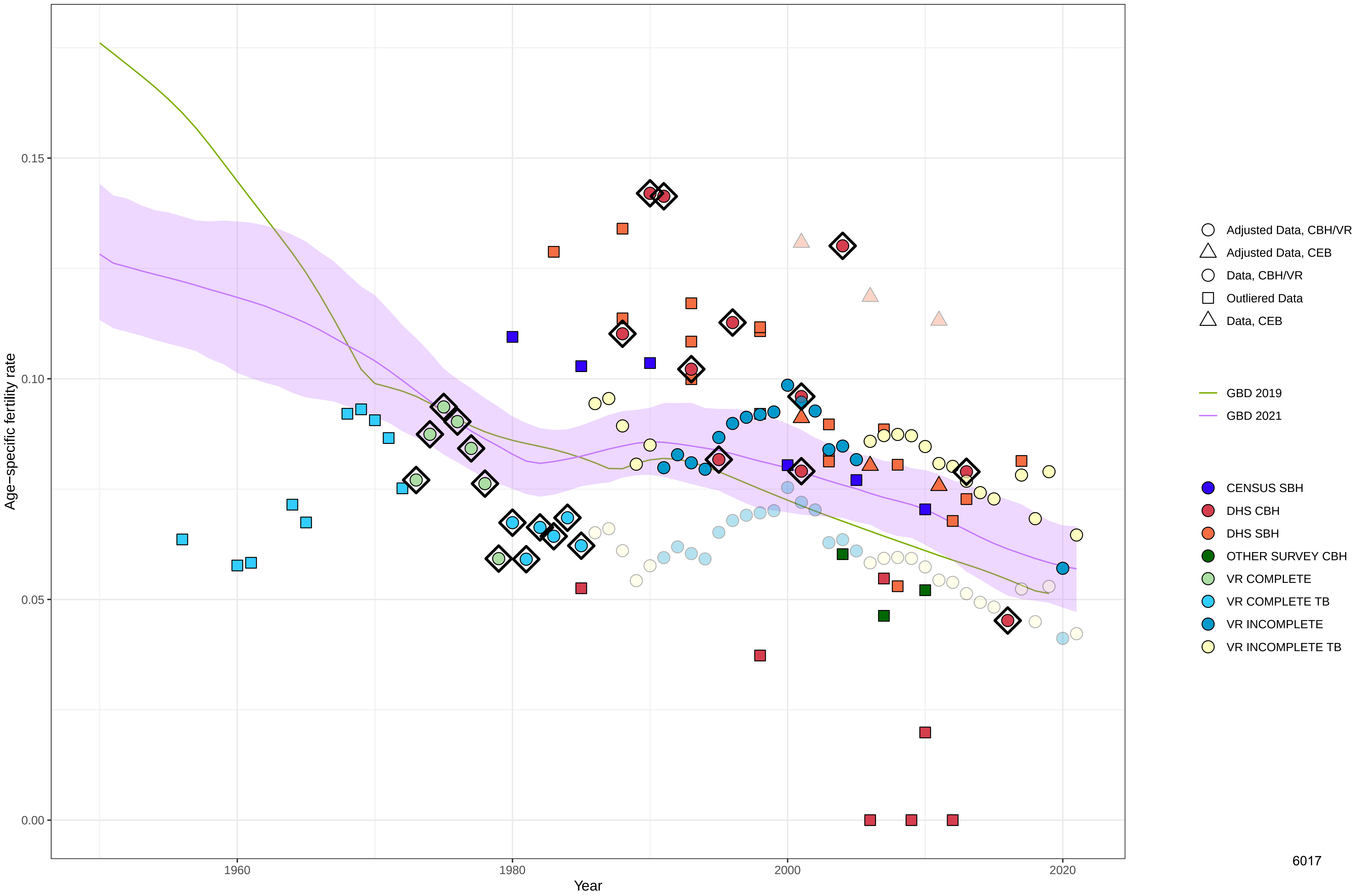

Age−specific fertility fitted trend lines, 1950−2021: Cavite, Ages: 40−44
Data points enclosed with a diamond are reference sources

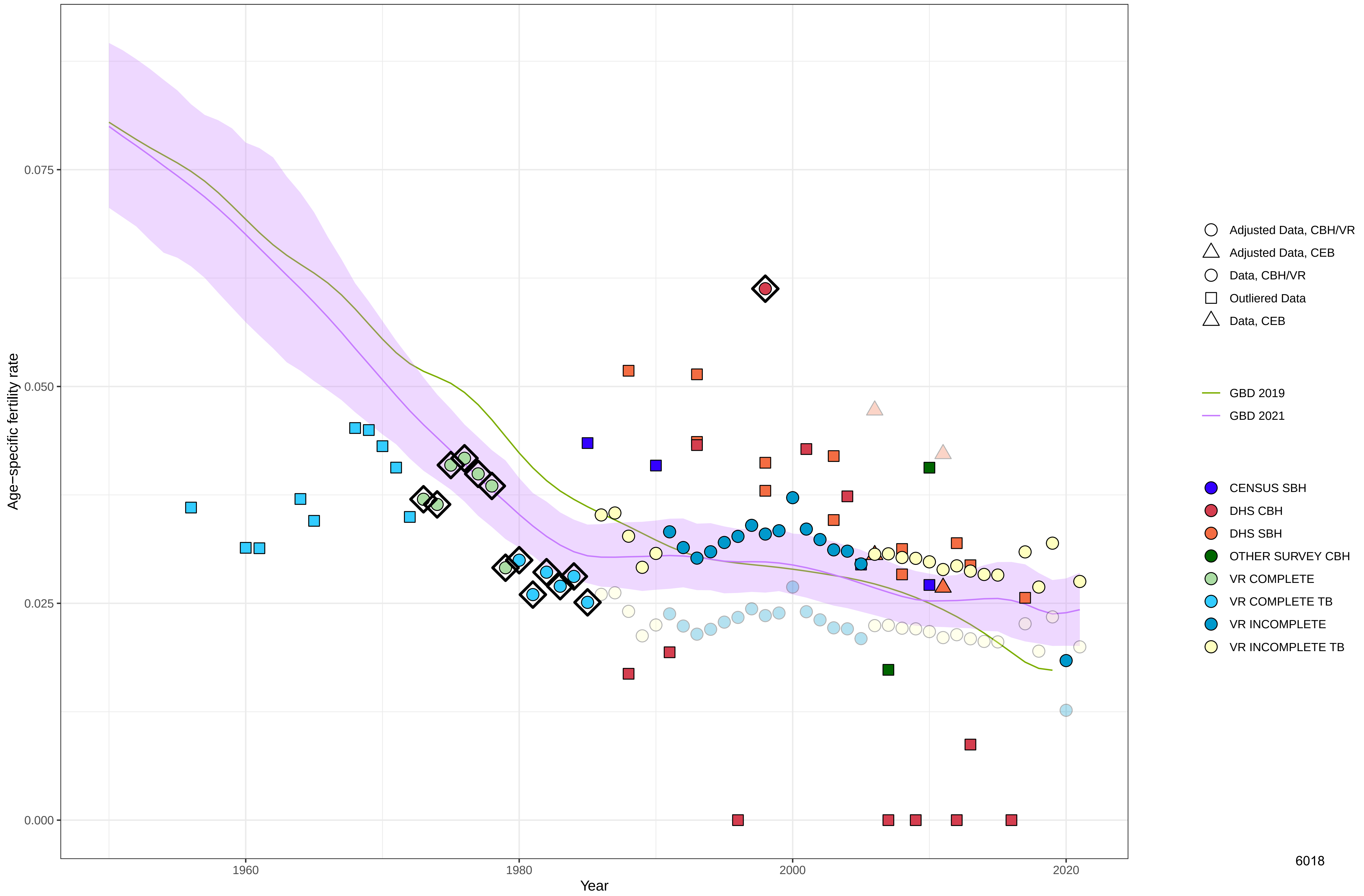

Age−specific fertility fitted trend lines, 1950−2021: Cavite, Ages: 45−49
Data points enclosed with a diamond are reference sources

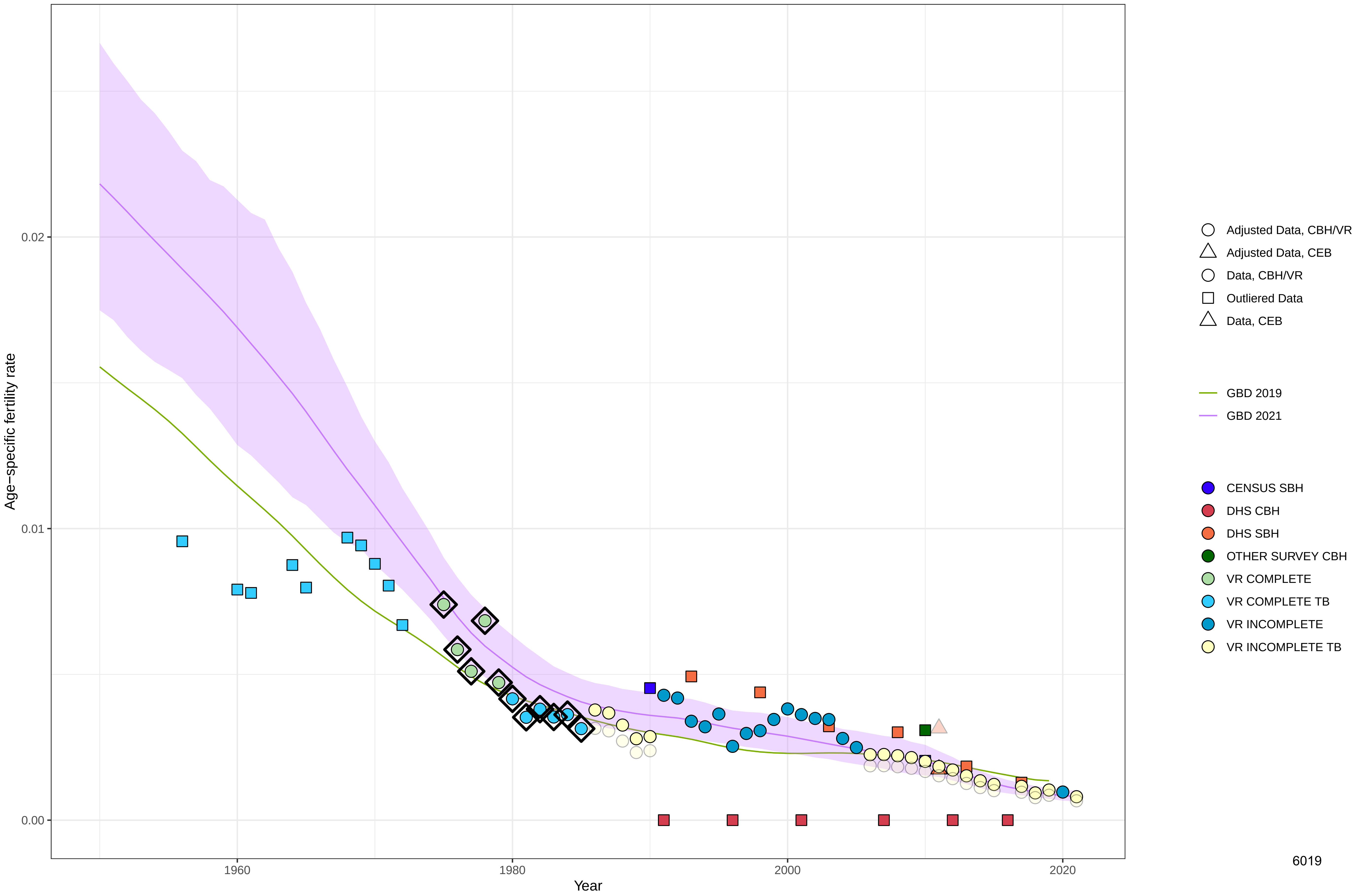

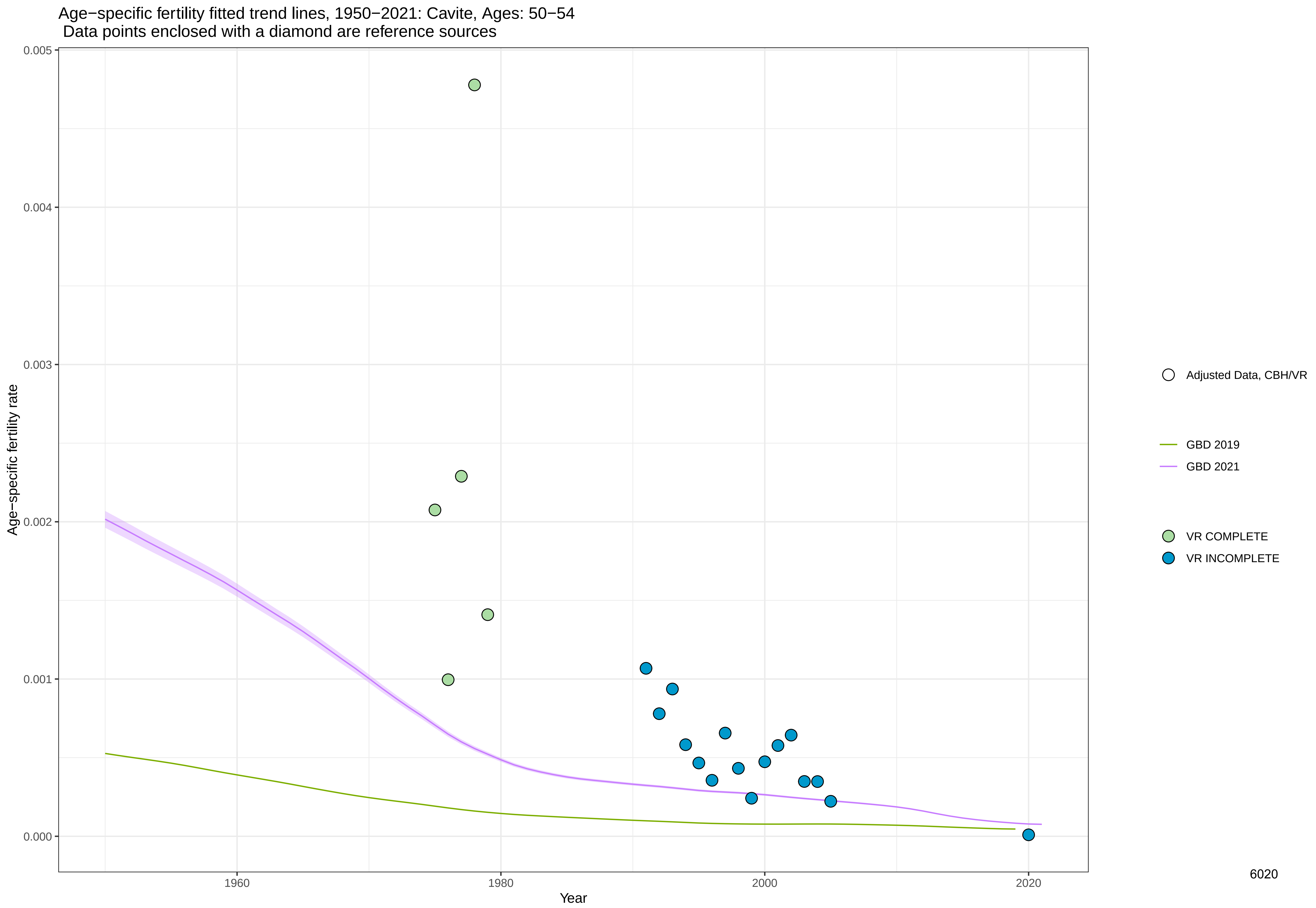

Age−specific fertility fitted trend lines, 1950−2021: Cavite, Ages: 50−54
Data points enclosed with a diamond are reference sources
Age−specific fertility rate
0.005
0.004
0.003
0.002
0.001
0.000
1960
1980
2000
2020
Year
Adjusted Data, CBH/VR
GBD 2019
GBD 2021
VR COMPLETE
VR INCOMPLETE

Total fertility rate fitted trend lines, 1950−2021: Cavite TFR
Data points enclosed with a diamond are reference sources

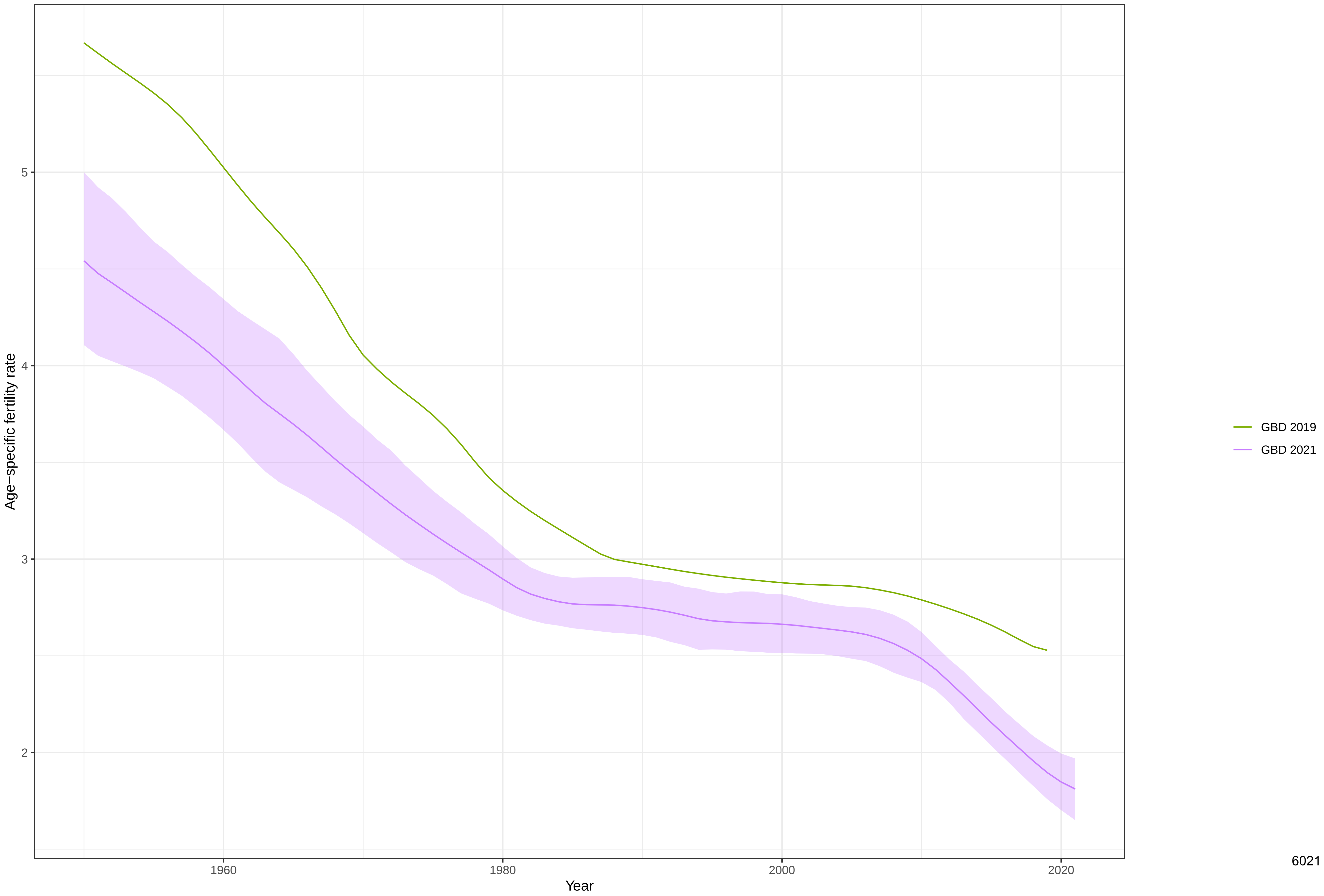

Age−specific fertility fitted trend lines, 1950−2021: Cebu, Ages: 10−14
Data points enclosed with a diamond are reference sources

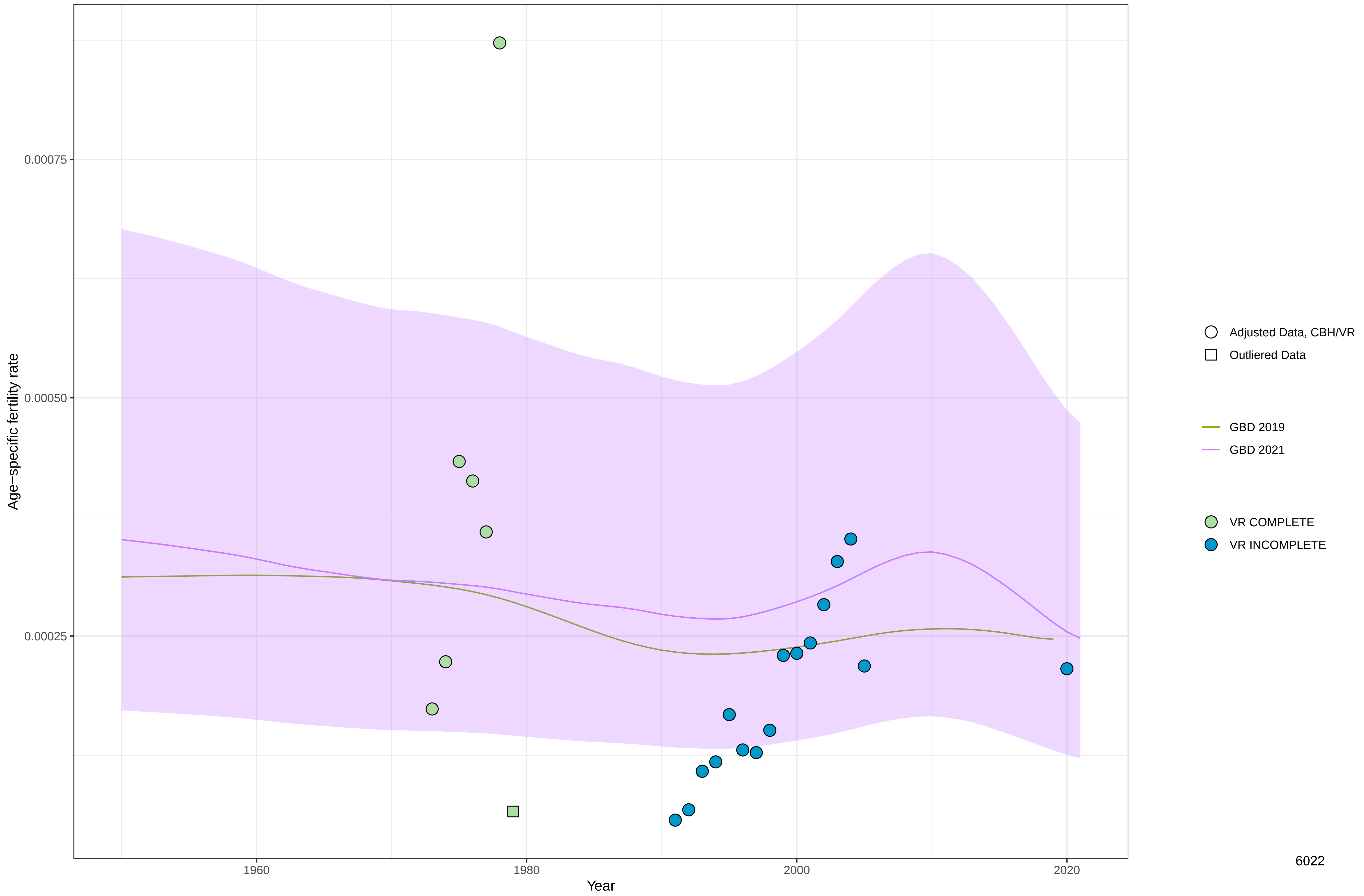

Age−specific fertility fitted trend lines, 1950−2021: Cebu, Ages: 15−19
Data points enclosed with a diamond are reference sources

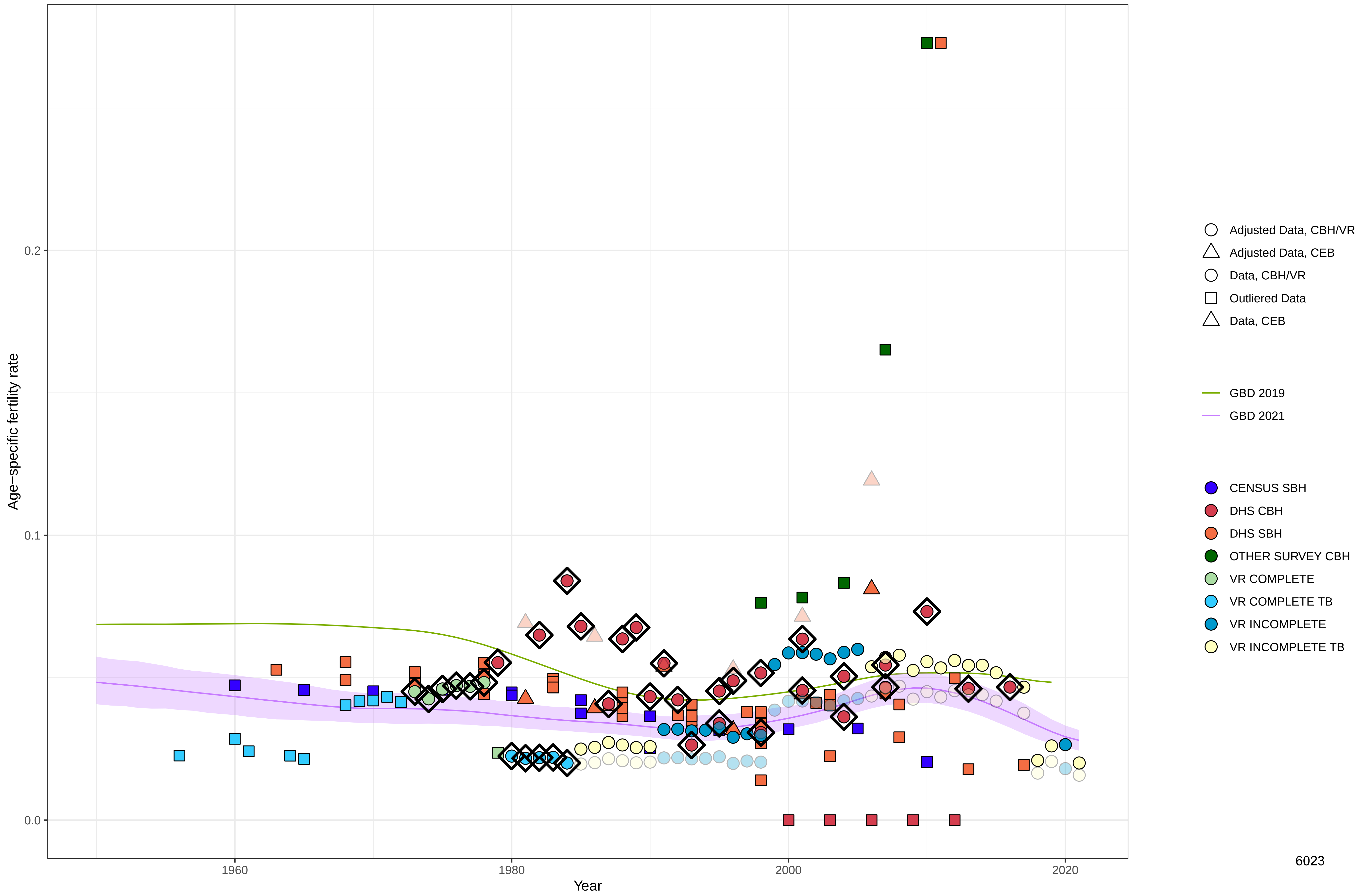

Age−specific fertility fitted trend lines, 1950−2021: Cebu, Ages: 20−24
Data points enclosed with a diamond are reference sources

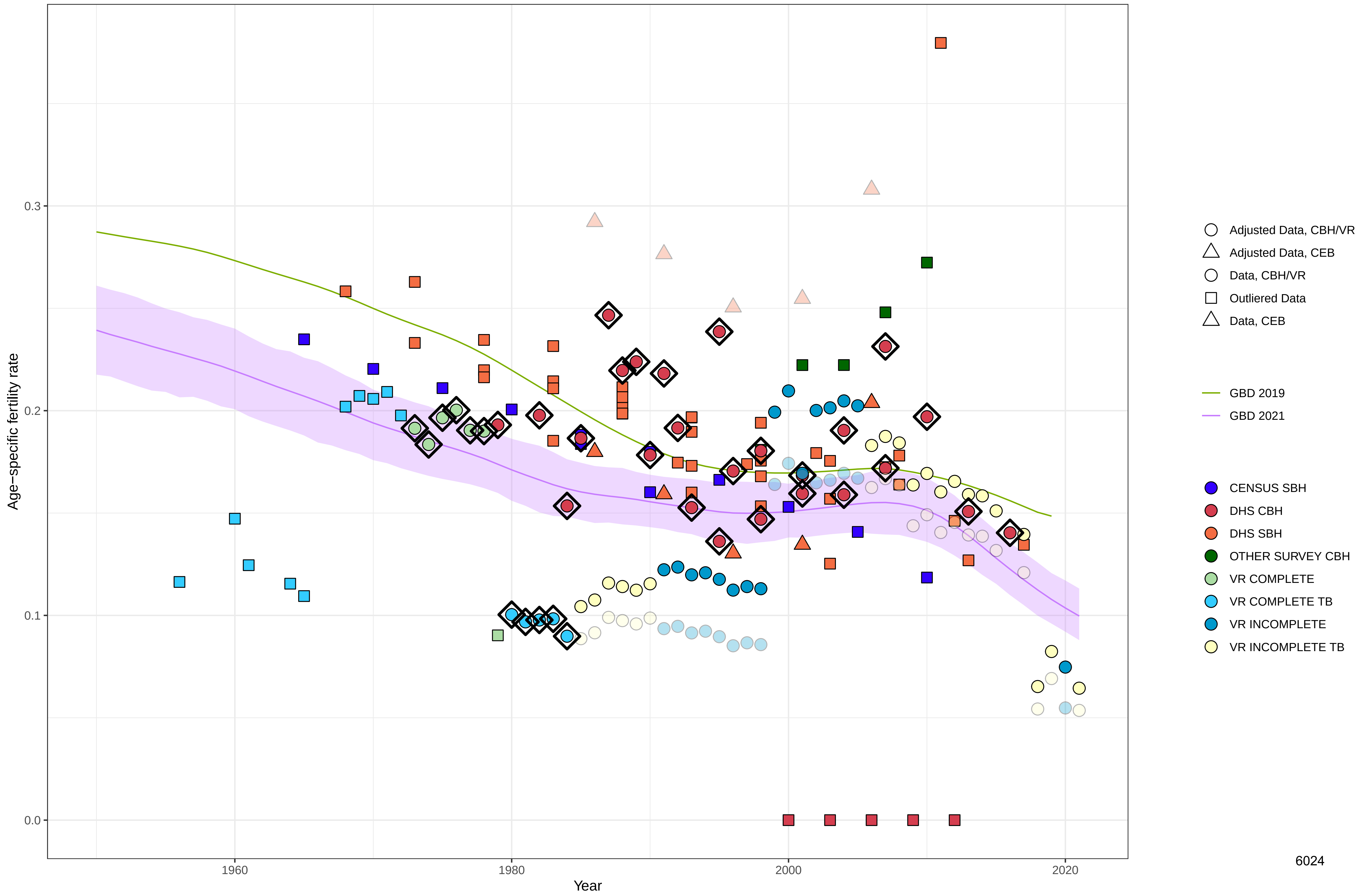

Age−specific fertility fitted trend lines, 1950−2021: Cebu, Ages: 25−29
Data points enclosed with a diamond are reference sources

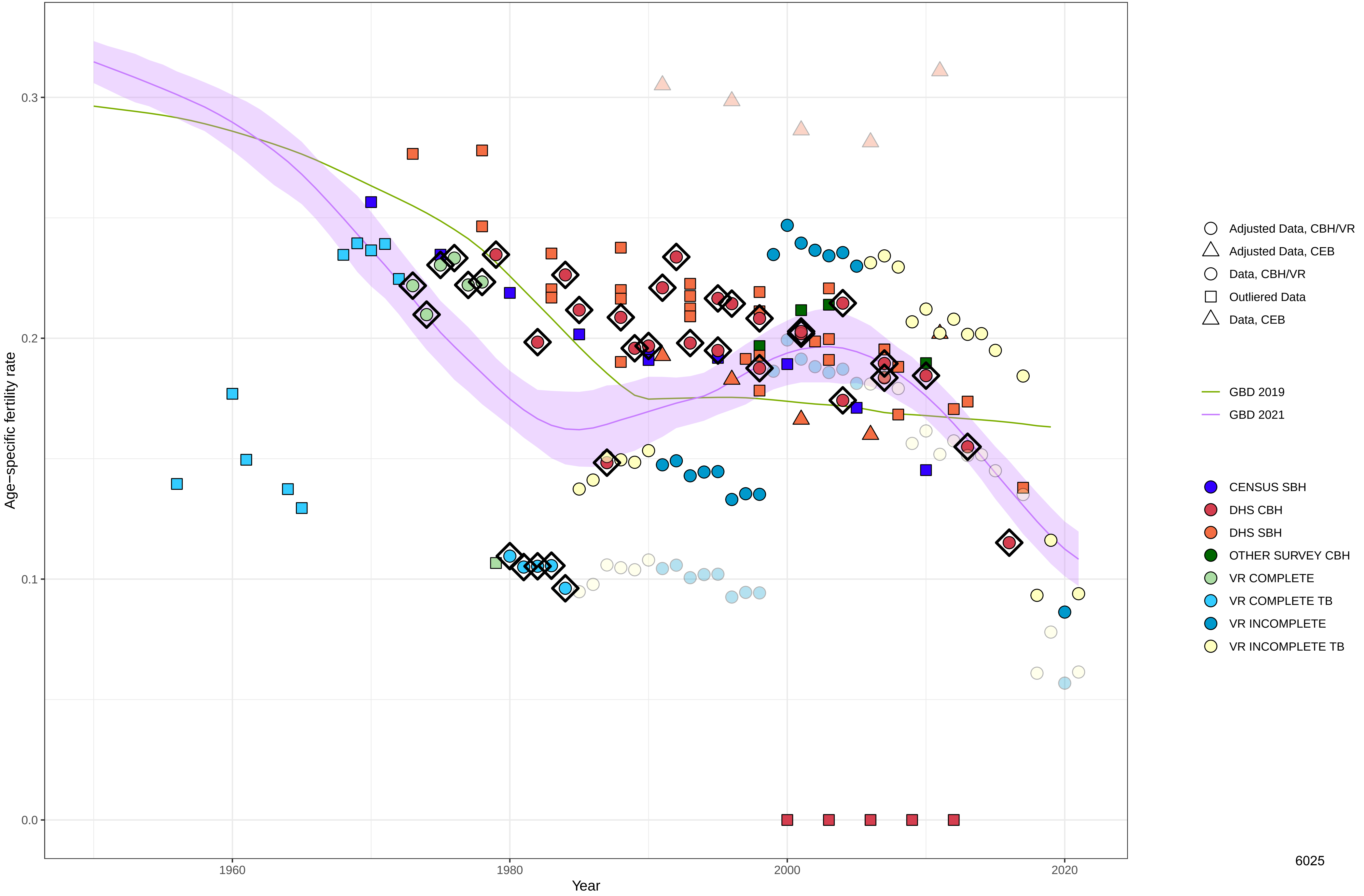

Age−specific fertility fitted trend lines, 1950−2021: Cebu, Ages: 30−34
Data points enclosed with a diamond are reference sources

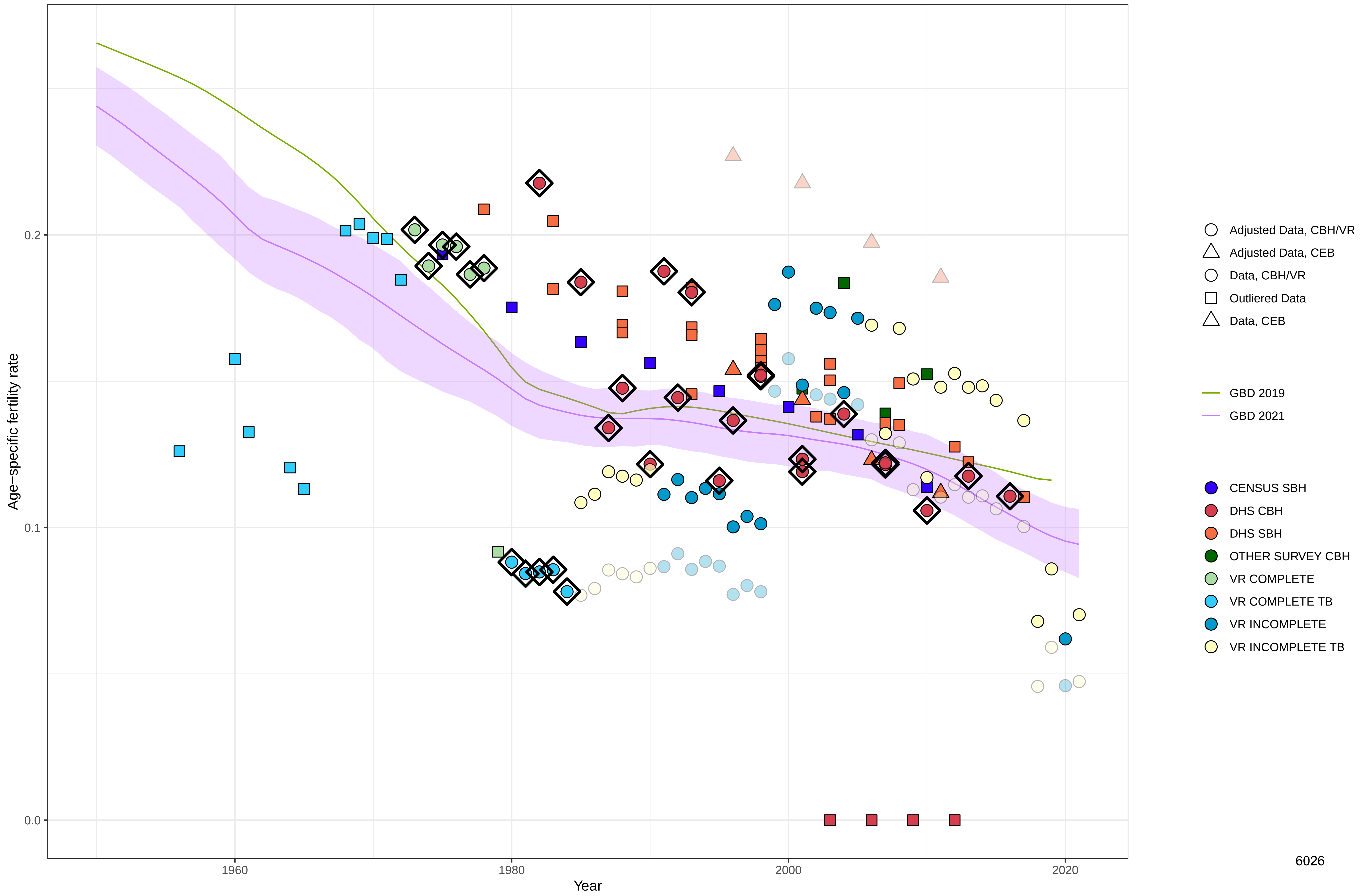

Age−specific fertility fitted trend lines, 1950−2021: Cebu, Ages: 35−39
Data points enclosed with a diamond are reference sources

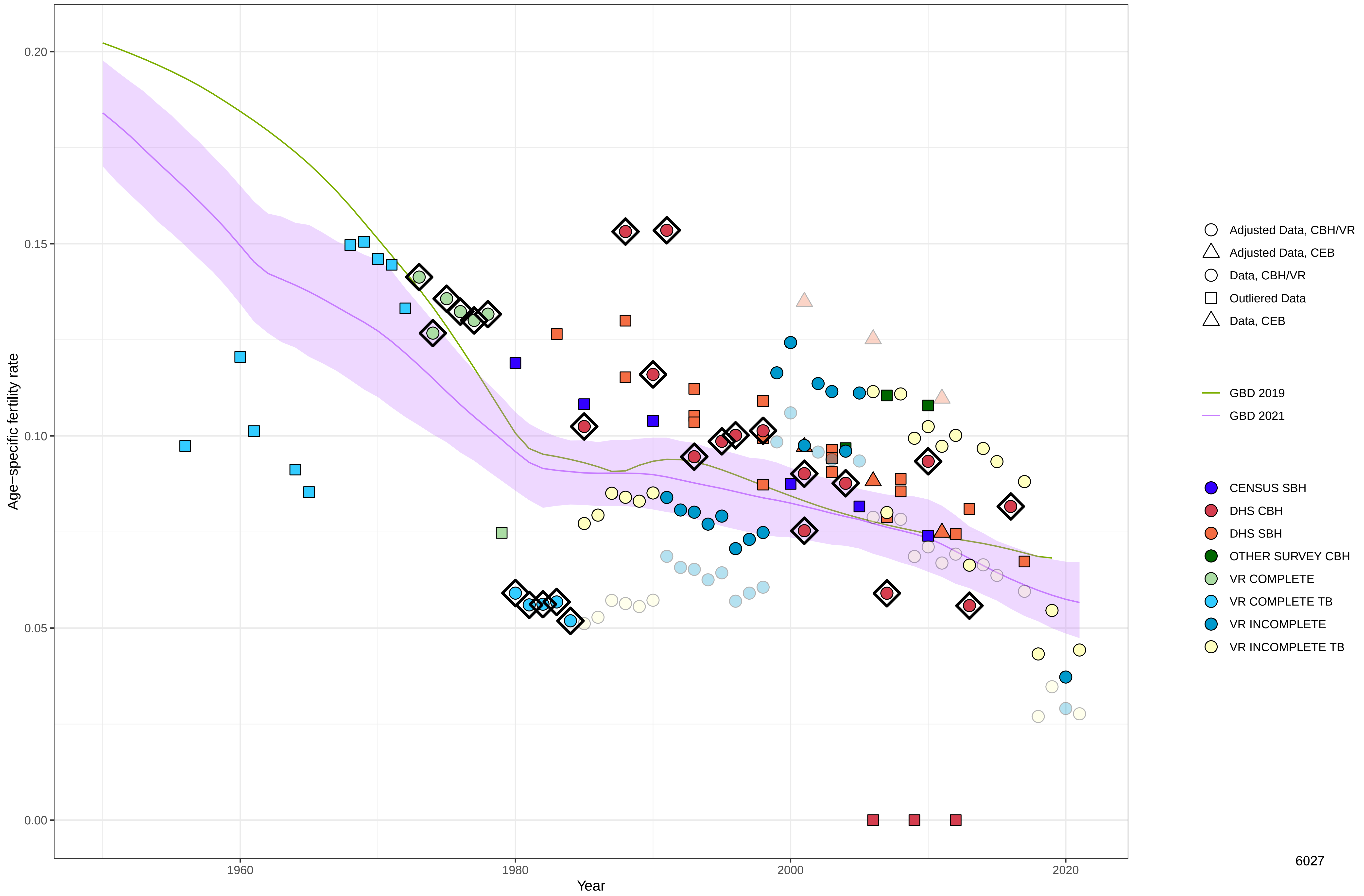

Age−specific fertility fitted trend lines, 1950−2021: Cebu, Ages: 40−44
Data points enclosed with a diamond are reference sources

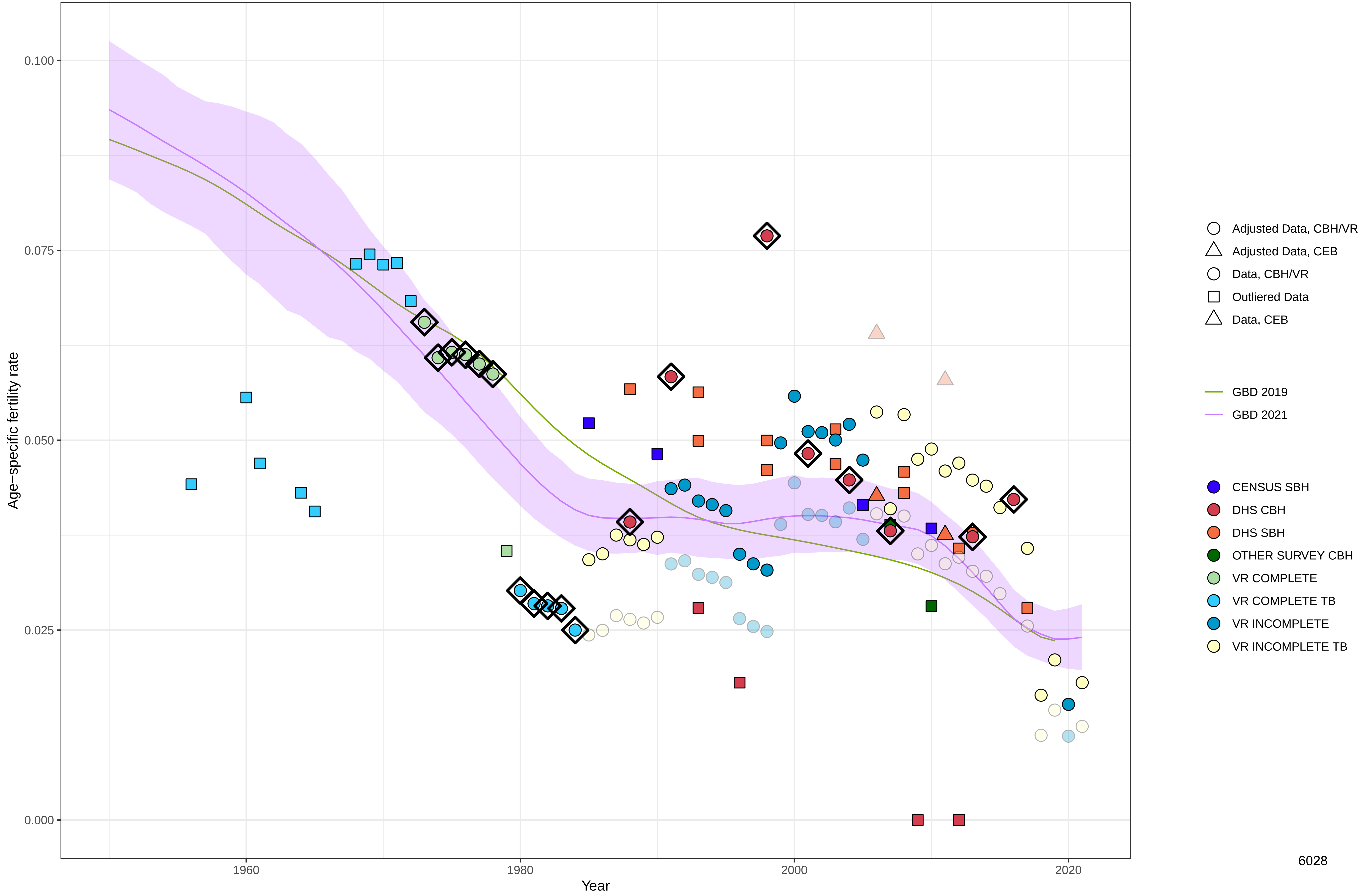

Age−specific fertility fitted trend lines, 1950−2021: Cebu, Ages: 45−49
Data points enclosed with a diamond are reference sources

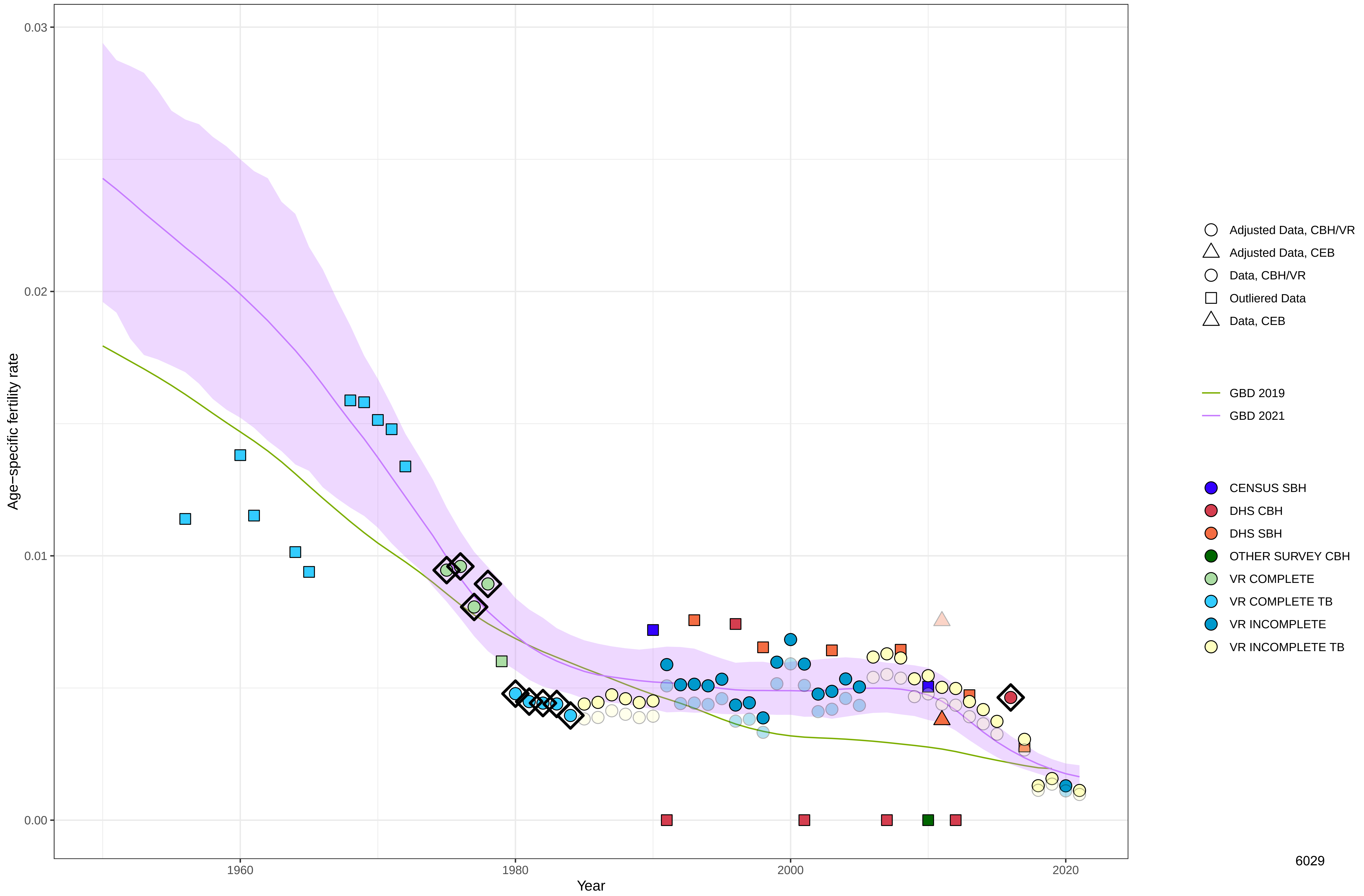

Age−specific fertility fitted trend lines, 1950−2021: Cebu, Ages: 50−54
Data points enclosed with a diamond are reference sources

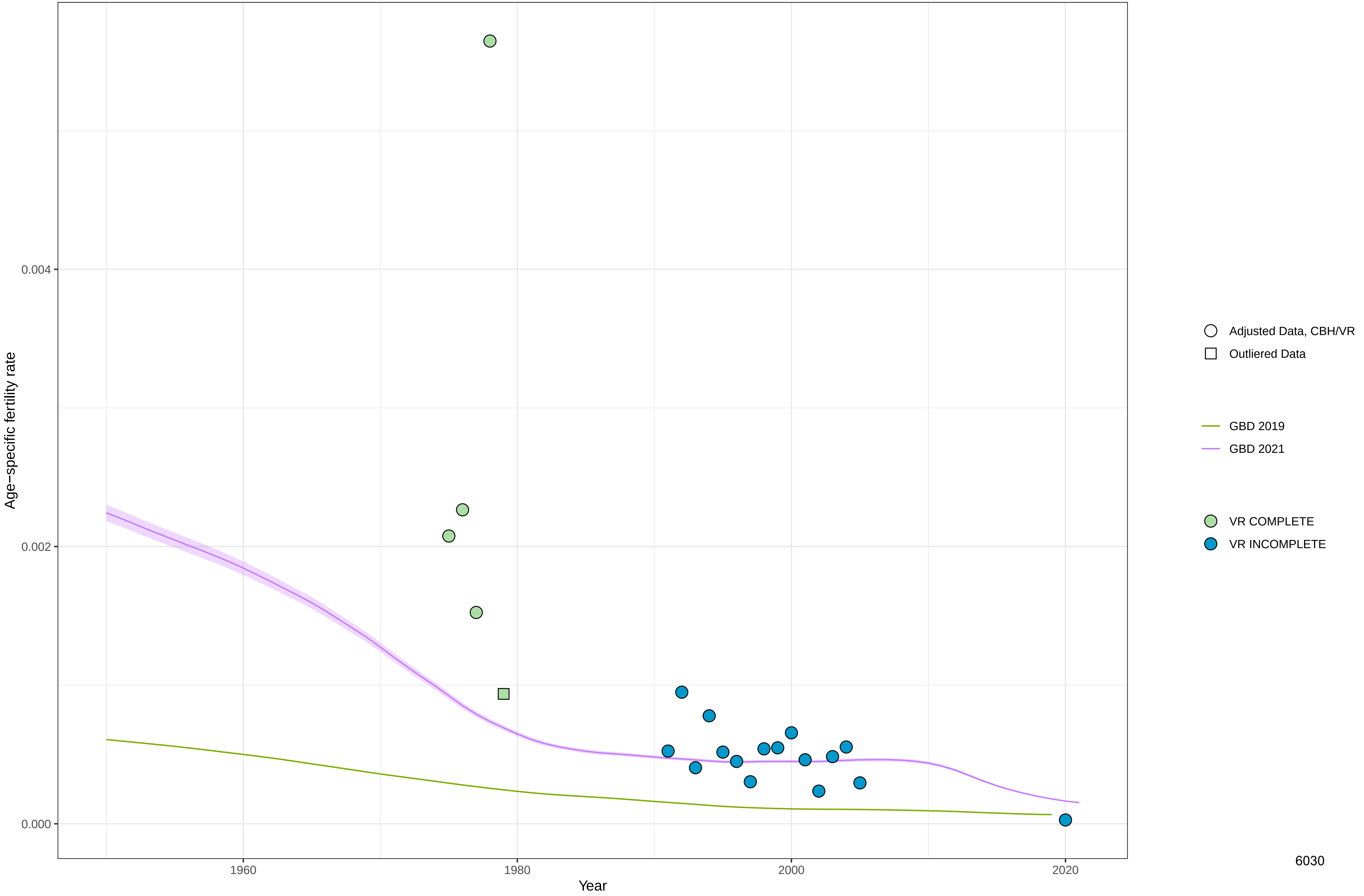

Total fertility rate fitted trend lines, 1950−2021: Cebu TFR
Data points enclosed with a diamond are reference sources

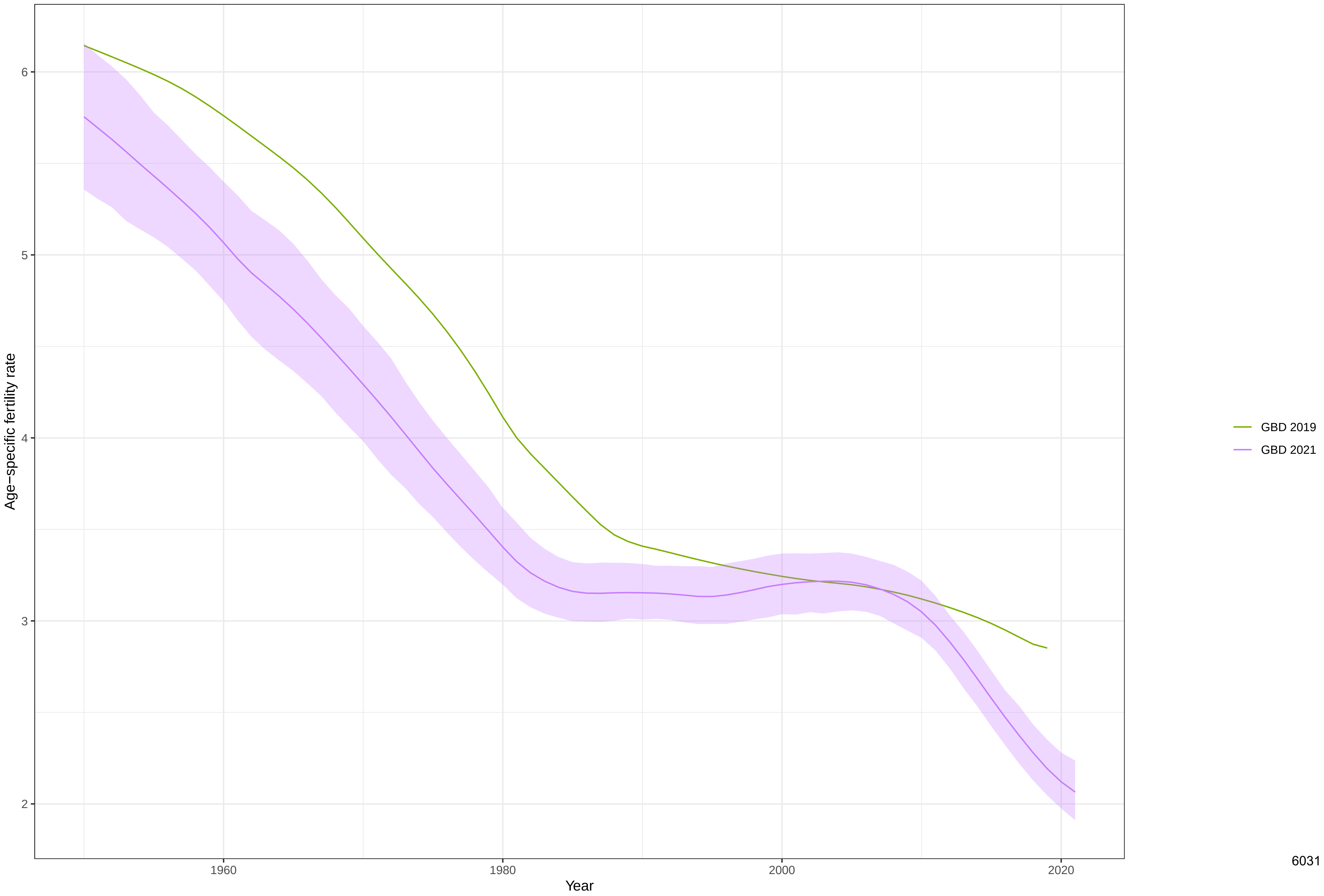

6031

Age−specific fertility fitted trend lines, 1950−2021: Cotabato (North Cotabato), Ages: 10−14
Data points enclosed with a diamond are reference sources

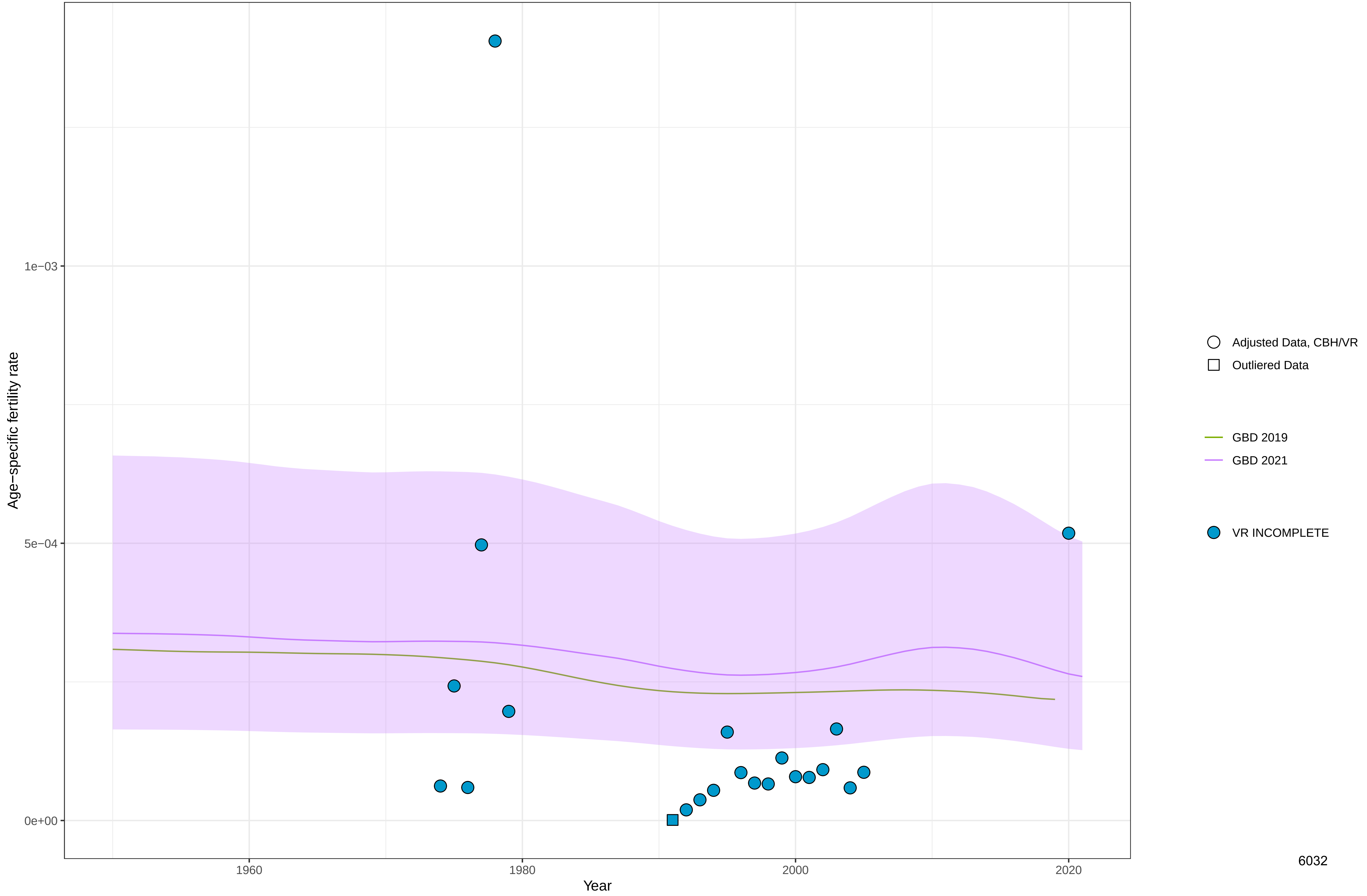

6032

Age−specific fertility fitted trend lines, 1950−2021: Cotabato (North Cotabato), Ages: 15−19
Data points enclosed with a diamond are reference sources

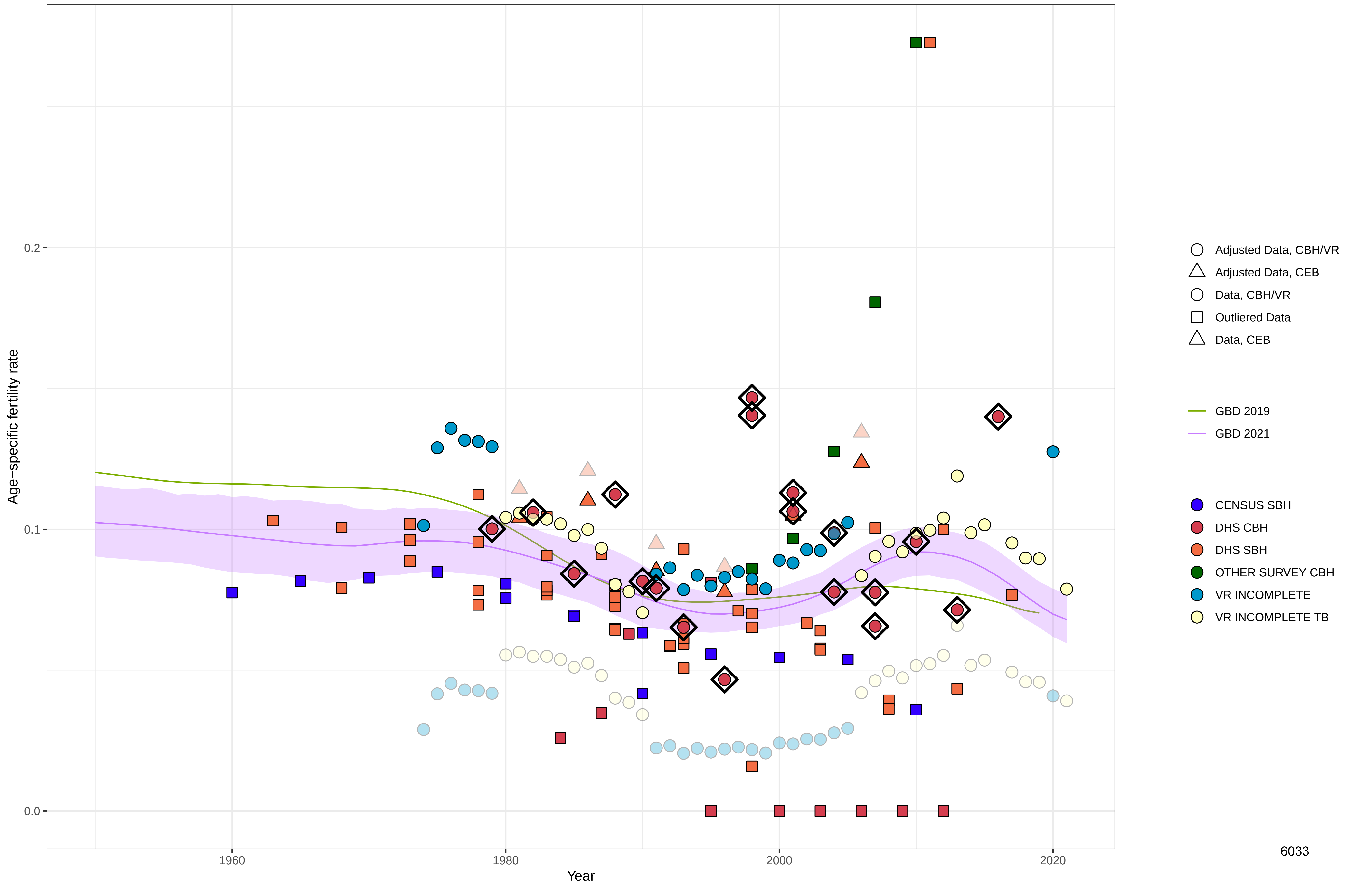

Age−specific fertility fitted trend lines, 1950−2021: Cotabato (North Cotabato), Ages: 20−24
Data points enclosed with a diamond are reference sources

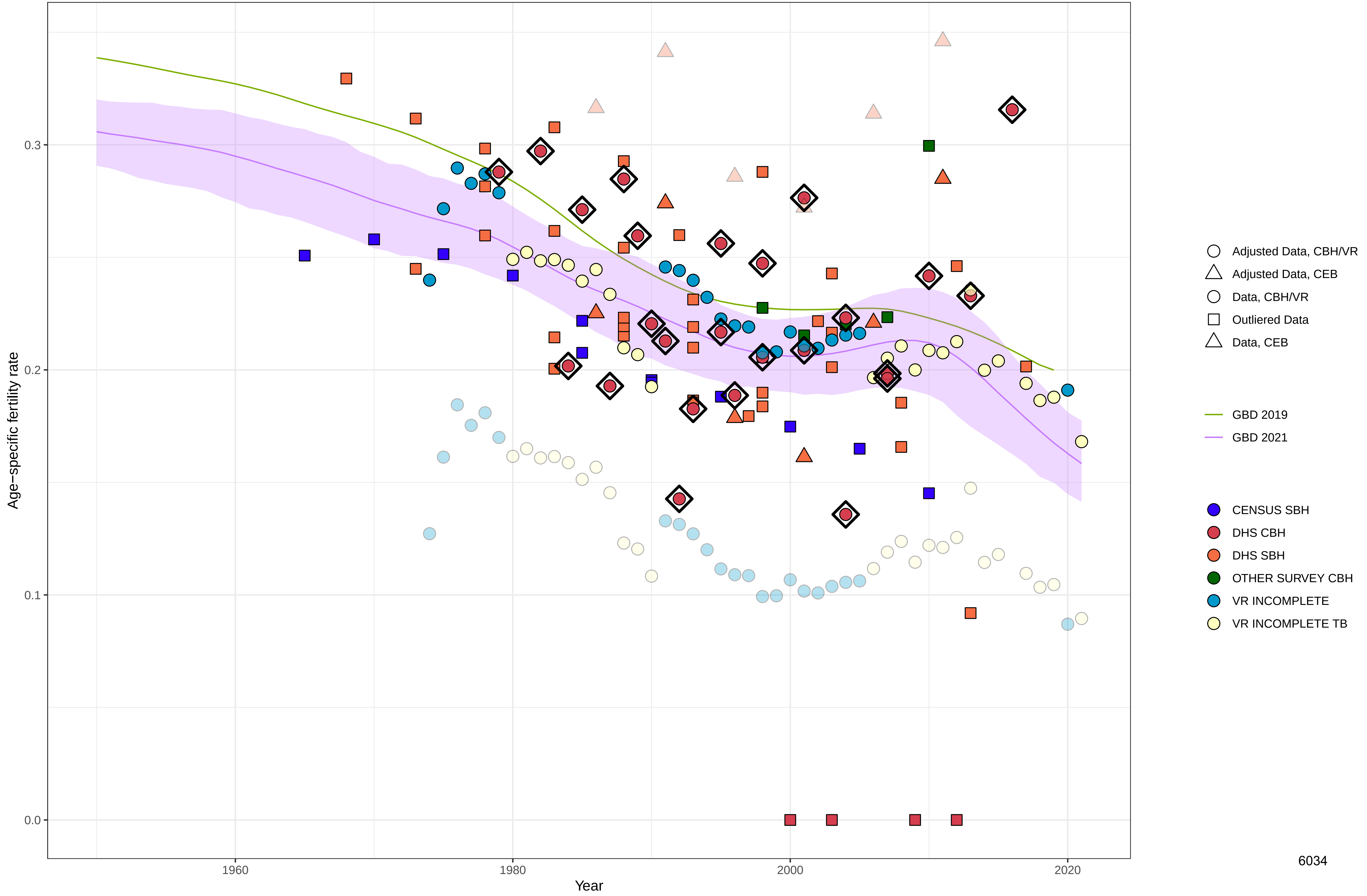

Age−specific fertility fitted trend lines, 1950−2021: Cotabato (North Cotabato), Ages: 25−29
Data points enclosed with a diamond are reference sources

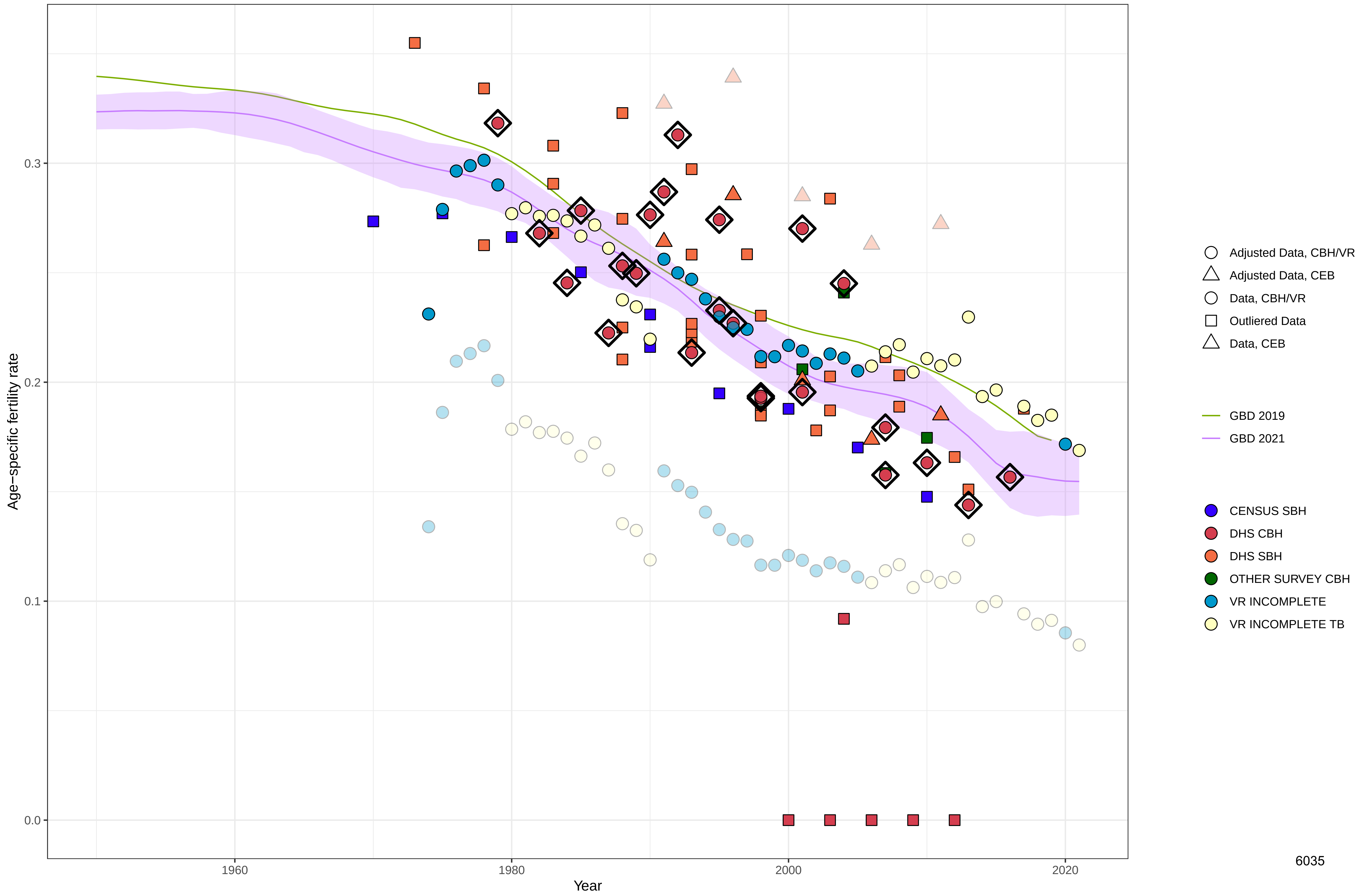

Age−specific fertility fitted trend lines, 1950−2021: Cotabato (North Cotabato), Ages: 30−34
Data points enclosed with a diamond are reference sources

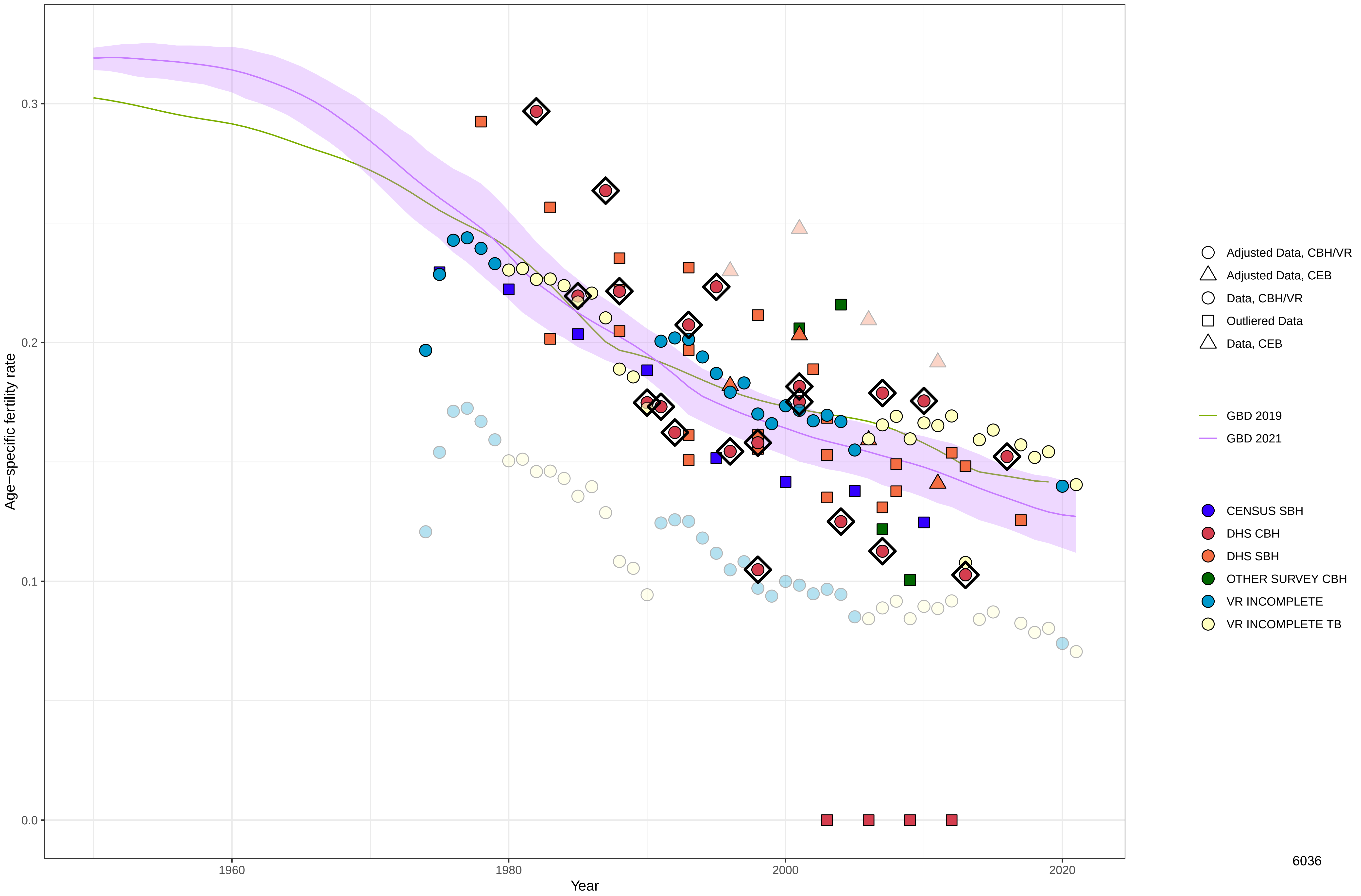

Age−specific fertility fitted trend lines, 1950−2021: Cotabato (North Cotabato), Ages: 35−39
Data points enclosed with a diamond are reference sources

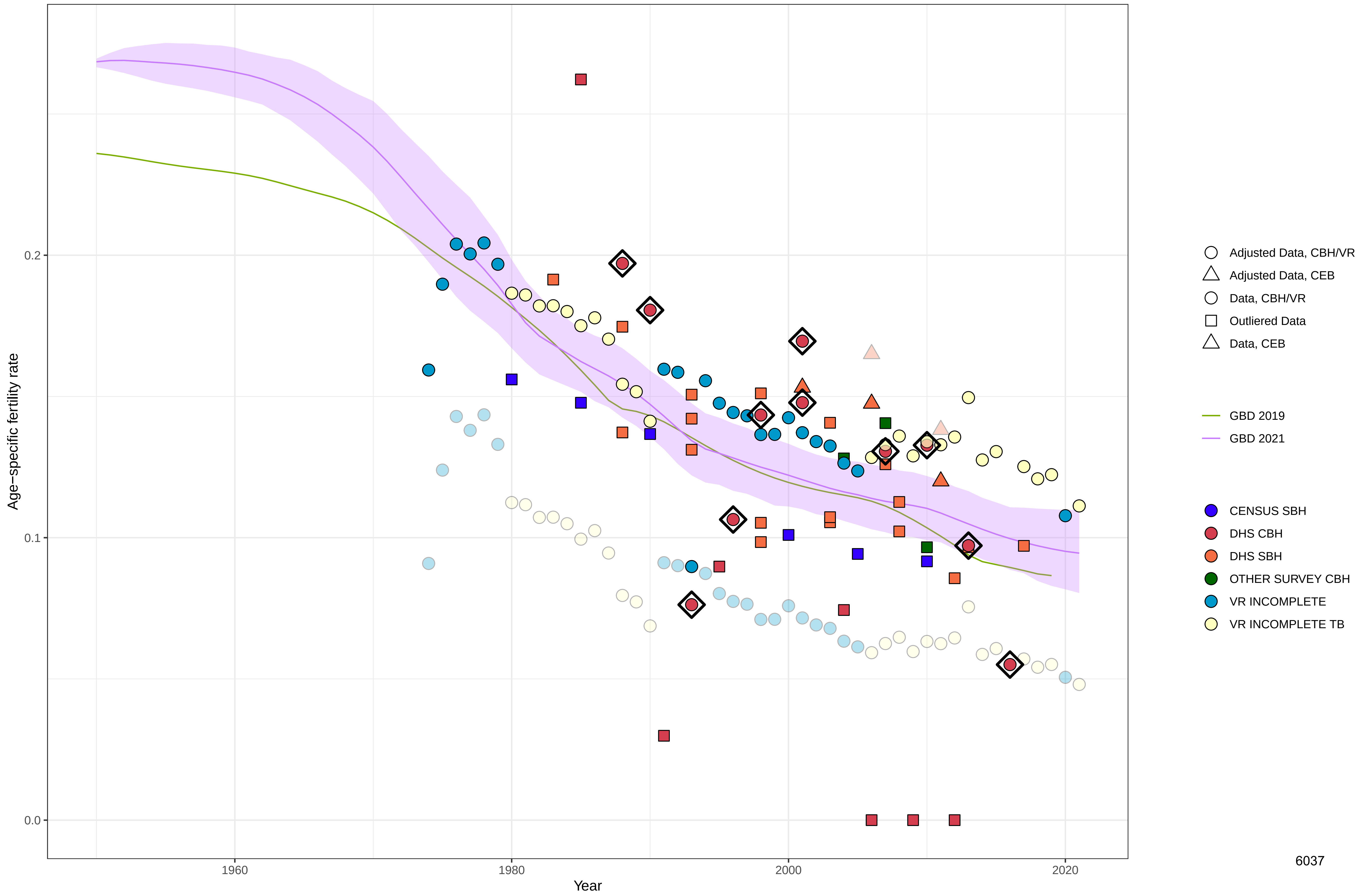

Age−specific fertility fitted trend lines, 1950−2021: Cotabato (North Cotabato), Ages: 40−44
Data points enclosed with a diamond are reference sources

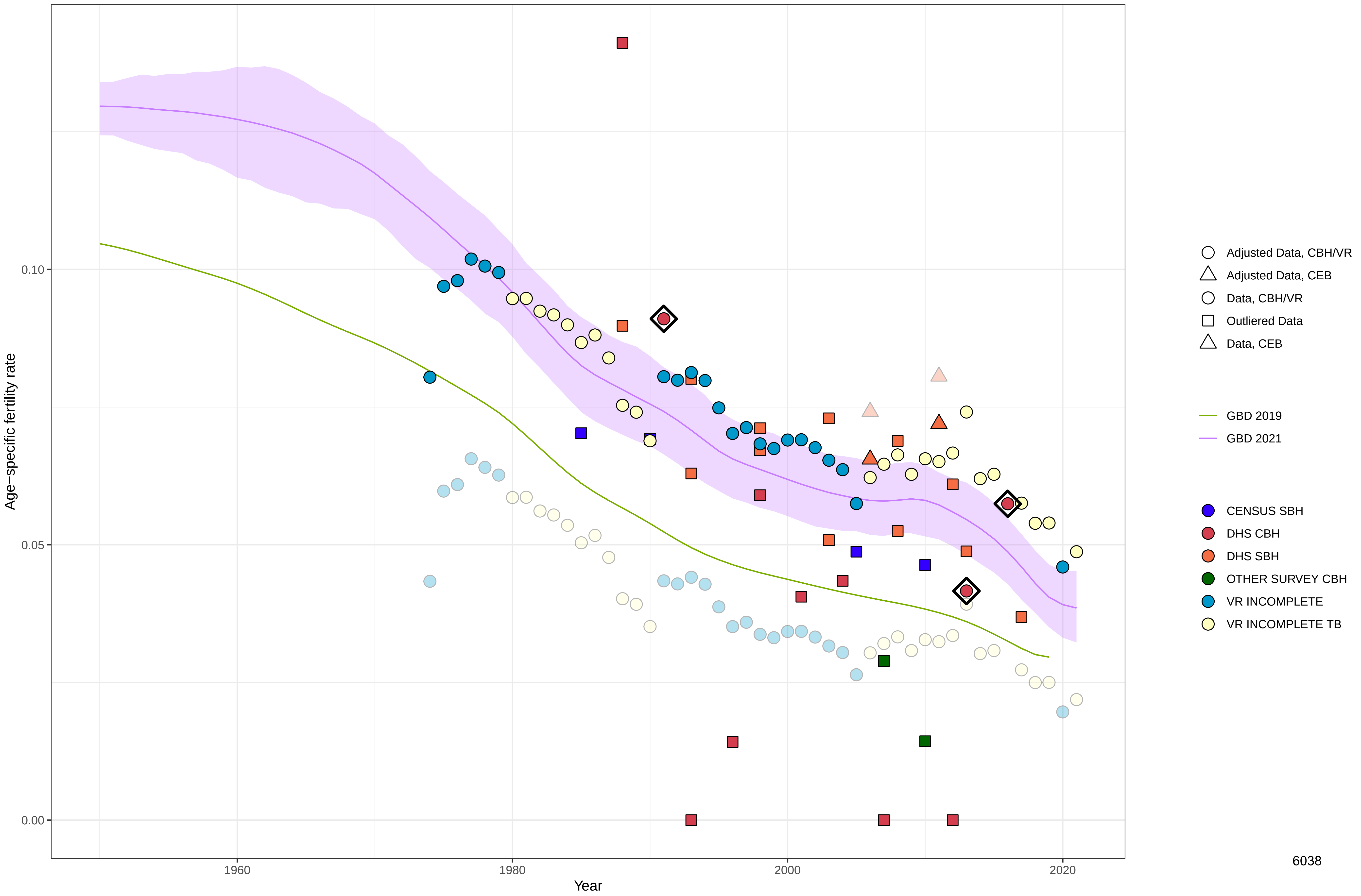

Age−specific fertility fitted trend lines, 1950−2021: Cotabato (North Cotabato), Ages: 45−49
Data points enclosed with a diamond are reference sources

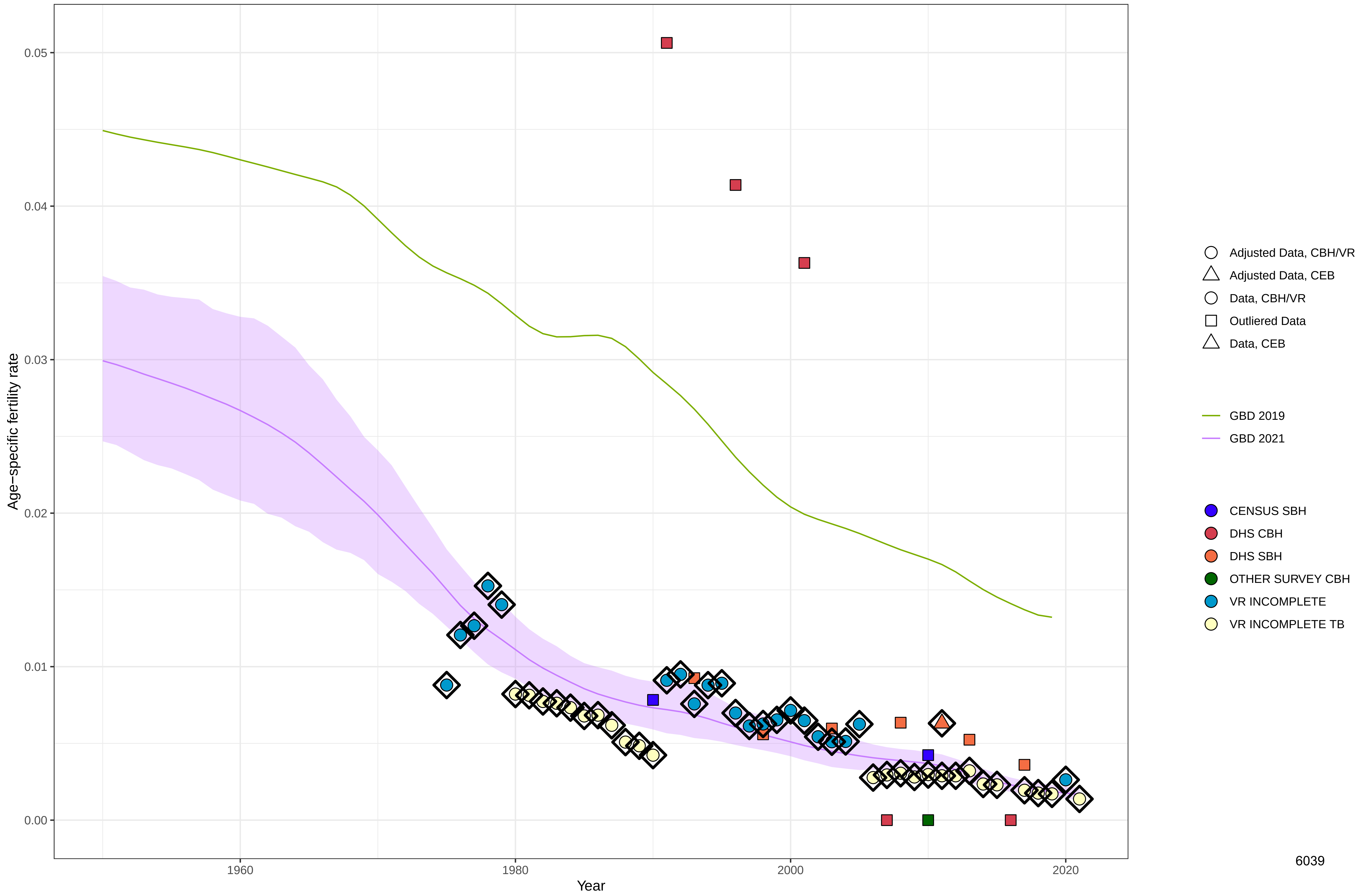

Age−specific fertility fitted trend lines, 1950−2021: Cotabato (North Cotabato), Ages: 50−54
Data points enclosed with a diamond are reference sources

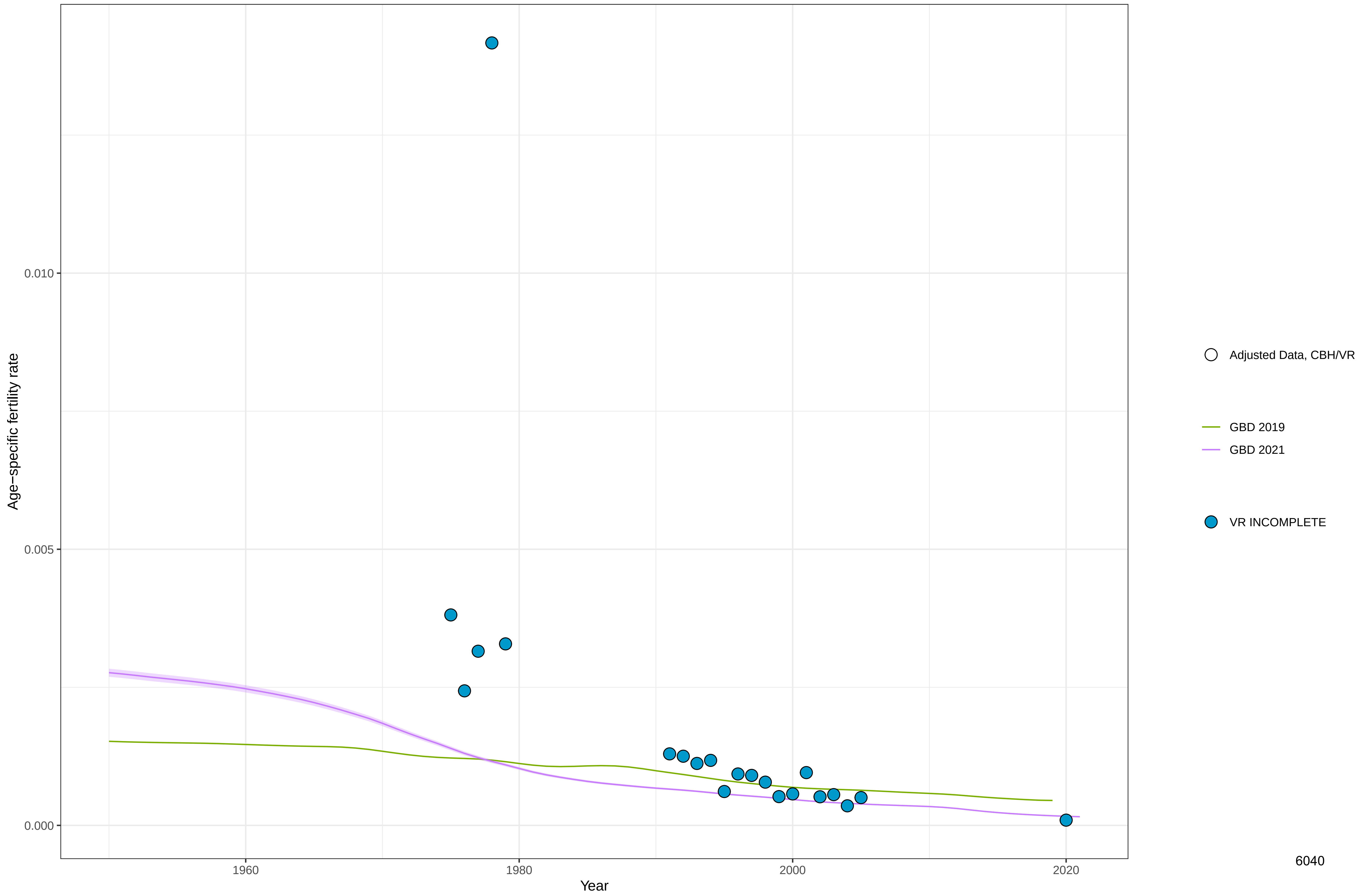

Total fertility rate fitted trend lines, 1950−2021: Cotabato (North Cotabato) TFR
Data points enclosed with a diamond are reference sources

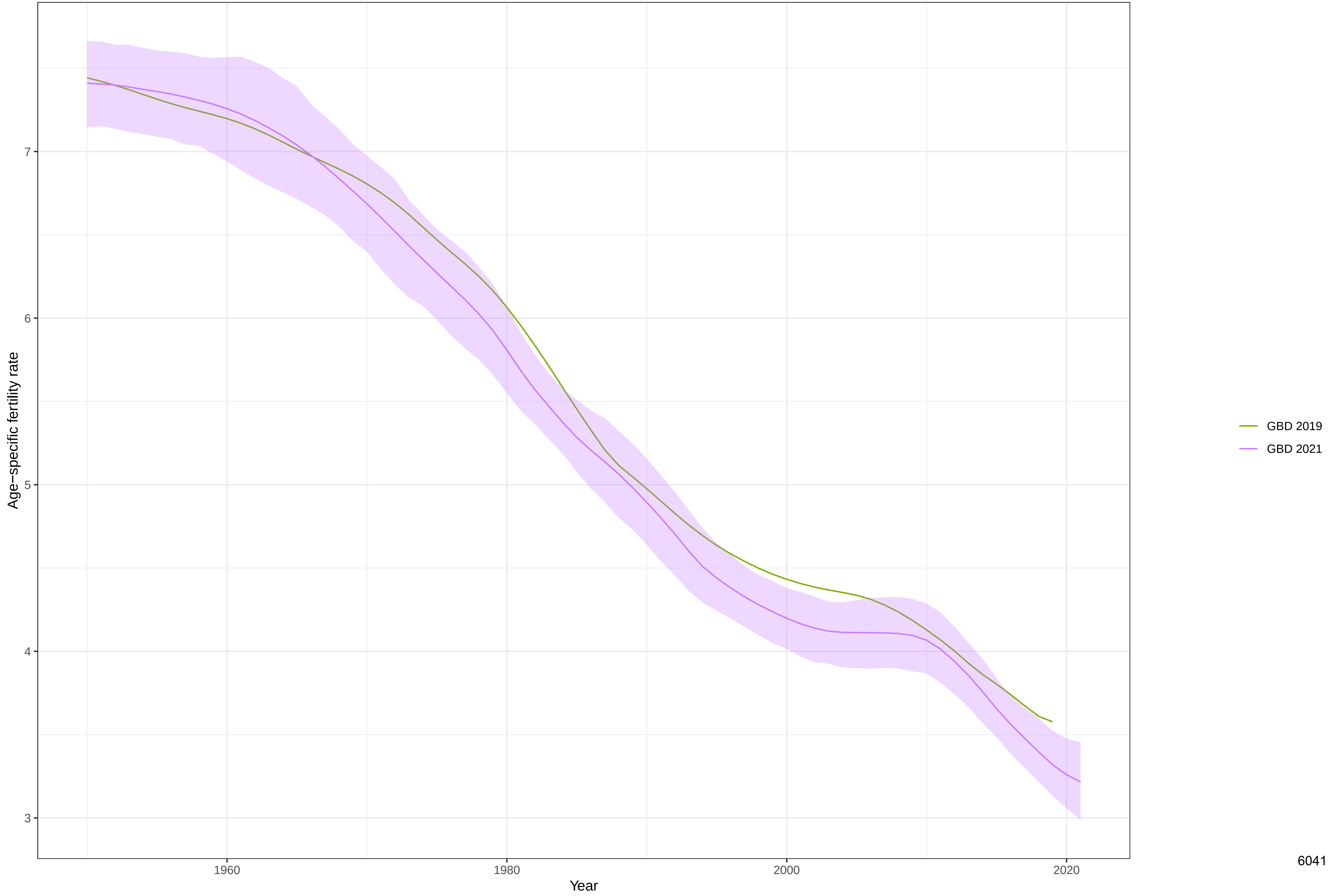

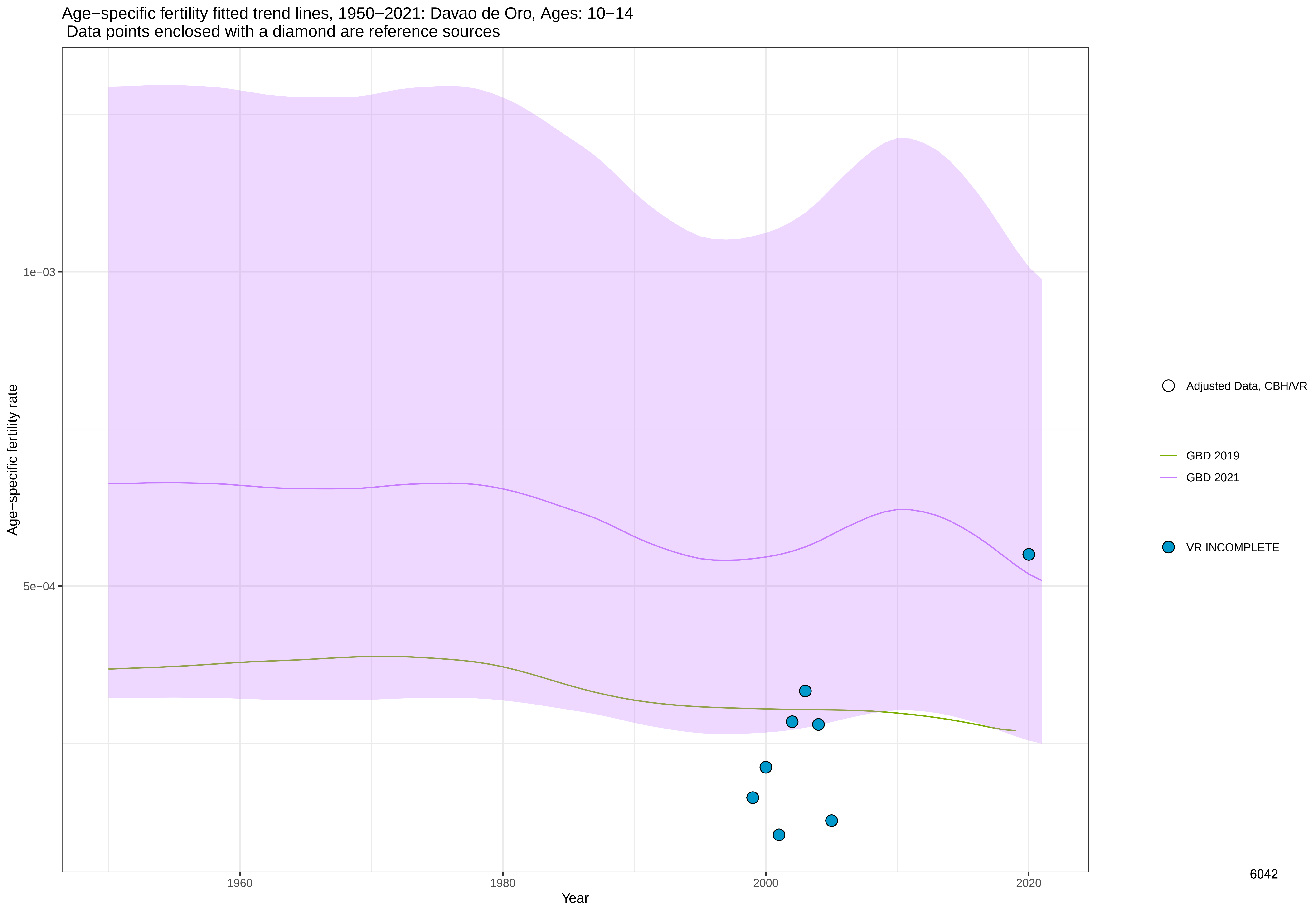

Age−specific fertility fitted trend lines, 1950−2021: Davao de Oro, Ages: 10−14
Data points enclosed with a diamond are reference sources
Age−specific fertility rate
1e−03
5e−04
1960
1980
2000
2020
Year
Adjusted Data, CBH/VR
GBD 2019
GBD 2021
VR INCOMPLETE

Age−specific fertility fitted trend lines, 1950−2021: Davao de Oro, Ages: 15−19
Data points enclosed with a diamond are reference sources

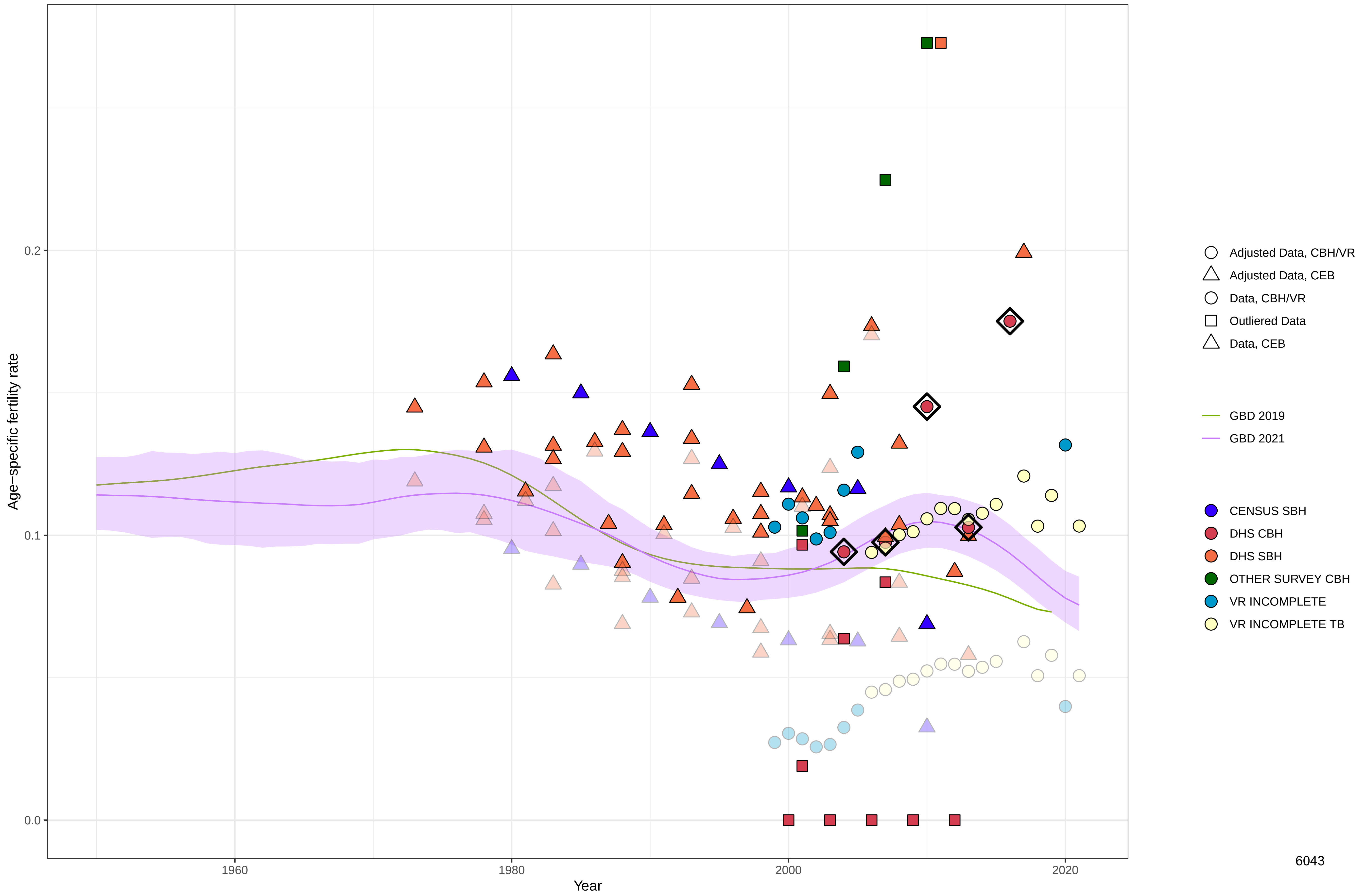

Age−specific fertility fitted trend lines, 1950−2021: Davao de Oro, Ages: 20−24
Data points enclosed with a diamond are reference sources

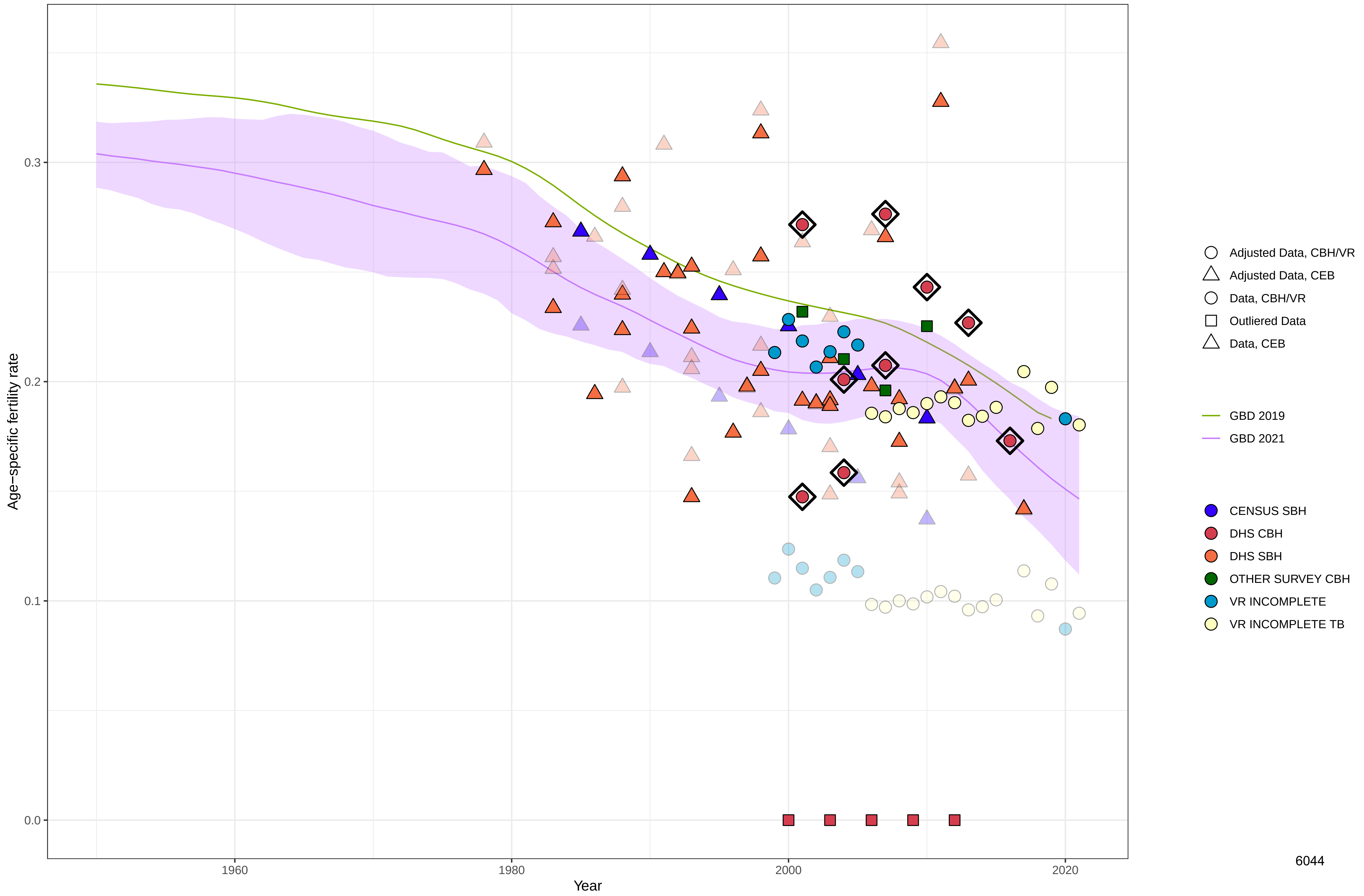

Age−specific fertility fitted trend lines, 1950−2021: Davao de Oro, Ages: 25−29
Data points enclosed with a diamond are reference sources

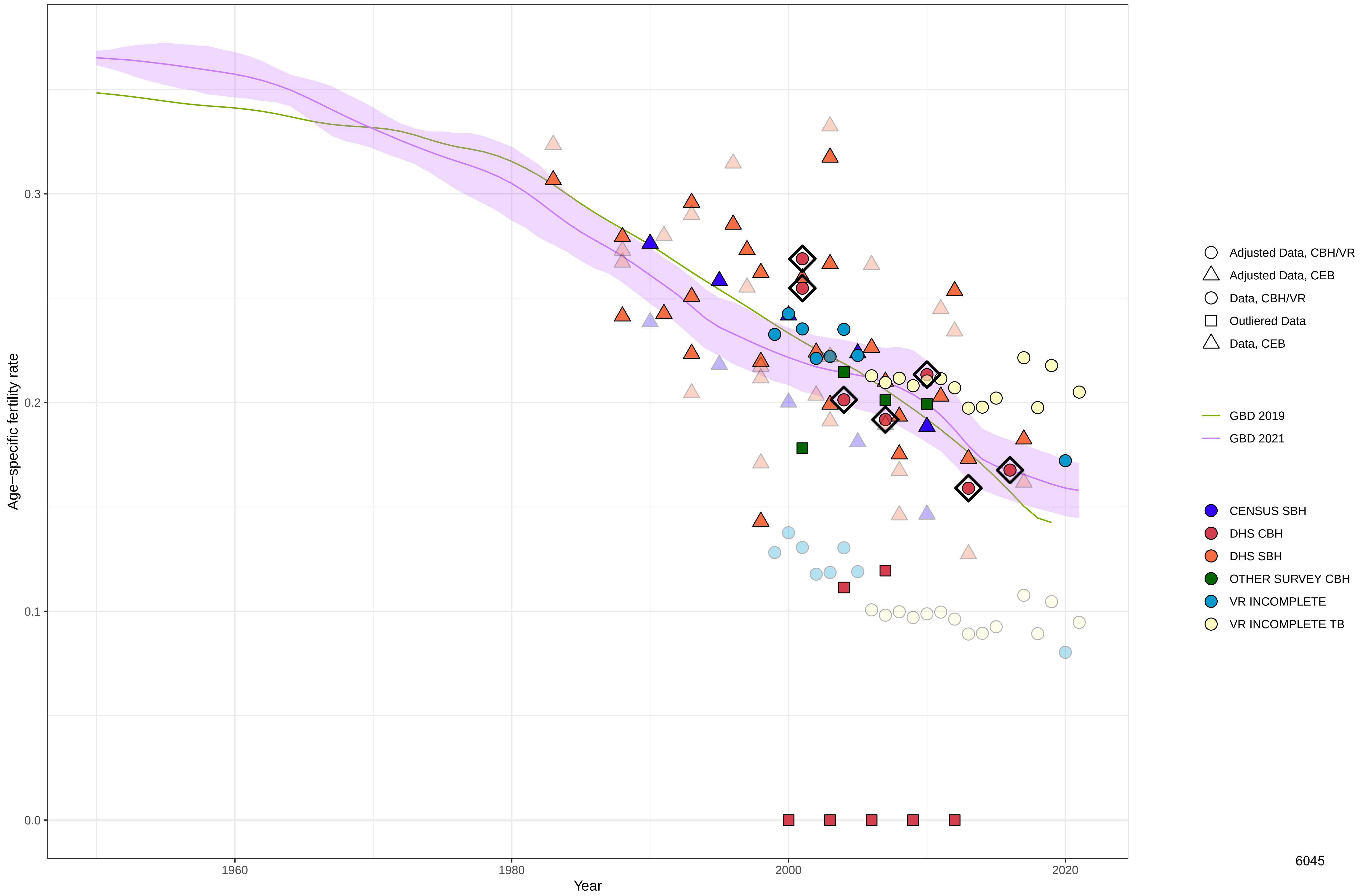

Age−specific fertility fitted trend lines, 1950−2021: Davao de Oro, Ages: 30−34
Data points enclosed with a diamond are reference sources

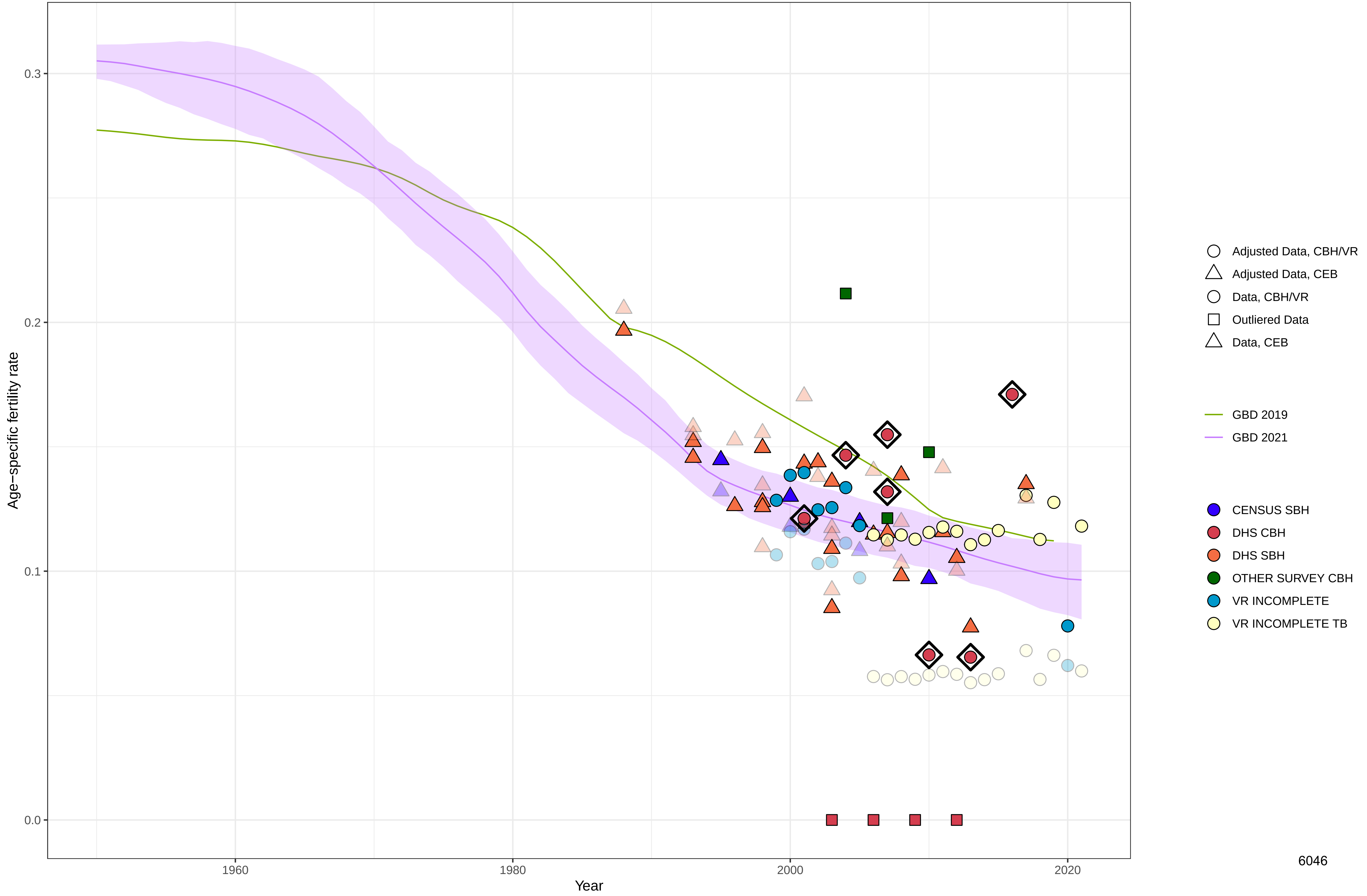

Age−specific fertility fitted trend lines, 1950−2021: Davao de Oro, Ages: 35−39
Data points enclosed with a diamond are reference sources

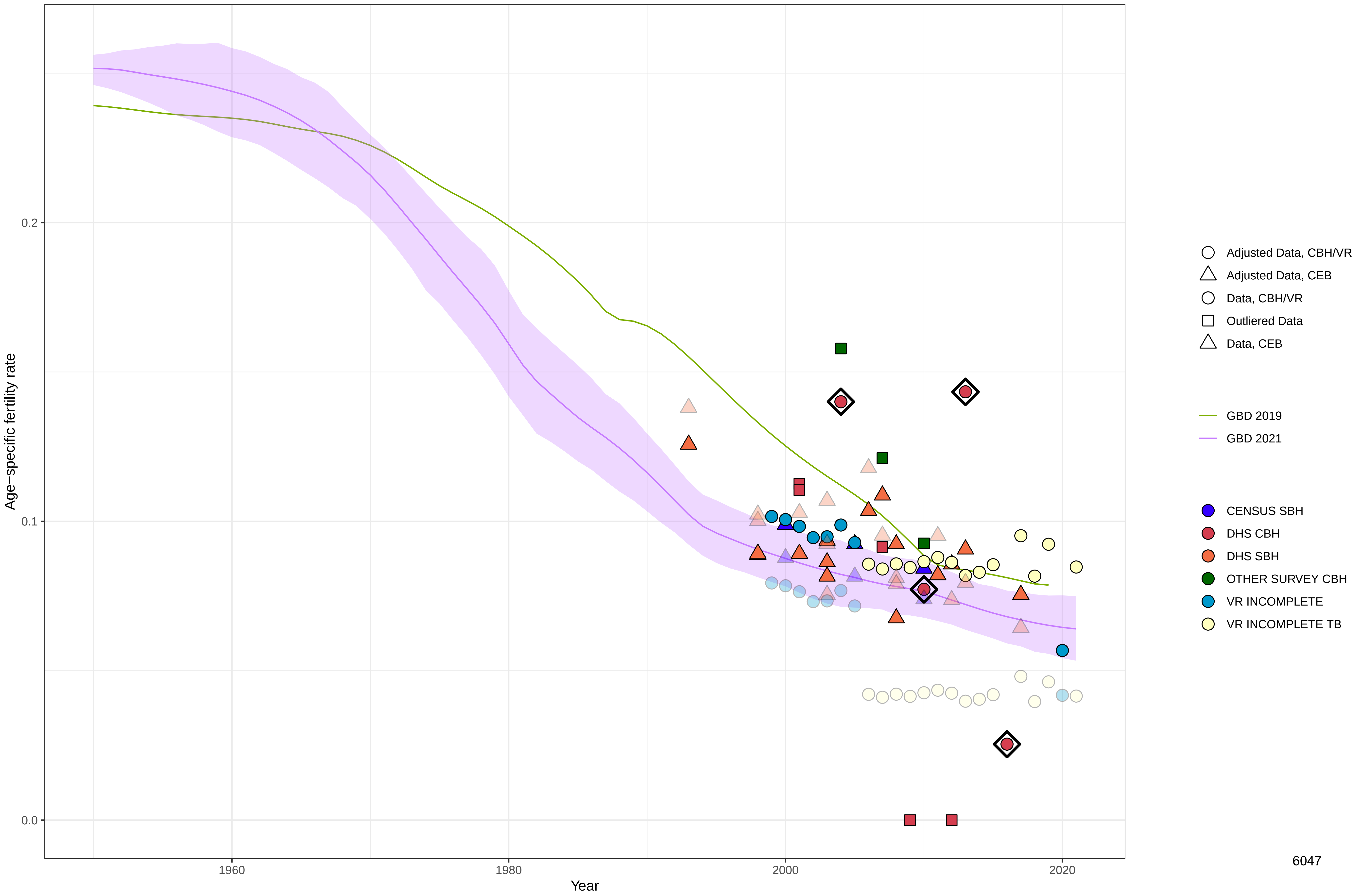

Age−specific fertility fitted trend lines, 1950−2021: Davao de Oro, Ages: 40−44
Data points enclosed with a diamond are reference sources

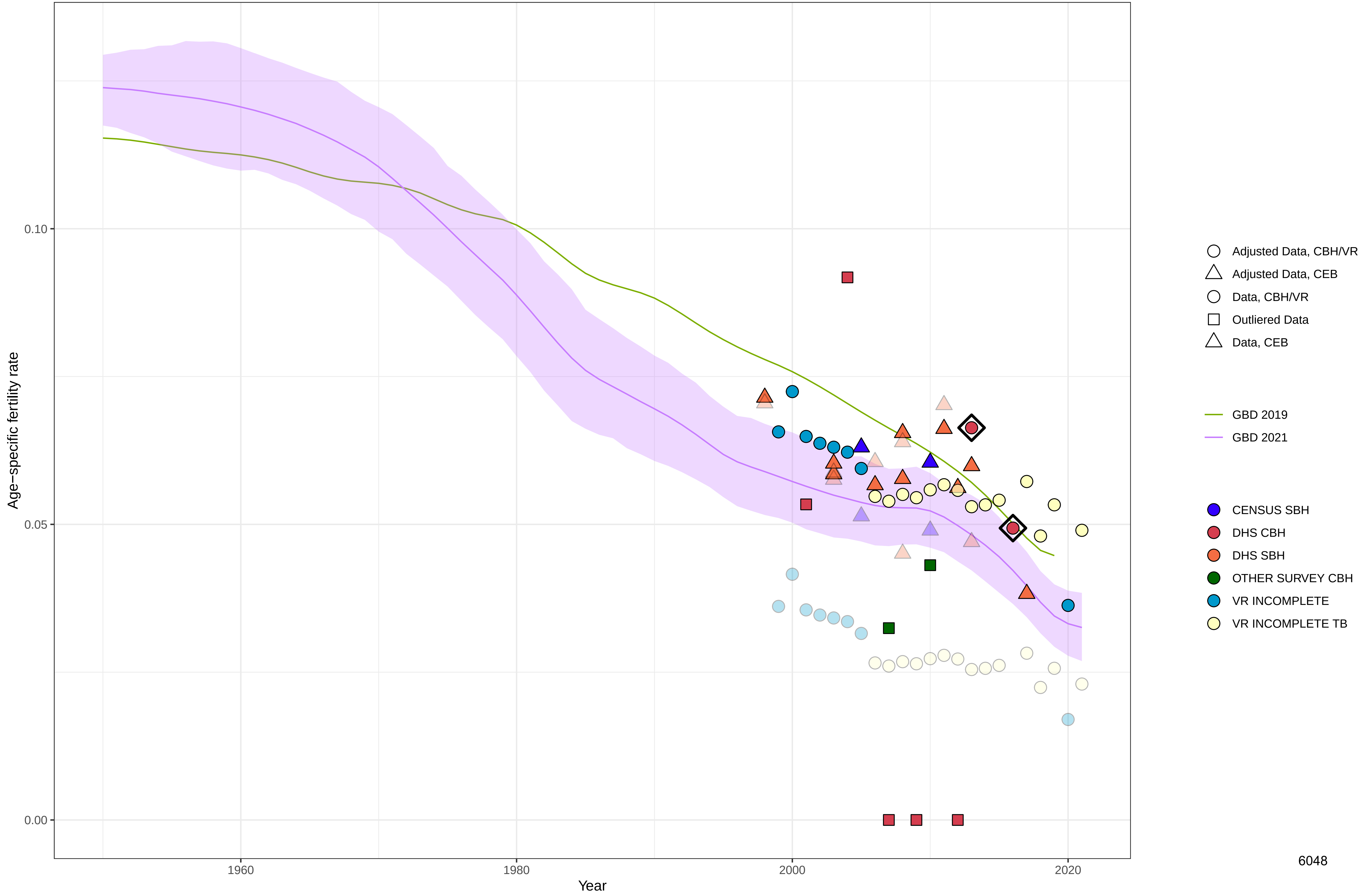

Age−specific fertility fitted trend lines, 1950−2021: Davao de Oro, Ages: 45−49
Data points enclosed with a diamond are reference sources

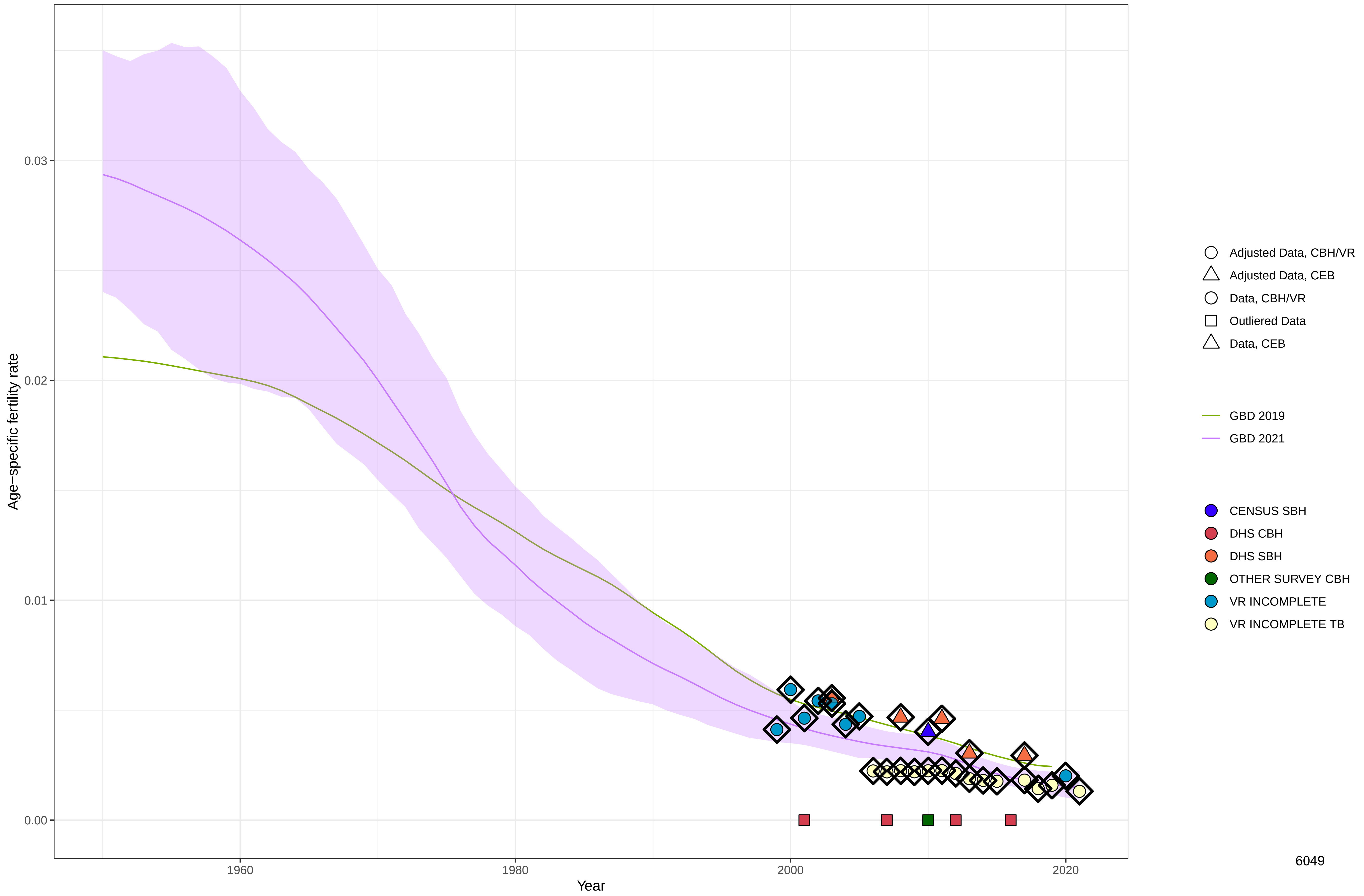

Age−specific fertility fitted trend lines, 1950−2021: Davao de Oro, Ages: 50−54
Data points enclosed with a diamond are reference sources

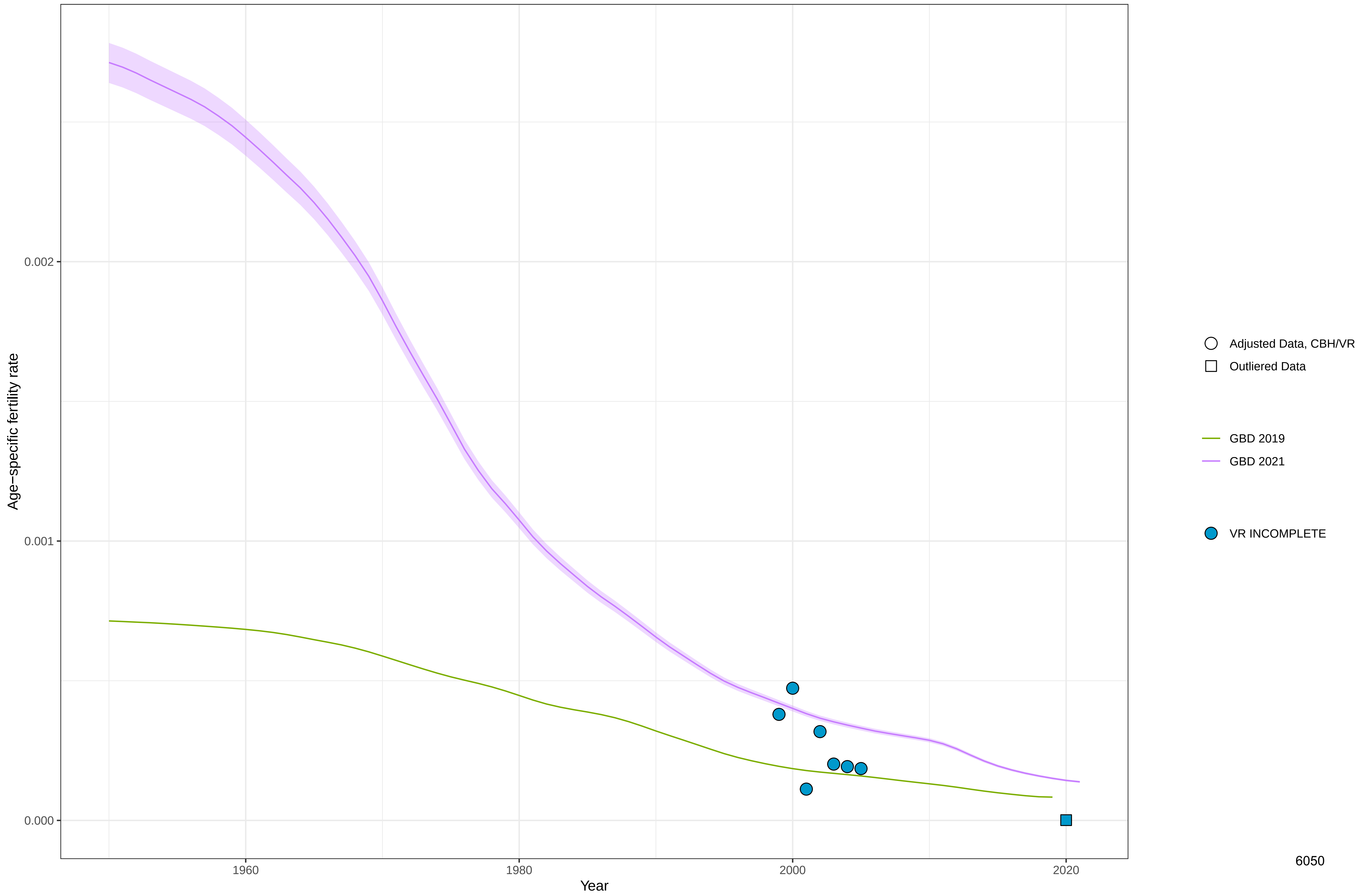

Total fertility rate fitted trend lines, 1950−2021: Davao de Oro TFR
Data points enclosed with a diamond are reference sources

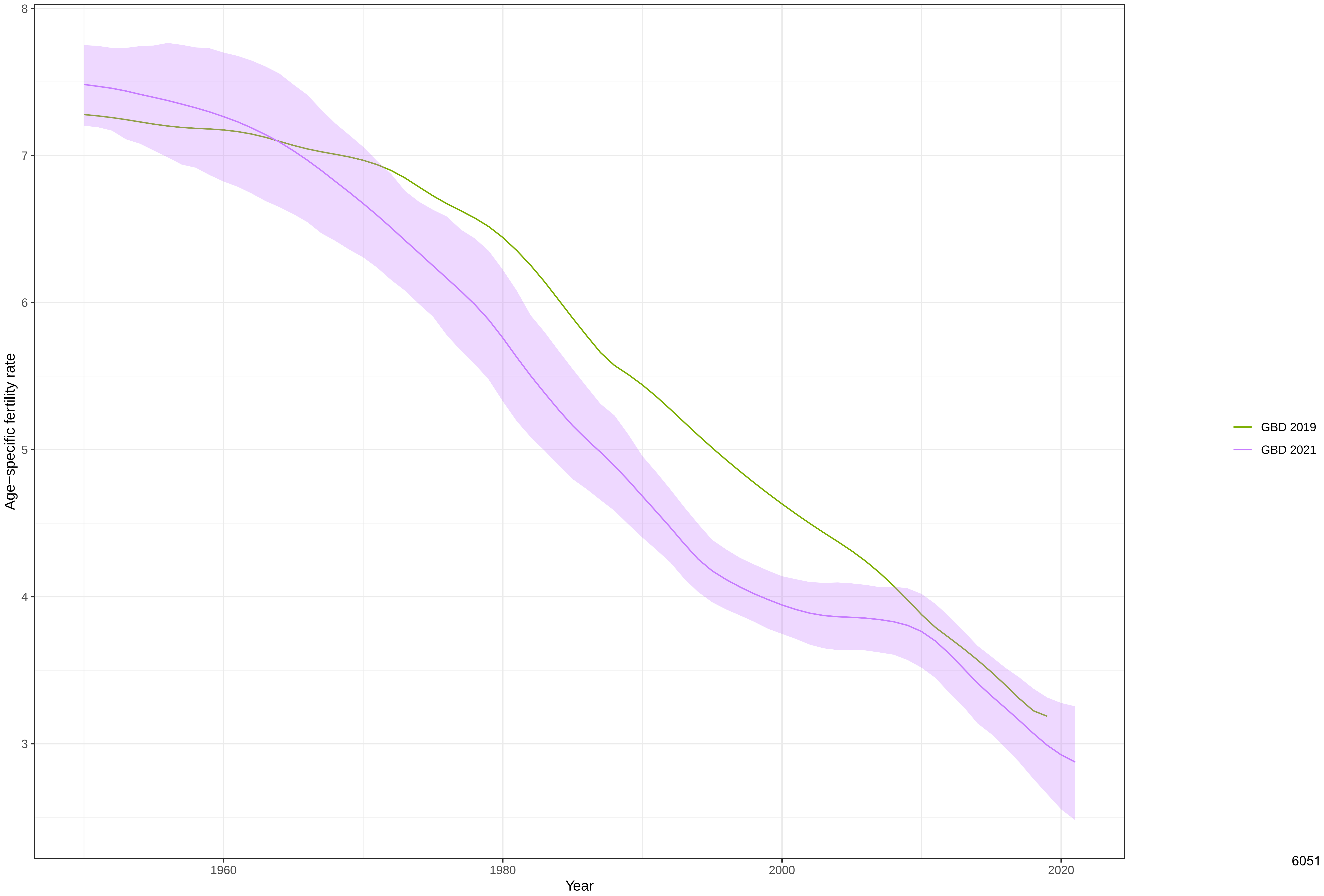

Age−specific fertility fitted trend lines, 1950−2021: Davao Del Norte, Ages: 10−14
Data points enclosed with a diamond are reference sources

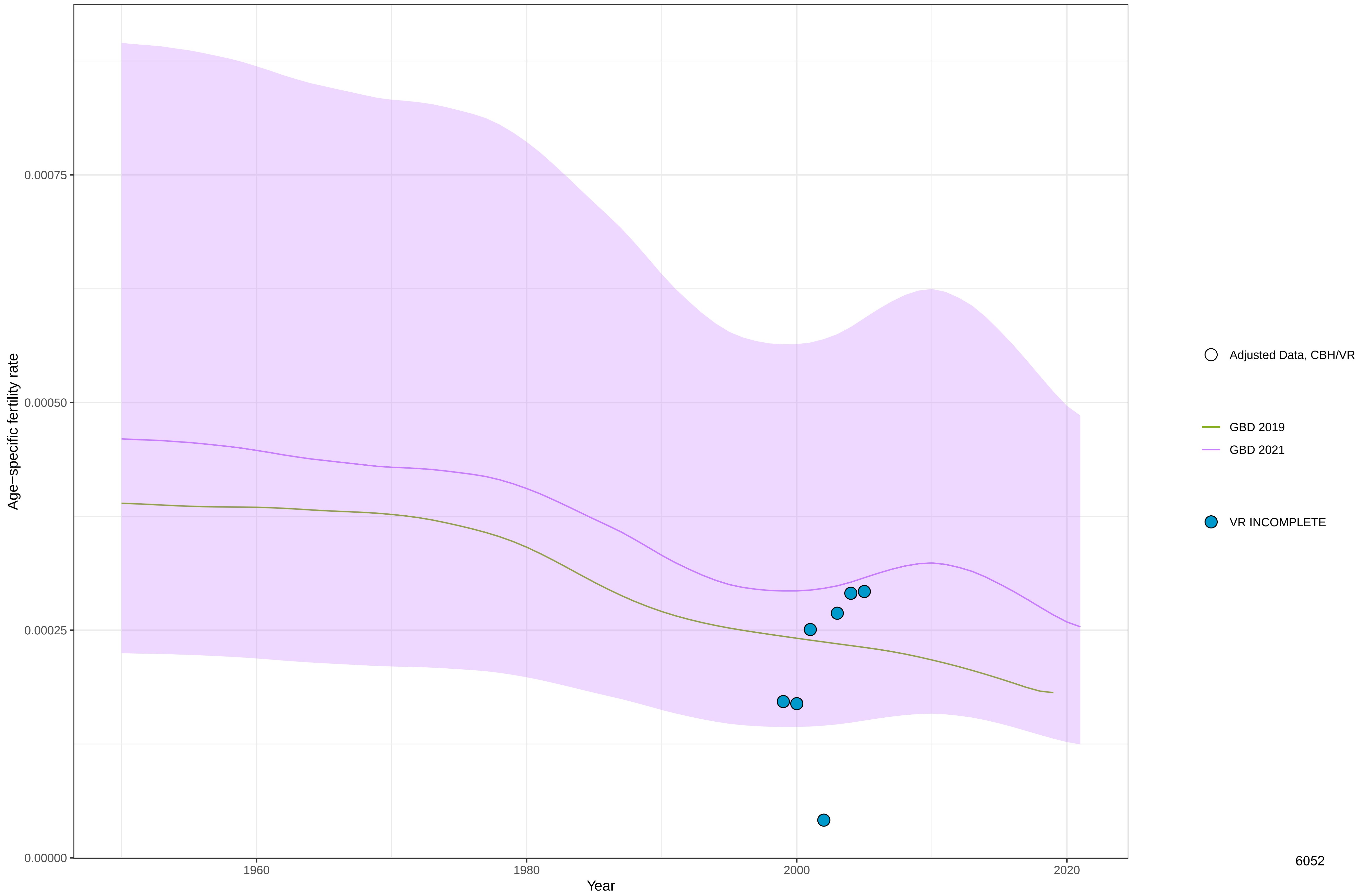

Age−specific fertility fitted trend lines, 1950−2021: Davao Del Norte, Ages: 15−19
Data points enclosed with a diamond are reference sources

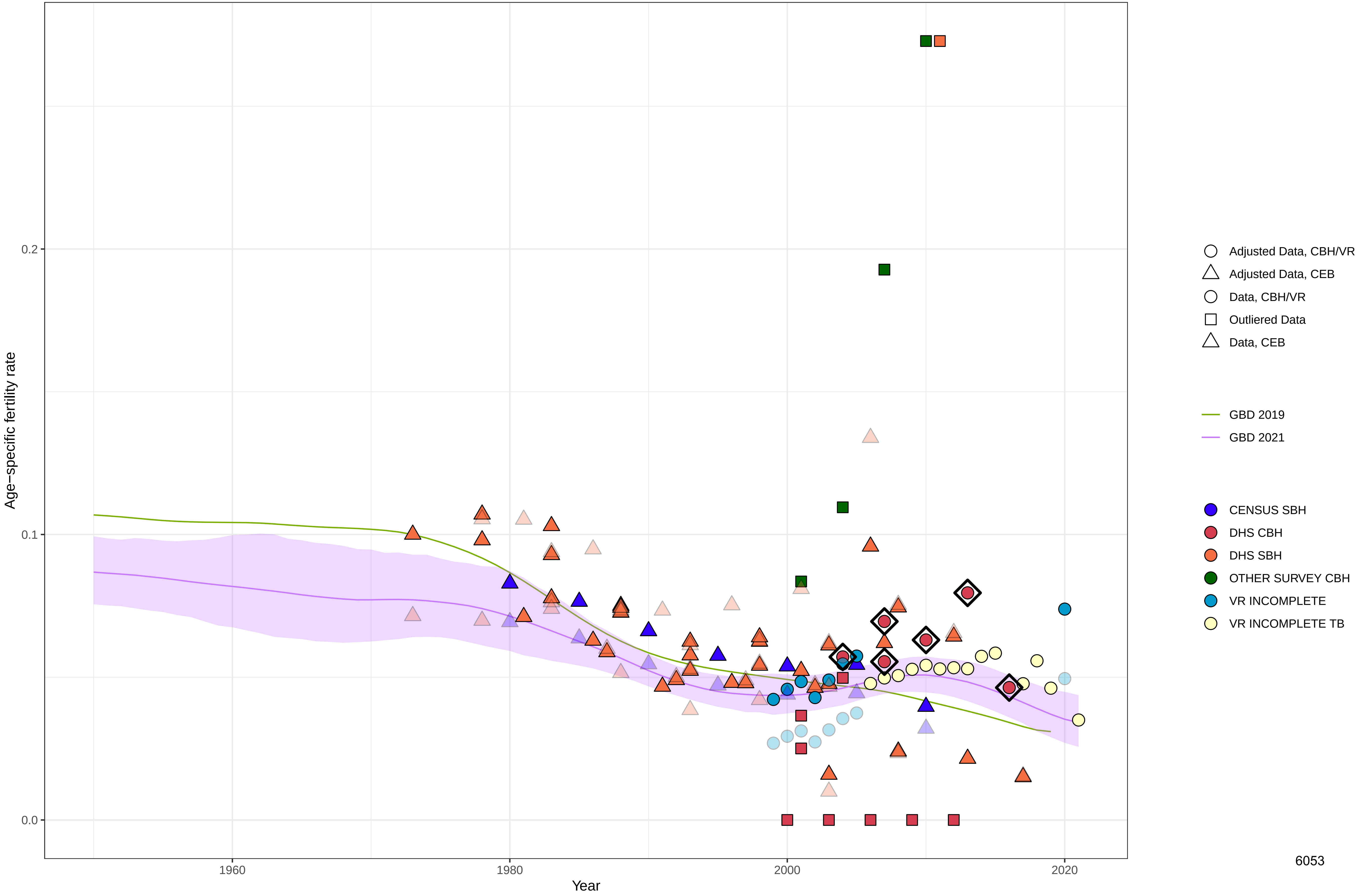

Age−specific fertility fitted trend lines, 1950−2021: Davao Del Norte, Ages: 20−24
Data points enclosed with a diamond are reference sources

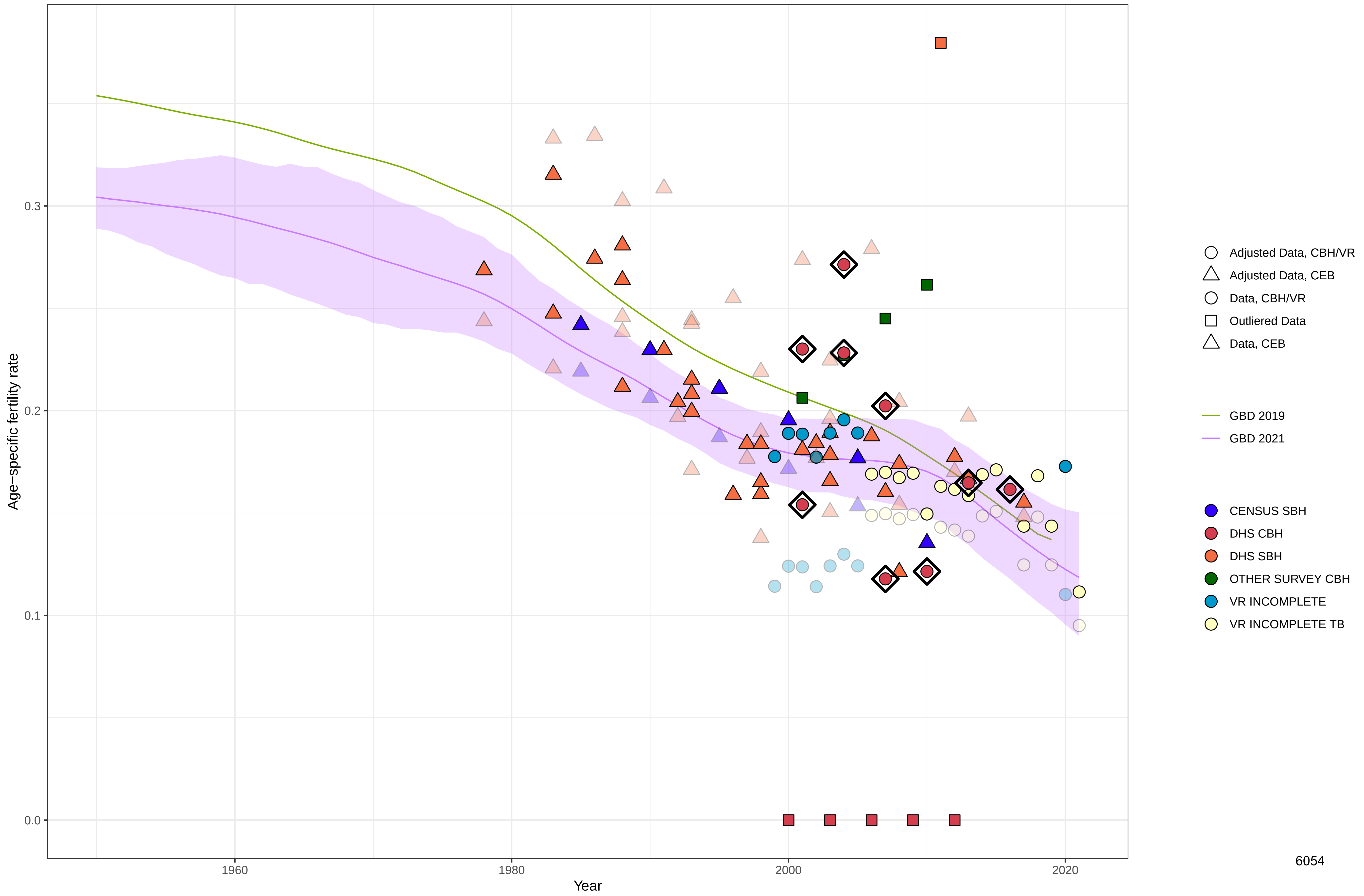

Age−specific fertility fitted trend lines, 1950−2021: Davao Del Norte, Ages: 25−29
Data points enclosed with a diamond are reference sources

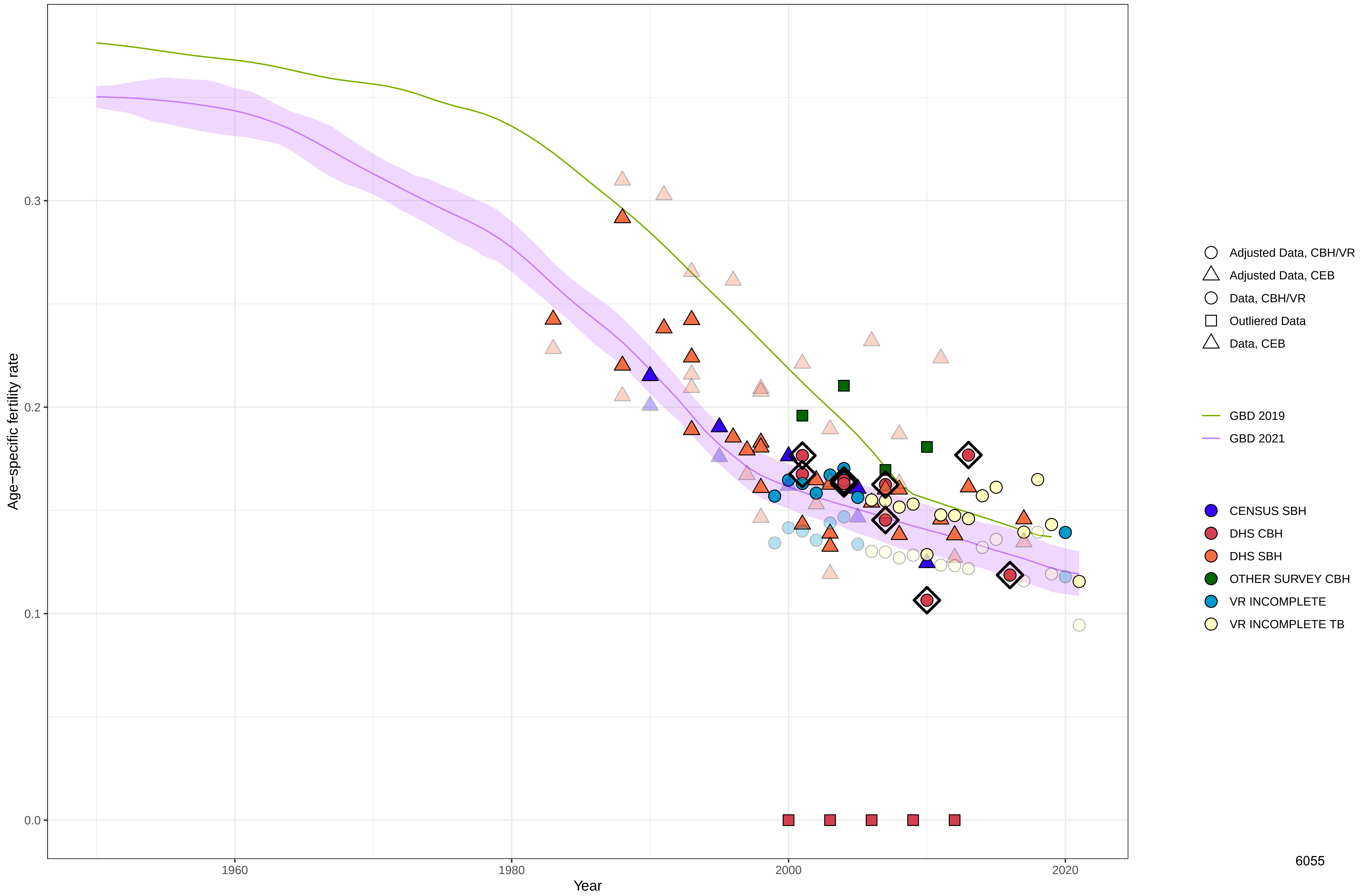

Age−specific fertility fitted trend lines, 1950−2021: Davao Del Norte, Ages: 30−34
Data points enclosed with a diamond are reference sources

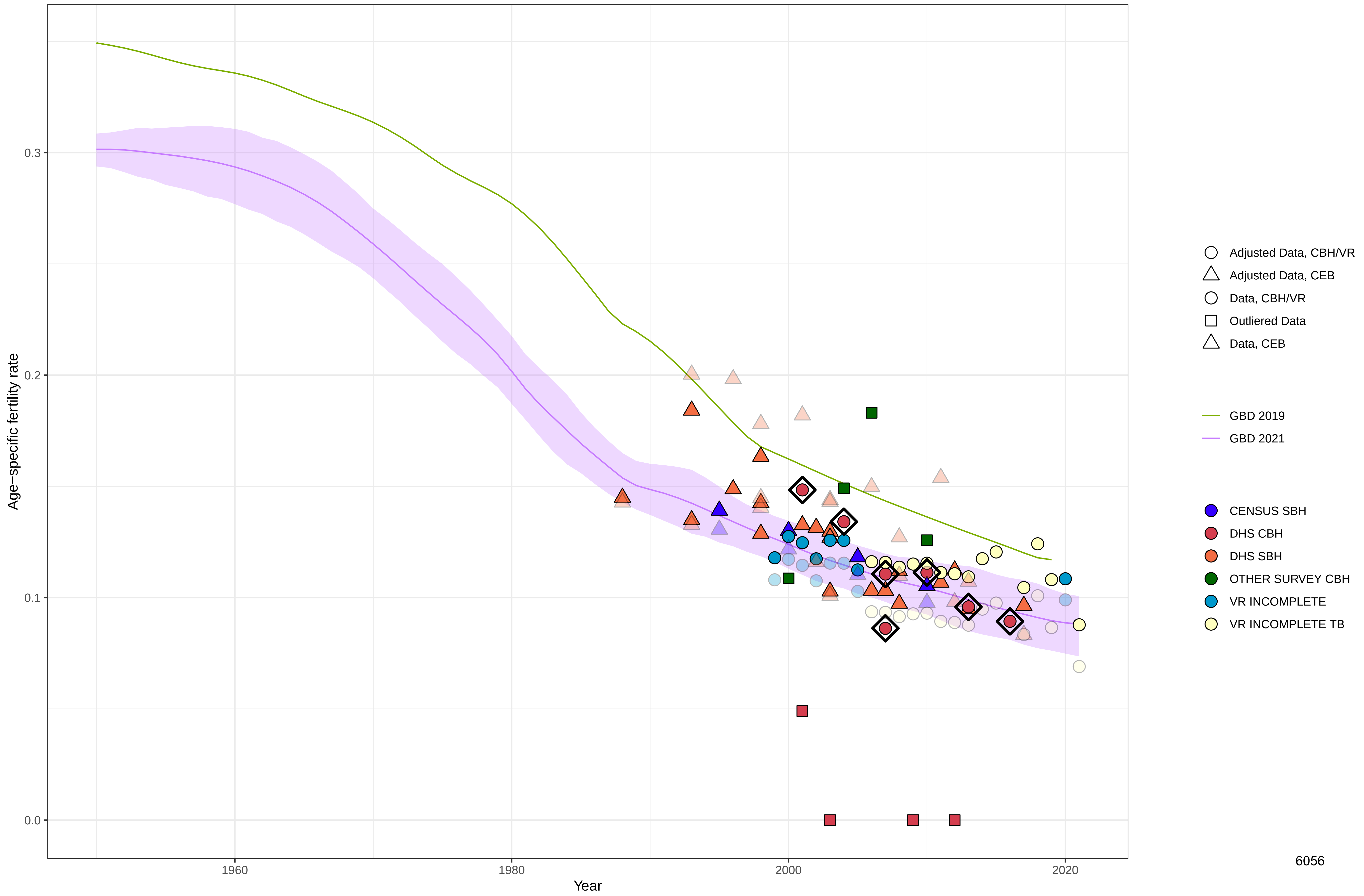

Age−specific fertility fitted trend lines, 1950−2021: Davao Del Norte, Ages: 35−39
Data points enclosed with a diamond are reference sources

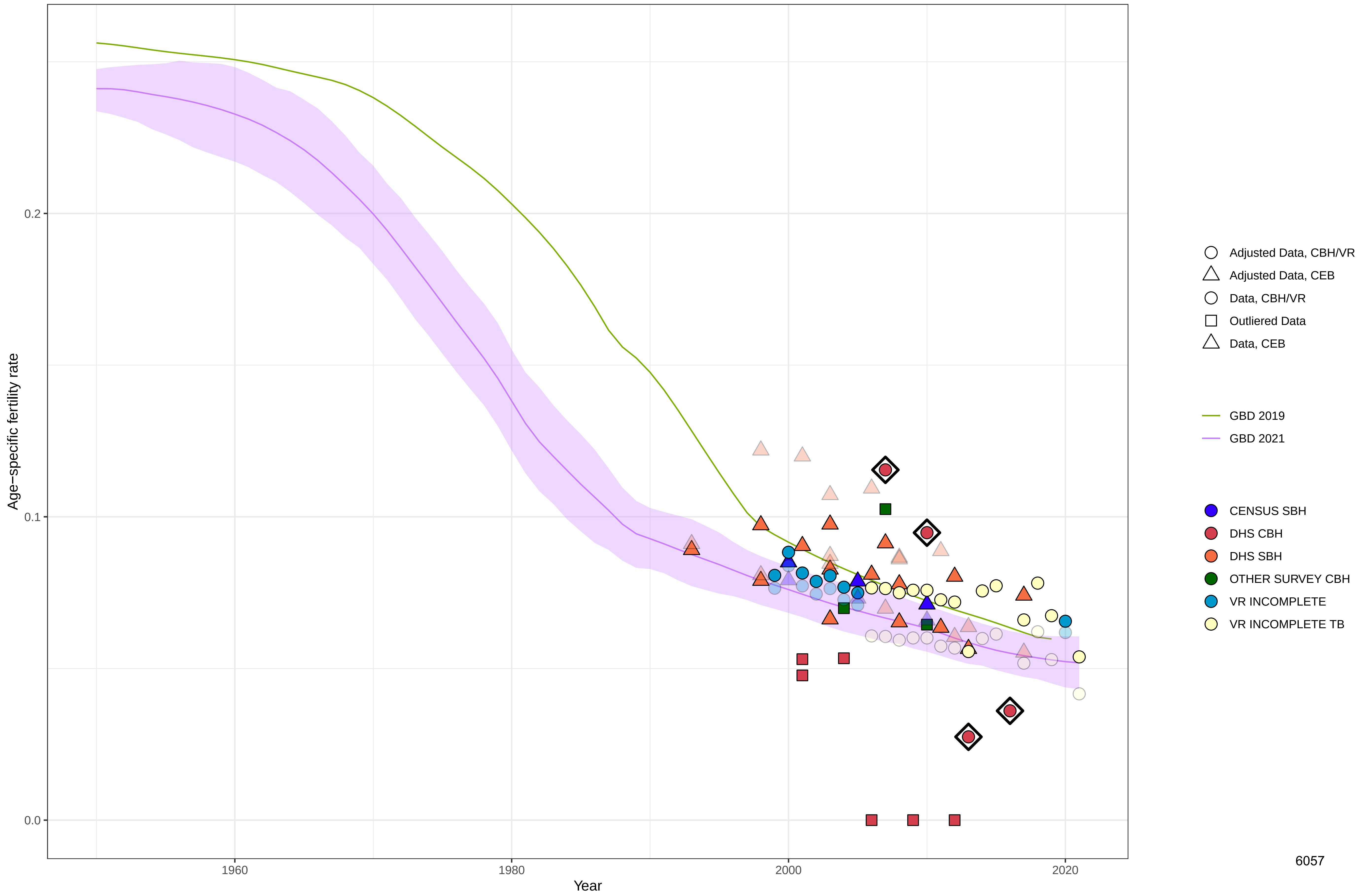

Age−specific fertility fitted trend lines, 1950−2021: Davao Del Norte, Ages: 40−44
Data points enclosed with a diamond are reference sources

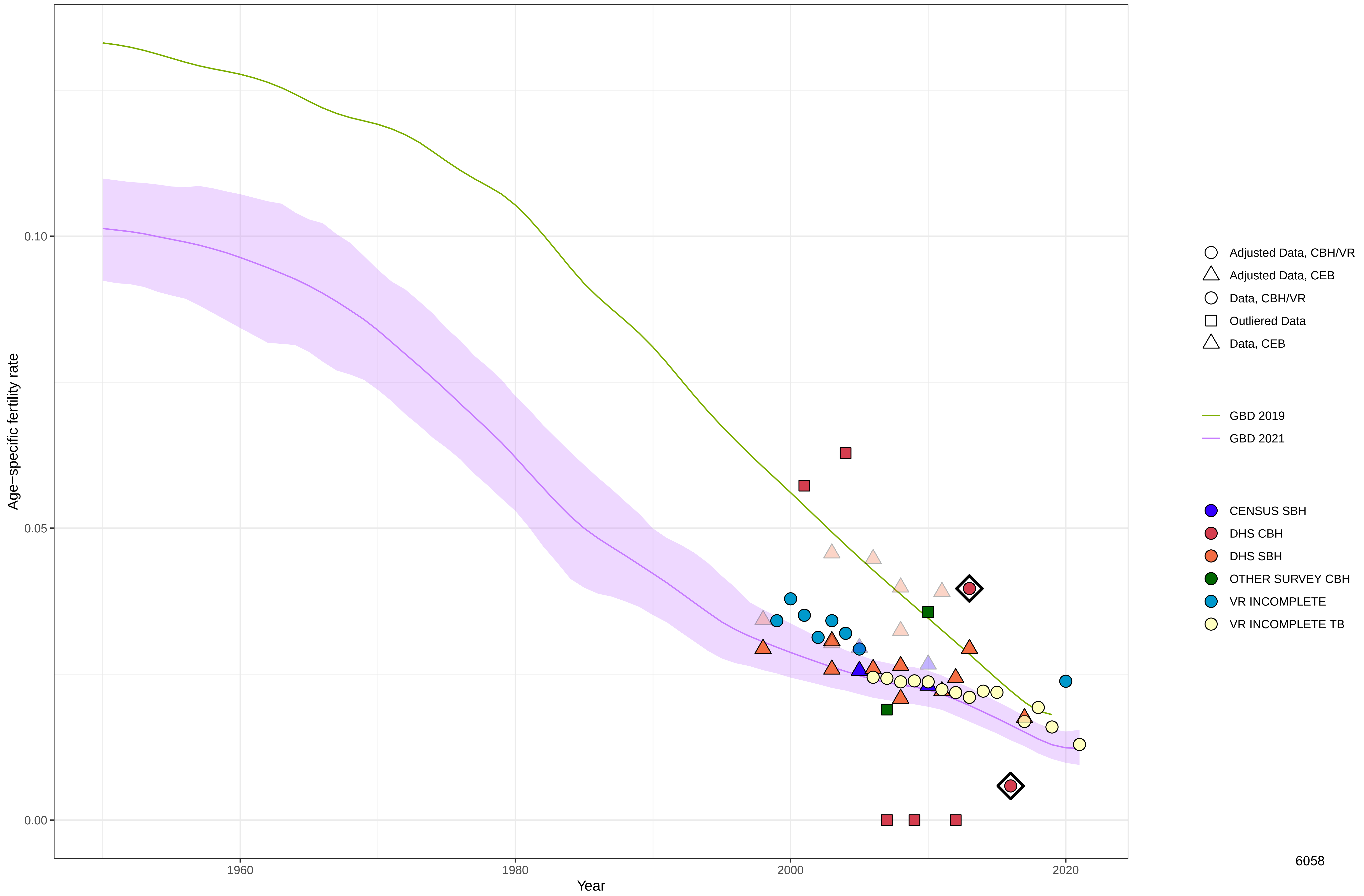

Age−specific fertility fitted trend lines, 1950−2021: Davao Del Norte, Ages: 45−49
Data points enclosed with a diamond are reference sources

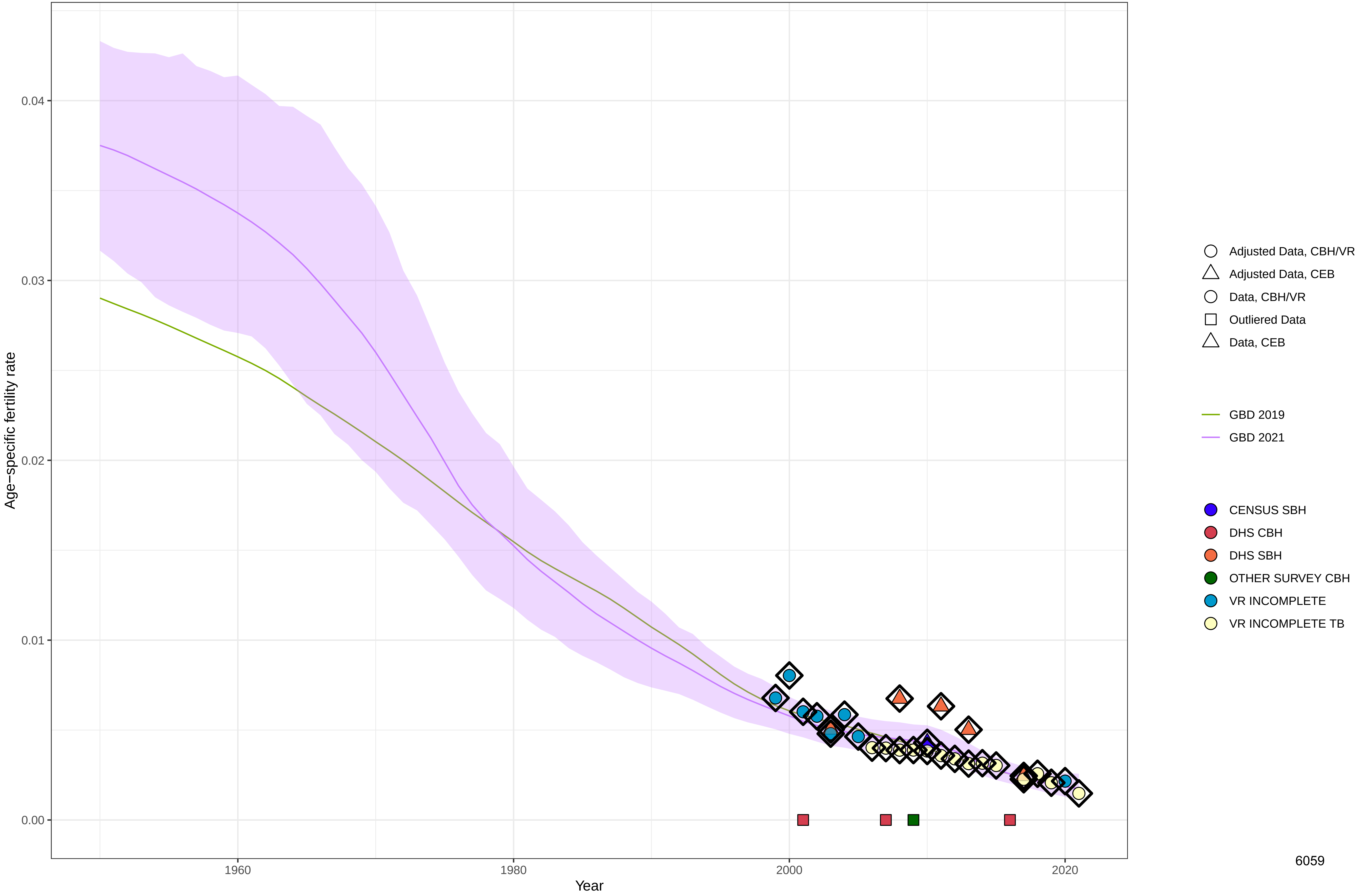

Age−specific fertility fitted trend lines, 1950−2021: Davao Del Norte, Ages: 50−54
Data points enclosed with a diamond are reference sources

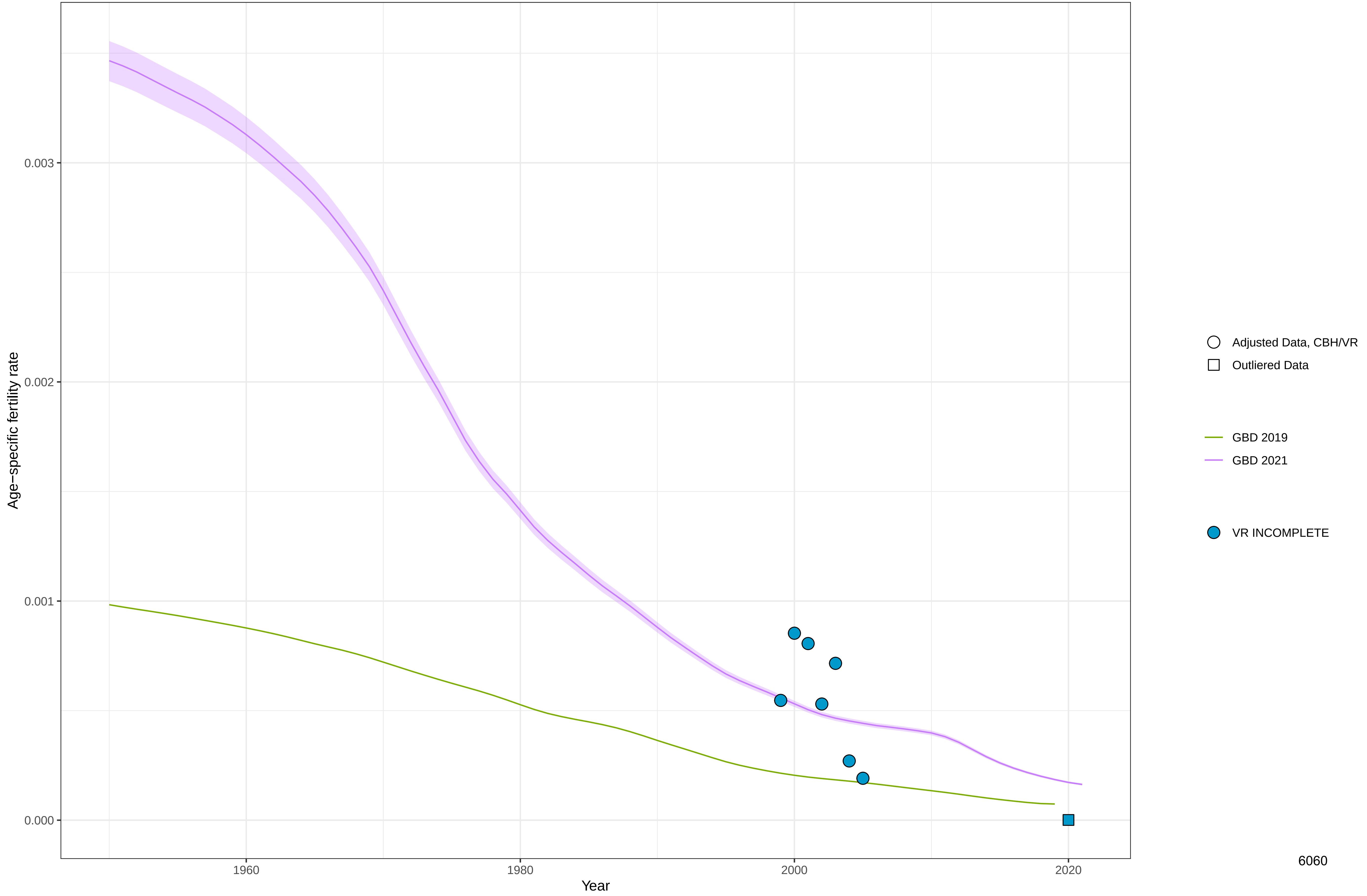

Total fertility rate fitted trend lines, 1950−2021: Davao Del Norte TFR
Data points enclosed with a diamond are reference sources

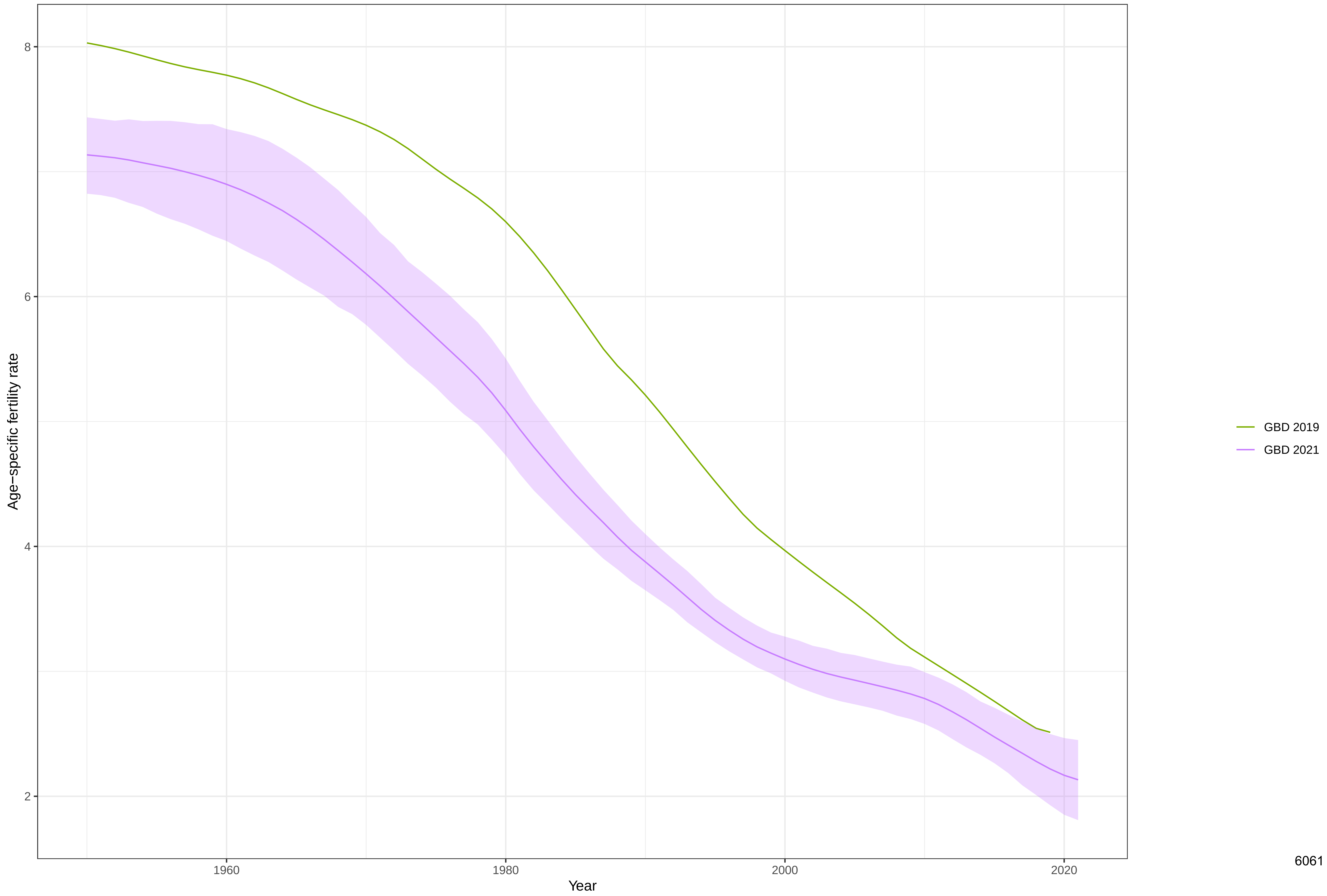

Age−specific fertility fitted trend lines, 1950−2021: Davao Del Sur, Ages: 10−14
Data points enclosed with a diamond are reference sources

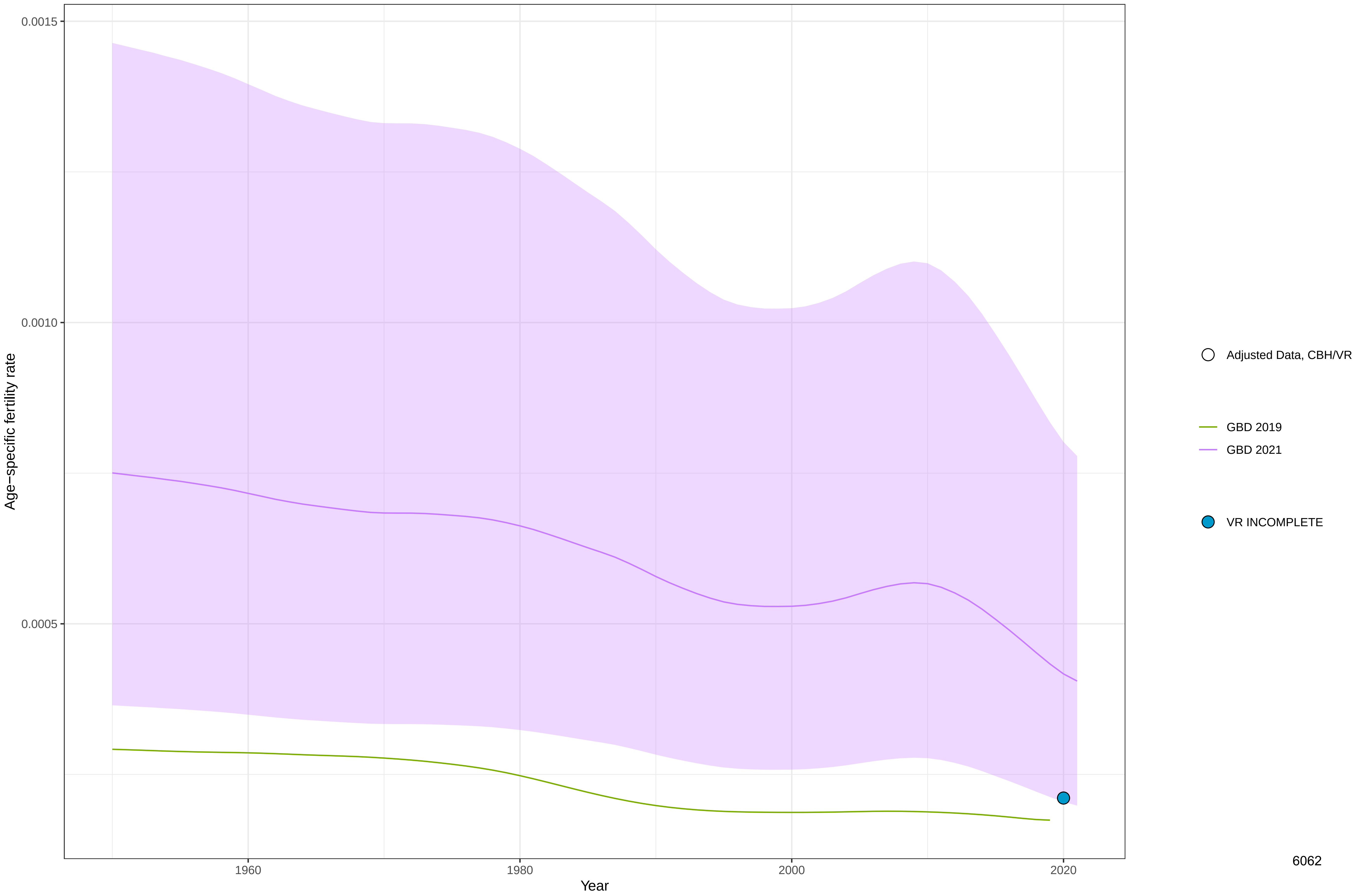

Age−specific fertility fitted trend lines, 1950−2021: Davao Del Sur, Ages: 15−19
Data points enclosed with a diamond are reference sources

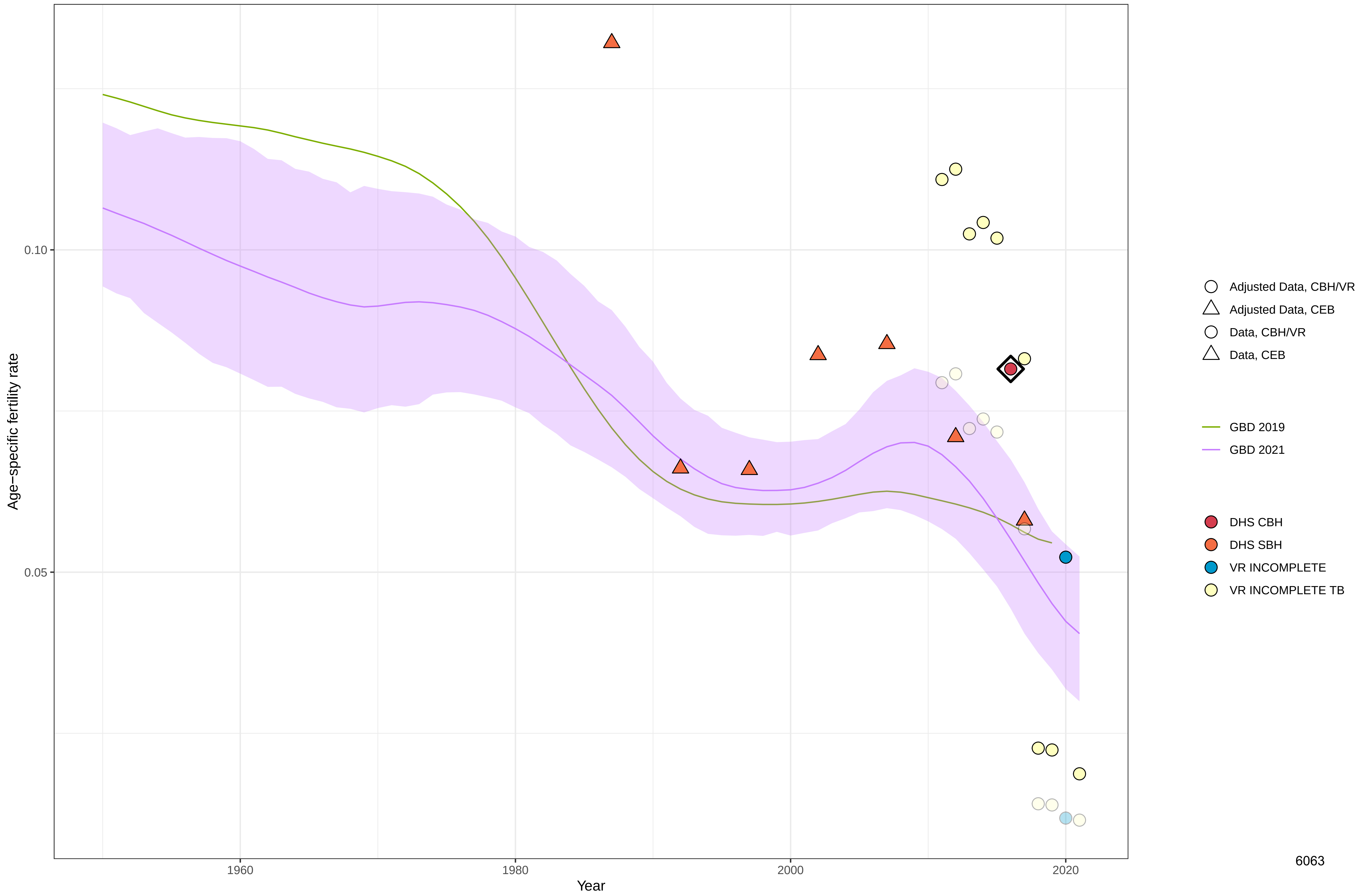

Age−specific fertility fitted trend lines, 1950−2021: Davao Del Sur, Ages: 20−24
Data points enclosed with a diamond are reference sources

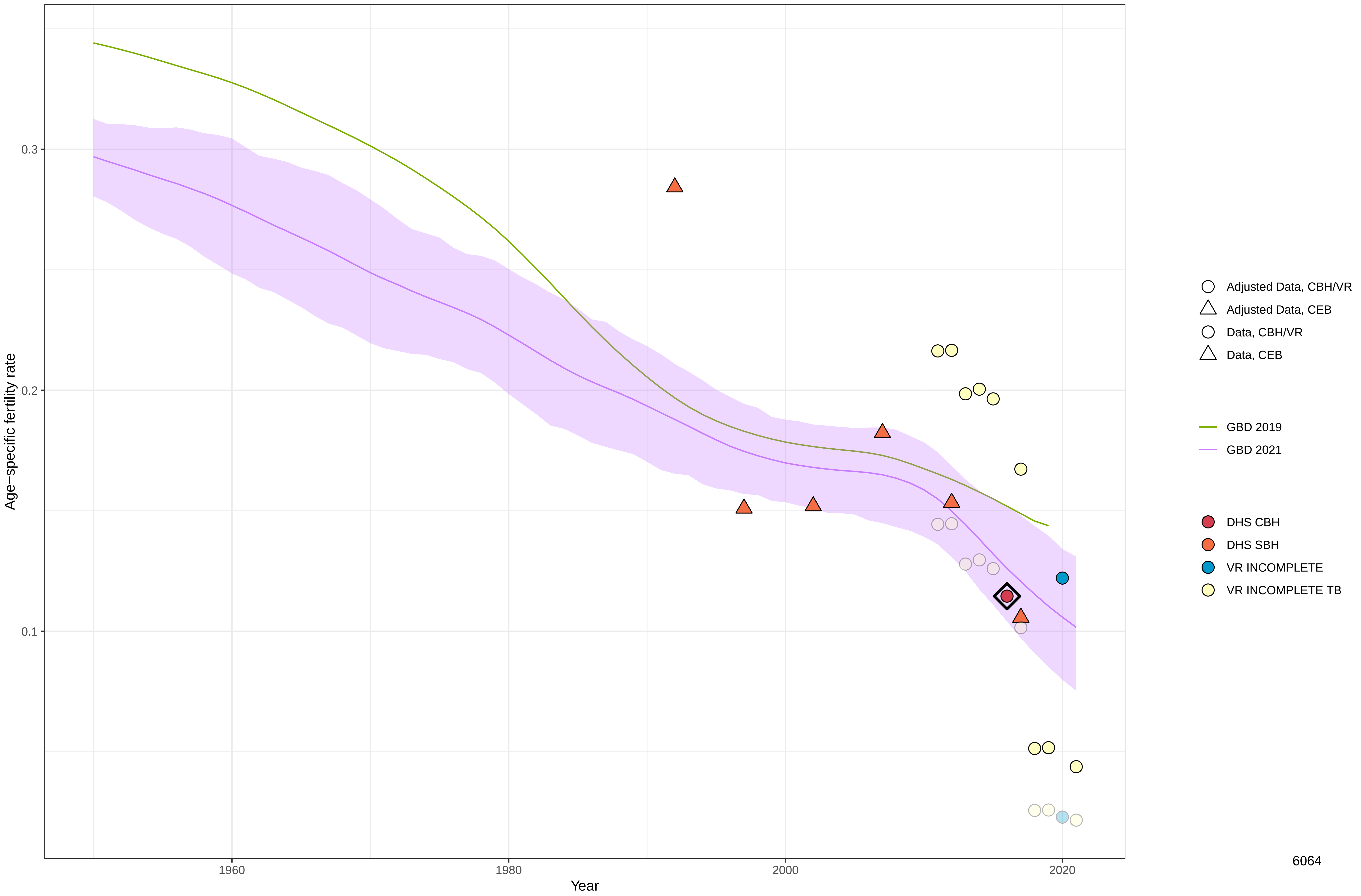

Age−specific fertility fitted trend lines, 1950−2021: Davao Del Sur, Ages: 25−29
Data points enclosed with a diamond are reference sources

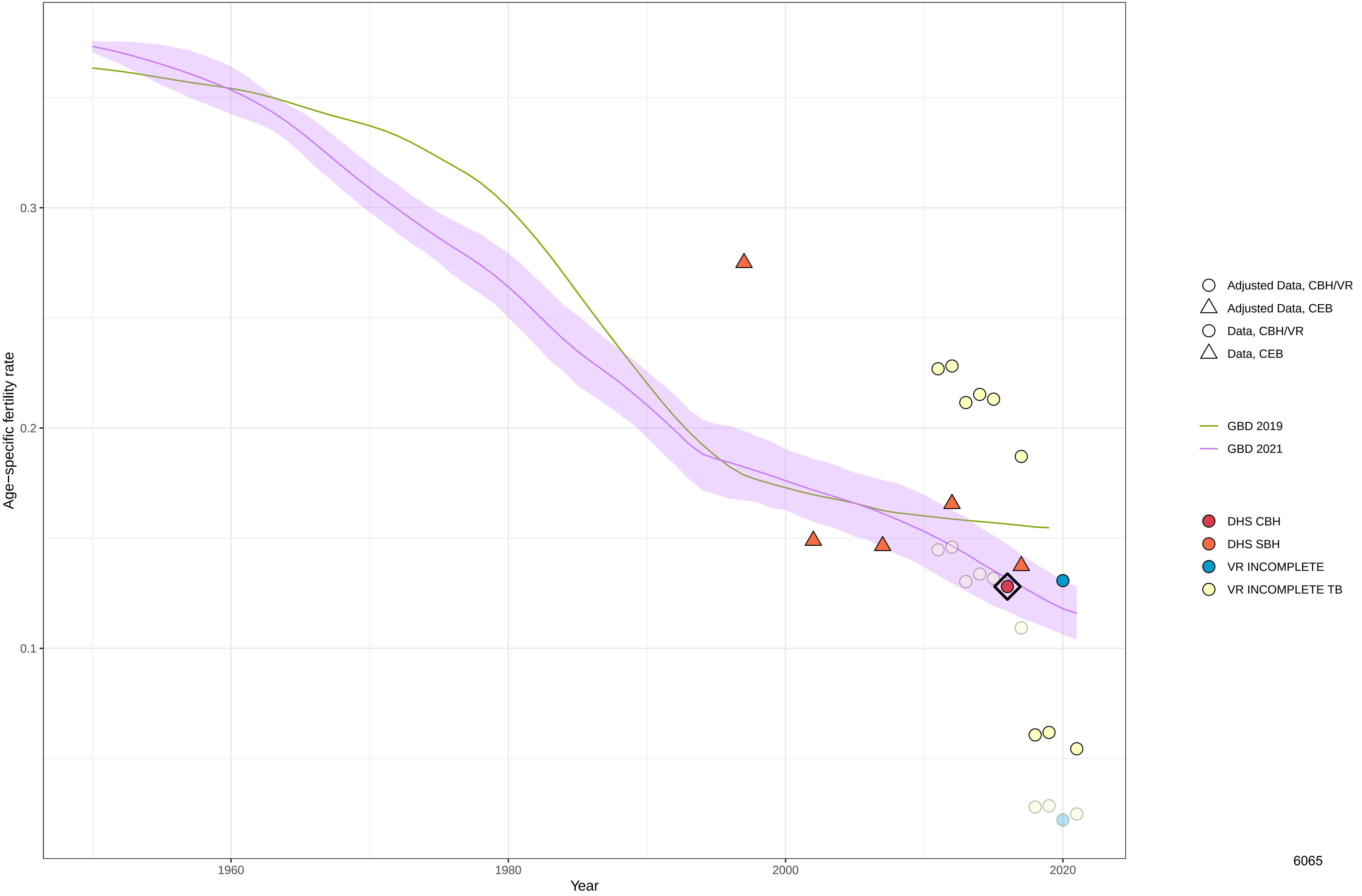

Age−specific fertility fitted trend lines, 1950−2021: Davao Del Sur, Ages: 30−34
Data points enclosed with a diamond are reference sources

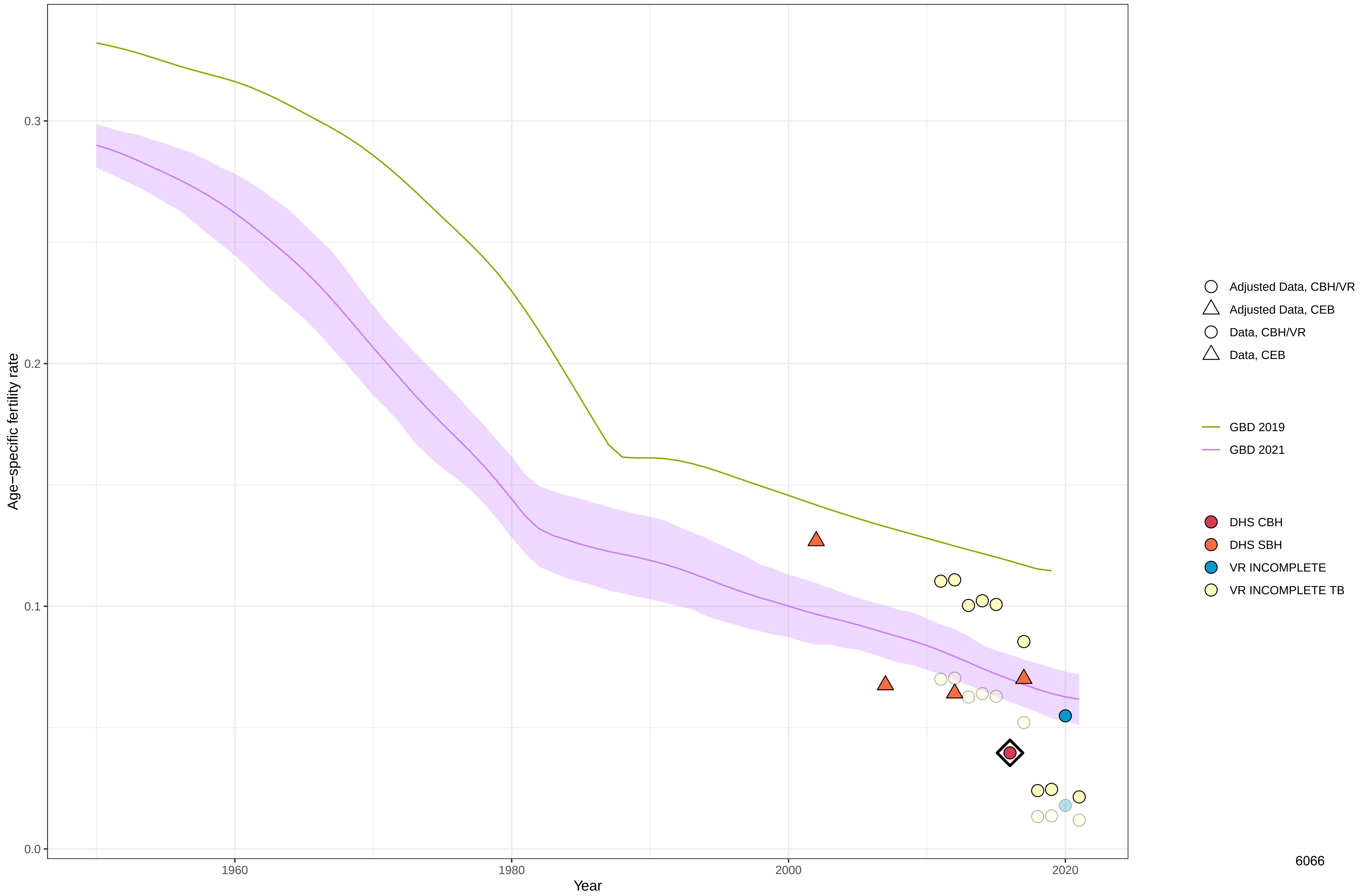

Age−specific fertility fitted trend lines, 1950−2021: Davao Del Sur, Ages: 35−39
Data points enclosed with a diamond are reference sources

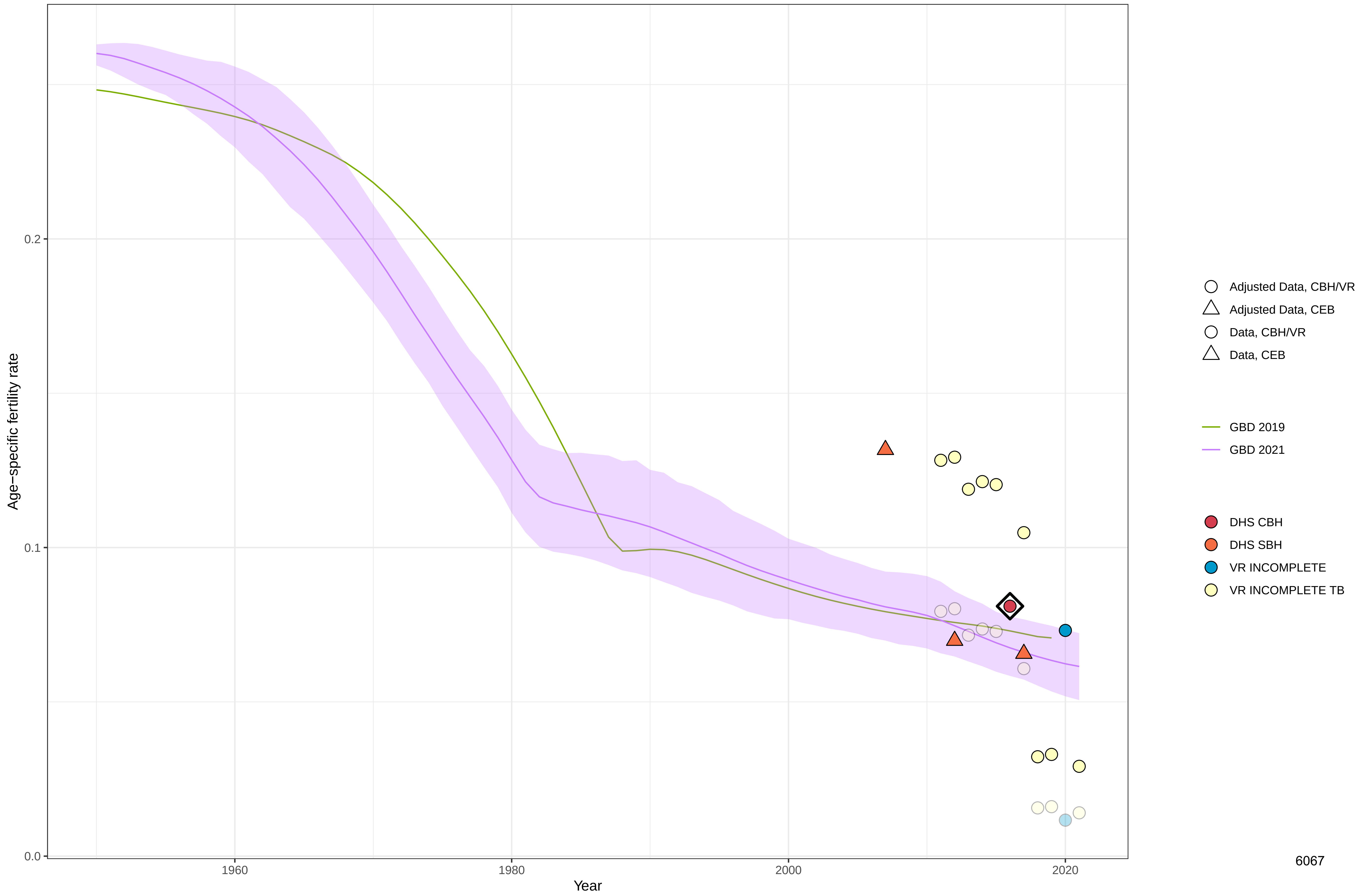

Age−specific fertility fitted trend lines, 1950−2021: Davao Del Sur, Ages: 40−44
Data points enclosed with a diamond are reference sources

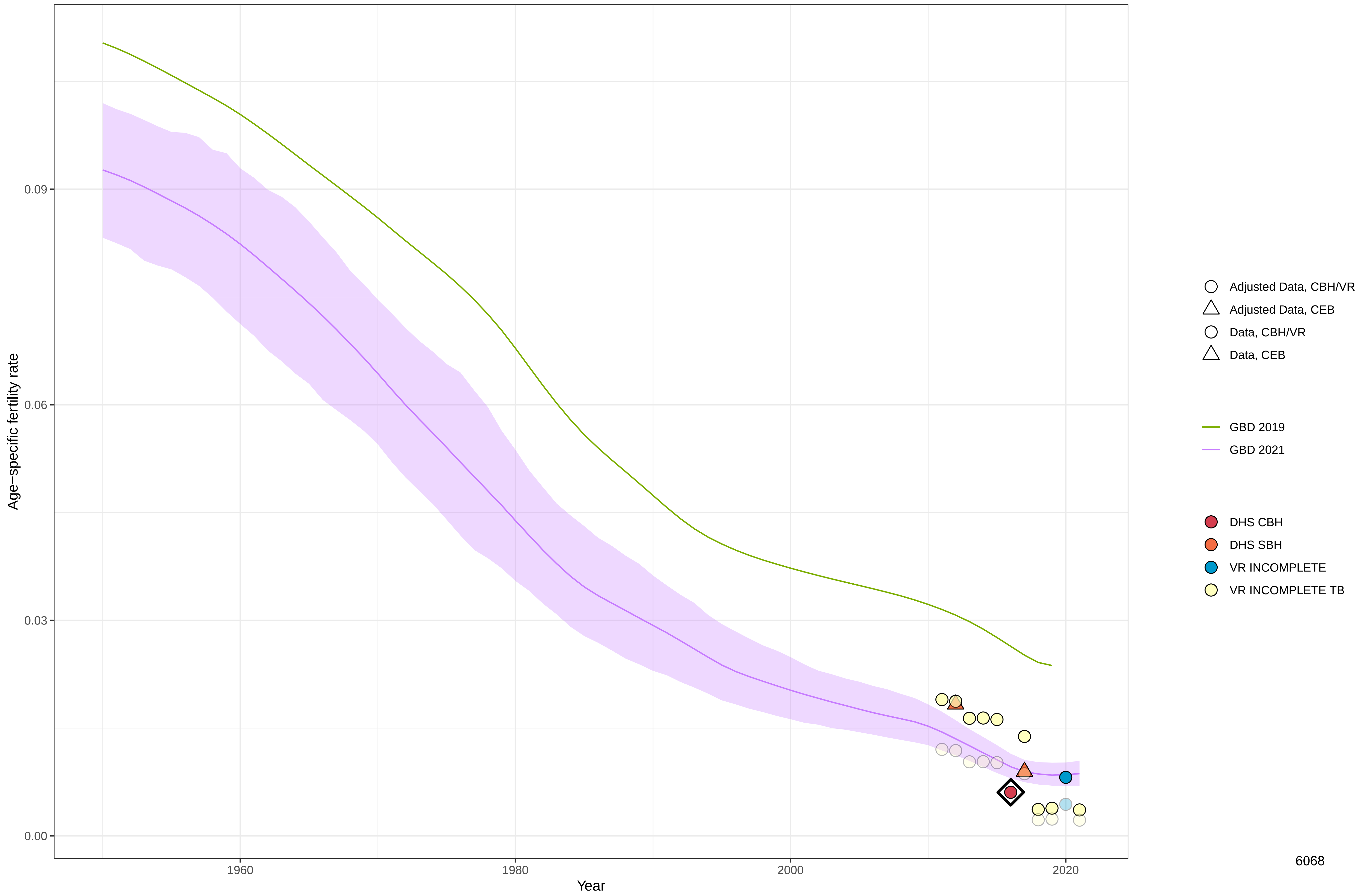

Age−specific fertility fitted trend lines, 1950−2021: Davao Del Sur, Ages: 45−49
Data points enclosed with a diamond are reference sources

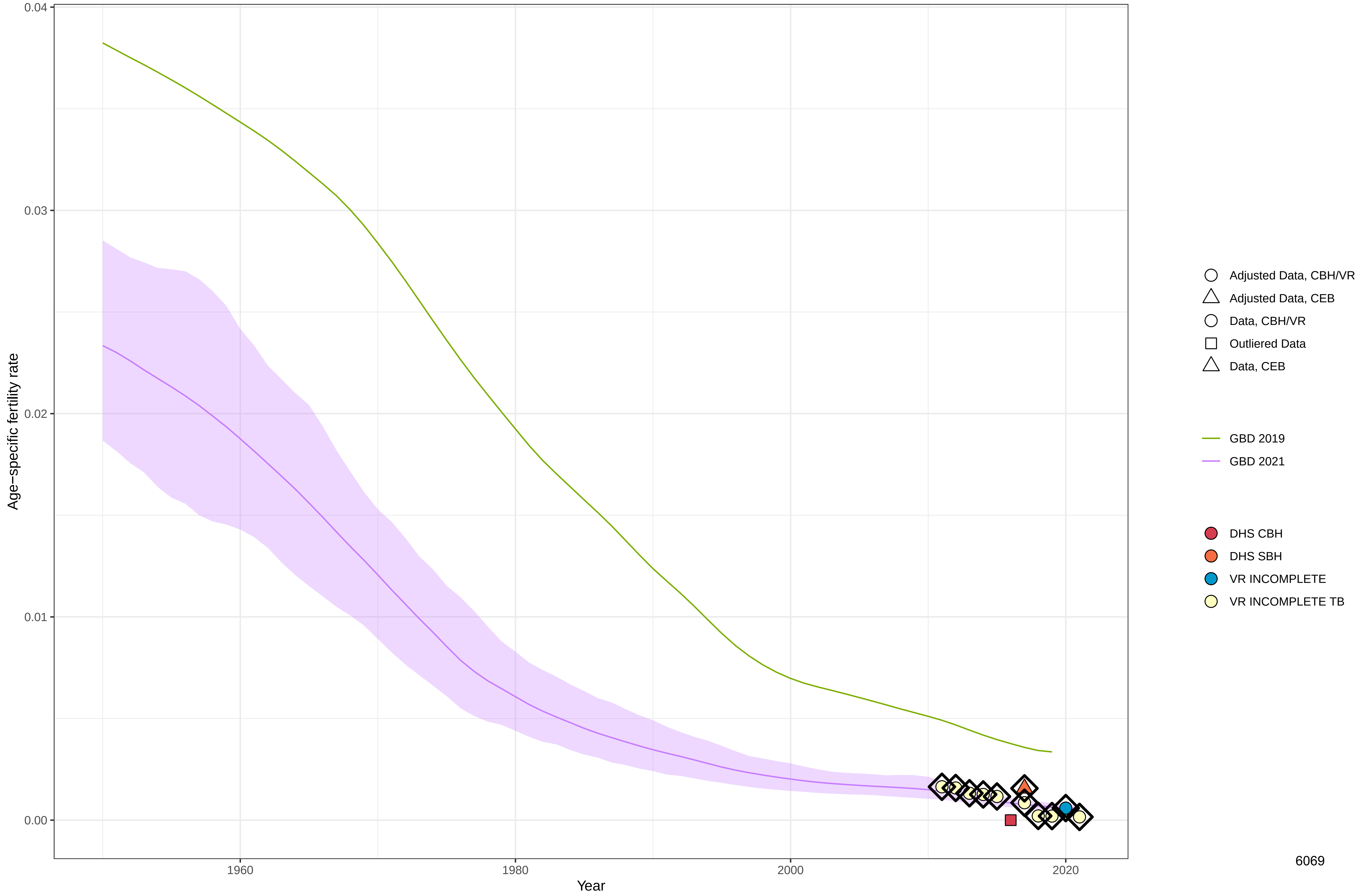

Age−specific fertility fitted trend lines, 1950−2021: Davao Del Sur, Ages: 50−54
Data points enclosed with a diamond are reference sources

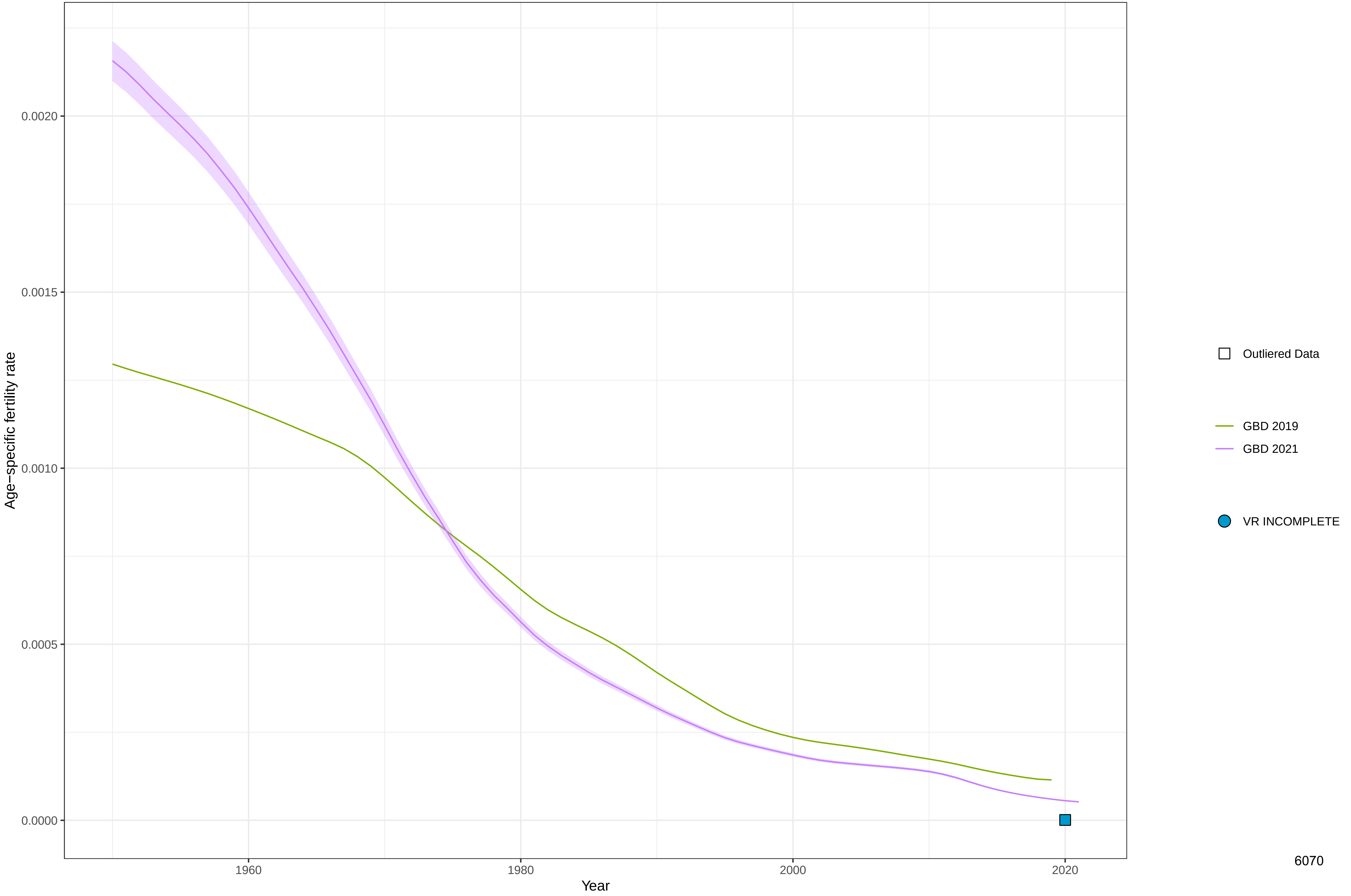

Total fertility rate fitted trend lines, 1950−2021: Davao Del Sur TFR
Data points enclosed with a diamond are reference sources

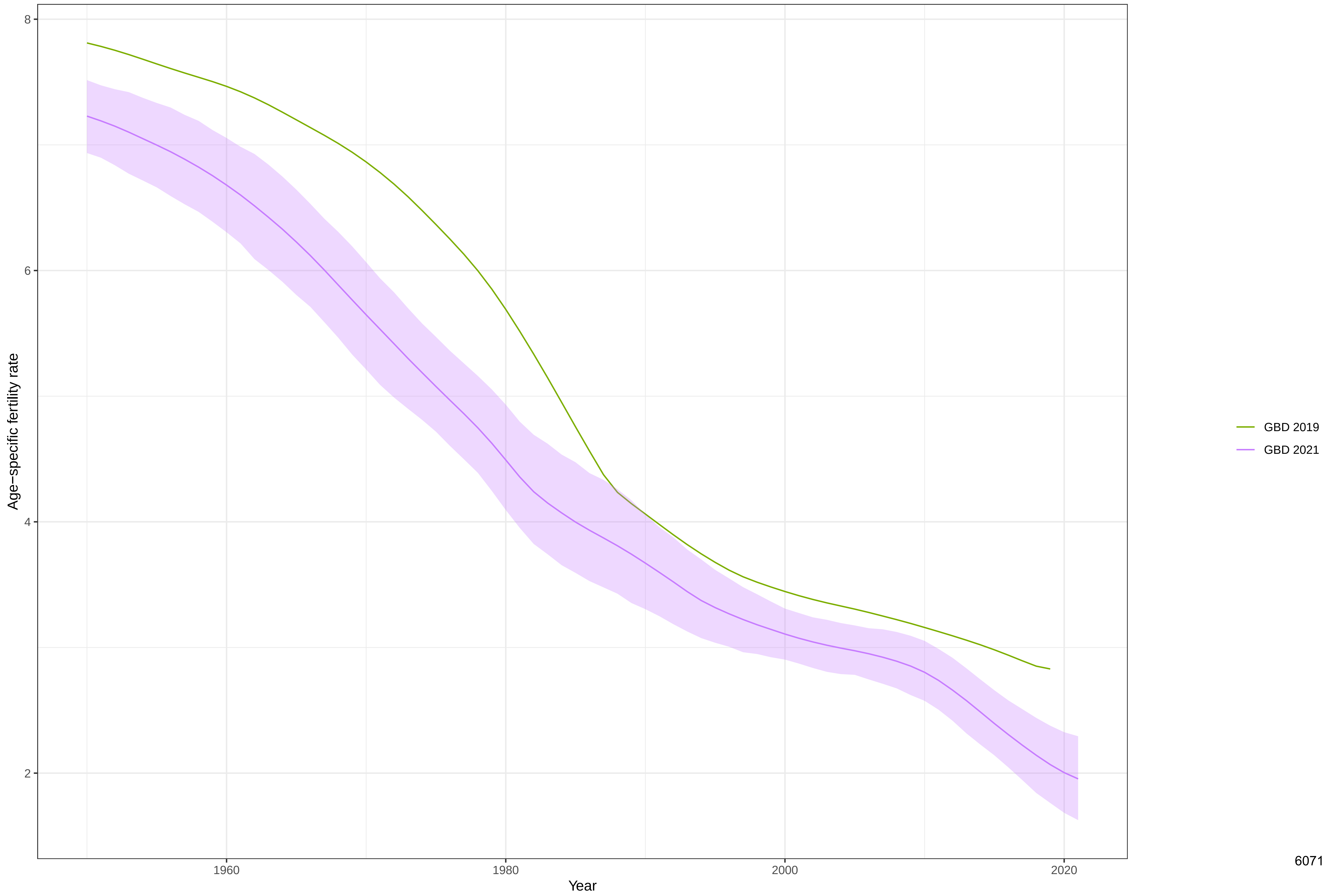

Age−specific fertility fitted trend lines, 1950−2021: Davao Occidental, Ages: 10−14
Data points enclosed with a diamond are reference sources

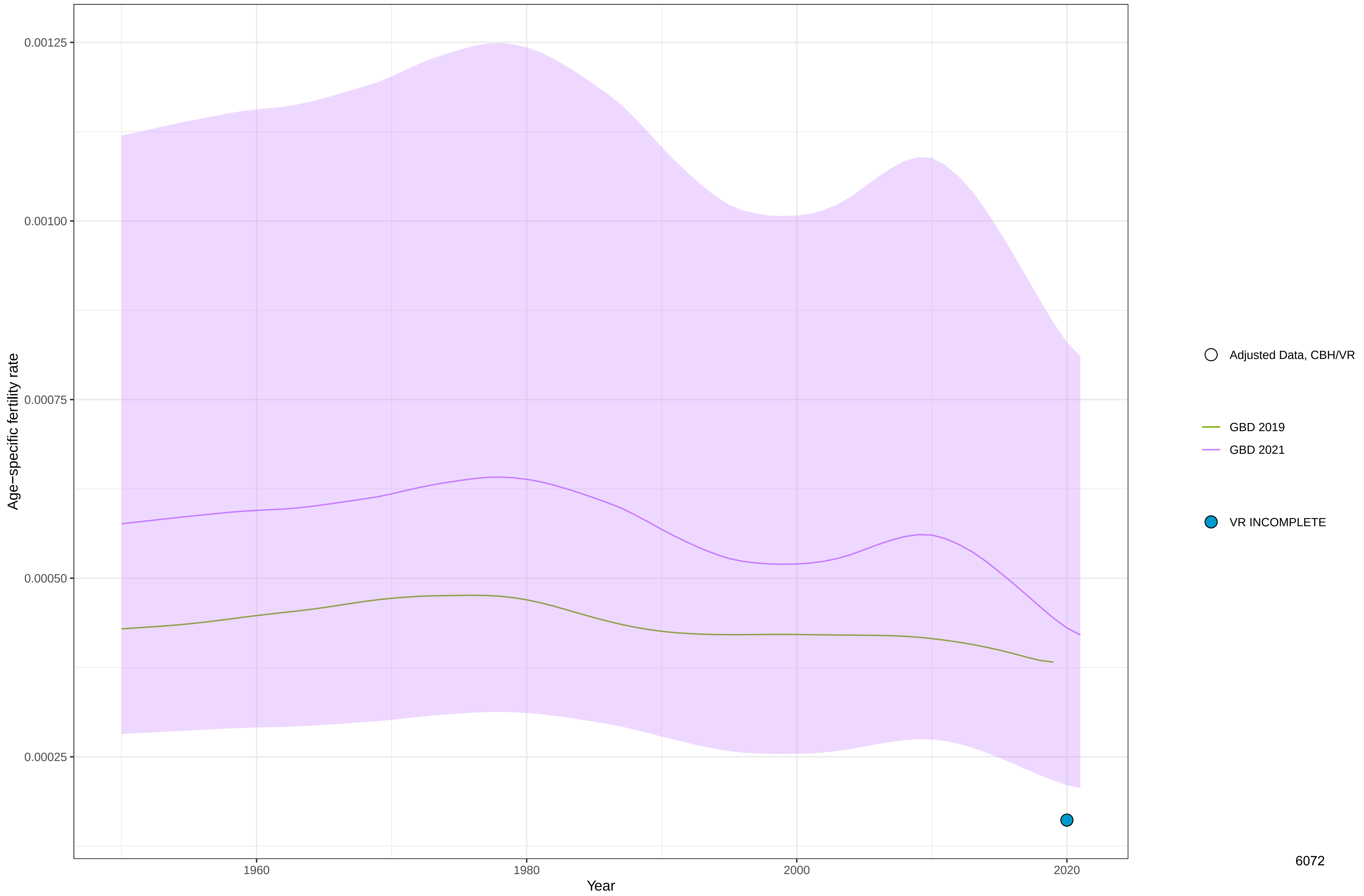

Age−specific fertility fitted trend lines, 1950−2021: Davao Occidental, Ages: 15−19
Data points enclosed with a diamond are reference sources

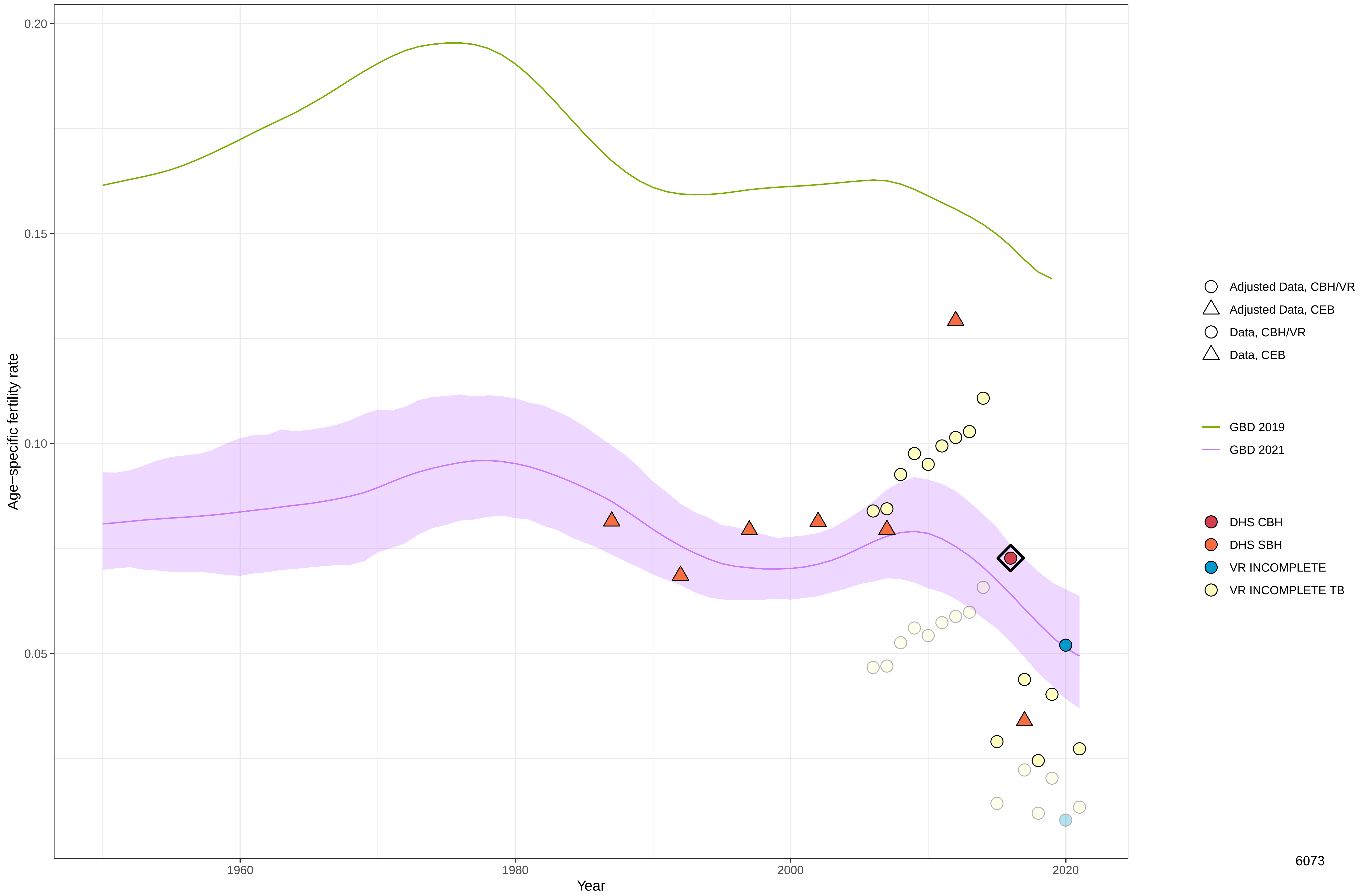

Age−specific fertility fitted trend lines, 1950−2021: Davao Occidental, Ages: 20−24
Data points enclosed with a diamond are reference sources

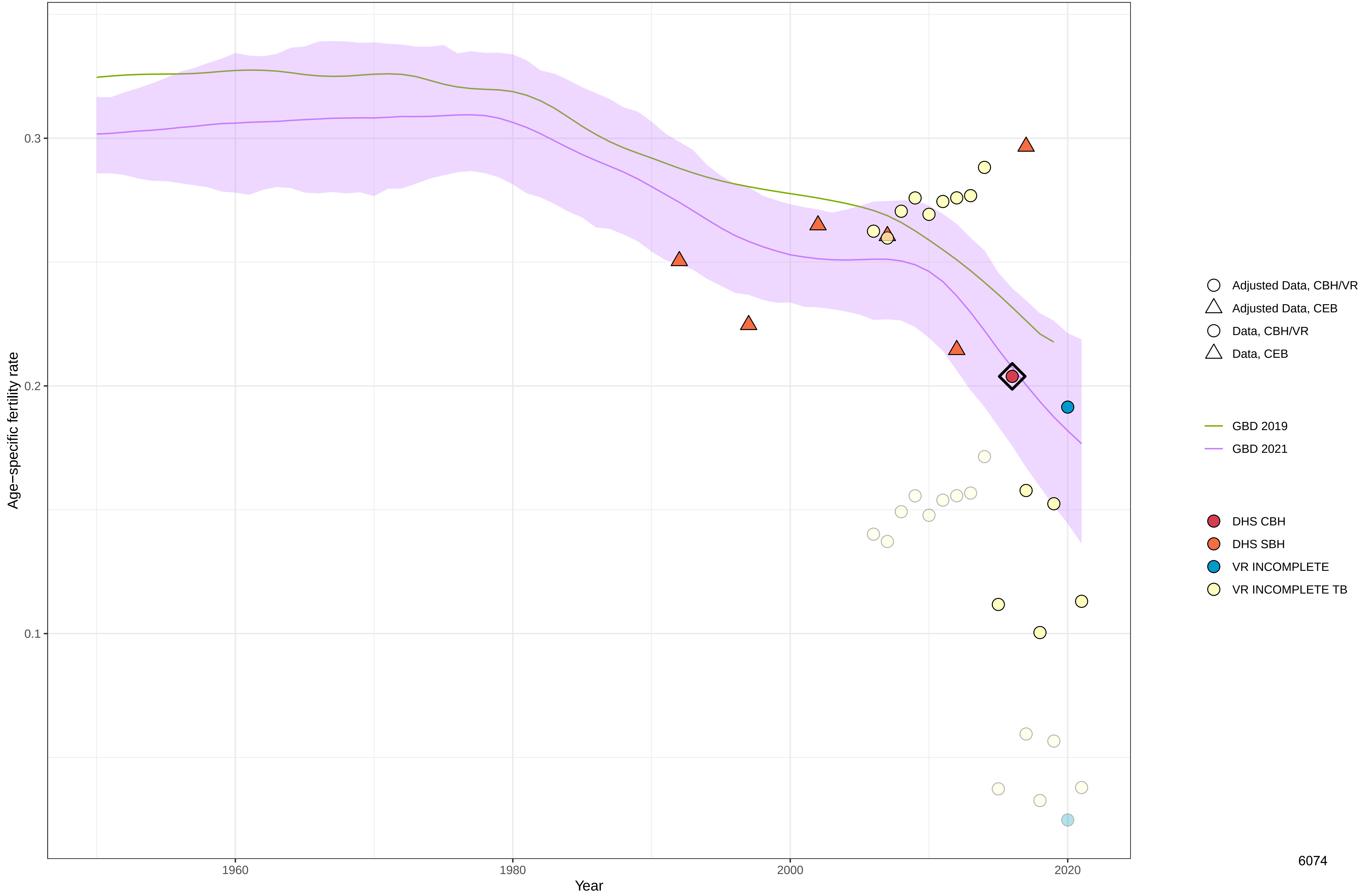

Age−specific fertility fitted trend lines, 1950−2021: Davao Occidental, Ages: 25−29
Data points enclosed with a diamond are reference sources

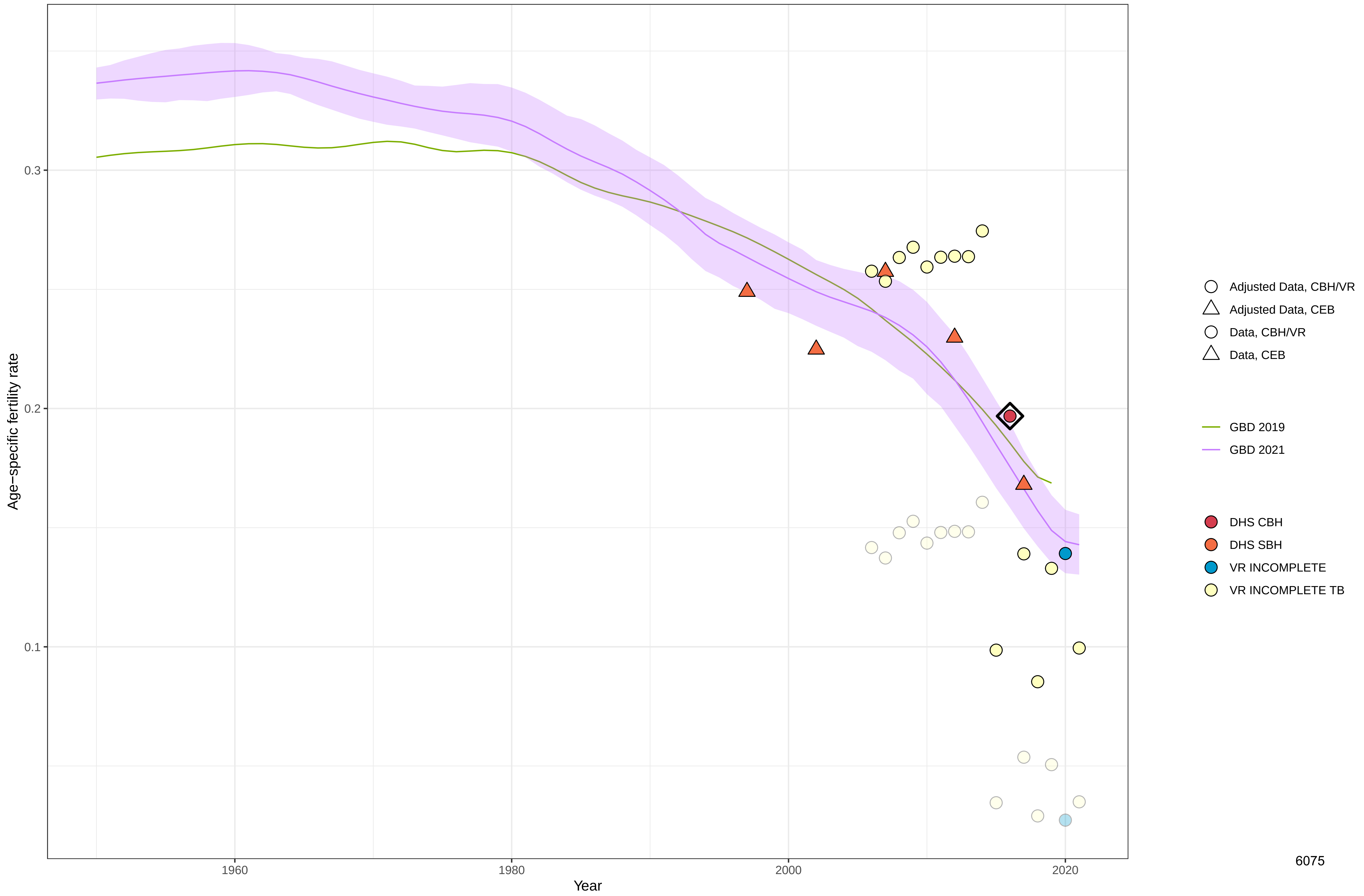

Age−specific fertility fitted trend lines, 1950−2021: Davao Occidental, Ages: 30−34
Data points enclosed with a diamond are reference sources

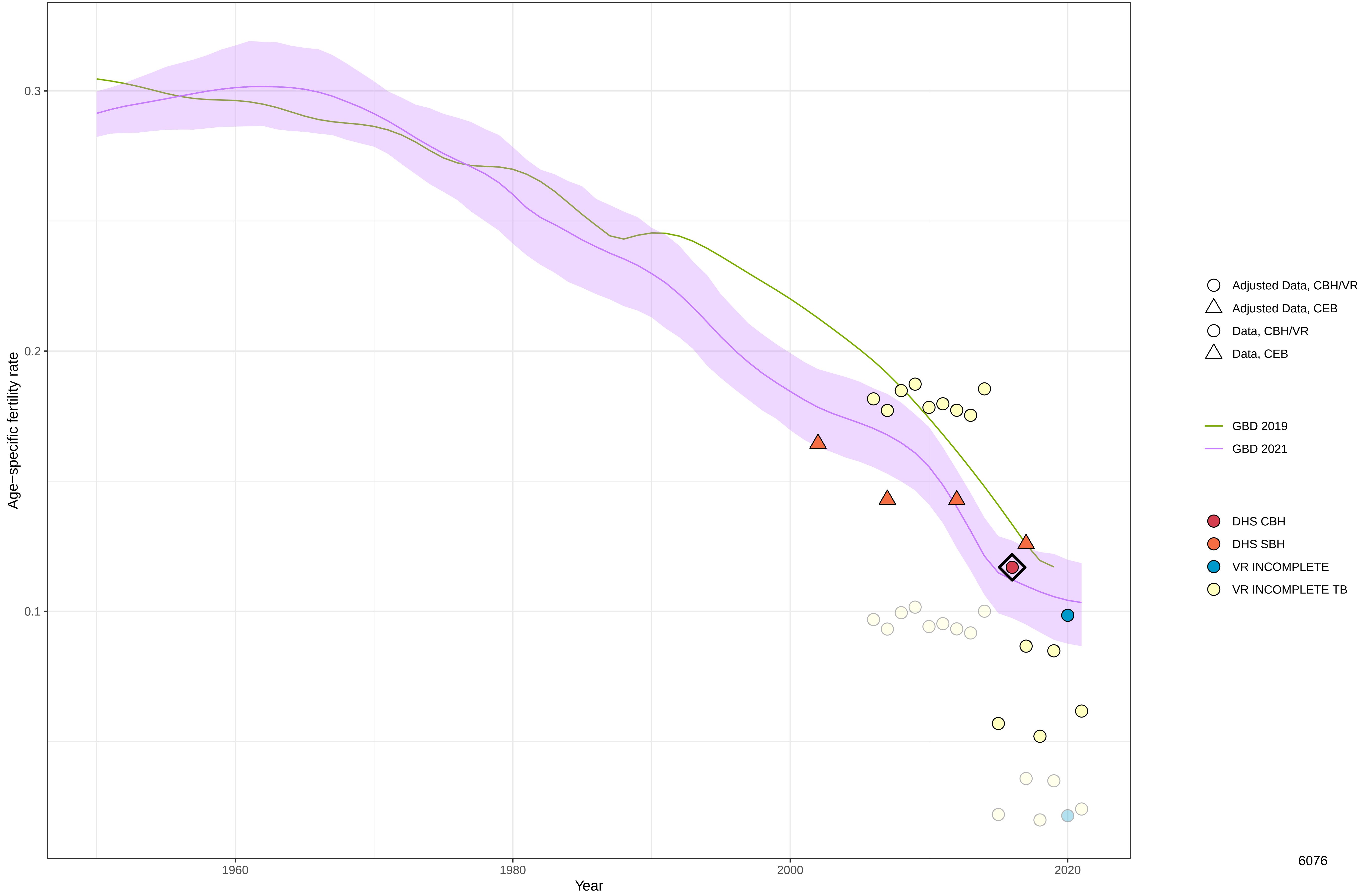

Age−specific fertility fitted trend lines, 1950−2021: Davao Occidental, Ages: 35−39
Data points enclosed with a diamond are reference sources

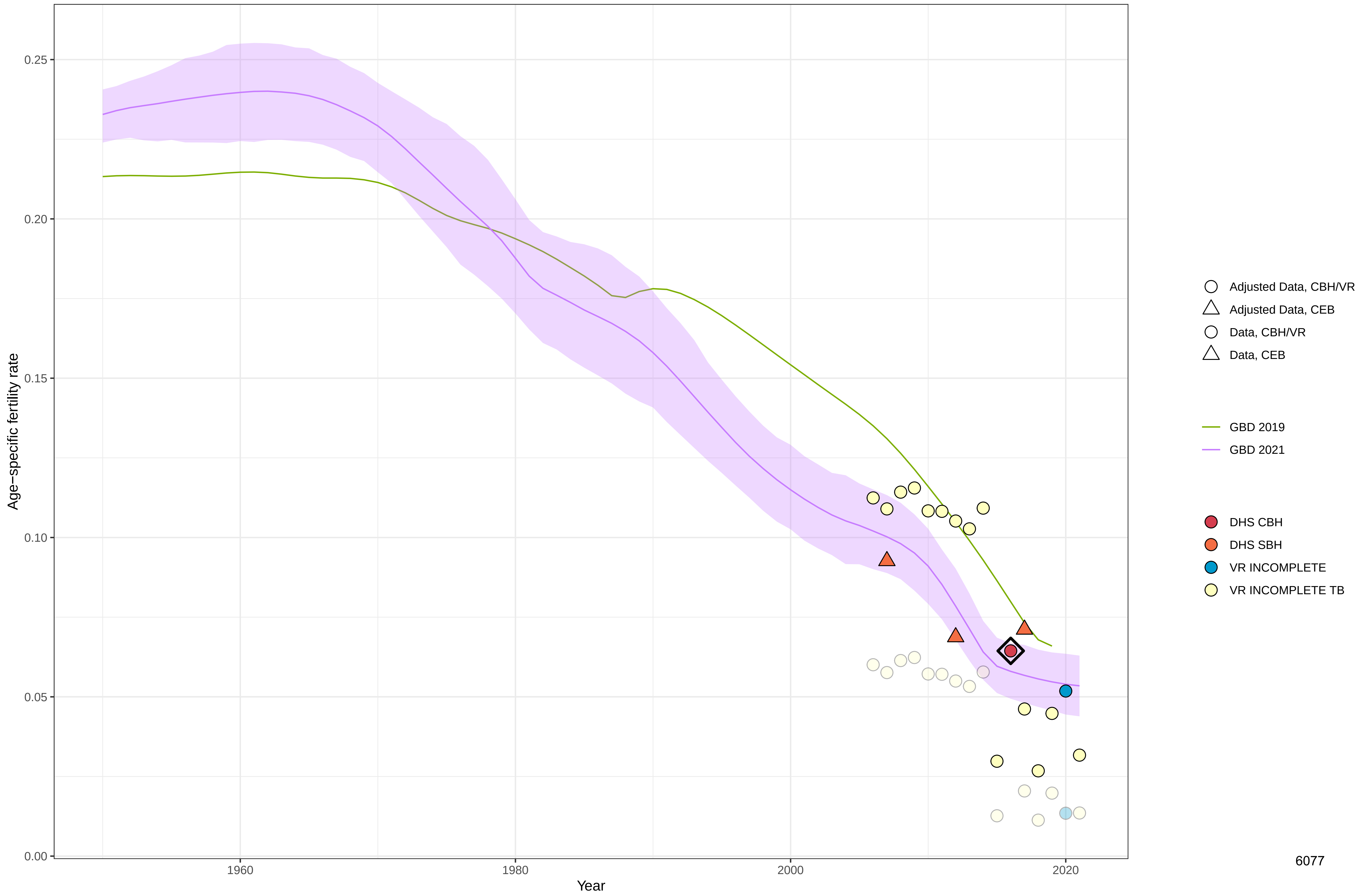

Age−specific fertility fitted trend lines, 1950−2021: Davao Occidental, Ages: 40−44
Data points enclosed with a diamond are reference sources

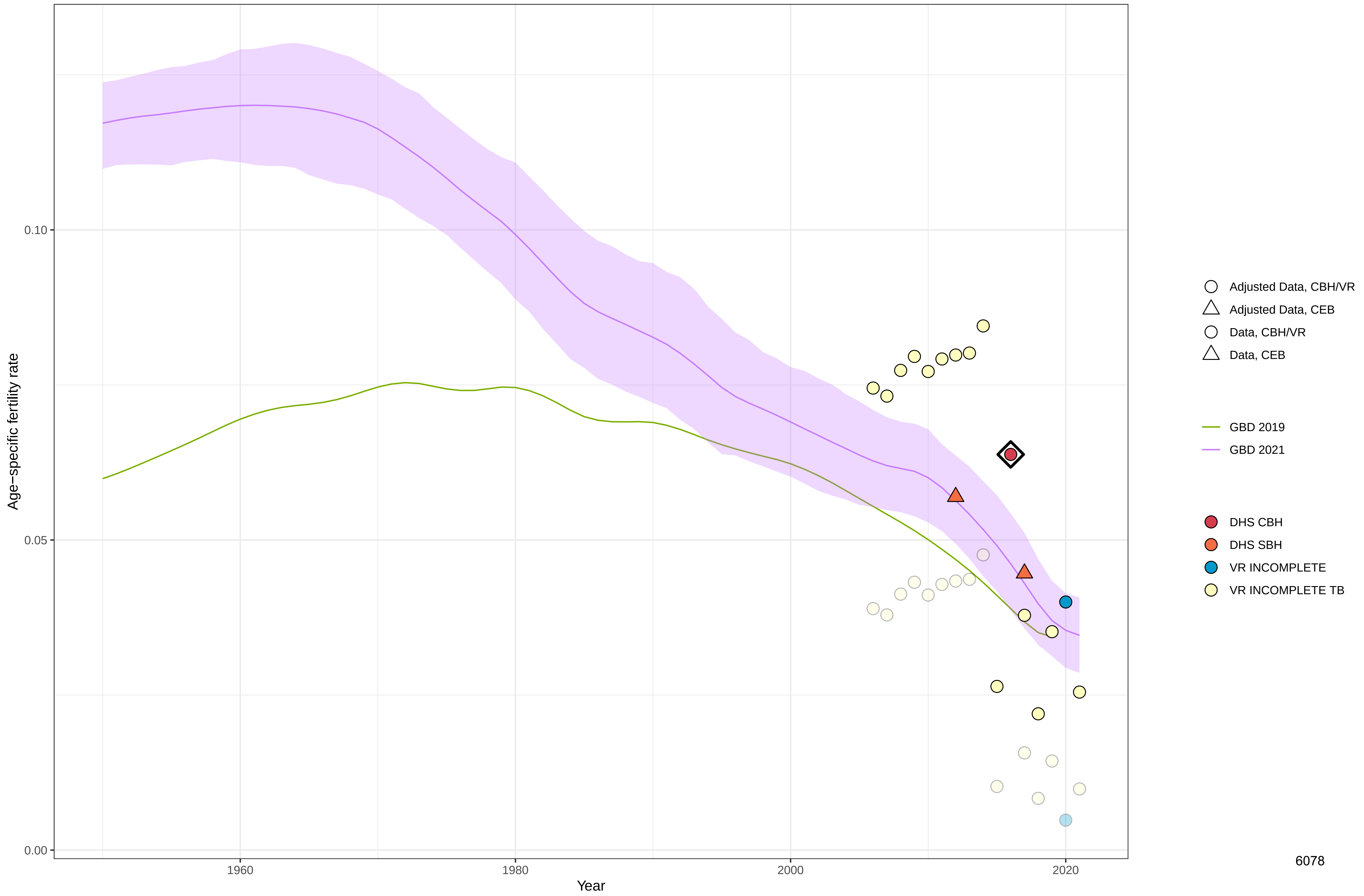

Age−specific fertility fitted trend lines, 1950−2021: Davao Occidental, Ages: 45−49
Data points enclosed with a diamond are reference sources

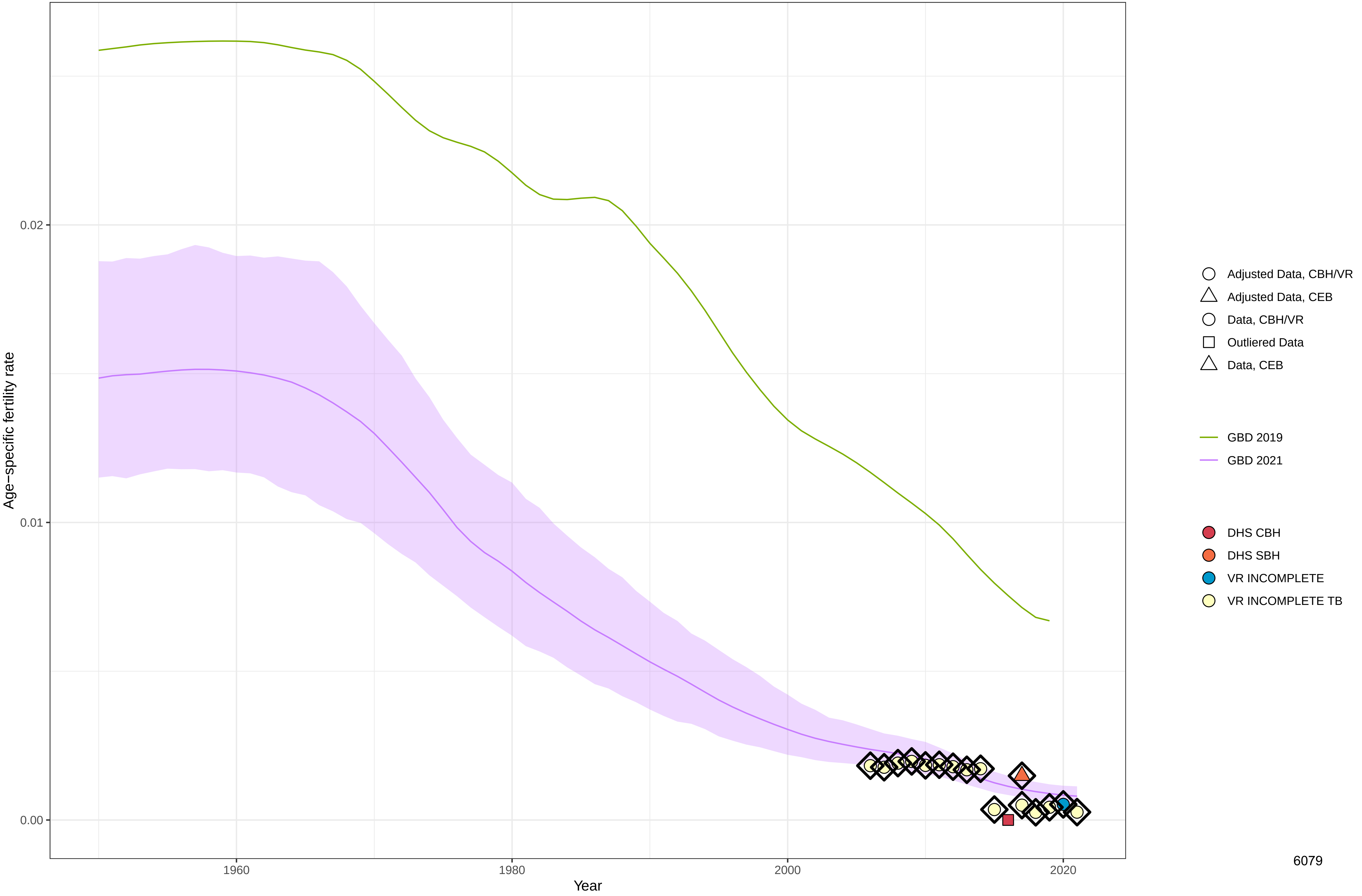

Age−specific fertility fitted trend lines, 1950−2021: Davao Occidental, Ages: 50−54
Data points enclosed with a diamond are reference sources

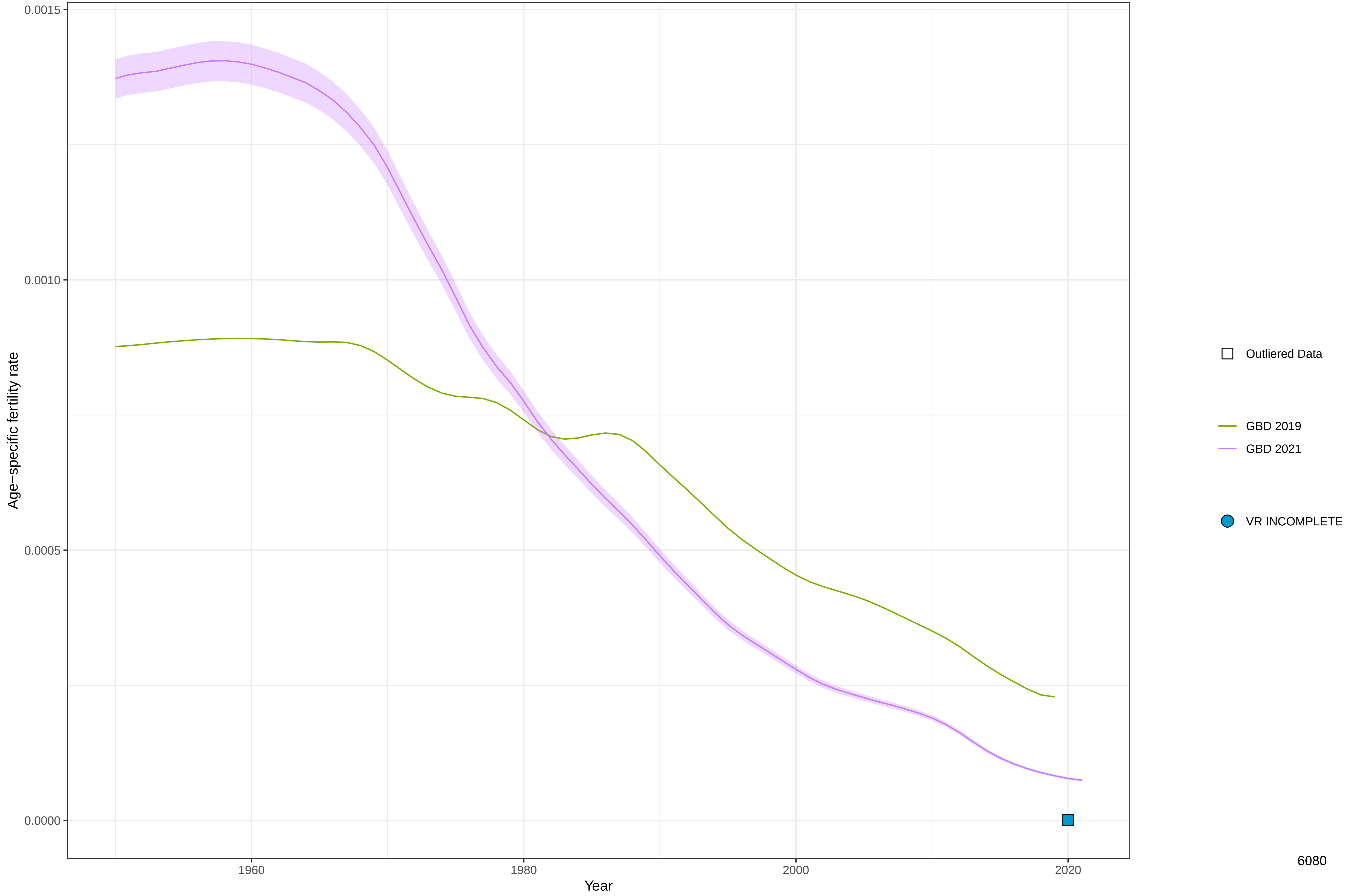

Total fertility rate fitted trend lines, 1950−2021: Davao Occidental TFR
Data points enclosed with a diamond are reference sources

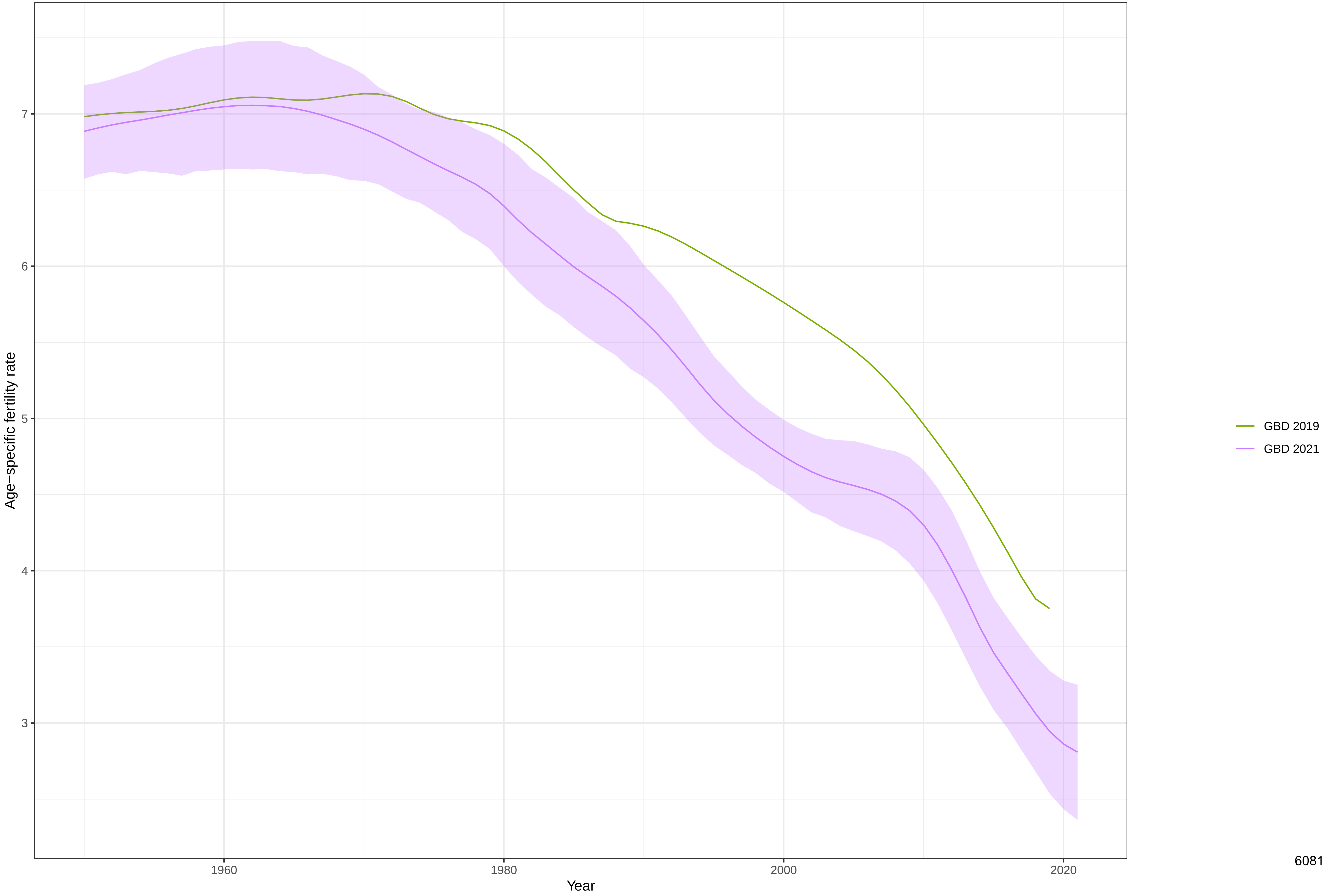

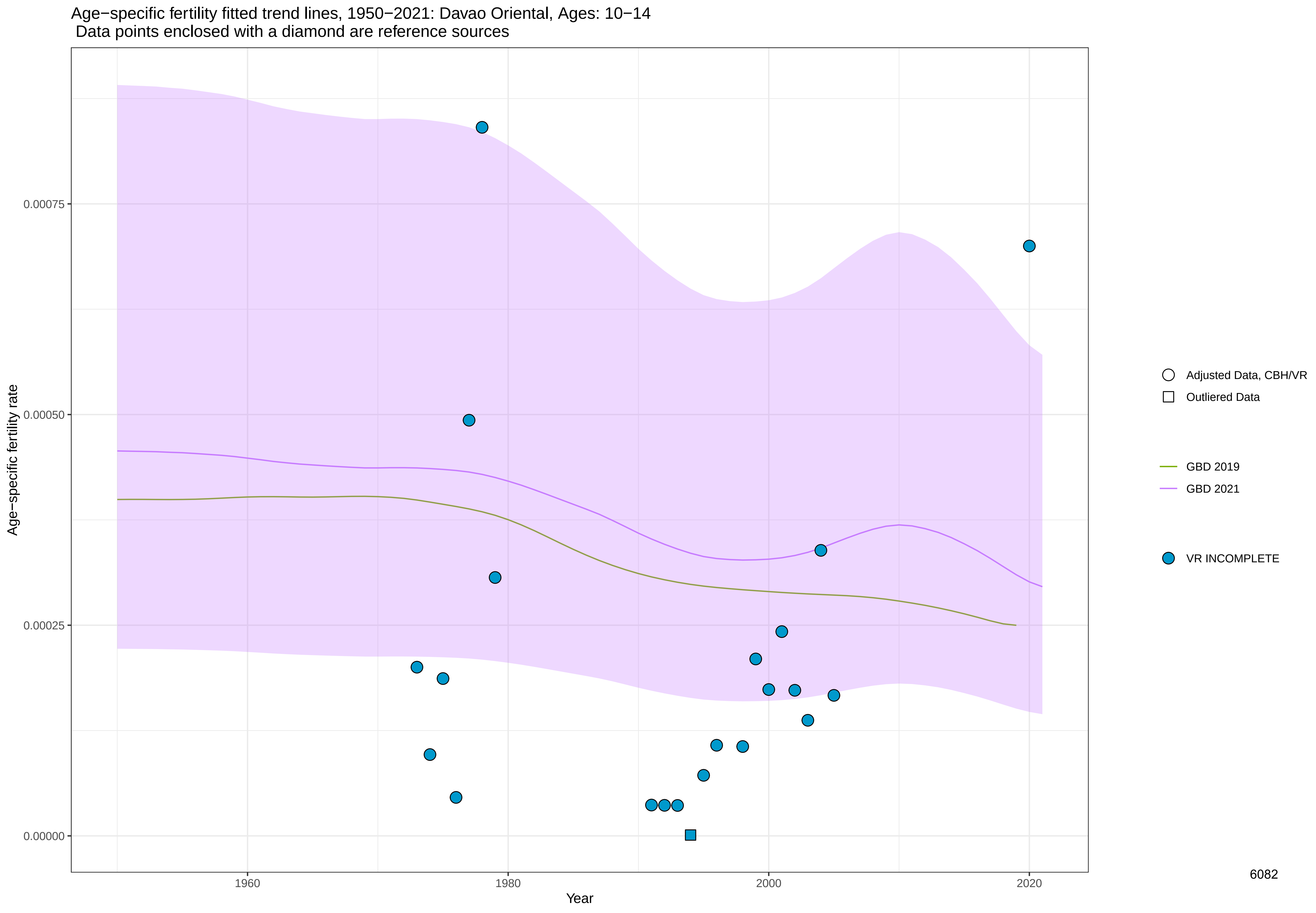

Age−specific fertility fitted trend lines, 1950−2021: Davao Oriental, Ages: 10−14
Data points enclosed with a diamond are reference sources
Age−specific fertility rate
0.00000
0.00025
0.00050
0.00075
1960
1980
2000
2020
Year
Adjusted Data, CBH/VR
Outliered Data
GBD 2019
GBD 2021
VR INCOMPLETE

6082

Age−specific fertility fitted trend lines, 1950−2021: Davao Oriental, Ages: 15−19
Data points enclosed with a diamond are reference sources

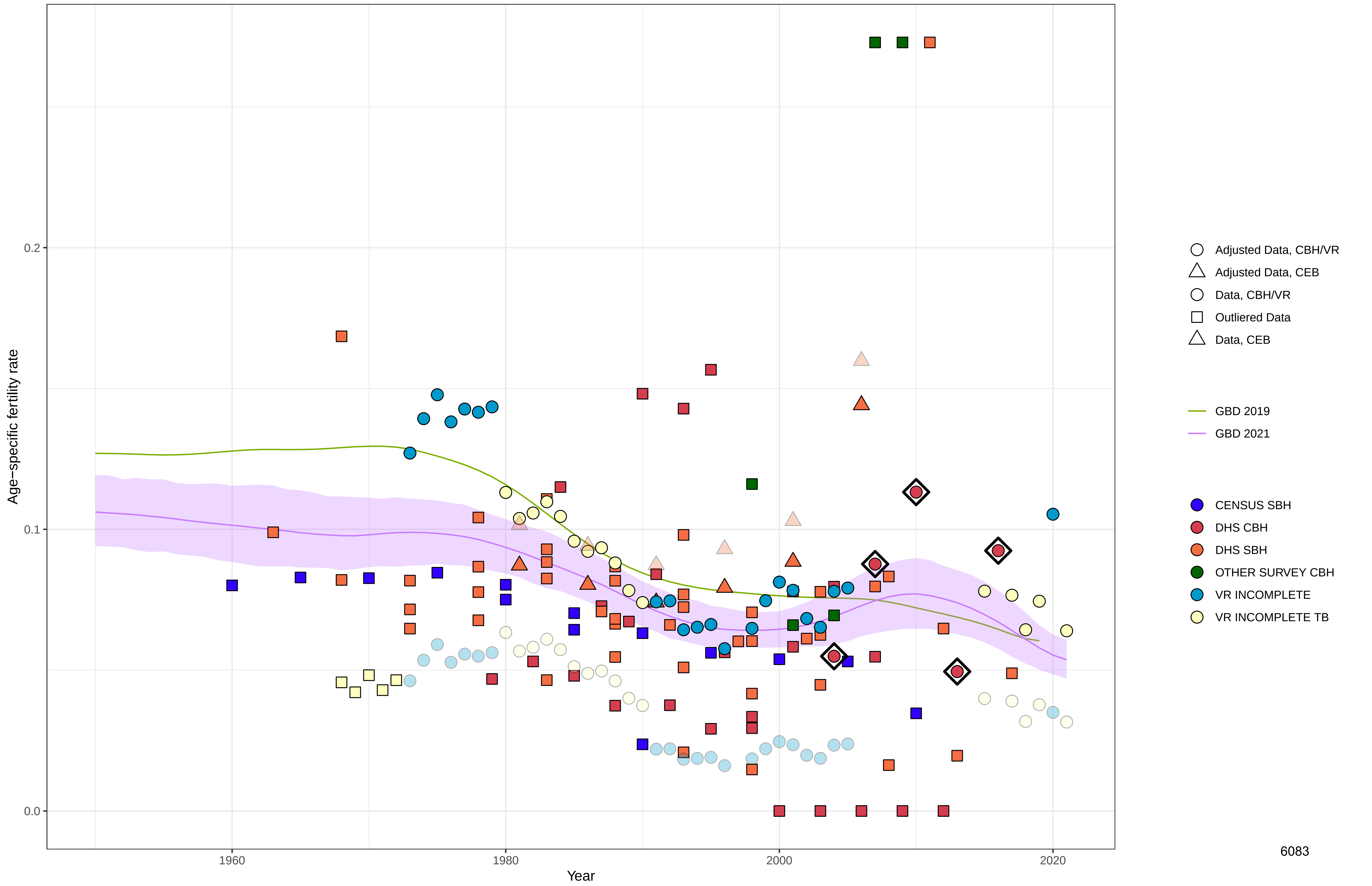

Age−specific fertility fitted trend lines, 1950−2021: Davao Oriental, Ages: 20−24
Data points enclosed with a diamond are reference sources

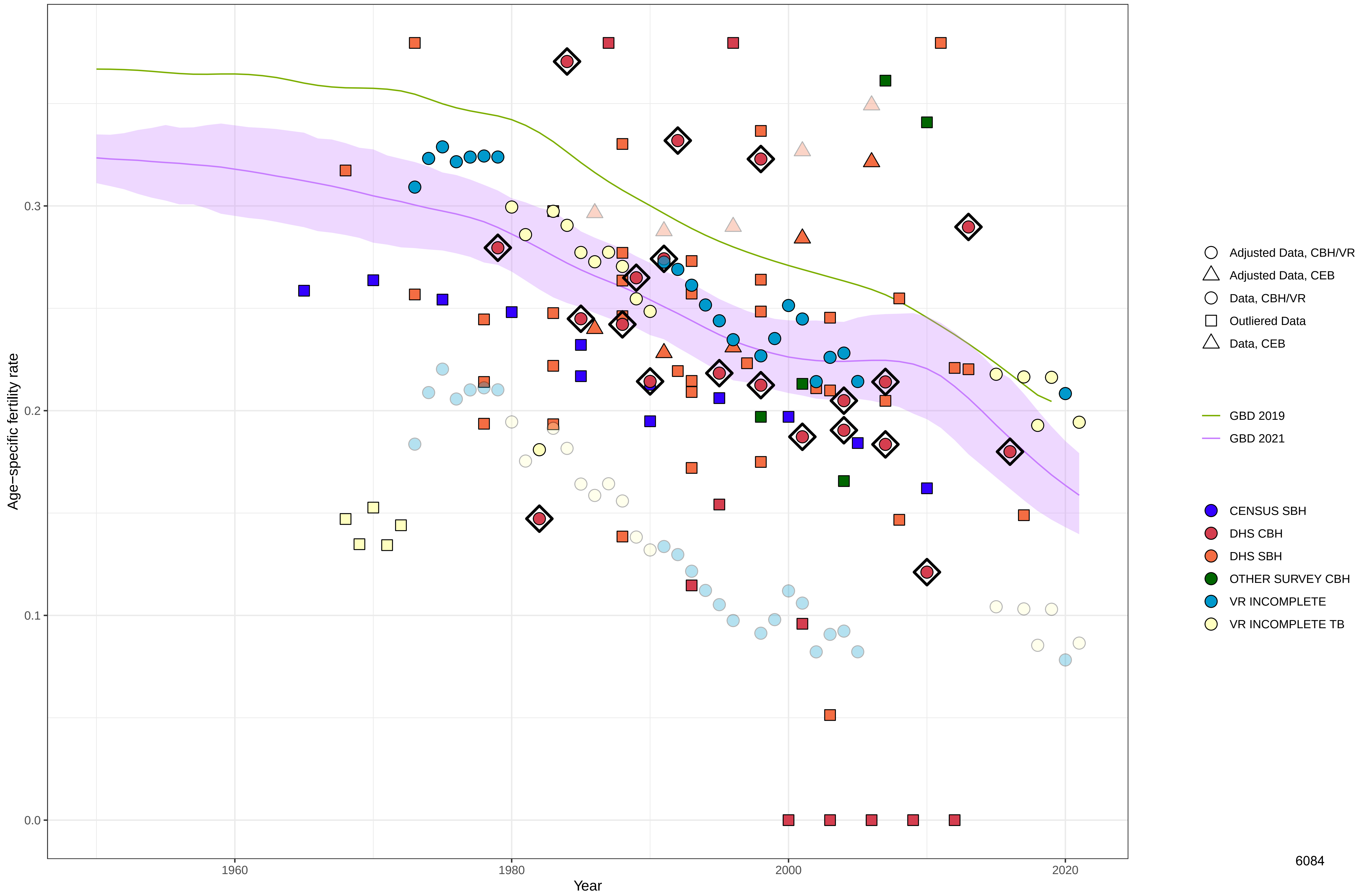

Age−specific fertility fitted trend lines, 1950−2021: Davao Oriental, Ages: 25−29
Data points enclosed with a diamond are reference sources

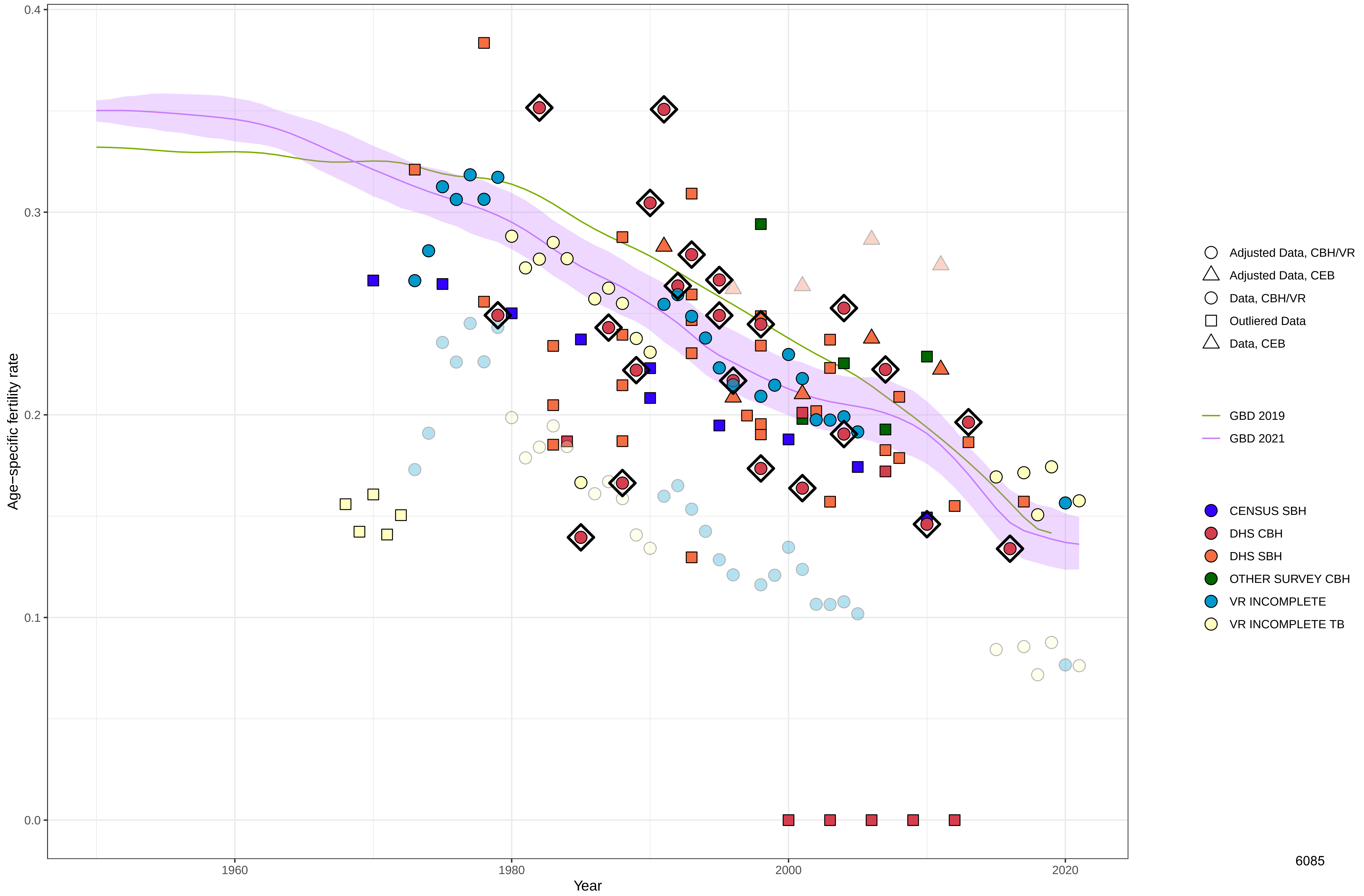

Age−specific fertility fitted trend lines, 1950−2021: Davao Oriental, Ages: 30−34
Data points enclosed with a diamond are reference sources

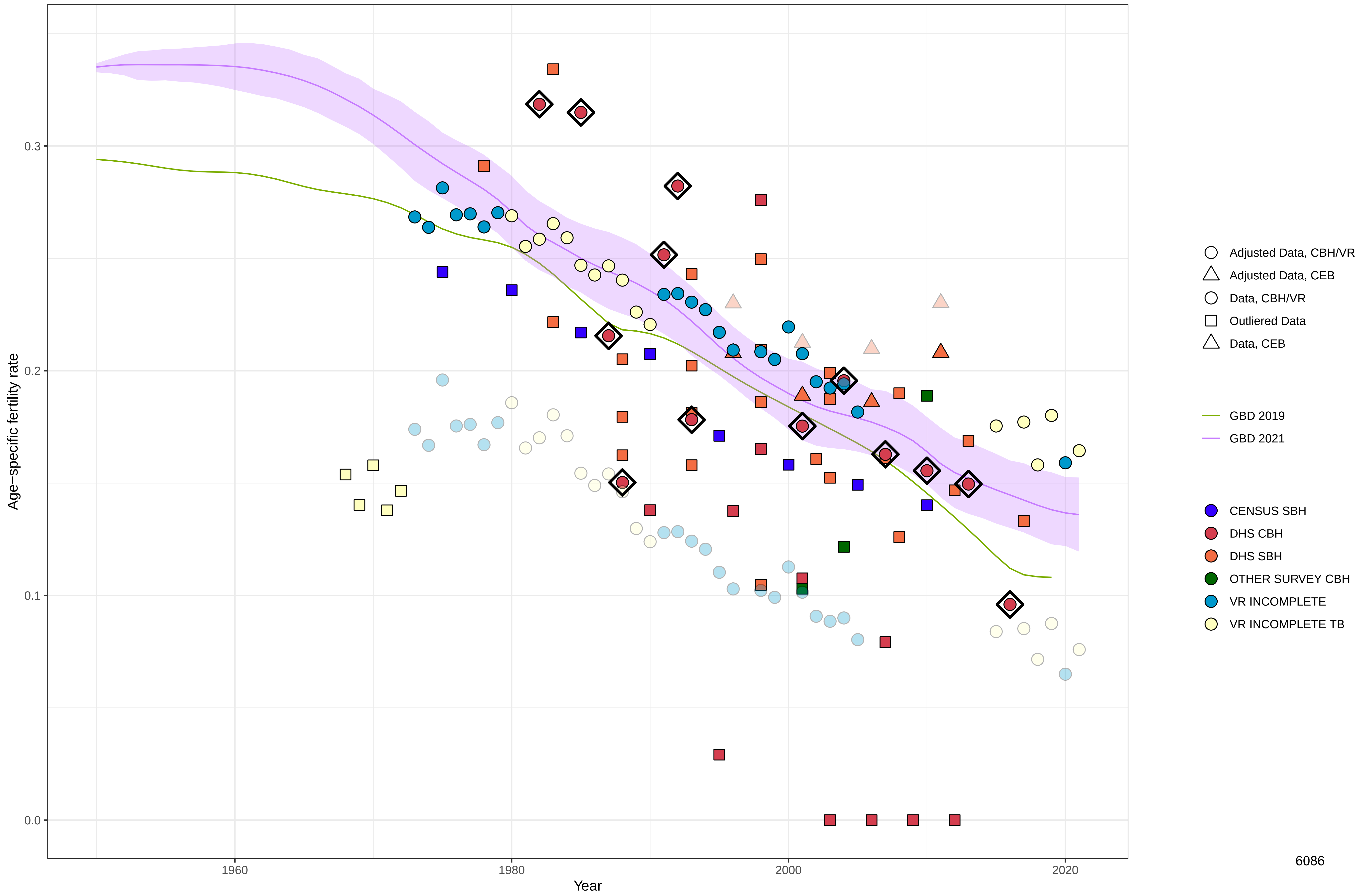

Age−specific fertility fitted trend lines, 1950−2021: Davao Oriental, Ages: 35−39
Data points enclosed with a diamond are reference sources

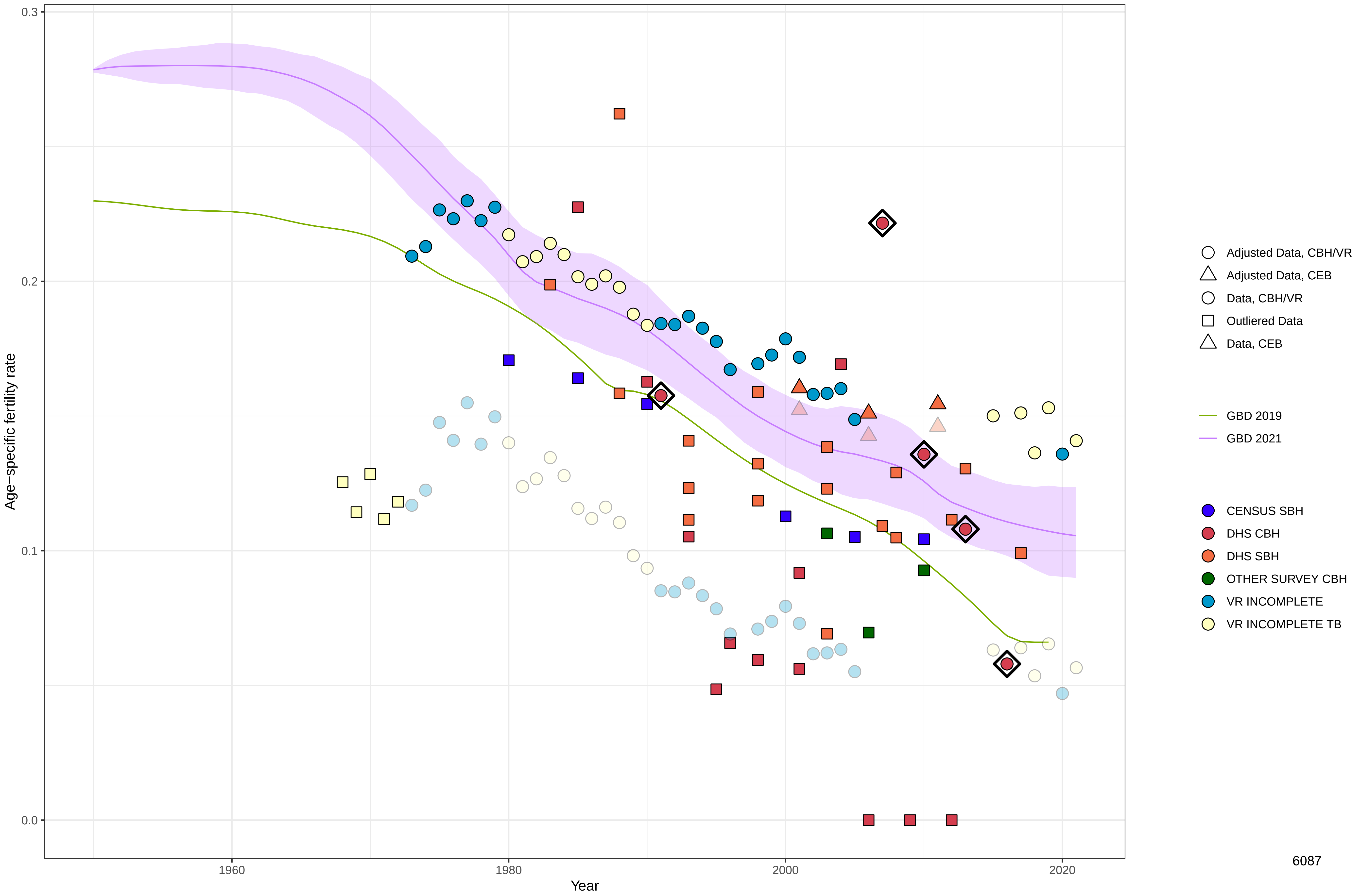

Age−specific fertility fitted trend lines, 1950−2021: Davao Oriental, Ages: 40−44
Data points enclosed with a diamond are reference sources

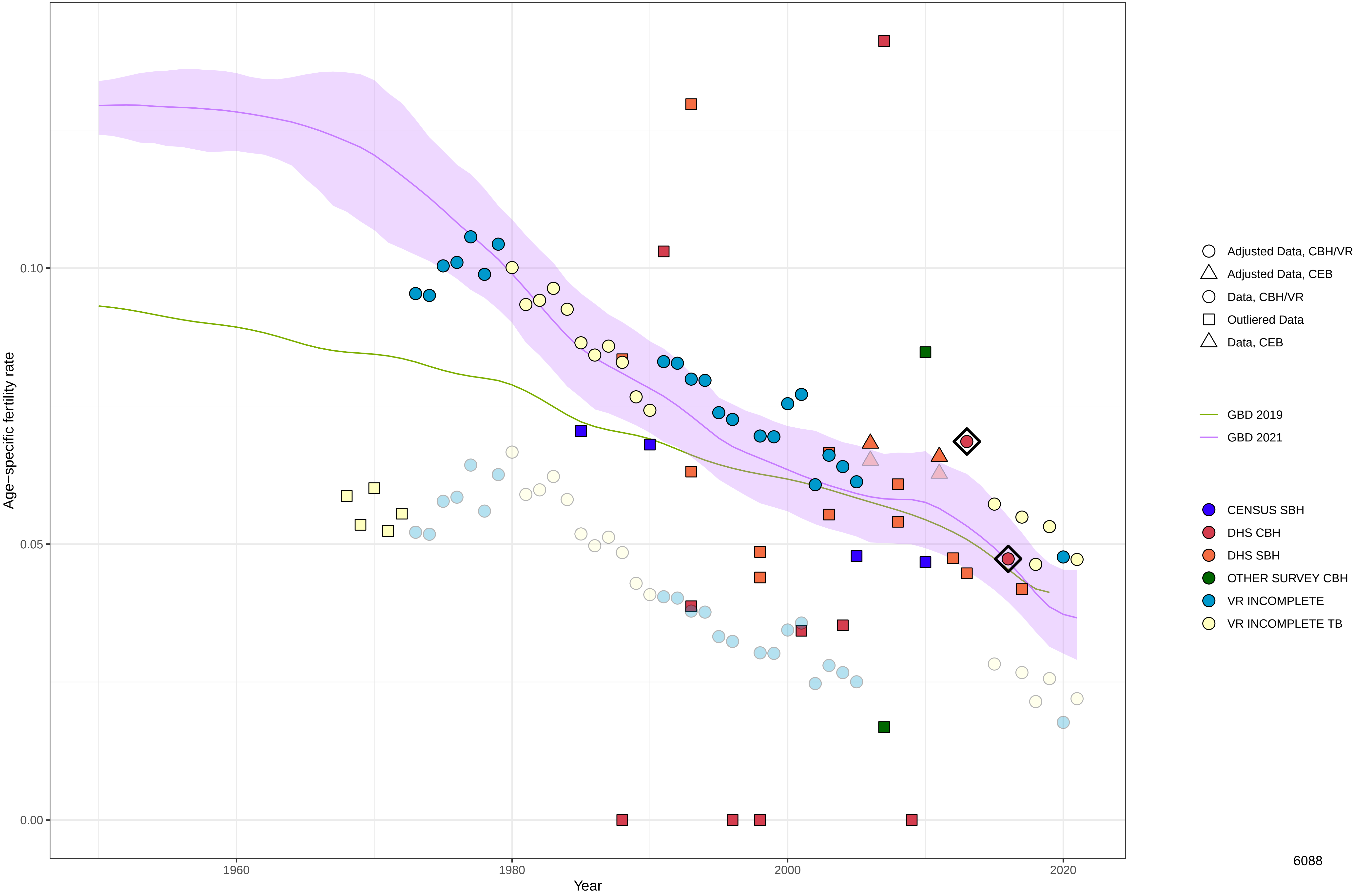

Age−specific fertility fitted trend lines, 1950−2021: Davao Oriental, Ages: 45−49
Data points enclosed with a diamond are reference sources

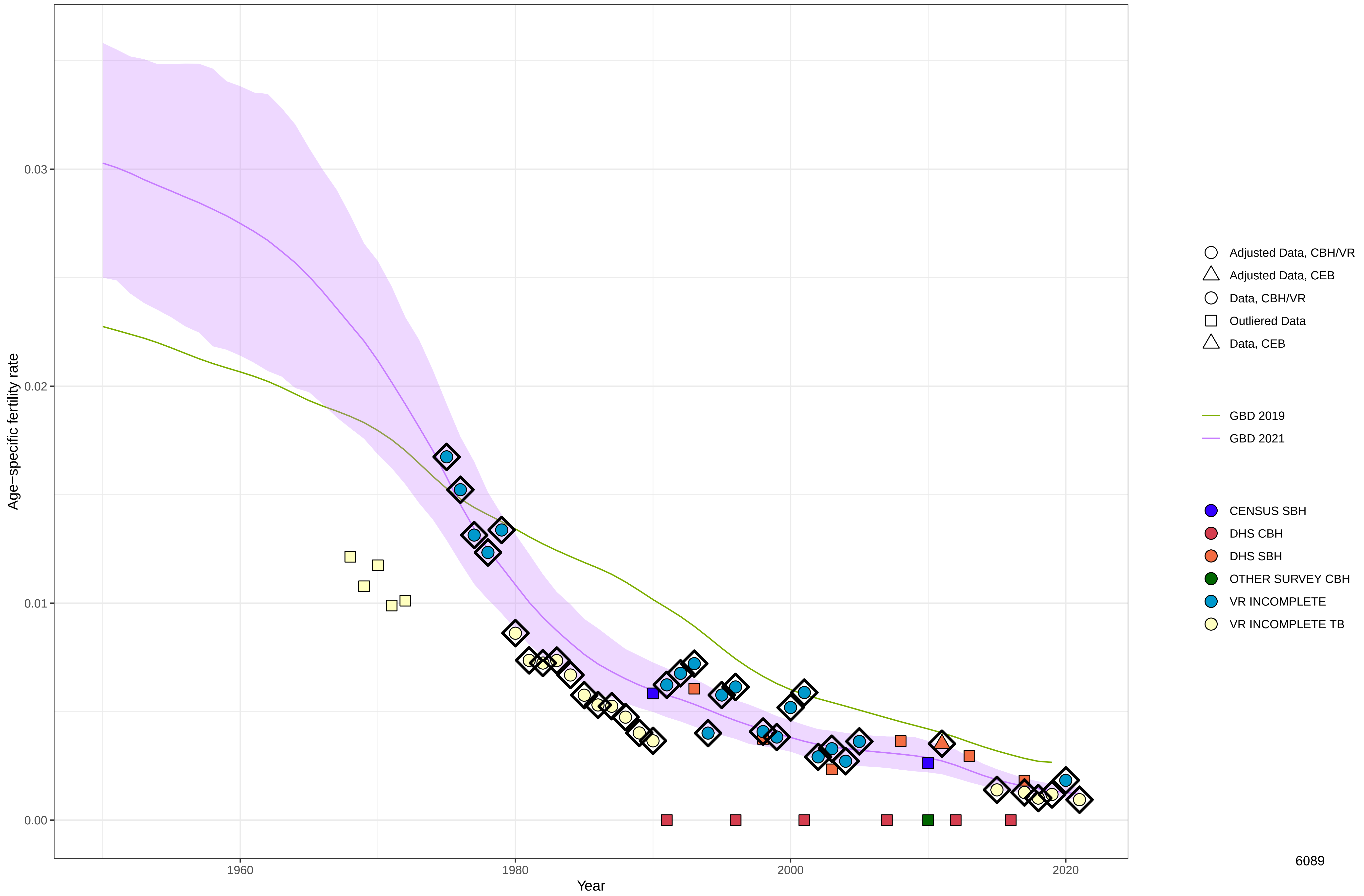

Age−specific fertility fitted trend lines, 1950−2021: Davao Oriental, Ages: 50−54
Data points enclosed with a diamond are reference sources

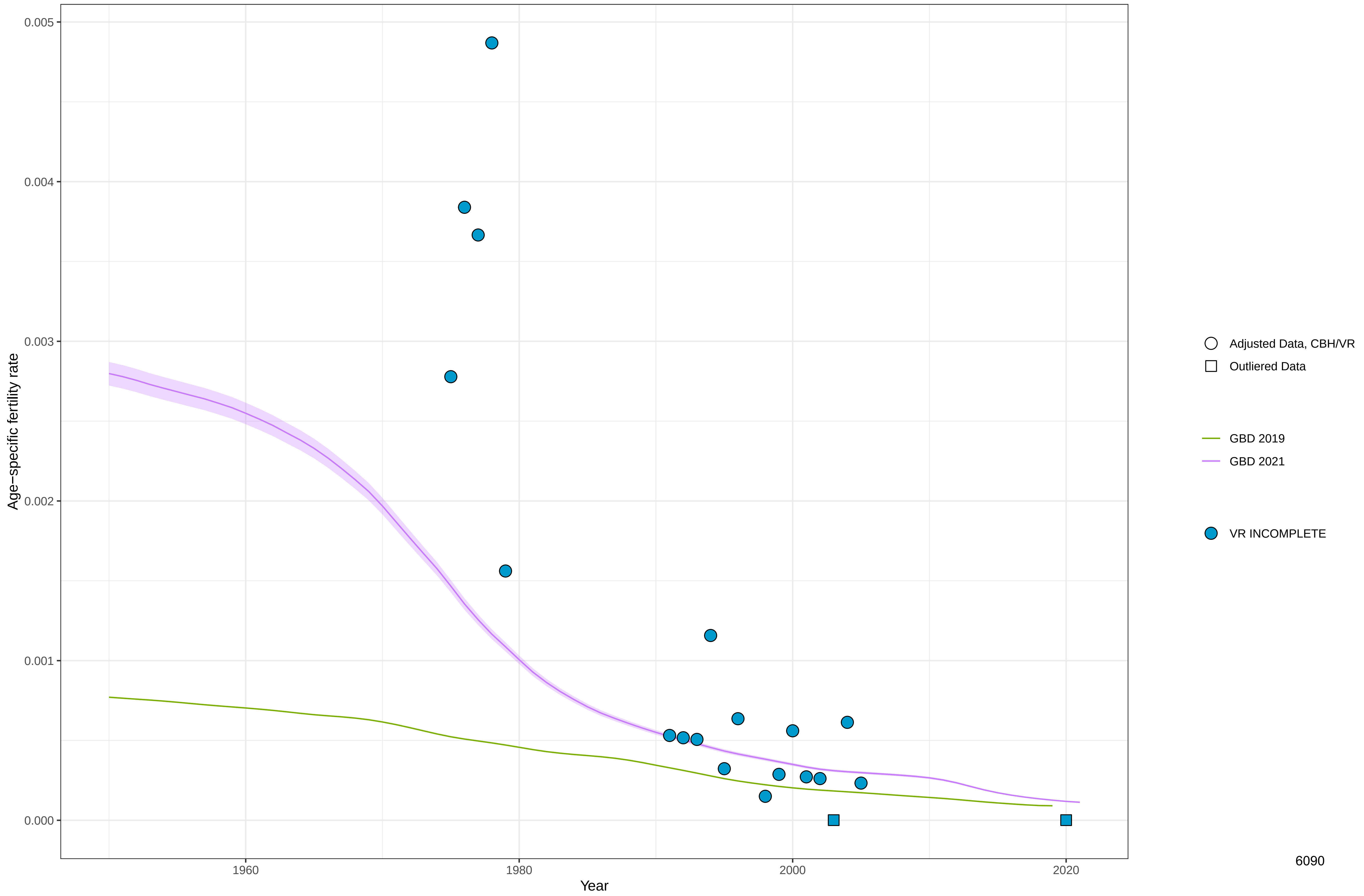

Total fertility rate fitted trend lines, 1950−2021: Davao Oriental TFR
Data points enclosed with a diamond are reference sources

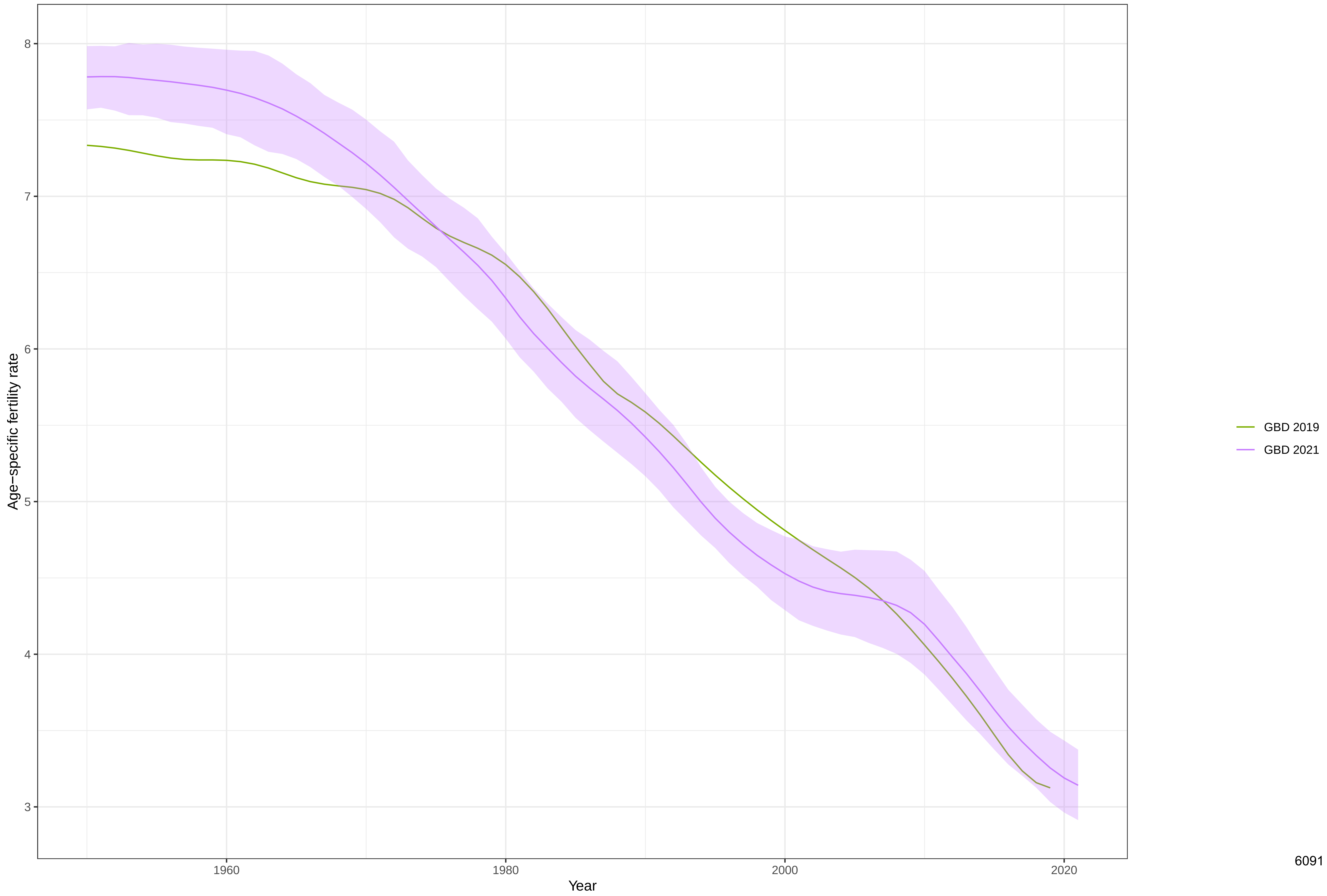

Age−specific fertility fitted trend lines, 1950−2021: Dinagat Islands, Ages: 10−14
Data points enclosed with a diamond are reference sources

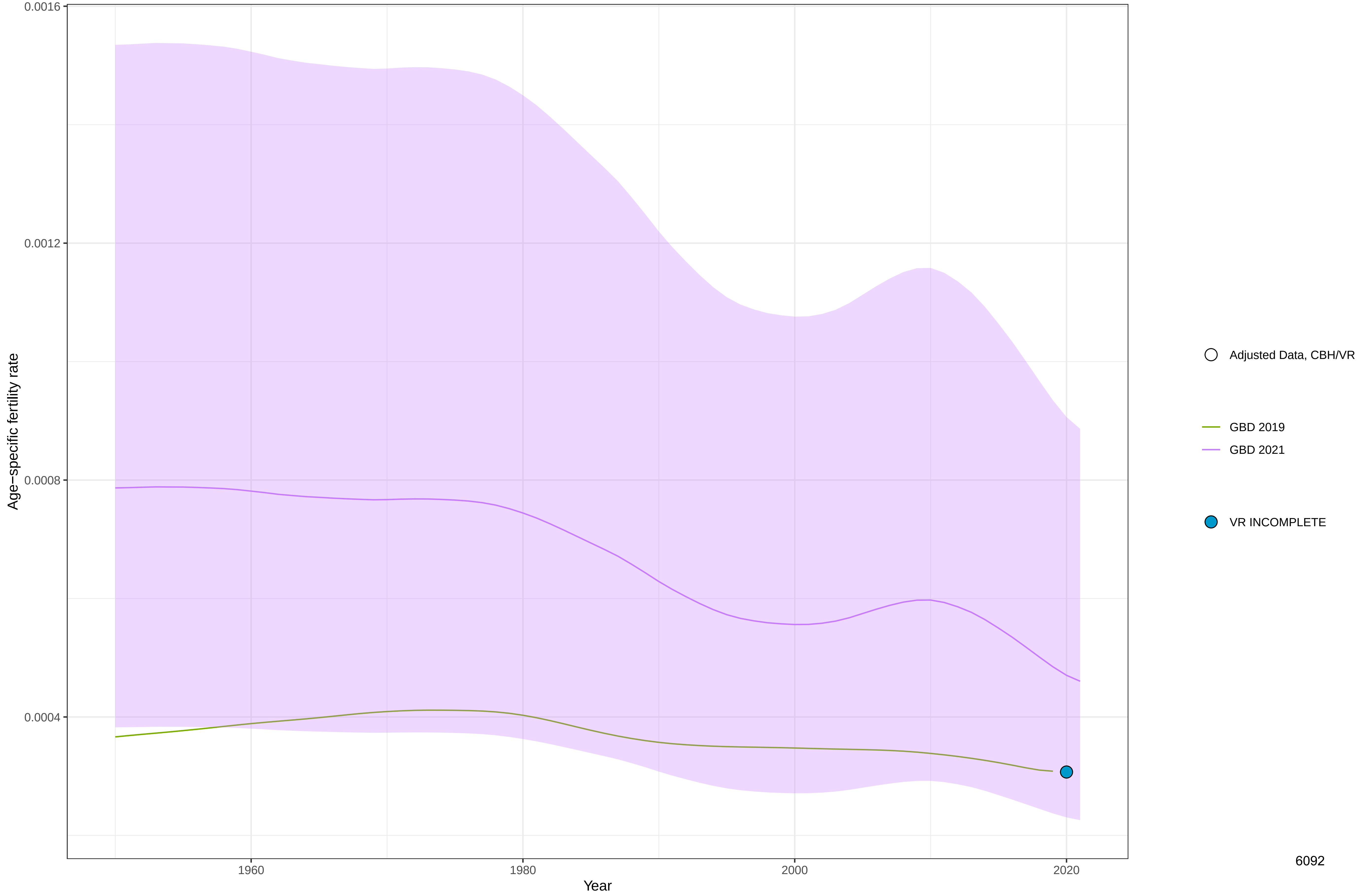

Age−specific fertility fitted trend lines, 1950−2021: Dinagat Islands, Ages: 15−19
Data points enclosed with a diamond are reference sources

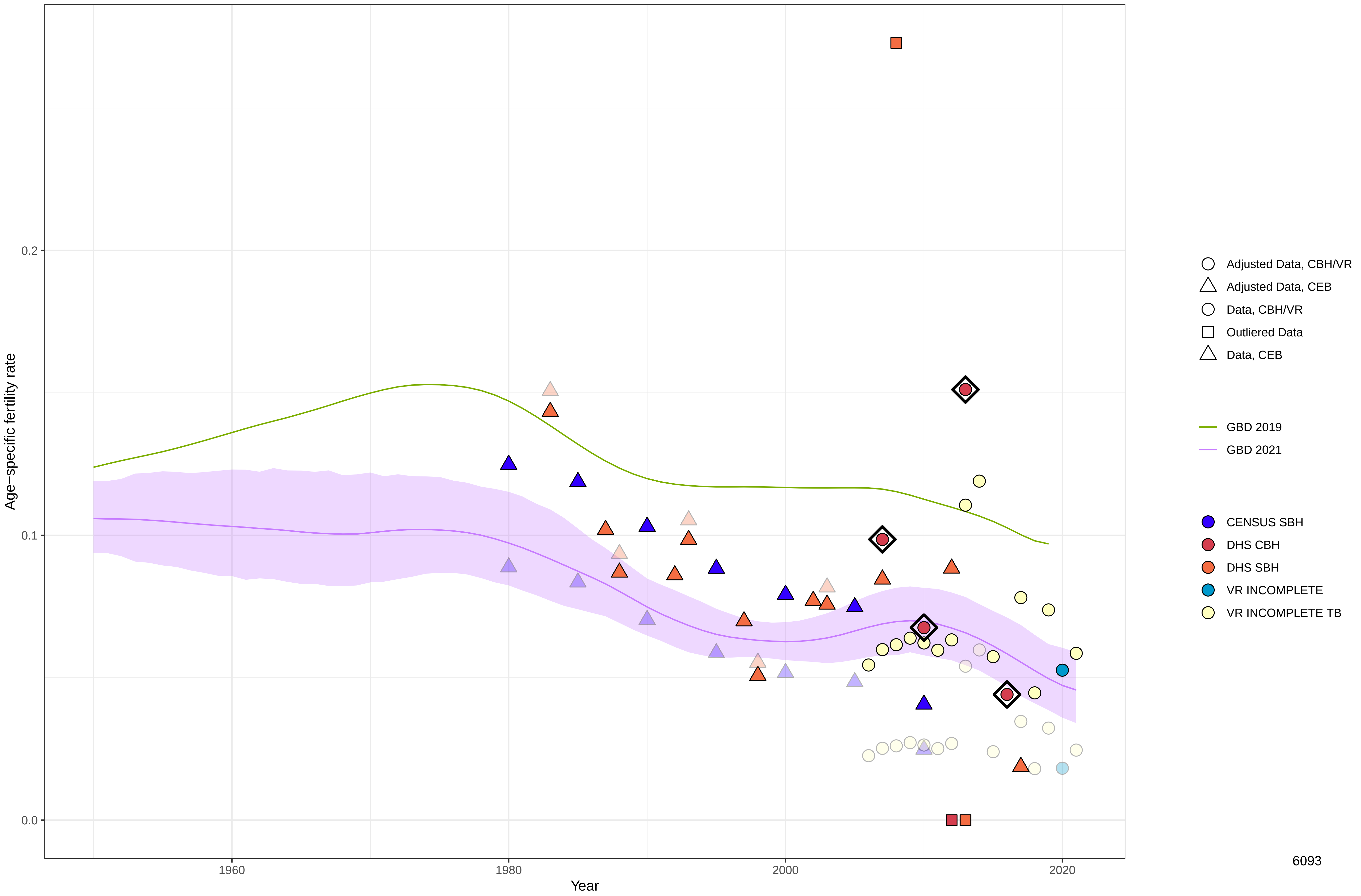

Age−specific fertility fitted trend lines, 1950−2021: Dinagat Islands, Ages: 20−24
Data points enclosed with a diamond are reference sources

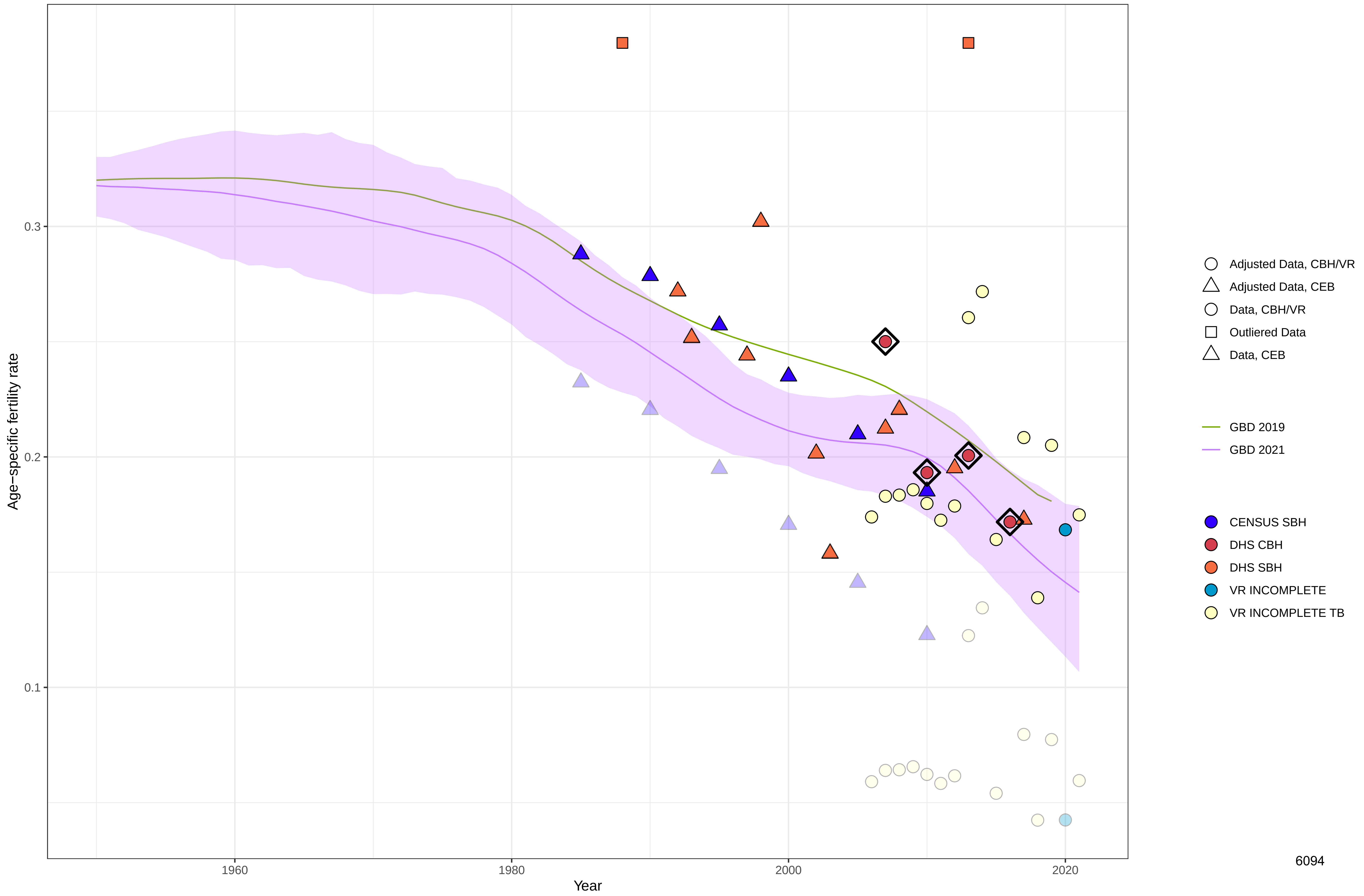

Age−specific fertility fitted trend lines, 1950−2021: Dinagat Islands, Ages: 25−29
Data points enclosed with a diamond are reference sources

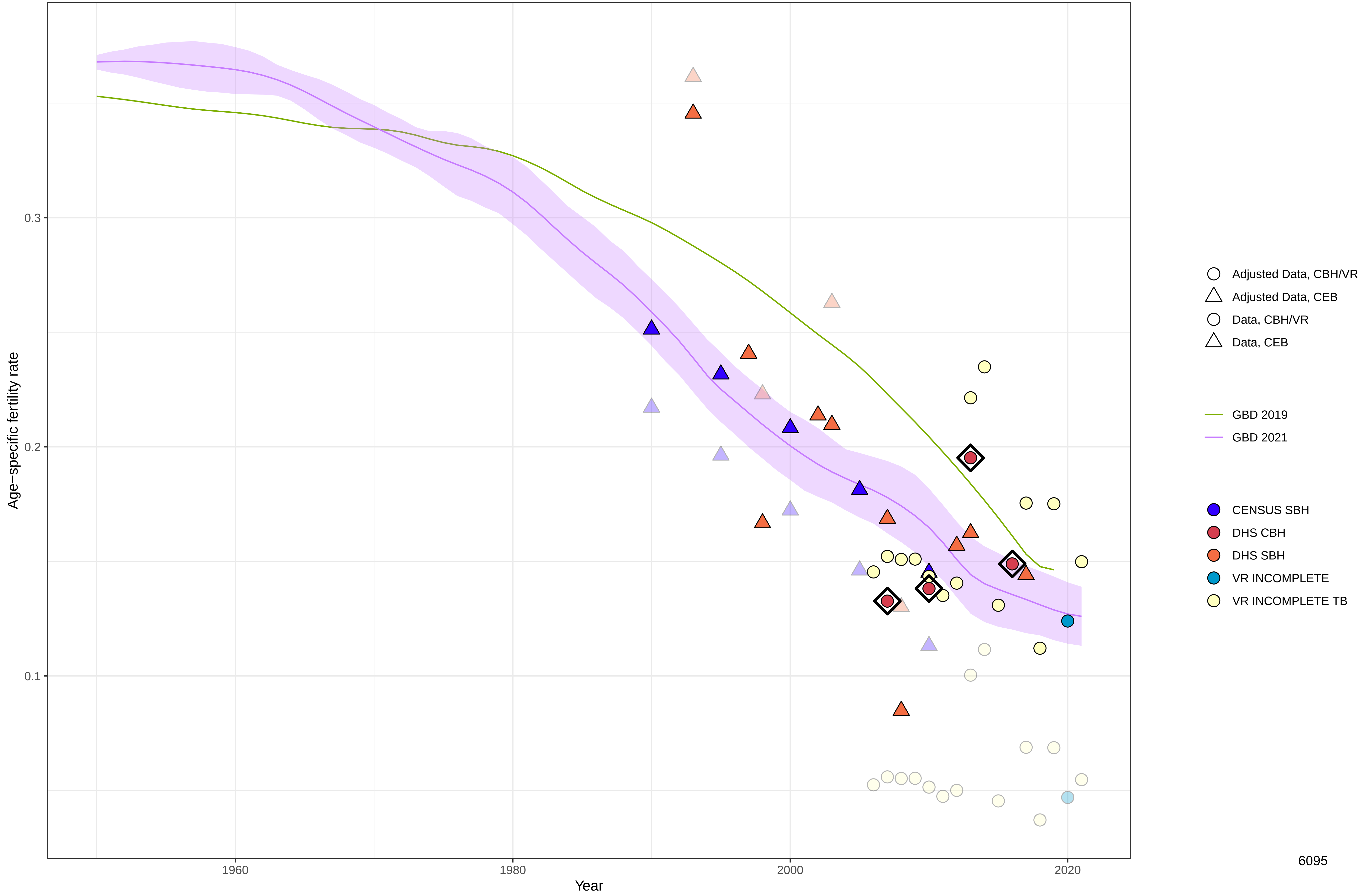

Age−specific fertility fitted trend lines, 1950−2021: Dinagat Islands, Ages: 30−34
Data points enclosed with a diamond are reference sources

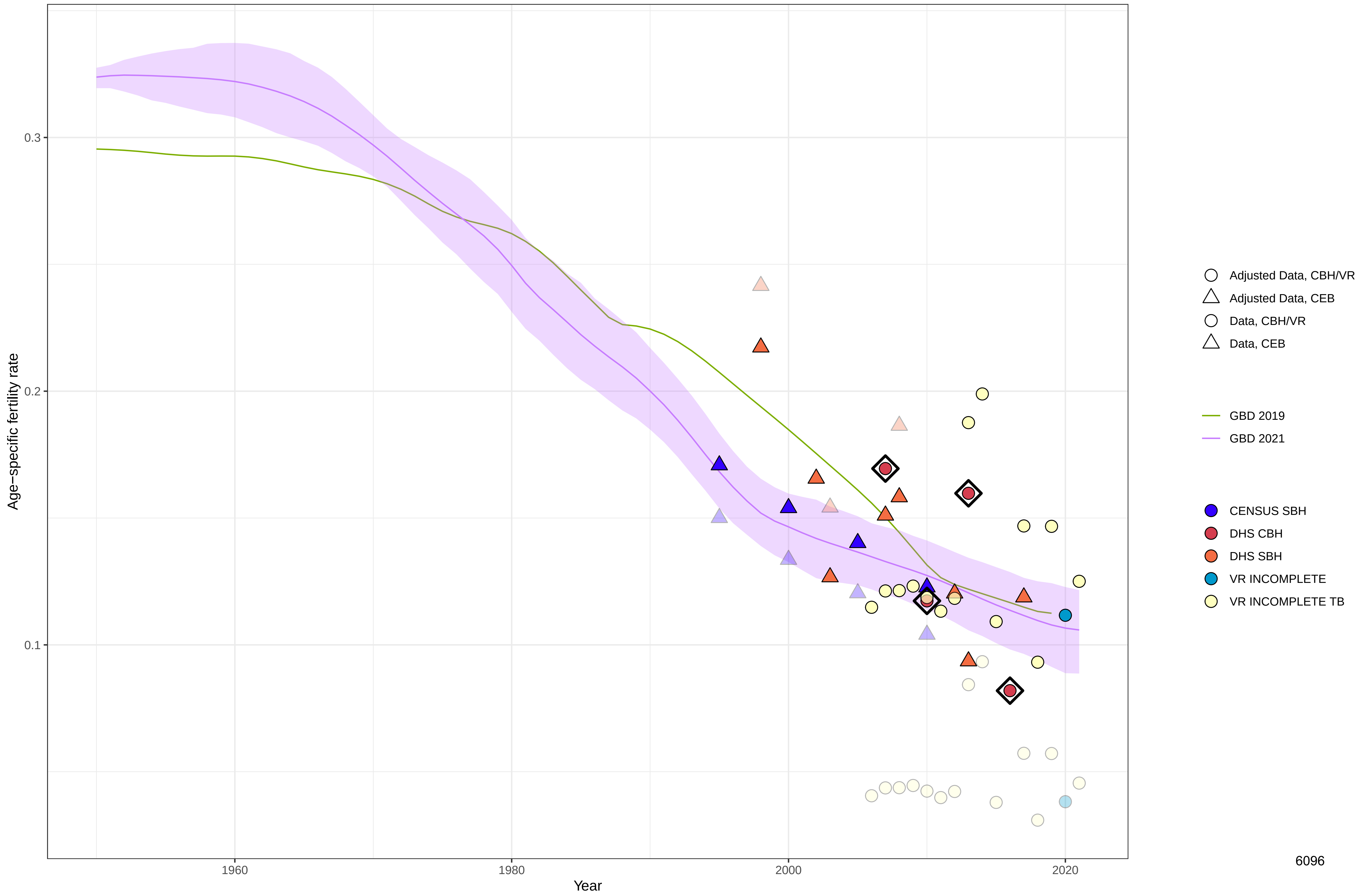

Age−specific fertility fitted trend lines, 1950−2021: Dinagat Islands, Ages: 35−39
Data points enclosed with a diamond are reference sources

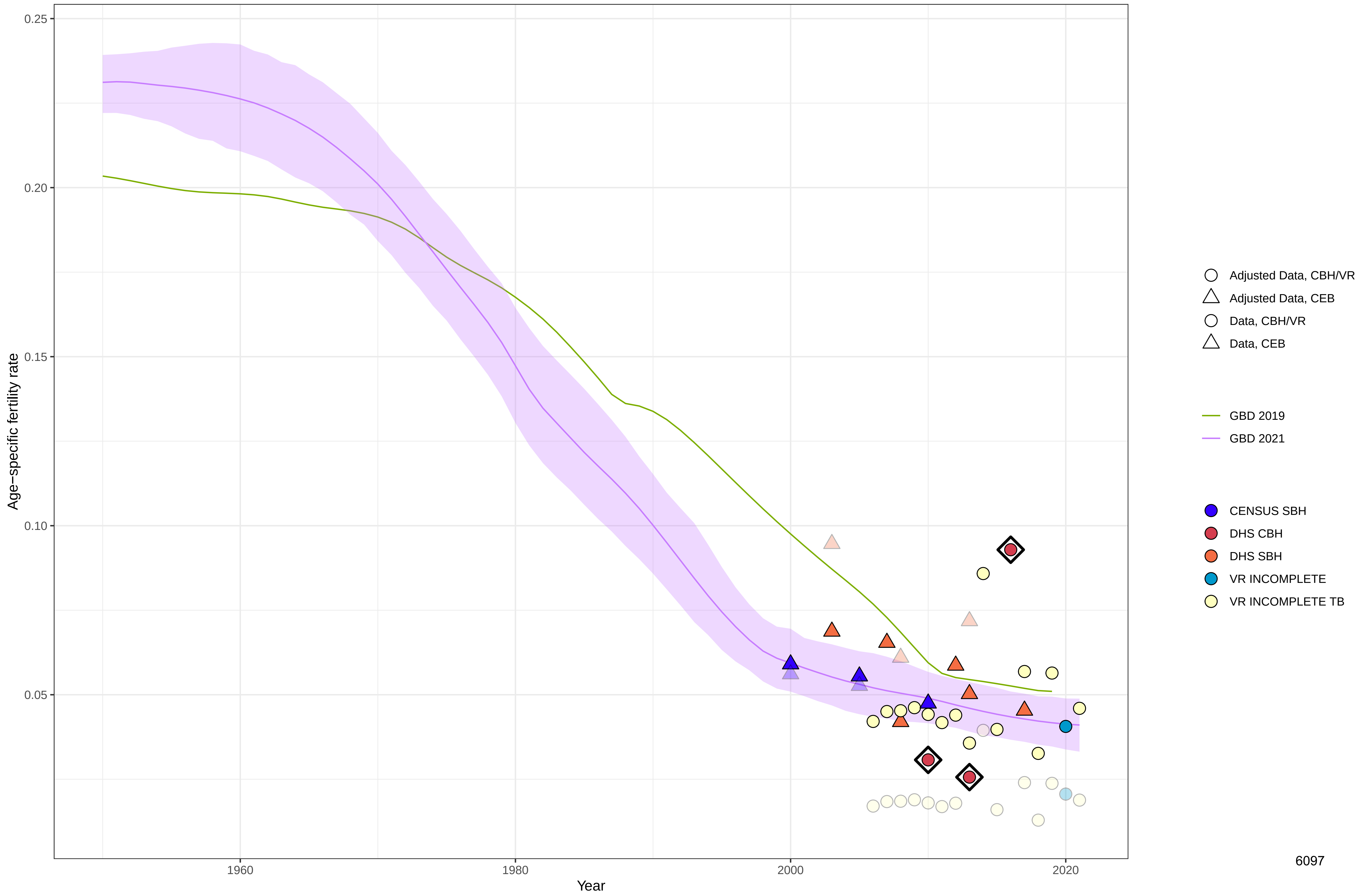

Age−specific fertility fitted trend lines, 1950−2021: Dinagat Islands, Ages: 40−44
Data points enclosed with a diamond are reference sources

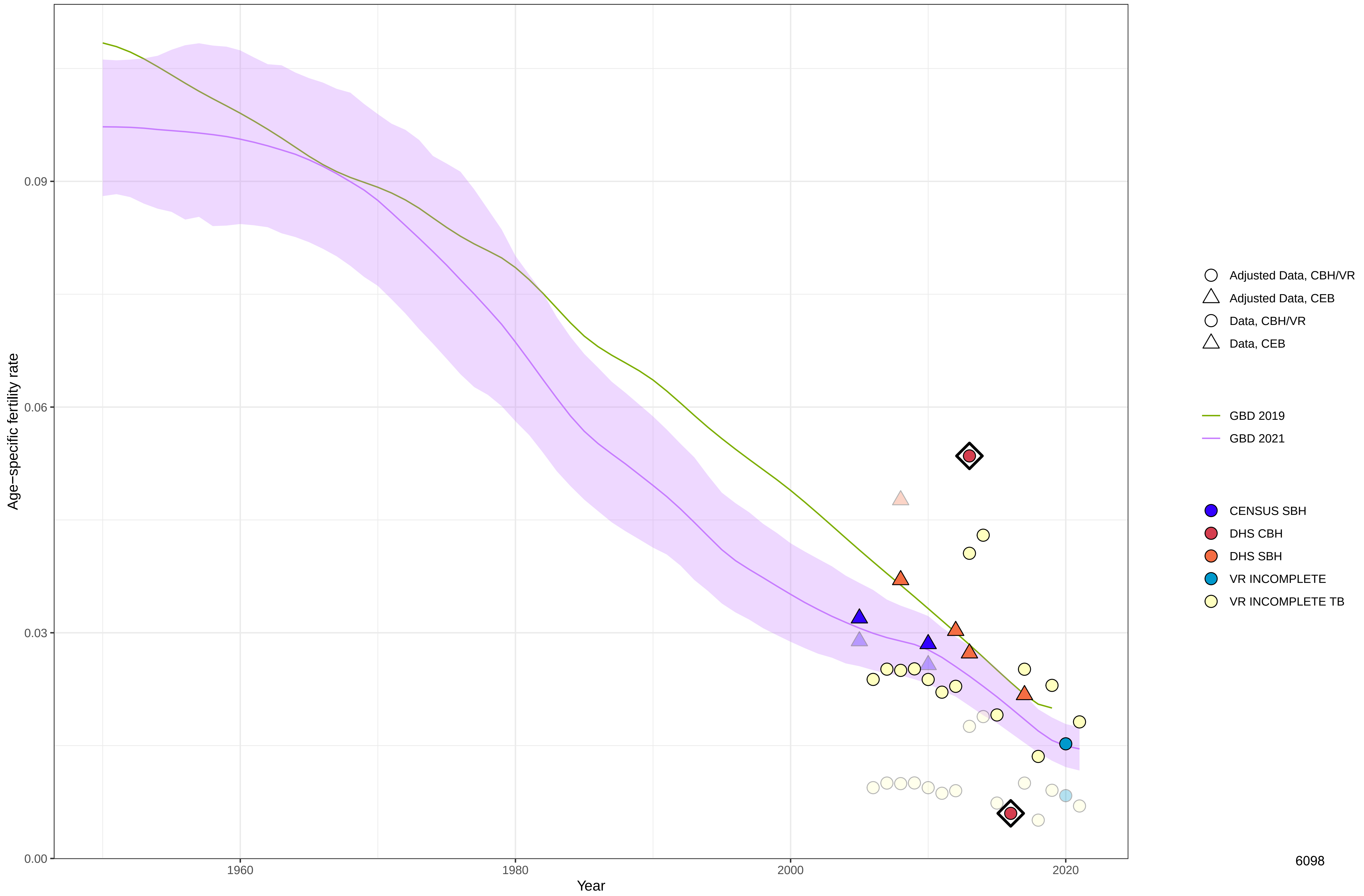

Age−specific fertility fitted trend lines, 1950−2021: Dinagat Islands, Ages: 45−49
Data points enclosed with a diamond are reference sources

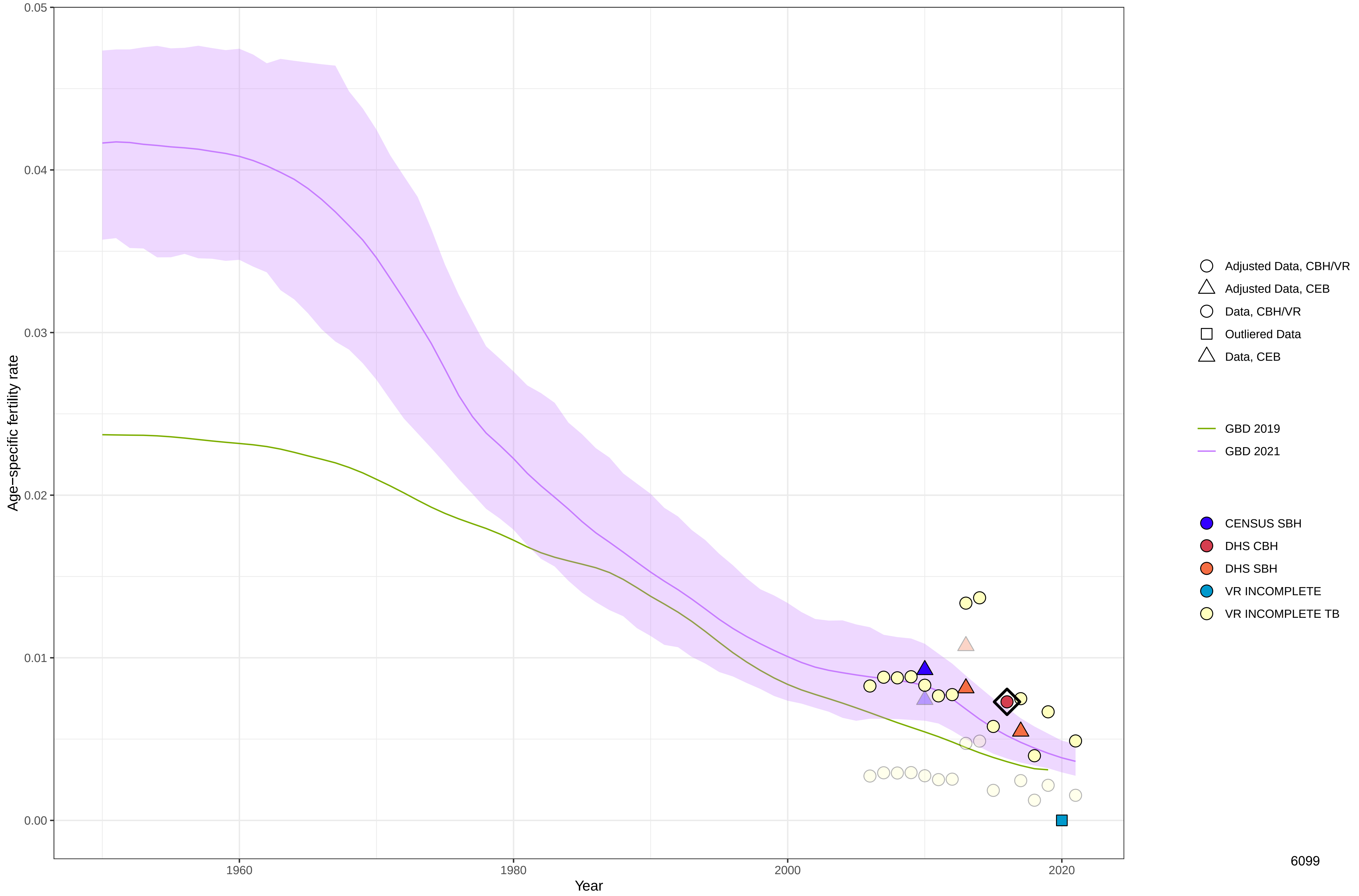

Age−specific fertility fitted trend lines, 1950−2021: Dinagat Islands, Ages: 50−54
Data points enclosed with a diamond are reference sources

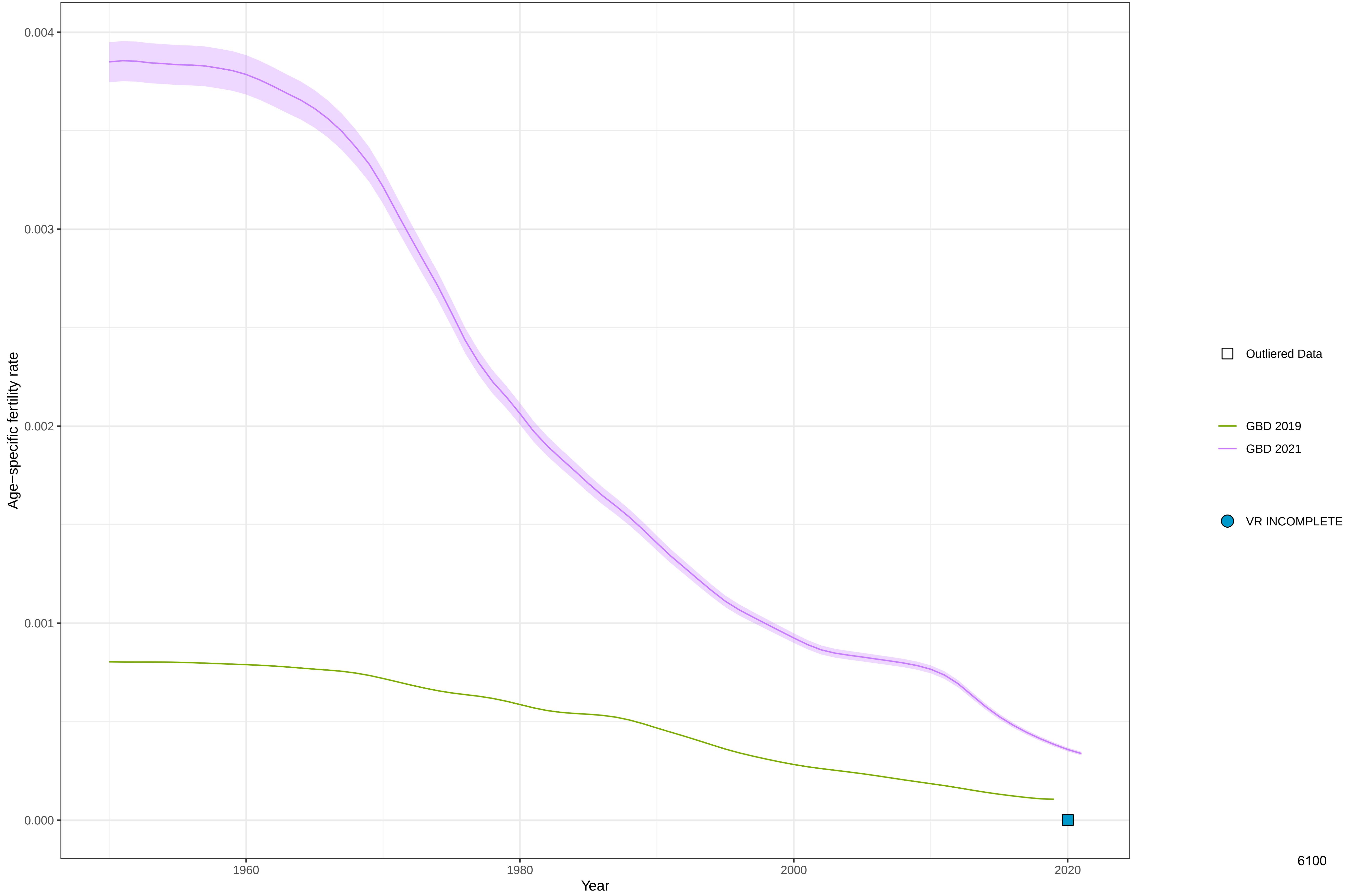

Total fertility rate fitted trend lines, 1950−2021: Dinagat Islands TFR
Data points enclosed with a diamond are reference sources

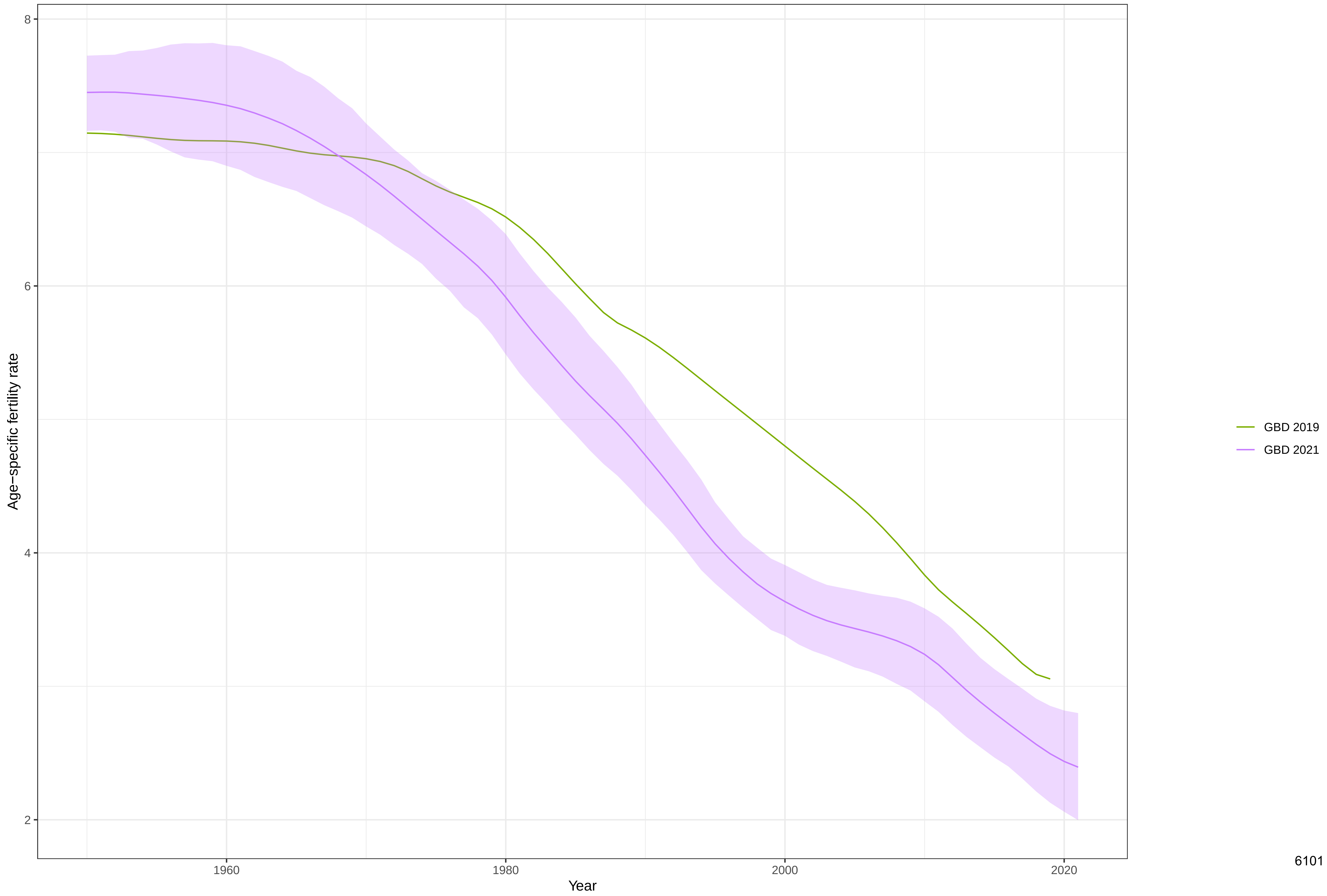

Age−specific fertility fitted trend lines, 1950−2021: Eastern Samar, Ages: 10−14
Data points enclosed with a diamond are reference sources

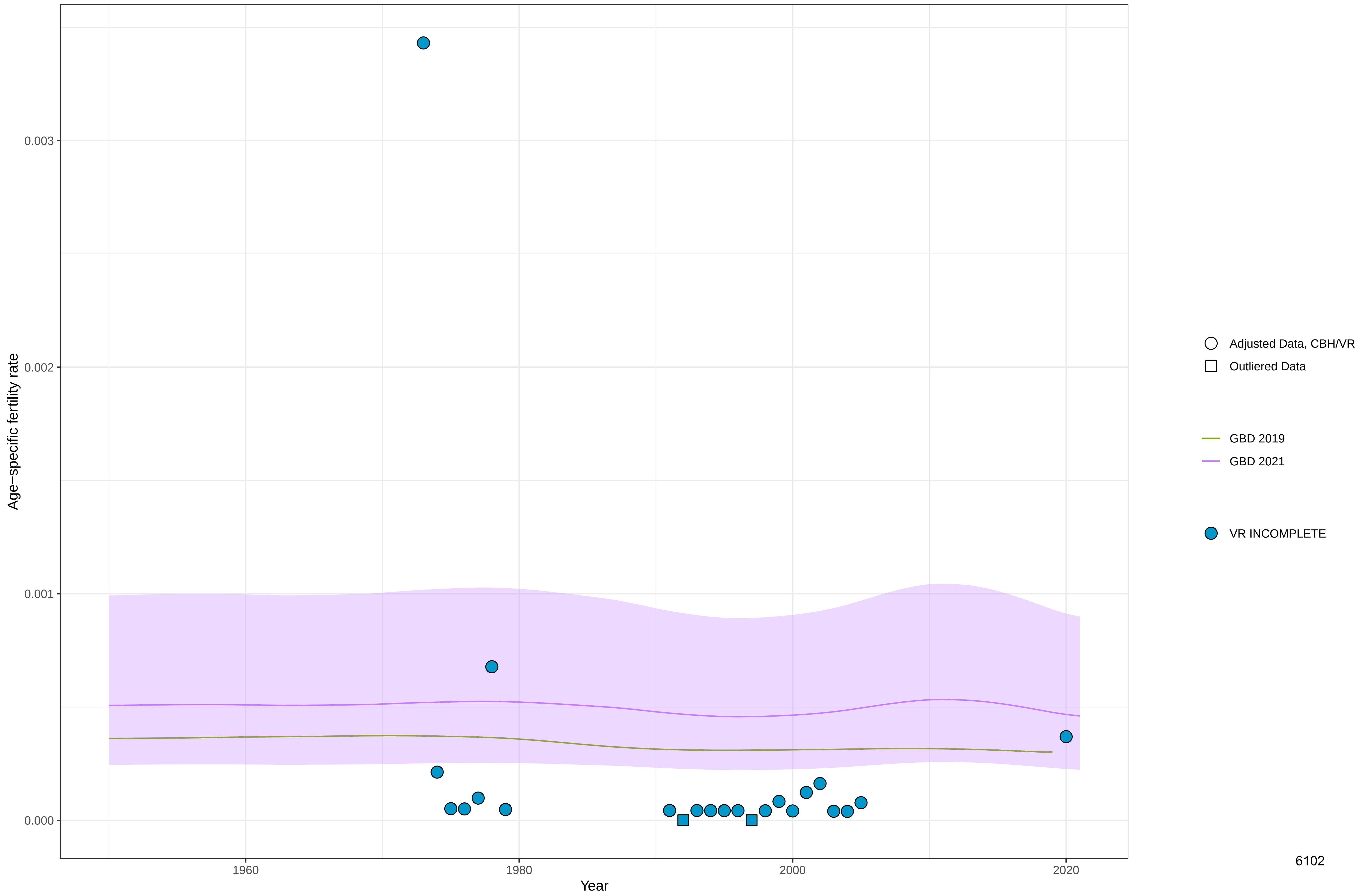

Age−specific fertility fitted trend lines, 1950−2021: Eastern Samar, Ages: 15−19
Data points enclosed with a diamond are reference sources

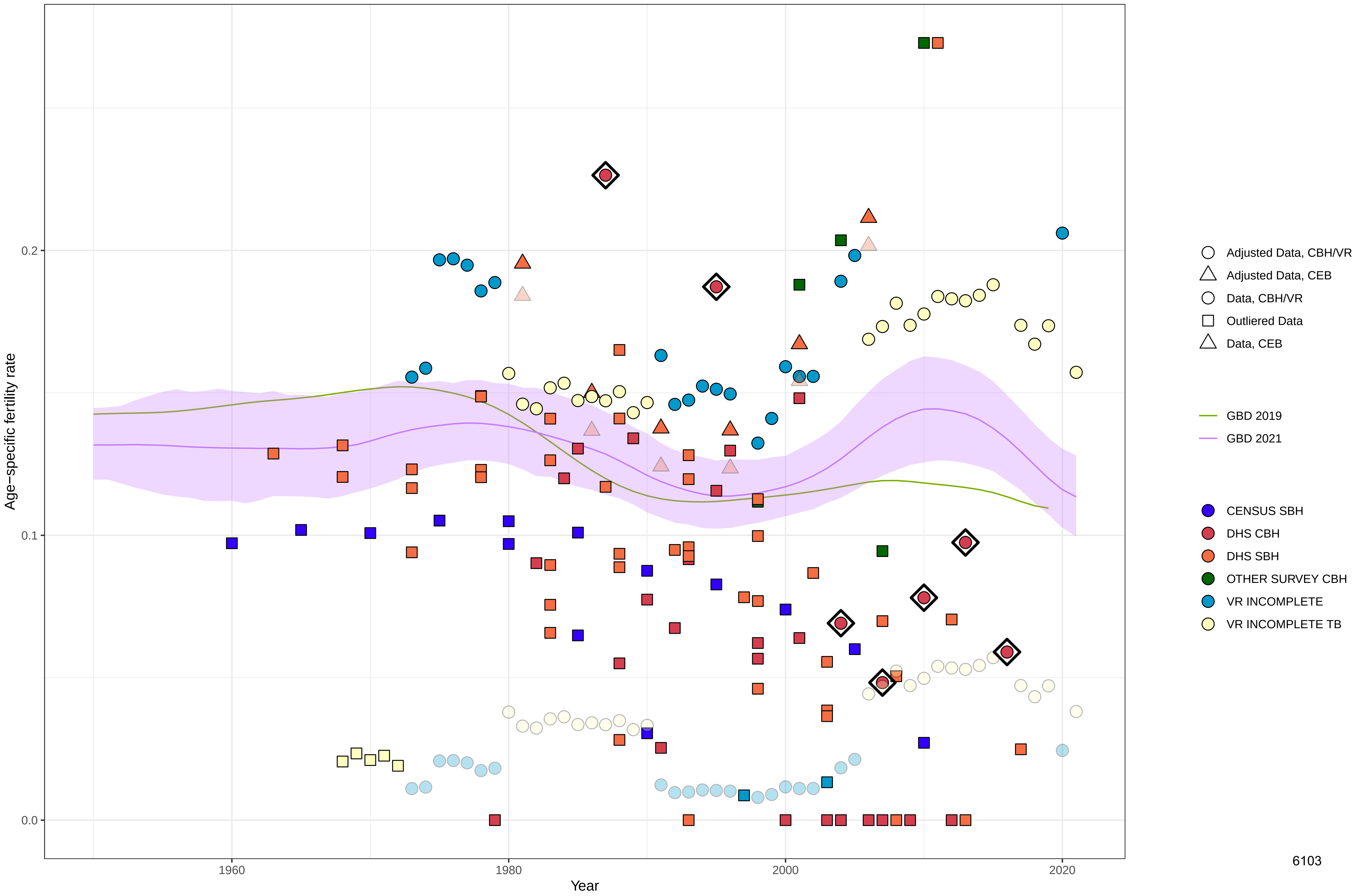

Age−specific fertility fitted trend lines, 1950−2021: Eastern Samar, Ages: 20−24
Data points enclosed with a diamond are reference sources

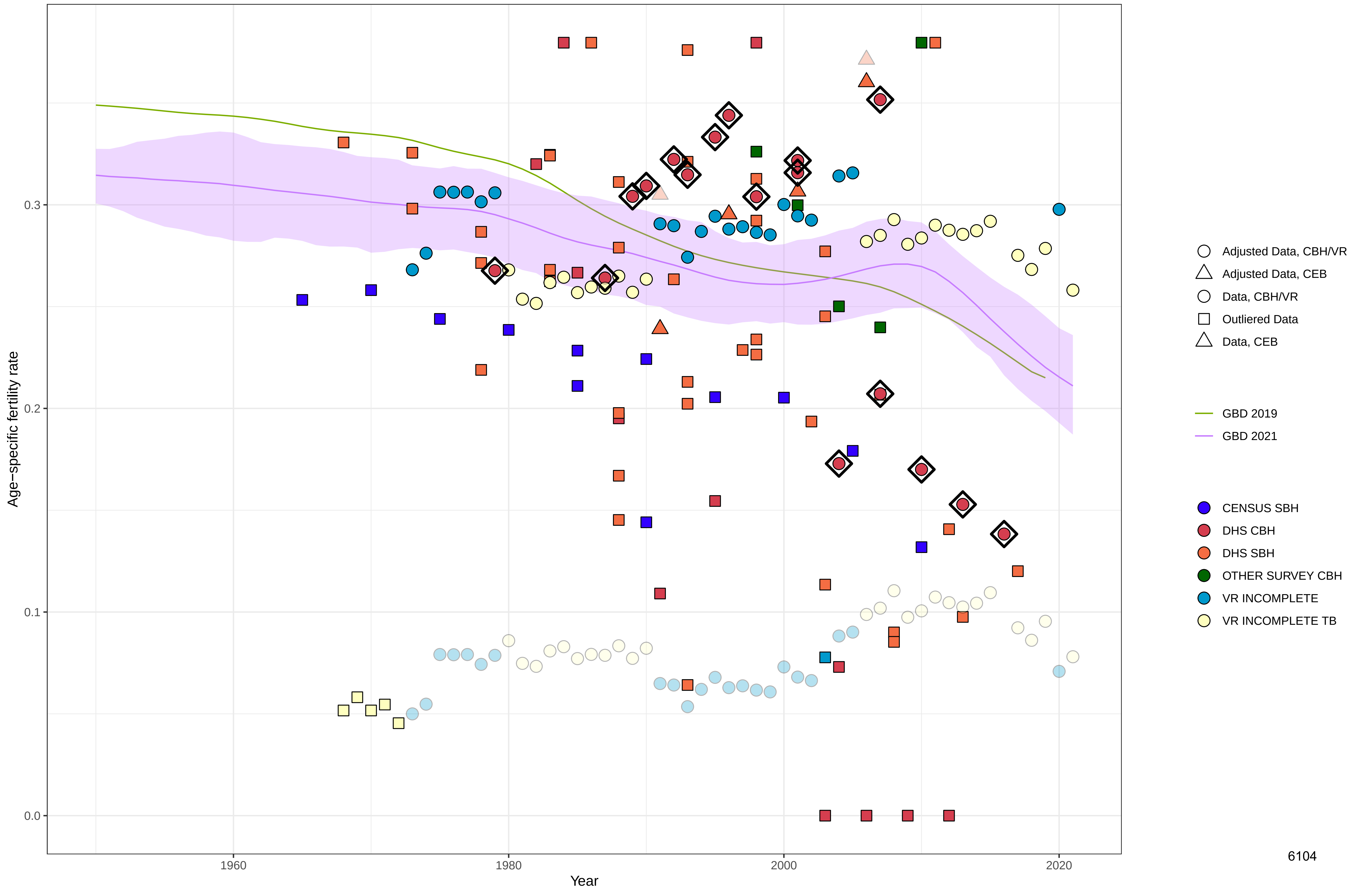

6104

Age−specific fertility fitted trend lines, 1950−2021: Eastern Samar, Ages: 25−29
Data points enclosed with a diamond are reference sources

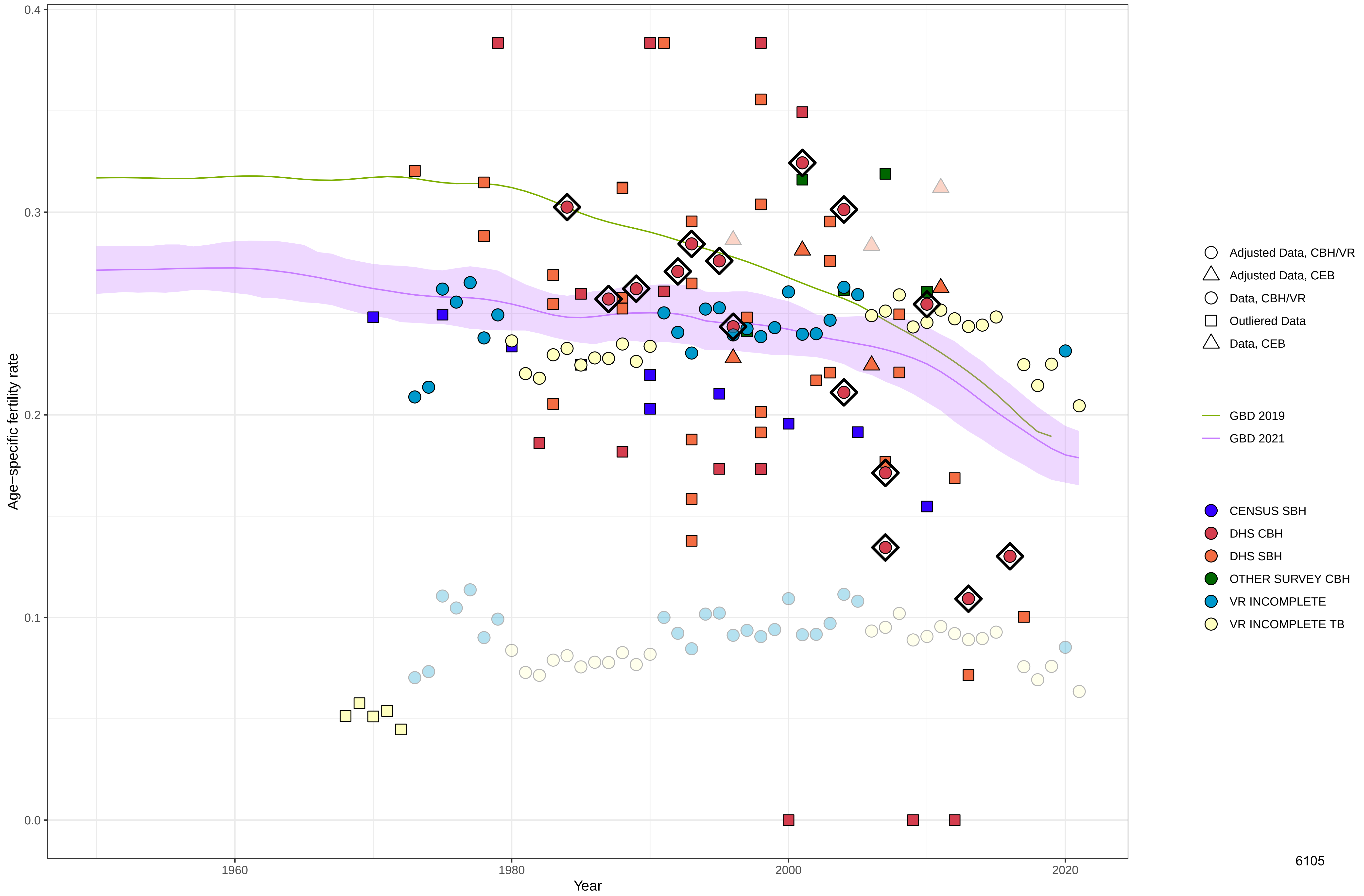

Age−specific fertility fitted trend lines, 1950−2021: Eastern Samar, Ages: 30−34
Data points enclosed with a diamond are reference sources

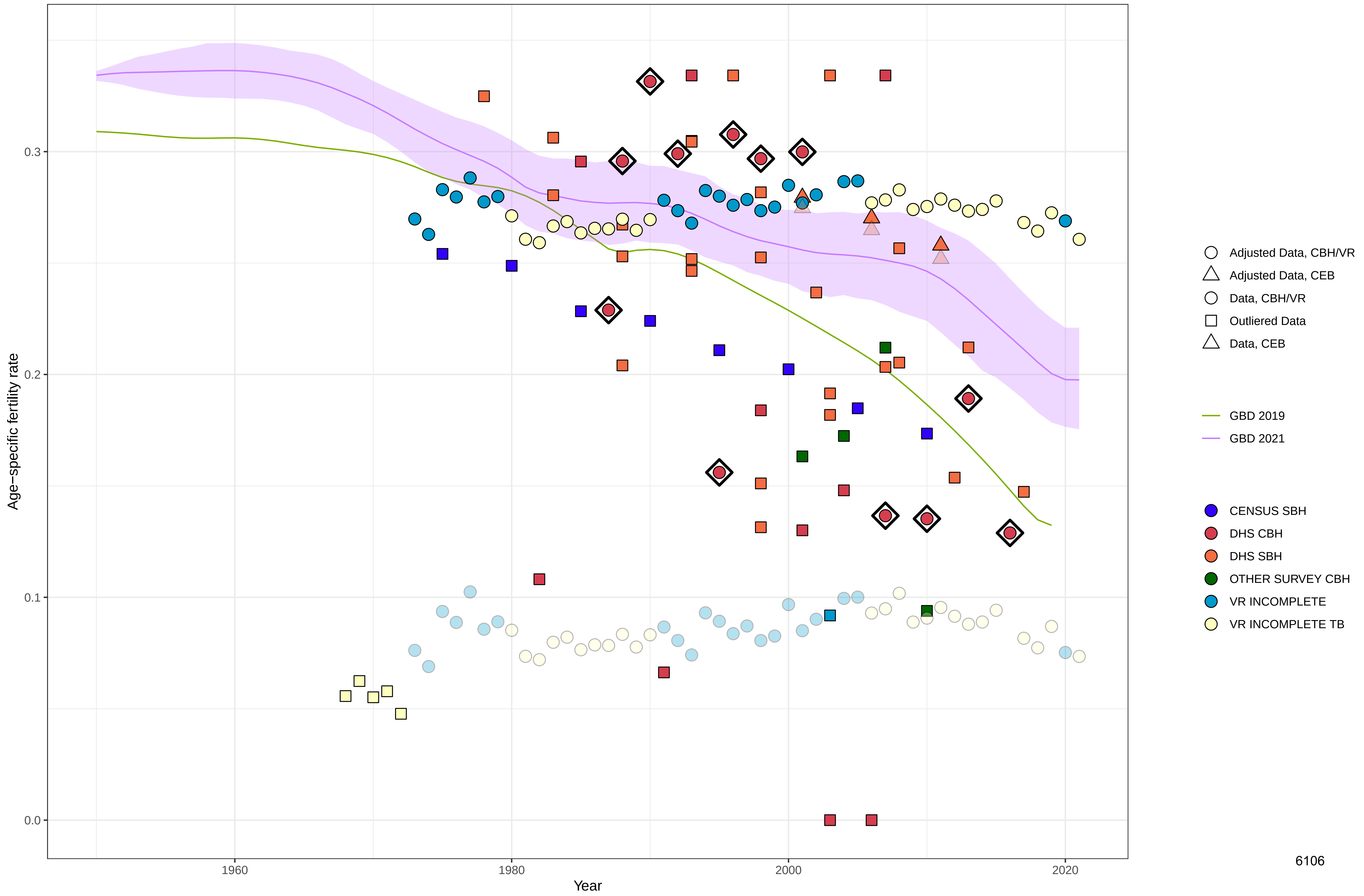

Age−specific fertility fitted trend lines, 1950−2021: Eastern Samar, Ages: 35−39
Data points enclosed with a diamond are reference sources

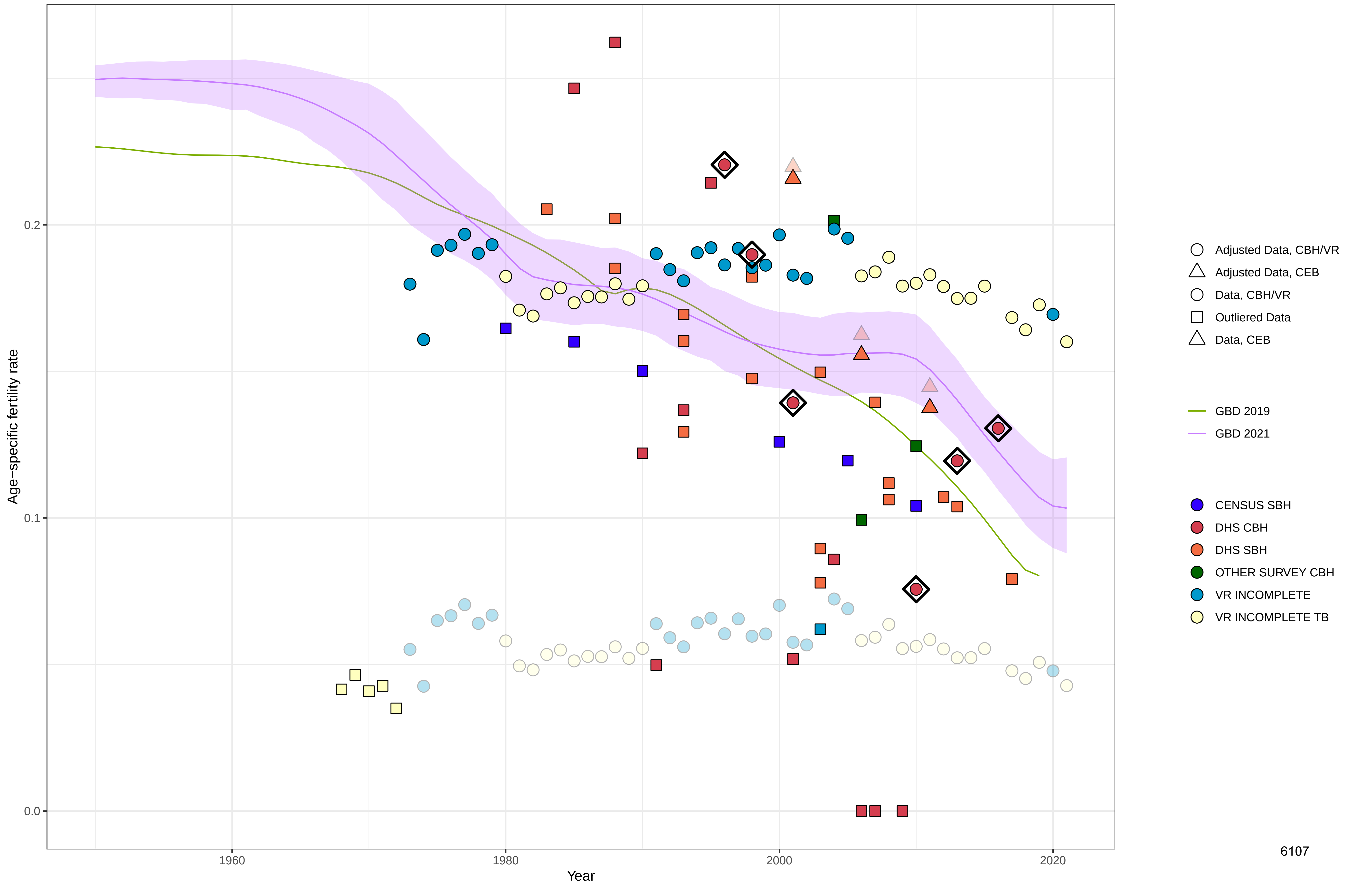

Age−specific fertility fitted trend lines, 1950−2021: Eastern Samar, Ages: 40−44
Data points enclosed with a diamond are reference sources

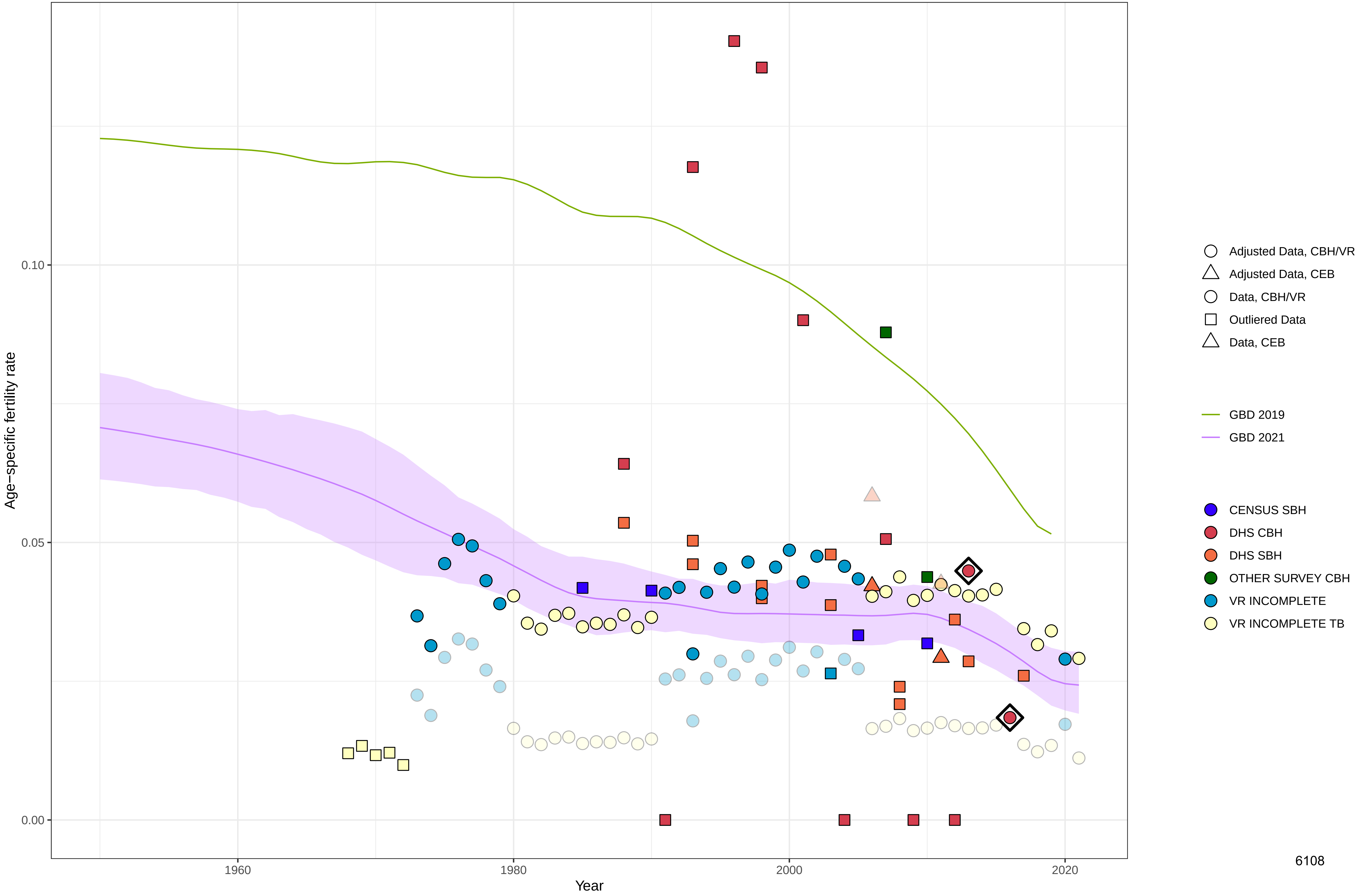

Age−specific fertility fitted trend lines, 1950−2021: Eastern Samar, Ages: 45−49
Data points enclosed with a diamond are reference sources

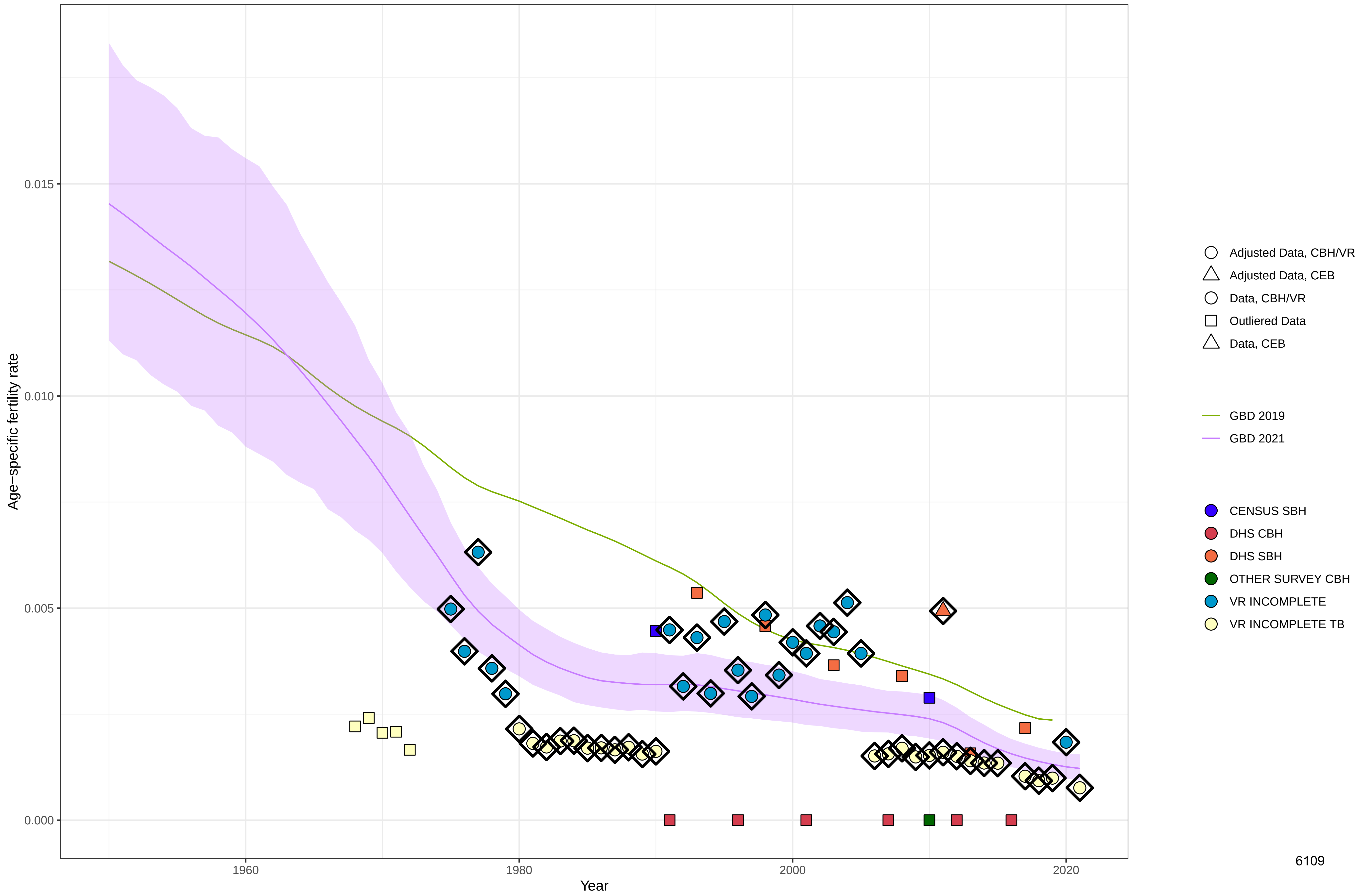

Age−specific fertility fitted trend lines, 1950−2021: Eastern Samar, Ages: 50−54
Data points enclosed with a diamond are reference sources

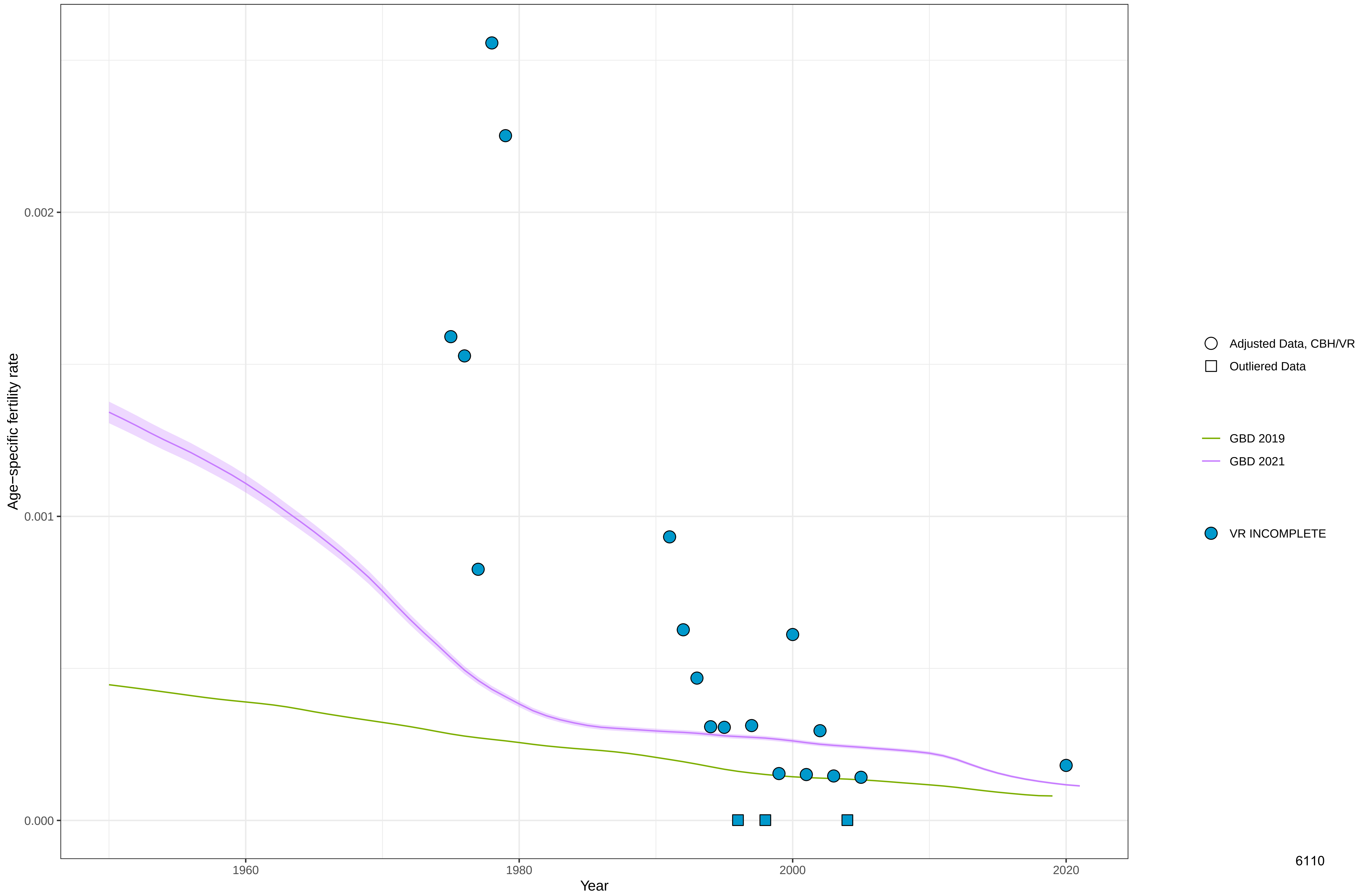

Total fertility rate fitted trend lines, 1950−2021: Eastern Samar TFR
Data points enclosed with a diamond are reference sources

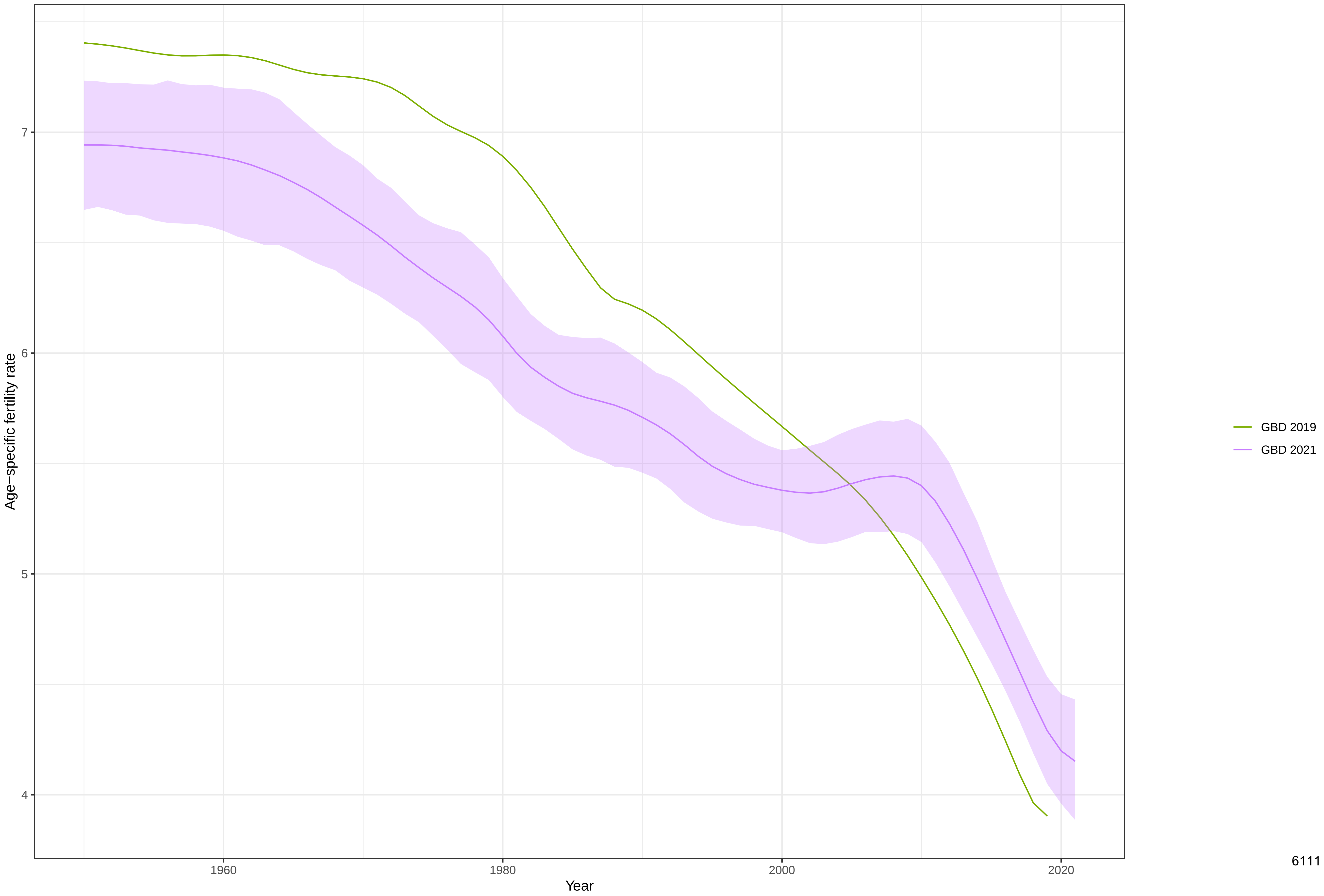

6111

Age−specific fertility fitted trend lines, 1950−2021: Guimaras, Ages: 10−14
Data points enclosed with a diamond are reference sources

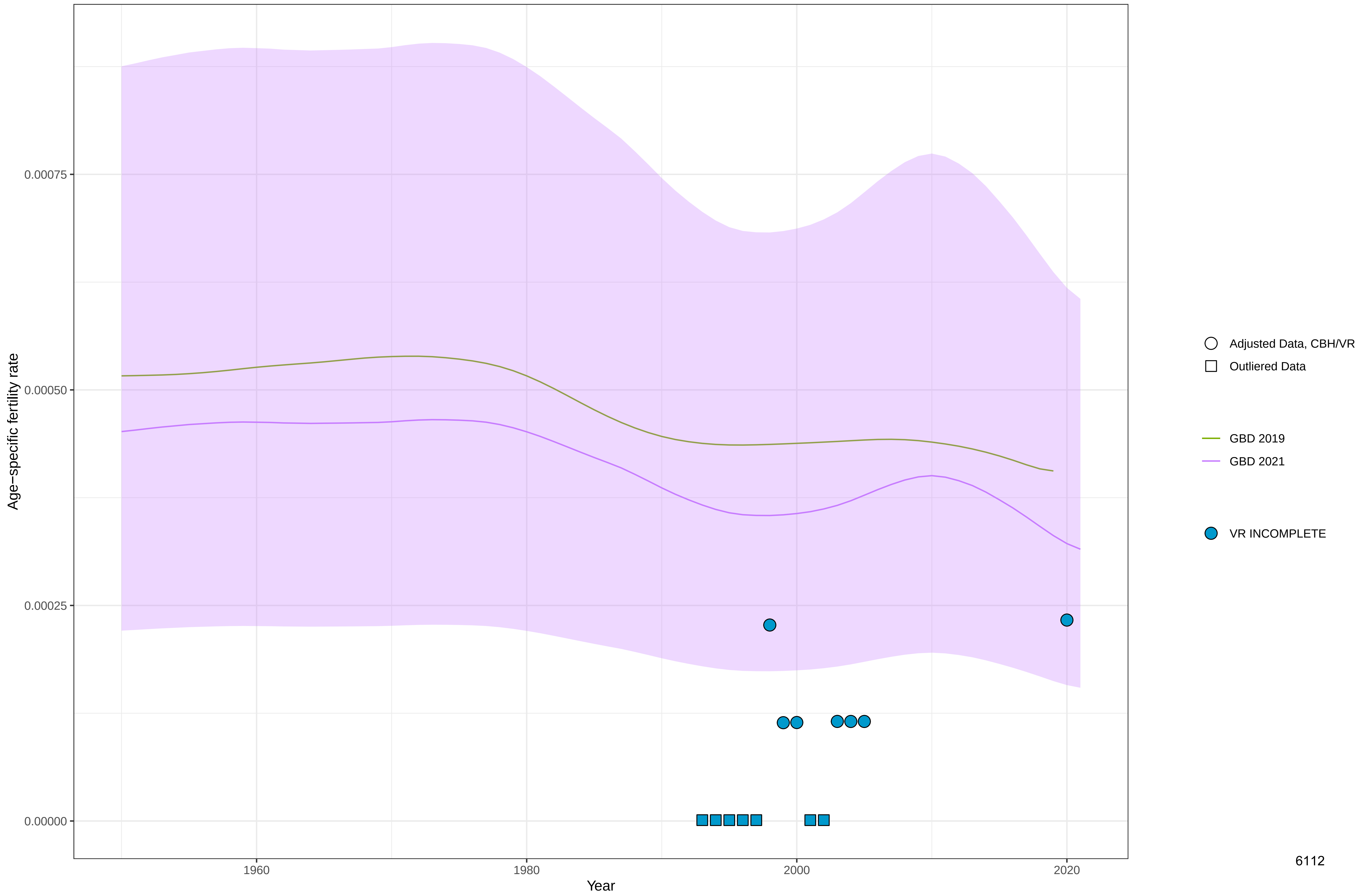

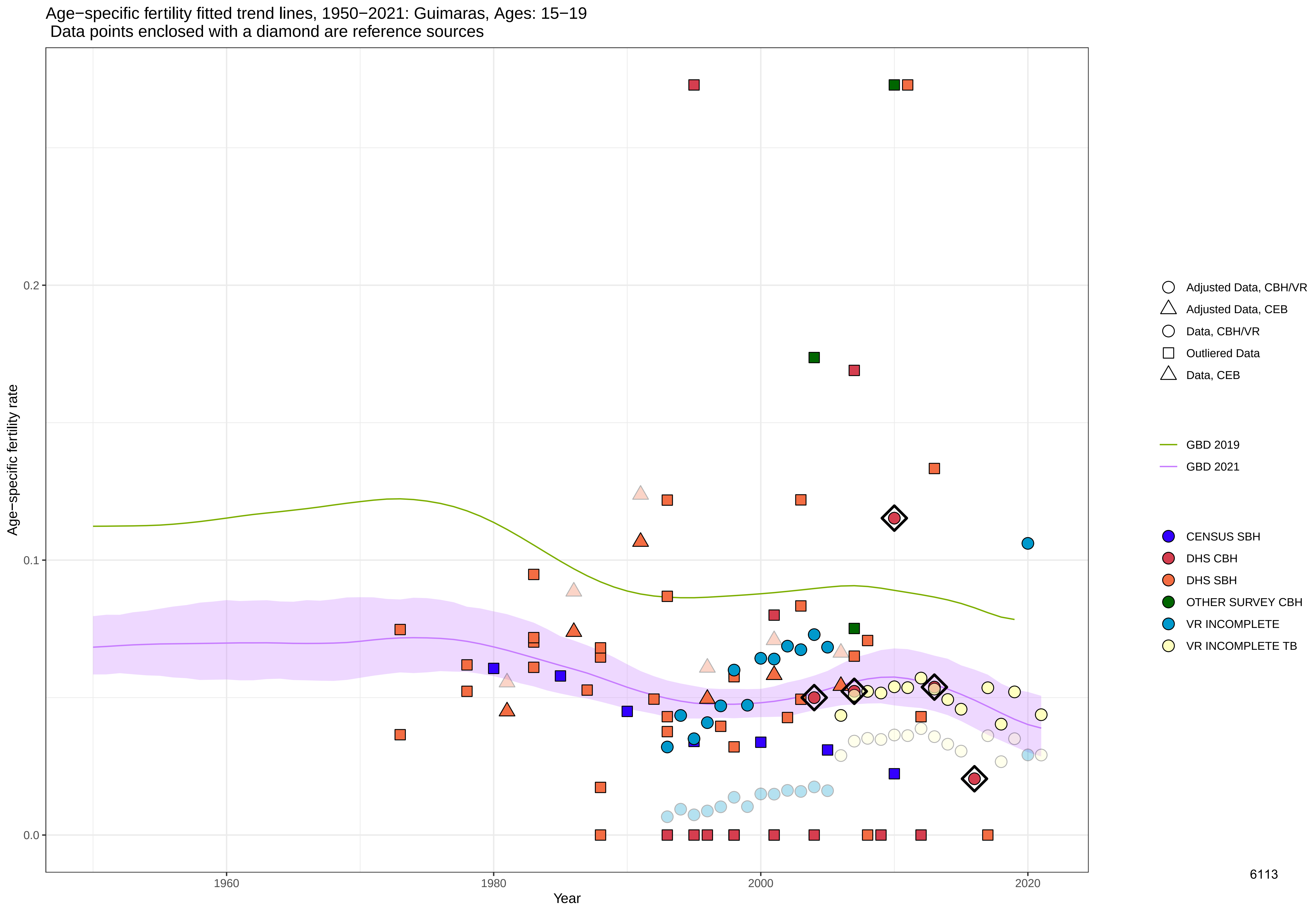
Age−specific fertility fitted trend lines, 1950−2021: Guimaras, Ages: 15−19
Data points enclosed with a diamond are reference sources
Age−specific fertility rate
0.0
0.1
0.2
1960
1980
2000
2020
Year
Adjusted Data, CBH/VR
Adjusted Data, CEB
Data, CBH/VR
Outliered Data
Data, CEB
GBD 2019
GBD 2021
CENSUS SBH
DHS CBH
DHS SBH
OTHER SURVEY CBH
VR INCOMPLETE
VR INCOMPLETE TB

Age−specific fertility fitted trend lines, 1950−2021: Guimaras, Ages: 20−24
Data points enclosed with a diamond are reference sources

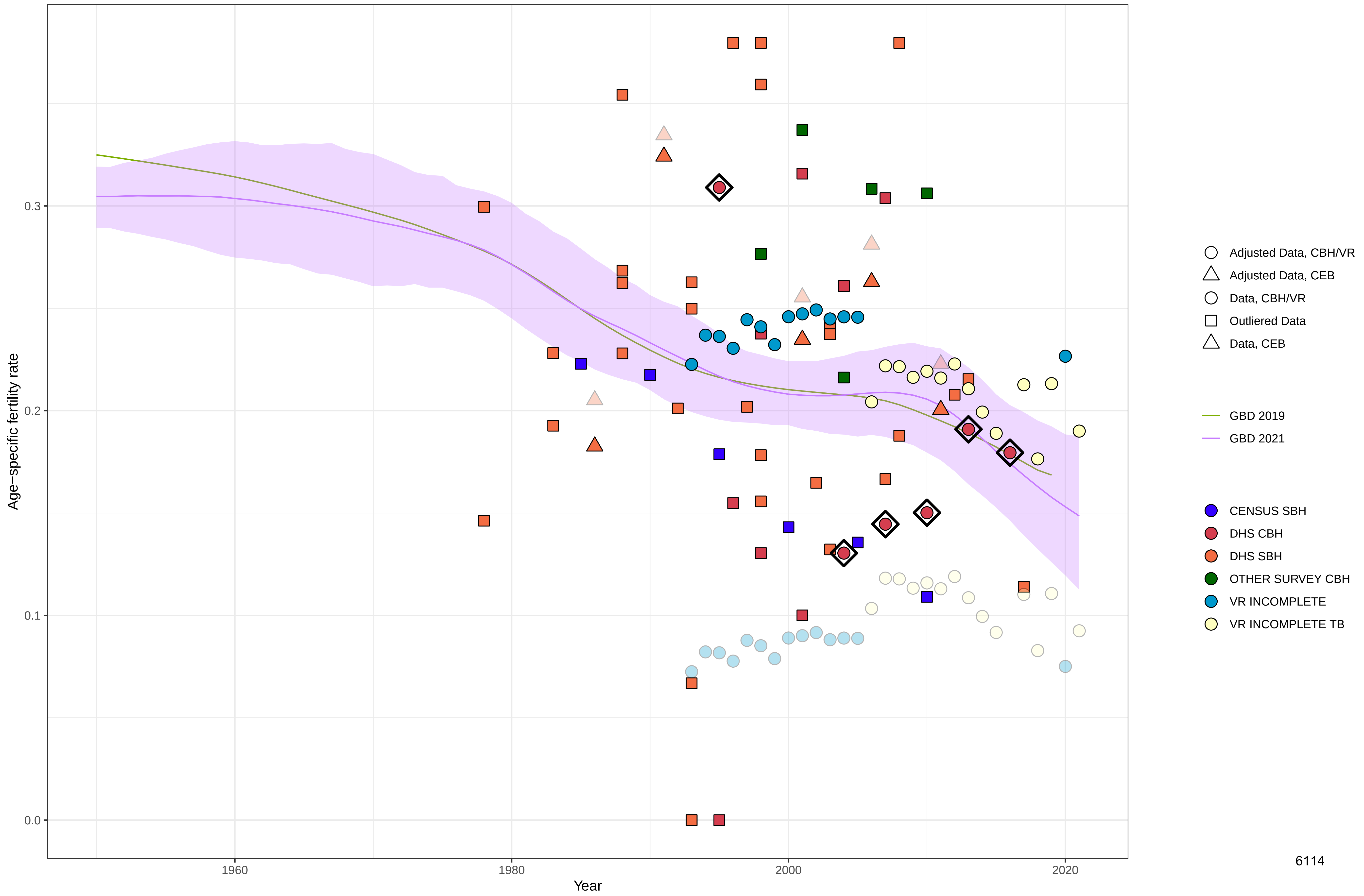

Age−specific fertility fitted trend lines, 1950−2021: Guimaras, Ages: 25−29
Data points enclosed with a diamond are reference sources

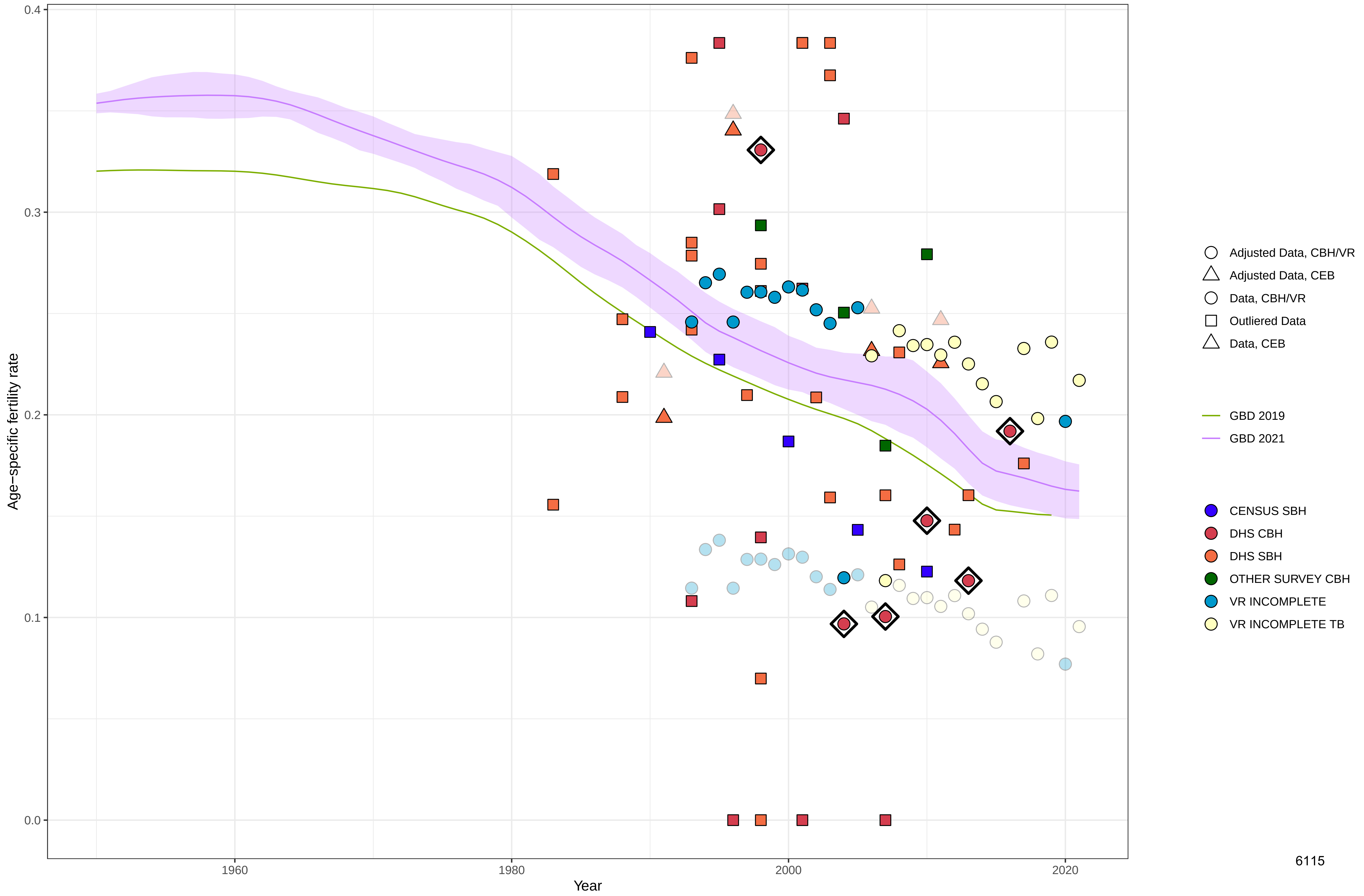

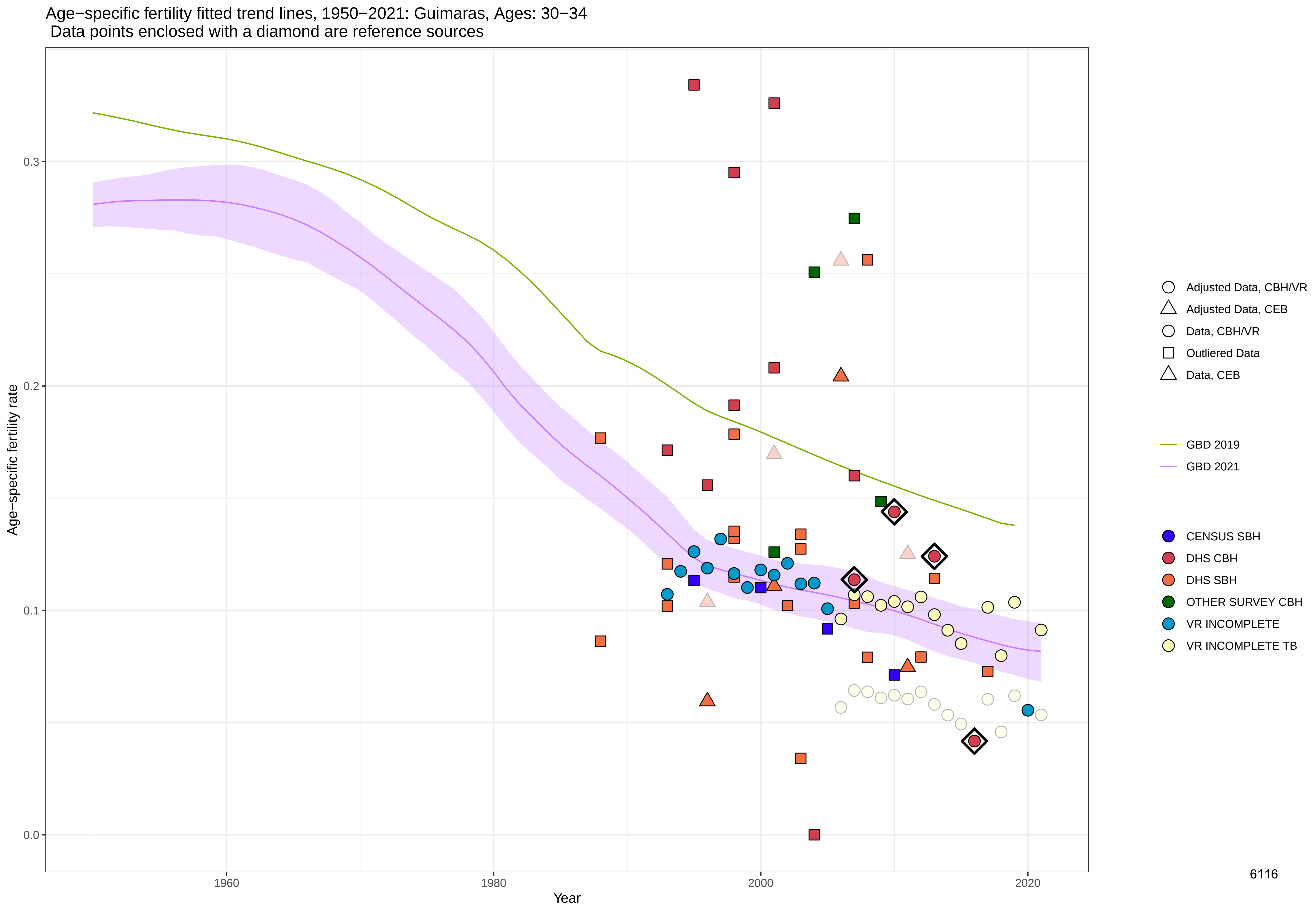

Age−specific fertility fitted trend lines, 1950−2021: Guimaras, Ages: 30−34
Data points enclosed with a diamond are reference sources
Age−specific fertility rate
Year
0.0
0.1
0.2
0.3
1960
1980
2000
2020
Adjusted Data, CBH/VR
Adjusted Data, CEB
Data, CBH/VR
Outliered Data
Data, CEB
GBD 2019
GBD 2021
CENSUS SBH
DHS CBH
DHS SBH
OTHER SURVEY CBH
VR INCOMPLETE
VR INCOMPLETE TB

Age−specific fertility fitted trend lines, 1950−2021: Guimaras, Ages: 35−39
Data points enclosed with a diamond are reference sources

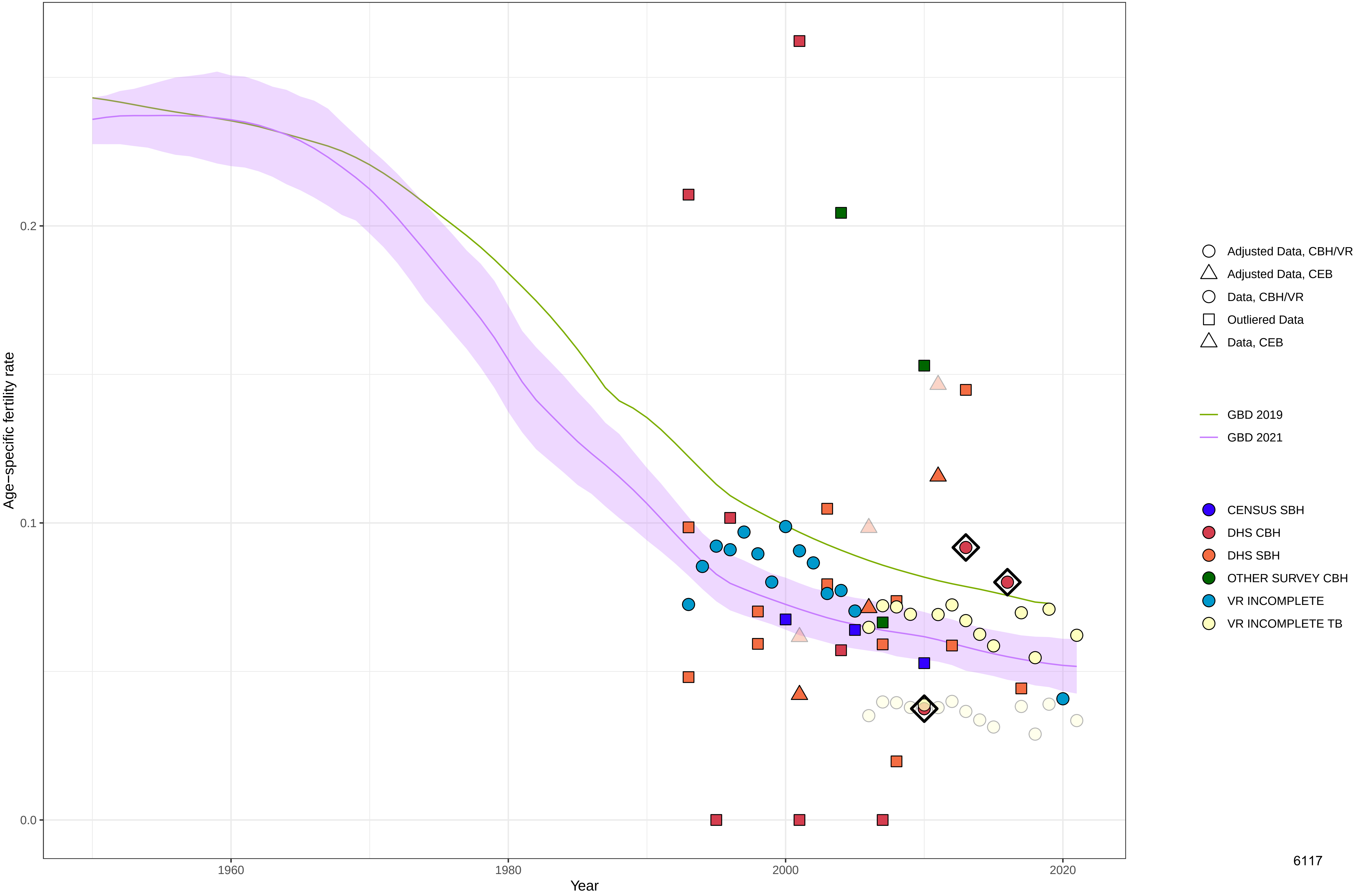

Age−specific fertility fitted trend lines, 1950−2021: Guimaras, Ages: 40−44
Data points enclosed with a diamond are reference sources

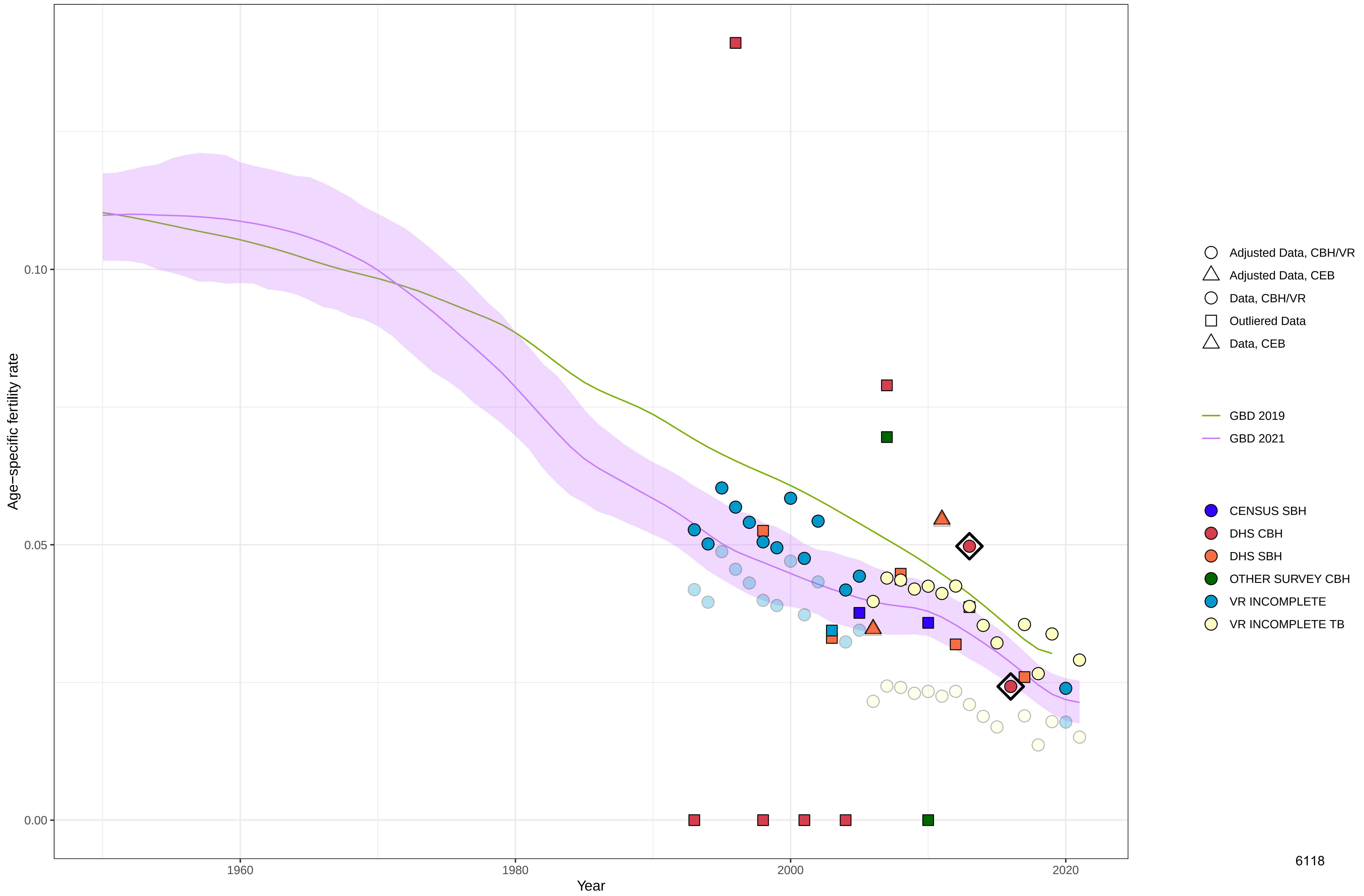

Age−specific fertility fitted trend lines, 1950−2021: Guimaras, Ages: 45−49
Data points enclosed with a diamond are reference sources

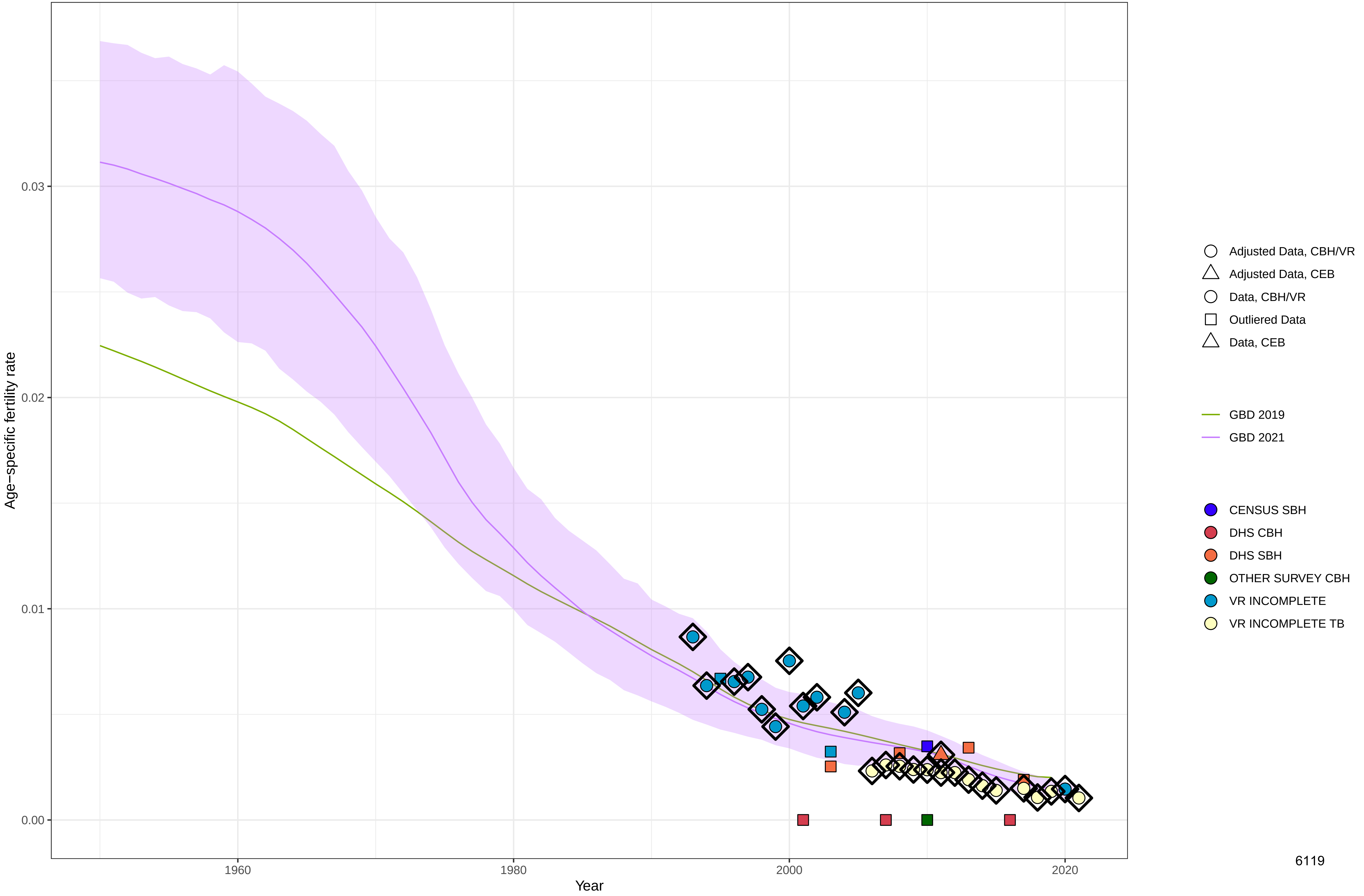

Age−specific fertility fitted trend lines, 1950−2021: Guimaras, Ages: 50−54
Data points enclosed with a diamond are reference sources

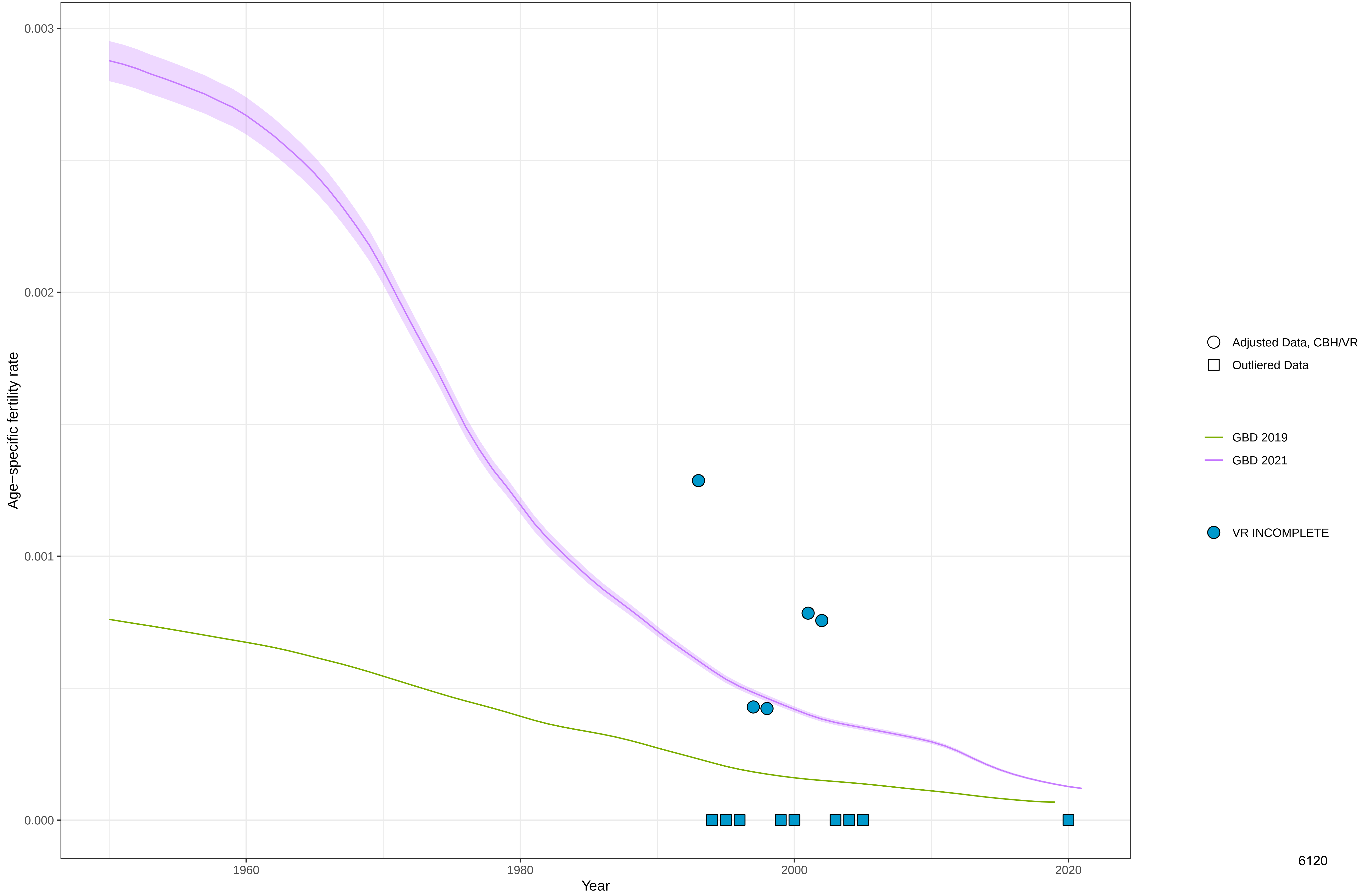

Total fertility rate fitted trend lines, 1950−2021: Guimaras TFR
Data points enclosed with a diamond are reference sources

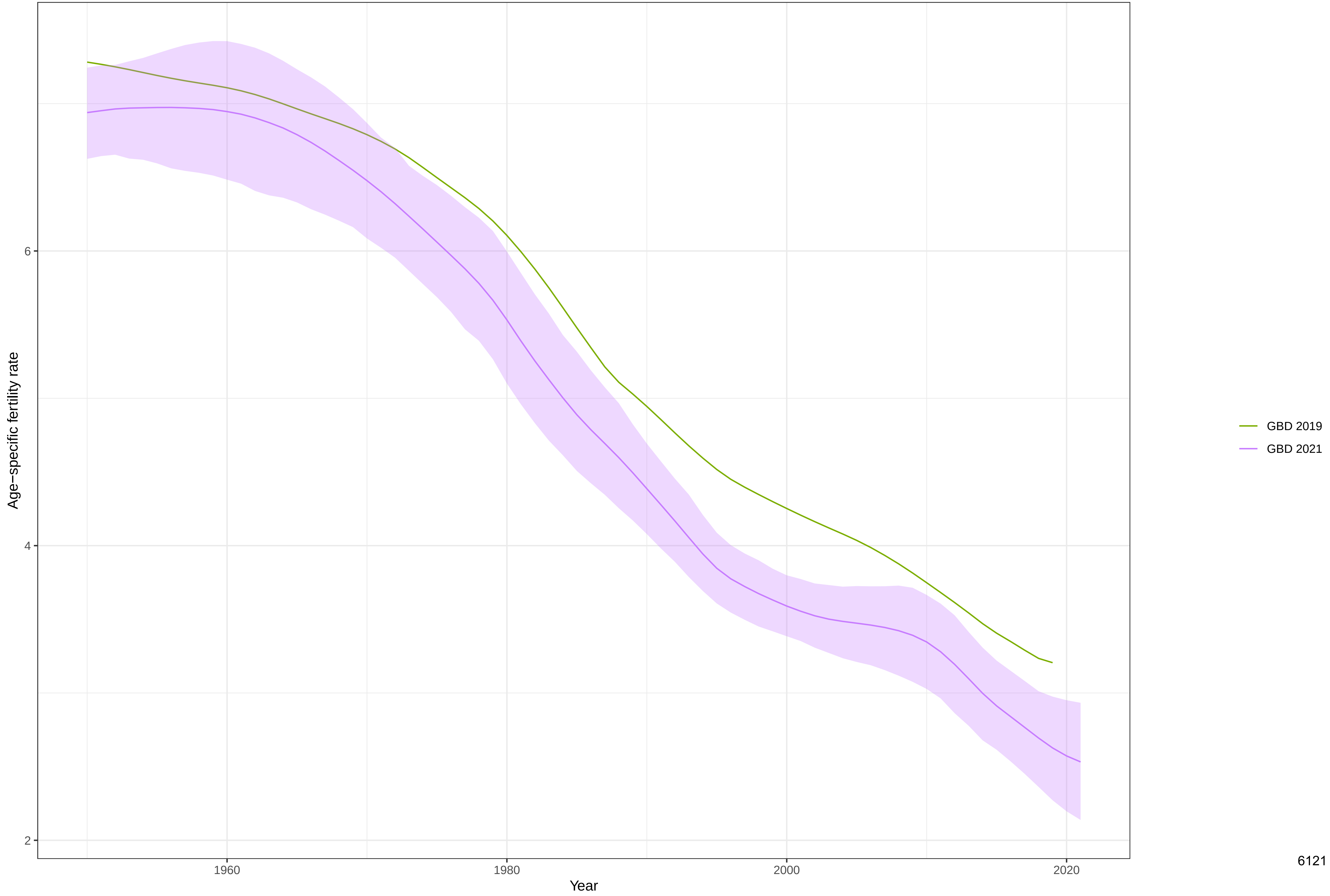

Age−specific fertility fitted trend lines, 1950−2021: Ifugao, Ages: 10−14
Data points enclosed with a diamond are reference sources

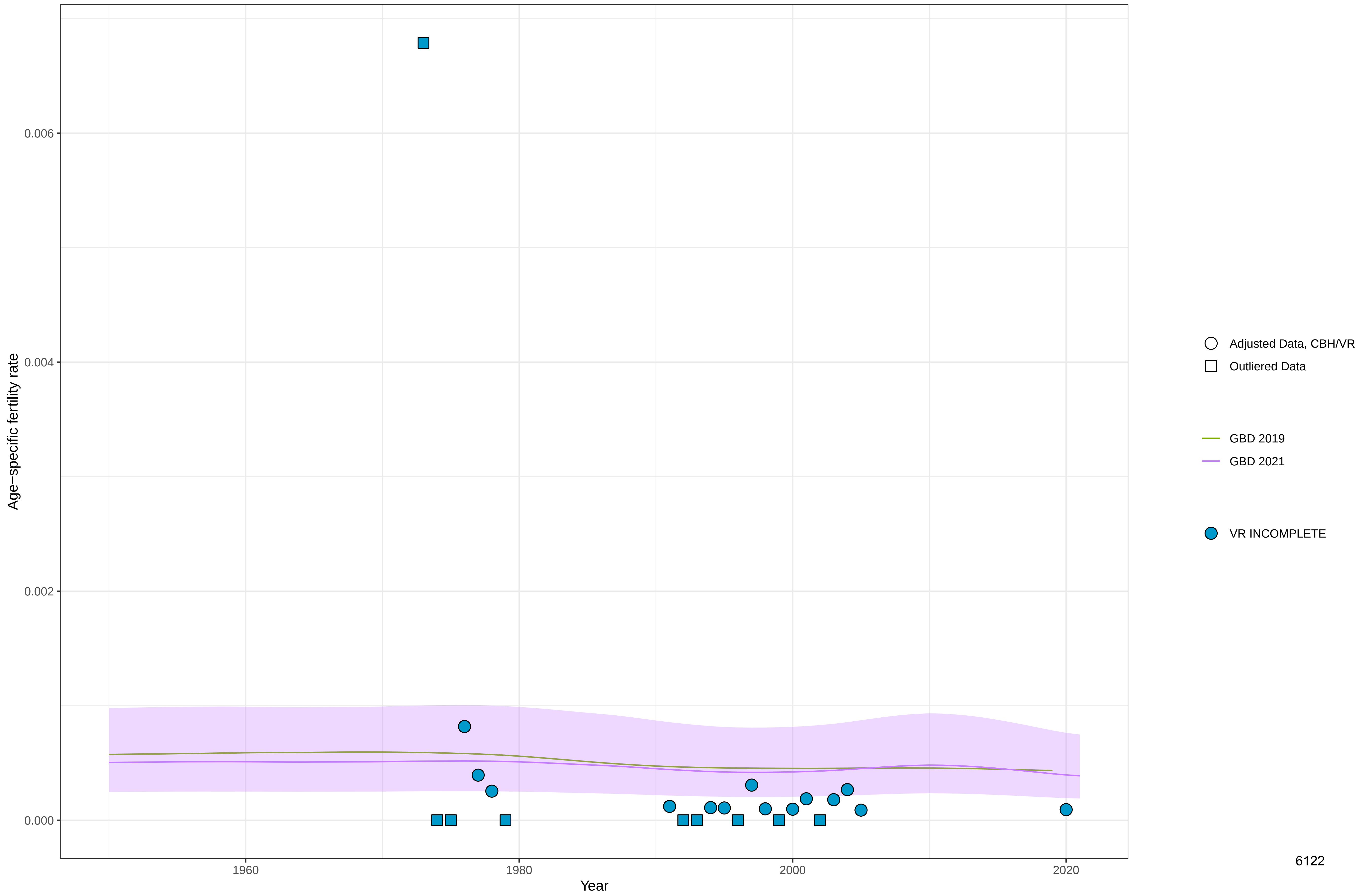

6122

Age−specific fertility fitted trend lines, 1950−2021: Ifugao, Ages: 15−19
Data points enclosed with a diamond are reference sources

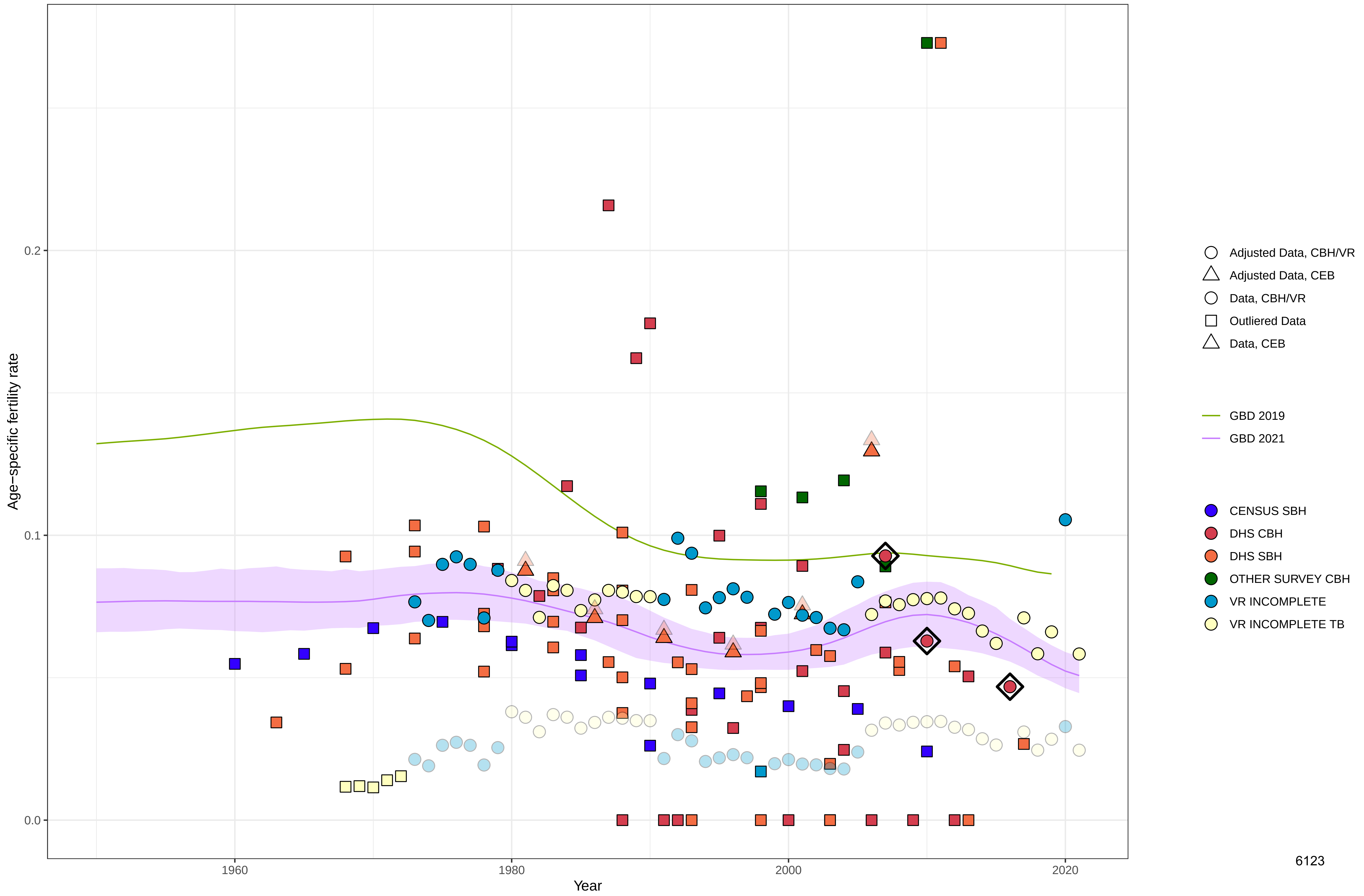

Age−specific fertility fitted trend lines, 1950−2021: Ifugao, Ages: 20−24
Data points enclosed with a diamond are reference sources

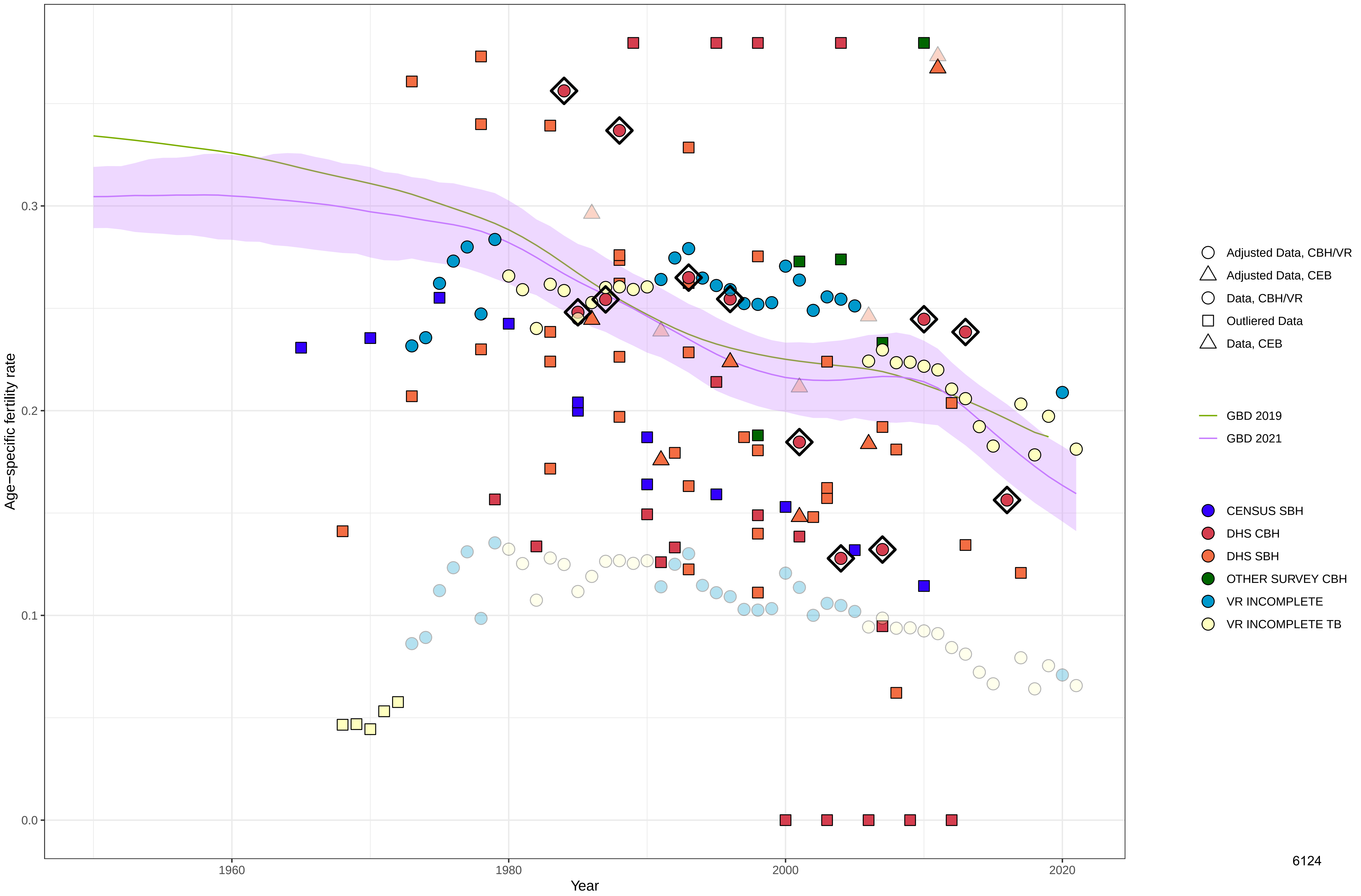

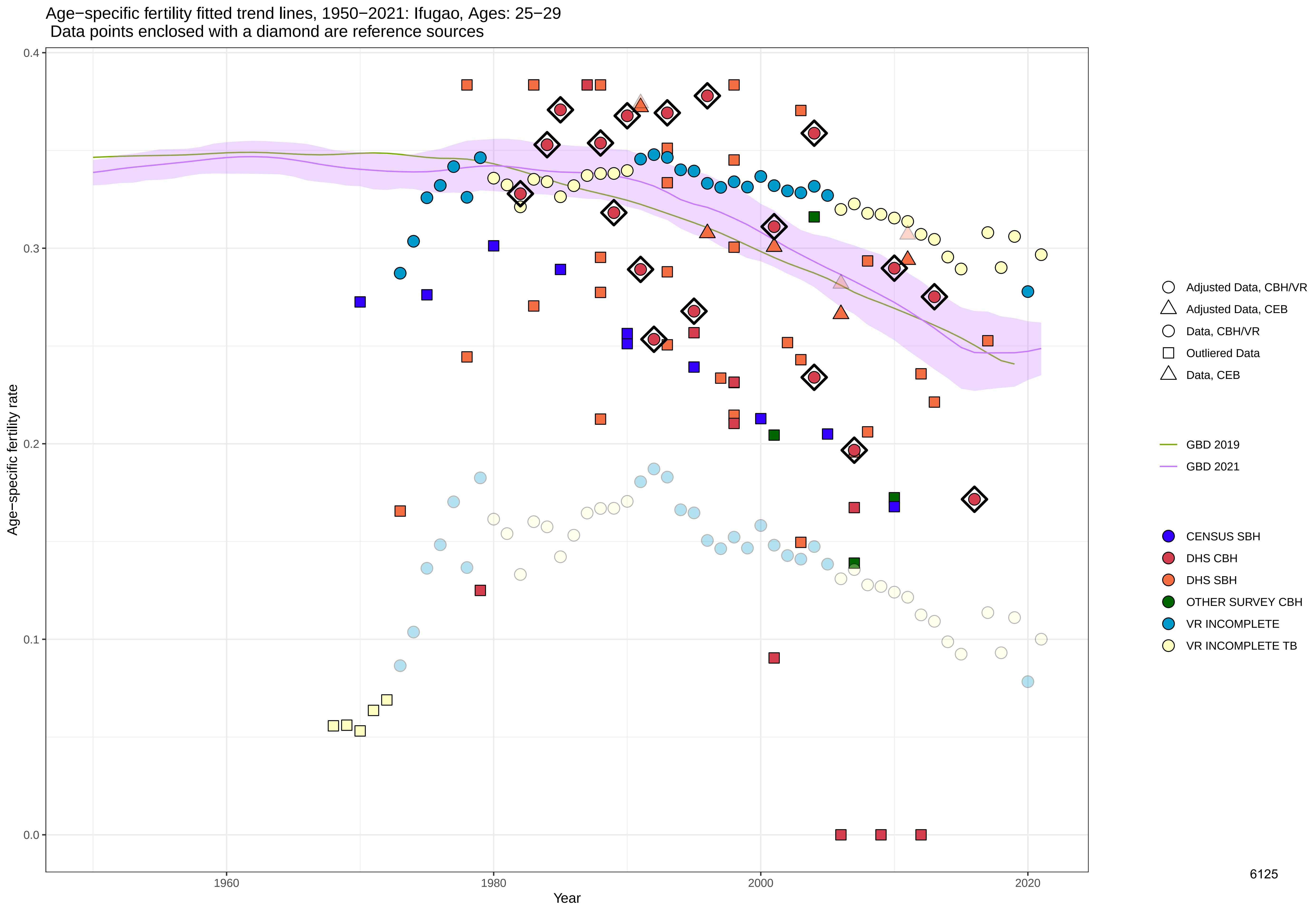

Age−specific fertility fitted trend lines, 1950−2021: Ifugao, Ages: 25−29
Data points enclosed with a diamond are reference sources
Age−specific fertility rate
Year
0.0
0.1
0.2
0.3
0.4
1960
1980
2000
2020
Adjusted Data, CBH/VR
Adjusted Data, CEB
Data, CBH/VR
Outliered Data
Data, CEB
GBD 2019
GBD 2021
CENSUS SBH
DHS CBH
DHS SBH
OTHER SURVEY CBH
VR INCOMPLETE
VR INCOMPLETE TB

Age−specific fertility fitted trend lines, 1950−2021: Ifugao, Ages: 30−34
Data points enclosed with a diamond are reference sources

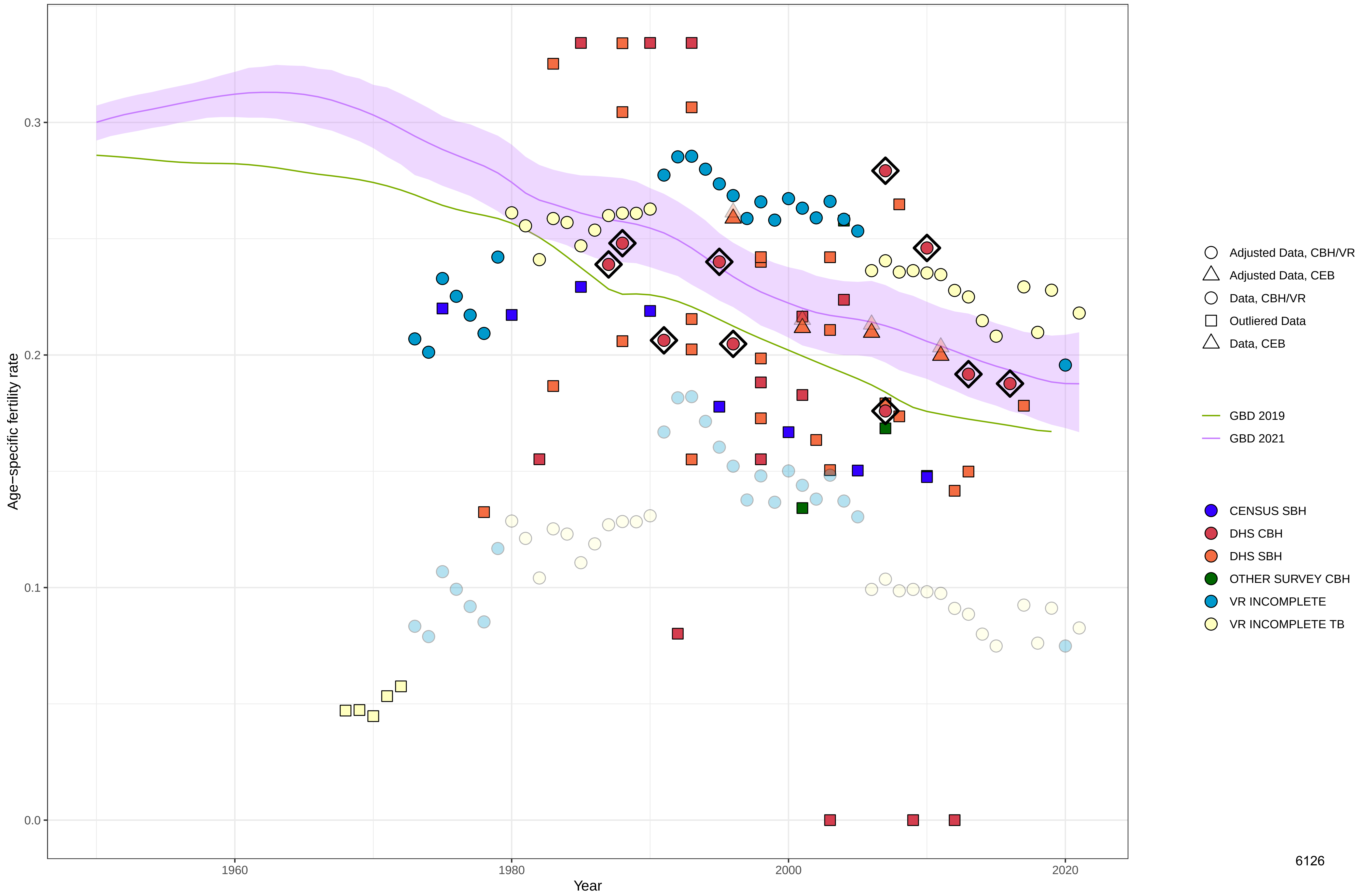

Age−specific fertility fitted trend lines, 1950−2021: Ifugao, Ages: 35−39
Data points enclosed with a diamond are reference sources

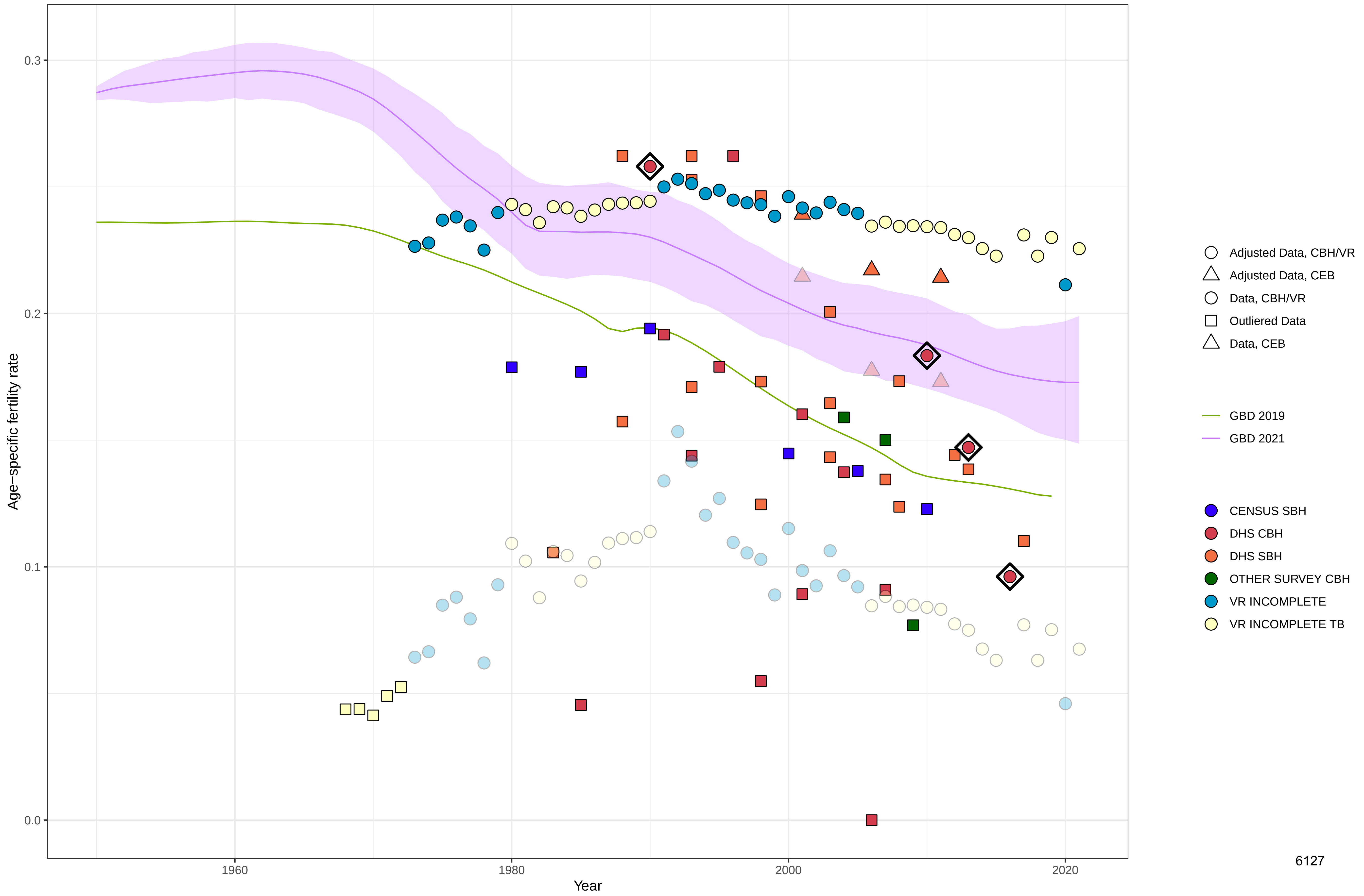

Age−specific fertility fitted trend lines, 1950−2021: Ifugao, Ages: 40−44
Data points enclosed with a diamond are reference sources

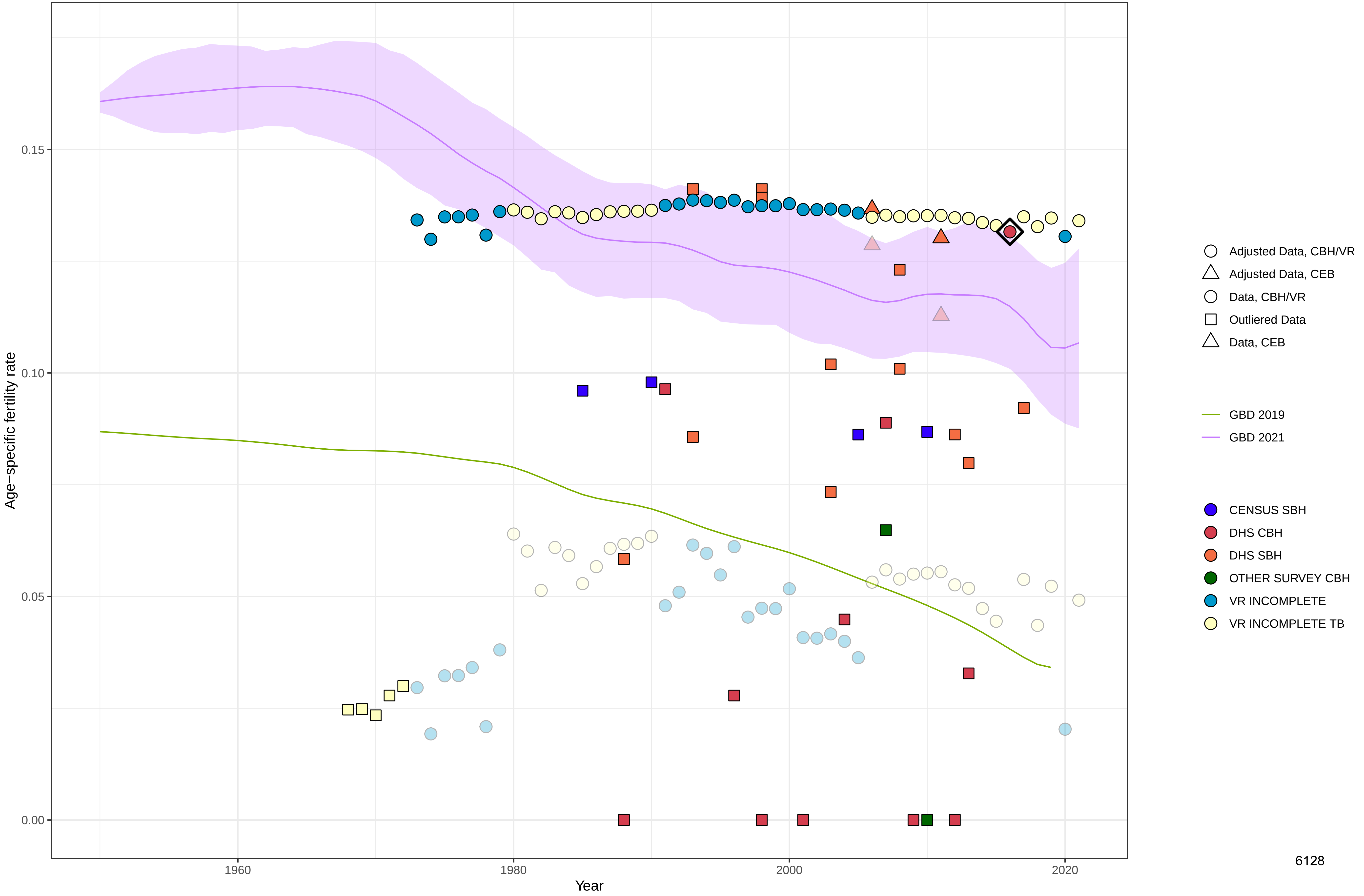

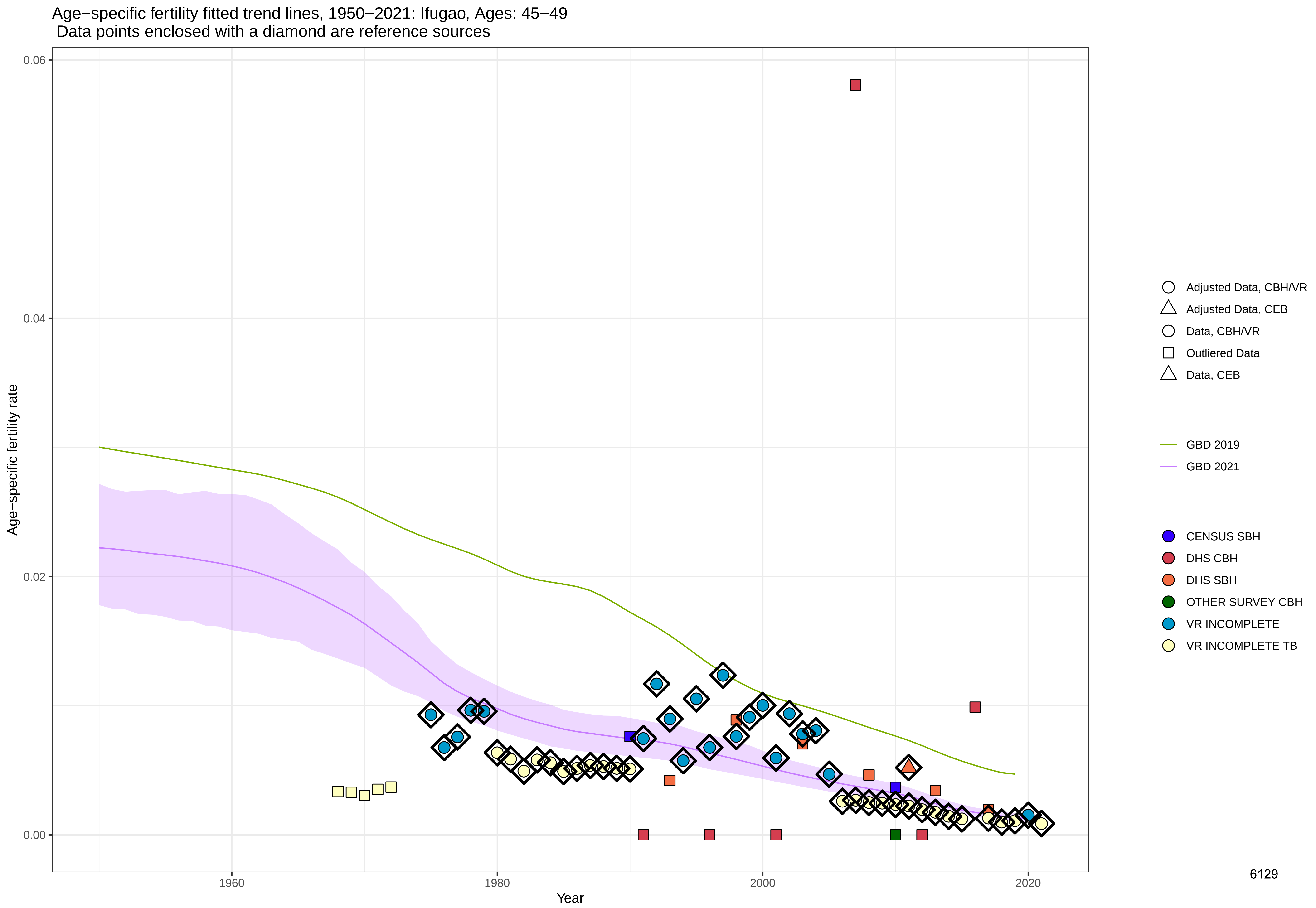

Age−specific fertility fitted trend lines, 1950−2021: Ifugao, Ages: 45−49
Data points enclosed with a diamond are reference sources
0.06
0.04
0.02
0.00
Age−specific fertility rate
1960
1980
2000
2020
Year
Adjusted Data, CBH/VR
Adjusted Data, CEB
Data, CBH/VR
Outliered Data
Data, CEB
GBD 2019
GBD 2021
CENSUS SBH
DHS CBH
DHS SBH
OTHER SURVEY CBH
VR INCOMPLETE
VR INCOMPLETE TB

6129

Age−specific fertility fitted trend lines, 1950−2021: Ifugao, Ages: 50−54
Data points enclosed with a diamond are reference sources

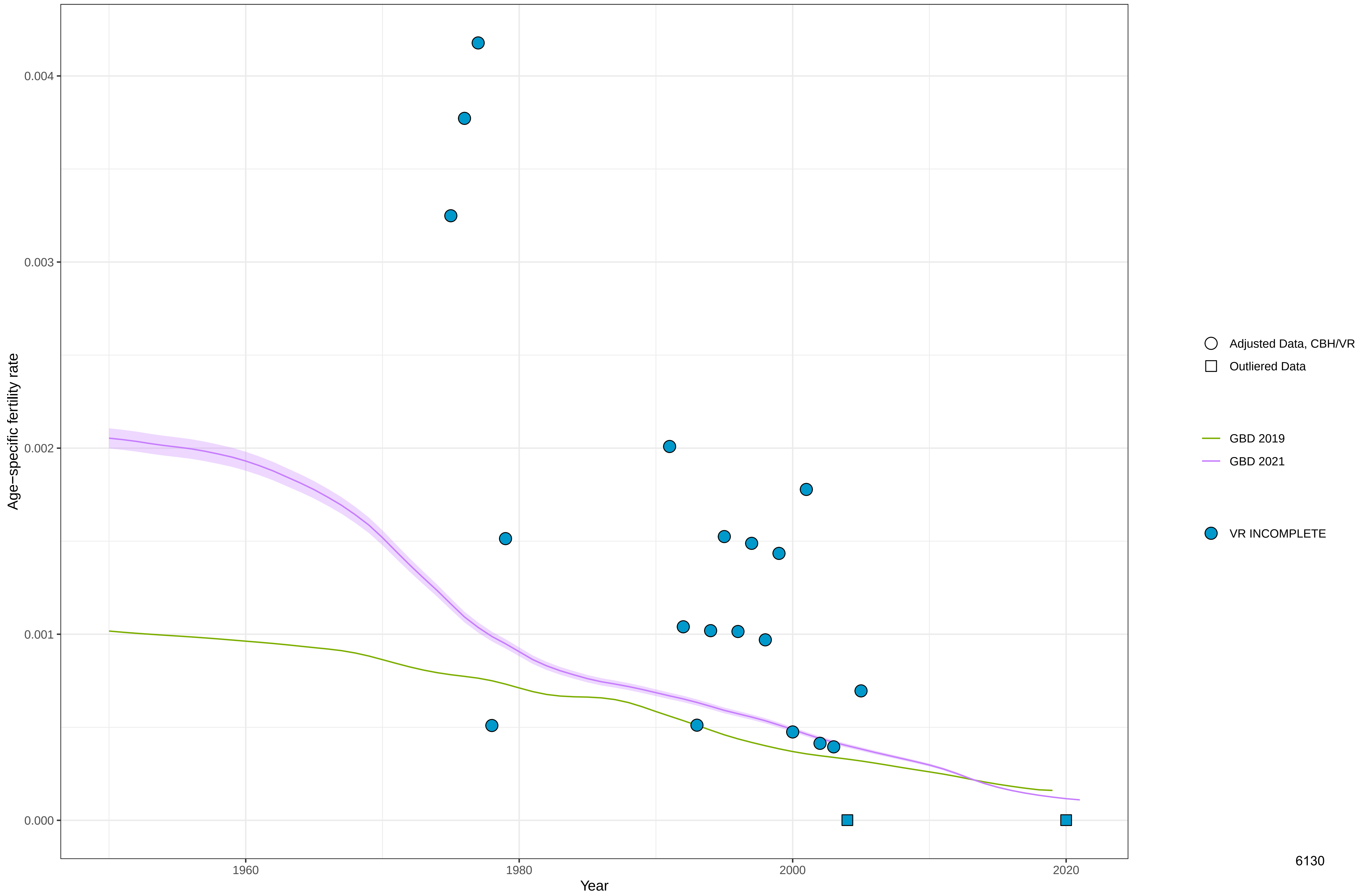

Total fertility rate fitted trend lines, 1950−2021: Ifugao TFR
Data points enclosed with a diamond are reference sources

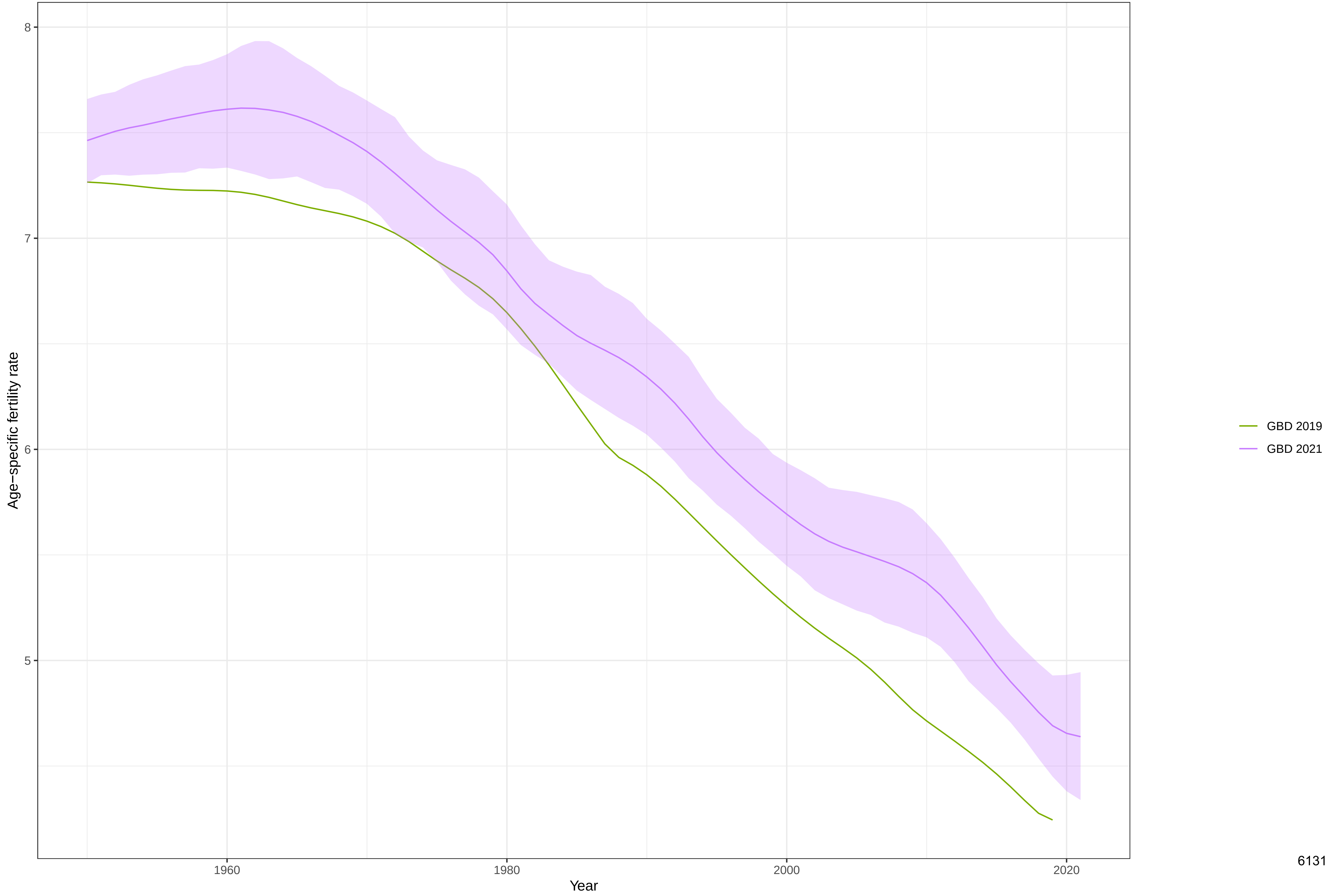

Age−specific fertility fitted trend lines, 1950−2021: Ilocos Norte, Ages: 10−14
Data points enclosed with a diamond are reference sources

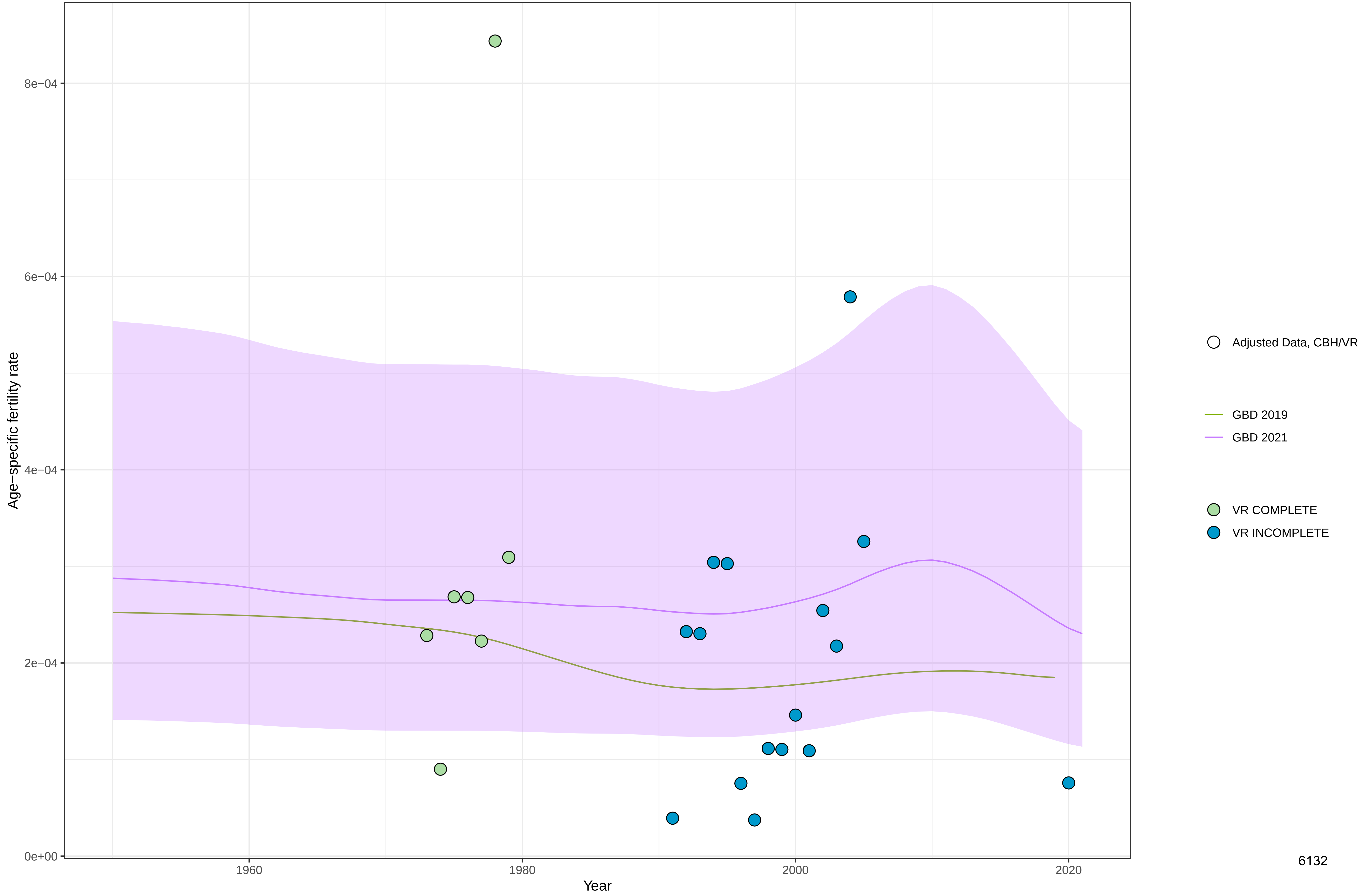

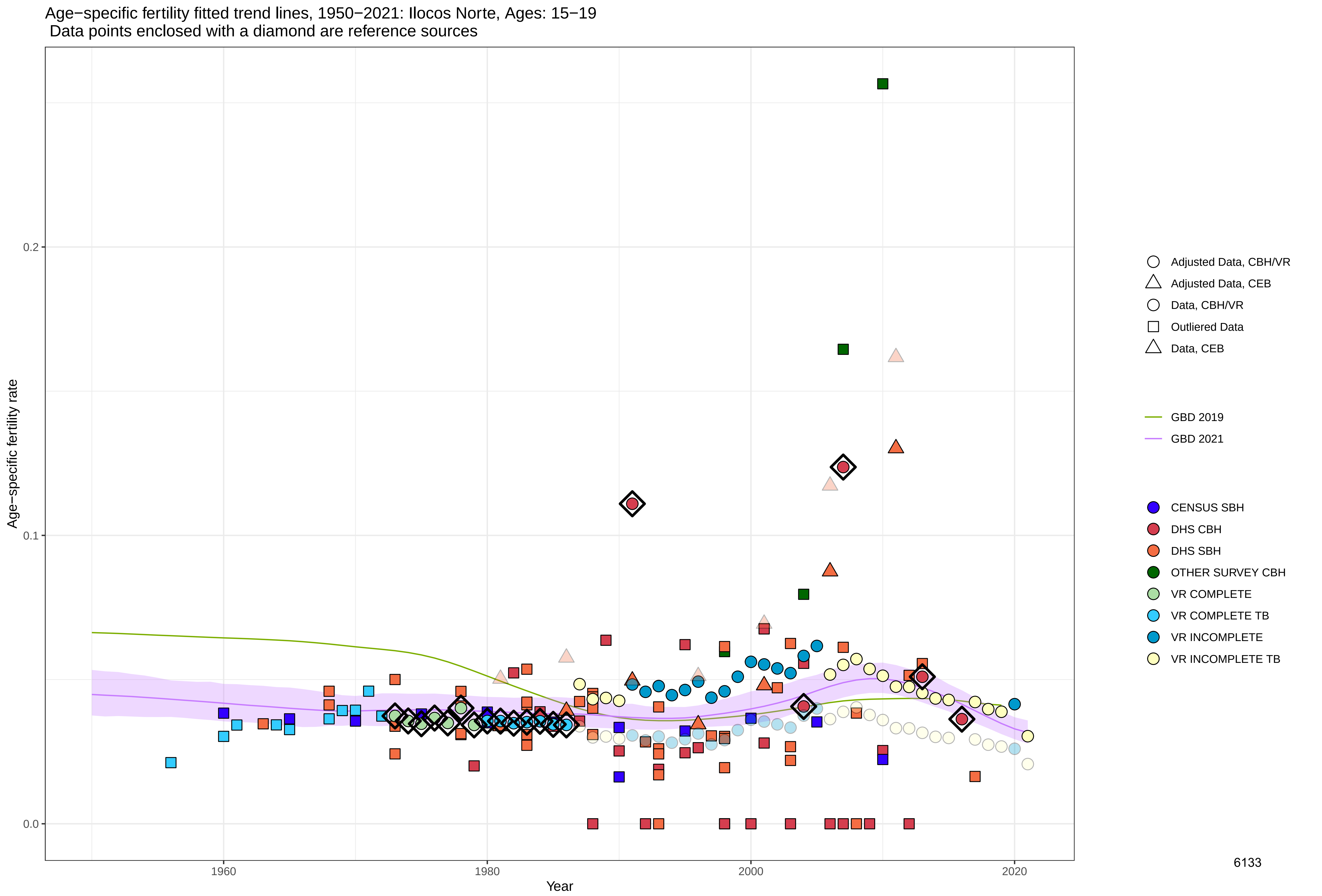

Age−specific fertility fitted trend lines, 1950−2021: Ilocos Norte, Ages: 15−19
Data points enclosed with a diamond are reference sources
Age−specific fertility rate
0.0
0.1
0.2
1960
1980
2000
2020
Year
Adjusted Data, CBH/VR
Adjusted Data, CEB
Data, CBH/VR
Outliered Data
Data, CEB
GBD 2019
GBD 2021
CENSUS SBH
DHS CBH
DHS SBH
OTHER SURVEY CBH
VR COMPLETE
VR COMPLETE TB
VR INCOMPLETE
VR INCOMPLETE TB

Age−specific fertility fitted trend lines, 1950−2021: Ilocos Norte, Ages: 20−24
Data points enclosed with a diamond are reference sources

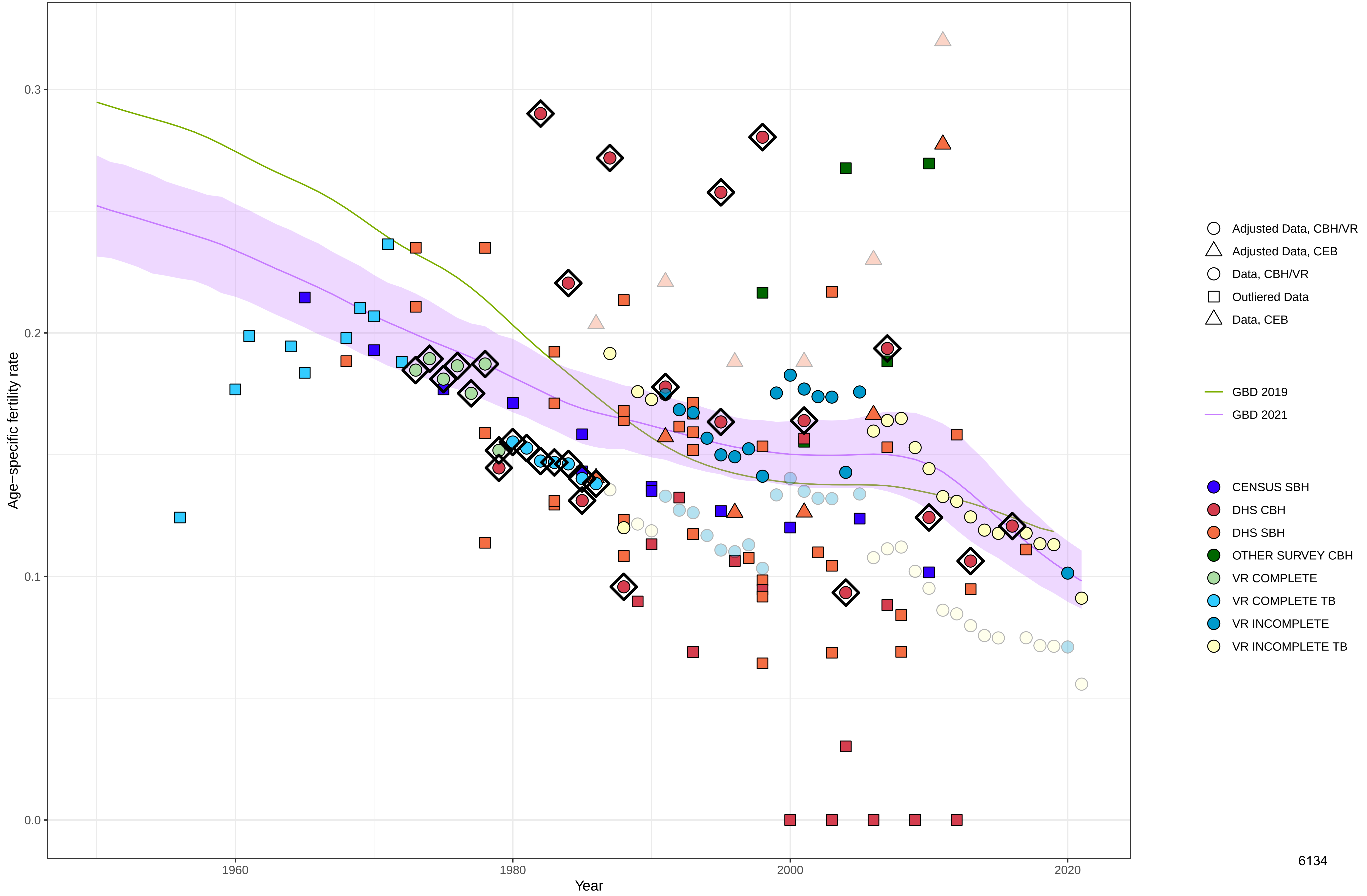

Age−specific fertility fitted trend lines, 1950−2021: Ilocos Norte, Ages: 25−29
Data points enclosed with a diamond are reference sources

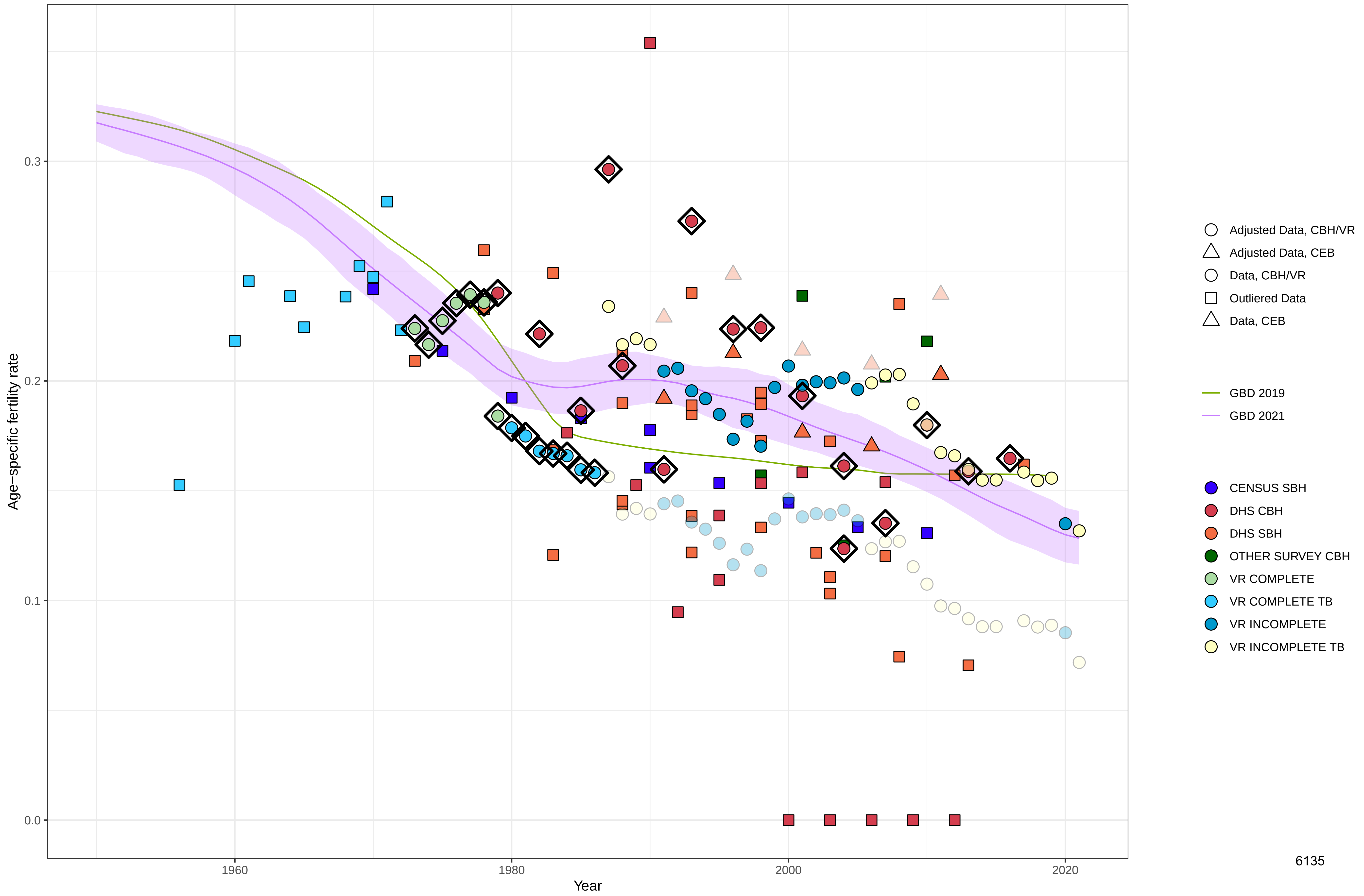

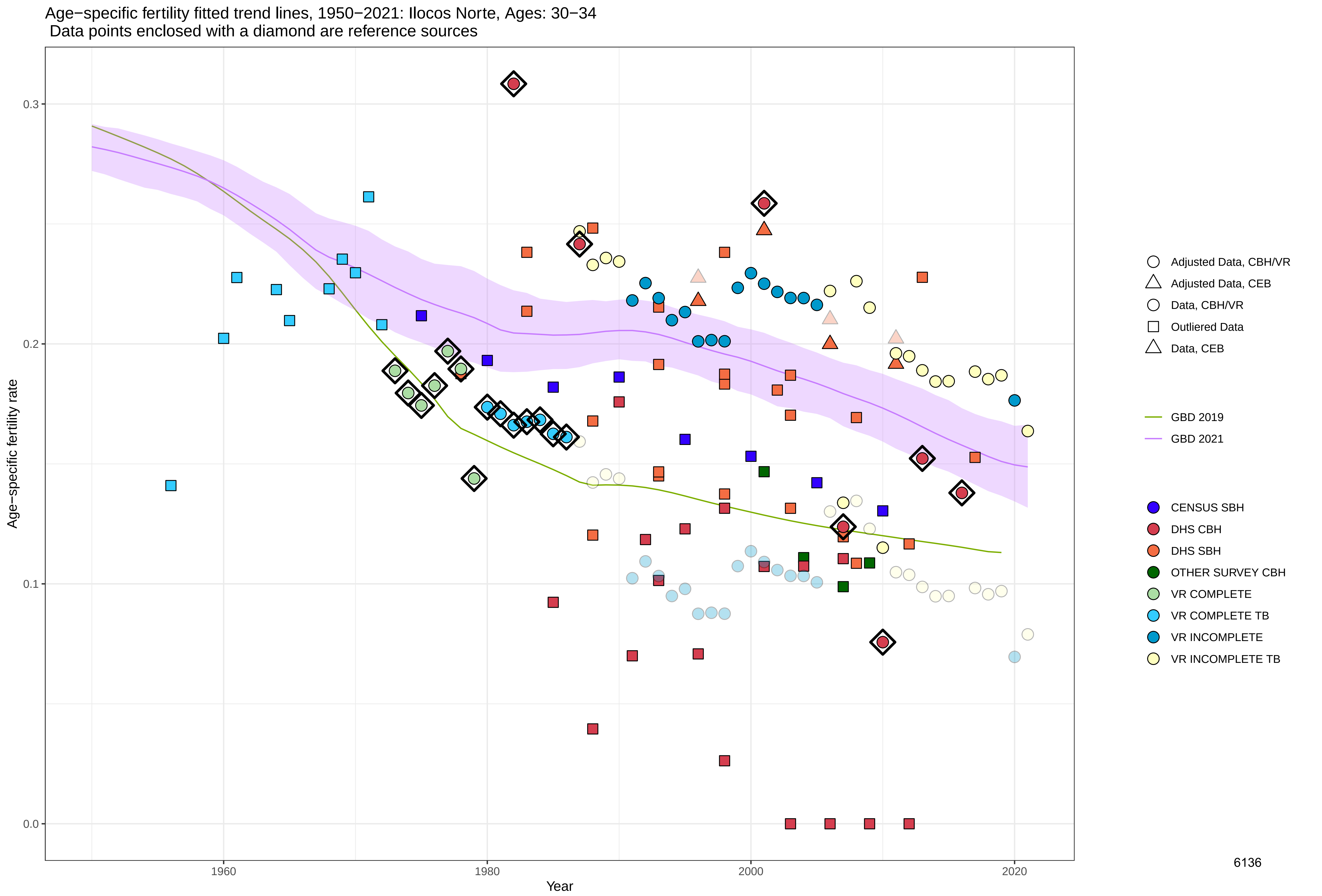

Age−specific fertility fitted trend lines, 1950−2021: Ilocos Norte, Ages: 30−34
Data points enclosed with a diamond are reference sources
Age−specific fertility rate
Year
0.0
0.1
0.2
0.3
1960
1980
2000
2020
Adjusted Data, CBH/VR
Adjusted Data, CEB
Data, CBH/VR
Outliered Data
Data, CEB
GBD 2019
GBD 2021
CENSUS SBH
DHS CBH
DHS SBH
OTHER SURVEY CBH
VR COMPLETE
VR COMPLETE TB
VR INCOMPLETE
VR INCOMPLETE TB

Age−specific fertility fitted trend lines, 1950−2021: Ilocos Norte, Ages: 35−39
Data points enclosed with a diamond are reference sources

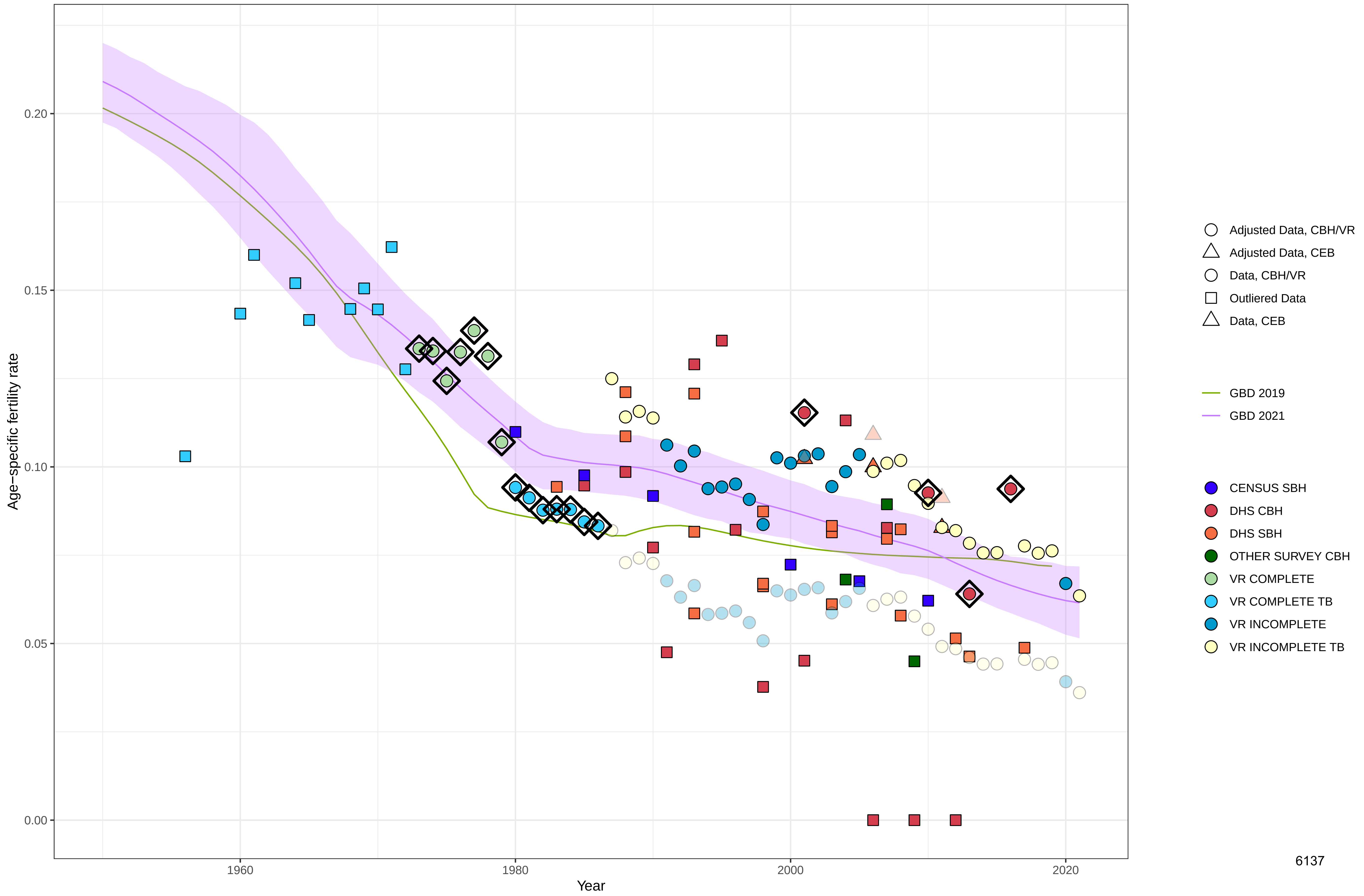

Age−specific fertility fitted trend lines, 1950−2021: Ilocos Norte, Ages: 40−44
Data points enclosed with a diamond are reference sources

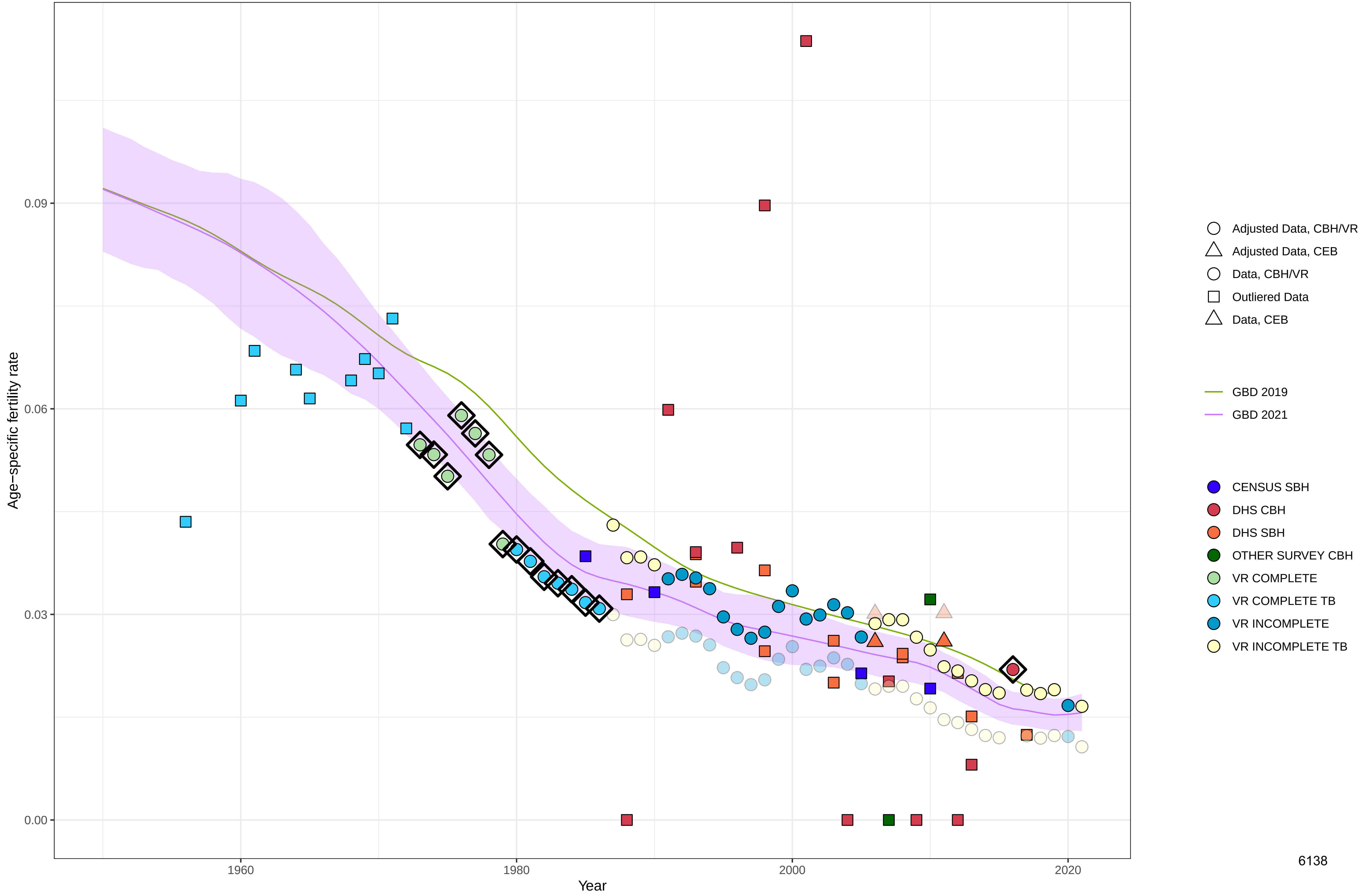

Age−specific fertility fitted trend lines, 1950−2021: Ilocos Norte, Ages: 45−49
Data points enclosed with a diamond are reference sources

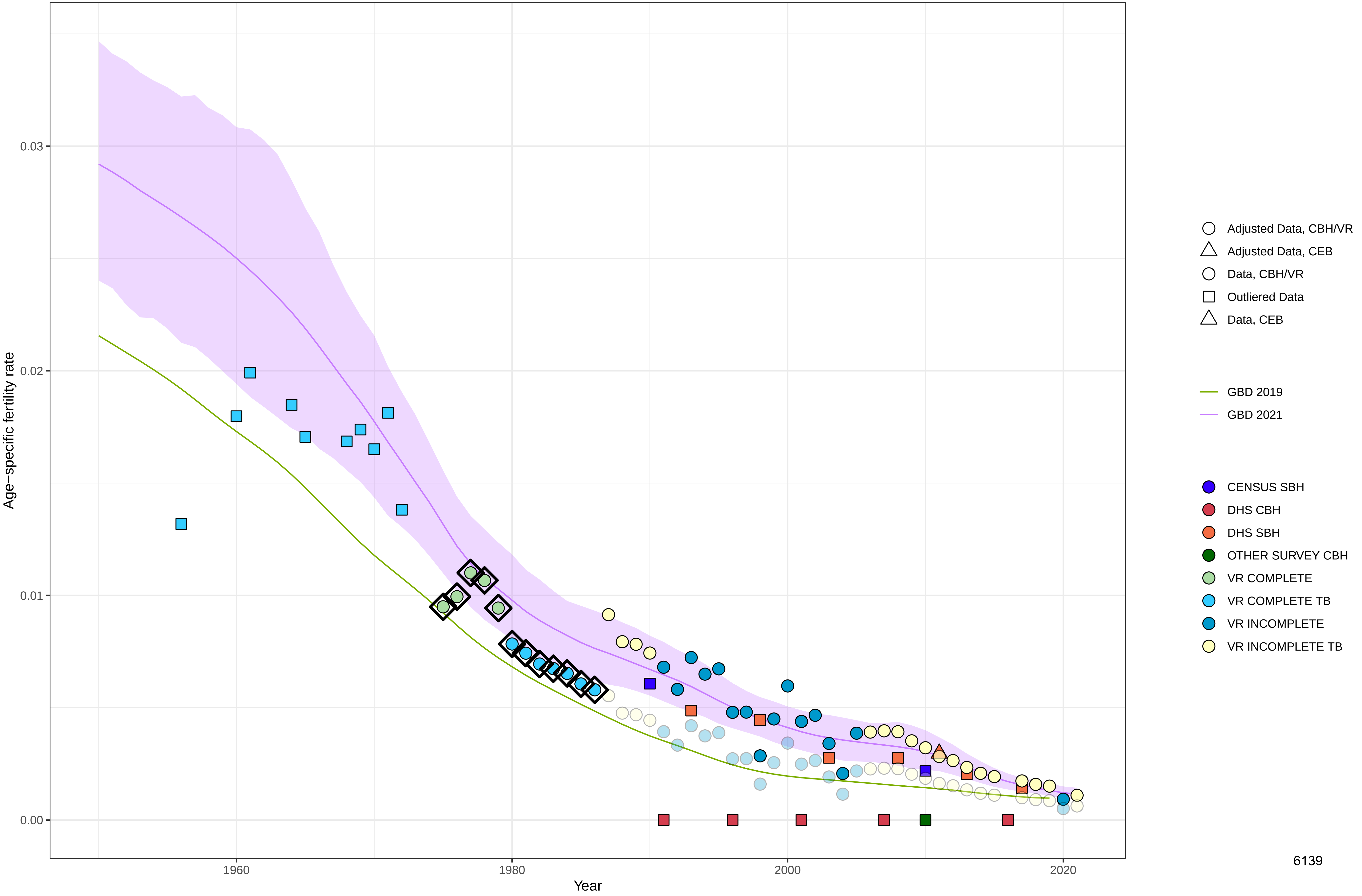

Age−specific fertility fitted trend lines, 1950−2021: Ilocos Norte, Ages: 50−54
Data points enclosed with a diamond are reference sources

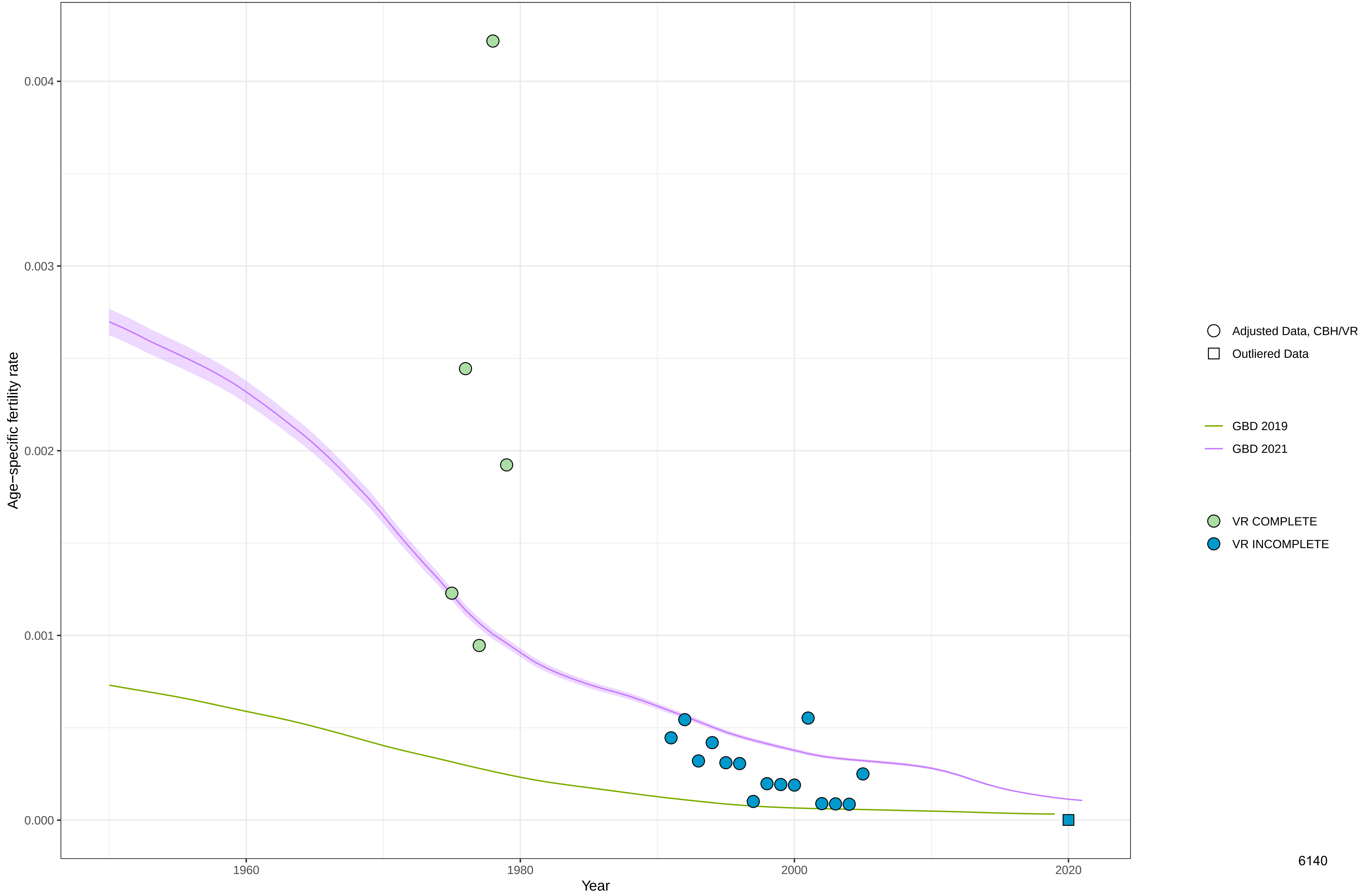

Total fertility rate fitted trend lines, 1950−2021: Ilocos Norte TFR
Data points enclosed with a diamond are reference sources

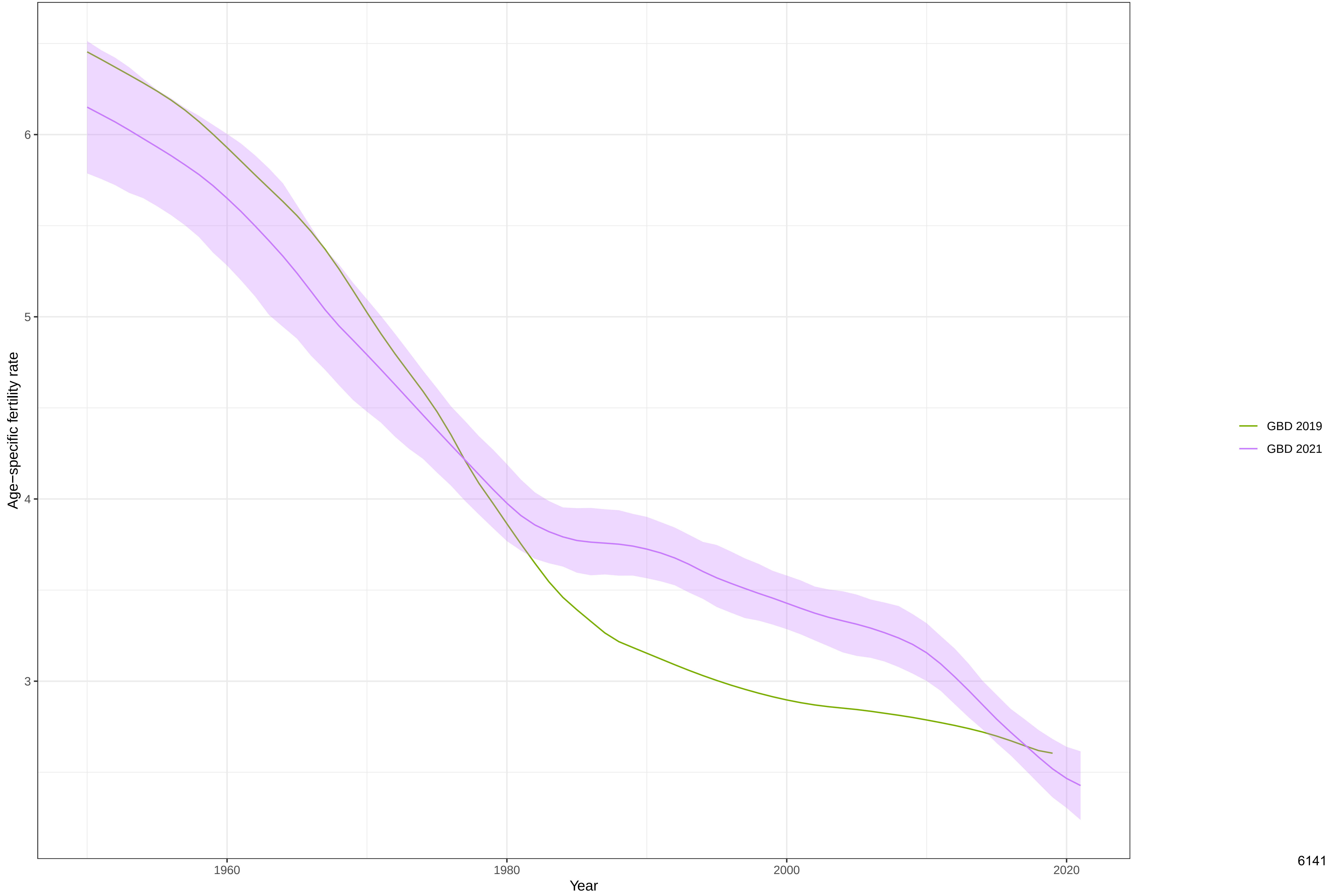

Age−specific fertility fitted trend lines, 1950−2021: Ilocos Sur, Ages: 10−14
Data points enclosed with a diamond are reference sources

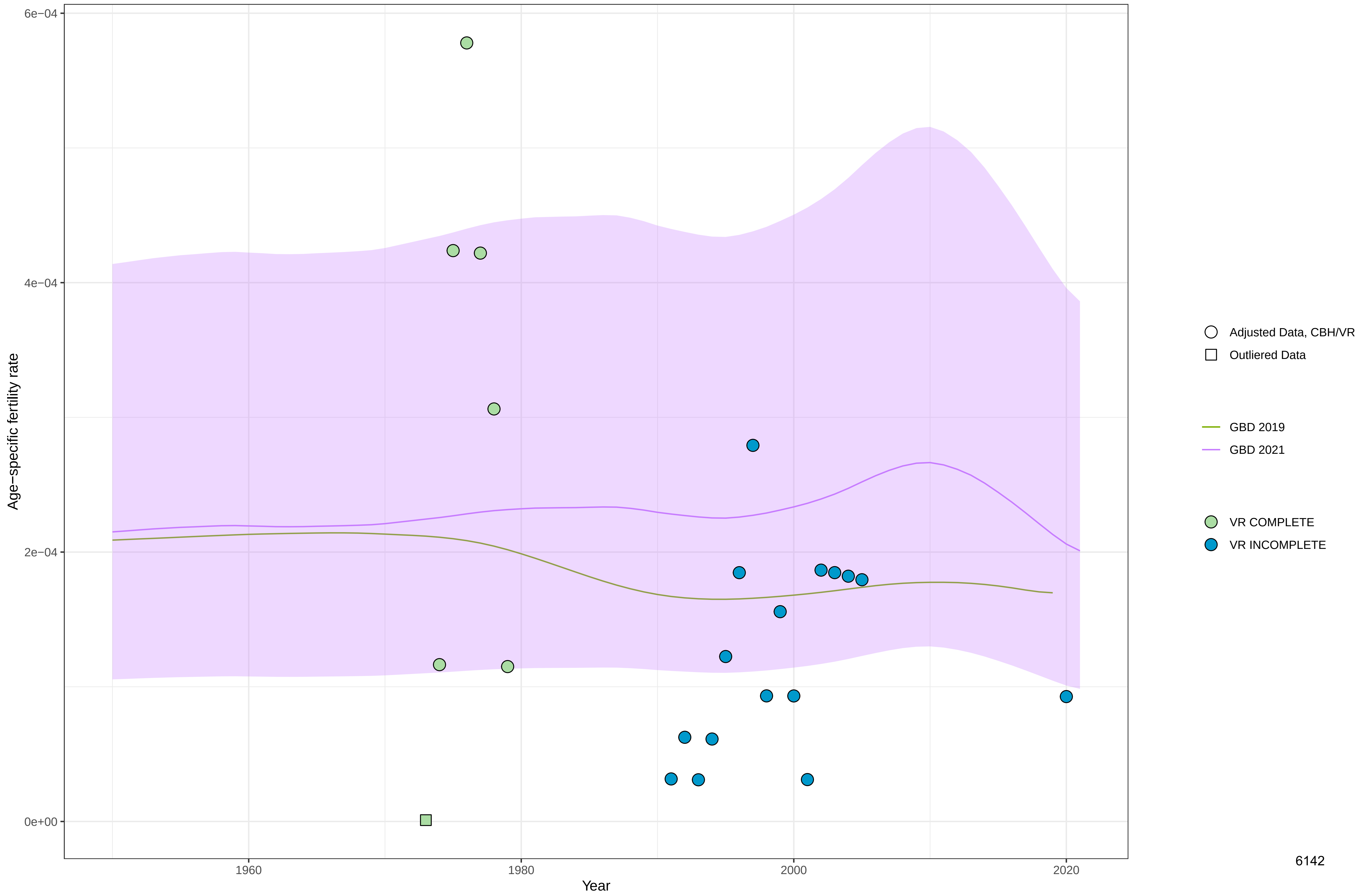

Age−specific fertility fitted trend lines, 1950−2021: Ilocos Sur, Ages: 15−19
Data points enclosed with a diamond are reference sources

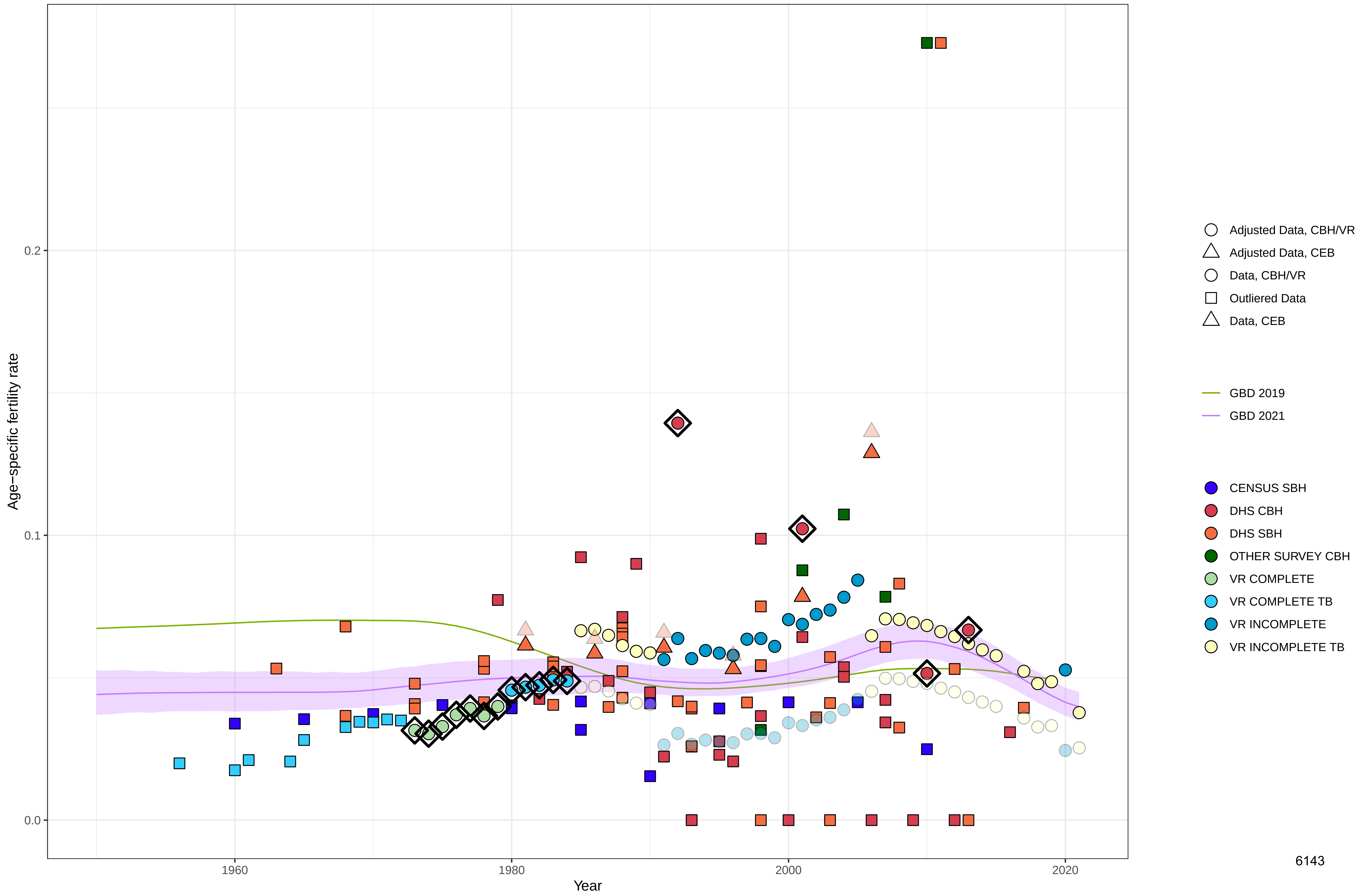

Age−specific fertility fitted trend lines, 1950−2021: Ilocos Sur, Ages: 20−24
Data points enclosed with a diamond are reference sources

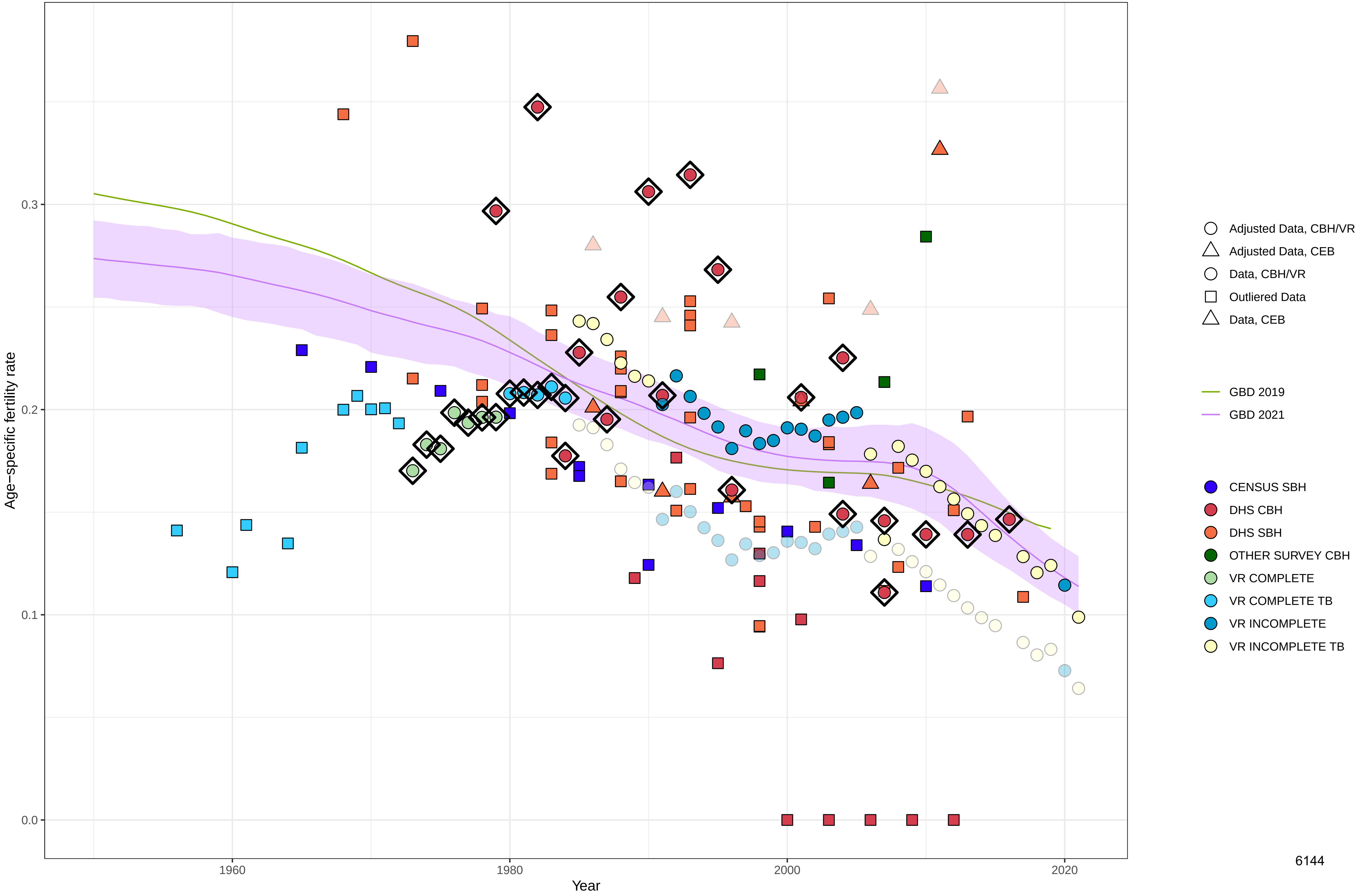

Age−specific fertility fitted trend lines, 1950−2021: Ilocos Sur, Ages: 25−29
Data points enclosed with a diamond are reference sources

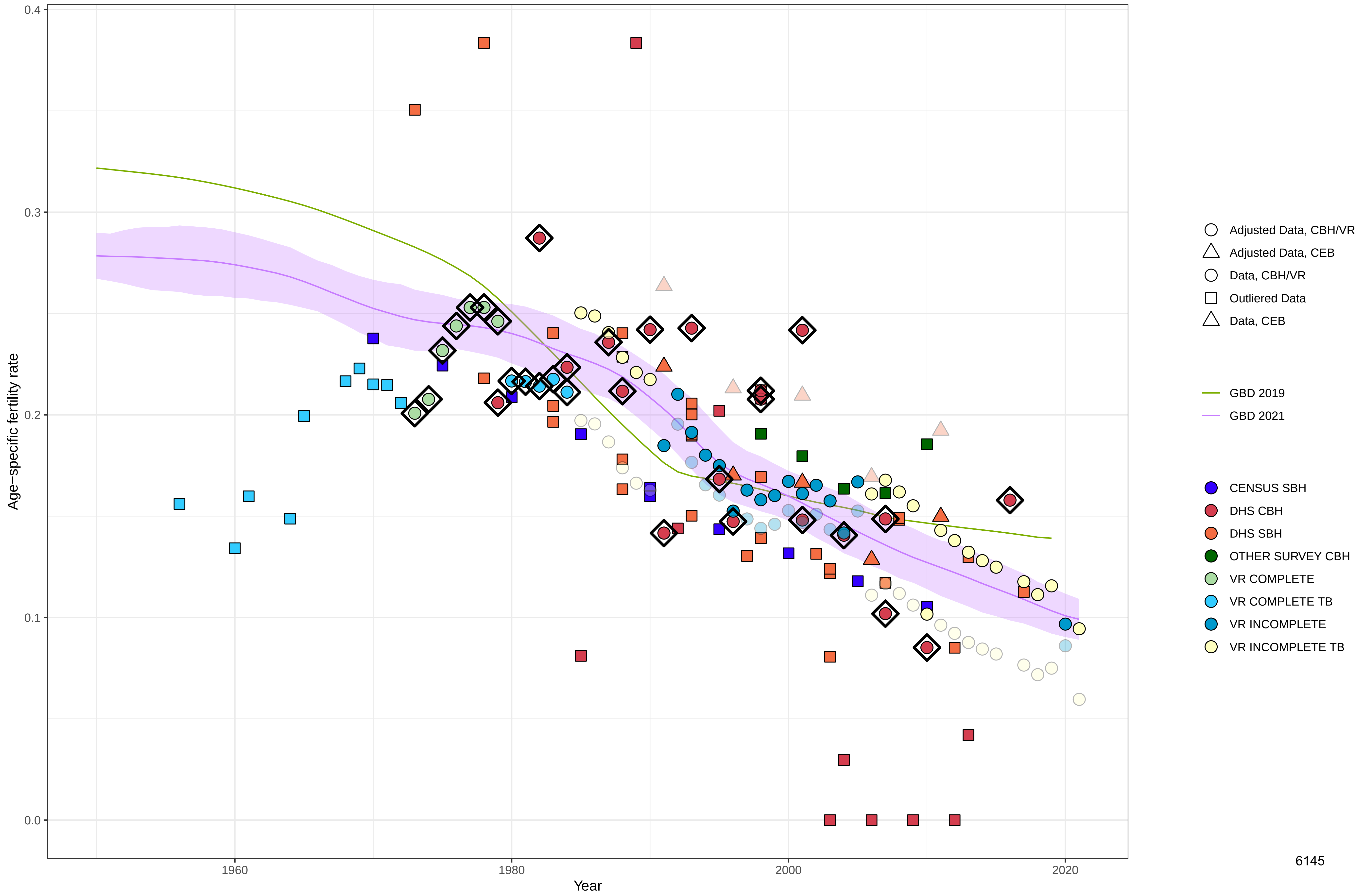

Age−specific fertility fitted trend lines, 1950−2021: Ilocos Sur, Ages: 30−34
Data points enclosed with a diamond are reference sources

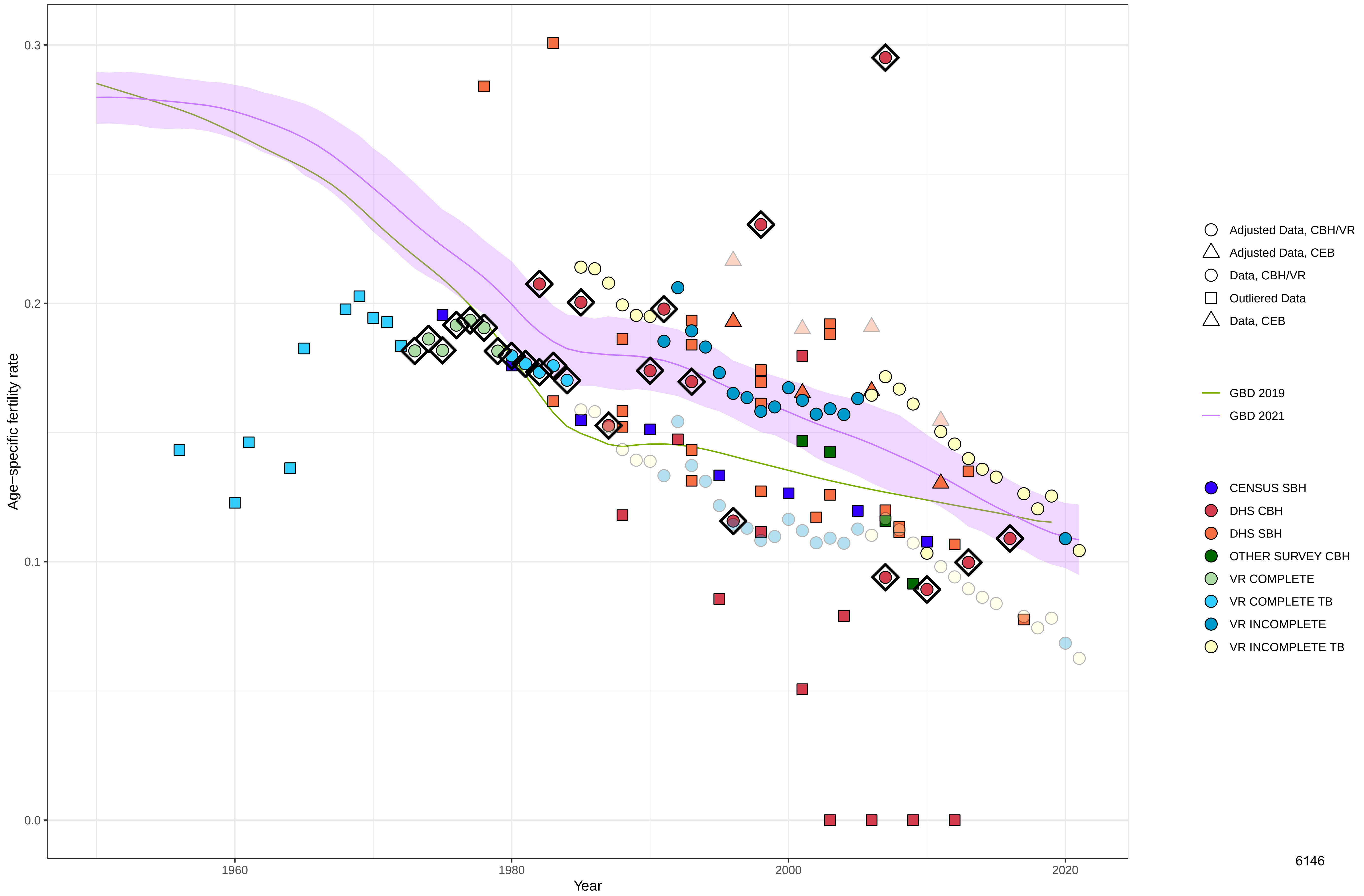

Age−specific fertility fitted trend lines, 1950−2021: Ilocos Sur, Ages: 35−39
Data points enclosed with a diamond are reference sources

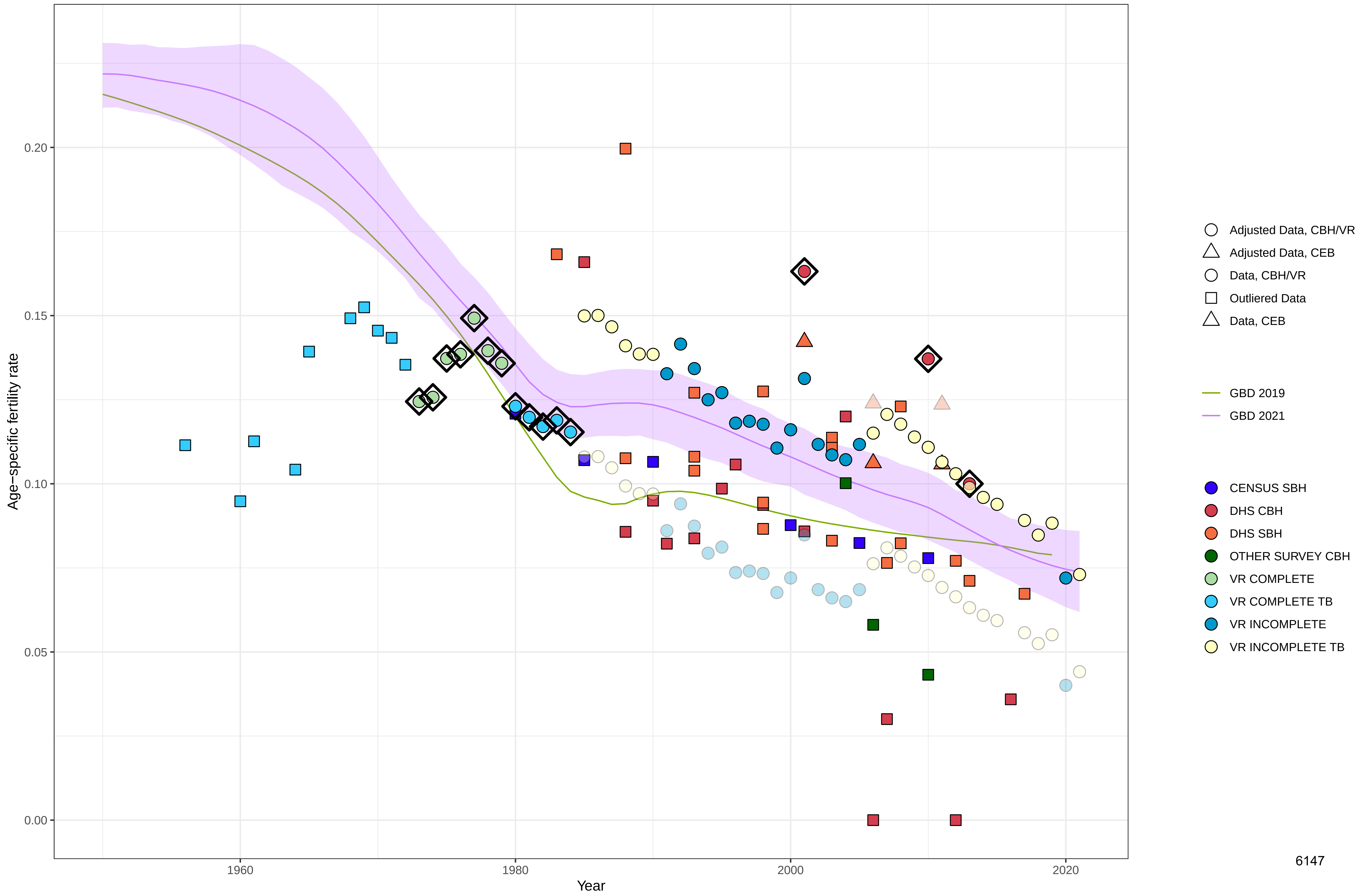

Age−specific fertility fitted trend lines, 1950−2021: Ilocos Sur, Ages: 40−44
Data points enclosed with a diamond are reference sources

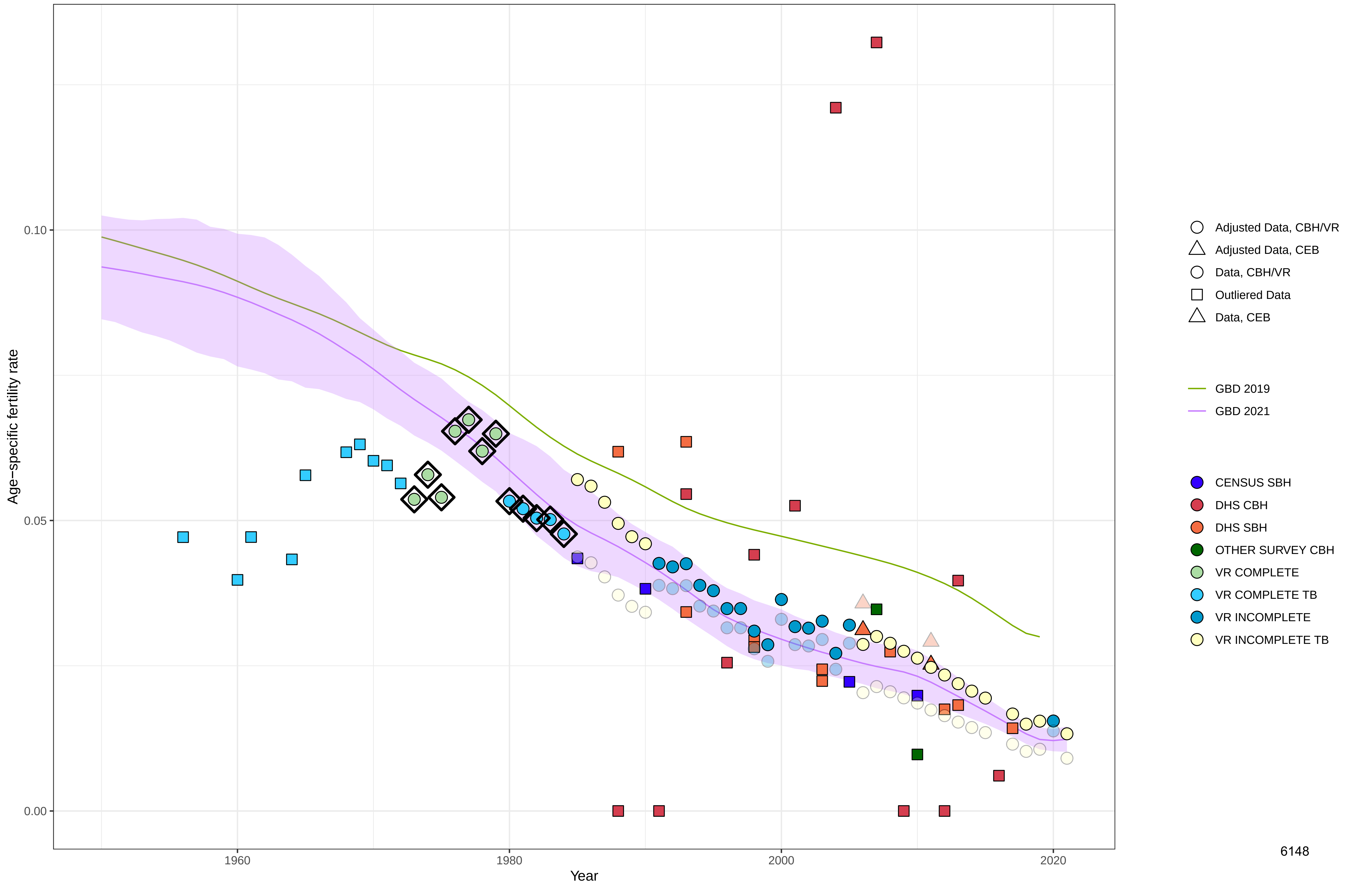

6148

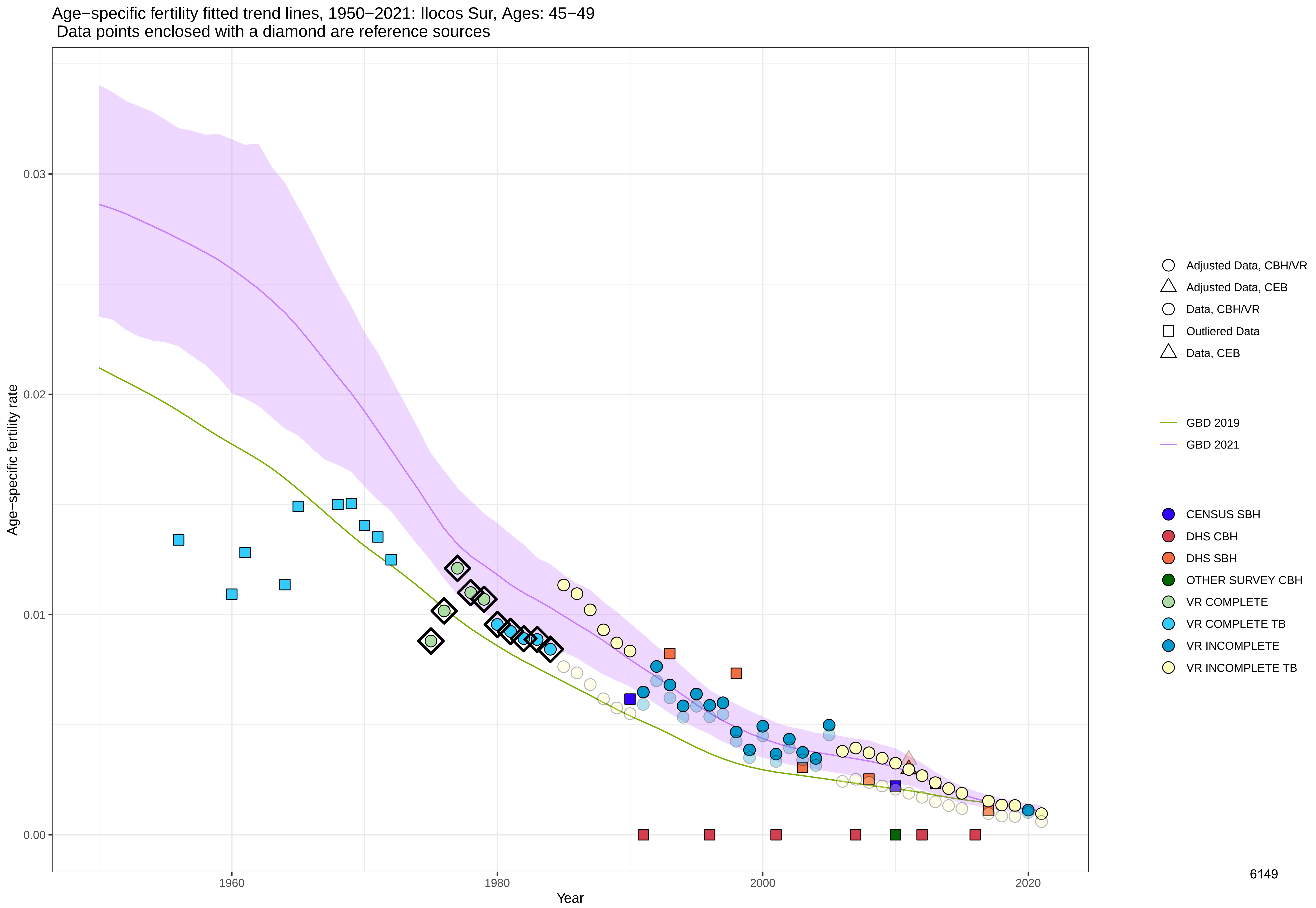
Age−specific fertility fitted trend lines, 1950−2021: Ilocos Sur, Ages: 45−49
Data points enclosed with a diamond are reference sources
Age−specific fertility rate
Year
0.00
0.01
0.02
0.03
1960
1980
2000
2020
Adjusted Data, CBH/VR
Adjusted Data, CEB
Data, CBH/VR
Outliered Data
Data, CEB
GBD 2019
GBD 2021
CENSUS SBH
DHS CBH
DHS SBH
OTHER SURVEY CBH
VR COMPLETE
VR COMPLETE TB
VR INCOMPLETE
VR INCOMPLETE TB

Age−specific fertility fitted trend lines, 1950−2021: Ilocos Sur, Ages: 50−54
Data points enclosed with a diamond are reference sources

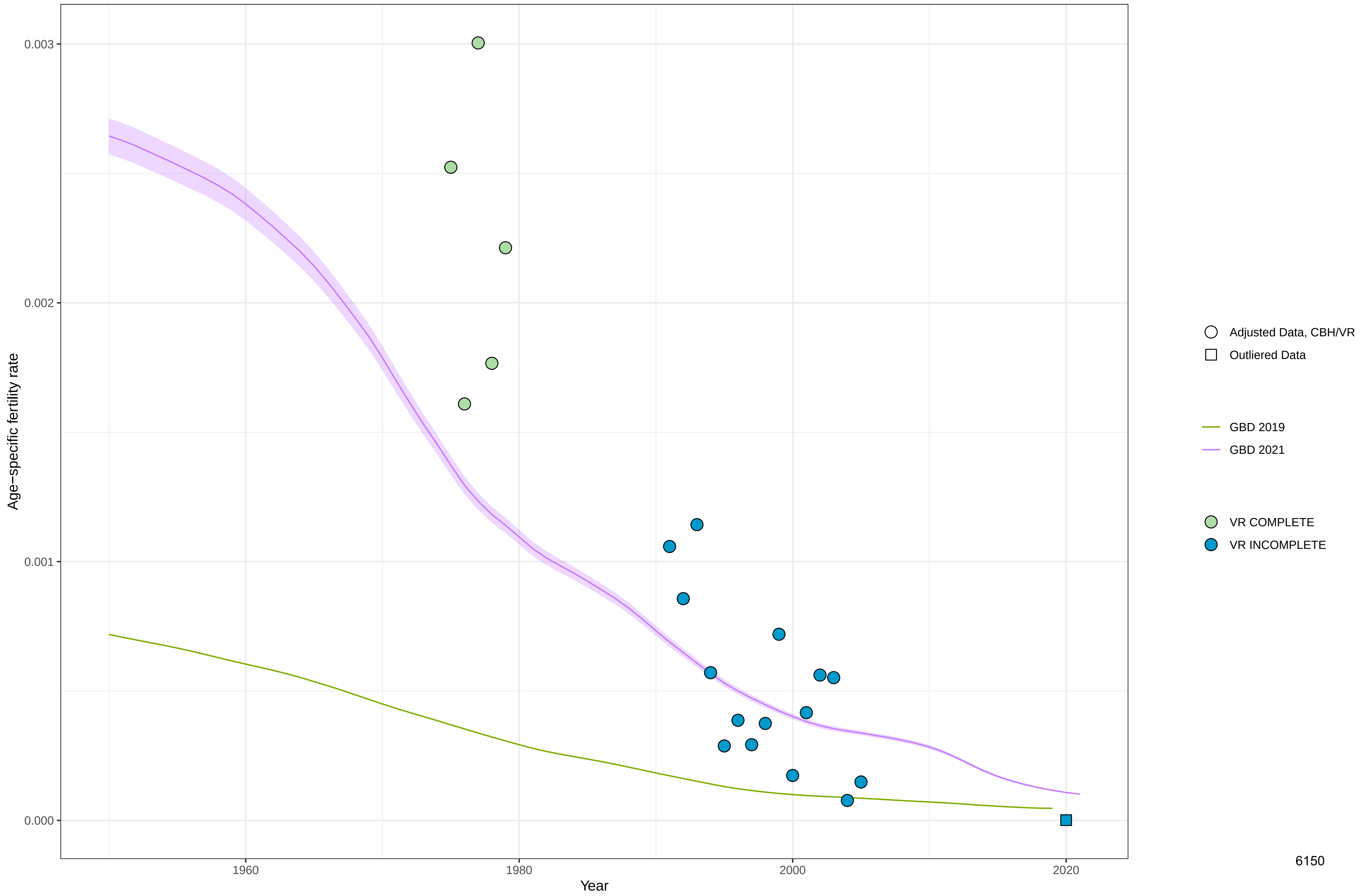

Total fertility rate fitted trend lines, 1950−2021: Ilocos Sur TFR
Data points enclosed with a diamond are reference sources

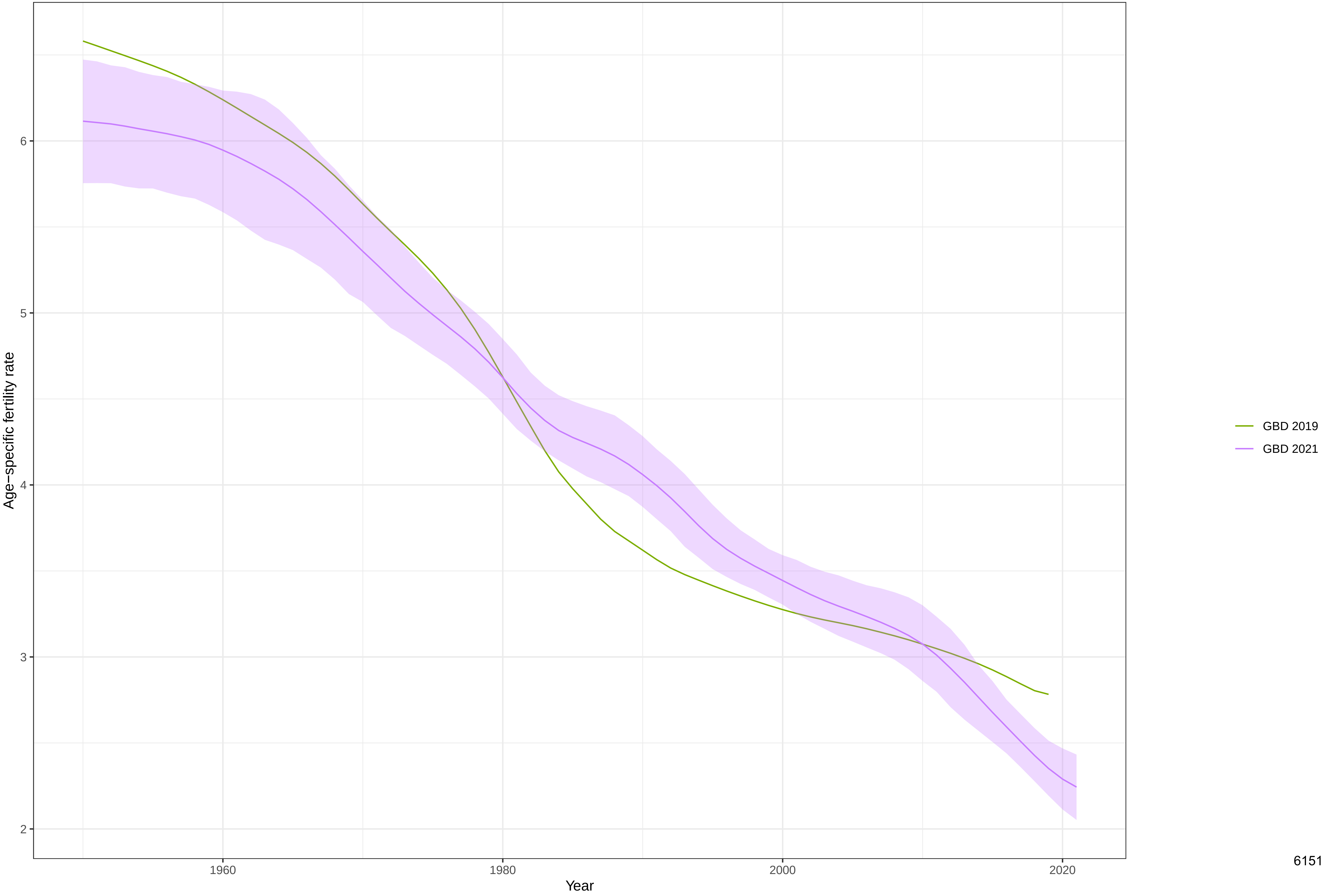

Age−specific fertility fitted trend lines, 1950−2021: Iloilo, Ages: 10−14
Data points enclosed with a diamond are reference sources

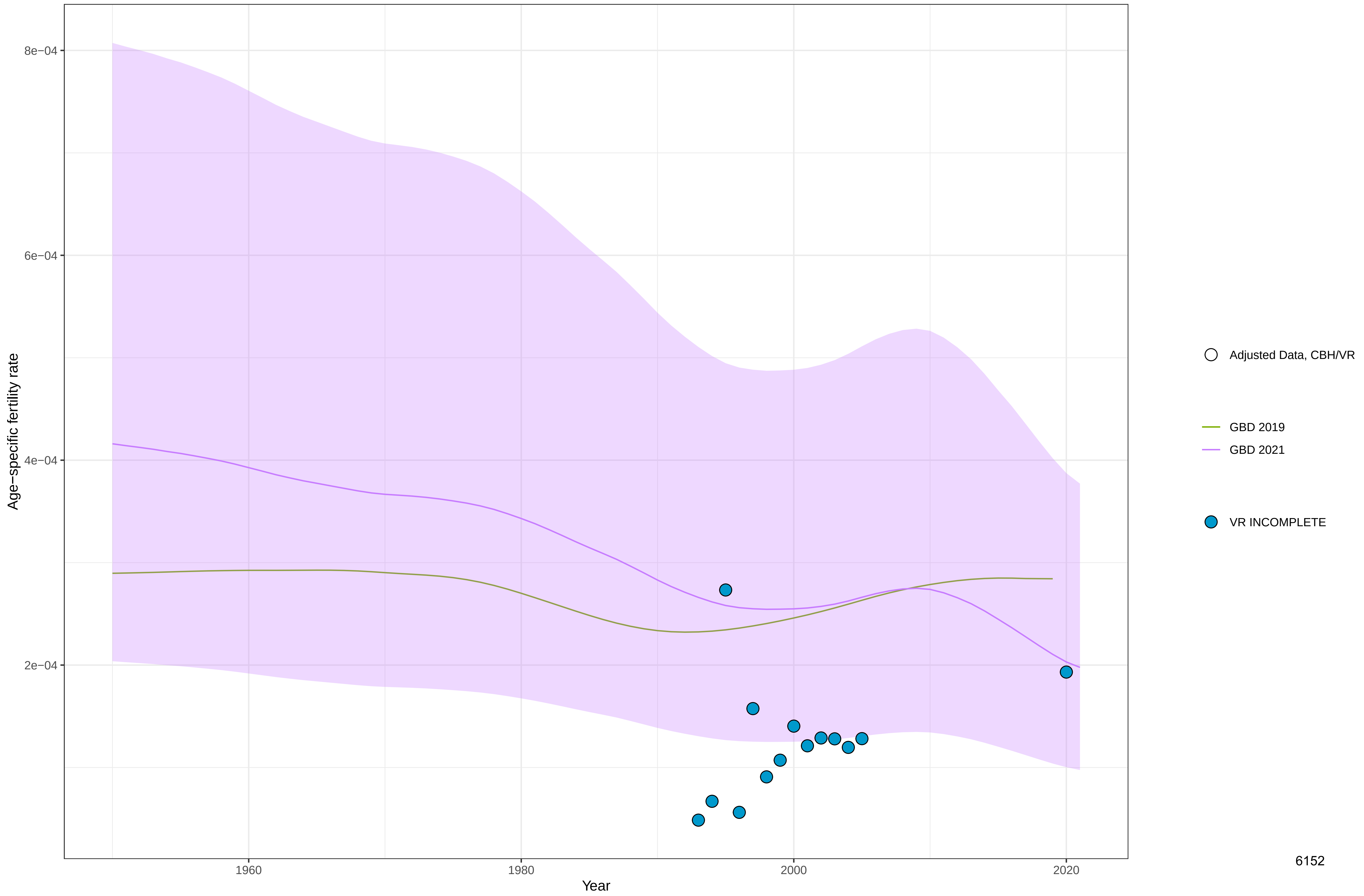

Age−specific fertility fitted trend lines, 1950−2021: Iloilo, Ages: 15−19
Data points enclosed with a diamond are reference sources

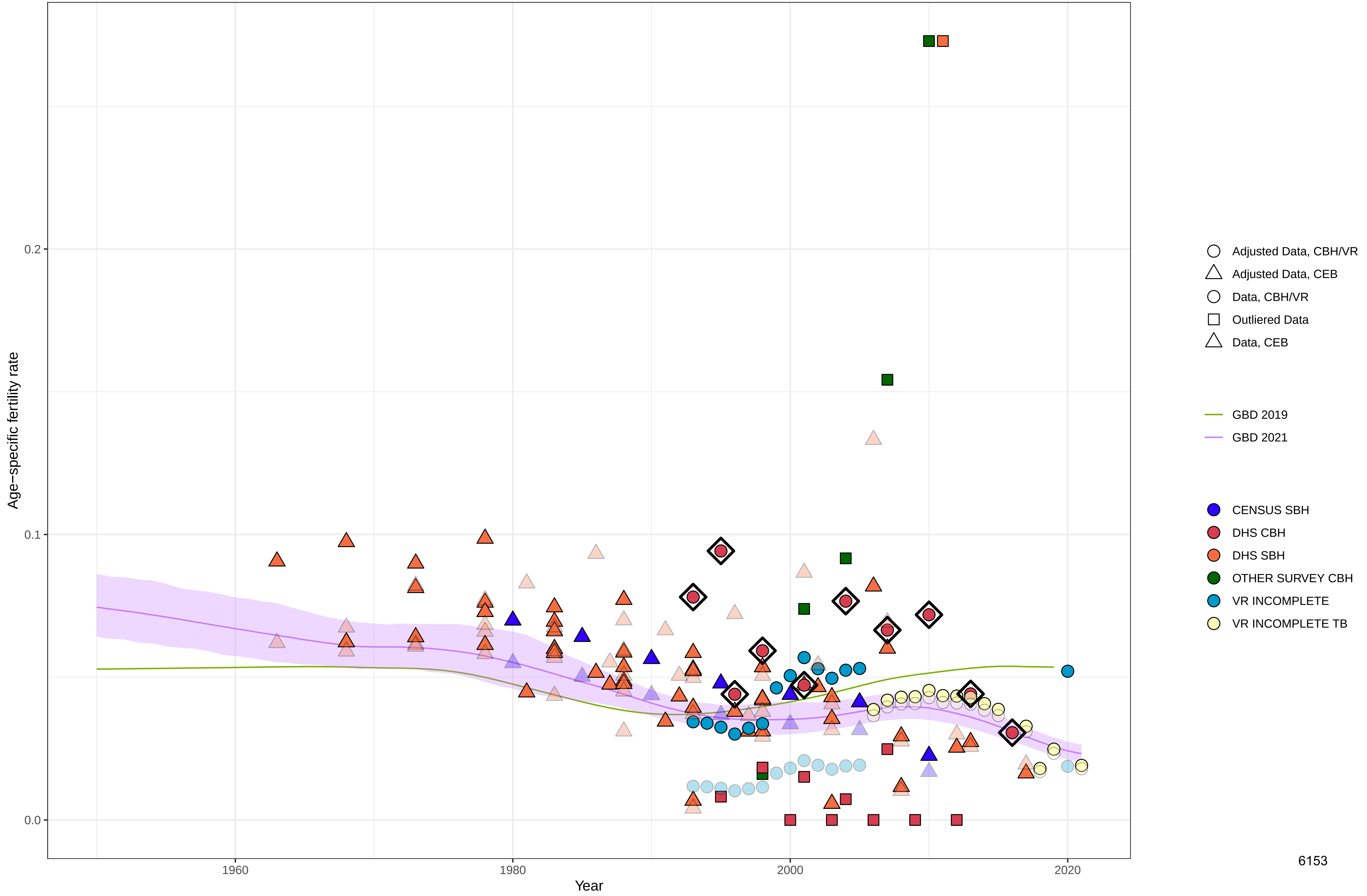

6153

Age−specific fertility fitted trend lines, 1950−2021: Iloilo, Ages: 20−24
Data points enclosed with a diamond are reference sources

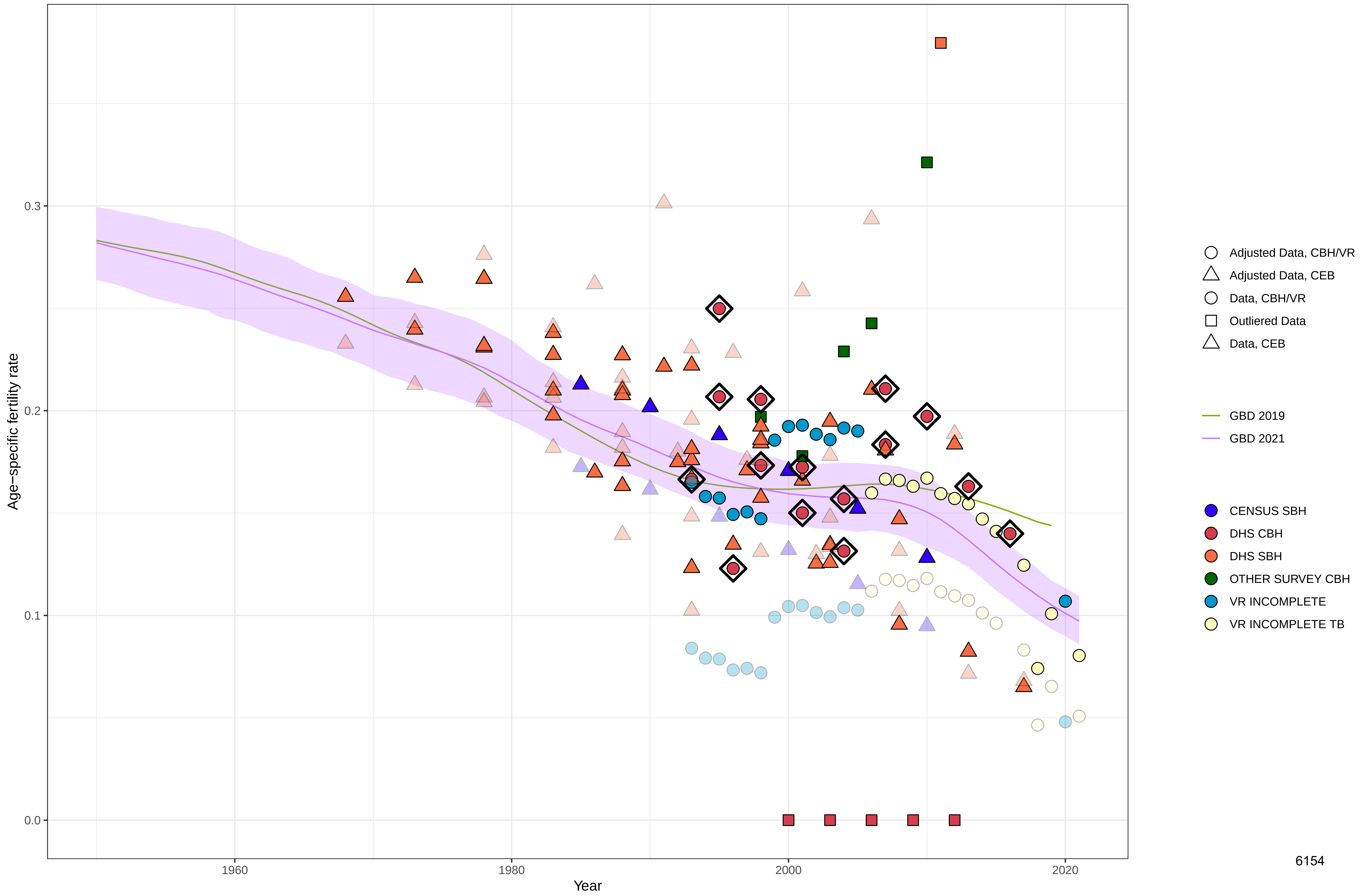

Age−specific fertility fitted trend lines, 1950−2021: Iloilo, Ages: 25−29
Data points enclosed with a diamond are reference sources

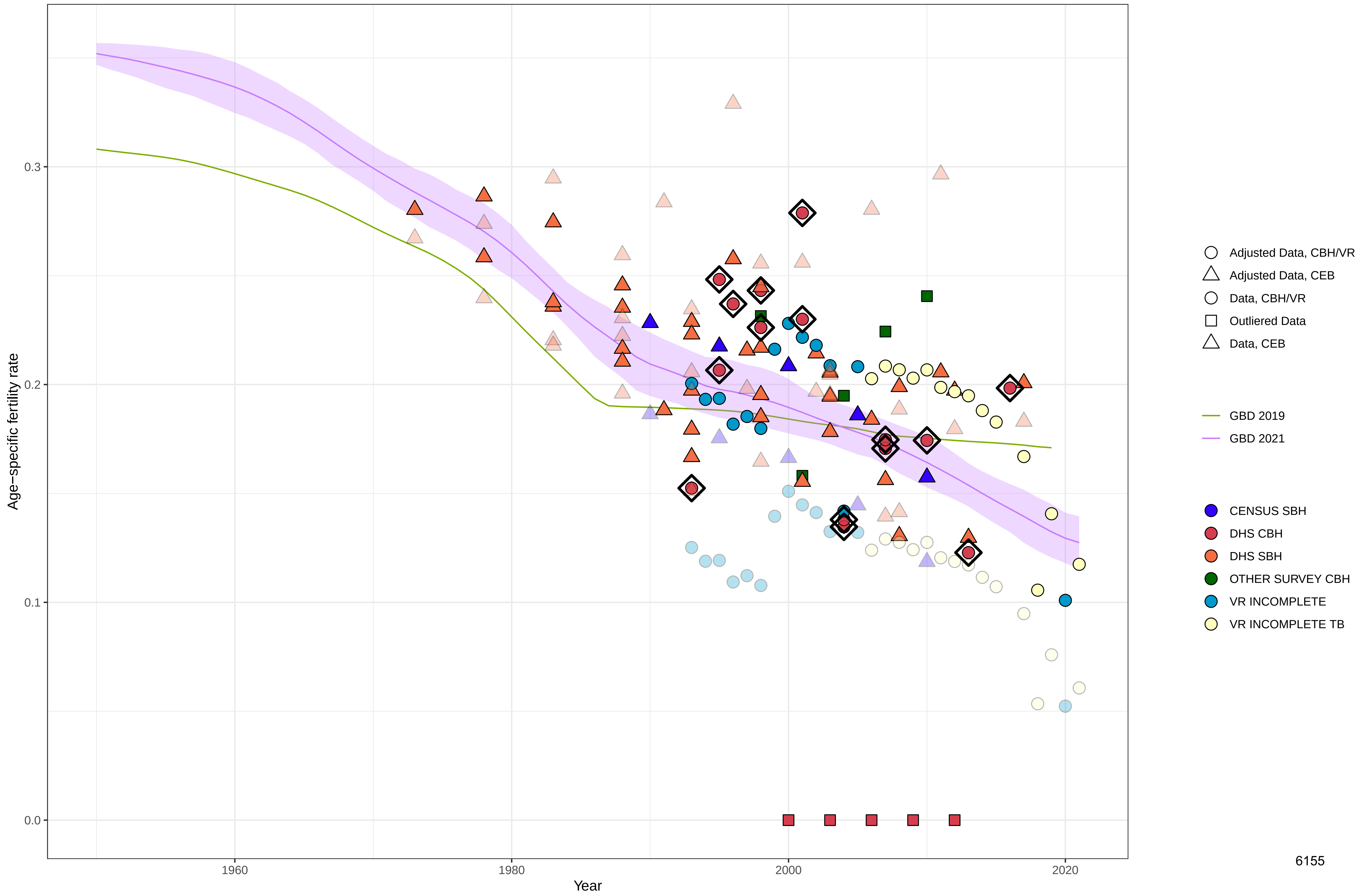

Age−specific fertility fitted trend lines, 1950−2021: Iloilo, Ages: 30−34
Data points enclosed with a diamond are reference sources

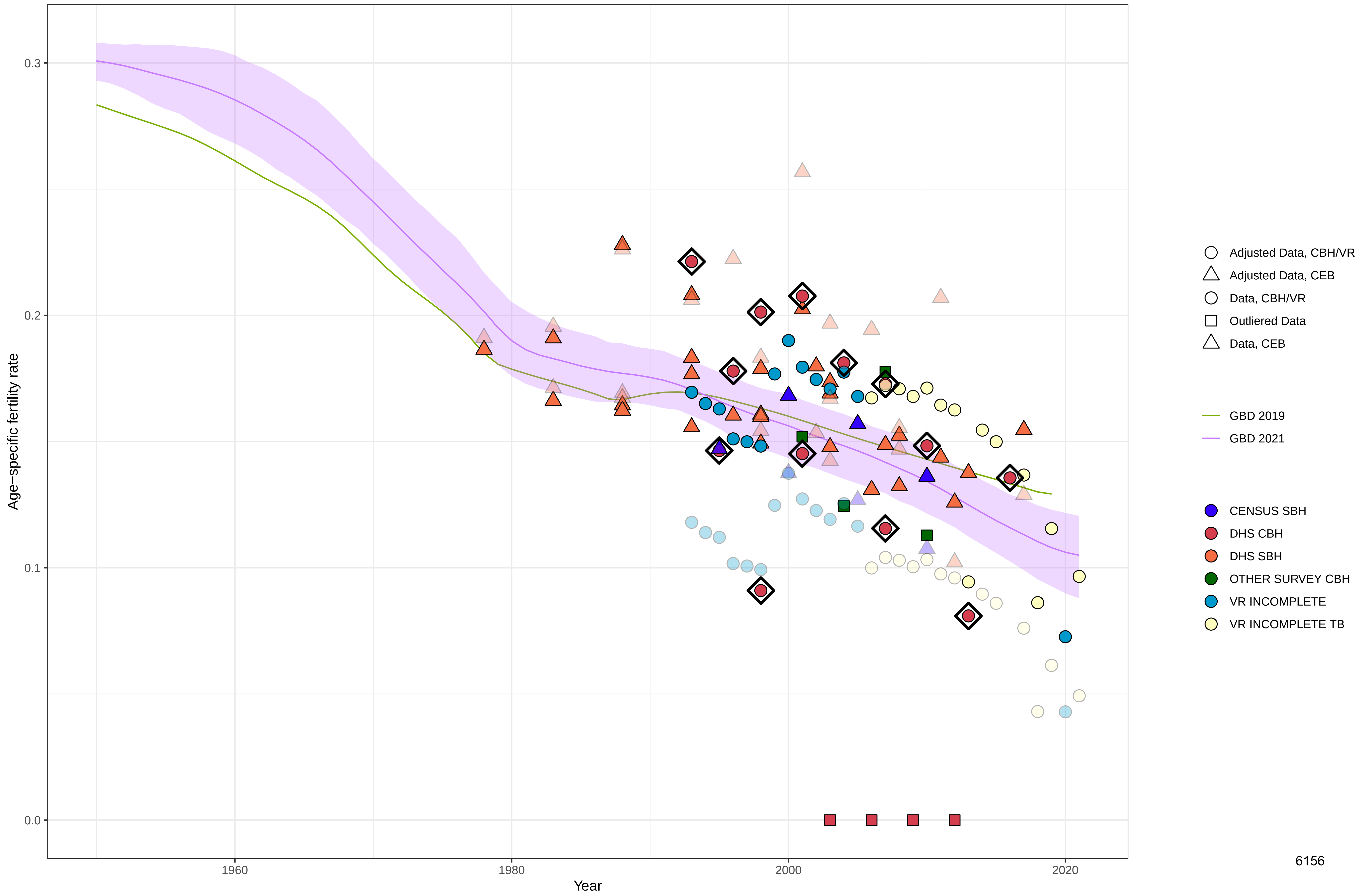

Age−specific fertility fitted trend lines, 1950−2021: Iloilo, Ages: 35−39
Data points enclosed with a diamond are reference sources

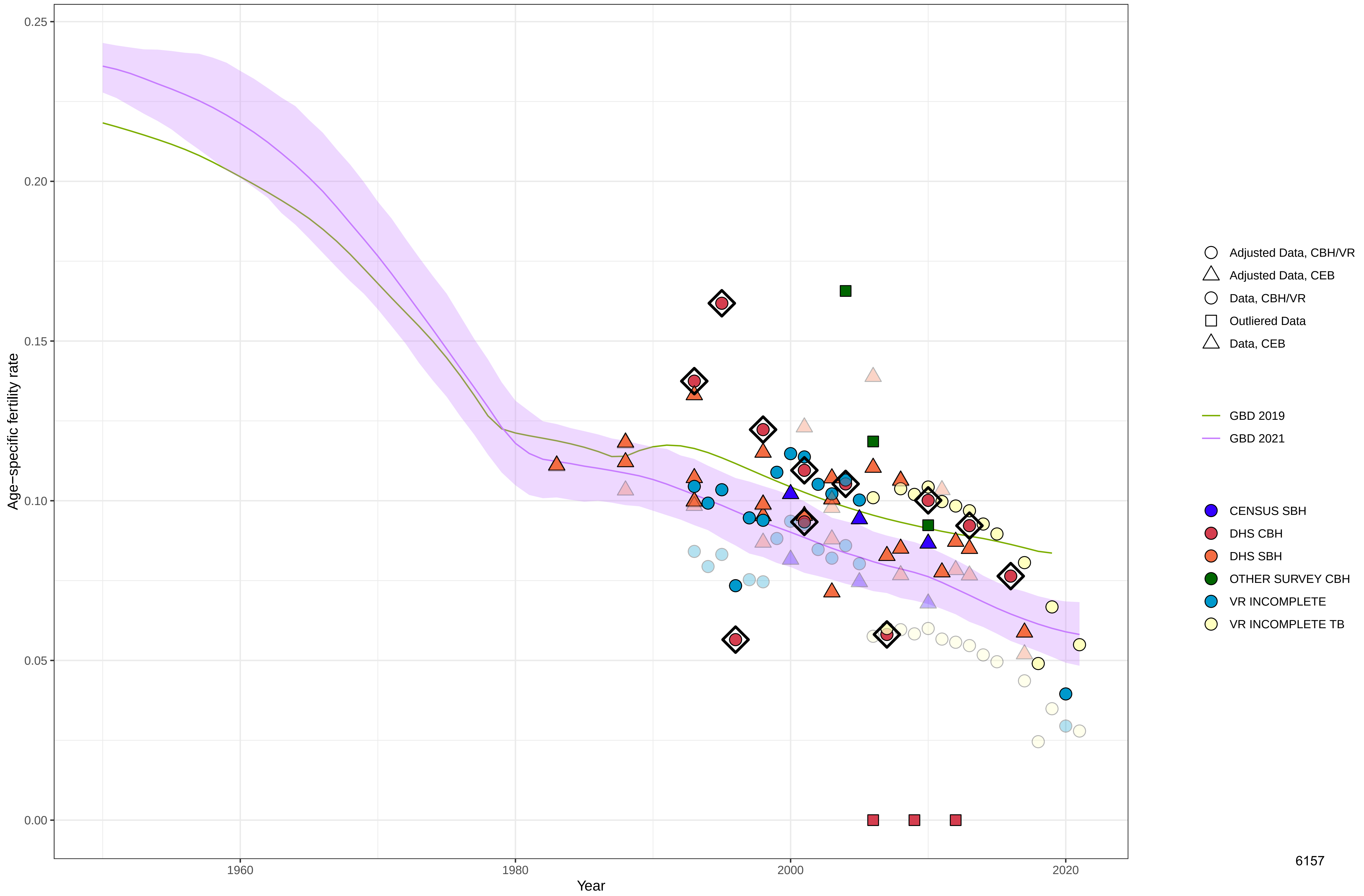

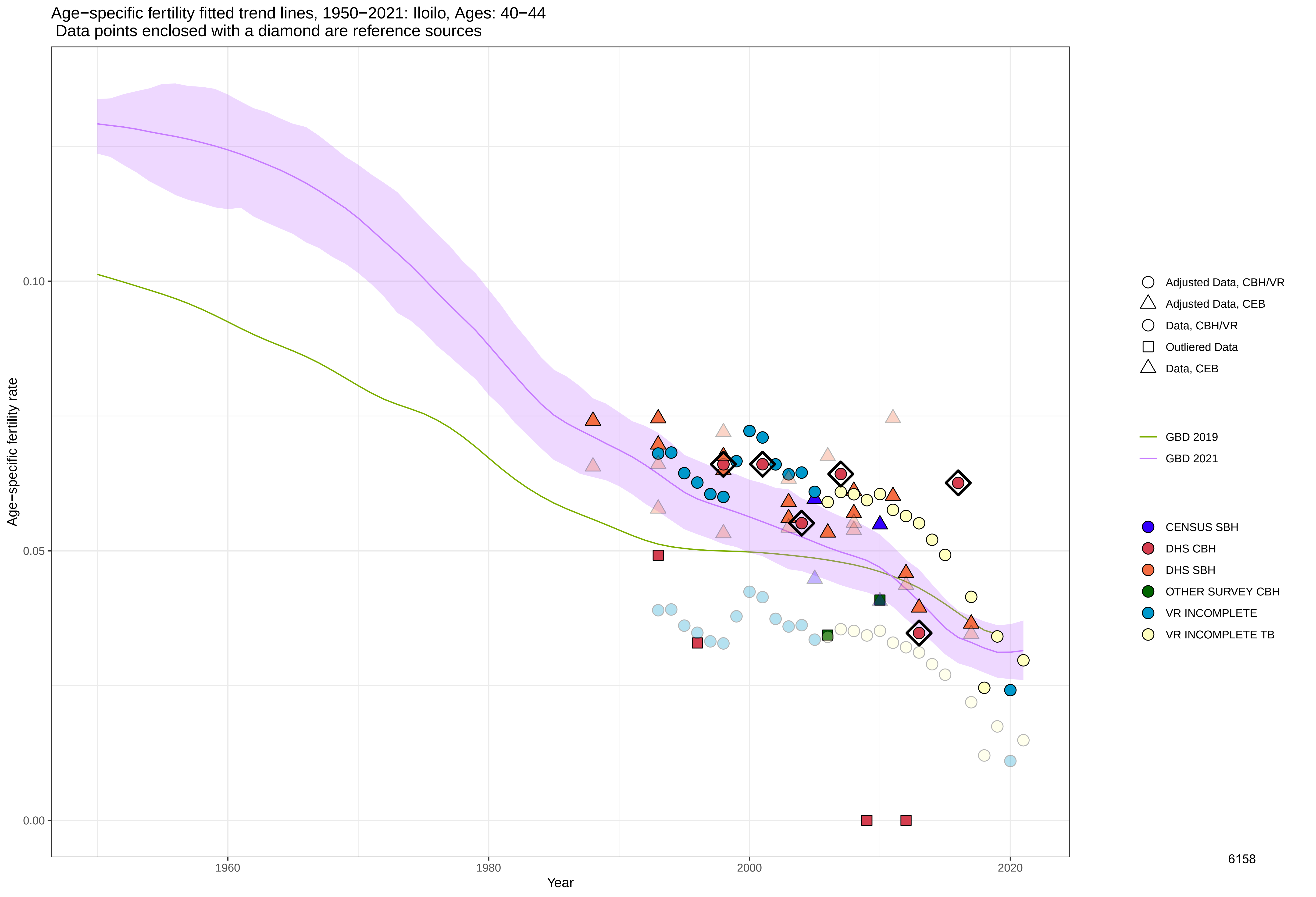

Age−specific fertility fitted trend lines, 1950−2021: Iloilo, Ages: 40−44
Data points enclosed with a diamond are reference sources
Age−specific fertility rate
0.00
0.05
0.10
1960
1980
2000
2020
Year
Adjusted Data, CBH/VR
Adjusted Data, CEB
Data, CBH/VR
Outliered Data
Data, CEB
GBD 2019
GBD 2021
CENSUS SBH
DHS CBH
DHS SBH
OTHER SURVEY CBH
VR INCOMPLETE
VR INCOMPLETE TB

6158

Age−specific fertility fitted trend lines, 1950−2021: Iloilo, Ages: 45−49
Data points enclosed with a diamond are reference sources

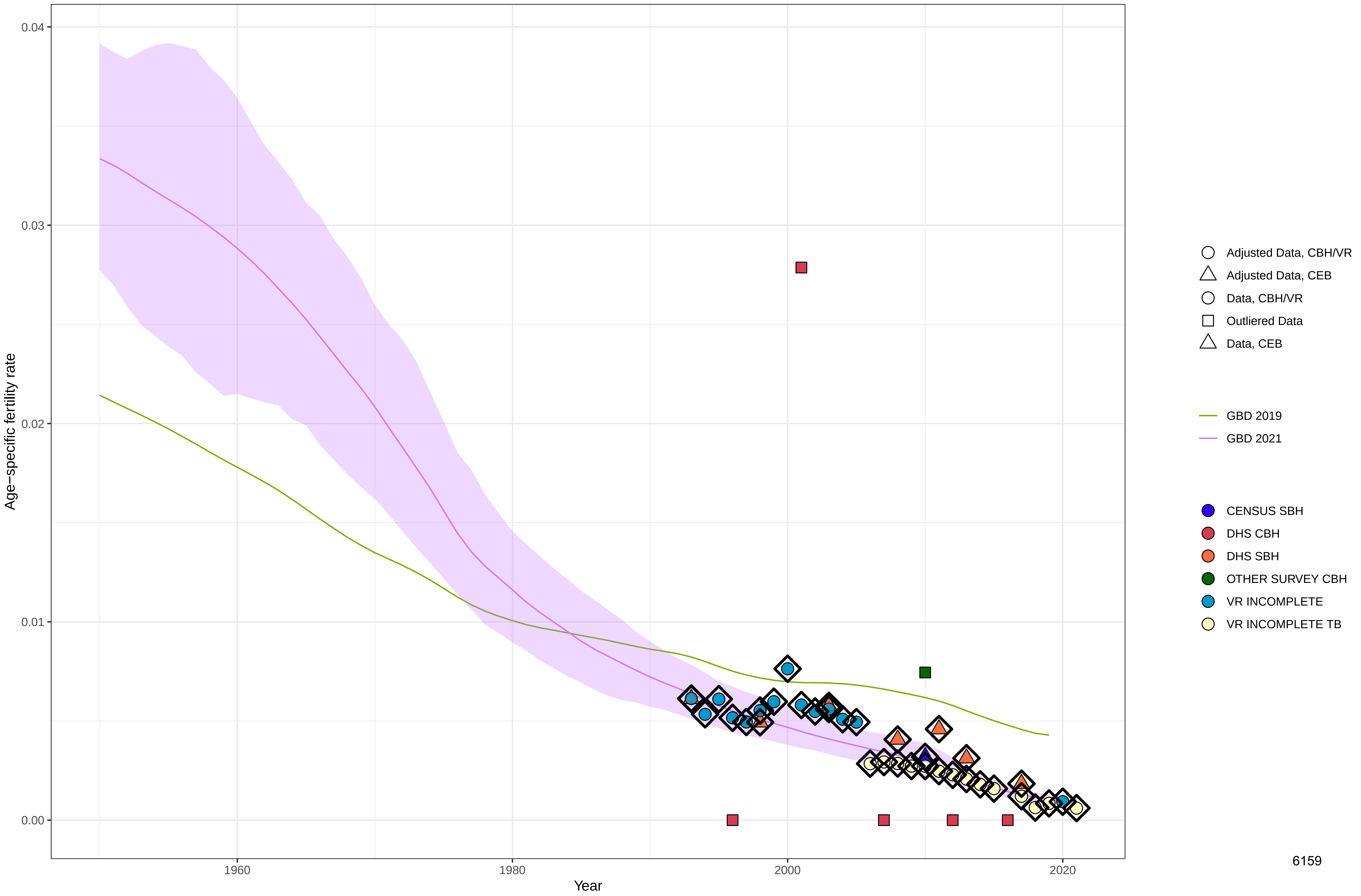

Age−specific fertility fitted trend lines, 1950−2021: Iloilo, Ages: 50−54
Data points enclosed with a diamond are reference sources

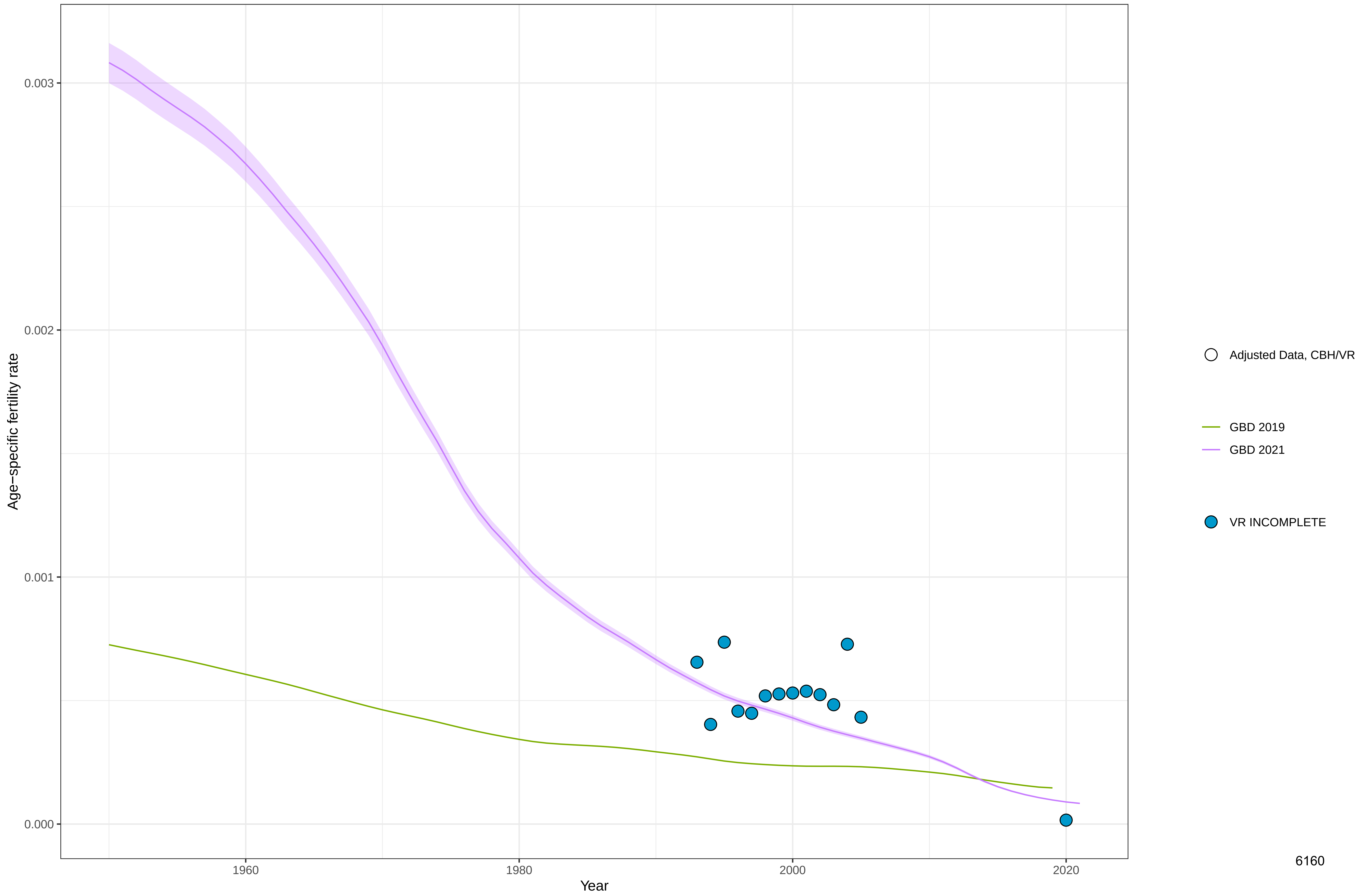

Total fertility rate fitted trend lines, 1950−2021: Iloilo TFR
Data points enclosed with a diamond are reference sources

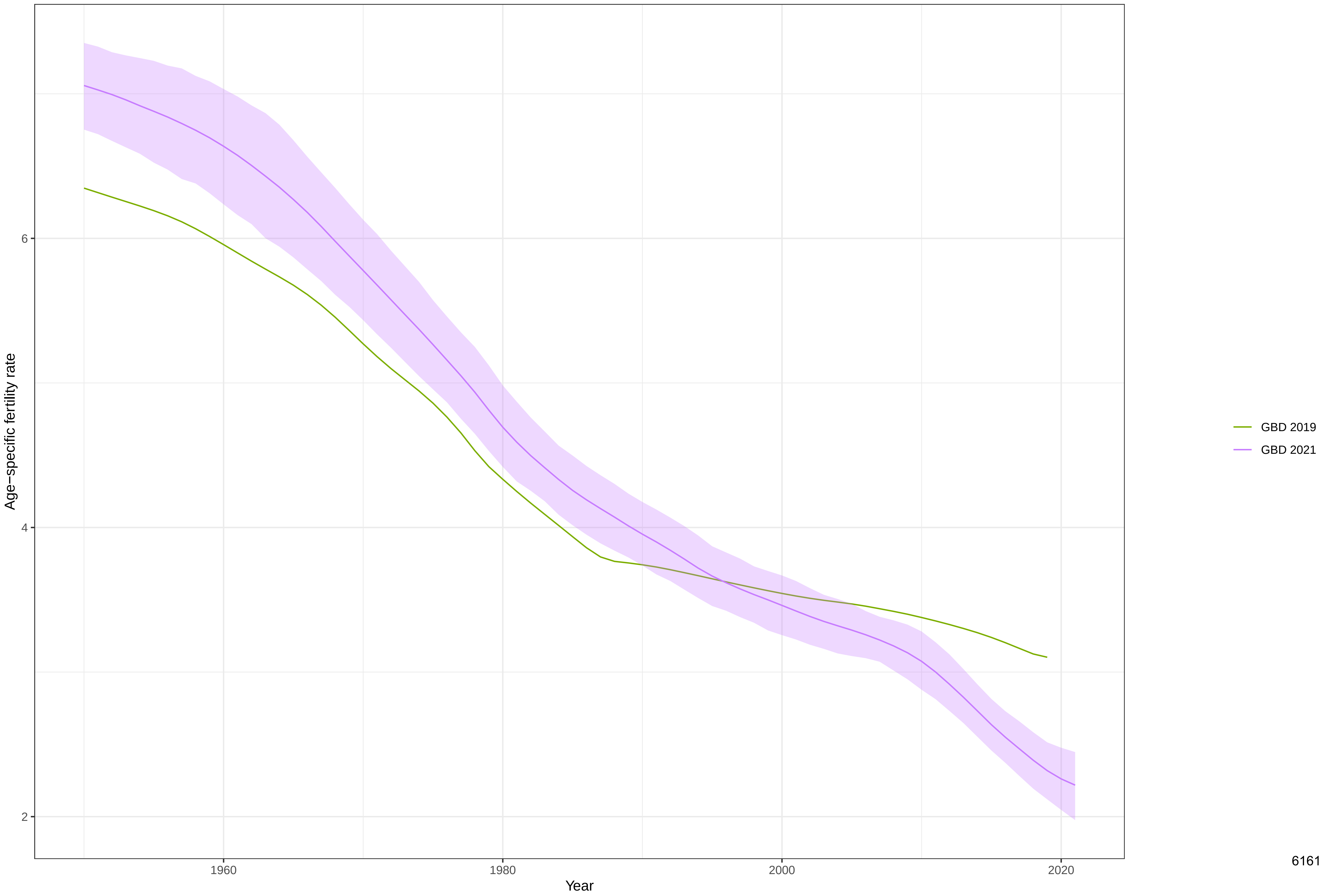

Age−specific fertility fitted trend lines, 1950−2021: Isabela, Ages: 10−14
Data points enclosed with a diamond are reference sources

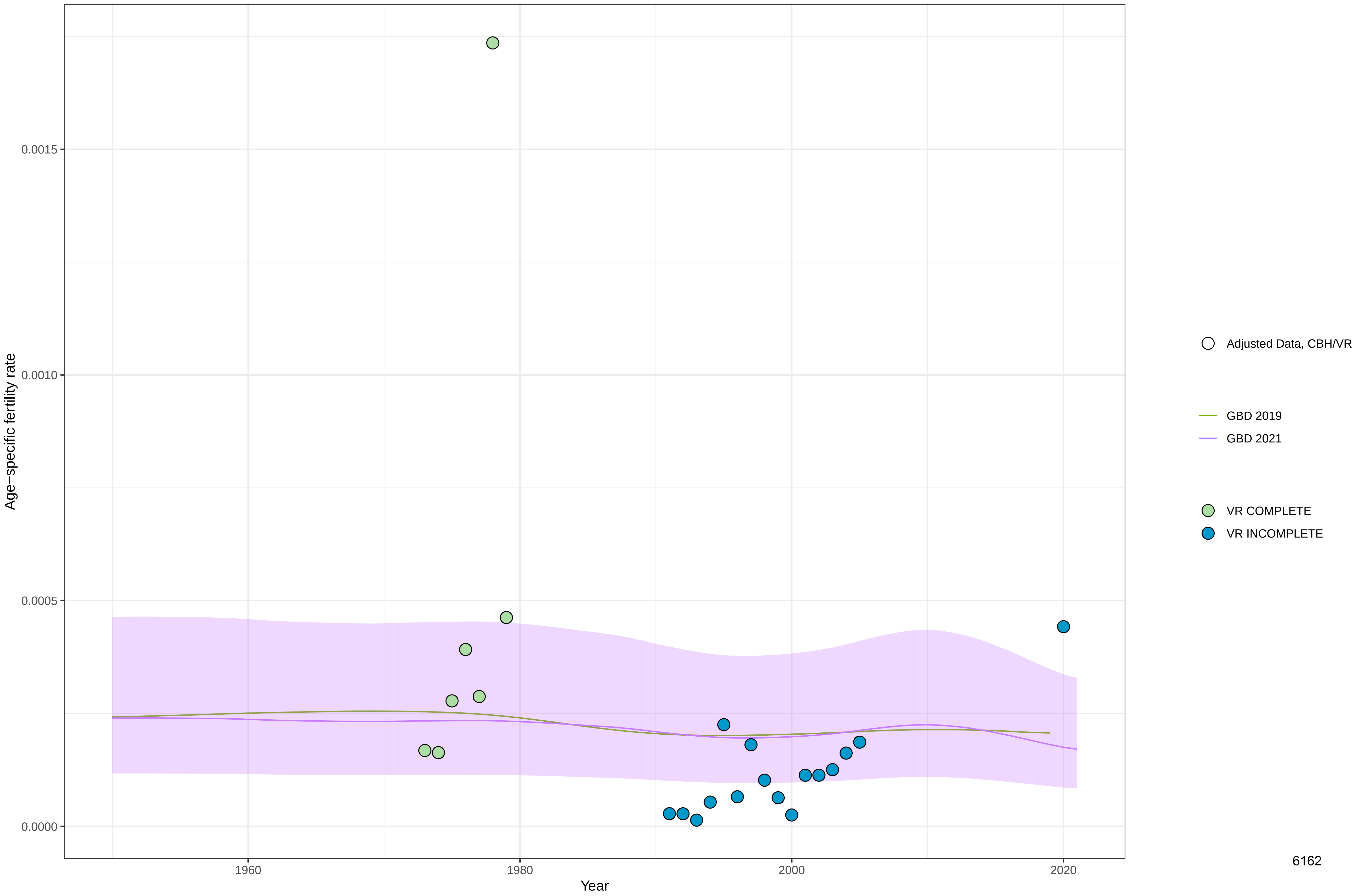

6162

Age−specific fertility fitted trend lines, 1950−2021: Isabela, Ages: 15−19
Data points enclosed with a diamond are reference sources

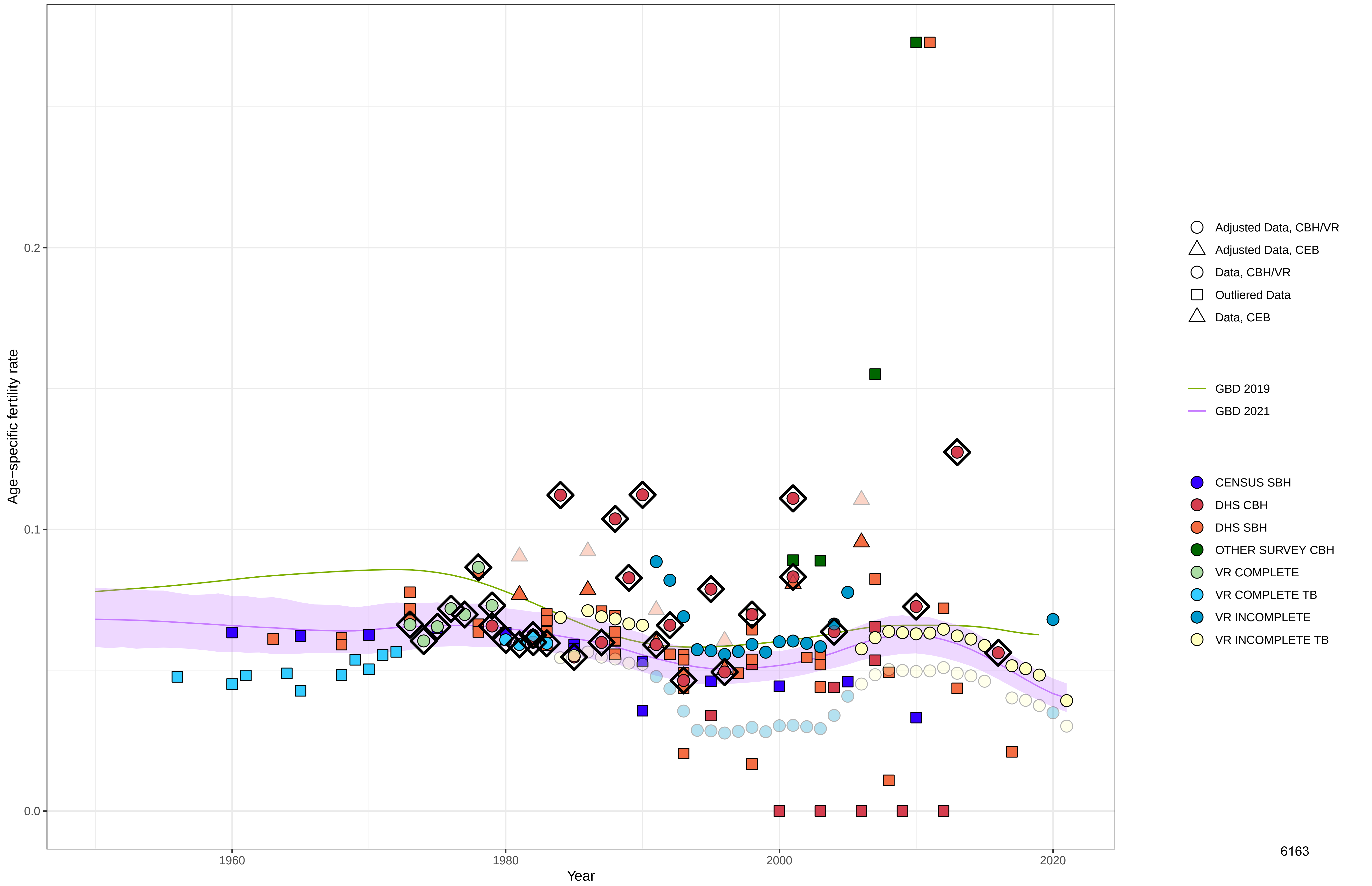

Age−specific fertility fitted trend lines, 1950−2021: Isabela, Ages: 20−24
Data points enclosed with a diamond are reference sources

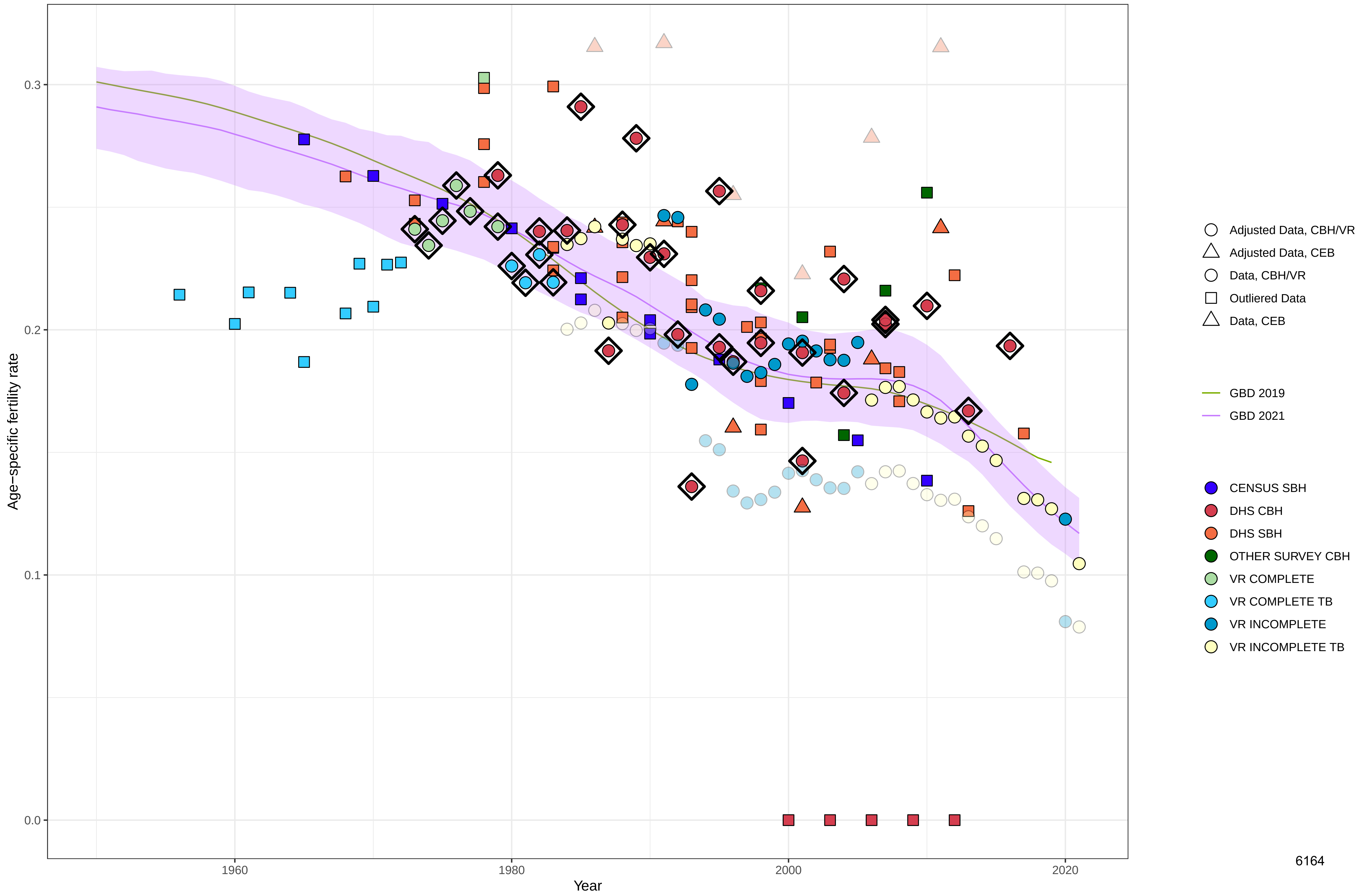

Age−specific fertility fitted trend lines, 1950−2021: Isabela, Ages: 25−29
Data points enclosed with a diamond are reference sources

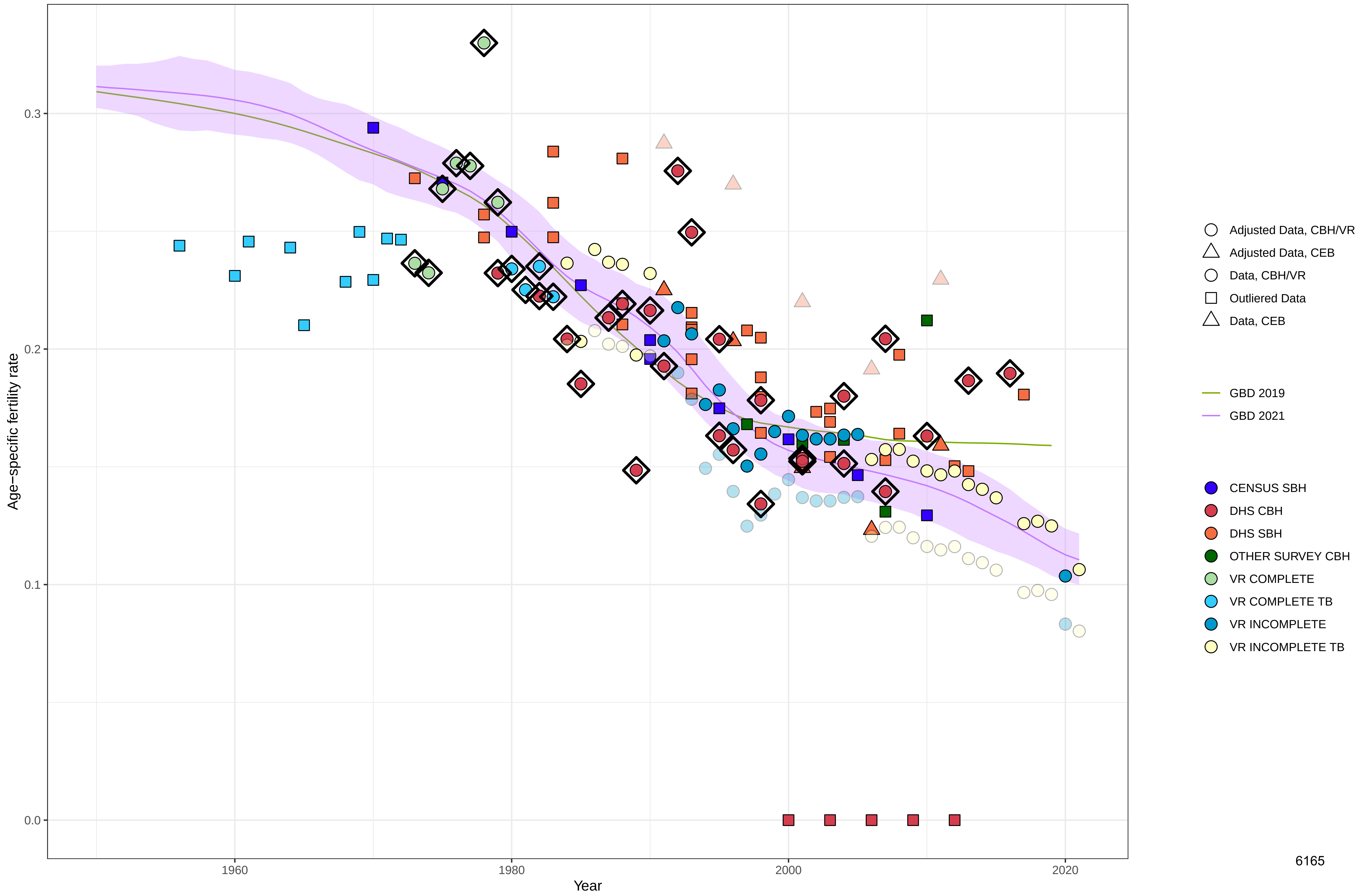

6165

Age−specific fertility fitted trend lines, 1950−2021: Isabela, Ages: 30−34
Data points enclosed with a diamond are reference sources

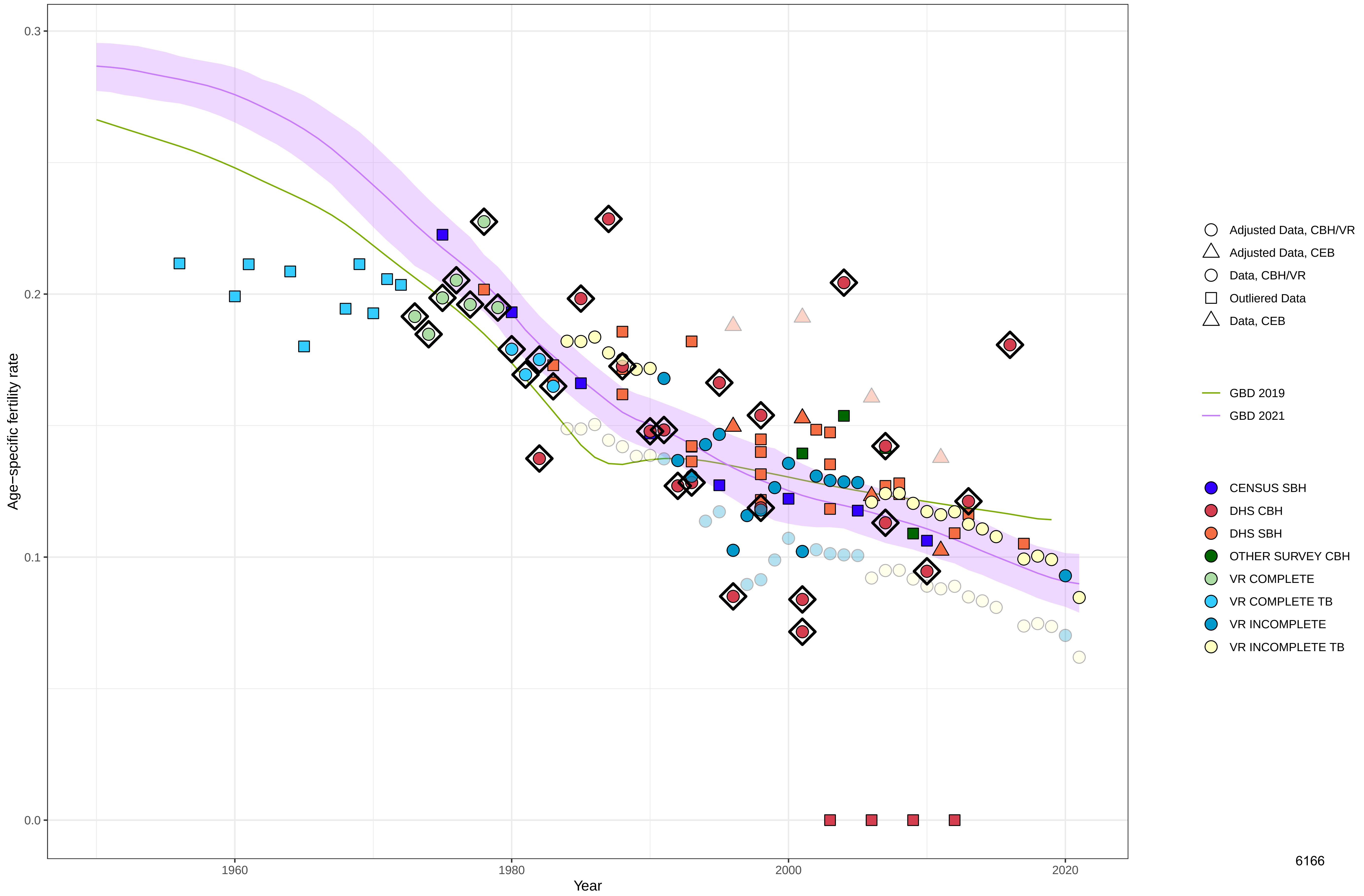

Age−specific fertility fitted trend lines, 1950−2021: Isabela, Ages: 35−39
Data points enclosed with a diamond are reference sources

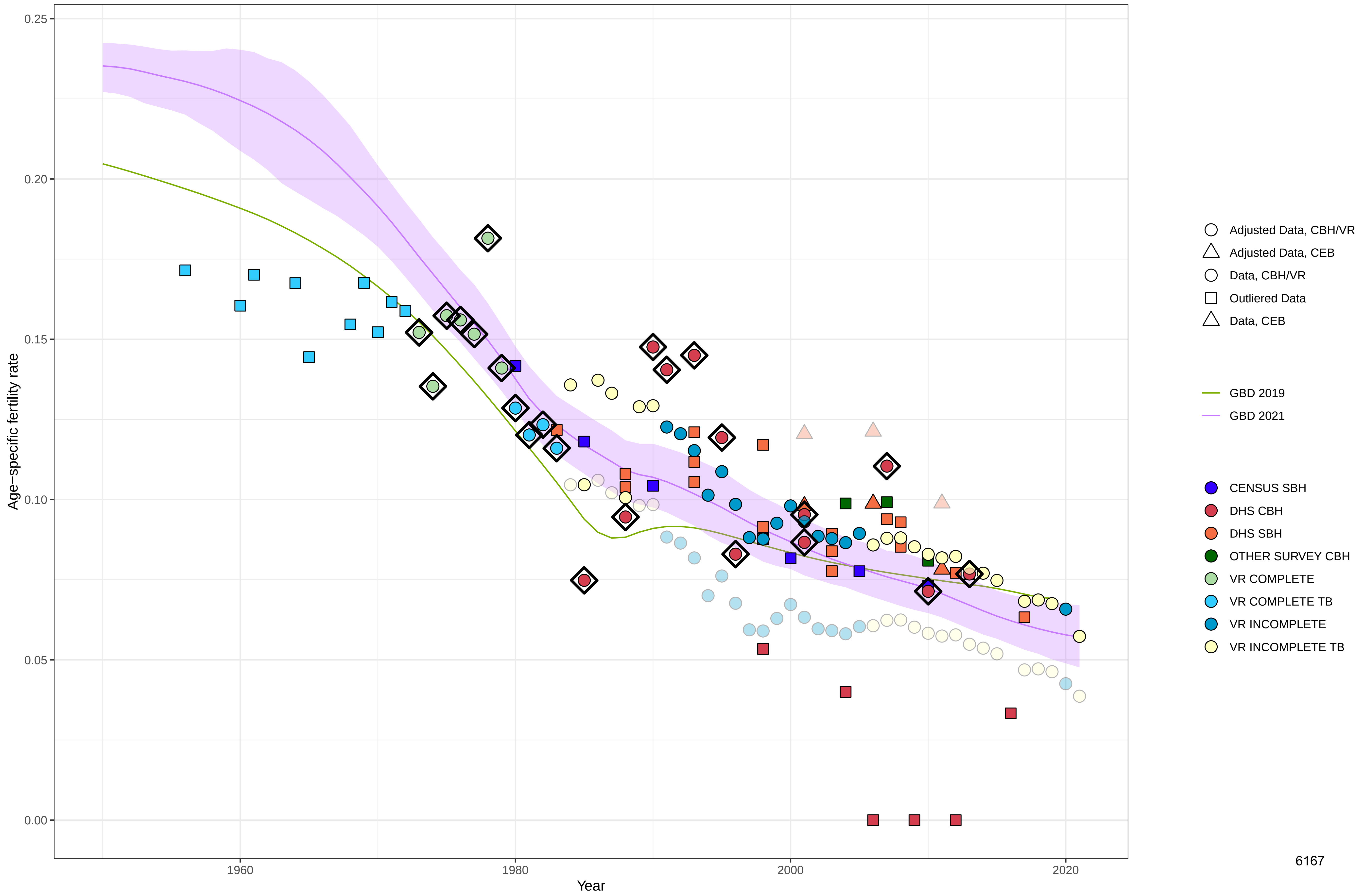

Age−specific fertility fitted trend lines, 1950−2021: Isabela, Ages: 40−44
Data points enclosed with a diamond are reference sources

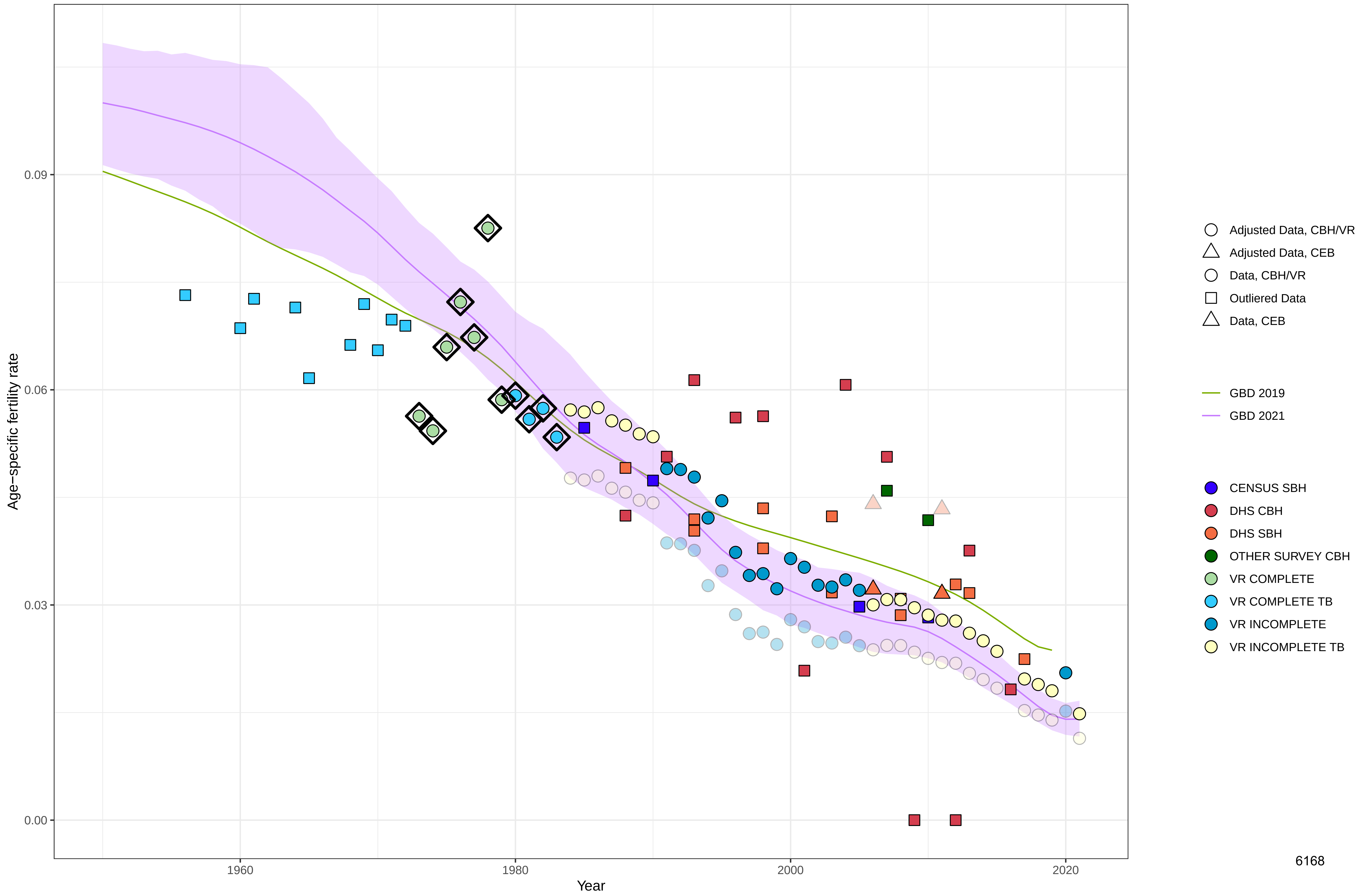

Age−specific fertility fitted trend lines, 1950−2021: Isabela, Ages: 45−49
Data points enclosed with a diamond are reference sources

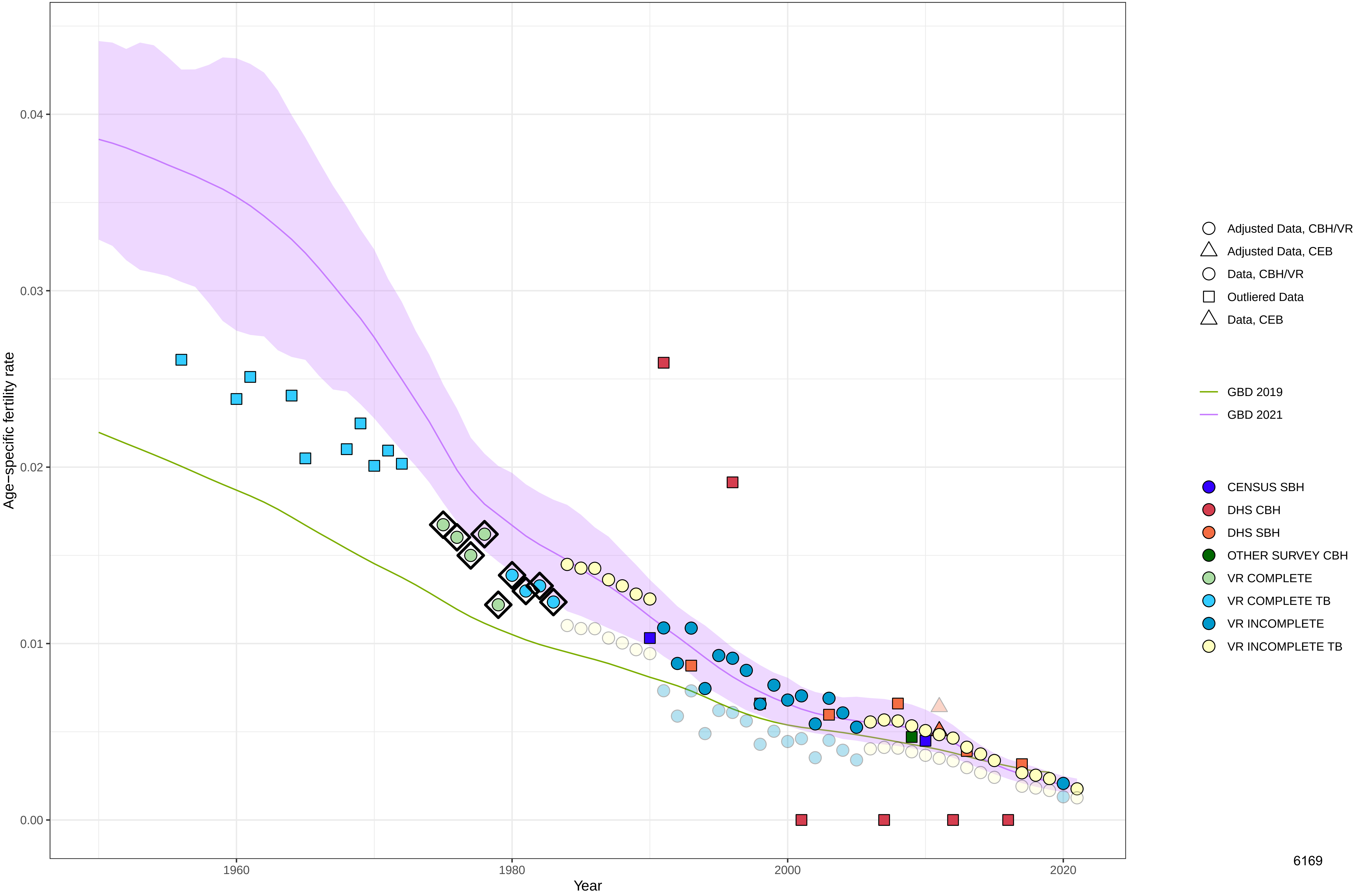

Age−specific fertility fitted trend lines, 1950−2021: Isabela, Ages: 50−54
Data points enclosed with a diamond are reference sources

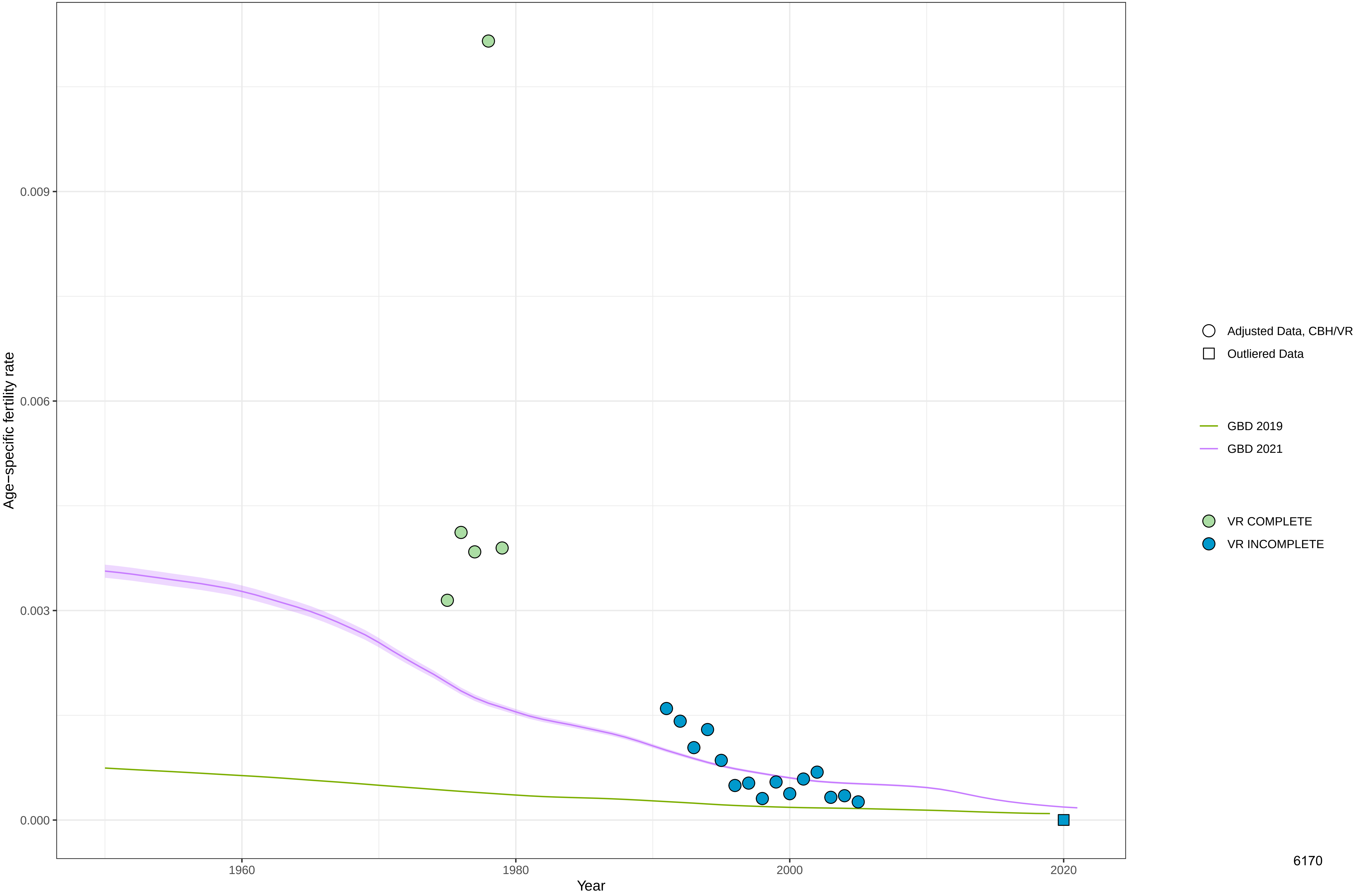

Total fertility rate fitted trend lines, 1950−2021: Isabela TFR
Data points enclosed with a diamond are reference sources

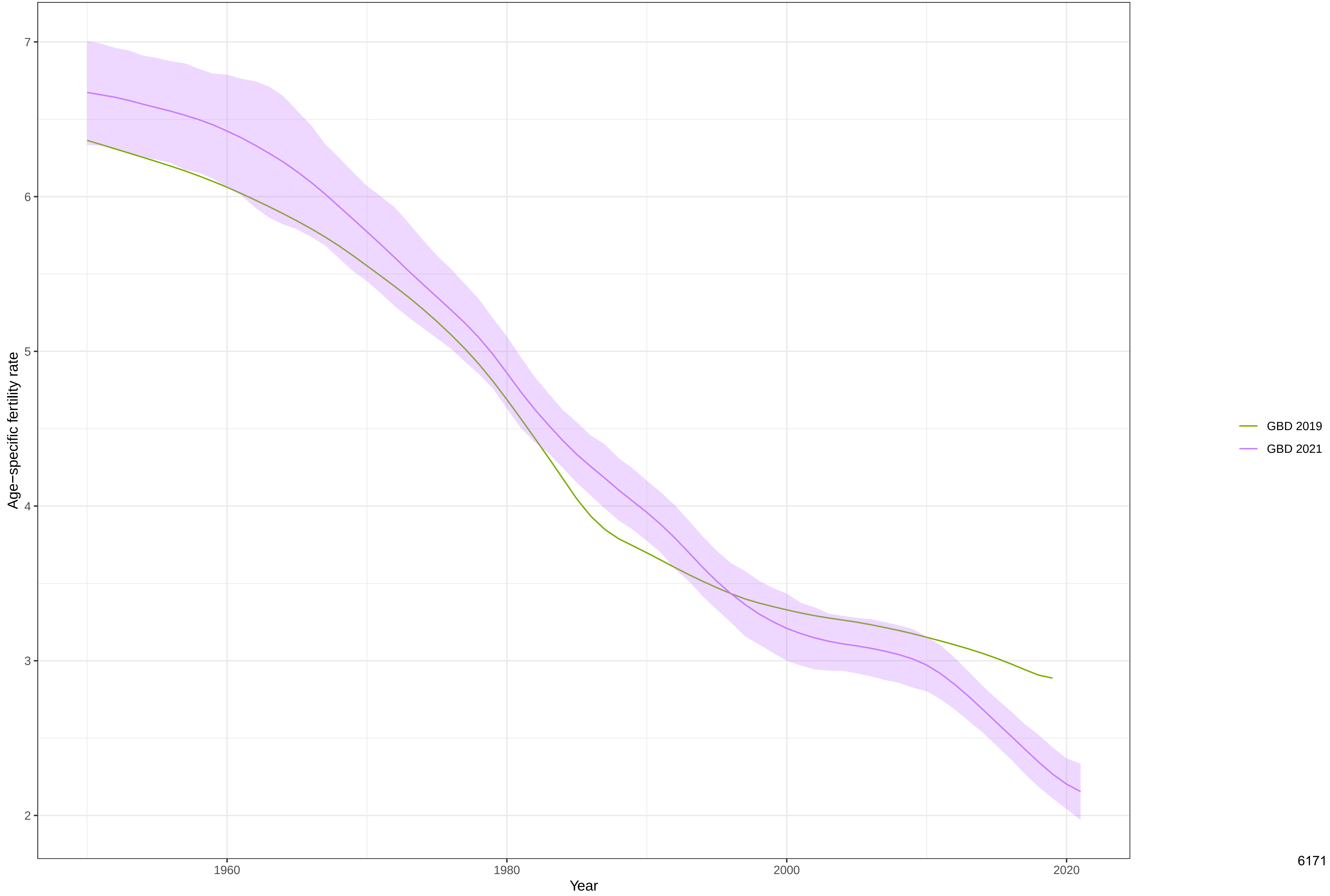

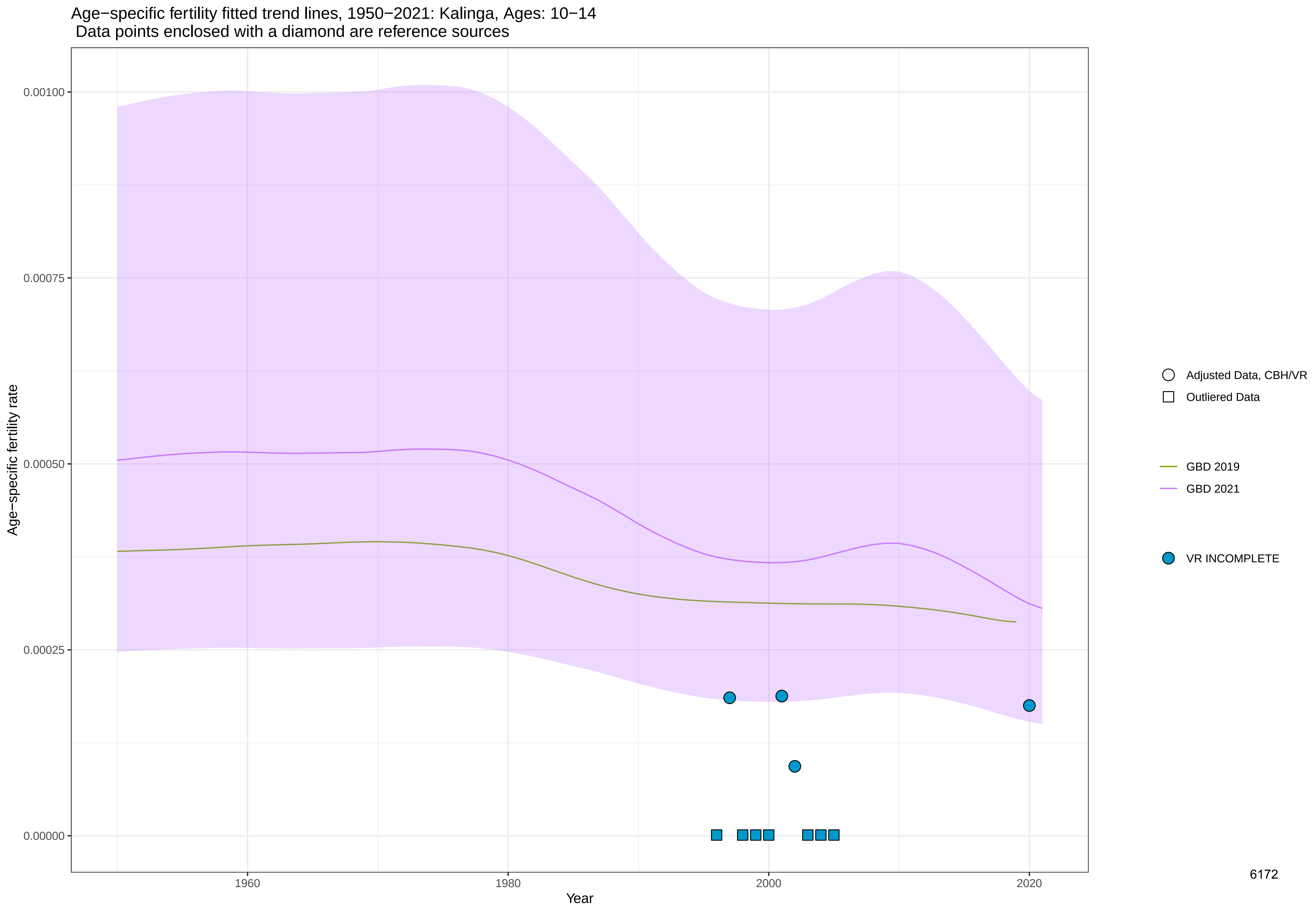

Age−specific fertility fitted trend lines, 1950−2021: Kalinga, Ages: 10−14
Data points enclosed with a diamond are reference sources
0.00100
0.00075
0.00050
0.00025
0.00000
Age−specific fertility rate
1960
1980
2000
2020
Year
Adjusted Data, CBH/VR
Outliered Data
GBD 2019
GBD 2021
VR INCOMPLETE

Age−specific fertility fitted trend lines, 1950−2021: Kalinga, Ages: 15−19
Data points enclosed with a diamond are reference sources

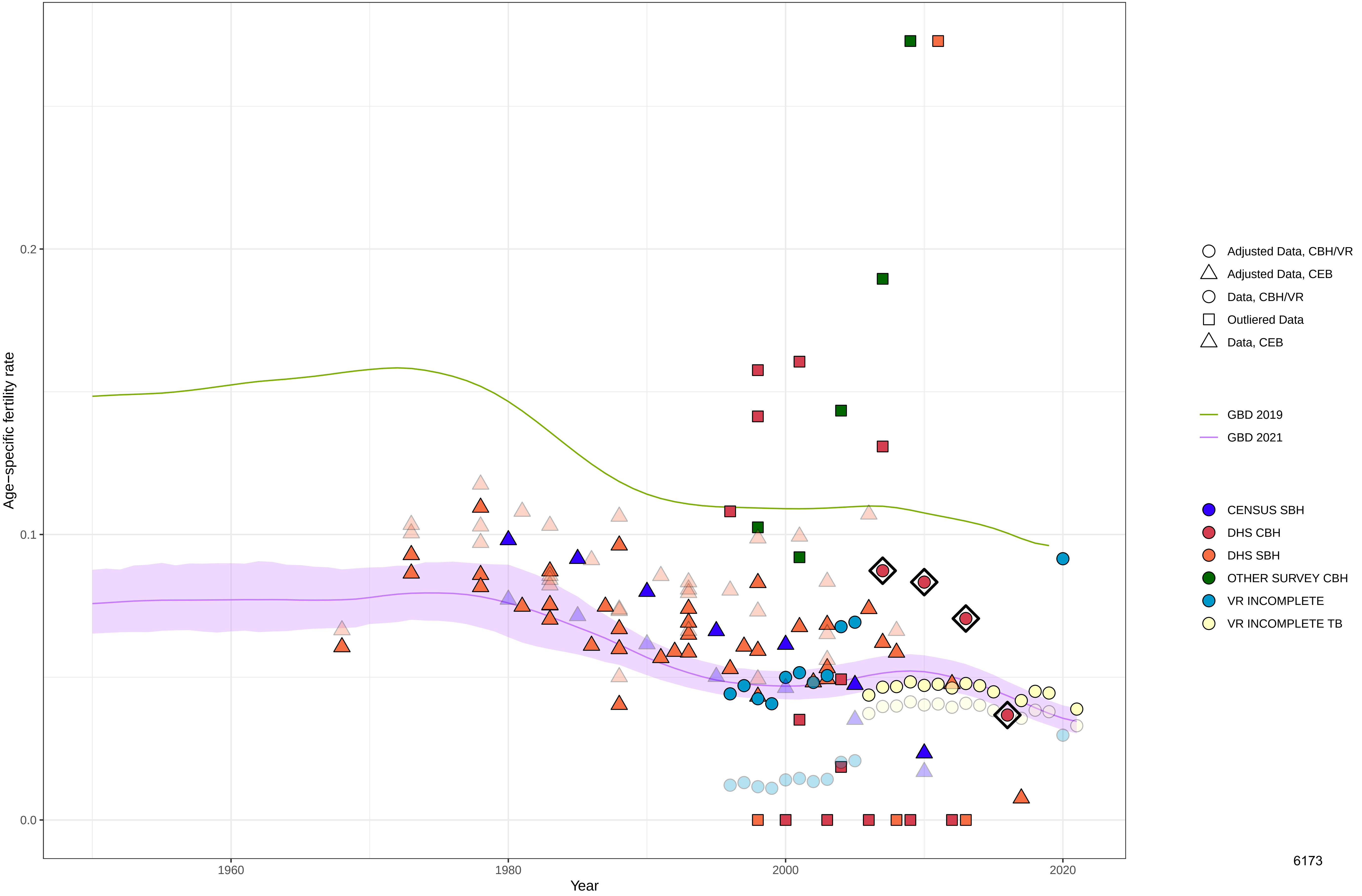

Age−specific fertility fitted trend lines, 1950−2021: Kalinga, Ages: 20−24
Data points enclosed with a diamond are reference sources

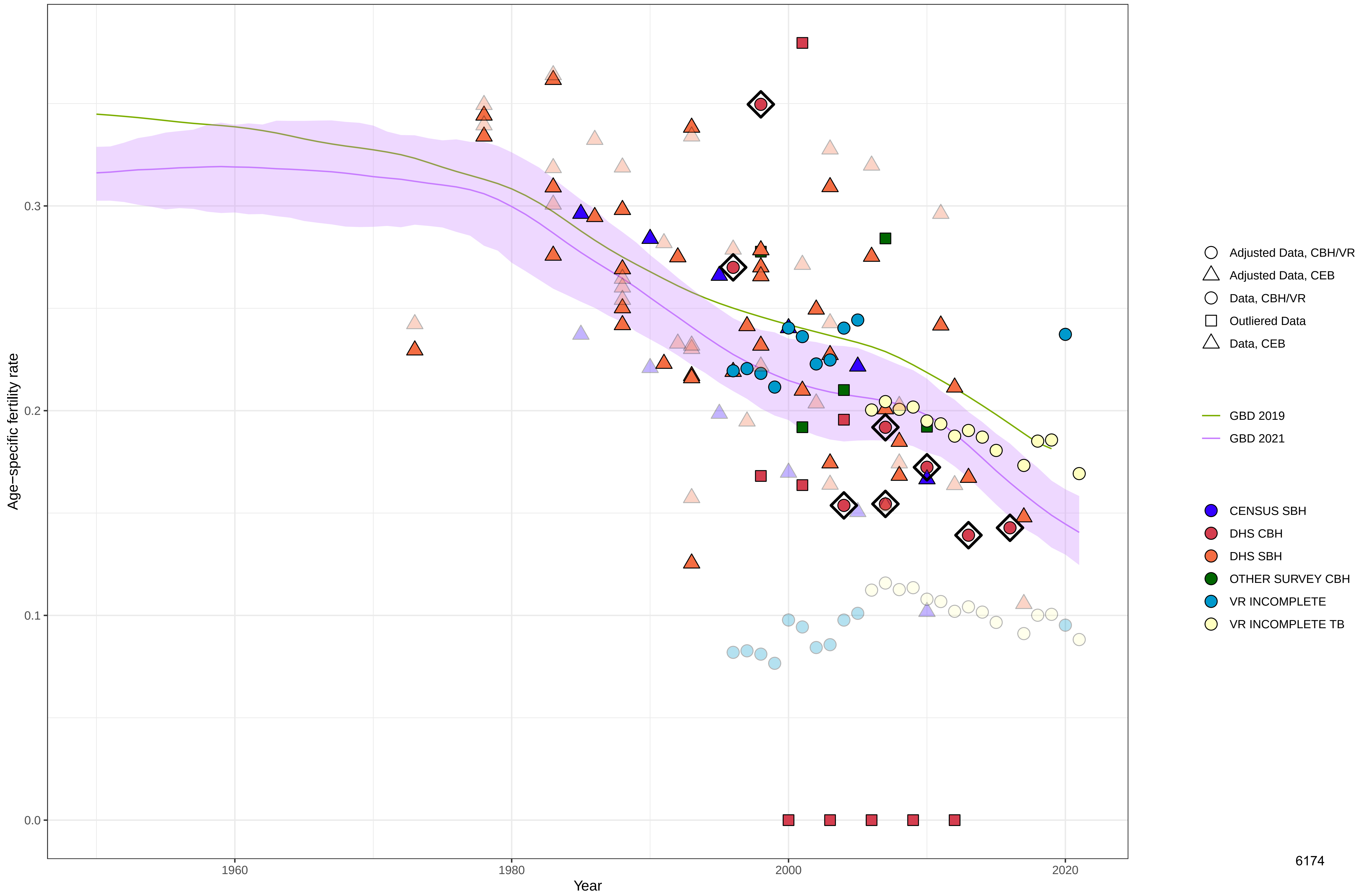

Age−specific fertility fitted trend lines, 1950−2021: Kalinga, Ages: 25−29
Data points enclosed with a diamond are reference sources

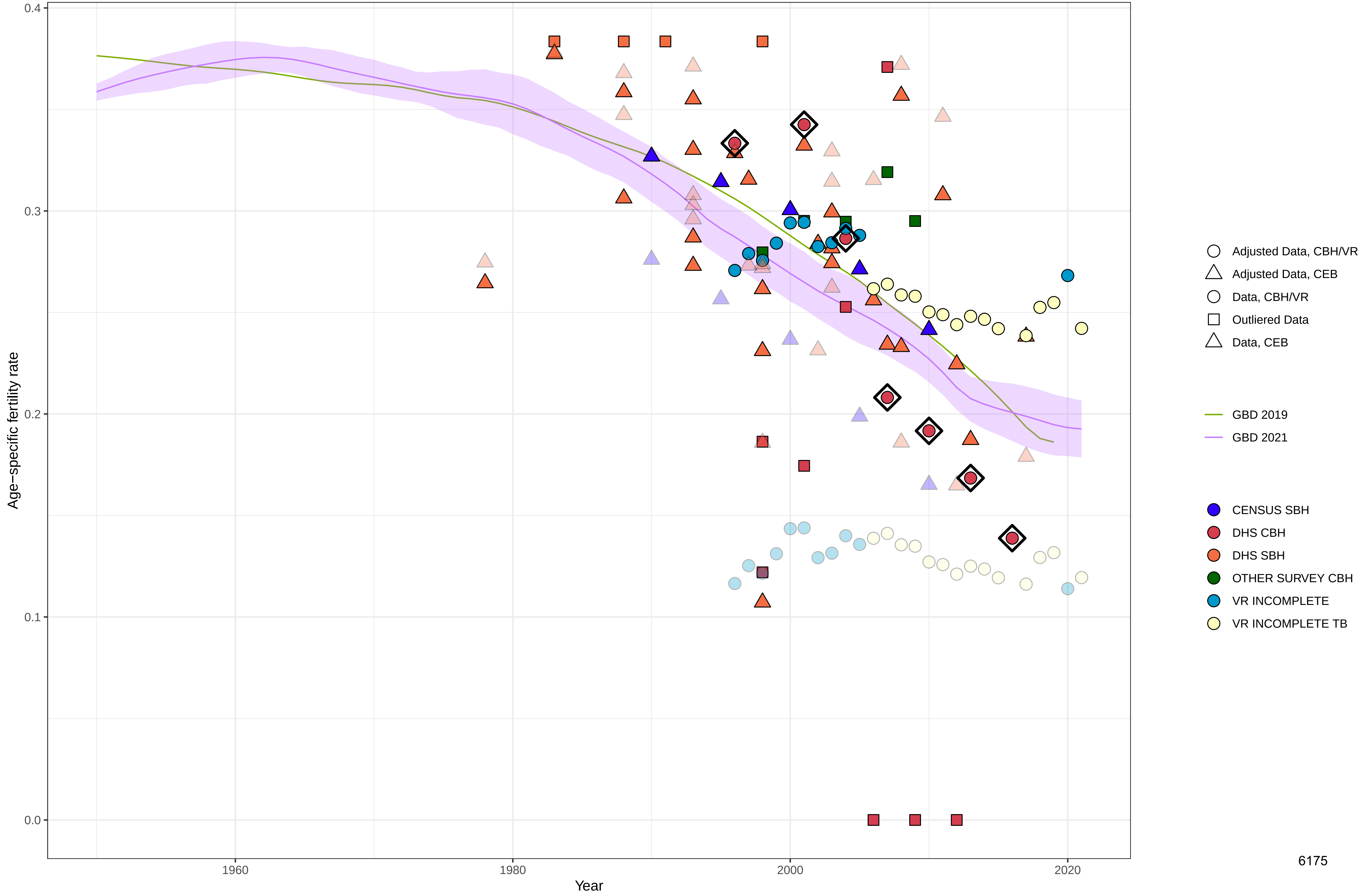

Age−specific fertility fitted trend lines, 1950−2021: Kalinga, Ages: 30−34
Data points enclosed with a diamond are reference sources

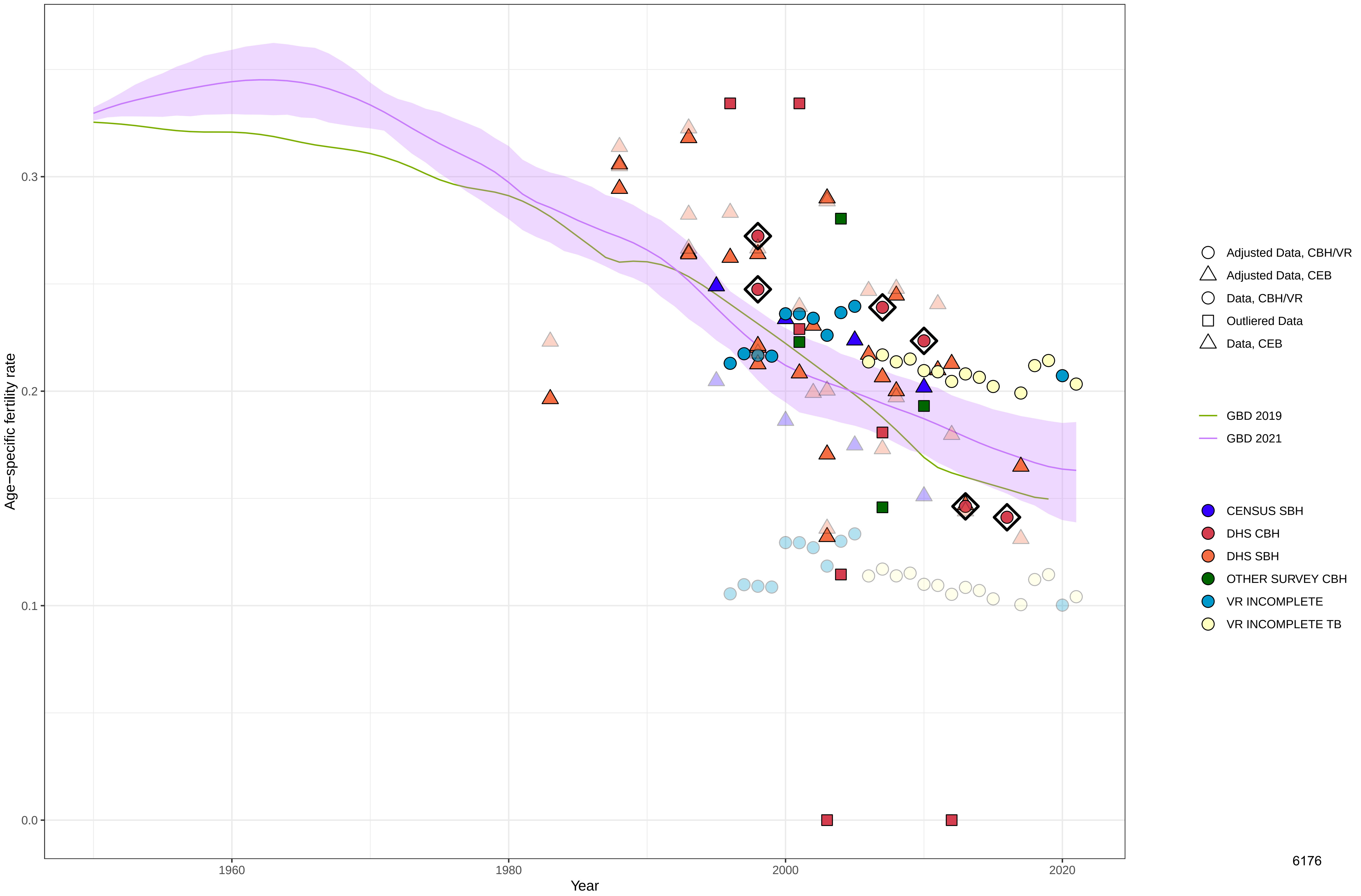

Age−specific fertility fitted trend lines, 1950−2021: Kalinga, Ages: 35−39
Data points enclosed with a diamond are reference sources

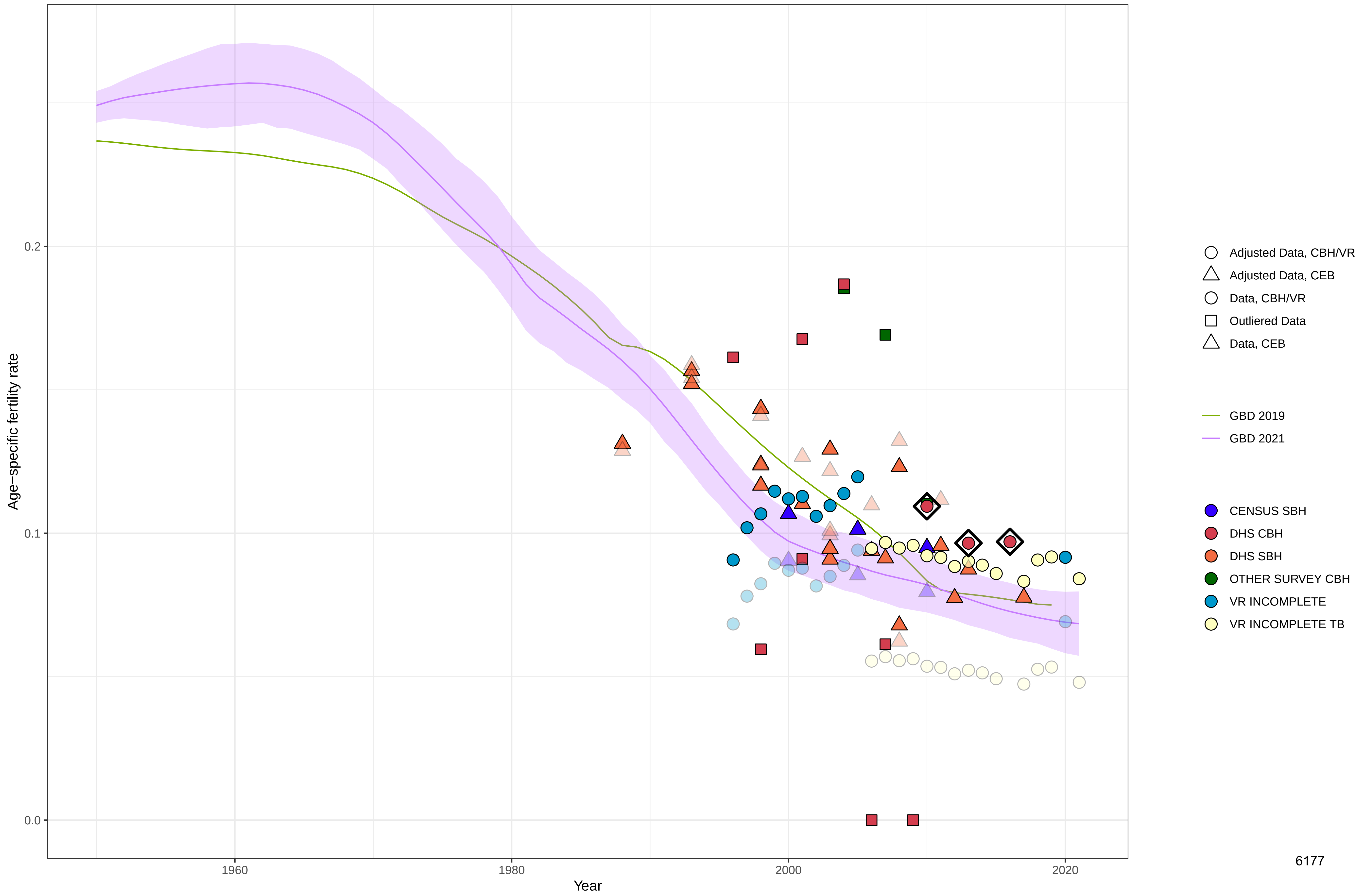

Age−specific fertility fitted trend lines, 1950−2021: Kalinga, Ages: 40−44
Data points enclosed with a diamond are reference sources

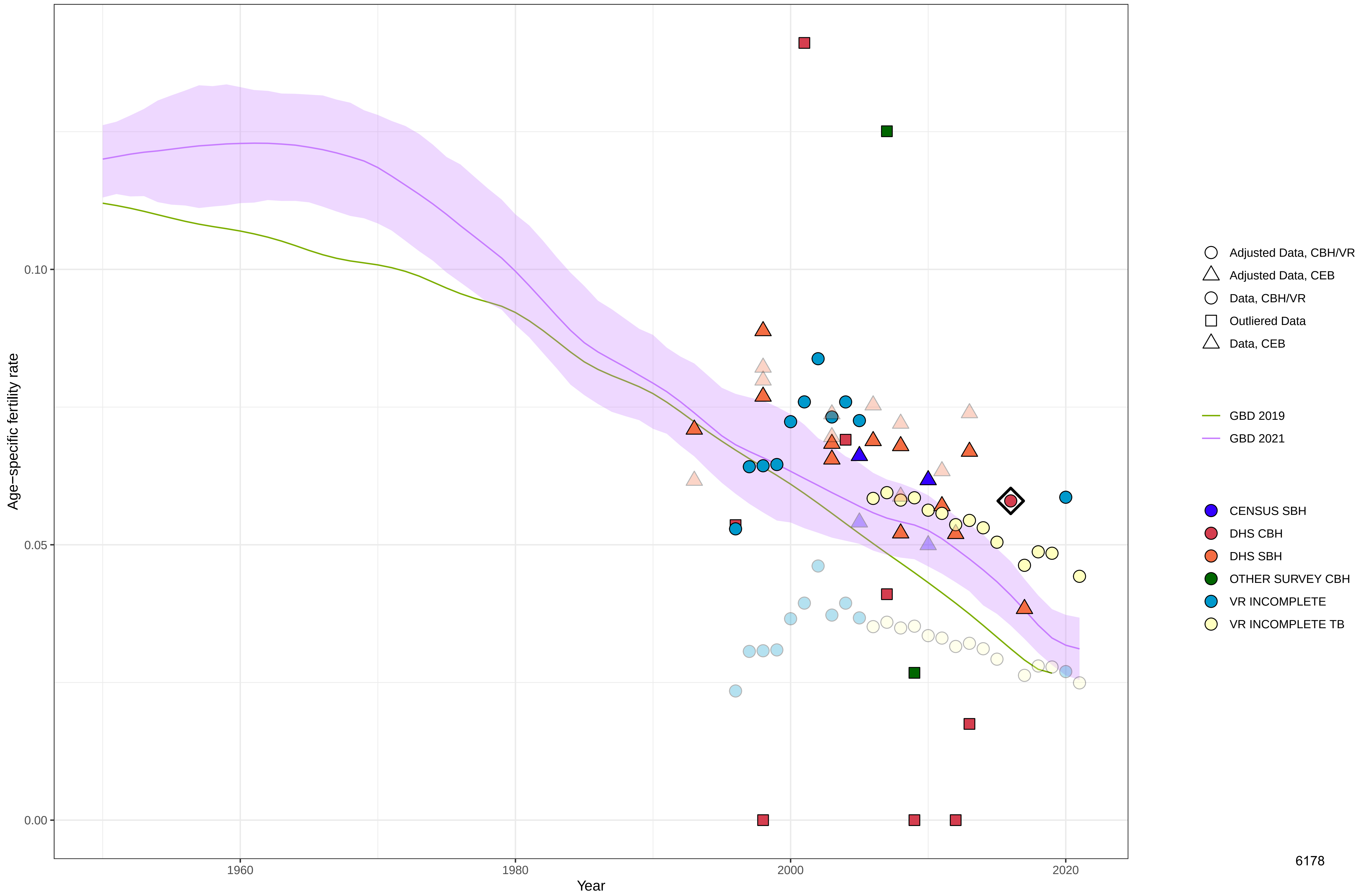

Age−specific fertility fitted trend lines, 1950−2021: Kalinga, Ages: 45−49
Data points enclosed with a diamond are reference sources

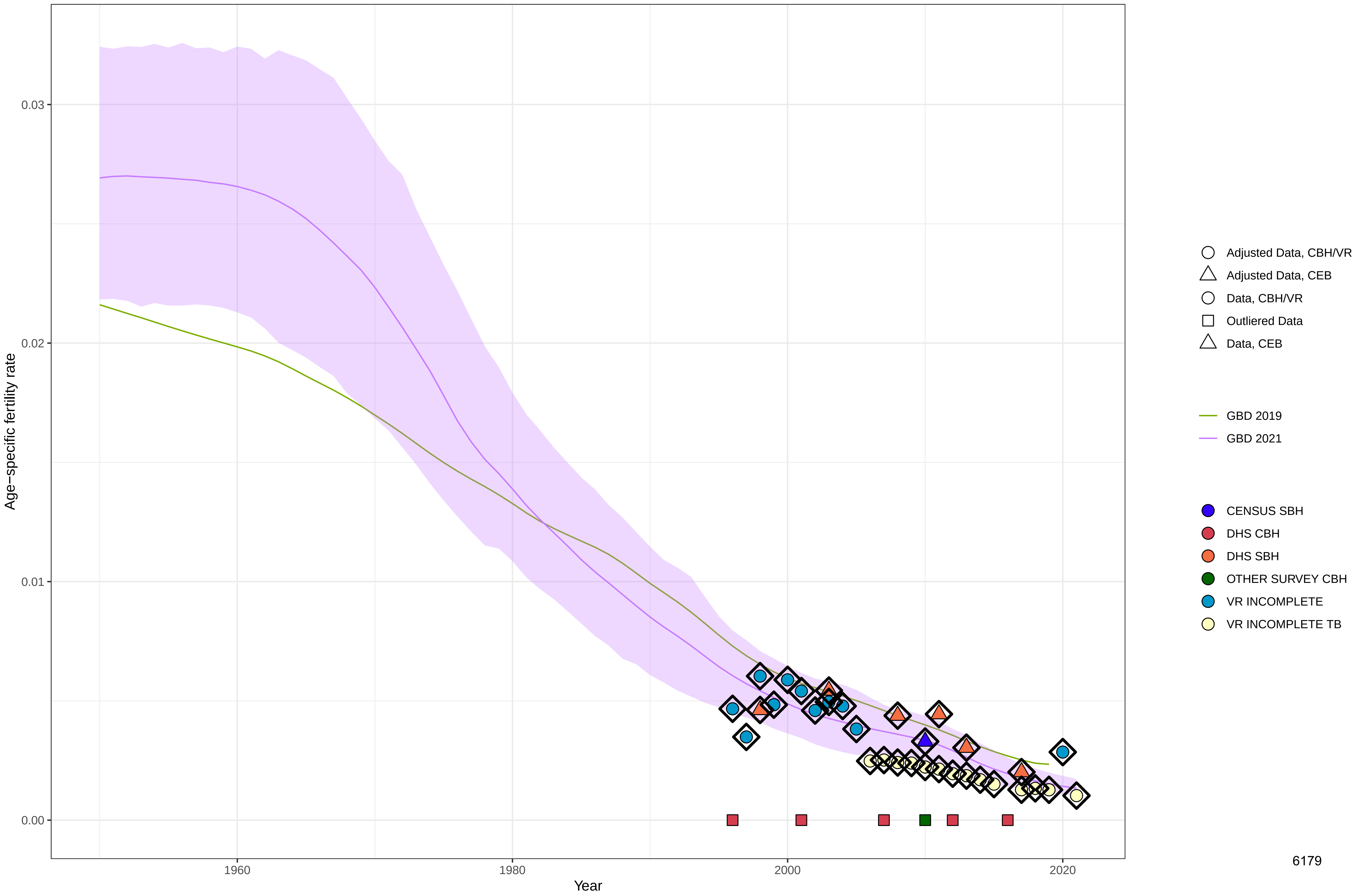

Age−specific fertility fitted trend lines, 1950−2021: Kalinga, Ages: 50−54
Data points enclosed with a diamond are reference sources

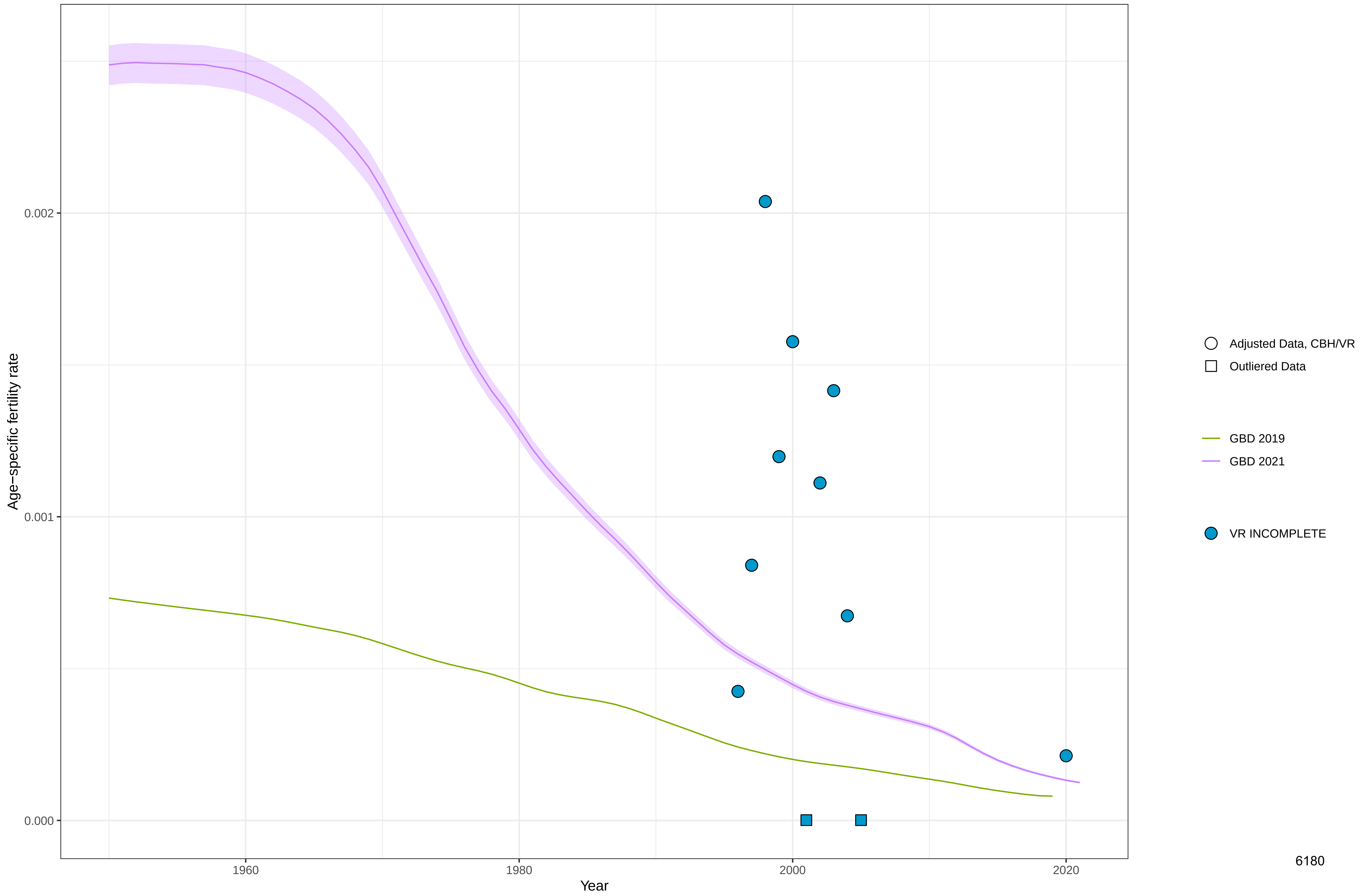

Total fertility rate fitted trend lines, 1950−2021: Kalinga TFR
Data points enclosed with a diamond are reference sources

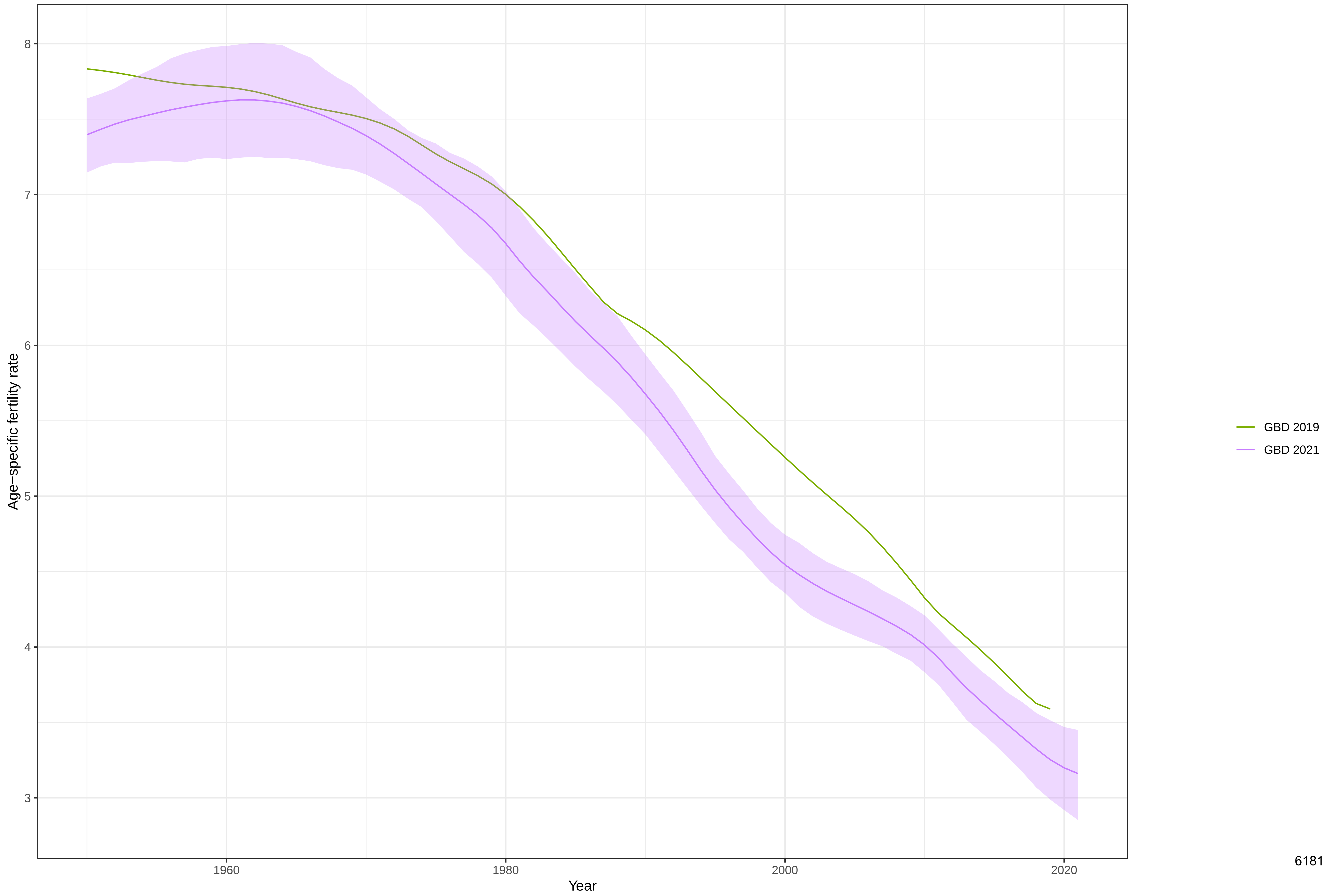

Age−specific fertility fitted trend lines, 1950−2021: La Union, Ages: 10−14
Data points enclosed with a diamond are reference sources

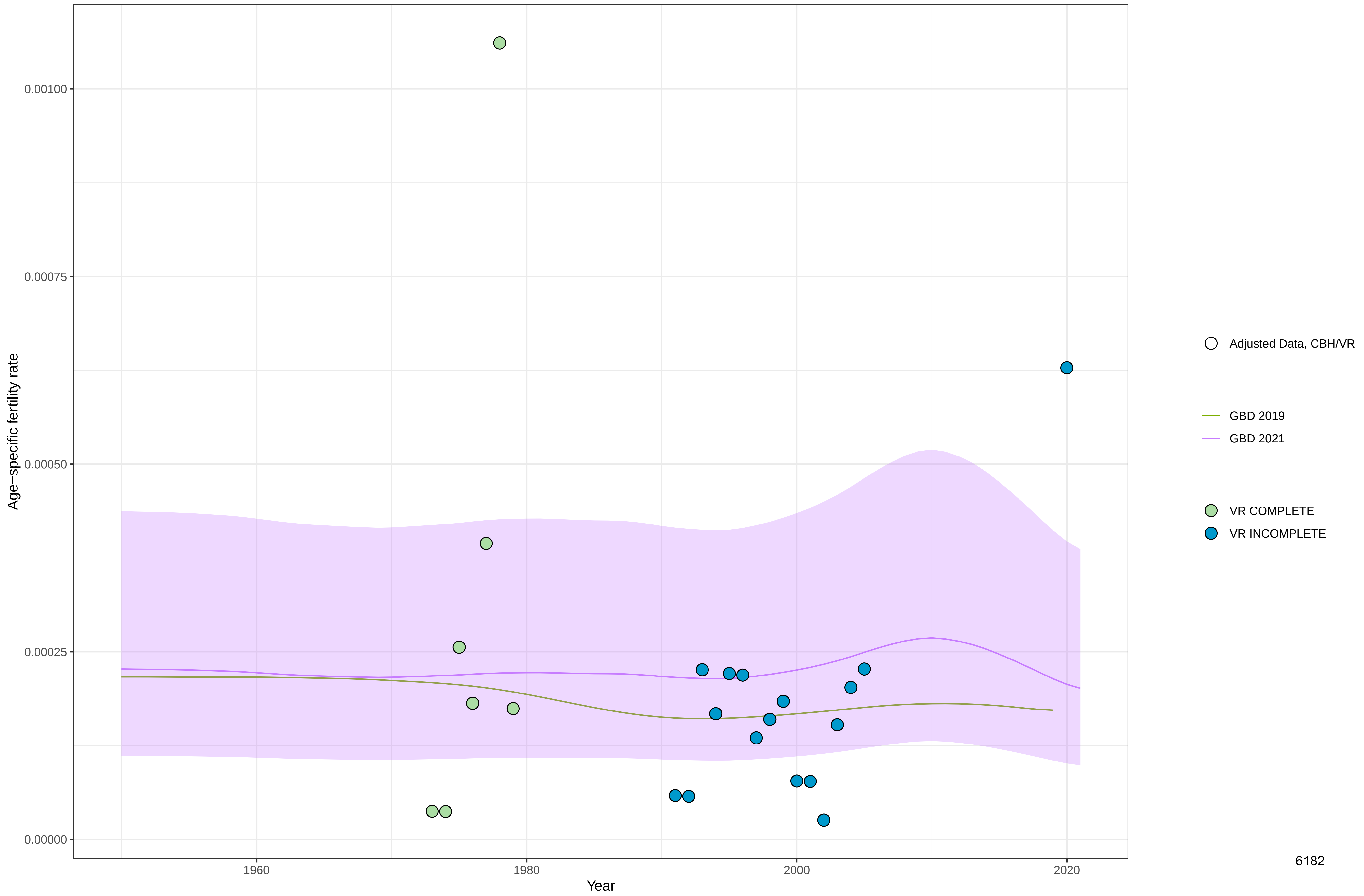

Age−specific fertility fitted trend lines, 1950−2021: La Union, Ages: 15−19
Data points enclosed with a diamond are reference sources

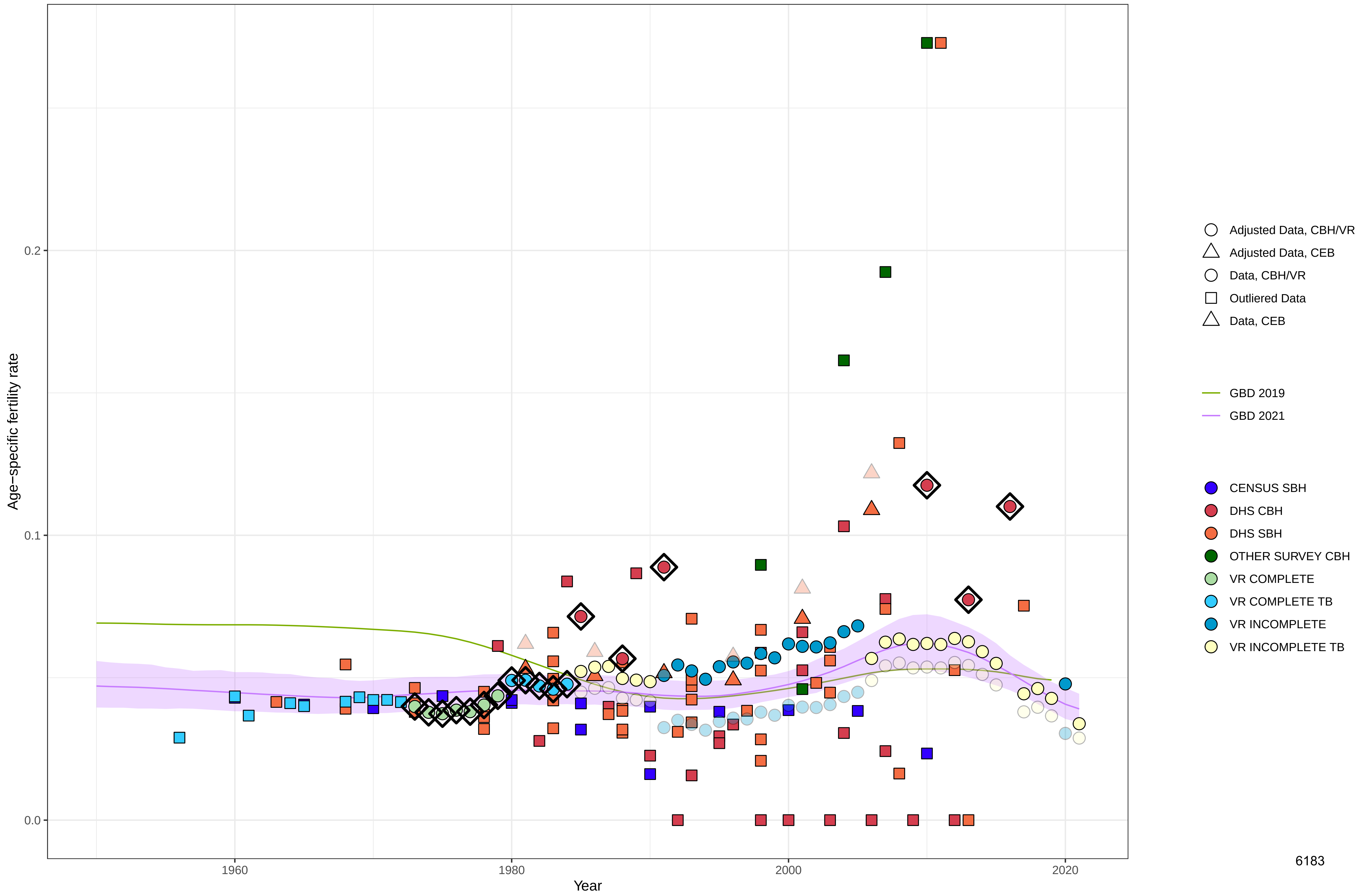

Age−specific fertility fitted trend lines, 1950−2021: La Union, Ages: 20−24
Data points enclosed with a diamond are reference sources

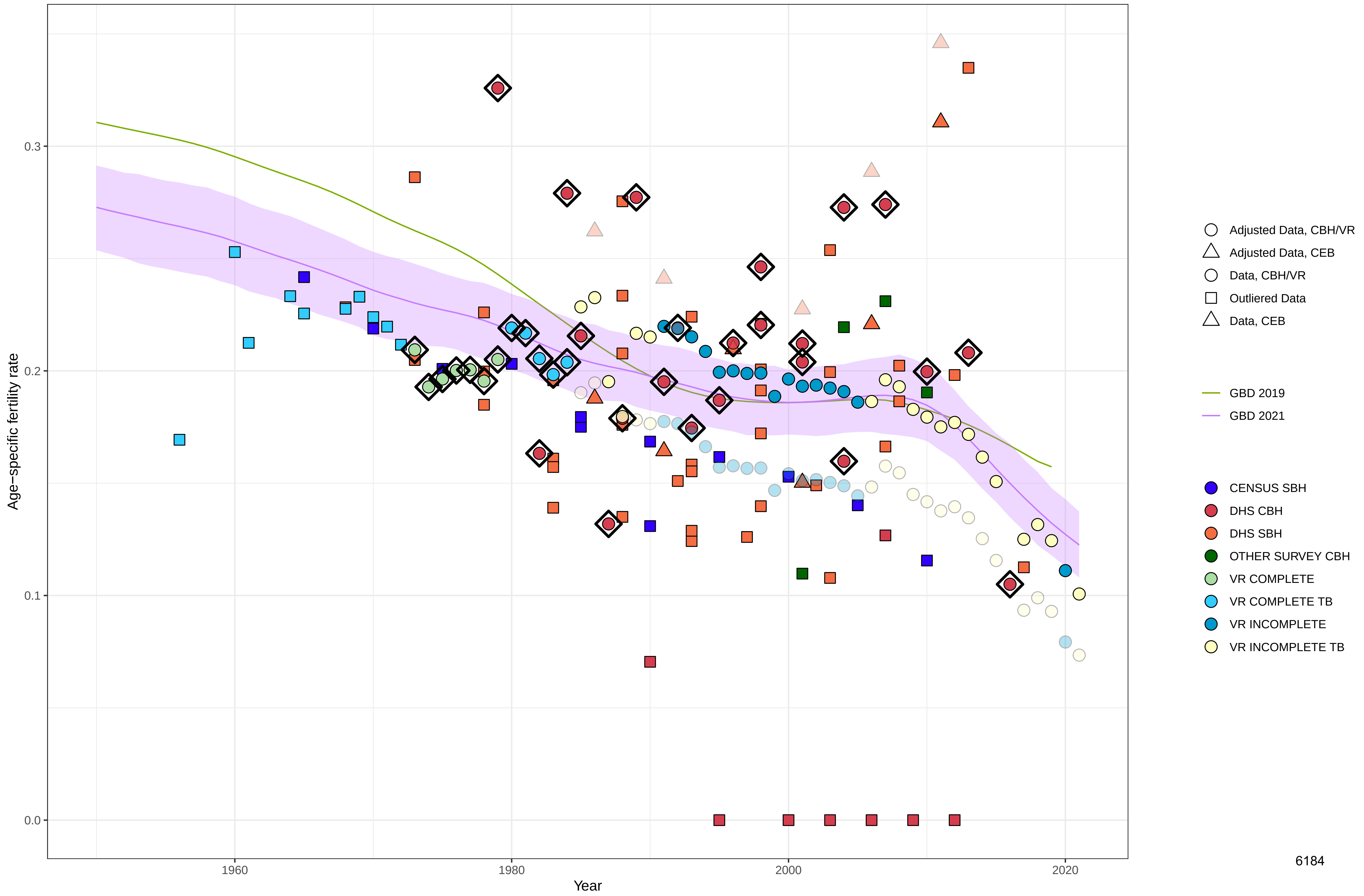

Age−specific fertility fitted trend lines, 1950−2021: La Union, Ages: 25−29
Data points enclosed with a diamond are reference sources

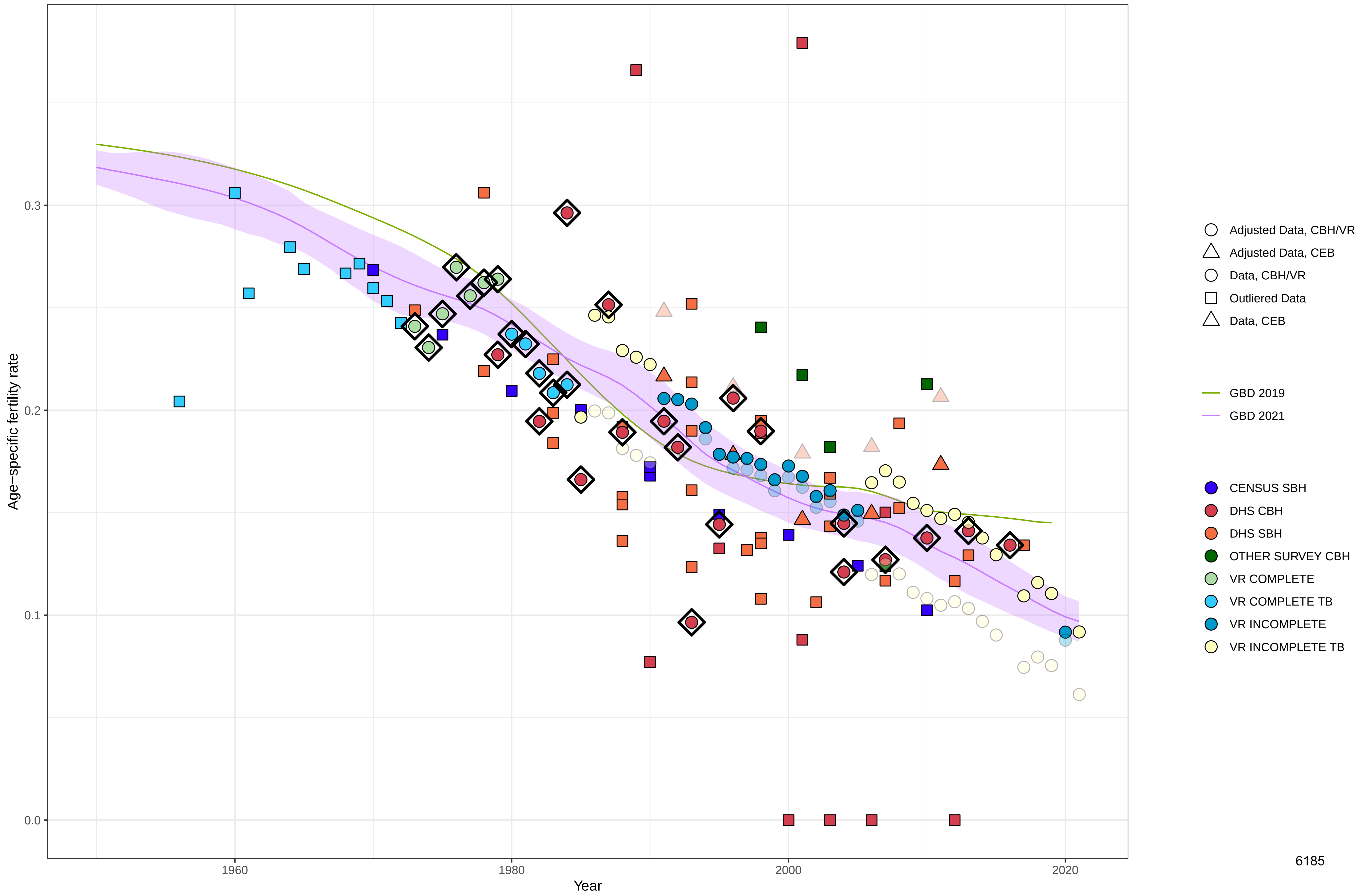

Age−specific fertility fitted trend lines, 1950−2021: La Union, Ages: 30−34
Data points enclosed with a diamond are reference sources

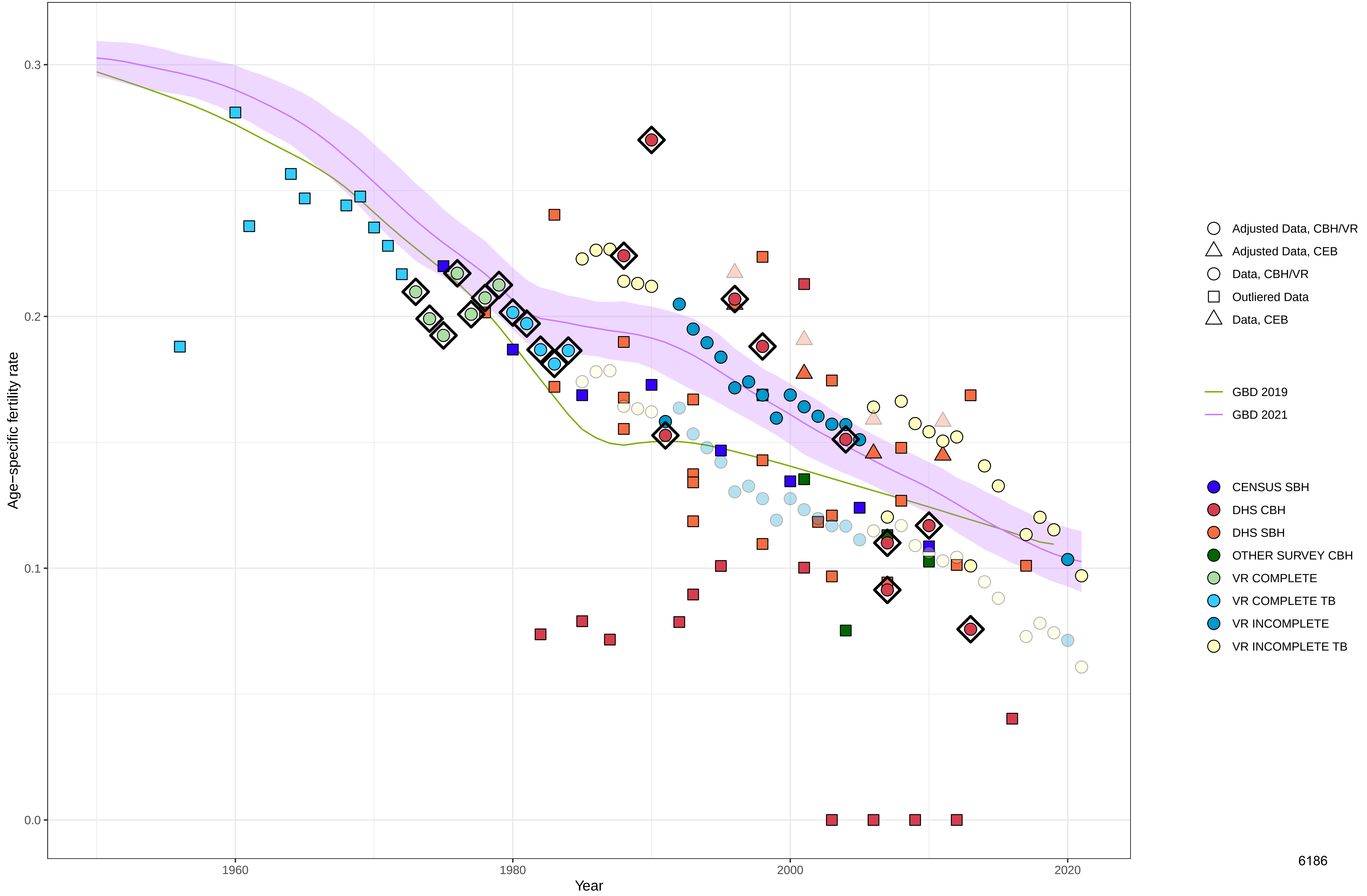

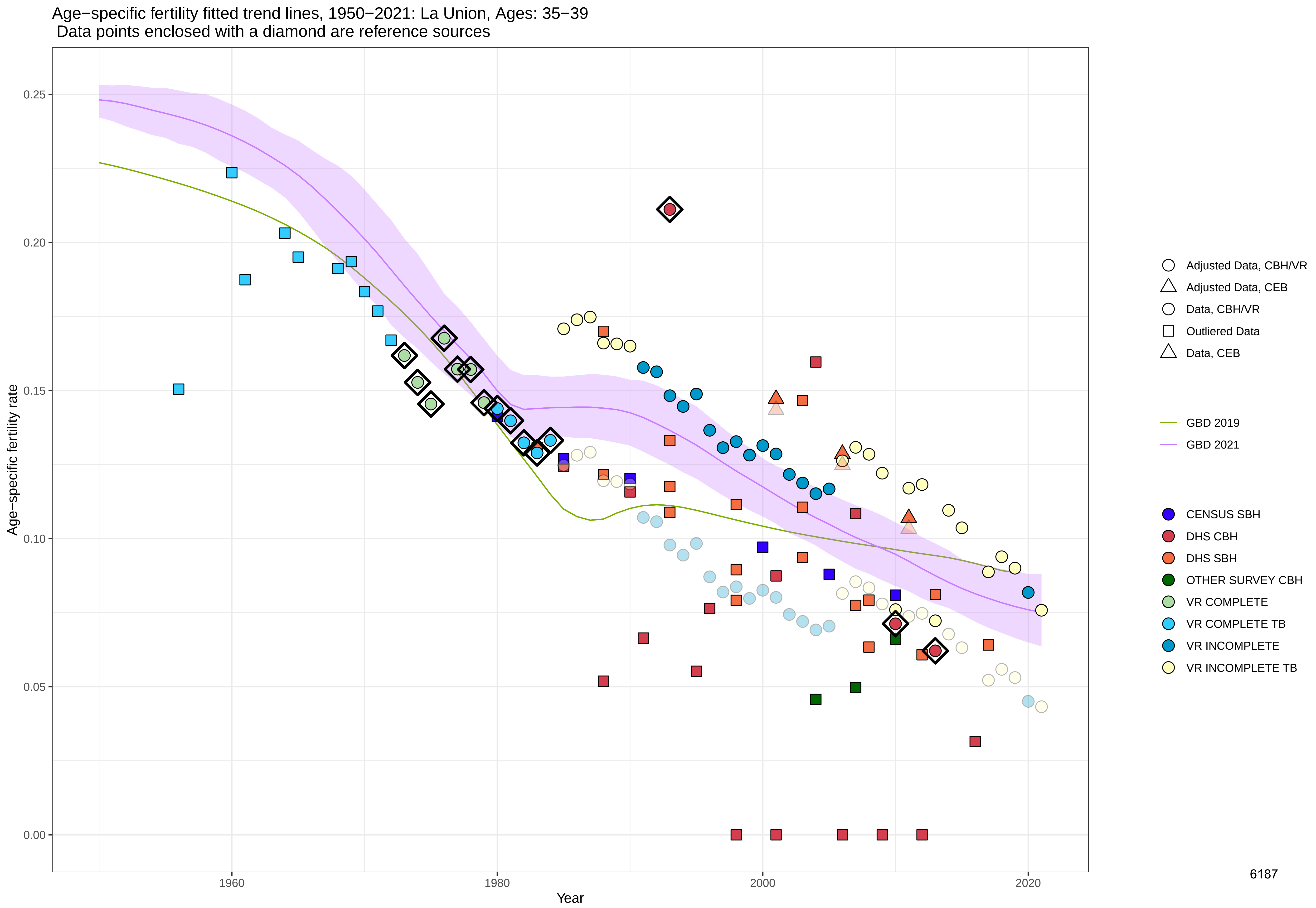

Age−specific fertility fitted trend lines, 1950−2021: La Union, Ages: 35−39
Data points enclosed with a diamond are reference sources
Age−specific fertility rate
0.25
0.20
0.15
0.10
0.05
0.00
Year
1960
1980
2000
2020
Adjusted Data, CBH/VR
Adjusted Data, CEB
Data, CBH/VR
Outliered Data
Data, CEB
GBD 2019
GBD 2021
CENSUS SBH
DHS CBH
DHS SBH
OTHER SURVEY CBH
VR COMPLETE
VR COMPLETE TB
VR INCOMPLETE
VR INCOMPLETE TB

Age−specific fertility fitted trend lines, 1950−2021: La Union, Ages: 40−44
Data points enclosed with a diamond are reference sources

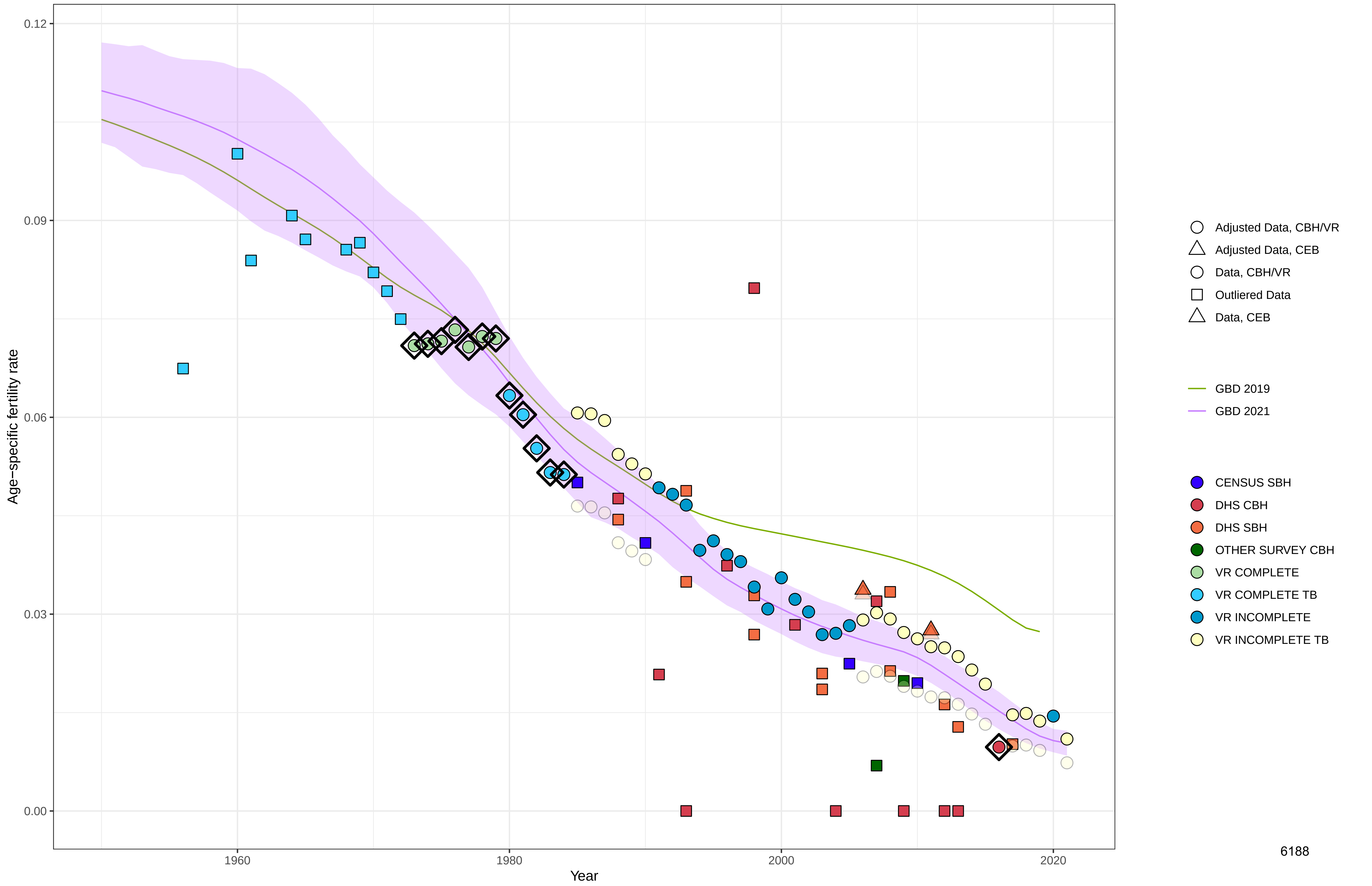

6188

Age−specific fertility fitted trend lines, 1950−2021: La Union, Ages: 45−49
Data points enclosed with a diamond are reference sources

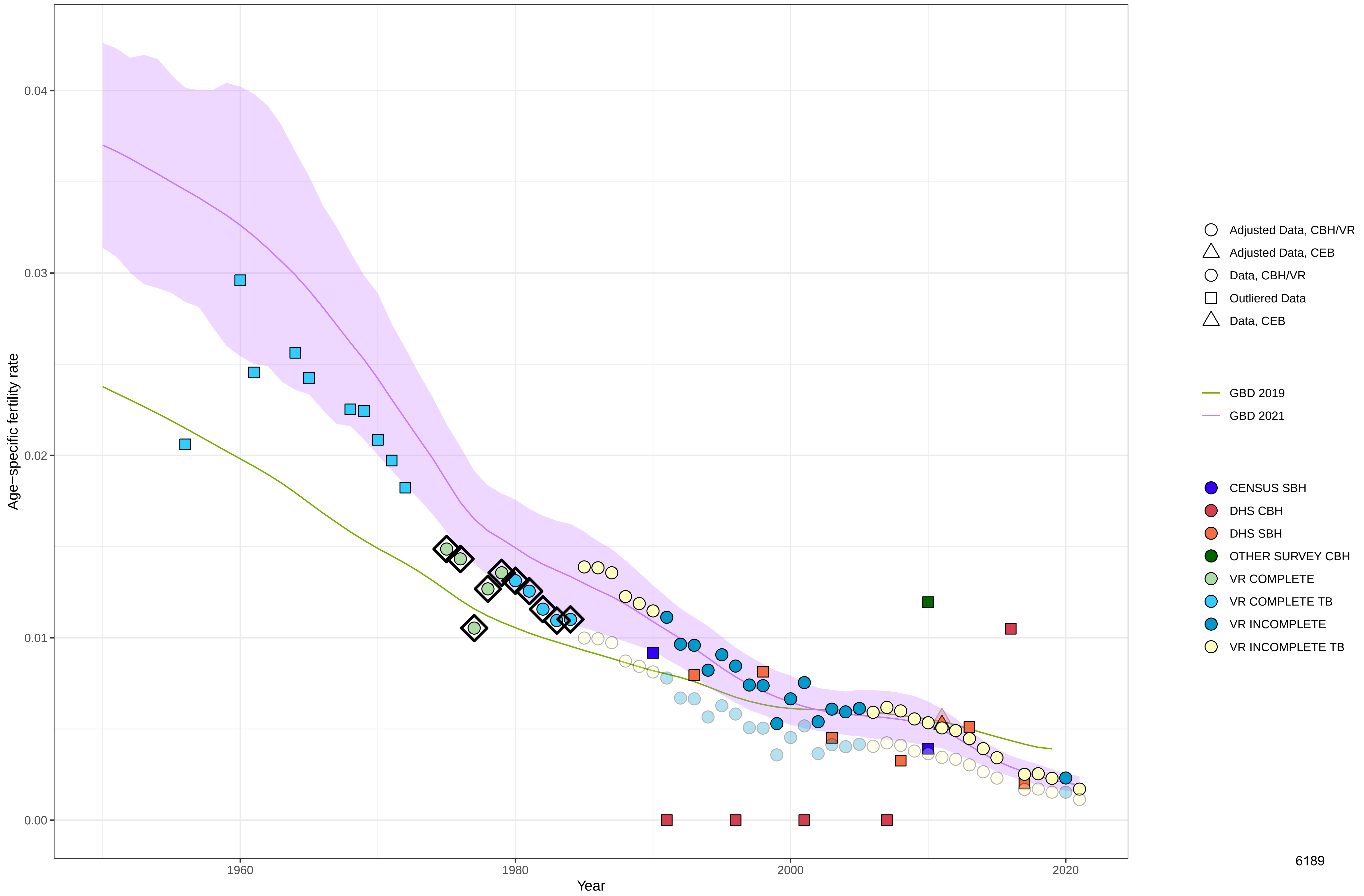

Age−specific fertility fitted trend lines, 1950−2021: La Union, Ages: 50−54
Data points enclosed with a diamond are reference sources

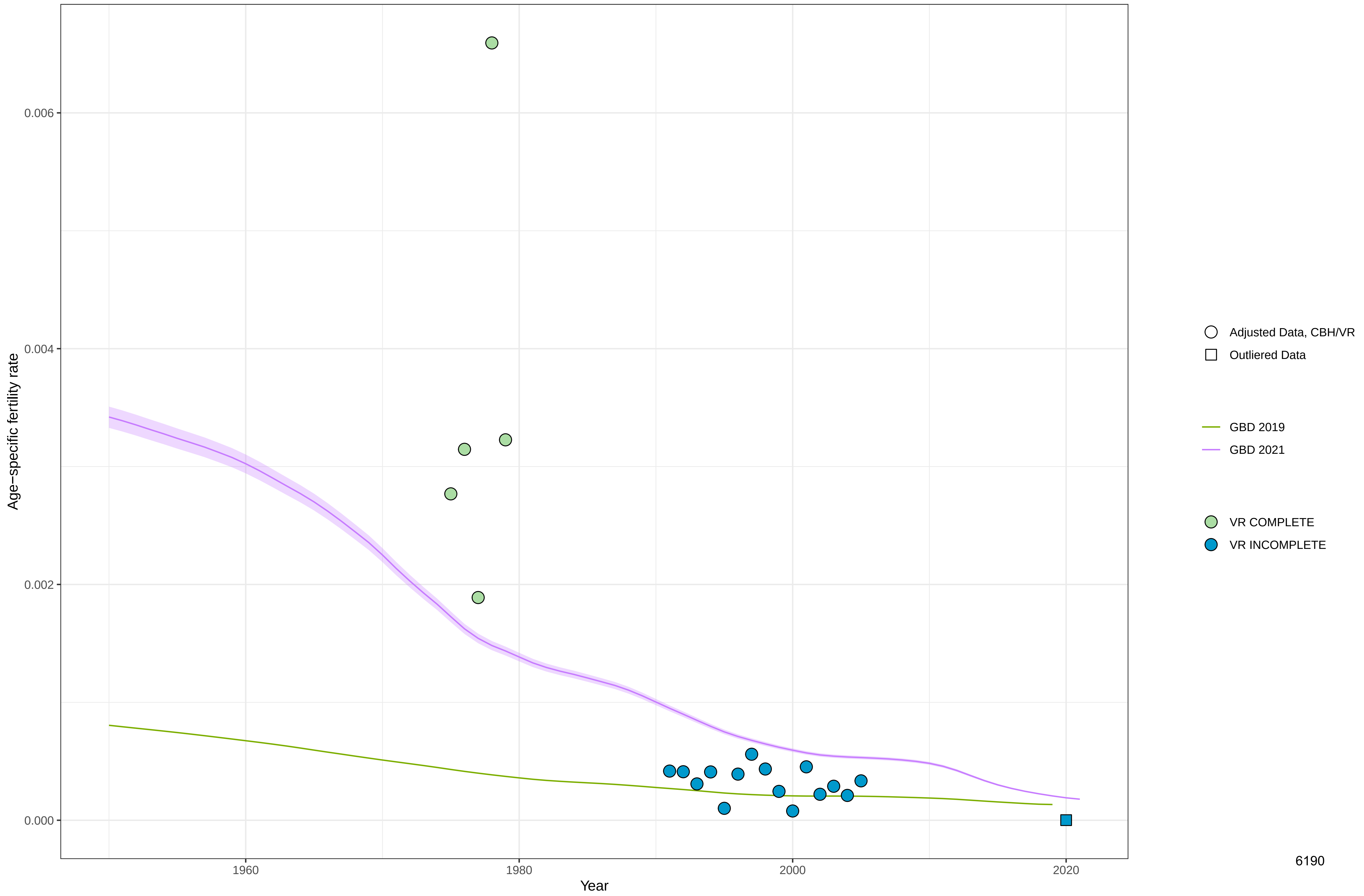

Total fertility rate fitted trend lines, 1950−2021: La Union TFR
Data points enclosed with a diamond are reference sources

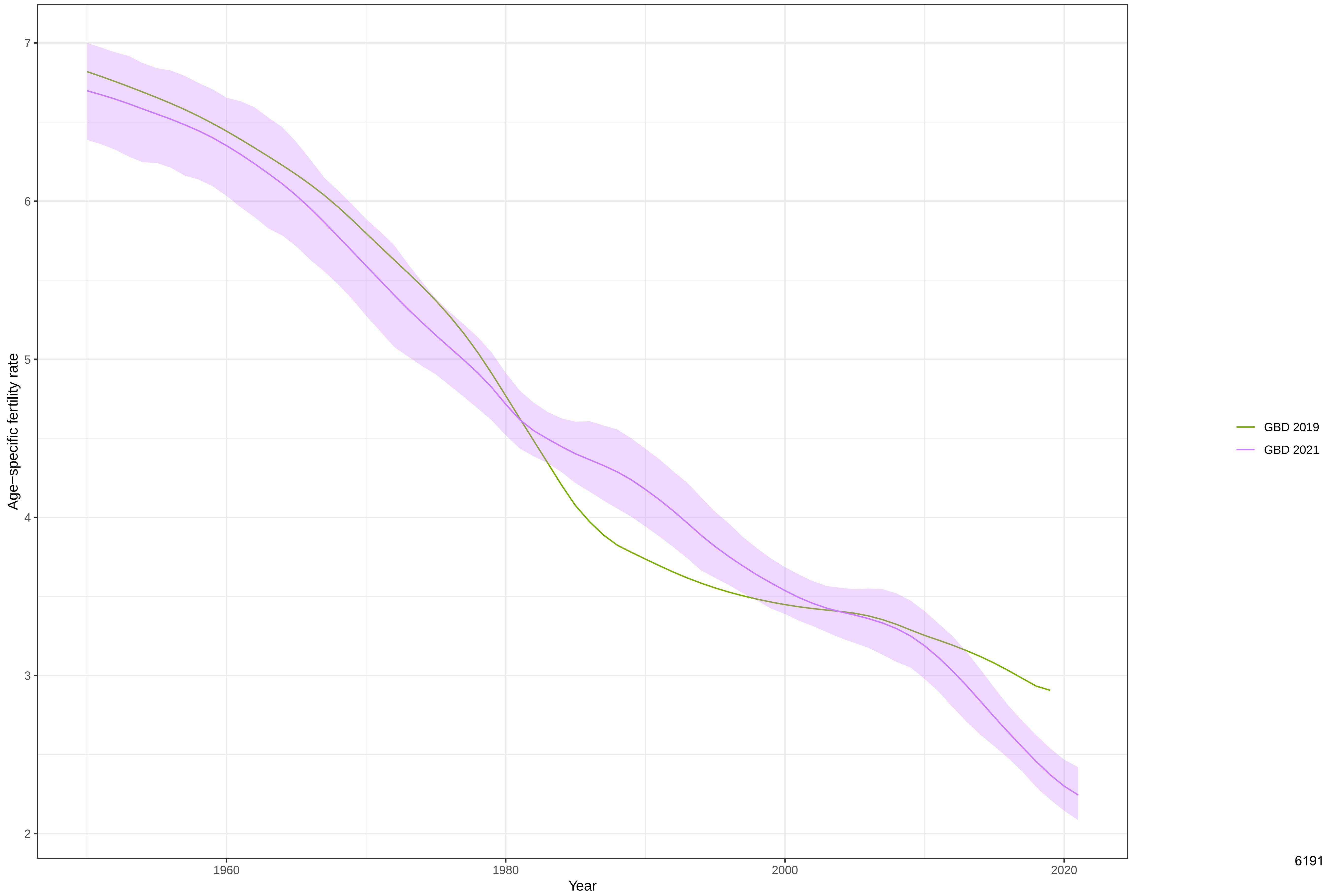

Age−specific fertility fitted trend lines, 1950−2021: Laguna, Ages: 10−14
Data points enclosed with a diamond are reference sources

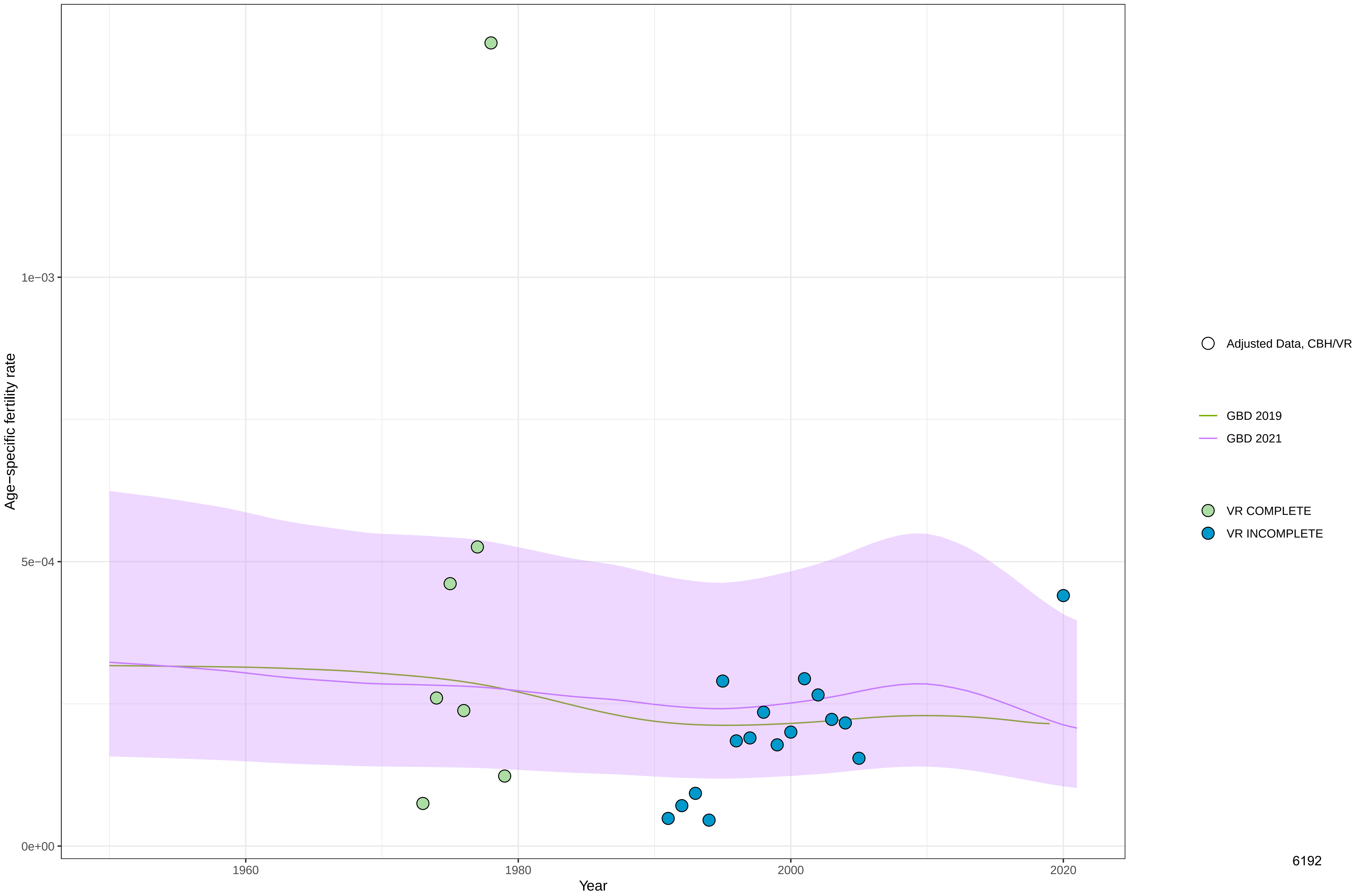

Age−specific fertility fitted trend lines, 1950−2021: Laguna, Ages: 15−19
Data points enclosed with a diamond are reference sources

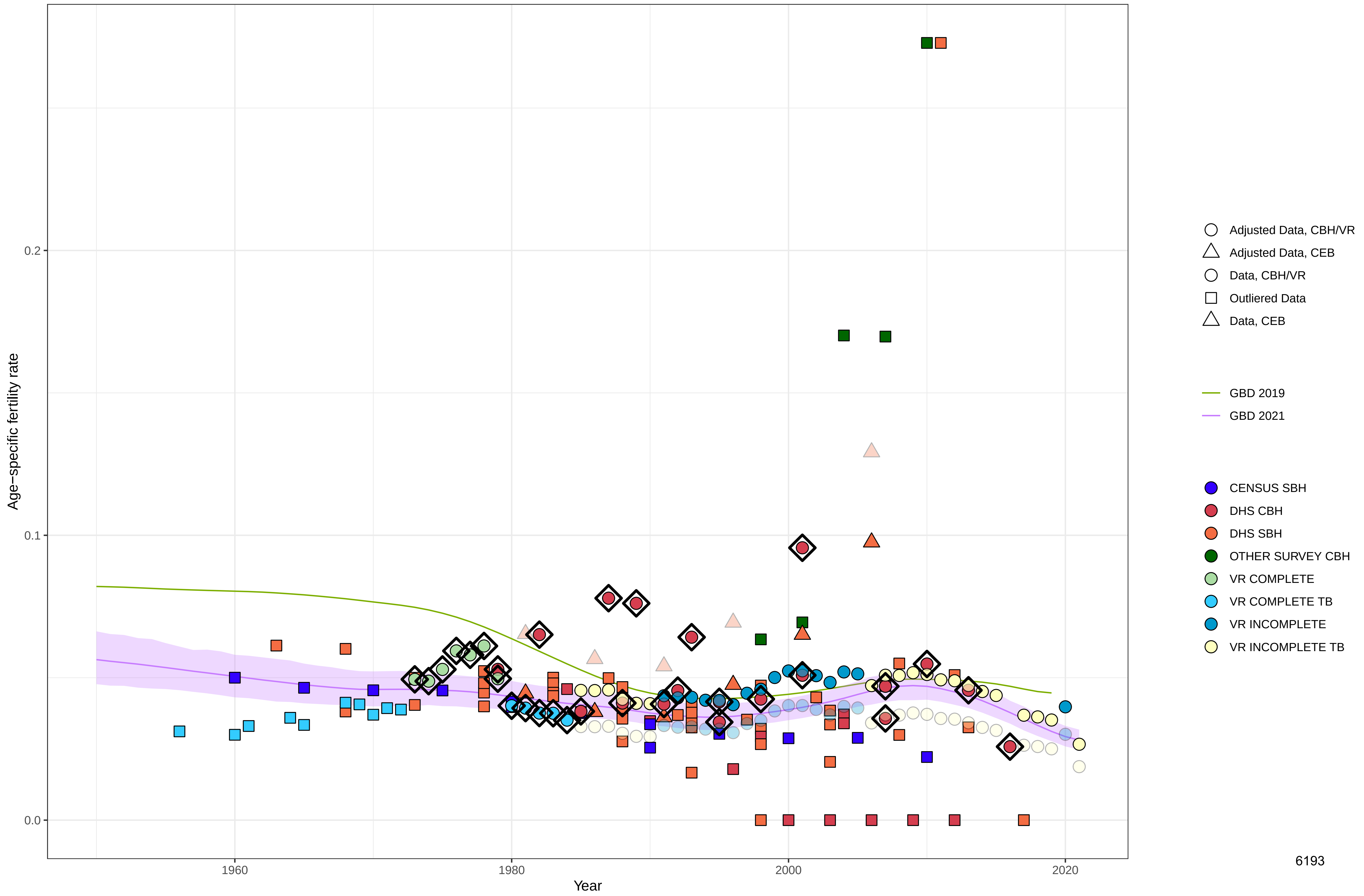

Age−specific fertility fitted trend lines, 1950−2021: Laguna, Ages: 20−24
Data points enclosed with a diamond are reference sources

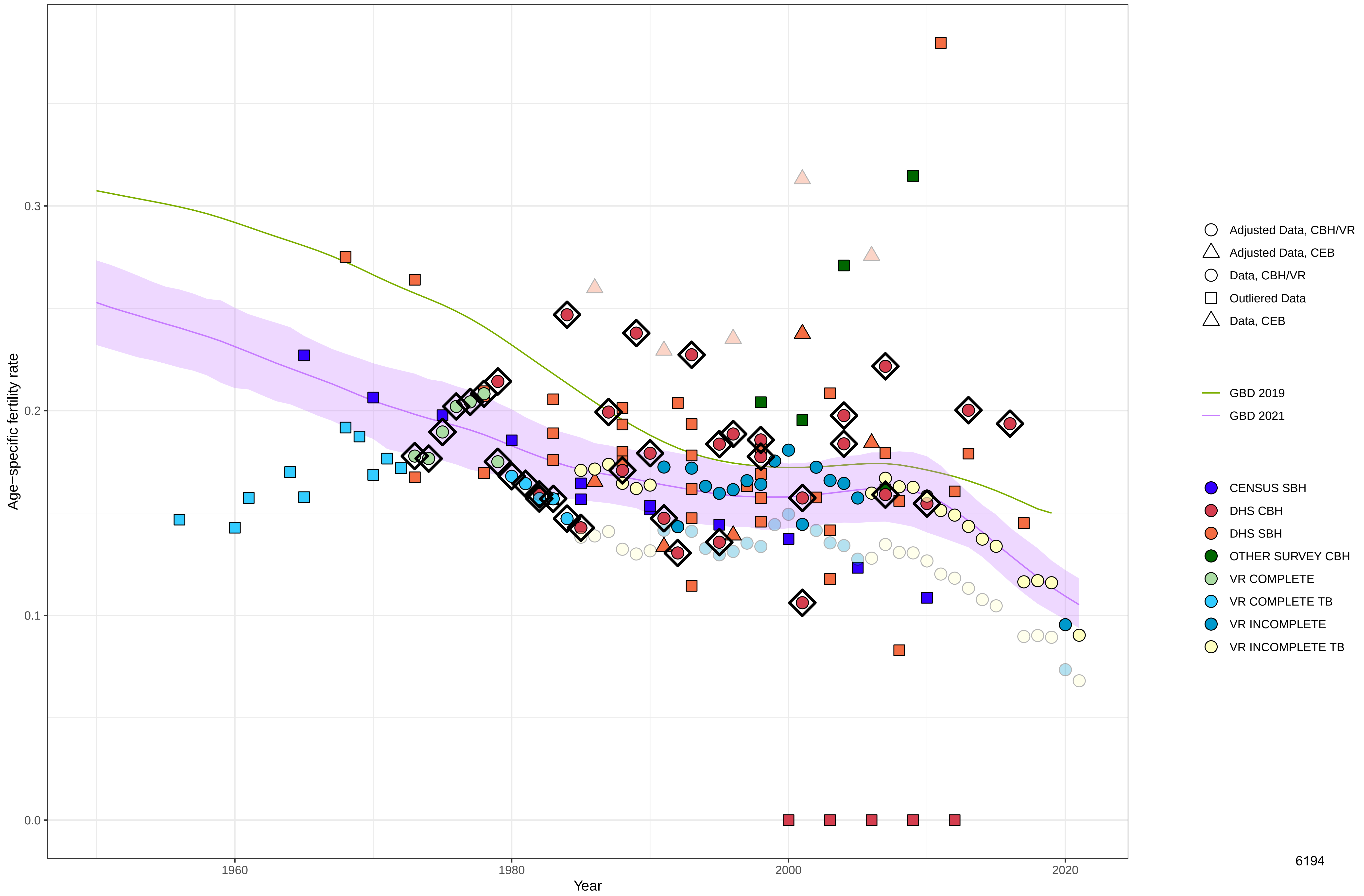

Age−specific fertility fitted trend lines, 1950−2021: Laguna, Ages: 25−29
Data points enclosed with a diamond are reference sources

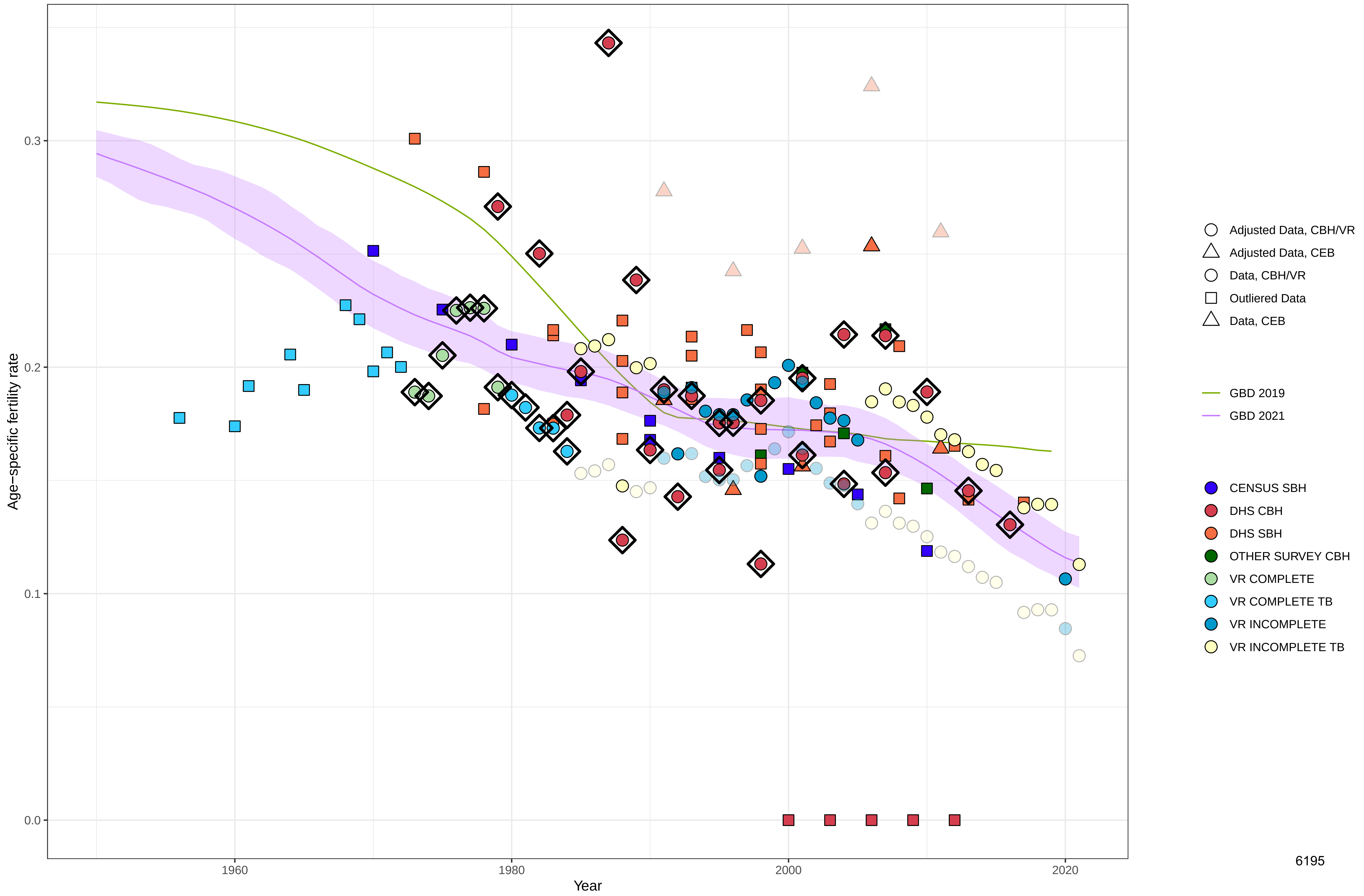

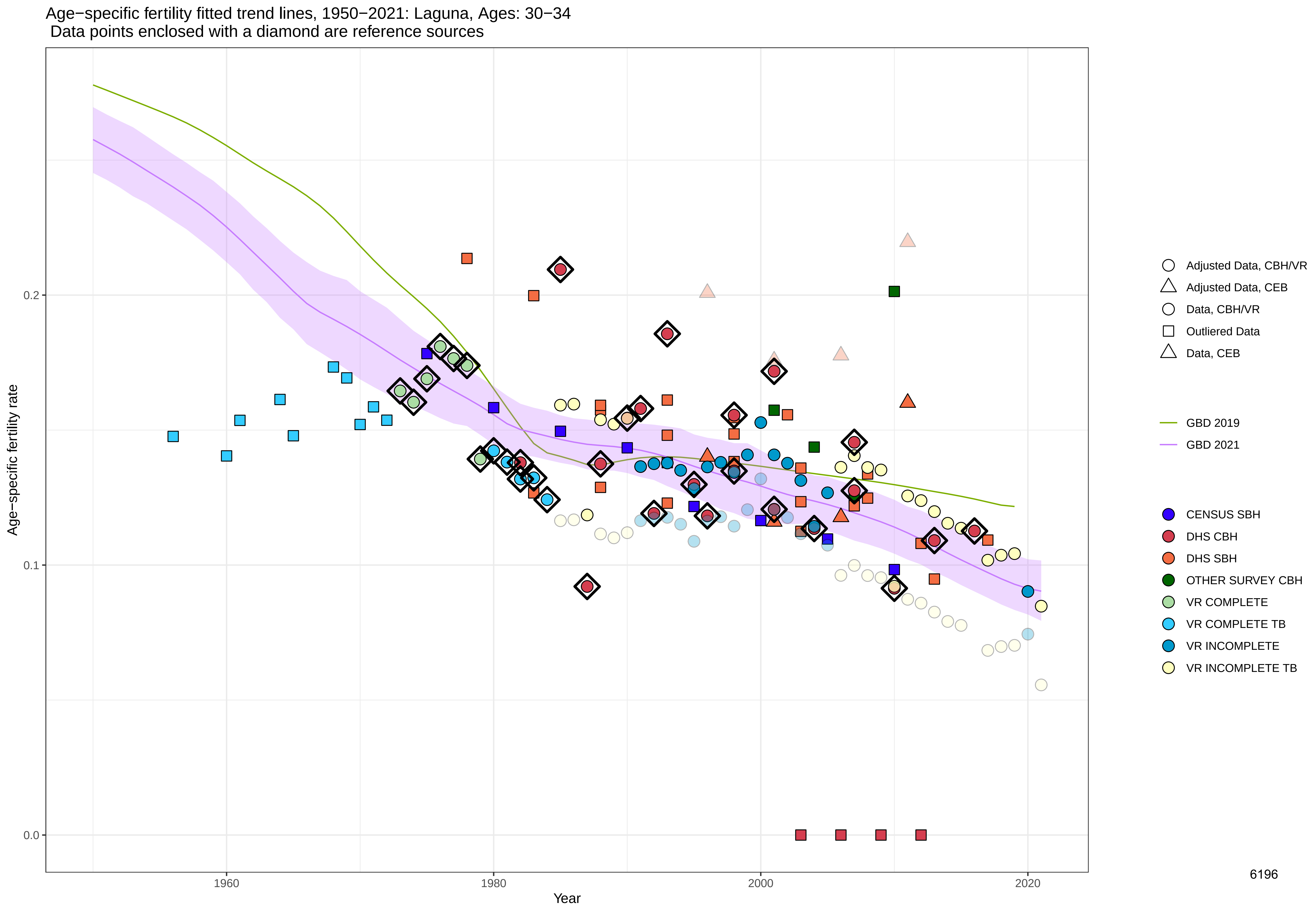

Age−specific fertility fitted trend lines, 1950−2021: Laguna, Ages: 30−34
Data points enclosed with a diamond are reference sources
Age−specific fertility rate
Year
0.0
0.1
0.2
1960
1980
2000
2020
Adjusted Data, CBH/VR
Adjusted Data, CEB
Data, CBH/VR
Outliered Data
Data, CEB
GBD 2019
GBD 2021
CENSUS SBH
DHS CBH
DHS SBH
OTHER SURVEY CBH
VR COMPLETE
VR COMPLETE TB
VR INCOMPLETE
VR INCOMPLETE TB

6196

Age−specific fertility fitted trend lines, 1950−2021: Laguna, Ages: 35−39
Data points enclosed with a diamond are reference sources

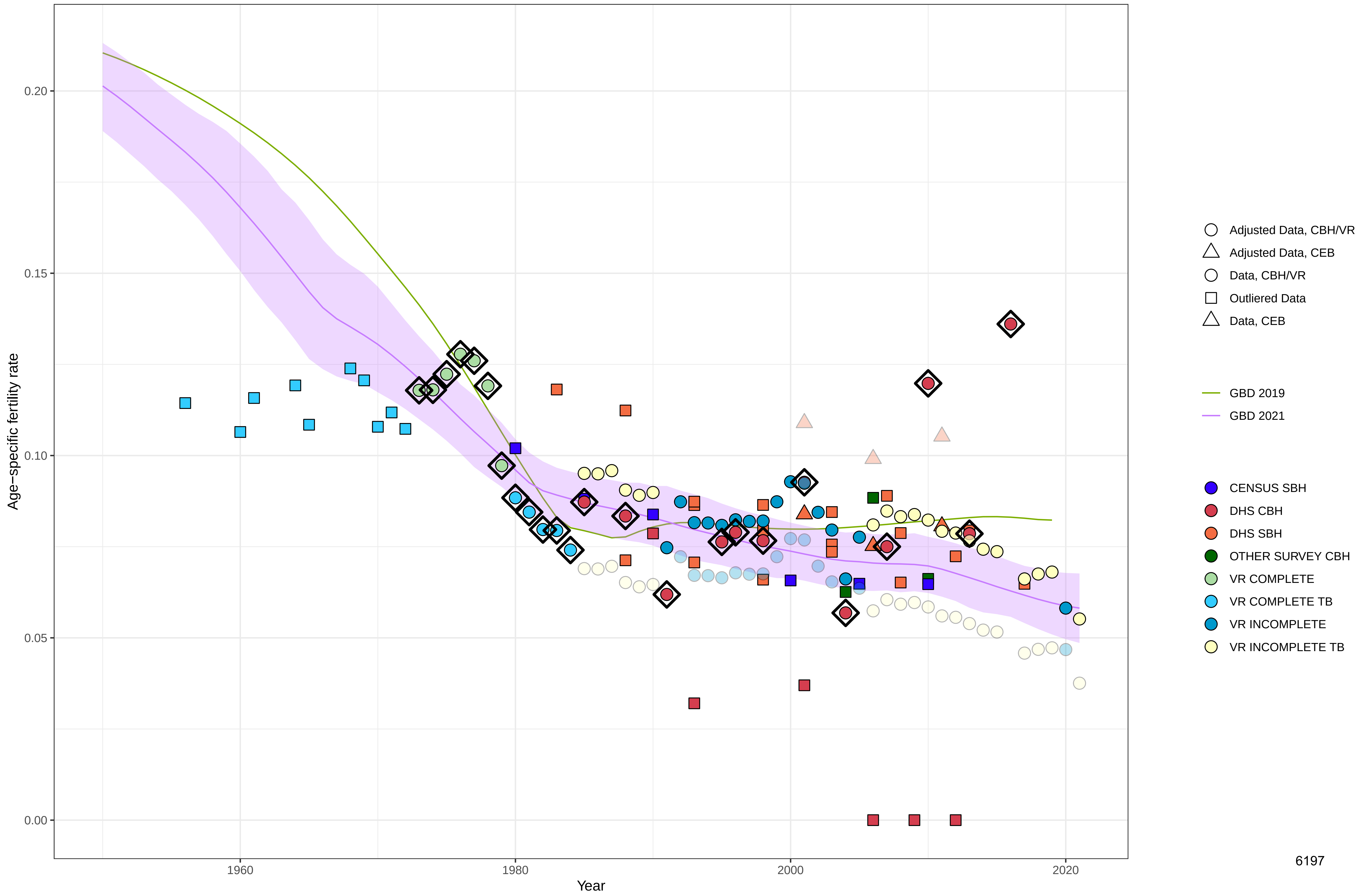

Age−specific fertility fitted trend lines, 1950−2021: Laguna, Ages: 40−44
Data points enclosed with a diamond are reference sources

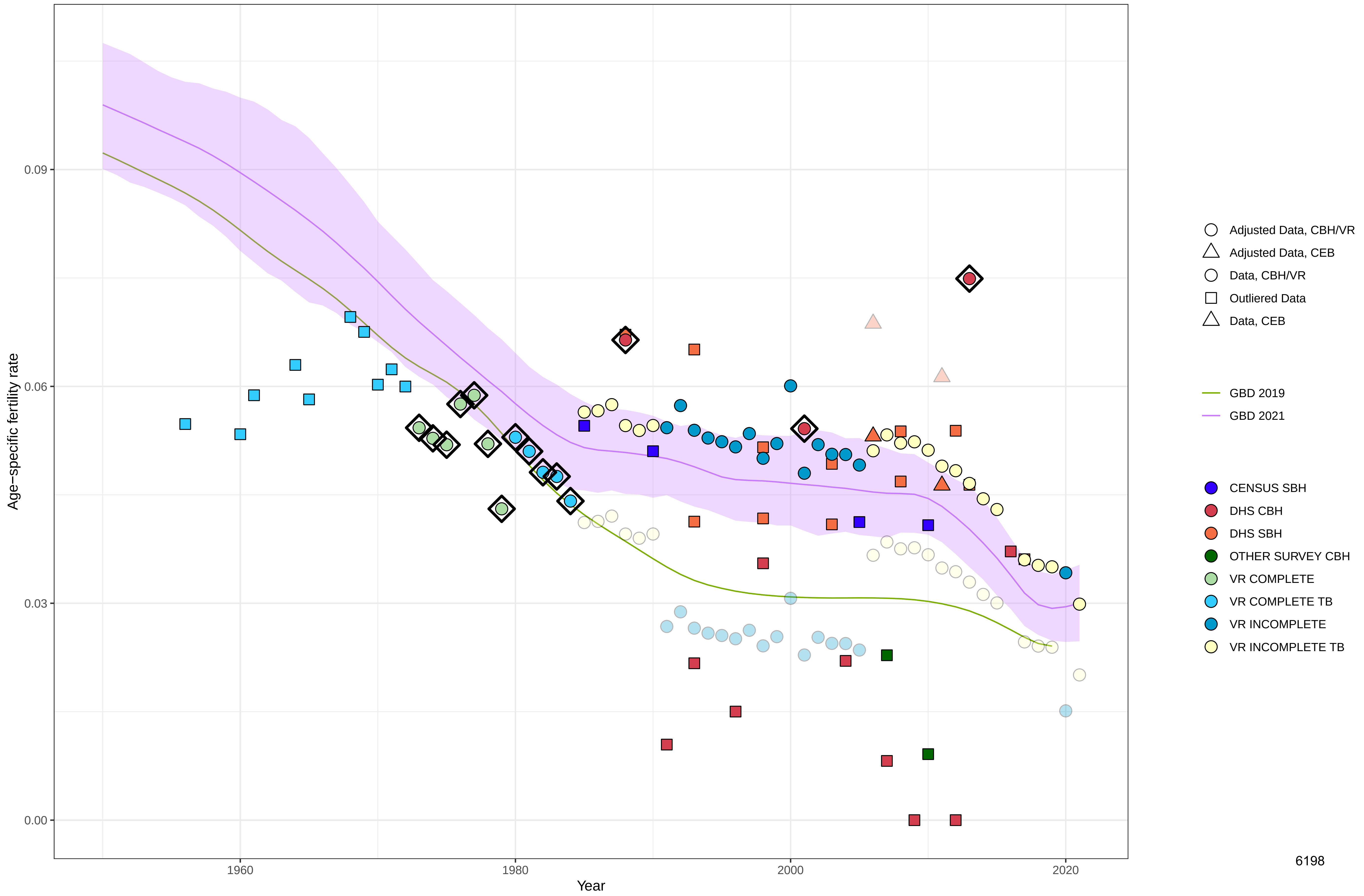

Age−specific fertility fitted trend lines, 1950−2021: Laguna, Ages: 45−49
Data points enclosed with a diamond are reference sources

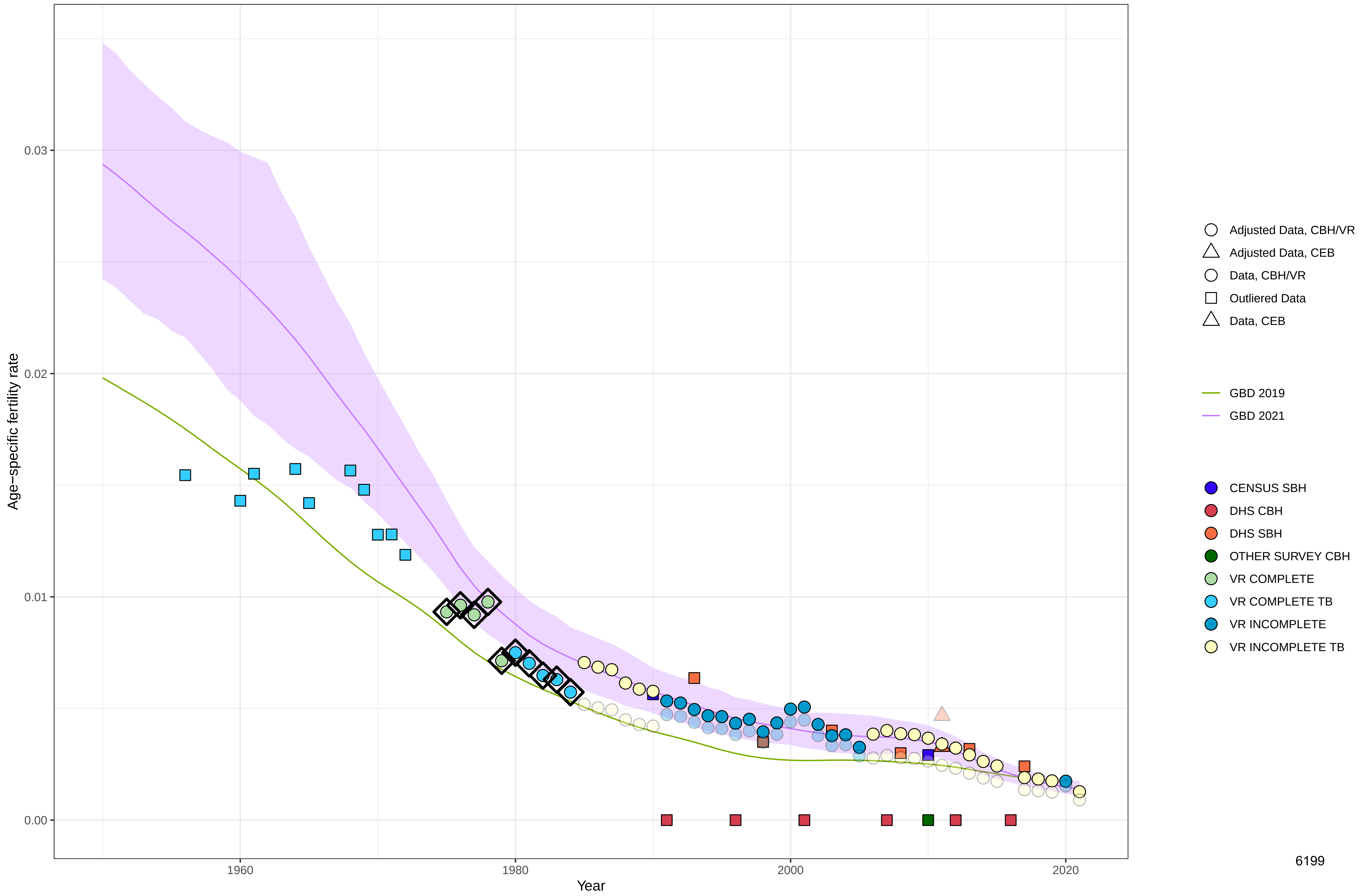

Age−specific fertility fitted trend lines, 1950−2021: Laguna, Ages: 50−54
Data points enclosed with a diamond are reference sources

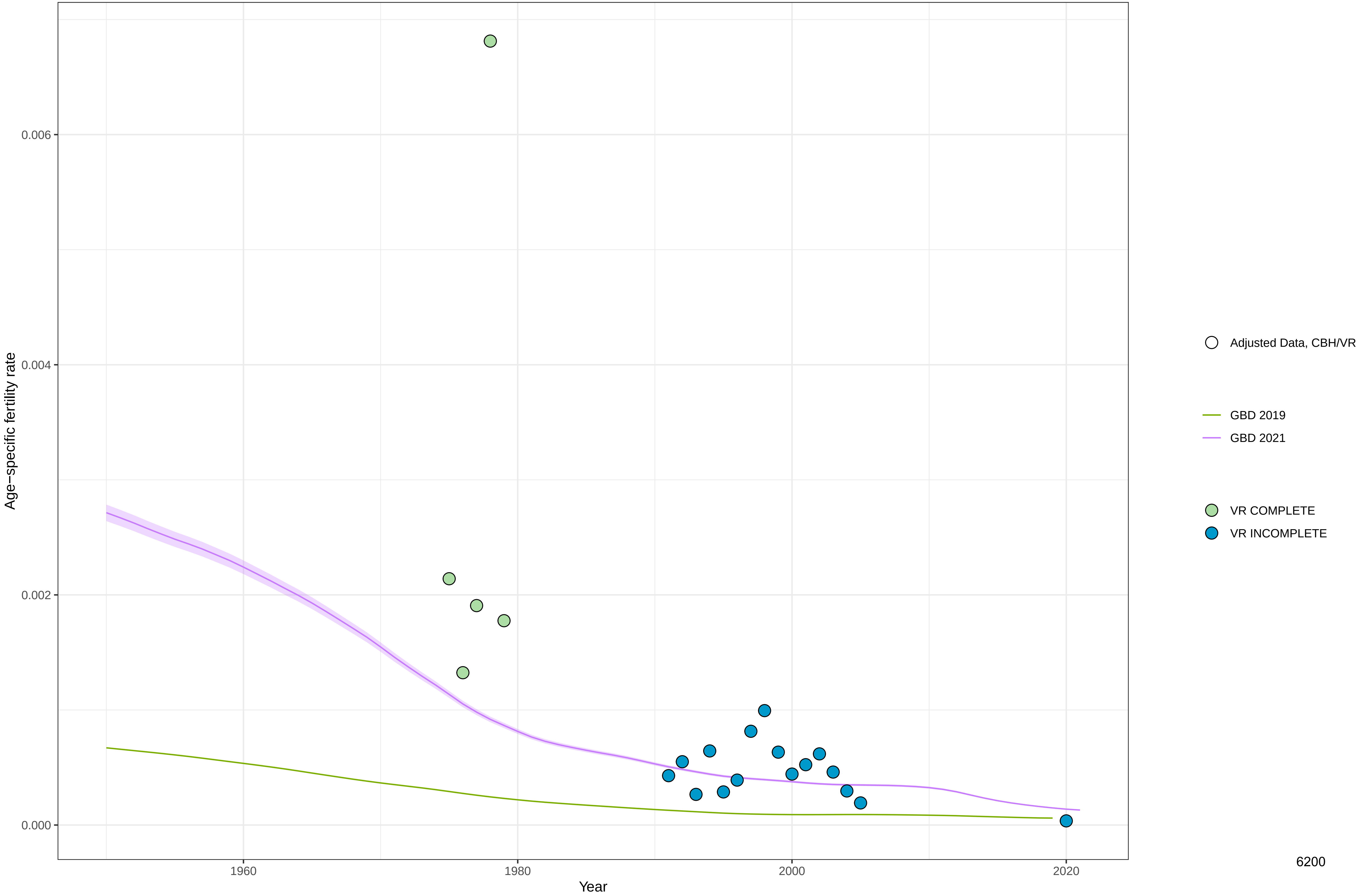

Total fertility rate fitted trend lines, 1950−2021: Laguna TFR
Data points enclosed with a diamond are reference sources

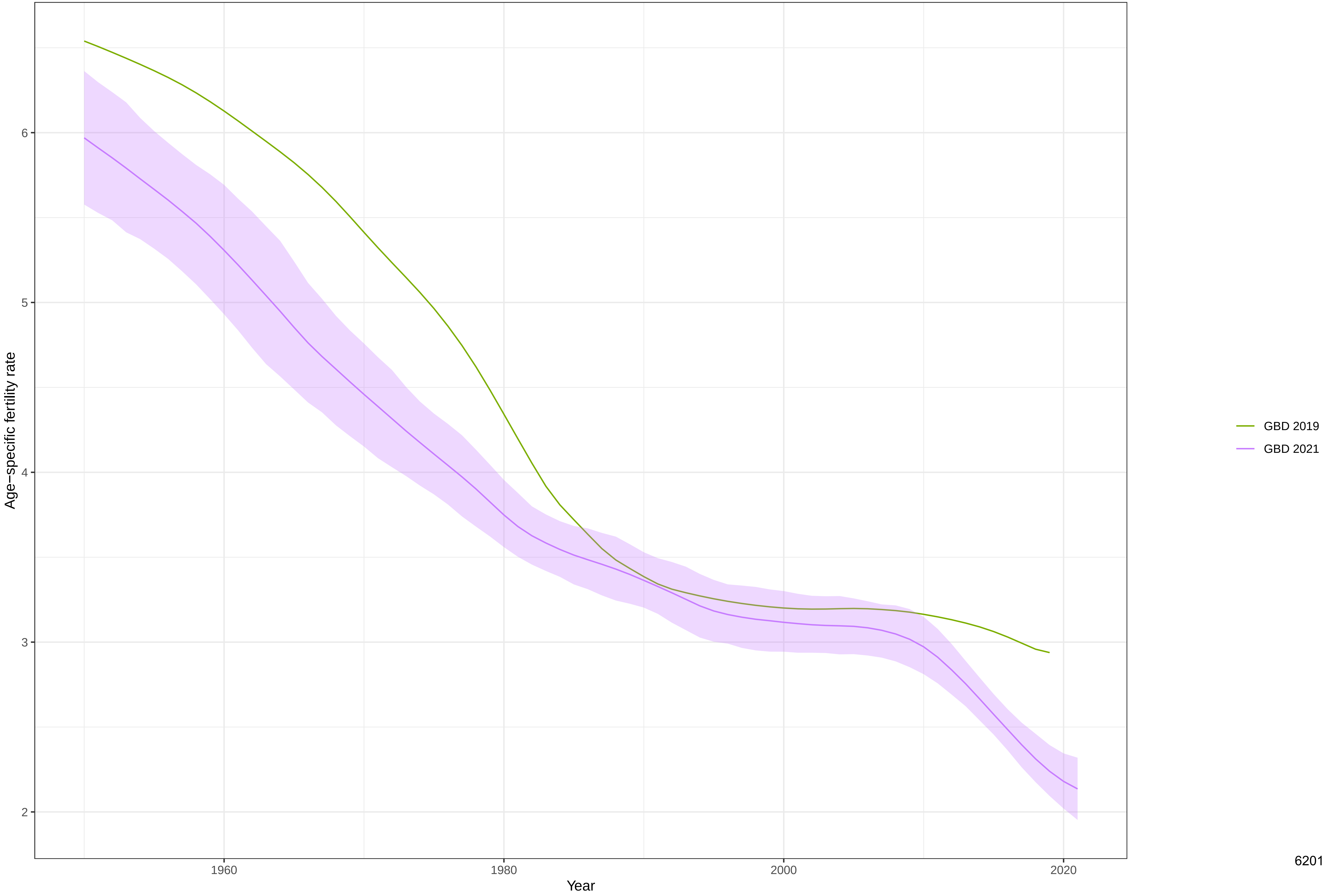

Age−specific fertility fitted trend lines, 1950−2021: Lanao Del Norte, Ages: 10−14
Data points enclosed with a diamond are reference sources

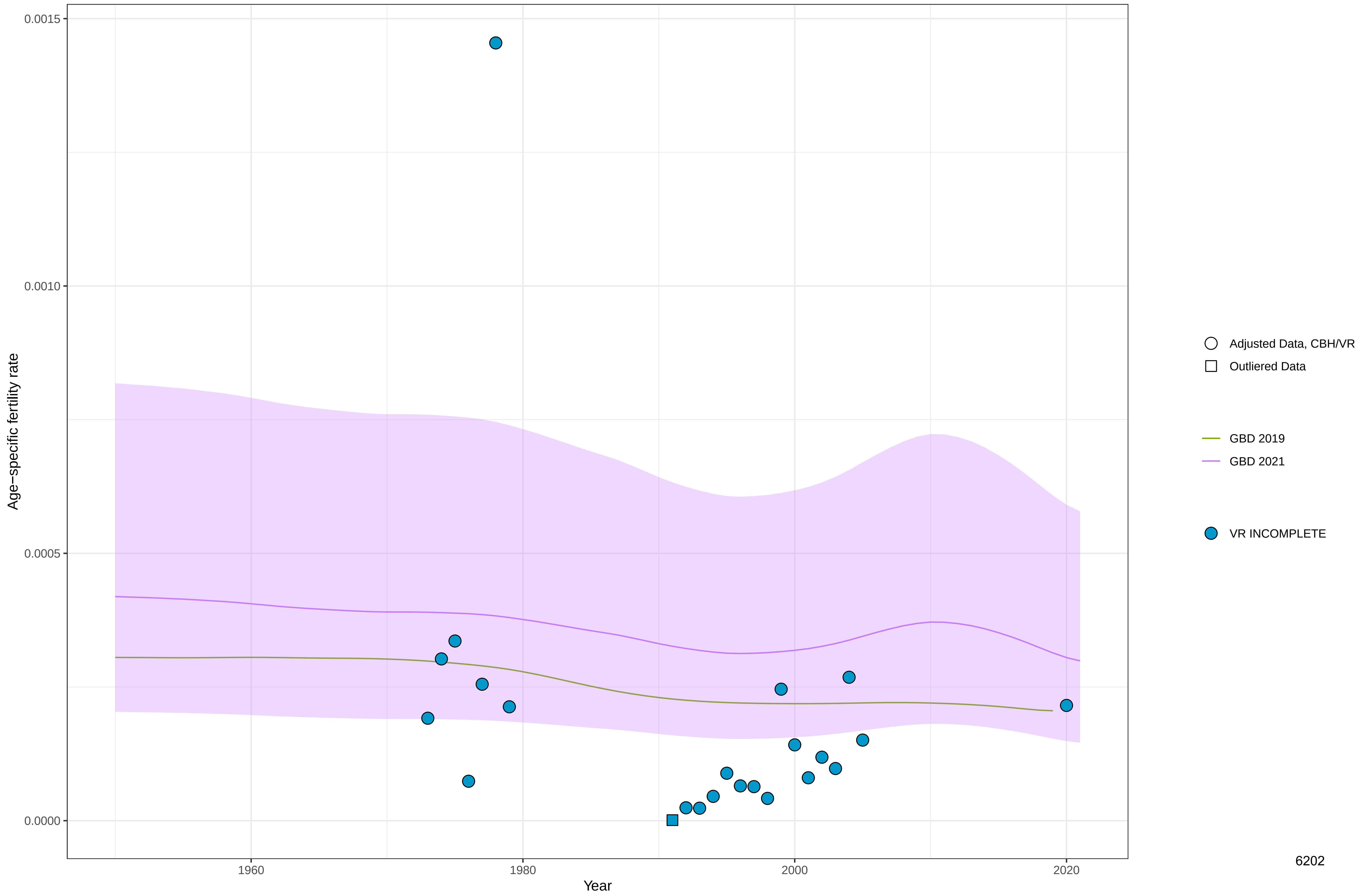

Age−specific fertility fitted trend lines, 1950−2021: Lanao Del Norte, Ages: 15−19
Data points enclosed with a diamond are reference sources

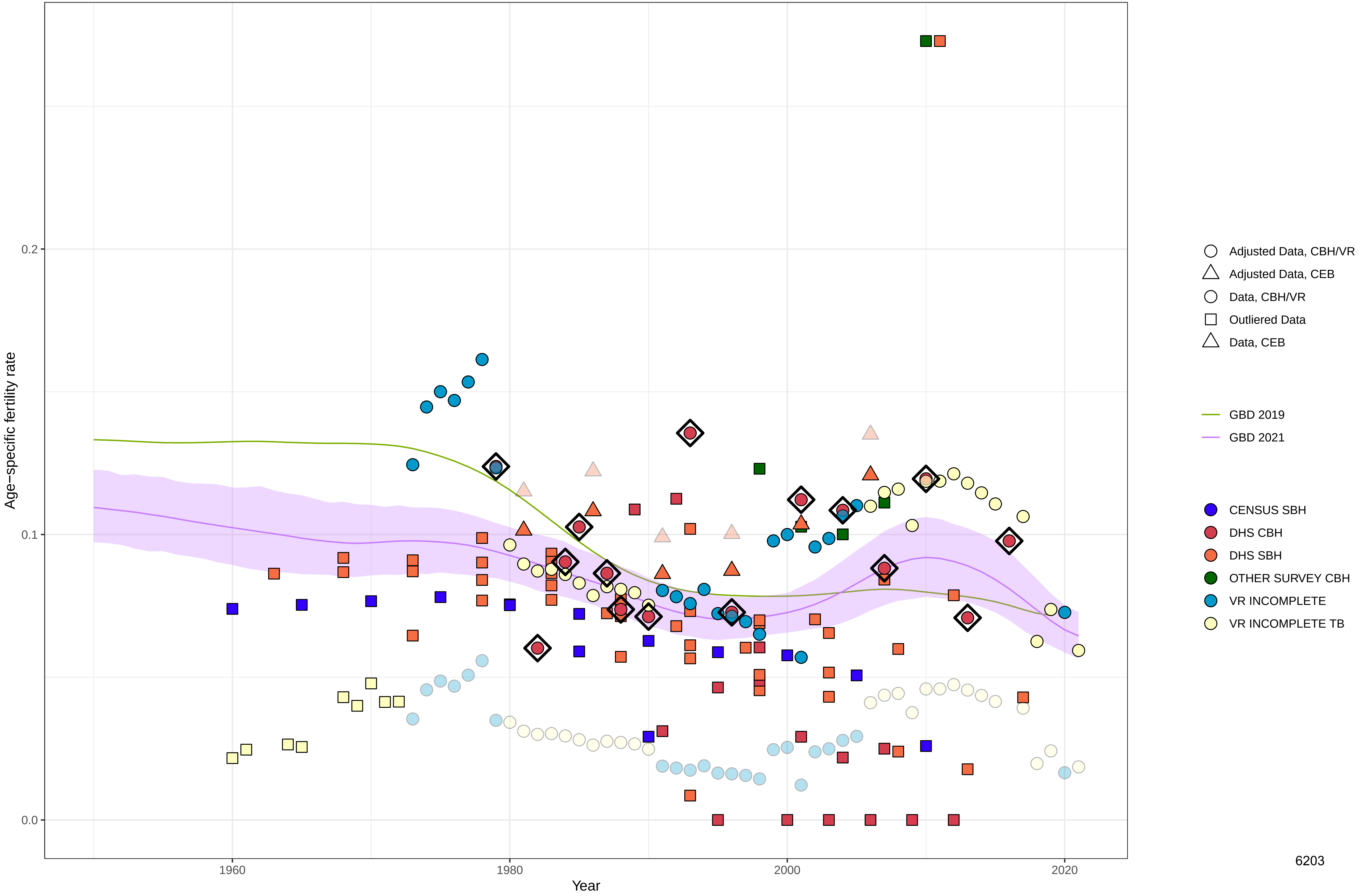

Age−specific fertility fitted trend lines, 1950−2021: Lanao Del Norte, Ages: 20−24
Data points enclosed with a diamond are reference sources

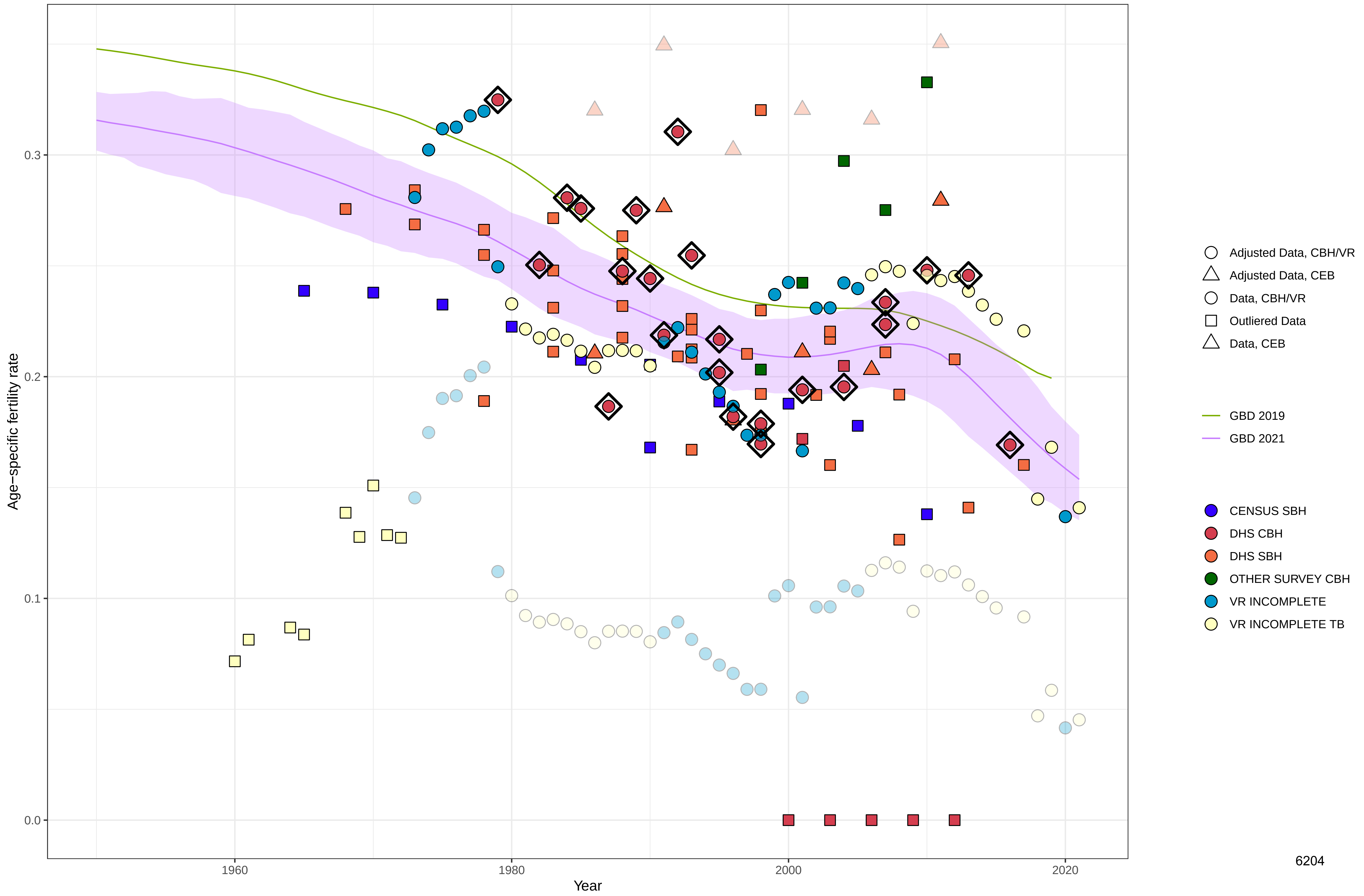

Age−specific fertility fitted trend lines, 1950−2021: Lanao Del Norte, Ages: 25−29
Data points enclosed with a diamond are reference sources

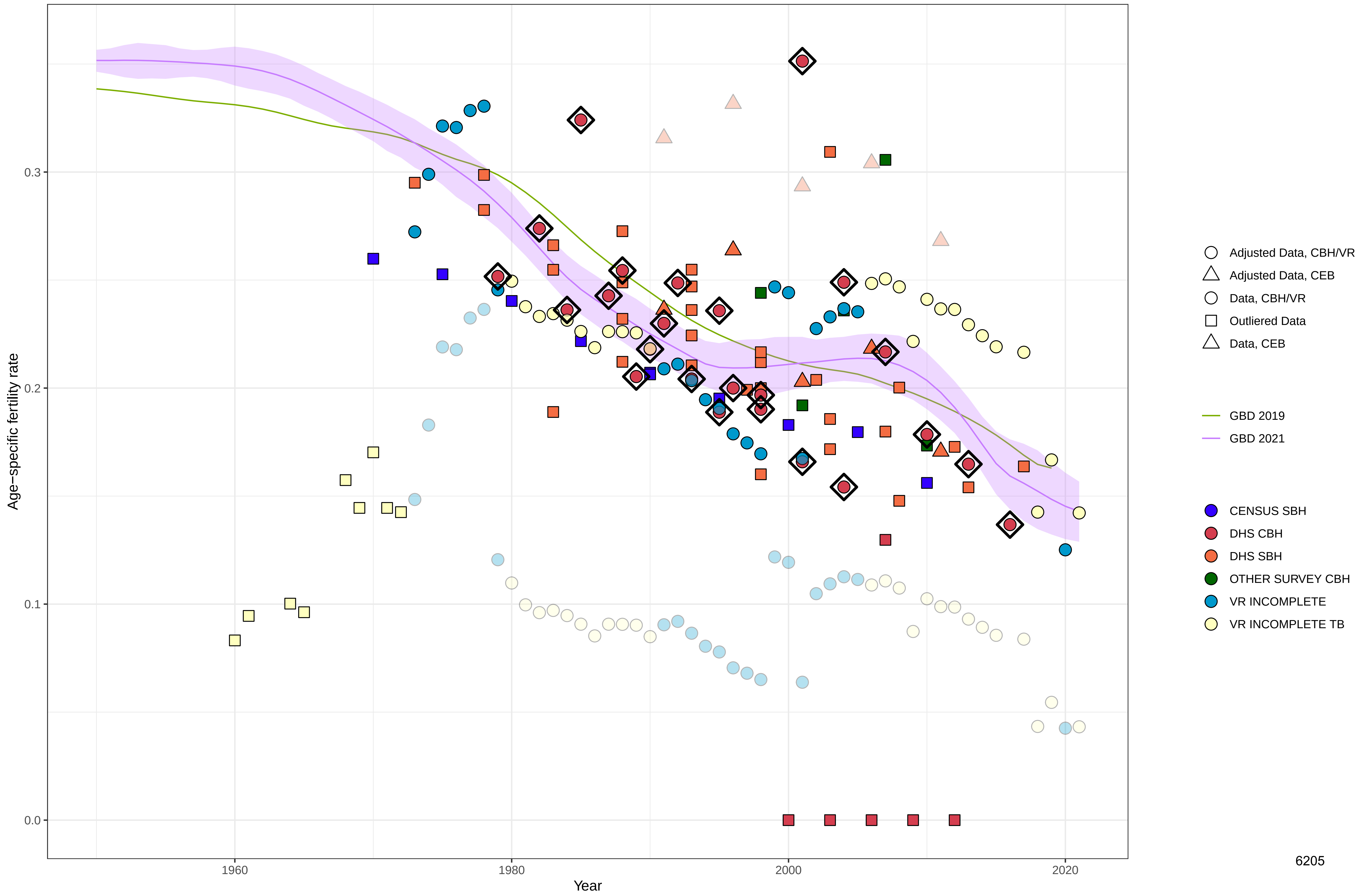

Age−specific fertility fitted trend lines, 1950−2021: Lanao Del Norte, Ages: 30−34
Data points enclosed with a diamond are reference sources

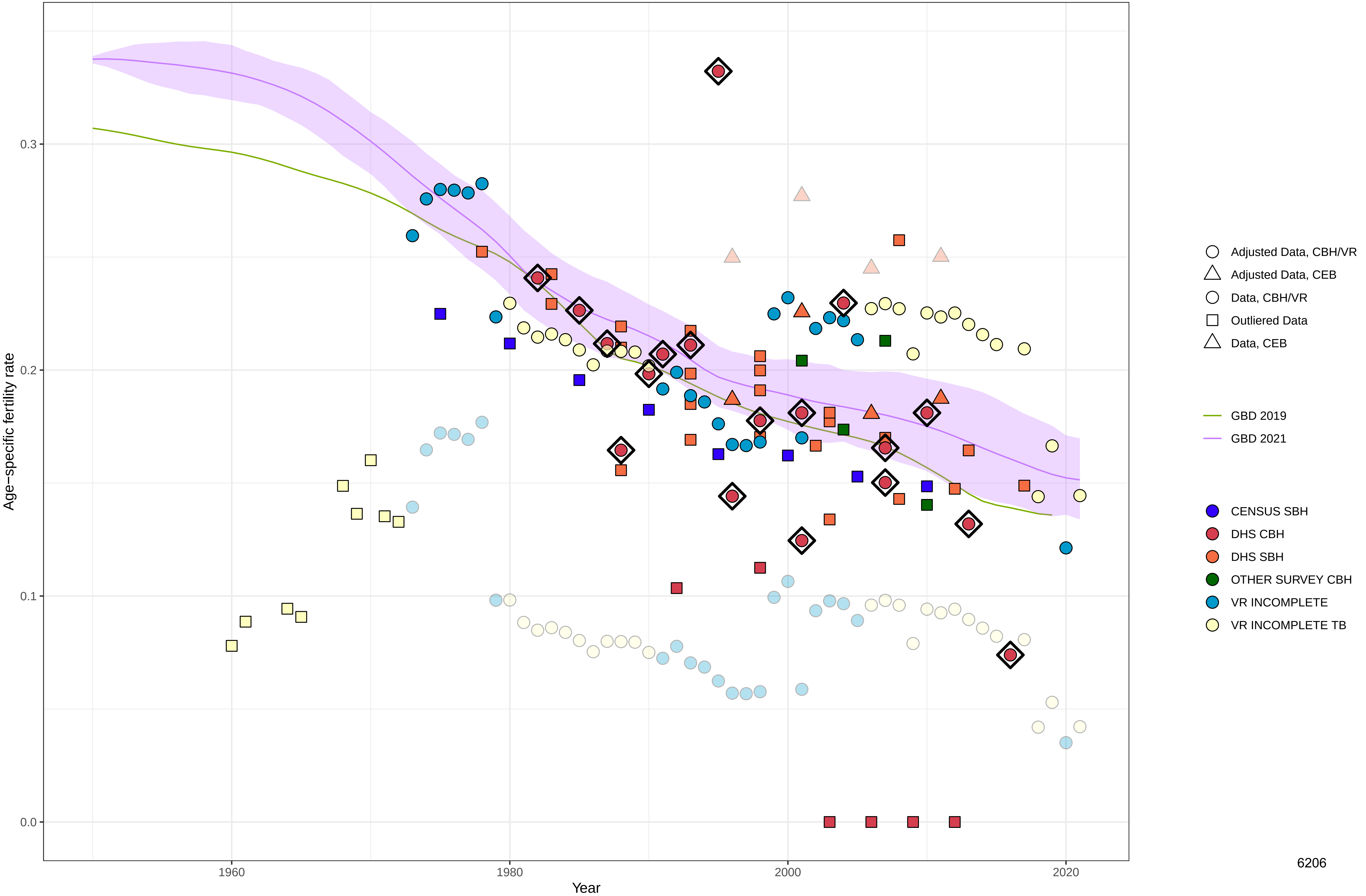

Age−specific fertility fitted trend lines, 1950−2021: Lanao Del Norte, Ages: 35−39
Data points enclosed with a diamond are reference sources

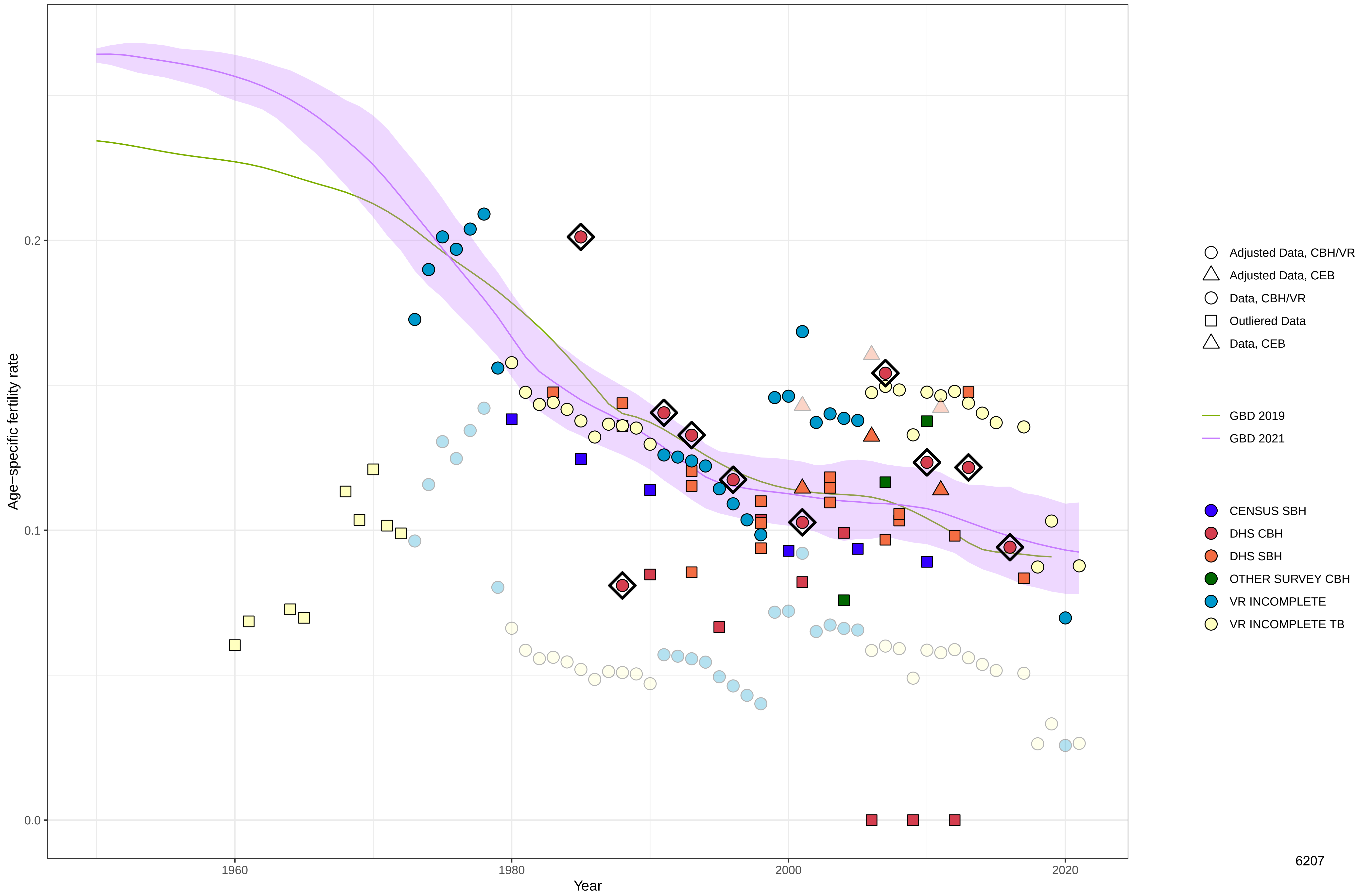

6207

Age−specific fertility fitted trend lines, 1950−2021: Lanao Del Norte, Ages: 40−44
Data points enclosed with a diamond are reference sources

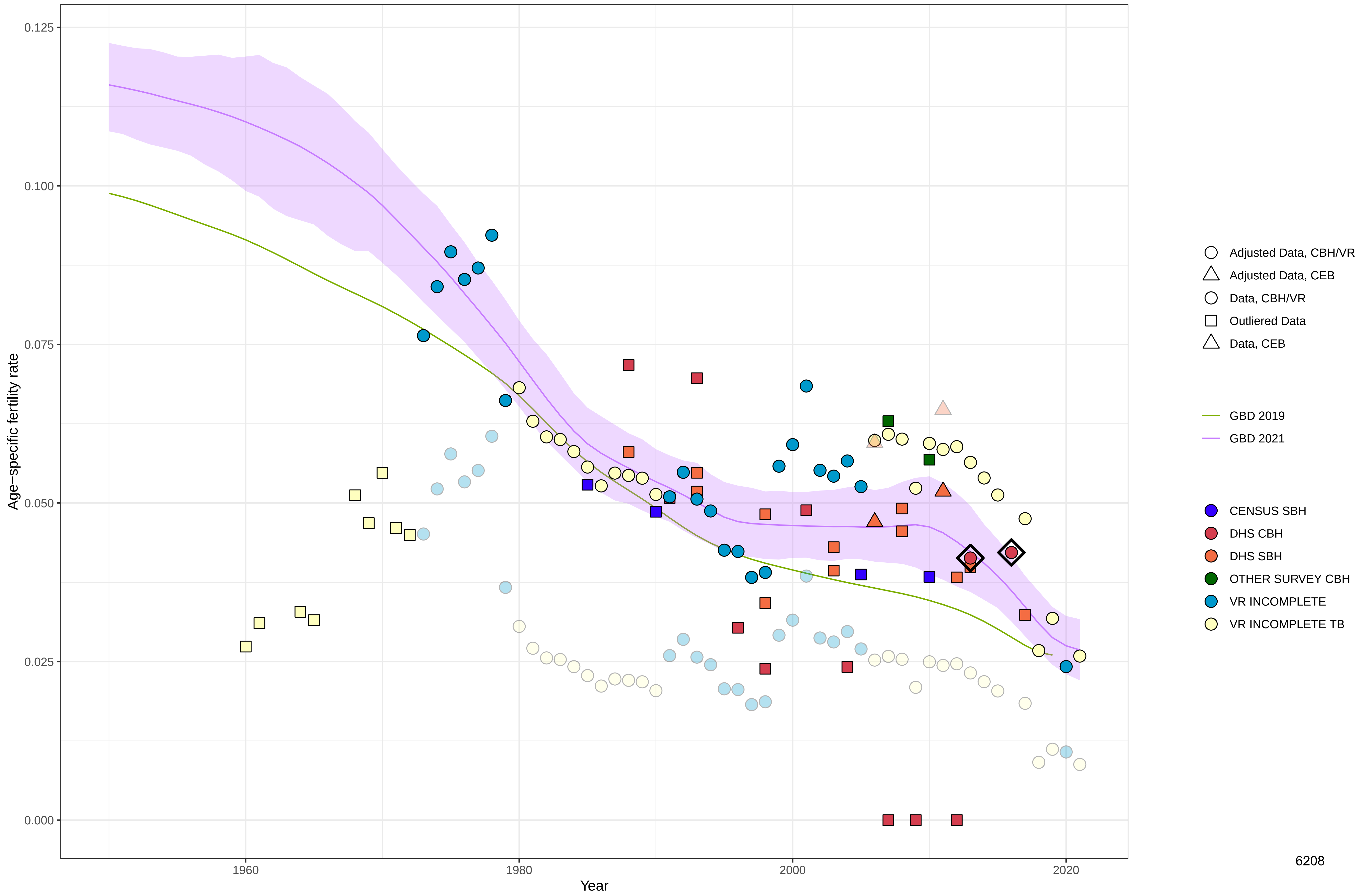

Age−specific fertility fitted trend lines, 1950−2021: Lanao Del Norte, Ages: 45−49
Data points enclosed with a diamond are reference sources

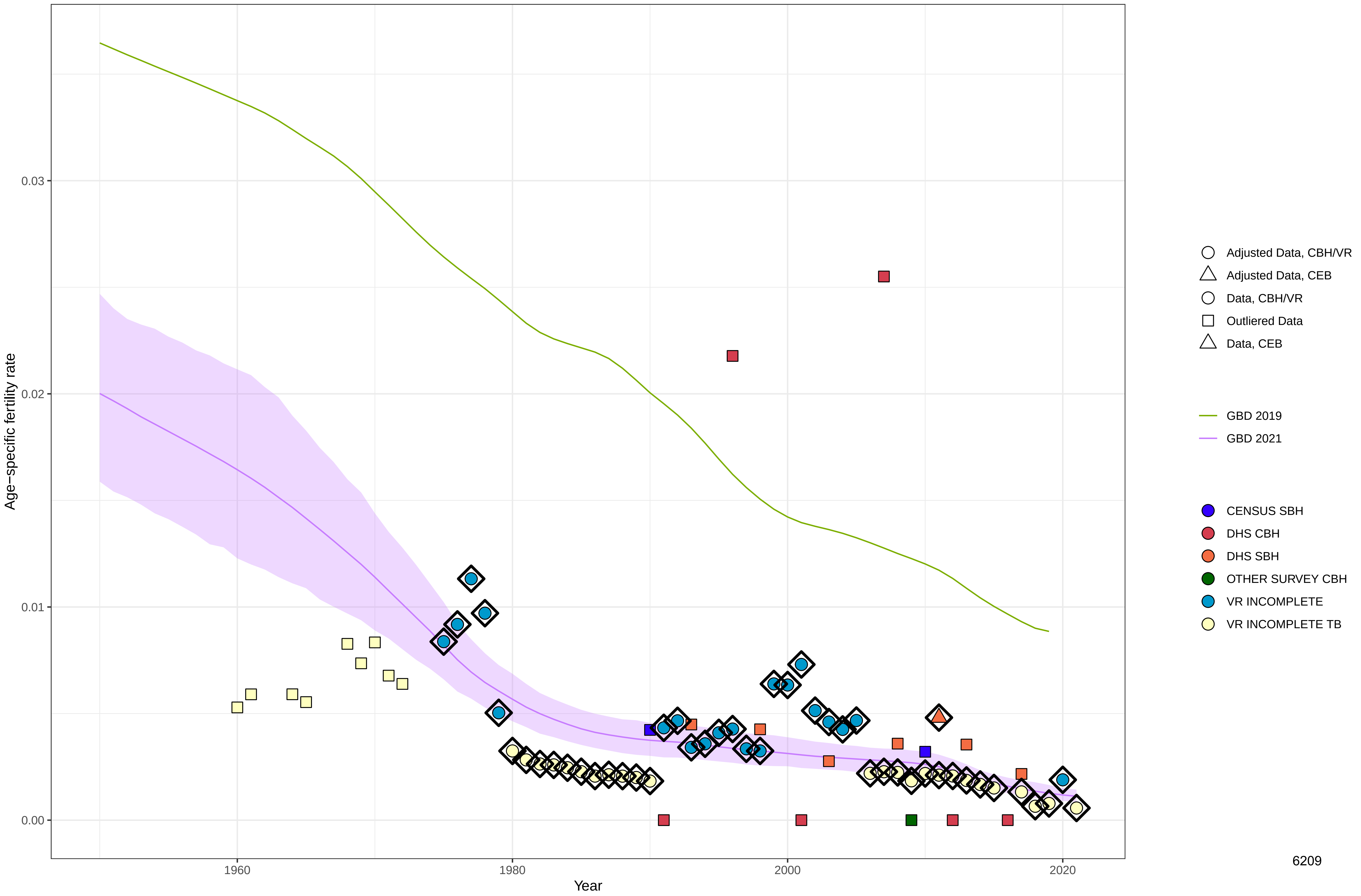

Age−specific fertility fitted trend lines, 1950−2021: Lanao Del Norte, Ages: 50−54
Data points enclosed with a diamond are reference sources

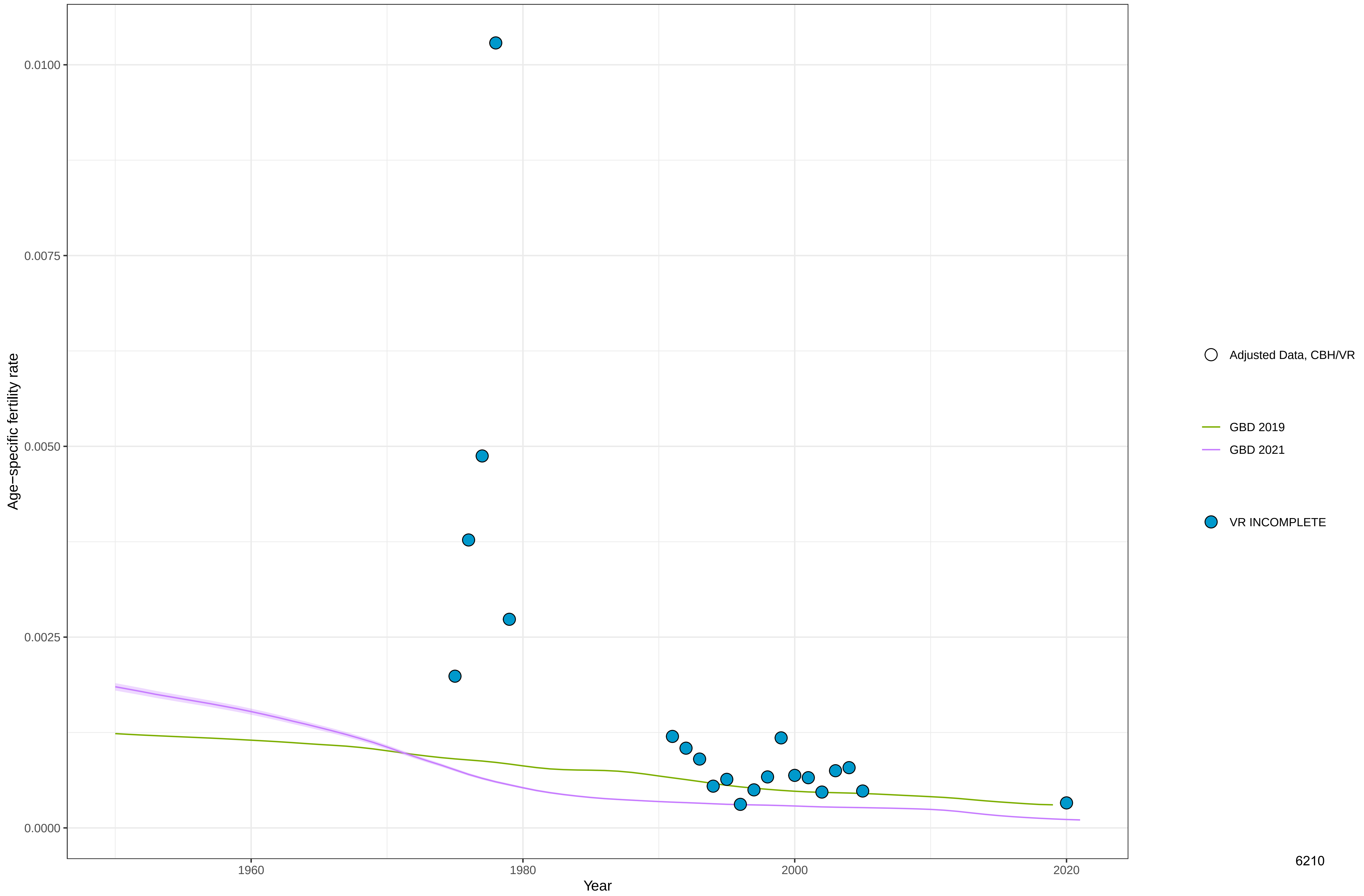

Total fertility rate fitted trend lines, 1950−2021: Lanao Del Norte TFR
Data points enclosed with a diamond are reference sources

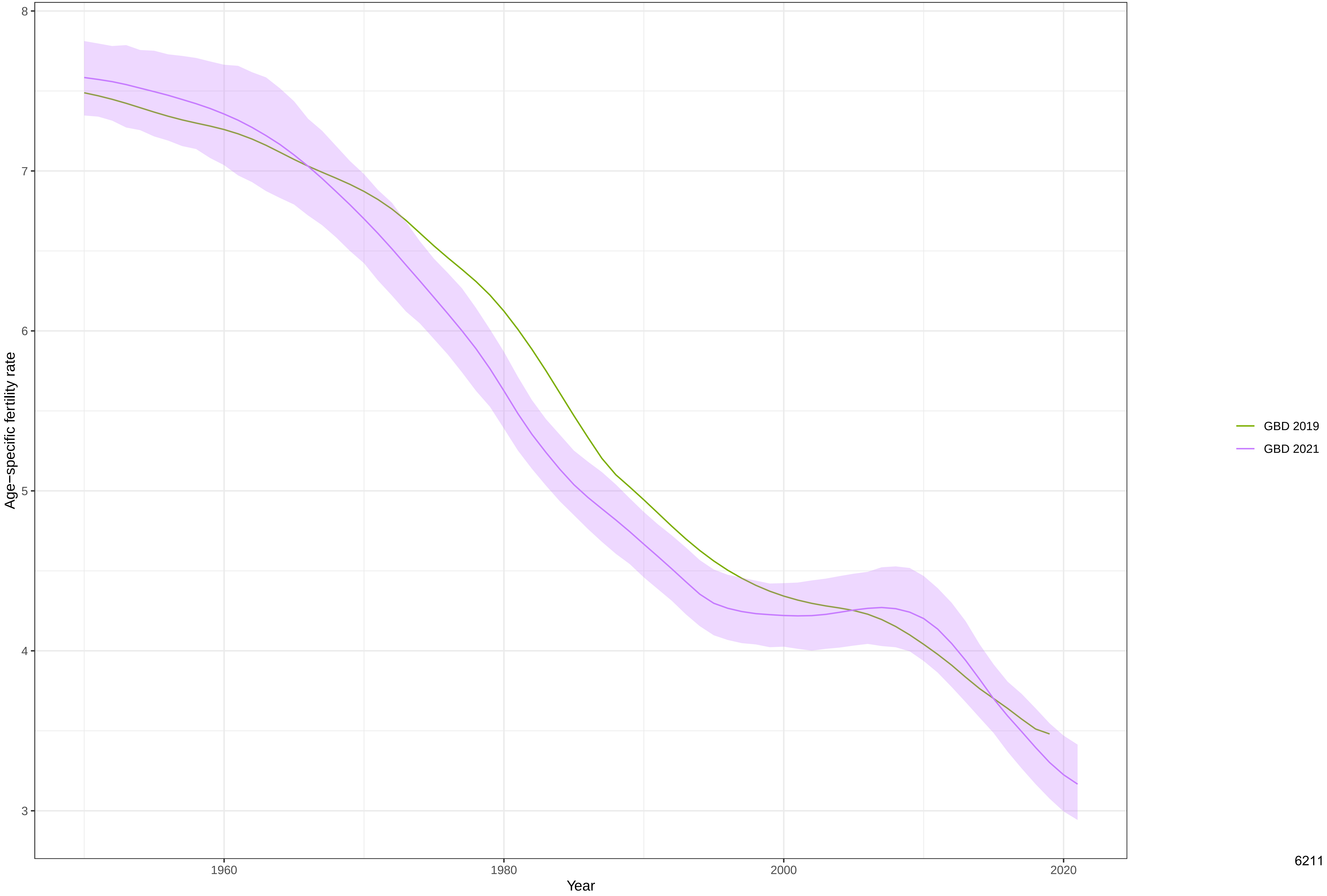

6211

Age−specific fertility fitted trend lines, 1950−2021: Lanao Del Sur, Ages: 10−14
Data points enclosed with a diamond are reference sources

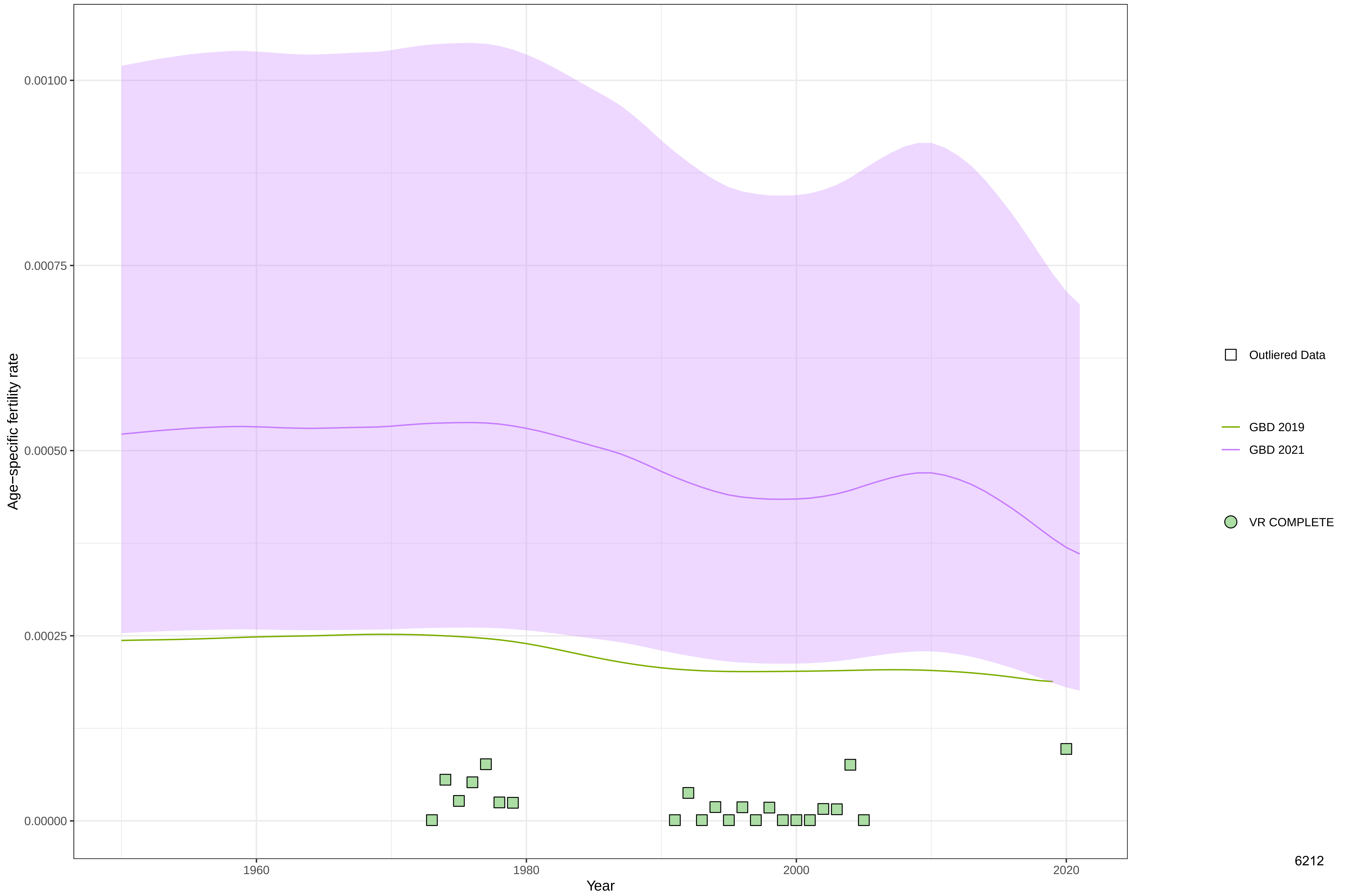

Age−specific fertility fitted trend lines, 1950−2021: Lanao Del Sur, Ages: 15−19
Data points enclosed with a diamond are reference sources

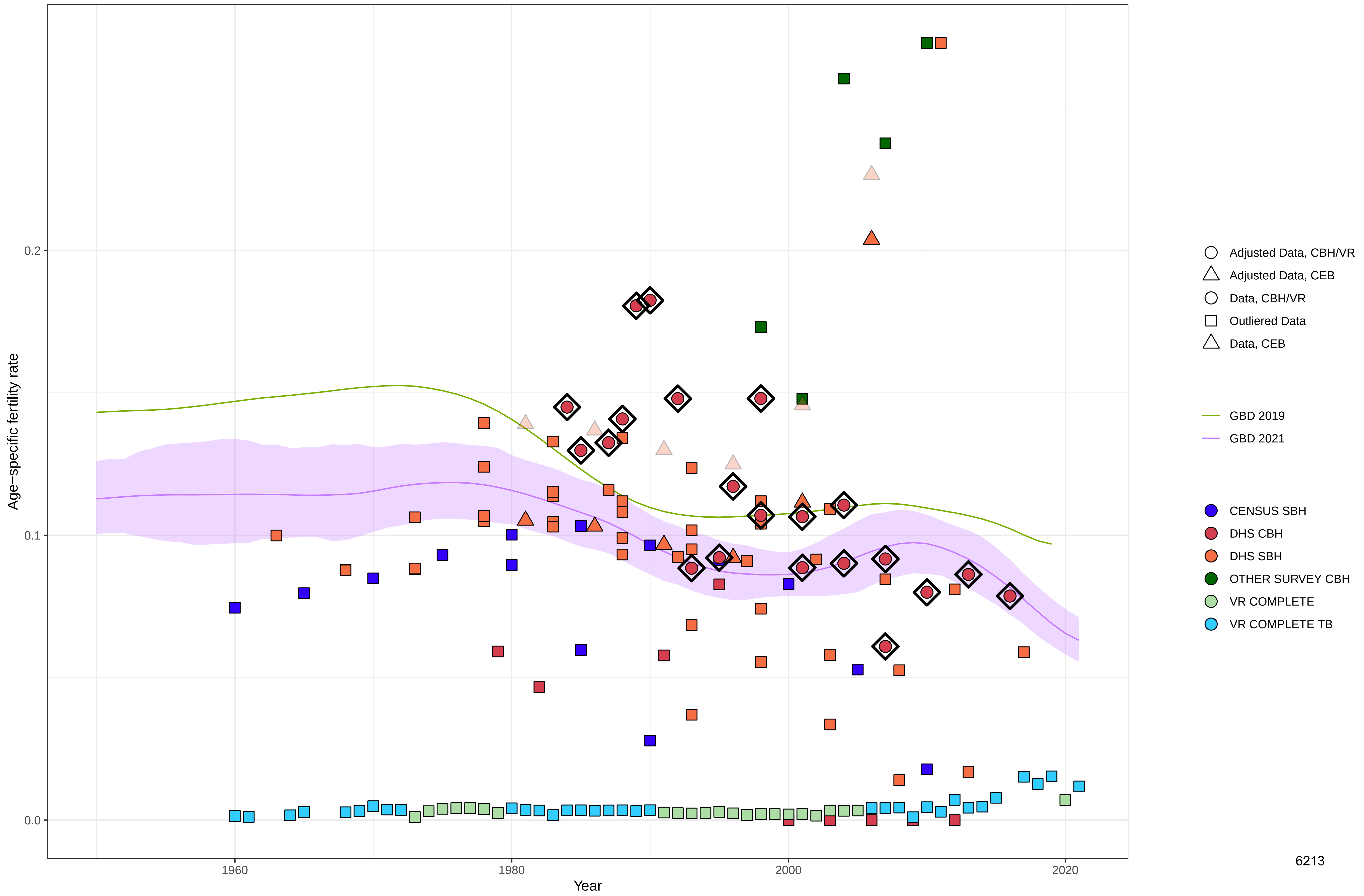

Age−specific fertility fitted trend lines, 1950−2021: Lanao Del Sur, Ages: 20−24
Data points enclosed with a diamond are reference sources

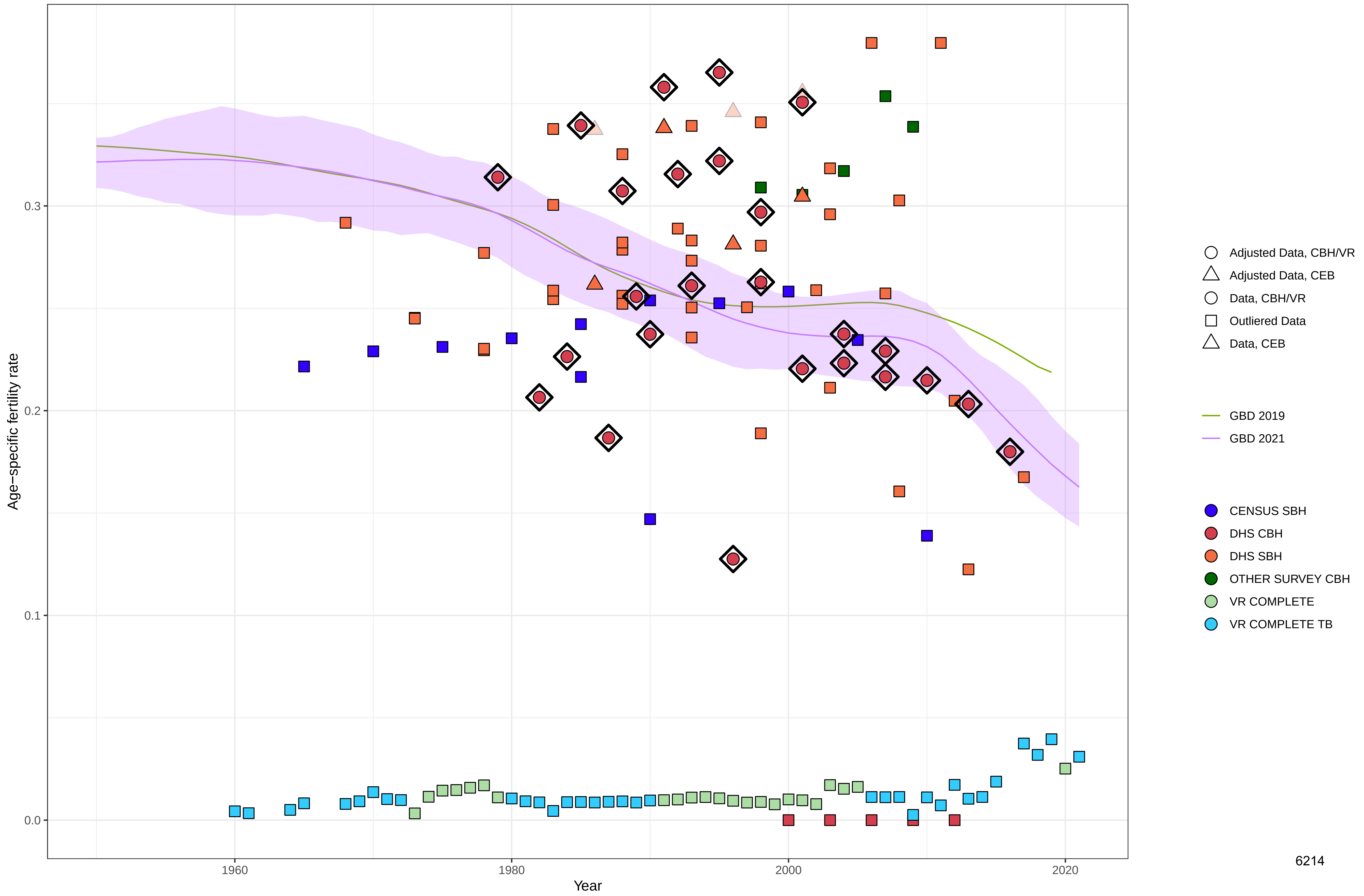

Age−specific fertility fitted trend lines, 1950−2021: Lanao Del Sur, Ages: 25−29
Data points enclosed with a diamond are reference sources

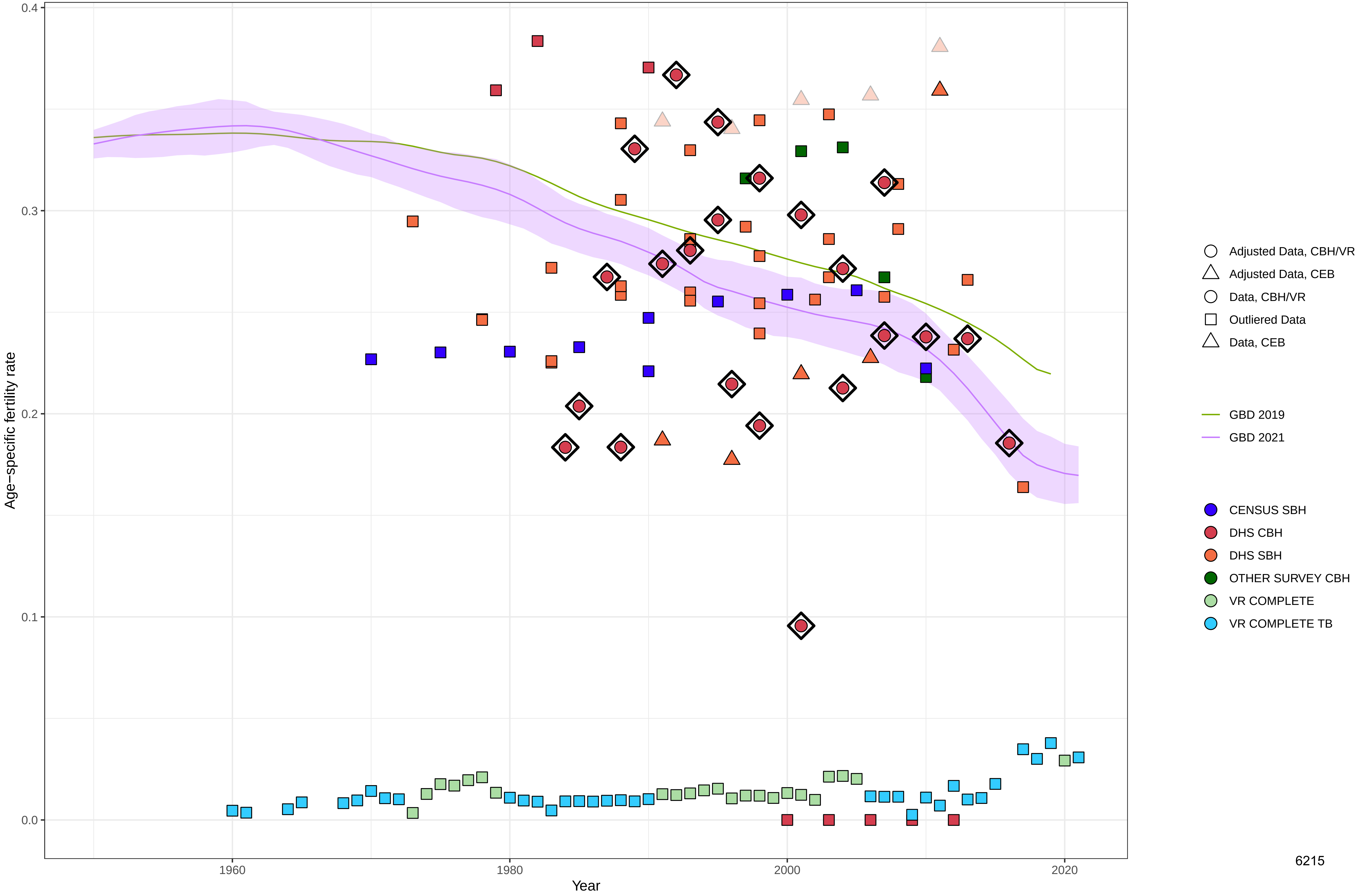

Age−specific fertility fitted trend lines, 1950−2021: Lanao Del Sur, Ages: 30−34
Data points enclosed with a diamond are reference sources

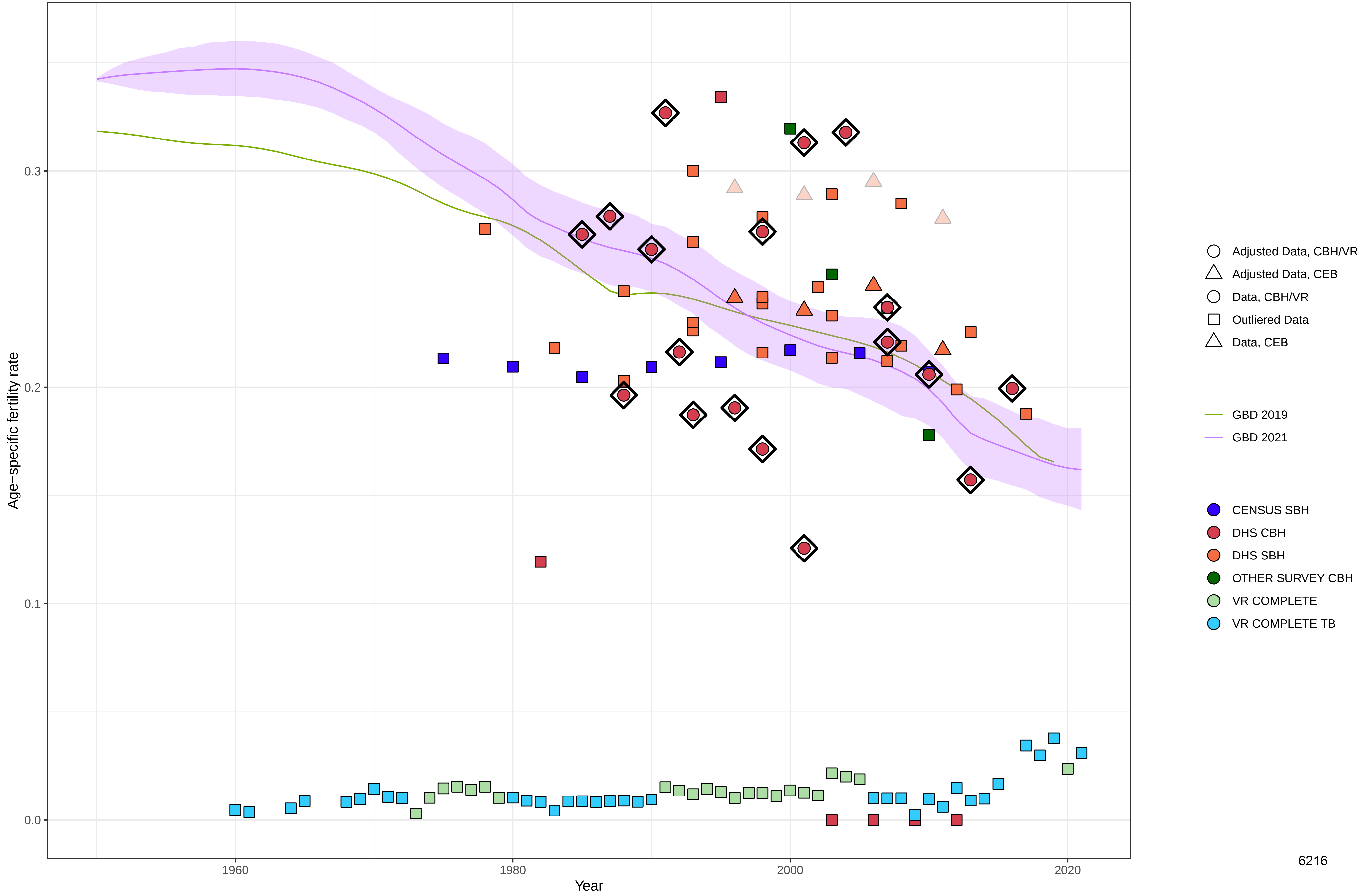

Age−specific fertility fitted trend lines, 1950−2021: Lanao Del Sur, Ages: 35−39
Data points enclosed with a diamond are reference sources

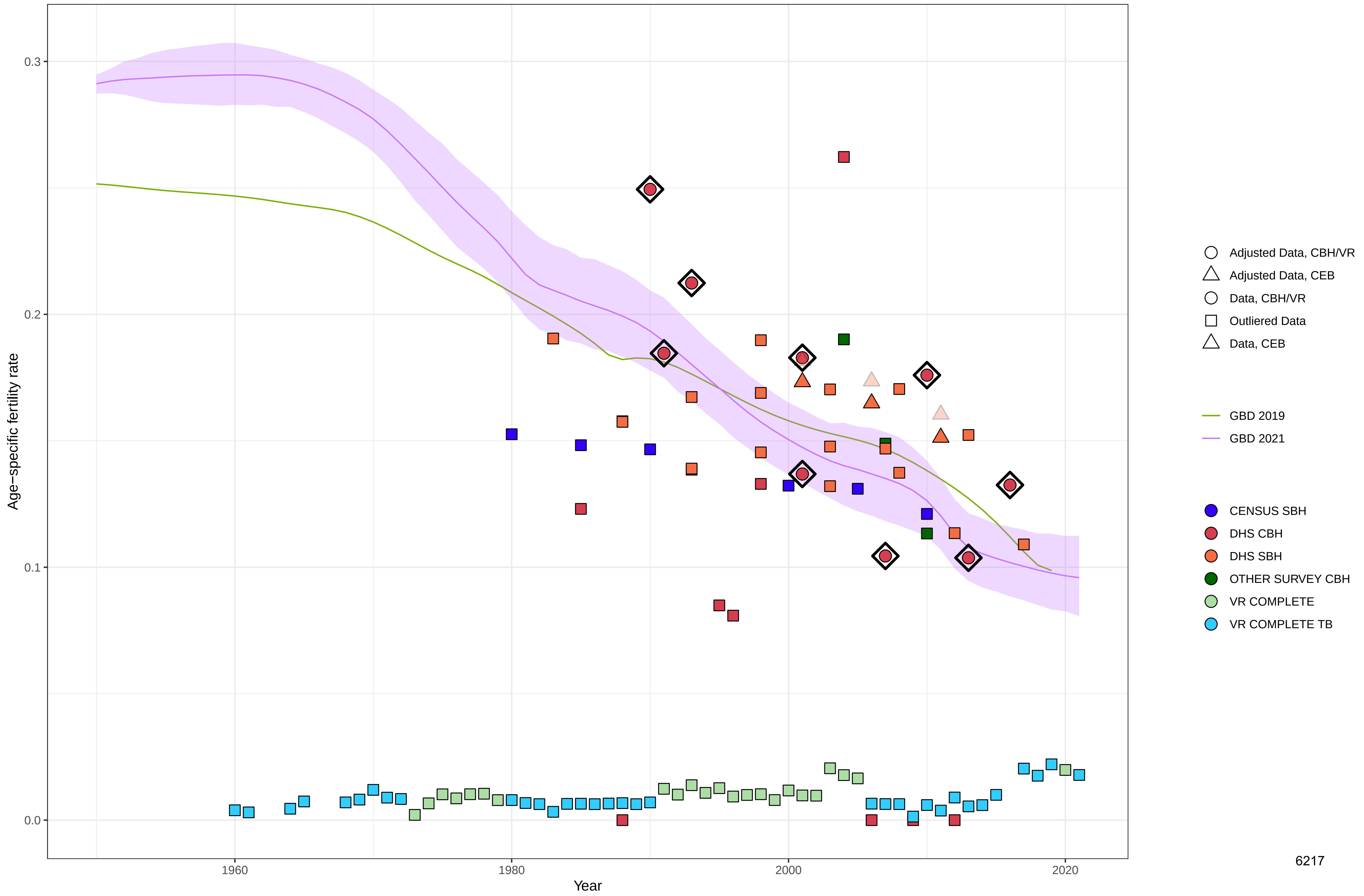

Age−specific fertility fitted trend lines, 1950−2021: Lanao Del Sur, Ages: 40−44
Data points enclosed with a diamond are reference sources

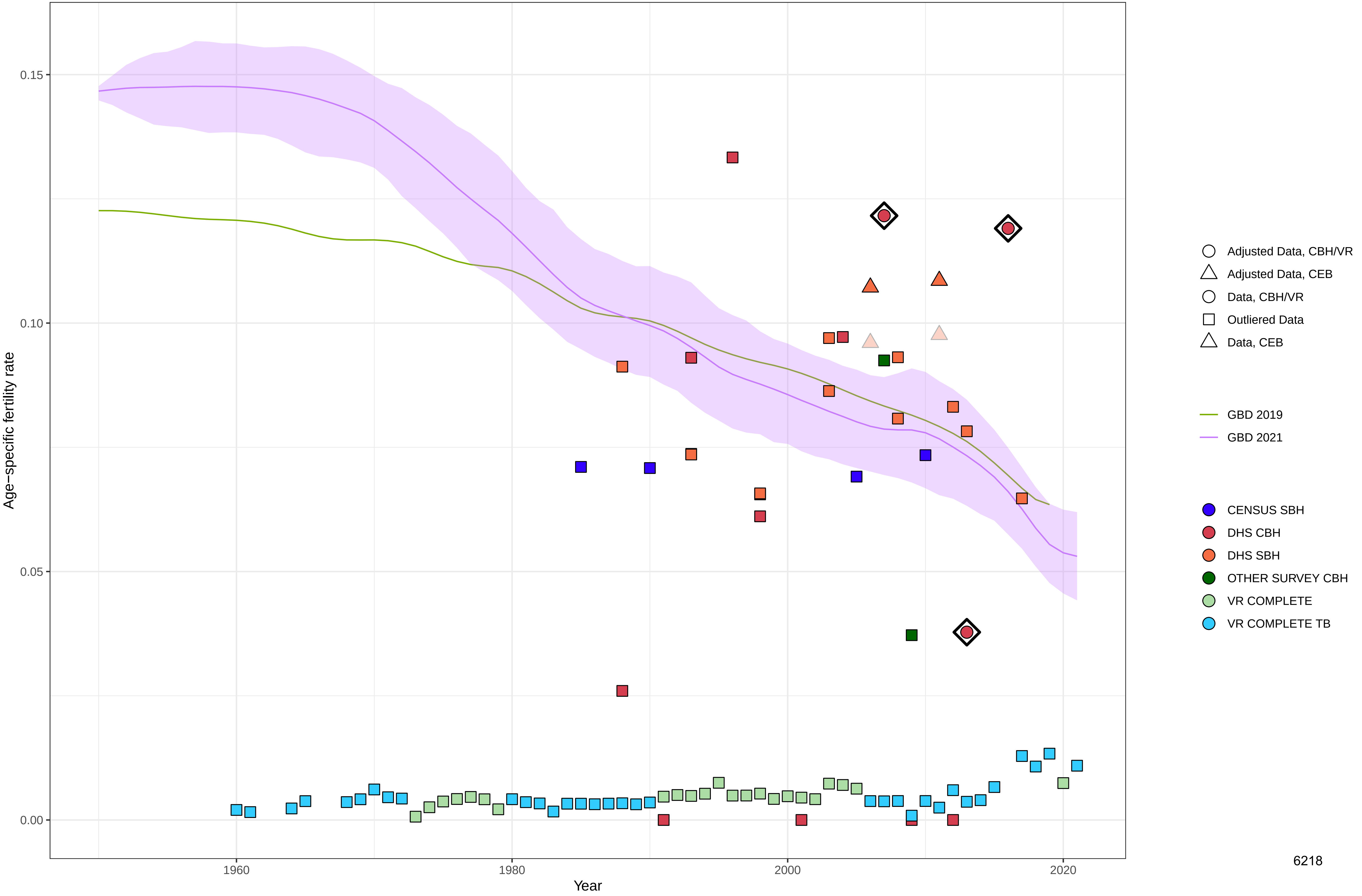

6218

Age−specific fertility fitted trend lines, 1950−2021: Lanao Del Sur, Ages: 45−49
Data points enclosed with a diamond are reference sources

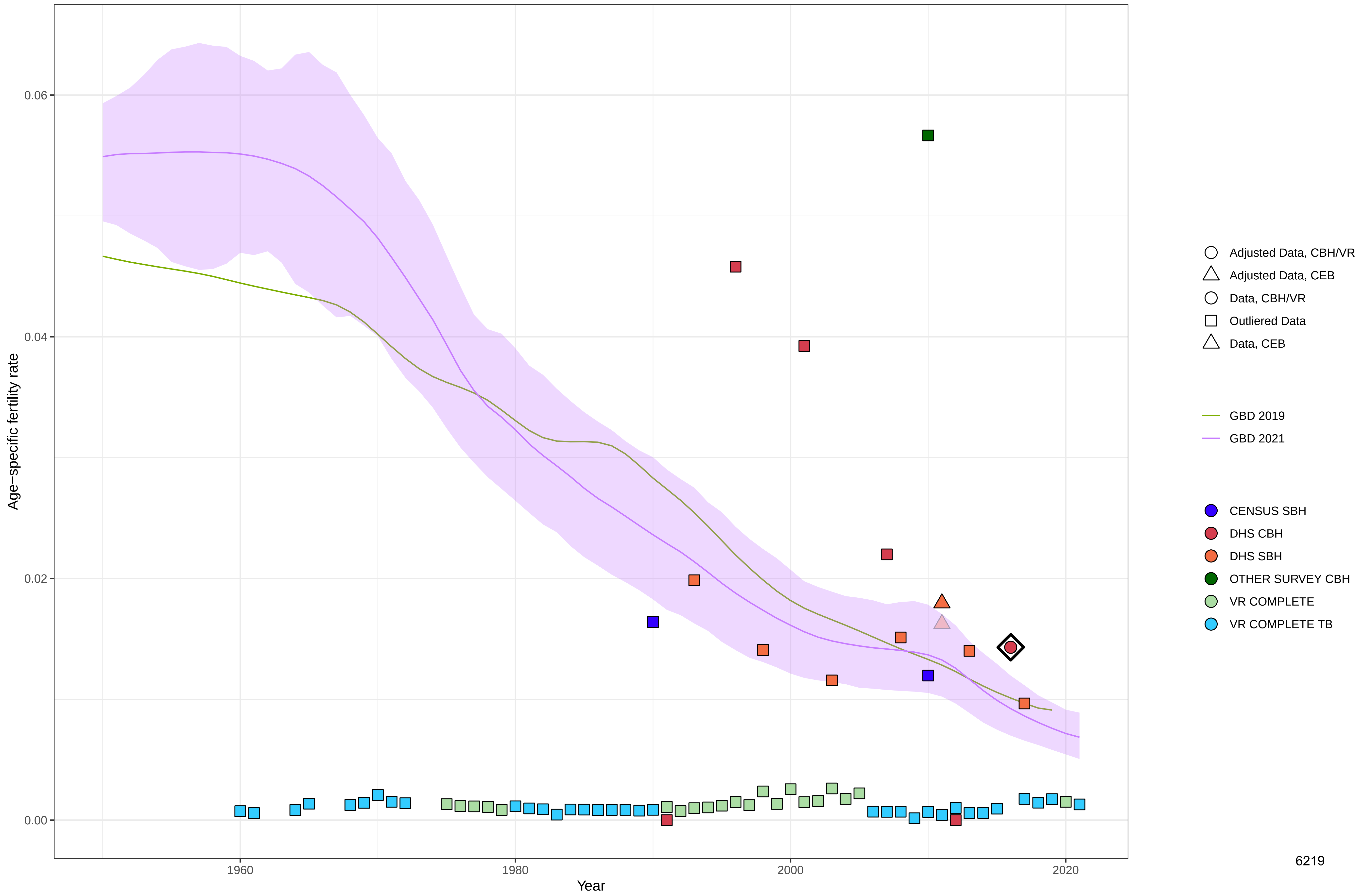

Age−specific fertility fitted trend lines, 1950−2021: Lanao Del Sur, Ages: 50−54
Data points enclosed with a diamond are reference sources

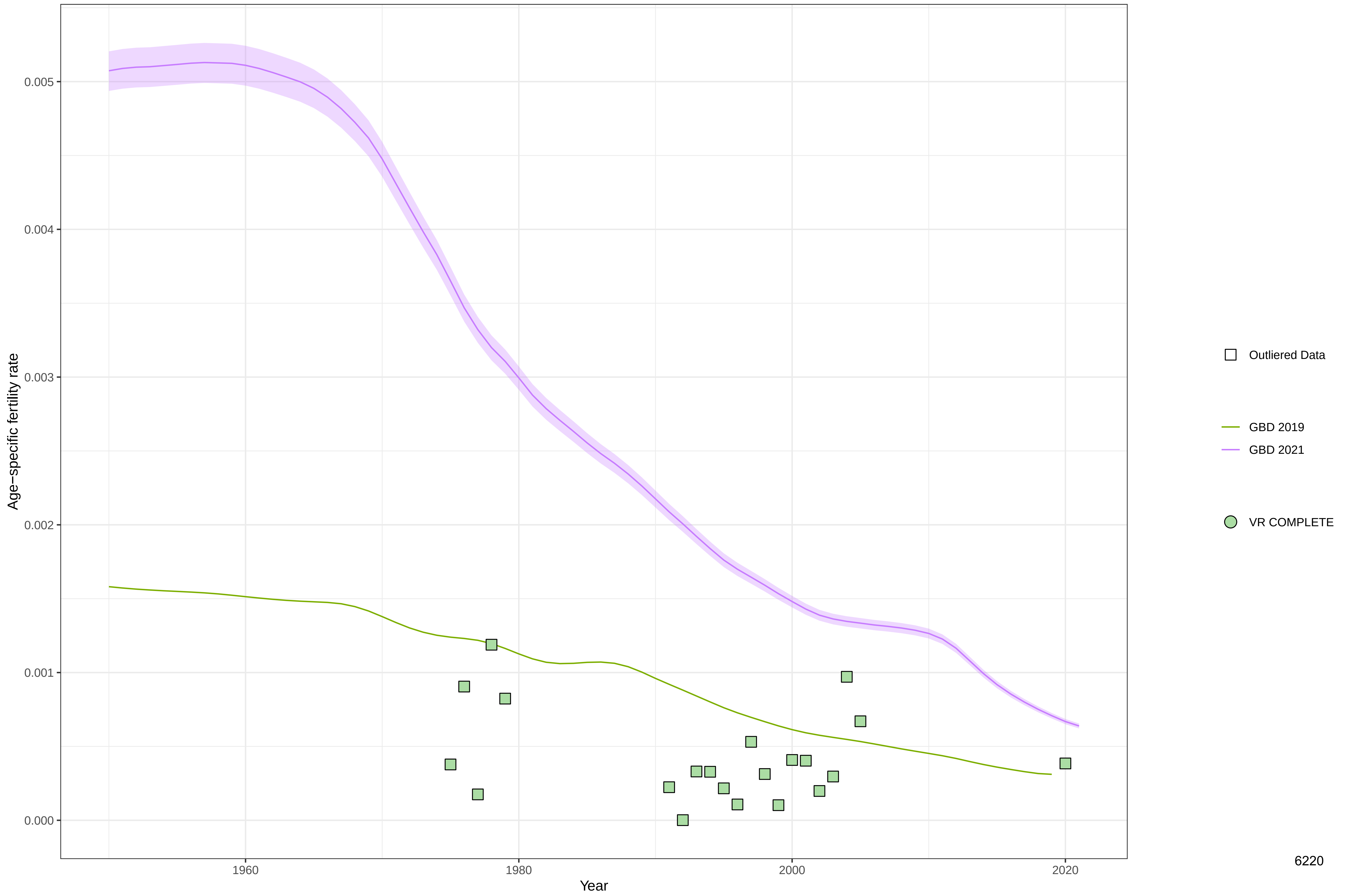

Total fertility rate fitted trend lines, 1950−2021: Lanao Del Sur TFR
Data points enclosed with a diamond are reference sources

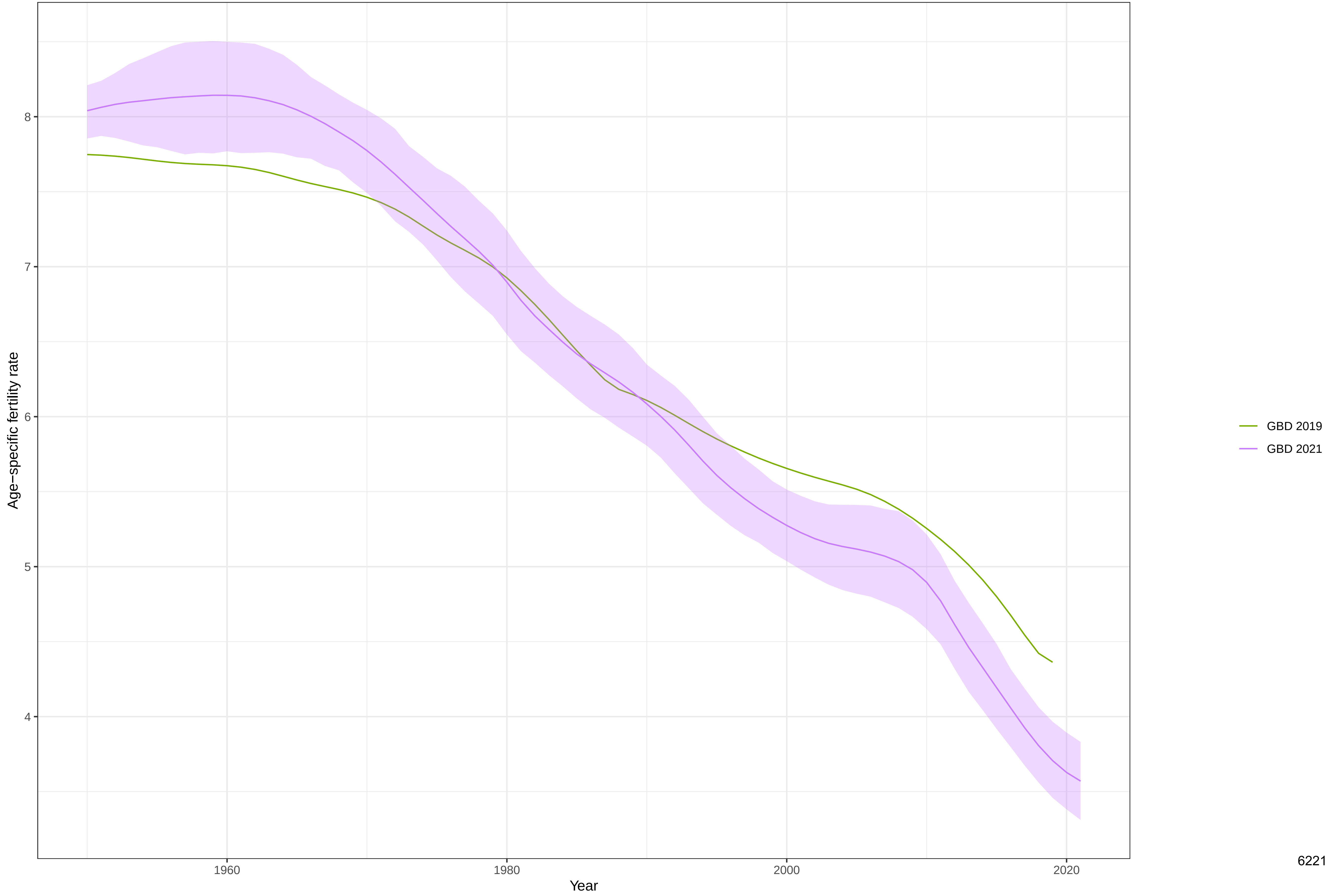

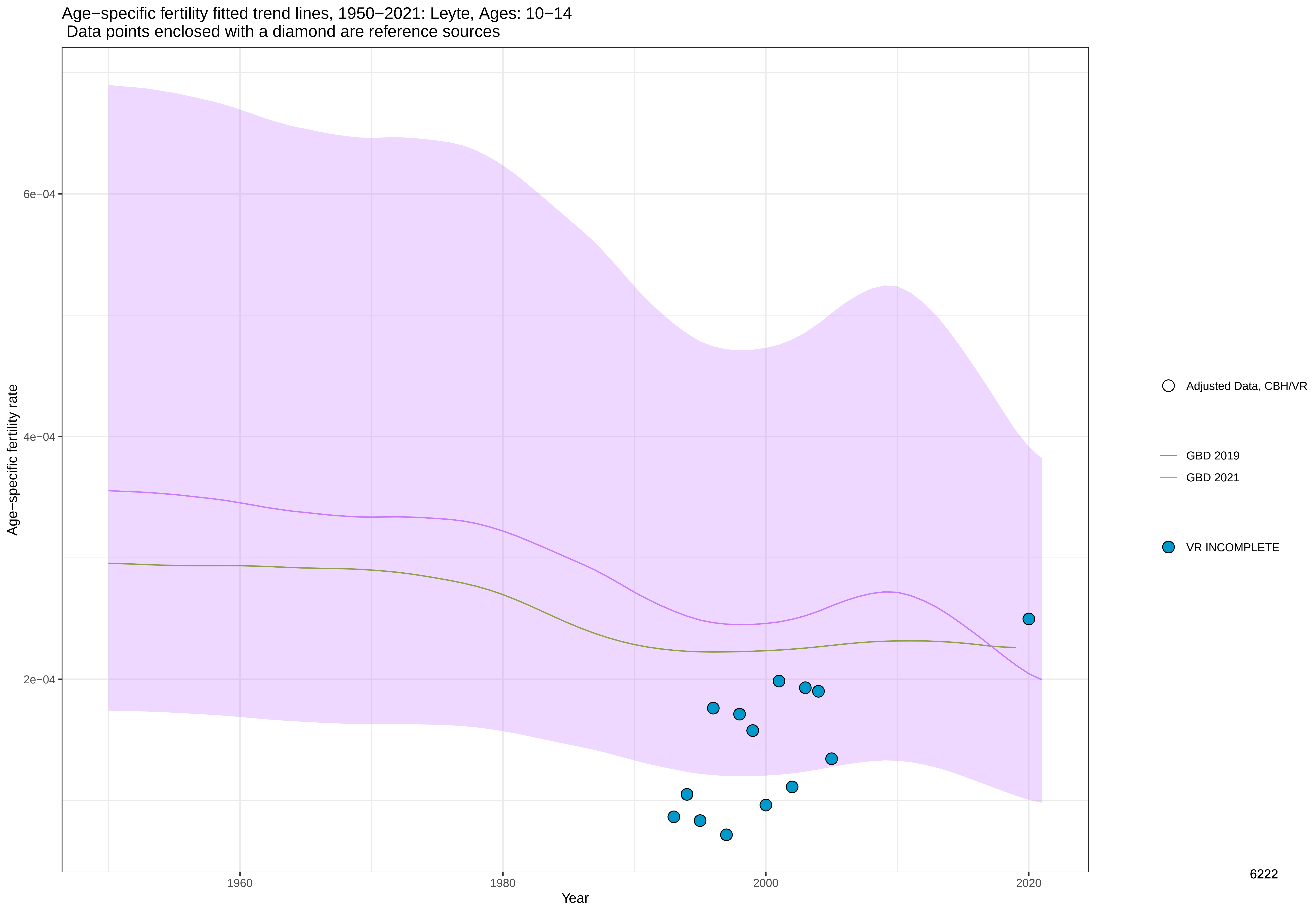

Age−specific fertility fitted trend lines, 1950−2021: Leyte, Ages: 10−14
Data points enclosed with a diamond are reference sources
Age−specific fertility rate
6e−04
4e−04
2e−04
Year
1960
1980
2000
2020
Adjusted Data, CBH/VR
GBD 2019
GBD 2021
VR INCOMPLETE

Age−specific fertility fitted trend lines, 1950−2021: Leyte, Ages: 15−19
Data points enclosed with a diamond are reference sources

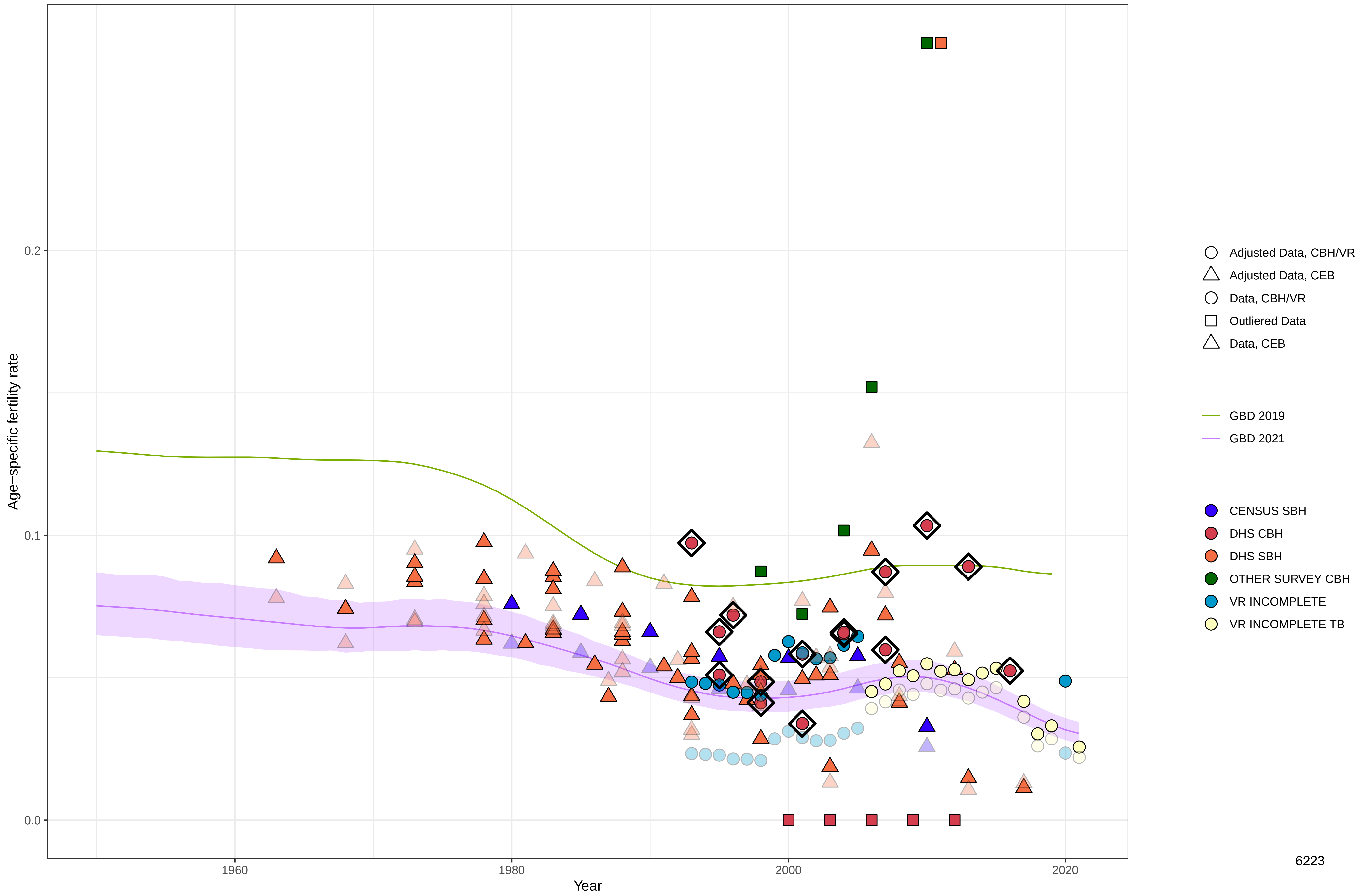

Age−specific fertility fitted trend lines, 1950−2021: Leyte, Ages: 20−24
Data points enclosed with a diamond are reference sources

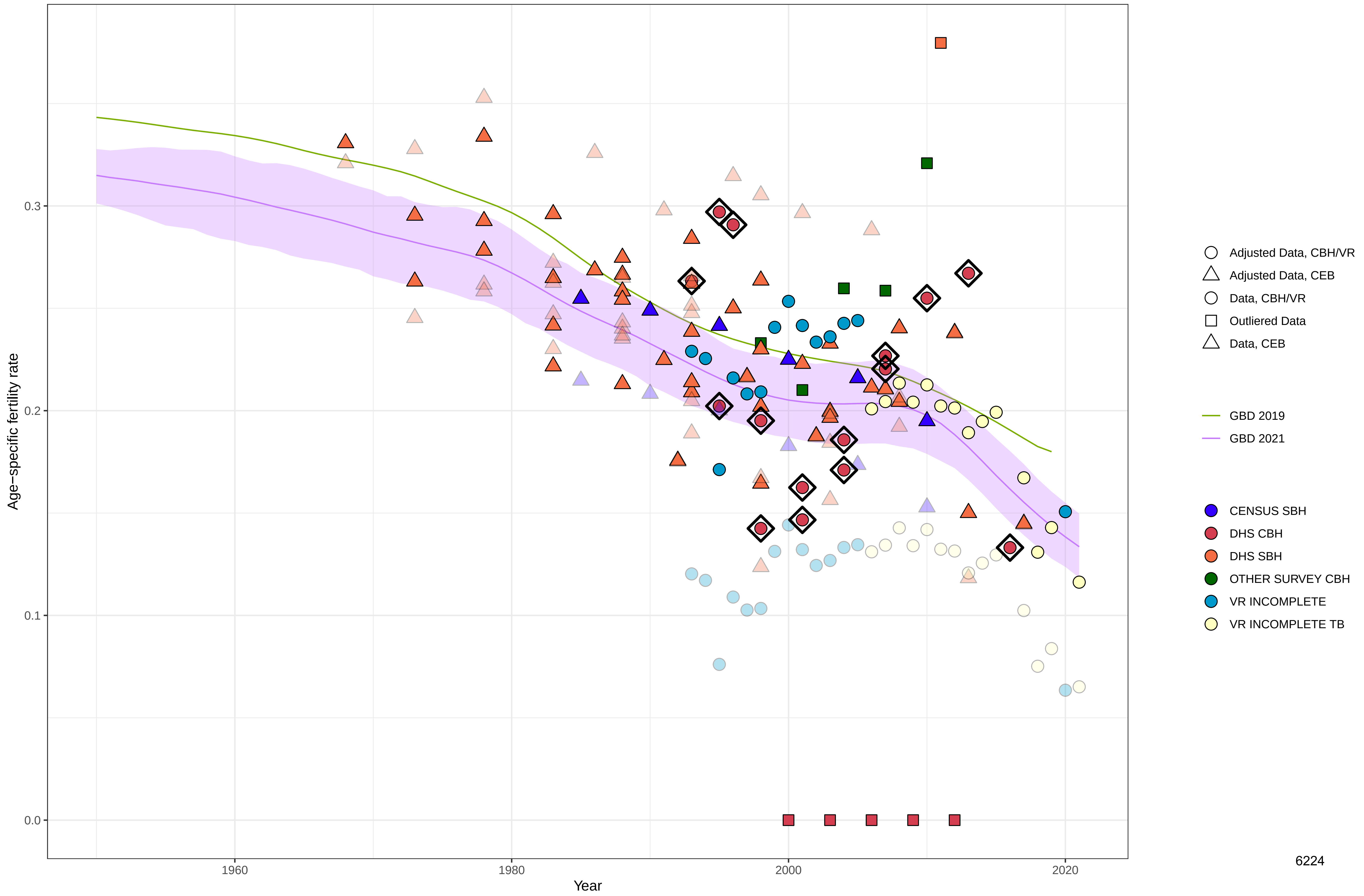

Age−specific fertility fitted trend lines, 1950−2021: Leyte, Ages: 25−29
Data points enclosed with a diamond are reference sources

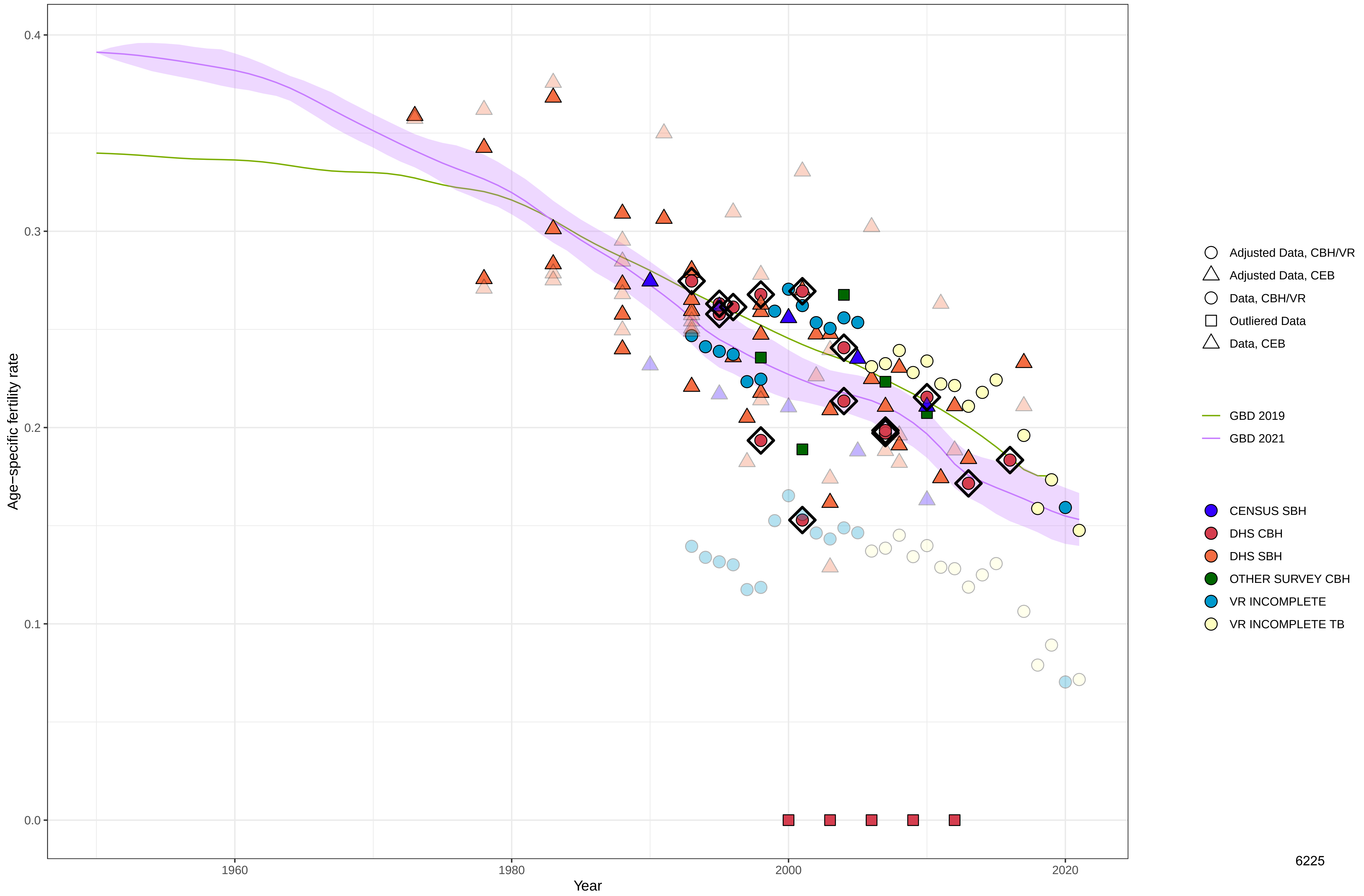

Age−specific fertility fitted trend lines, 1950−2021: Leyte, Ages: 30−34
Data points enclosed with a diamond are reference sources

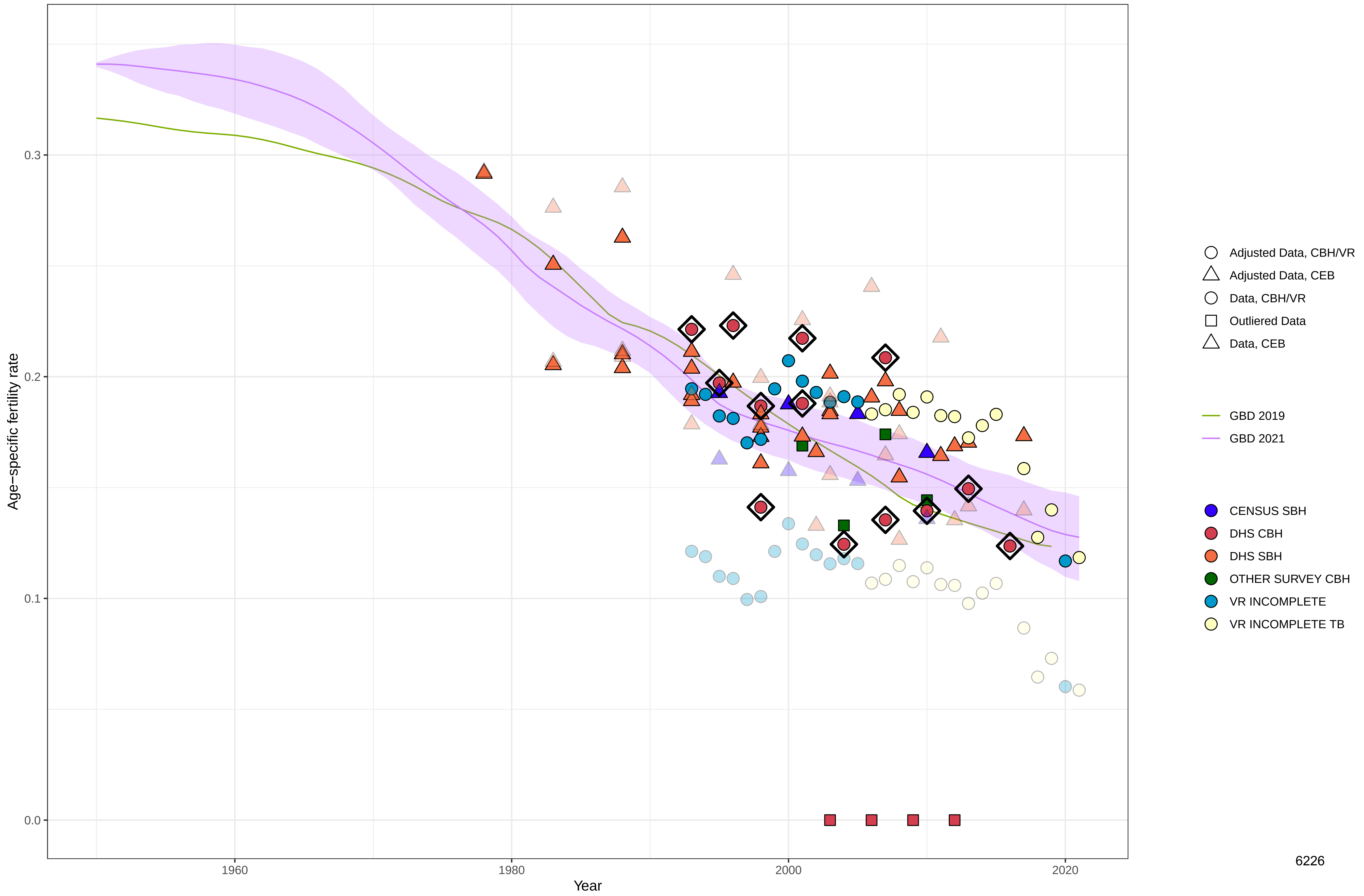

6226

Age−specific fertility fitted trend lines, 1950−2021: Leyte, Ages: 35−39
Data points enclosed with a diamond are reference sources

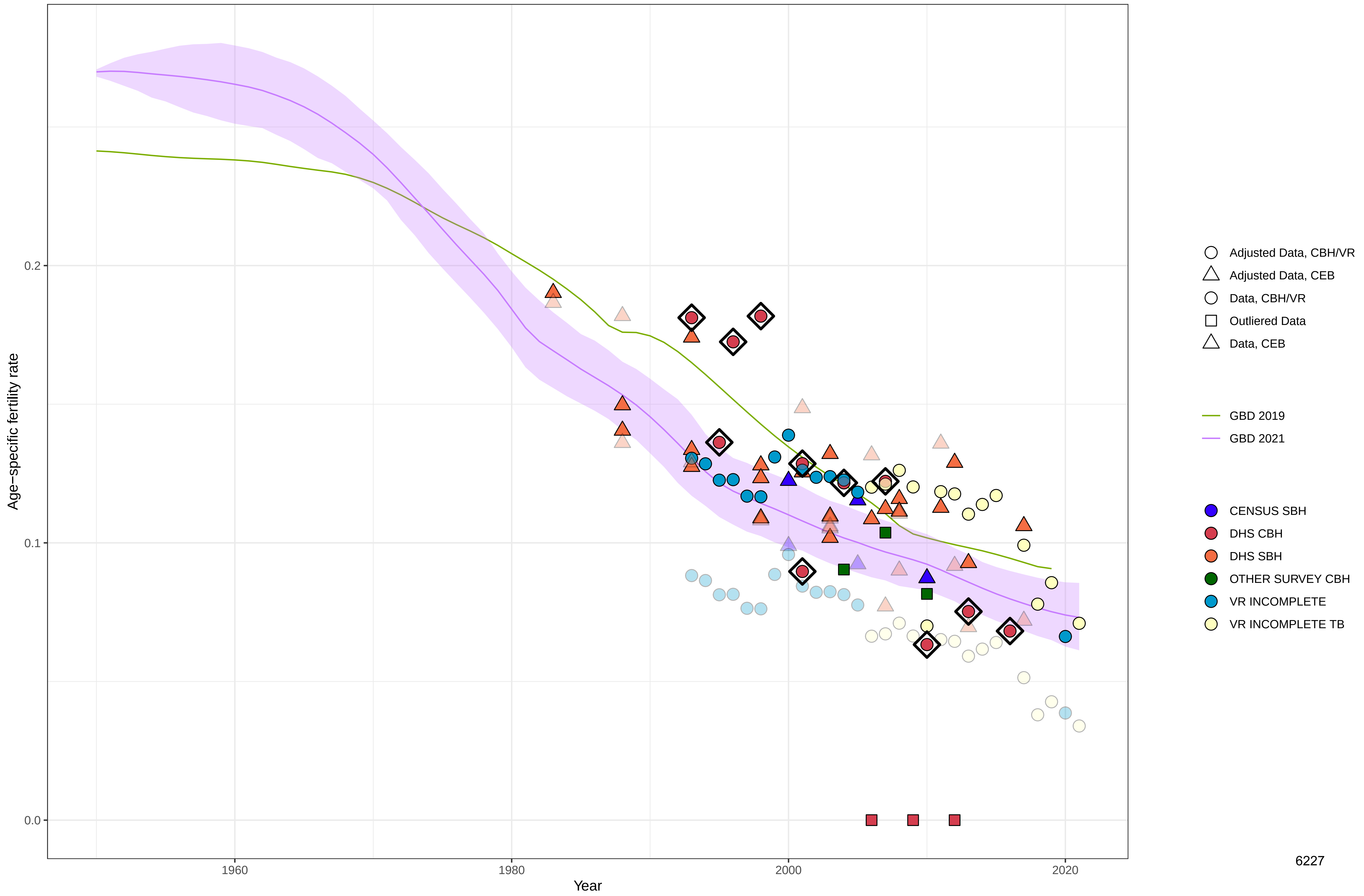

Age−specific fertility fitted trend lines, 1950−2021: Leyte, Ages: 40−44
Data points enclosed with a diamond are reference sources

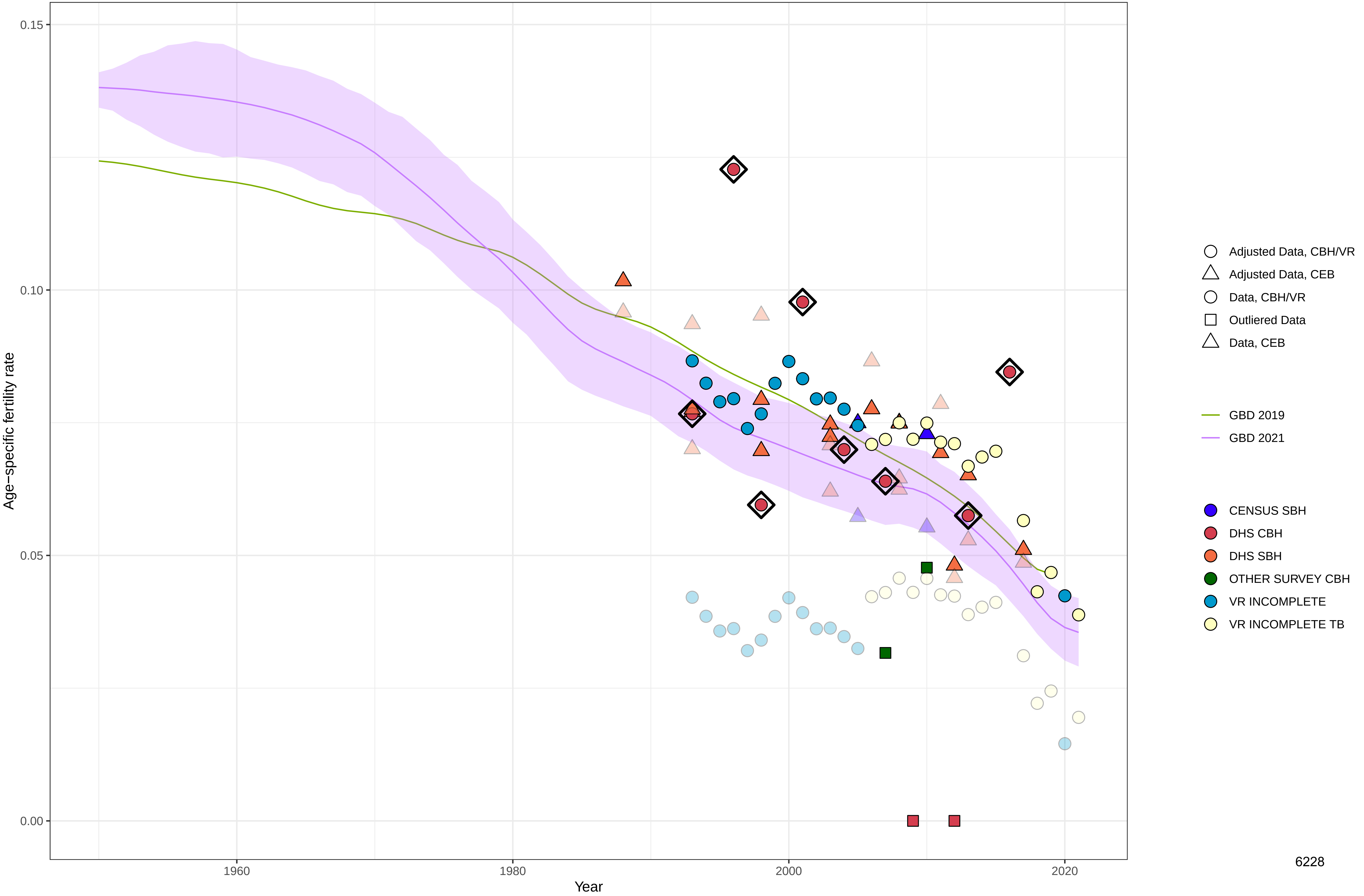

Age−specific fertility fitted trend lines, 1950−2021: Leyte, Ages: 45−49
Data points enclosed with a diamond are reference sources

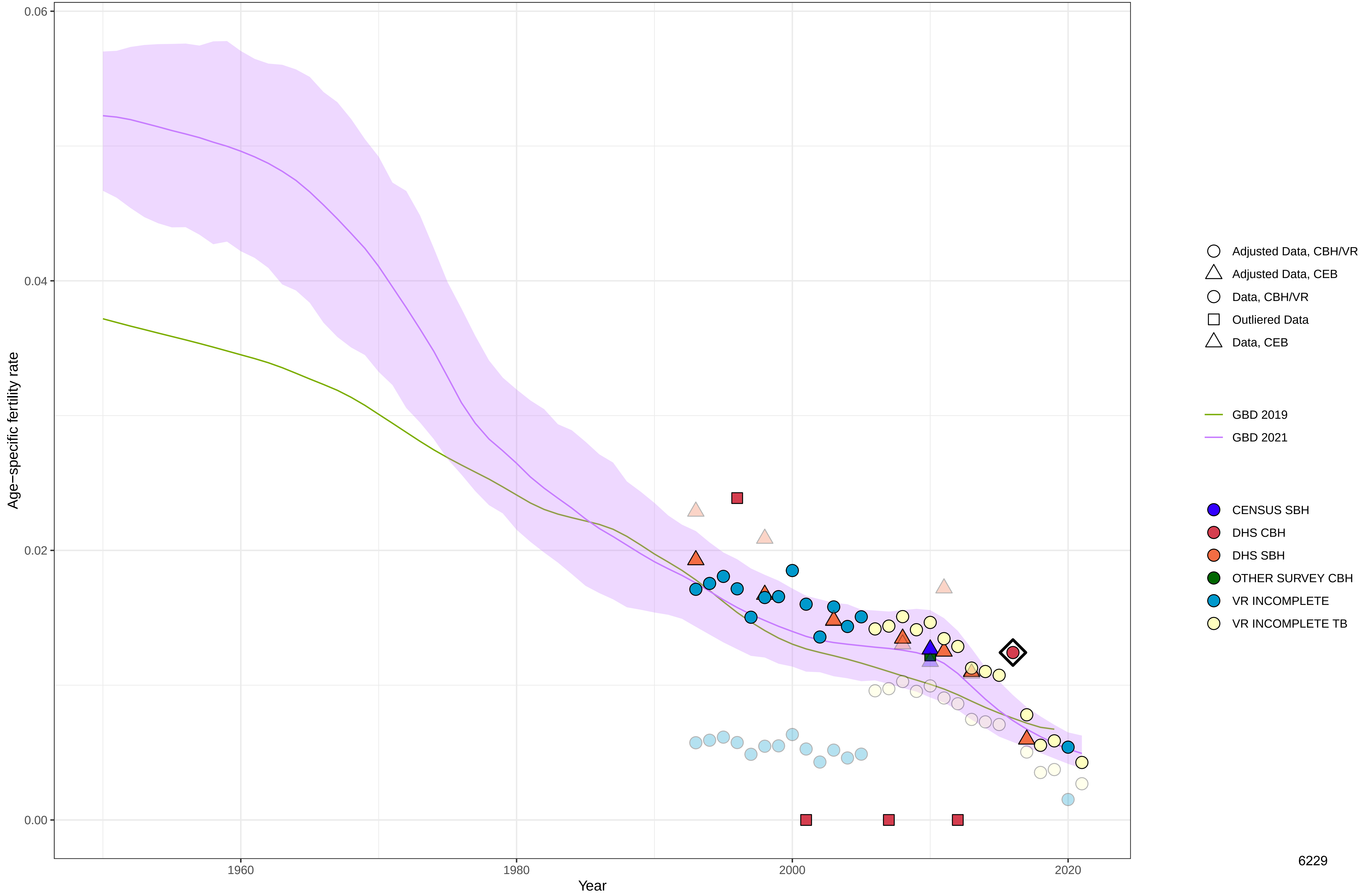

6229

Age−specific fertility fitted trend lines, 1950−2021: Leyte, Ages: 50−54
Data points enclosed with a diamond are reference sources

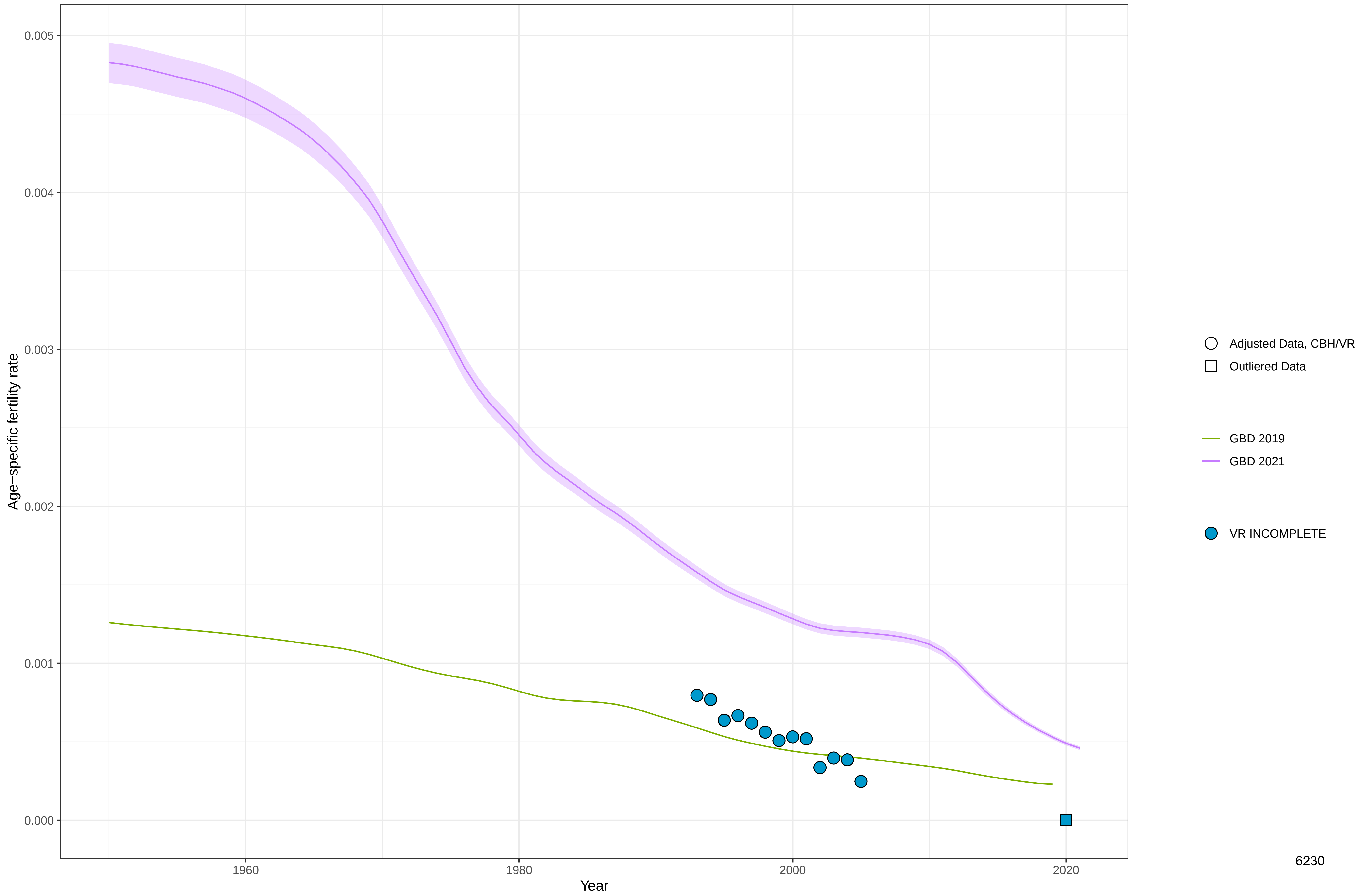

Total fertility rate fitted trend lines, 1950−2021: Leyte TFR
Data points enclosed with a diamond are reference sources

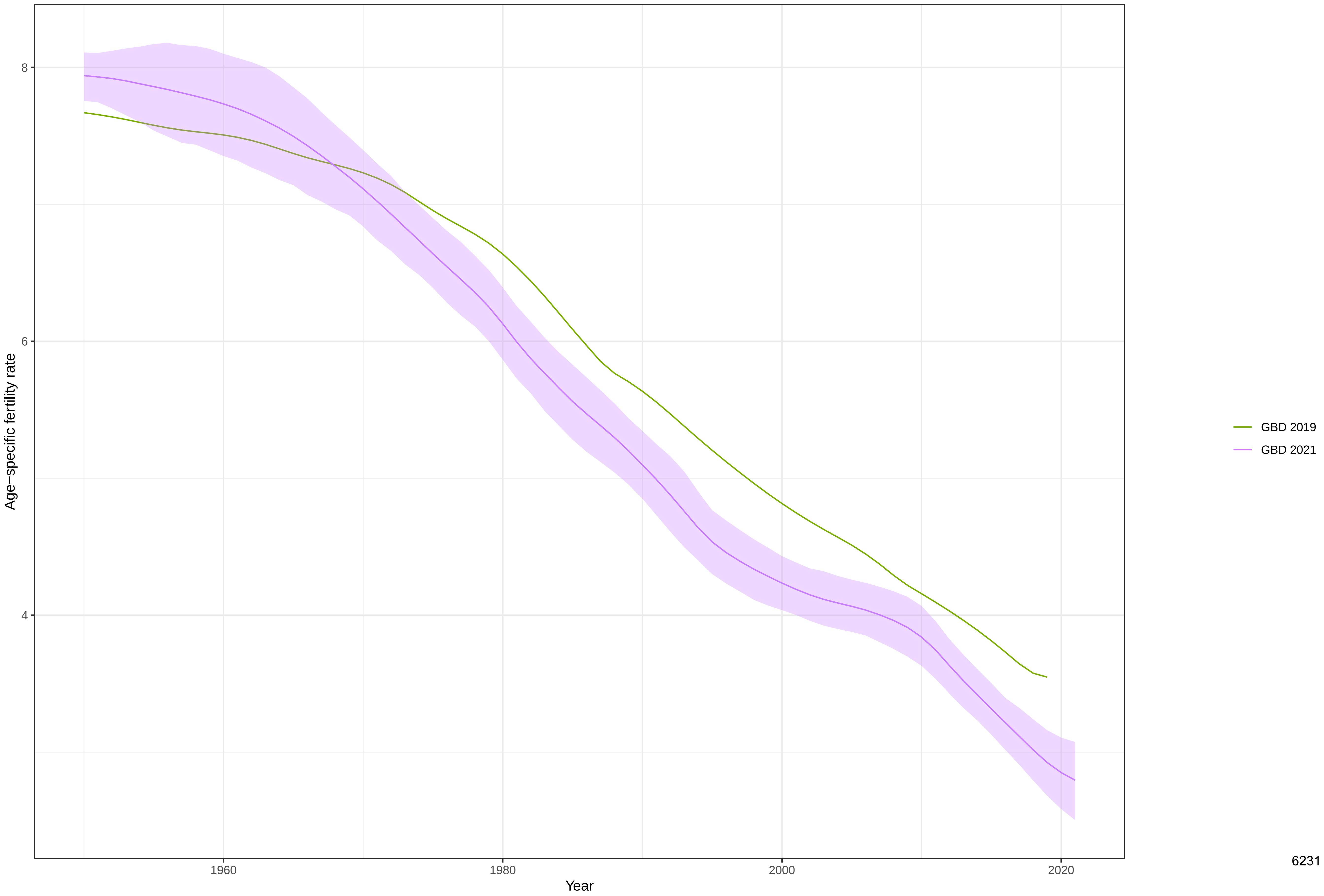

Age−specific fertility fitted trend lines, 1950−2021: Maguindanao, Ages: 10−14
Data points enclosed with a diamond are reference sources

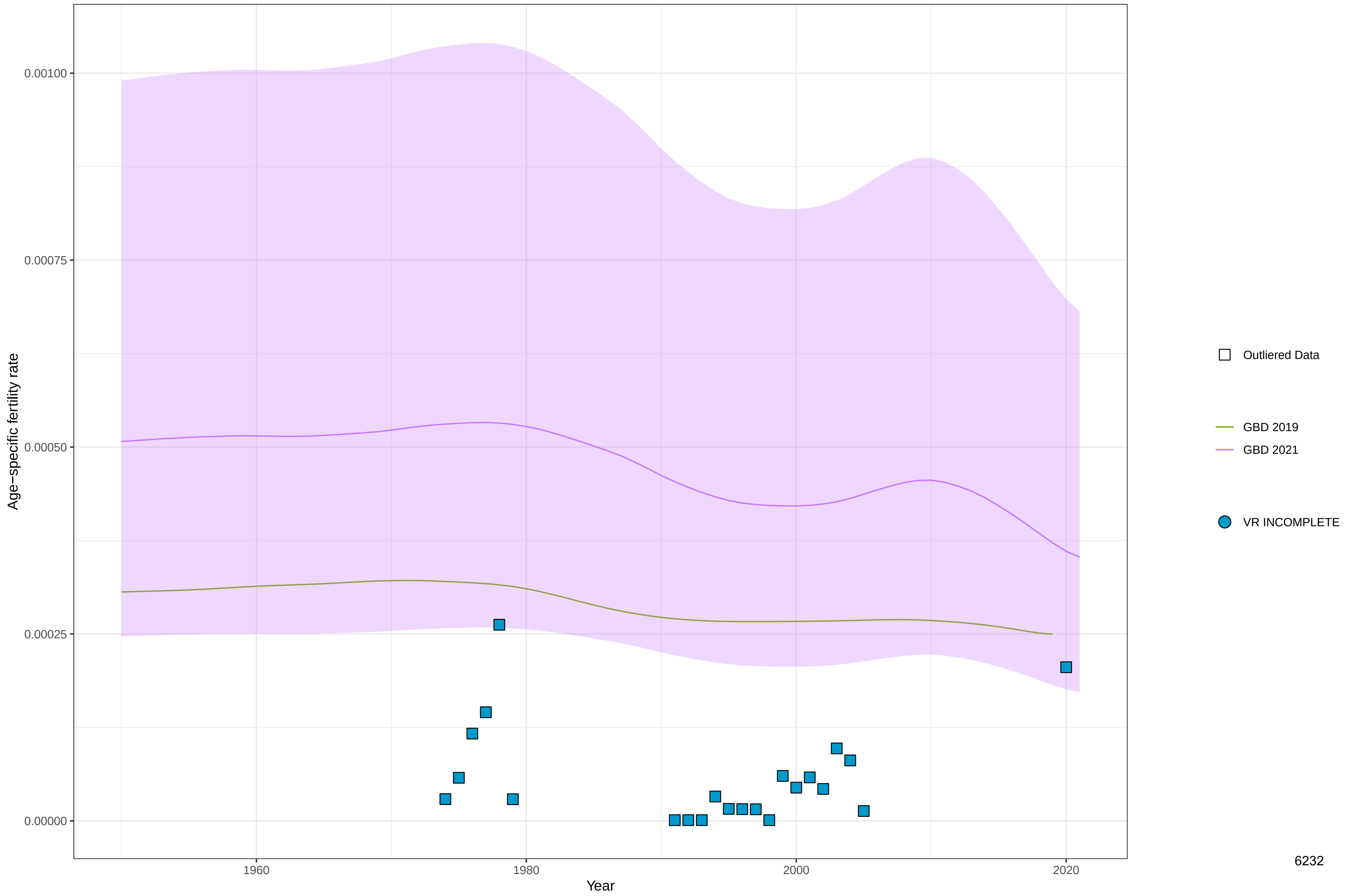

Age−specific fertility fitted trend lines, 1950−2021: Maguindanao, Ages: 15−19
Data points enclosed with a diamond are reference sources

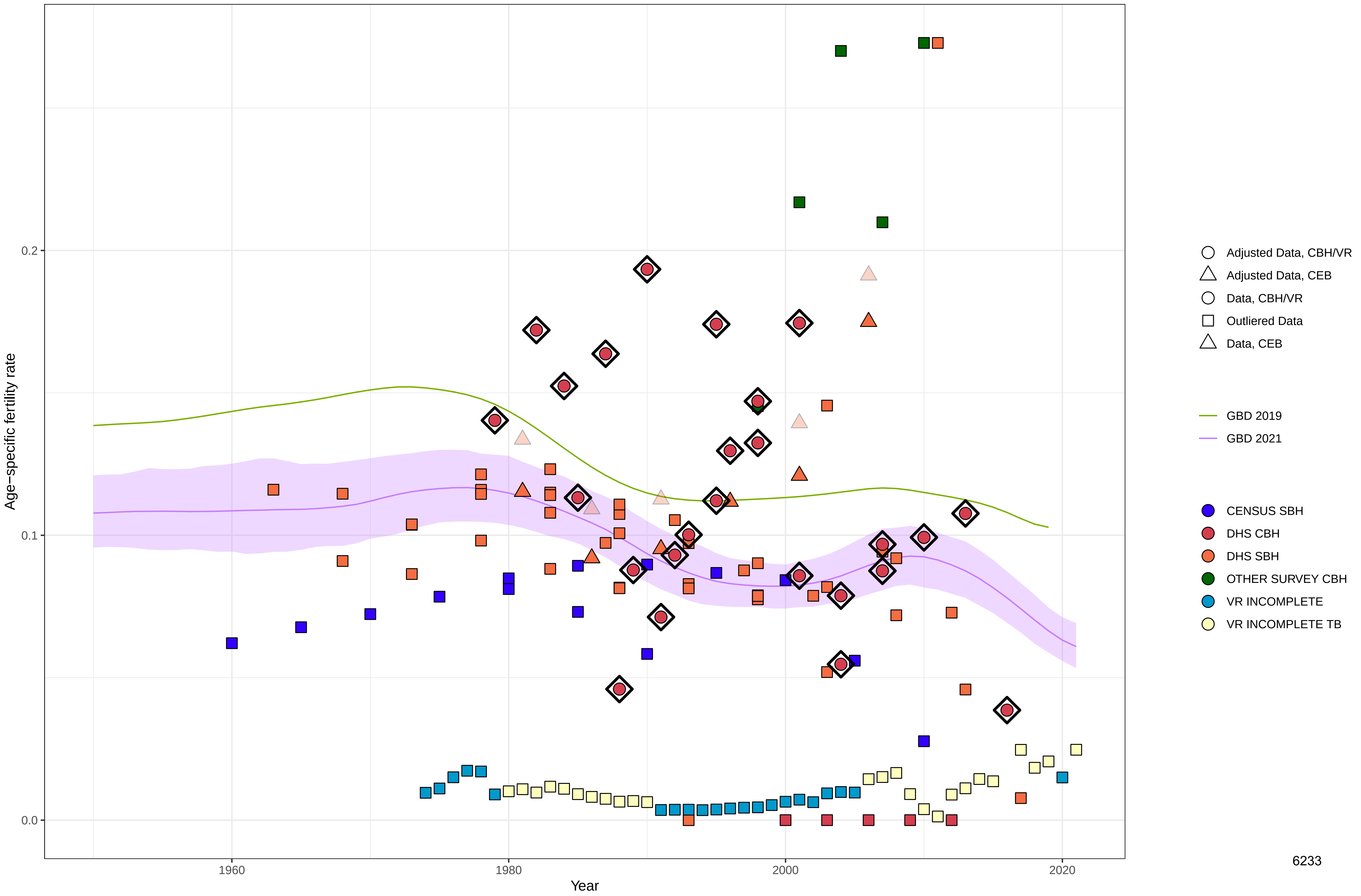

Age−specific fertility fitted trend lines, 1950−2021: Maguindanao, Ages: 20−24
Data points enclosed with a diamond are reference sources

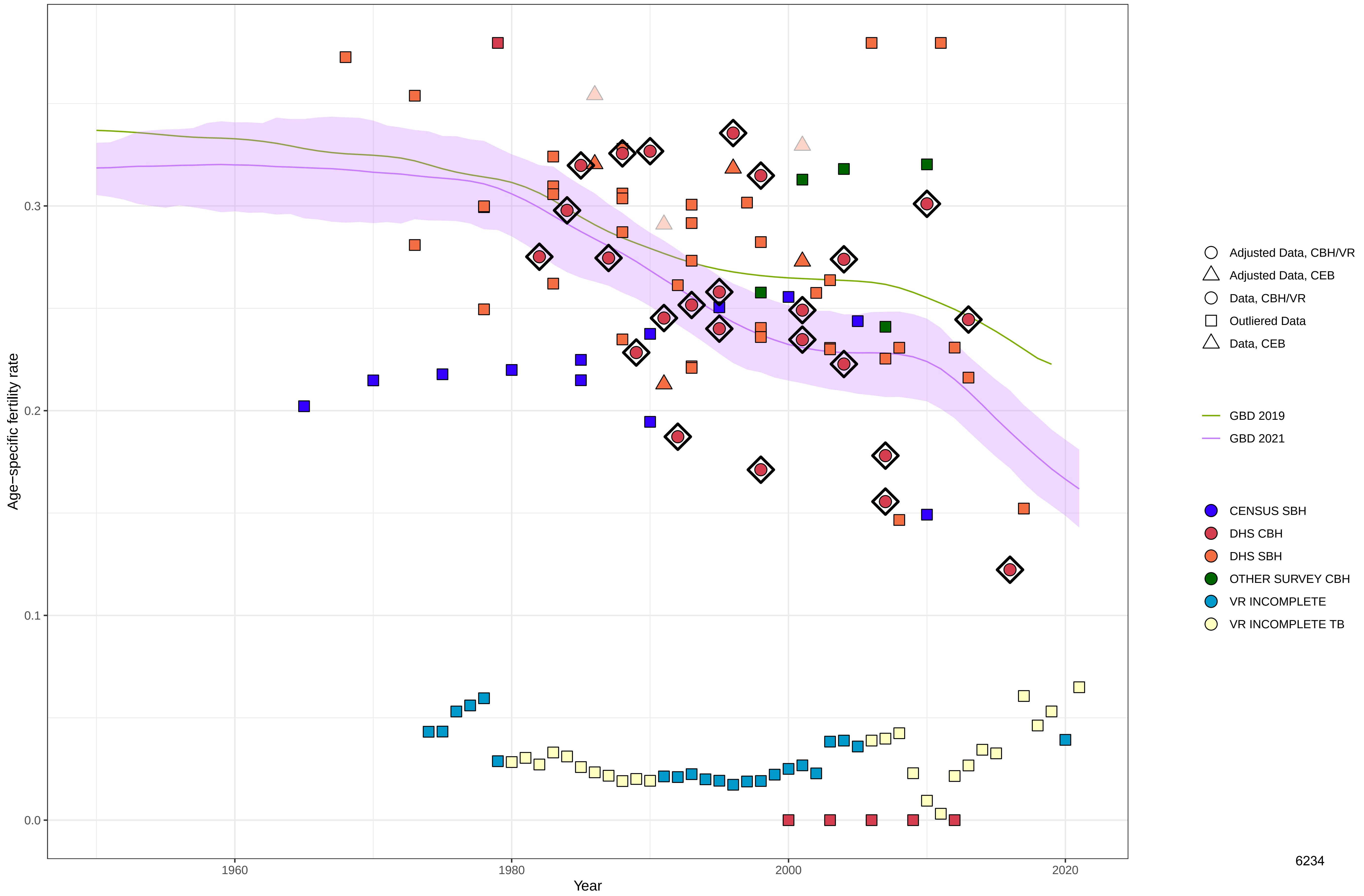

Age−specific fertility fitted trend lines, 1950−2021: Maguindanao, Ages: 25−29
Data points enclosed with a diamond are reference sources

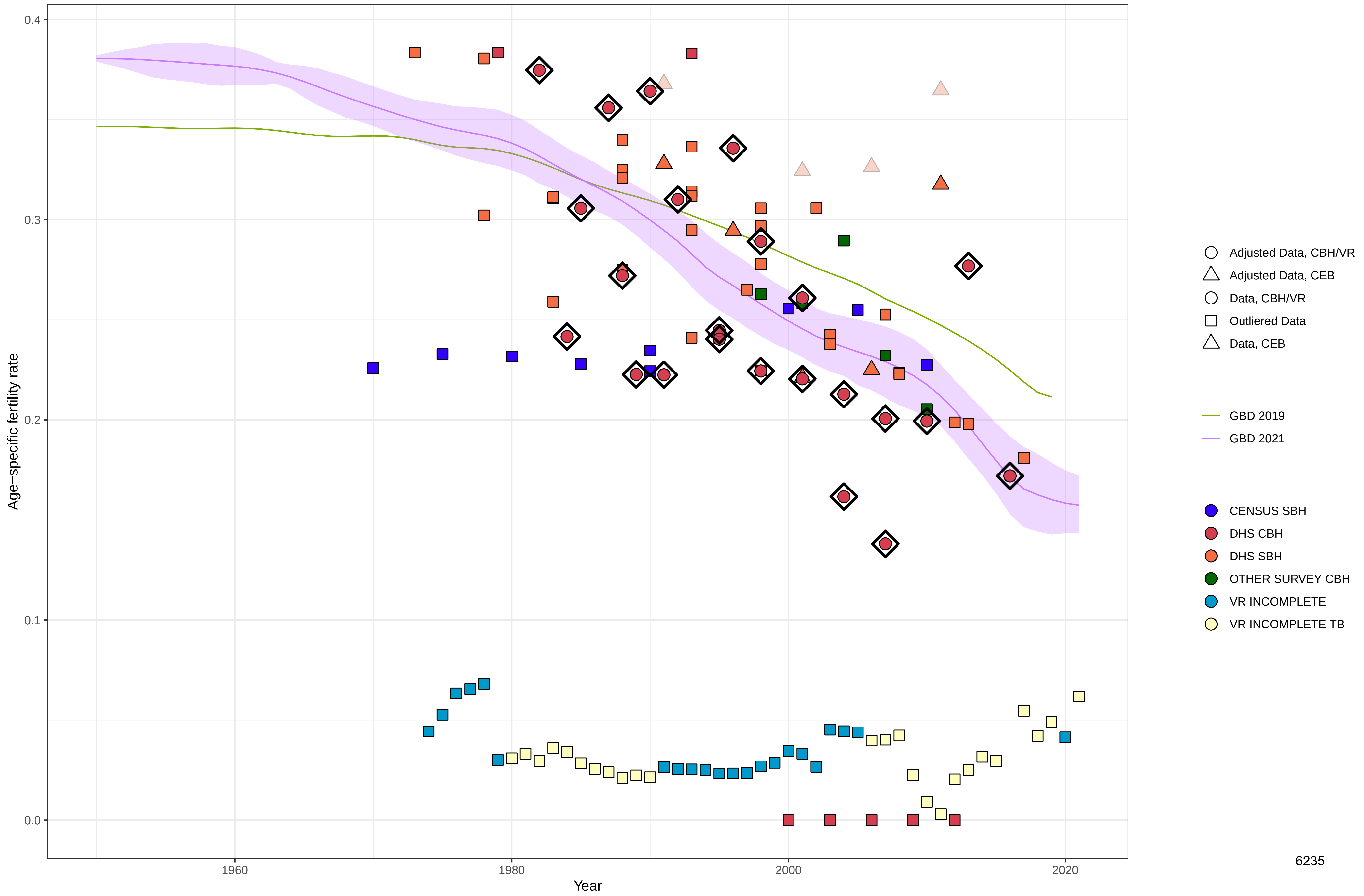

Age−specific fertility fitted trend lines, 1950−2021: Maguindanao, Ages: 30−34
Data points enclosed with a diamond are reference sources

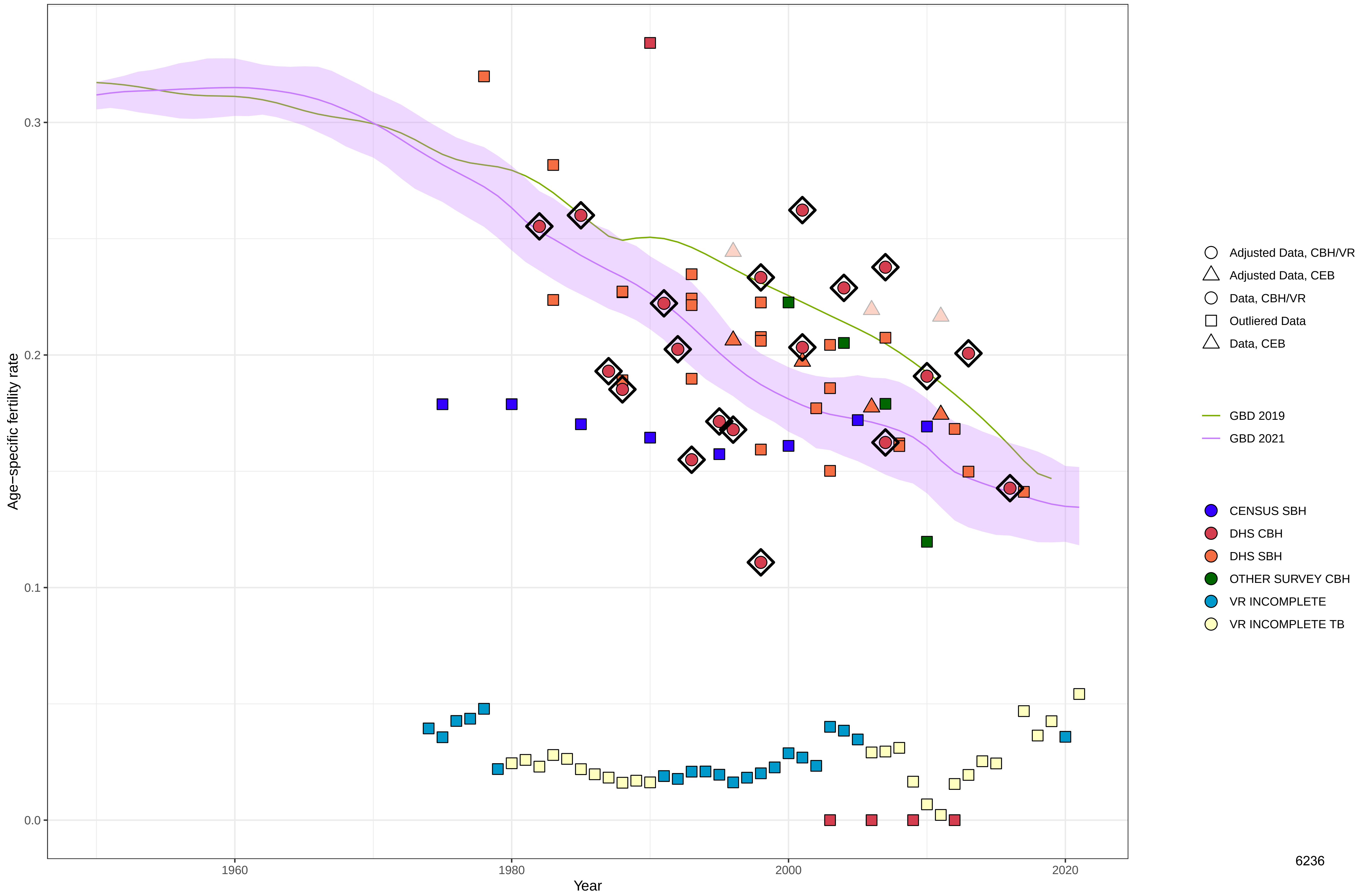

Age−specific fertility fitted trend lines, 1950−2021: Maguindanao, Ages: 35−39
Data points enclosed with a diamond are reference sources

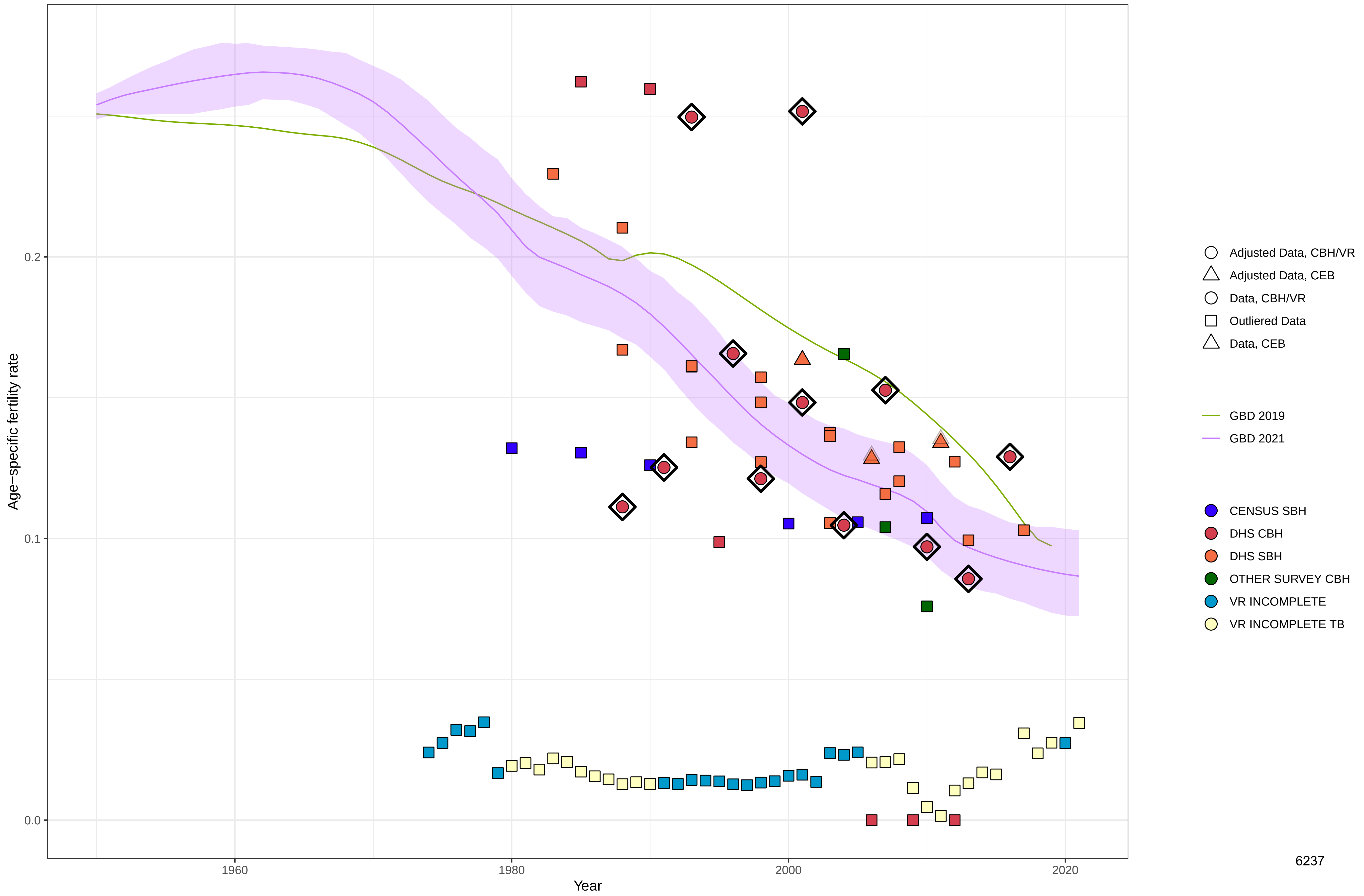

Age−specific fertility fitted trend lines, 1950−2021: Maguindanao, Ages: 40−44
Data points enclosed with a diamond are reference sources

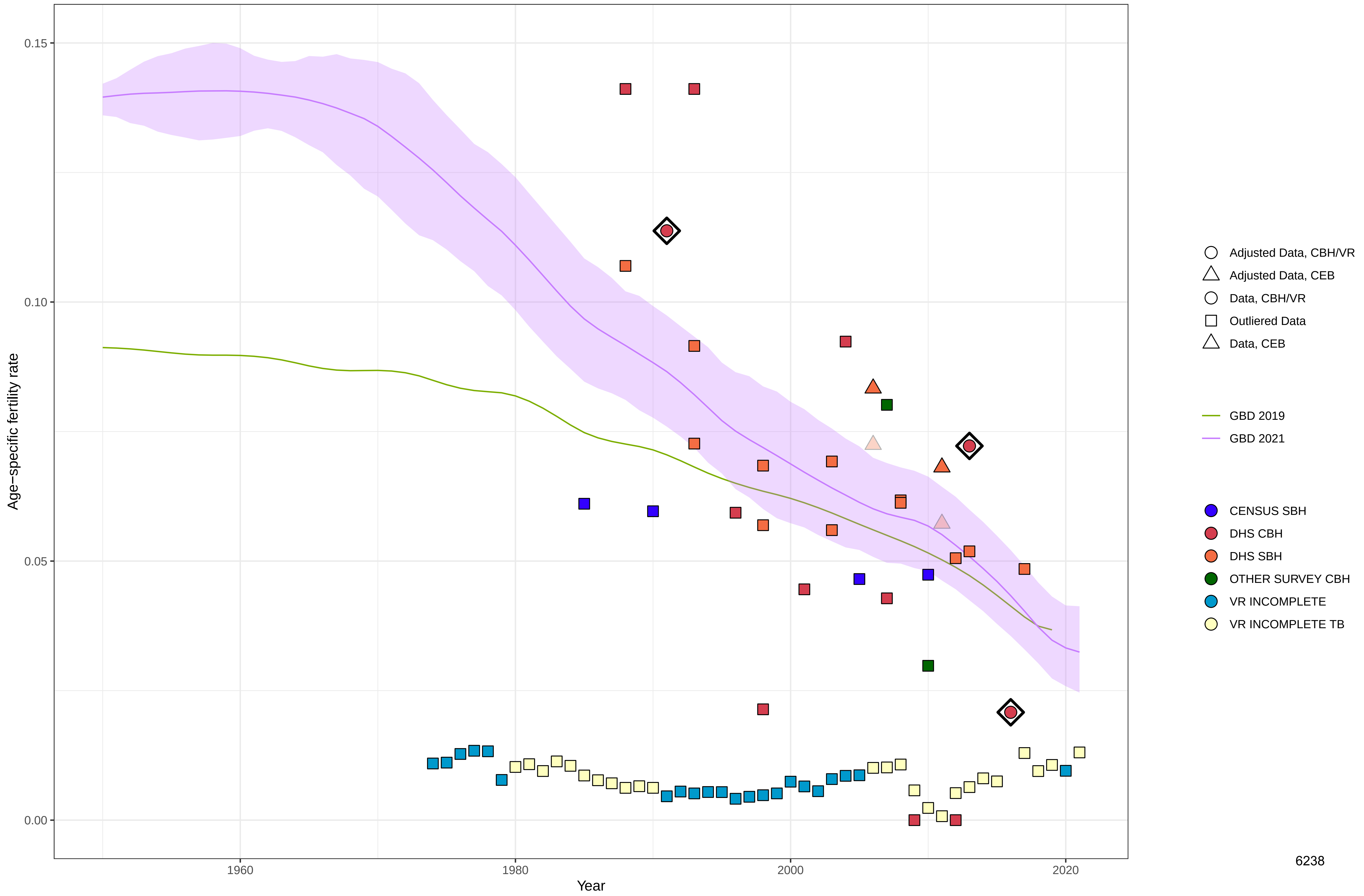

Age−specific fertility fitted trend lines, 1950−2021: Maguindanao, Ages: 45−49
Data points enclosed with a diamond are reference sources

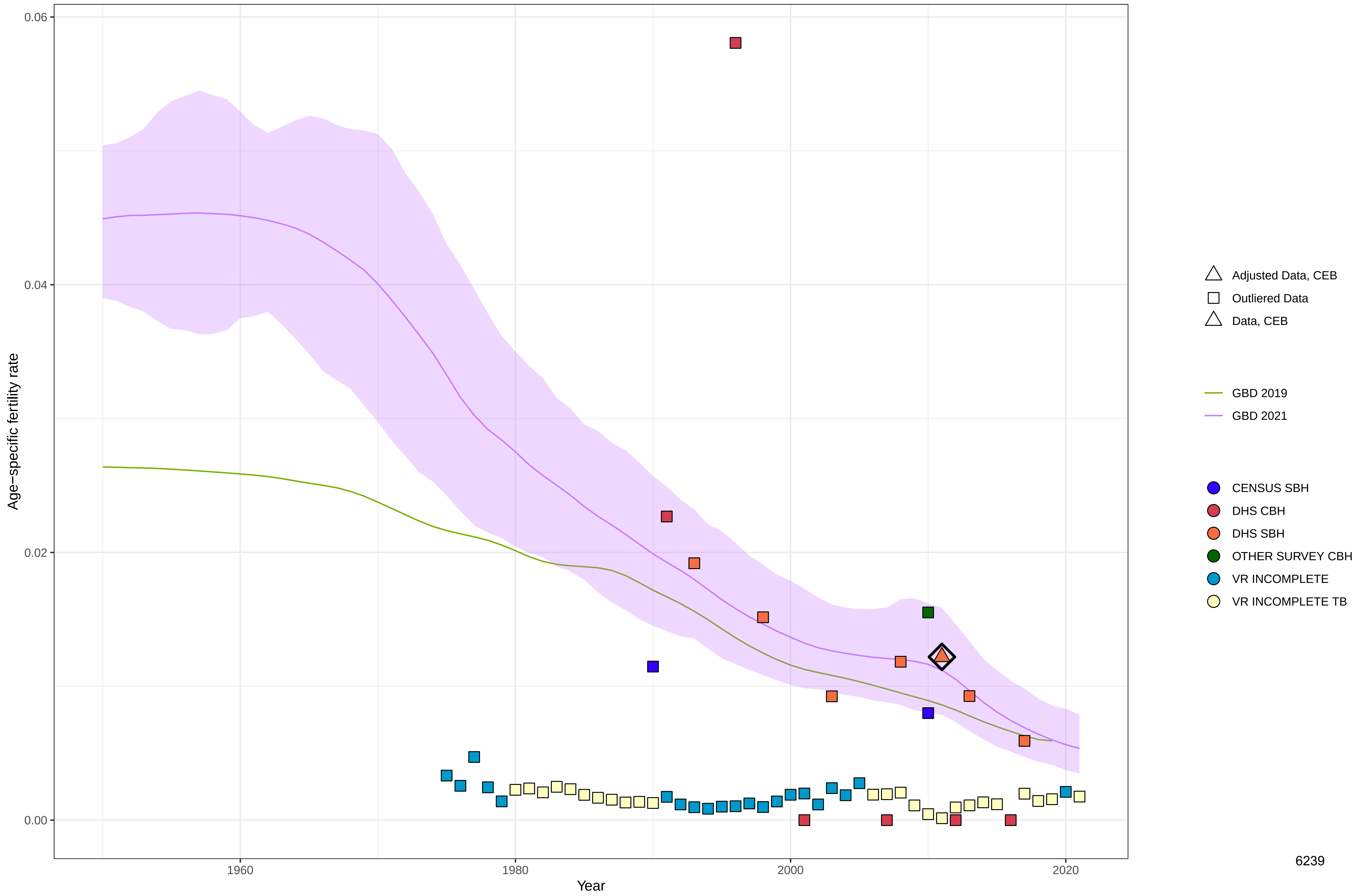

6239

Age−specific fertility fitted trend lines, 1950−2021: Maguindanao, Ages: 50−54
Data points enclosed with a diamond are reference sources

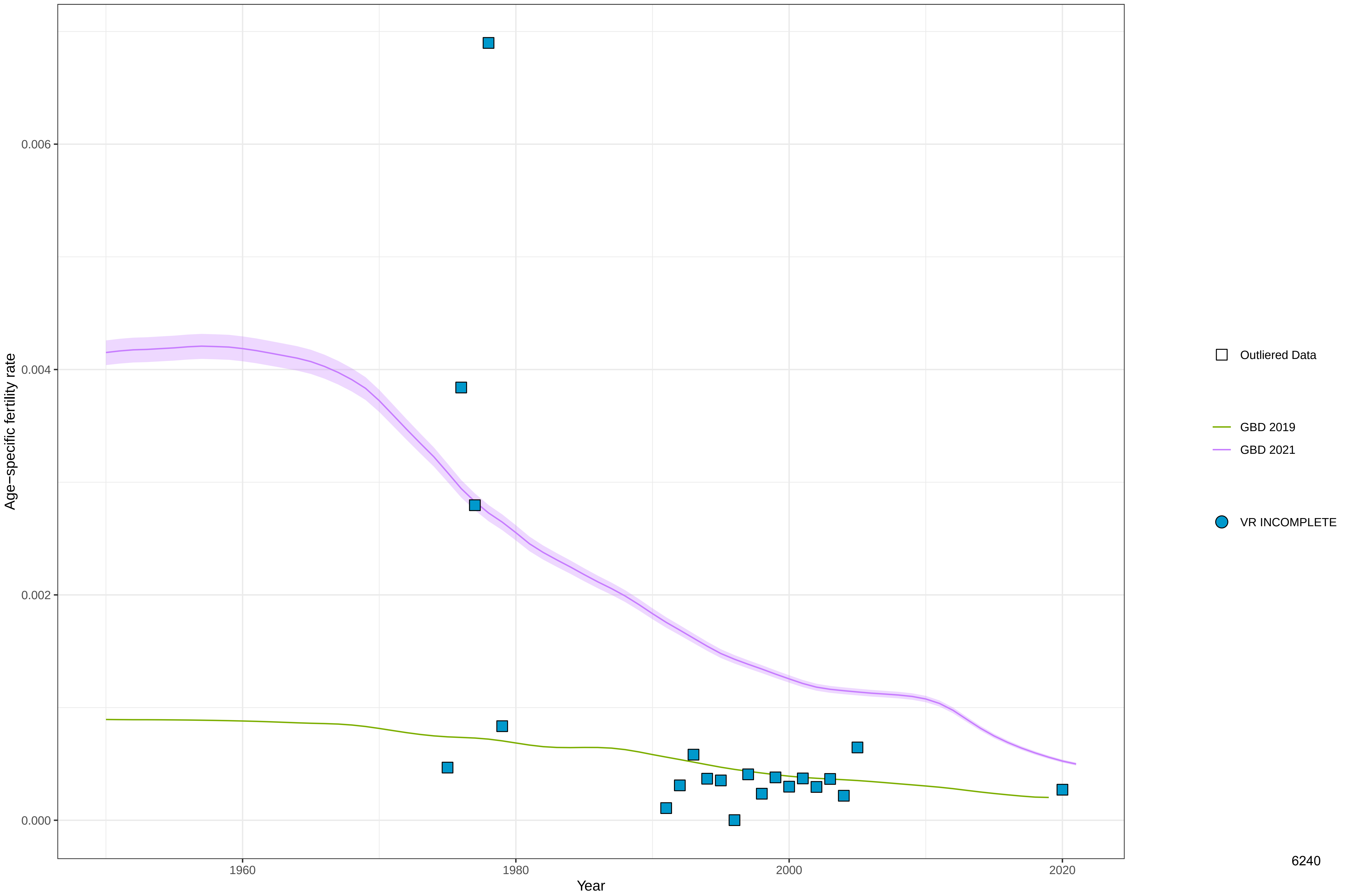

Total fertility rate fitted trend lines, 1950−2021: Maguindanao TFR
Data points enclosed with a diamond are reference sources

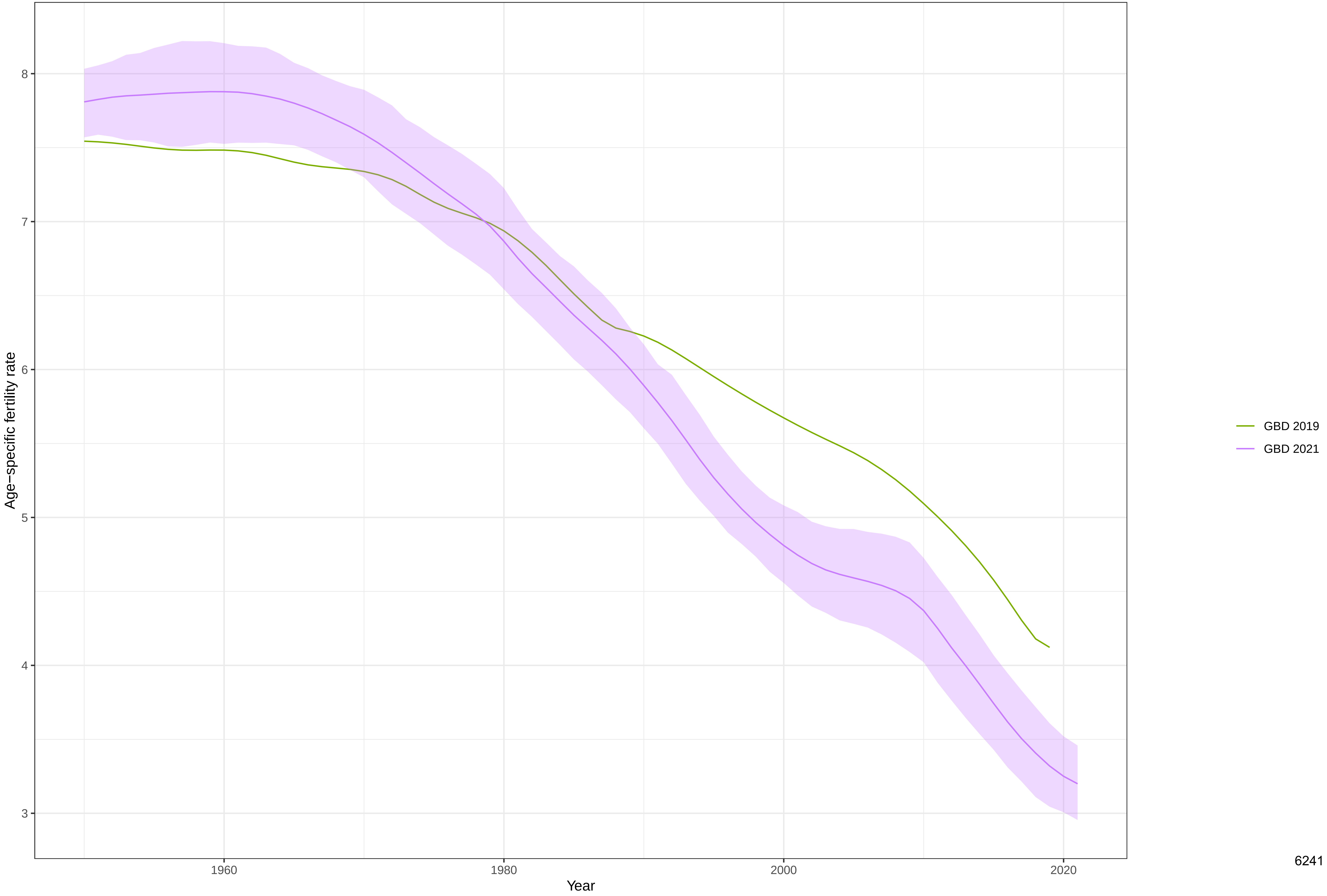

6241

Age−specific fertility fitted trend lines, 1950−2021: Marinduque, Ages: 10−14
Data points enclosed with a diamond are reference sources

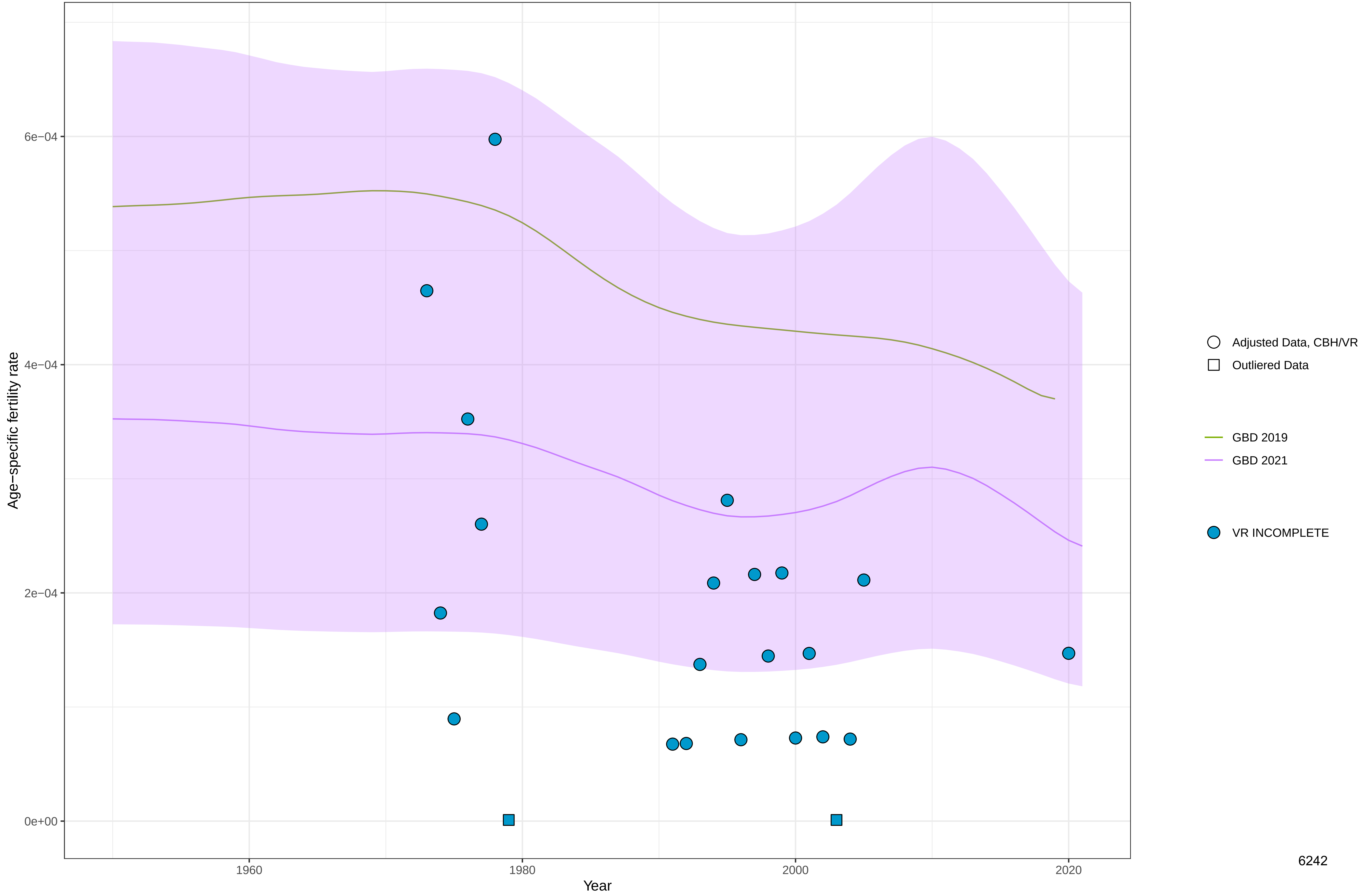

Age−specific fertility fitted trend lines, 1950−2021: Marinduque, Ages: 15−19
Data points enclosed with a diamond are reference sources

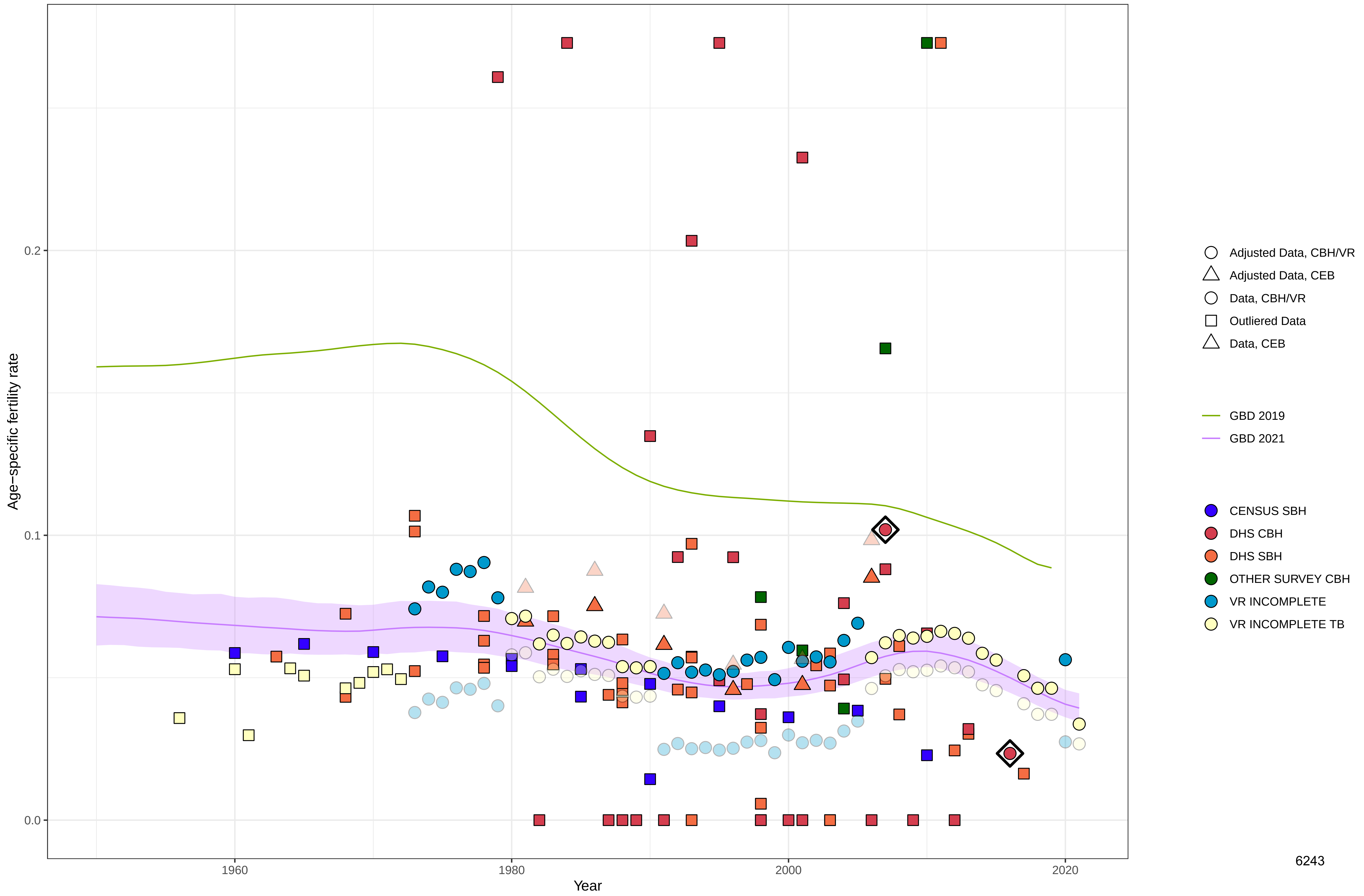

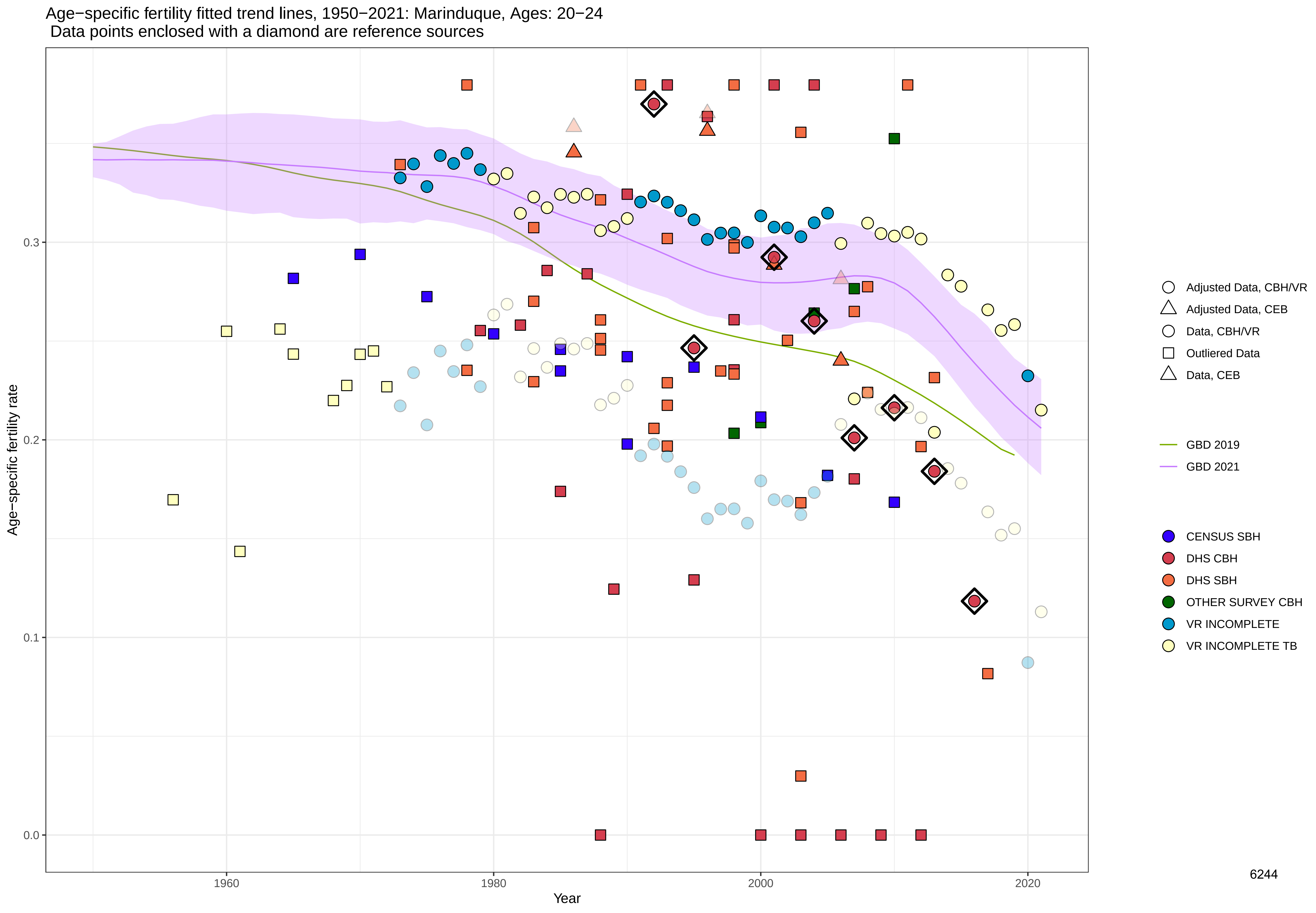

Age−specific fertility fitted trend lines, 1950−2021: Marinduque, Ages: 20−24
Data points enclosed with a diamond are reference sources
Age−specific fertility rate
Year
0.0
0.1
0.2
0.3
1960
1980
2000
2020
Adjusted Data, CBH/VR
Adjusted Data, CEB
Data, CBH/VR
Outliered Data
Data, CEB
GBD 2019
GBD 2021
CENSUS SBH
DHS CBH
DHS SBH
OTHER SURVEY CBH
VR INCOMPLETE
VR INCOMPLETE TB

Age−specific fertility fitted trend lines, 1950−2021: Marinduque, Ages: 25−29
Data points enclosed with a diamond are reference sources

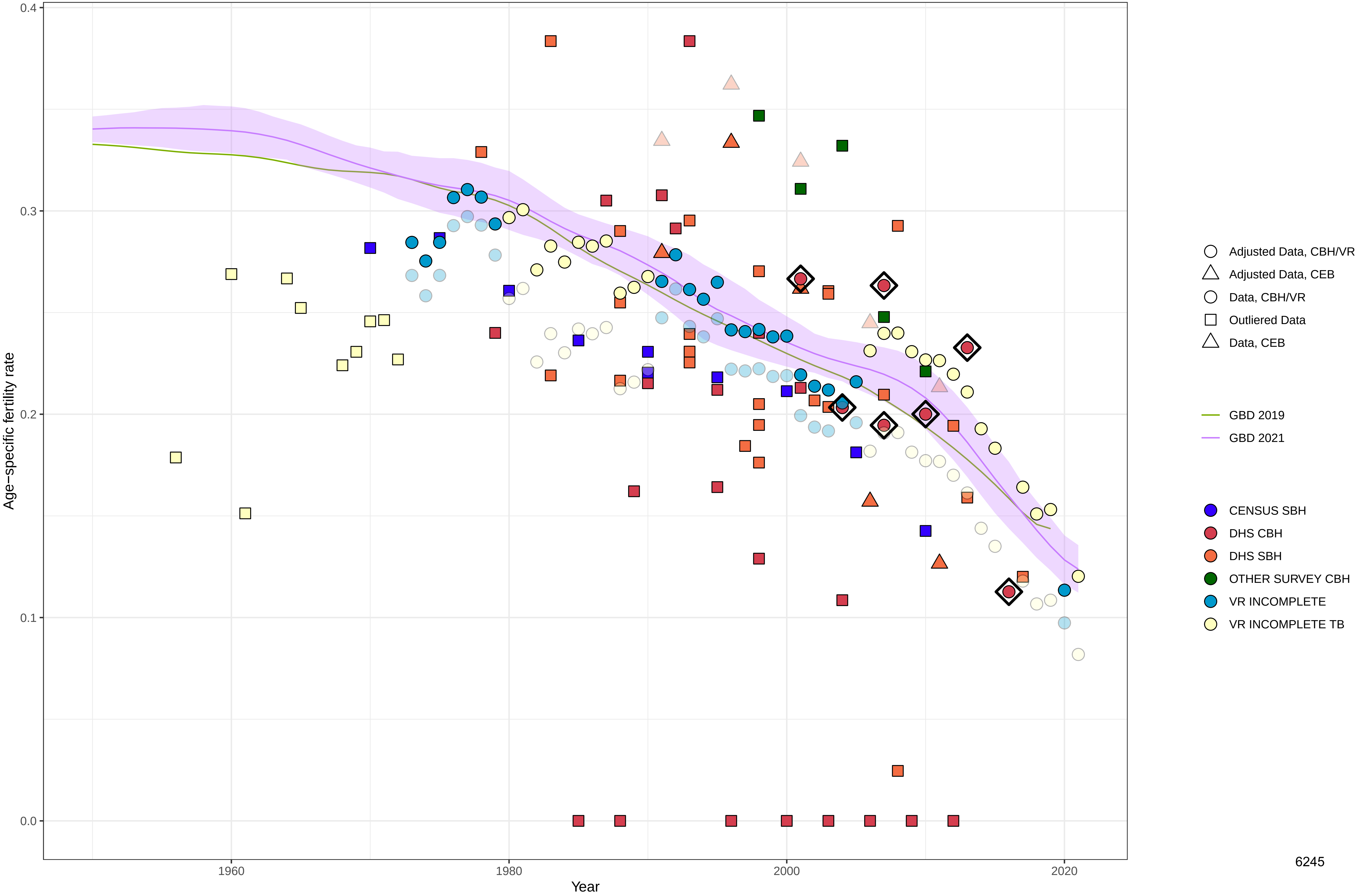

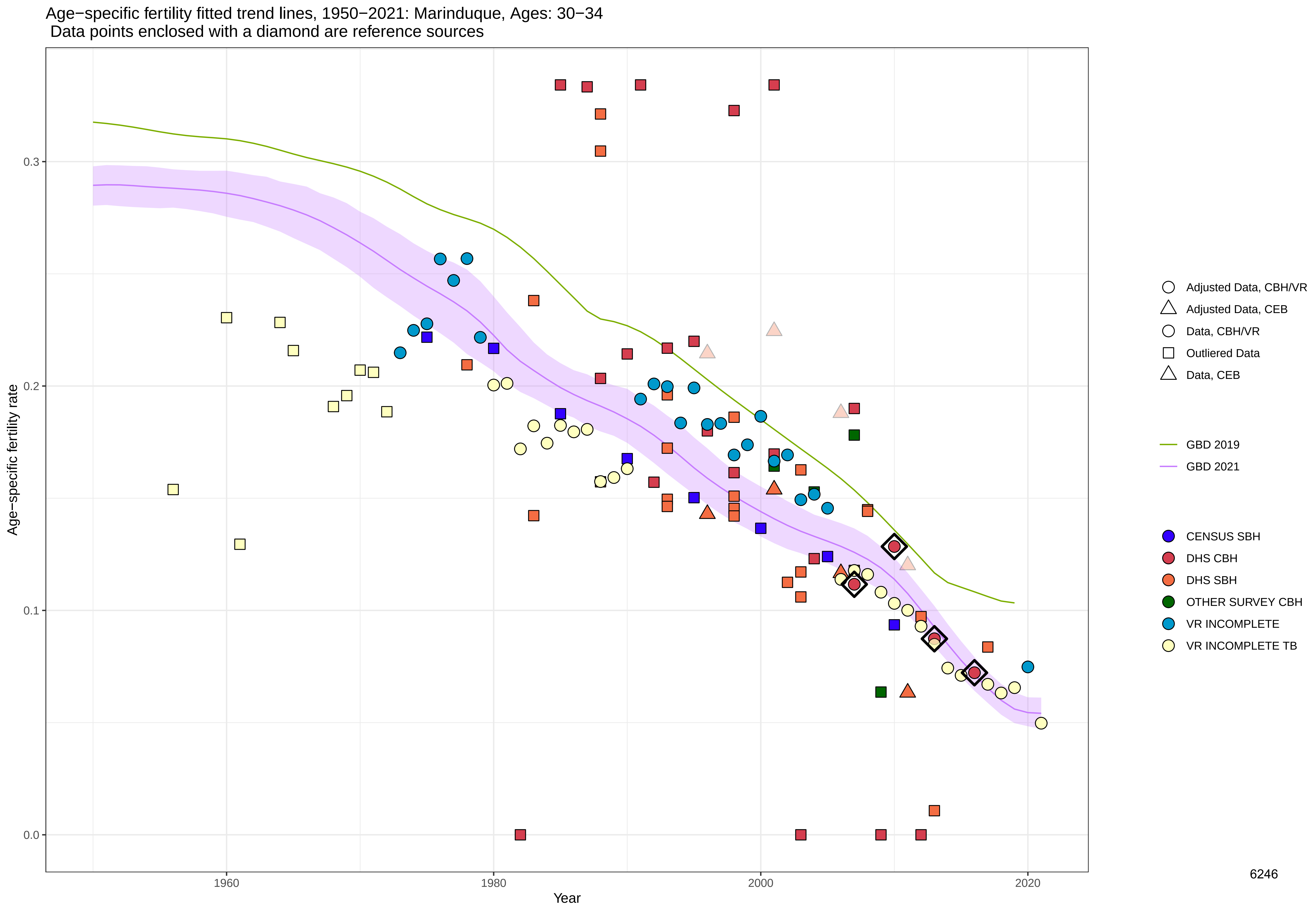

Age−specific fertility fitted trend lines, 1950−2021: Marinduque, Ages: 30−34
Data points enclosed with a diamond are reference sources
Age−specific fertility rate
0.0
0.1
0.2
0.3
1960
1980
2000
2020
Year
Adjusted Data, CBH/VR
Adjusted Data, CEB
Data, CBH/VR
Outliered Data
Data, CEB
GBD 2019
GBD 2021
CENSUS SBH
DHS CBH
DHS SBH
OTHER SURVEY CBH
VR INCOMPLETE
VR INCOMPLETE TB

Age−specific fertility fitted trend lines, 1950−2021: Marinduque, Ages: 35−39
Data points enclosed with a diamond are reference sources

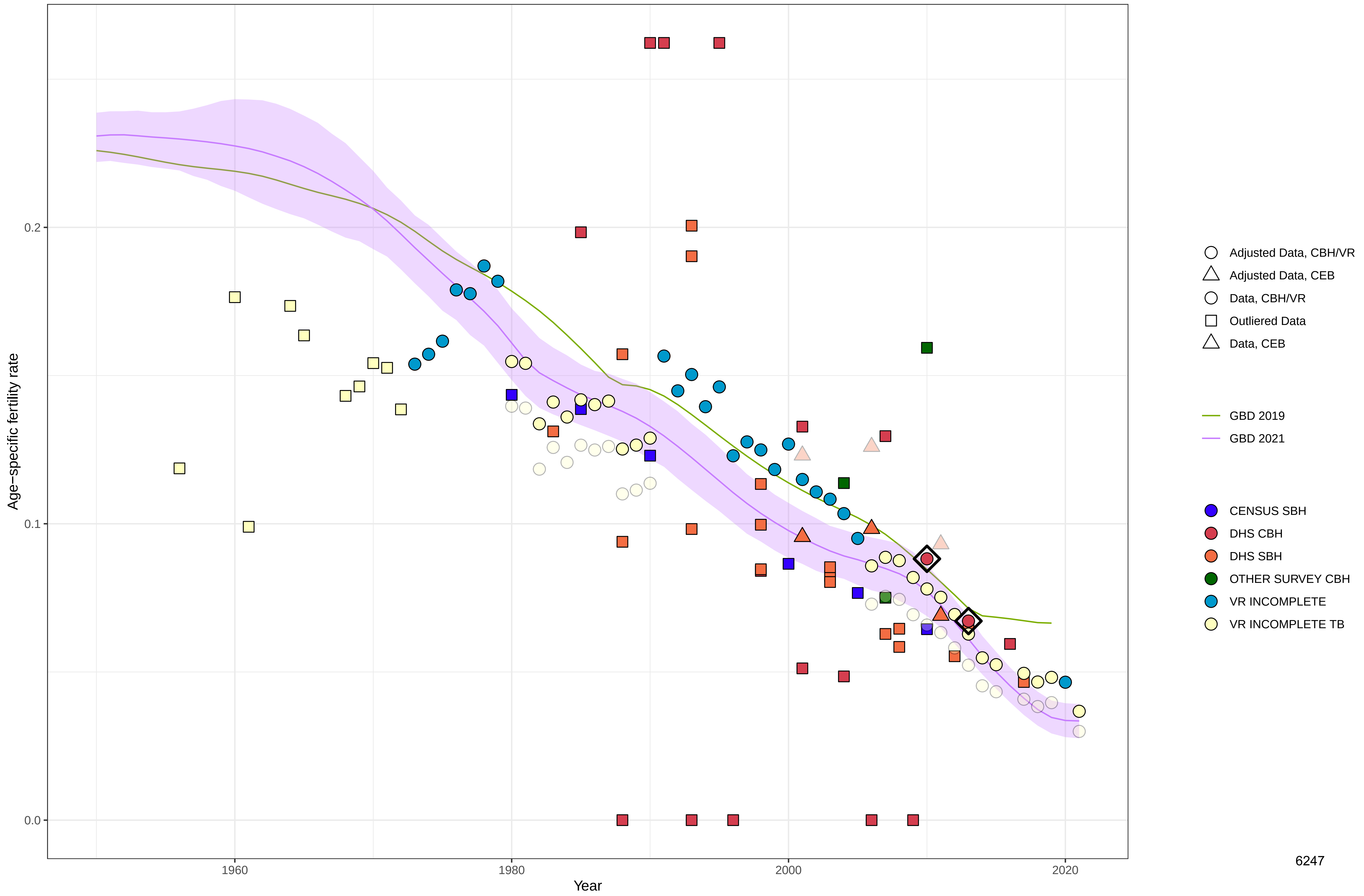

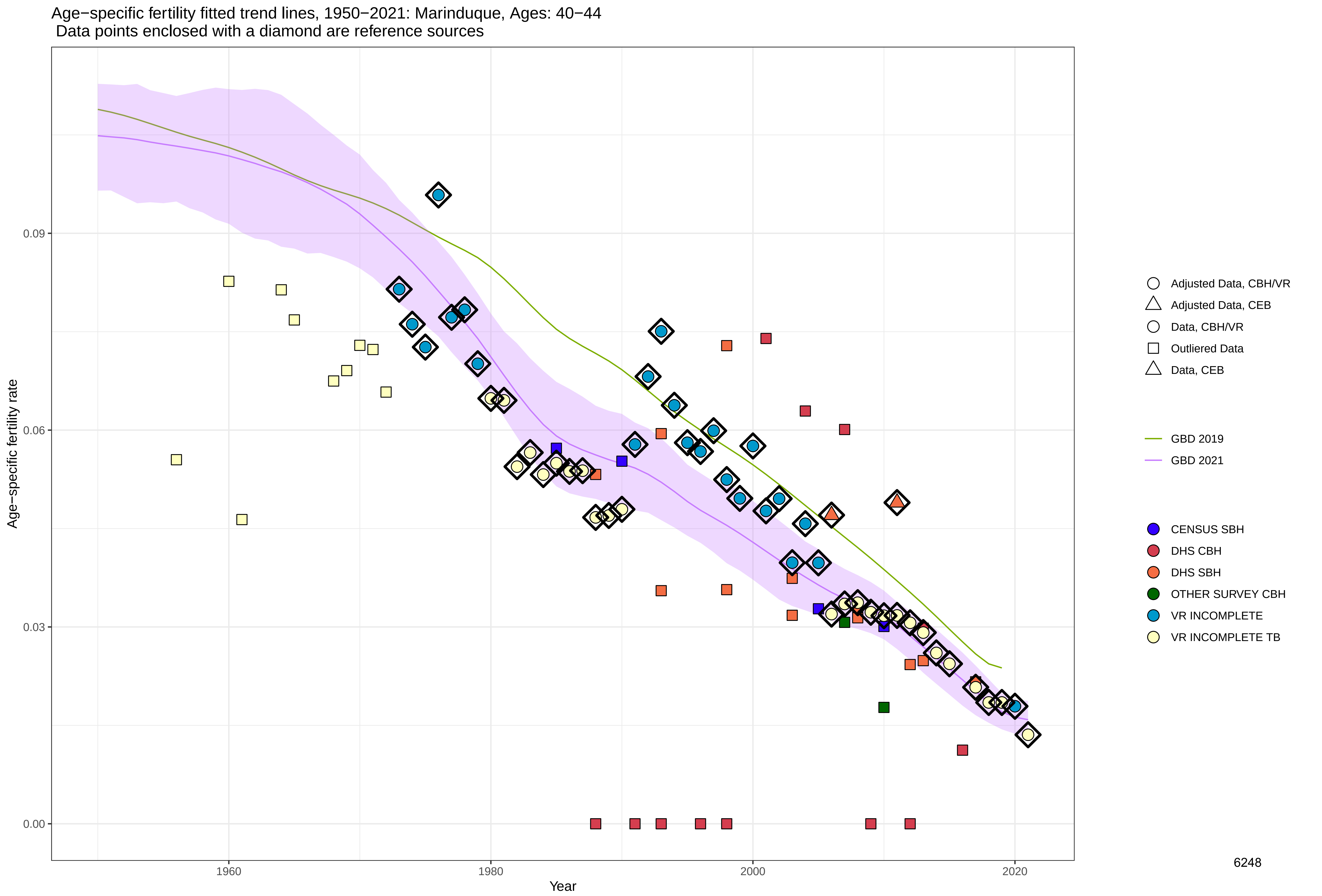

Age−specific fertility fitted trend lines, 1950−2021: Marinduque, Ages: 40−44
Data points enclosed with a diamond are reference sources
Age−specific fertility rate
Year
0.00
0.03
0.06
0.09
1960
1980
2000
2020
Adjusted Data, CBH/VR
Adjusted Data, CEB
Data, CBH/VR
Outliered Data
Data, CEB
GBD 2019
GBD 2021
CENSUS SBH
DHS CBH
DHS SBH
OTHER SURVEY CBH
VR INCOMPLETE
VR INCOMPLETE TB

Age−specific fertility fitted trend lines, 1950−2021: Marinduque, Ages: 45−49
Data points enclosed with a diamond are reference sources

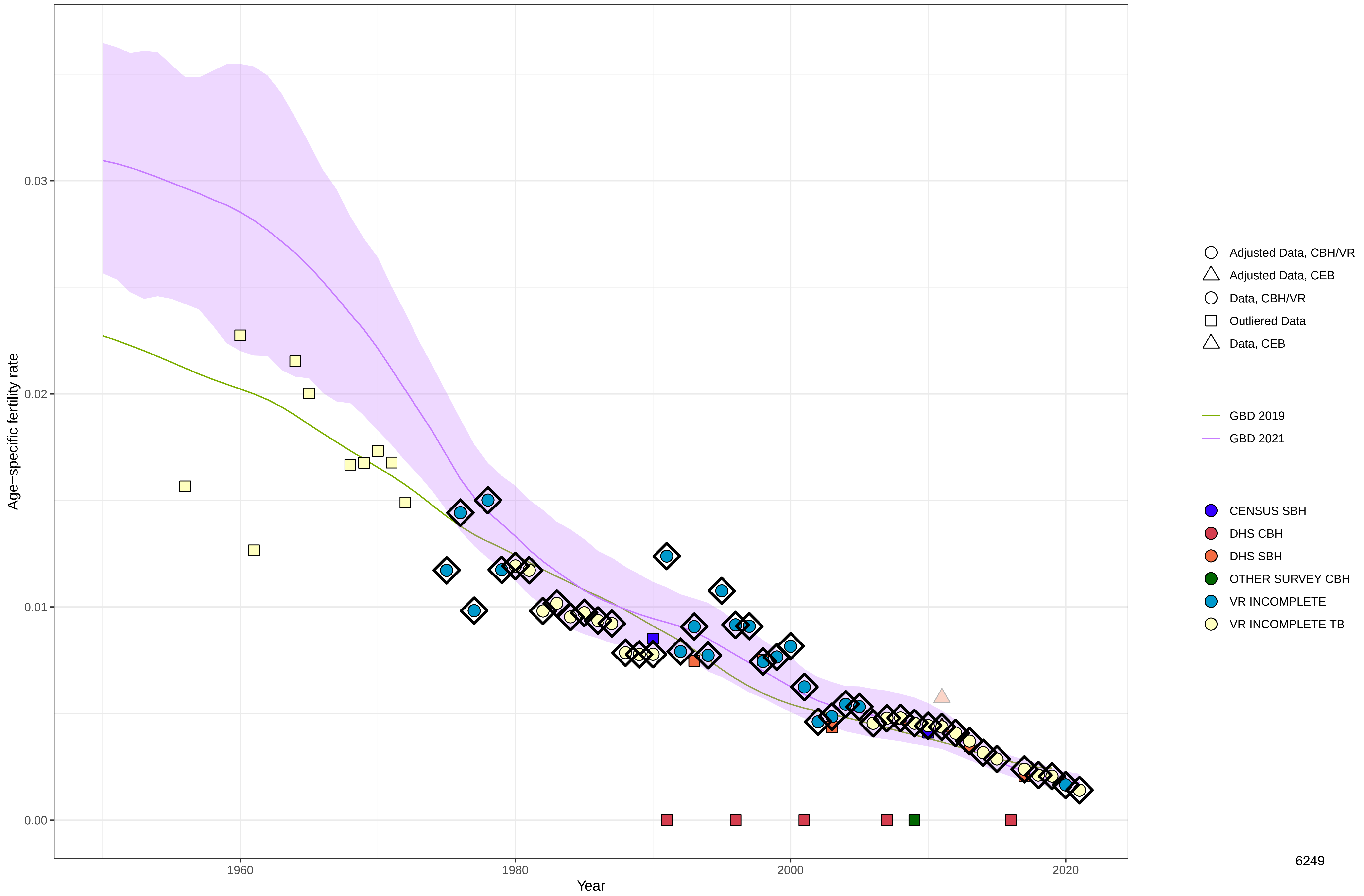

Age−specific fertility fitted trend lines, 1950−2021: Marinduque, Ages: 50−54
Data points enclosed with a diamond are reference sources

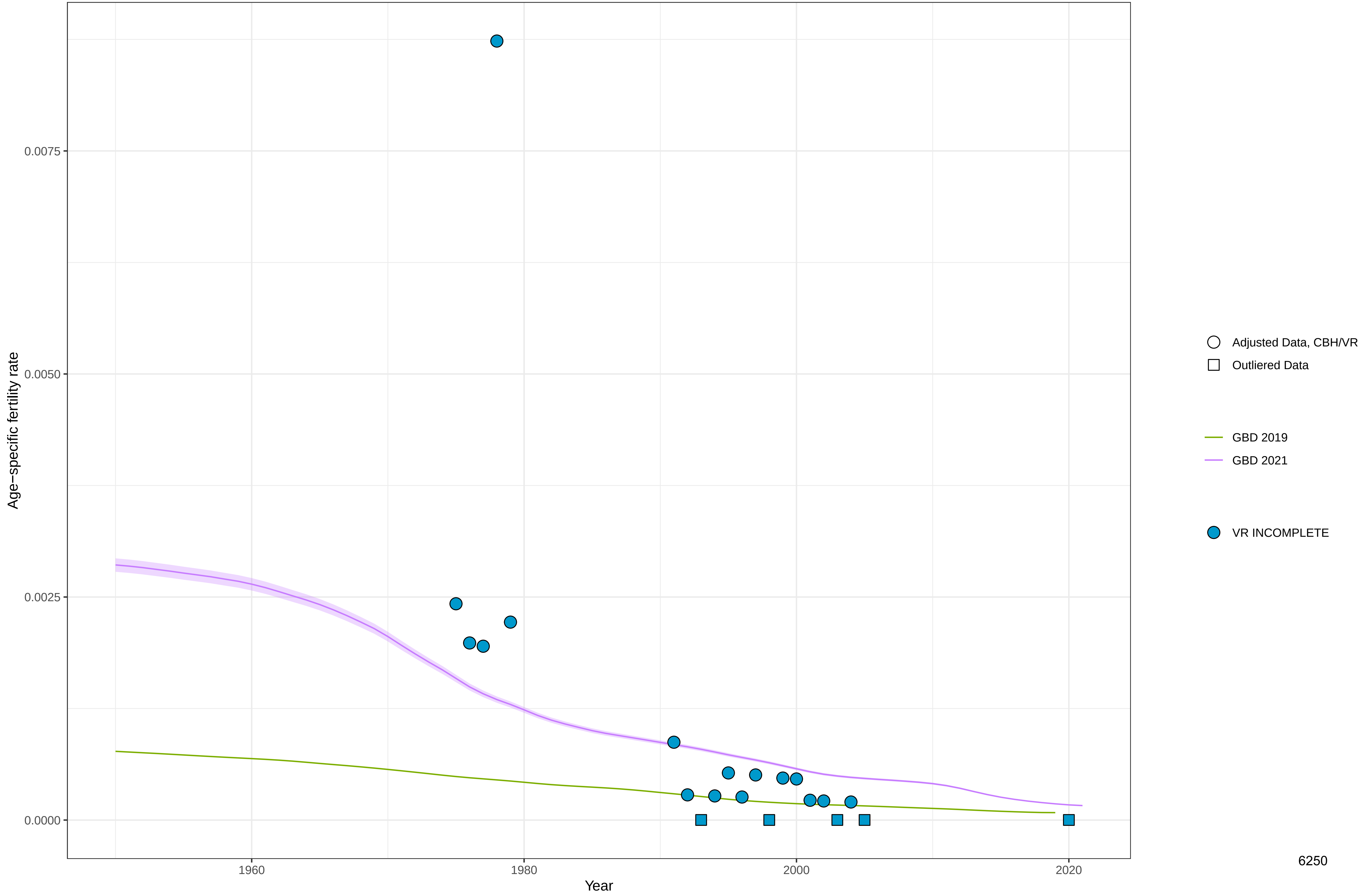

Total fertility rate fitted trend lines, 1950−2021: Marinduque TFR
Data points enclosed with a diamond are reference sources

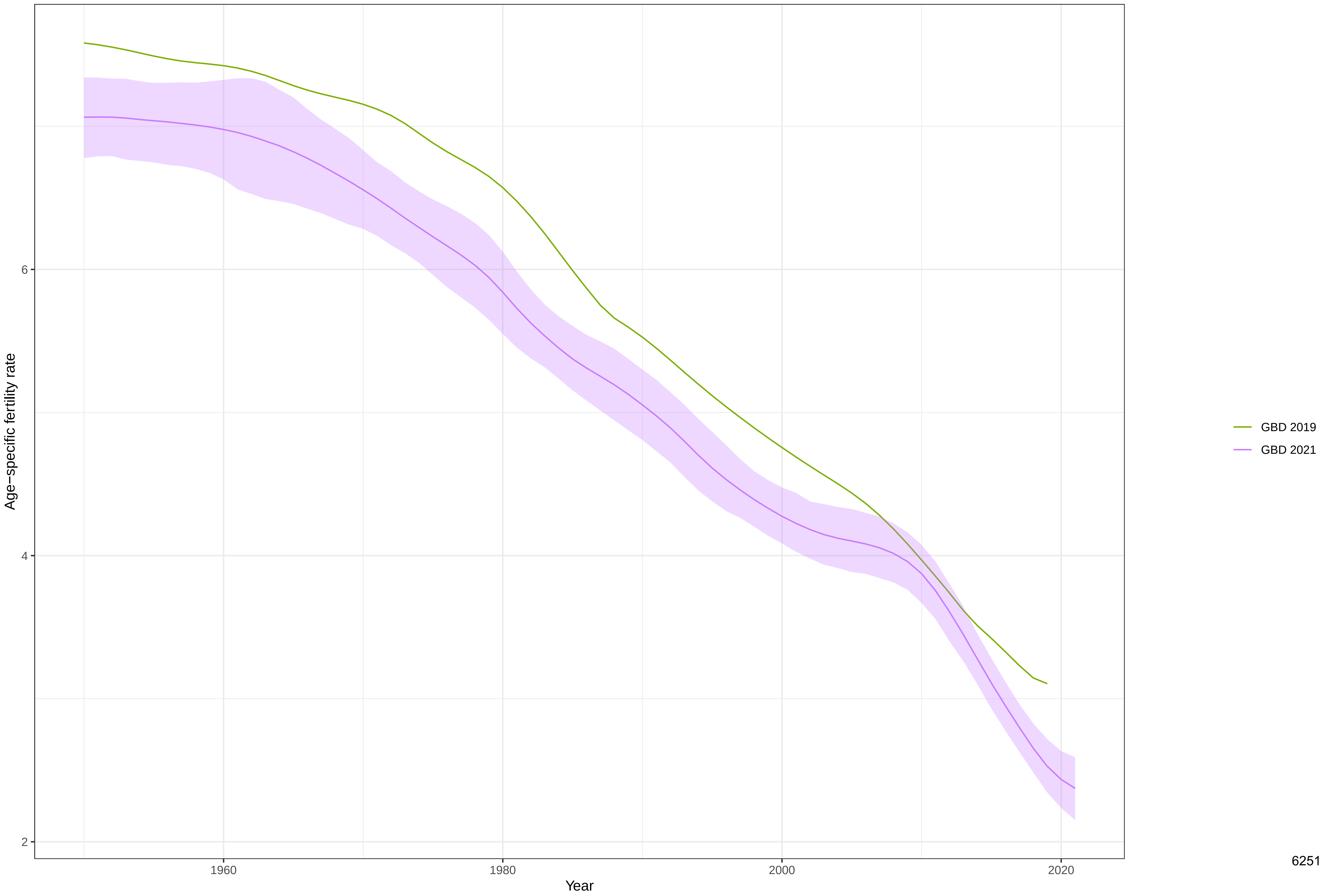

Age−specific fertility fitted trend lines, 1950−2021: Masbate, Ages: 10−14
Data points enclosed with a diamond are reference sources

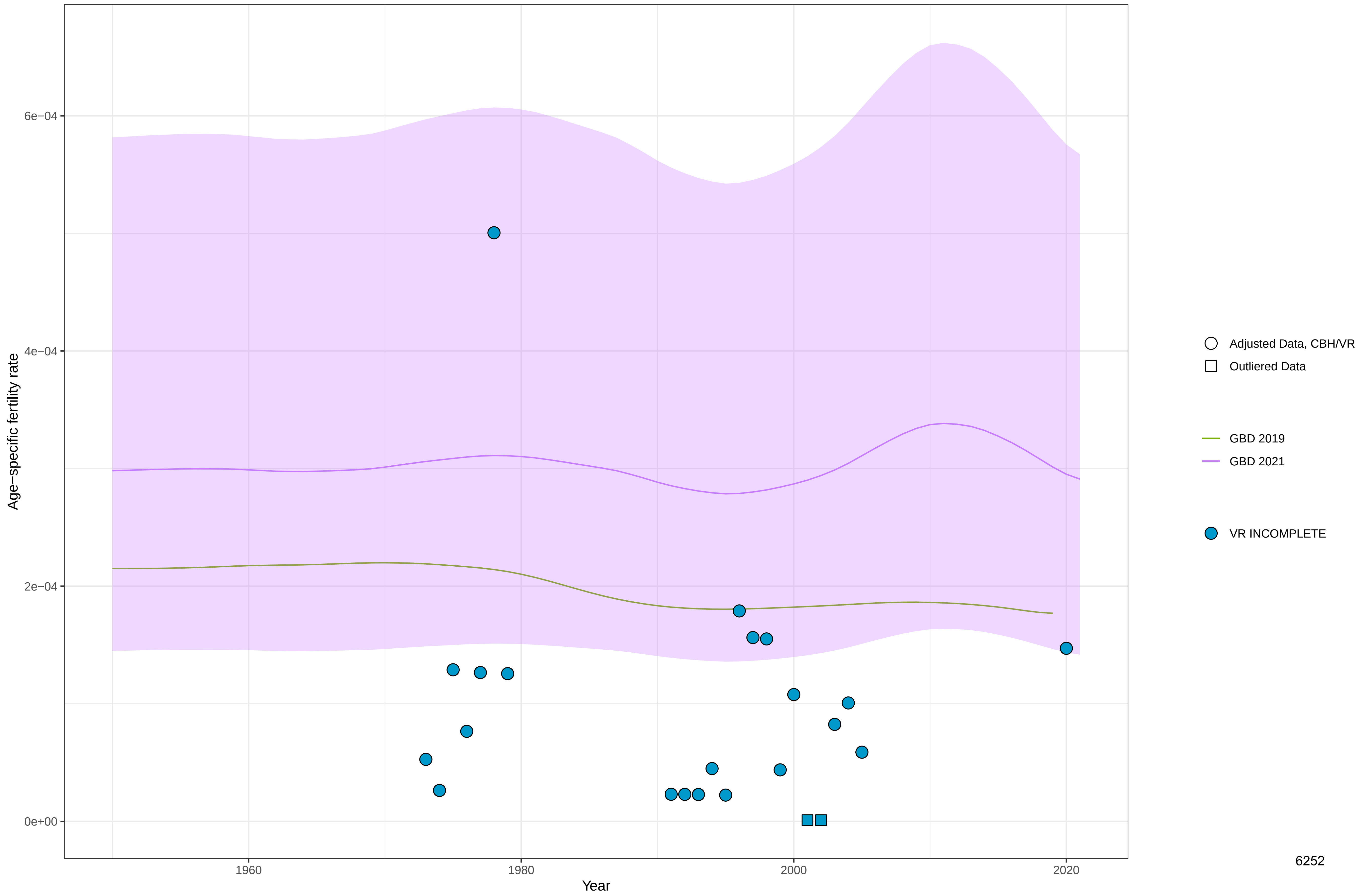

Age−specific fertility fitted trend lines, 1950−2021: Masbate, Ages: 15−19
Data points enclosed with a diamond are reference sources

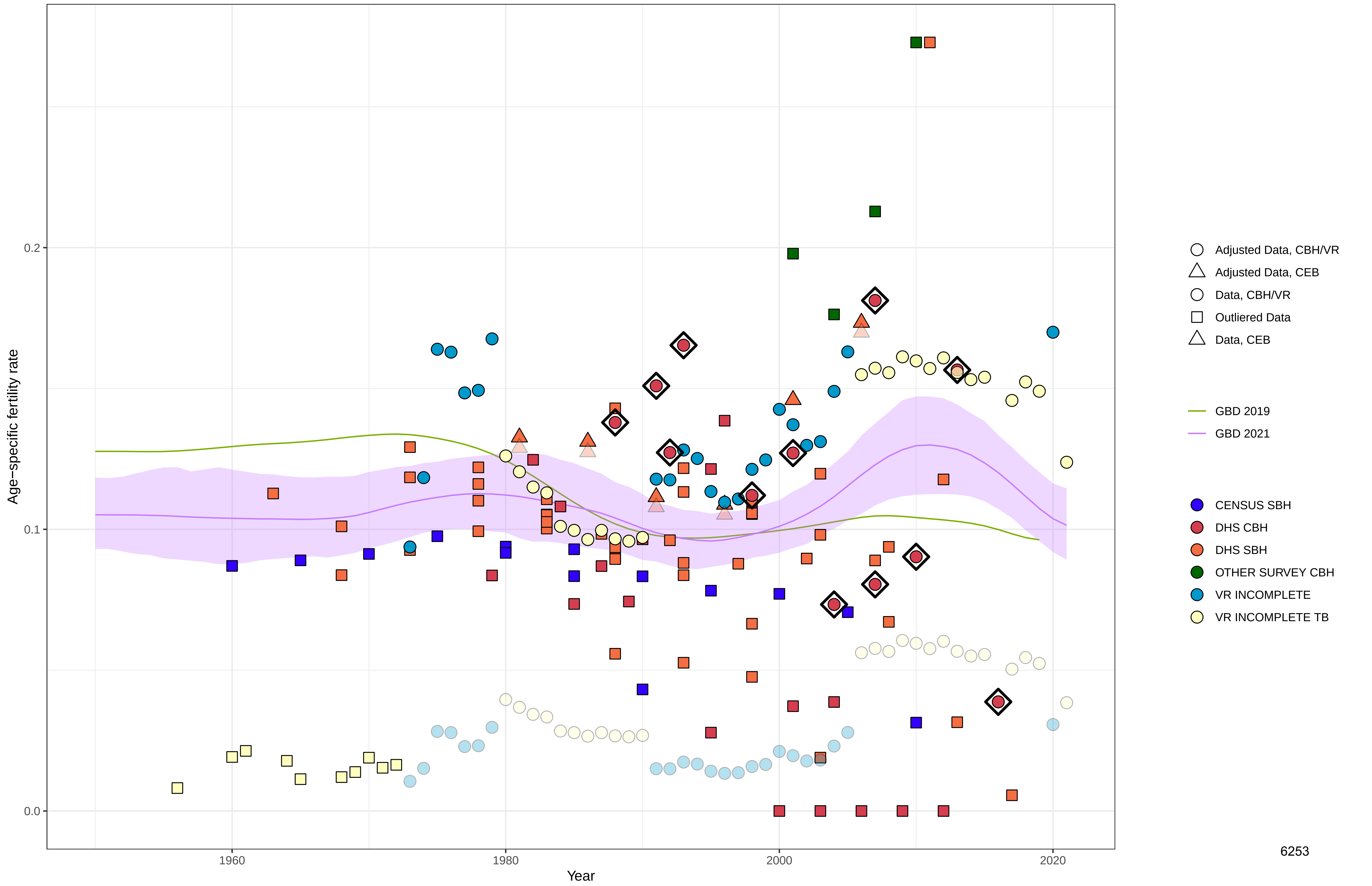

Age−specific fertility fitted trend lines, 1950−2021: Masbate, Ages: 20−24
Data points enclosed with a diamond are reference sources

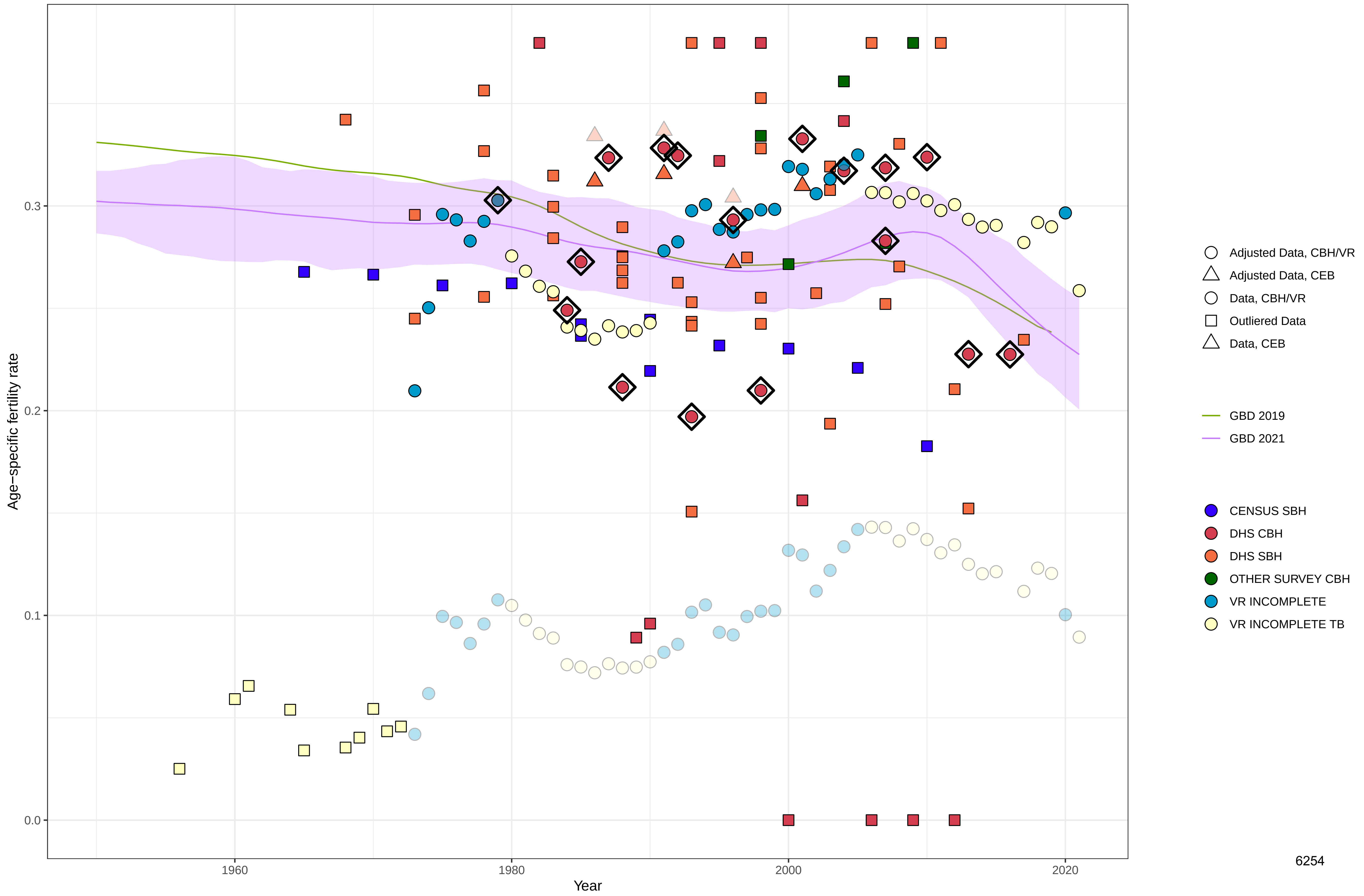

Age−specific fertility fitted trend lines, 1950−2021: Masbate, Ages: 25−29
Data points enclosed with a diamond are reference sources

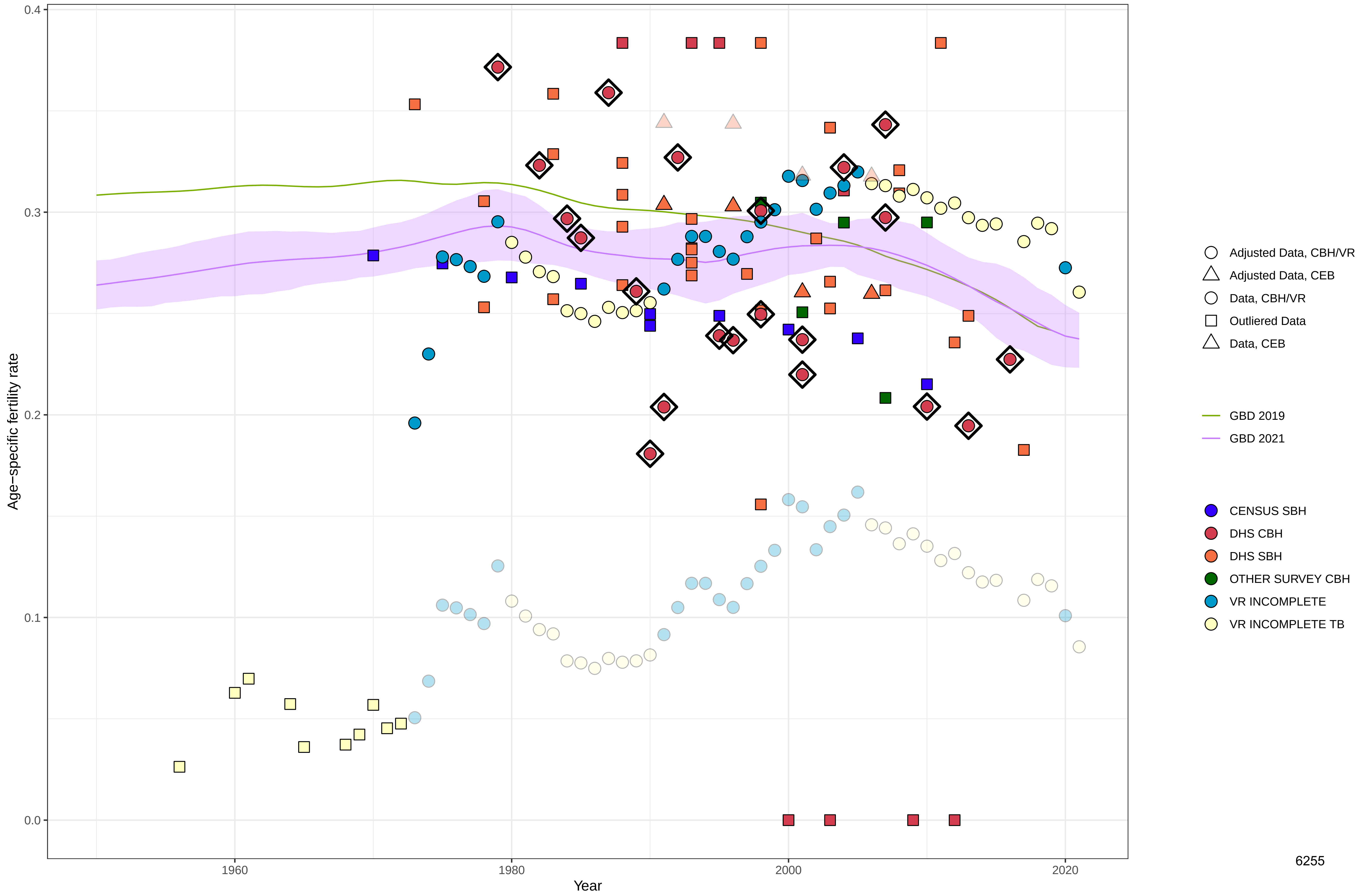

Age−specific fertility fitted trend lines, 1950−2021: Masbate, Ages: 30−34
Data points enclosed with a diamond are reference sources

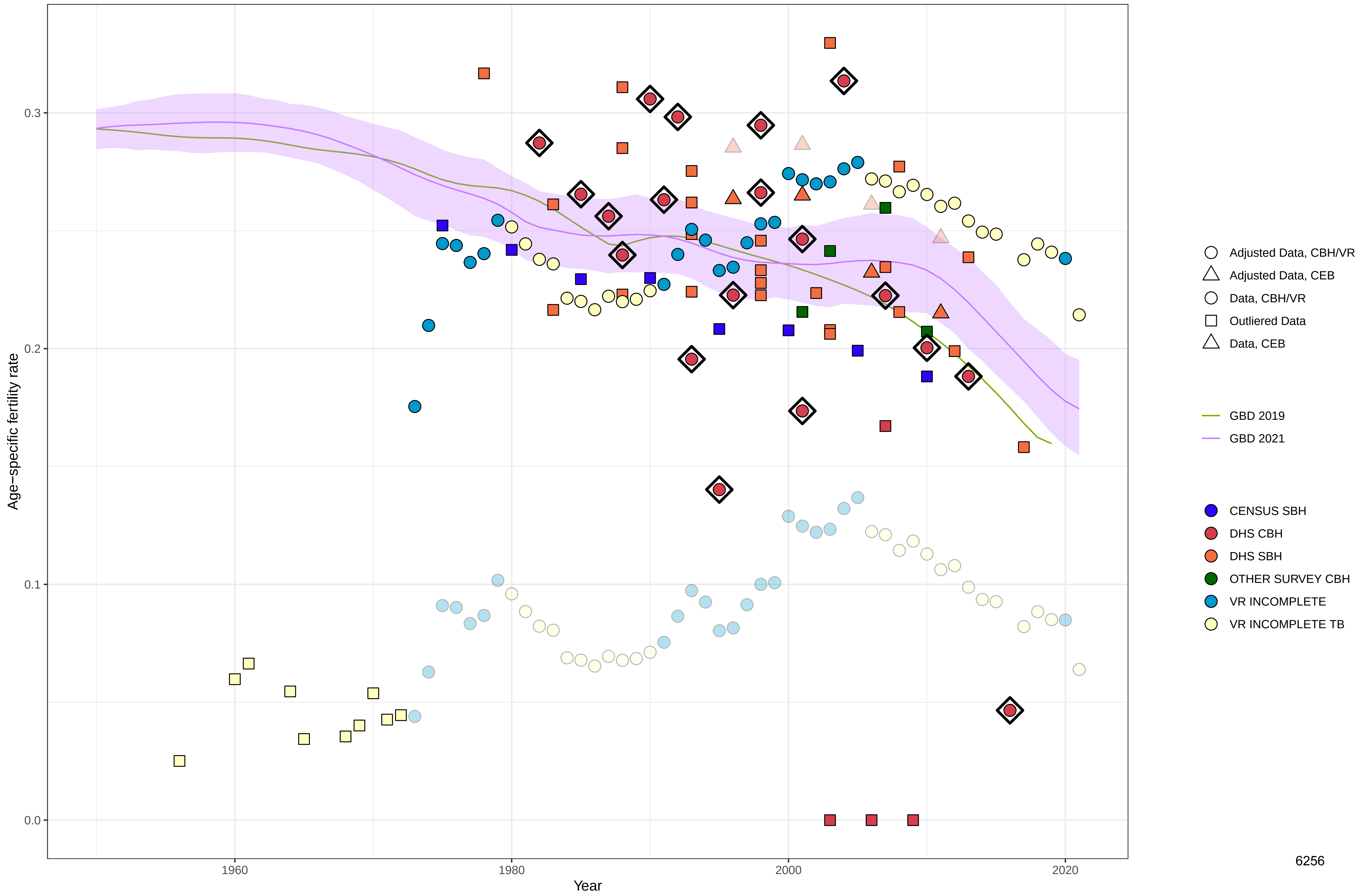

Age−specific fertility fitted trend lines, 1950−2021: Masbate, Ages: 35−39
Data points enclosed with a diamond are reference sources

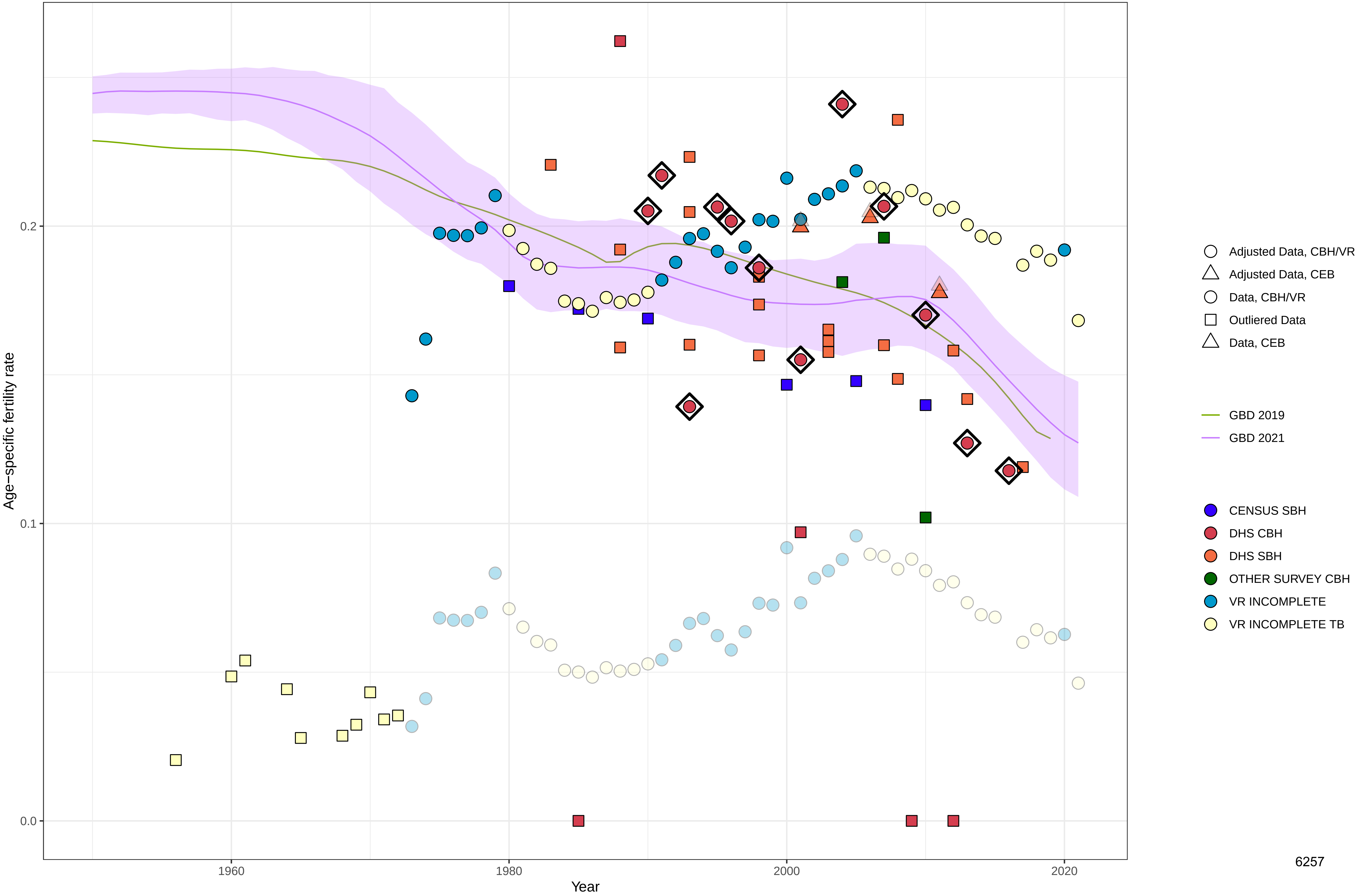

Age−specific fertility fitted trend lines, 1950−2021: Masbate, Ages: 40−44
Data points enclosed with a diamond are reference sources

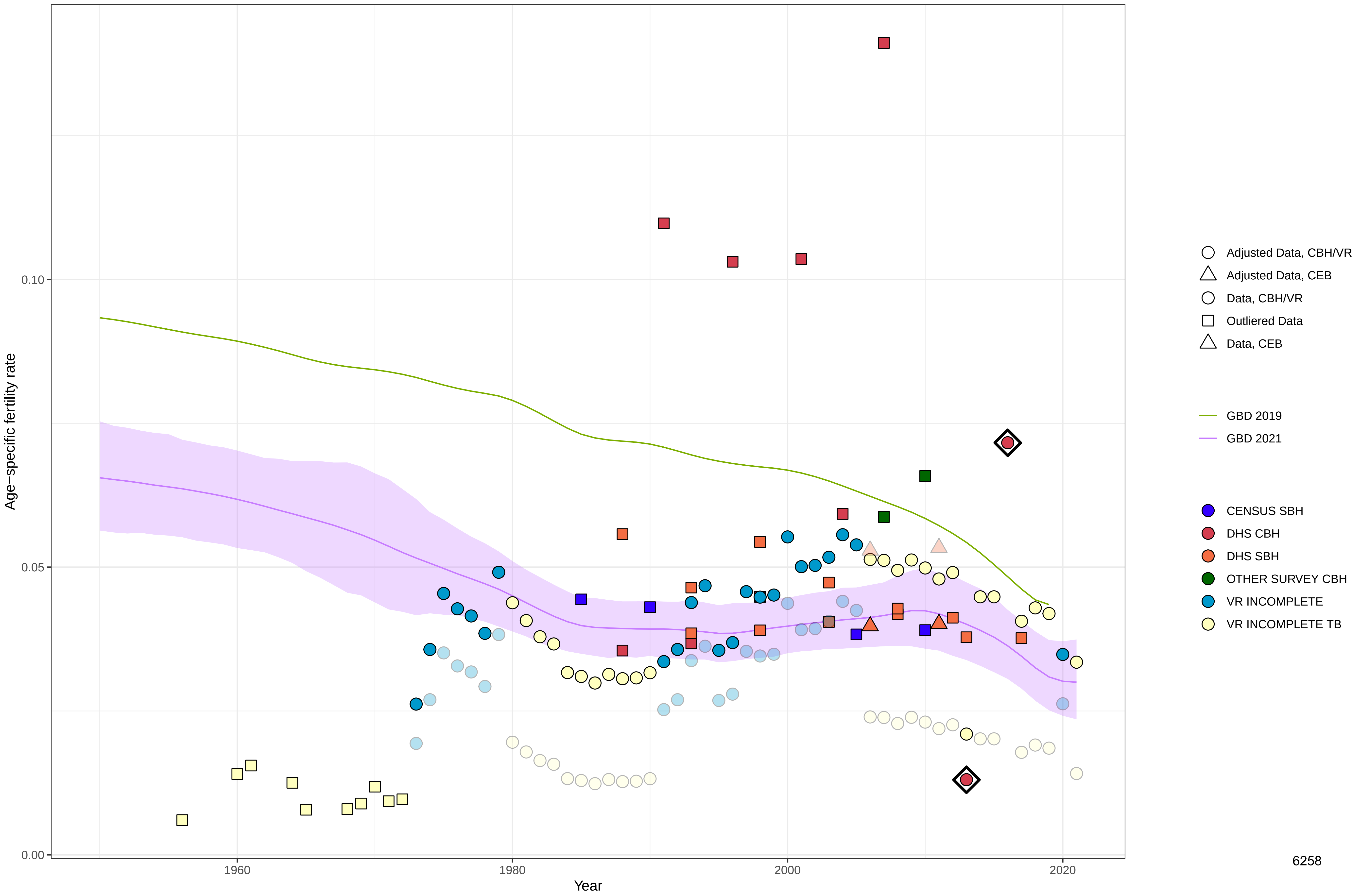

Age−specific fertility fitted trend lines, 1950−2021: Masbate, Ages: 45−49
Data points enclosed with a diamond are reference sources

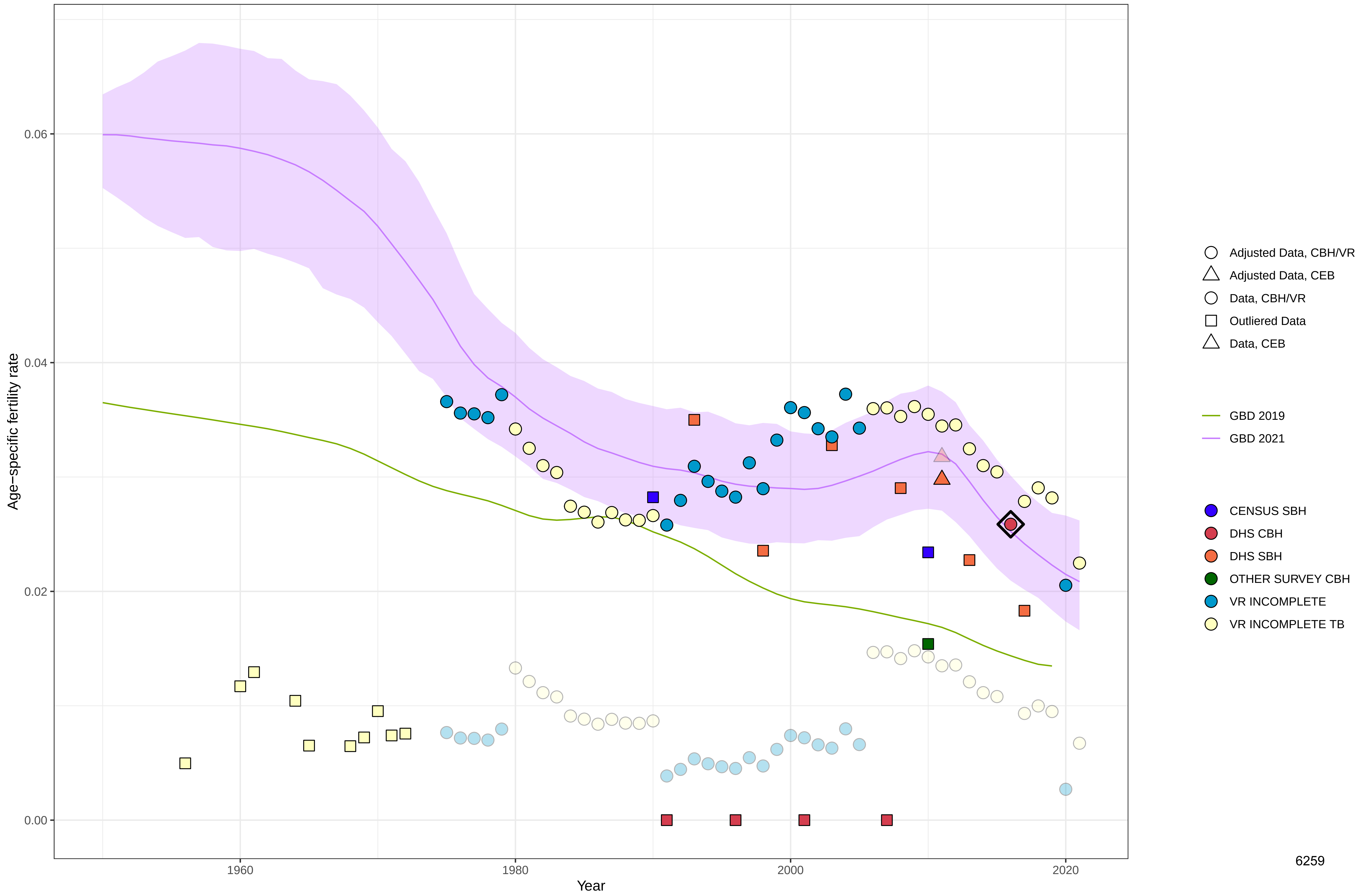

Age−specific fertility fitted trend lines, 1950−2021: Masbate, Ages: 50−54
Data points enclosed with a diamond are reference sources

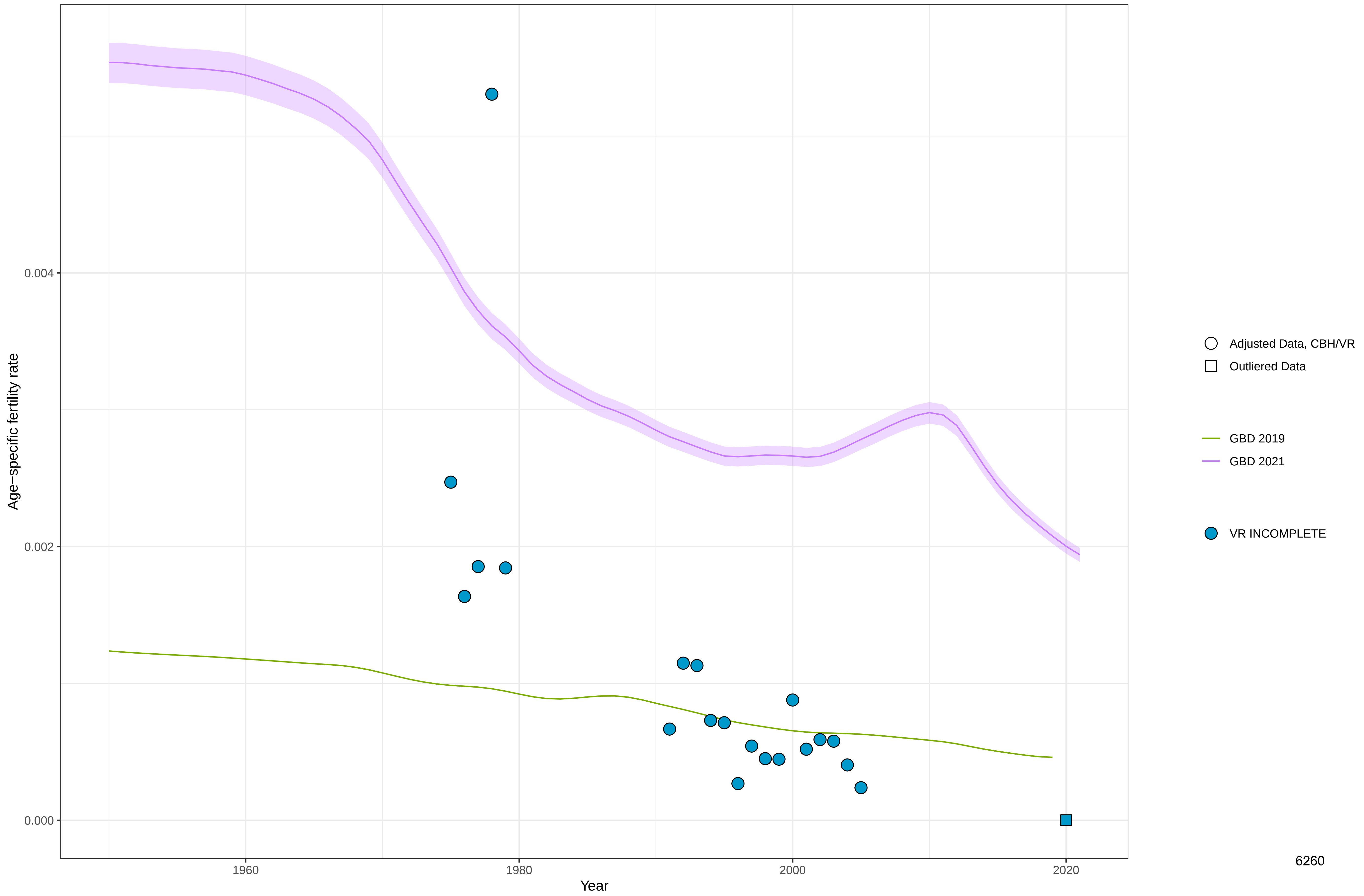

6260

Total fertility rate fitted trend lines, 1950−2021: Masbate TFR
Data points enclosed with a diamond are reference sources

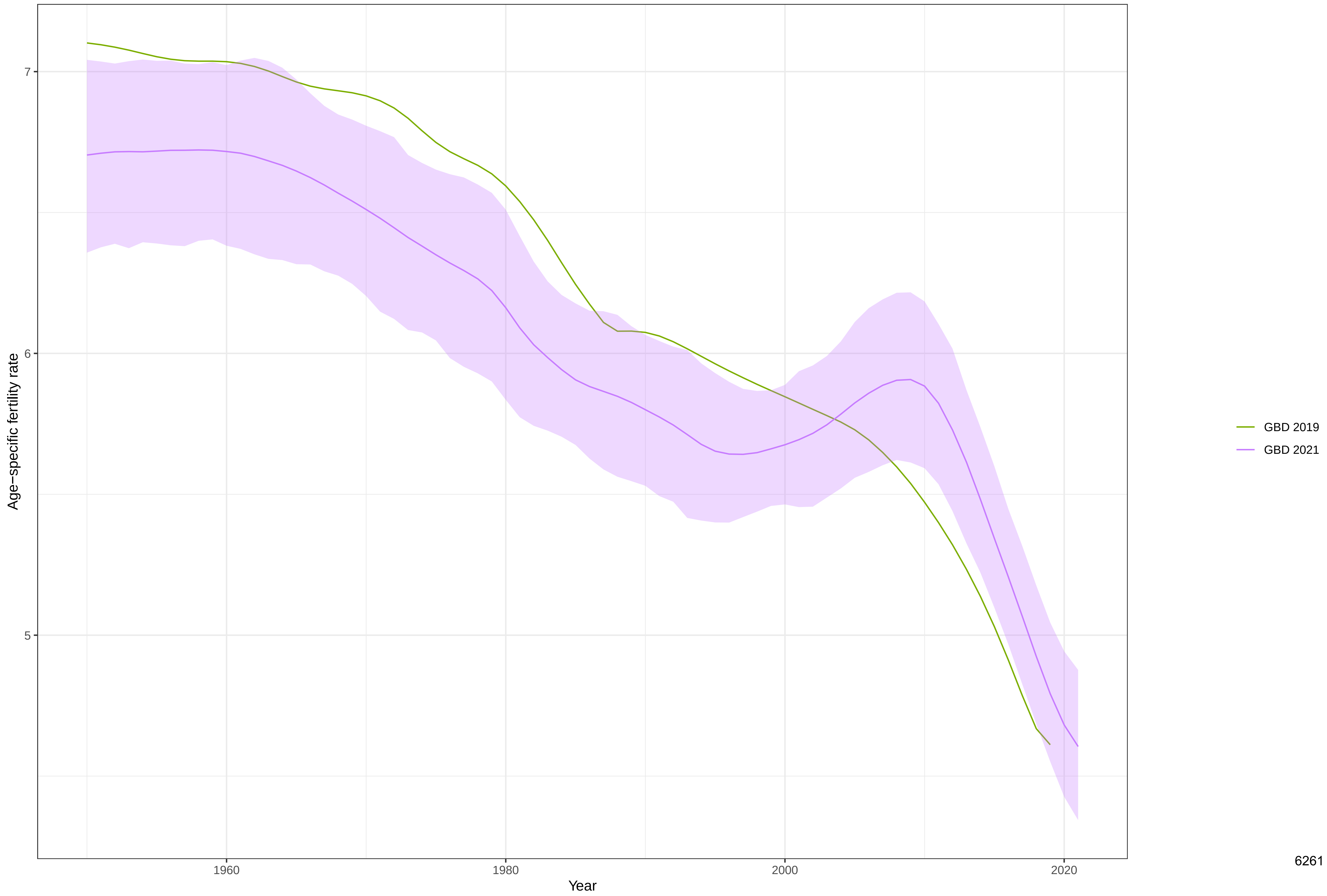

6261

Age−specific fertility fitted trend lines, 1950−2021: Misamis Occidental, Ages: 10−14
Data points enclosed with a diamond are reference sources

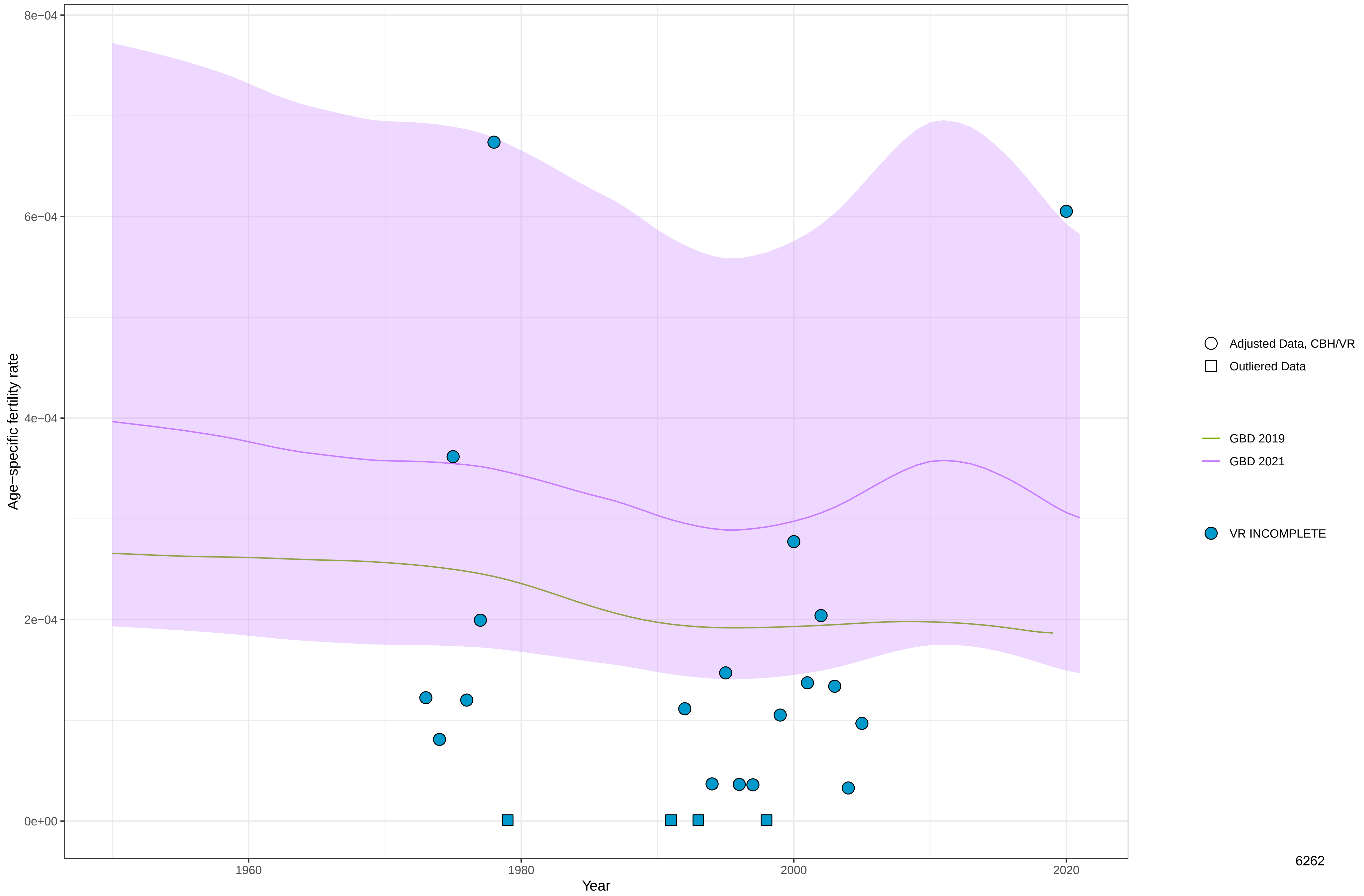

Age−specific fertility fitted trend lines, 1950−2021: Misamis Occidental, Ages: 15−19
Data points enclosed with a diamond are reference sources

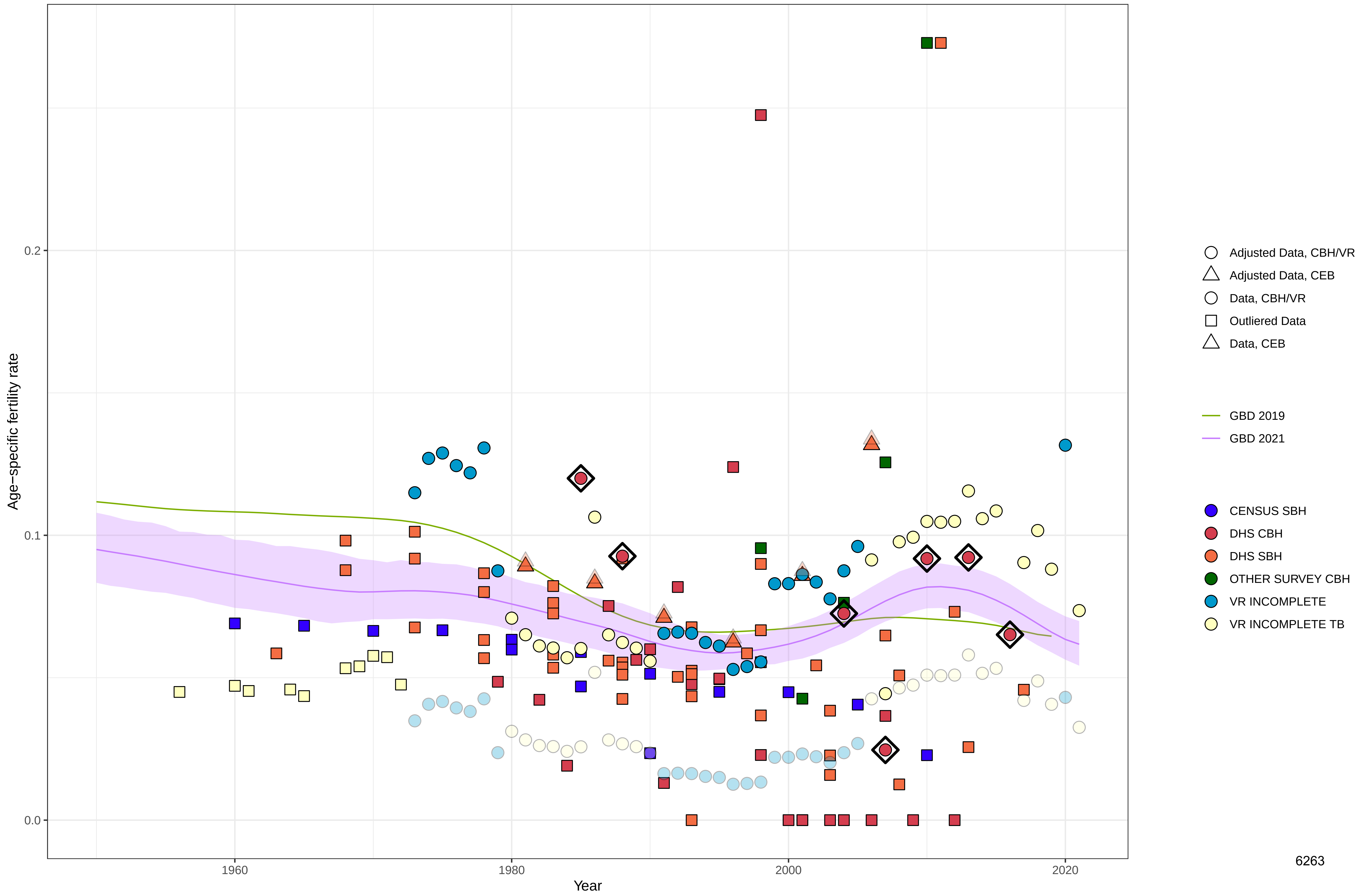

Age−specific fertility fitted trend lines, 1950−2021: Misamis Occidental, Ages: 20−24
Data points enclosed with a diamond are reference sources

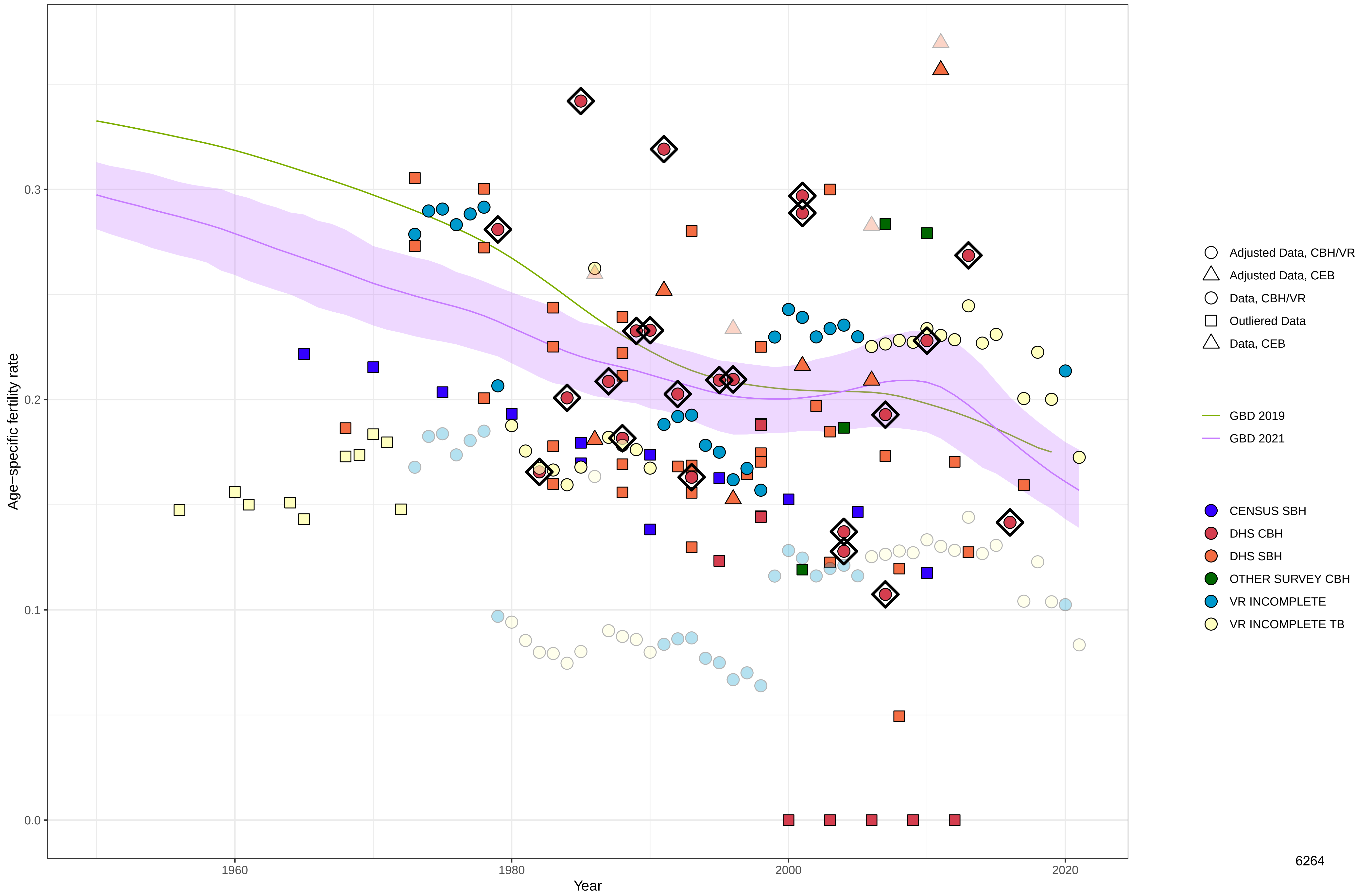

Age−specific fertility fitted trend lines, 1950−2021: Misamis Occidental, Ages: 25−29
Data points enclosed with a diamond are reference sources

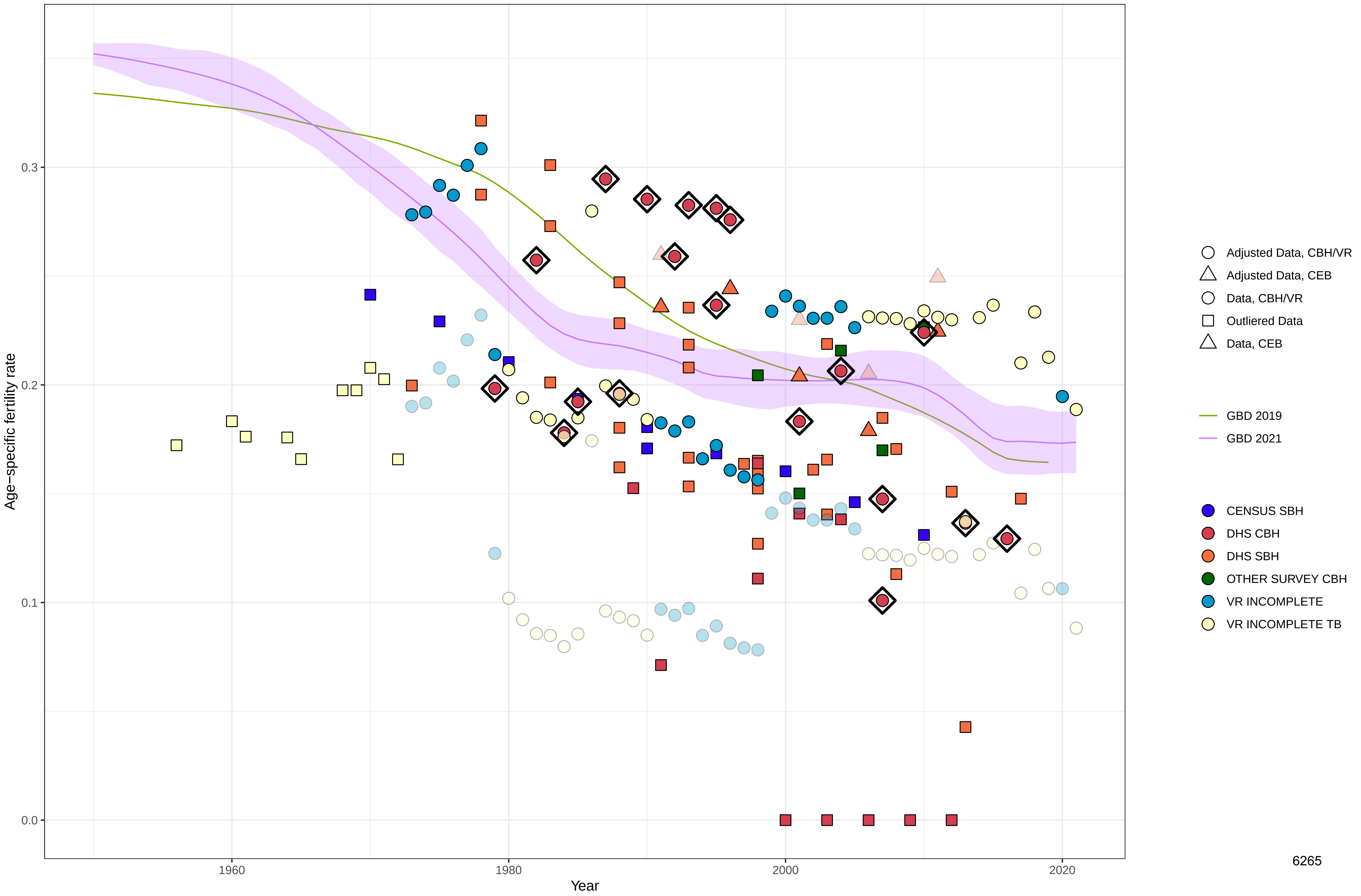

Age−specific fertility fitted trend lines, 1950−2021: Misamis Occidental, Ages: 30−34
Data points enclosed with a diamond are reference sources

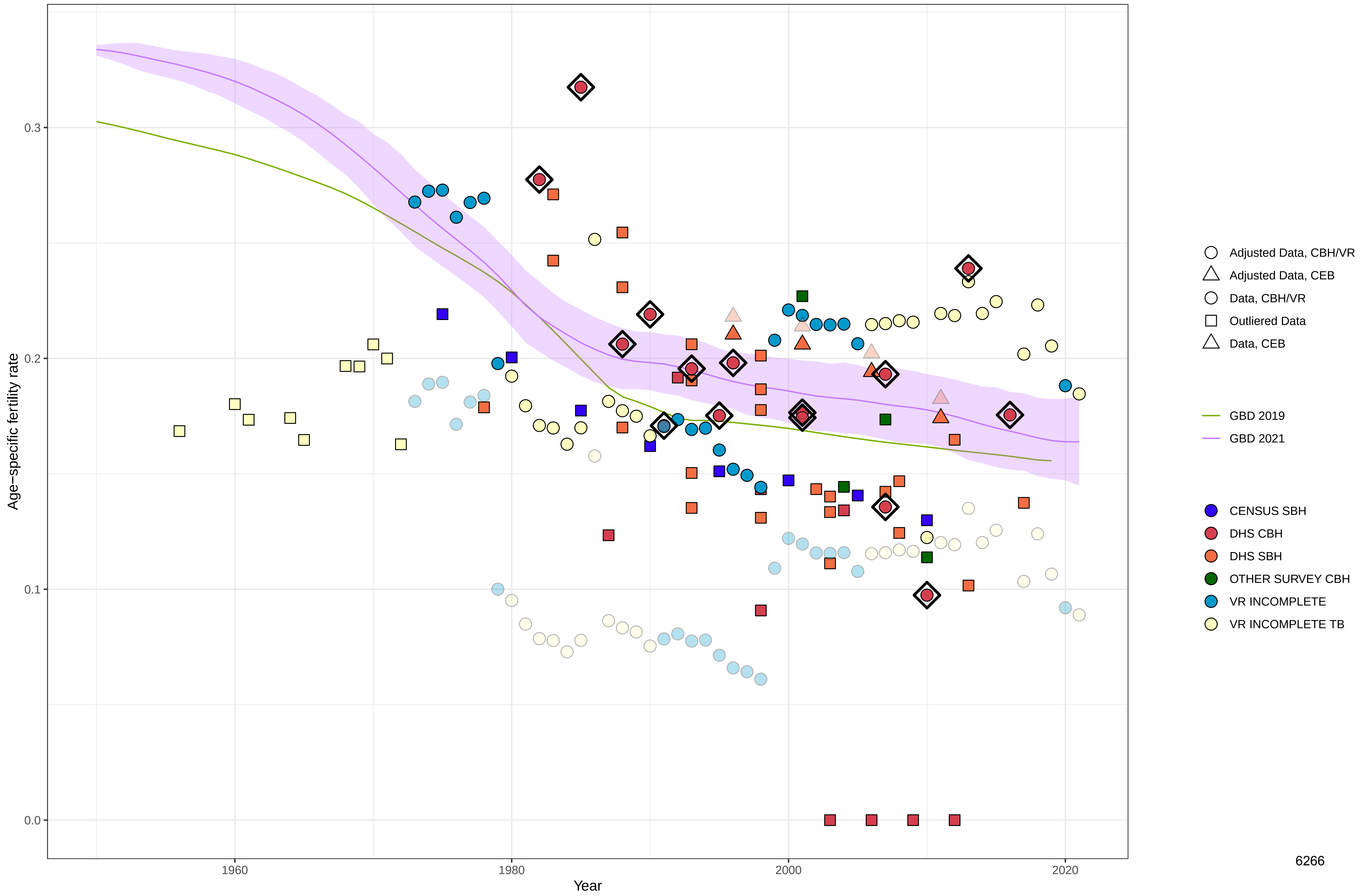

Age−specific fertility fitted trend lines, 1950−2021: Misamis Occidental, Ages: 35−39
Data points enclosed with a diamond are reference sources

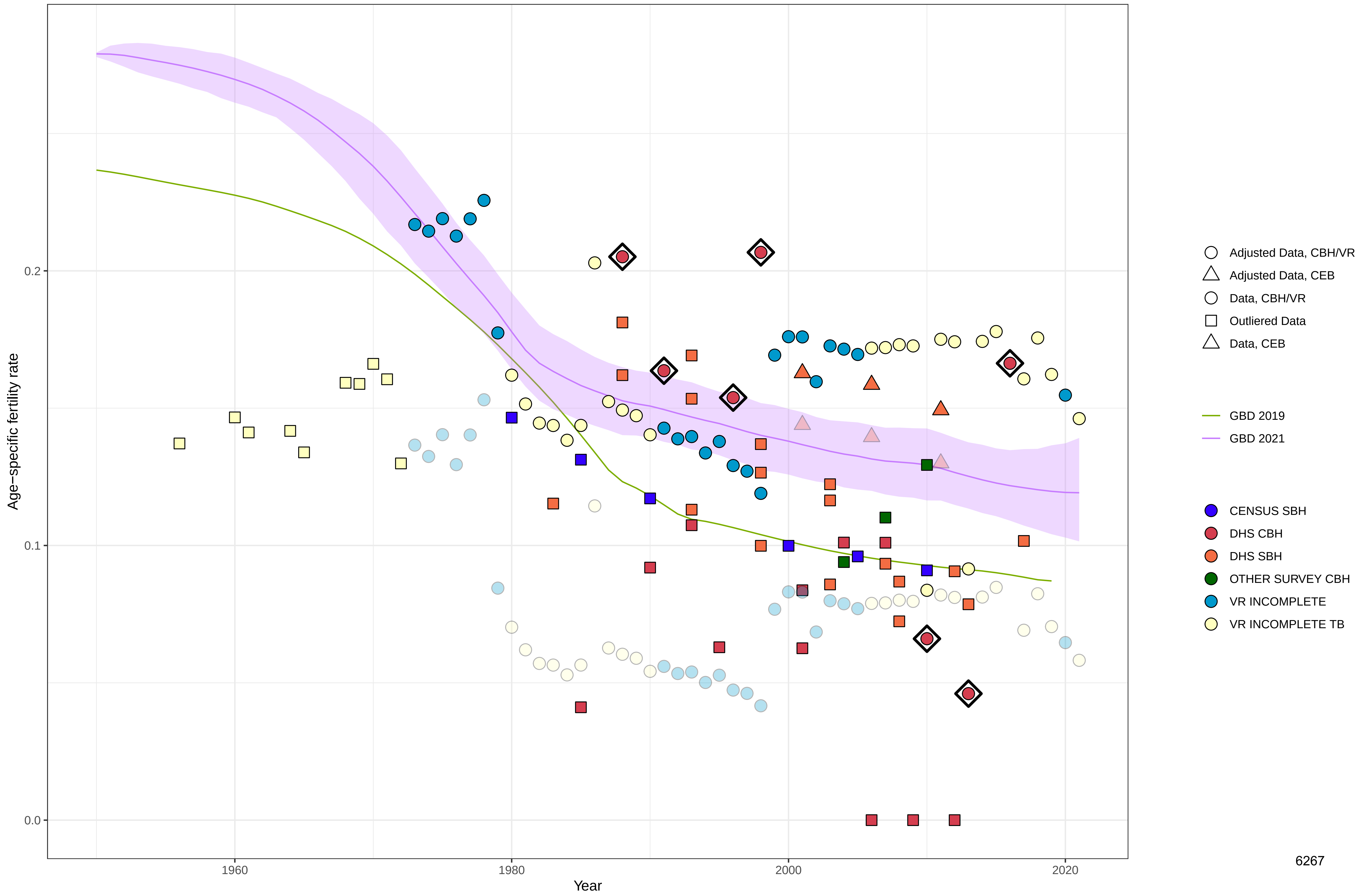

Age−specific fertility fitted trend lines, 1950−2021: Misamis Occidental, Ages: 40−44
Data points enclosed with a diamond are reference sources

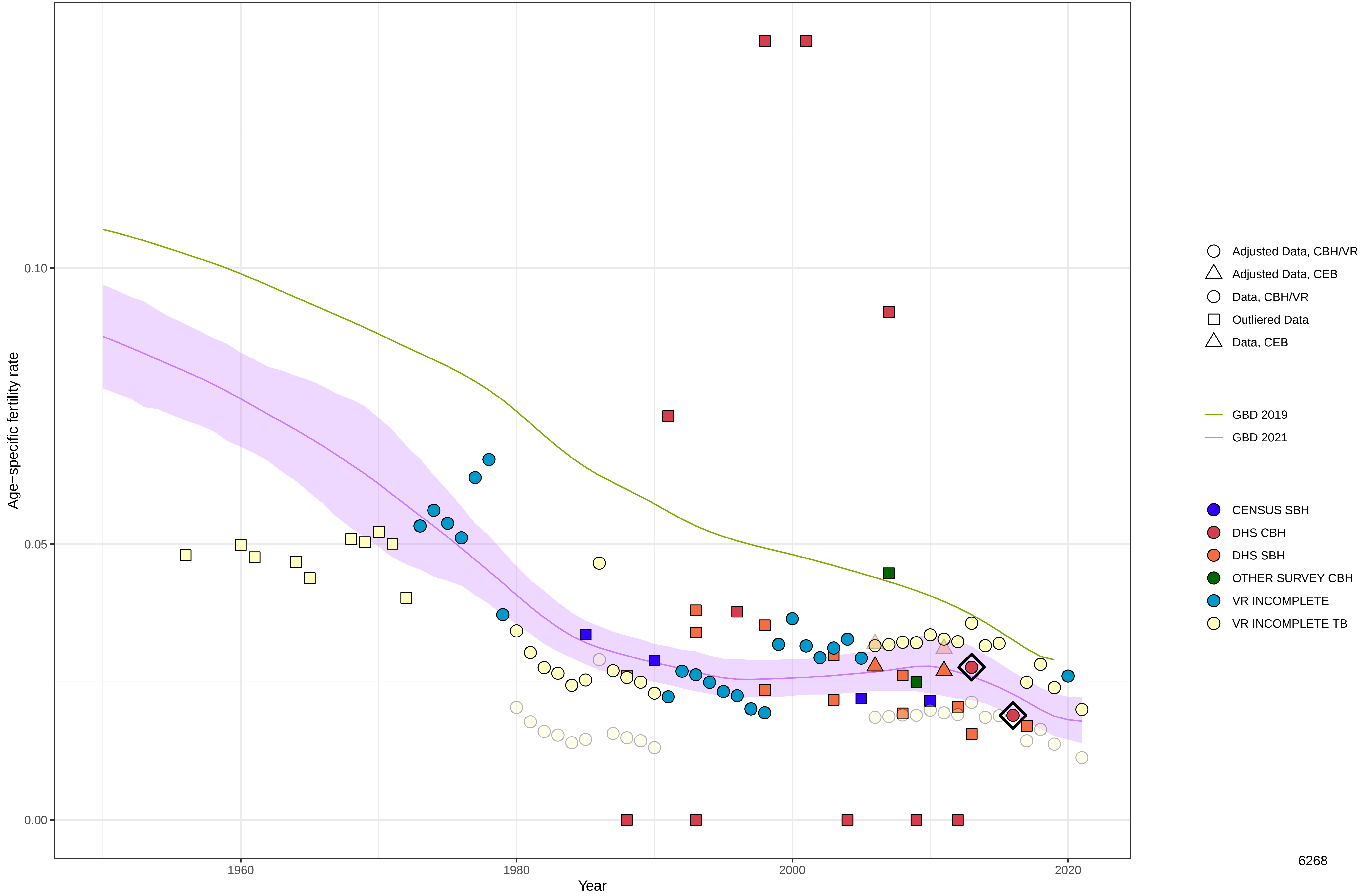

Age−specific fertility fitted trend lines, 1950−2021: Misamis Occidental, Ages: 45−49
Data points enclosed with a diamond are reference sources

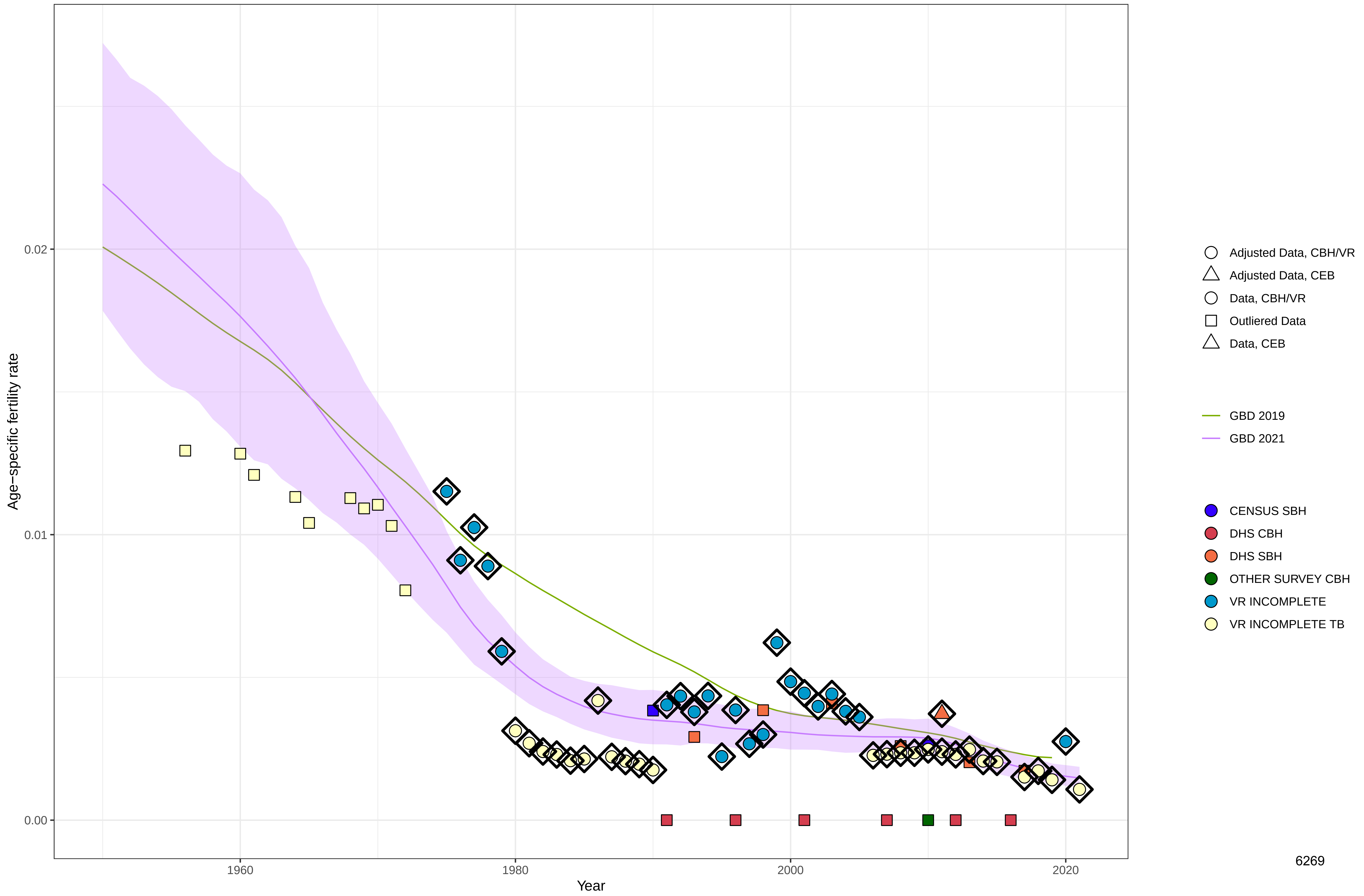

Age−specific fertility fitted trend lines, 1950−2021: Misamis Occidental, Ages: 50−54
Data points enclosed with a diamond are reference sources

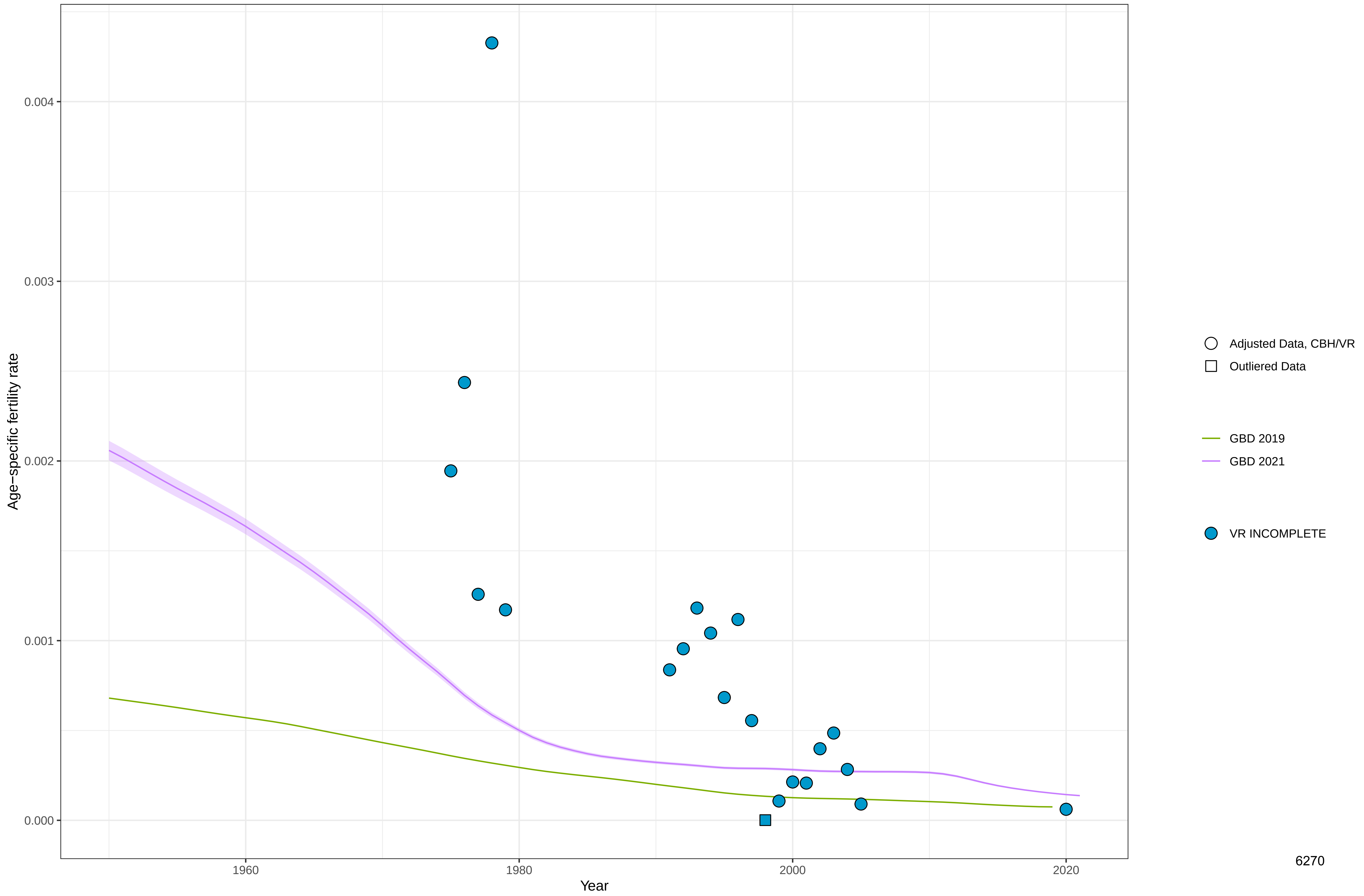

Total fertility rate fitted trend lines, 1950−2021: Misamis Occidental TFR
Data points enclosed with a diamond are reference sources

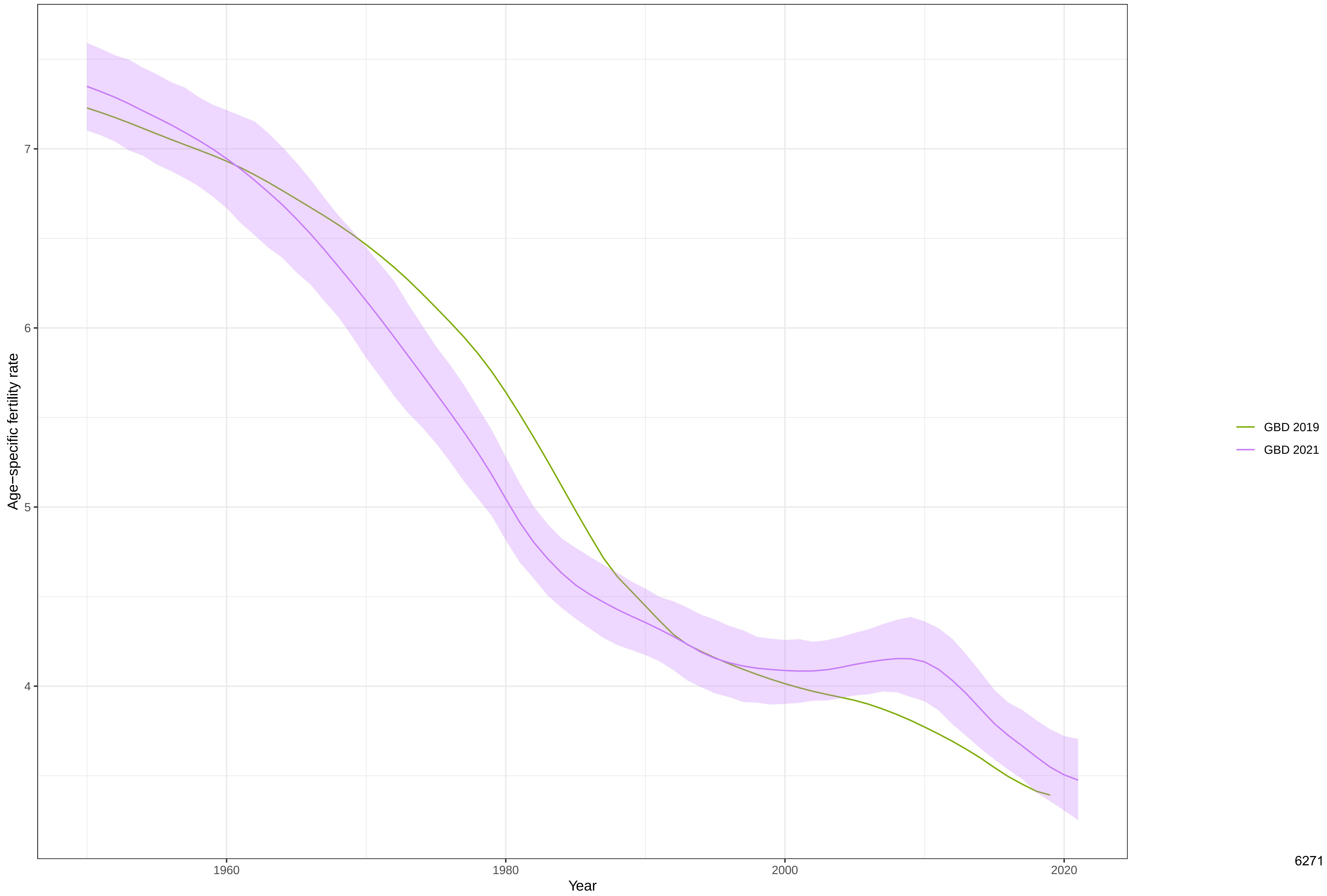

Age−specific fertility fitted trend lines, 1950−2021: Misamis Oriental, Ages: 10−14
Data points enclosed with a diamond are reference sources

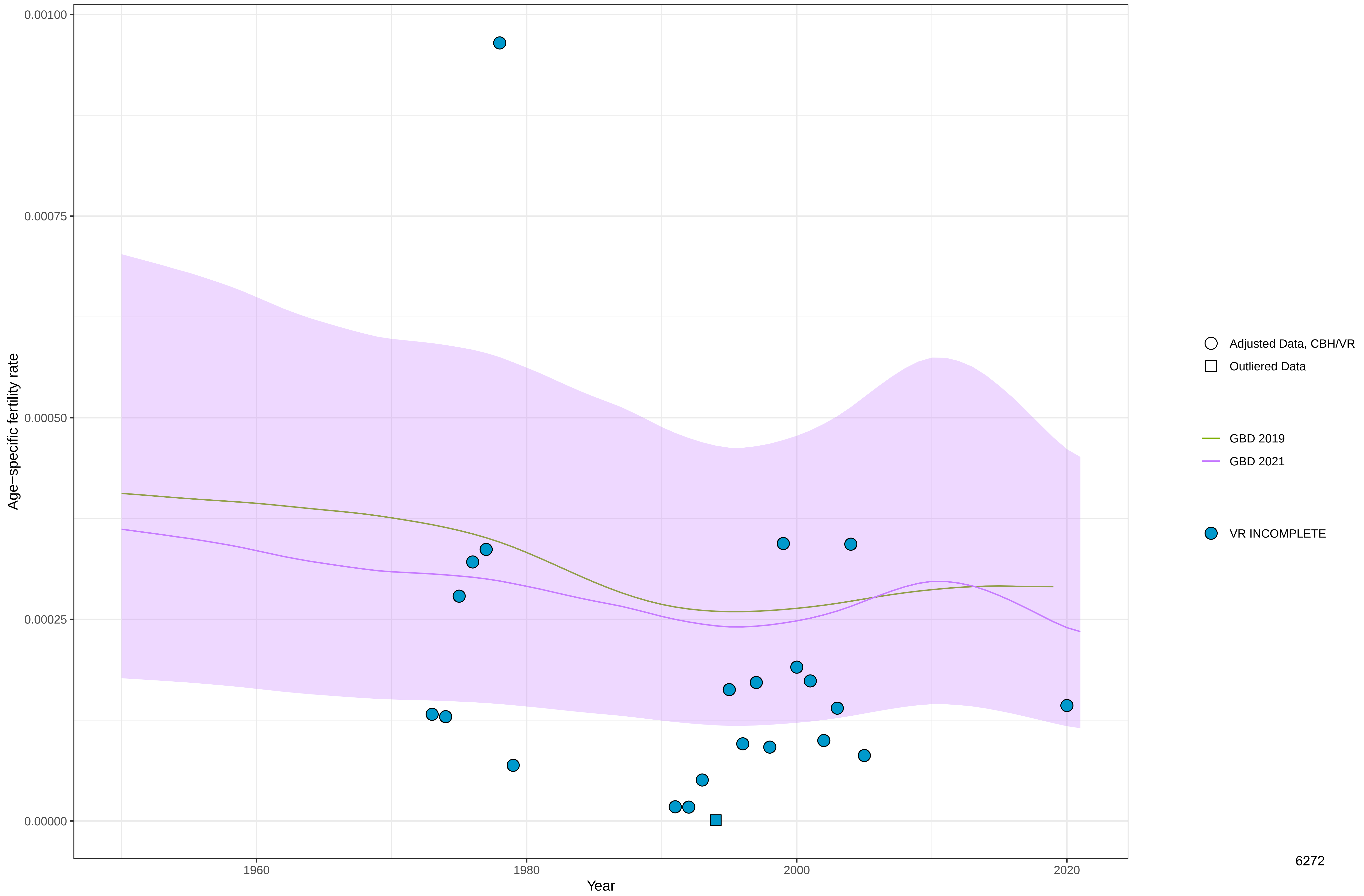

Age−specific fertility fitted trend lines, 1950−2021: Misamis Oriental, Ages: 15−19
Data points enclosed with a diamond are reference sources

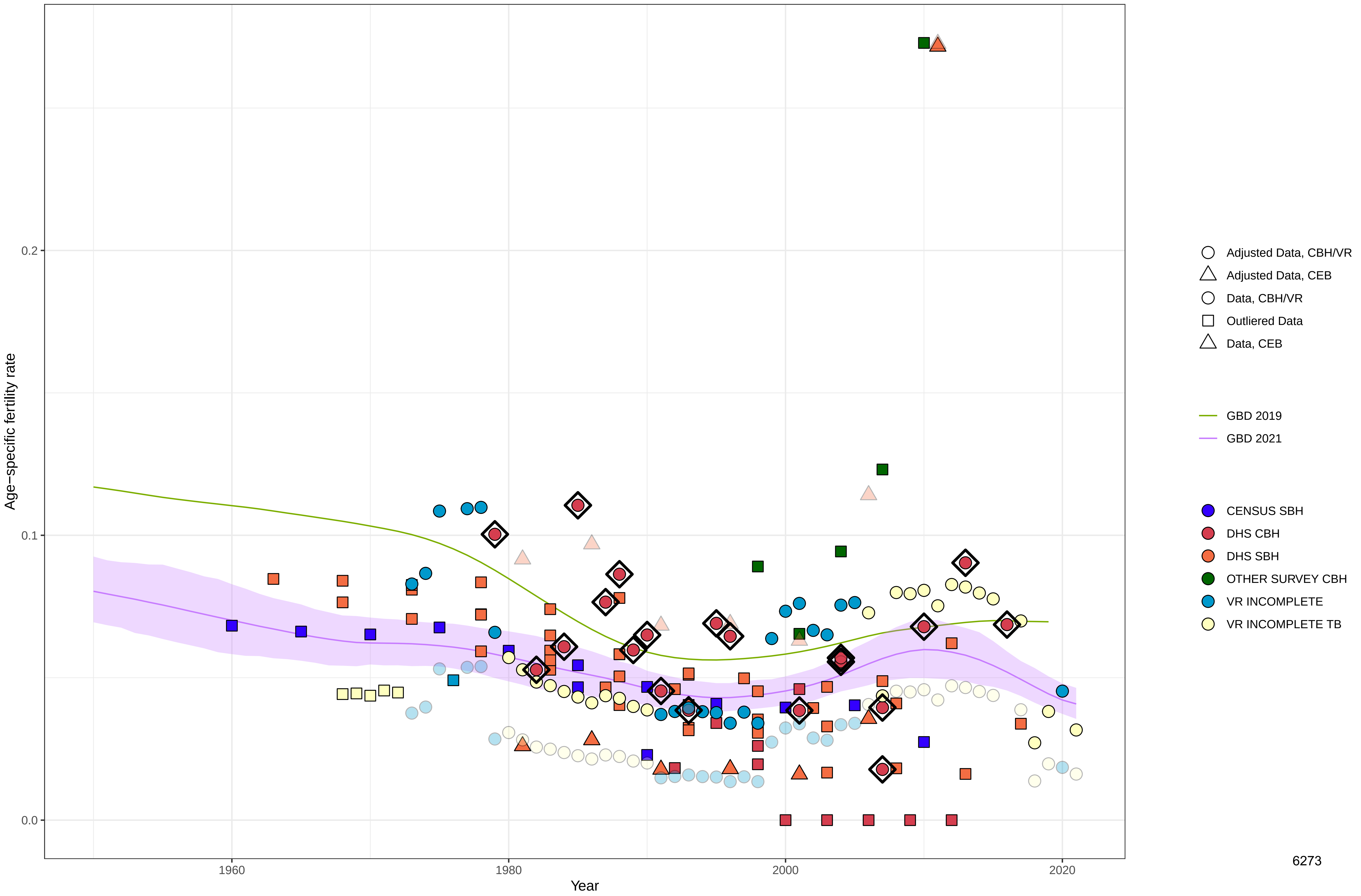

Age−specific fertility fitted trend lines, 1950−2021: Misamis Oriental, Ages: 20−24
Data points enclosed with a diamond are reference sources

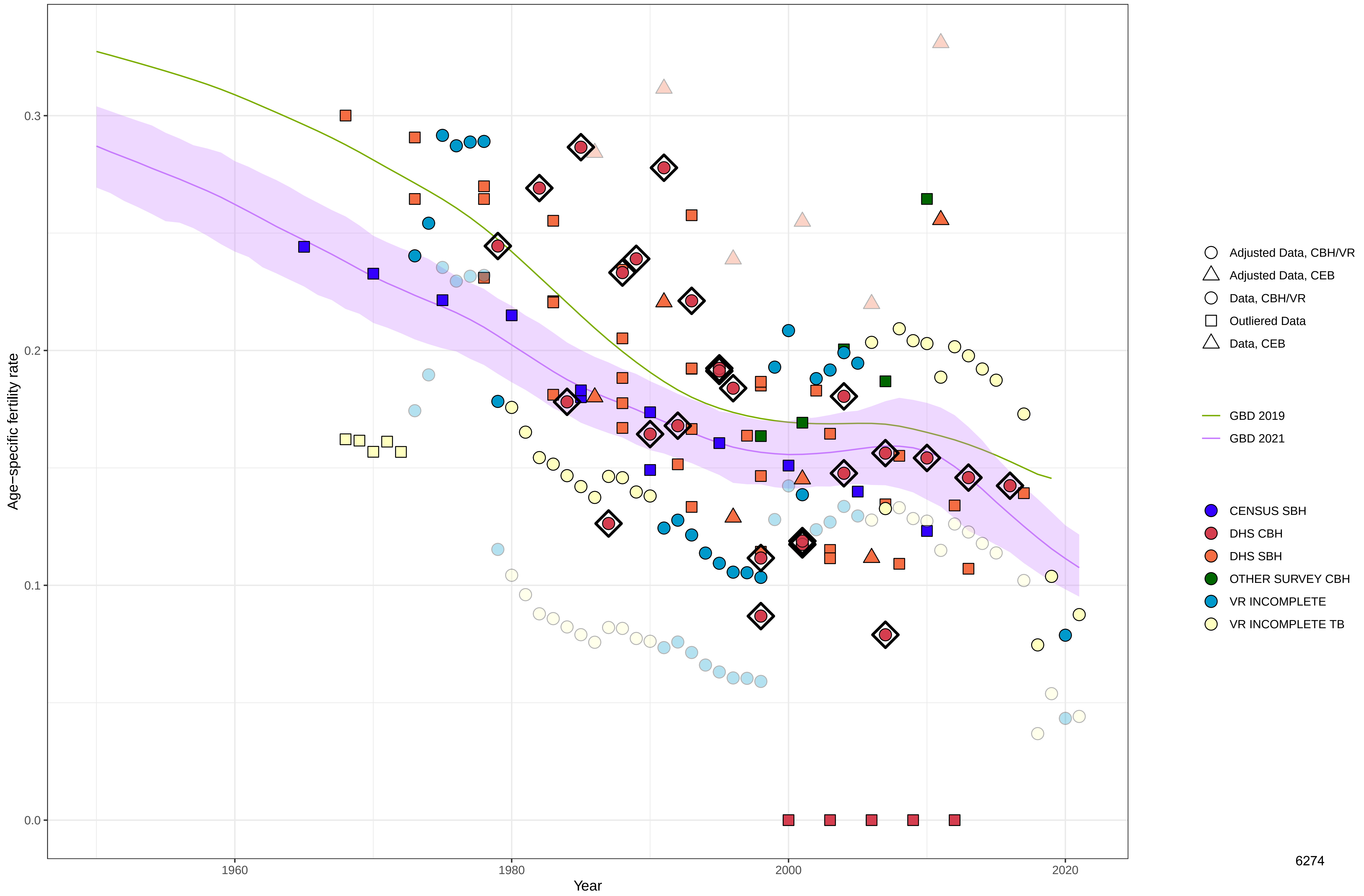

Age−specific fertility fitted trend lines, 1950−2021: Misamis Oriental, Ages: 25−29
Data points enclosed with a diamond are reference sources

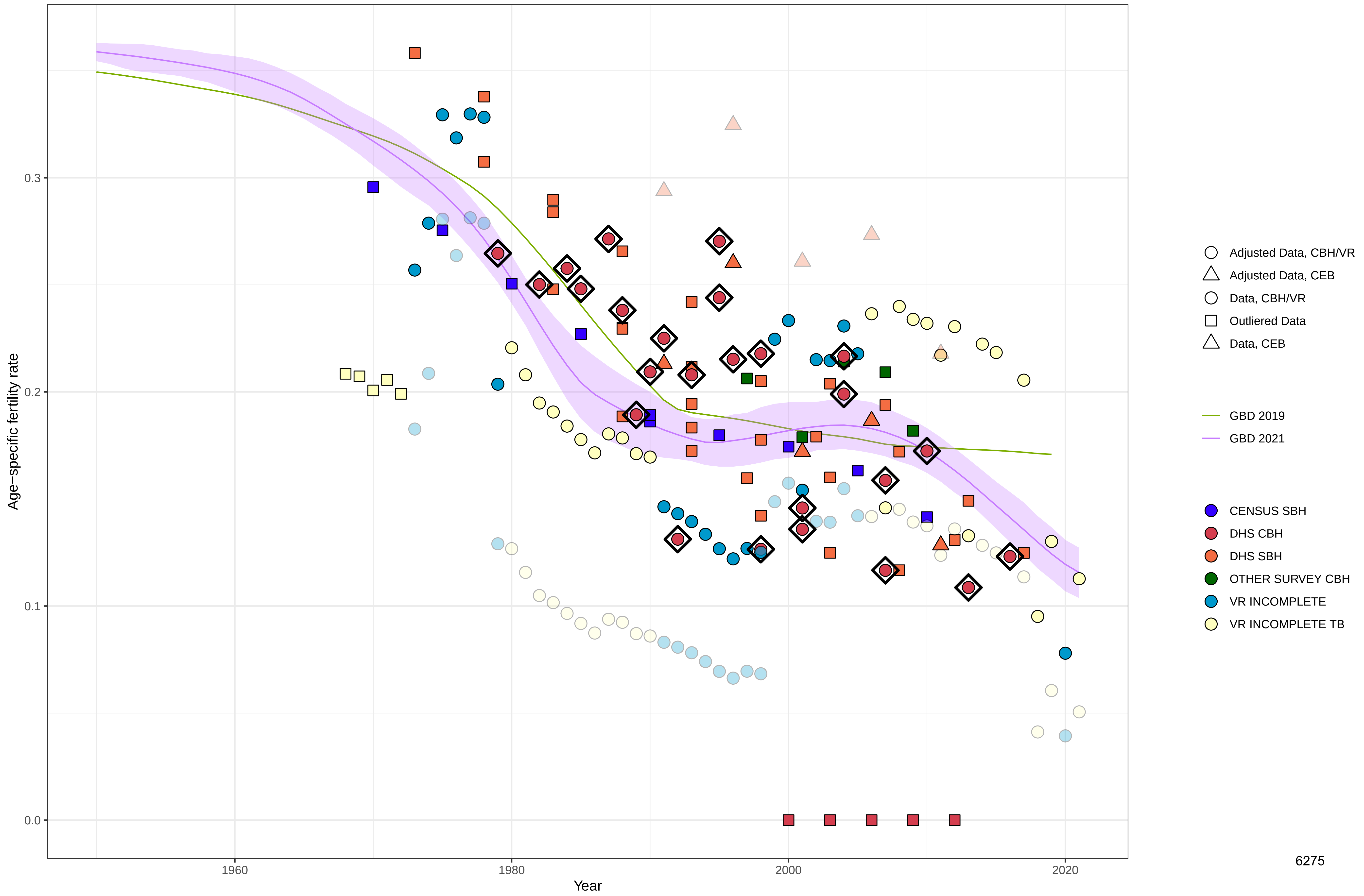

Age−specific fertility fitted trend lines, 1950−2021: Misamis Oriental, Ages: 30−34
Data points enclosed with a diamond are reference sources

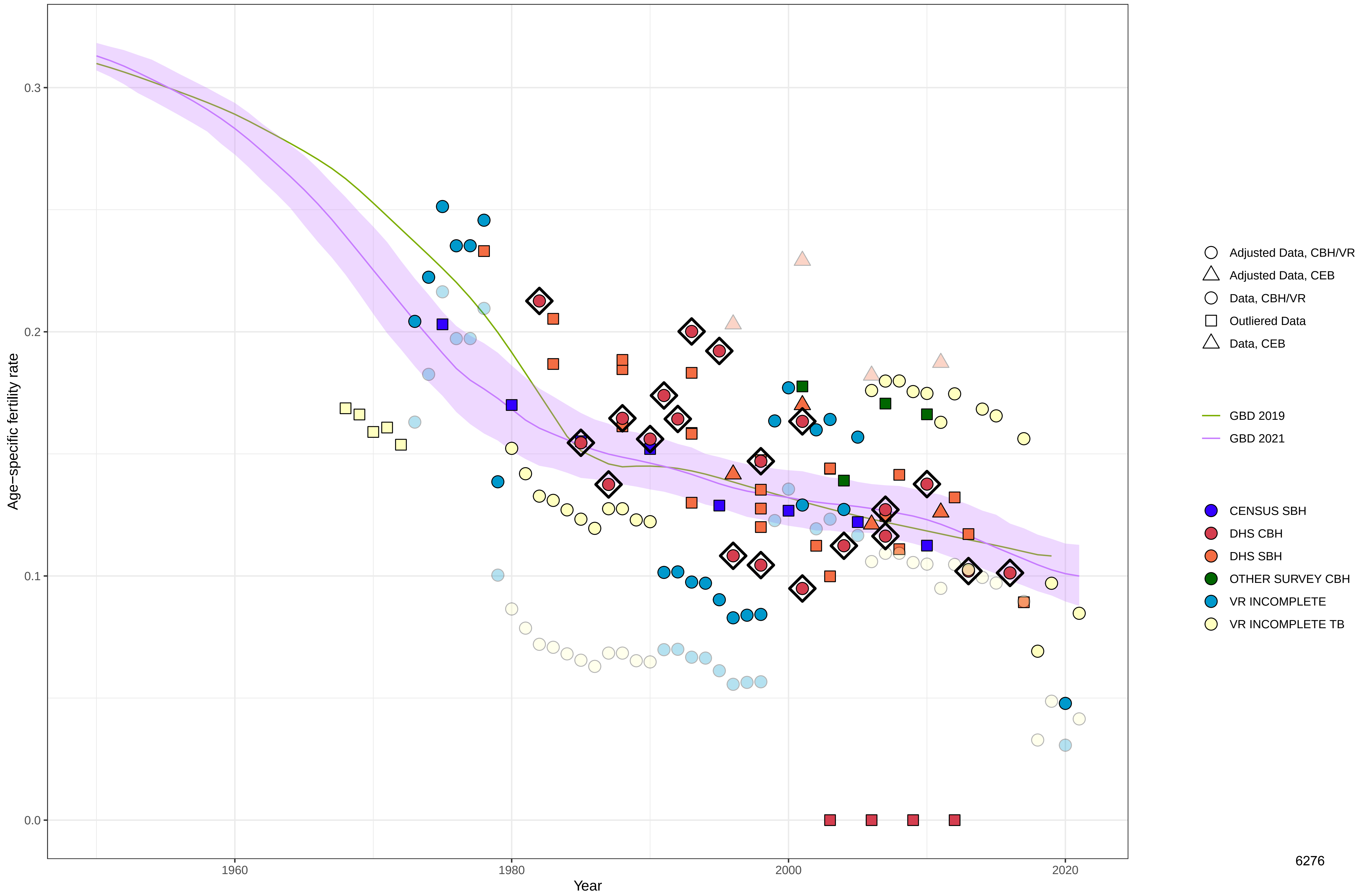

Age−specific fertility fitted trend lines, 1950−2021: Misamis Oriental, Ages: 35−39
Data points enclosed with a diamond are reference sources

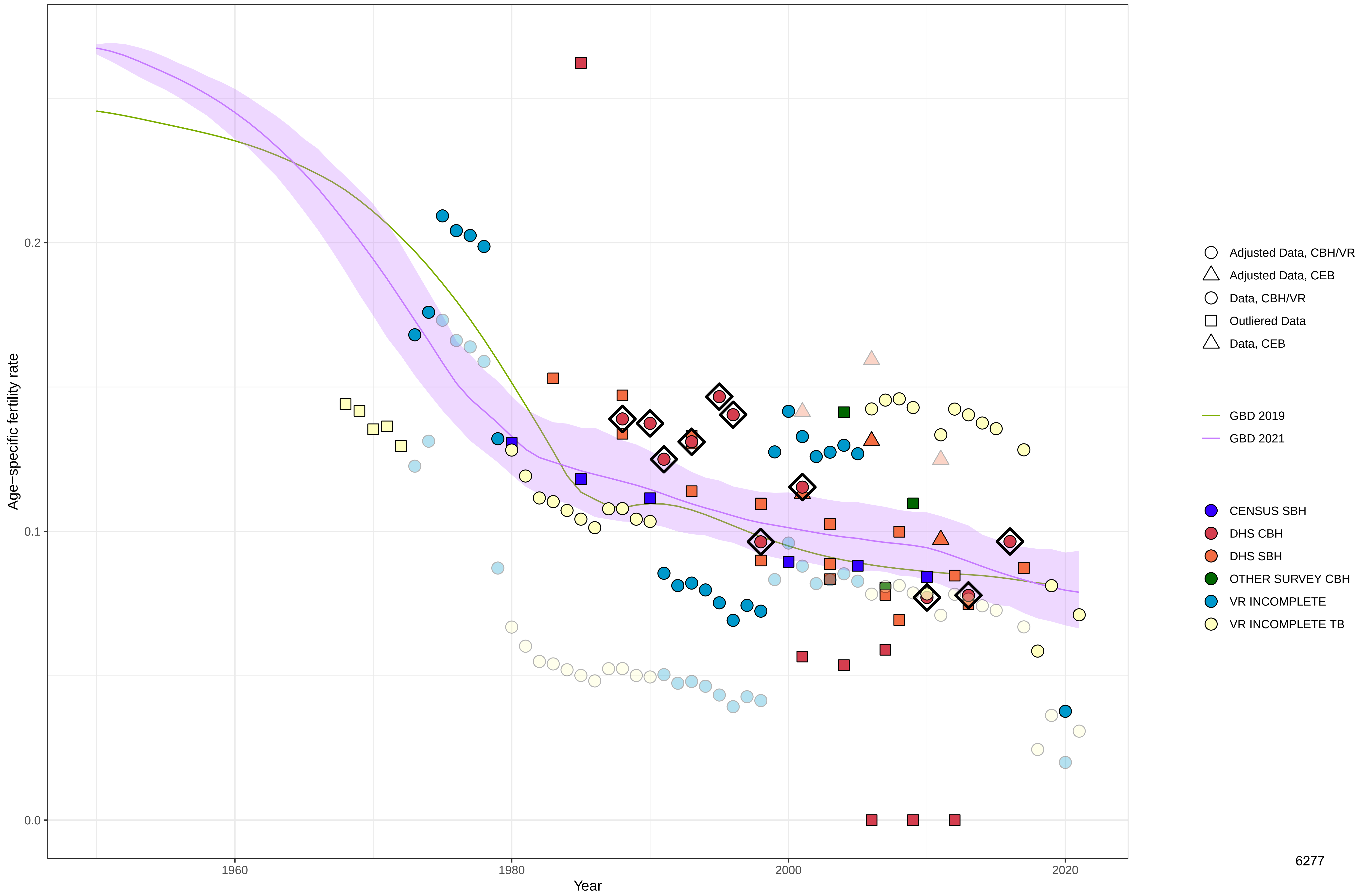

6277

Age−specific fertility fitted trend lines, 1950−2021: Misamis Oriental, Ages: 40−44
Data points enclosed with a diamond are reference sources

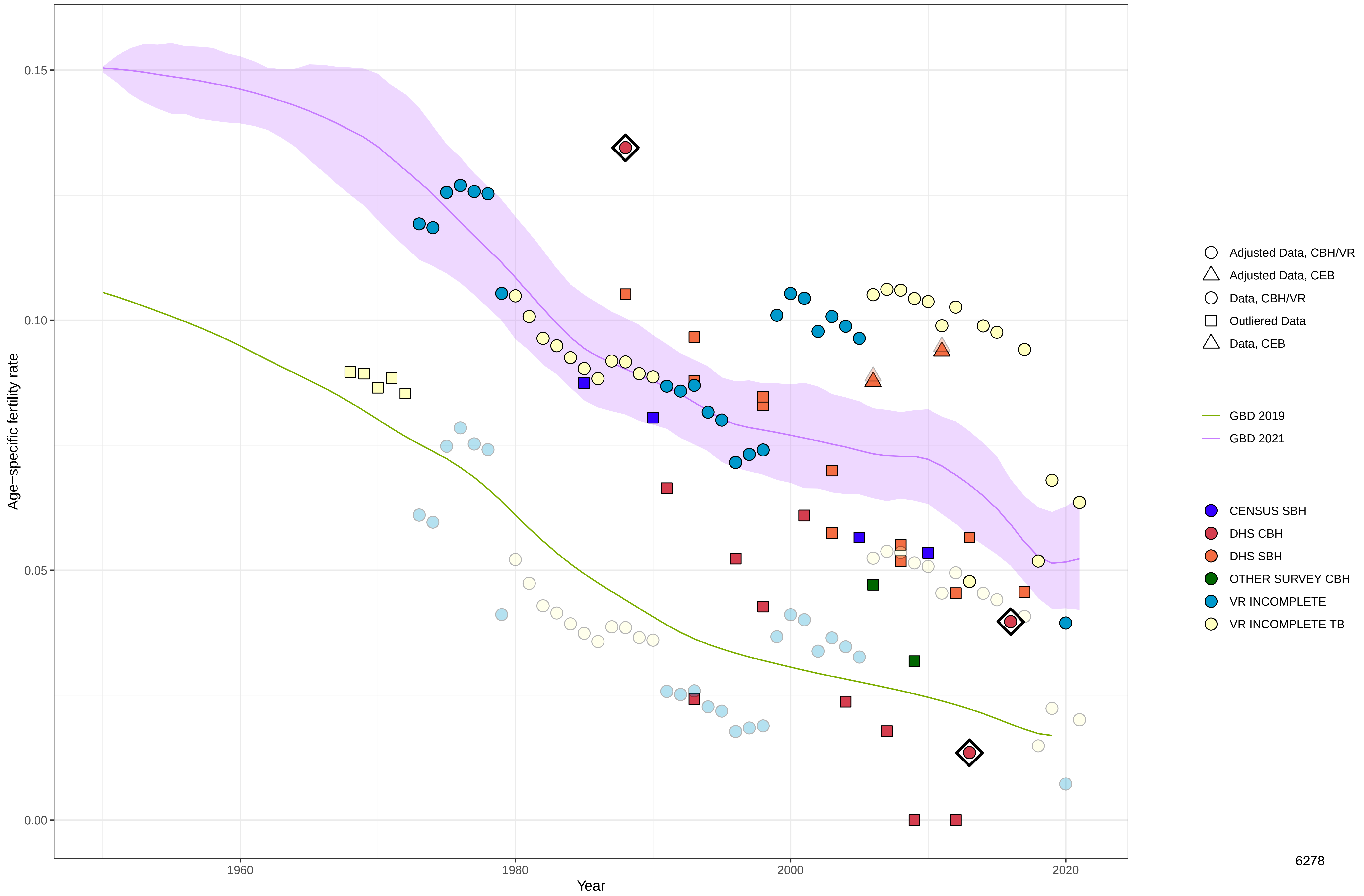

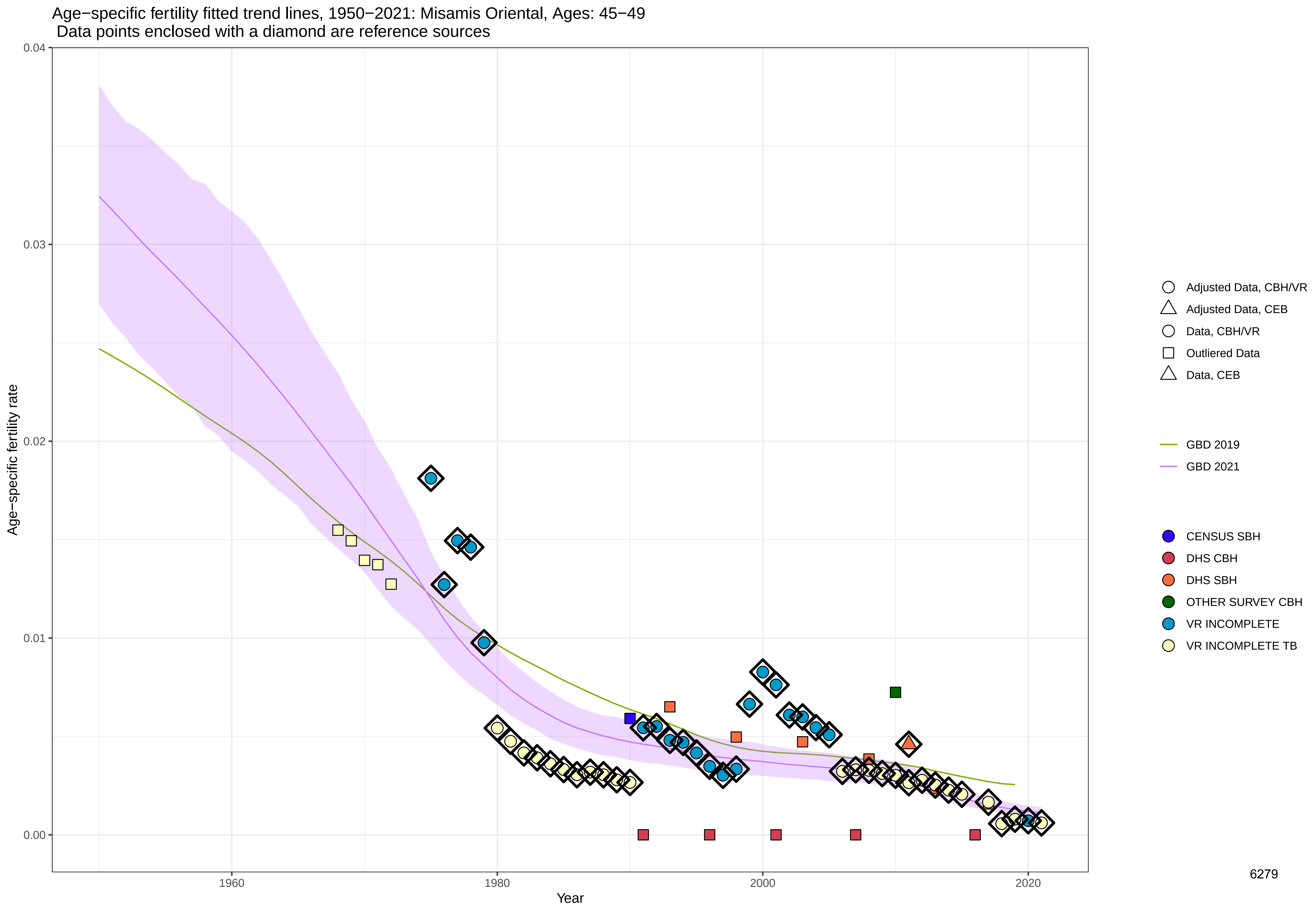

Age−specific fertility fitted trend lines, 1950−2021: Misamis Oriental, Ages: 45−49
Data points enclosed with a diamond are reference sources
Age−specific fertility rate
Year
0.00
0.01
0.02
0.03
0.04
1960
1980
2000
2020
Adjusted Data, CBH/VR
Adjusted Data, CEB
Data, CBH/VR
Outliered Data
Data, CEB
GBD 2019
GBD 2021
CENSUS SBH
DHS CBH
DHS SBH
OTHER SURVEY CBH
VR INCOMPLETE
VR INCOMPLETE TB

6279

Age−specific fertility fitted trend lines, 1950−2021: Misamis Oriental, Ages: 50−54
Data points enclosed with a diamond are reference sources

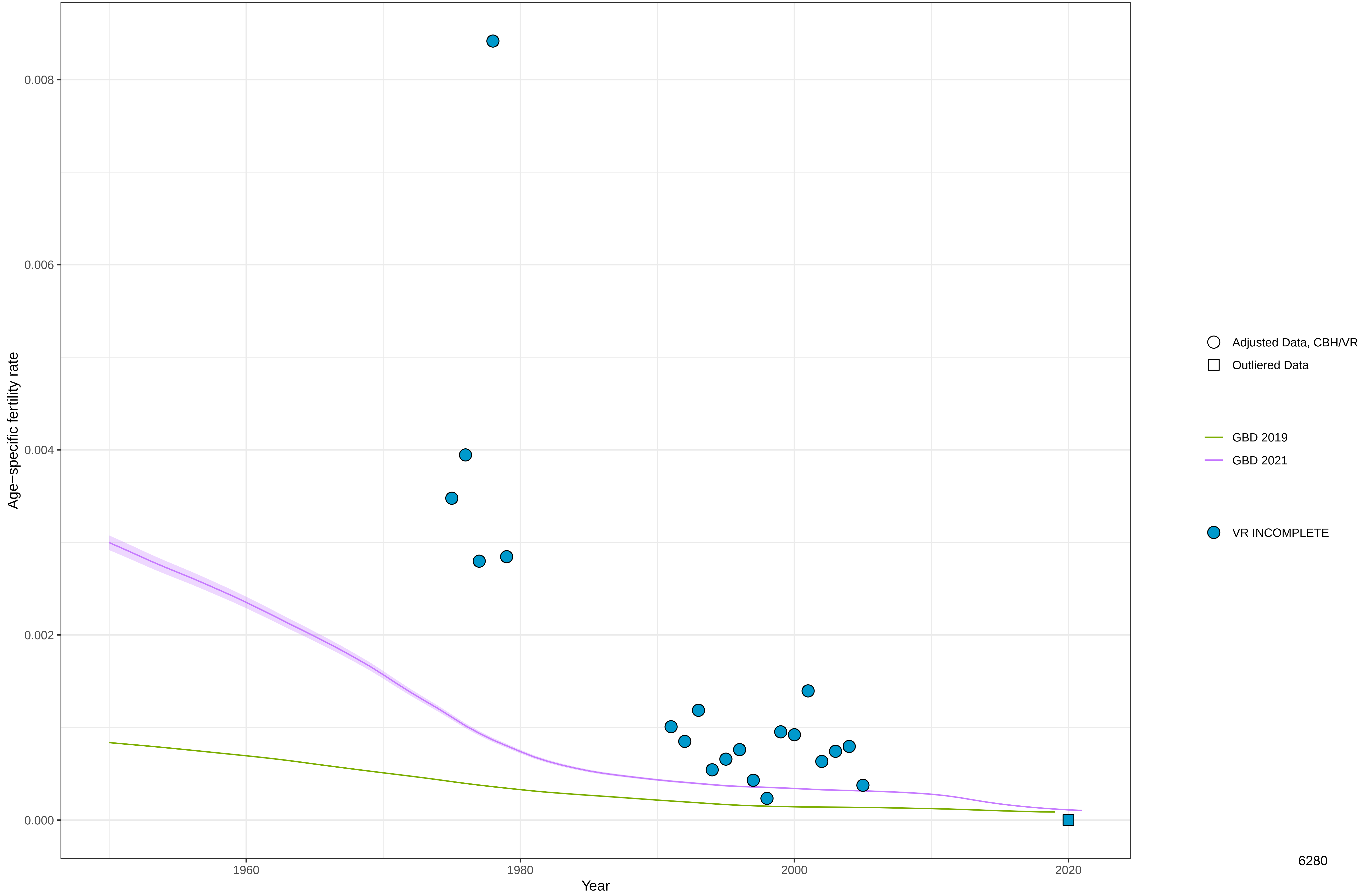

Total fertility rate fitted trend lines, 1950−2021: Misamis Oriental TFR
Data points enclosed with a diamond are reference sources

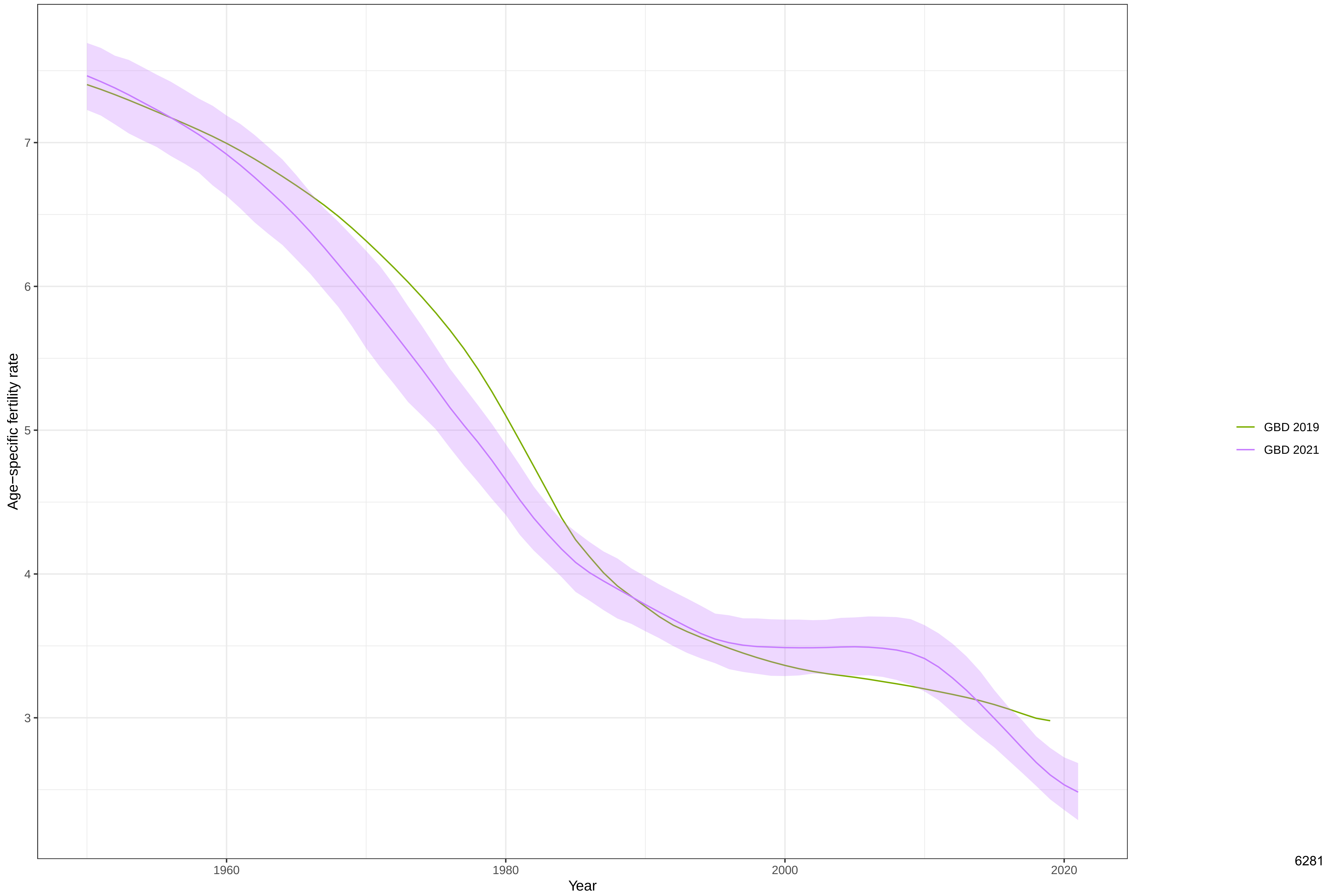

6281

Age−specific fertility fitted trend lines, 1950−2021: Mountain Province, Ages: 10−14
Data points enclosed with a diamond are reference sources

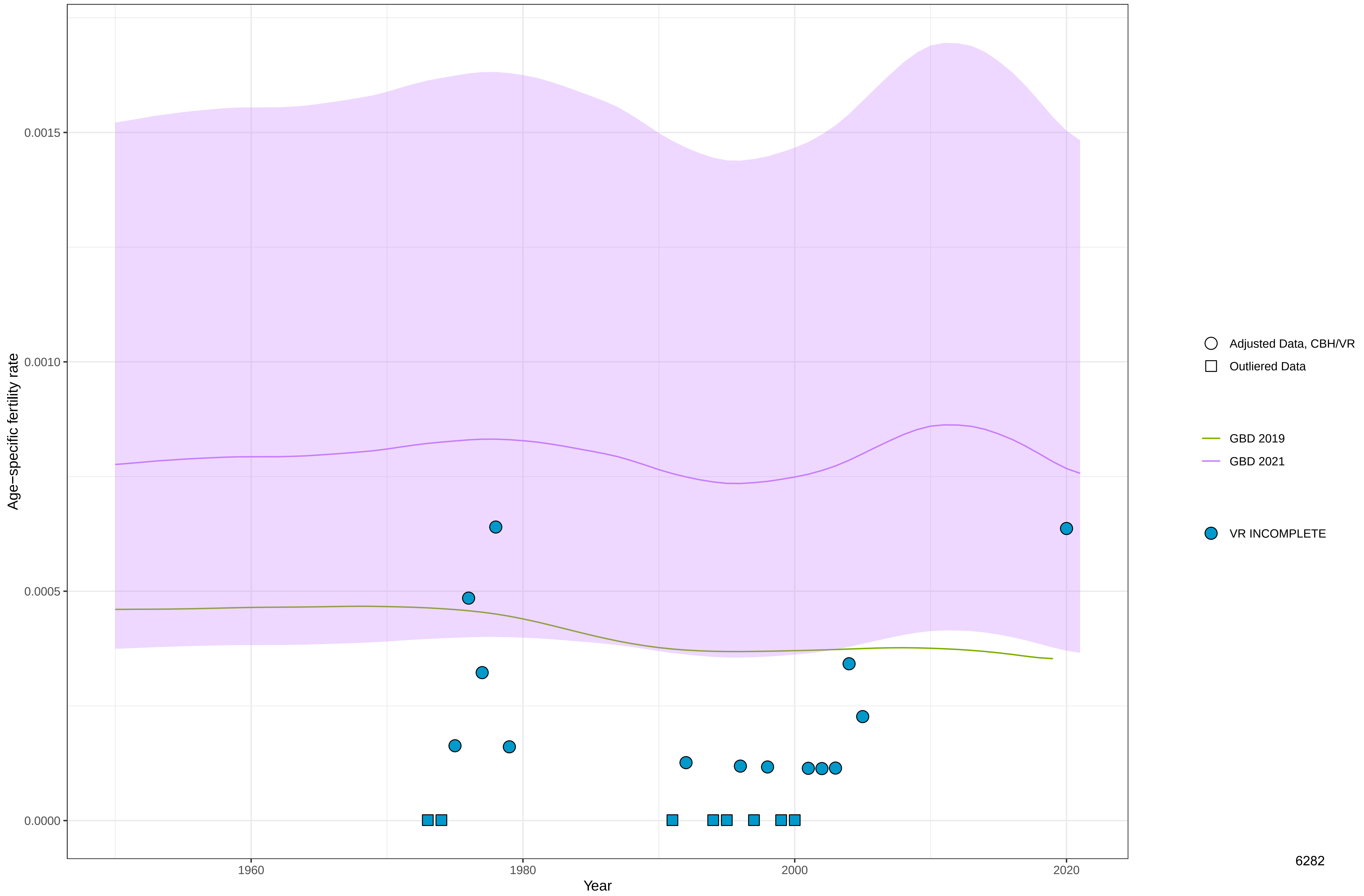

Age−specific fertility fitted trend lines, 1950−2021: Mountain Province, Ages: 15−19
Data points enclosed with a diamond are reference sources

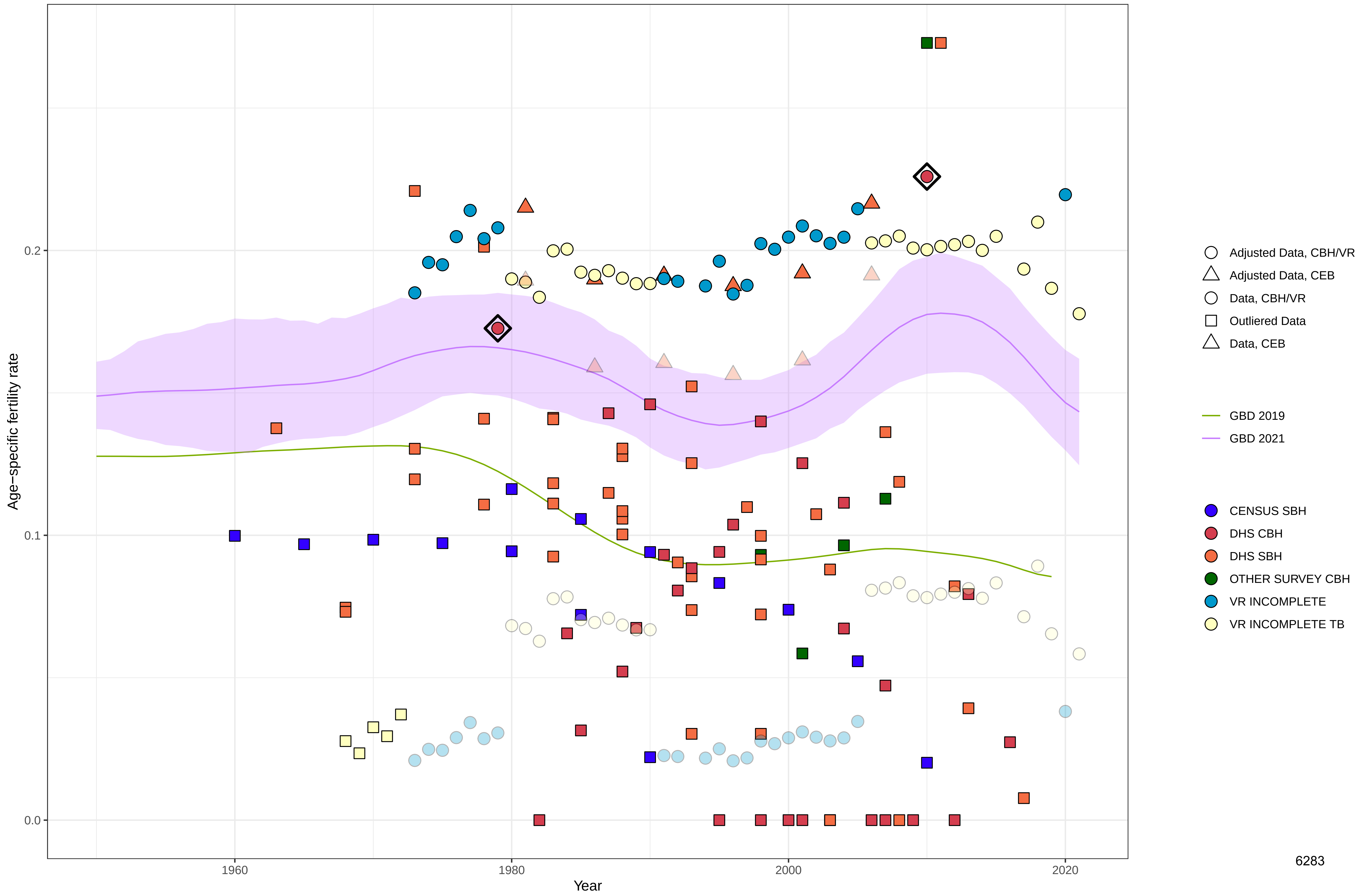

Age−specific fertility fitted trend lines, 1950−2021: Mountain Province, Ages: 20−24
Data points enclosed with a diamond are reference sources

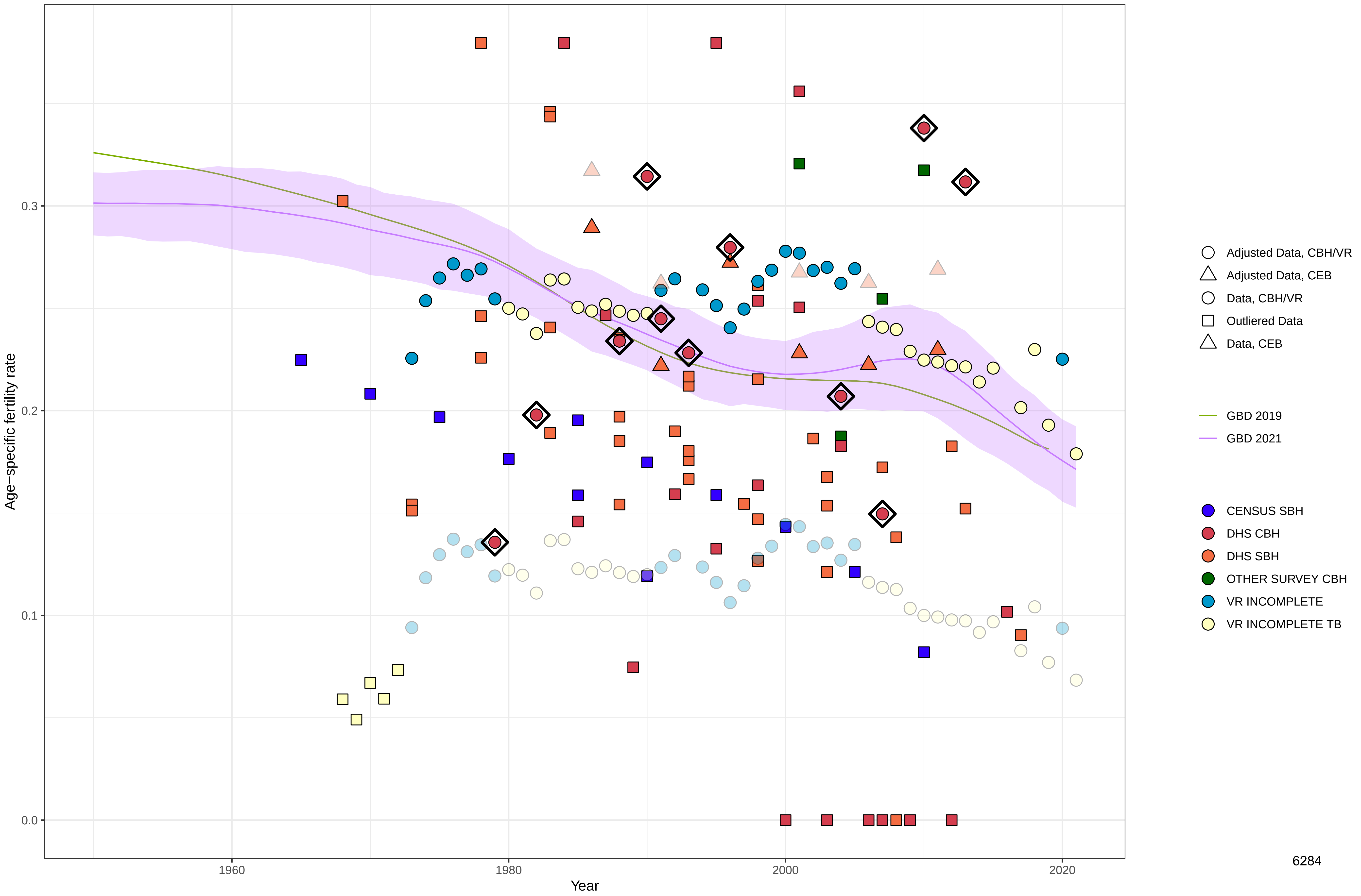

Age−specific fertility fitted trend lines, 1950−2021: Mountain Province, Ages: 25−29
Data points enclosed with a diamond are reference sources

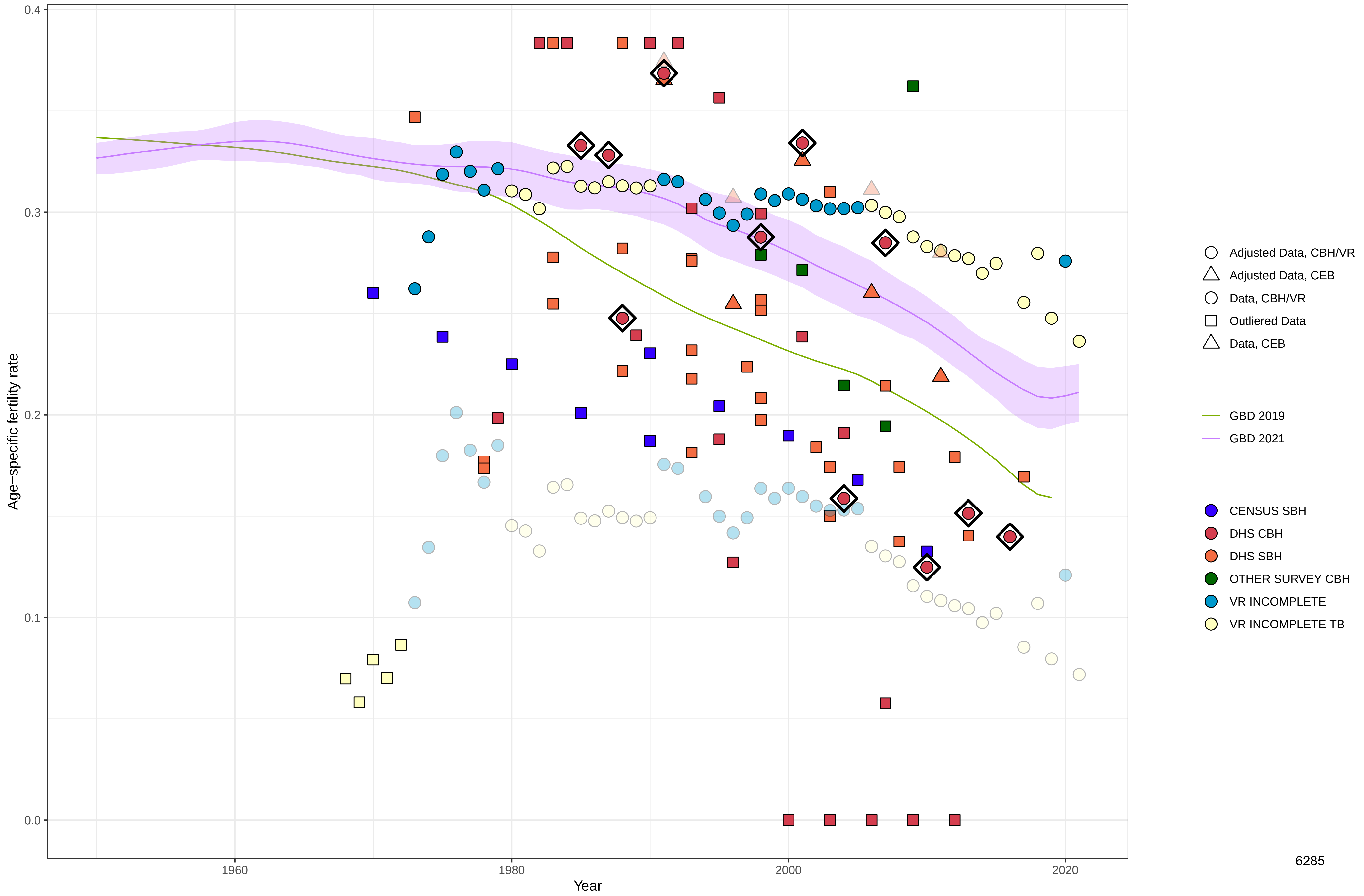

Age−specific fertility fitted trend lines, 1950−2021: Mountain Province, Ages: 30−34
Data points enclosed with a diamond are reference sources

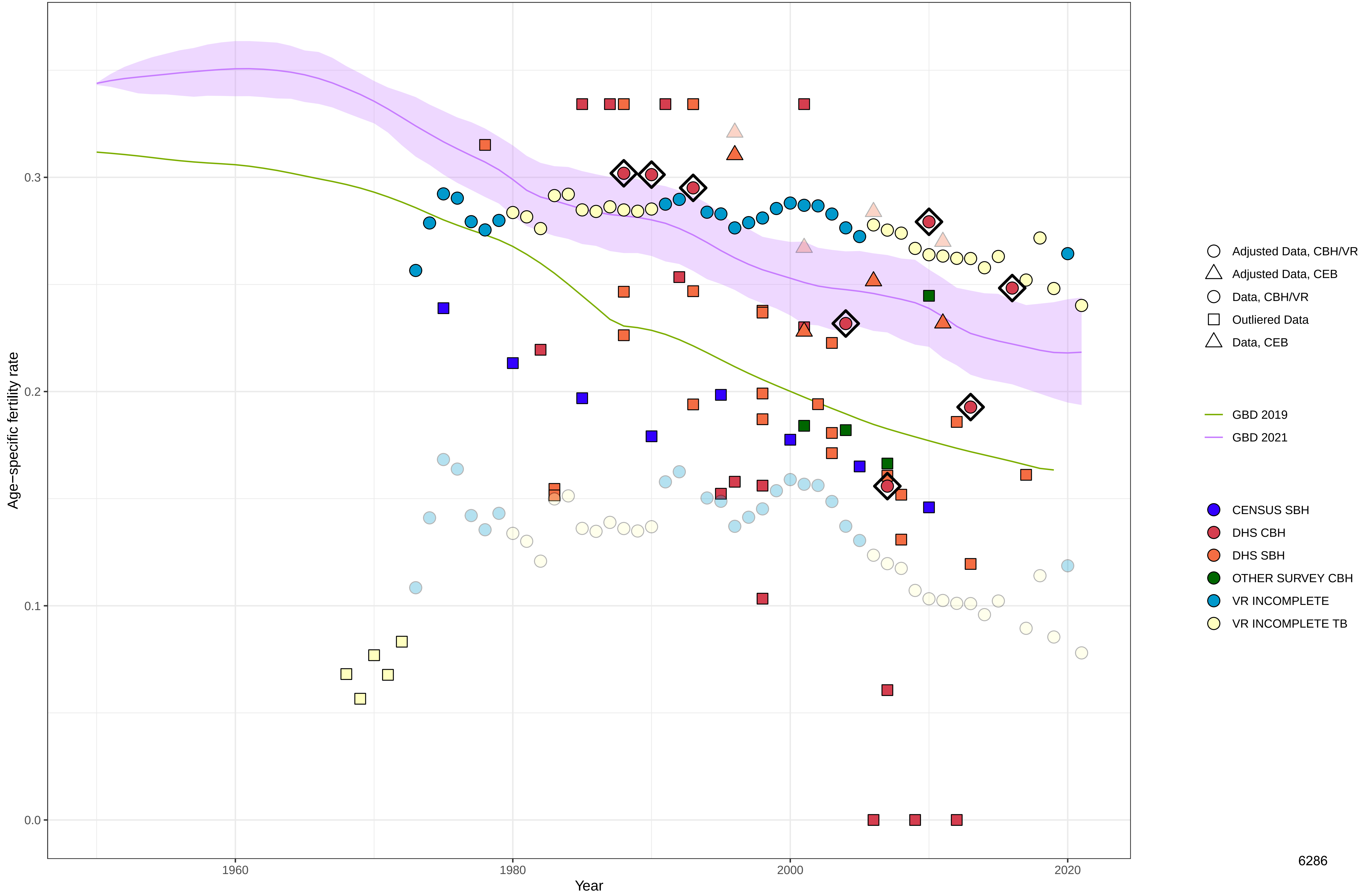

Age−specific fertility fitted trend lines, 1950−2021: Mountain Province, Ages: 35−39
Data points enclosed with a diamond are reference sources

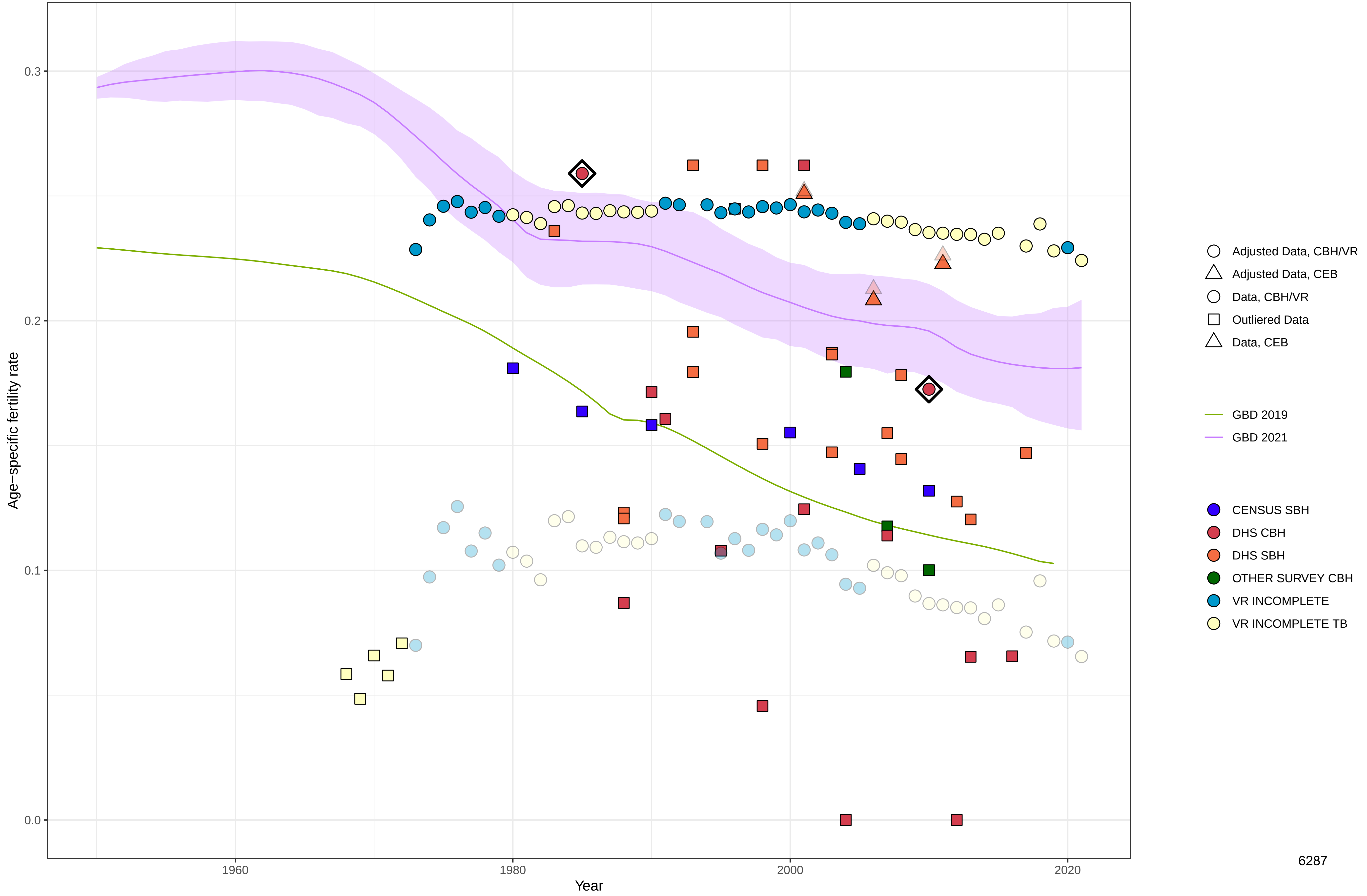

Age−specific fertility fitted trend lines, 1950−2021: Mountain Province, Ages: 40−44
Data points enclosed with a diamond are reference sources

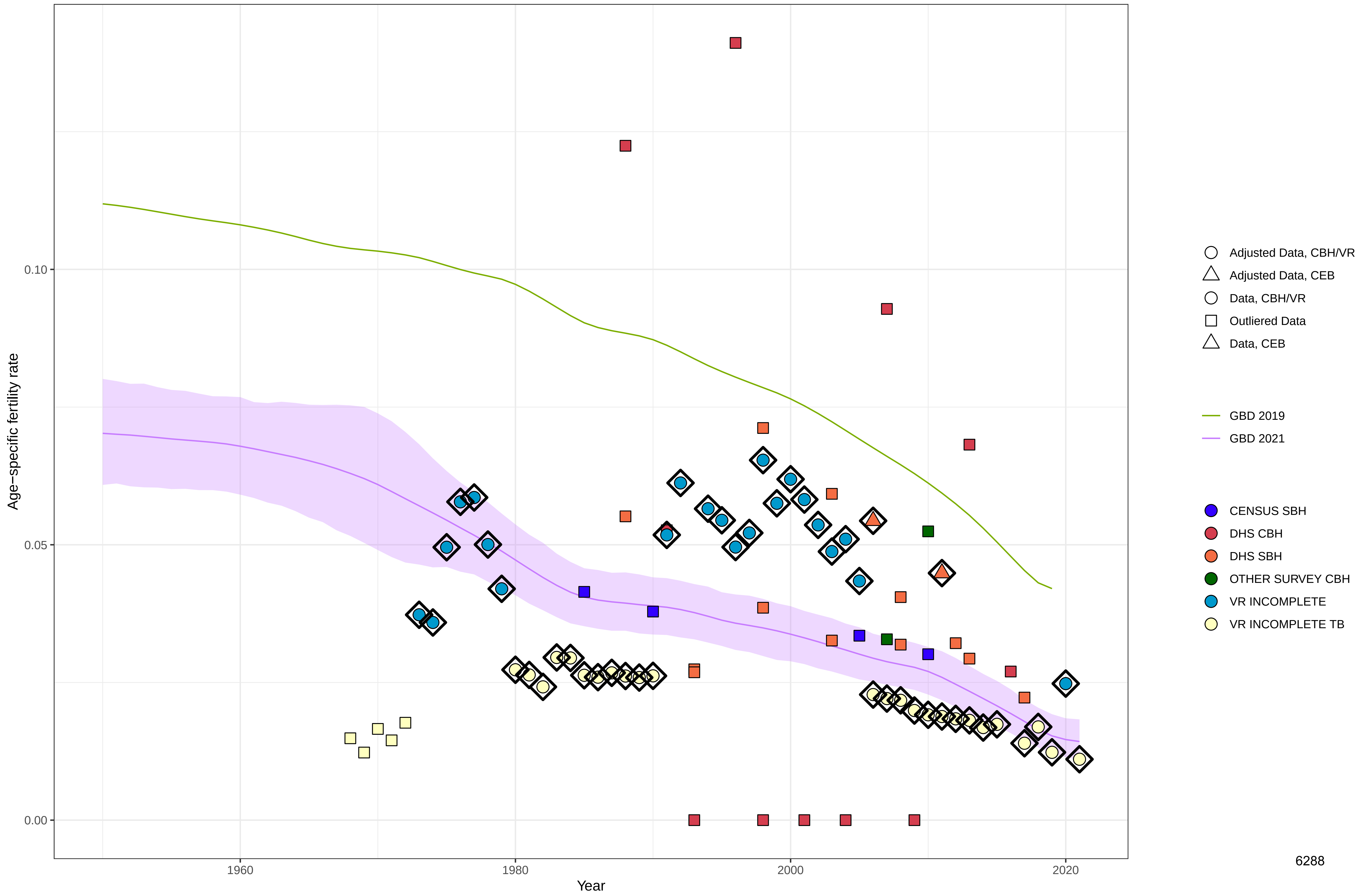

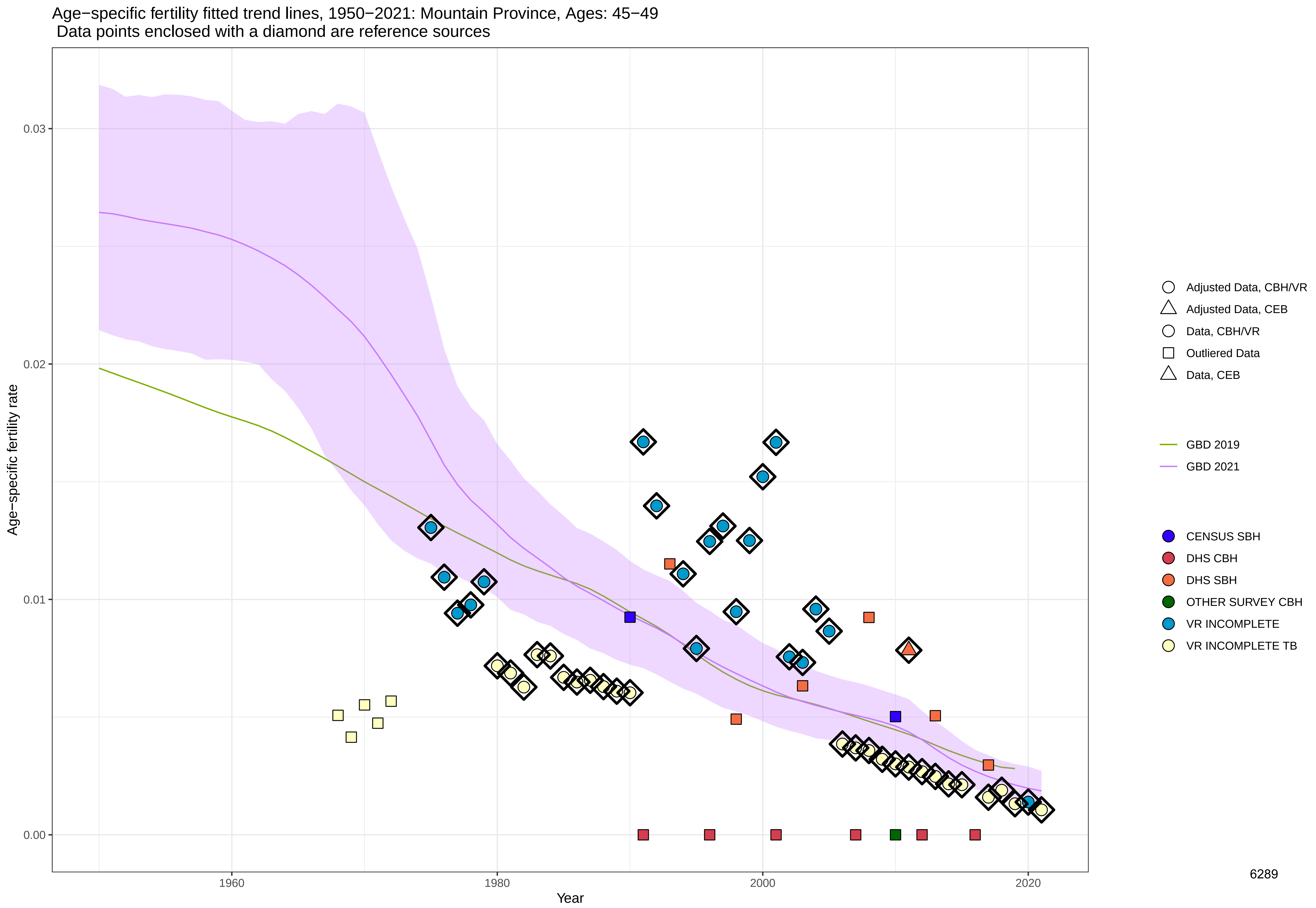

Age−specific fertility fitted trend lines, 1950−2021: Mountain Province, Ages: 45−49
Data points enclosed with a diamond are reference sources
Age−specific fertility rate
Year
0.00
0.01
0.02
0.03
1960
1980
2000
2020
Adjusted Data, CBH/VR
Adjusted Data, CEB
Data, CBH/VR
Outliered Data
Data, CEB
GBD 2019
GBD 2021
CENSUS SBH
DHS CBH
DHS SBH
OTHER SURVEY CBH
VR INCOMPLETE
VR INCOMPLETE TB

Age−specific fertility fitted trend lines, 1950−2021: Mountain Province, Ages: 50−54
Data points enclosed with a diamond are reference sources

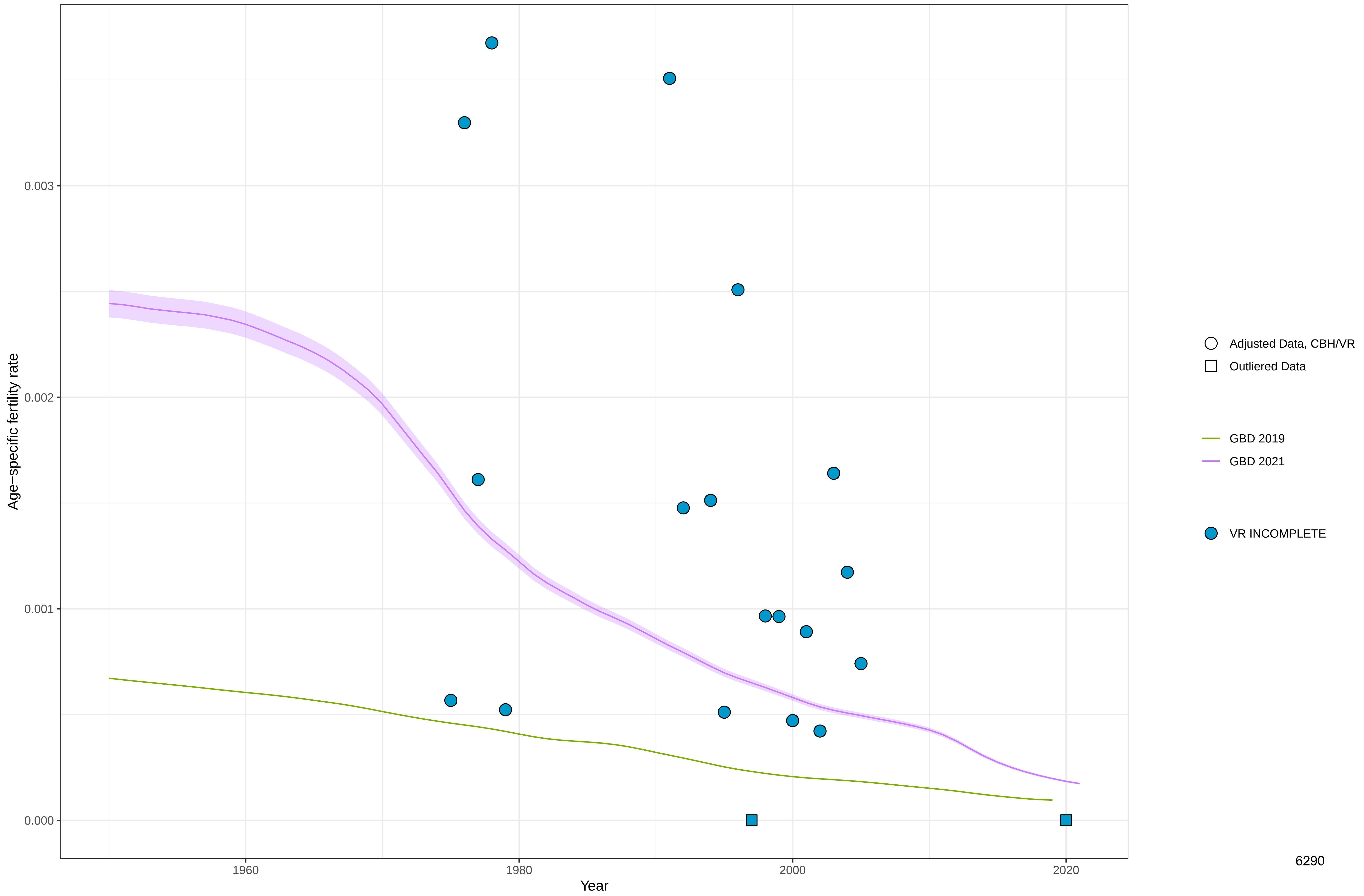

Total fertility rate fitted trend lines, 1950−2021: Mountain Province TFR
Data points enclosed with a diamond are reference sources

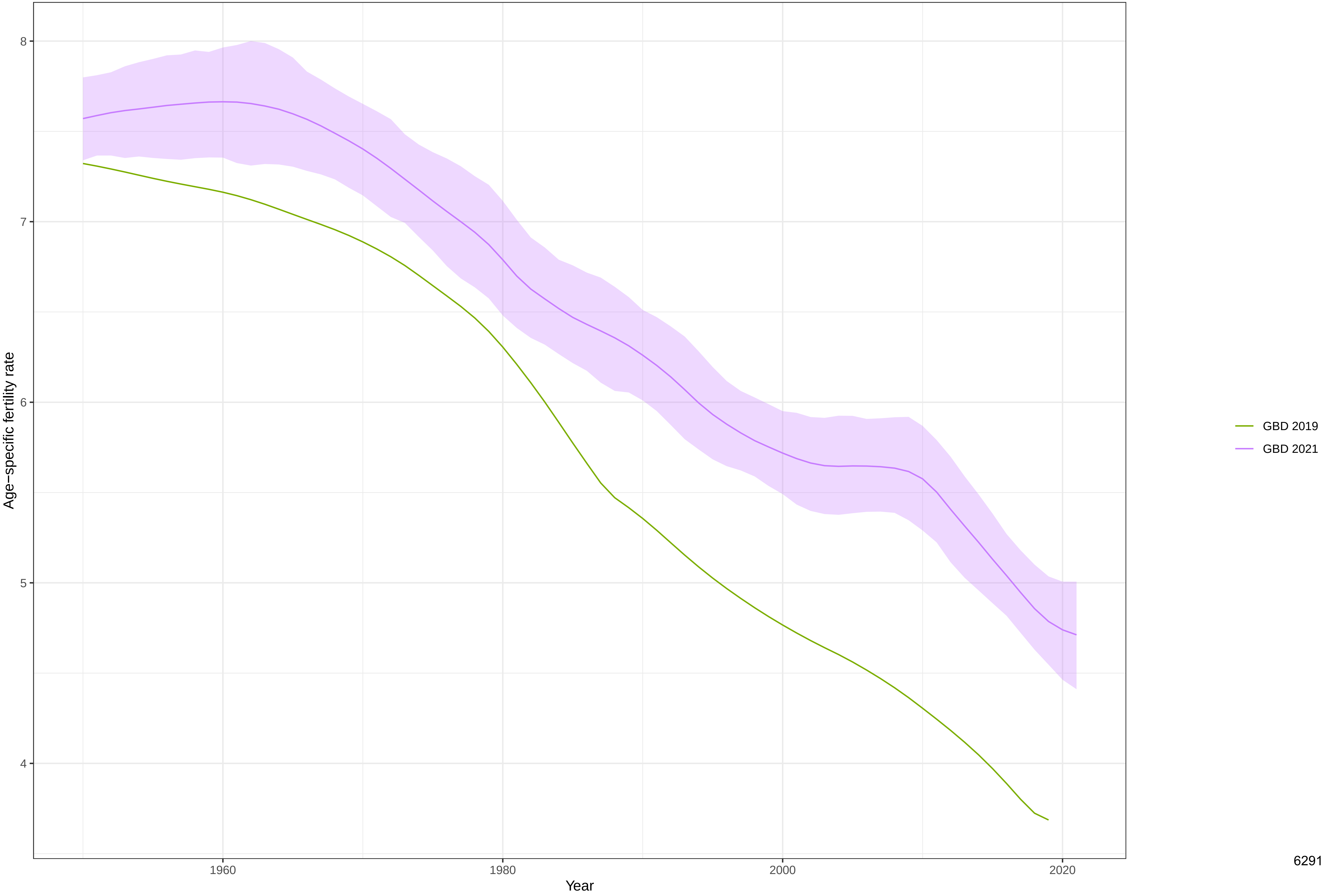

6291

Age−specific fertility fitted trend lines, 1950−2021: National Capital Region, Ages: 10−14
Data points enclosed with a diamond are reference sources

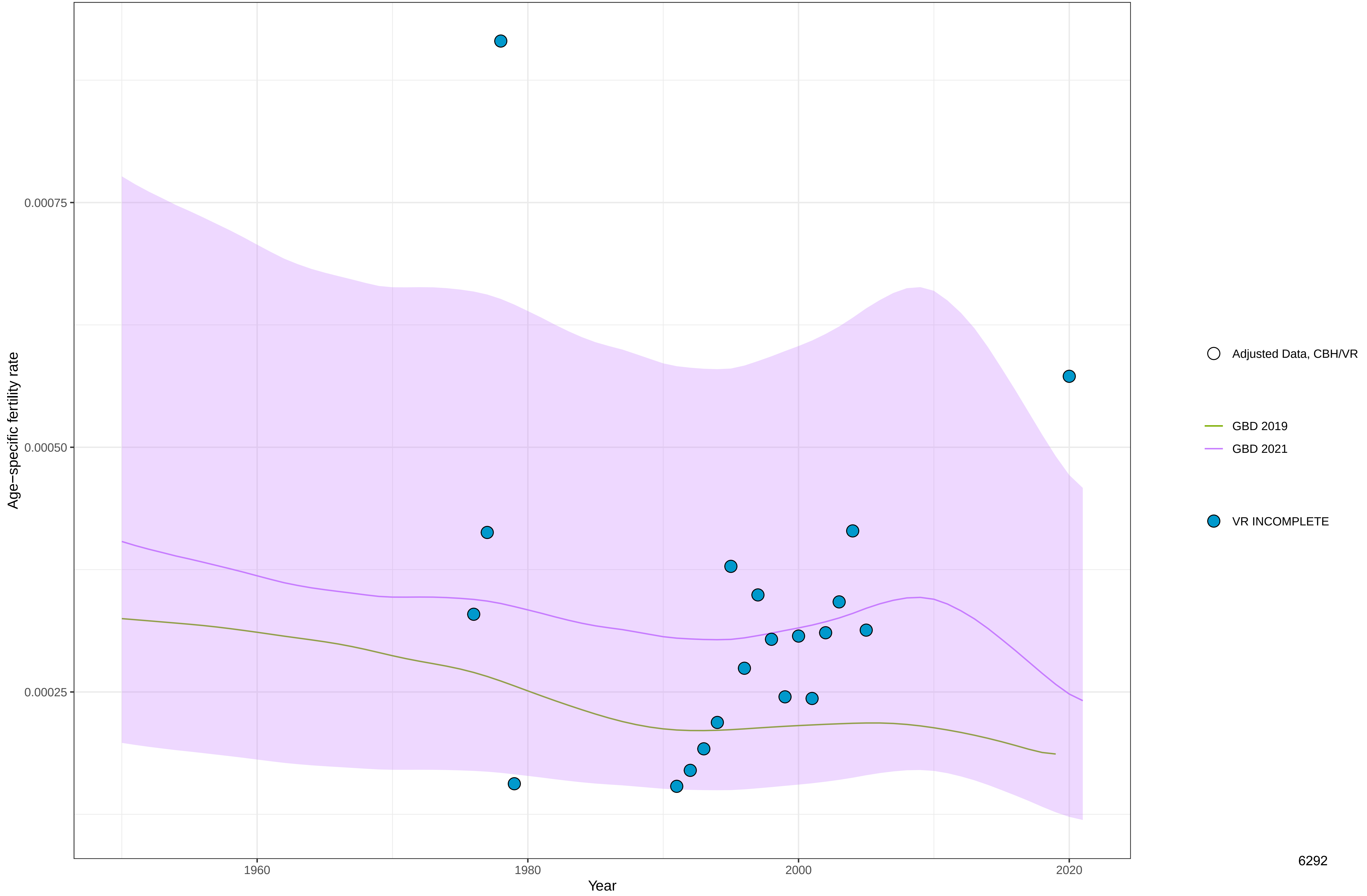

6292

Age−specific fertility fitted trend lines, 1950−2021: National Capital Region, Ages: 15−19
Data points enclosed with a diamond are reference sources

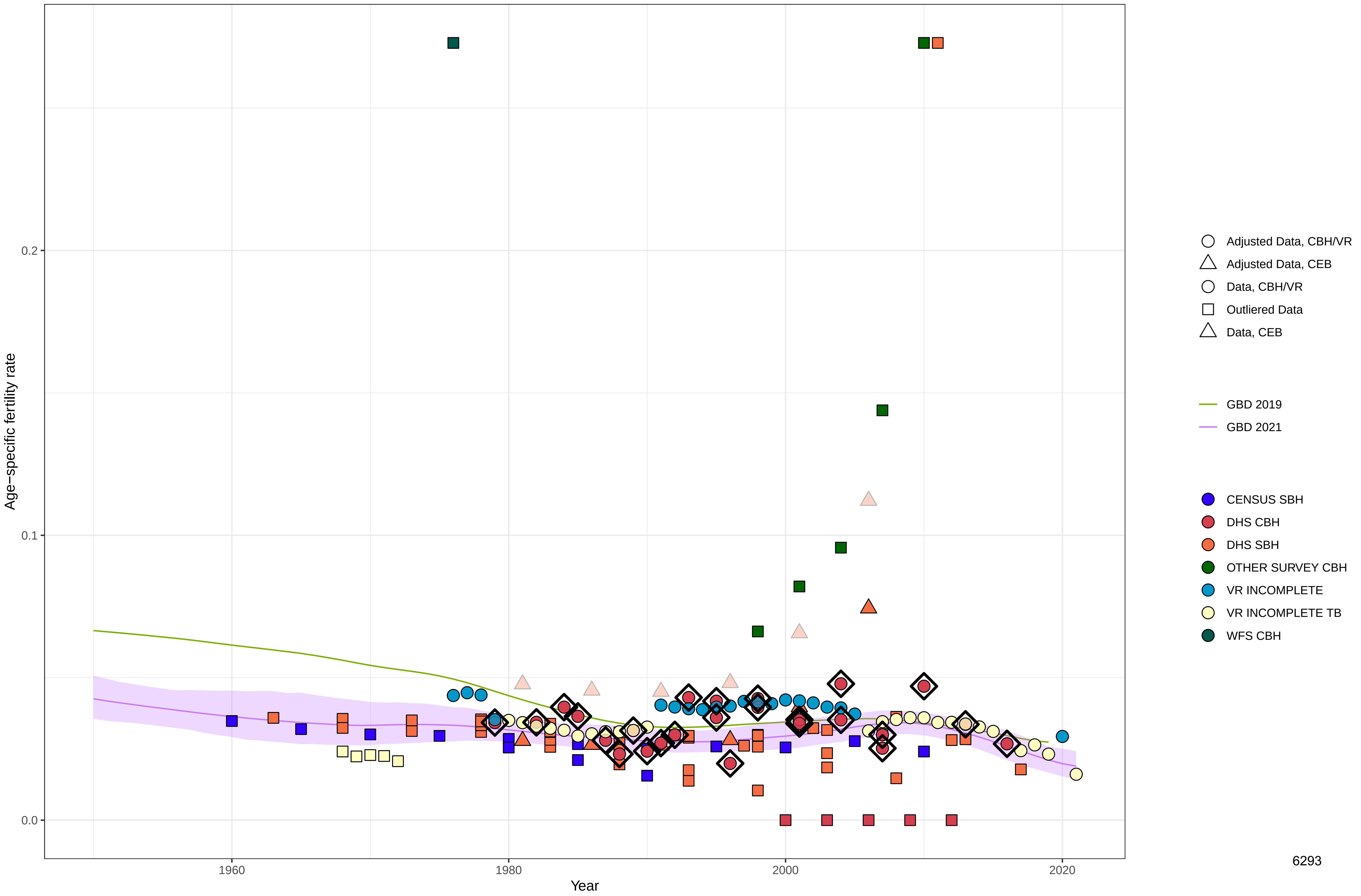

Age−specific fertility fitted trend lines, 1950−2021: National Capital Region, Ages: 20−24
Data points enclosed with a diamond are reference sources

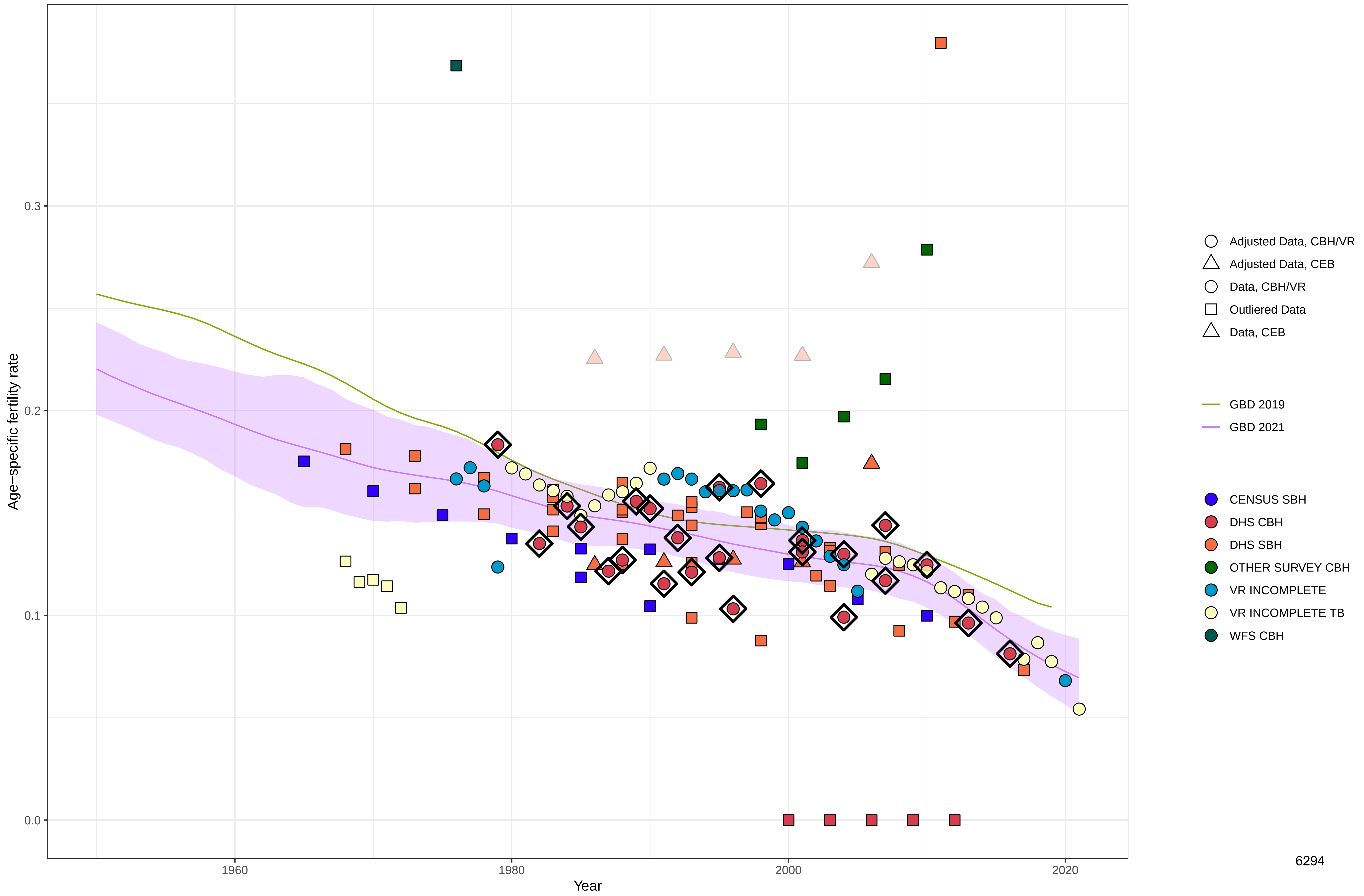

Age−specific fertility fitted trend lines, 1950−2021: National Capital Region, Ages: 25−29
Data points enclosed with a diamond are reference sources

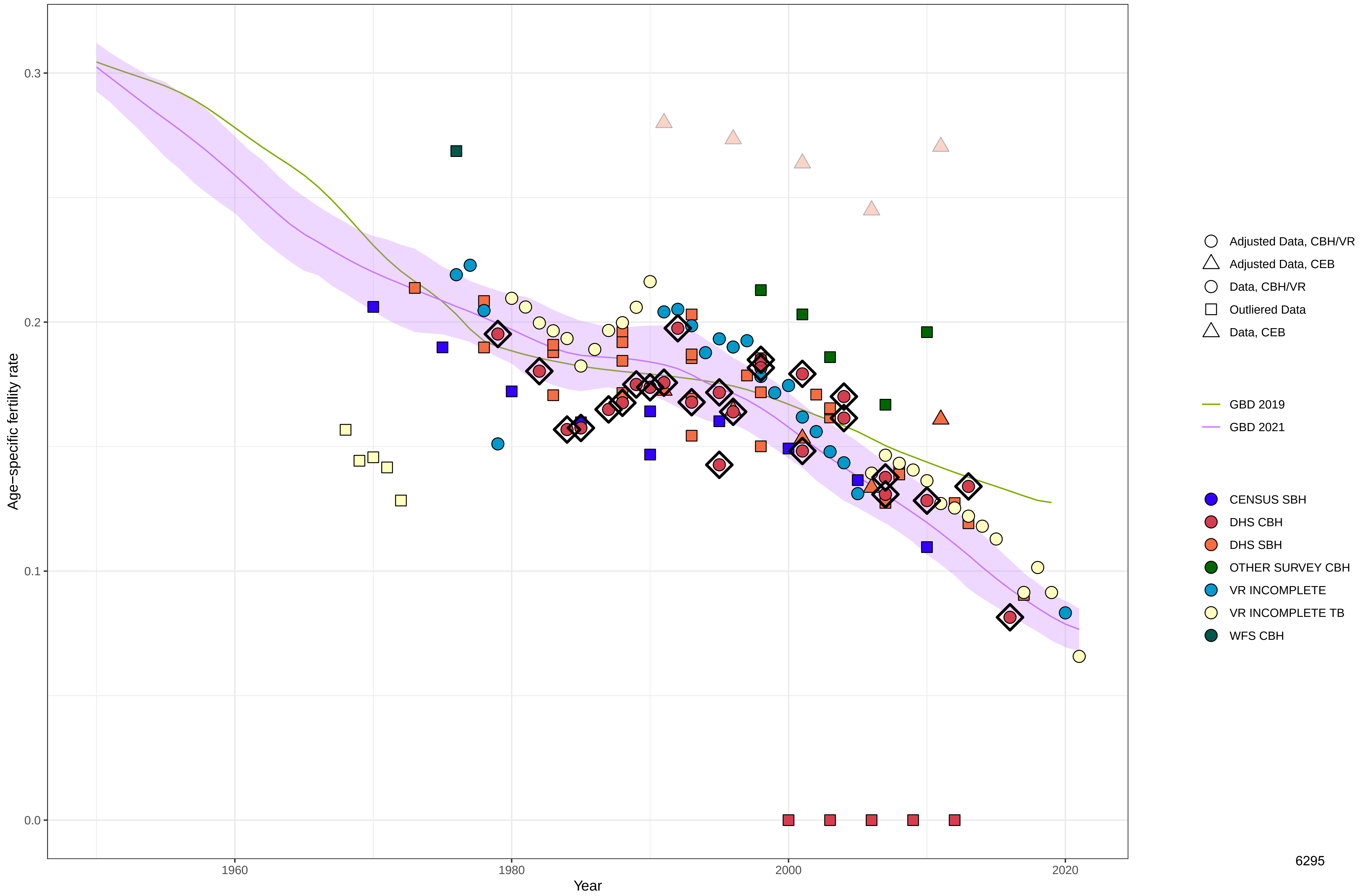

Age−specific fertility fitted trend lines, 1950−2021: National Capital Region, Ages: 30−34
Data points enclosed with a diamond are reference sources

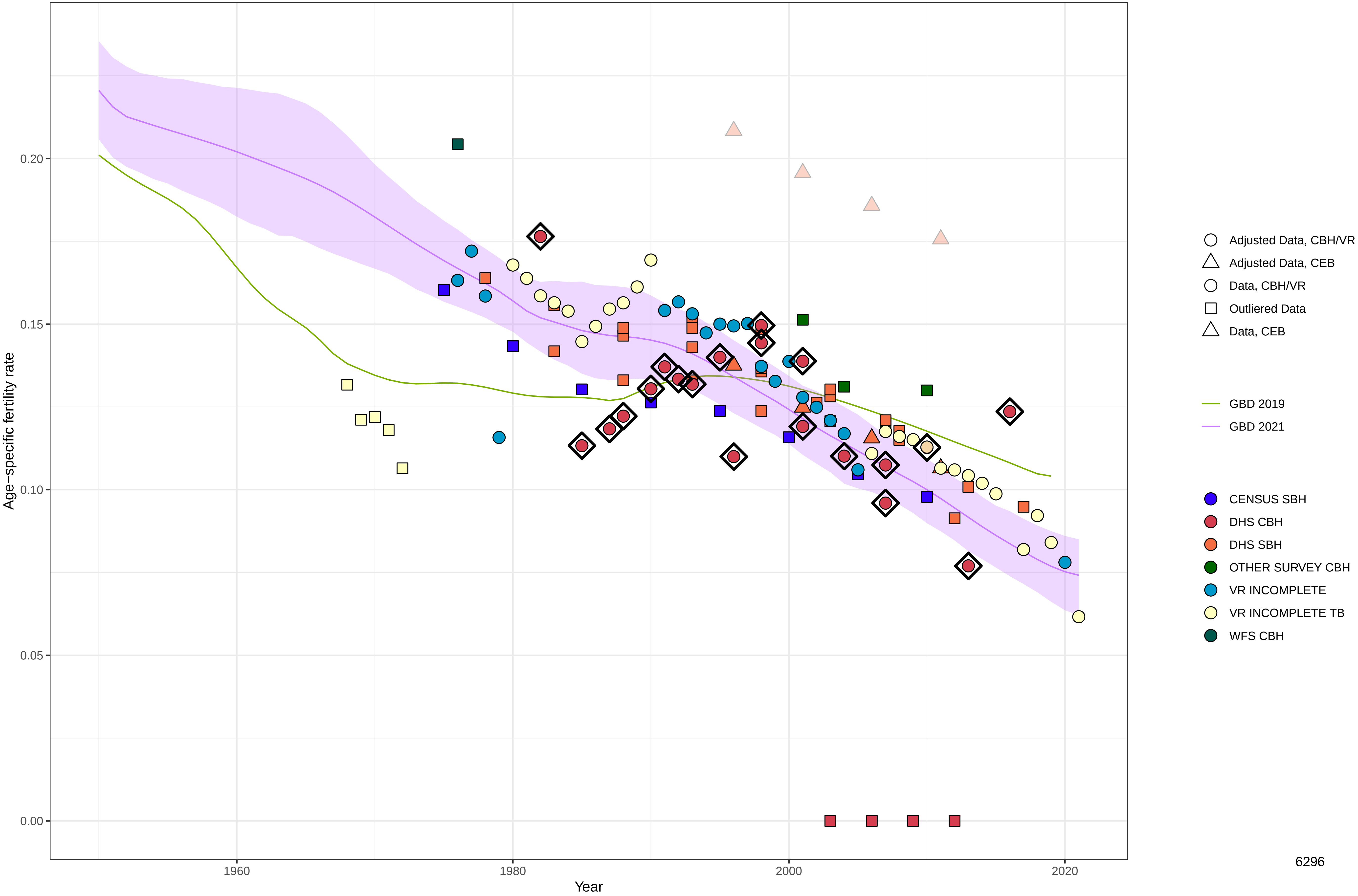

6296

Age−specific fertility fitted trend lines, 1950−2021: National Capital Region, Ages: 35−39
Data points enclosed with a diamond are reference sources

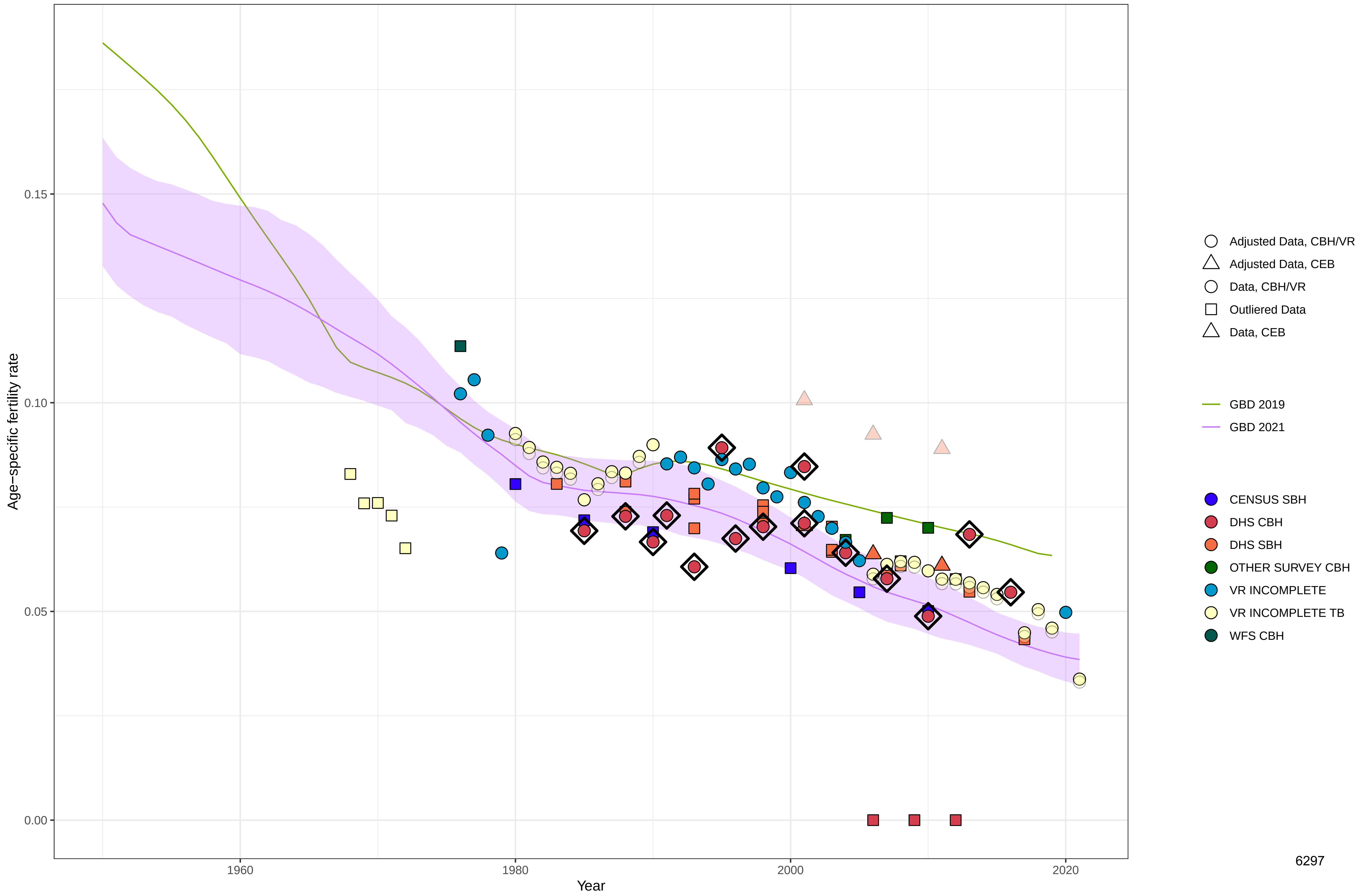

Age−specific fertility fitted trend lines, 1950−2021: National Capital Region, Ages: 40−44
Data points enclosed with a diamond are reference sources

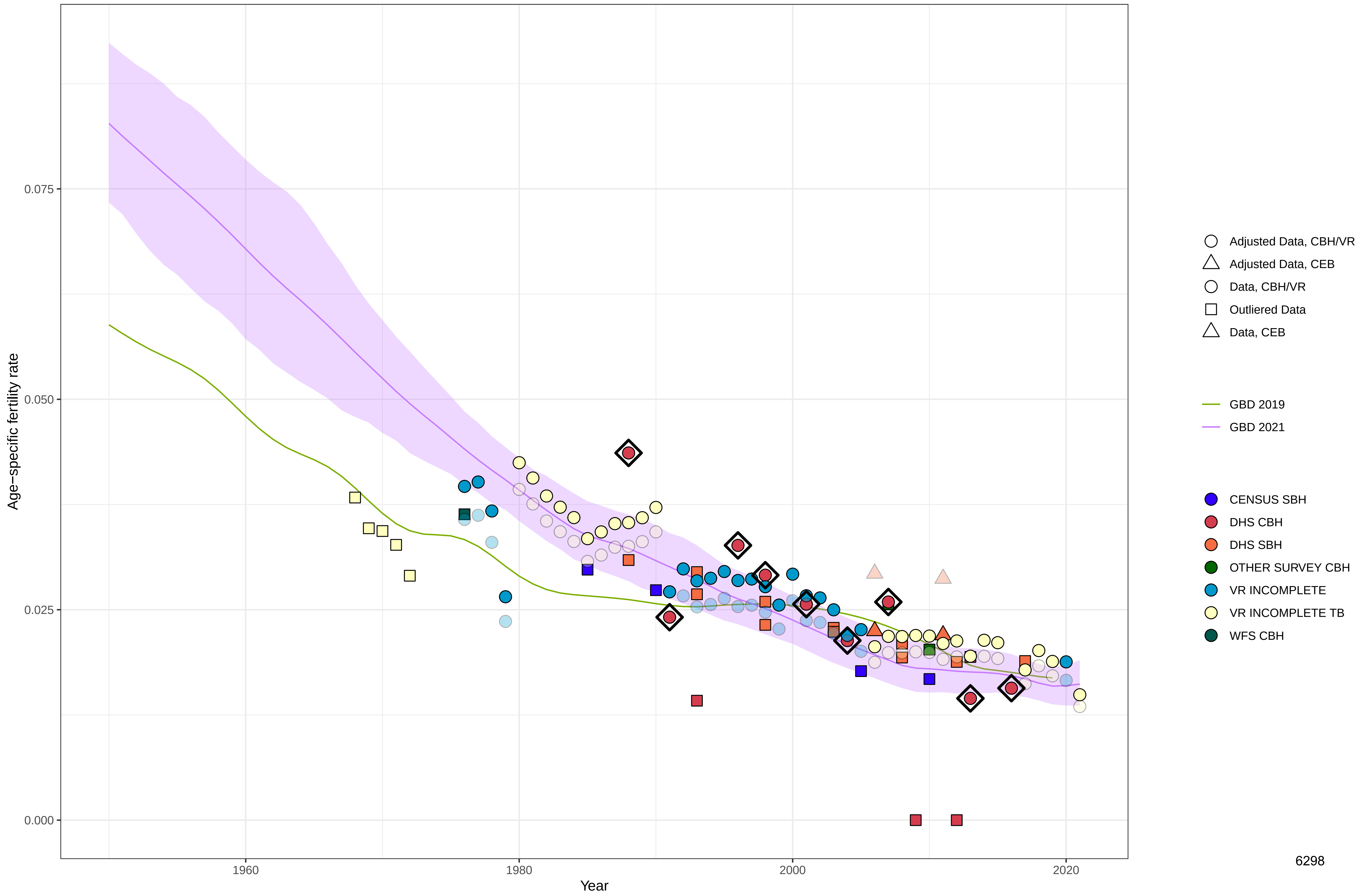

6298

Age−specific fertility fitted trend lines, 1950−2021: National Capital Region, Ages: 45−49
Data points enclosed with a diamond are reference sources

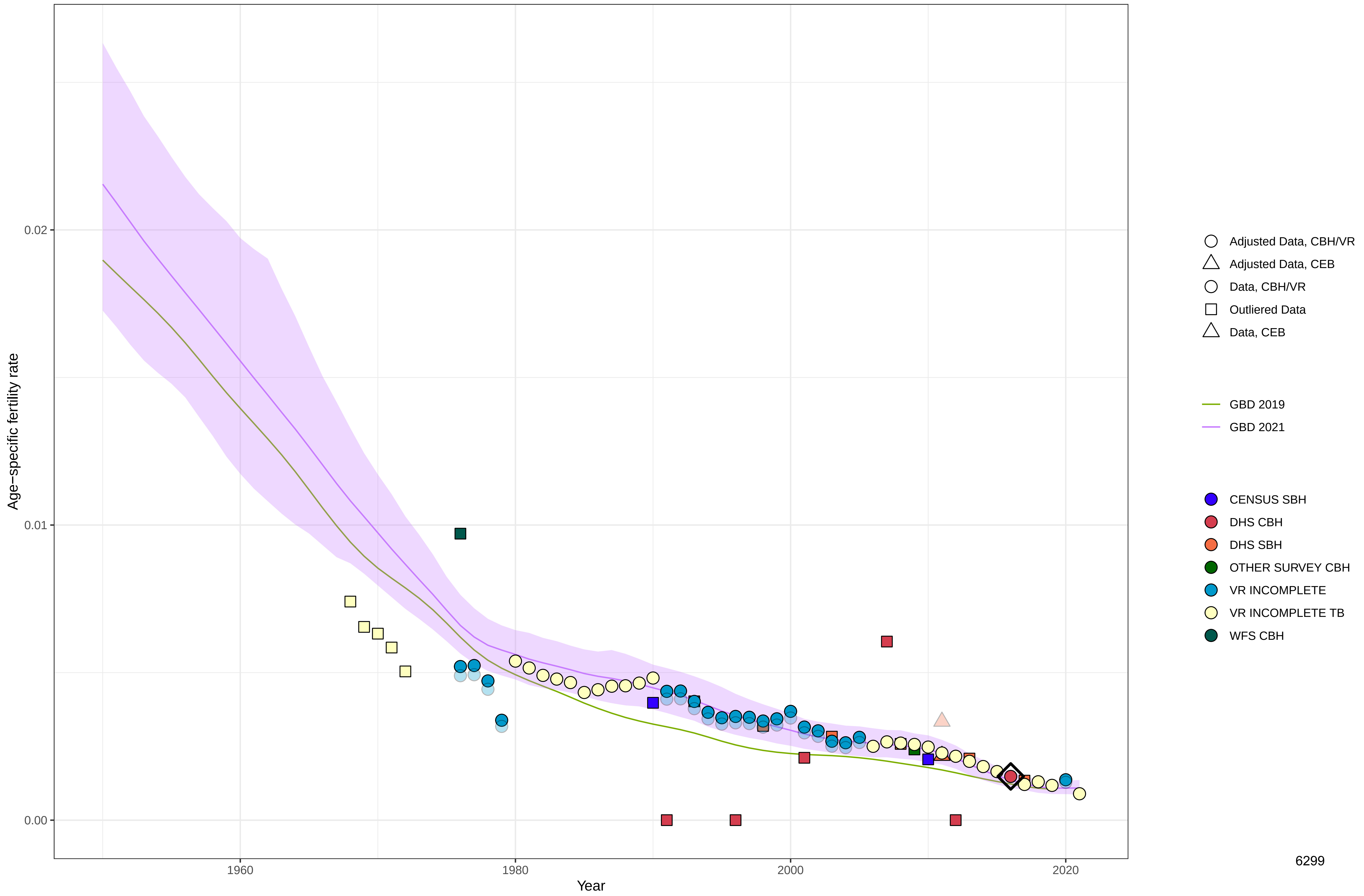

Age−specific fertility fitted trend lines, 1950−2021: National Capital Region, Ages: 50−54
Data points enclosed with a diamond are reference sources

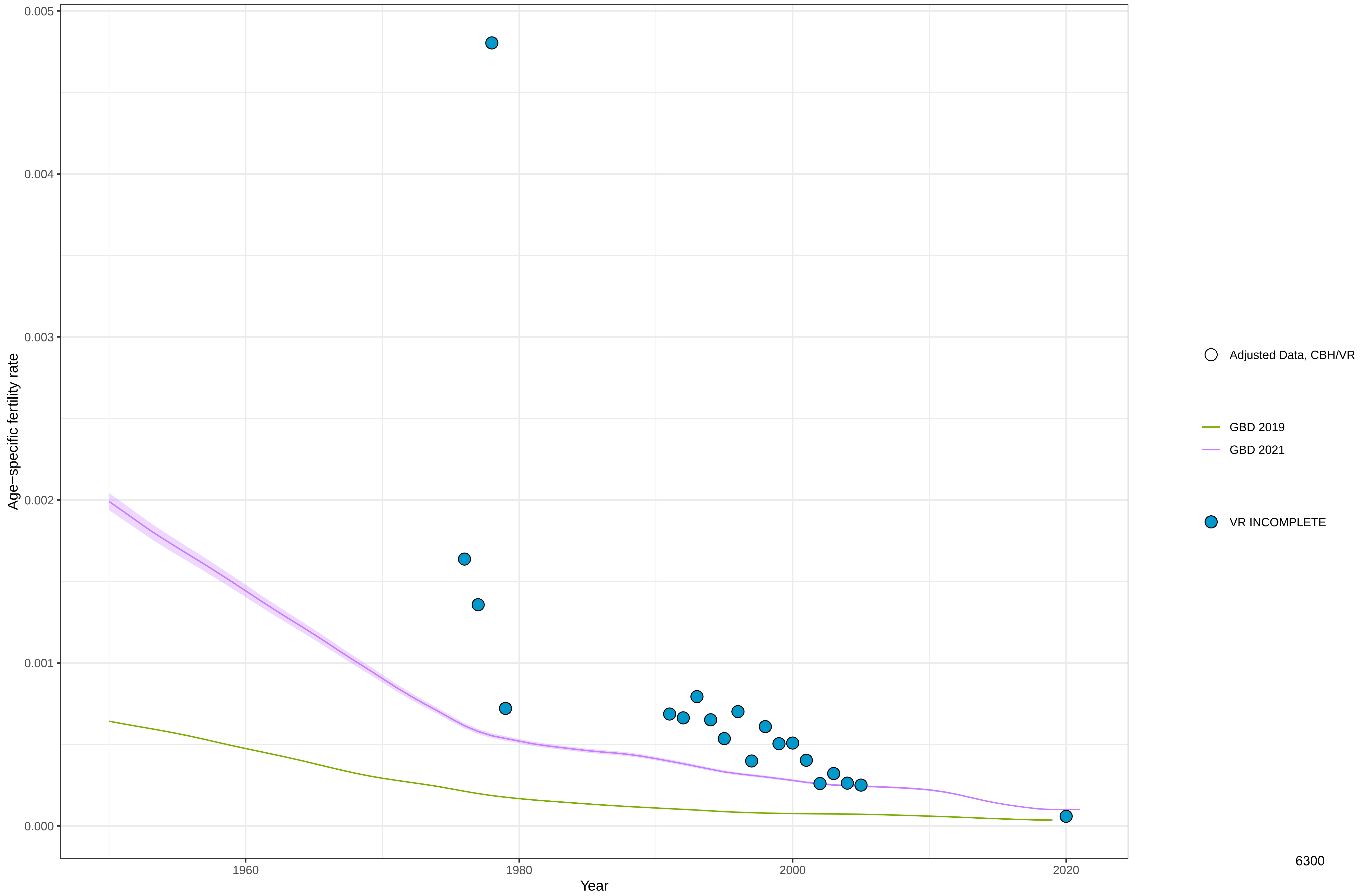

Total fertility rate fitted trend lines, 1950−2021: National Capital Region TFR
Data points enclosed with a diamond are reference sources

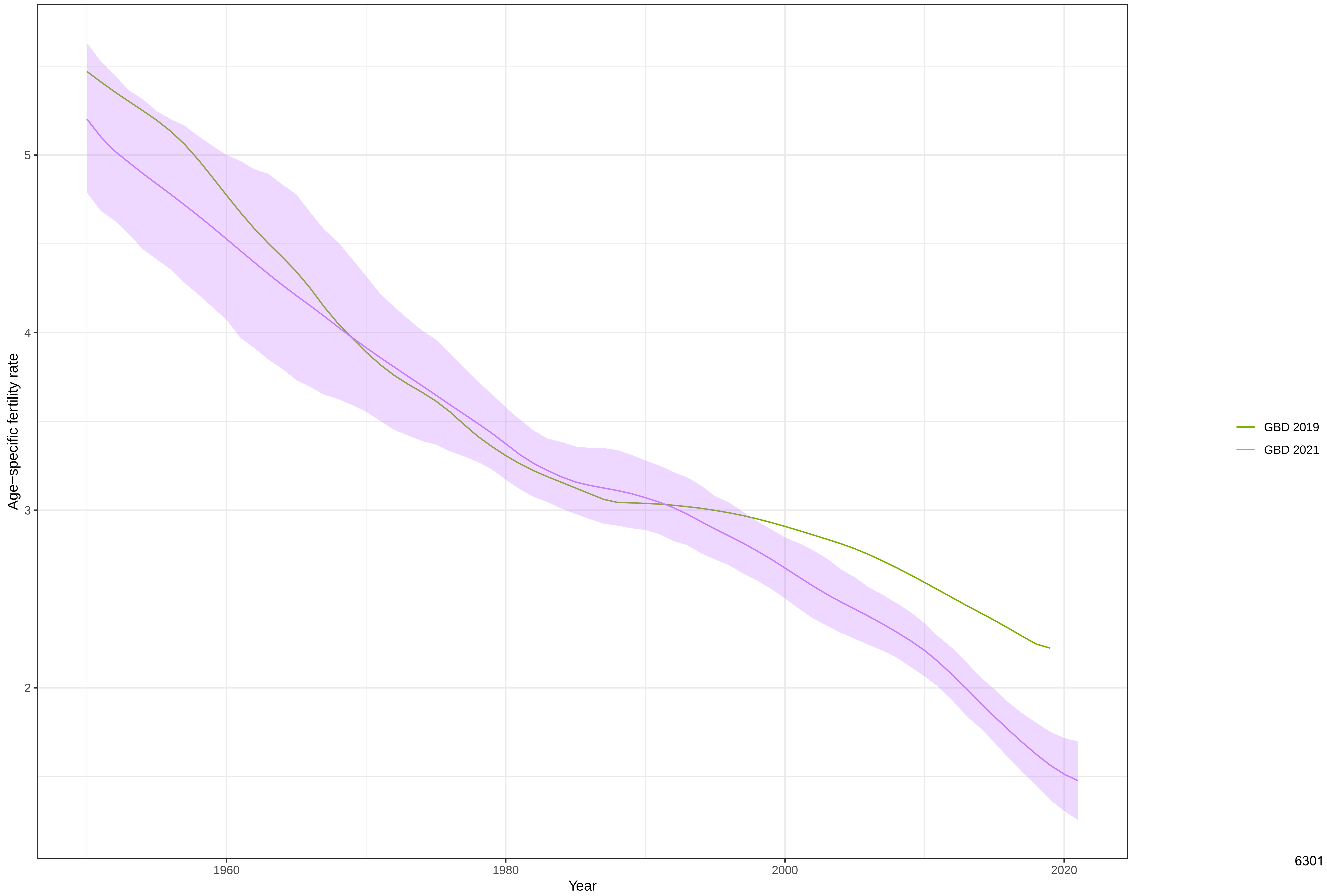

Age−specific fertility fitted trend lines, 1950−2021: Negros Occidental, Ages: 10−14
Data points enclosed with a diamond are reference sources

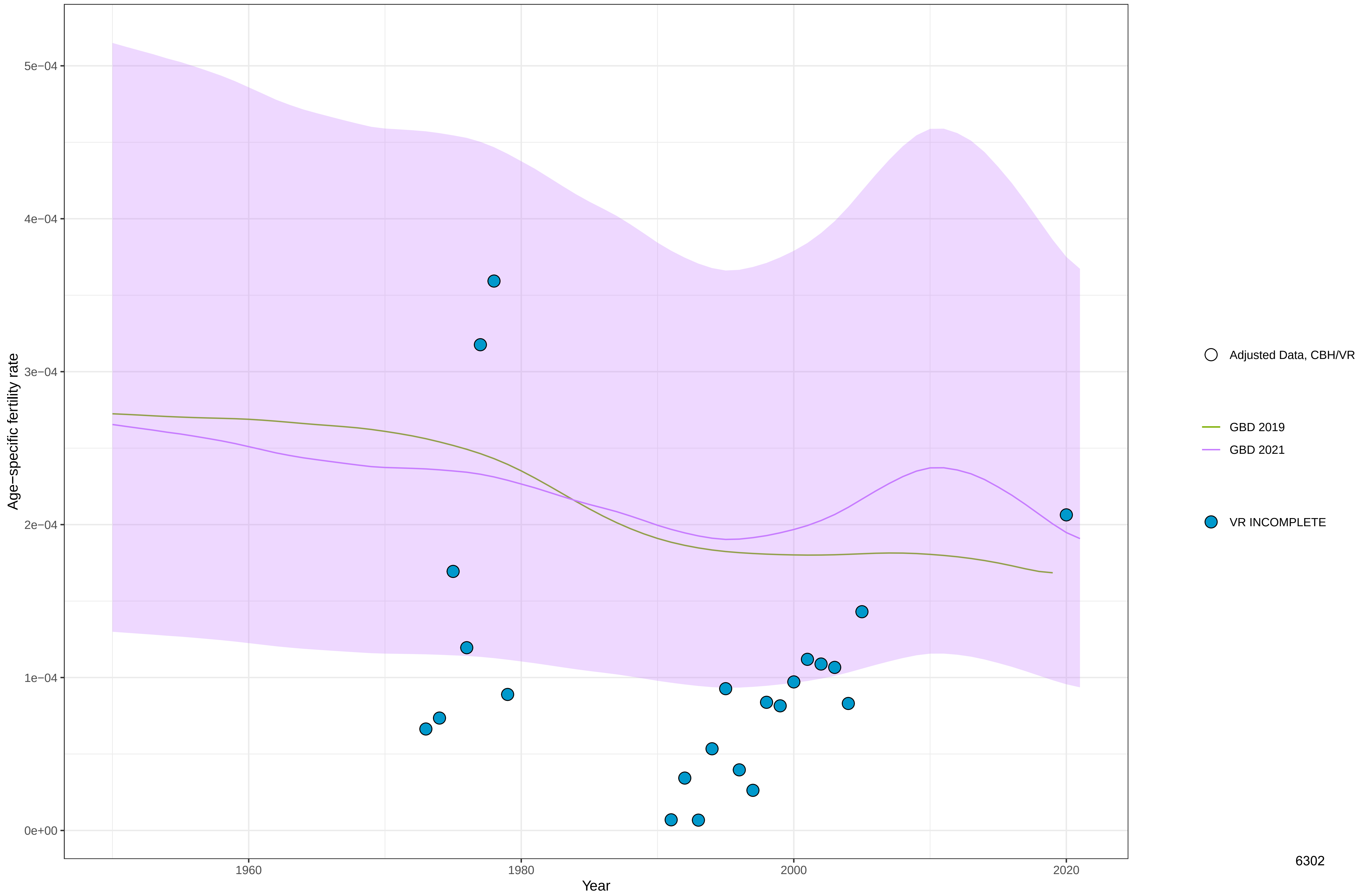

6302

Age−specific fertility fitted trend lines, 1950−2021: Negros Occidental, Ages: 15−19
Data points enclosed with a diamond are reference sources

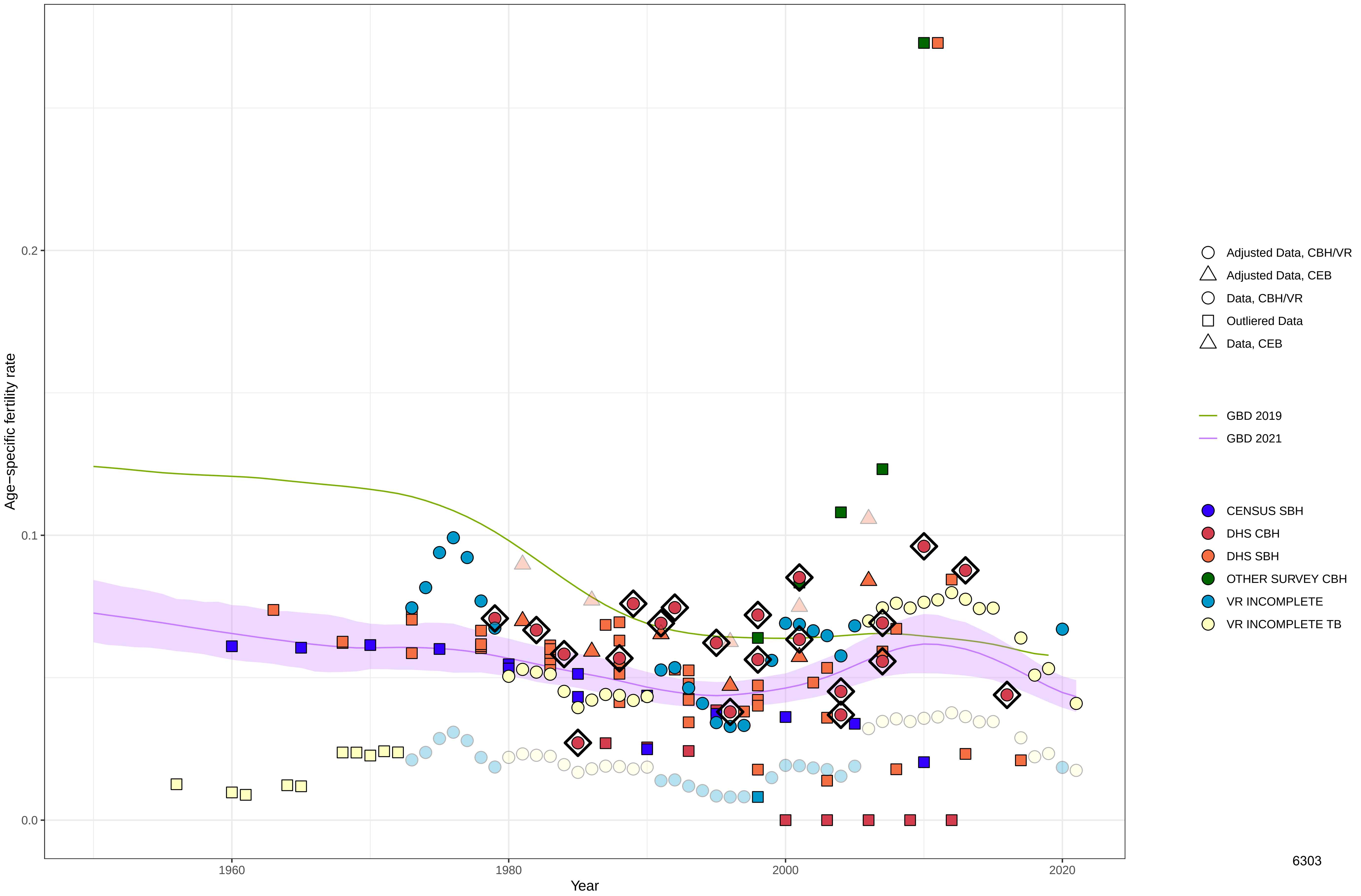

Age−specific fertility fitted trend lines, 1950−2021: Negros Occidental, Ages: 20−24
Data points enclosed with a diamond are reference sources

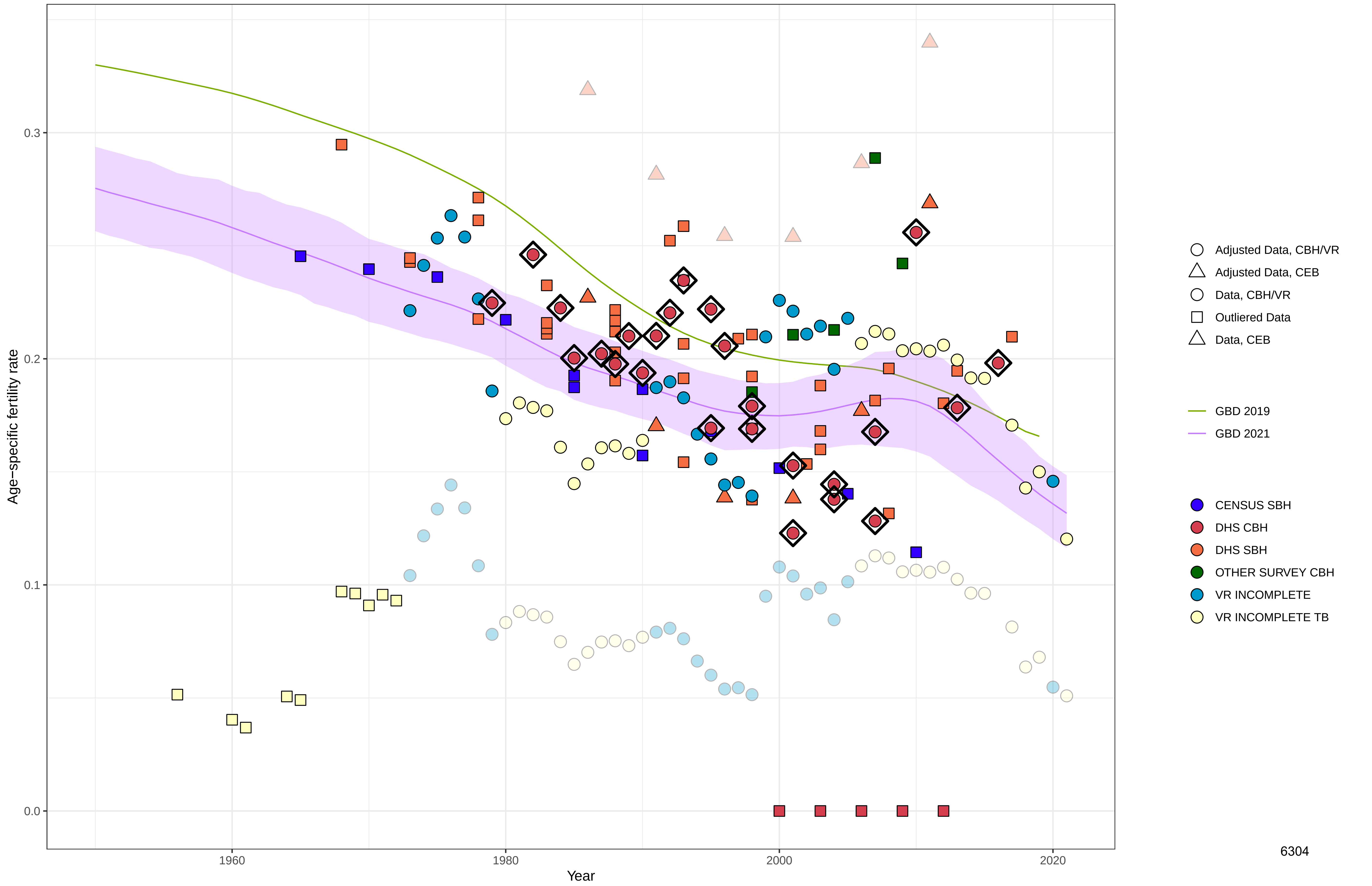

Age−specific fertility fitted trend lines, 1950−2021: Negros Occidental, Ages: 25−29
Data points enclosed with a diamond are reference sources

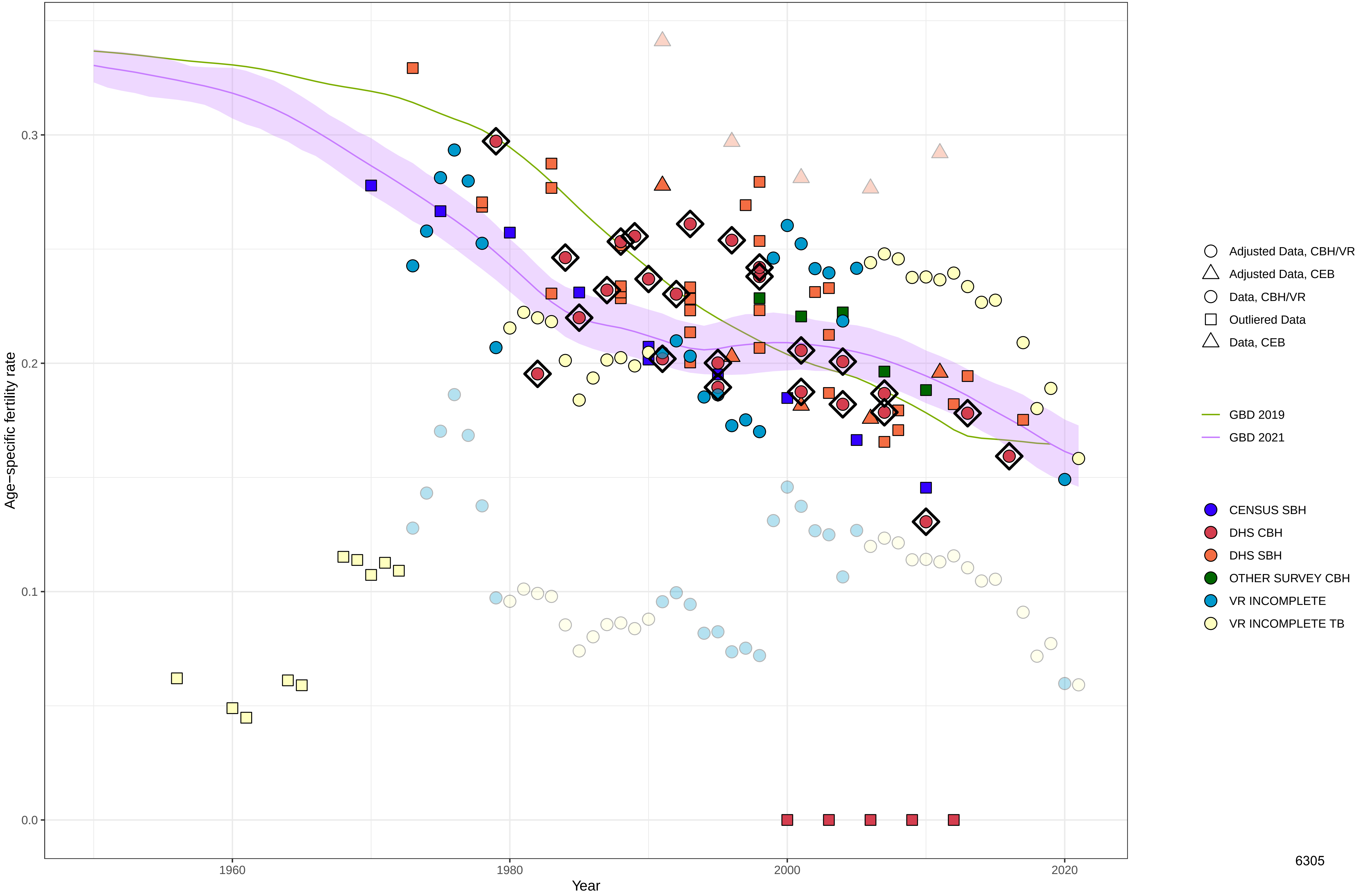

Age−specific fertility fitted trend lines, 1950−2021: Negros Occidental, Ages: 30−34
Data points enclosed with a diamond are reference sources

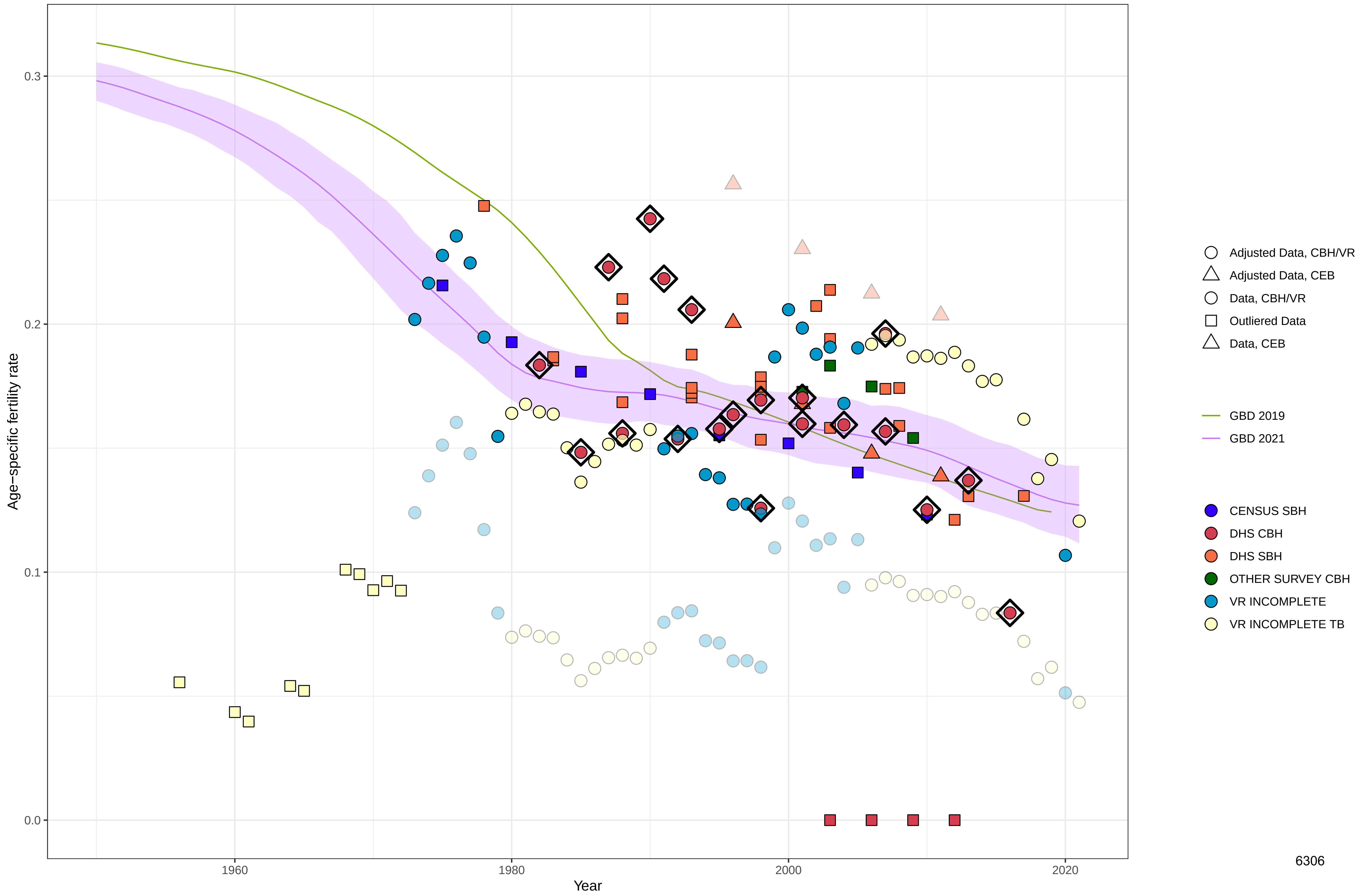

Age−specific fertility fitted trend lines, 1950−2021: Negros Occidental, Ages: 35−39
Data points enclosed with a diamond are reference sources

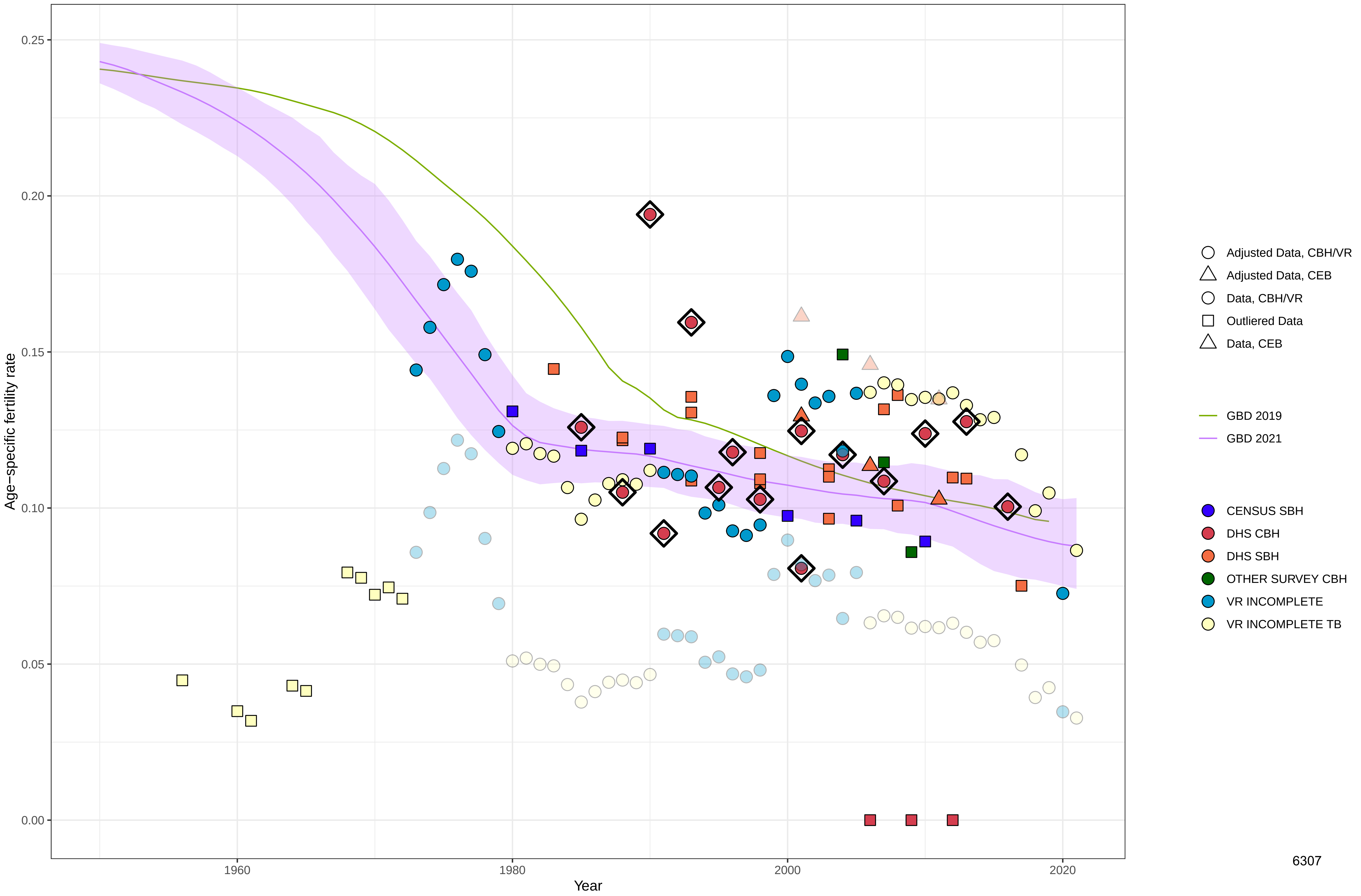

Age−specific fertility fitted trend lines, 1950−2021: Negros Occidental, Ages: 40−44
Data points enclosed with a diamond are reference sources

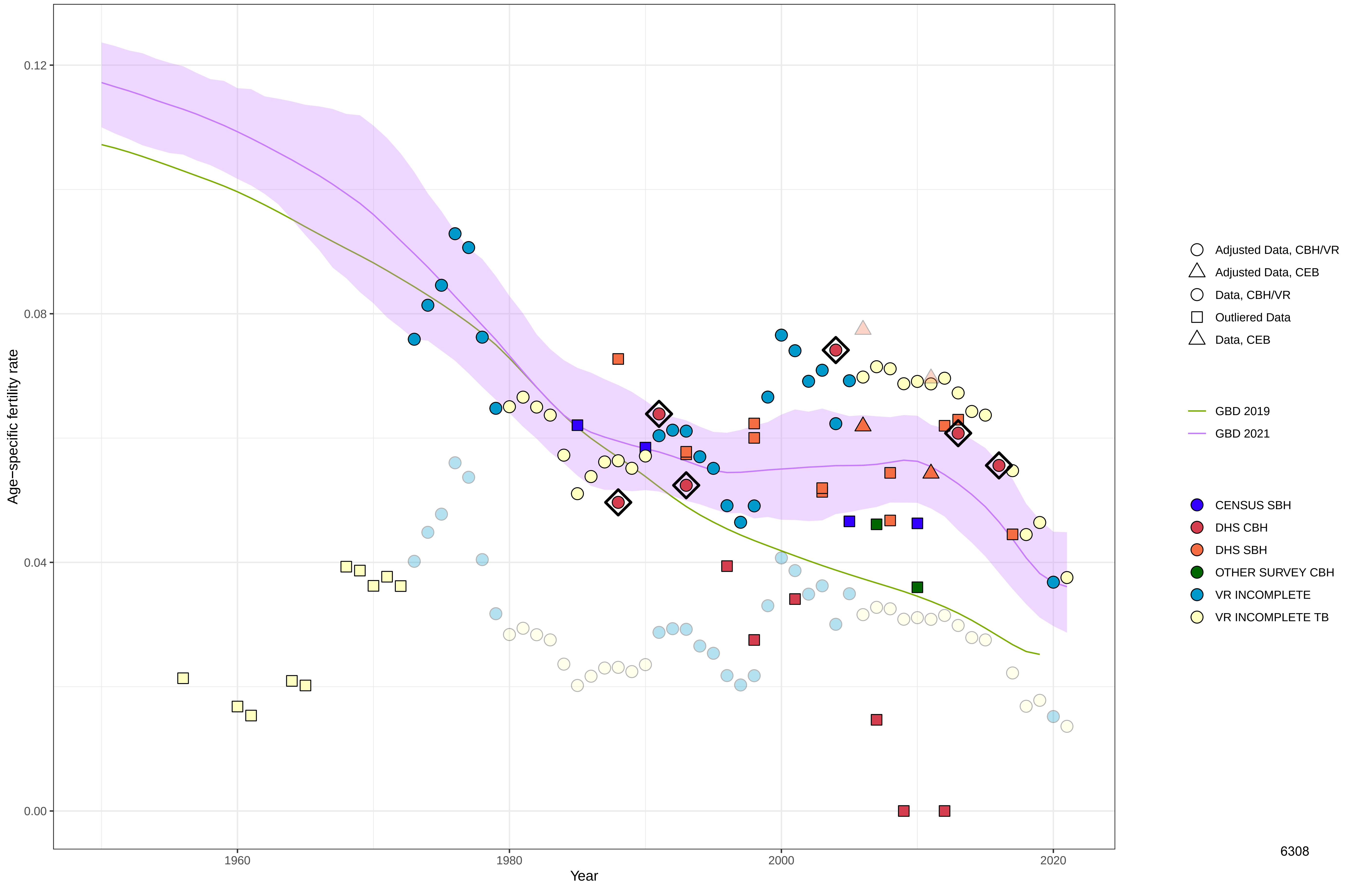

Age−specific fertility fitted trend lines, 1950−2021: Negros Occidental, Ages: 45−49
Data points enclosed with a diamond are reference sources

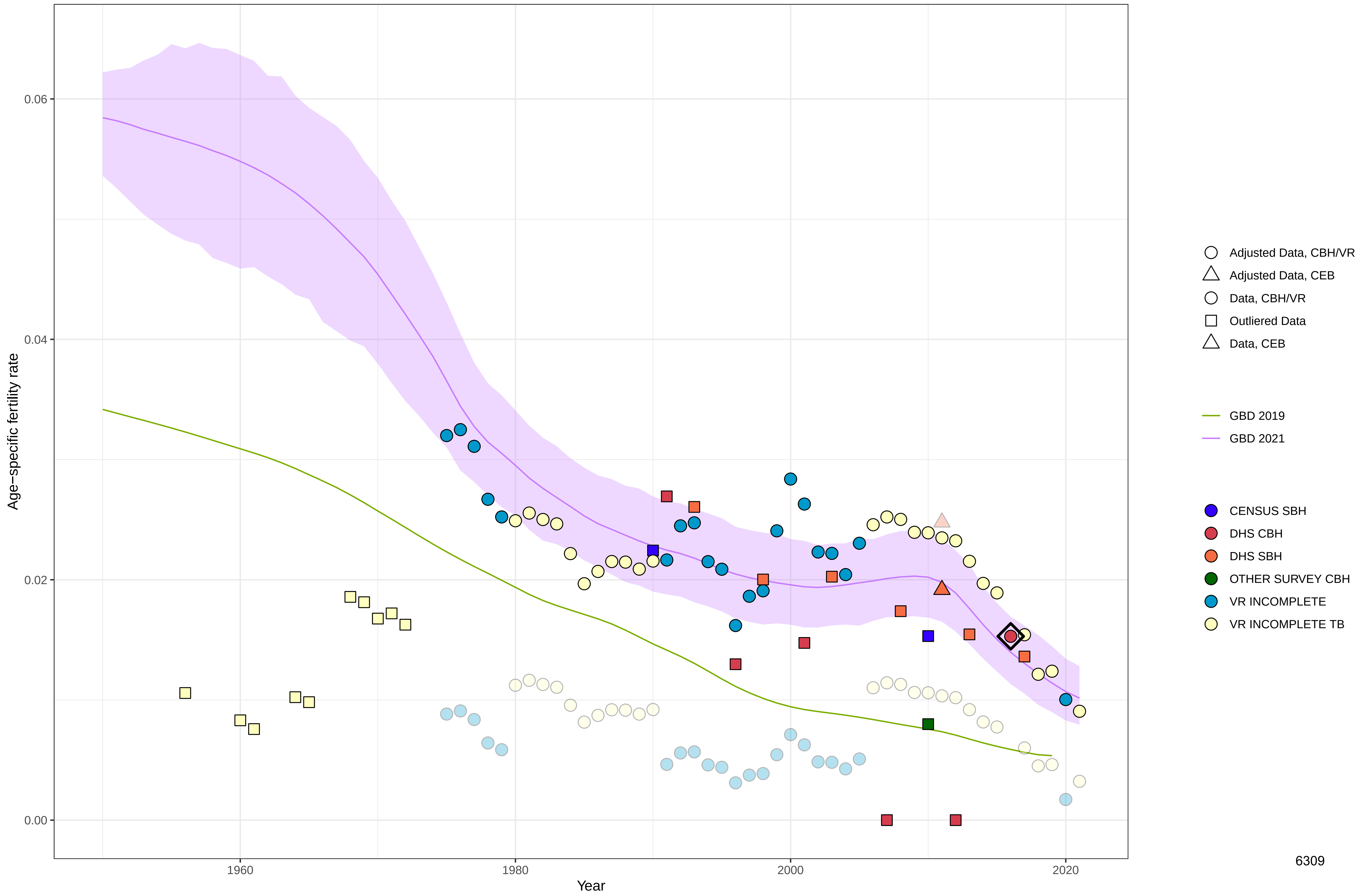

Age−specific fertility fitted trend lines, 1950−2021: Negros Occidental, Ages: 50−54
Data points enclosed with a diamond are reference sources

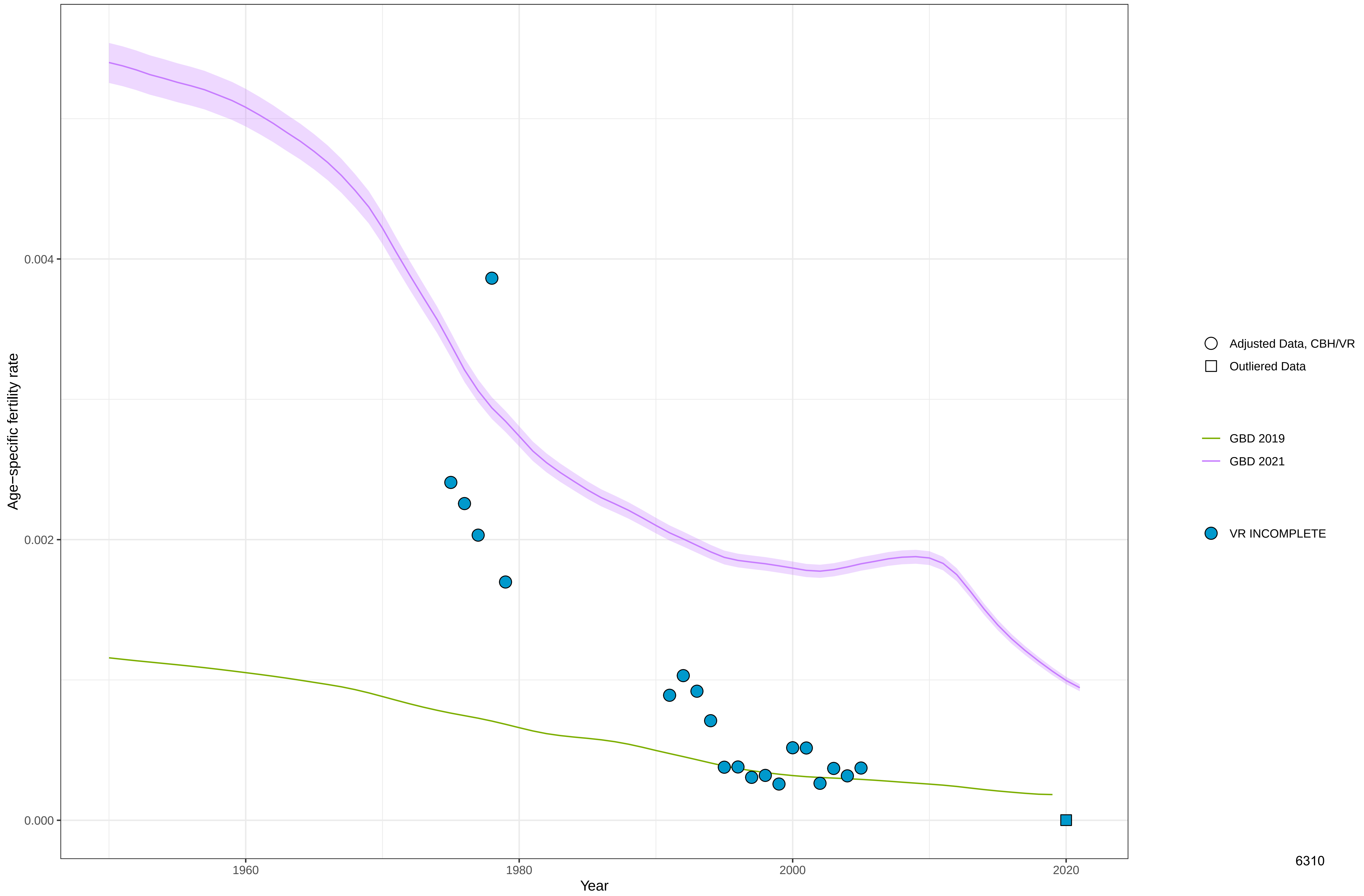

Total fertility rate fitted trend lines, 1950−2021: Negros Occidental TFR
Data points enclosed with a diamond are reference sources

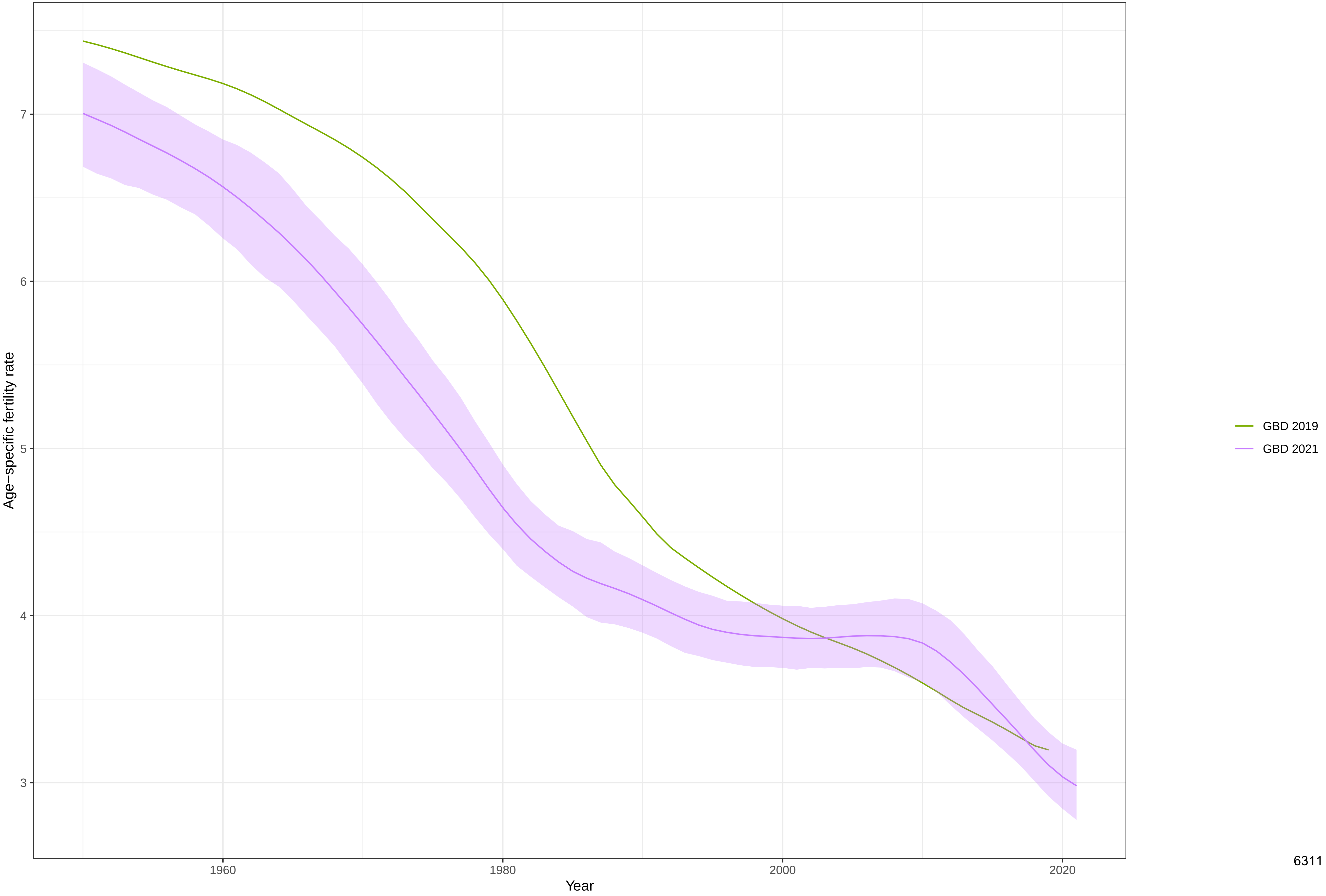

Age−specific fertility fitted trend lines, 1950−2021: Negros Oriental, Ages: 10−14
Data points enclosed with a diamond are reference sources

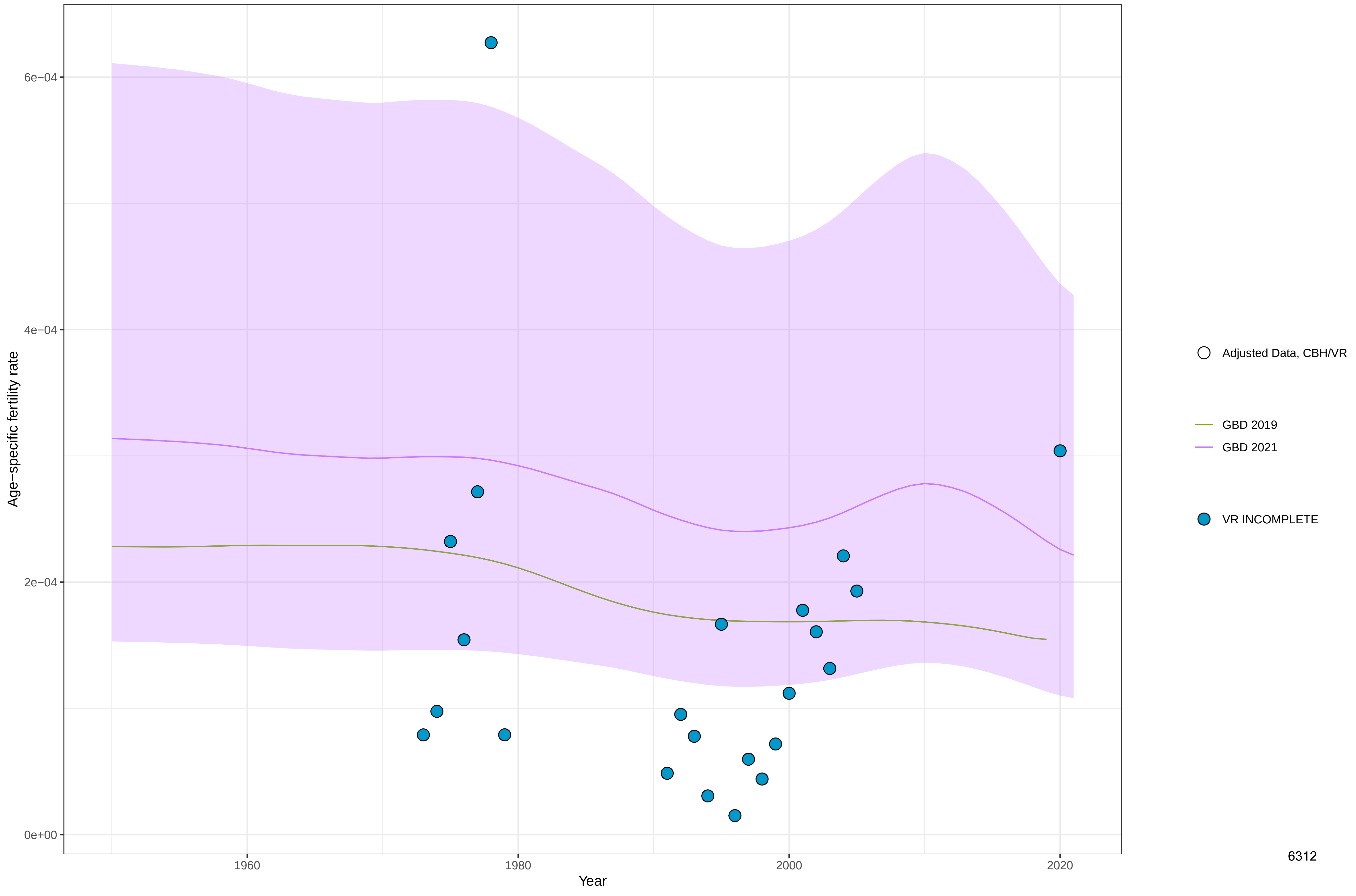

6312

Age−specific fertility fitted trend lines, 1950−2021: Negros Oriental, Ages: 15−19
Data points enclosed with a diamond are reference sources

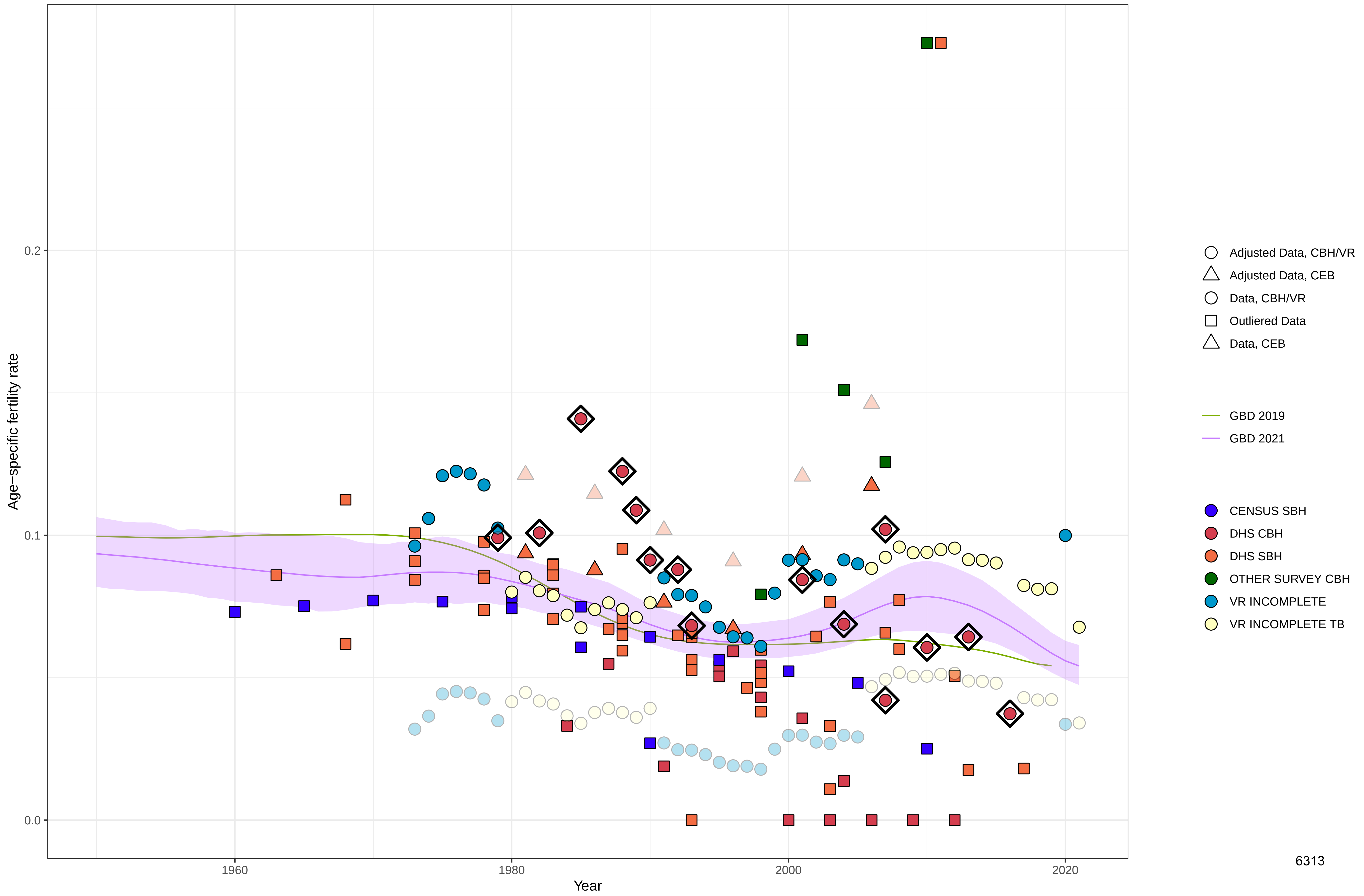

Age−specific fertility fitted trend lines, 1950−2021: Negros Oriental, Ages: 20−24
Data points enclosed with a diamond are reference sources

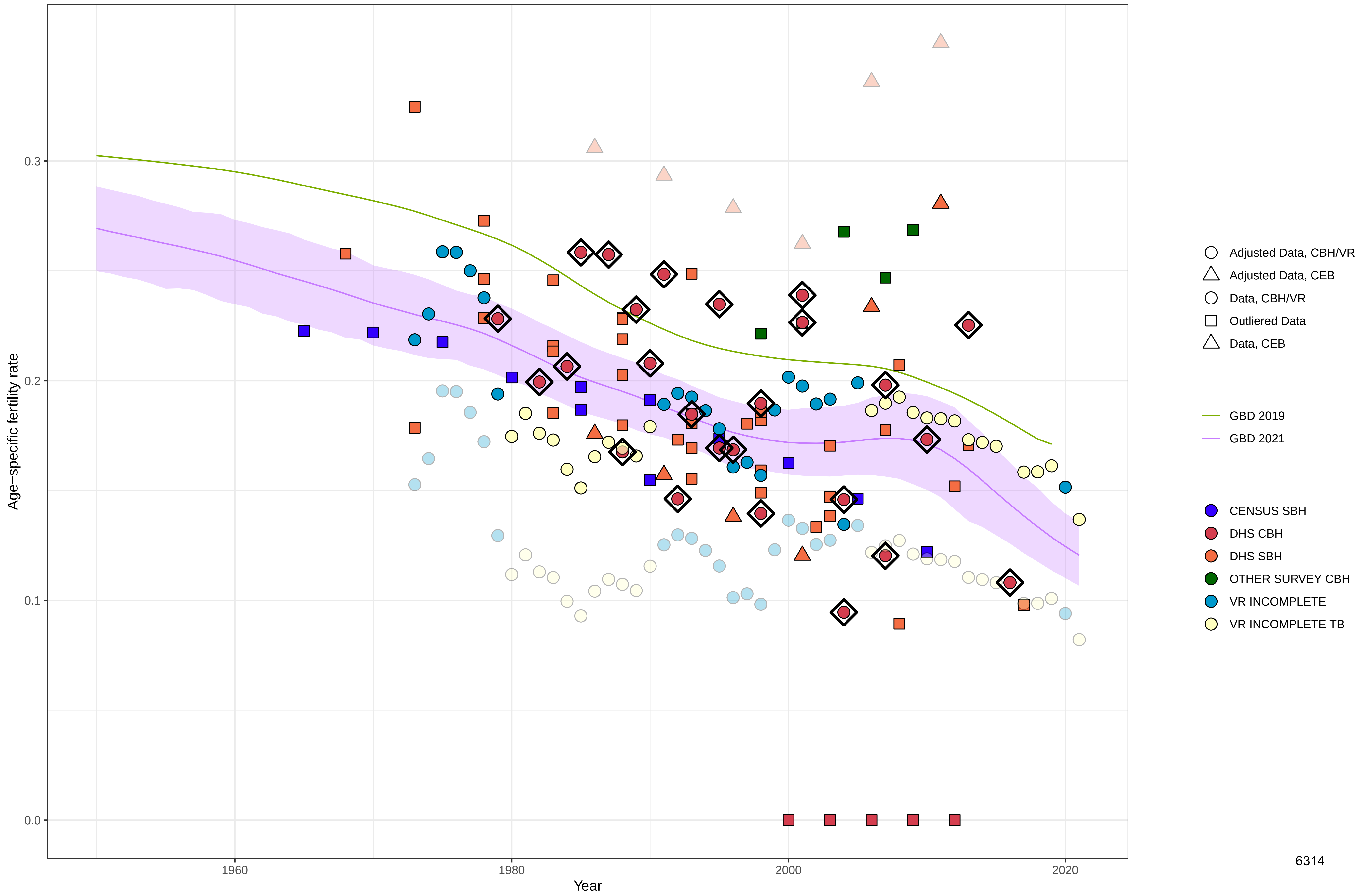

Age−specific fertility fitted trend lines, 1950−2021: Negros Oriental, Ages: 25−29
Data points enclosed with a diamond are reference sources

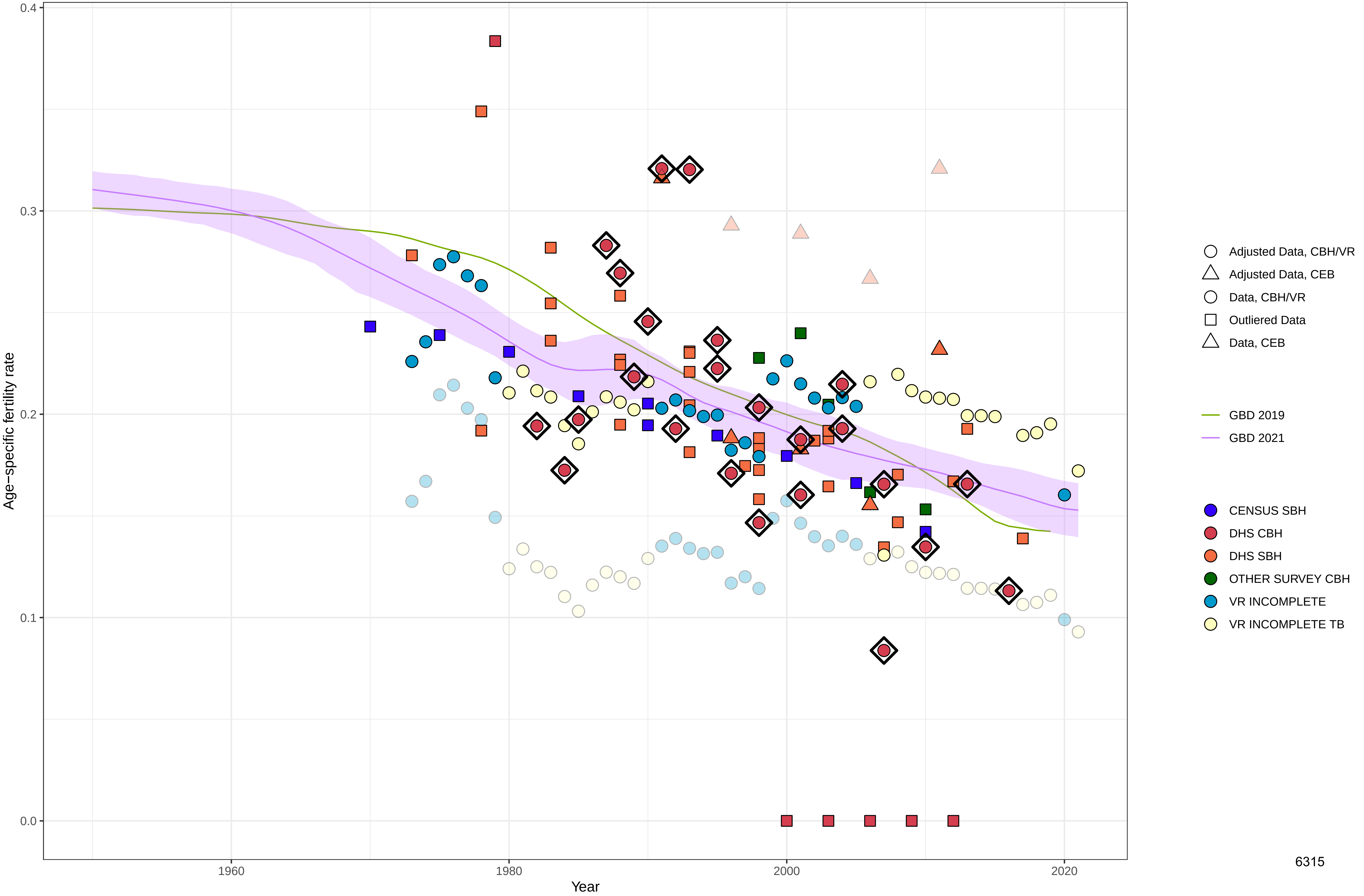

Age−specific fertility fitted trend lines, 1950−2021: Negros Oriental, Ages: 30−34
Data points enclosed with a diamond are reference sources

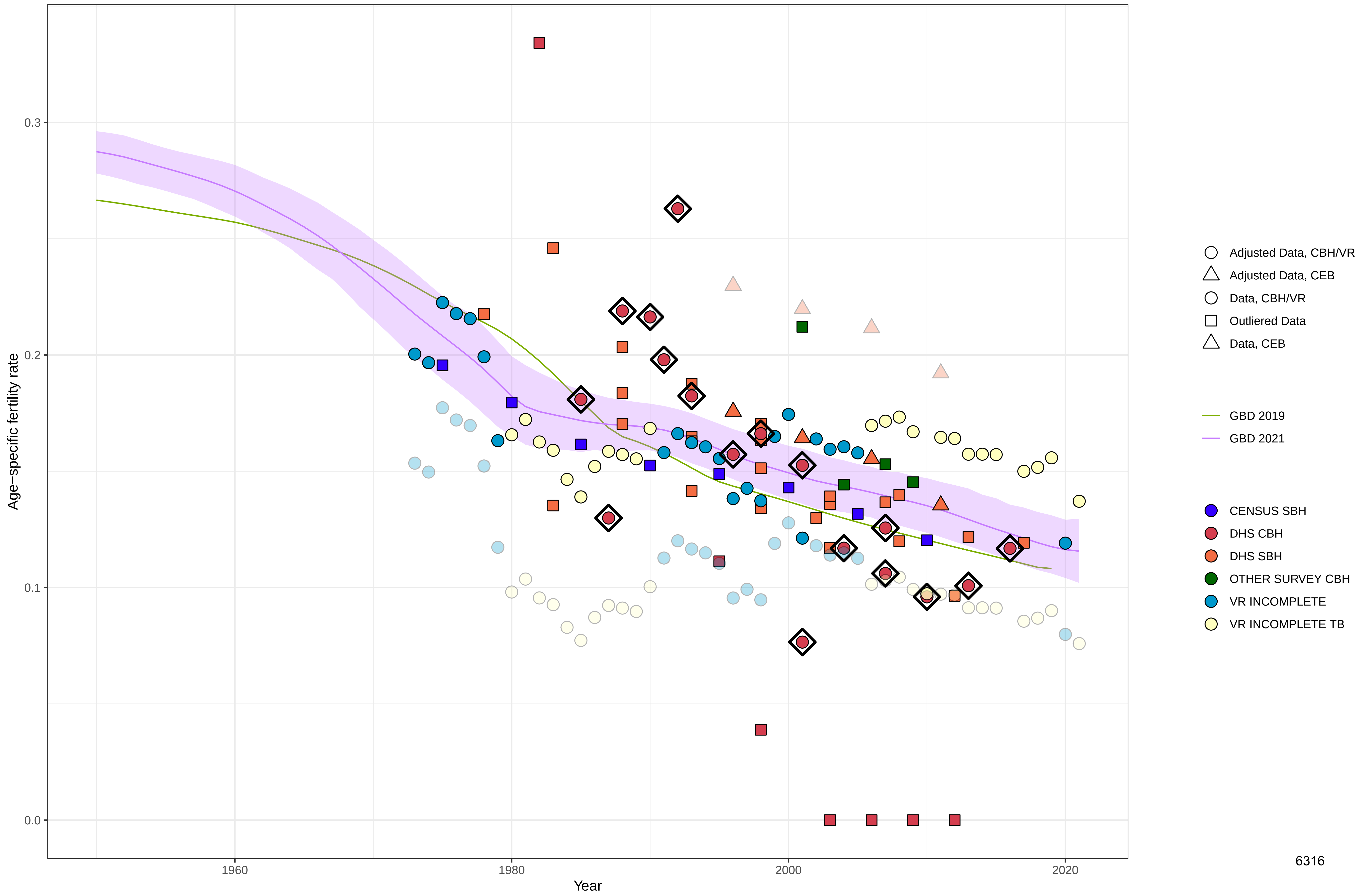

Age−specific fertility fitted trend lines, 1950−2021: Negros Oriental, Ages: 35−39
Data points enclosed with a diamond are reference sources

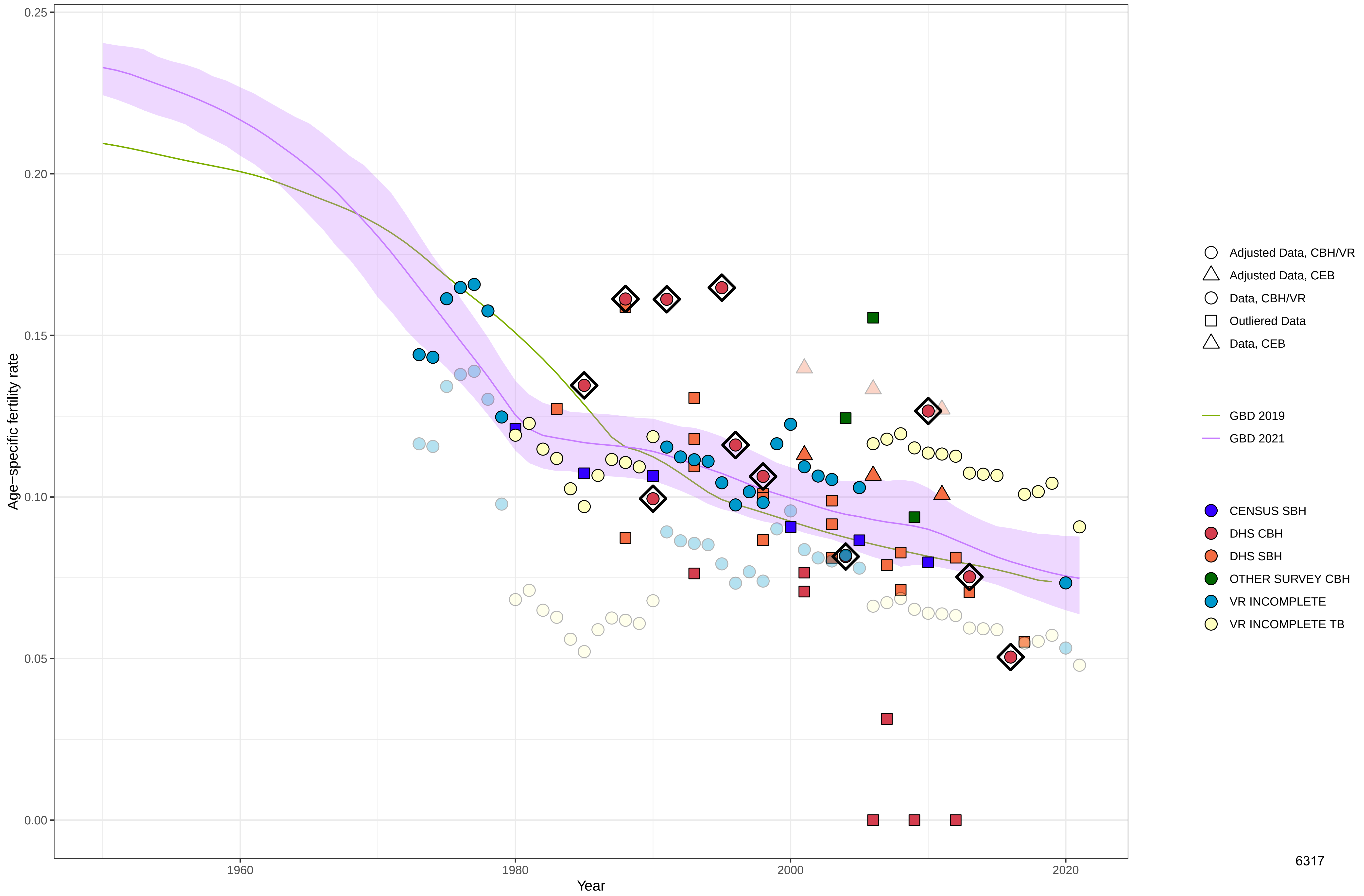

Age−specific fertility fitted trend lines, 1950−2021: Negros Oriental, Ages: 40−44
Data points enclosed with a diamond are reference sources

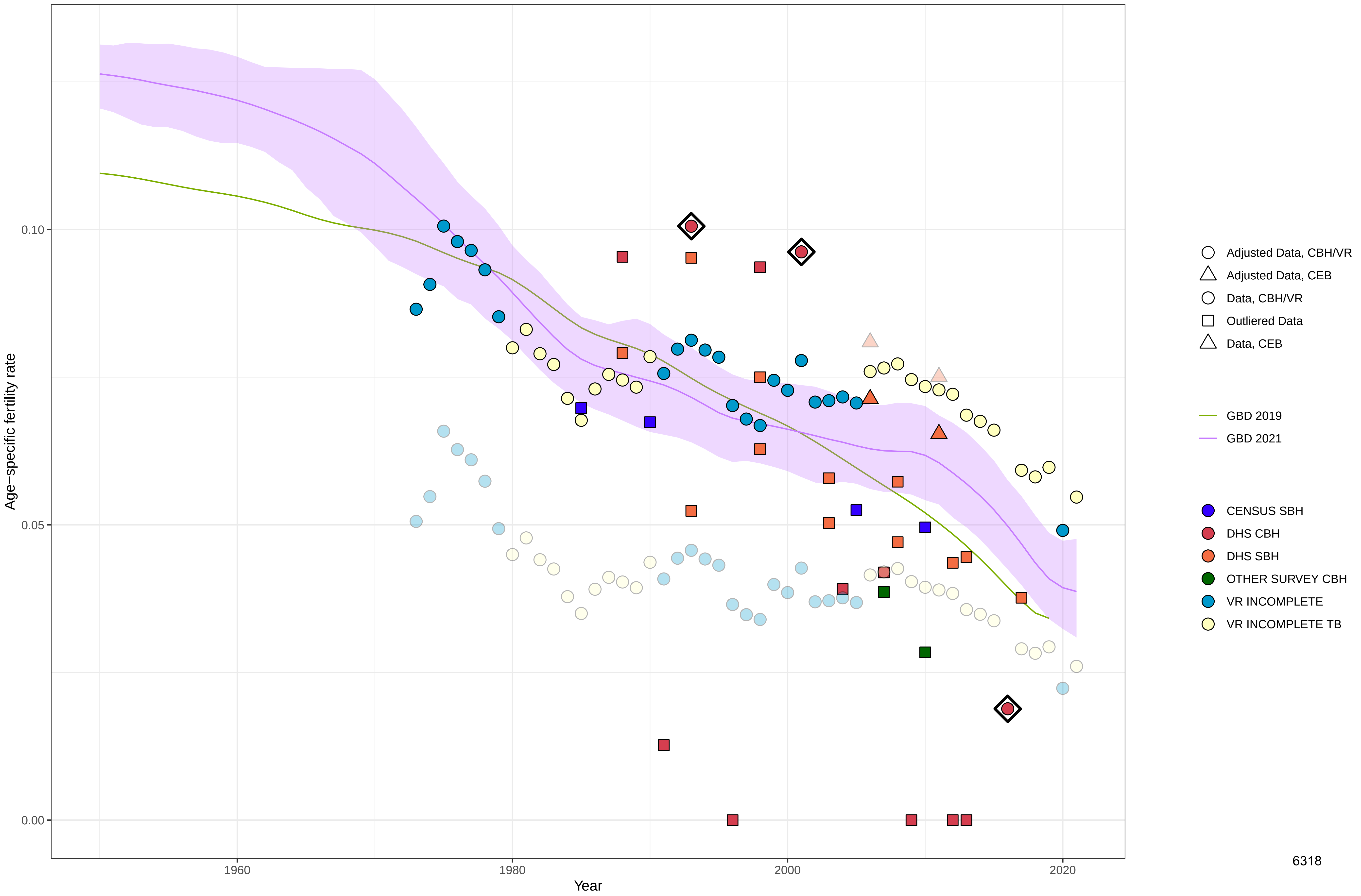

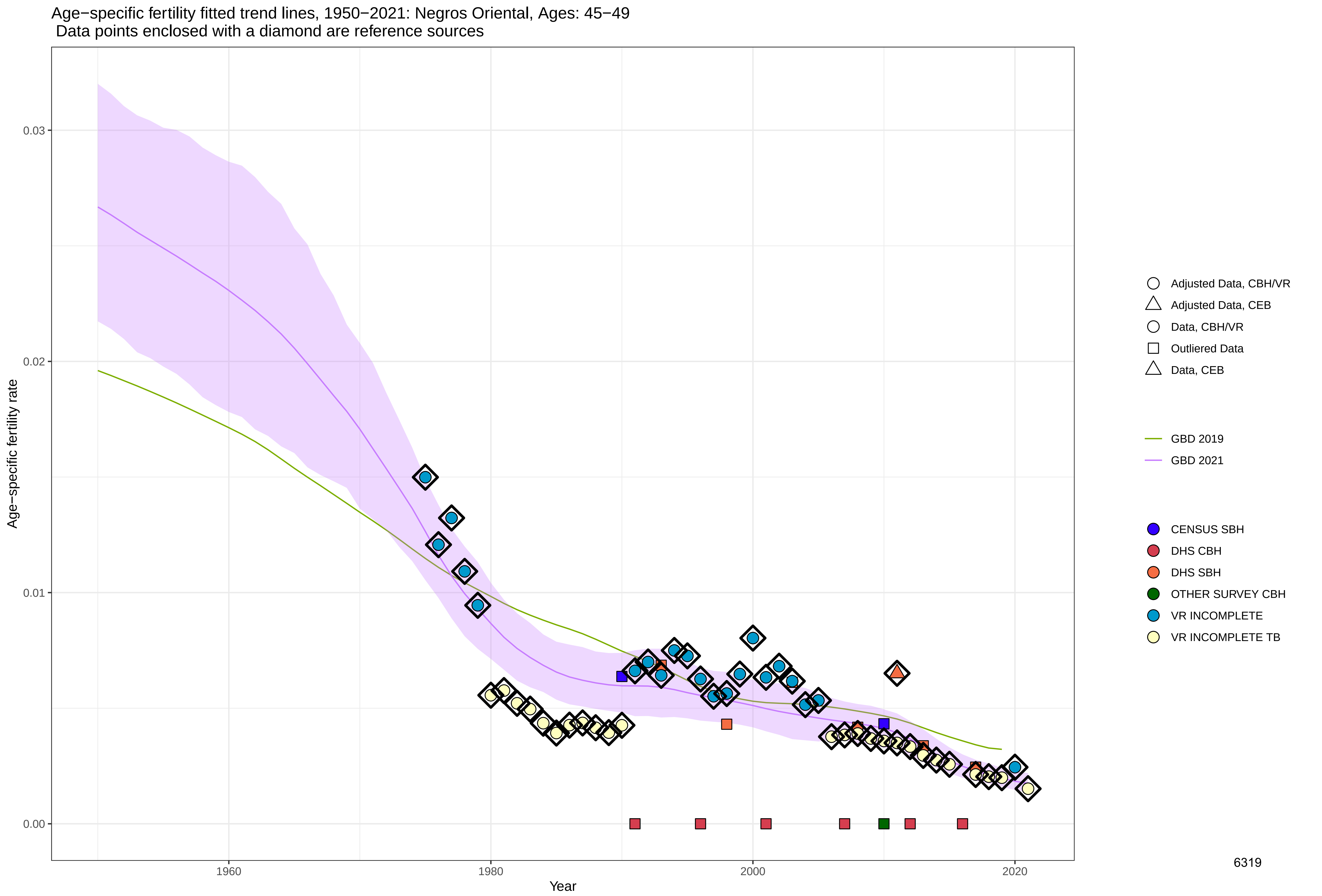

Age−specific fertility fitted trend lines, 1950−2021: Negros Oriental, Ages: 45−49
Data points enclosed with a diamond are reference sources
0.00
0.01
0.02
0.03
Age−specific fertility rate
1960
1980
2000
2020
Year
Adjusted Data, CBH/VR
Adjusted Data, CEB
Data, CBH/VR
Outliered Data
Data, CEB
GBD 2019
GBD 2021
CENSUS SBH
DHS CBH
DHS SBH
OTHER SURVEY CBH
VR INCOMPLETE
VR INCOMPLETE TB

Age−specific fertility fitted trend lines, 1950−2021: Negros Oriental, Ages: 50−54
Data points enclosed with a diamond are reference sources

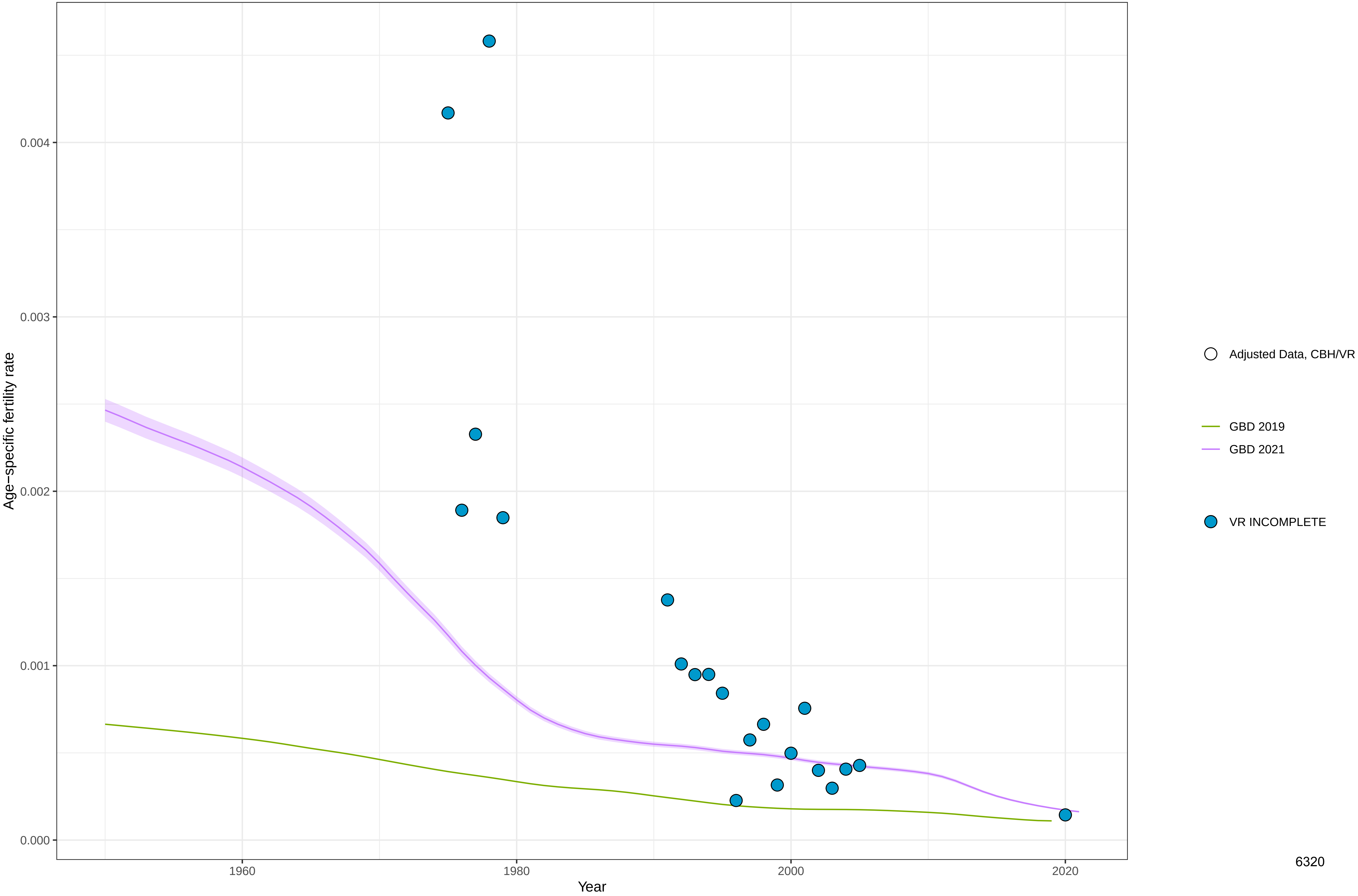

Total fertility rate fitted trend lines, 1950−2021: Negros Oriental TFR
Data points enclosed with a diamond are reference sources

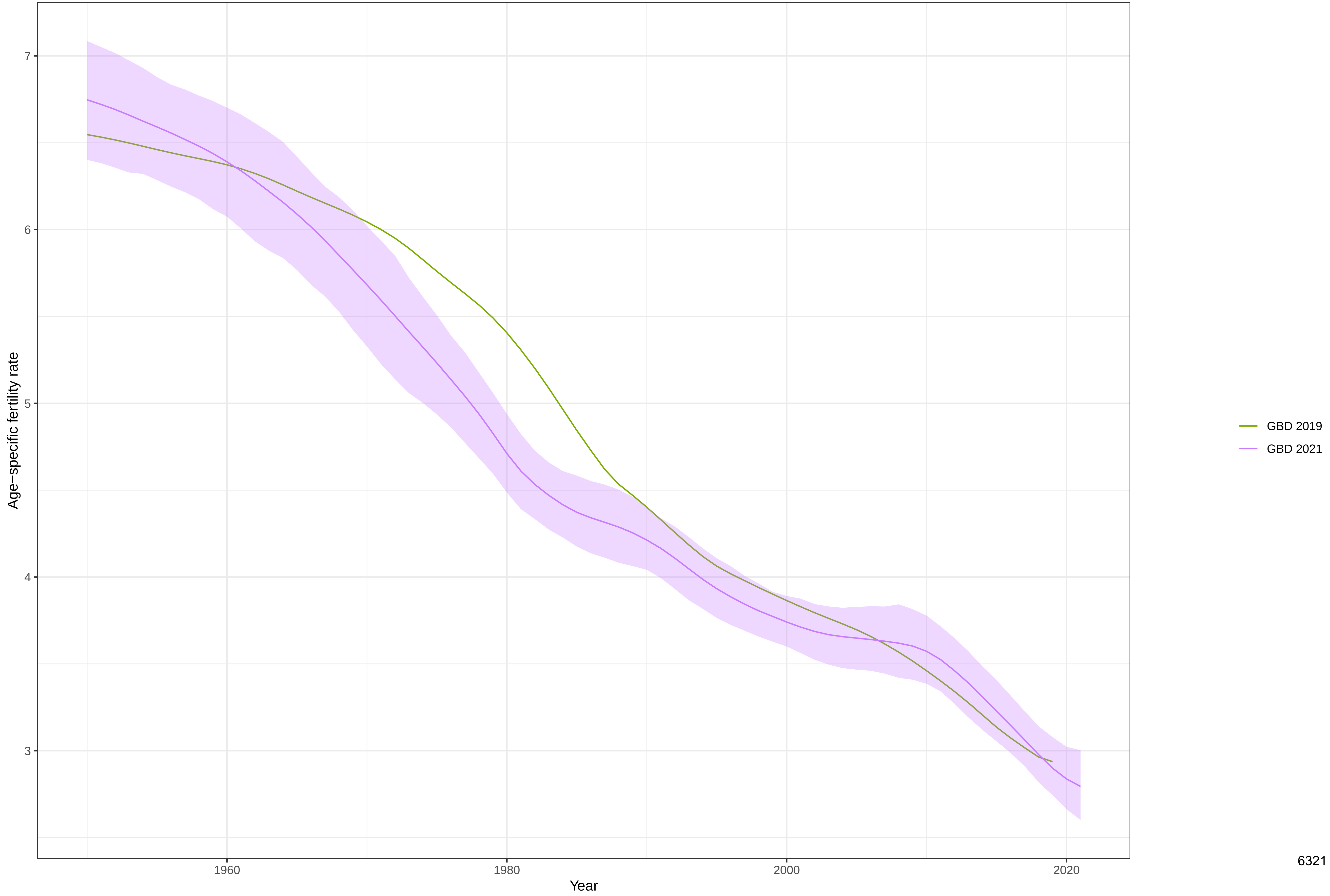

Age−specific fertility fitted trend lines, 1950−2021: Northern Samar, Ages: 10−14
Data points enclosed with a diamond are reference sources

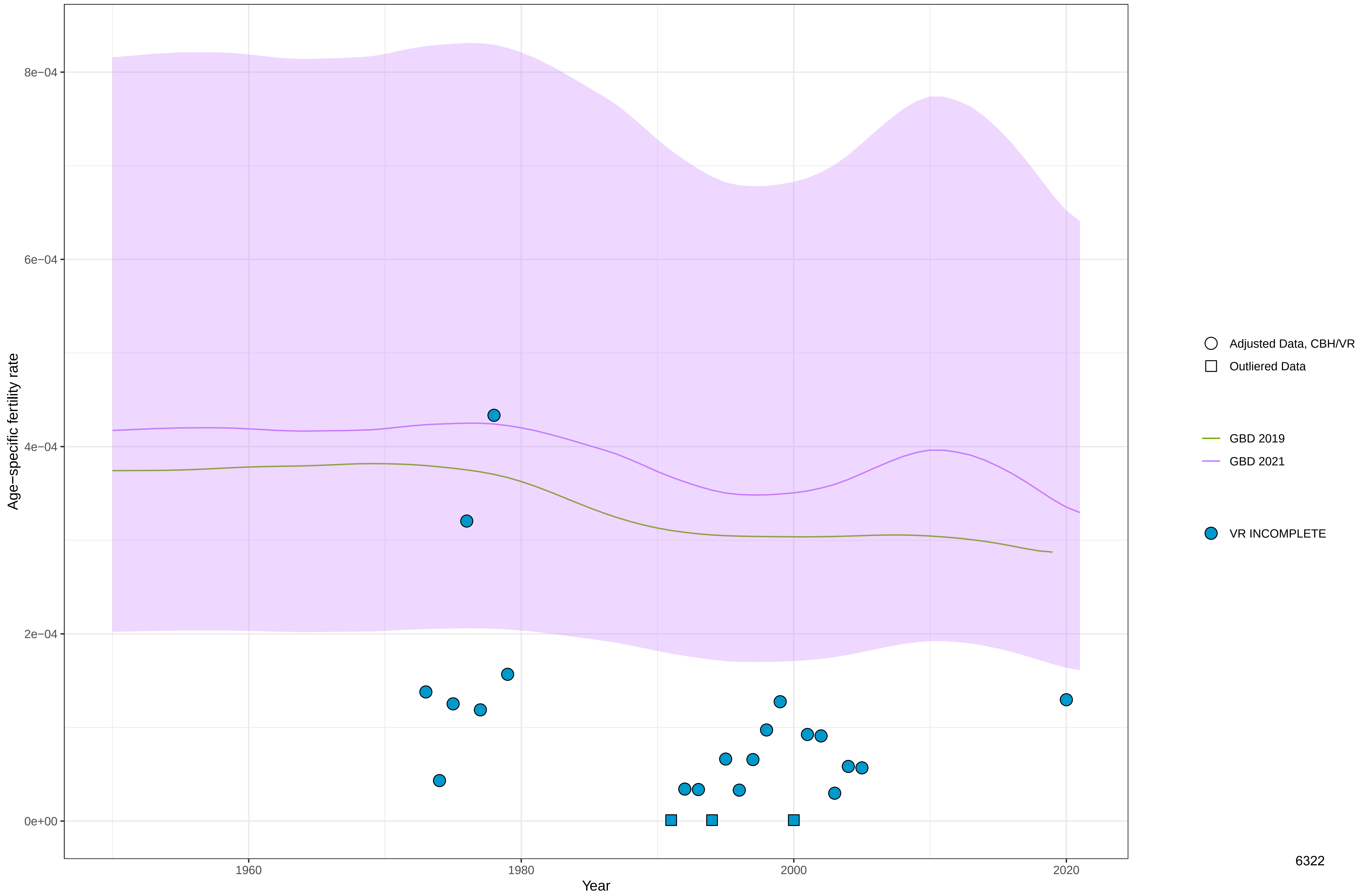

Age−specific fertility fitted trend lines, 1950−2021: Northern Samar, Ages: 15−19
Data points enclosed with a diamond are reference sources

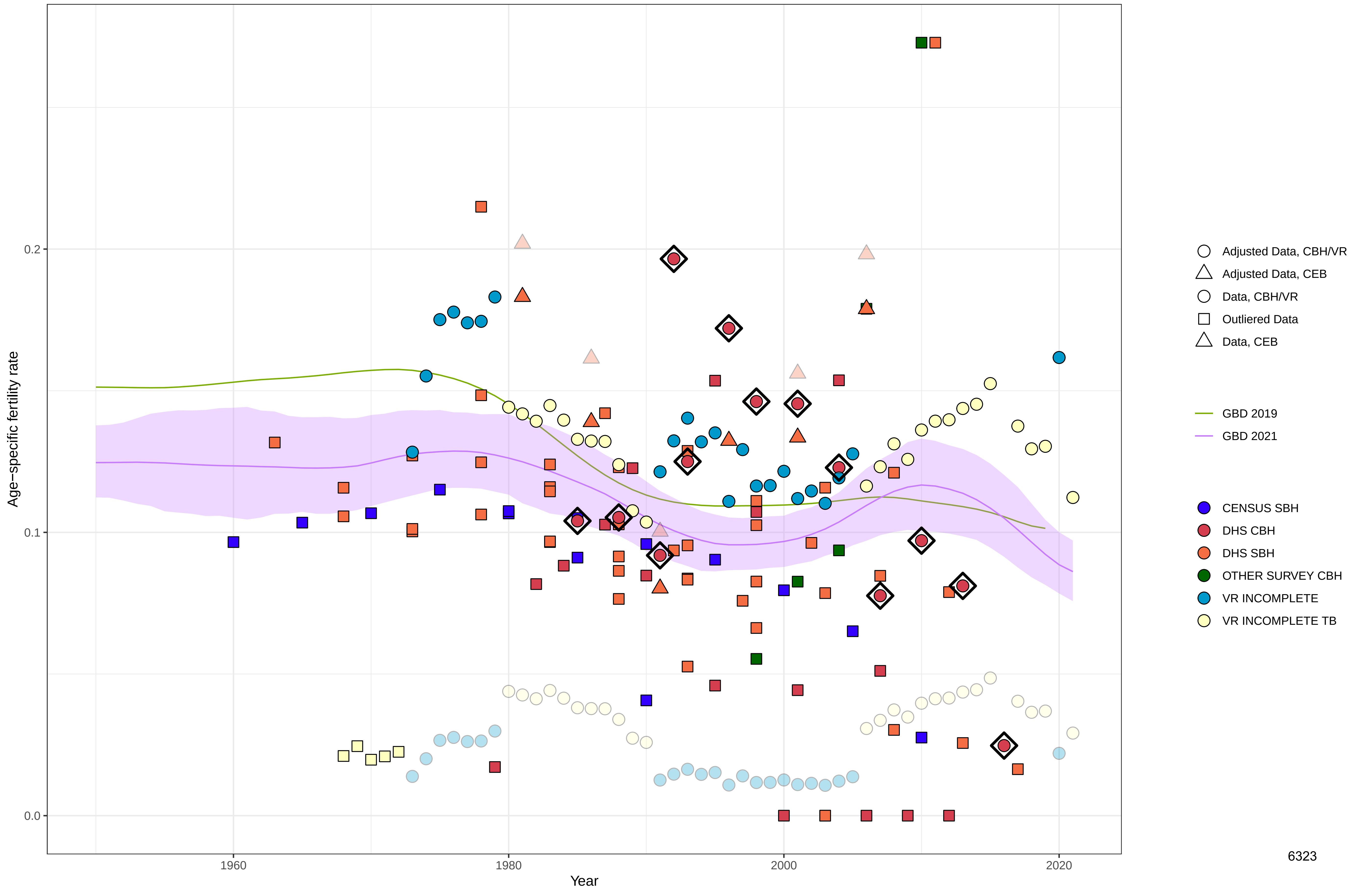

Age−specific fertility fitted trend lines, 1950−2021: Northern Samar, Ages: 20−24
Data points enclosed with a diamond are reference sources

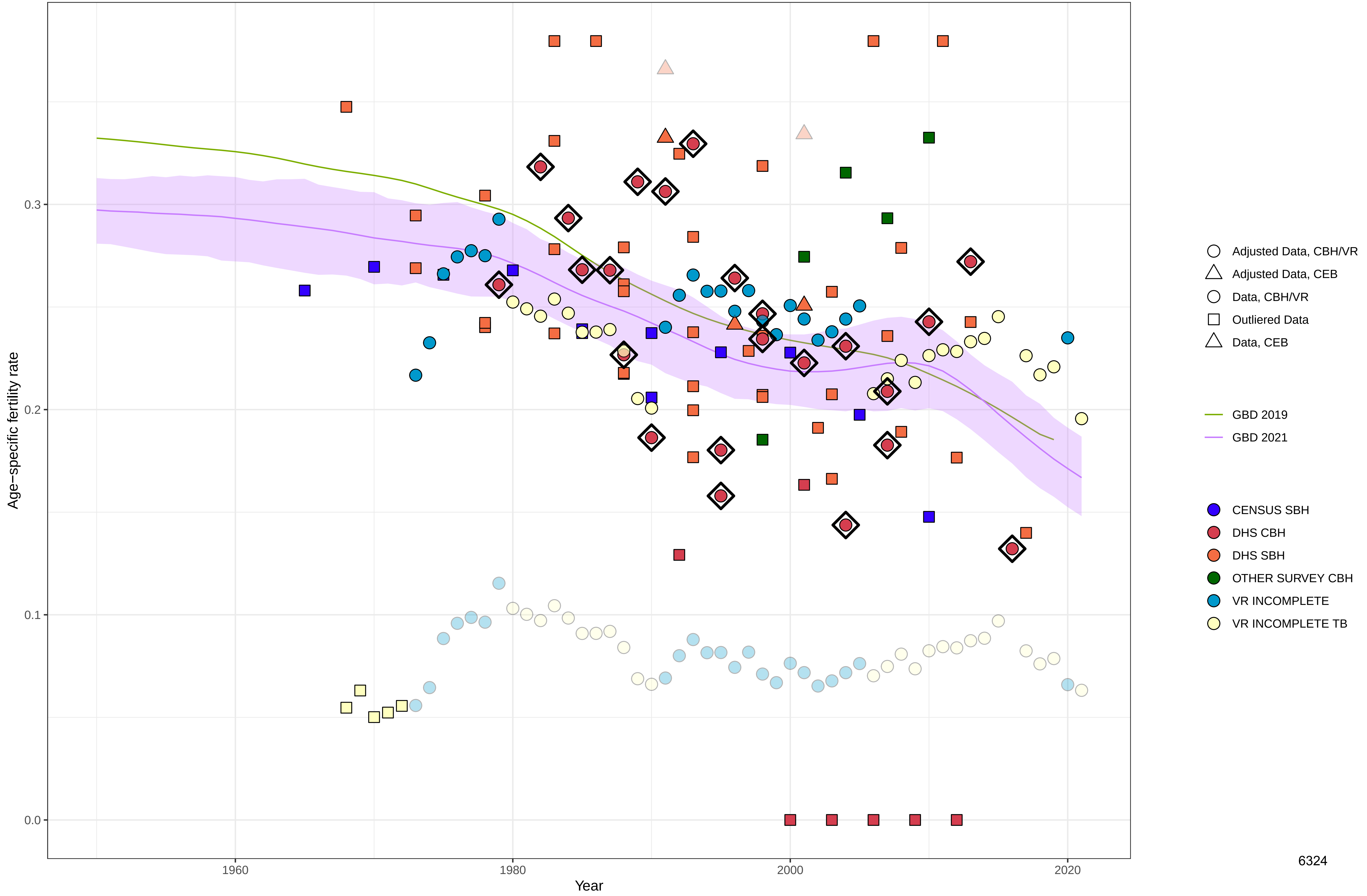

Age−specific fertility fitted trend lines, 1950−2021: Northern Samar, Ages: 25−29
Data points enclosed with a diamond are reference sources

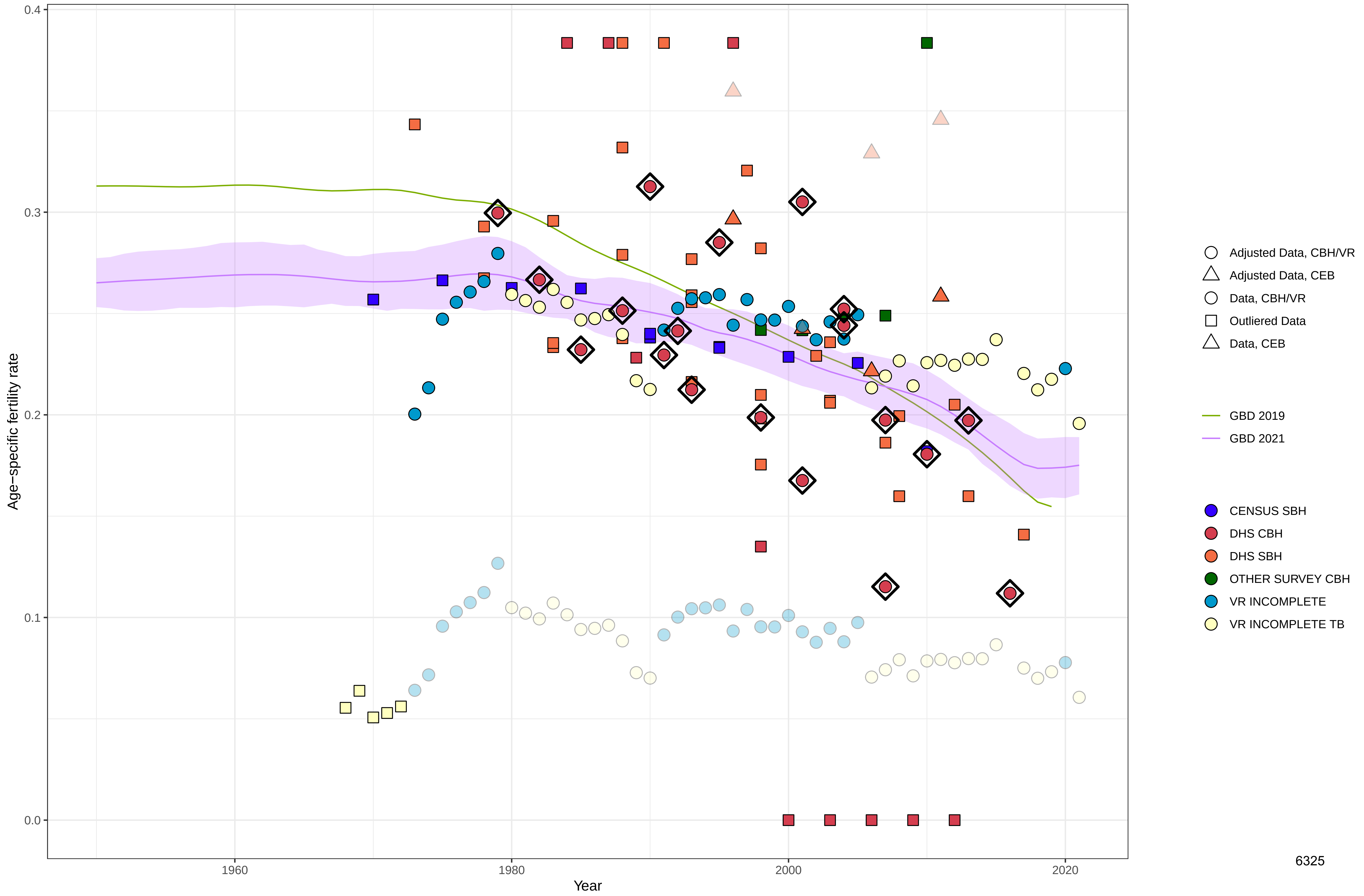

Age−specific fertility fitted trend lines, 1950−2021: Northern Samar, Ages: 30−34
Data points enclosed with a diamond are reference sources

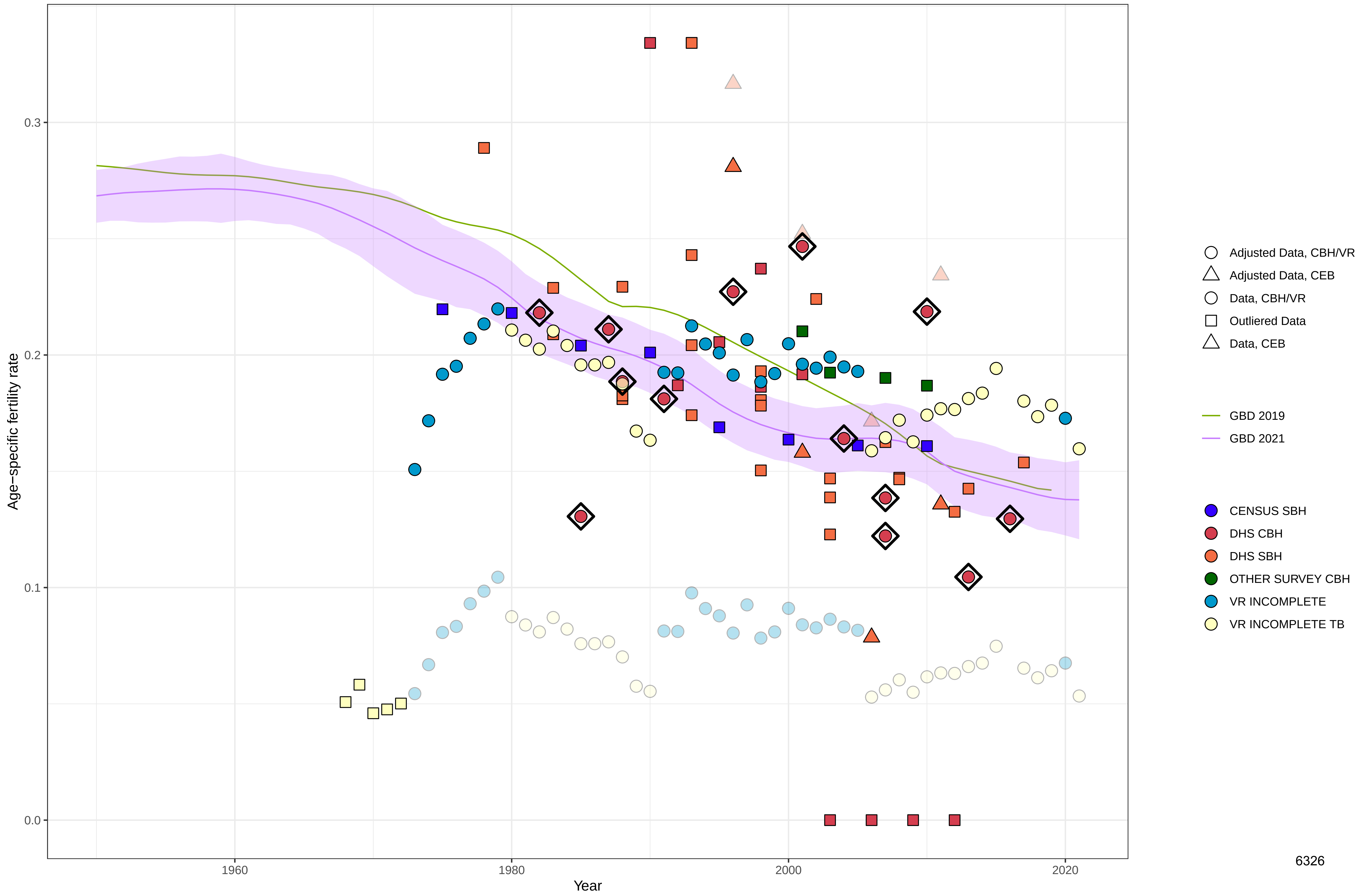

Age−specific fertility fitted trend lines, 1950−2021: Northern Samar, Ages: 35−39
Data points enclosed with a diamond are reference sources

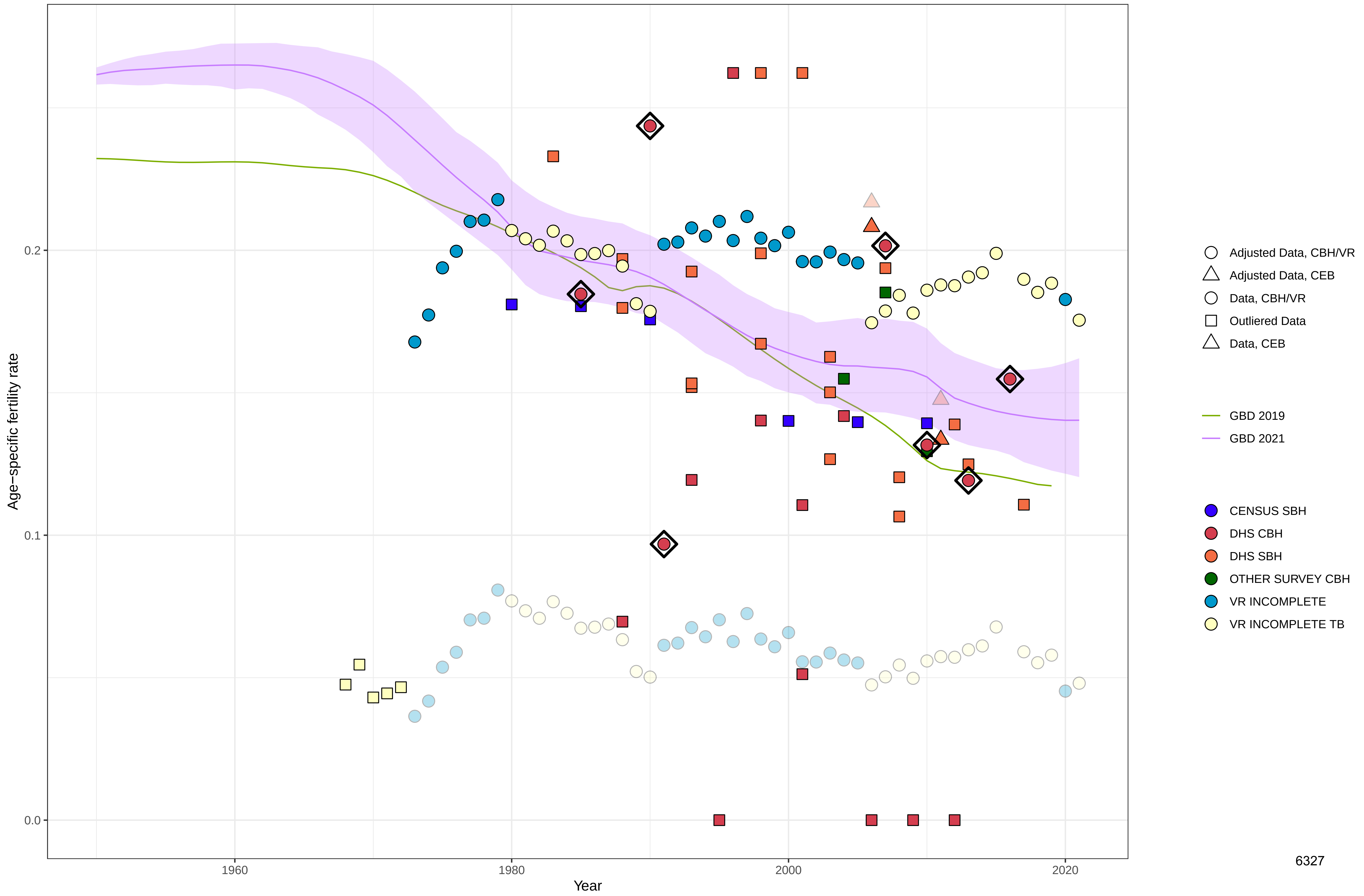

Age−specific fertility fitted trend lines, 1950−2021: Northern Samar, Ages: 40−44
Data points enclosed with a diamond are reference sources

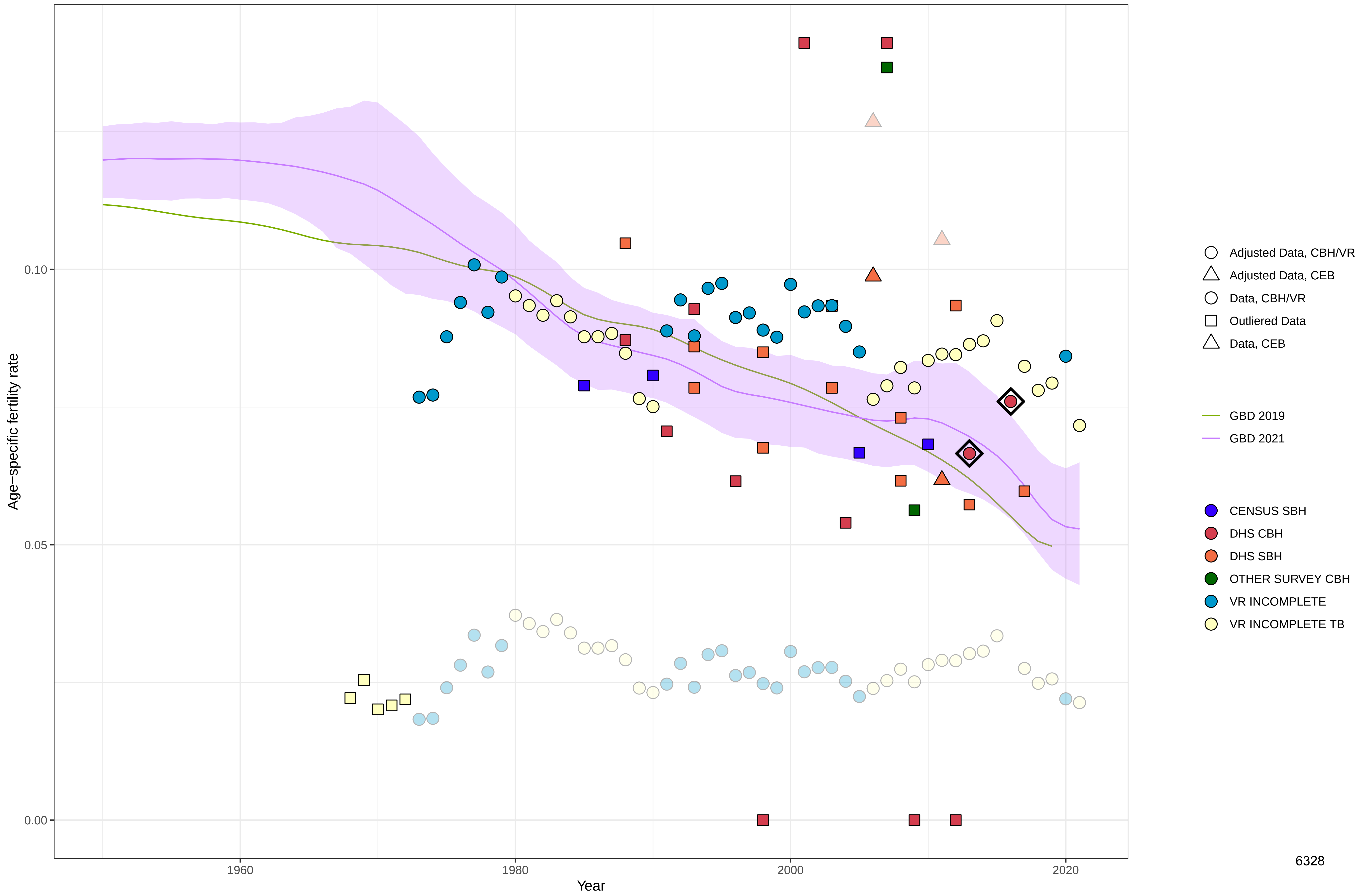

Age−specific fertility fitted trend lines, 1950−2021: Northern Samar, Ages: 45−49
Data points enclosed with a diamond are reference sources

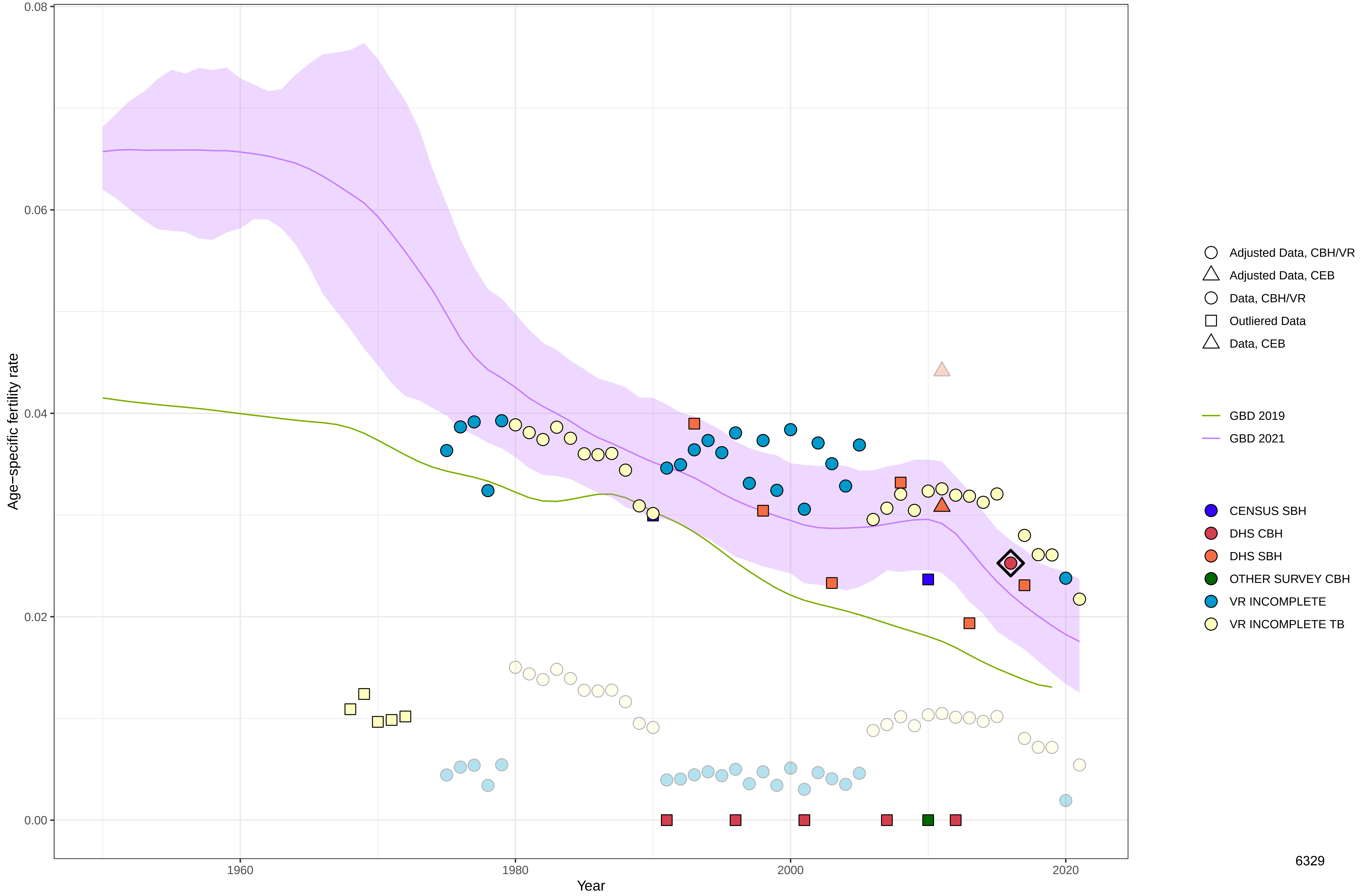

Age−specific fertility fitted trend lines, 1950−2021: Northern Samar, Ages: 50−54
Data points enclosed with a diamond are reference sources

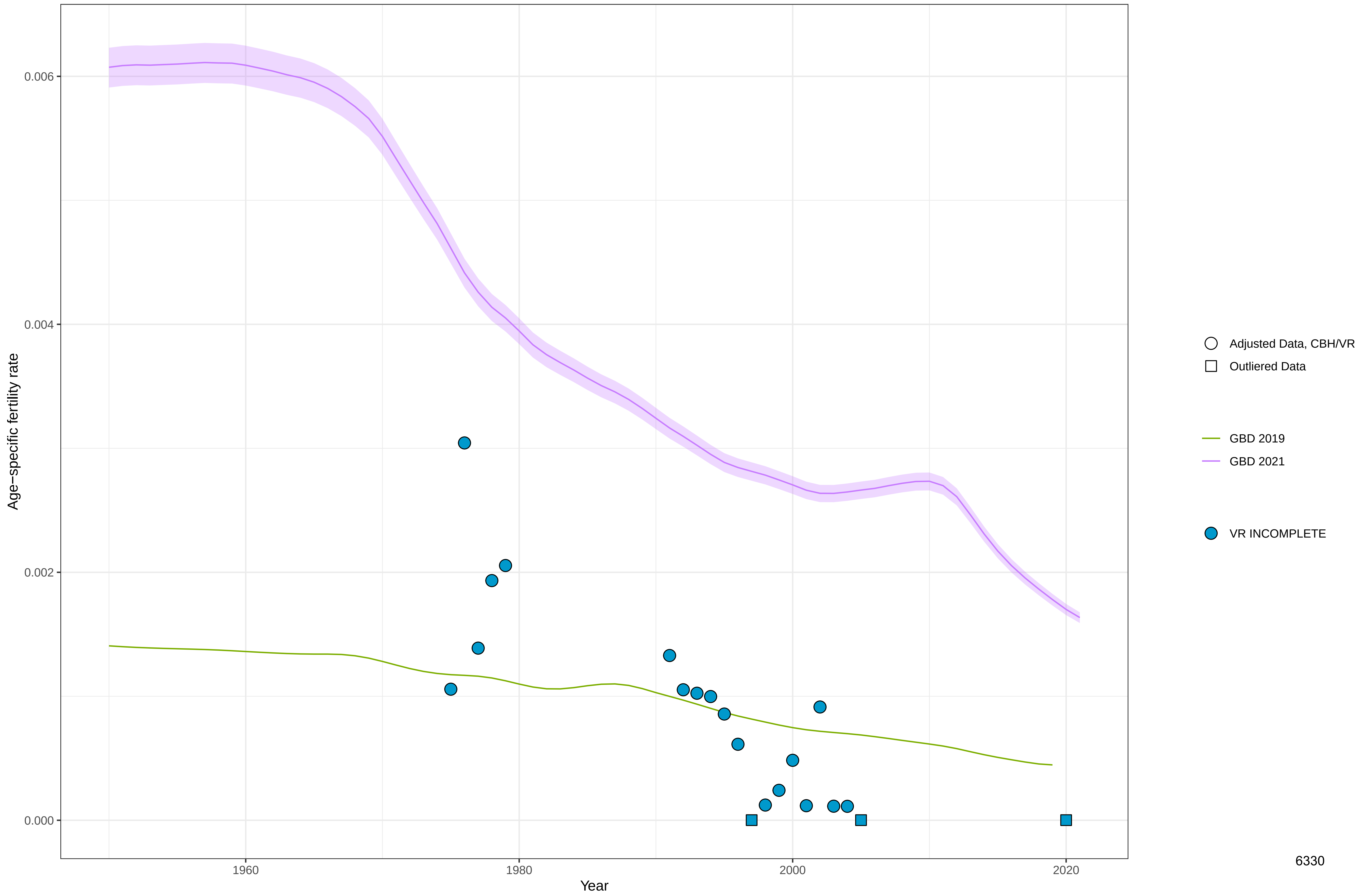

Total fertility rate fitted trend lines, 1950−2021: Northern Samar TFR
Data points enclosed with a diamond are reference sources

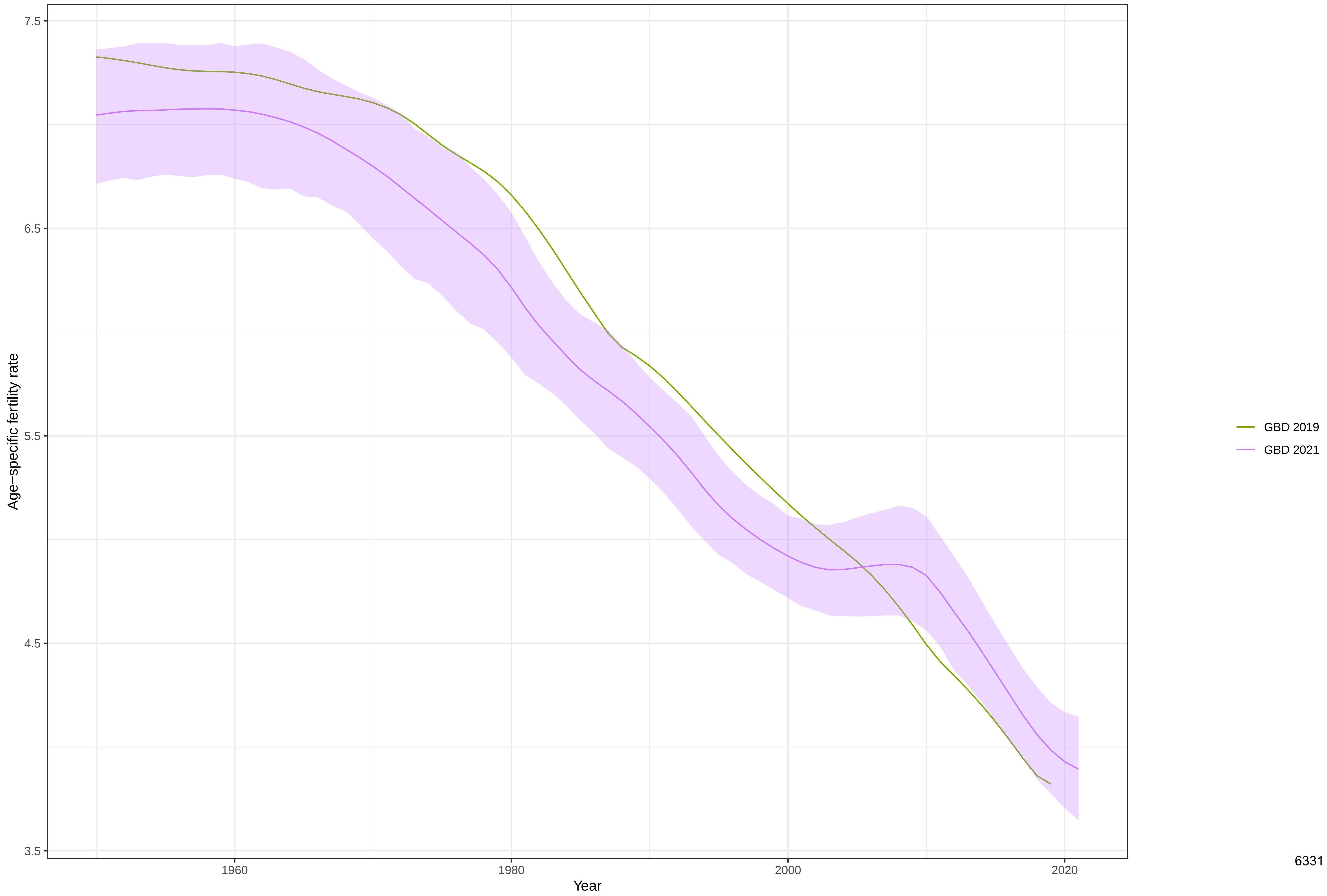

6331

Age−specific fertility fitted trend lines, 1950−2021: Nueva Ecija, Ages: 10−14
Data points enclosed with a diamond are reference sources

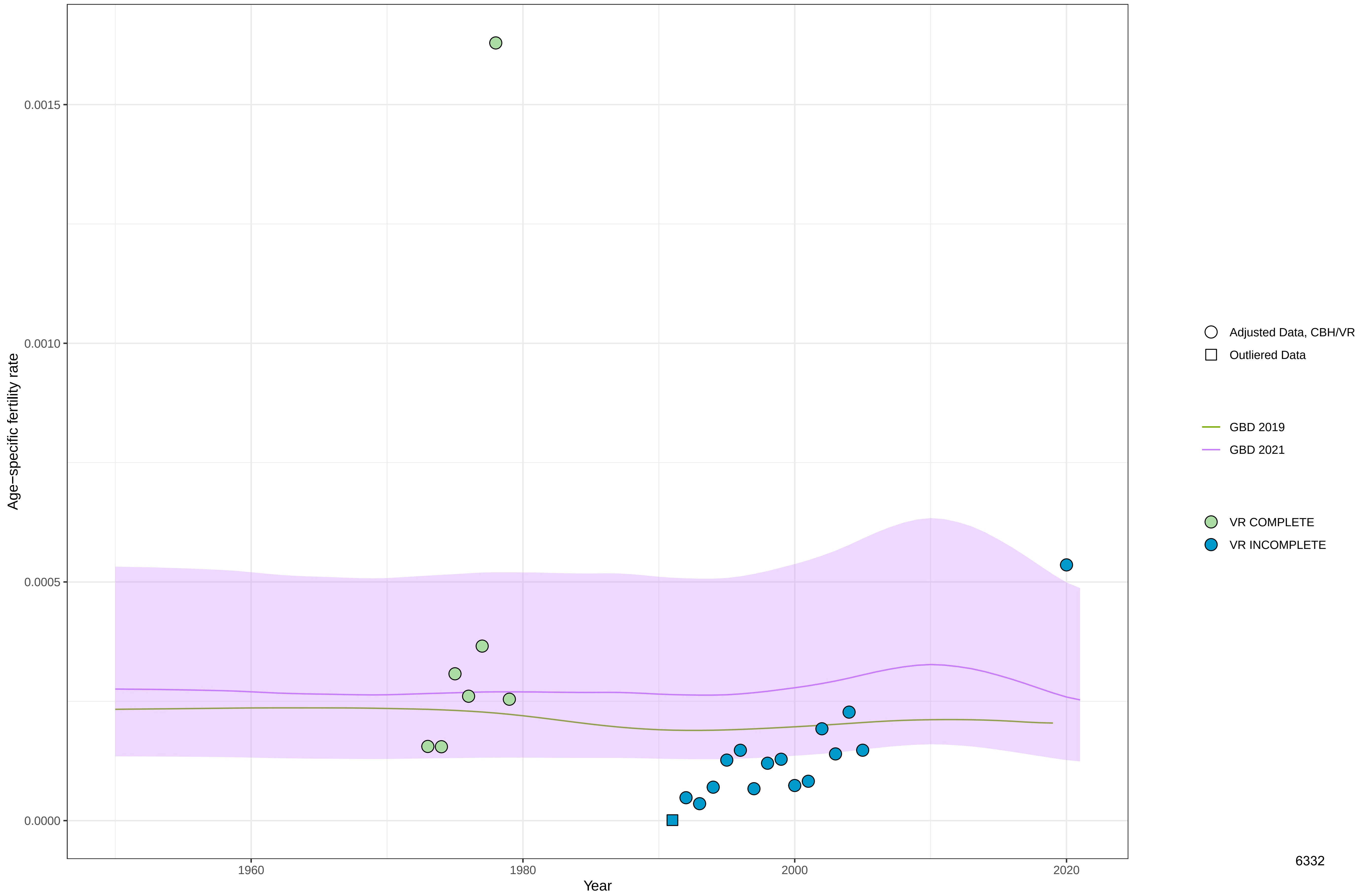

Age−specific fertility fitted trend lines, 1950−2021: Nueva Ecija, Ages: 15−19
Data points enclosed with a diamond are reference sources

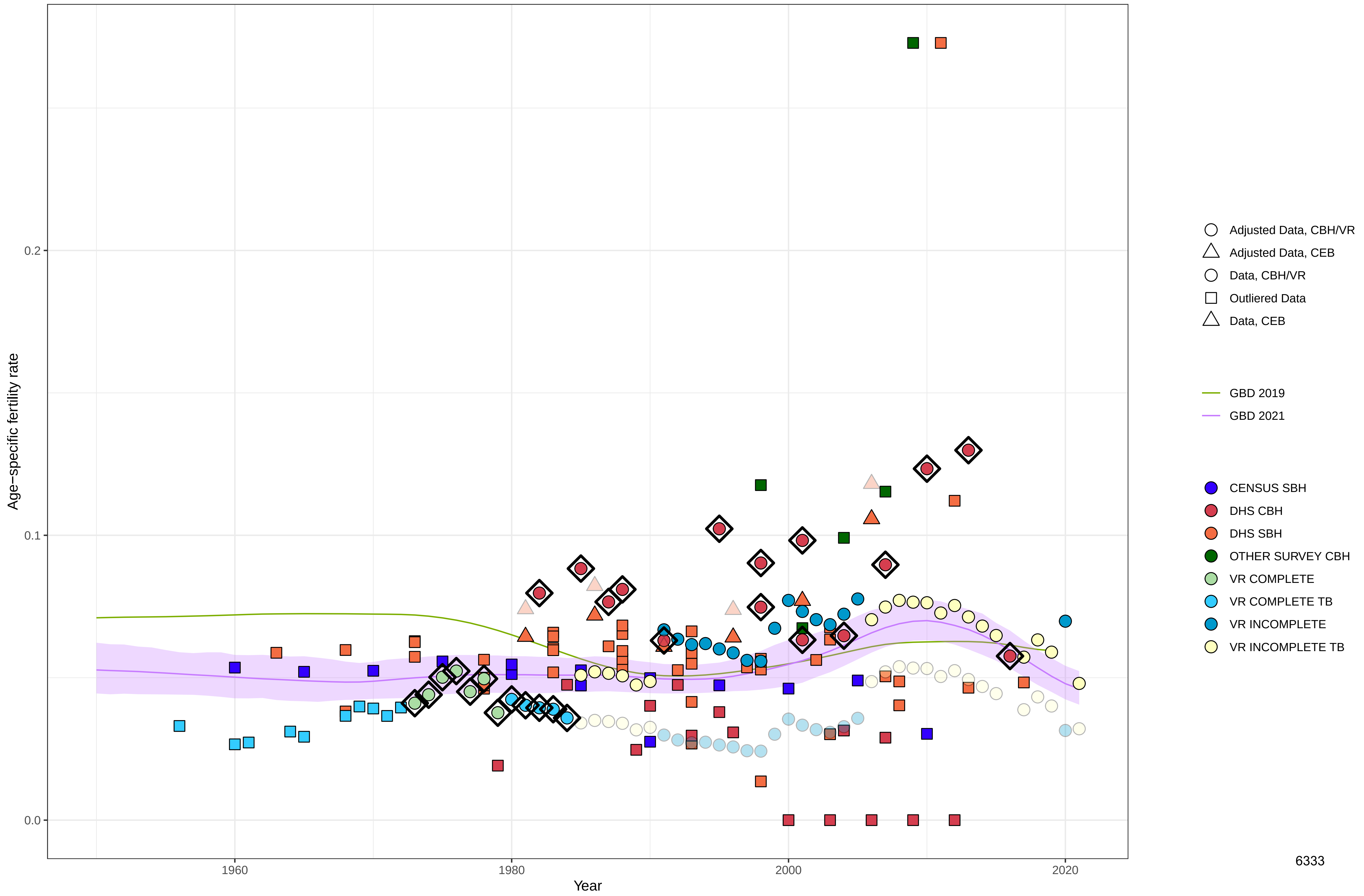

Age−specific fertility fitted trend lines, 1950−2021: Nueva Ecija, Ages: 20−24
Data points enclosed with a diamond are reference sources

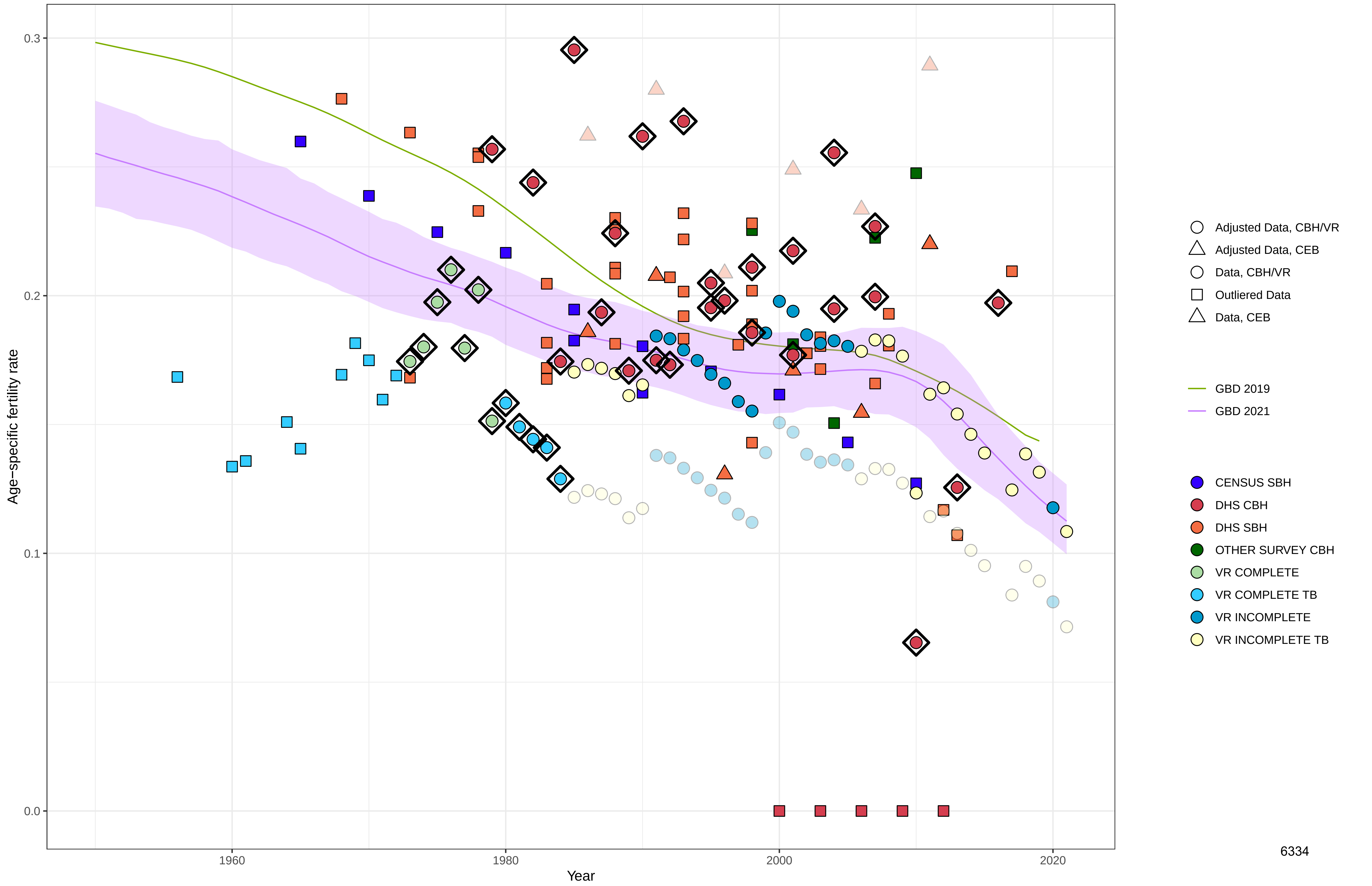

Age−specific fertility fitted trend lines, 1950−2021: Nueva Ecija, Ages: 25−29
Data points enclosed with a diamond are reference sources

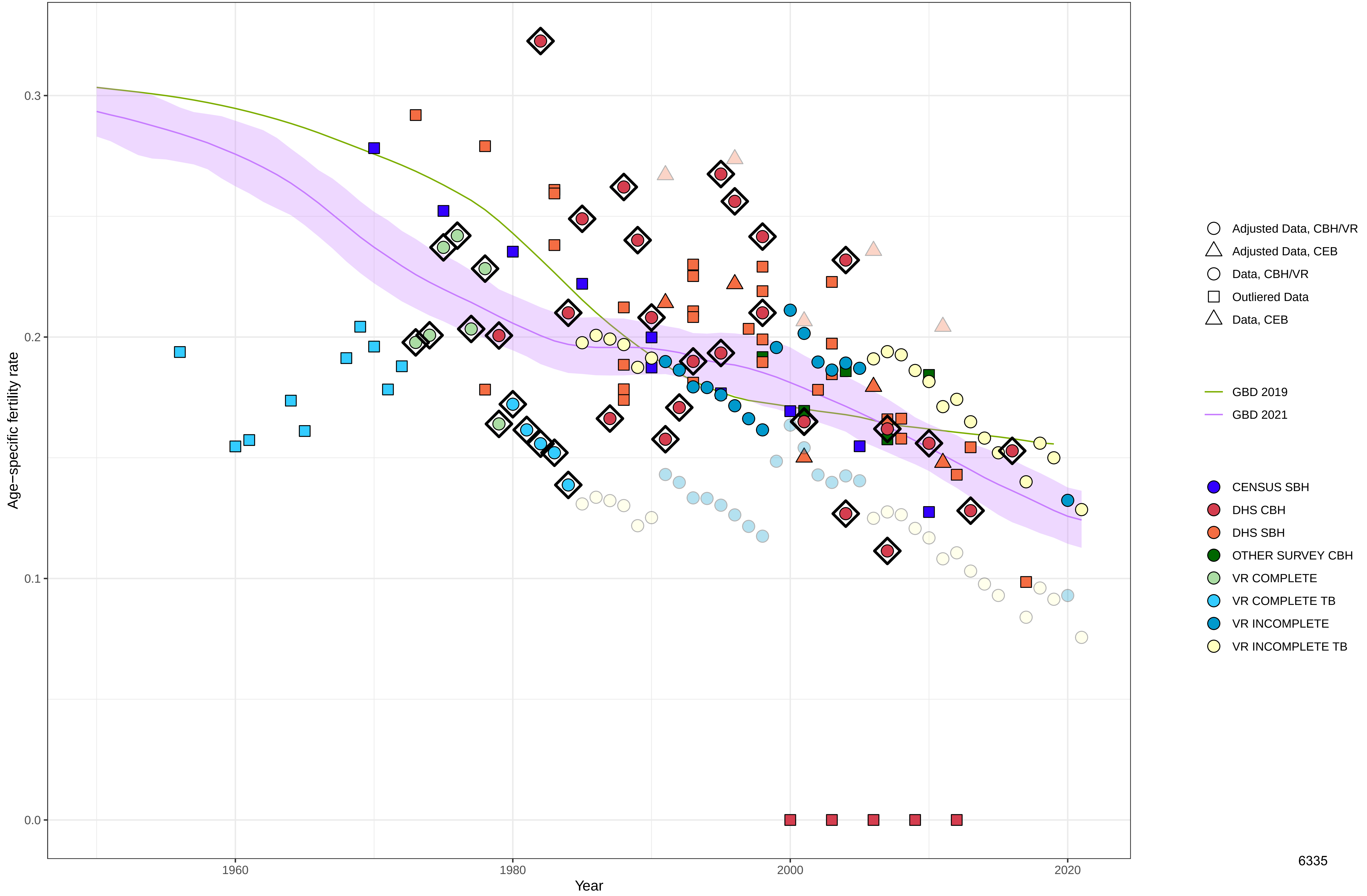

Age−specific fertility fitted trend lines, 1950−2021: Nueva Ecija, Ages: 30−34
Data points enclosed with a diamond are reference sources

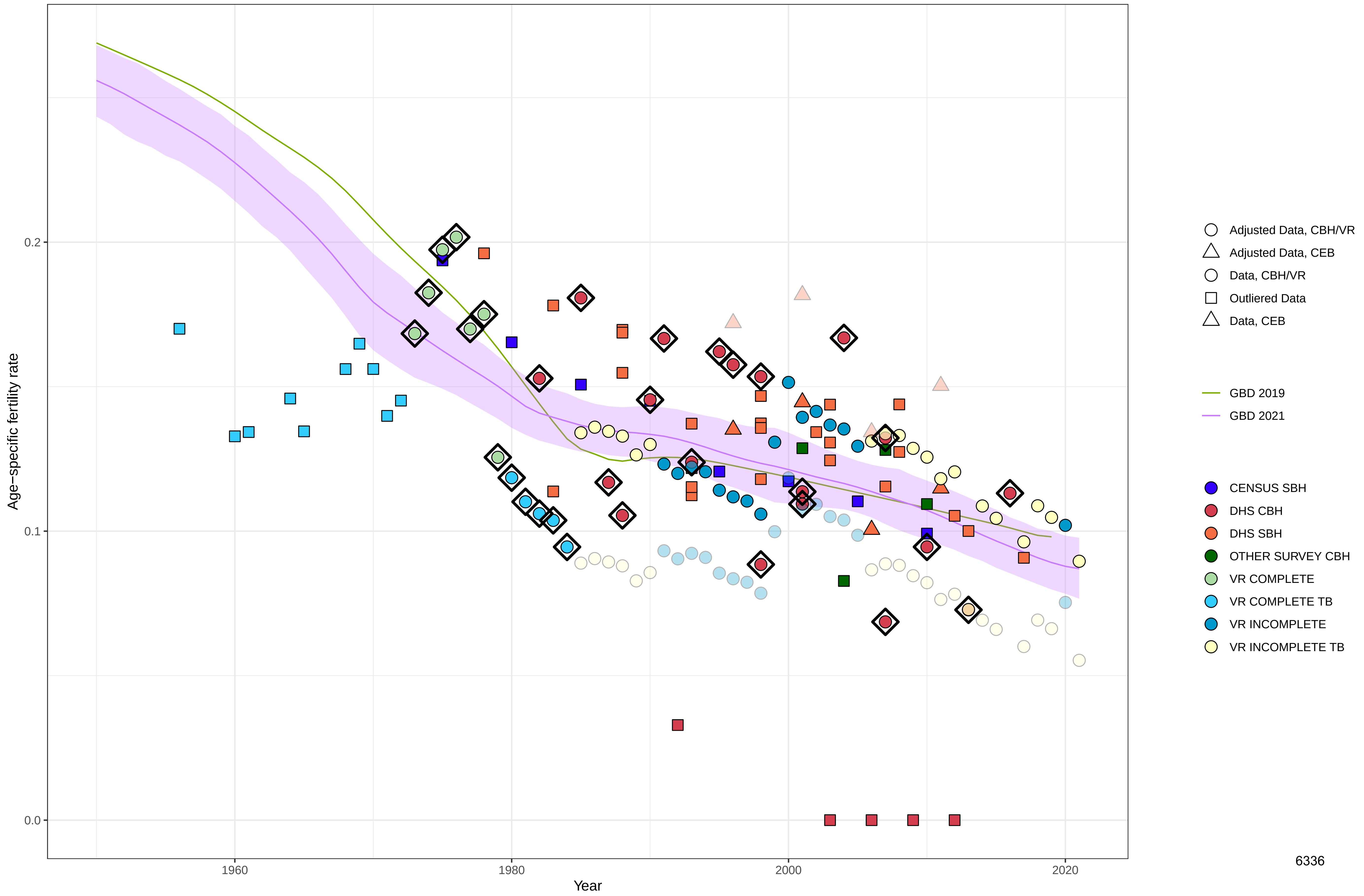

Age−specific fertility fitted trend lines, 1950−2021: Nueva Ecija, Ages: 35−39
Data points enclosed with a diamond are reference sources

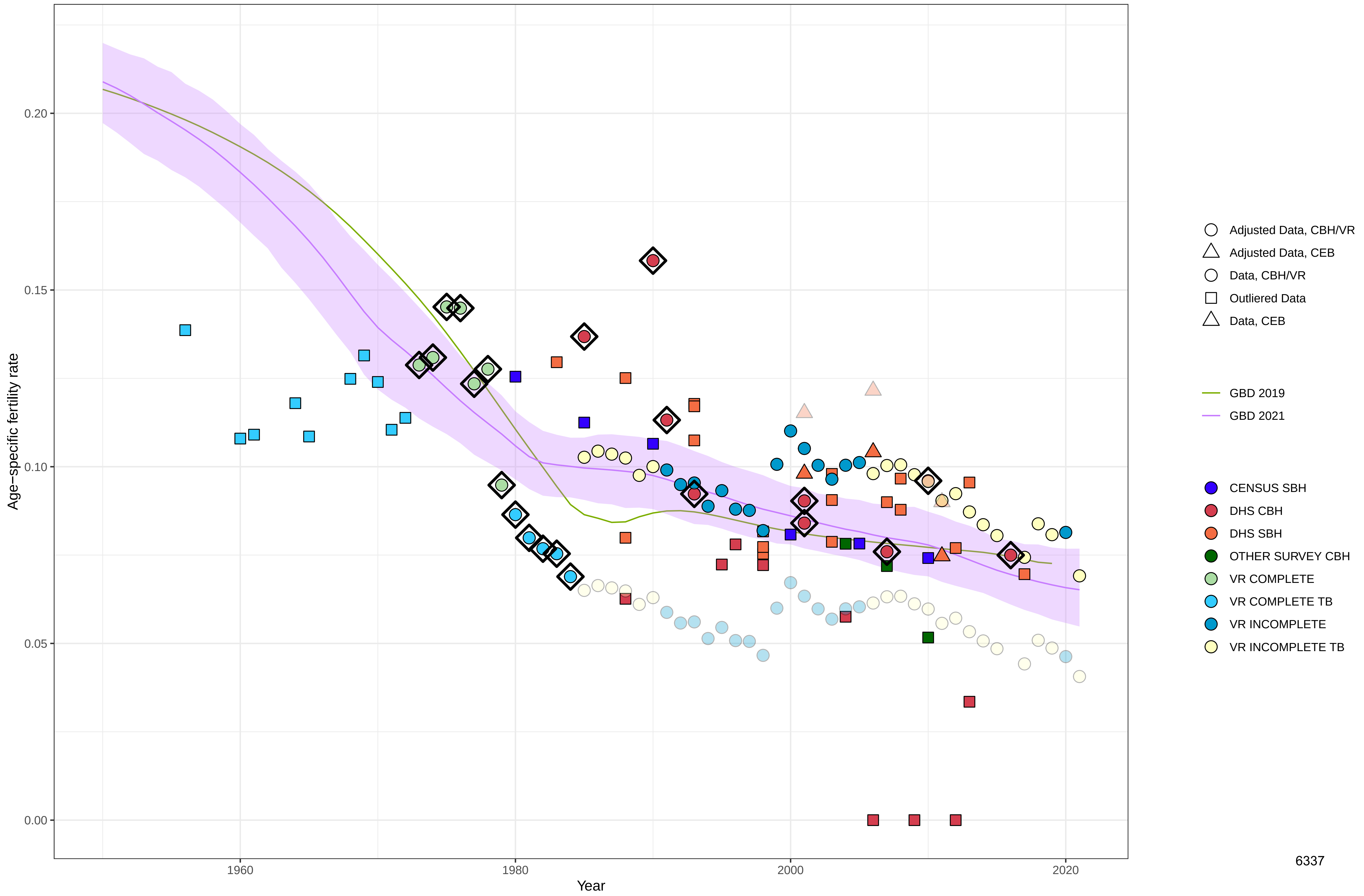

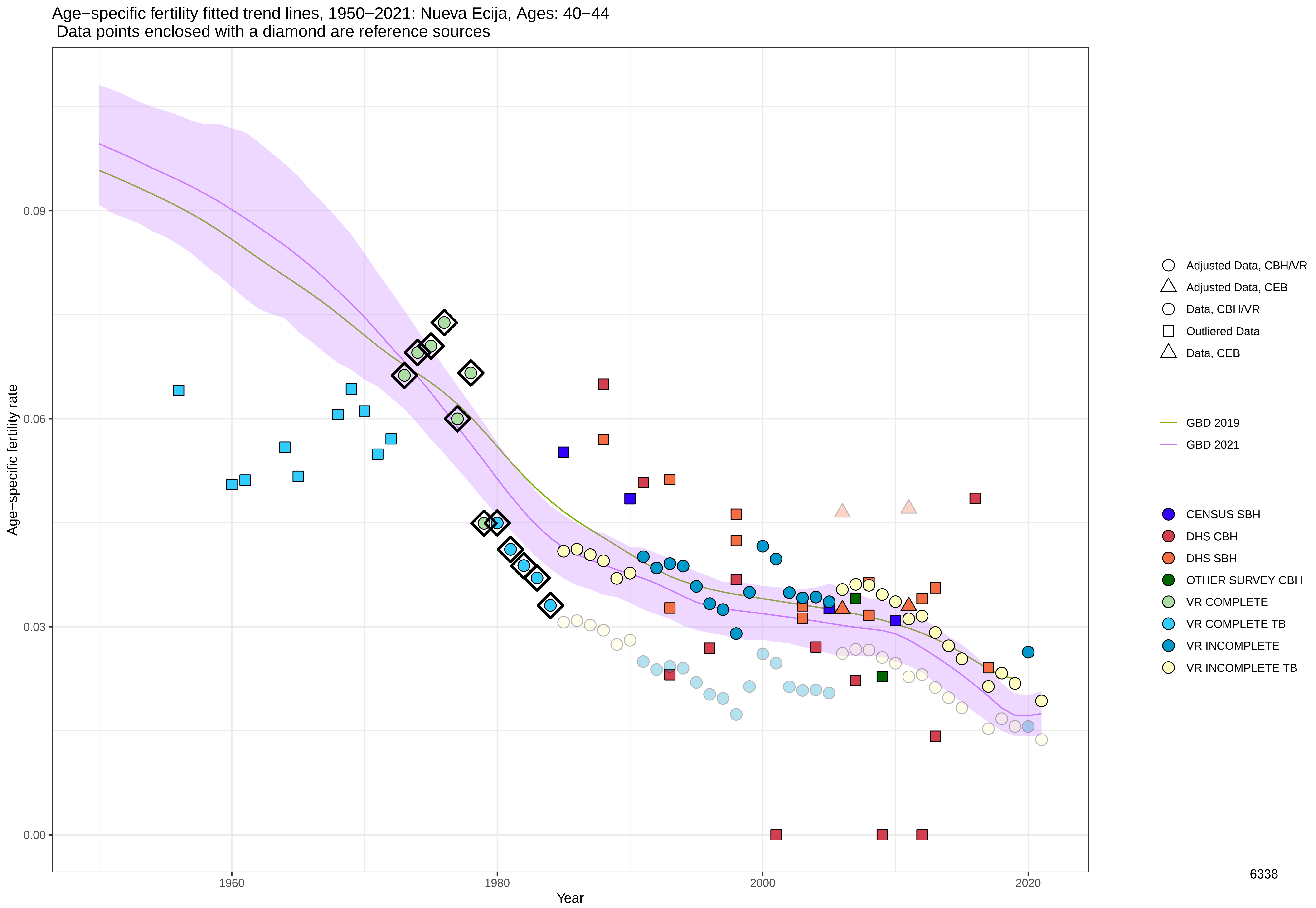

Age−specific fertility fitted trend lines, 1950−2021: Nueva Ecija, Ages: 40−44
Data points enclosed with a diamond are reference sources
Age−specific fertility rate
0.09
0.06
0.03
0.00
1960
1980
2000
2020
Year
Adjusted Data, CBH/VR
Adjusted Data, CEB
Data, CBH/VR
Outliered Data
Data, CEB
GBD 2019
GBD 2021
CENSUS SBH
DHS CBH
DHS SBH
OTHER SURVEY CBH
VR COMPLETE
VR COMPLETE TB
VR INCOMPLETE
VR INCOMPLETE TB

Age−specific fertility fitted trend lines, 1950−2021: Nueva Ecija, Ages: 45−49
Data points enclosed with a diamond are reference sources

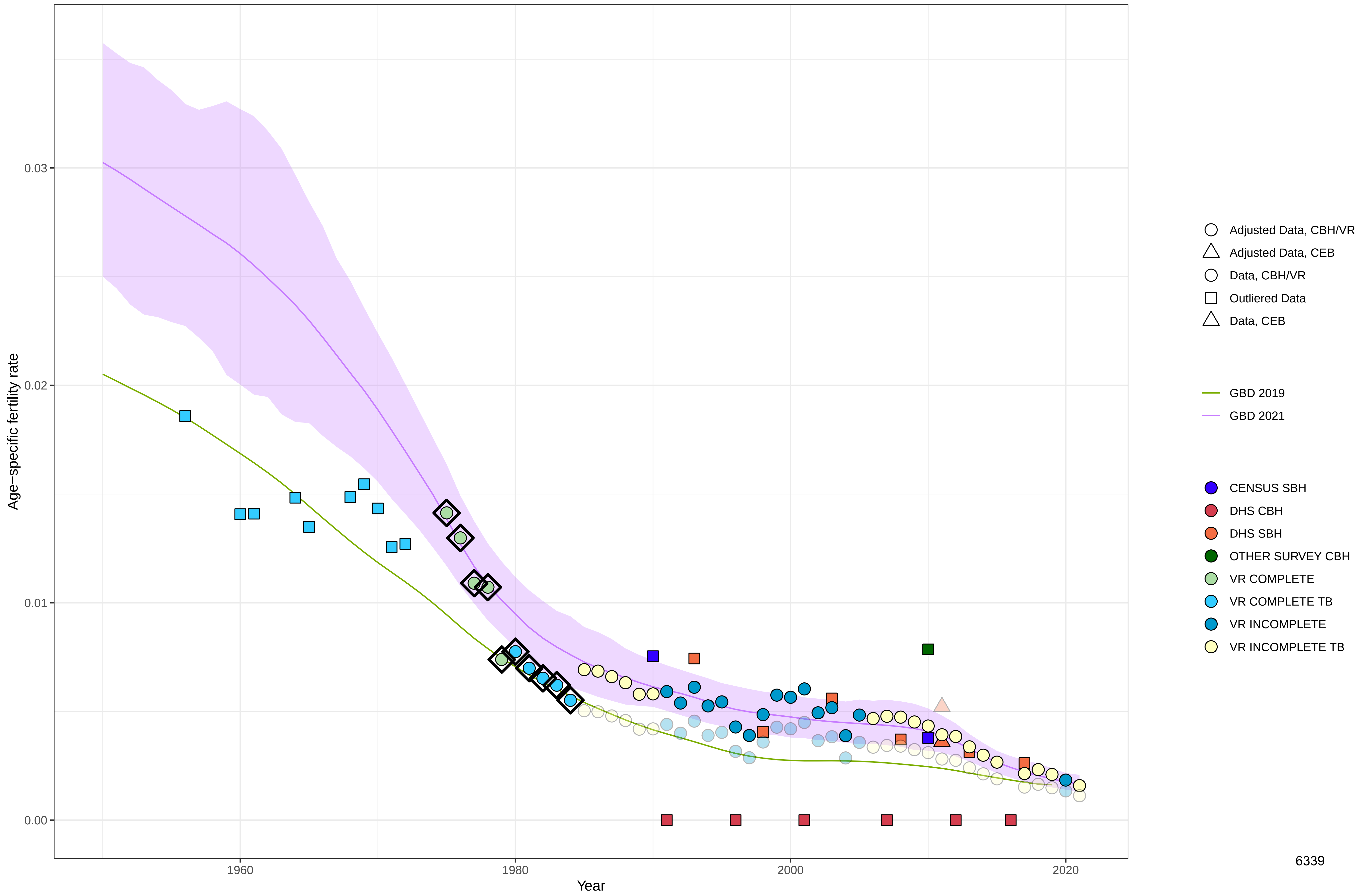

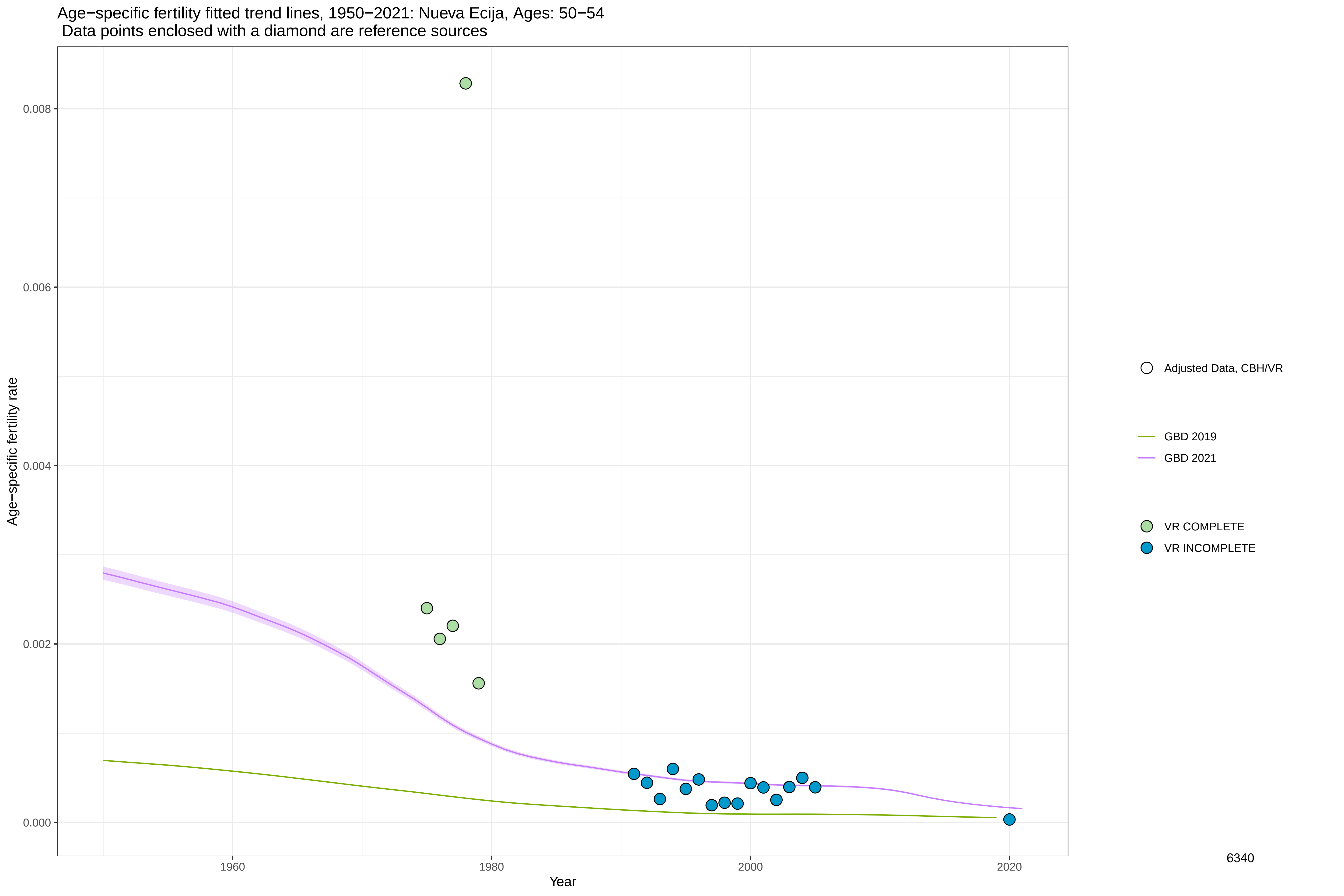

Age−specific fertility fitted trend lines, 1950−2021: Nueva Ecija, Ages: 50−54
Data points enclosed with a diamond are reference sources
Age−specific fertility rate
0.000
0.002
0.004
0.006
0.008
Year
1960
1980
2000
2020
Adjusted Data, CBH/VR
GBD 2019
GBD 2021
VR COMPLETE
VR INCOMPLETE
6340

Total fertility rate fitted trend lines, 1950−2021: Nueva Ecija TFR
Data points enclosed with a diamond are reference sources

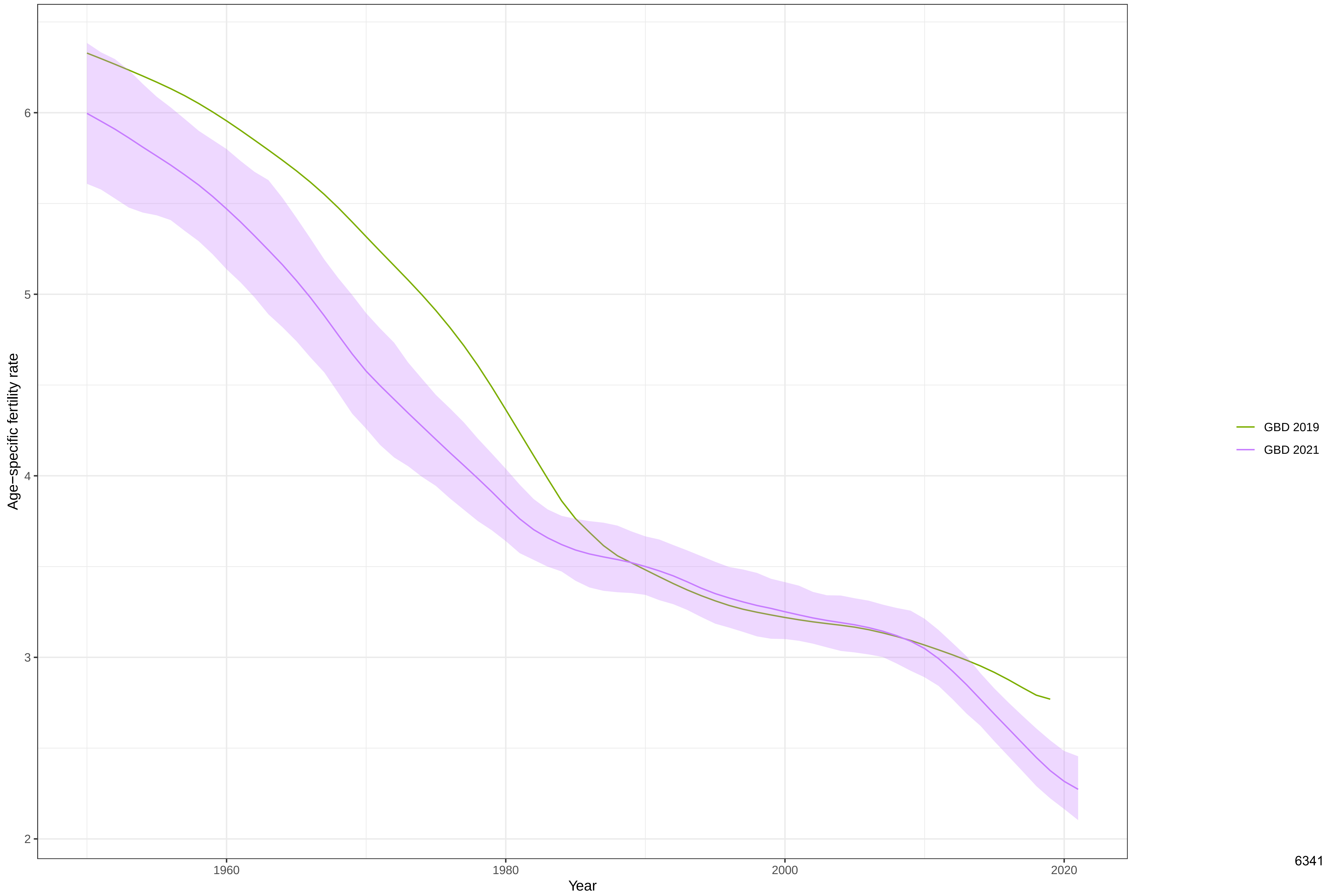

Age−specific fertility fitted trend lines, 1950−2021: Nueva Vizcaya, Ages: 10−14
Data points enclosed with a diamond are reference sources

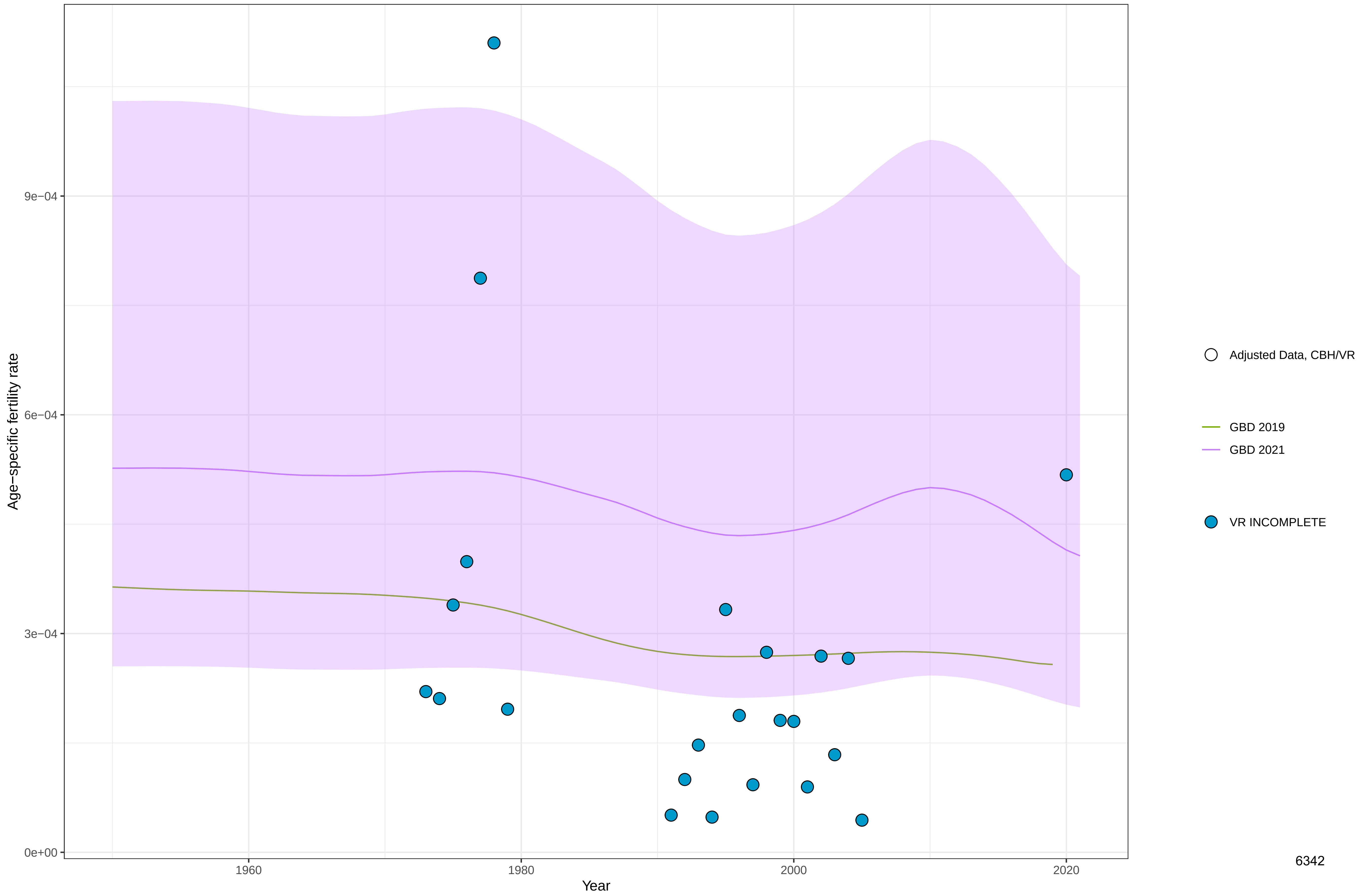

Age−specific fertility fitted trend lines, 1950−2021: Nueva Vizcaya, Ages: 15−19
Data points enclosed with a diamond are reference sources

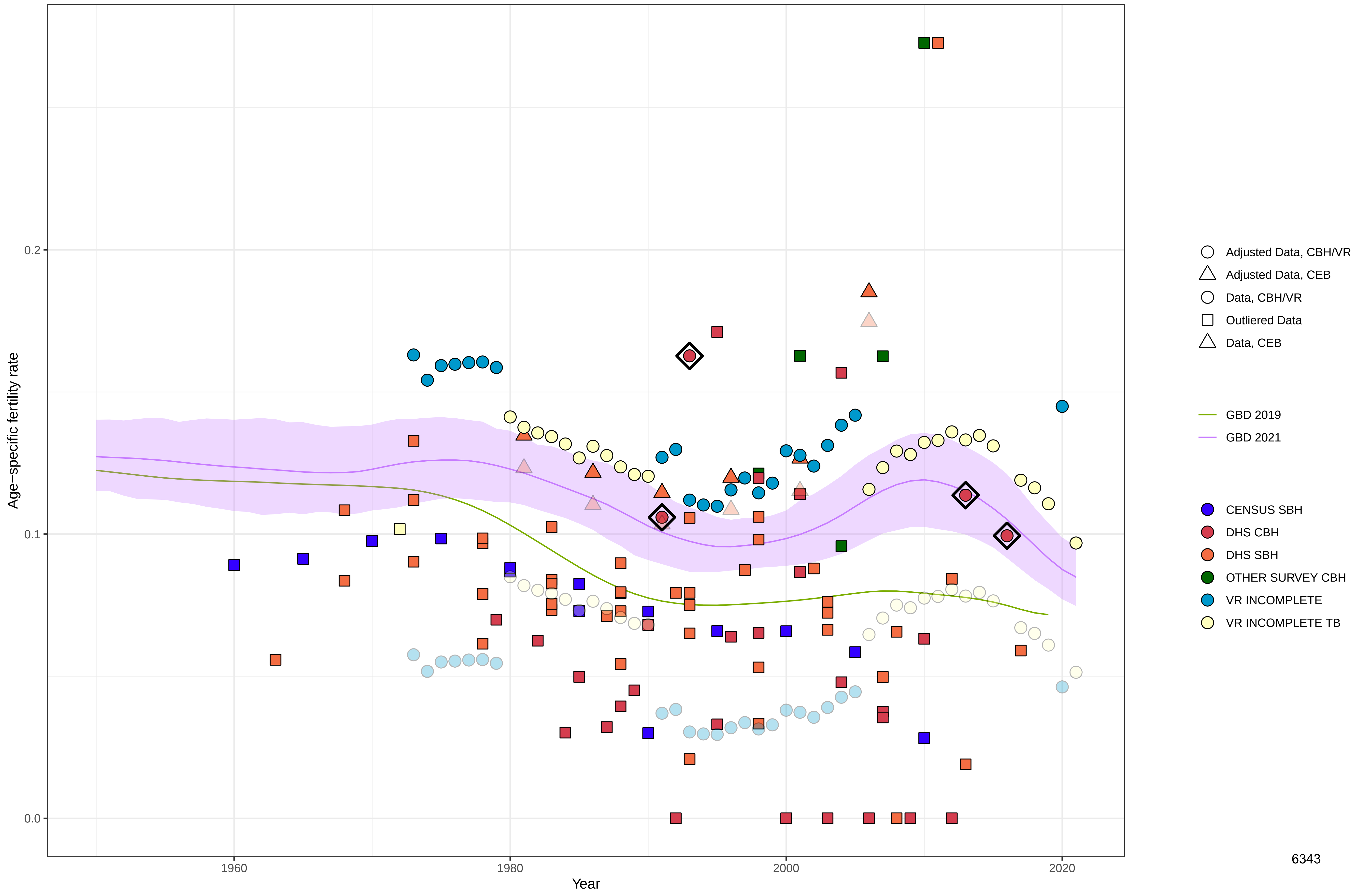

Age−specific fertility fitted trend lines, 1950−2021: Nueva Vizcaya, Ages: 20−24
Data points enclosed with a diamond are reference sources

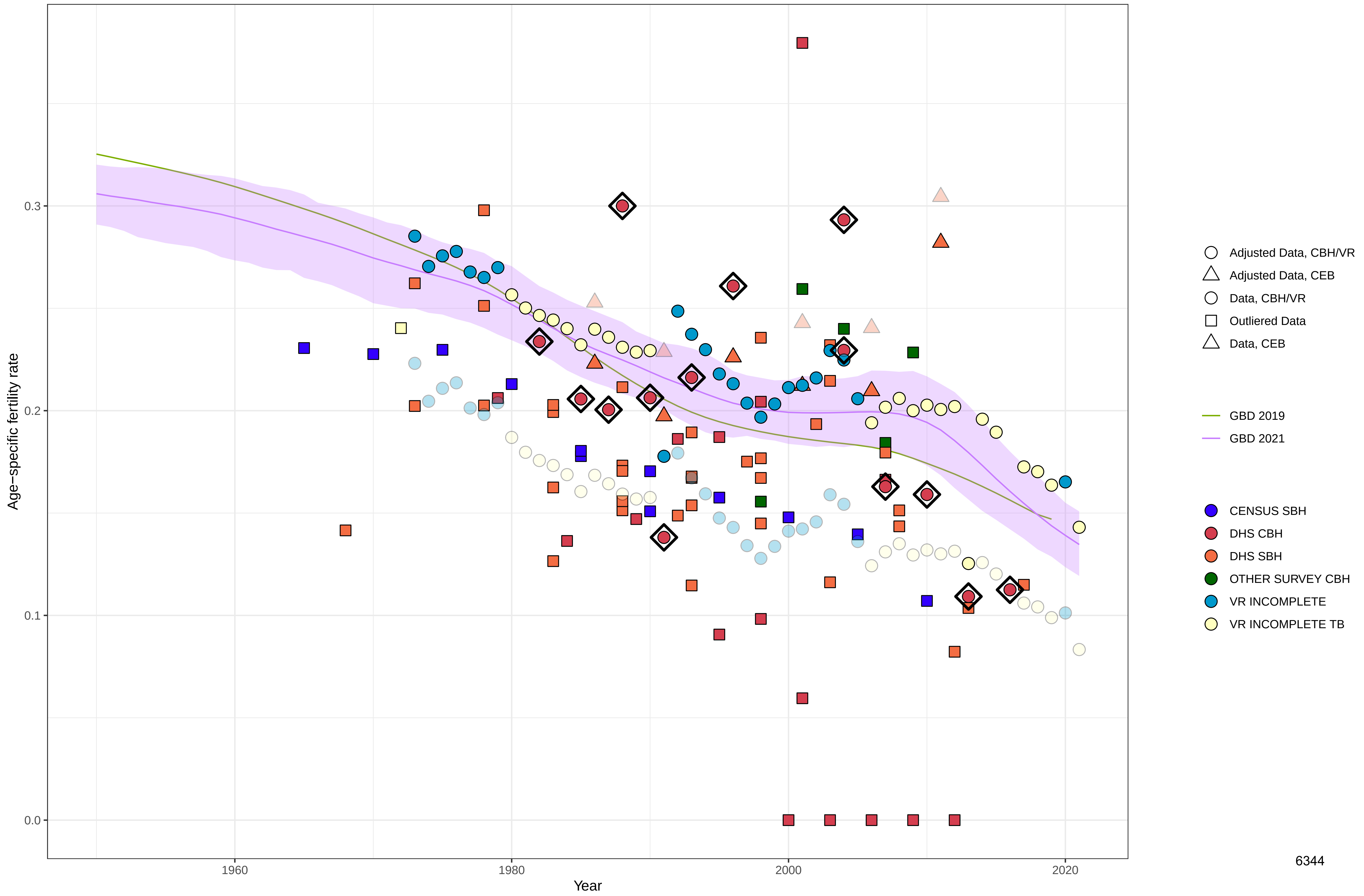

Age−specific fertility fitted trend lines, 1950−2021: Nueva Vizcaya, Ages: 25−29
Data points enclosed with a diamond are reference sources

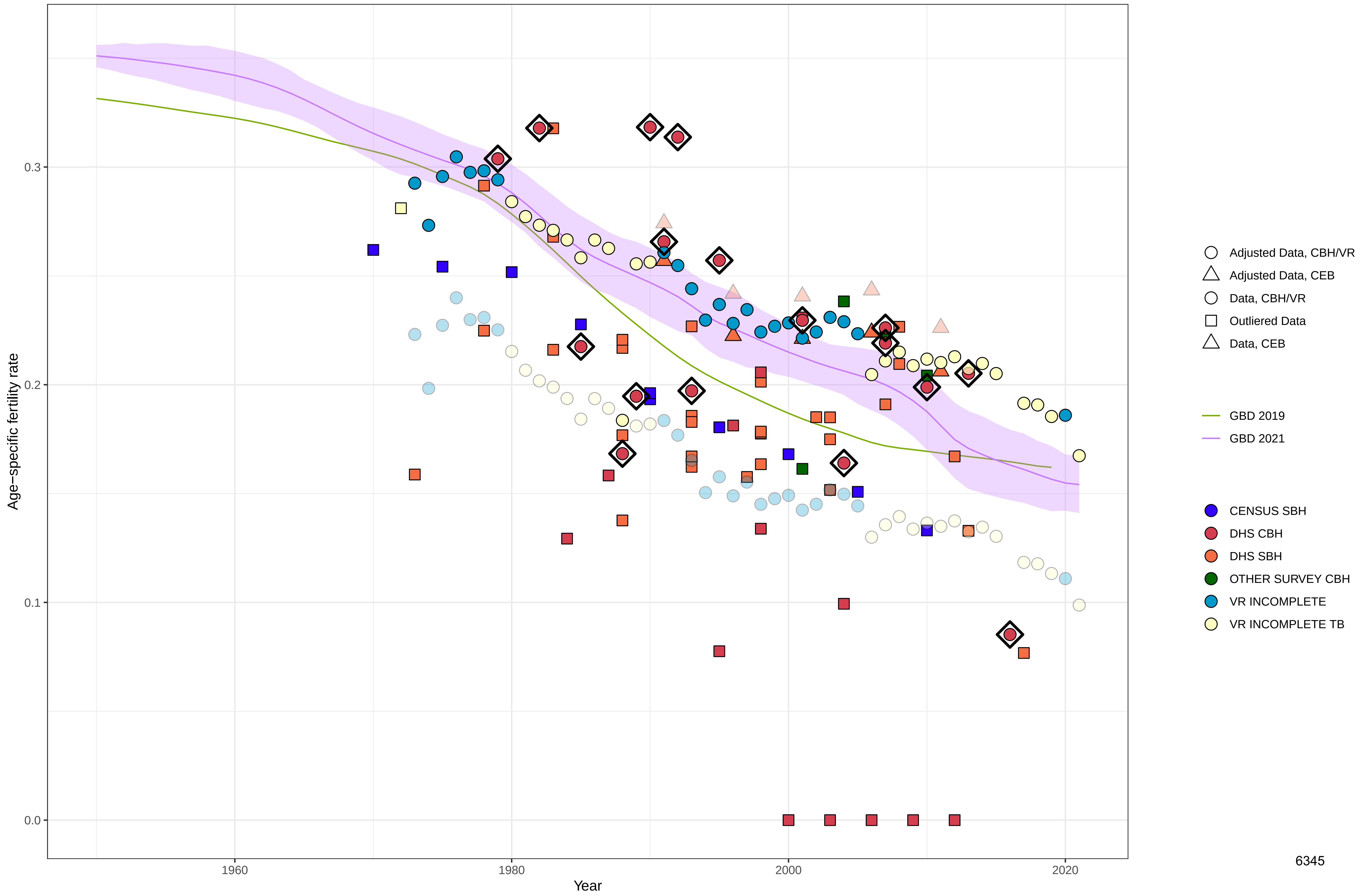

Age−specific fertility fitted trend lines, 1950−2021: Nueva Vizcaya, Ages: 30−34
Data points enclosed with a diamond are reference sources

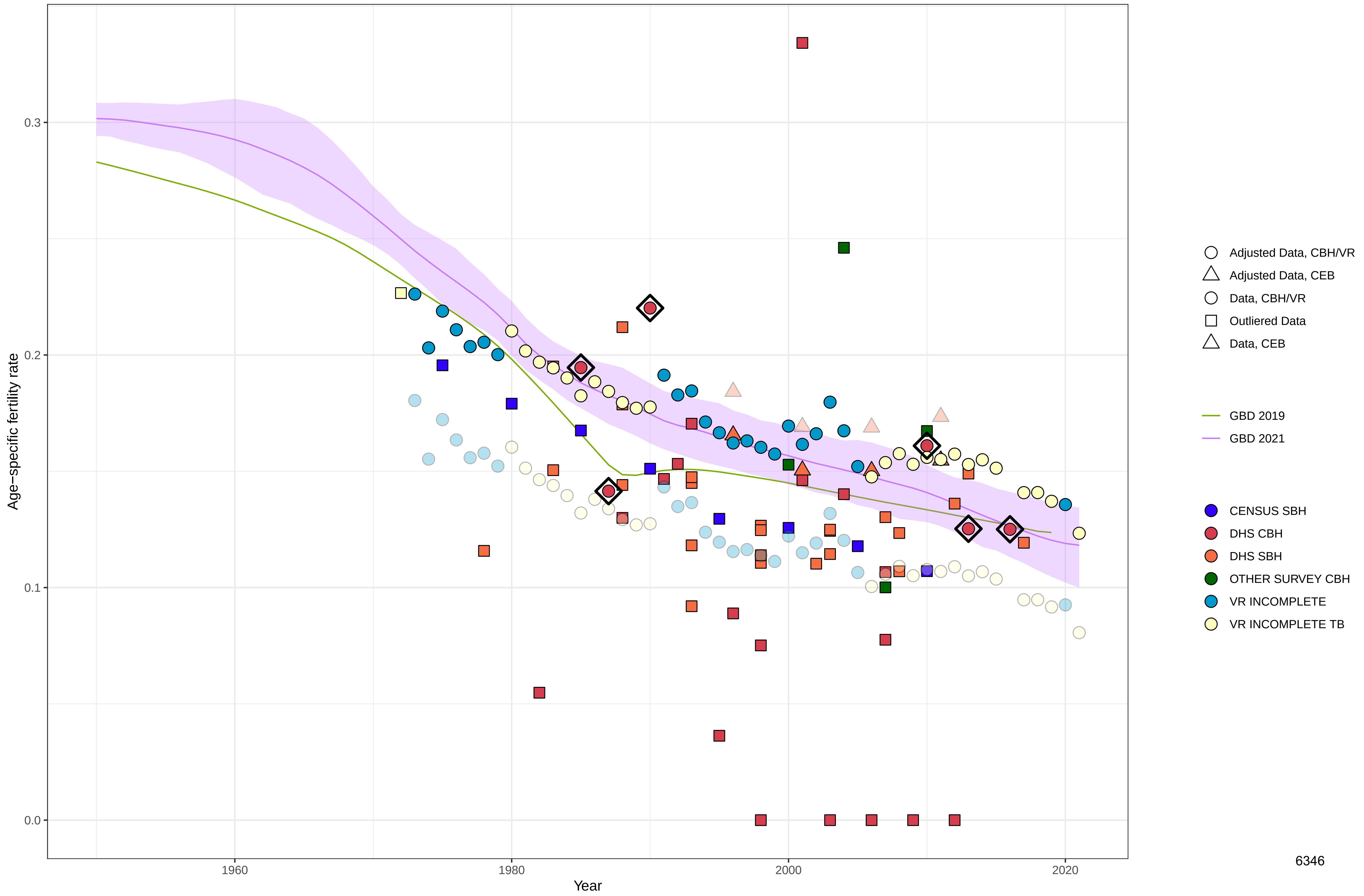

Age−specific fertility fitted trend lines, 1950−2021: Nueva Vizcaya, Ages: 35−39
Data points enclosed with a diamond are reference sources

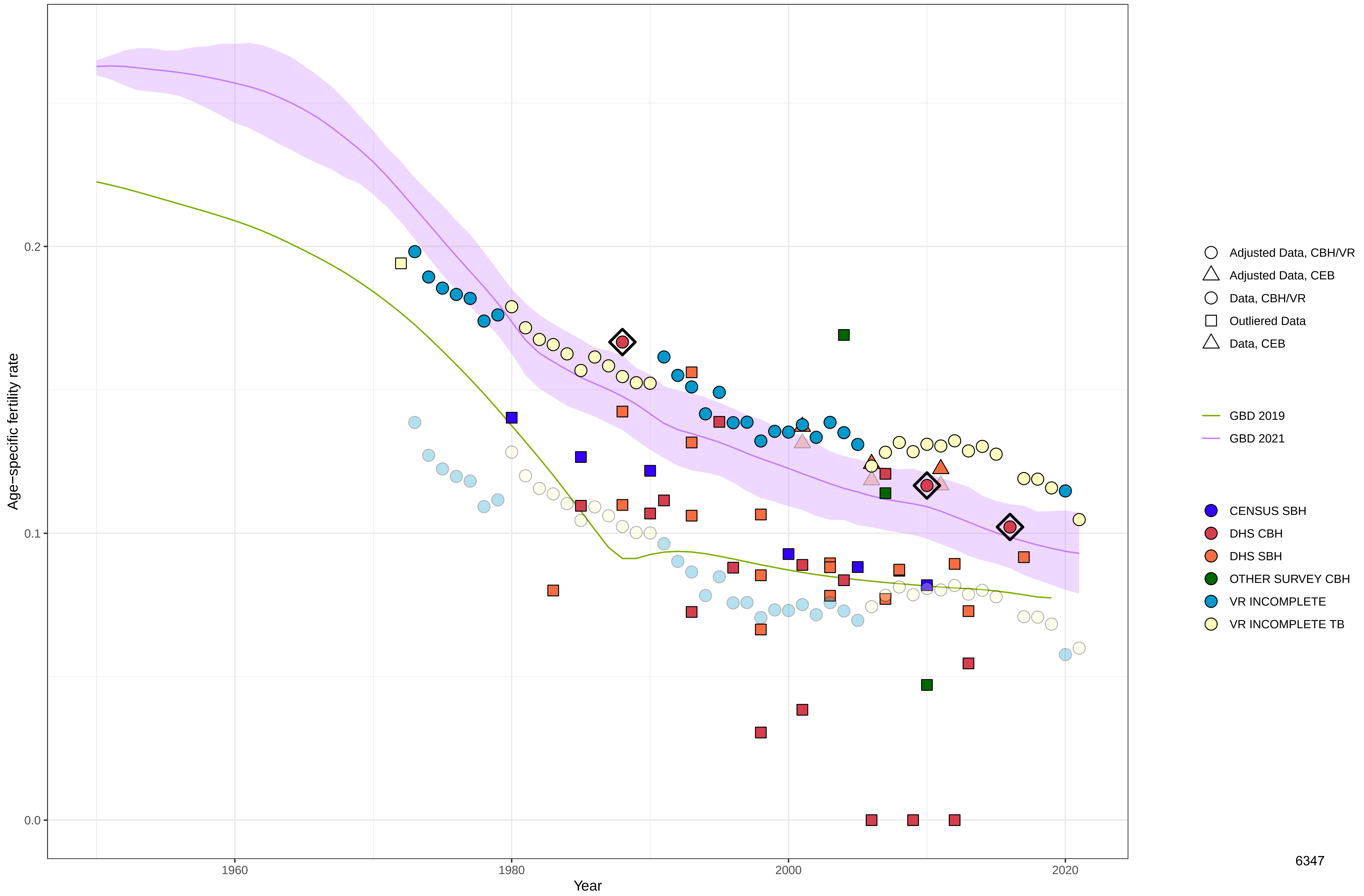

Age−specific fertility fitted trend lines, 1950−2021: Nueva Vizcaya, Ages: 40−44
Data points enclosed with a diamond are reference sources

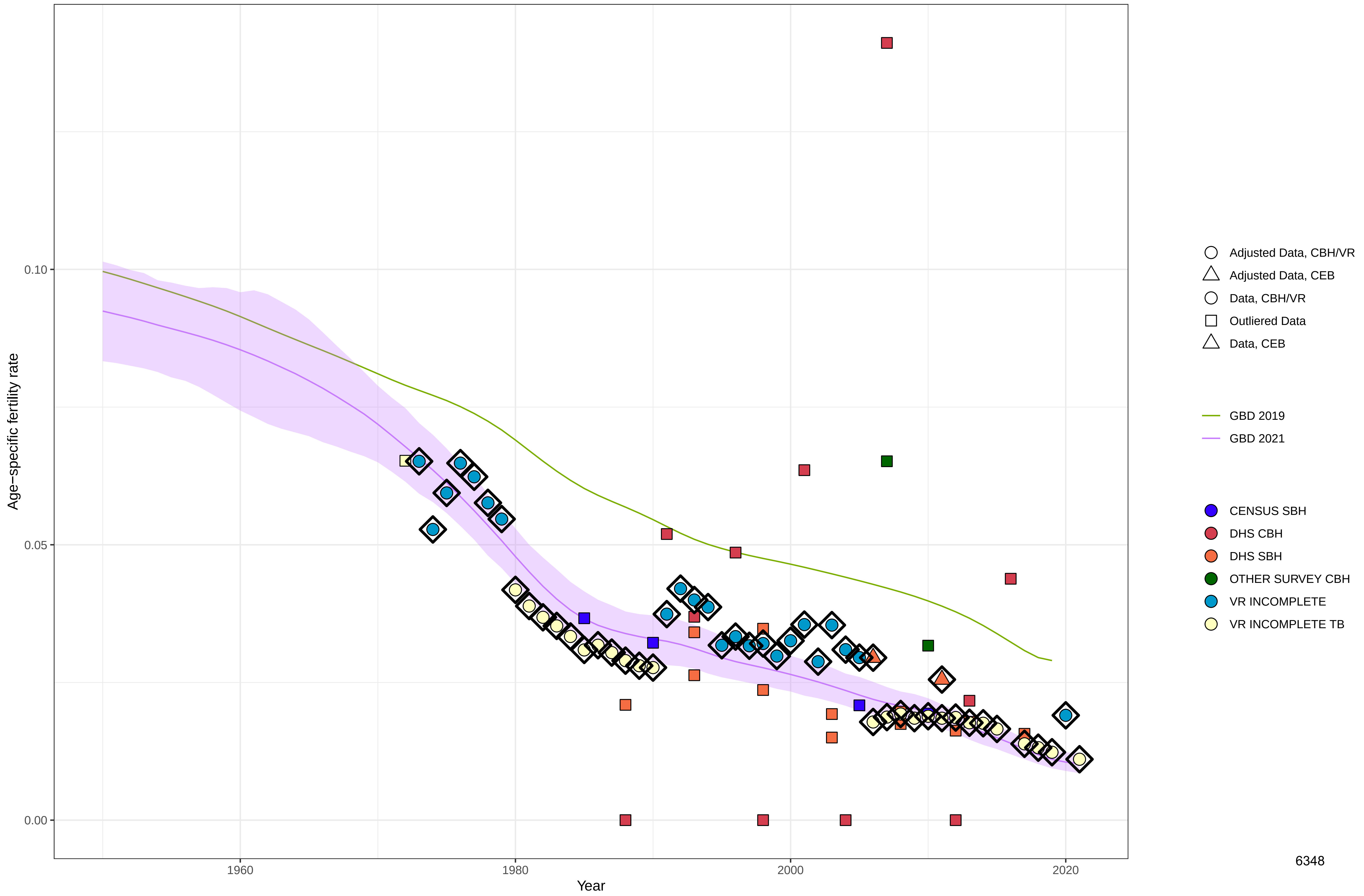

Age−specific fertility fitted trend lines, 1950−2021: Nueva Vizcaya, Ages: 45−49
Data points enclosed with a diamond are reference sources

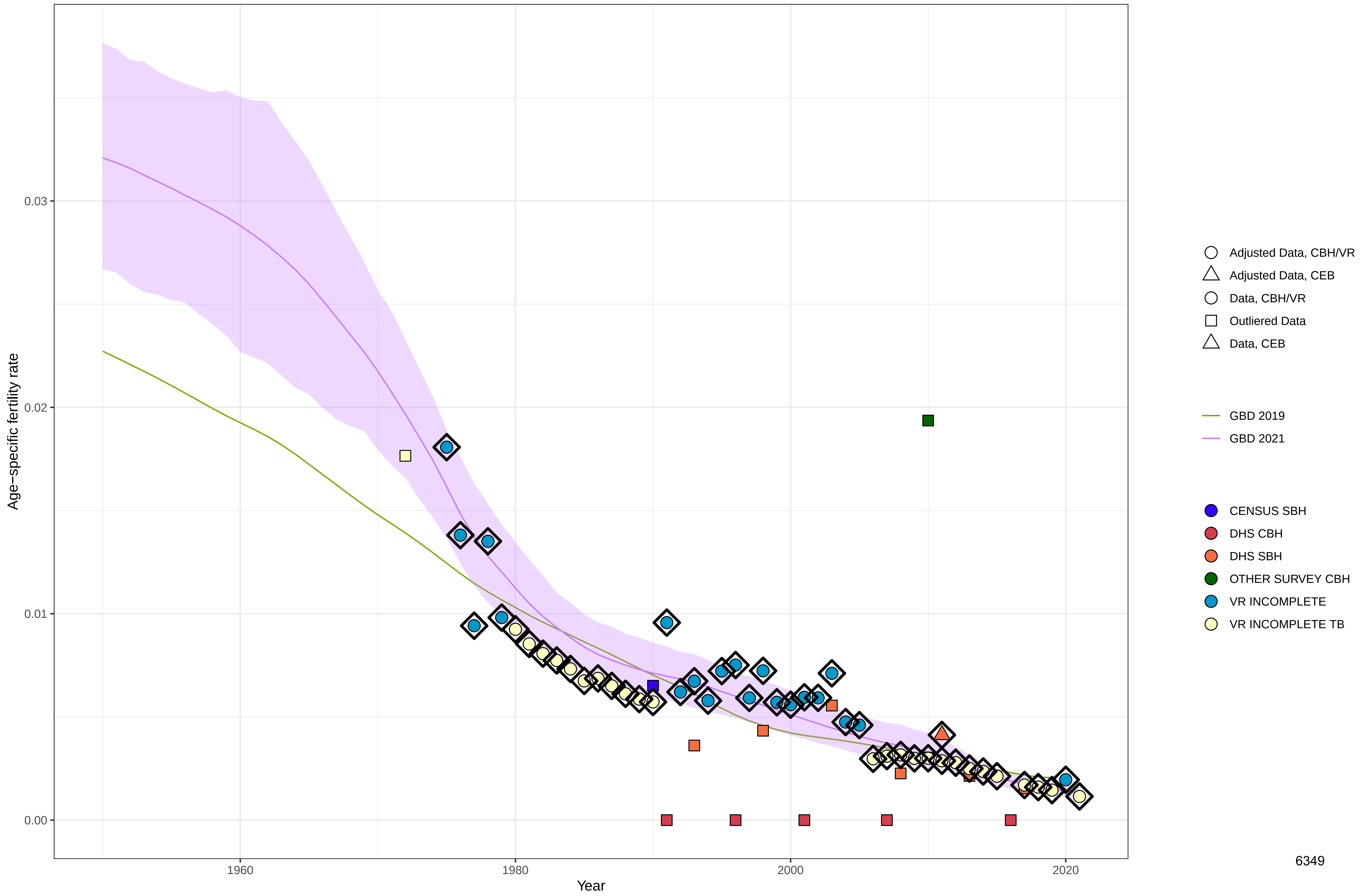

Age−specific fertility fitted trend lines, 1950−2021: Nueva Vizcaya, Ages: 50−54
Data points enclosed with a diamond are reference sources

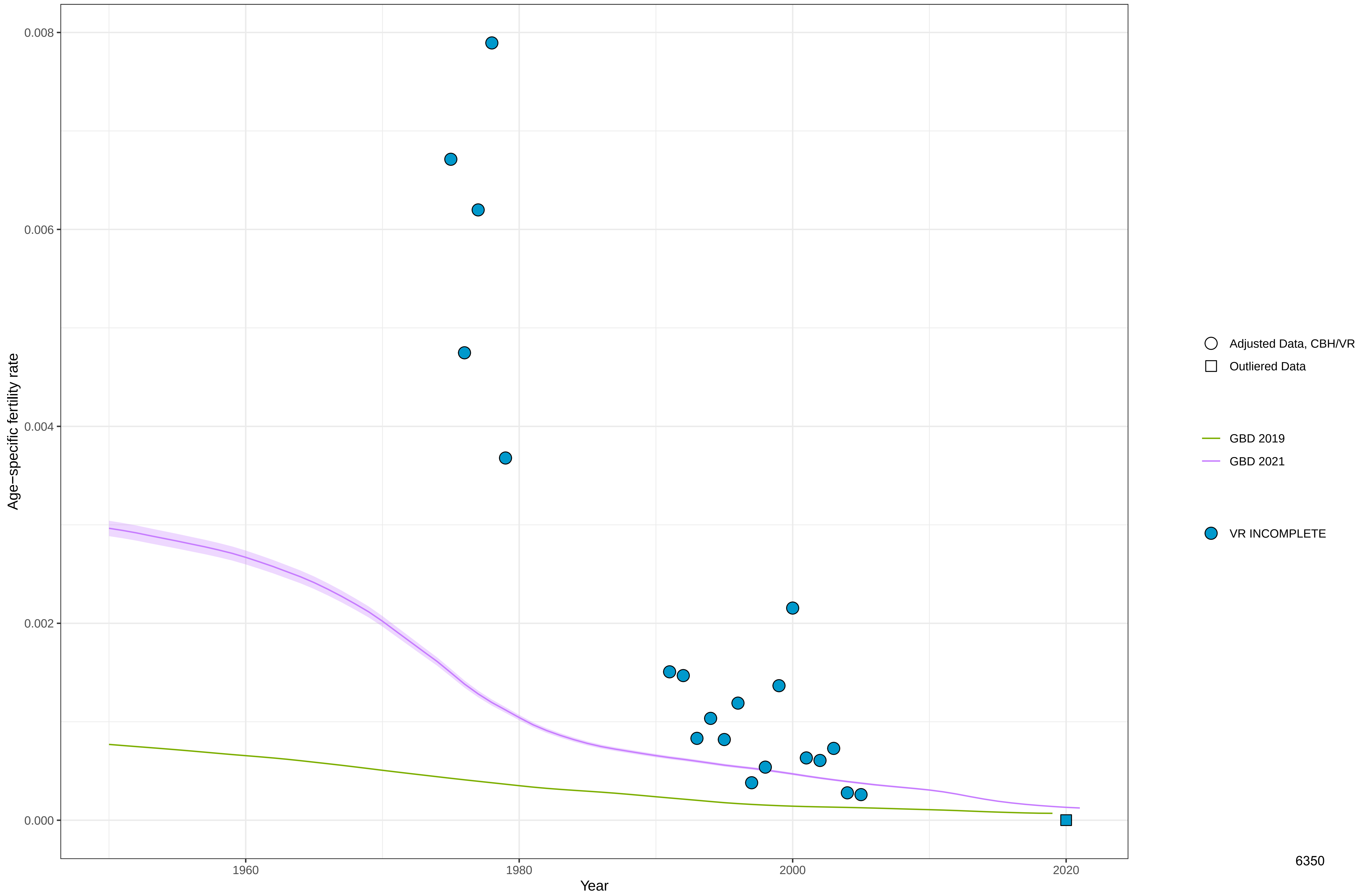

Total fertility rate fitted trend lines, 1950−2021: Nueva Vizcaya TFR
Data points enclosed with a diamond are reference sources

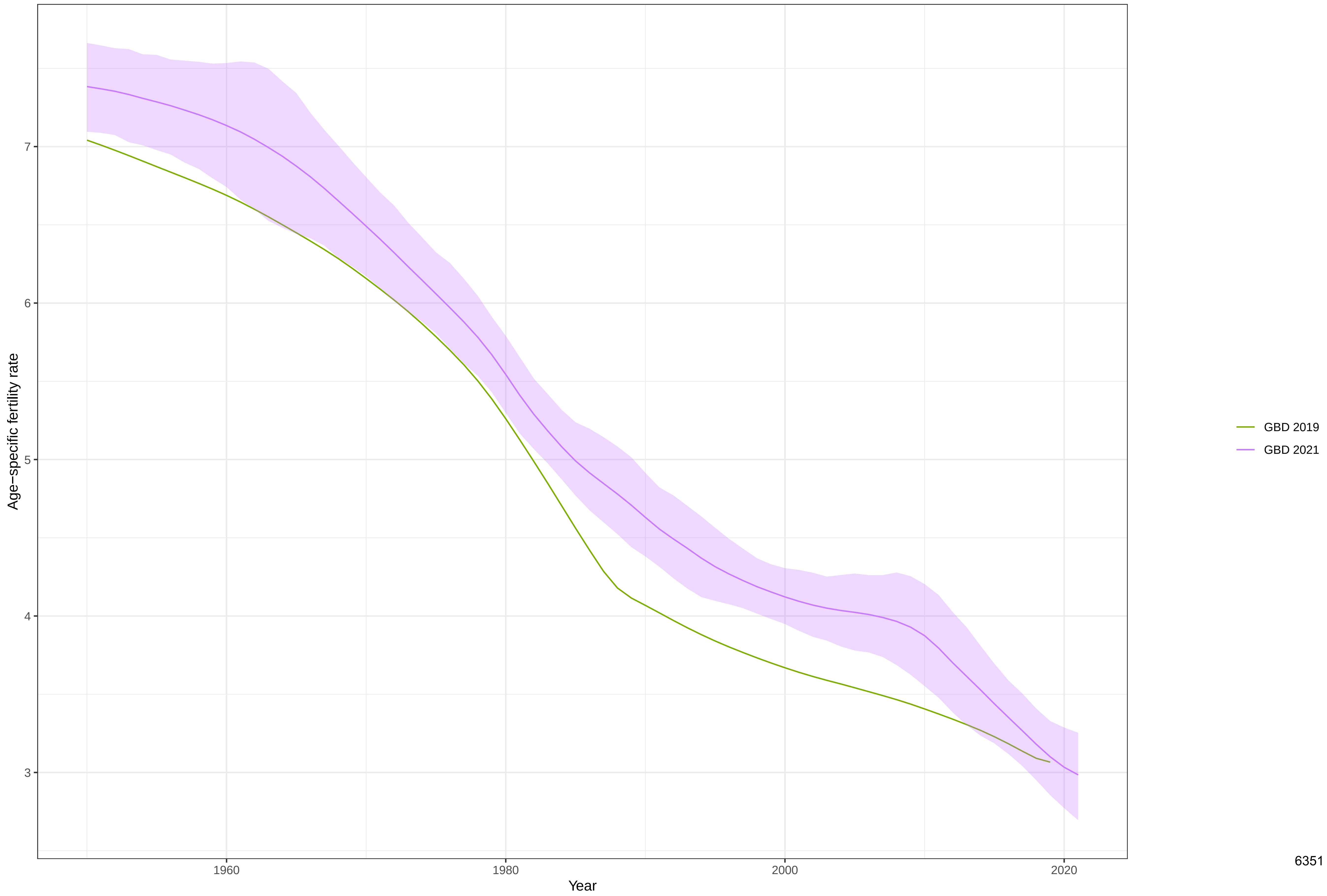

Age−specific fertility fitted trend lines, 1950−2021: Occidental Mindoro, Ages: 10−14
Data points enclosed with a diamond are reference sources

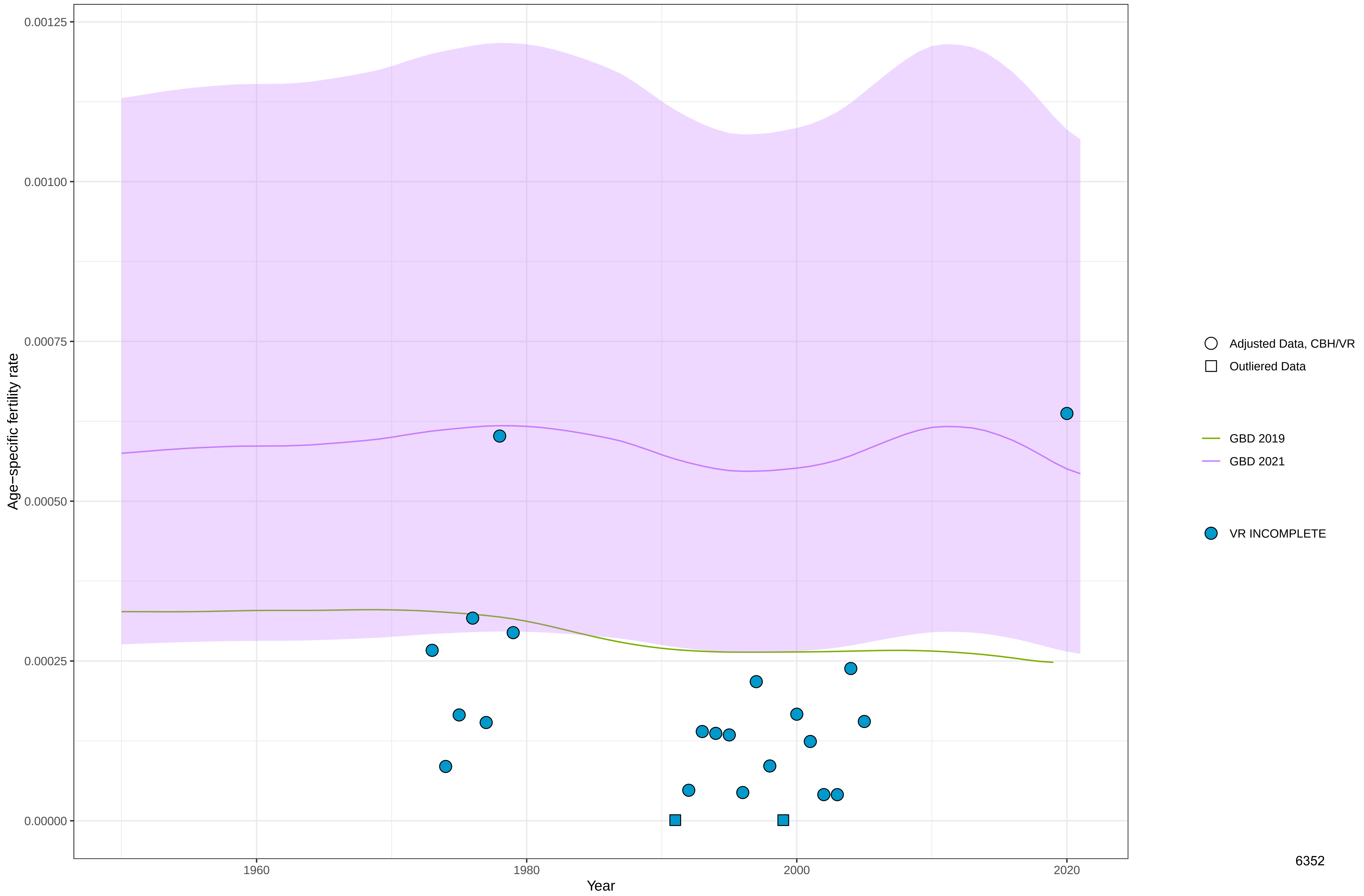

Age−specific fertility fitted trend lines, 1950−2021: Occidental Mindoro, Ages: 15−19
Data points enclosed with a diamond are reference sources

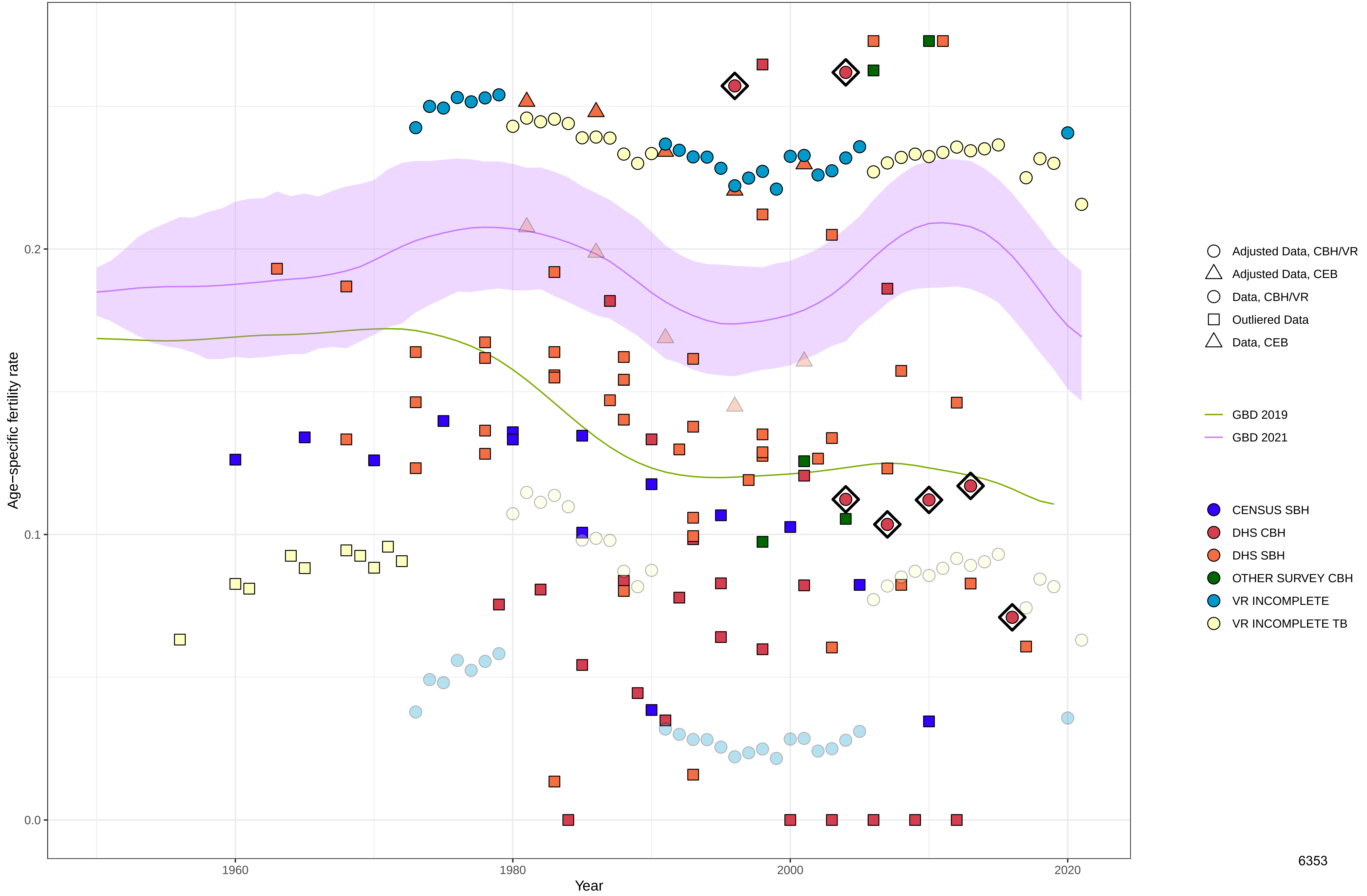

Age−specific fertility fitted trend lines, 1950−2021: Occidental Mindoro, Ages: 20−24
Data points enclosed with a diamond are reference sources

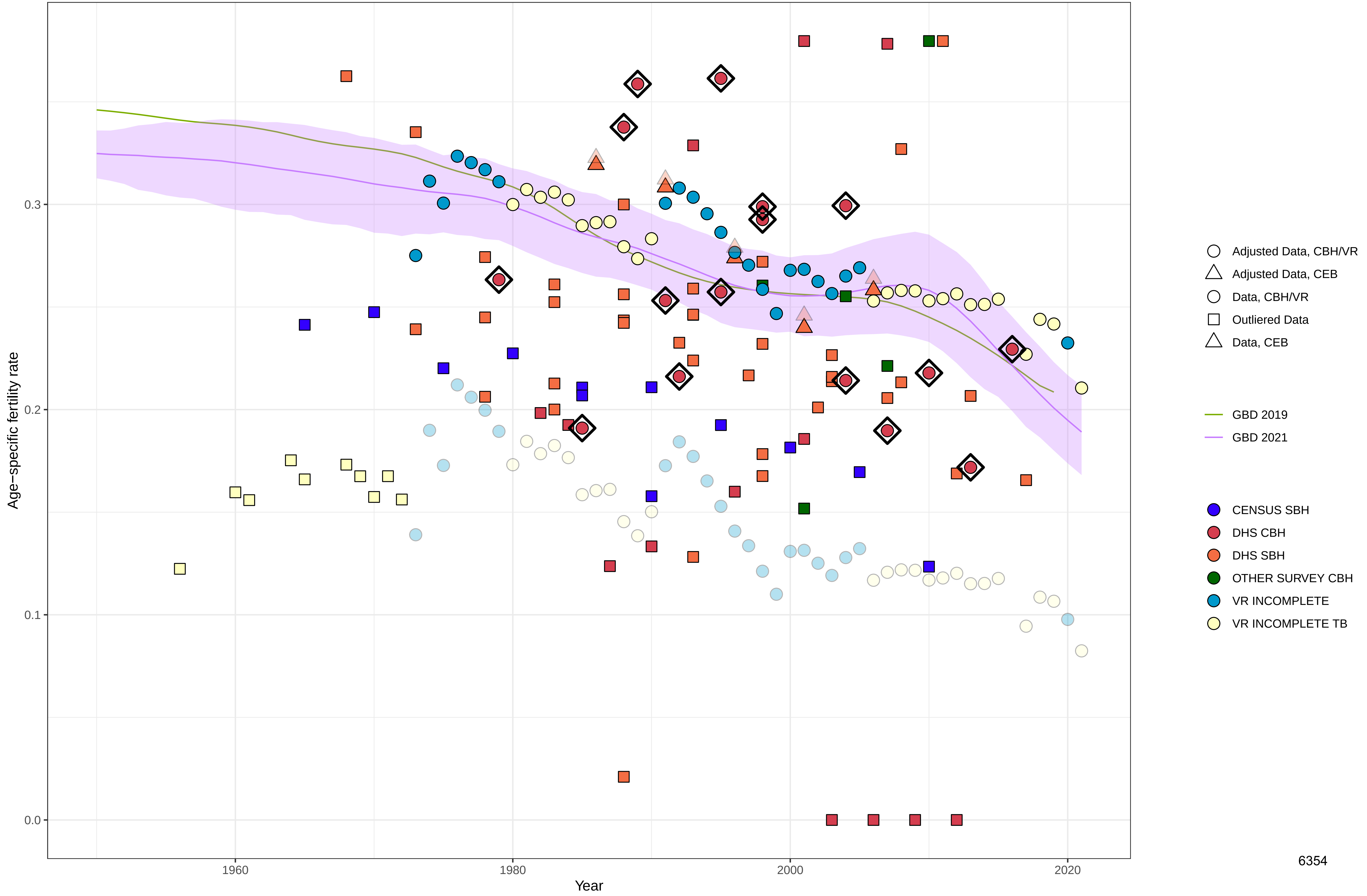

Age−specific fertility fitted trend lines, 1950−2021: Occidental Mindoro, Ages: 25−29
Data points enclosed with a diamond are reference sources

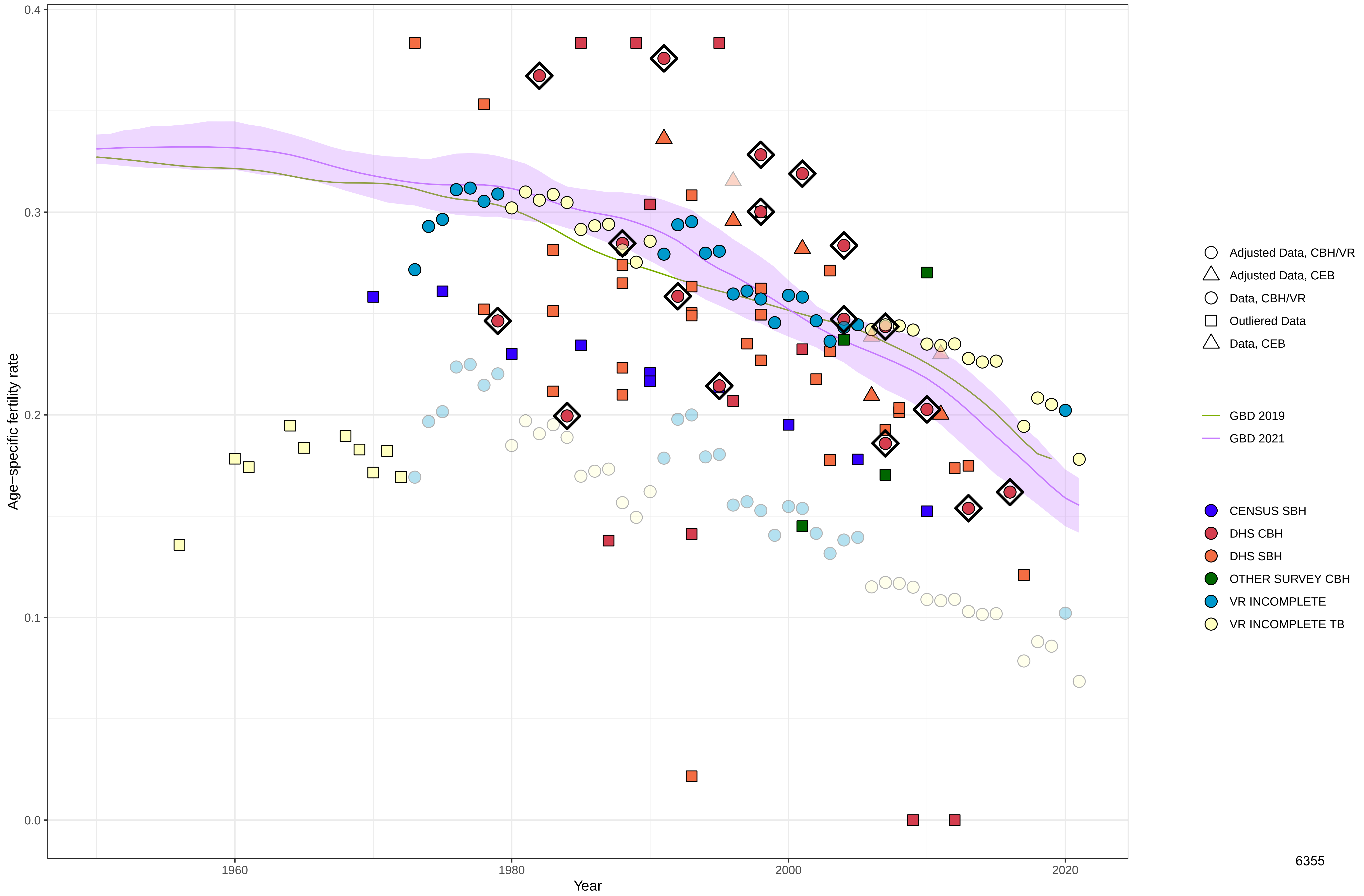

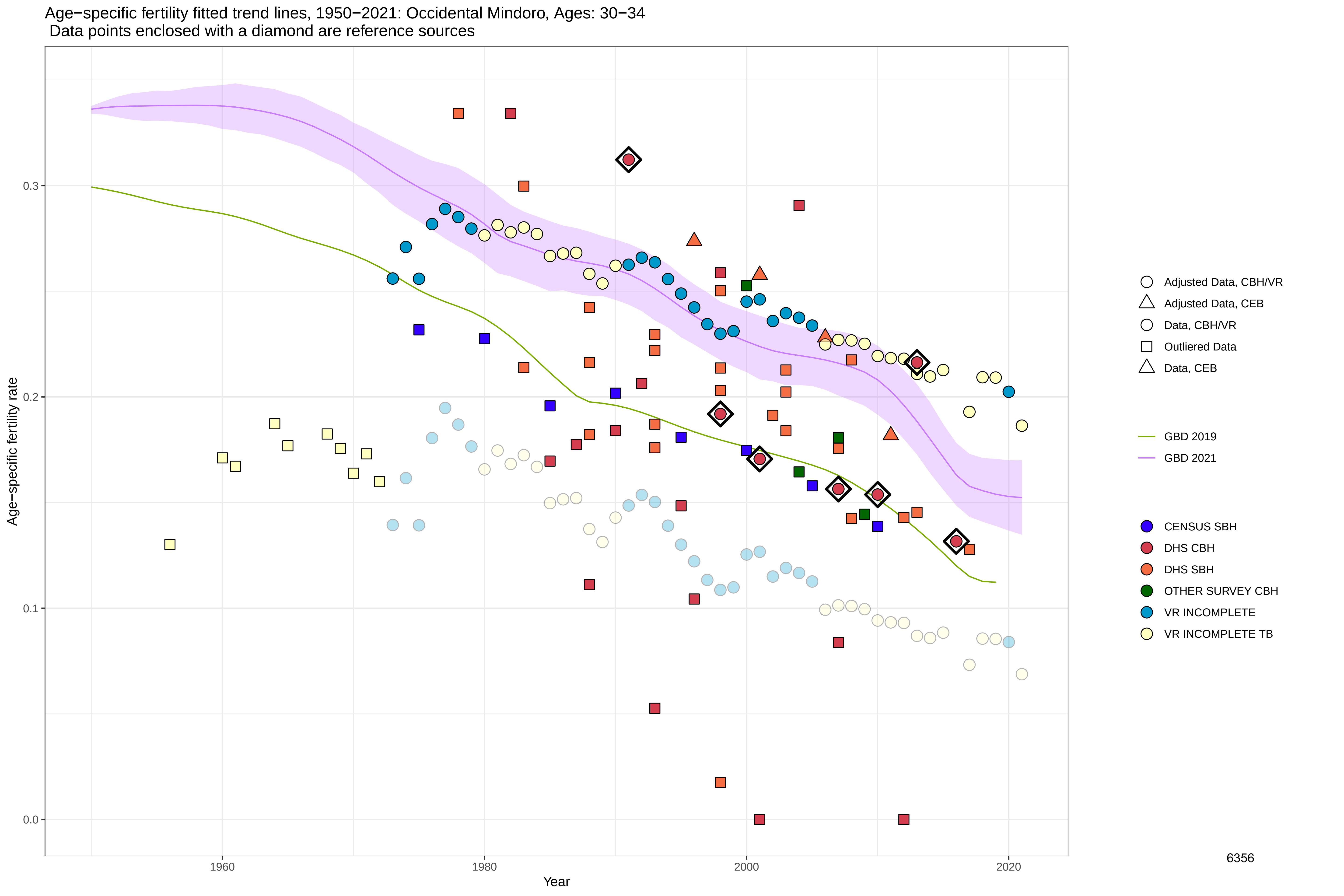

Age−specific fertility fitted trend lines, 1950−2021: Occidental Mindoro, Ages: 30−34
Data points enclosed with a diamond are reference sources
Age−specific fertility rate
Year
0.0
0.1
0.2
0.3
1960
1980
2000
2020
Adjusted Data, CBH/VR
Adjusted Data, CEB
Data, CBH/VR
Outliered Data
Data, CEB
GBD 2019
GBD 2021
CENSUS SBH
DHS CBH
DHS SBH
OTHER SURVEY CBH
VR INCOMPLETE
VR INCOMPLETE TB

Age−specific fertility fitted trend lines, 1950−2021: Occidental Mindoro, Ages: 35−39
Data points enclosed with a diamond are reference sources

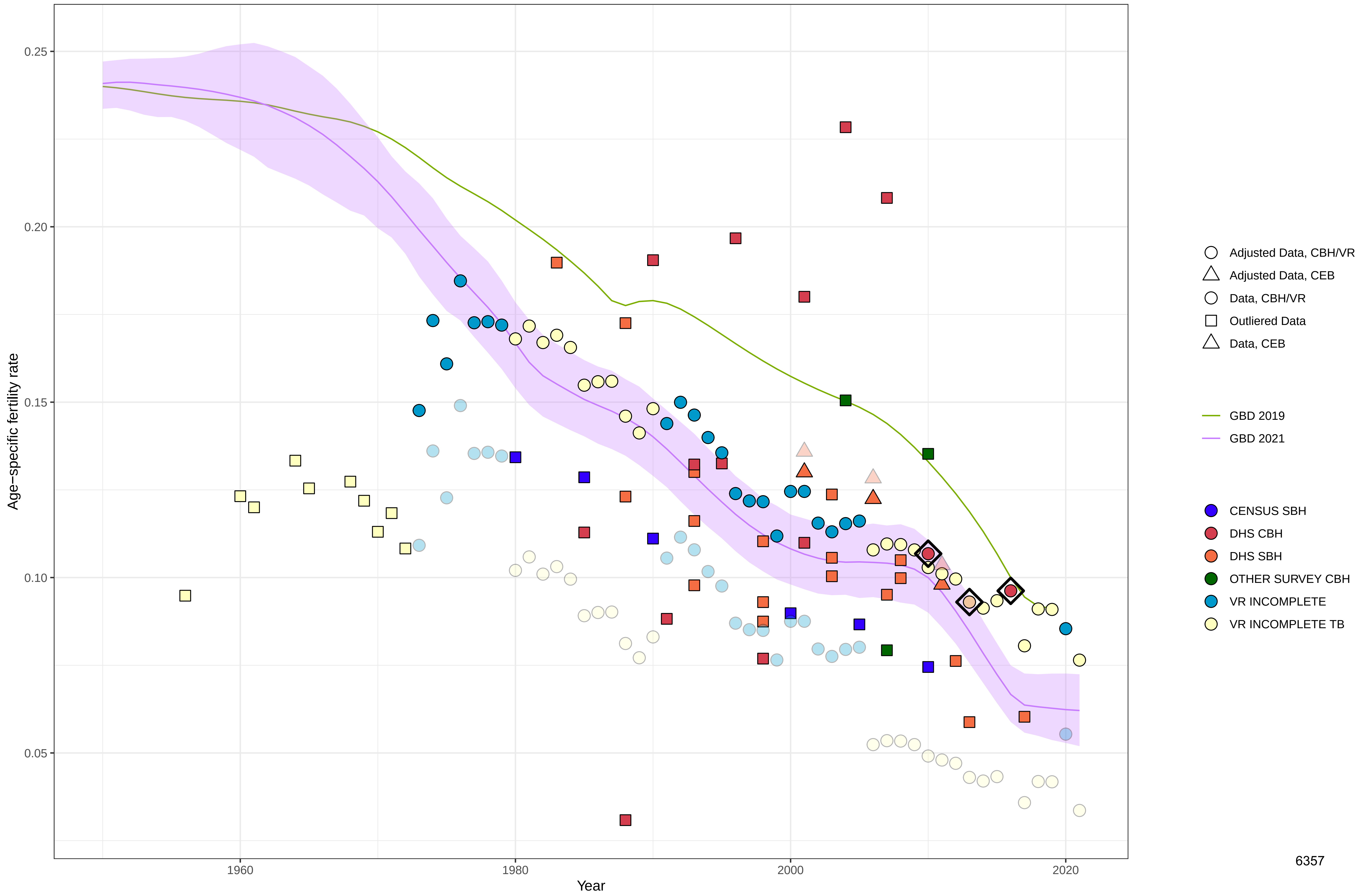

Age−specific fertility fitted trend lines, 1950−2021: Occidental Mindoro, Ages: 40−44
Data points enclosed with a diamond are reference sources

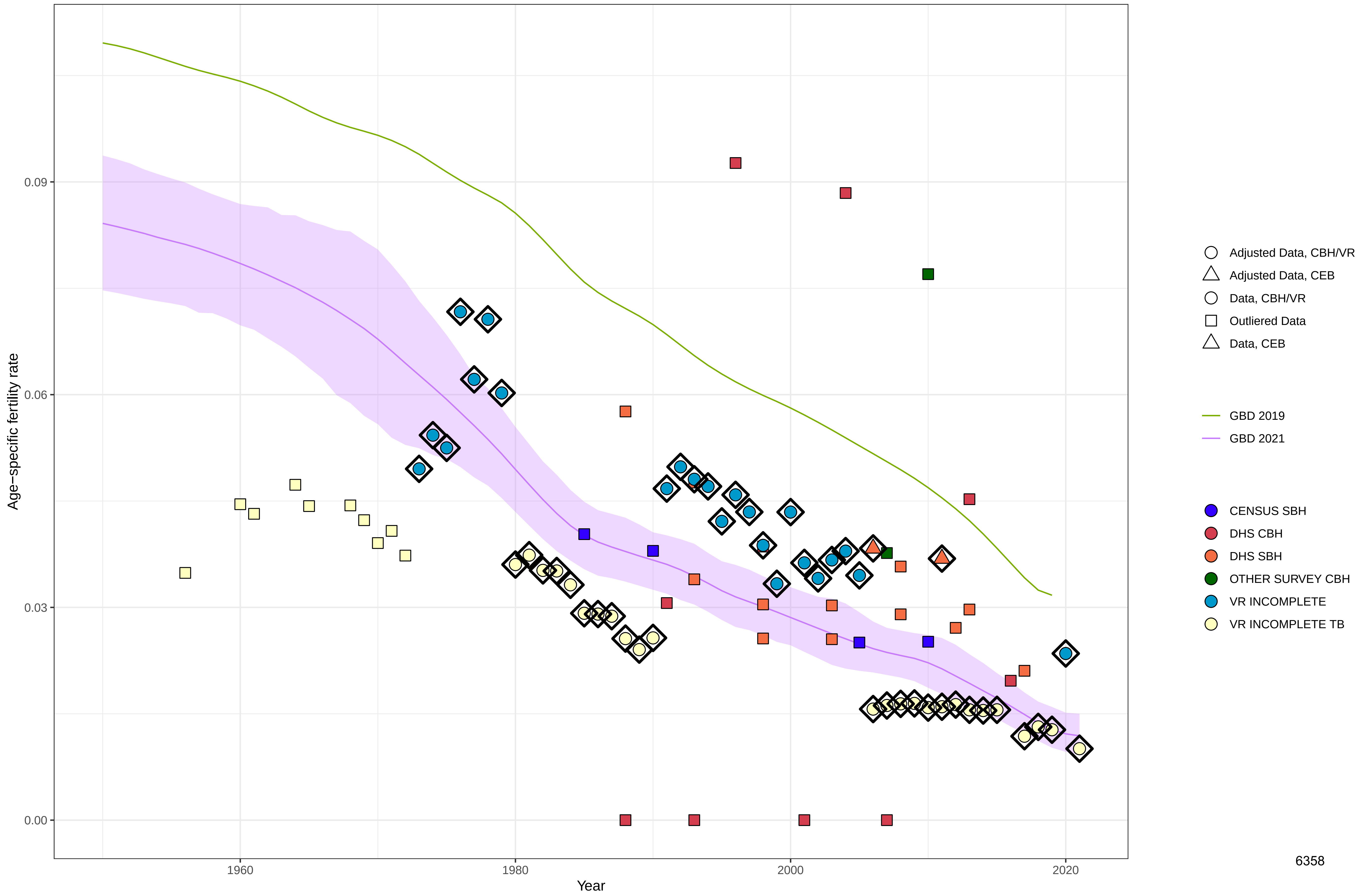

Age−specific fertility fitted trend lines, 1950−2021: Occidental Mindoro, Ages: 45−49
Data points enclosed with a diamond are reference sources

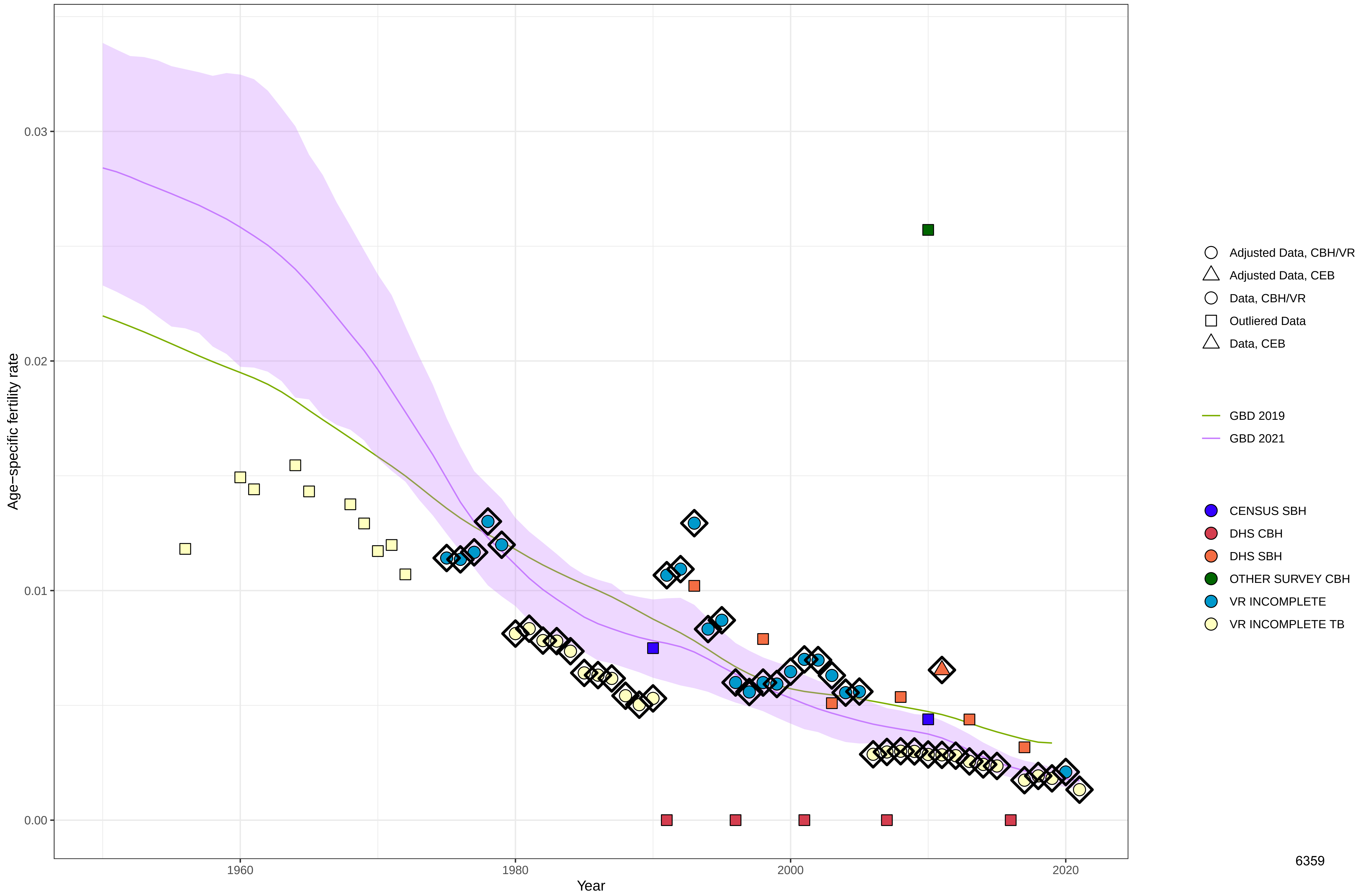

Age−specific fertility fitted trend lines, 1950−2021: Occidental Mindoro, Ages: 50−54
Data points enclosed with a diamond are reference sources

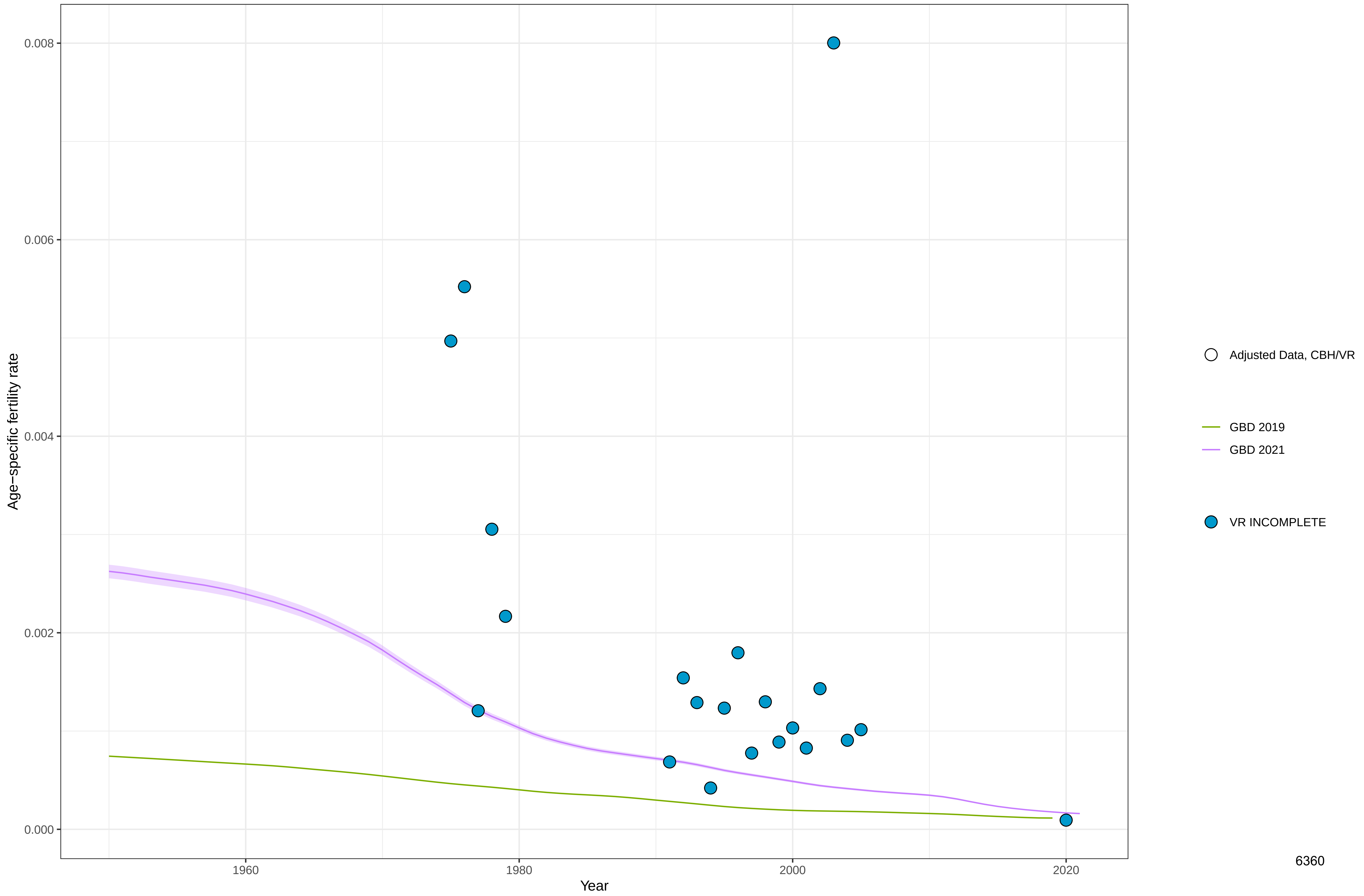

Total fertility rate fitted trend lines, 1950−2021: Occidental Mindoro TFR
Data points enclosed with a diamond are reference sources

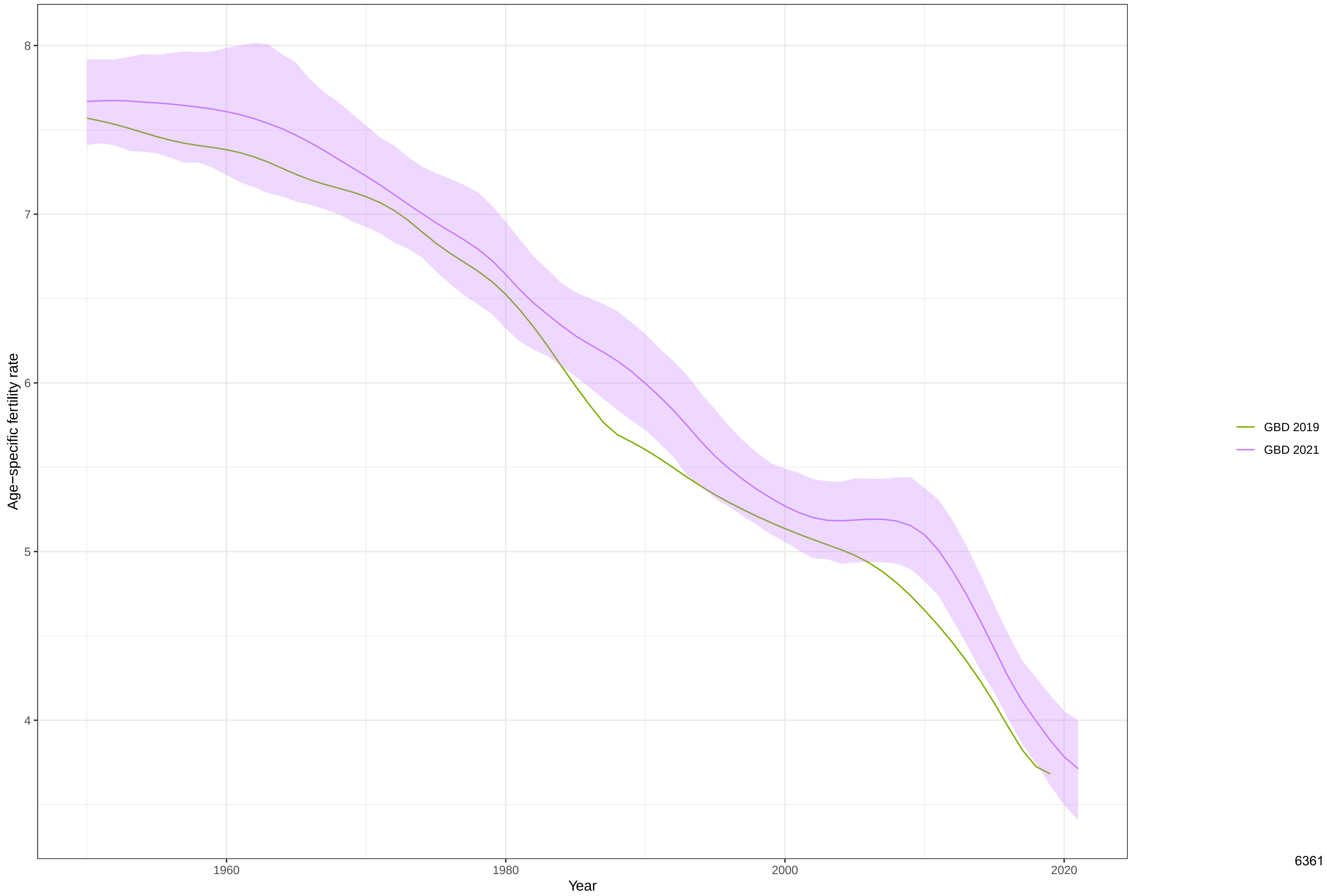

6361

Age−specific fertility fitted trend lines, 1950−2021: Oriental Mindoro, Ages: 10−14
Data points enclosed with a diamond are reference sources

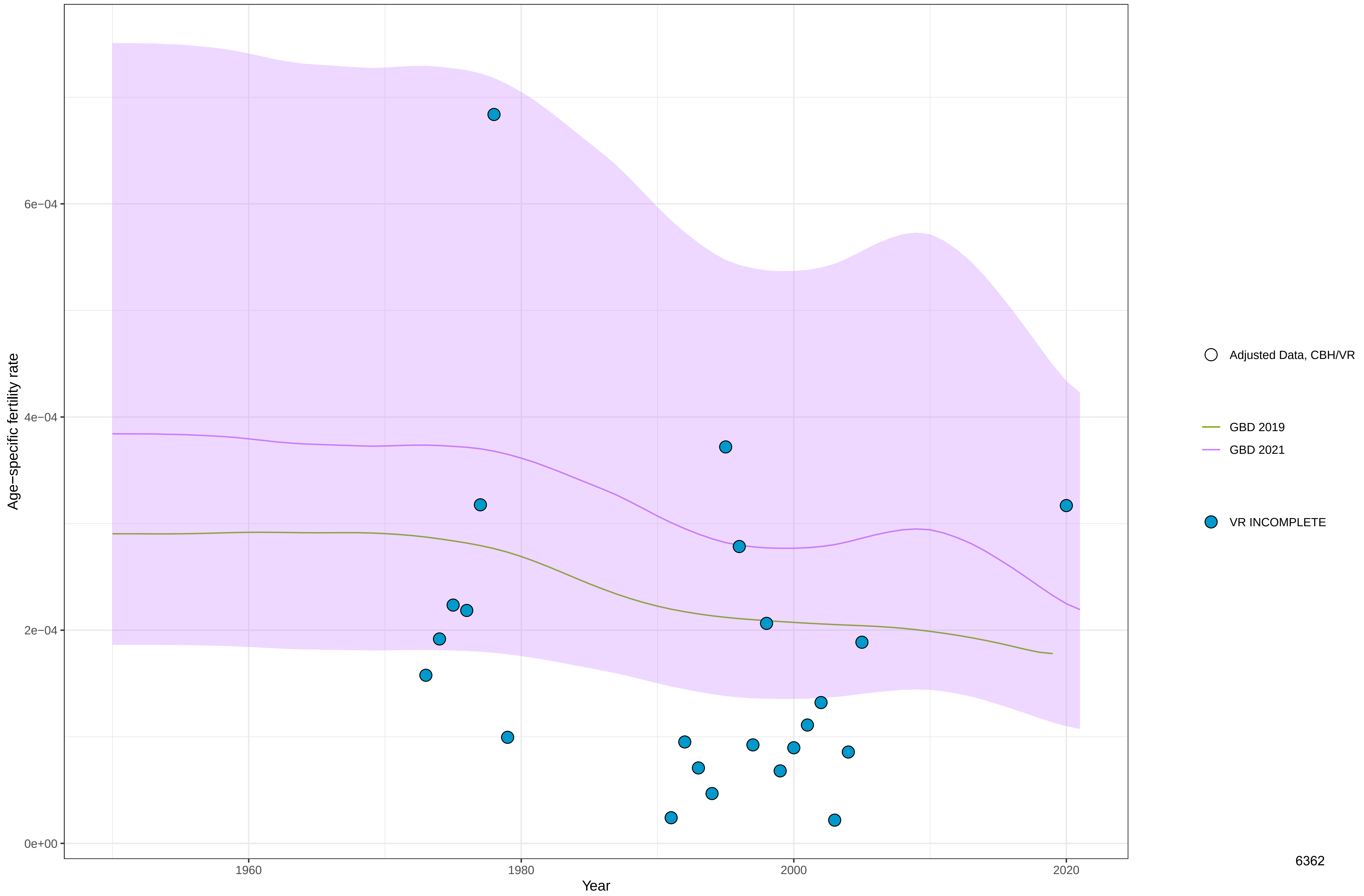

6362

Age−specific fertility fitted trend lines, 1950−2021: Oriental Mindoro, Ages: 15−19
Data points enclosed with a diamond are reference sources

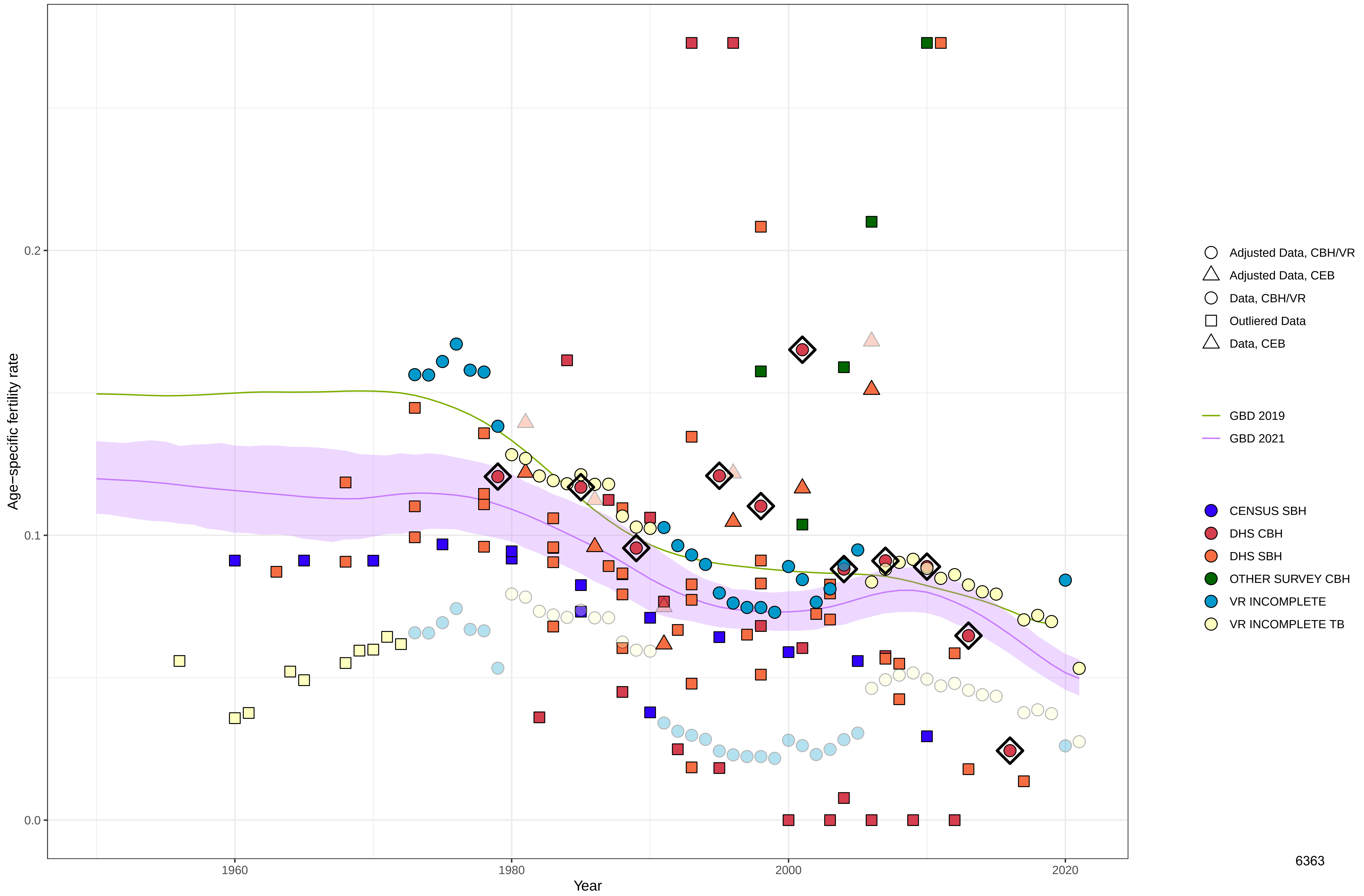

Age−specific fertility fitted trend lines, 1950−2021: Oriental Mindoro, Ages: 20−24
Data points enclosed with a diamond are reference sources

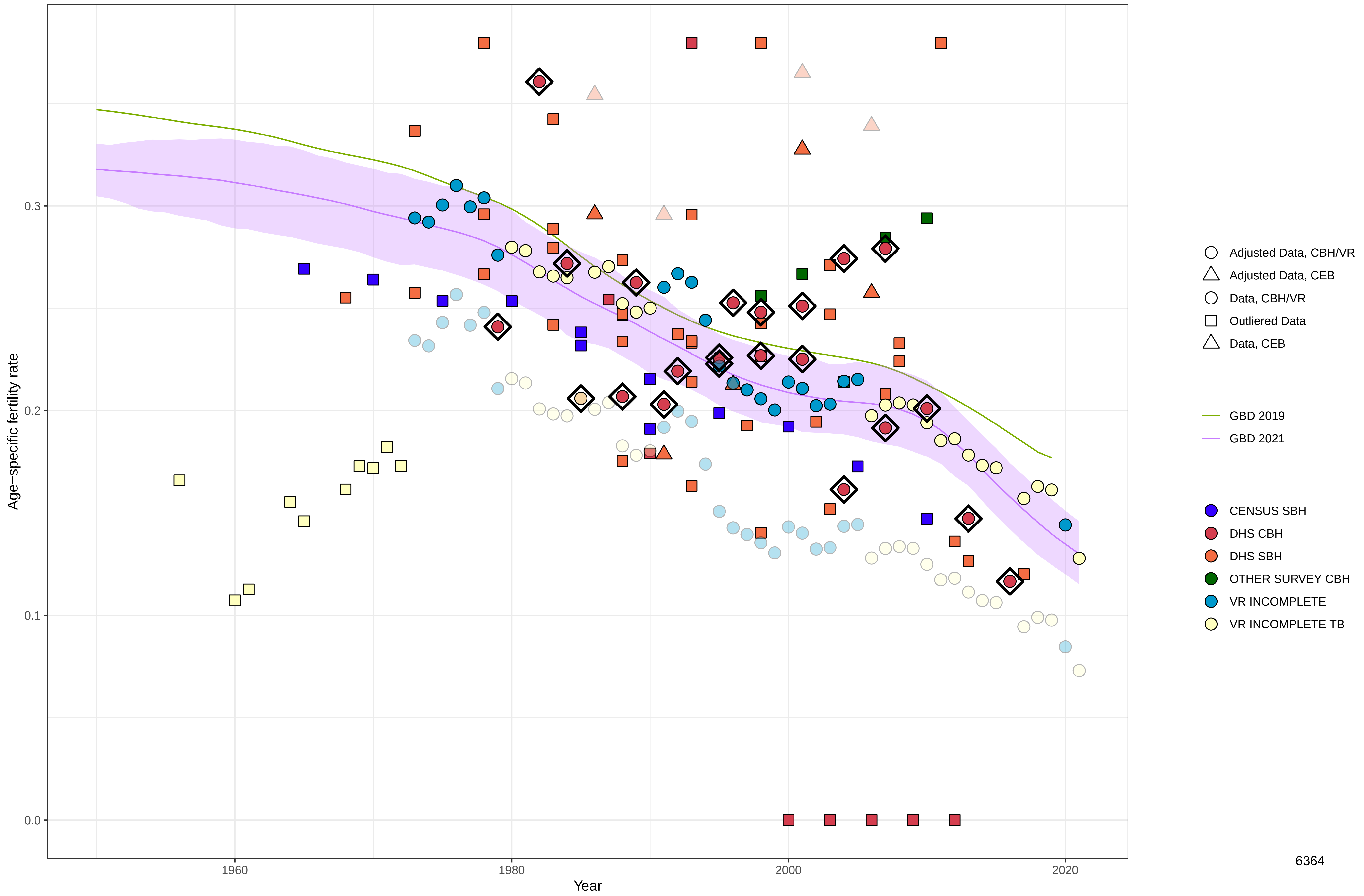

Age−specific fertility fitted trend lines, 1950−2021: Oriental Mindoro, Ages: 25−29
Data points enclosed with a diamond are reference sources

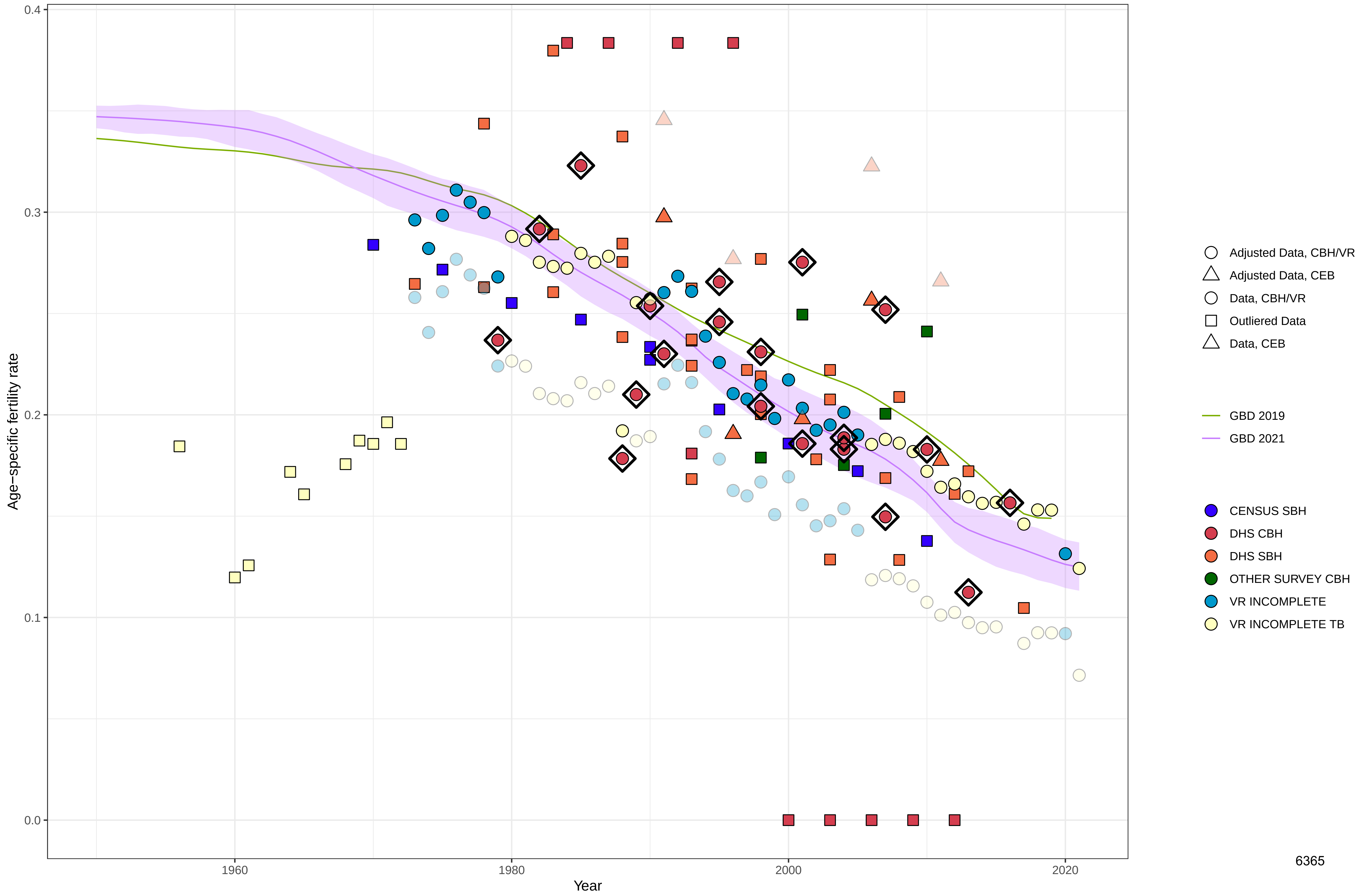

Age−specific fertility fitted trend lines, 1950−2021: Oriental Mindoro, Ages: 30−34
Data points enclosed with a diamond are reference sources

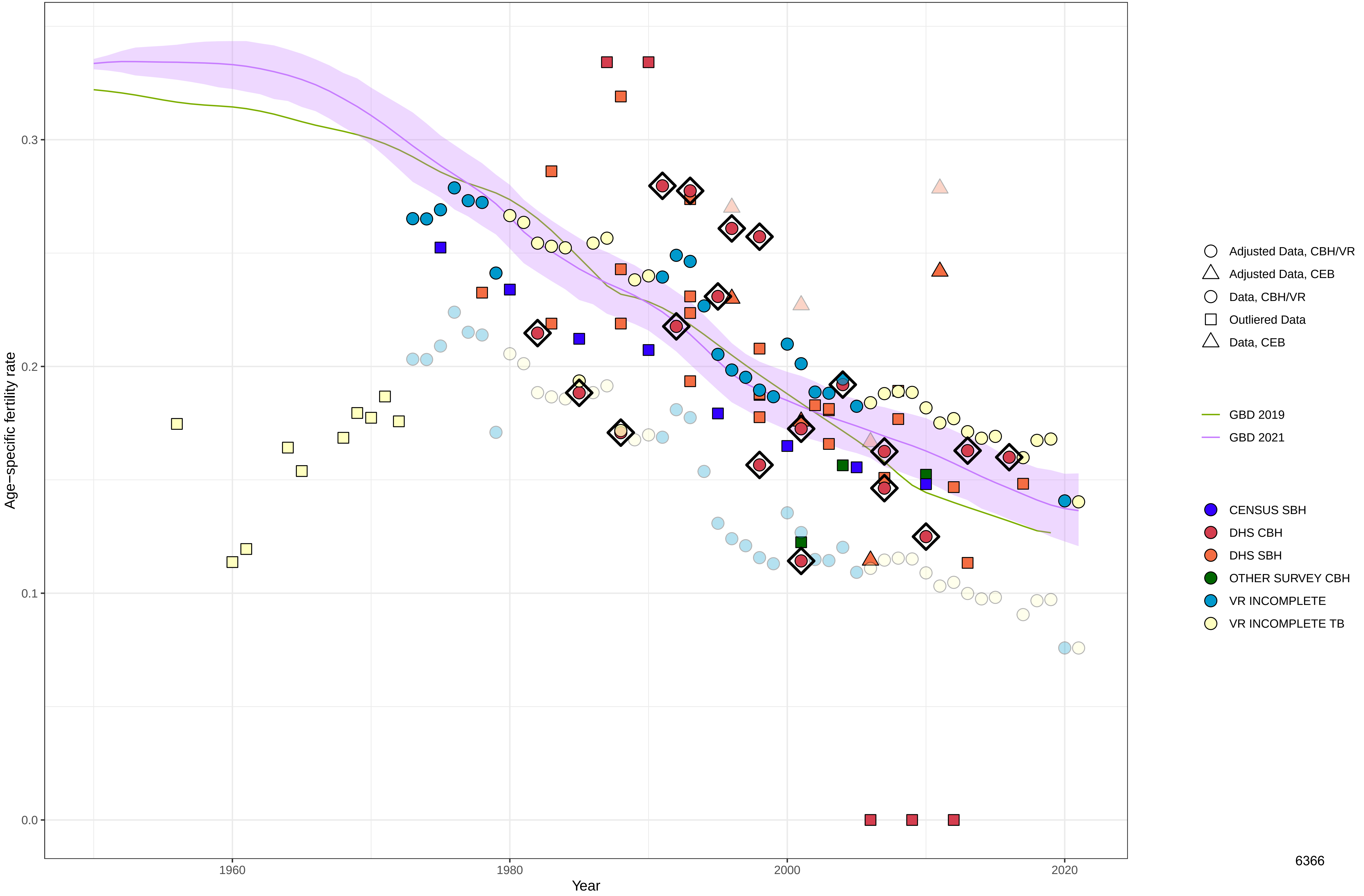

Age−specific fertility fitted trend lines, 1950−2021: Oriental Mindoro, Ages: 35−39
Data points enclosed with a diamond are reference sources

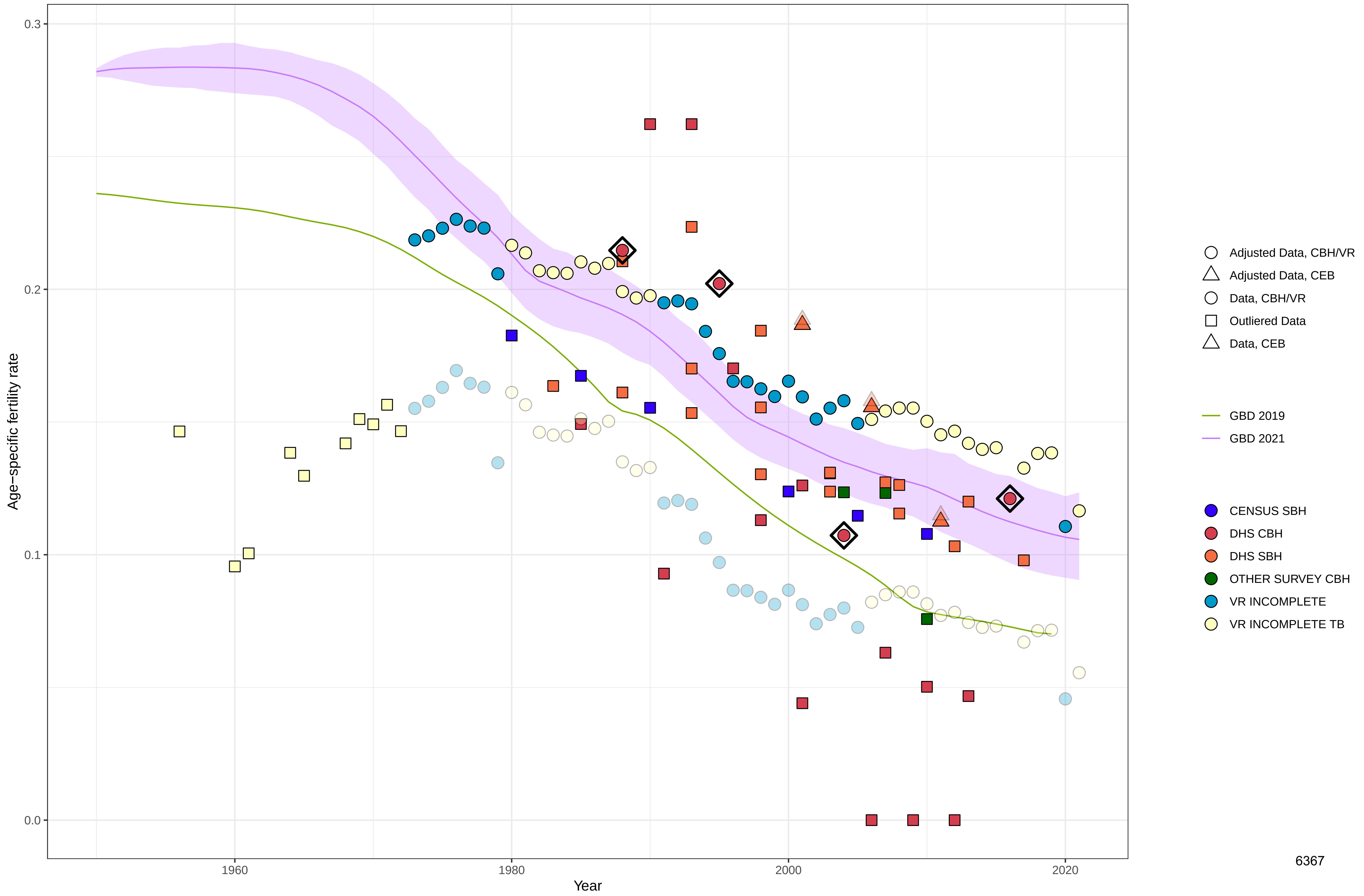

Age−specific fertility fitted trend lines, 1950−2021: Oriental Mindoro, Ages: 40−44
Data points enclosed with a diamond are reference sources

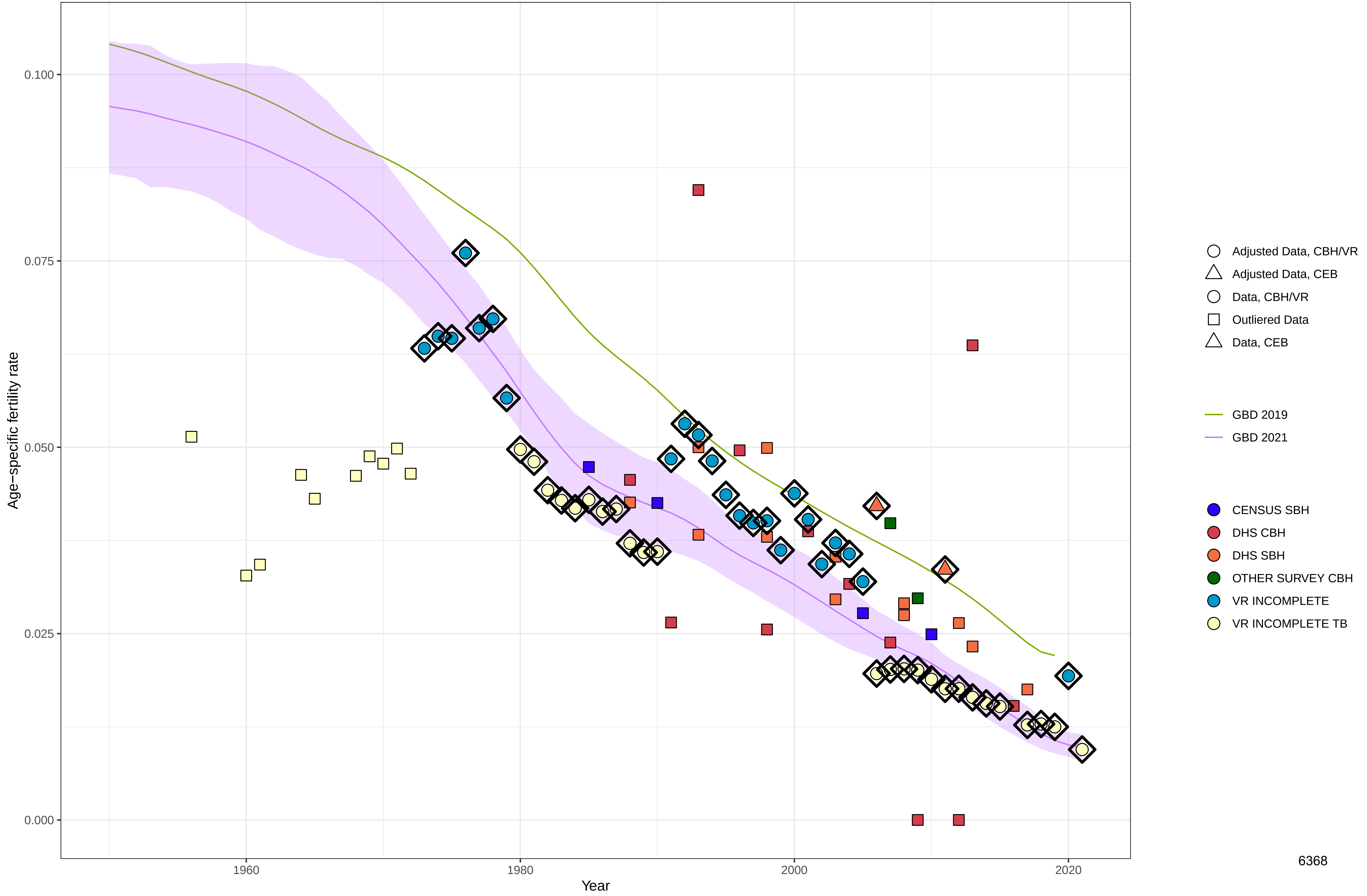

6368

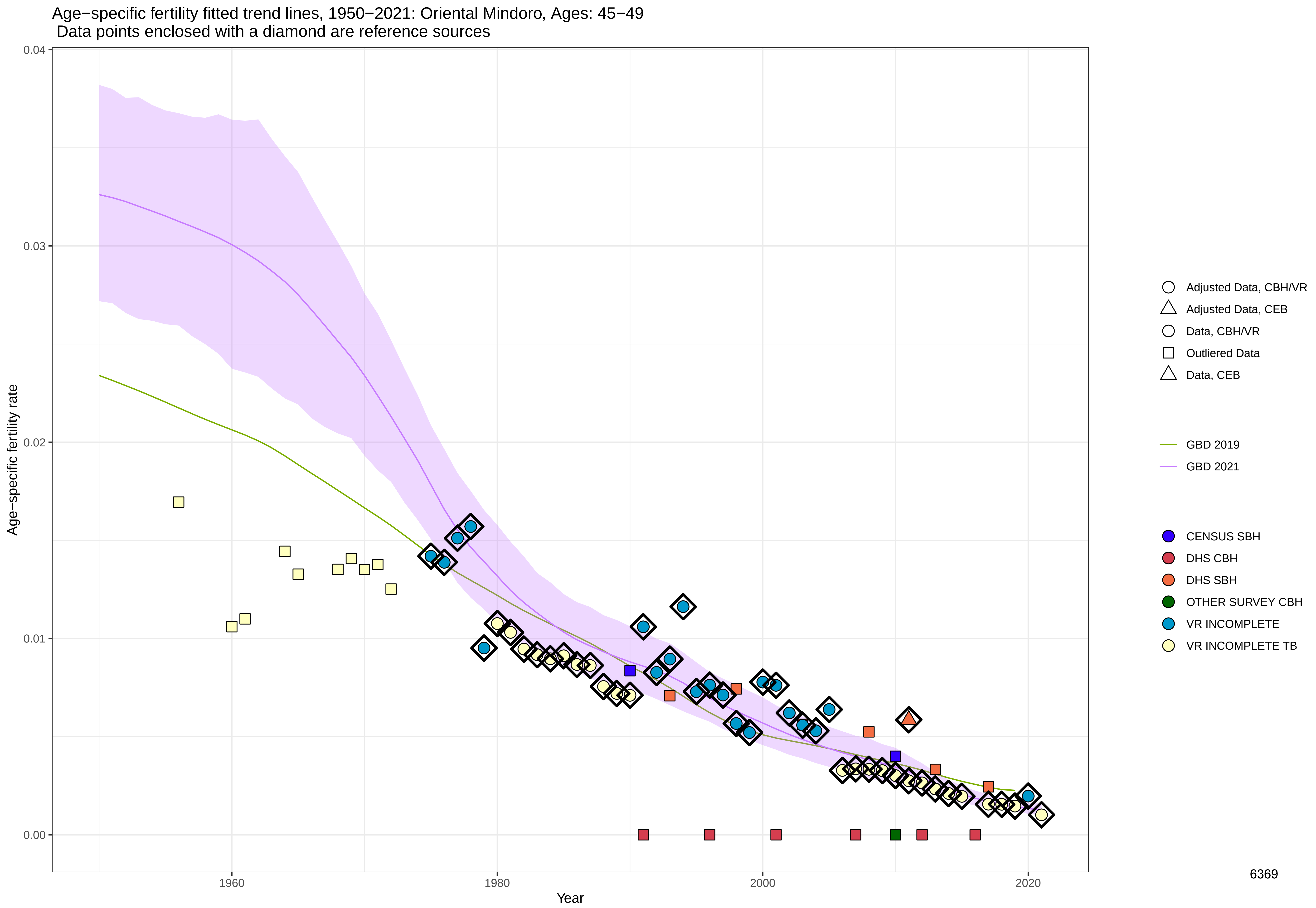
Age−specific fertility fitted trend lines, 1950−2021: Oriental Mindoro, Ages: 45−49
Data points enclosed with a diamond are reference sources
Age−specific fertility rate
Year
0.00
0.01
0.02
0.03
0.04
1960
1980
2000
2020
Adjusted Data, CBH/VR
Adjusted Data, CEB
Data, CBH/VR
Outliered Data
Data, CEB
GBD 2019
GBD 2021
CENSUS SBH
DHS CBH
DHS SBH
OTHER SURVEY CBH
VR INCOMPLETE
VR INCOMPLETE TB

Age−specific fertility fitted trend lines, 1950−2021: Oriental Mindoro, Ages: 50−54
Data points enclosed with a diamond are reference sources

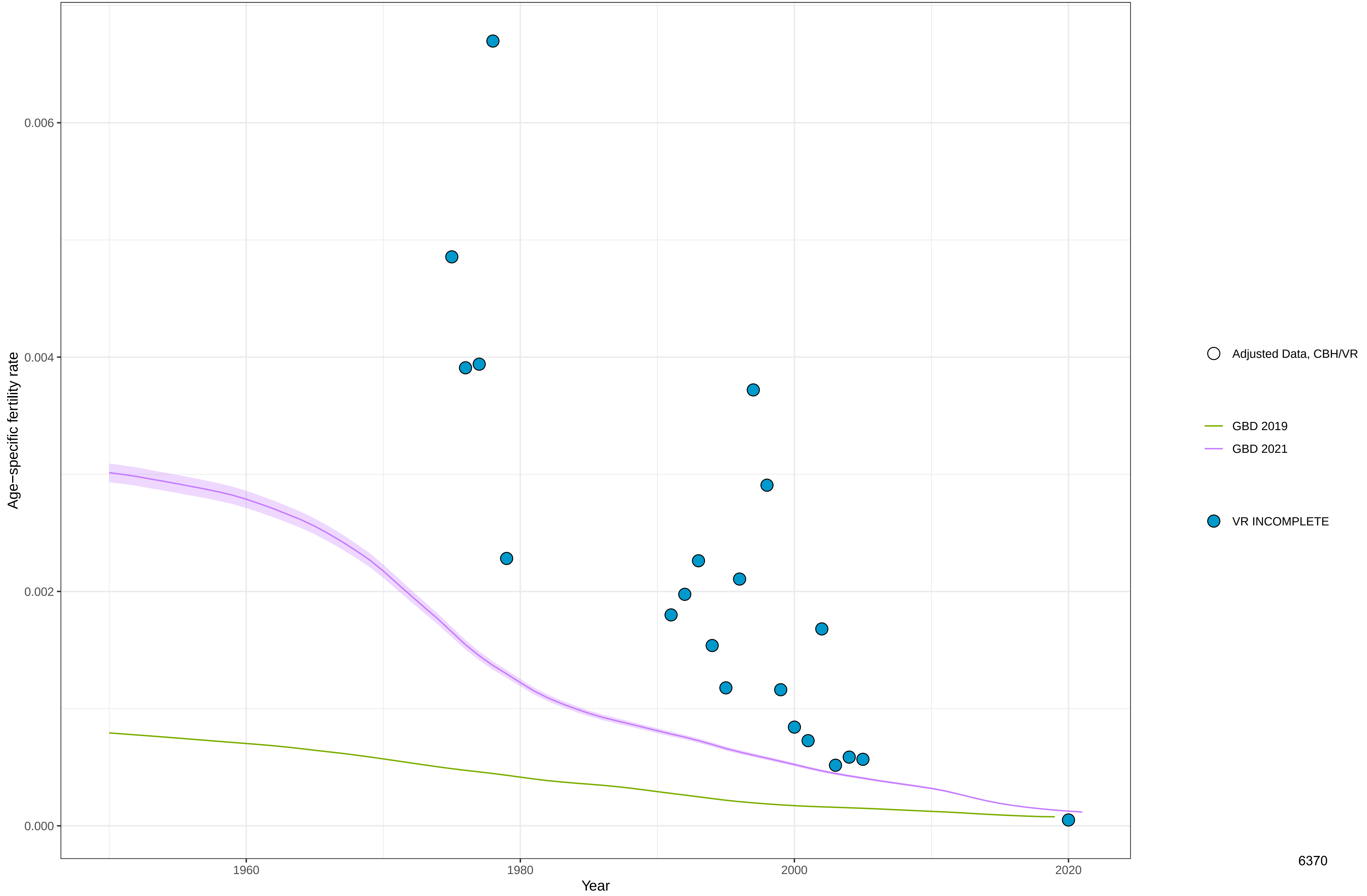

Total fertility rate fitted trend lines, 1950−2021: Oriental Mindoro TFR
Data points enclosed with a diamond are reference sources

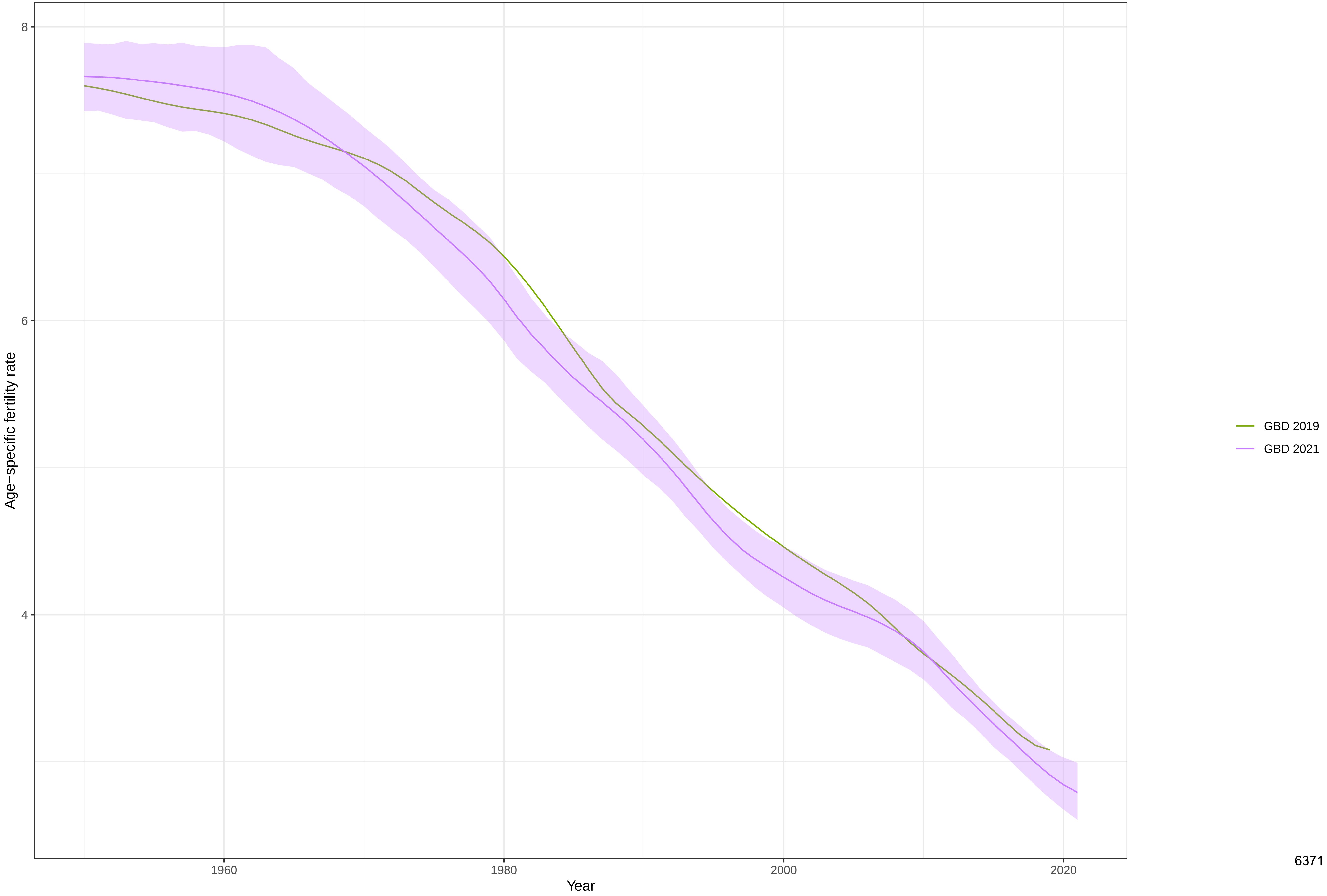

Age−specific fertility fitted trend lines, 1950−2021: Palawan, Ages: 10−14
Data points enclosed with a diamond are reference sources

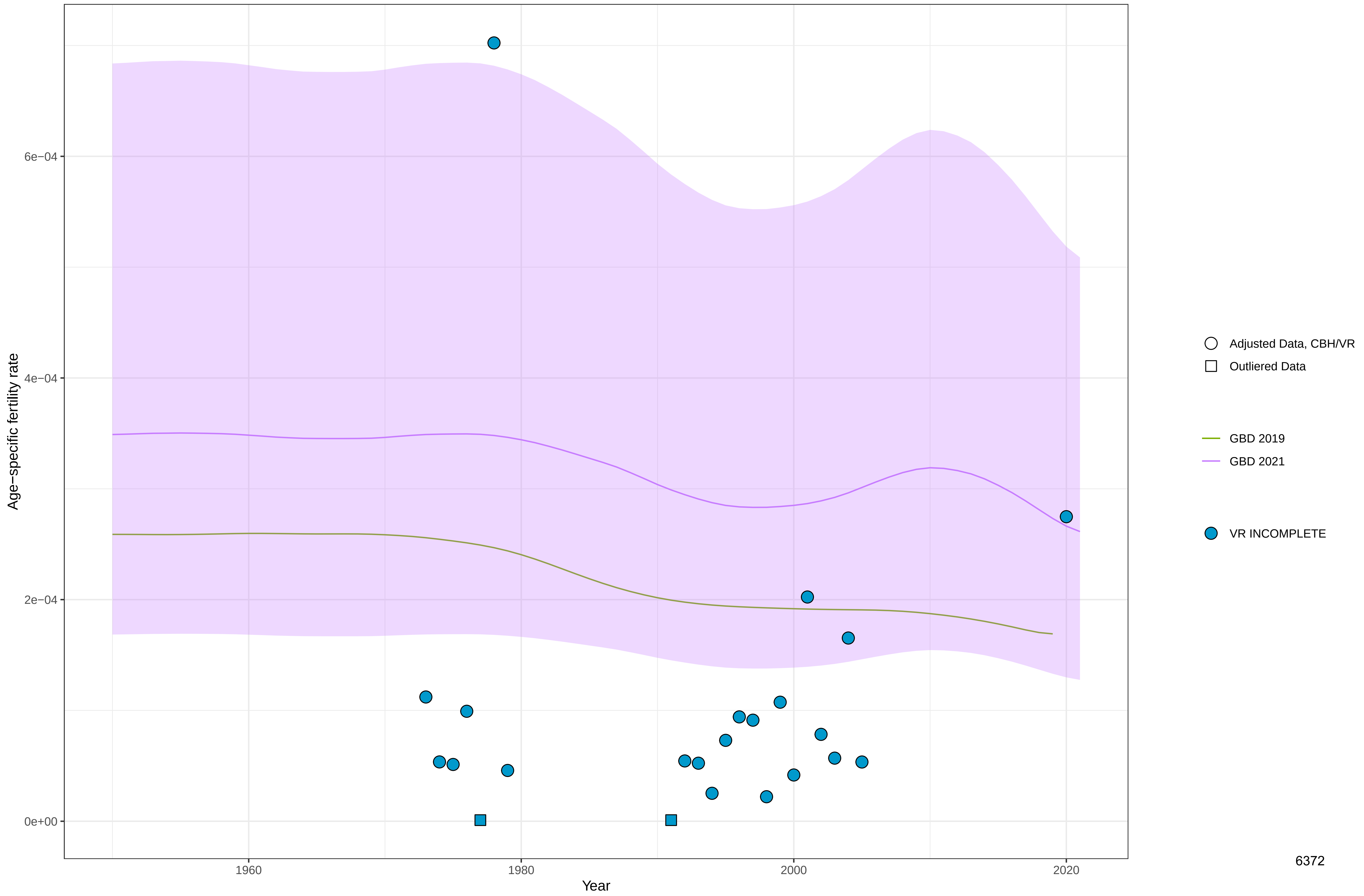

Age−specific fertility fitted trend lines, 1950−2021: Palawan, Ages: 15−19
Data points enclosed with a diamond are reference sources

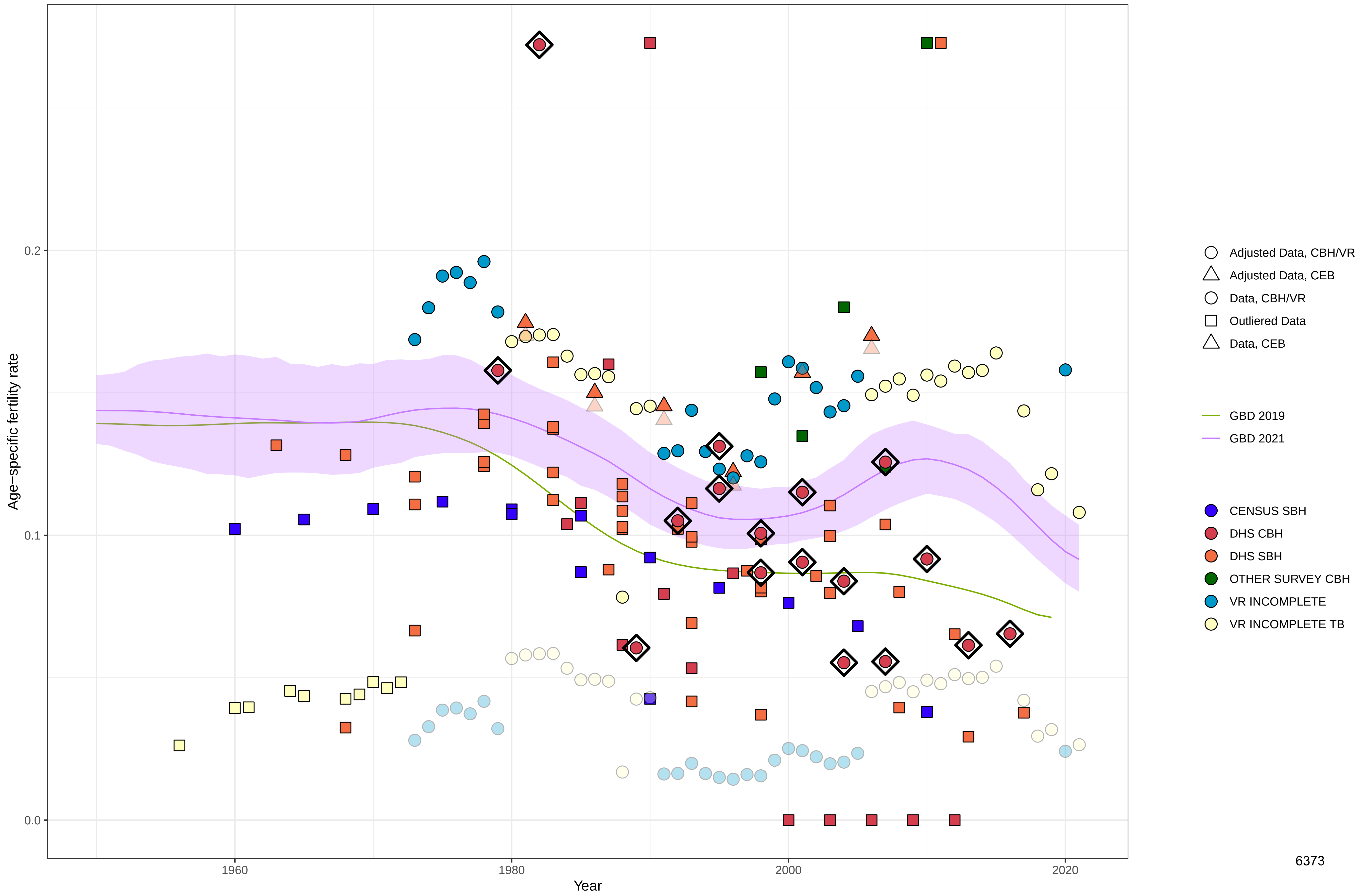

Age−specific fertility fitted trend lines, 1950−2021: Palawan, Ages: 20−24
Data points enclosed with a diamond are reference sources

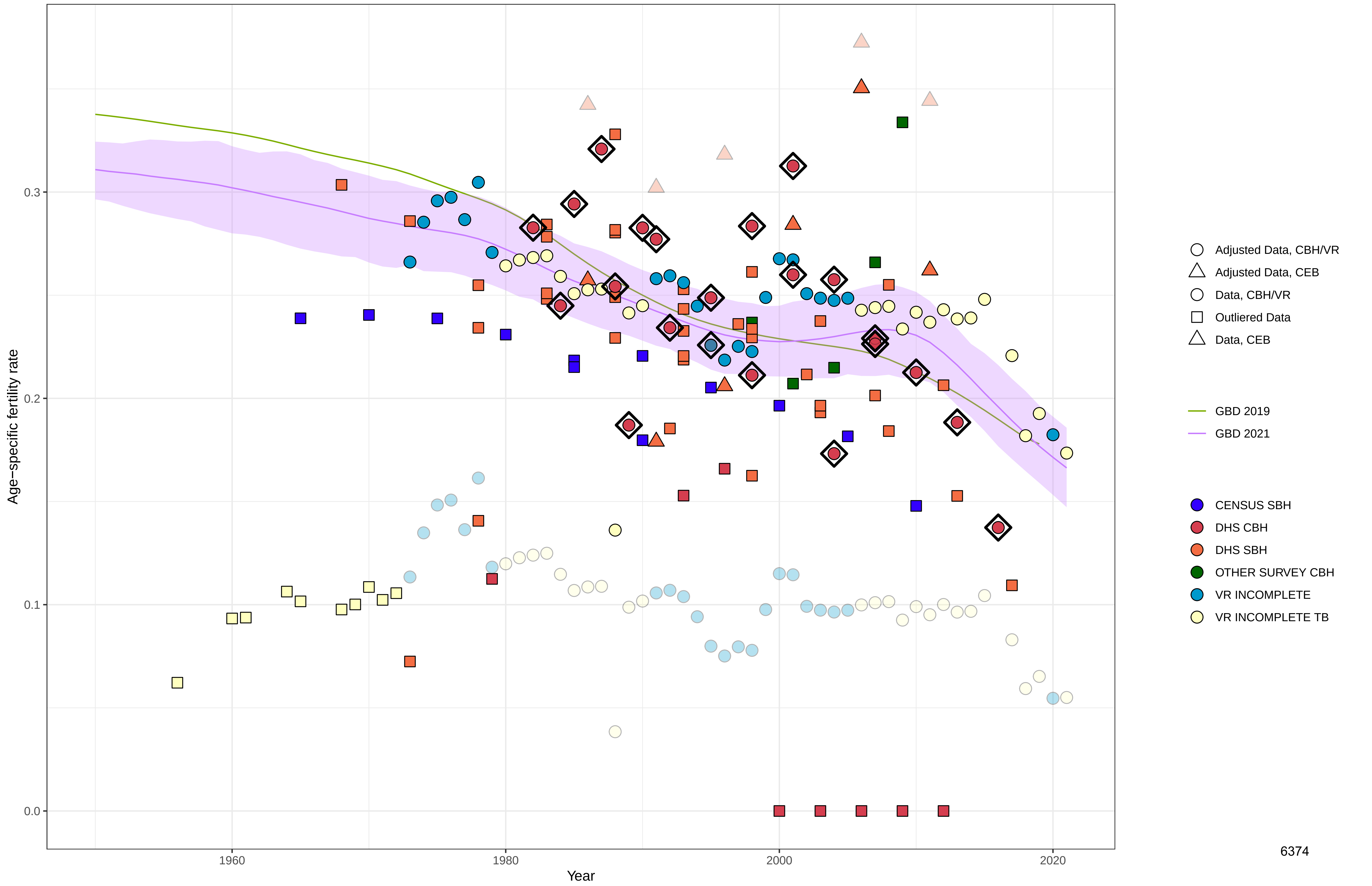

Age−specific fertility fitted trend lines, 1950−2021: Palawan, Ages: 25−29
Data points enclosed with a diamond are reference sources

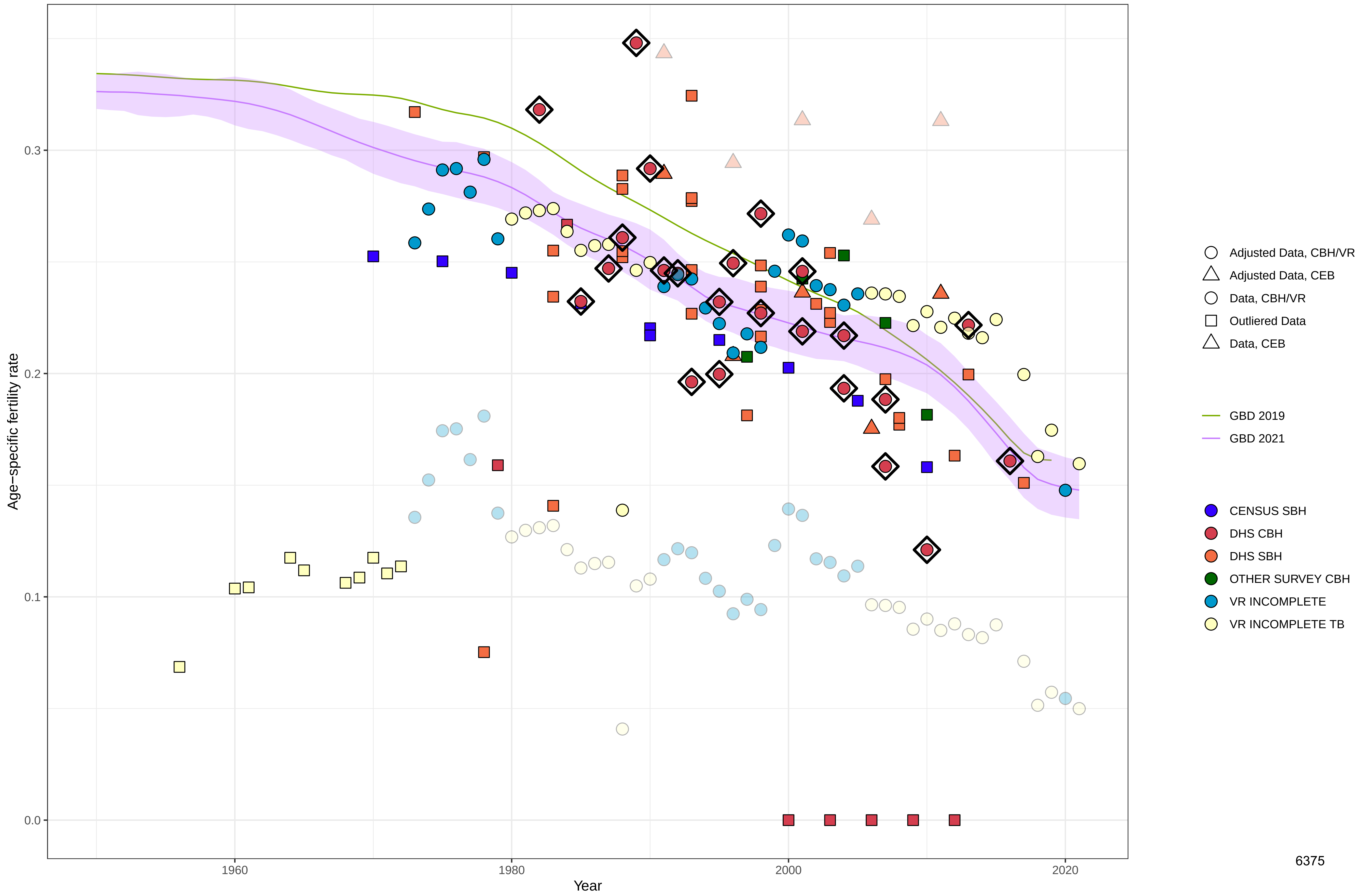

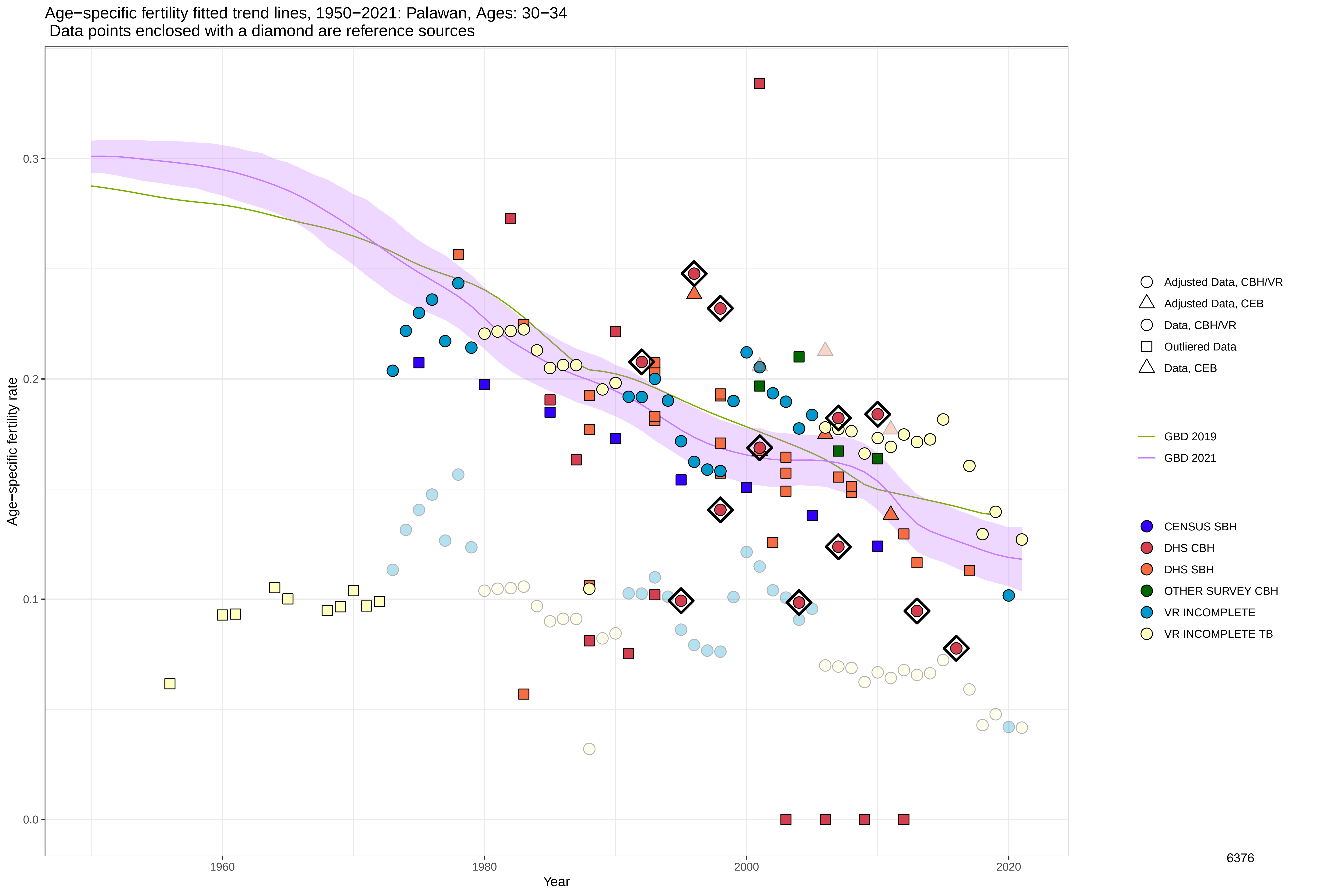

Age−specific fertility fitted trend lines, 1950−2021: Palawan, Ages: 30−34
Data points enclosed with a diamond are reference sources
Age−specific fertility rate
0.0
0.1
0.2
0.3
1960
1980
2000
2020
Year
Adjusted Data, CBH/VR
Adjusted Data, CEB
Data, CBH/VR
Outliered Data
Data, CEB
GBD 2019
GBD 2021
CENSUS SBH
DHS CBH
DHS SBH
OTHER SURVEY CBH
VR INCOMPLETE
VR INCOMPLETE TB

Age−specific fertility fitted trend lines, 1950−2021: Palawan, Ages: 35−39
Data points enclosed with a diamond are reference sources

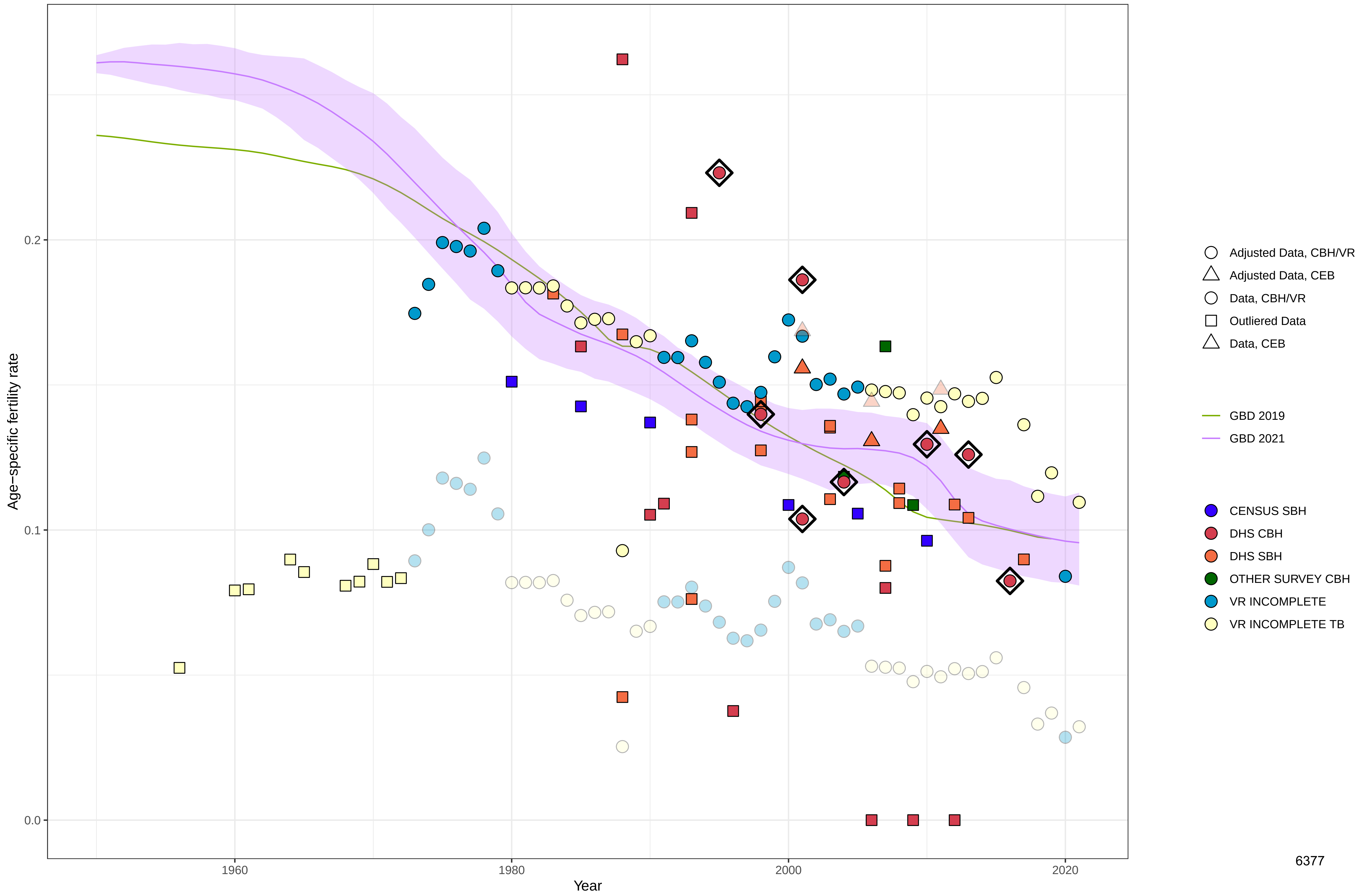

6377

Age−specific fertility fitted trend lines, 1950−2021: Palawan, Ages: 40−44
Data points enclosed with a diamond are reference sources

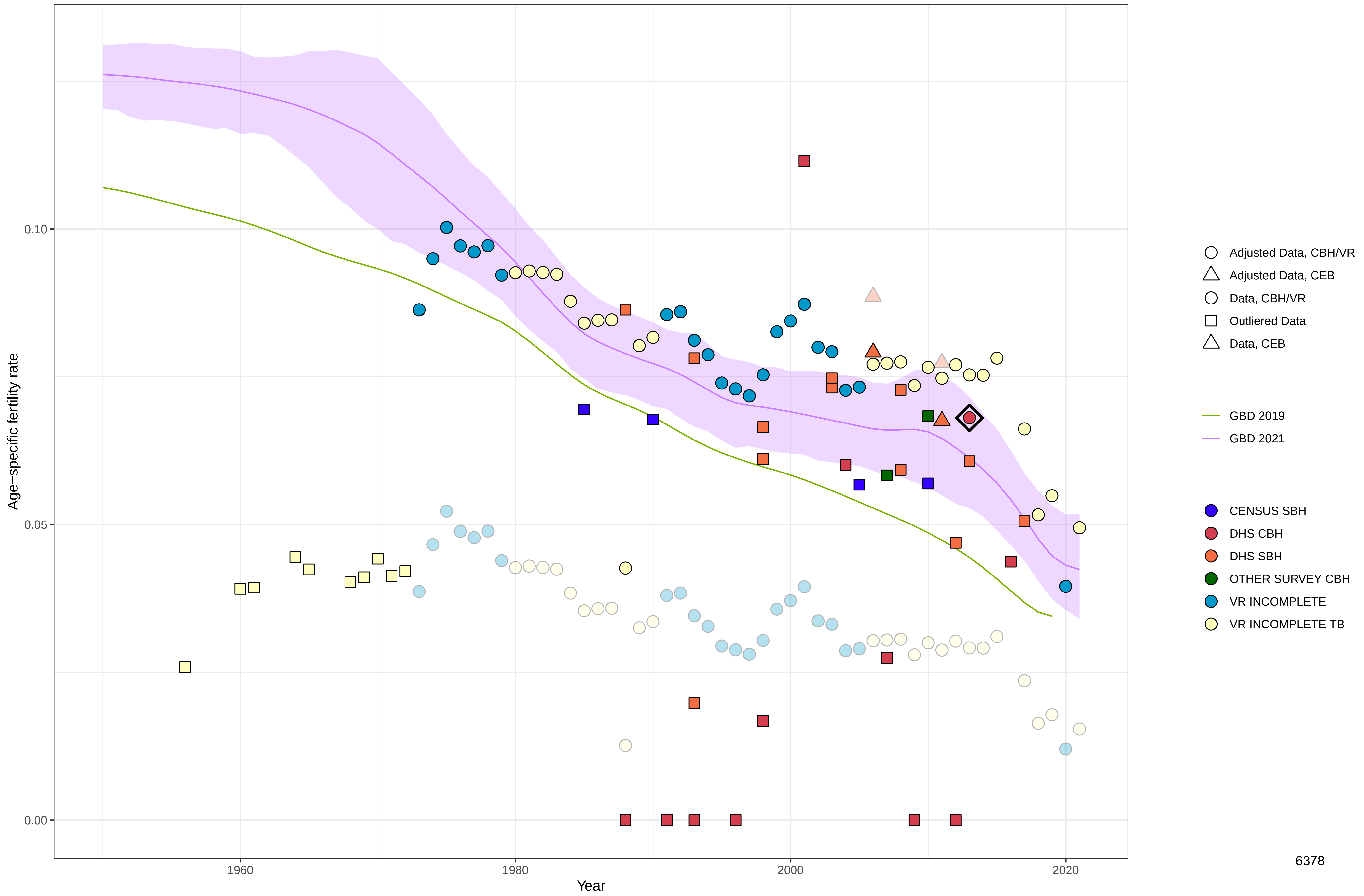

Age−specific fertility fitted trend lines, 1950−2021: Palawan, Ages: 45−49
Data points enclosed with a diamond are reference sources

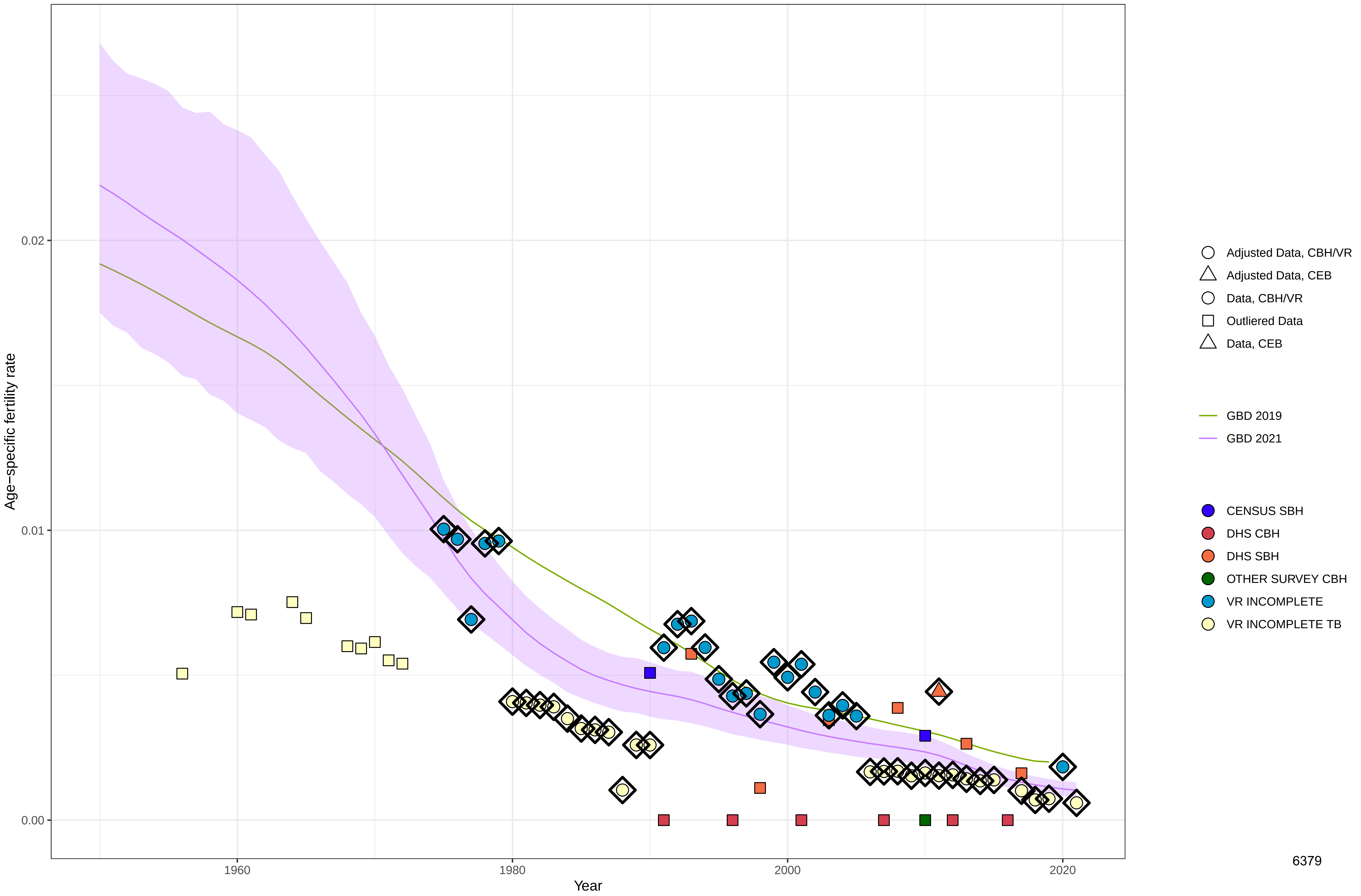

Age−specific fertility fitted trend lines, 1950−2021: Palawan, Ages: 50−54
Data points enclosed with a diamond are reference sources

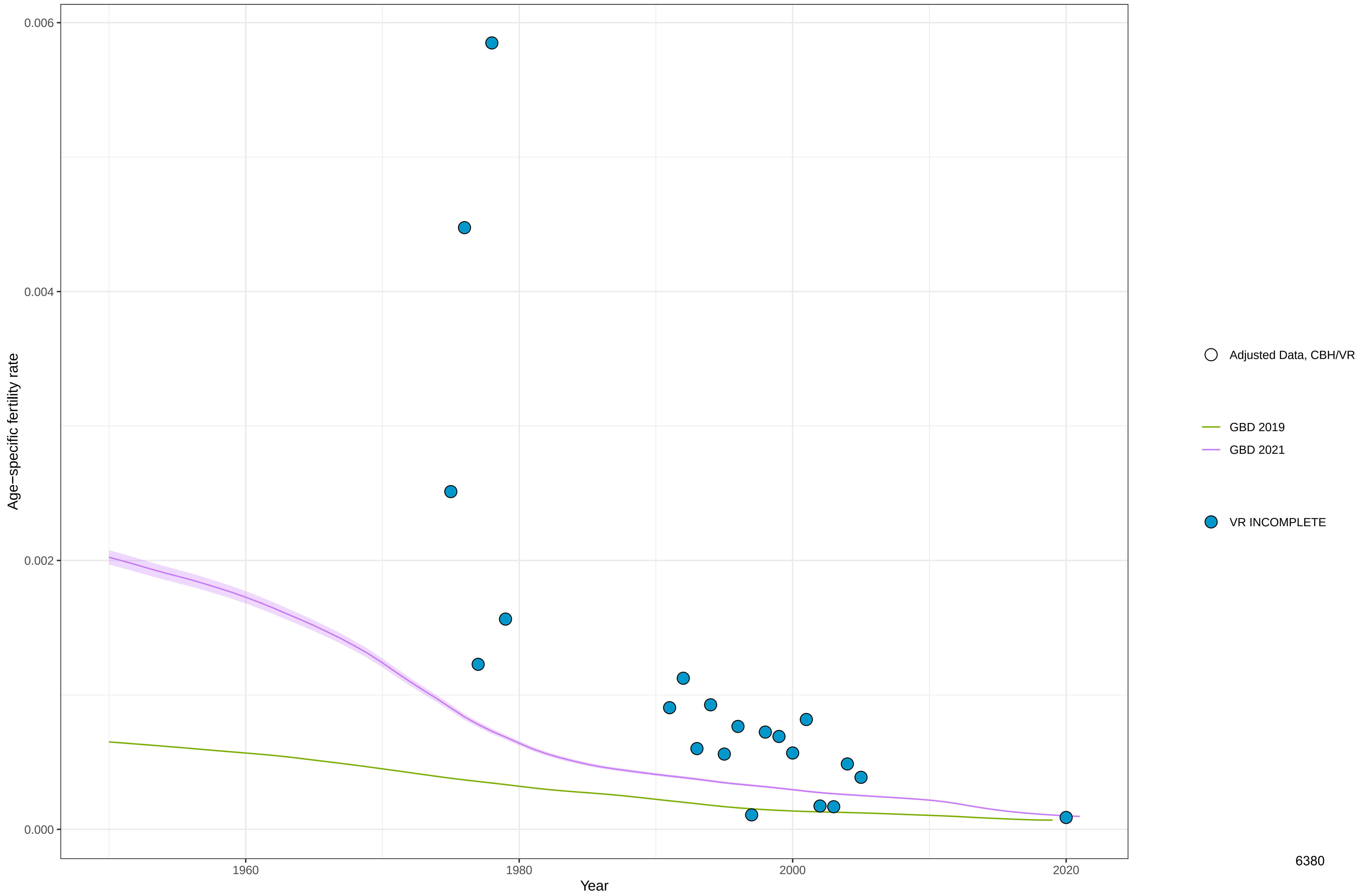

Total fertility rate fitted trend lines, 1950−2021: Palawan TFR
Data points enclosed with a diamond are reference sources

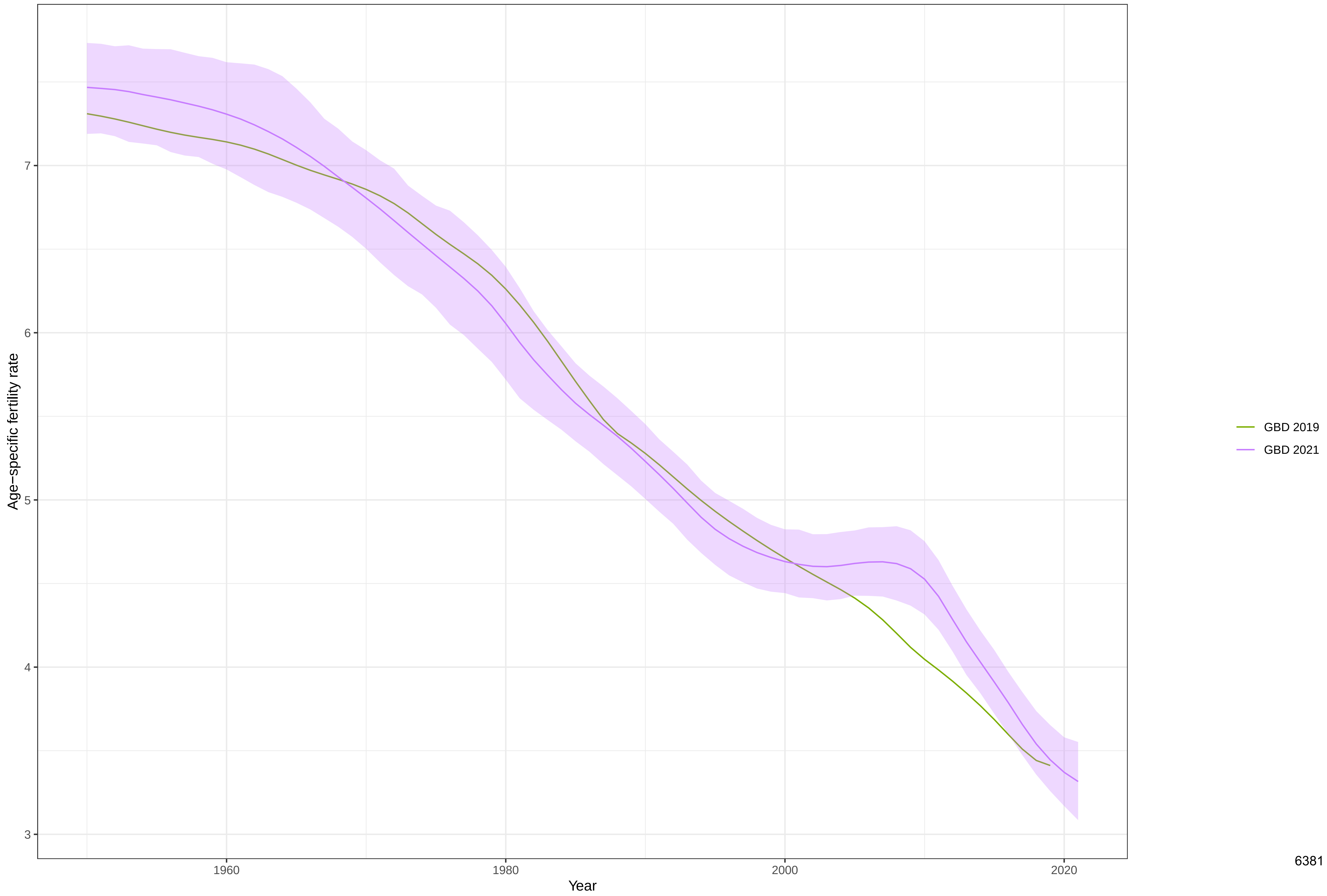

Age−specific fertility fitted trend lines, 1950−2021: Pampanga, Ages: 10−14
Data points enclosed with a diamond are reference sources

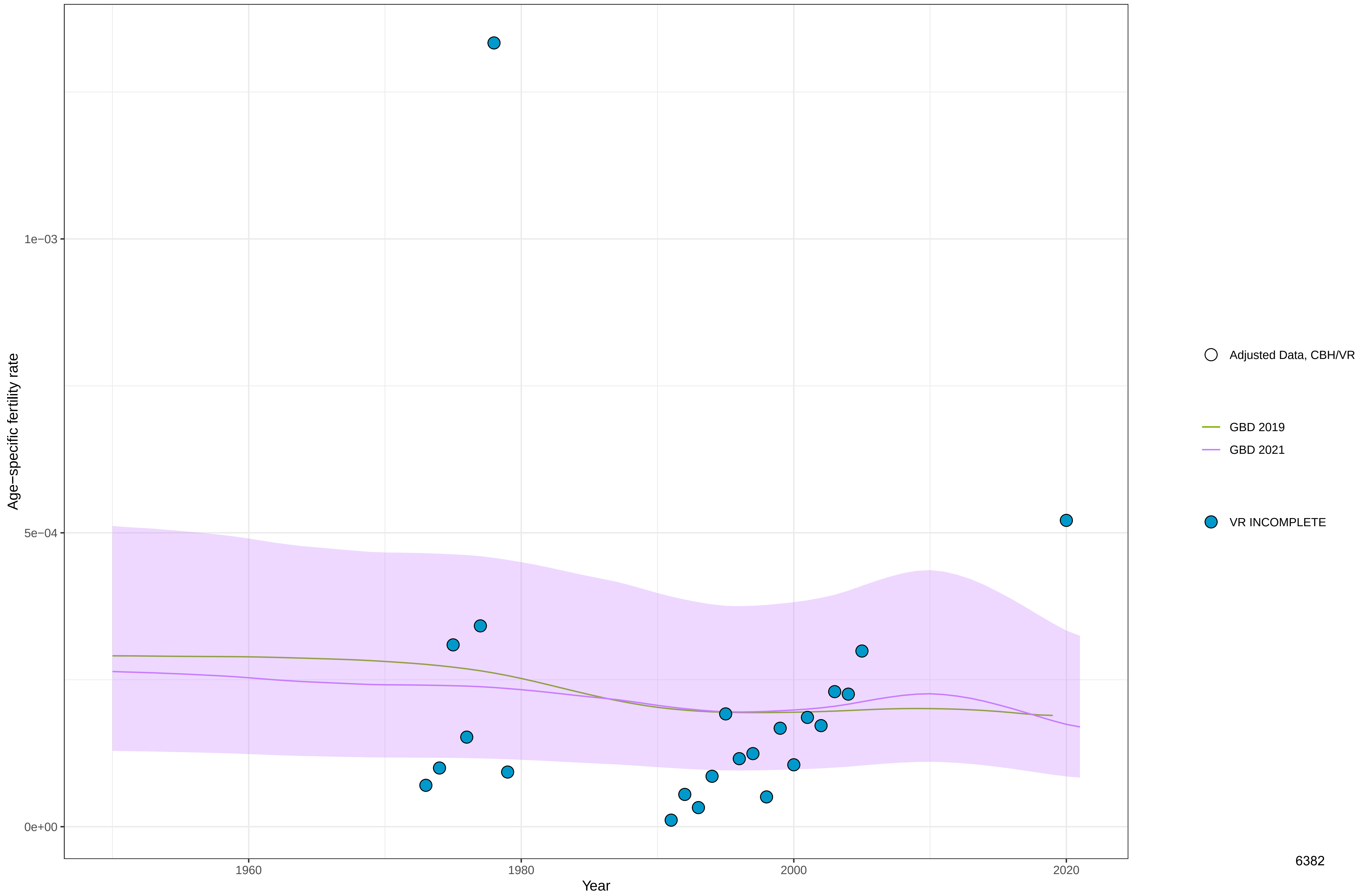

6382

Age−specific fertility fitted trend lines, 1950−2021: Pampanga, Ages: 15−19
Data points enclosed with a diamond are reference sources

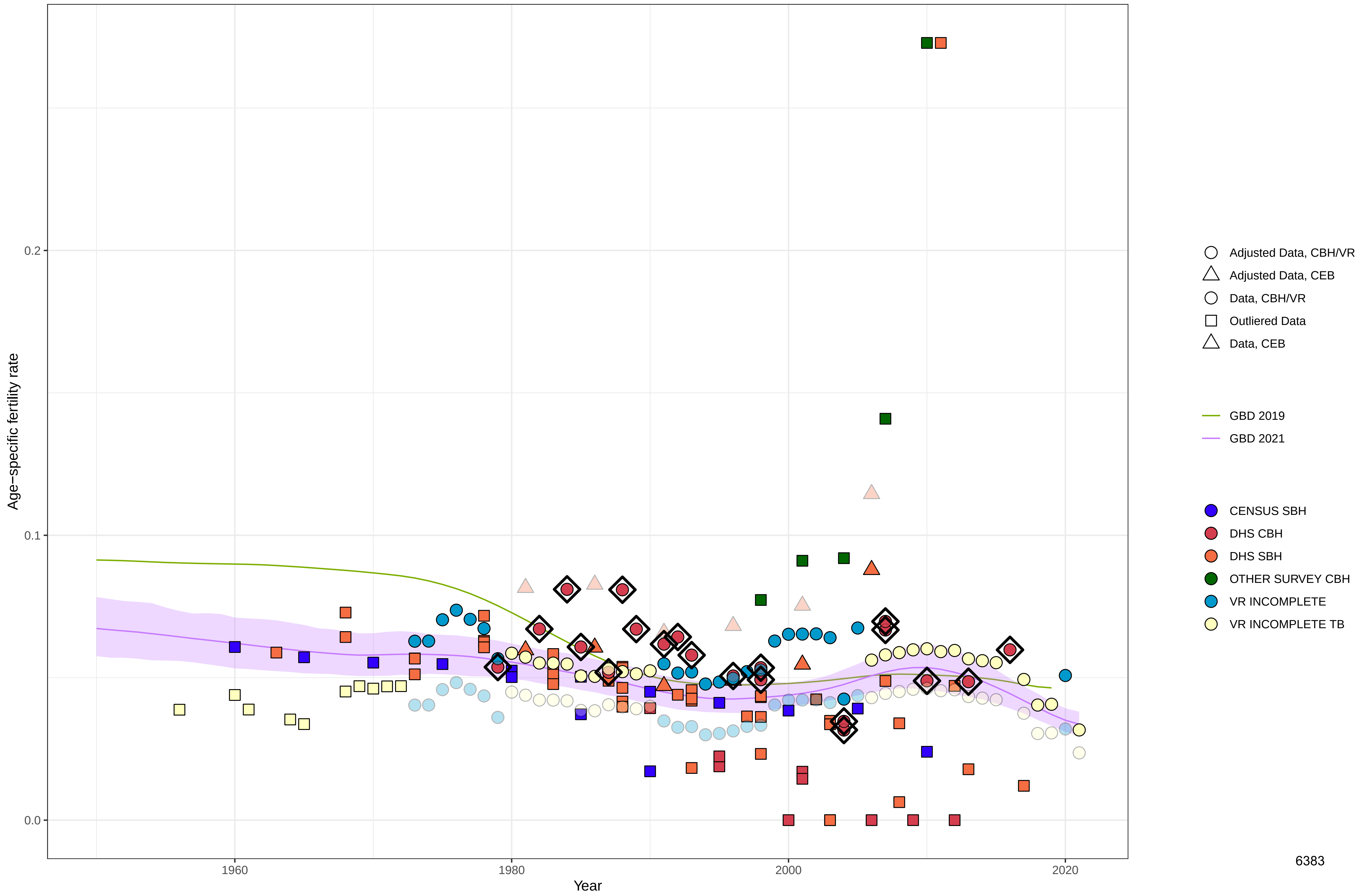

Age−specific fertility fitted trend lines, 1950−2021: Pampanga, Ages: 20−24
Data points enclosed with a diamond are reference sources

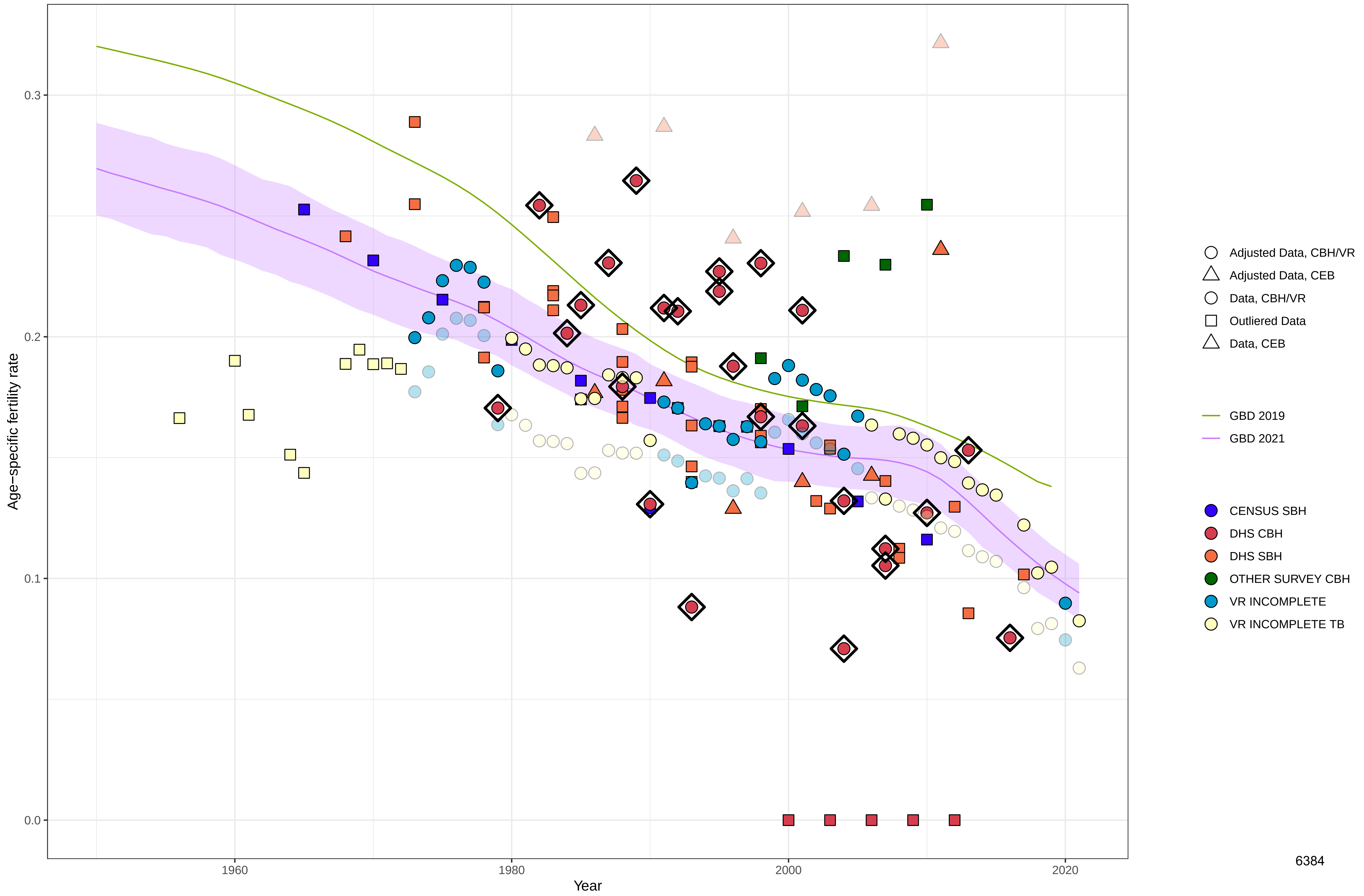

Age−specific fertility fitted trend lines, 1950−2021: Pampanga, Ages: 25−29
Data points enclosed with a diamond are reference sources

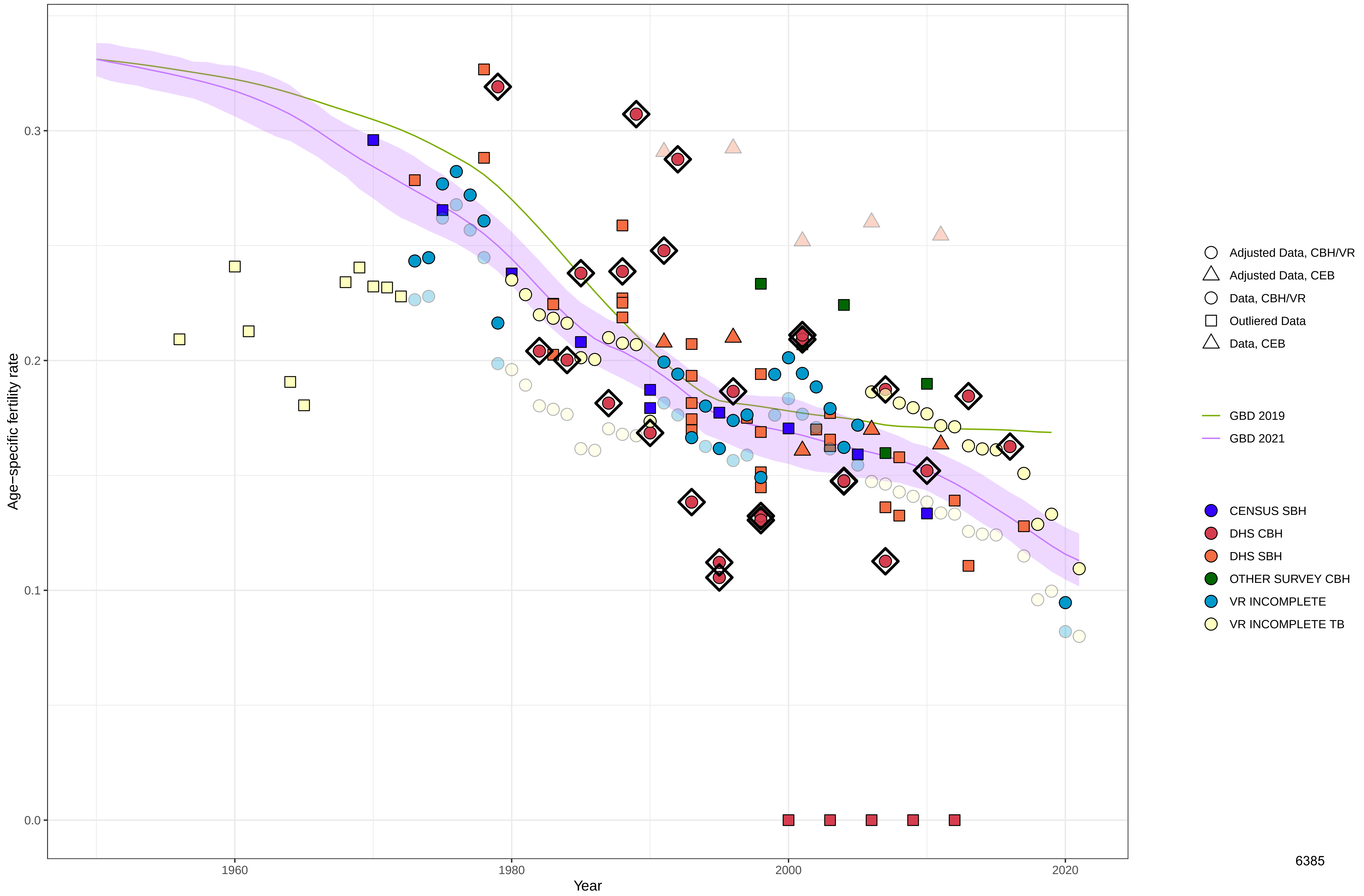

Age−specific fertility fitted trend lines, 1950−2021: Pampanga, Ages: 30−34
Data points enclosed with a diamond are reference sources

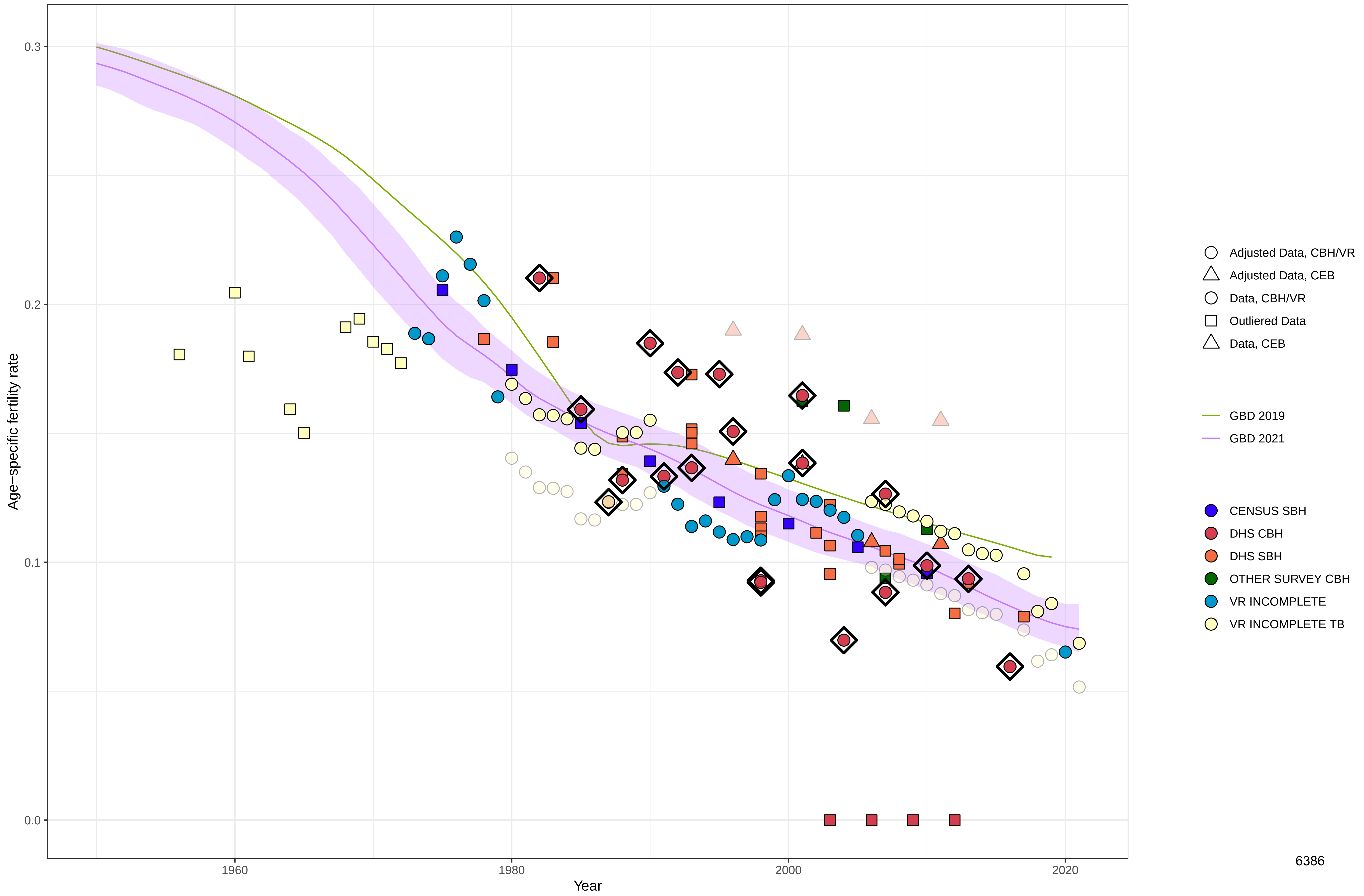

Age−specific fertility fitted trend lines, 1950−2021: Pampanga, Ages: 35−39
Data points enclosed with a diamond are reference sources

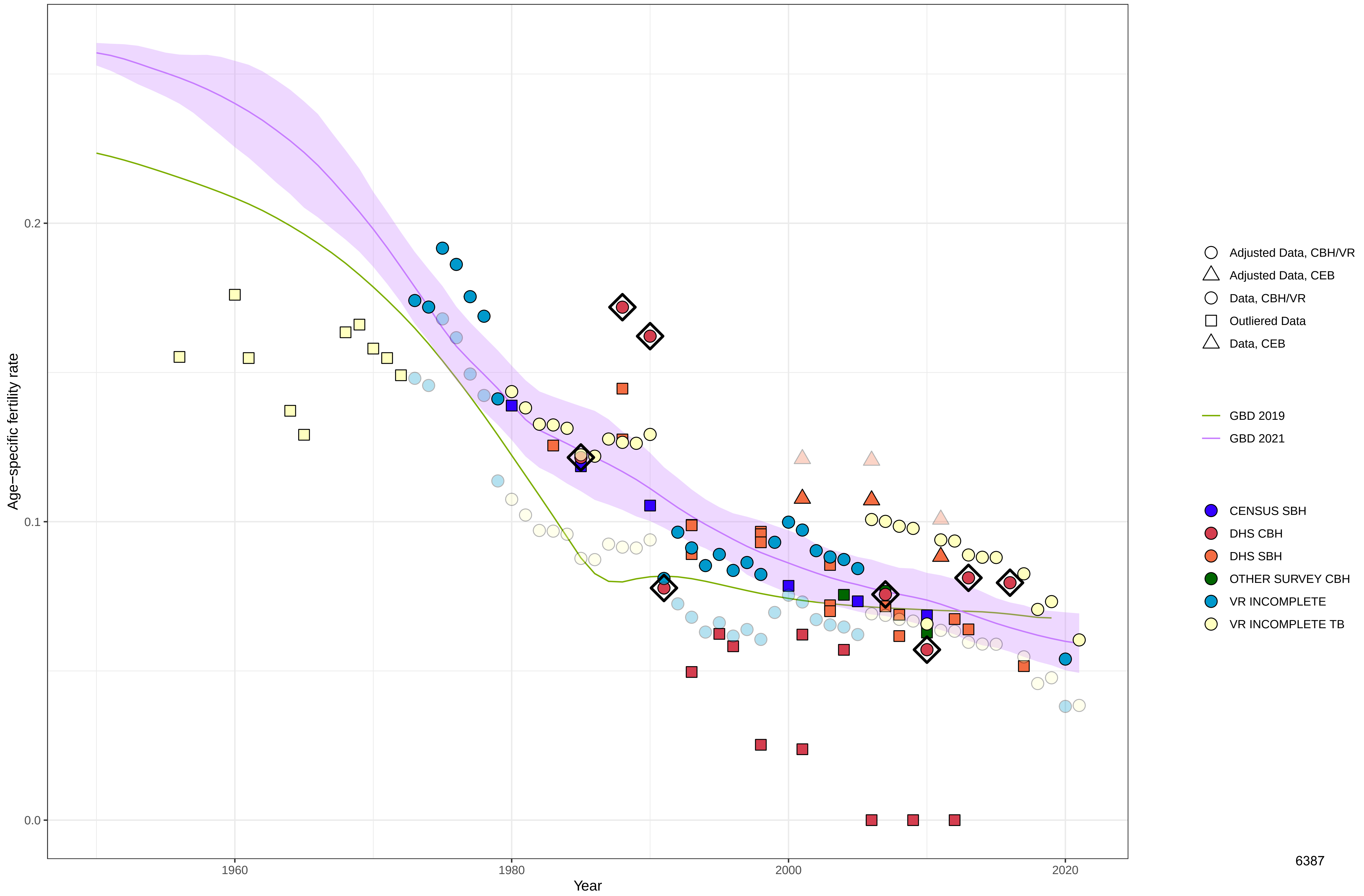

Age−specific fertility fitted trend lines, 1950−2021: Pampanga, Ages: 40−44
Data points enclosed with a diamond are reference sources

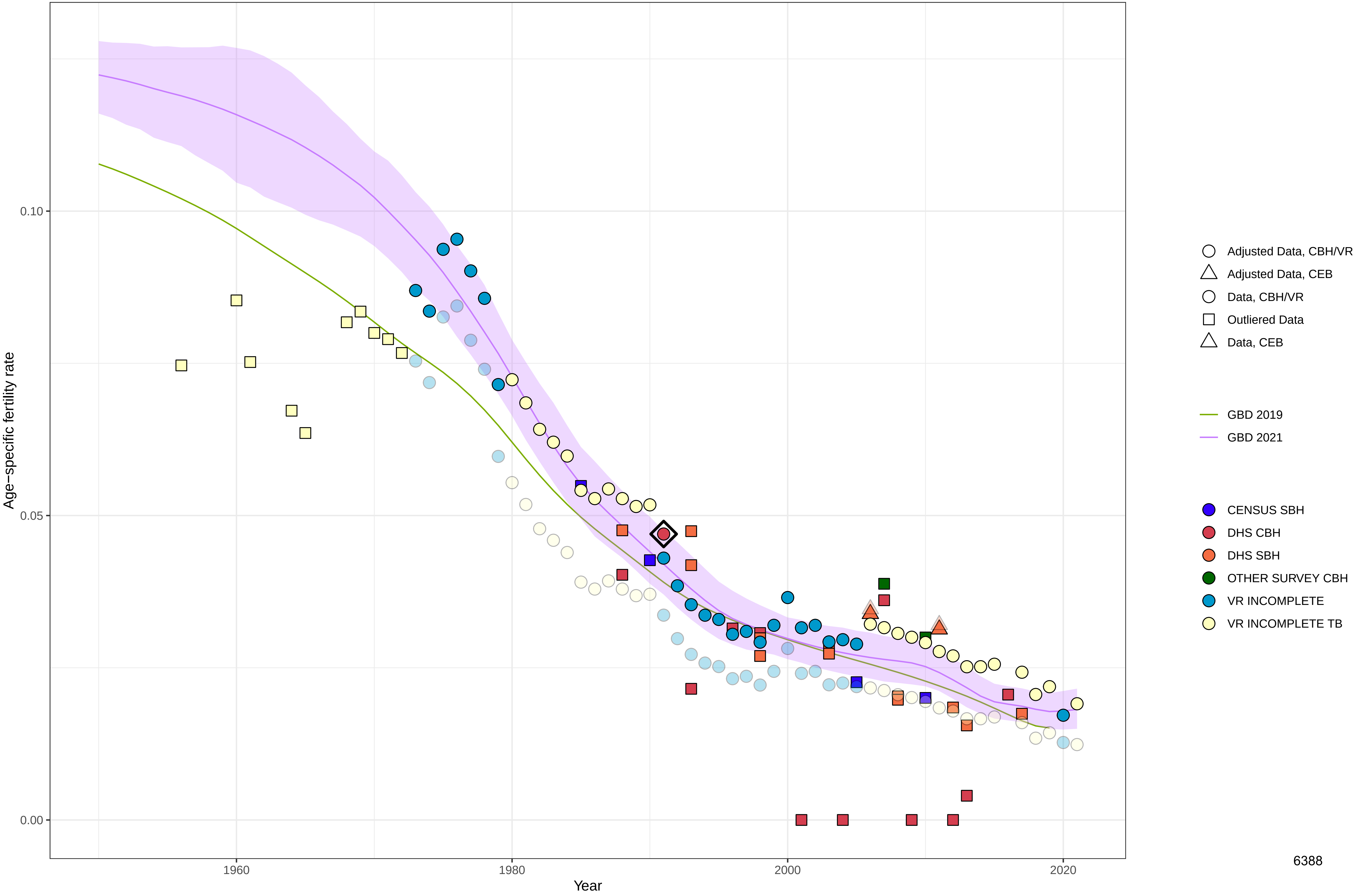

Age−specific fertility fitted trend lines, 1950−2021: Pampanga, Ages: 45−49
Data points enclosed with a diamond are reference sources

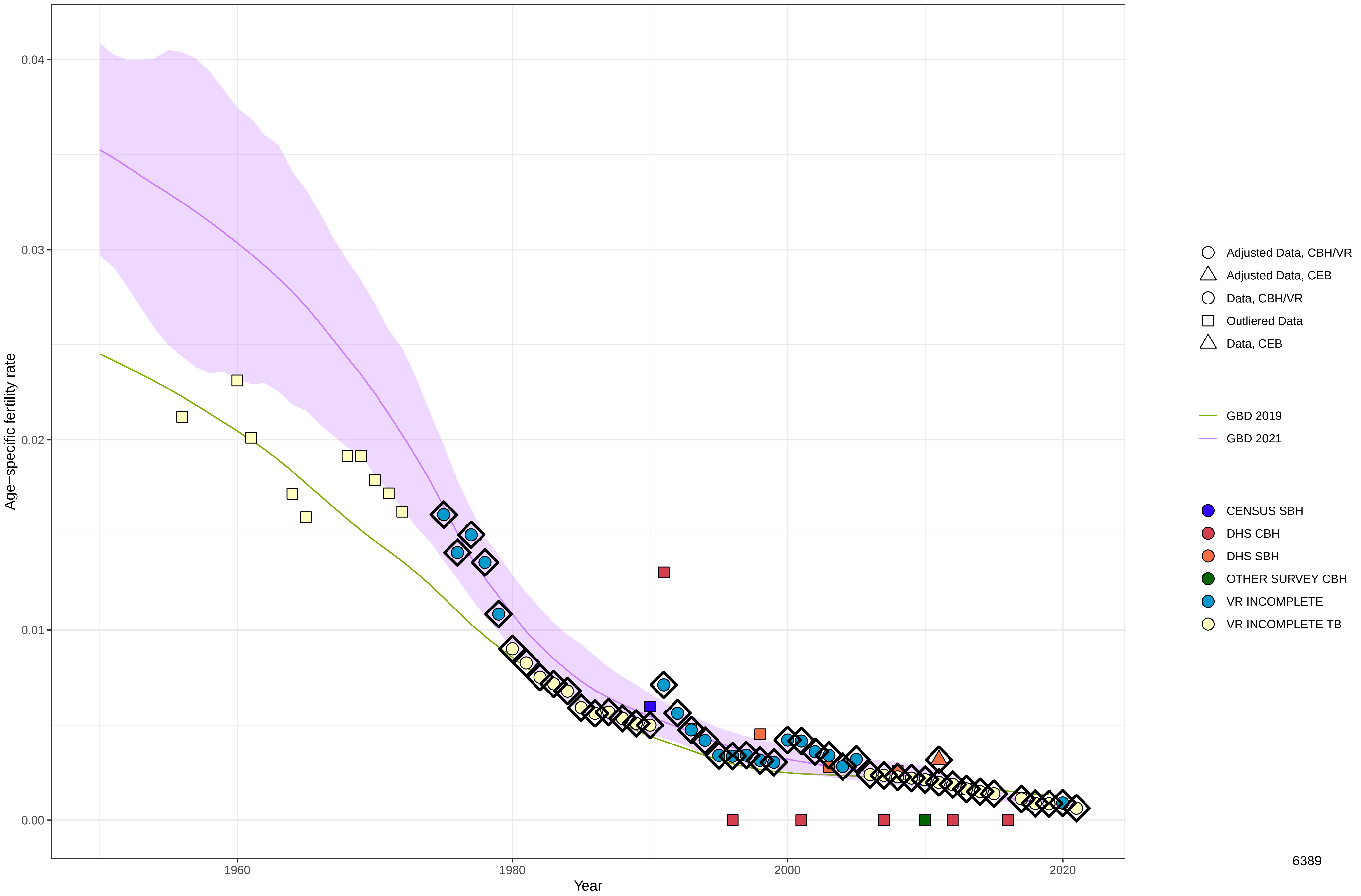

Age−specific fertility fitted trend lines, 1950−2021: Pampanga, Ages: 50−54
Data points enclosed with a diamond are reference sources

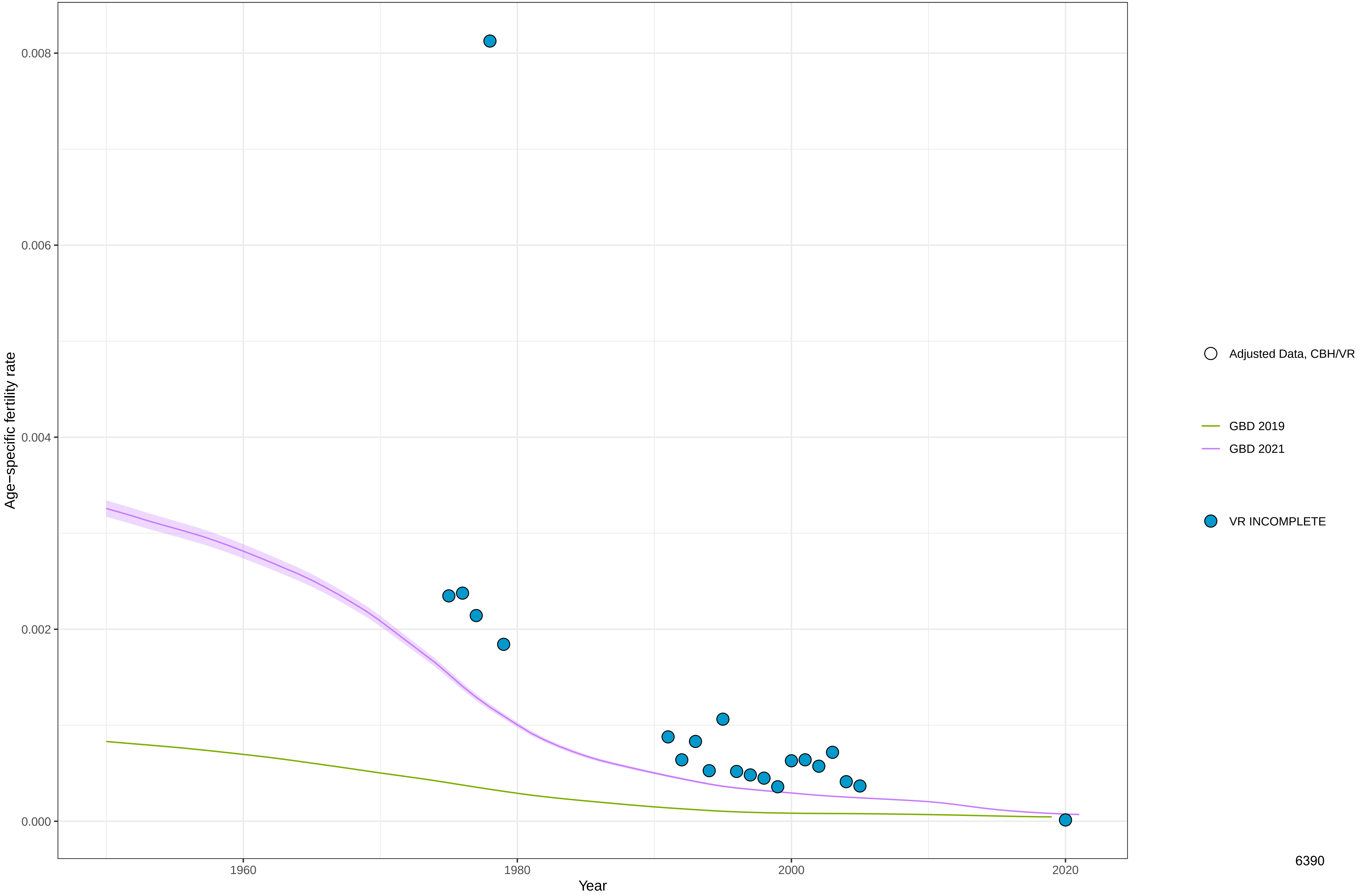

Total fertility rate fitted trend lines, 1950−2021: Pampanga TFR
Data points enclosed with a diamond are reference sources

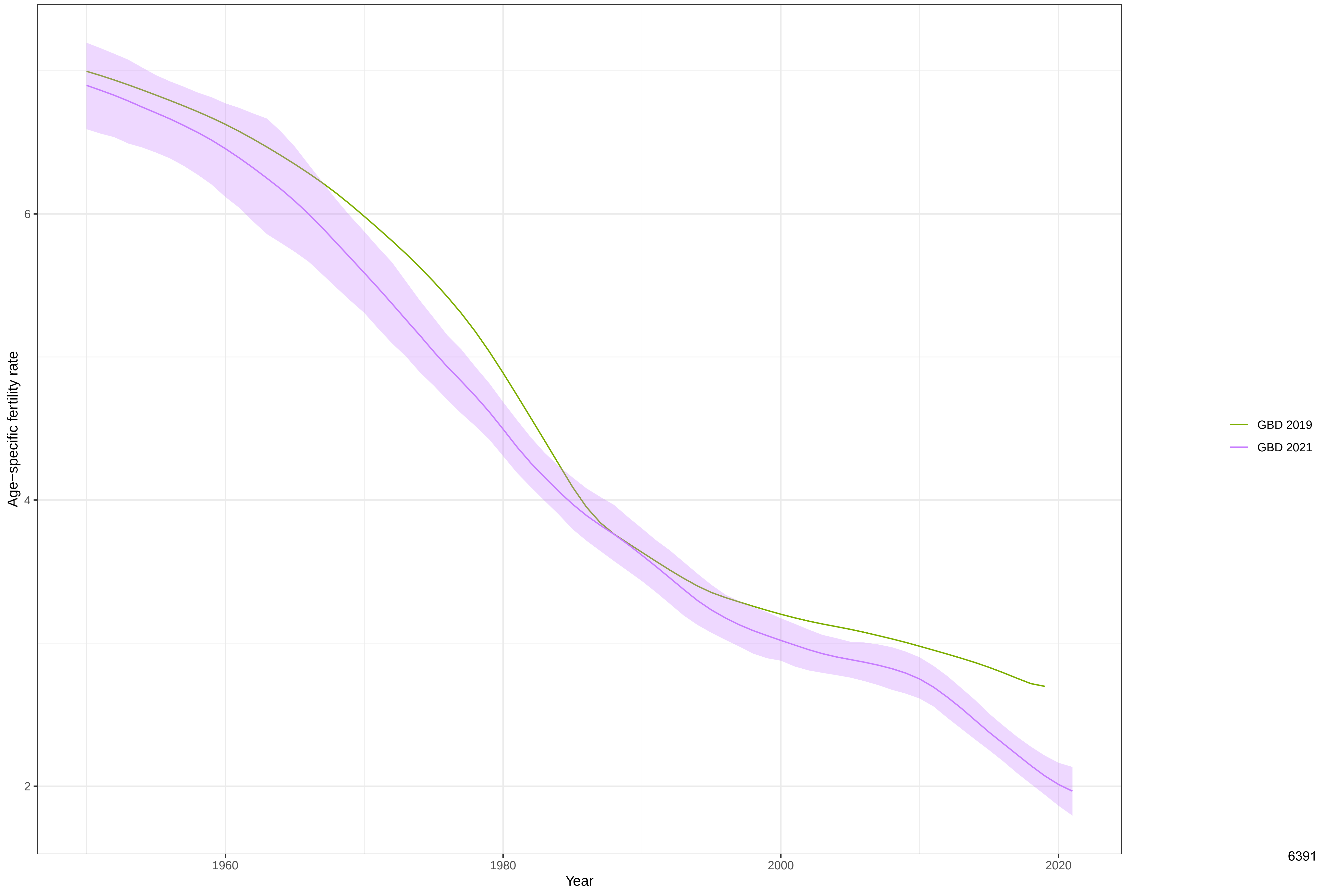

Age−specific fertility fitted trend lines, 1950−2021: Pangasinan, Ages: 10−14
Data points enclosed with a diamond are reference sources

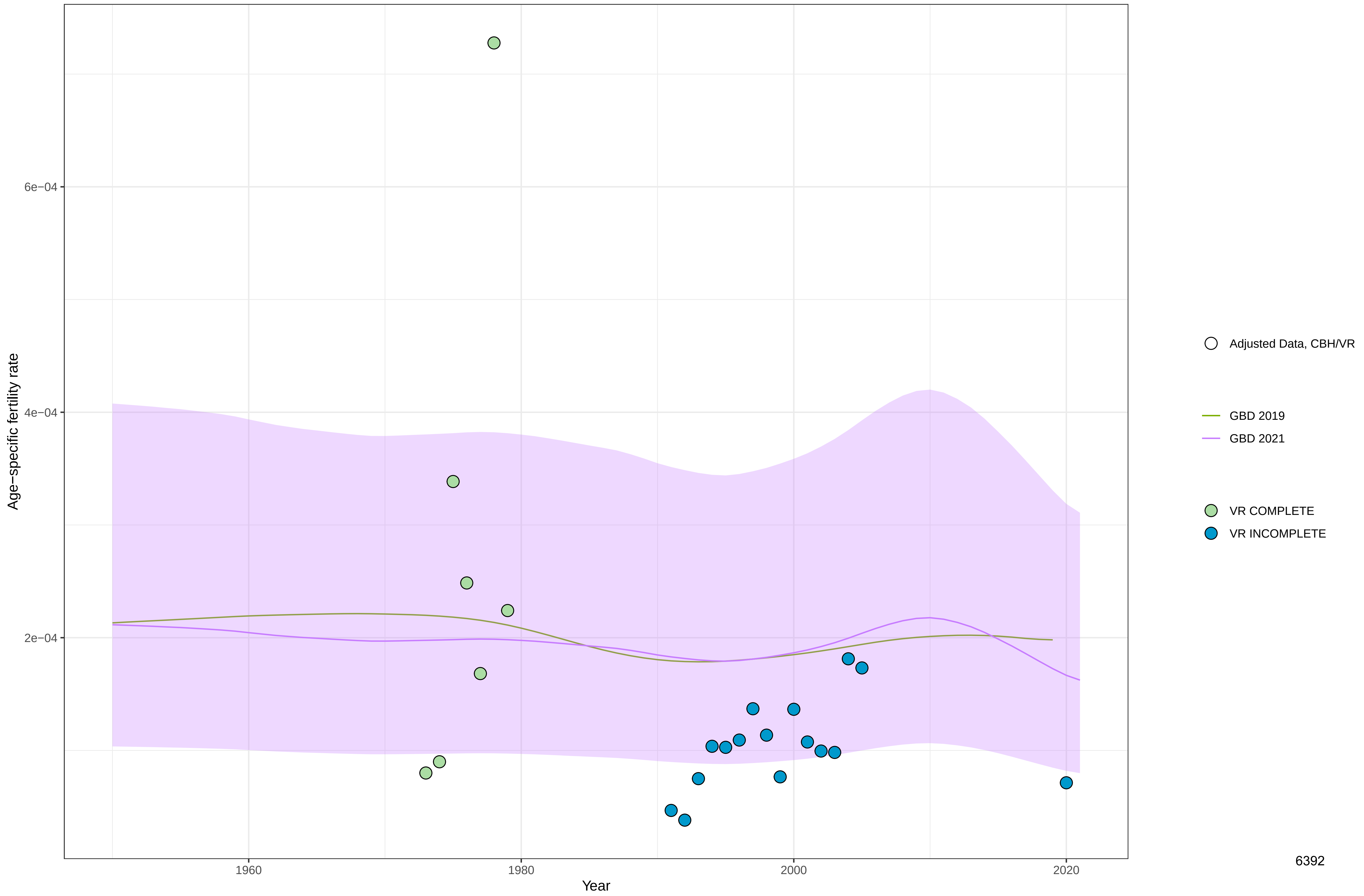

6392

Age−specific fertility fitted trend lines, 1950−2021: Pangasinan, Ages: 15−19
Data points enclosed with a diamond are reference sources

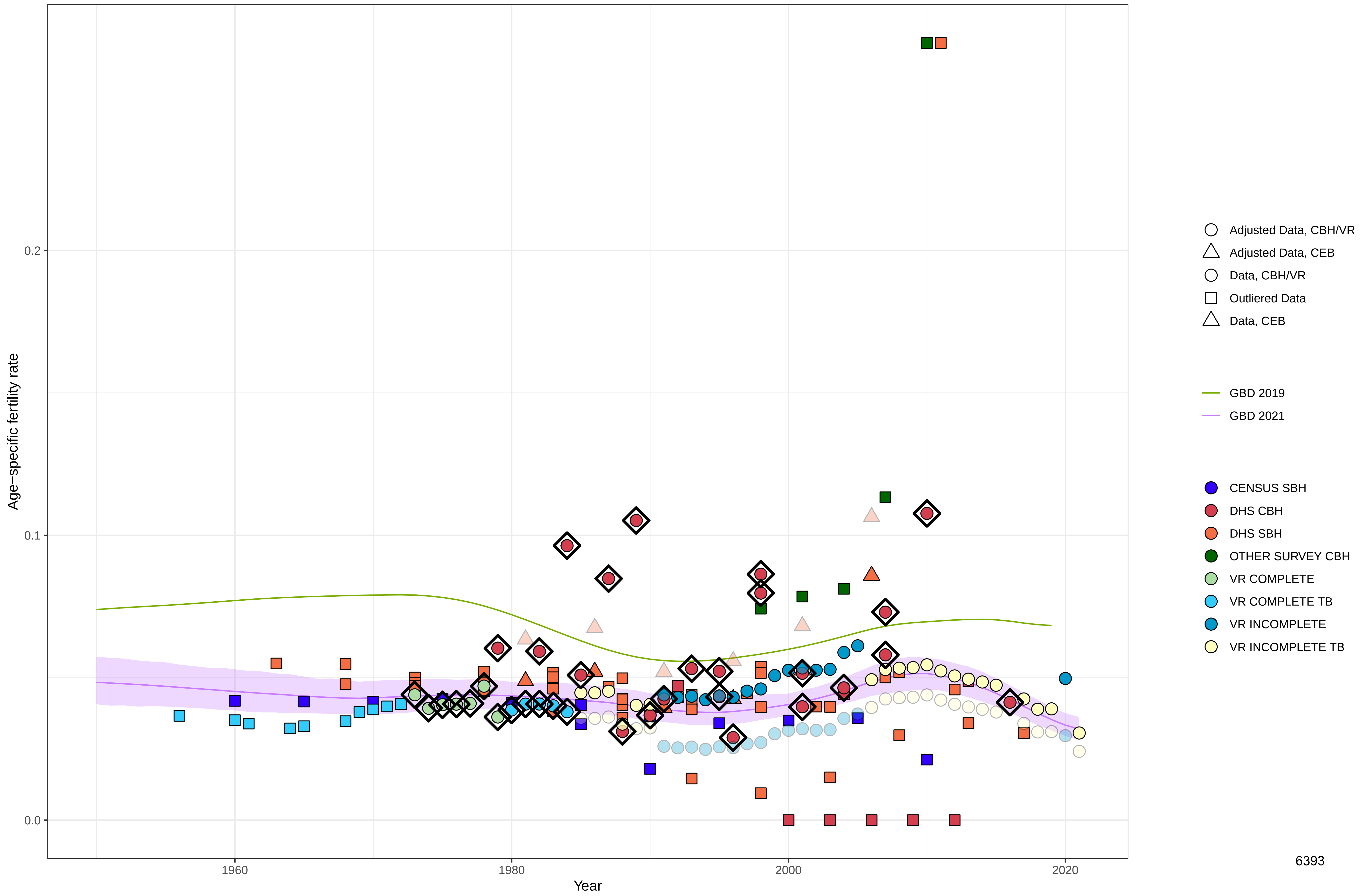

Age−specific fertility fitted trend lines, 1950−2021: Pangasinan, Ages: 20−24
Data points enclosed with a diamond are reference sources

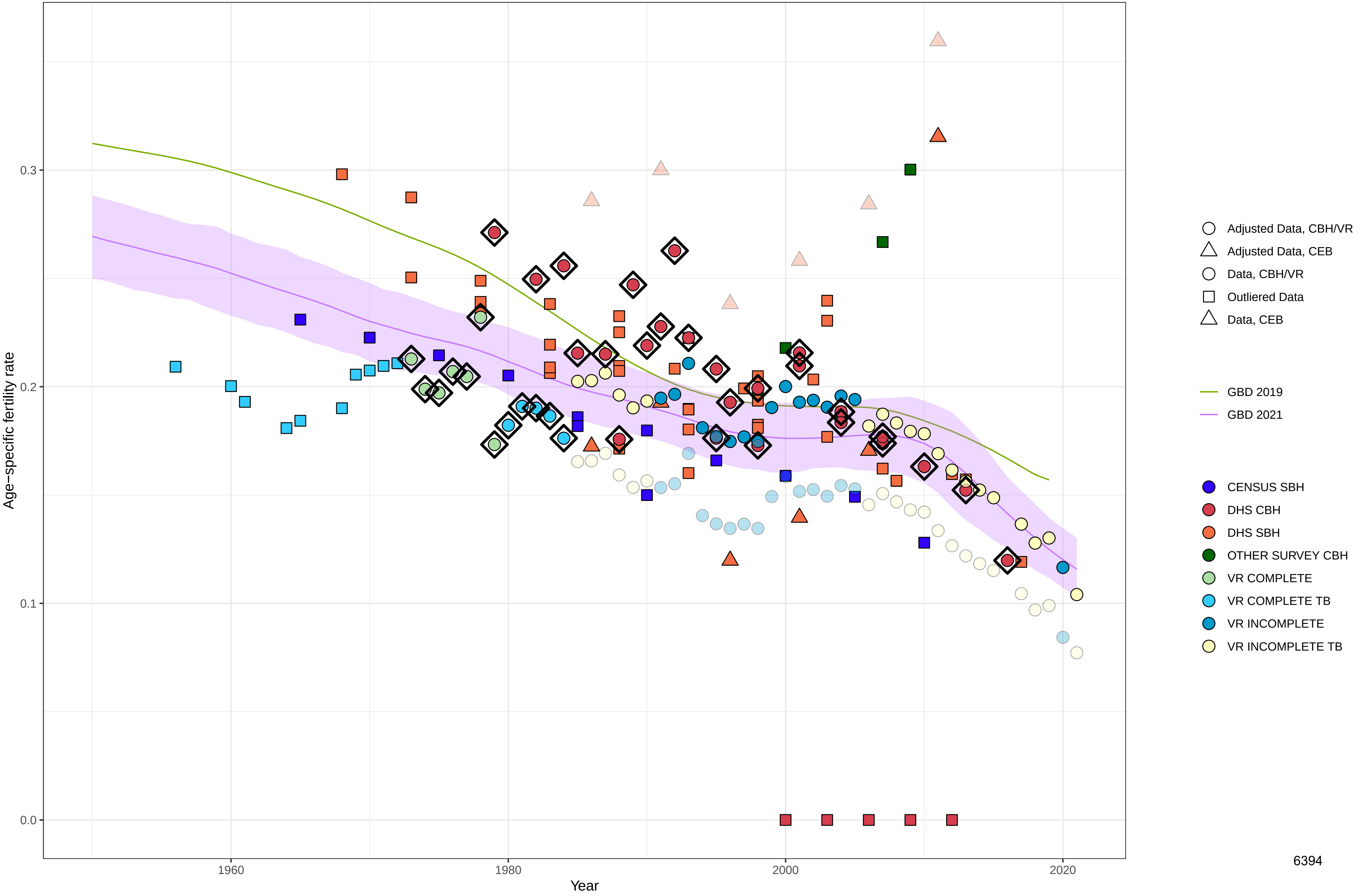

Age−specific fertility fitted trend lines, 1950−2021: Pangasinan, Ages: 25−29
Data points enclosed with a diamond are reference sources

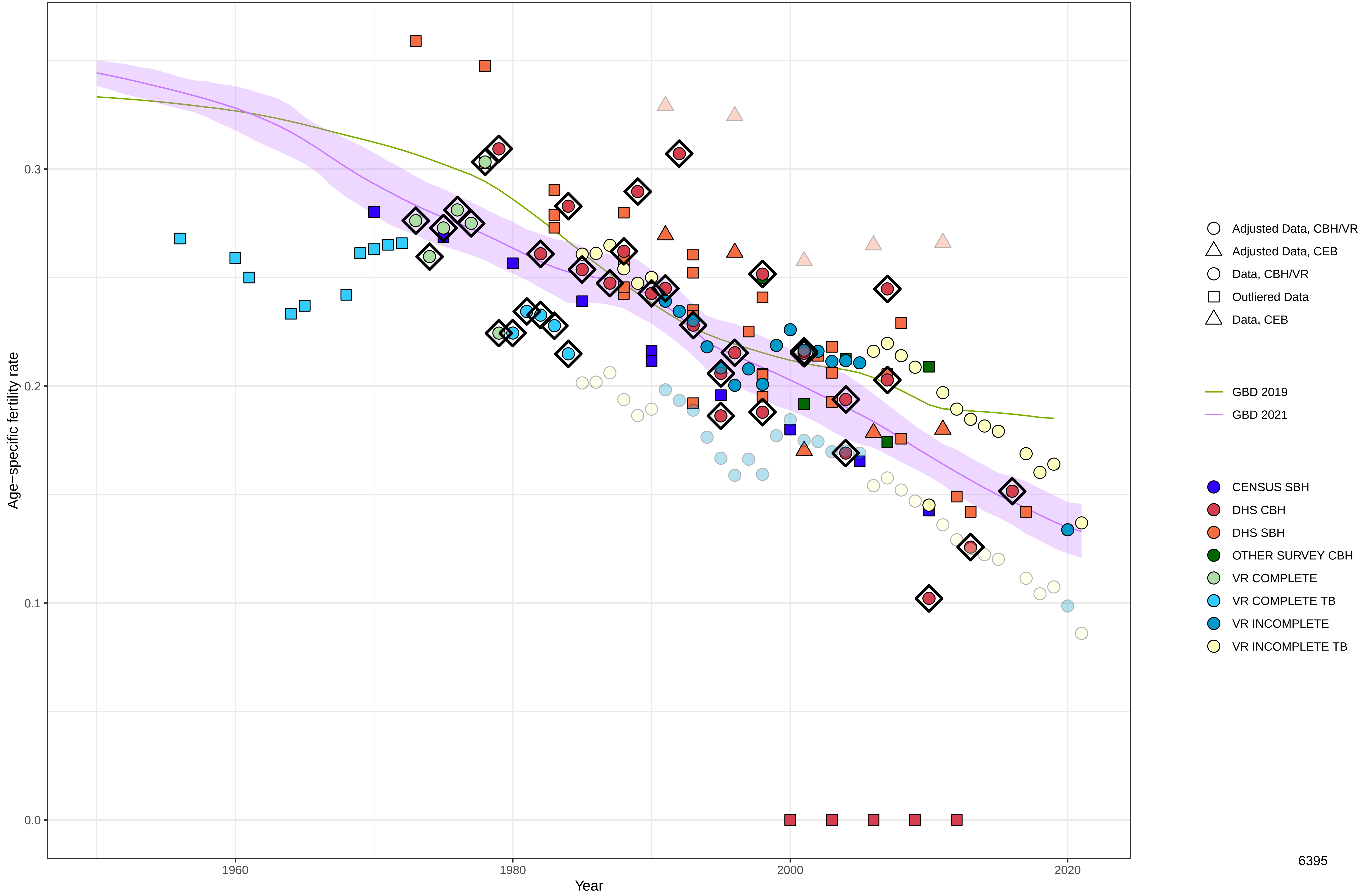

Age−specific fertility fitted trend lines, 1950−2021: Pangasinan, Ages: 30−34
Data points enclosed with a diamond are reference sources

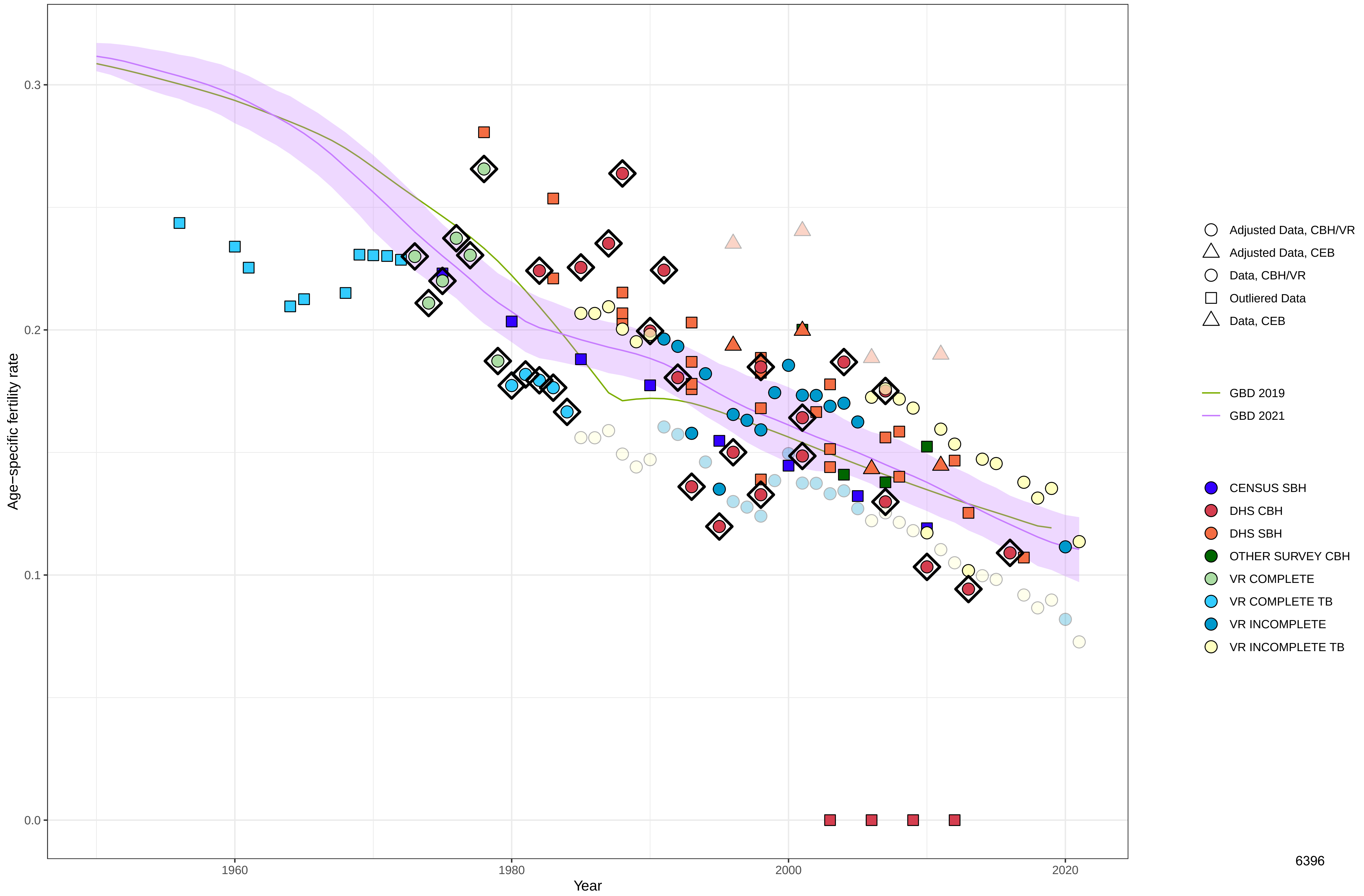

Age−specific fertility fitted trend lines, 1950−2021: Pangasinan, Ages: 35−39
Data points enclosed with a diamond are reference sources

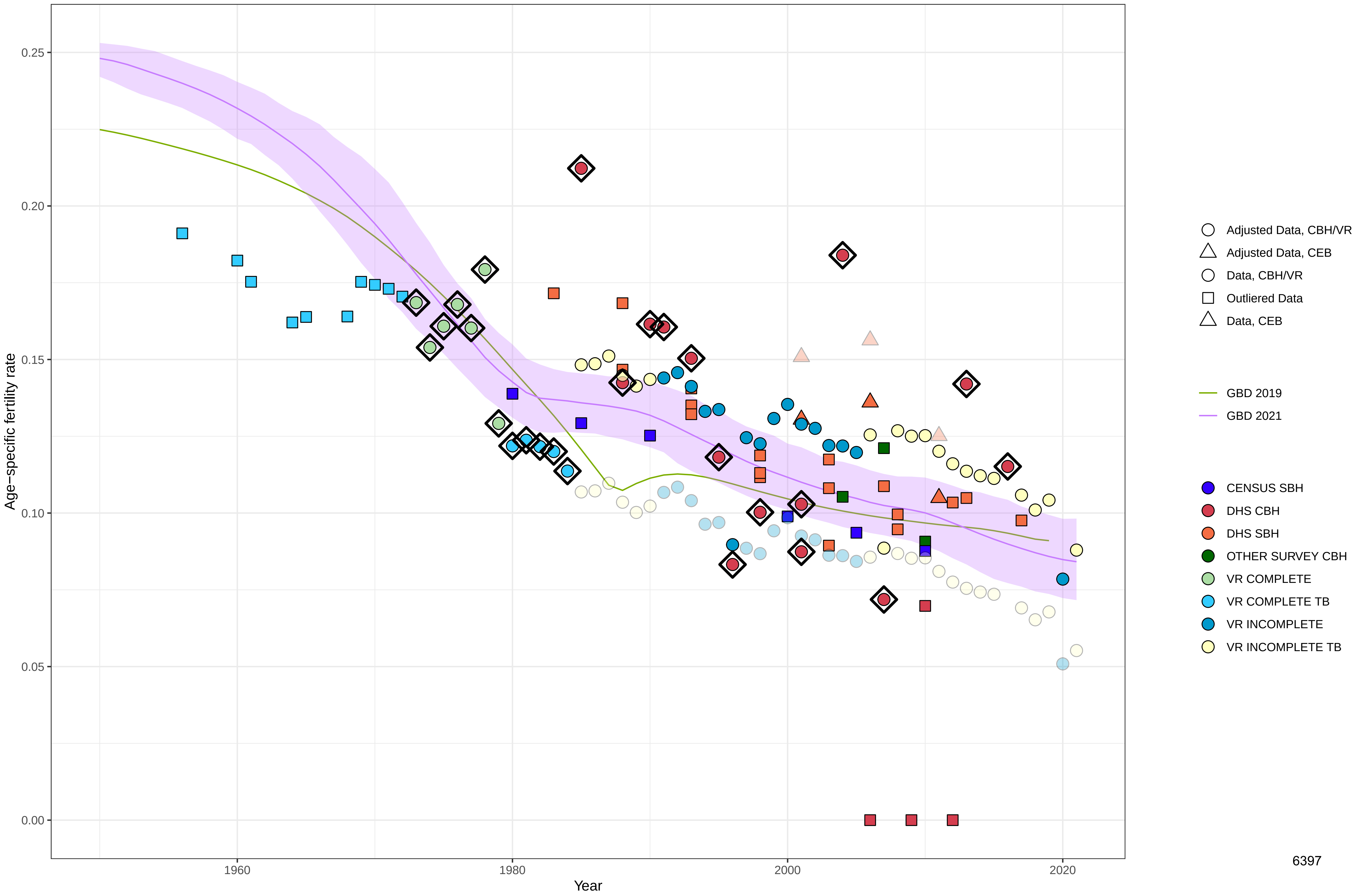

Age−specific fertility fitted trend lines, 1950−2021: Pangasinan, Ages: 40−44
Data points enclosed with a diamond are reference sources

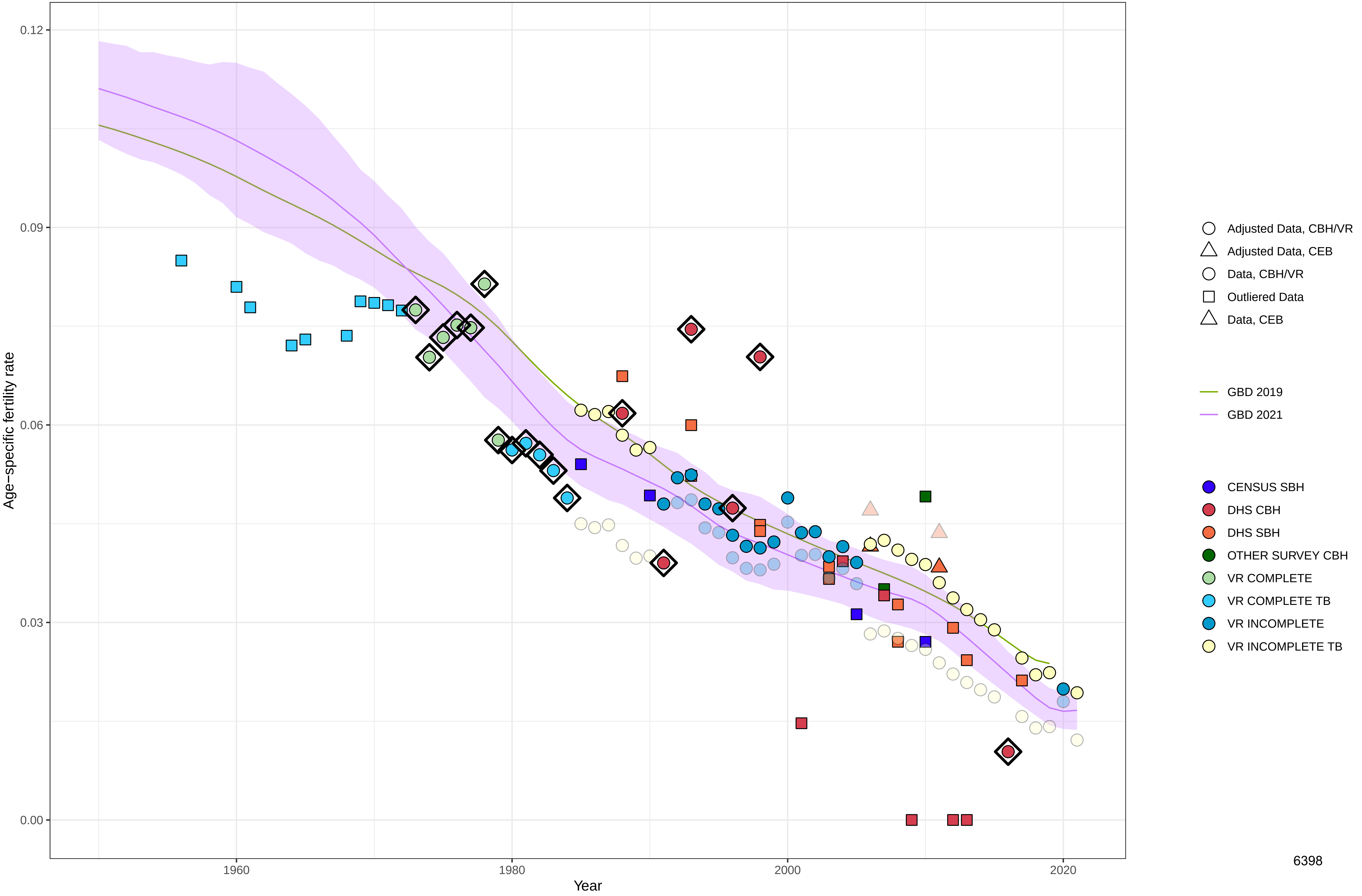

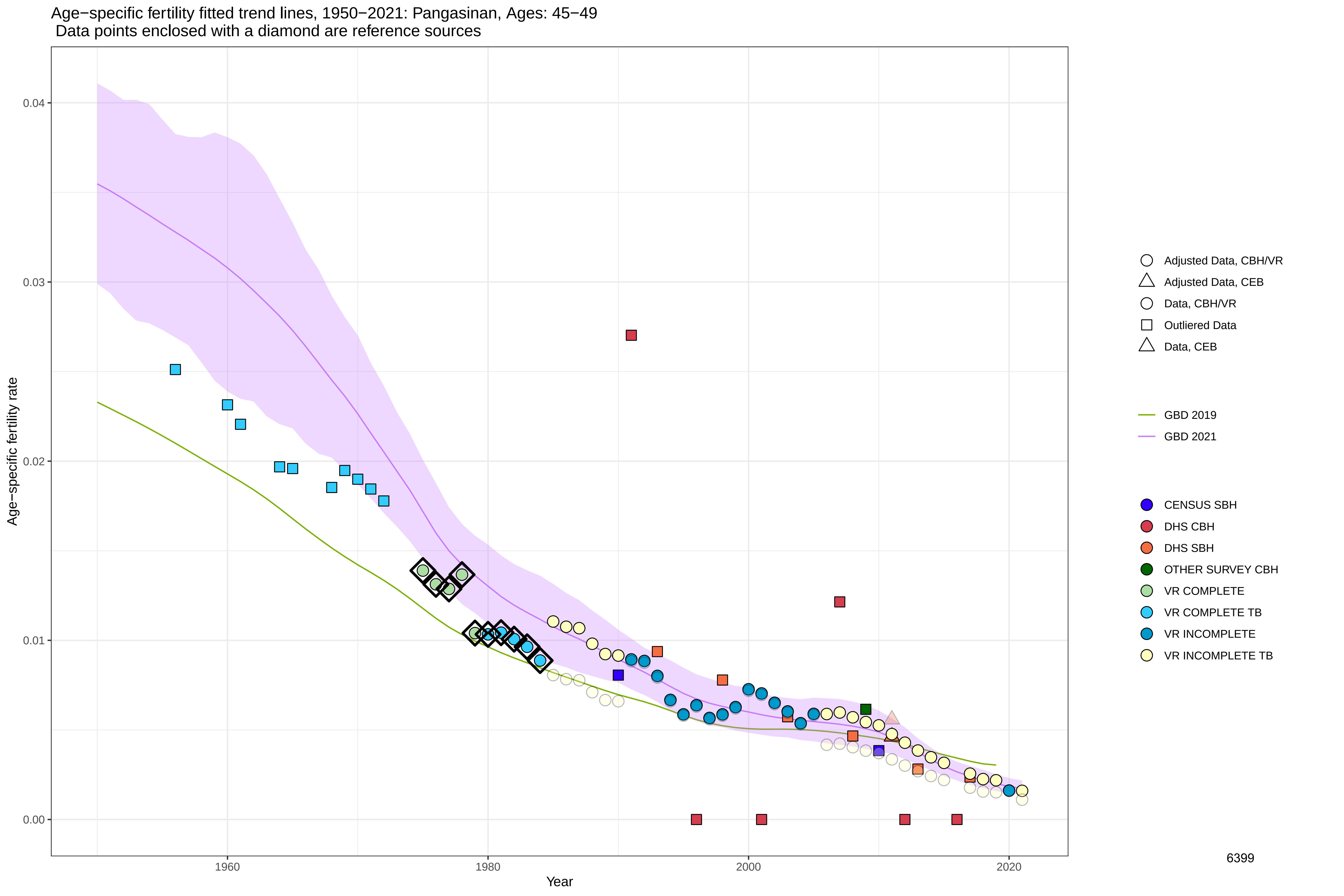

Age−specific fertility fitted trend lines, 1950−2021: Pangasinan, Ages: 45−49
Data points enclosed with a diamond are reference sources
Age−specific fertility rate
Year
0.00
0.01
0.02
0.03
0.04
1960
1980
2000
2020
Adjusted Data, CBH/VR
Adjusted Data, CEB
Data, CBH/VR
Outliered Data
Data, CEB
GBD 2019
GBD 2021
CENSUS SBH
DHS CBH
DHS SBH
OTHER SURVEY CBH
VR COMPLETE
VR COMPLETE TB
VR INCOMPLETE
VR INCOMPLETE TB
6399

Age−specific fertility fitted trend lines, 1950−2021: Pangasinan, Ages: 50−54
Data points enclosed with a diamond are reference sources

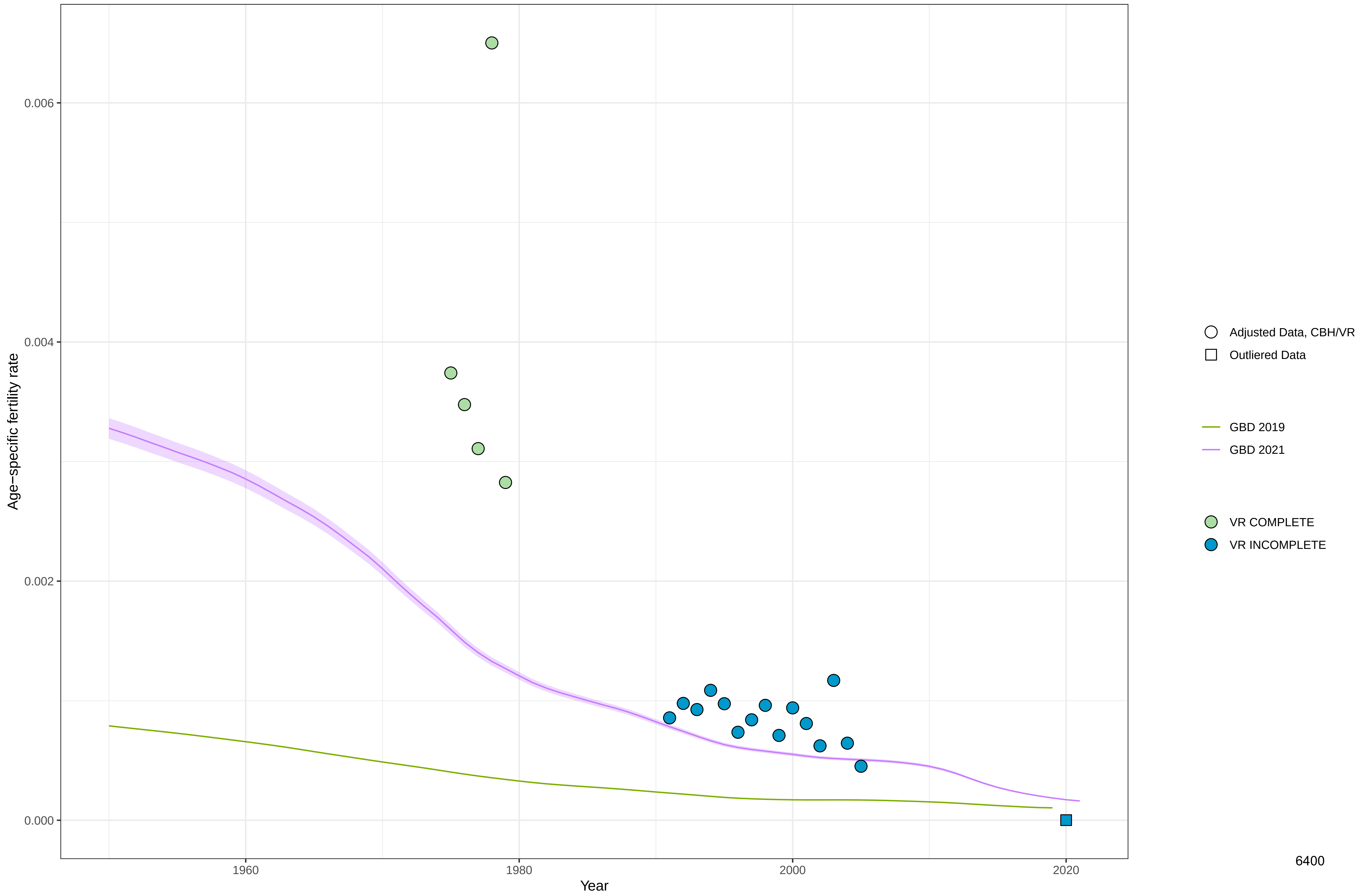

Total fertility rate fitted trend lines, 1950−2021: Pangasinan TFR
Data points enclosed with a diamond are reference sources

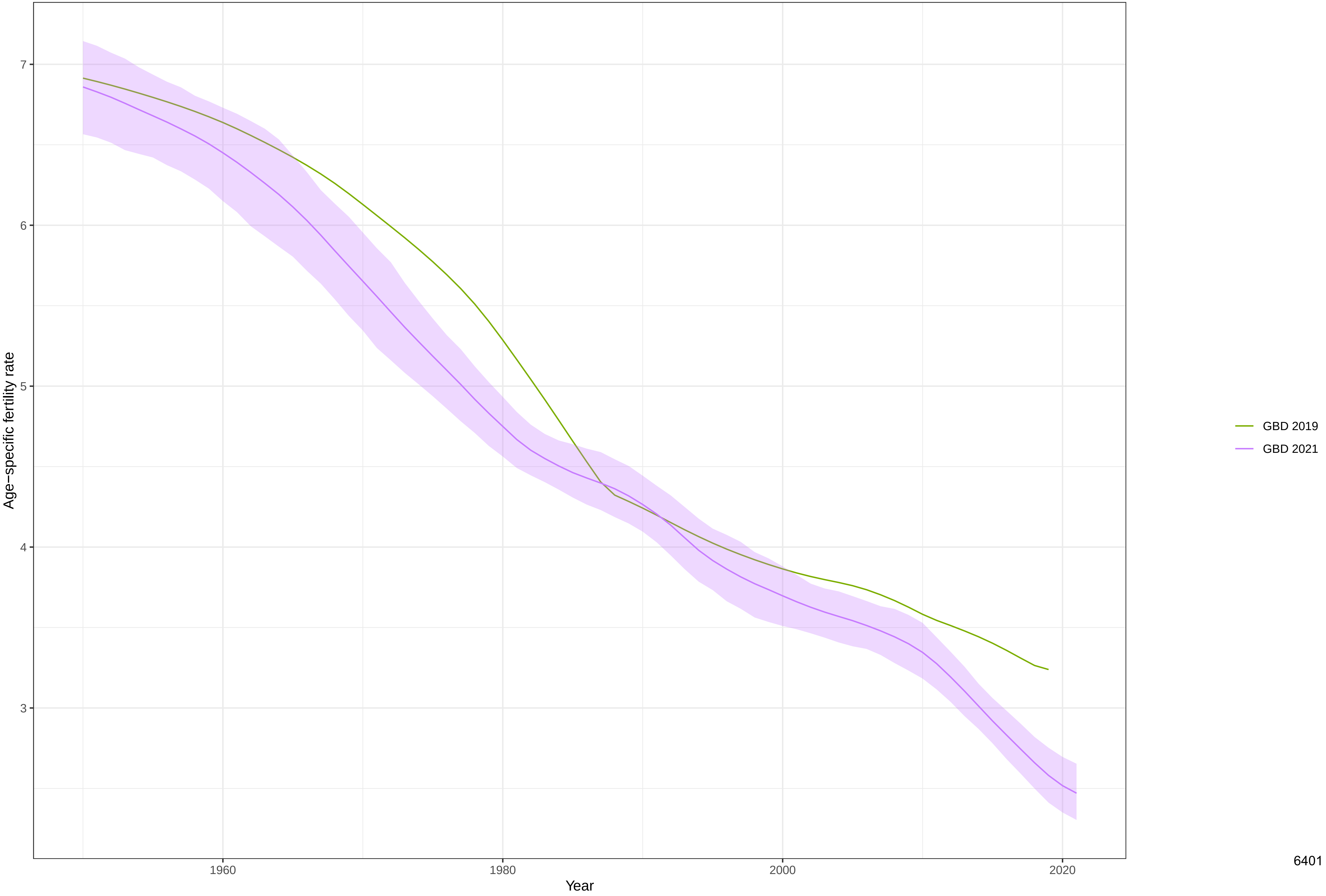

Age−specific fertility fitted trend lines, 1950−2021: Quezon, Ages: 10−14
Data points enclosed with a diamond are reference sources

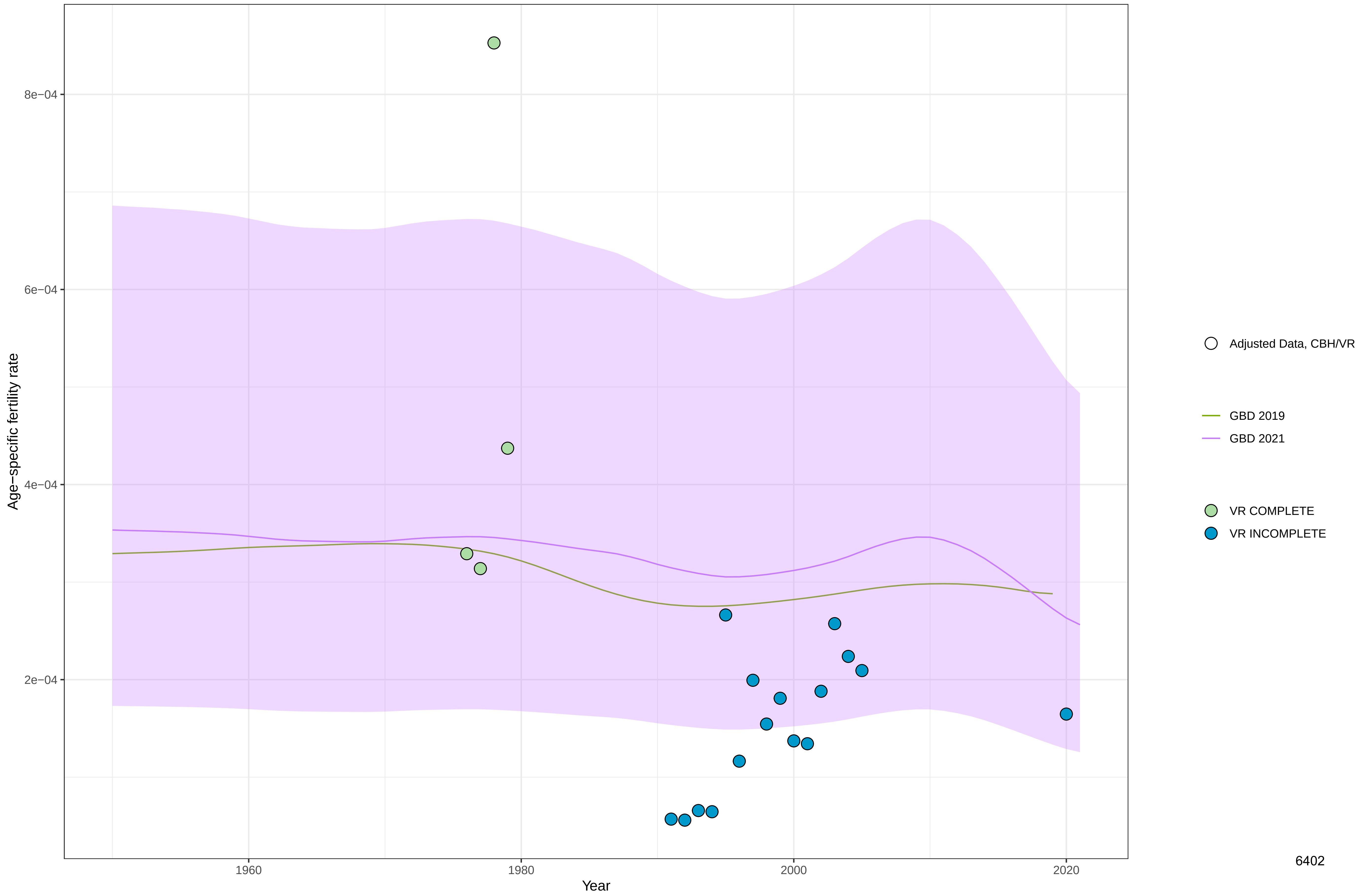

6402

Age−specific fertility fitted trend lines, 1950−2021: Quezon, Ages: 15−19
Data points enclosed with a diamond are reference sources

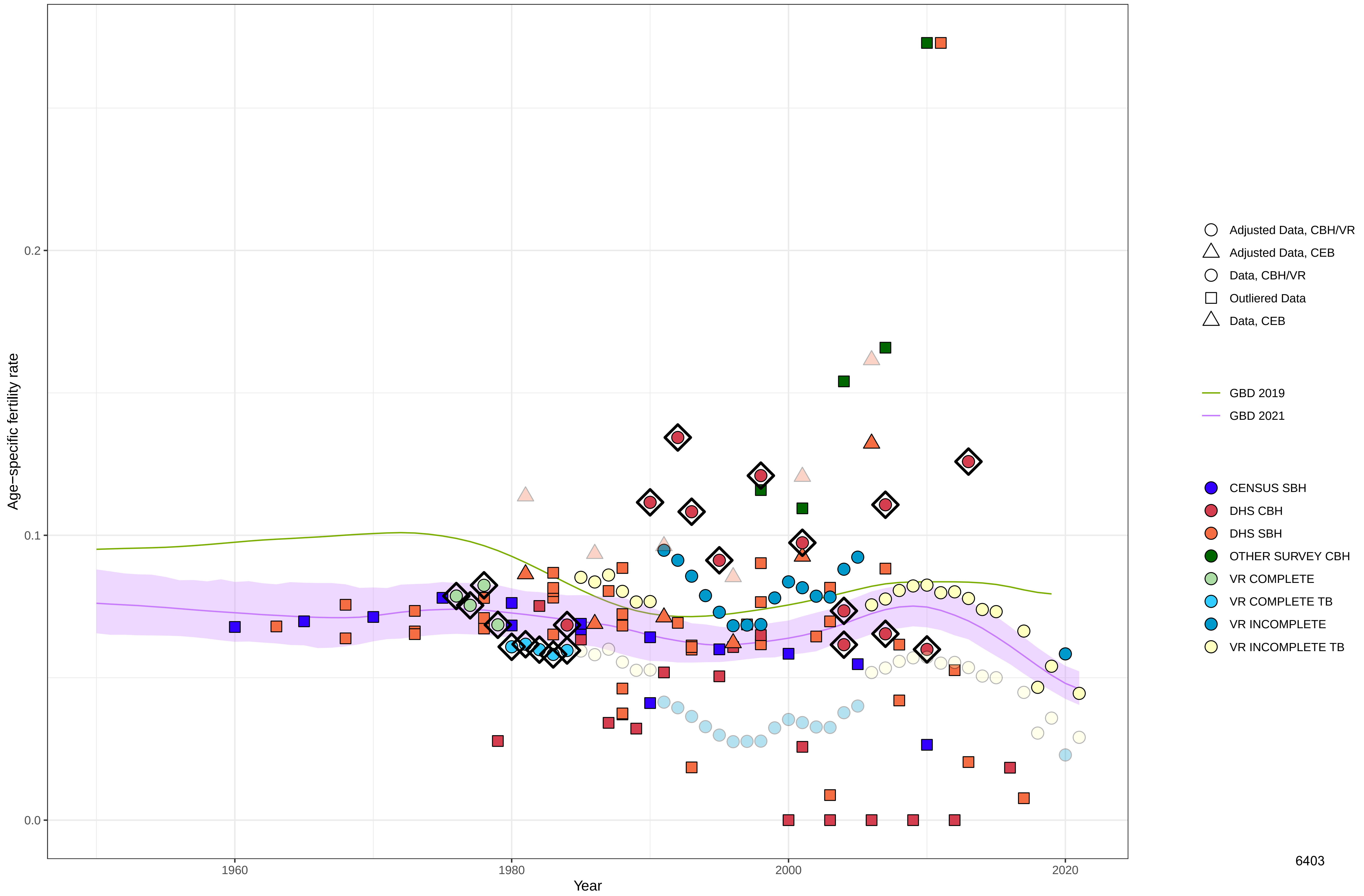

Age−specific fertility fitted trend lines, 1950−2021: Quezon, Ages: 20−24
Data points enclosed with a diamond are reference sources

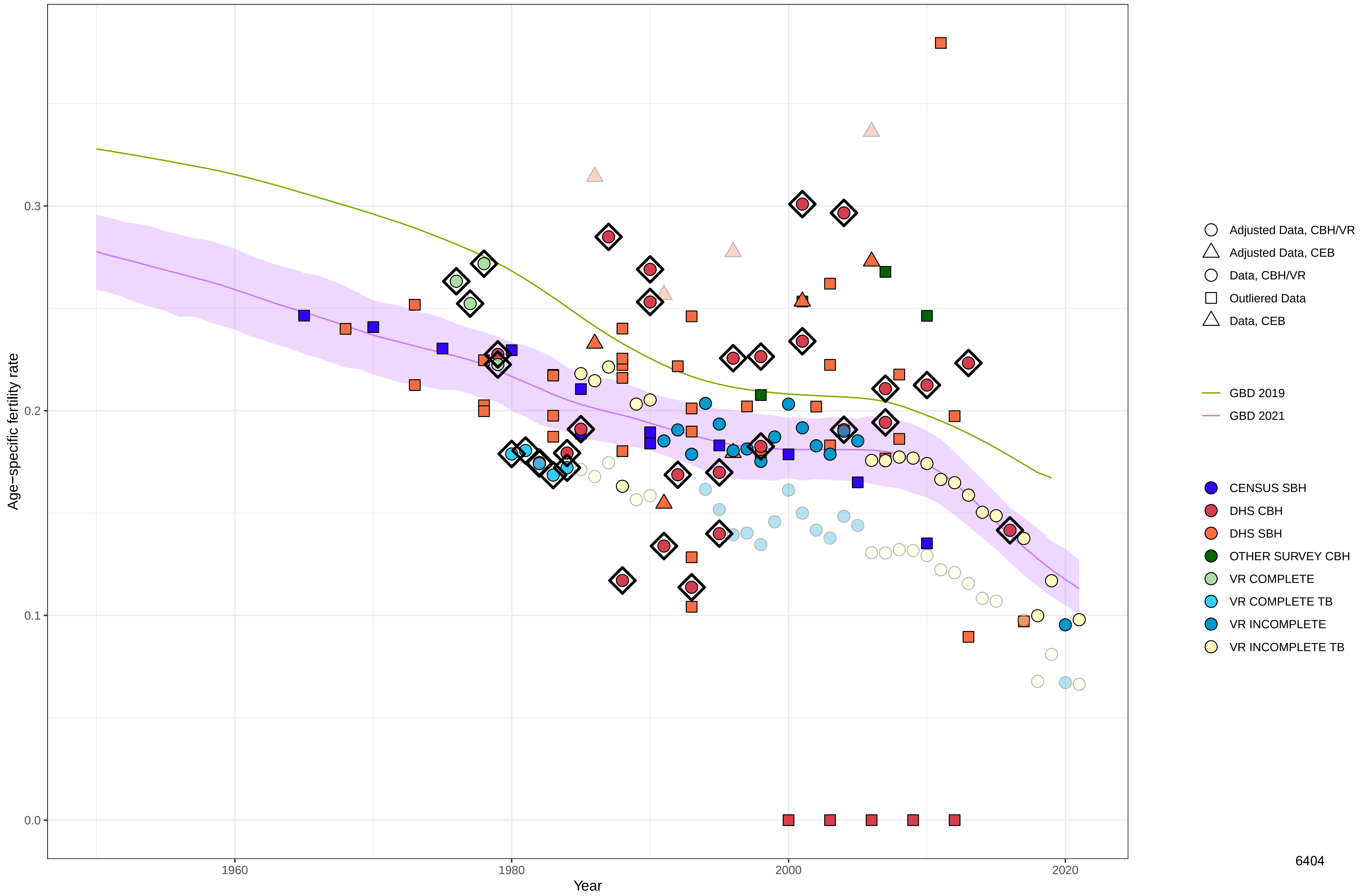

Age−specific fertility fitted trend lines, 1950−2021: Quezon, Ages: 25−29
Data points enclosed with a diamond are reference sources

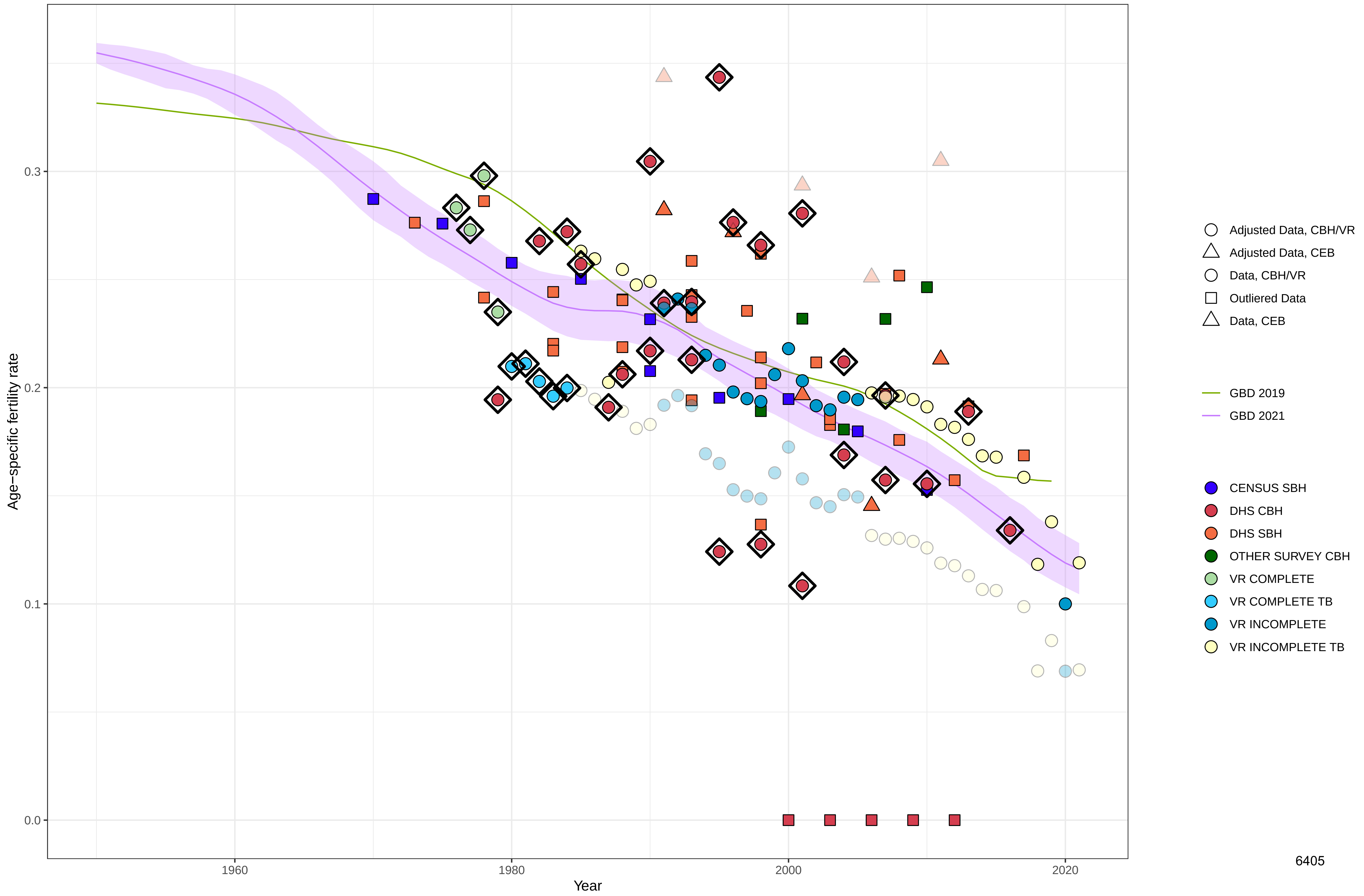

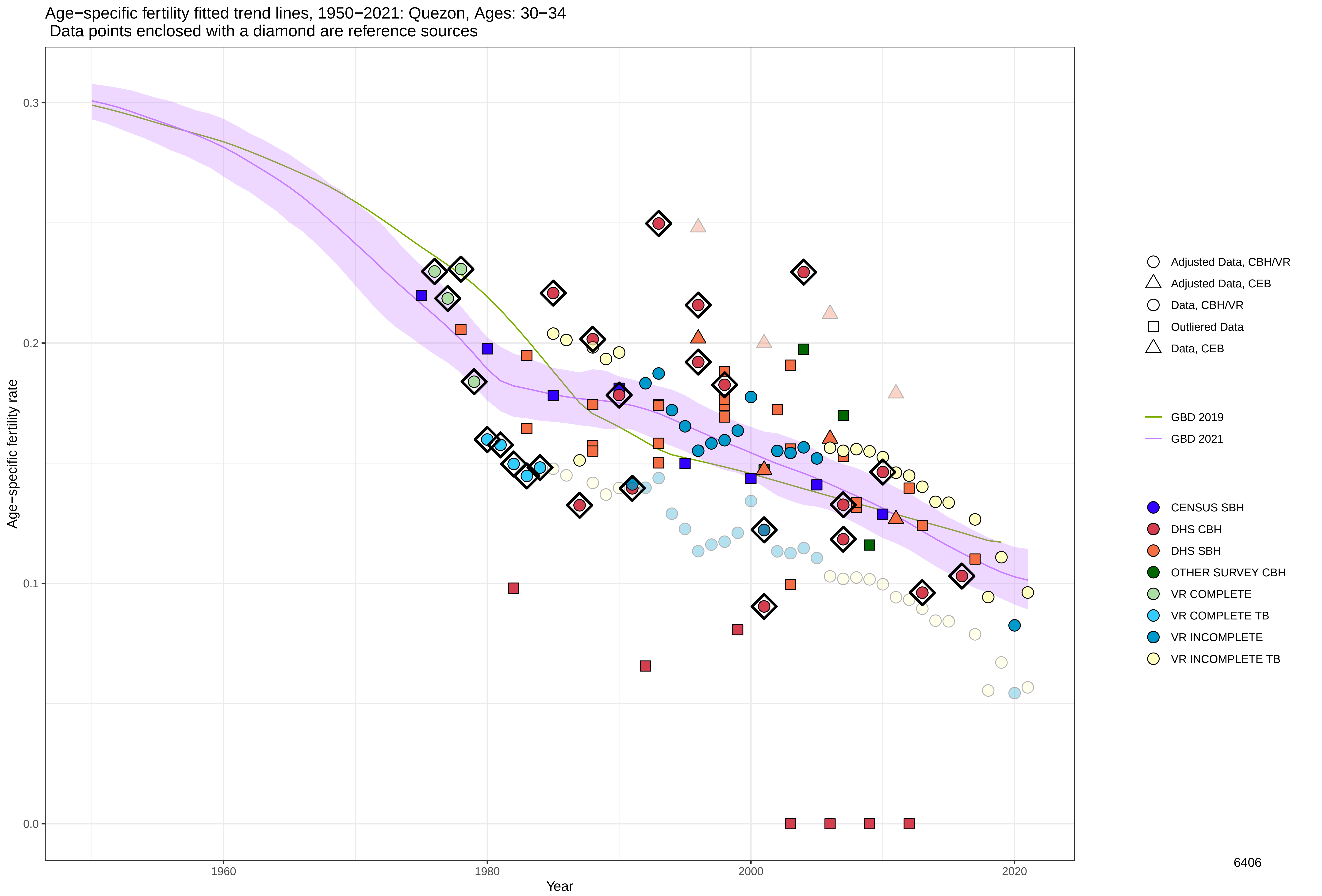

Age−specific fertility fitted trend lines, 1950−2021: Quezon, Ages: 30−34
Data points enclosed with a diamond are reference sources
Age−specific fertility rate
Year
0.0
0.1
0.2
0.3
1960
1980
2000
2020
Adjusted Data, CBH/VR
Adjusted Data, CEB
Data, CBH/VR
Outliered Data
Data, CEB
GBD 2019
GBD 2021
CENSUS SBH
DHS CBH
DHS SBH
OTHER SURVEY CBH
VR COMPLETE
VR COMPLETE TB
VR INCOMPLETE
VR INCOMPLETE TB

Age−specific fertility fitted trend lines, 1950−2021: Quezon, Ages: 35−39
Data points enclosed with a diamond are reference sources

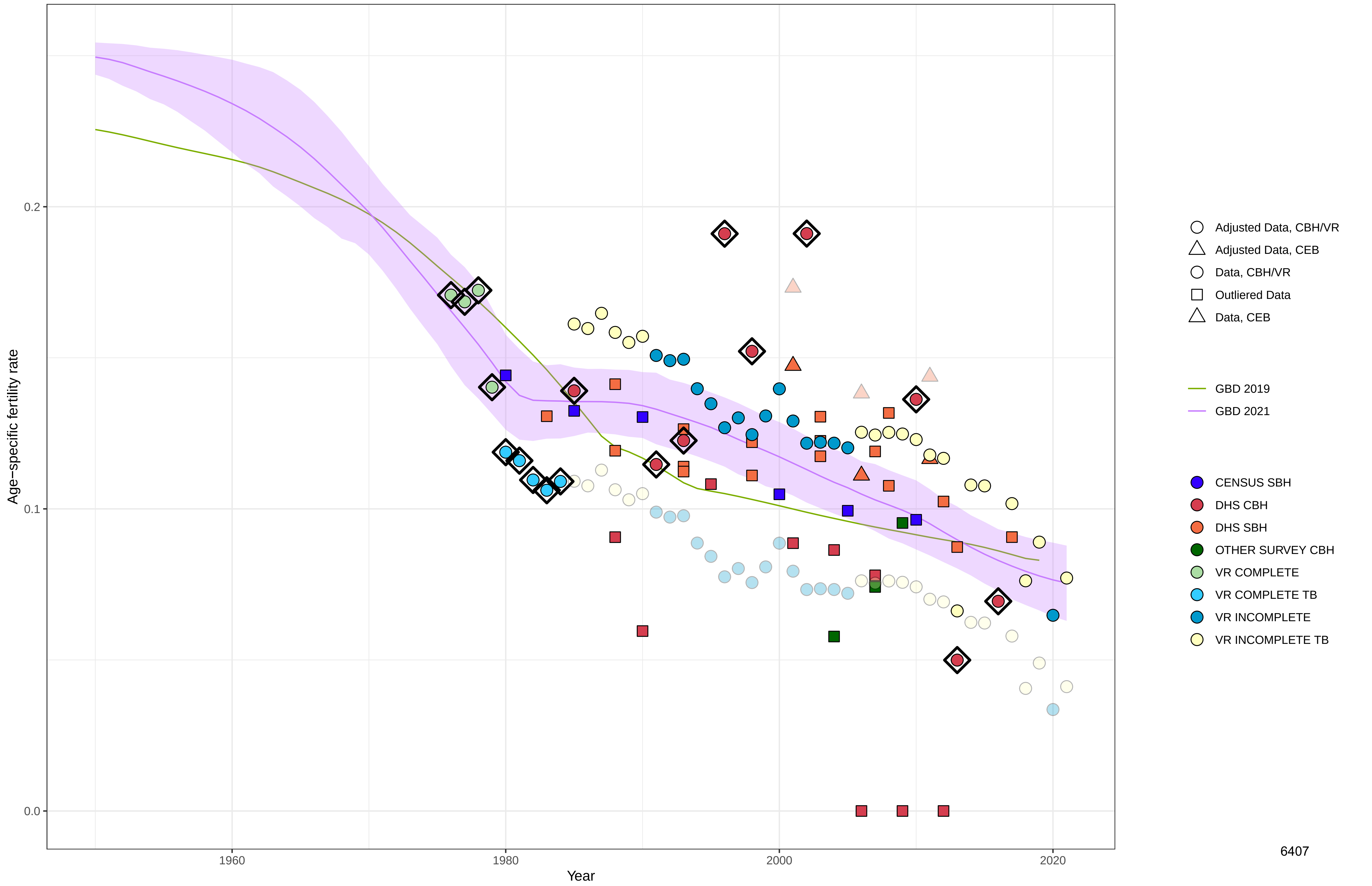

Age−specific fertility fitted trend lines, 1950−2021: Quezon, Ages: 40−44
Data points enclosed with a diamond are reference sources

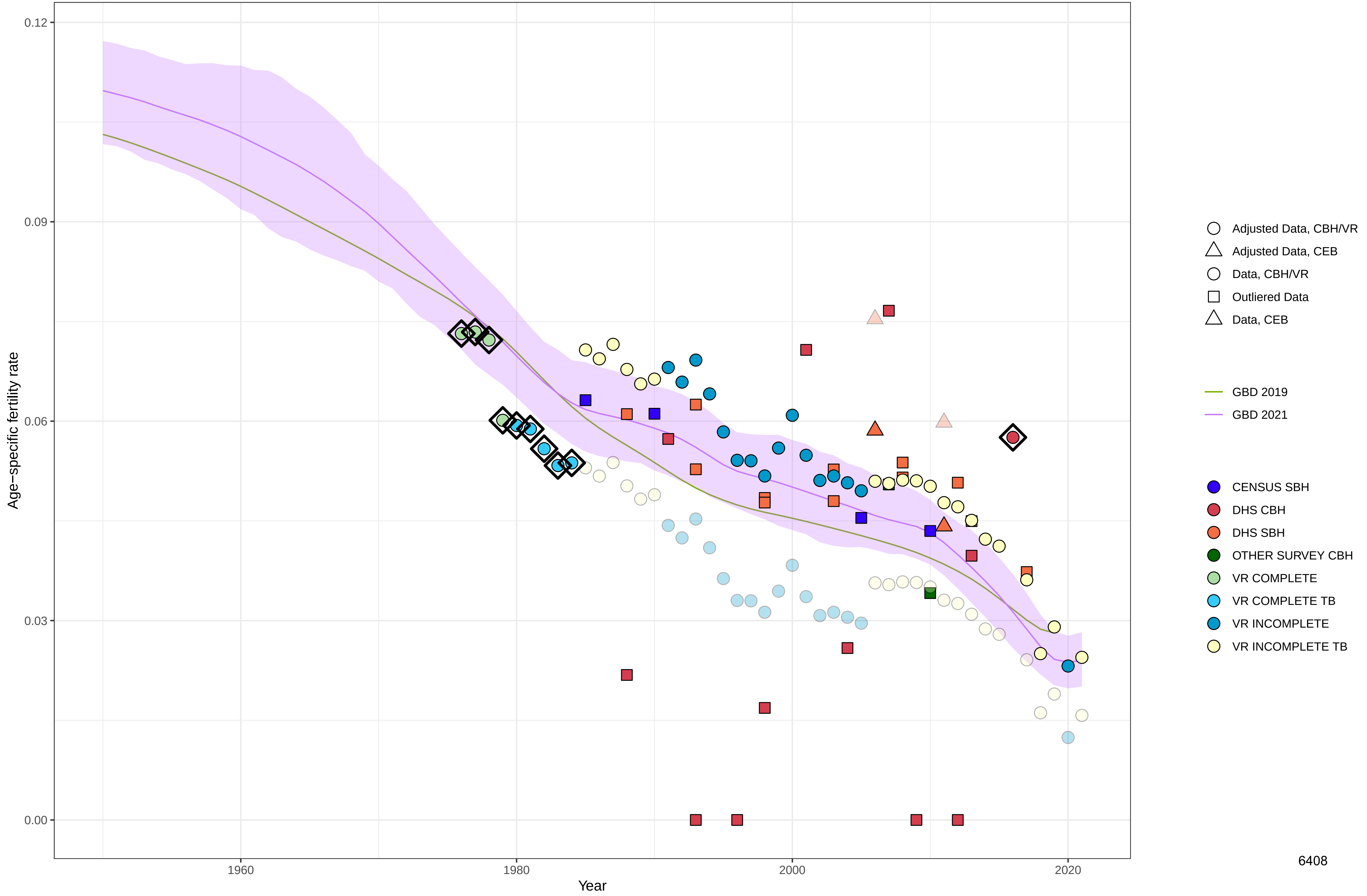

Age−specific fertility fitted trend lines, 1950−2021: Quezon, Ages: 45−49
Data points enclosed with a diamond are reference sources

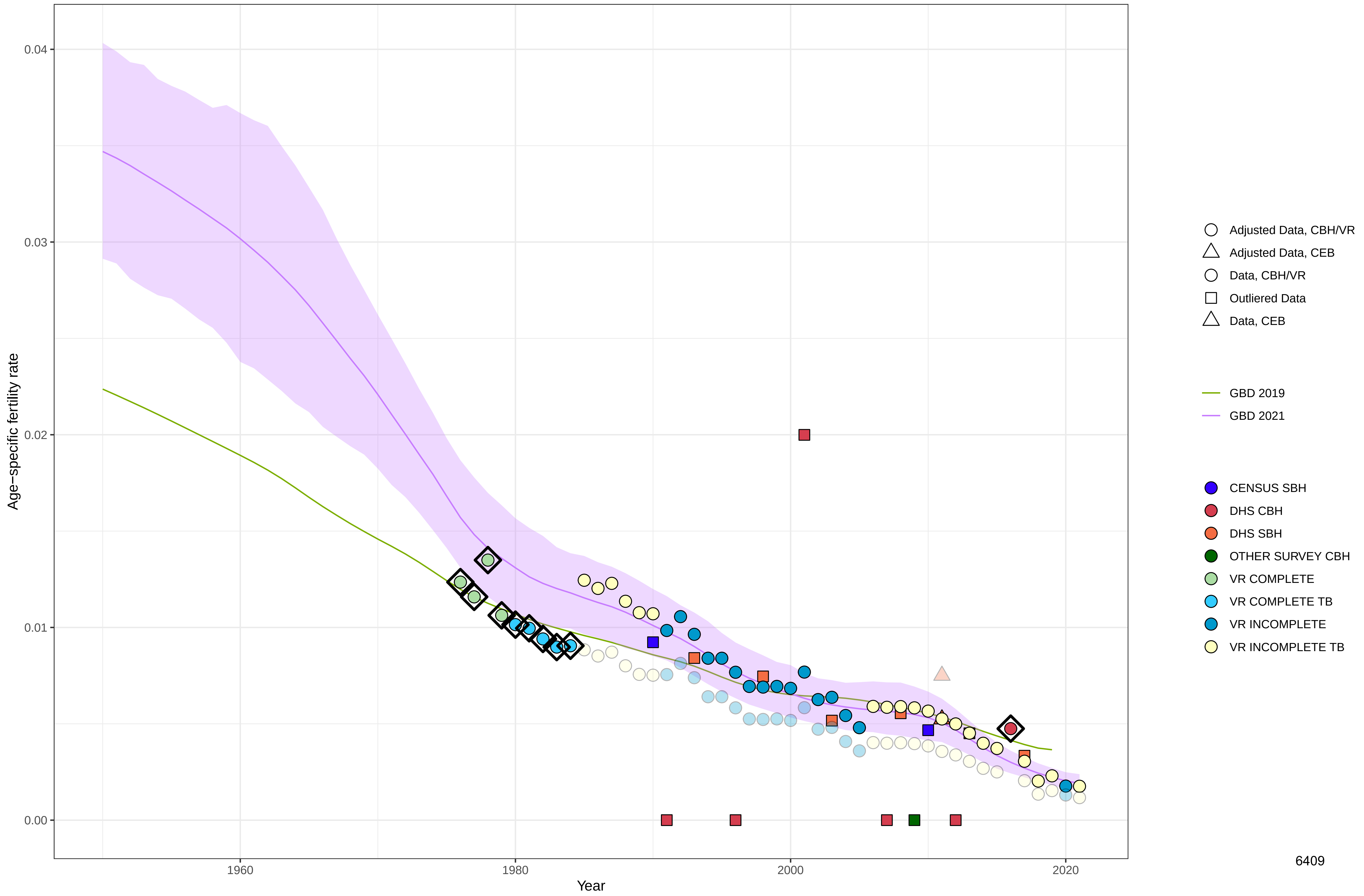

Age−specific fertility fitted trend lines, 1950−2021: Quezon, Ages: 50−54
Data points enclosed with a diamond are reference sources

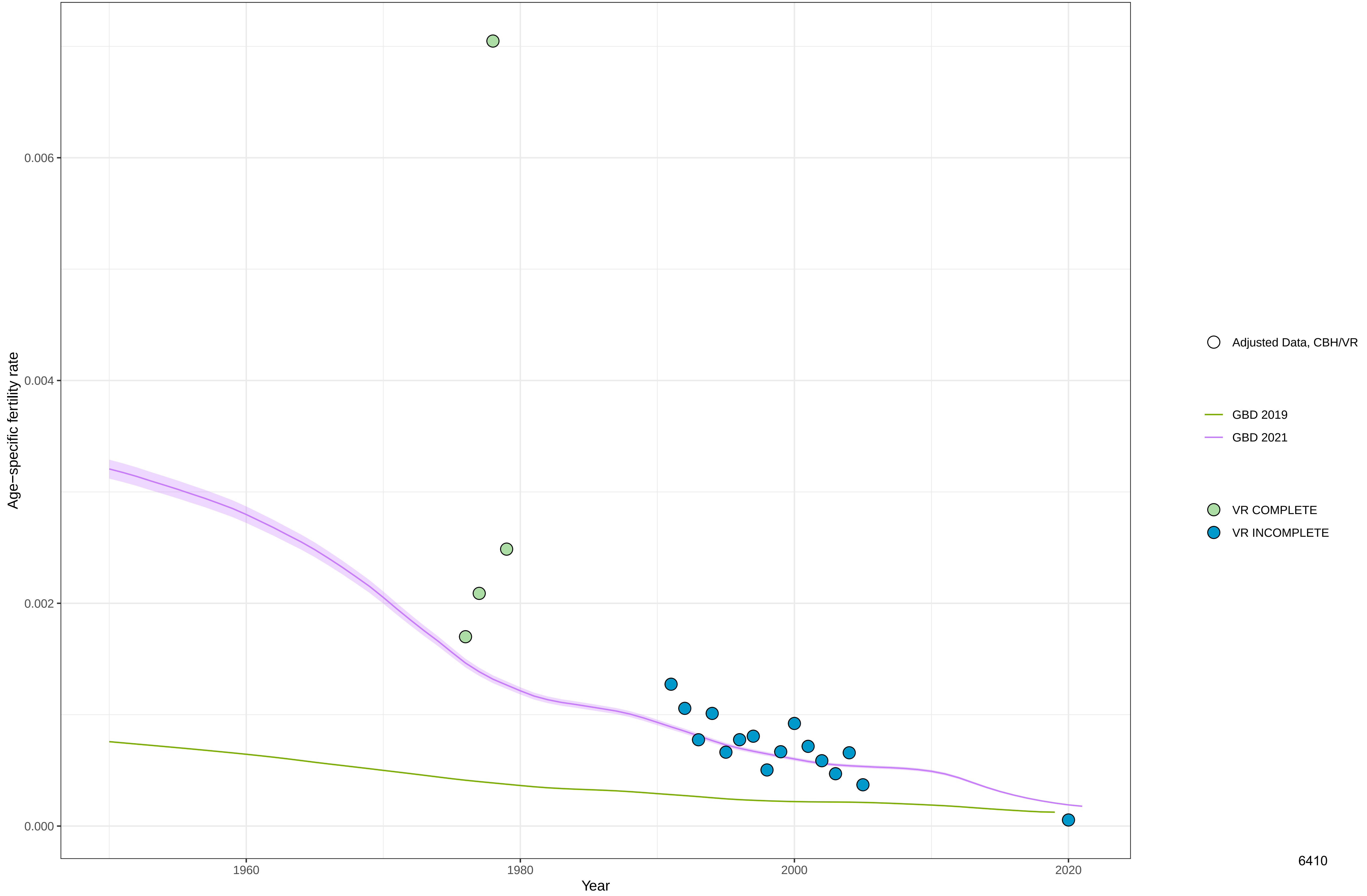

Total fertility rate fitted trend lines, 1950−2021: Quezon TFR
Data points enclosed with a diamond are reference sources

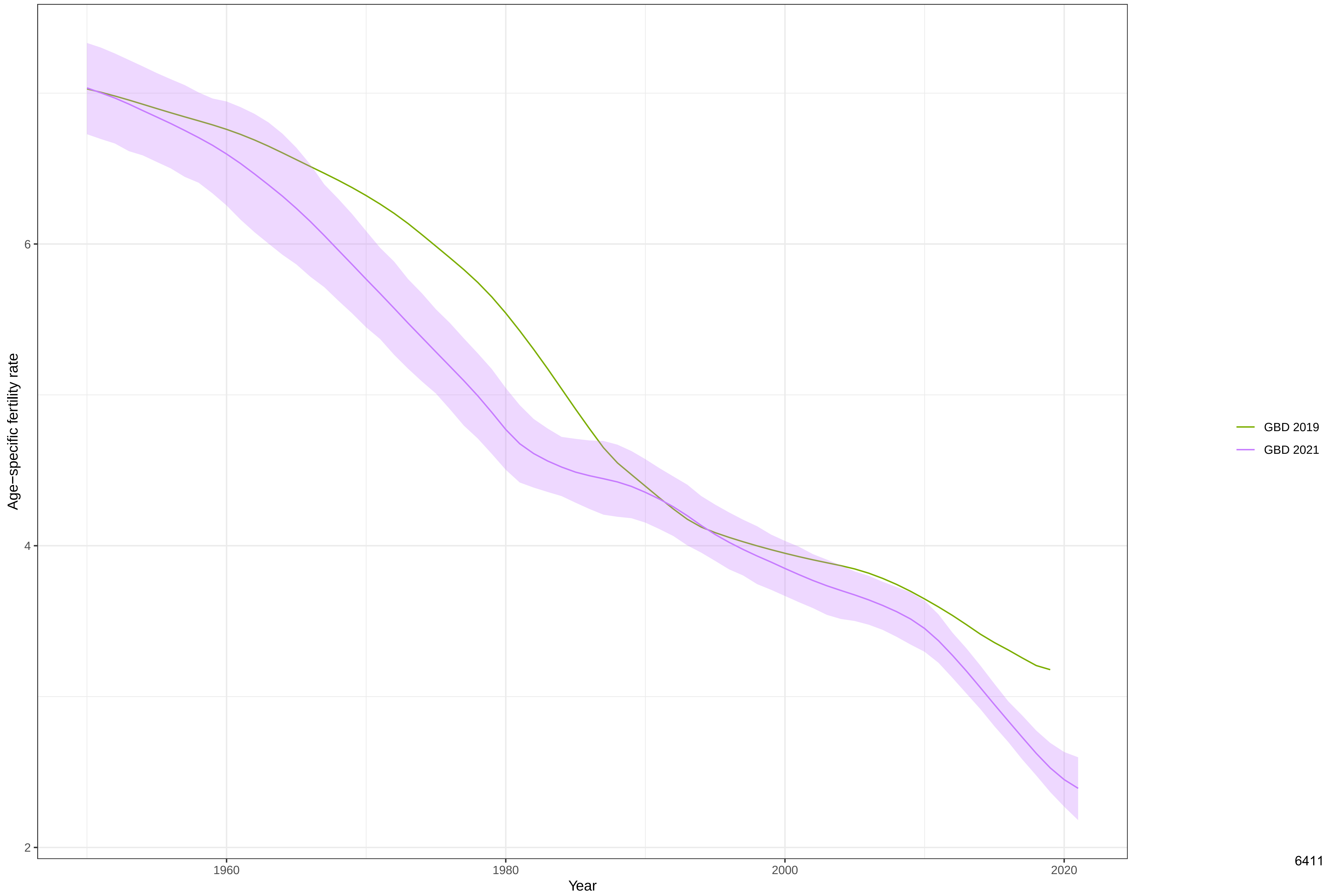

Age−specific fertility fitted trend lines, 1950−2021: Quirino, Ages: 10−14
Data points enclosed with a diamond are reference sources

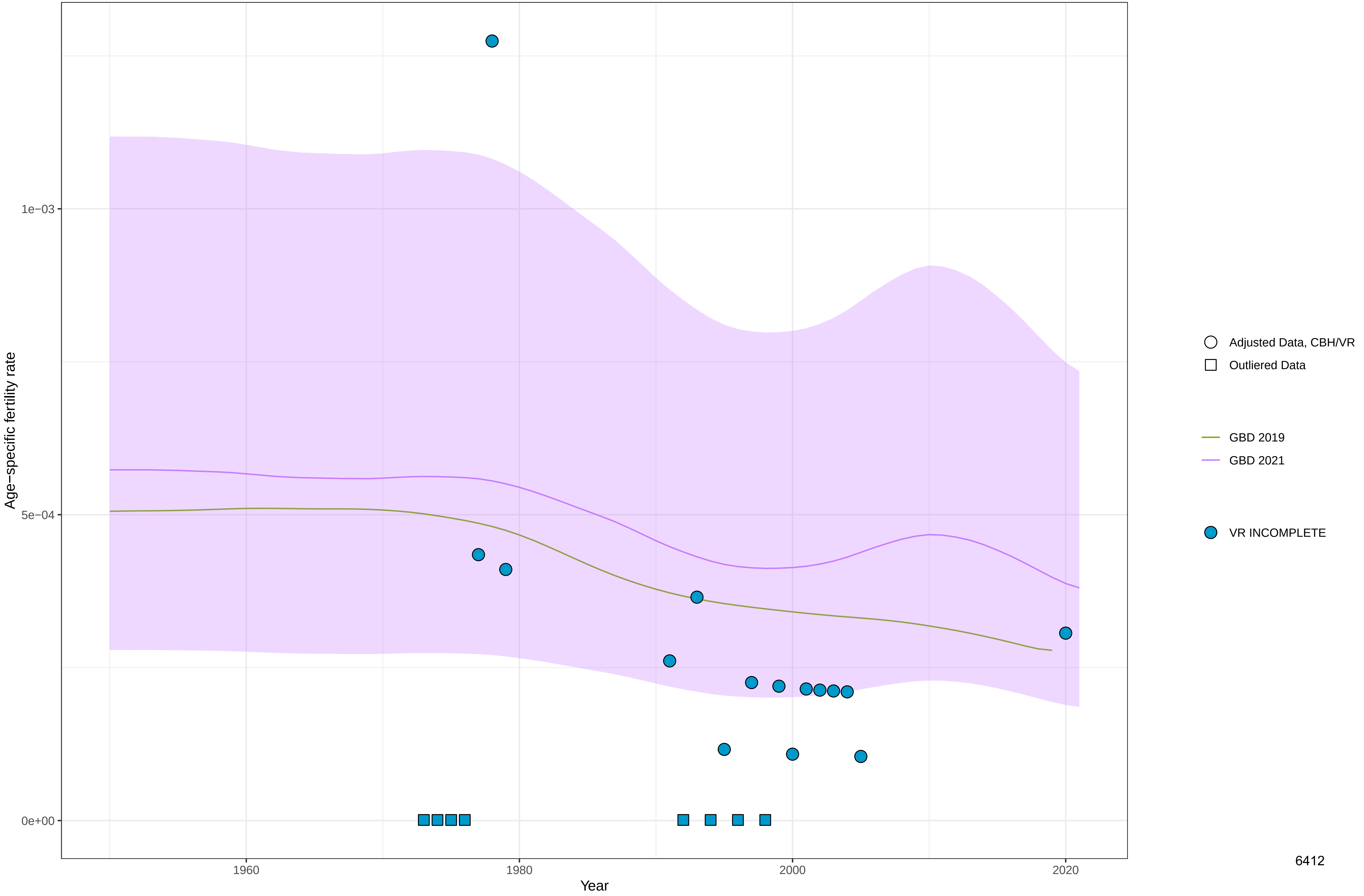

6412

Age−specific fertility fitted trend lines, 1950−2021: Quirino, Ages: 15−19
Data points enclosed with a diamond are reference sources

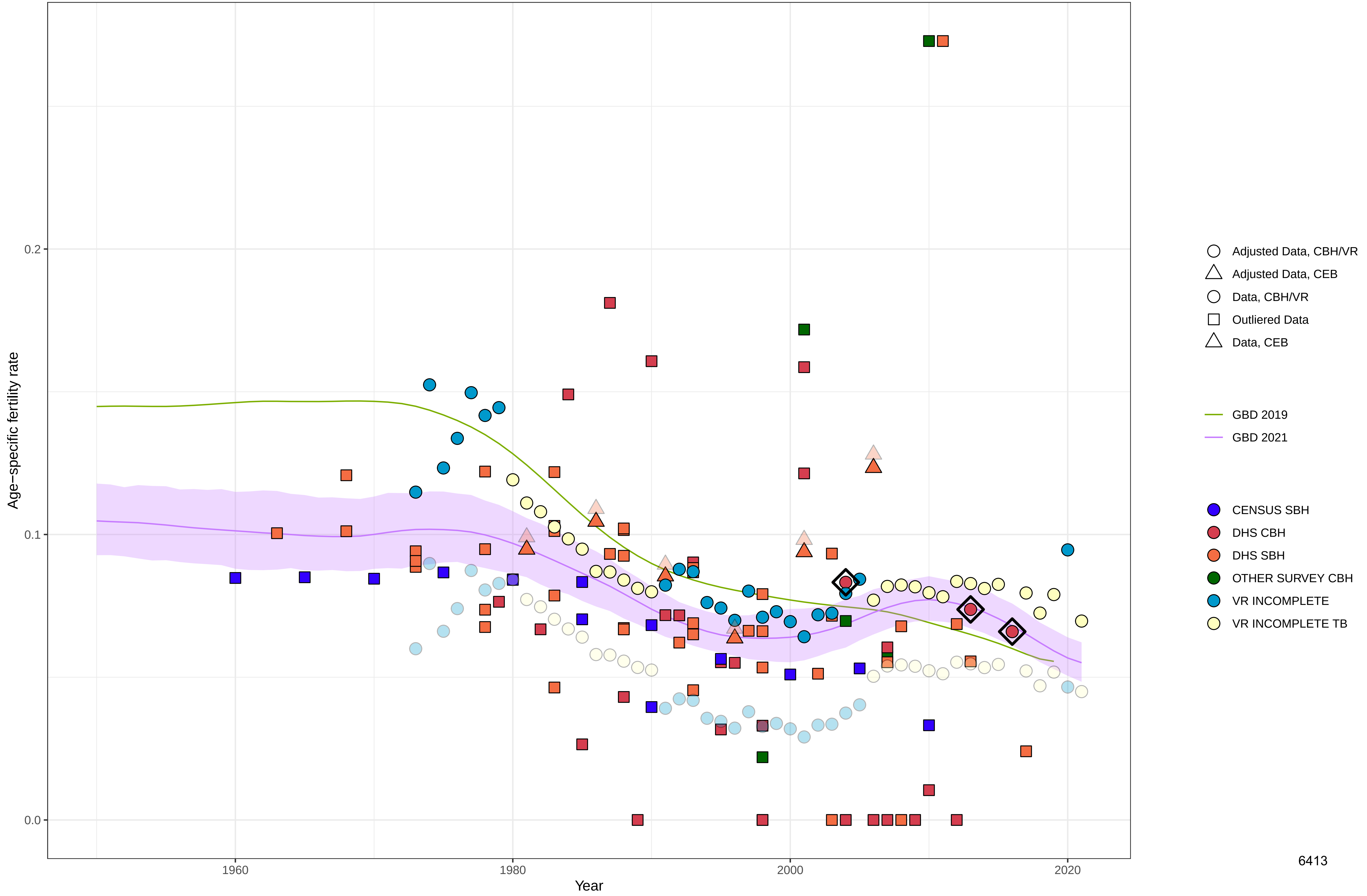

6413

Age−specific fertility fitted trend lines, 1950−2021: Quirino, Ages: 20−24
Data points enclosed with a diamond are reference sources

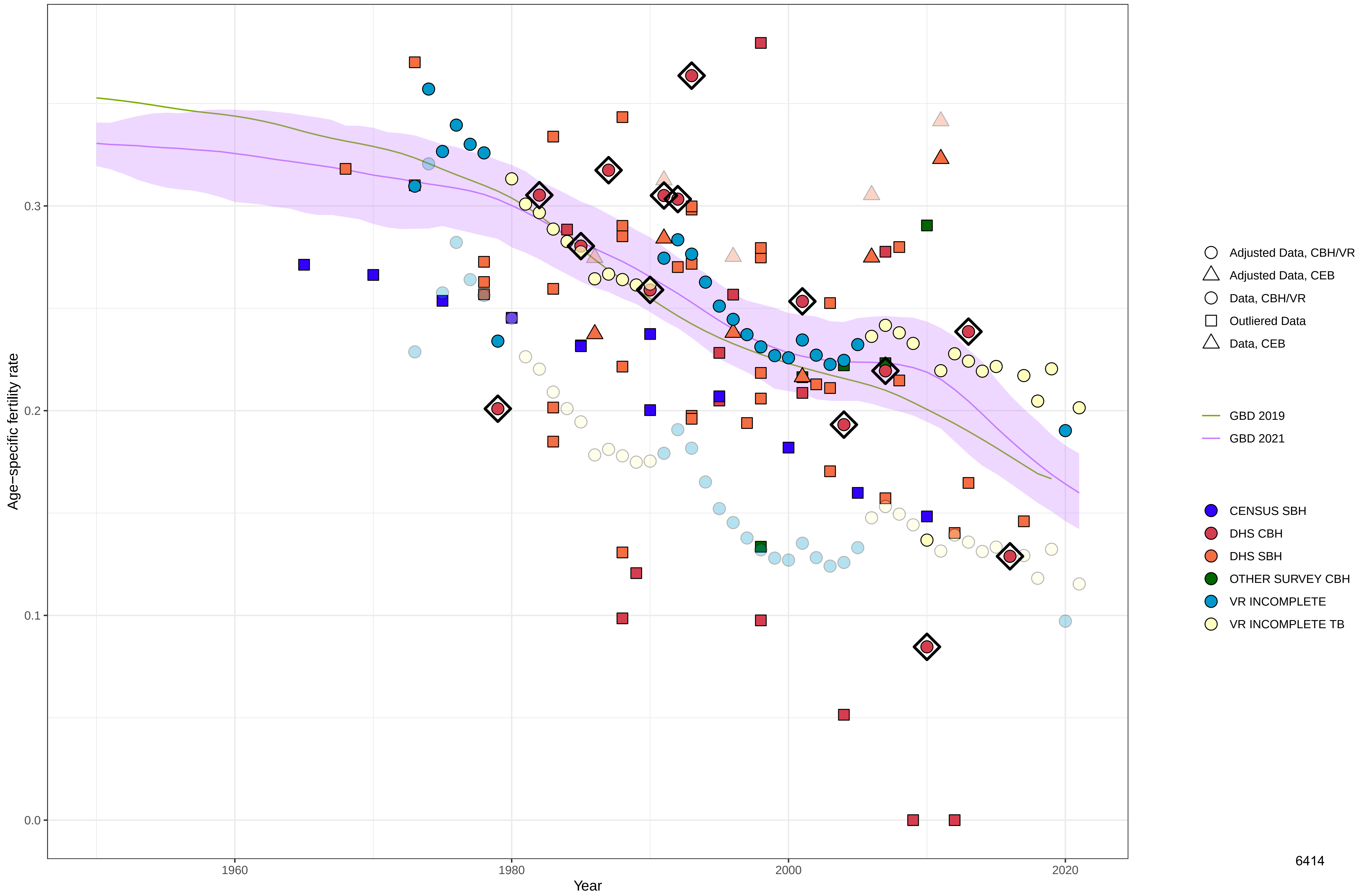

Age−specific fertility fitted trend lines, 1950−2021: Quirino, Ages: 25−29
Data points enclosed with a diamond are reference sources

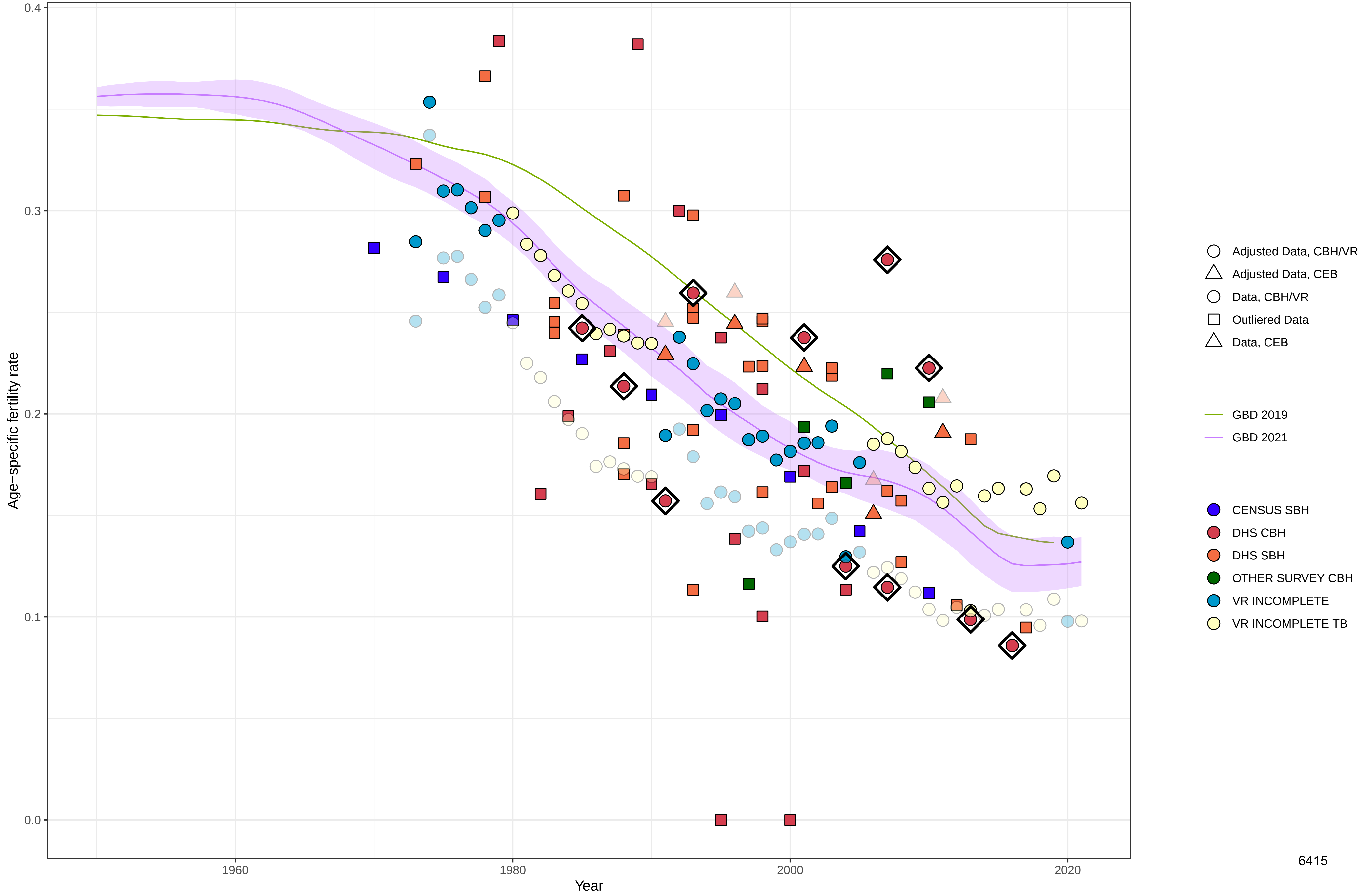

Age−specific fertility fitted trend lines, 1950−2021: Quirino, Ages: 30−34
Data points enclosed with a diamond are reference sources

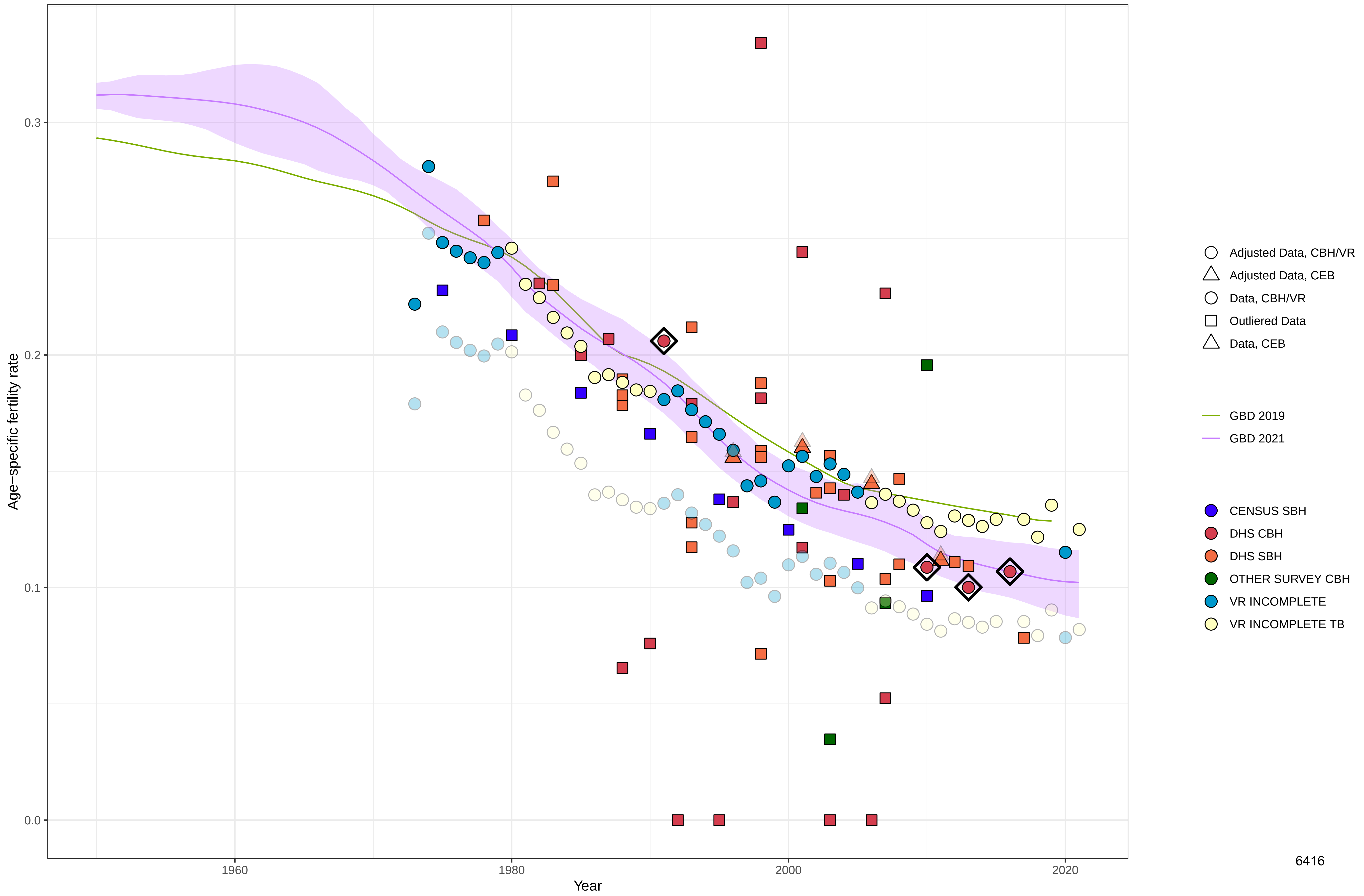

Age−specific fertility fitted trend lines, 1950−2021: Quirino, Ages: 35−39
Data points enclosed with a diamond are reference sources

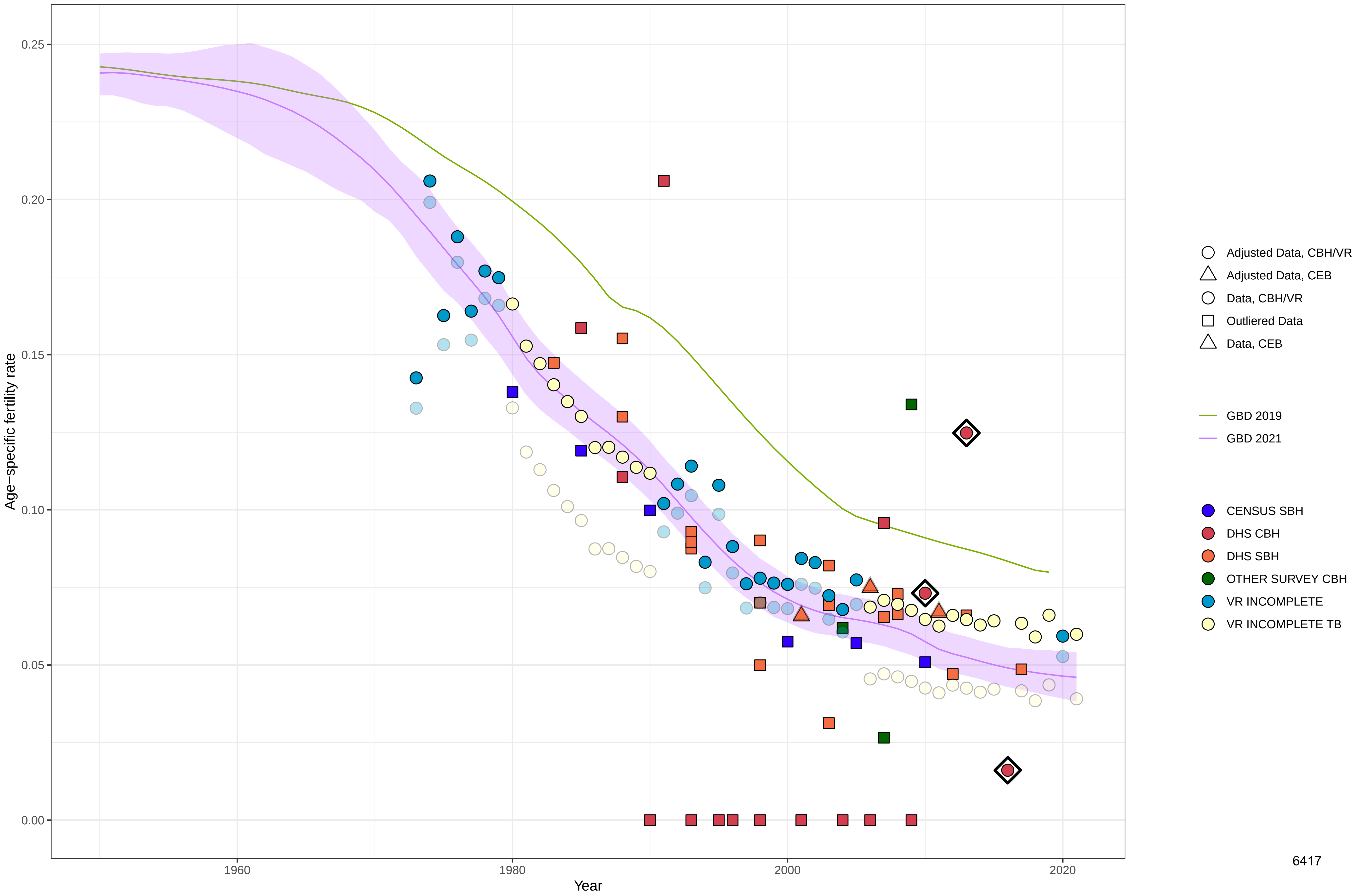

Age−specific fertility fitted trend lines, 1950−2021: Quirino, Ages: 40−44
Data points enclosed with a diamond are reference sources

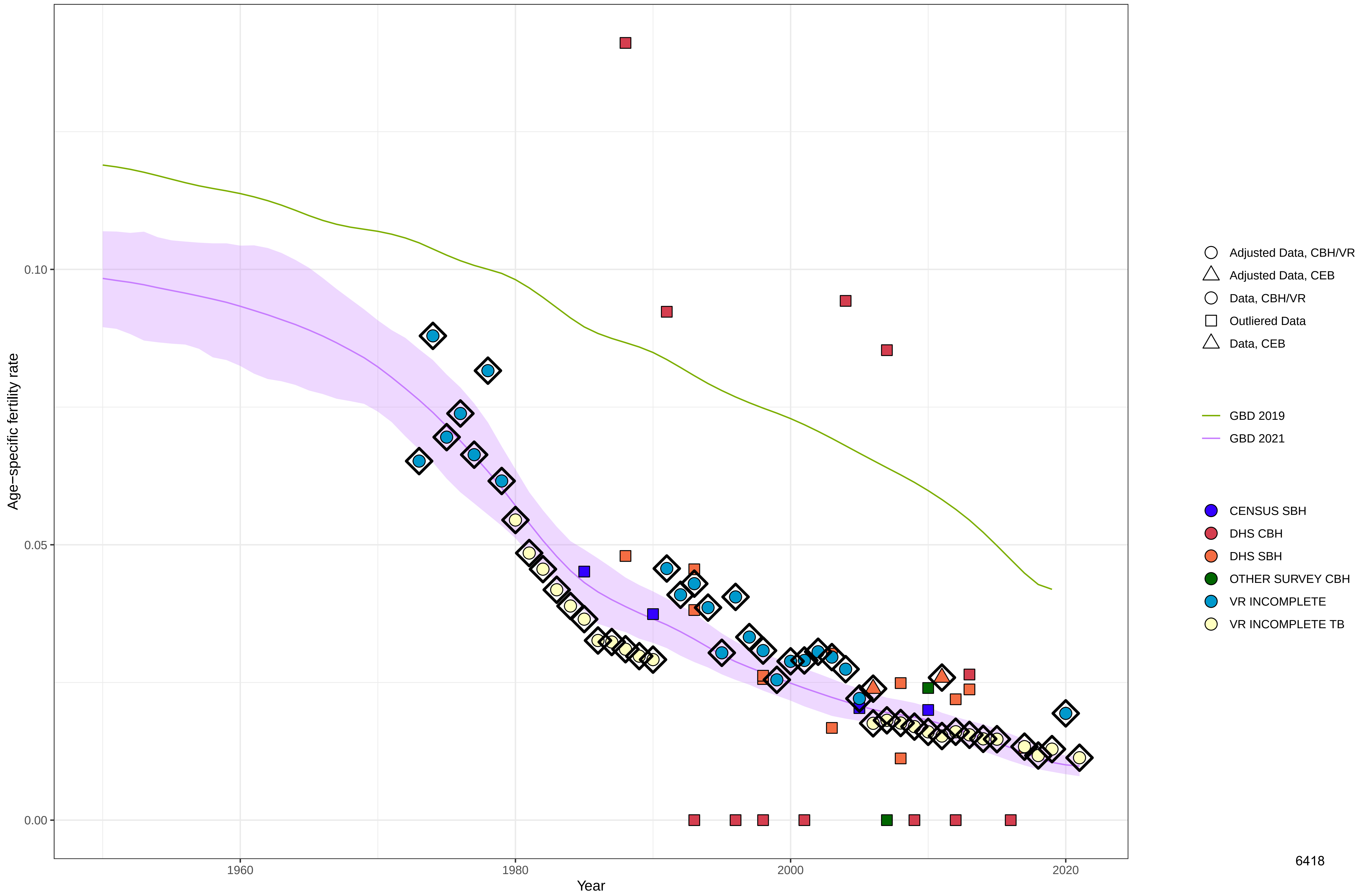

6418

Age−specific fertility fitted trend lines, 1950−2021: Quirino, Ages: 45−49
Data points enclosed with a diamond are reference sources

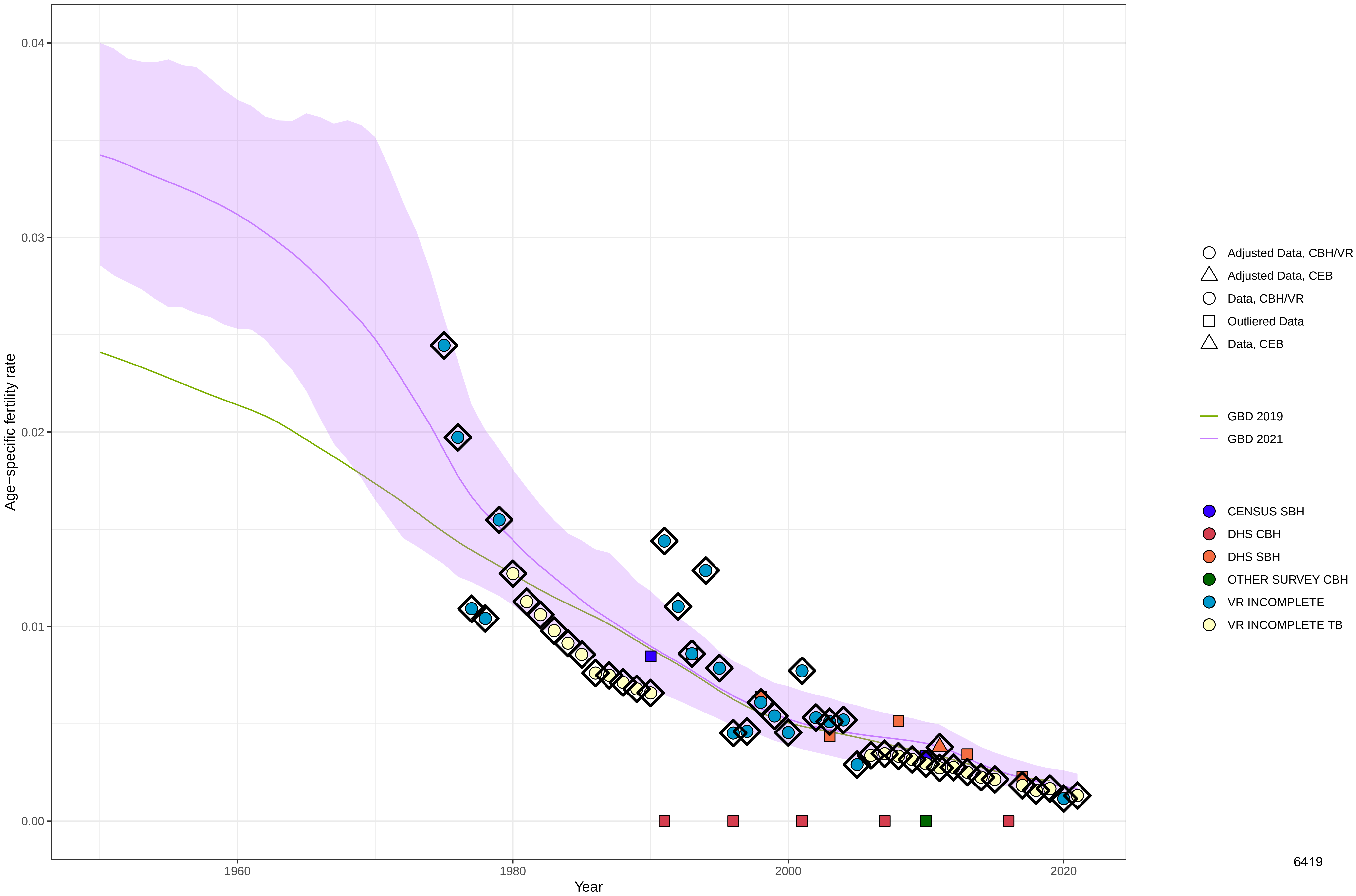

Age−specific fertility fitted trend lines, 1950−2021: Quirino, Ages: 50−54
Data points enclosed with a diamond are reference sources

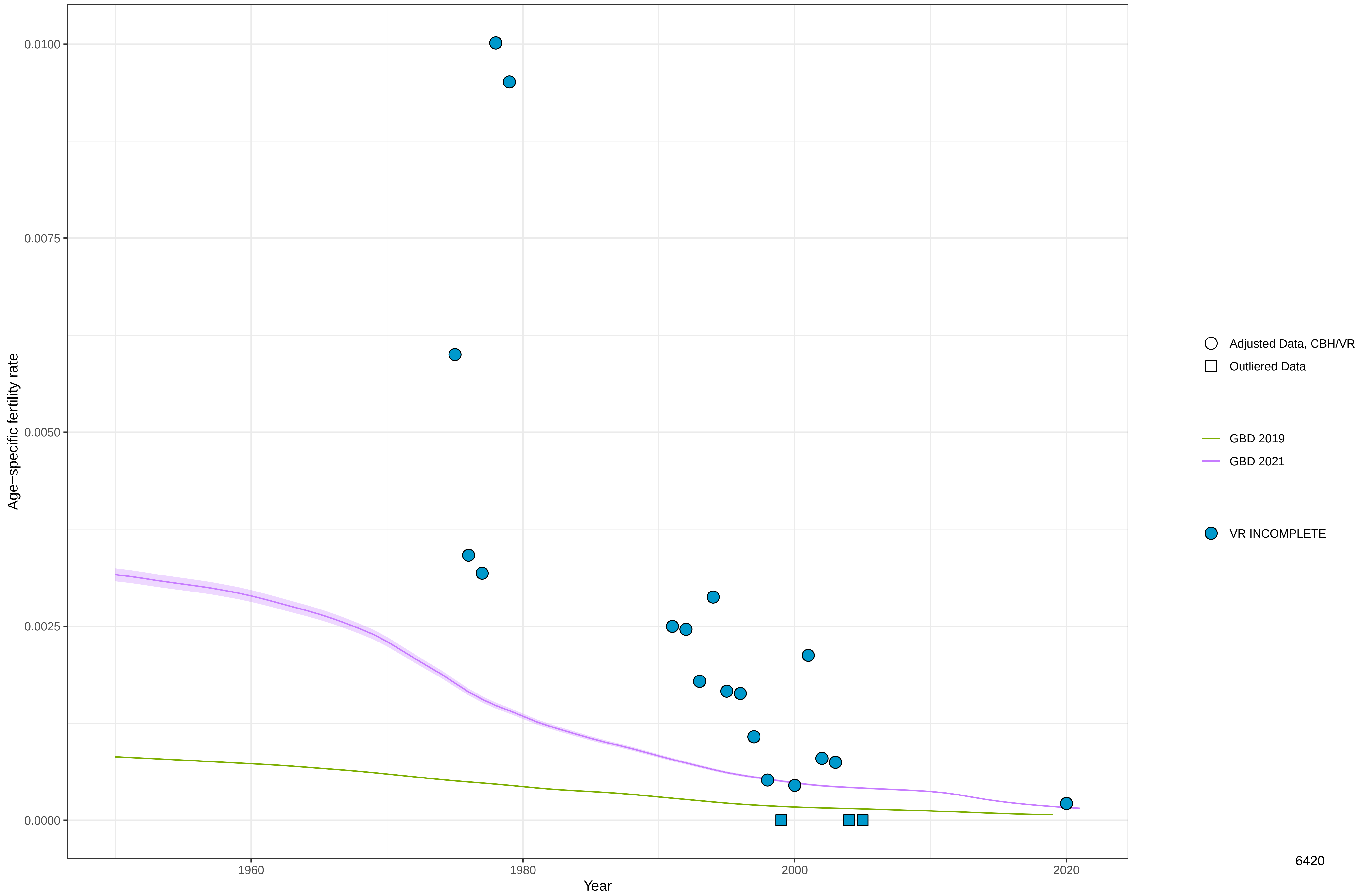

Total fertility rate fitted trend lines, 1950−2021: Quirino TFR
Data points enclosed with a diamond are reference sources

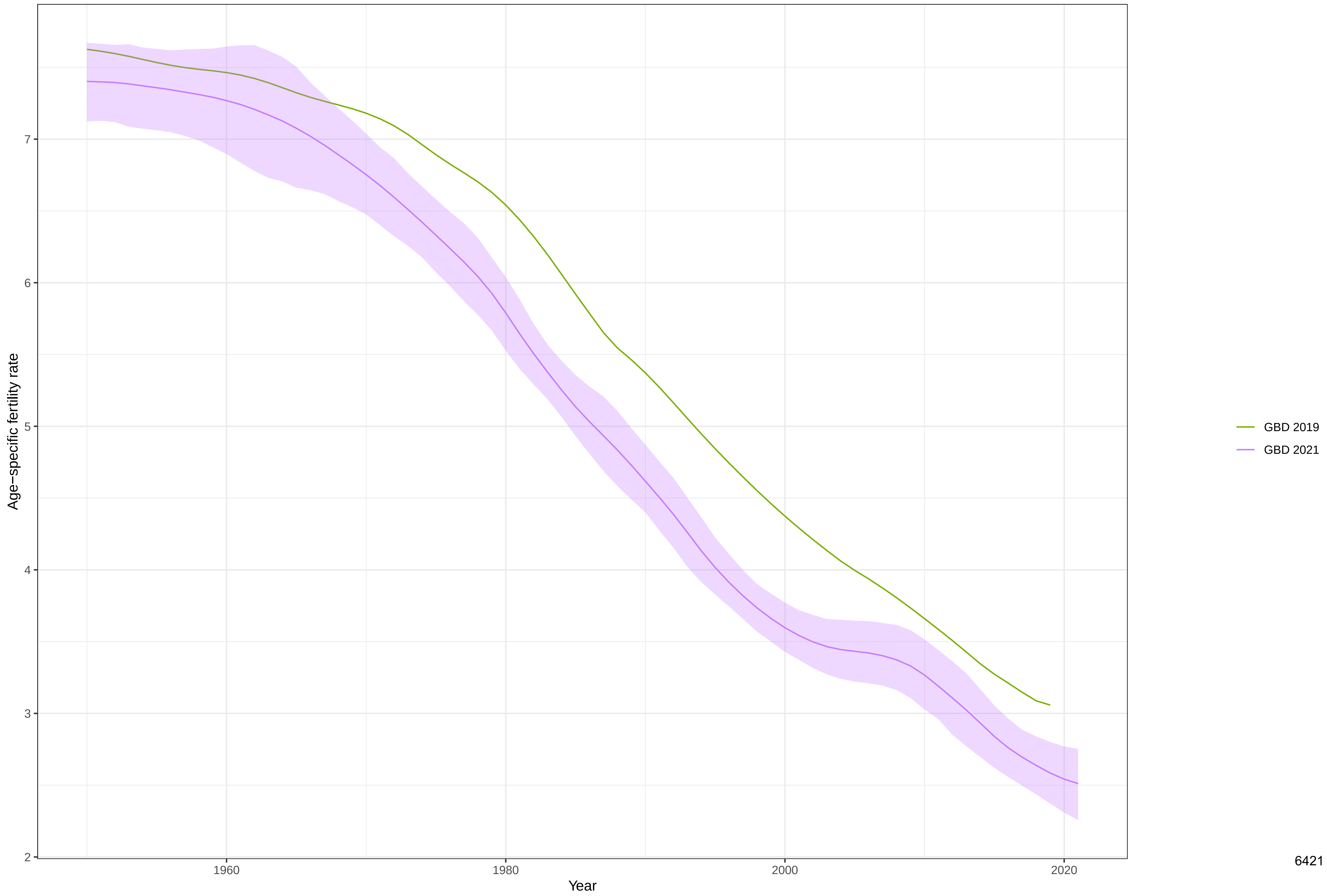

6421

Age−specific fertility fitted trend lines, 1950−2021: Rizal, Ages: 10−14
Data points enclosed with a diamond are reference sources

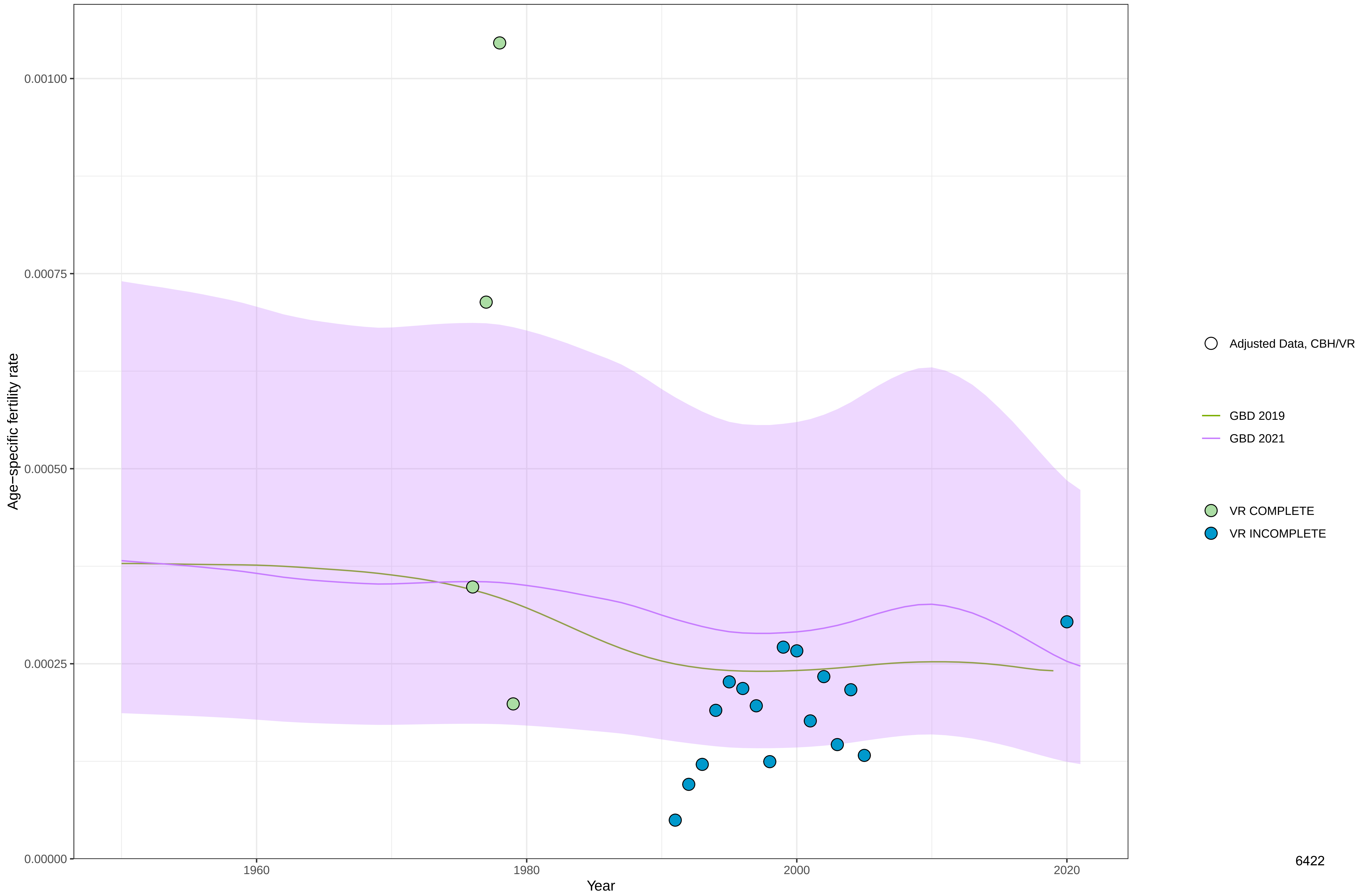

Age−specific fertility fitted trend lines, 1950−2021: Rizal, Ages: 15−19
Data points enclosed with a diamond are reference sources

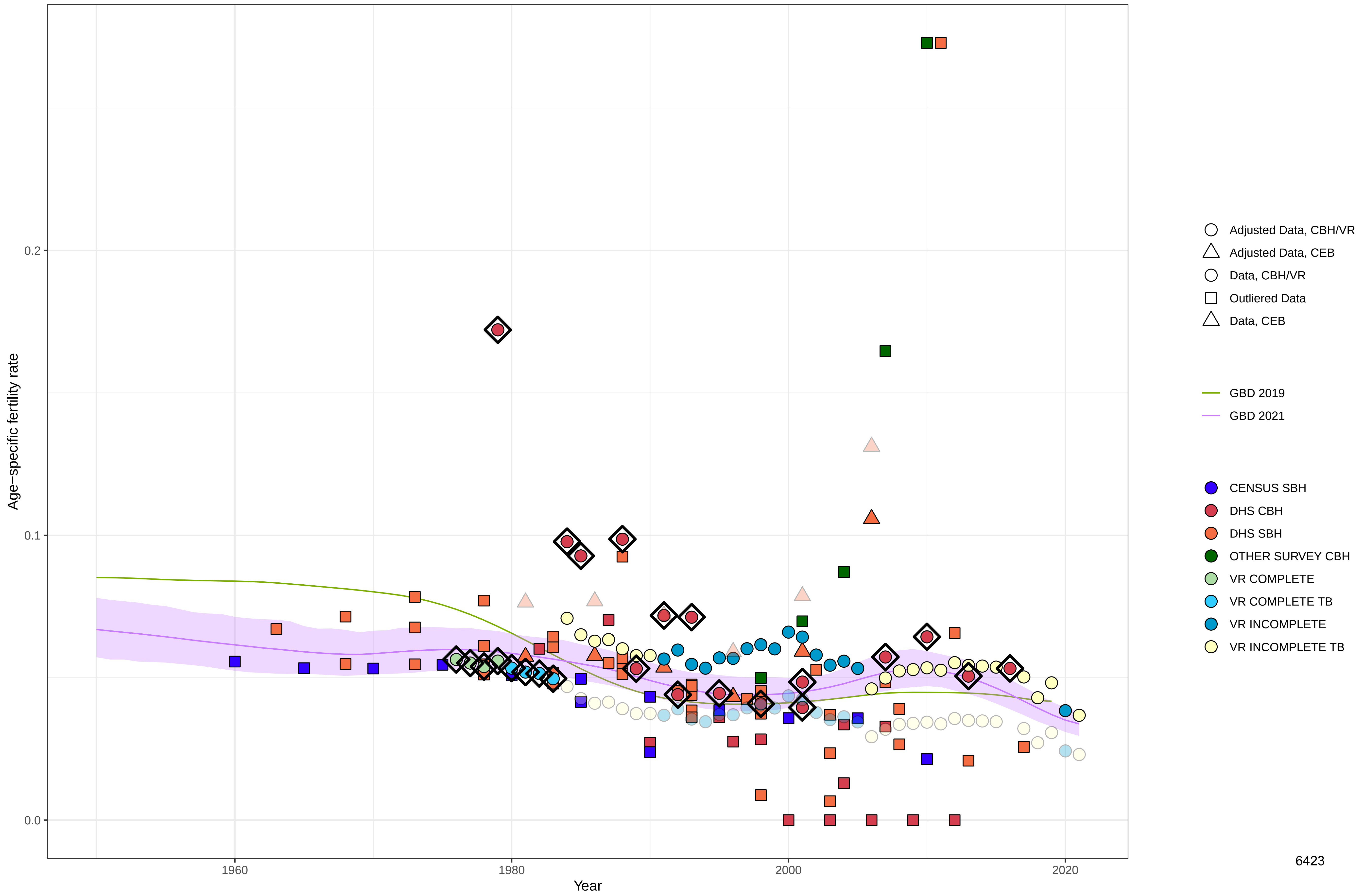

Age−specific fertility fitted trend lines, 1950−2021: Rizal, Ages: 20−24
Data points enclosed with a diamond are reference sources

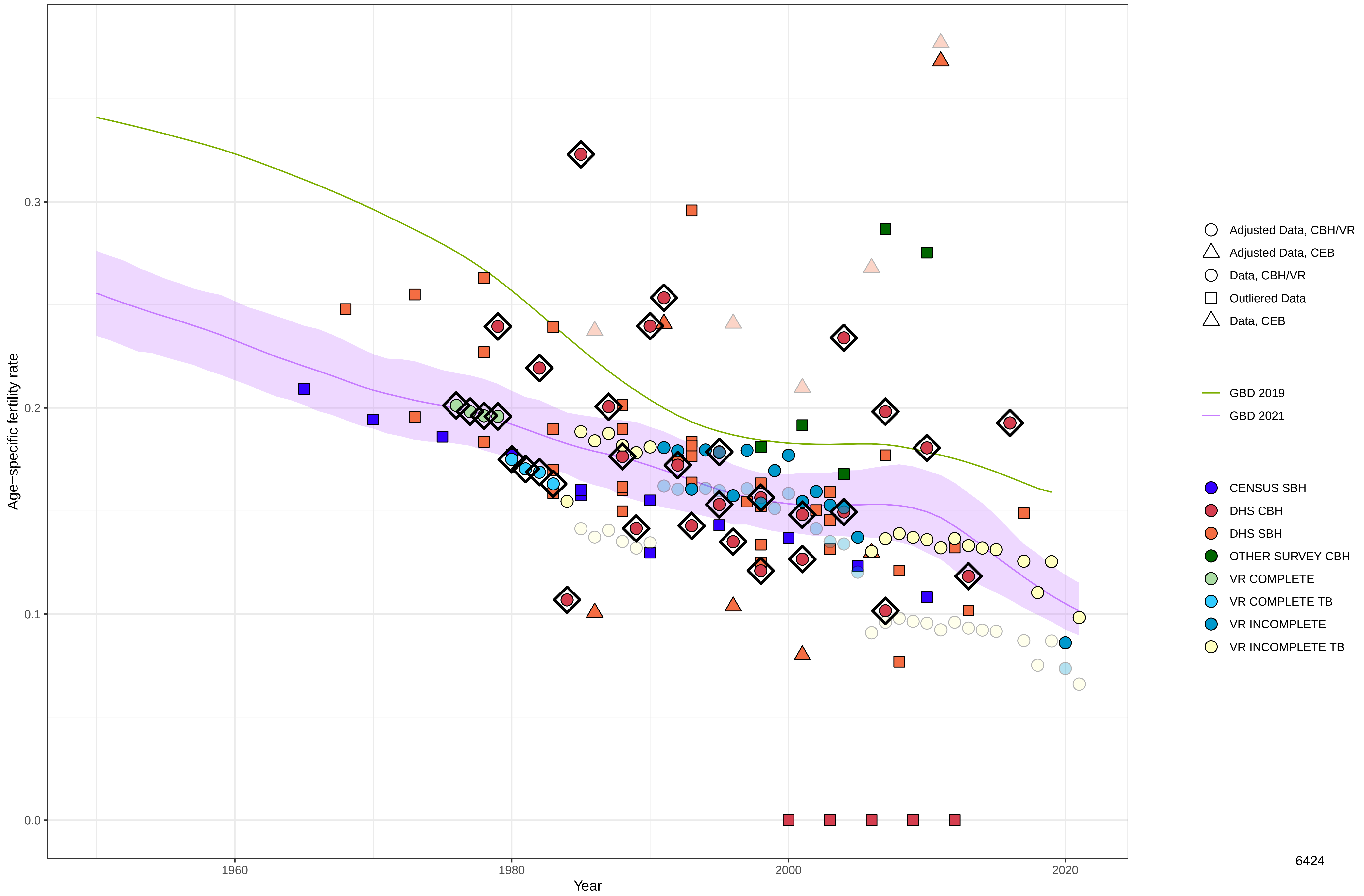

Age−specific fertility fitted trend lines, 1950−2021: Rizal, Ages: 25−29
Data points enclosed with a diamond are reference sources

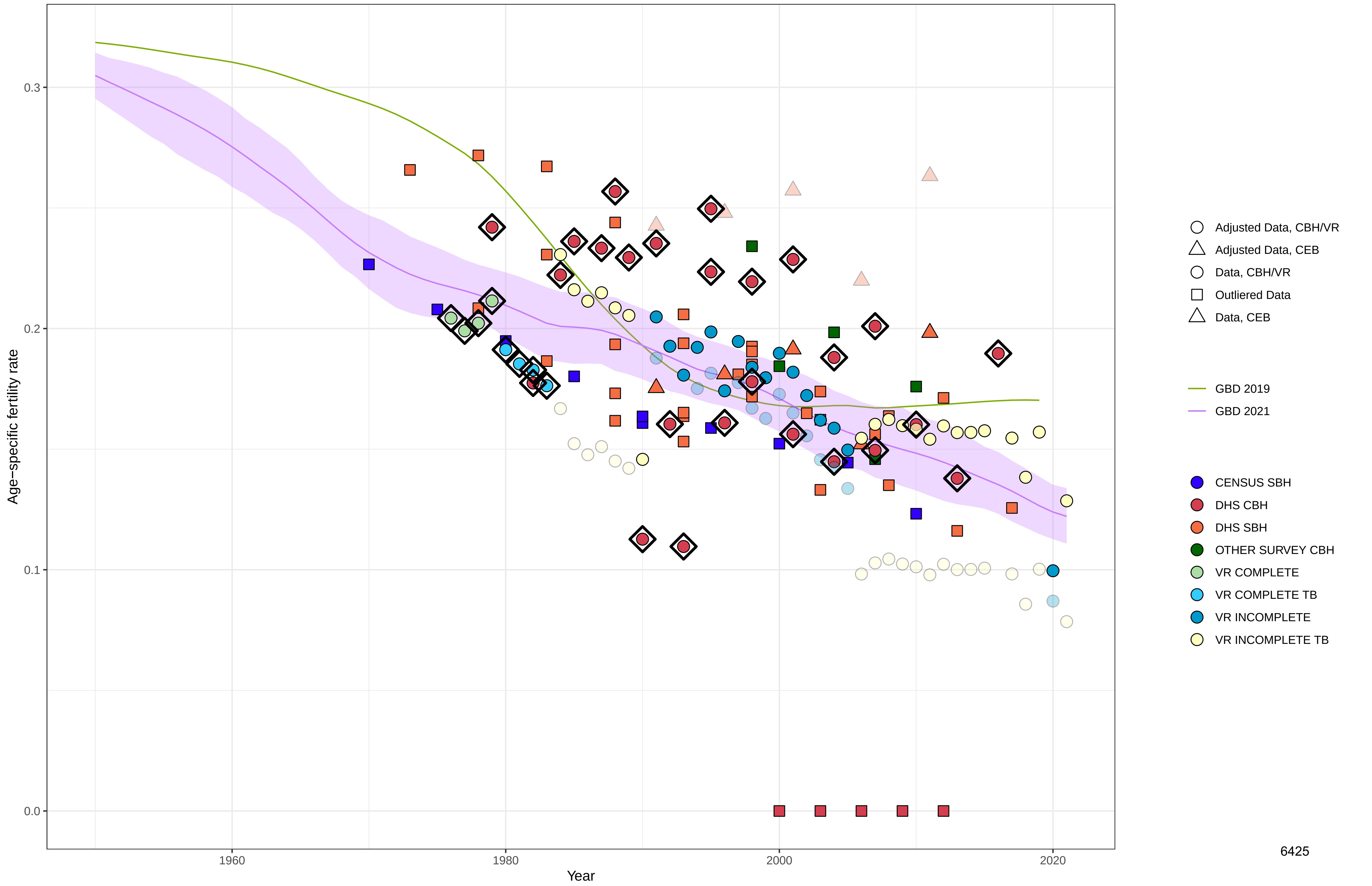

Age−specific fertility fitted trend lines, 1950−2021: Rizal, Ages: 30−34
Data points enclosed with a diamond are reference sources

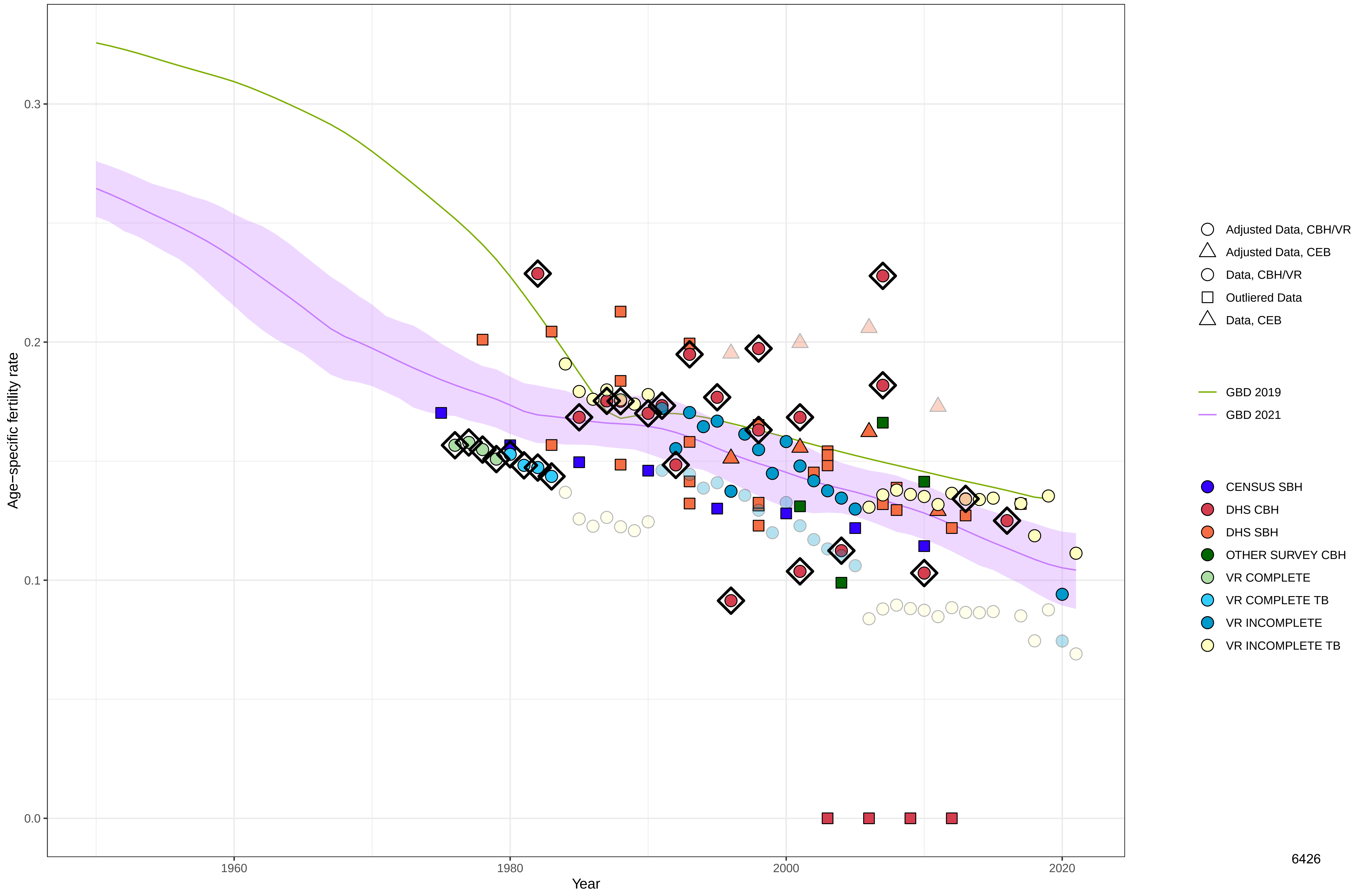

Age−specific fertility fitted trend lines, 1950−2021: Rizal, Ages: 35−39
Data points enclosed with a diamond are reference sources

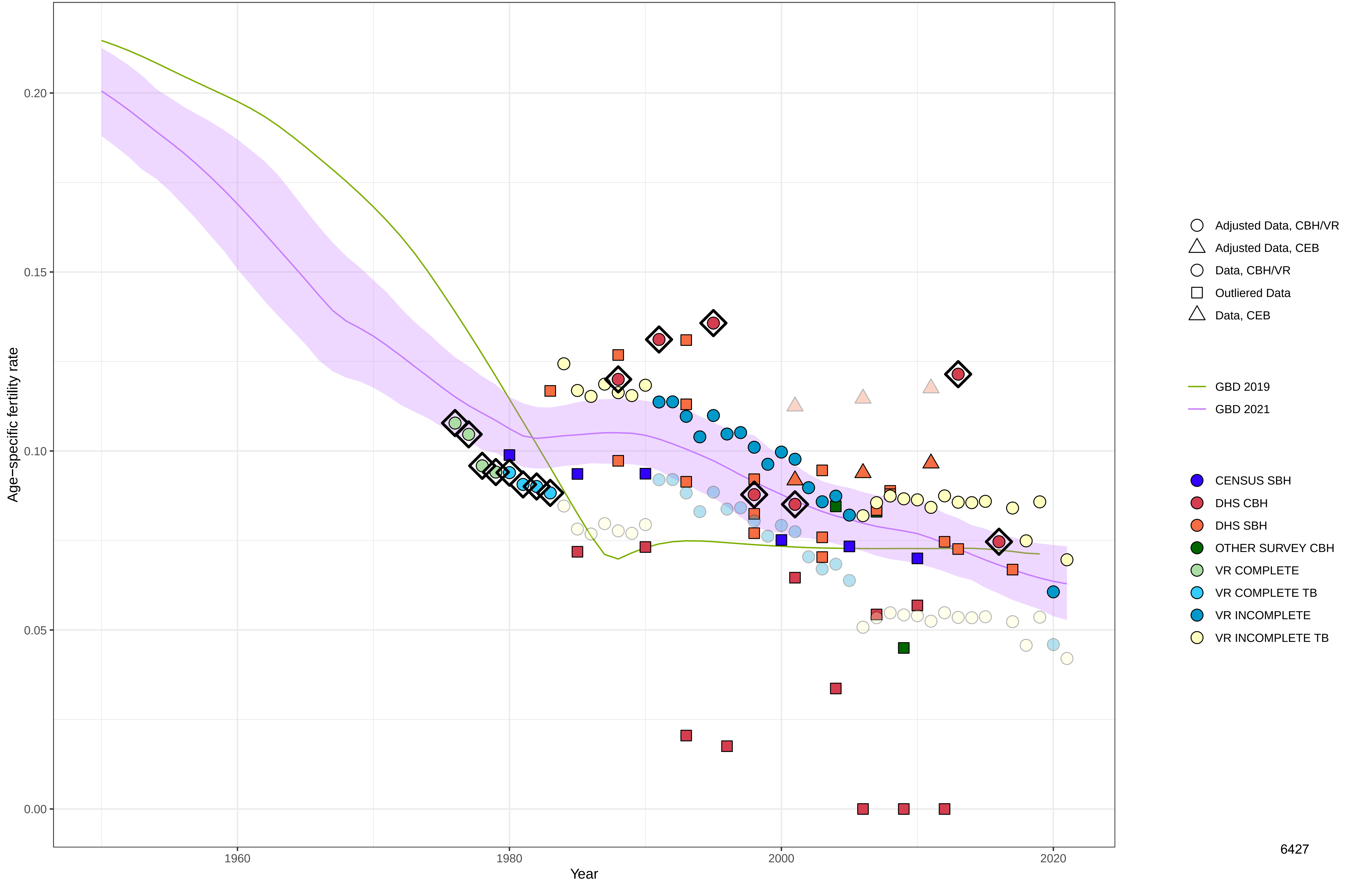

Age−specific fertility fitted trend lines, 1950−2021: Rizal, Ages: 40−44
Data points enclosed with a diamond are reference sources

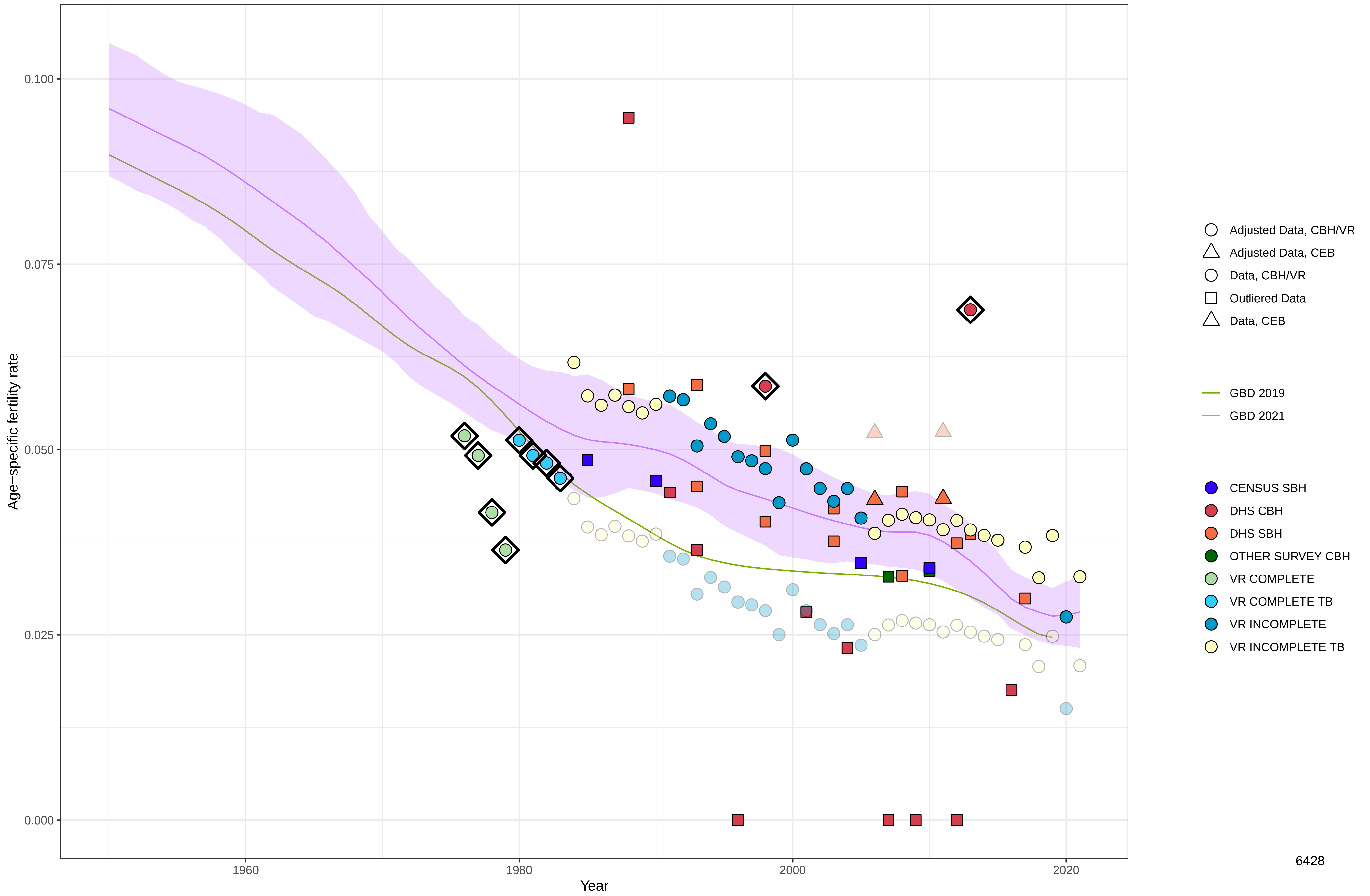

Age−specific fertility fitted trend lines, 1950−2021: Rizal, Ages: 45−49
Data points enclosed with a diamond are reference sources

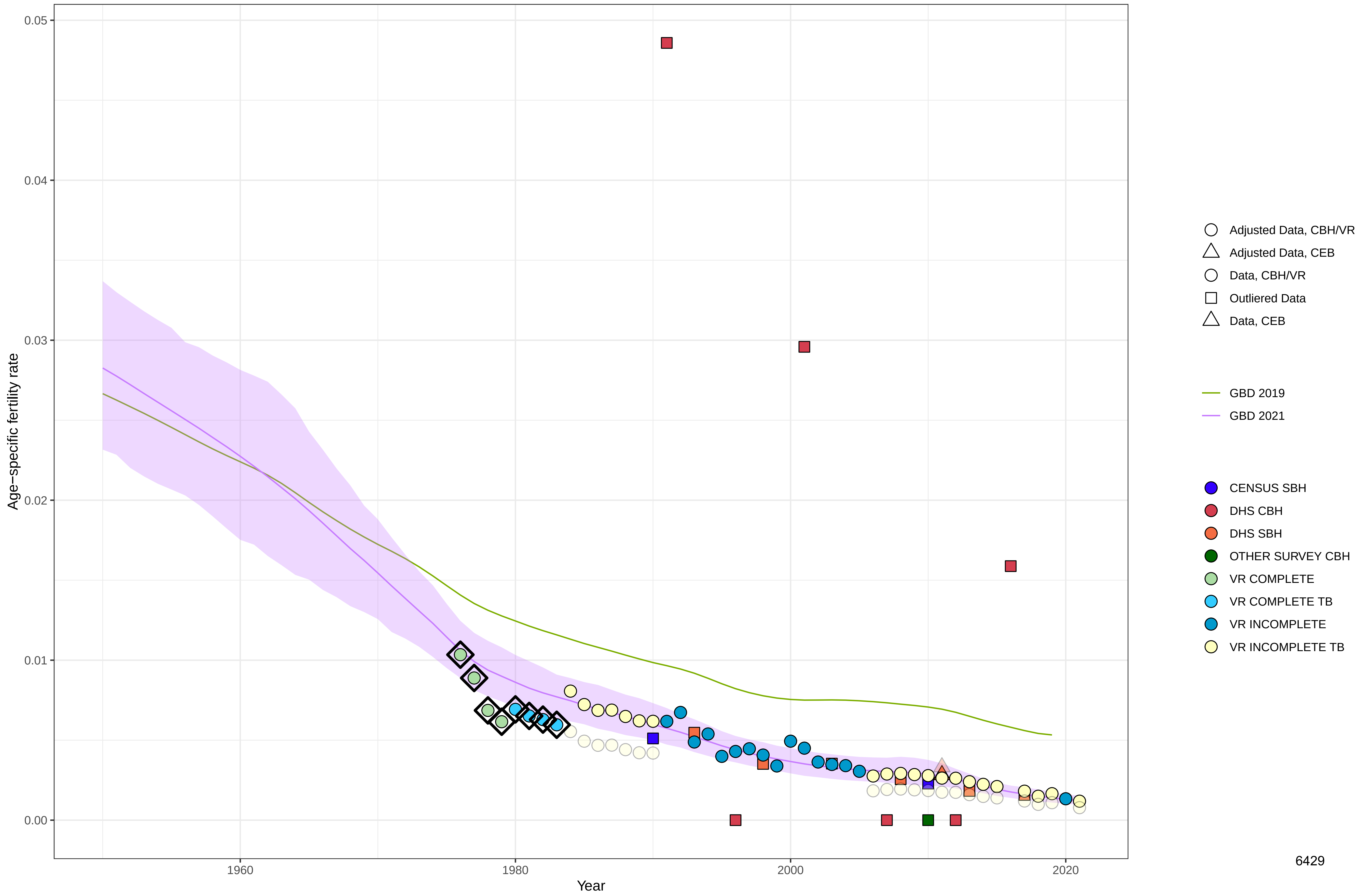

Age−specific fertility fitted trend lines, 1950−2021: Rizal, Ages: 50−54
Data points enclosed with a diamond are reference sources

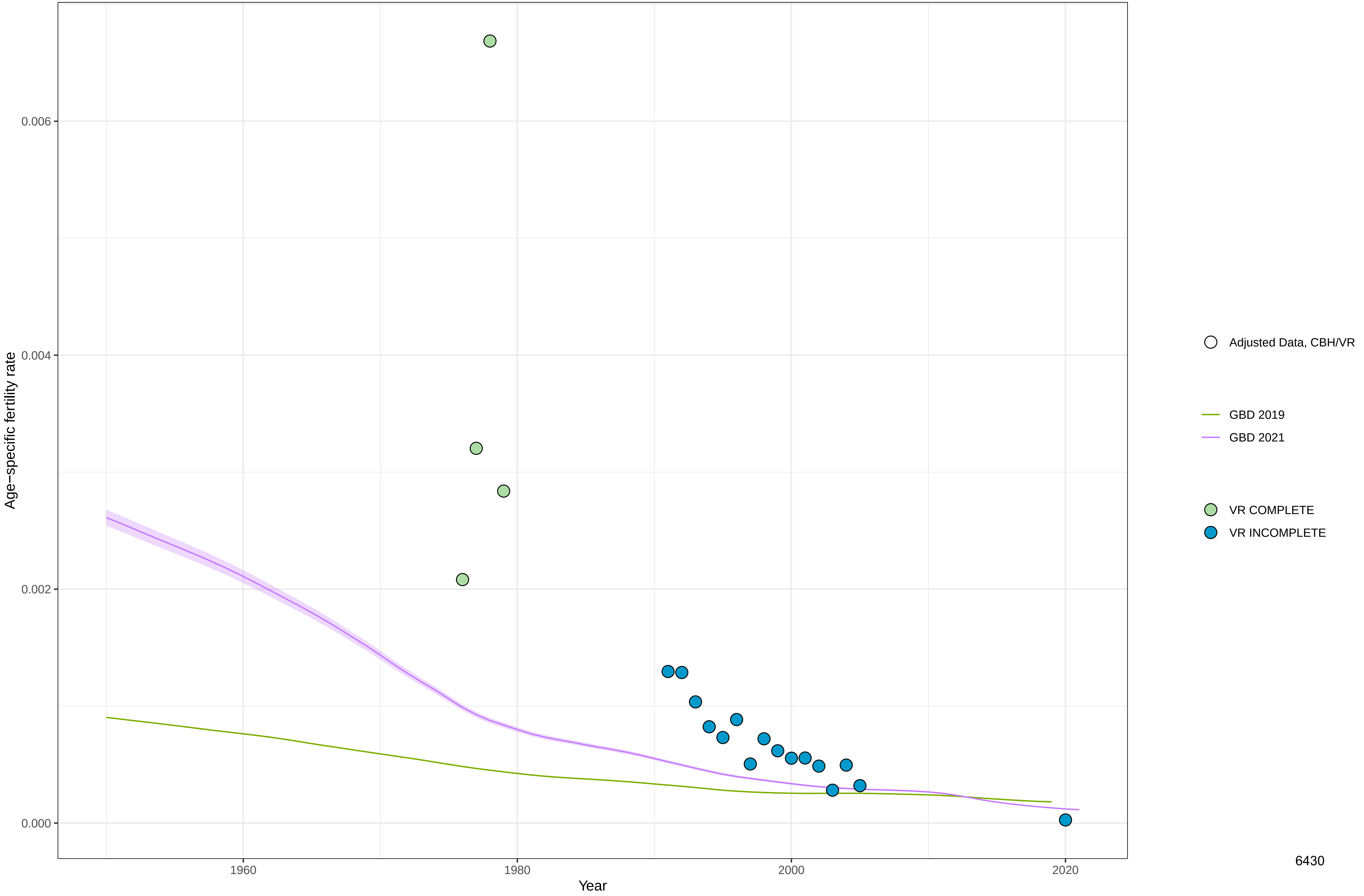

Total fertility rate fitted trend lines, 1950−2021: Rizal TFR
Data points enclosed with a diamond are reference sources

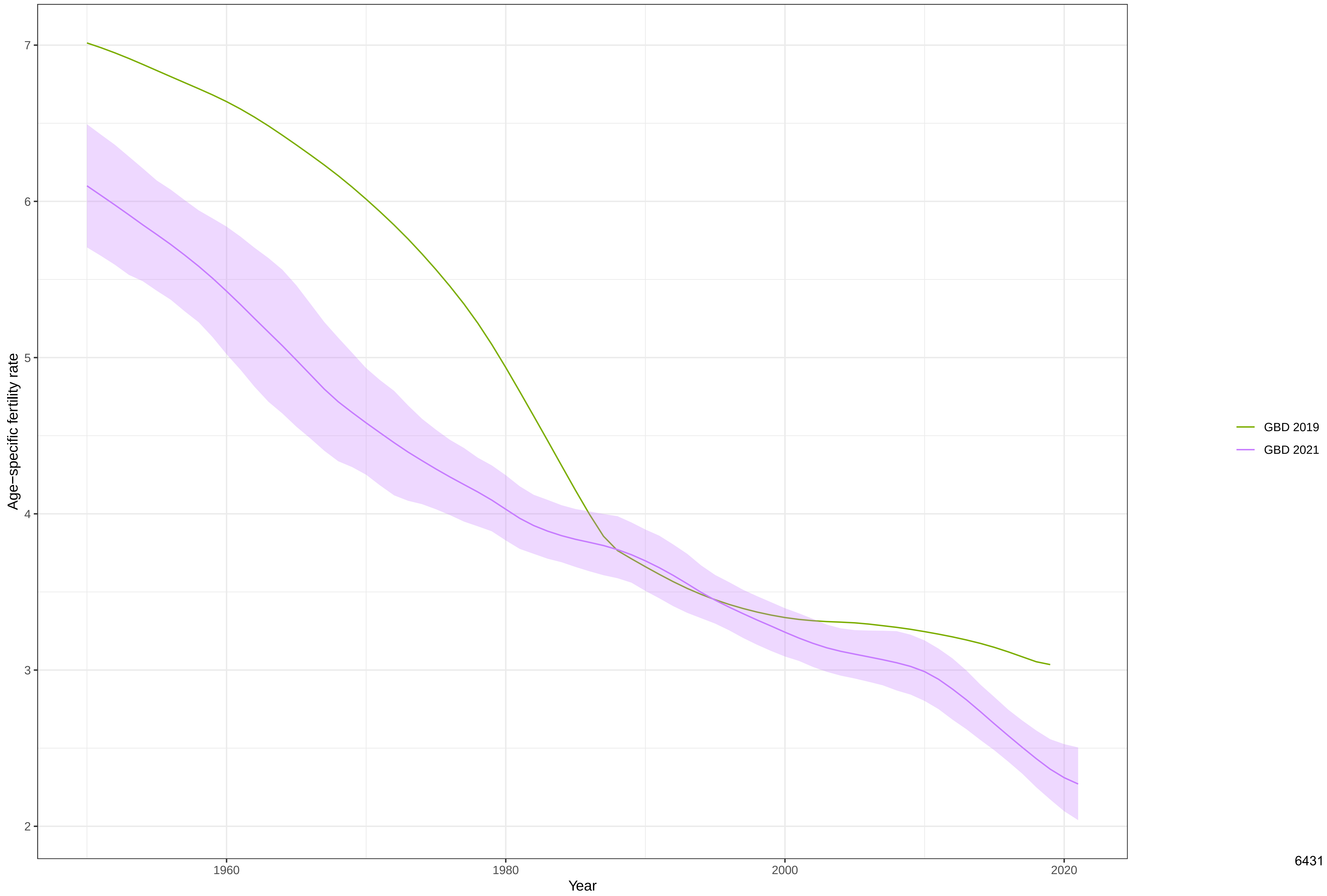

Age−specific fertility fitted trend lines, 1950−2021: Romblon, Ages: 10−14
Data points enclosed with a diamond are reference sources

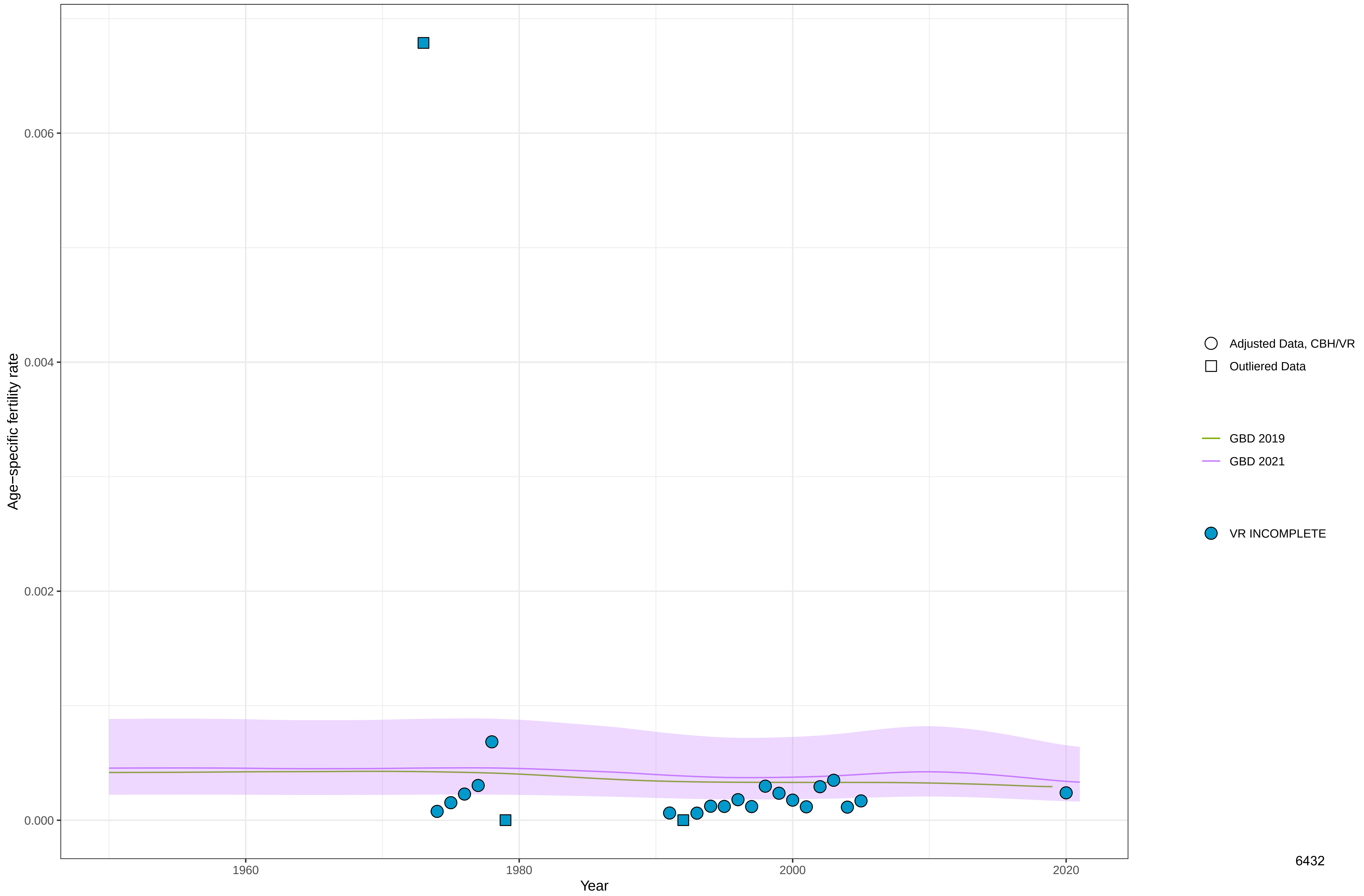

Age−specific fertility fitted trend lines, 1950−2021: Romblon, Ages: 15−19
Data points enclosed with a diamond are reference sources

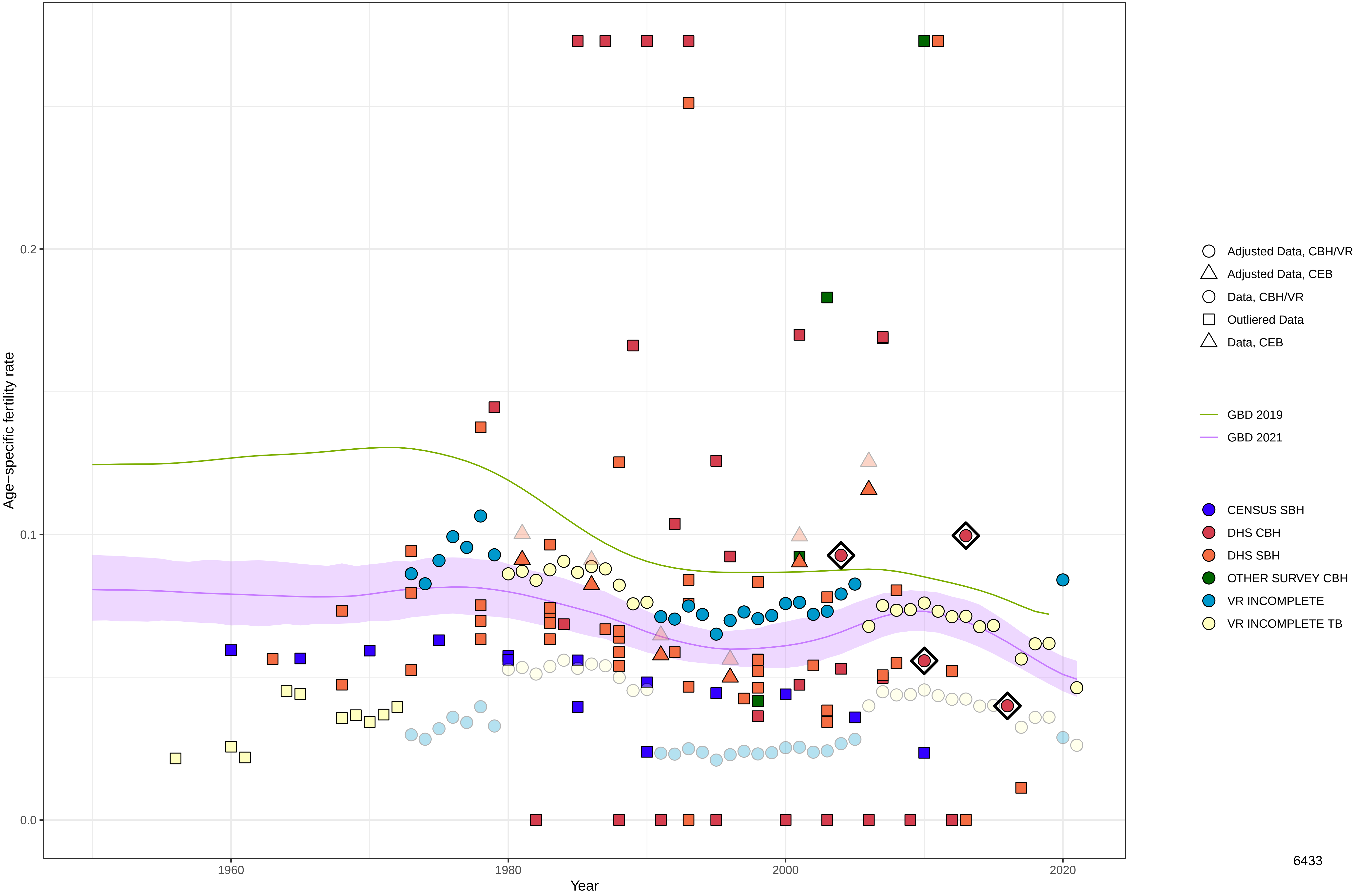

Age−specific fertility fitted trend lines, 1950−2021: Romblon, Ages: 20−24
Data points enclosed with a diamond are reference sources

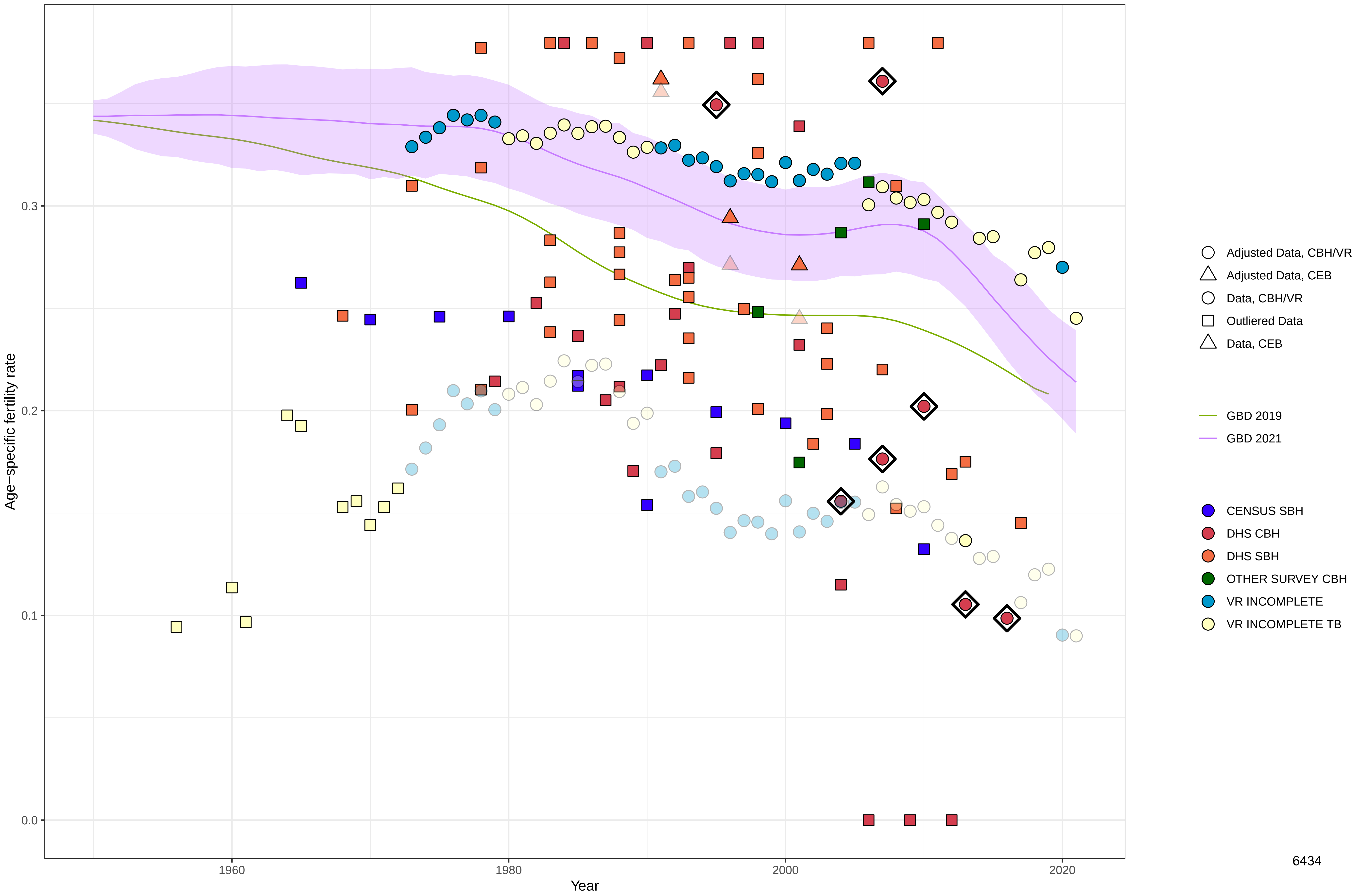

Age−specific fertility fitted trend lines, 1950−2021: Romblon, Ages: 25−29
Data points enclosed with a diamond are reference sources

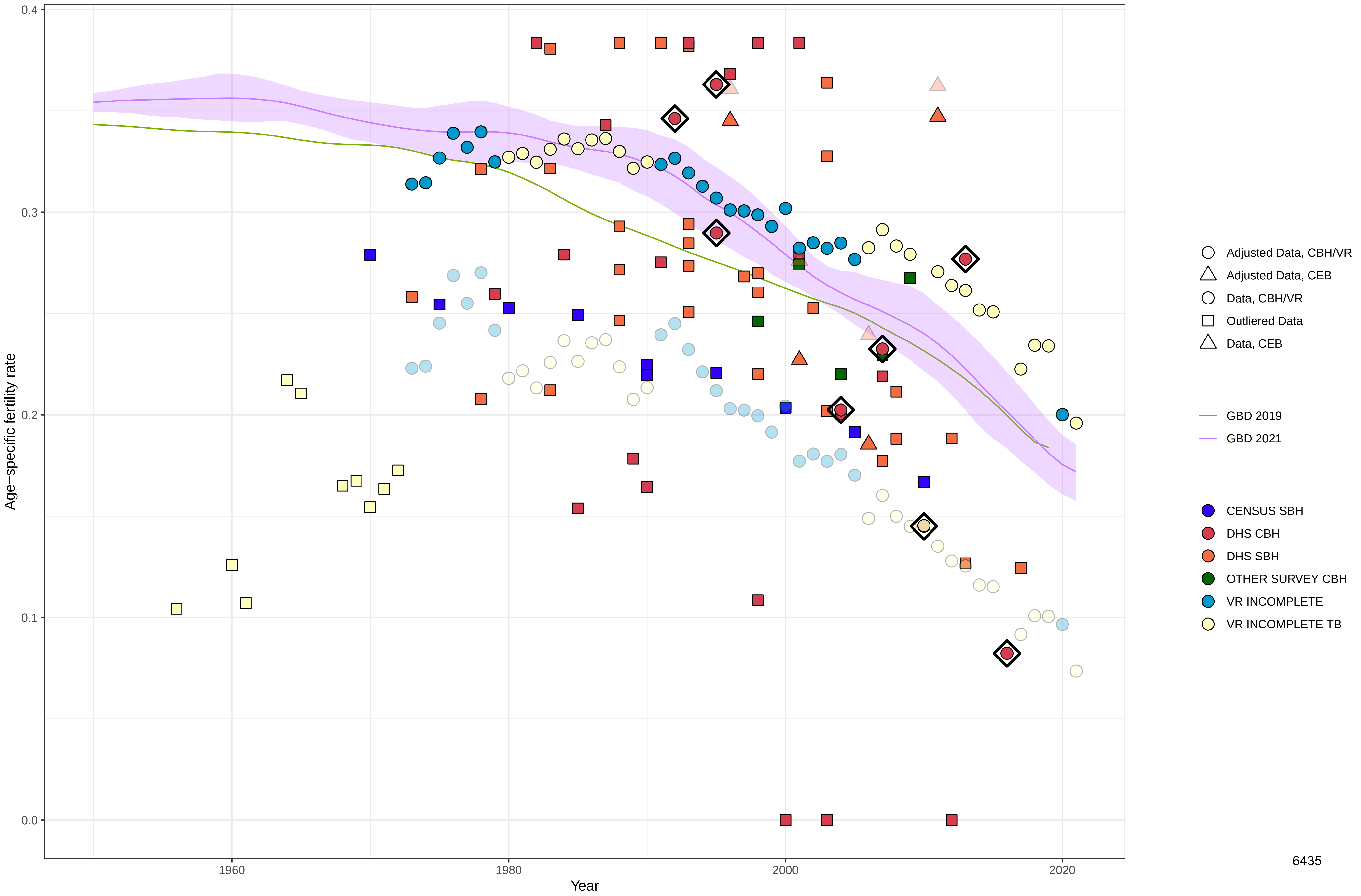

Age−specific fertility fitted trend lines, 1950−2021: Romblon, Ages: 30−34
Data points enclosed with a diamond are reference sources

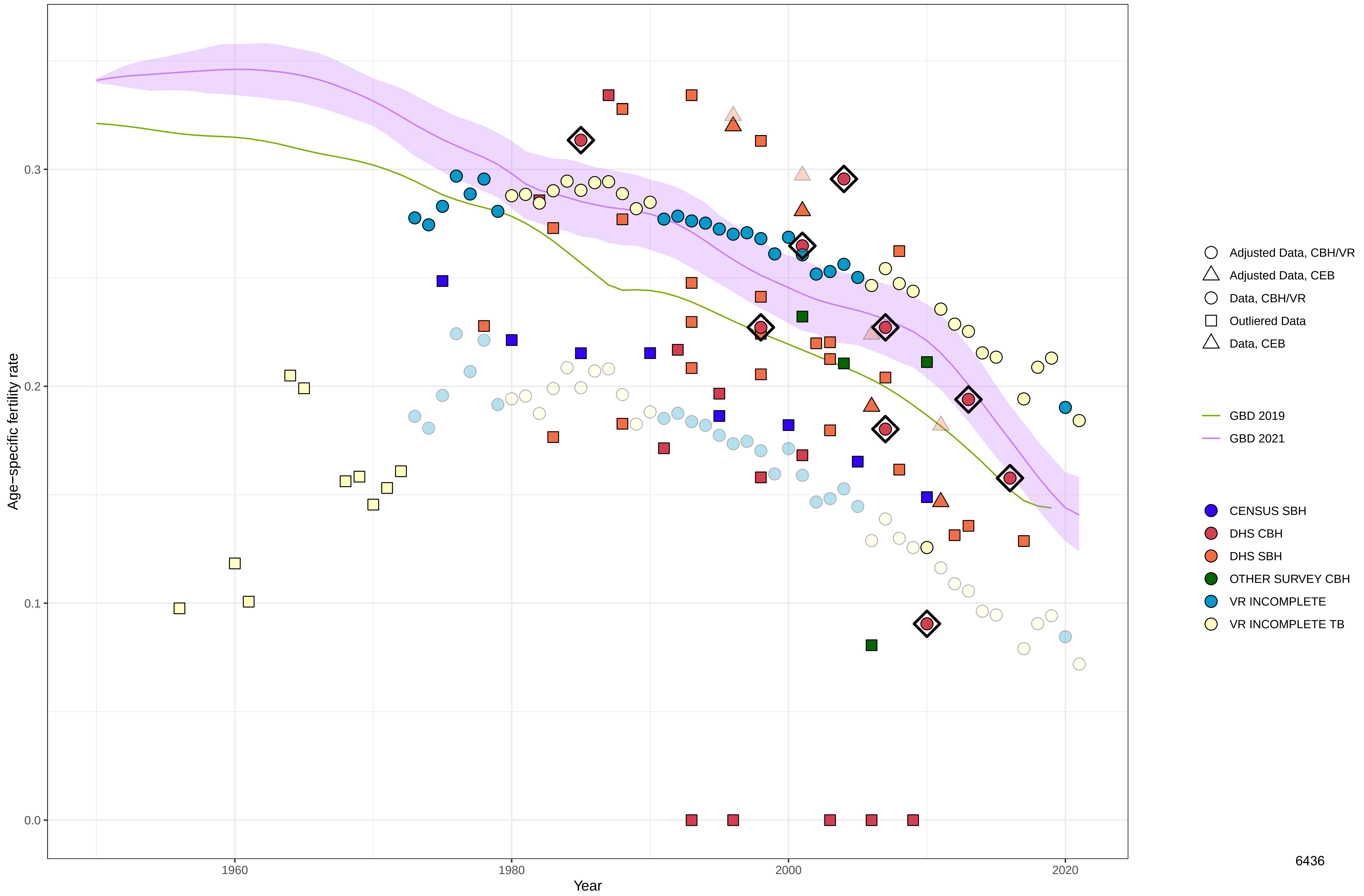

Age−specific fertility fitted trend lines, 1950−2021: Romblon, Ages: 35−39
Data points enclosed with a diamond are reference sources

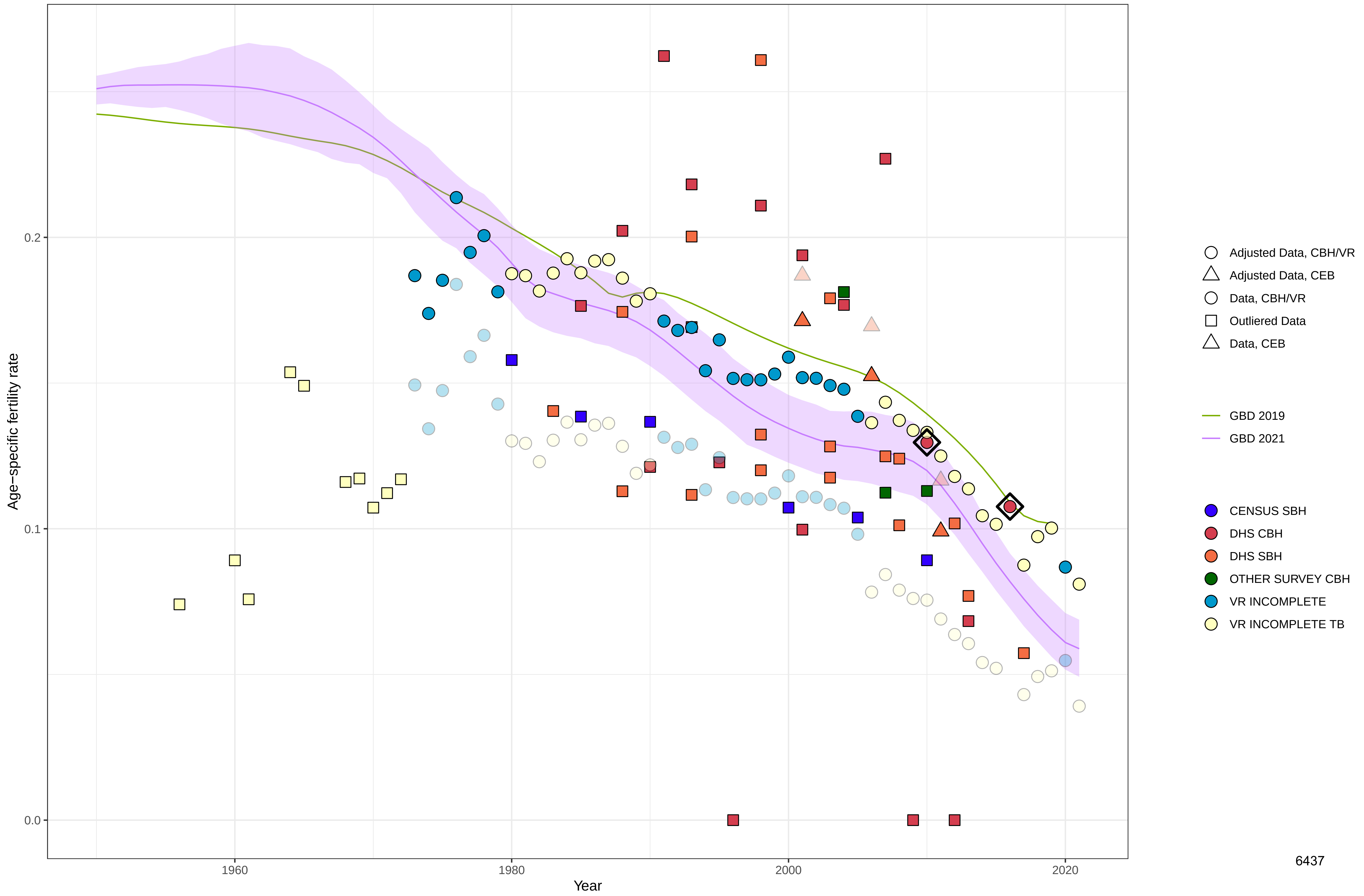

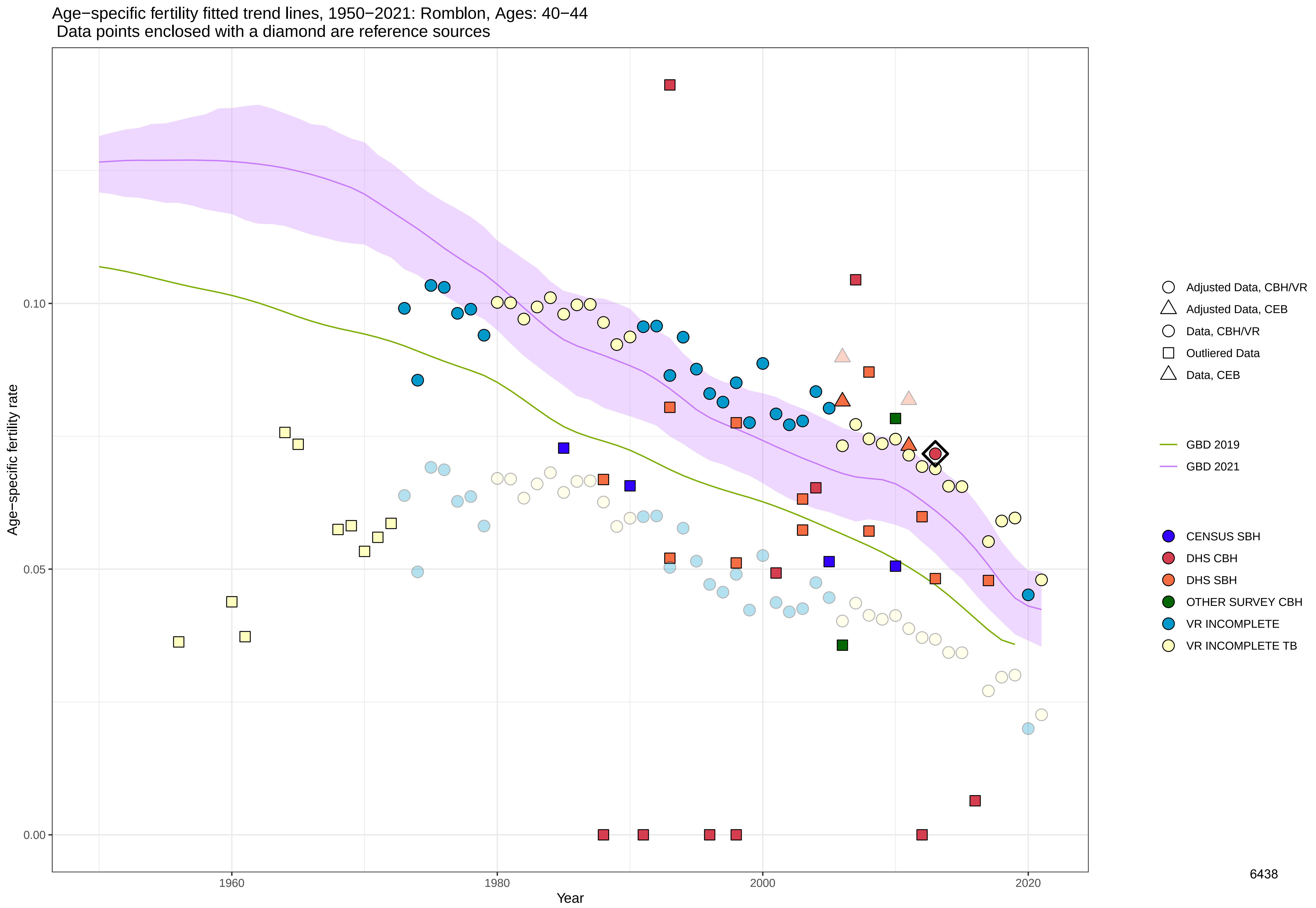
Age−specific fertility fitted trend lines, 1950−2021: Romblon, Ages: 40−44
Data points enclosed with a diamond are reference sources
Age−specific fertility rate
0.00
0.05
0.10
Year
1960
1980
2000
2020
Adjusted Data, CBH/VR
Adjusted Data, CEB
Data, CBH/VR
Outliered Data
Data, CEB
GBD 2019
GBD 2021
CENSUS SBH
DHS CBH
DHS SBH
OTHER SURVEY CBH
VR INCOMPLETE
VR INCOMPLETE TB

6438

Age−specific fertility fitted trend lines, 1950−2021: Romblon, Ages: 45−49
Data points enclosed with a diamond are reference sources

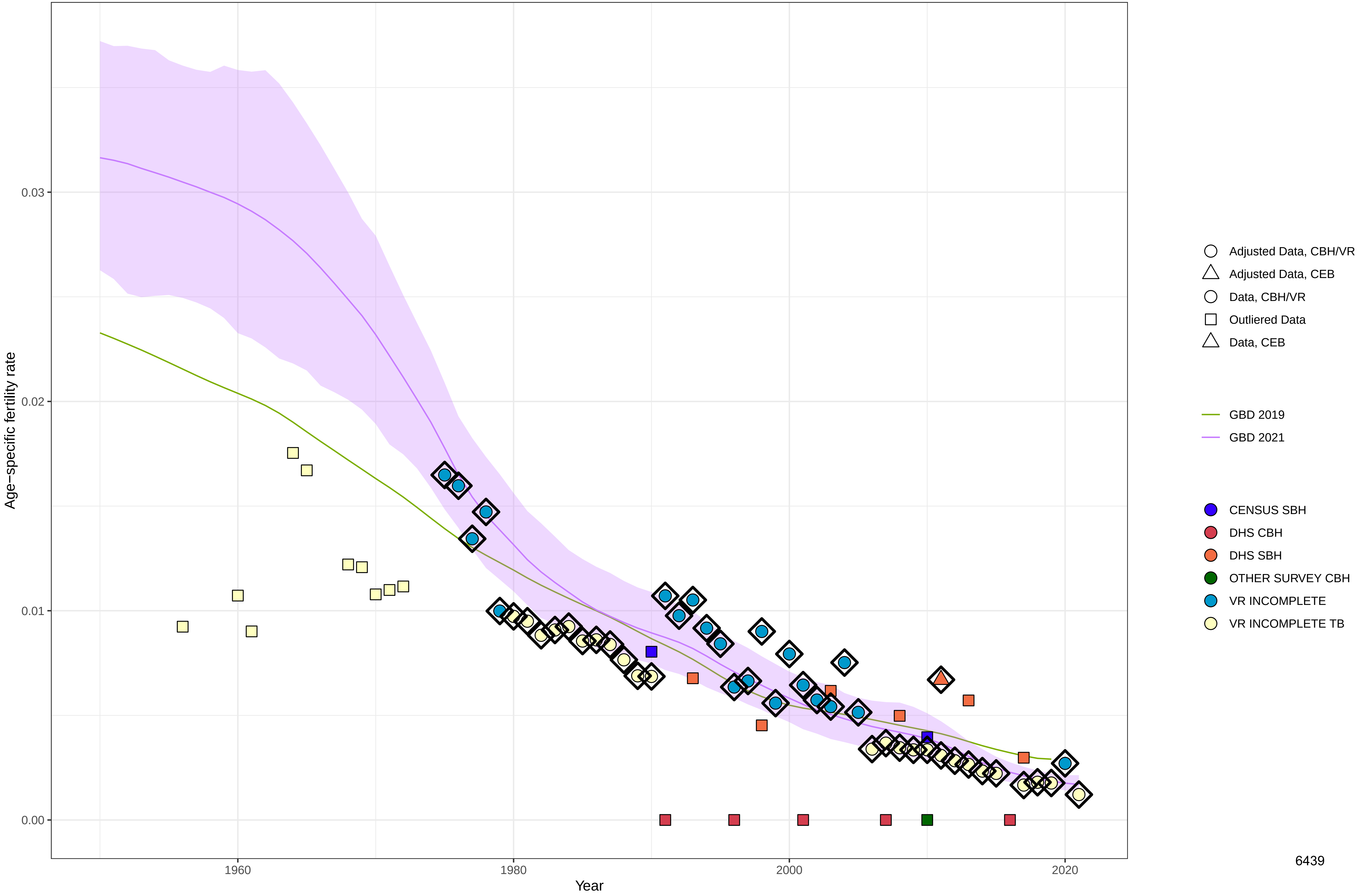

Age−specific fertility fitted trend lines, 1950−2021: Romblon, Ages: 50−54
Data points enclosed with a diamond are reference sources

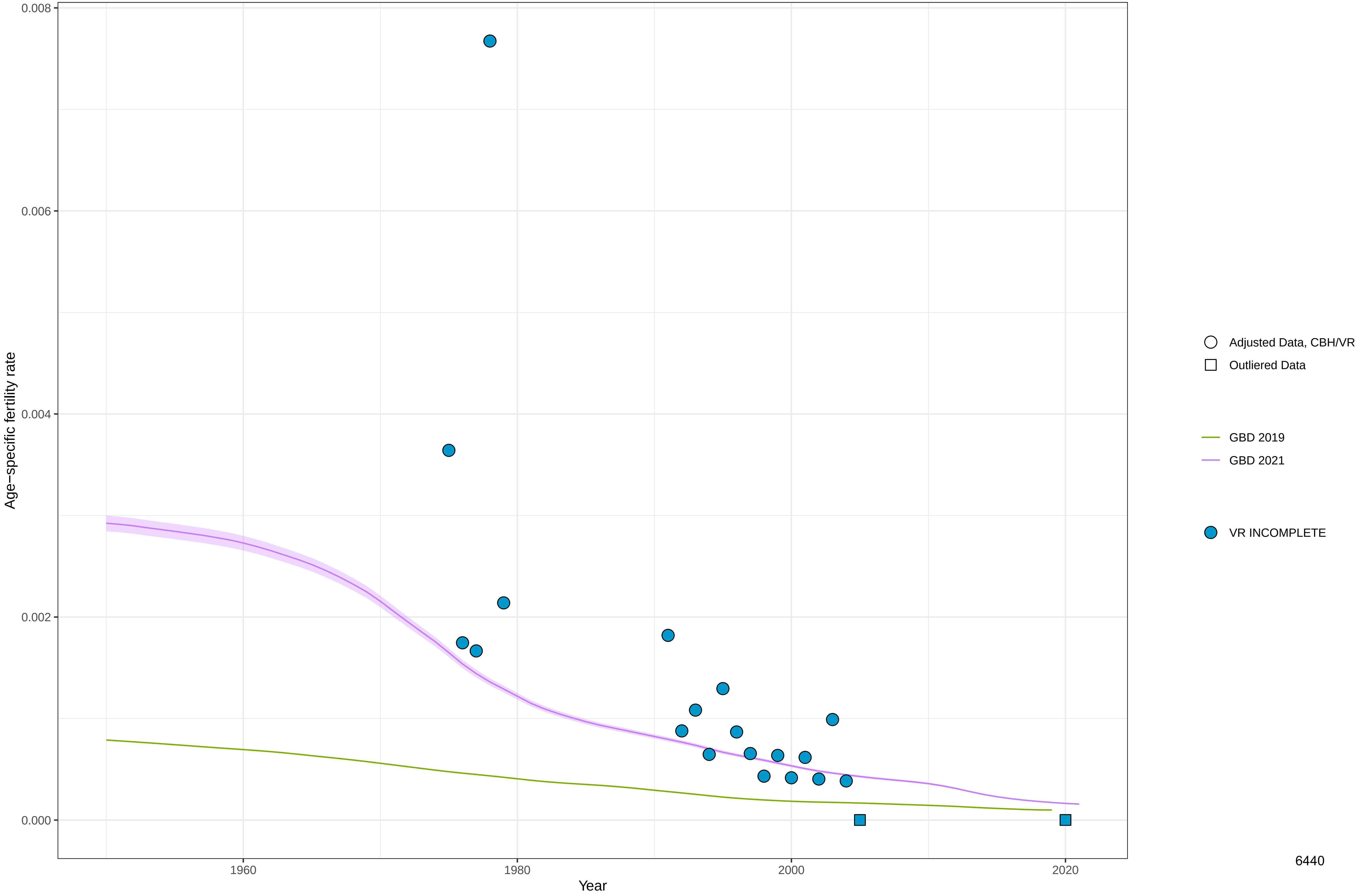

Total fertility rate fitted trend lines, 1950−2021: Romblon TFR
Data points enclosed with a diamond are reference sources

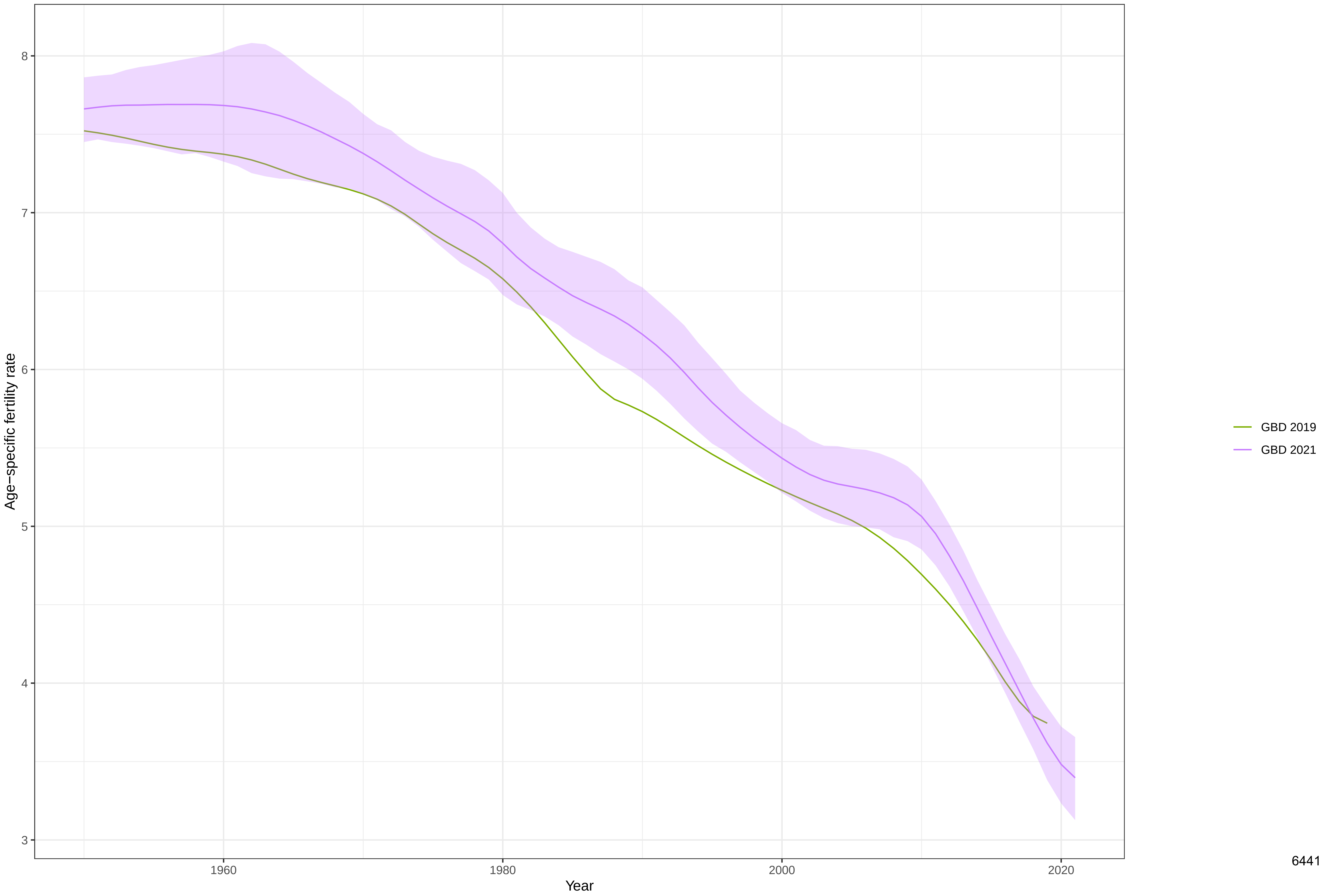

Age−specific fertility fitted trend lines, 1950−2021: Samar (Western Samar), Ages: 10−14
Data points enclosed with a diamond are reference sources

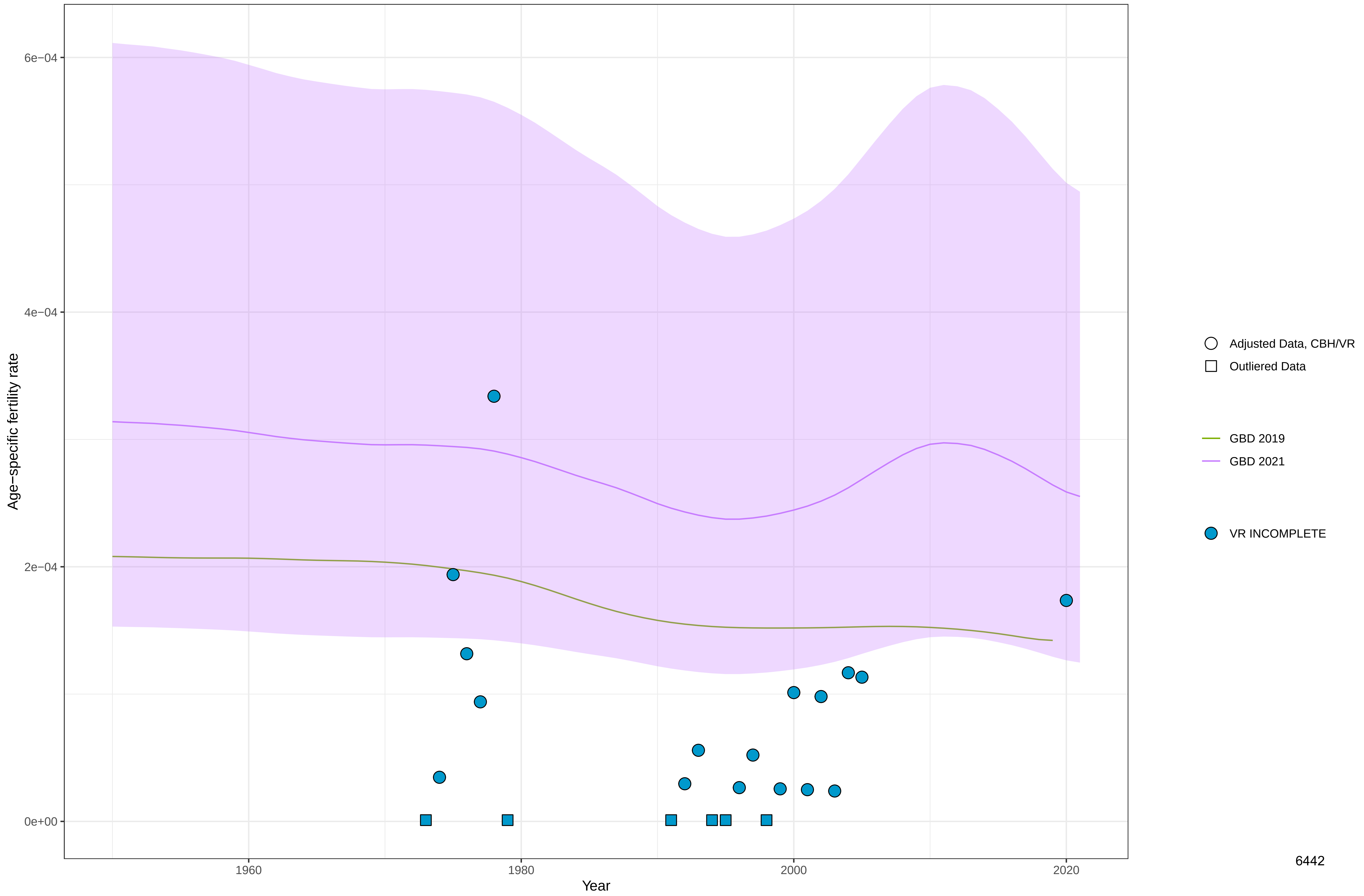

Age−specific fertility fitted trend lines, 1950−2021: Samar (Western Samar), Ages: 15−19
Data points enclosed with a diamond are reference sources

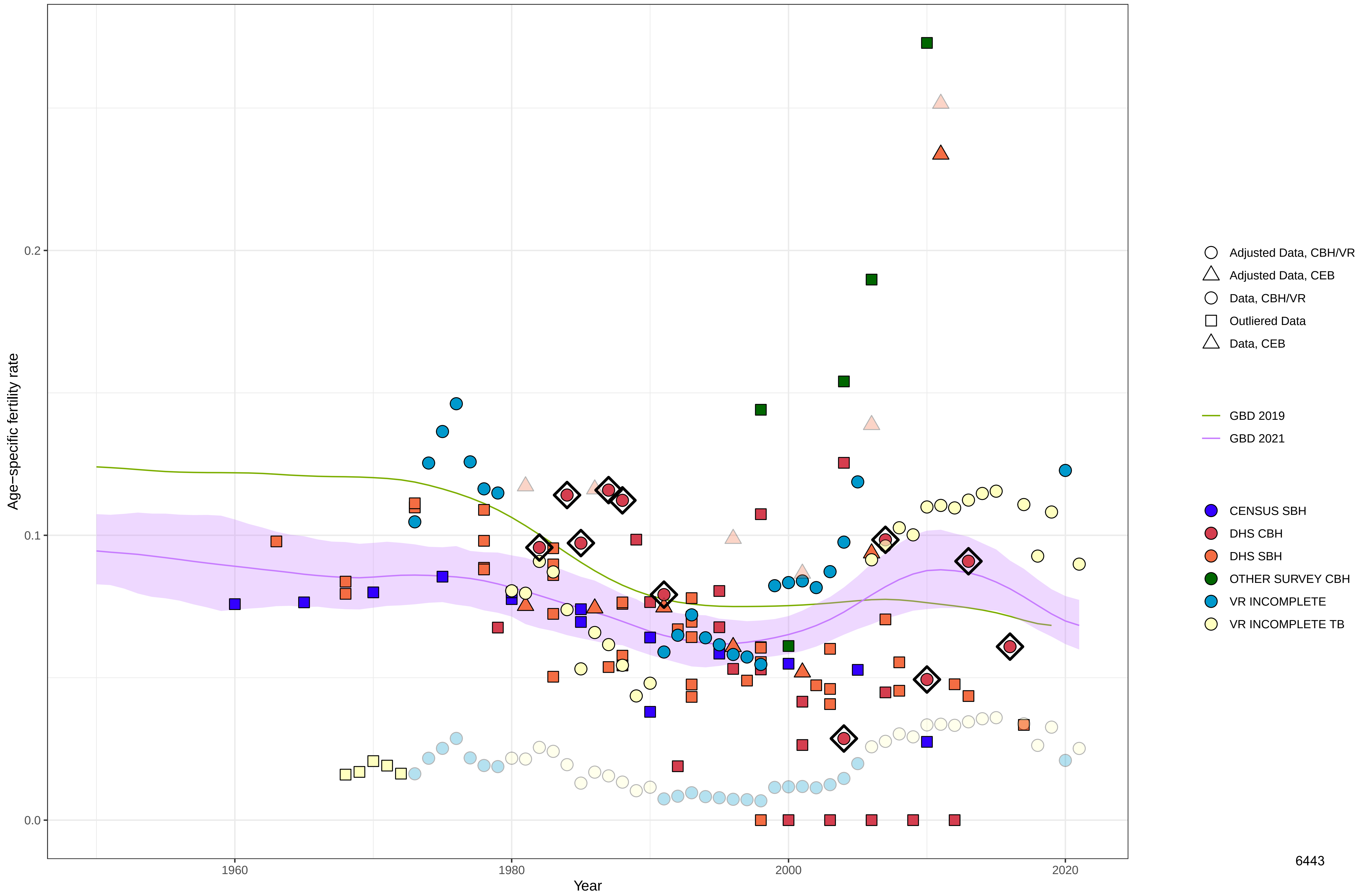

Age−specific fertility fitted trend lines, 1950−2021: Samar (Western Samar), Ages: 20−24
Data points enclosed with a diamond are reference sources

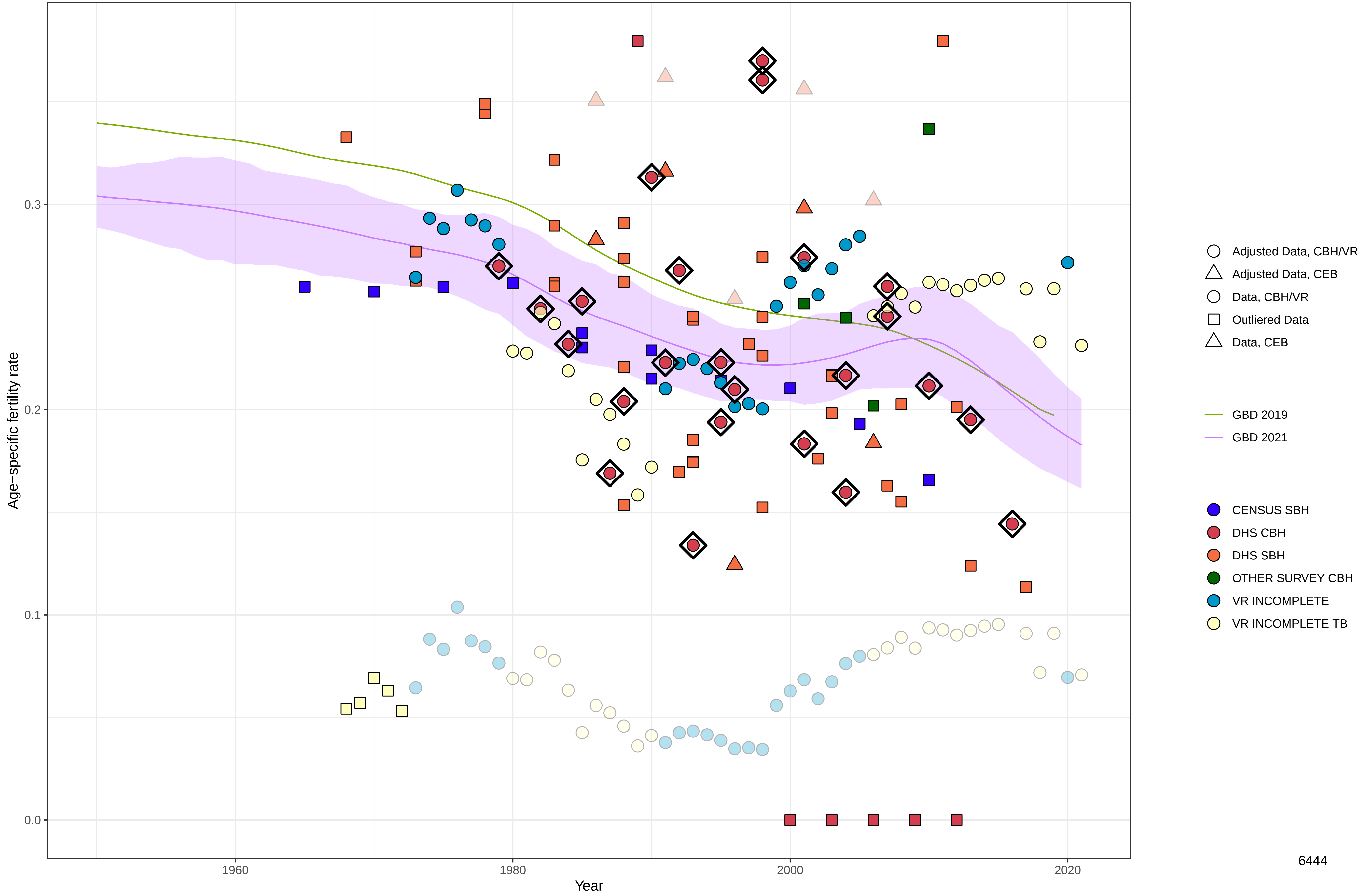

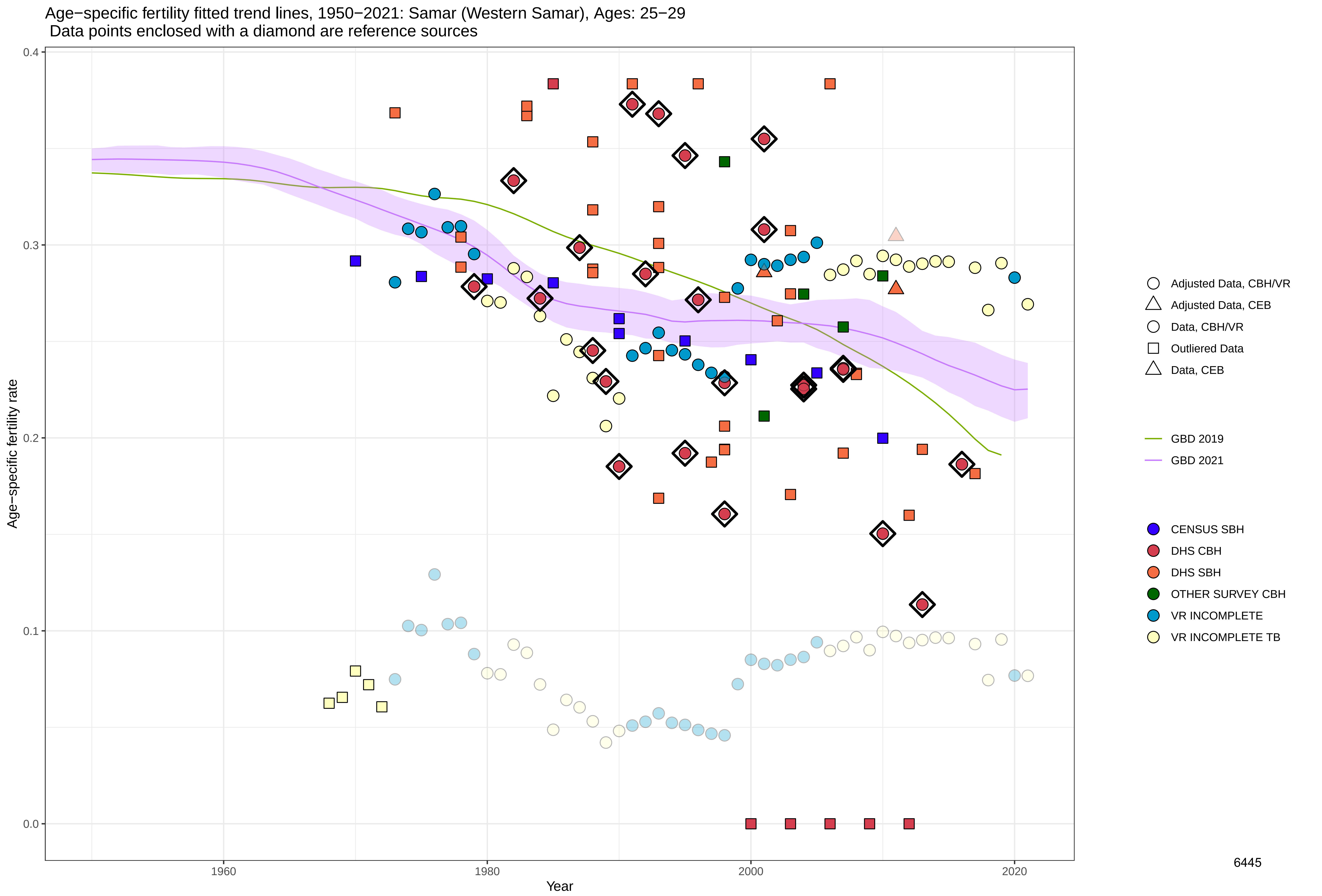

Age−specific fertility fitted trend lines, 1950−2021: Samar (Western Samar), Ages: 25−29
Data points enclosed with a diamond are reference sources
Age−specific fertility rate
Year
0.0
0.1
0.2
0.3
0.4
1960
1980
2000
2020
Adjusted Data, CBH/VR
Adjusted Data, CEB
Data, CBH/VR
Outliered Data
Data, CEB
GBD 2019
GBD 2021
CENSUS SBH
DHS CBH
DHS SBH
OTHER SURVEY CBH
VR INCOMPLETE
VR INCOMPLETE TB

Age−specific fertility fitted trend lines, 1950−2021: Samar (Western Samar), Ages: 30−34
Data points enclosed with a diamond are reference sources

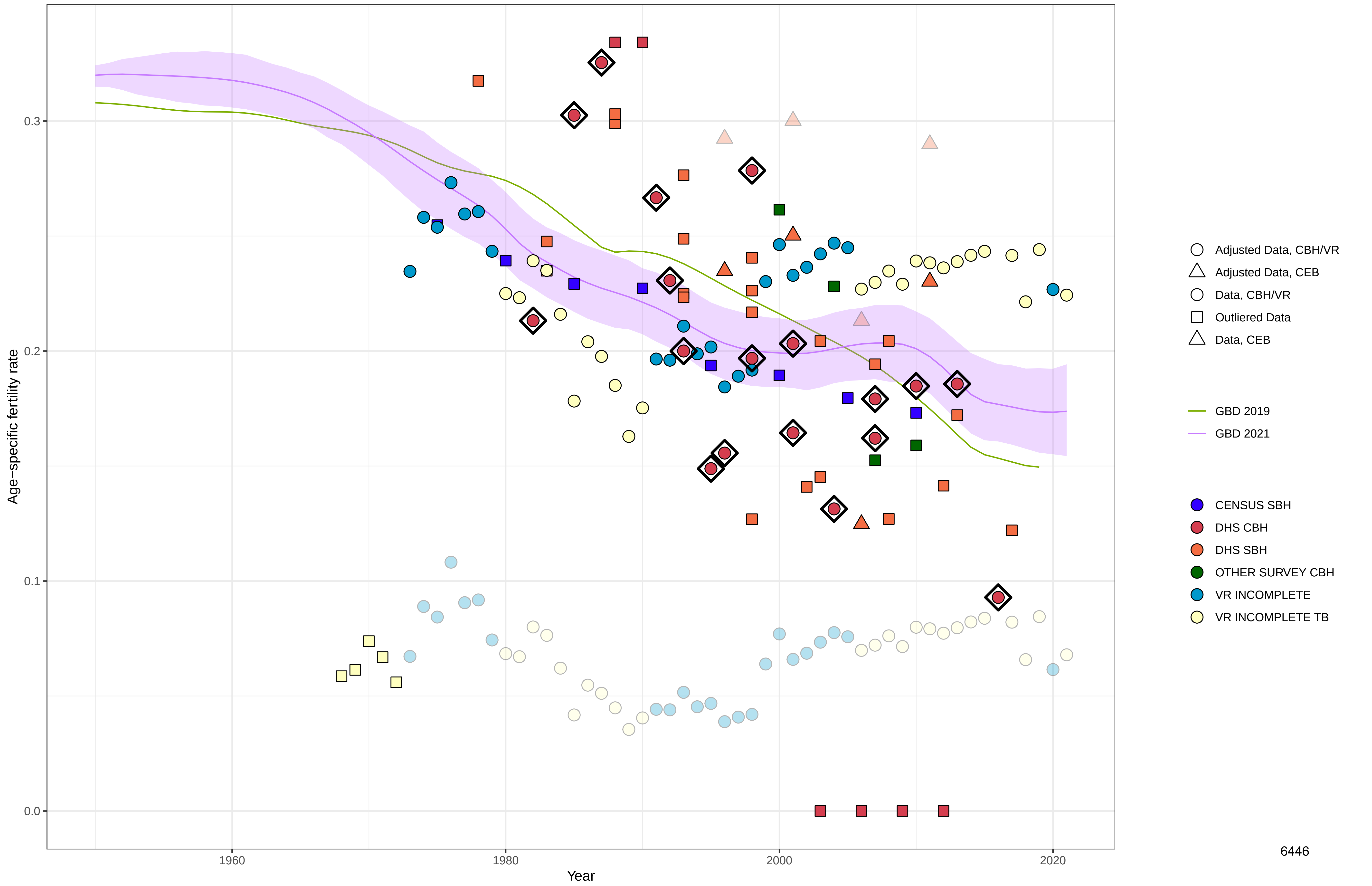

6446

Age−specific fertility fitted trend lines, 1950−2021: Samar (Western Samar), Ages: 35−39
Data points enclosed with a diamond are reference sources

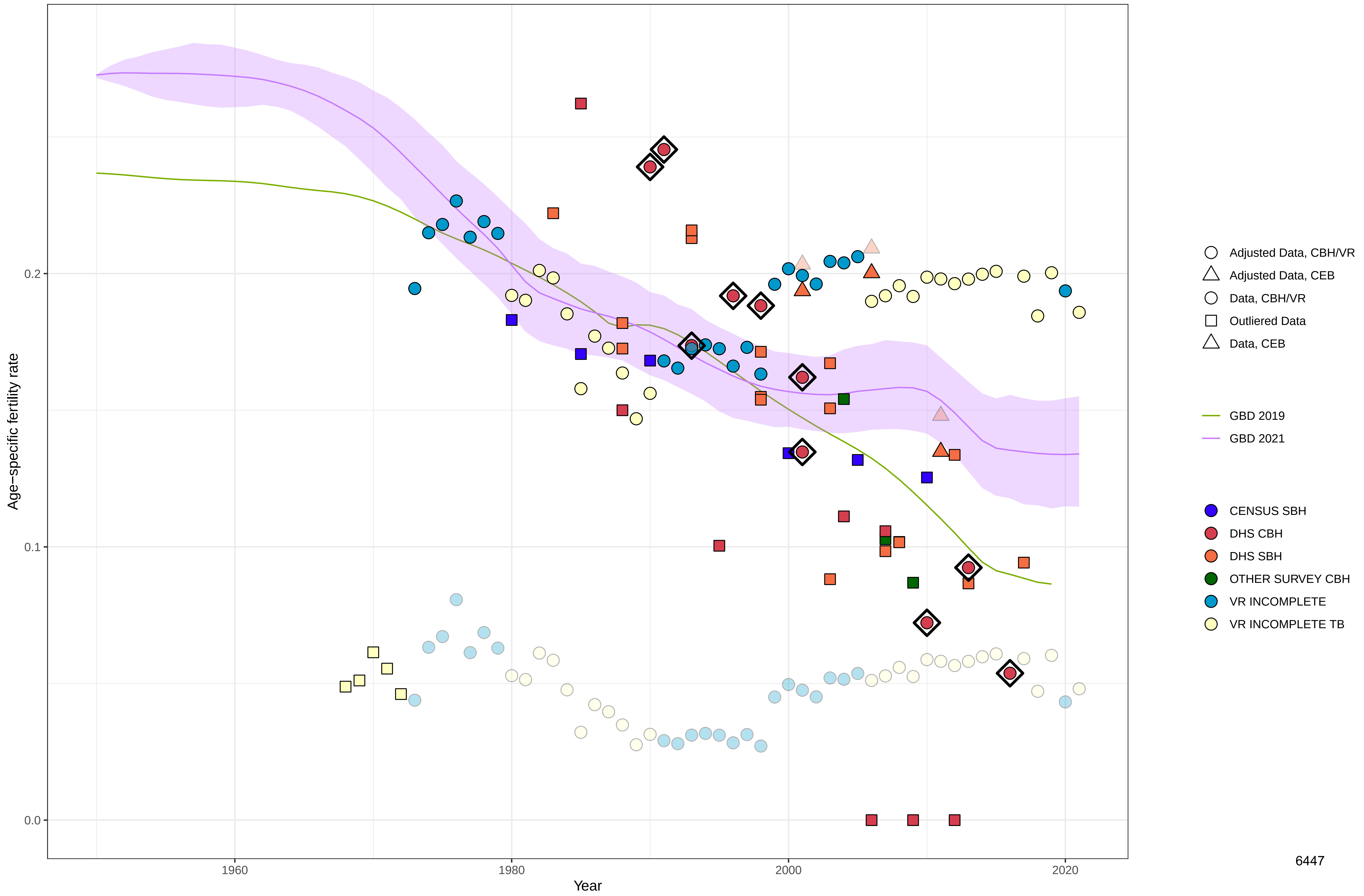

Age−specific fertility fitted trend lines, 1950−2021: Samar (Western Samar), Ages: 40−44
Data points enclosed with a diamond are reference sources

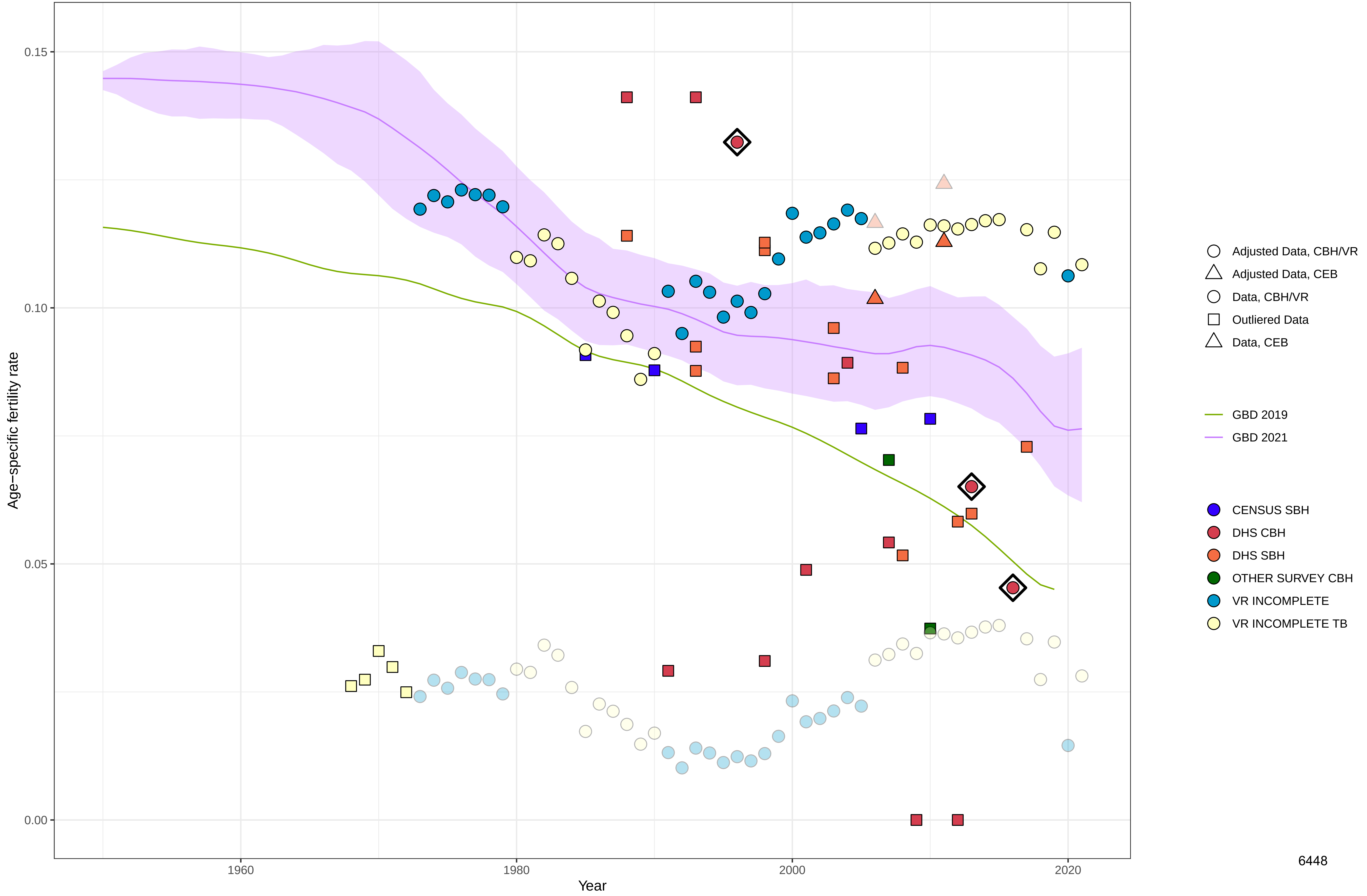

Age−specific fertility fitted trend lines, 1950−2021: Samar (Western Samar), Ages: 45−49
Data points enclosed with a diamond are reference sources

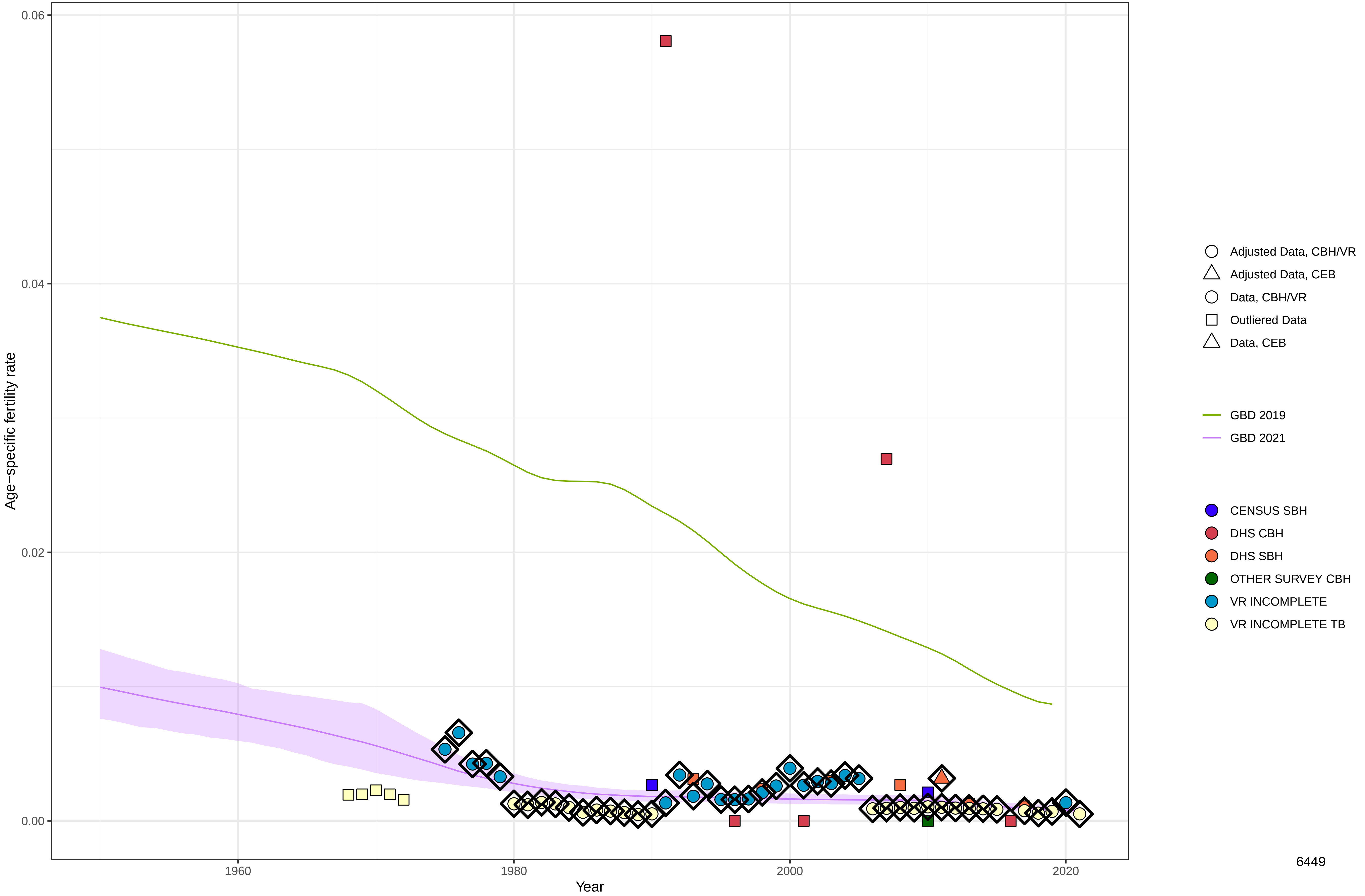

Age−specific fertility fitted trend lines, 1950−2021: Samar (Western Samar), Ages: 50−54
Data points enclosed with a diamond are reference sources

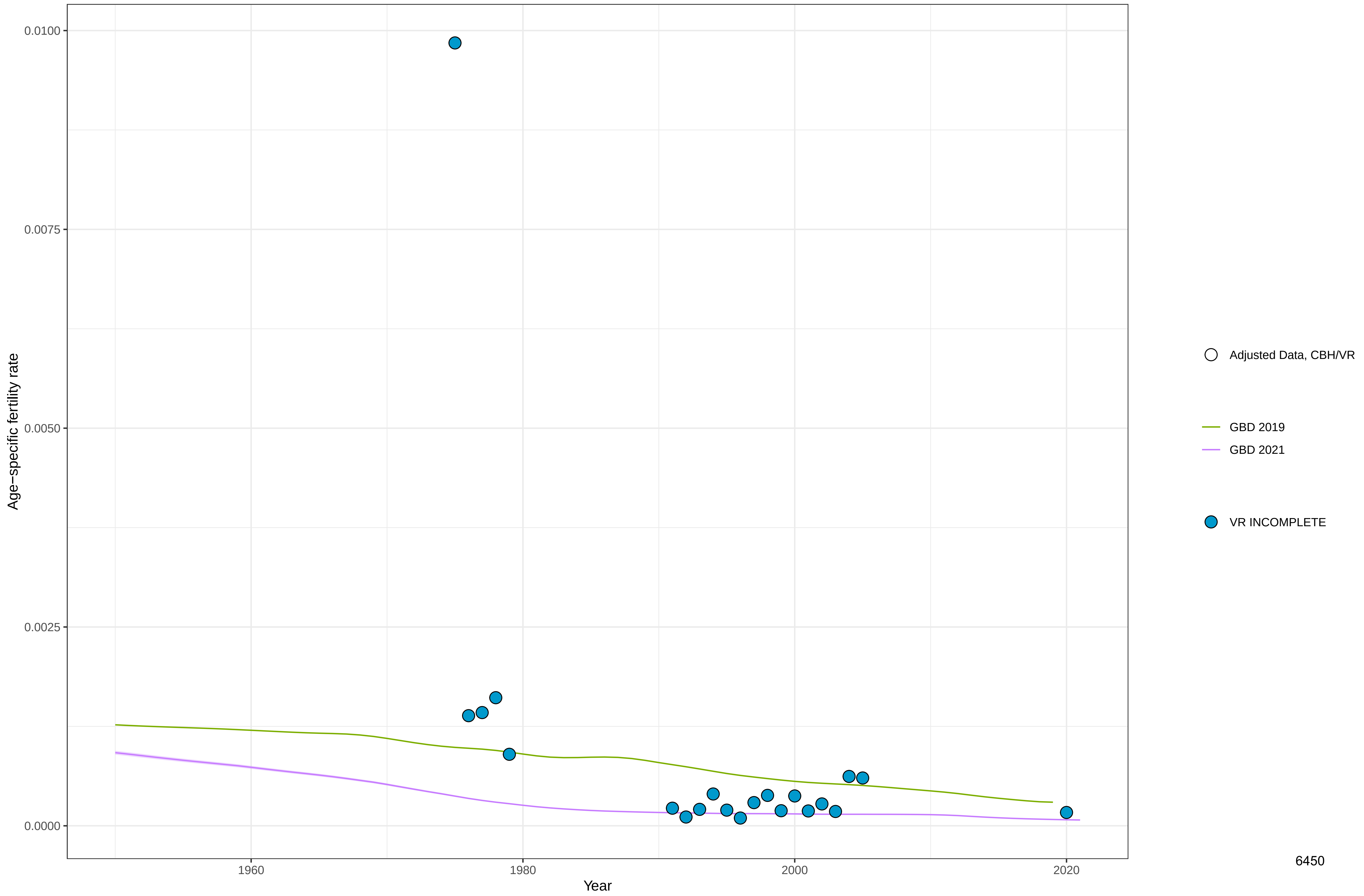

Total fertility rate fitted trend lines, 1950−2021: Samar (Western Samar) TFR
Data points enclosed with a diamond are reference sources

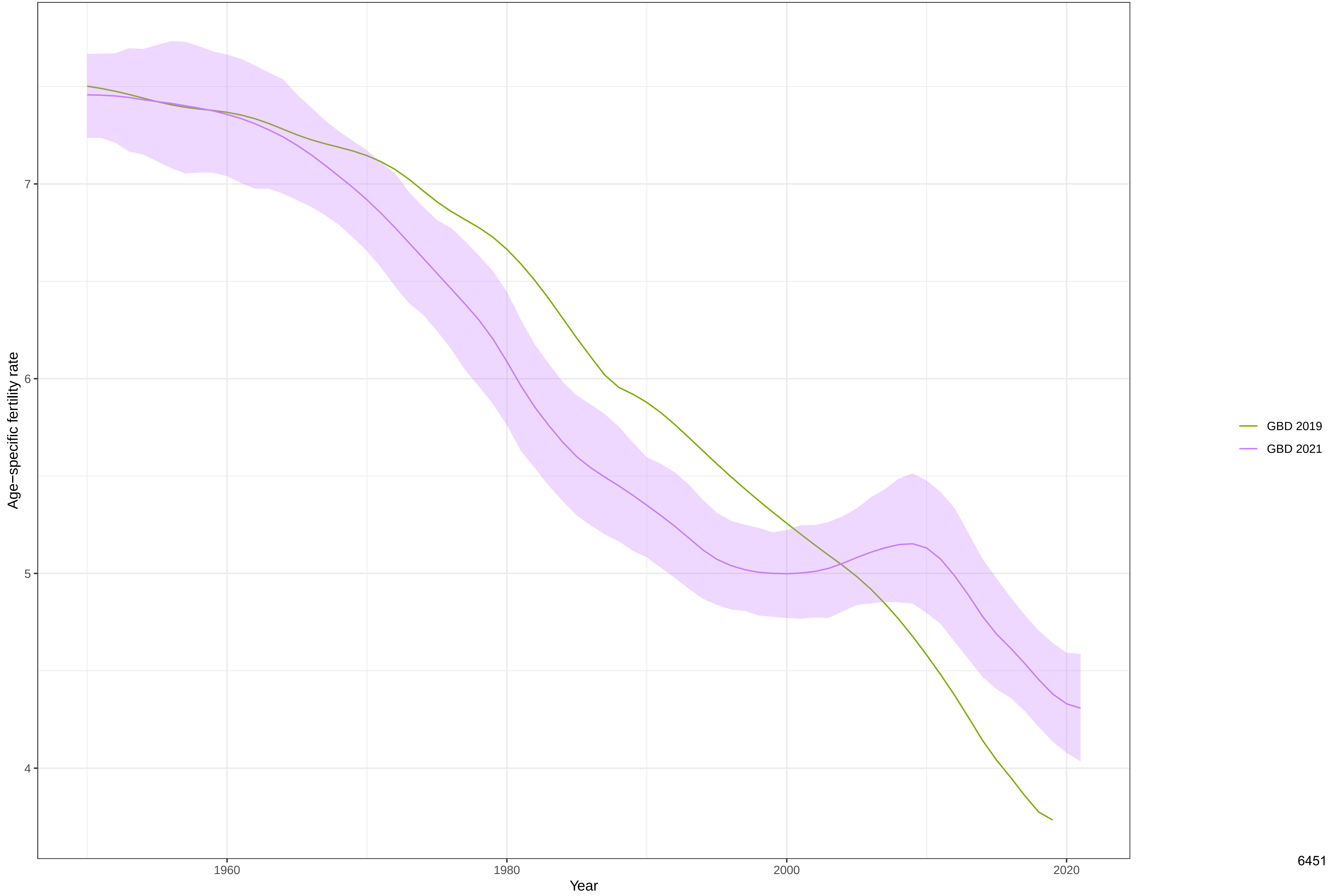

Age−specific fertility fitted trend lines, 1950−2021: Sarangani, Ages: 10−14
Data points enclosed with a diamond are reference sources

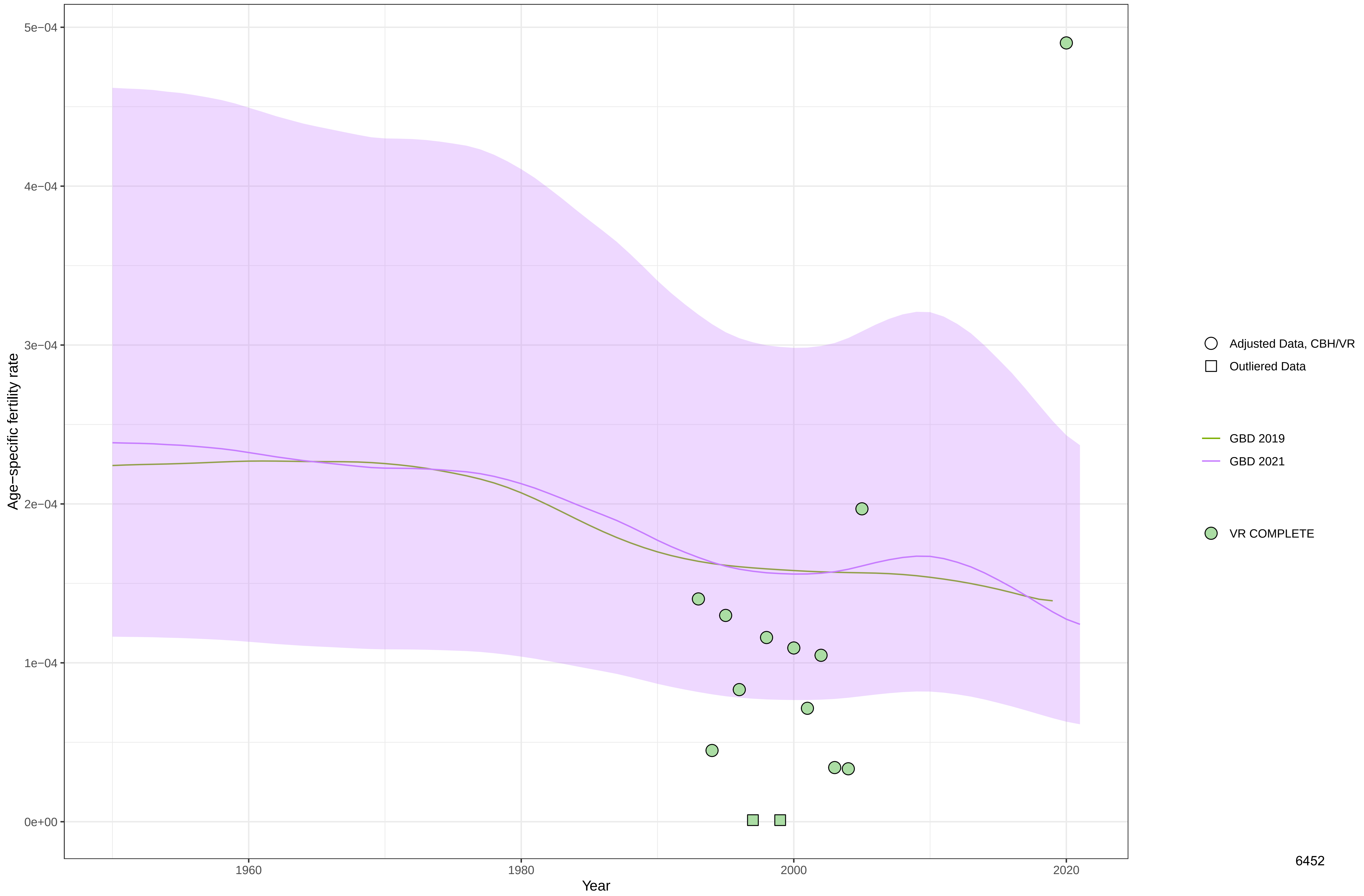

Age−specific fertility fitted trend lines, 1950−2021: Sarangani, Ages: 15−19
Data points enclosed with a diamond are reference sources

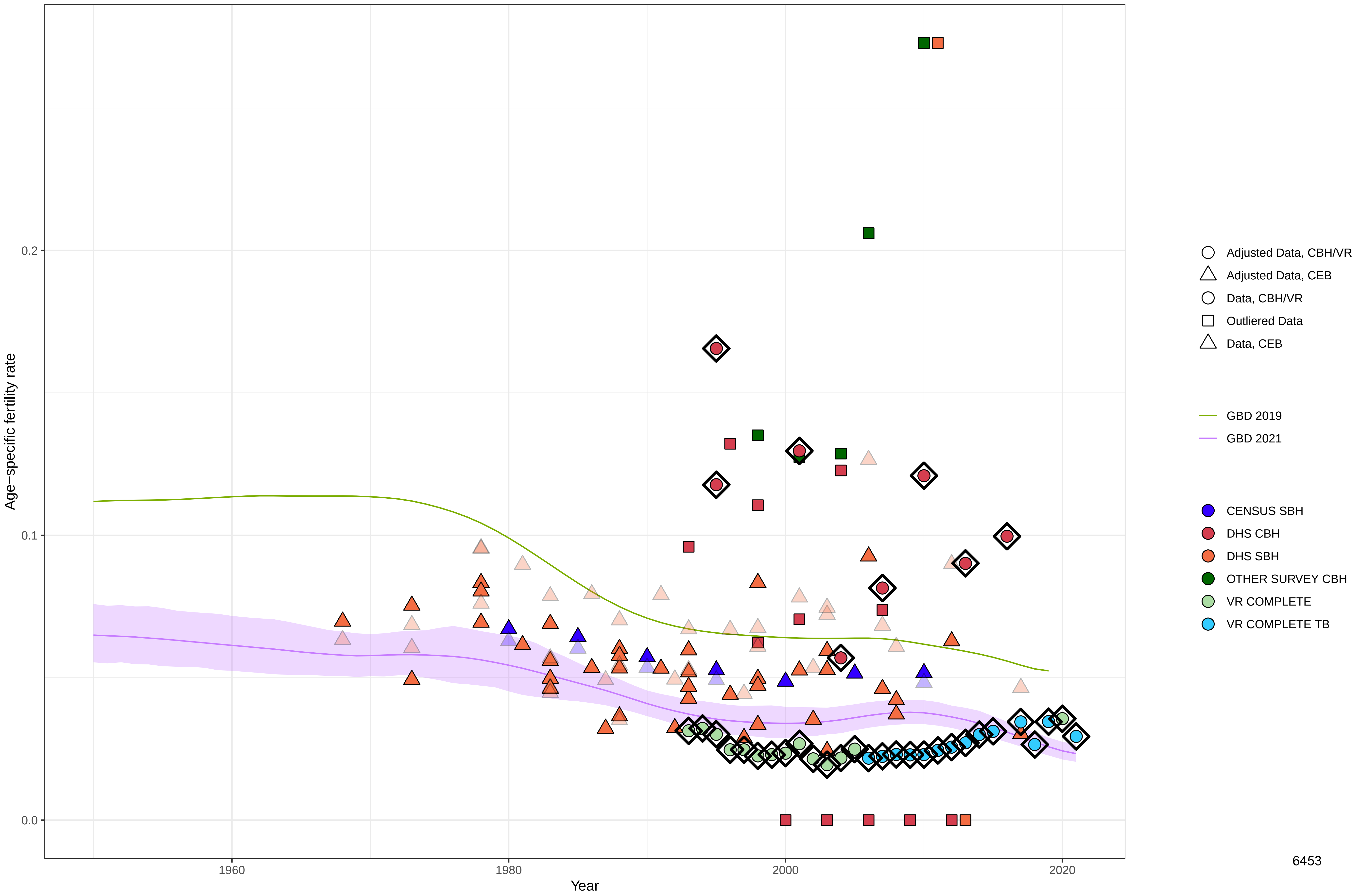

Age−specific fertility fitted trend lines, 1950−2021: Sarangani, Ages: 20−24
Data points enclosed with a diamond are reference sources

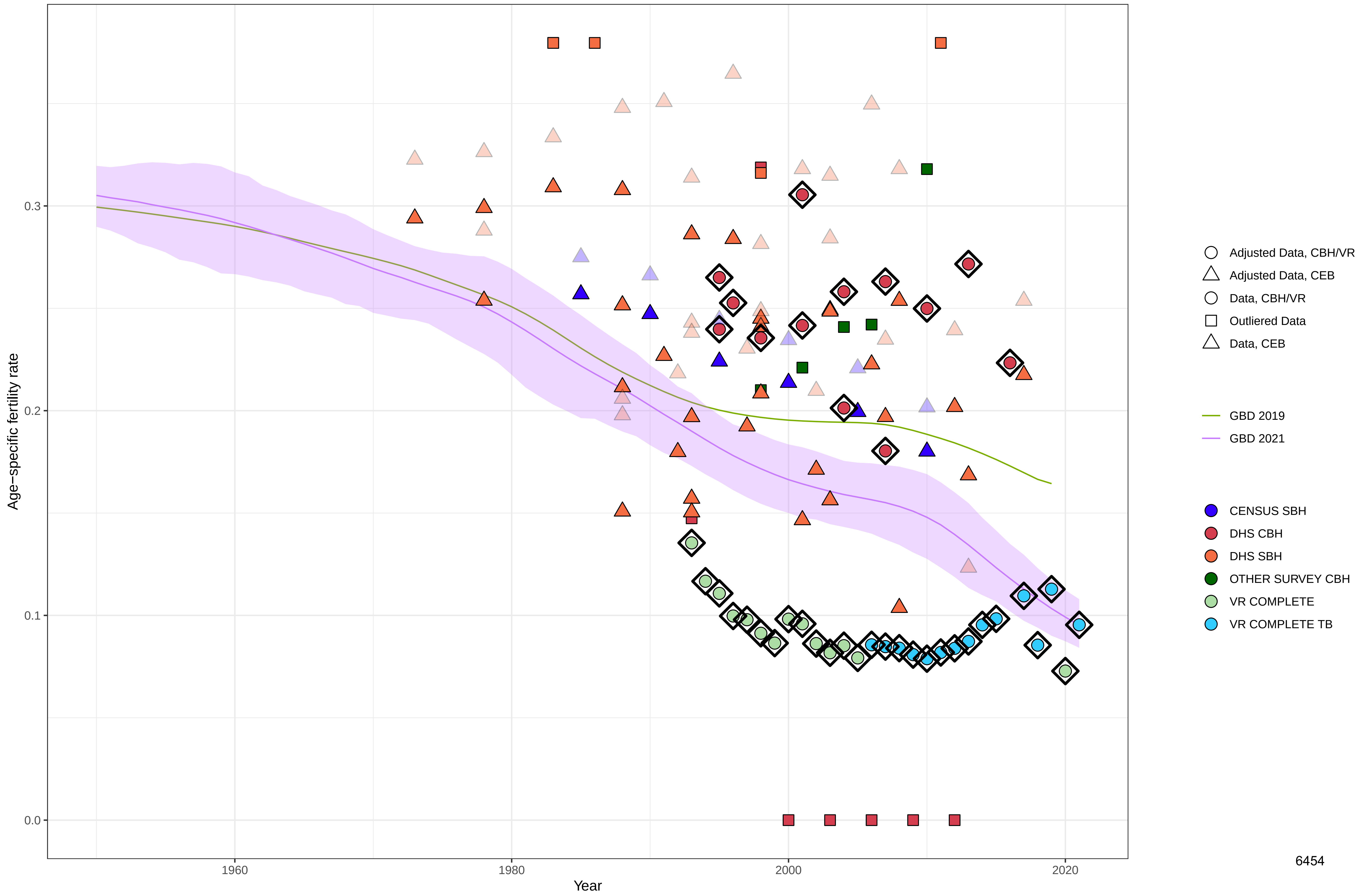

6454

Age−specific fertility fitted trend lines, 1950−2021: Sarangani, Ages: 25−29
Data points enclosed with a diamond are reference sources

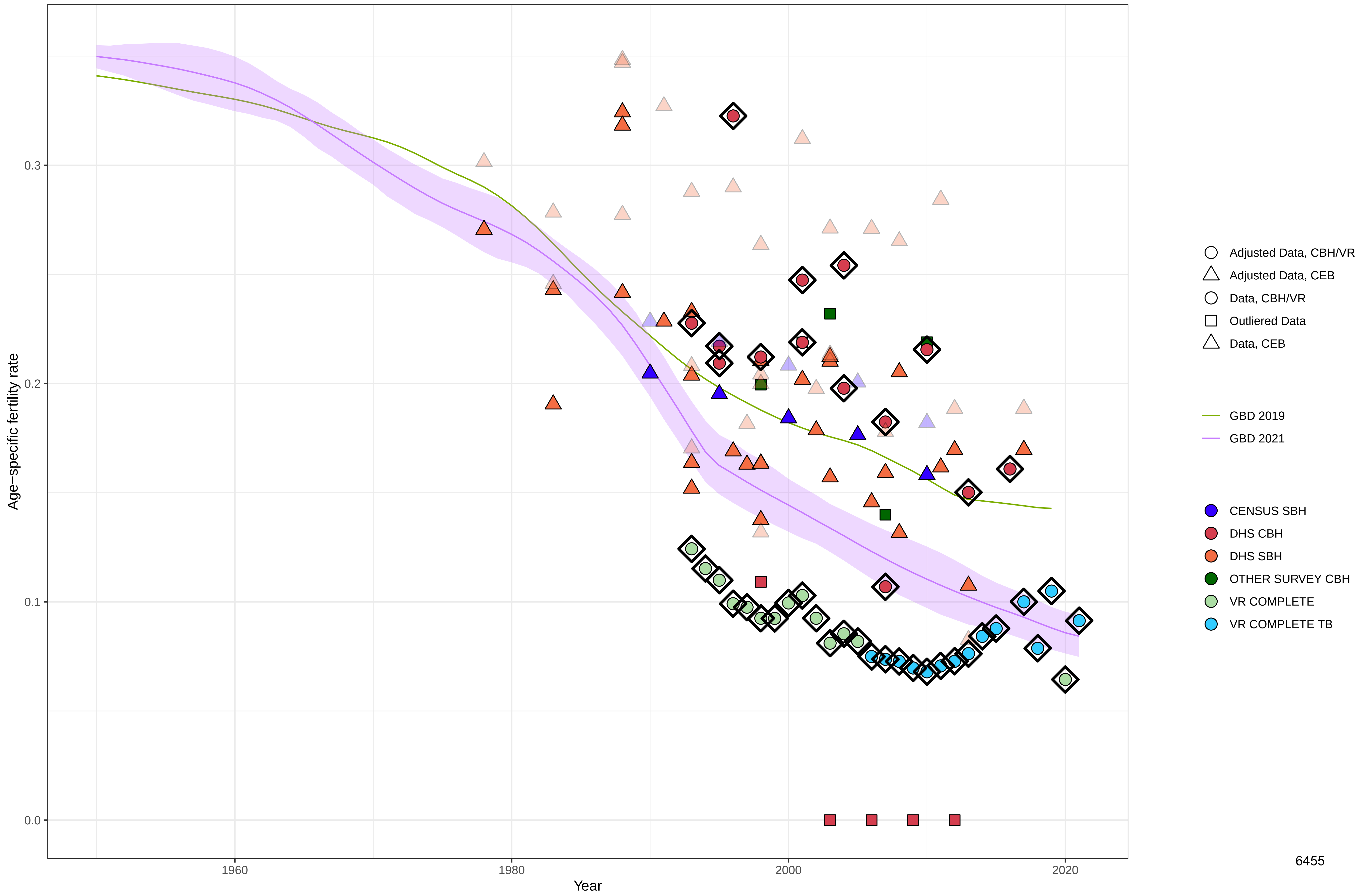

6455

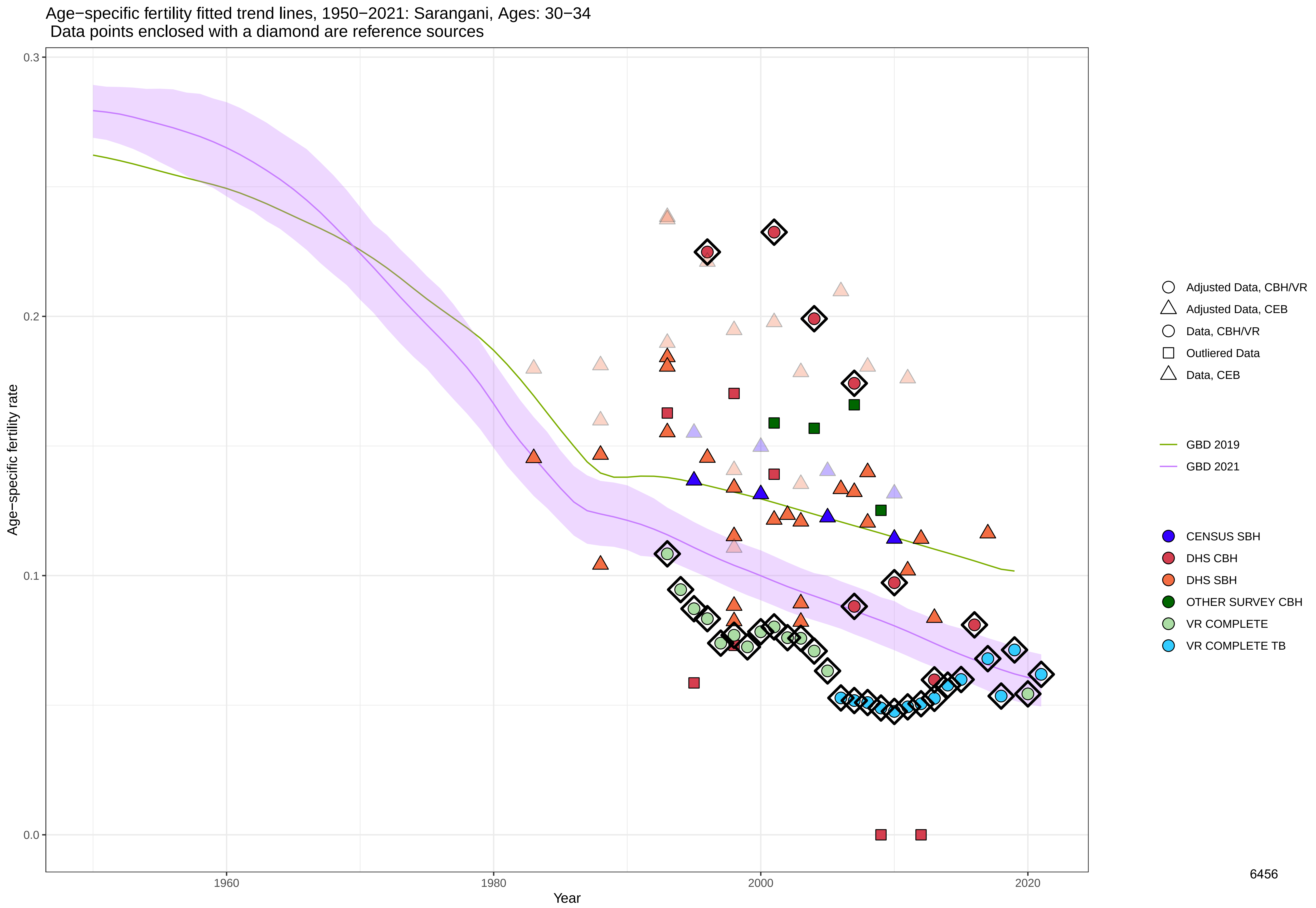

Age−specific fertility fitted trend lines, 1950−2021: Sarangani, Ages: 30−34
Data points enclosed with a diamond are reference sources
Age−specific fertility rate
Year
0.0
0.1
0.2
0.3
1960
1980
2000
2020
Adjusted Data, CBH/VR
Adjusted Data, CEB
Data, CBH/VR
Outliered Data
Data, CEB
GBD 2019
GBD 2021
CENSUS SBH
DHS CBH
DHS SBH
OTHER SURVEY CBH
VR COMPLETE
VR COMPLETE TB

6456

Age−specific fertility fitted trend lines, 1950−2021: Sarangani, Ages: 35−39
Data points enclosed with a diamond are reference sources

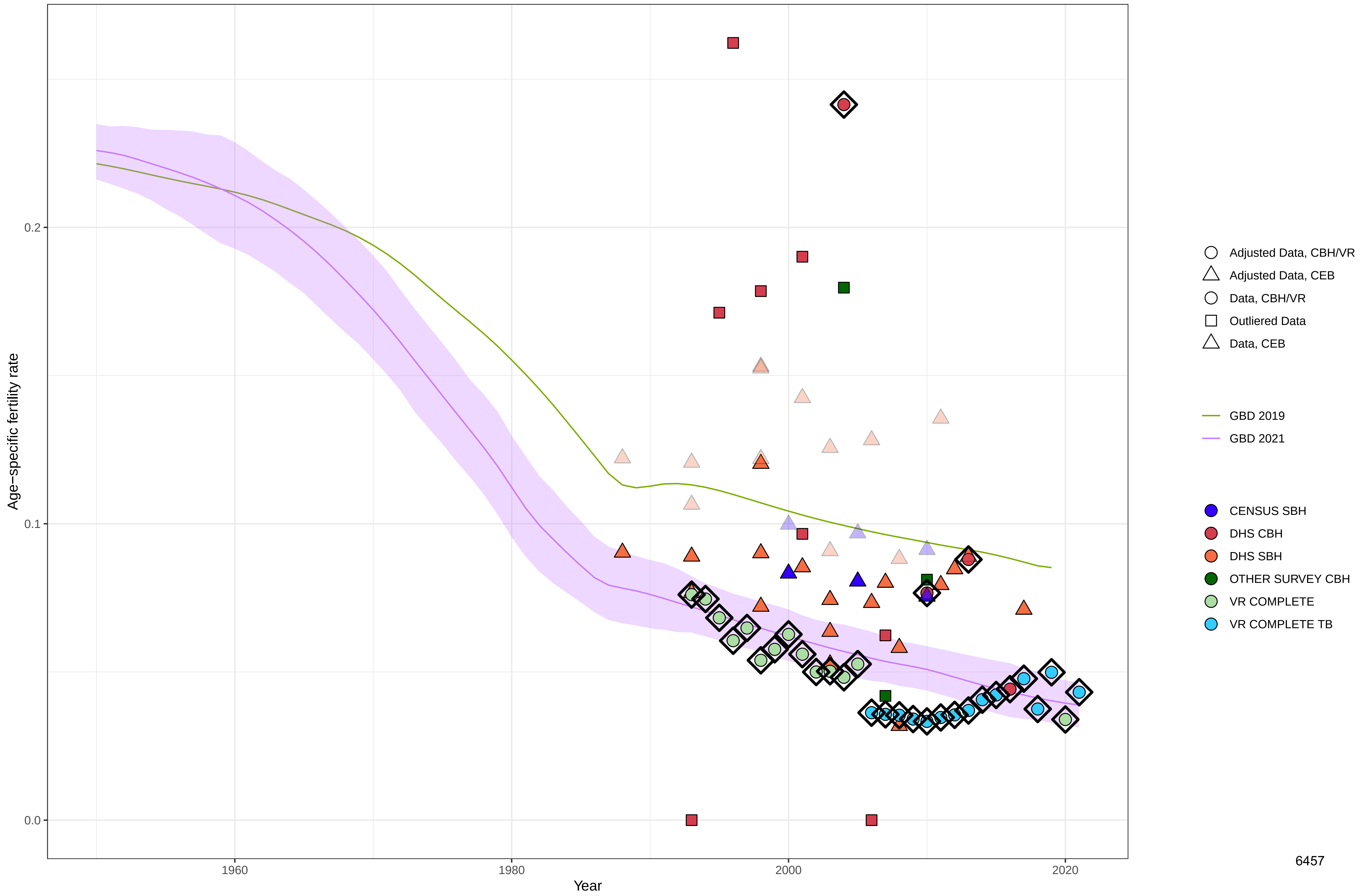

Age−specific fertility fitted trend lines, 1950−2021: Sarangani, Ages: 40−44
Data points enclosed with a diamond are reference sources

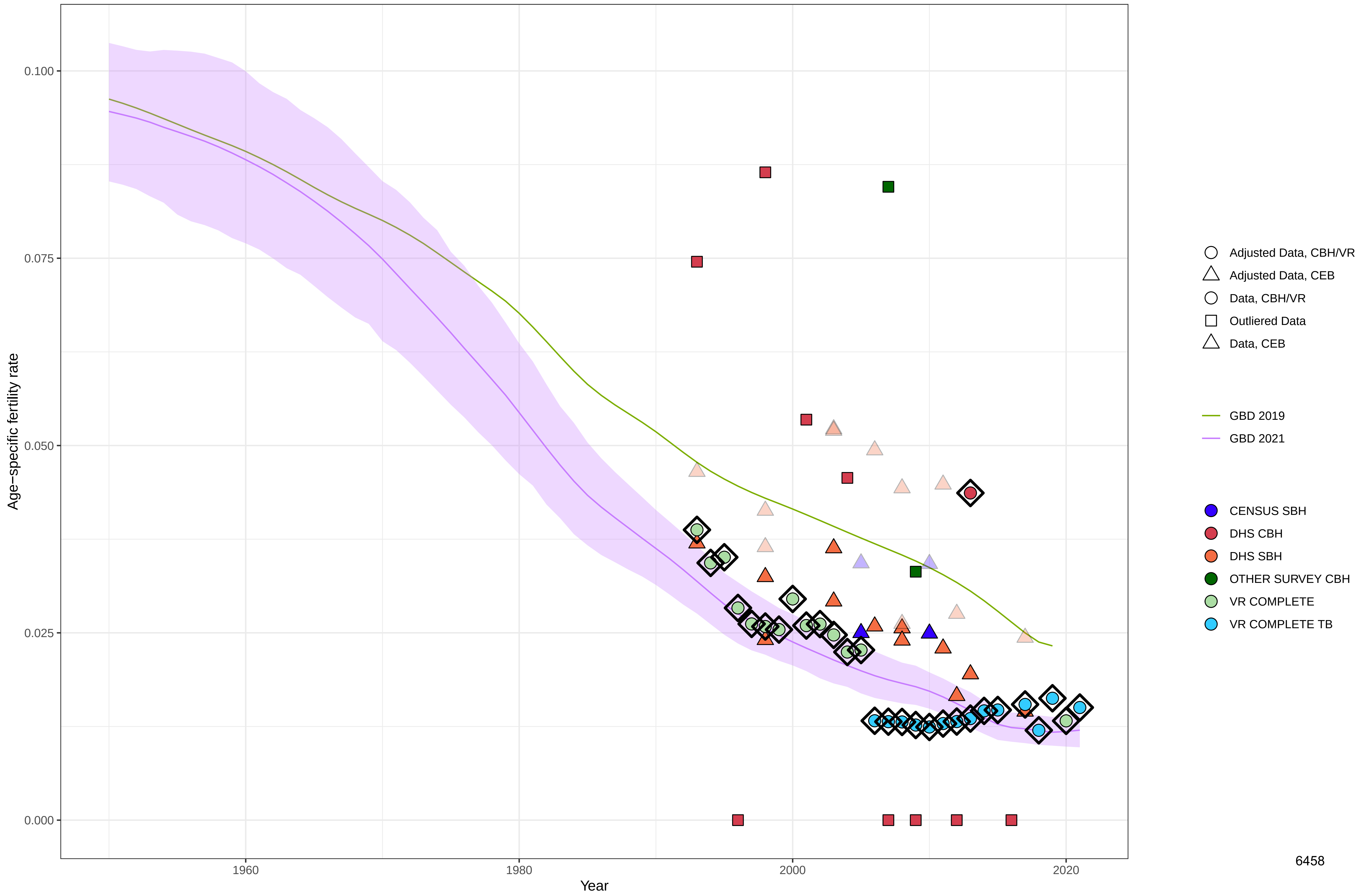

Age−specific fertility fitted trend lines, 1950−2021: Sarangani, Ages: 45−49
Data points enclosed with a diamond are reference sources

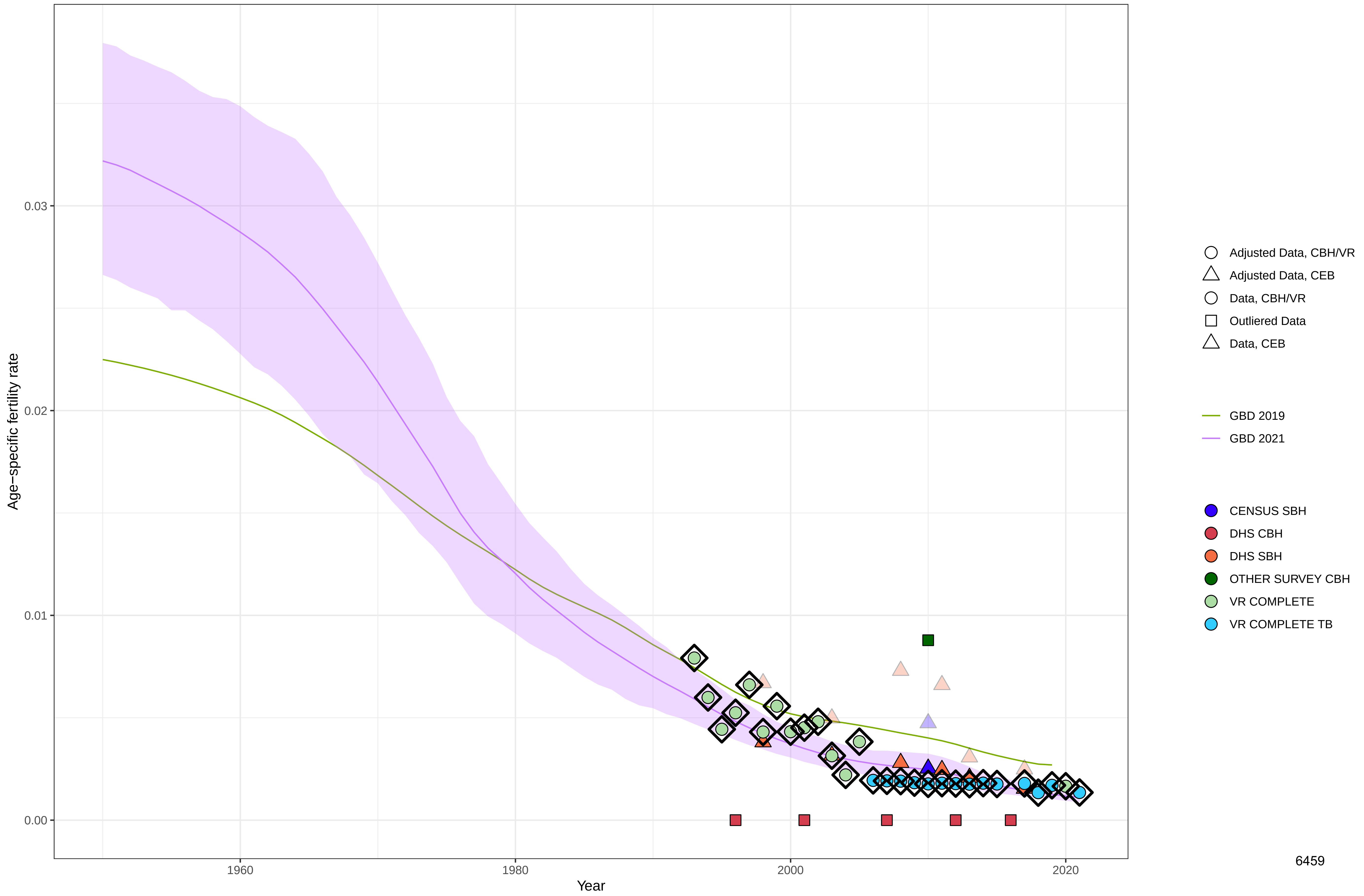

Age−specific fertility fitted trend lines, 1950−2021: Sarangani, Ages: 50−54
Data points enclosed with a diamond are reference sources

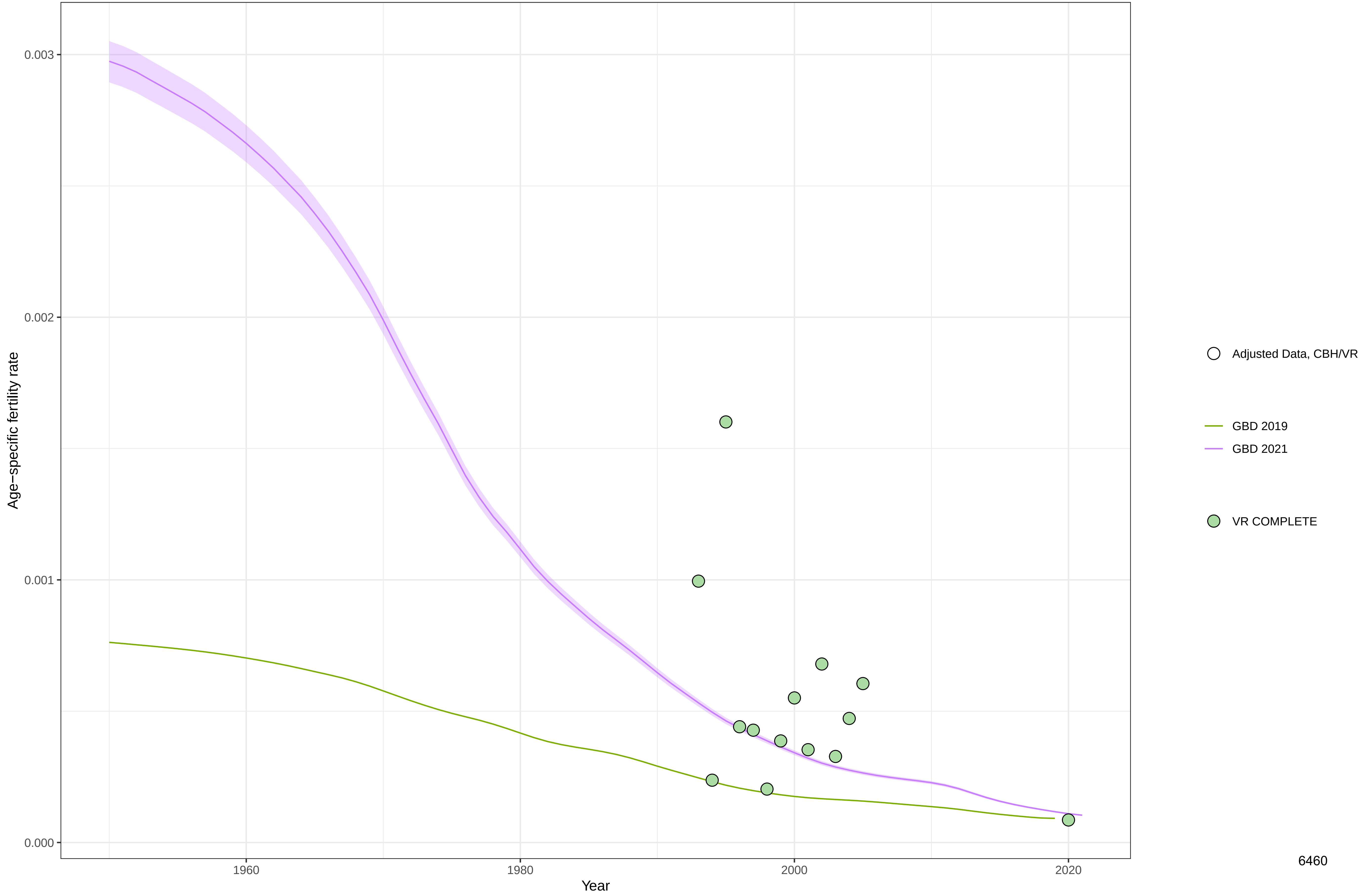

Total fertility rate fitted trend lines, 1950−2021: Sarangani TFR
Data points enclosed with a diamond are reference sources

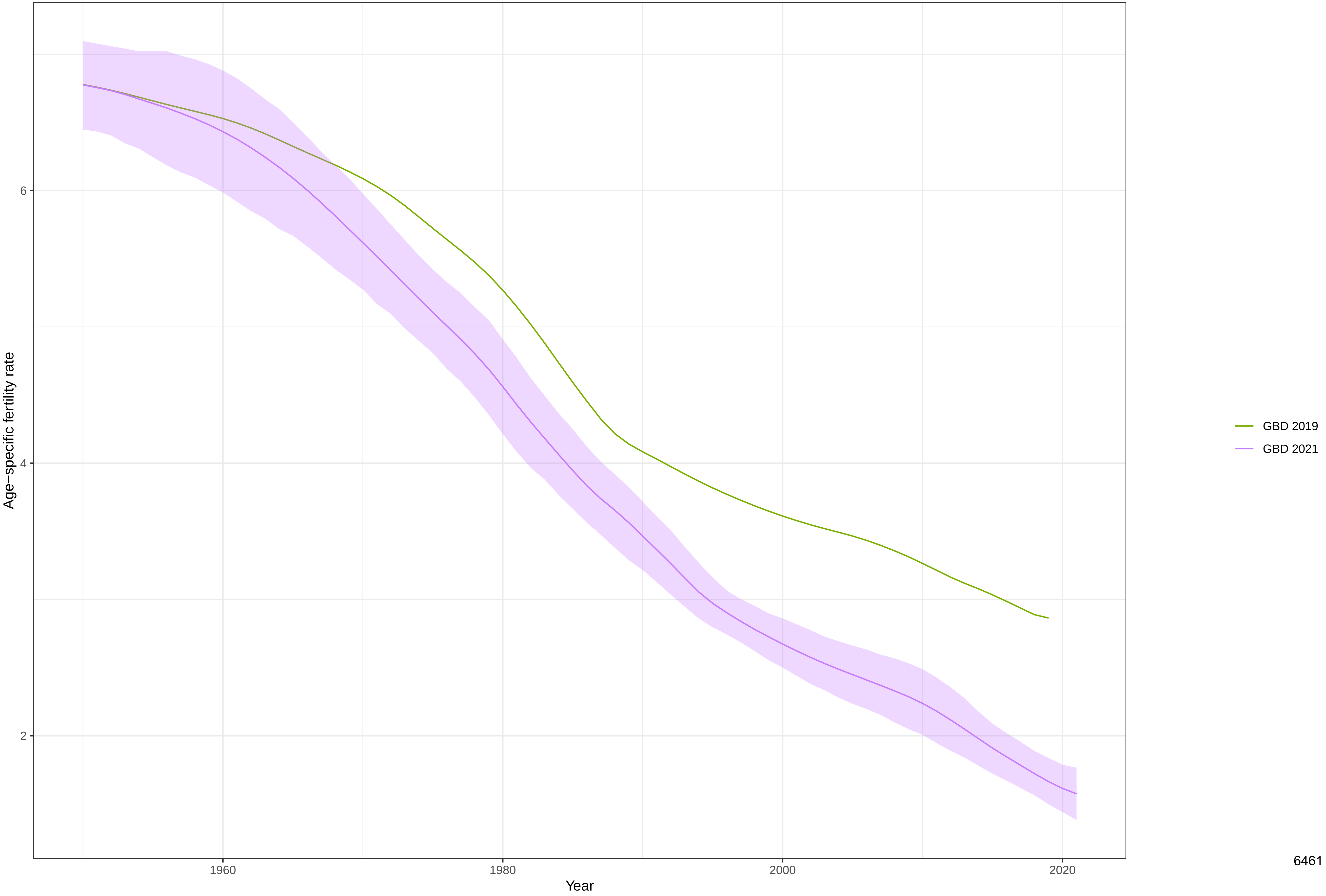

Age−specific fertility fitted trend lines, 1950−2021: Siquijor, Ages: 10−14
Data points enclosed with a diamond are reference sources

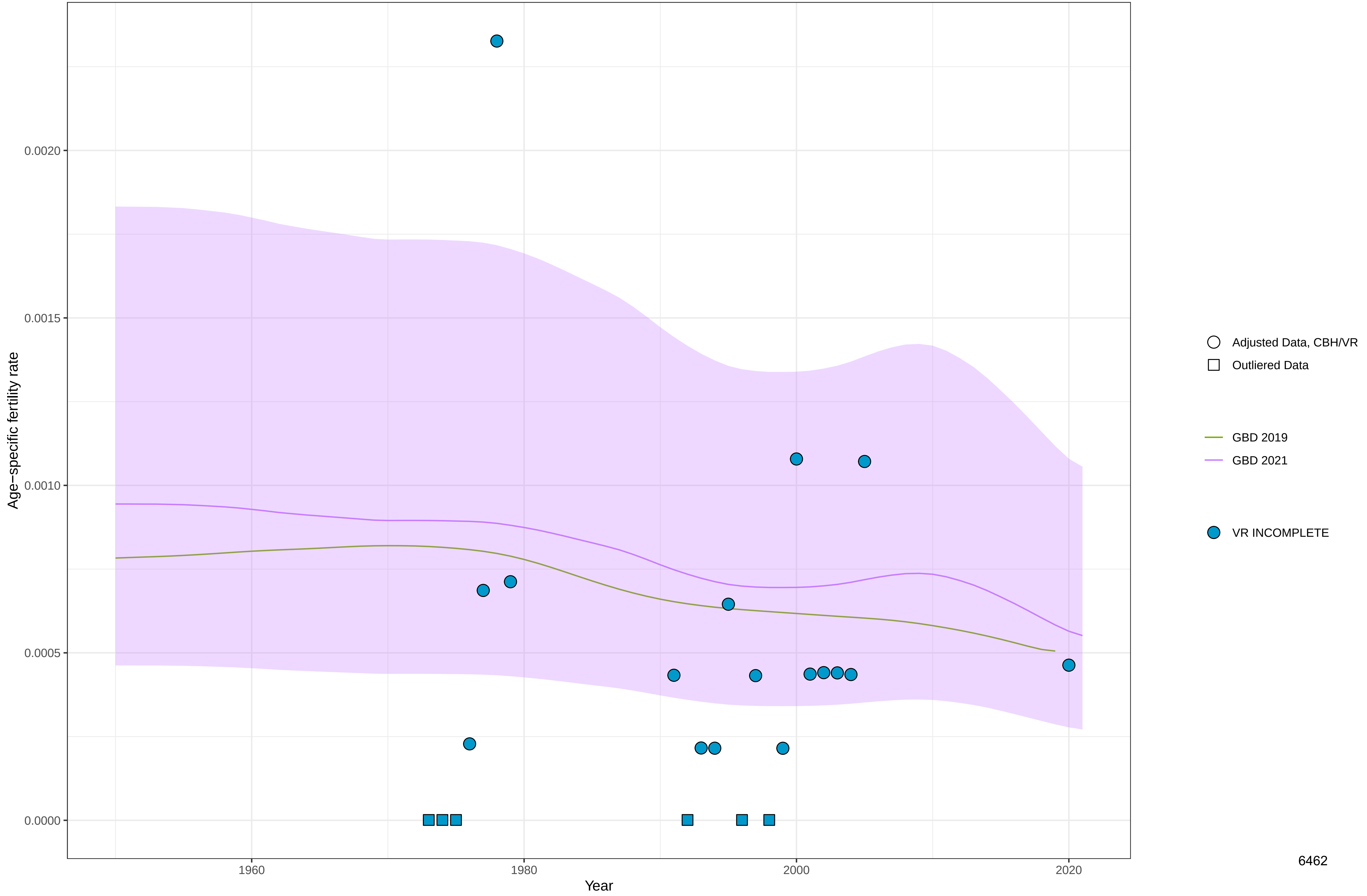

Age−specific fertility fitted trend lines, 1950−2021: Siquijor, Ages: 15−19
Data points enclosed with a diamond are reference sources

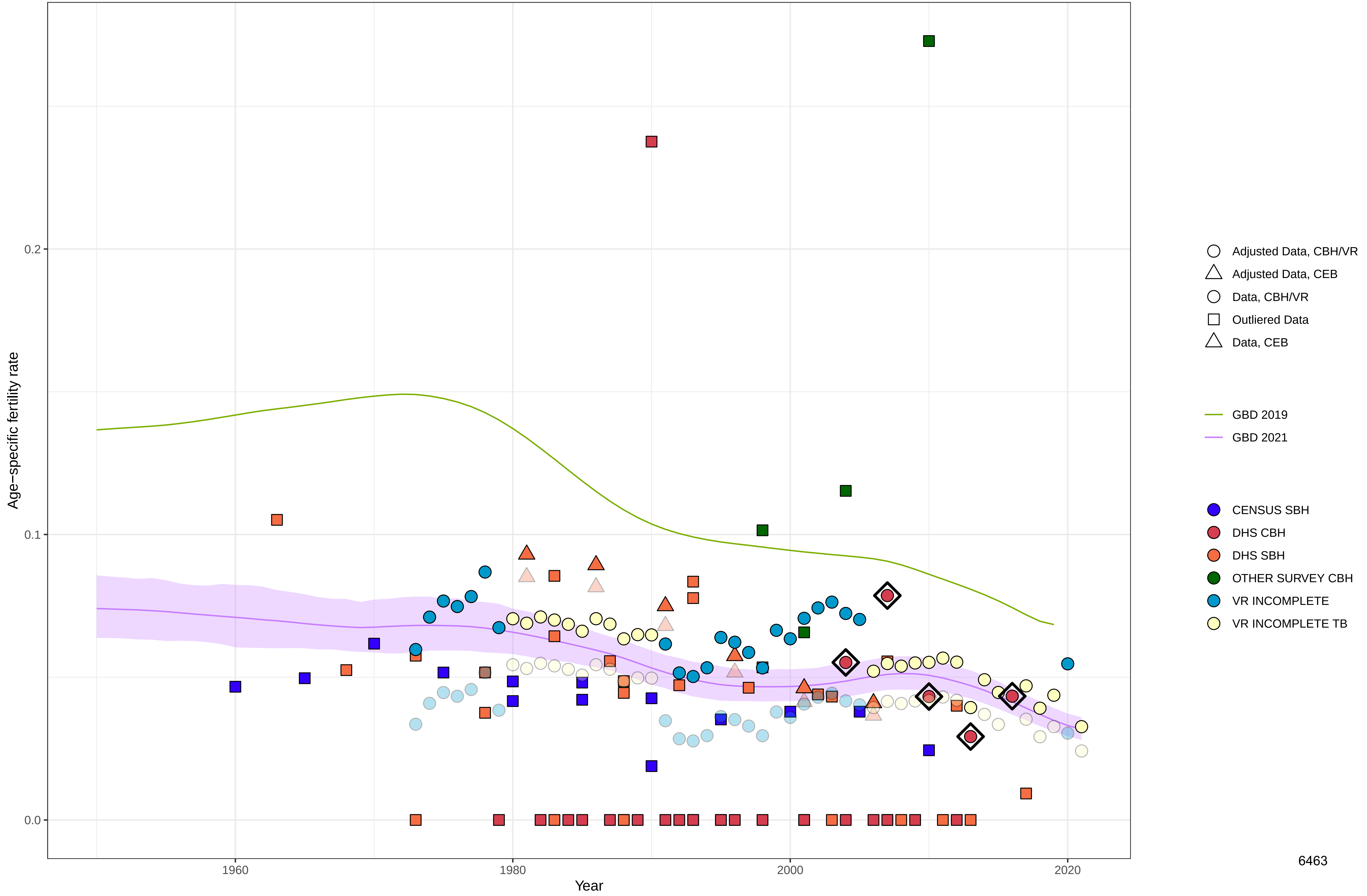

Age−specific fertility fitted trend lines, 1950−2021: Siquijor, Ages: 20−24
Data points enclosed with a diamond are reference sources

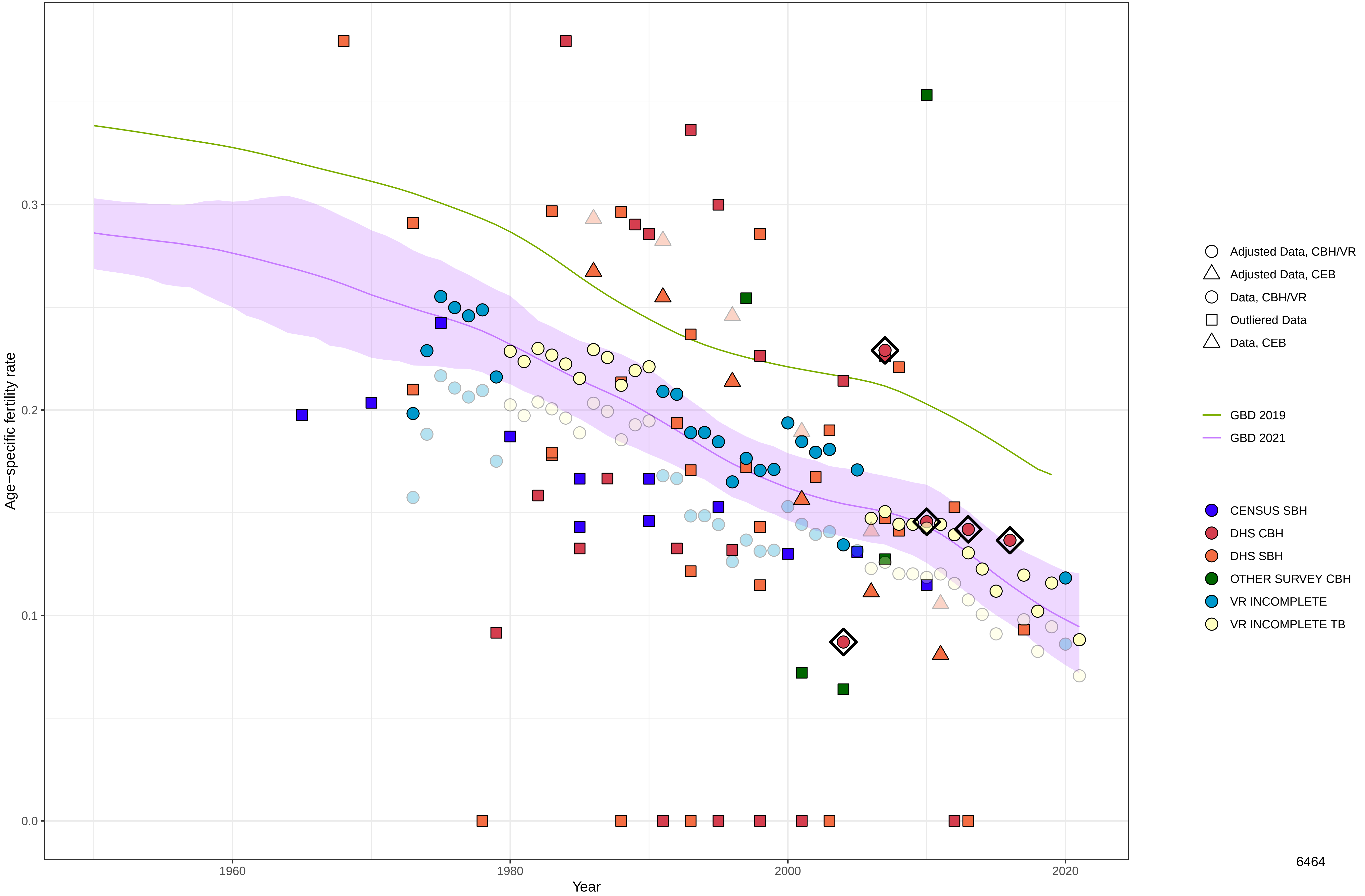

Age−specific fertility fitted trend lines, 1950−2021: Siquijor, Ages: 25−29
Data points enclosed with a diamond are reference sources

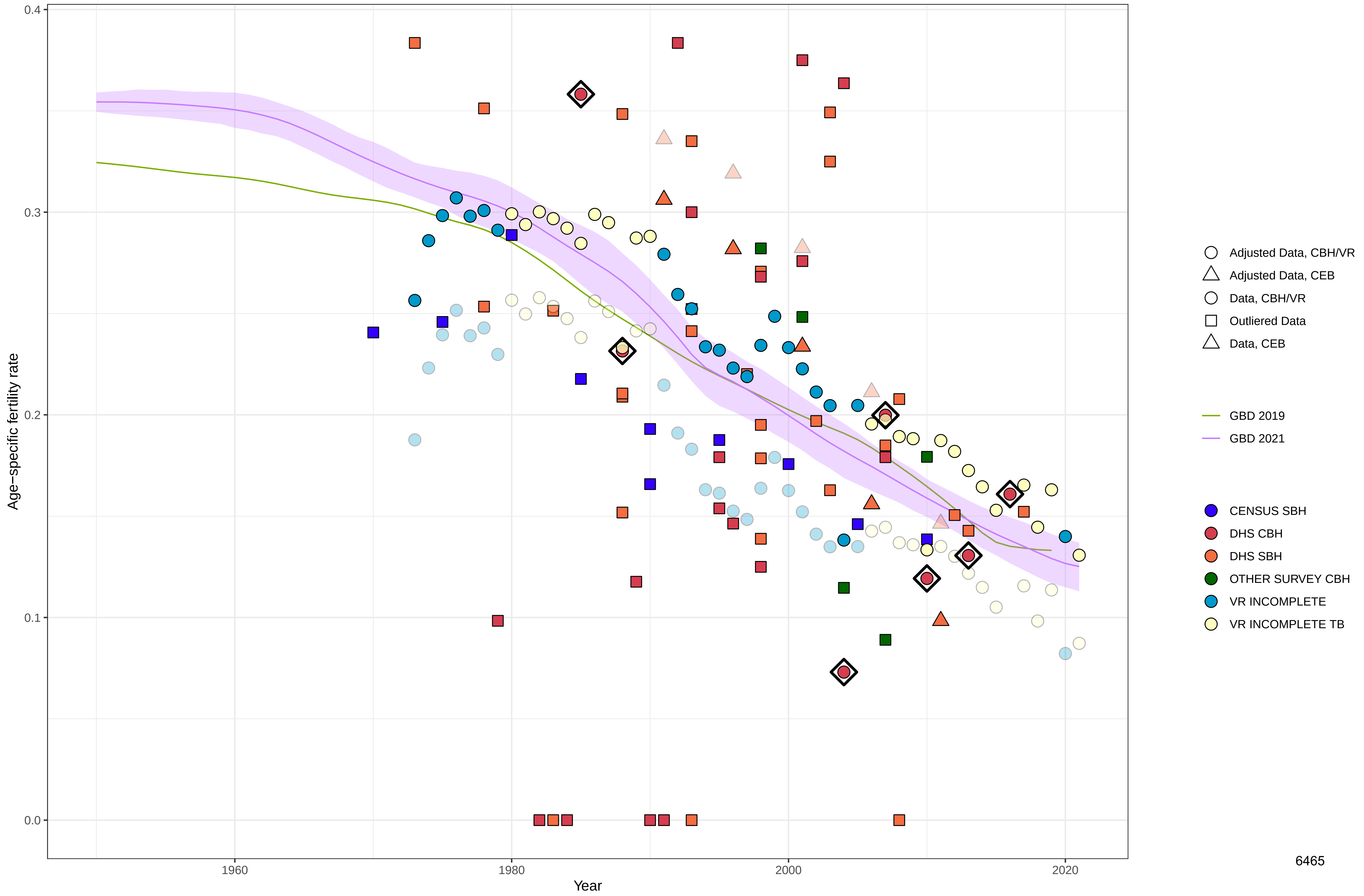

Age−specific fertility fitted trend lines, 1950−2021: Siquijor, Ages: 30−34
Data points enclosed with a diamond are reference sources

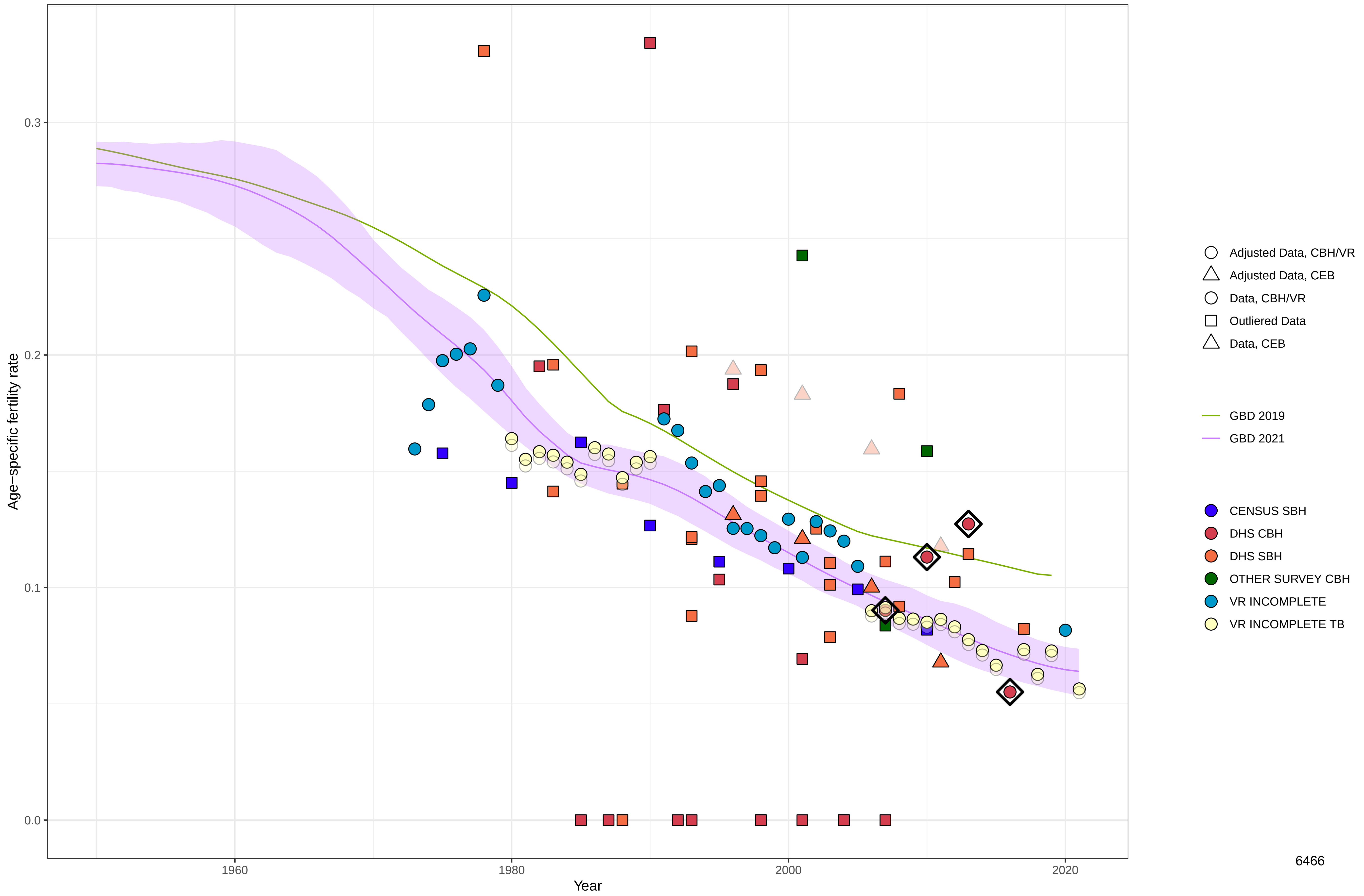

6466

Age−specific fertility fitted trend lines, 1950−2021: Siquijor, Ages: 35−39
Data points enclosed with a diamond are reference sources

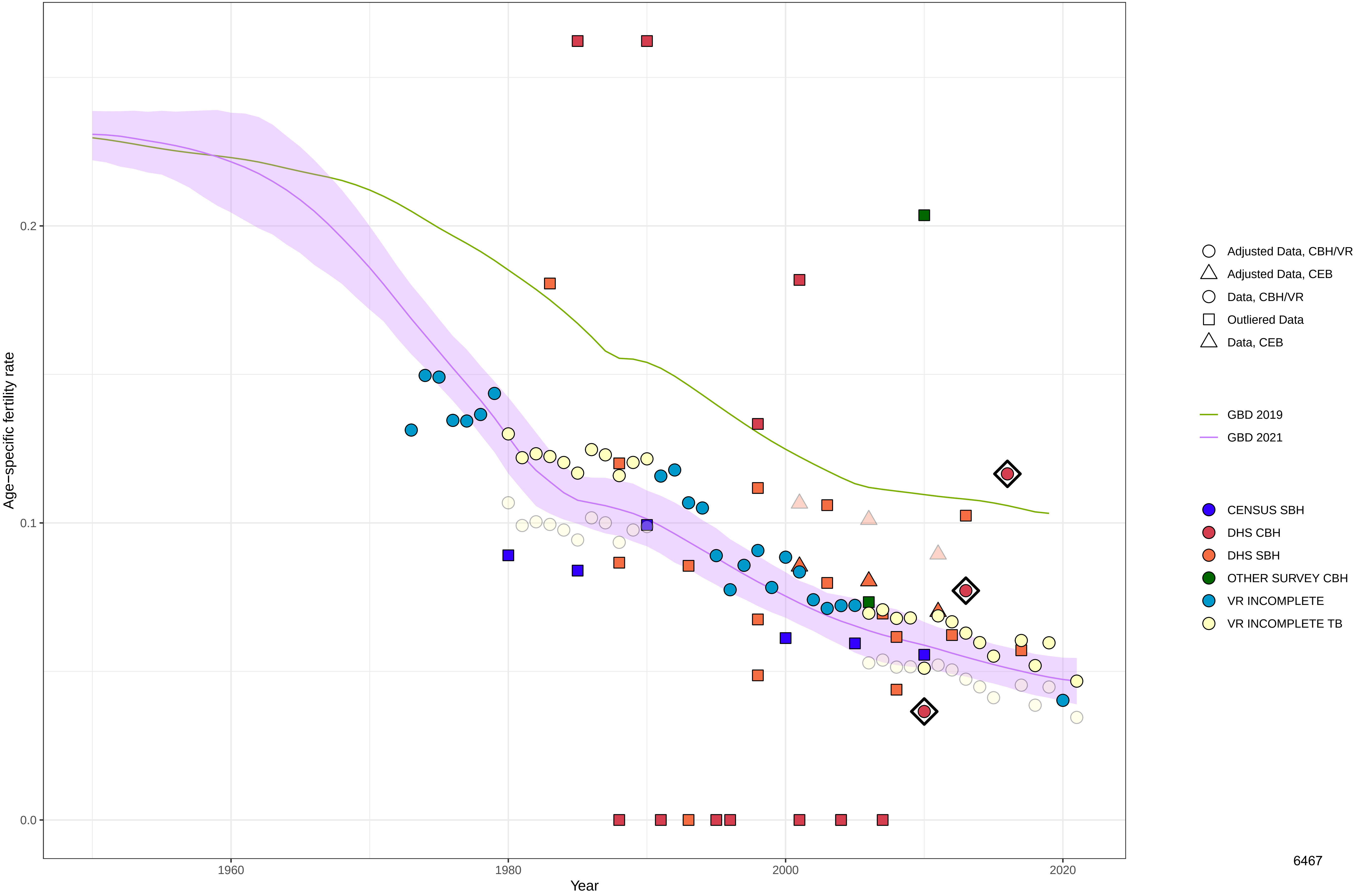

Age−specific fertility fitted trend lines, 1950−2021: Siquijor, Ages: 40−44
Data points enclosed with a diamond are reference sources

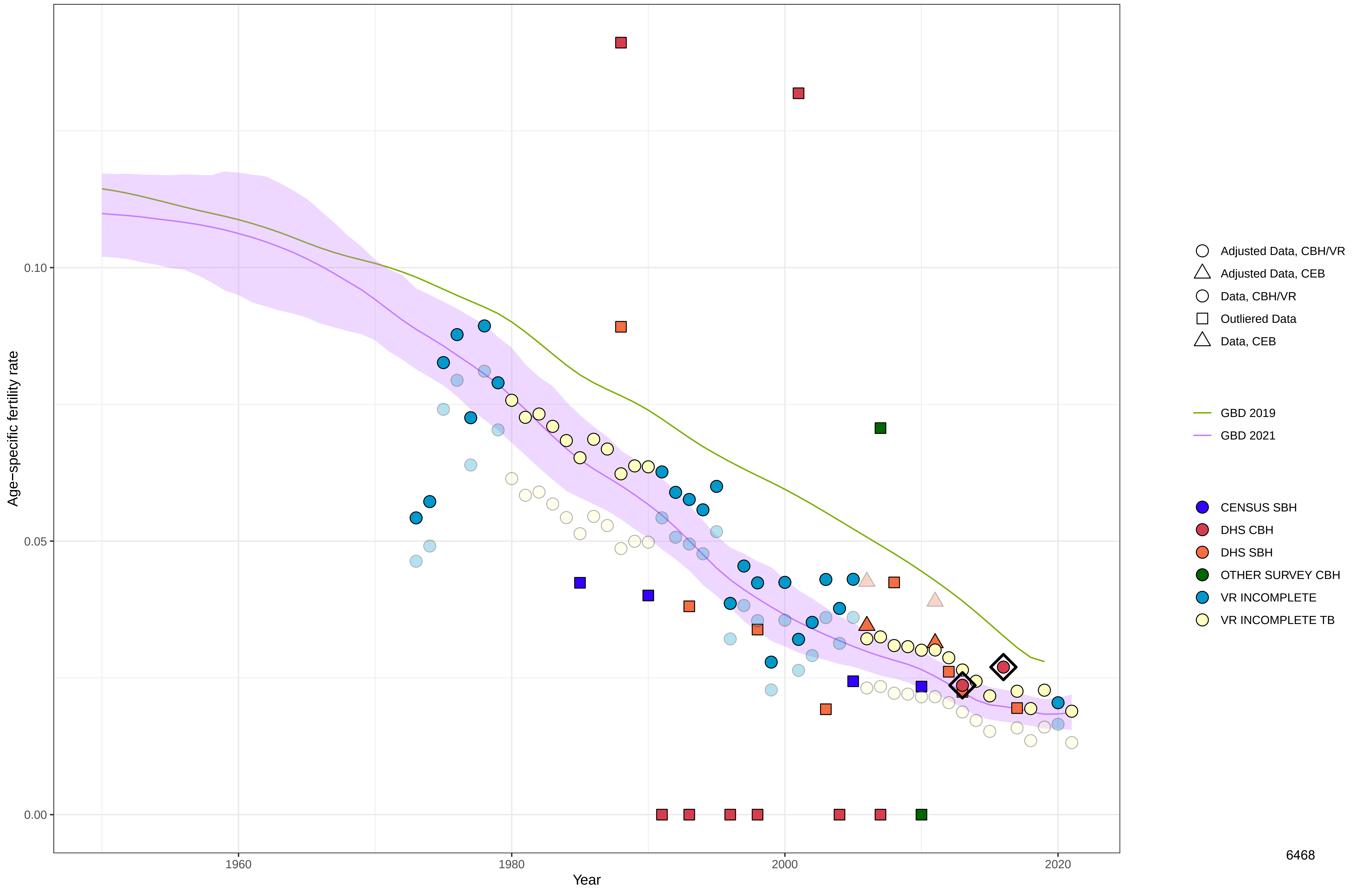

6468

Age−specific fertility fitted trend lines, 1950−2021: Siquijor, Ages: 45−49
Data points enclosed with a diamond are reference sources

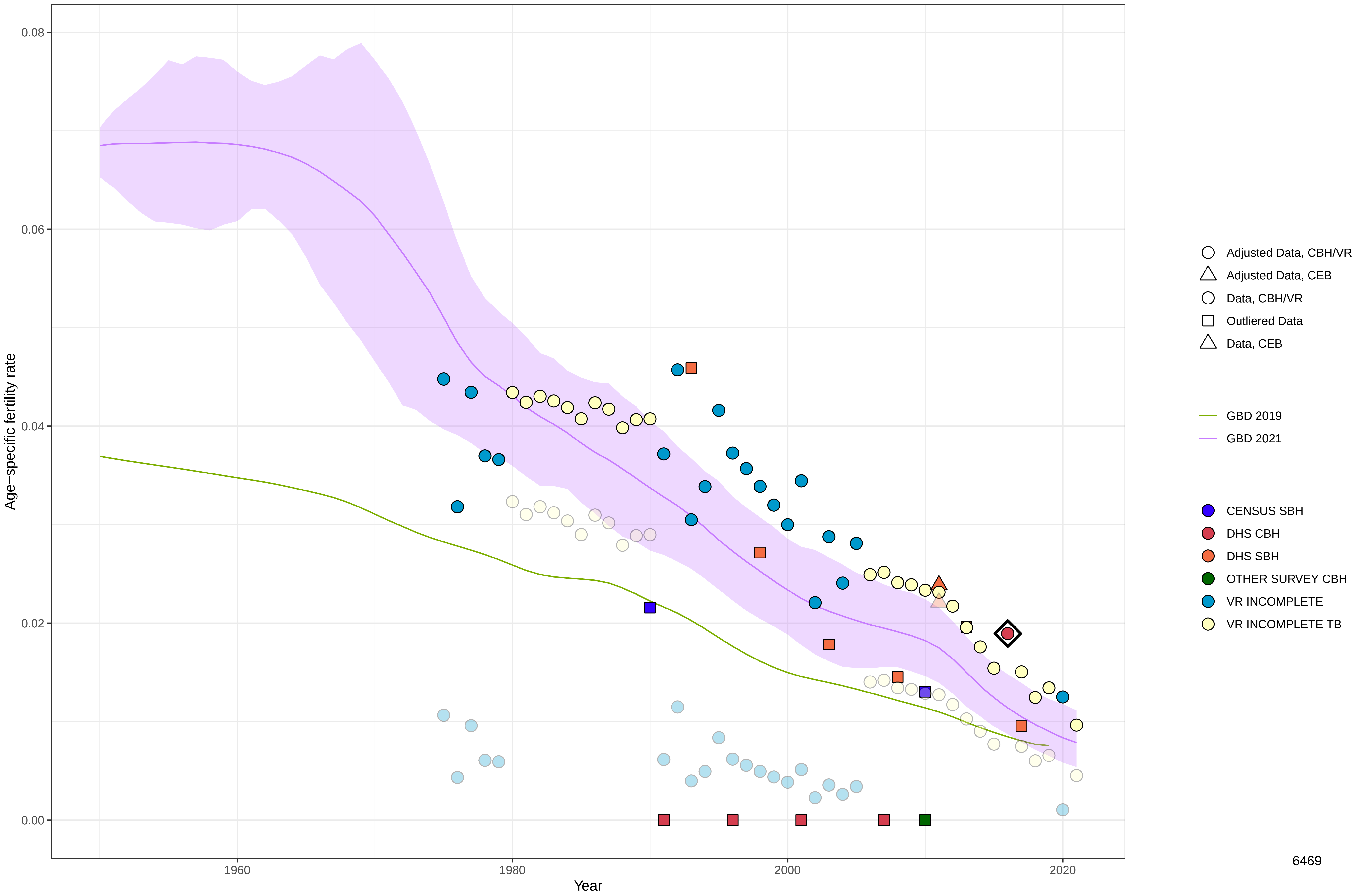

6469

Age−specific fertility fitted trend lines, 1950−2021: Siquijor, Ages: 50−54
Data points enclosed with a diamond are reference sources

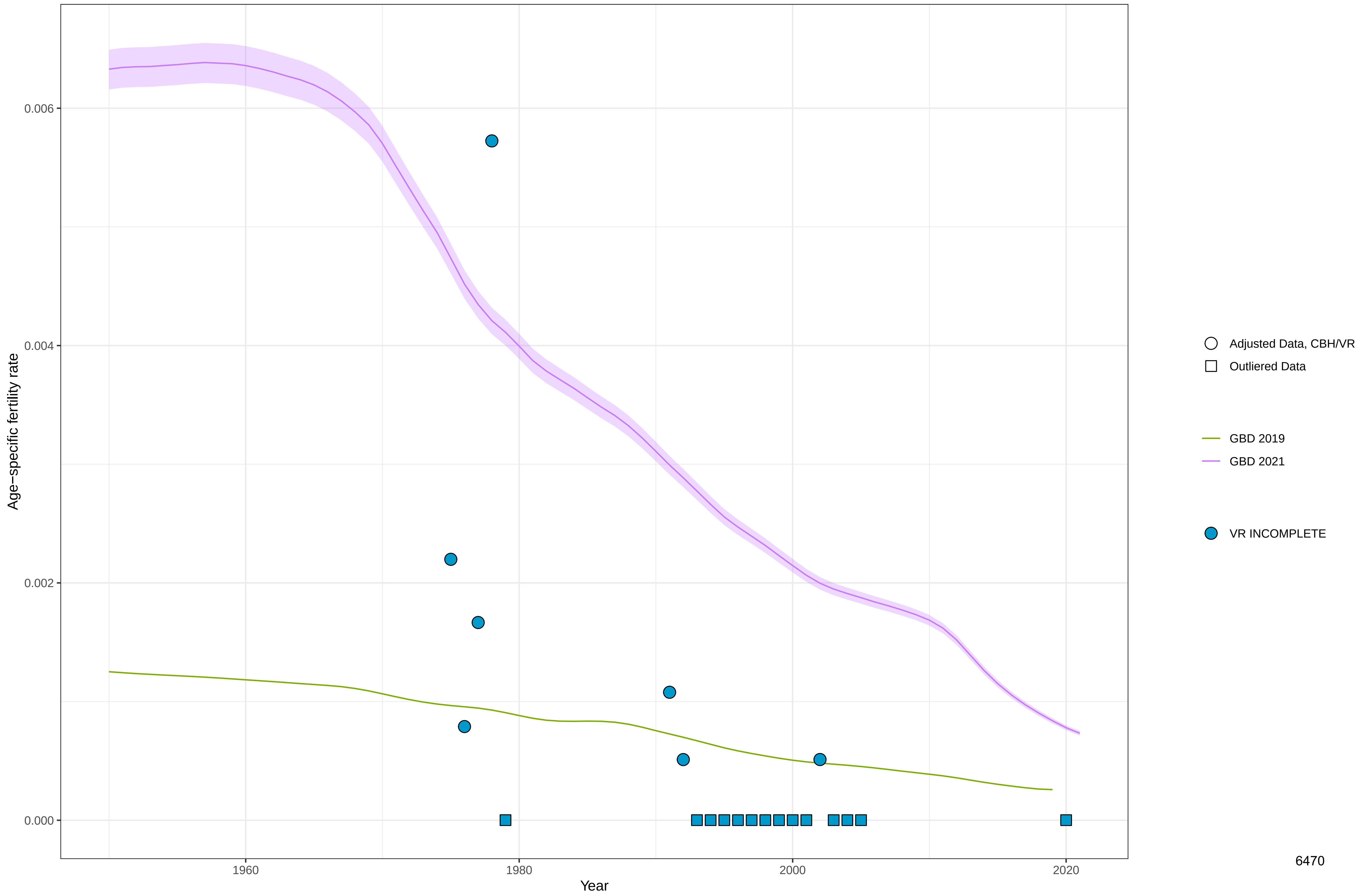

Total fertility rate fitted trend lines, 1950−2021: Siquijor TFR
Data points enclosed with a diamond are reference sources

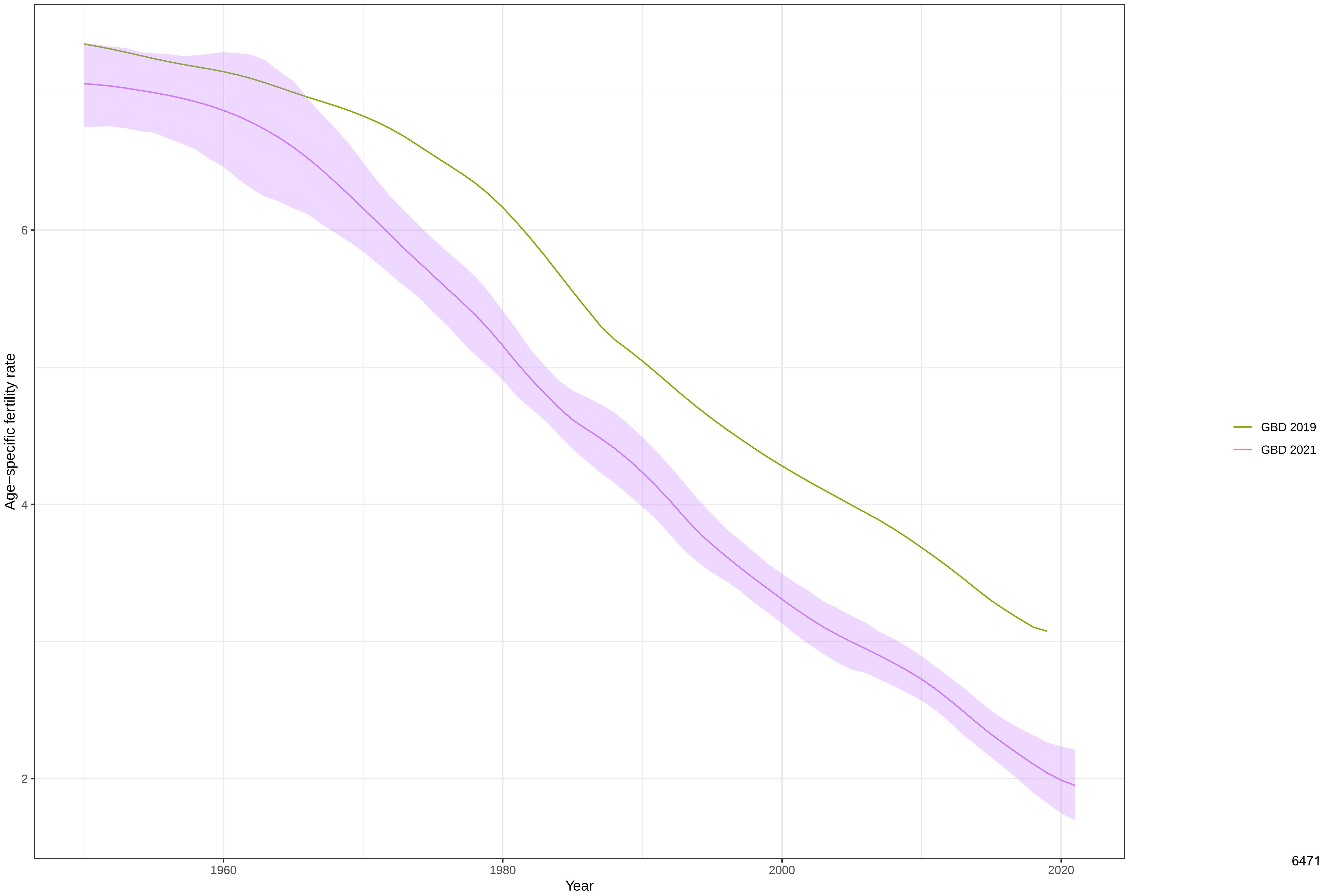

6471

Age−specific fertility fitted trend lines, 1950−2021: Sorsogon, Ages: 10−14
Data points enclosed with a diamond are reference sources

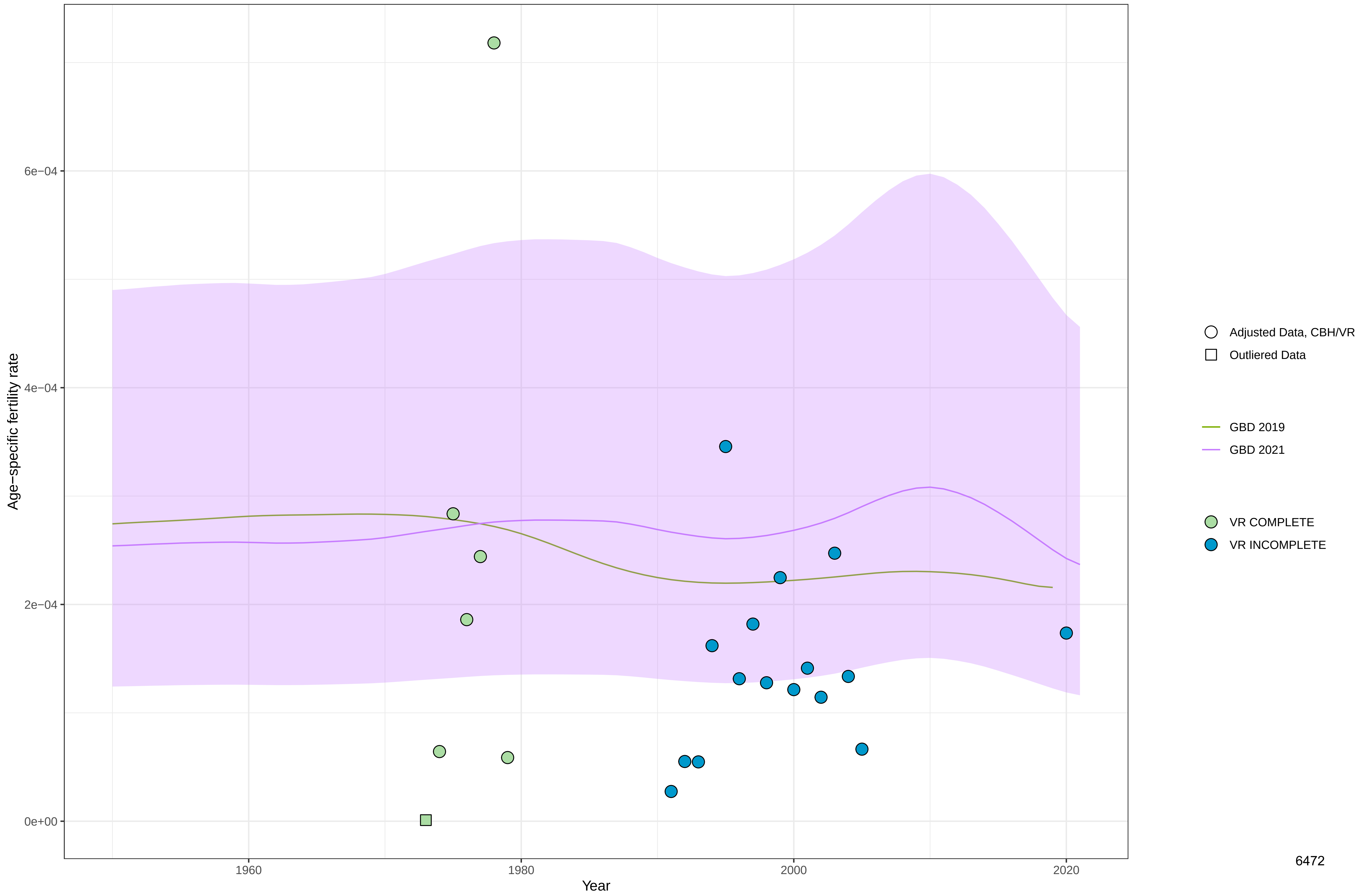

Age−specific fertility fitted trend lines, 1950−2021: Sorsogon, Ages: 15−19
Data points enclosed with a diamond are reference sources

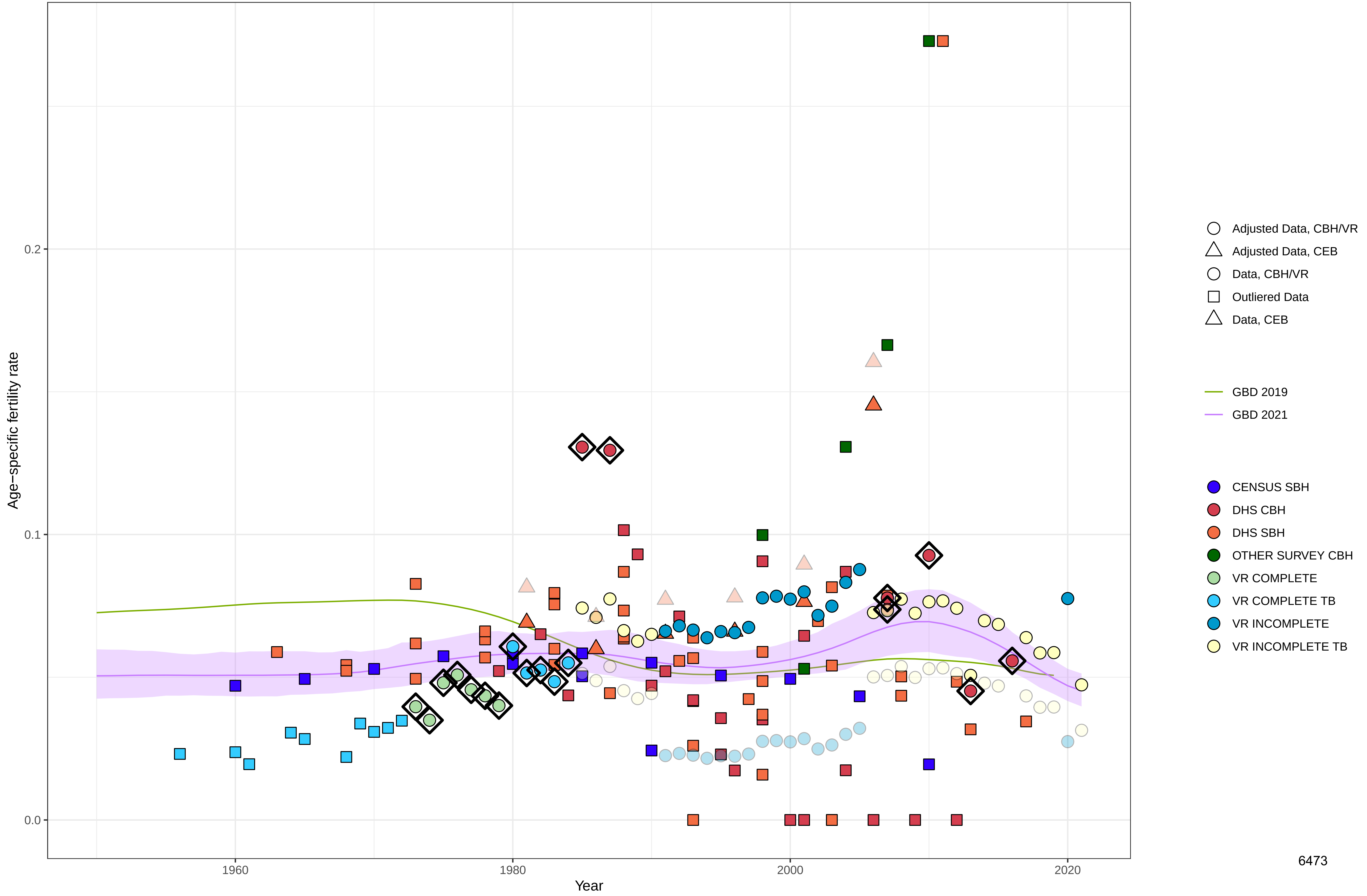

Age−specific fertility fitted trend lines, 1950−2021: Sorsogon, Ages: 20−24
Data points enclosed with a diamond are reference sources

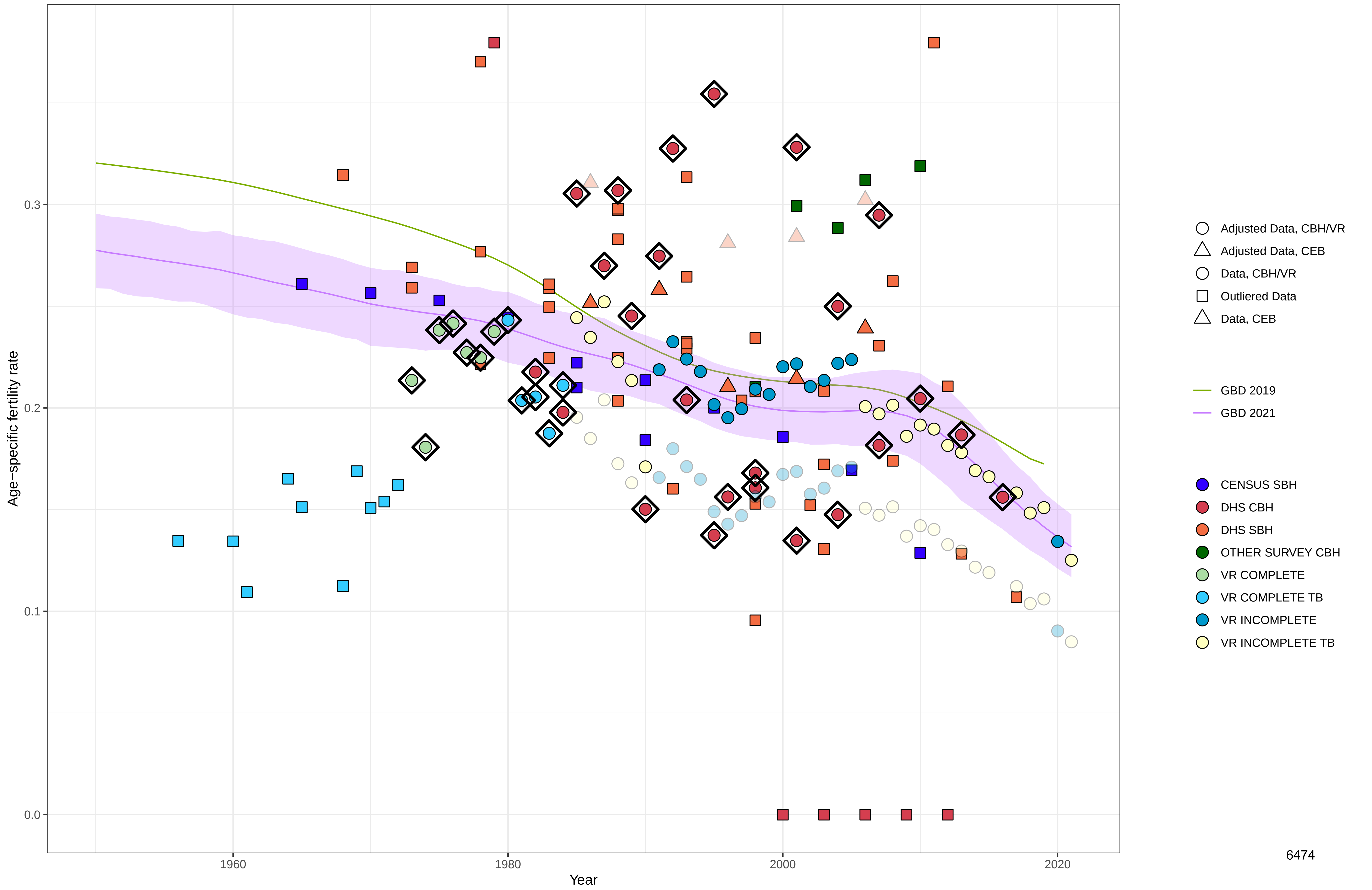

Age−specific fertility fitted trend lines, 1950−2021: Sorsogon, Ages: 25−29
Data points enclosed with a diamond are reference sources

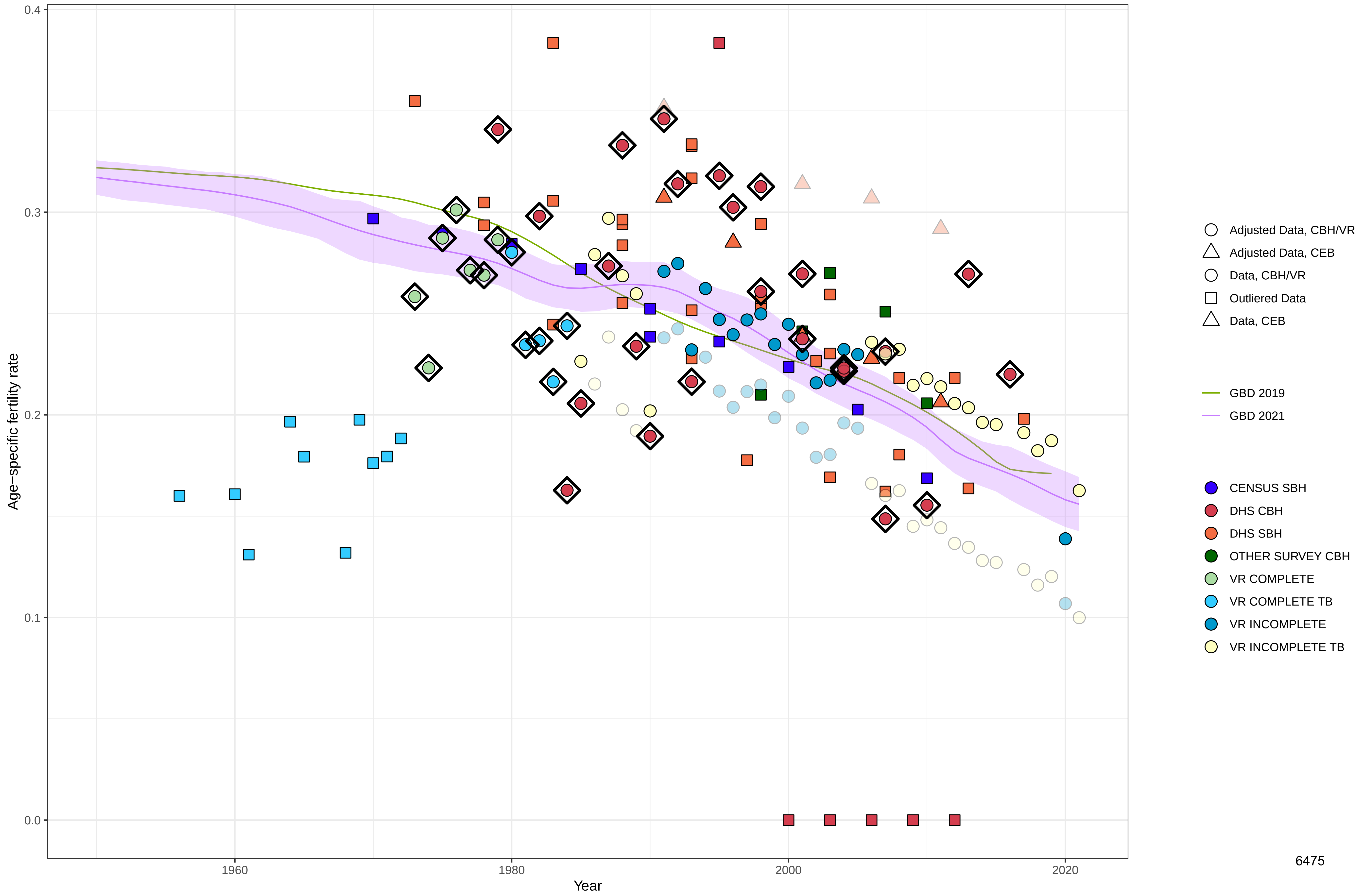

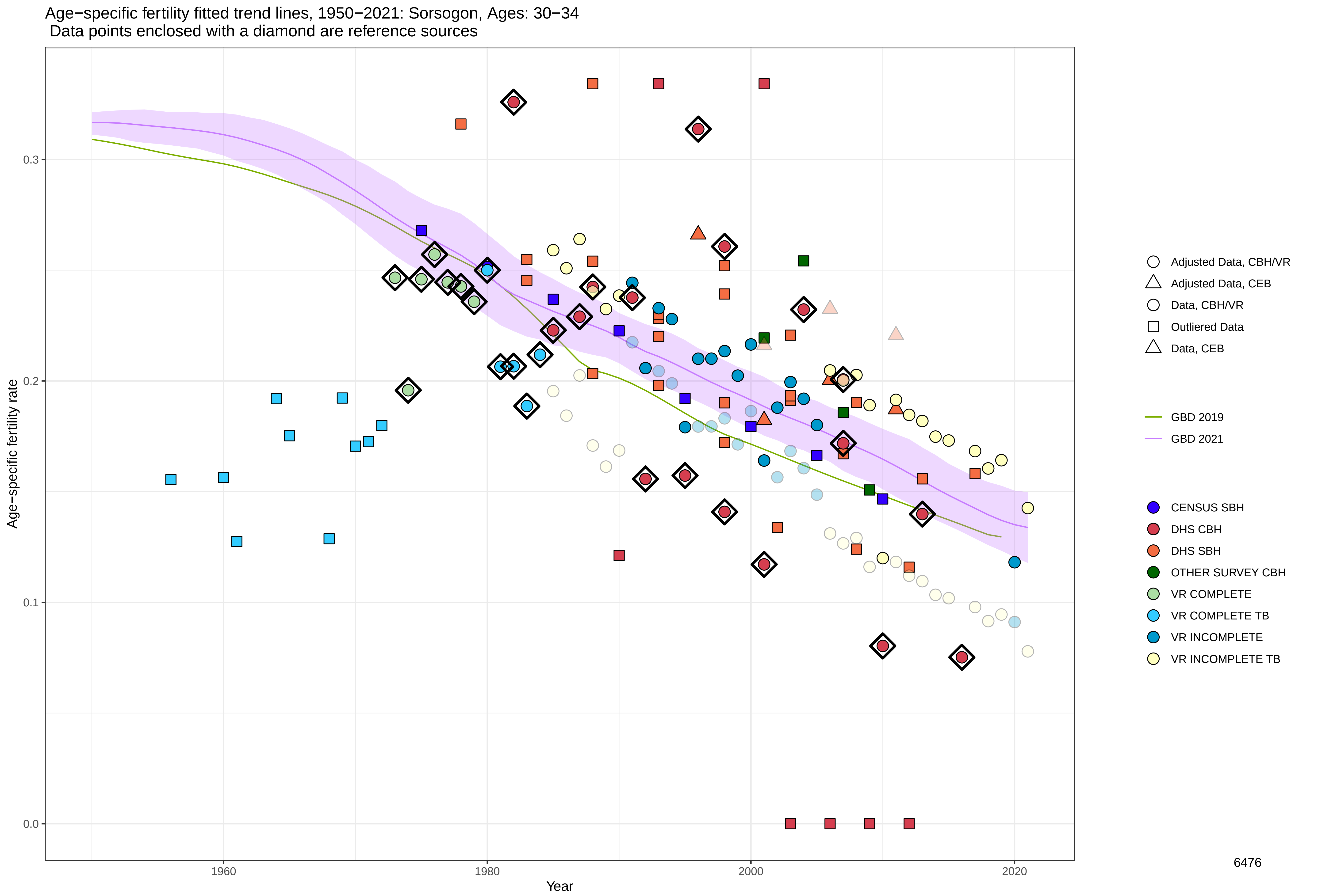
Age−specific fertility fitted trend lines, 1950−2021: Sorsogon, Ages: 30−34
Data points enclosed with a diamond are reference sources
Age−specific fertility rate
Year
0.0
0.1
0.2
0.3
1960
1980
2000
2020
Adjusted Data, CBH/VR
Adjusted Data, CEB
Data, CBH/VR
Outliered Data
Data, CEB
GBD 2019
GBD 2021
CENSUS SBH
DHS CBH
DHS SBH
OTHER SURVEY CBH
VR COMPLETE
VR COMPLETE TB
VR INCOMPLETE
VR INCOMPLETE TB

Age−specific fertility fitted trend lines, 1950−2021: Sorsogon, Ages: 35−39
Data points enclosed with a diamond are reference sources

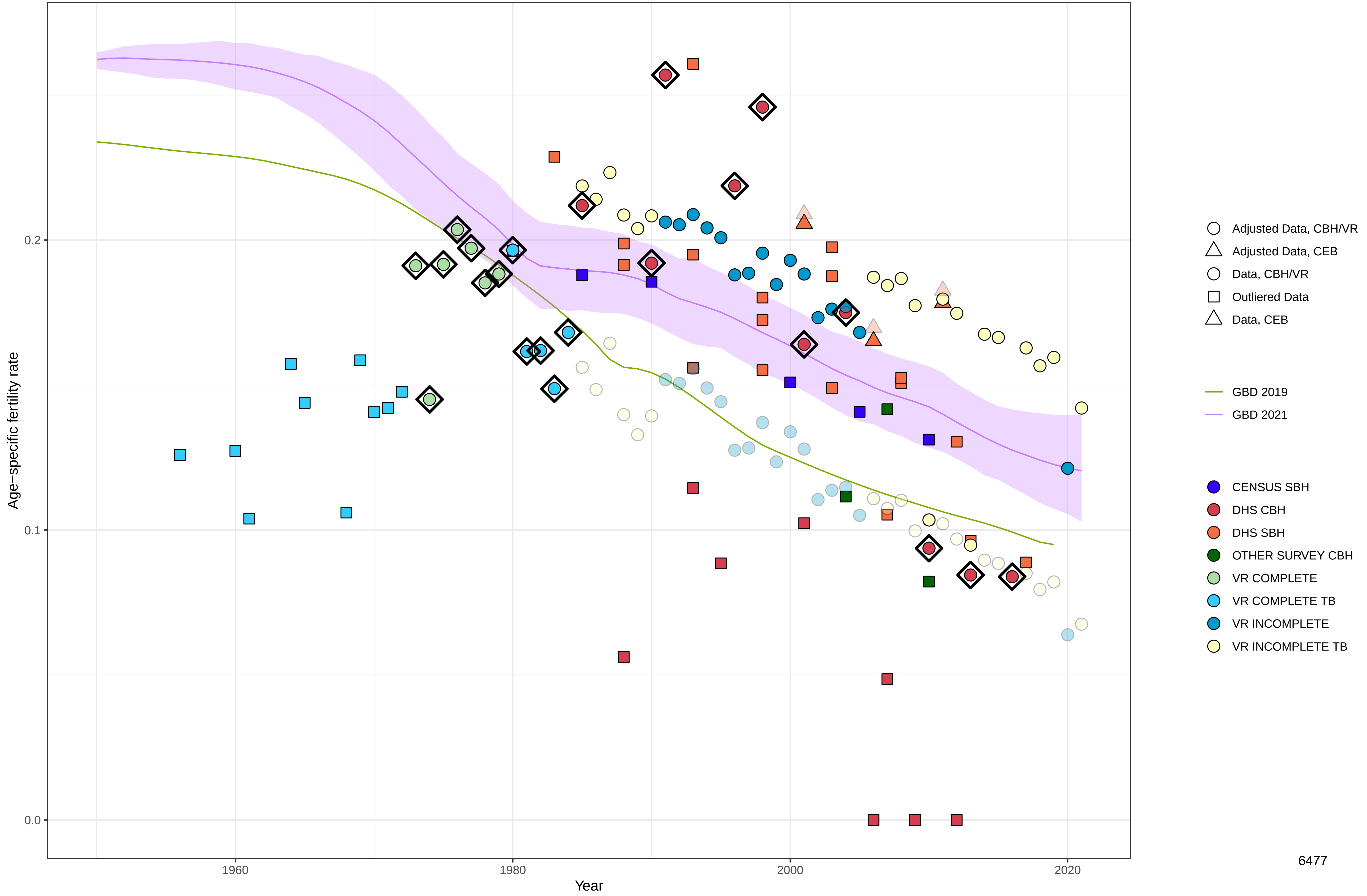

Age−specific fertility fitted trend lines, 1950−2021: Sorsogon, Ages: 40−44
Data points enclosed with a diamond are reference sources

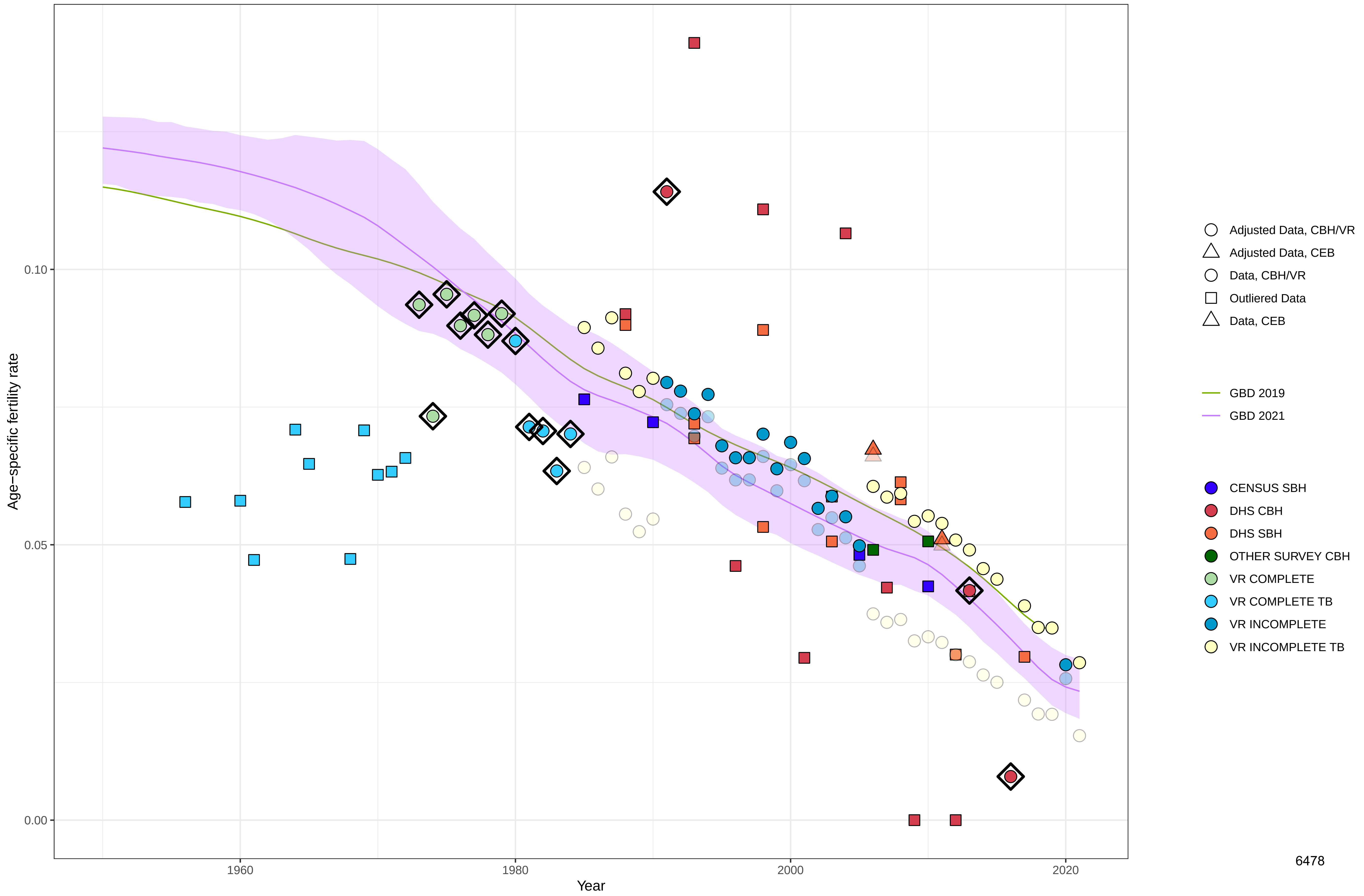

Age−specific fertility fitted trend lines, 1950−2021: Sorsogon, Ages: 45−49
Data points enclosed with a diamond are reference sources

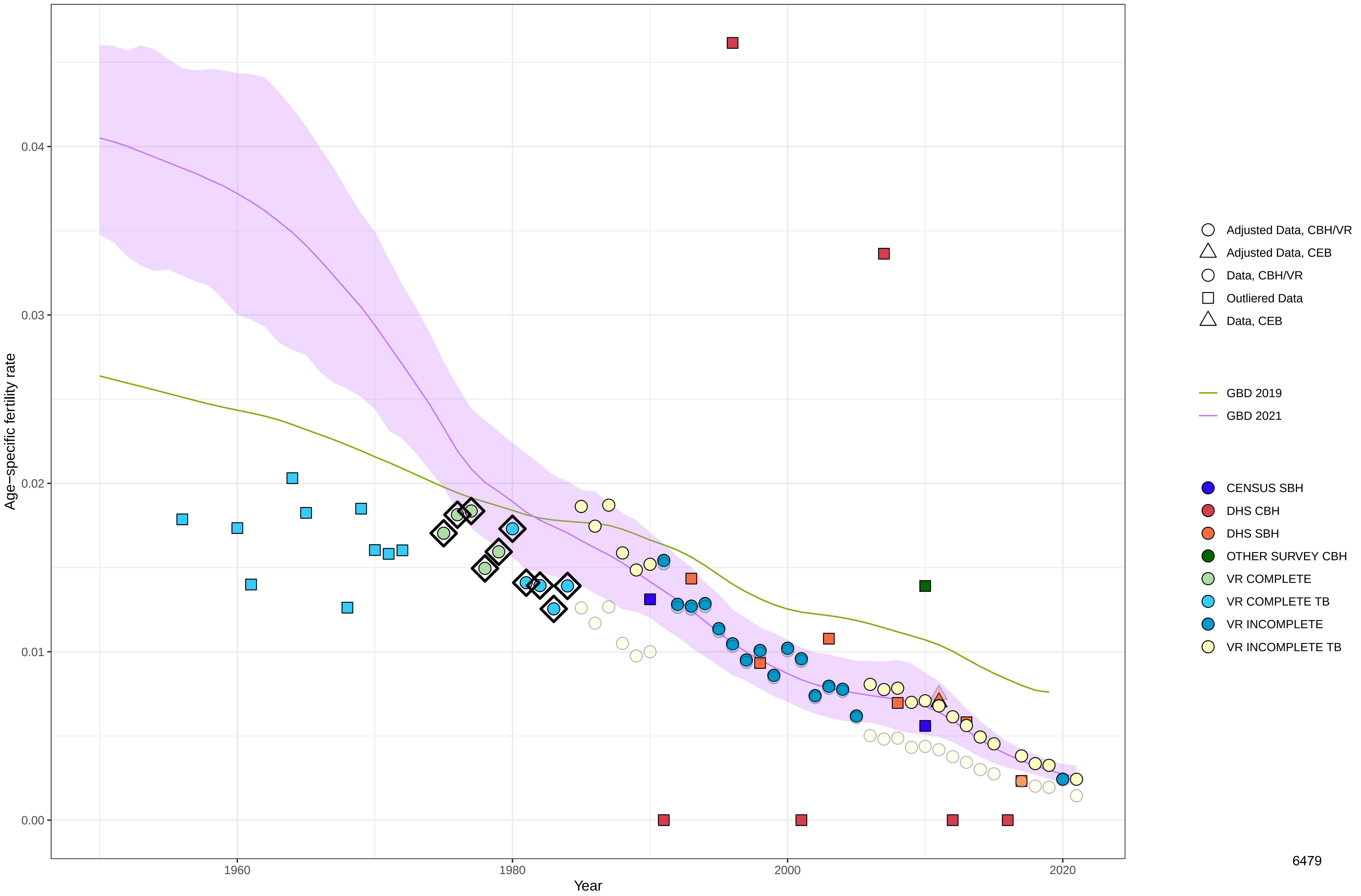

Age−specific fertility fitted trend lines, 1950−2021: Sorsogon, Ages: 50−54
Data points enclosed with a diamond are reference sources

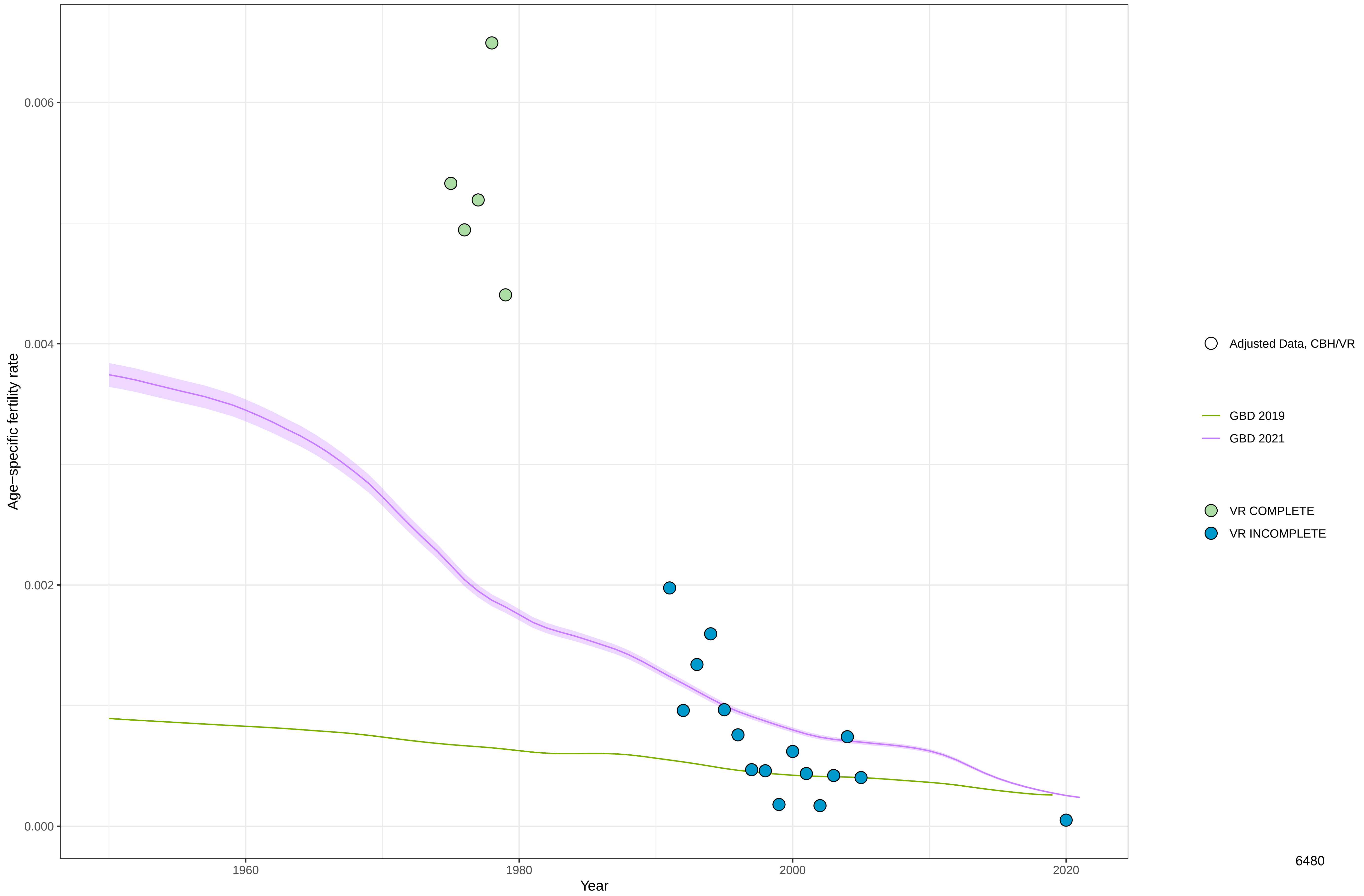

Total fertility rate fitted trend lines, 1950−2021: Sorsogon TFR
Data points enclosed with a diamond are reference sources

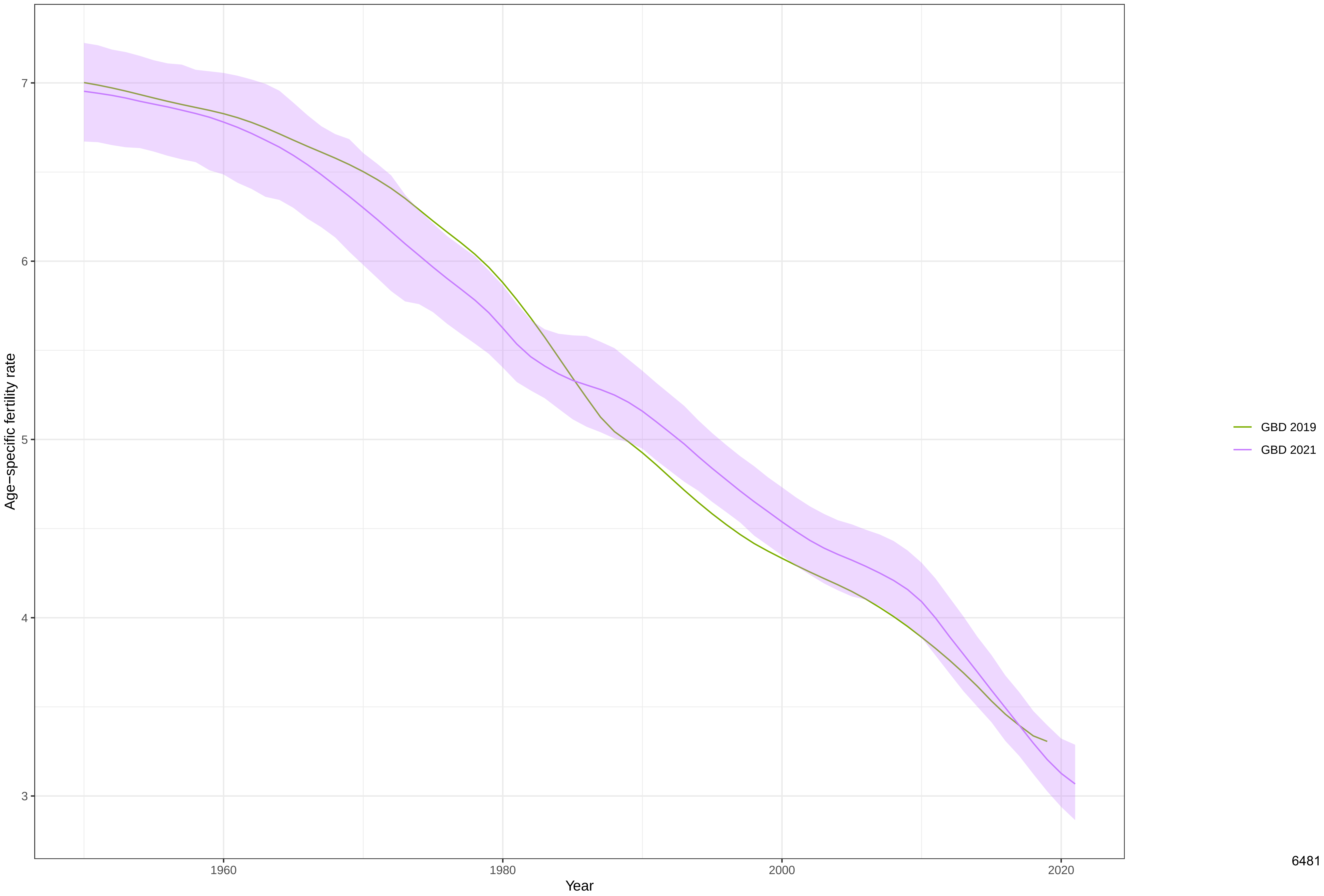

Age−specific fertility fitted trend lines, 1950−2021: South Cotabato, Ages: 10−14
Data points enclosed with a diamond are reference sources

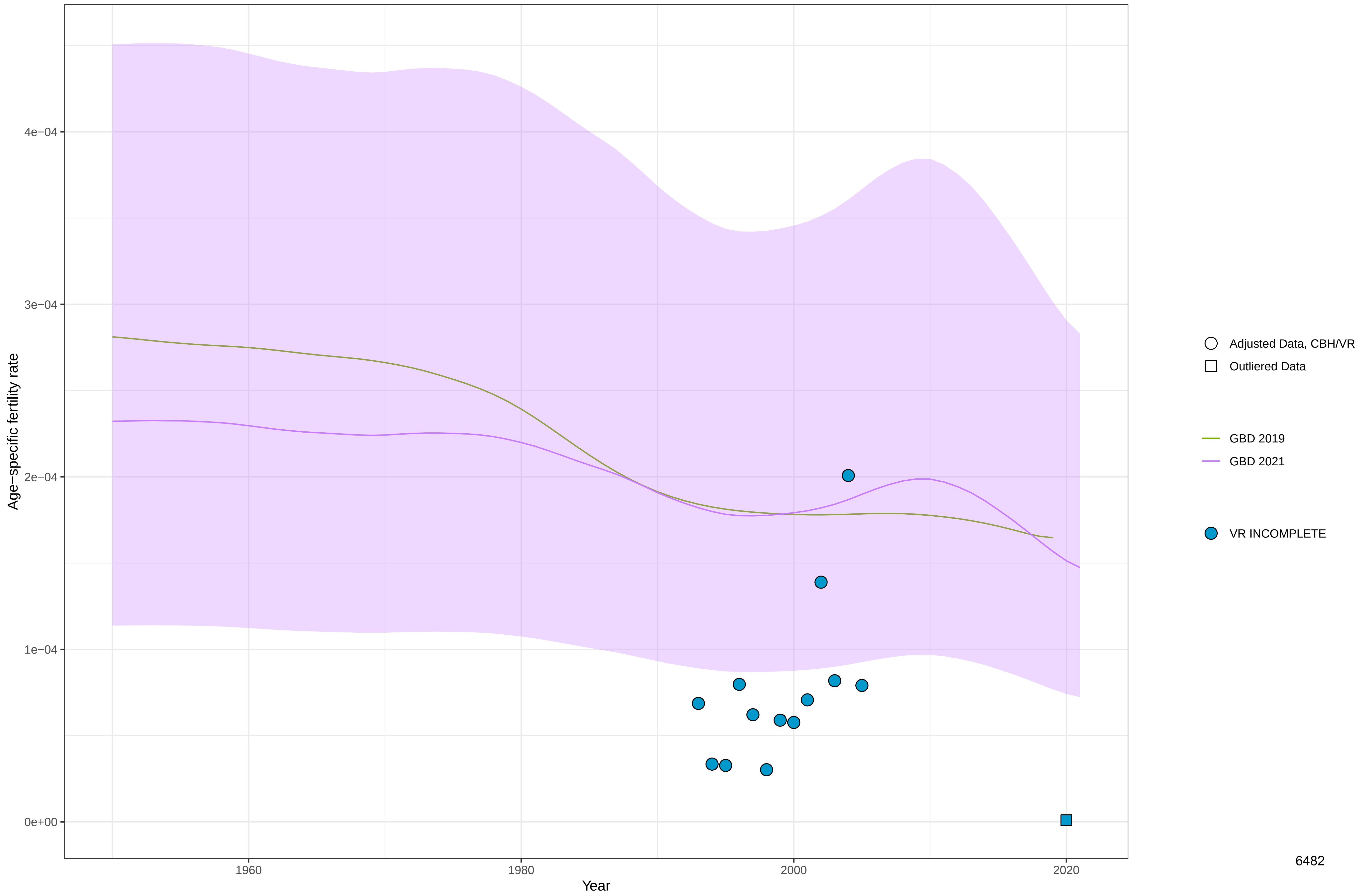

6482

Age−specific fertility fitted trend lines, 1950−2021: South Cotabato, Ages: 15−19
Data points enclosed with a diamond are reference sources

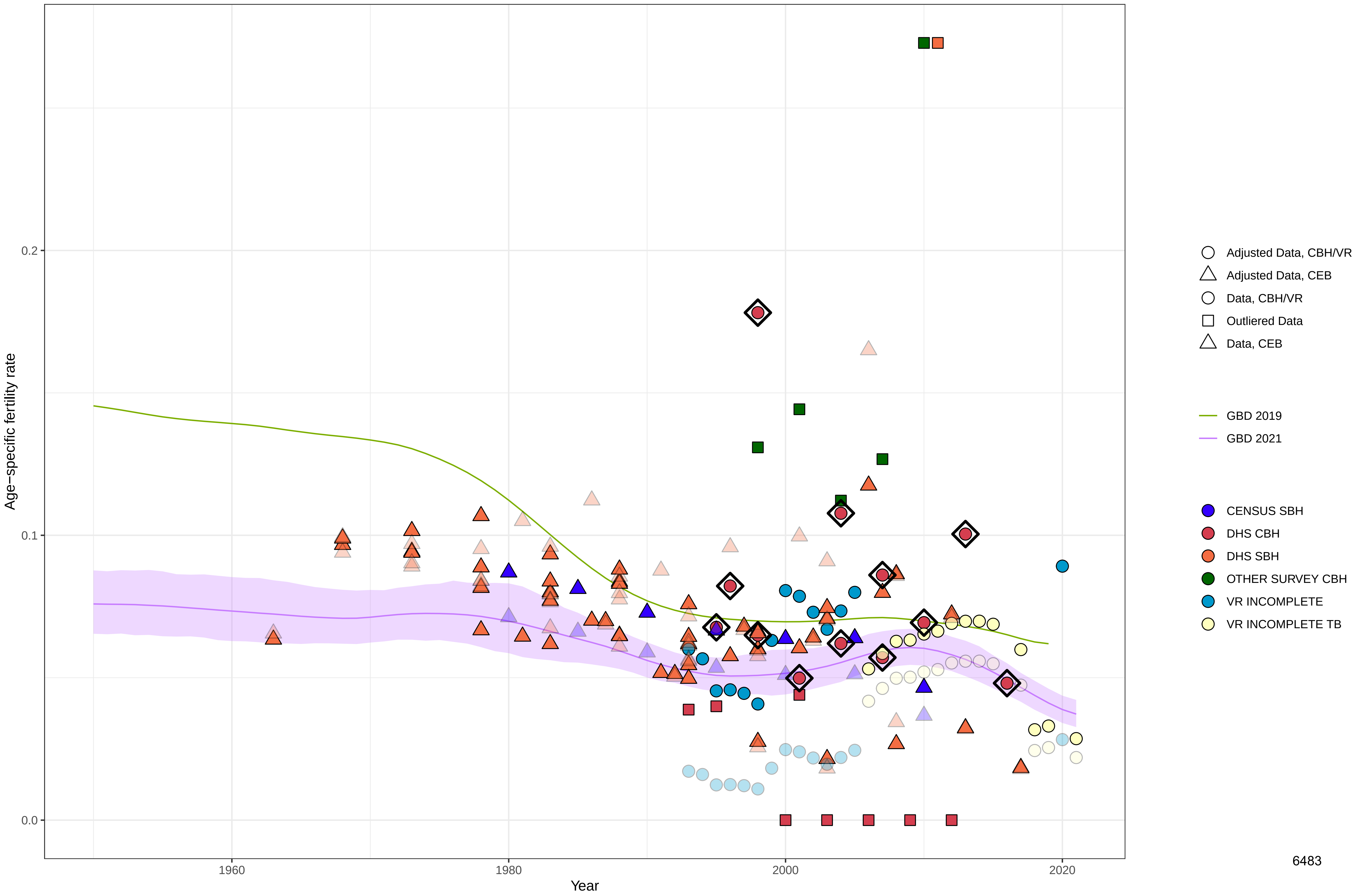

Age−specific fertility fitted trend lines, 1950−2021: South Cotabato, Ages: 20−24
Data points enclosed with a diamond are reference sources

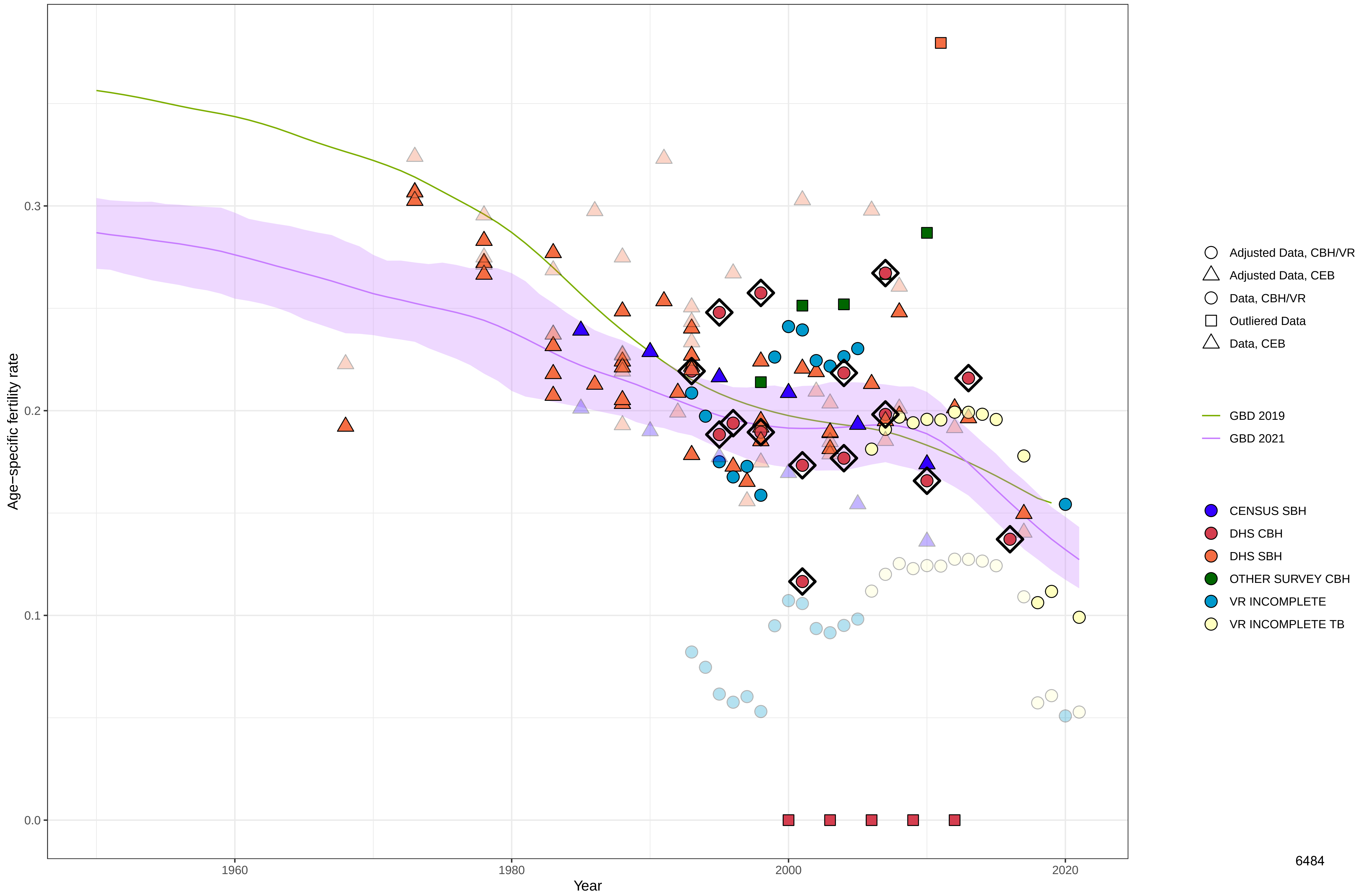

Age−specific fertility fitted trend lines, 1950−2021: South Cotabato, Ages: 25−29
Data points enclosed with a diamond are reference sources

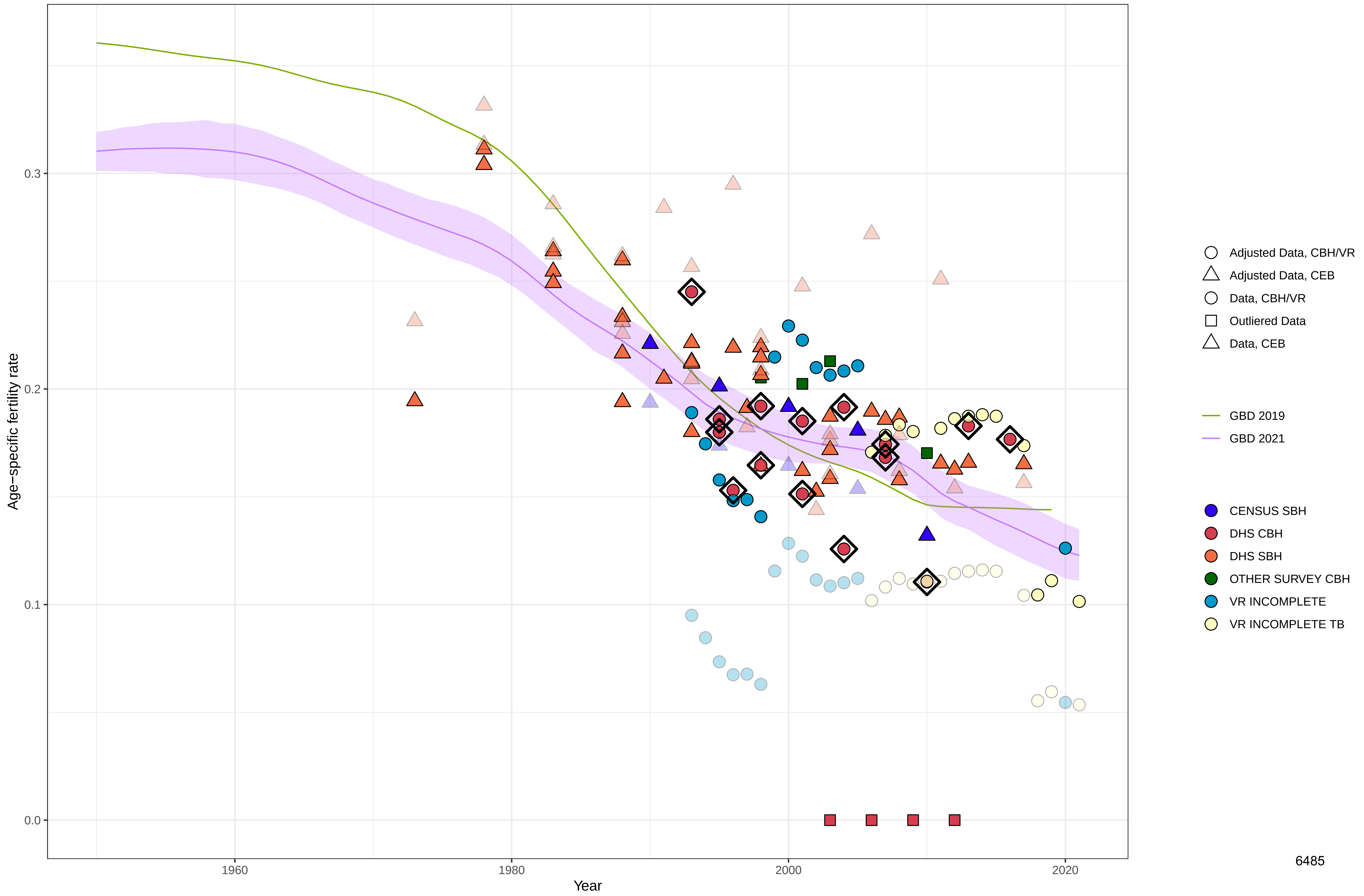

Age−specific fertility fitted trend lines, 1950−2021: South Cotabato, Ages: 30−34
Data points enclosed with a diamond are reference sources

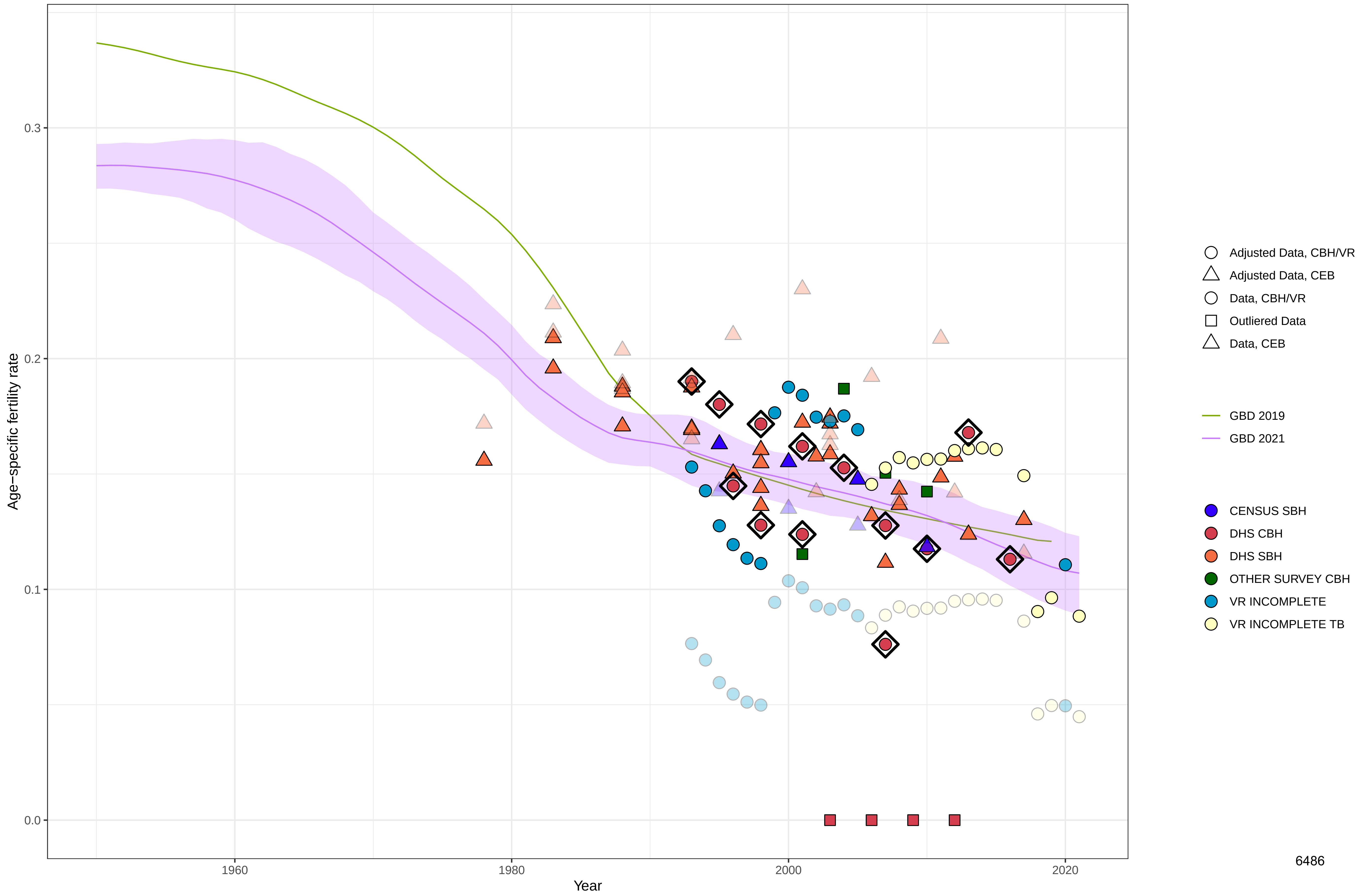

Age−specific fertility fitted trend lines, 1950−2021: South Cotabato, Ages: 35−39
Data points enclosed with a diamond are reference sources

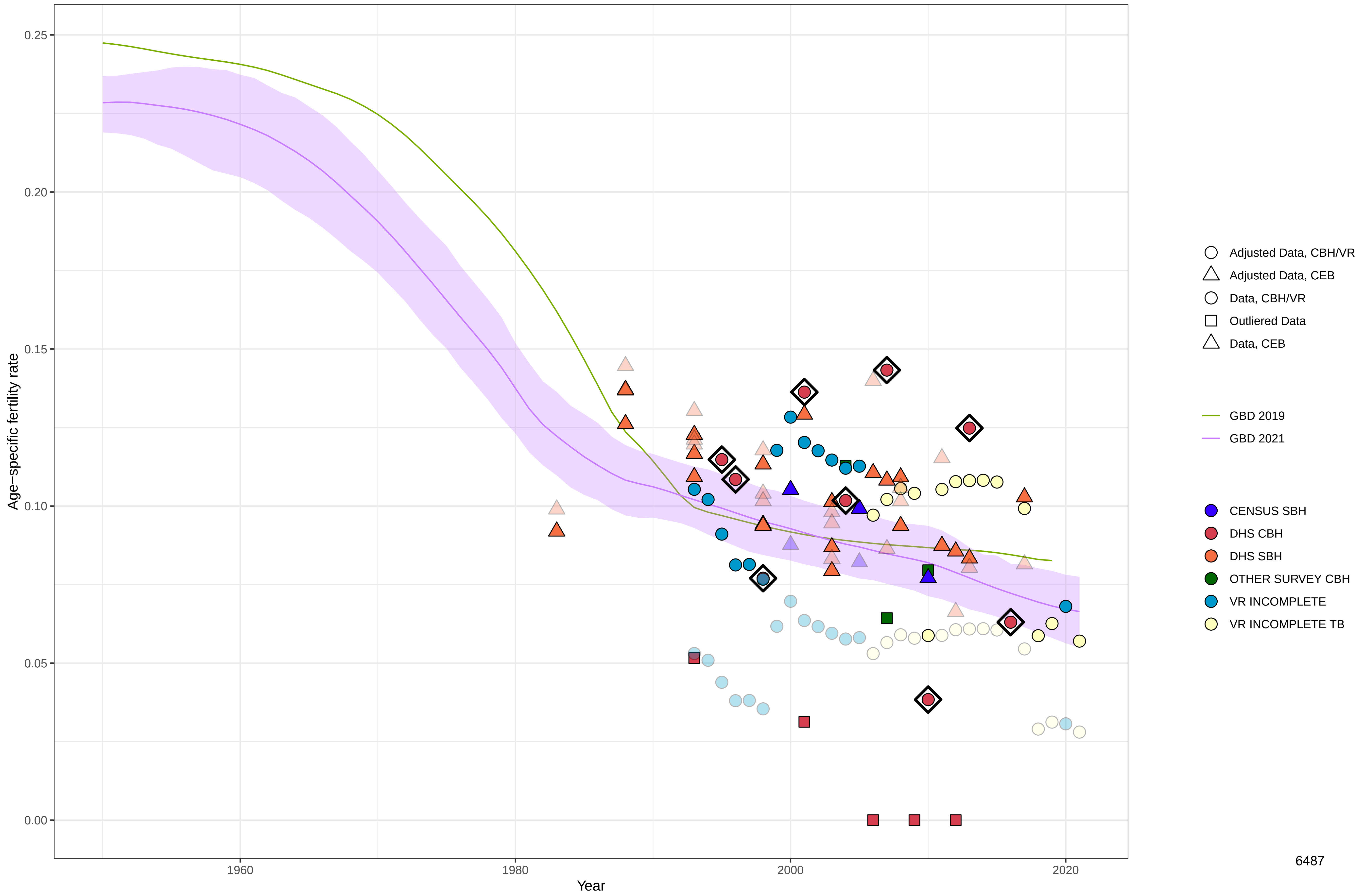

Age−specific fertility fitted trend lines, 1950−2021: South Cotabato, Ages: 40−44
Data points enclosed with a diamond are reference sources

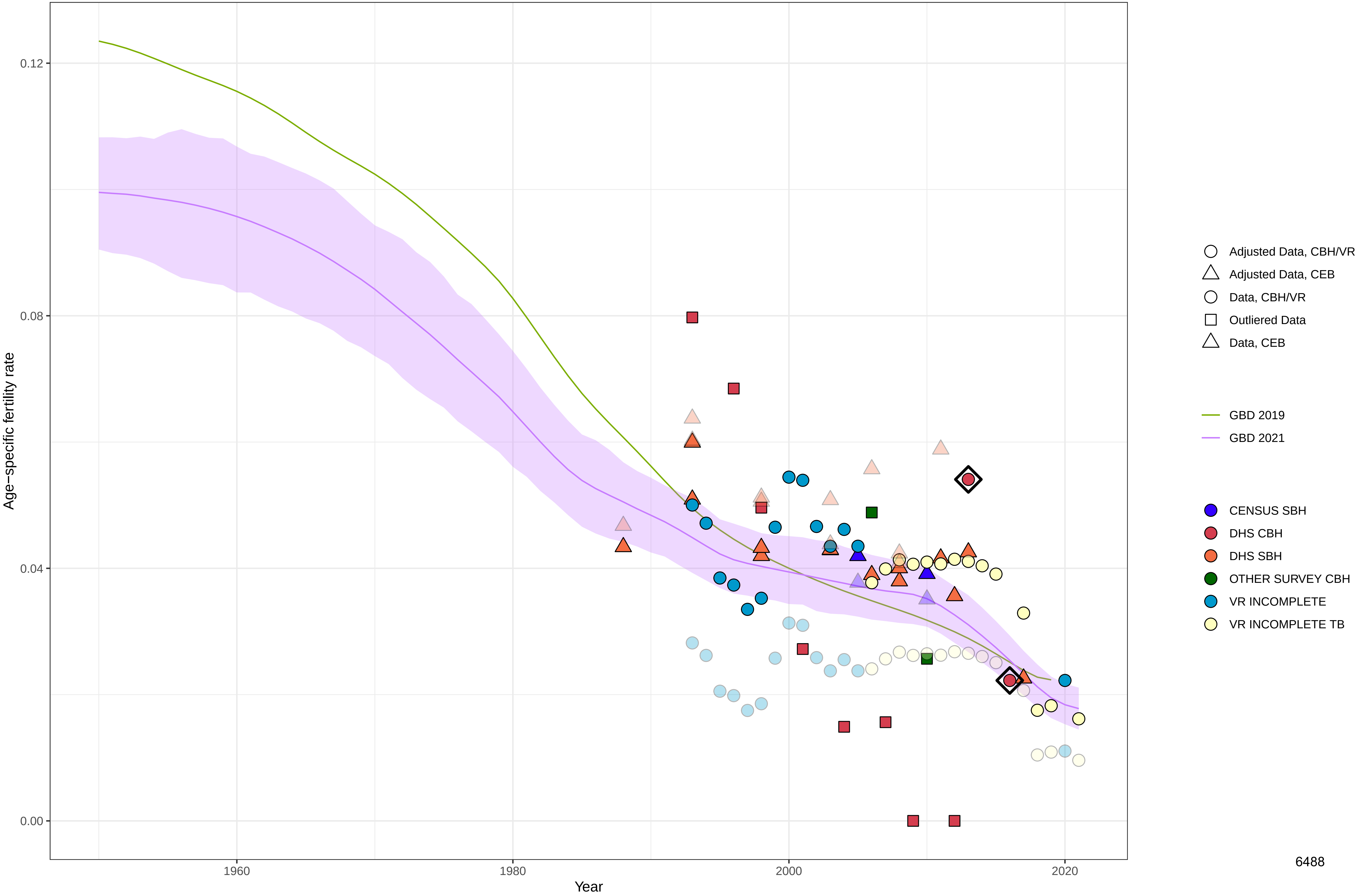

Age−specific fertility fitted trend lines, 1950−2021: South Cotabato, Ages: 45−49
Data points enclosed with a diamond are reference sources

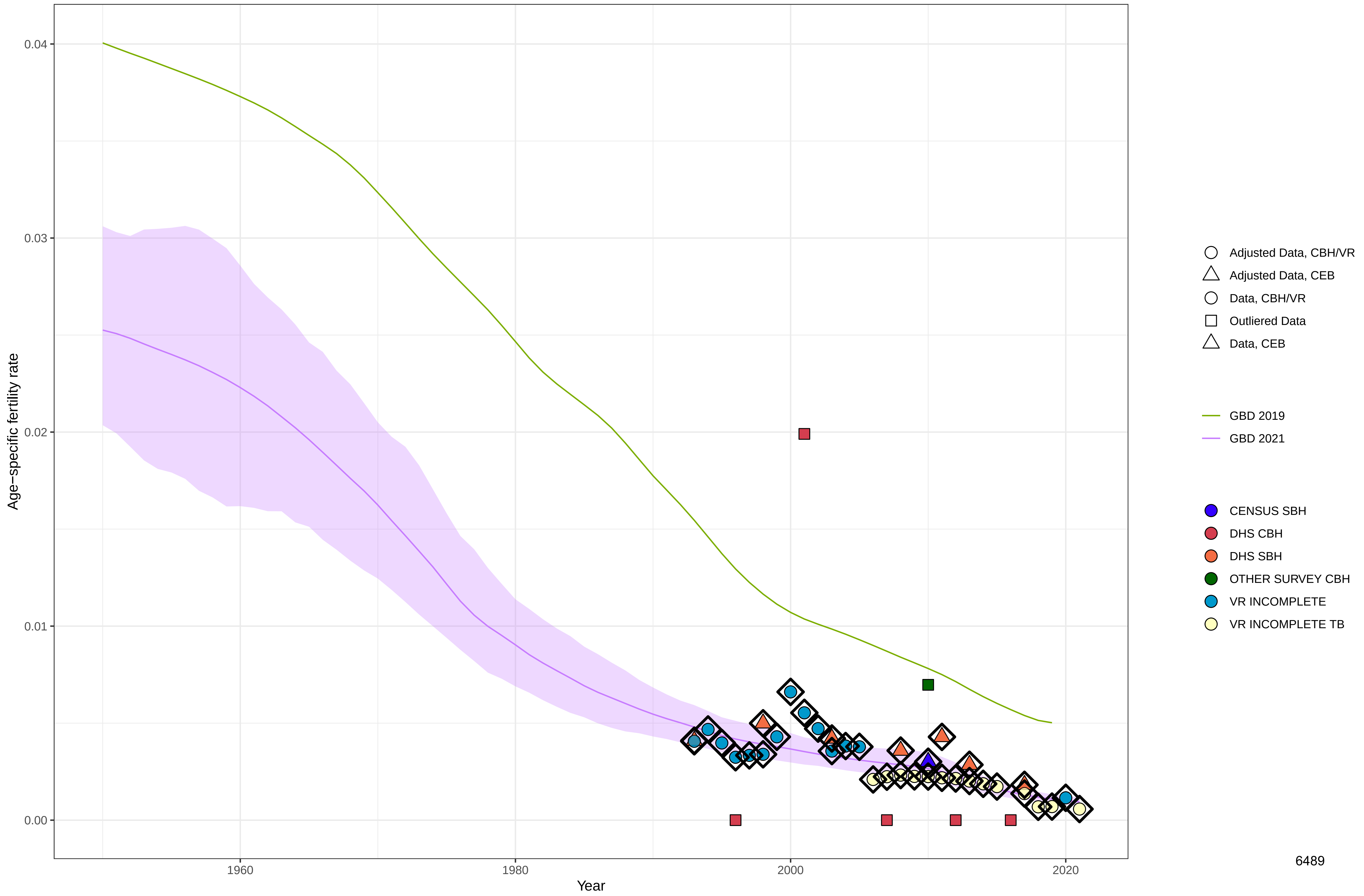

6489

Age−specific fertility fitted trend lines, 1950−2021: South Cotabato, Ages: 50−54
Data points enclosed with a diamond are reference sources

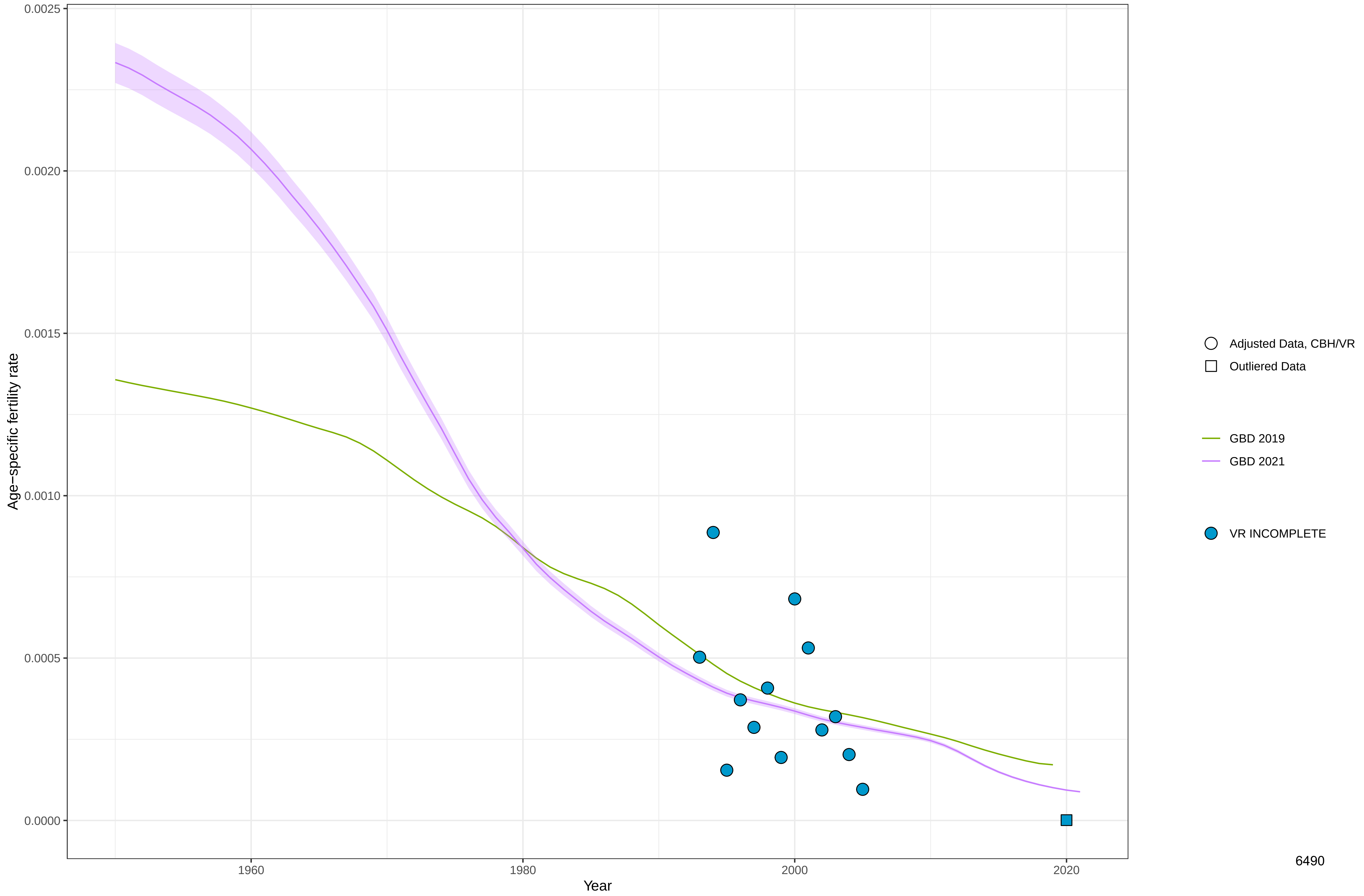

Total fertility rate fitted trend lines, 1950−2021: South Cotabato TFR
Data points enclosed with a diamond are reference sources

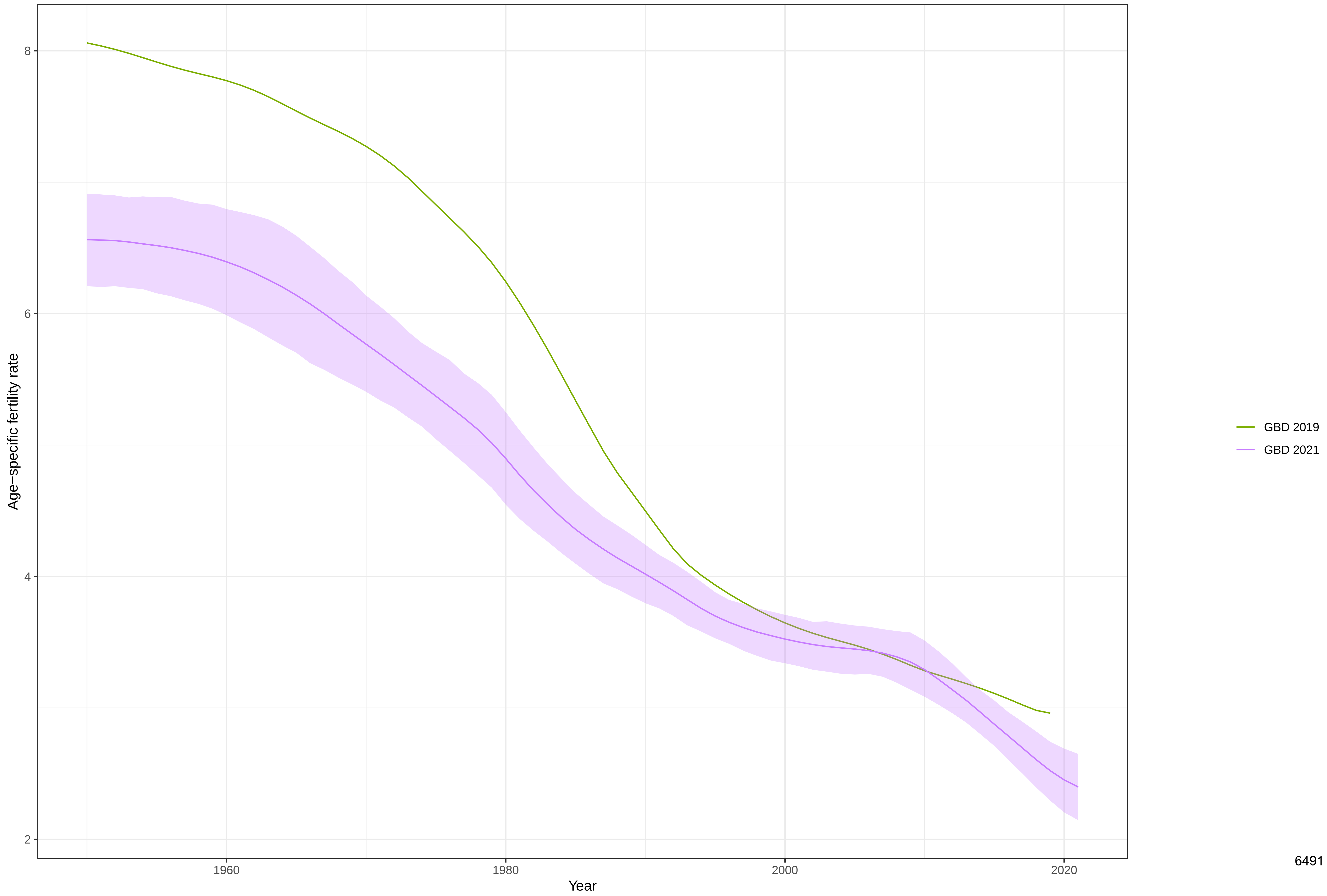

6491

Age−specific fertility fitted trend lines, 1950−2021: Southern Leyte, Ages: 10−14
Data points enclosed with a diamond are reference sources

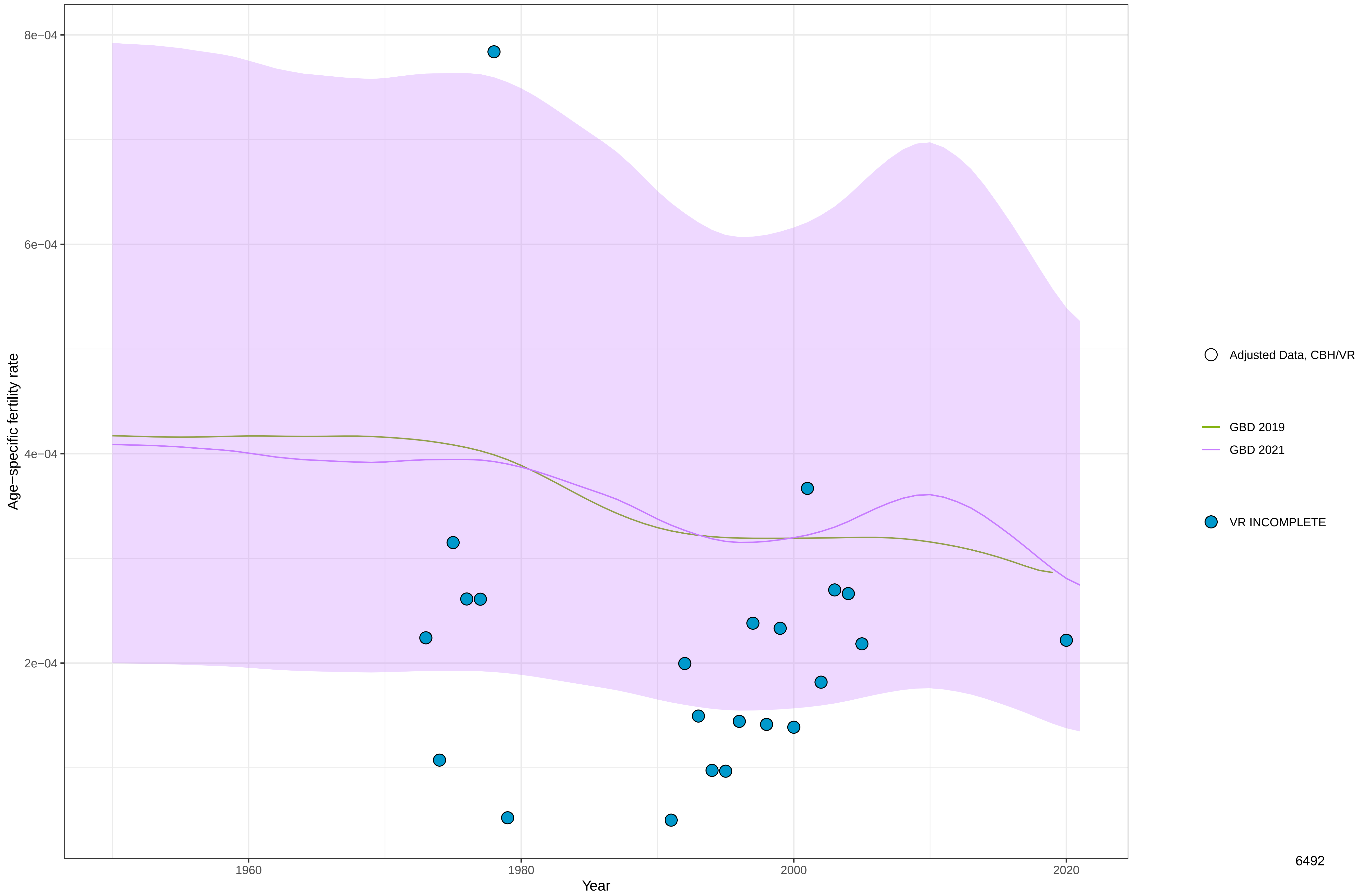

6492

Age−specific fertility fitted trend lines, 1950−2021: Southern Leyte, Ages: 15−19
Data points enclosed with a diamond are reference sources

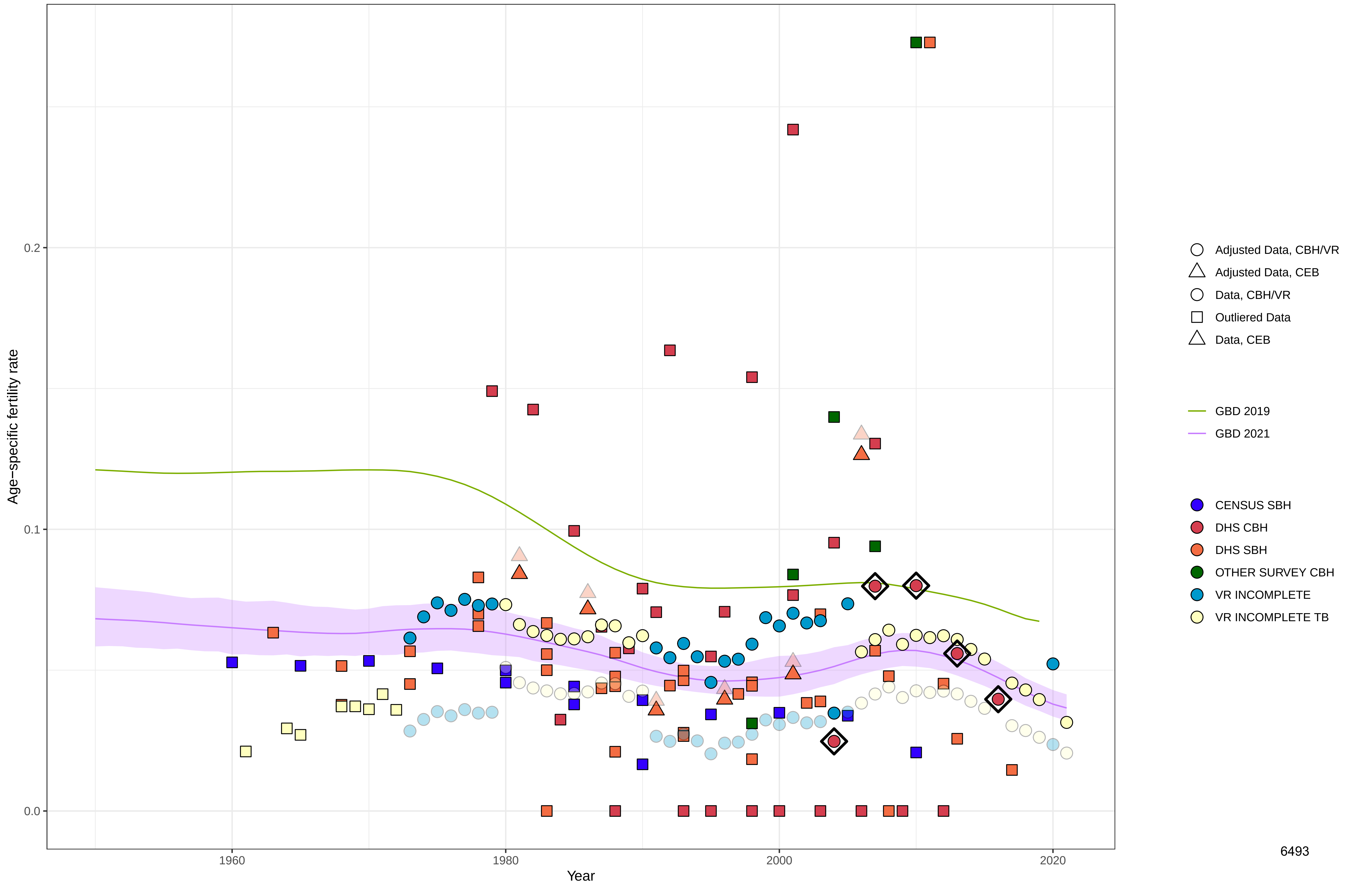

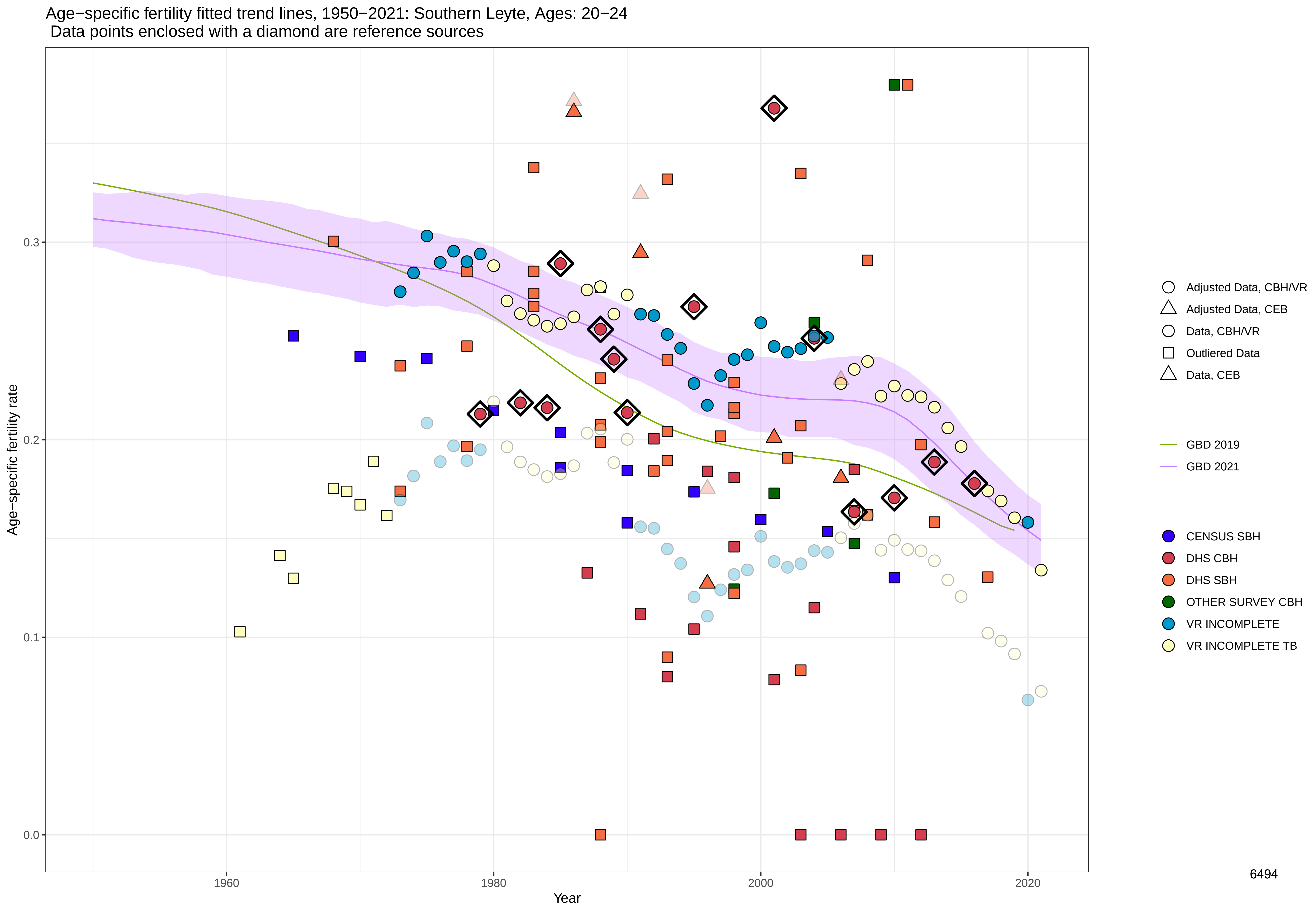

Age−specific fertility fitted trend lines, 1950−2021: Southern Leyte, Ages: 20−24
Data points enclosed with a diamond are reference sources
Age−specific fertility rate
Year
0.0
0.1
0.2
0.3
1960
1980
2000
2020
Adjusted Data, CBH/VR
Adjusted Data, CEB
Data, CBH/VR
Outliered Data
Data, CEB
GBD 2019
GBD 2021
CENSUS SBH
DHS CBH
DHS SBH
OTHER SURVEY CBH
VR INCOMPLETE
VR INCOMPLETE TB

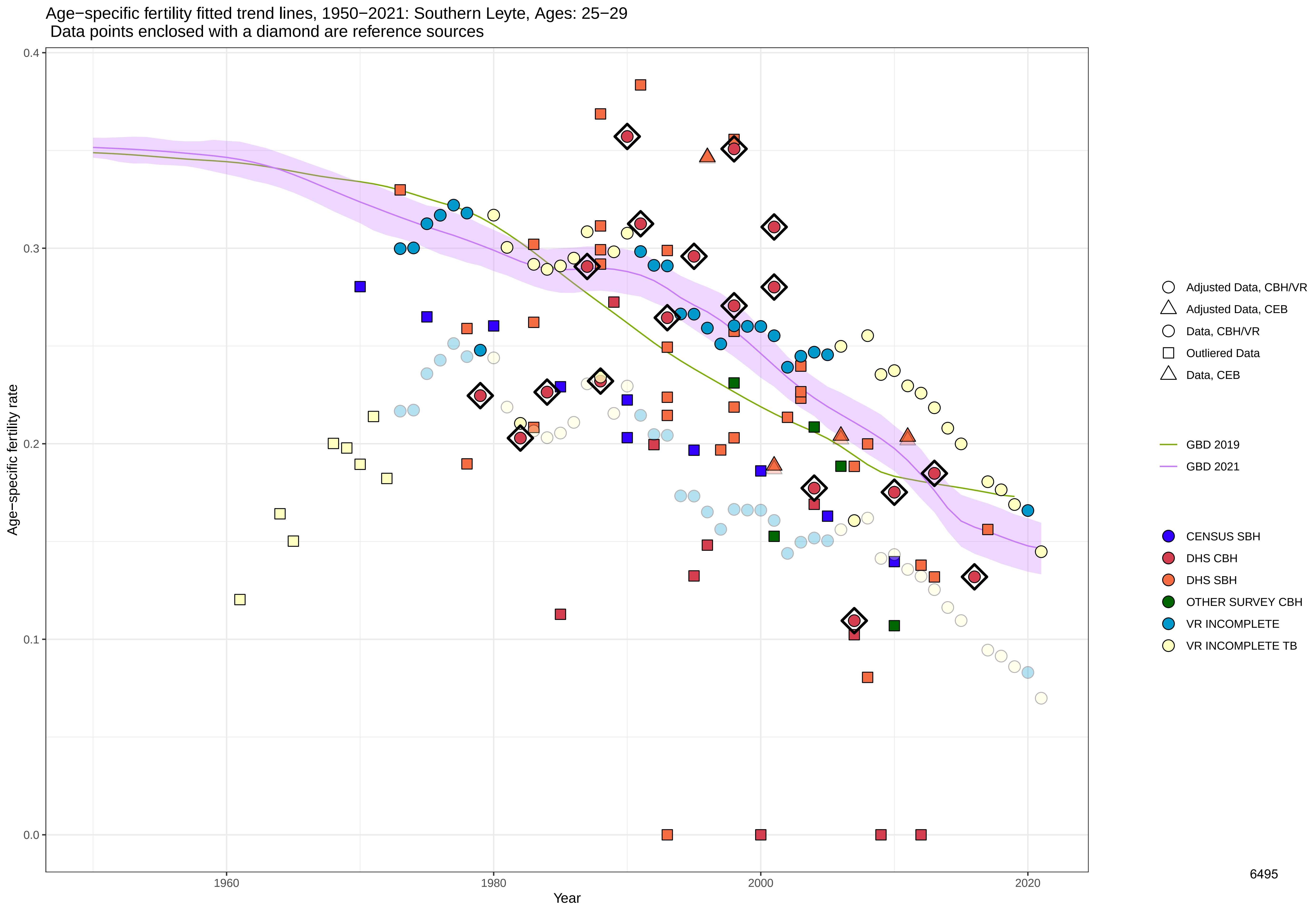

Age−specific fertility fitted trend lines, 1950−2021: Southern Leyte, Ages: 25−29
Data points enclosed with a diamond are reference sources
Age−specific fertility rate
Year
0.0
0.1
0.2
0.3
0.4
1960
1980
2000
2020
Adjusted Data, CBH/VR
Adjusted Data, CEB
Data, CBH/VR
Outliered Data
Data, CEB
GBD 2019
GBD 2021
CENSUS SBH
DHS CBH
DHS SBH
OTHER SURVEY CBH
VR INCOMPLETE
VR INCOMPLETE TB

6495

Age−specific fertility fitted trend lines, 1950−2021: Southern Leyte, Ages: 30−34
Data points enclosed with a diamond are reference sources

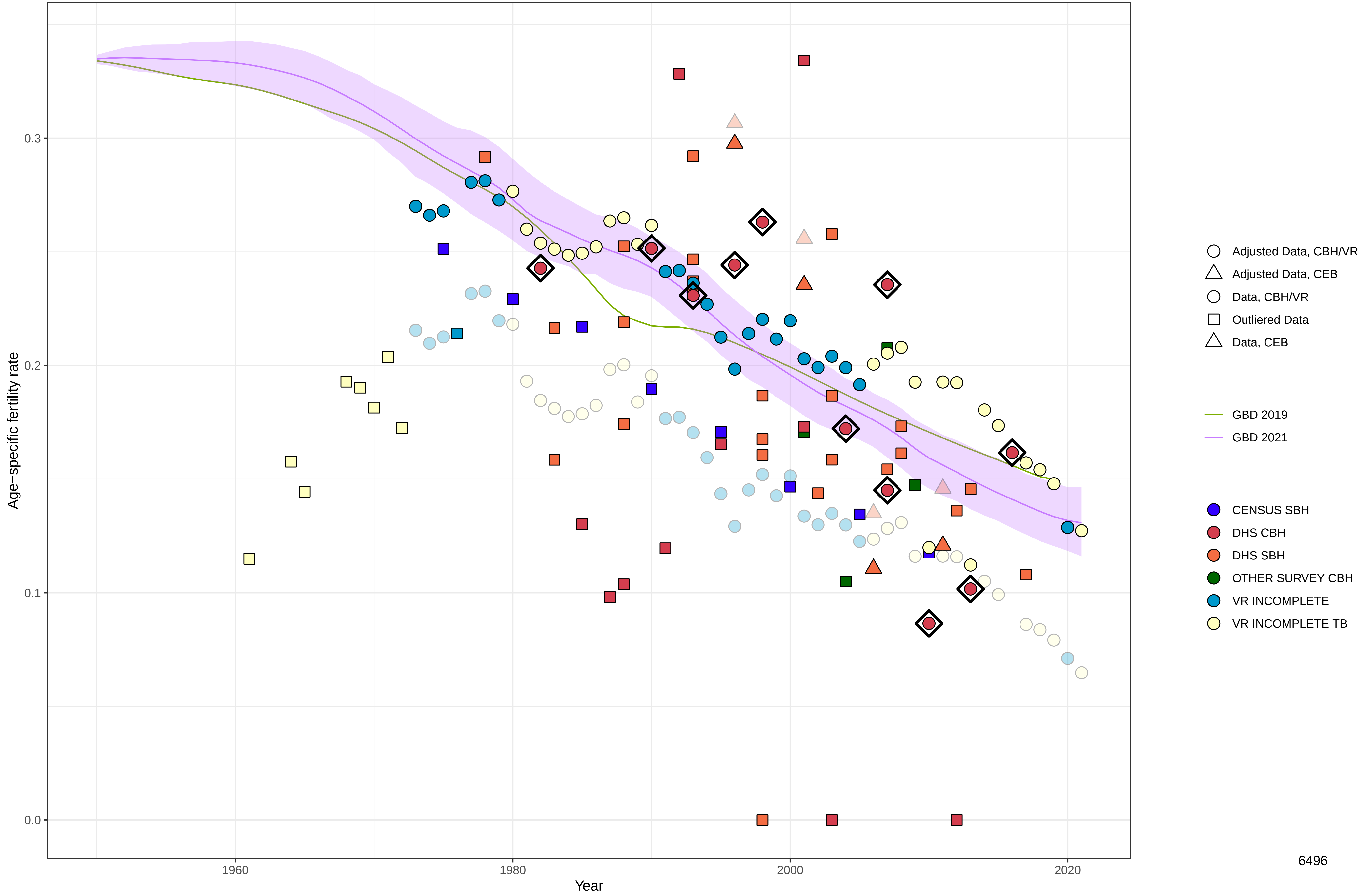

Age−specific fertility fitted trend lines, 1950−2021: Southern Leyte, Ages: 35−39
Data points enclosed with a diamond are reference sources

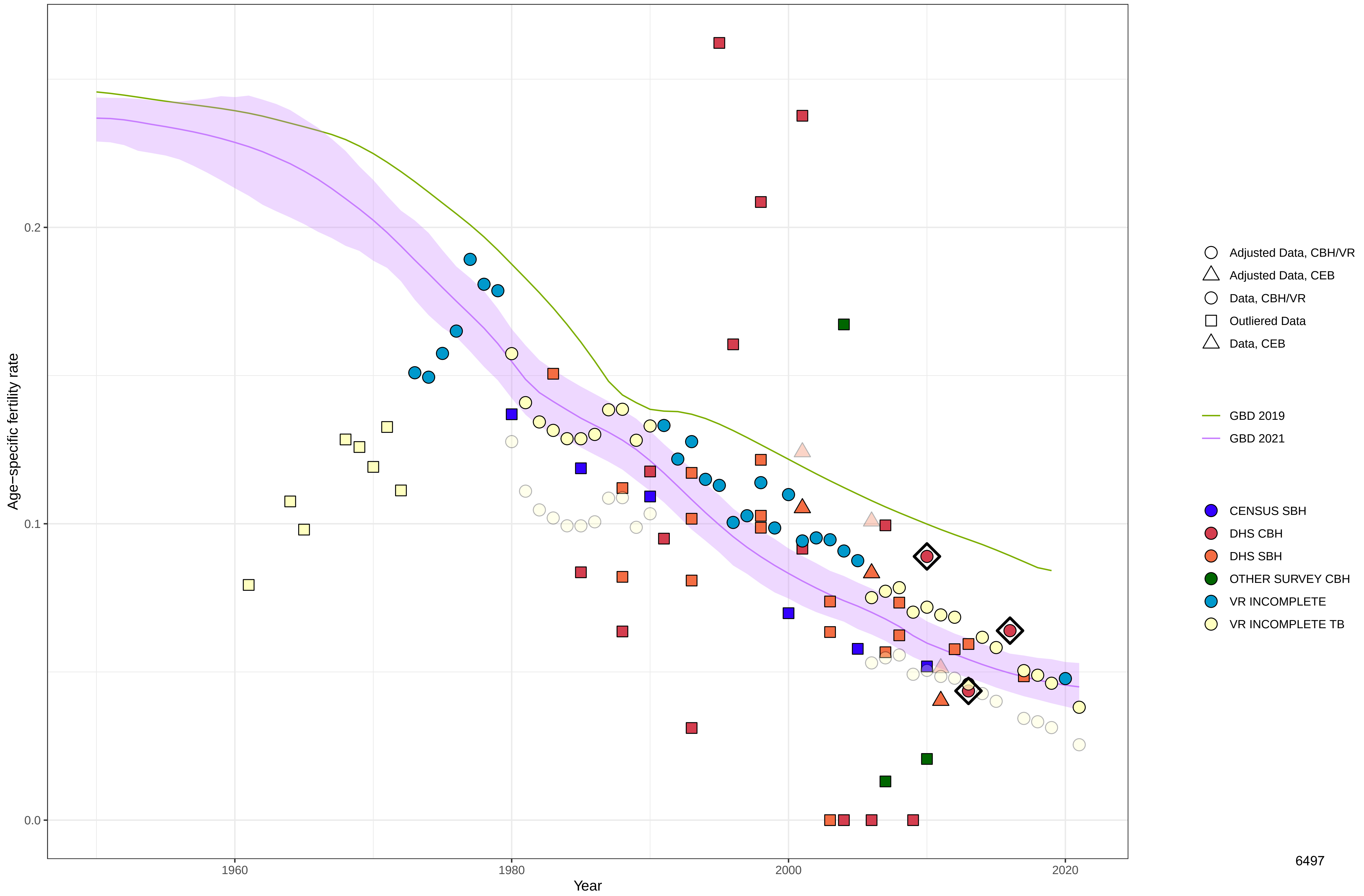

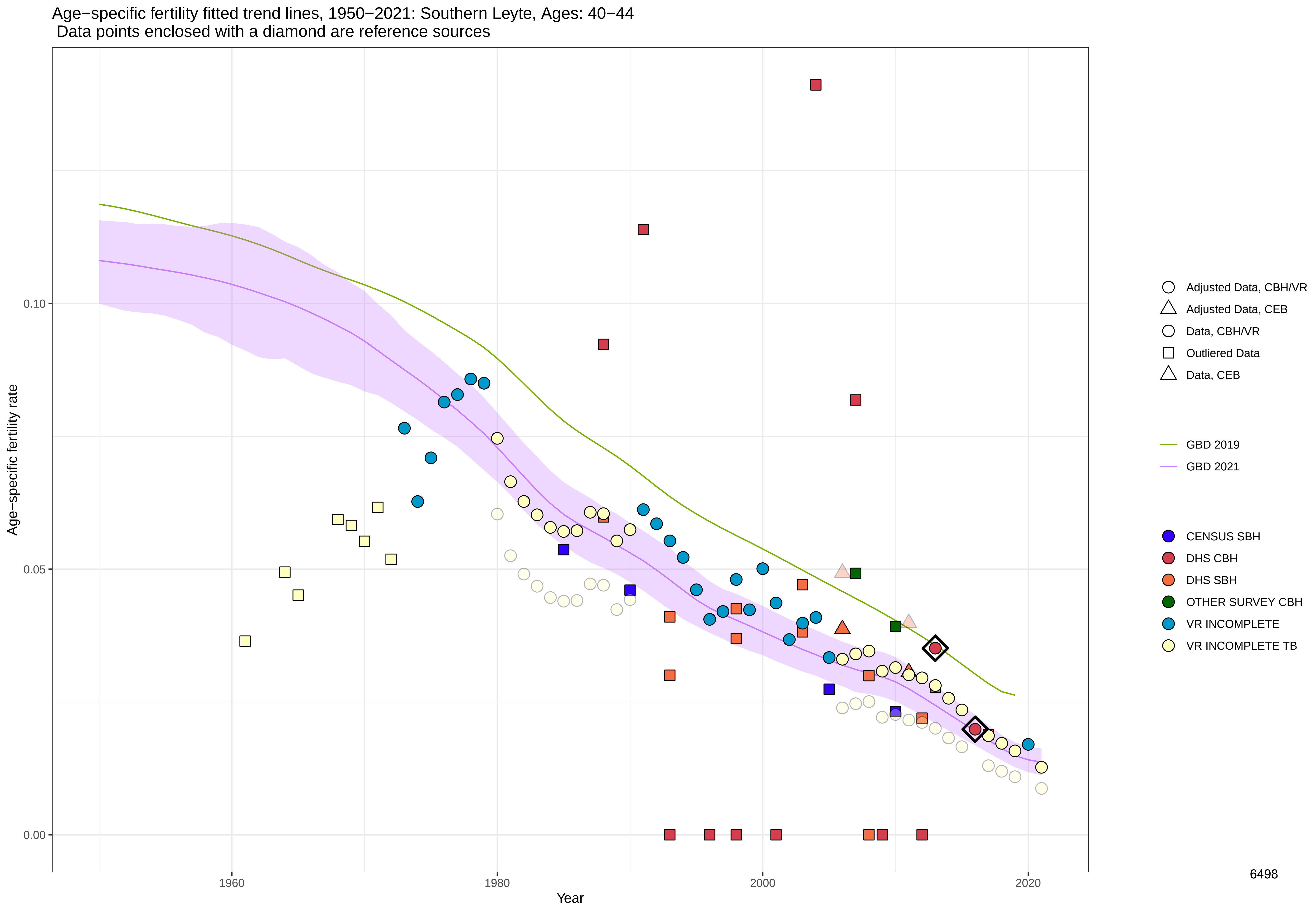

Age−specific fertility fitted trend lines, 1950−2021: Southern Leyte, Ages: 40−44
Data points enclosed with a diamond are reference sources
Age−specific fertility rate
0.00
0.05
0.10
1960
1980
2000
2020
Year
Adjusted Data, CBH/VR
Adjusted Data, CEB
Data, CBH/VR
Outliered Data
Data, CEB
GBD 2019
GBD 2021
CENSUS SBH
DHS CBH
DHS SBH
OTHER SURVEY CBH
VR INCOMPLETE
VR INCOMPLETE TB

Age−specific fertility fitted trend lines, 1950−2021: Southern Leyte, Ages: 45−49
Data points enclosed with a diamond are reference sources

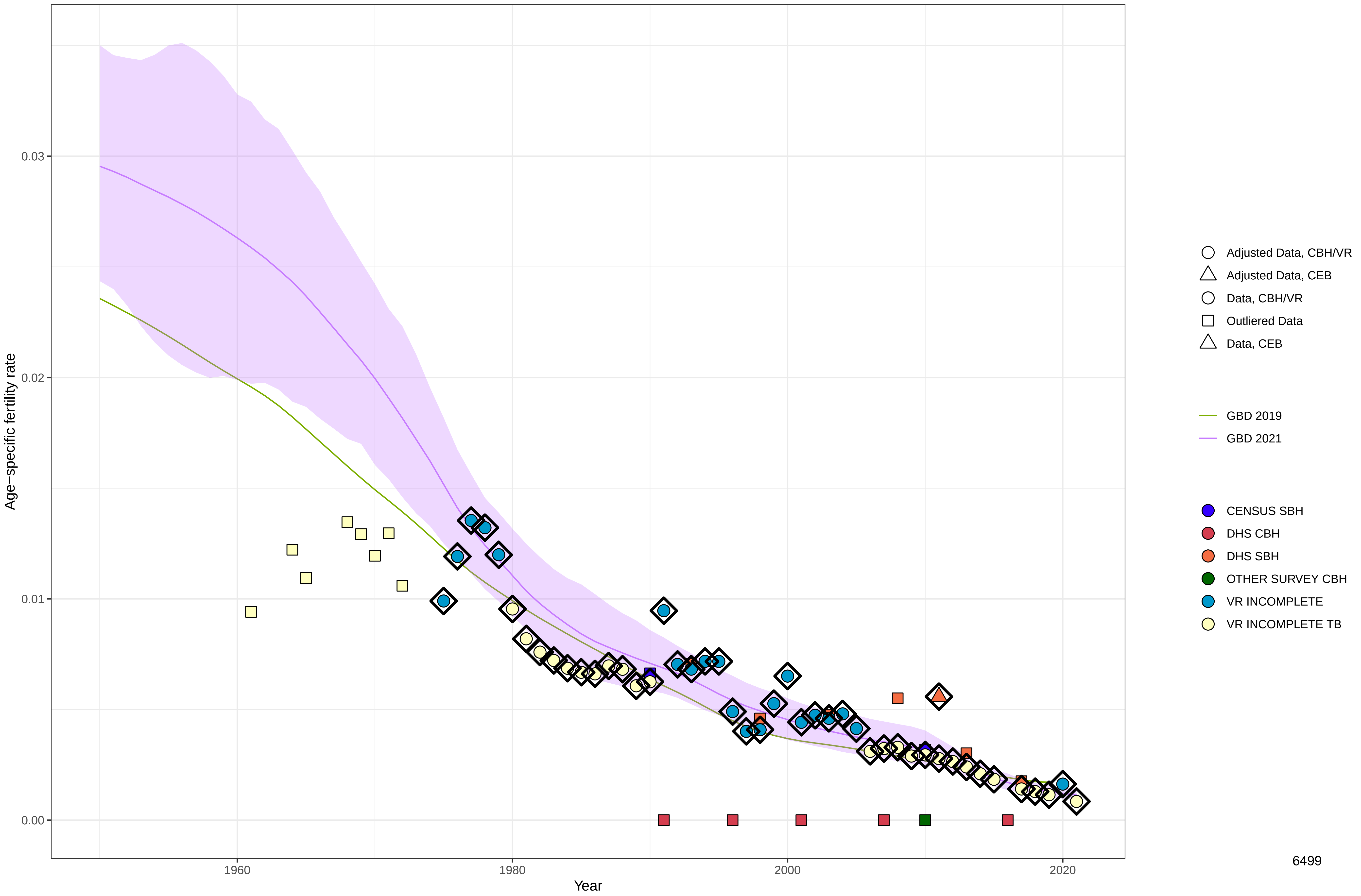

Age−specific fertility fitted trend lines, 1950−2021: Southern Leyte, Ages: 50−54
Data points enclosed with a diamond are reference sources

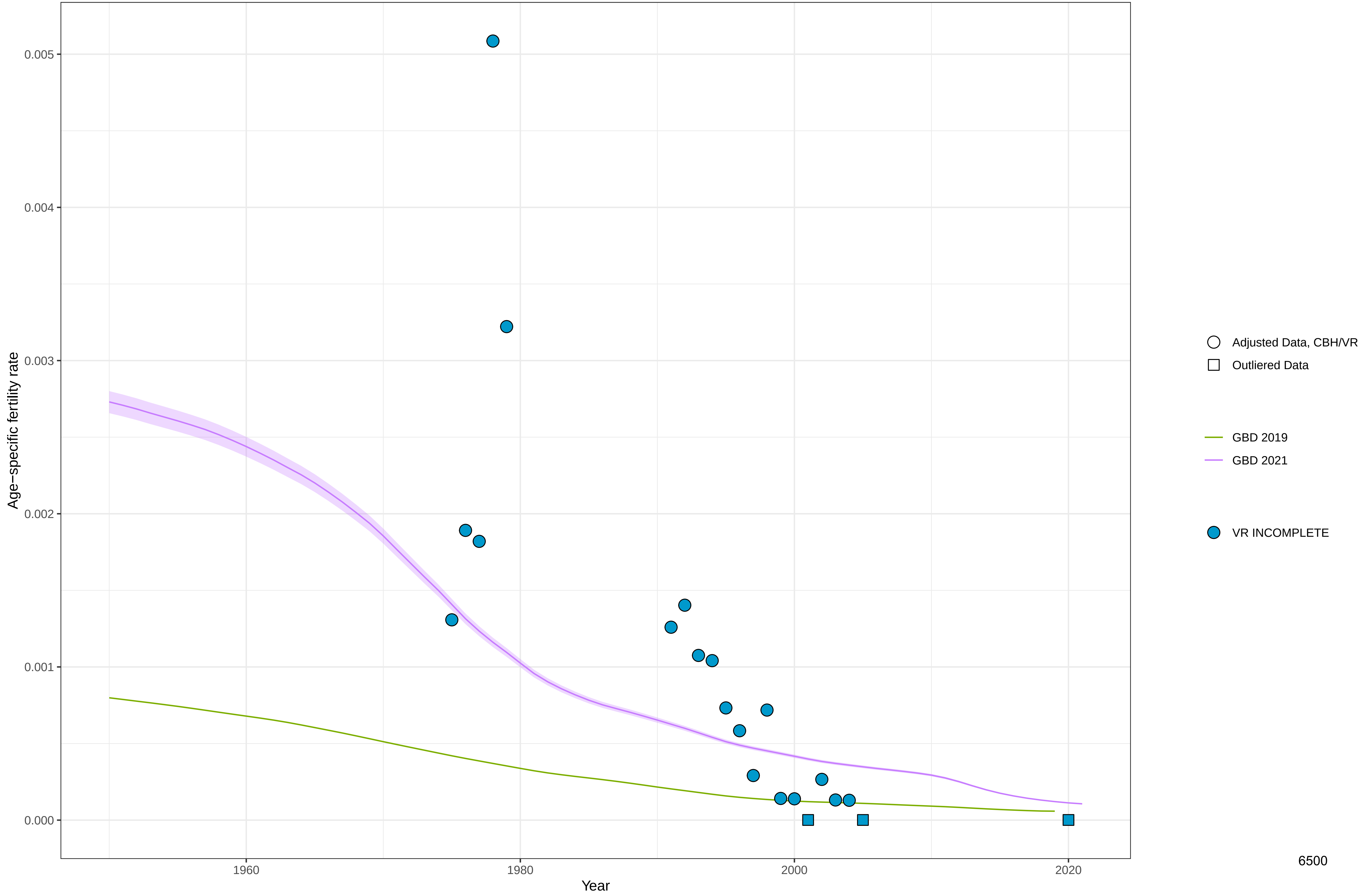

Total fertility rate fitted trend lines, 1950−2021: Southern Leyte TFR
Data points enclosed with a diamond are reference sources

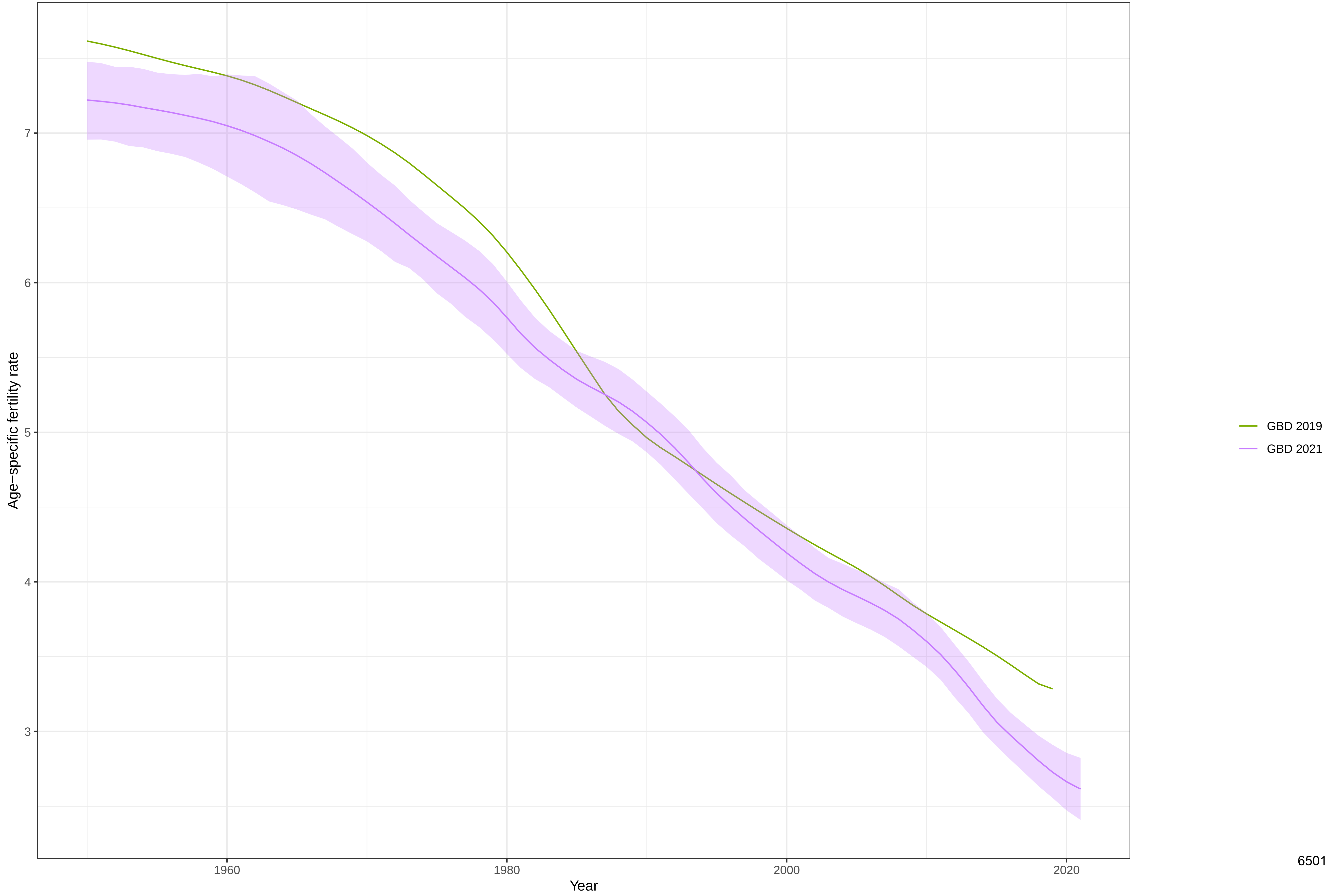

Age−specific fertility fitted trend lines, 1950−2021: Sultan Kudarat, Ages: 10−14
Data points enclosed with a diamond are reference sources

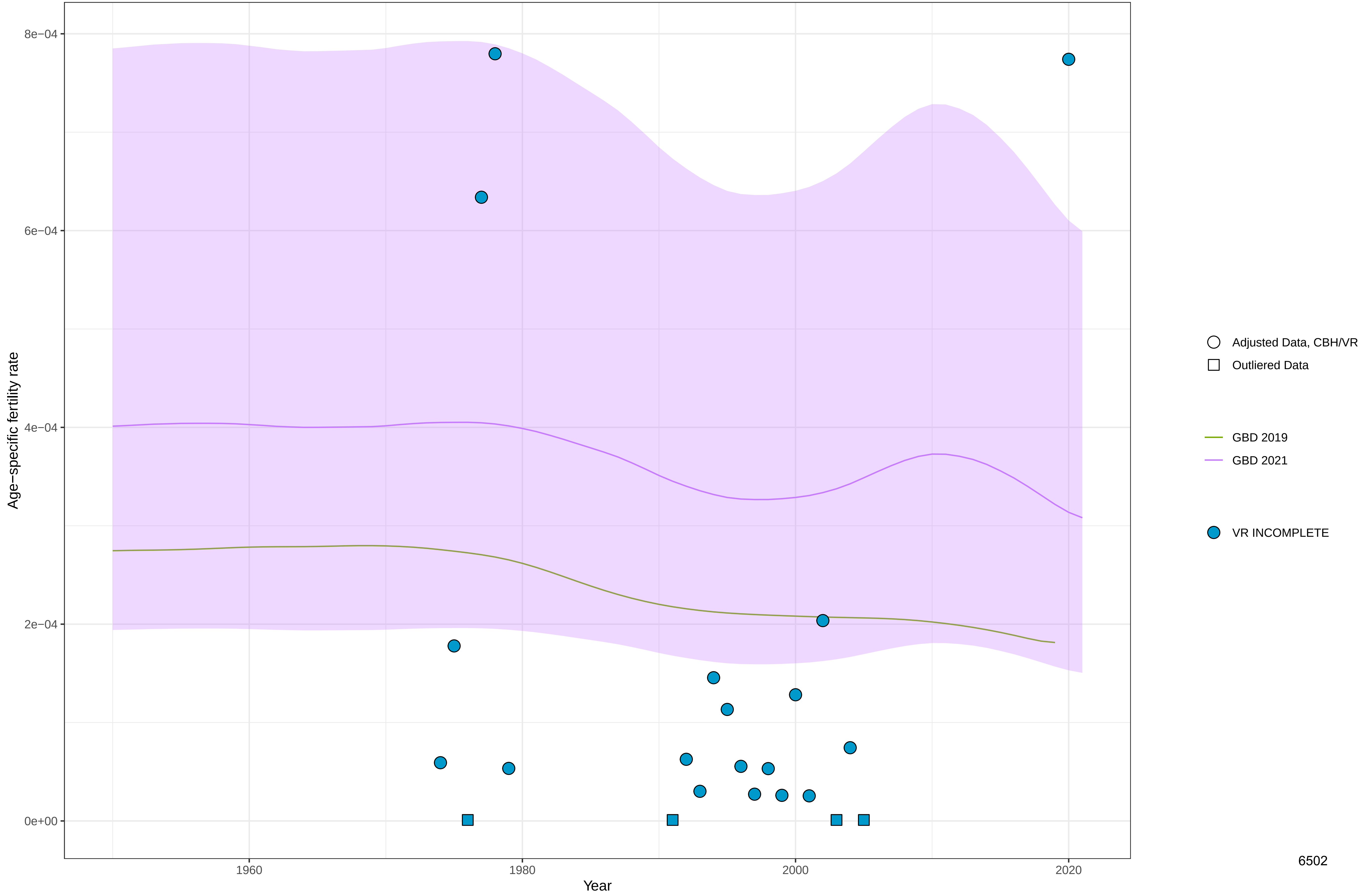

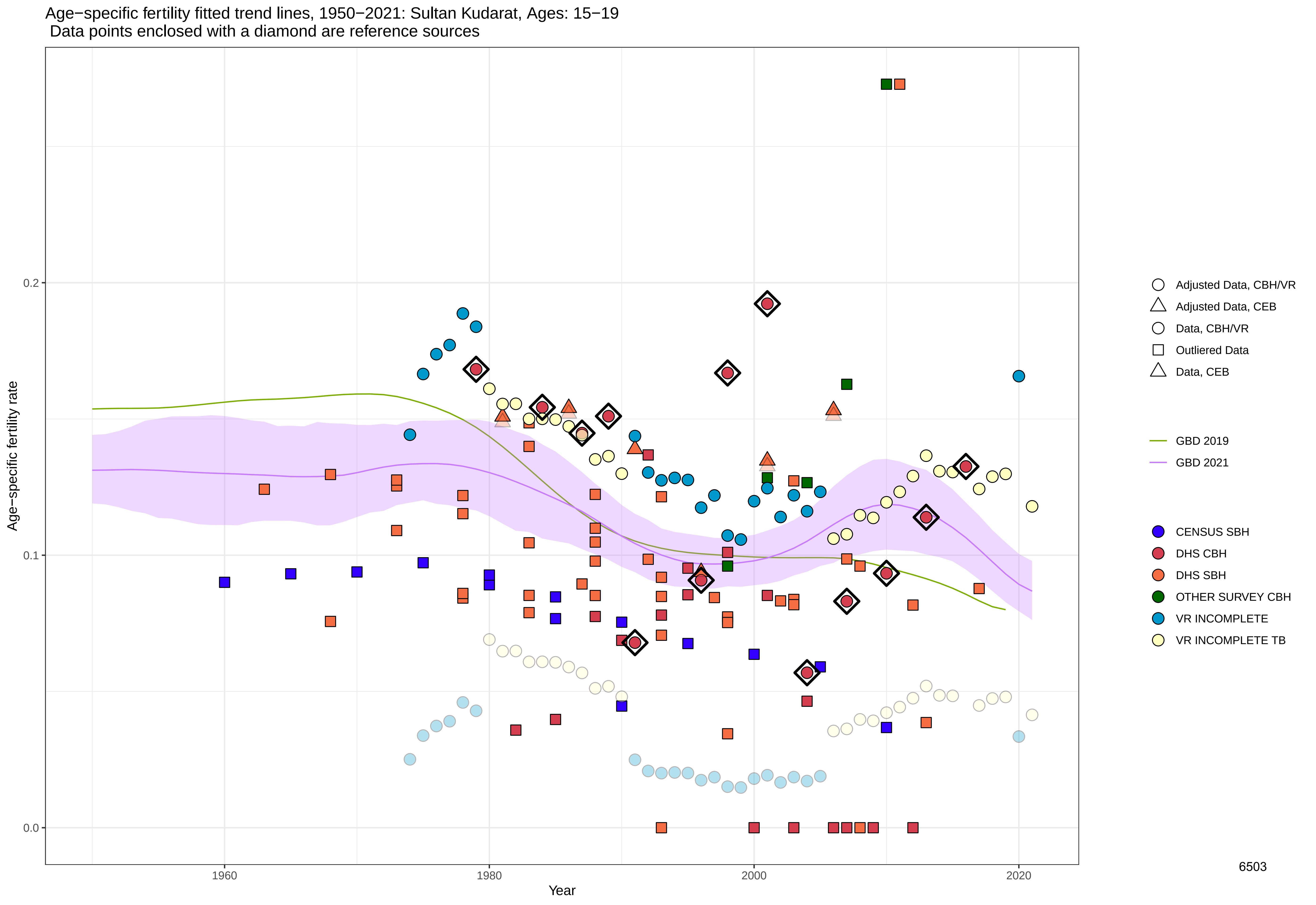

Age−specific fertility fitted trend lines, 1950−2021: Sultan Kudarat, Ages: 15−19
Data points enclosed with a diamond are reference sources
Age−specific fertility rate
0.0
0.1
0.2
1960
1980
2000
2020
Year
Adjusted Data, CBH/VR
Adjusted Data, CEB
Data, CBH/VR
Outliered Data
Data, CEB
GBD 2019
GBD 2021
CENSUS SBH
DHS CBH
DHS SBH
OTHER SURVEY CBH
VR INCOMPLETE
VR INCOMPLETE TB

Age−specific fertility fitted trend lines, 1950−2021: Sultan Kudarat, Ages: 20−24
Data points enclosed with a diamond are reference sources

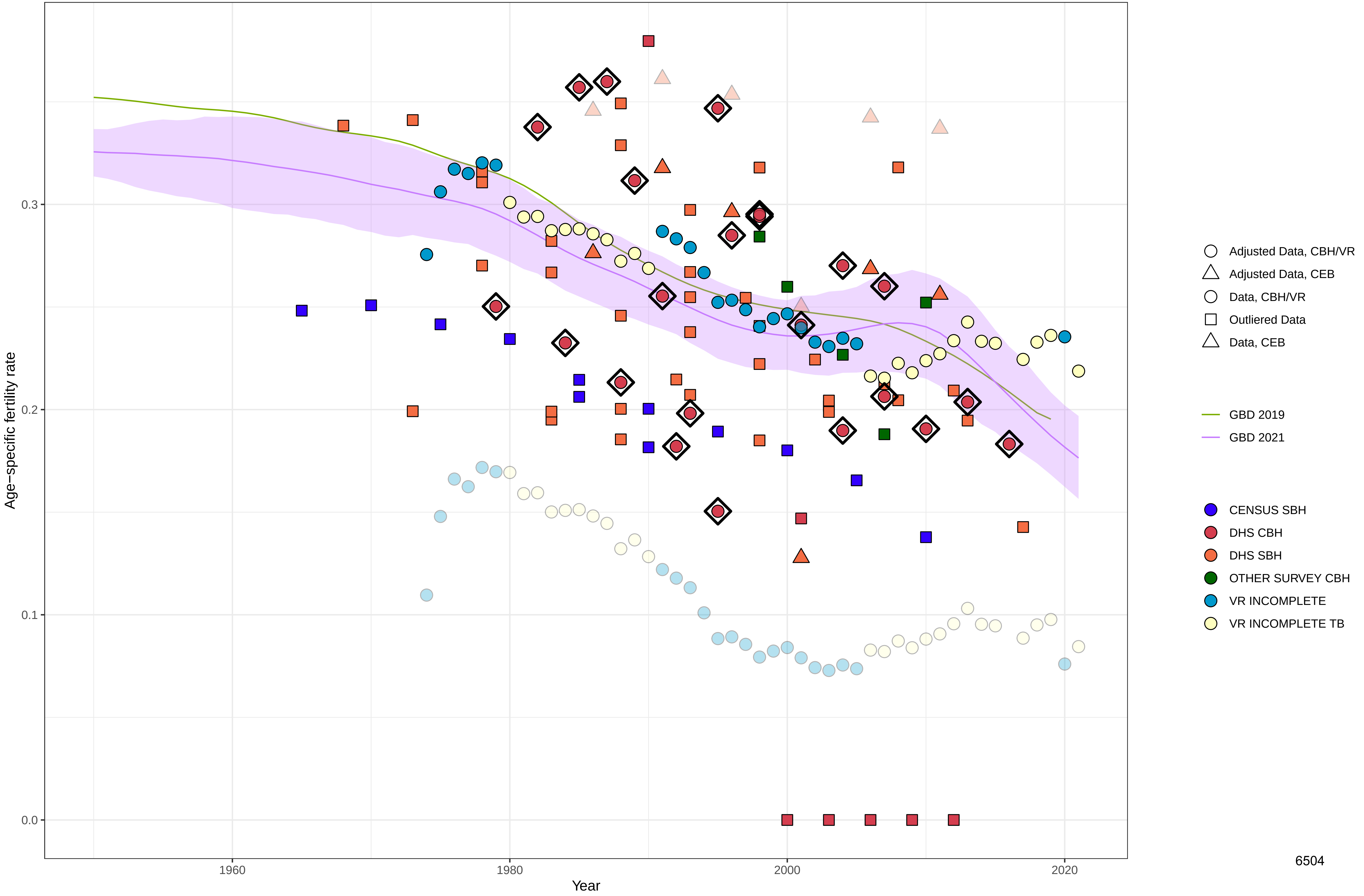

Age−specific fertility fitted trend lines, 1950−2021: Sultan Kudarat, Ages: 25−29
Data points enclosed with a diamond are reference sources

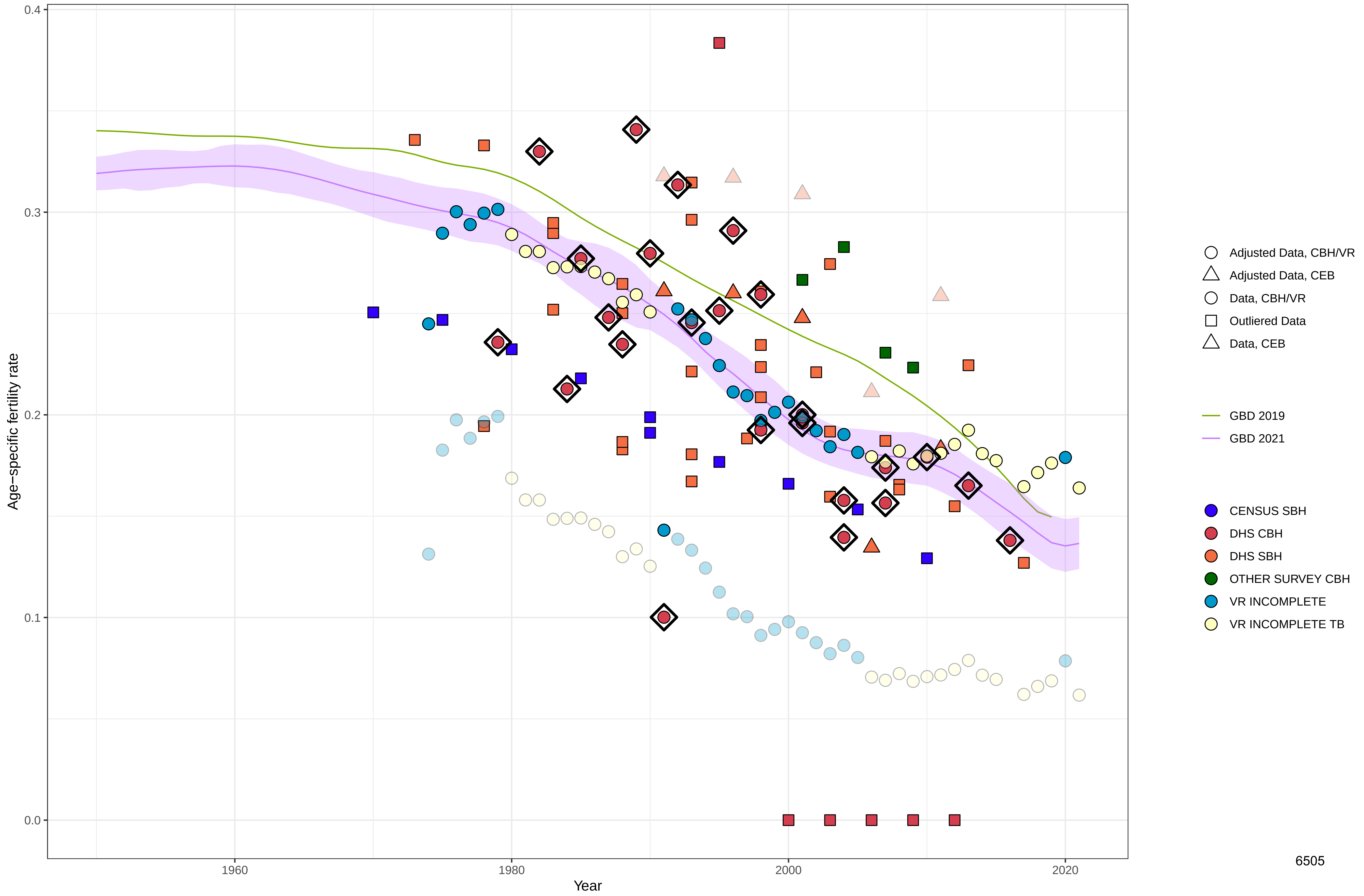

Age−specific fertility fitted trend lines, 1950−2021: Sultan Kudarat, Ages: 30−34
Data points enclosed with a diamond are reference sources

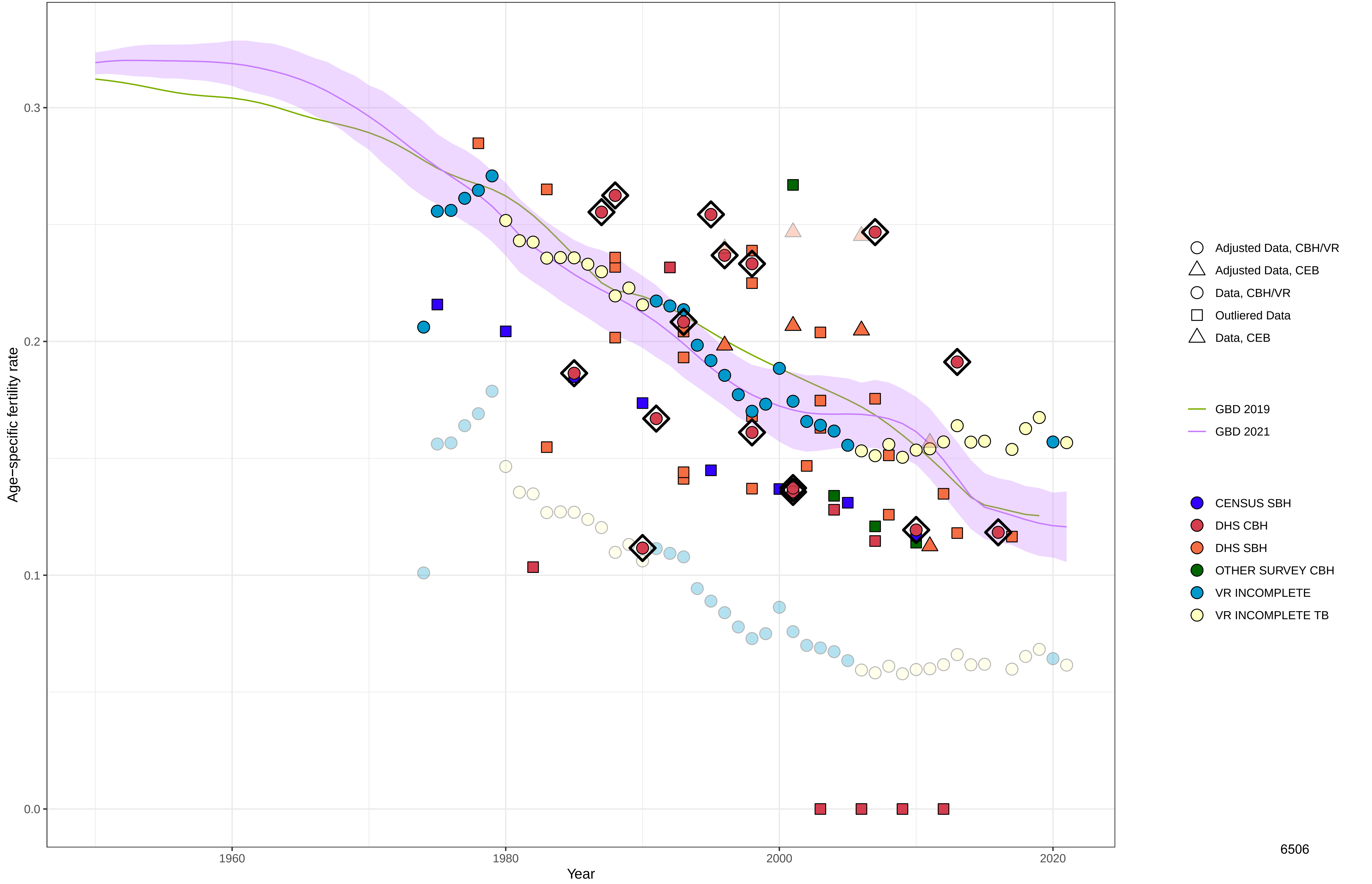

Age−specific fertility fitted trend lines, 1950−2021: Sultan Kudarat, Ages: 35−39
Data points enclosed with a diamond are reference sources

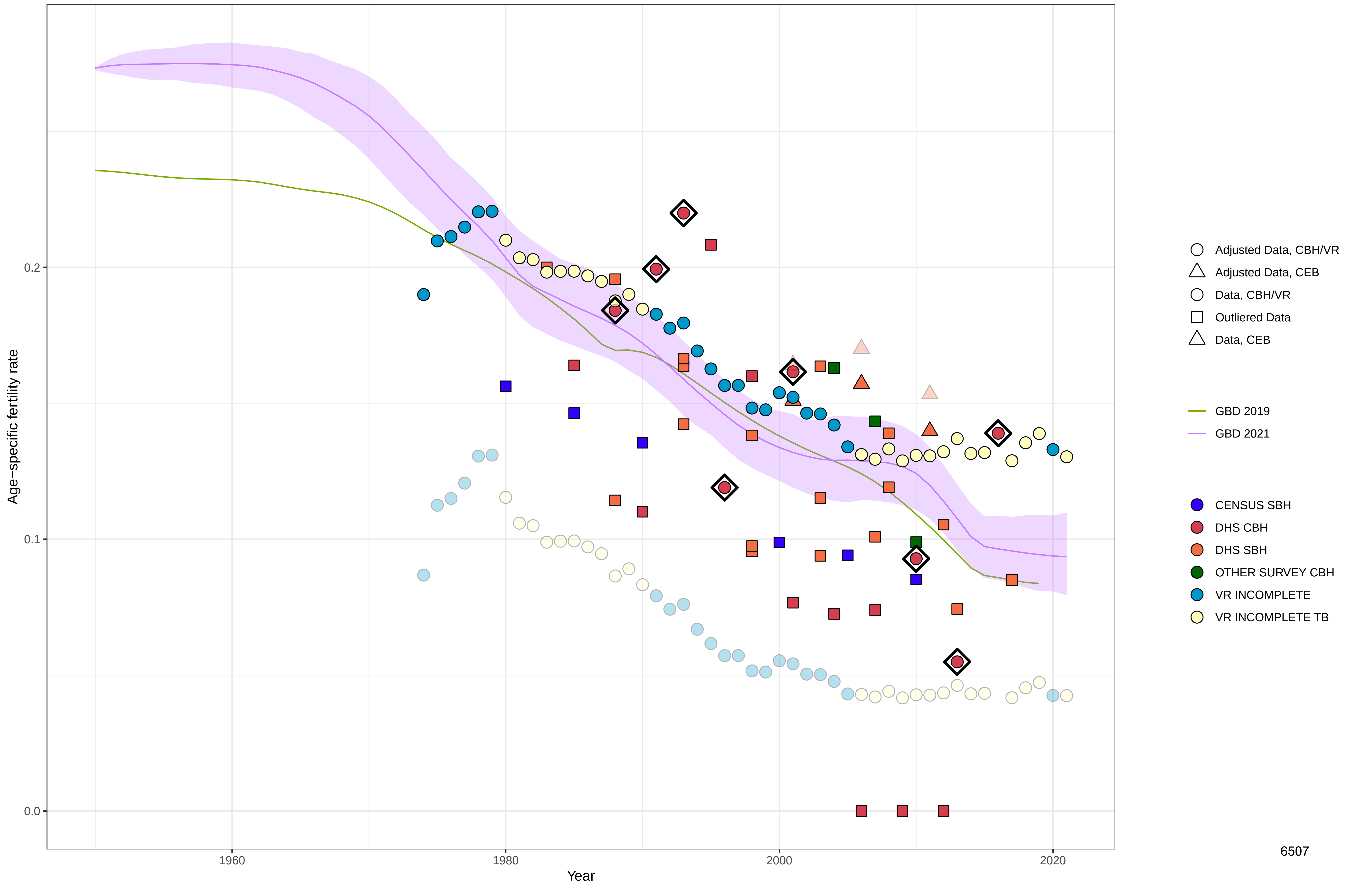

6507

Age−specific fertility fitted trend lines, 1950−2021: Sultan Kudarat, Ages: 40−44
Data points enclosed with a diamond are reference sources

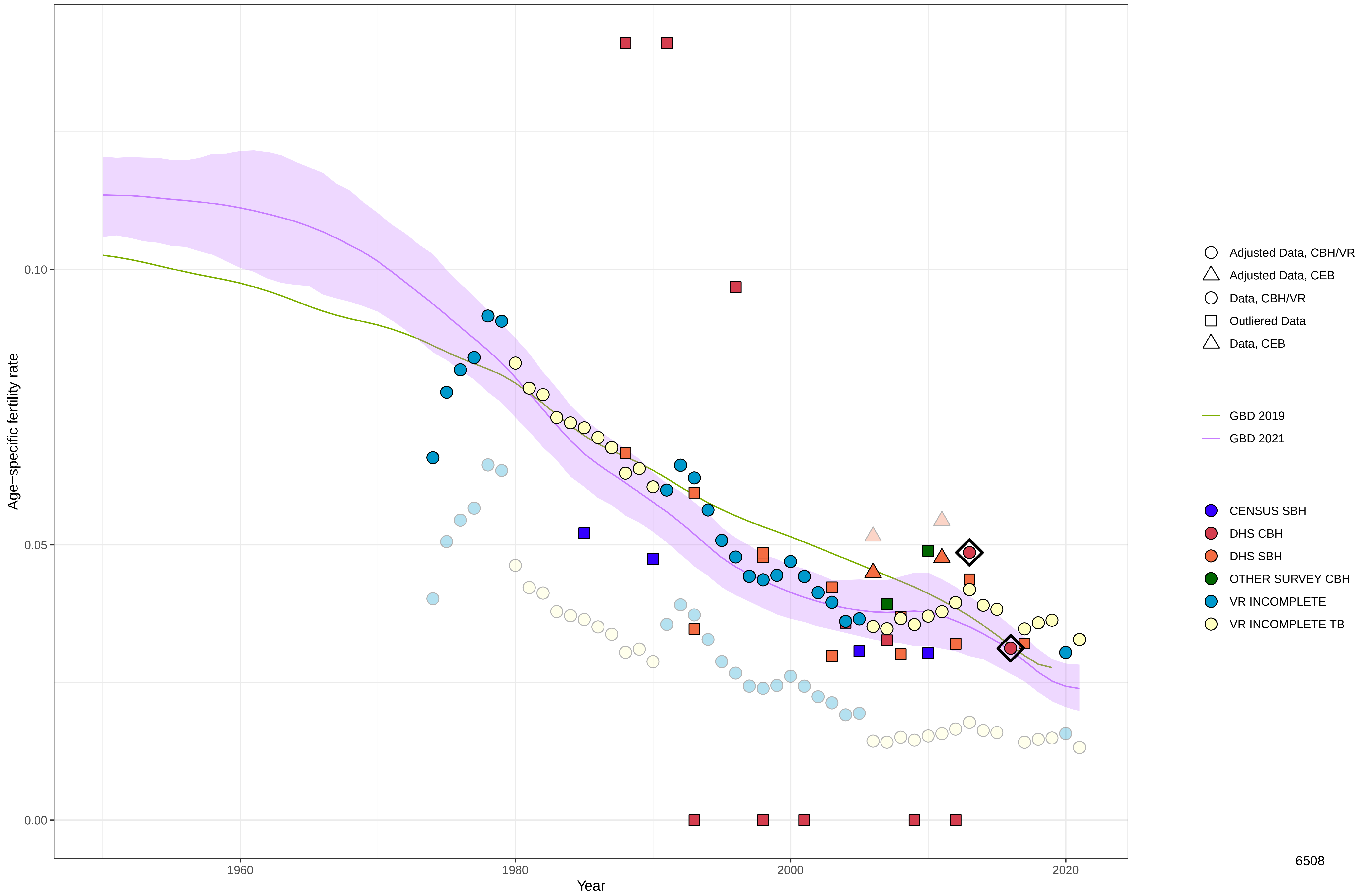

Age−specific fertility fitted trend lines, 1950−2021: Sultan Kudarat, Ages: 45−49
Data points enclosed with a diamond are reference sources

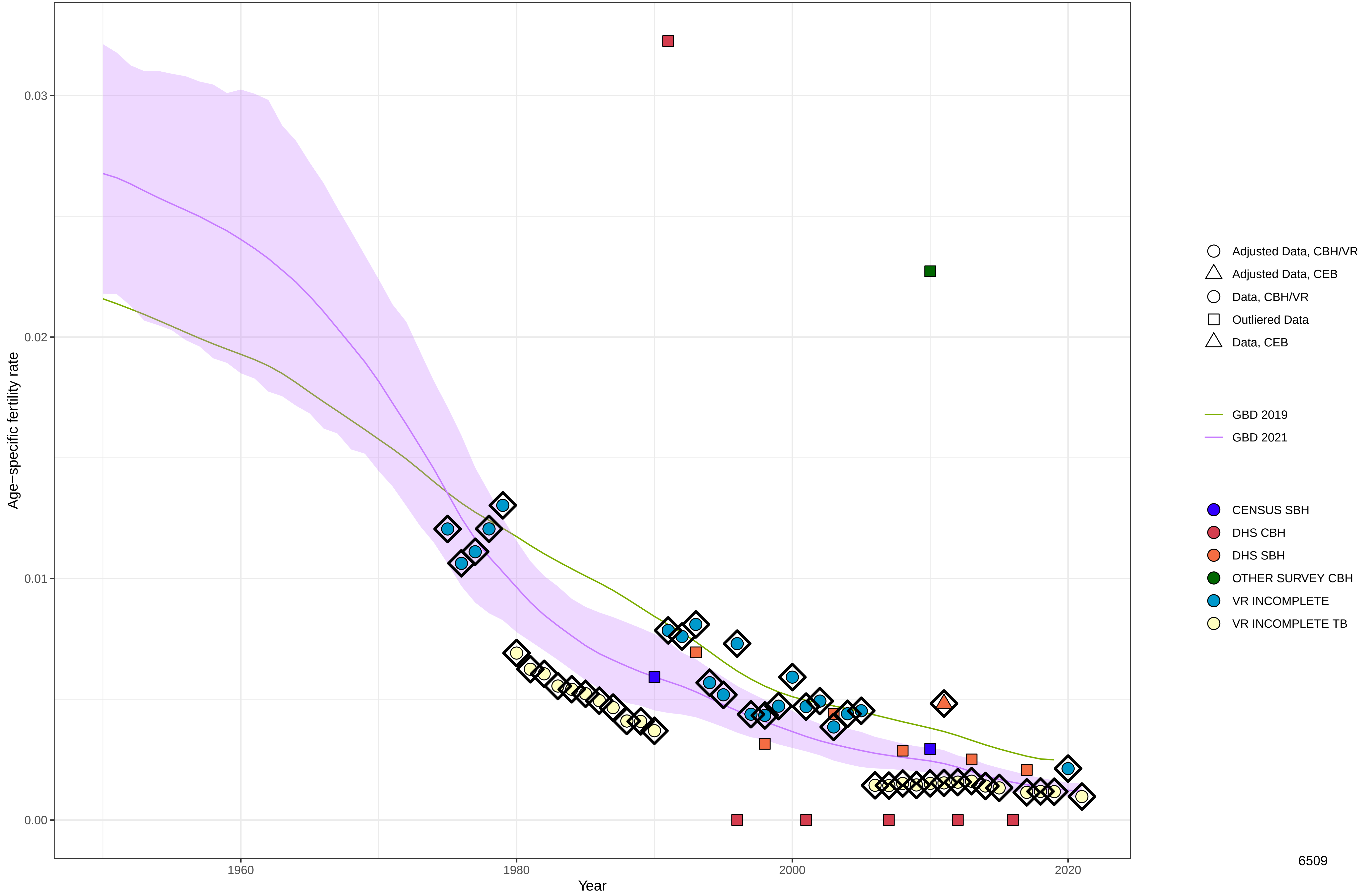

Age−specific fertility fitted trend lines, 1950−2021: Sultan Kudarat, Ages: 50−54
Data points enclosed with a diamond are reference sources

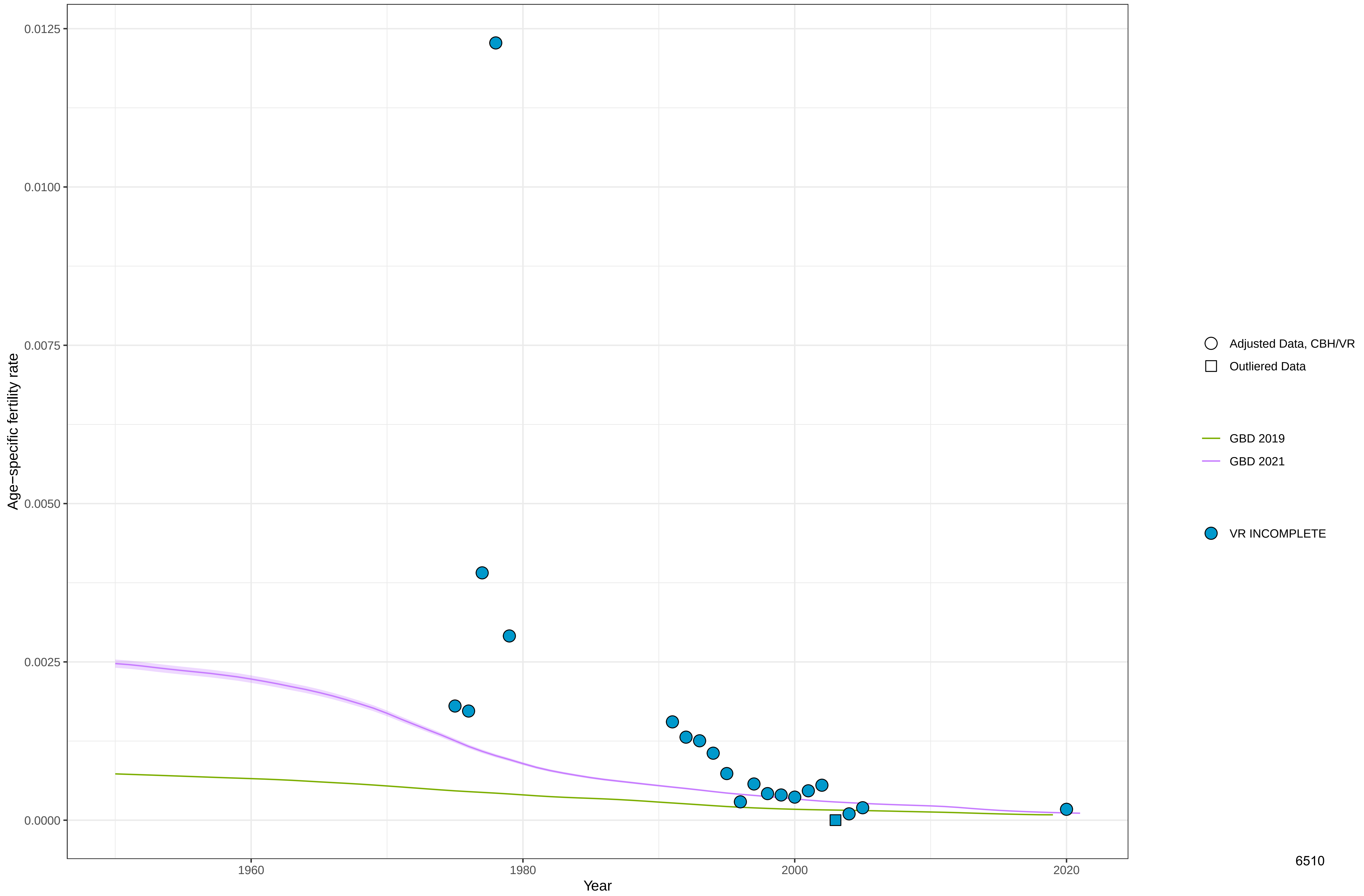

Total fertility rate fitted trend lines, 1950−2021: Sultan Kudarat TFR
Data points enclosed with a diamond are reference sources

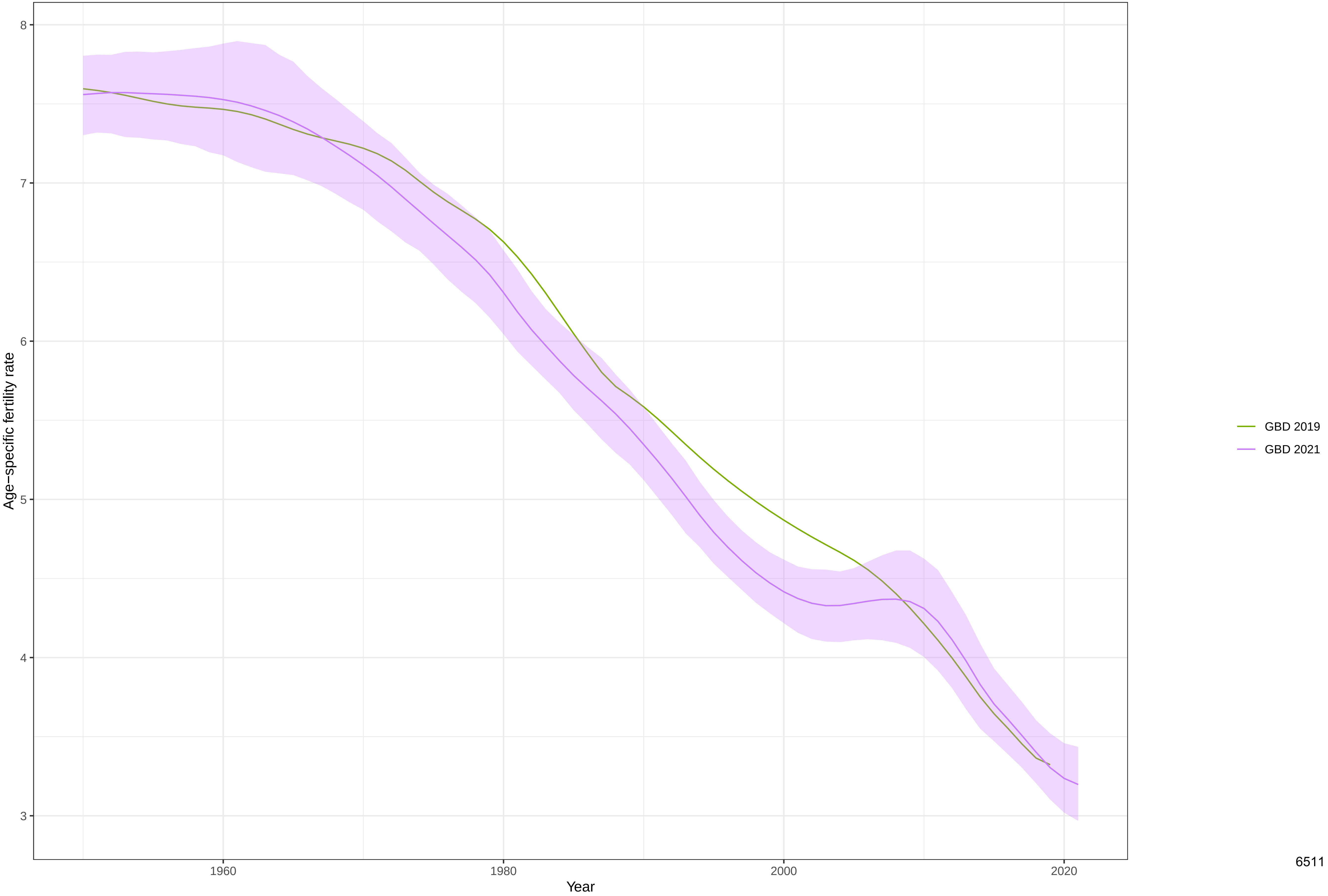

Age−specific fertility fitted trend lines, 1950−2021: Sulu, Ages: 10−14
Data points enclosed with a diamond are reference sources

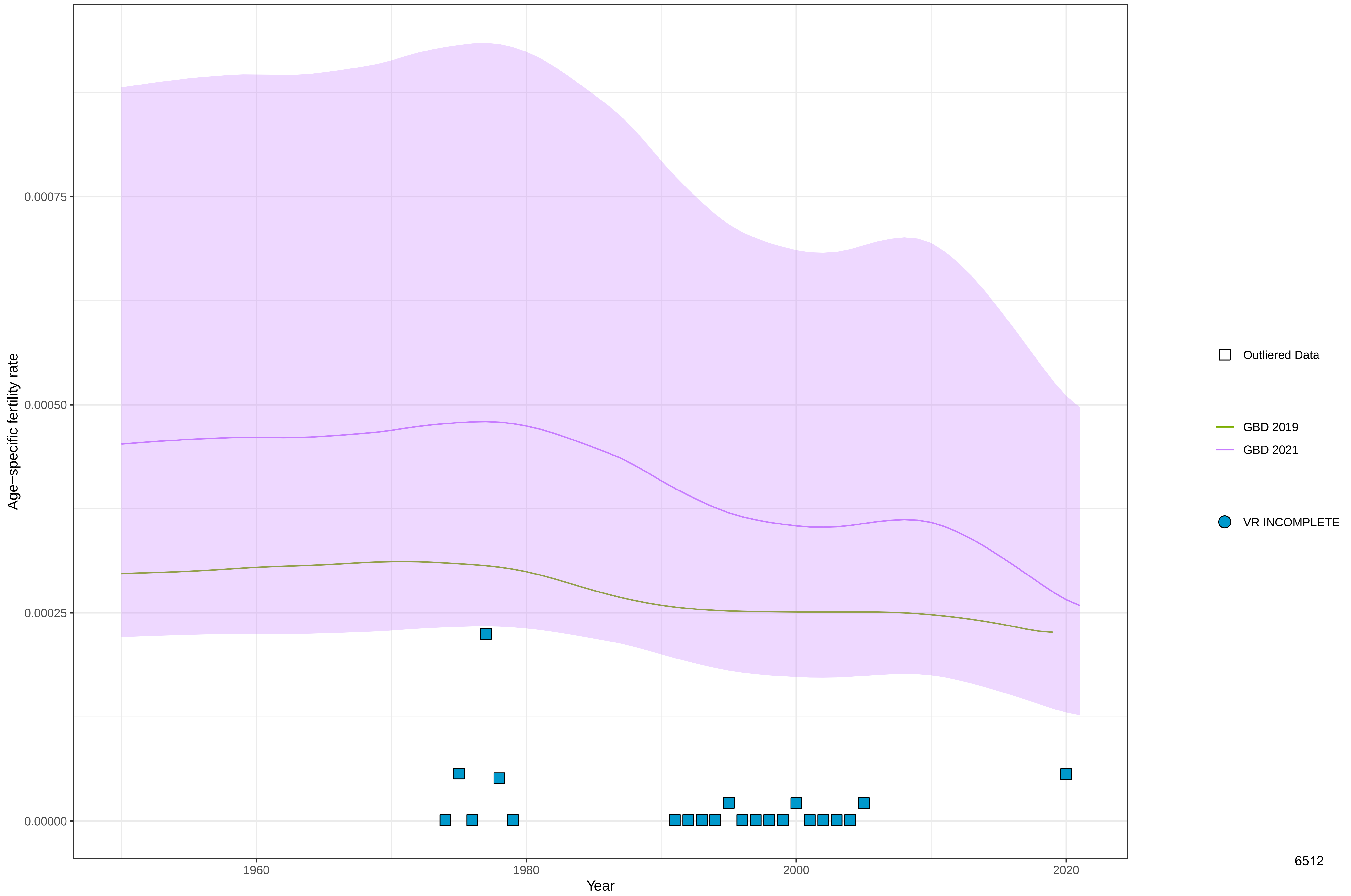

Age−specific fertility fitted trend lines, 1950−2021: Sulu, Ages: 15−19
Data points enclosed with a diamond are reference sources

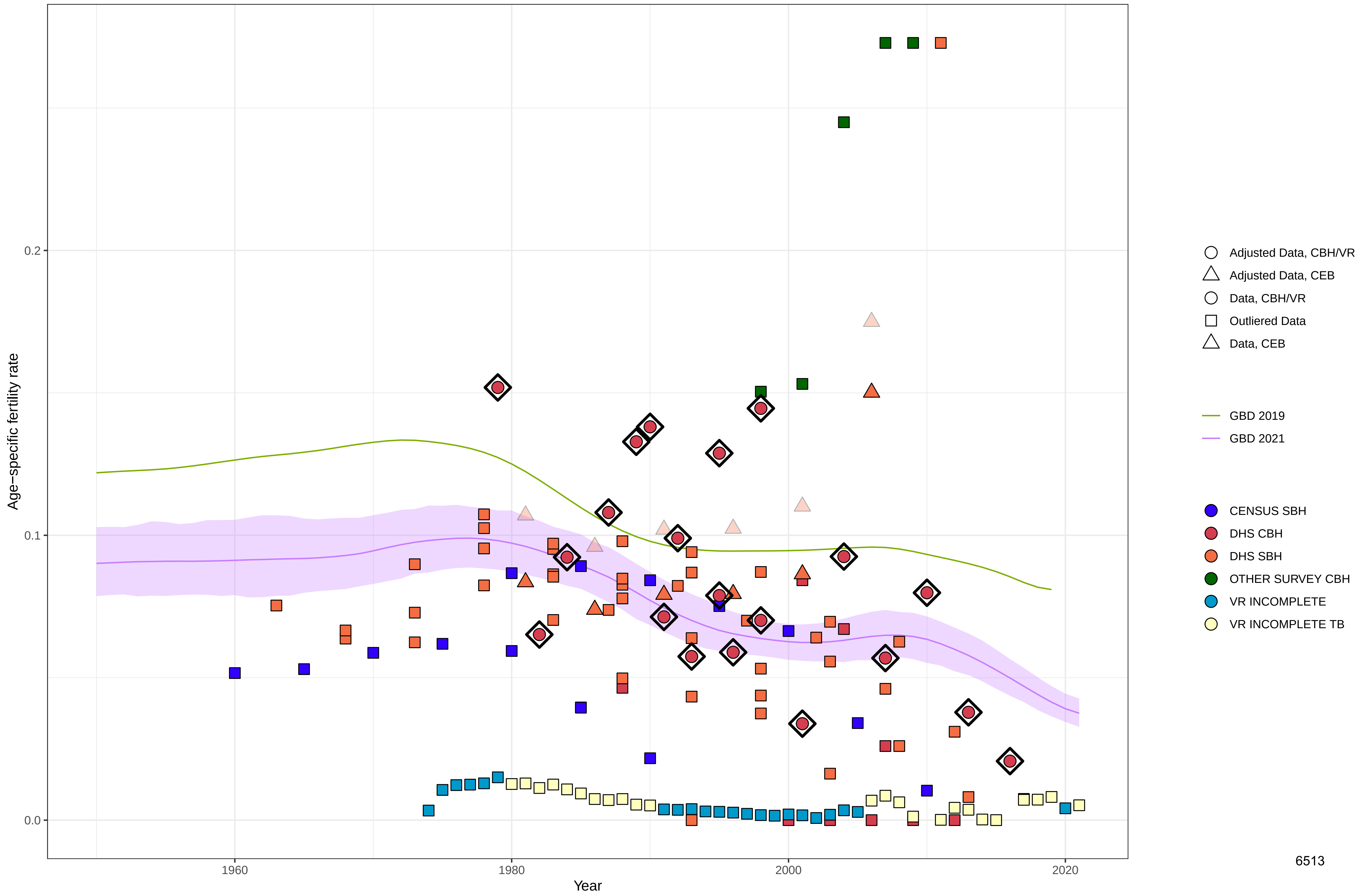

Age−specific fertility fitted trend lines, 1950−2021: Sulu, Ages: 20−24
Data points enclosed with a diamond are reference sources

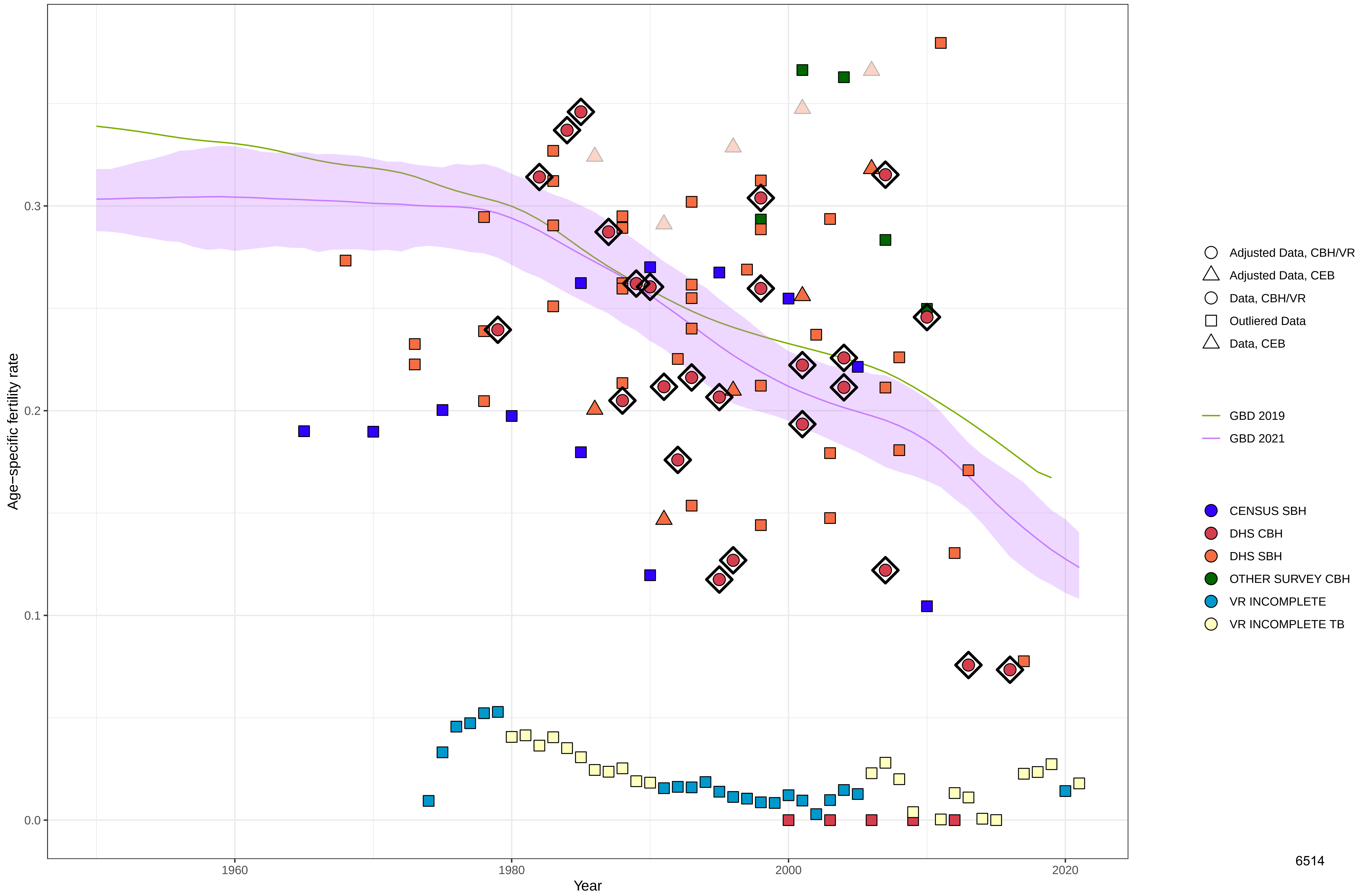

6514

Age−specific fertility fitted trend lines, 1950−2021: Sulu, Ages: 25−29
Data points enclosed with a diamond are reference sources

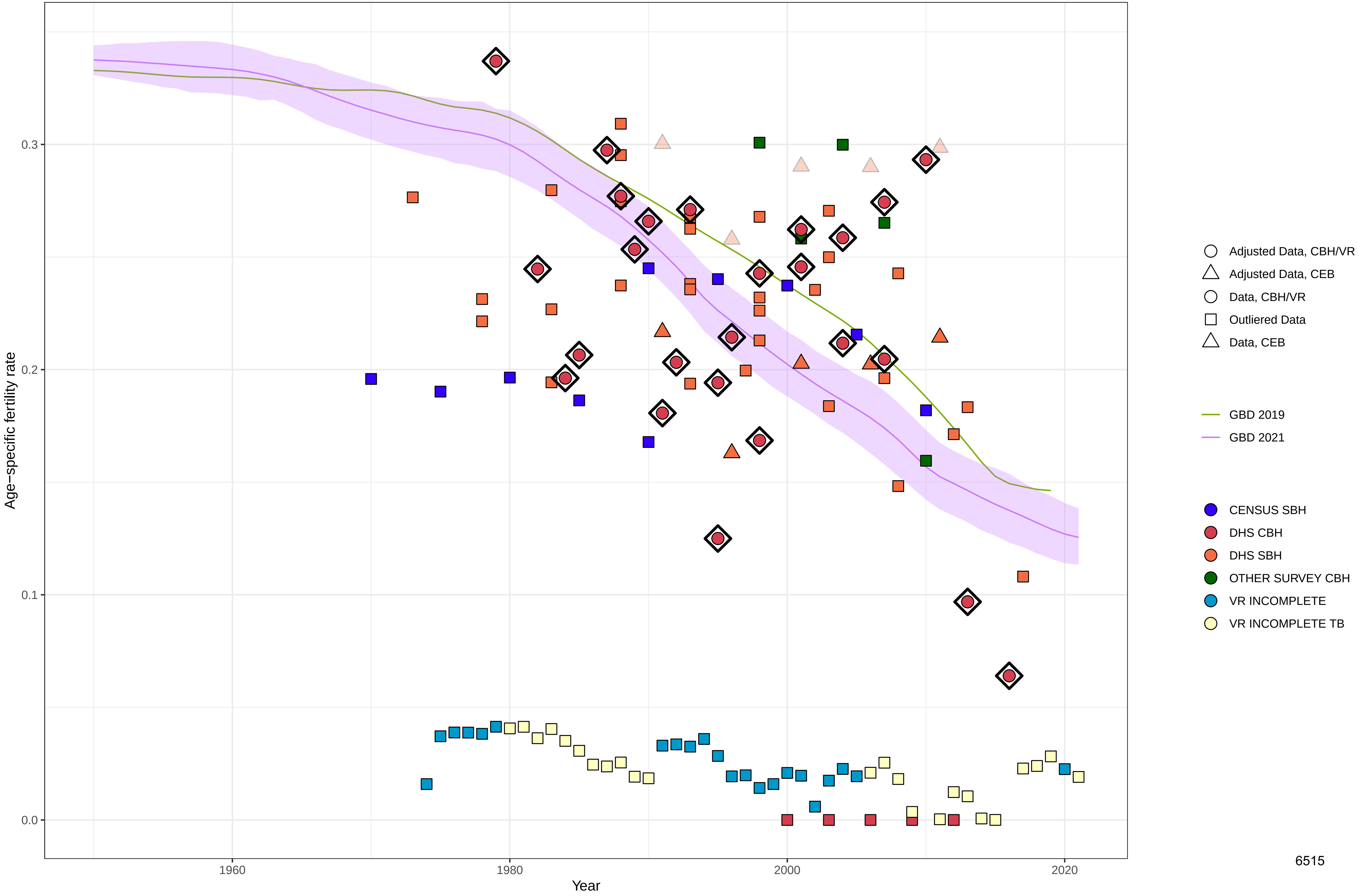

Age−specific fertility fitted trend lines, 1950−2021: Sulu, Ages: 30−34
Data points enclosed with a diamond are reference sources

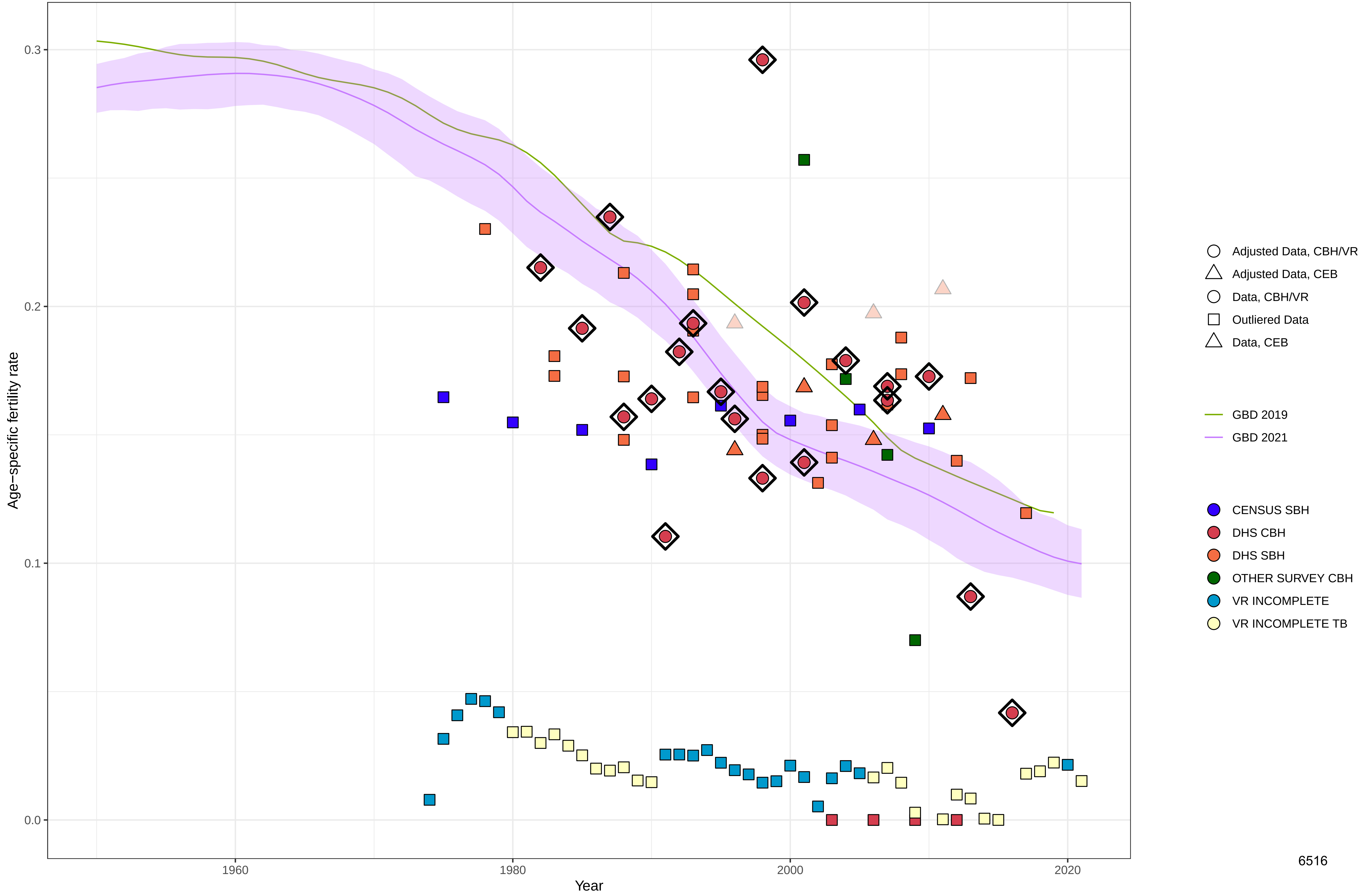

Age−specific fertility fitted trend lines, 1950−2021: Sulu, Ages: 35−39
Data points enclosed with a diamond are reference sources

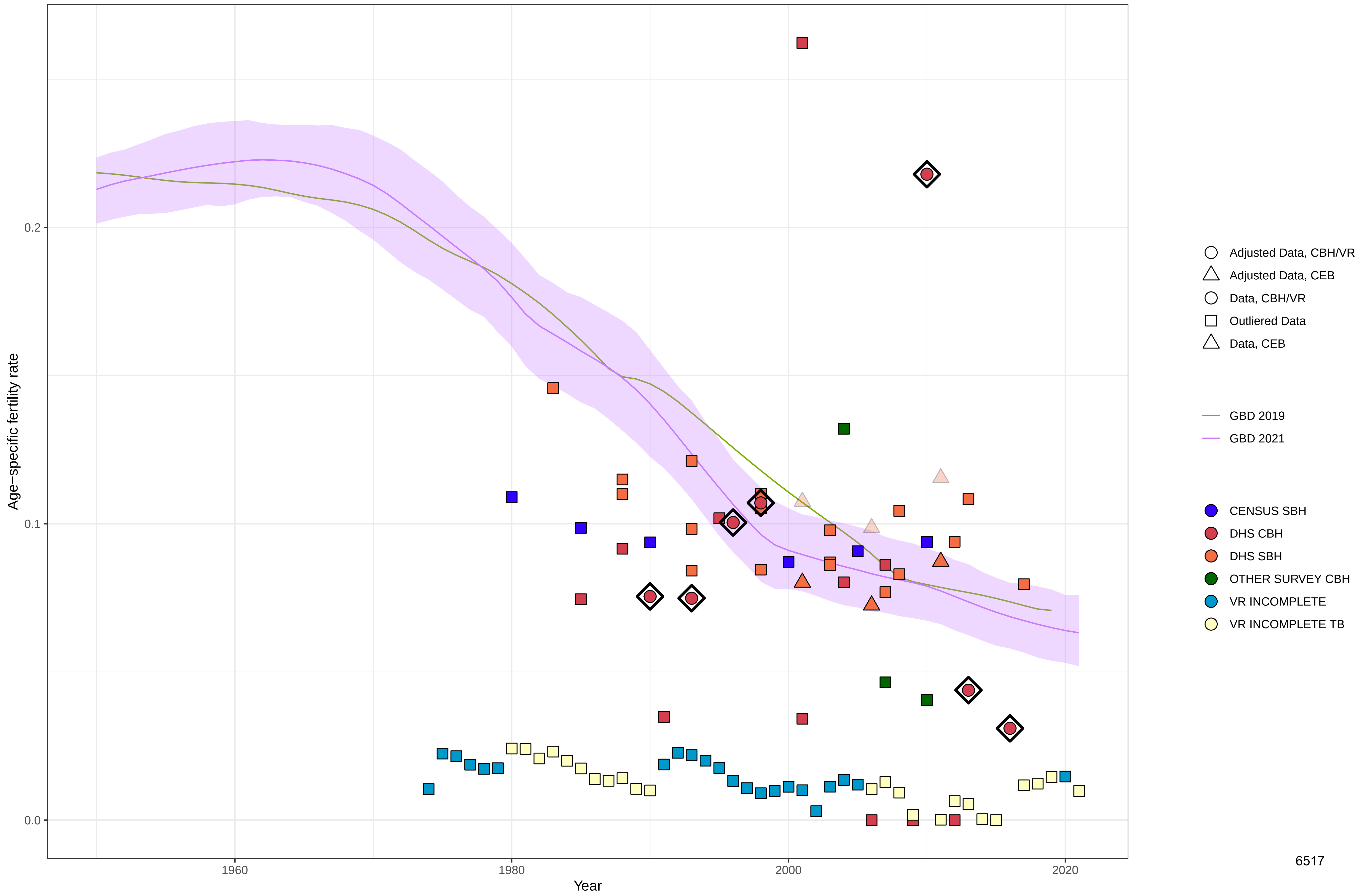

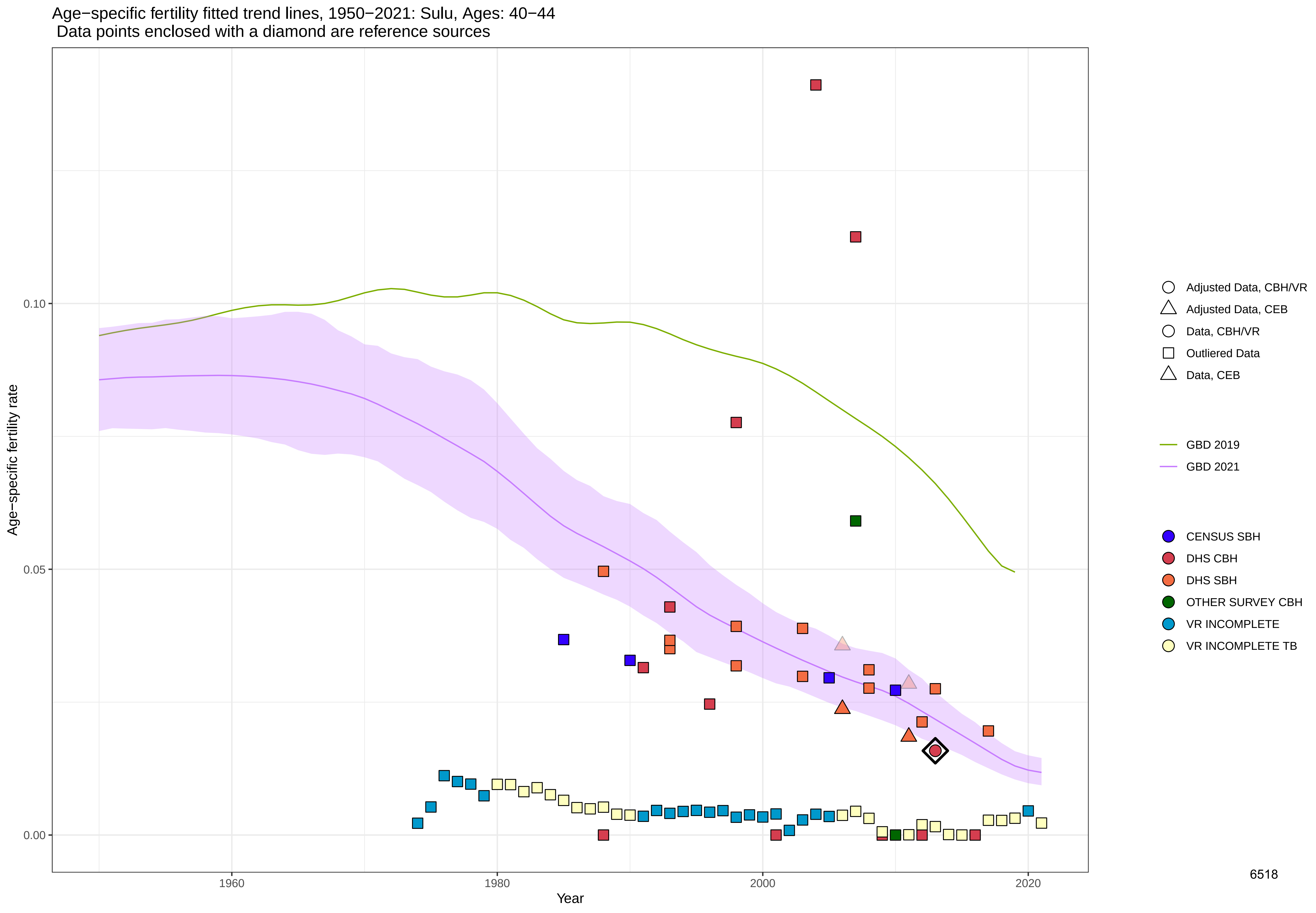

Age−specific fertility fitted trend lines, 1950−2021: Sulu, Ages: 40−44
Data points enclosed with a diamond are reference sources
Age−specific fertility rate
0.10
0.05
0.00
1960
1980
2000
2020
Year
Adjusted Data, CBH/VR
Adjusted Data, CEB
Data, CBH/VR
Outliered Data
Data, CEB
GBD 2019
GBD 2021
CENSUS SBH
DHS CBH
DHS SBH
OTHER SURVEY CBH
VR INCOMPLETE
VR INCOMPLETE TB

Age−specific fertility fitted trend lines, 1950−2021: Sulu, Ages: 45−49
Data points enclosed with a diamond are reference sources

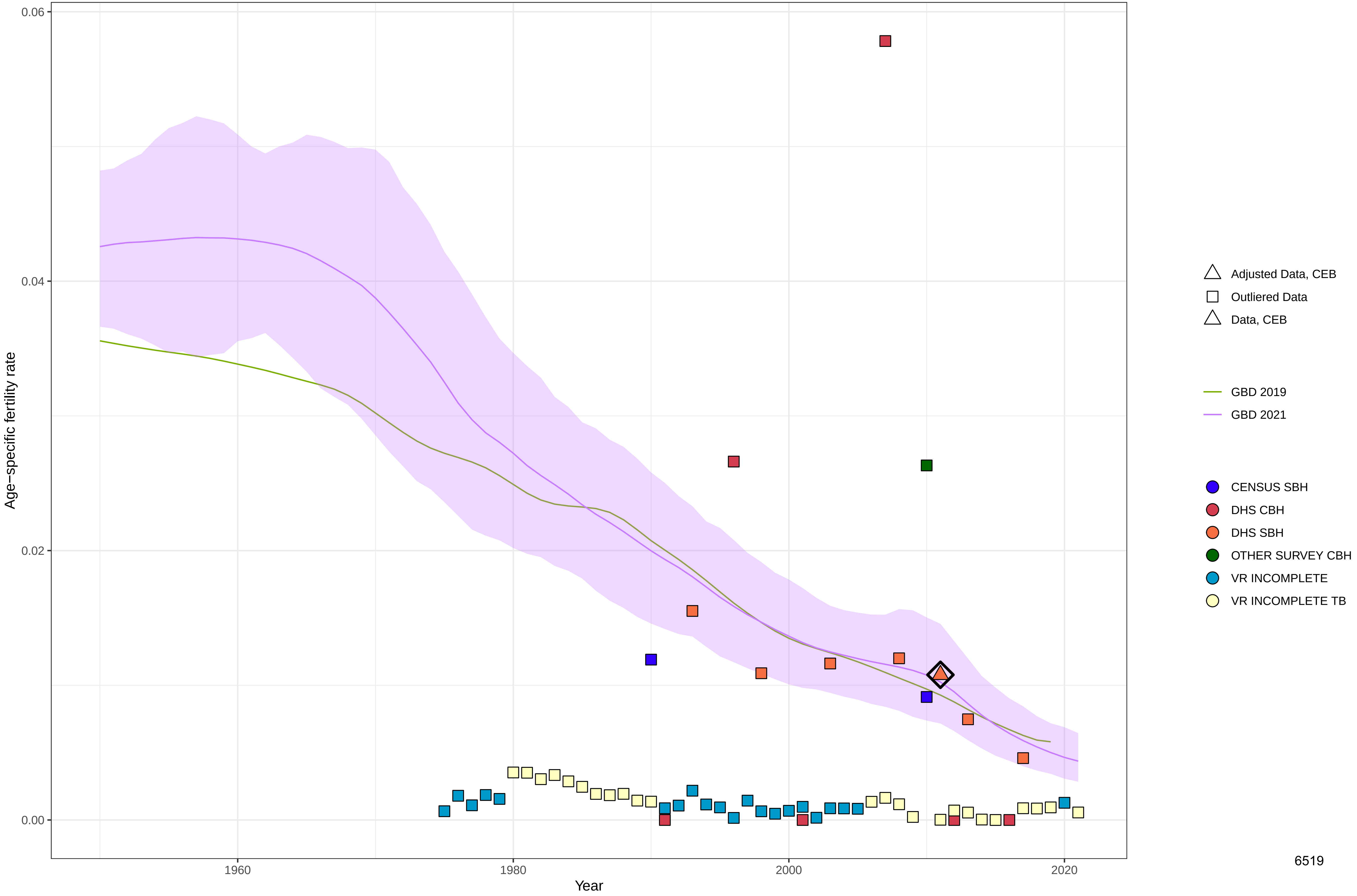

Age−specific fertility fitted trend lines, 1950−2021: Sulu, Ages: 50−54
Data points enclosed with a diamond are reference sources

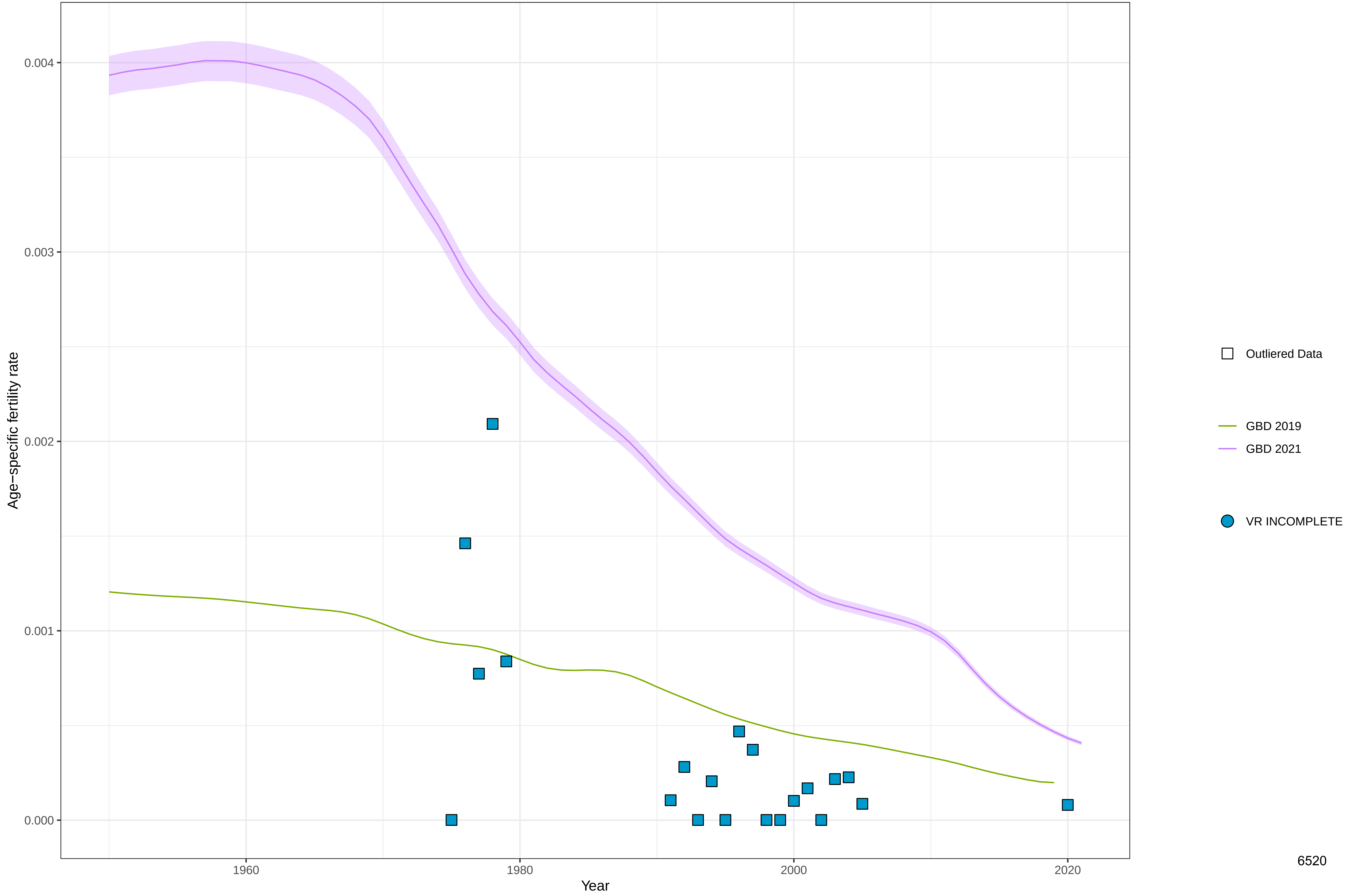

Total fertility rate fitted trend lines, 1950−2021: Sulu TFR
Data points enclosed with a diamond are reference sources

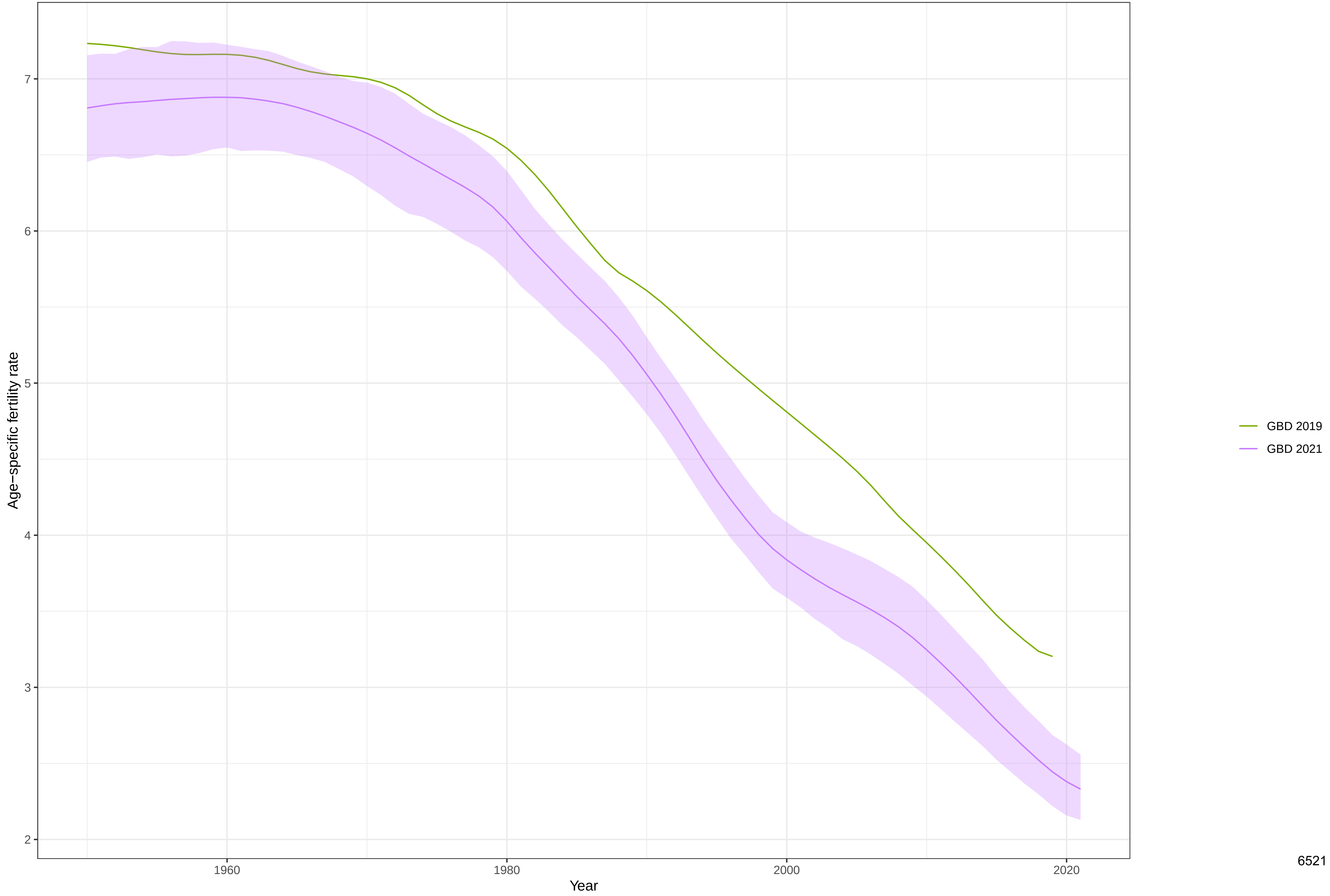

Age−specific fertility fitted trend lines, 1950−2021: Surigao Del Norte, Ages: 10−14
Data points enclosed with a diamond are reference sources

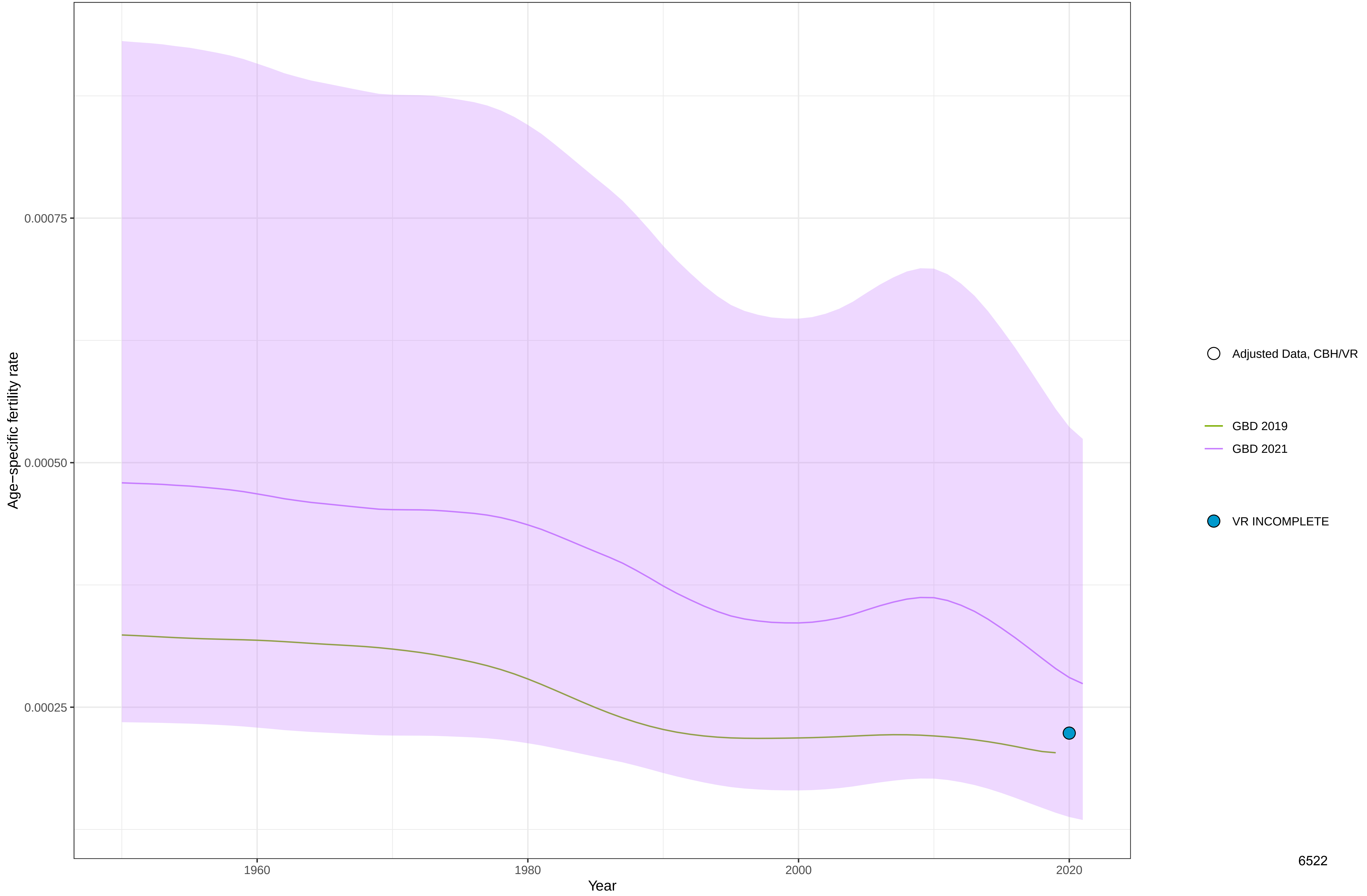

6522

Age−specific fertility fitted trend lines, 1950−2021: Surigao Del Norte, Ages: 15−19
Data points enclosed with a diamond are reference sources

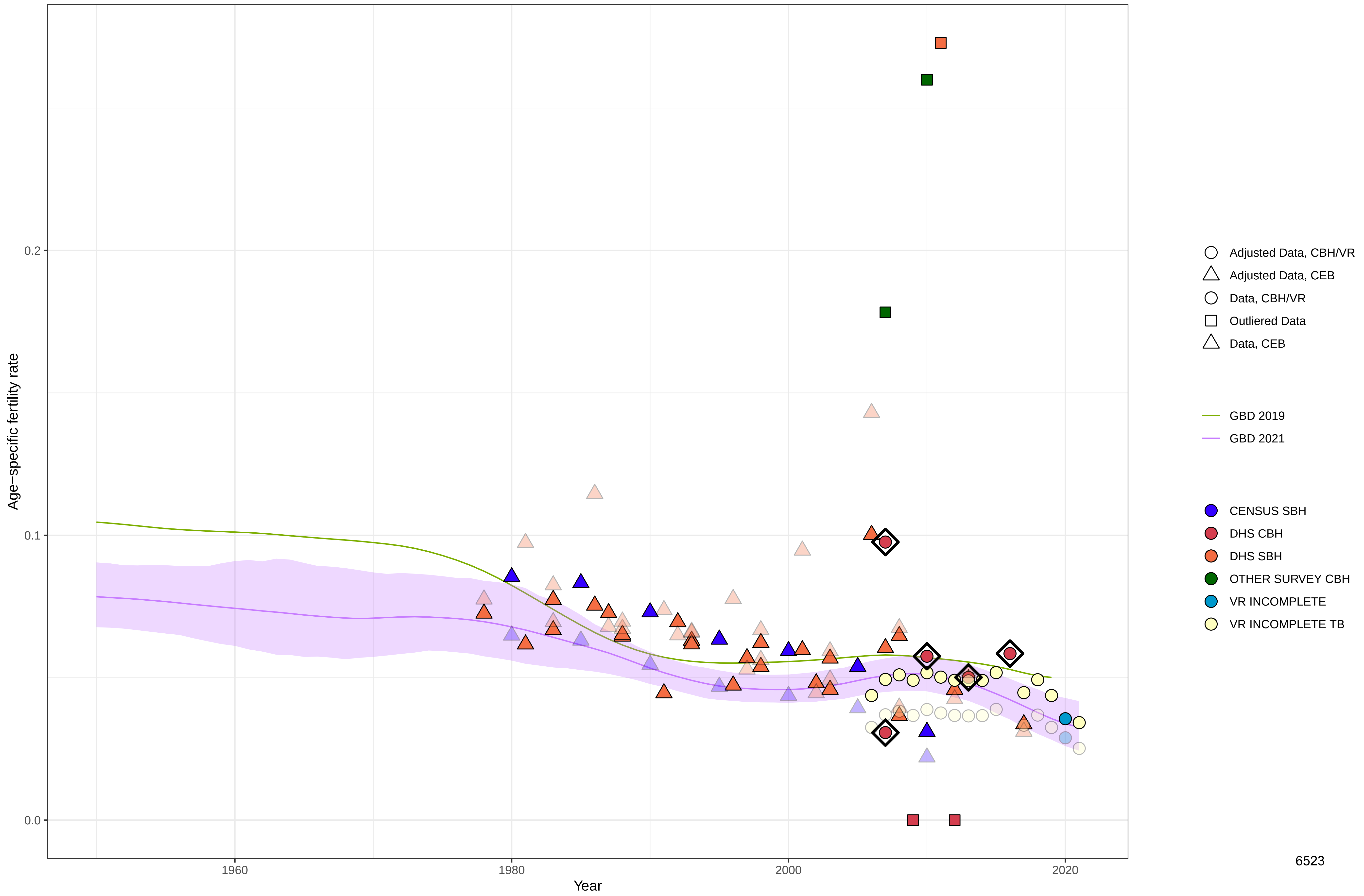

Age−specific fertility fitted trend lines, 1950−2021: Surigao Del Norte, Ages: 20−24
Data points enclosed with a diamond are reference sources

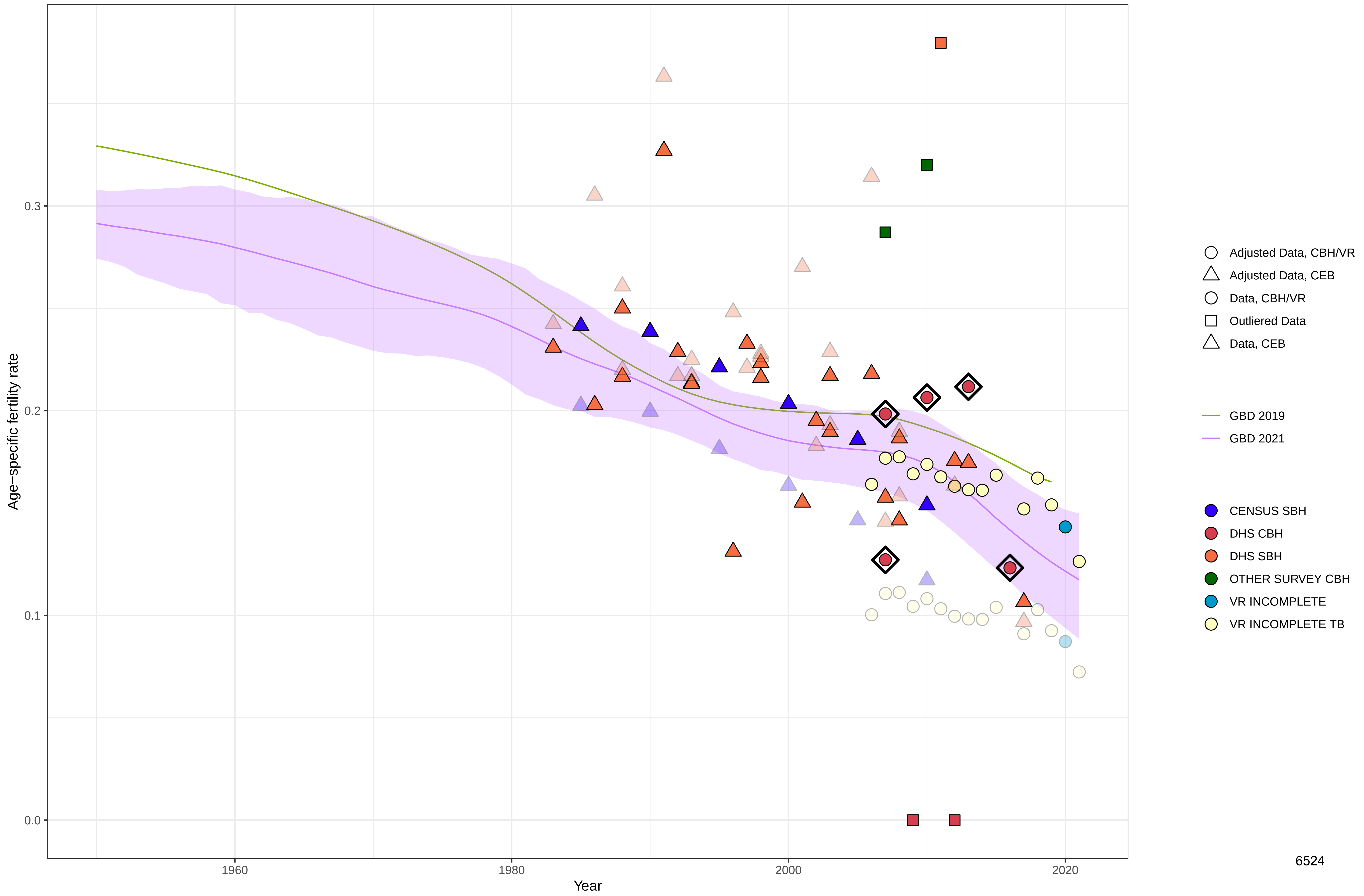

Age−specific fertility fitted trend lines, 1950−2021: Surigao Del Norte, Ages: 25−29
Data points enclosed with a diamond are reference sources

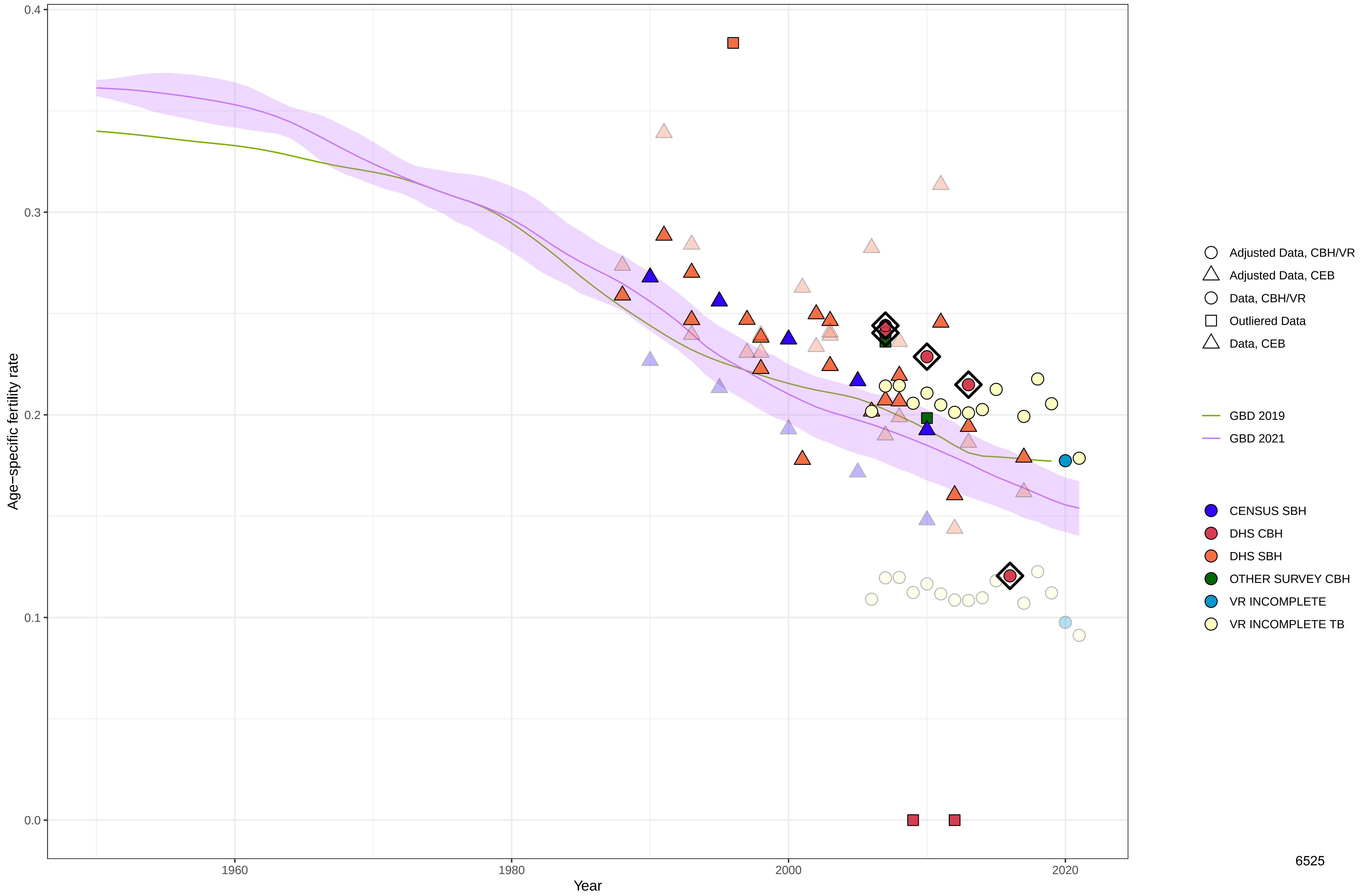

Age−specific fertility fitted trend lines, 1950−2021: Surigao Del Norte, Ages: 30−34
Data points enclosed with a diamond are reference sources

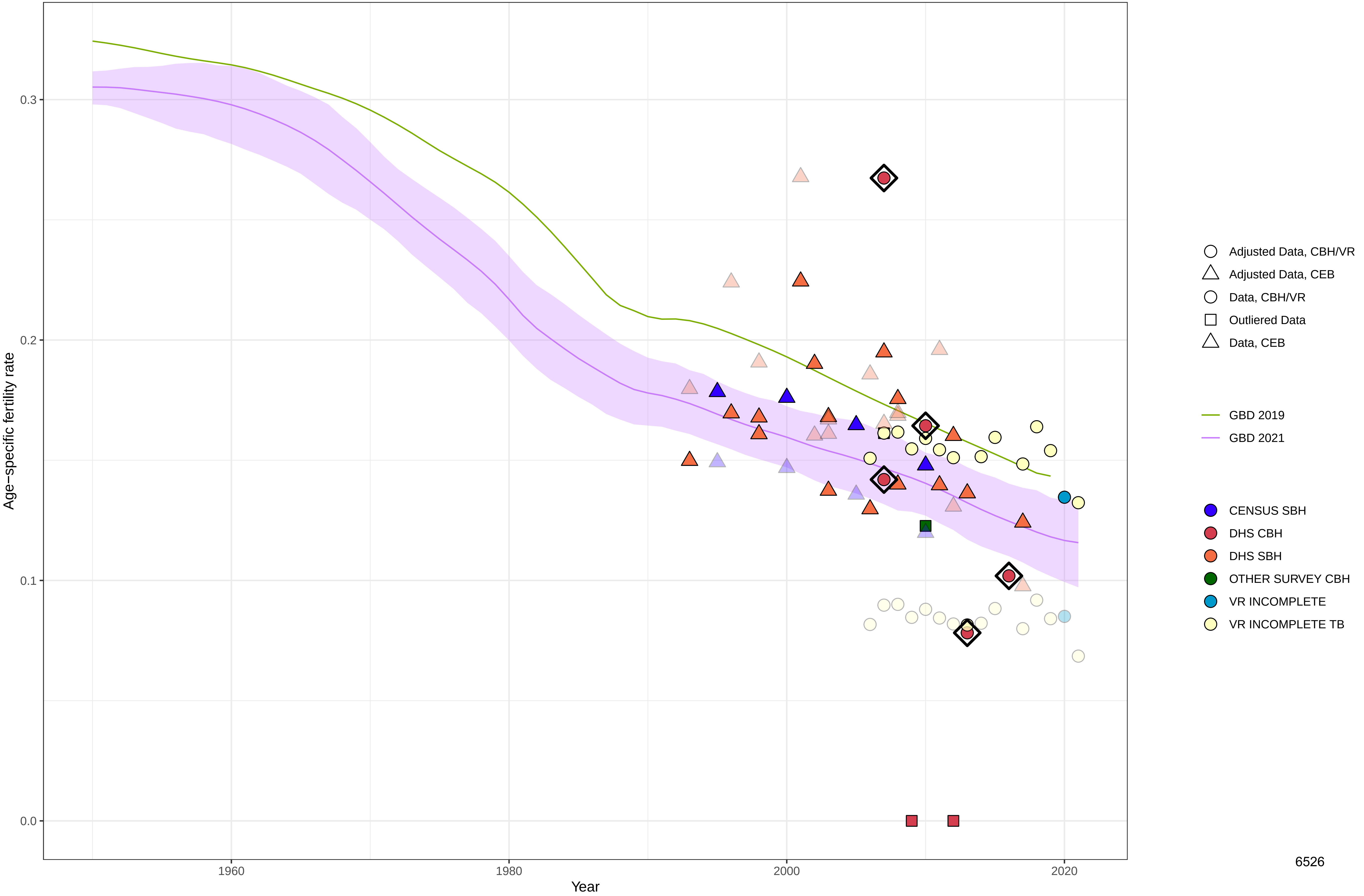

Age−specific fertility fitted trend lines, 1950−2021: Surigao Del Norte, Ages: 35−39
Data points enclosed with a diamond are reference sources

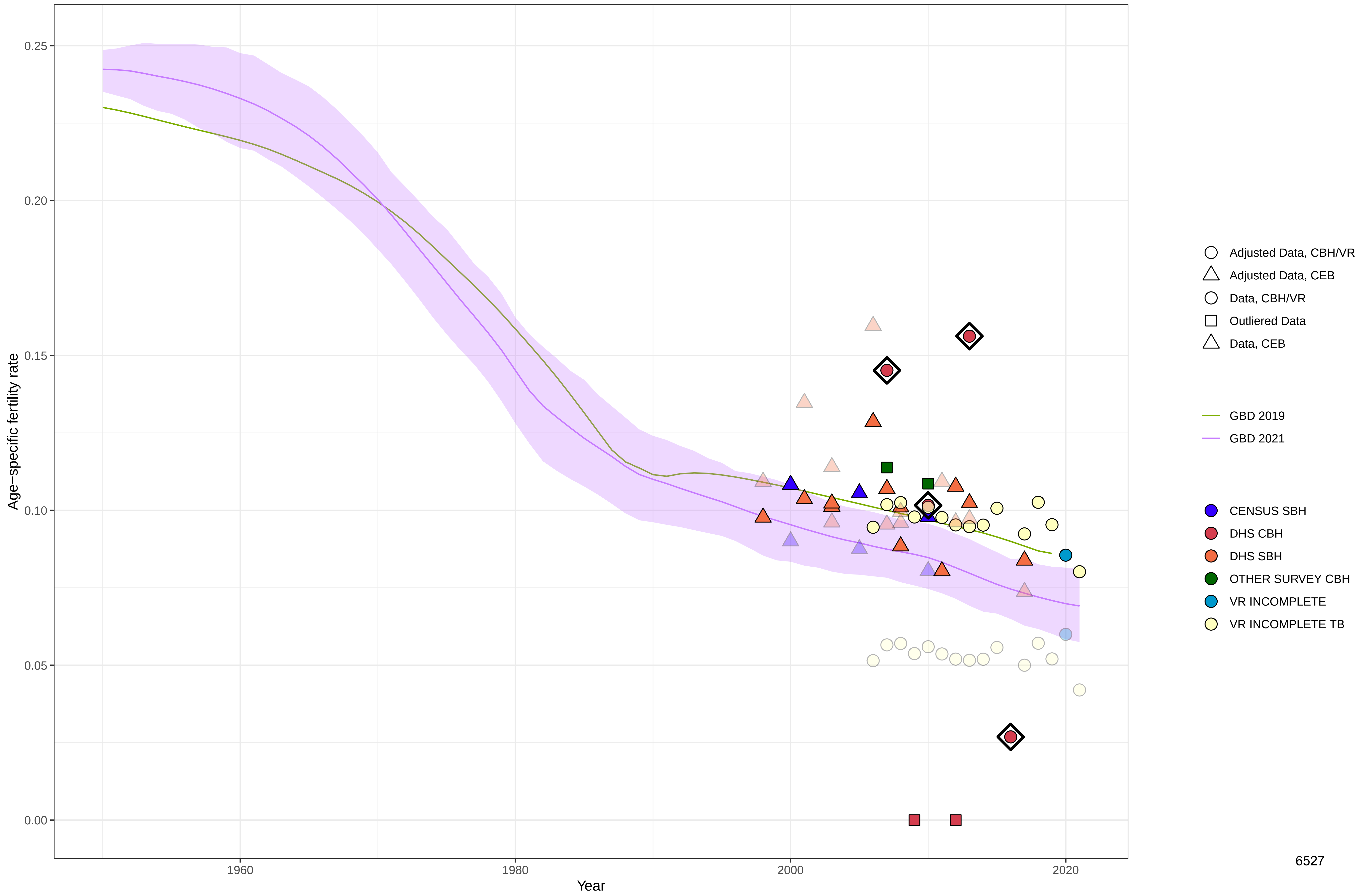

Age−specific fertility fitted trend lines, 1950−2021: Surigao Del Norte, Ages: 40−44
Data points enclosed with a diamond are reference sources

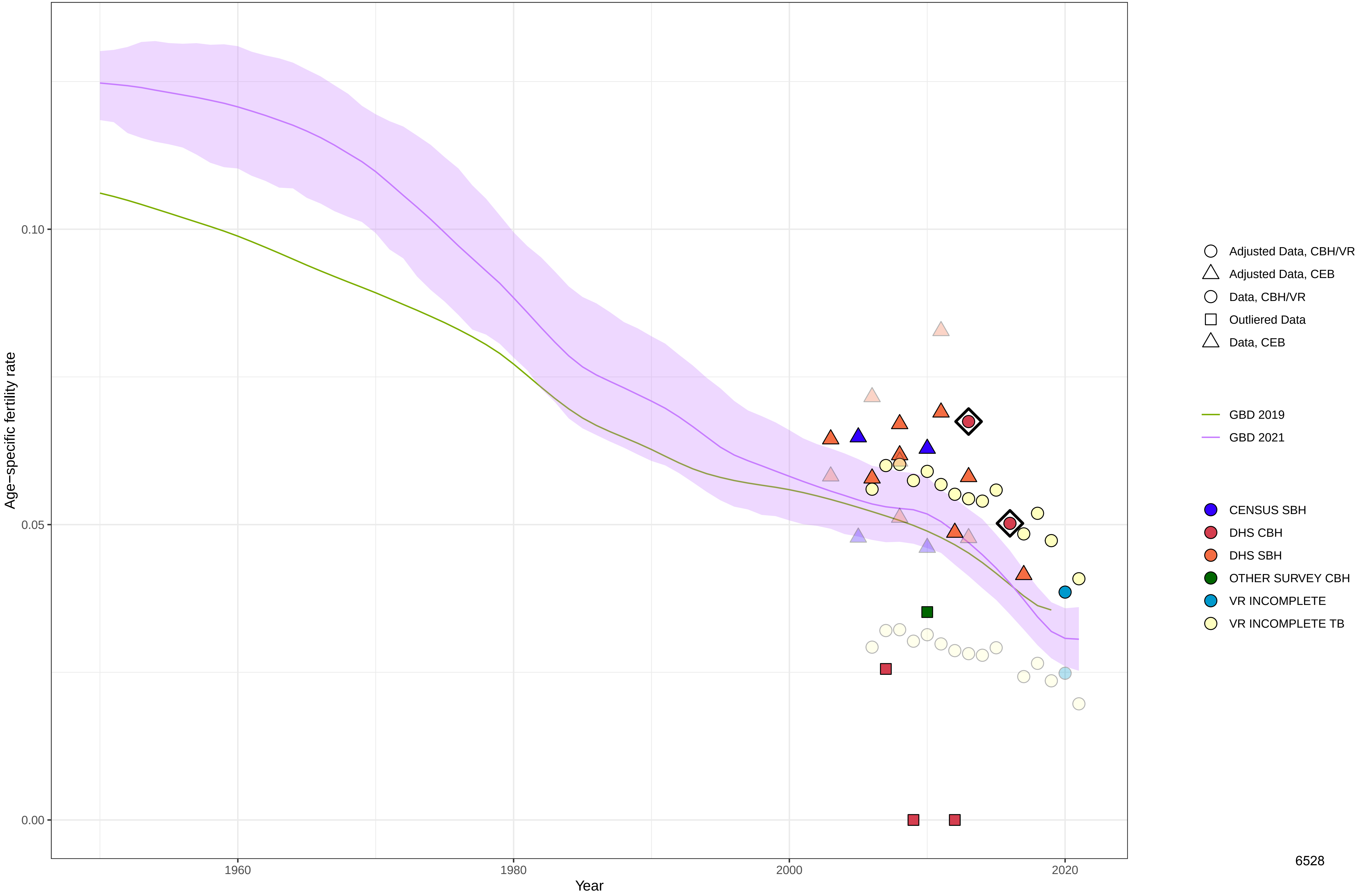

Age−specific fertility fitted trend lines, 1950−2021: Surigao Del Norte, Ages: 45−49
Data points enclosed with a diamond are reference sources

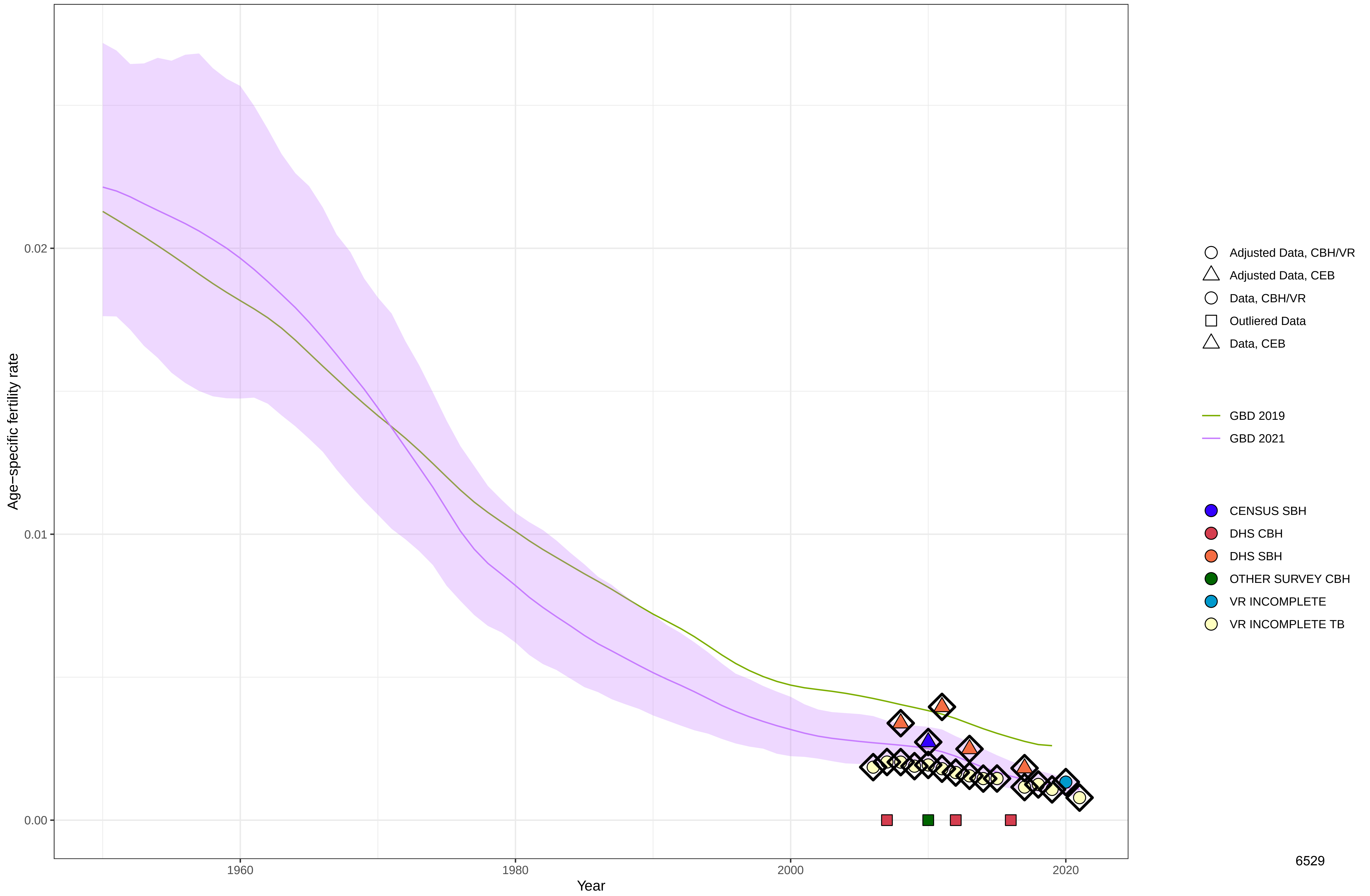

Age−specific fertility fitted trend lines, 1950−2021: Surigao Del Norte, Ages: 50−54
Data points enclosed with a diamond are reference sources

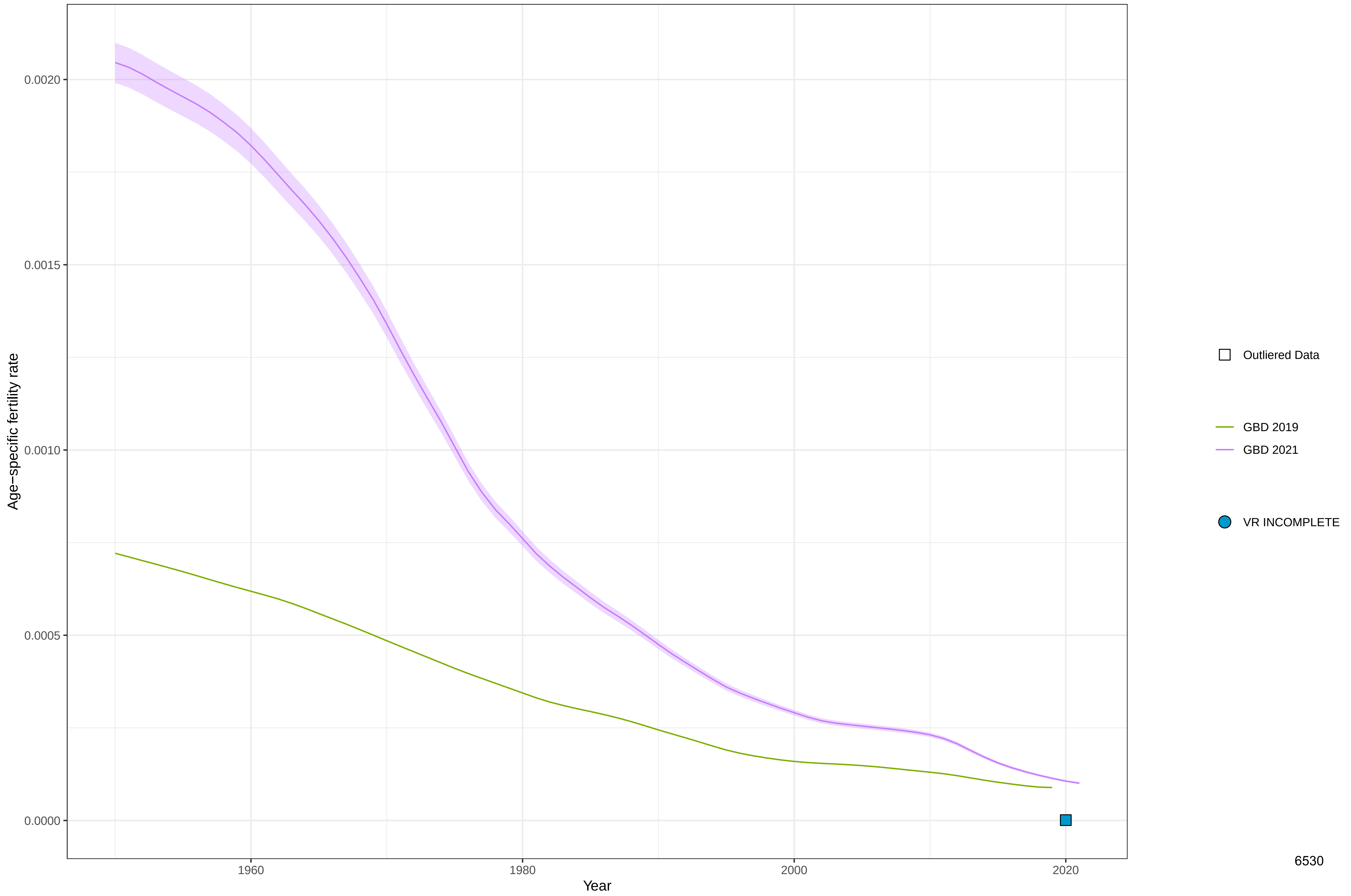

Total fertility rate fitted trend lines, 1950−2021: Surigao Del Norte TFR
Data points enclosed with a diamond are reference sources

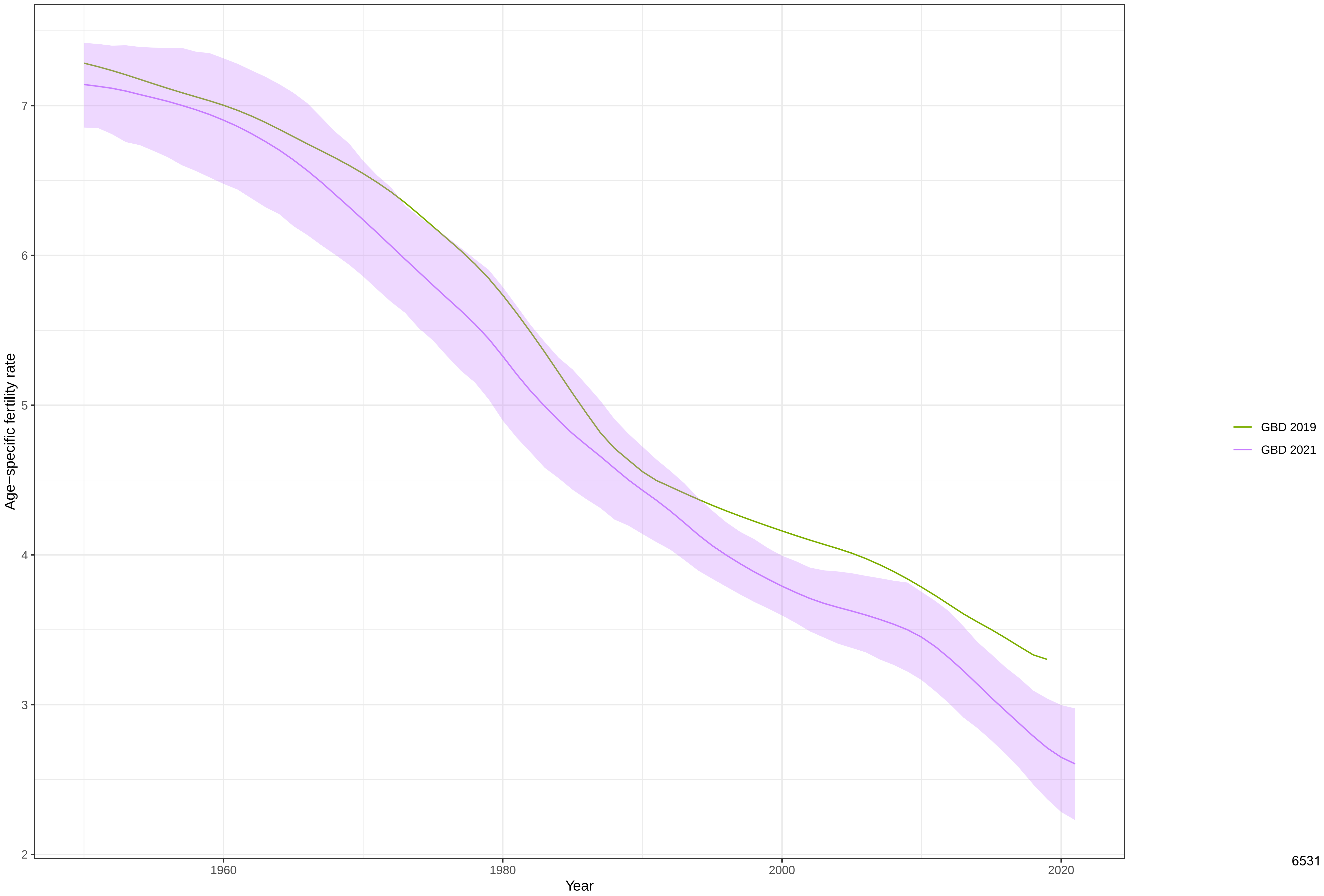

6531

Age−specific fertility fitted trend lines, 1950−2021: Surigao Del Sur, Ages: 10−14
Data points enclosed with a diamond are reference sources

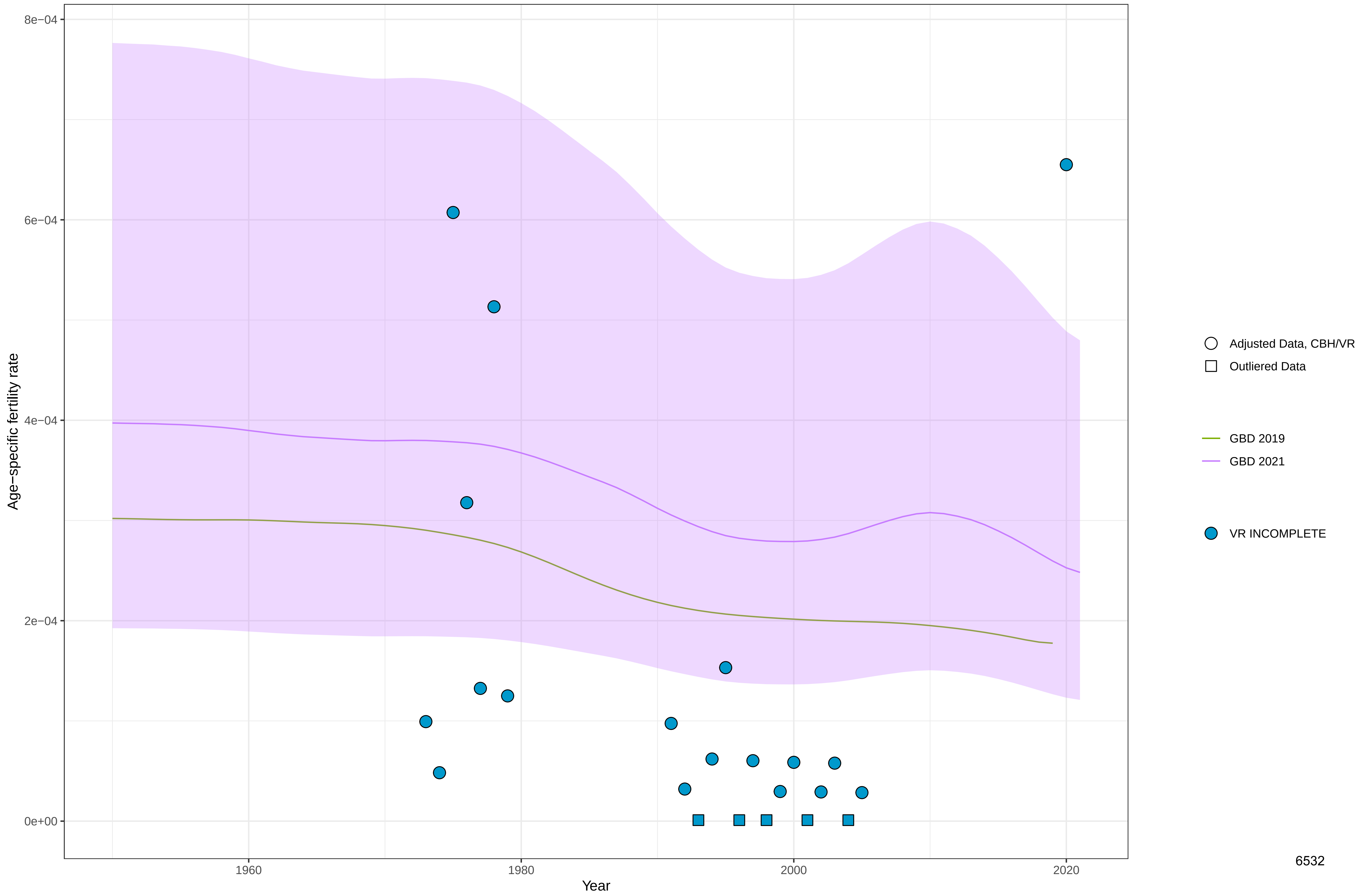

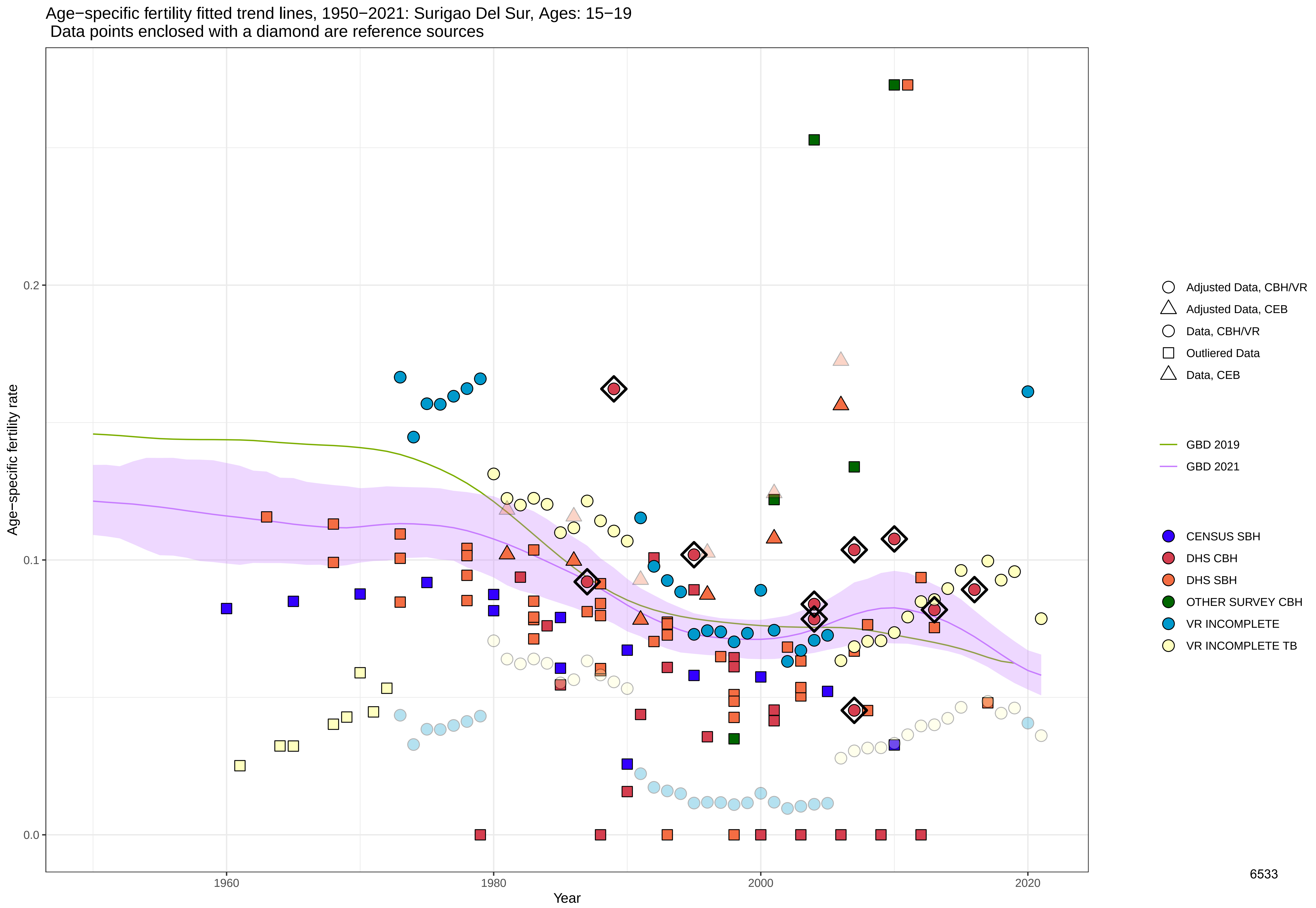

Age−specific fertility fitted trend lines, 1950−2021: Surigao Del Sur, Ages: 15−19
Data points enclosed with a diamond are reference sources
Age−specific fertility rate
0.0
0.1
0.2
1960
1980
2000
2020
Year
Adjusted Data, CBH/VR
Adjusted Data, CEB
Data, CBH/VR
Outliered Data
Data, CEB
GBD 2019
GBD 2021
CENSUS SBH
DHS CBH
DHS SBH
OTHER SURVEY CBH
VR INCOMPLETE
VR INCOMPLETE TB

Age−specific fertility fitted trend lines, 1950−2021: Surigao Del Sur, Ages: 20−24
Data points enclosed with a diamond are reference sources

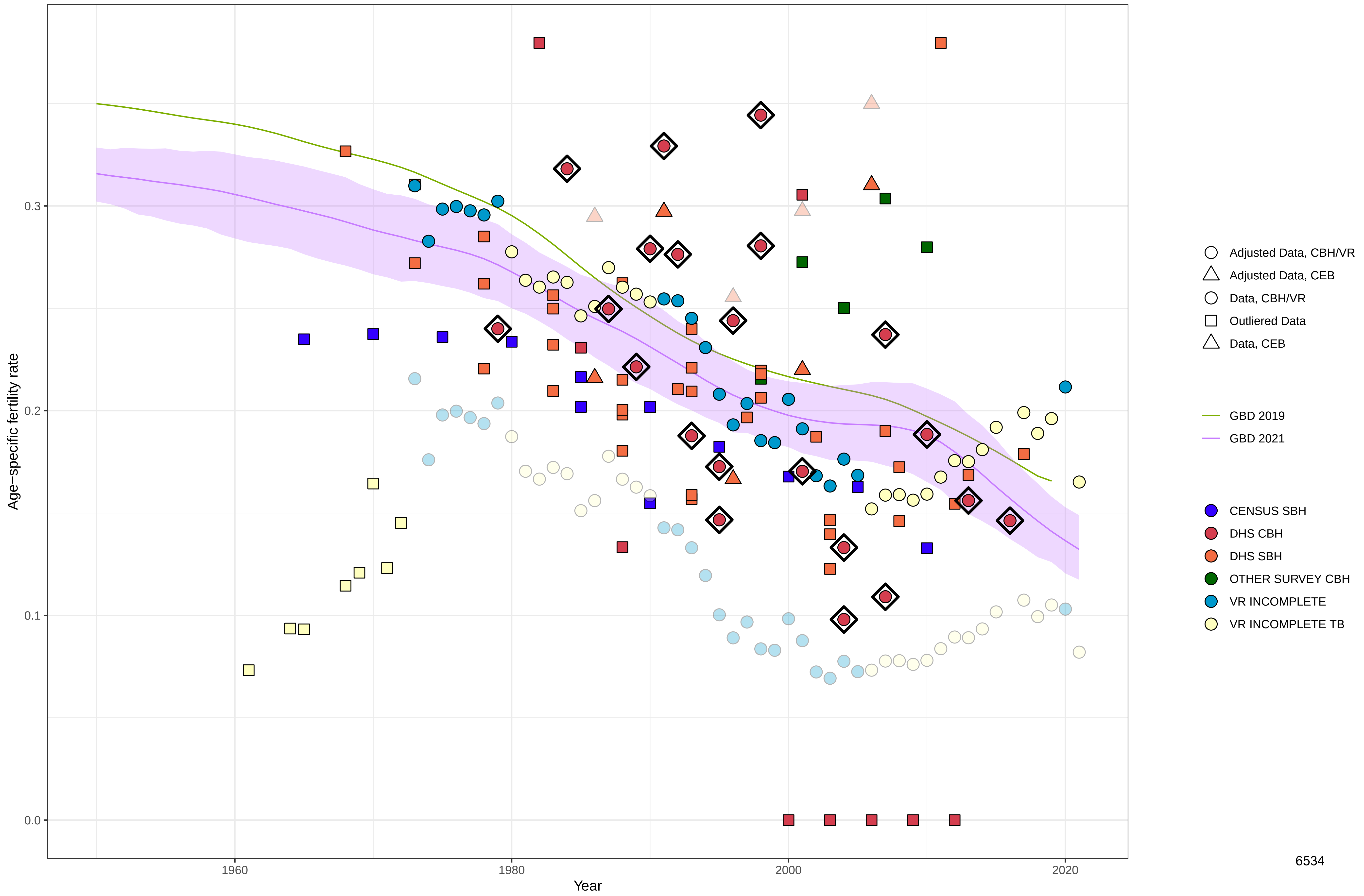

Age−specific fertility fitted trend lines, 1950−2021: Surigao Del Sur, Ages: 25−29
Data points enclosed with a diamond are reference sources

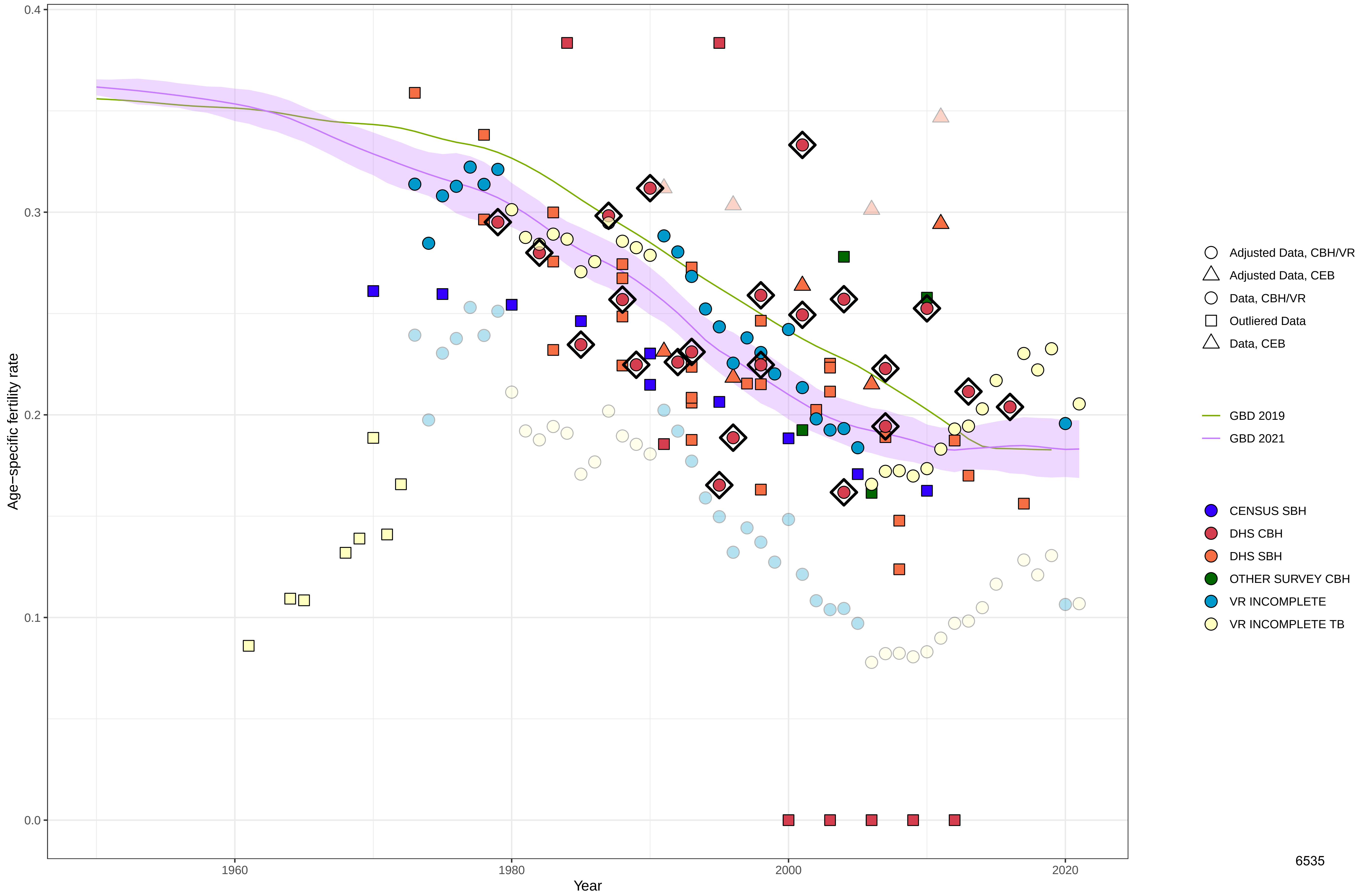

Age−specific fertility fitted trend lines, 1950−2021: Surigao Del Sur, Ages: 30−34
Data points enclosed with a diamond are reference sources

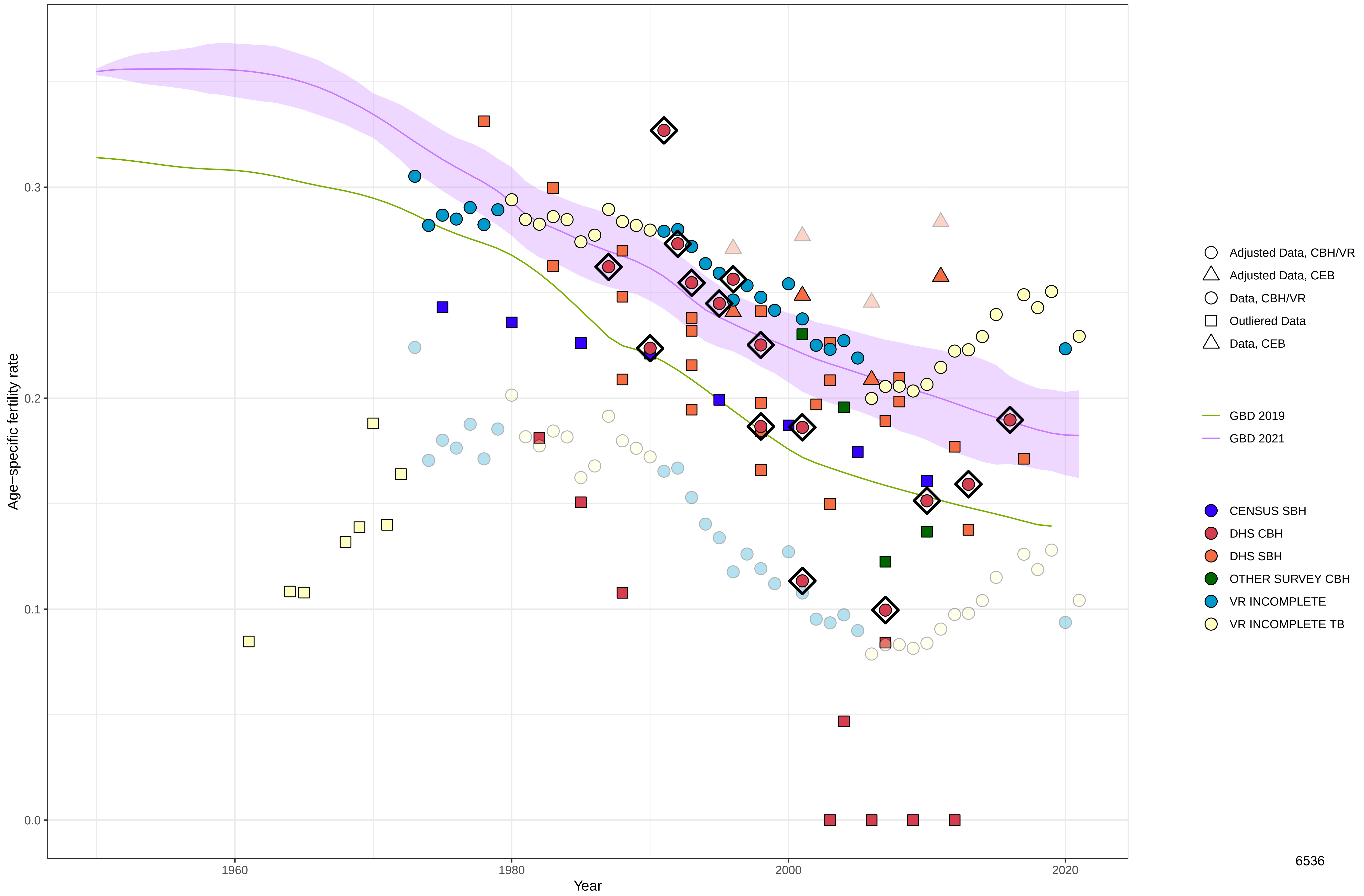

Age−specific fertility fitted trend lines, 1950−2021: Surigao Del Sur, Ages: 35−39
Data points enclosed with a diamond are reference sources

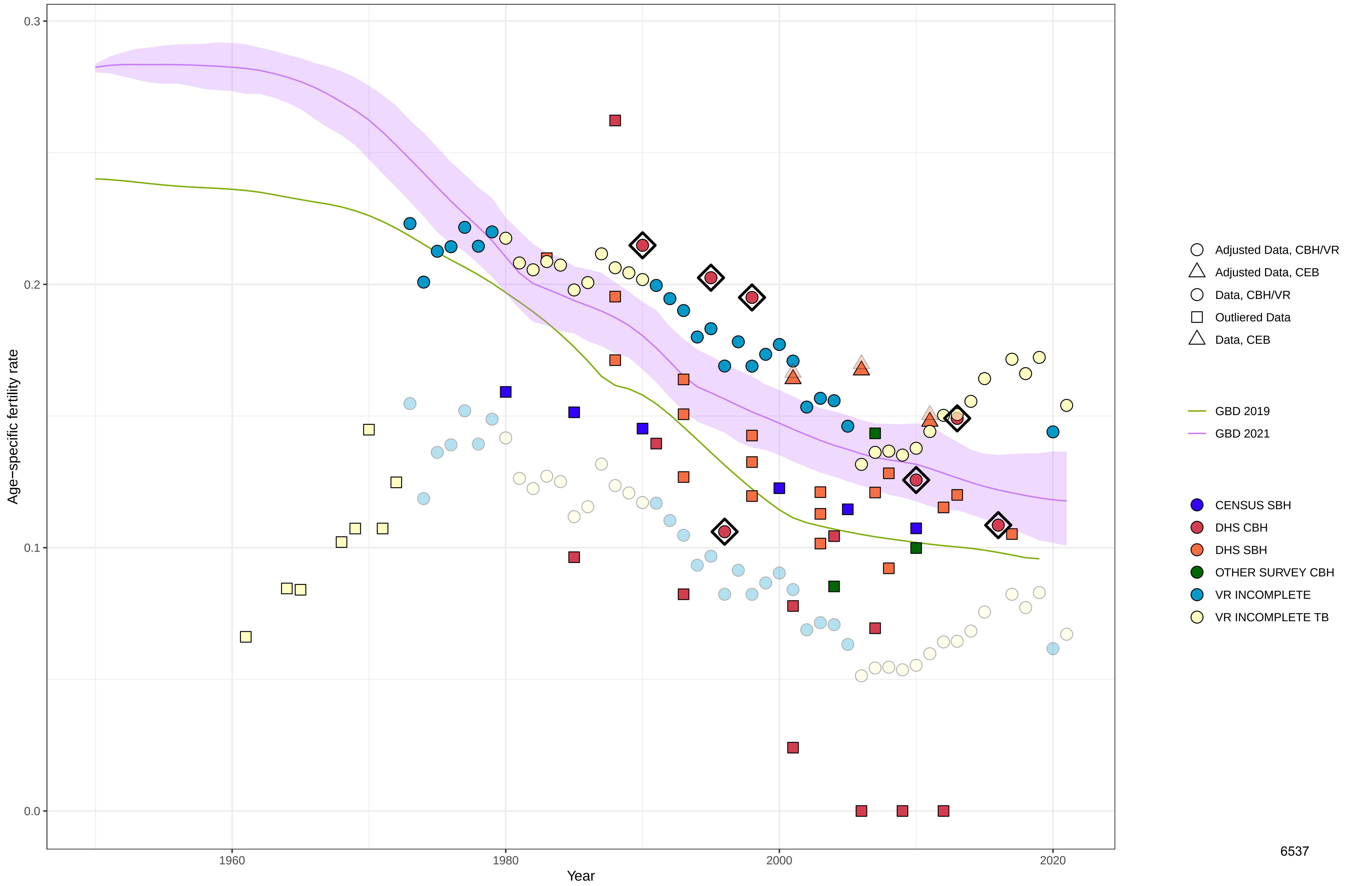

Age−specific fertility fitted trend lines, 1950−2021: Surigao Del Sur, Ages: 40−44
Data points enclosed with a diamond are reference sources

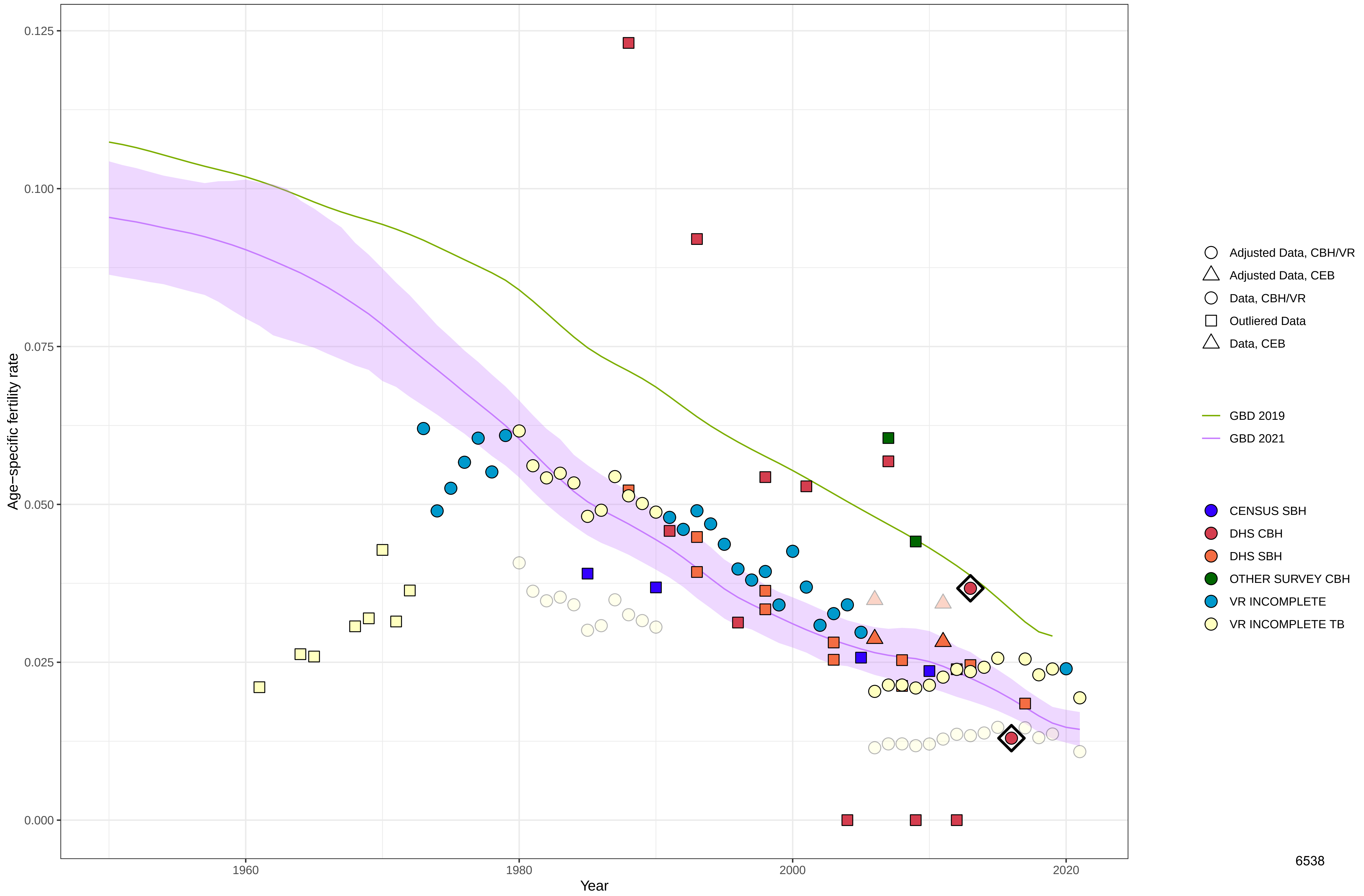

Age−specific fertility fitted trend lines, 1950−2021: Surigao Del Sur, Ages: 45−49
Data points enclosed with a diamond are reference sources

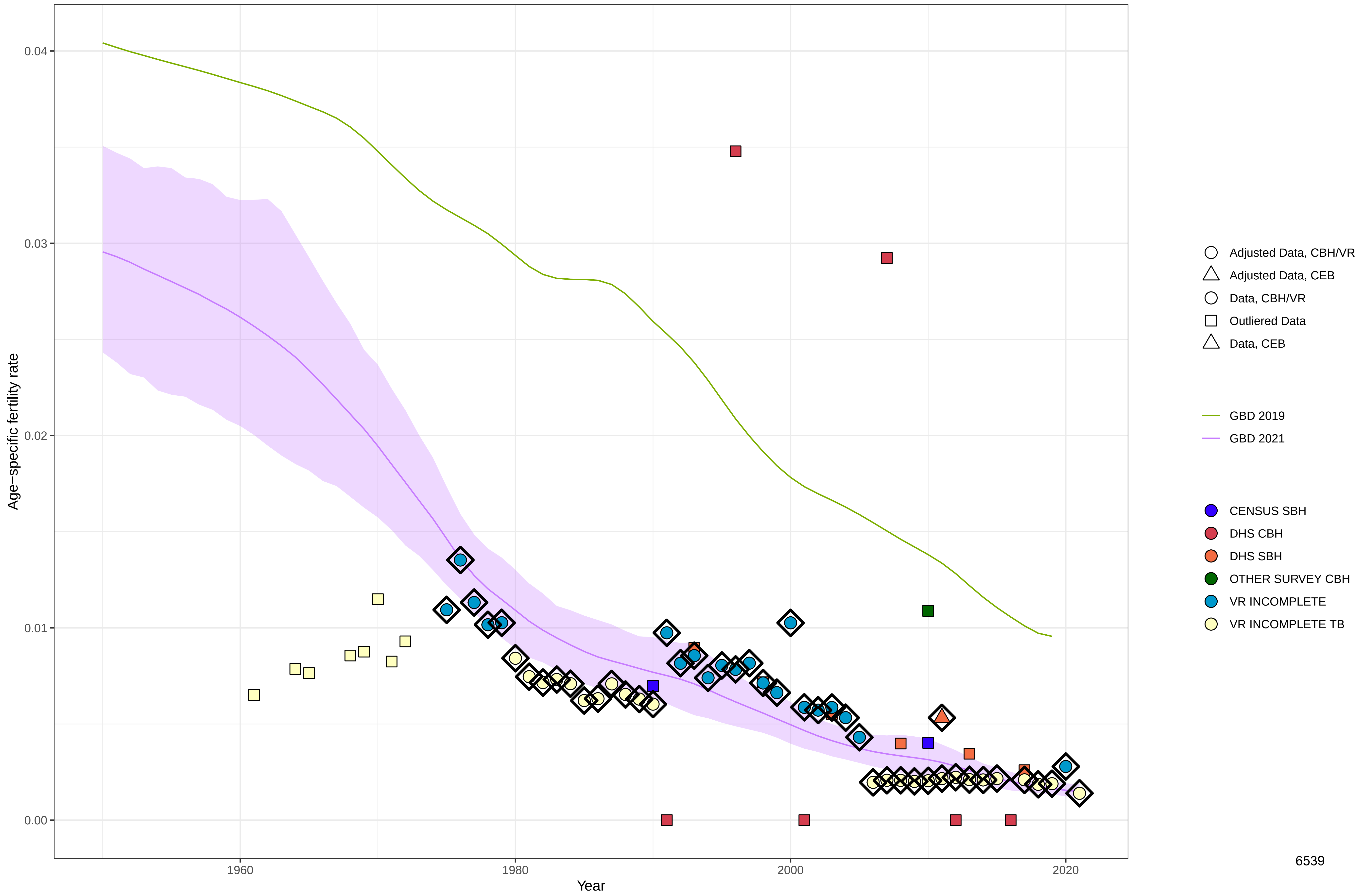

Age−specific fertility fitted trend lines, 1950−2021: Surigao Del Sur, Ages: 50−54
Data points enclosed with a diamond are reference sources

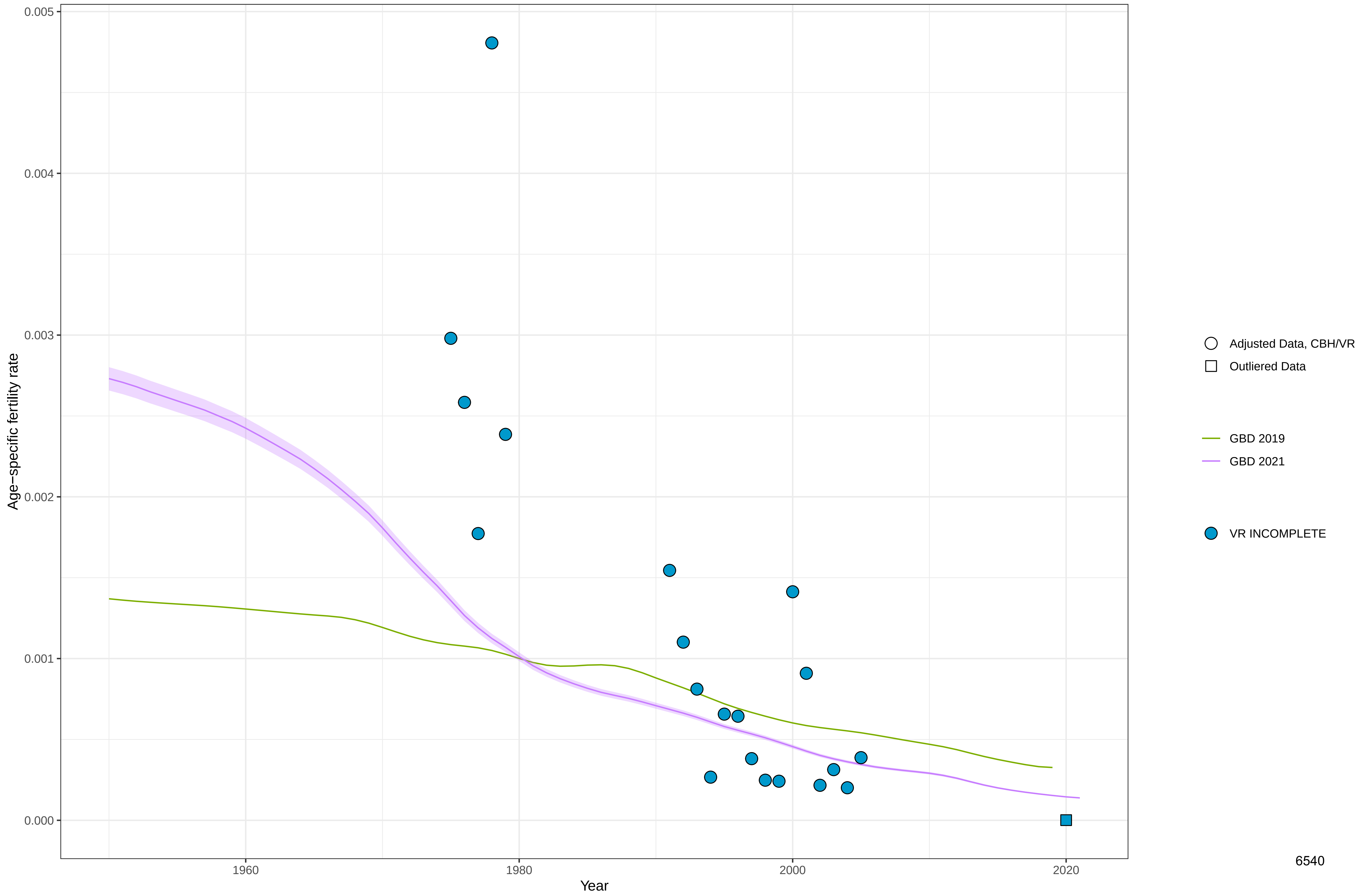

Total fertility rate fitted trend lines, 1950−2021: Surigao Del Sur TFR
Data points enclosed with a diamond are reference sources

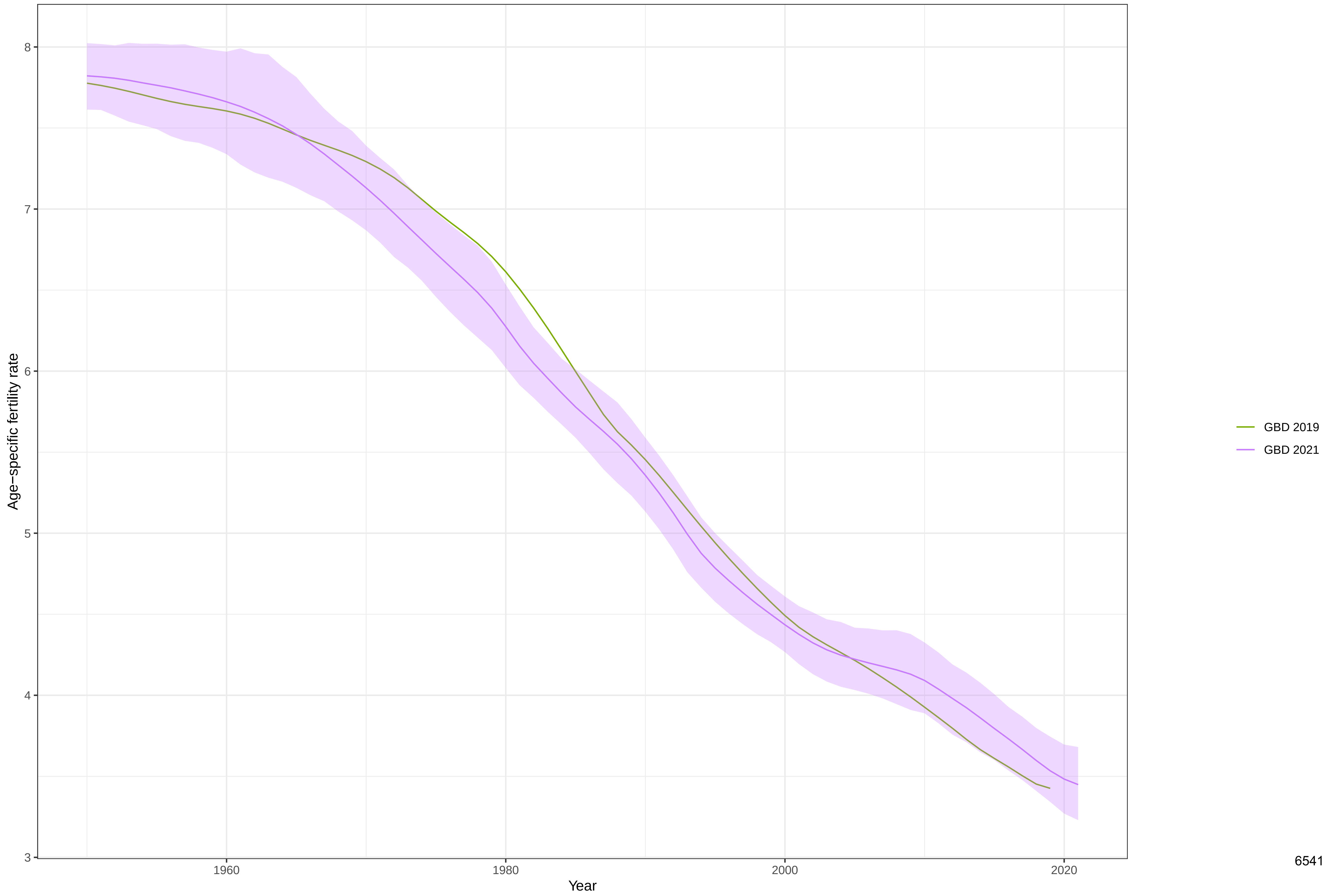

Age−specific fertility fitted trend lines, 1950−2021: Tarlac, Ages: 10−14
Data points enclosed with a diamond are reference sources

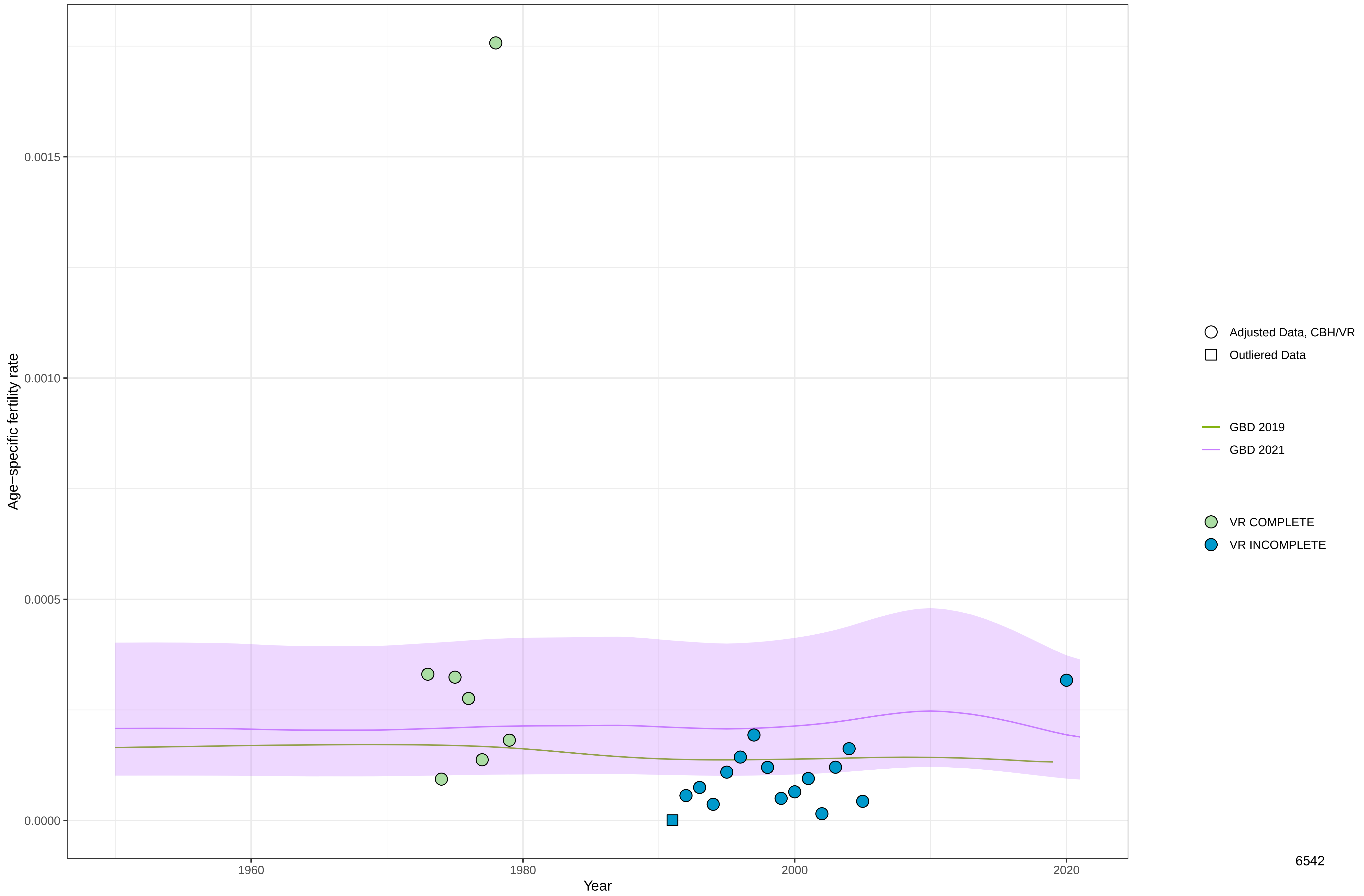

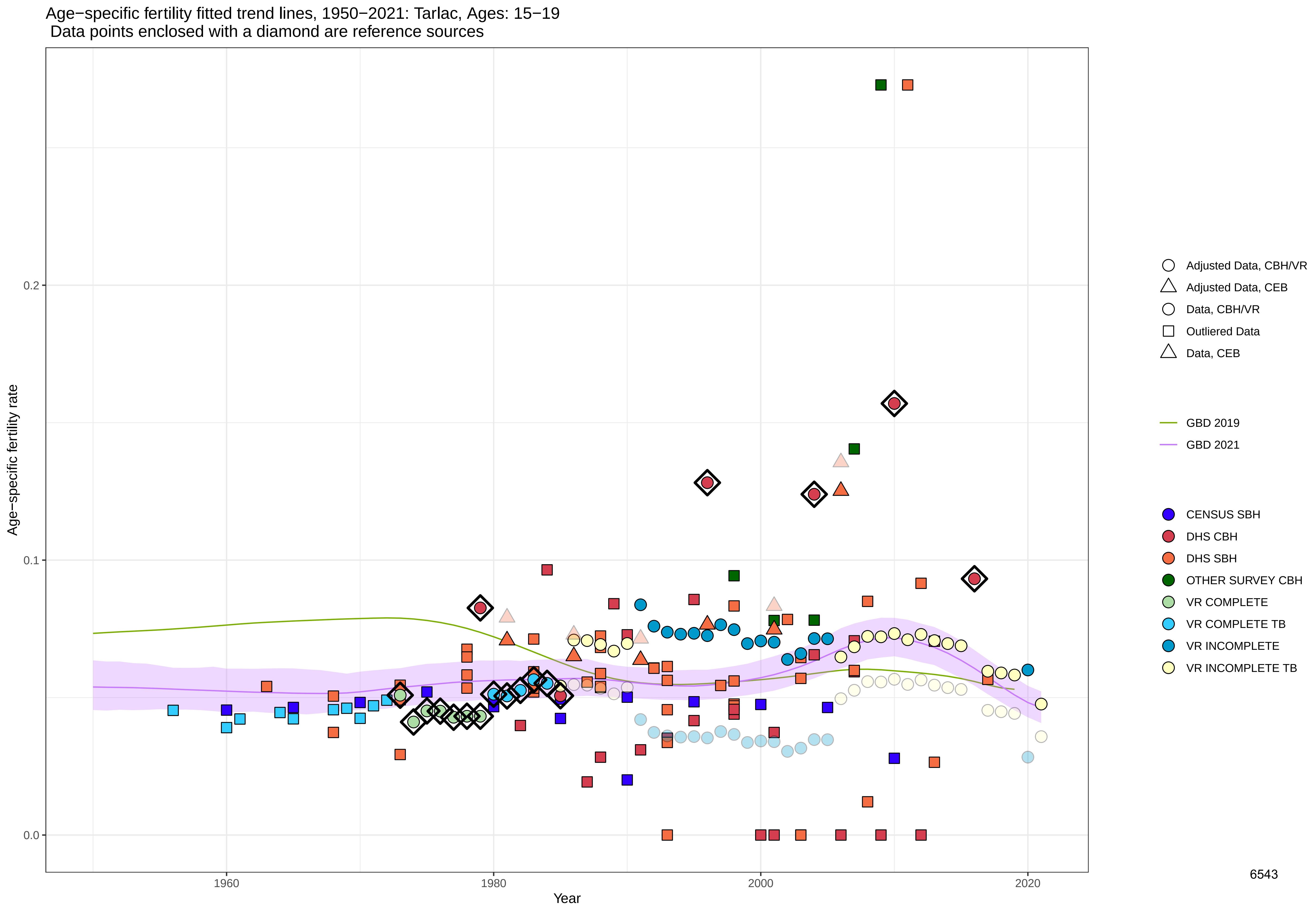

Age−specific fertility fitted trend lines, 1950−2021: Tarlac, Ages: 15−19
Data points enclosed with a diamond are reference sources
Age−specific fertility rate
Year
0.0
0.1
0.2
1960
1980
2000
2020
Adjusted Data, CBH/VR
Adjusted Data, CEB
Data, CBH/VR
Outliered Data
Data, CEB
GBD 2019
GBD 2021
CENSUS SBH
DHS CBH
DHS SBH
OTHER SURVEY CBH
VR COMPLETE
VR COMPLETE TB
VR INCOMPLETE
VR INCOMPLETE TB

Age−specific fertility fitted trend lines, 1950−2021: Tarlac, Ages: 20−24
Data points enclosed with a diamond are reference sources

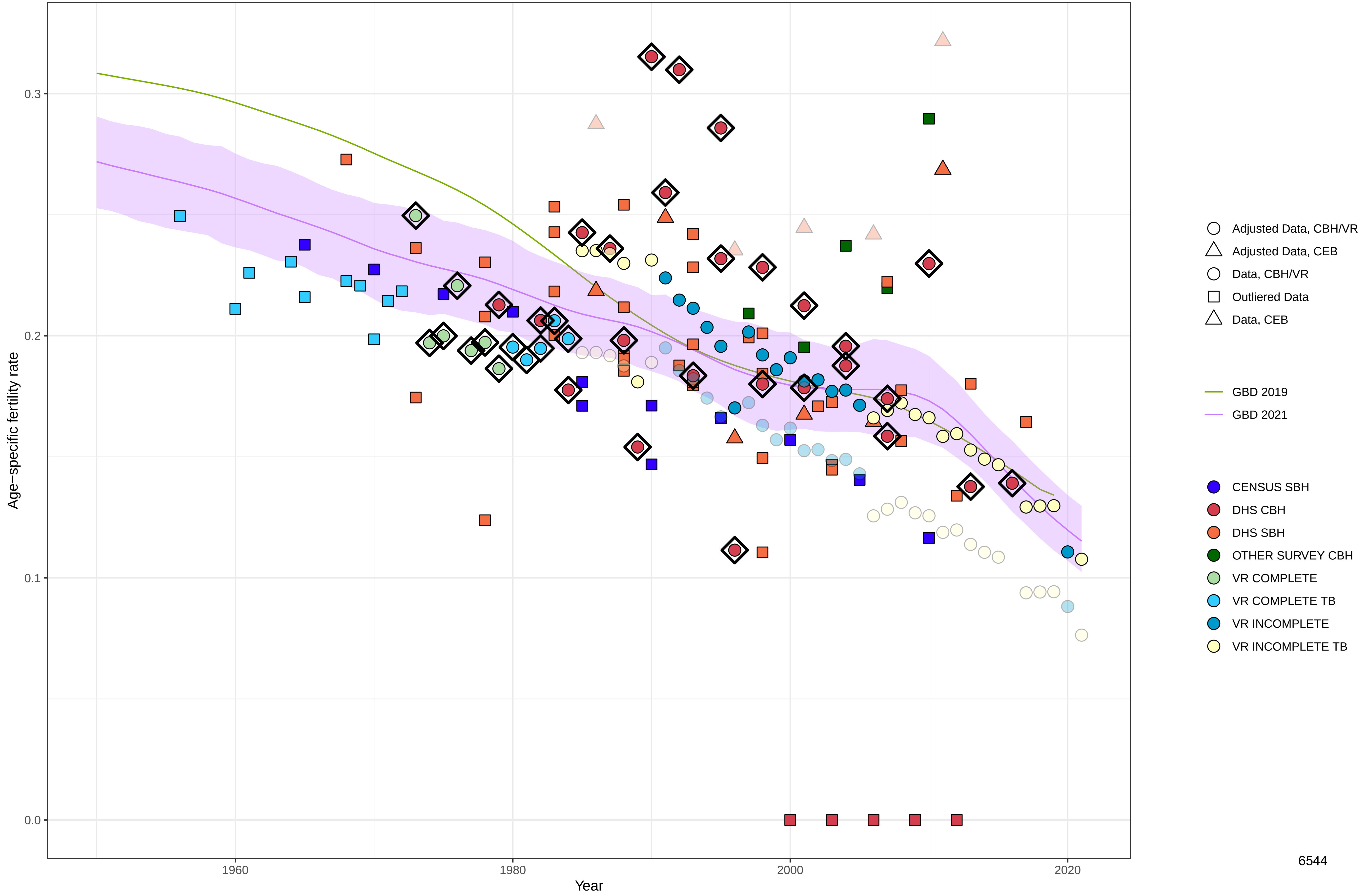

Age−specific fertility fitted trend lines, 1950−2021: Tarlac, Ages: 25−29
Data points enclosed with a diamond are reference sources

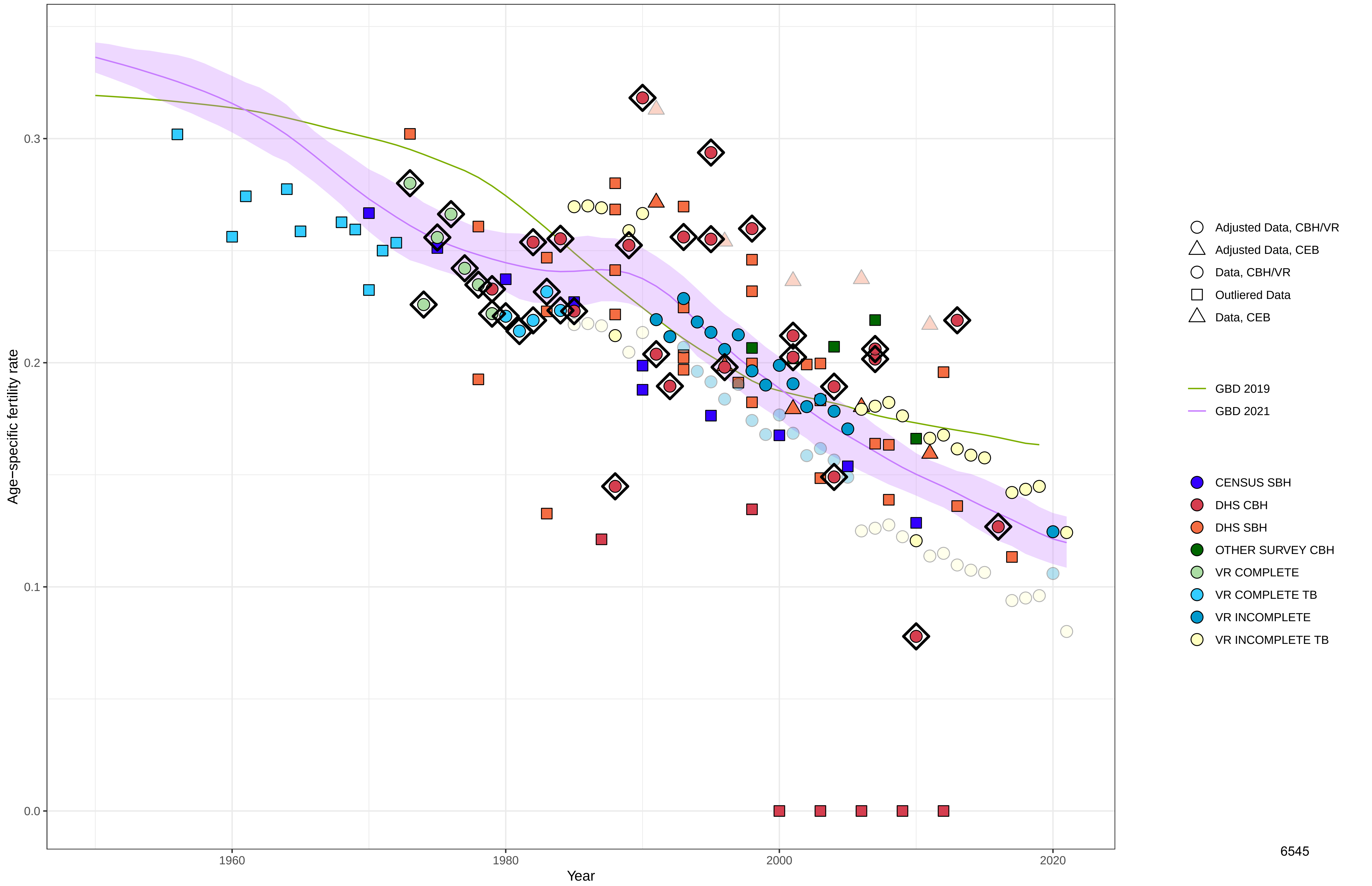

Age−specific fertility fitted trend lines, 1950−2021: Tarlac, Ages: 30−34
Data points enclosed with a diamond are reference sources

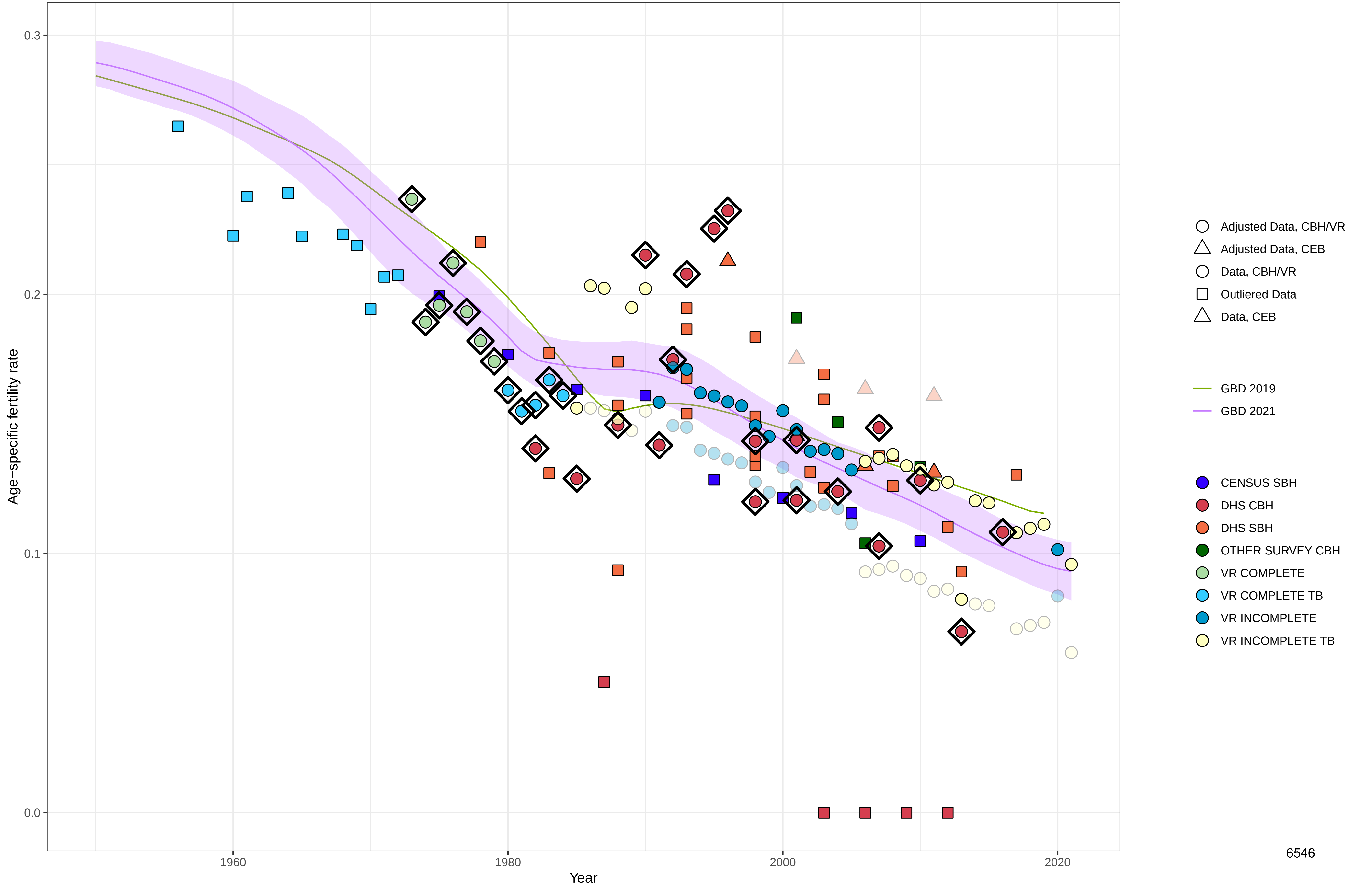

Age−specific fertility fitted trend lines, 1950−2021: Tarlac, Ages: 35−39
Data points enclosed with a diamond are reference sources

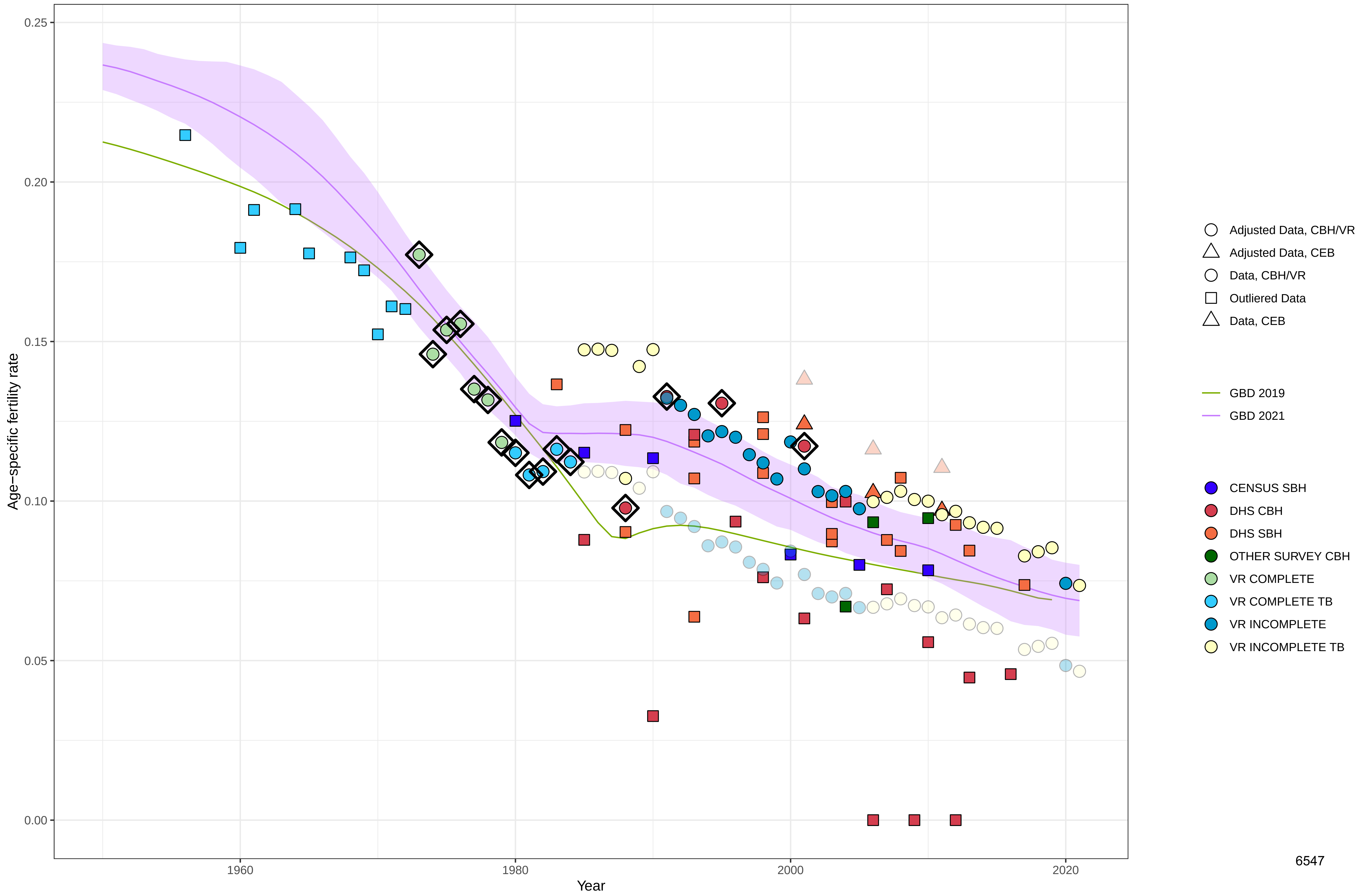

Age−specific fertility fitted trend lines, 1950−2021: Tarlac, Ages: 40−44
Data points enclosed with a diamond are reference sources

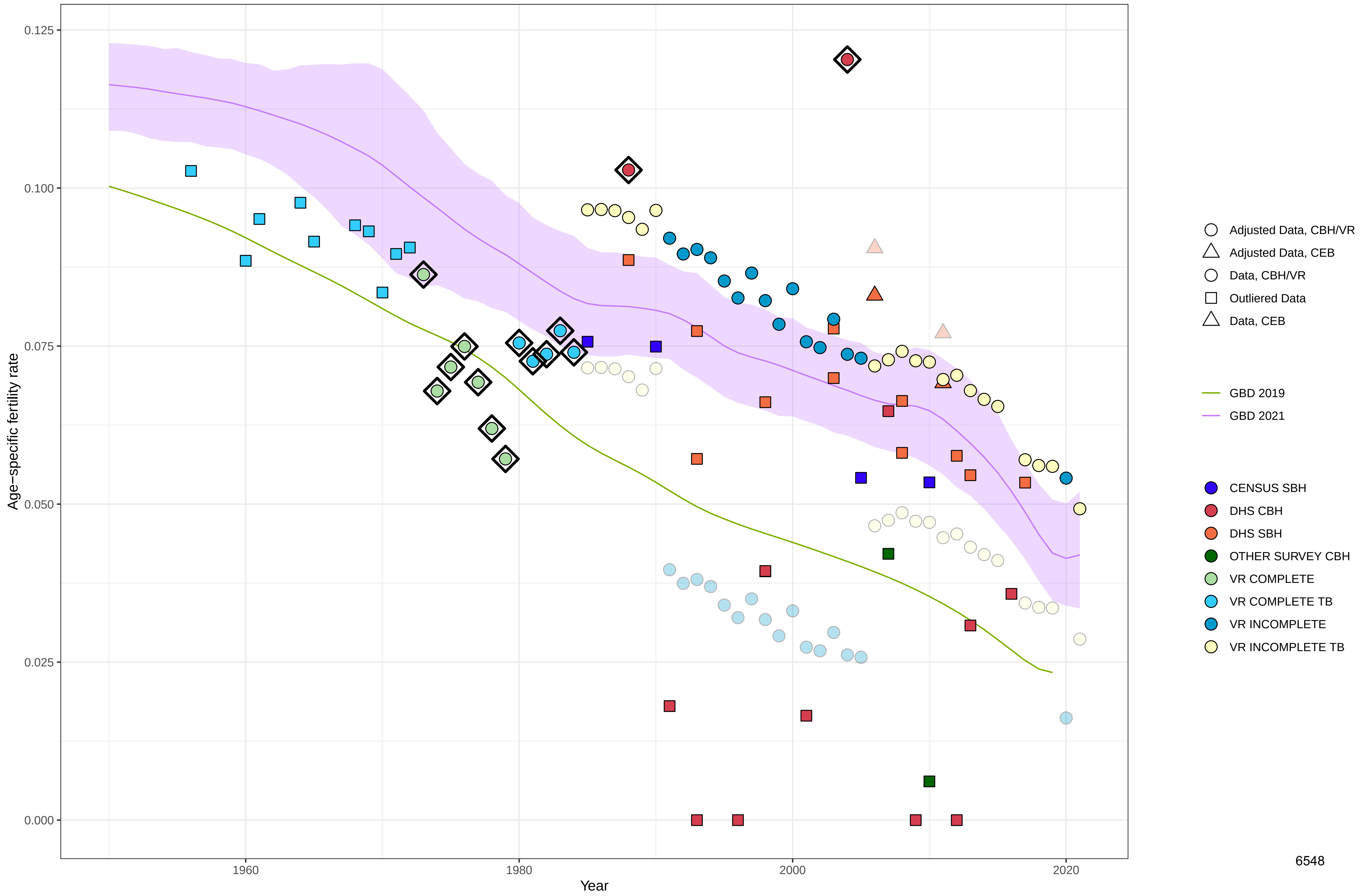

Age−specific fertility fitted trend lines, 1950−2021: Tarlac, Ages: 45−49
Data points enclosed with a diamond are reference sources

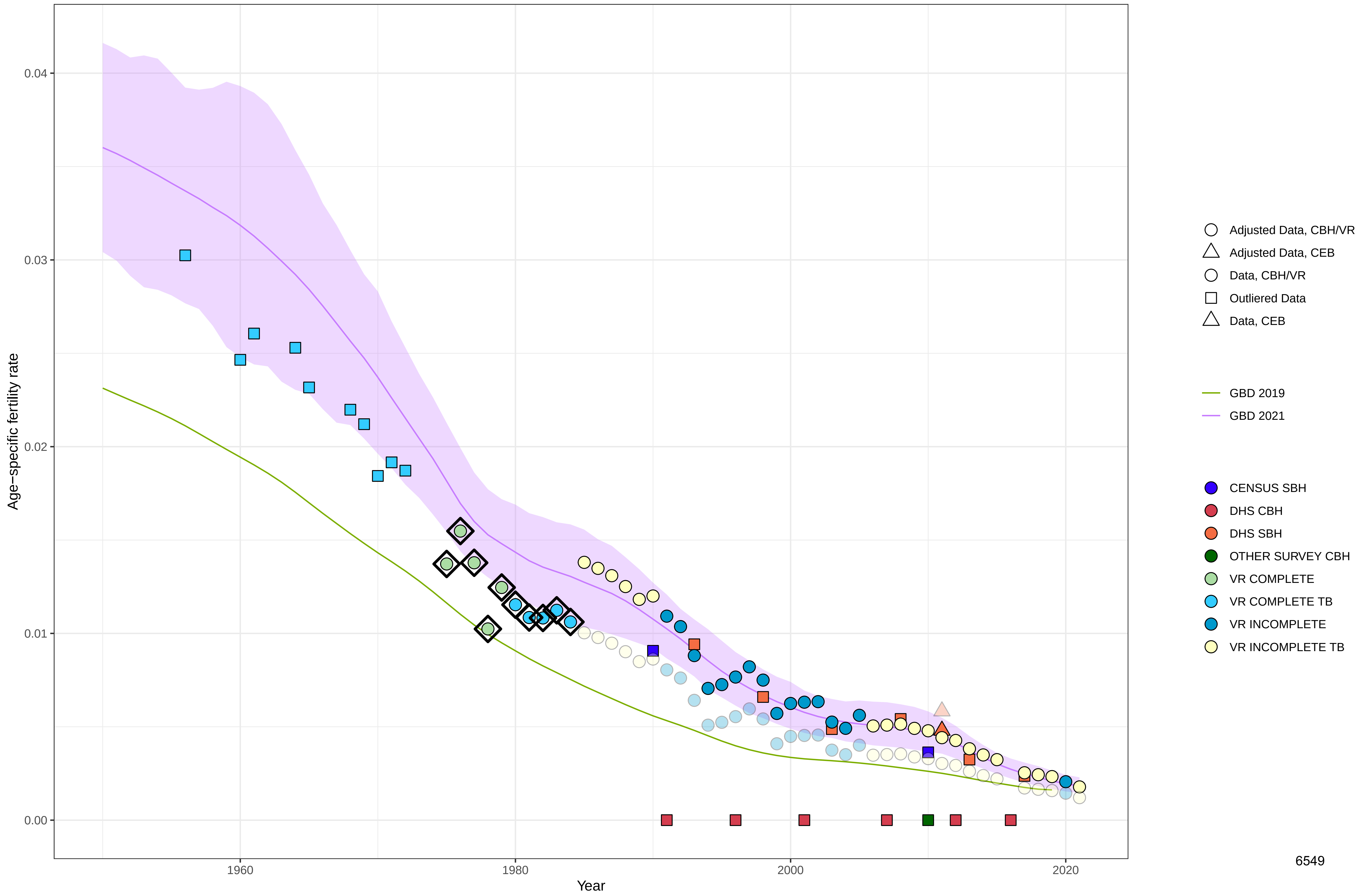

Age−specific fertility fitted trend lines, 1950−2021: Tarlac, Ages: 50−54
Data points enclosed with a diamond are reference sources

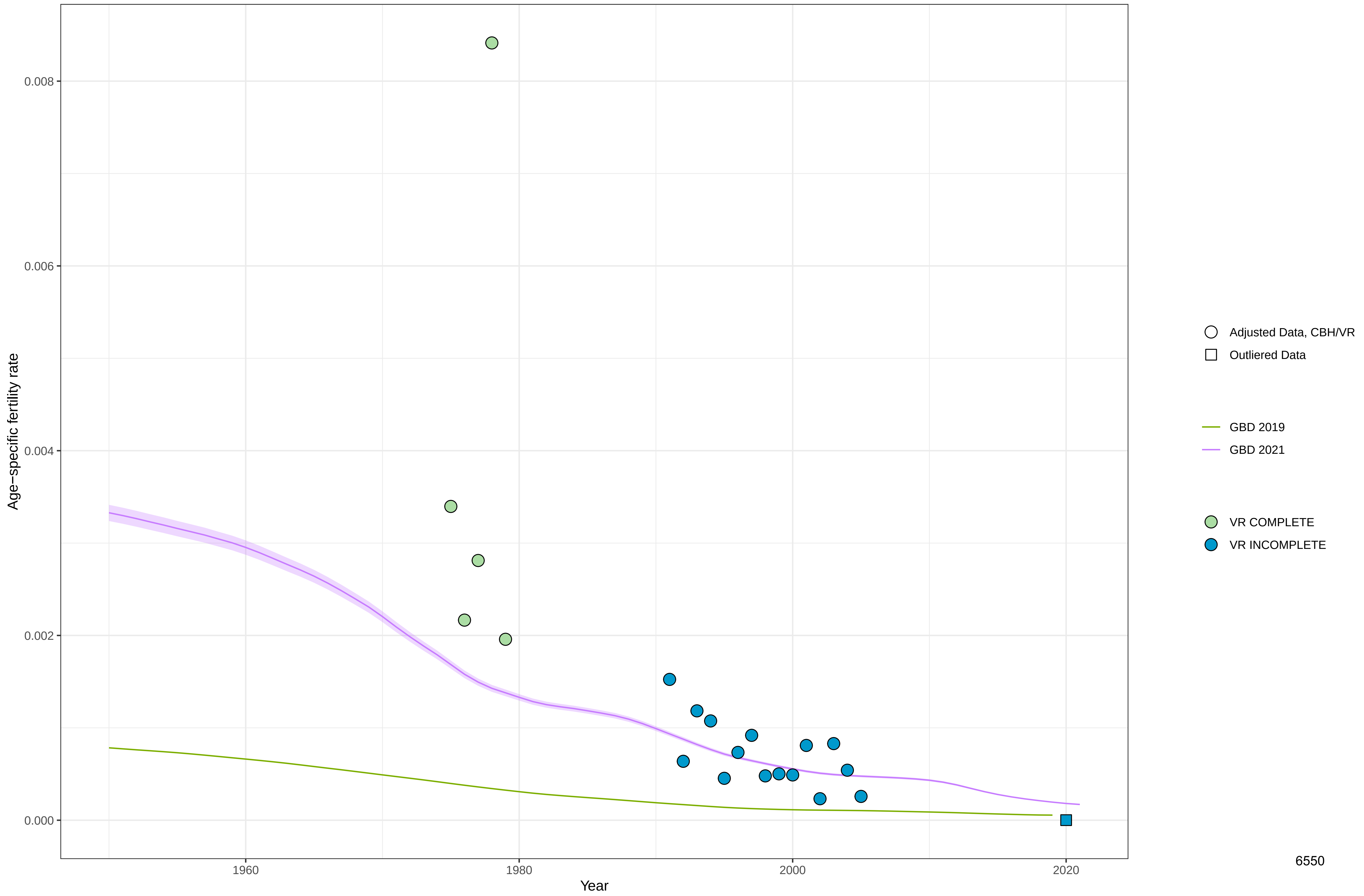

Total fertility rate fitted trend lines, 1950−2021: Tarlac TFR
Data points enclosed with a diamond are reference sources

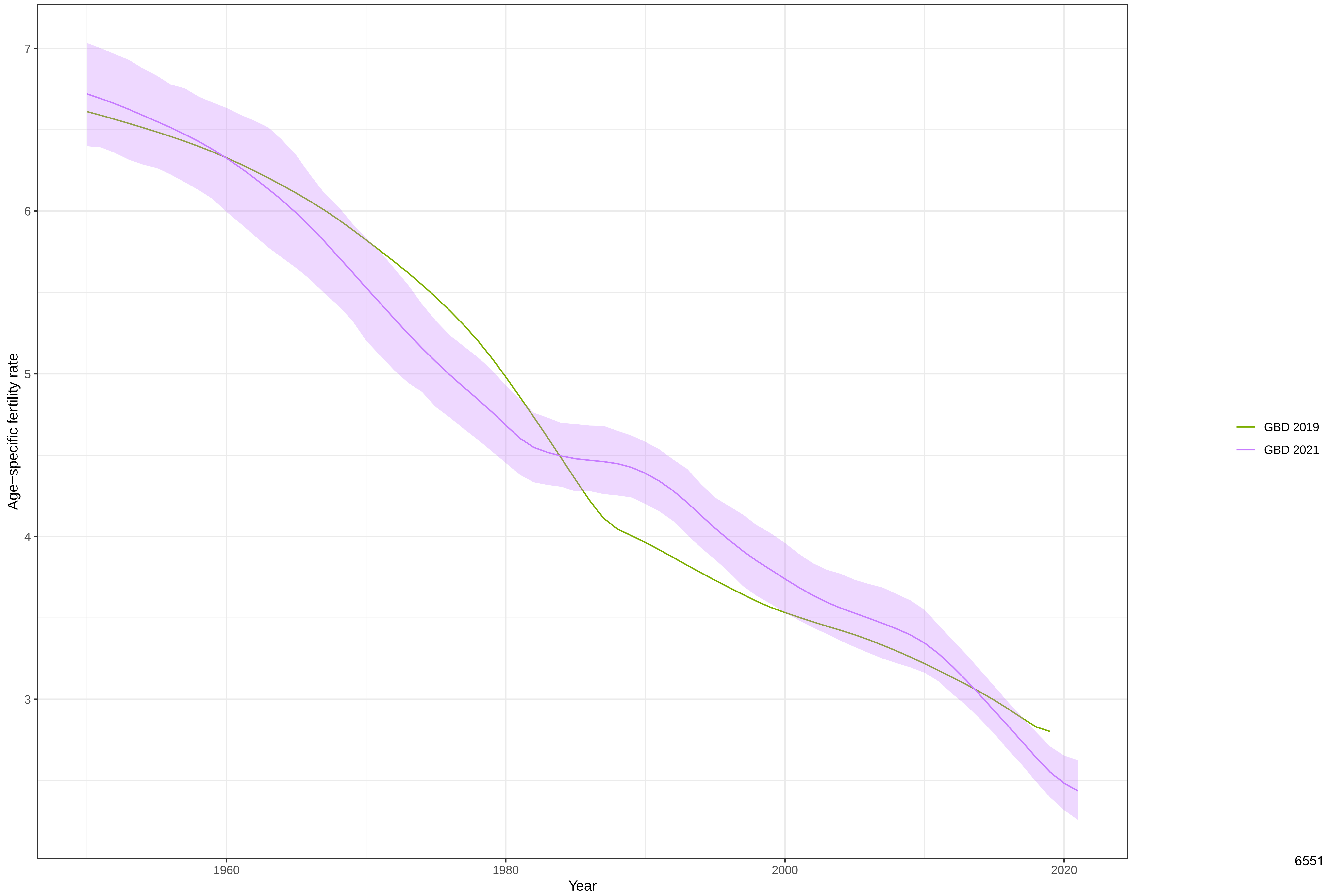

Age−specific fertility fitted trend lines, 1950−2021: Tawi−Tawi, Ages: 10−14
Data points enclosed with a diamond are reference sources

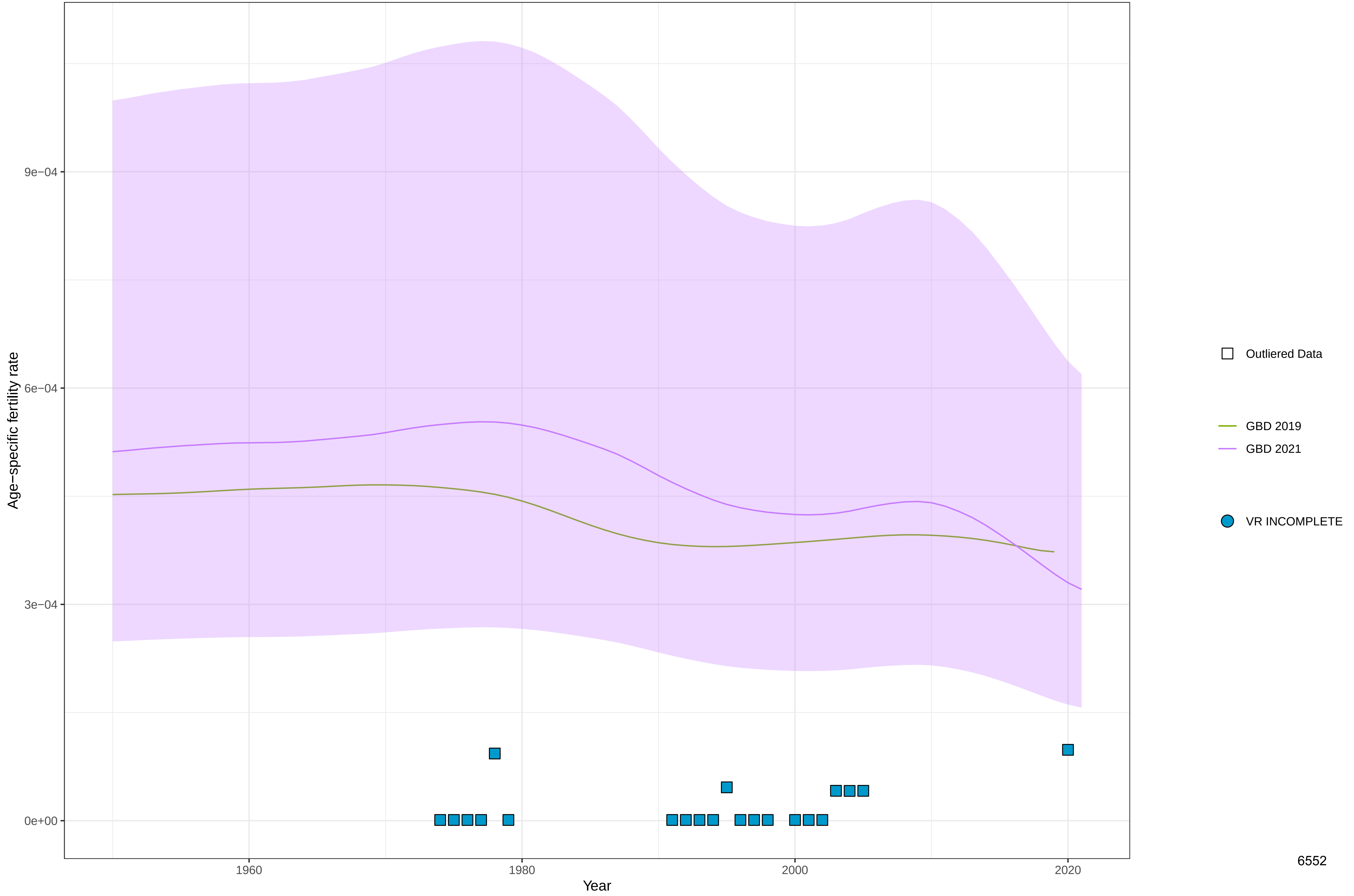

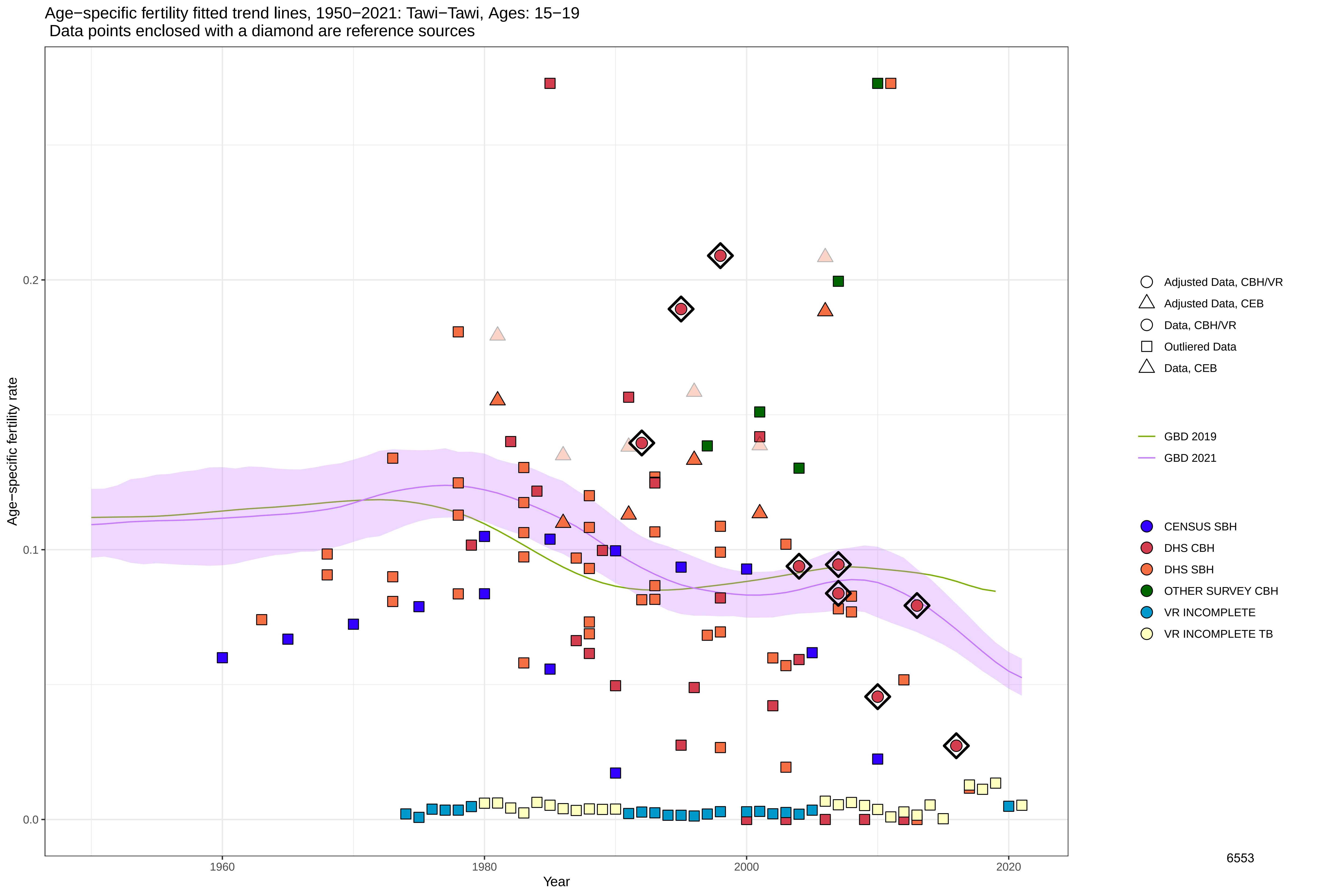
Age−specific fertility fitted trend lines, 1950−2021: Tawi−Tawi, Ages: 15−19
Data points enclosed with a diamond are reference sources
Age−specific fertility rate
0.0
0.1
0.2
1960
1980
2000
2020
Year
Adjusted Data, CBH/VR
Adjusted Data, CEB
Data, CBH/VR
Outliered Data
Data, CEB
GBD 2019
GBD 2021
CENSUS SBH
DHS CBH
DHS SBH
OTHER SURVEY CBH
VR INCOMPLETE
VR INCOMPLETE TB

Age−specific fertility fitted trend lines, 1950−2021: Tawi−Tawi, Ages: 20−24
Data points enclosed with a diamond are reference sources

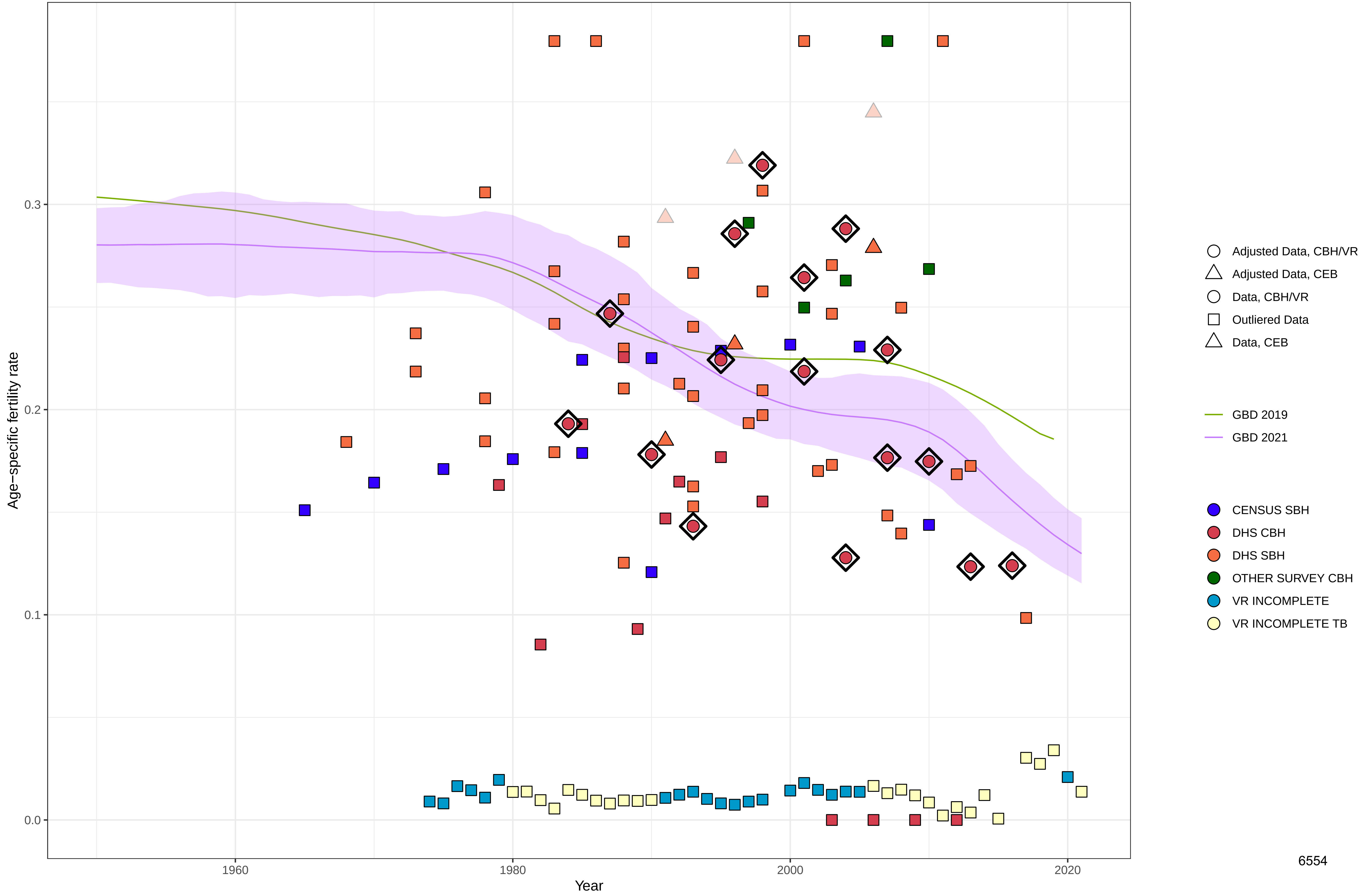

Age−specific fertility fitted trend lines, 1950−2021: Tawi−Tawi, Ages: 25−29
Data points enclosed with a diamond are reference sources

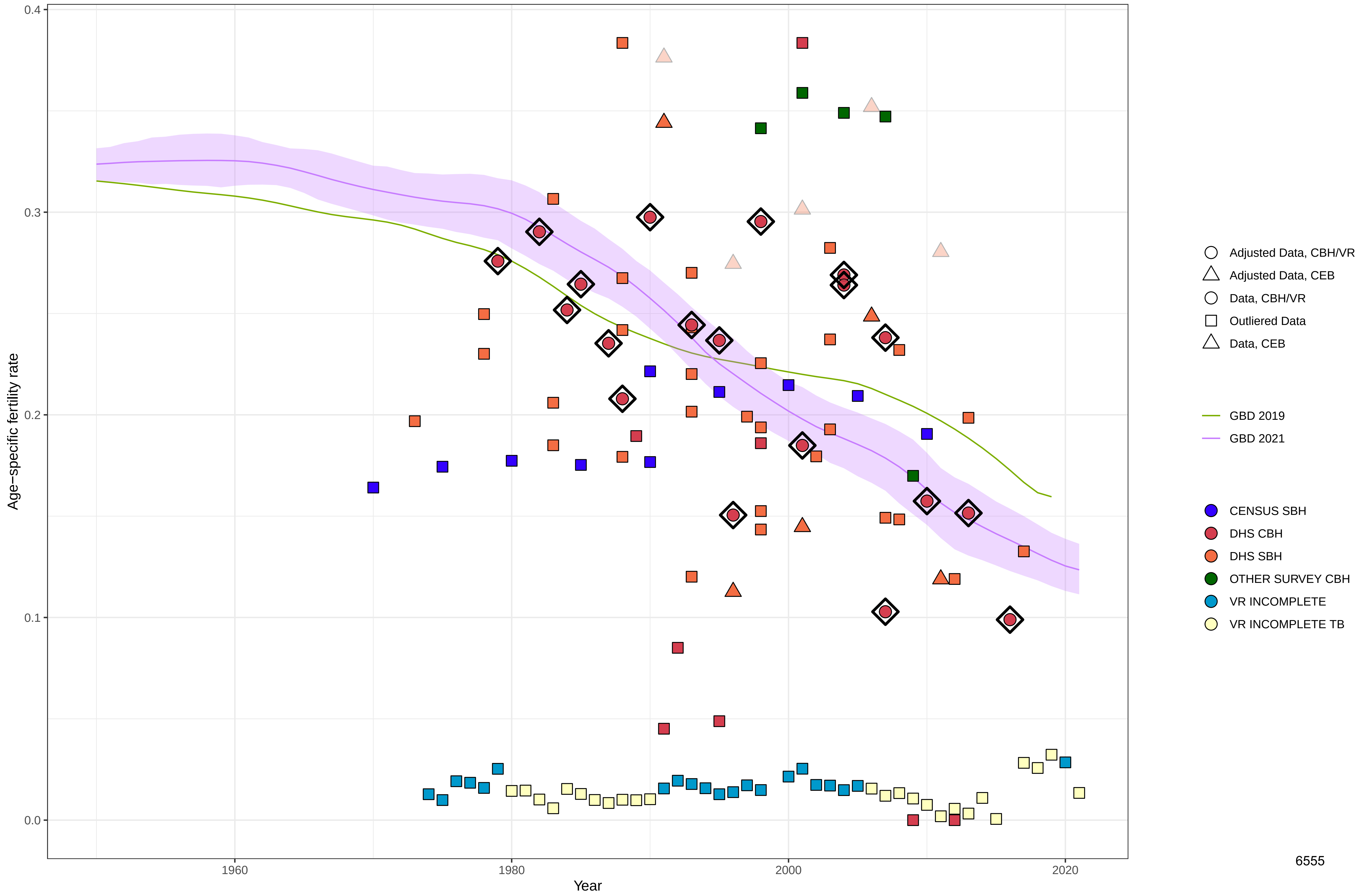

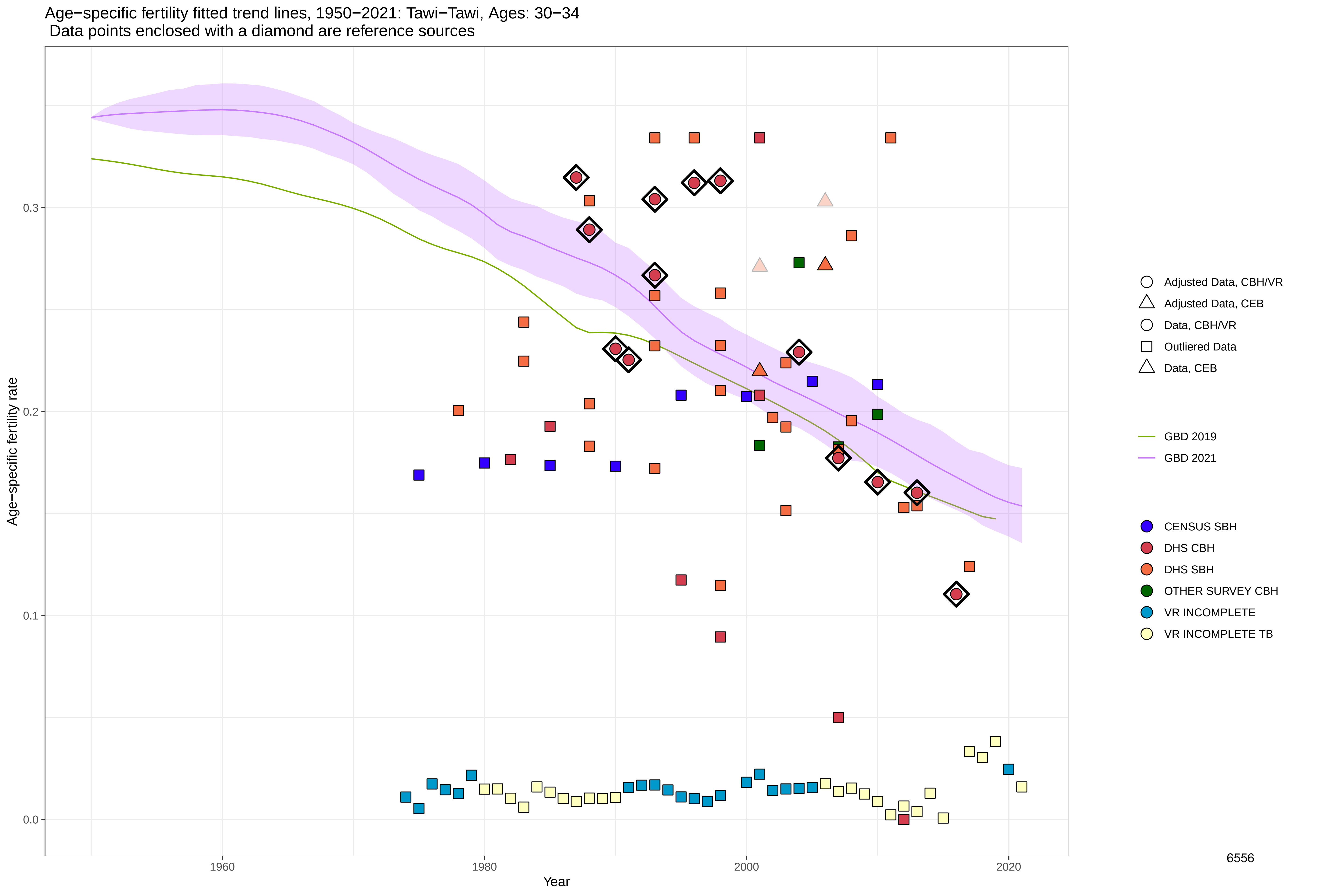
Age−specific fertility fitted trend lines, 1950−2021: Tawi−Tawi, Ages: 30−34
Data points enclosed with a diamond are reference sources
Age−specific fertility rate
Year
0.0
0.1
0.2
0.3
1960
1980
2000
2020
Adjusted Data, CBH/VR
Adjusted Data, CEB
Data, CBH/VR
Outliered Data
Data, CEB
GBD 2019
GBD 2021
CENSUS SBH
DHS CBH
DHS SBH
OTHER SURVEY CBH
VR INCOMPLETE
VR INCOMPLETE TB

Age−specific fertility fitted trend lines, 1950−2021: Tawi−Tawi, Ages: 35−39
Data points enclosed with a diamond are reference sources

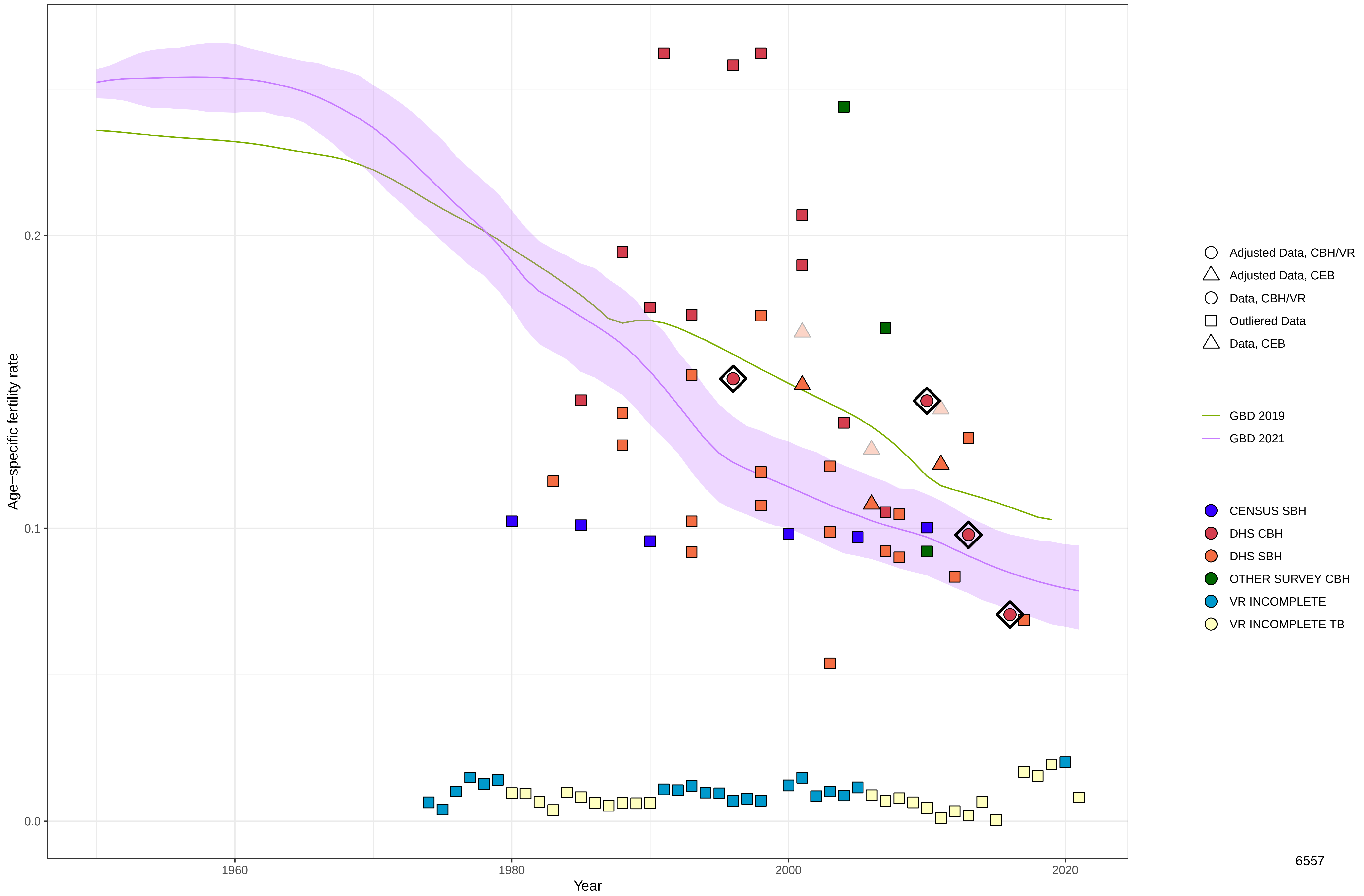

Age−specific fertility fitted trend lines, 1950−2021: Tawi−Tawi, Ages: 40−44
Data points enclosed with a diamond are reference sources

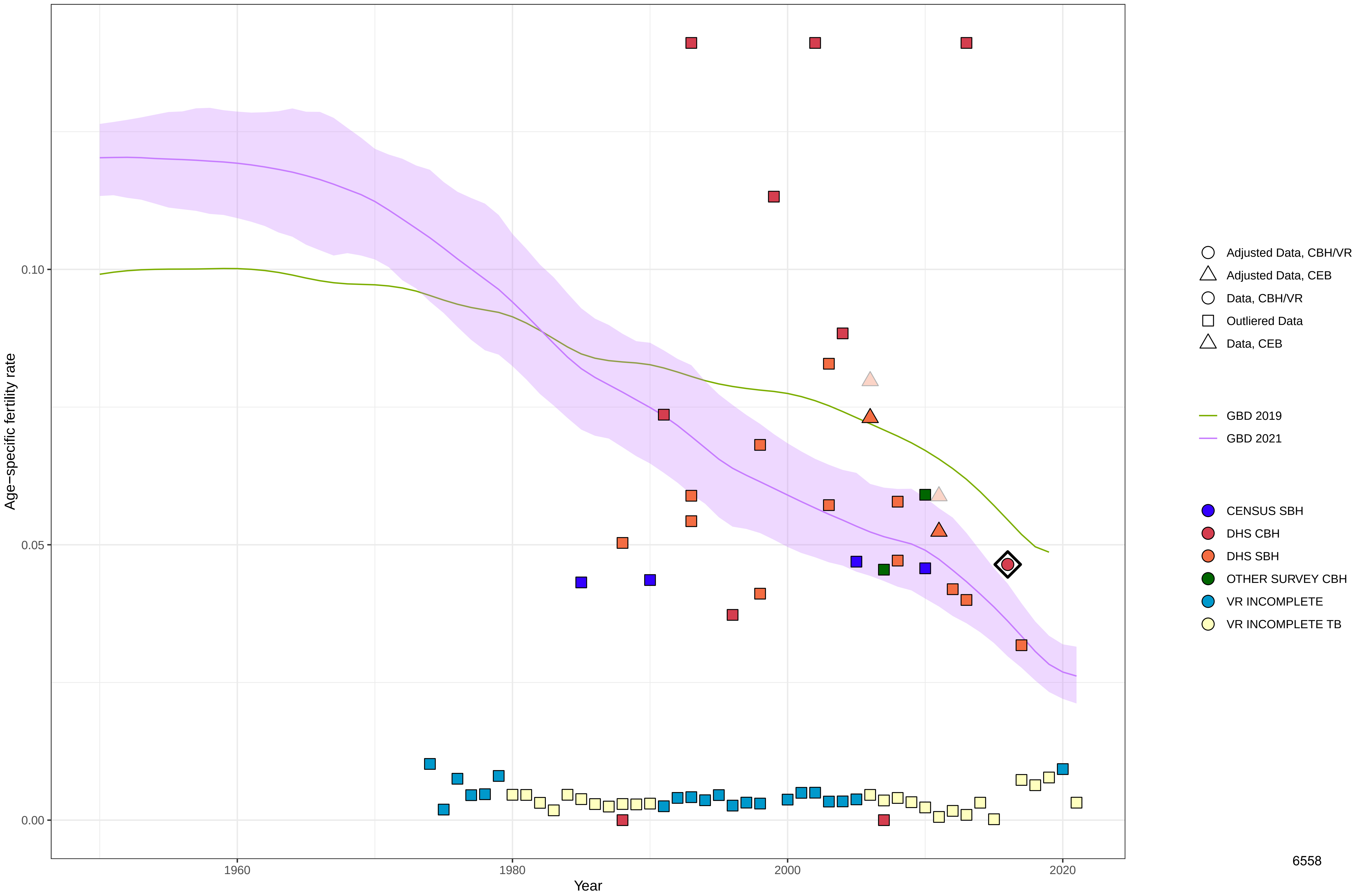

Age−specific fertility fitted trend lines, 1950−2021: Tawi−Tawi, Ages: 45−49
Data points enclosed with a diamond are reference sources

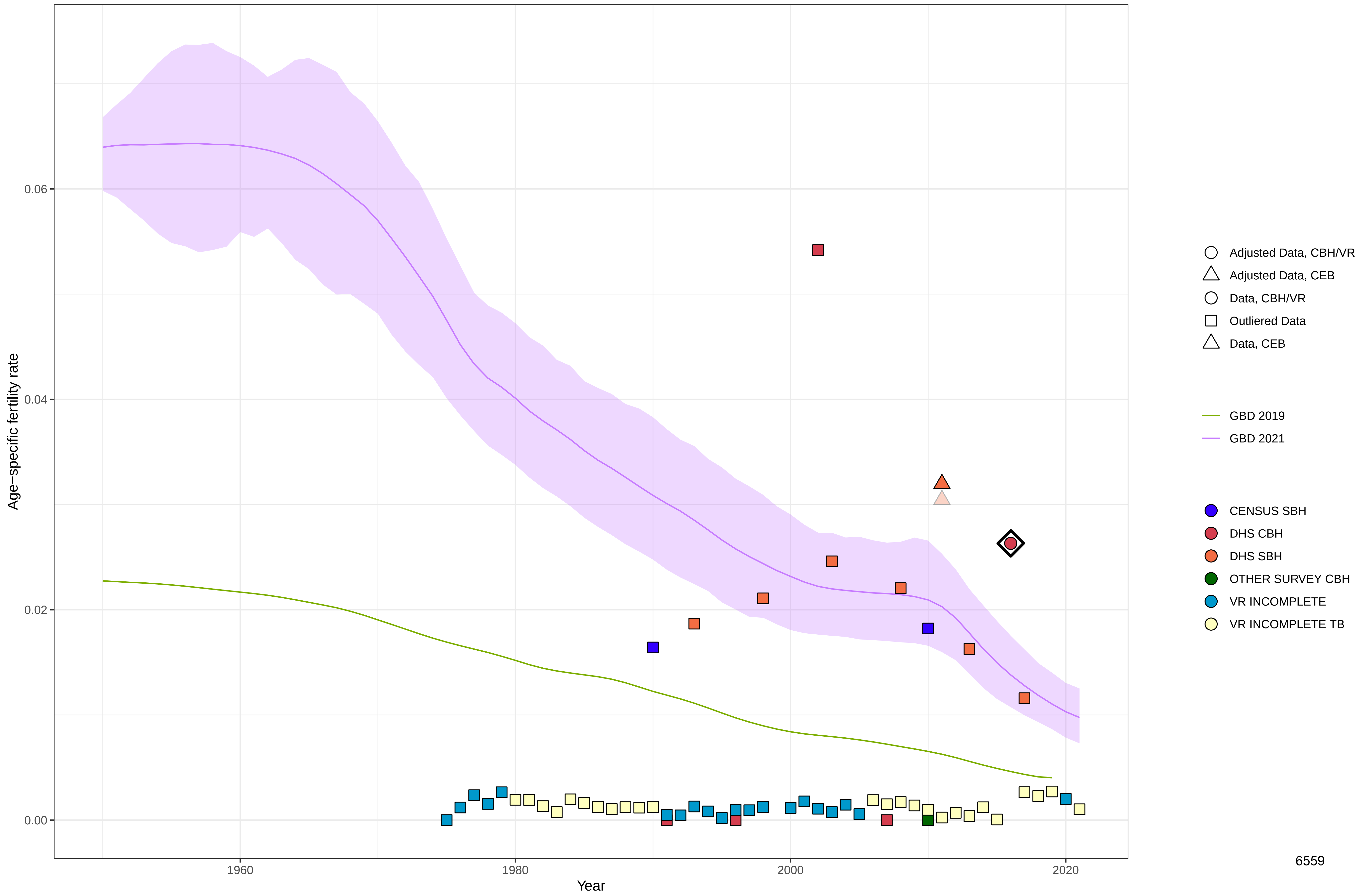

Age−specific fertility fitted trend lines, 1950−2021: Tawi−Tawi, Ages: 50−54
Data points enclosed with a diamond are reference sources

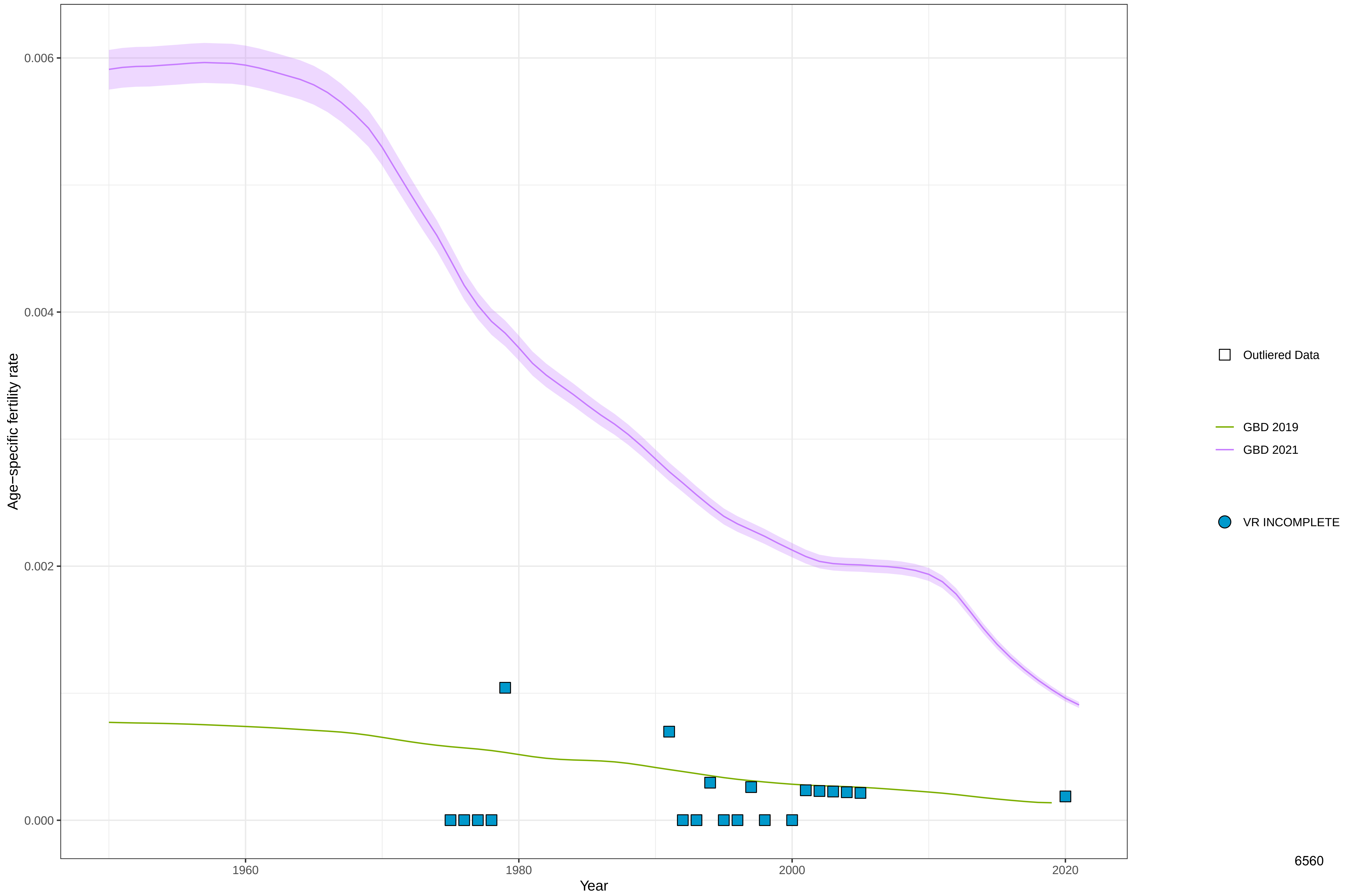

Total fertility rate fitted trend lines, 1950−2021: Tawi−Tawi TFR
Data points enclosed with a diamond are reference sources

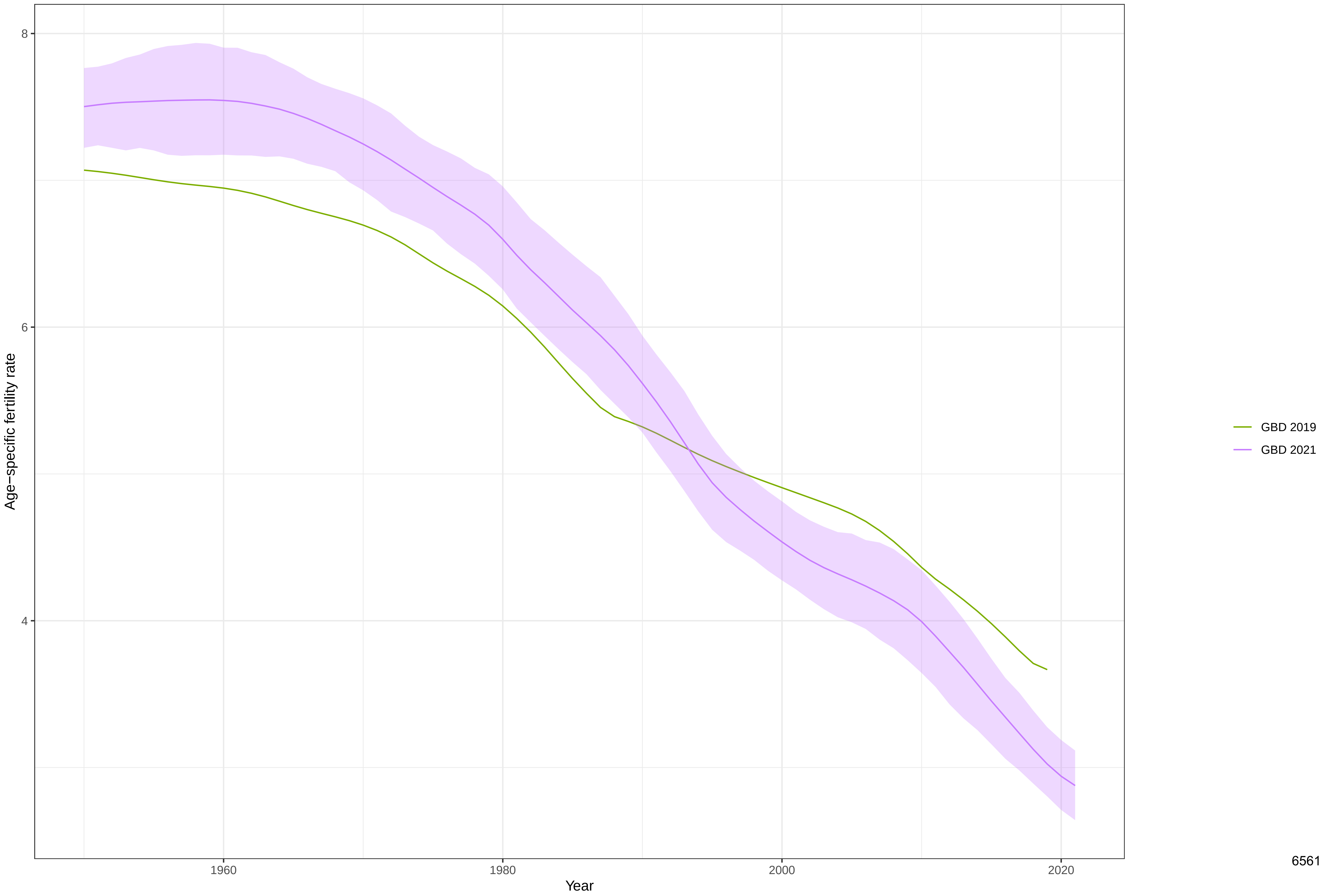

Age−specific fertility fitted trend lines, 1950−2021: Zambales, Ages: 10−14
Data points enclosed with a diamond are reference sources

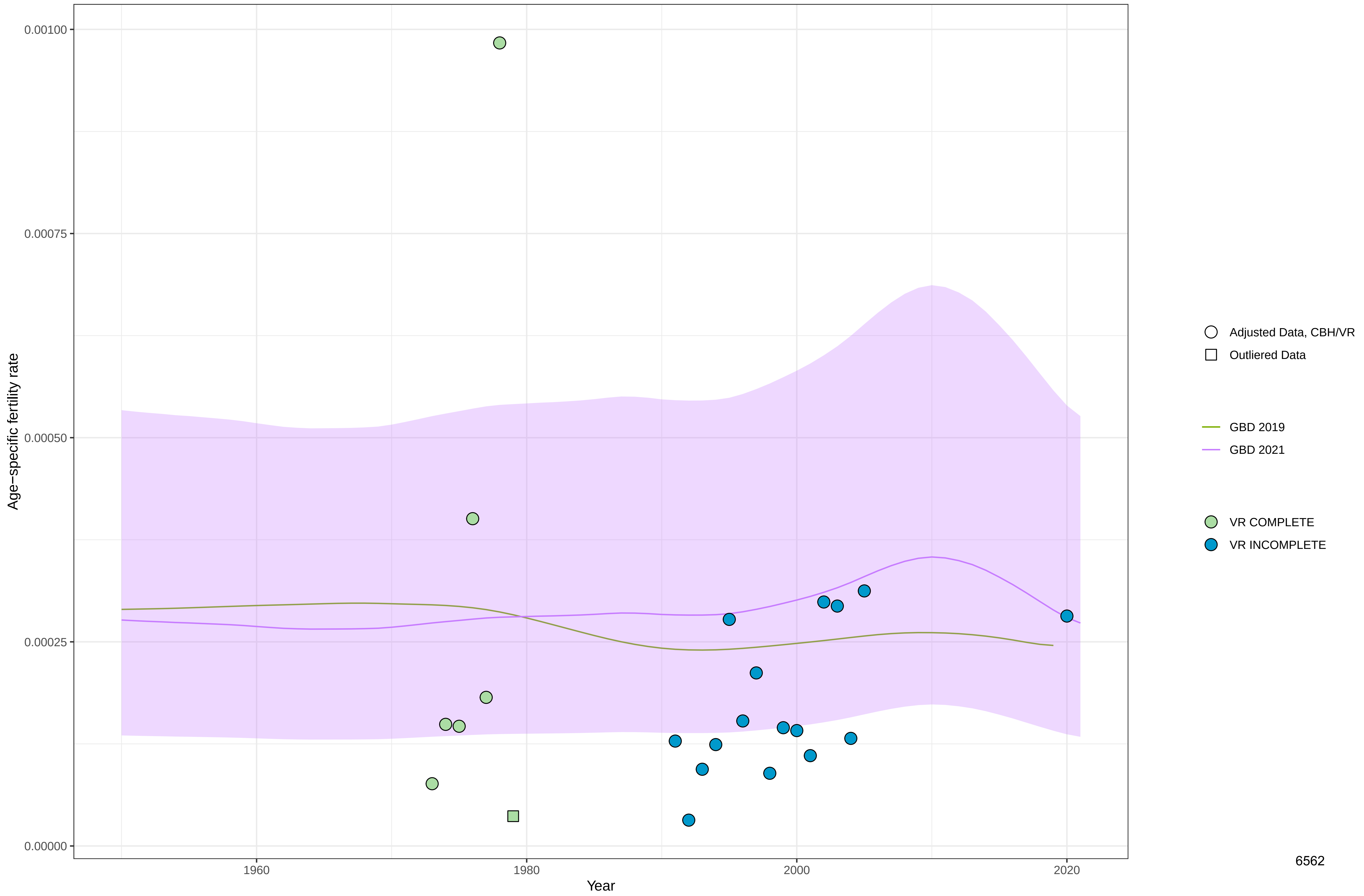

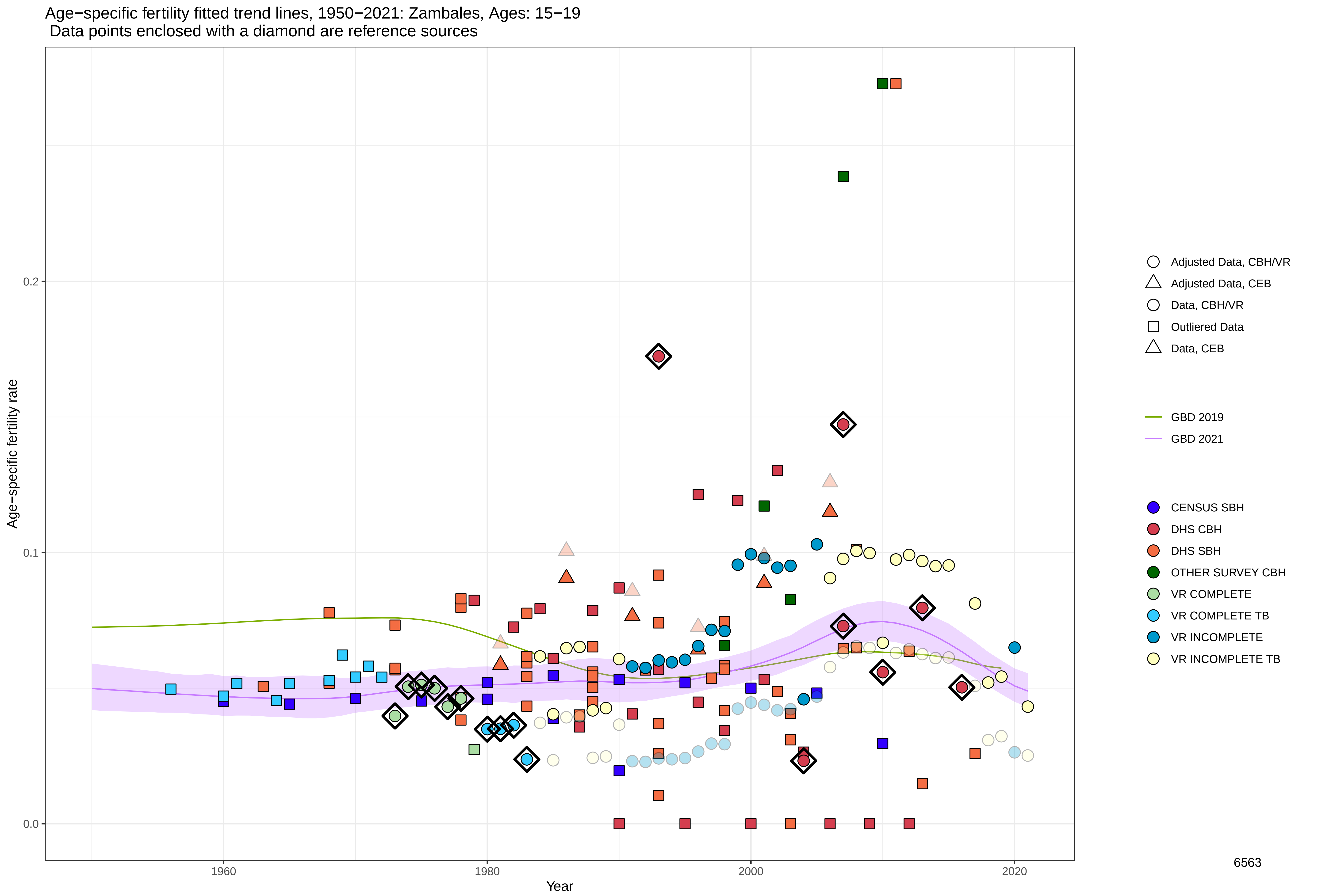
Age−specific fertility fitted trend lines, 1950−2021: Zambales, Ages: 15−19
Data points enclosed with a diamond are reference sources
Age−specific fertility rate
Year
0.0
0.1
0.2
1960
1980
2000
2020
Adjusted Data, CBH/VR
Adjusted Data, CEB
Data, CBH/VR
Outliered Data
Data, CEB
GBD 2019
GBD 2021
CENSUS SBH
DHS CBH
DHS SBH
OTHER SURVEY CBH
VR COMPLETE
VR COMPLETE TB
VR INCOMPLETE
VR INCOMPLETE TB

6563

Age−specific fertility fitted trend lines, 1950−2021: Zambales, Ages: 20−24
Data points enclosed with a diamond are reference sources

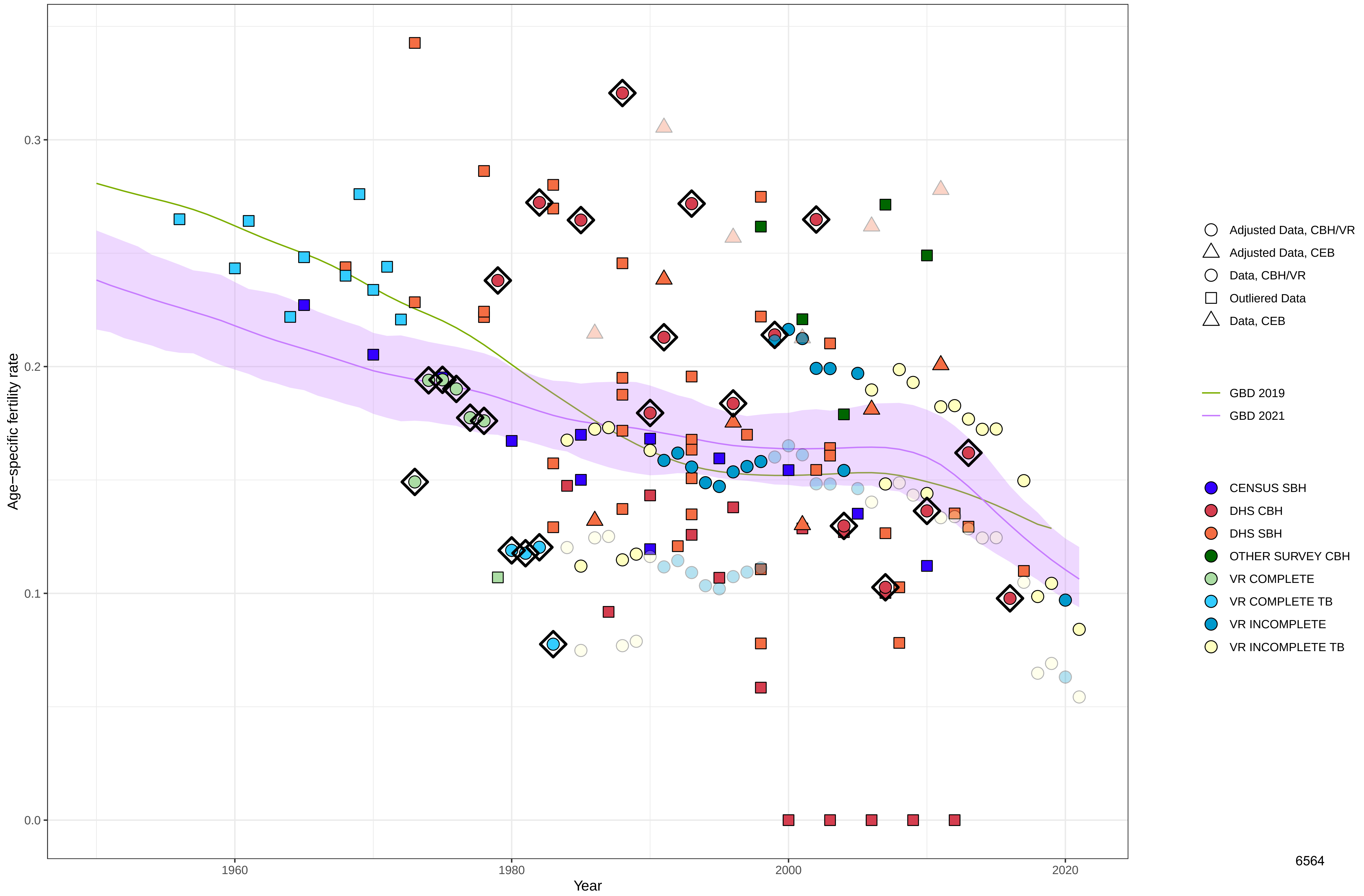

Age−specific fertility fitted trend lines, 1950−2021: Zambales, Ages: 25−29
Data points enclosed with a diamond are reference sources

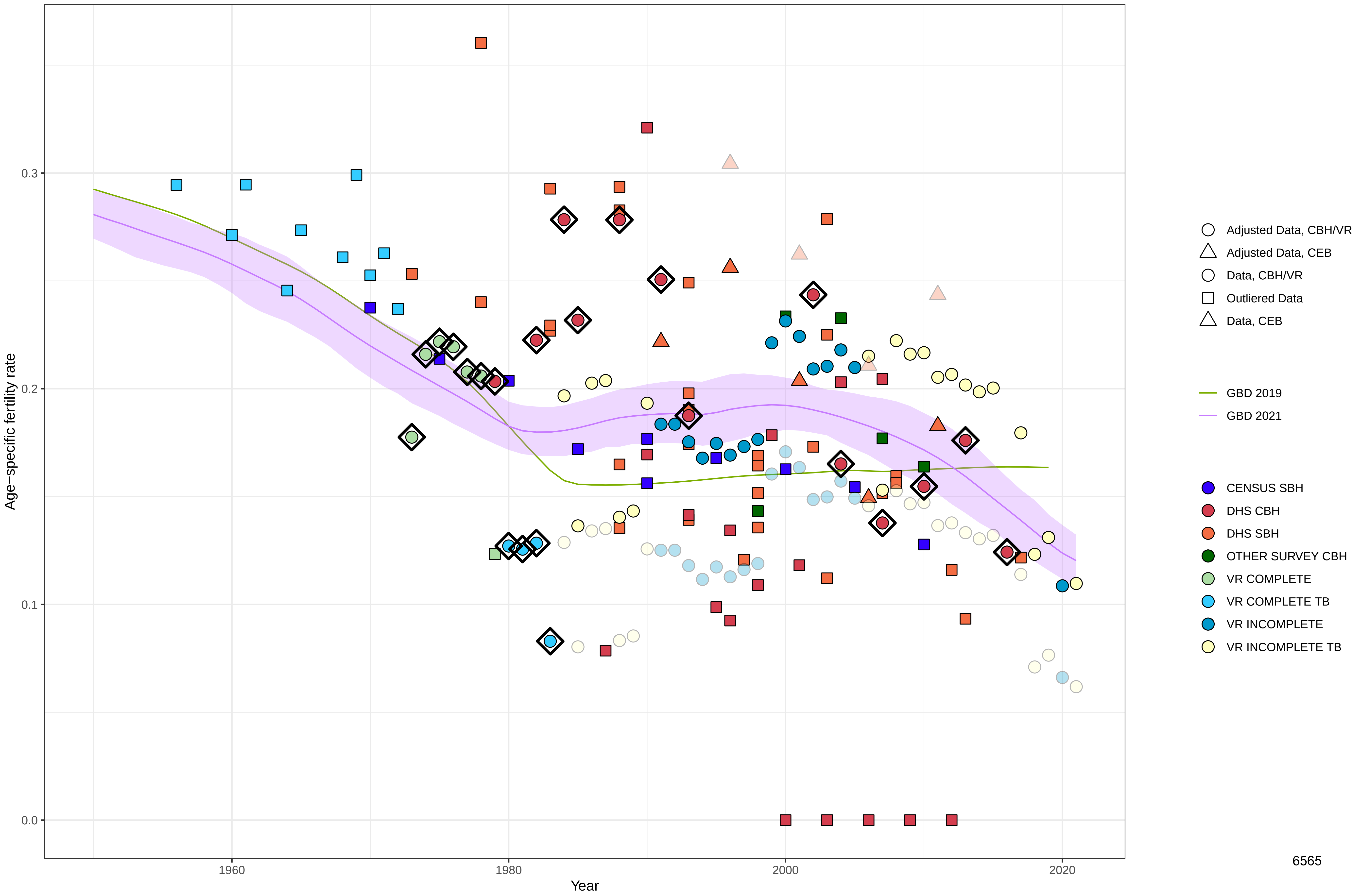

Age−specific fertility fitted trend lines, 1950−2021: Zambales, Ages: 30−34
Data points enclosed with a diamond are reference sources

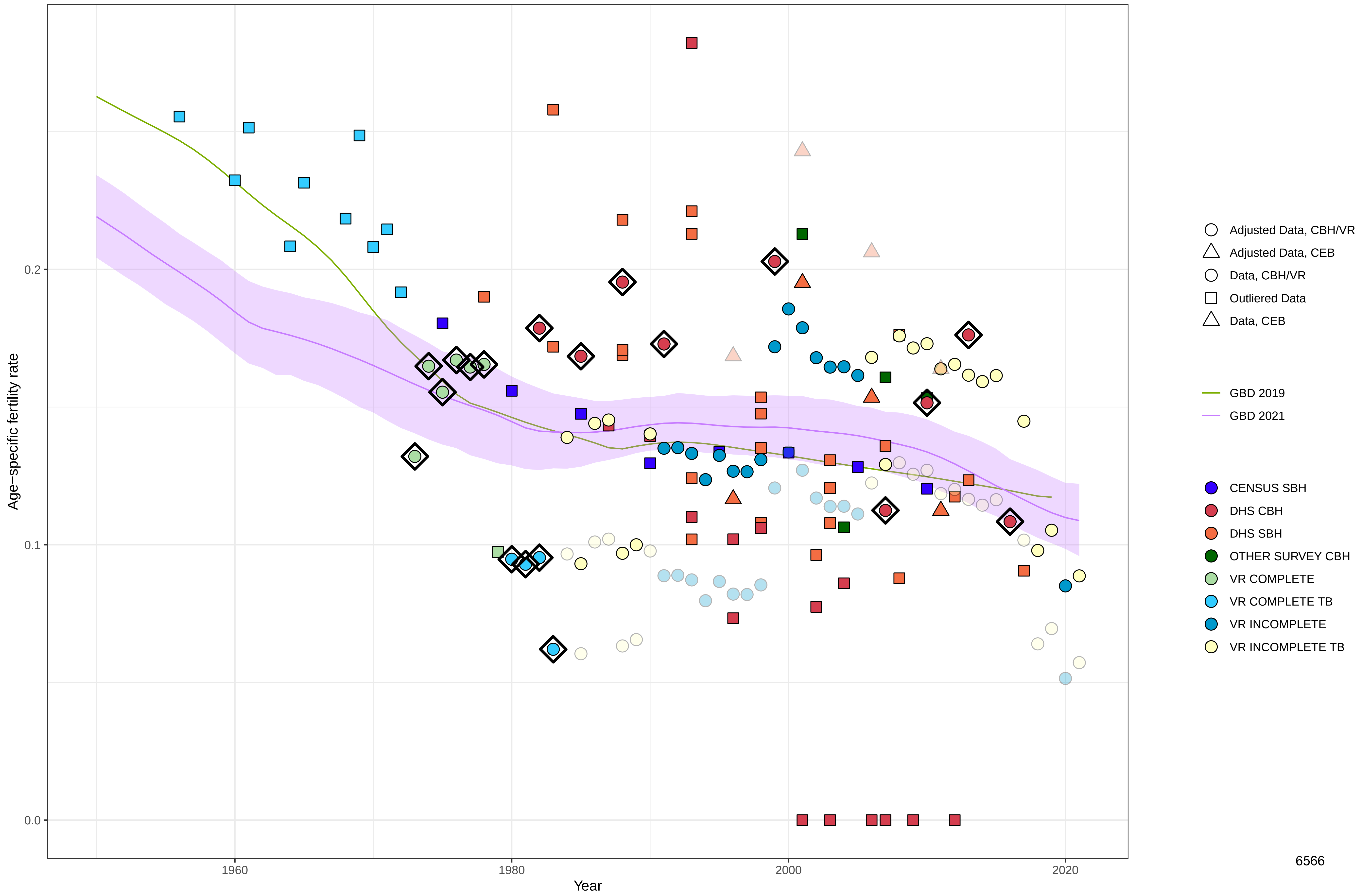

Age−specific fertility fitted trend lines, 1950−2021: Zambales, Ages: 35−39
Data points enclosed with a diamond are reference sources

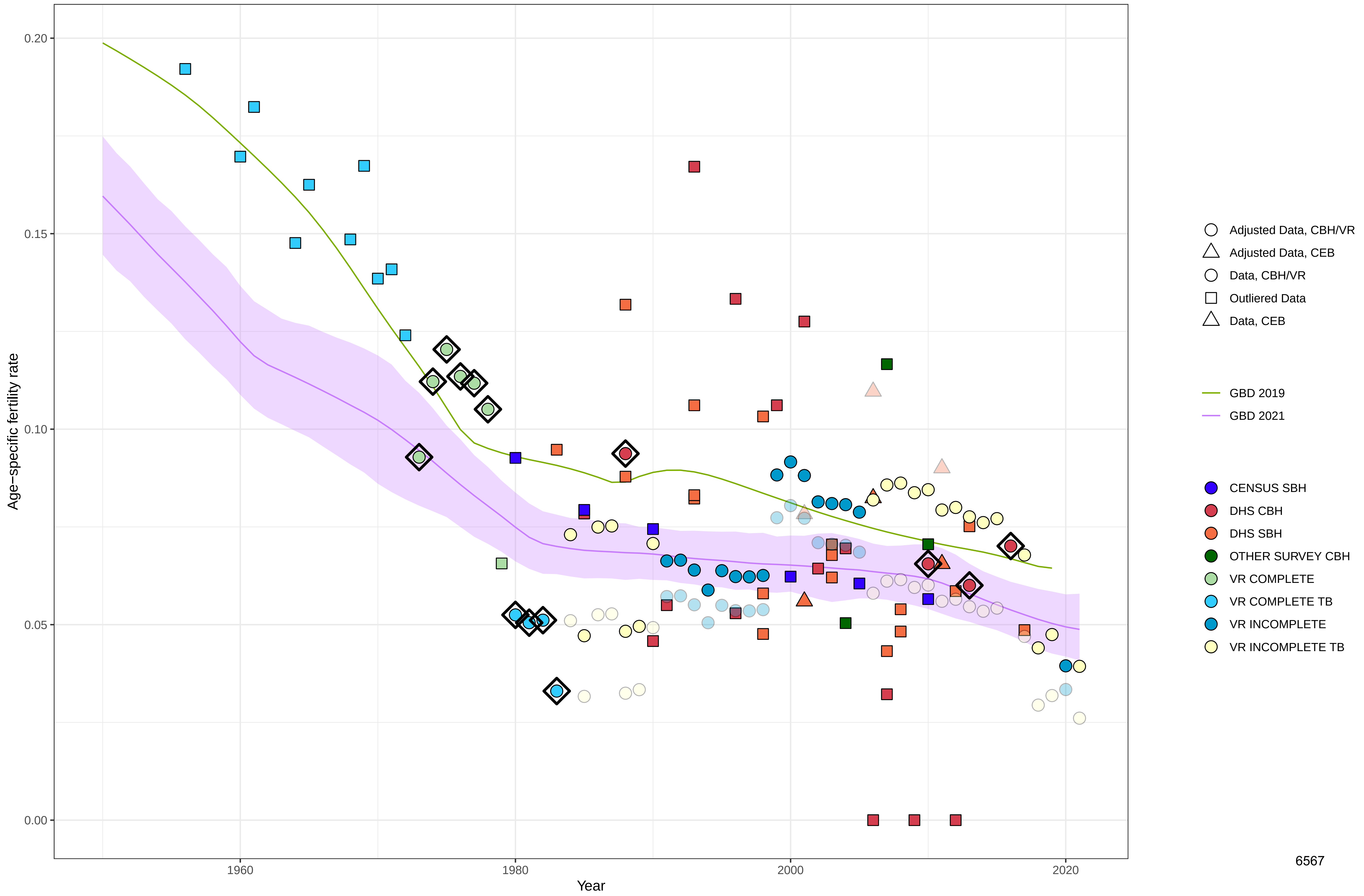

Age−specific fertility fitted trend lines, 1950−2021: Zambales, Ages: 40−44
Data points enclosed with a diamond are reference sources

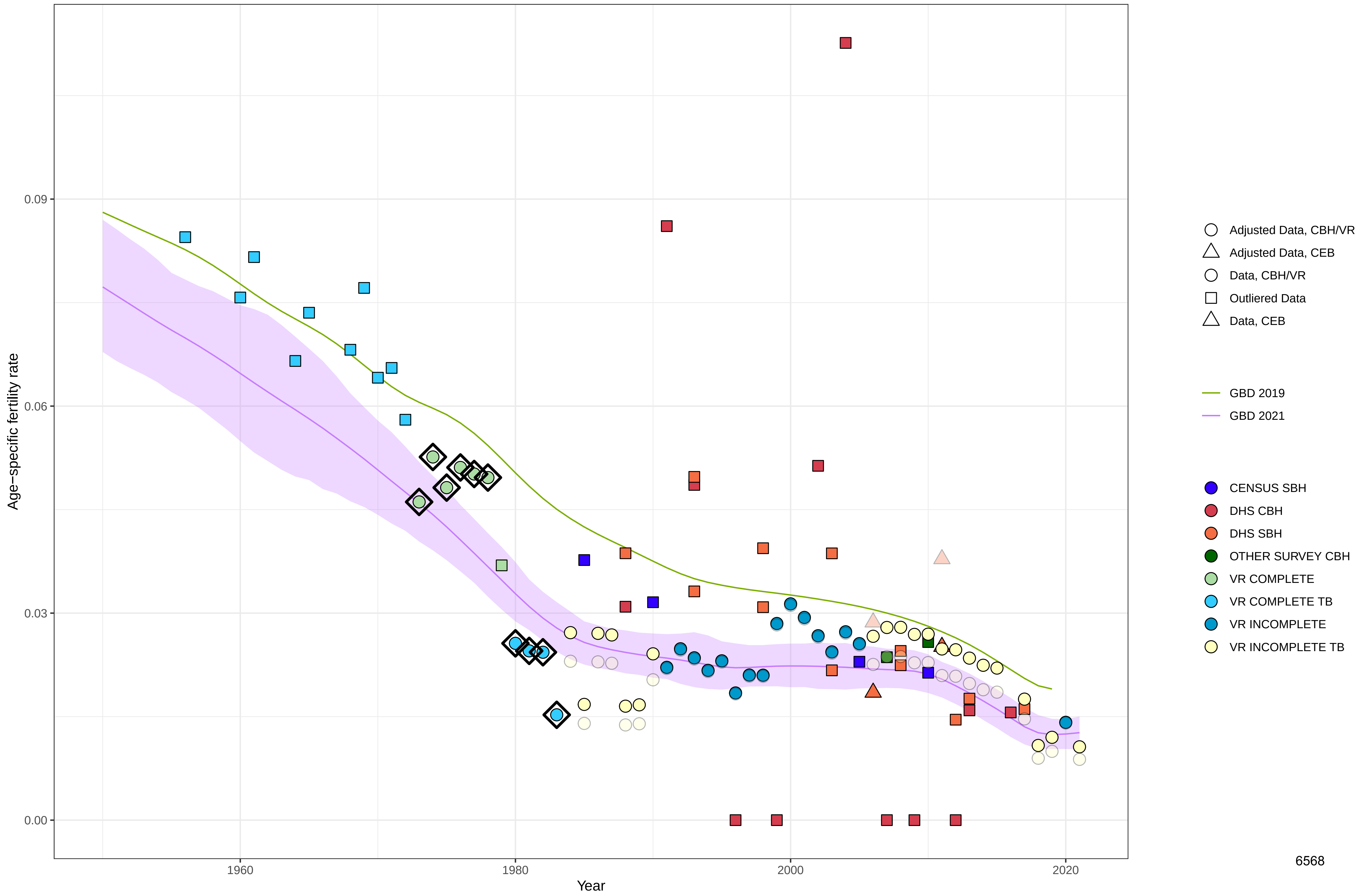

Age−specific fertility fitted trend lines, 1950−2021: Zambales, Ages: 45−49
Data points enclosed with a diamond are reference sources

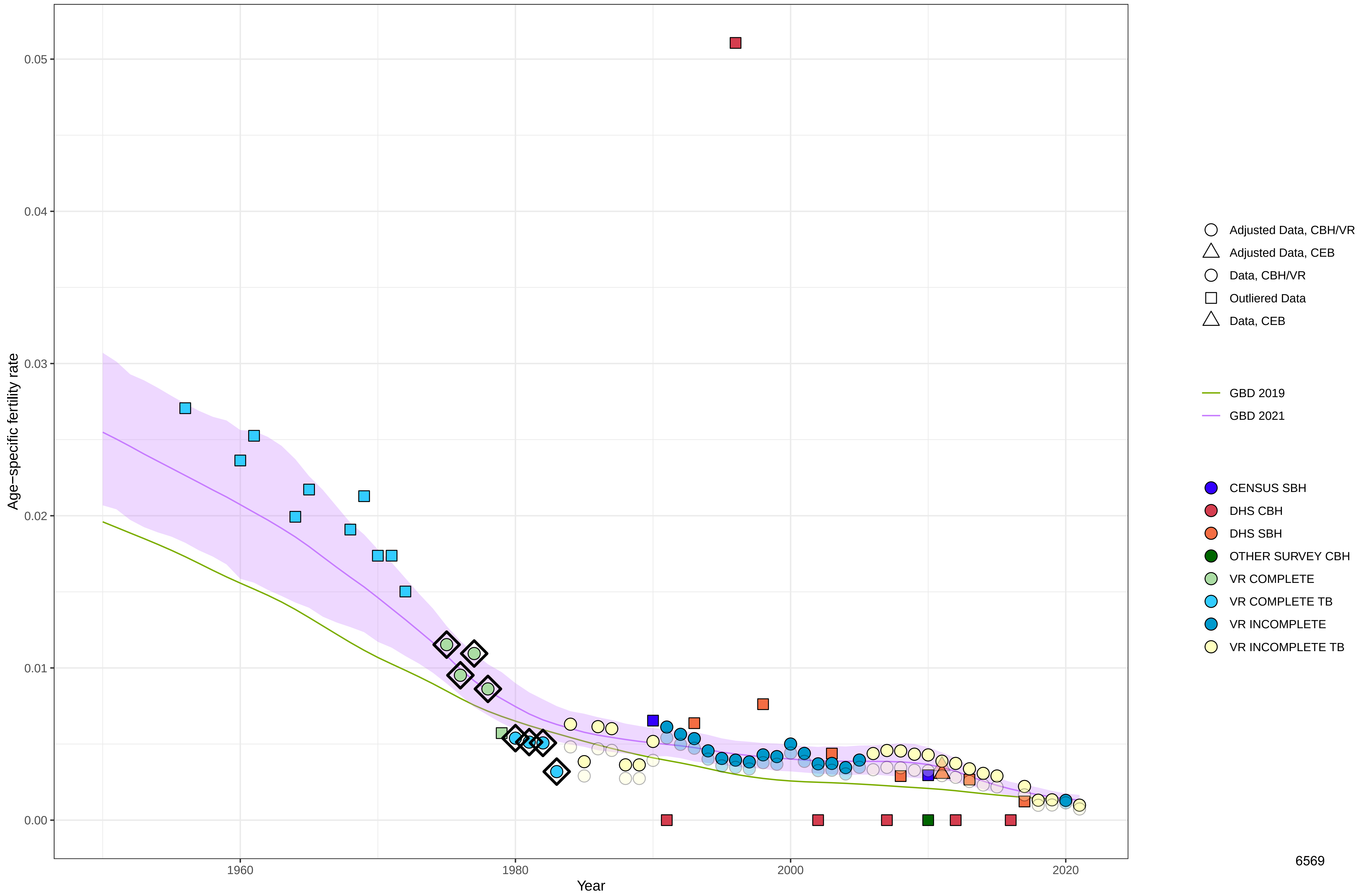

Age−specific fertility fitted trend lines, 1950−2021: Zambales, Ages: 50−54
Data points enclosed with a diamond are reference sources

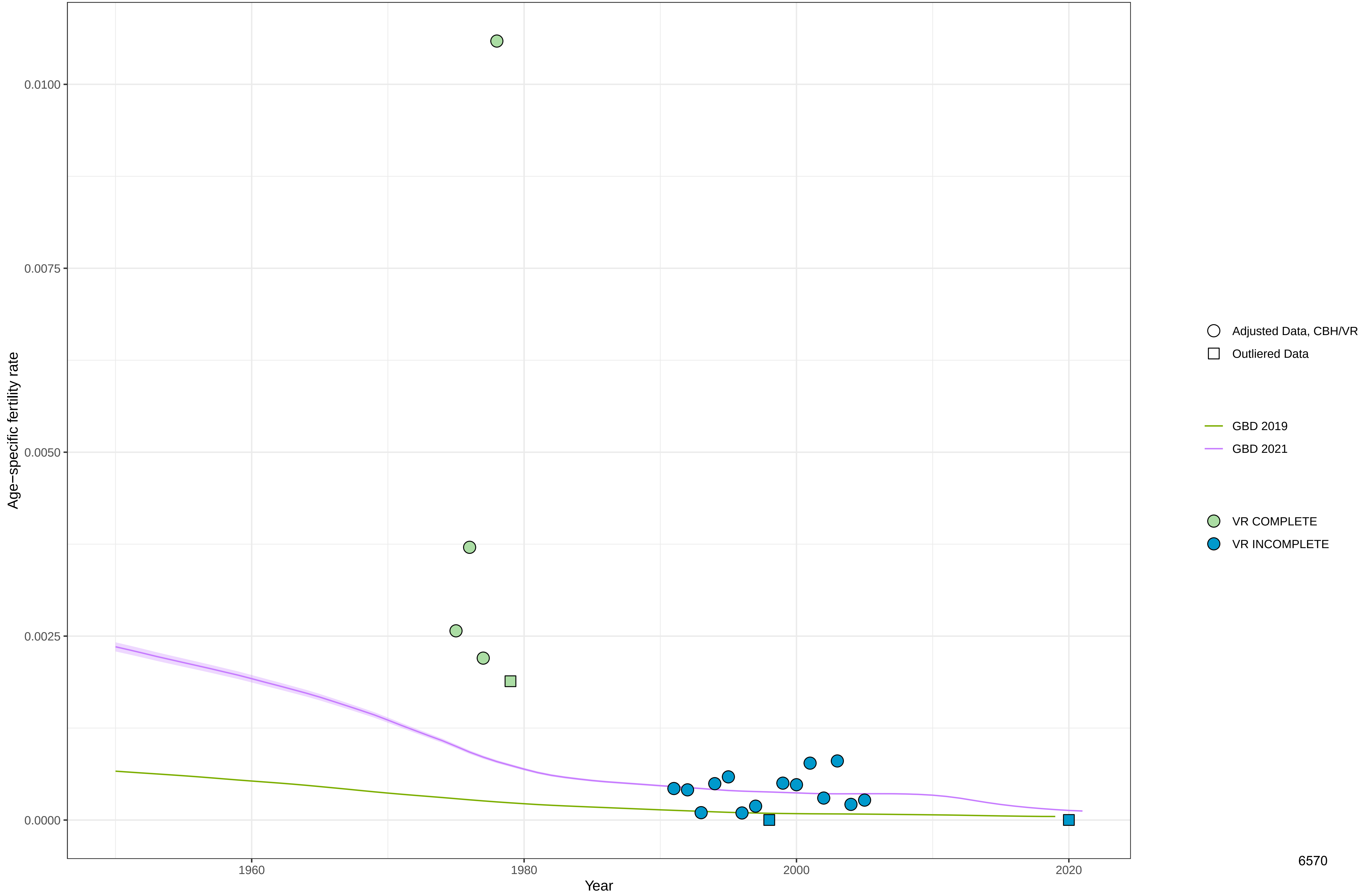

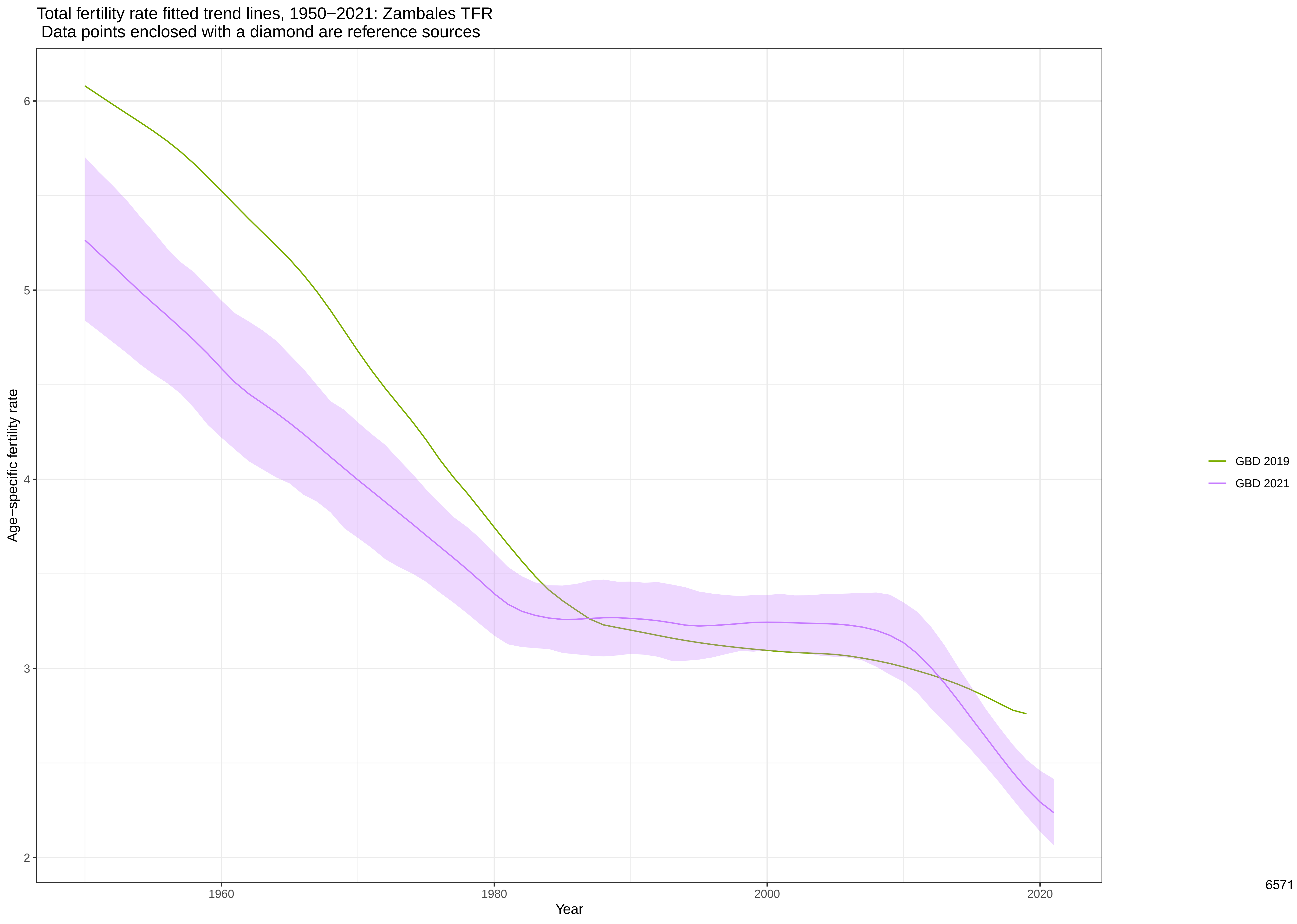
Total fertility rate fitted trend lines, 1950−2021: Zambales TFR
Data points enclosed with a diamond are reference sources
Age−specific fertility rate
Year
6
5
4
3
2
1960
1980
2000
2020
GBD 2019
GBD 2021

6571

Age−specific fertility fitted trend lines, 1950−2021: Zamboanga Del Norte, Ages: 10−14
Data points enclosed with a diamond are reference sources

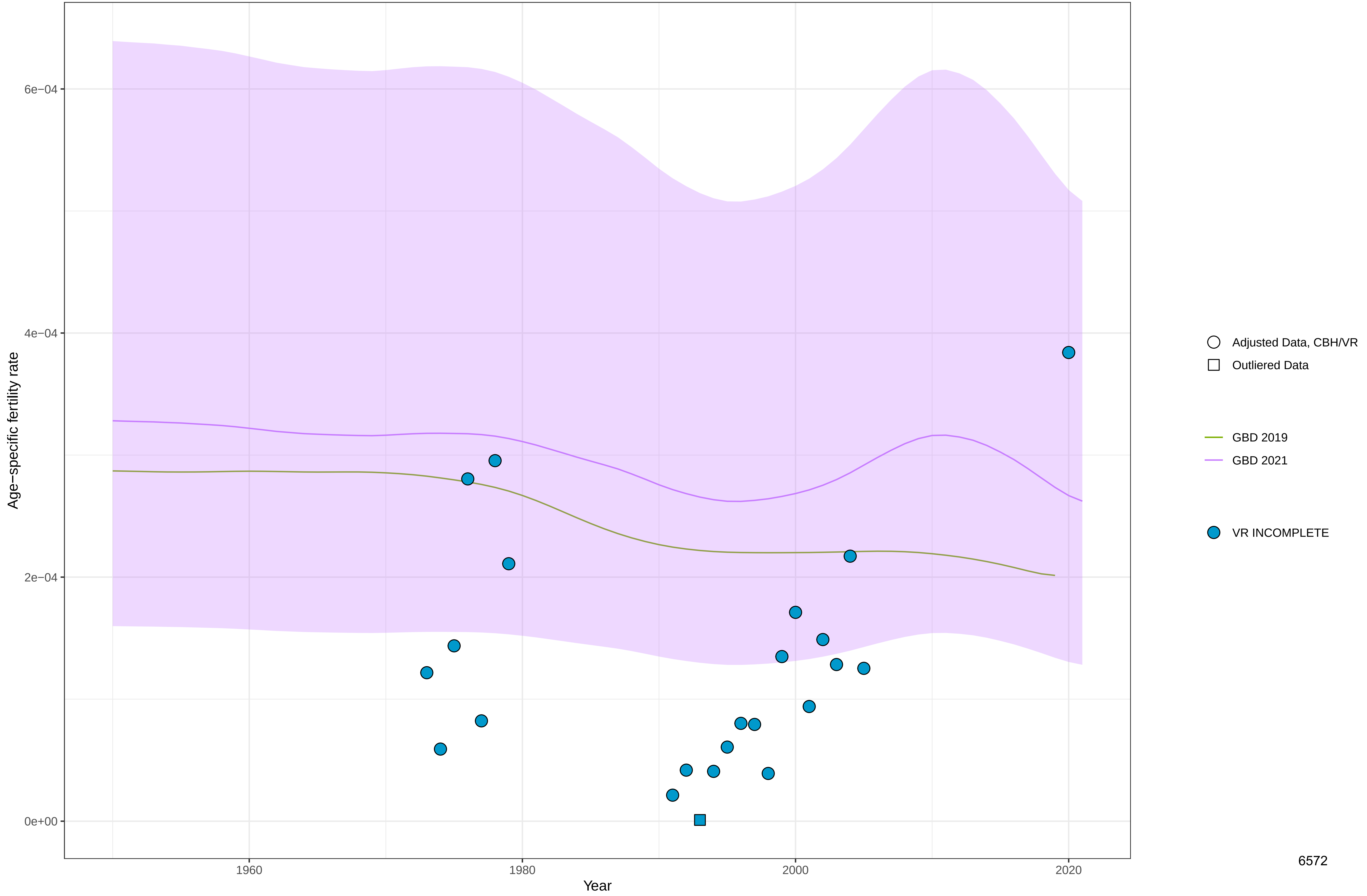

Age−specific fertility fitted trend lines, 1950−2021: Zamboanga Del Norte, Ages: 15−19
Data points enclosed with a diamond are reference sources

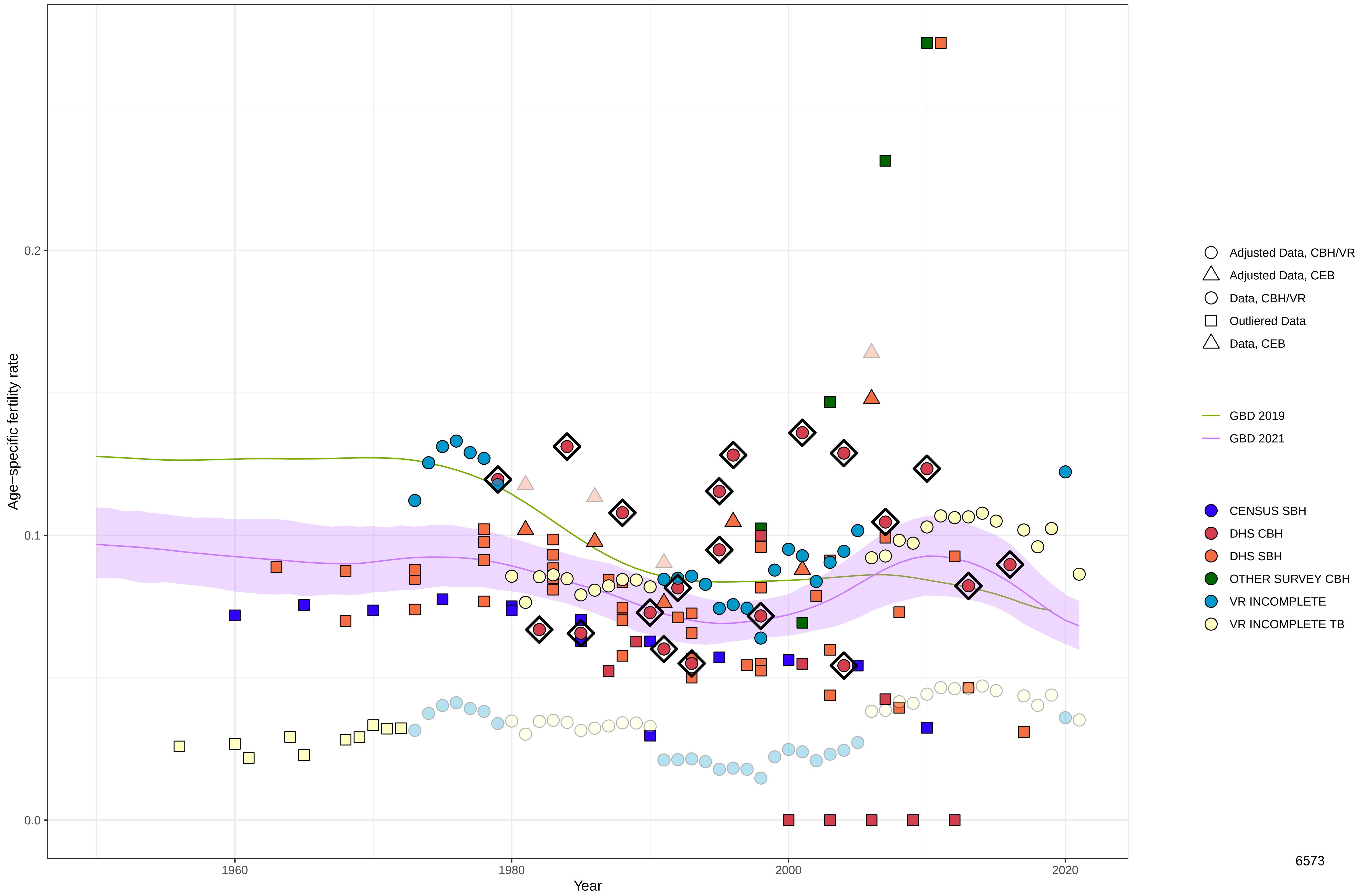

Age−specific fertility fitted trend lines, 1950−2021: Zamboanga Del Norte, Ages: 20−24
Data points enclosed with a diamond are reference sources

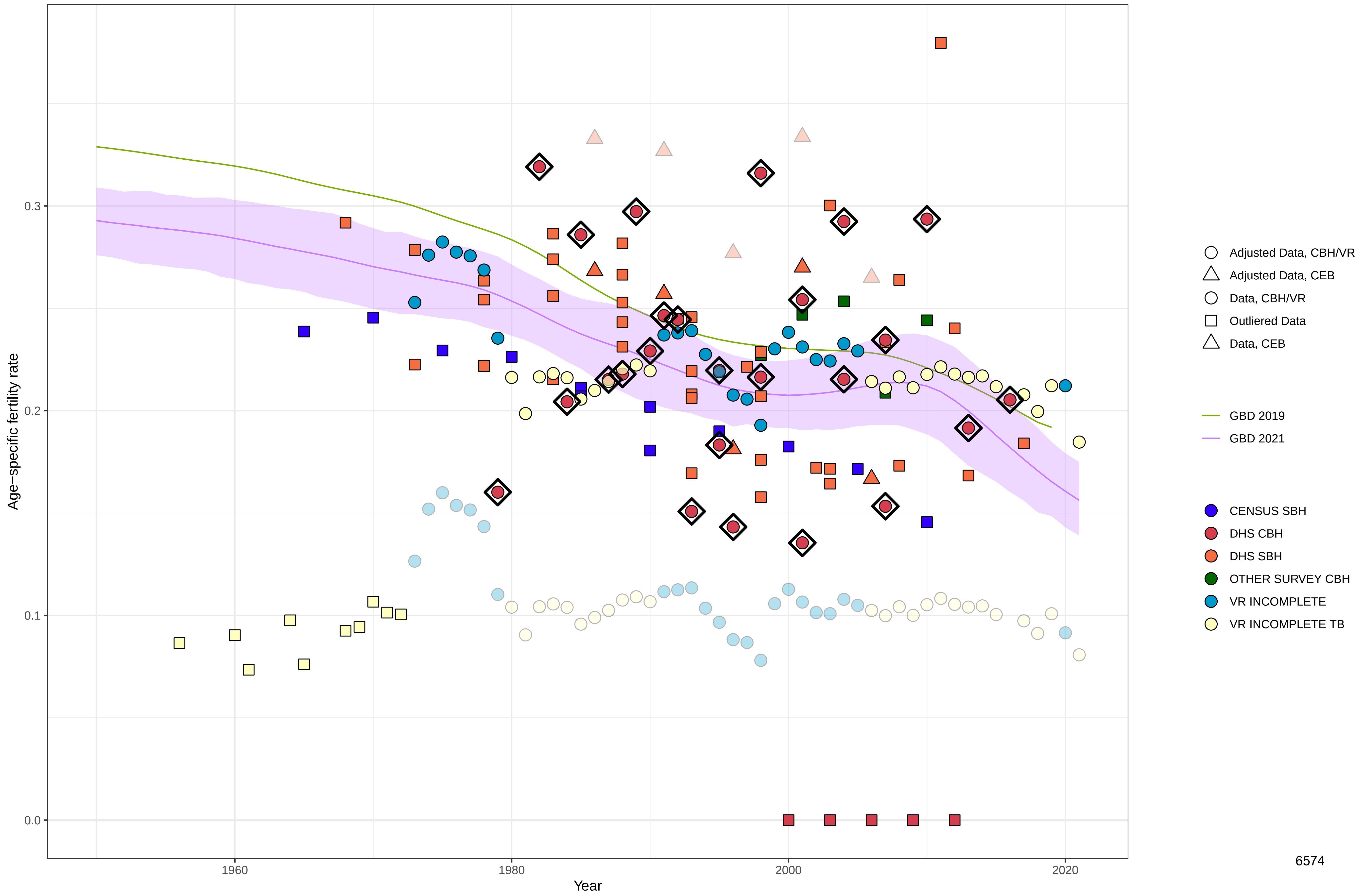

Age−specific fertility fitted trend lines, 1950−2021: Zamboanga Del Norte, Ages: 25−29
Data points enclosed with a diamond are reference sources

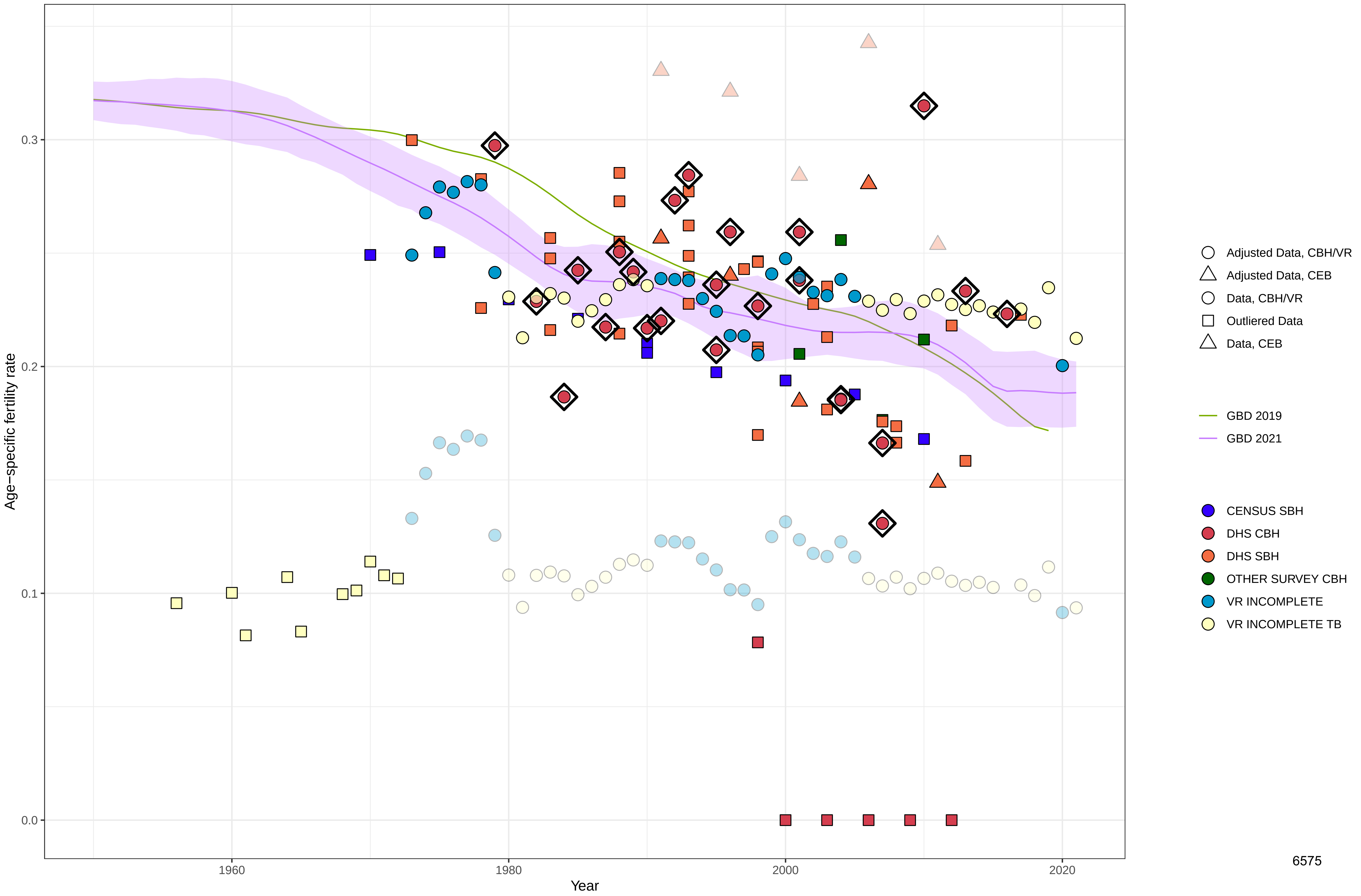

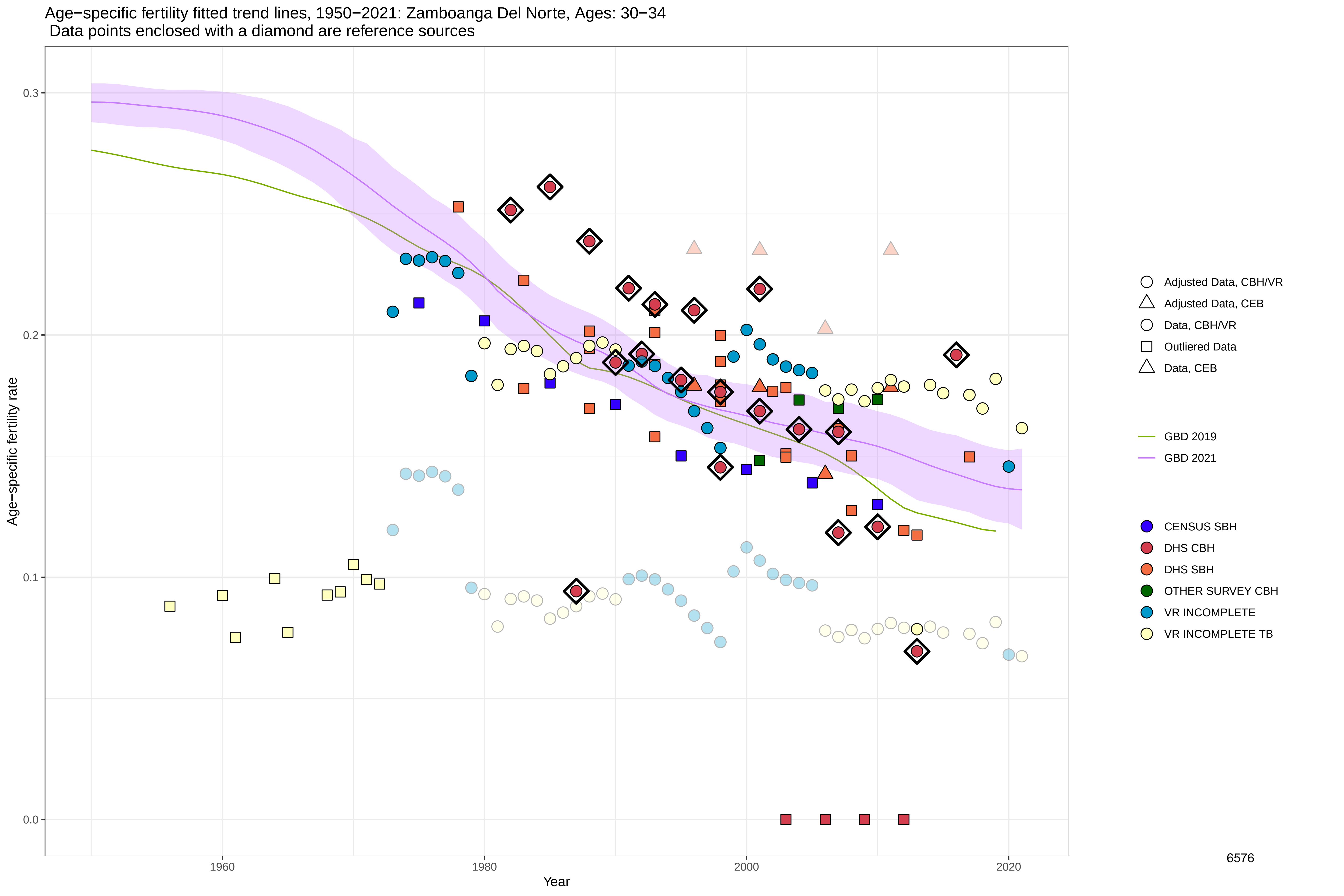

Age−specific fertility fitted trend lines, 1950−2021: Zamboanga Del Norte, Ages: 30−34
Data points enclosed with a diamond are reference sources
Age−specific fertility rate
Year
0.0
0.1
0.2
0.3
1960
1980
2000
2020
Adjusted Data, CBH/VR
Adjusted Data, CEB
Data, CBH/VR
Outliered Data
Data, CEB
GBD 2019
GBD 2021
CENSUS SBH
DHS CBH
DHS SBH
OTHER SURVEY CBH
VR INCOMPLETE
VR INCOMPLETE TB

Age−specific fertility fitted trend lines, 1950−2021: Zamboanga Del Norte, Ages: 35−39
Data points enclosed with a diamond are reference sources

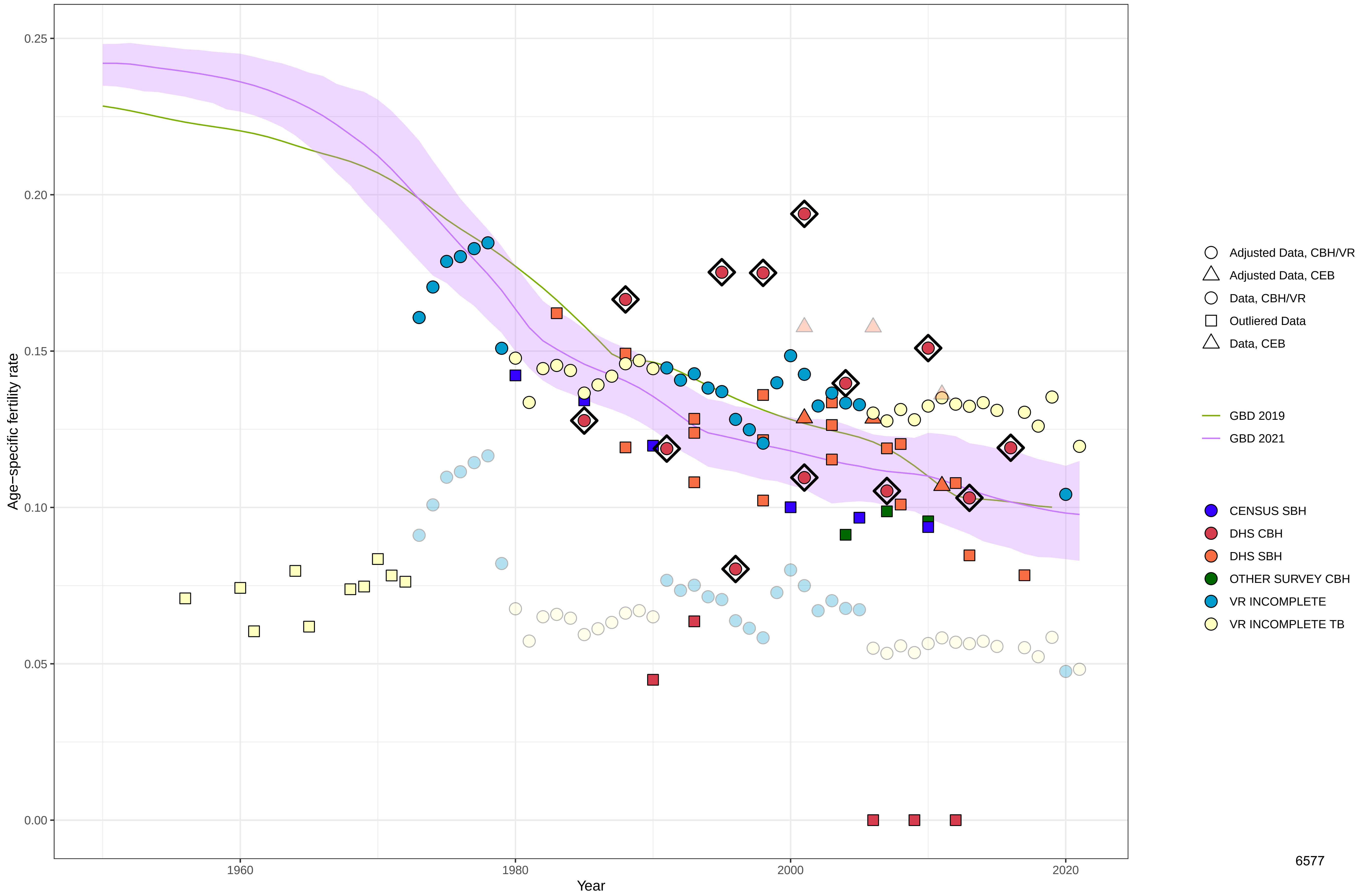

6577

Age−specific fertility fitted trend lines, 1950−2021: Zamboanga Del Norte, Ages: 40−44
Data points enclosed with a diamond are reference sources

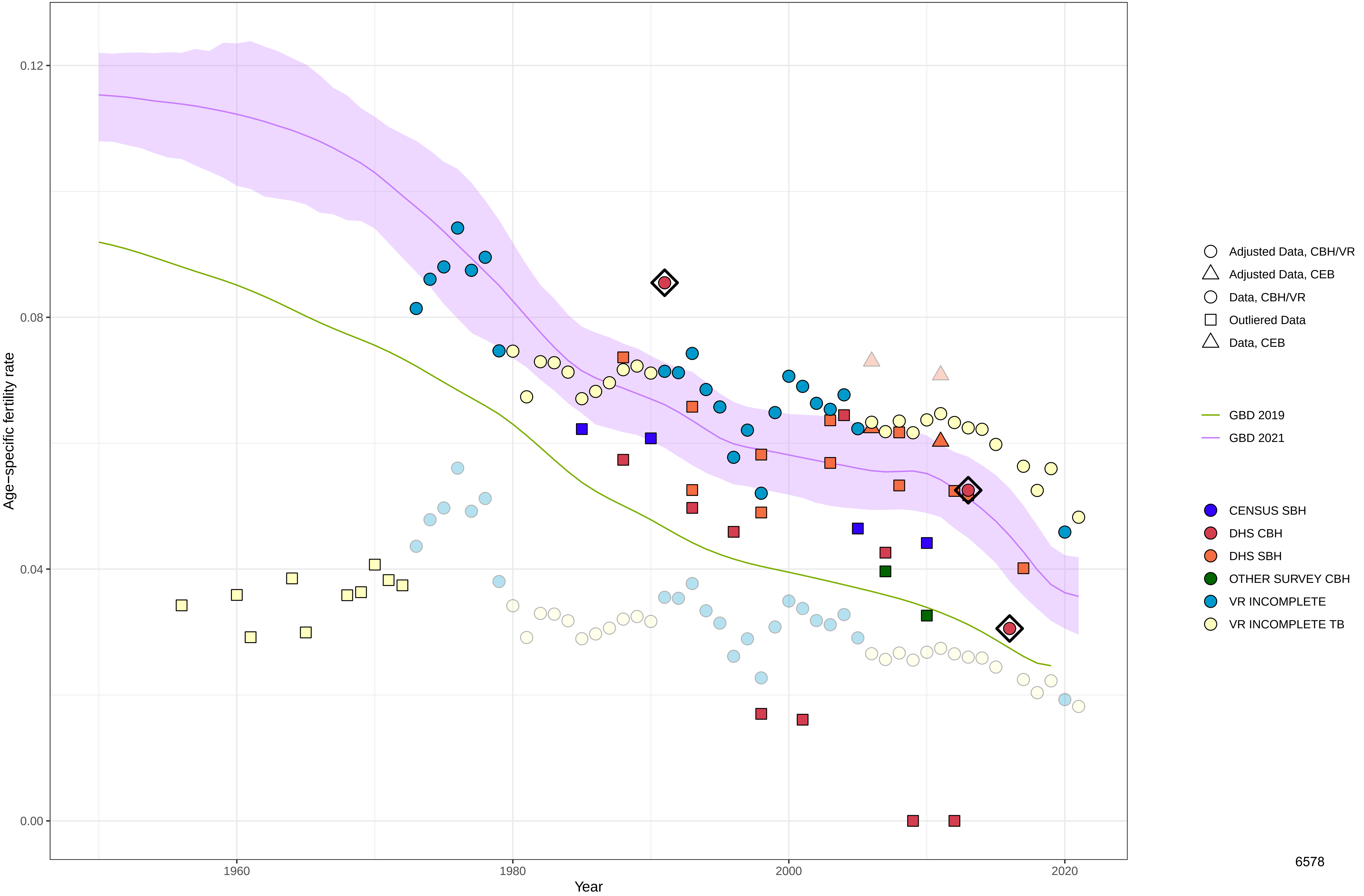

Age−specific fertility fitted trend lines, 1950−2021: Zamboanga Del Norte, Ages: 45−49
Data points enclosed with a diamond are reference sources

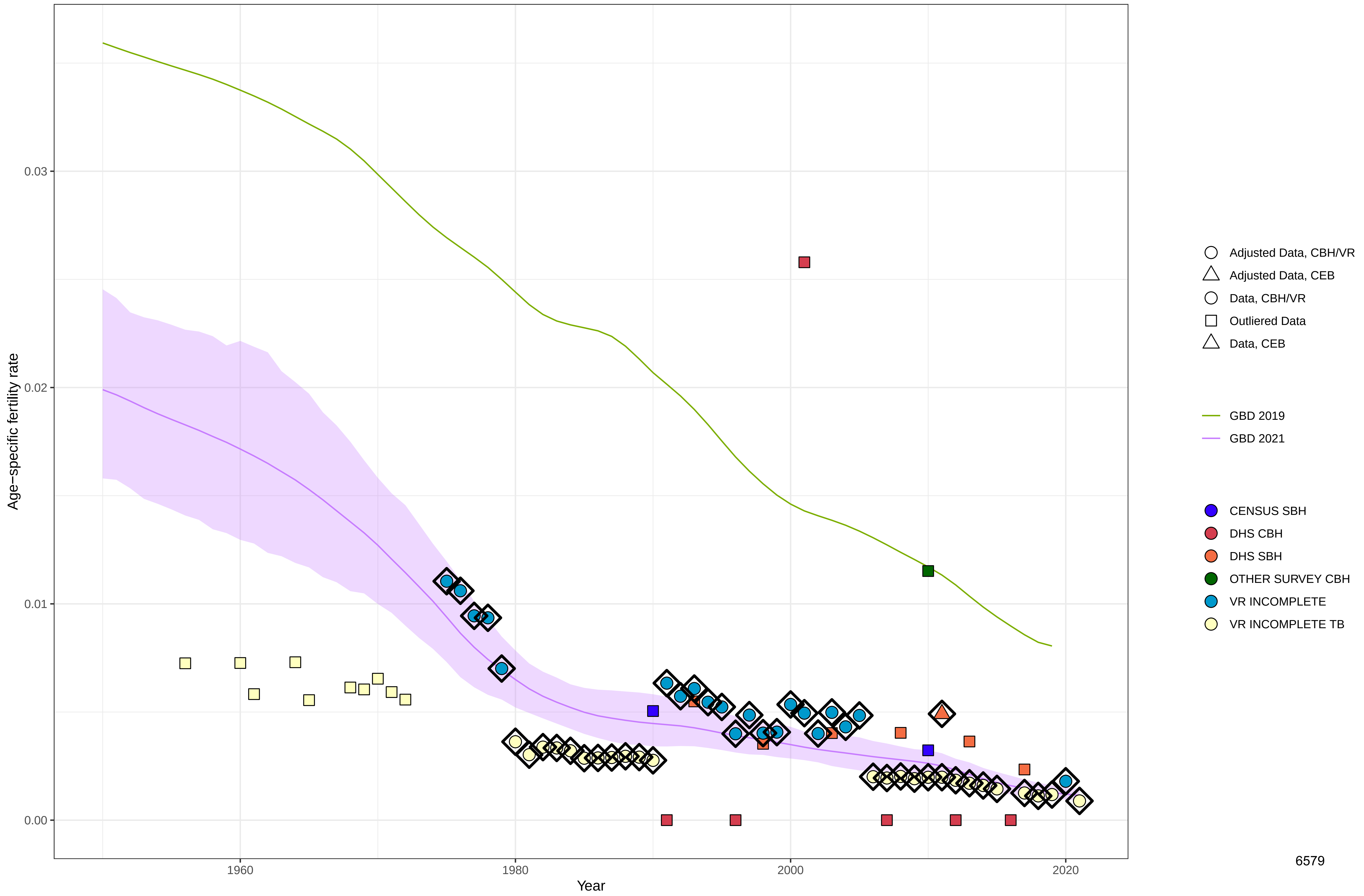

Age−specific fertility fitted trend lines, 1950−2021: Zamboanga Del Norte, Ages: 50−54
Data points enclosed with a diamond are reference sources

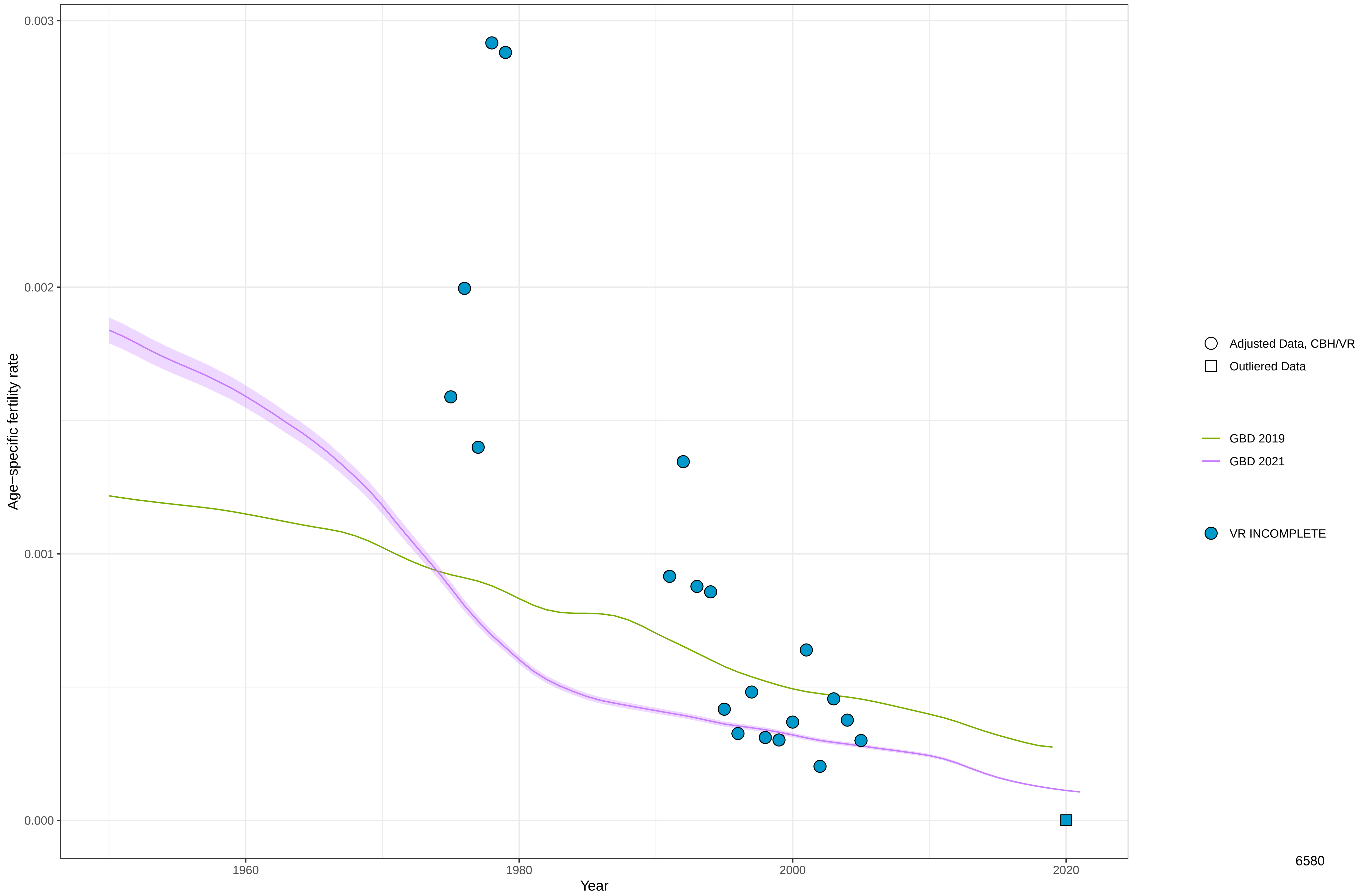

Total fertility rate fitted trend lines, 1950−2021: Zamboanga Del Norte TFR
Data points enclosed with a diamond are reference sources

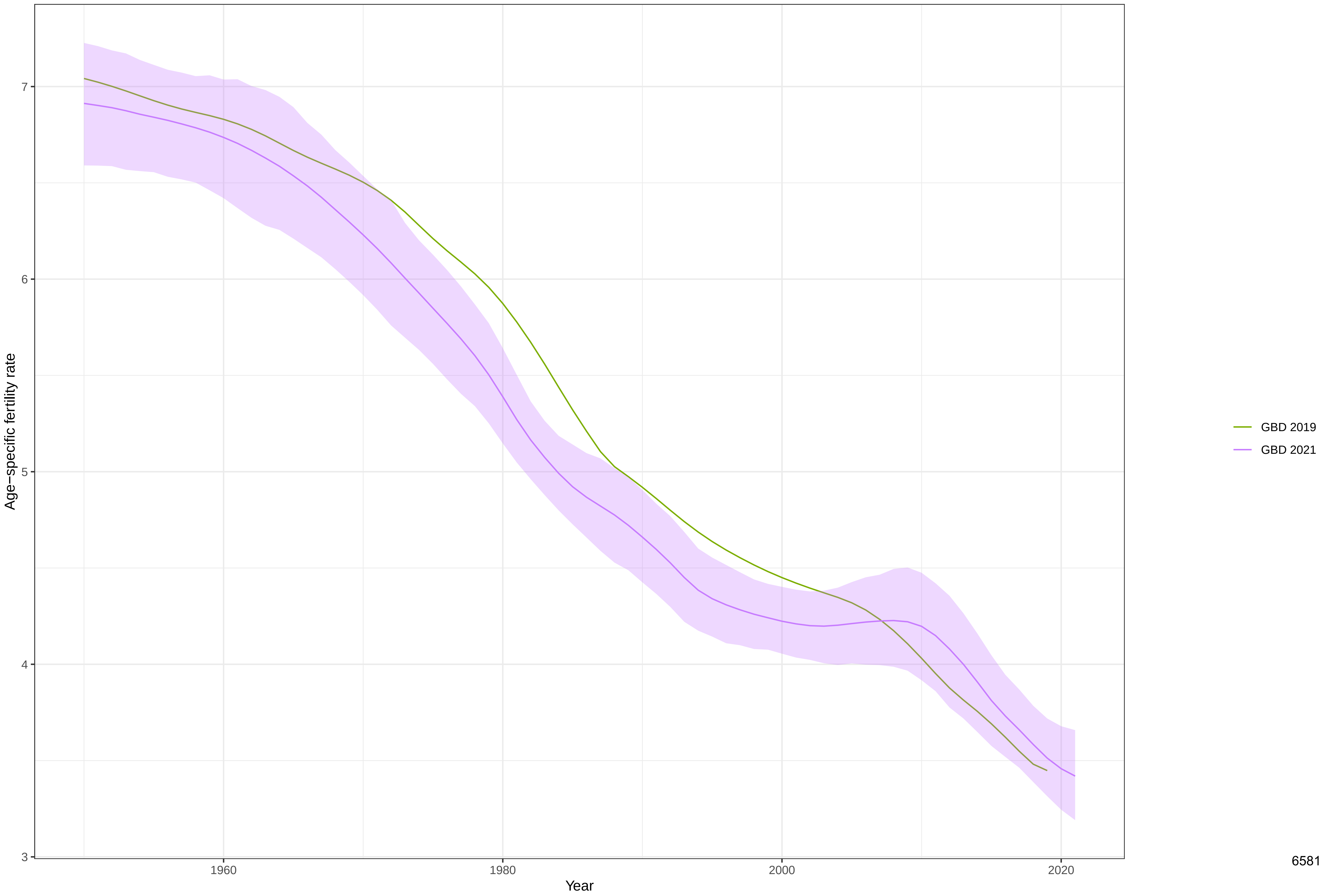

Age−specific fertility fitted trend lines, 1950−2021: Zamboanga Del Sur, Ages: 10−14
Data points enclosed with a diamond are reference sources

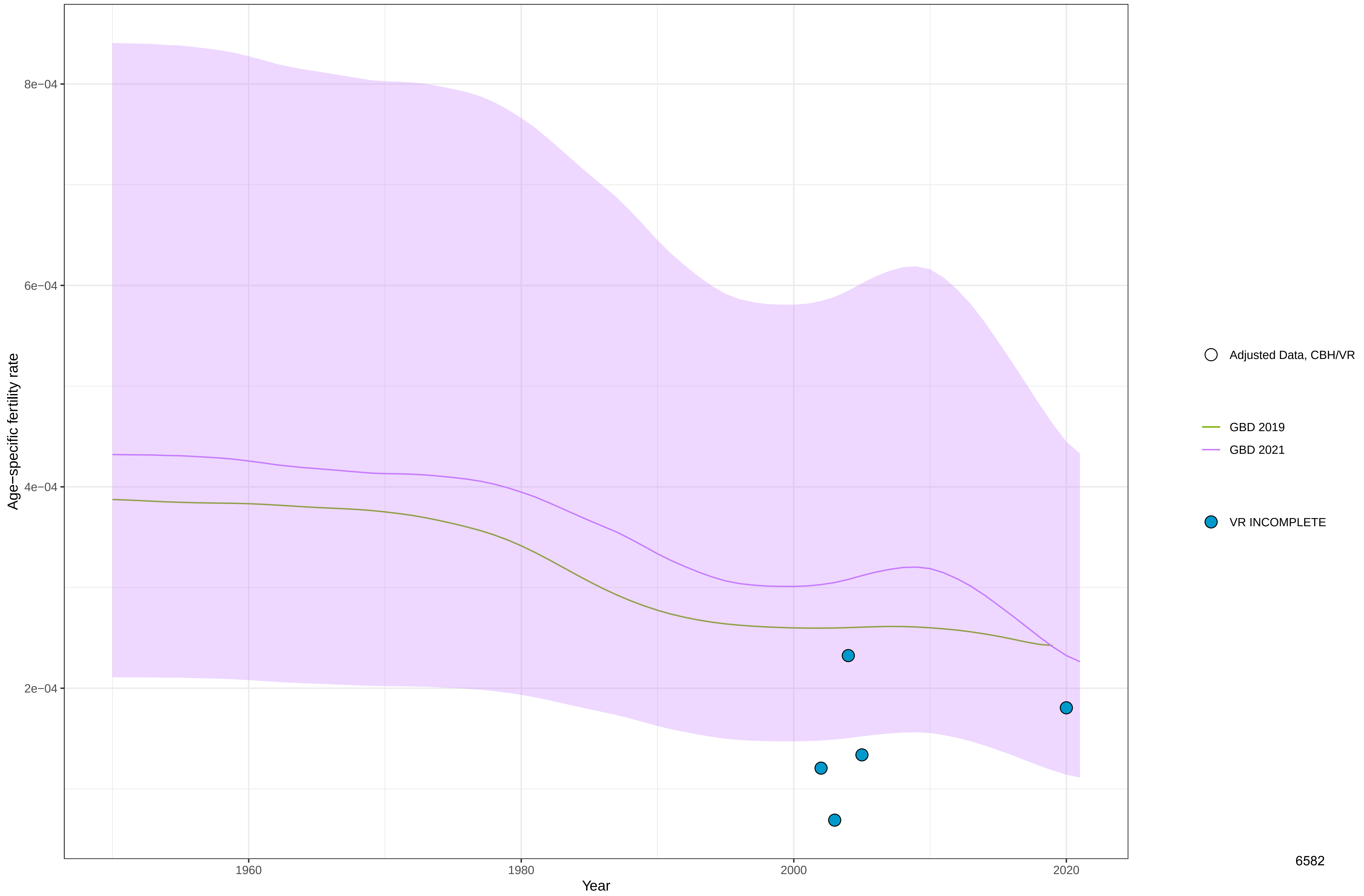

Age−specific fertility fitted trend lines, 1950−2021: Zamboanga Del Sur, Ages: 15−19
Data points enclosed with a diamond are reference sources

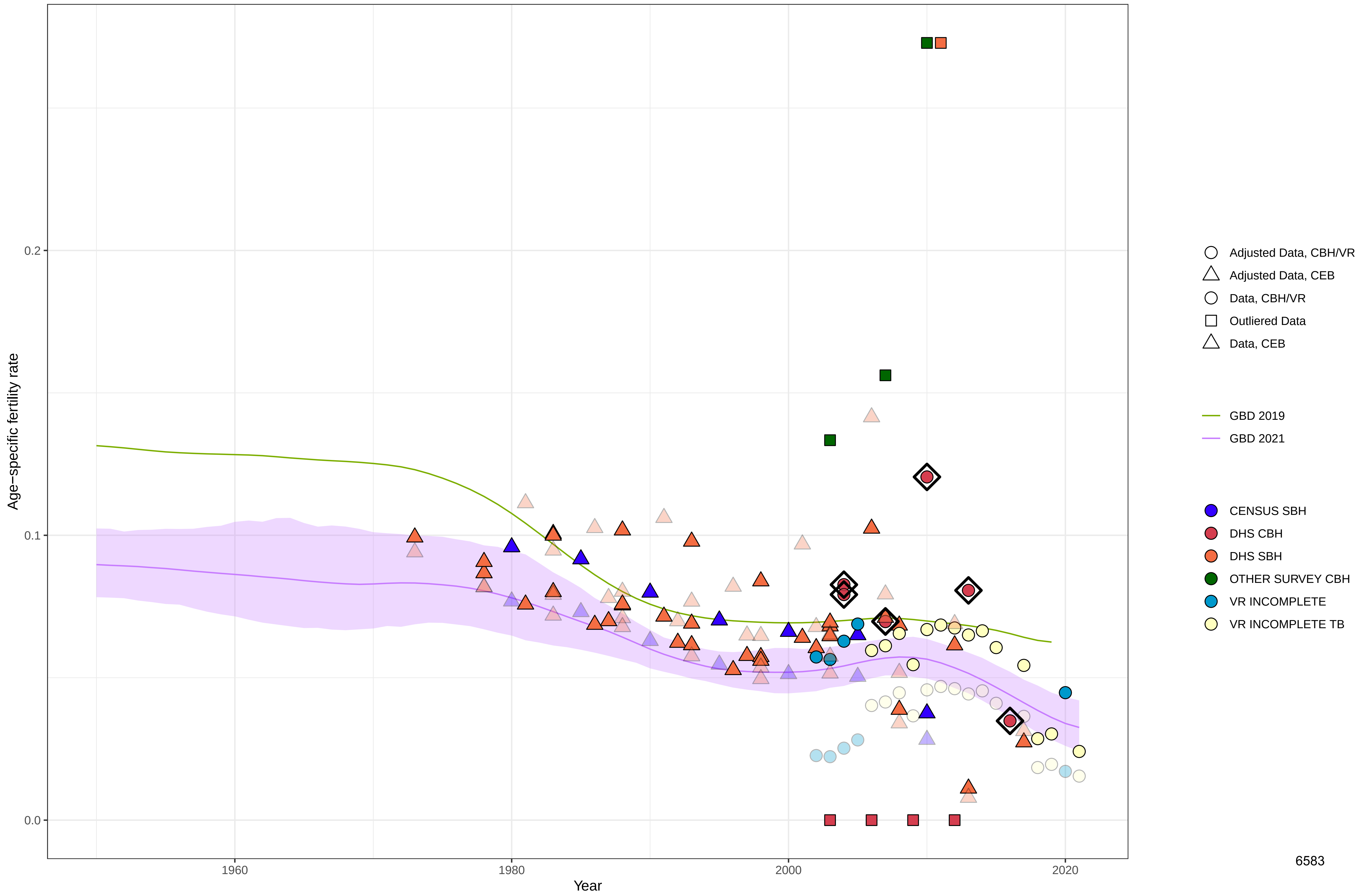

Age−specific fertility fitted trend lines, 1950−2021: Zamboanga Del Sur, Ages: 20−24
Data points enclosed with a diamond are reference sources

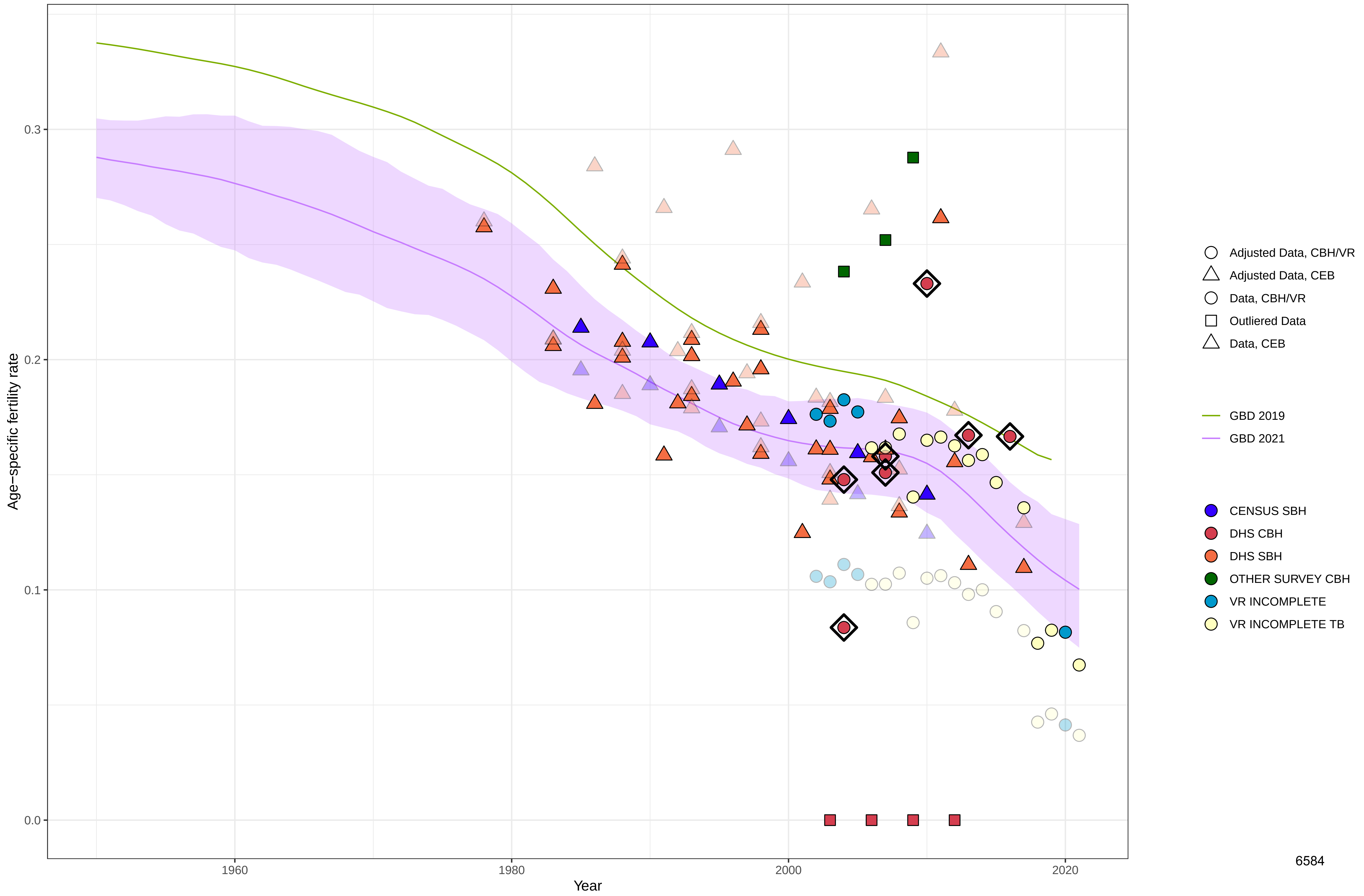

Age−specific fertility fitted trend lines, 1950−2021: Zamboanga Del Sur, Ages: 25−29
Data points enclosed with a diamond are reference sources

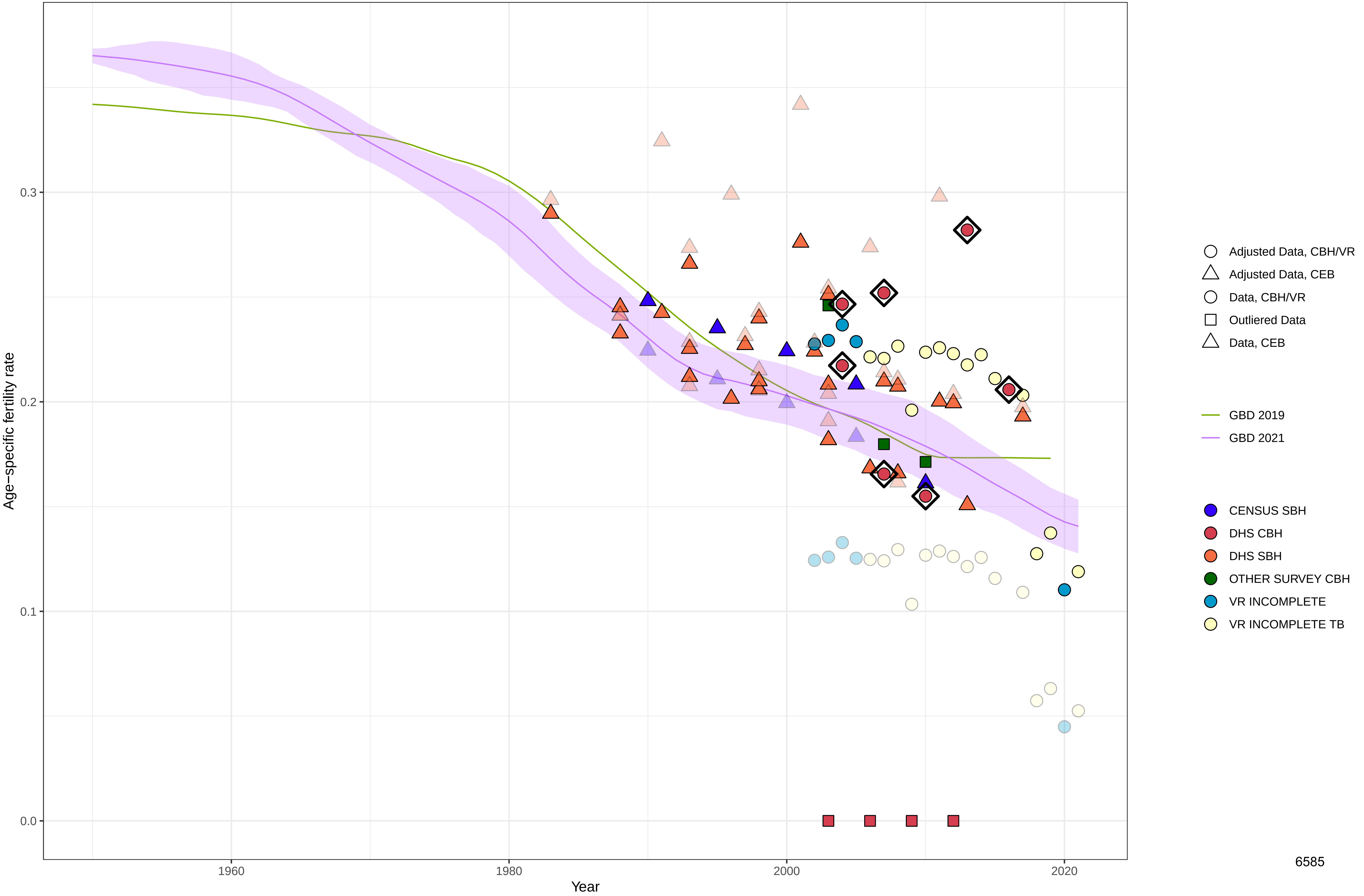

Age−specific fertility fitted trend lines, 1950−2021: Zamboanga Del Sur, Ages: 30−34
Data points enclosed with a diamond are reference sources

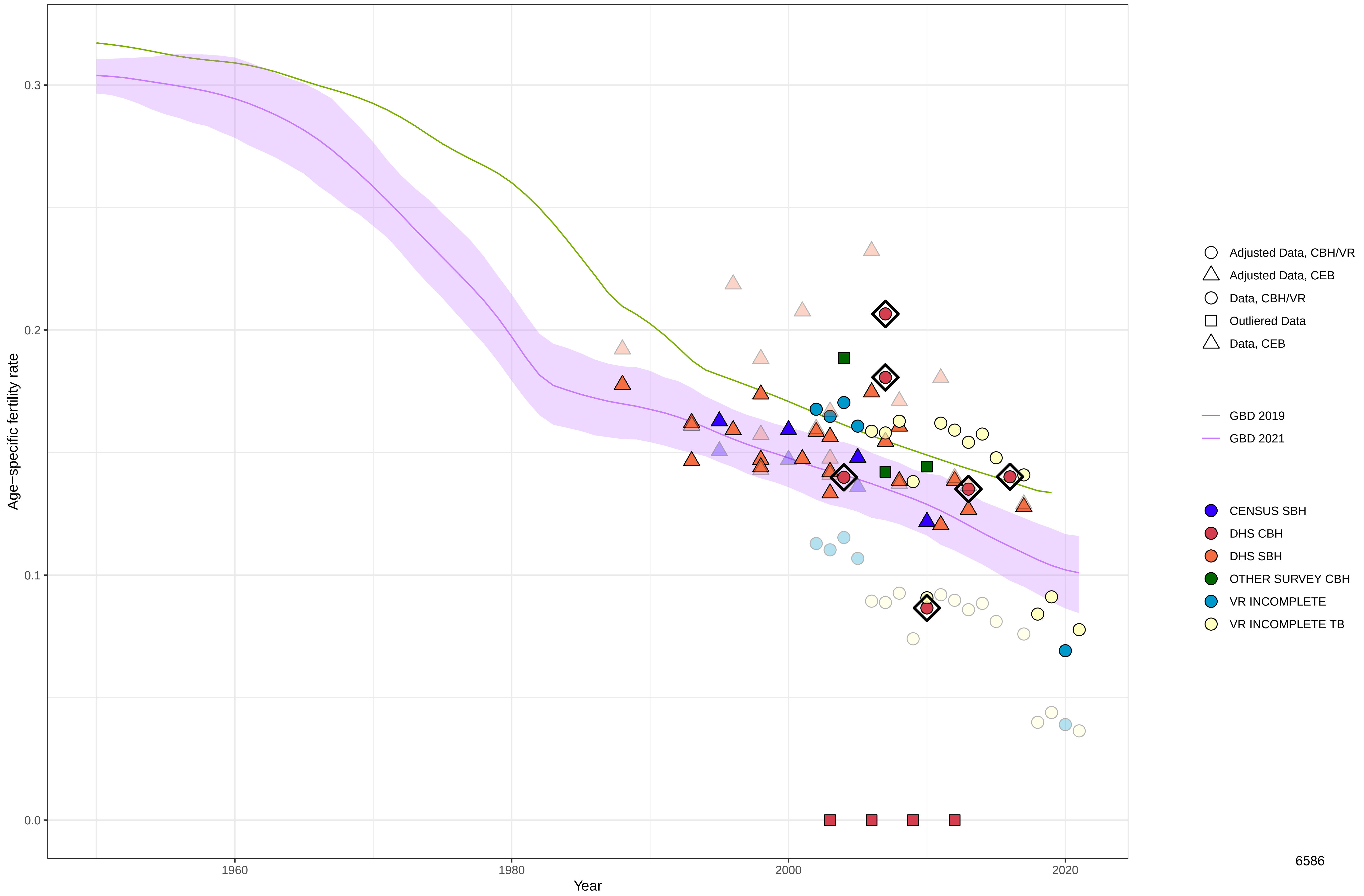

Age−specific fertility fitted trend lines, 1950−2021: Zamboanga Del Sur, Ages: 35−39
Data points enclosed with a diamond are reference sources

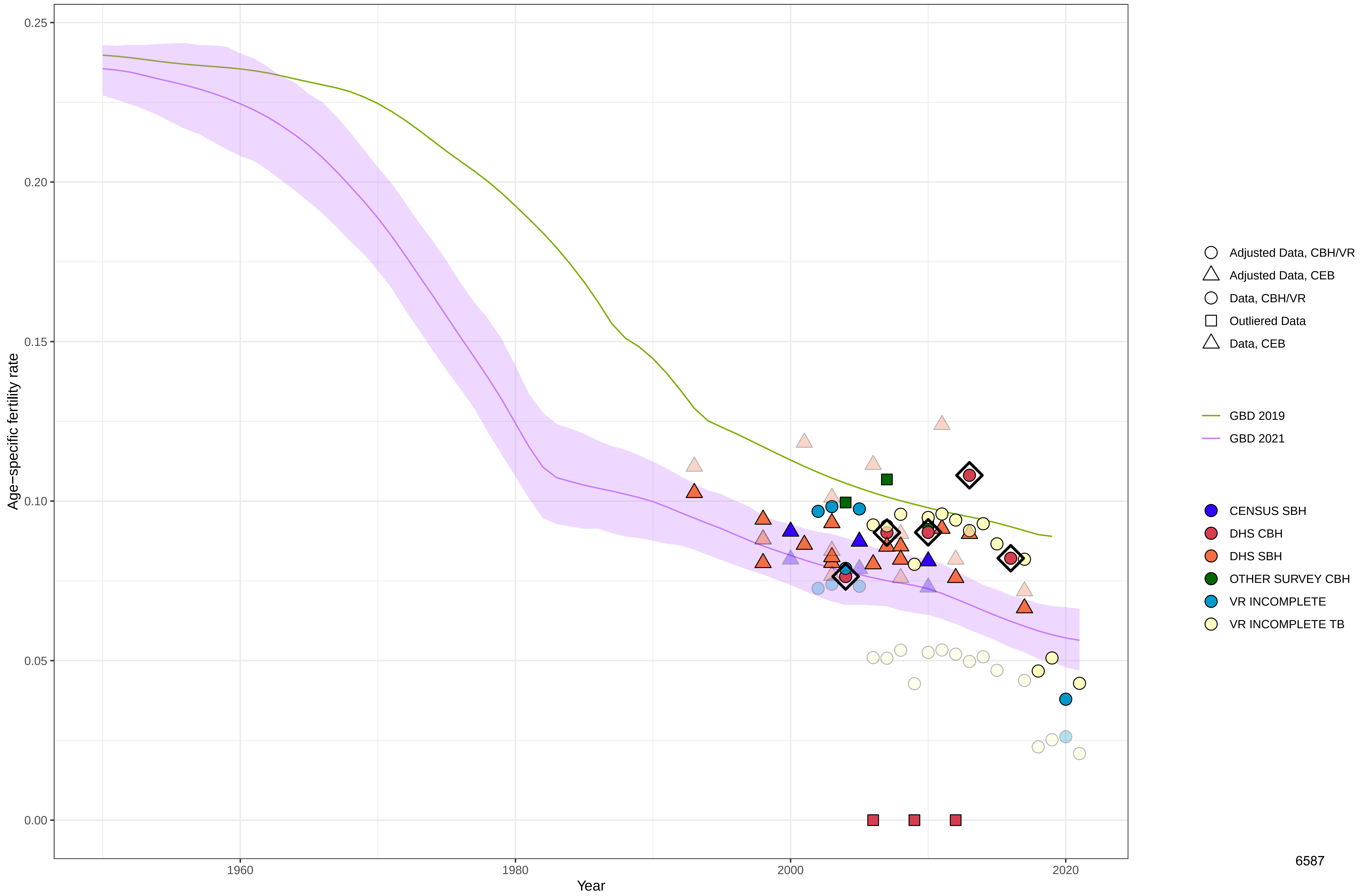

Age−specific fertility fitted trend lines, 1950−2021: Zamboanga Del Sur, Ages: 40−44
Data points enclosed with a diamond are reference sources

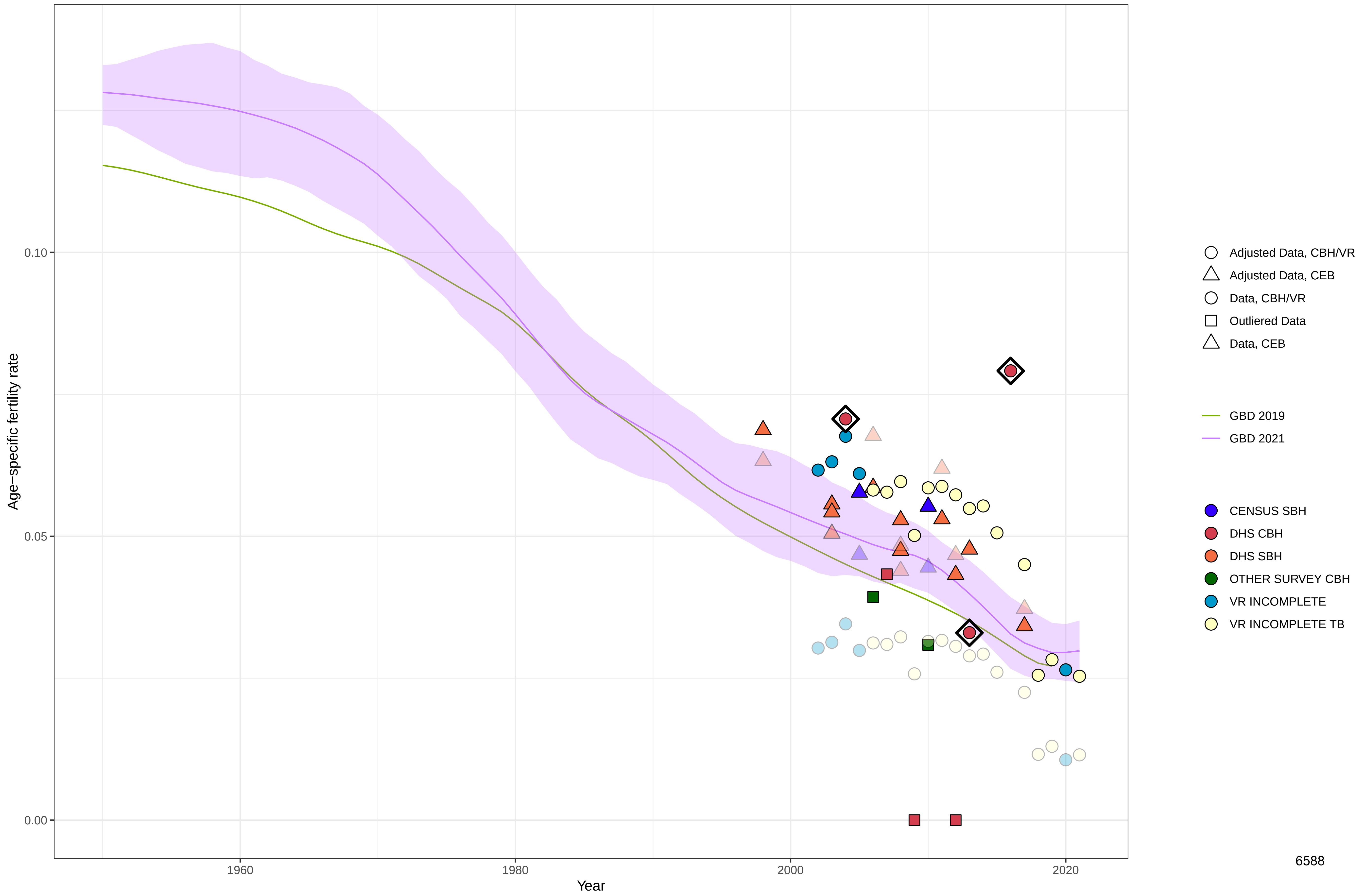

Age−specific fertility fitted trend lines, 1950−2021: Zamboanga Del Sur, Ages: 45−49
Data points enclosed with a diamond are reference sources

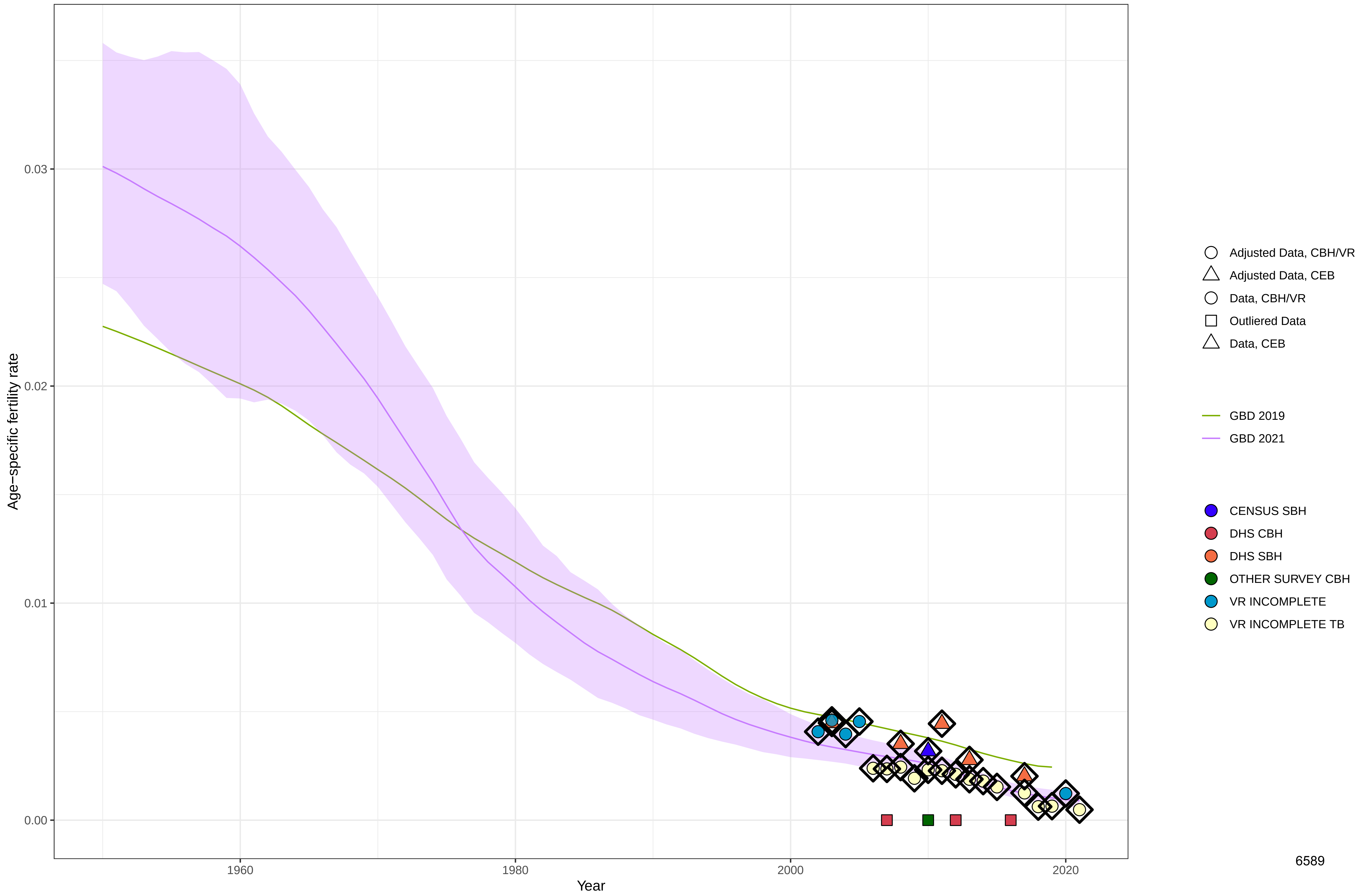

Age−specific fertility fitted trend lines, 1950−2021: Zamboanga Del Sur, Ages: 50−54
Data points enclosed with a diamond are reference sources

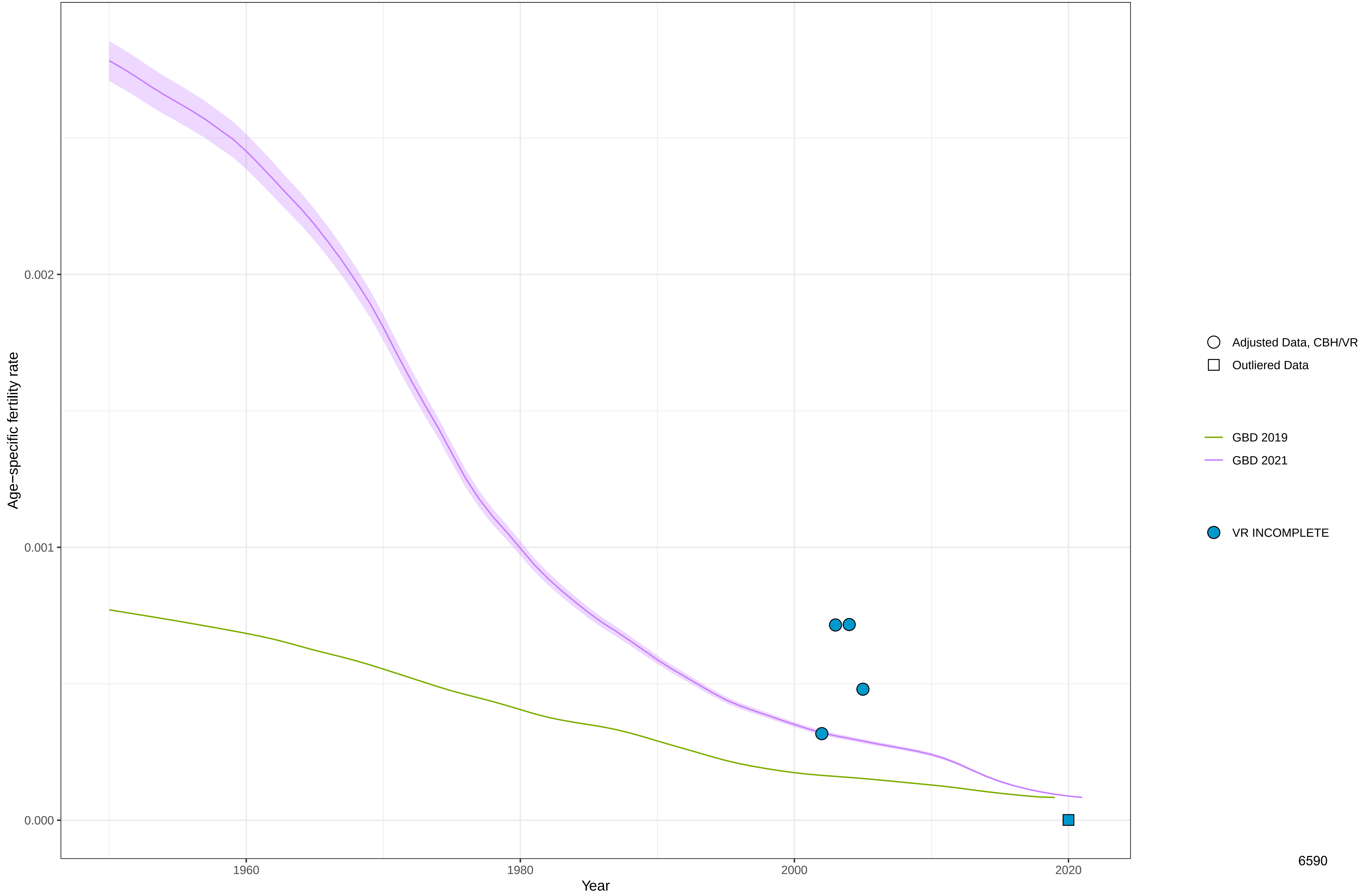

Total fertility rate fitted trend lines, 1950−2021: Zamboanga Del Sur TFR
Data points enclosed with a diamond are reference sources

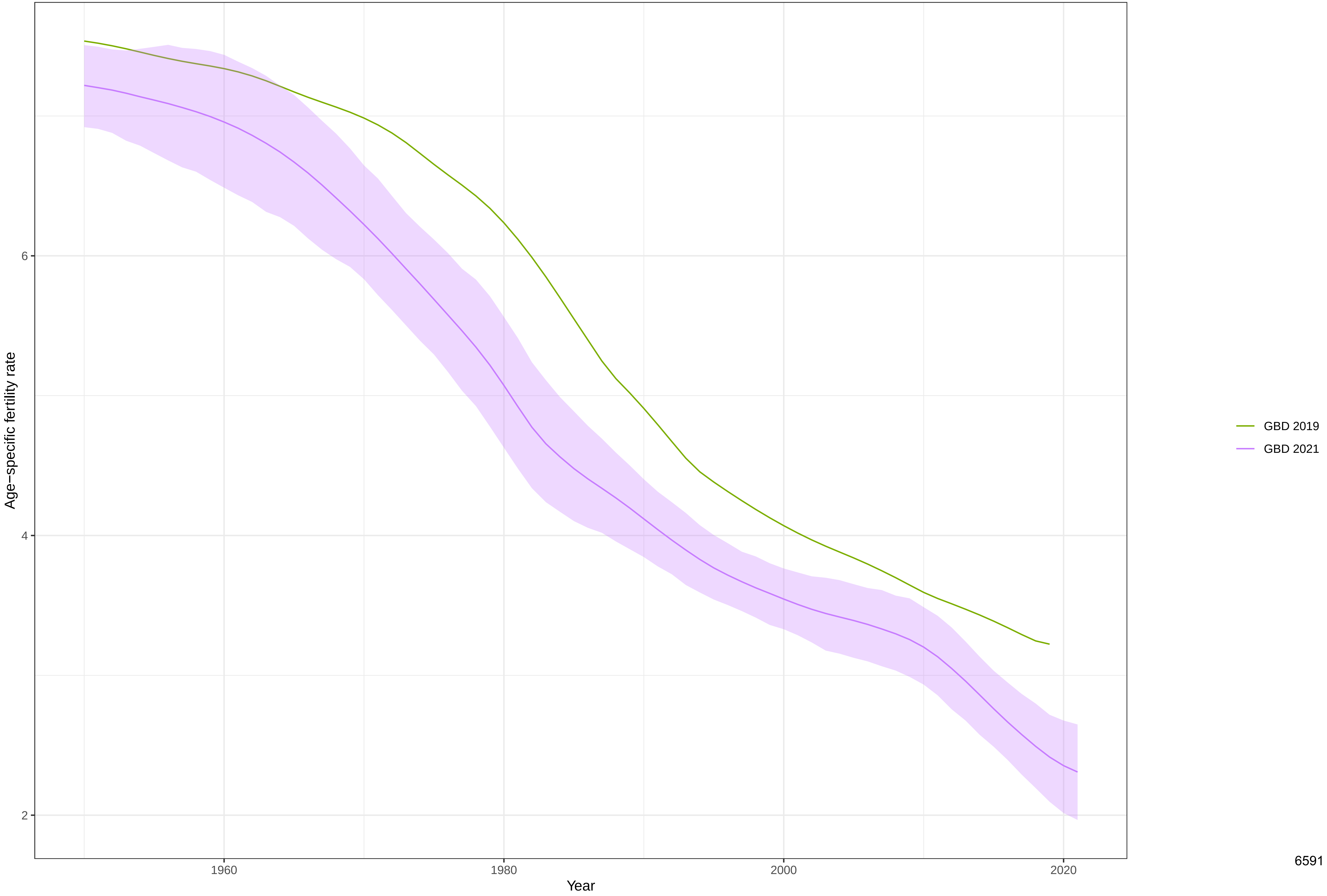

Age−specific fertility fitted trend lines, 1950−2021: Zamboanga Sibugay, Ages: 10−14
Data points enclosed with a diamond are reference sources

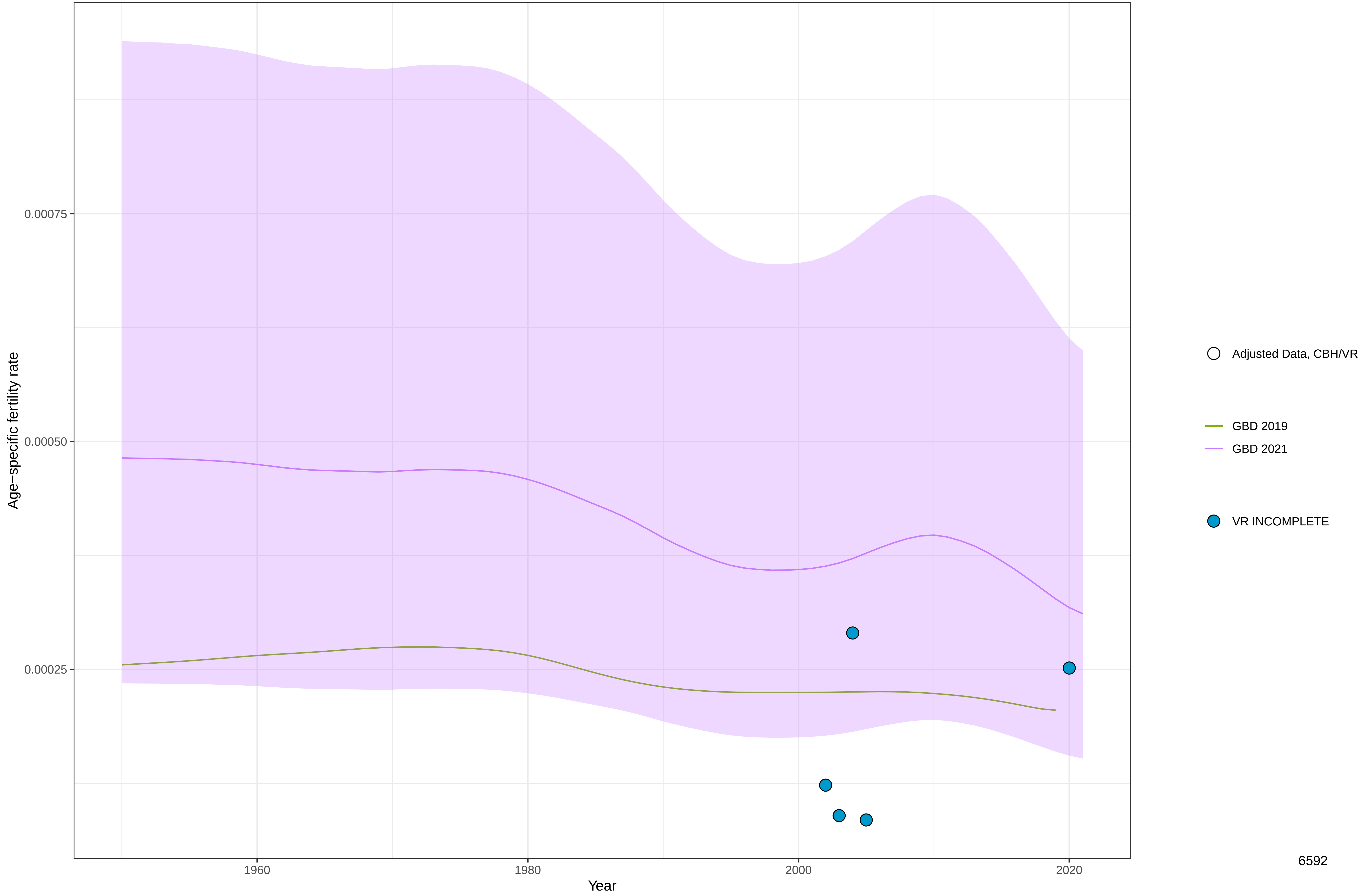

6592

Age−specific fertility fitted trend lines, 1950−2021: Zamboanga Sibugay, Ages: 15−19
Data points enclosed with a diamond are reference sources

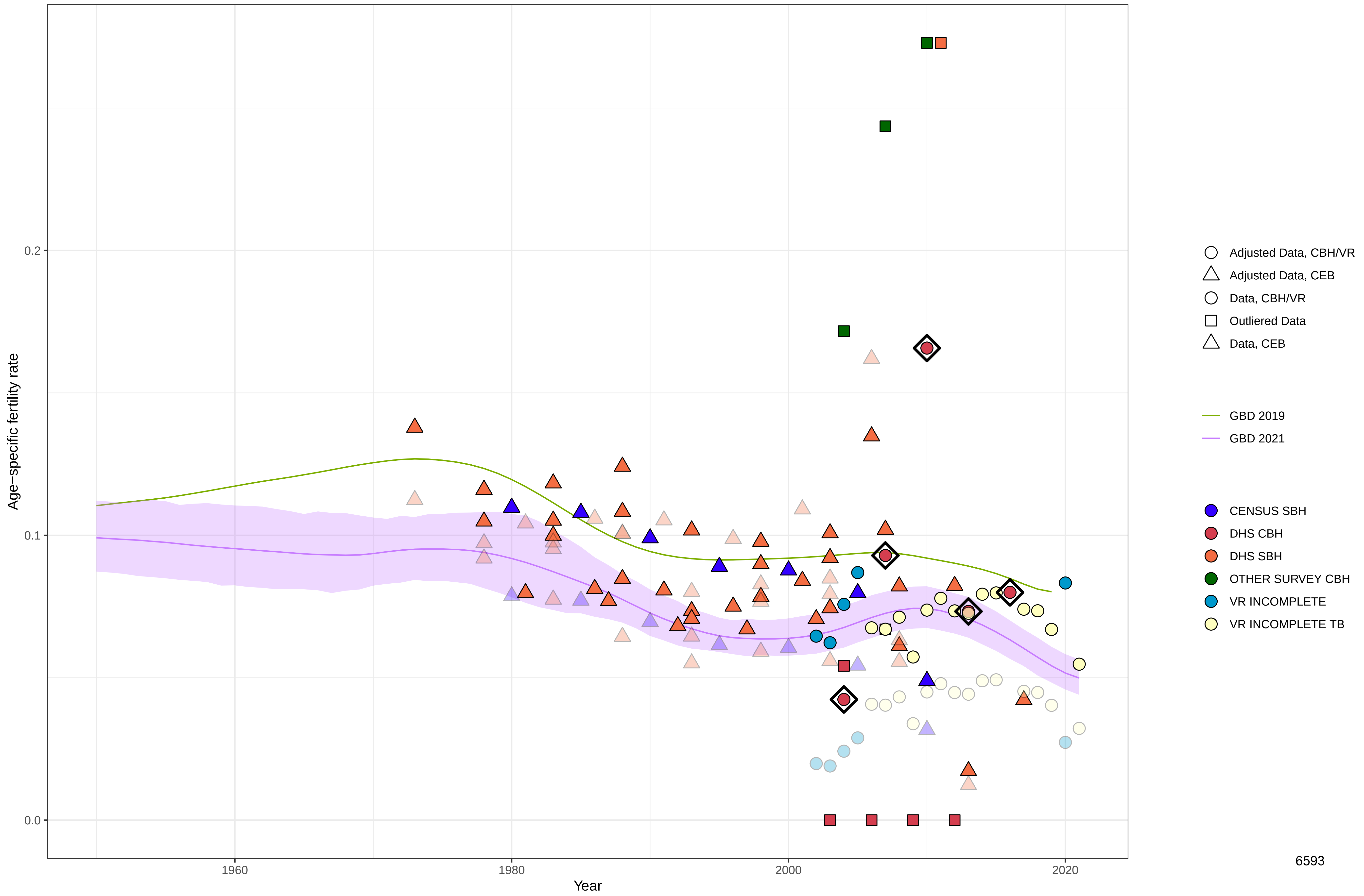

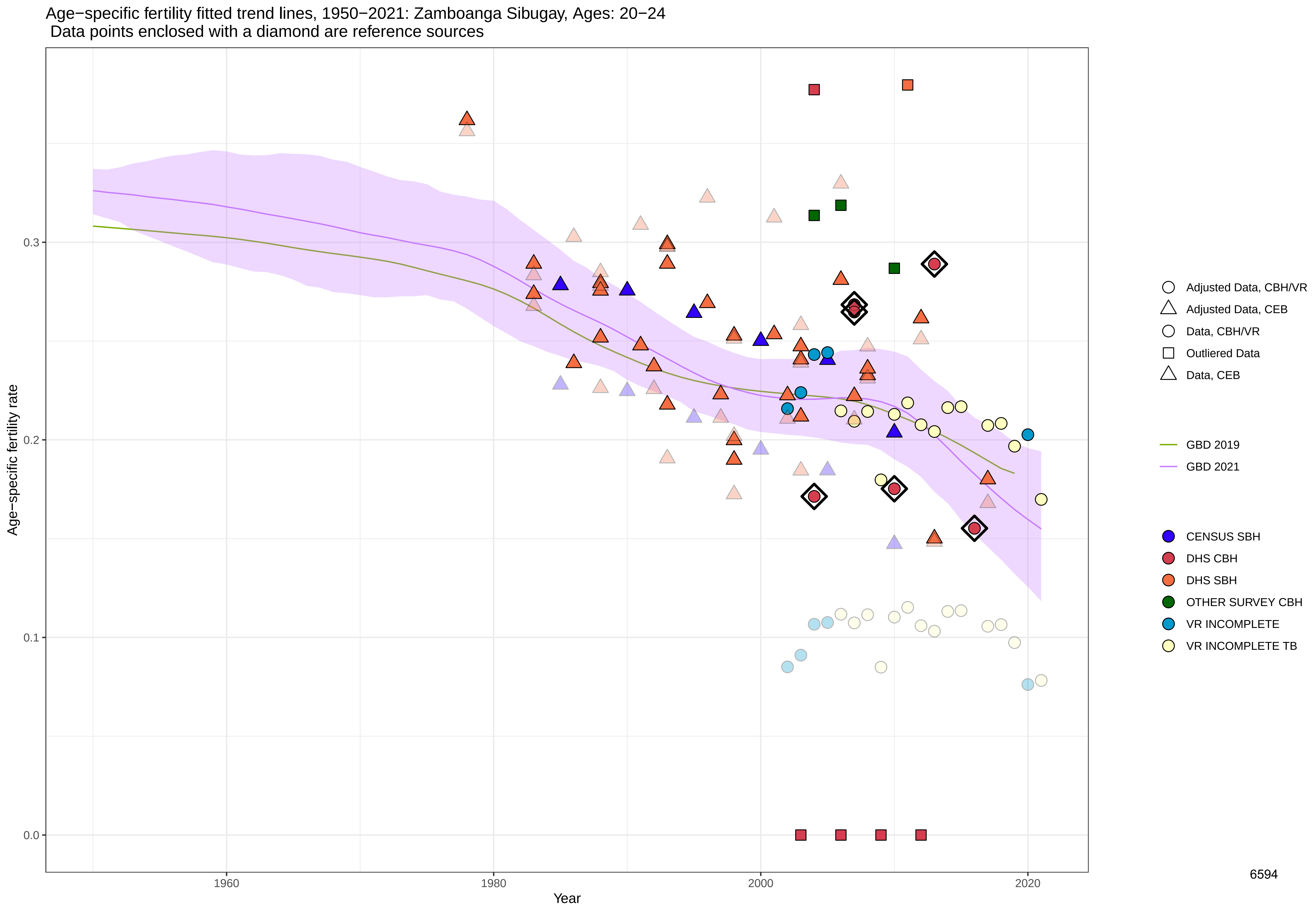

Age−specific fertility fitted trend lines, 1950−2021: Zamboanga Sibugay, Ages: 20−24
Data points enclosed with a diamond are reference sources
Age−specific fertility rate
Year
0.0
0.1
0.2
0.3
1960
1980
2000
2020
Adjusted Data, CBH/VR
Adjusted Data, CEB
Data, CBH/VR
Outliered Data
Data, CEB
GBD 2019
GBD 2021
CENSUS SBH
DHS CBH
DHS SBH
OTHER SURVEY CBH
VR INCOMPLETE
VR INCOMPLETE TB

6594

Age−specific fertility fitted trend lines, 1950−2021: Zamboanga Sibugay, Ages: 25−29
Data points enclosed with a diamond are reference sources

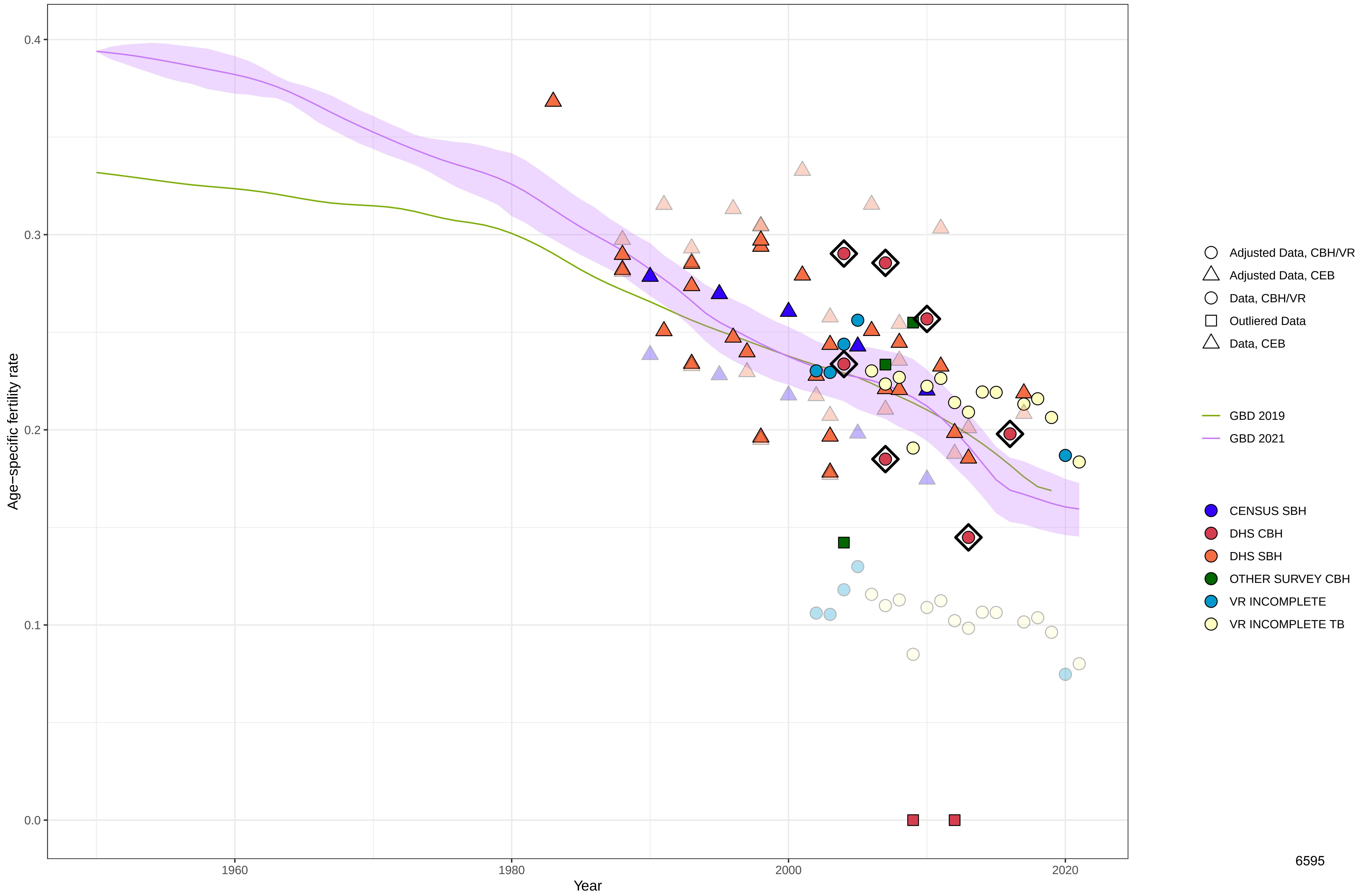

6595

Age−specific fertility fitted trend lines, 1950−2021: Zamboanga Sibugay, Ages: 30−34
Data points enclosed with a diamond are reference sources

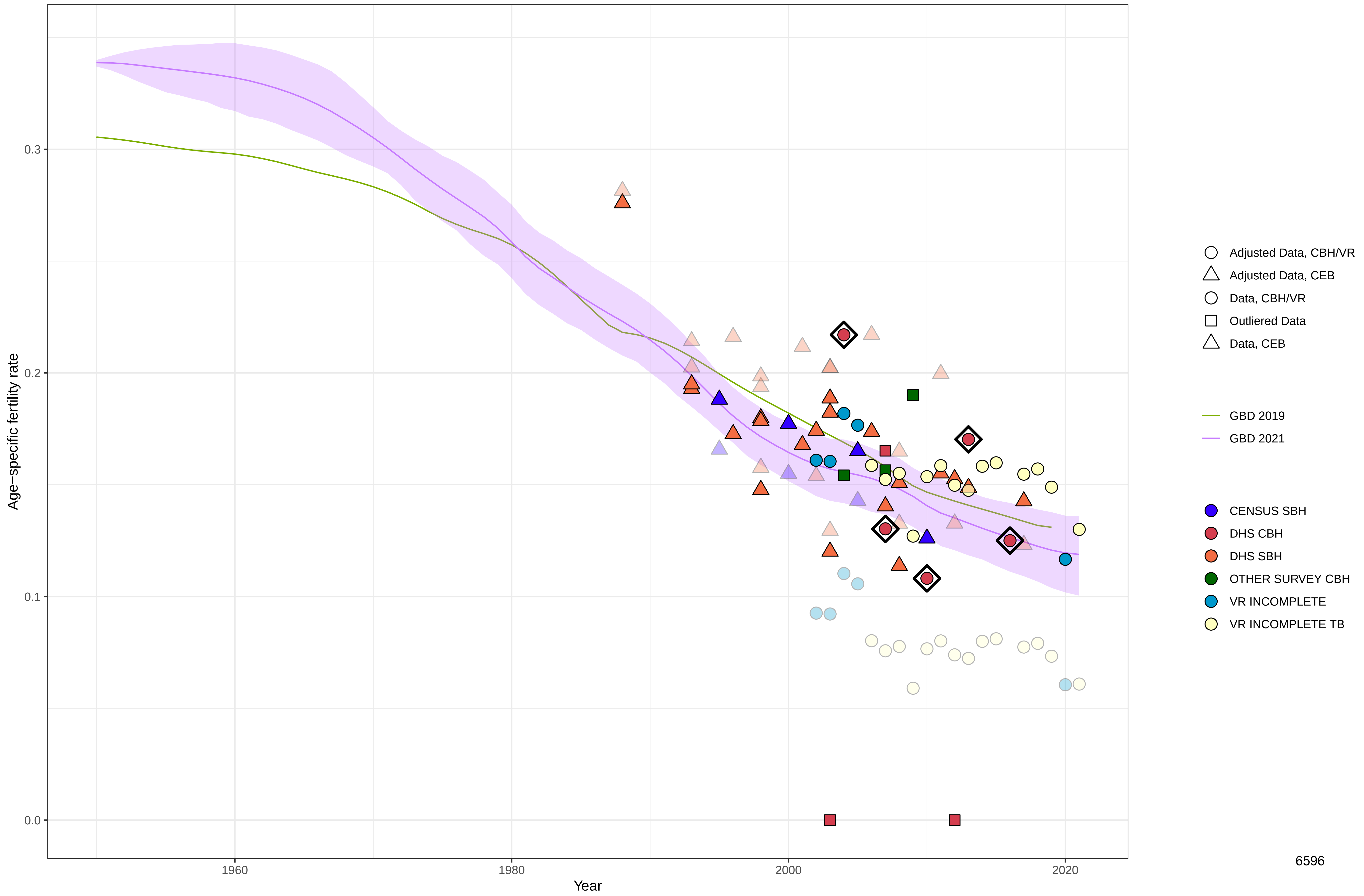

Age−specific fertility fitted trend lines, 1950−2021: Zamboanga Sibugay, Ages: 35−39
Data points enclosed with a diamond are reference sources

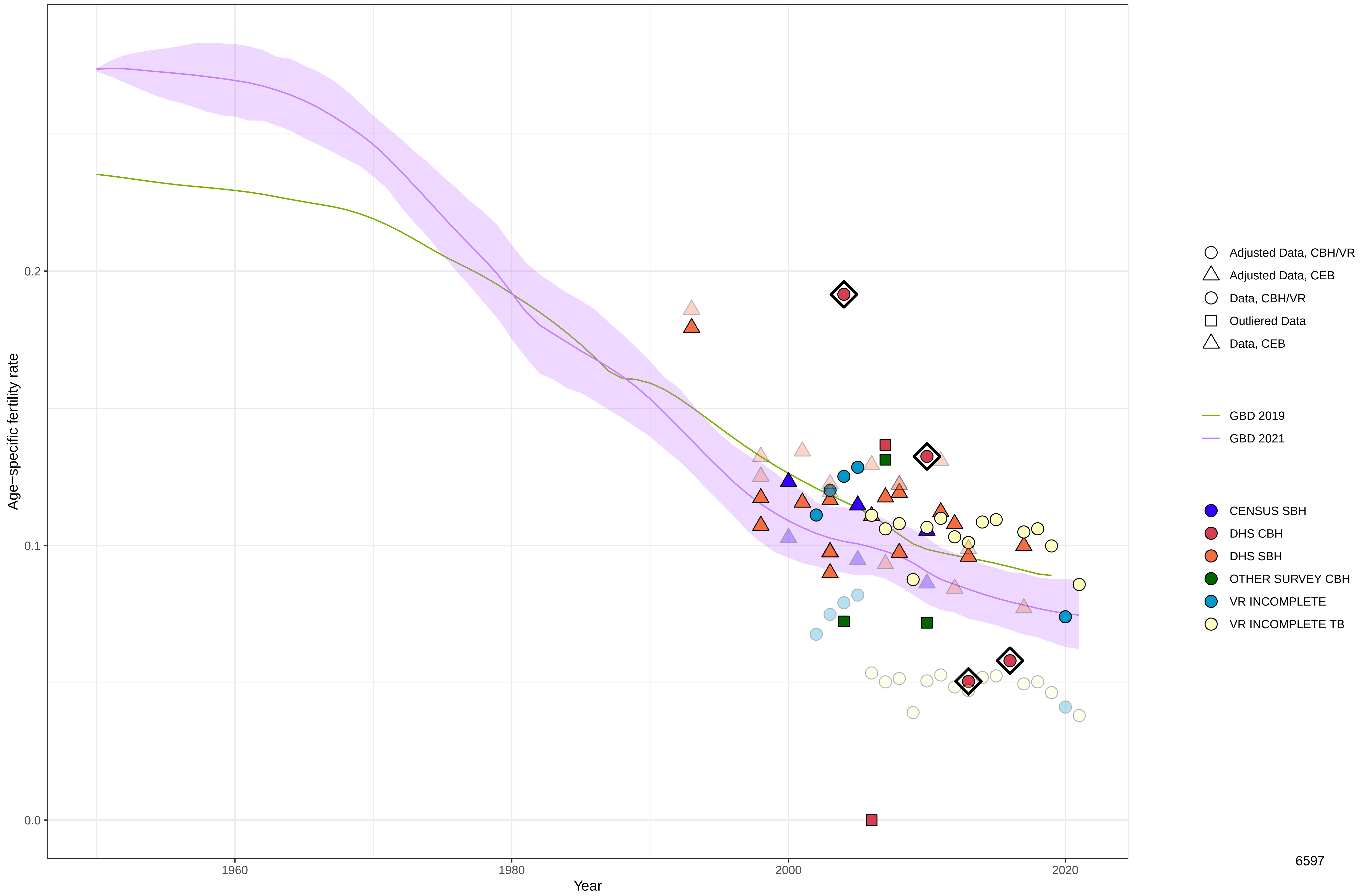

Age−specific fertility fitted trend lines, 1950−2021: Zamboanga Sibugay, Ages: 40−44
Data points enclosed with a diamond are reference sources

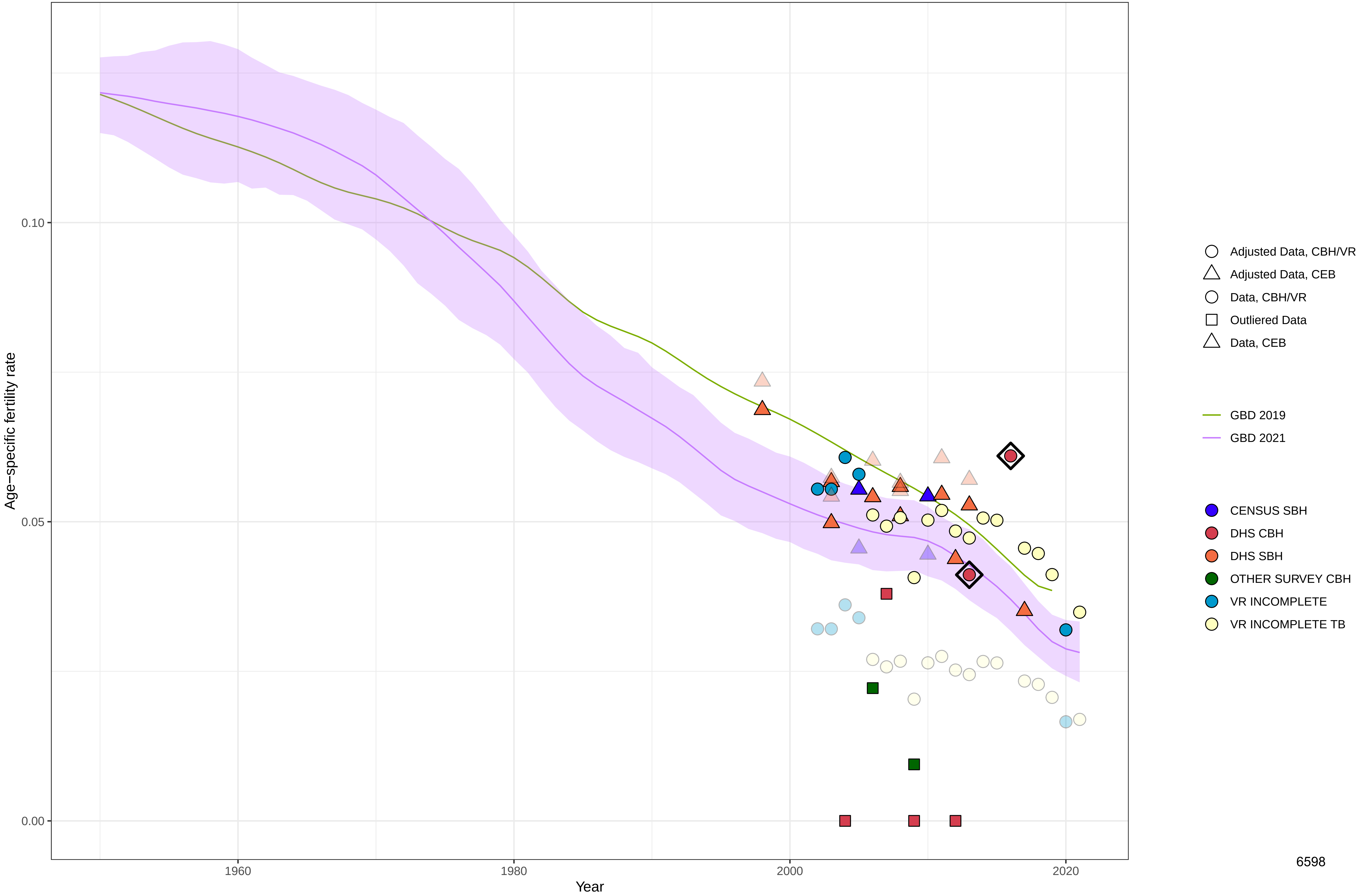

Age−specific fertility fitted trend lines, 1950−2021: Zamboanga Sibugay, Ages: 45−49
Data points enclosed with a diamond are reference sources

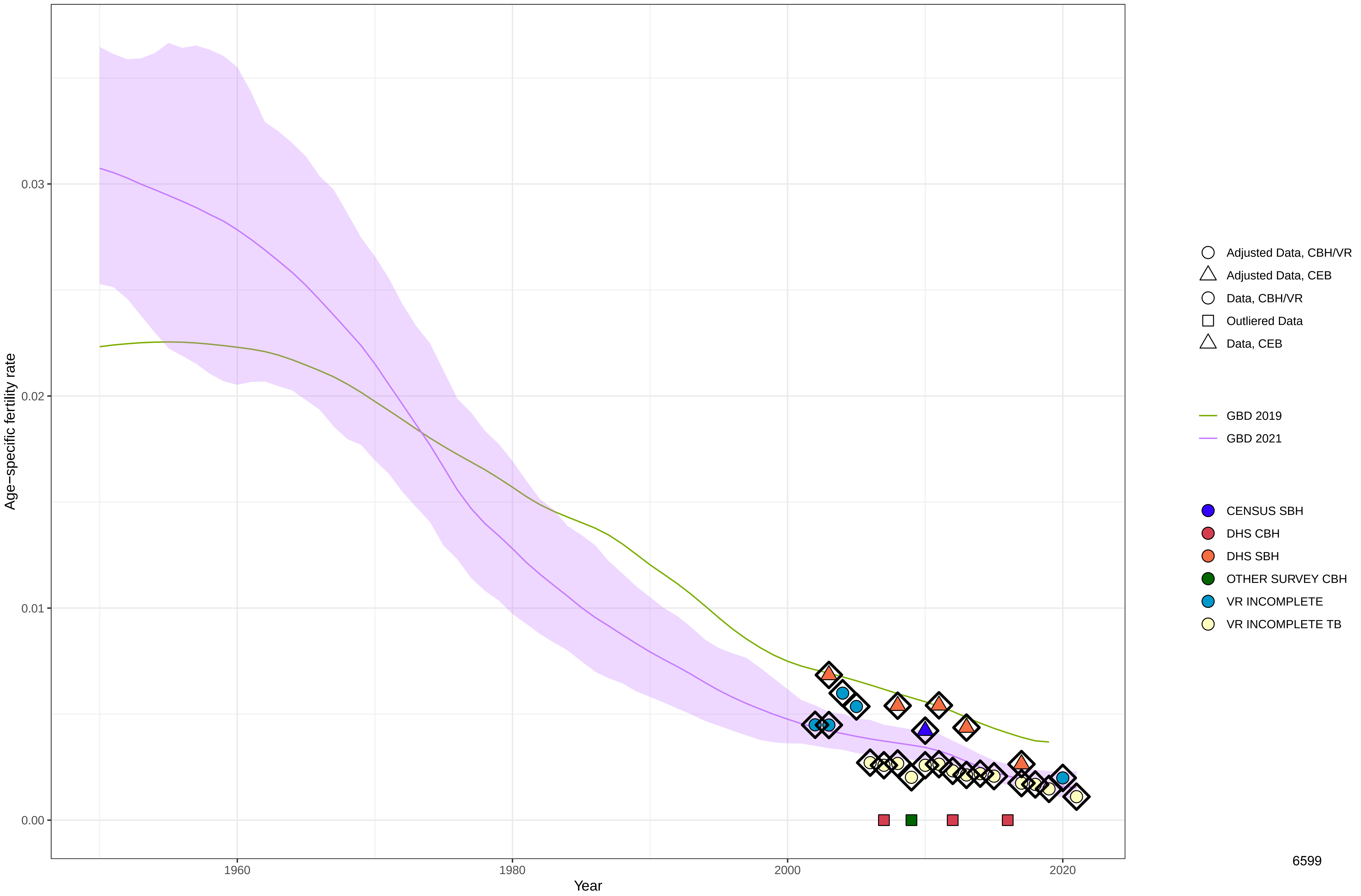

Age−specific fertility fitted trend lines, 1950−2021: Zamboanga Sibugay, Ages: 50−54
Data points enclosed with a diamond are reference sources

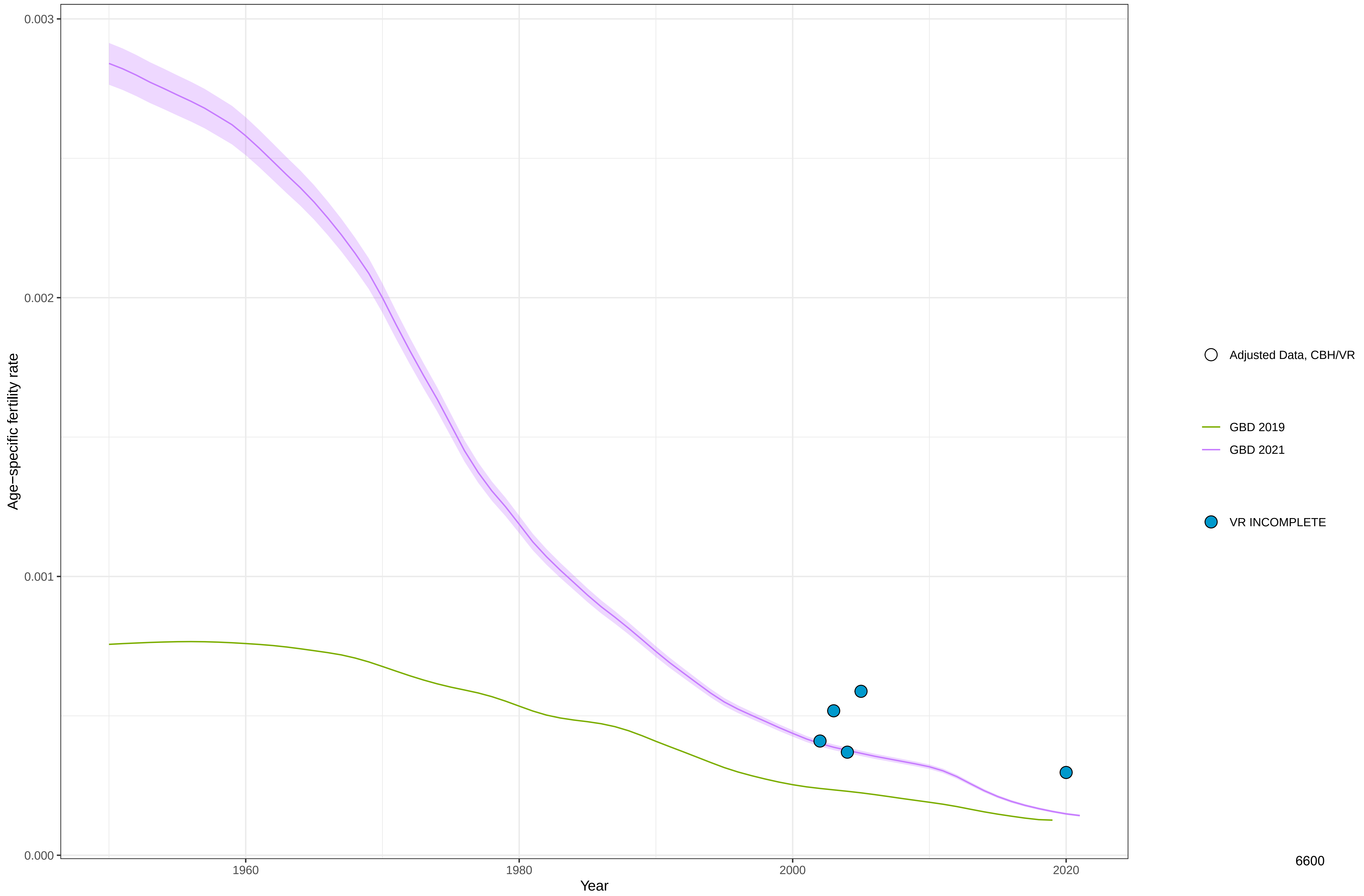

Total fertility rate fitted trend lines, 1950−2021: Zamboanga Sibugay TFR
Data points enclosed with a diamond are reference sources

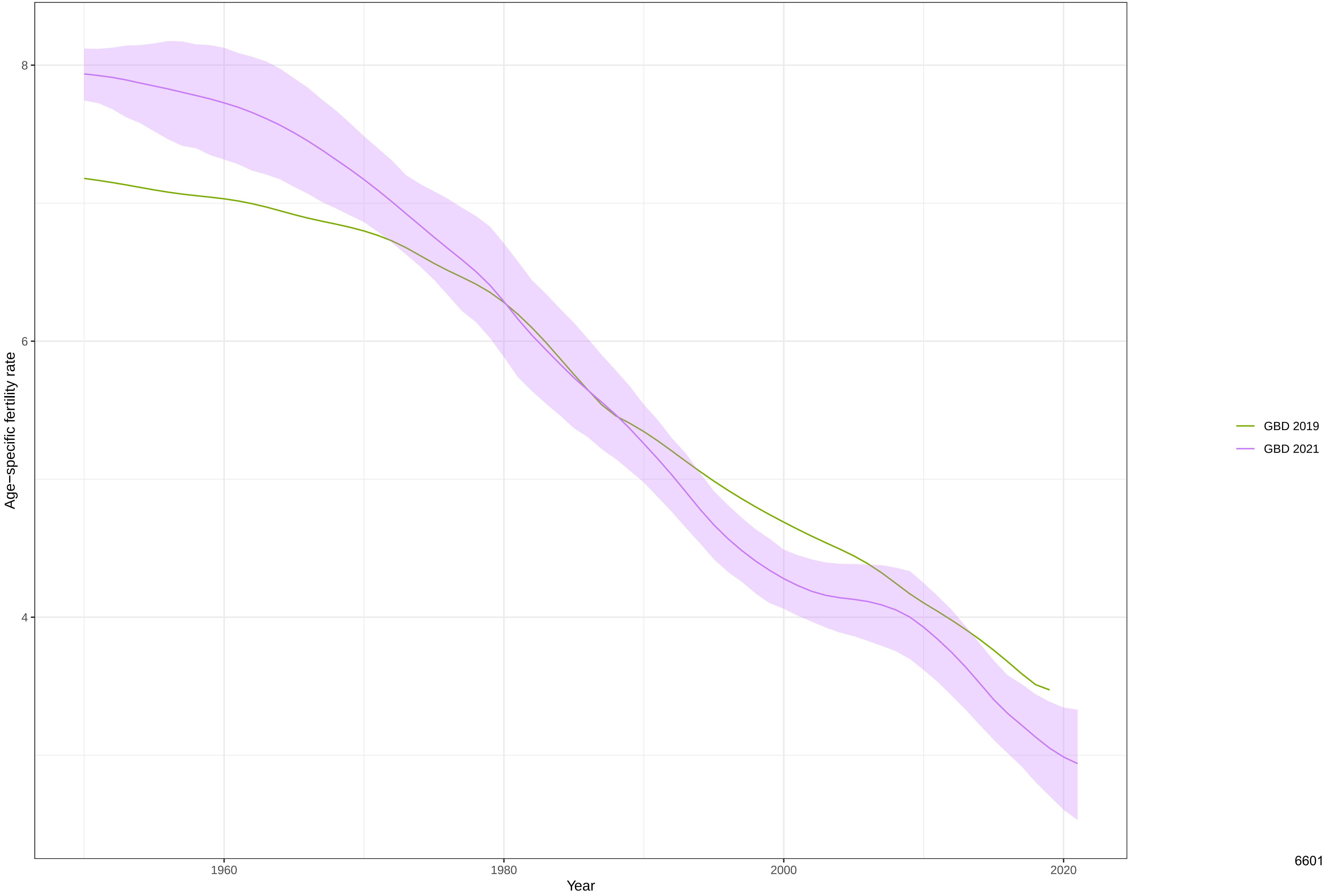

Age−specific fertility fitted trend lines, 1950−2021: Seychelles, Ages: 10−14
Data points enclosed with a diamond are reference sources

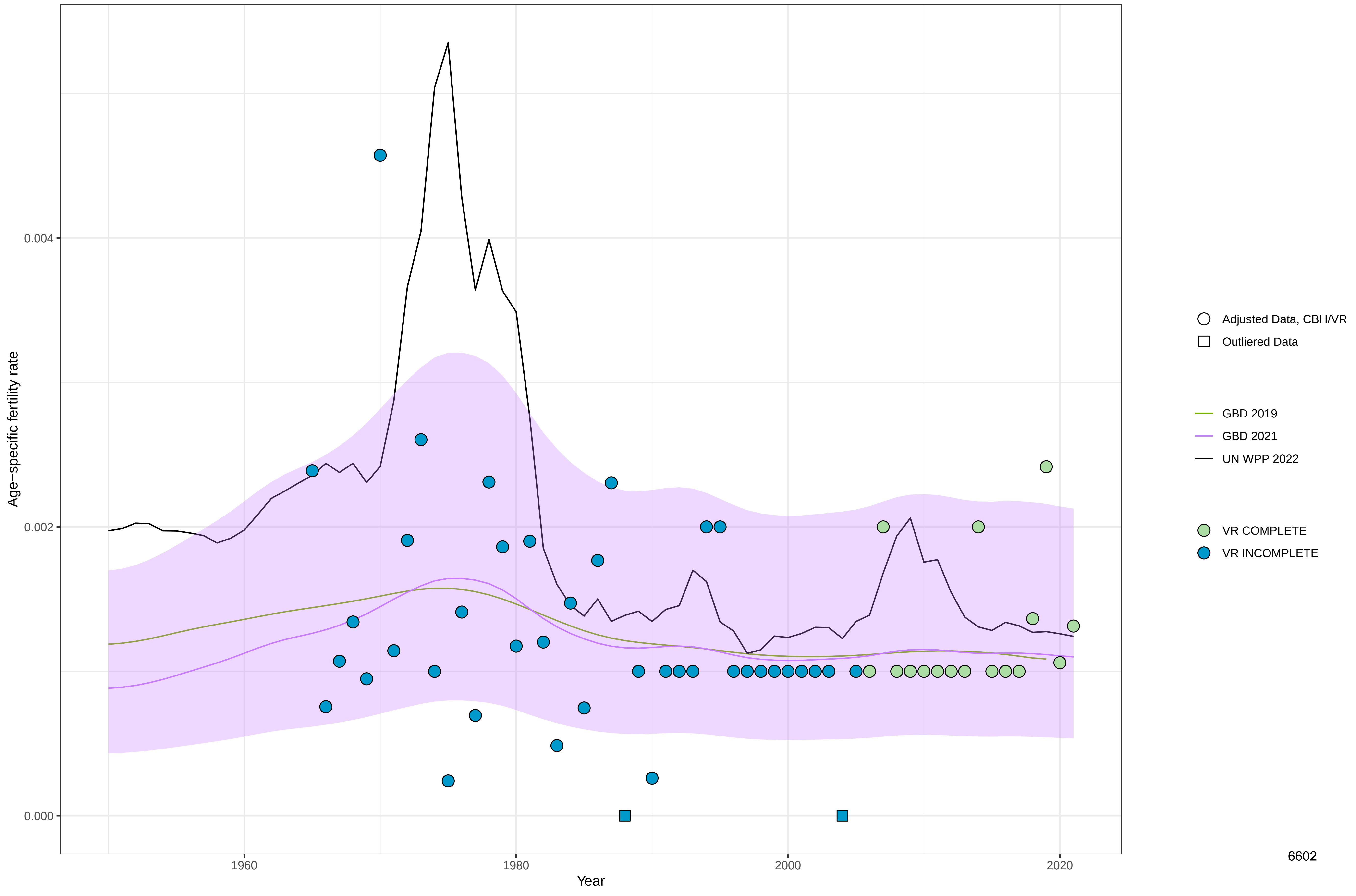

Age−specific fertility fitted trend lines, 1950−2021: Seychelles, Ages: 15−19
Data points enclosed with a diamond are reference sources

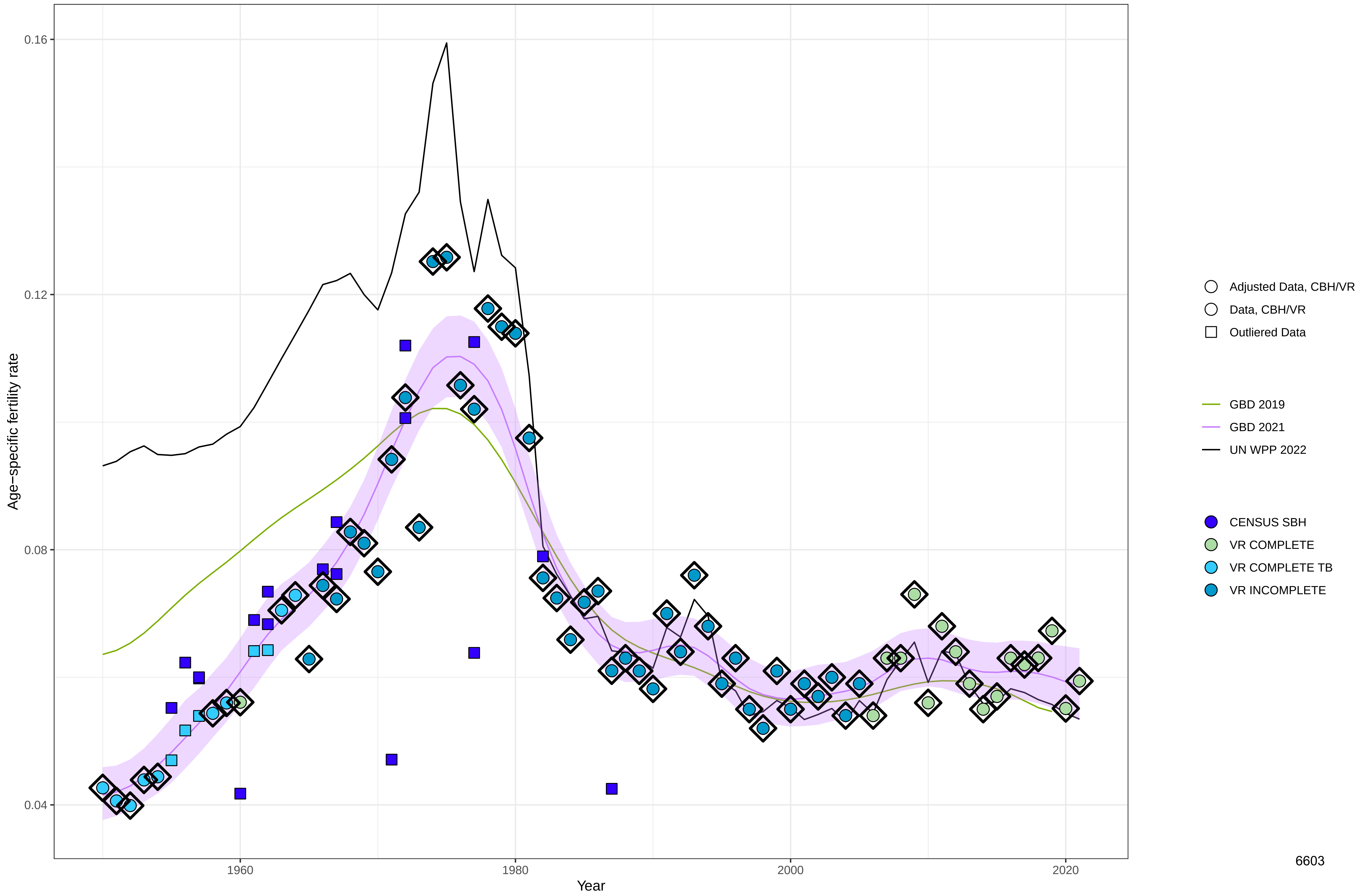

Age−specific fertility fitted trend lines, 1950−2021: Seychelles, Ages: 20−24
Data points enclosed with a diamond are reference sources

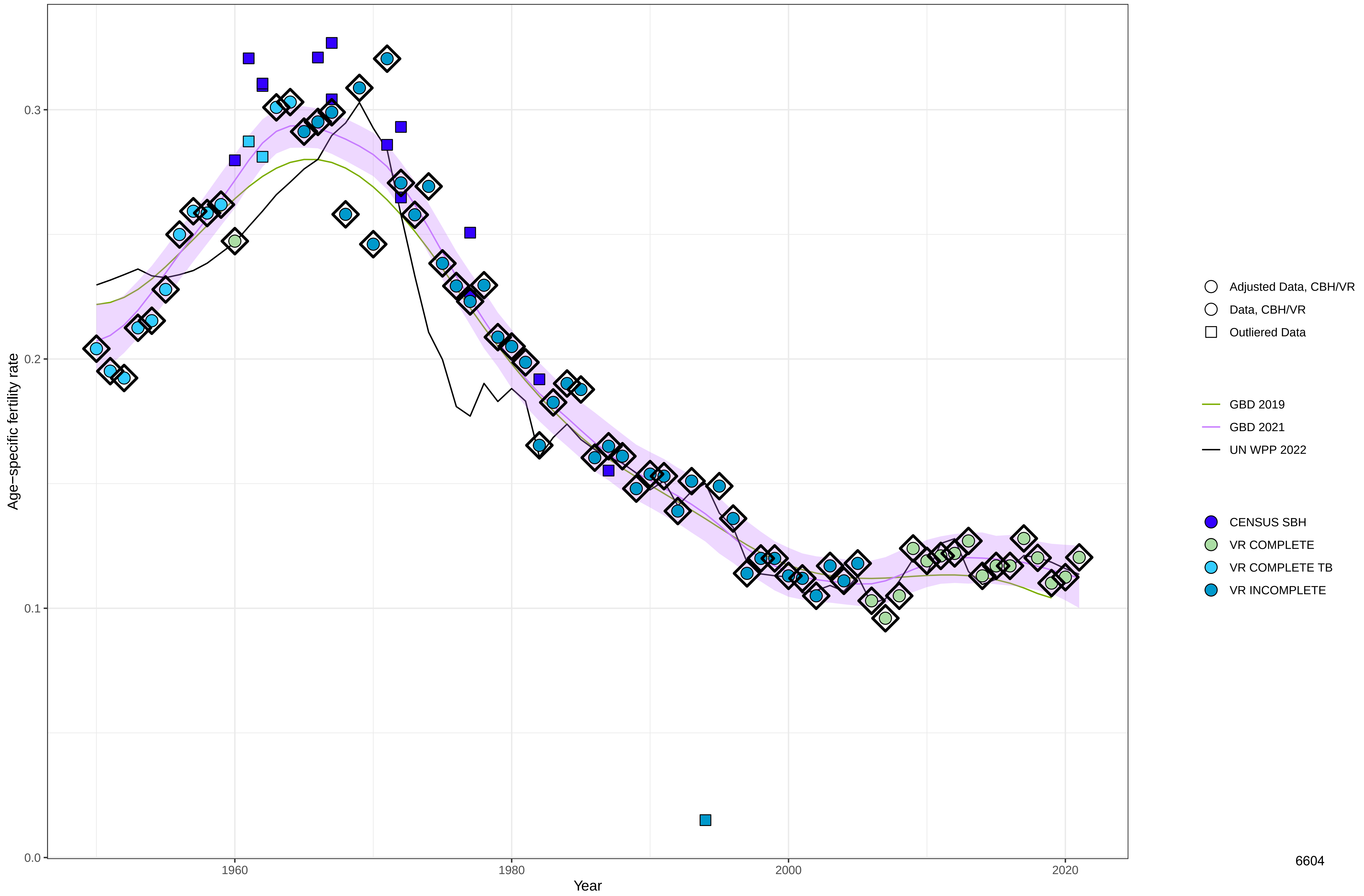

Age−specific fertility fitted trend lines, 1950−2021: Seychelles, Ages: 25−29
Data points enclosed with a diamond are reference sources

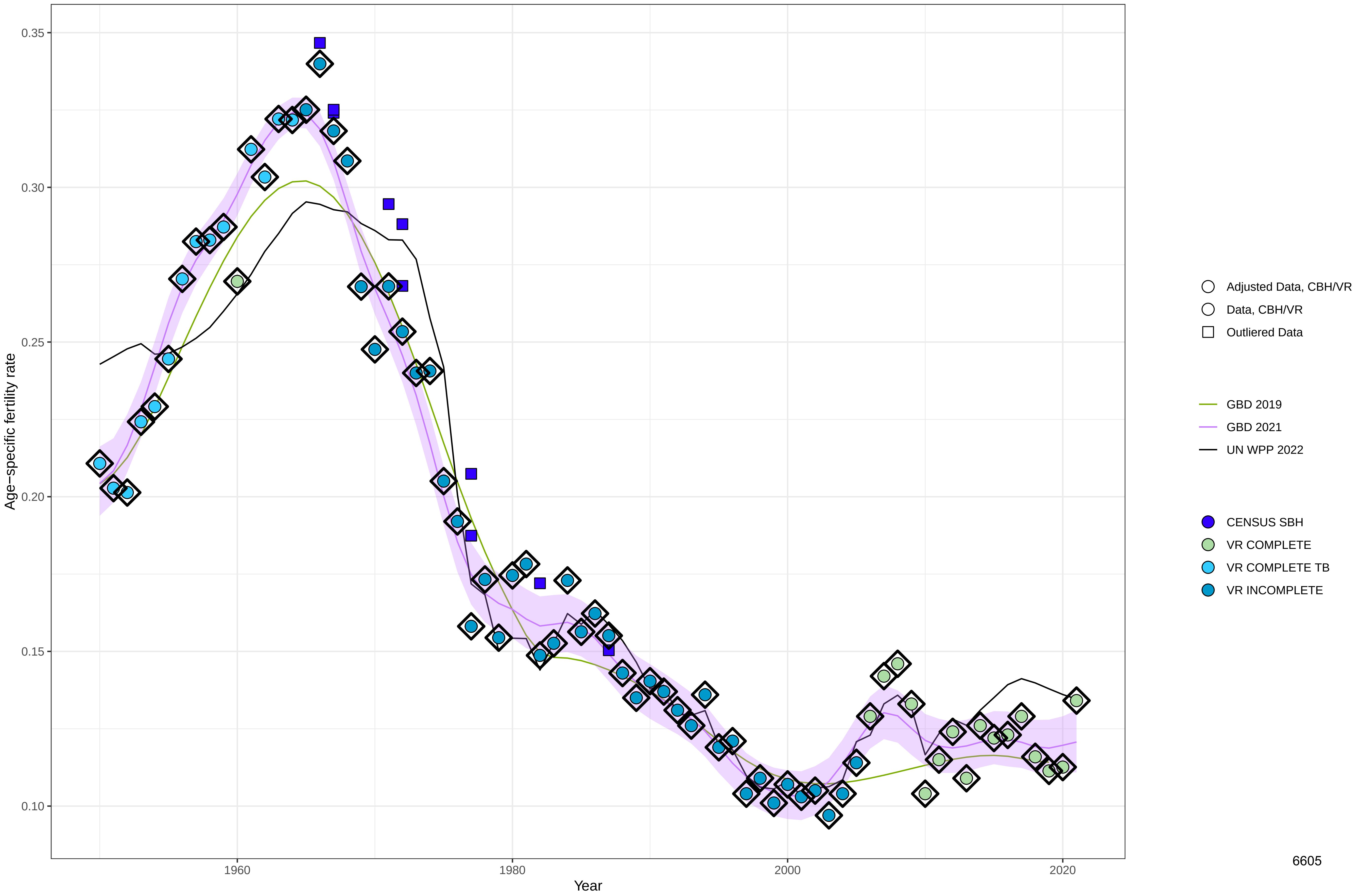

Age−specific fertility fitted trend lines, 1950−2021: Seychelles, Ages: 30−34
Data points enclosed with a diamond are reference sources

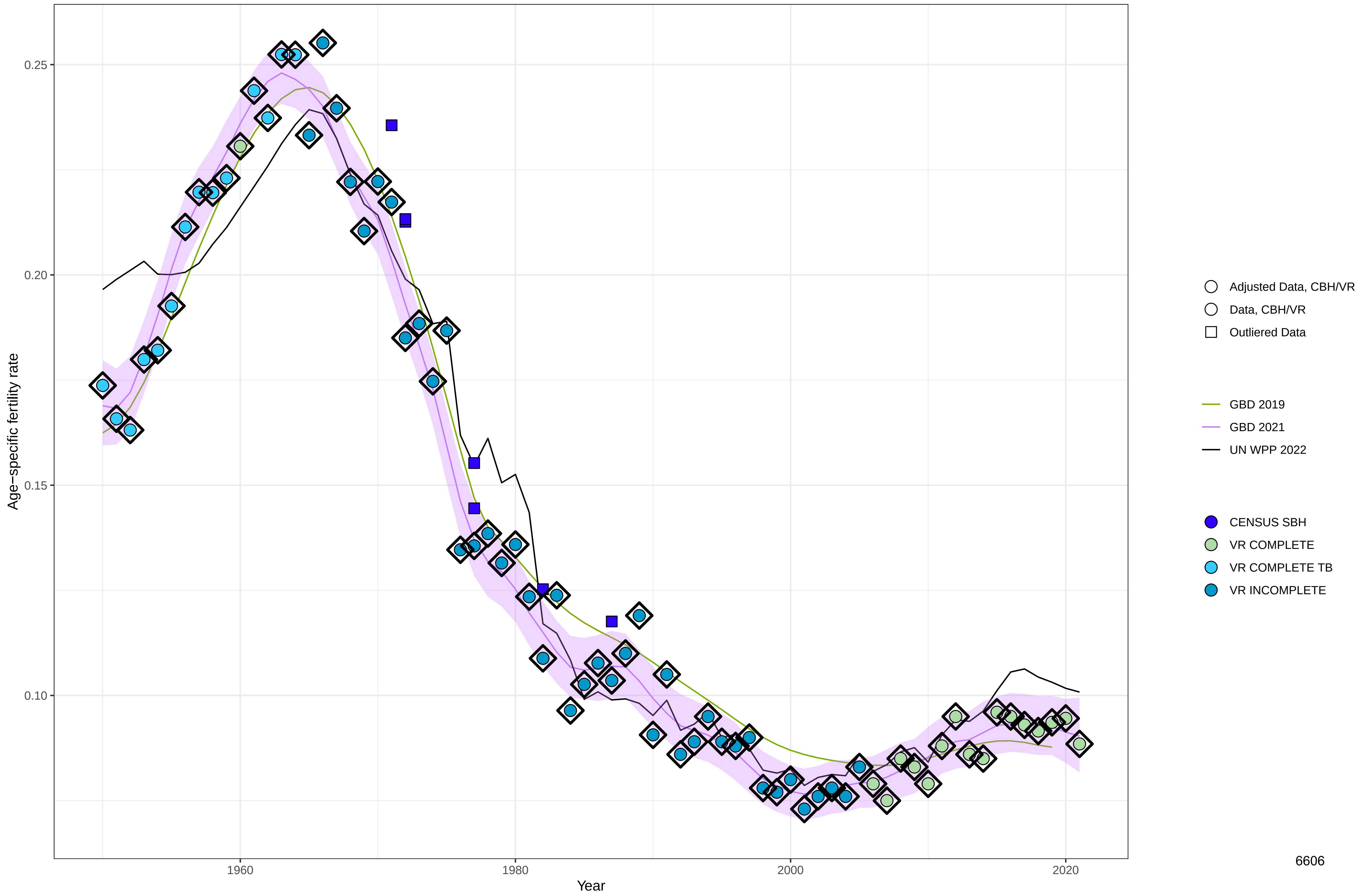

Age−specific fertility fitted trend lines, 1950−2021: Seychelles, Ages: 35−39
Data points enclosed with a diamond are reference sources

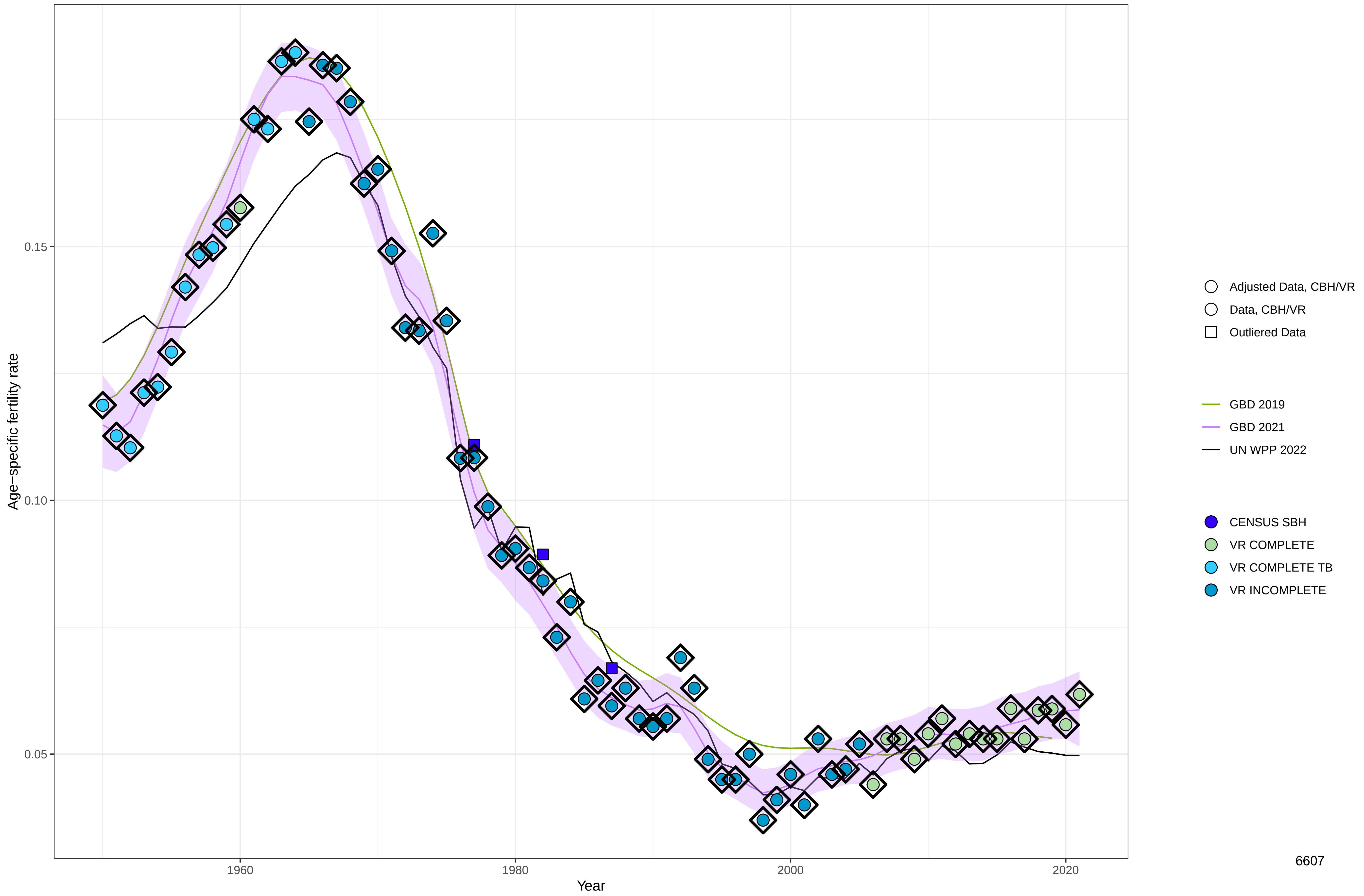

Age−specific fertility fitted trend lines, 1950−2021: Seychelles, Ages: 40−44
Data points enclosed with a diamond are reference sources

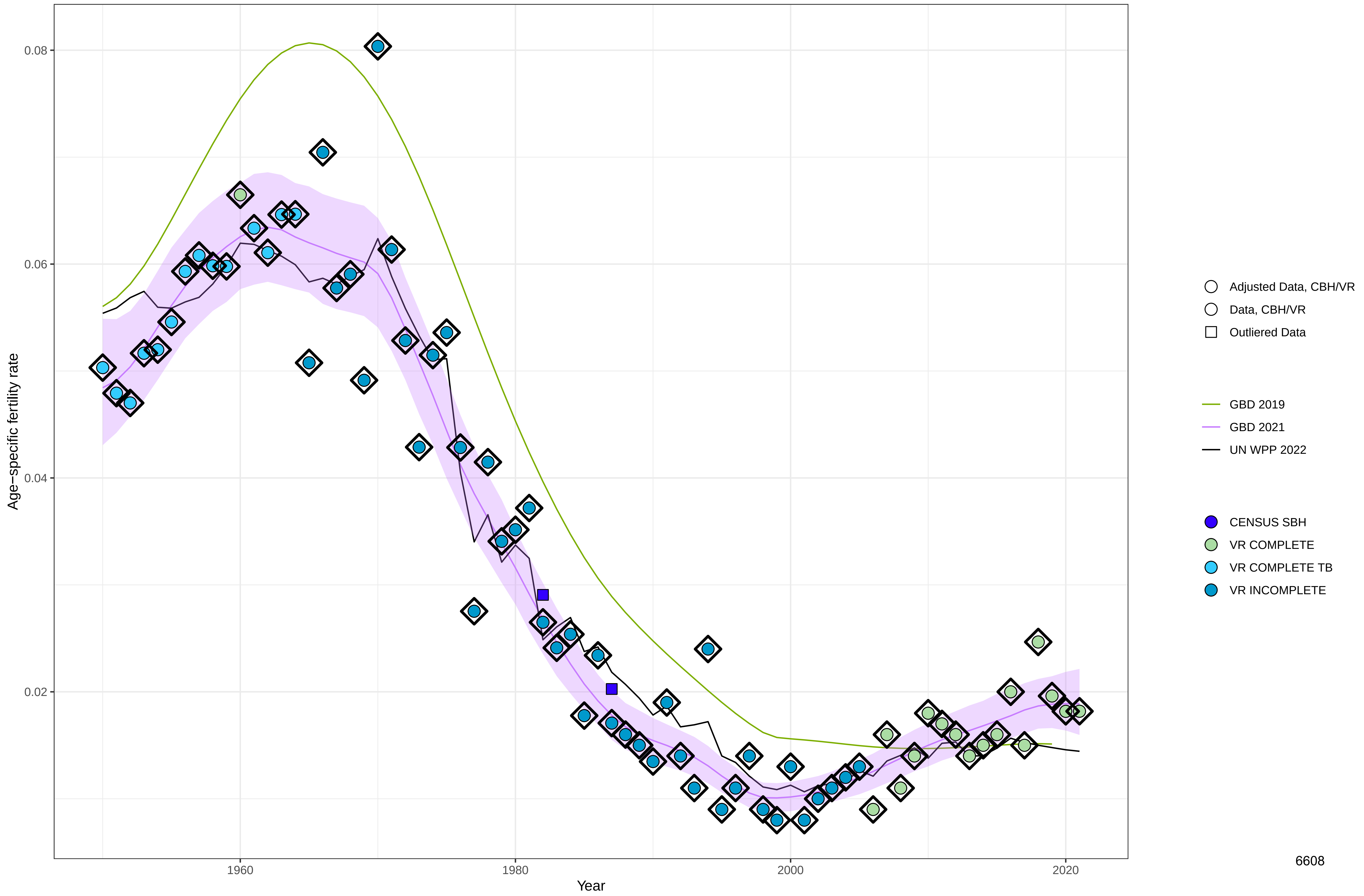

Age−specific fertility fitted trend lines, 1950−2021: Seychelles, Ages: 45−49
Data points enclosed with a diamond are reference sources

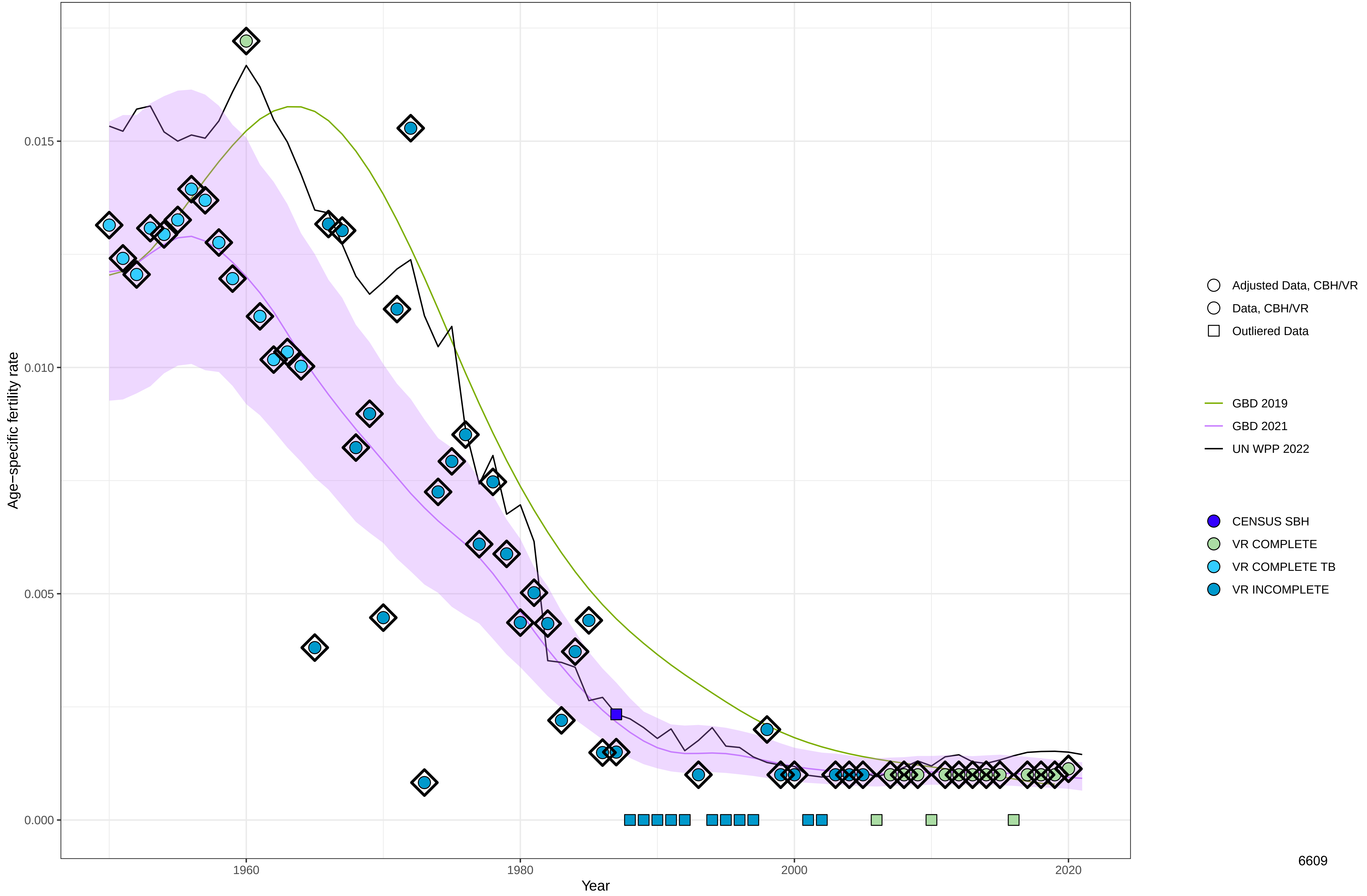

Age−specific fertility fitted trend lines, 1950−2021: Seychelles, Ages: 50−54
Data points enclosed with a diamond are reference sources

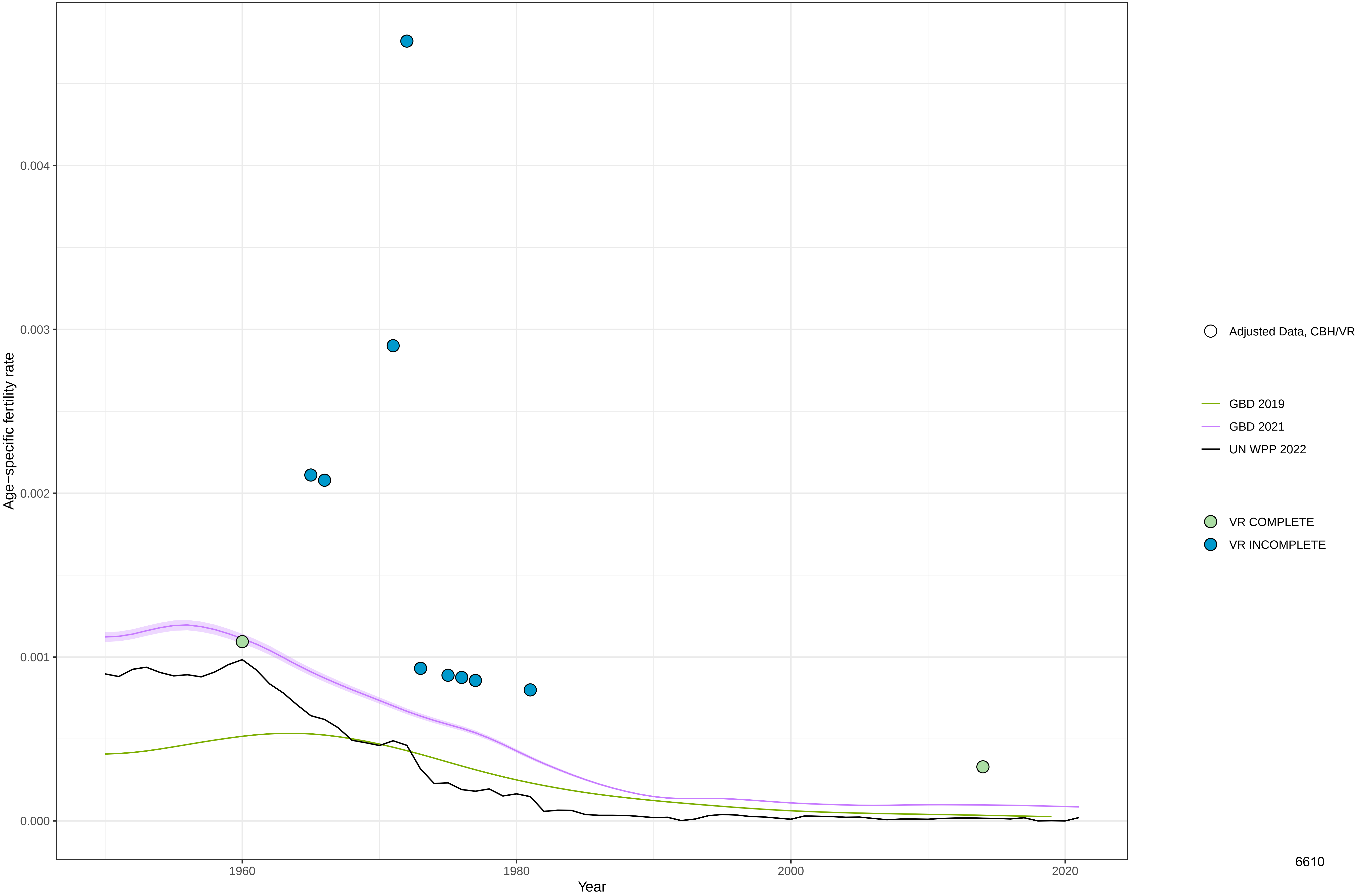

Total fertility rate fitted trend lines, 1950−2021: Seychelles TFR
Data points enclosed with a diamond are reference sources

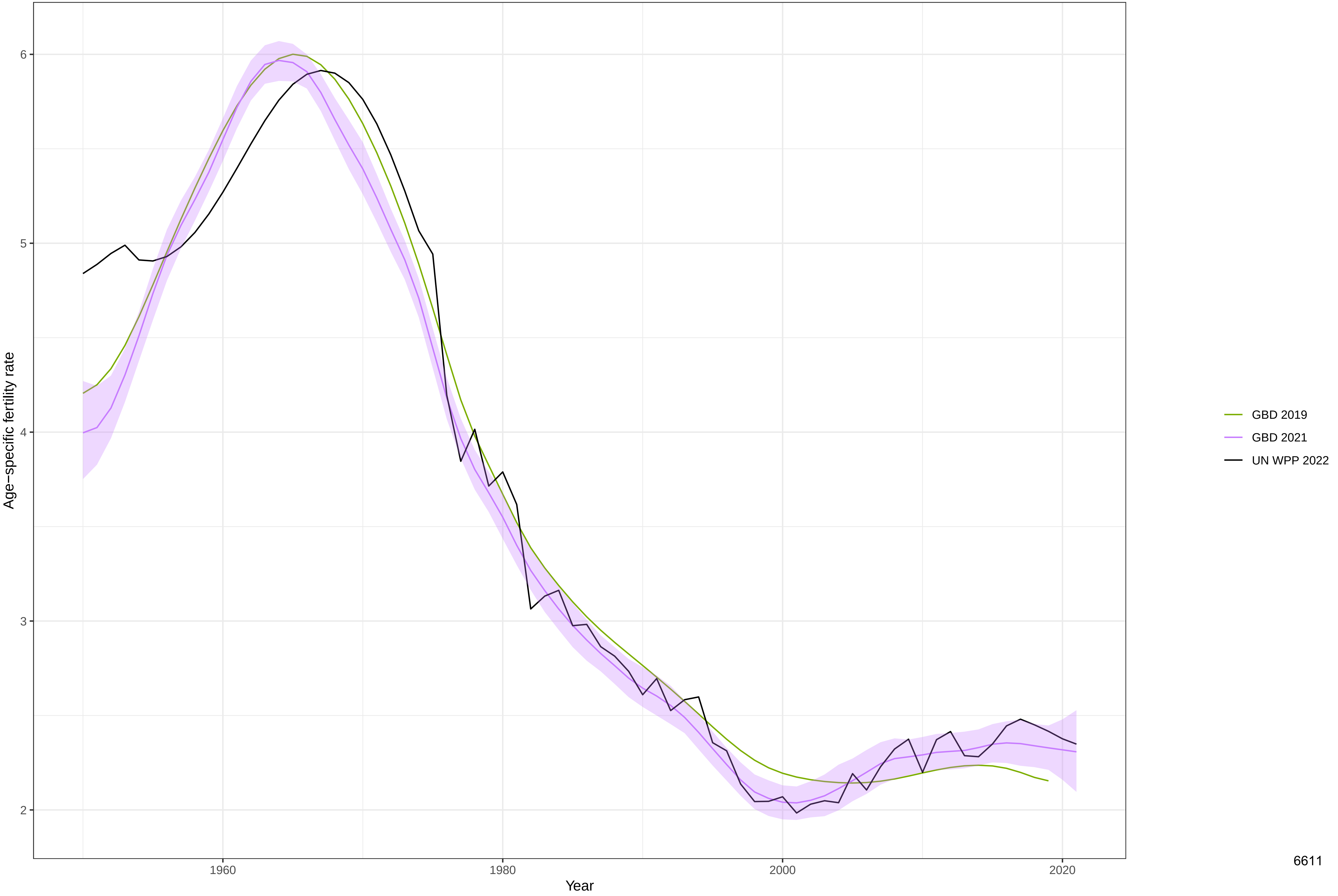

Age−specific fertility fitted trend lines, 1950−2021: Sri Lanka, Ages: 10−14
Data points enclosed with a diamond are reference sources

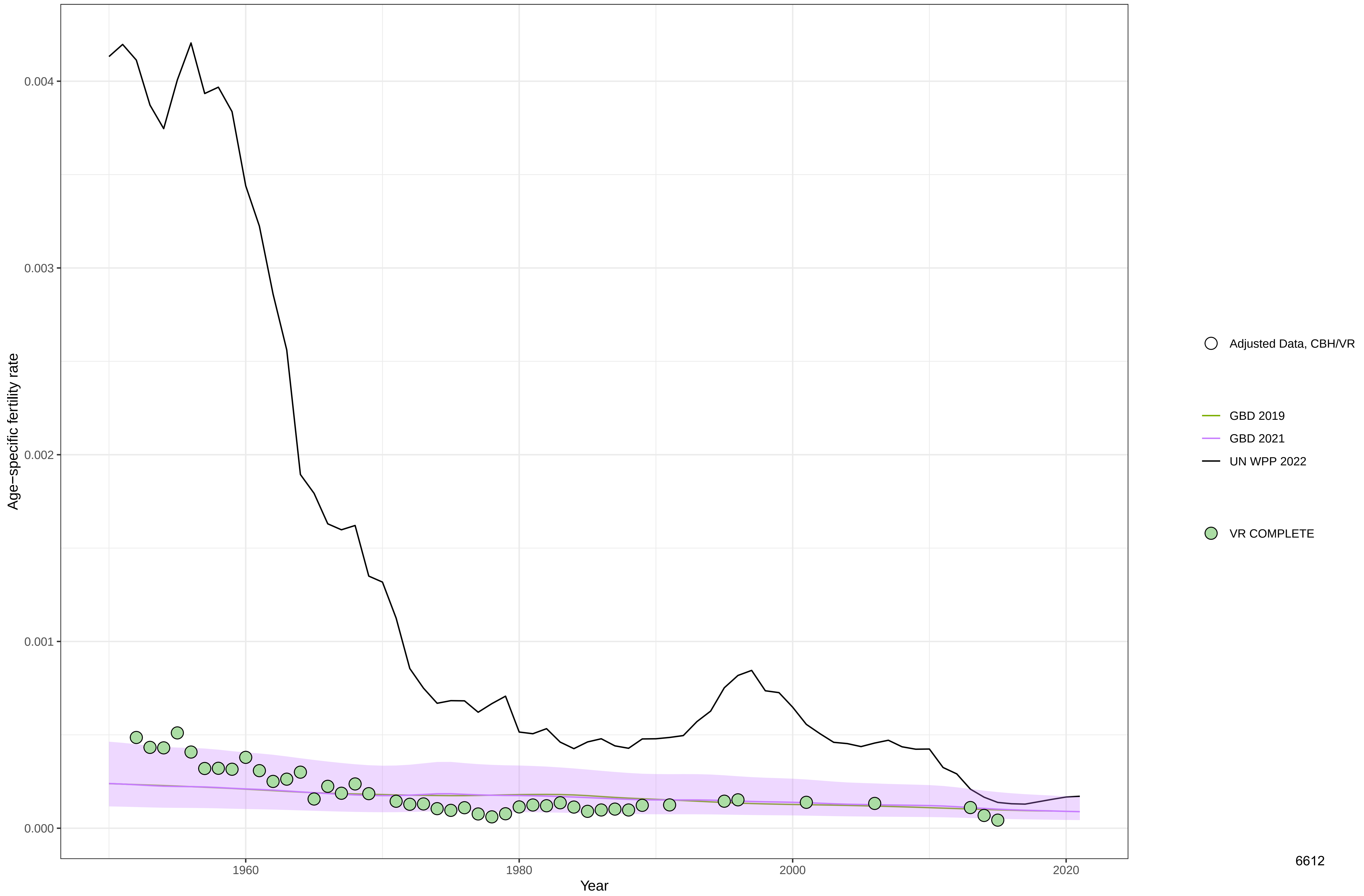

Age−specific fertility fitted trend lines, 1950−2021: Sri Lanka, Ages: 15−19
Data points enclosed with a diamond are reference sources

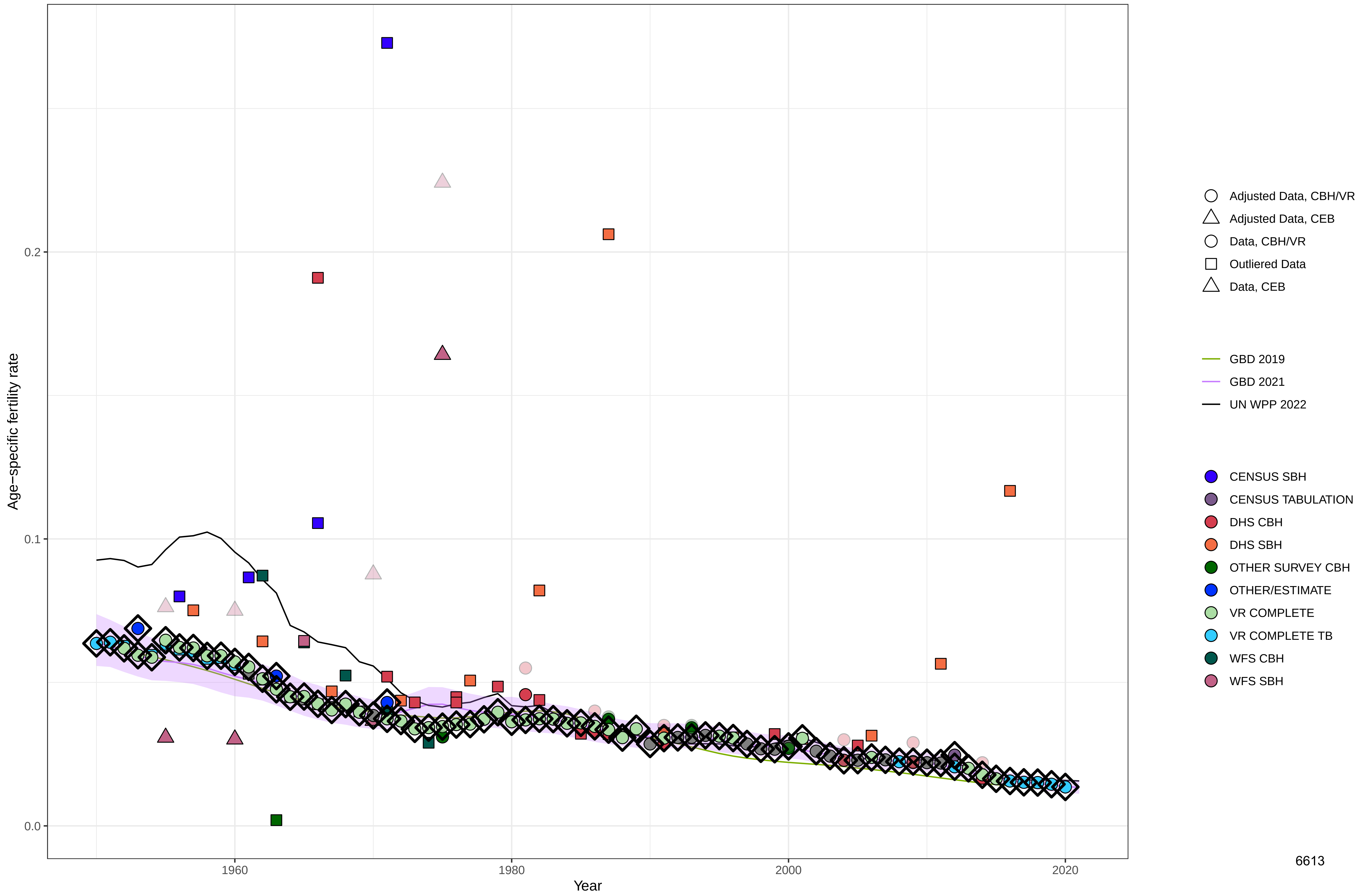

Age−specific fertility fitted trend lines, 1950−2021: Sri Lanka, Ages: 20−24
Data points enclosed with a diamond are reference sources

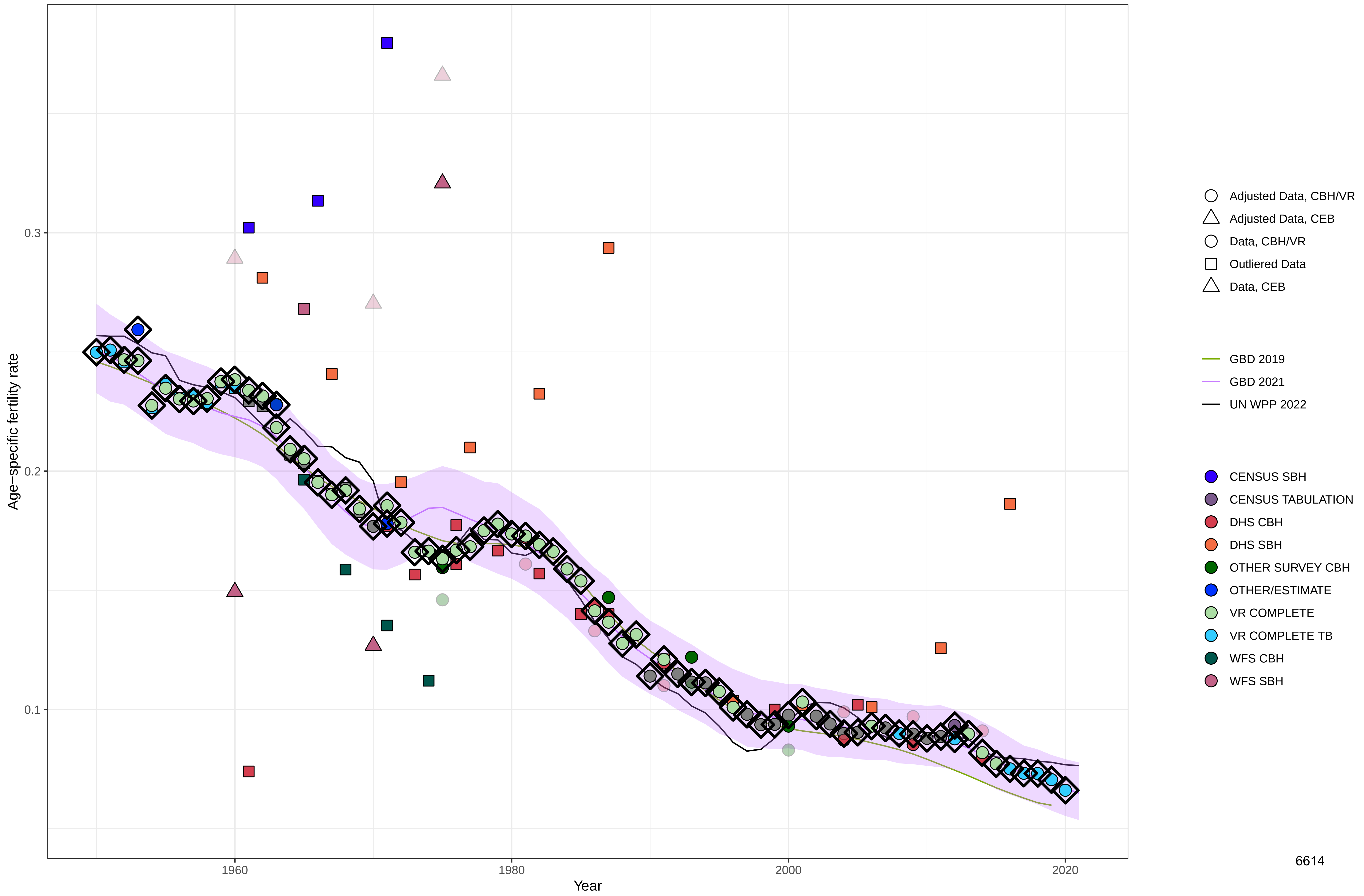

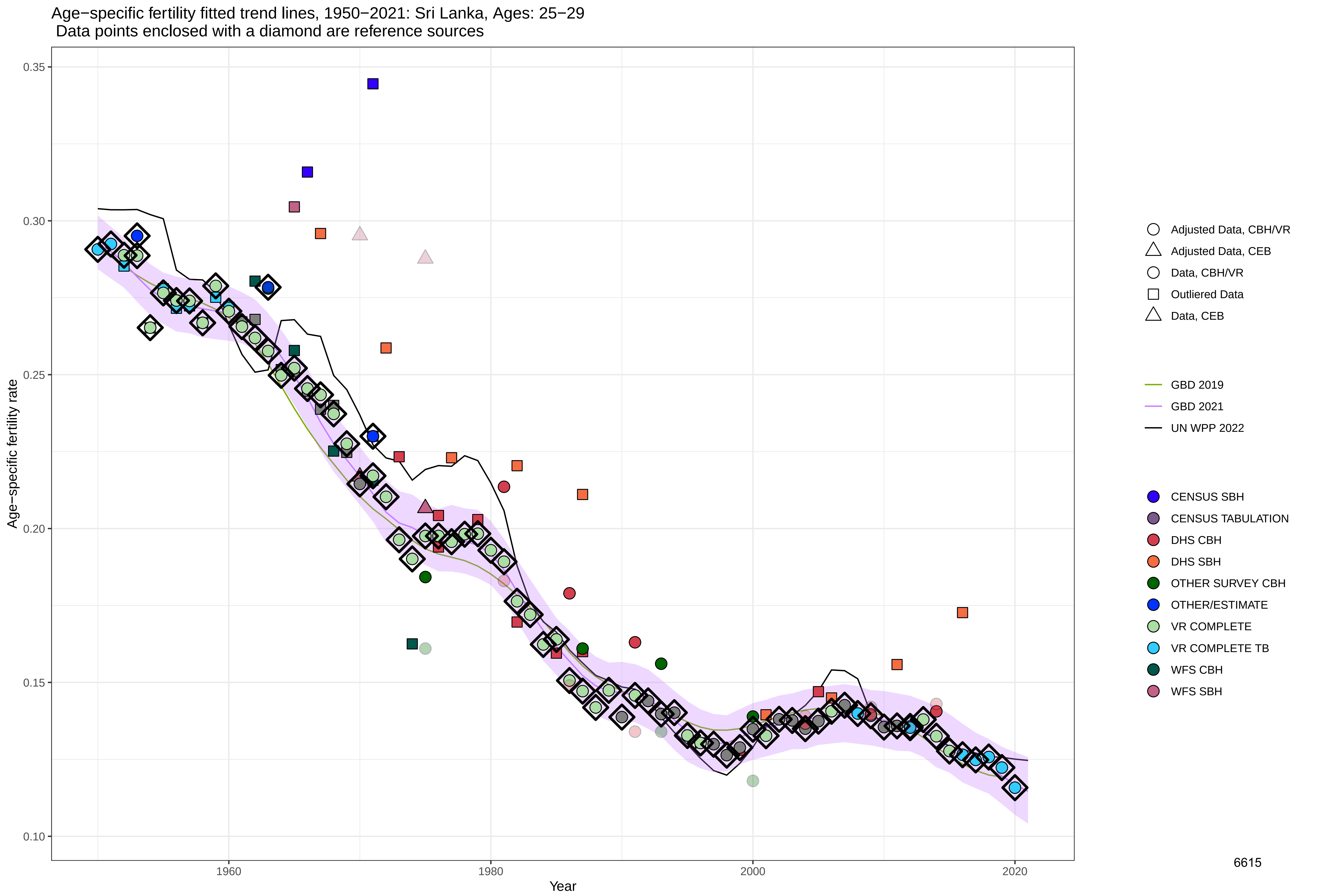
Age−specific fertility fitted trend lines, 1950−2021: Sri Lanka, Ages: 25−29
Data points enclosed with a diamond are reference sources
Age−specific fertility rate
Year
0.35
0.30
0.25
0.20
0.15
0.10
1960
1980
2000
2020
Adjusted Data, CBH/VR
Adjusted Data, CEB
Data, CBH/VR
Outliered Data
Data, CEB
GBD 2019
GBD 2021
UN WPP 2022
CENSUS SBH
CENSUS TABULATION
DHS CBH
DHS SBH
OTHER SURVEY CBH
OTHER/ESTIMATE
VR COMPLETE
VR COMPLETE TB
WFS CBH
WFS SBH

Age−specific fertility fitted trend lines, 1950−2021: Sri Lanka, Ages: 30−34
Data points enclosed with a diamond are reference sources

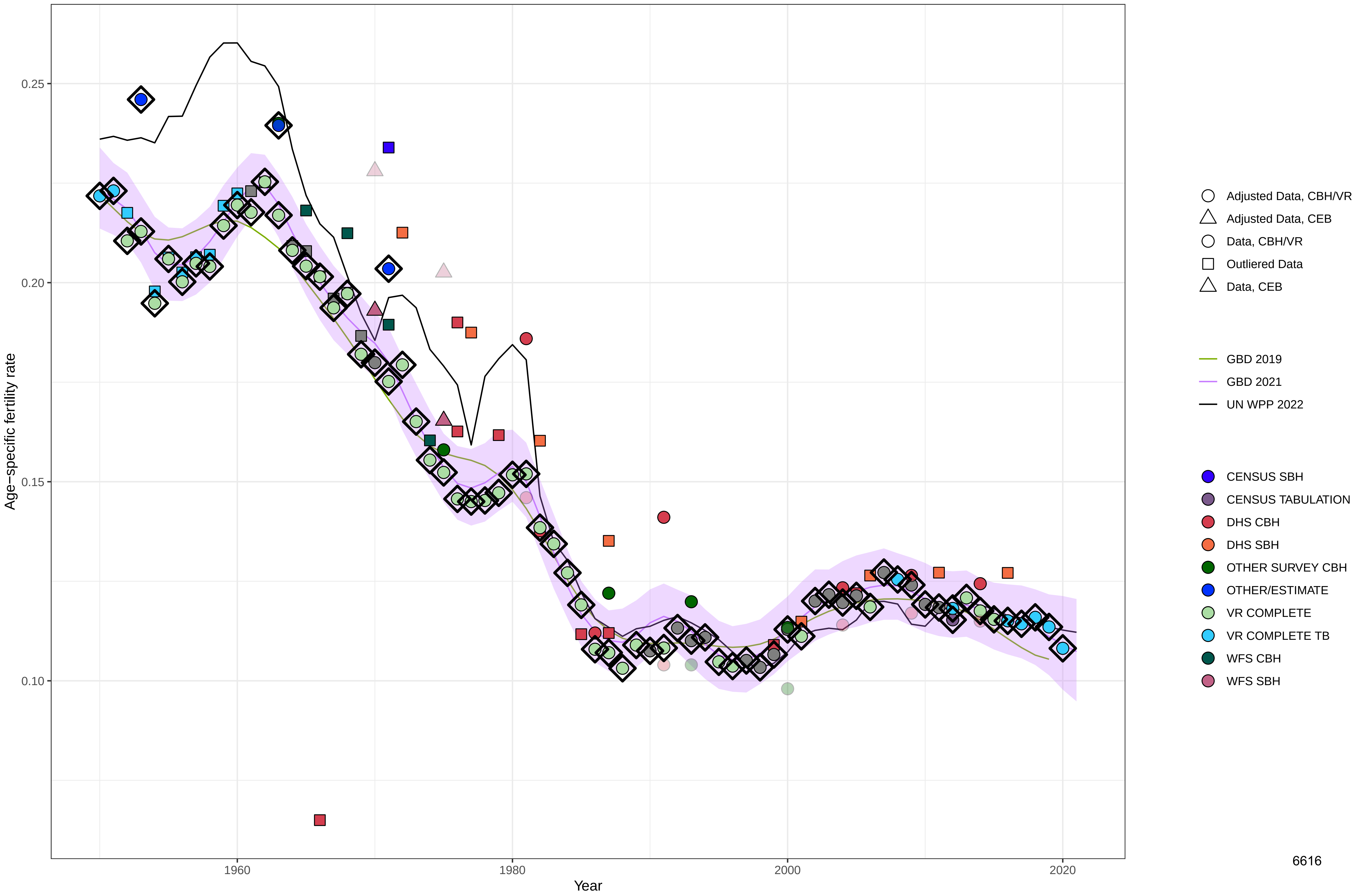

Age−specific fertility fitted trend lines, 1950−2021: Sri Lanka, Ages: 35−39
Data points enclosed with a diamond are reference sources

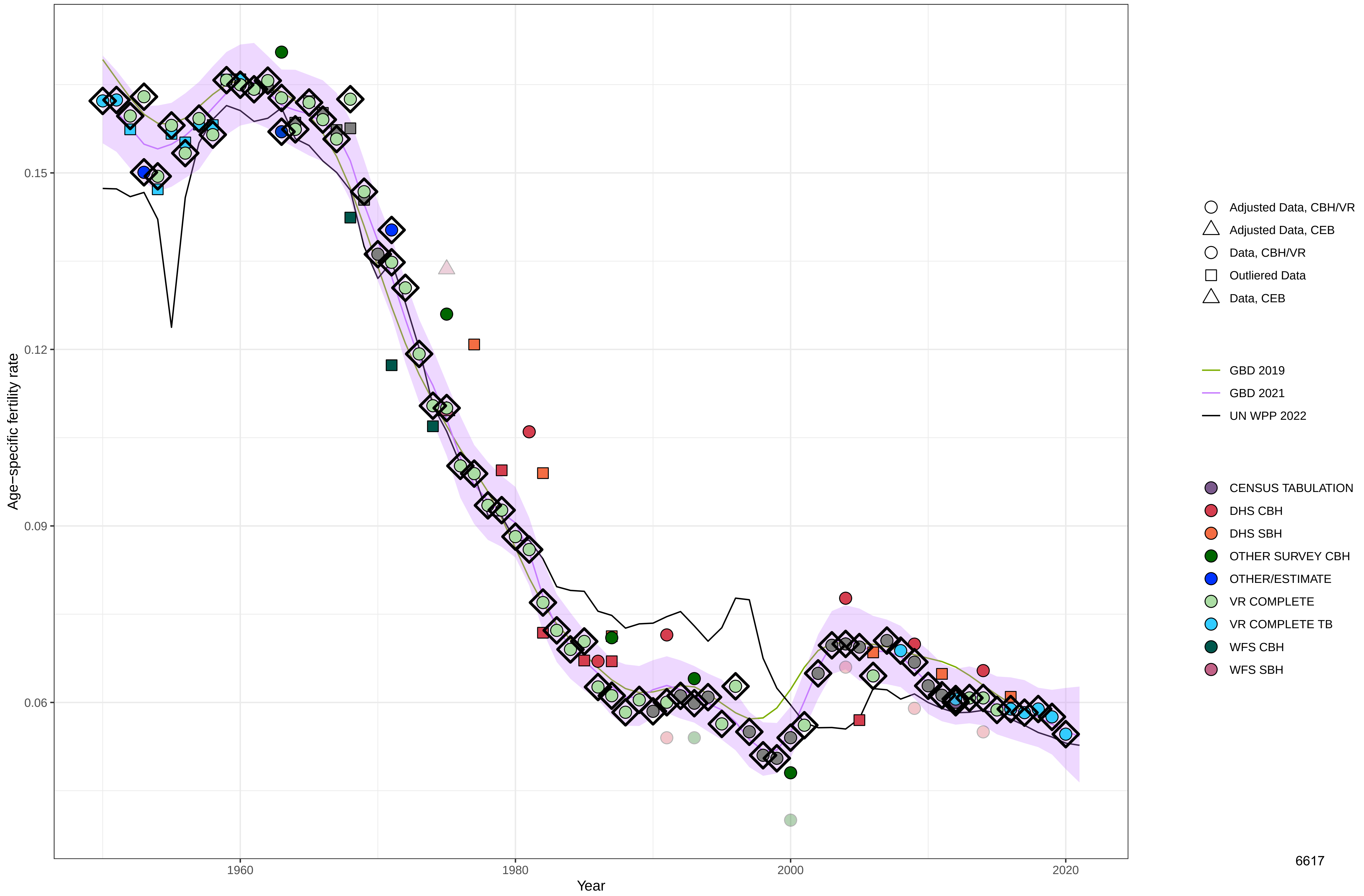

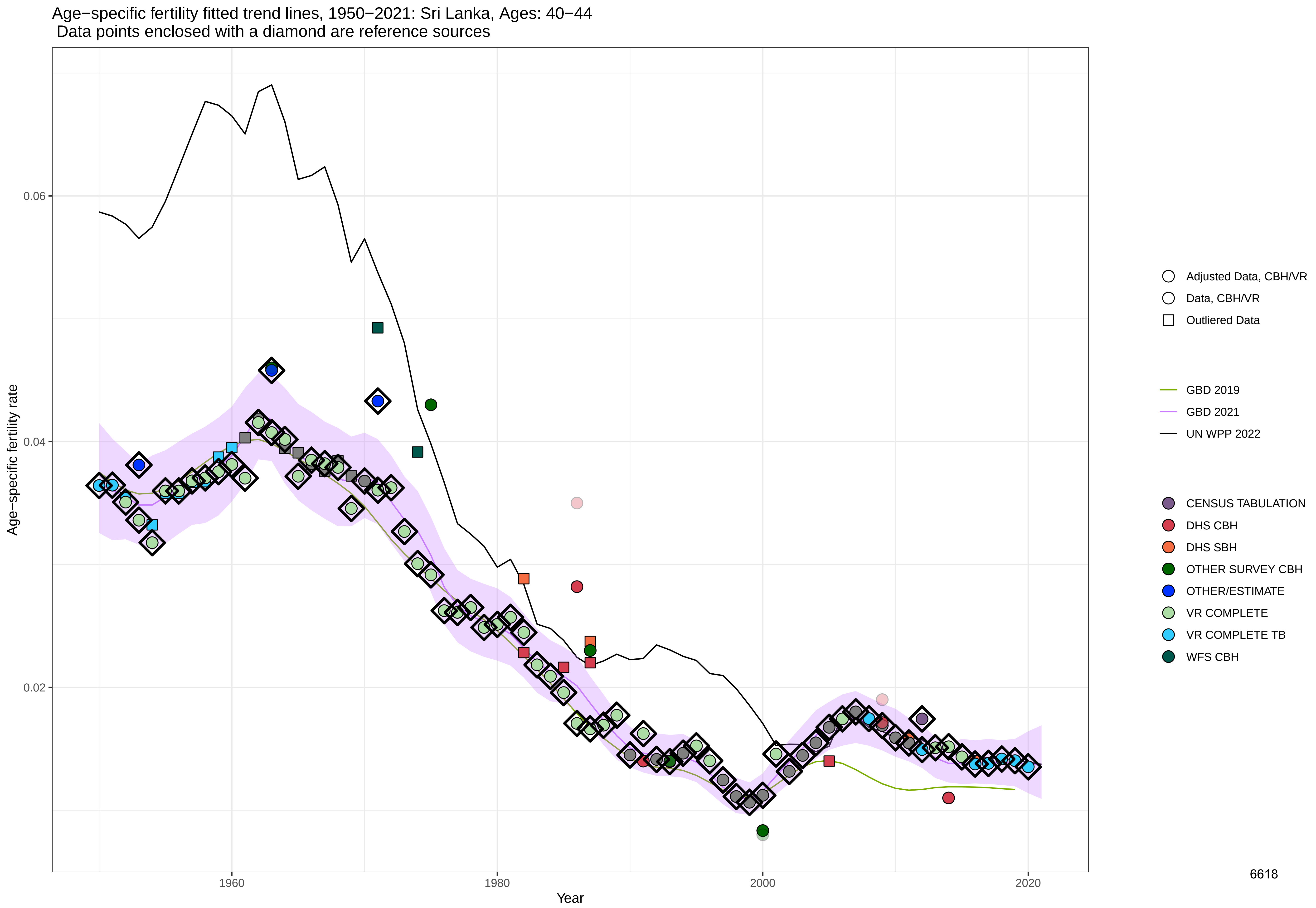

Age−specific fertility fitted trend lines, 1950−2021: Sri Lanka, Ages: 40−44
Data points enclosed with a diamond are reference sources
Age−specific fertility rate
0.06
0.04
0.02
1960
1980
2000
2020
Year
Adjusted Data, CBH/VR
Data, CBH/VR
Outliered Data
GBD 2019
GBD 2021
UN WPP 2022
CENSUS TABULATION
DHS CBH
DHS SBH
OTHER SURVEY CBH
OTHER/ESTIMATE
VR COMPLETE
VR COMPLETE TB
WFS CBH

Age−specific fertility fitted trend lines, 1950−2021: Sri Lanka, Ages: 45−49
Data points enclosed with a diamond are reference sources

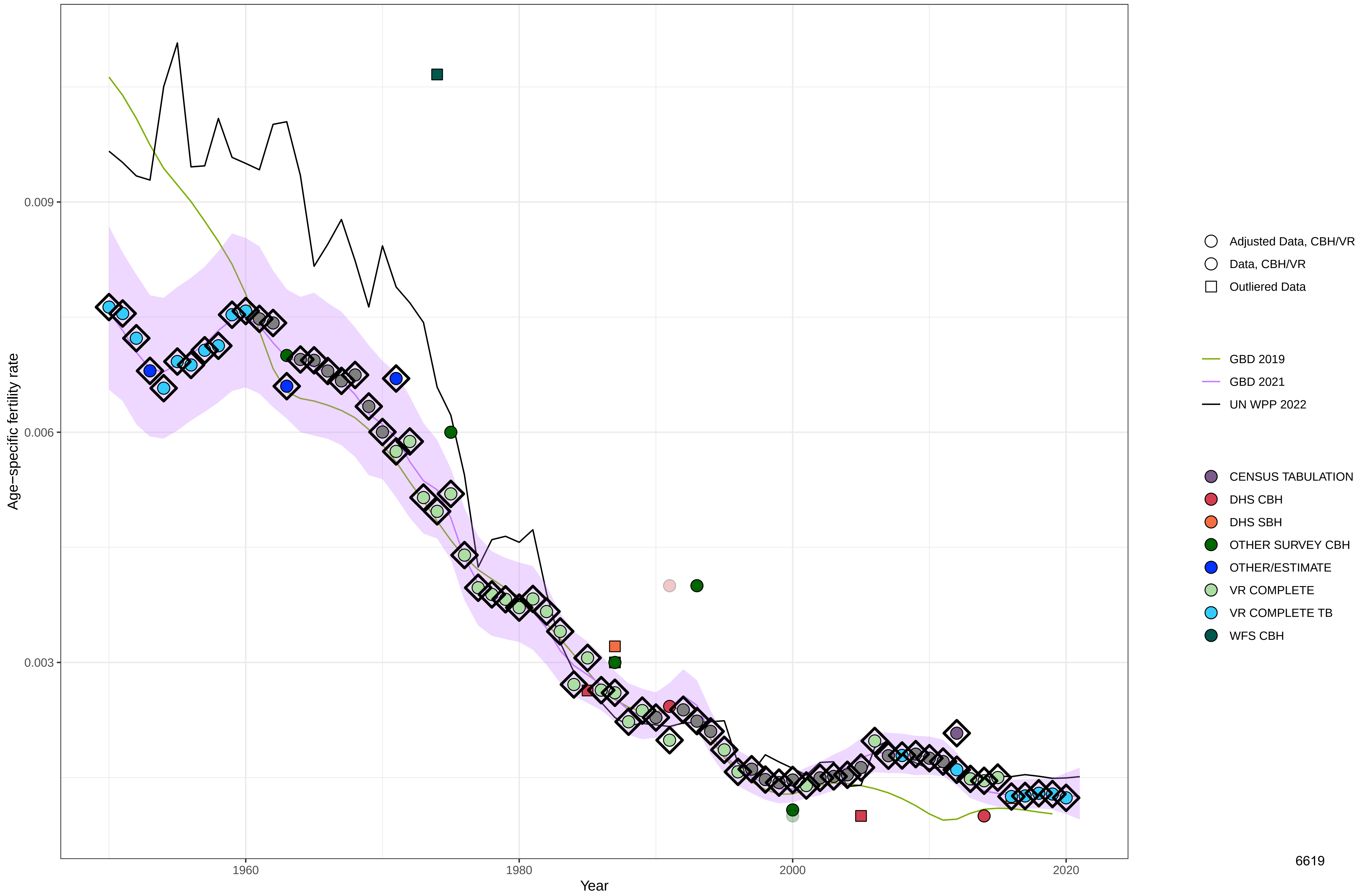

Age−specific fertility fitted trend lines, 1950−2021: Sri Lanka, Ages: 50−54
 Data points enclosed with a diamond are reference sources

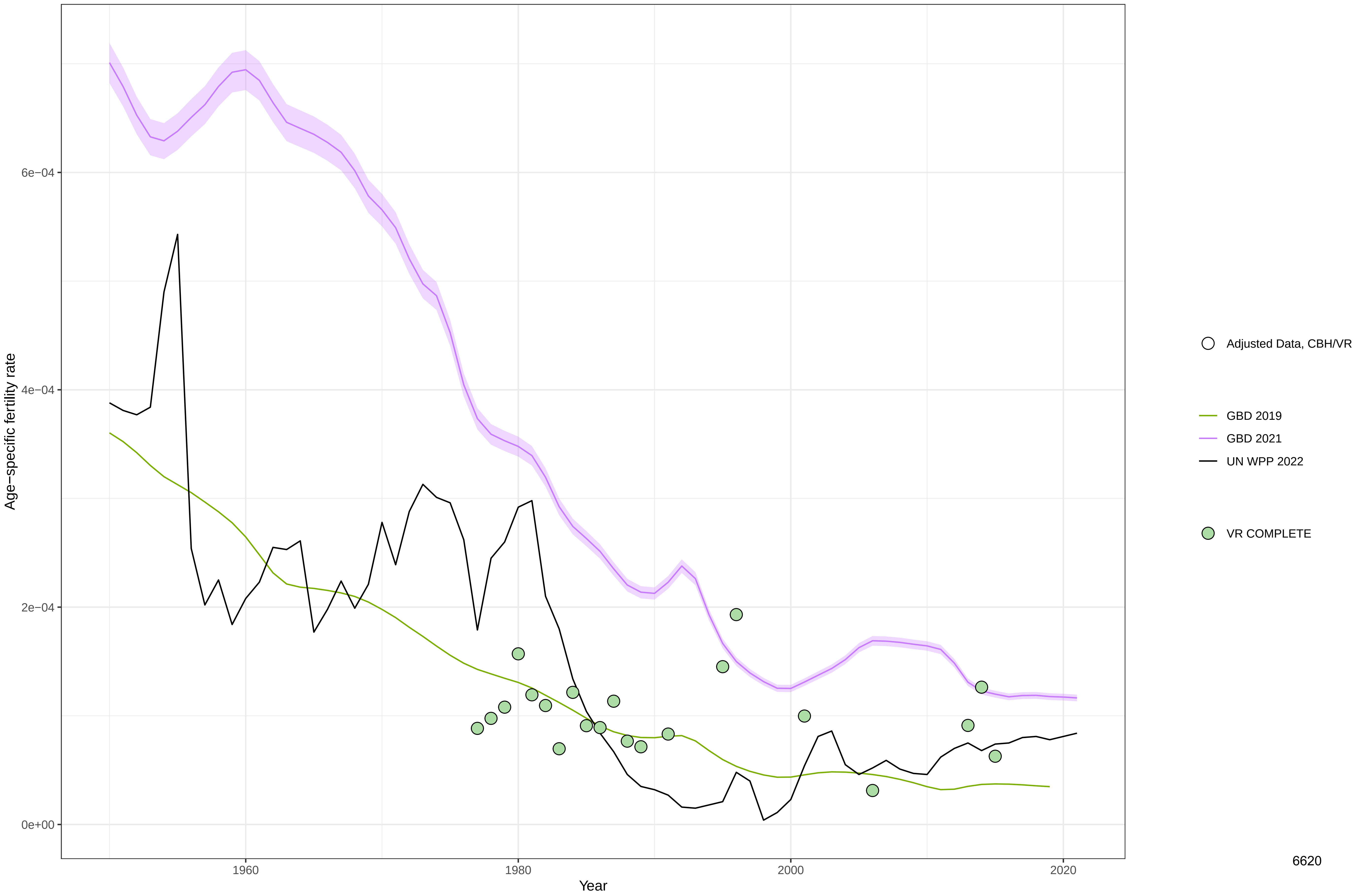

Total fertility rate fitted trend lines, 1950−2021: Sri Lanka TFR
Data points enclosed with a diamond are reference sources

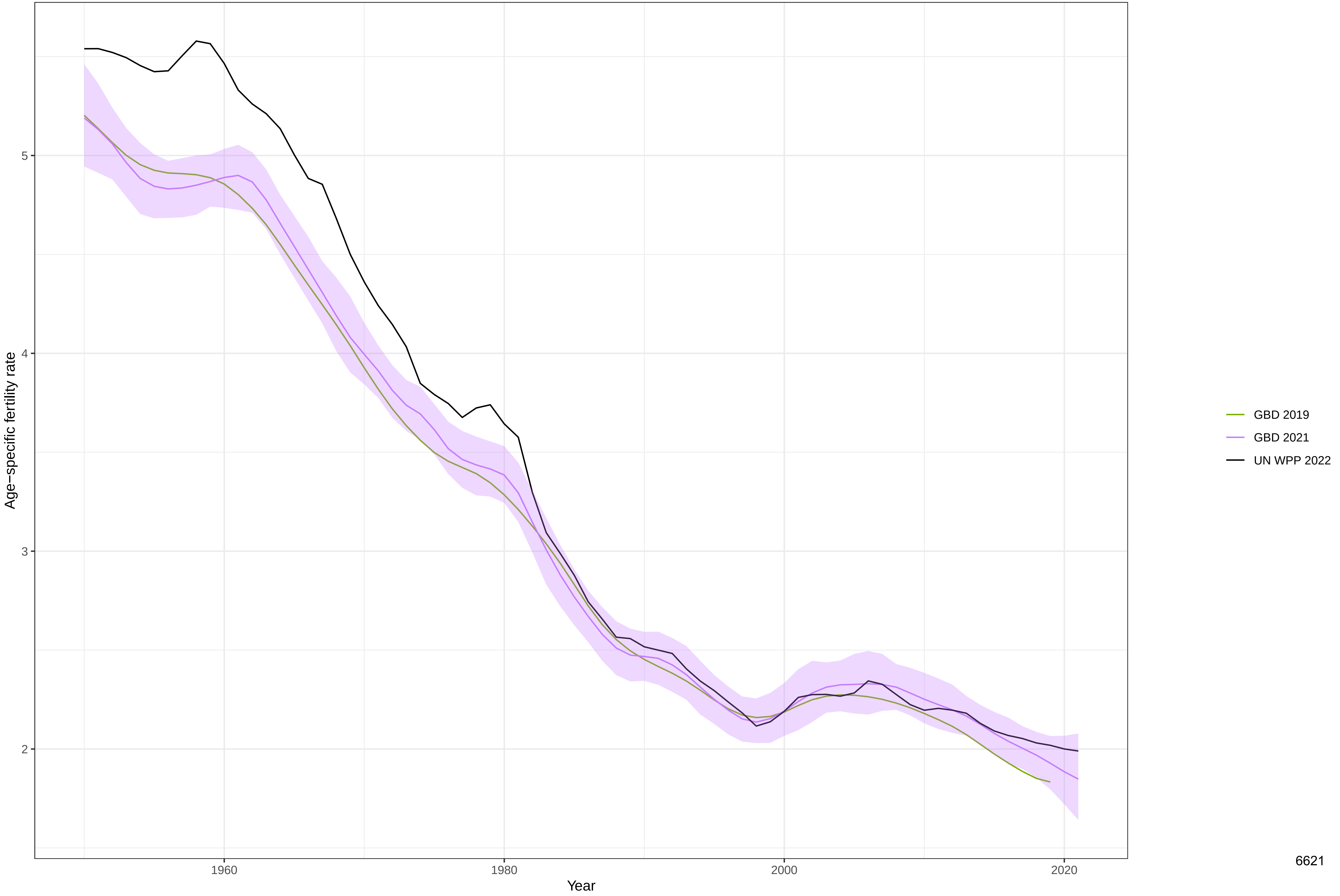

Age−specific fertility fitted trend lines, 1950−2021: Thailand, Ages: 10−14
Data points enclosed with a diamond are reference sources

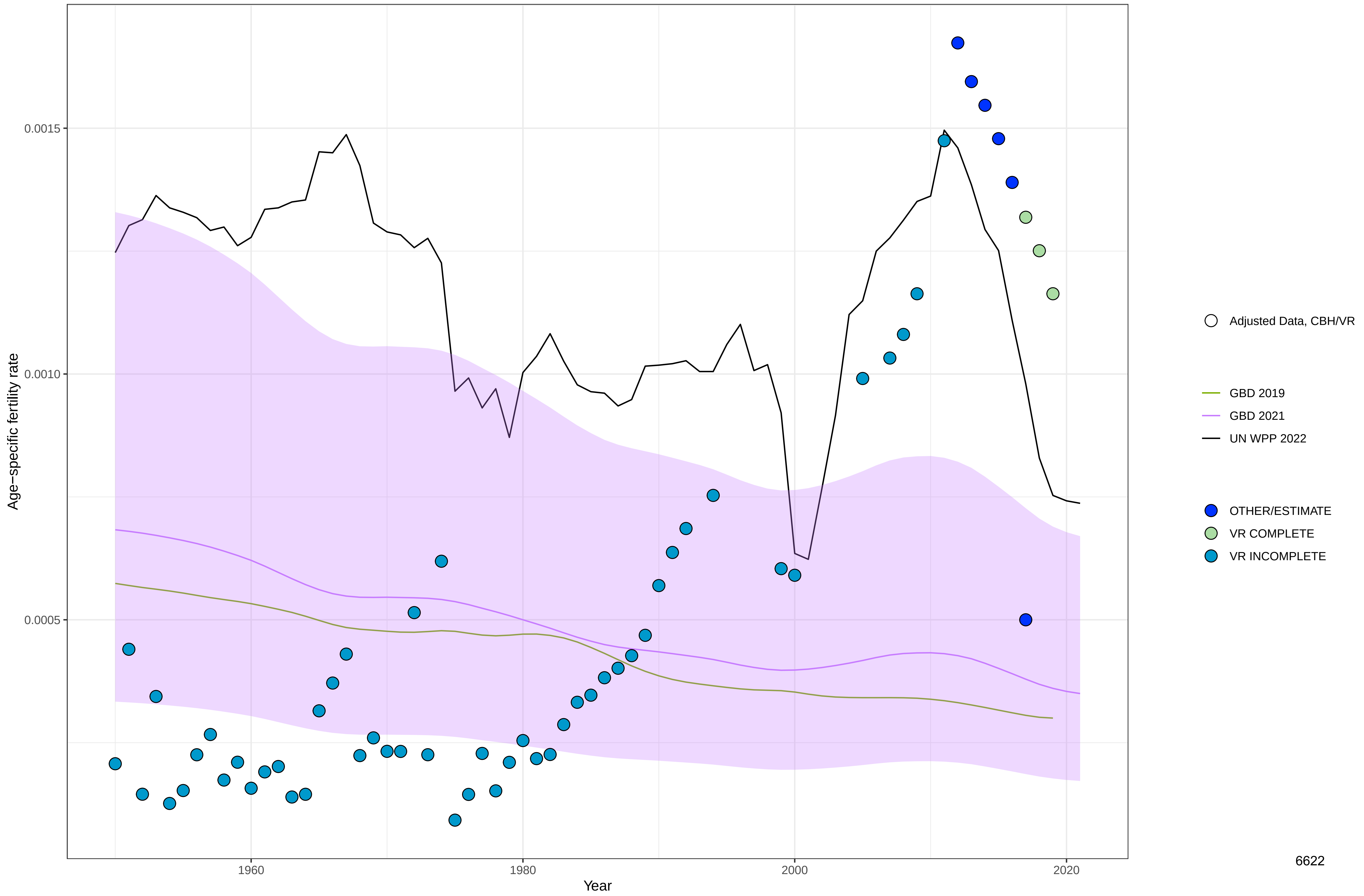

Age−specific fertility fitted trend lines, 1950−2021: Thailand, Ages: 15−19
Data points enclosed with a diamond are reference sources

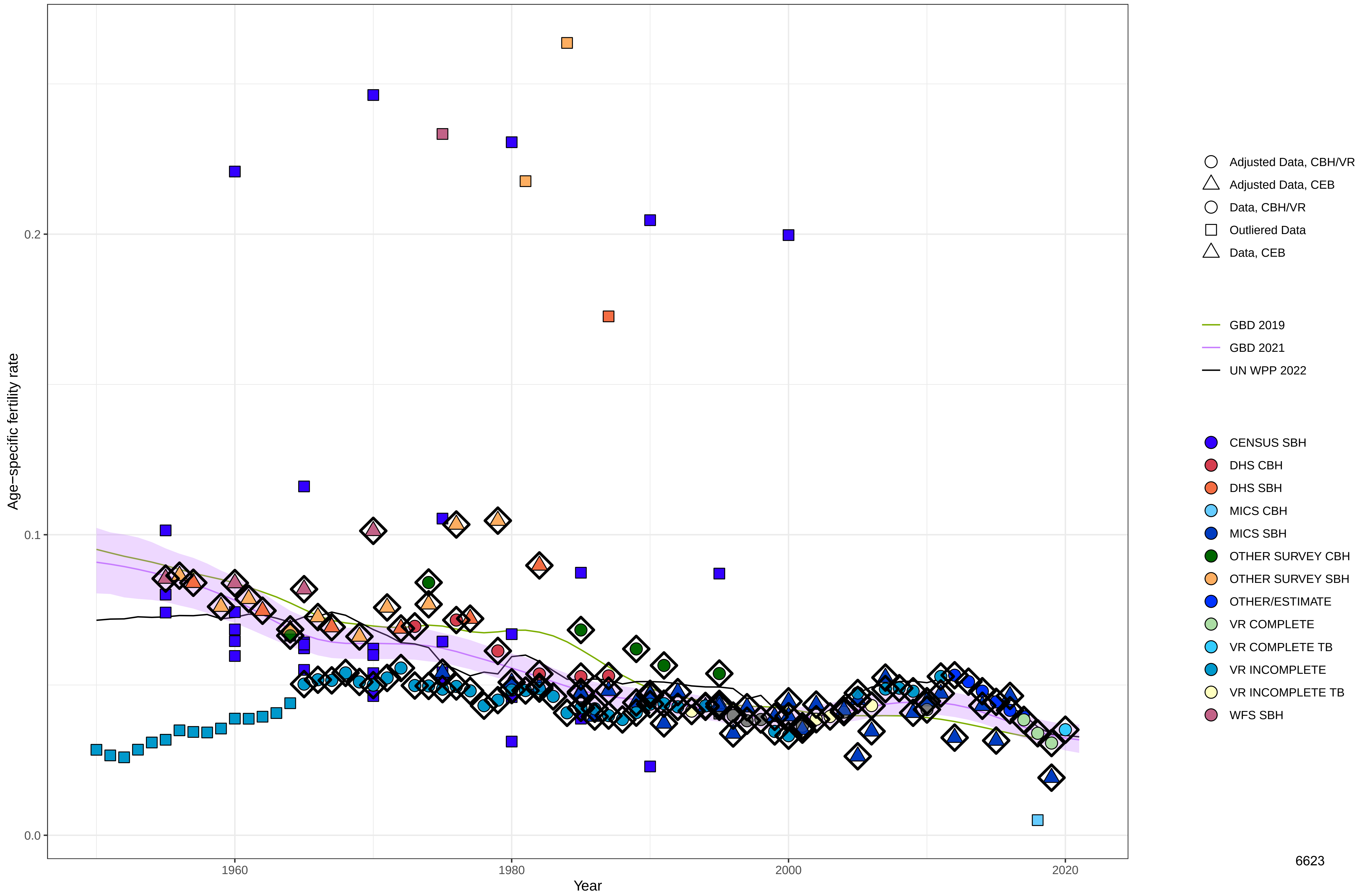

Age−specific fertility fitted trend lines, 1950−2021: Thailand, Ages: 20−24
Data points enclosed with a diamond are reference sources

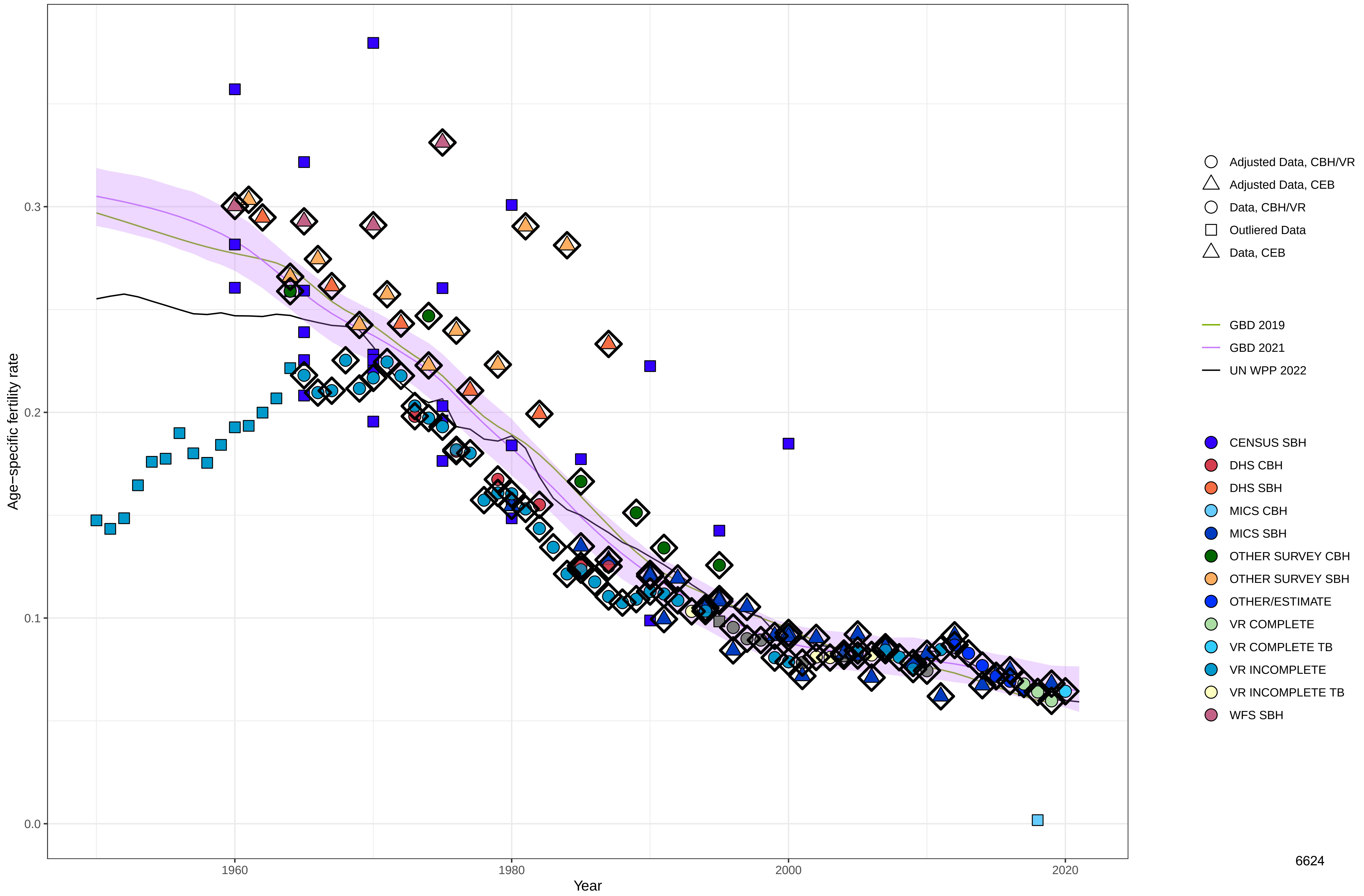

Age−specific fertility fitted trend lines, 1950−2021: Thailand, Ages: 25−29
Data points enclosed with a diamond are reference sources

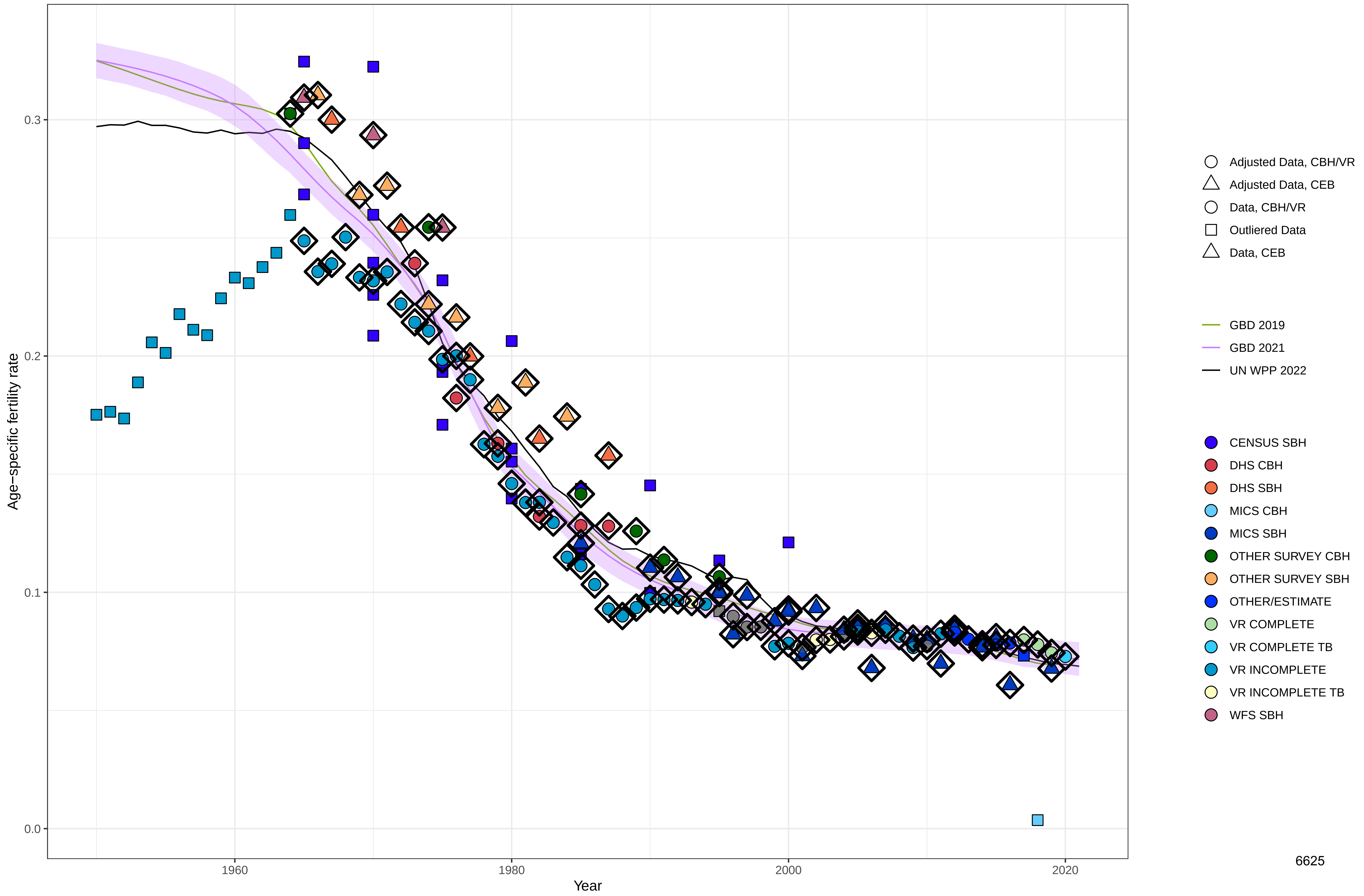

Age−specific fertility fitted trend lines, 1950−2021: Thailand, Ages: 30−34
Data points enclosed with a diamond are reference sources

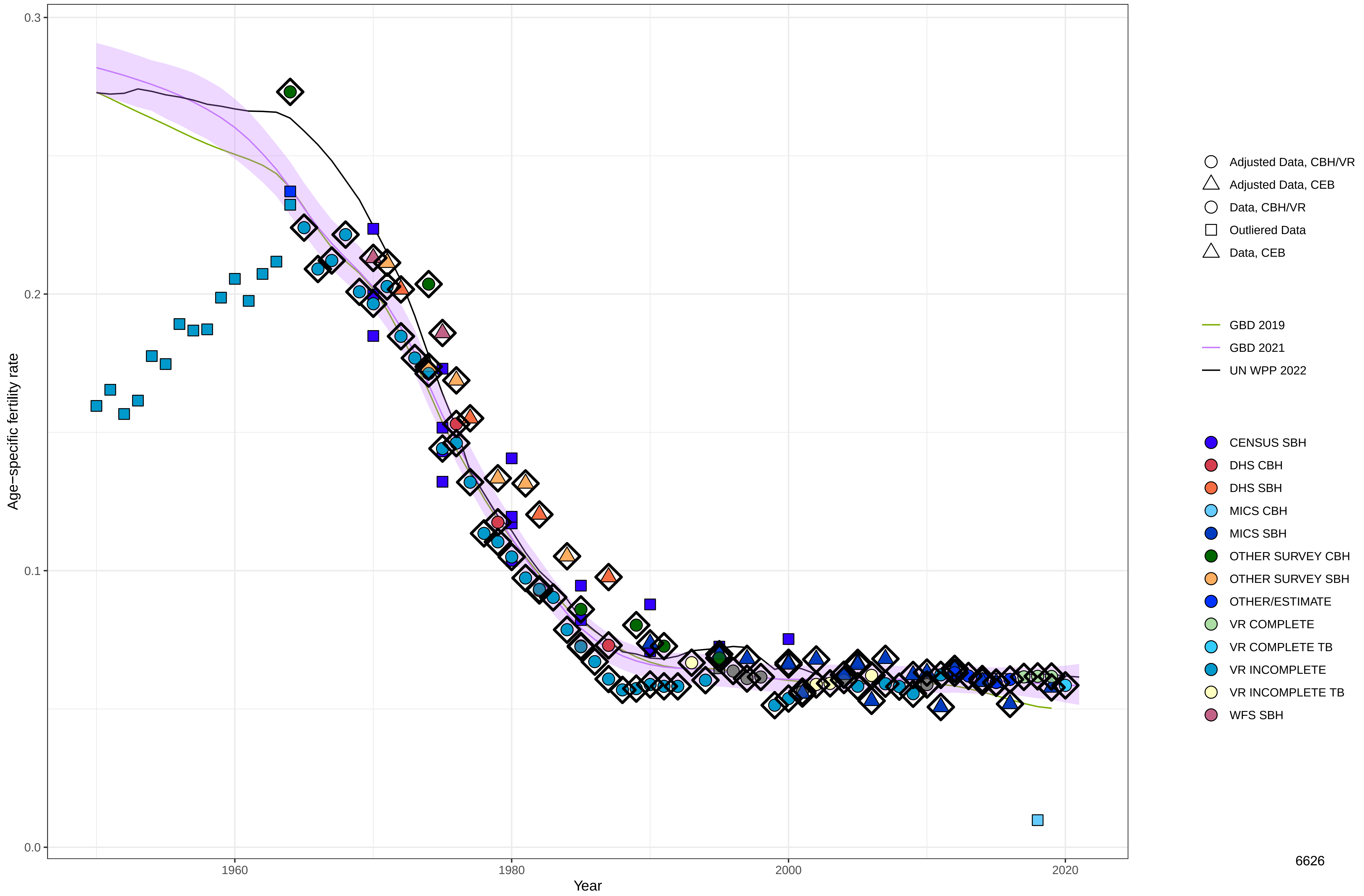

Age−specific fertility fitted trend lines, 1950−2021: Thailand, Ages: 35−39
Data points enclosed with a diamond are reference sources

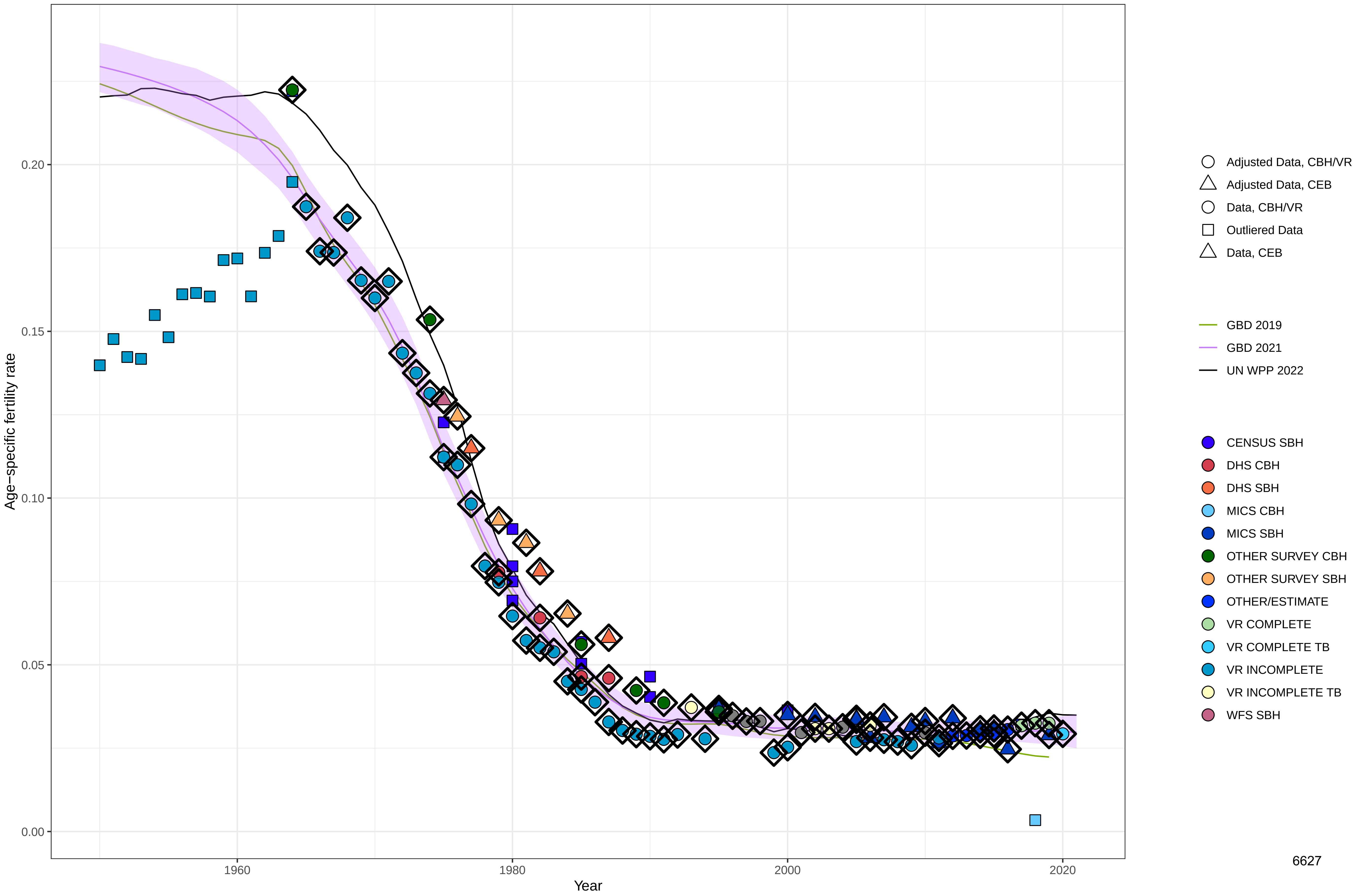

Age−specific fertility fitted trend lines, 1950−2021: Thailand, Ages: 40−44
Data points enclosed with a diamond are reference sources

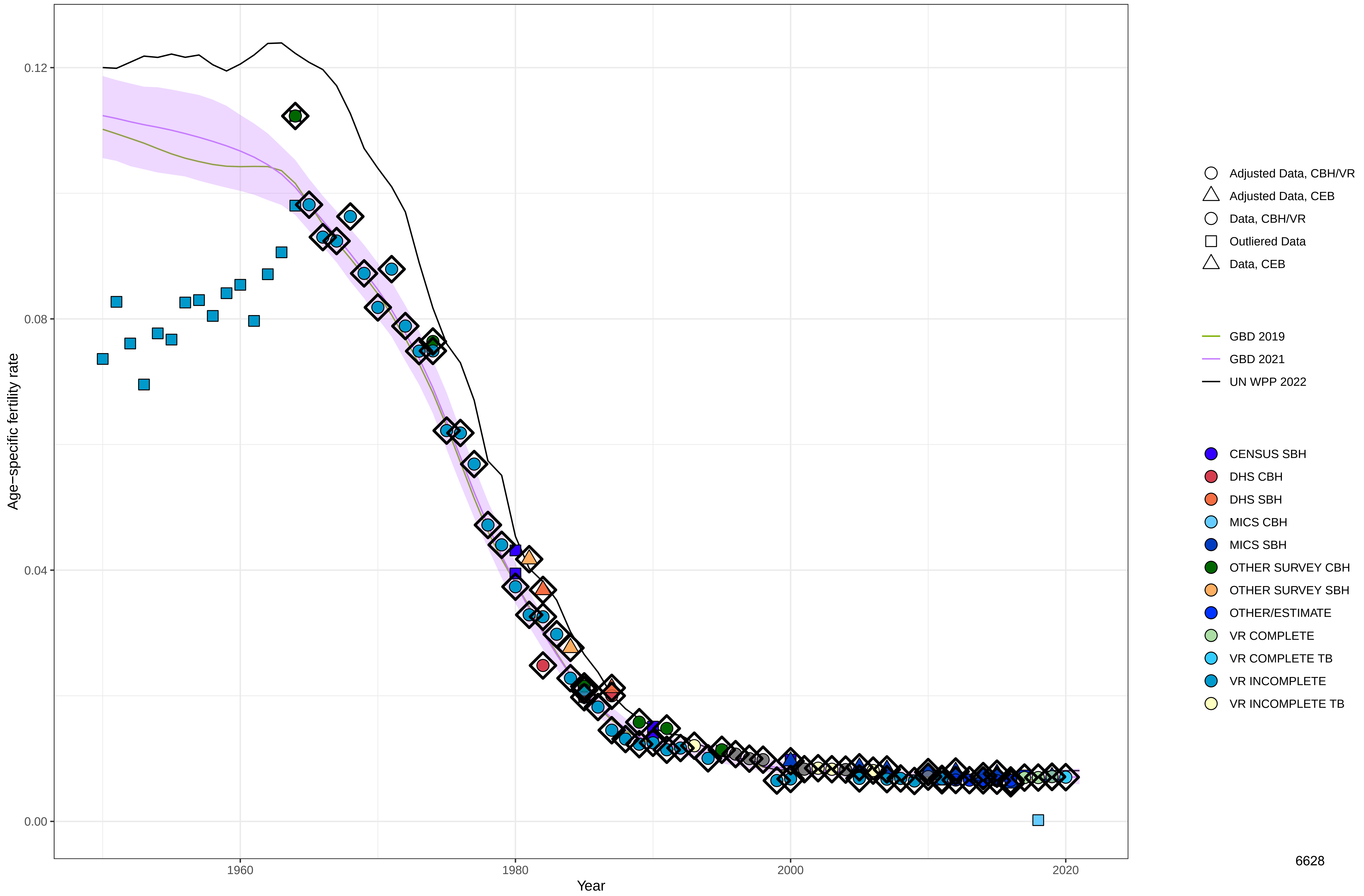

Age−specific fertility fitted trend lines, 1950−2021: Thailand, Ages: 45−49
Data points enclosed with a diamond are reference sources

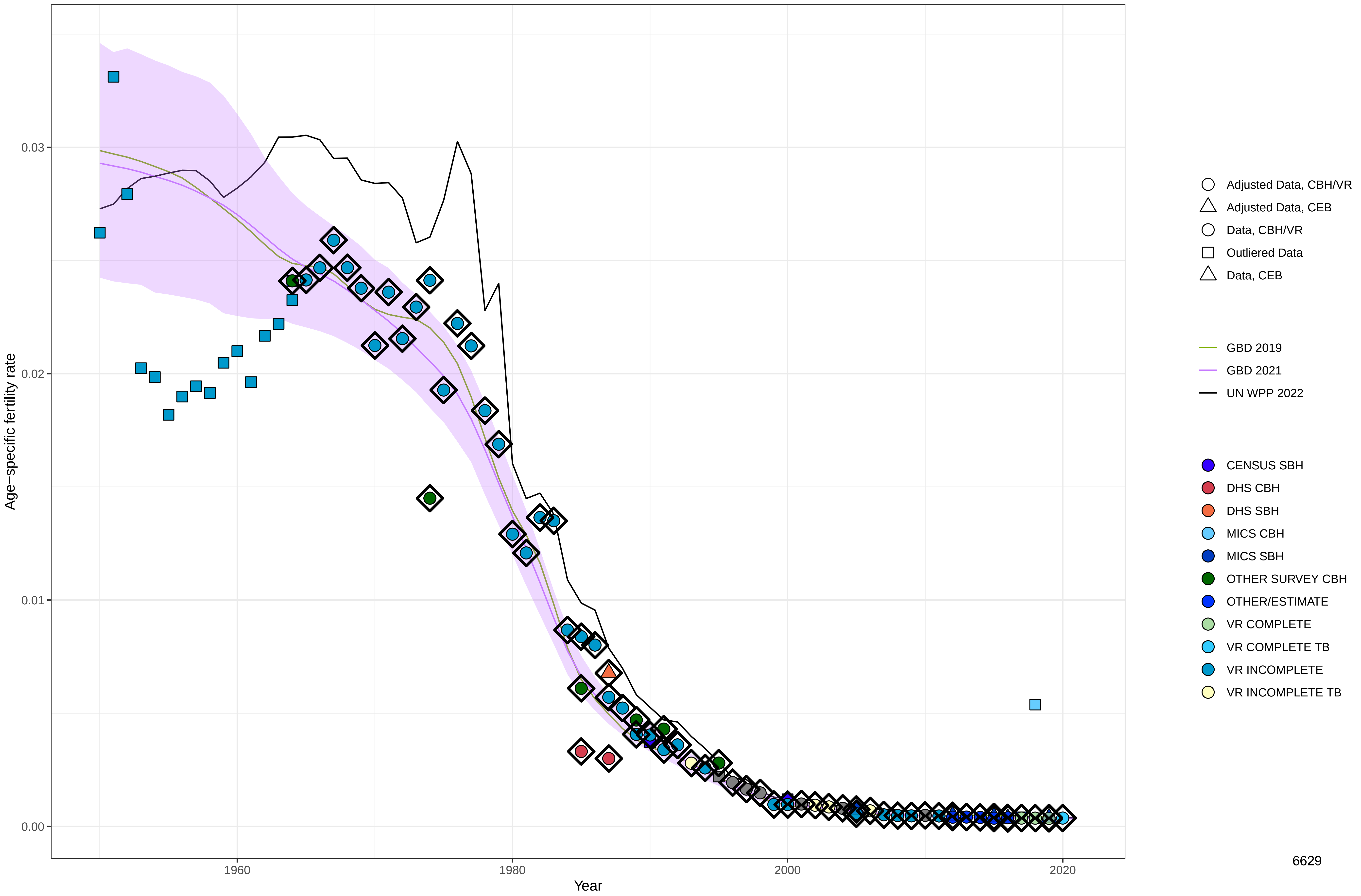

Age−specific fertility fitted trend lines, 1950−2021: Thailand, Ages: 50−54
Data points enclosed with a diamond are reference sources

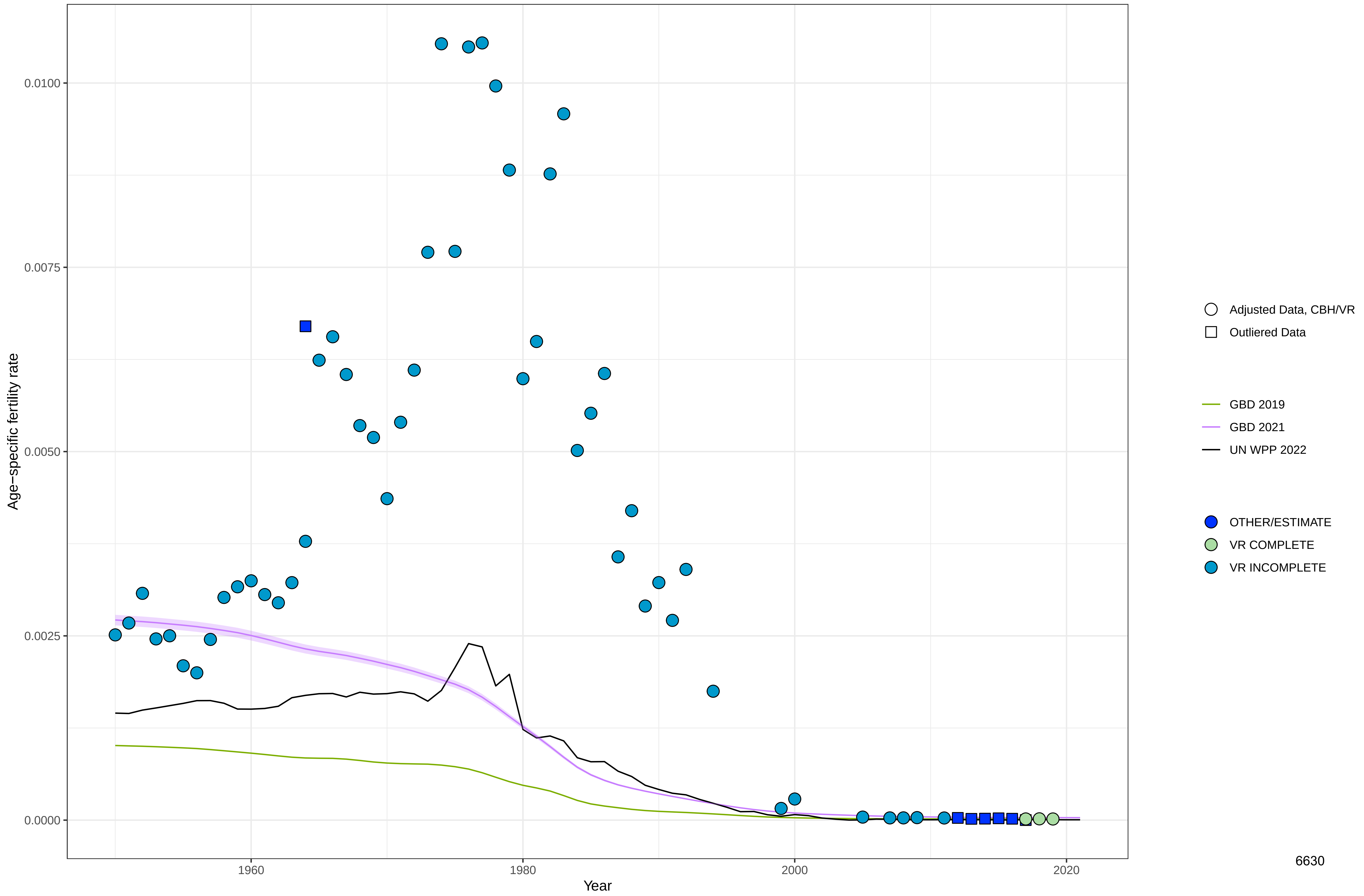

Total fertility rate fitted trend lines, 1950−2021: Thailand TFR
Data points enclosed with a diamond are reference sources

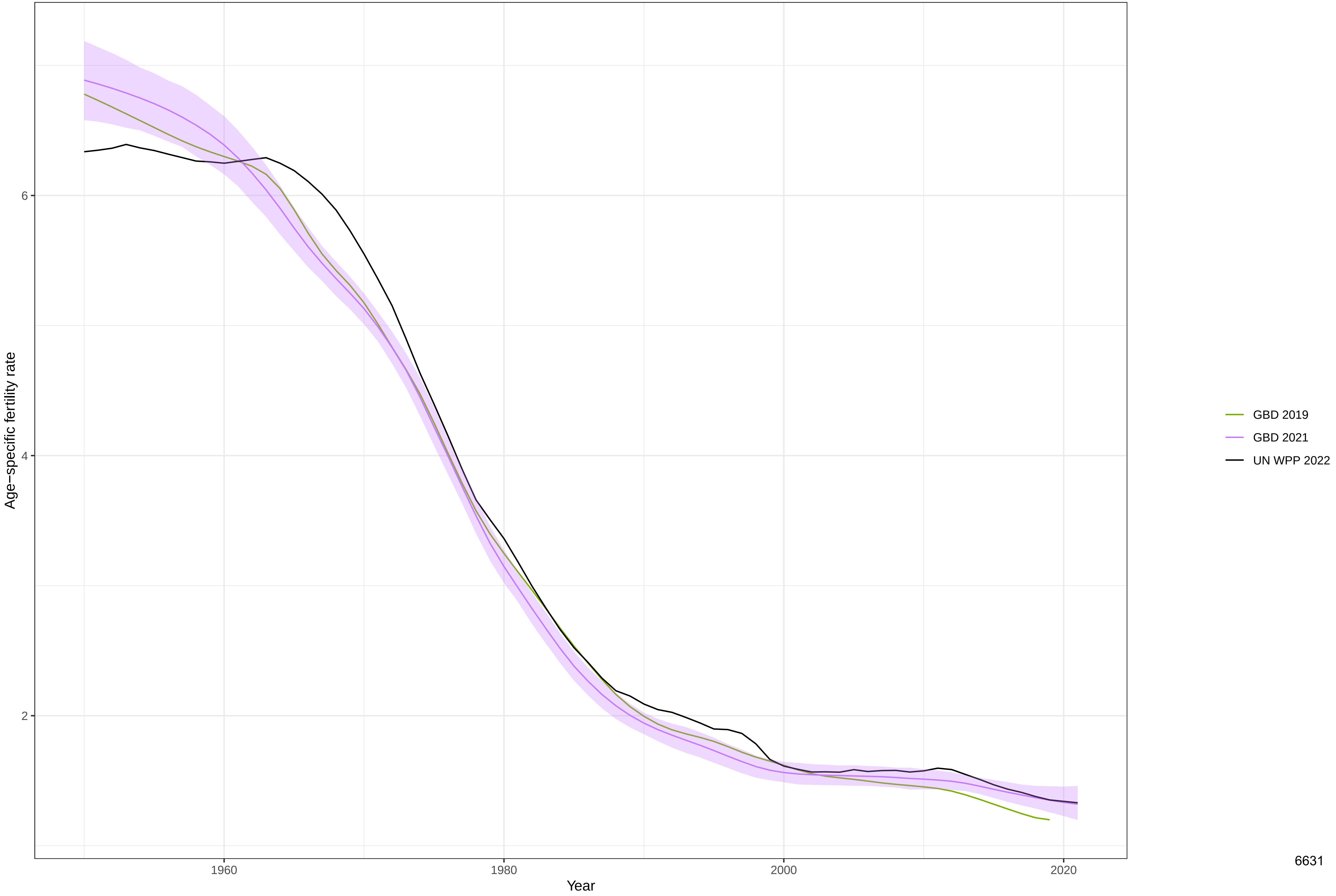

Age−specific fertility fitted trend lines, 1950−2021: Timor−Leste, Ages: 10−14
Data points enclosed with a diamond are reference sources

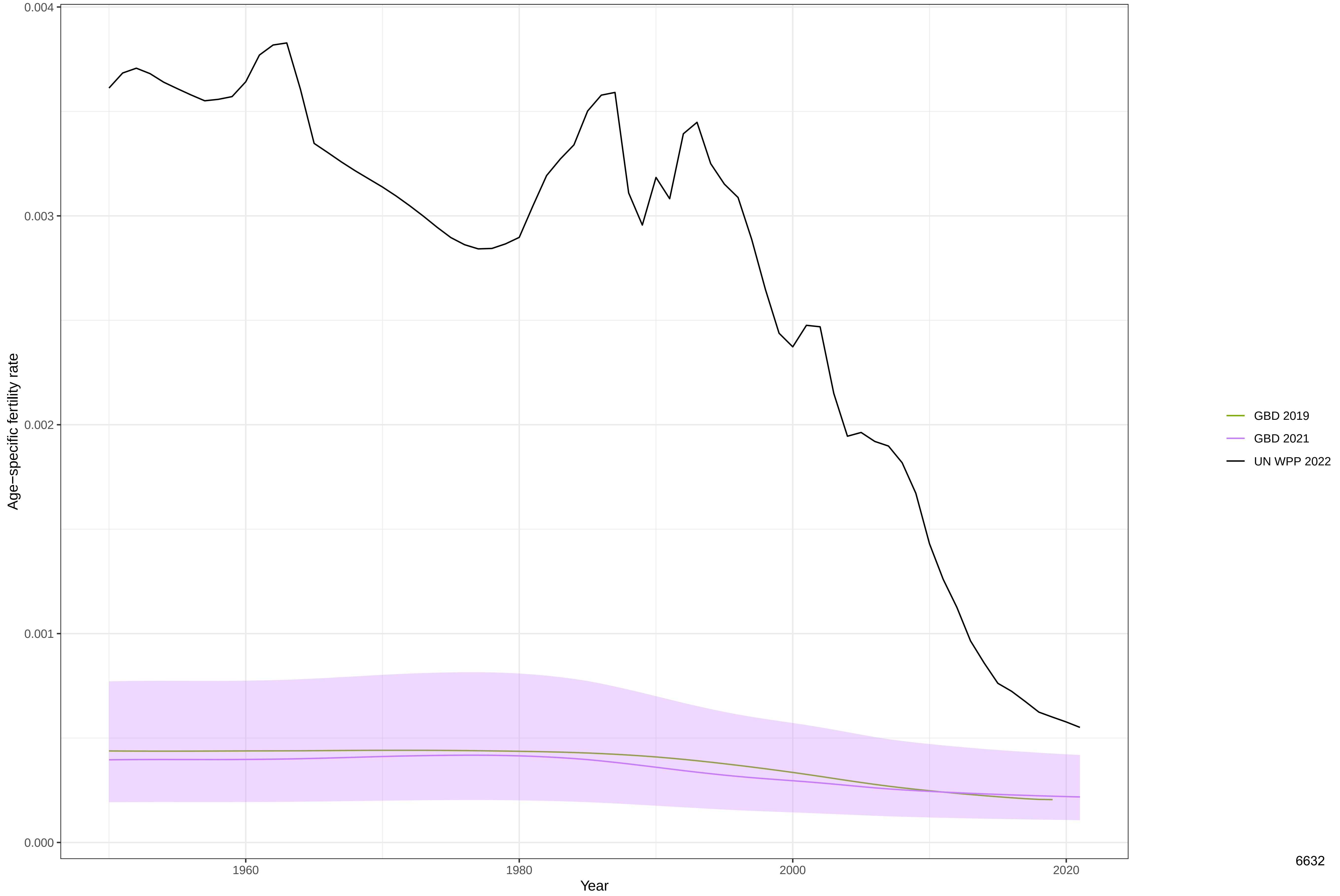

Age−specific fertility fitted trend lines, 1950−2021: Timor−Leste, Ages: 15−19
Data points enclosed with a diamond are reference sources

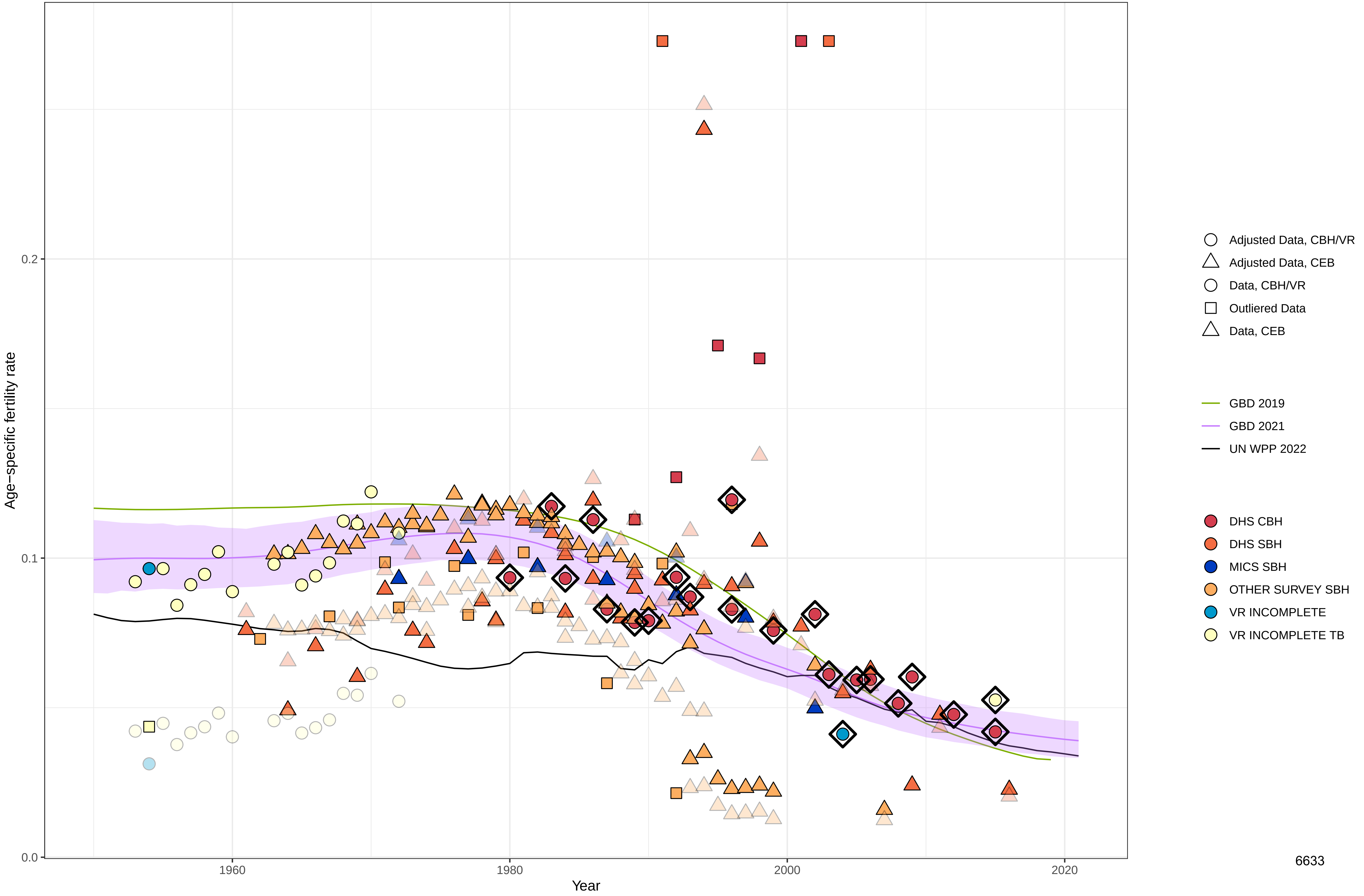

Age−specific fertility fitted trend lines, 1950−2021: Timor−Leste, Ages: 20−24
Data points enclosed with a diamond are reference sources

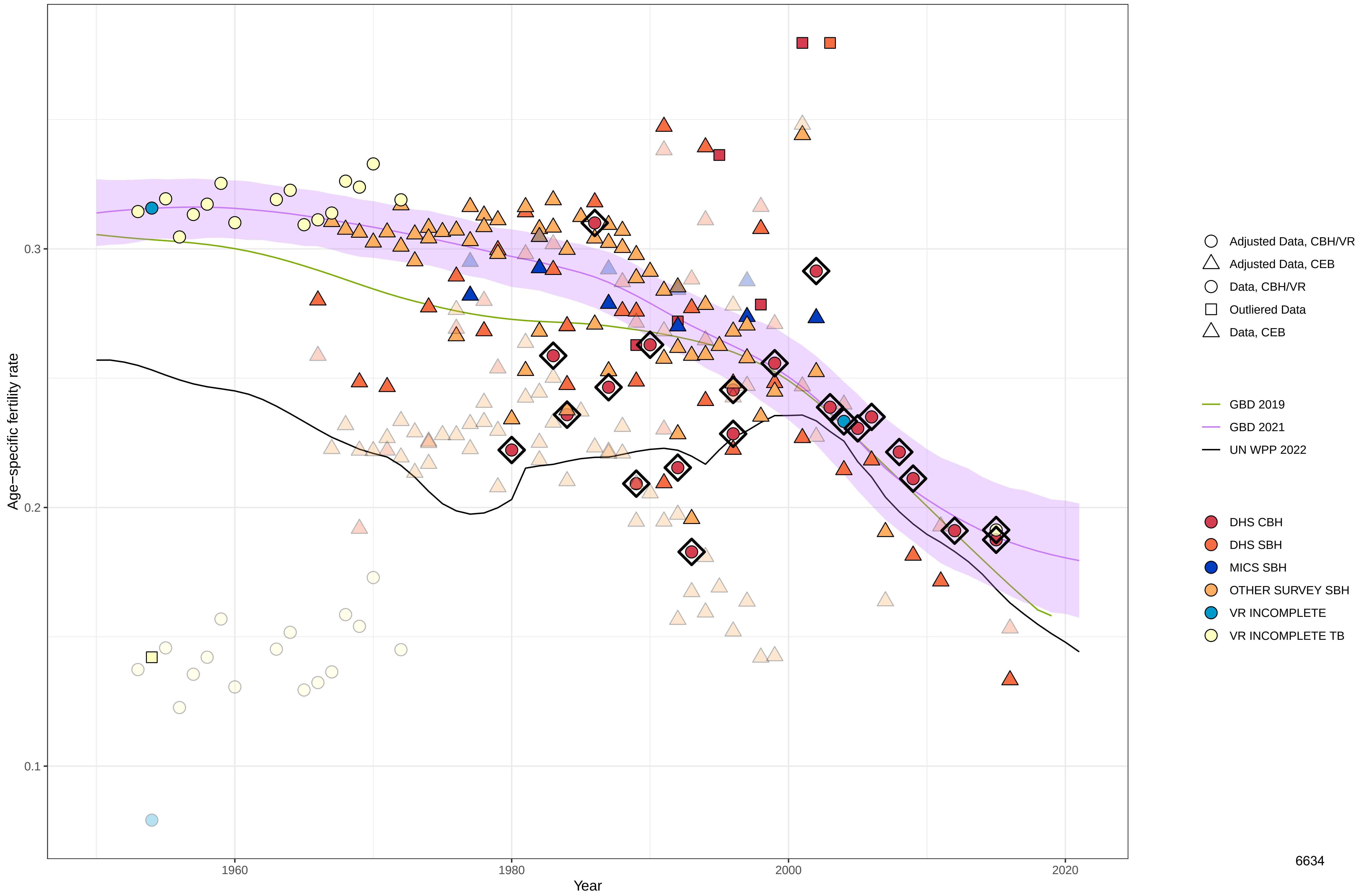

Age−specific fertility fitted trend lines, 1950−2021: Timor−Leste, Ages: 25−29
Data points enclosed with a diamond are reference sources

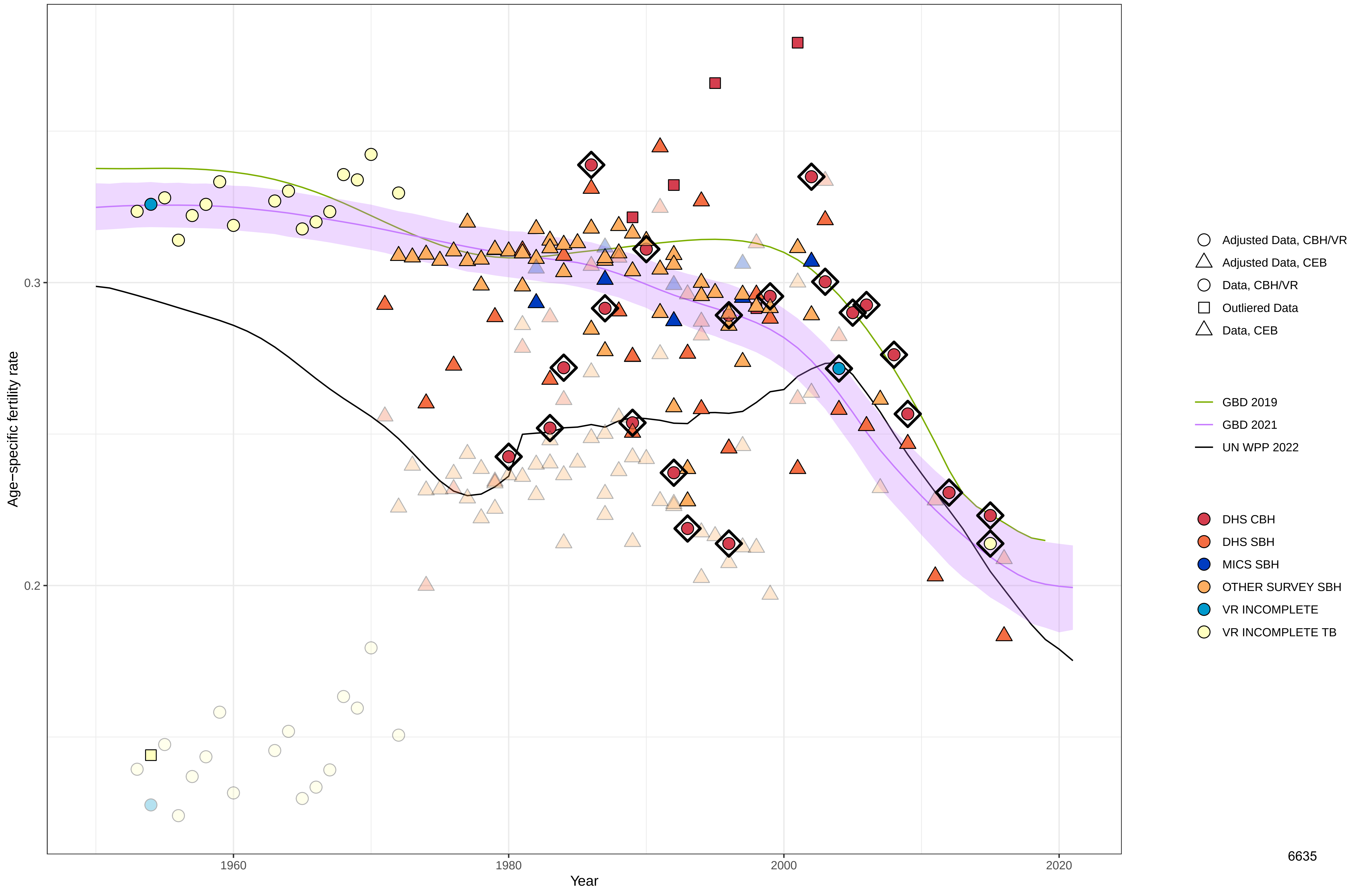

Age−specific fertility fitted trend lines, 1950−2021: Timor−Leste, Ages: 30−34
Data points enclosed with a diamond are reference sources

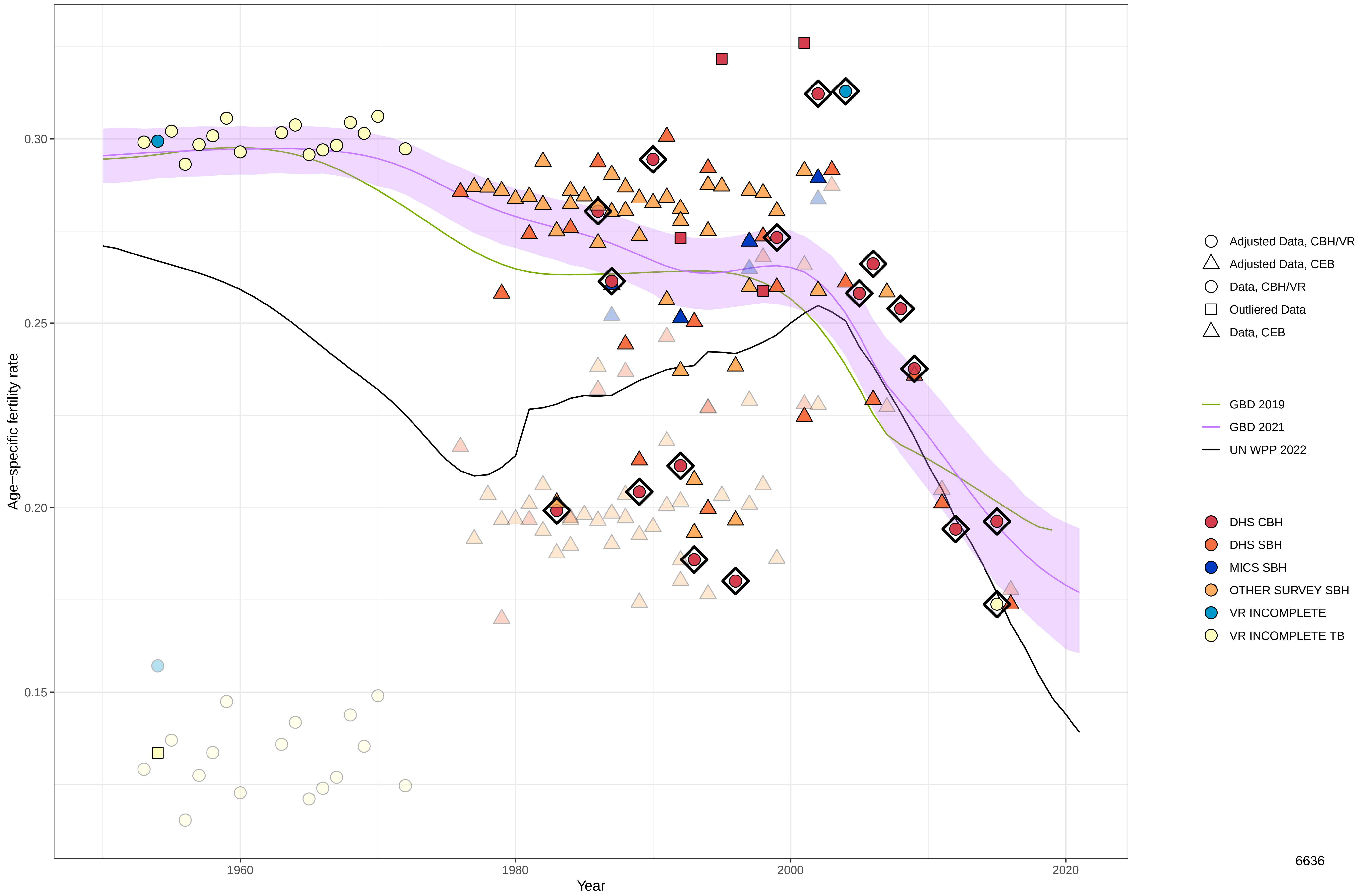

6636

Age−specific fertility fitted trend lines, 1950−2021: Timor−Leste, Ages: 35−39
Data points enclosed with a diamond are reference sources

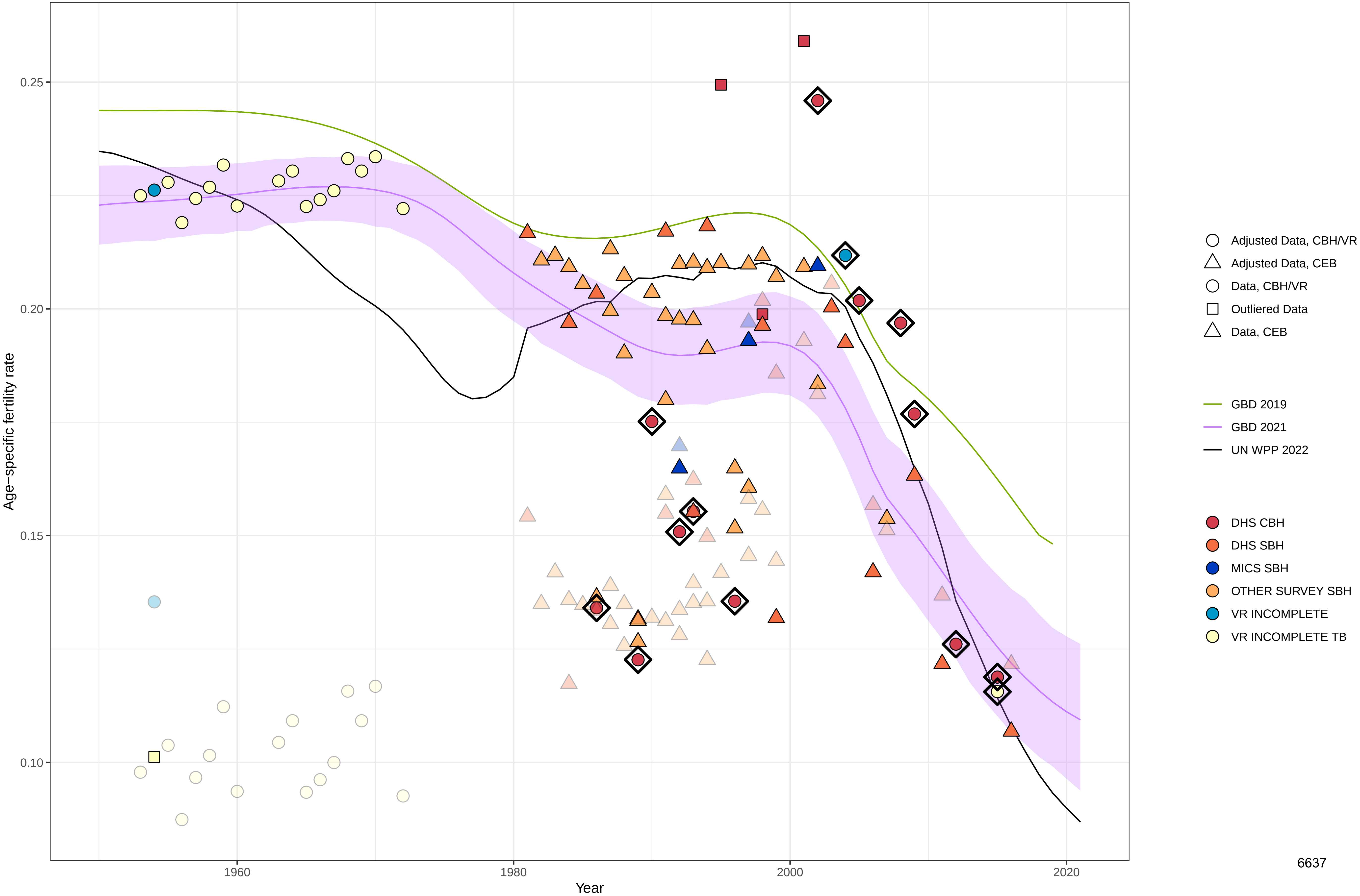

Age−specific fertility fitted trend lines, 1950−2021: Timor−Leste, Ages: 40−44
Data points enclosed with a diamond are reference sources

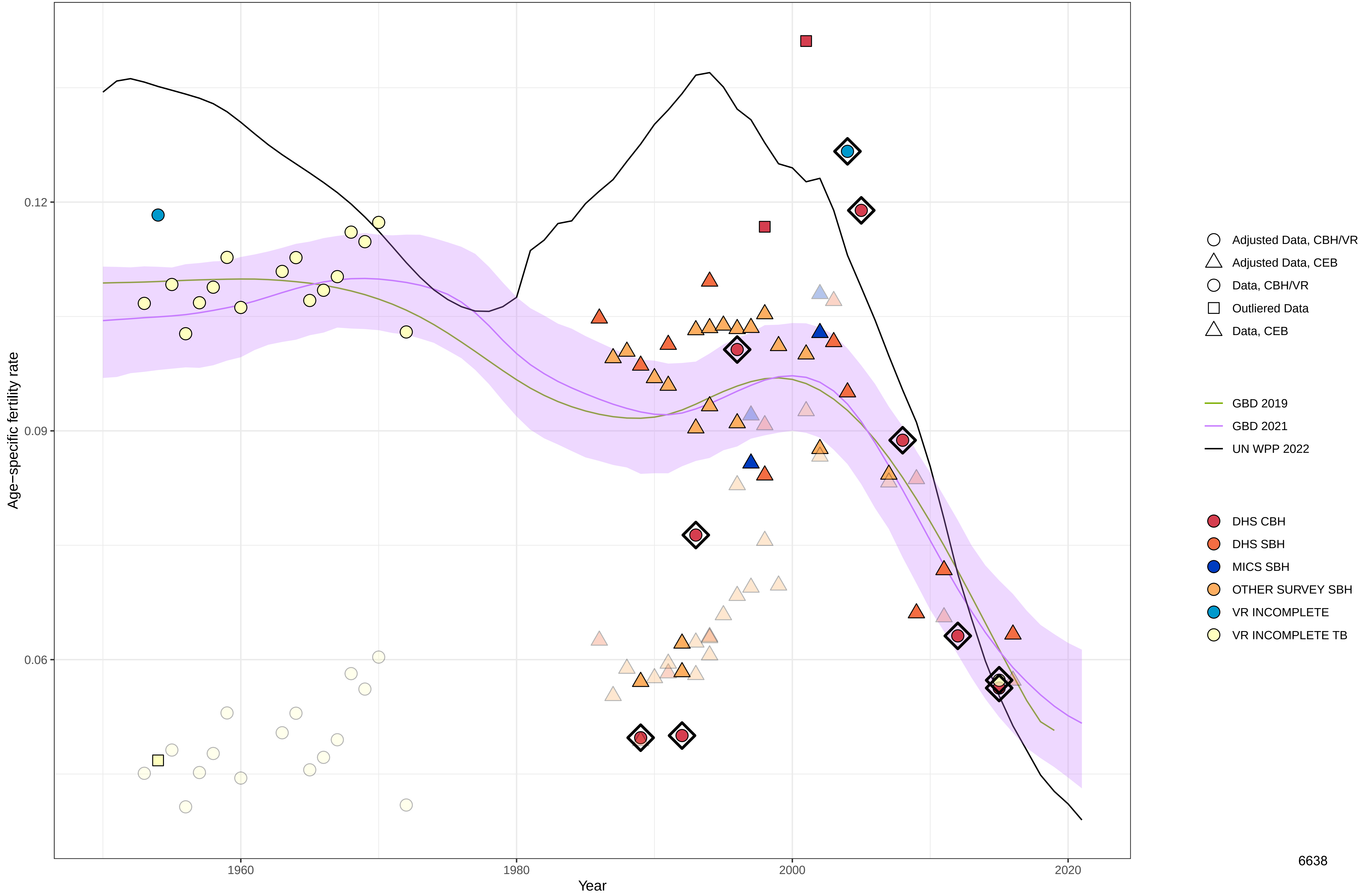

Age−specific fertility fitted trend lines, 1950−2021: Timor−Leste, Ages: 45−49
Data points enclosed with a diamond are reference sources

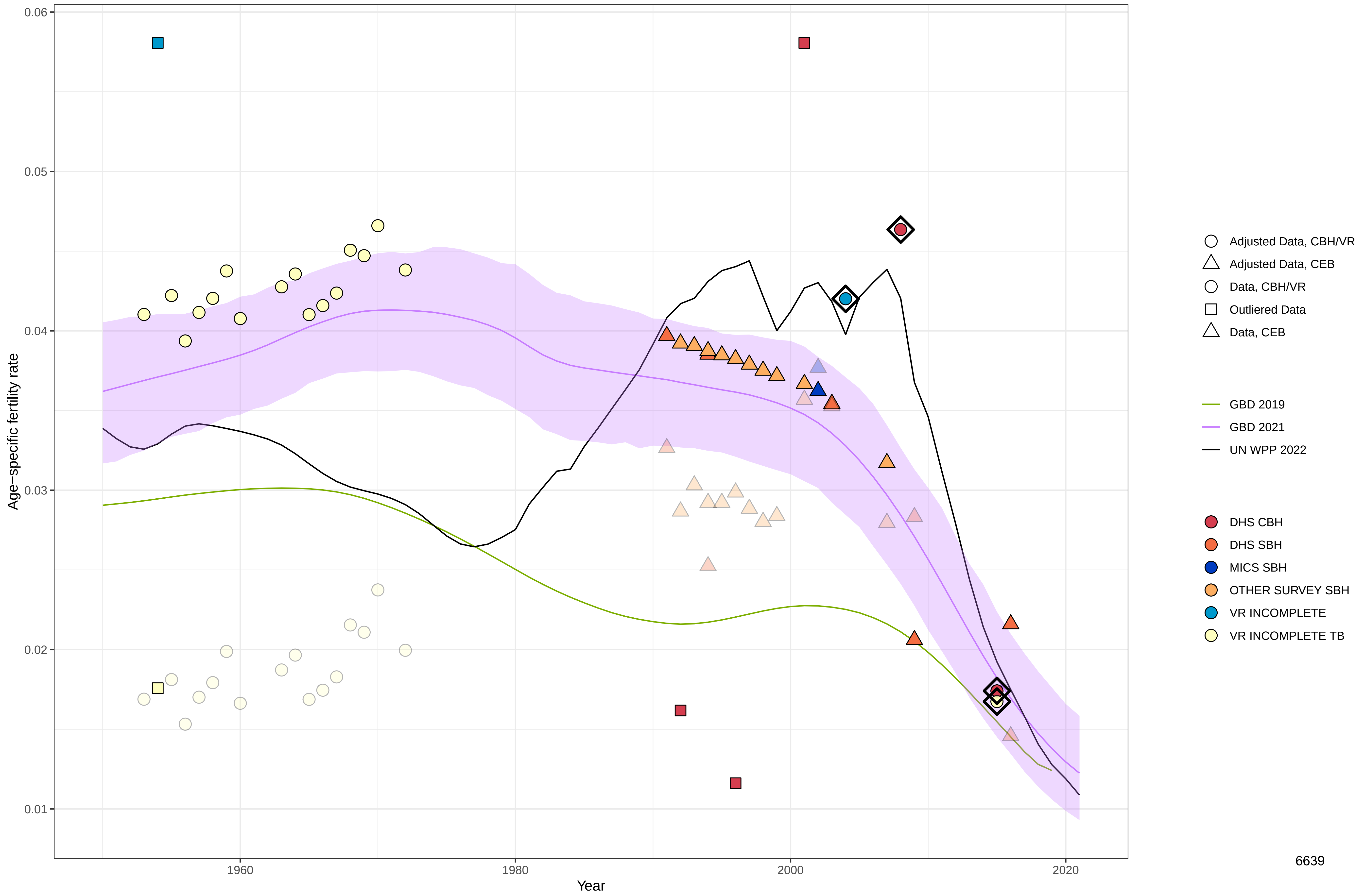

Age−specific fertility fitted trend lines, 1950−2021: Timor−Leste, Ages: 50−54
Data points enclosed with a diamond are reference sources

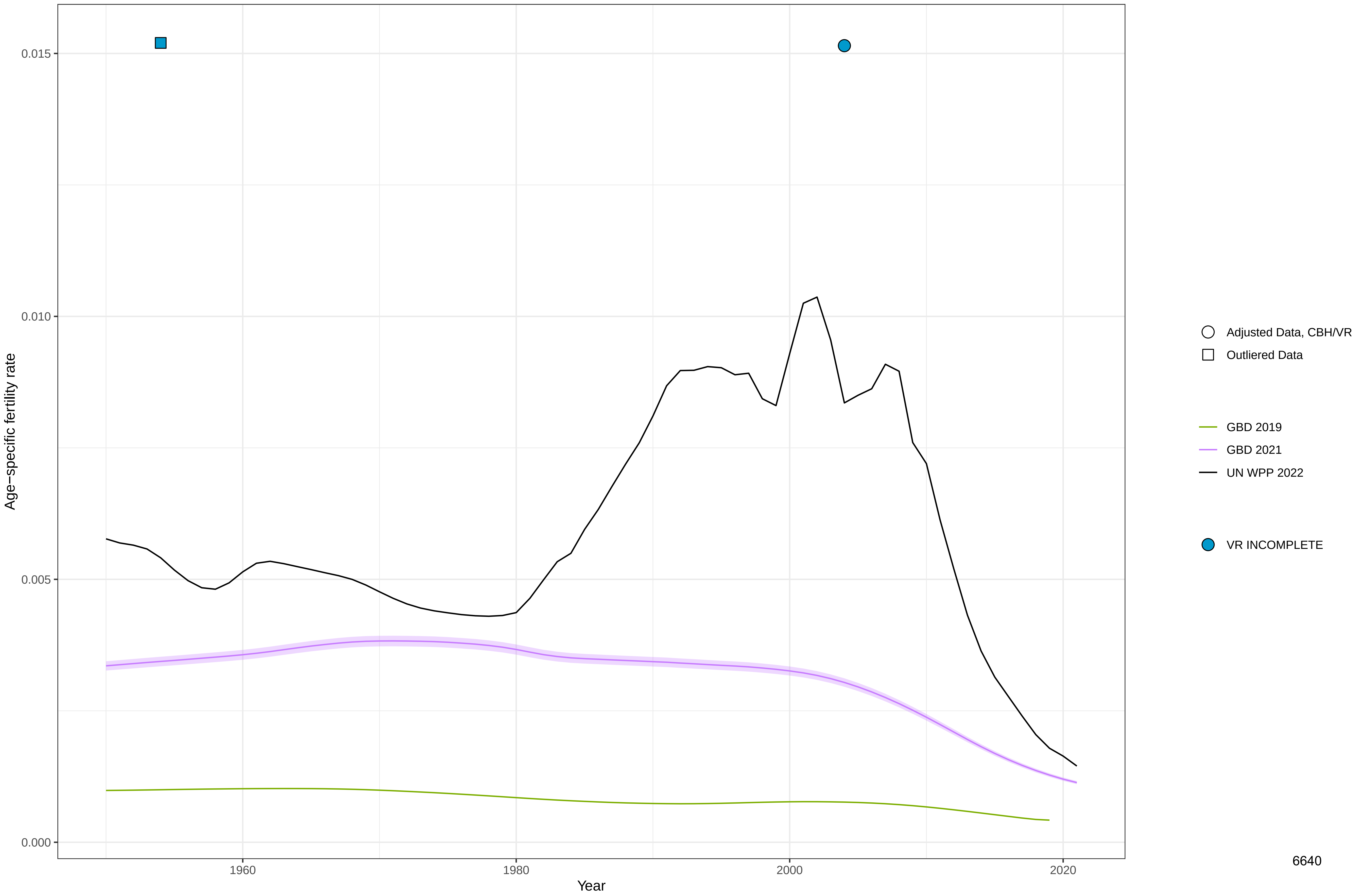

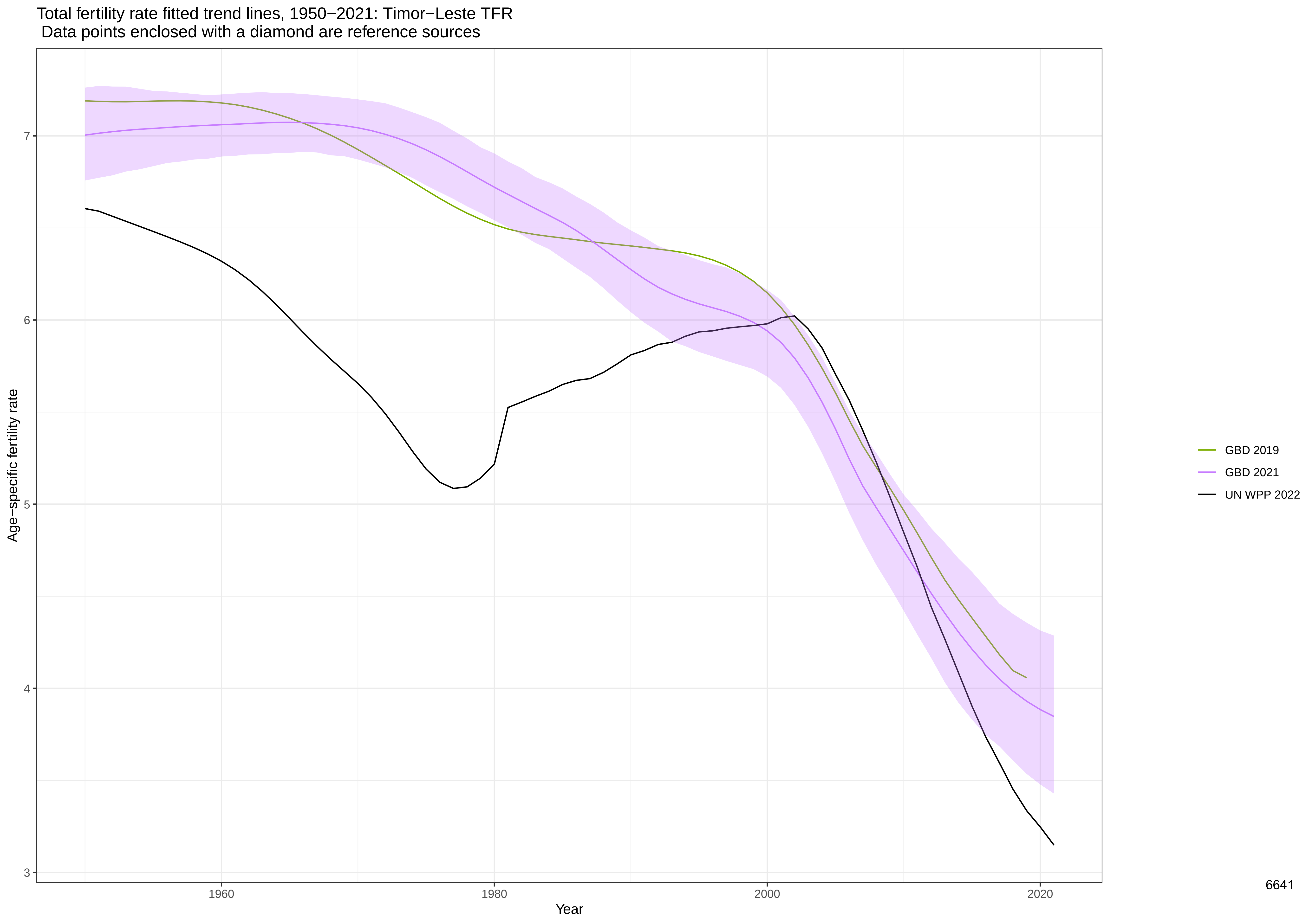

Total fertility rate fitted trend lines, 1950−2021: Timor−Leste TFR
Data points enclosed with a diamond are reference sources
Age−specific fertility rate
3
4
5
6
7
1960
1980
2000
2020
Year
GBD 2019
GBD 2021
UN WPP 2022

6641

Age−specific fertility fitted trend lines, 1950−2021: Viet Nam, Ages: 10−14
Data points enclosed with a diamond are reference sources

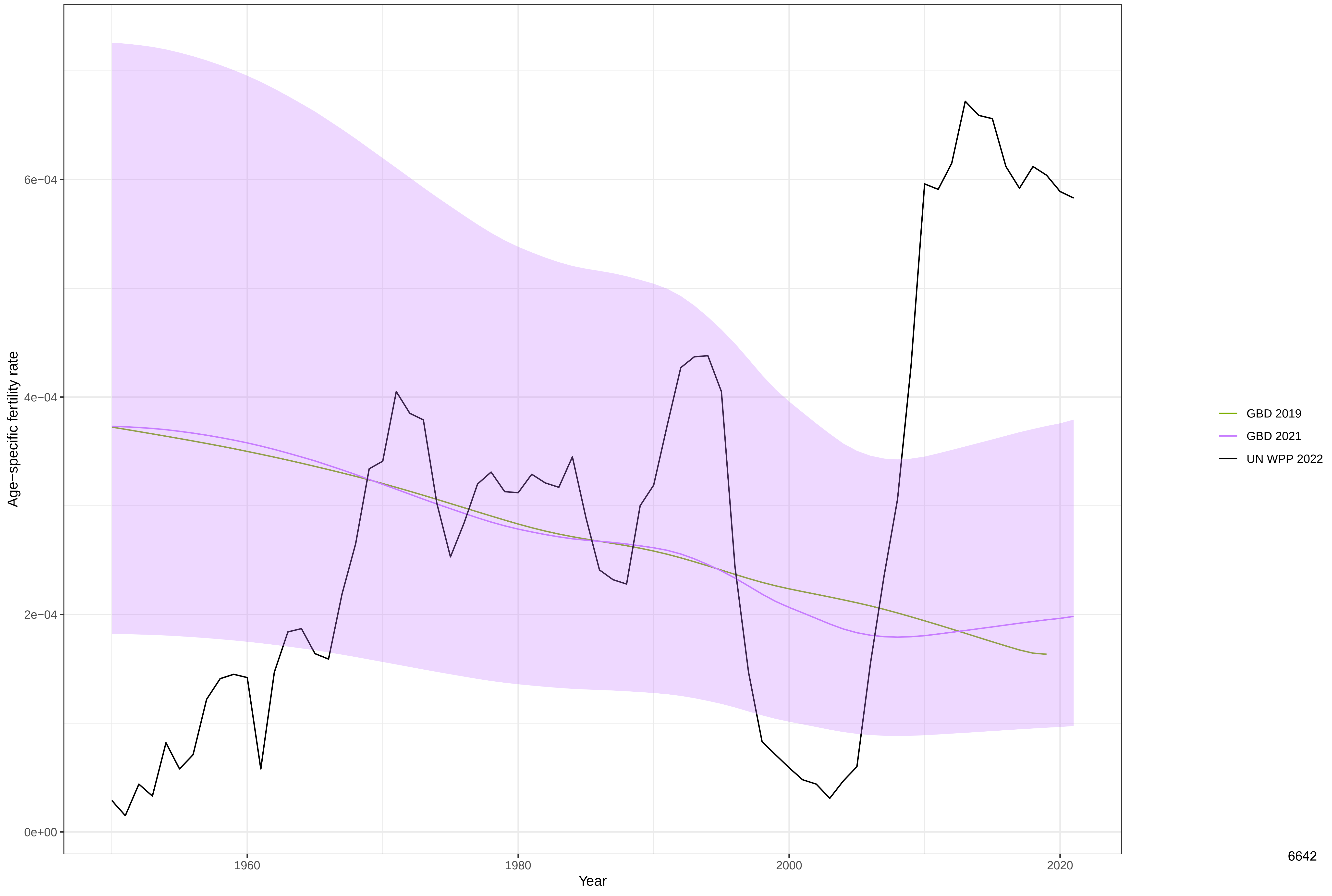

Age−specific fertility fitted trend lines, 1950−2021: Viet Nam, Ages: 15−19
Data points enclosed with a diamond are reference sources

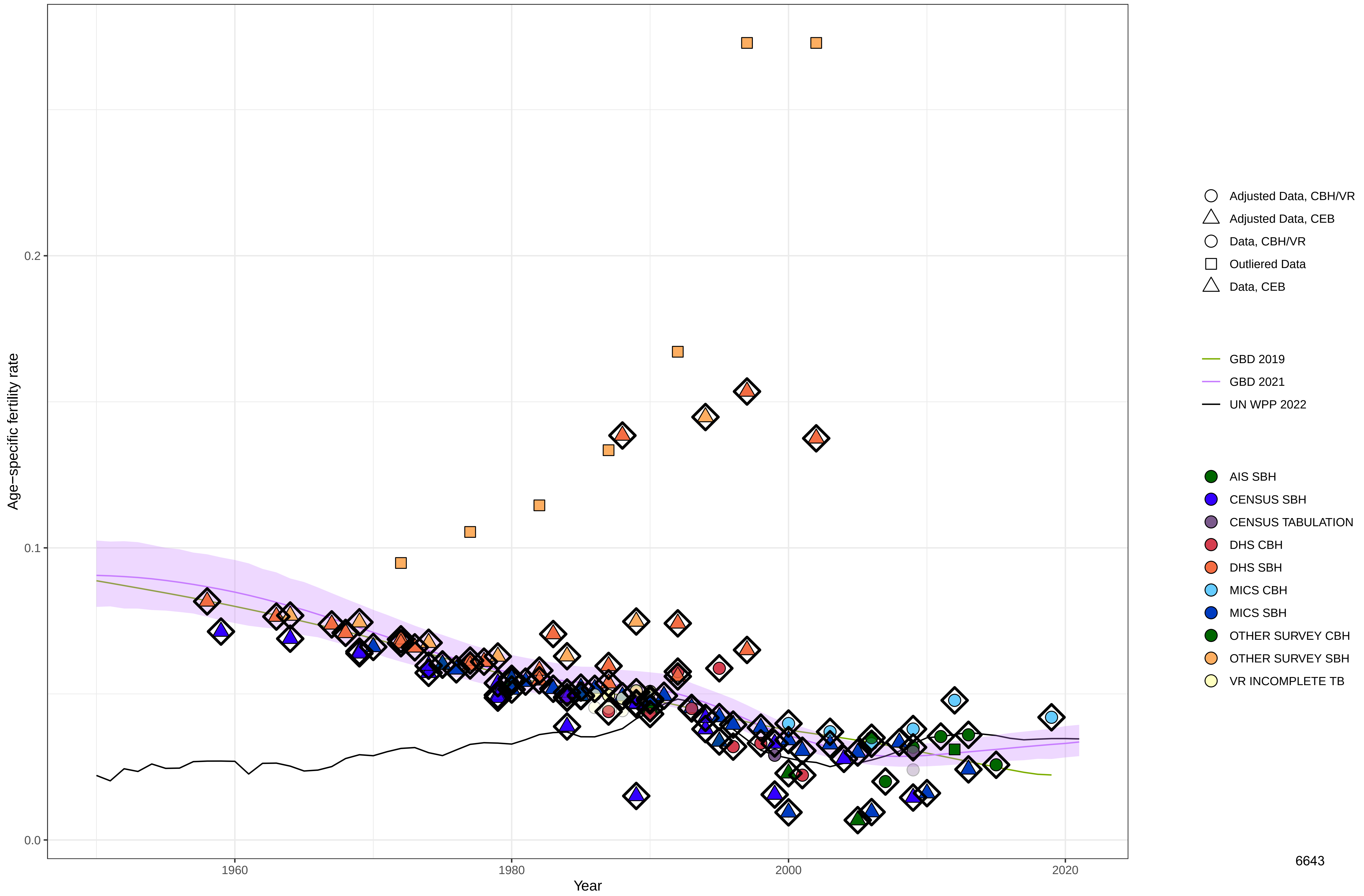

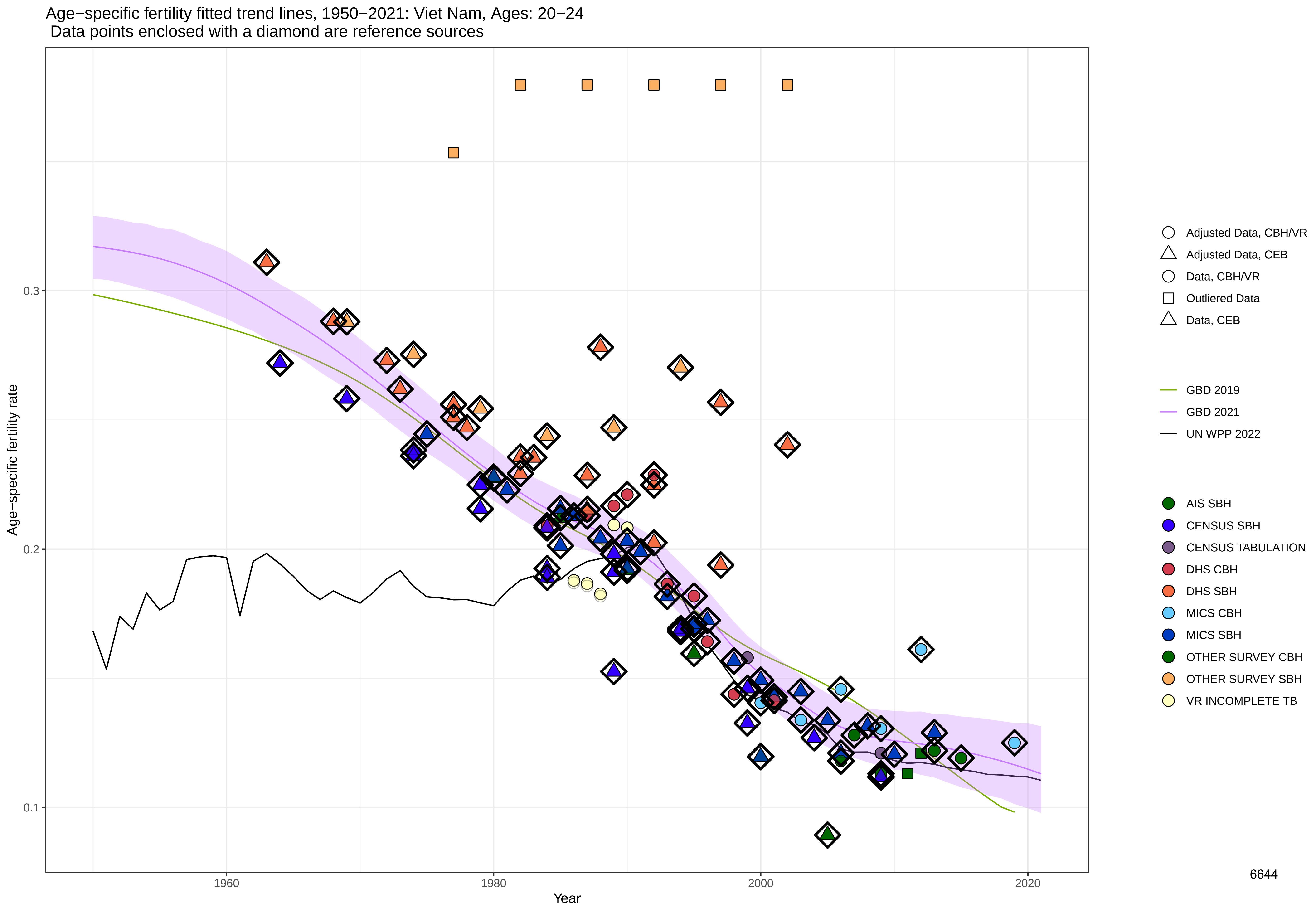

Age−specific fertility fitted trend lines, 1950−2021: Viet Nam, Ages: 20−24
Data points enclosed with a diamond are reference sources
Age−specific fertility rate
0.1
0.2
0.3
Year
1960
1980
2000
2020
Adjusted Data, CBH/VR
Adjusted Data, CEB
Data, CBH/VR
Outliered Data
Data, CEB
GBD 2019
GBD 2021
UN WPP 2022
AIS SBH
CENSUS SBH
CENSUS TABULATION
DHS CBH
DHS SBH
MICS CBH
MICS SBH
OTHER SURVEY CBH
OTHER SURVEY SBH
VR INCOMPLETE TB

Age−specific fertility fitted trend lines, 1950−2021: Viet Nam, Ages: 25−29
Data points enclosed with a diamond are reference sources

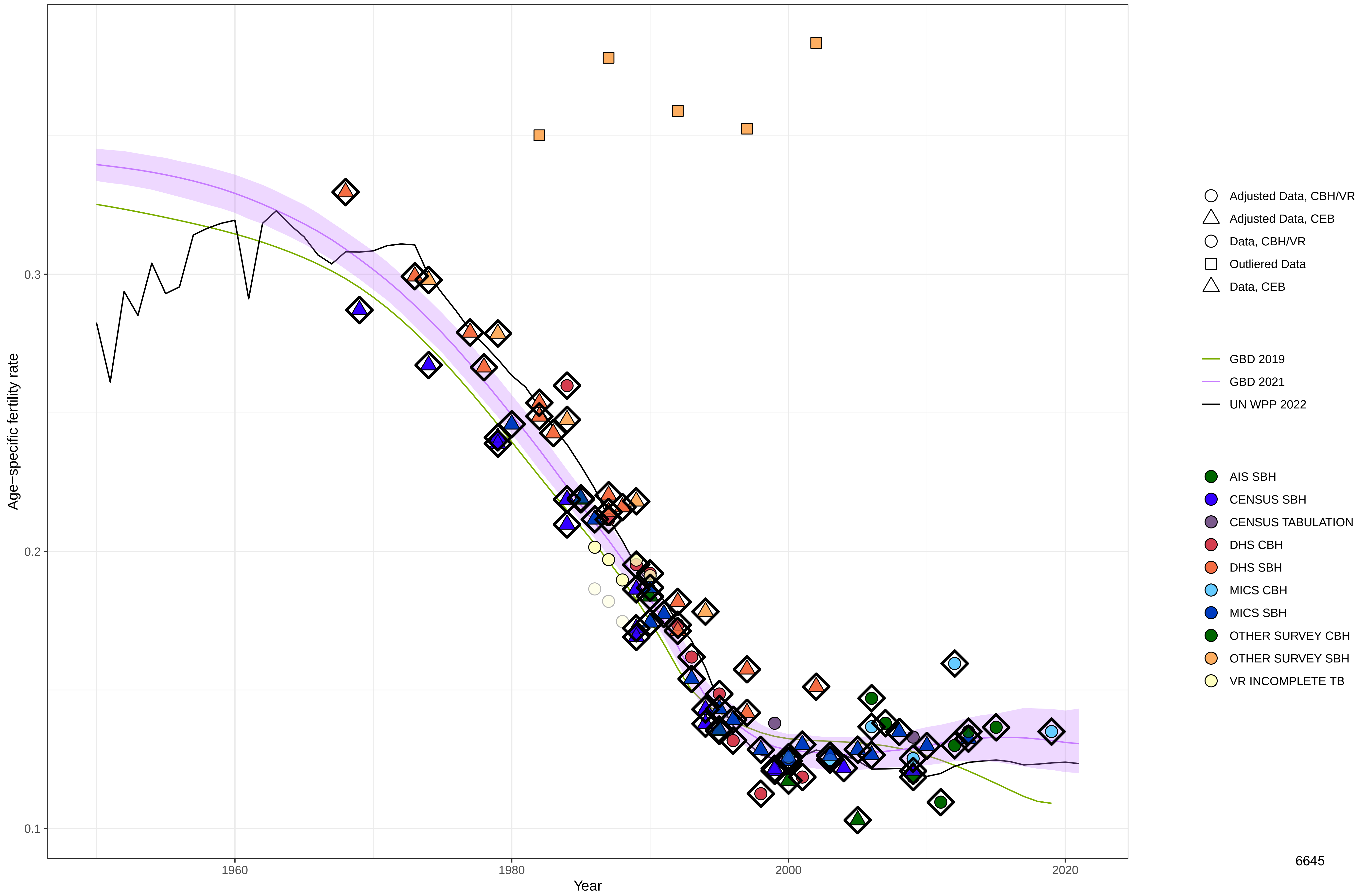

Age−specific fertility fitted trend lines, 1950−2021: Viet Nam, Ages: 30−34
Data points enclosed with a diamond are reference sources

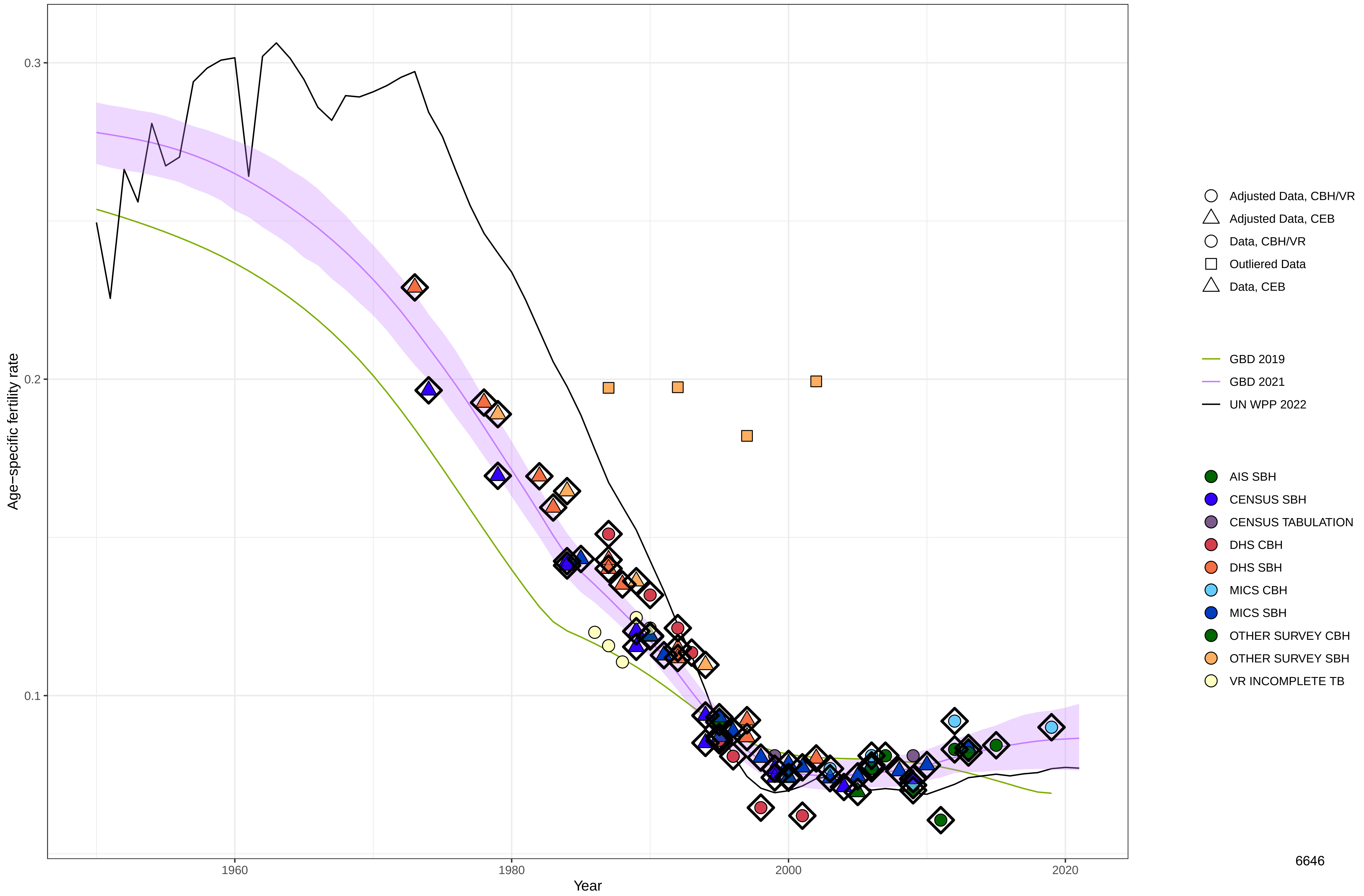

Age−specific fertility fitted trend lines, 1950−2021: Viet Nam, Ages: 35−39
Data points enclosed with a diamond are reference sources

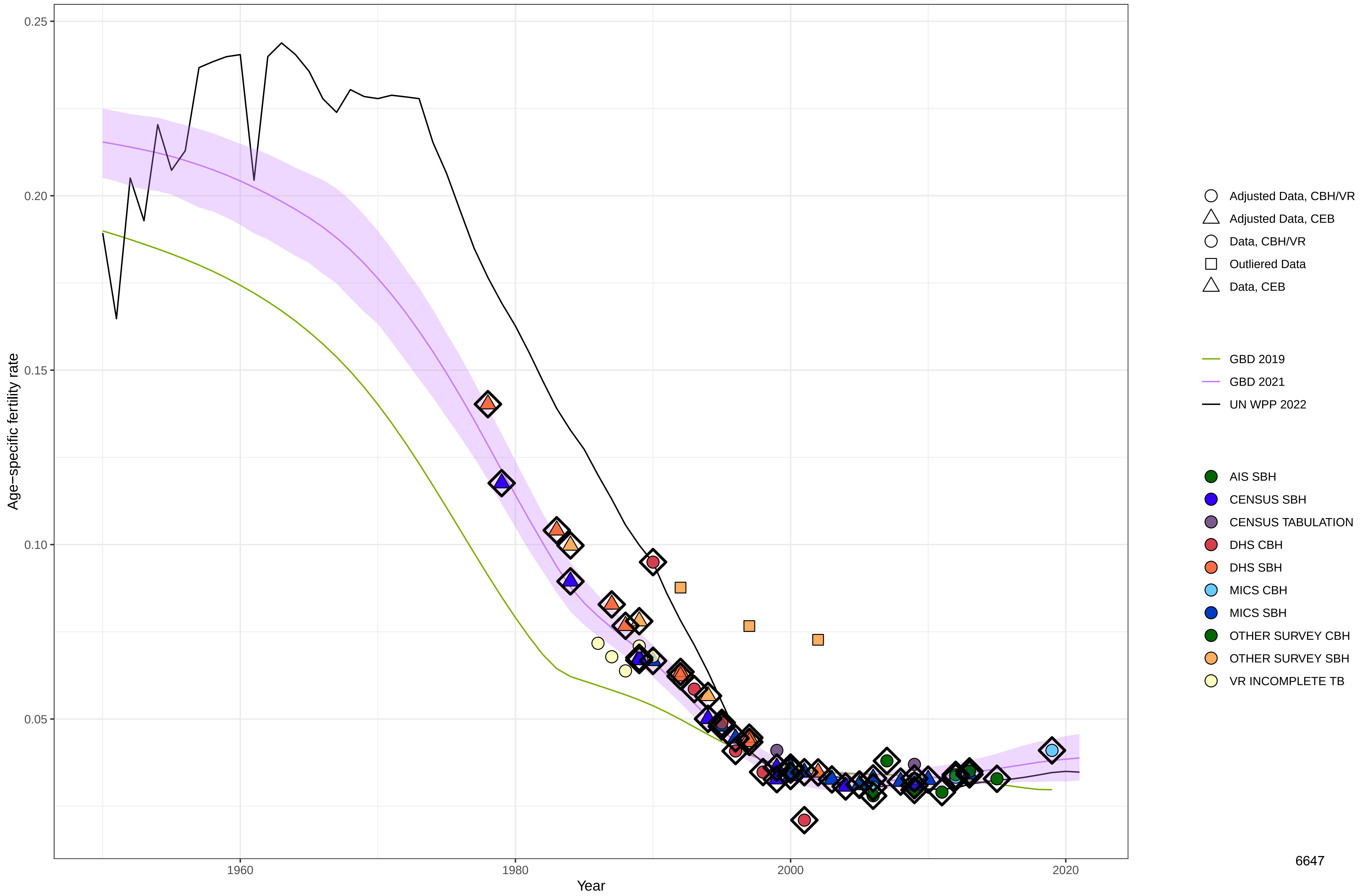

Age−specific fertility fitted trend lines, 1950−2021: Viet Nam, Ages: 40−44
Data points enclosed with a diamond are reference sources

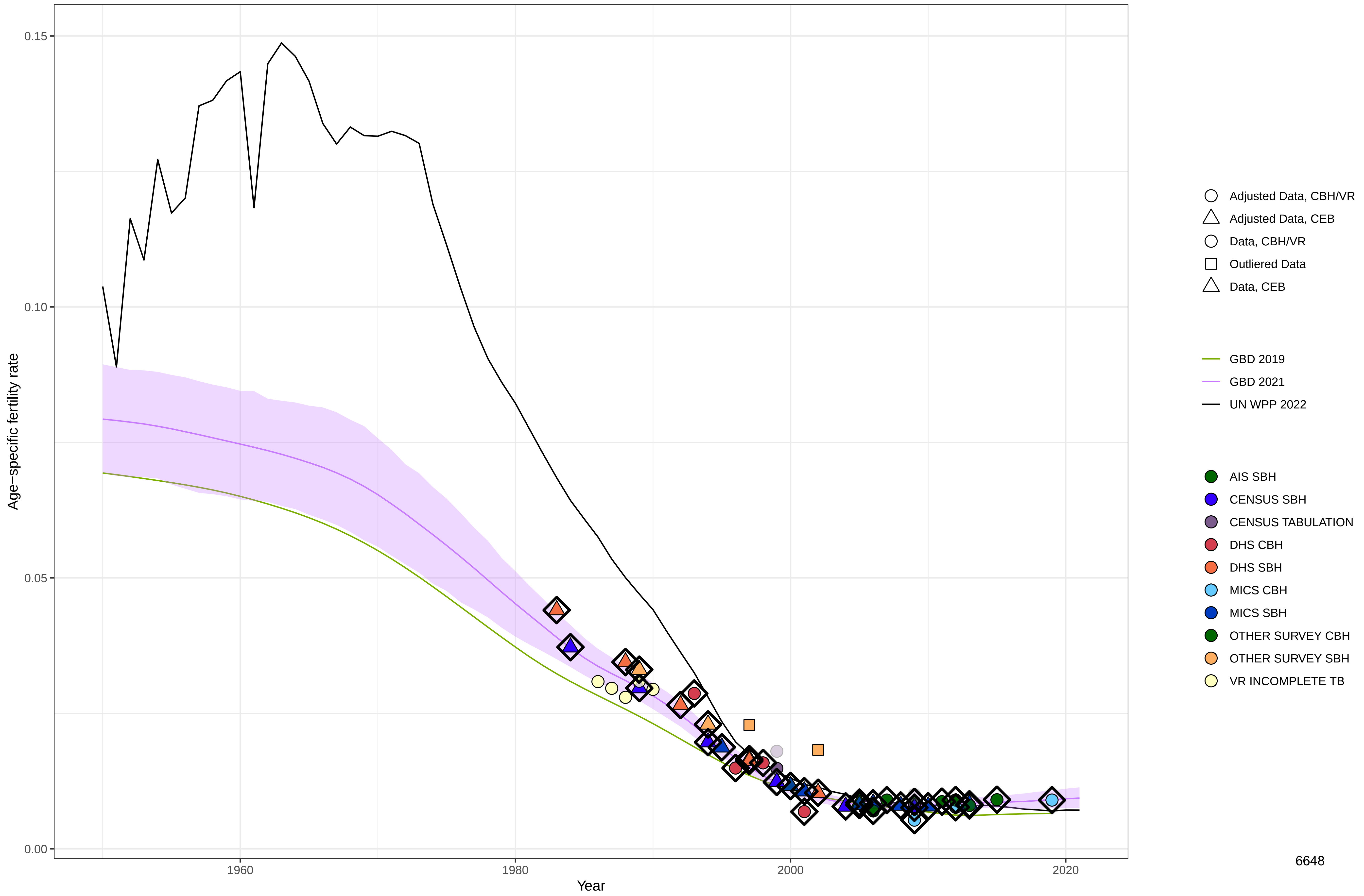

Age−specific fertility fitted trend lines, 1950−2021: Viet Nam, Ages: 45−49
Data points enclosed with a diamond are reference sources

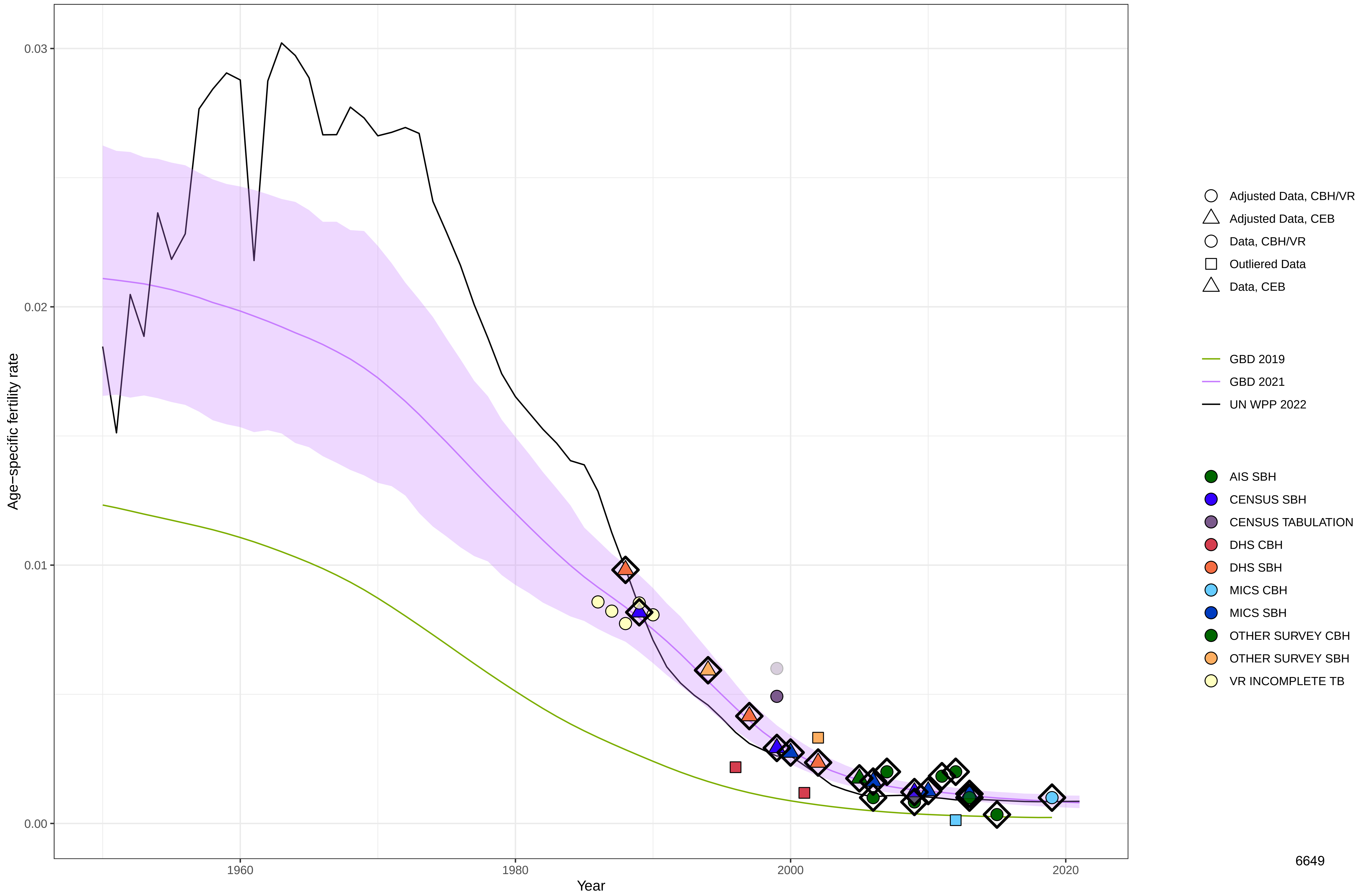

Age−specific fertility fitted trend lines, 1950−2021: Viet Nam, Ages: 50−54
Data points enclosed with a diamond are reference sources

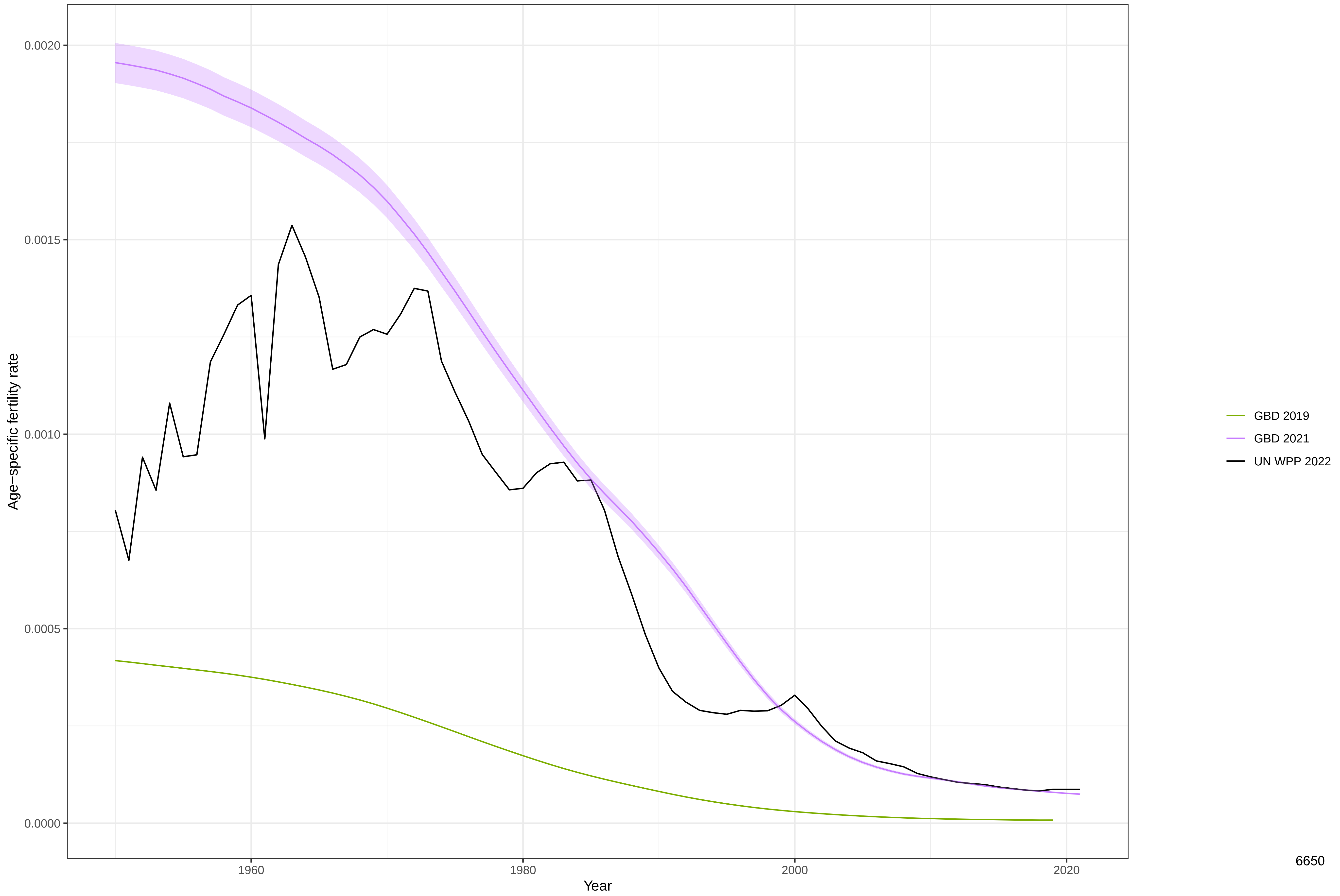

Total fertility rate fitted trend lines, 1950−2021: Viet Nam TFR
Data points enclosed with a diamond are reference sources

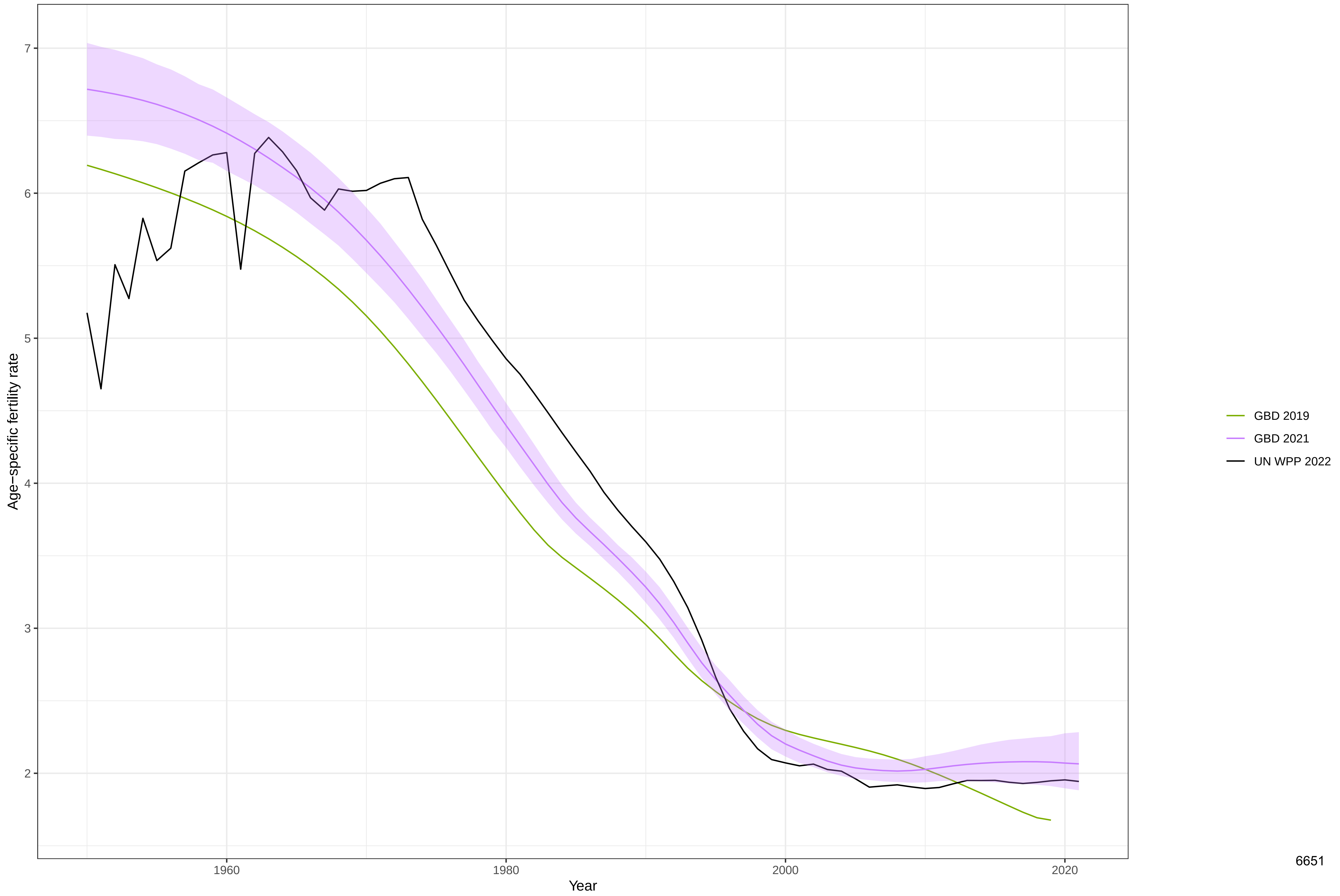

Age−specific fertility fitted trend lines, 1950−2021: Angola, Ages: 10−14
Data points enclosed with a diamond are reference sources

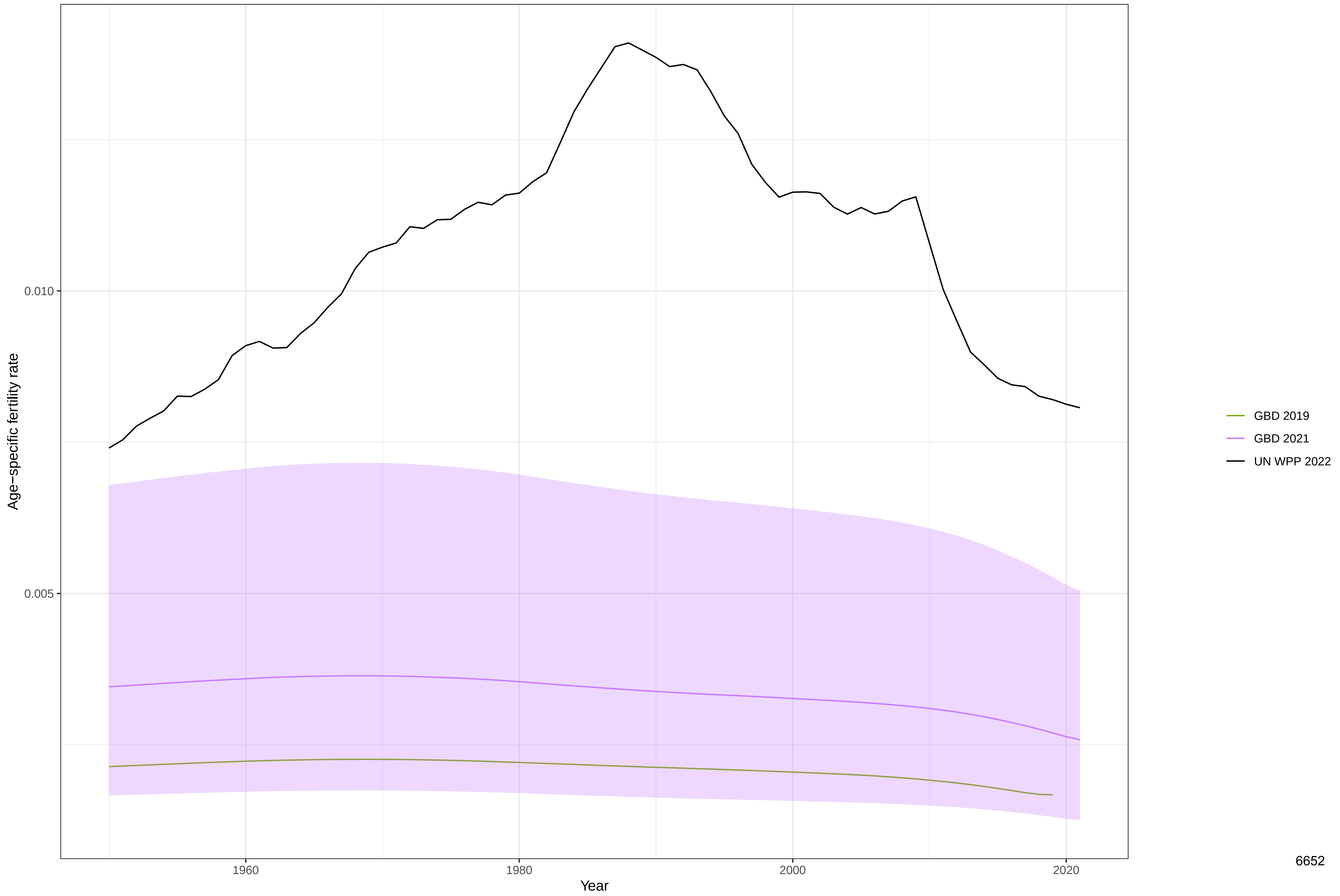

Age−specific fertility fitted trend lines, 1950−2021: Angola, Ages: 15−19
Data points enclosed with a diamond are reference sources

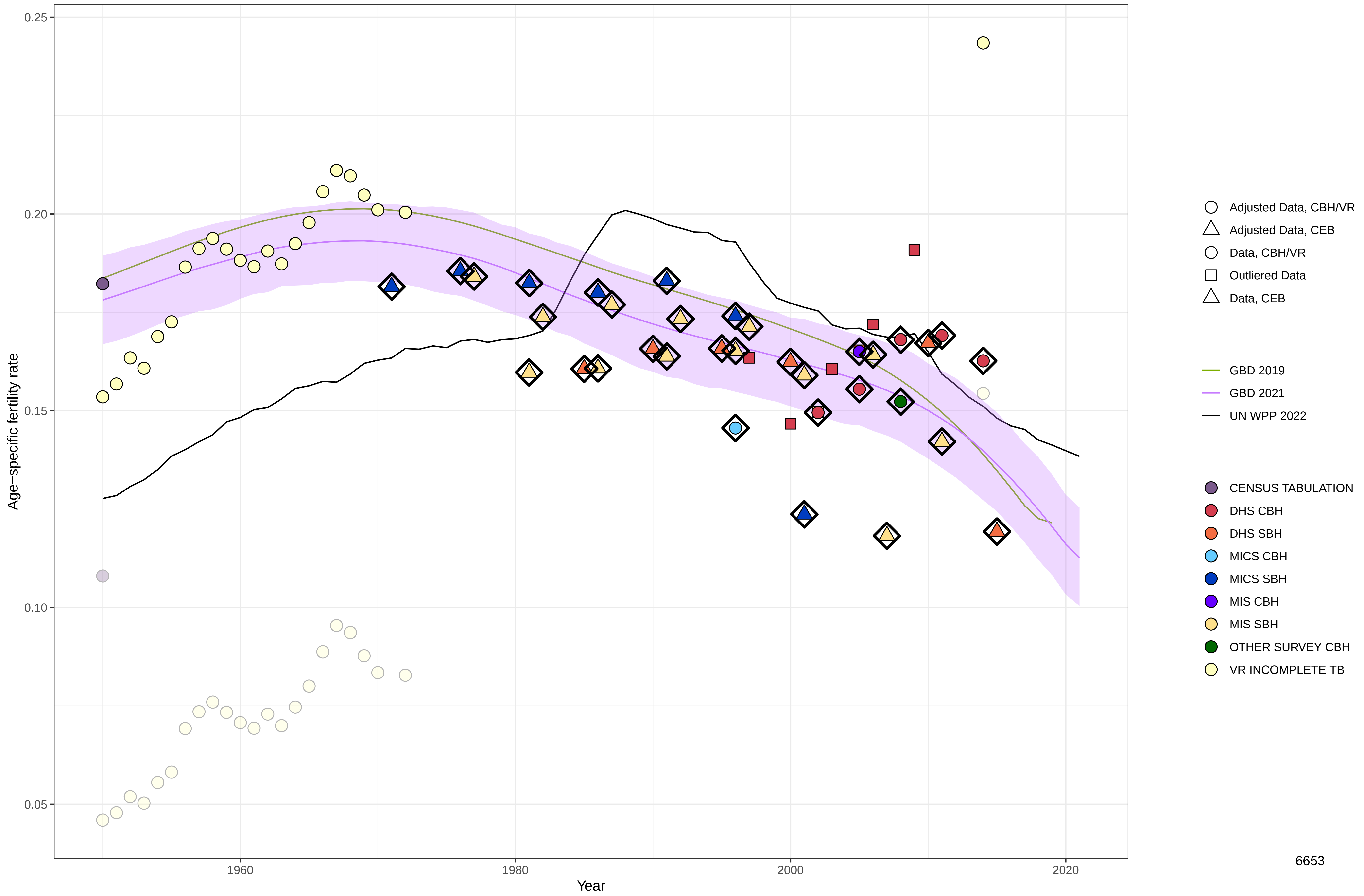

Age−specific fertility fitted trend lines, 1950−2021: Angola, Ages: 20−24
Data points enclosed with a diamond are reference sources

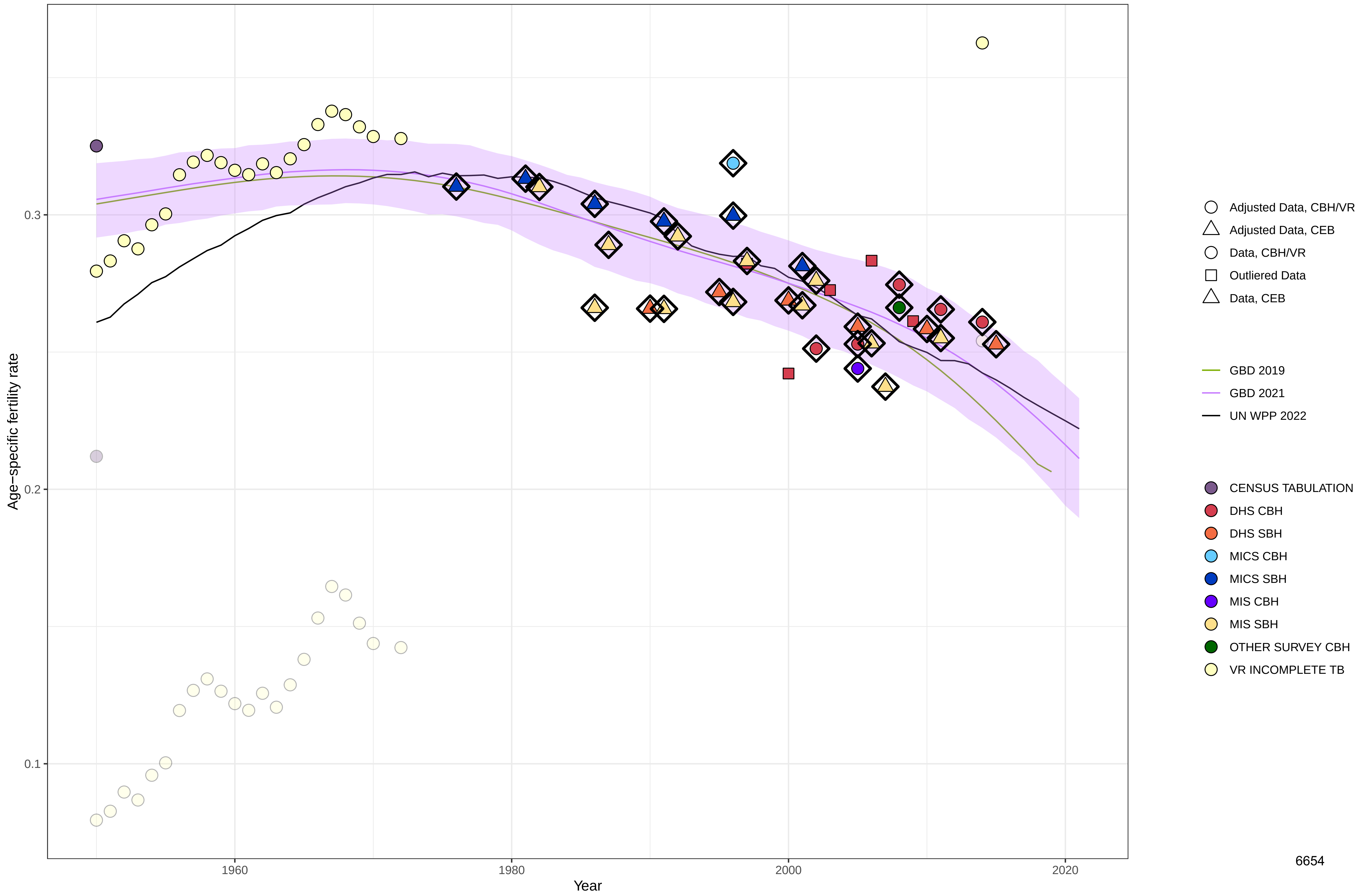

Age−specific fertility fitted trend lines, 1950−2021: Angola, Ages: 25−29
Data points enclosed with a diamond are reference sources

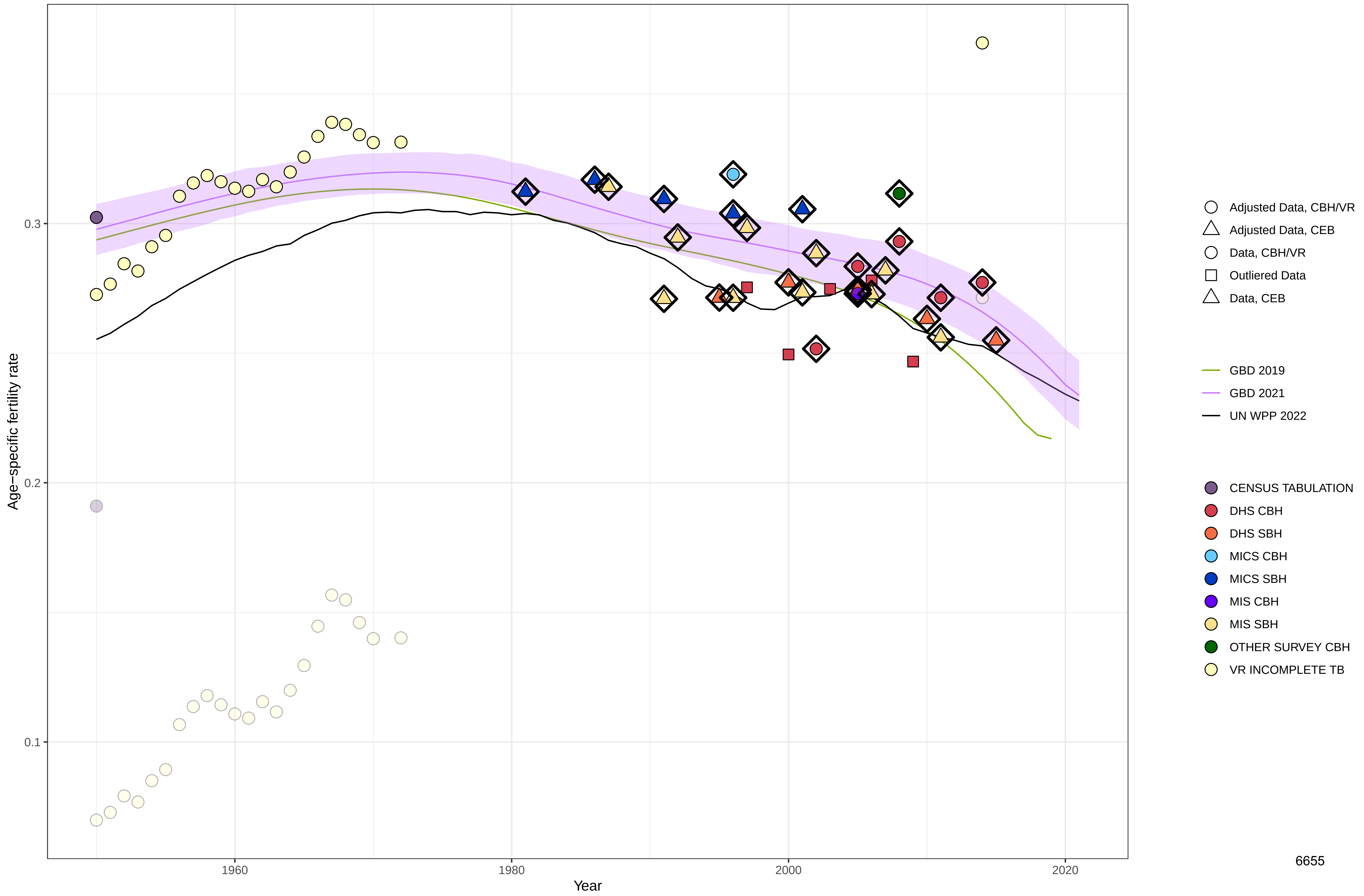

Age−specific fertility fitted trend lines, 1950−2021: Angola, Ages: 30−34
Data points enclosed with a diamond are reference sources

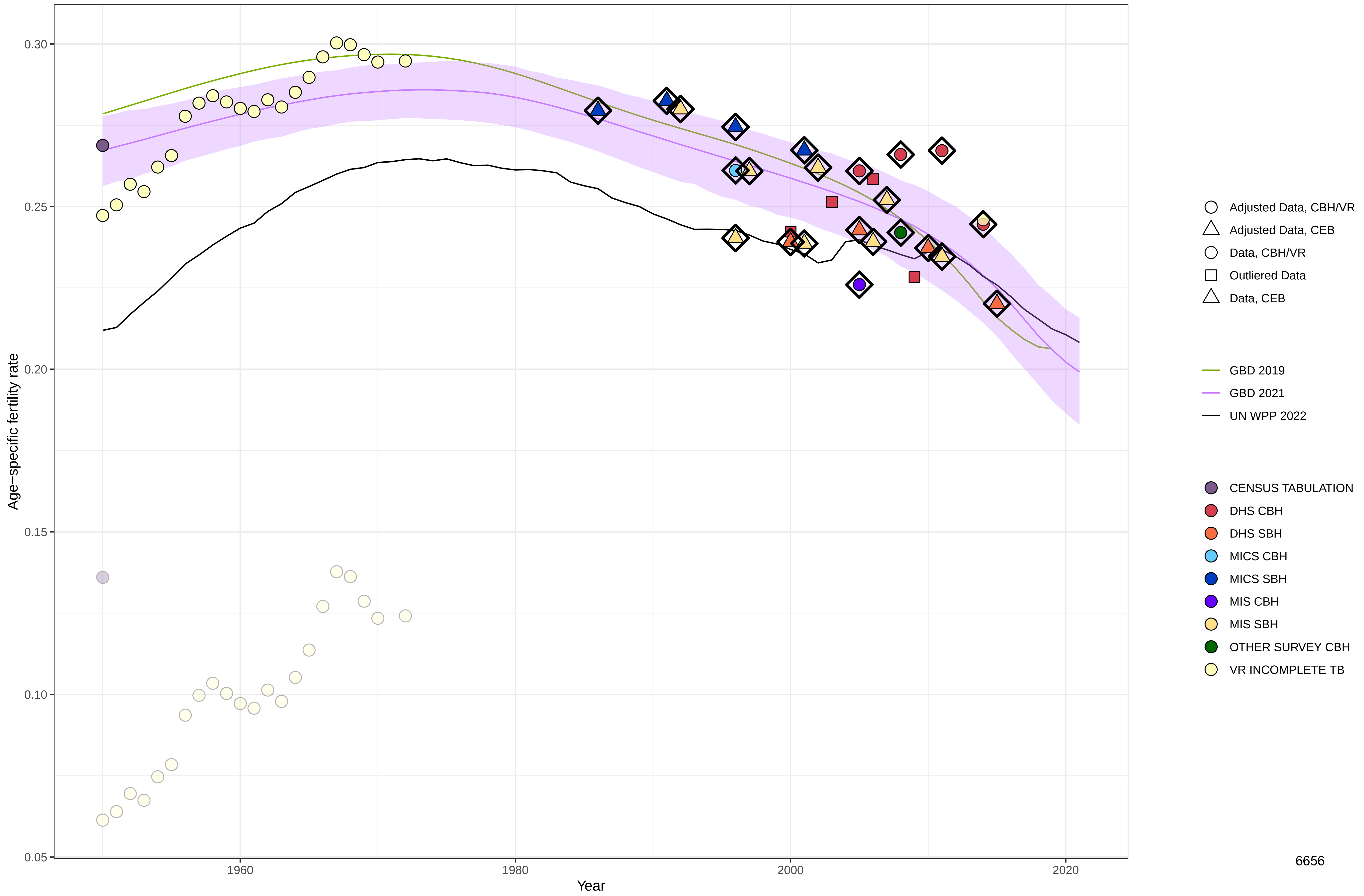

Age−specific fertility fitted trend lines, 1950−2021: Angola, Ages: 35−39
Data points enclosed with a diamond are reference sources

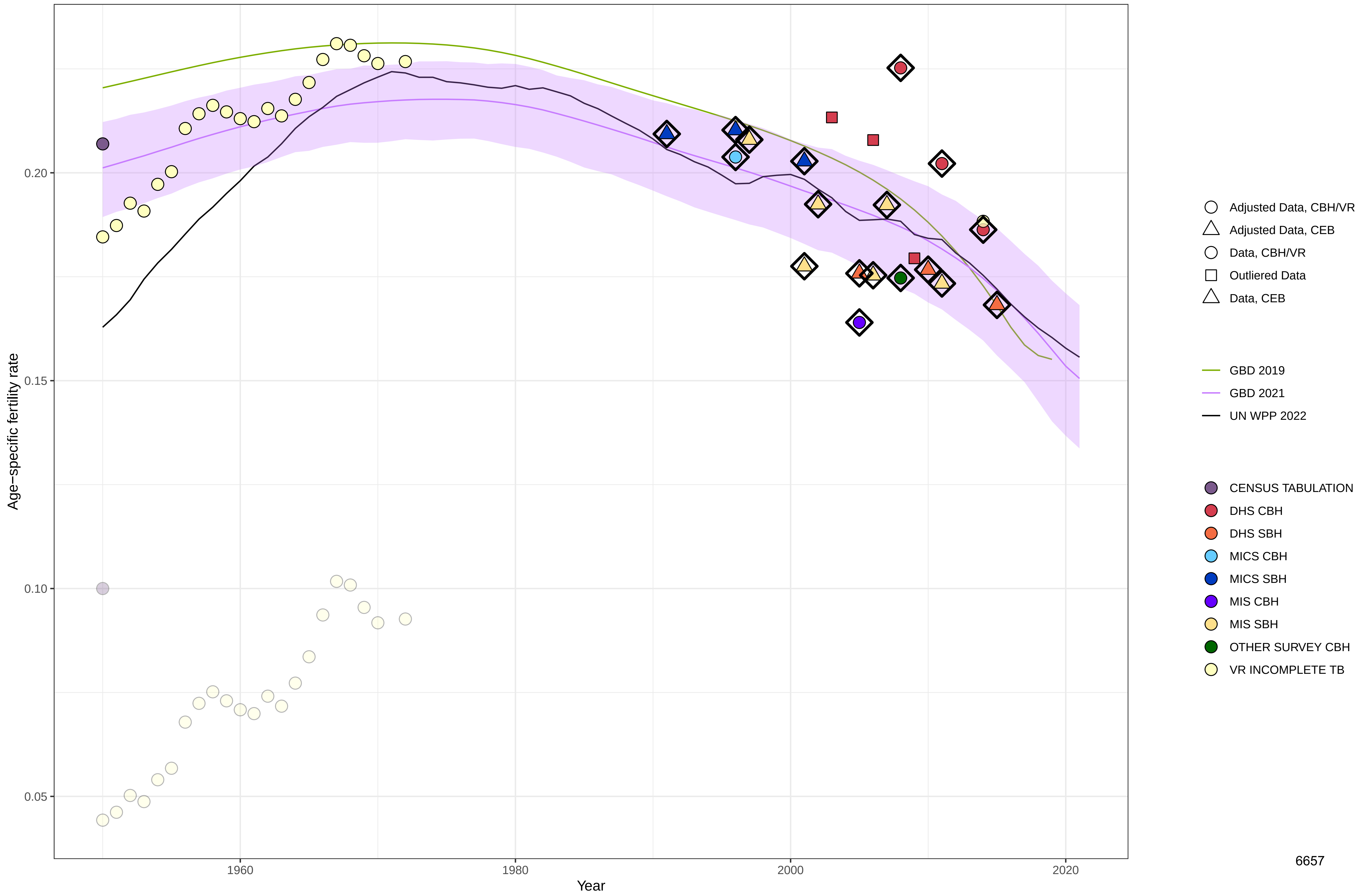

Age−specific fertility fitted trend lines, 1950−2021: Angola, Ages: 40−44
Data points enclosed with a diamond are reference sources

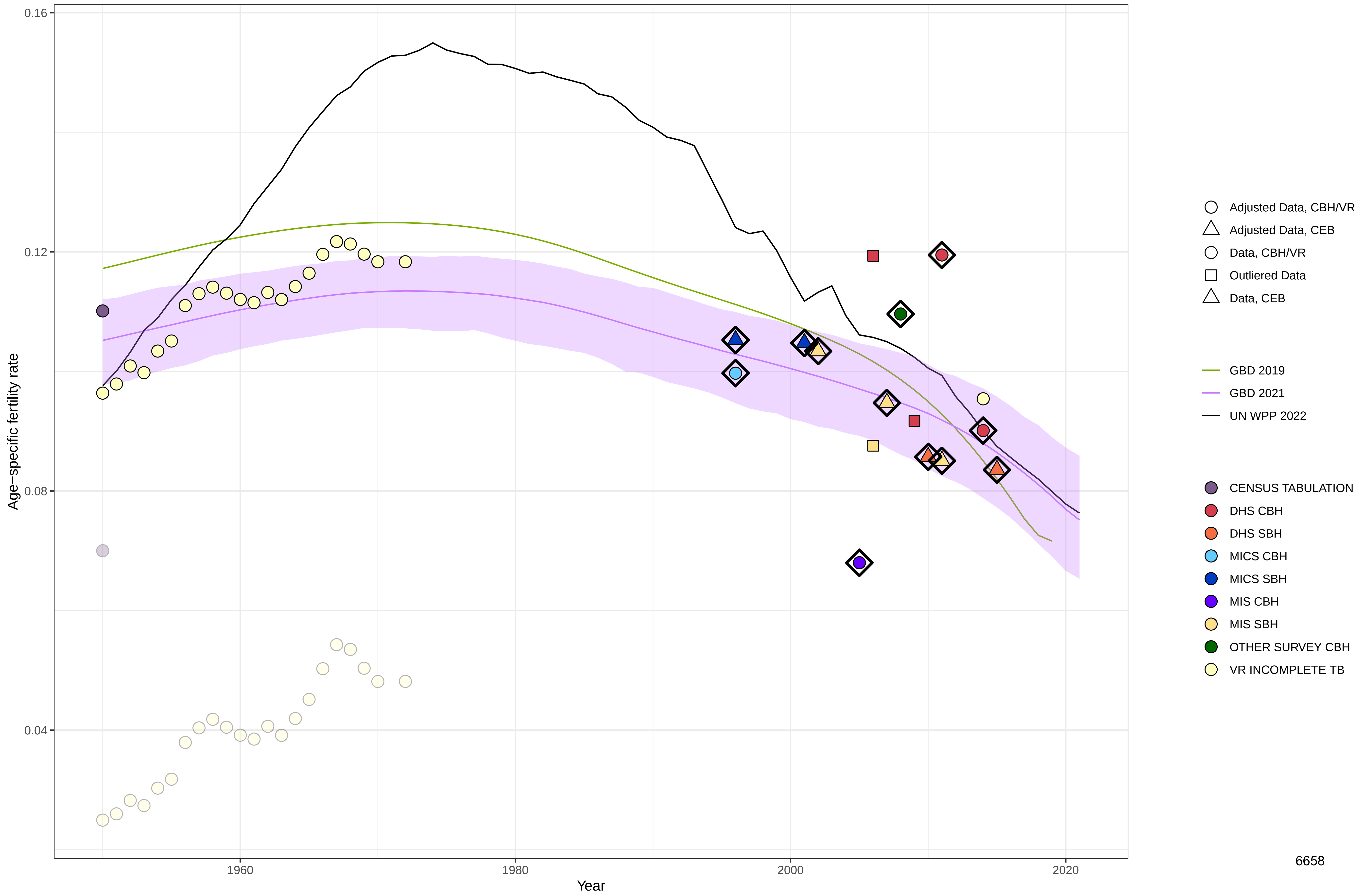

Age−specific fertility fitted trend lines, 1950−2021: Angola, Ages: 45−49
Data points enclosed with a diamond are reference sources

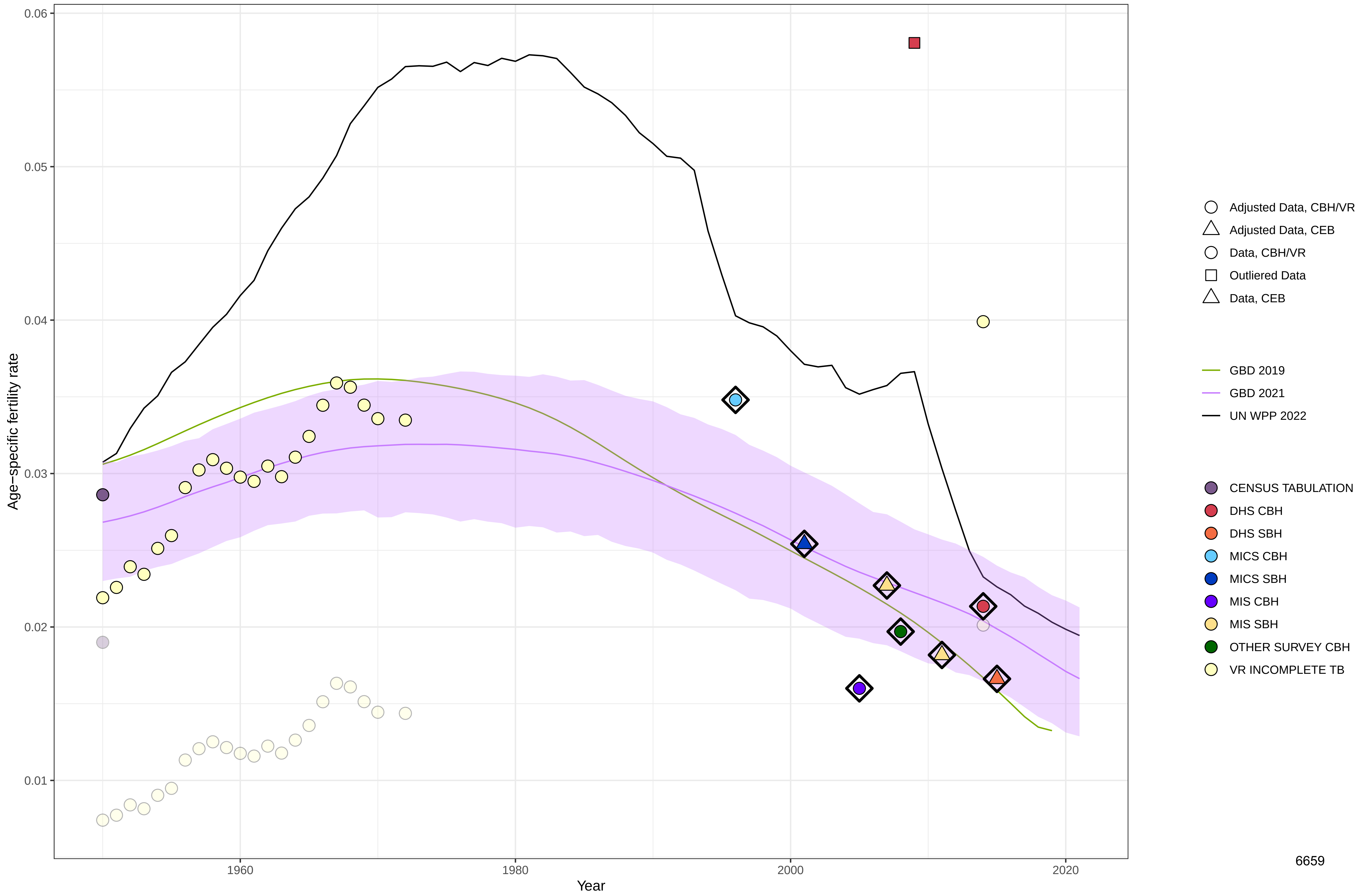

Age−specific fertility fitted trend lines, 1950−2021: Angola, Ages: 50−54
Data points enclosed with a diamond are reference sources

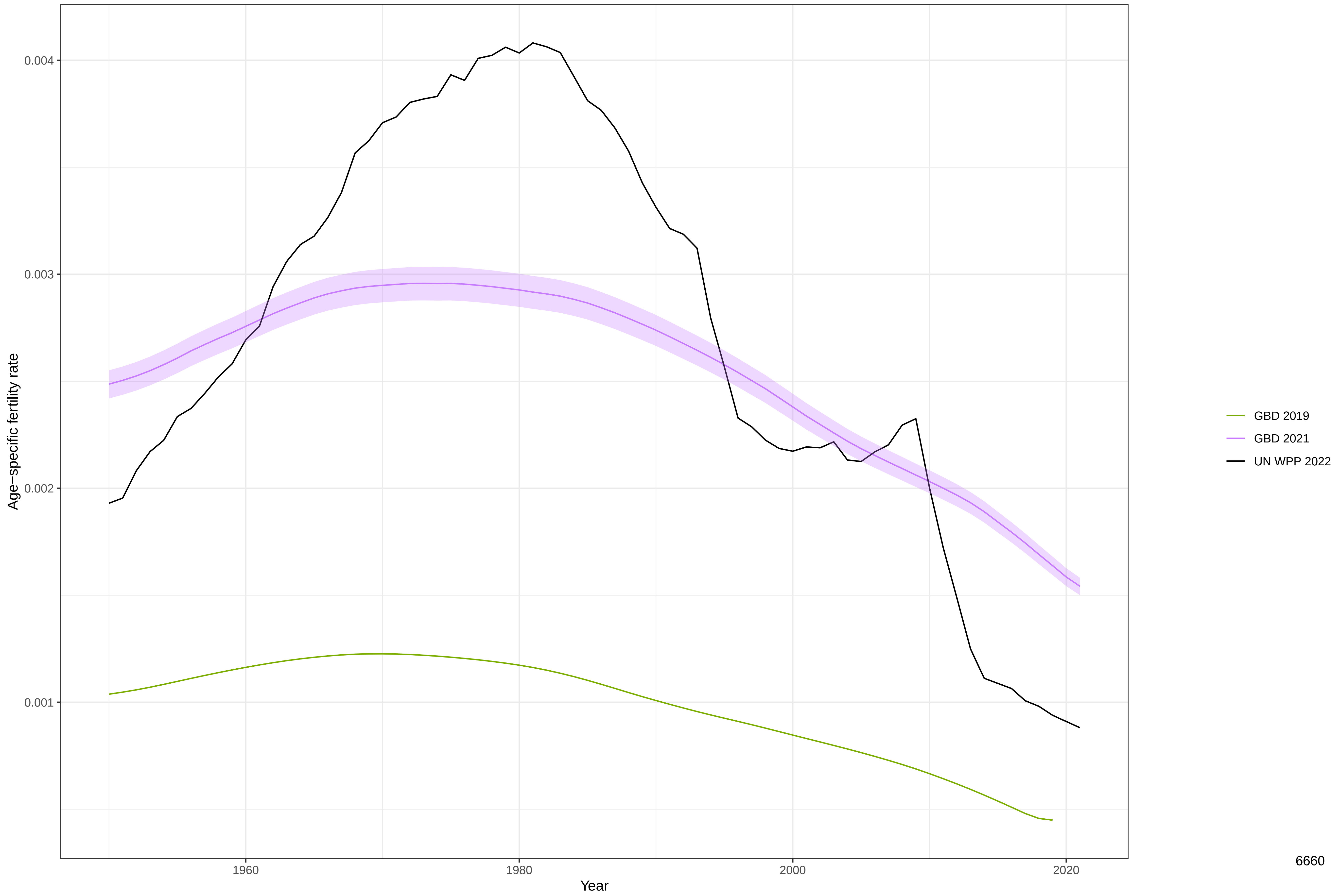

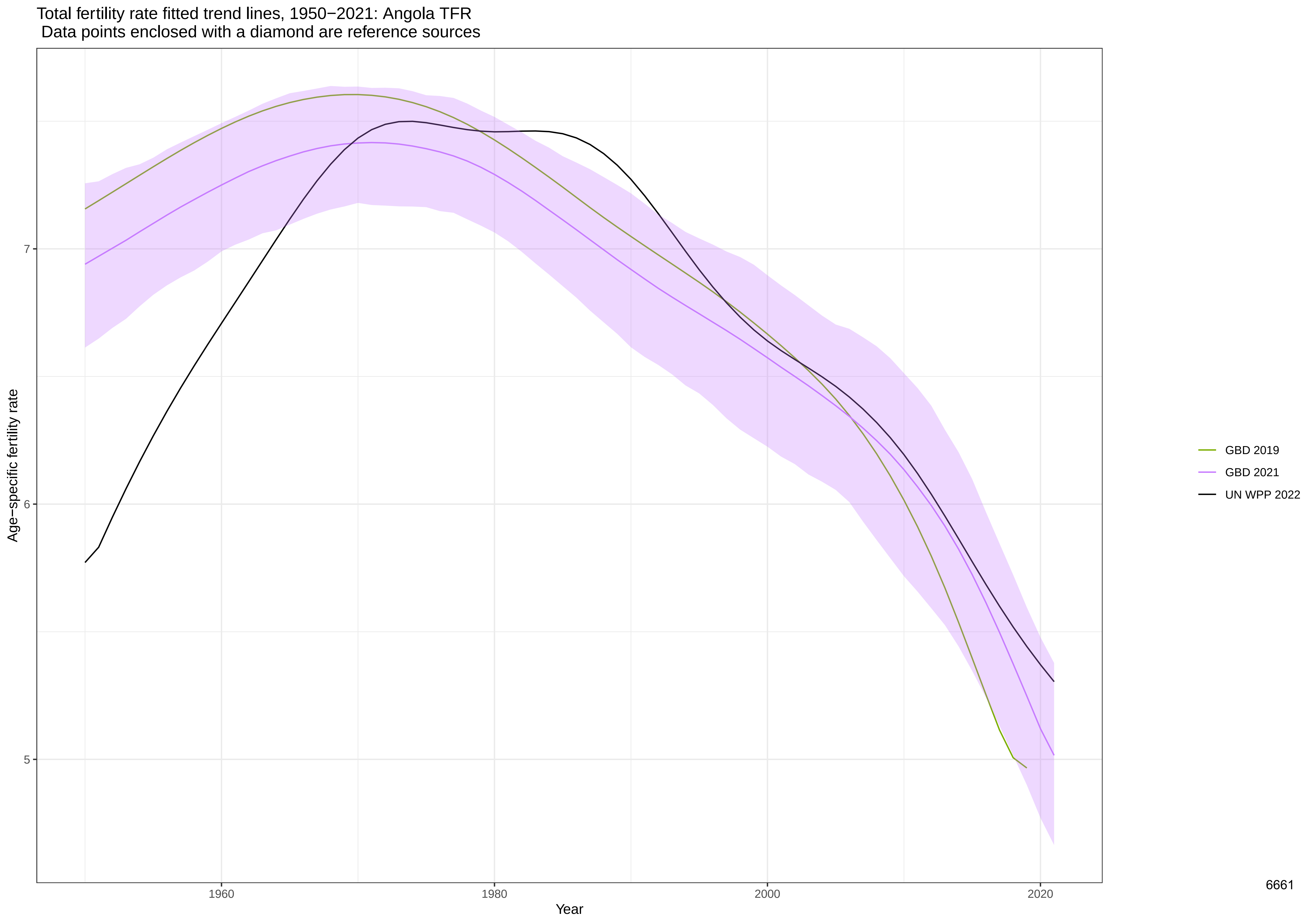
Total fertility rate fitted trend lines, 1950−2021: Angola TFR
Data points enclosed with a diamond are reference sources
Age−specific fertility rate
7
6
5
1960
1980
2000
2020
Year
GBD 2019
GBD 2021
UN WPP 2022

Age−specific fertility fitted trend lines, 1950−2021: Central African Republic, Ages: 10−14
Data points enclosed with a diamond are reference sources

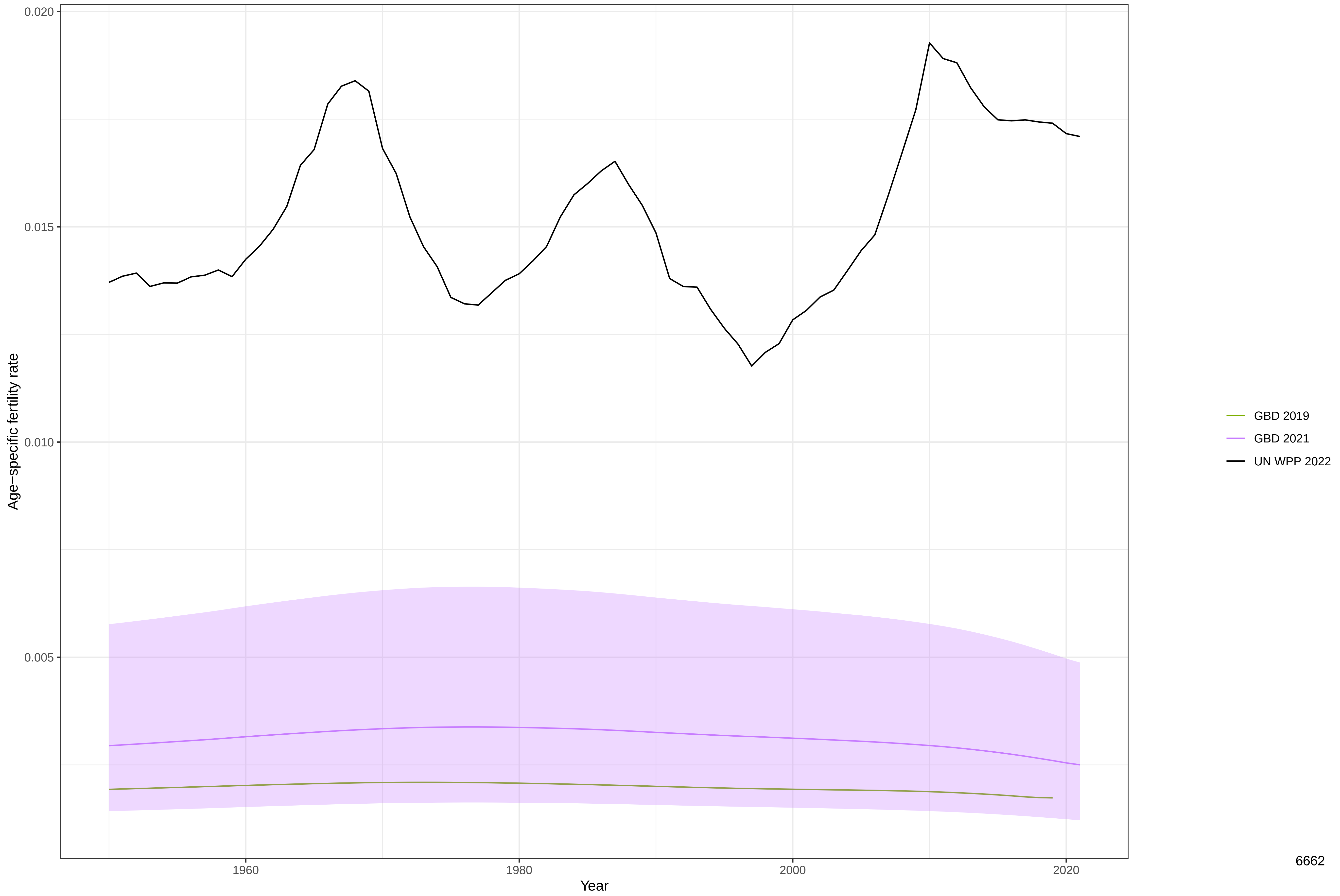

Age−specific fertility fitted trend lines, 1950−2021: Central African Republic, Ages: 15−19
Data points enclosed with a diamond are reference sources

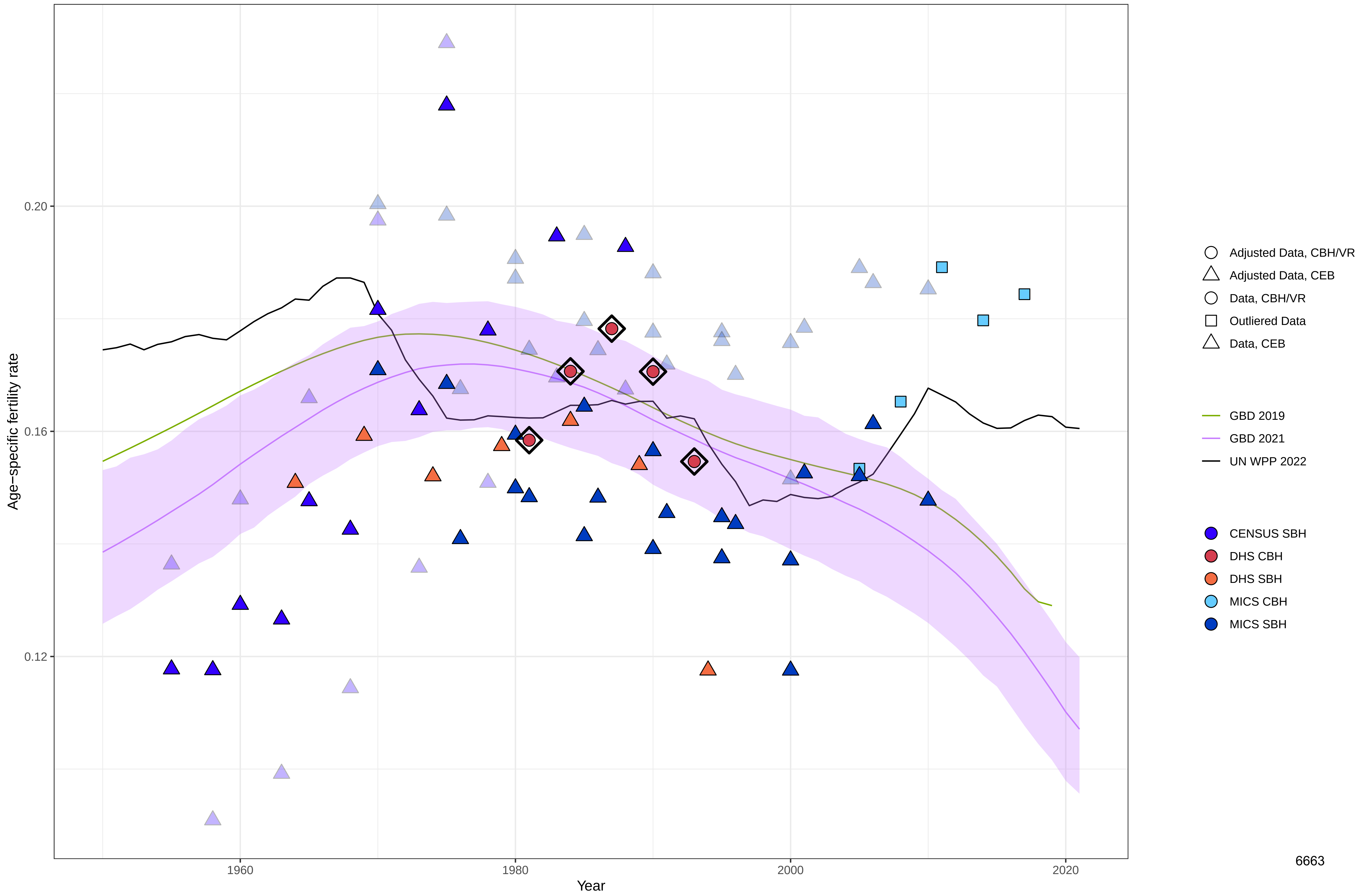

Age−specific fertility fitted trend lines, 1950−2021: Central African Republic, Ages: 20−24
Data points enclosed with a diamond are reference sources

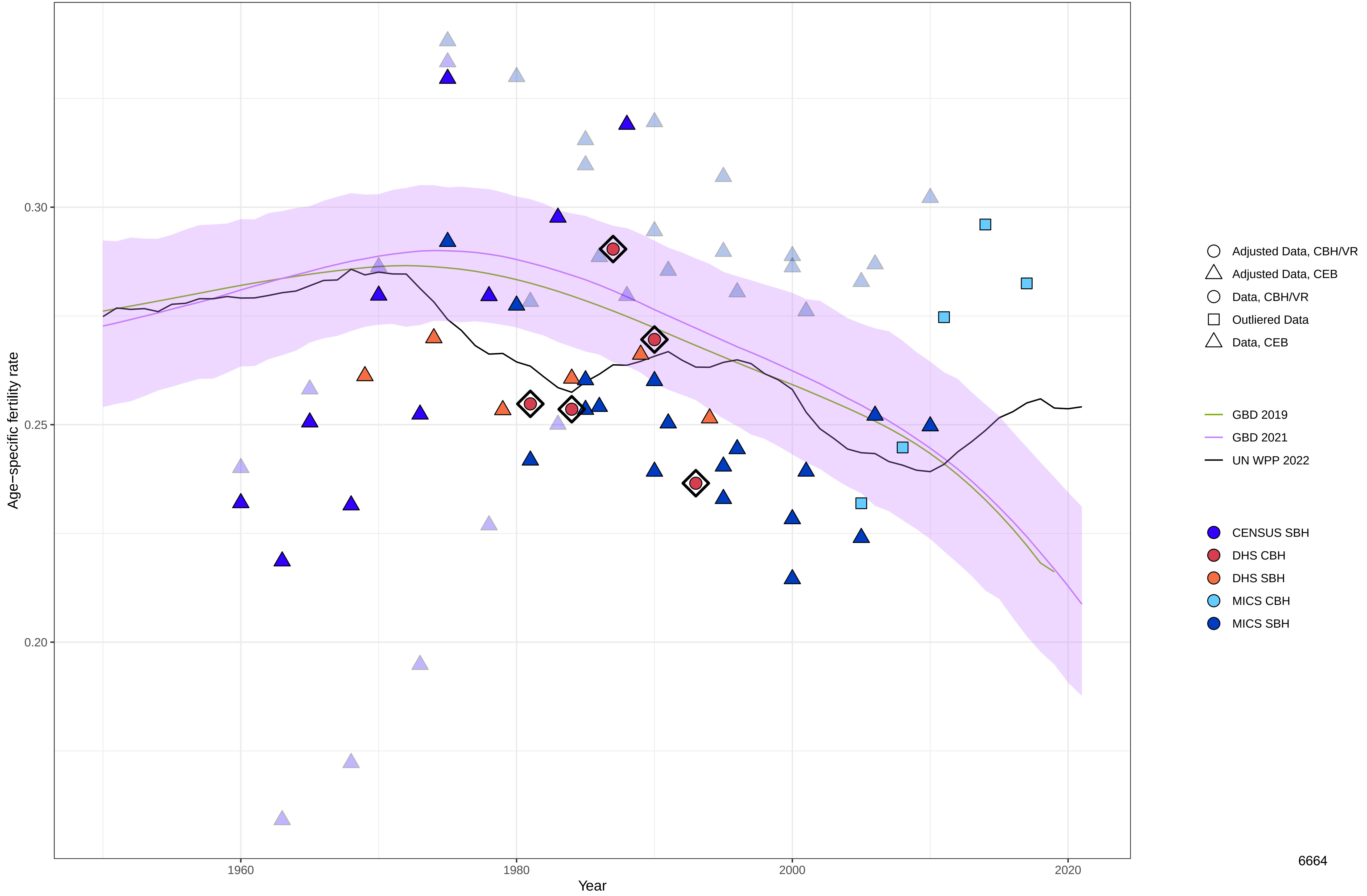

Age−specific fertility fitted trend lines, 1950−2021: Central African Republic, Ages: 25−29
Data points enclosed with a diamond are reference sources

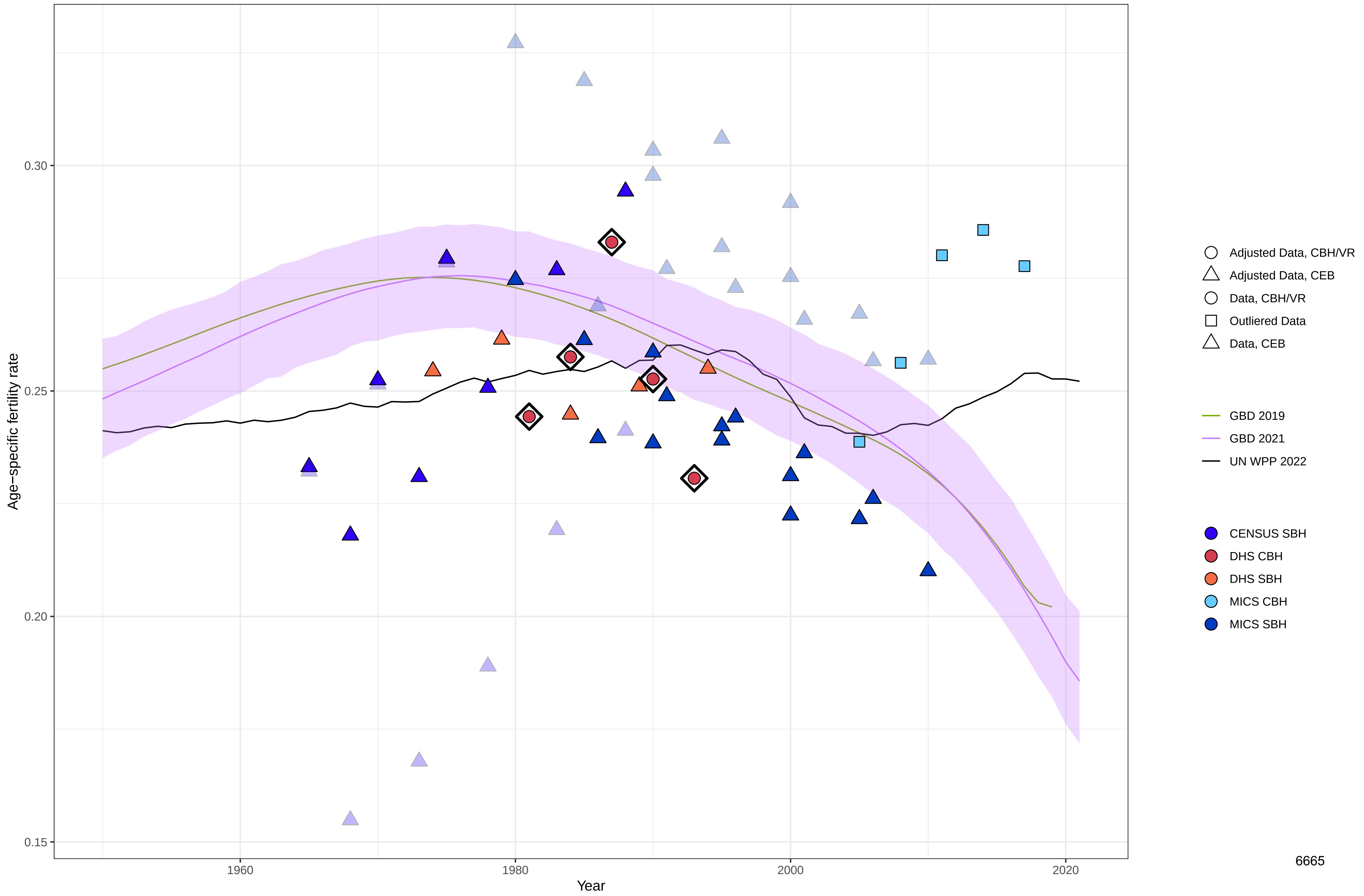

Age−specific fertility fitted trend lines, 1950−2021: Central African Republic, Ages: 30−34
Data points enclosed with a diamond are reference sources

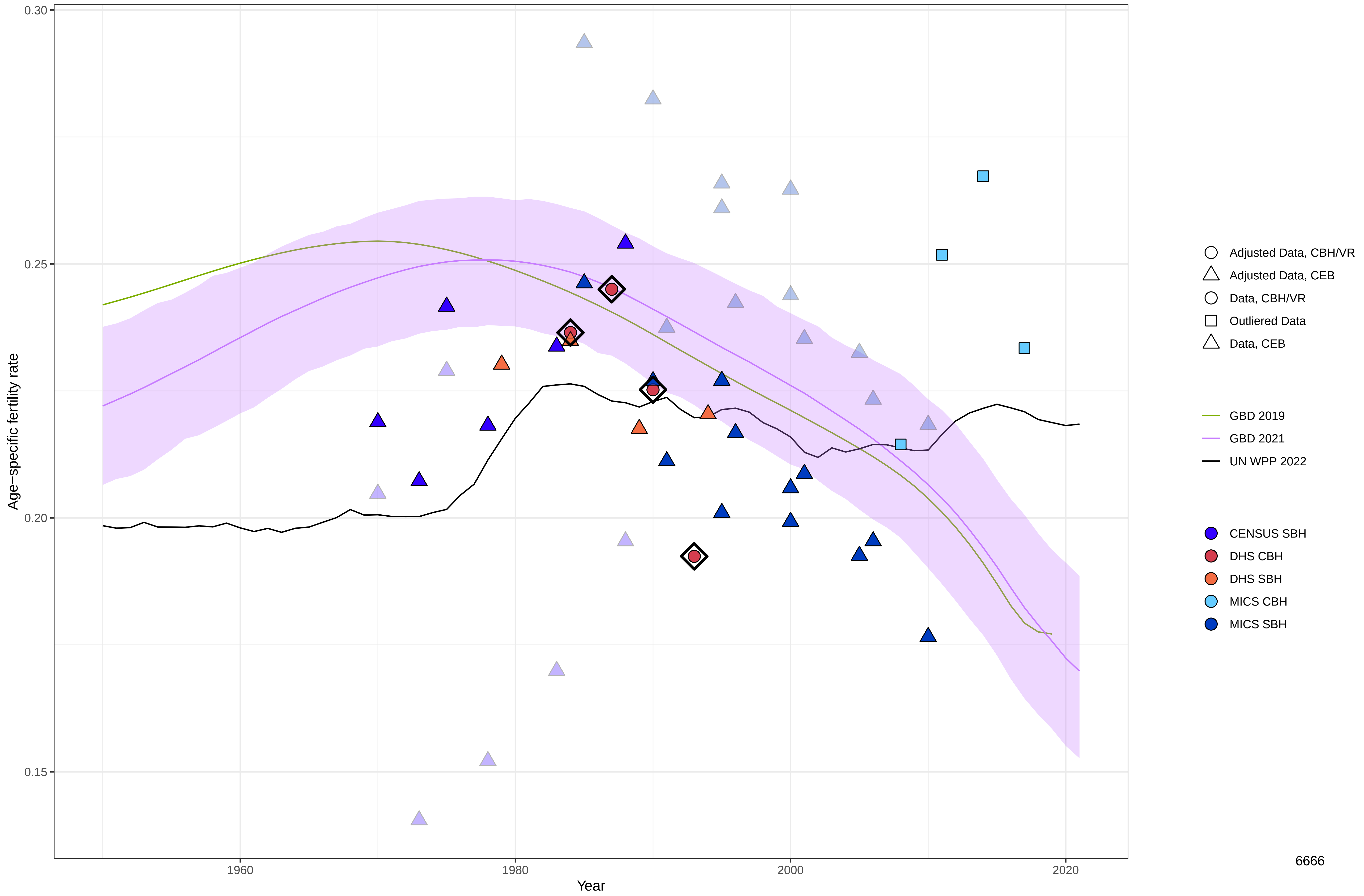

Age−specific fertility fitted trend lines, 1950−2021: Central African Republic, Ages: 35−39
Data points enclosed with a diamond are reference sources

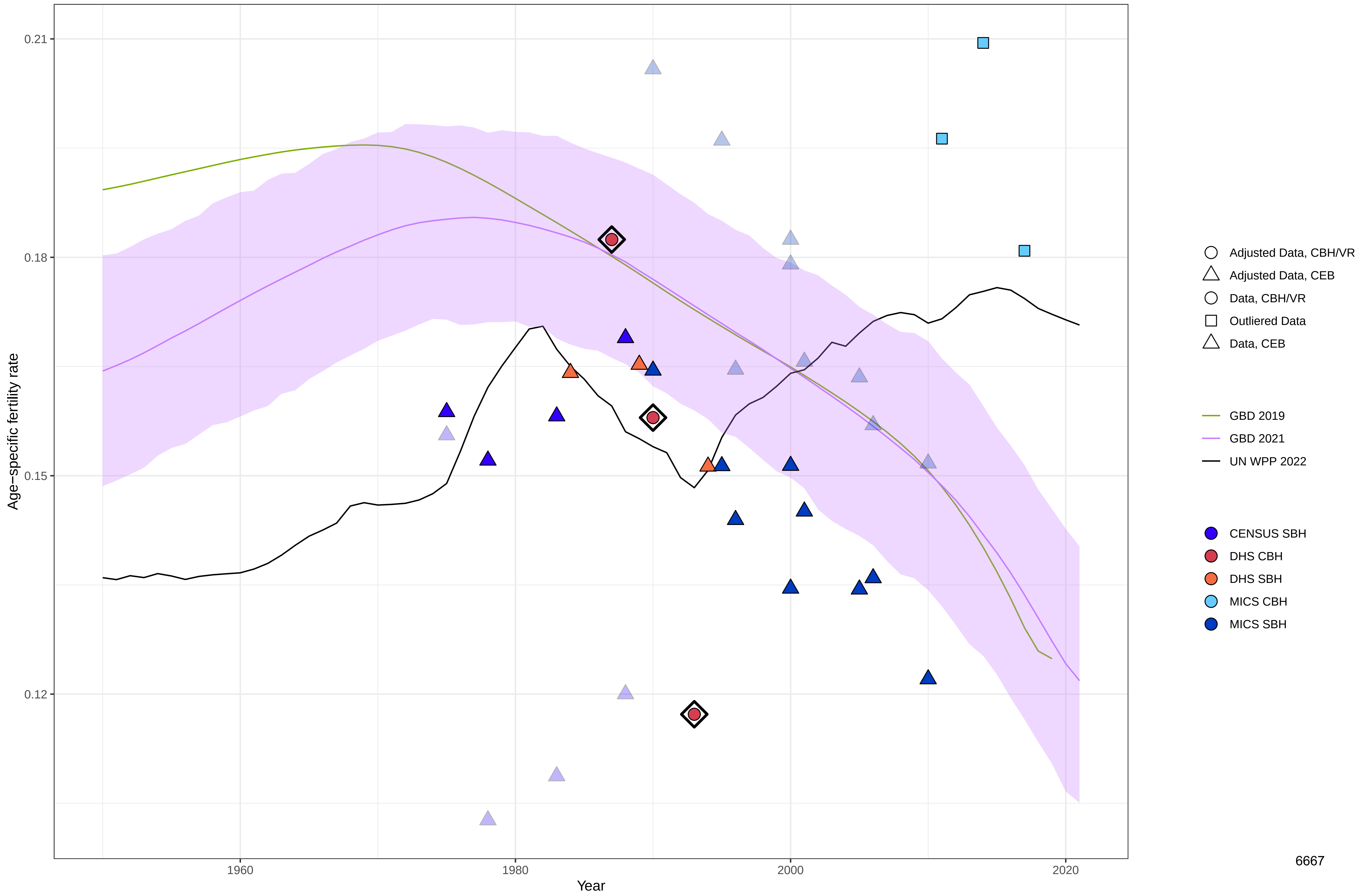

Age−specific fertility fitted trend lines, 1950−2021: Central African Republic, Ages: 40−44
Data points enclosed with a diamond are reference sources

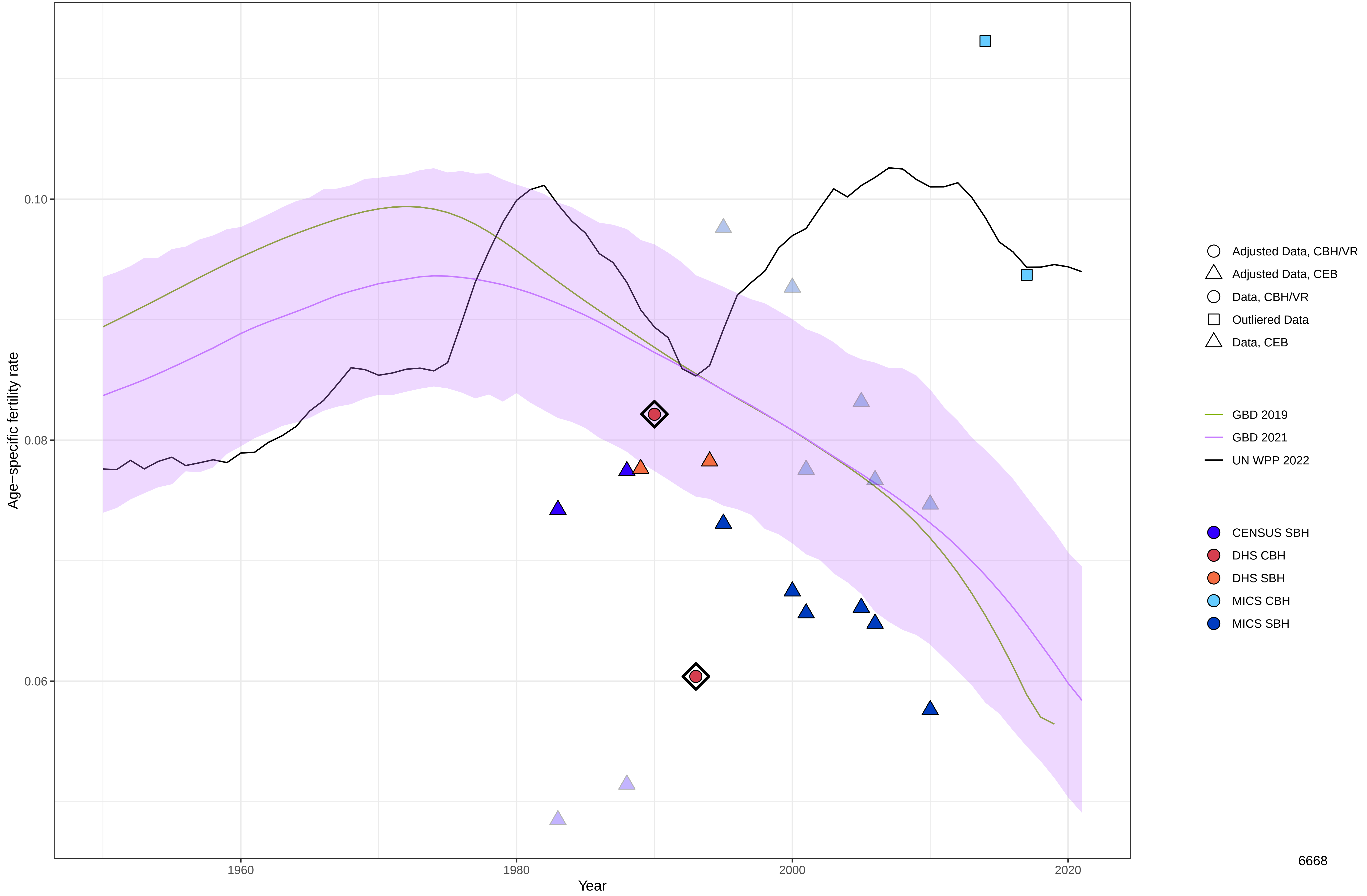

Age−specific fertility fitted trend lines, 1950−2021: Central African Republic, Ages: 45−49
Data points enclosed with a diamond are reference sources

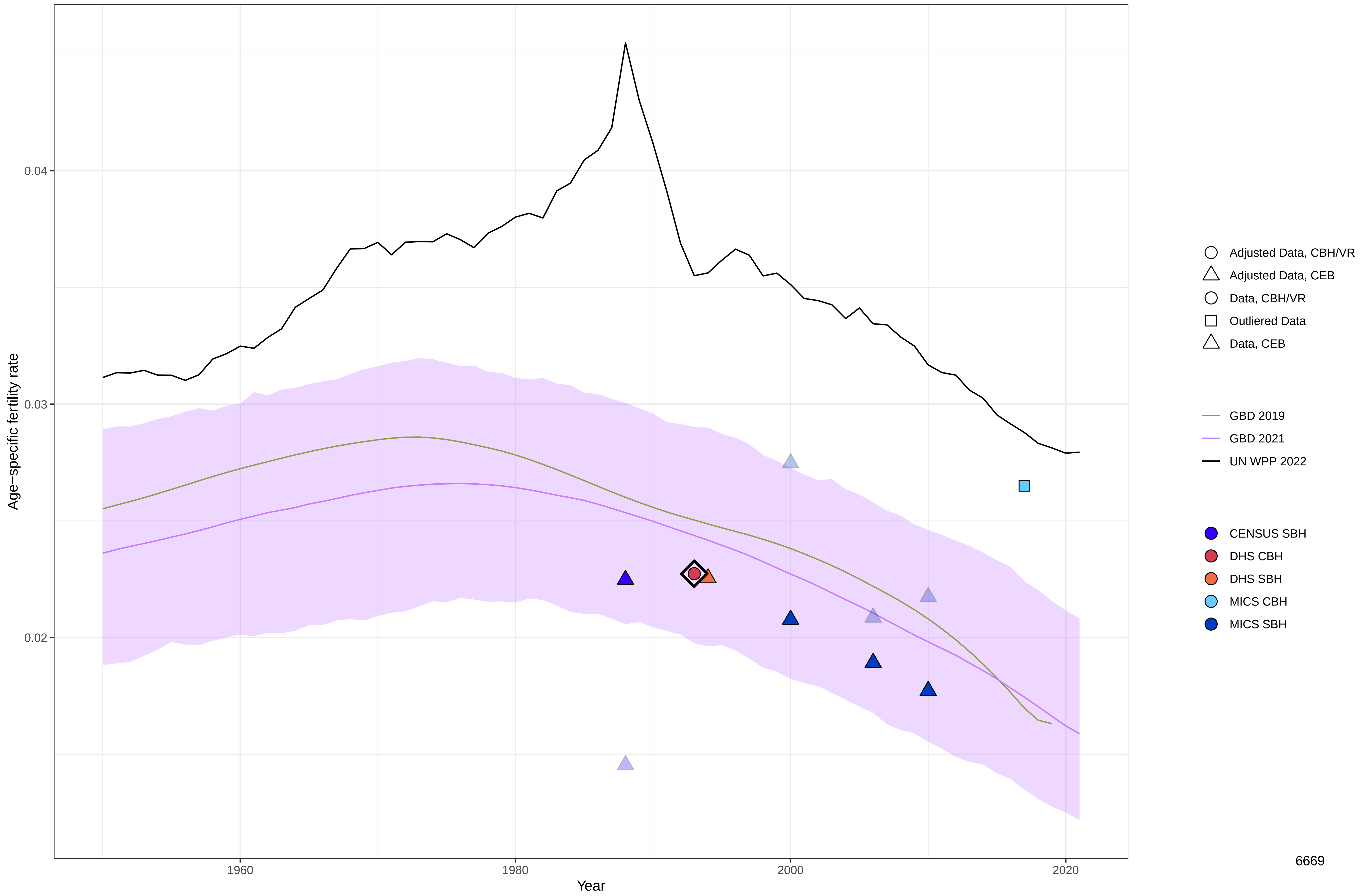

Age−specific fertility fitted trend lines, 1950−2021: Central African Republic, Ages: 50−54
Data points enclosed with a diamond are reference sources

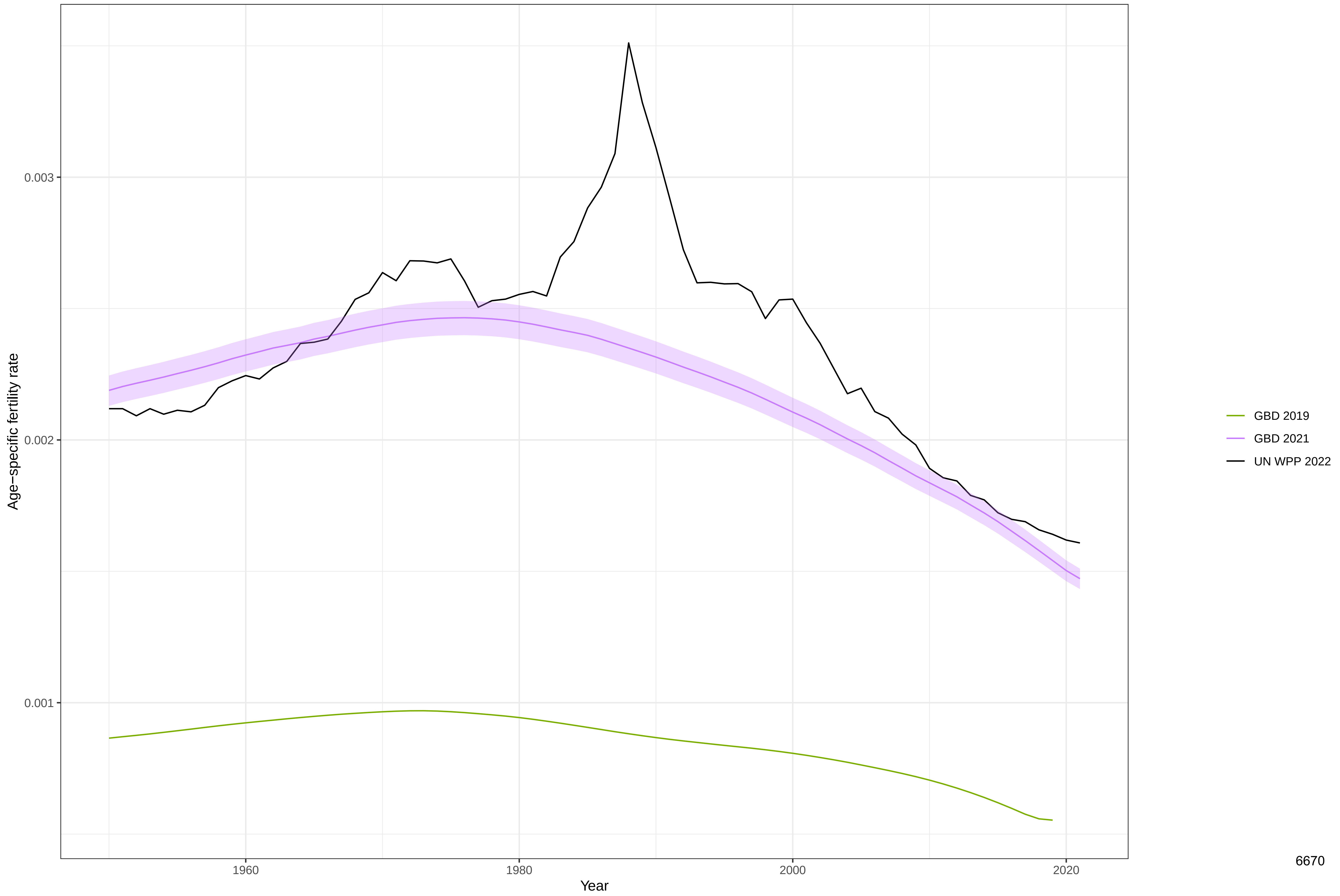

Total fertility rate fitted trend lines, 1950−2021: Central African Republic TFR
Data points enclosed with a diamond are reference sources

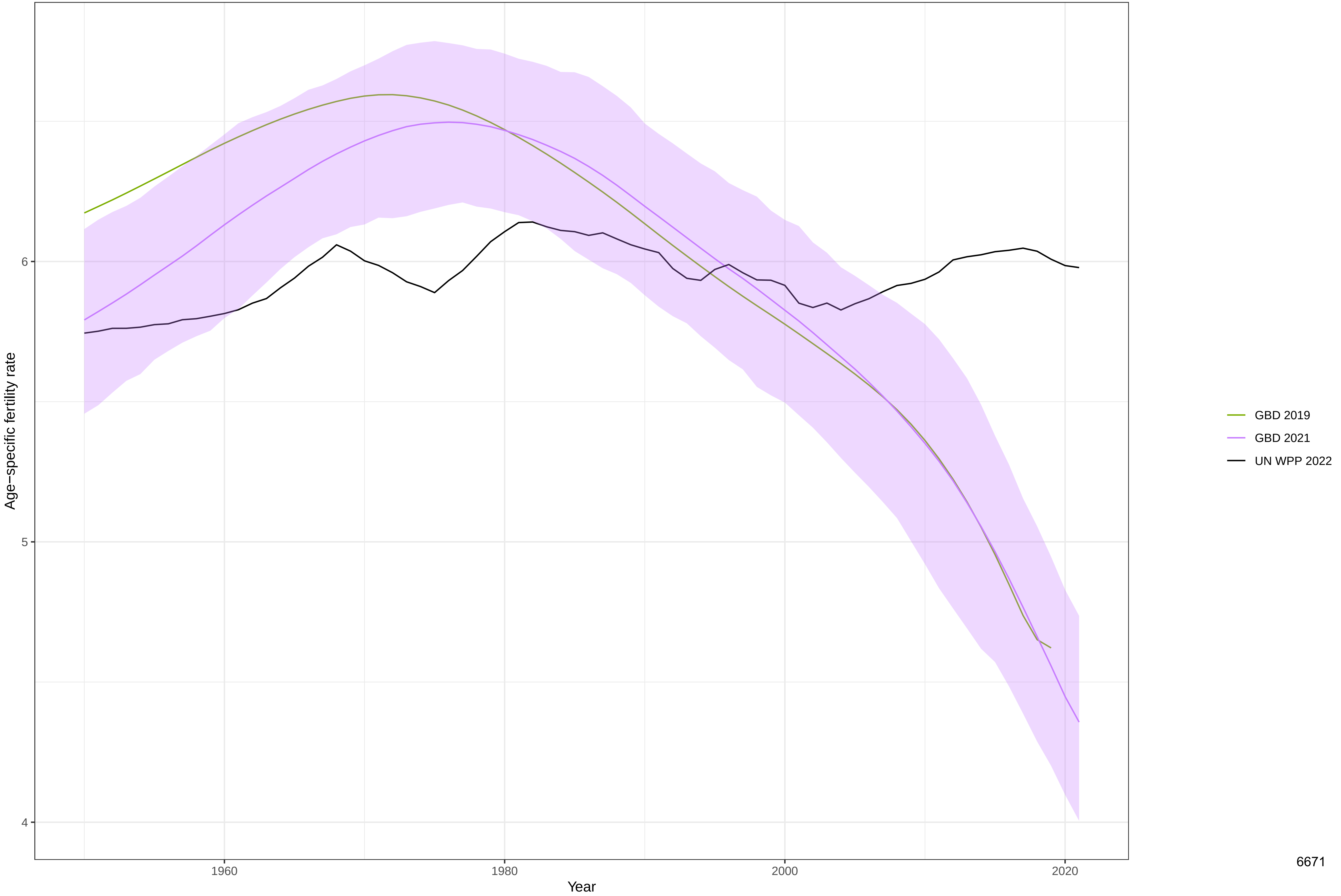

Age−specific fertility fitted trend lines, 1950−2021: Congo (Brazzaville), Ages: 10−14
Data points enclosed with a diamond are reference sources

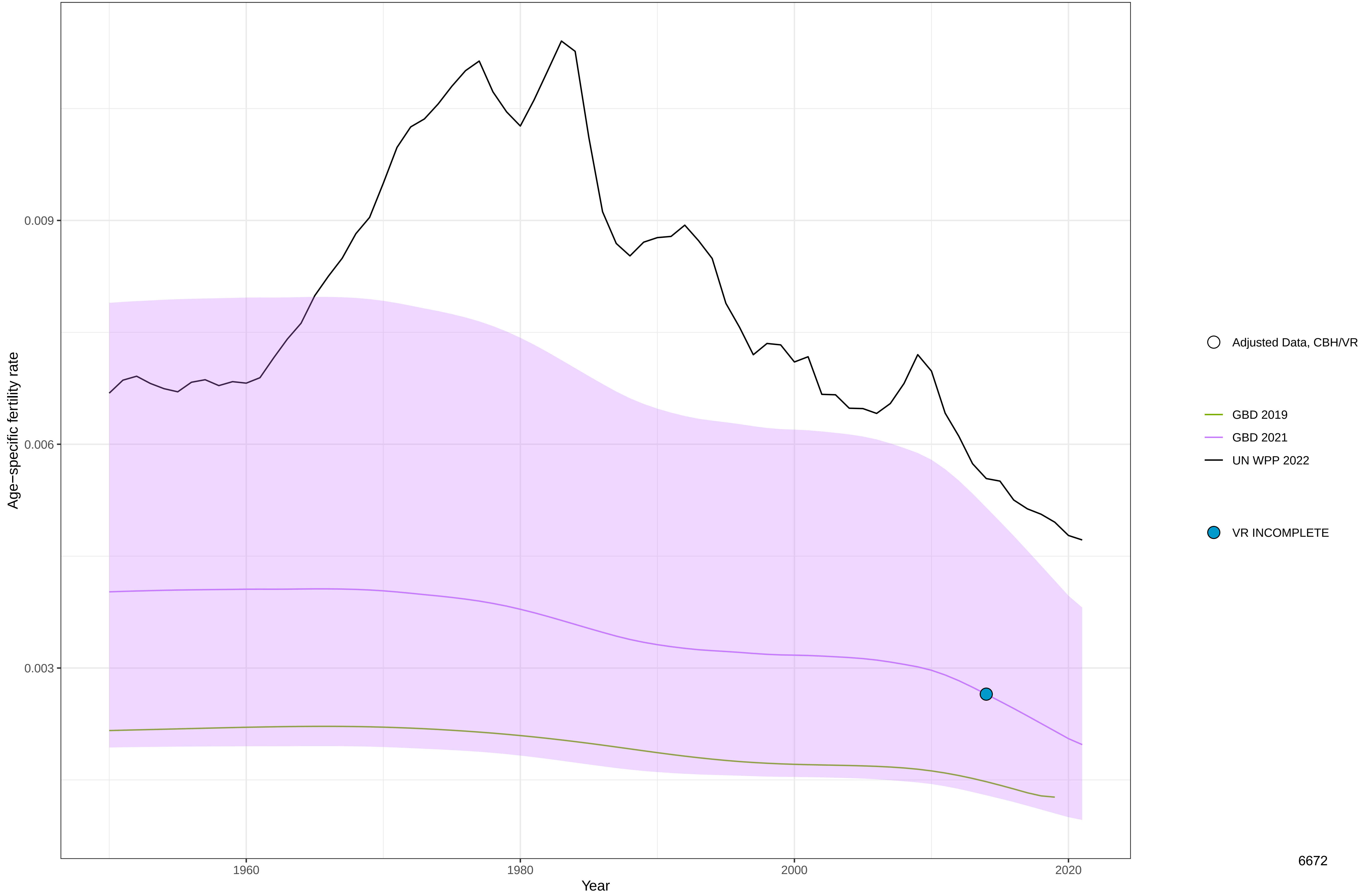

Age−specific fertility fitted trend lines, 1950−2021: Congo (Brazzaville), Ages: 15−19
Data points enclosed with a diamond are reference sources

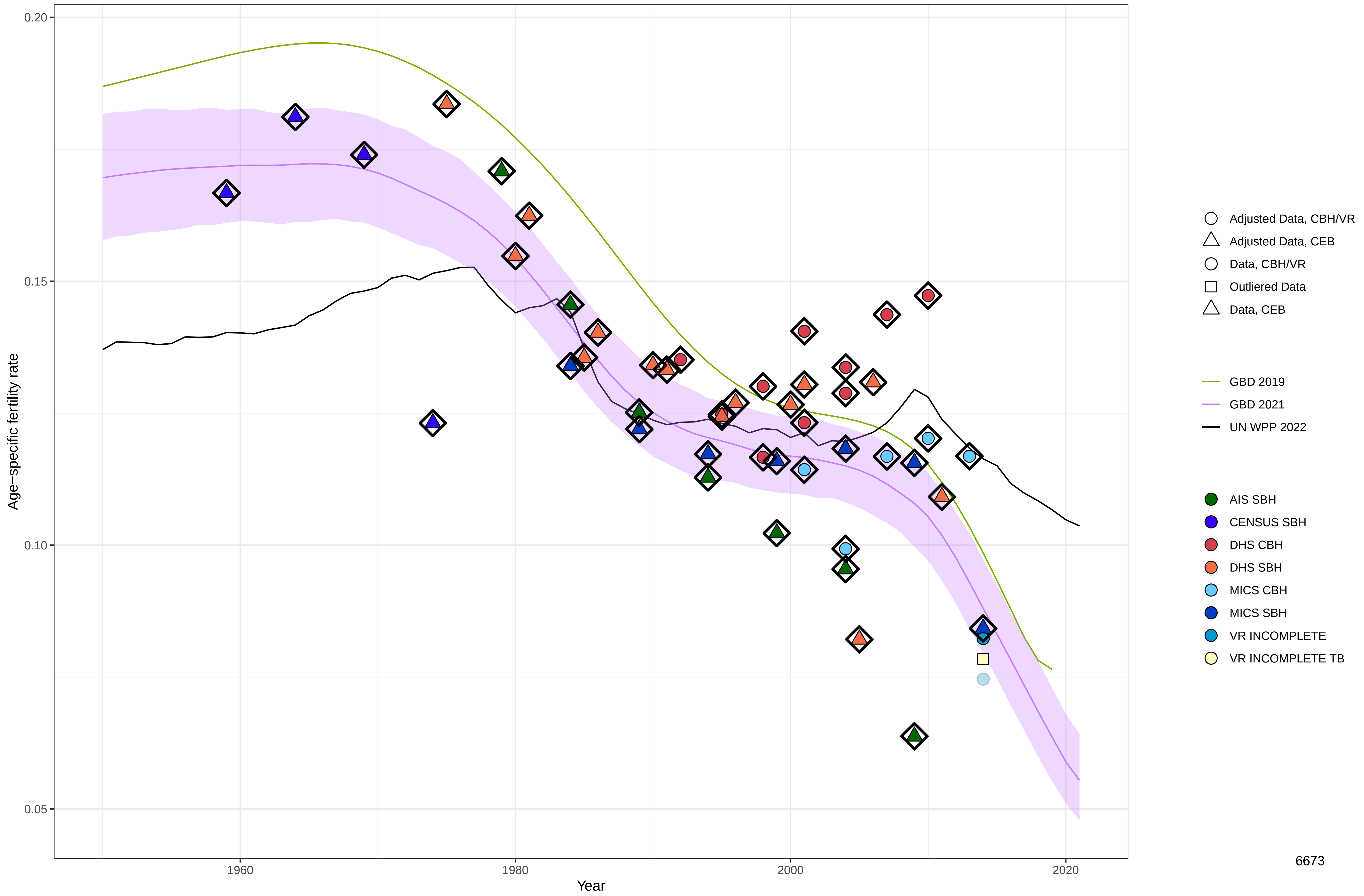

Age−specific fertility fitted trend lines, 1950−2021: Congo (Brazzaville), Ages: 20−24
Data points enclosed with a diamond are reference sources

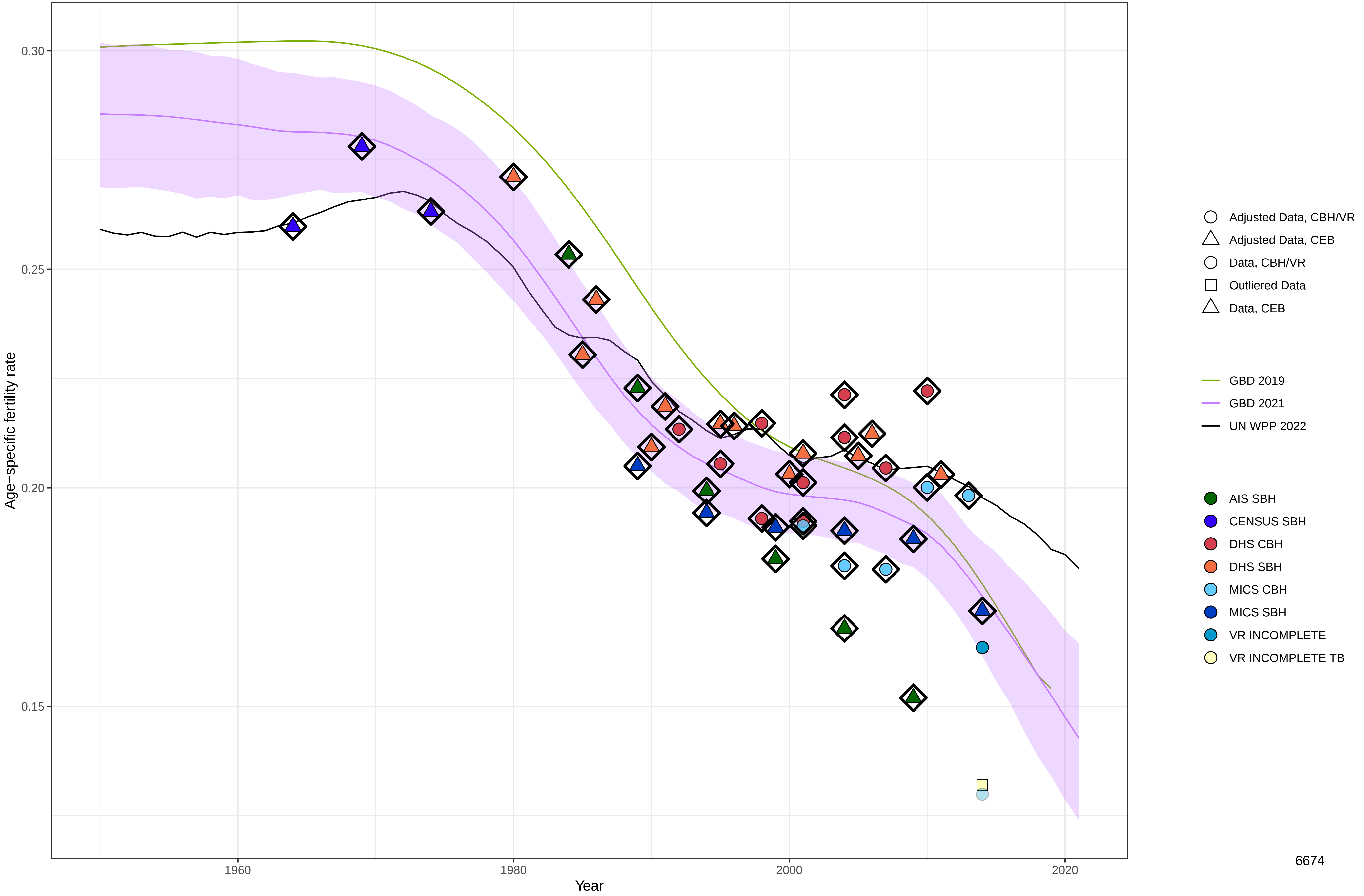

Age−specific fertility fitted trend lines, 1950−2021: Congo (Brazzaville), Ages: 25−29
Data points enclosed with a diamond are reference sources

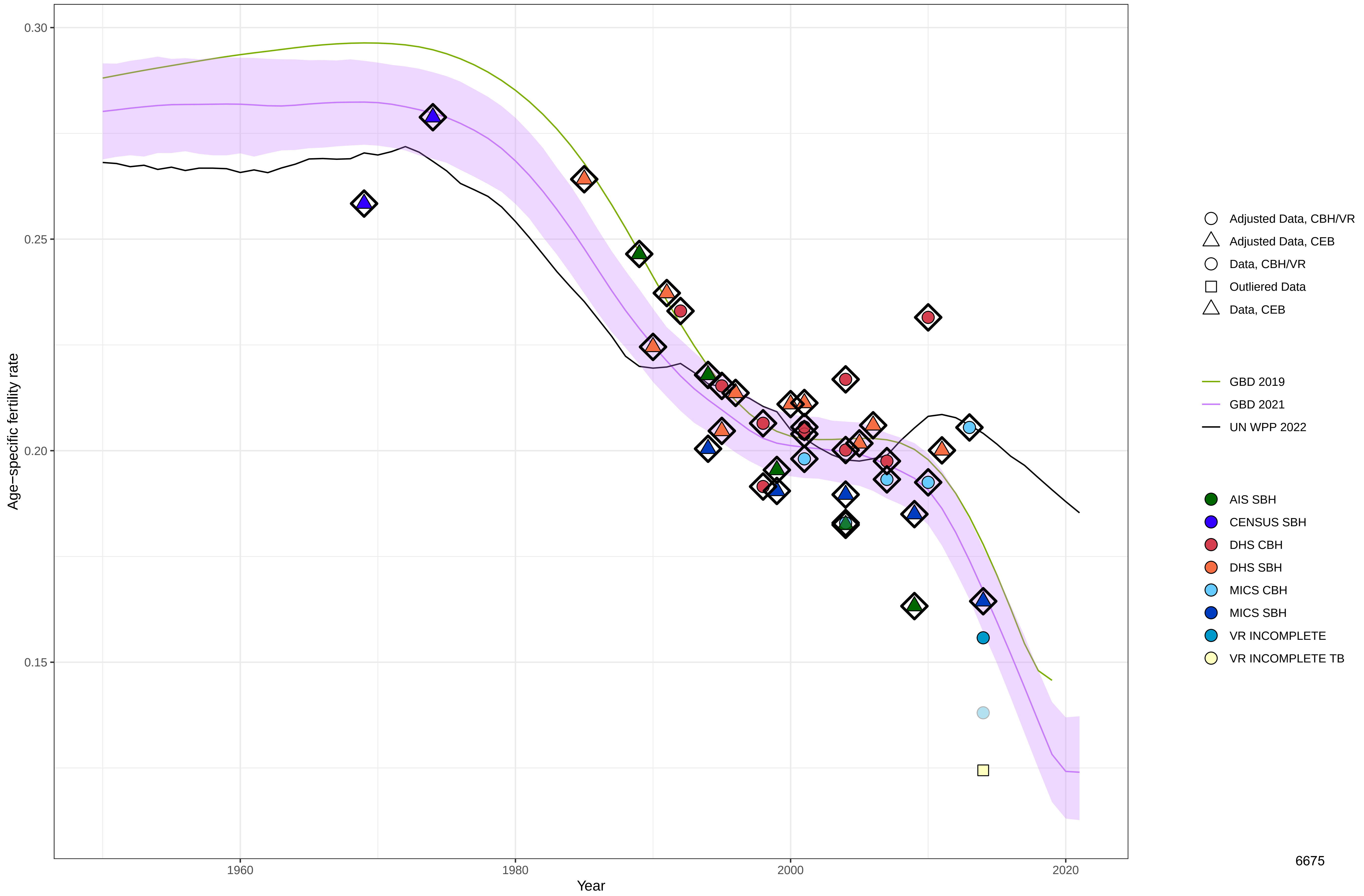

Age−specific fertility fitted trend lines, 1950−2021: Congo (Brazzaville), Ages: 30−34
Data points enclosed with a diamond are reference sources

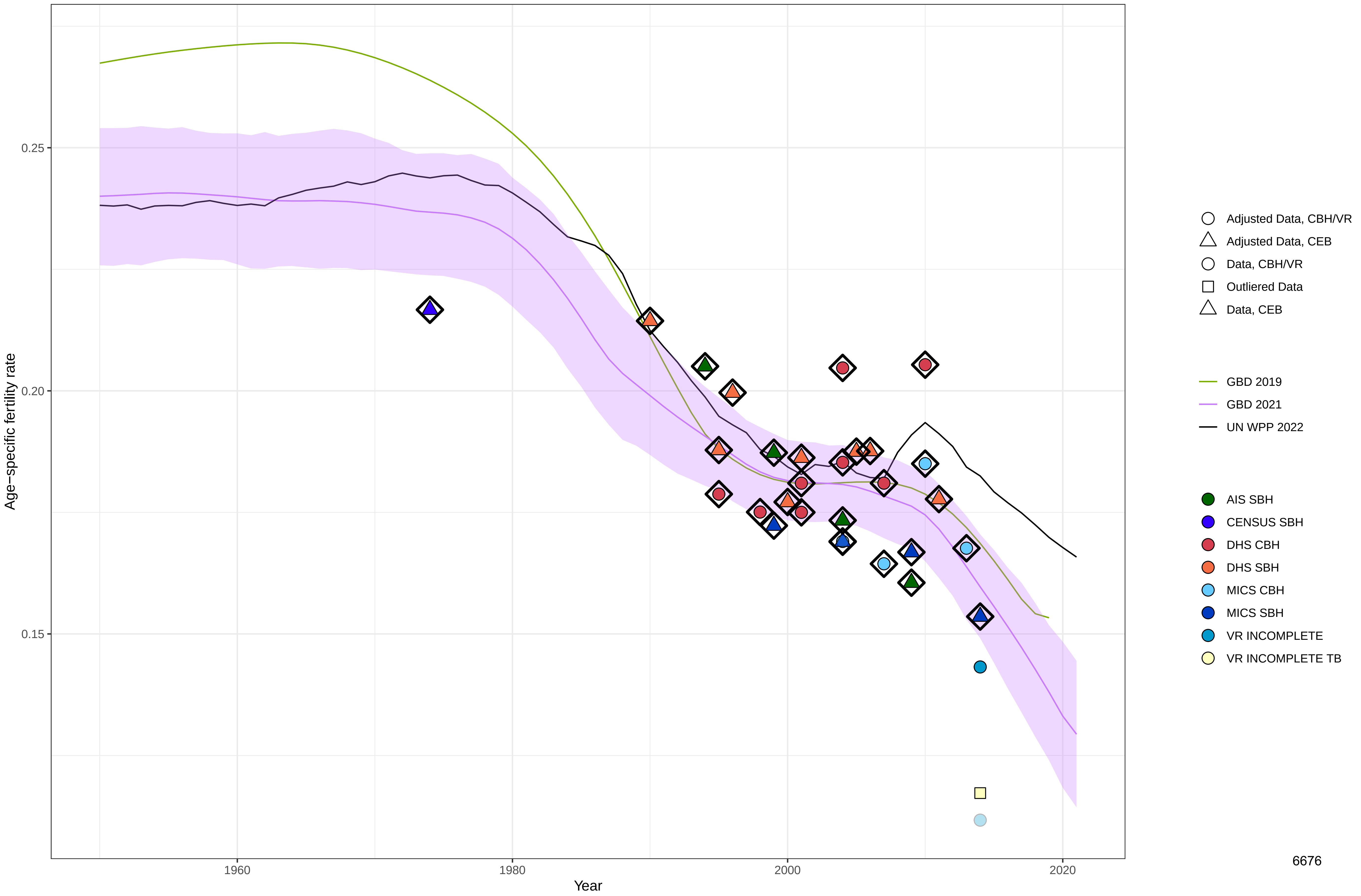

Age−specific fertility fitted trend lines, 1950−2021: Congo (Brazzaville), Ages: 35−39
Data points enclosed with a diamond are reference sources

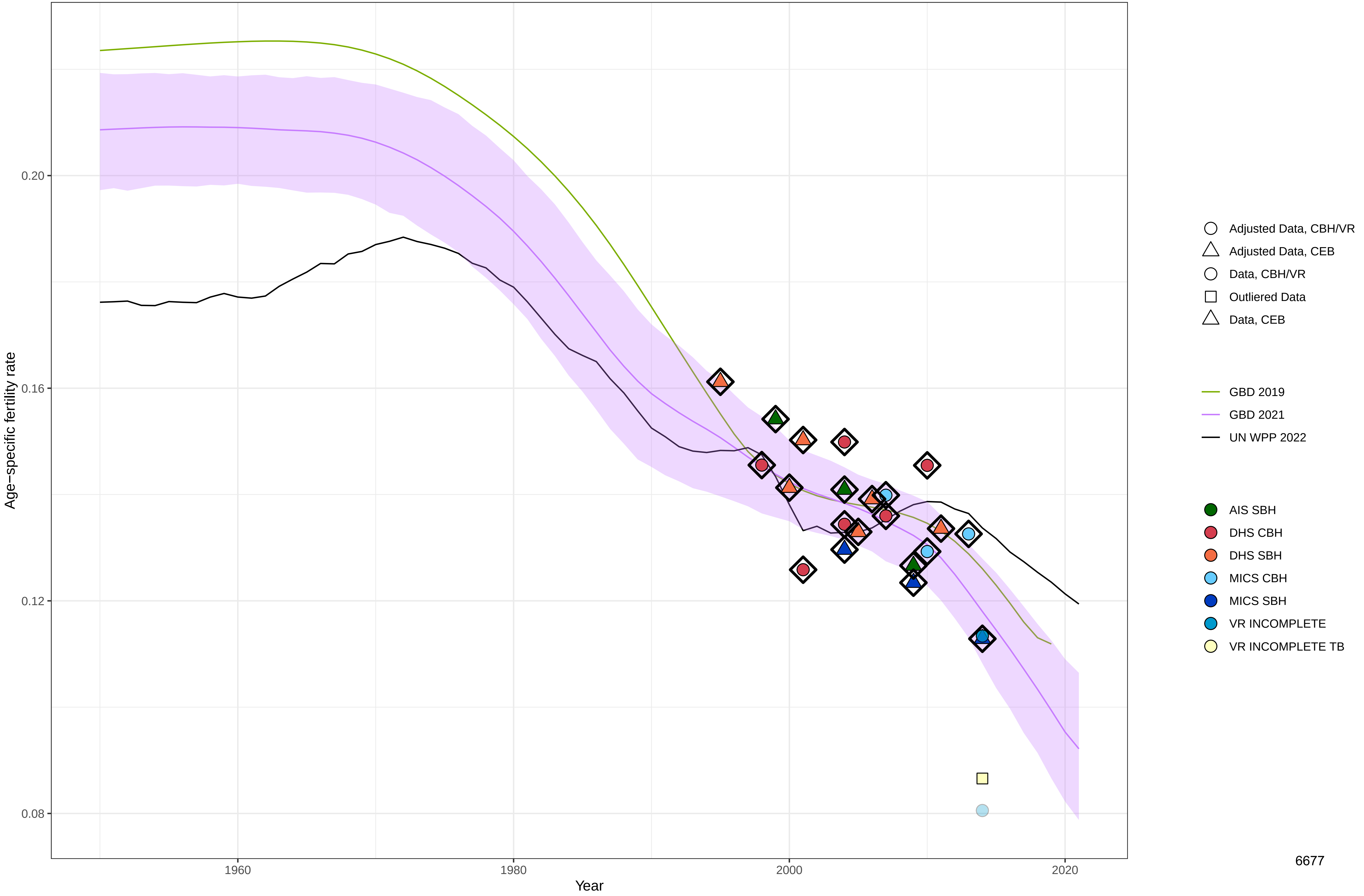

Age−specific fertility fitted trend lines, 1950−2021: Congo (Brazzaville), Ages: 40−44
Data points enclosed with a diamond are reference sources

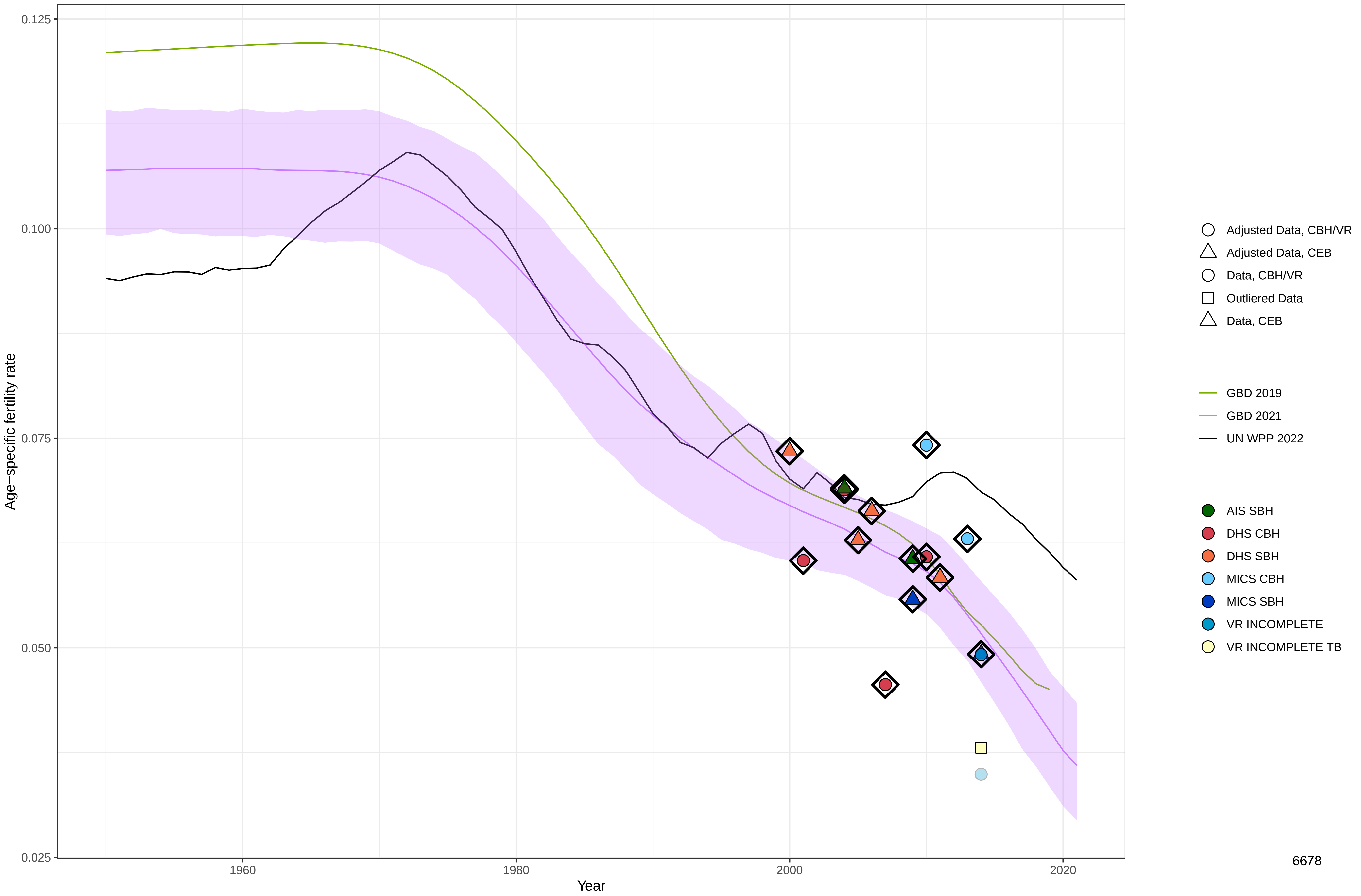

Age−specific fertility fitted trend lines, 1950−2021: Congo (Brazzaville), Ages: 45−49
Data points enclosed with a diamond are reference sources

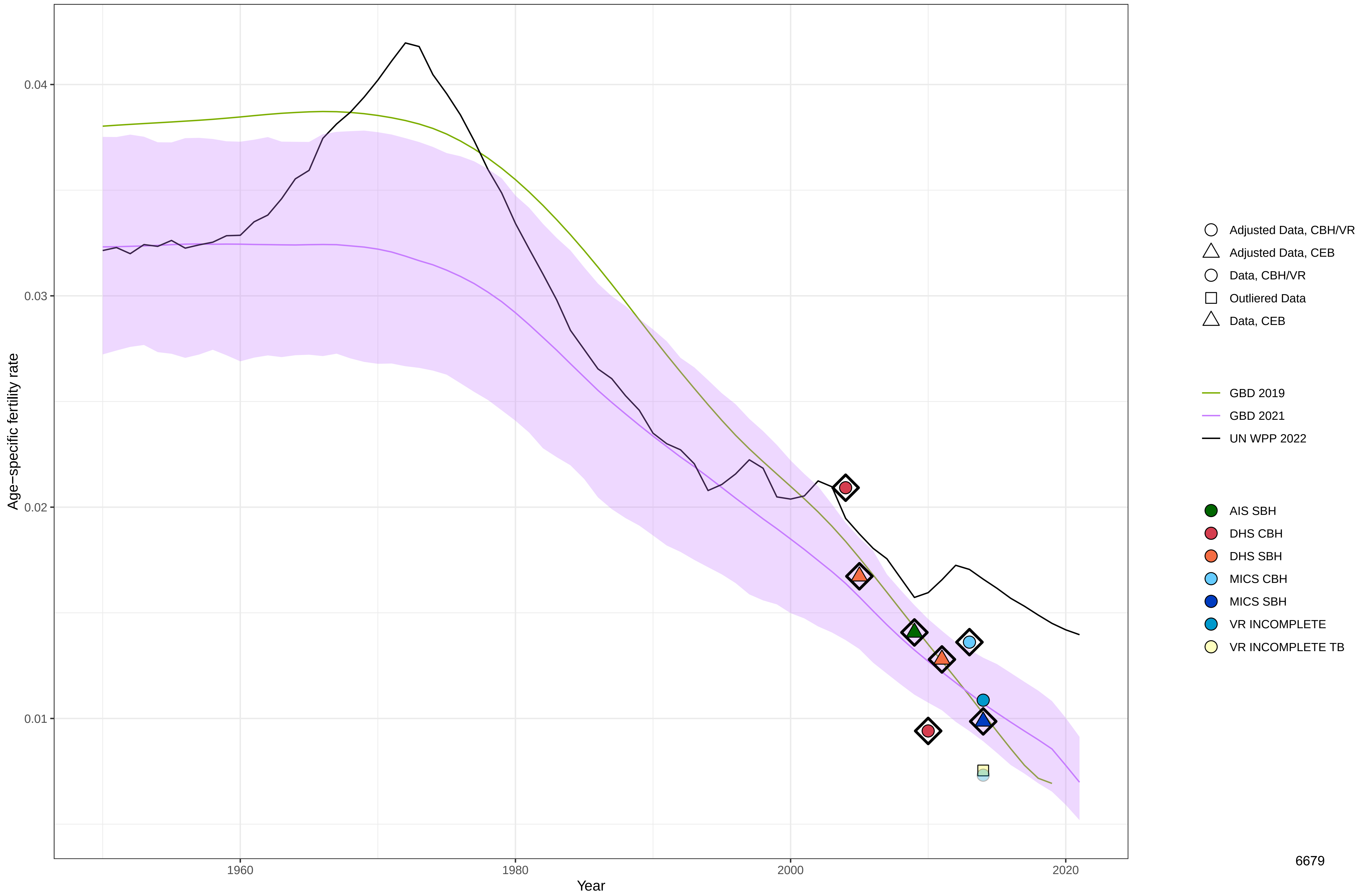

Age−specific fertility fitted trend lines, 1950−2021: Congo (Brazzaville), Ages: 50−54
Data points enclosed with a diamond are reference sources

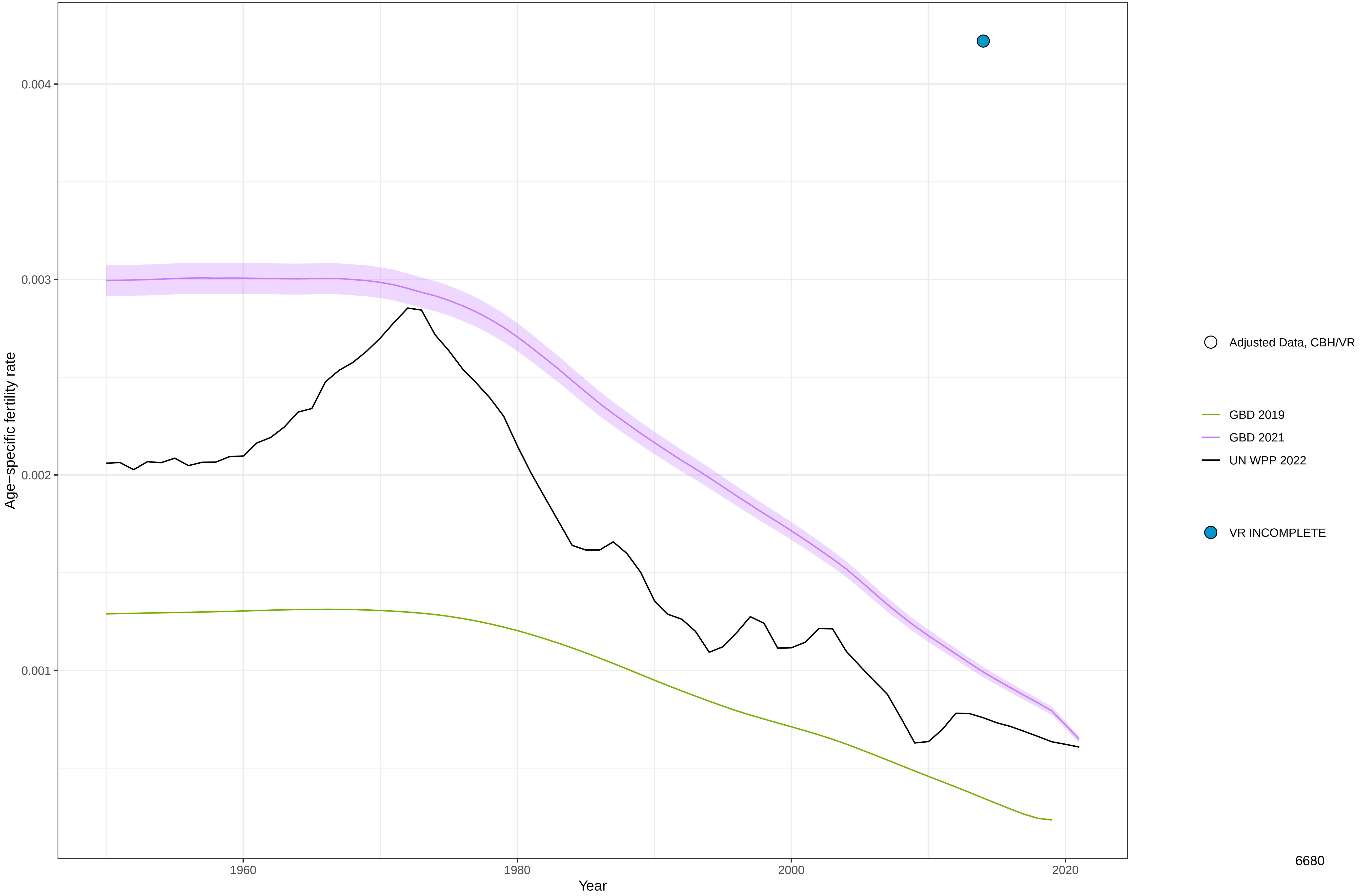

Total fertility rate fitted trend lines, 1950−2021: Congo (Brazzaville) TFR
 Data points enclosed with a diamond are reference sources

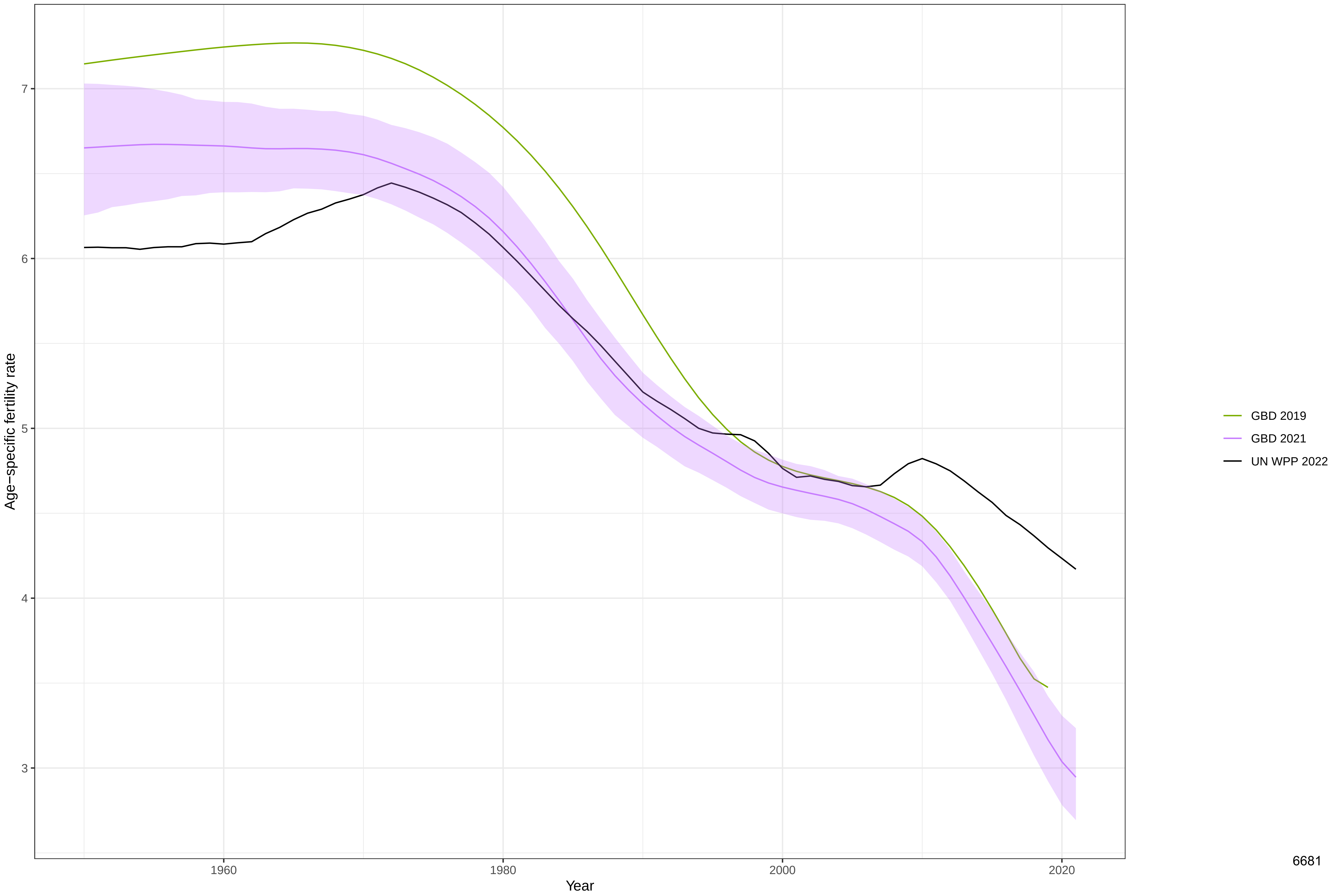

Age−specific fertility fitted trend lines, 1950−2021: DR Congo, Ages: 10−14
Data points enclosed with a diamond are reference sources

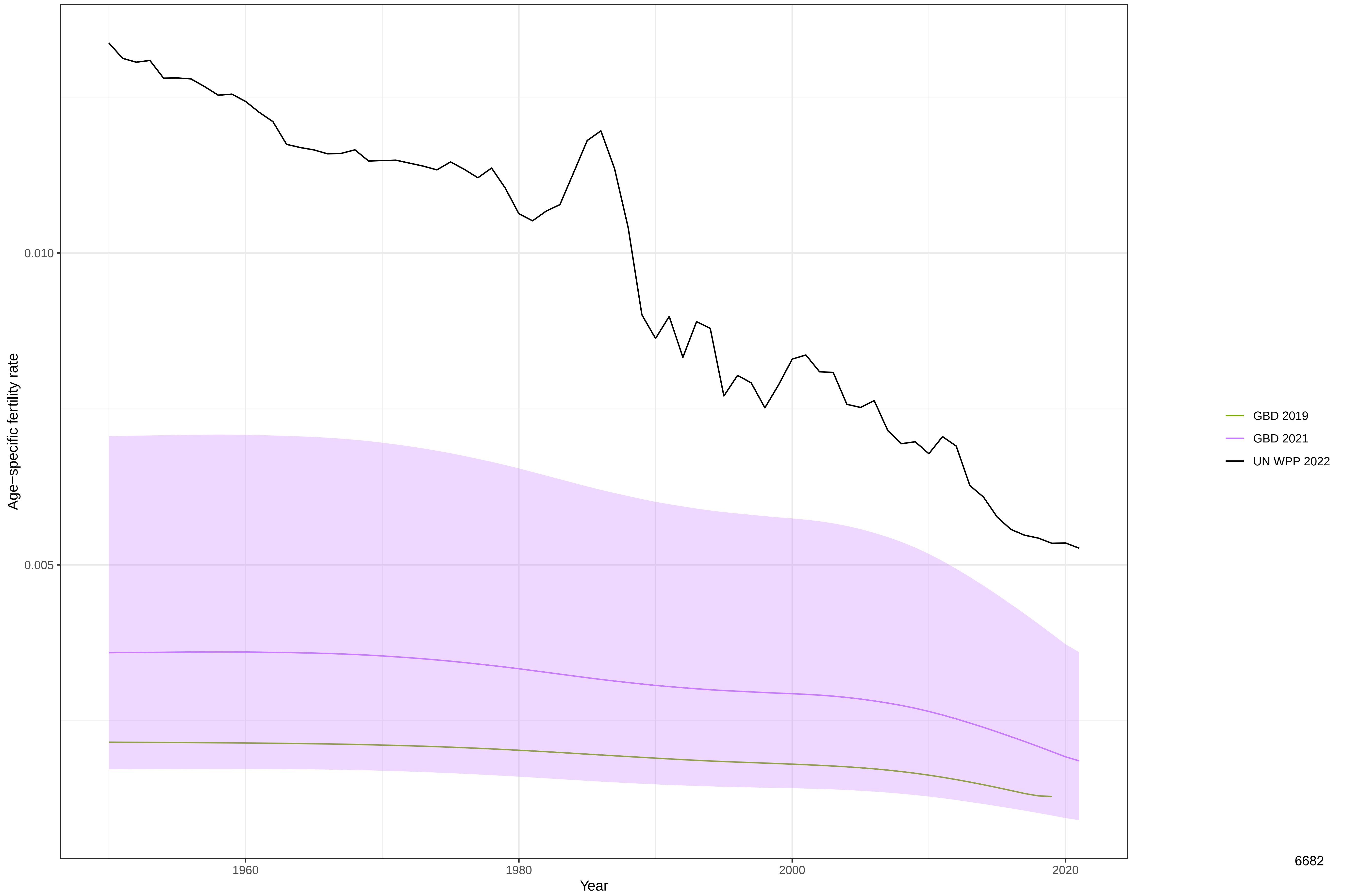

Age−specific fertility fitted trend lines, 1950−2021: DR Congo, Ages: 15−19
Data points enclosed with a diamond are reference sources

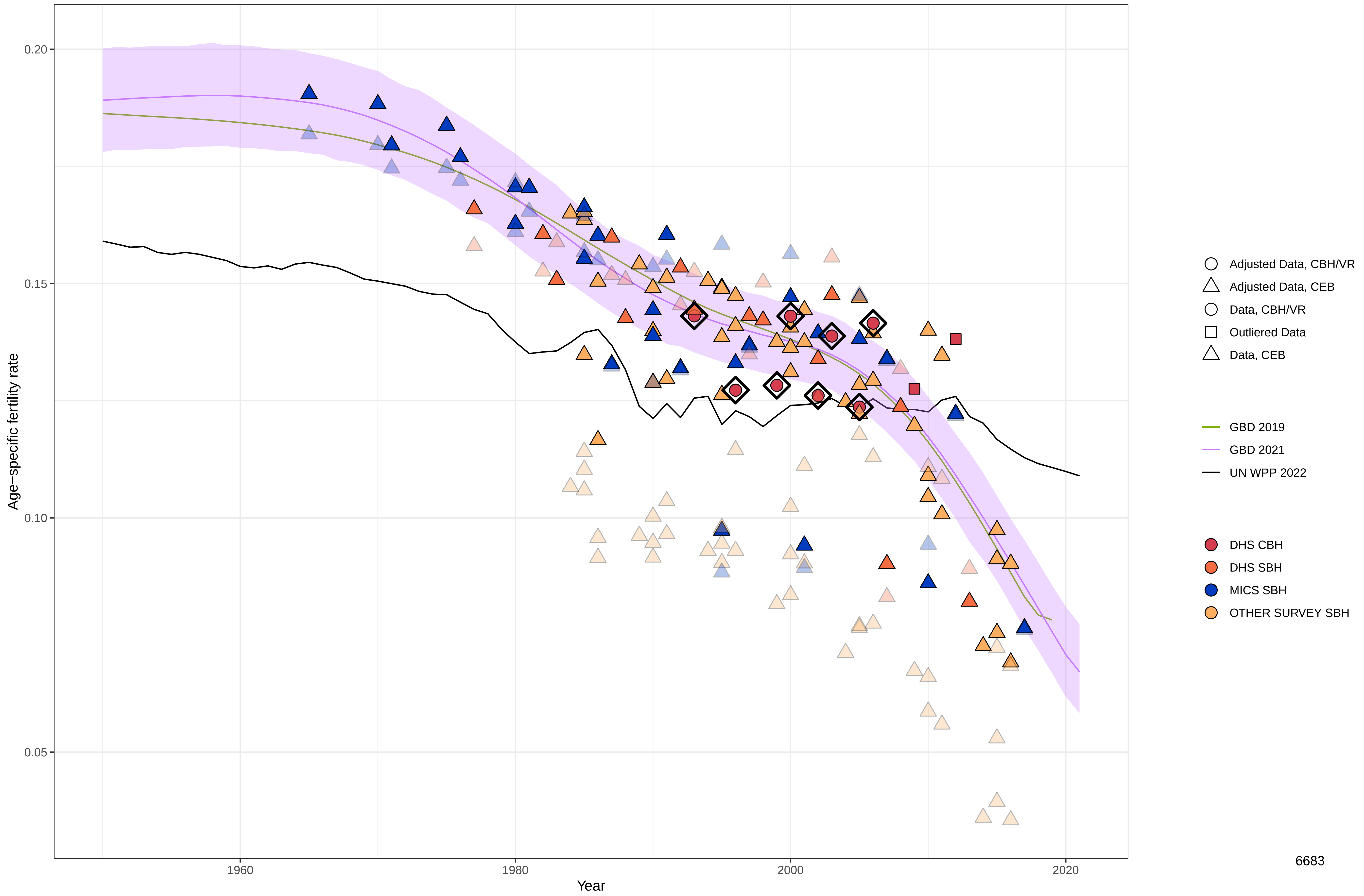

Age−specific fertility fitted trend lines, 1950−2021: DR Congo, Ages: 20−24
Data points enclosed with a diamond are reference sources

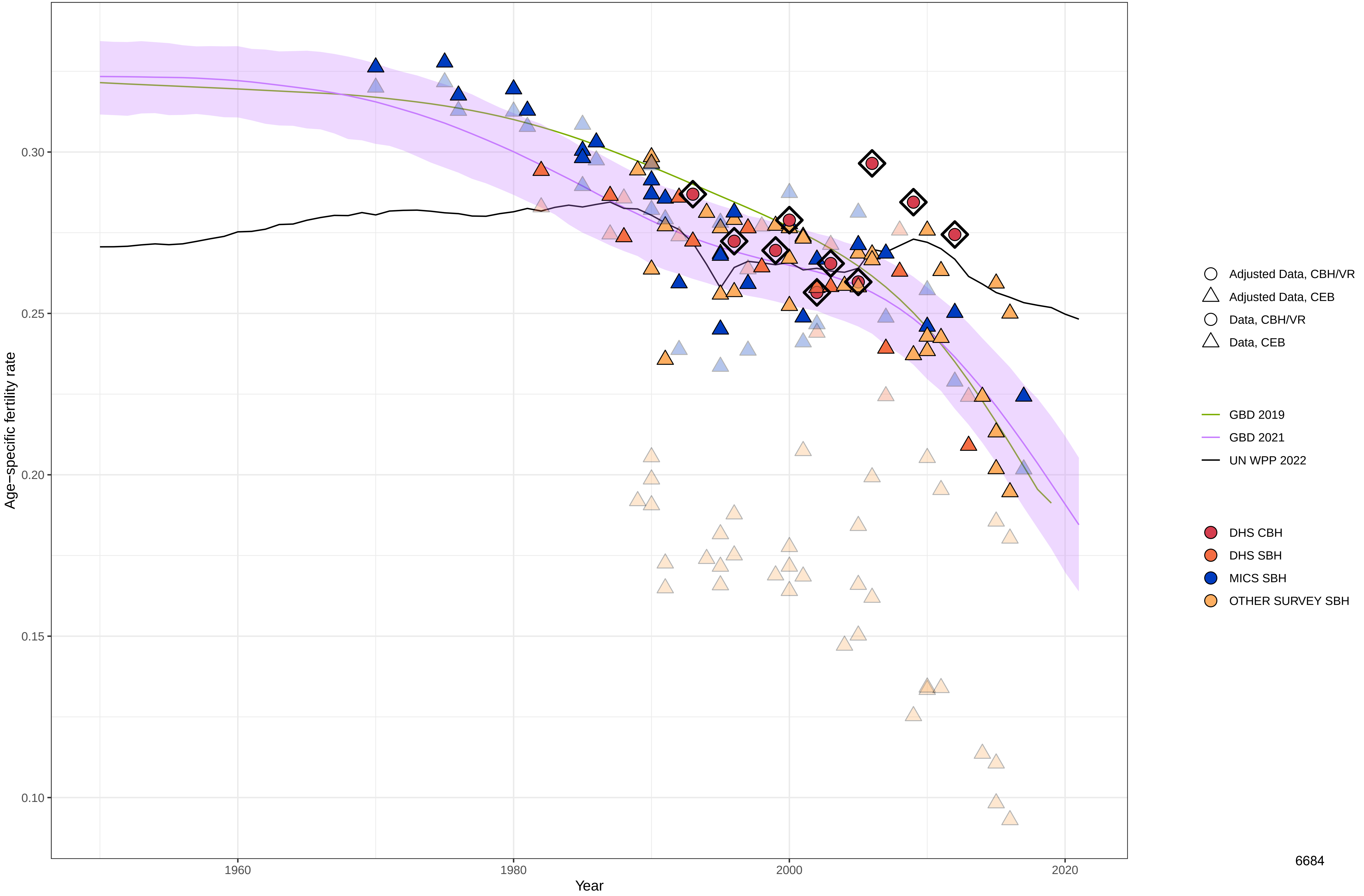

Age−specific fertility fitted trend lines, 1950−2021: DR Congo, Ages: 25−29
Data points enclosed with a diamond are reference sources

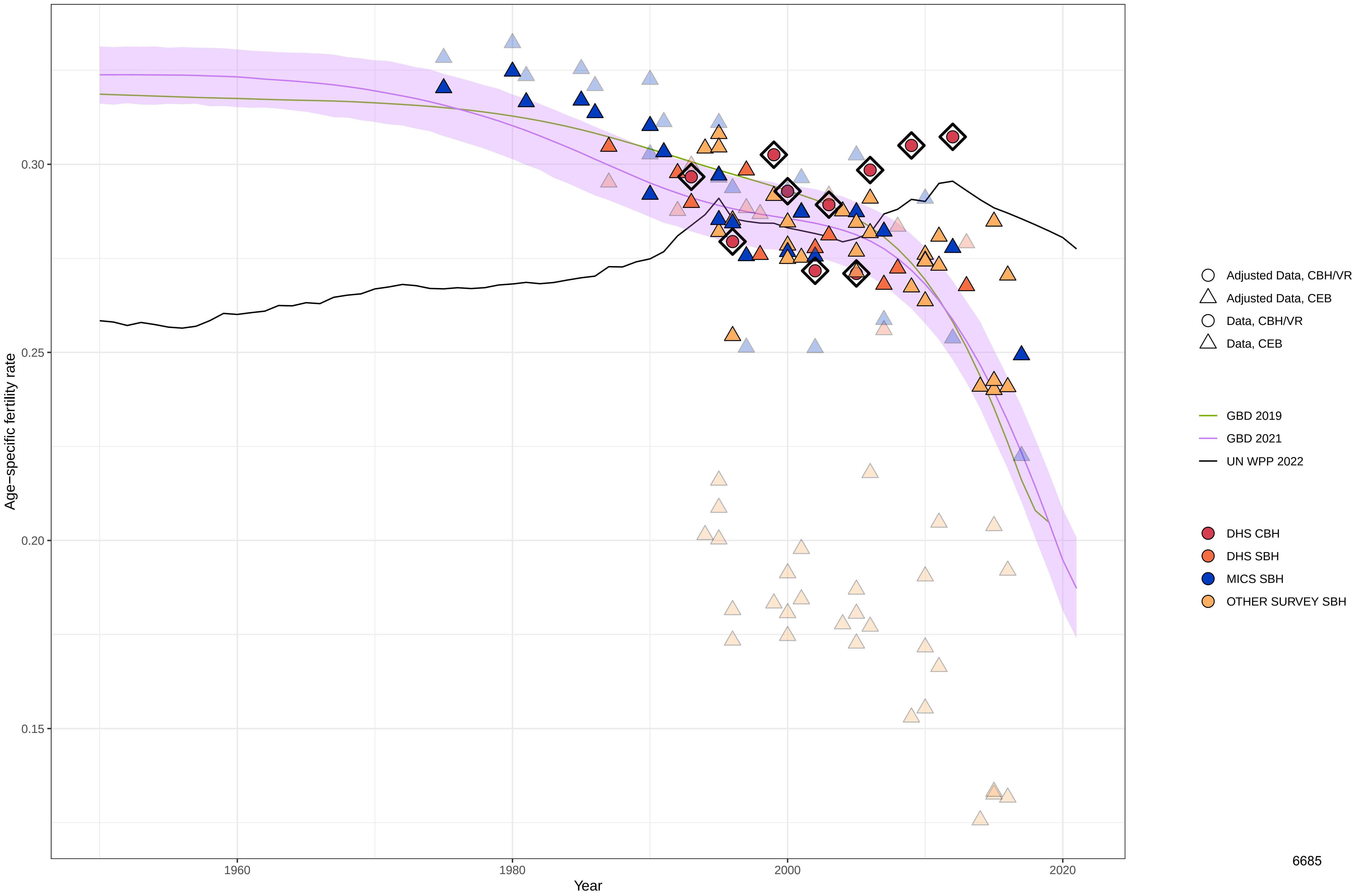

Age−specific fertility fitted trend lines, 1950−2021: DR Congo, Ages: 30−34
Data points enclosed with a diamond are reference sources

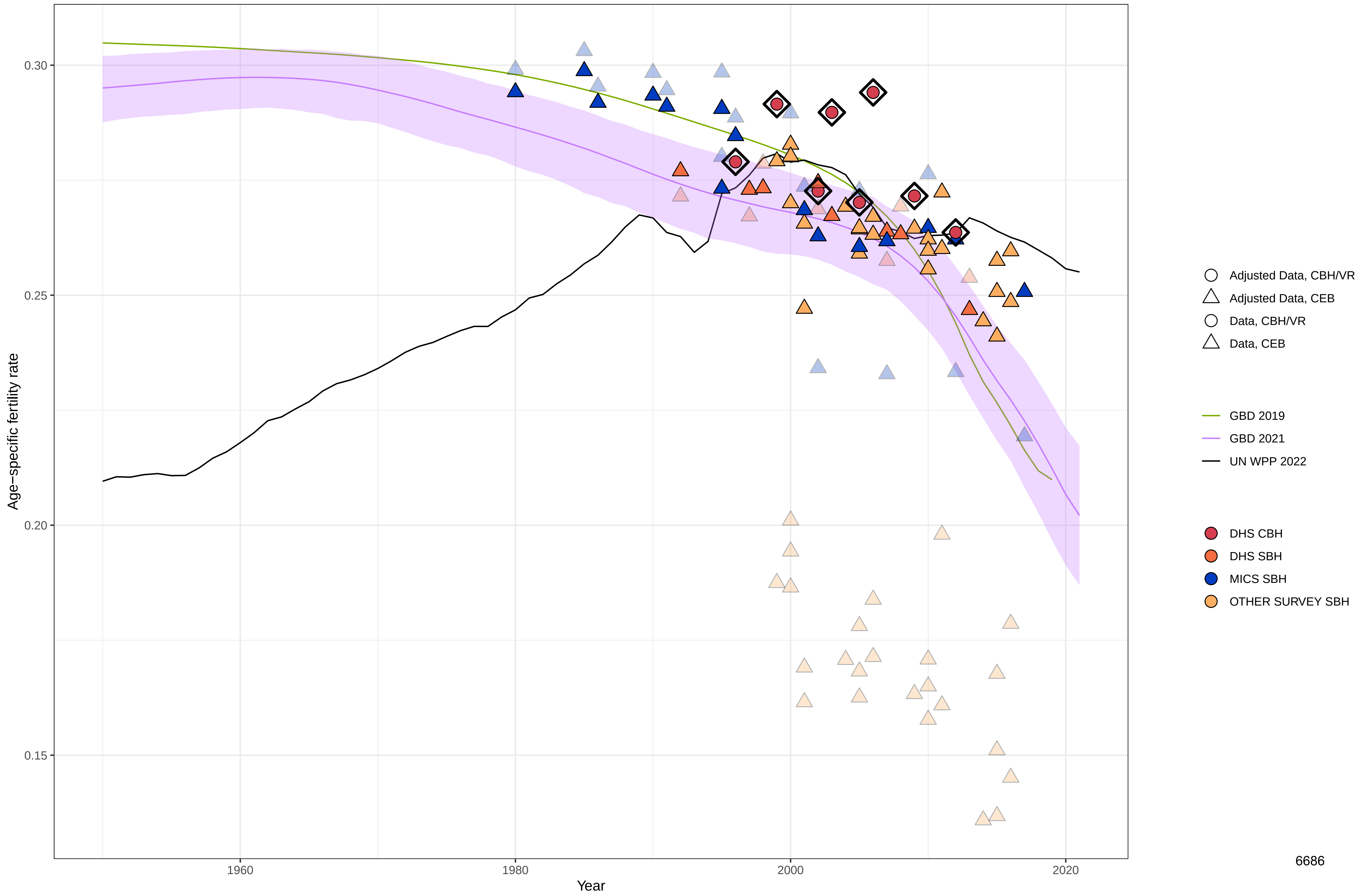

6686

Age−specific fertility fitted trend lines, 1950−2021: DR Congo, Ages: 35−39
Data points enclosed with a diamond are reference sources

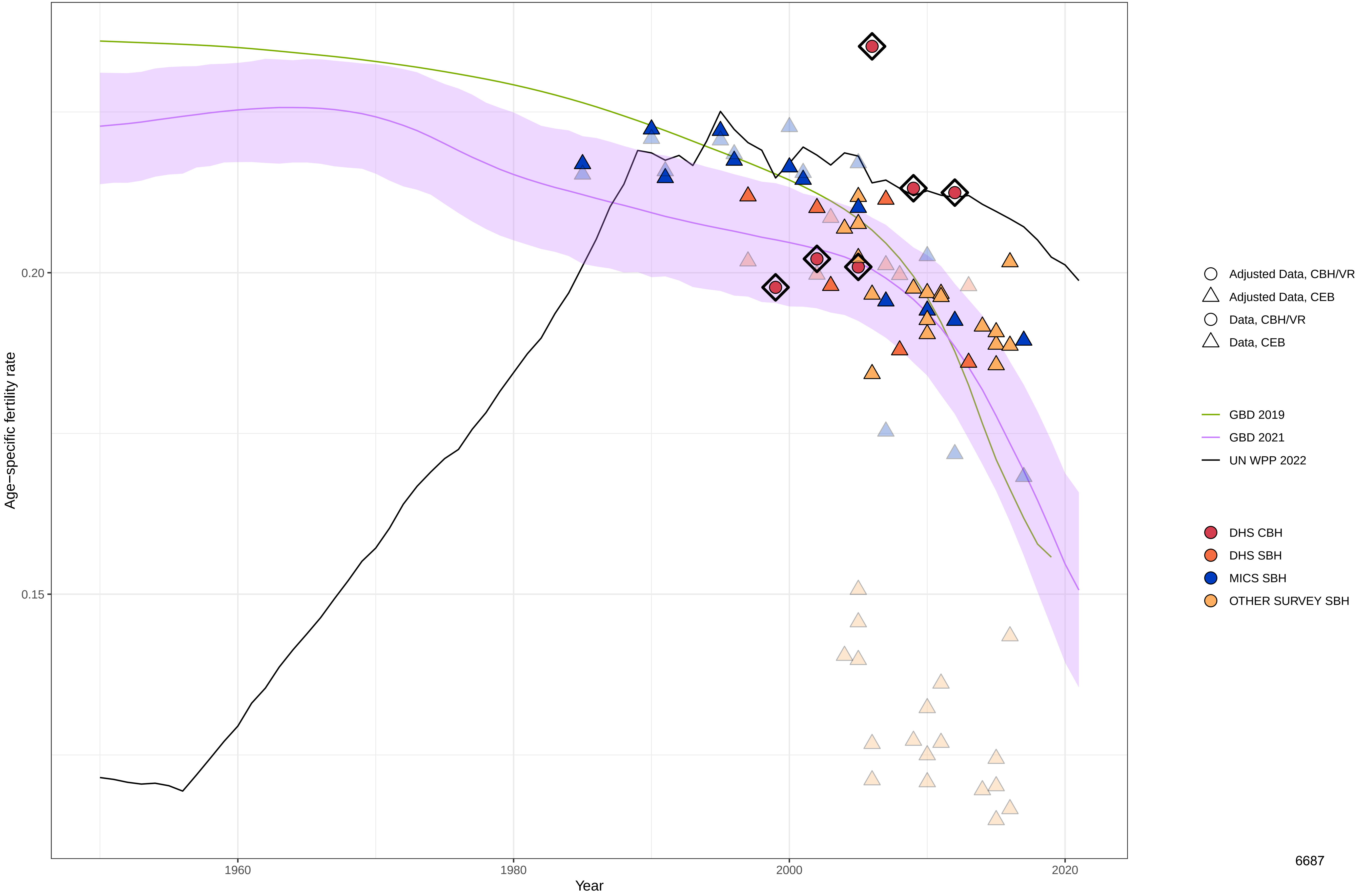

Age−specific fertility fitted trend lines, 1950−2021: DR Congo, Ages: 40−44
Data points enclosed with a diamond are reference sources

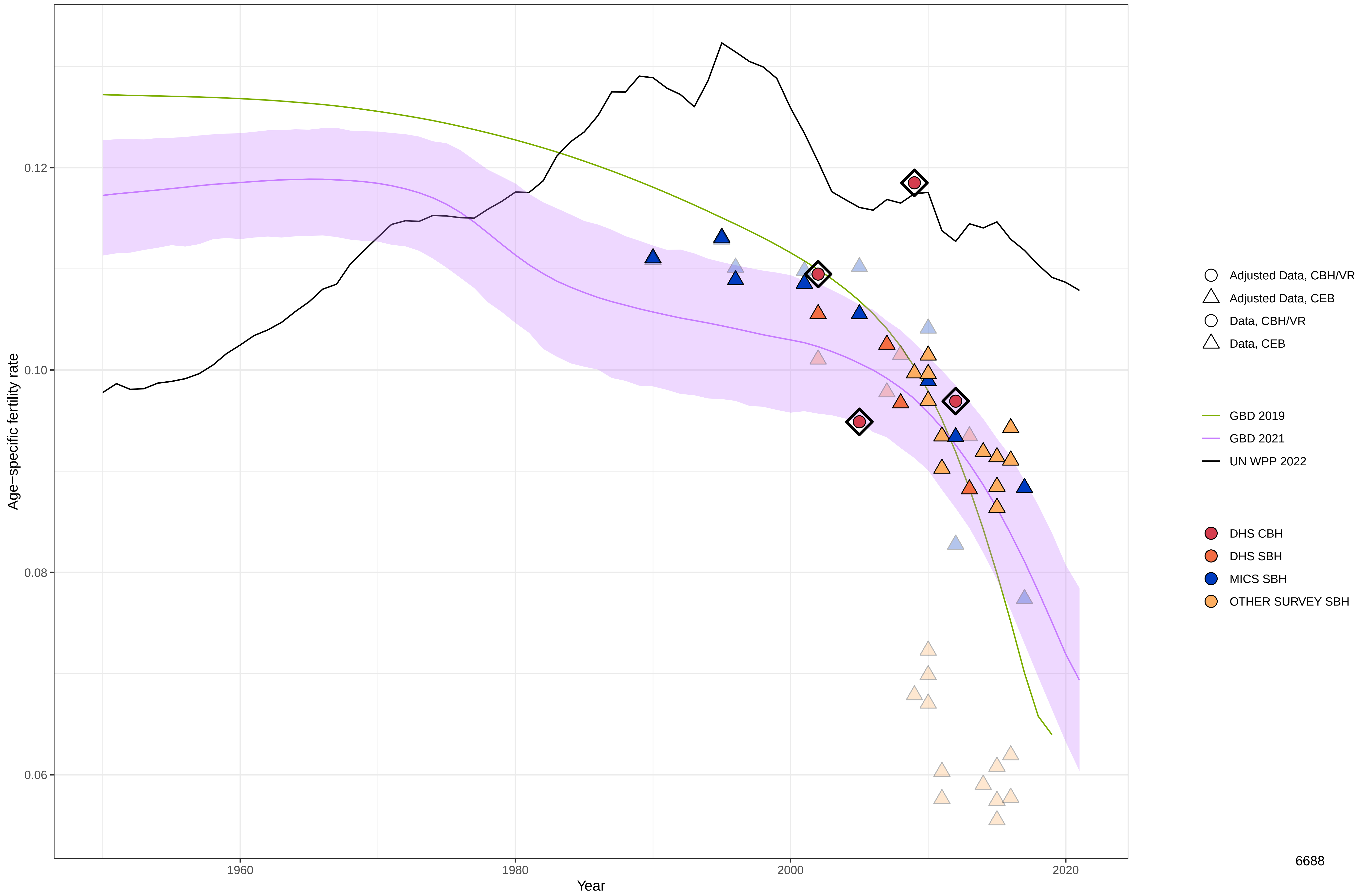

Age−specific fertility fitted trend lines, 1950−2021: DR Congo, Ages: 45−49
Data points enclosed with a diamond are reference sources

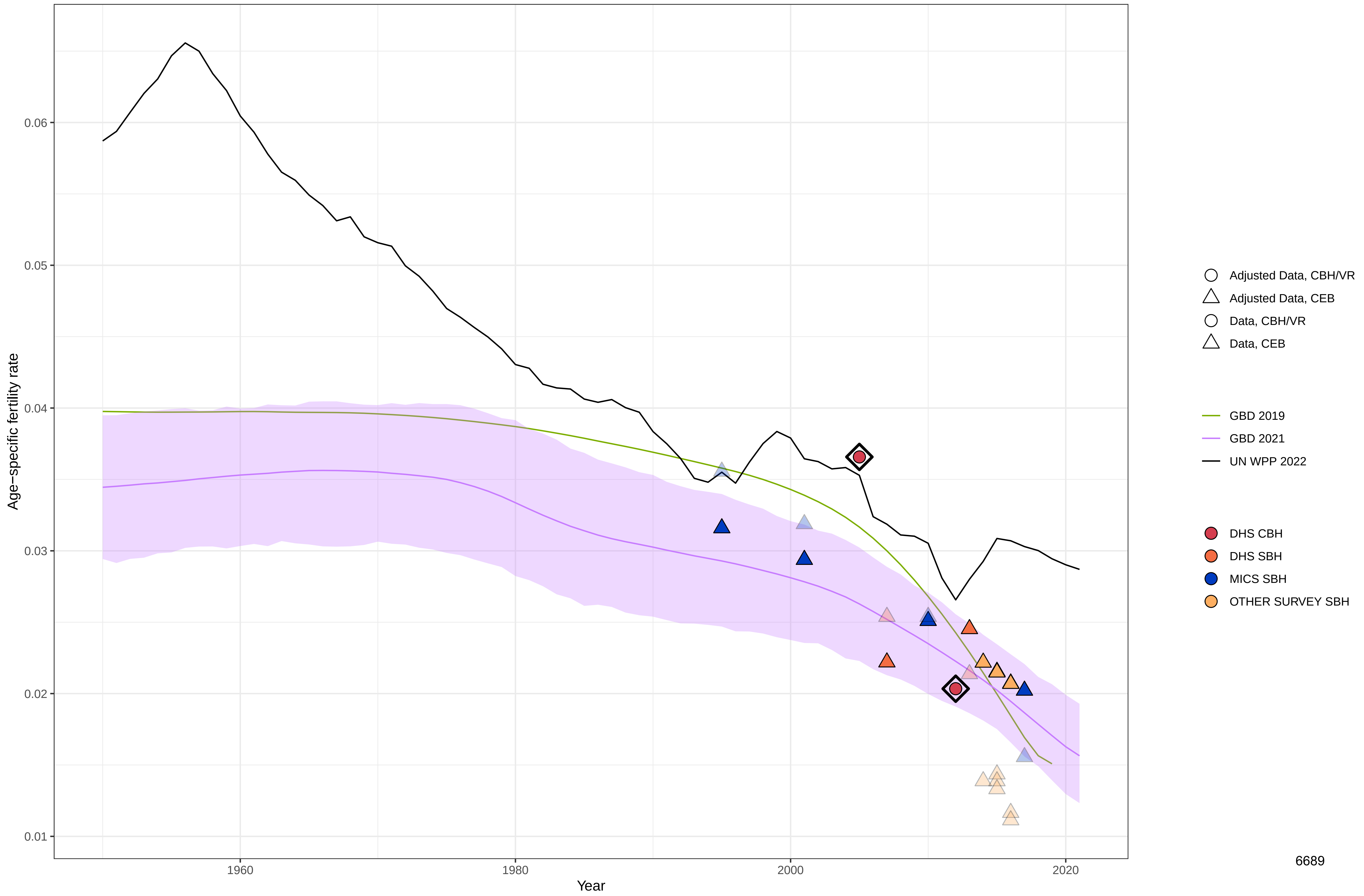

Age−specific fertility fitted trend lines, 1950−2021: DR Congo, Ages: 50−54
Data points enclosed with a diamond are reference sources

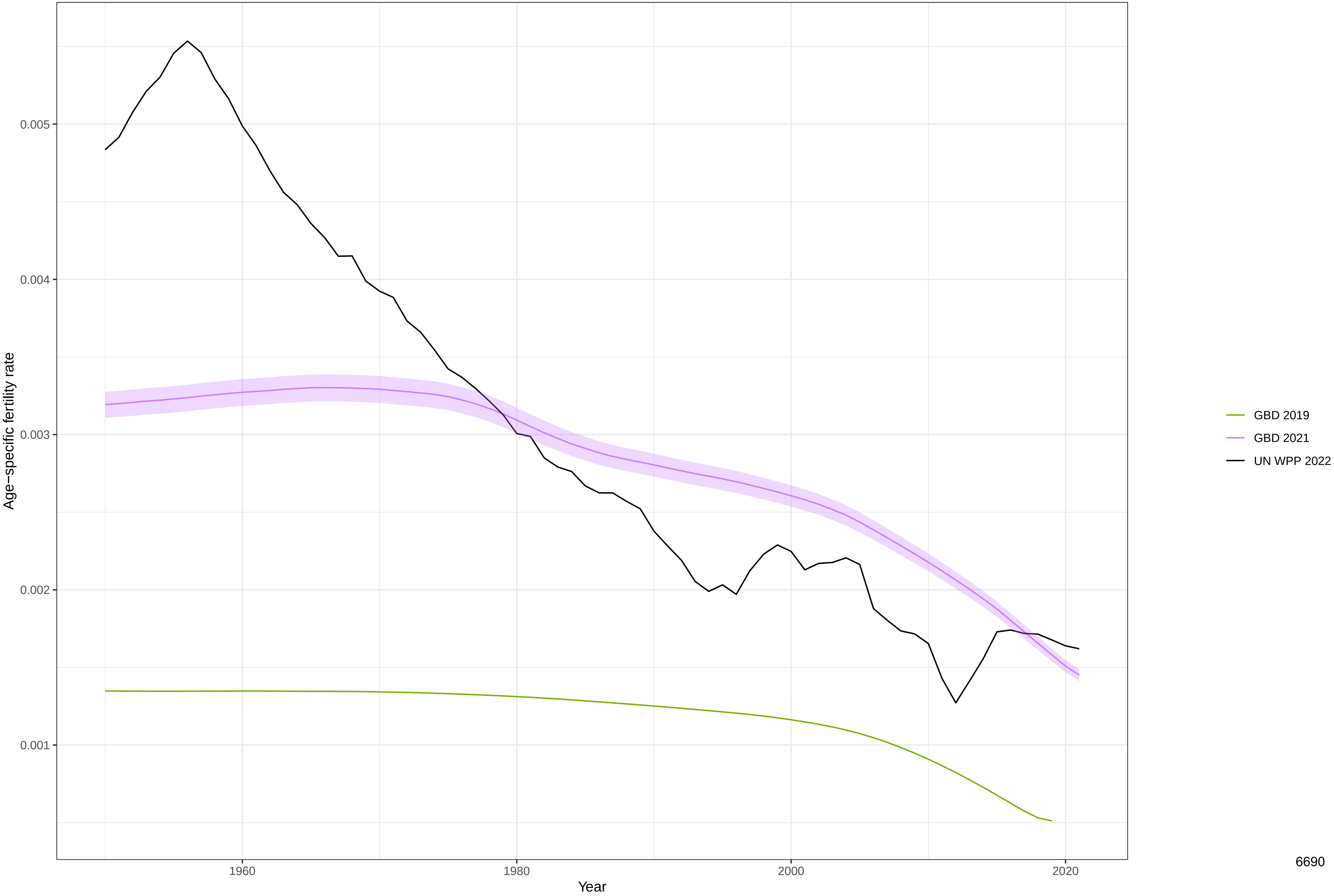

Total fertility rate fitted trend lines, 1950−2021: DR Congo TFR
Data points enclosed with a diamond are reference sources

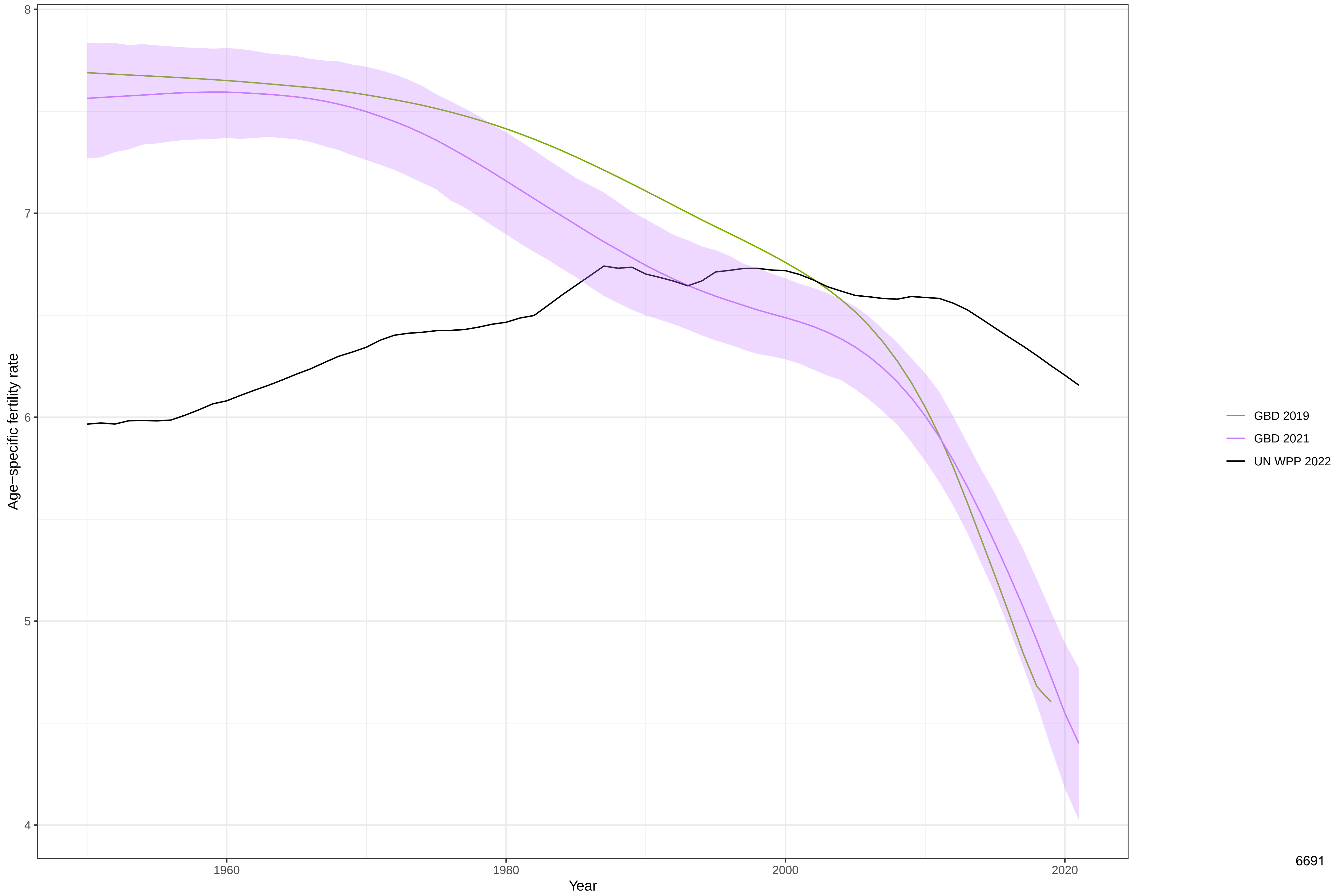

Age−specific fertility fitted trend lines, 1950−2021: Equatorial Guinea, Ages: 10−14
Data points enclosed with a diamond are reference sources

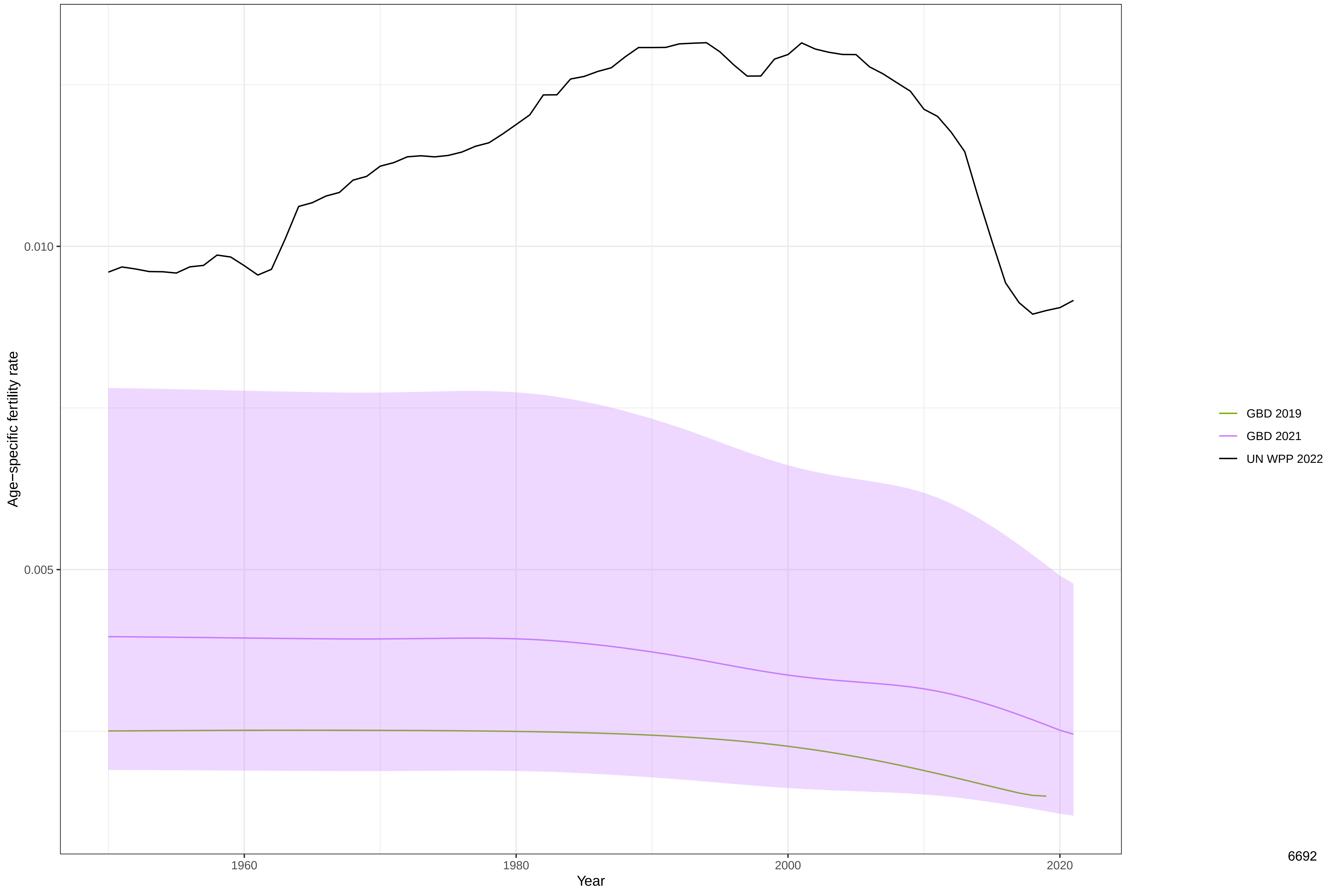

6692

Age−specific fertility fitted trend lines, 1950−2021: Equatorial Guinea, Ages: 15−19
Data points enclosed with a diamond are reference sources

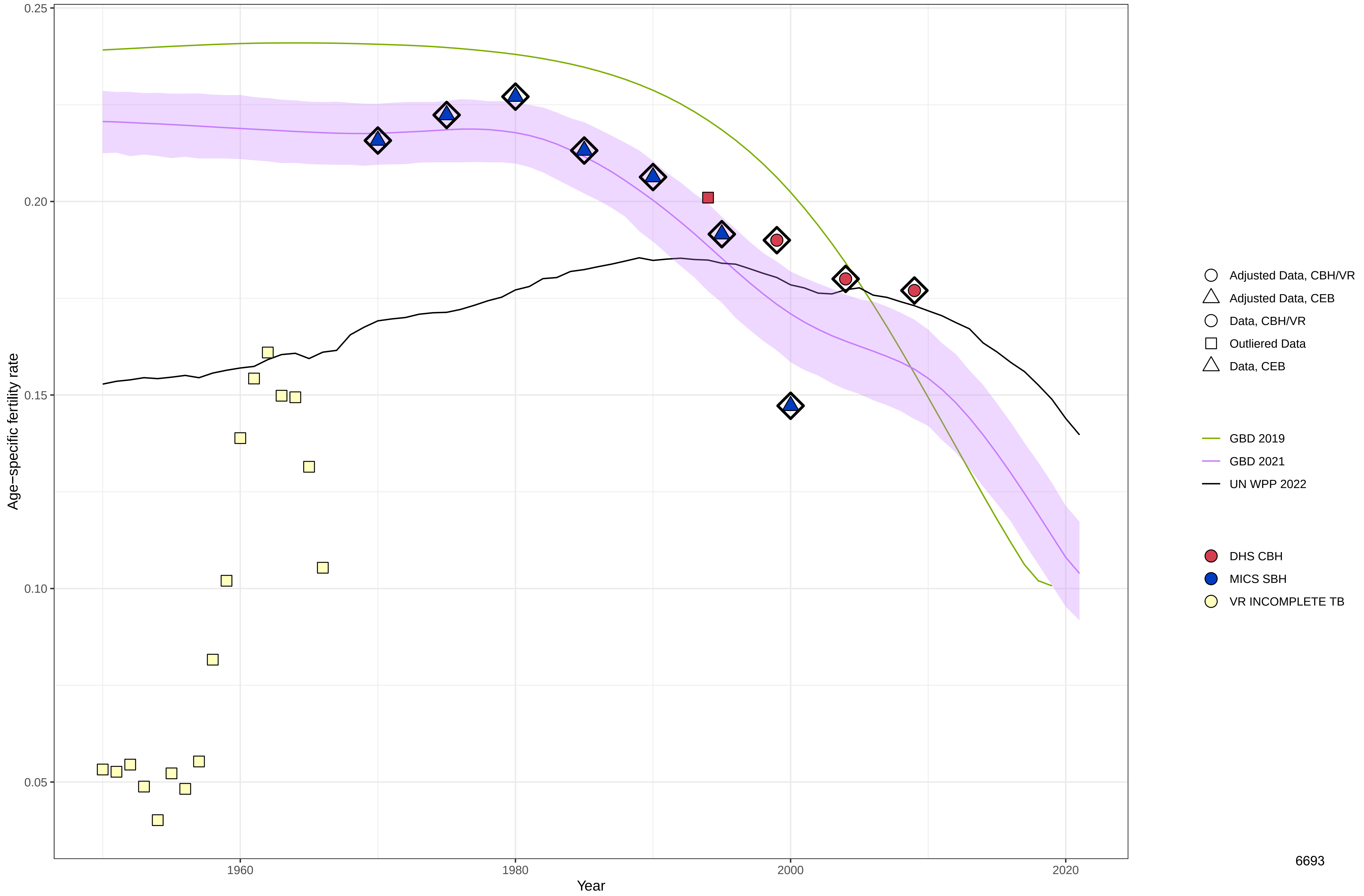

Age−specific fertility fitted trend lines, 1950−2021: Equatorial Guinea, Ages: 20−24
Data points enclosed with a diamond are reference sources

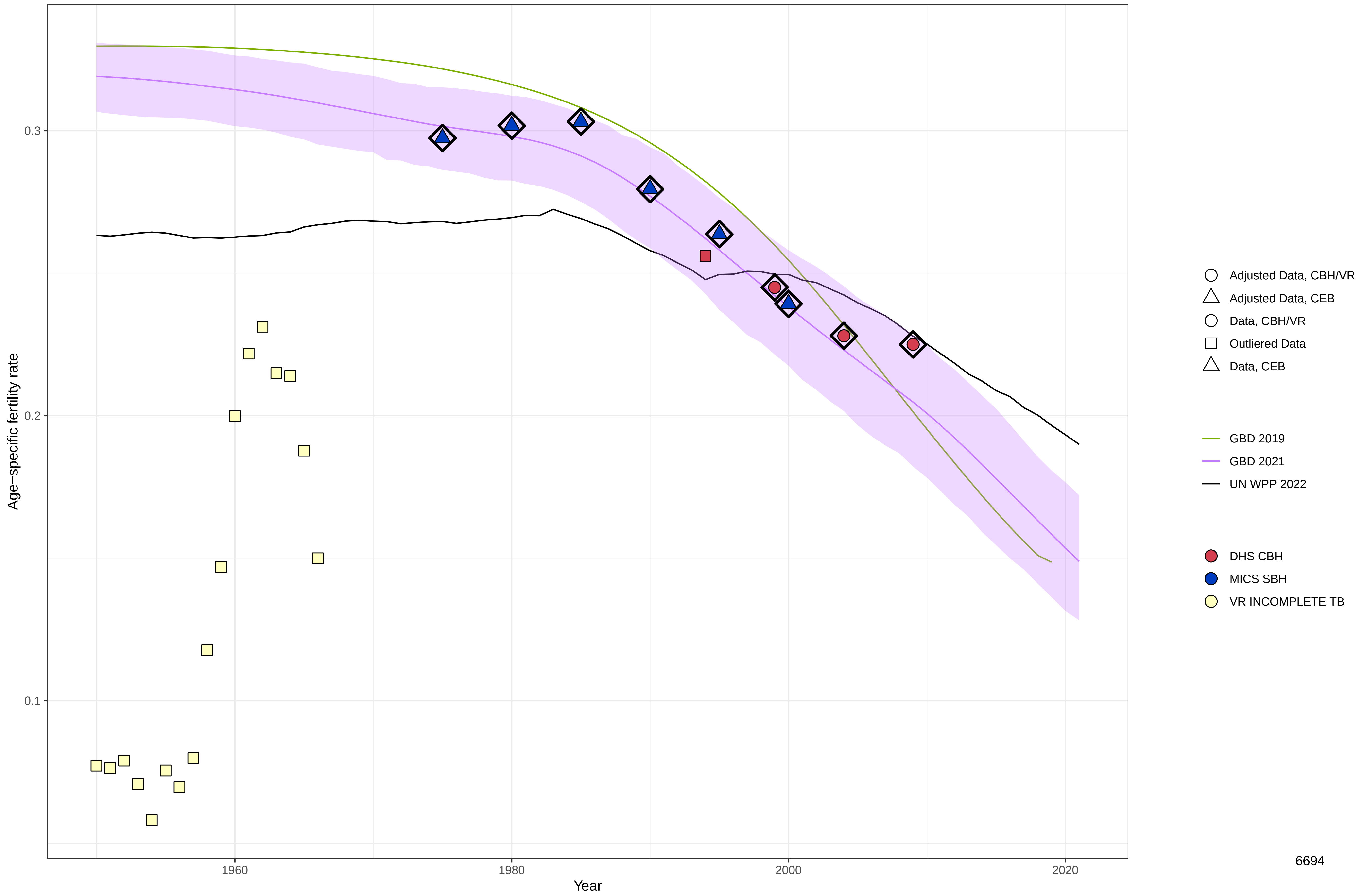

Age−specific fertility fitted trend lines, 1950−2021: Equatorial Guinea, Ages: 25−29
Data points enclosed with a diamond are reference sources

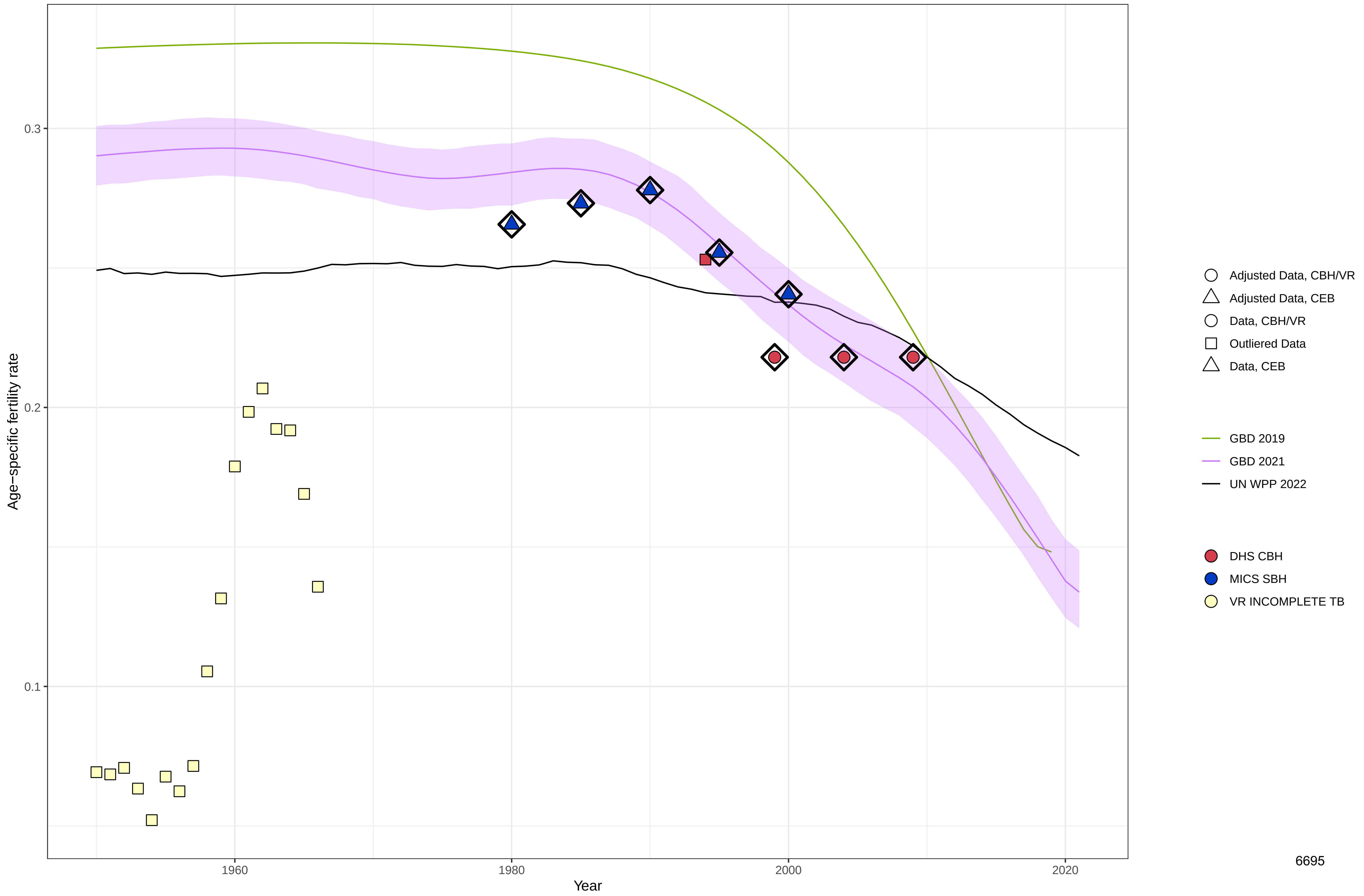

Age−specific fertility fitted trend lines, 1950−2021: Equatorial Guinea, Ages: 30−34
Data points enclosed with a diamond are reference sources

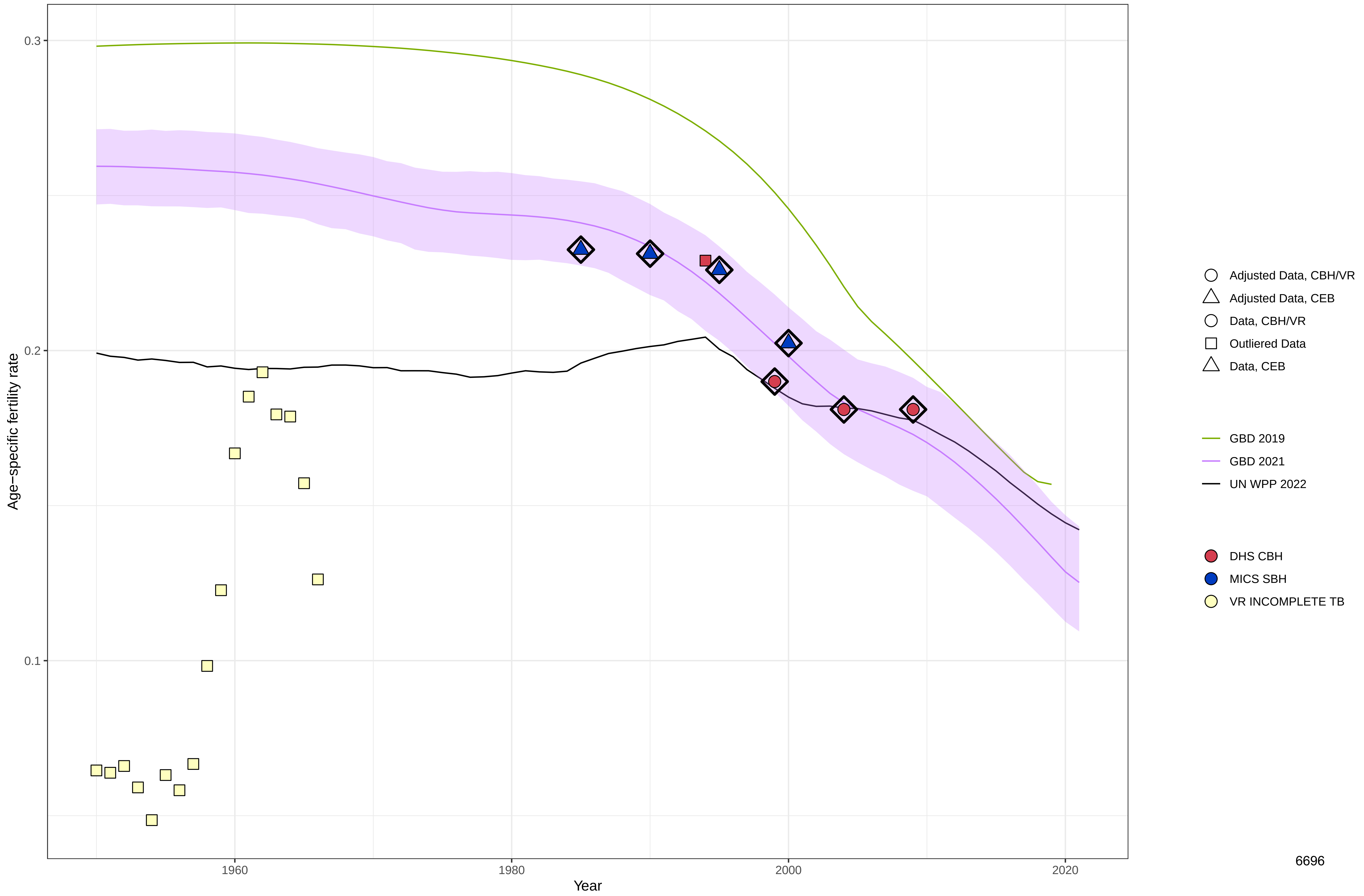

Age−specific fertility fitted trend lines, 1950−2021: Equatorial Guinea, Ages: 35−39
Data points enclosed with a diamond are reference sources

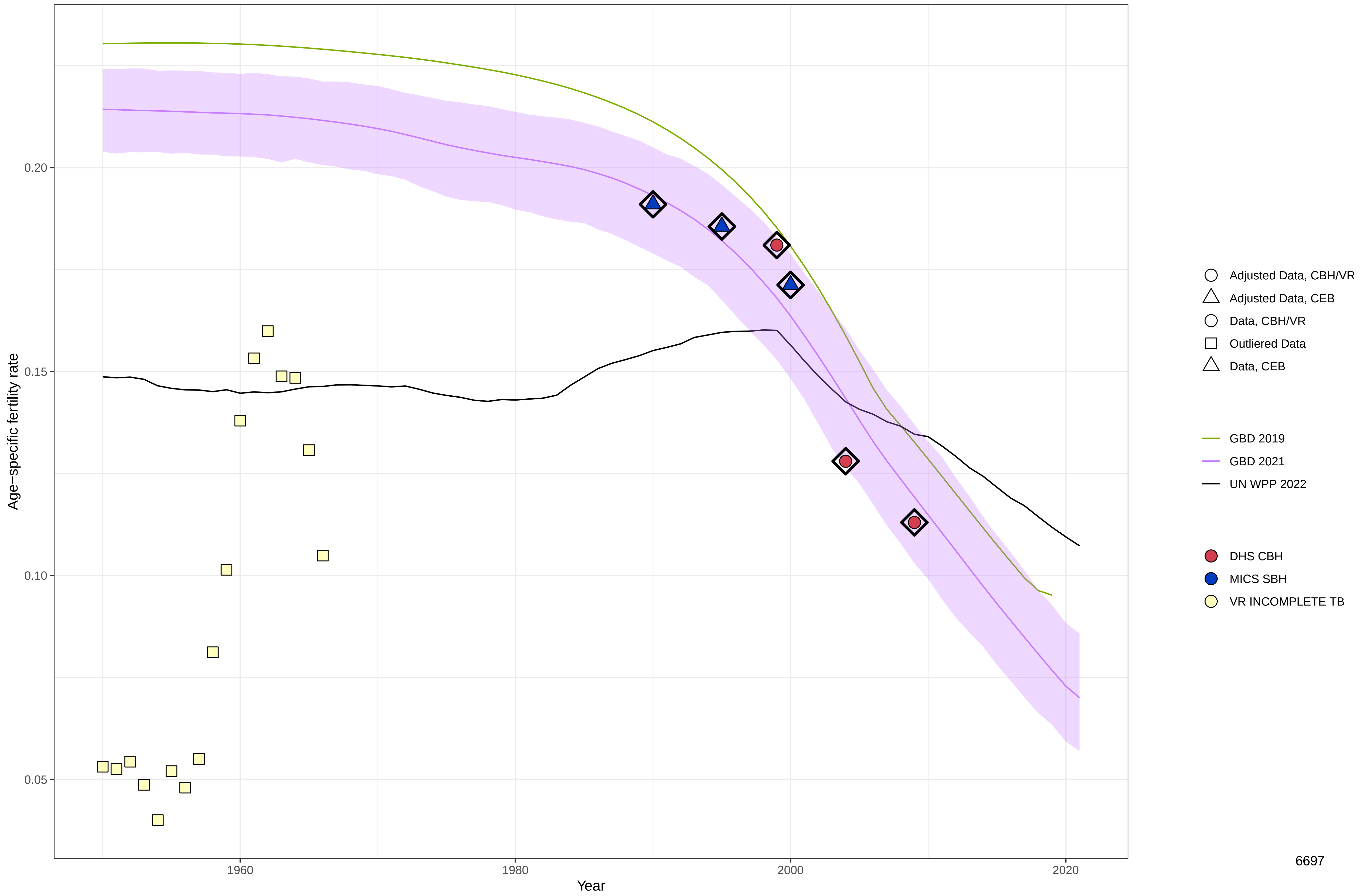

Age−specific fertility fitted trend lines, 1950−2021: Equatorial Guinea, Ages: 40−44
Data points enclosed with a diamond are reference sources

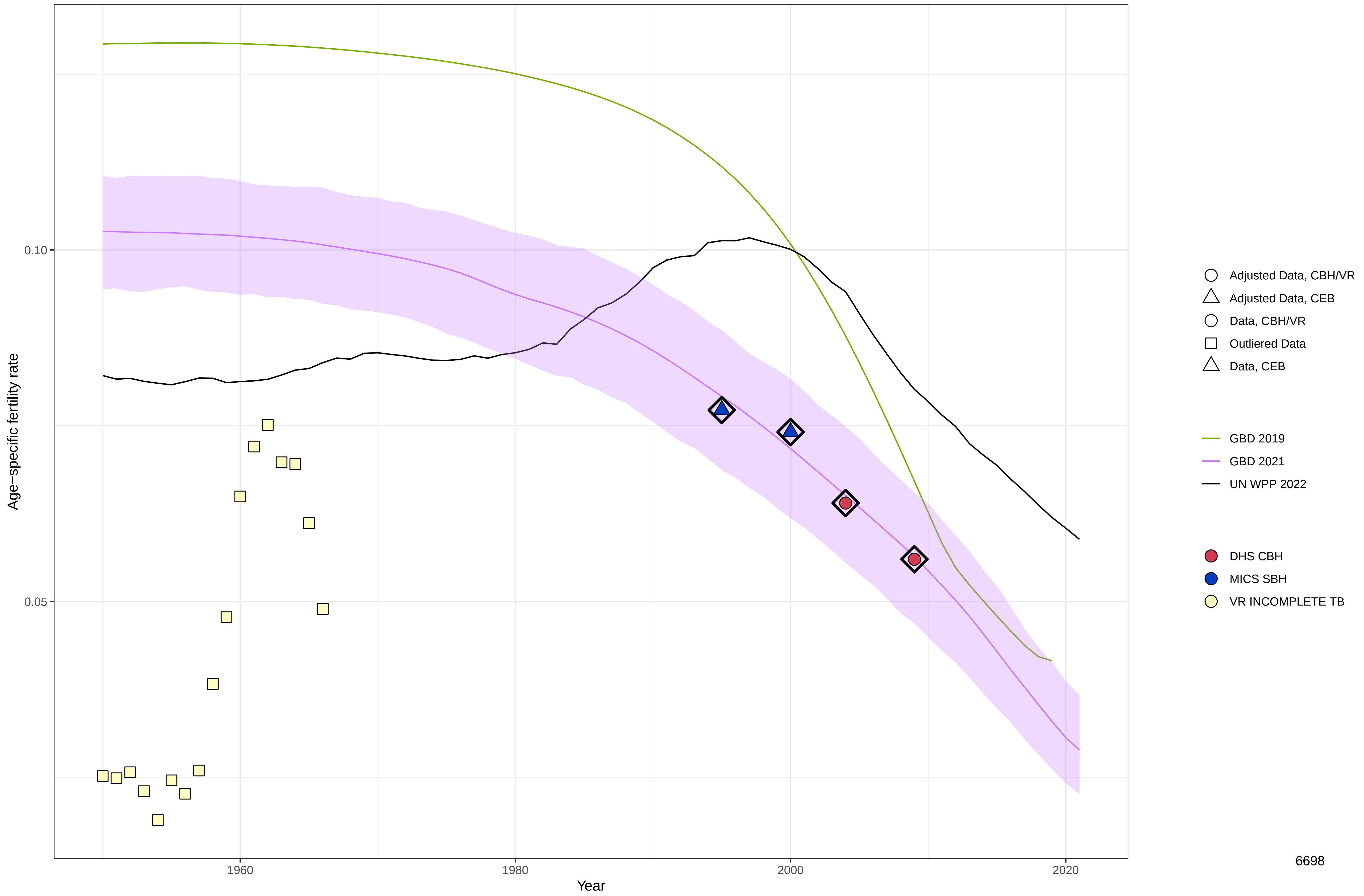

Age−specific fertility fitted trend lines, 1950−2021: Equatorial Guinea, Ages: 45−49
Data points enclosed with a diamond are reference sources

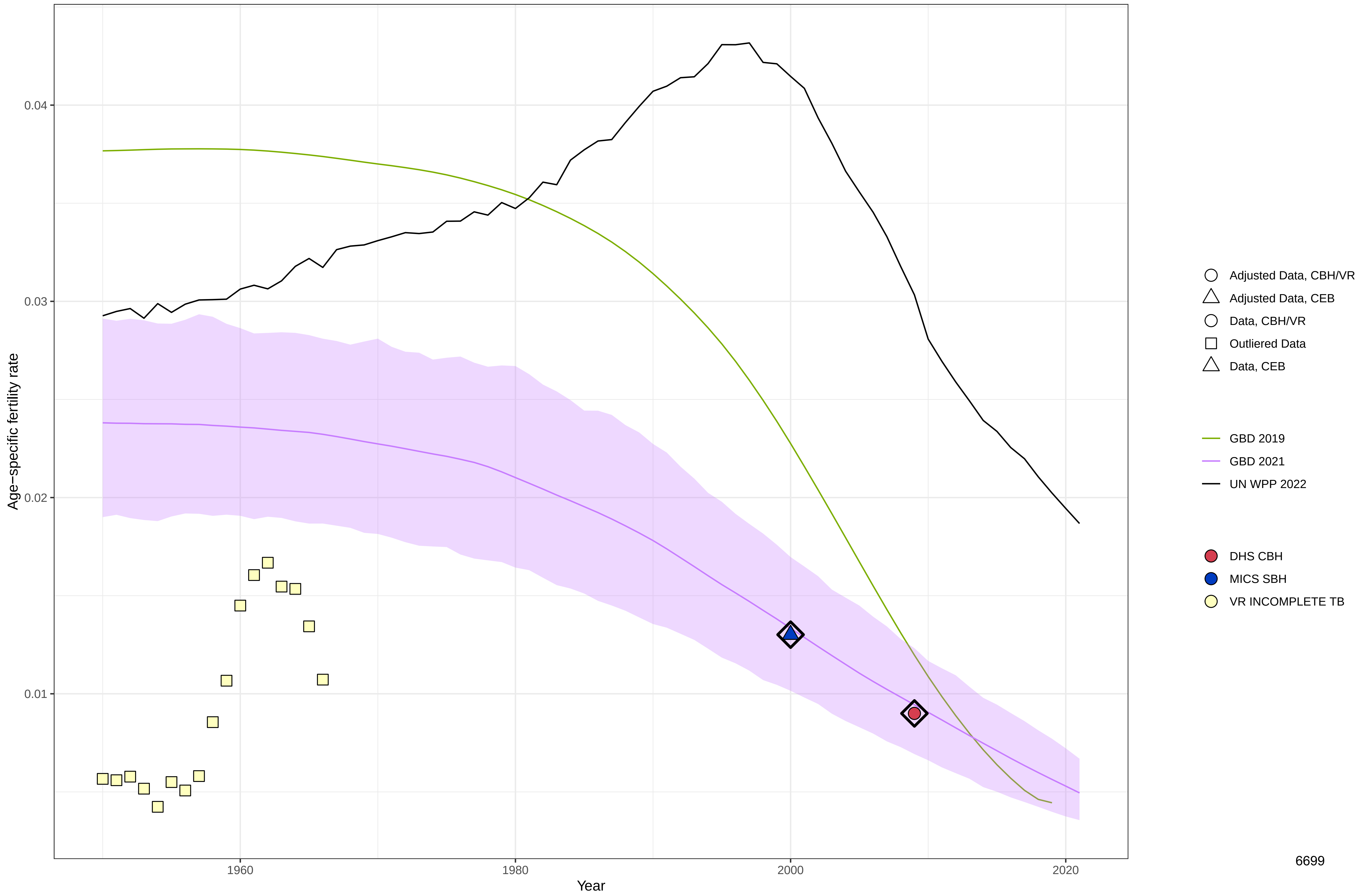

Age−specific fertility fitted trend lines, 1950−2021: Equatorial Guinea, Ages: 50−54
Data points enclosed with a diamond are reference sources

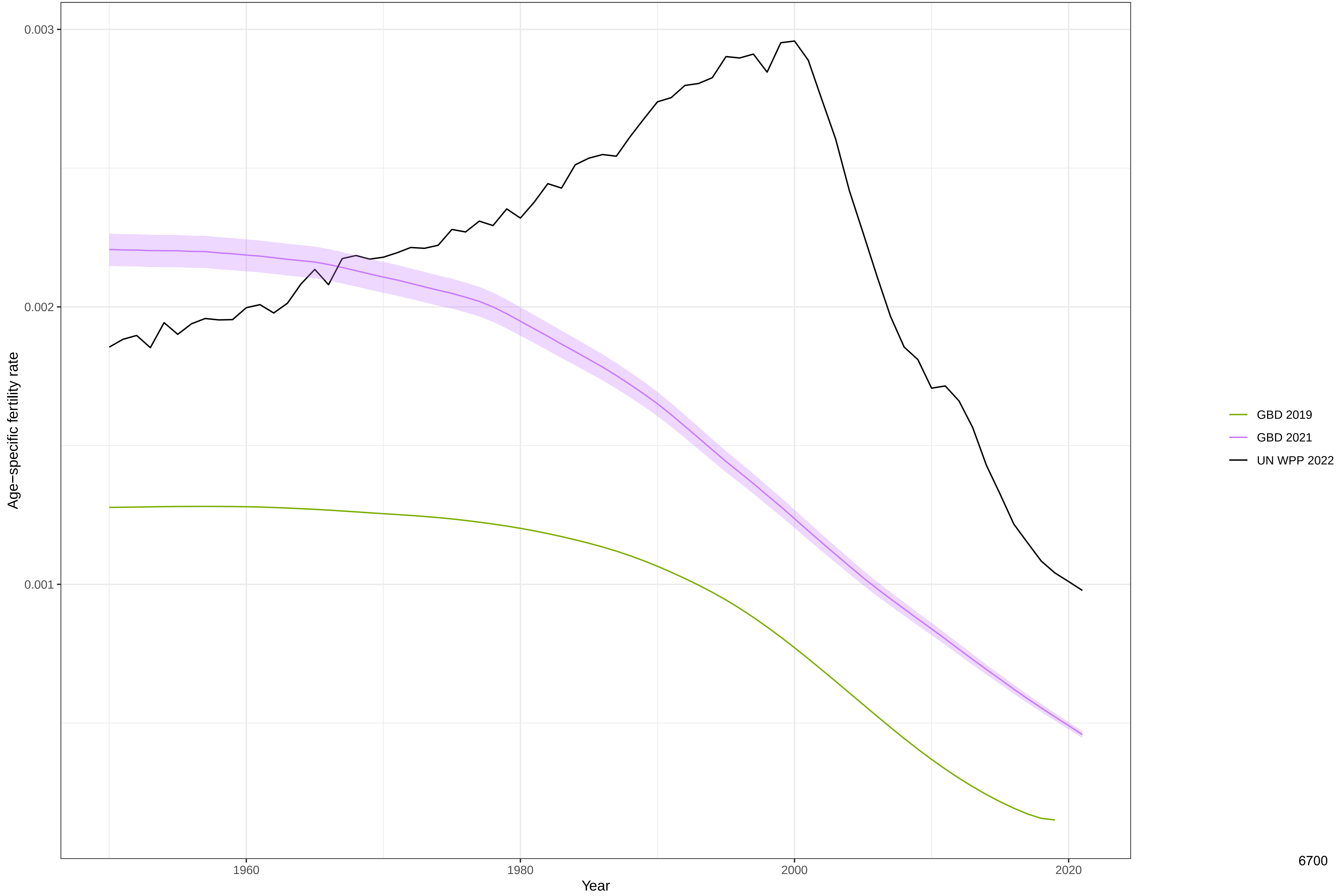

Total fertility rate fitted trend lines, 1950−2021: Equatorial Guinea TFR
Data points enclosed with a diamond are reference sources

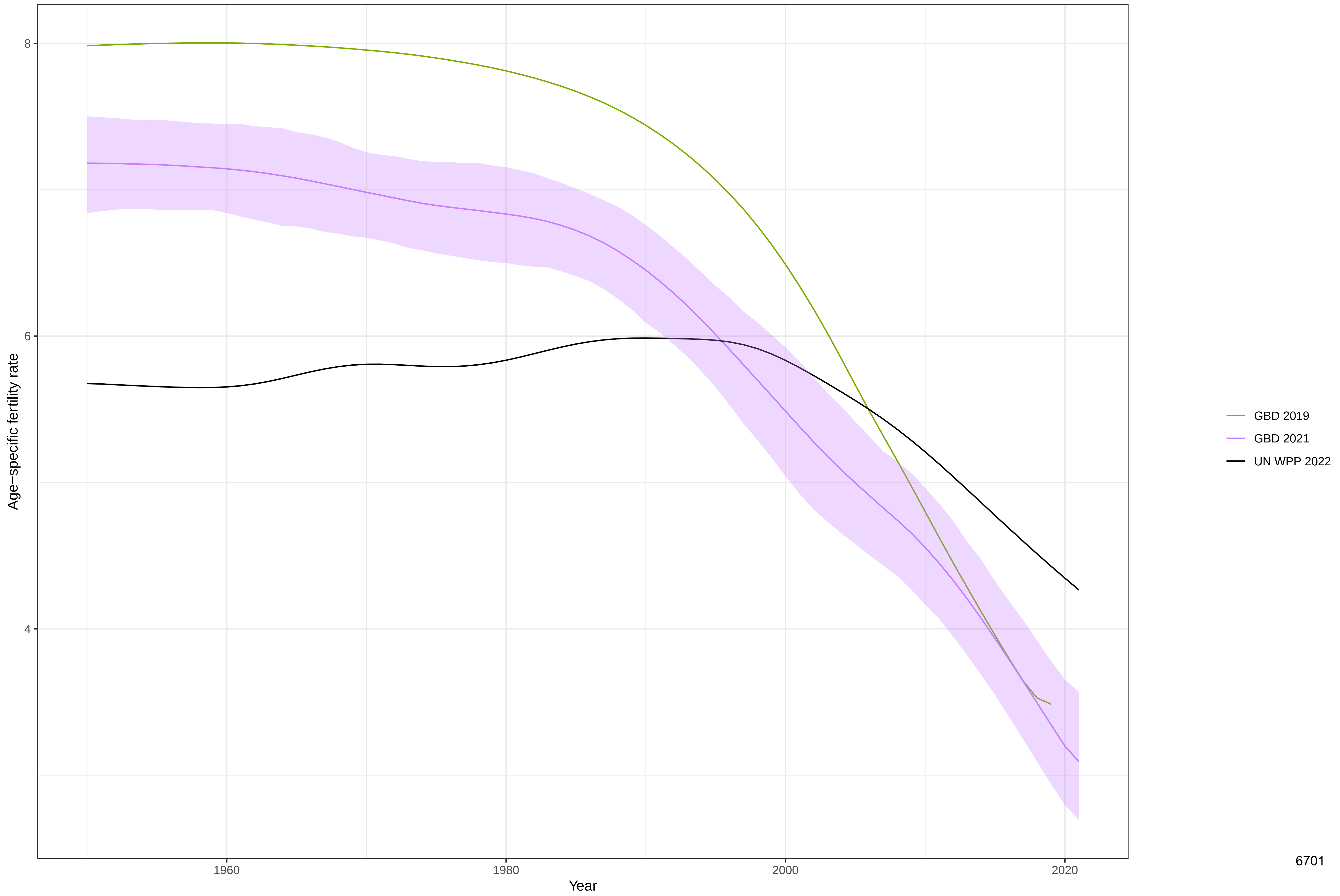

Age−specific fertility fitted trend lines, 1950−2021: Gabon, Ages: 10−14
Data points enclosed with a diamond are reference sources

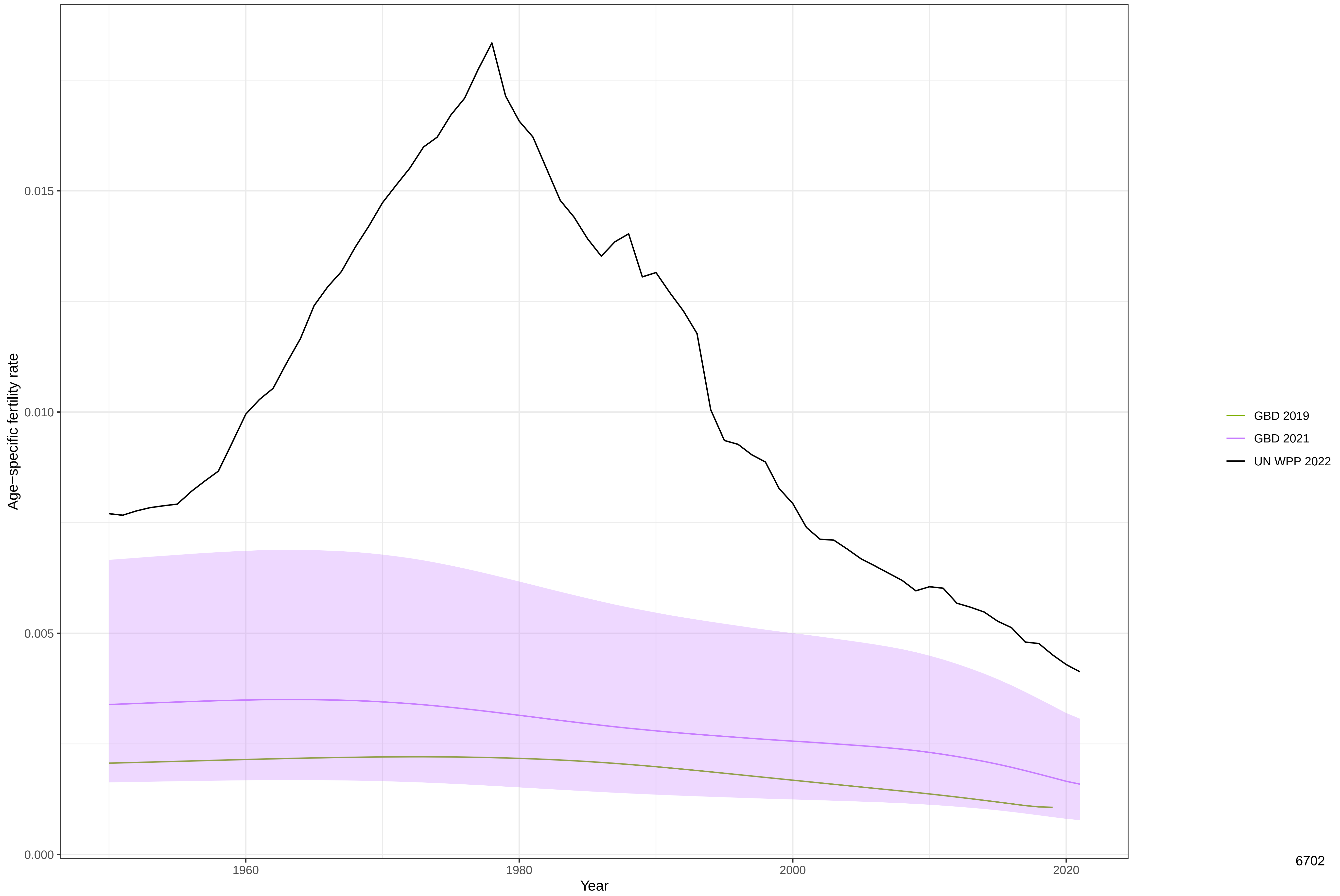

Age−specific fertility fitted trend lines, 1950−2021: Gabon, Ages: 15−19
Data points enclosed with a diamond are reference sources

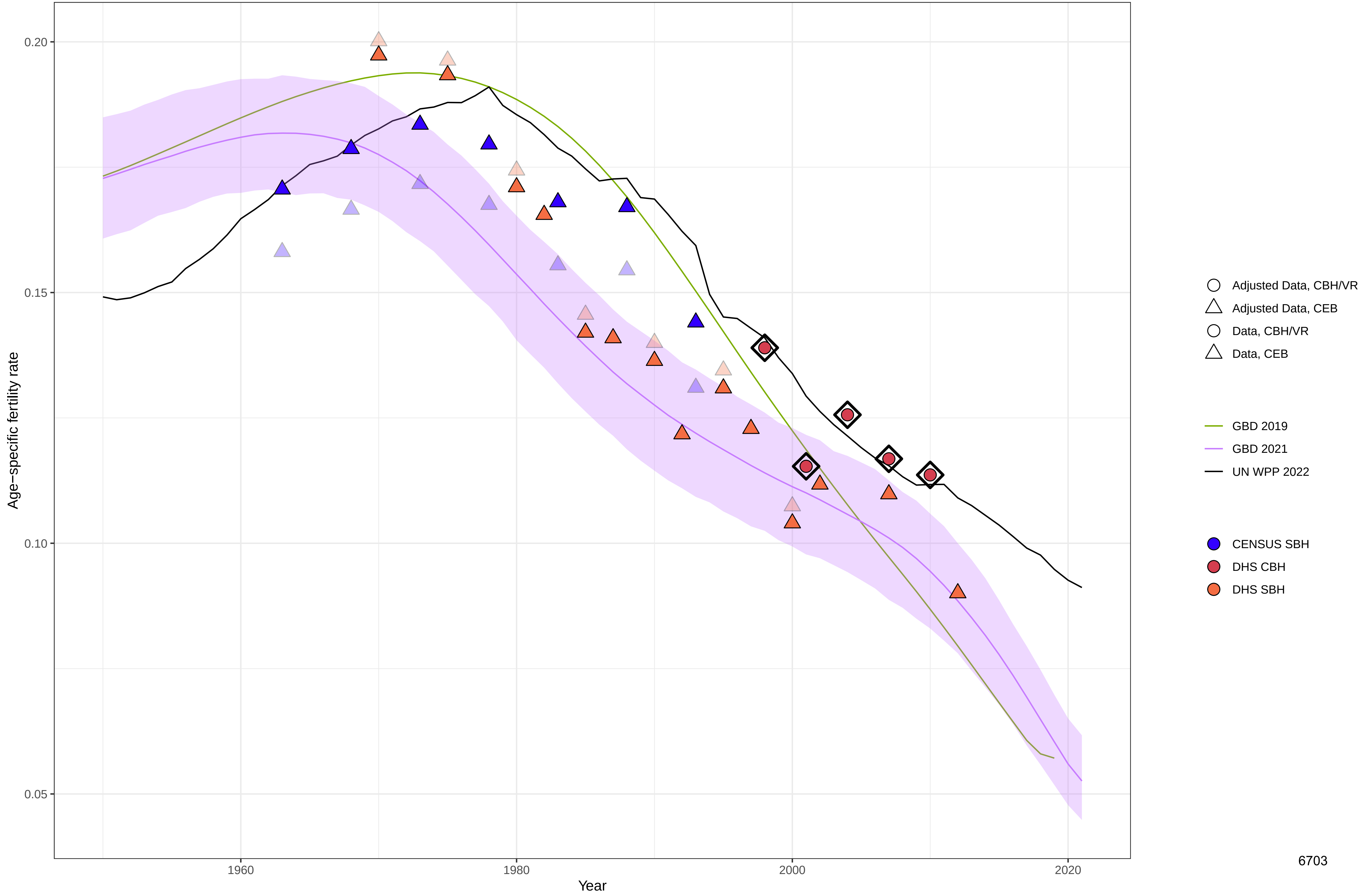

Age−specific fertility fitted trend lines, 1950−2021: Gabon, Ages: 20−24
Data points enclosed with a diamond are reference sources

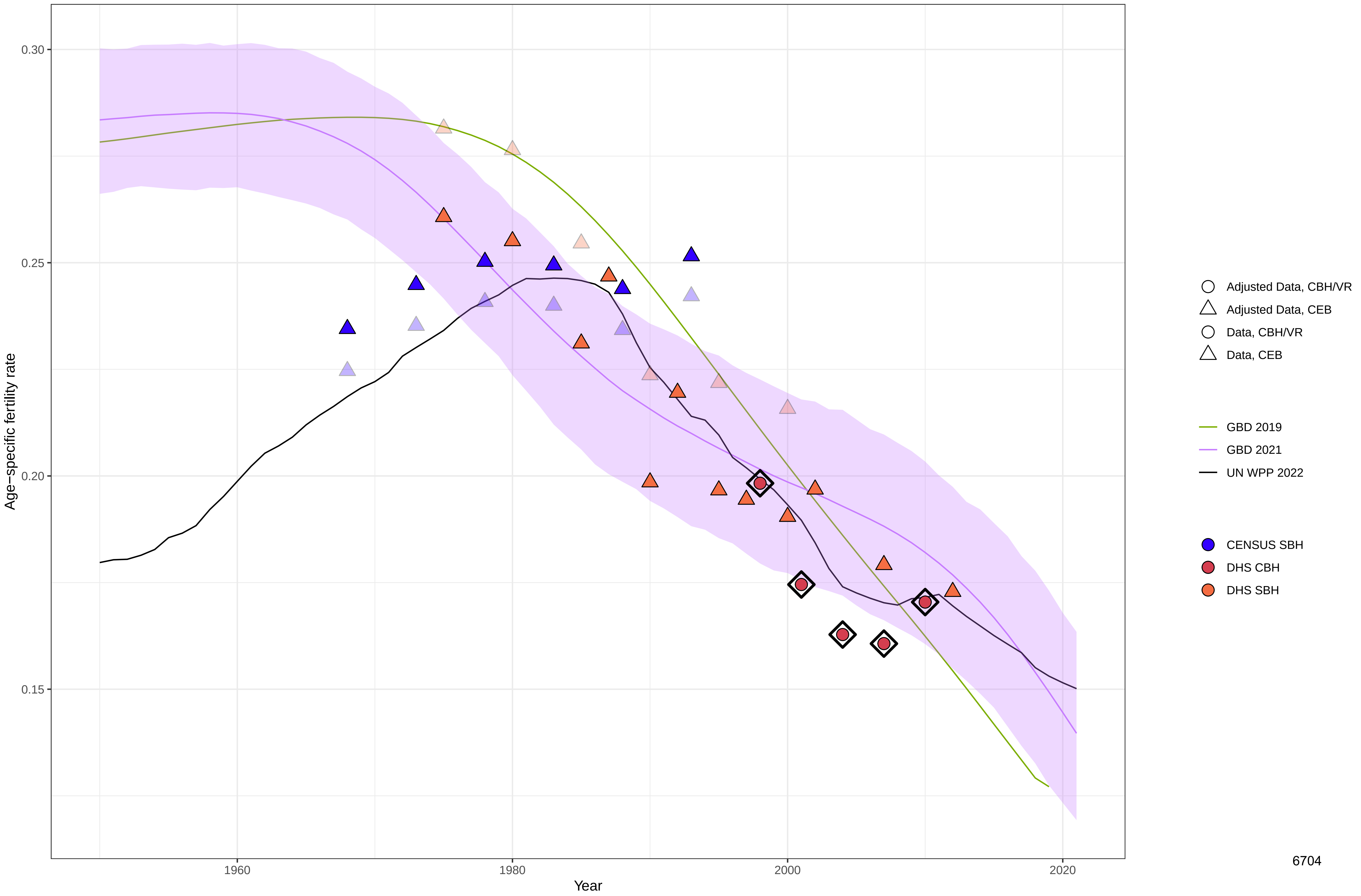

Age−specific fertility fitted trend lines, 1950−2021: Gabon, Ages: 25−29
Data points enclosed with a diamond are reference sources

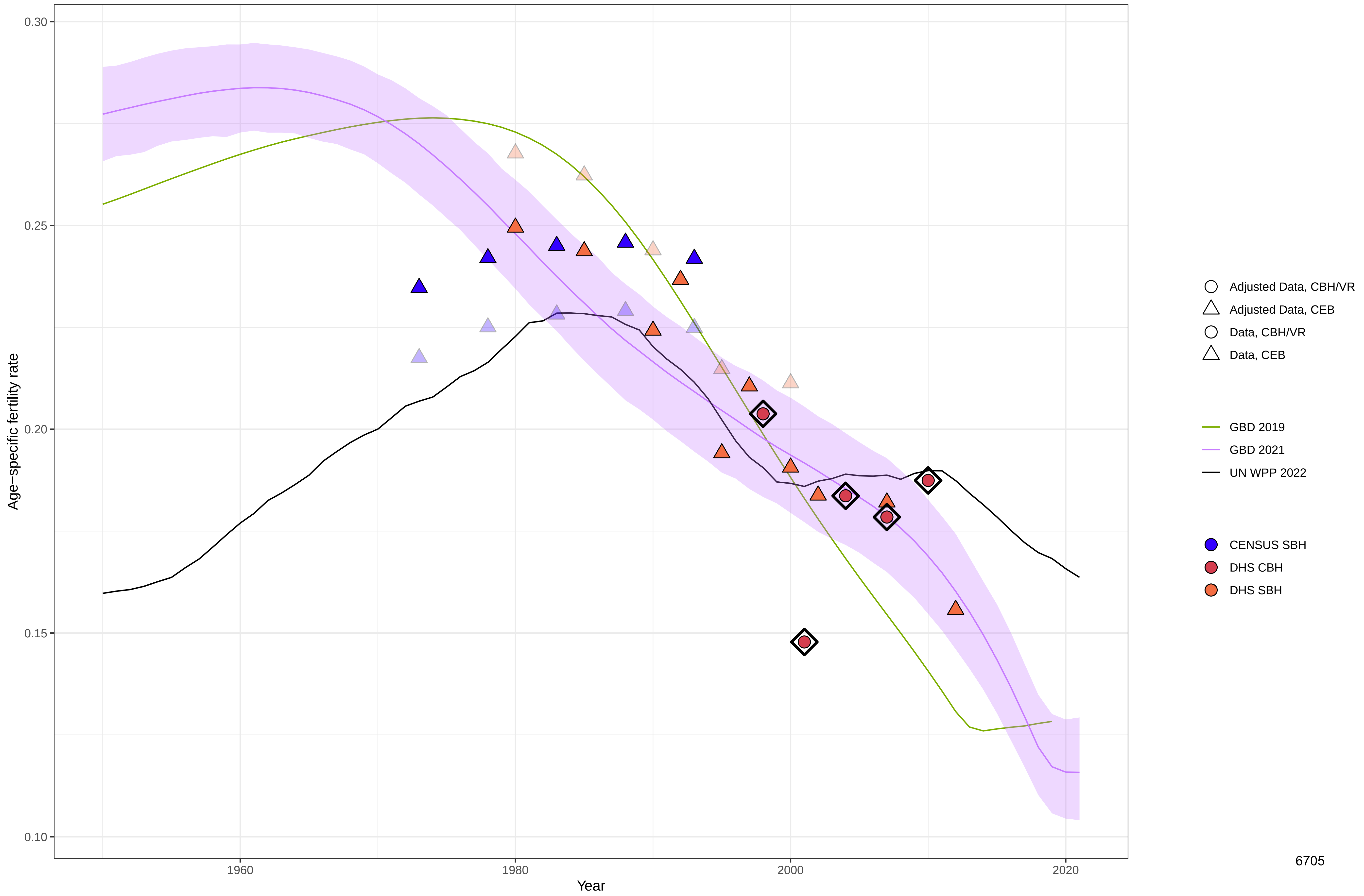

Age−specific fertility fitted trend lines, 1950−2021: Gabon, Ages: 30−34
Data points enclosed with a diamond are reference sources

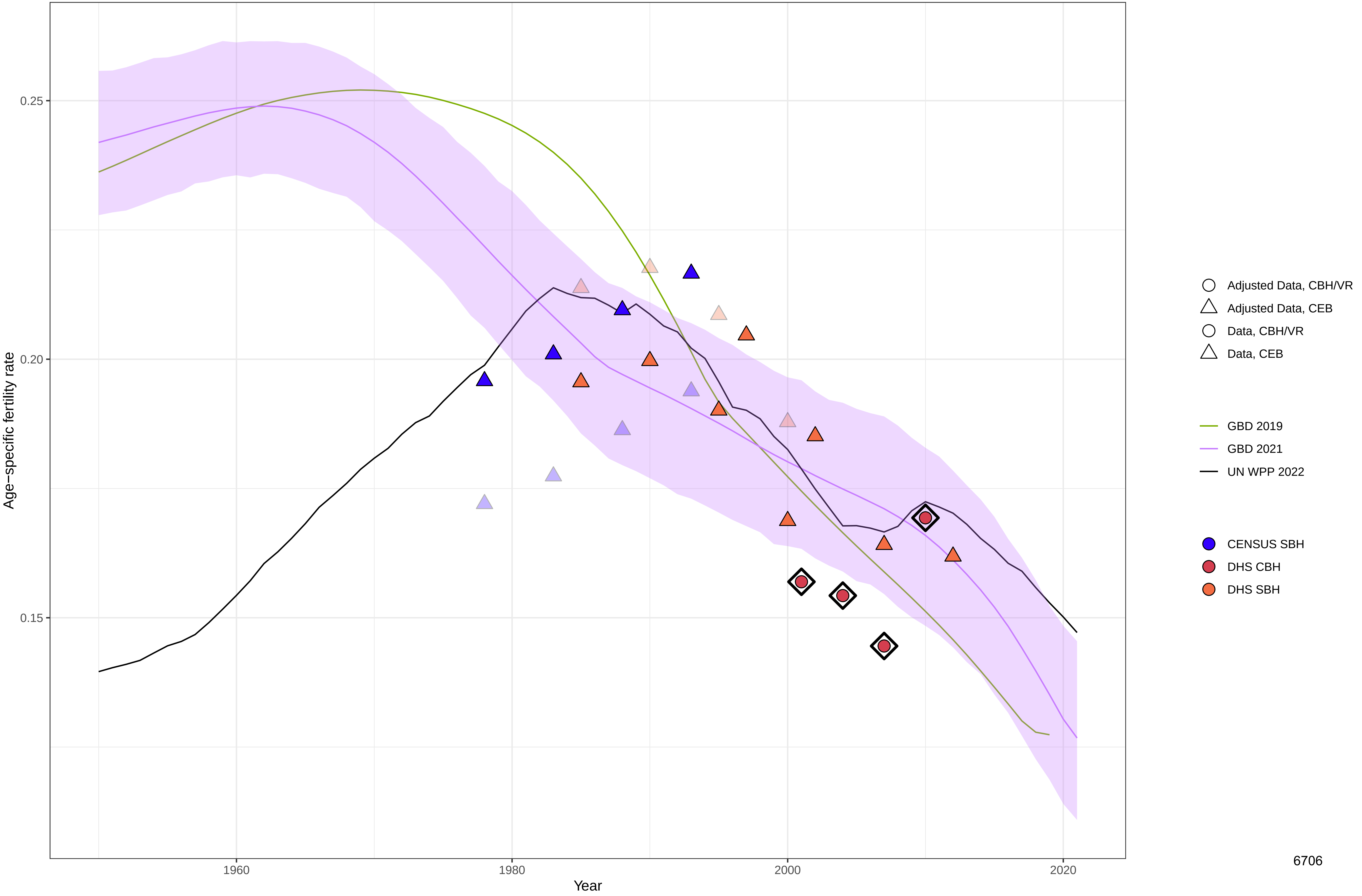

Age−specific fertility fitted trend lines, 1950−2021: Gabon, Ages: 35−39
Data points enclosed with a diamond are reference sources

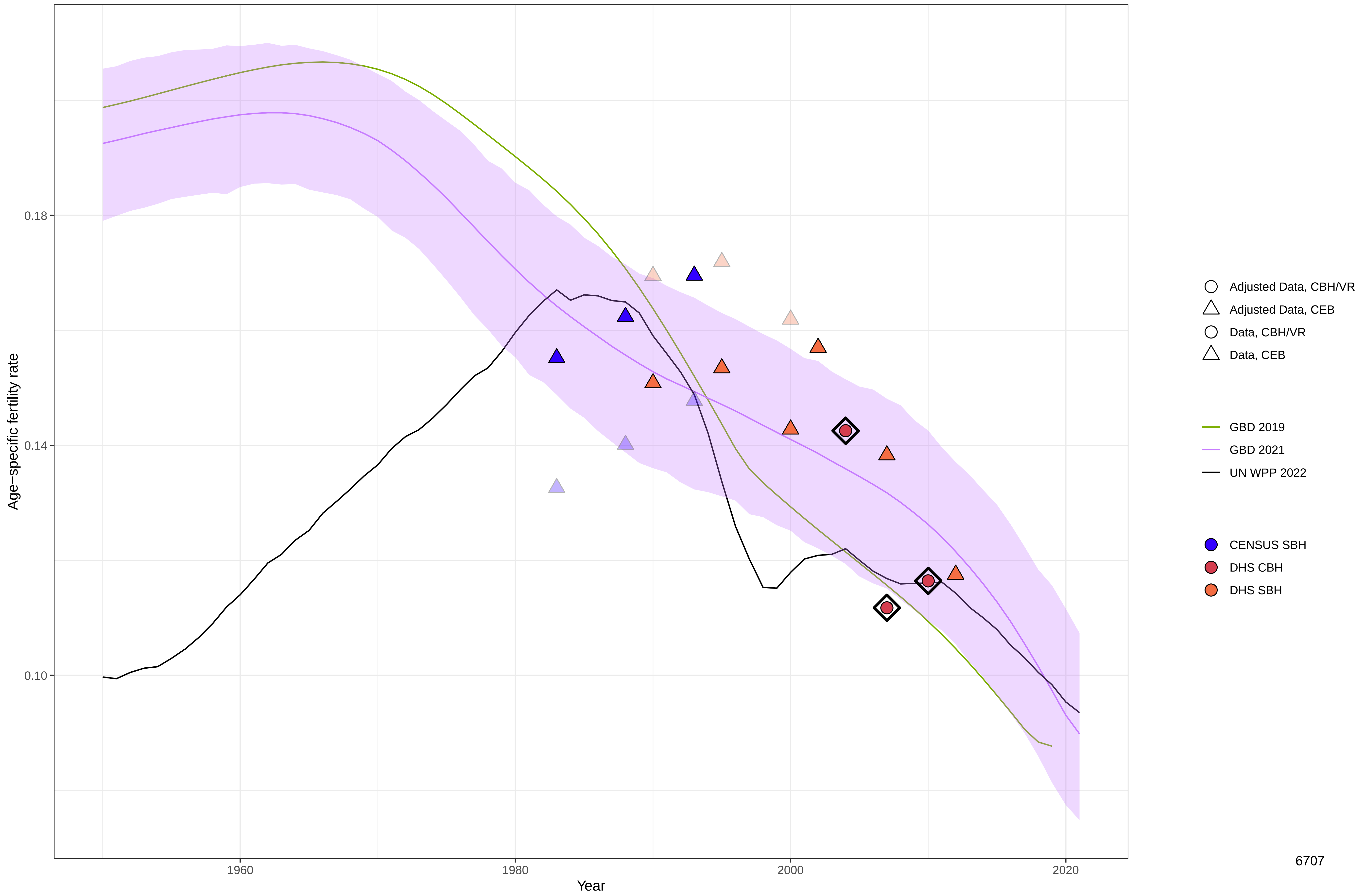

Age−specific fertility fitted trend lines, 1950−2021: Gabon, Ages: 40−44
Data points enclosed with a diamond are reference sources

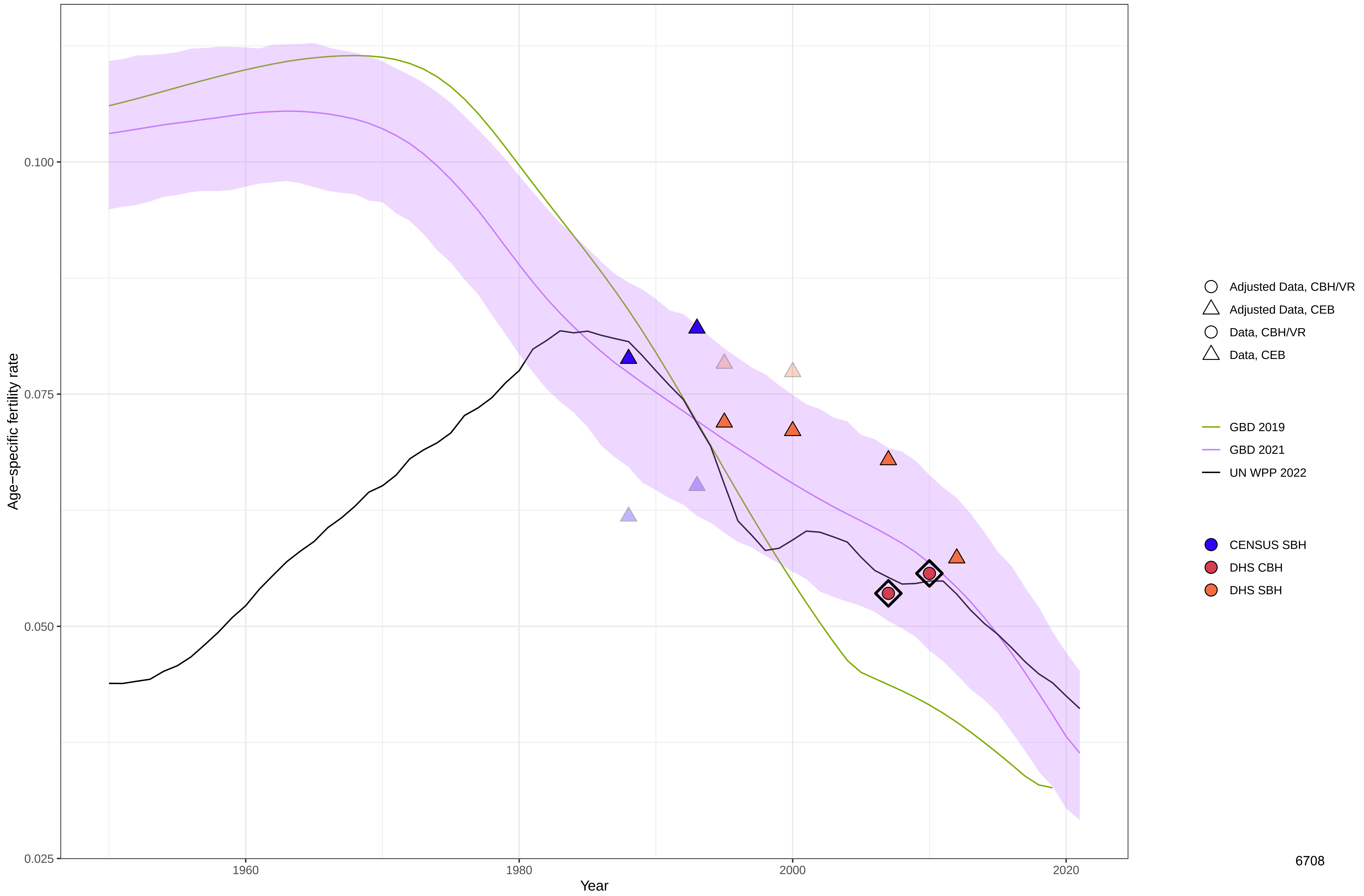

Age−specific fertility fitted trend lines, 1950−2021: Gabon, Ages: 45−49
Data points enclosed with a diamond are reference sources

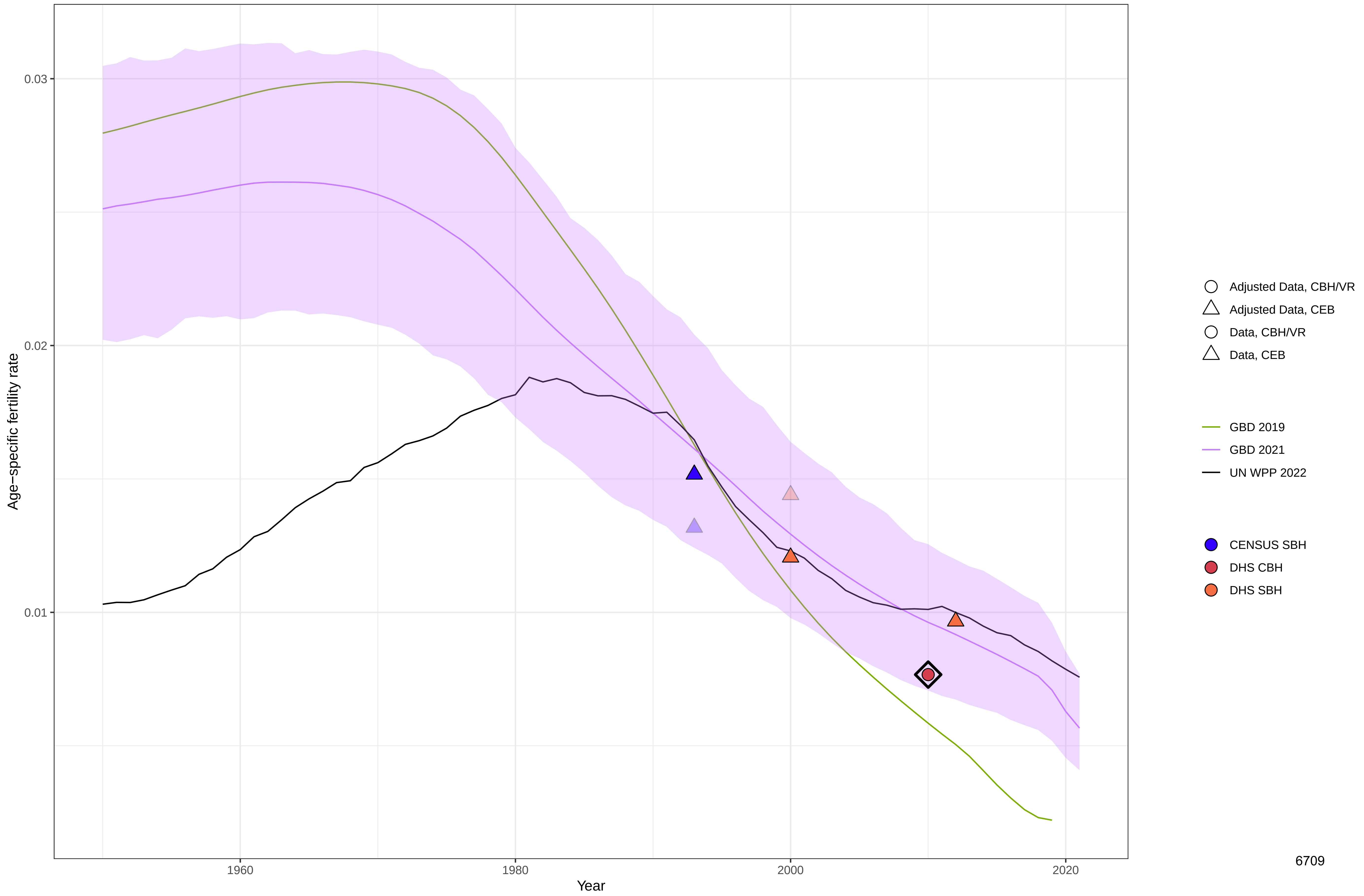

Age−specific fertility fitted trend lines, 1950−2021: Gabon, Ages: 50−54
Data points enclosed with a diamond are reference sources

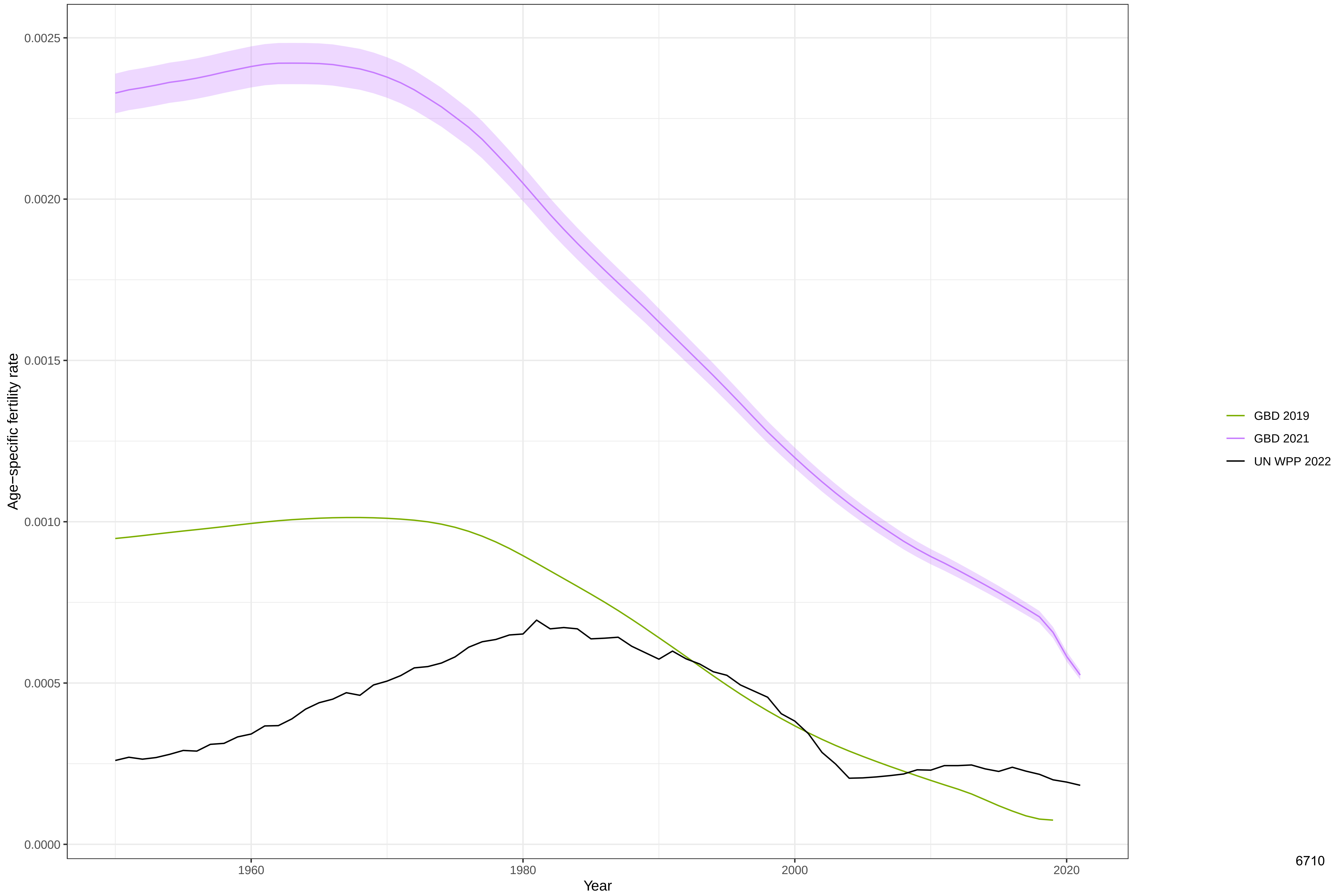

Total fertility rate fitted trend lines, 1950−2021: Gabon TFR
Data points enclosed with a diamond are reference sources

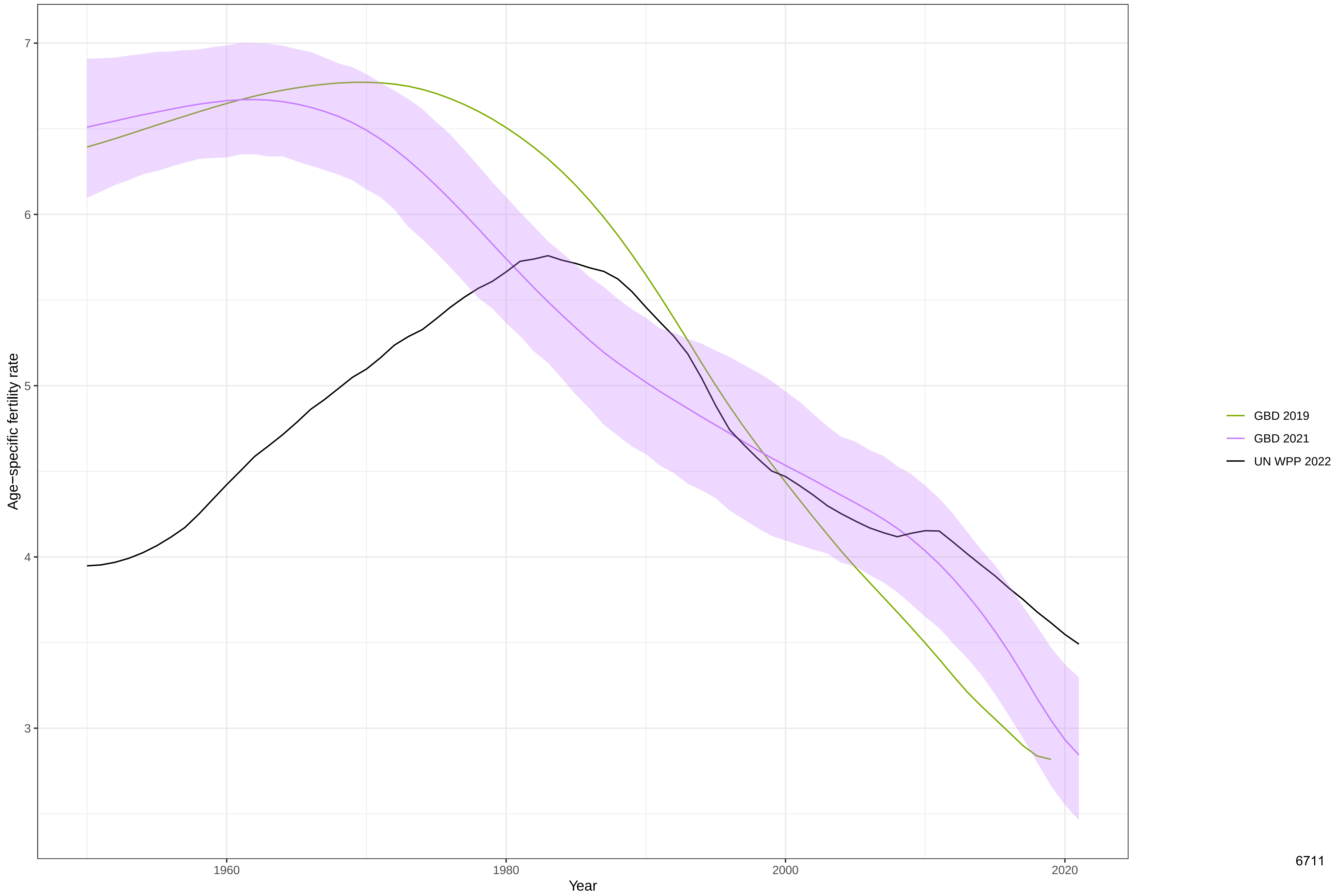

Age−specific fertility fitted trend lines, 1950−2021: Burundi, Ages: 10−14
Data points enclosed with a diamond are reference sources

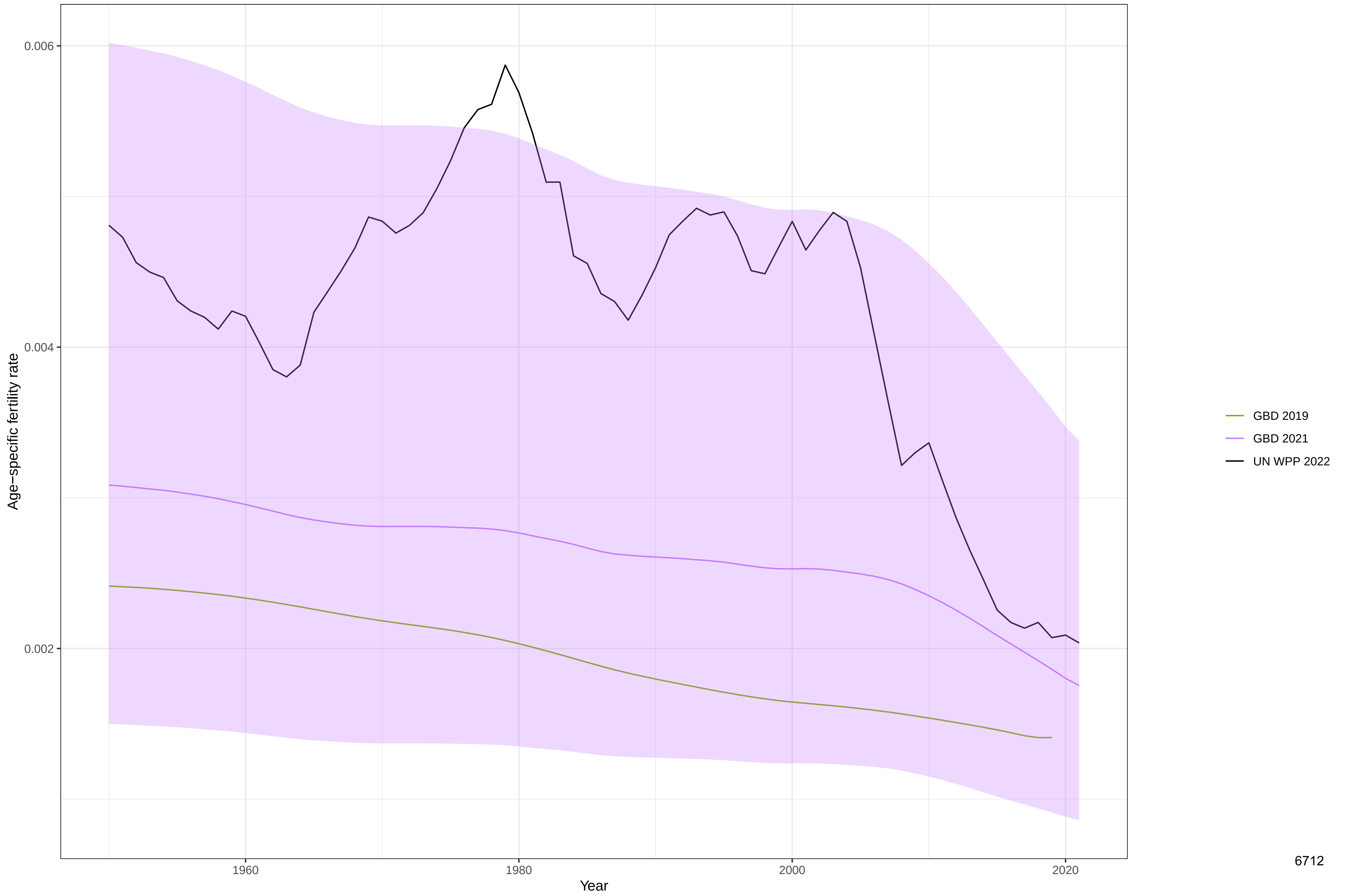

Age−specific fertility fitted trend lines, 1950−2021: Burundi, Ages: 15−19
Data points enclosed with a diamond are reference sources

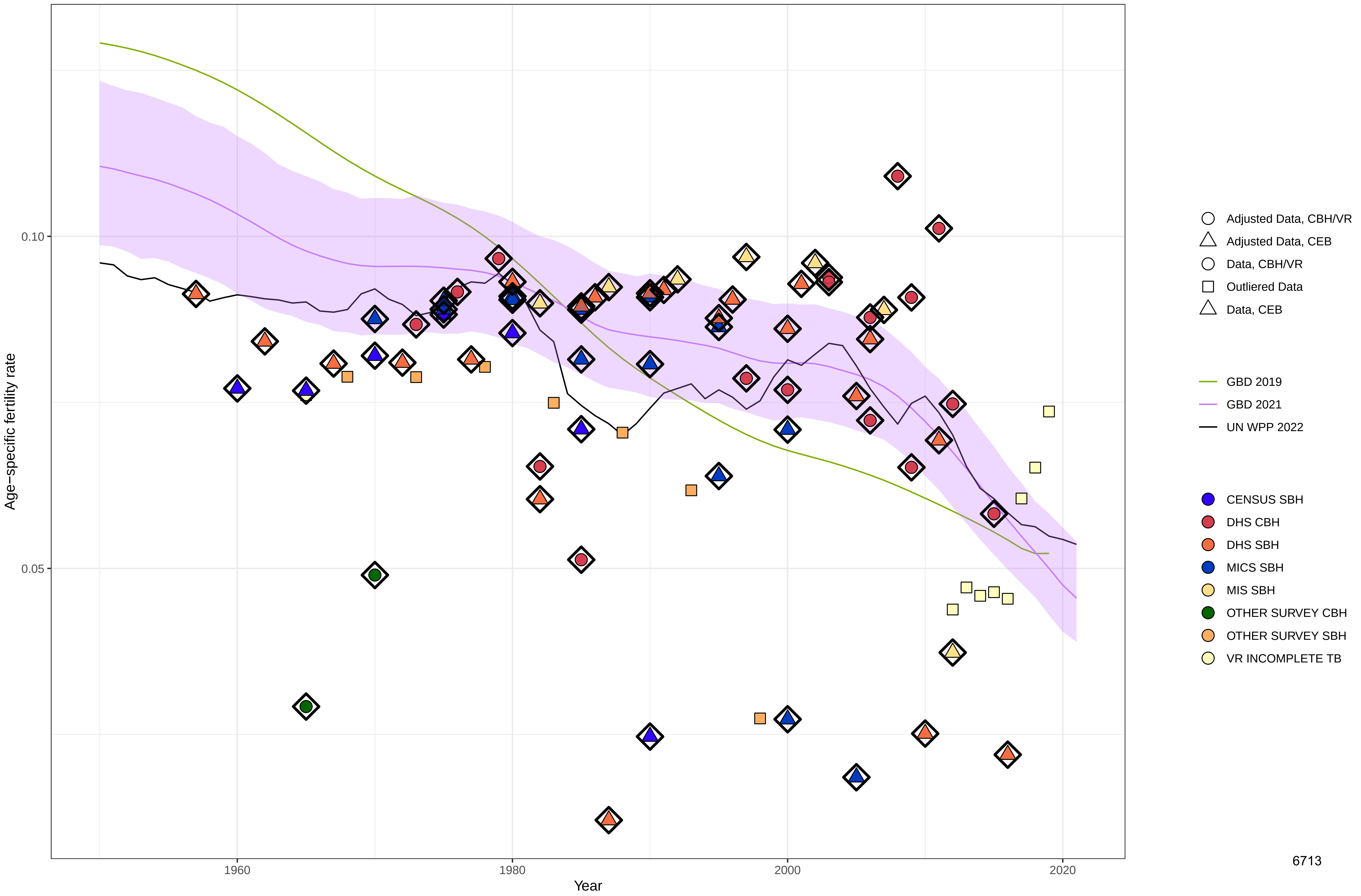

Age−specific fertility fitted trend lines, 1950−2021: Burundi, Ages: 20−24
Data points enclosed with a diamond are reference sources

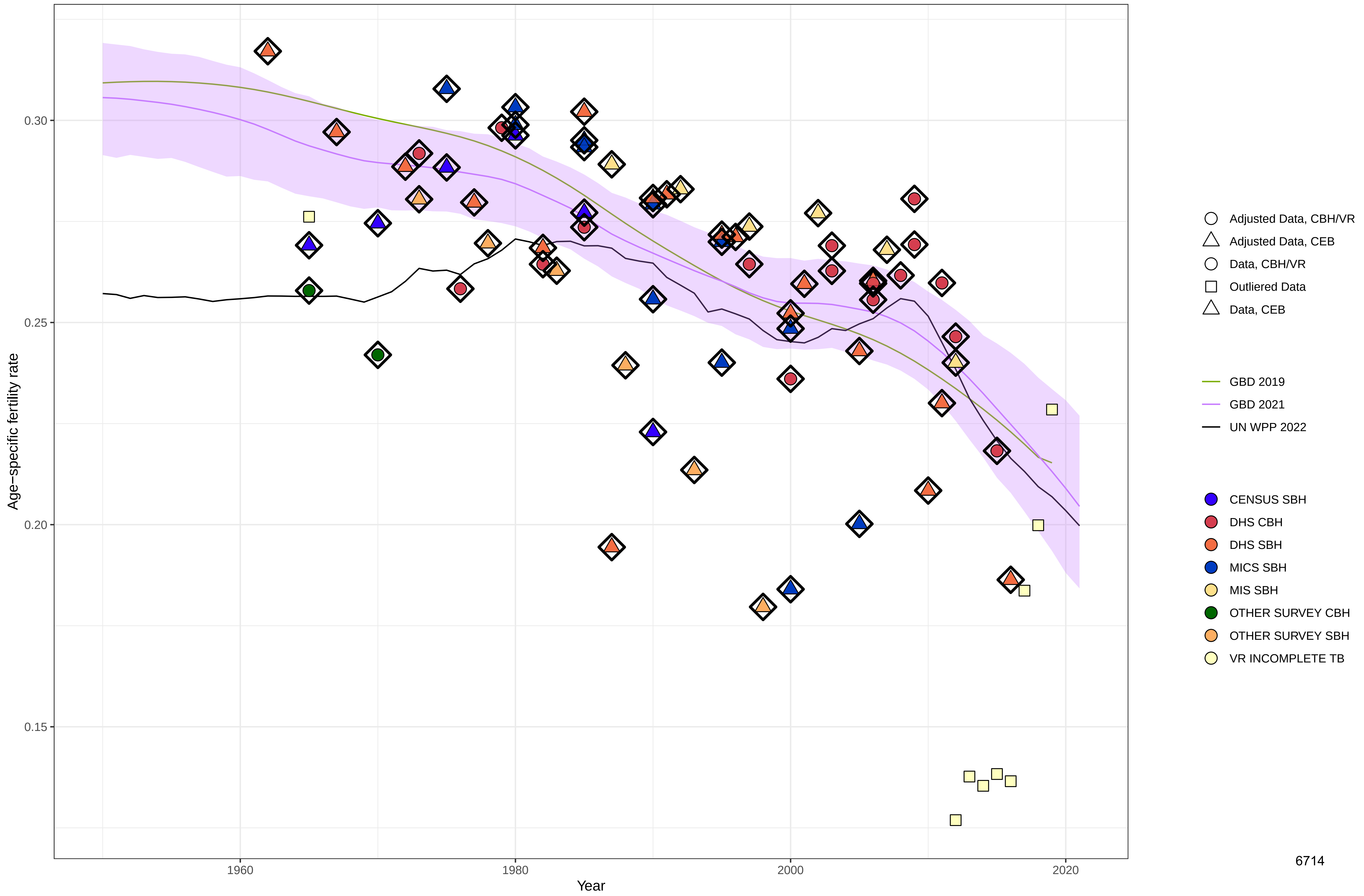

Age−specific fertility fitted trend lines, 1950−2021: Burundi, Ages: 25−29
Data points enclosed with a diamond are reference sources

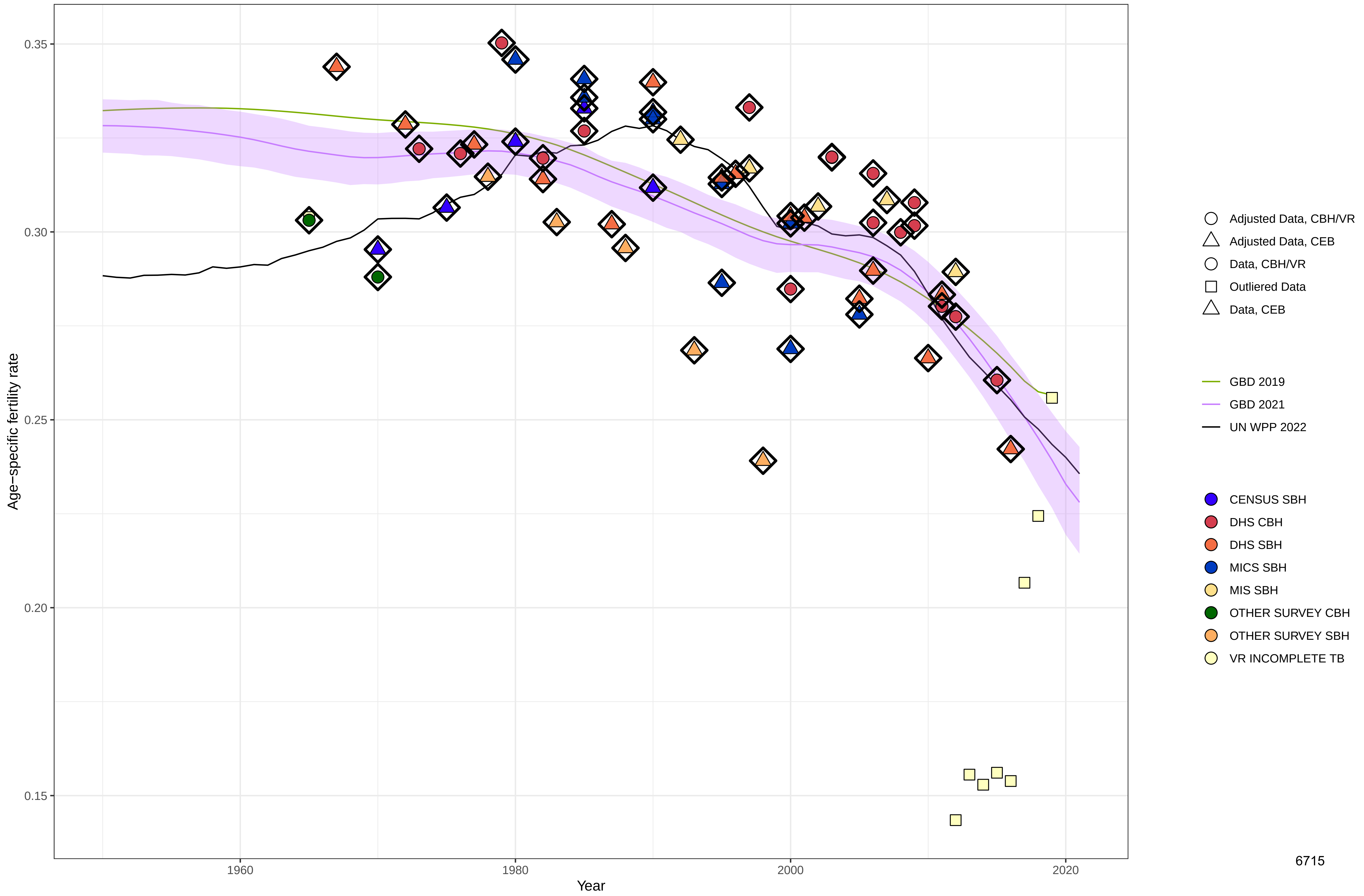

Age−specific fertility fitted trend lines, 1950−2021: Burundi, Ages: 30−34
Data points enclosed with a diamond are reference sources

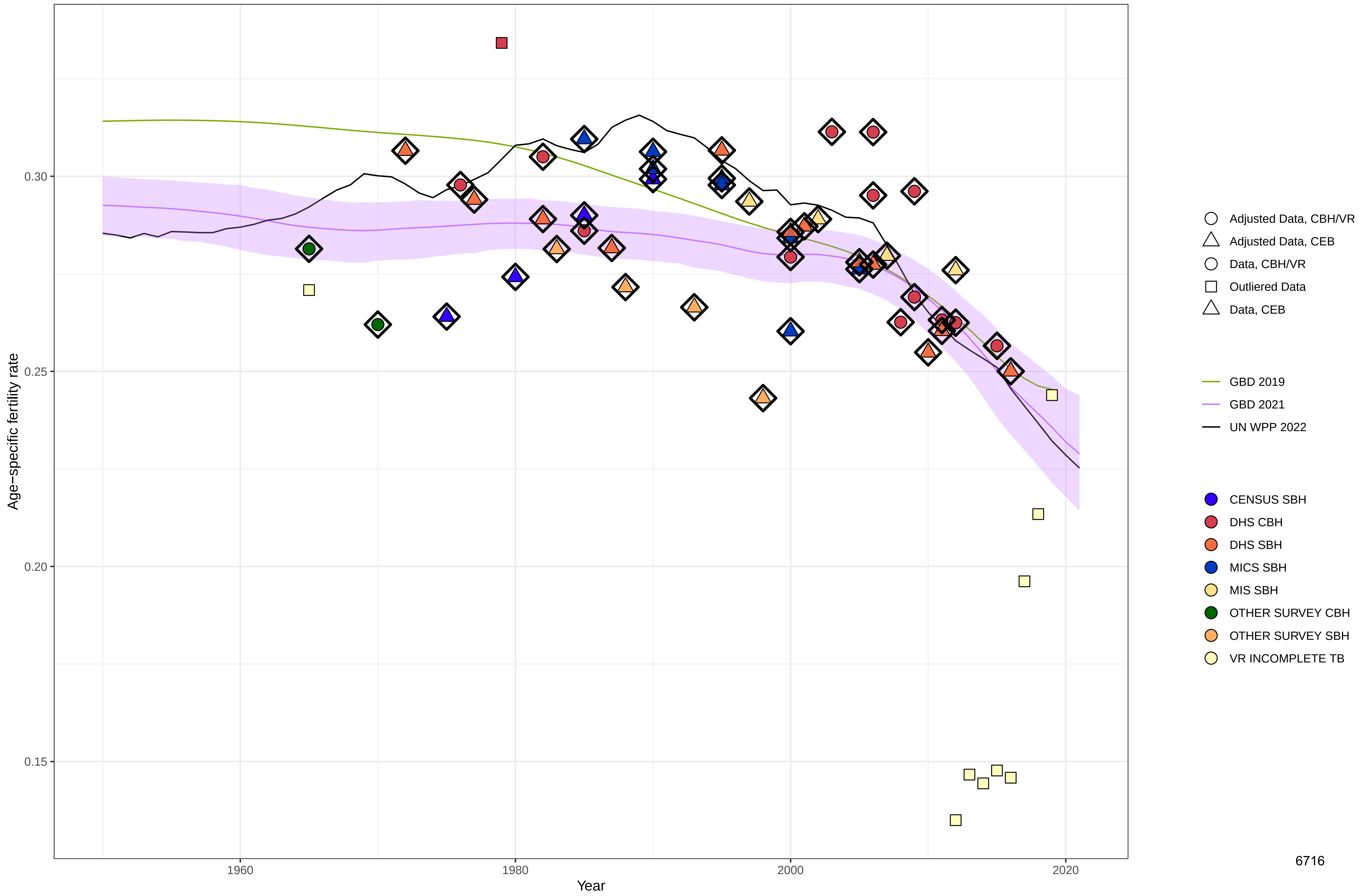

Age−specific fertility fitted trend lines, 1950−2021: Burundi, Ages: 35−39
Data points enclosed with a diamond are reference sources

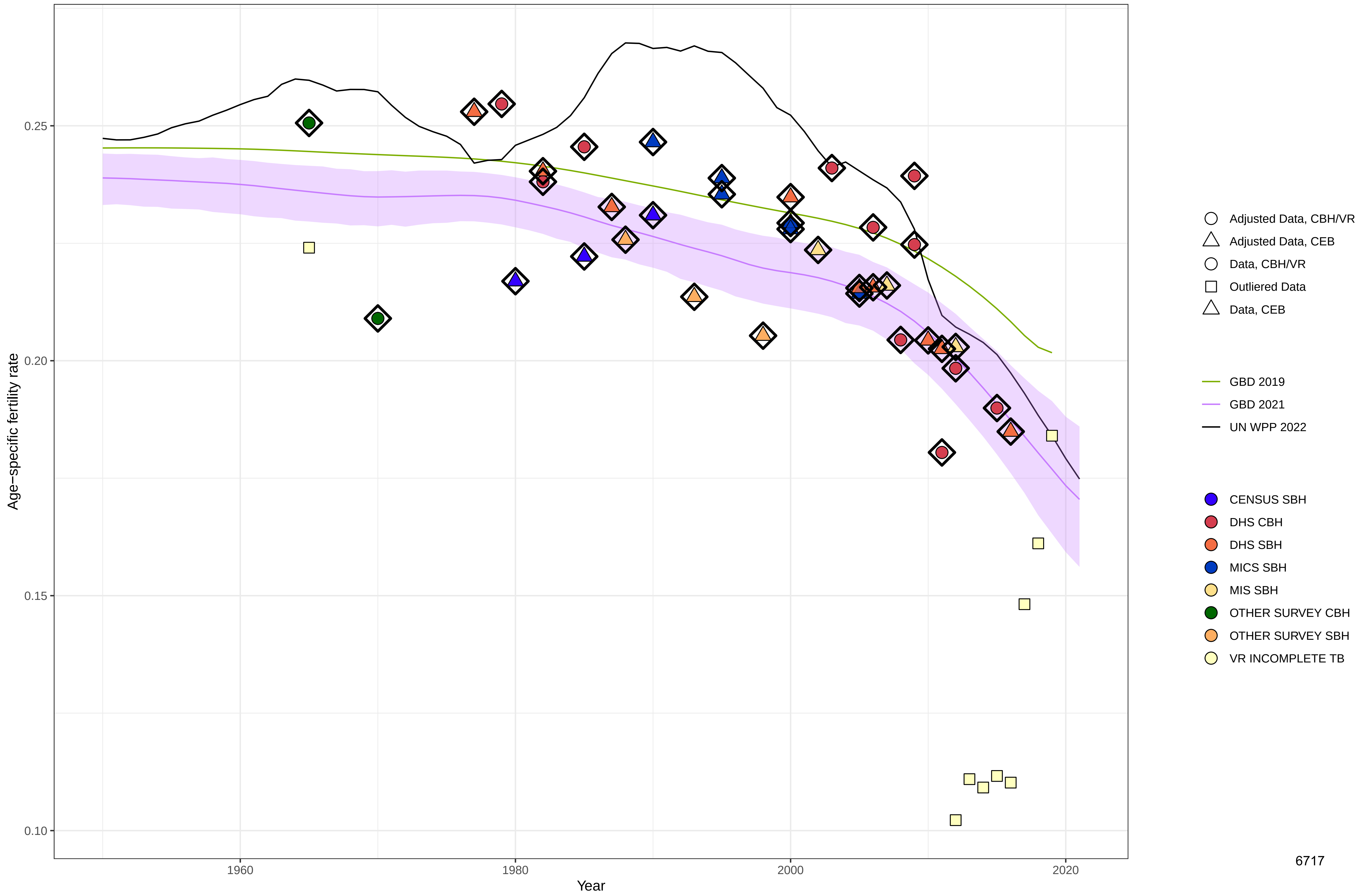

Age−specific fertility fitted trend lines, 1950−2021: Burundi, Ages: 40−44
Data points enclosed with a diamond are reference sources

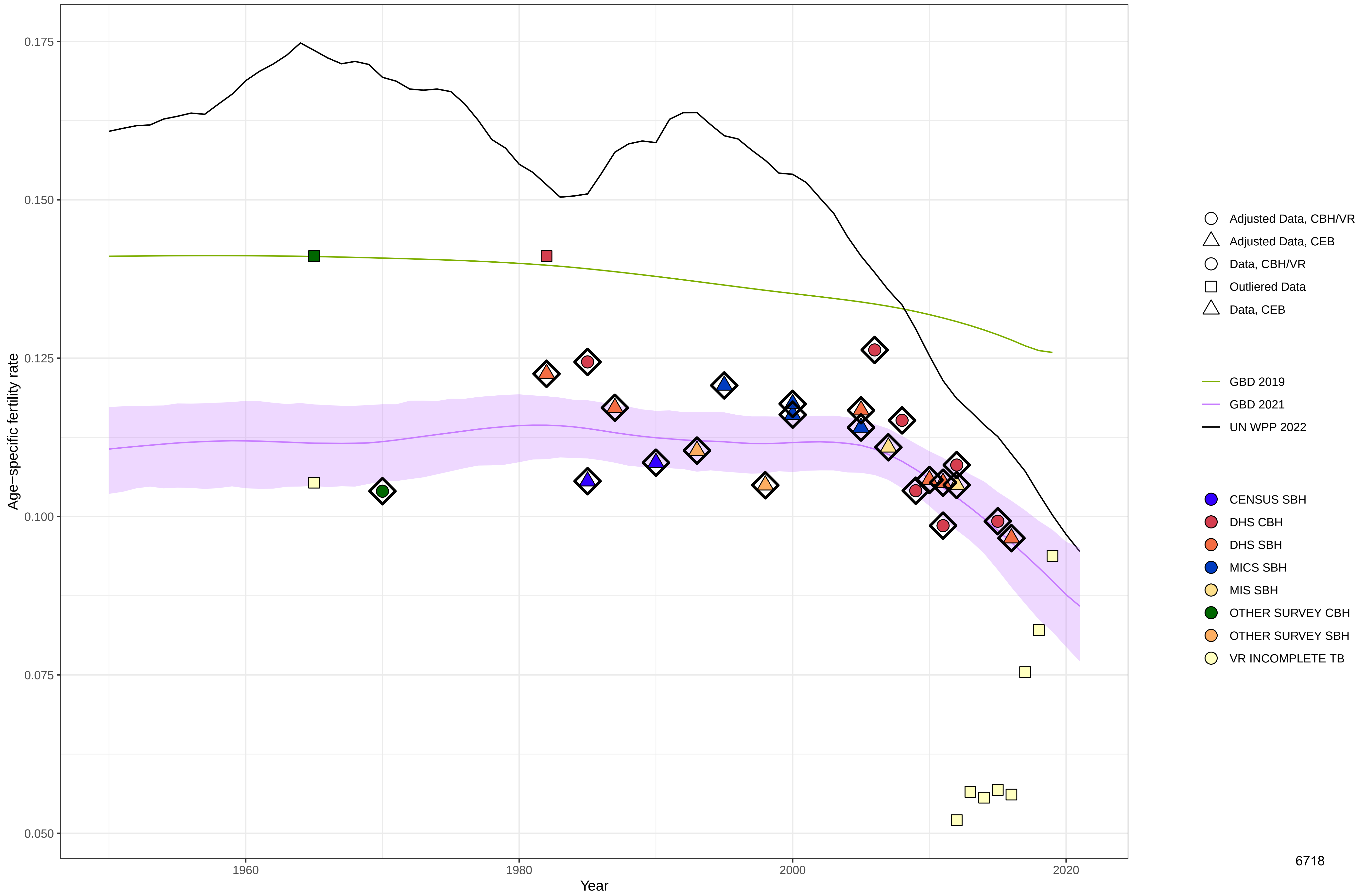

Age−specific fertility fitted trend lines, 1950−2021: Burundi, Ages: 45−49
Data points enclosed with a diamond are reference sources

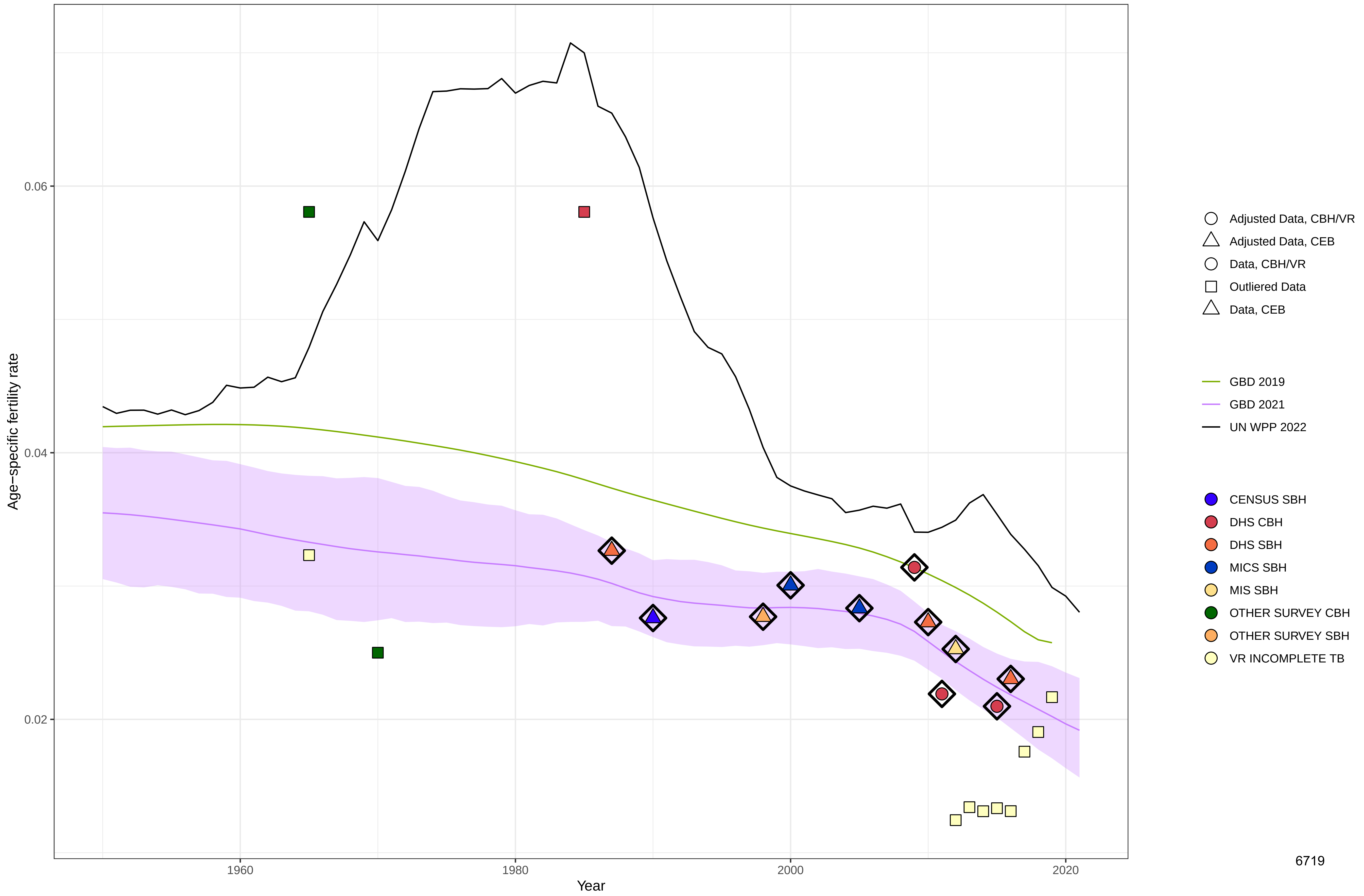

Age−specific fertility fitted trend lines, 1950−2021: Burundi, Ages: 50−54
Data points enclosed with a diamond are reference sources

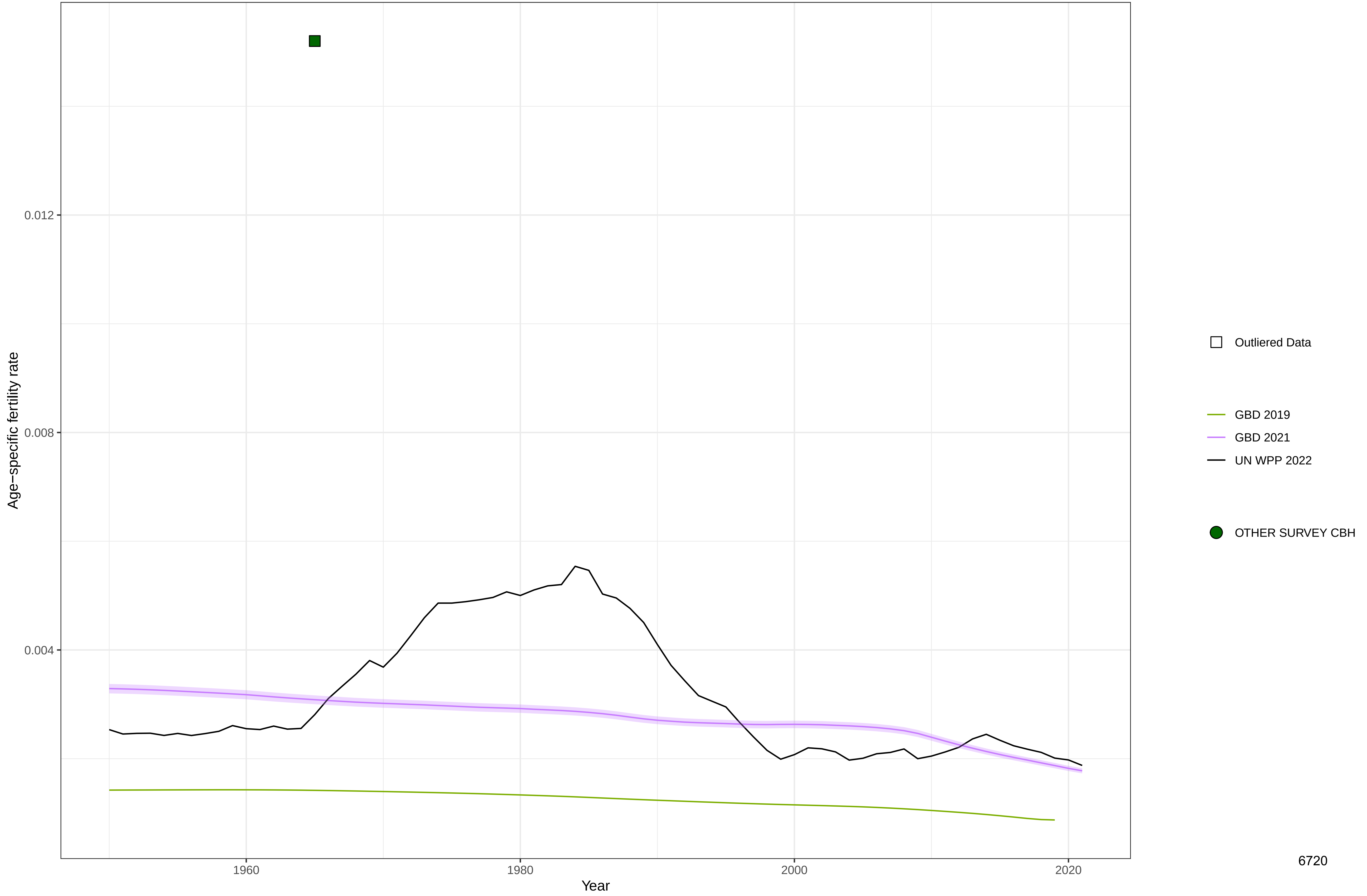

Total fertility rate fitted trend lines, 1950−2021: Burundi TFR
Data points enclosed with a diamond are reference sources

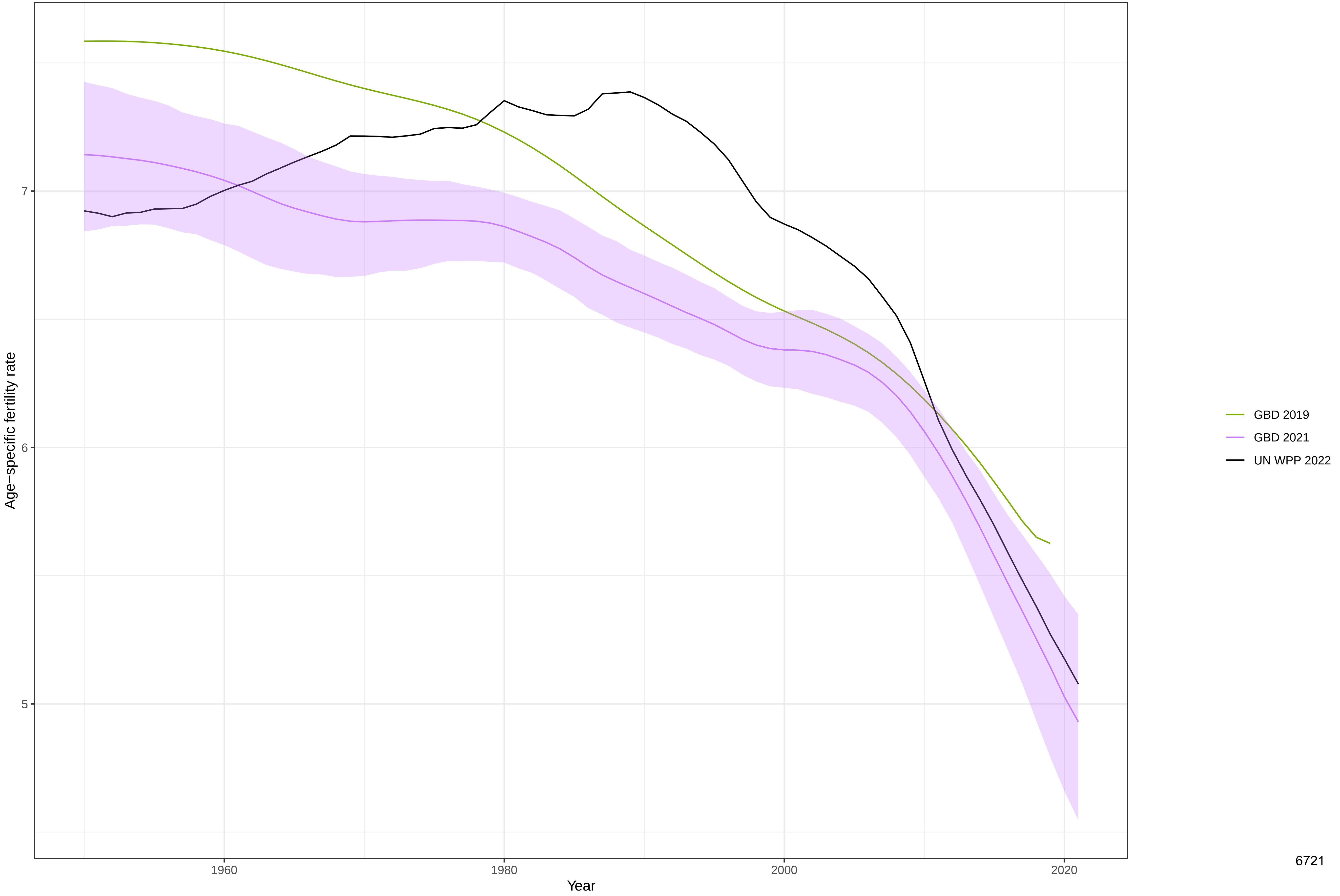

Age−specific fertility fitted trend lines, 1950−2021: Comoros, Ages: 10−14
Data points enclosed with a diamond are reference sources

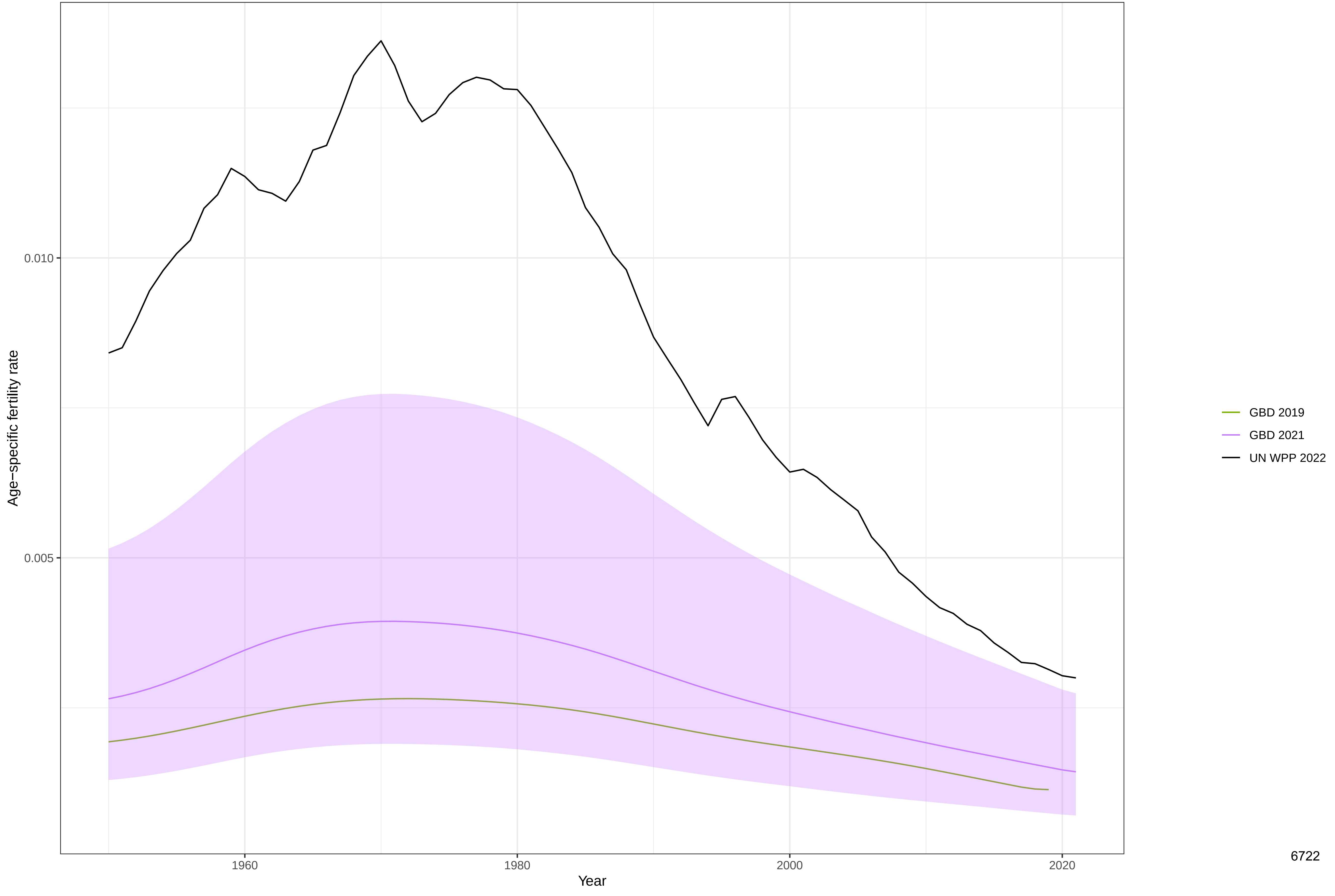

Age−specific fertility fitted trend lines, 1950−2021: Comoros, Ages: 15−19
Data points enclosed with a diamond are reference sources

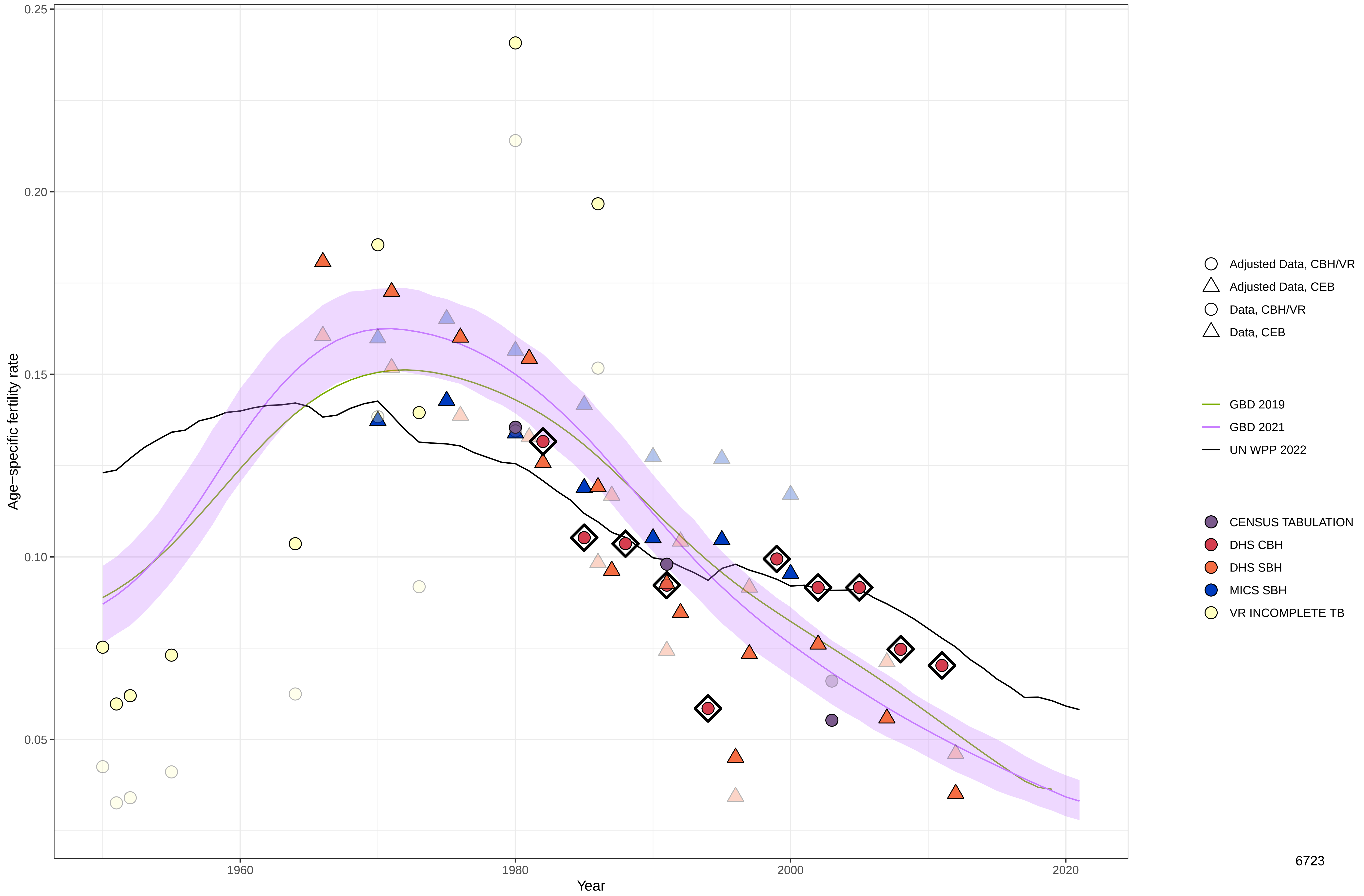

Age−specific fertility fitted trend lines, 1950−2021: Comoros, Ages: 20−24
Data points enclosed with a diamond are reference sources

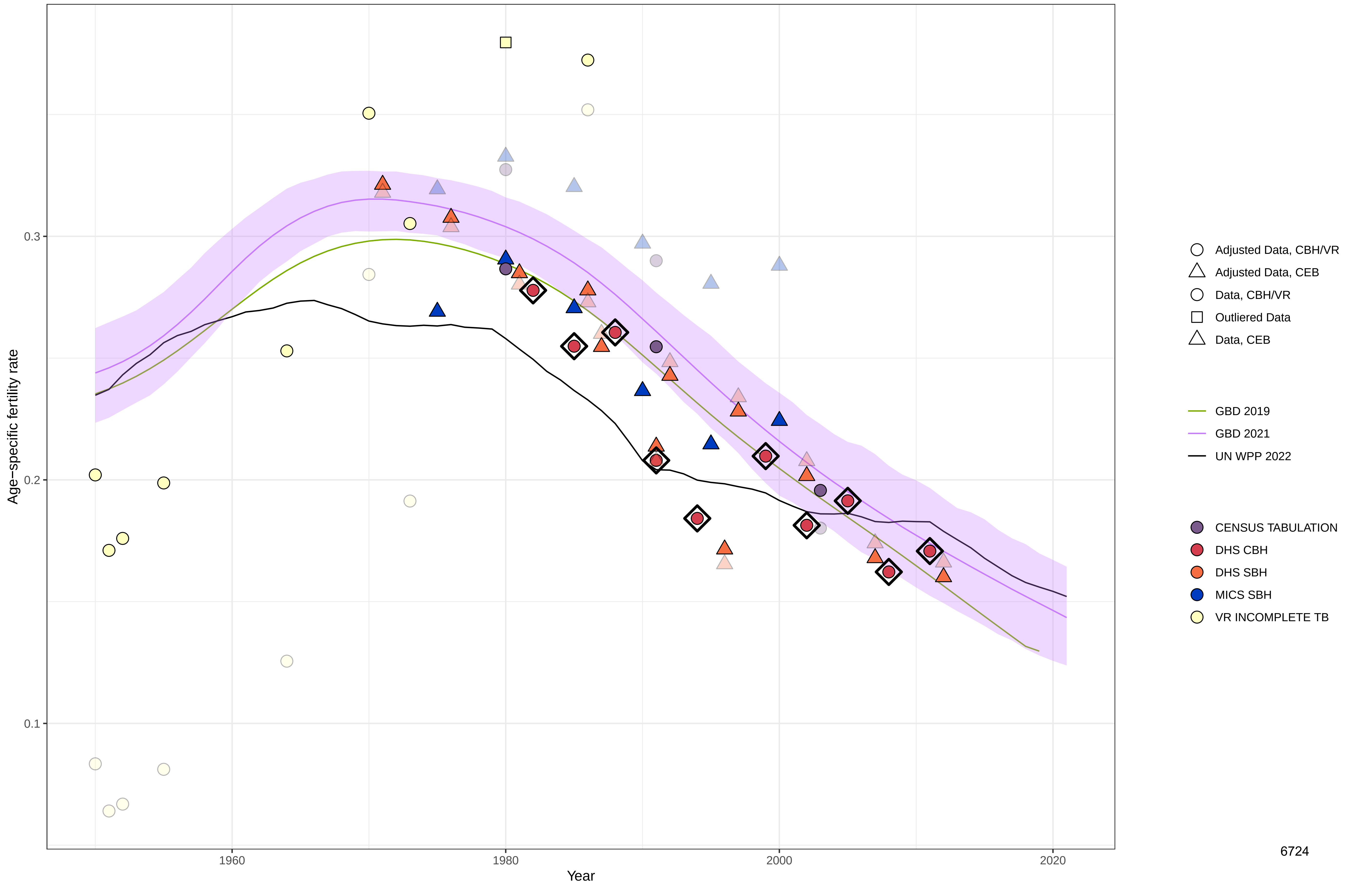

Age−specific fertility fitted trend lines, 1950−2021: Comoros, Ages: 25−29
Data points enclosed with a diamond are reference sources

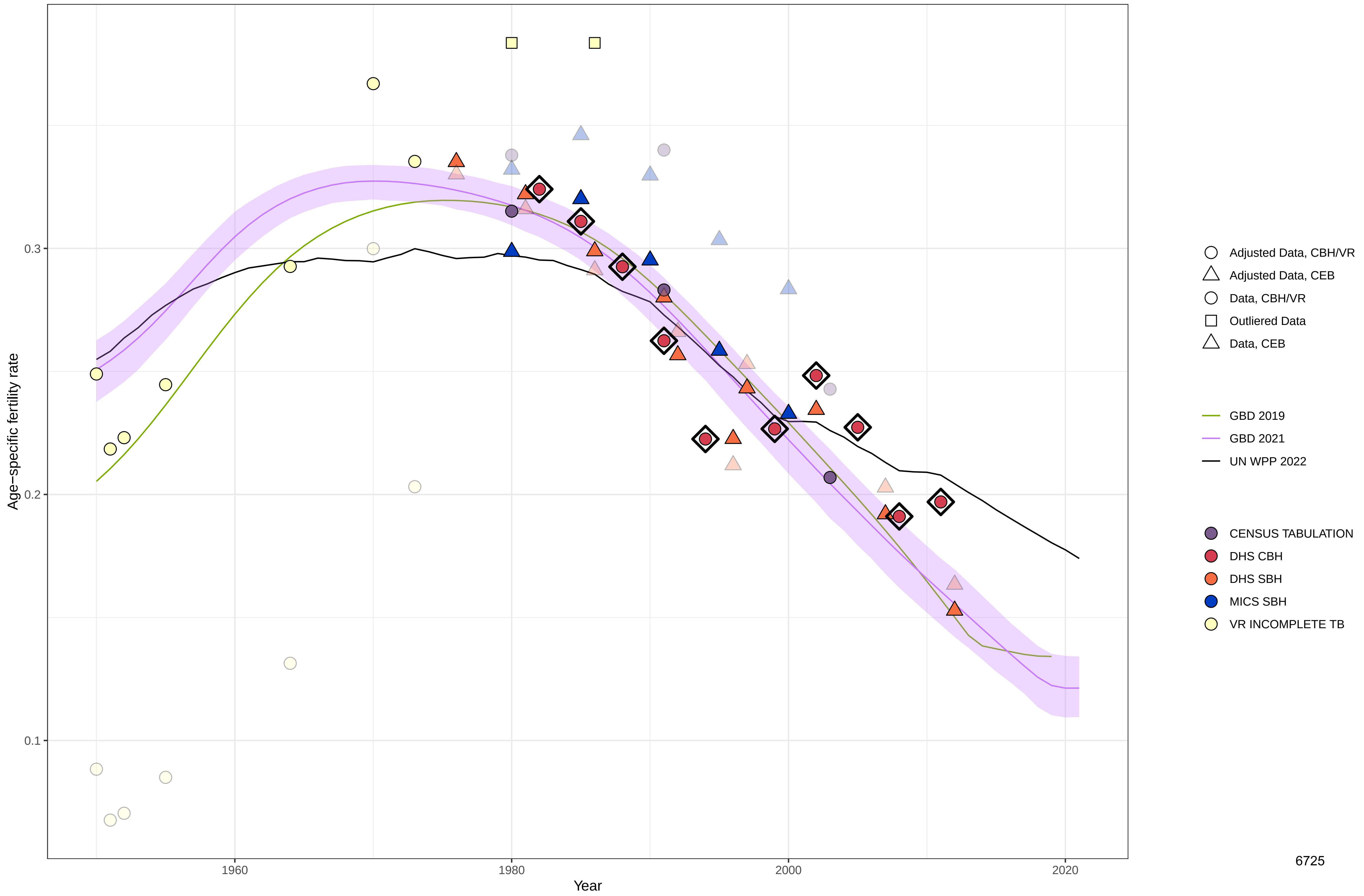

Age−specific fertility fitted trend lines, 1950−2021: Comoros, Ages: 30−34
Data points enclosed with a diamond are reference sources

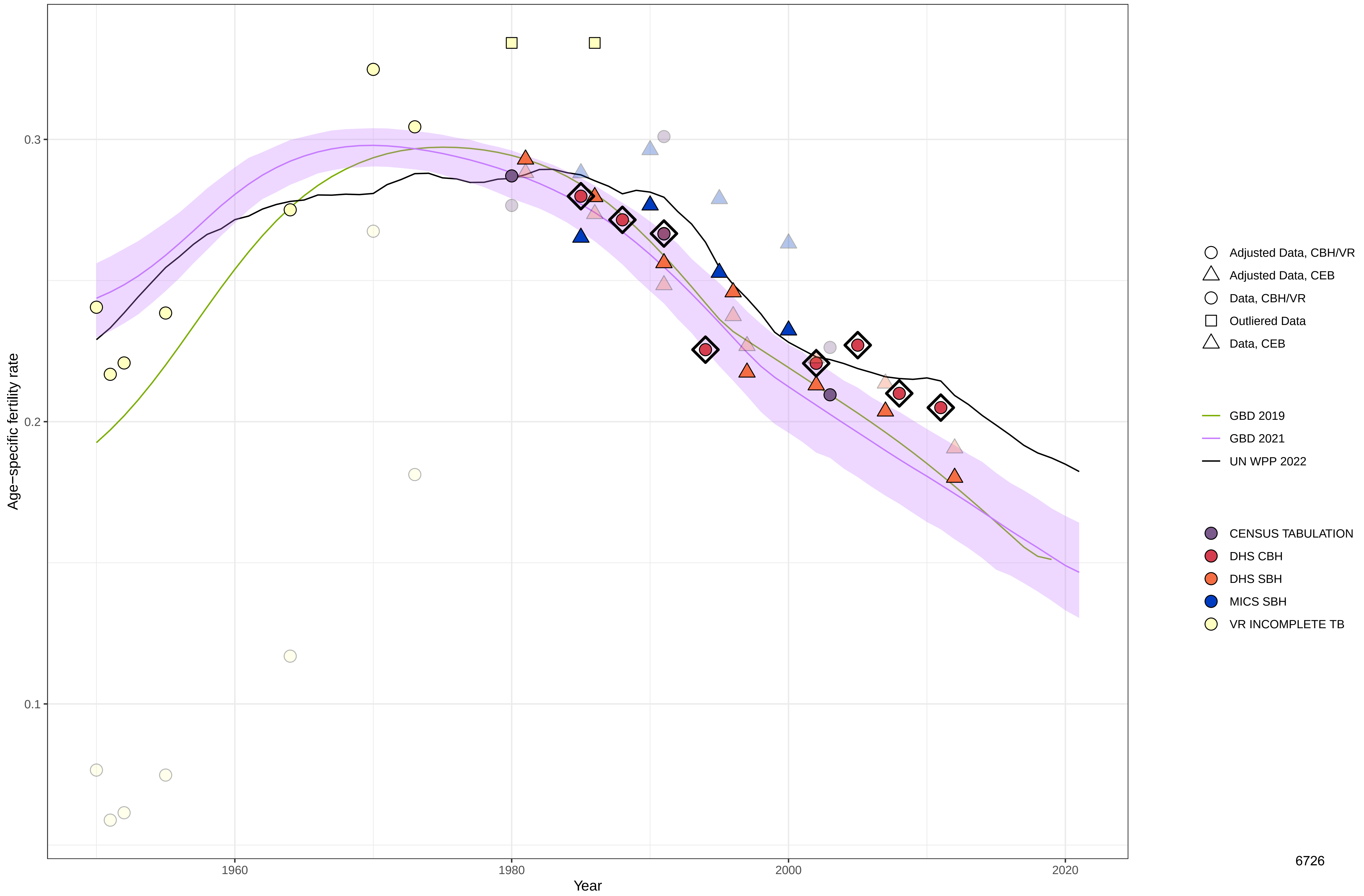

Age−specific fertility fitted trend lines, 1950−2021: Comoros, Ages: 35−39
Data points enclosed with a diamond are reference sources

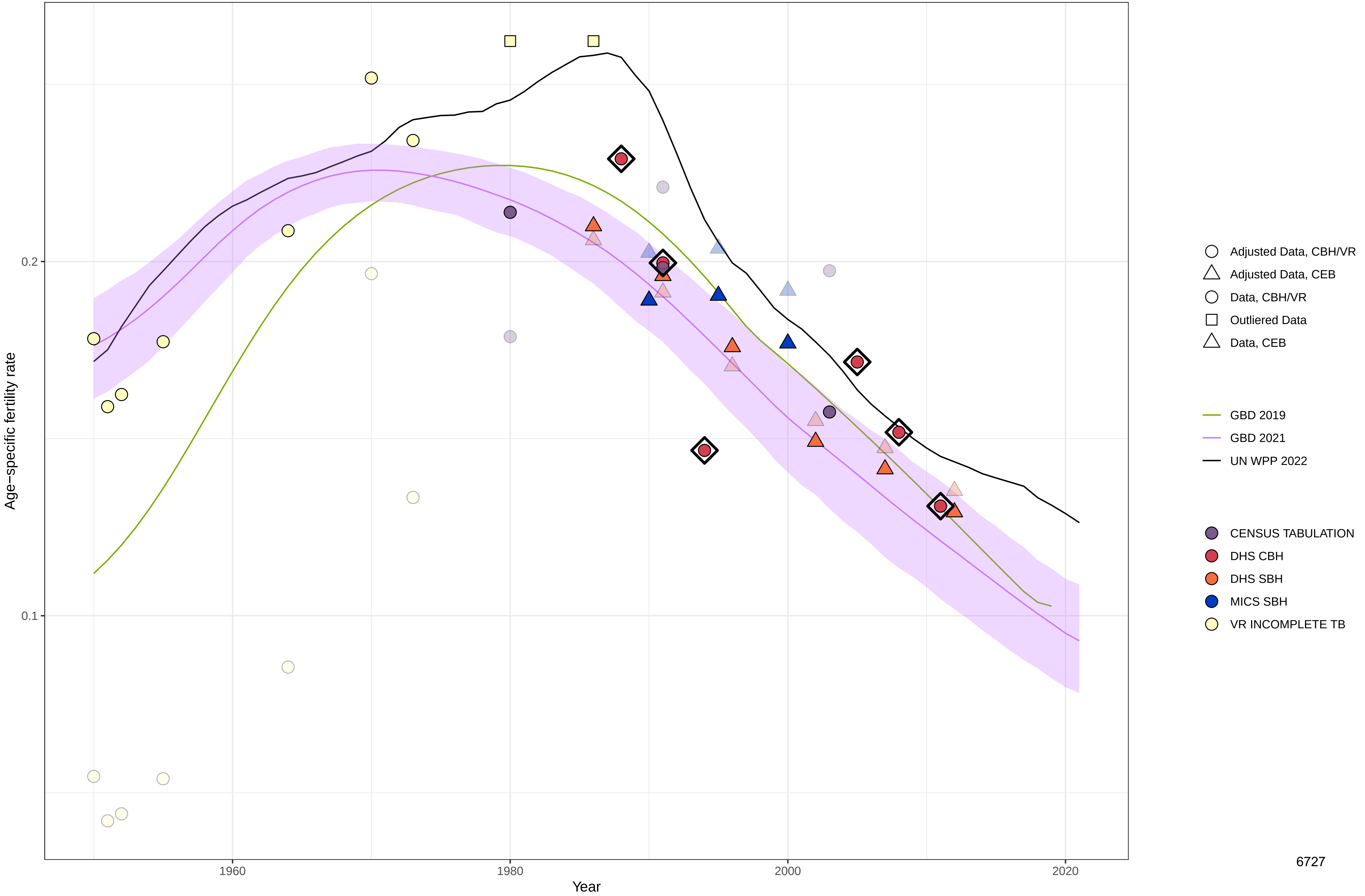

Age−specific fertility fitted trend lines, 1950−2021: Comoros, Ages: 40−44
Data points enclosed with a diamond are reference sources

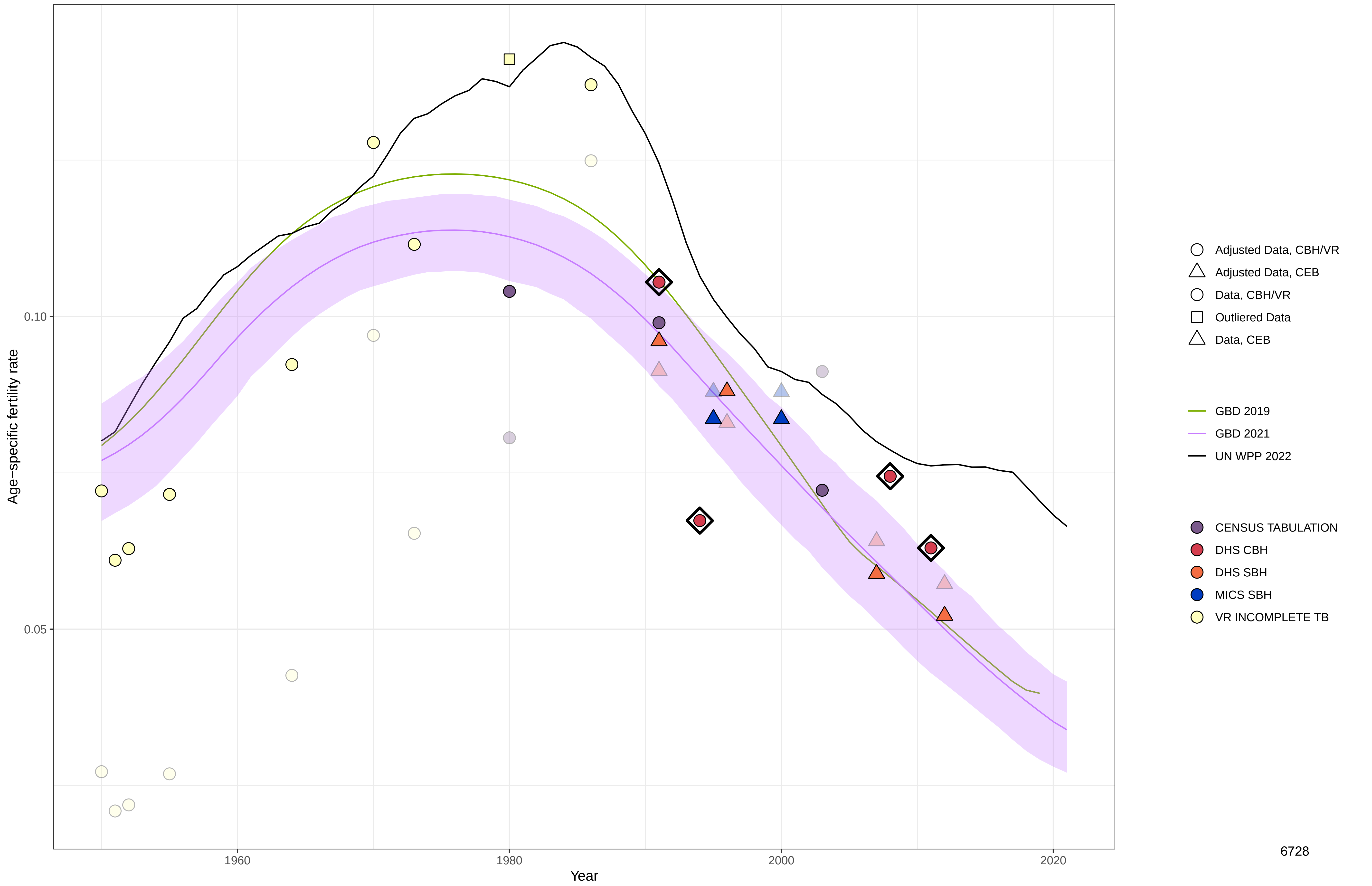

Age−specific fertility fitted trend lines, 1950−2021: Comoros, Ages: 45−49
Data points enclosed with a diamond are reference sources

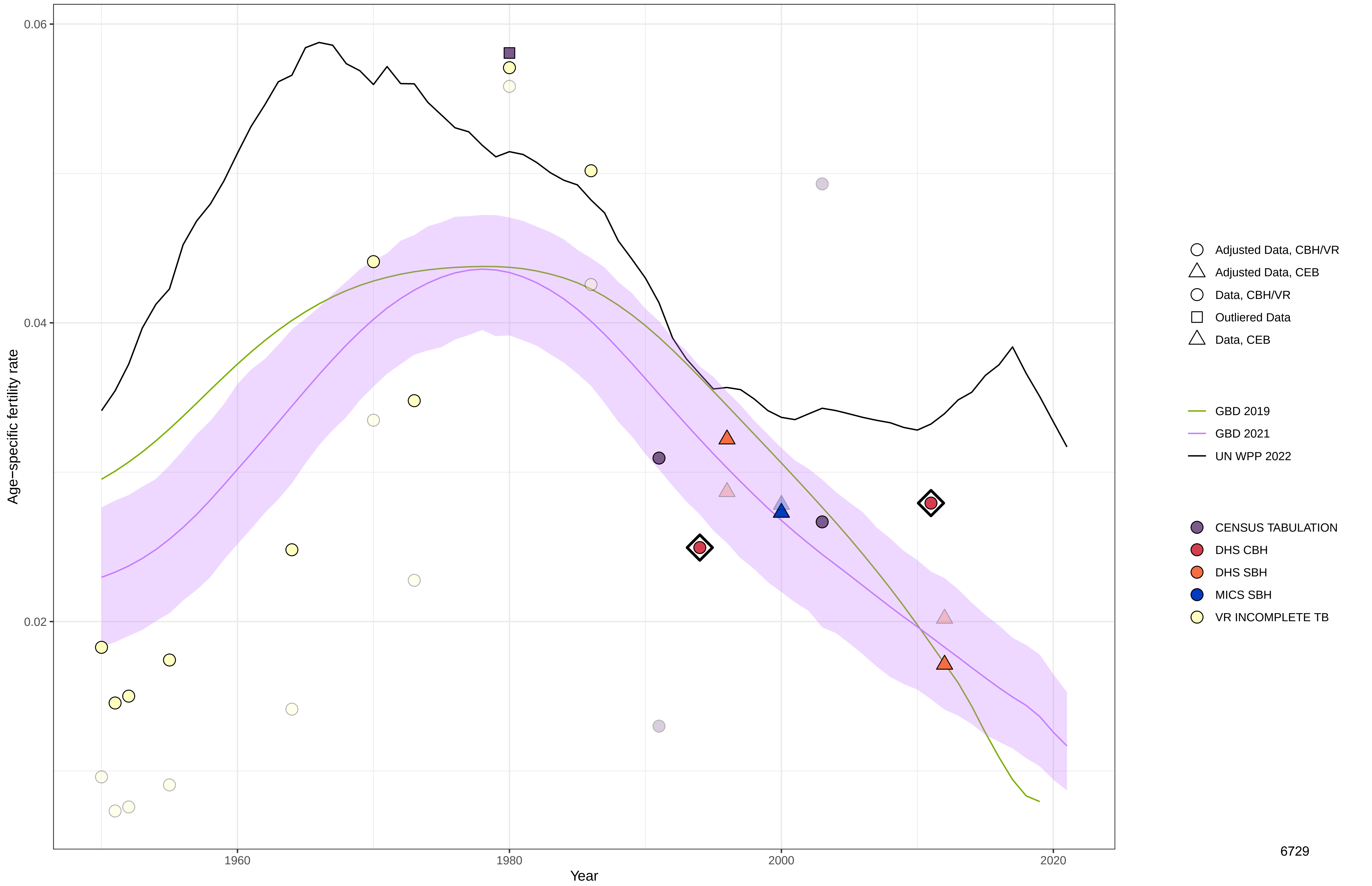

Age−specific fertility fitted trend lines, 1950−2021: Comoros, Ages: 50−54
Data points enclosed with a diamond are reference sources

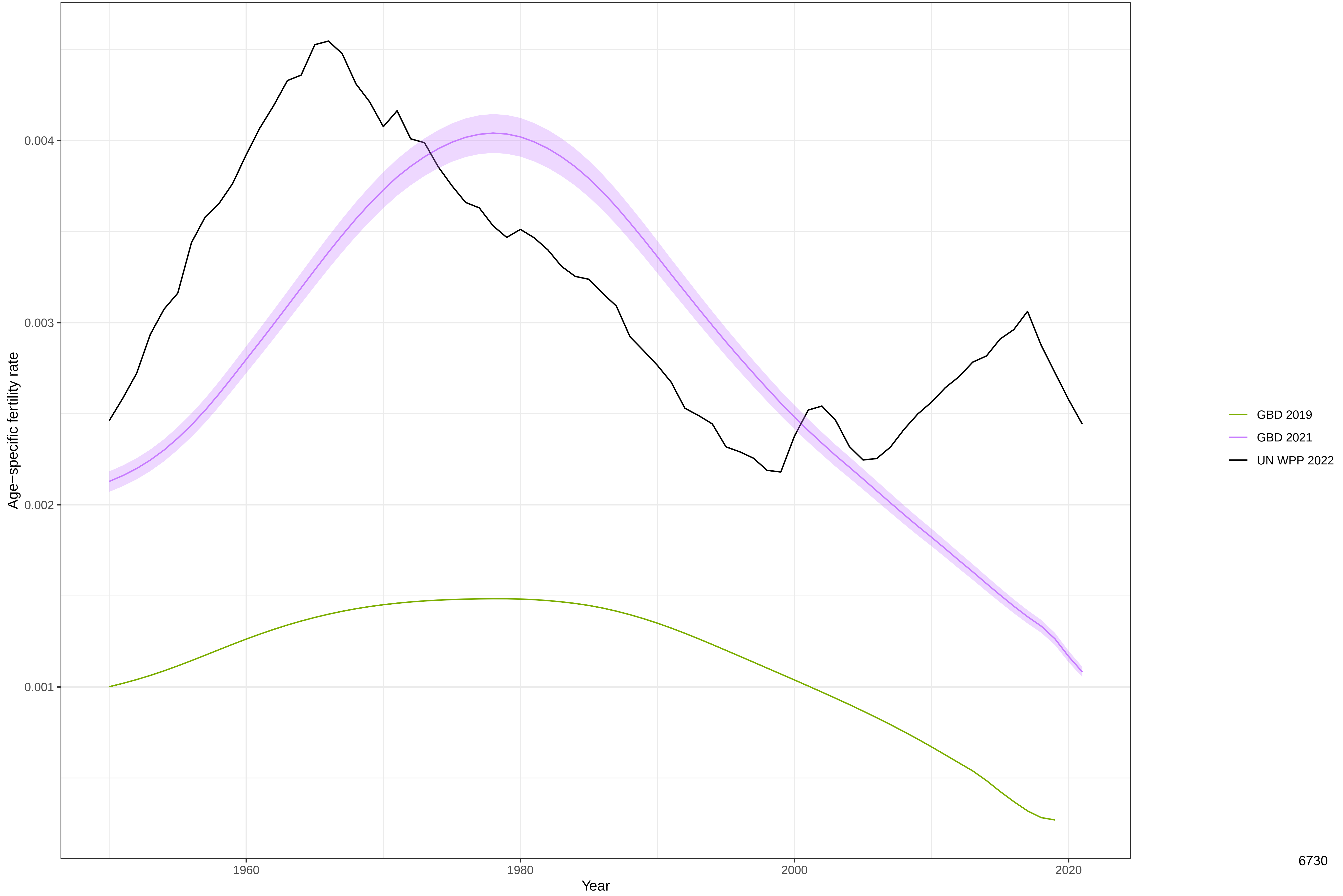

Total fertility rate fitted trend lines, 1950−2021: Comoros TFR
Data points enclosed with a diamond are reference sources

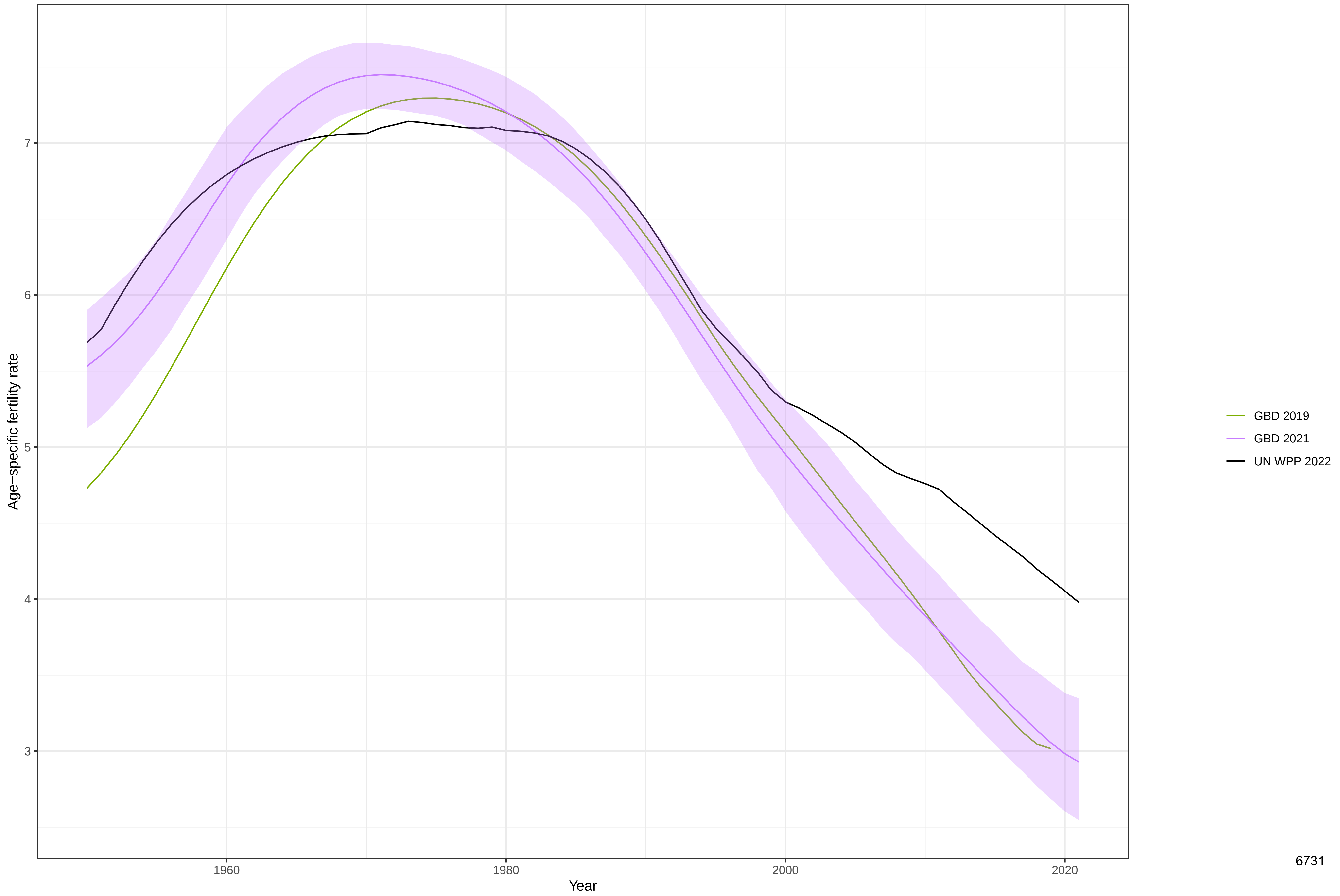

6731

Age−specific fertility fitted trend lines, 1950−2021: Djibouti, Ages: 10−14
Data points enclosed with a diamond are reference sources

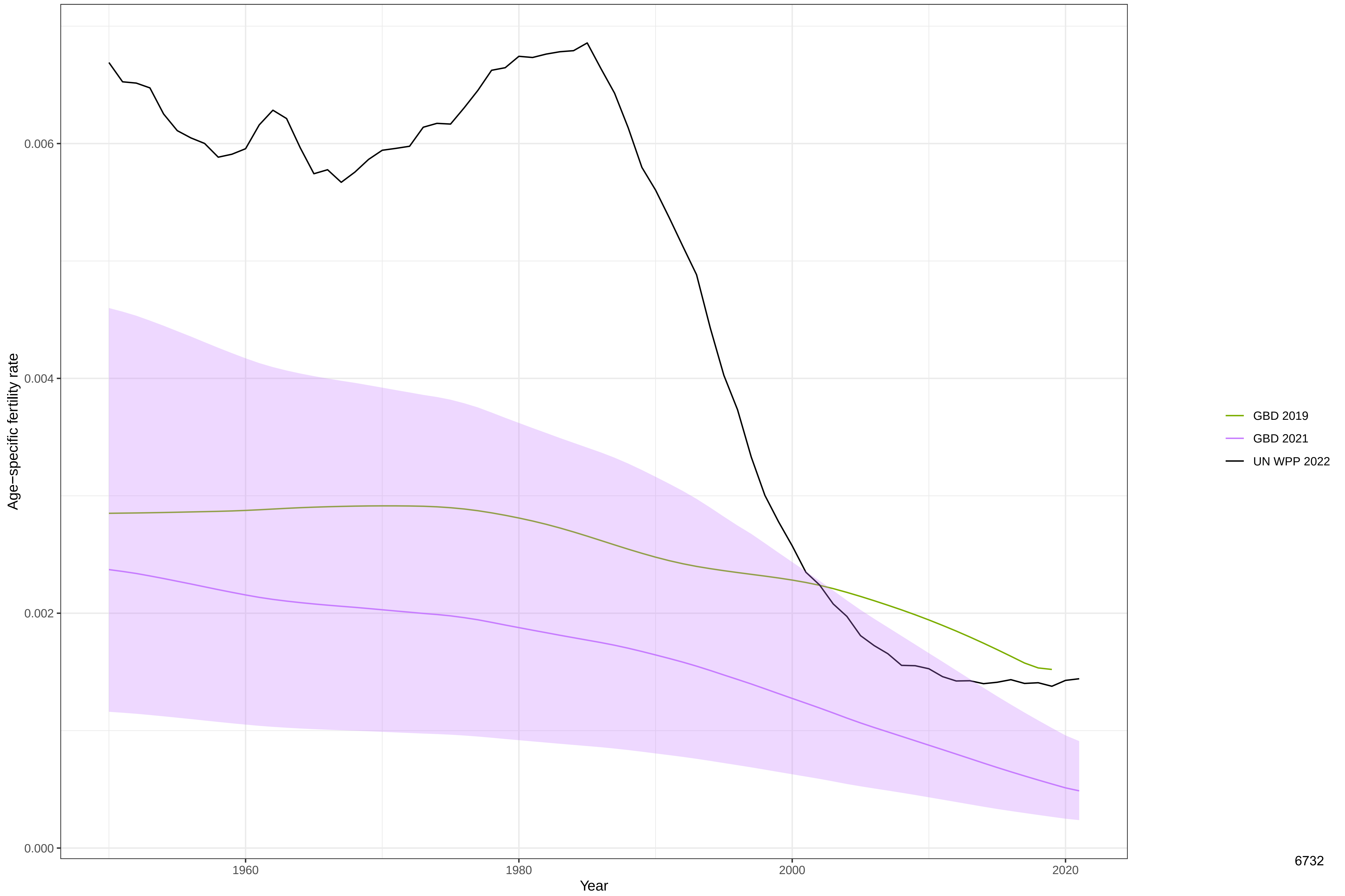

Age−specific fertility fitted trend lines, 1950−2021: Djibouti, Ages: 15−19
Data points enclosed with a diamond are reference sources

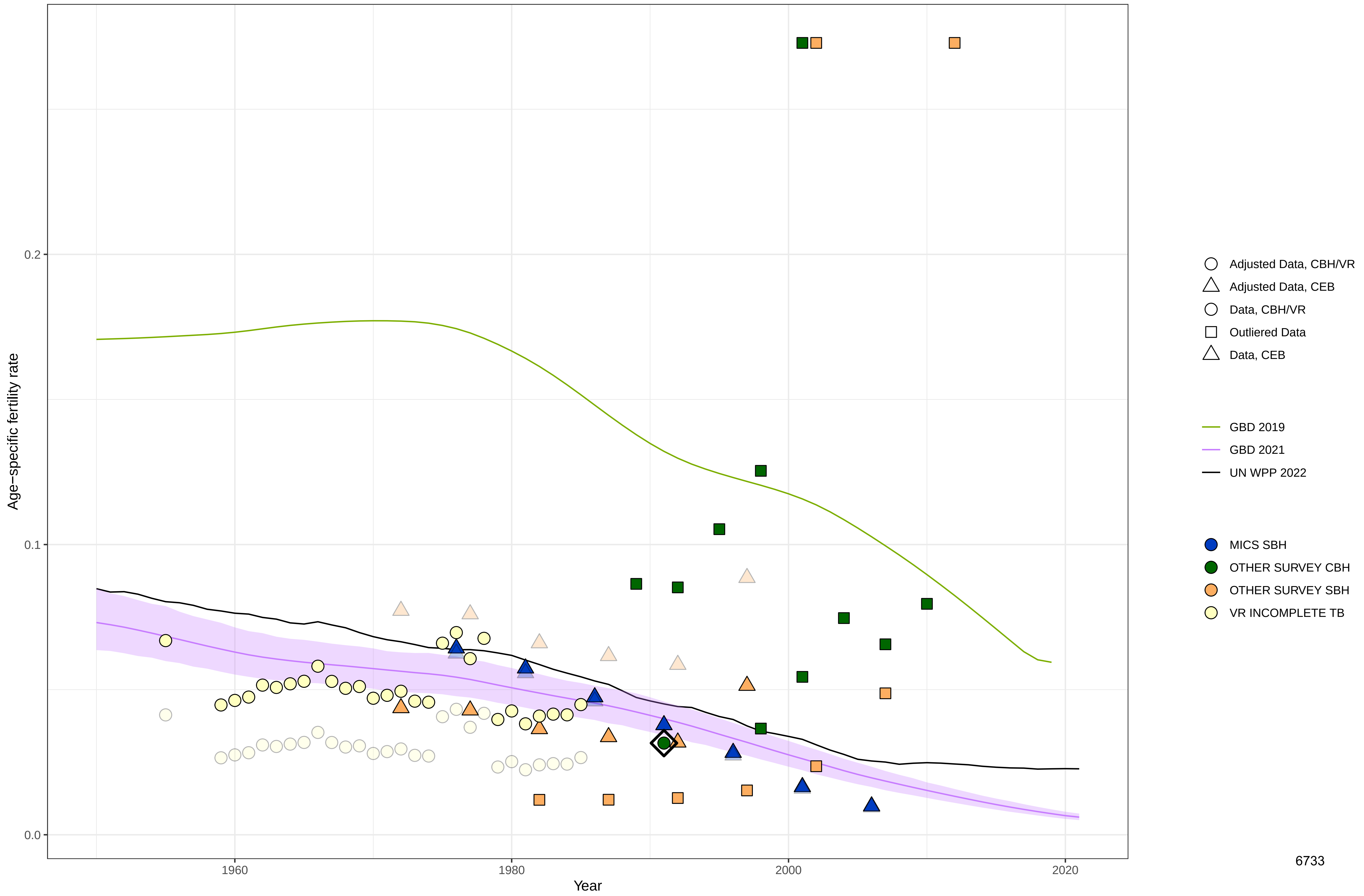

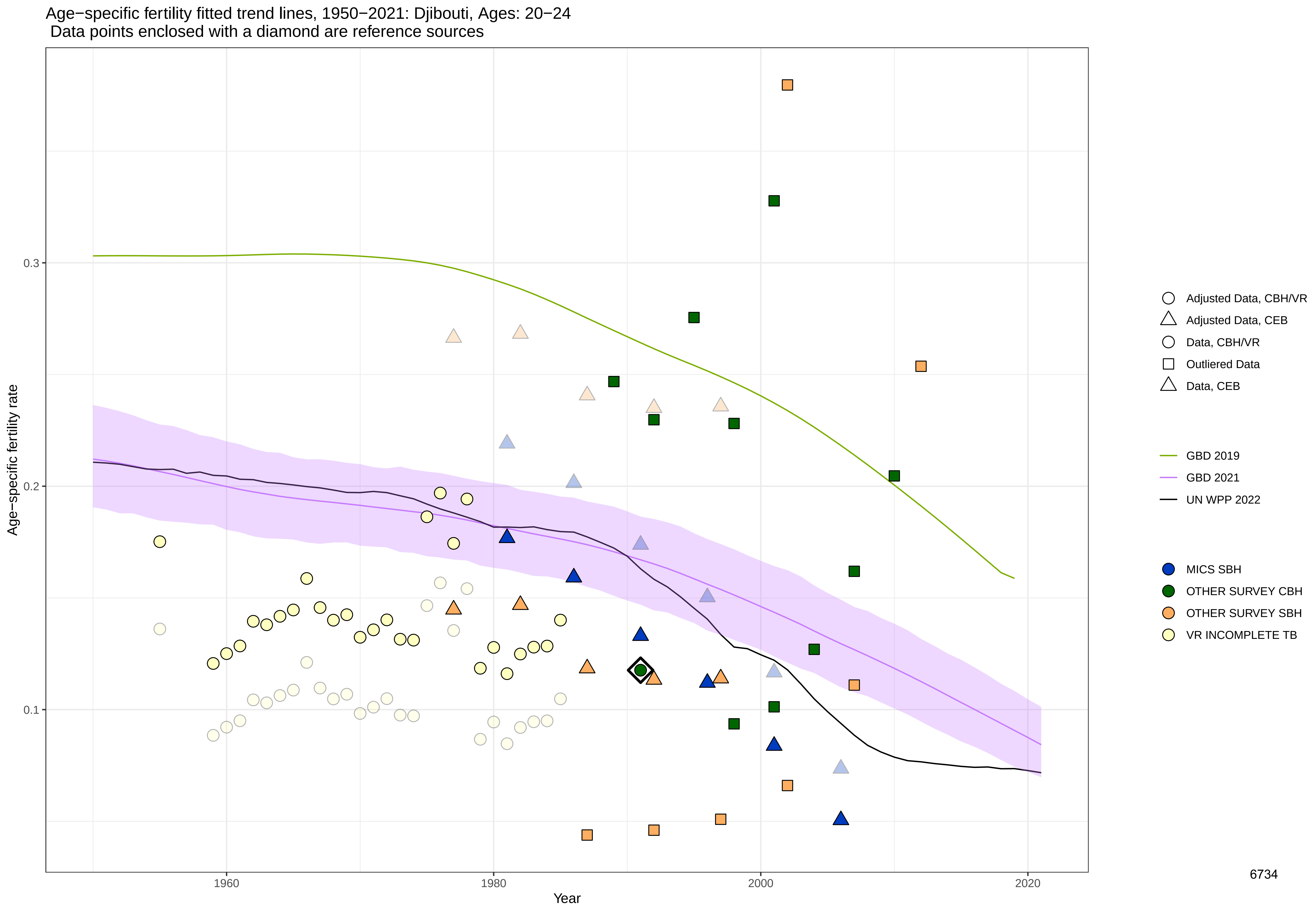
Age−specific fertility fitted trend lines, 1950−2021: Djibouti, Ages: 20−24
Data points enclosed with a diamond are reference sources
Age−specific fertility rate
0.1
0.2
0.3
Year
1960
1980
2000
2020
Adjusted Data, CBH/VR
Adjusted Data, CEB
Data, CBH/VR
Outliered Data
Data, CEB
GBD 2019
GBD 2021
UN WPP 2022
MICS SBH
OTHER SURVEY CBH
OTHER SURVEY SBH
VR INCOMPLETE TB

Age−specific fertility fitted trend lines, 1950−2021: Djibouti, Ages: 25−29
Data points enclosed with a diamond are reference sources

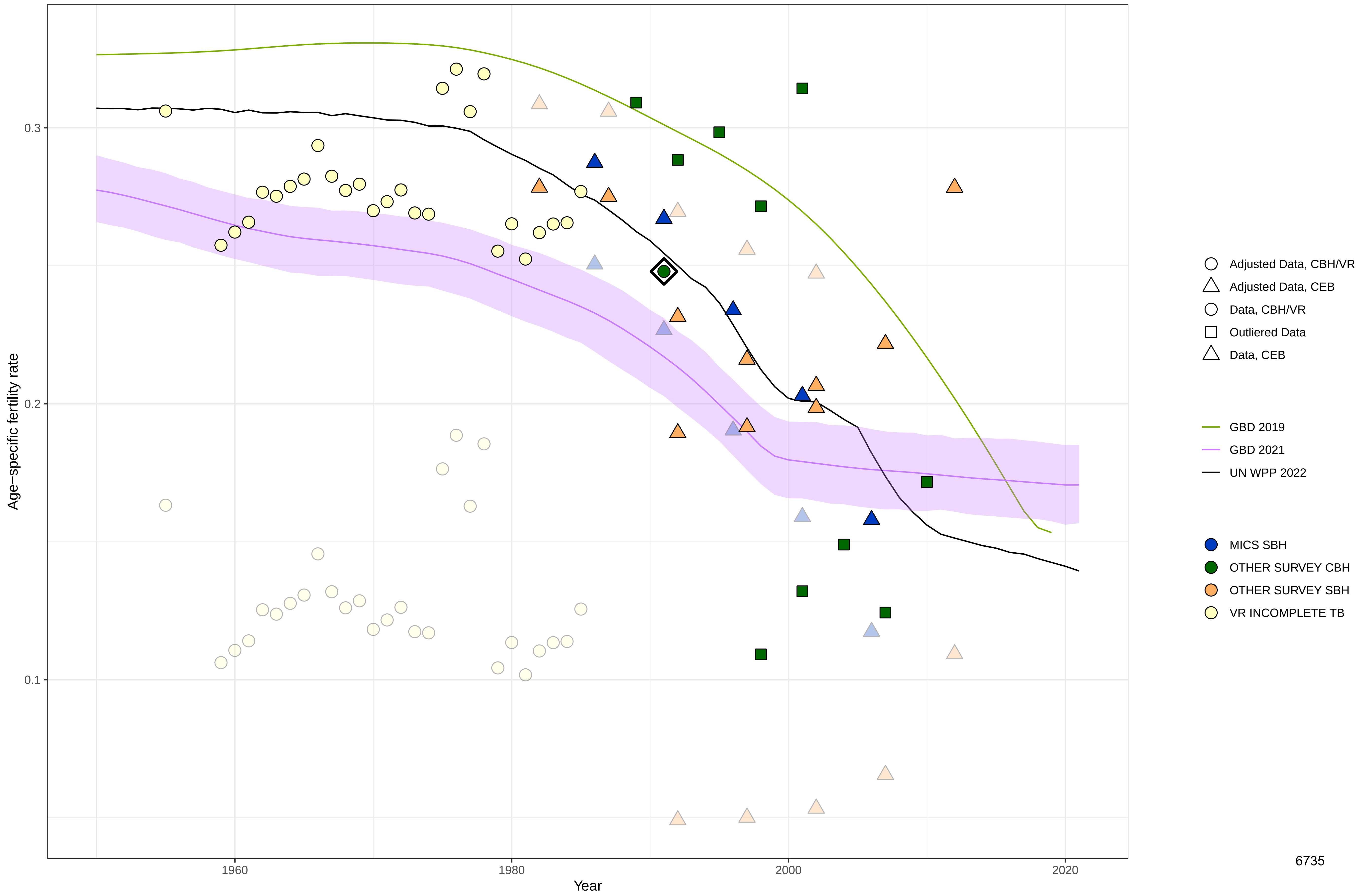

Age−specific fertility fitted trend lines, 1950−2021: Djibouti, Ages: 30−34
Data points enclosed with a diamond are reference sources

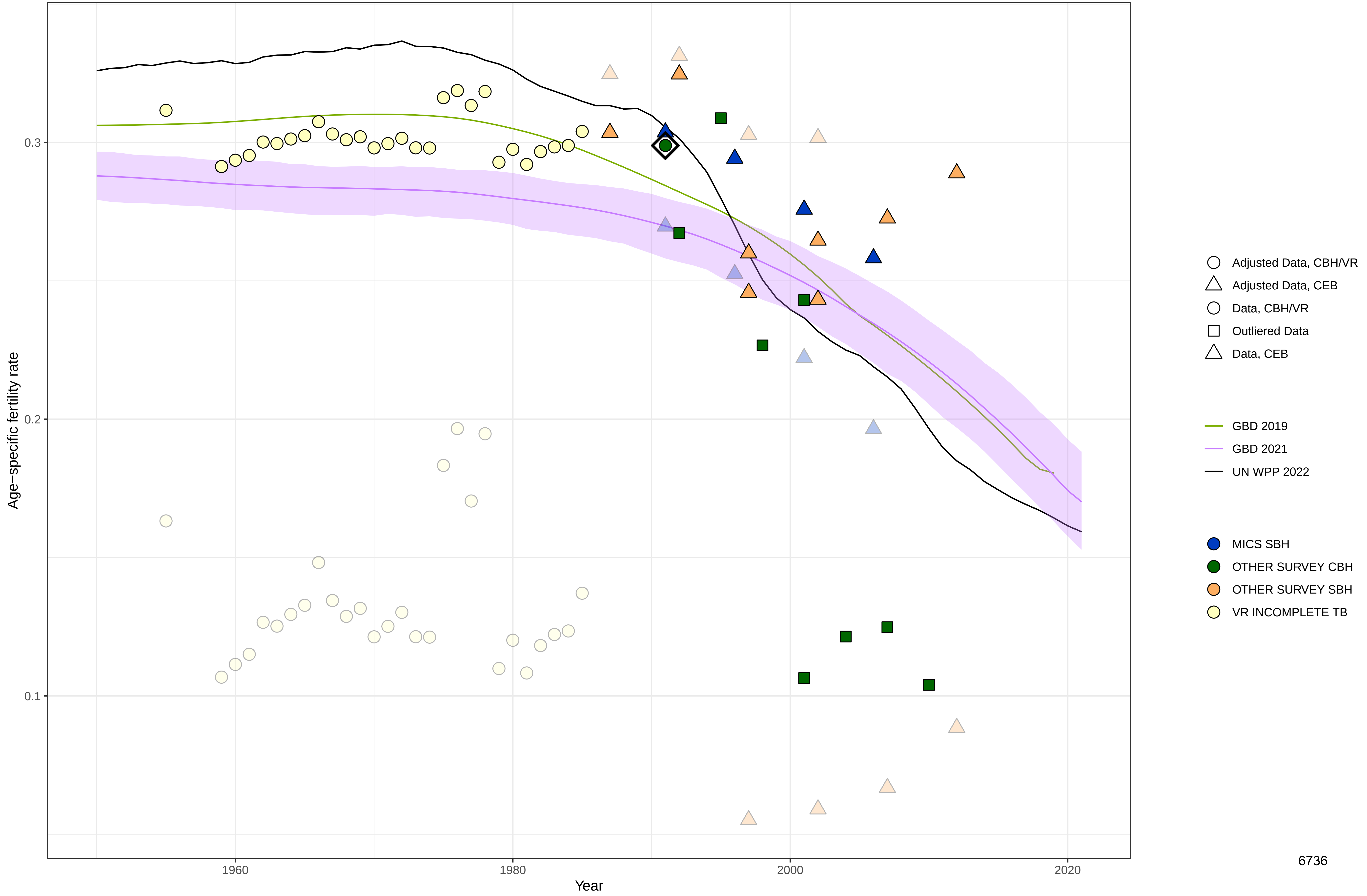

Age−specific fertility fitted trend lines, 1950−2021: Djibouti, Ages: 35−39
Data points enclosed with a diamond are reference sources

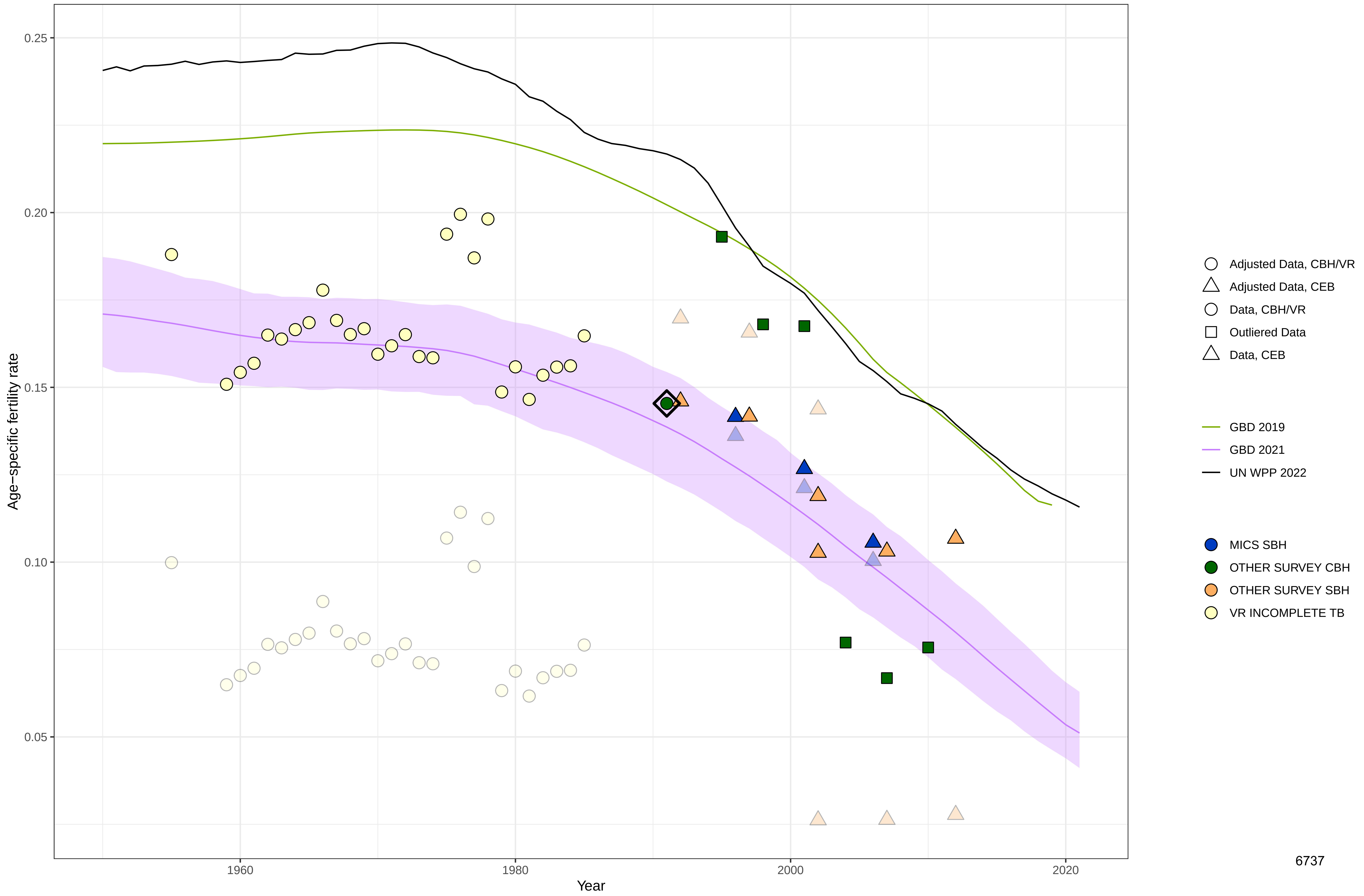

Age−specific fertility fitted trend lines, 1950−2021: Djibouti, Ages: 40−44
Data points enclosed with a diamond are reference sources

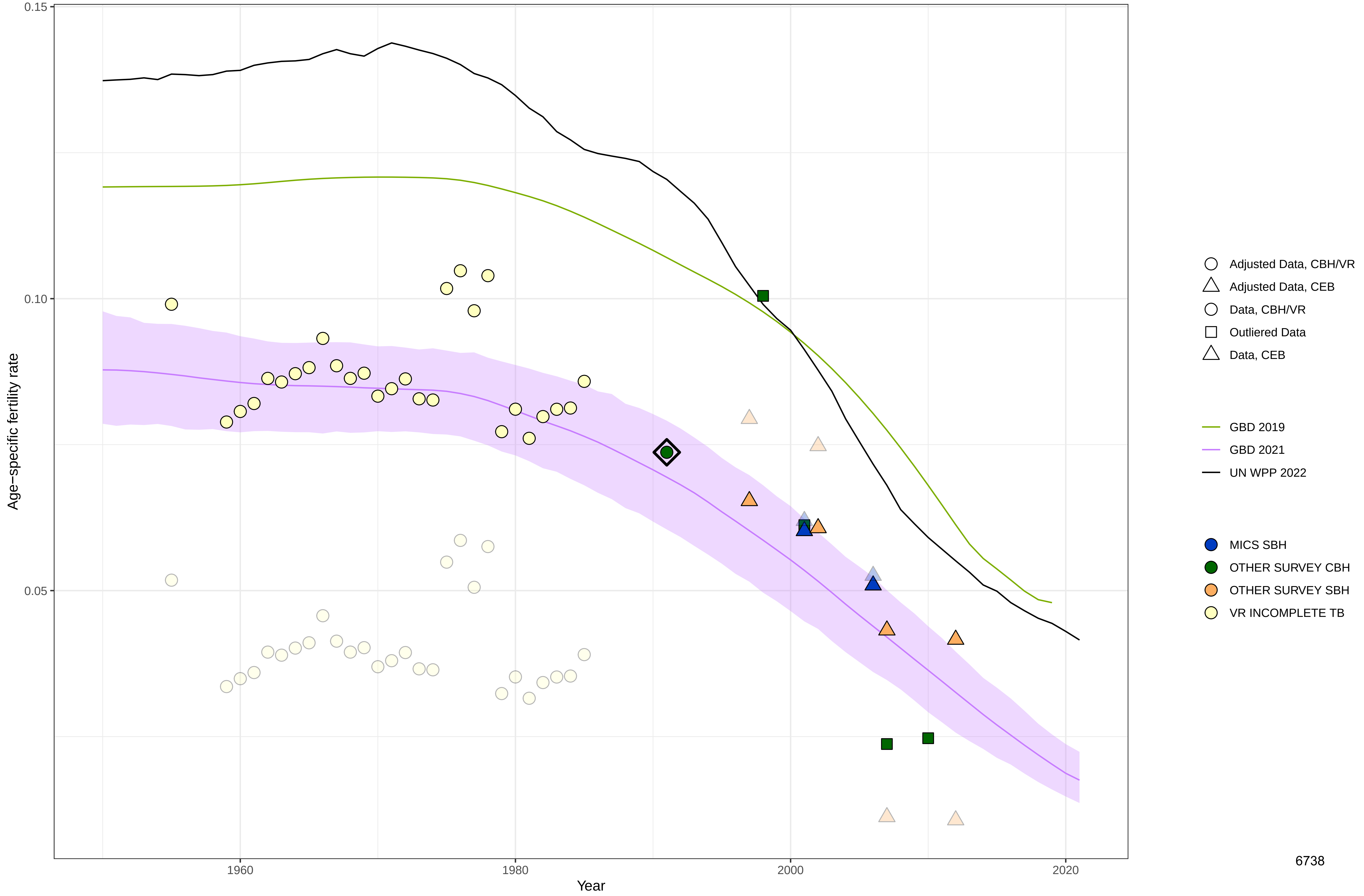

Age−specific fertility fitted trend lines, 1950−2021: Djibouti, Ages: 45−49
Data points enclosed with a diamond are reference sources

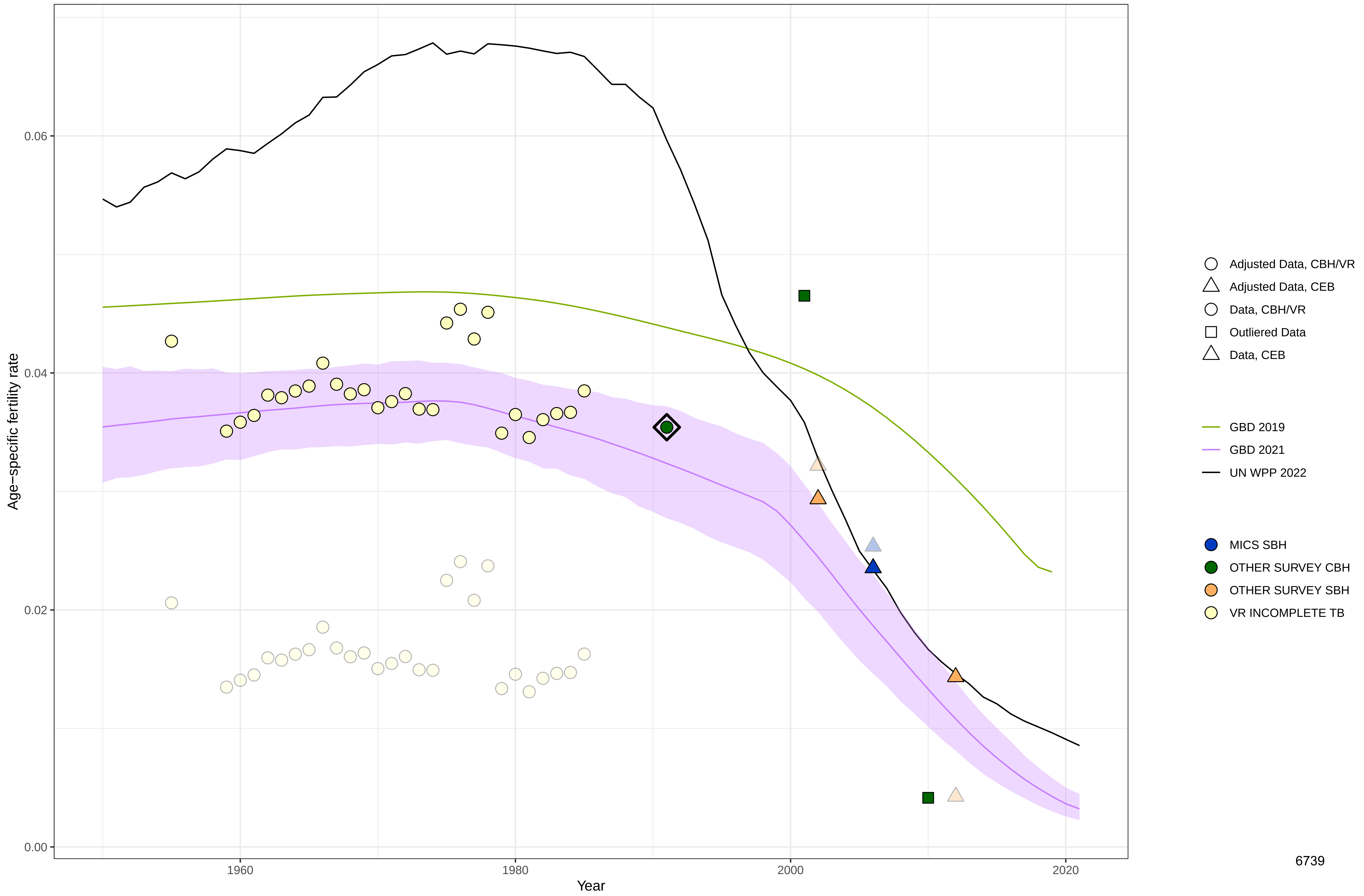

Age−specific fertility fitted trend lines, 1950−2021: Djibouti, Ages: 50−54
Data points enclosed with a diamond are reference sources

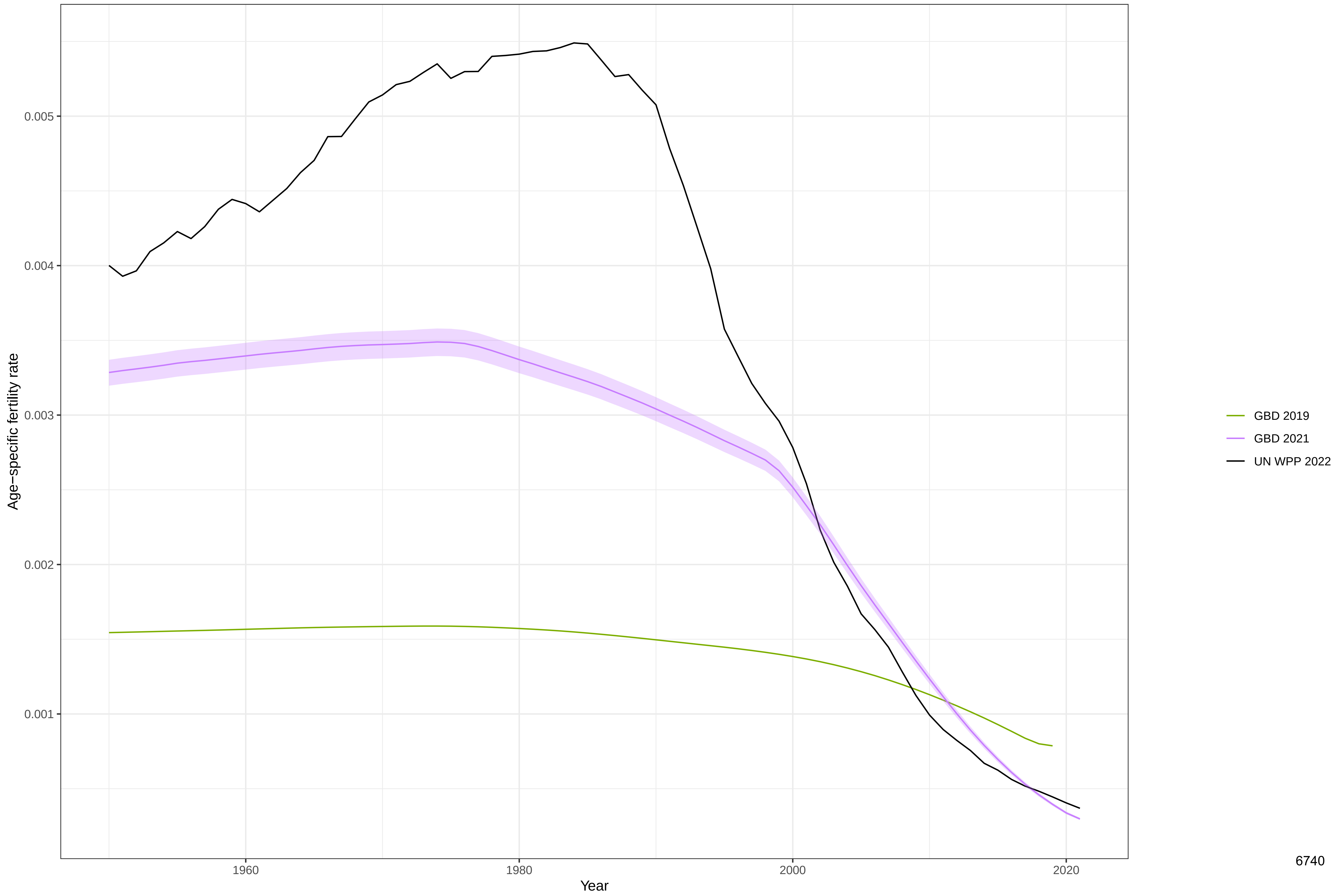

Total fertility rate fitted trend lines, 1950−2021: Djibouti TFR
Data points enclosed with a diamond are reference sources

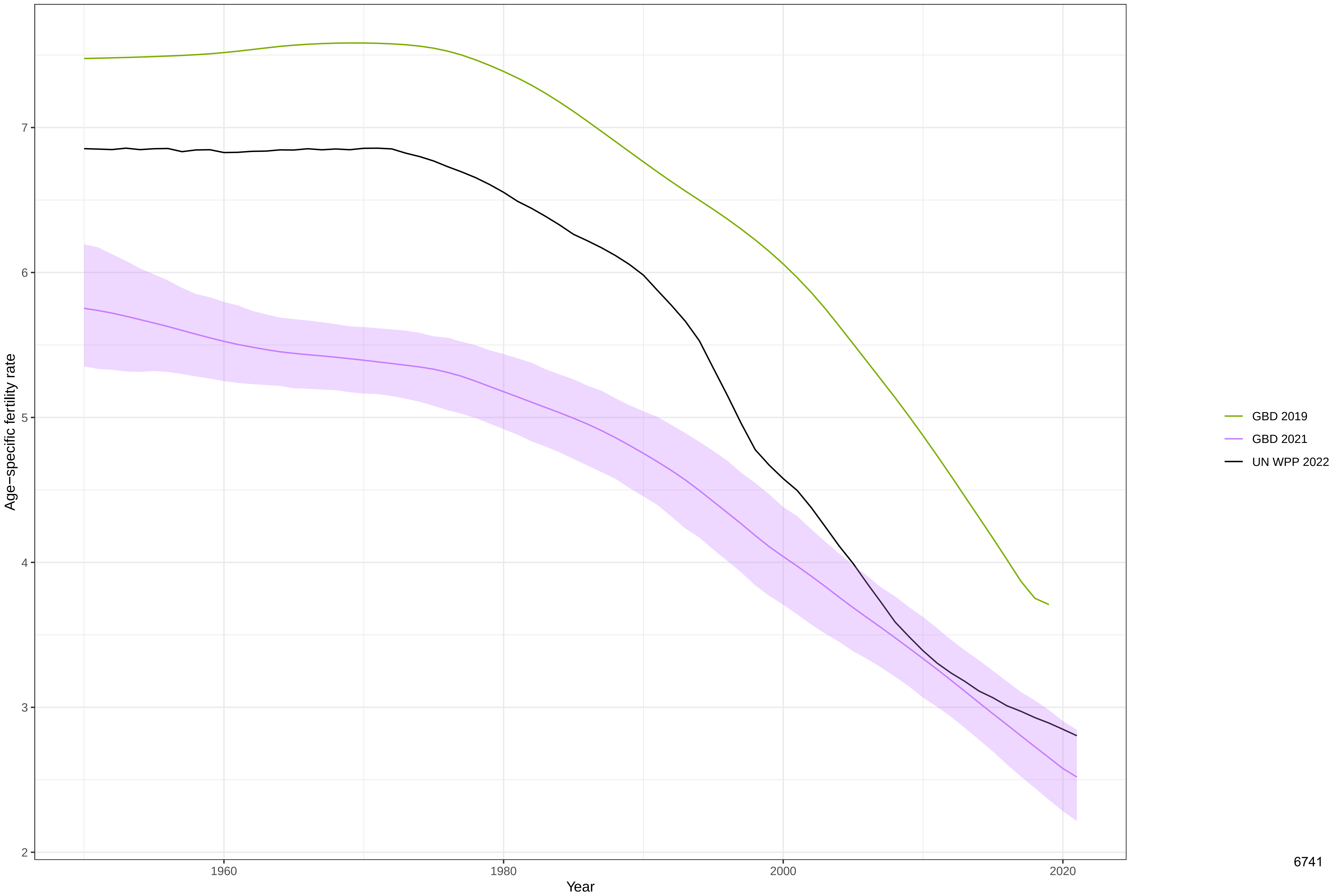

Age−specific fertility fitted trend lines, 1950−2021: Eritrea, Ages: 10−14
Data points enclosed with a diamond are reference sources

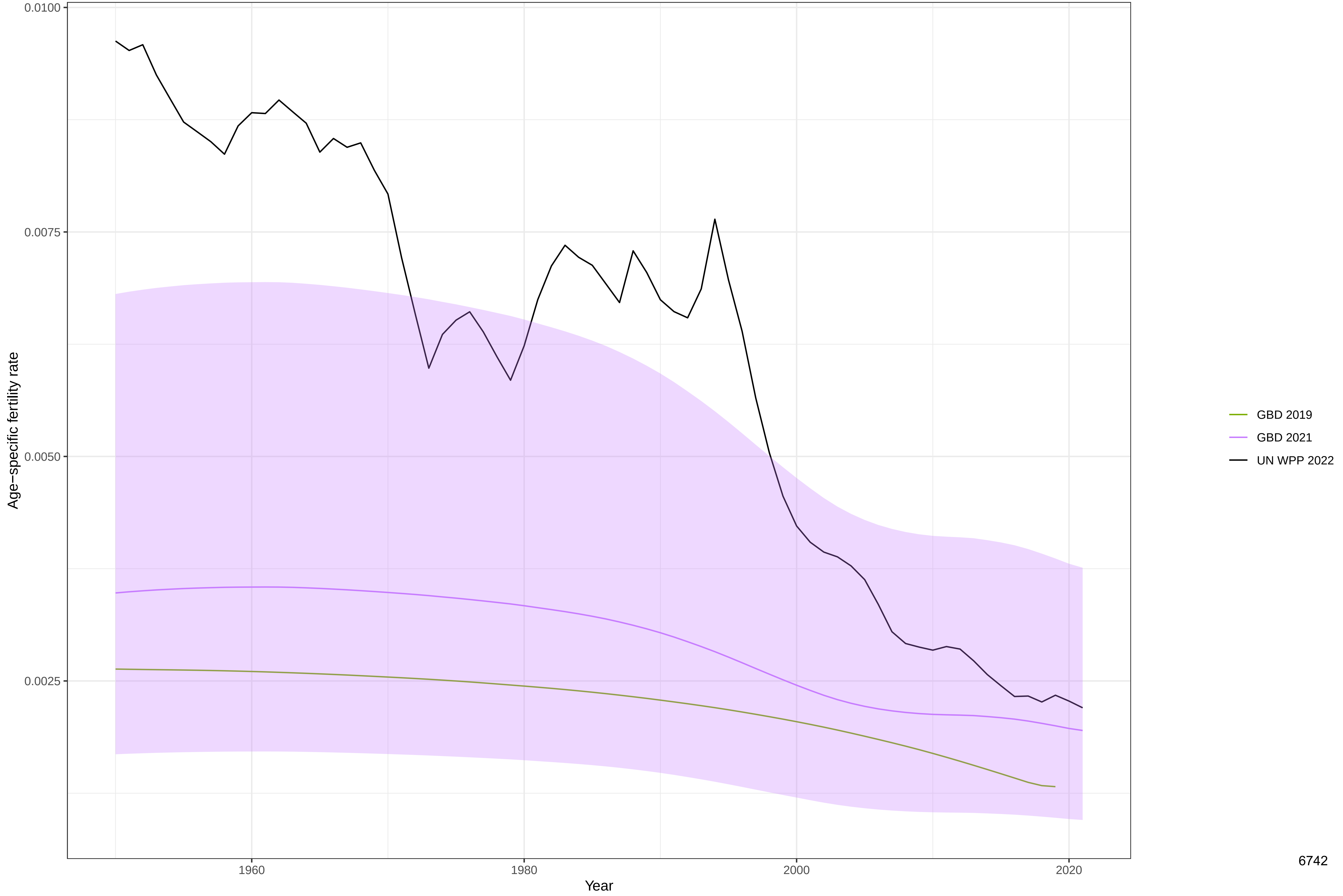

Age−specific fertility fitted trend lines, 1950−2021: Eritrea, Ages: 15−19
Data points enclosed with a diamond are reference sources

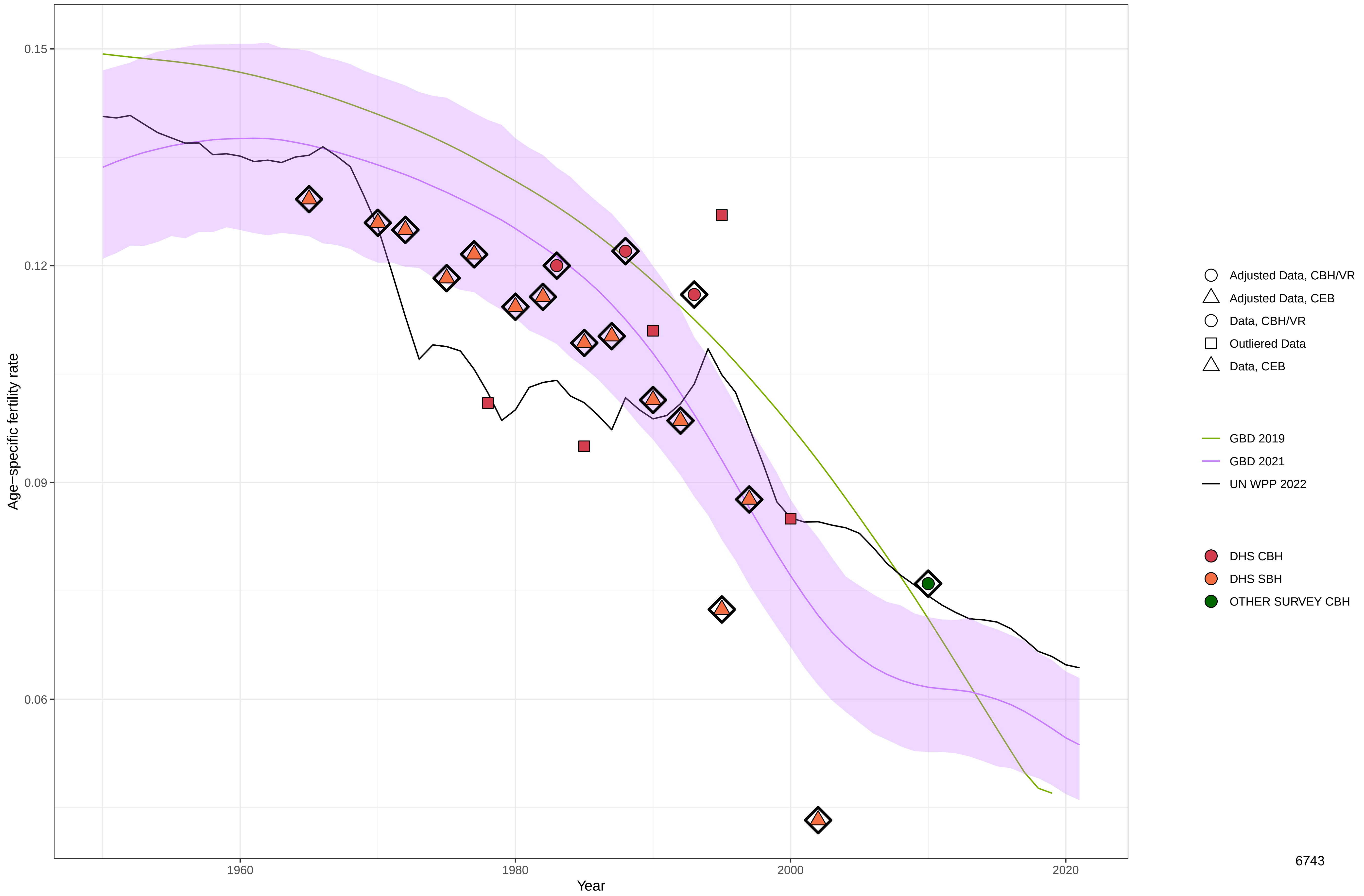

Age−specific fertility fitted trend lines, 1950−2021: Eritrea, Ages: 20−24
Data points enclosed with a diamond are reference sources

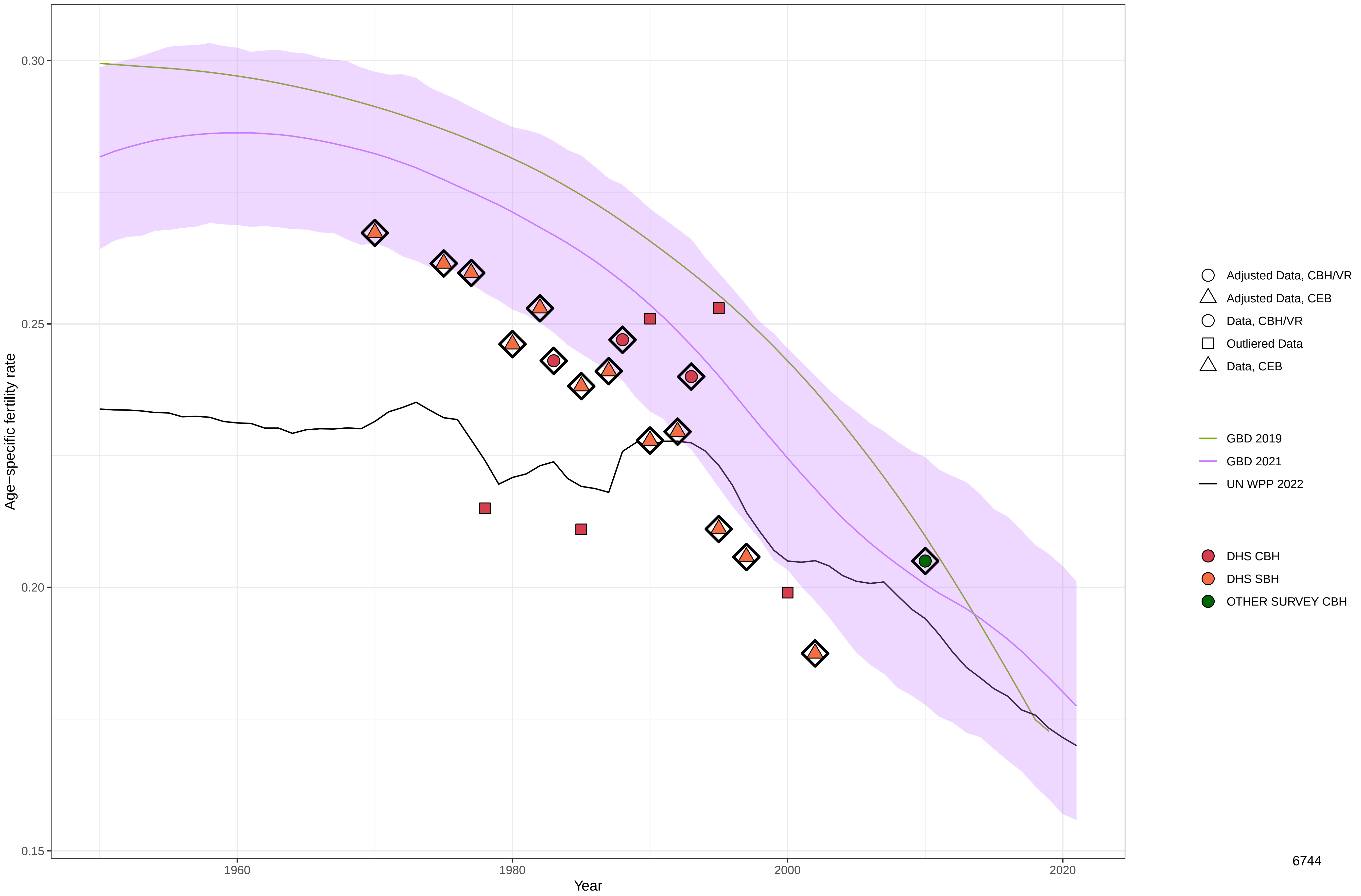

Age−specific fertility fitted trend lines, 1950−2021: Eritrea, Ages: 25−29
Data points enclosed with a diamond are reference sources

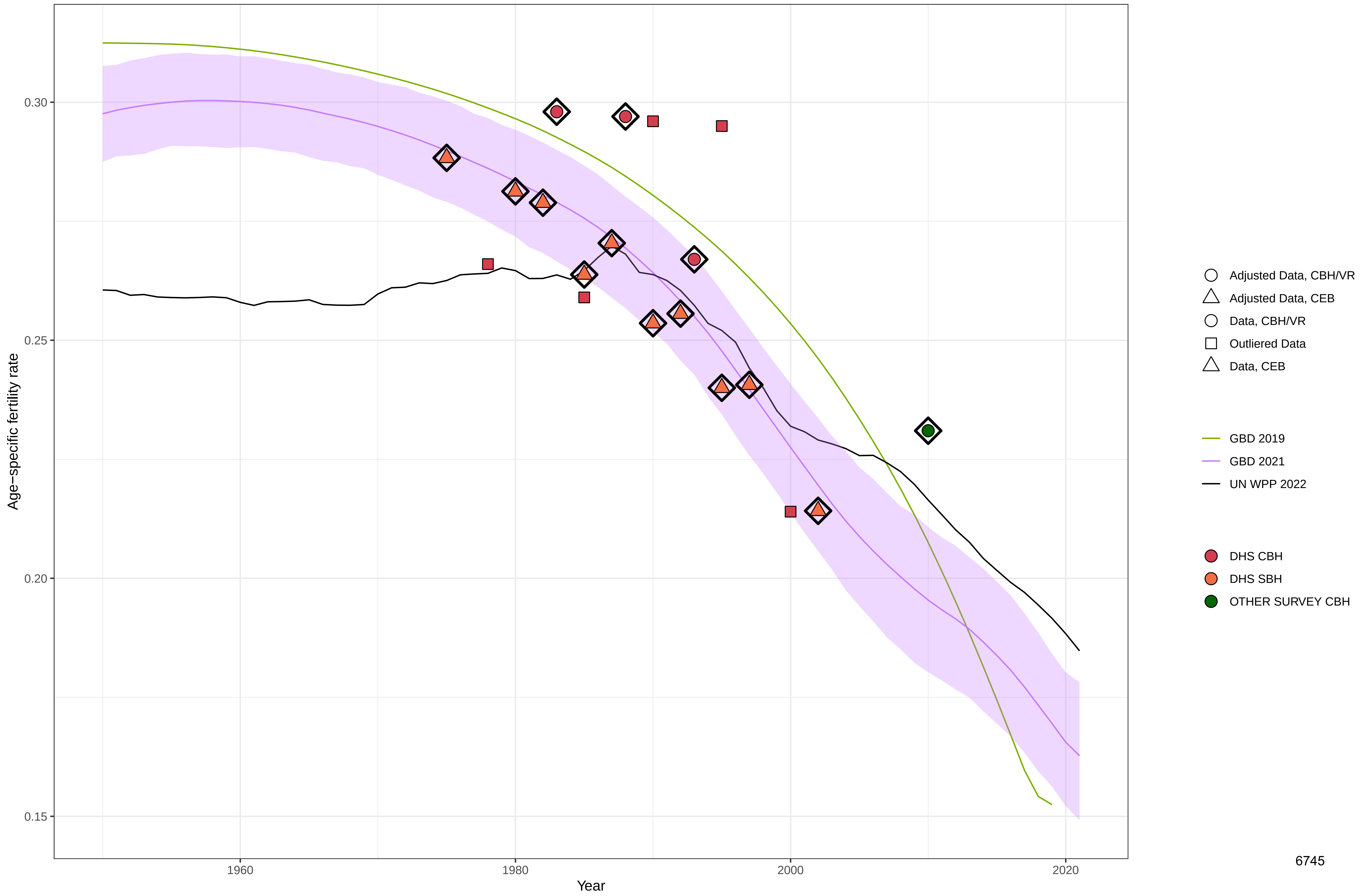

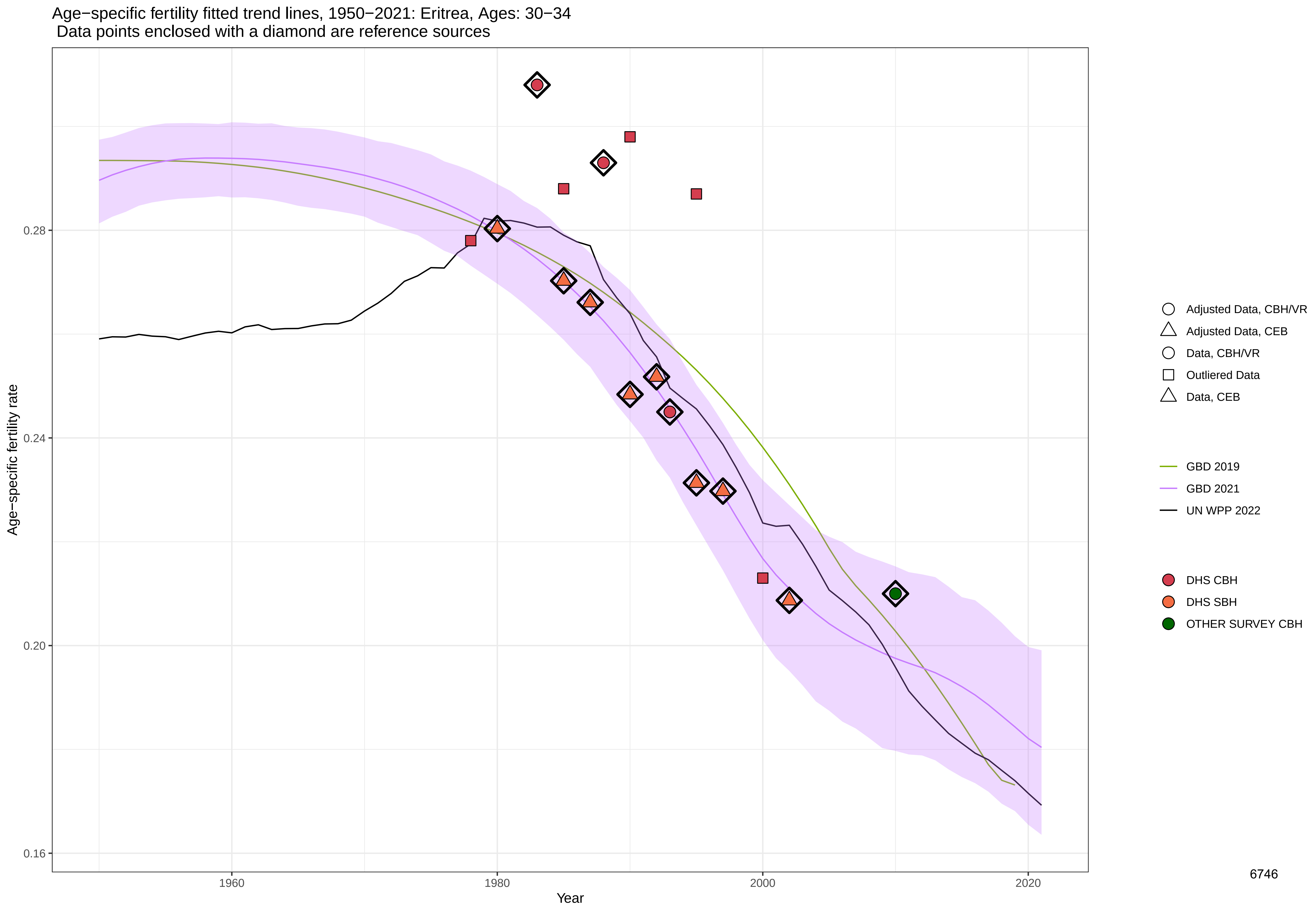

Age−specific fertility fitted trend lines, 1950−2021: Eritrea, Ages: 30−34
Data points enclosed with a diamond are reference sources
Age−specific fertility rate
0.28
0.24
0.20
0.16
1960
1980
2000
2020
Year
Adjusted Data, CBH/VR
Adjusted Data, CEB
Data, CBH/VR
Outliered Data
Data, CEB
GBD 2019
GBD 2021
UN WPP 2022
DHS CBH
DHS SBH
OTHER SURVEY CBH

6746

Age−specific fertility fitted trend lines, 1950−2021: Eritrea, Ages: 35−39
Data points enclosed with a diamond are reference sources

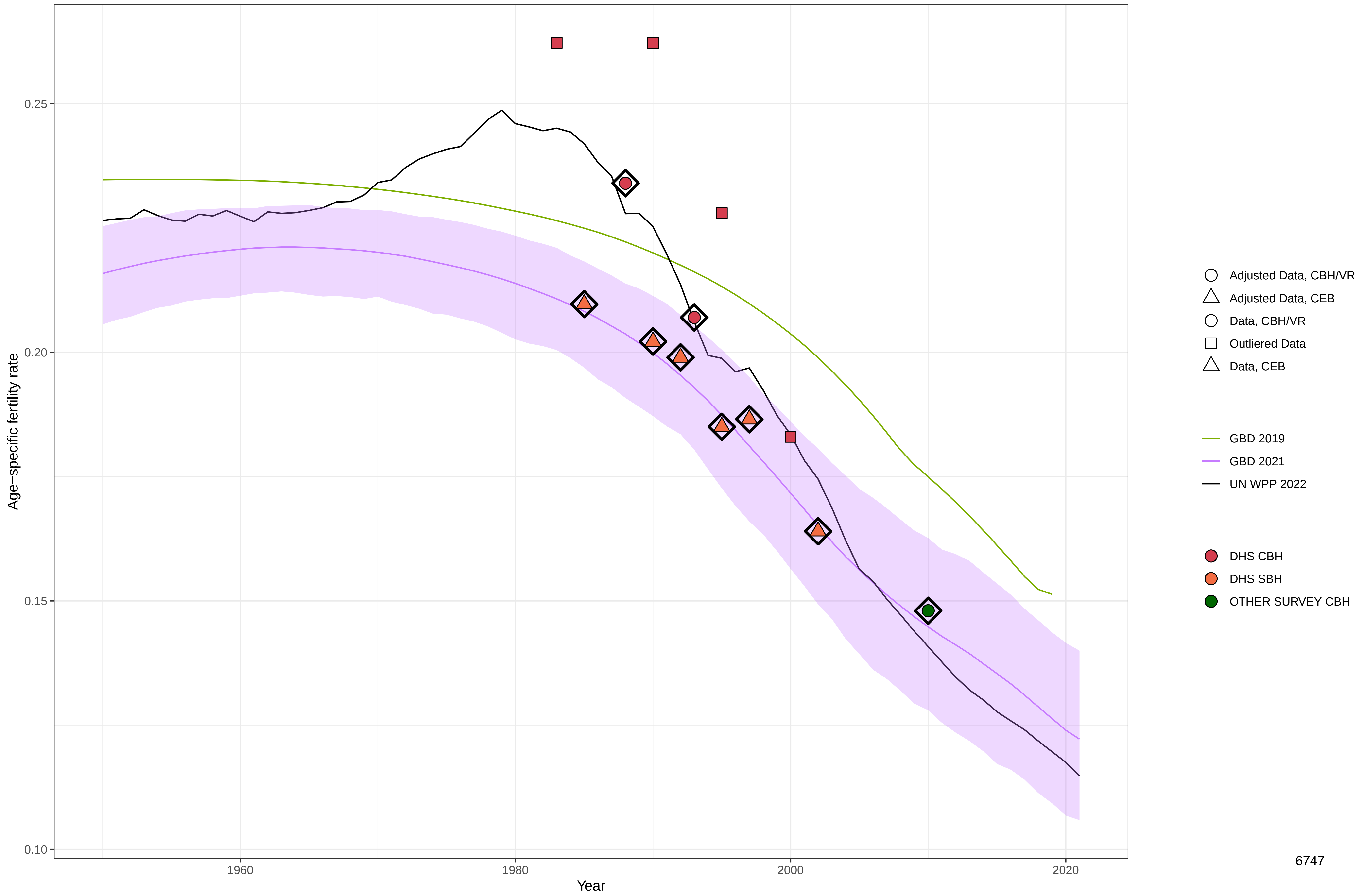

Age−specific fertility fitted trend lines, 1950−2021: Eritrea, Ages: 40−44
Data points enclosed with a diamond are reference sources

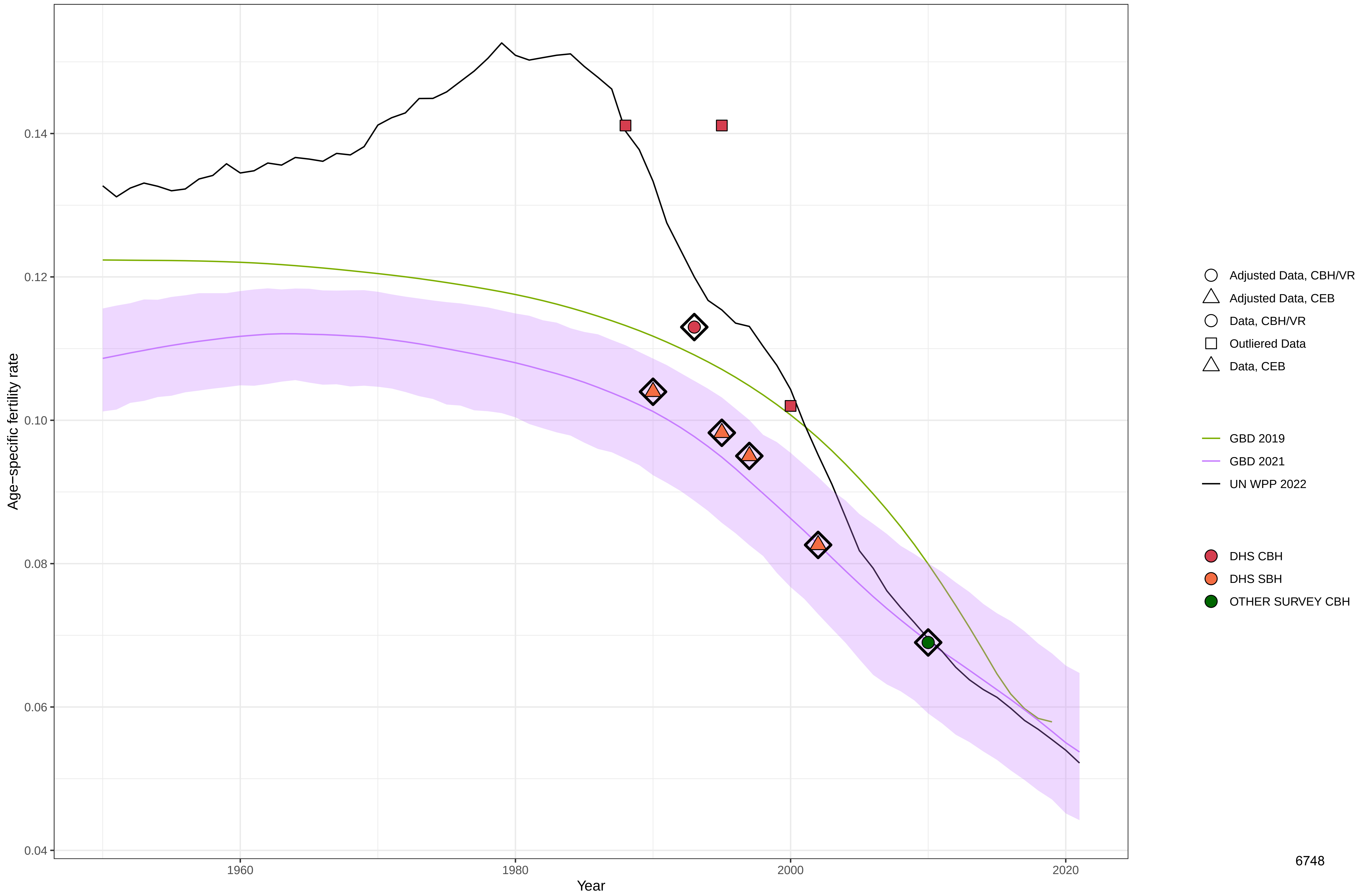

Age−specific fertility fitted trend lines, 1950−2021: Eritrea, Ages: 45−49
Data points enclosed with a diamond are reference sources

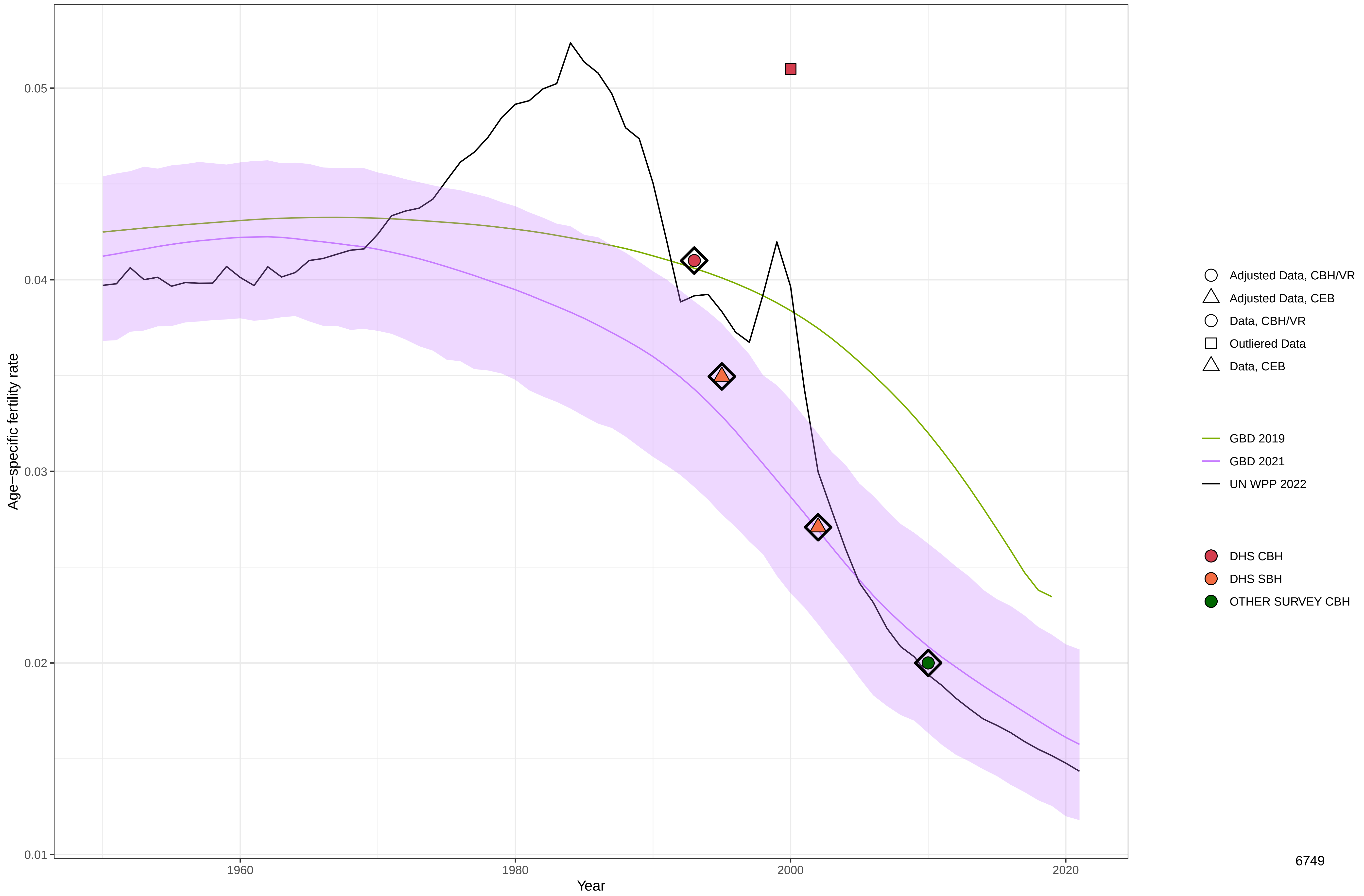

Age−specific fertility fitted trend lines, 1950−2021: Eritrea, Ages: 50−54
Data points enclosed with a diamond are reference sources

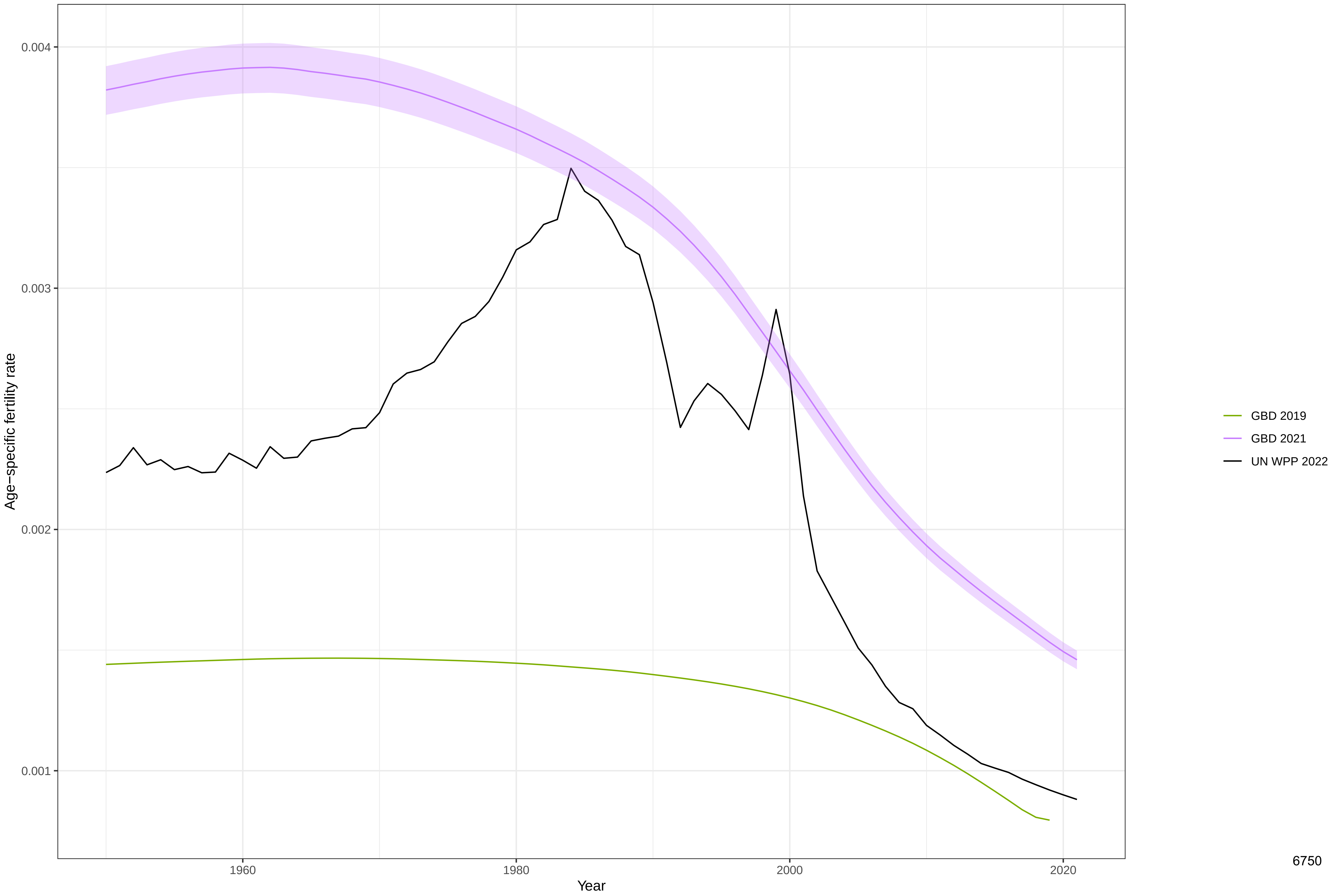

Total fertility rate fitted trend lines, 1950−2021: Eritrea TFR
Data points enclosed with a diamond are reference sources

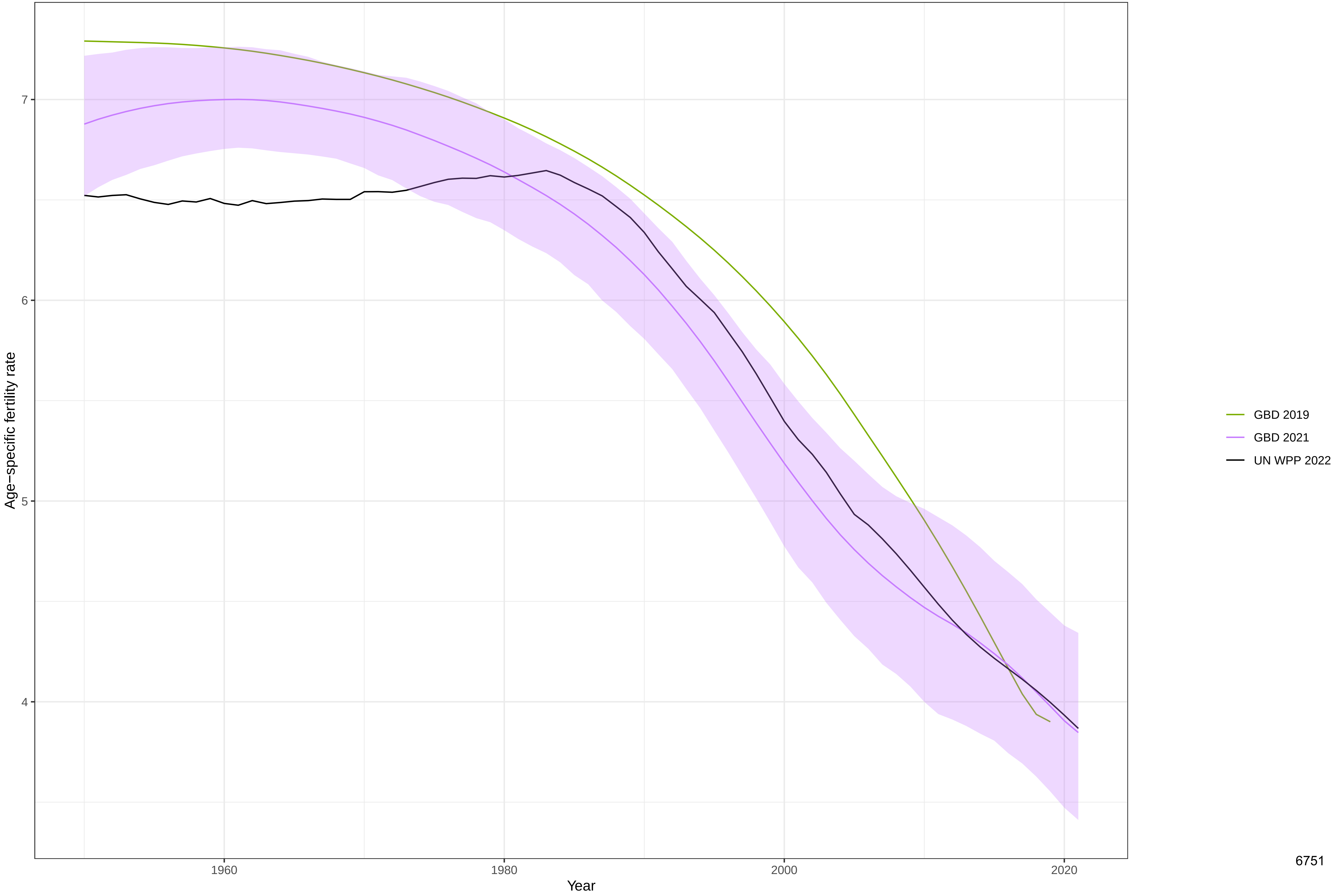

Age−specific fertility fitted trend lines, 1950−2021: Ethiopia, Ages: 10−14
Data points enclosed with a diamond are reference sources

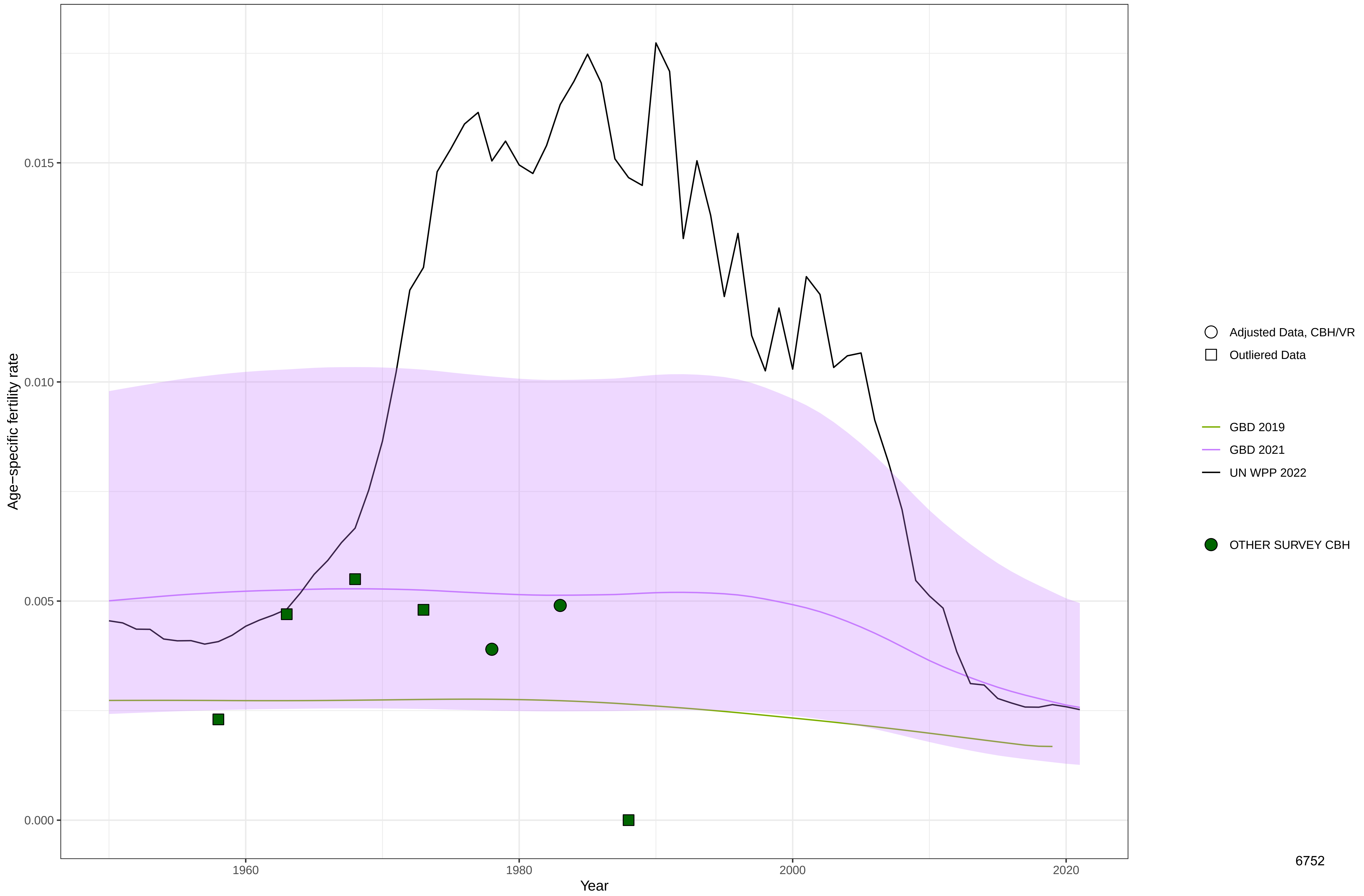

Age−specific fertility fitted trend lines, 1950−2021: Ethiopia, Ages: 15−19
Data points enclosed with a diamond are reference sources

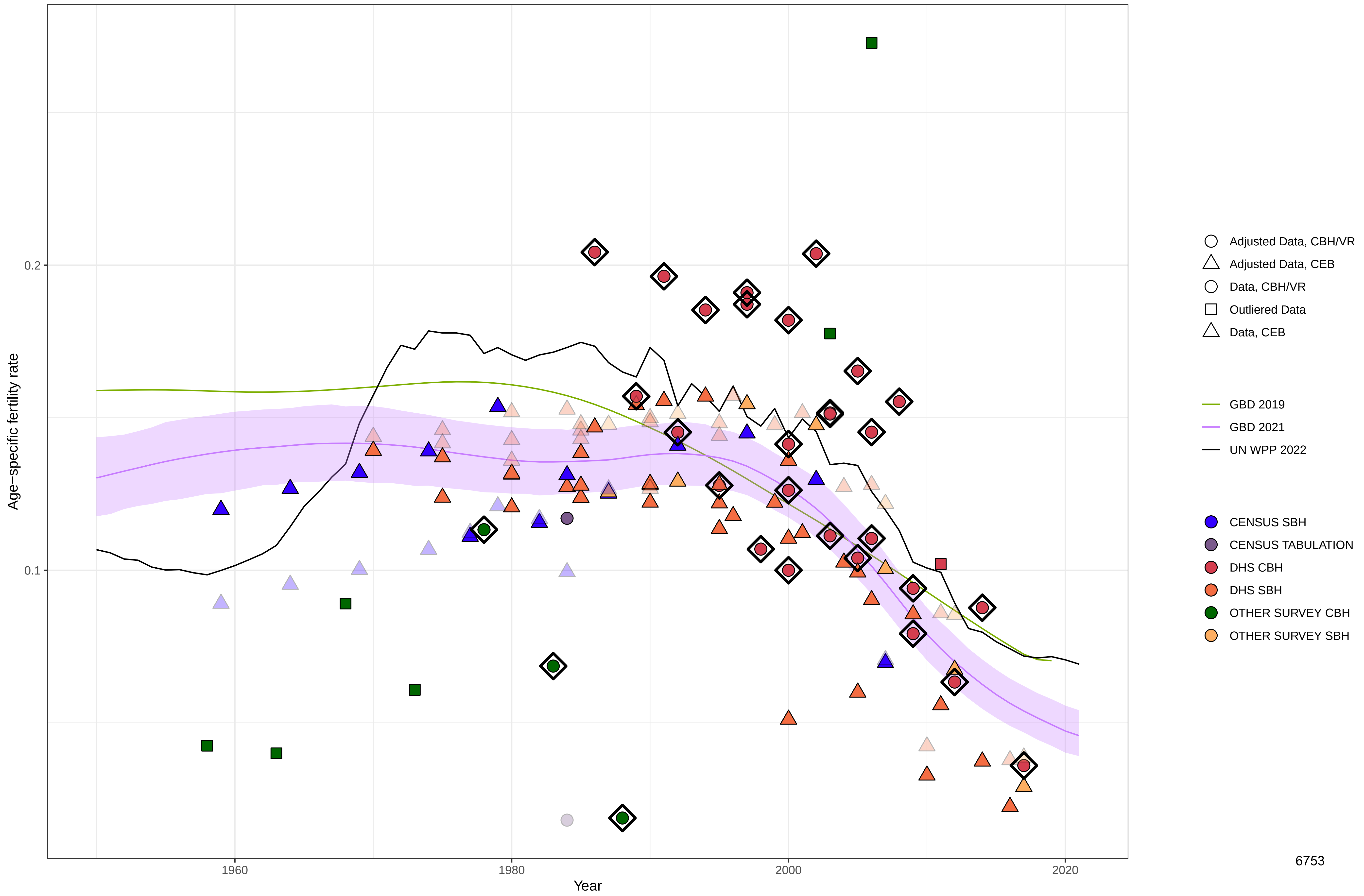

Age−specific fertility fitted trend lines, 1950−2021: Ethiopia, Ages: 20−24
Data points enclosed with a diamond are reference sources

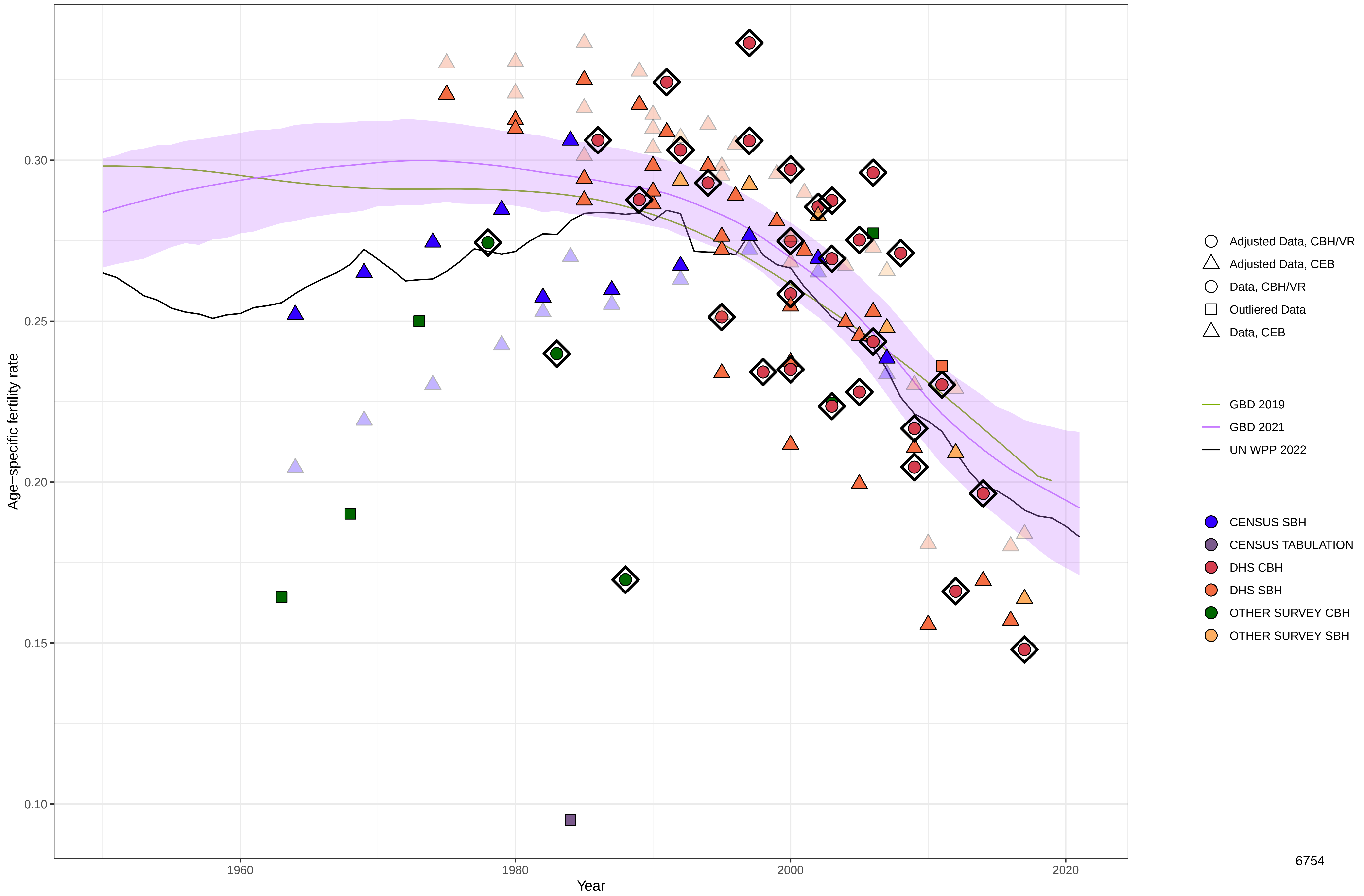

Age−specific fertility fitted trend lines, 1950−2021: Ethiopia, Ages: 25−29
Data points enclosed with a diamond are reference sources

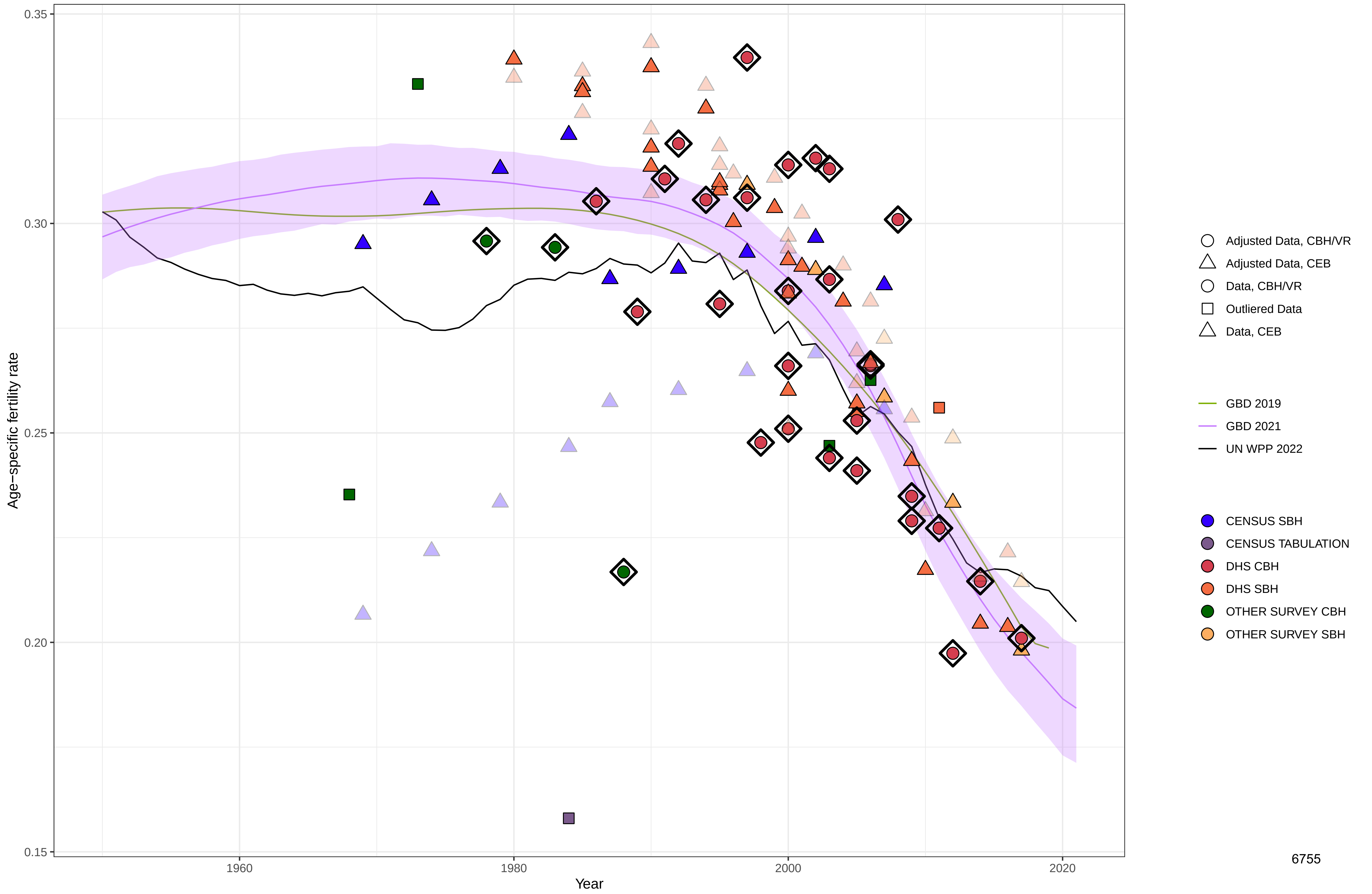

Age−specific fertility fitted trend lines, 1950−2021: Ethiopia, Ages: 30−34
Data points enclosed with a diamond are reference sources

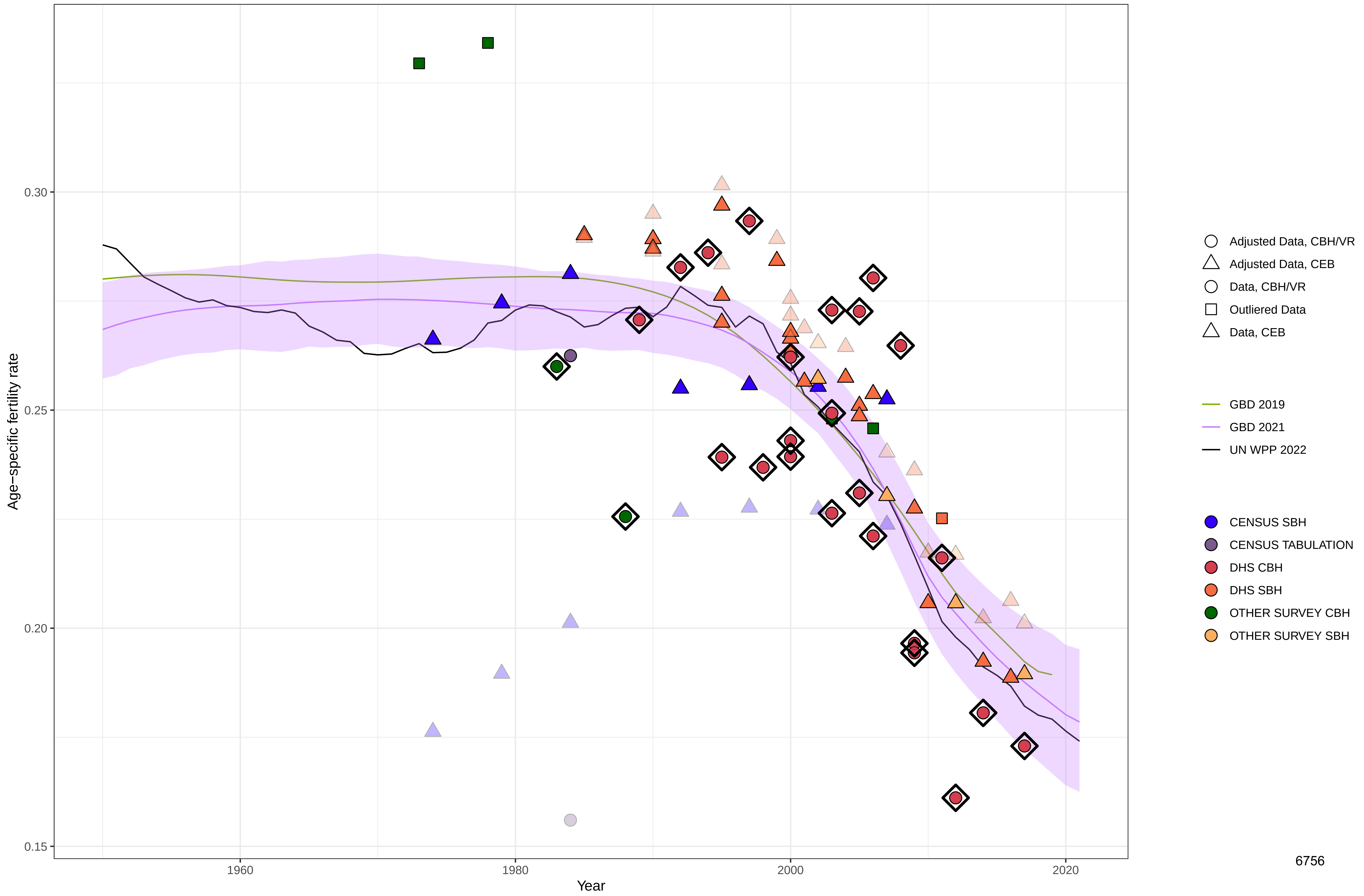

Age−specific fertility fitted trend lines, 1950−2021: Ethiopia, Ages: 35−39
Data points enclosed with a diamond are reference sources

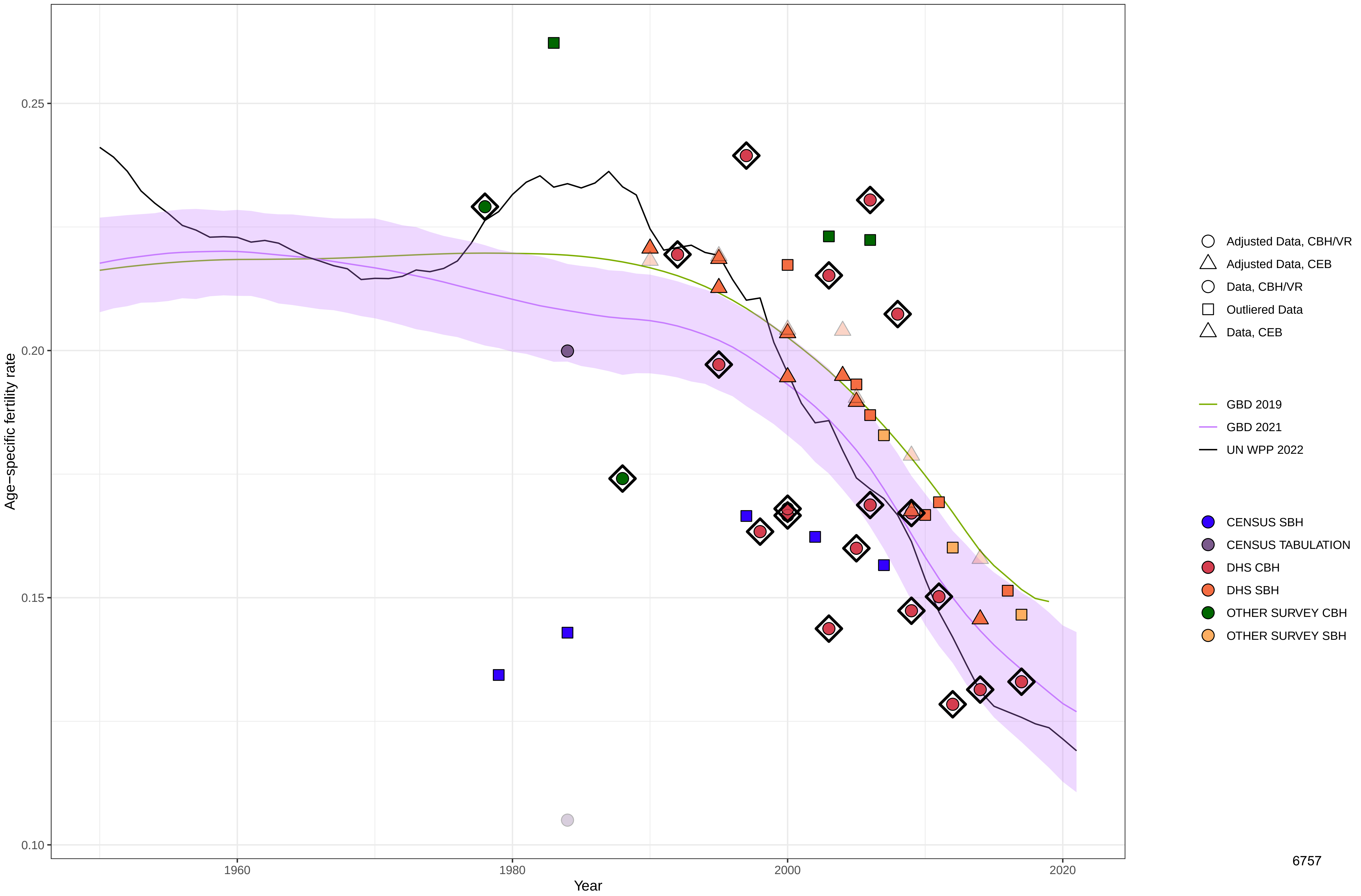

Age−specific fertility fitted trend lines, 1950−2021: Ethiopia, Ages: 40−44
Data points enclosed with a diamond are reference sources

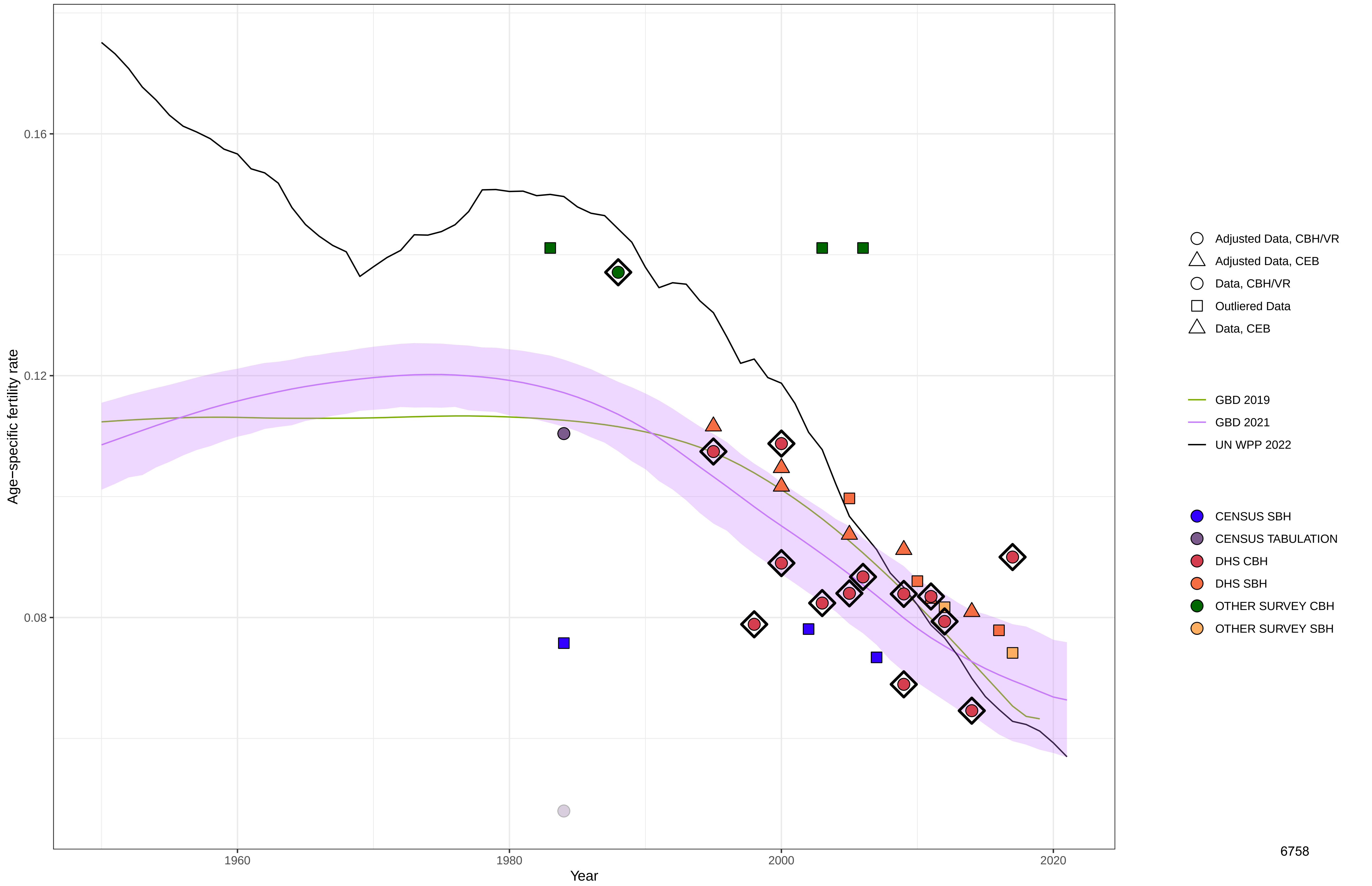

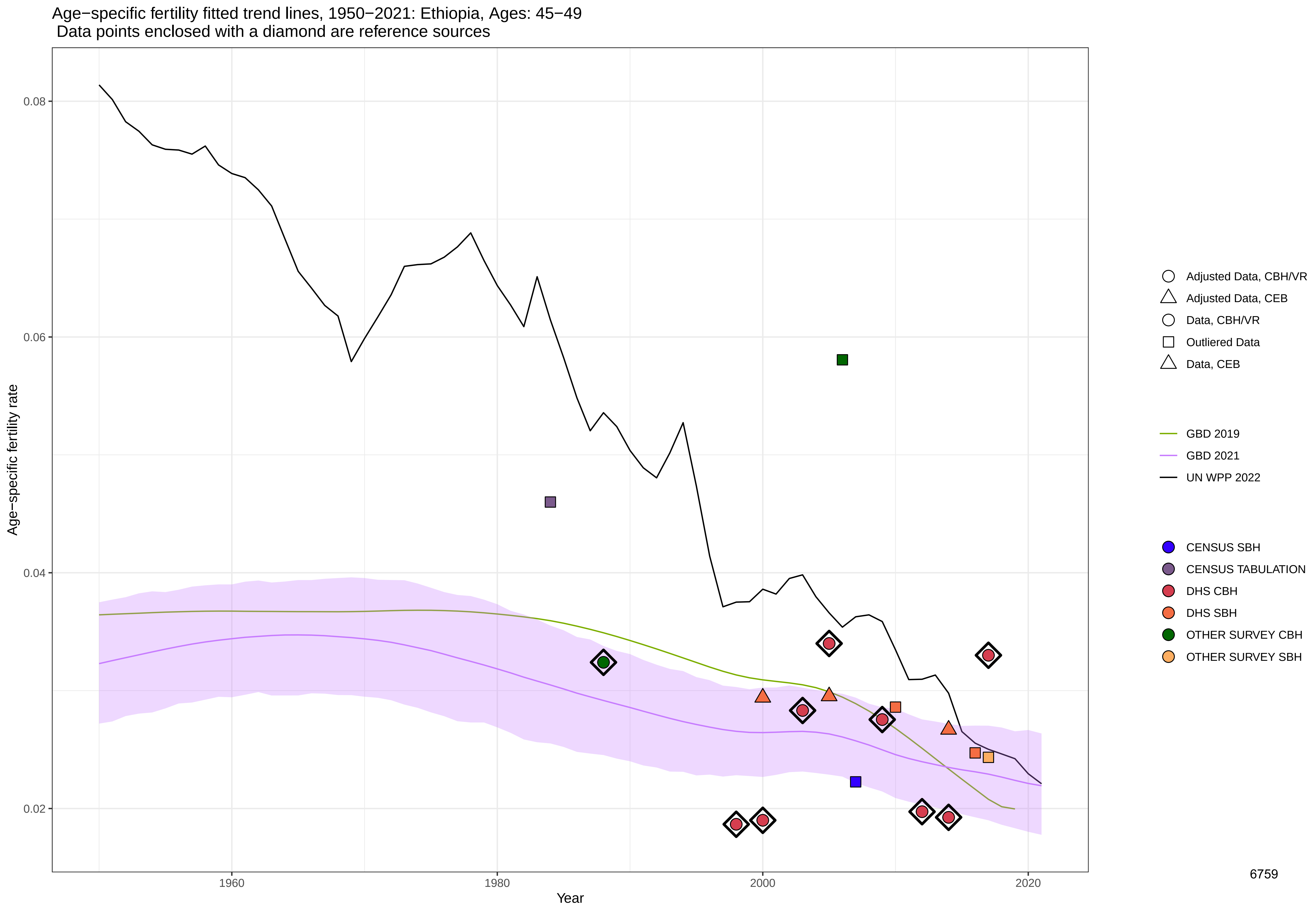
Age−specific fertility fitted trend lines, 1950−2021: Ethiopia, Ages: 45−49
Data points enclosed with a diamond are reference sources
Age−specific fertility rate
0.08
0.06
0.04
0.02
1960
1980
2000
2020
Year
Adjusted Data, CBH/VR
Adjusted Data, CEB
Data, CBH/VR
Outliered Data
Data, CEB
GBD 2019
GBD 2021
UN WPP 2022
CENSUS SBH
CENSUS TABULATION
DHS CBH
DHS SBH
OTHER SURVEY CBH
OTHER SURVEY SBH

Age−specific fertility fitted trend lines, 1950−2021: Ethiopia, Ages: 50−54
Data points enclosed with a diamond are reference sources

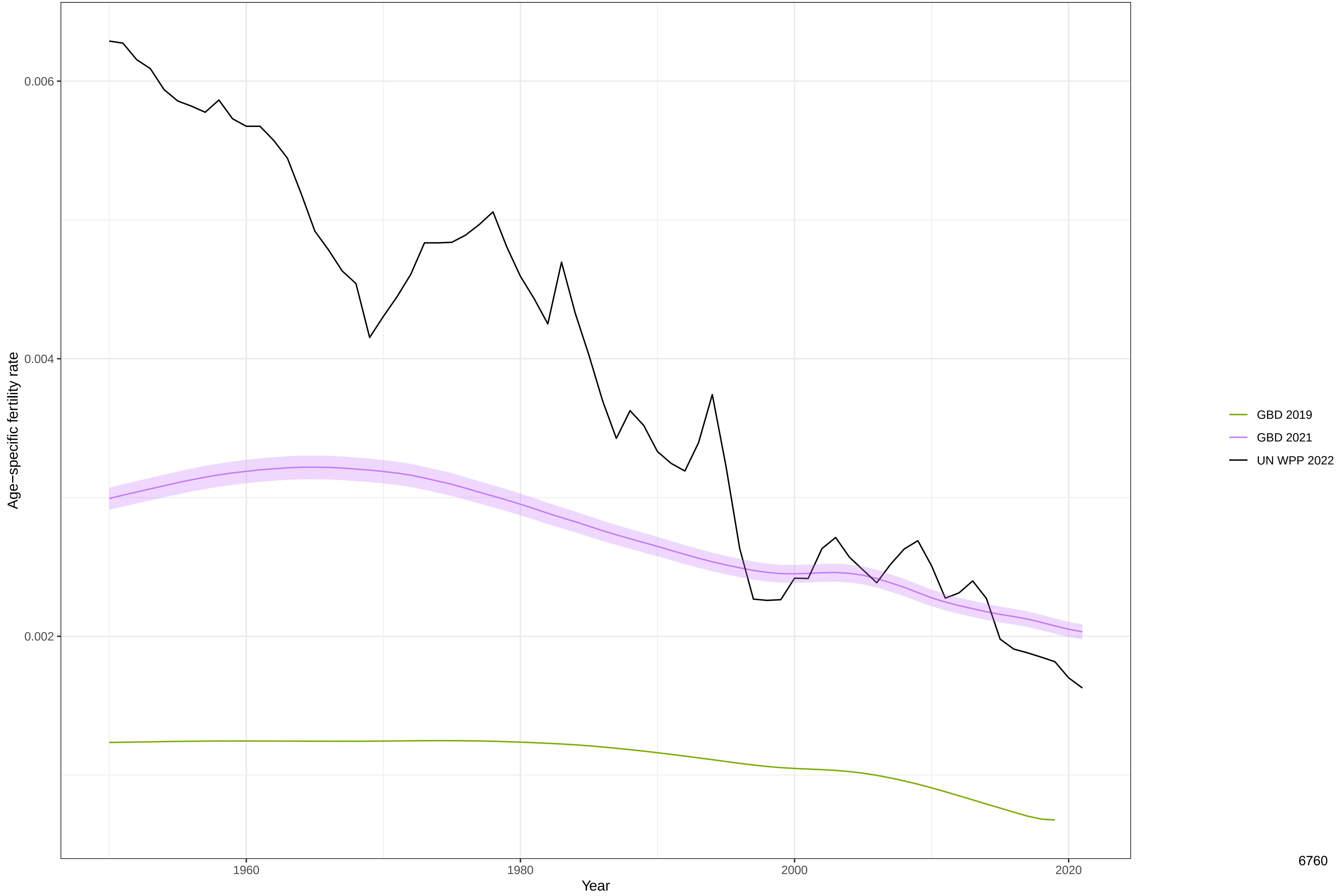

Total fertility rate fitted trend lines, 1950−2021: Ethiopia TFR
Data points enclosed with a diamond are reference sources

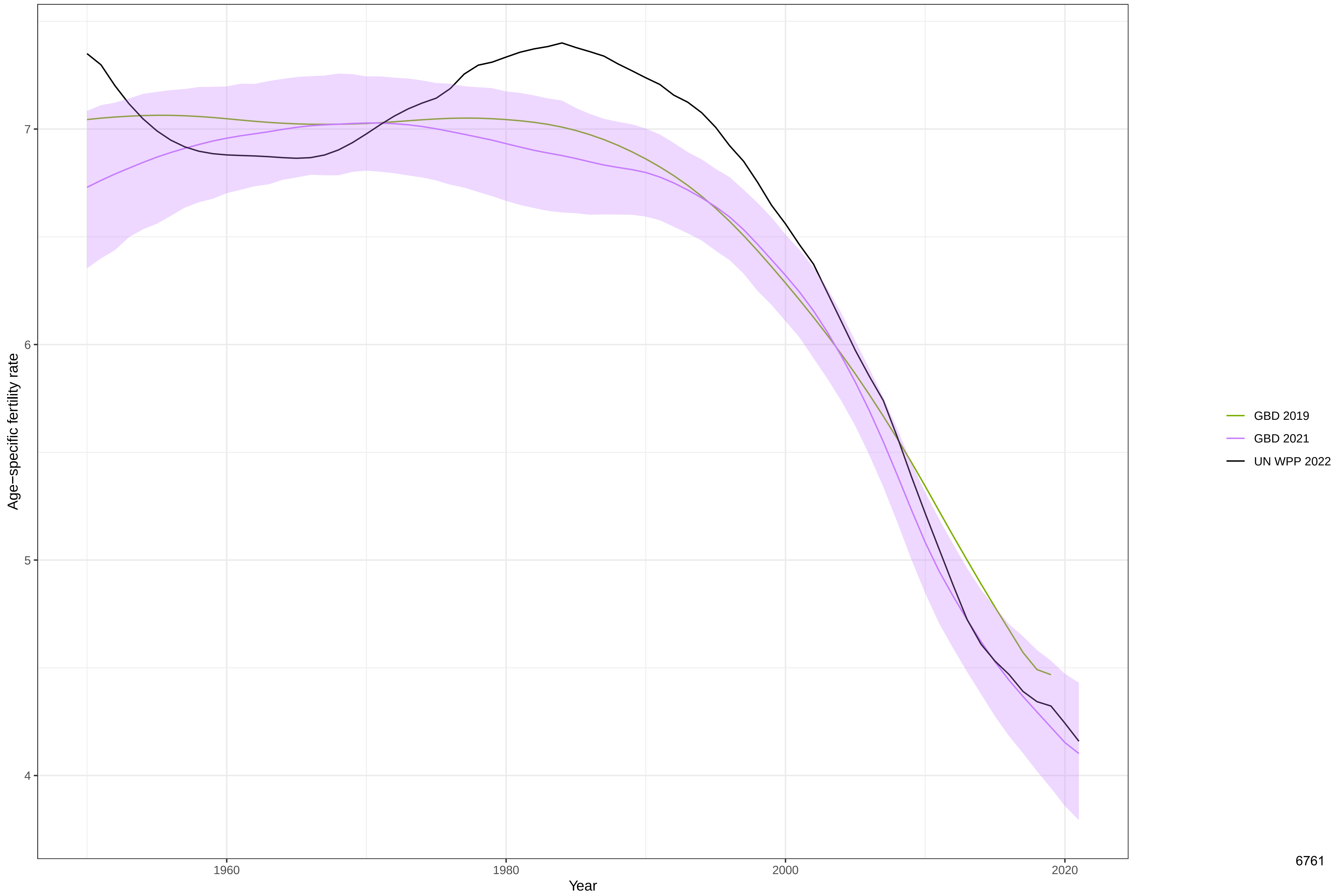

Age−specific fertility fitted trend lines, 1950−2021: Addis Ababa, Ages: 10−14
Data points enclosed with a diamond are reference sources

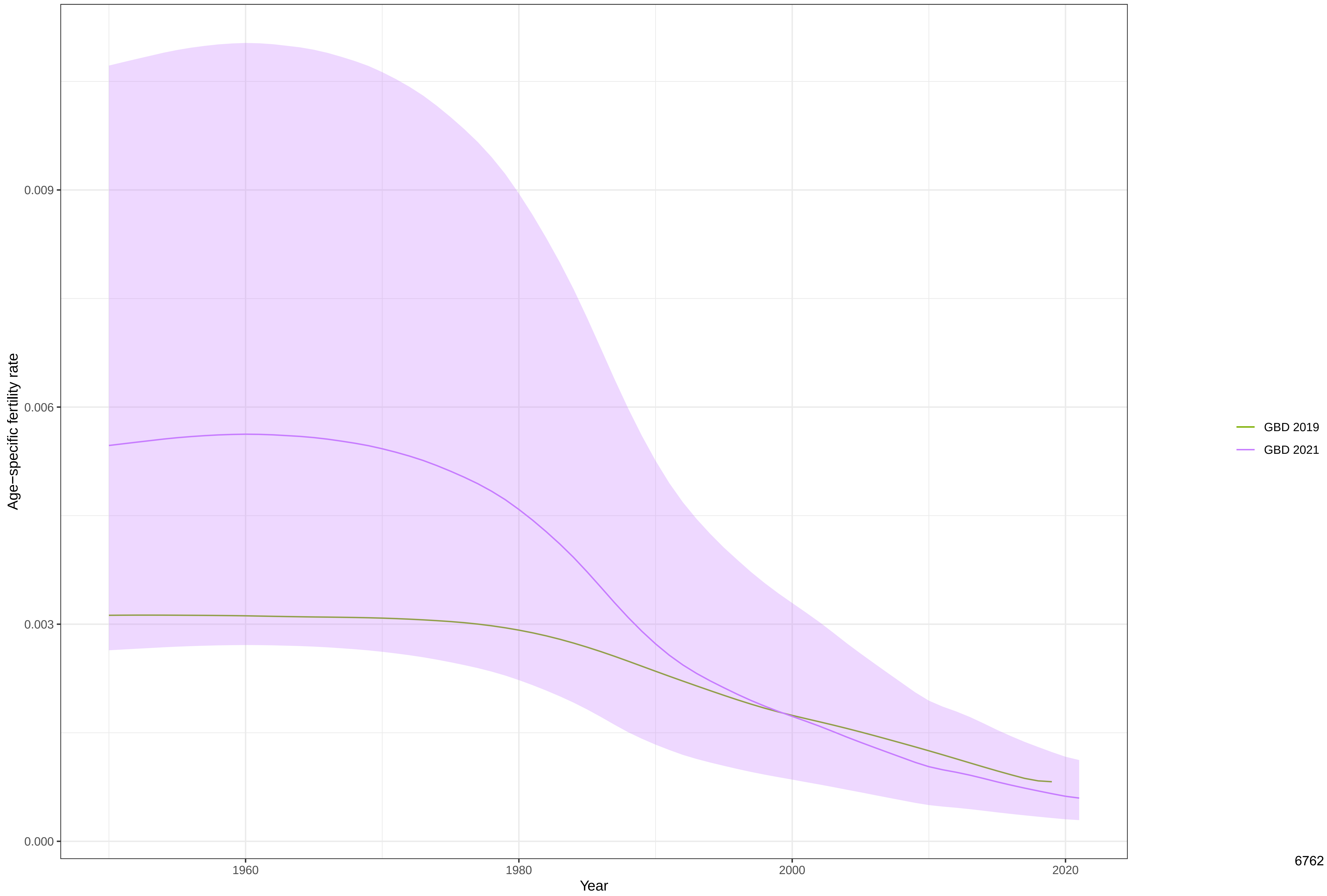

Age−specific fertility fitted trend lines, 1950−2021: Addis Ababa, Ages: 15−19
Data points enclosed with a diamond are reference sources

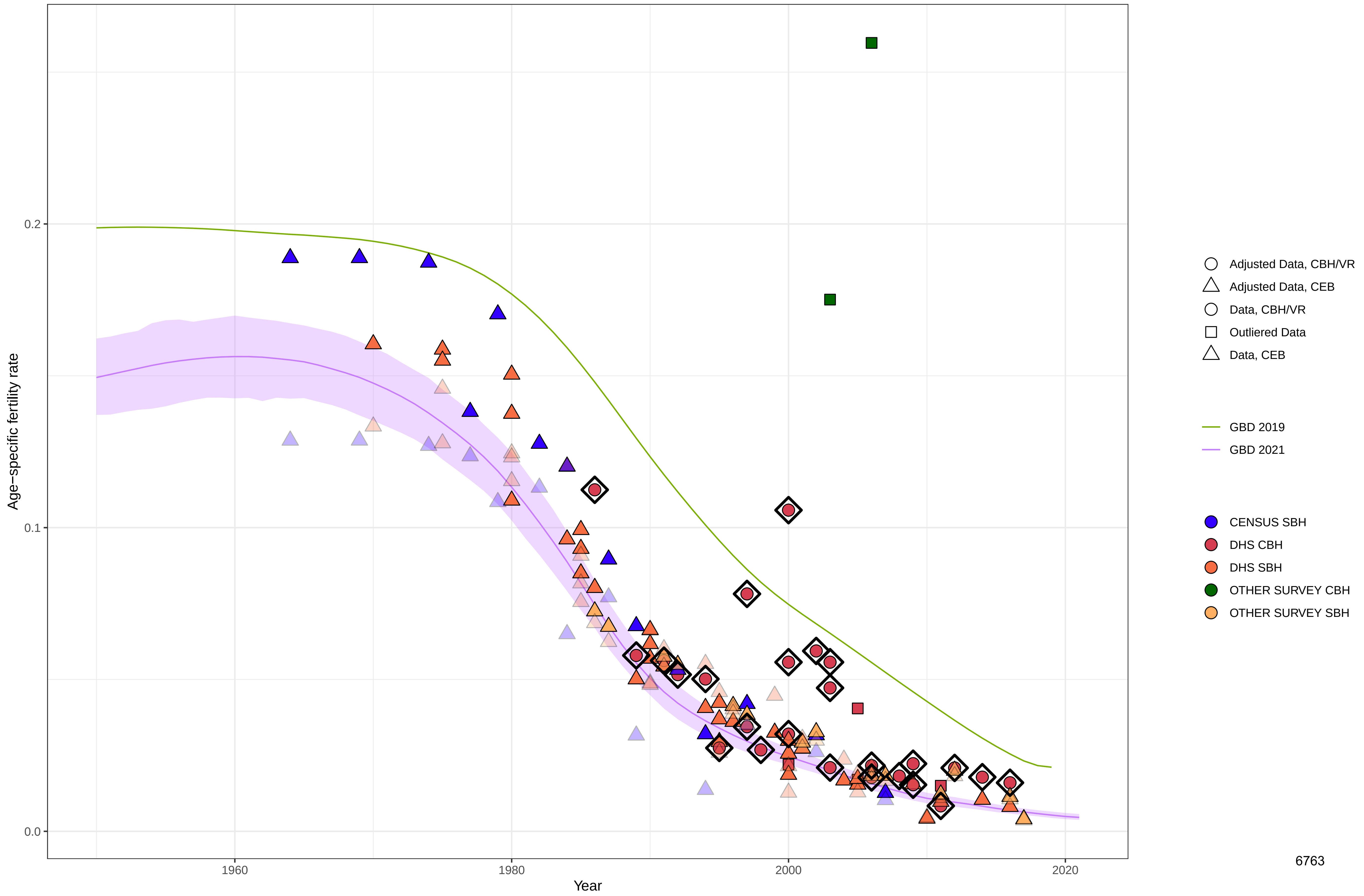

Age−specific fertility fitted trend lines, 1950−2021: Addis Ababa, Ages: 20−24
Data points enclosed with a diamond are reference sources

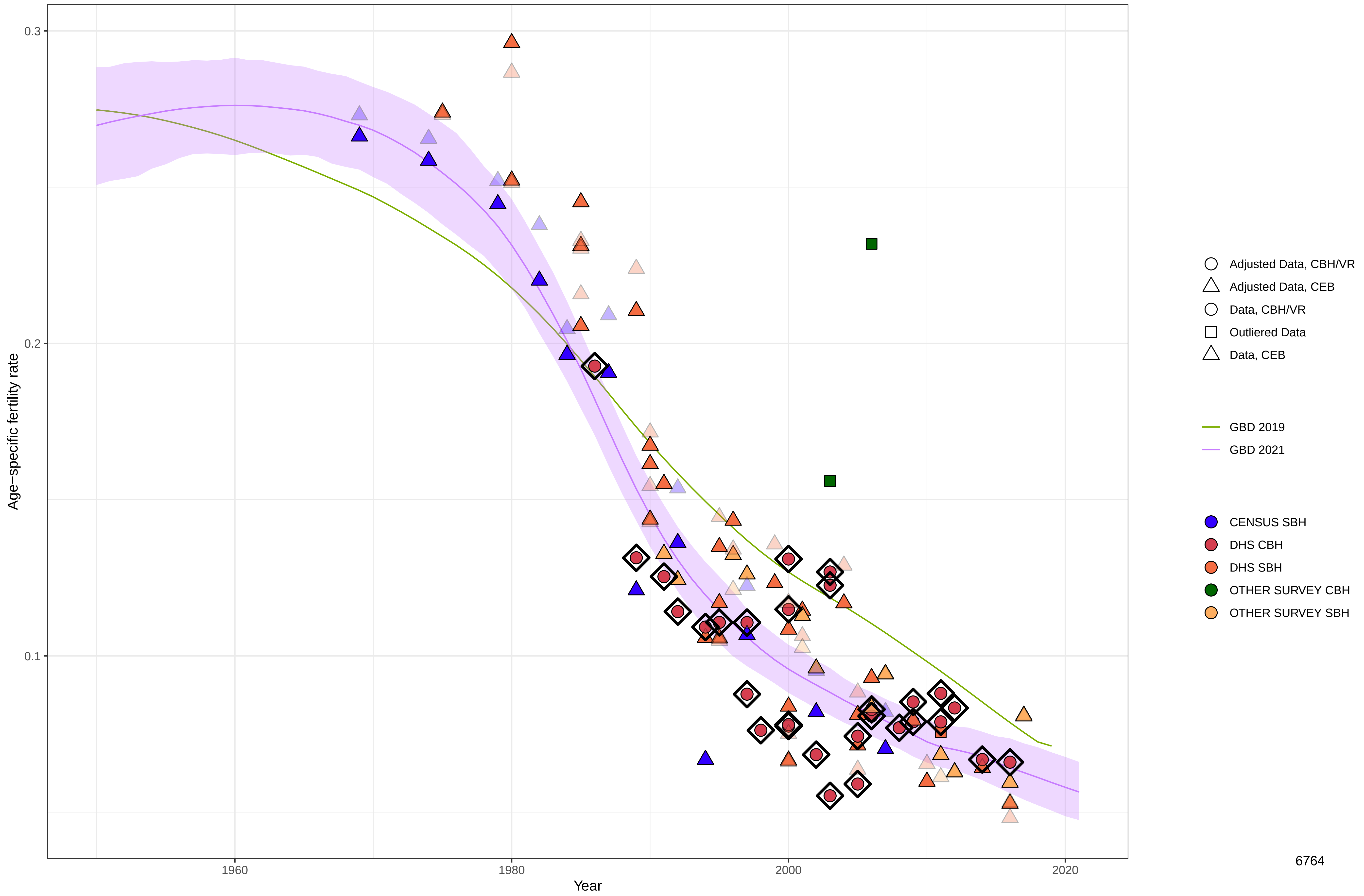

Age−specific fertility fitted trend lines, 1950−2021: Addis Ababa, Ages: 25−29
Data points enclosed with a diamond are reference sources

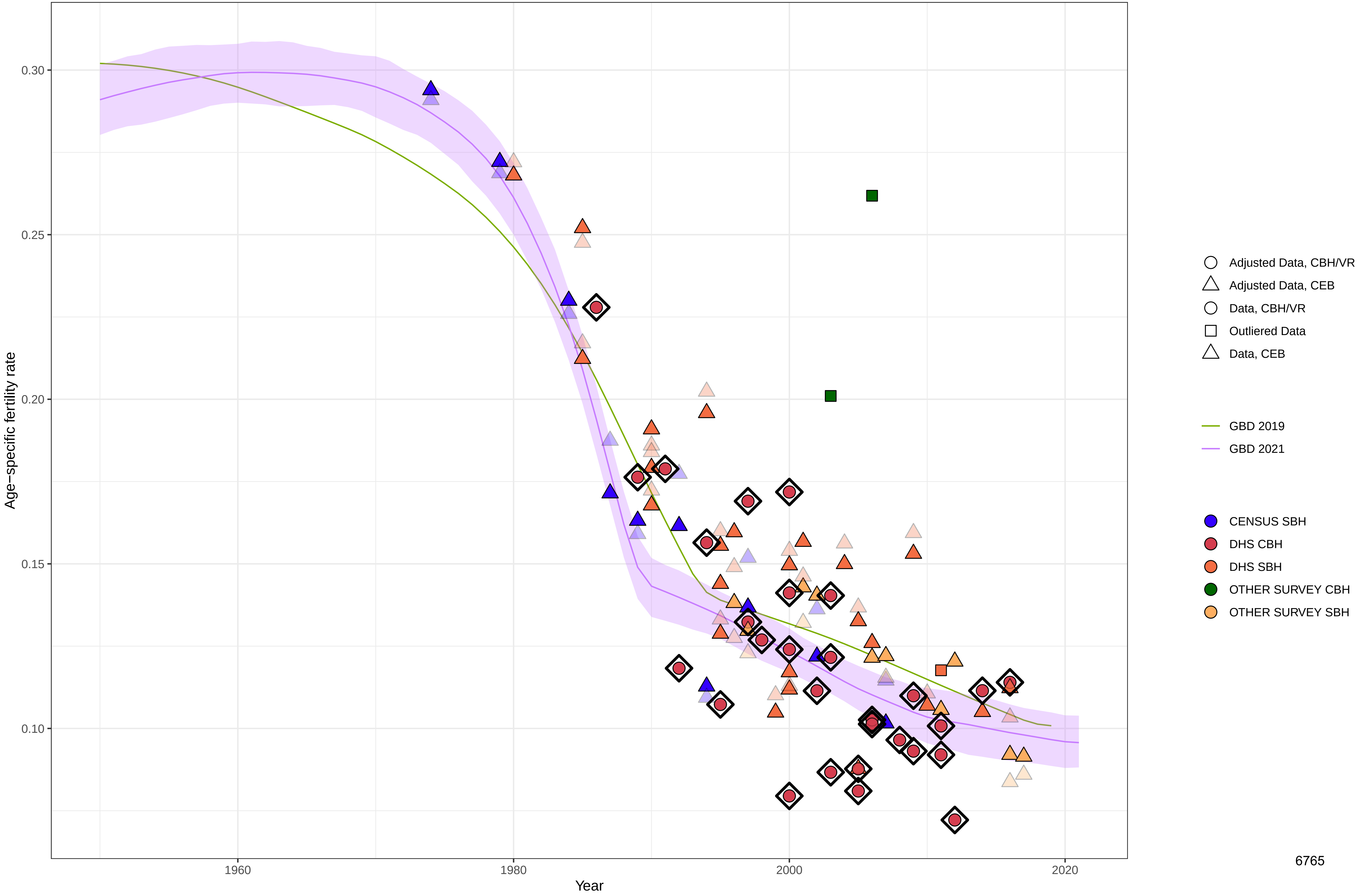

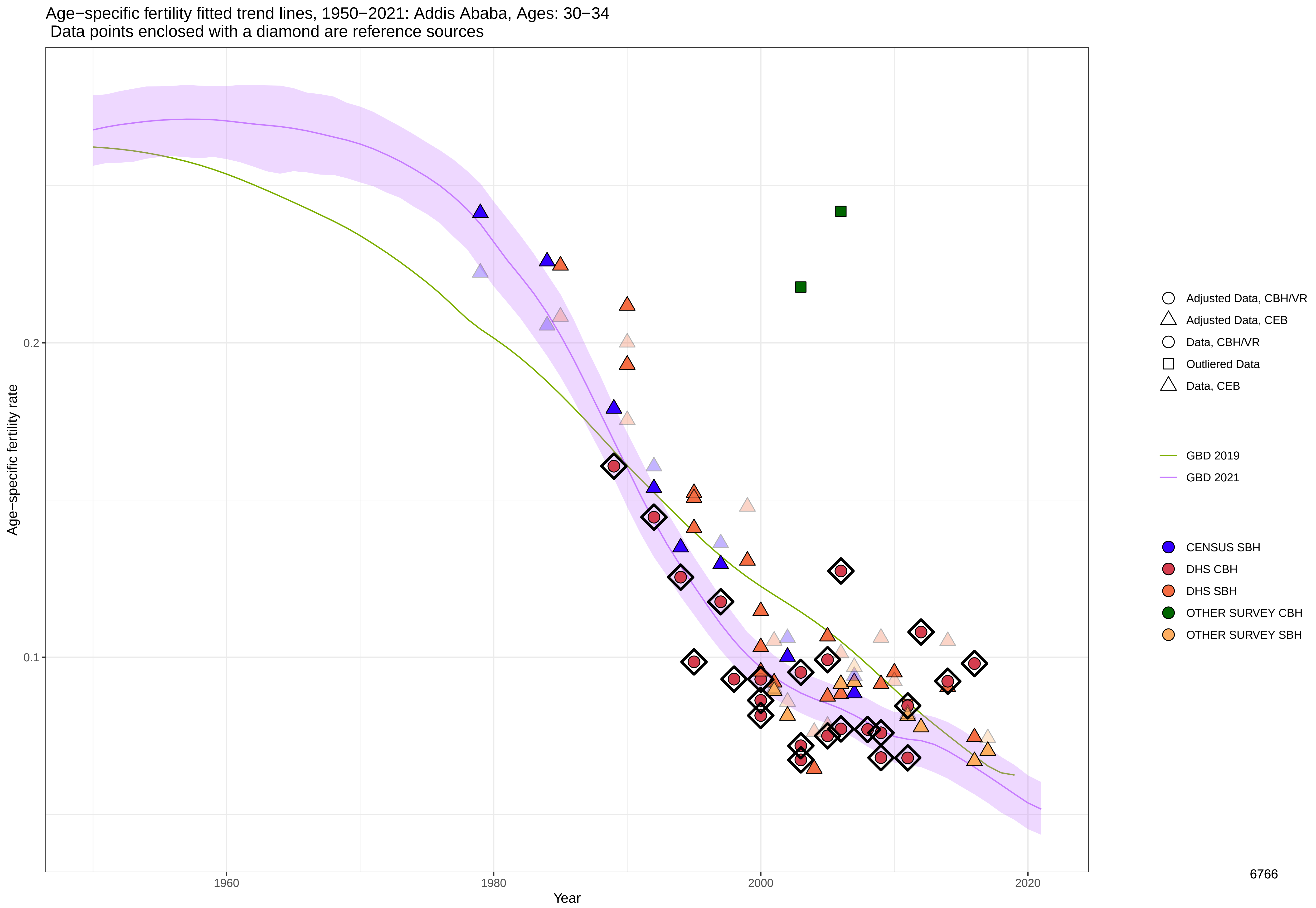

Age−specific fertility fitted trend lines, 1950−2021: Addis Ababa, Ages: 30−34
Data points enclosed with a diamond are reference sources
Age−specific fertility rate
0.2
0.1
1960
1980
2000
2020
Year
Adjusted Data, CBH/VR
Adjusted Data, CEB
Data, CBH/VR
Outliered Data
Data, CEB
GBD 2019
GBD 2021
CENSUS SBH
DHS CBH
DHS SBH
OTHER SURVEY CBH
OTHER SURVEY SBH

Age−specific fertility fitted trend lines, 1950−2021: Addis Ababa, Ages: 35−39
Data points enclosed with a diamond are reference sources

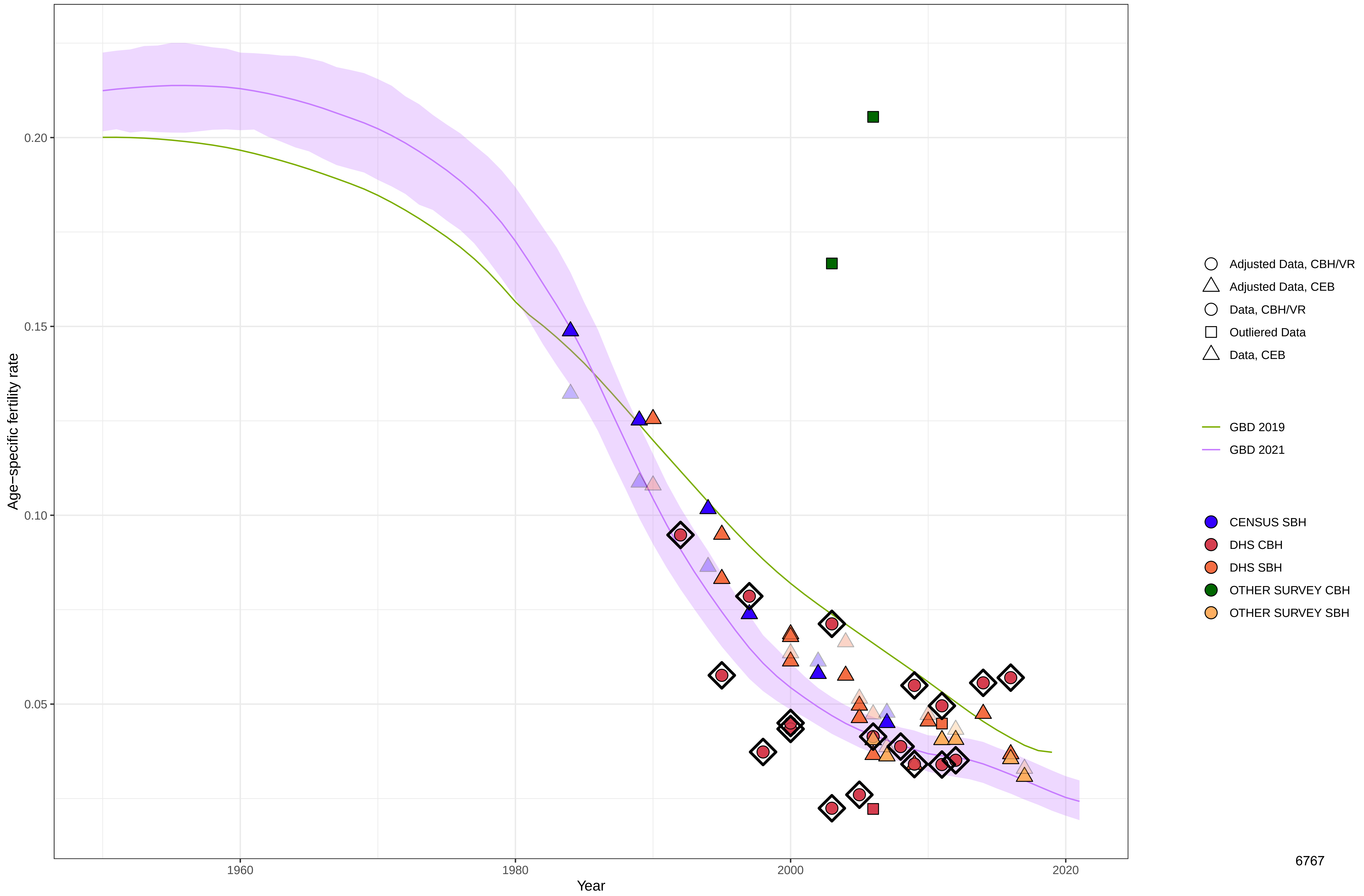

Age−specific fertility fitted trend lines, 1950−2021: Addis Ababa, Ages: 40−44
Data points enclosed with a diamond are reference sources

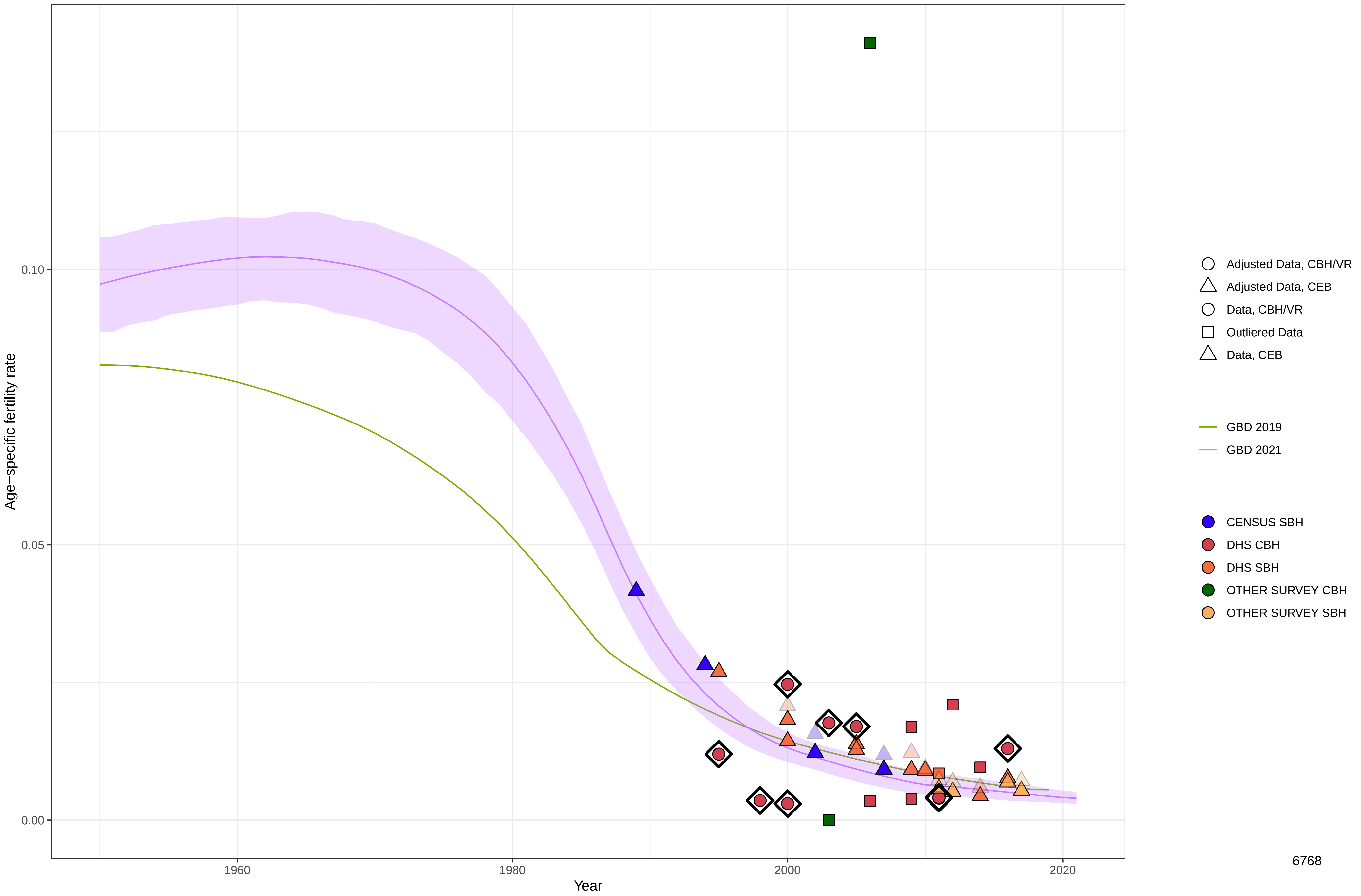

Age−specific fertility fitted trend lines, 1950−2021: Addis Ababa, Ages: 45−49
Data points enclosed with a diamond are reference sources

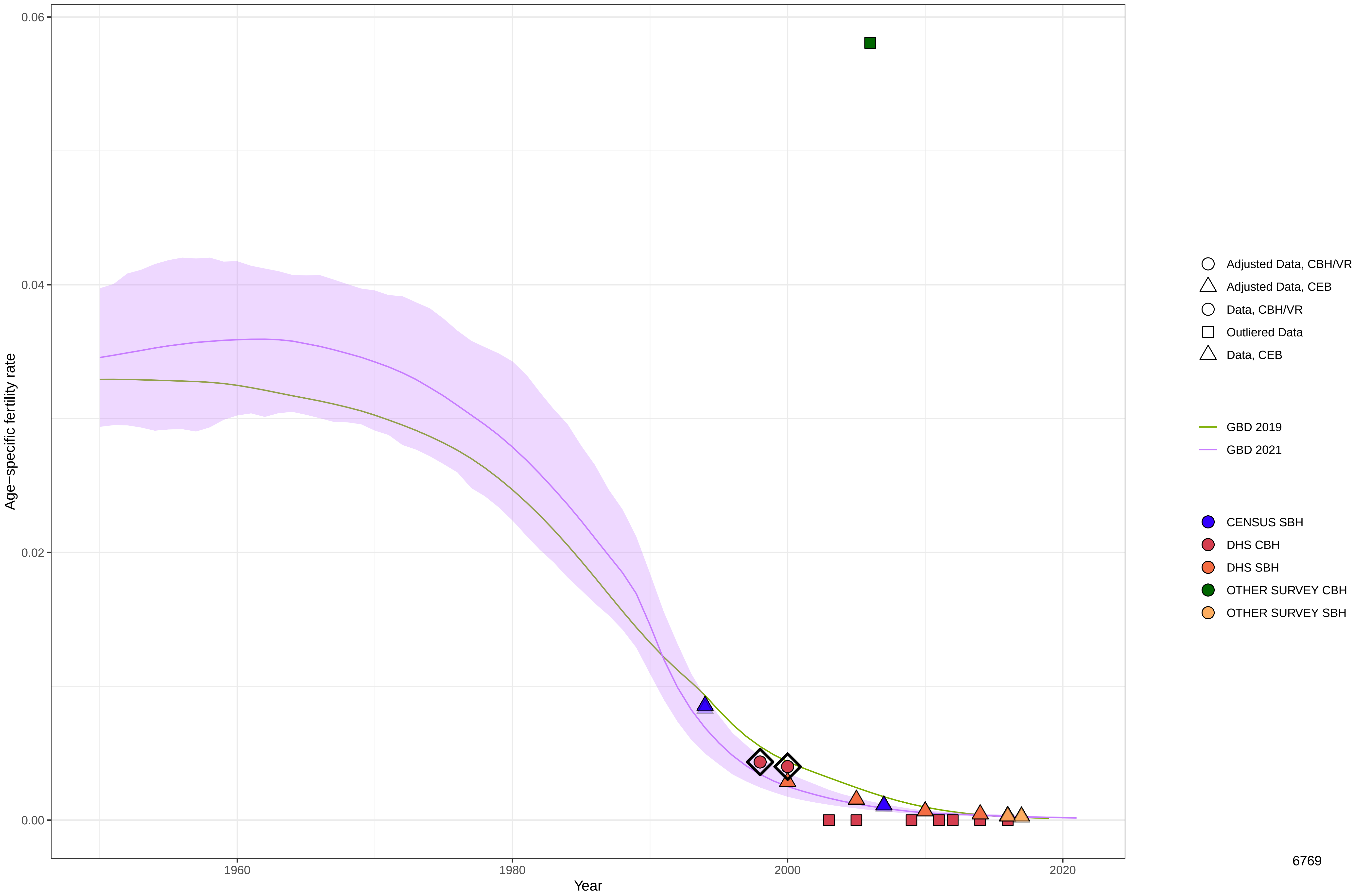

Age−specific fertility fitted trend lines, 1950−2021: Addis Ababa, Ages: 50−54
Data points enclosed with a diamond are reference sources

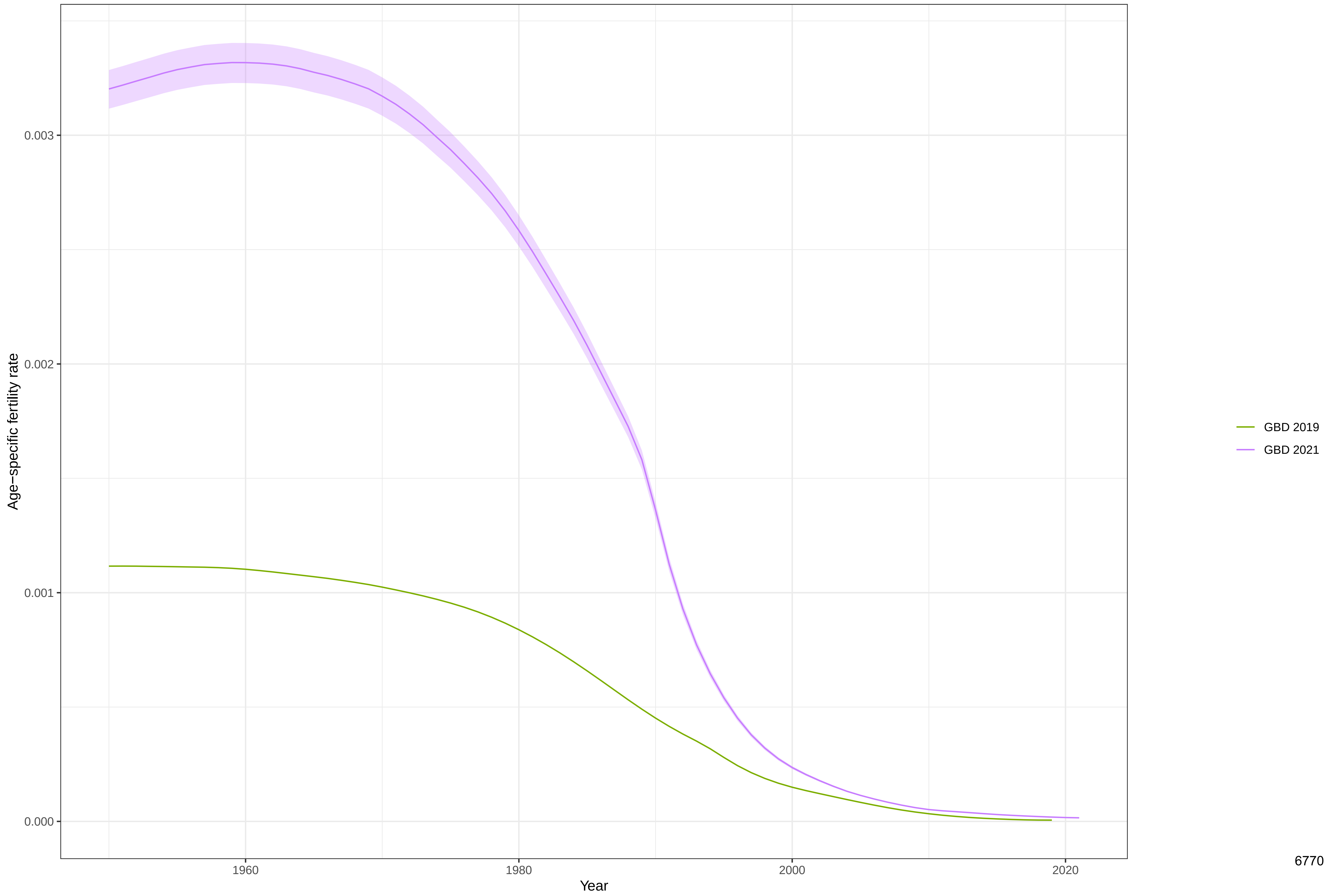

Total fertility rate fitted trend lines, 1950−2021: Addis Ababa TFR
Data points enclosed with a diamond are reference sources

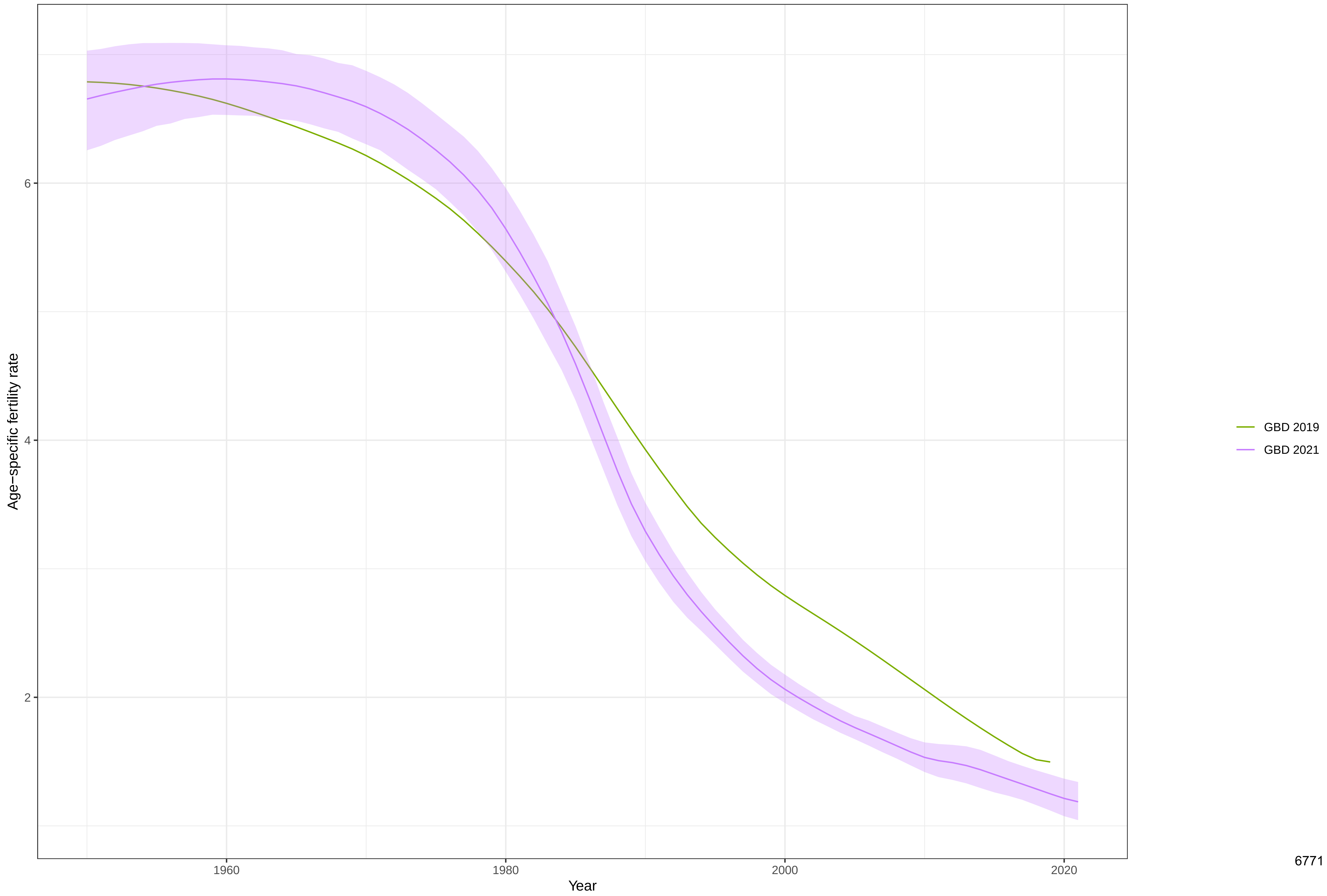

Age−specific fertility fitted trend lines, 1950−2021: Afar, Ages: 10−14
Data points enclosed with a diamond are reference sources

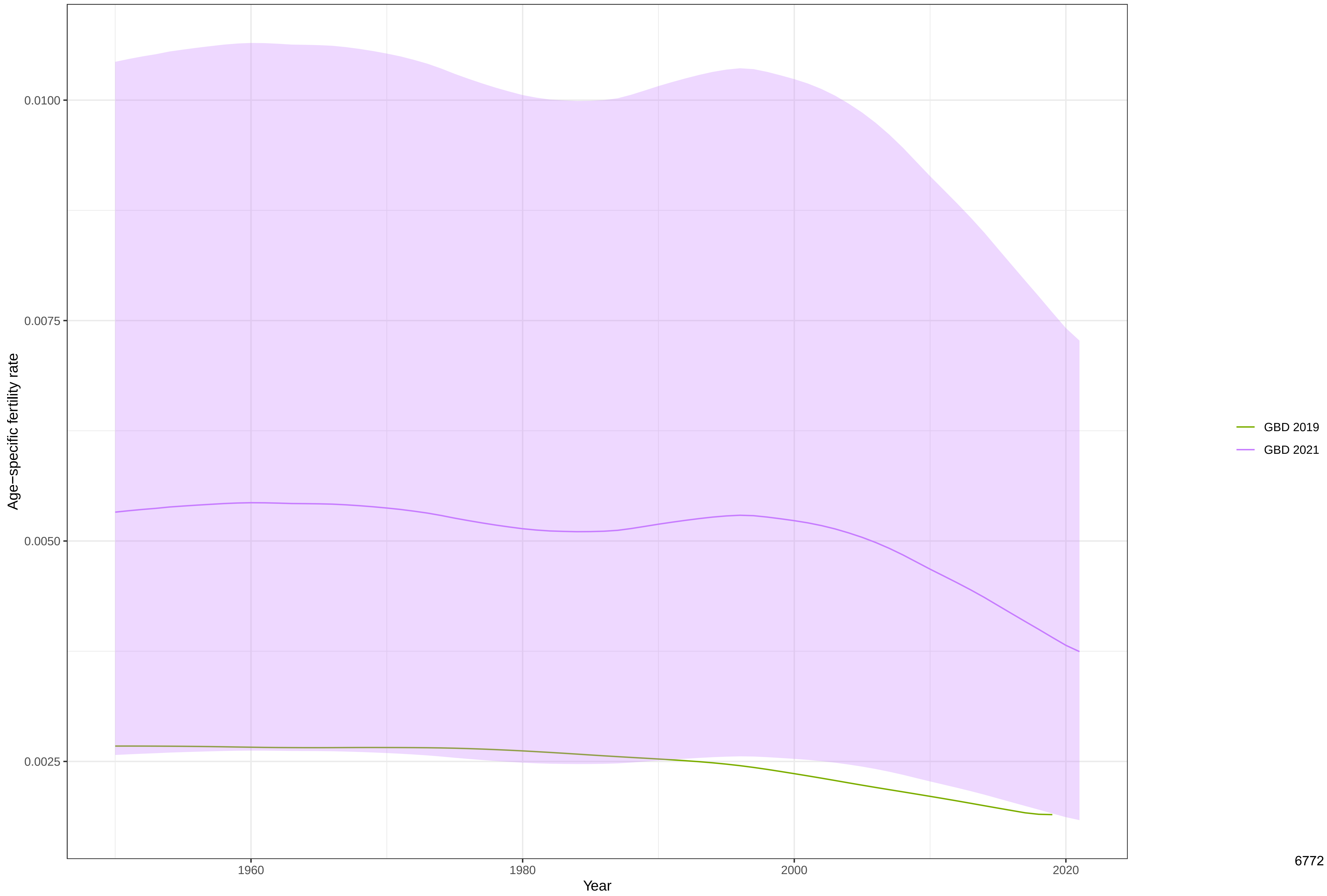

6772

Age−specific fertility fitted trend lines, 1950−2021: Afar, Ages: 15−19
Data points enclosed with a diamond are reference sources

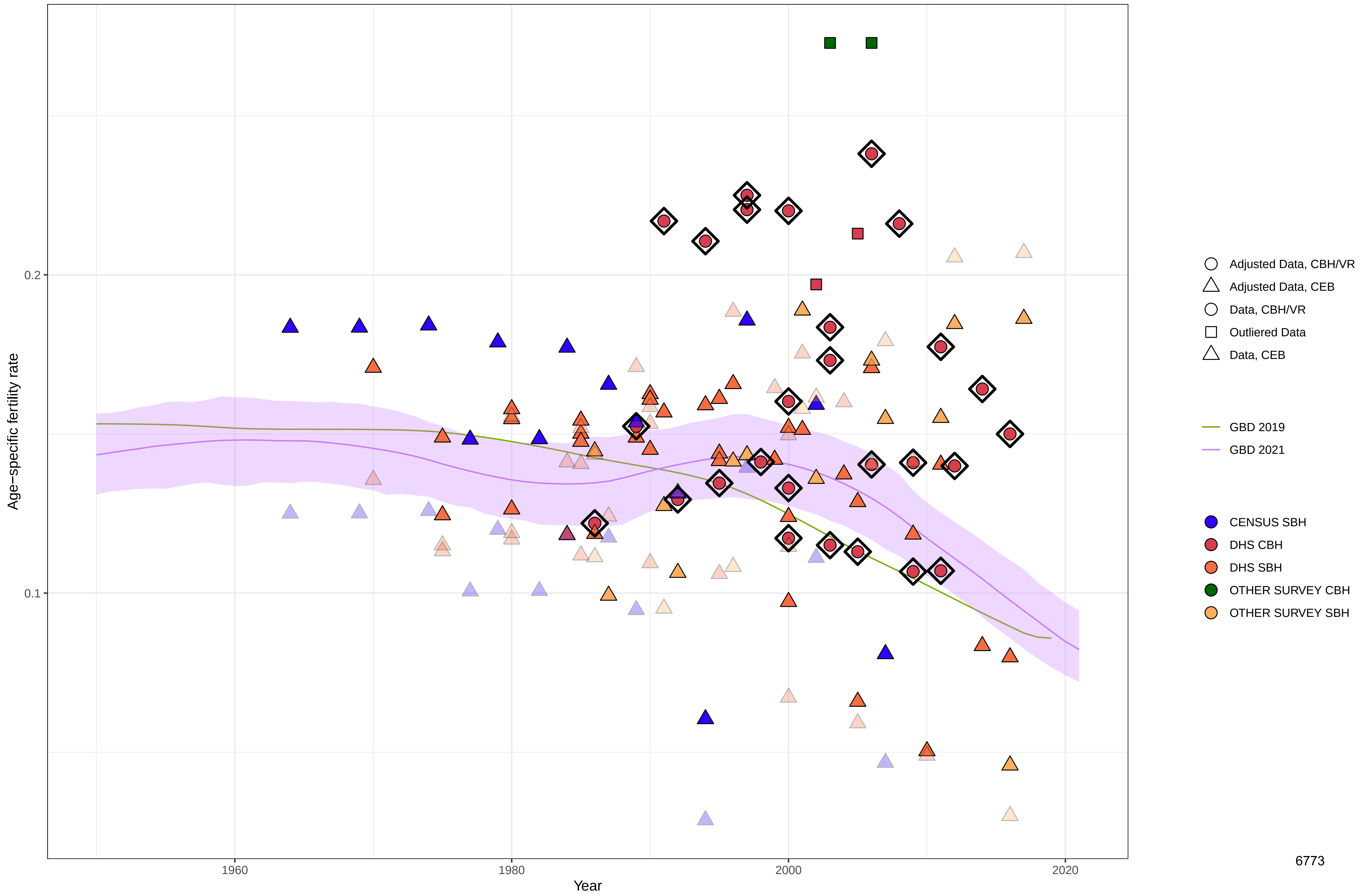

6773

Age−specific fertility fitted trend lines, 1950−2021: Afar, Ages: 20−24
Data points enclosed with a diamond are reference sources

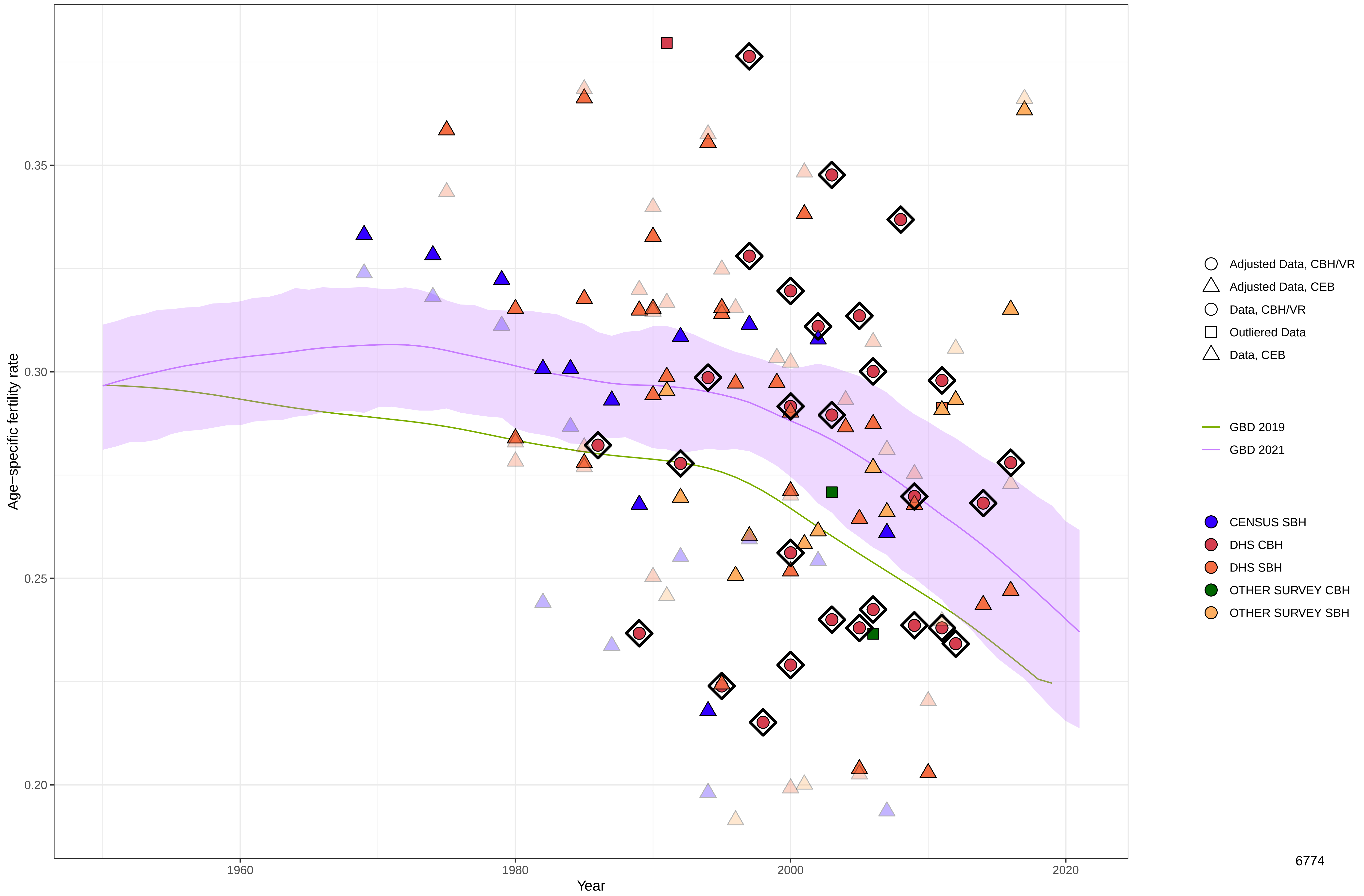

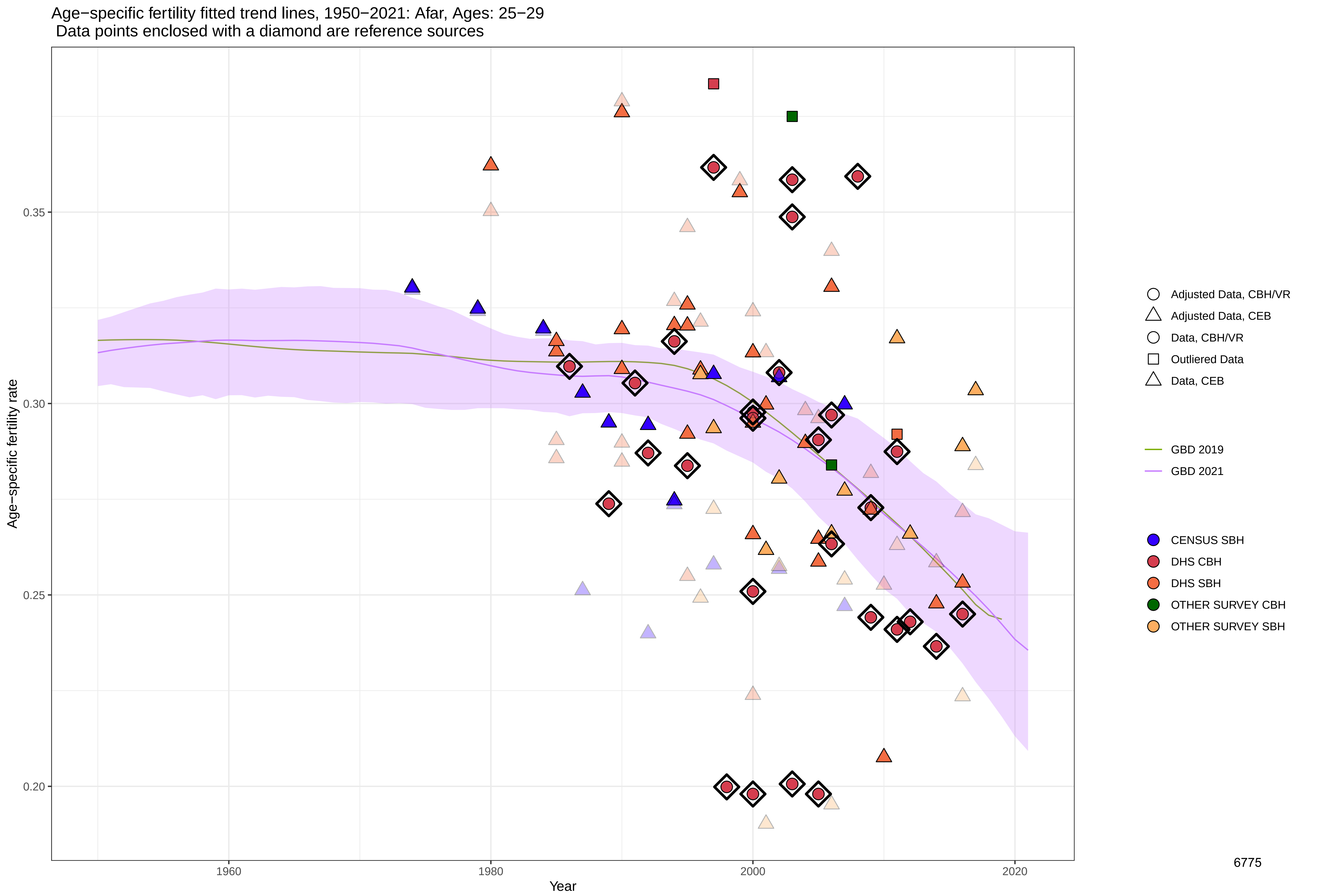

Age−specific fertility fitted trend lines, 1950−2021: Afar, Ages: 25−29
Data points enclosed with a diamond are reference sources
Age−specific fertility rate
Year
0.20
0.25
0.30
0.35
1960
1980
2000
2020
Adjusted Data, CBH/VR
Adjusted Data, CEB
Data, CBH/VR
Outliered Data
Data, CEB
GBD 2019
GBD 2021
CENSUS SBH
DHS CBH
DHS SBH
OTHER SURVEY CBH
OTHER SURVEY SBH

Age−specific fertility fitted trend lines, 1950−2021: Afar, Ages: 30−34
Data points enclosed with a diamond are reference sources

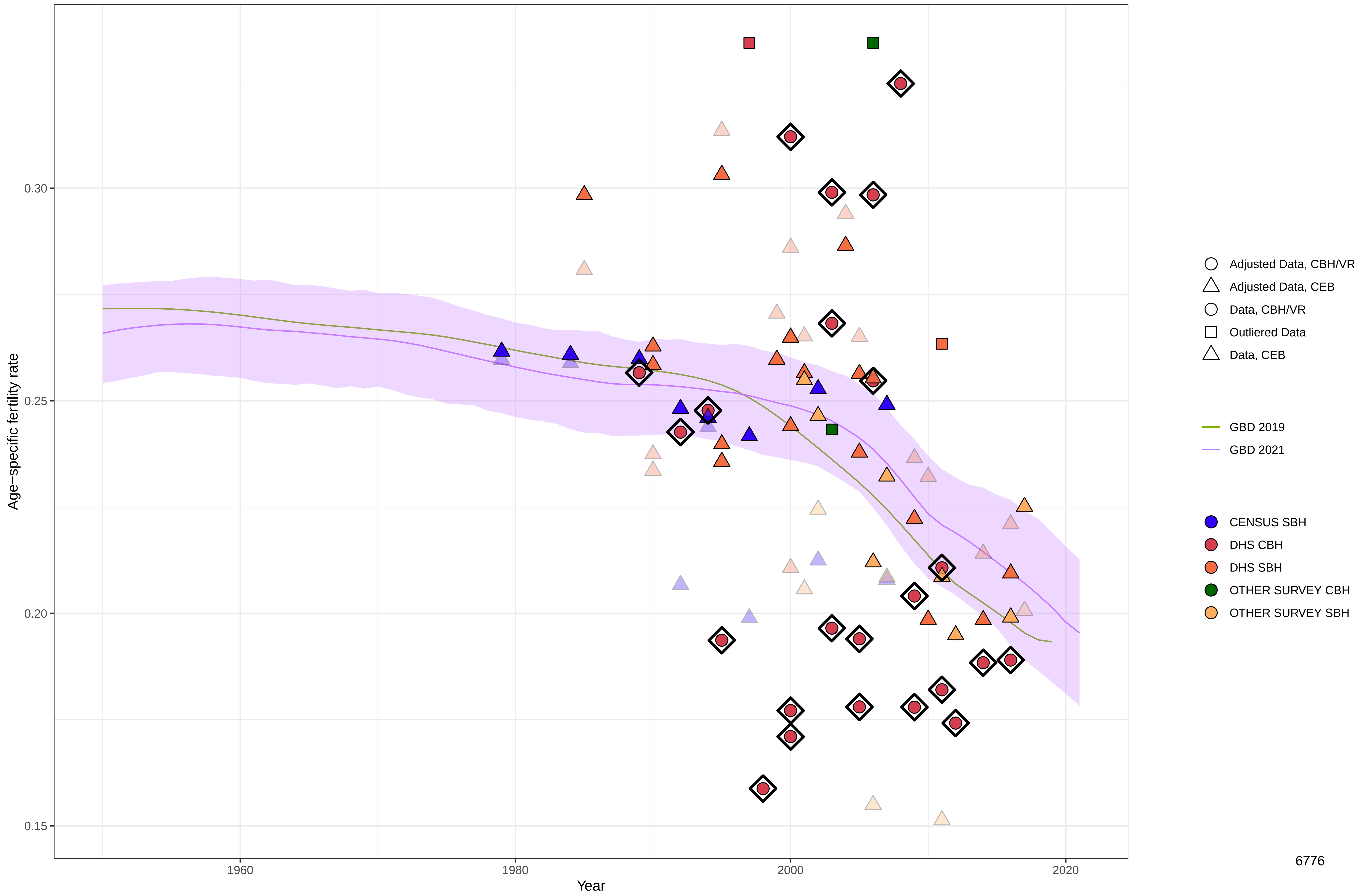

Age−specific fertility fitted trend lines, 1950−2021: Afar, Ages: 35−39
Data points enclosed with a diamond are reference sources

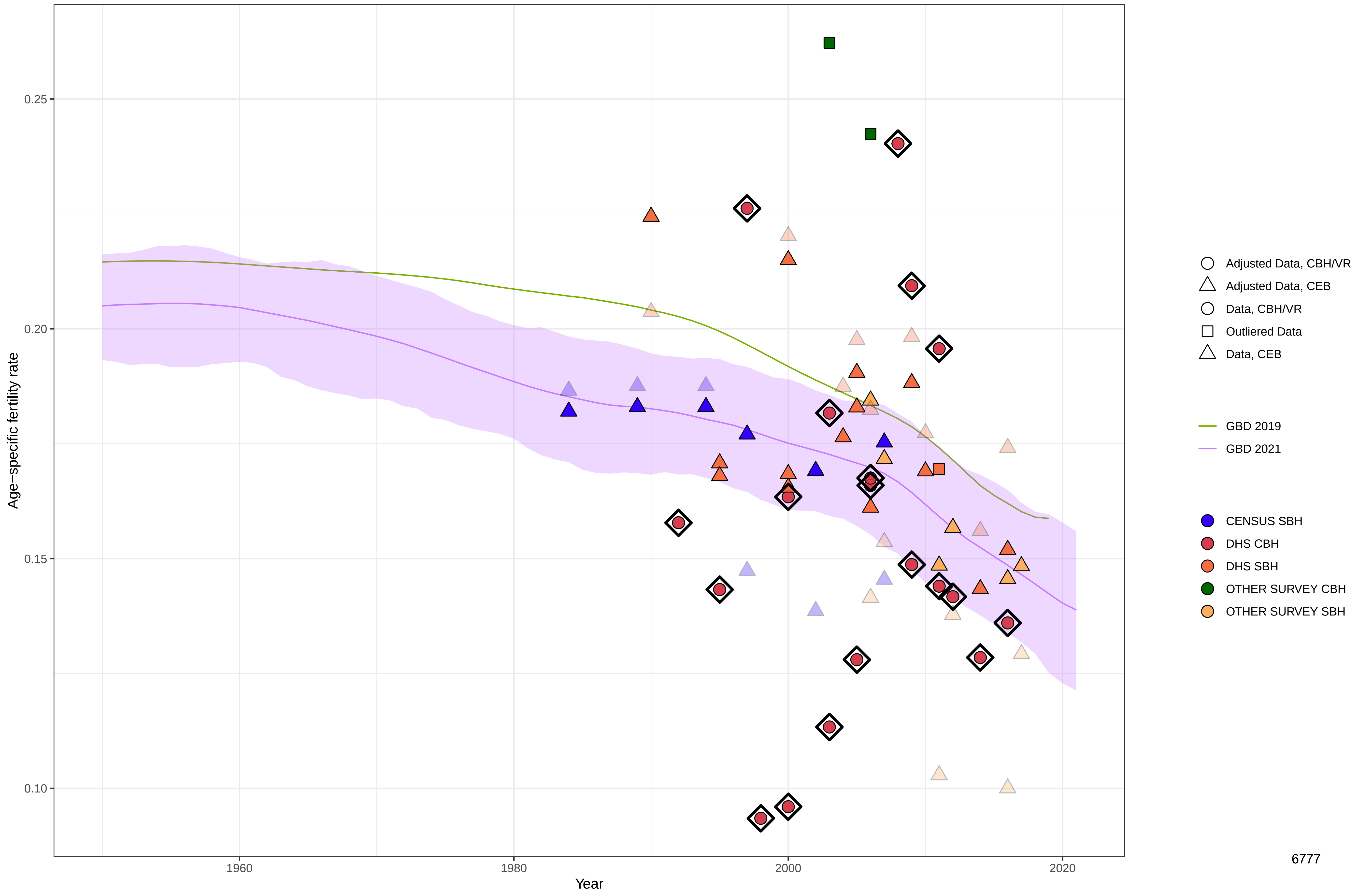

Age−specific fertility fitted trend lines, 1950−2021: Afar, Ages: 40−44
Data points enclosed with a diamond are reference sources

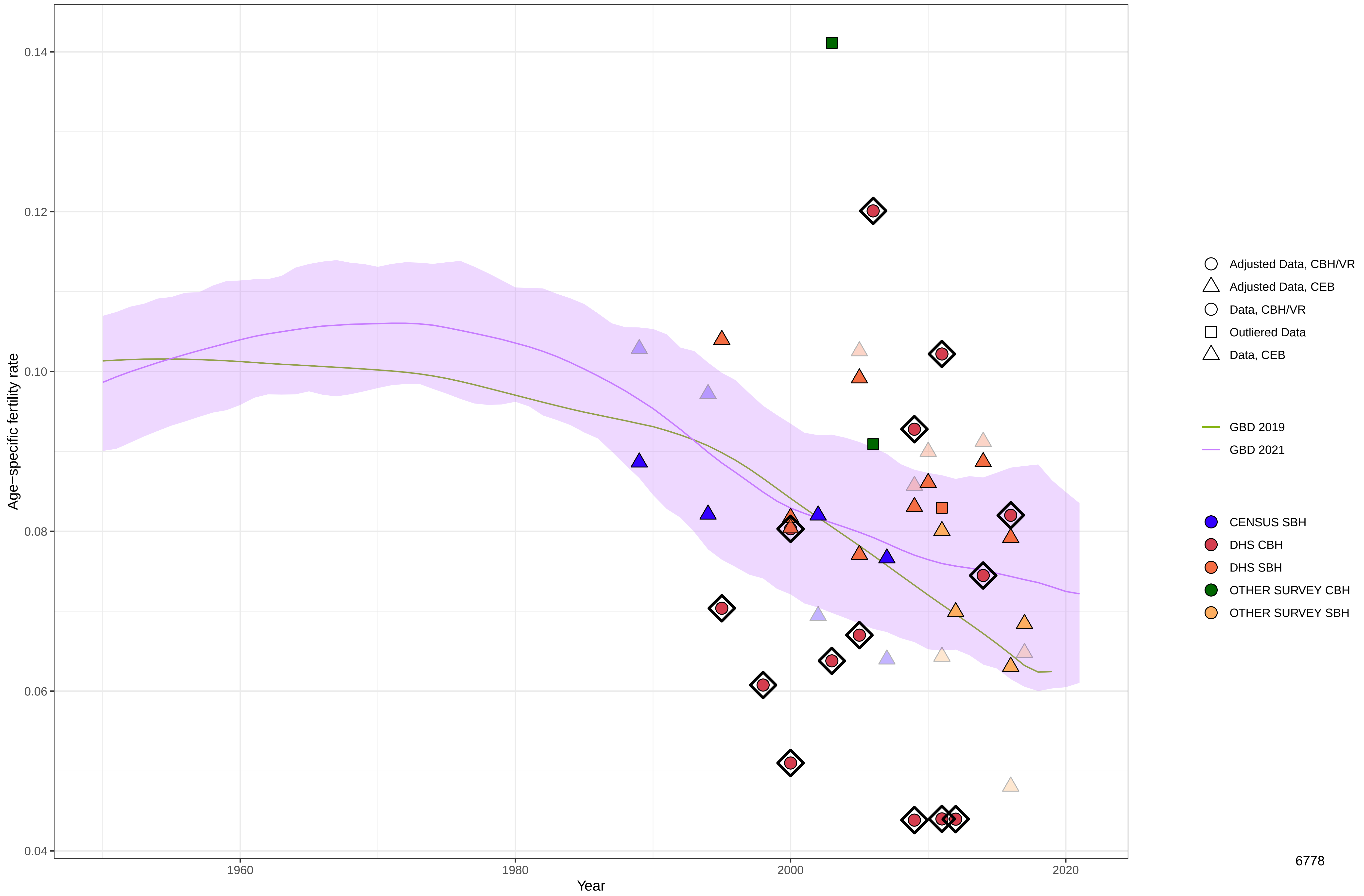

Age−specific fertility fitted trend lines, 1950−2021: Afar, Ages: 45−49
Data points enclosed with a diamond are reference sources

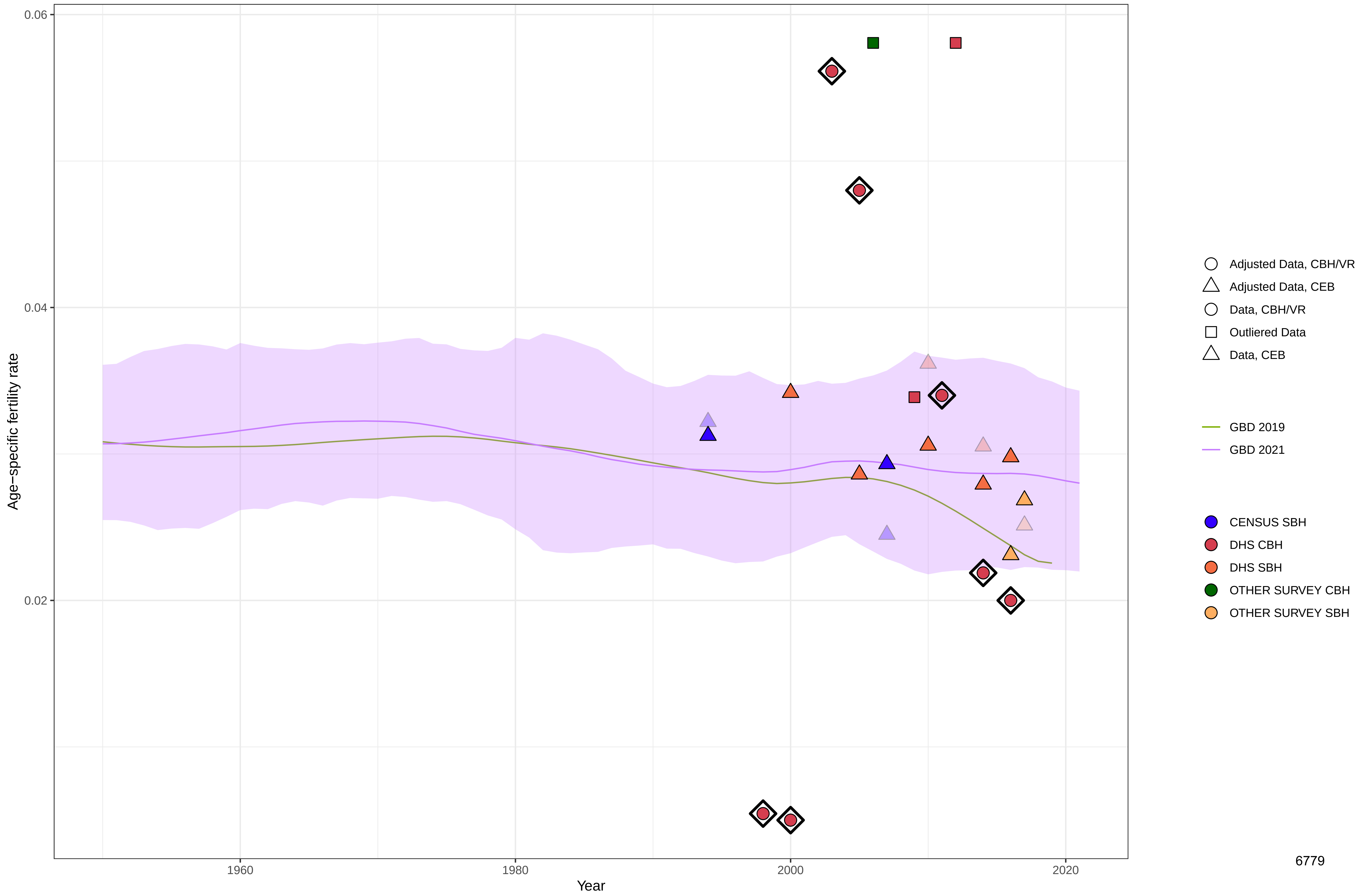

Age−specific fertility fitted trend lines, 1950−2021: Afar, Ages: 50−54
Data points enclosed with a diamond are reference sources

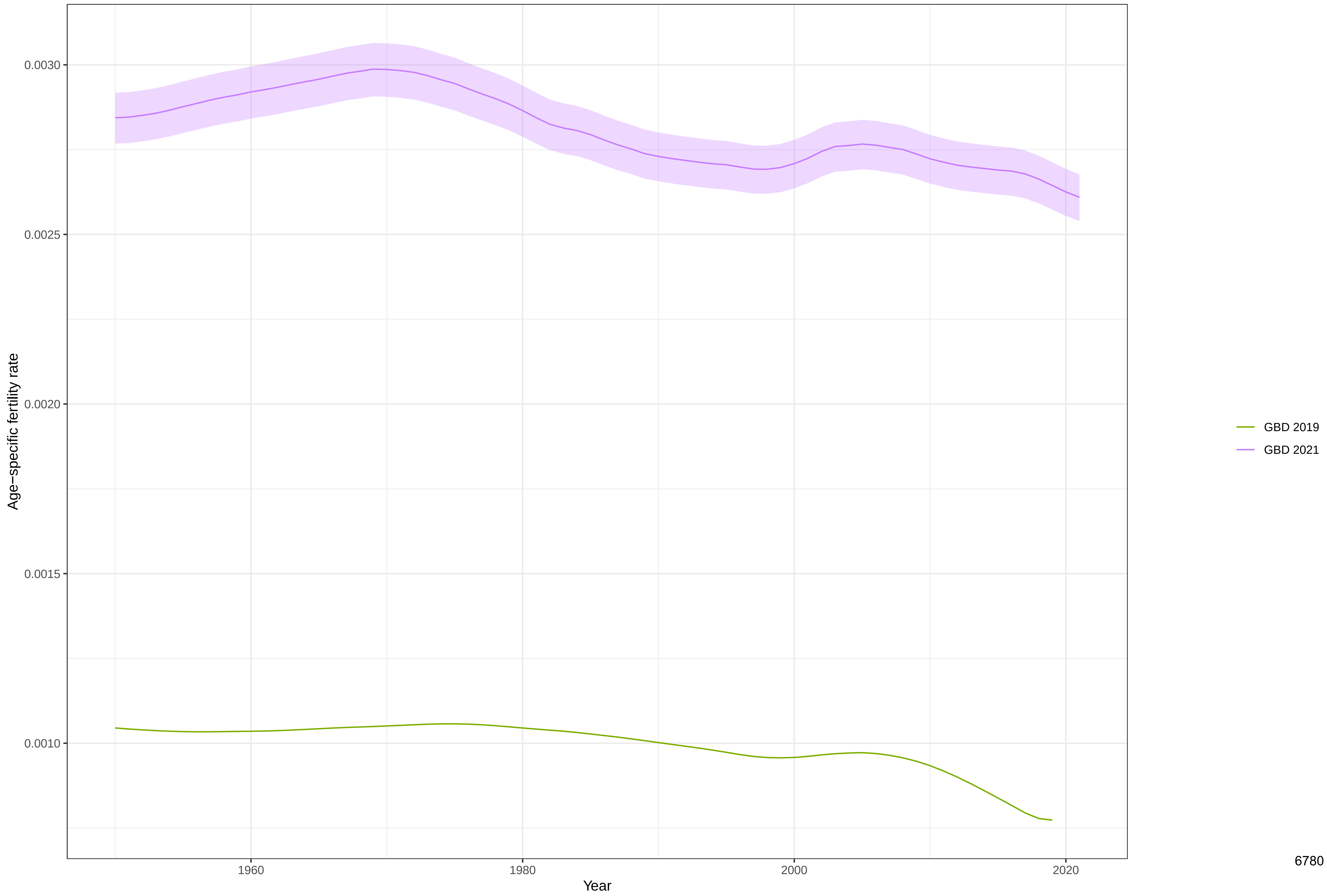

Total fertility rate fitted trend lines, 1950−2021: Afar TFR
Data points enclosed with a diamond are reference sources

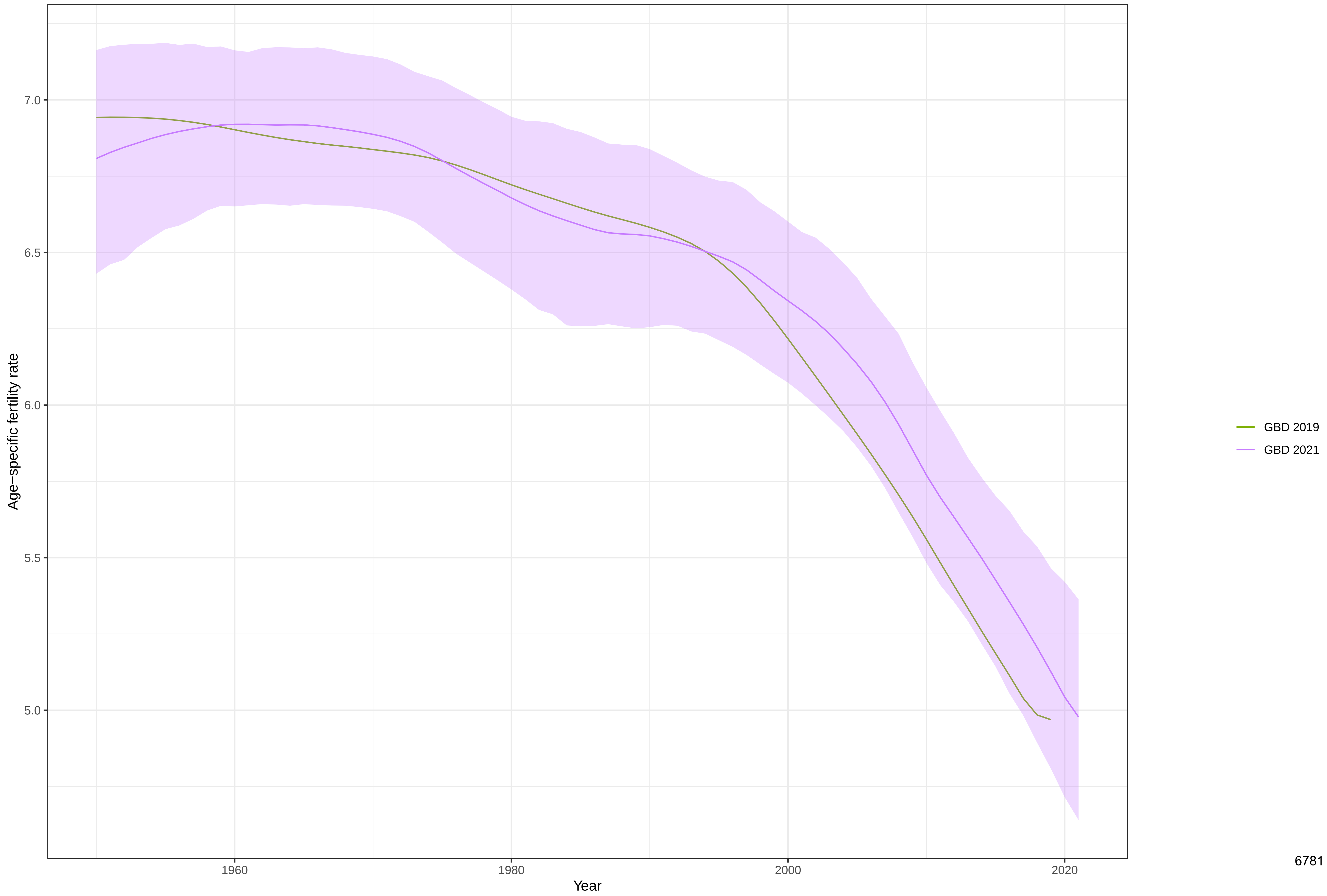

Age−specific fertility fitted trend lines, 1950−2021: Amhara, Ages: 10−14
Data points enclosed with a diamond are reference sources

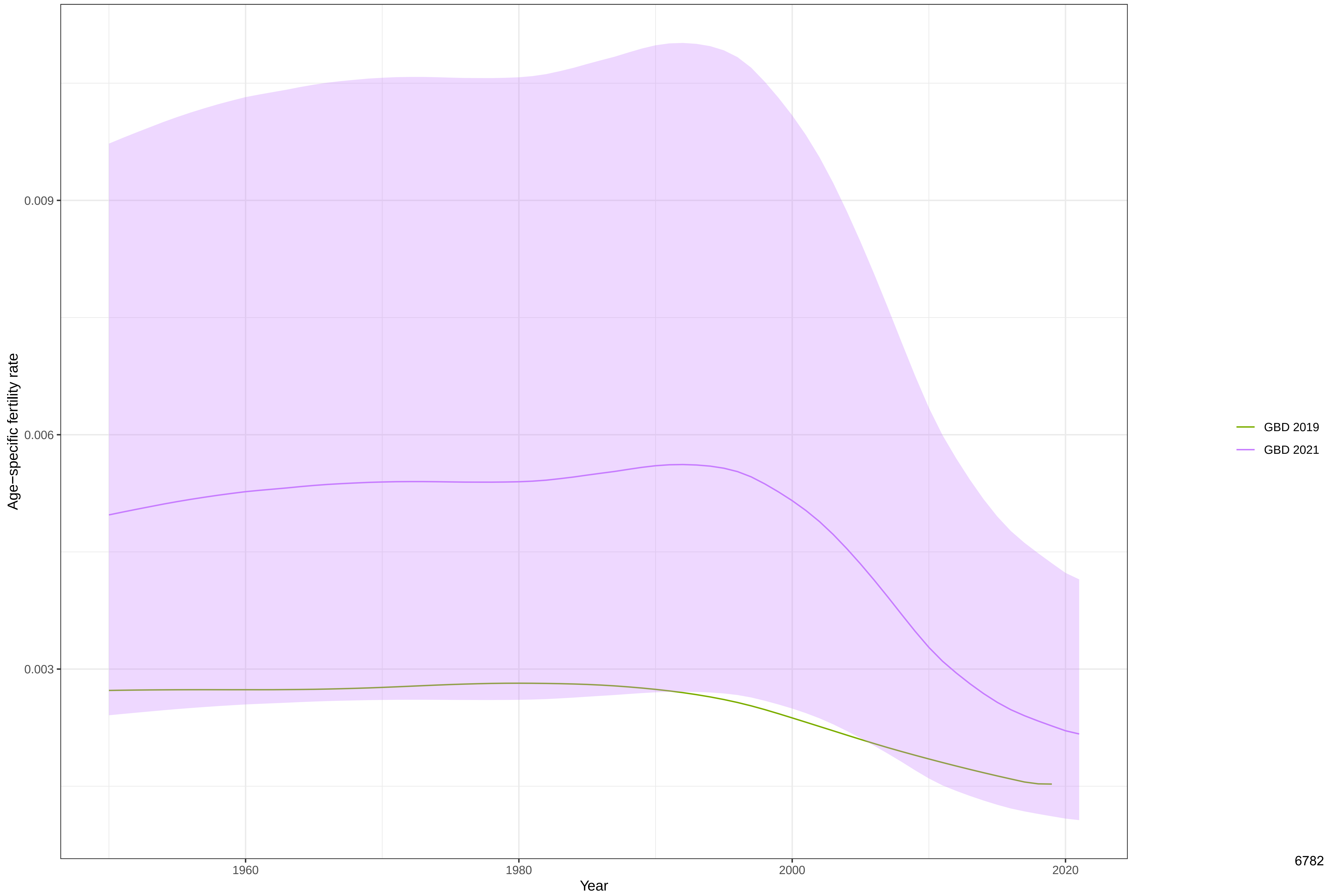

Age−specific fertility fitted trend lines, 1950−2021: Amhara, Ages: 15−19
Data points enclosed with a diamond are reference sources

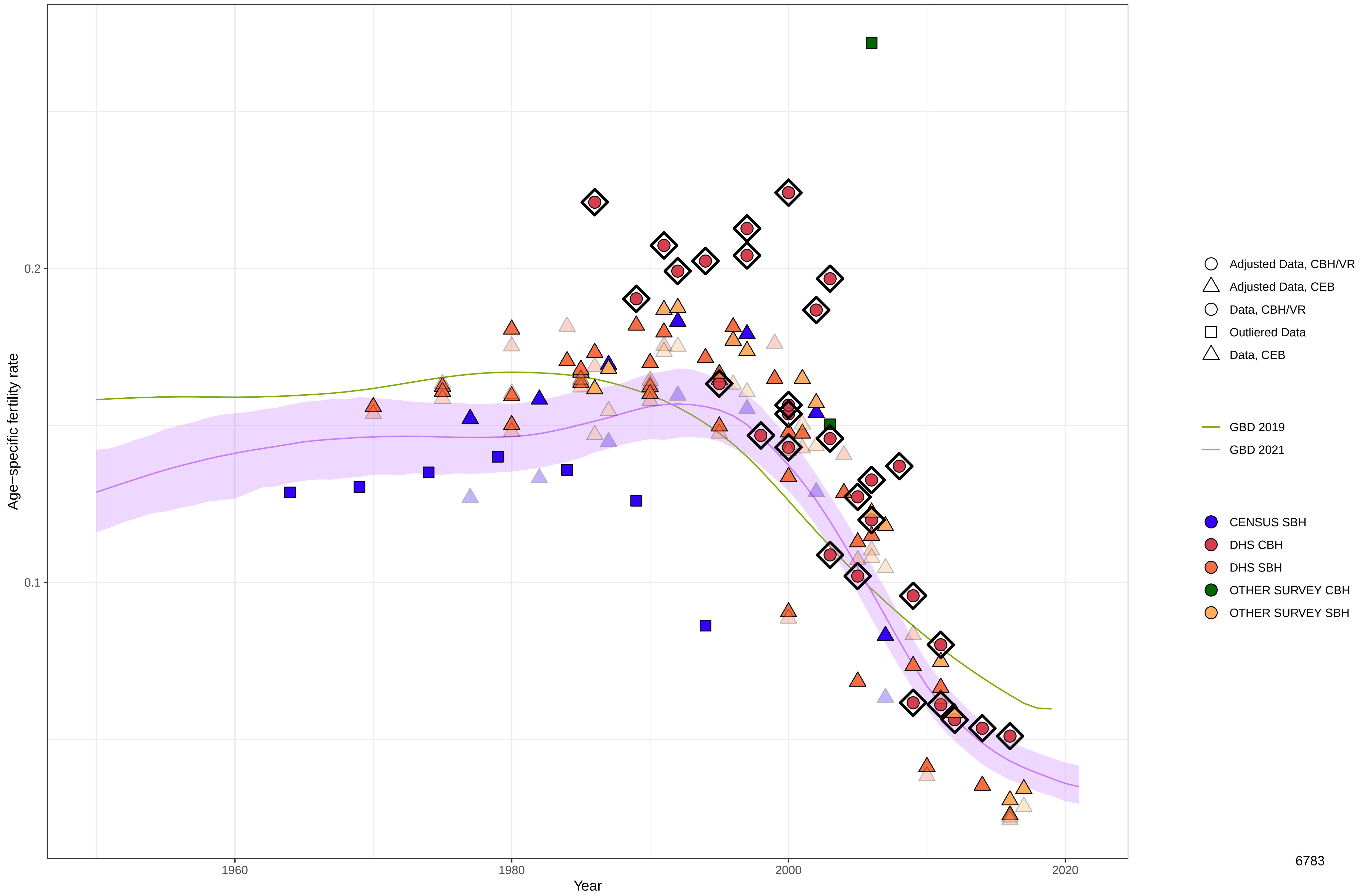

Age−specific fertility fitted trend lines, 1950−2021: Amhara, Ages: 20−24
Data points enclosed with a diamond are reference sources

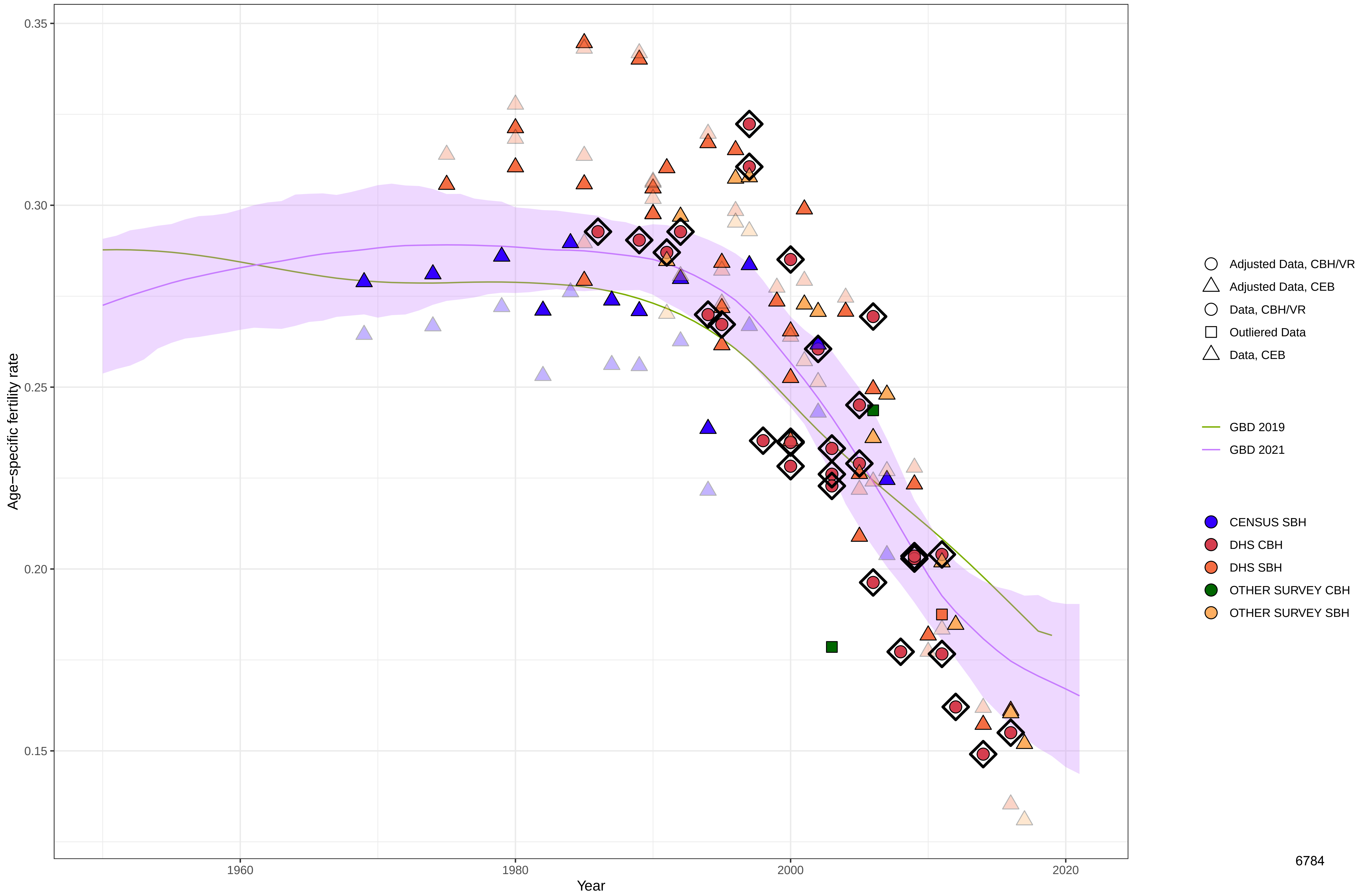

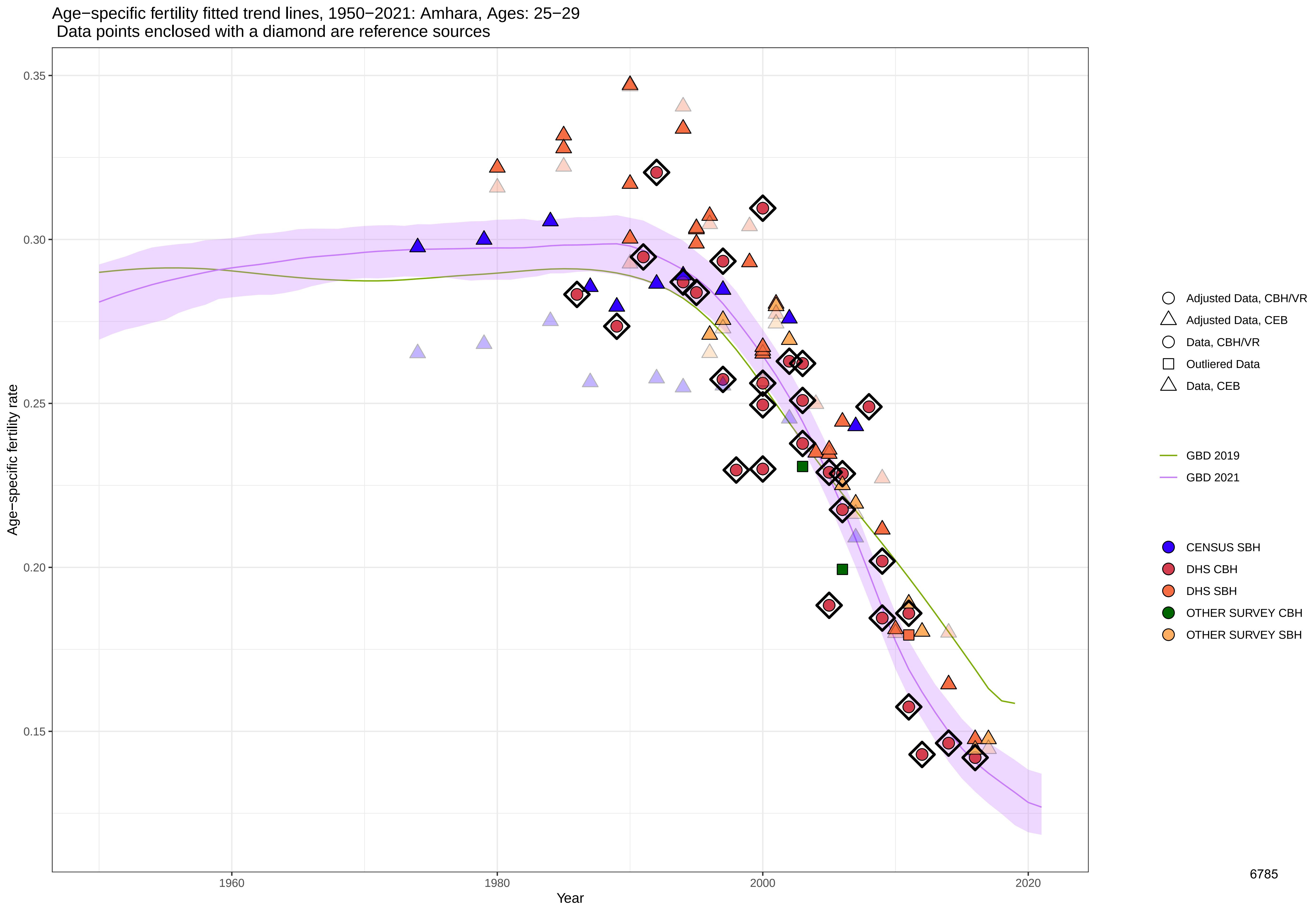

Age−specific fertility fitted trend lines, 1950−2021: Amhara, Ages: 25−29
Data points enclosed with a diamond are reference sources
0.35
0.30
0.25
0.20
0.15
1960
1980
2000
2020
Year
Age−specific fertility rate
Adjusted Data, CBH/VR
Adjusted Data, CEB
Data, CBH/VR
Outliered Data
Data, CEB
GBD 2019
GBD 2021
CENSUS SBH
DHS CBH
DHS SBH
OTHER SURVEY CBH
OTHER SURVEY SBH

Age−specific fertility fitted trend lines, 1950−2021: Amhara, Ages: 30−34
Data points enclosed with a diamond are reference sources

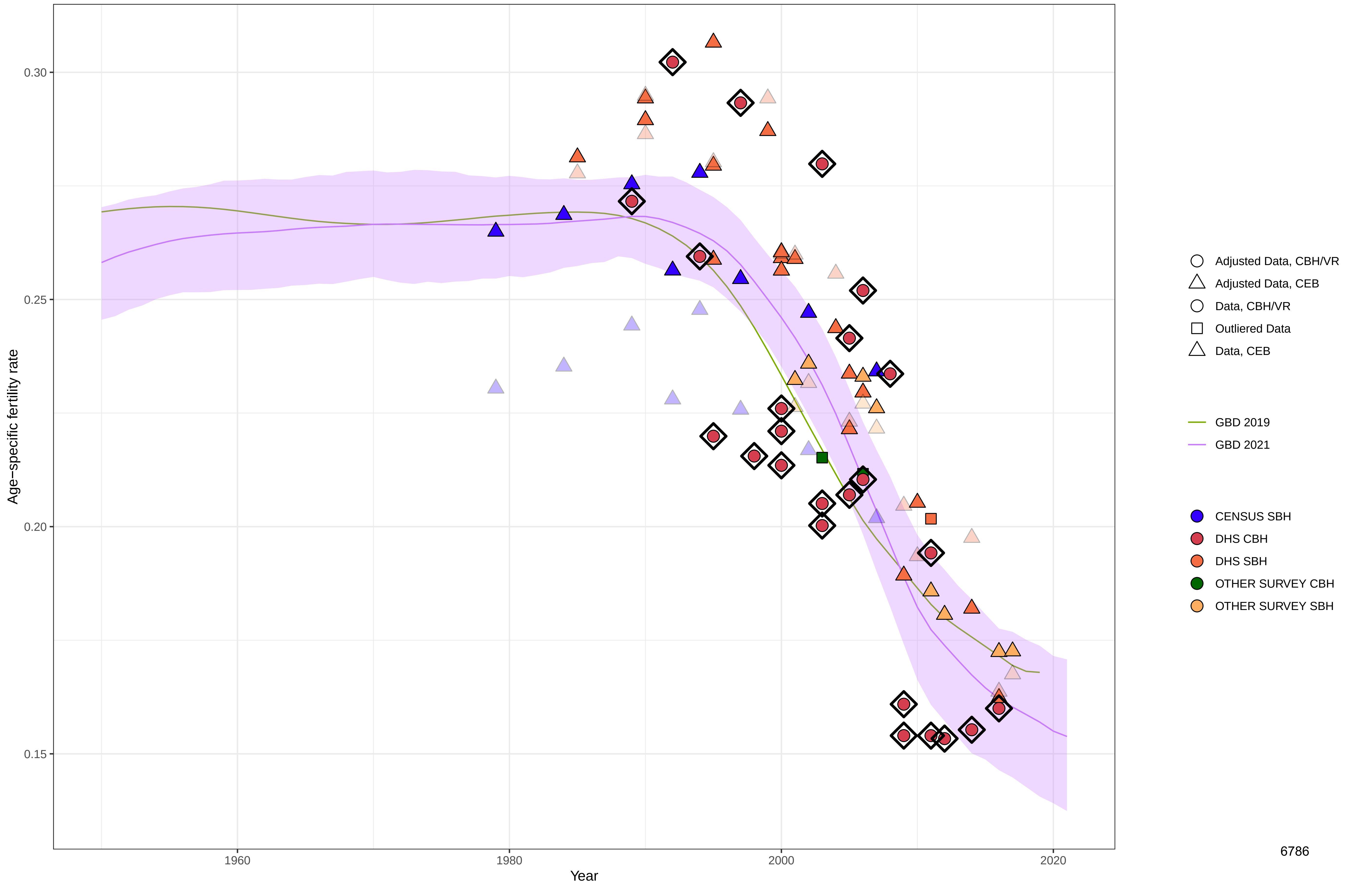

6786

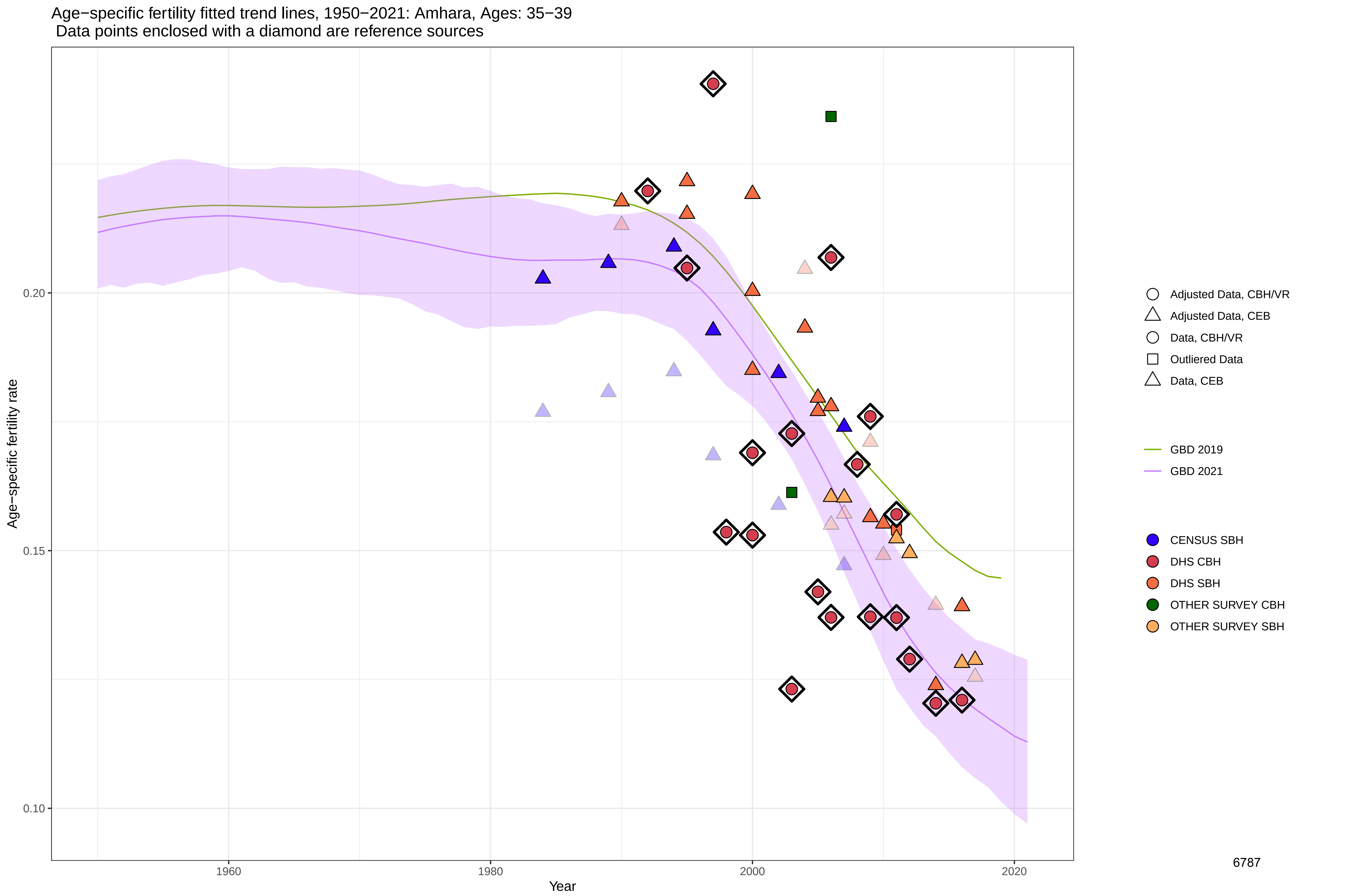

Age−specific fertility fitted trend lines, 1950−2021: Amhara, Ages: 35−39
Data points enclosed with a diamond are reference sources
Age−specific fertility rate
0.10
0.15
0.20
1960
1980
2000
2020
Year
Adjusted Data, CBH/VR
Adjusted Data, CEB
Data, CBH/VR
Outliered Data
Data, CEB
GBD 2019
GBD 2021
CENSUS SBH
DHS CBH
DHS SBH
OTHER SURVEY CBH
OTHER SURVEY SBH

Age−specific fertility fitted trend lines, 1950−2021: Amhara, Ages: 40−44
Data points enclosed with a diamond are reference sources

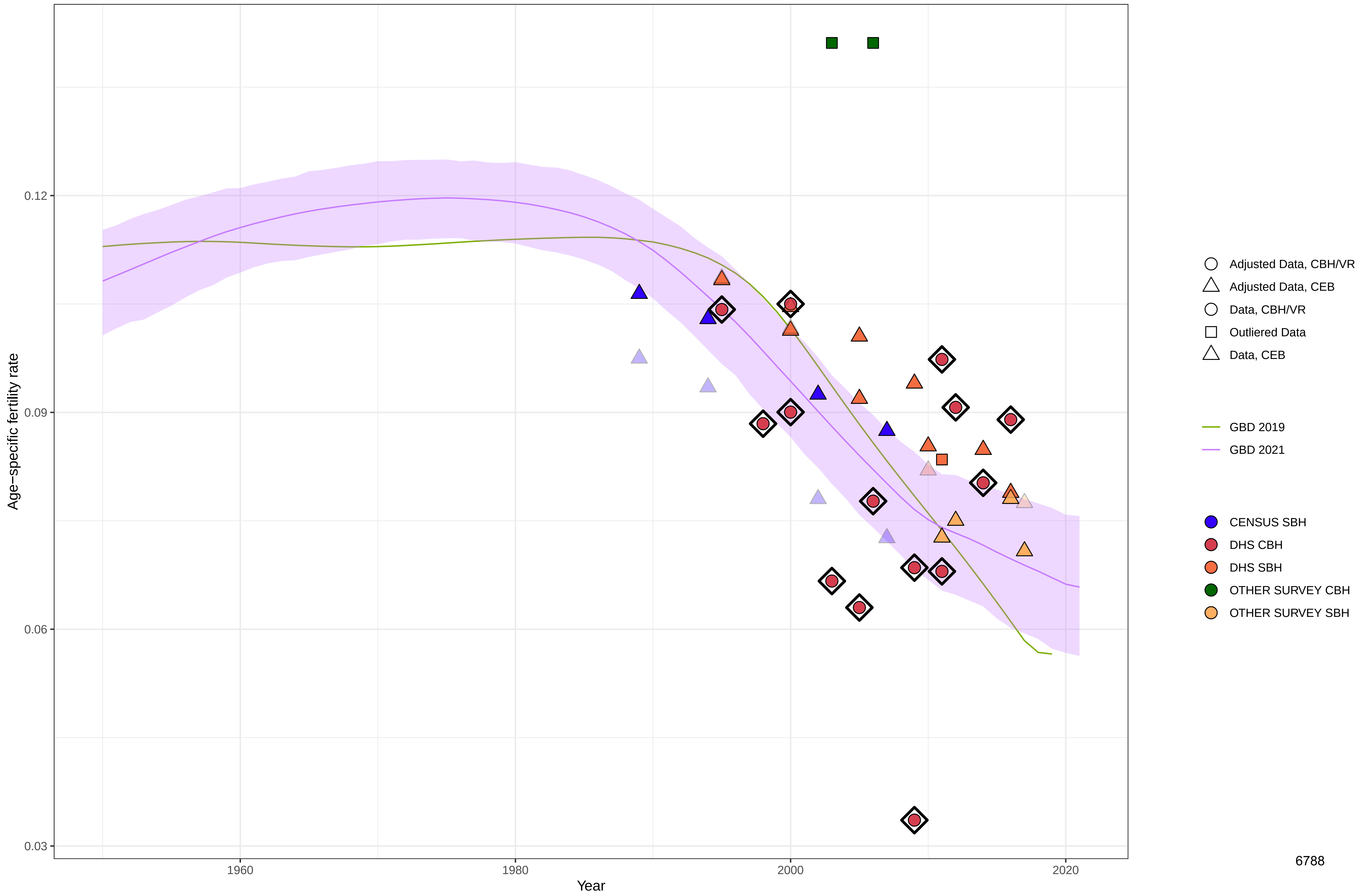

Age−specific fertility fitted trend lines, 1950−2021: Amhara, Ages: 45−49
Data points enclosed with a diamond are reference sources

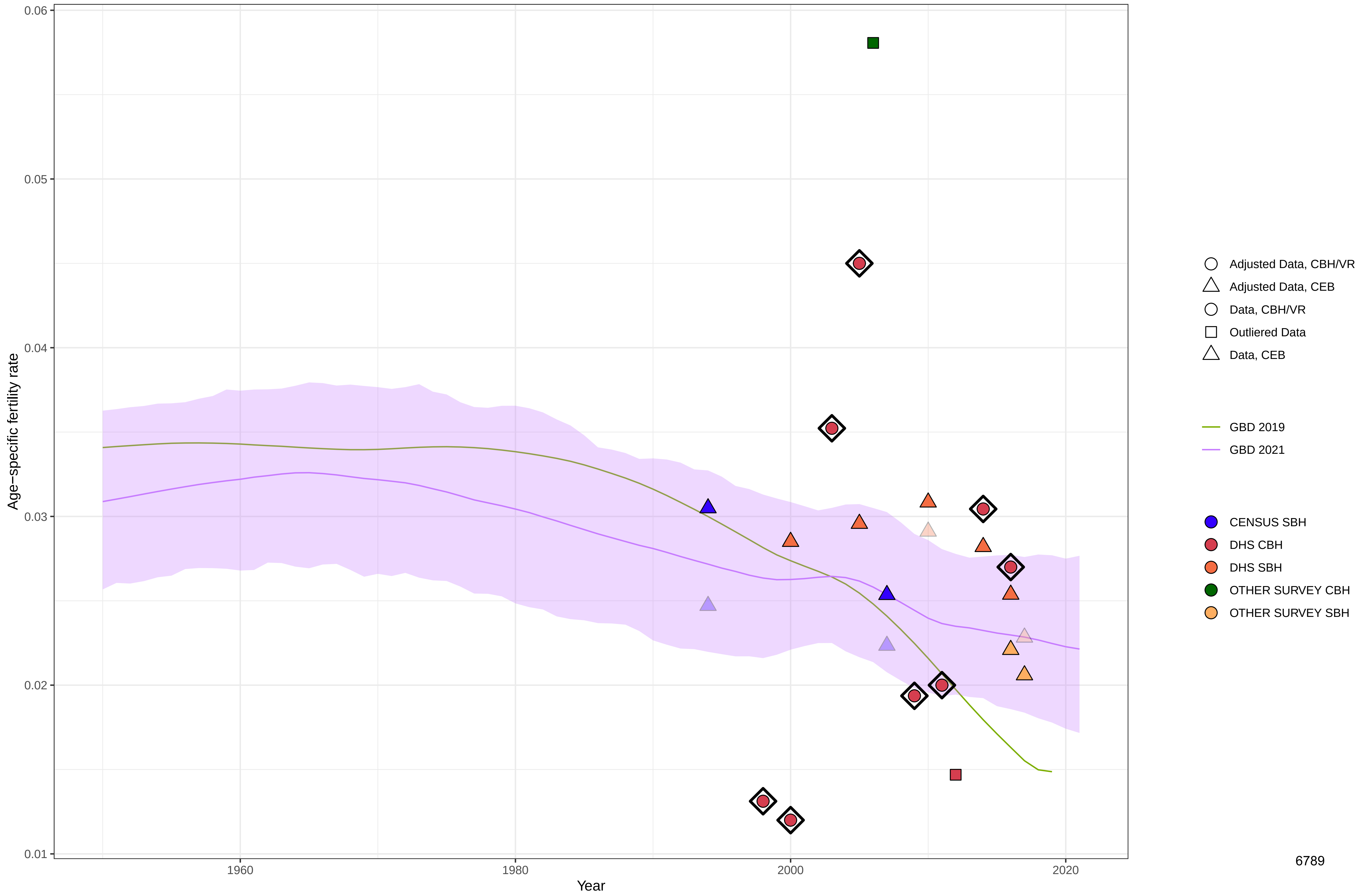

Age−specific fertility fitted trend lines, 1950−2021: Amhara, Ages: 50−54
Data points enclosed with a diamond are reference sources

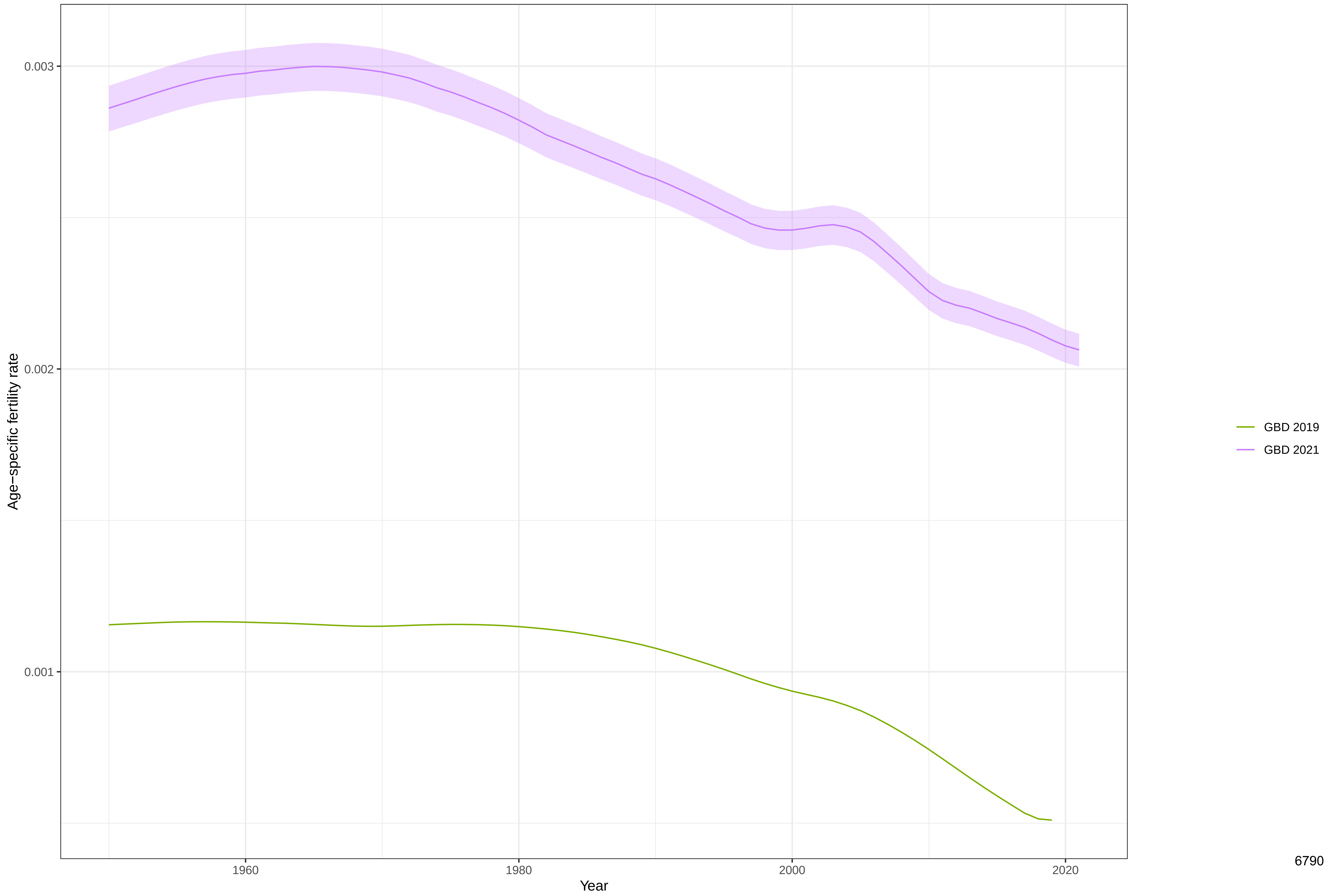

Total fertility rate fitted trend lines, 1950−2021: Amhara TFR
Data points enclosed with a diamond are reference sources

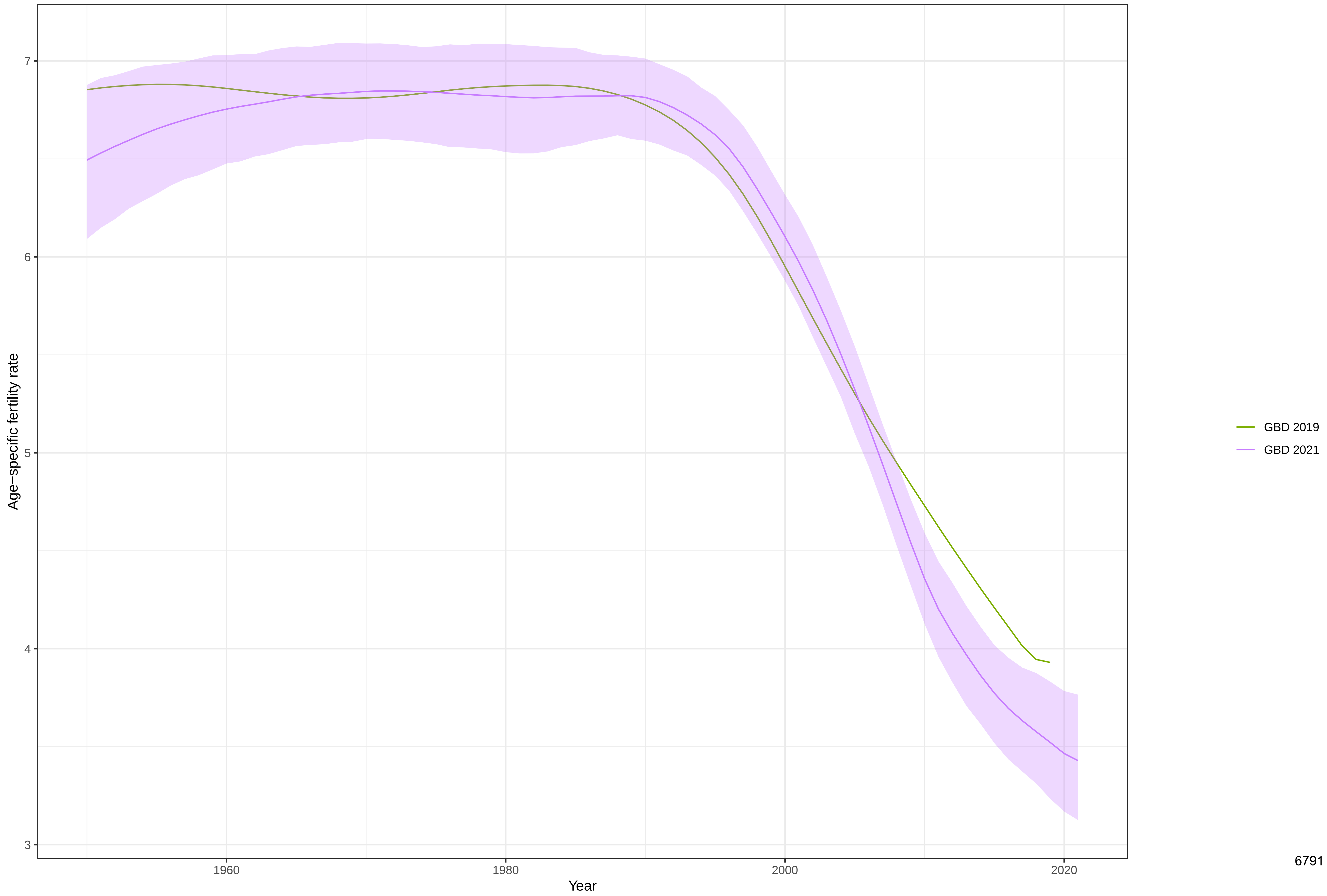

6791

Age−specific fertility fitted trend lines, 1950−2021: Benishangul−Gumuz, Ages: 10−14
Data points enclosed with a diamond are reference sources

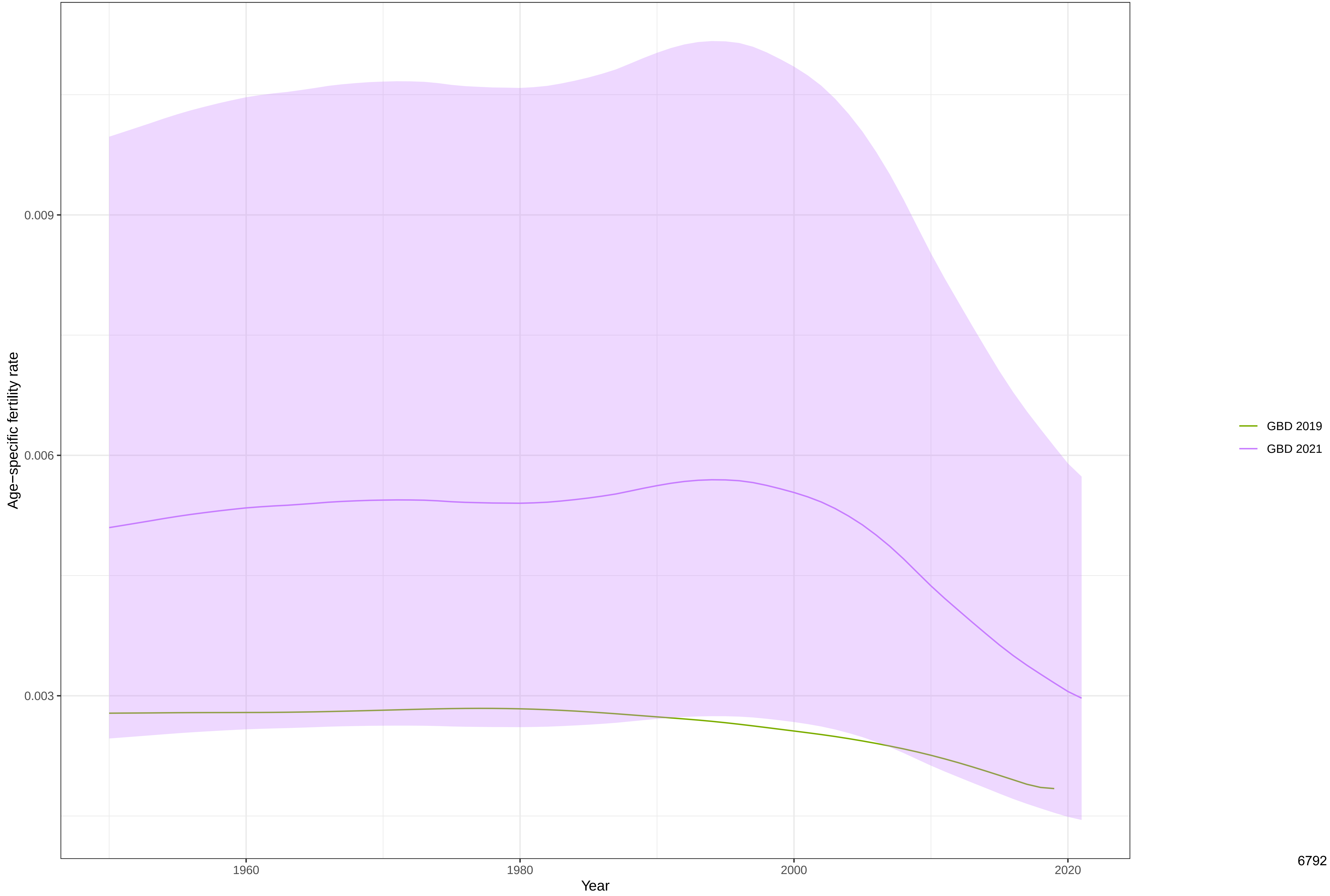

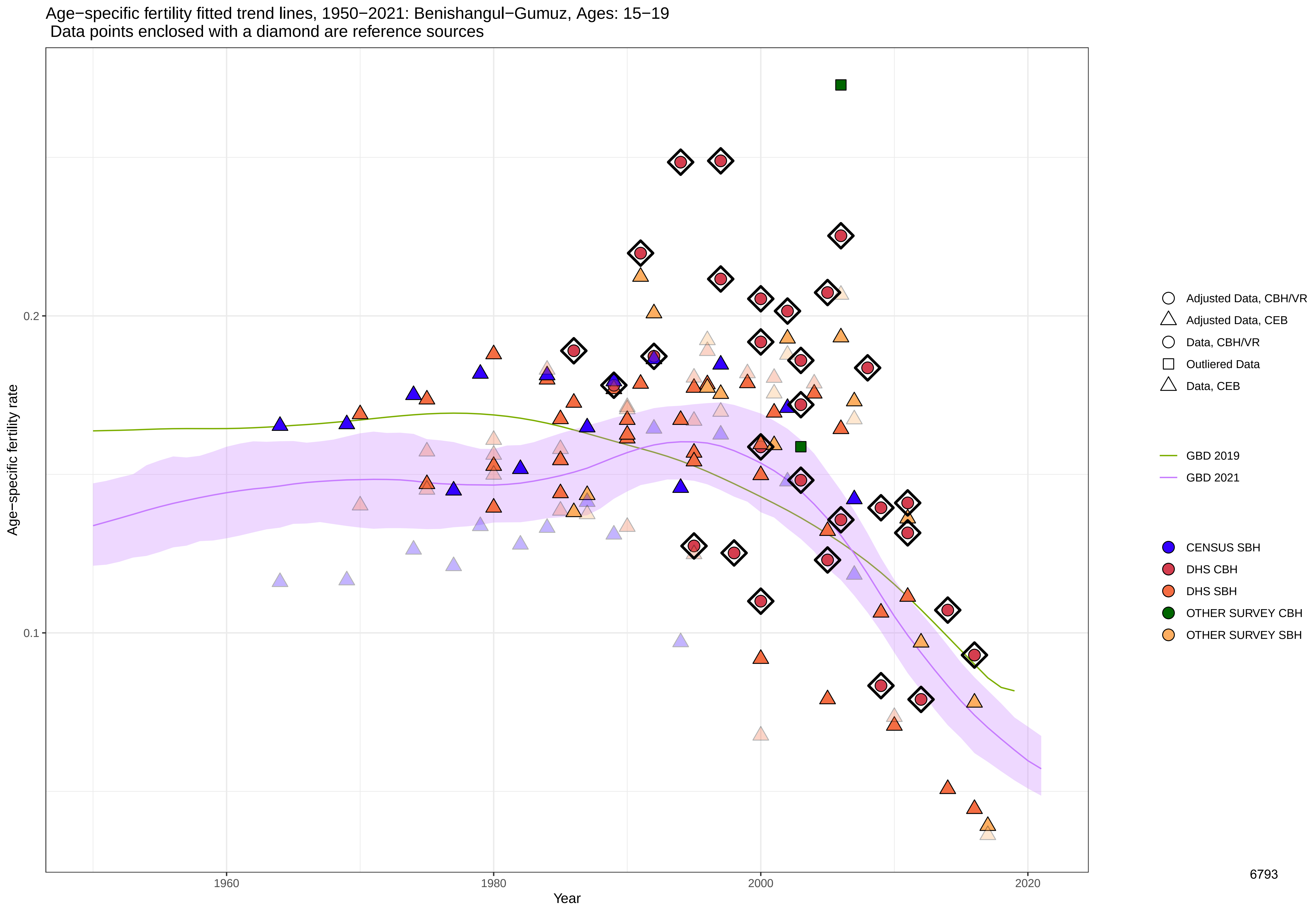

Age−specific fertility fitted trend lines, 1950−2021: Benishangul−Gumuz, Ages: 15−19
Data points enclosed with a diamond are reference sources
Age−specific fertility rate
0.1
0.2
1960
1980
2000
2020
Year
Adjusted Data, CBH/VR
Adjusted Data, CEB
Data, CBH/VR
Outliered Data
Data, CEB
GBD 2019
GBD 2021
CENSUS SBH
DHS CBH
DHS SBH
OTHER SURVEY CBH
OTHER SURVEY SBH

Age−specific fertility fitted trend lines, 1950−2021: Benishangul−Gumuz, Ages: 20−24
Data points enclosed with a diamond are reference sources

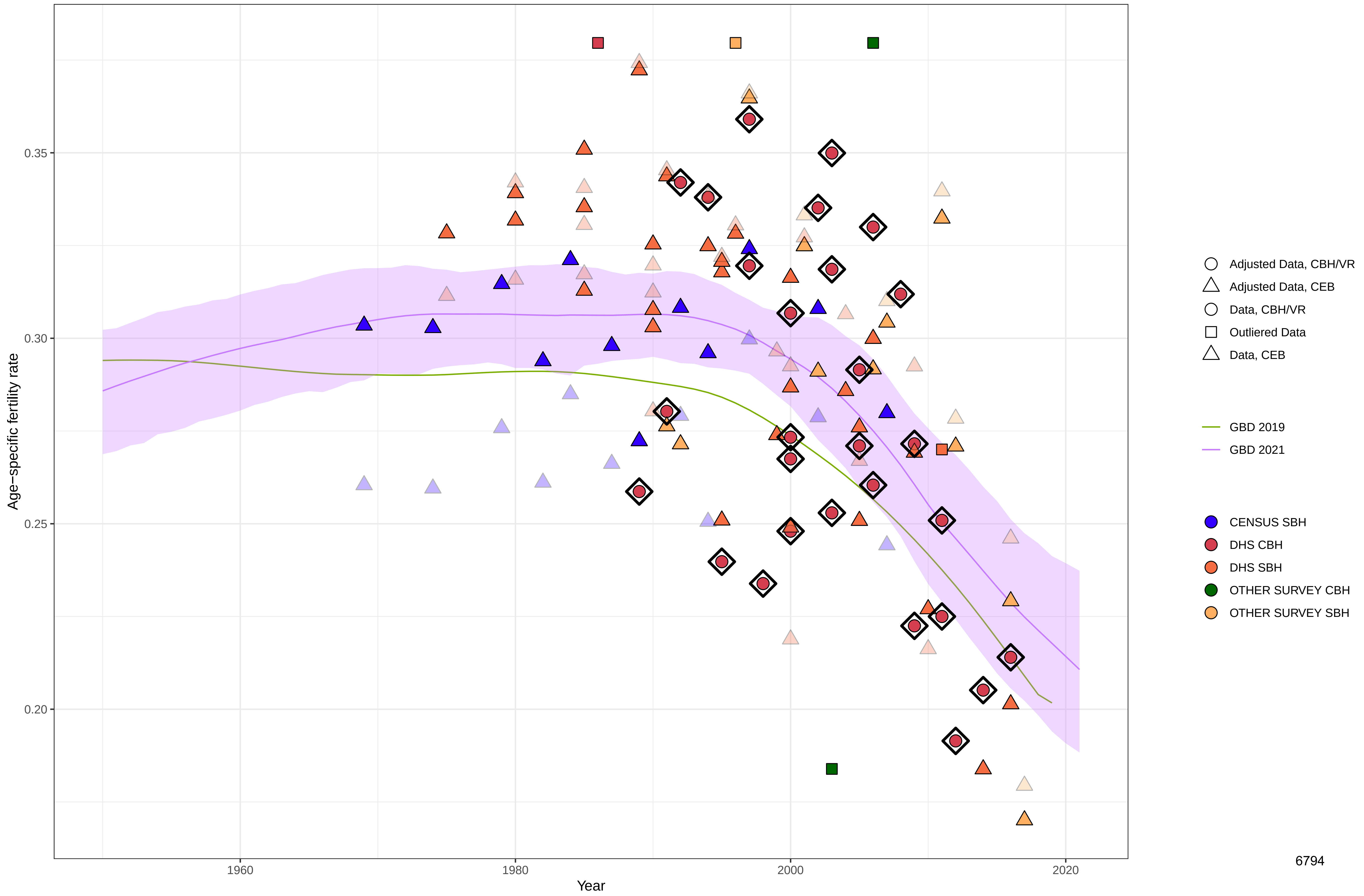

Age−specific fertility fitted trend lines, 1950−2021: Benishangul−Gumuz, Ages: 25−29
Data points enclosed with a diamond are reference sources

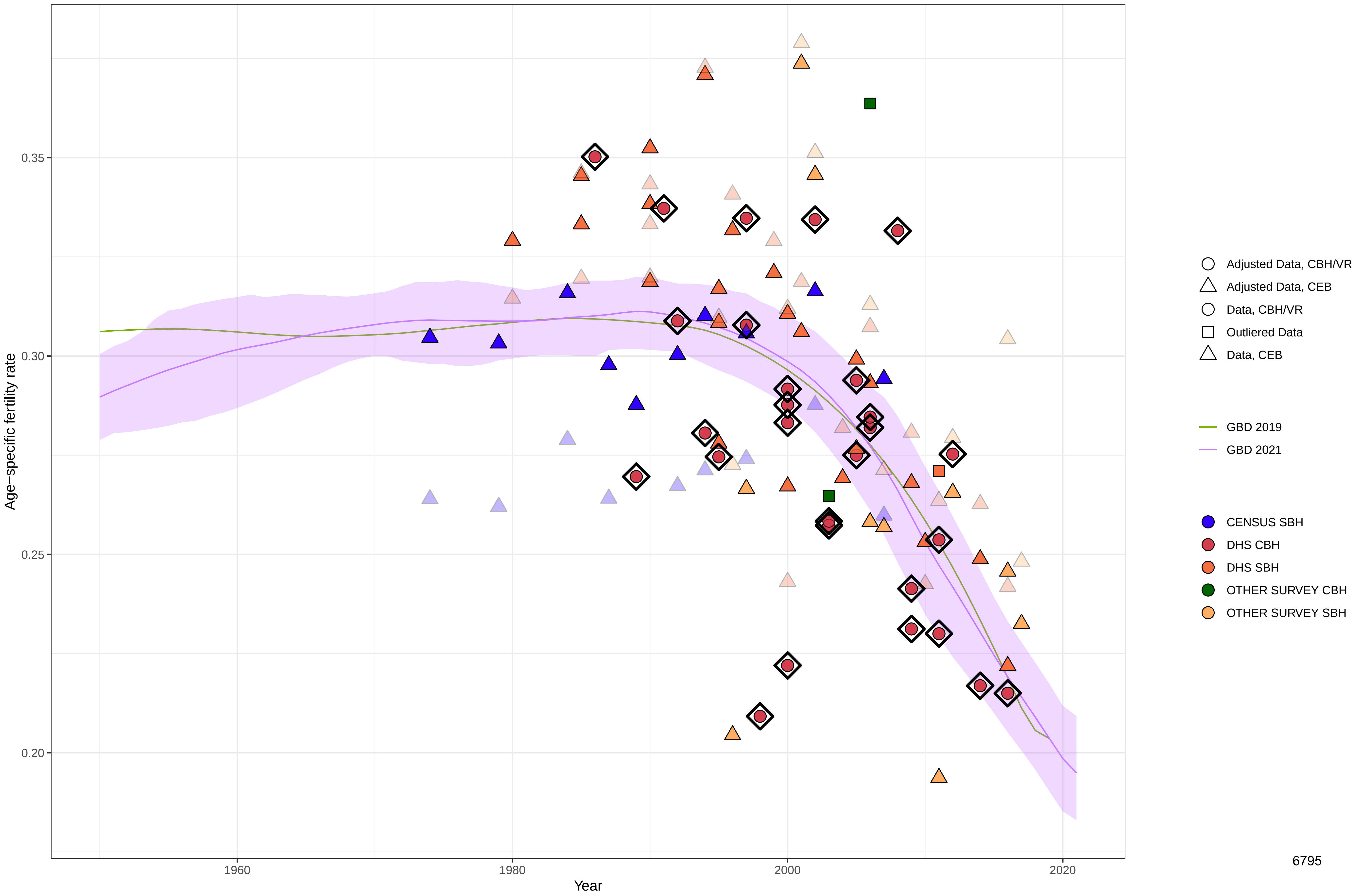

Age−specific fertility fitted trend lines, 1950−2021: Benishangul−Gumuz, Ages: 30−34
Data points enclosed with a diamond are reference sources

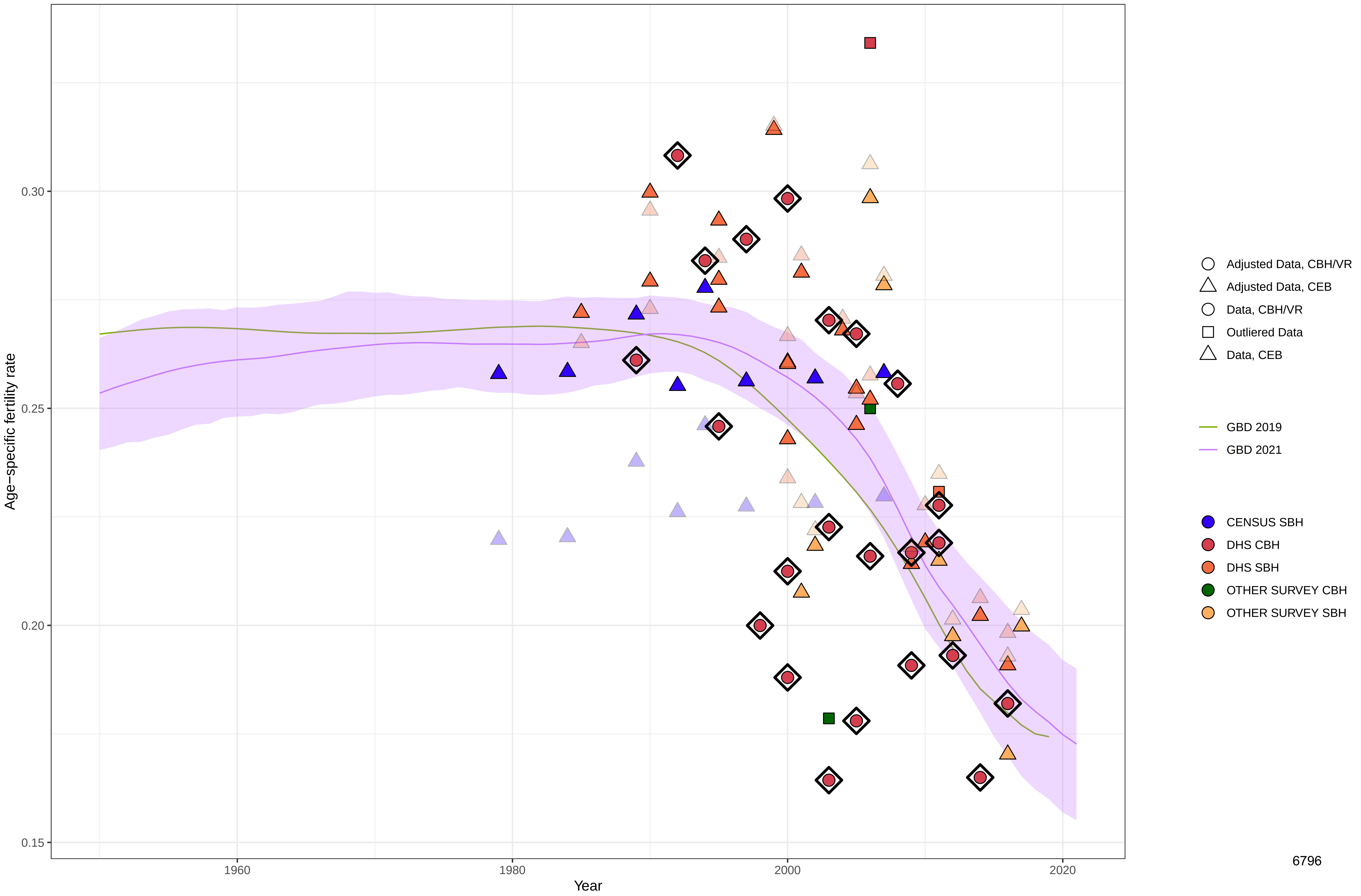

Age−specific fertility fitted trend lines, 1950−2021: Benishangul−Gumuz, Ages: 35−39
Data points enclosed with a diamond are reference sources

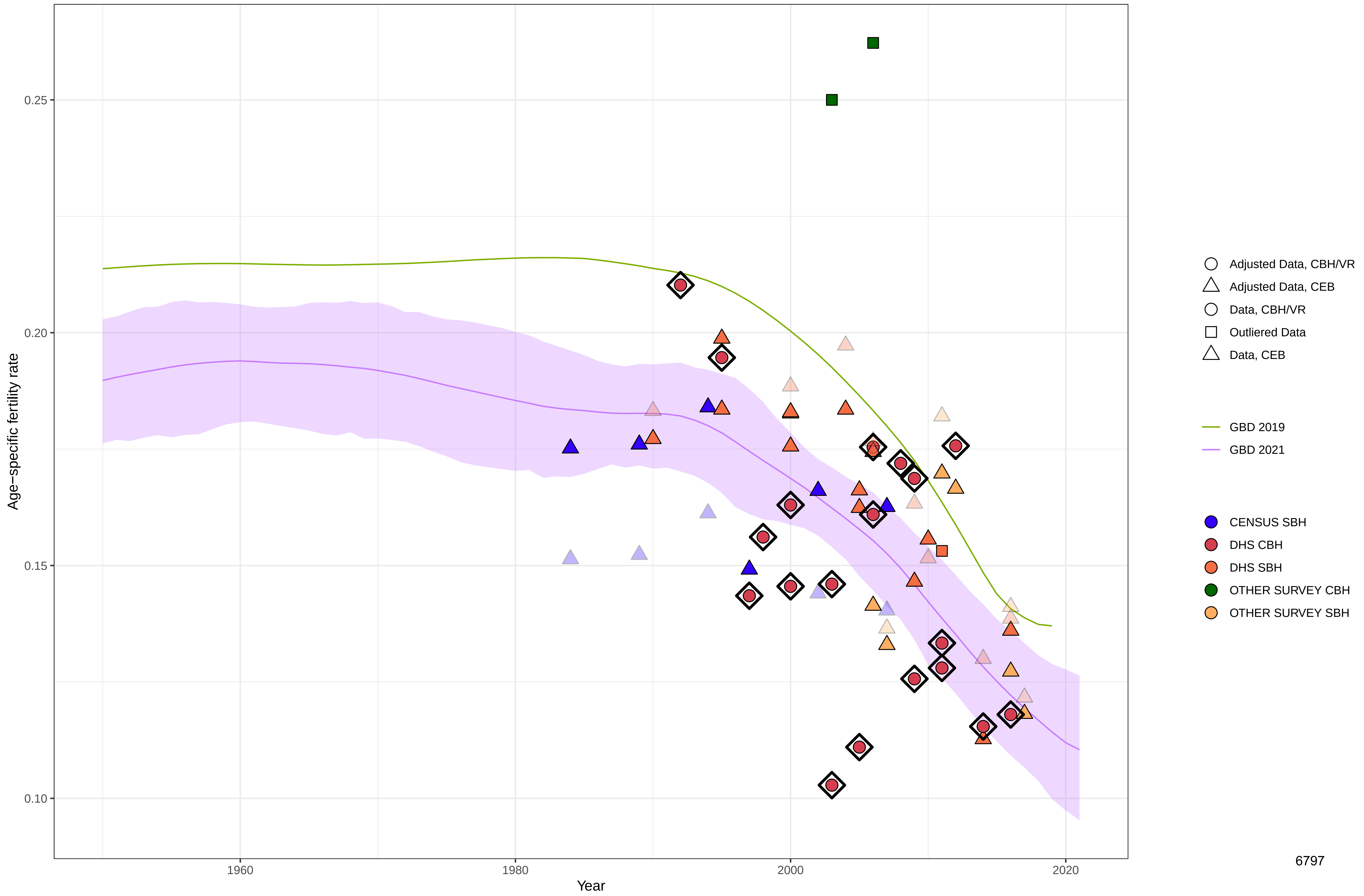

Age−specific fertility fitted trend lines, 1950−2021: Benishangul−Gumuz, Ages: 40−44
Data points enclosed with a diamond are reference sources

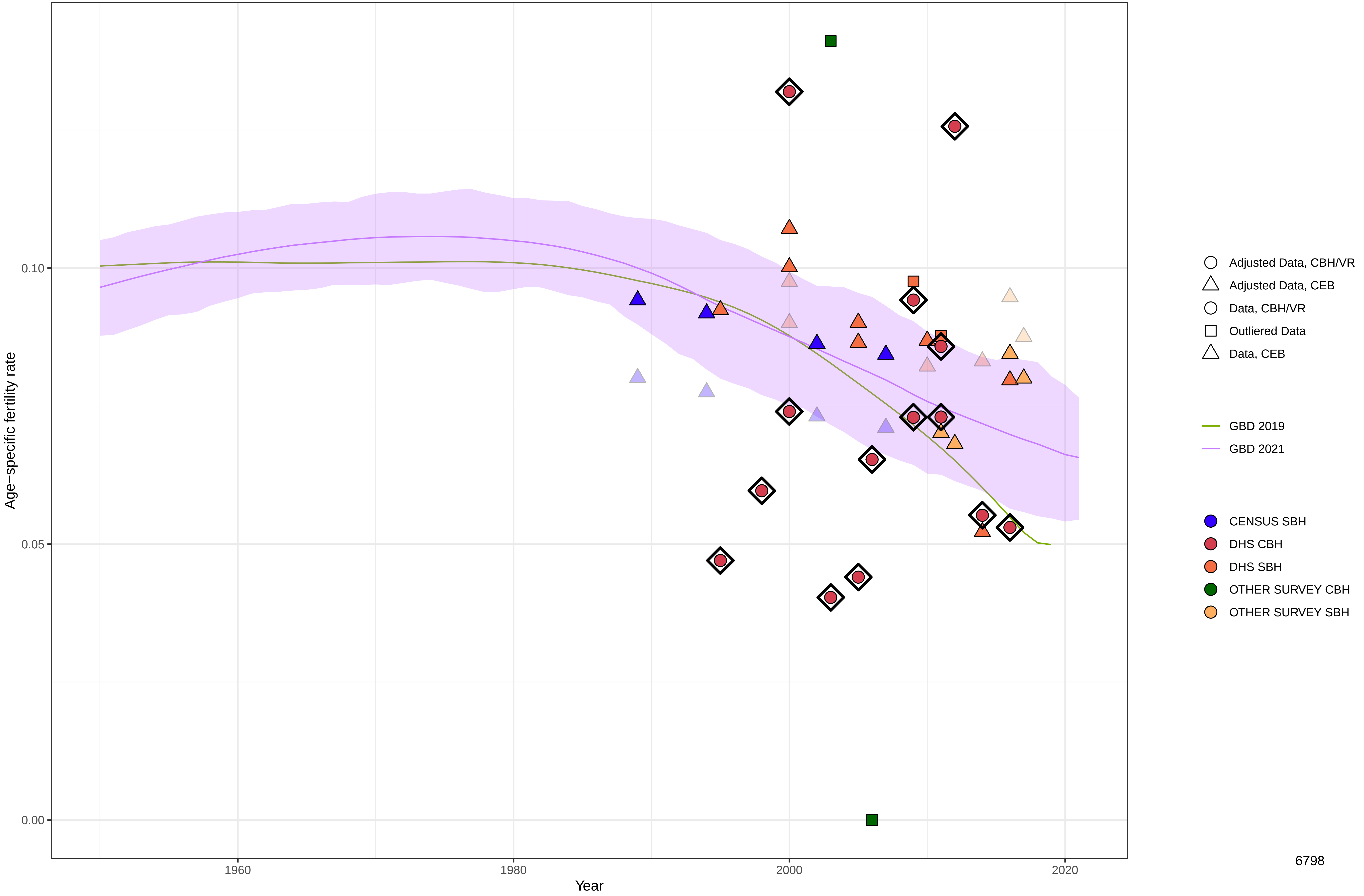

Age−specific fertility fitted trend lines, 1950−2021: Benishangul−Gumuz, Ages: 45−49
Data points enclosed with a diamond are reference sources

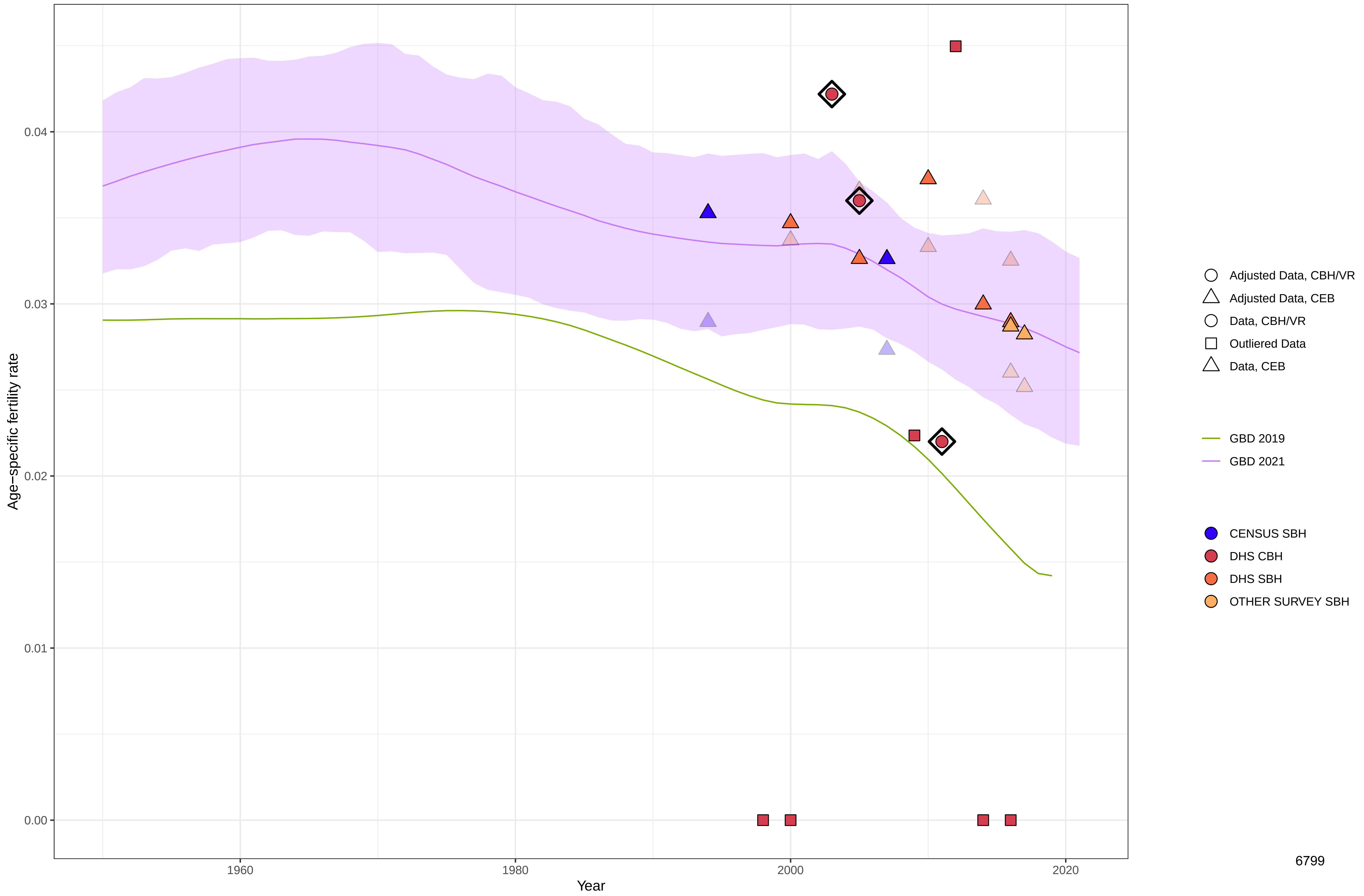

Age−specific fertility fitted trend lines, 1950−2021: Benishangul−Gumuz, Ages: 50−54
Data points enclosed with a diamond are reference sources

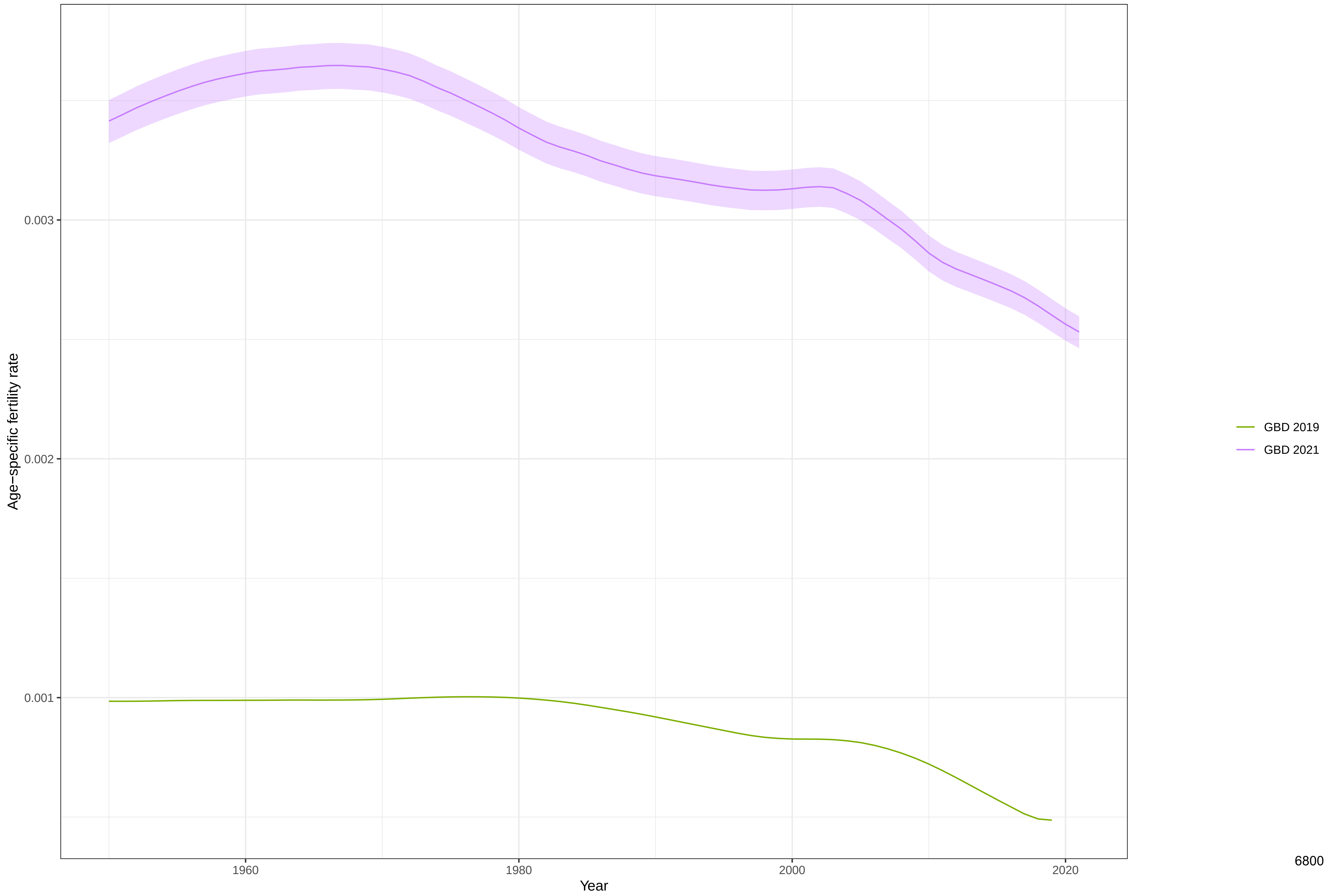

Total fertility rate fitted trend lines, 1950−2021: Benishangul−Gumuz TFR
Data points enclosed with a diamond are reference sources

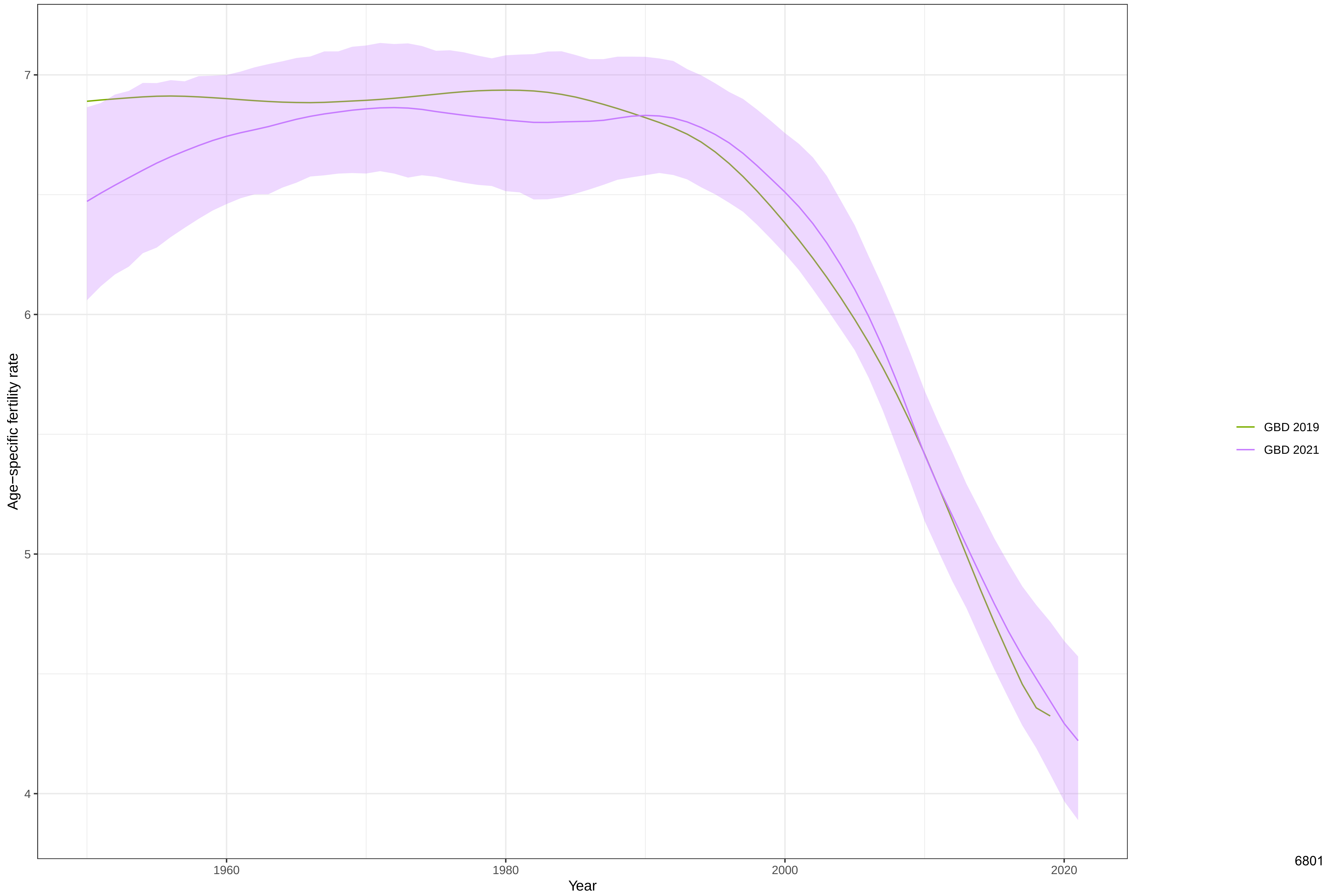

Age−specific fertility fitted trend lines, 1950−2021: Dire Dawa, Ages: 10−14
Data points enclosed with a diamond are reference sources

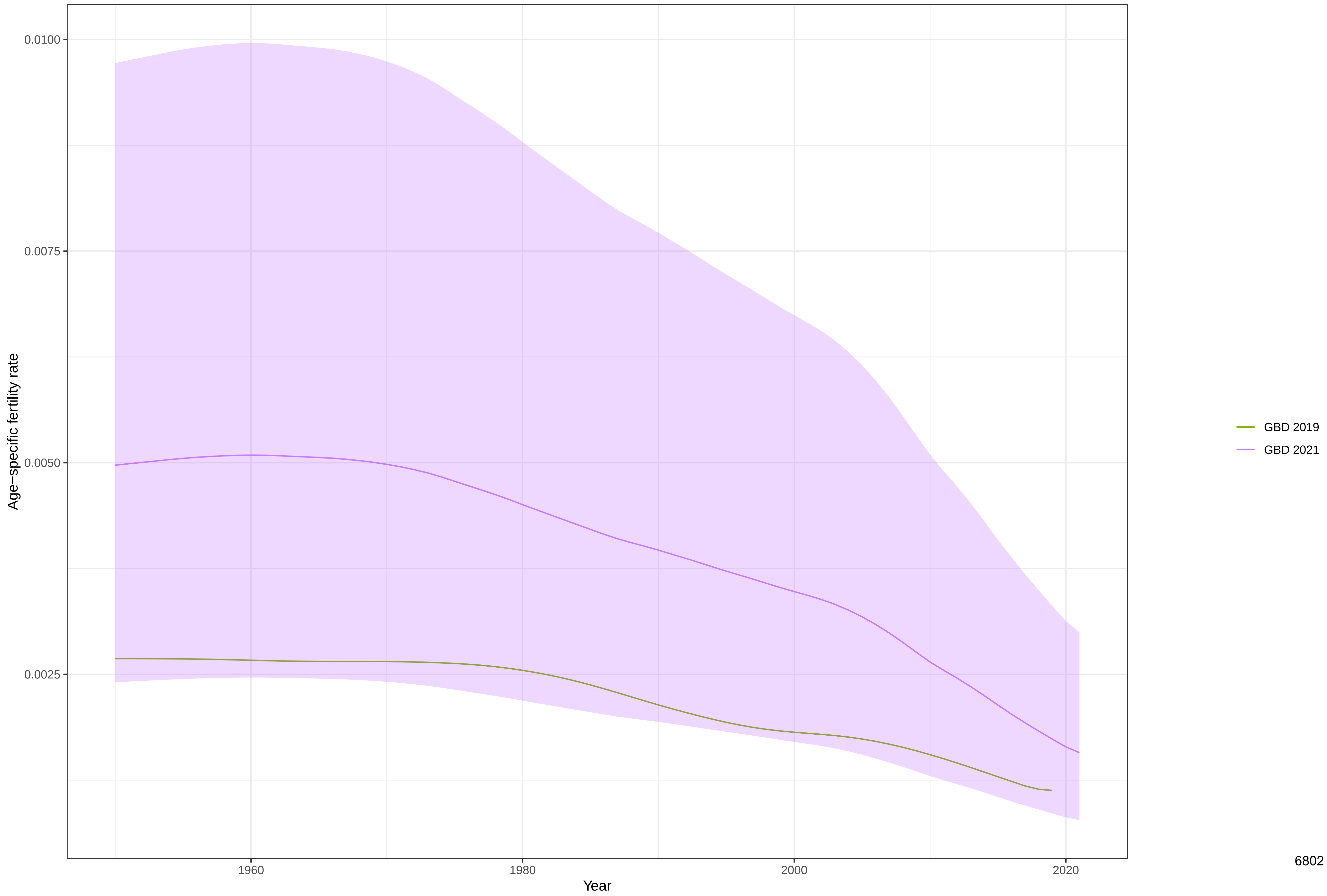

Age−specific fertility fitted trend lines, 1950−2021: Dire Dawa, Ages: 15−19
Data points enclosed with a diamond are reference sources

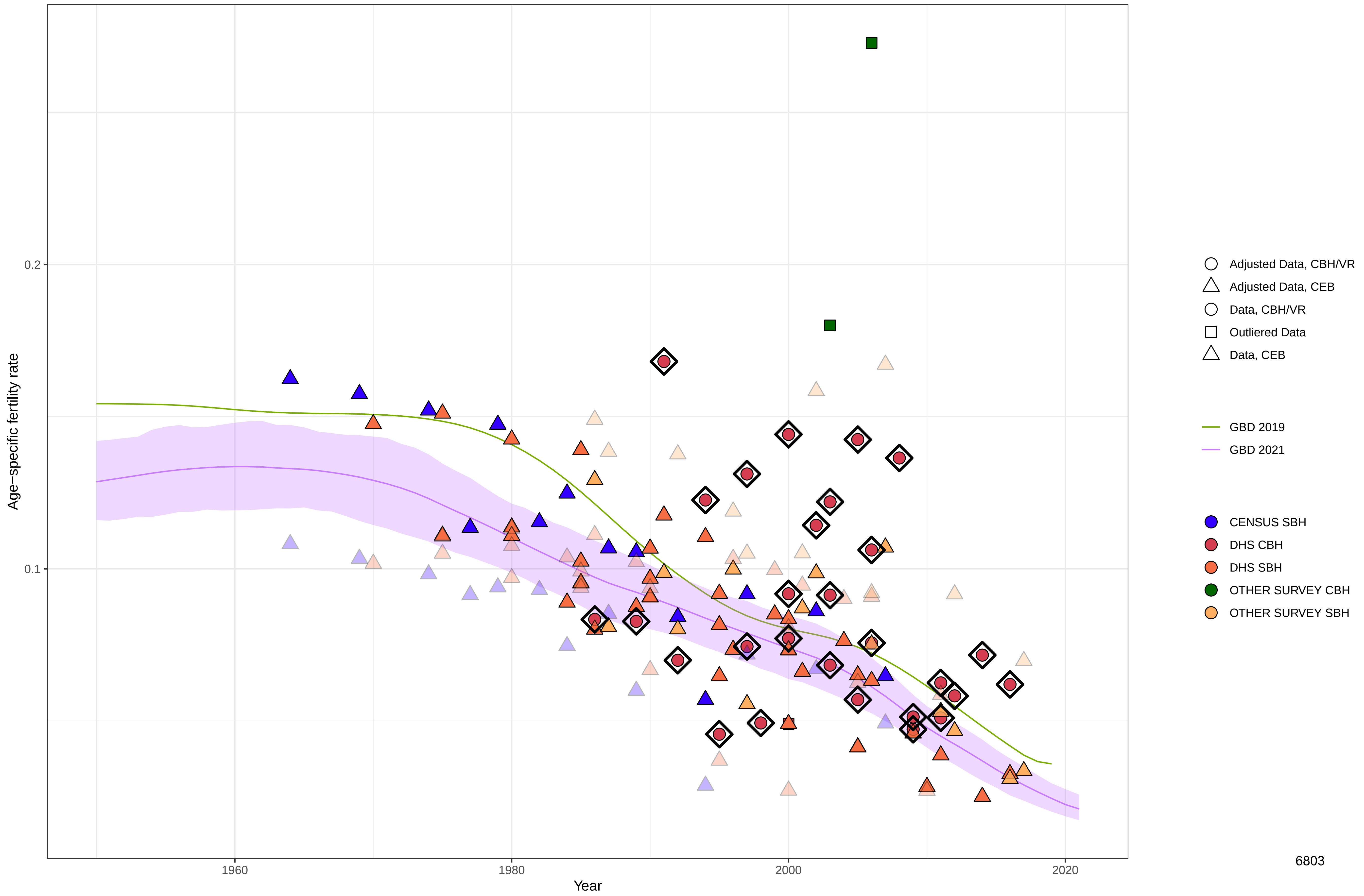

Age−specific fertility fitted trend lines, 1950−2021: Dire Dawa, Ages: 20−24
Data points enclosed with a diamond are reference sources

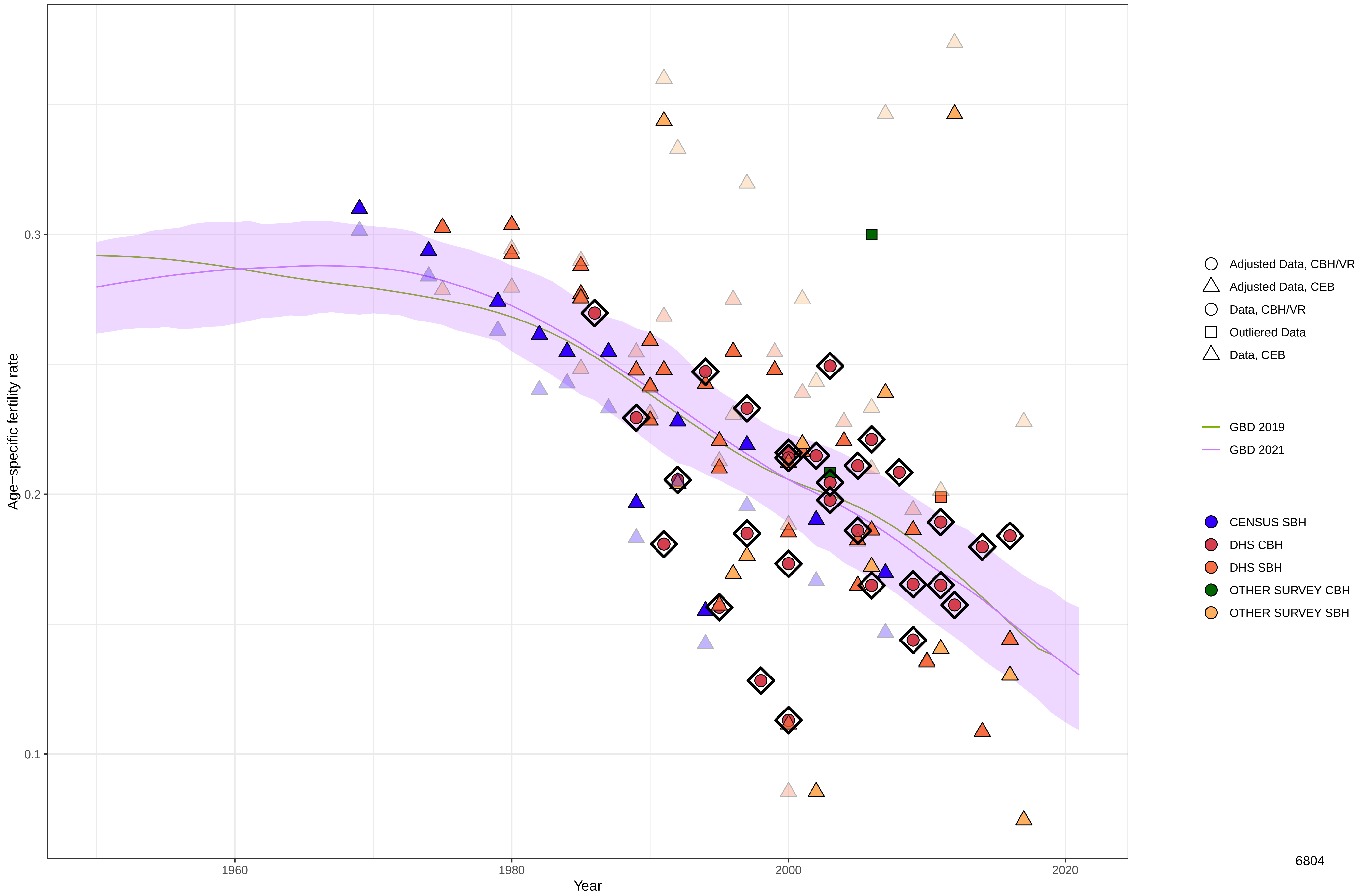

Age−specific fertility fitted trend lines, 1950−2021: Dire Dawa, Ages: 25−29
Data points enclosed with a diamond are reference sources

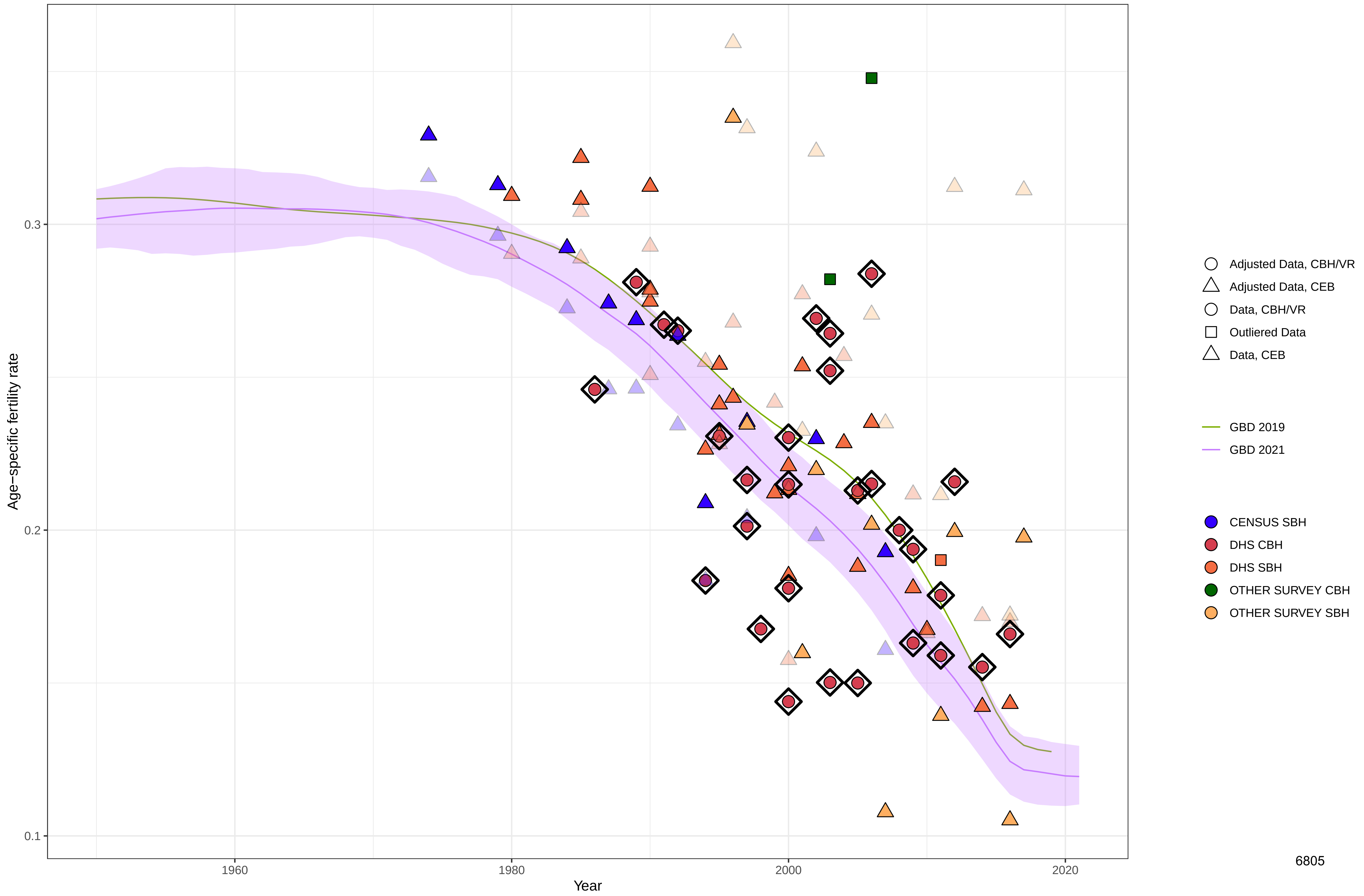

Age−specific fertility fitted trend lines, 1950−2021: Dire Dawa, Ages: 30−34
Data points enclosed with a diamond are reference sources

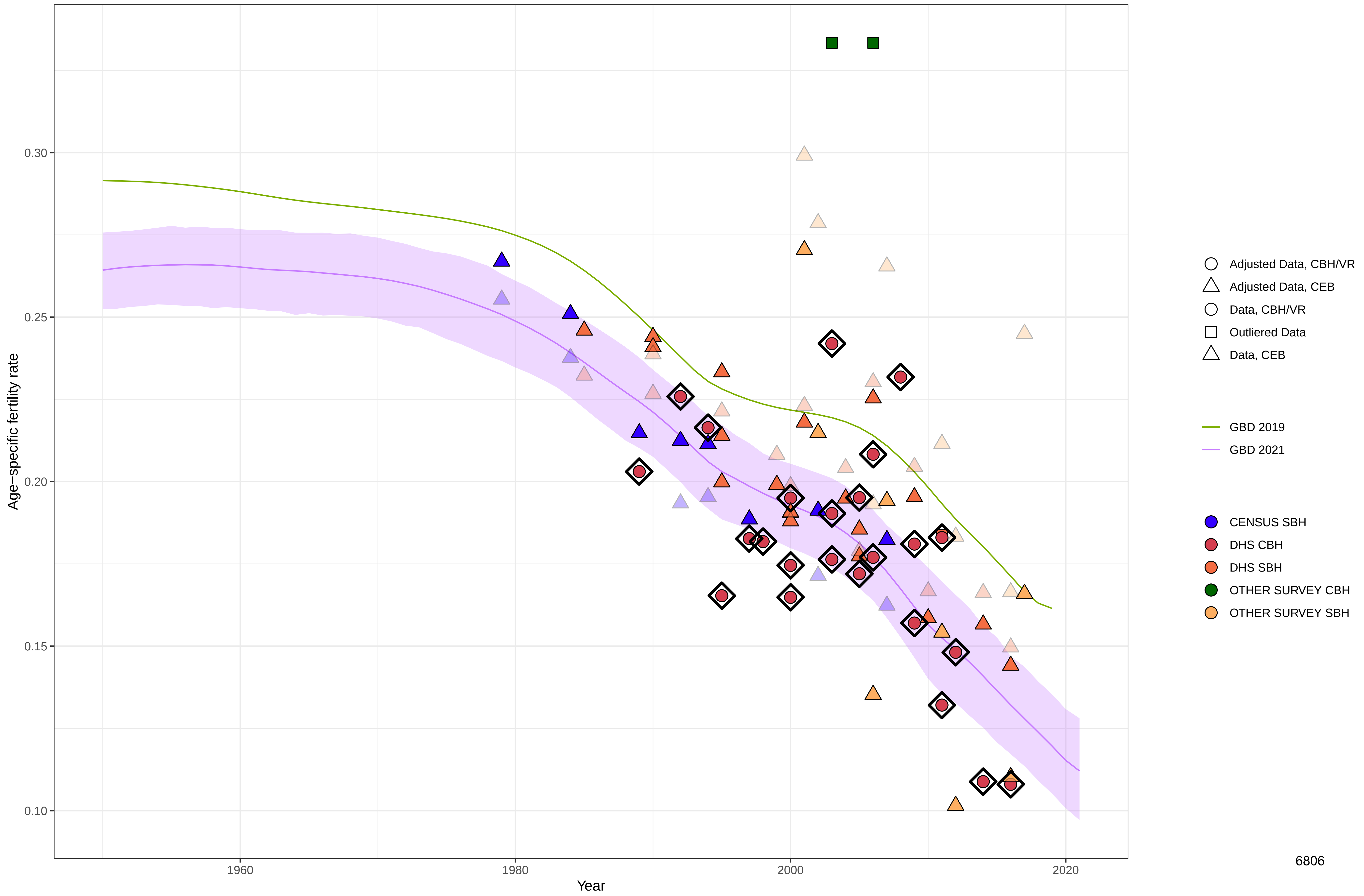

Age−specific fertility fitted trend lines, 1950−2021: Dire Dawa, Ages: 35−39
Data points enclosed with a diamond are reference sources

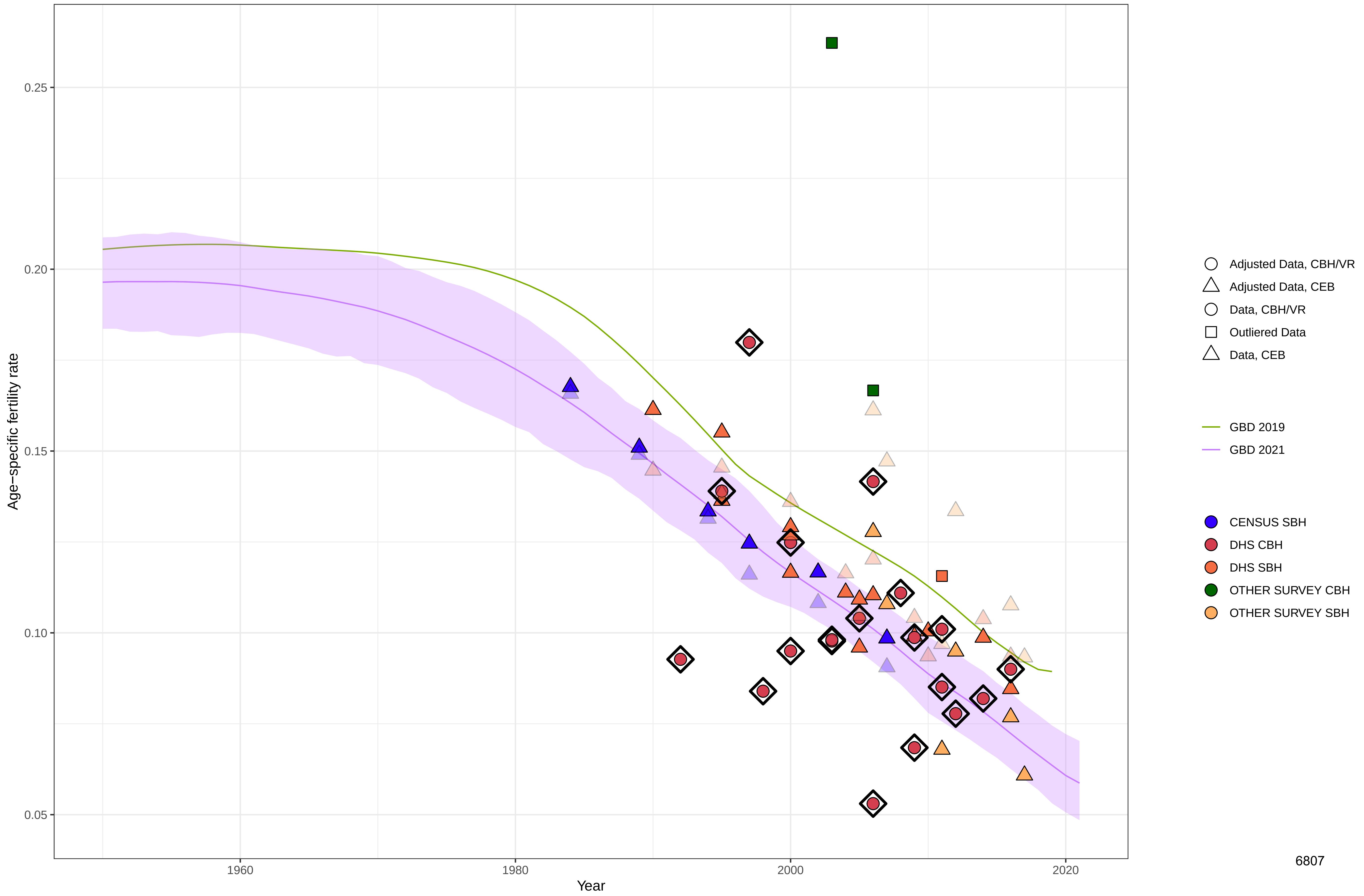

Age−specific fertility fitted trend lines, 1950−2021: Dire Dawa, Ages: 40−44
Data points enclosed with a diamond are reference sources

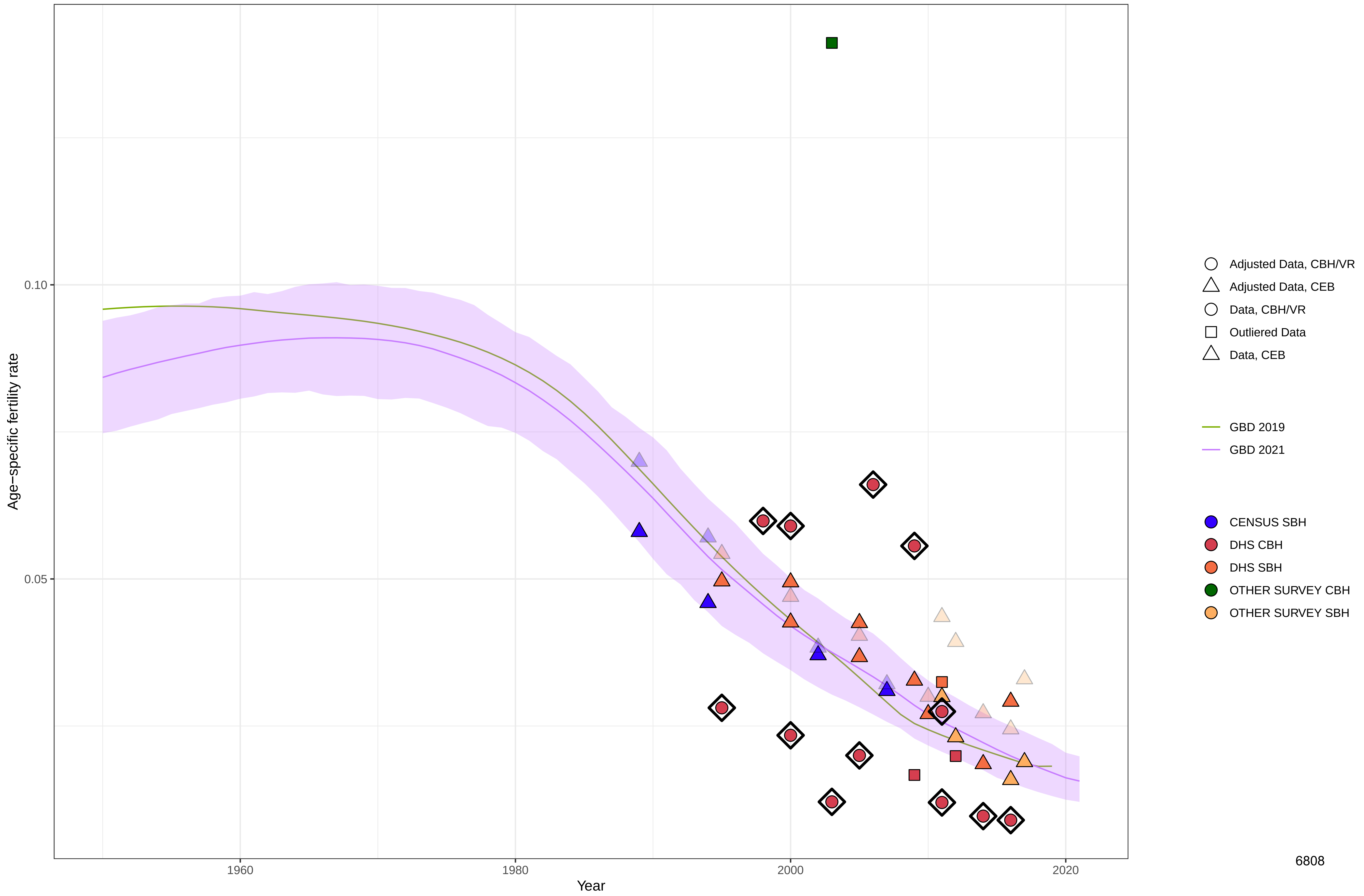

6808

Age−specific fertility fitted trend lines, 1950−2021: Dire Dawa, Ages: 45−49
Data points enclosed with a diamond are reference sources

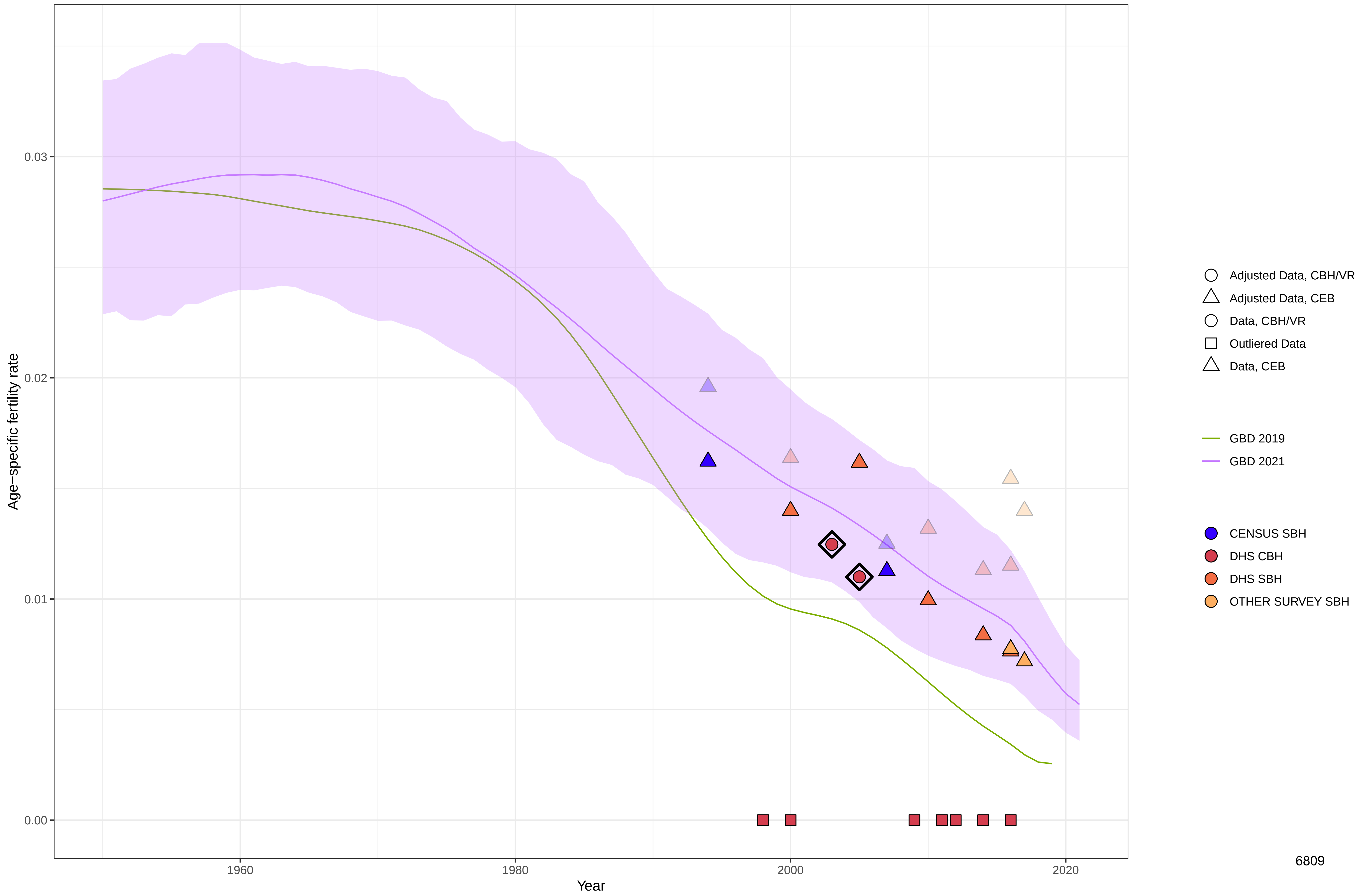

Age−specific fertility fitted trend lines, 1950−2021: Dire Dawa, Ages: 50−54
Data points enclosed with a diamond are reference sources

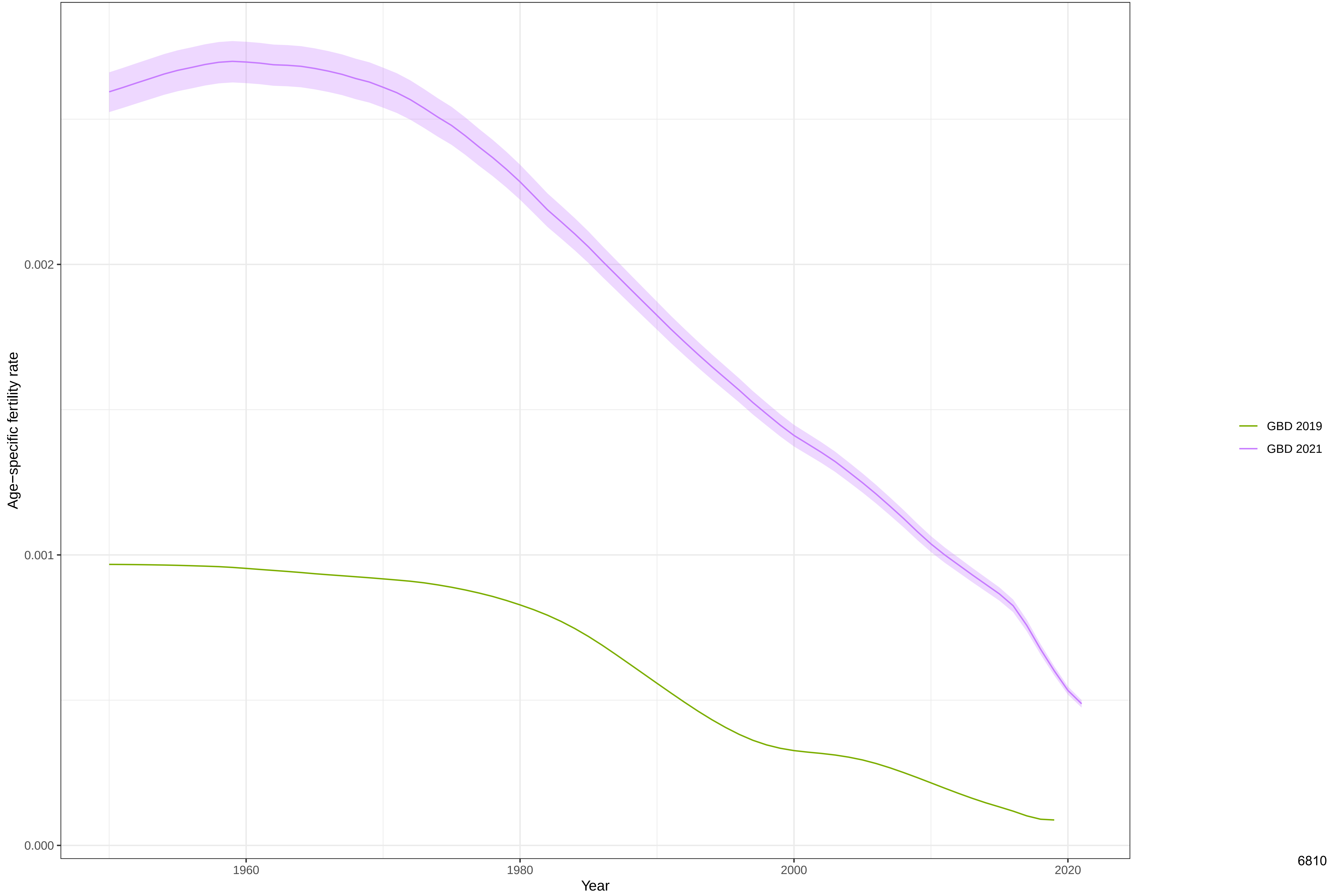

Total fertility rate fitted trend lines, 1950−2021: Dire Dawa TFR
Data points enclosed with a diamond are reference sources

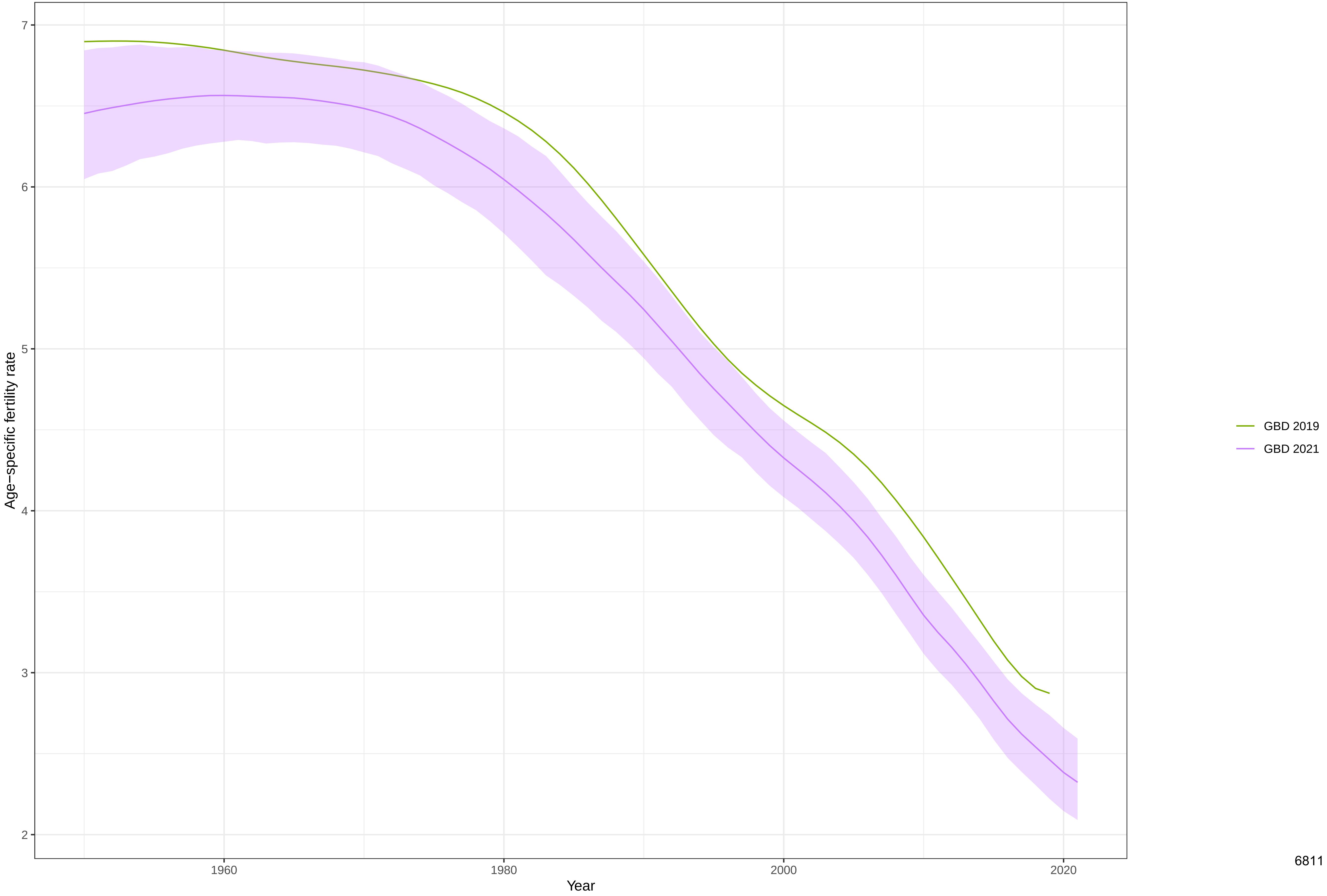

Age−specific fertility fitted trend lines, 1950−2021: Gambella, Ages: 10−14
Data points enclosed with a diamond are reference sources

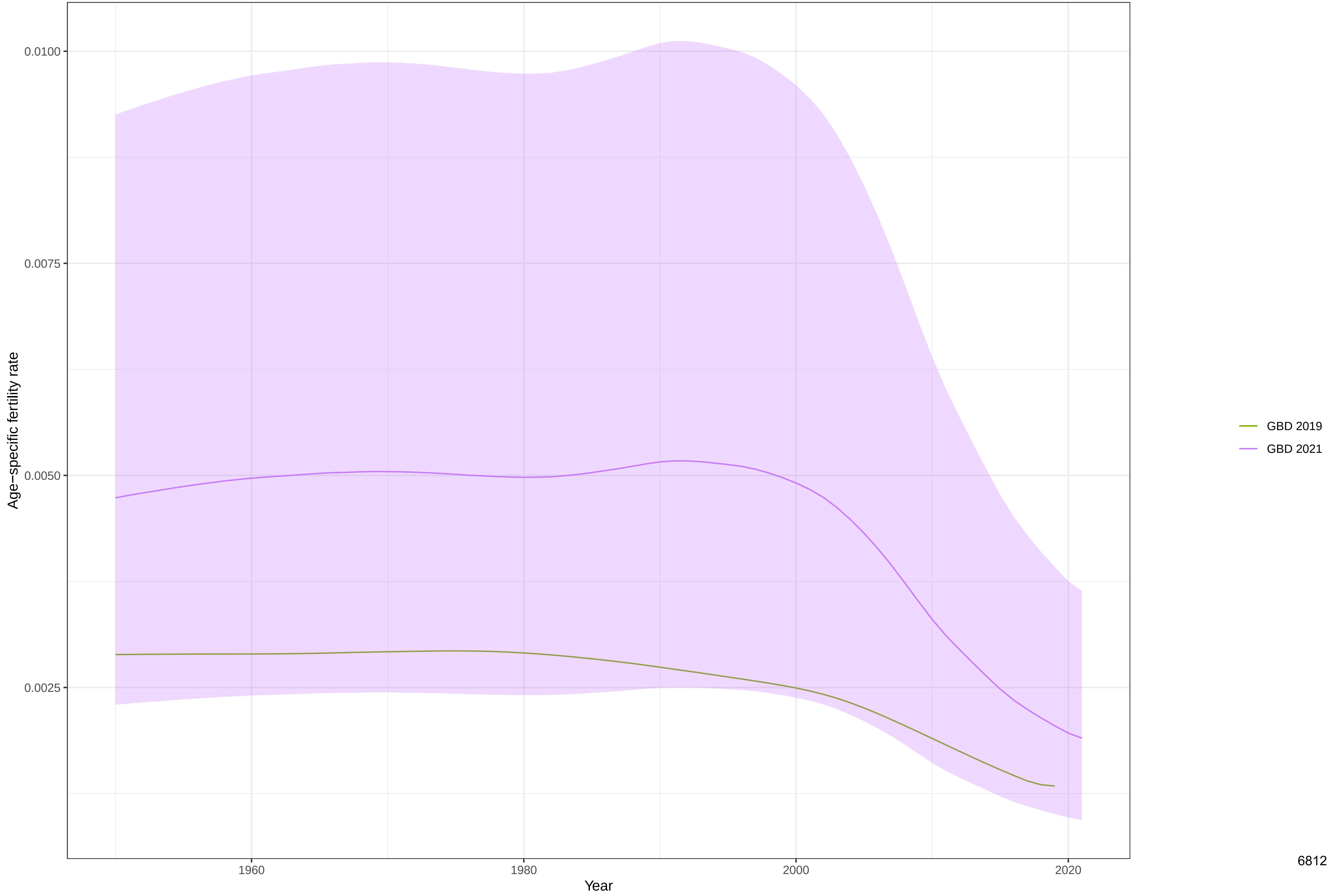

Age−specific fertility fitted trend lines, 1950−2021: Gambella, Ages: 15−19
Data points enclosed with a diamond are reference sources

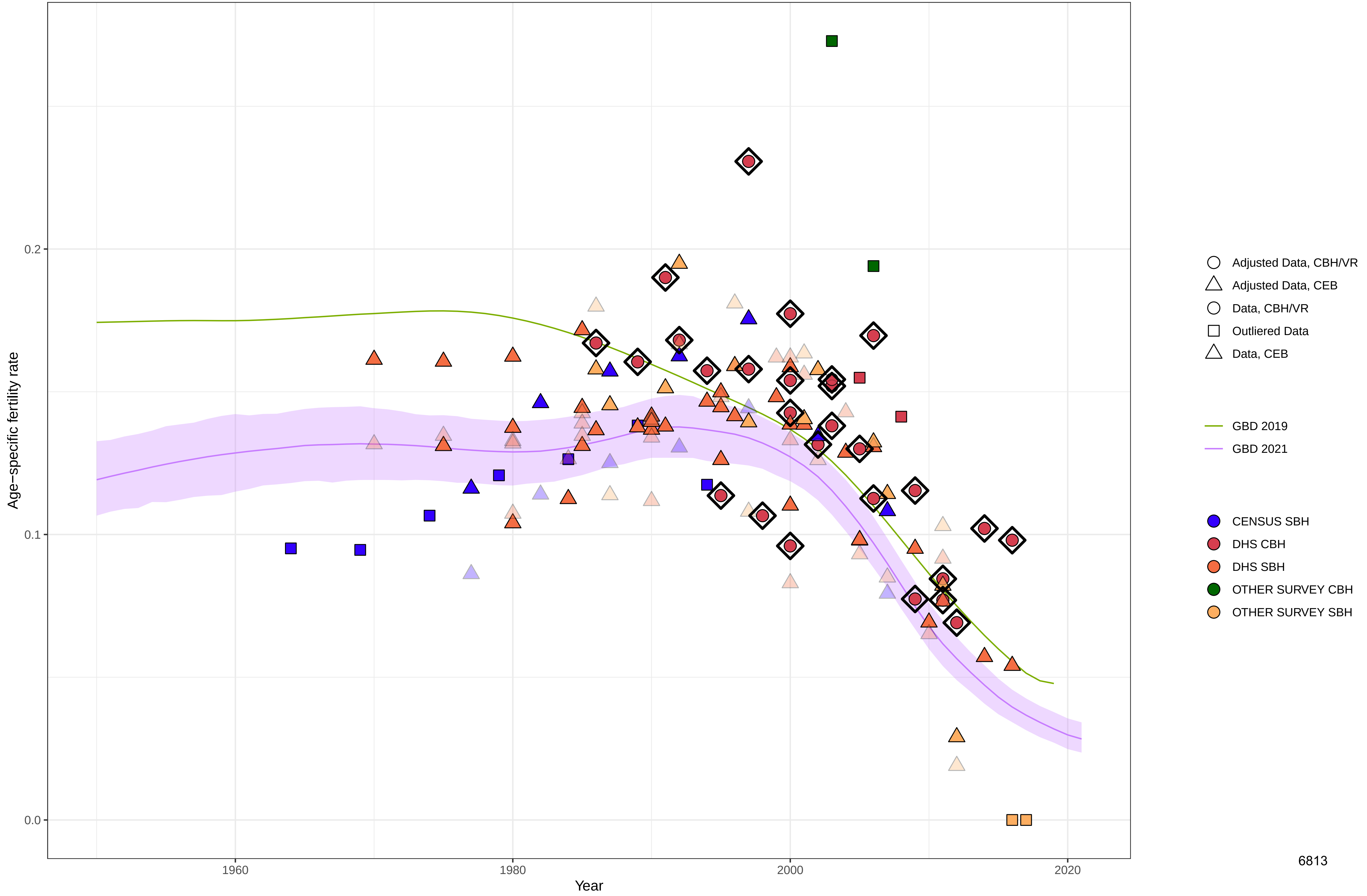

Age−specific fertility fitted trend lines, 1950−2021: Gambella, Ages: 20−24
Data points enclosed with a diamond are reference sources

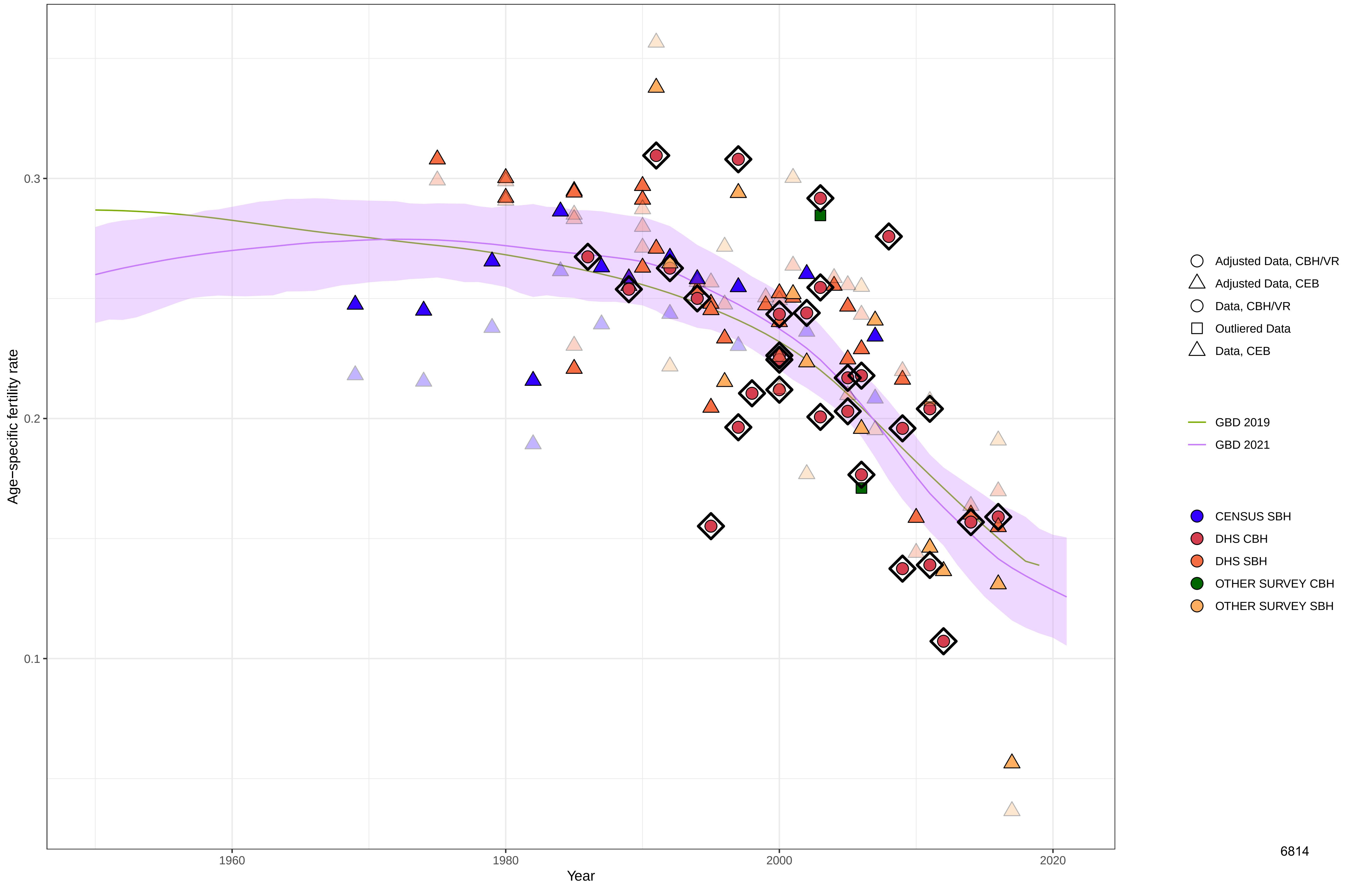

Age−specific fertility fitted trend lines, 1950−2021: Gambella, Ages: 25−29
Data points enclosed with a diamond are reference sources

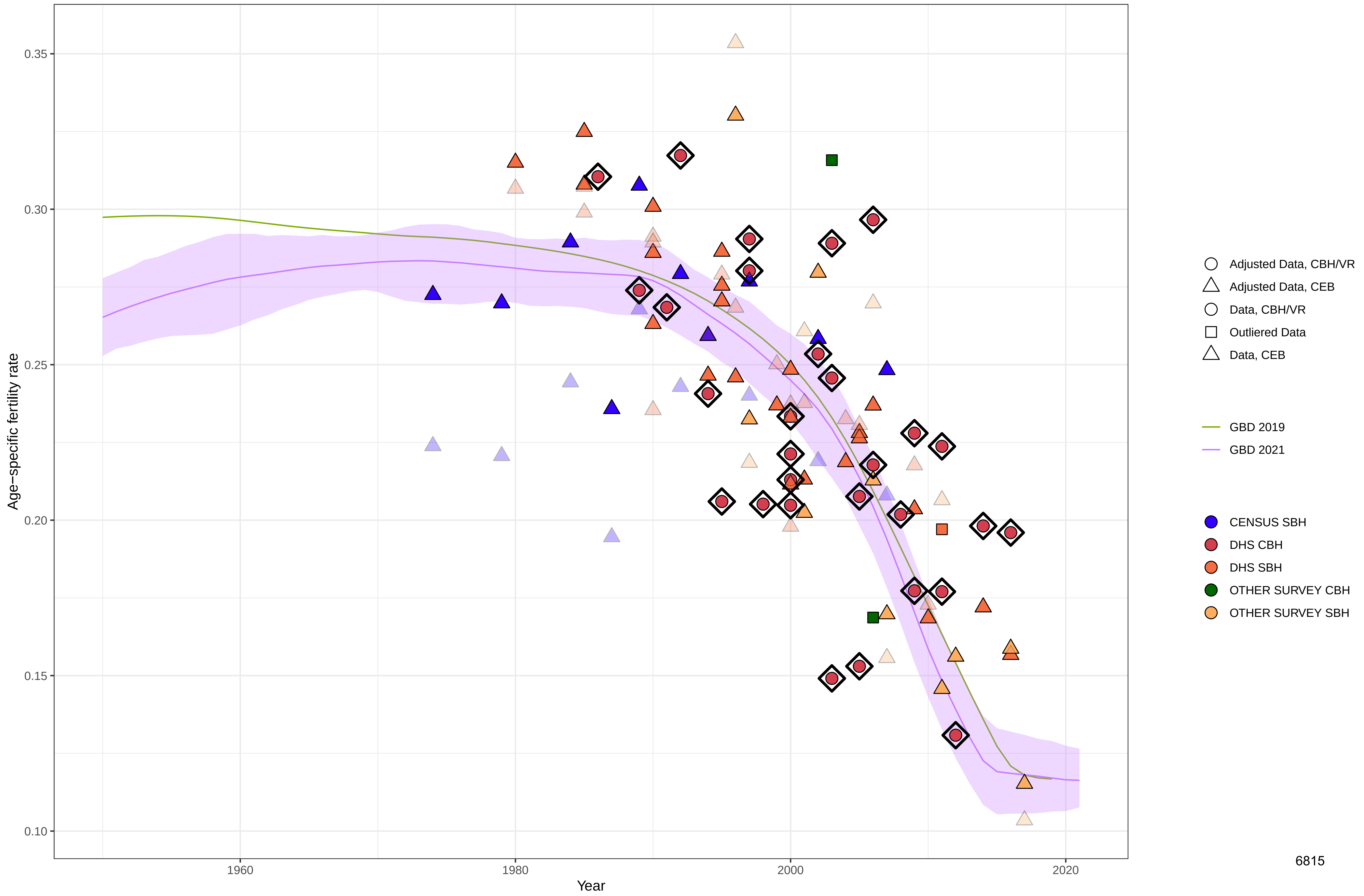

Age−specific fertility fitted trend lines, 1950−2021: Gambella, Ages: 30−34
Data points enclosed with a diamond are reference sources

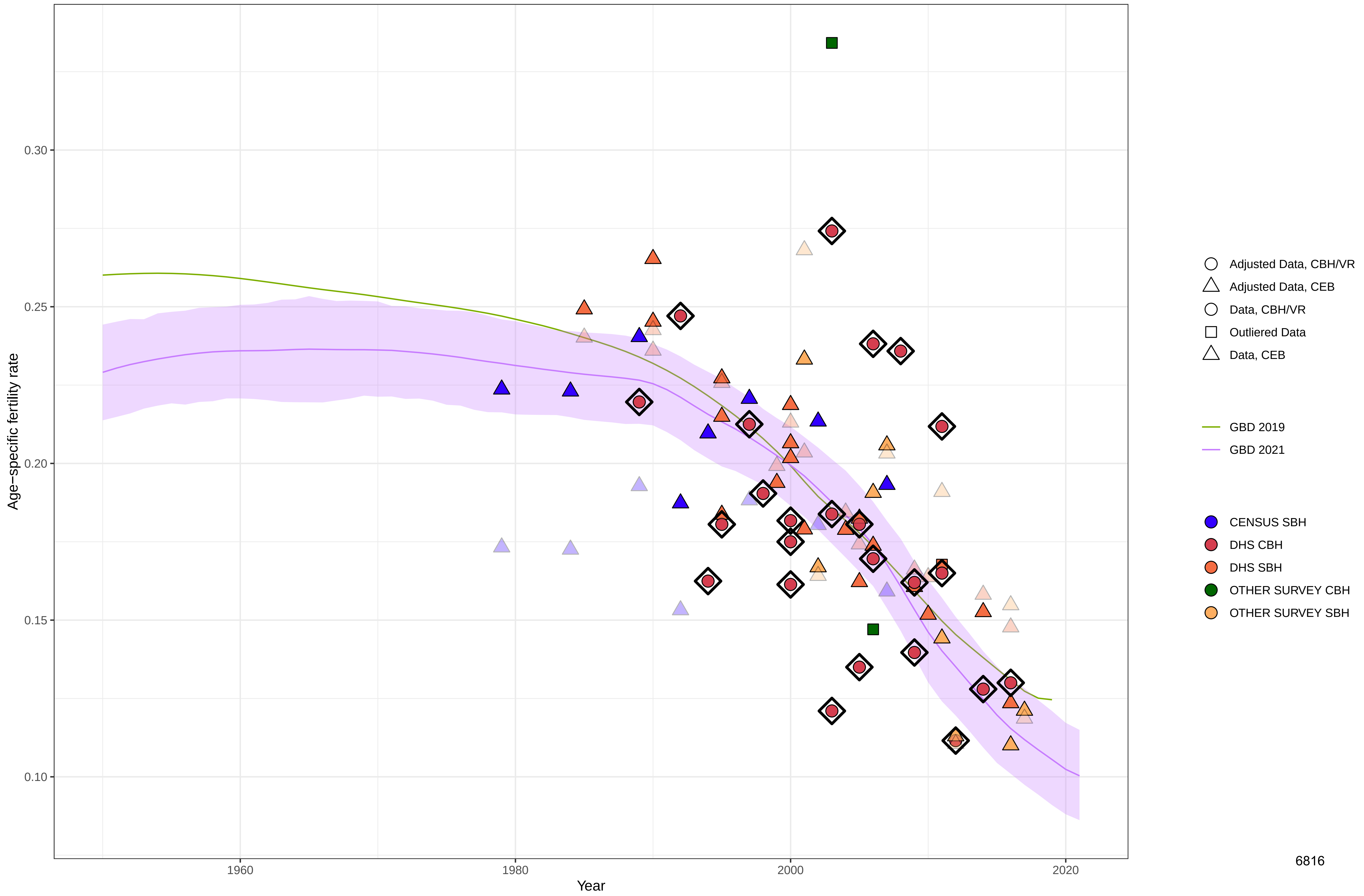

Age−specific fertility fitted trend lines, 1950−2021: Gambella, Ages: 35−39
Data points enclosed with a diamond are reference sources

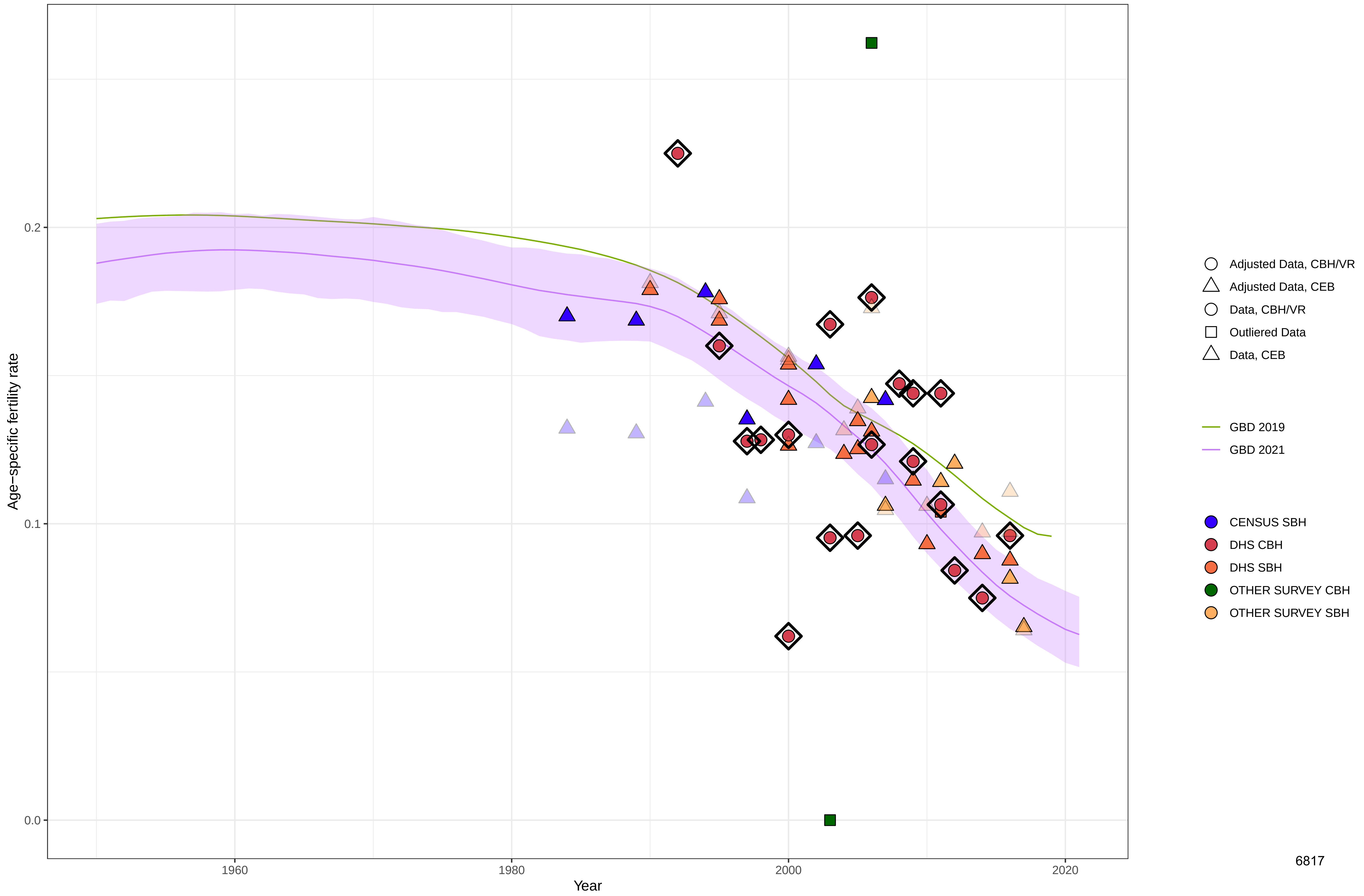

Age−specific fertility fitted trend lines, 1950−2021: Gambella, Ages: 40−44
Data points enclosed with a diamond are reference sources

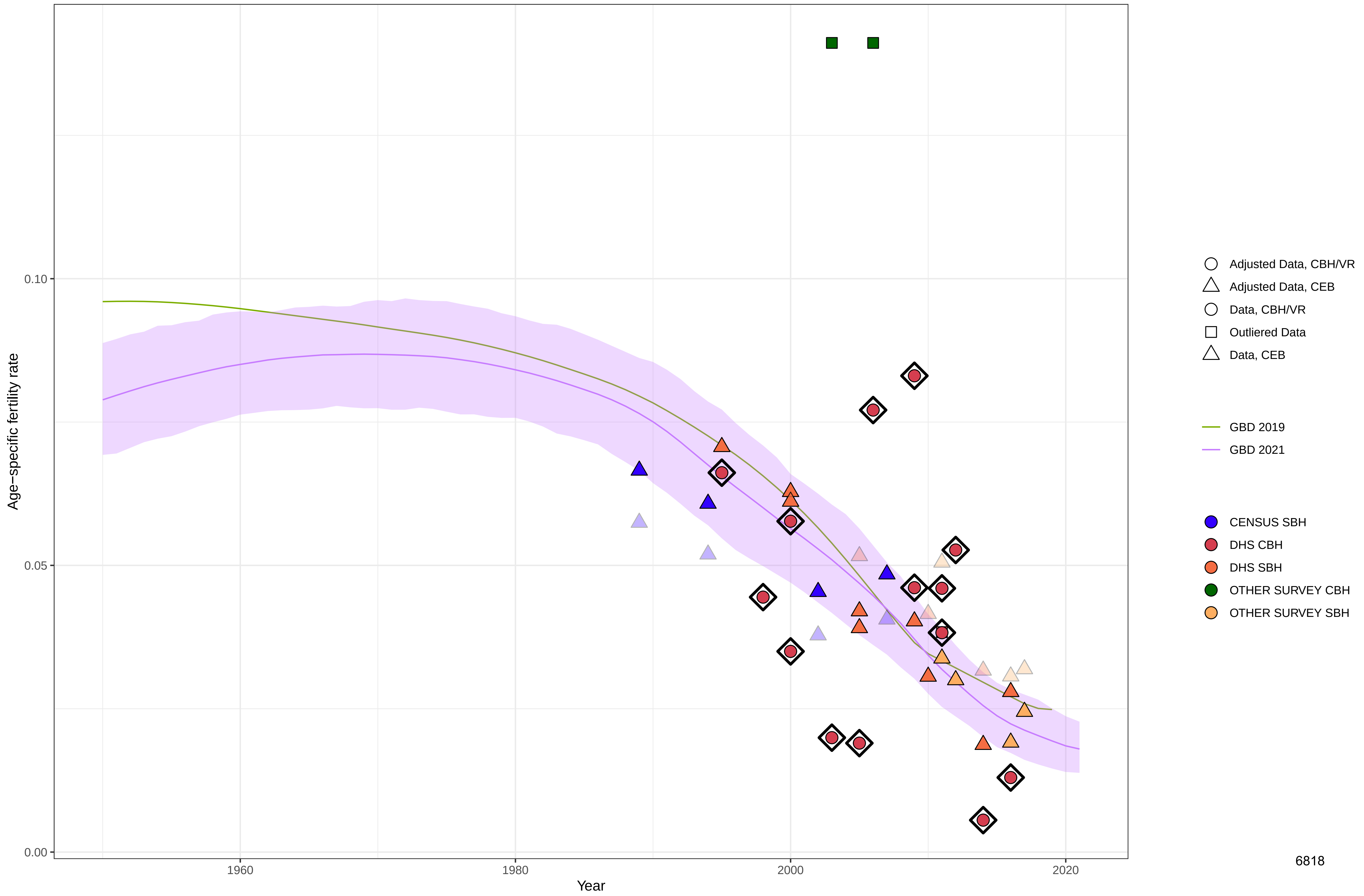

Age−specific fertility fitted trend lines, 1950−2021: Gambella, Ages: 45−49
Data points enclosed with a diamond are reference sources

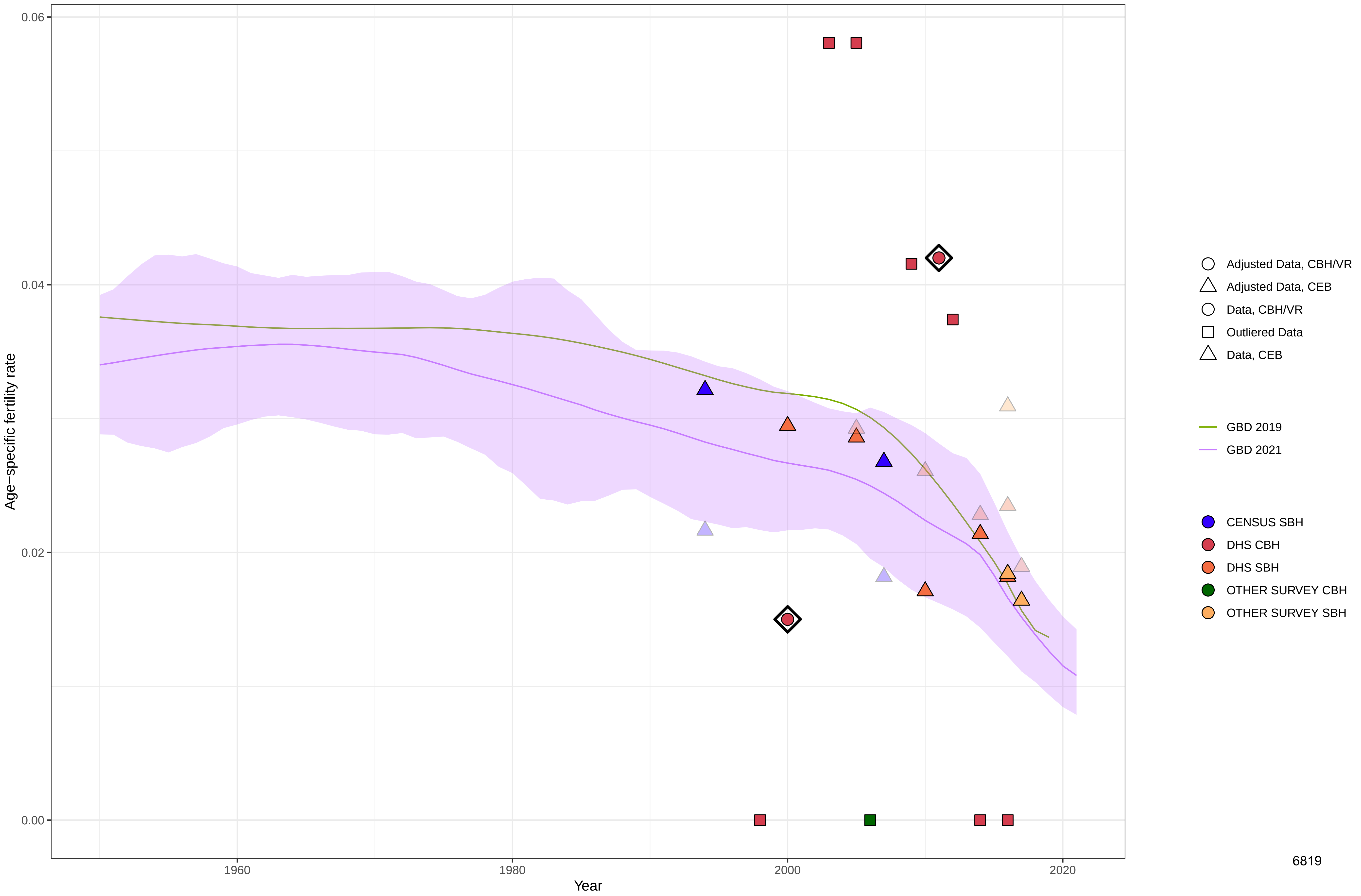

Age−specific fertility fitted trend lines, 1950−2021: Gambella, Ages: 50−54
Data points enclosed with a diamond are reference sources

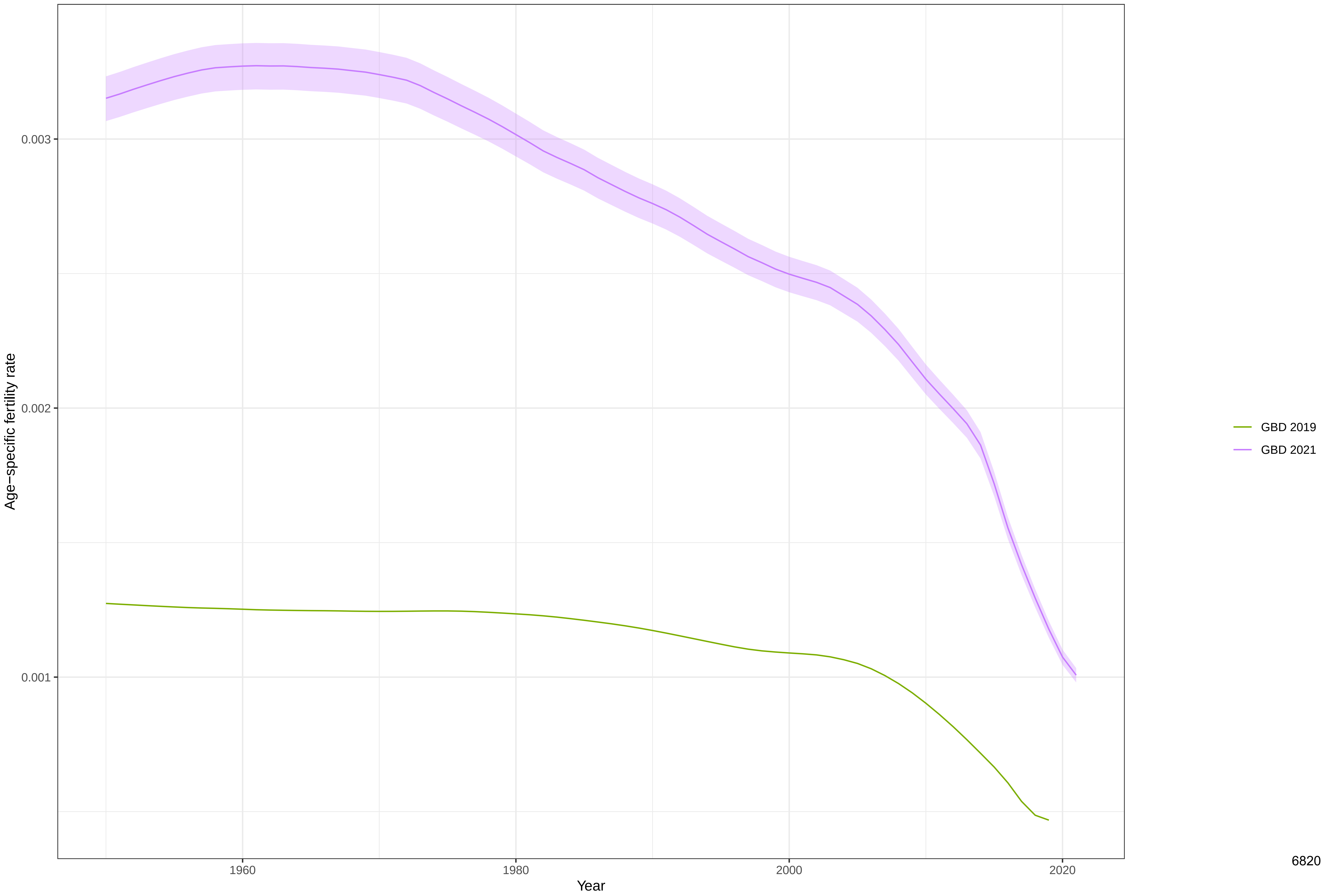

Total fertility rate fitted trend lines, 1950−2021: Gambella TFR
Data points enclosed with a diamond are reference sources

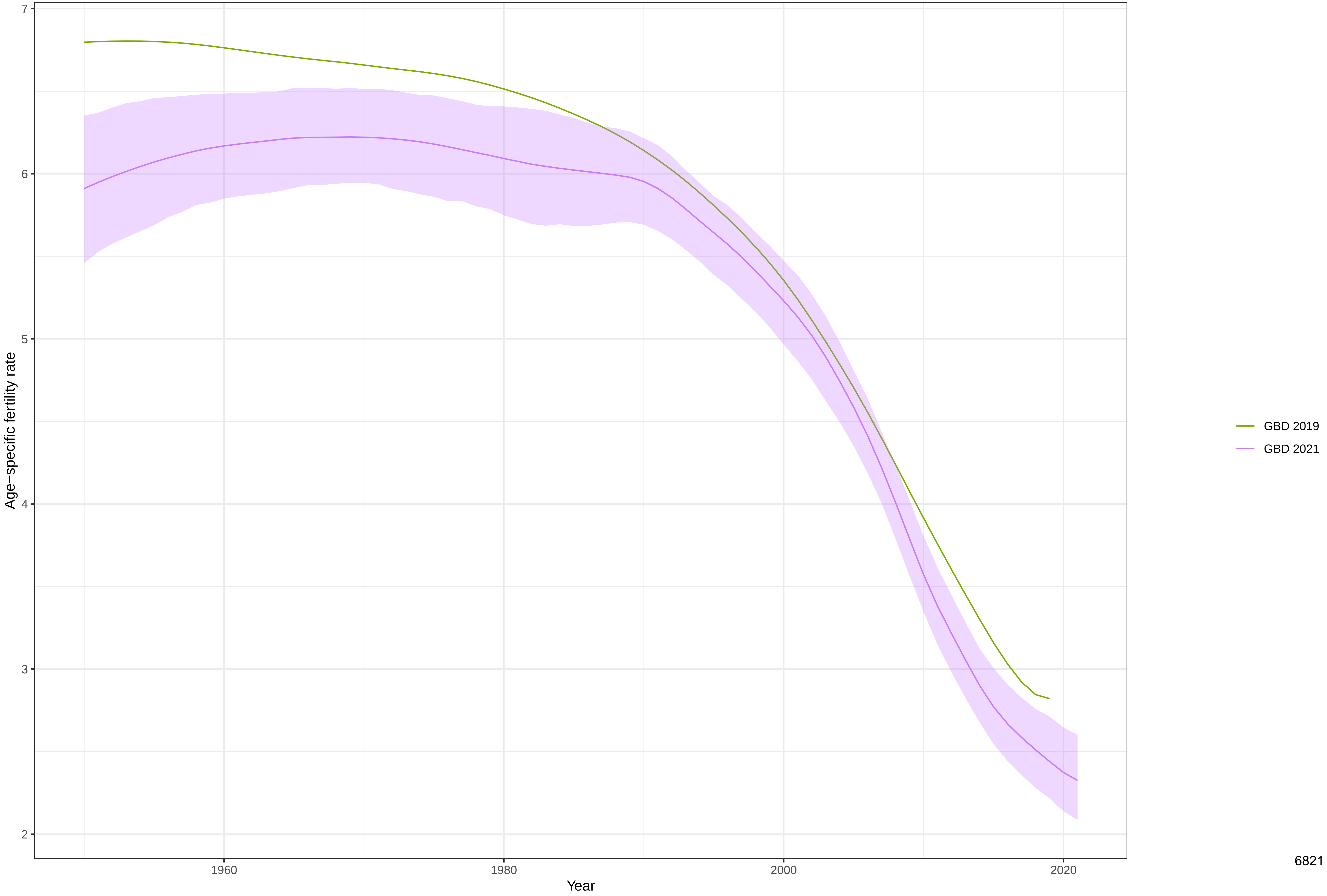

Age−specific fertility fitted trend lines, 1950−2021: Harari, Ages: 10−14
Data points enclosed with a diamond are reference sources

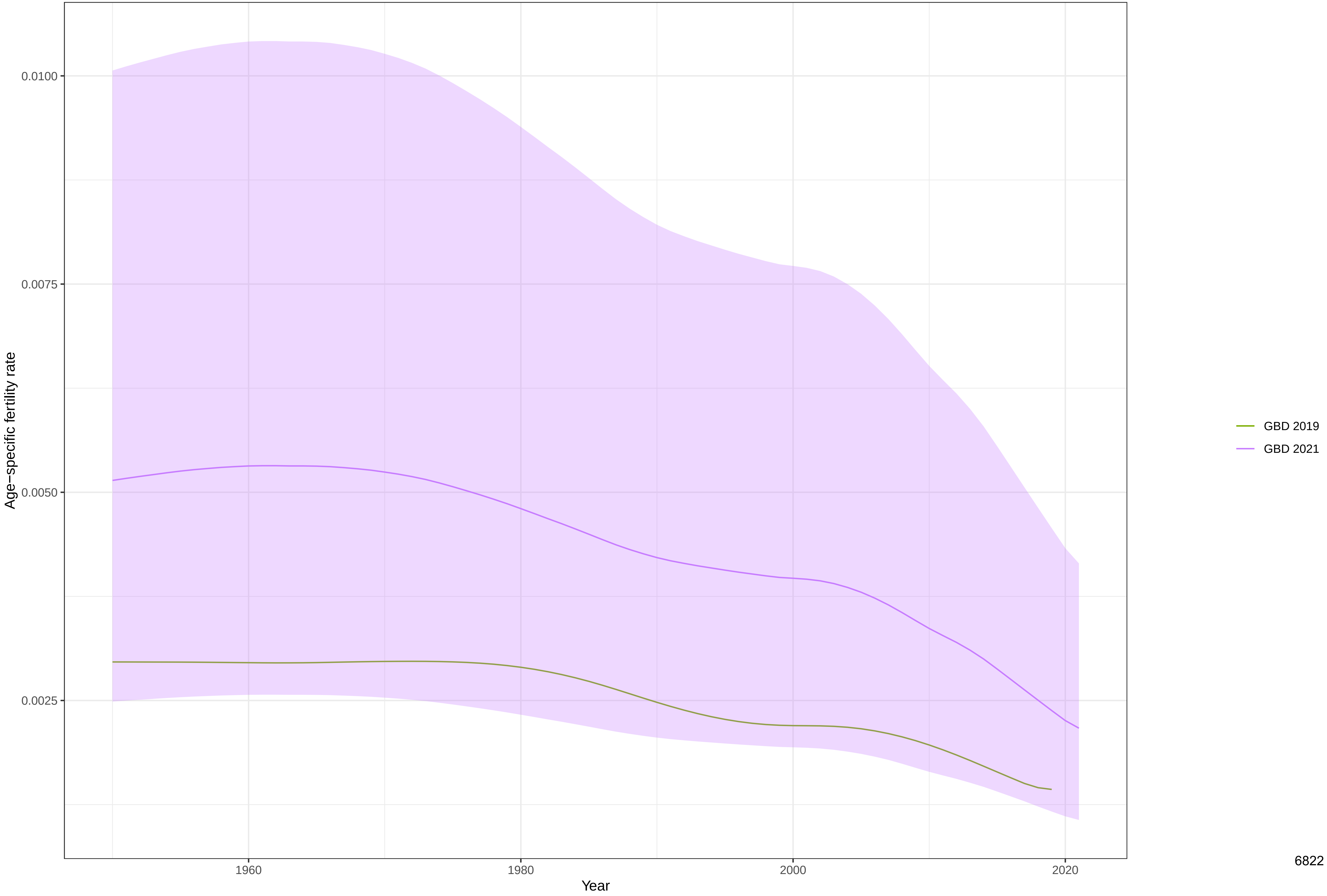

Age−specific fertility fitted trend lines, 1950−2021: Harari, Ages: 15−19
Data points enclosed with a diamond are reference sources

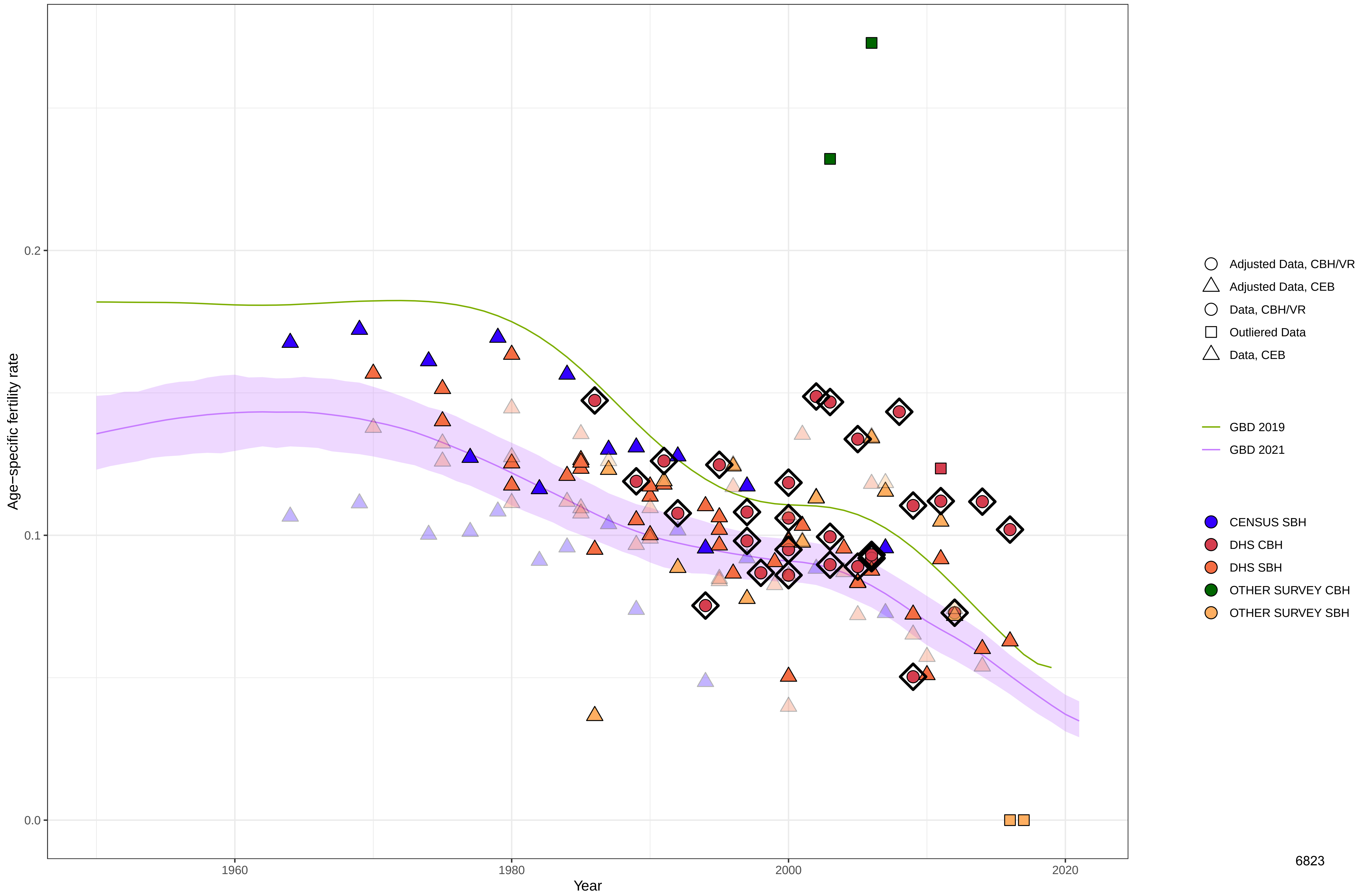

Age−specific fertility fitted trend lines, 1950−2021: Harari, Ages: 20−24
Data points enclosed with a diamond are reference sources

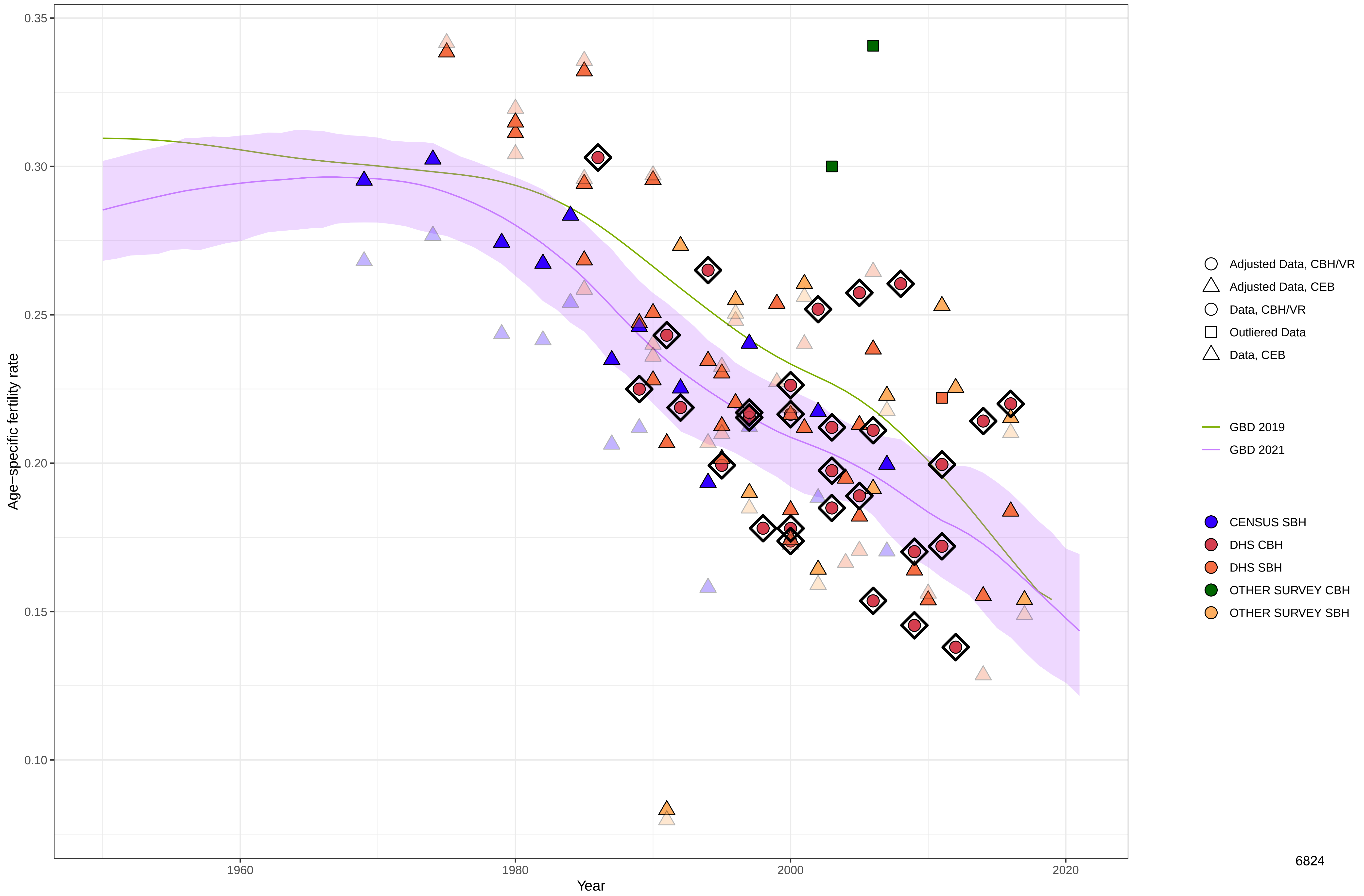

Age−specific fertility fitted trend lines, 1950−2021: Harari, Ages: 25−29
Data points enclosed with a diamond are reference sources

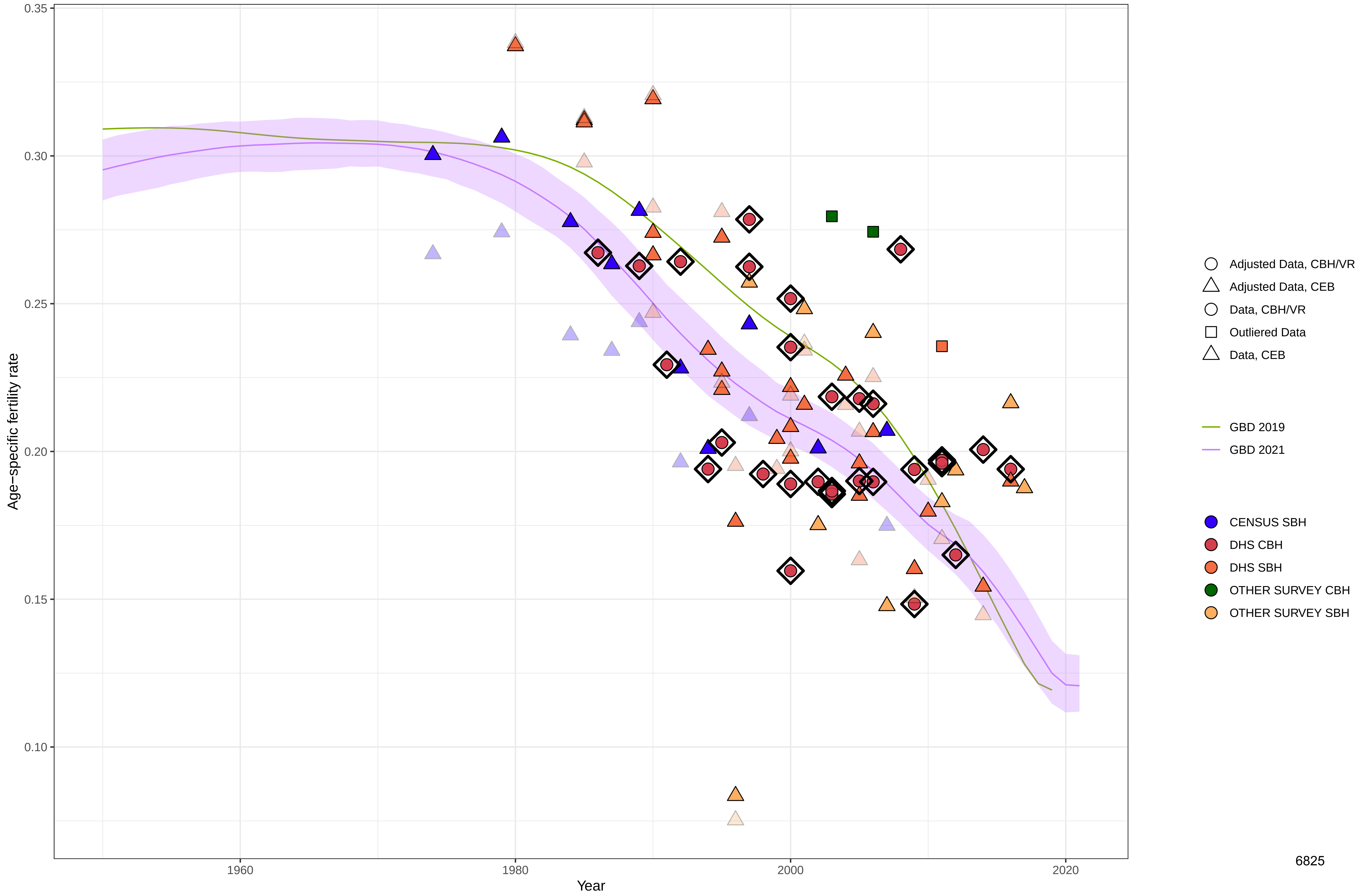

Age−specific fertility fitted trend lines, 1950−2021: Harari, Ages: 30−34
Data points enclosed with a diamond are reference sources

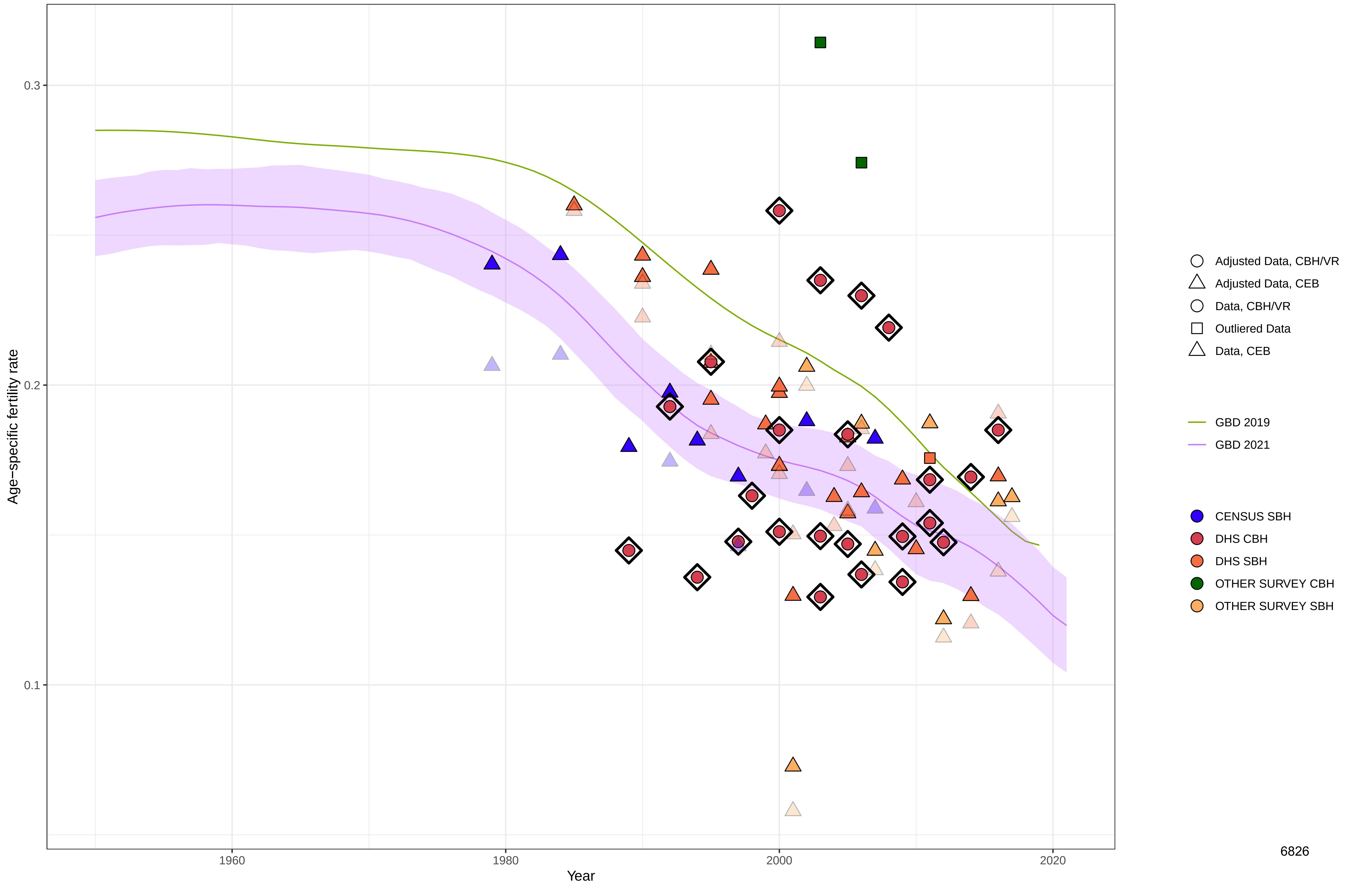

Age−specific fertility fitted trend lines, 1950−2021: Harari, Ages: 35−39
Data points enclosed with a diamond are reference sources

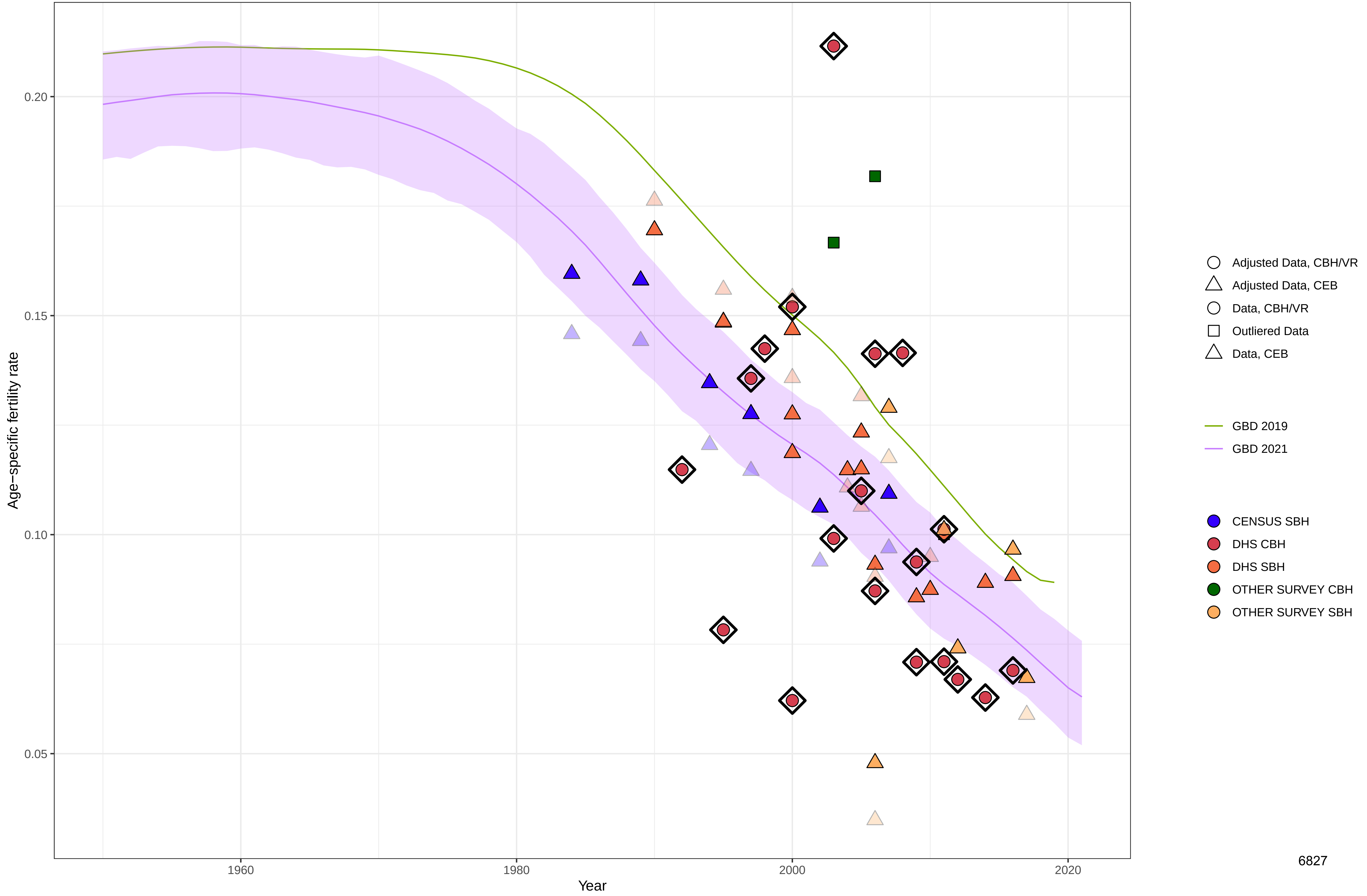

6827

Age−specific fertility fitted trend lines, 1950−2021: Harari, Ages: 40−44
Data points enclosed with a diamond are reference sources

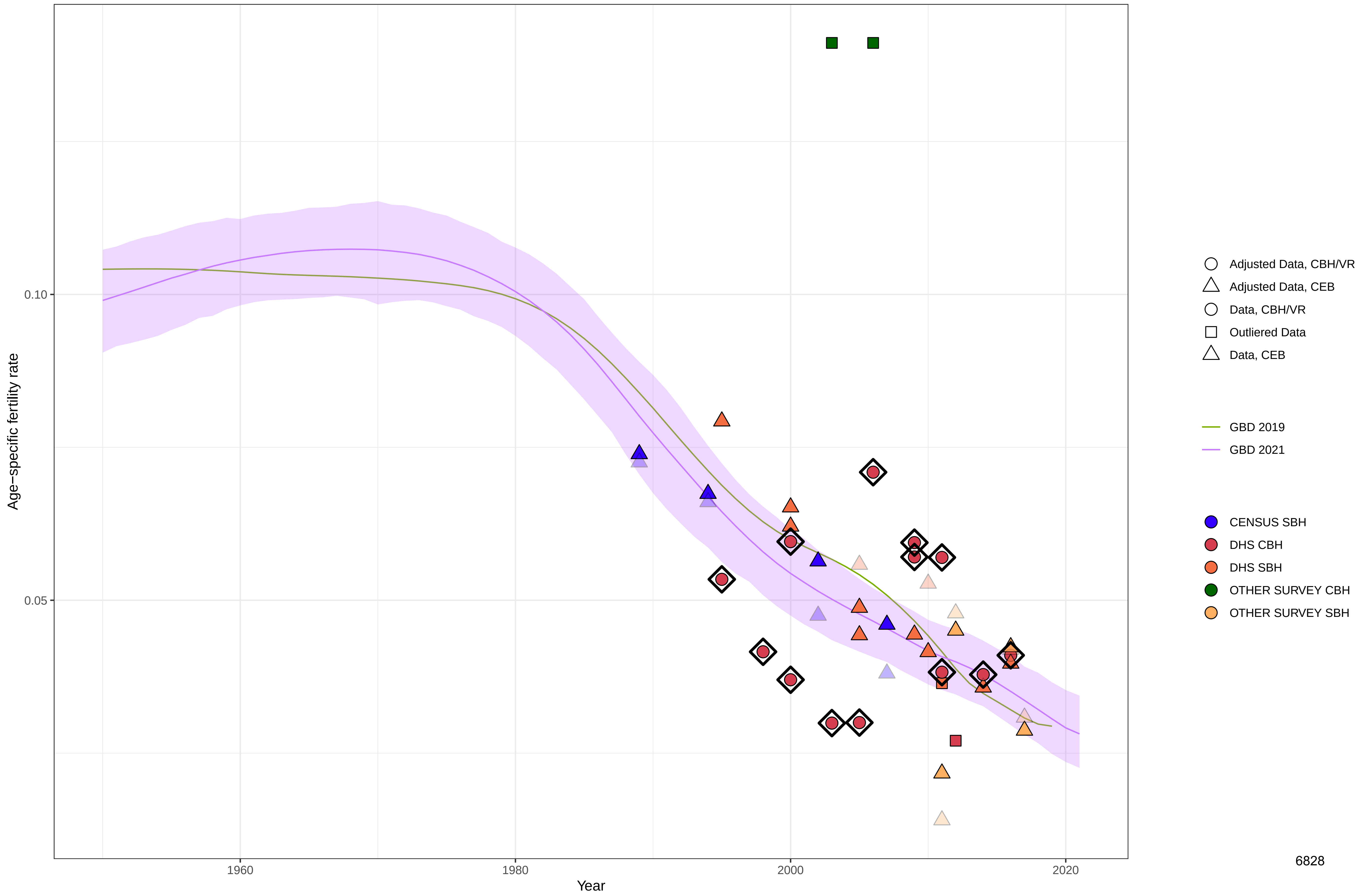

Age−specific fertility fitted trend lines, 1950−2021: Harari, Ages: 45−49
Data points enclosed with a diamond are reference sources

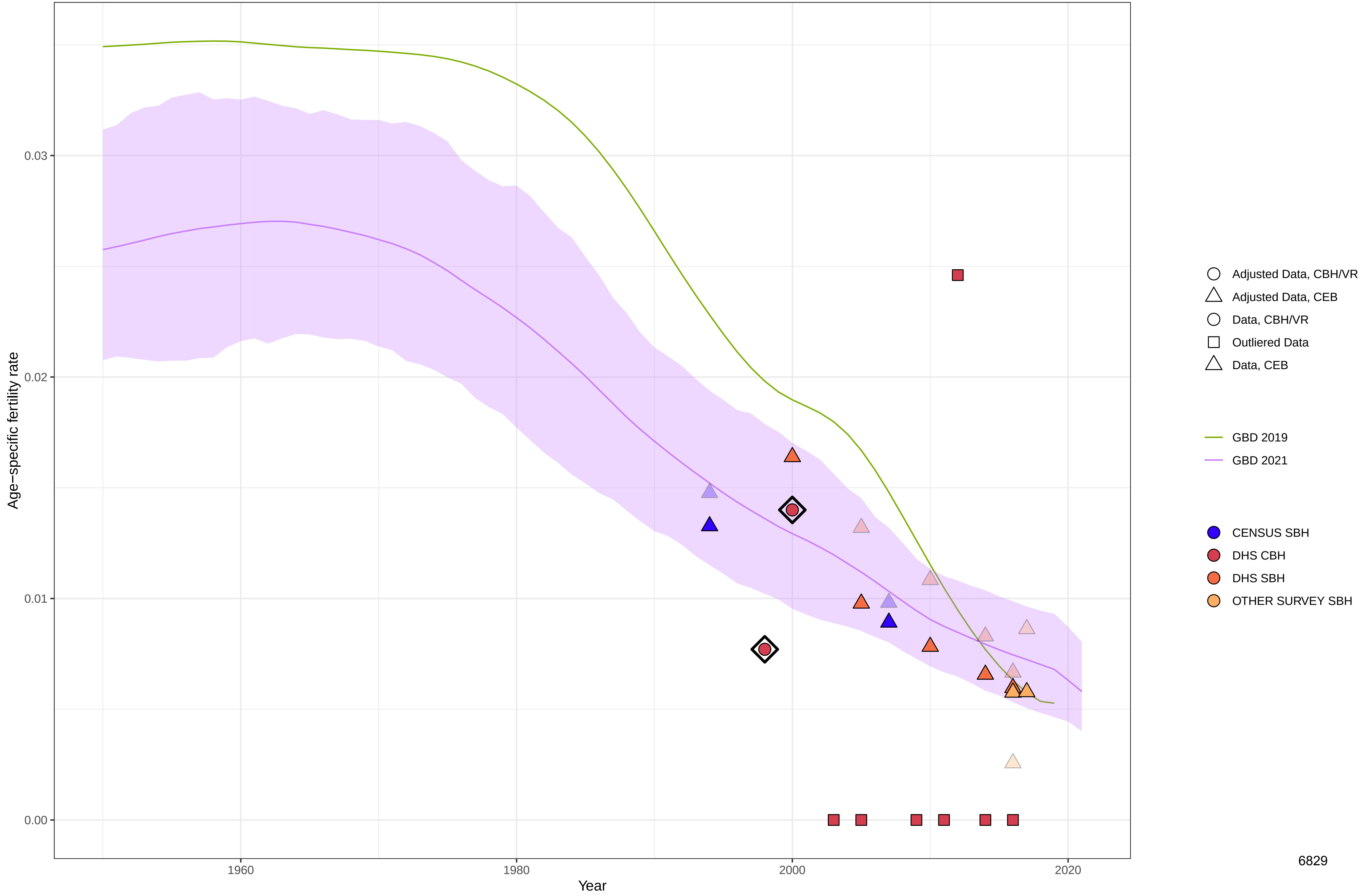

Age−specific fertility fitted trend lines, 1950−2021: Harari, Ages: 50−54
Data points enclosed with a diamond are reference sources

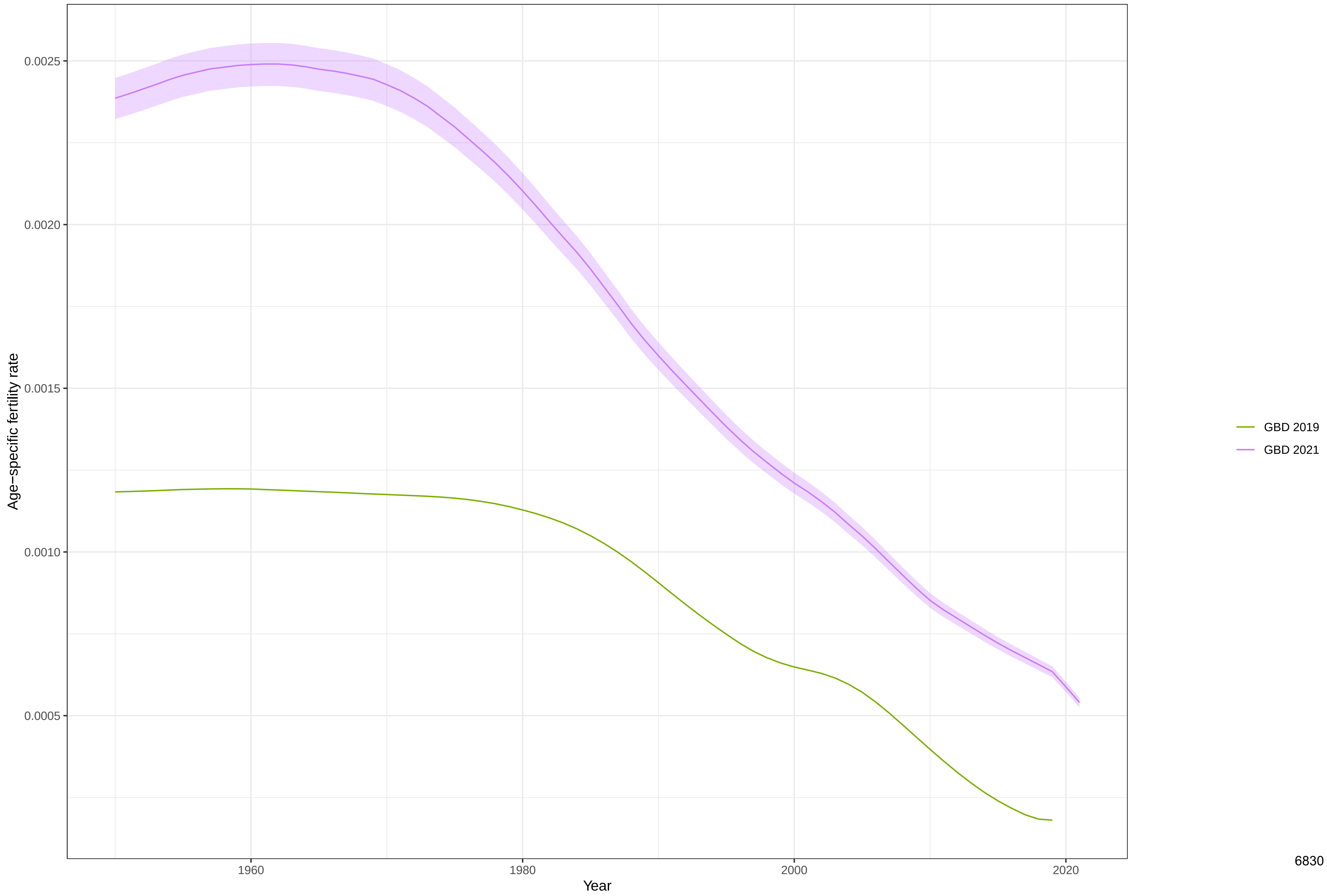

Total fertility rate fitted trend lines, 1950−2021: Harari TFR
Data points enclosed with a diamond are reference sources

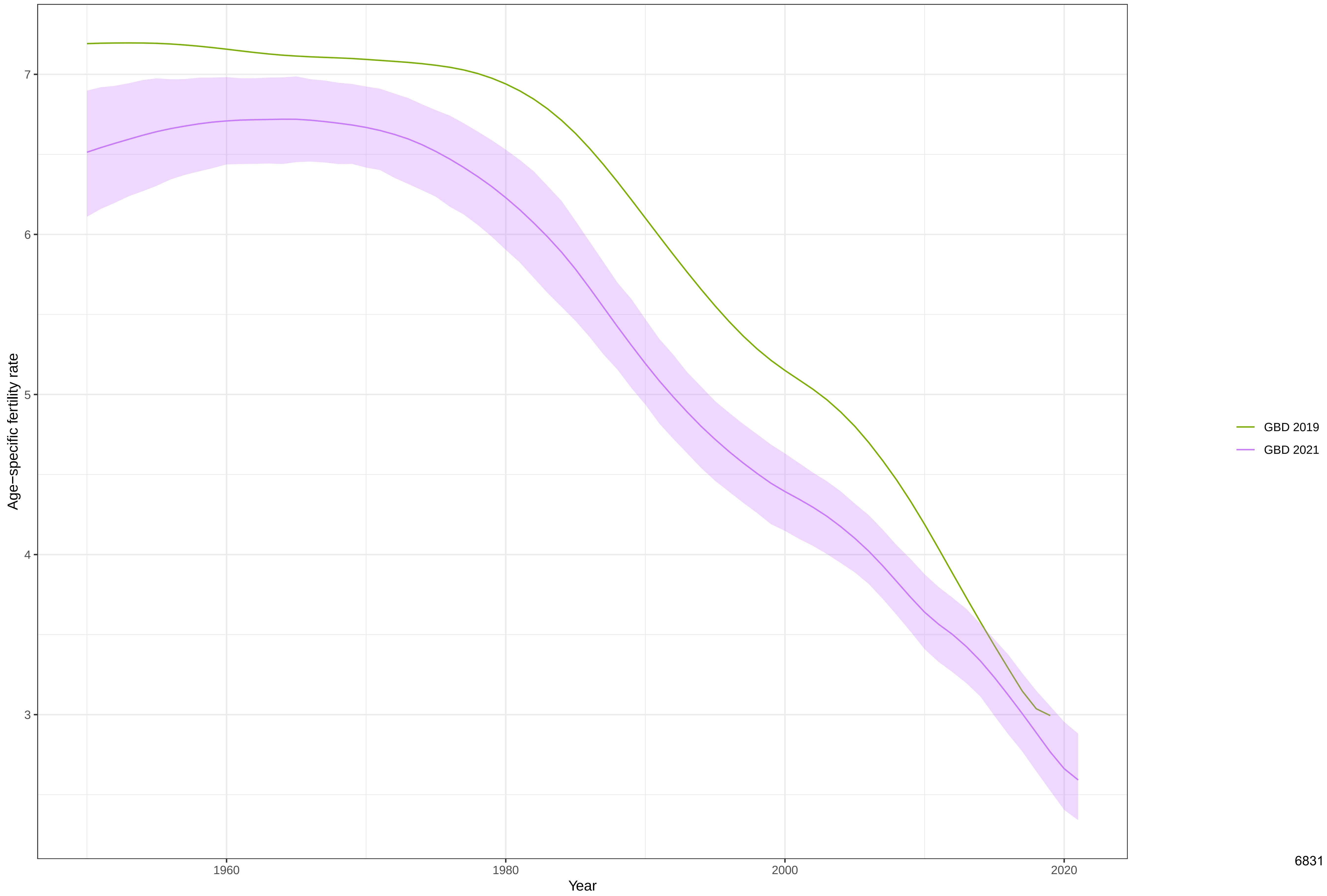

Age−specific fertility fitted trend lines, 1950−2021: Oromia, Ages: 10−14
Data points enclosed with a diamond are reference sources

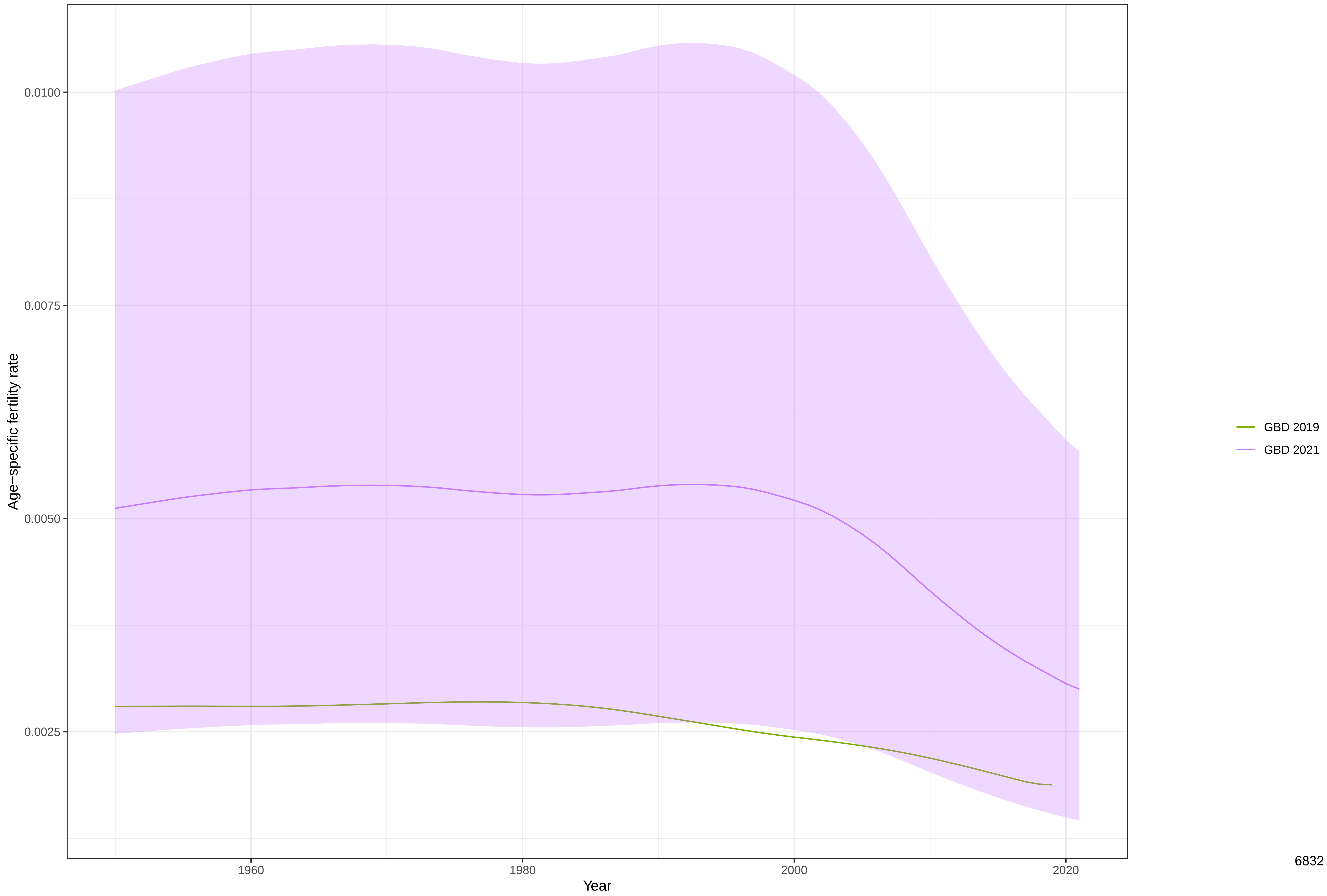

Age−specific fertility fitted trend lines, 1950−2021: Oromia, Ages: 15−19
Data points enclosed with a diamond are reference sources

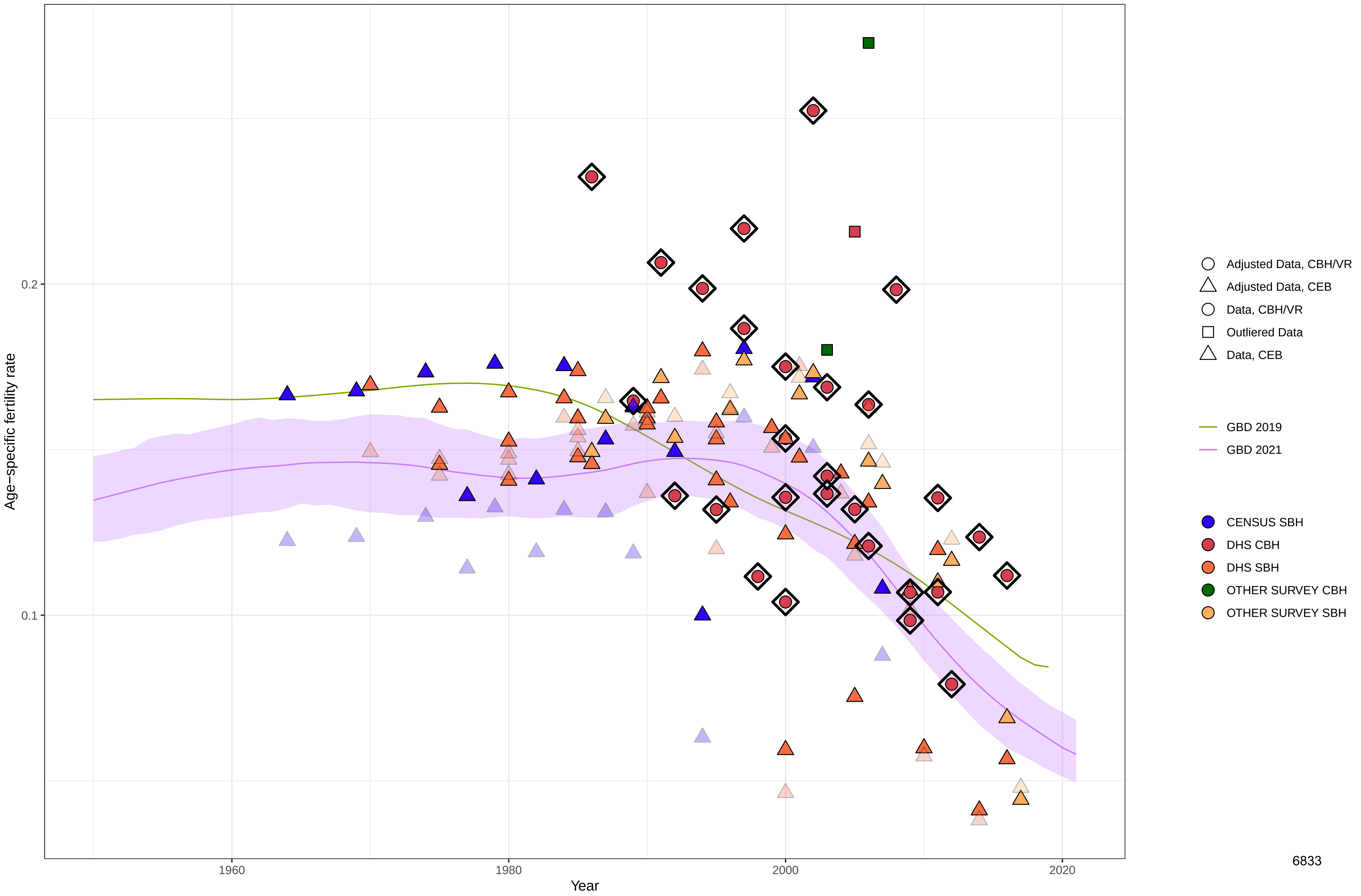

Age−specific fertility fitted trend lines, 1950−2021: Oromia, Ages: 20−24
Data points enclosed with a diamond are reference sources

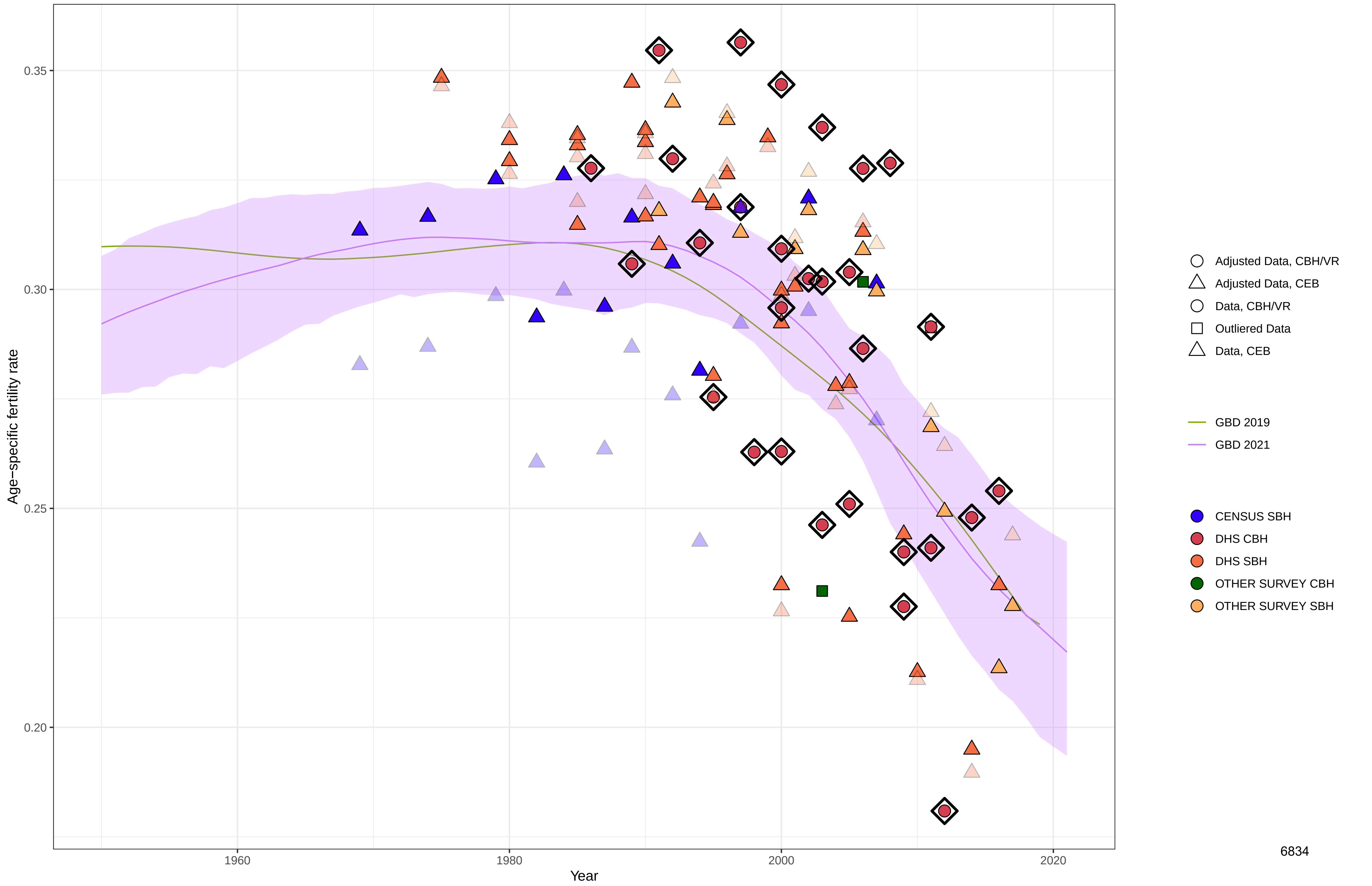

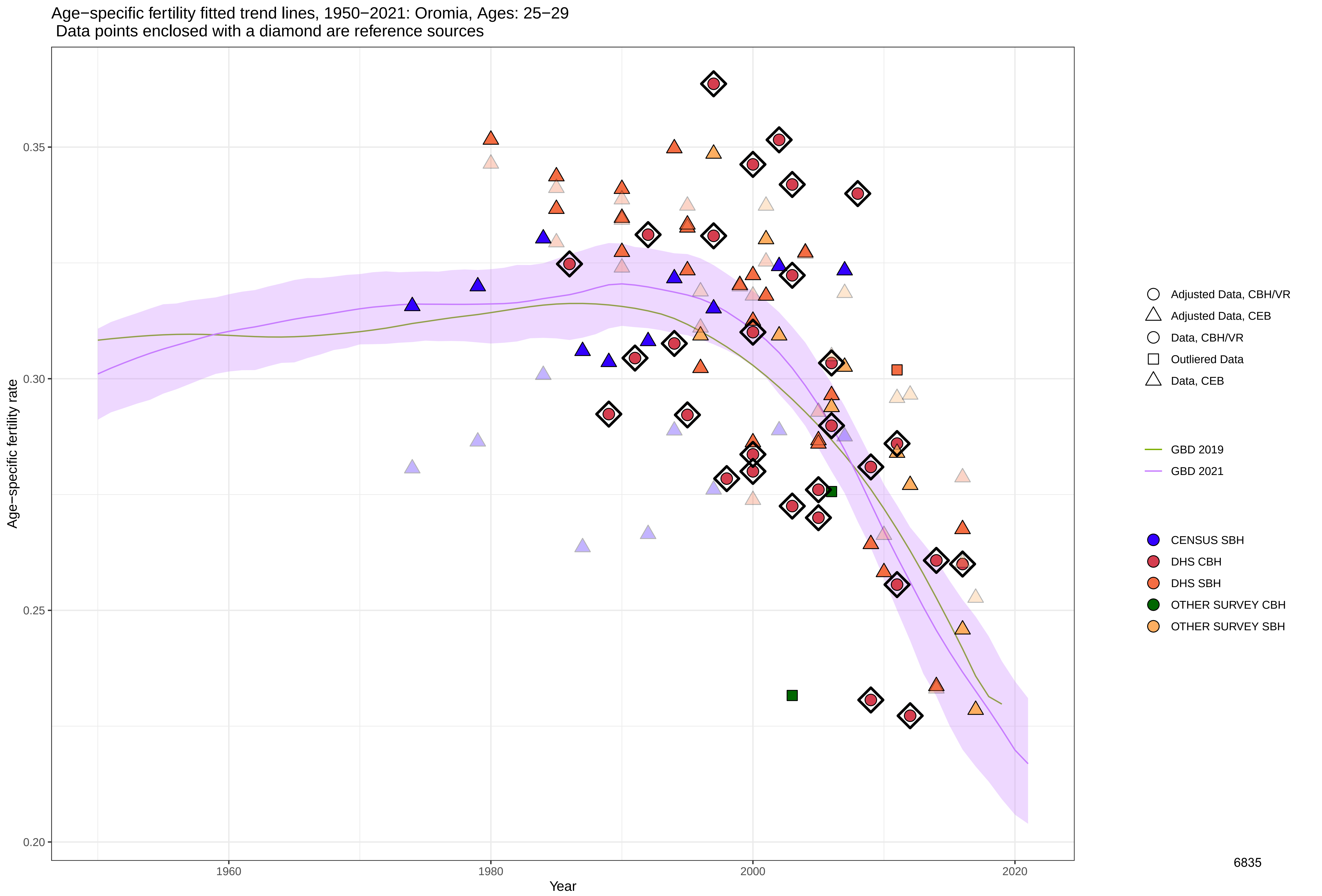

Age−specific fertility fitted trend lines, 1950−2021: Oromia, Ages: 25−29
Data points enclosed with a diamond are reference sources
Age−specific fertility rate
Year
0.20
0.25
0.30
0.35
1960
1980
2000
2020
Adjusted Data, CBH/VR
Adjusted Data, CEB
Data, CBH/VR
Outliered Data
Data, CEB
GBD 2019
GBD 2021
CENSUS SBH
DHS CBH
DHS SBH
OTHER SURVEY CBH
OTHER SURVEY SBH

6835

Age−specific fertility fitted trend lines, 1950−2021: Oromia, Ages: 30−34
Data points enclosed with a diamond are reference sources

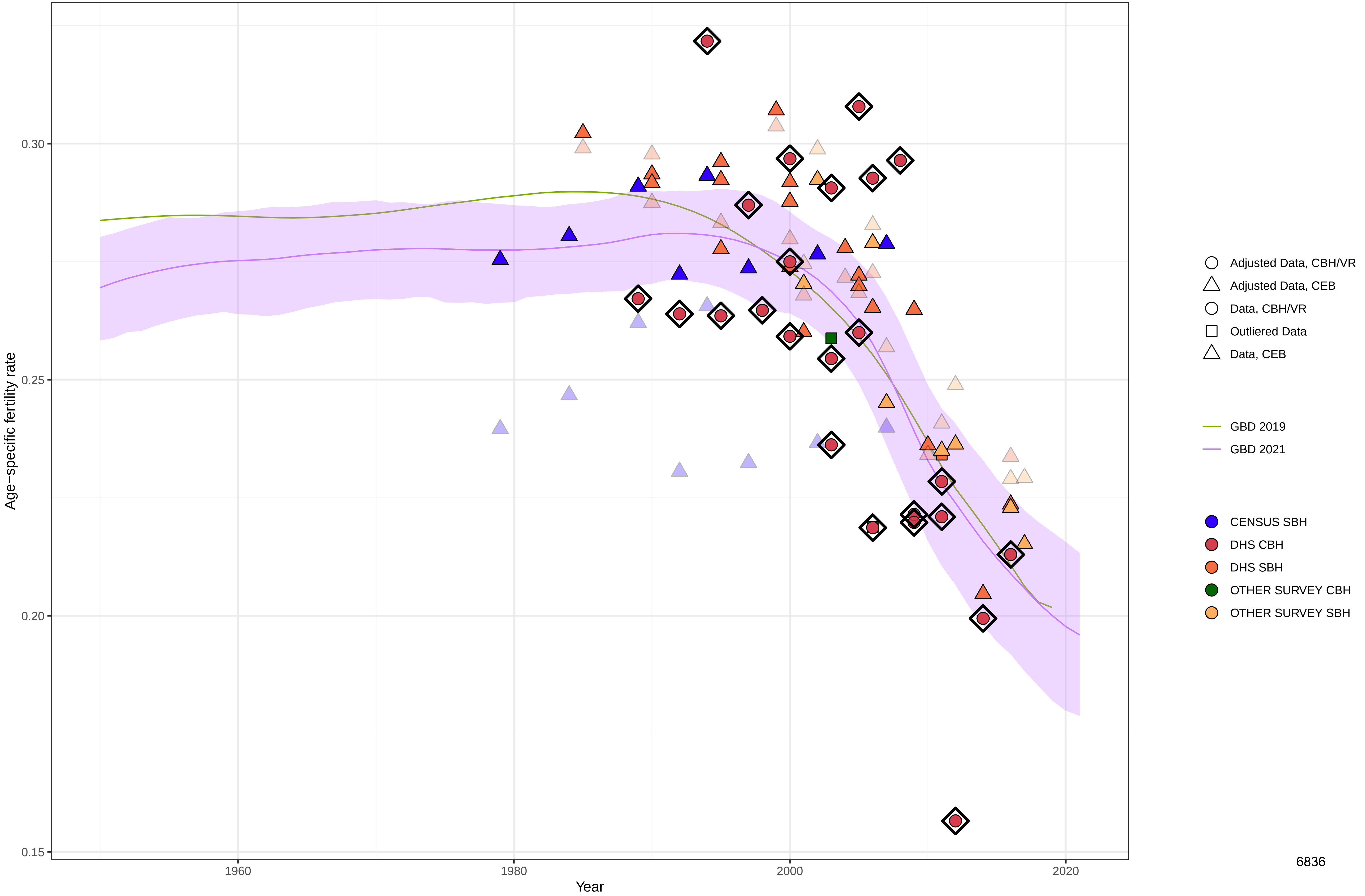

Age−specific fertility fitted trend lines, 1950−2021: Oromia, Ages: 35−39
Data points enclosed with a diamond are reference sources

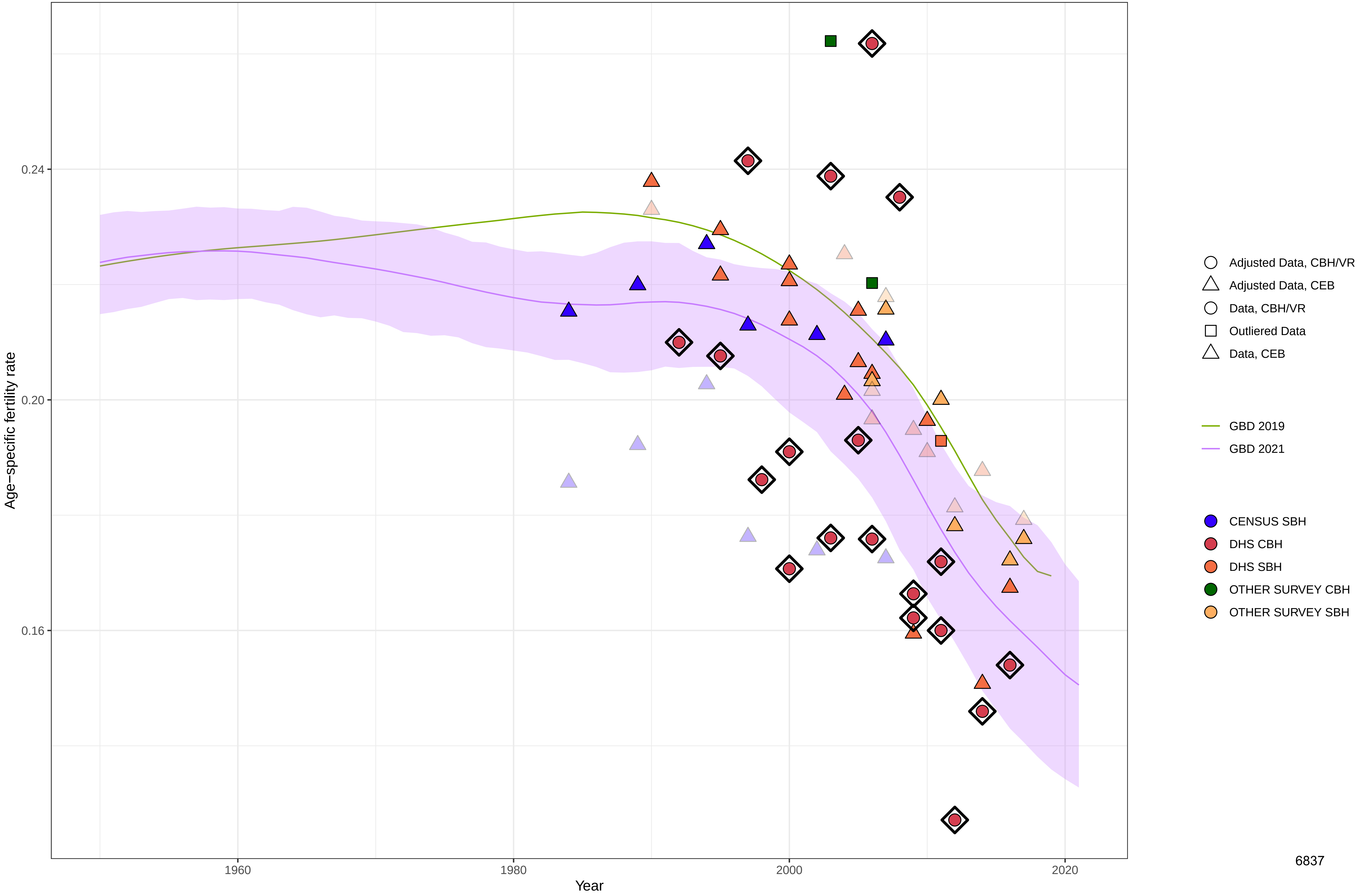

Age−specific fertility fitted trend lines, 1950−2021: Oromia, Ages: 40−44
Data points enclosed with a diamond are reference sources

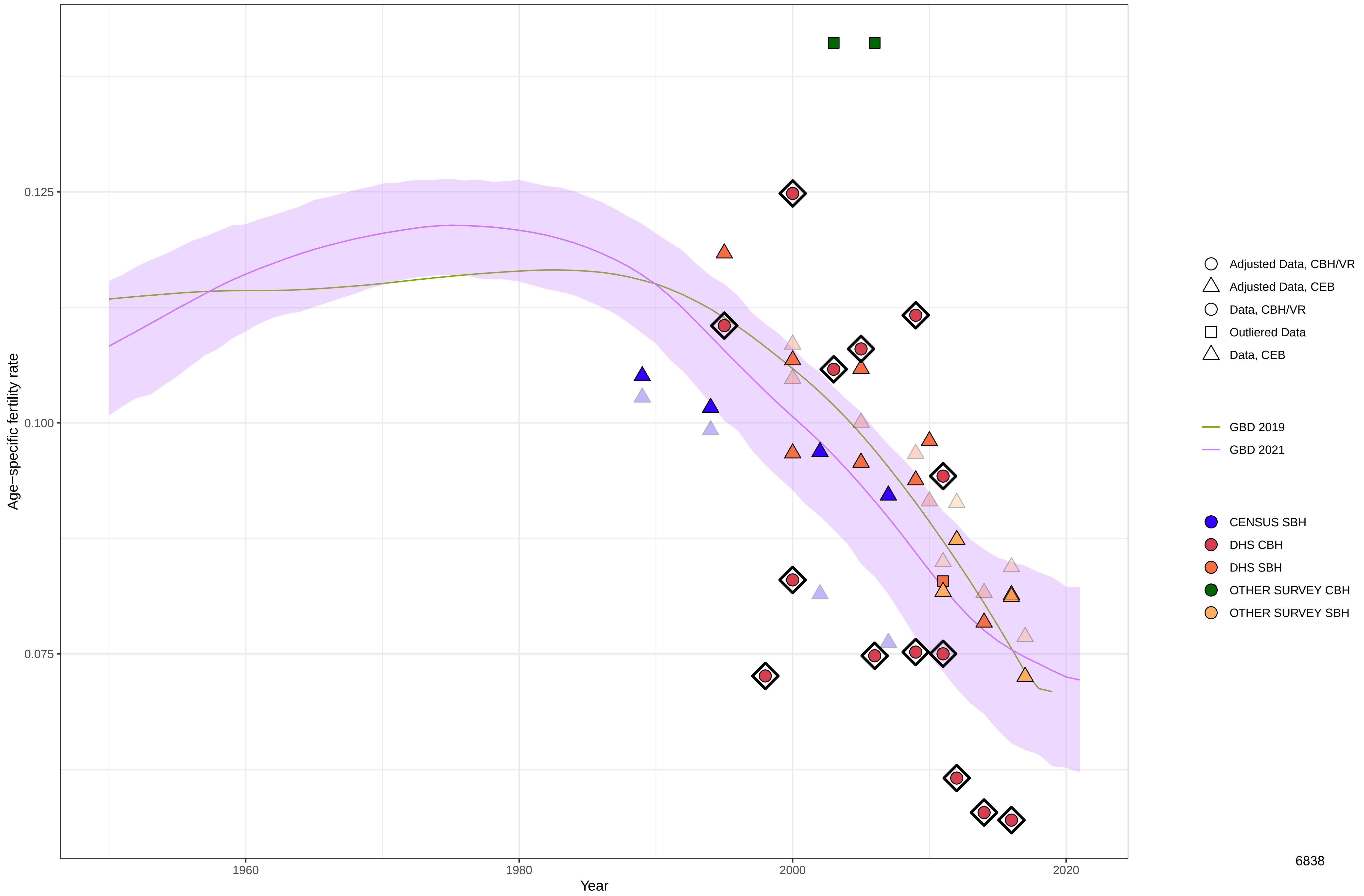

Age−specific fertility fitted trend lines, 1950−2021: Oromia, Ages: 45−49
Data points enclosed with a diamond are reference sources

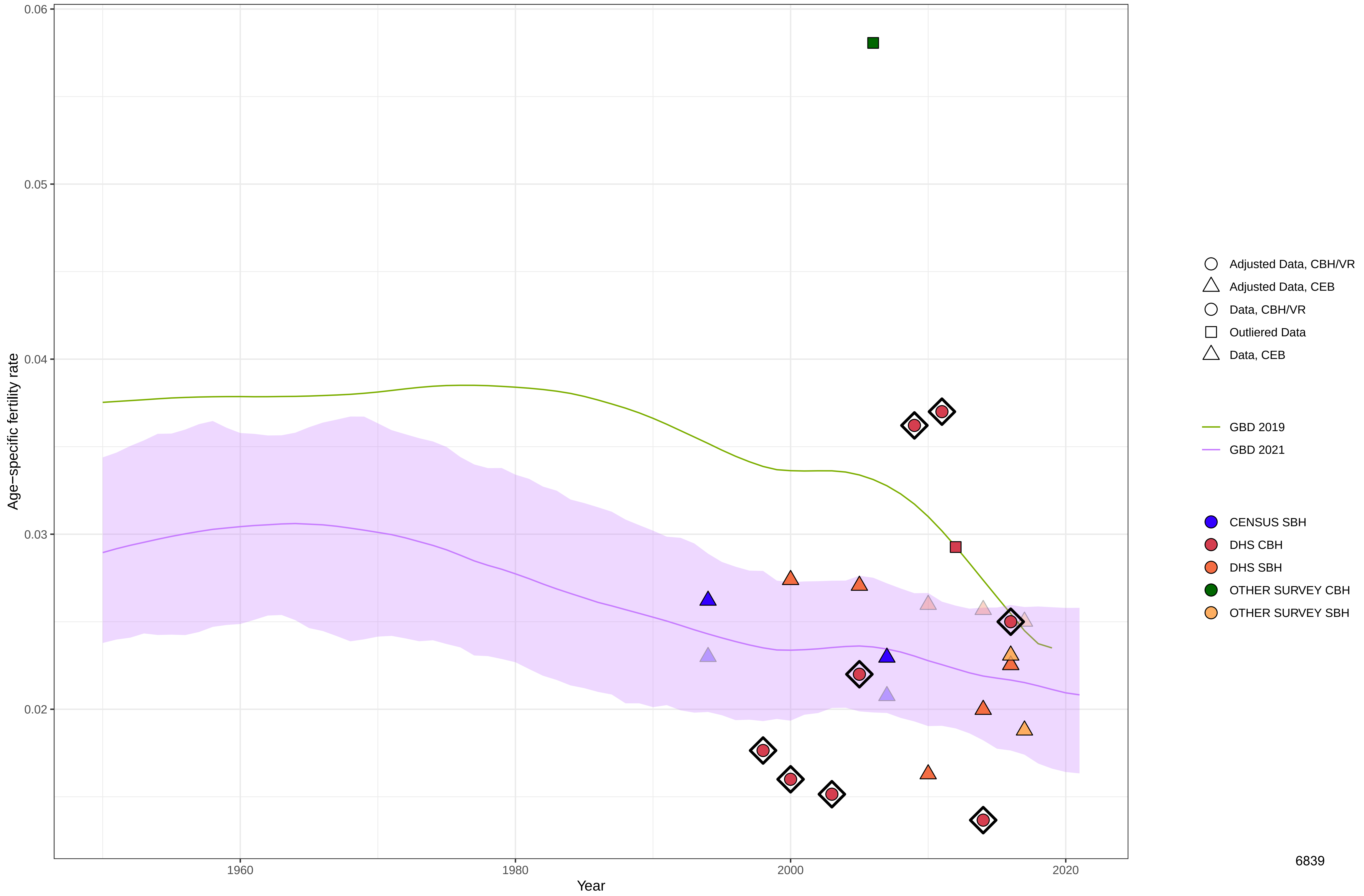

Age−specific fertility fitted trend lines, 1950−2021: Oromia, Ages: 50−54
Data points enclosed with a diamond are reference sources

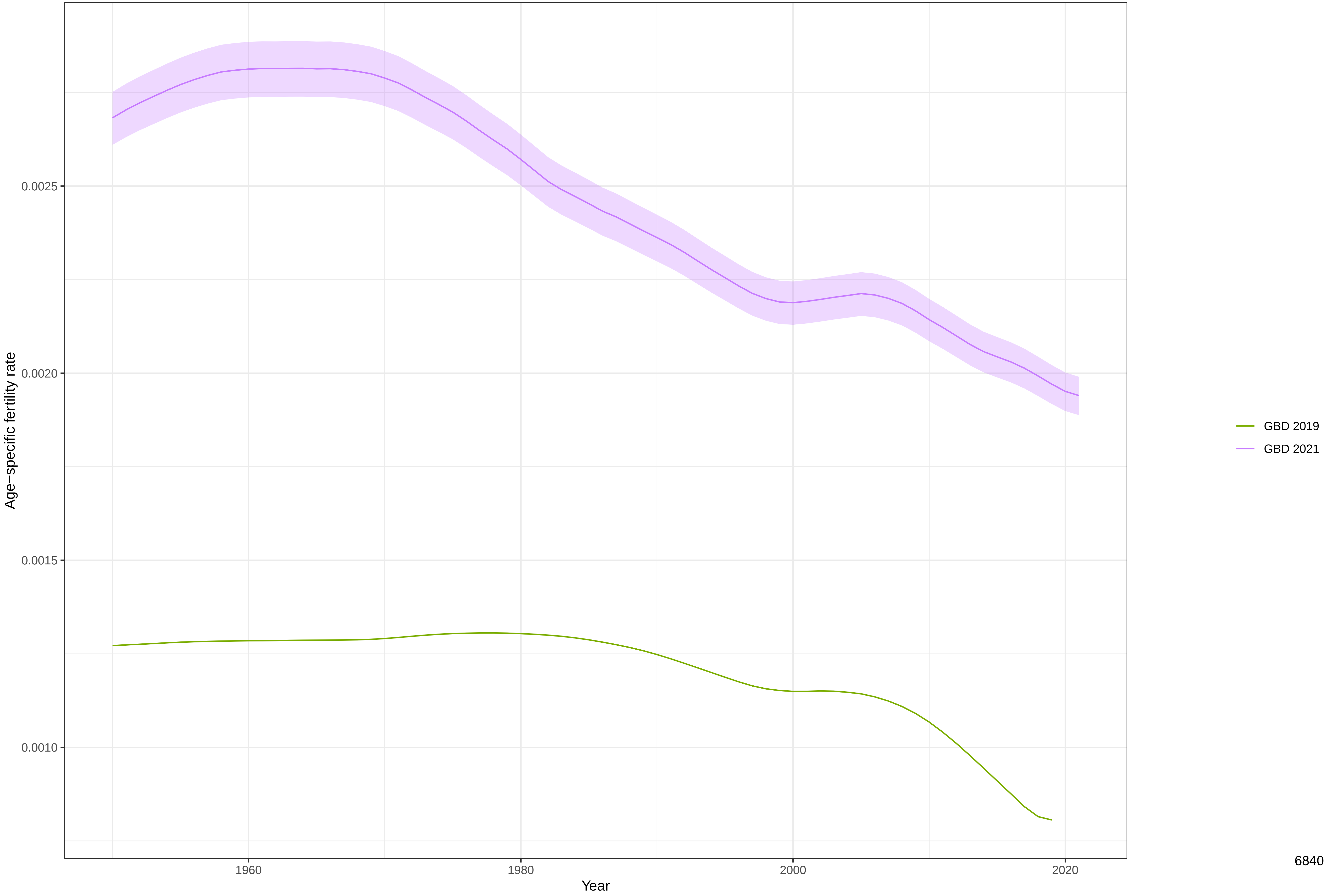

Total fertility rate fitted trend lines, 1950−2021: Oromia TFR
Data points enclosed with a diamond are reference sources

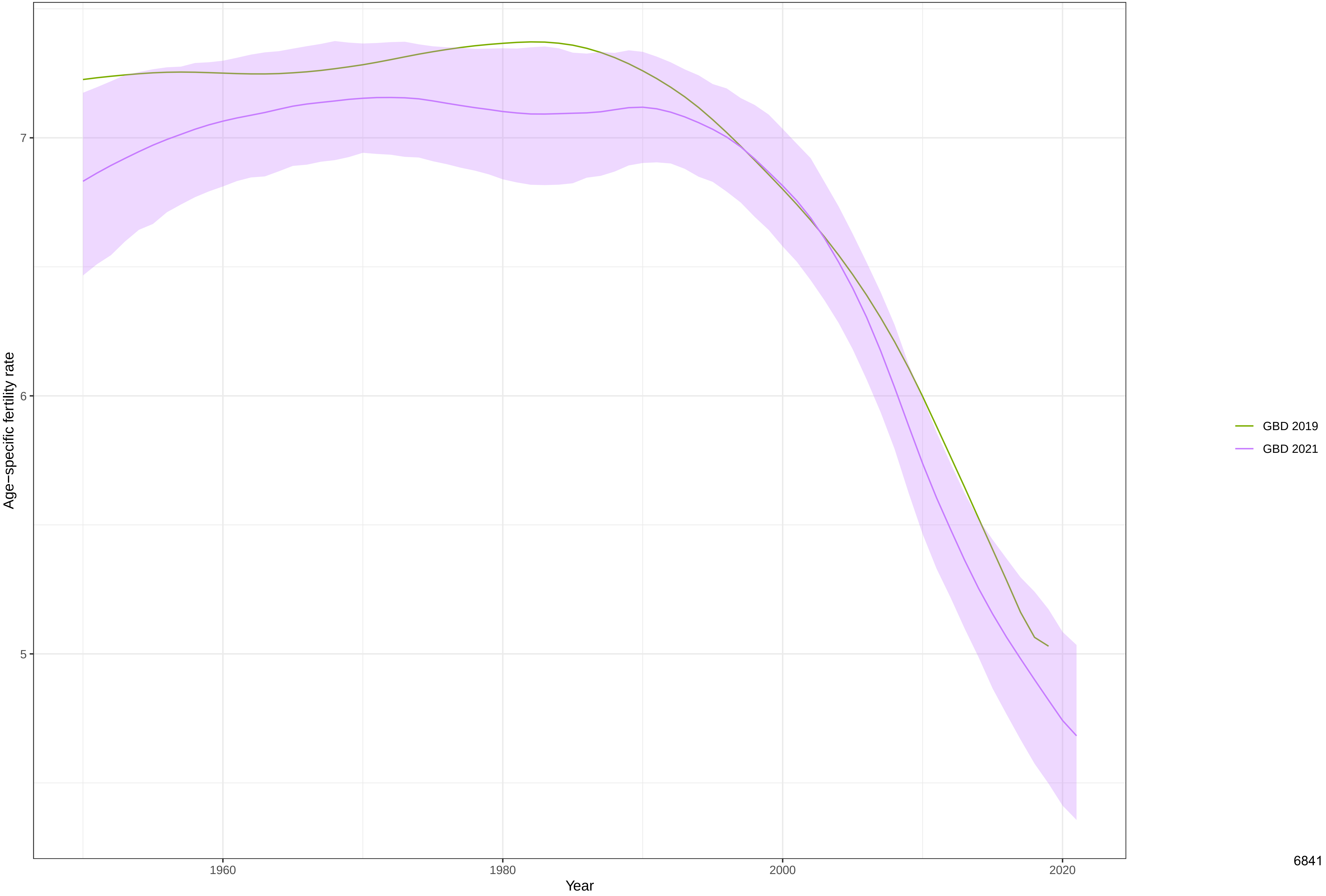

Age−specific fertility fitted trend lines, 1950−2021: Somali, Ages: 10−14
Data points enclosed with a diamond are reference sources

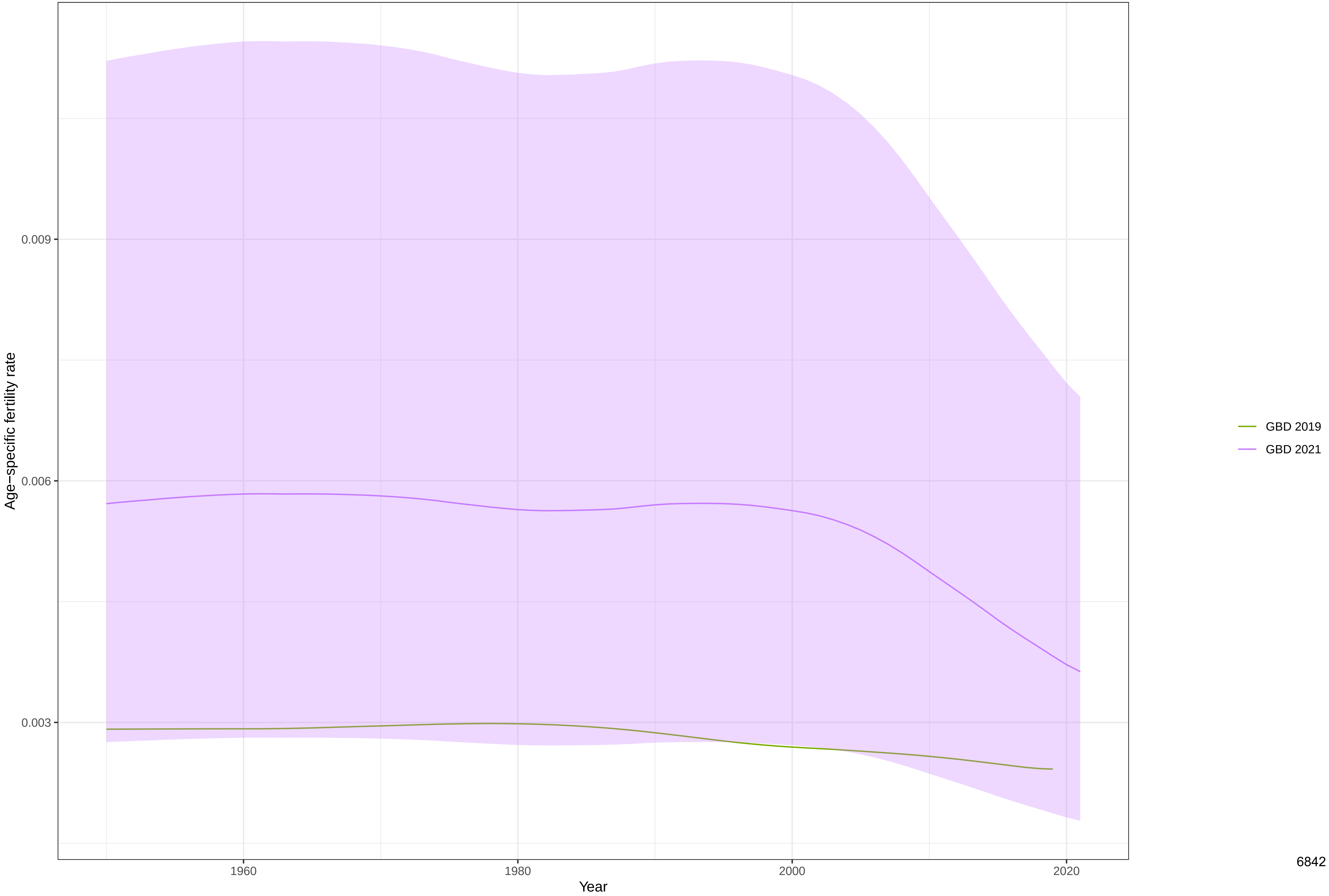

Age−specific fertility fitted trend lines, 1950−2021: Somali, Ages: 15−19
Data points enclosed with a diamond are reference sources

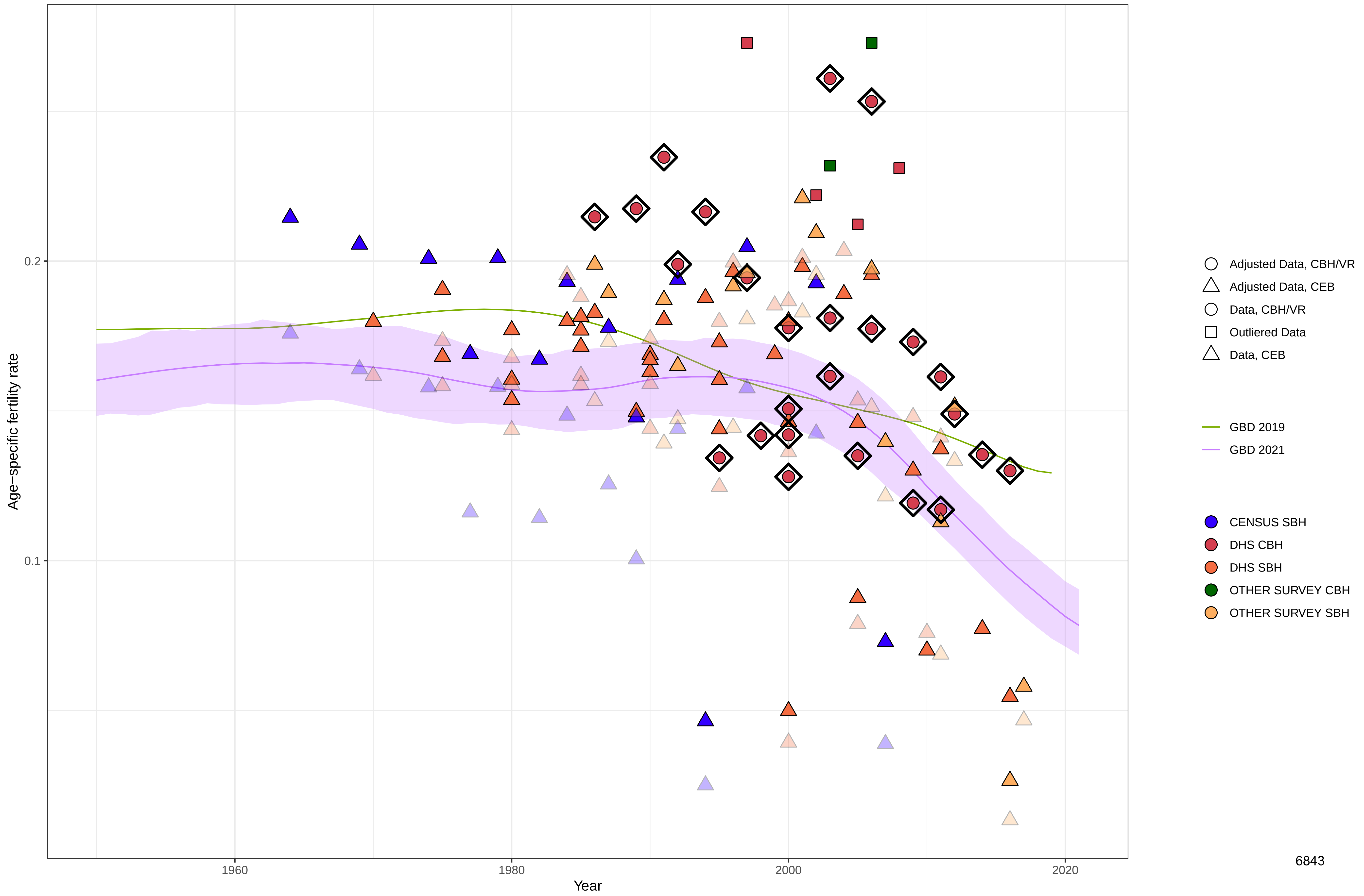

Age−specific fertility fitted trend lines, 1950−2021: Somali, Ages: 20−24
Data points enclosed with a diamond are reference sources

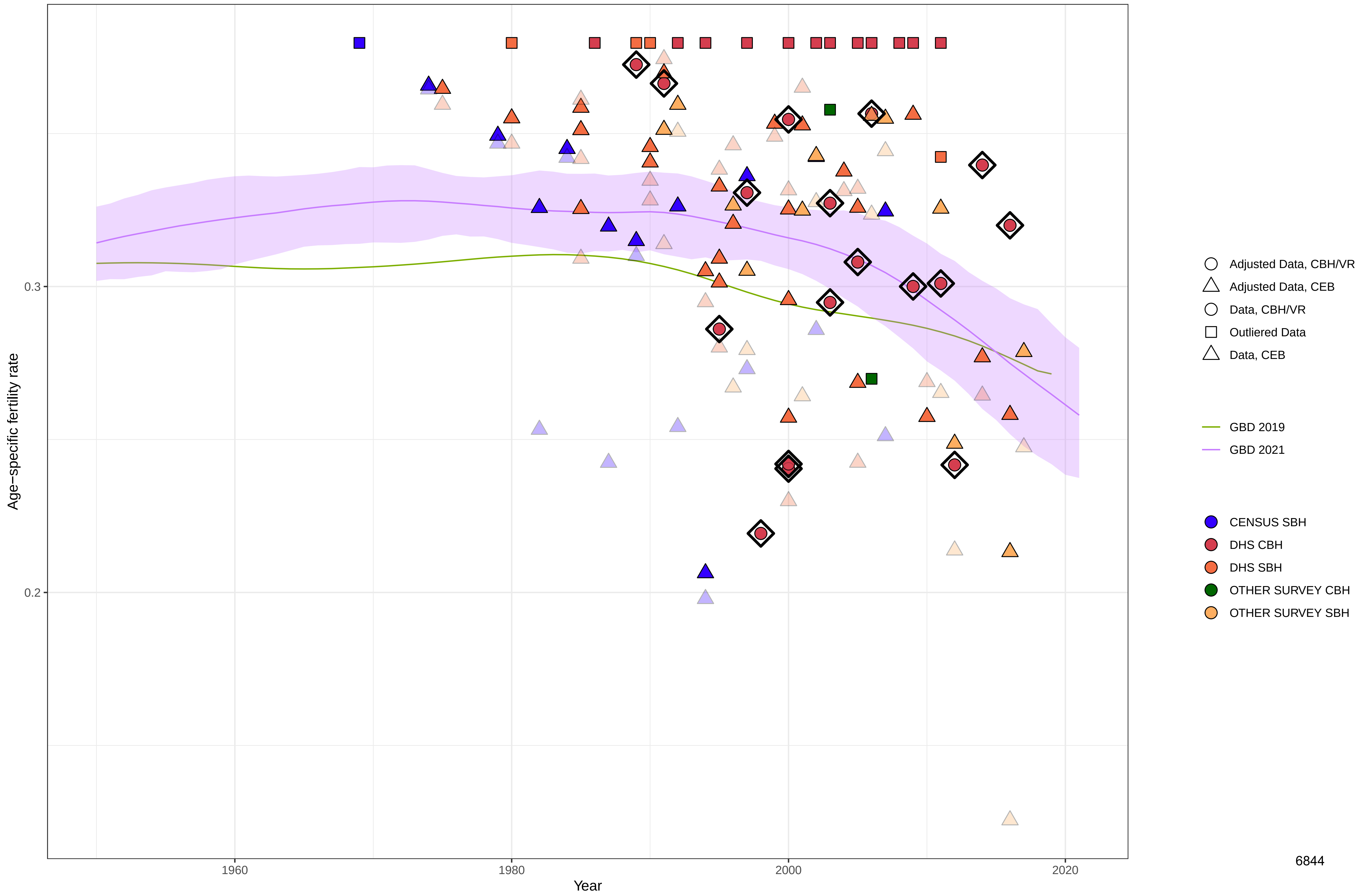

Age−specific fertility fitted trend lines, 1950−2021: Somali, Ages: 25−29
Data points enclosed with a diamond are reference sources

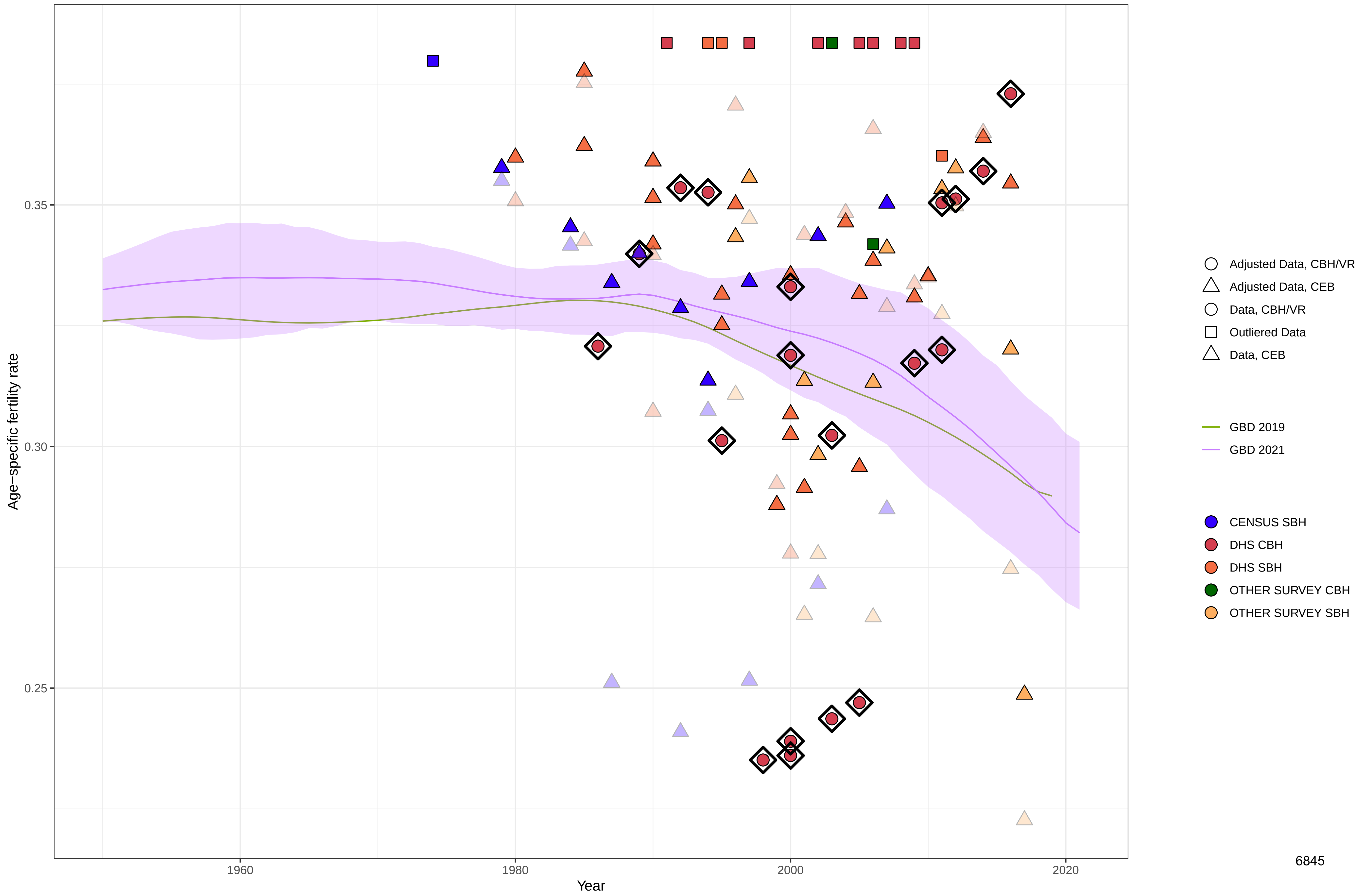

Age−specific fertility fitted trend lines, 1950−2021: Somali, Ages: 30−34
Data points enclosed with a diamond are reference sources

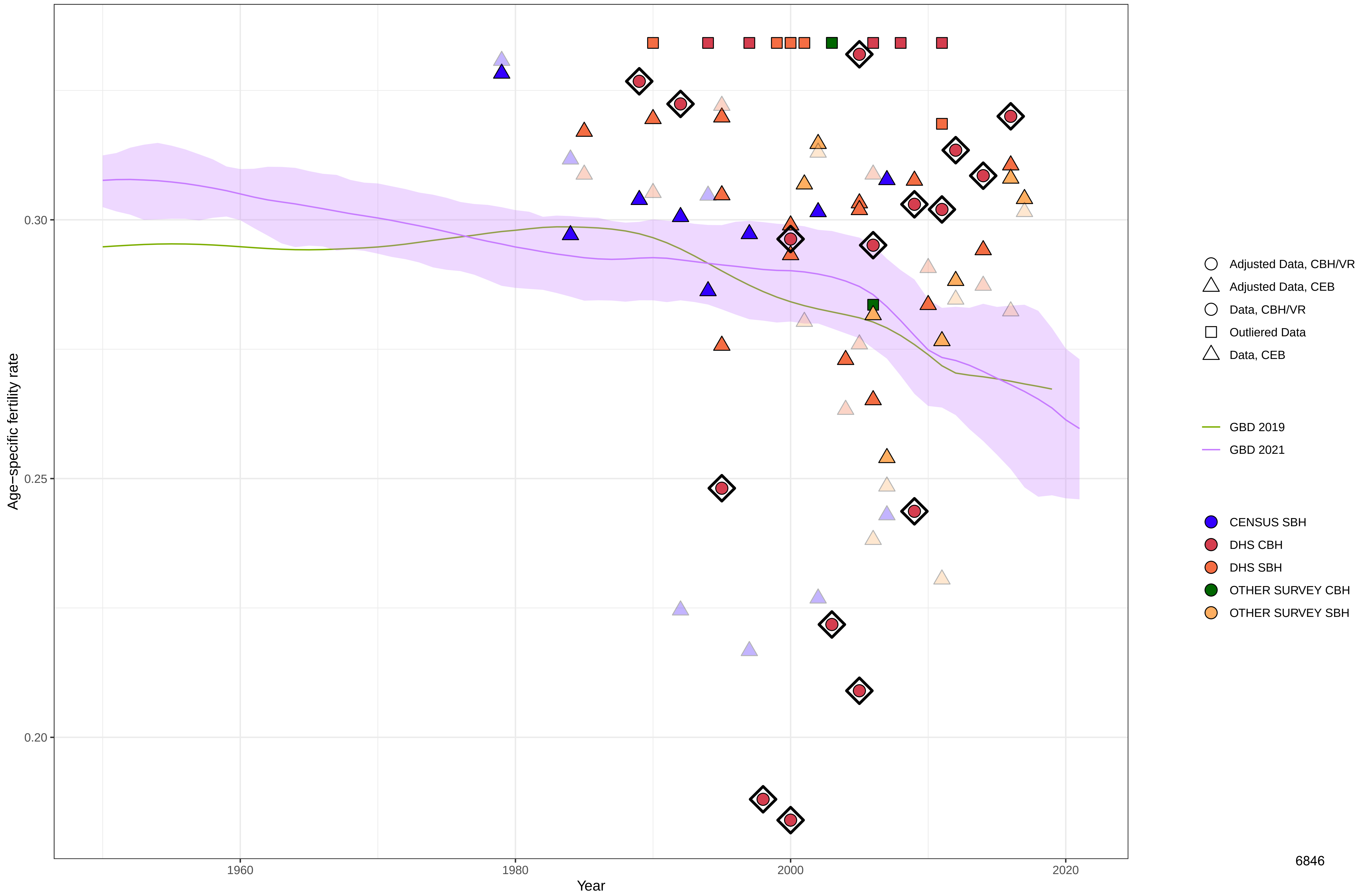

Age−specific fertility fitted trend lines, 1950−2021: Somali, Ages: 35−39
Data points enclosed with a diamond are reference sources

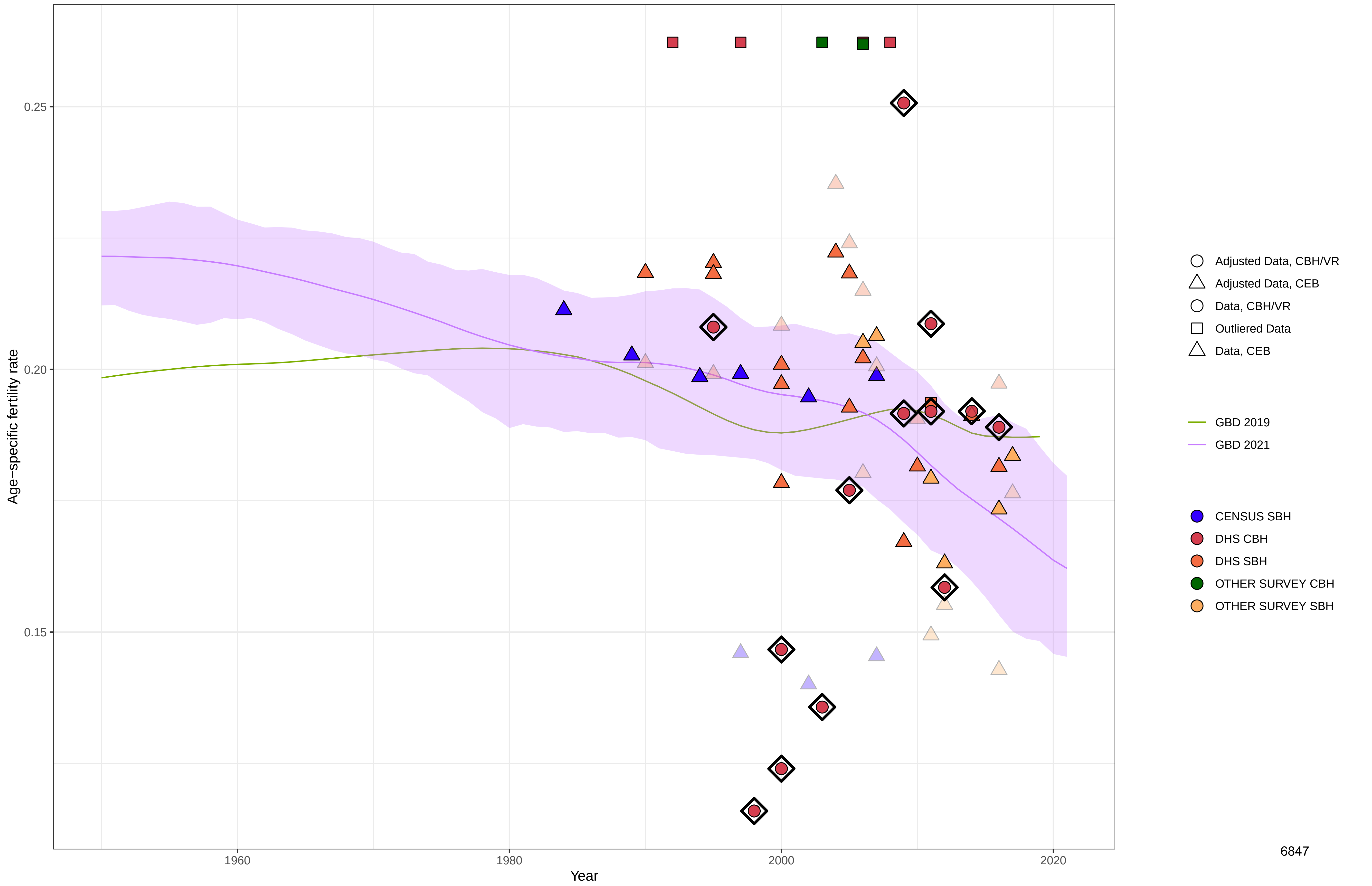

Age−specific fertility fitted trend lines, 1950−2021: Somali, Ages: 40−44
Data points enclosed with a diamond are reference sources

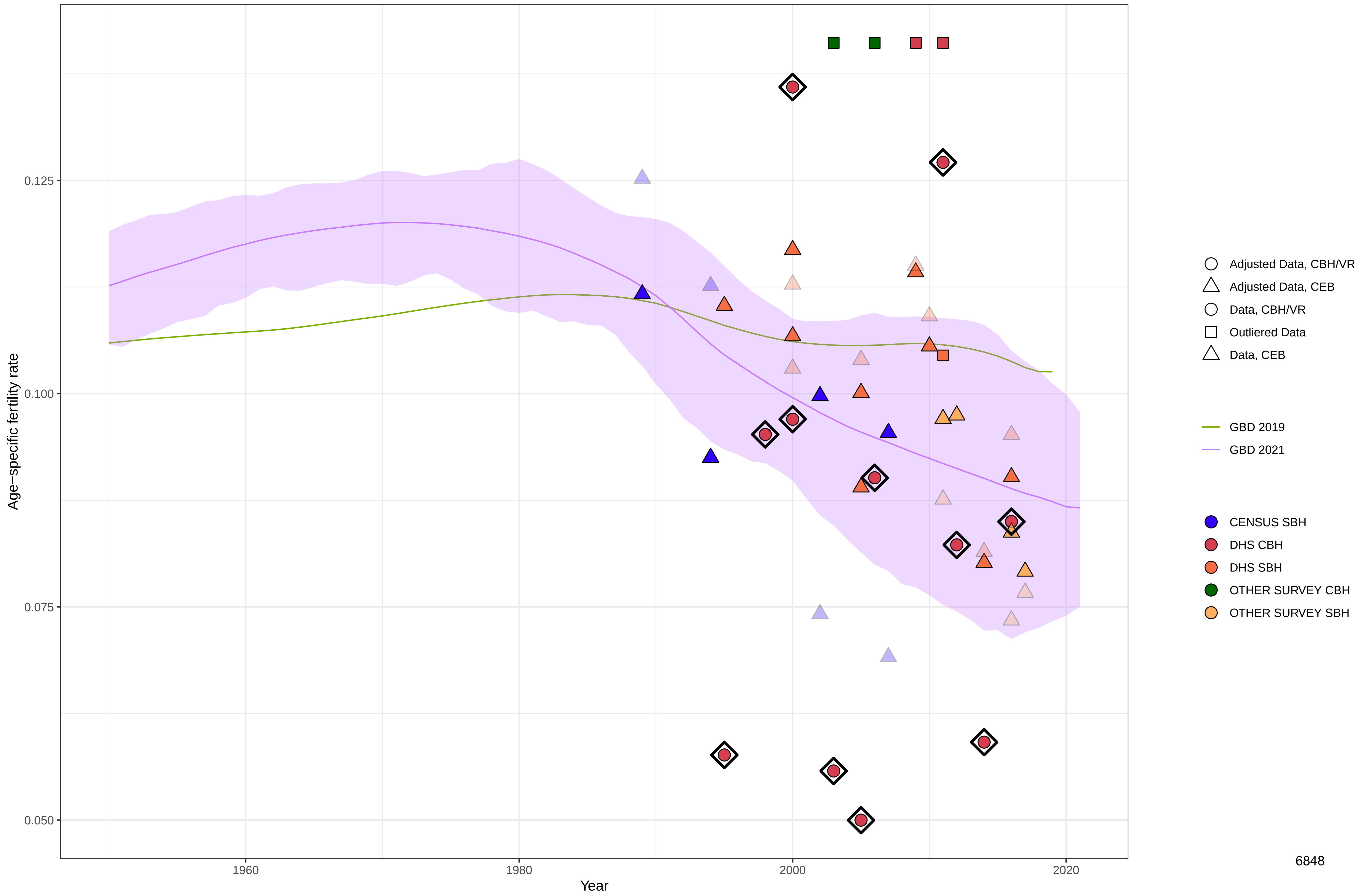

Age−specific fertility fitted trend lines, 1950−2021: Somali, Ages: 45−49
Data points enclosed with a diamond are reference sources

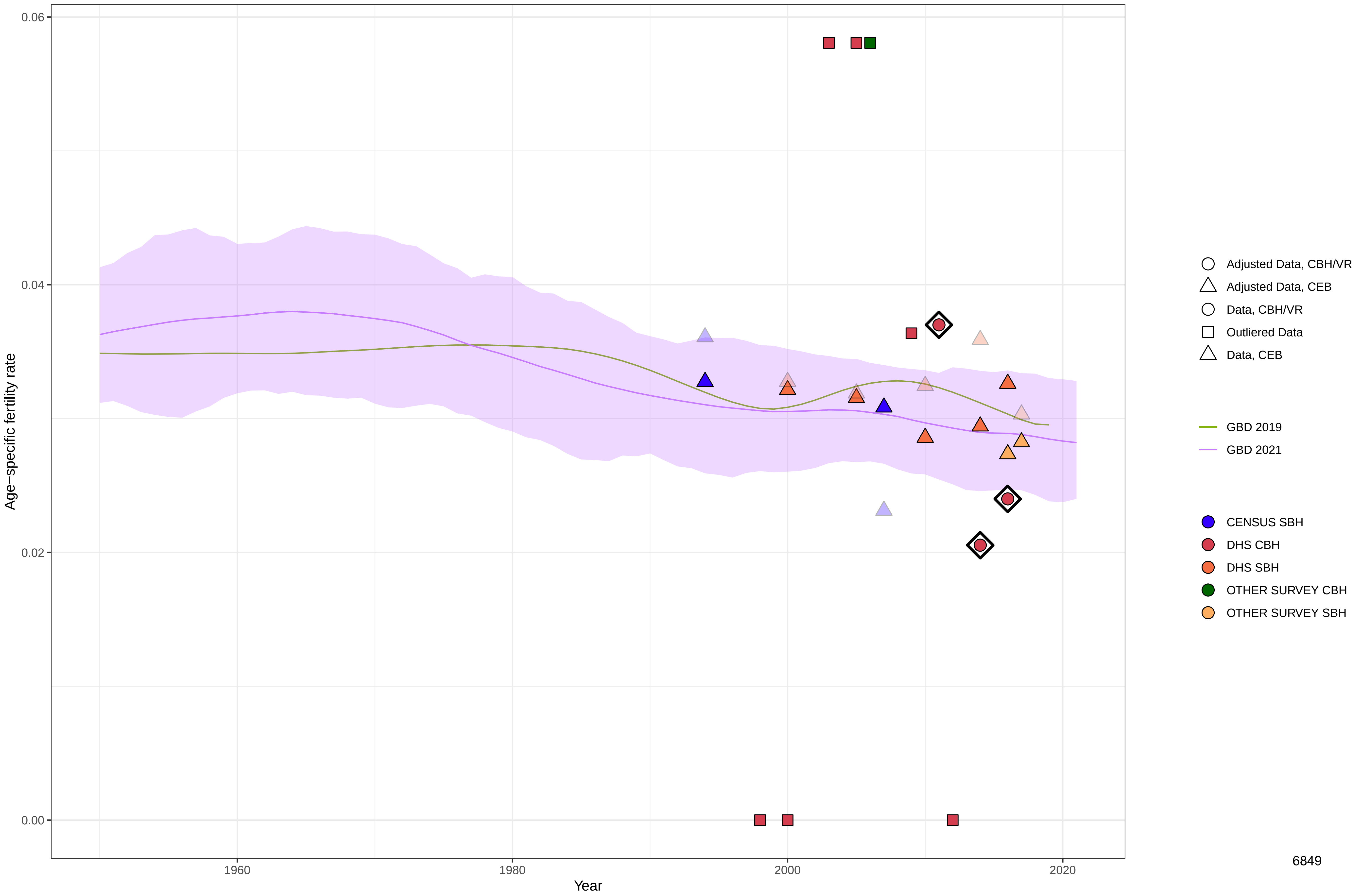

Age−specific fertility fitted trend lines, 1950−2021: Somali, Ages: 50−54
Data points enclosed with a diamond are reference sources

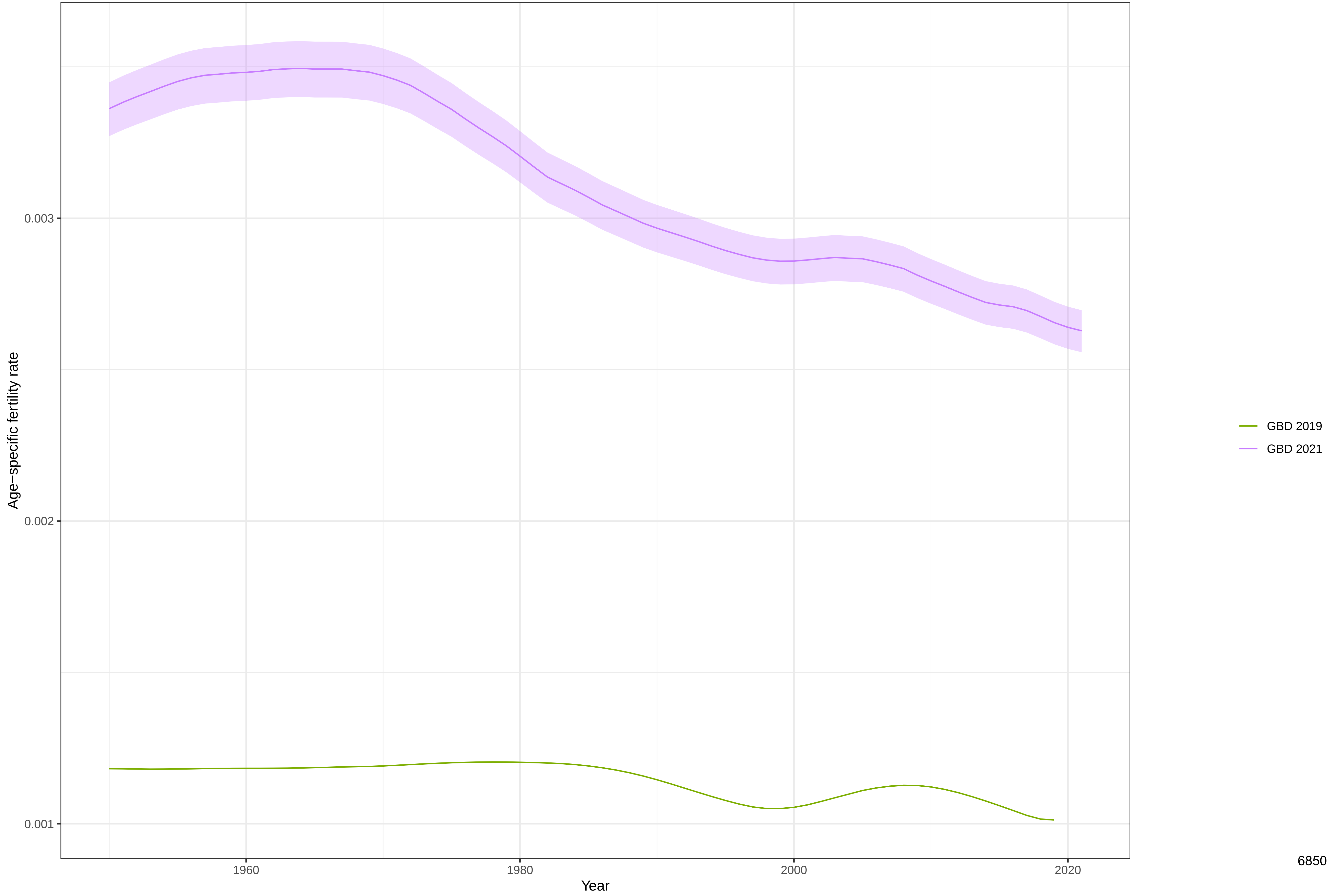

Total fertility rate fitted trend lines, 1950−2021: Somali TFR
Data points enclosed with a diamond are reference sources

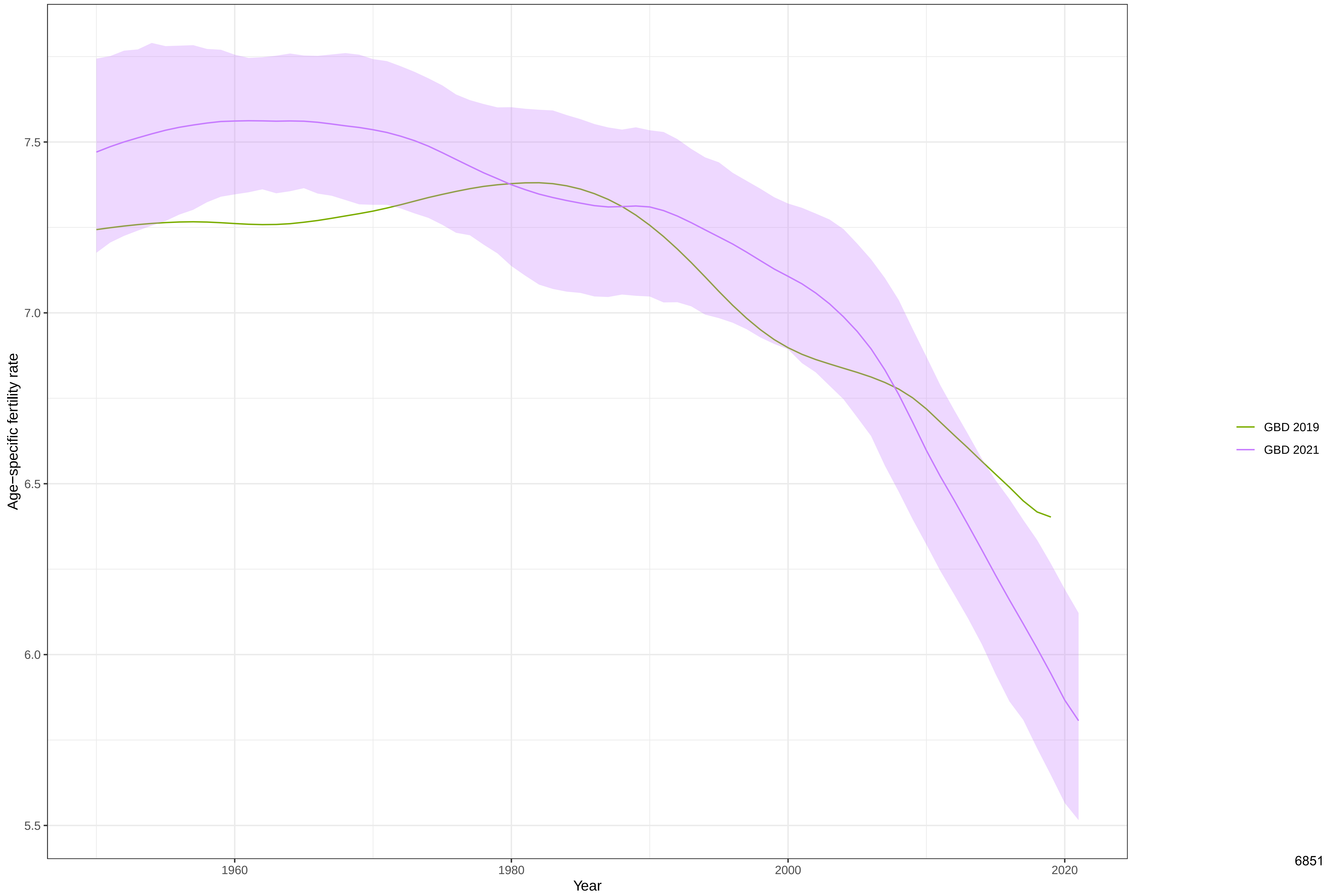

Age−specific fertility fitted trend lines, 1950−2021: Southern Nations, Nationalities, and Peoples, Ages: 10−14
Data points enclosed with a diamond are reference sources

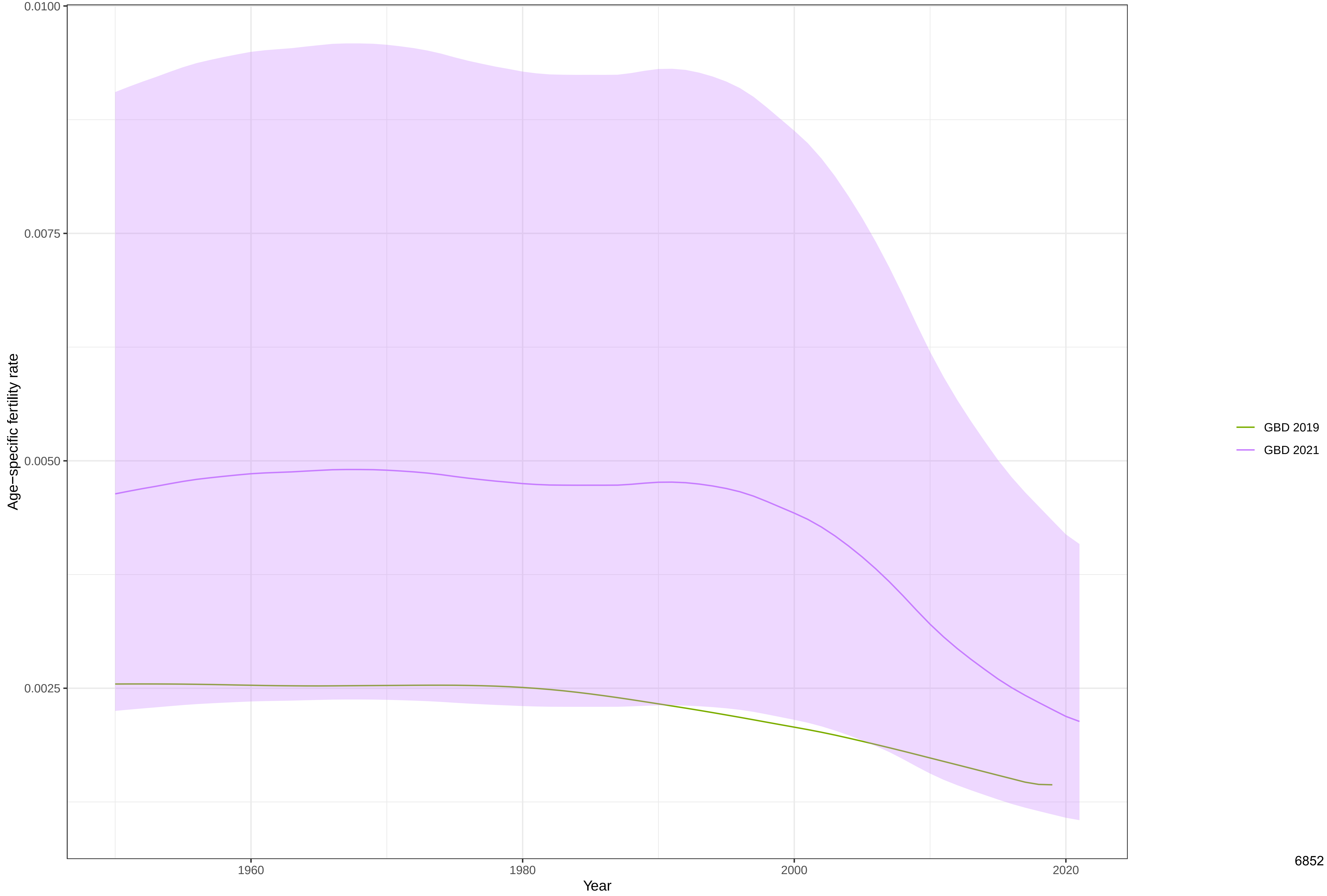

Age−specific fertility fitted trend lines, 1950−2021: Southern Nations, Nationalities, and Peoples, Ages: 15−19
Data points enclosed with a diamond are reference sources

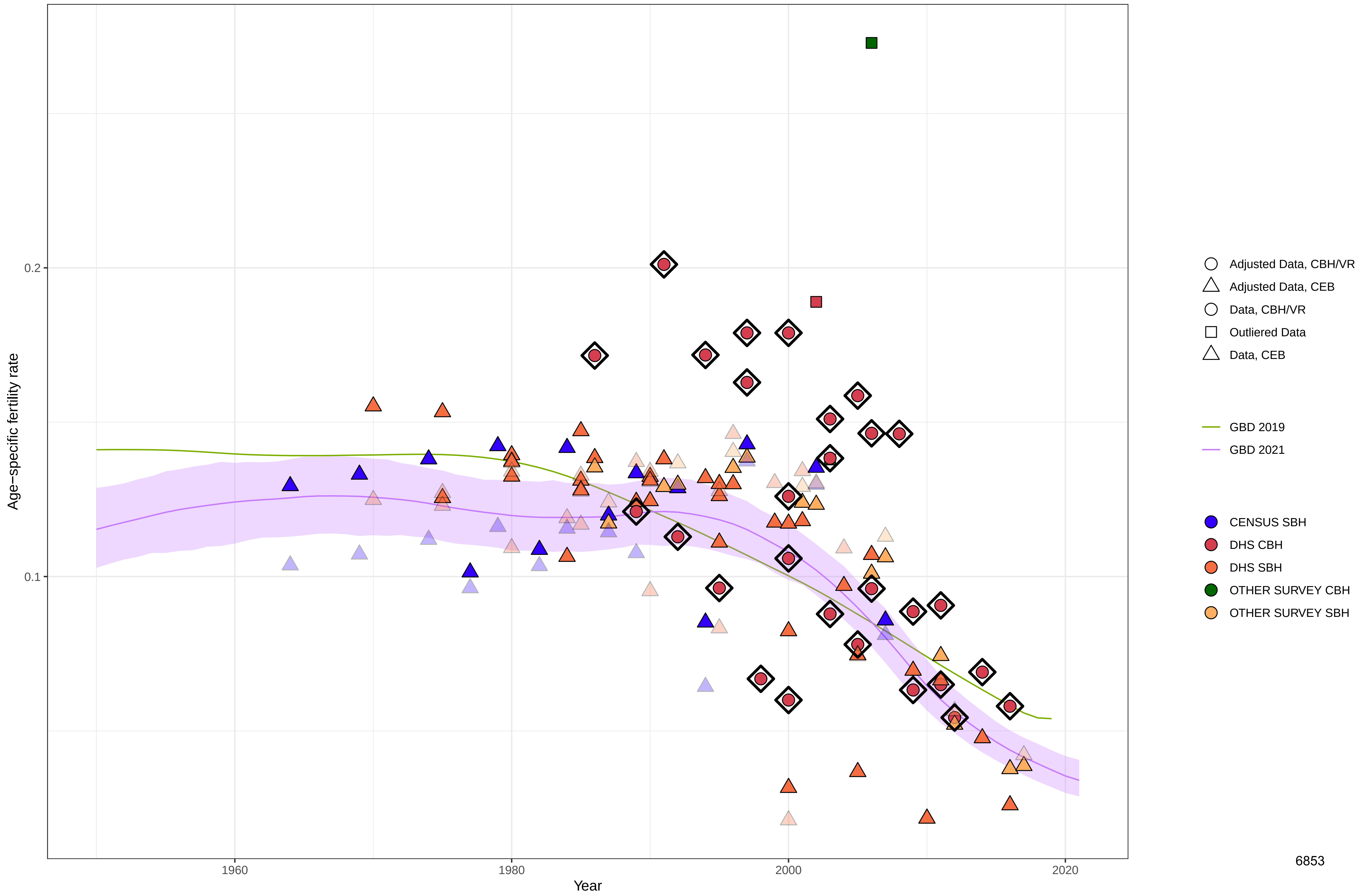

Age−specific fertility fitted trend lines, 1950−2021: Southern Nations, Nationalities, and Peoples, Ages: 20−24
Data points enclosed with a diamond are reference sources

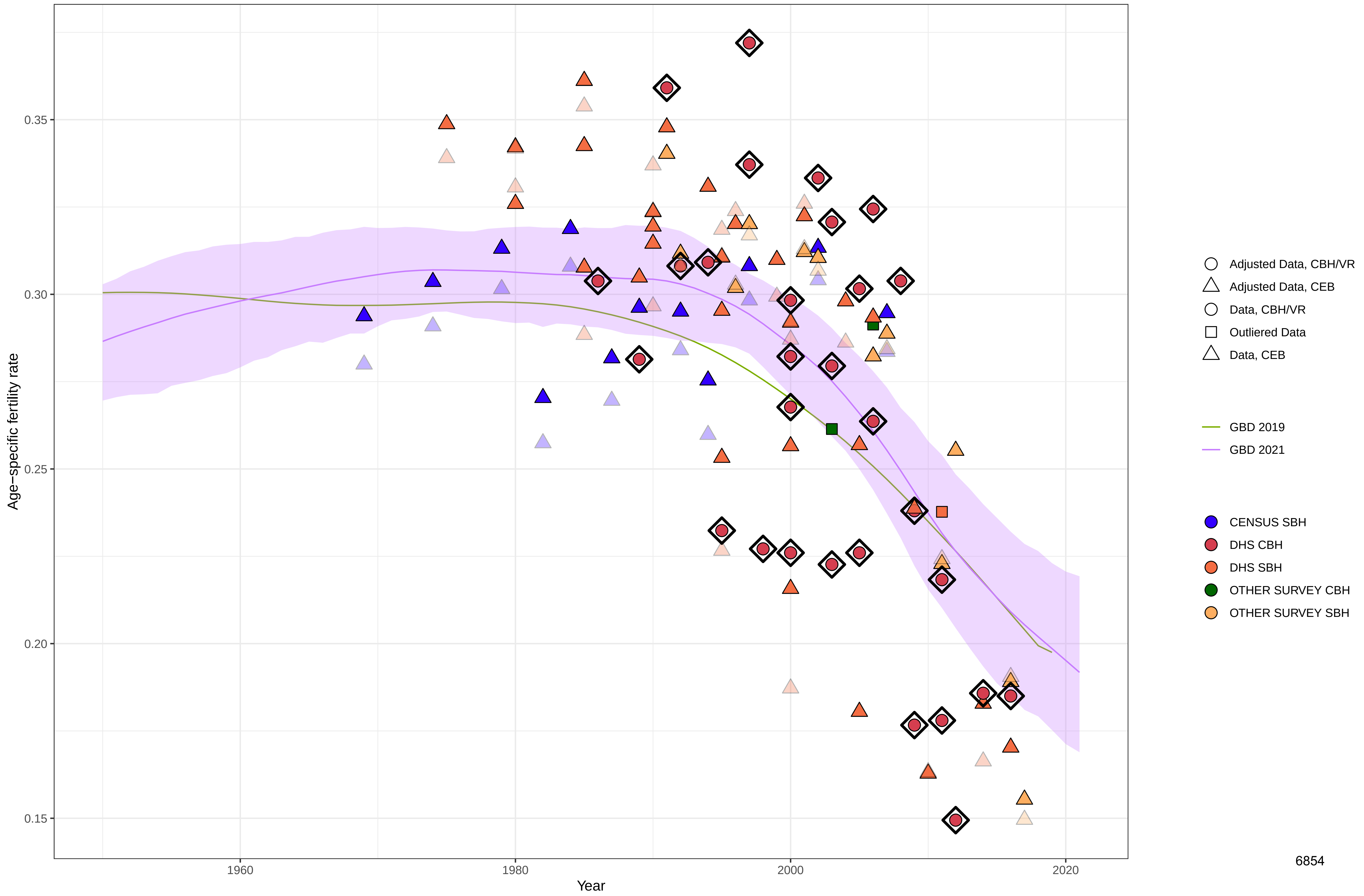

Age−specific fertility fitted trend lines, 1950−2021: Southern Nations, Nationalities, and Peoples, Ages: 25−29
Data points enclosed with a diamond are reference sources

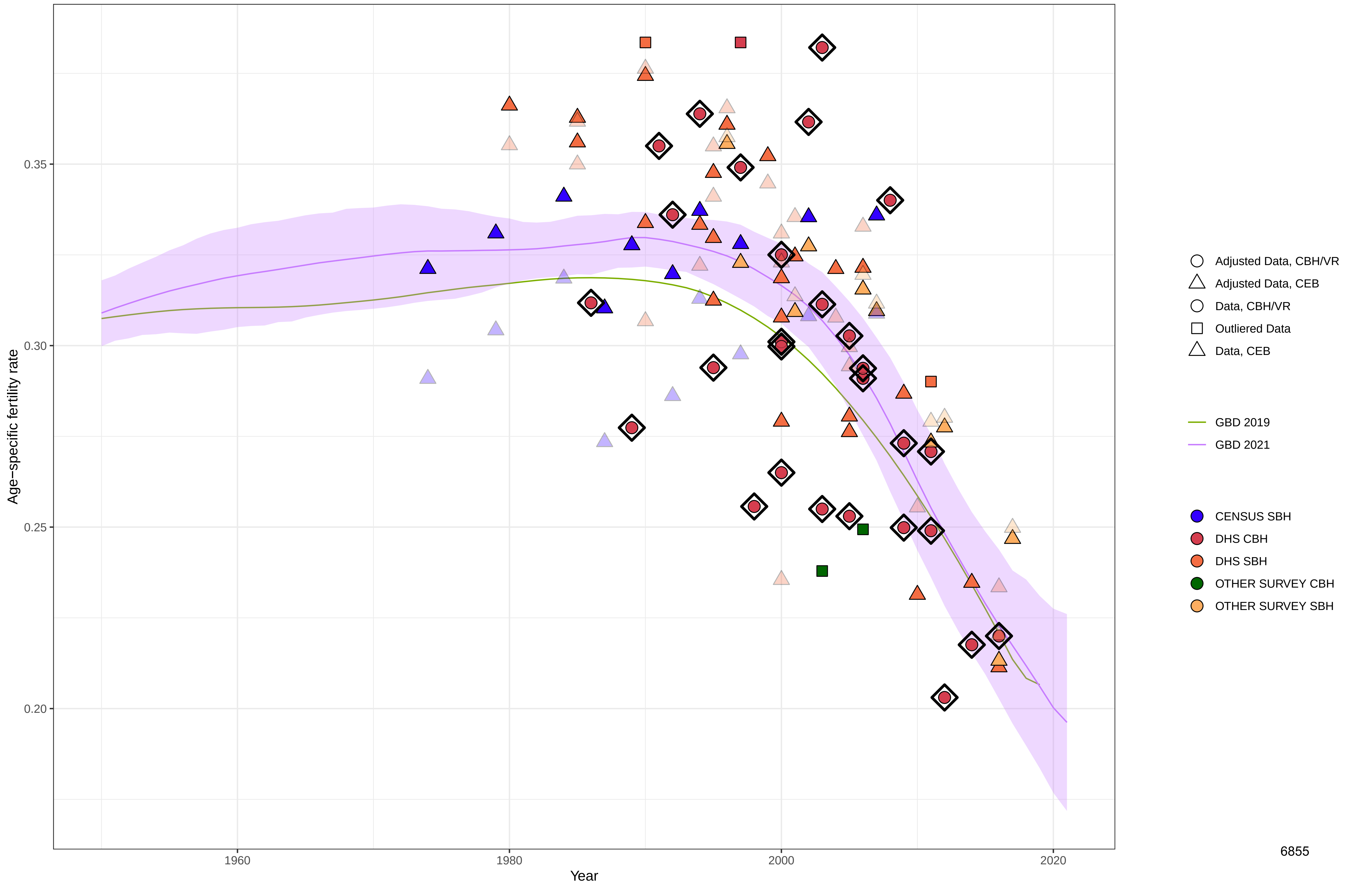

Age−specific fertility fitted trend lines, 1950−2021: Southern Nations, Nationalities, and Peoples, Ages: 30−34
Data points enclosed with a diamond are reference sources

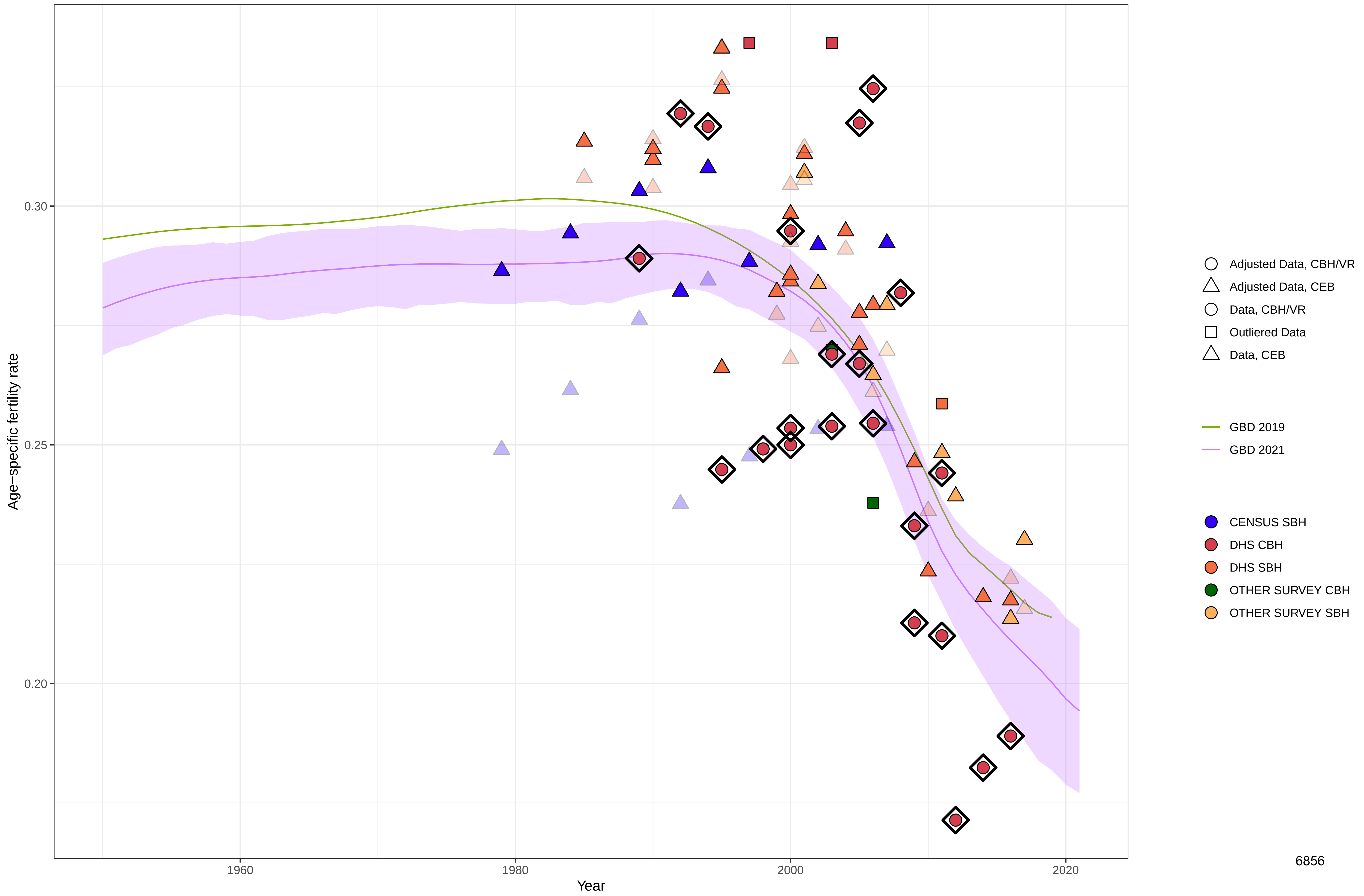

Age−specific fertility fitted trend lines, 1950−2021: Southern Nations, Nationalities, and Peoples, Ages: 35−39
Data points enclosed with a diamond are reference sources

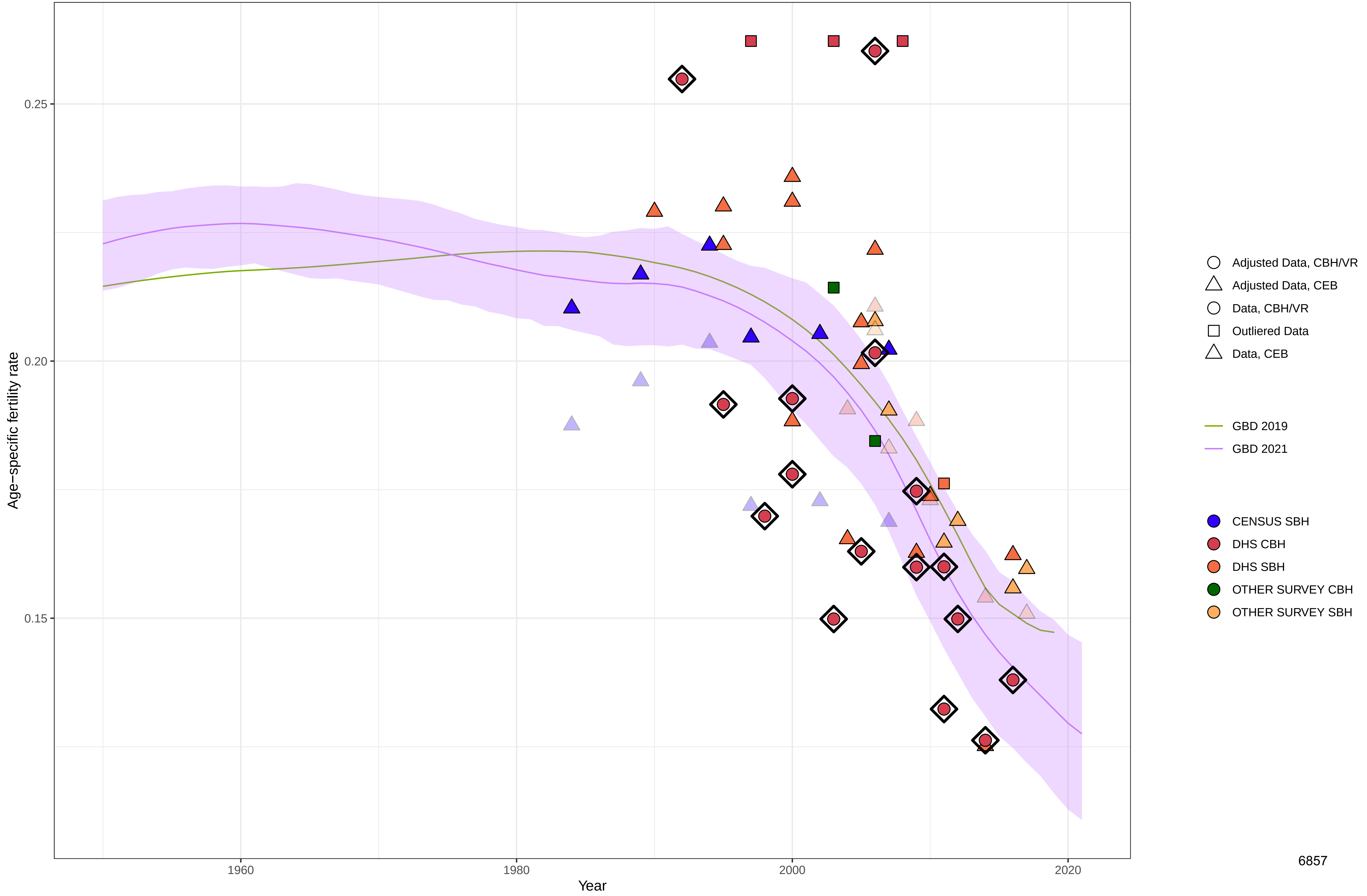

Age−specific fertility fitted trend lines, 1950−2021: Southern Nations, Nationalities, and Peoples, Ages: 40−44
Data points enclosed with a diamond are reference sources

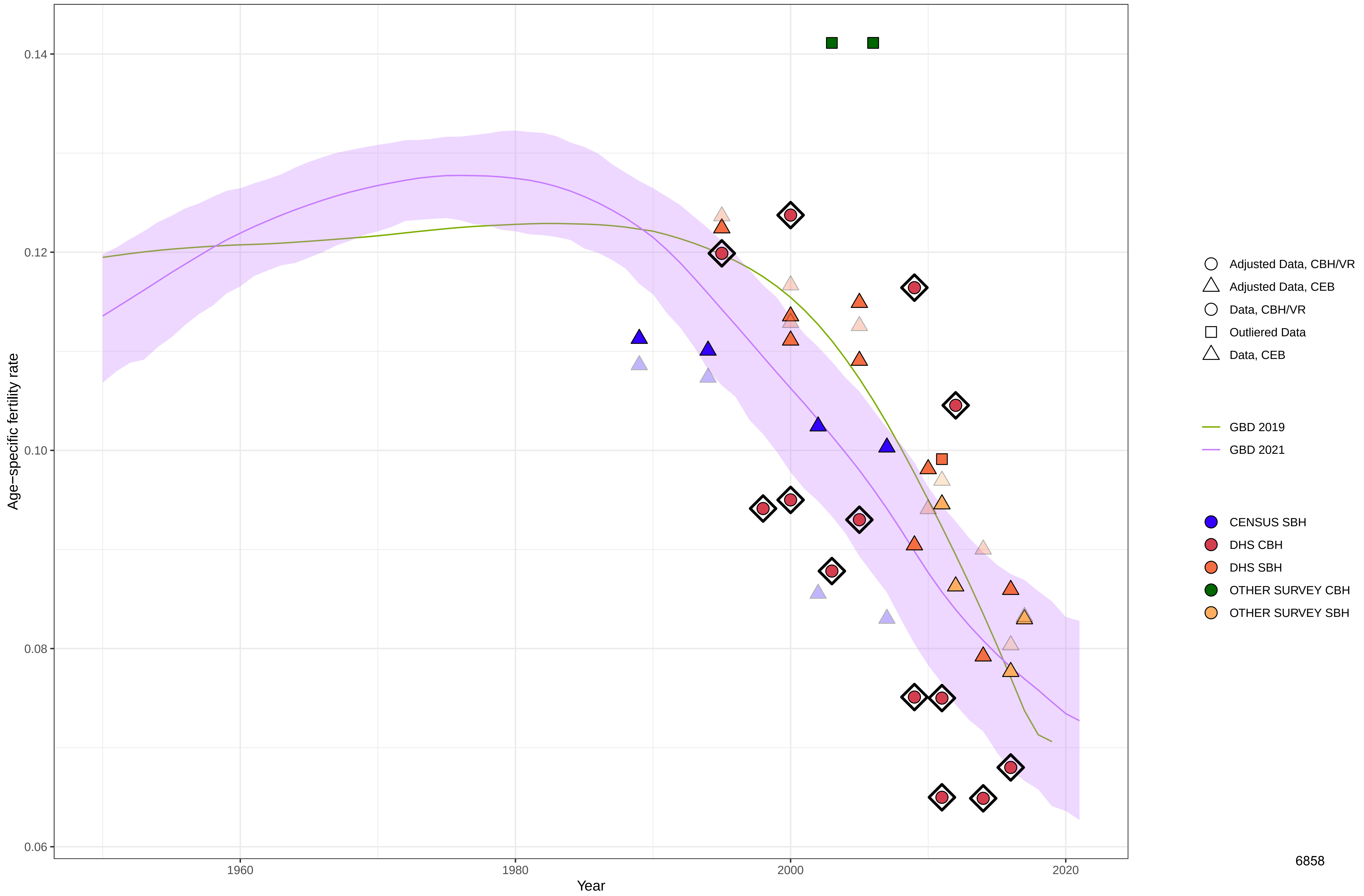

Age−specific fertility fitted trend lines, 1950−2021: Southern Nations, Nationalities, and Peoples, Ages: 45−49
Data points enclosed with a diamond are reference sources

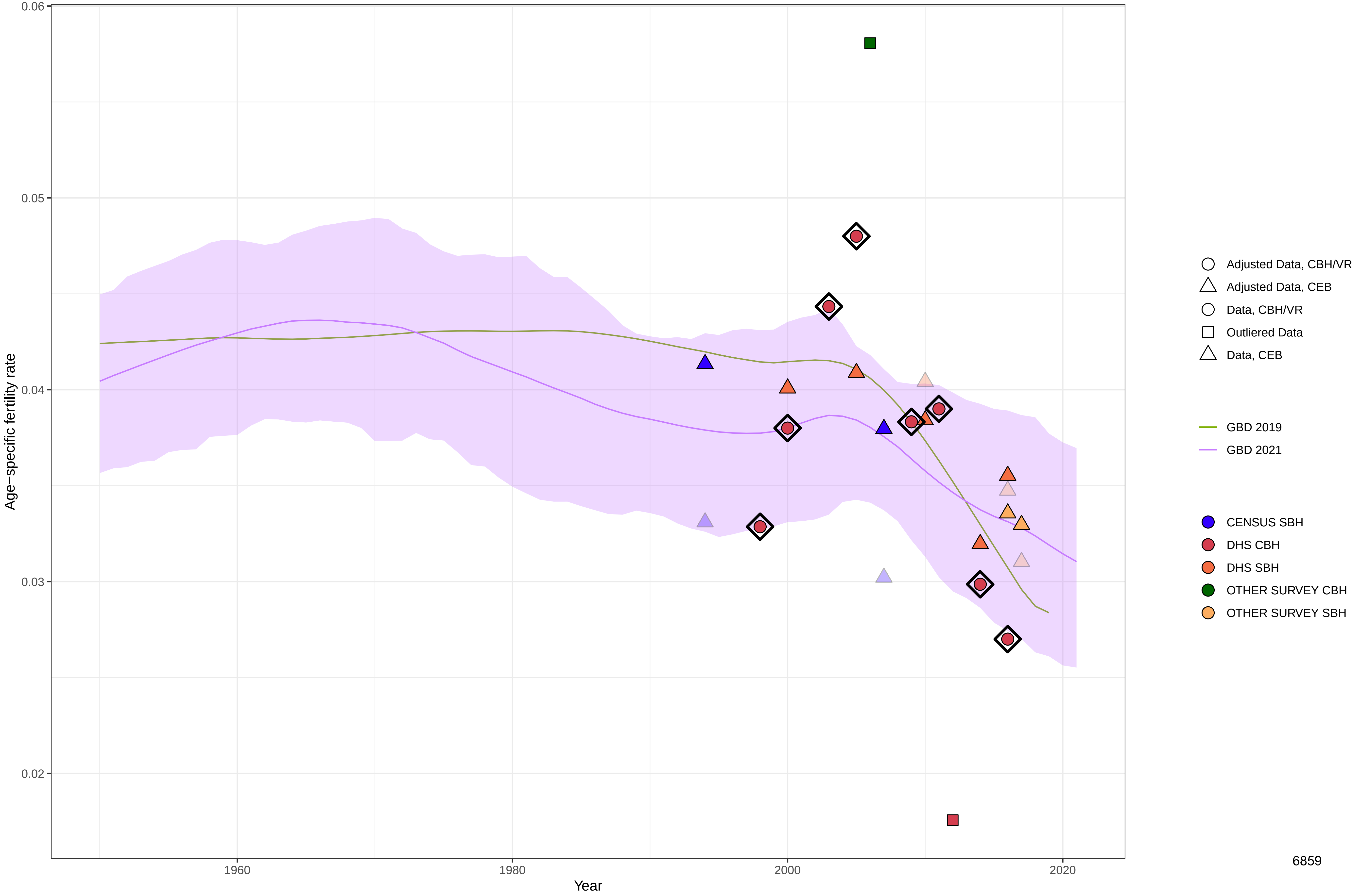

Age−specific fertility fitted trend lines, 1950−2021: Southern Nations, Nationalities, and Peoples, Ages: 50−54
Data points enclosed with a diamond are reference sources

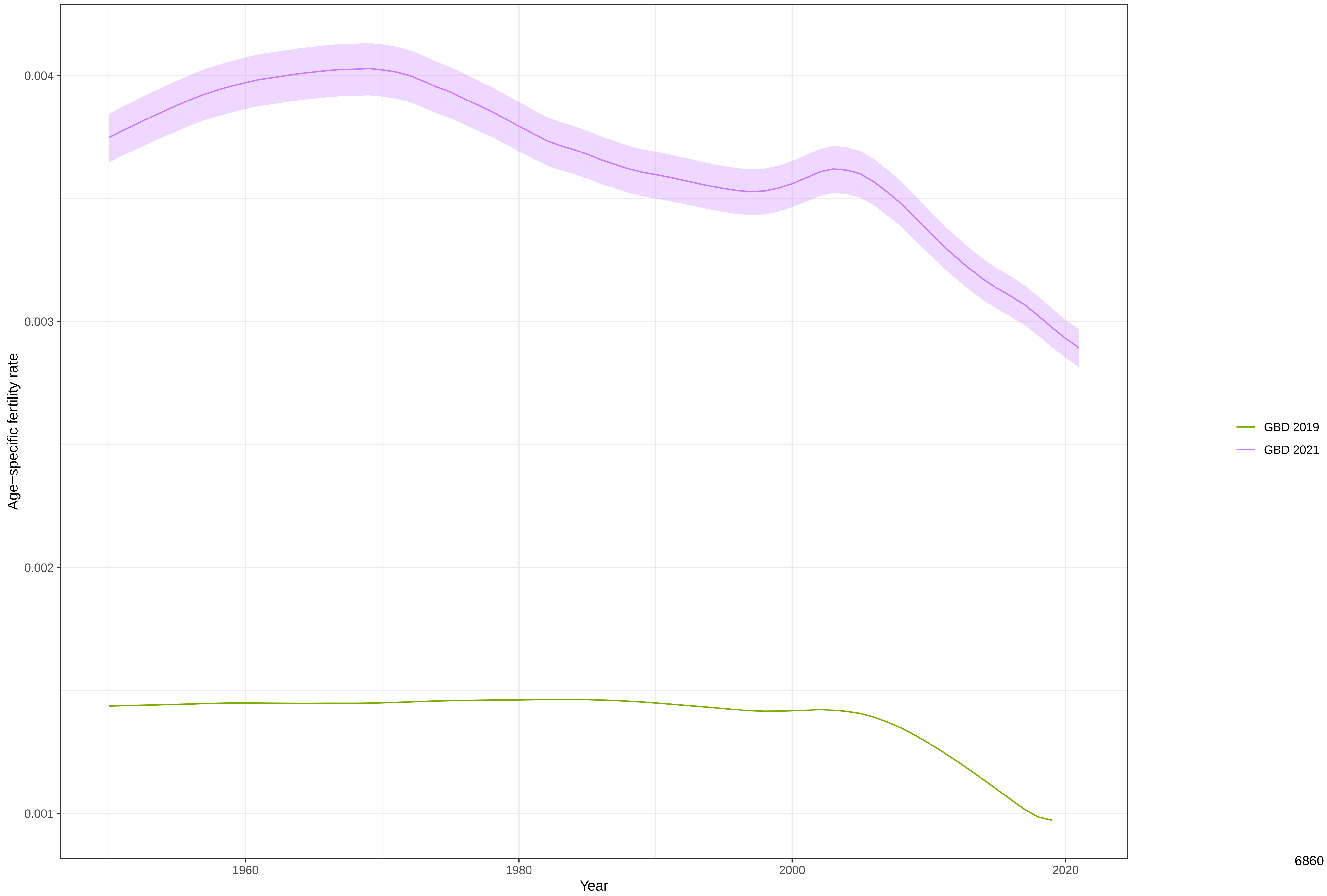

Total fertility rate fitted trend lines, 1950−2021: Southern Nations, Nationalities, and Peoples TFR
Data points enclosed with a diamond are reference sources

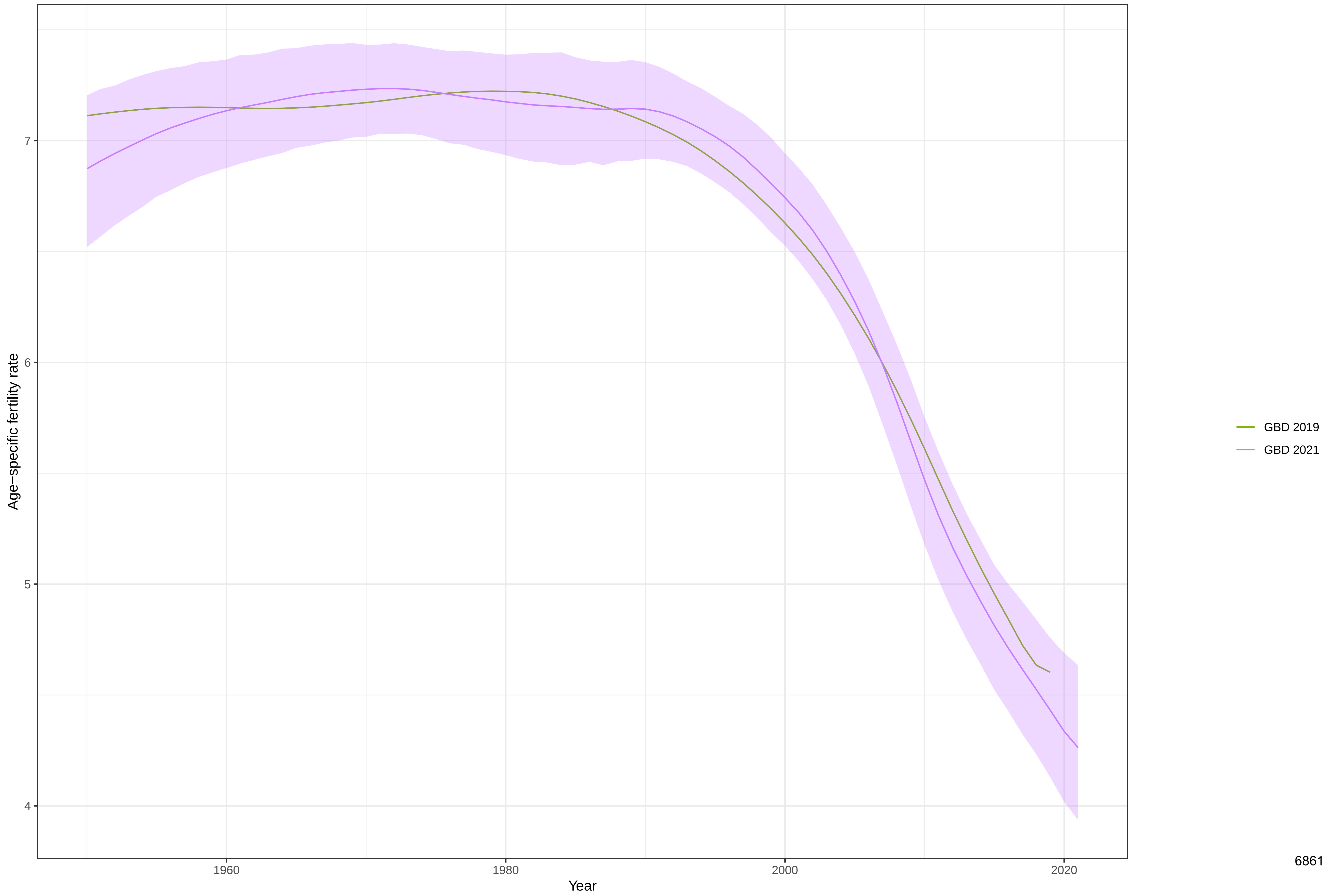

Age−specific fertility fitted trend lines, 1950−2021: Tigray, Ages: 10−14
Data points enclosed with a diamond are reference sources

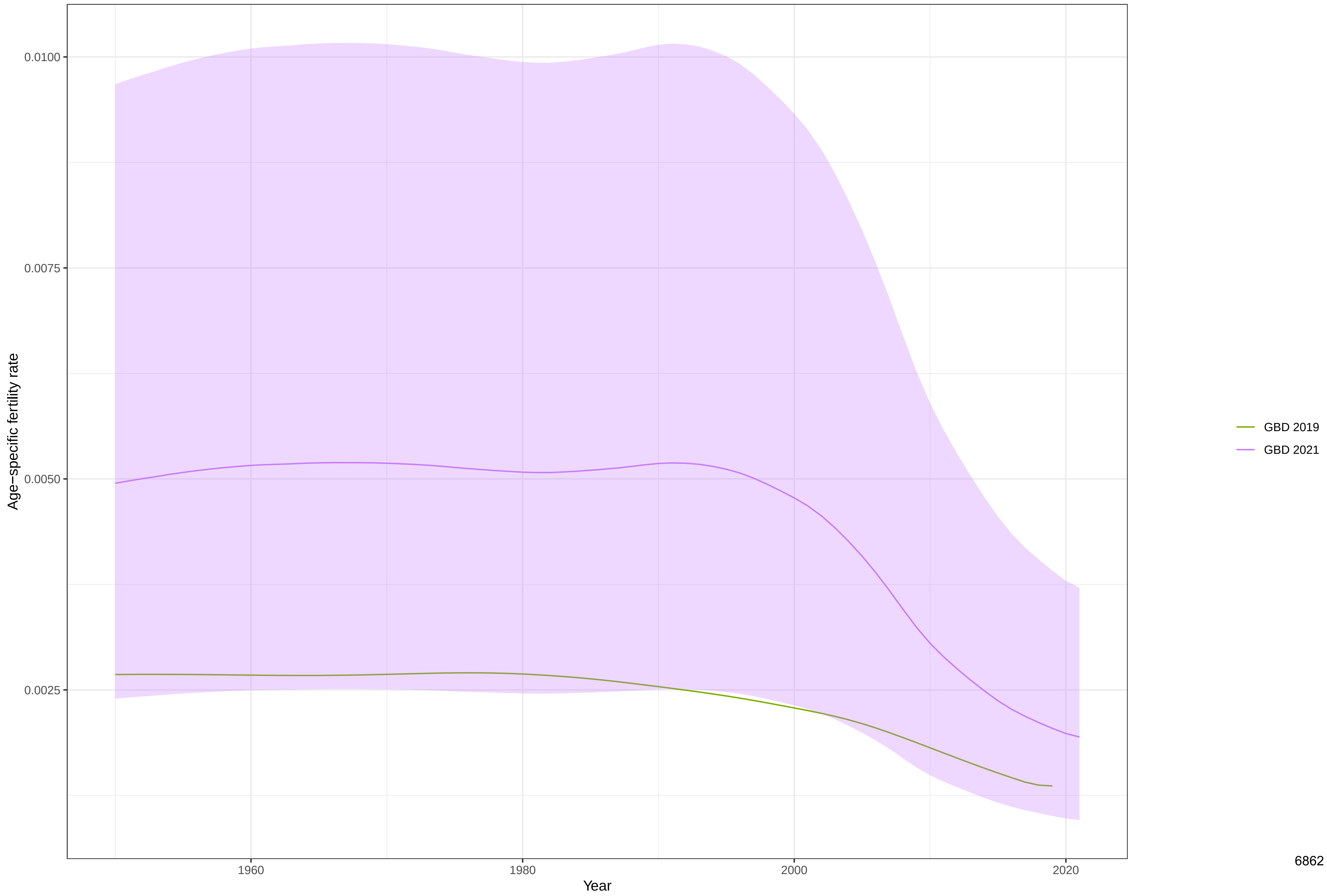

Age−specific fertility fitted trend lines, 1950−2021: Tigray, Ages: 15−19
Data points enclosed with a diamond are reference sources

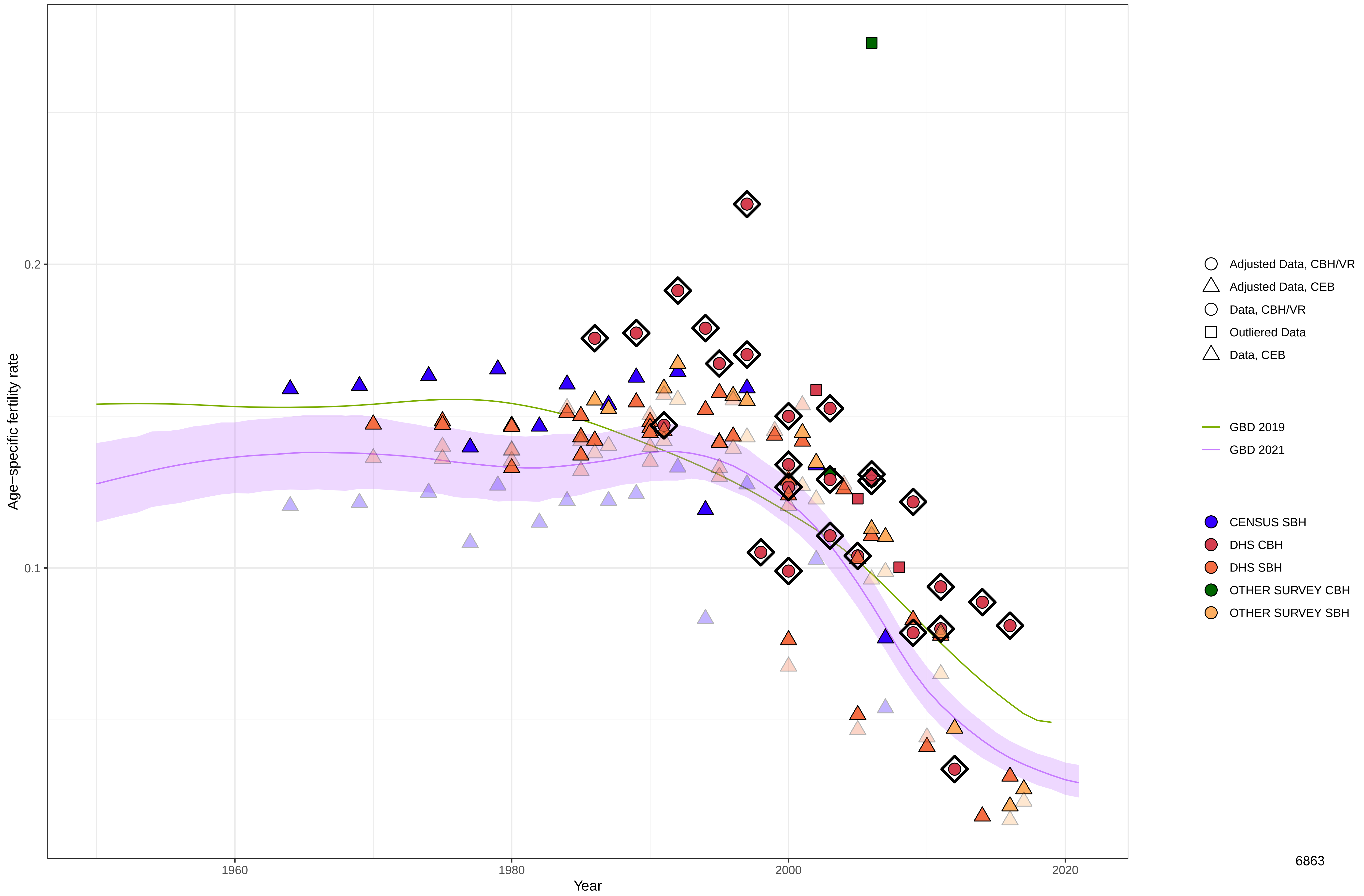

Age−specific fertility fitted trend lines, 1950−2021: Tigray, Ages: 20−24
Data points enclosed with a diamond are reference sources

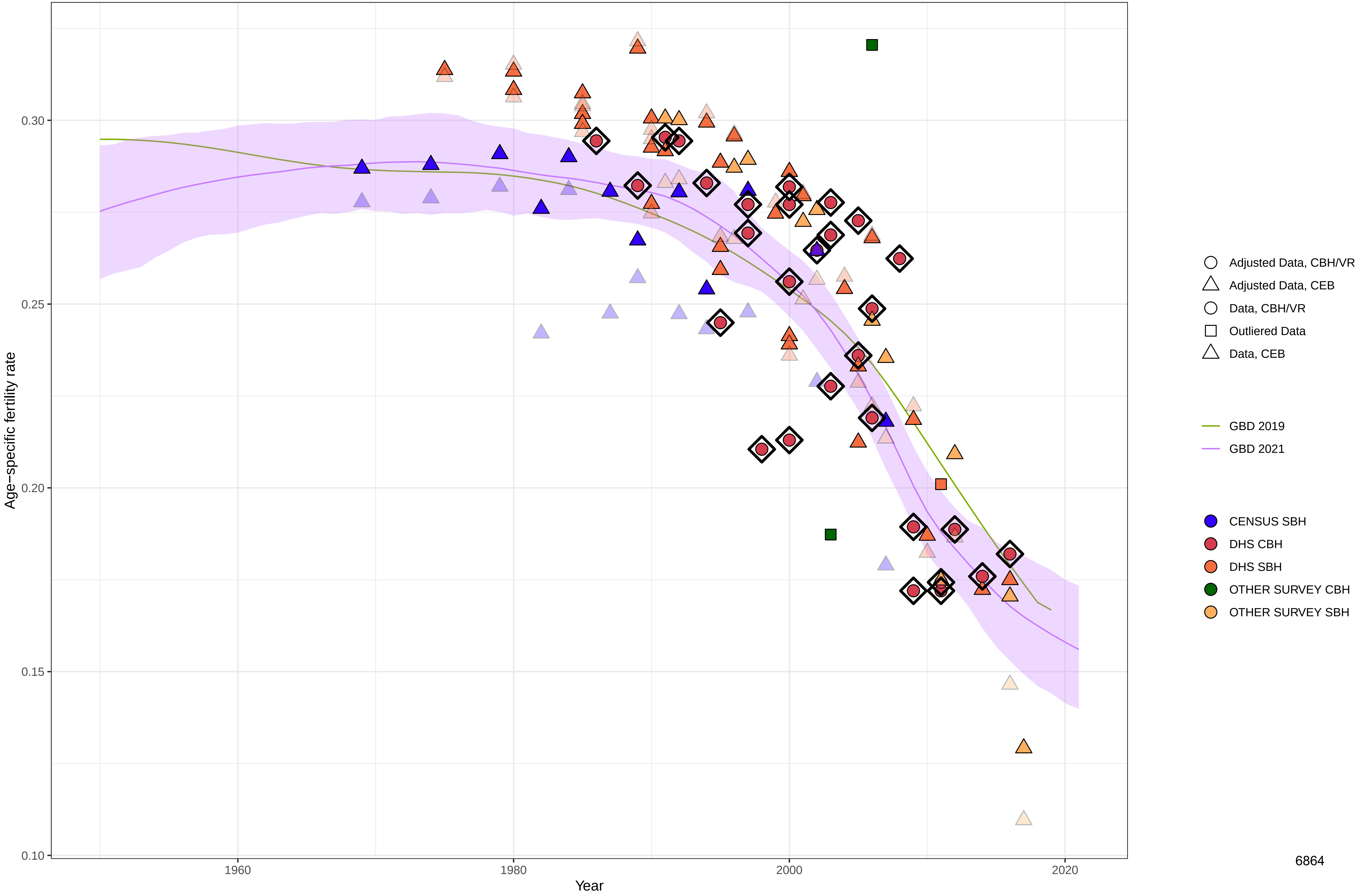

Age−specific fertility fitted trend lines, 1950−2021: Tigray, Ages: 25−29
Data points enclosed with a diamond are reference sources

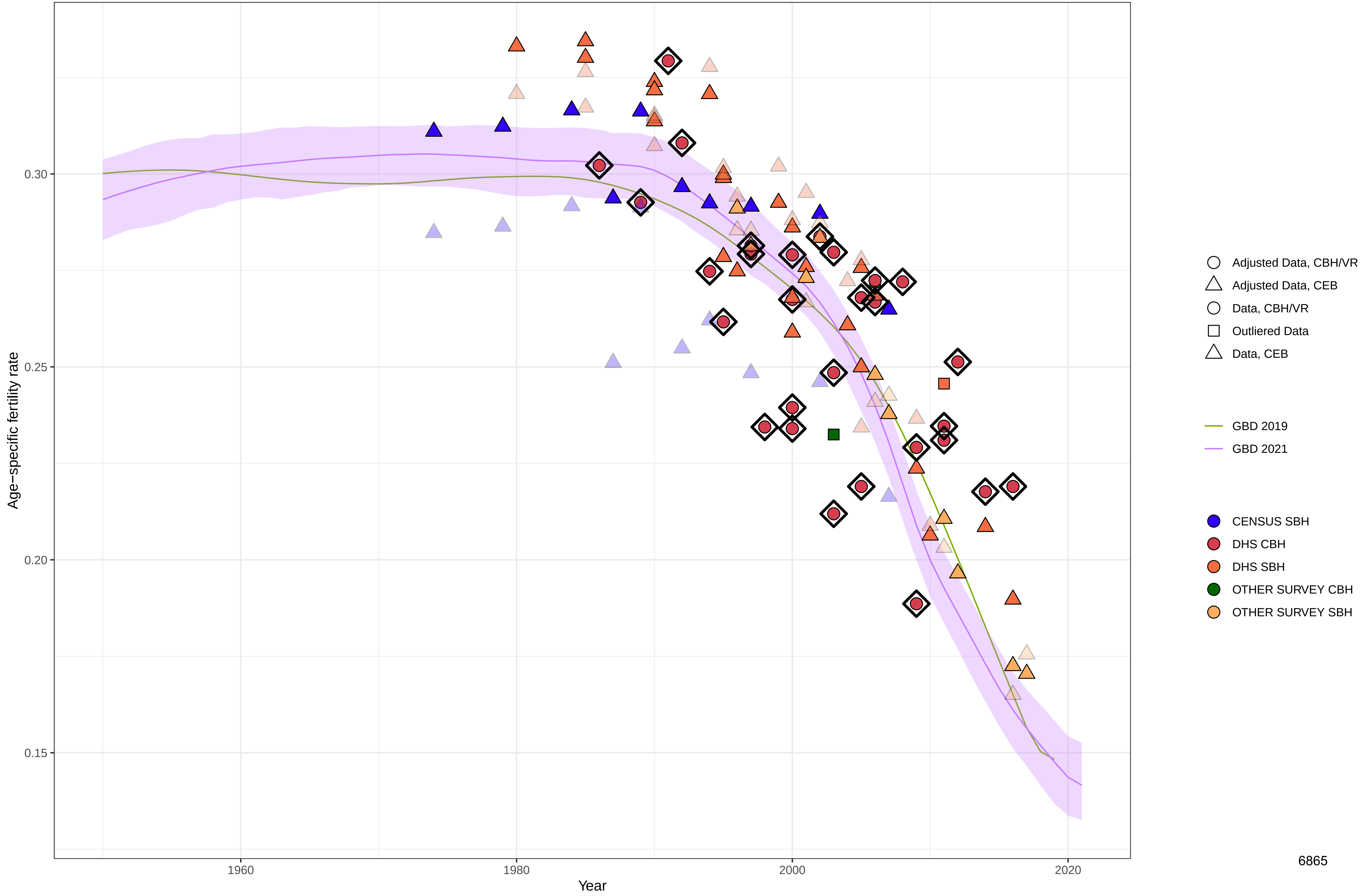

Age−specific fertility fitted trend lines, 1950−2021: Tigray, Ages: 30−34
Data points enclosed with a diamond are reference sources

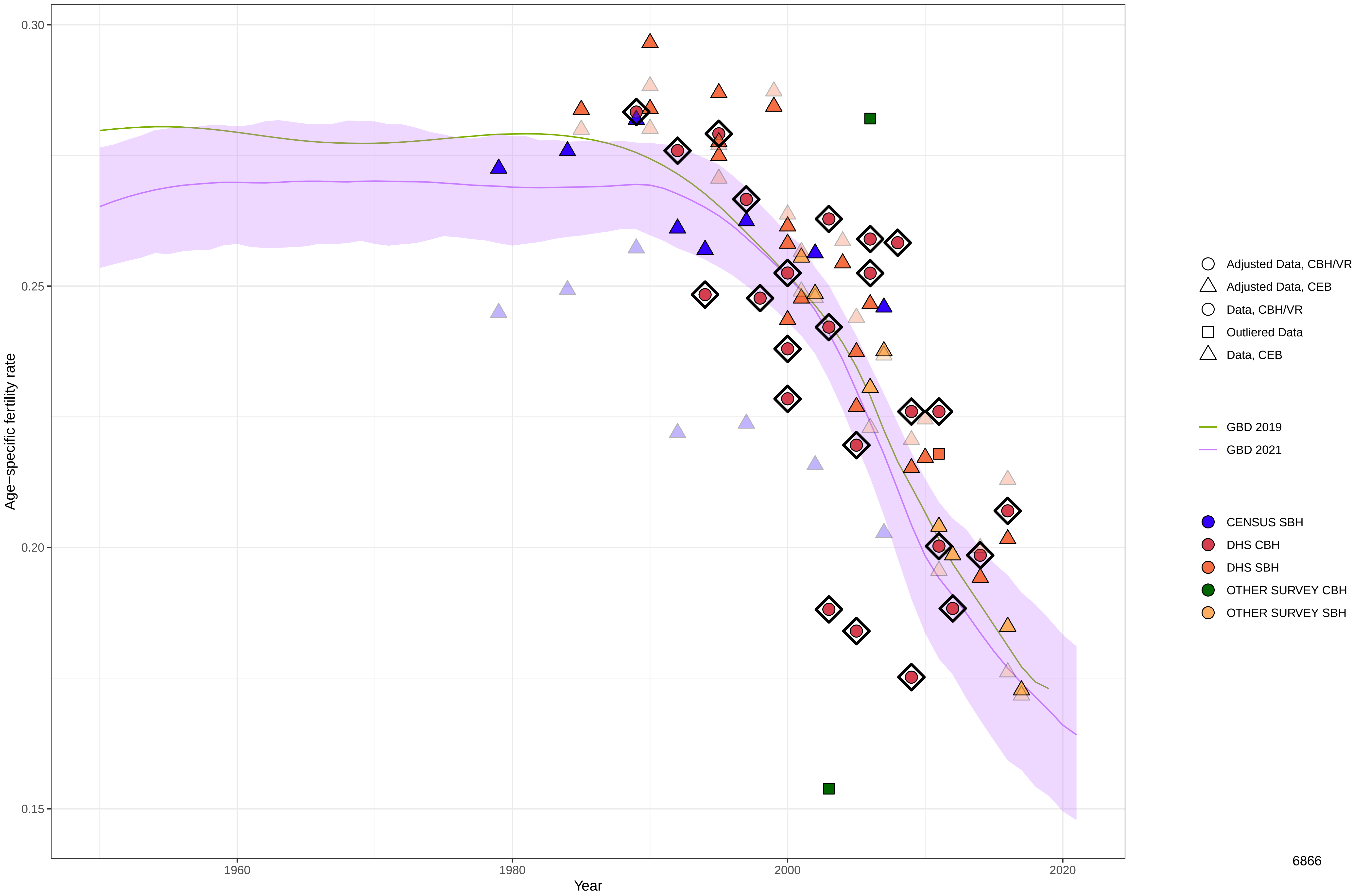

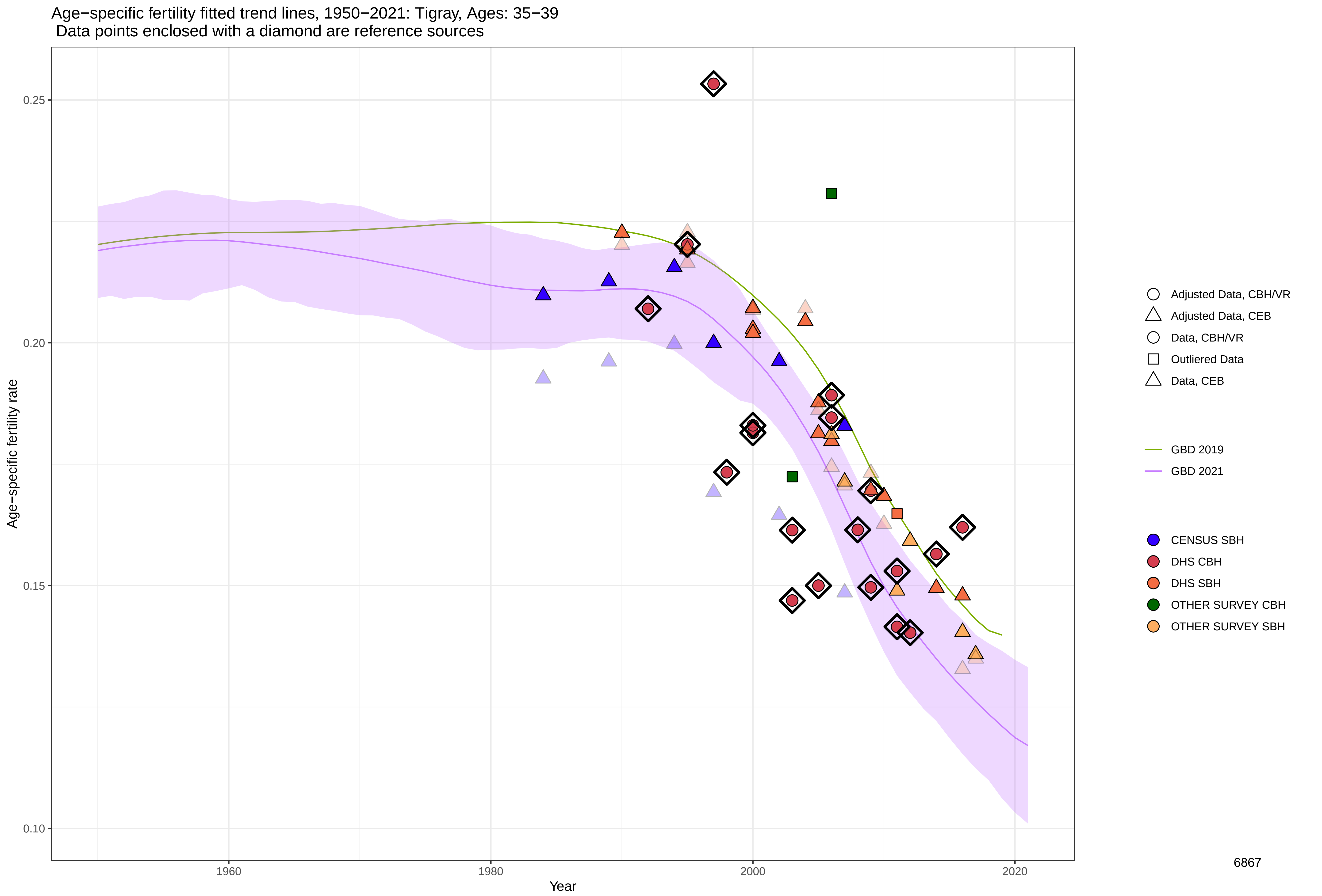

Age−specific fertility fitted trend lines, 1950−2021: Tigray, Ages: 35−39
Data points enclosed with a diamond are reference sources
Age−specific fertility rate
Year
0.25
0.20
0.15
0.10
1960
1980
2000
2020
Adjusted Data, CBH/VR
Adjusted Data, CEB
Data, CBH/VR
Outliered Data
Data, CEB
GBD 2019
GBD 2021
CENSUS SBH
DHS CBH
DHS SBH
OTHER SURVEY CBH
OTHER SURVEY SBH

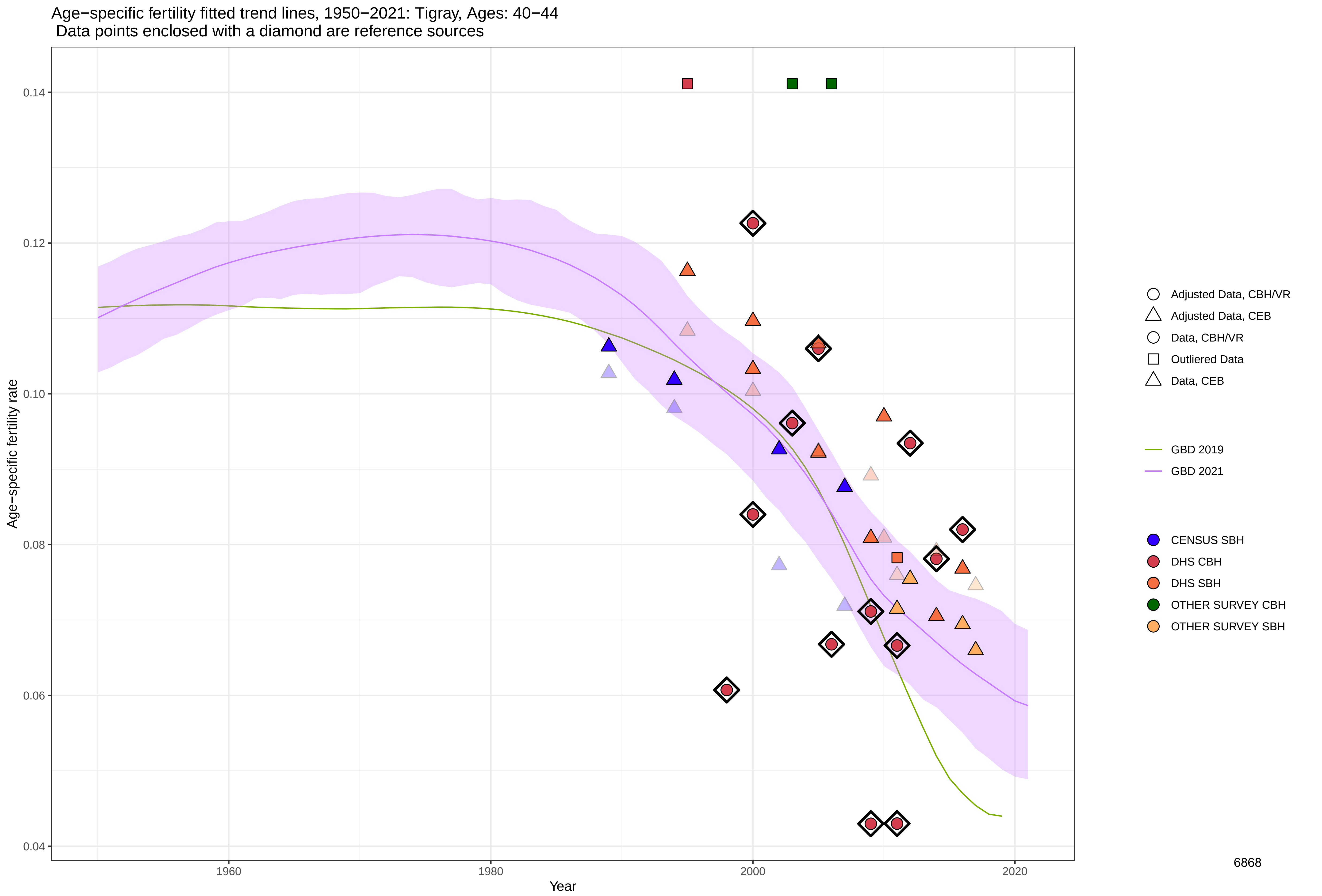

Age−specific fertility fitted trend lines, 1950−2021: Tigray, Ages: 40−44
Data points enclosed with a diamond are reference sources
Age−specific fertility rate
Year
0.04
0.06
0.08
0.10
0.12
0.14
1960
1980
2000
2020
Adjusted Data, CBH/VR
Adjusted Data, CEB
Data, CBH/VR
Outliered Data
Data, CEB
GBD 2019
GBD 2021
CENSUS SBH
DHS CBH
DHS SBH
OTHER SURVEY CBH
OTHER SURVEY SBH

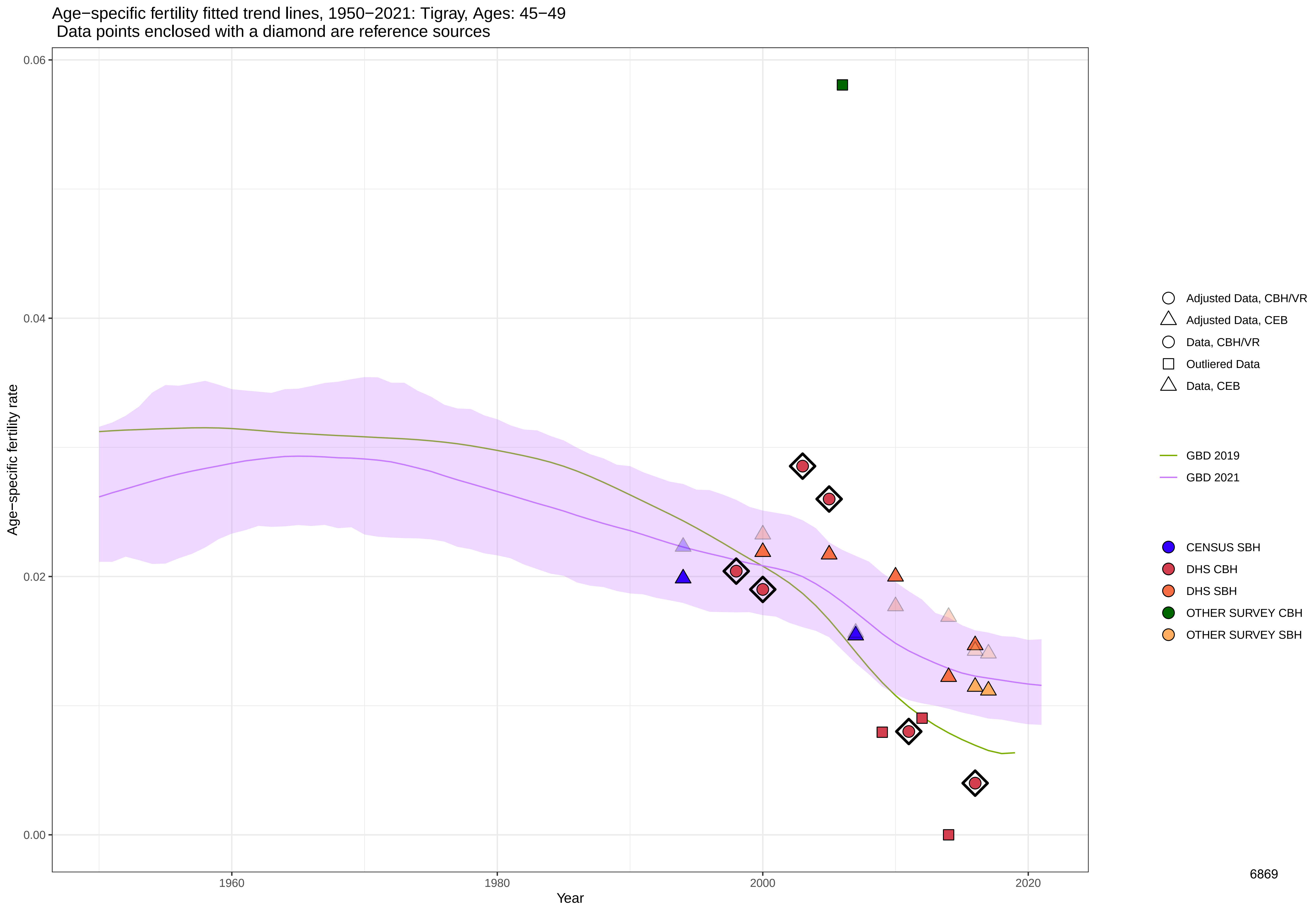

Age−specific fertility fitted trend lines, 1950−2021: Tigray, Ages: 45−49
Data points enclosed with a diamond are reference sources
Age−specific fertility rate
0.06
0.04
0.02
0.00
1960
1980
2000
2020
Year
Adjusted Data, CBH/VR
Adjusted Data, CEB
Data, CBH/VR
Outliered Data
Data, CEB
GBD 2019
GBD 2021
CENSUS SBH
DHS CBH
DHS SBH
OTHER SURVEY CBH
OTHER SURVEY SBH

Age−specific fertility fitted trend lines, 1950−2021: Tigray, Ages: 50−54
Data points enclosed with a diamond are reference sources

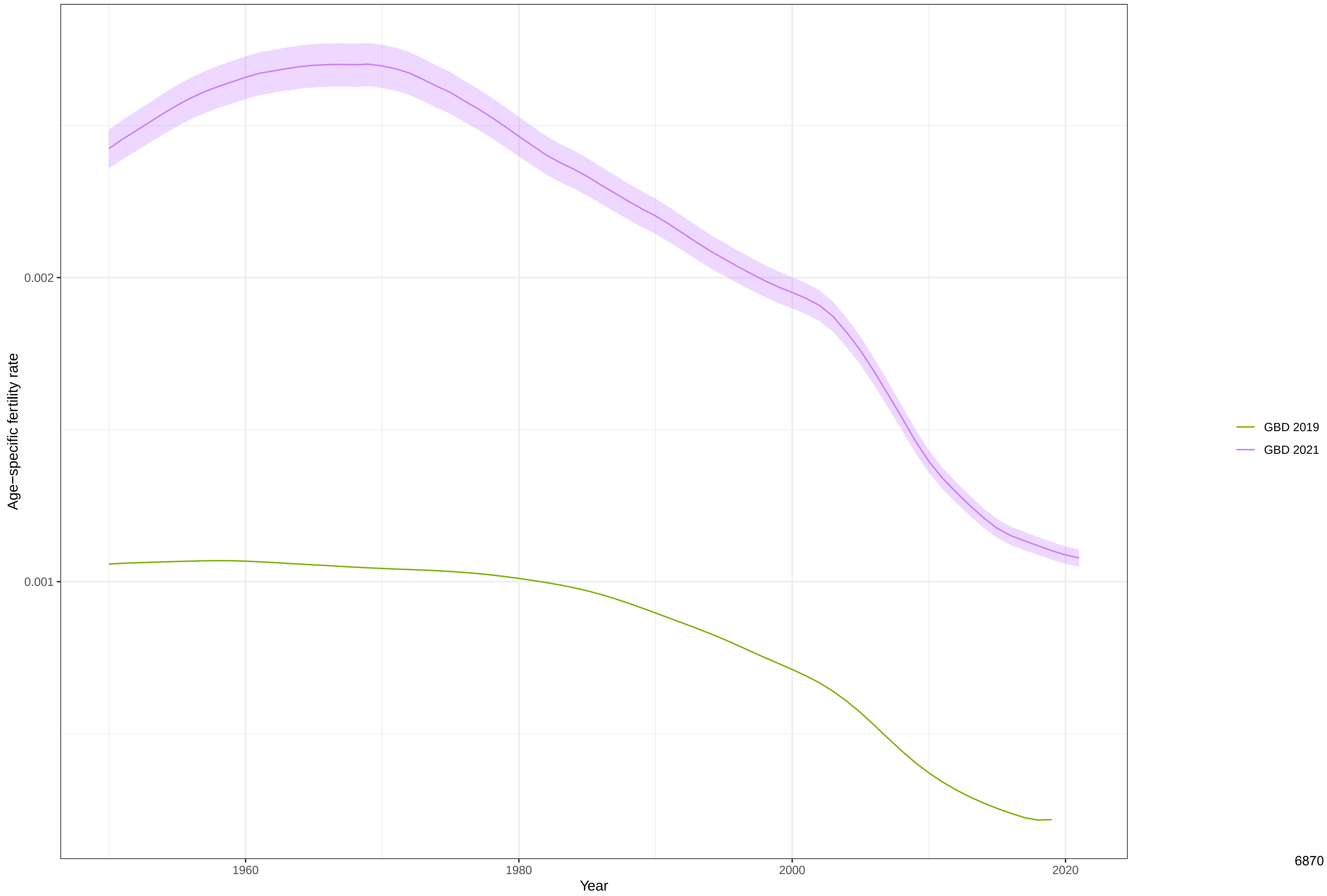

Total fertility rate fitted trend lines, 1950−2021: Tigray TFR
Data points enclosed with a diamond are reference sources

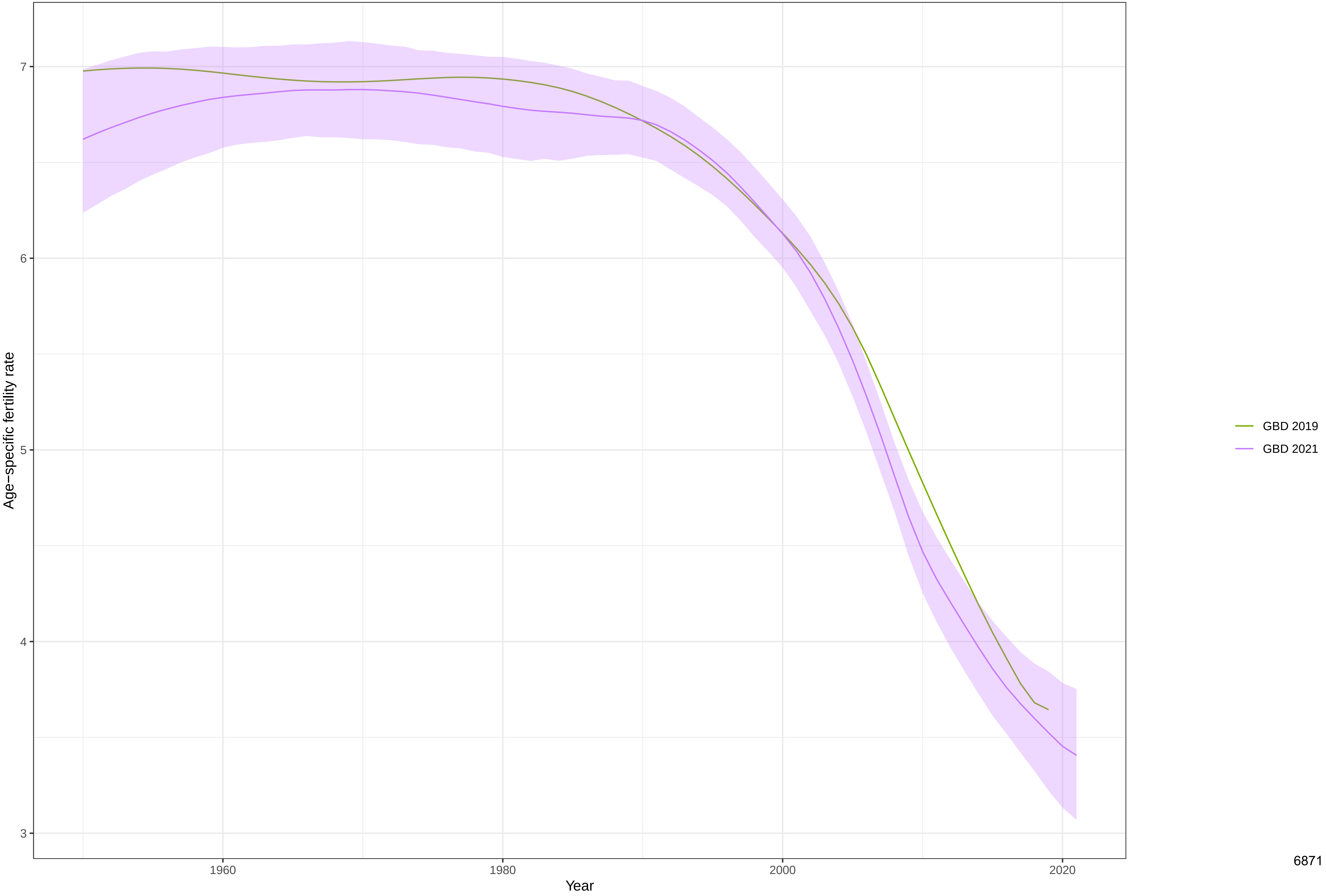

Age−specific fertility fitted trend lines, 1950−2021: Kenya, Ages: 10−14
Data points enclosed with a diamond are reference sources

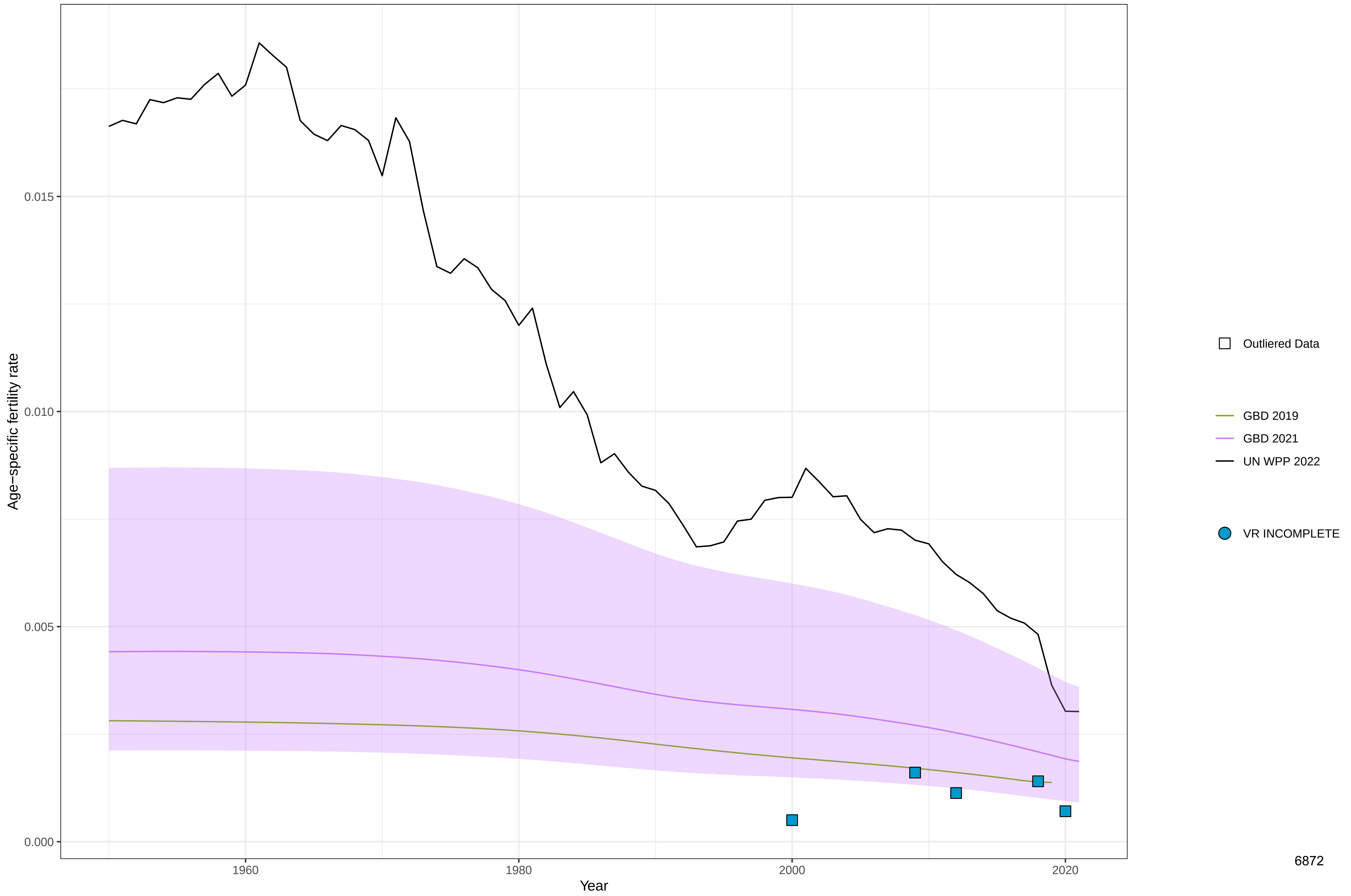

Age−specific fertility fitted trend lines, 1950−2021: Kenya, Ages: 15−19
Data points enclosed with a diamond are reference sources

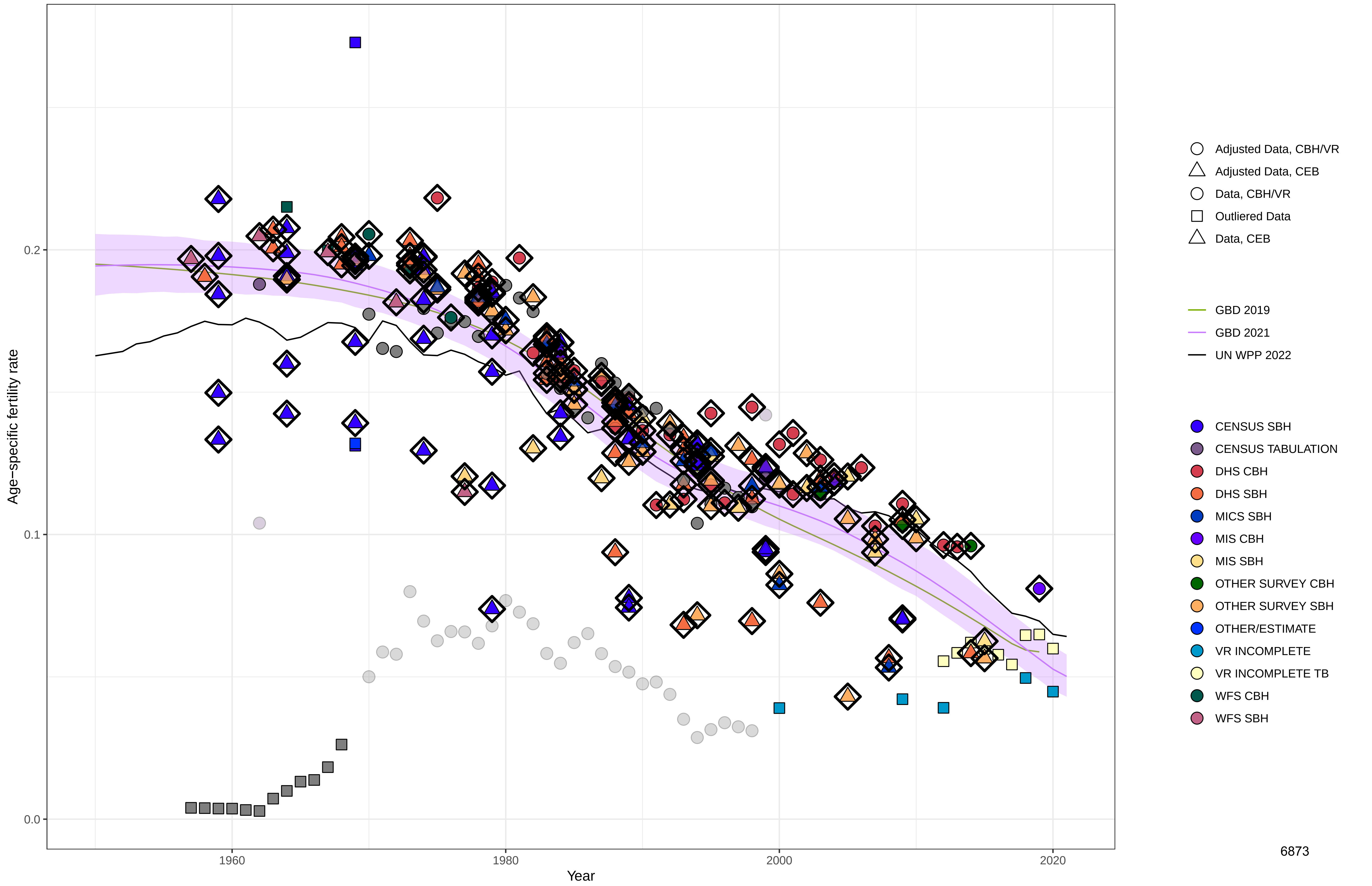

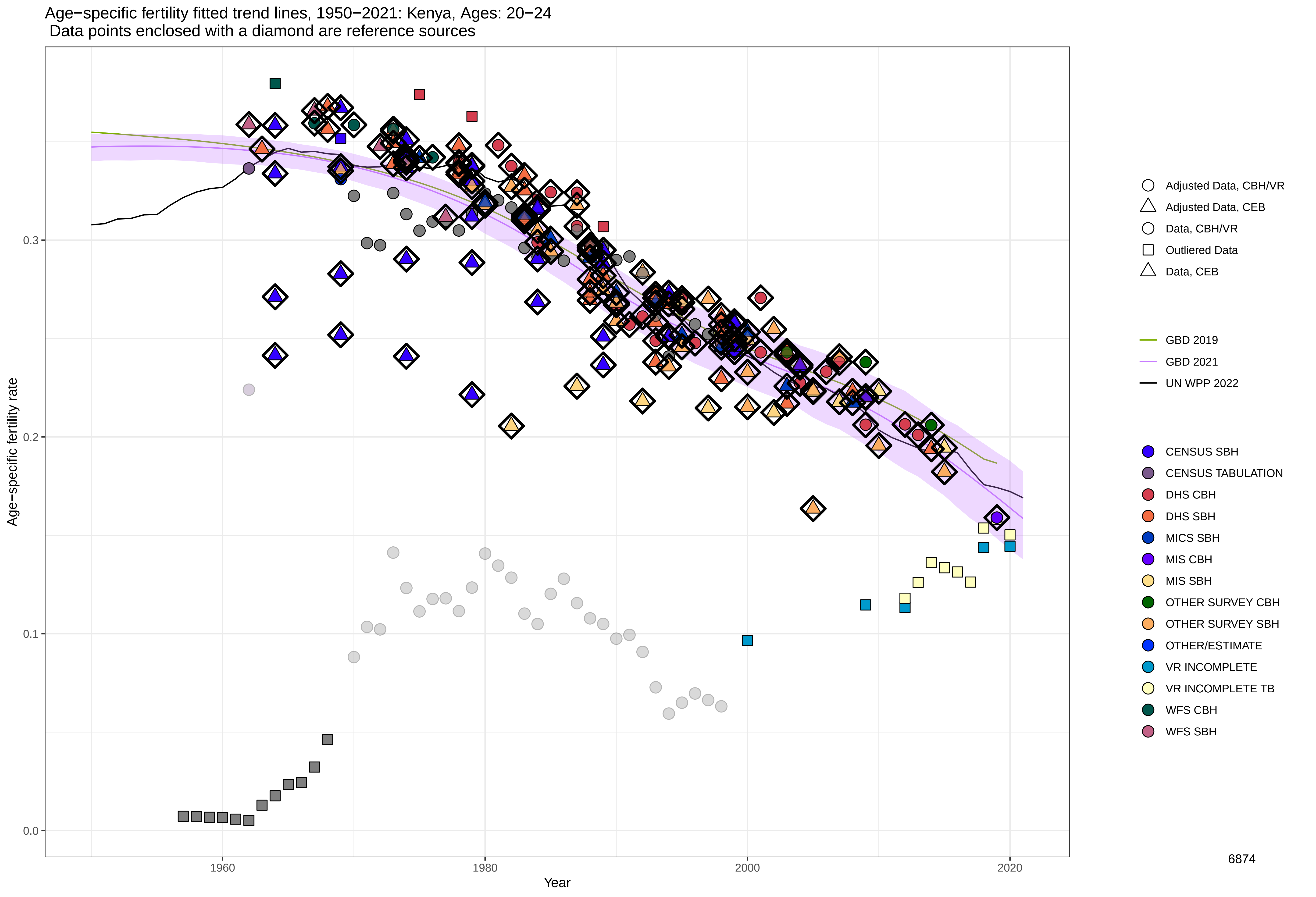

Age−specific fertility fitted trend lines, 1950−2021: Kenya, Ages: 20−24
Data points enclosed with a diamond are reference sources
Age−specific fertility rate
Year
0.0
0.1
0.2
0.3
1960
1980
2000
2020
Adjusted Data, CBH/VR
Adjusted Data, CEB
Data, CBH/VR
Outliered Data
Data, CEB
GBD 2019
GBD 2021
UN WPP 2022
CENSUS SBH
CENSUS TABULATION
DHS CBH
DHS SBH
MICS SBH
MIS CBH
MIS SBH
OTHER SURVEY CBH
OTHER SURVEY SBH
OTHER/ESTIMATE
VR INCOMPLETE
VR INCOMPLETE TB
WFS CBH
WFS SBH

Age−specific fertility fitted trend lines, 1950−2021: Kenya, Ages: 25−29
Data points enclosed with a diamond are reference sources

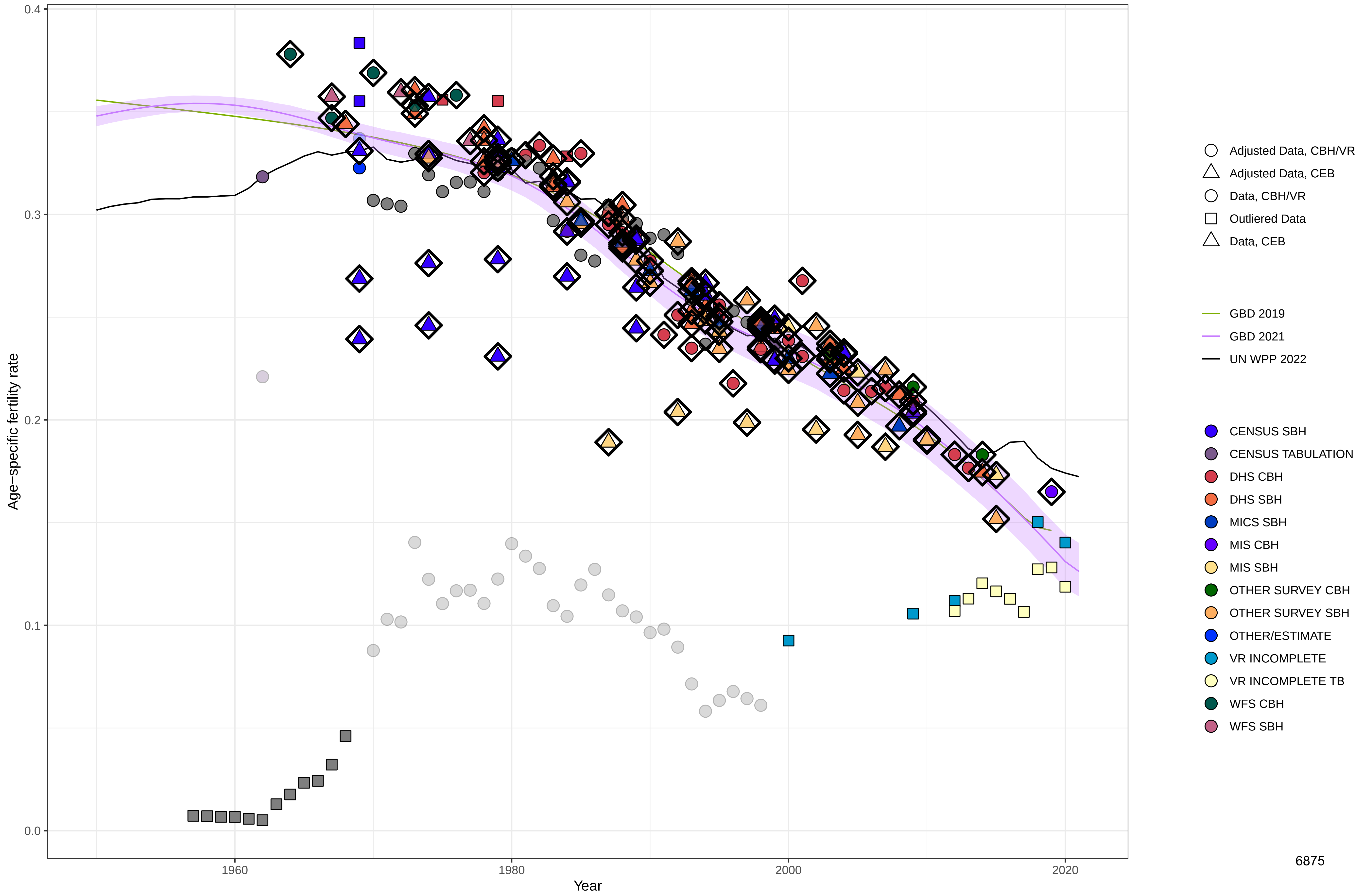

Age−specific fertility fitted trend lines, 1950−2021: Kenya, Ages: 30−34
Data points enclosed with a diamond are reference sources

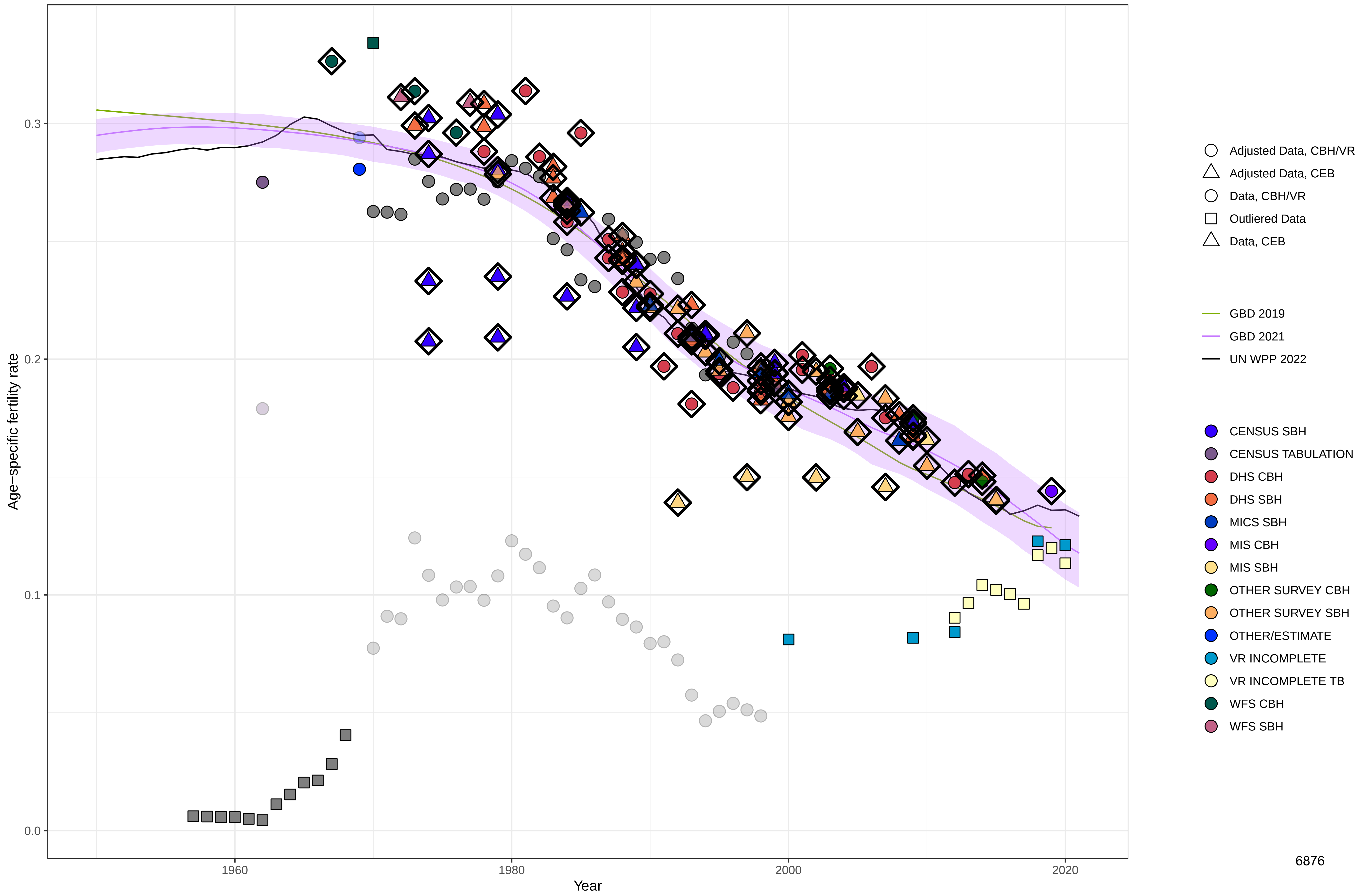

Age−specific fertility fitted trend lines, 1950−2021: Kenya, Ages: 35−39
Data points enclosed with a diamond are reference sources

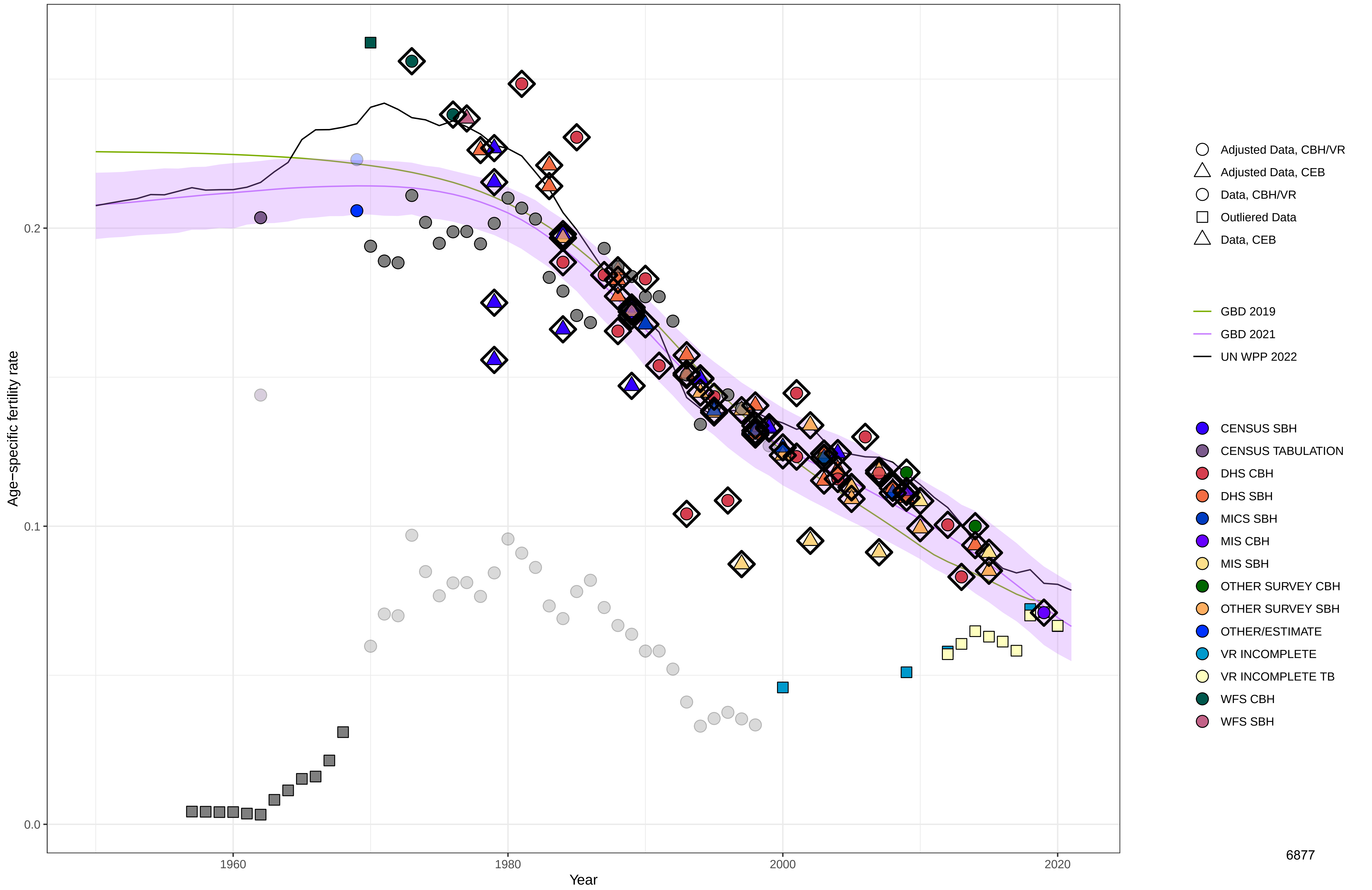

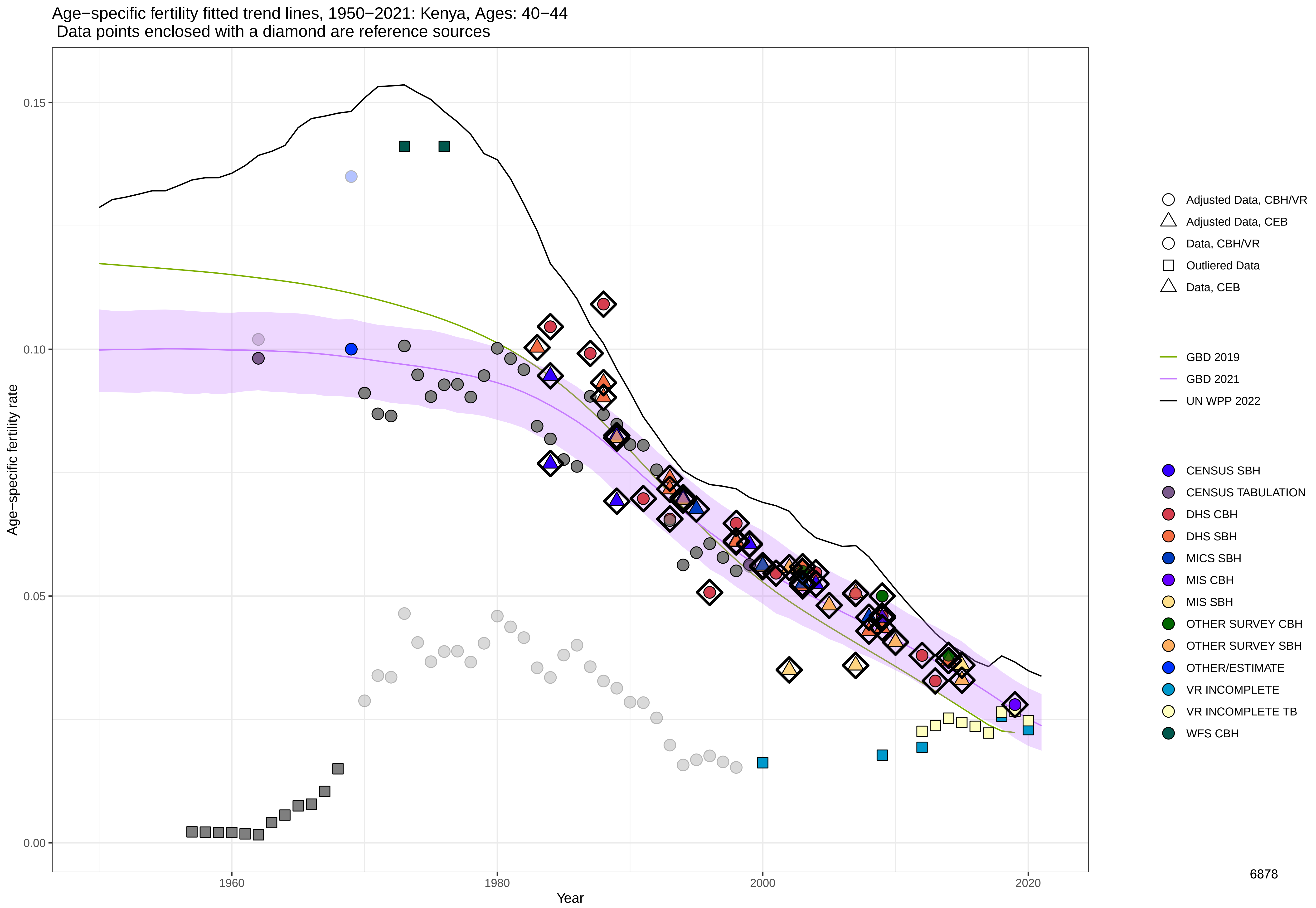

Age−specific fertility fitted trend lines, 1950−2021: Kenya, Ages: 40−44
Data points enclosed with a diamond are reference sources
Age−specific fertility rate
Year
0.00
0.05
0.10
0.15
1960
1980
2000
2020
Adjusted Data, CBH/VR
Adjusted Data, CEB
Data, CBH/VR
Outliered Data
Data, CEB
GBD 2019
GBD 2021
UN WPP 2022
CENSUS SBH
CENSUS TABULATION
DHS CBH
DHS SBH
MICS SBH
MIS CBH
MIS SBH
OTHER SURVEY CBH
OTHER SURVEY SBH
OTHER/ESTIMATE
VR INCOMPLETE
VR INCOMPLETE TB
WFS CBH

Age−specific fertility fitted trend lines, 1950−2021: Kenya, Ages: 45−49
Data points enclosed with a diamond are reference sources

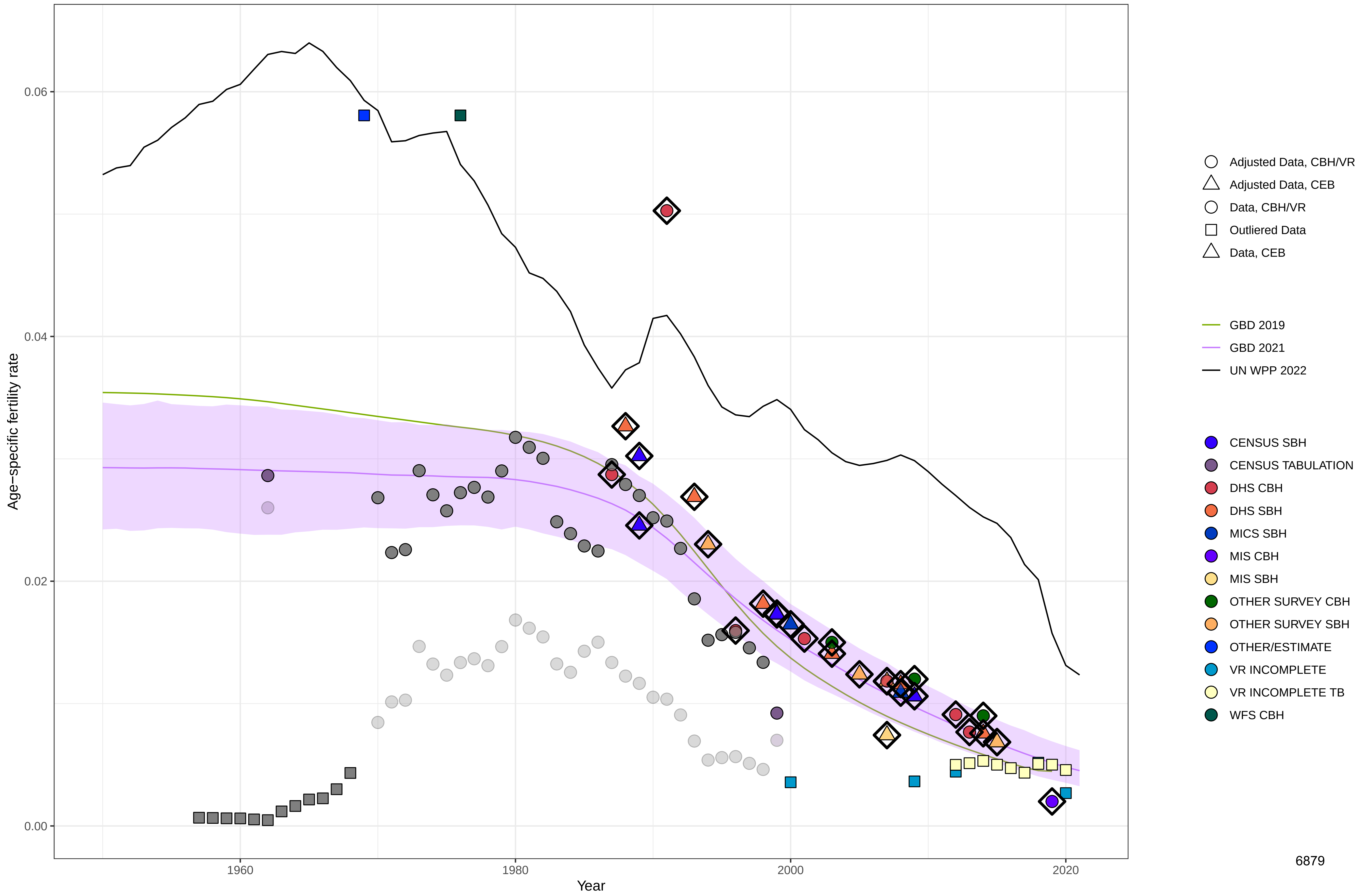

Age−specific fertility fitted trend lines, 1950−2021: Kenya, Ages: 50−54
Data points enclosed with a diamond are reference sources

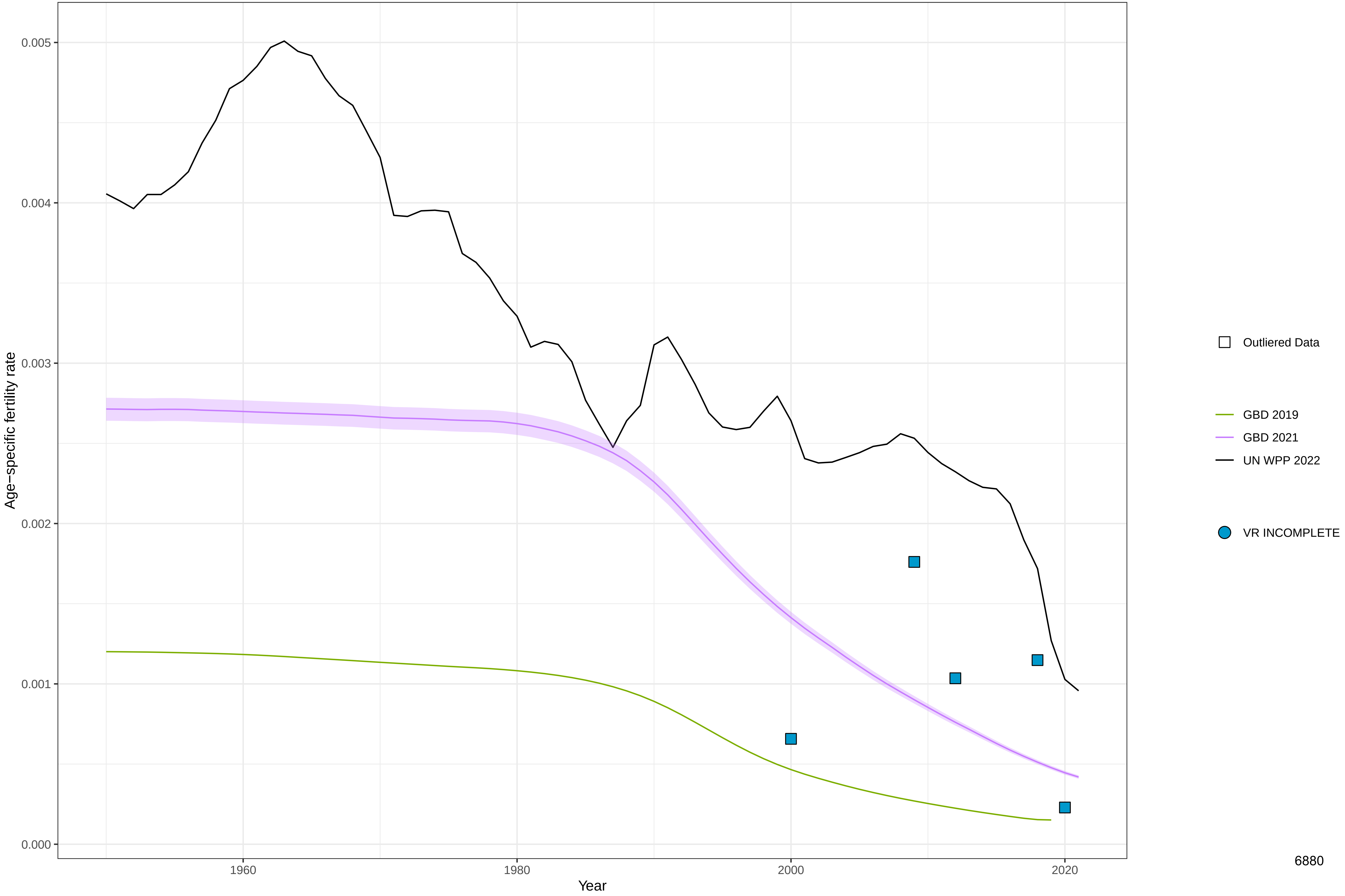

Total fertility rate fitted trend lines, 1950−2021: Kenya TFR
Data points enclosed with a diamond are reference sources

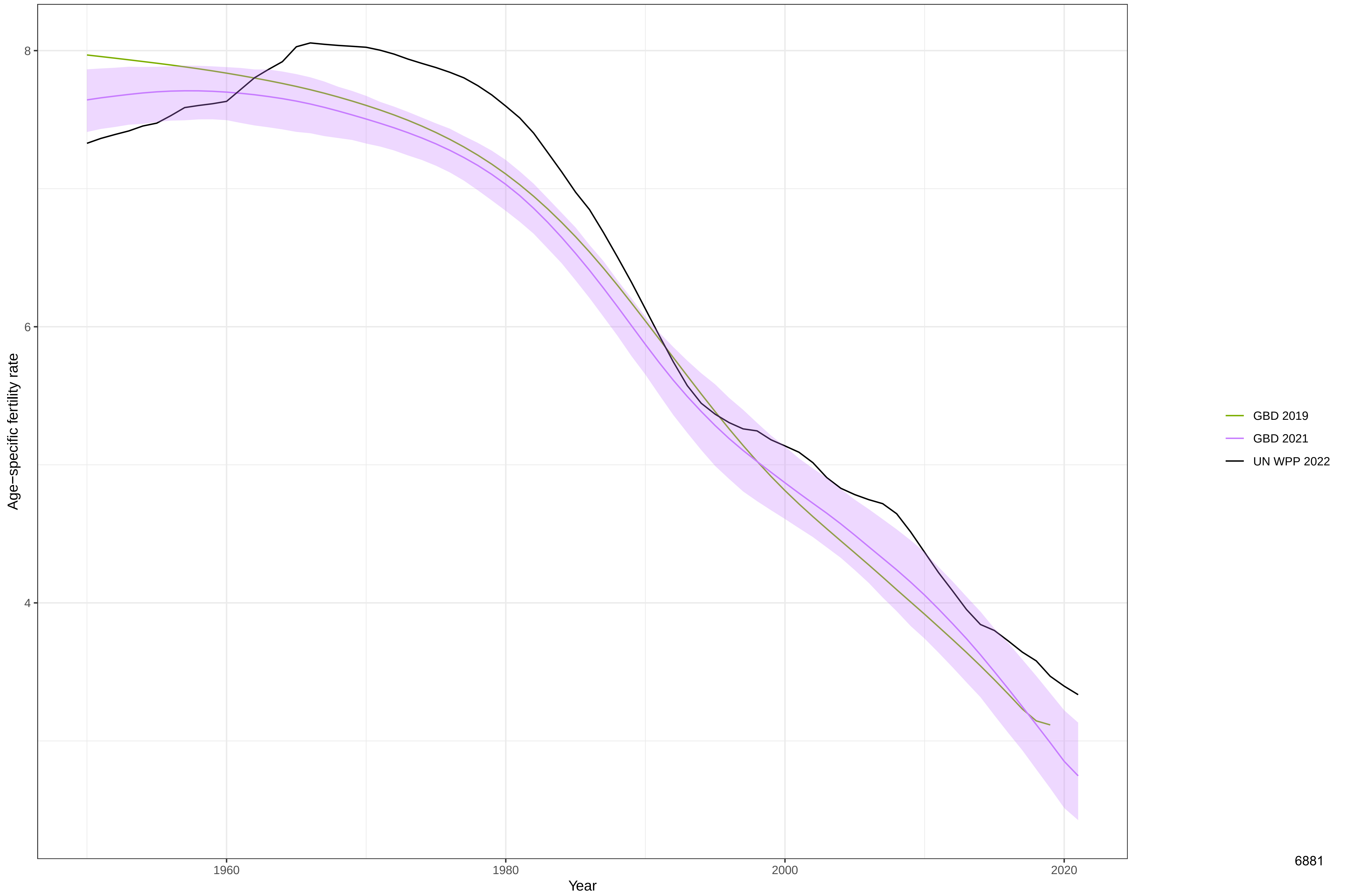

Age−specific fertility fitted trend lines, 1950−2021: Baringo, Ages: 10−14
Data points enclosed with a diamond are reference sources

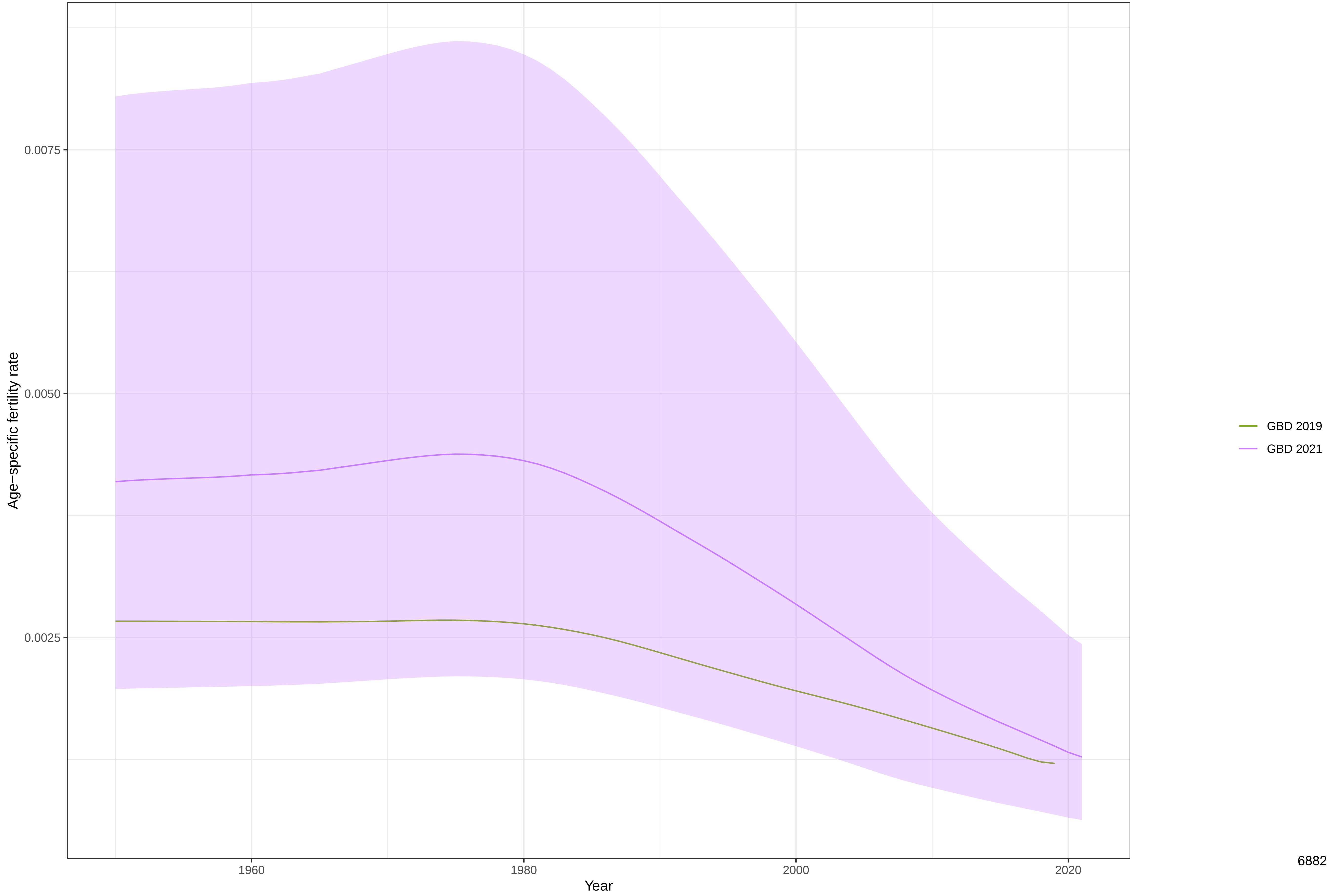

Age−specific fertility fitted trend lines, 1950−2021: Baringo, Ages: 15−19
Data points enclosed with a diamond are reference sources

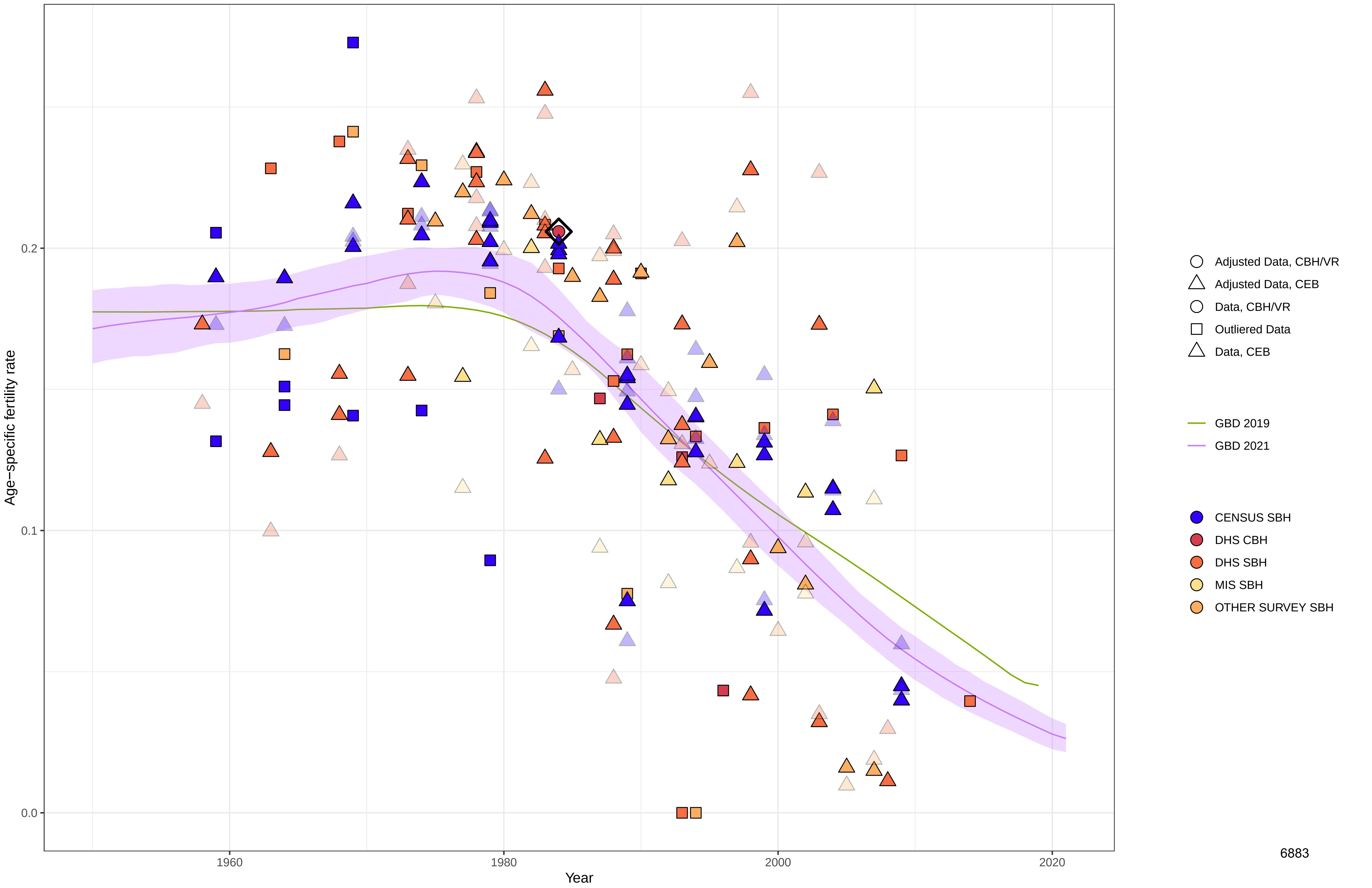

Age−specific fertility fitted trend lines, 1950−2021: Baringo, Ages: 20−24
Data points enclosed with a diamond are reference sources

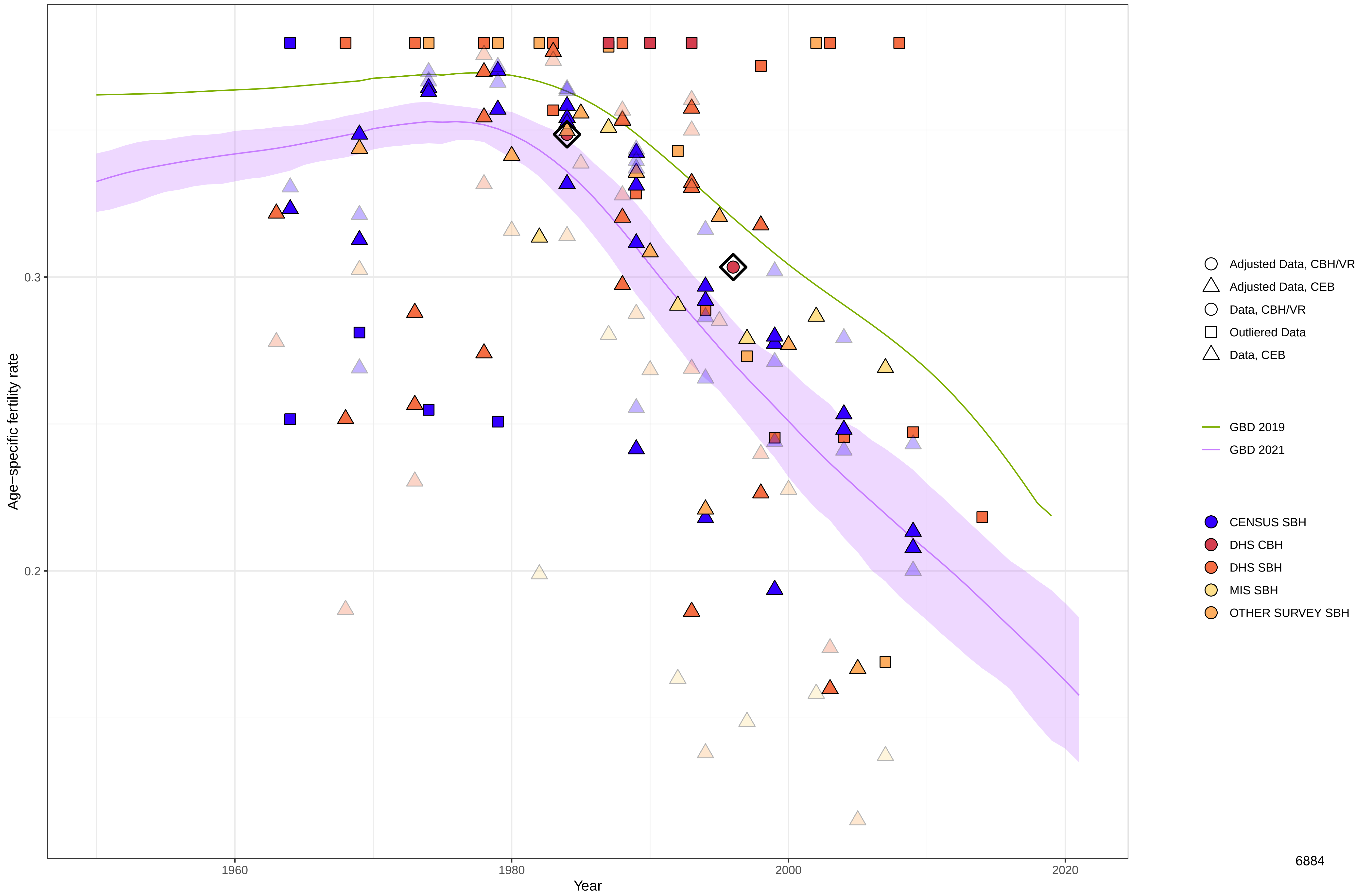

Age−specific fertility fitted trend lines, 1950−2021: Baringo, Ages: 25−29
Data points enclosed with a diamond are reference sources

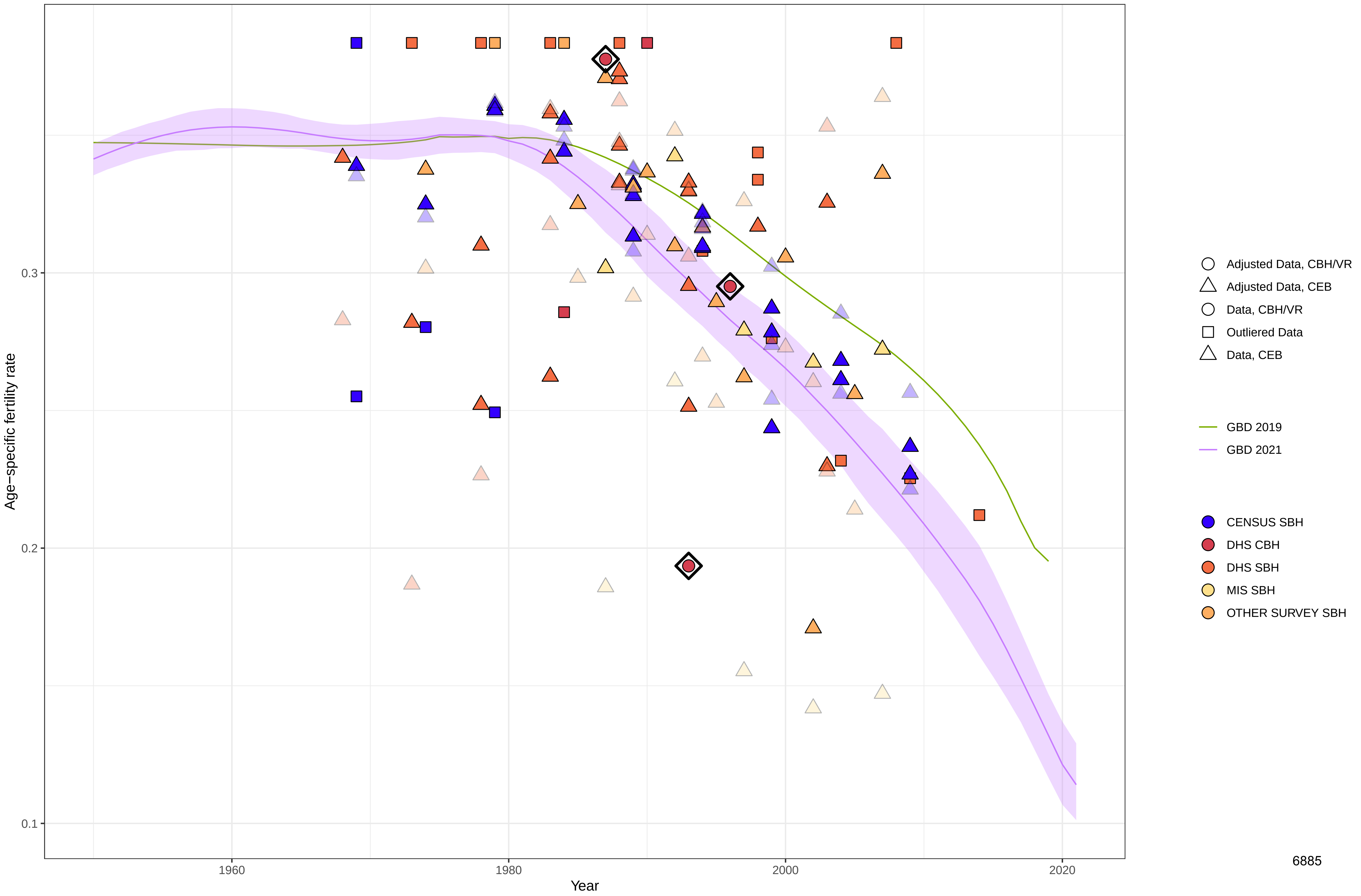

Age−specific fertility fitted trend lines, 1950−2021: Baringo, Ages: 30−34
Data points enclosed with a diamond are reference sources

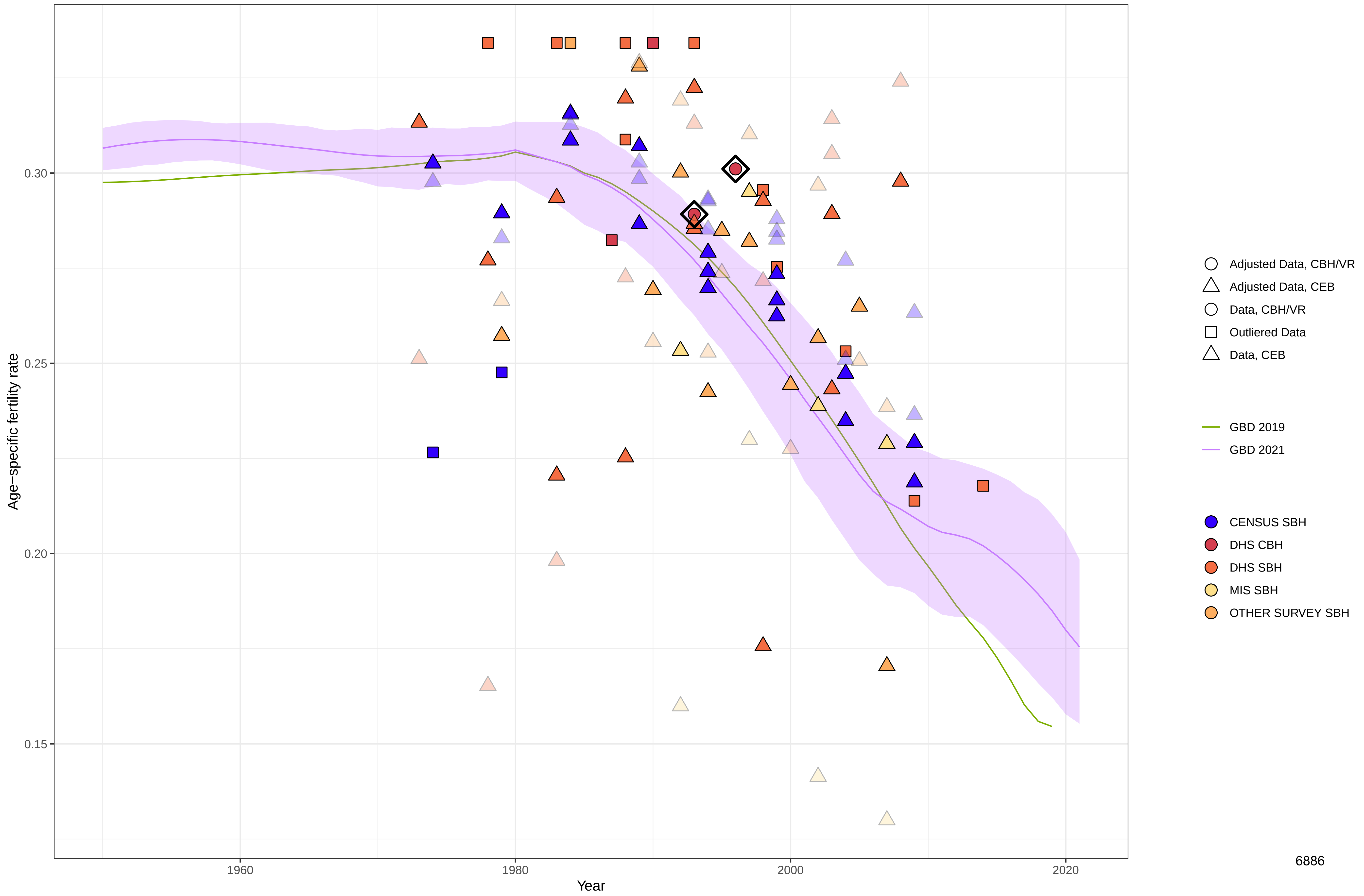

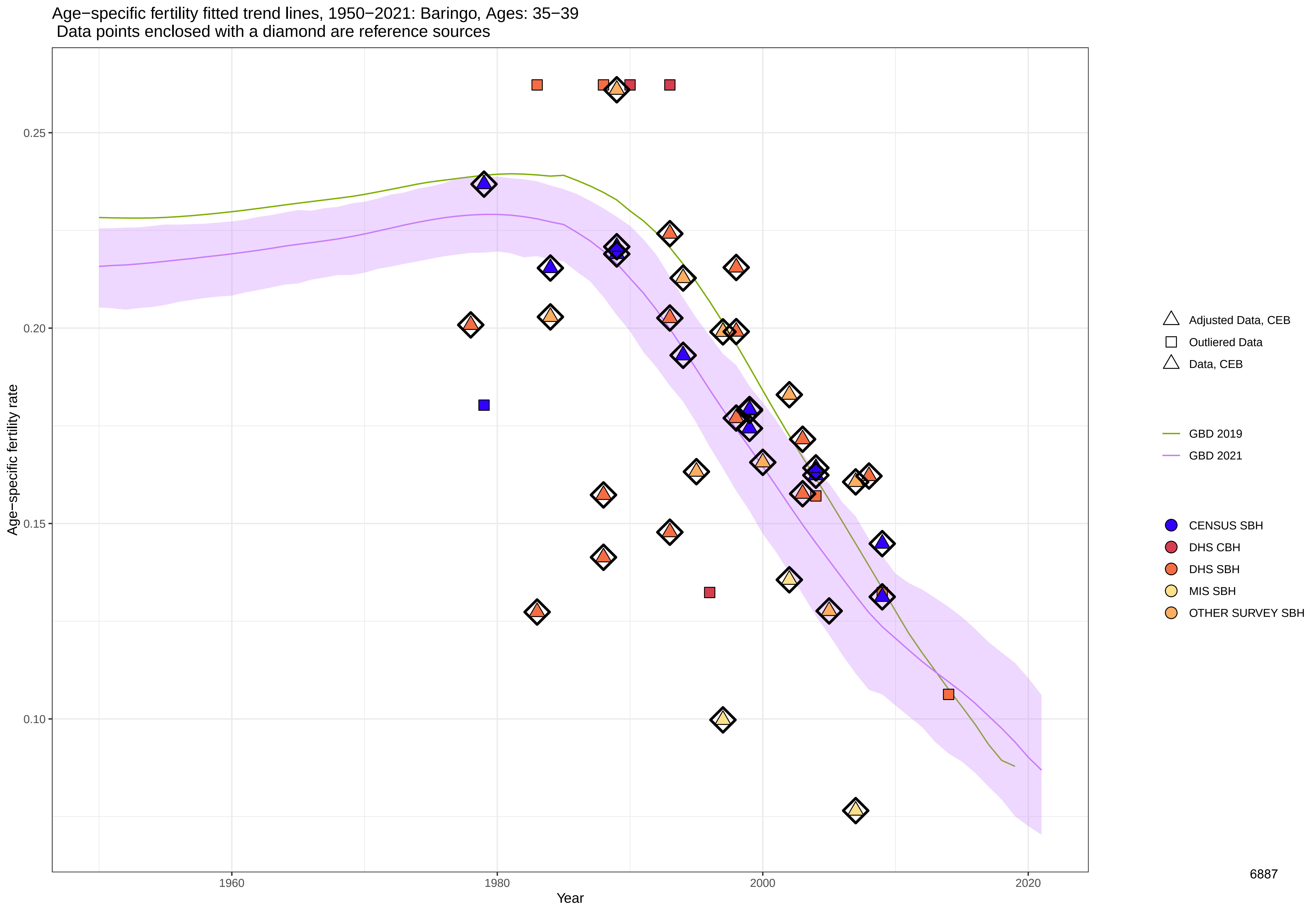

Age−specific fertility fitted trend lines, 1950−2021: Baringo, Ages: 35−39
Data points enclosed with a diamond are reference sources
Age−specific fertility rate
Year
0.25
0.20
0.15
0.10
1960
1980
2000
2020
Adjusted Data, CEB
Outliered Data
Data, CEB
GBD 2019
GBD 2021
CENSUS SBH
DHS CBH
DHS SBH
MIS SBH
OTHER SURVEY SBH

6887

Age−specific fertility fitted trend lines, 1950−2021: Baringo, Ages: 40−44
Data points enclosed with a diamond are reference sources

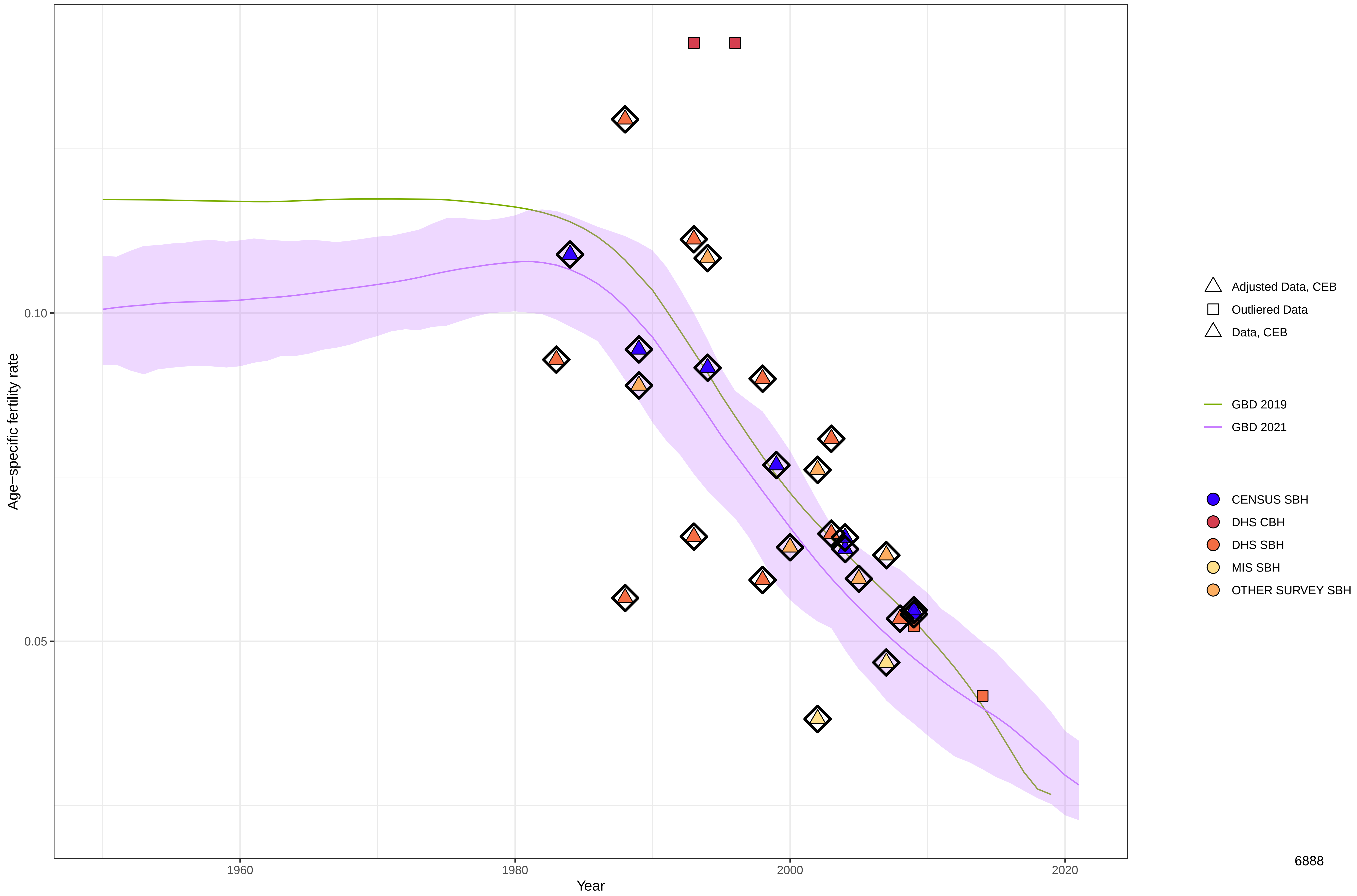

Age−specific fertility fitted trend lines, 1950−2021: Baringo, Ages: 45−49
Data points enclosed with a diamond are reference sources

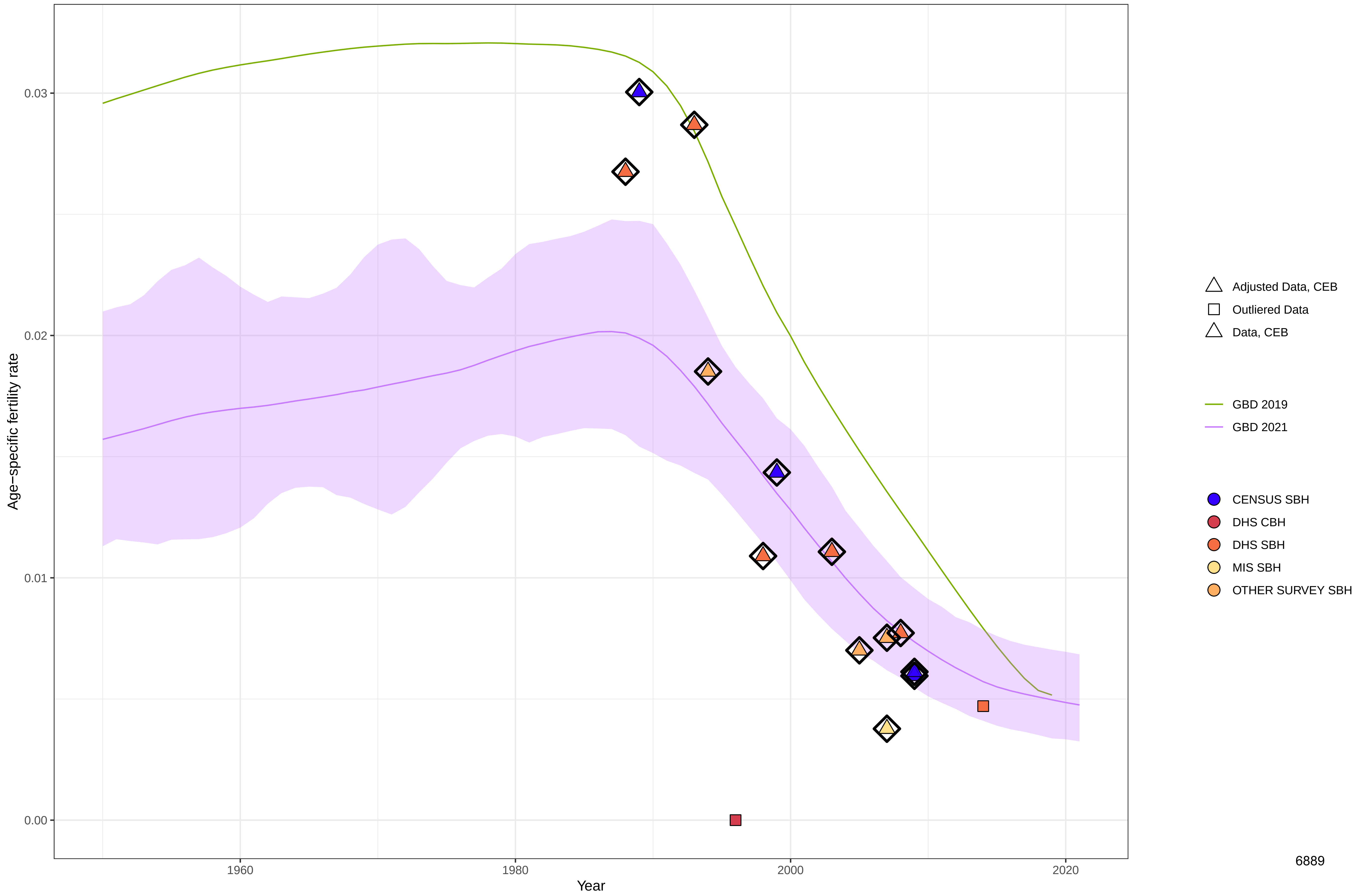

Age−specific fertility fitted trend lines, 1950−2021: Baringo, Ages: 50−54
Data points enclosed with a diamond are reference sources

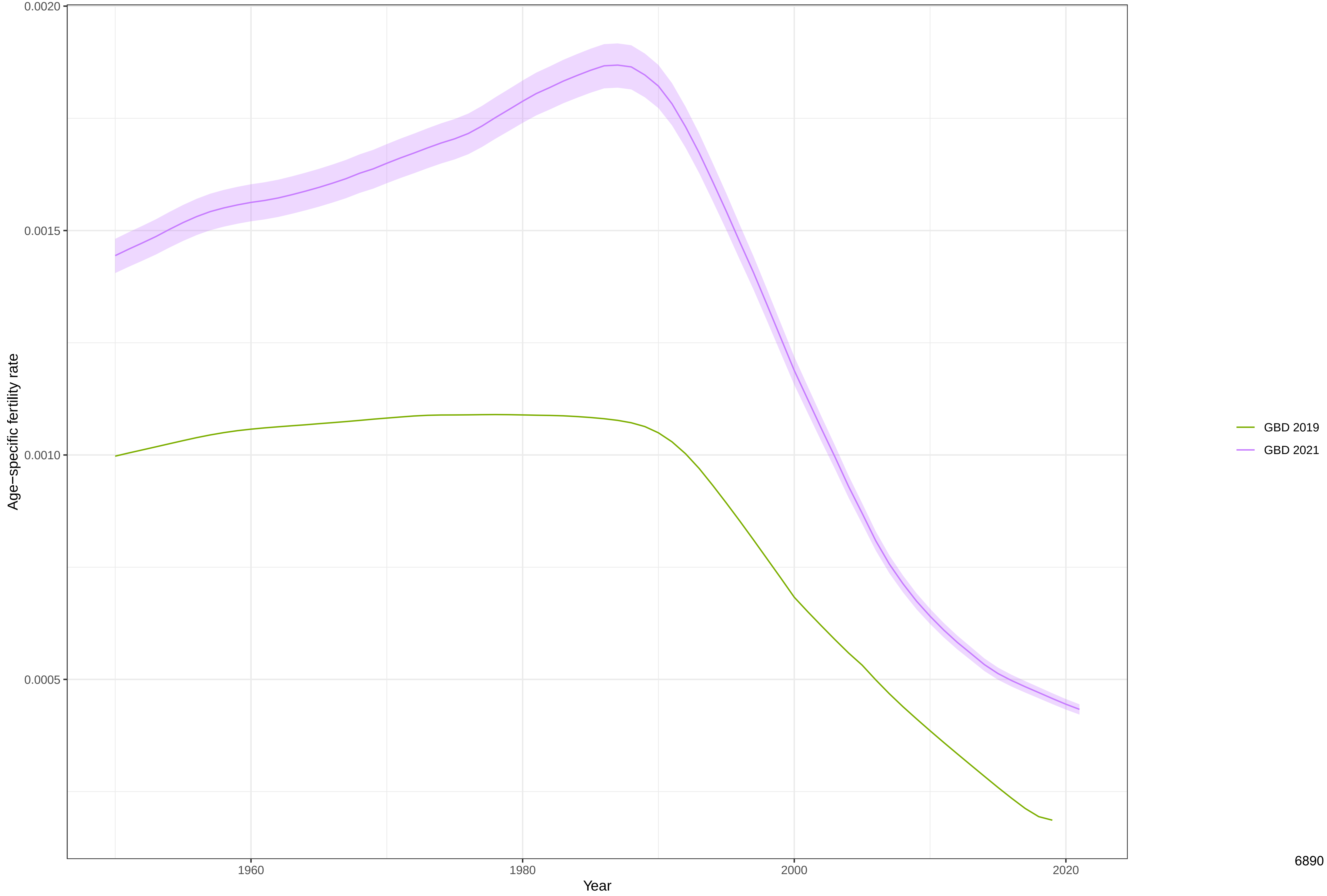

Total fertility rate fitted trend lines, 1950−2021: Baringo TFR
Data points enclosed with a diamond are reference sources

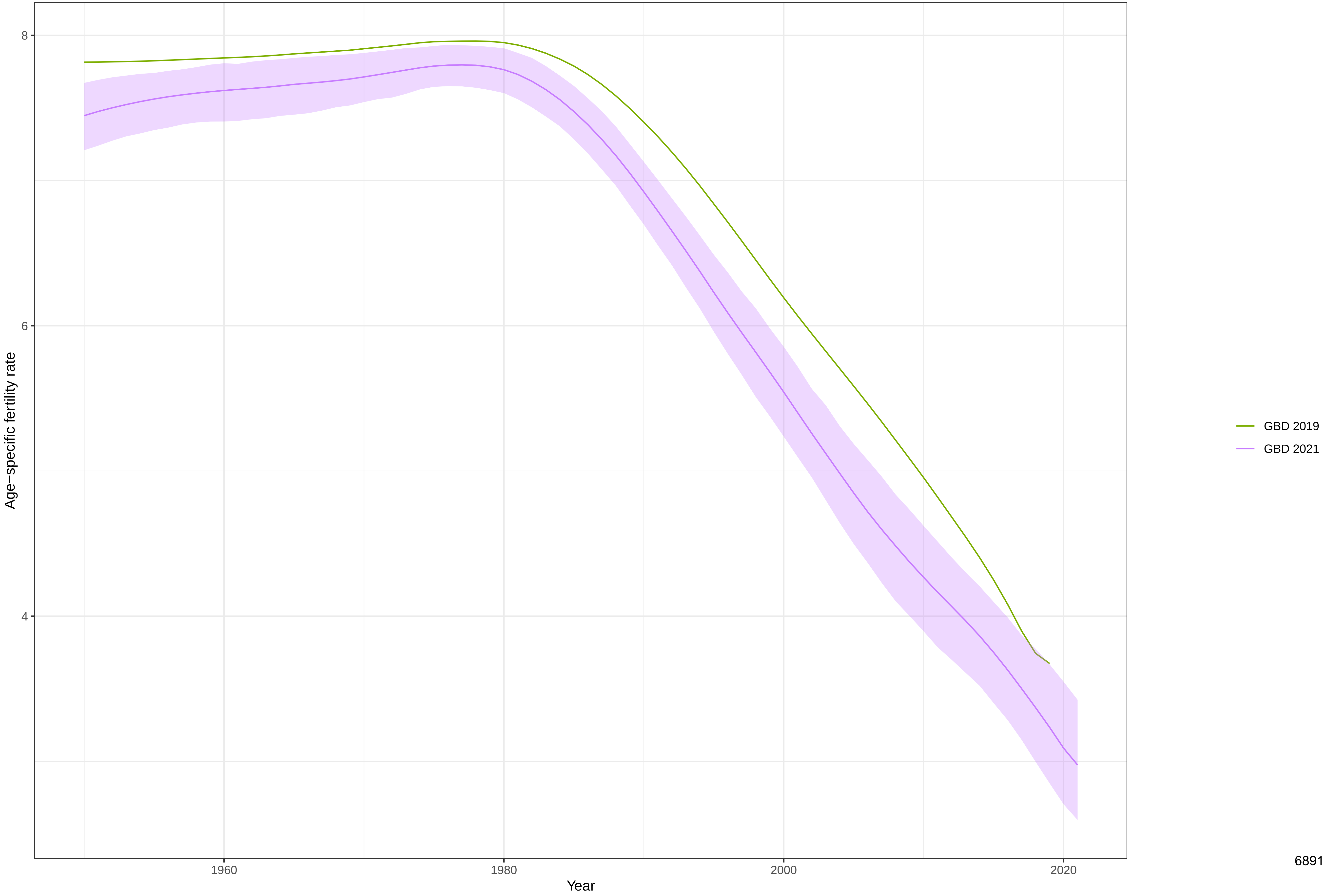

Age−specific fertility fitted trend lines, 1950−2021: Bomet, Ages: 10−14
Data points enclosed with a diamond are reference sources

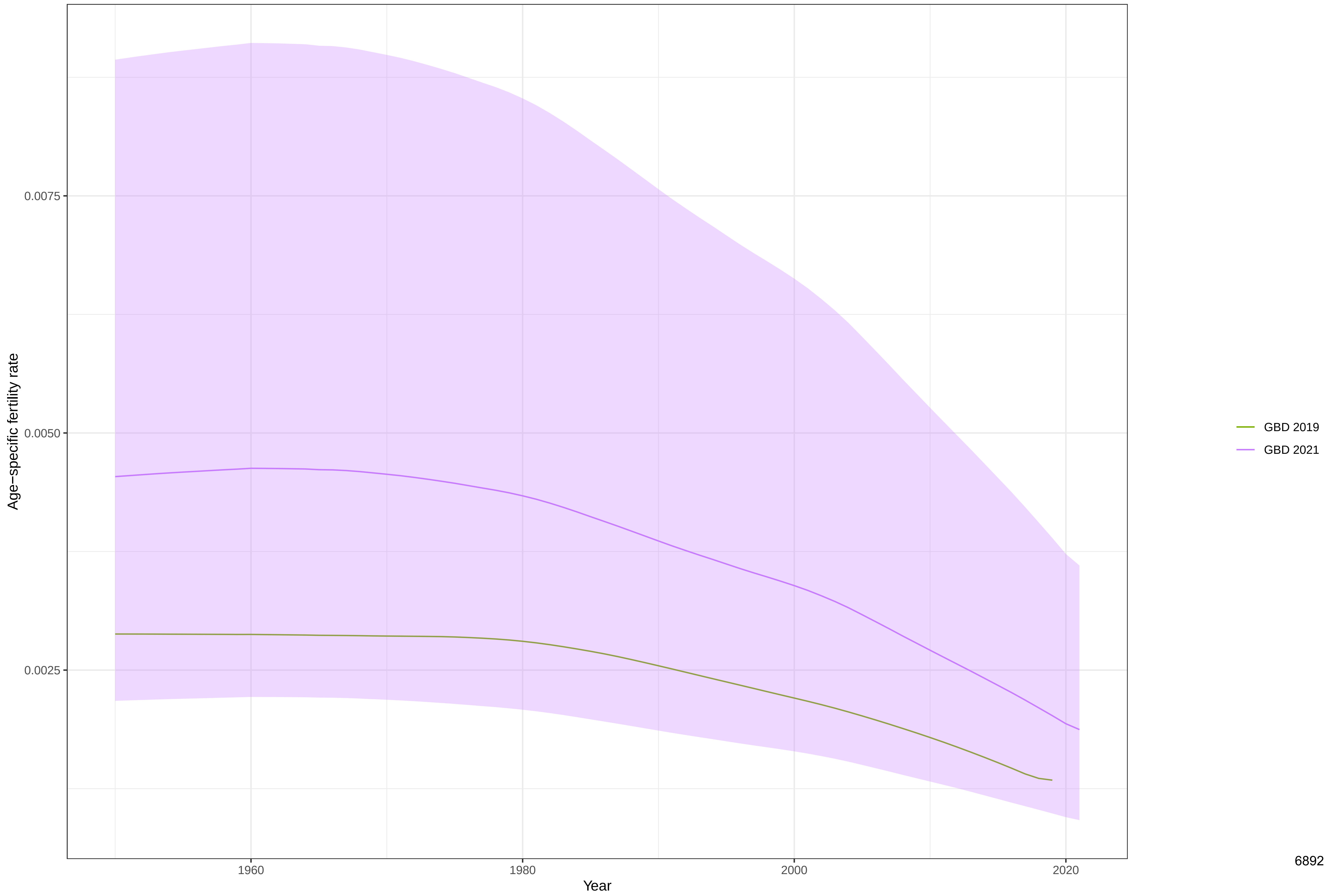

Age−specific fertility fitted trend lines, 1950−2021: Bomet, Ages: 15−19
Data points enclosed with a diamond are reference sources

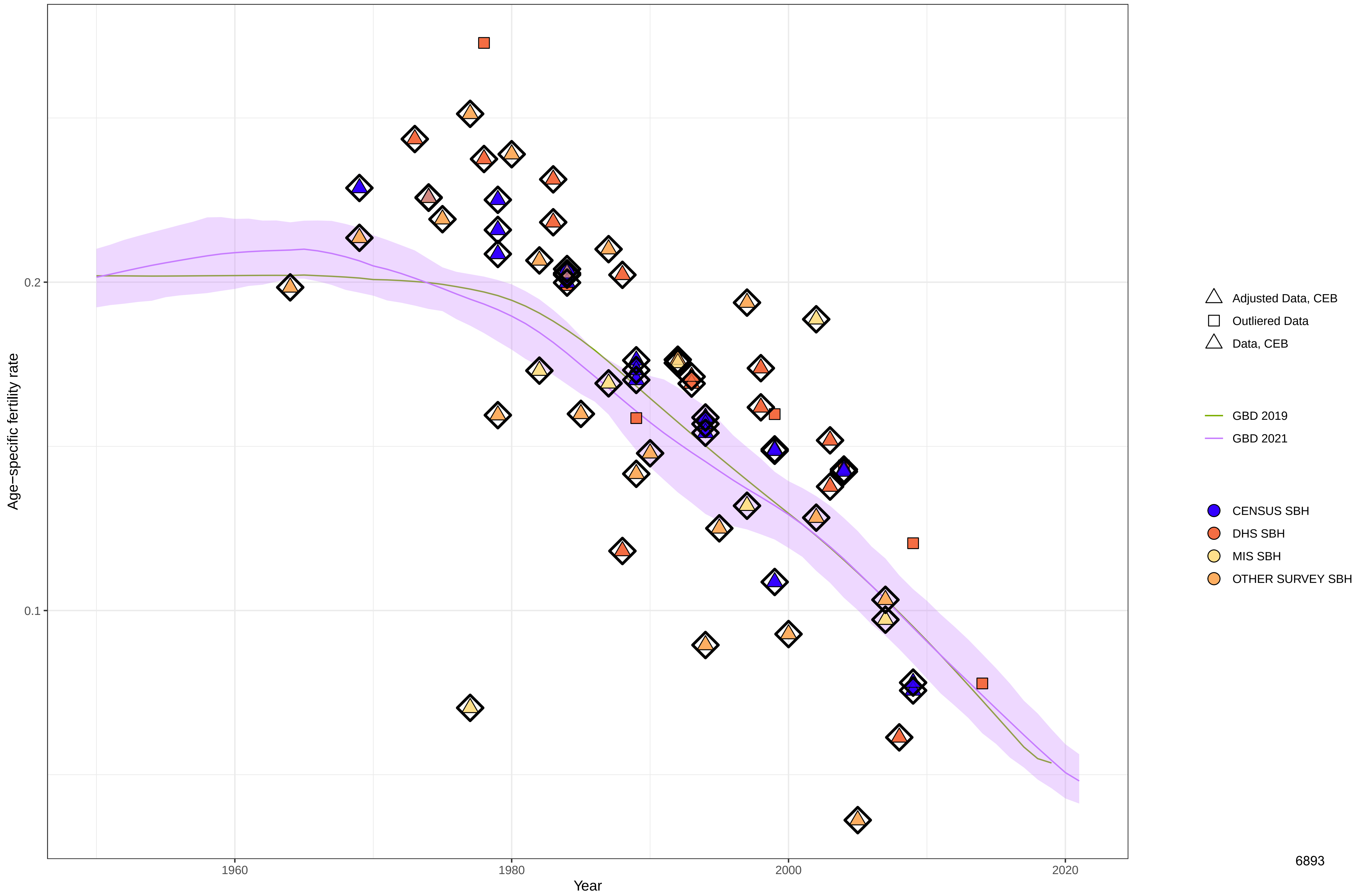

Age−specific fertility fitted trend lines, 1950−2021: Bomet, Ages: 20−24
Data points enclosed with a diamond are reference sources

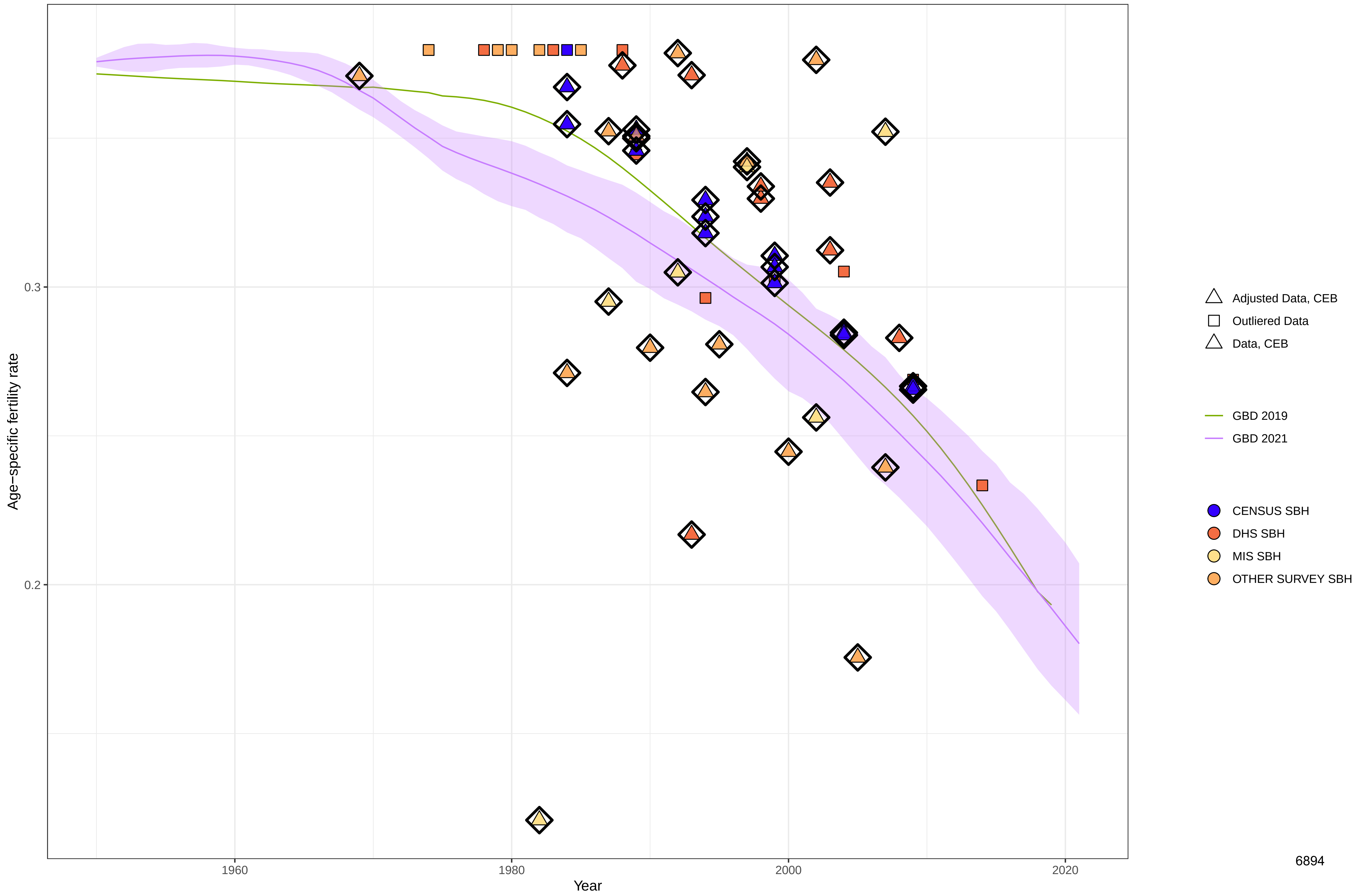

Age−specific fertility fitted trend lines, 1950−2021: Bomet, Ages: 25−29
Data points enclosed with a diamond are reference sources

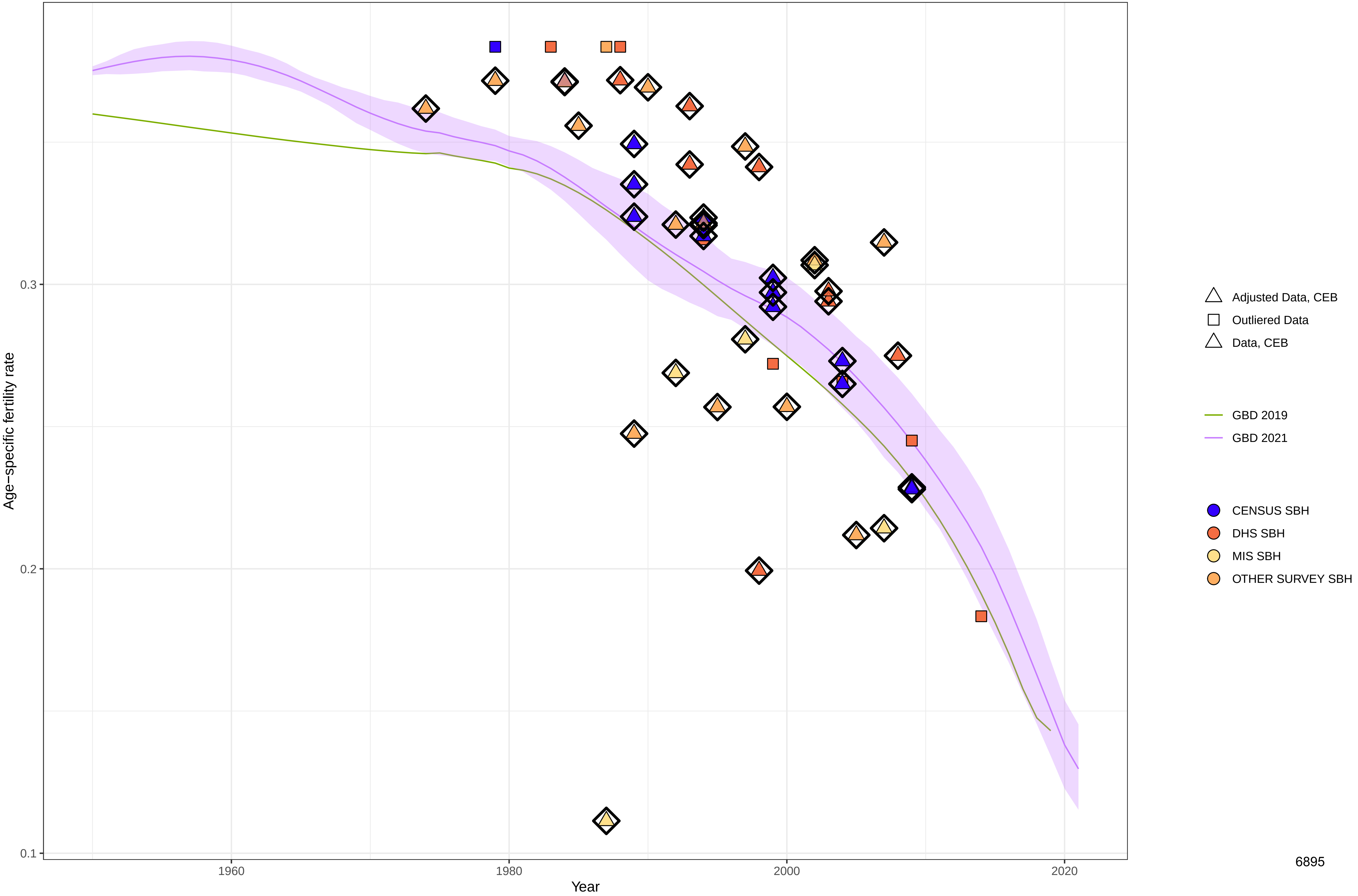

Age−specific fertility fitted trend lines, 1950−2021: Bomet, Ages: 30−34
Data points enclosed with a diamond are reference sources

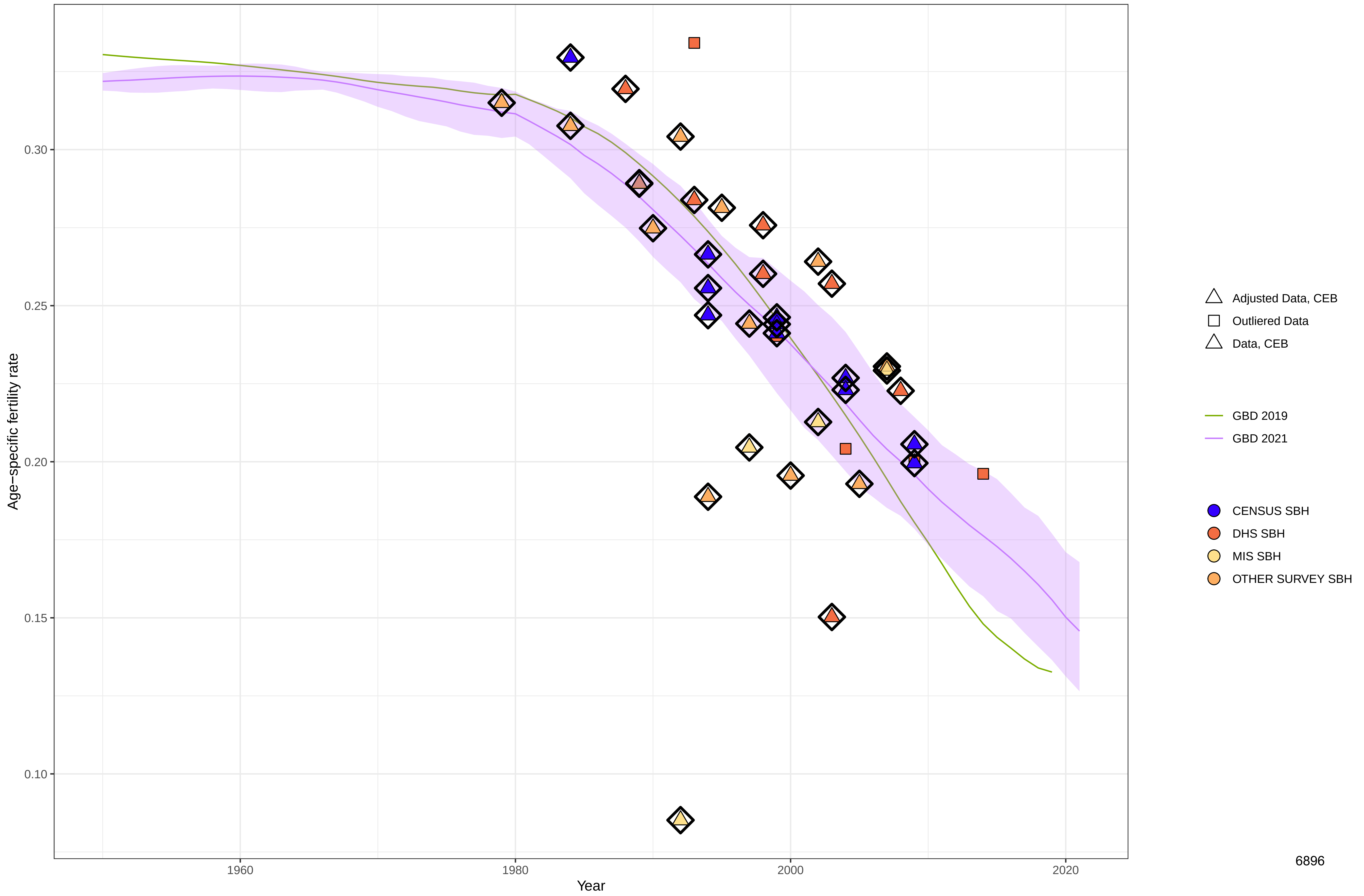

Age−specific fertility fitted trend lines, 1950−2021: Bomet, Ages: 35−39
Data points enclosed with a diamond are reference sources

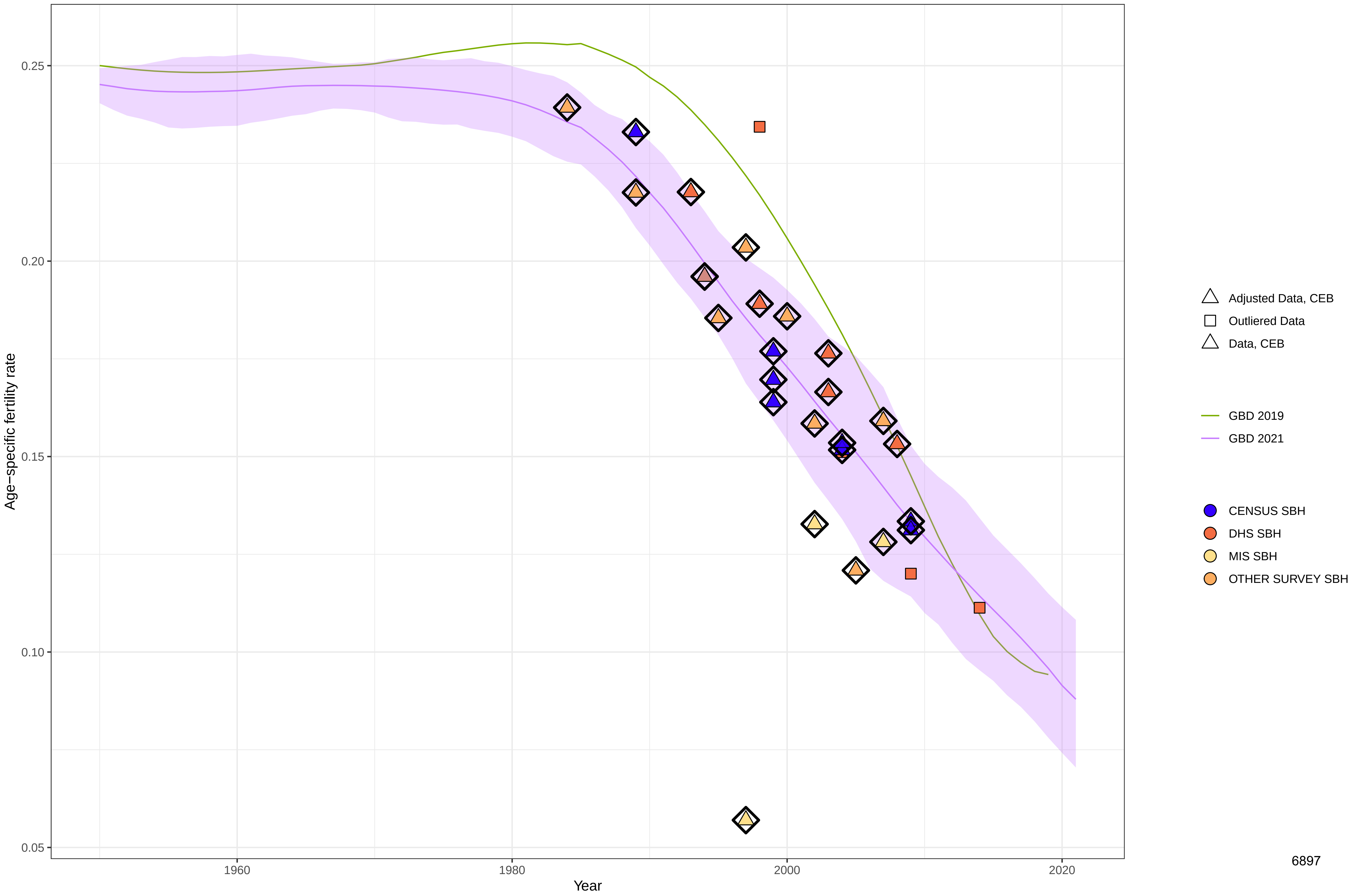

6897

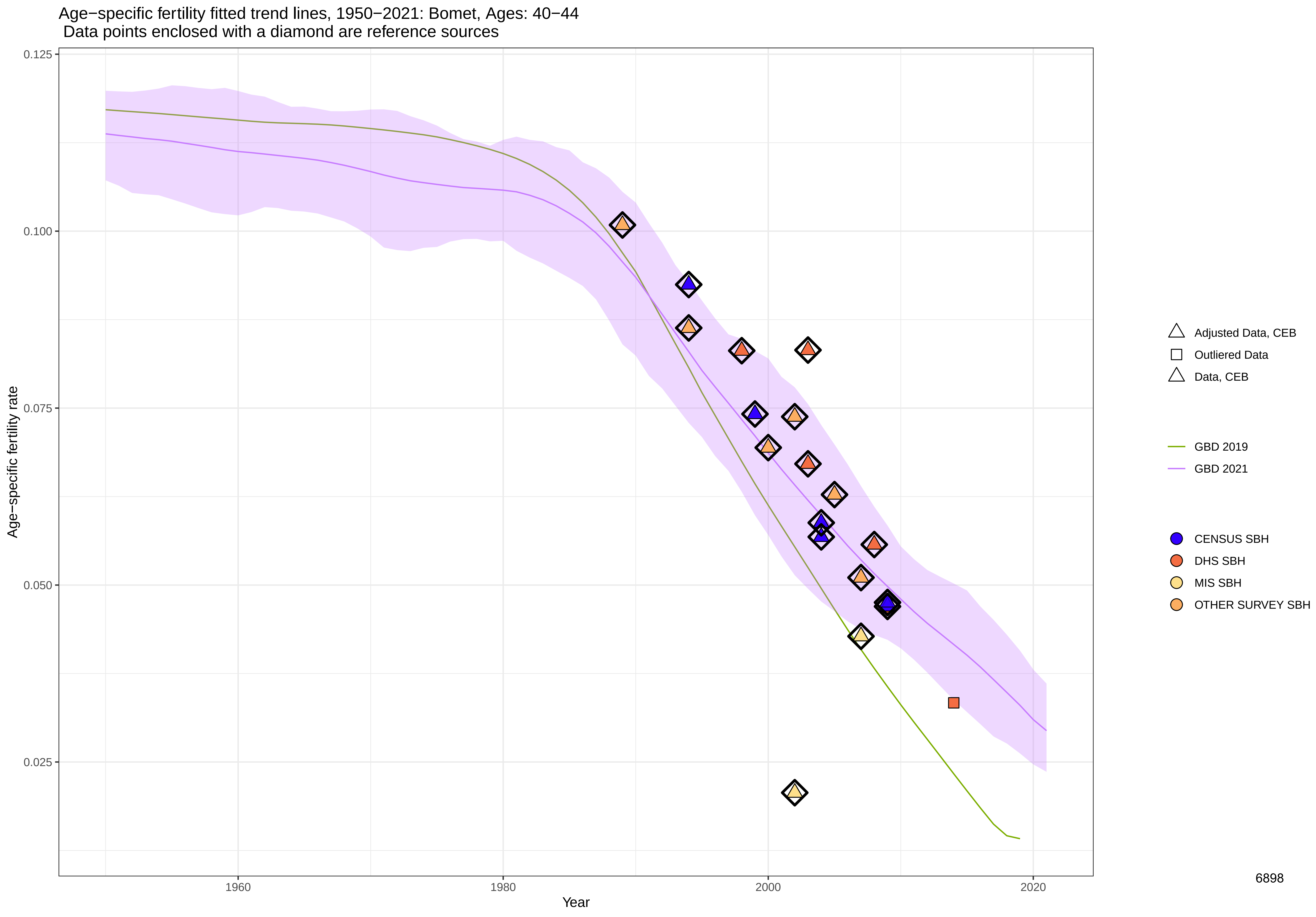

Age−specific fertility fitted trend lines, 1950−2021: Bomet, Ages: 40−44
Data points enclosed with a diamond are reference sources
Age−specific fertility rate
0.125
0.100
0.075
0.050
0.025
1960
1980
2000
2020
Year
Adjusted Data, CEB
Outliered Data
Data, CEB
GBD 2019
GBD 2021
CENSUS SBH
DHS SBH
MIS SBH
OTHER SURVEY SBH

6898

Age−specific fertility fitted trend lines, 1950−2021: Bomet, Ages: 45−49
Data points enclosed with a diamond are reference sources

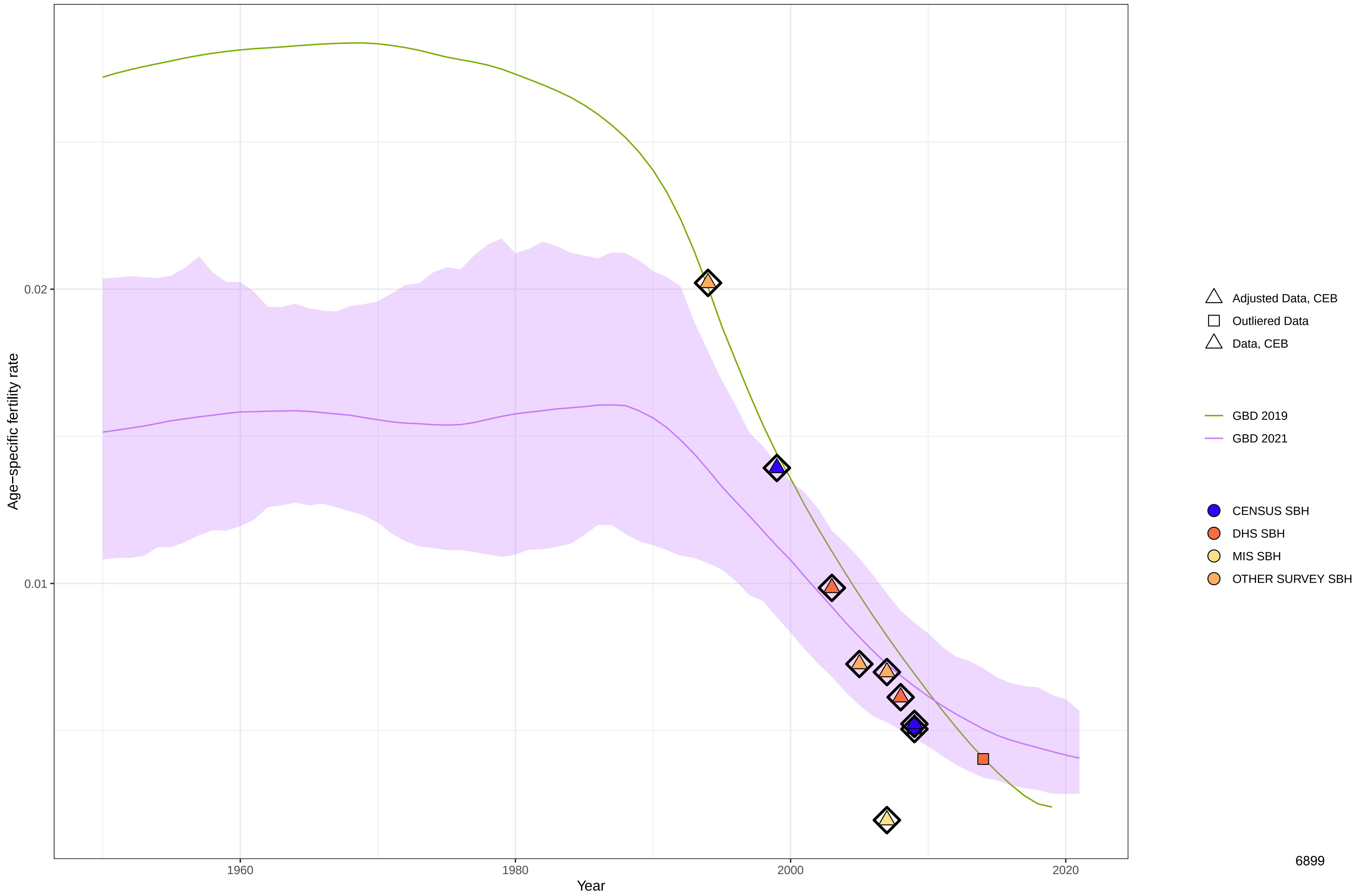

Age−specific fertility fitted trend lines, 1950−2021: Bomet, Ages: 50−54
Data points enclosed with a diamond are reference sources

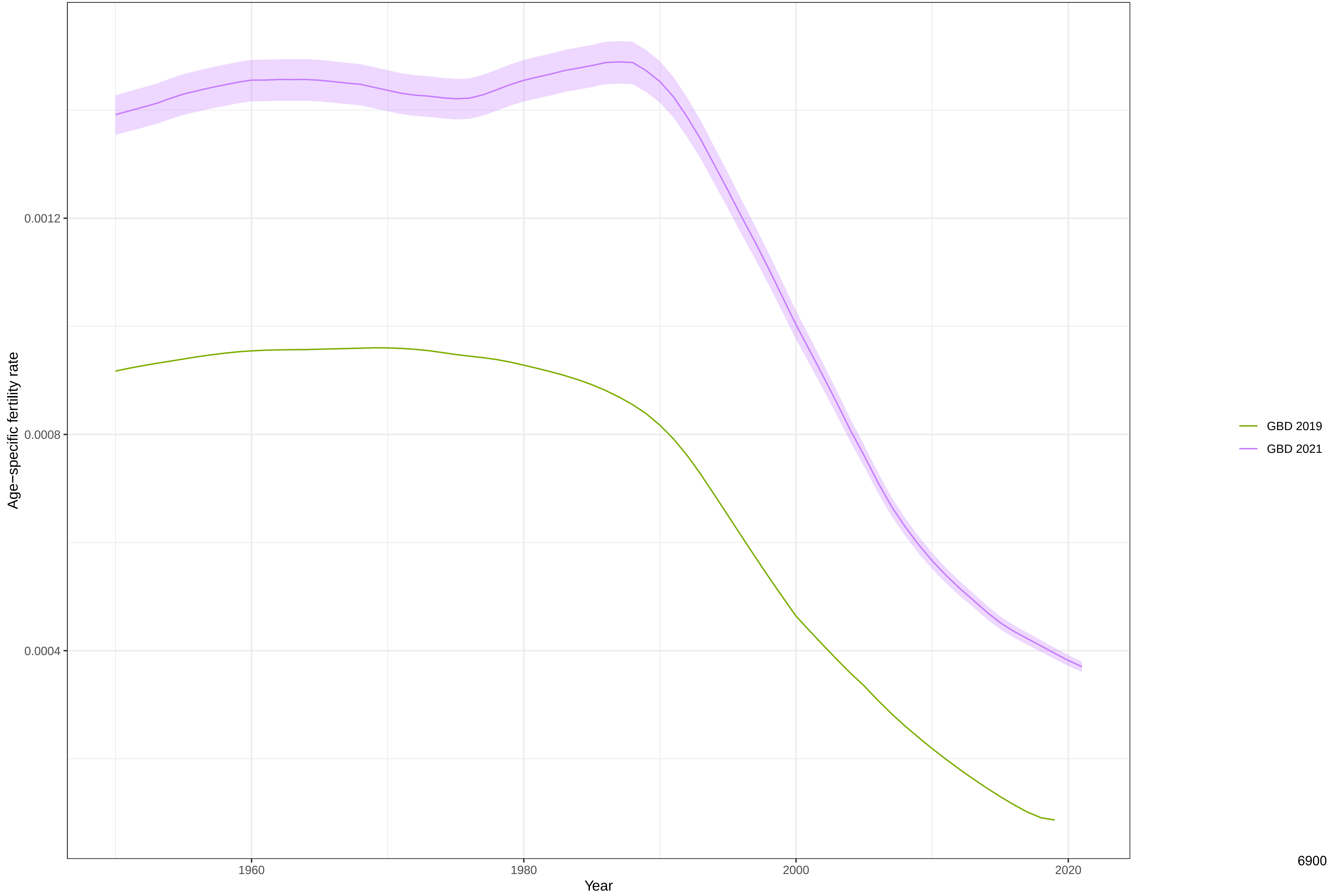

Total fertility rate fitted trend lines, 1950−2021: Bomet TFR
Data points enclosed with a diamond are reference sources

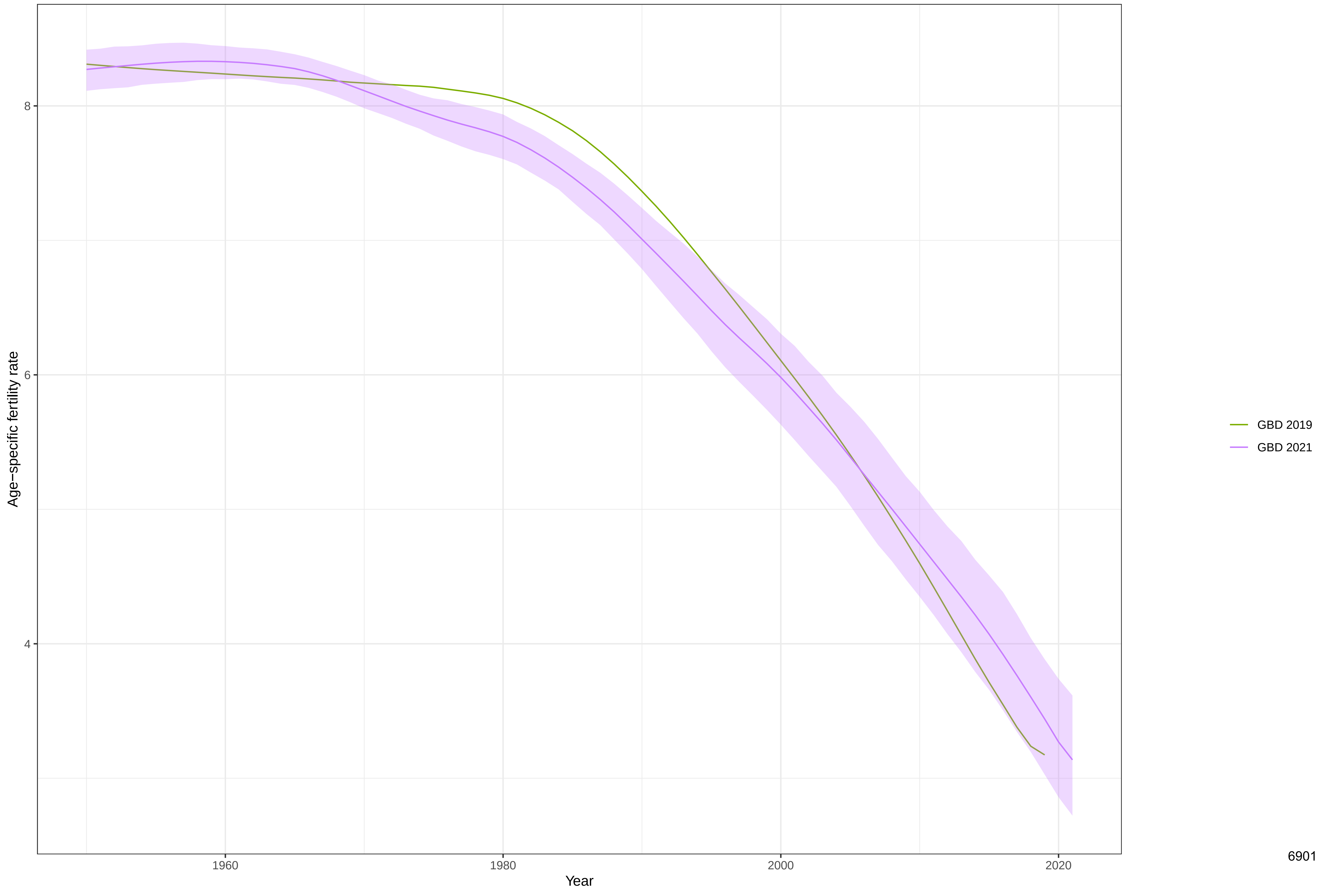

Age−specific fertility fitted trend lines, 1950−2021: Bungoma, Ages: 10−14
Data points enclosed with a diamond are reference sources

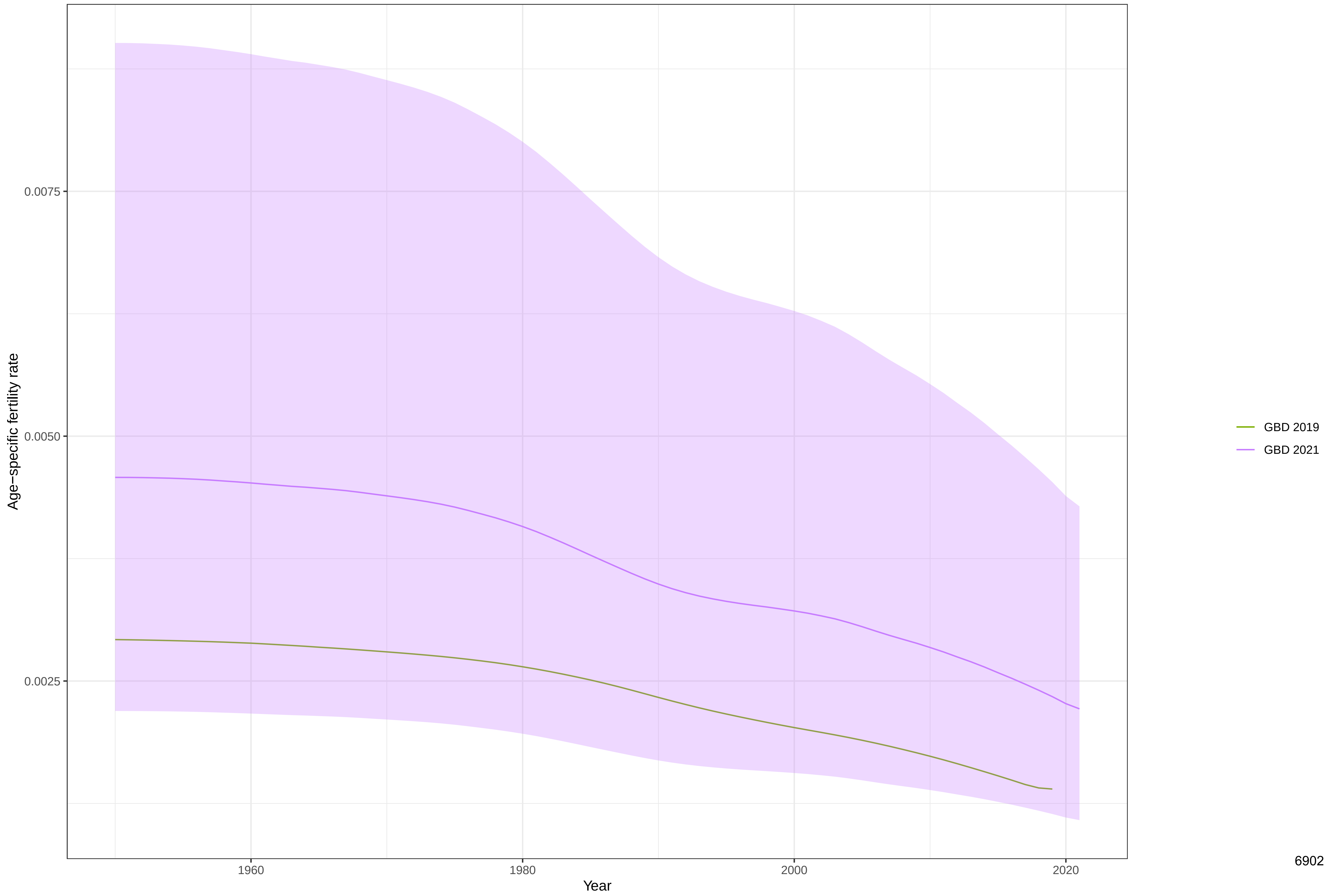

Age−specific fertility fitted trend lines, 1950−2021: Bungoma, Ages: 15−19
Data points enclosed with a diamond are reference sources

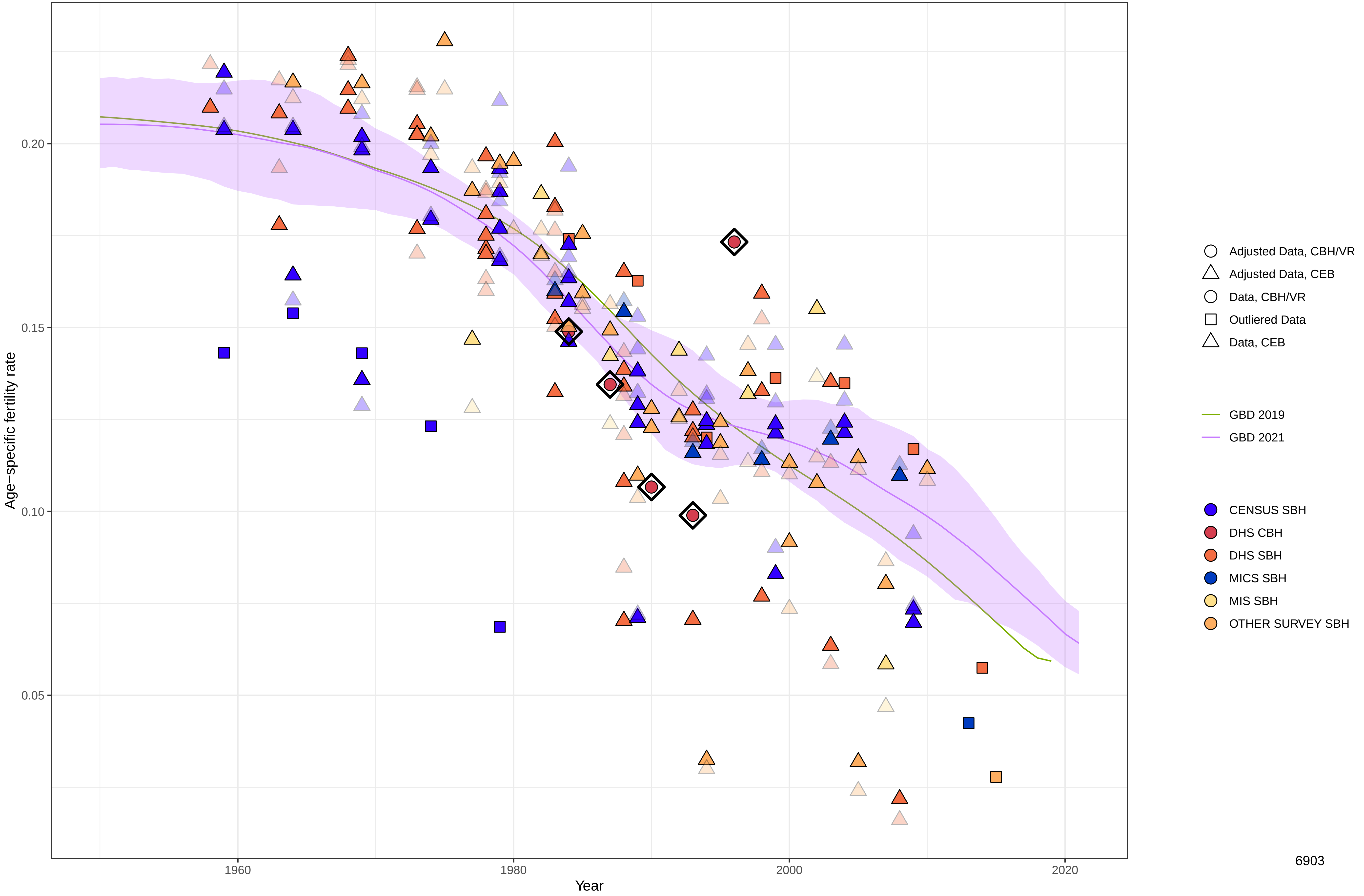

6903

Age−specific fertility fitted trend lines, 1950−2021: Bungoma, Ages: 20−24
Data points enclosed with a diamond are reference sources

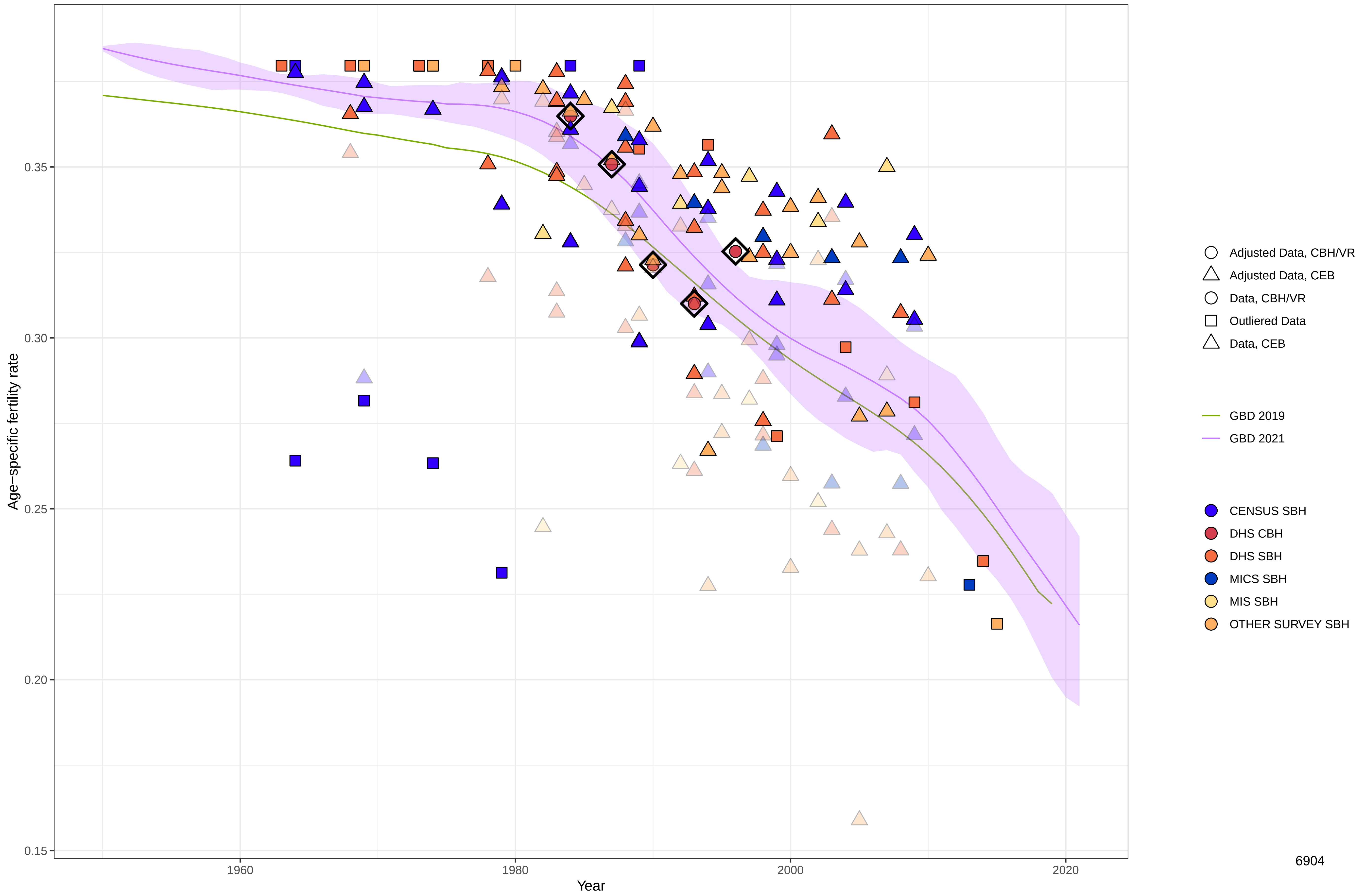

Age−specific fertility fitted trend lines, 1950−2021: Bungoma, Ages: 25−29
Data points enclosed with a diamond are reference sources

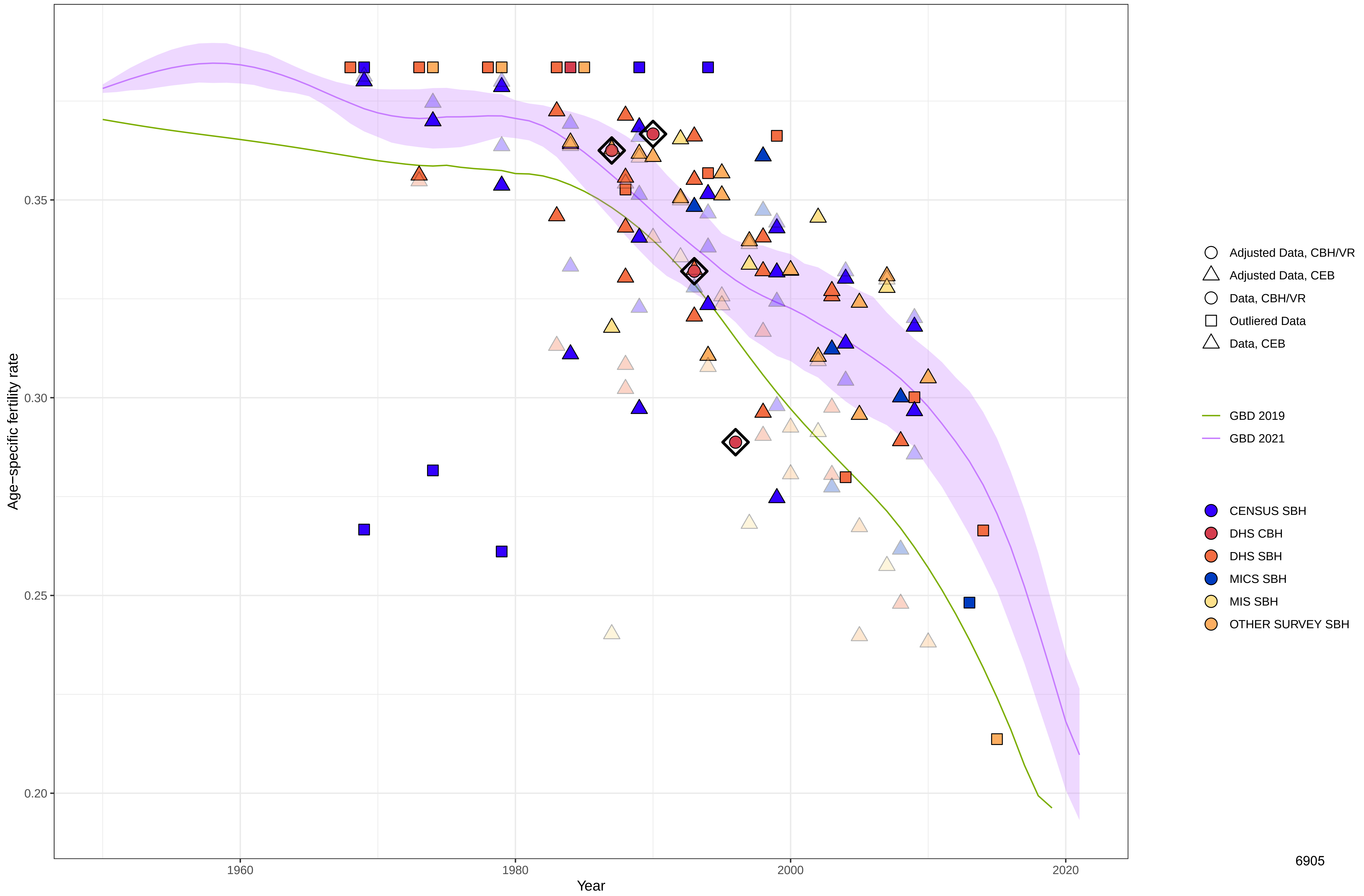

Age−specific fertility fitted trend lines, 1950−2021: Bungoma, Ages: 30−34
Data points enclosed with a diamond are reference sources

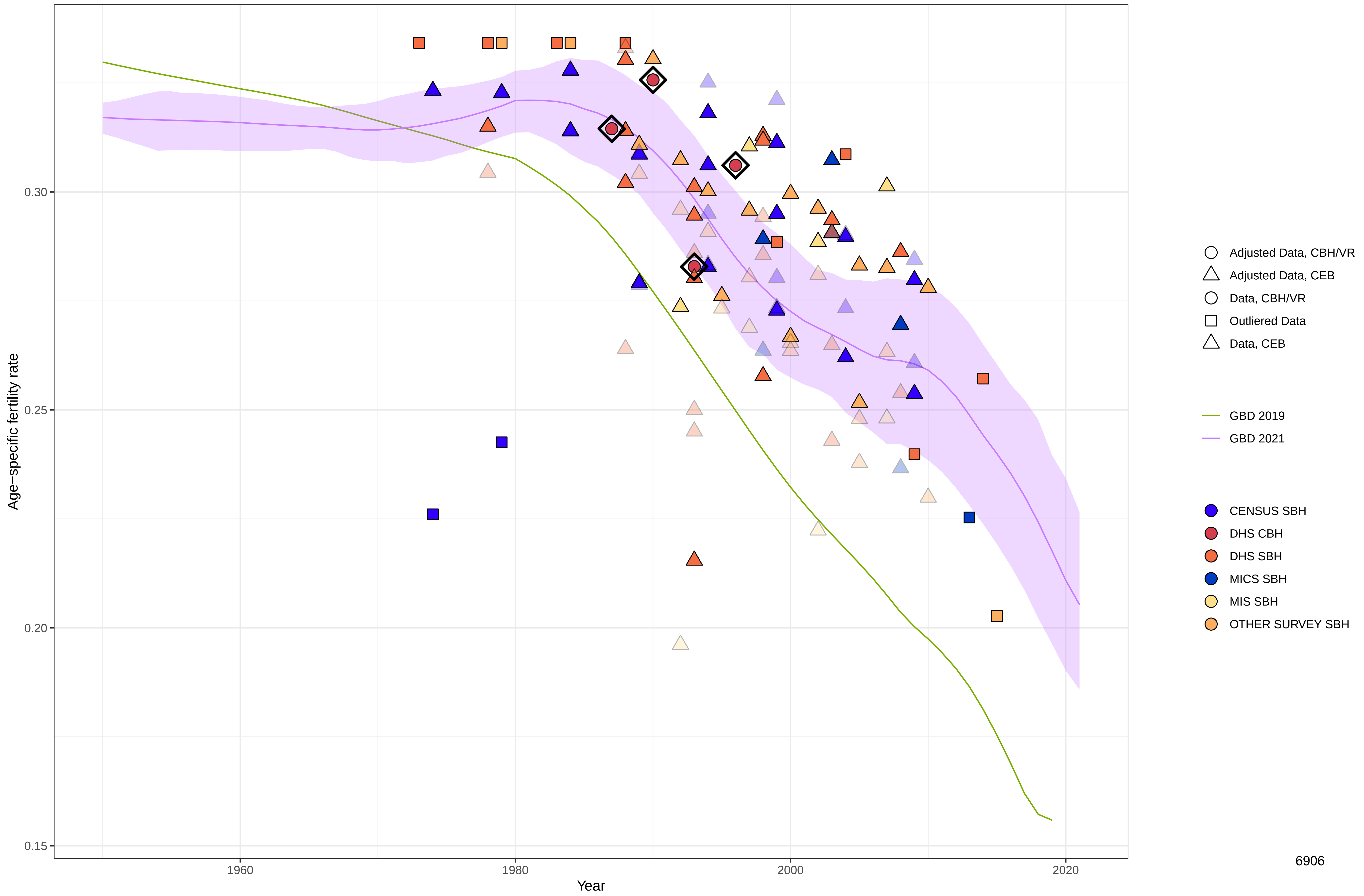

Age−specific fertility fitted trend lines, 1950−2021: Bungoma, Ages: 35−39
Data points enclosed with a diamond are reference sources

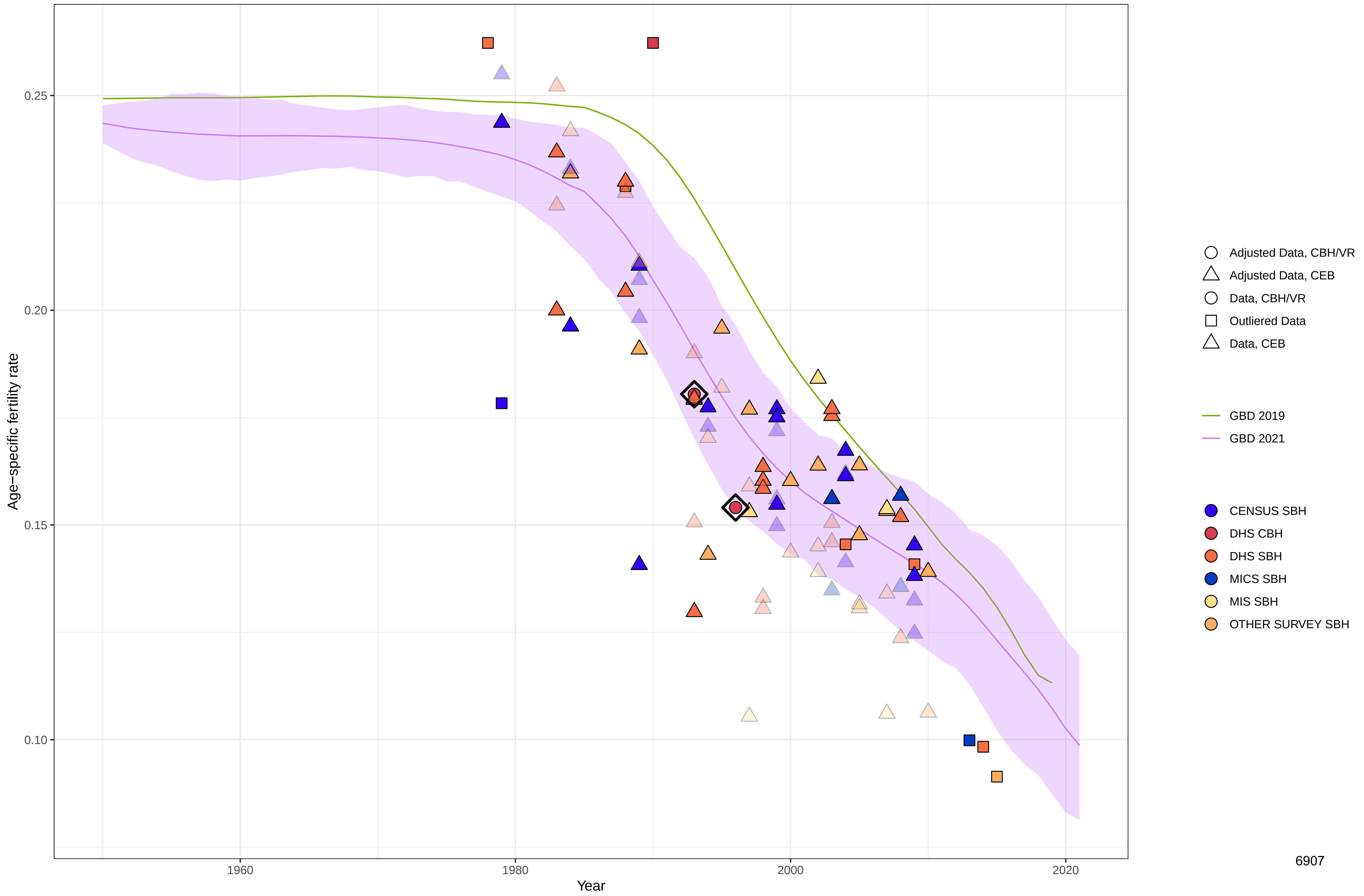

6907

Age−specific fertility fitted trend lines, 1950−2021: Bungoma, Ages: 40−44
Data points enclosed with a diamond are reference sources

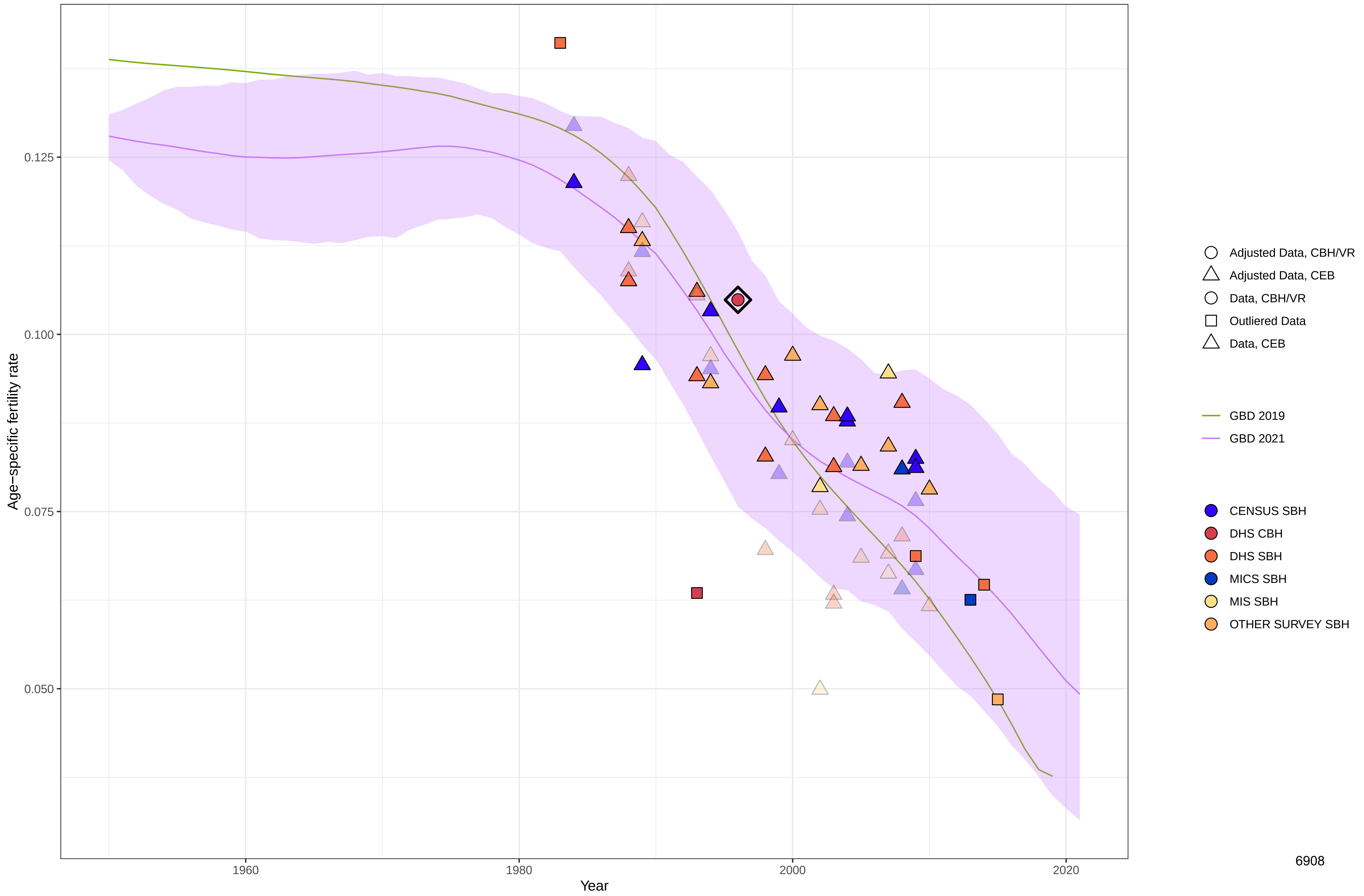

6908

Age−specific fertility fitted trend lines, 1950−2021: Bungoma, Ages: 45−49
Data points enclosed with a diamond are reference sources

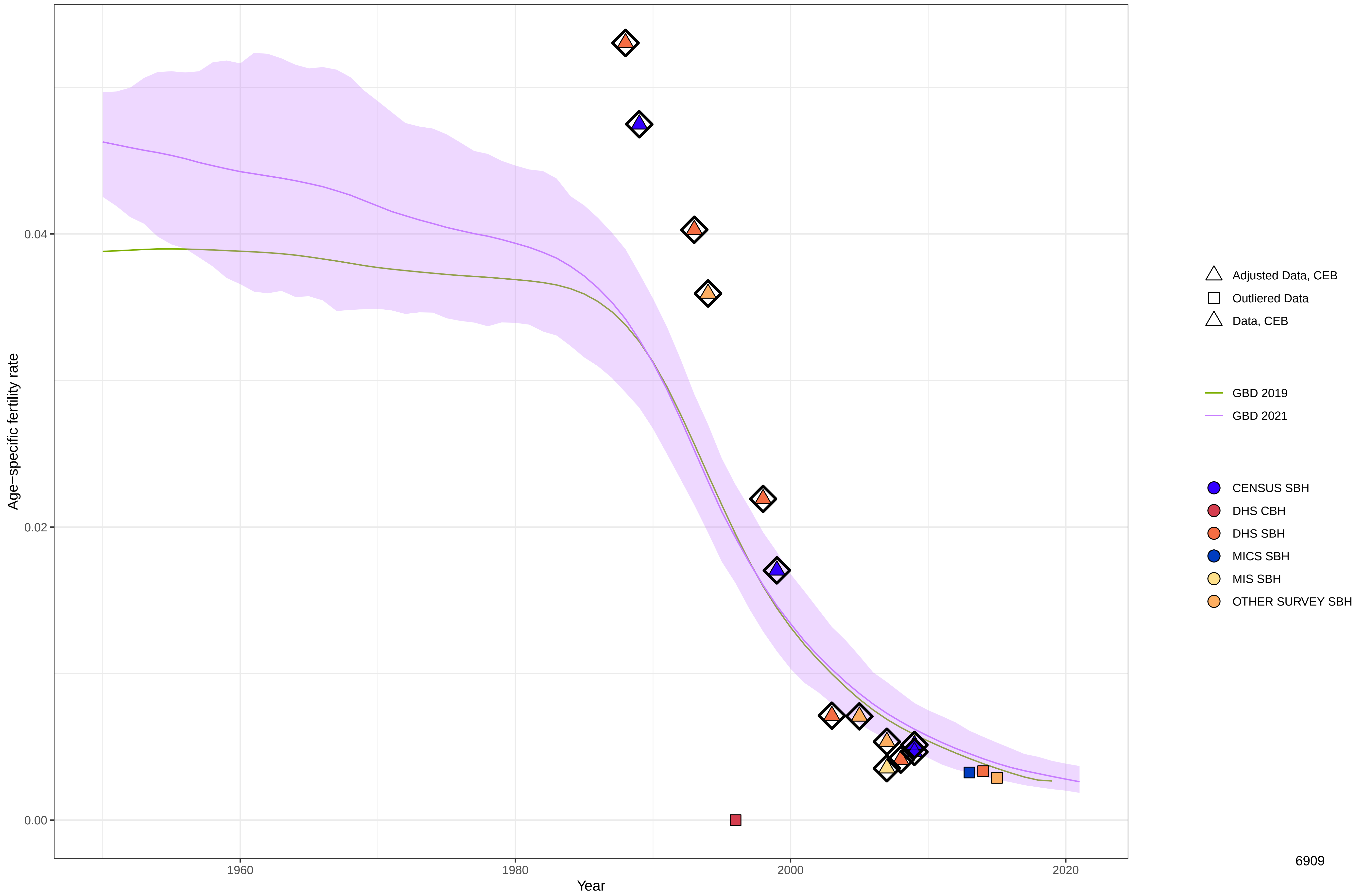

Age−specific fertility fitted trend lines, 1950−2021: Bungoma, Ages: 50−54
Data points enclosed with a diamond are reference sources

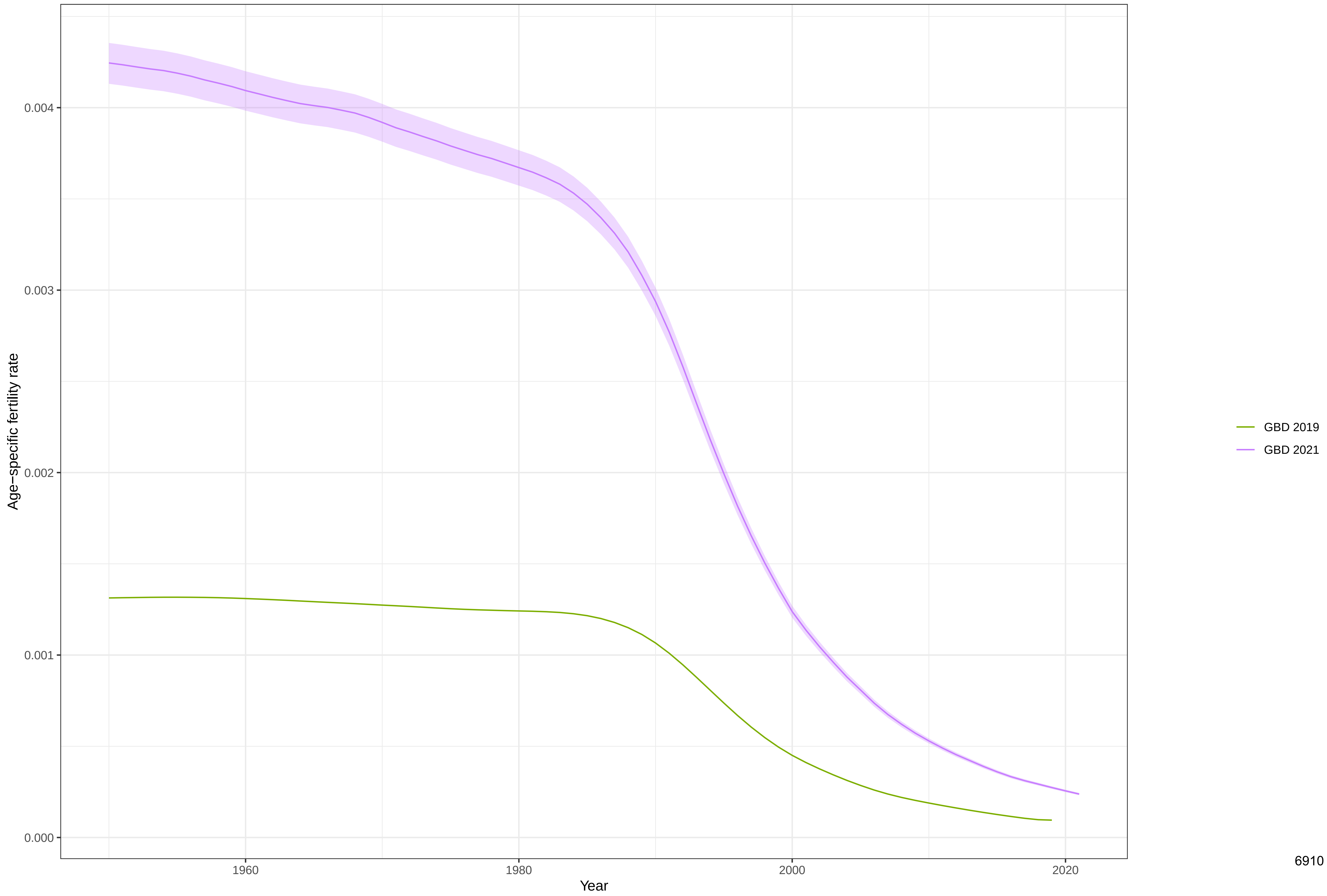

Total fertility rate fitted trend lines, 1950−2021: Bungoma TFR
Data points enclosed with a diamond are reference sources

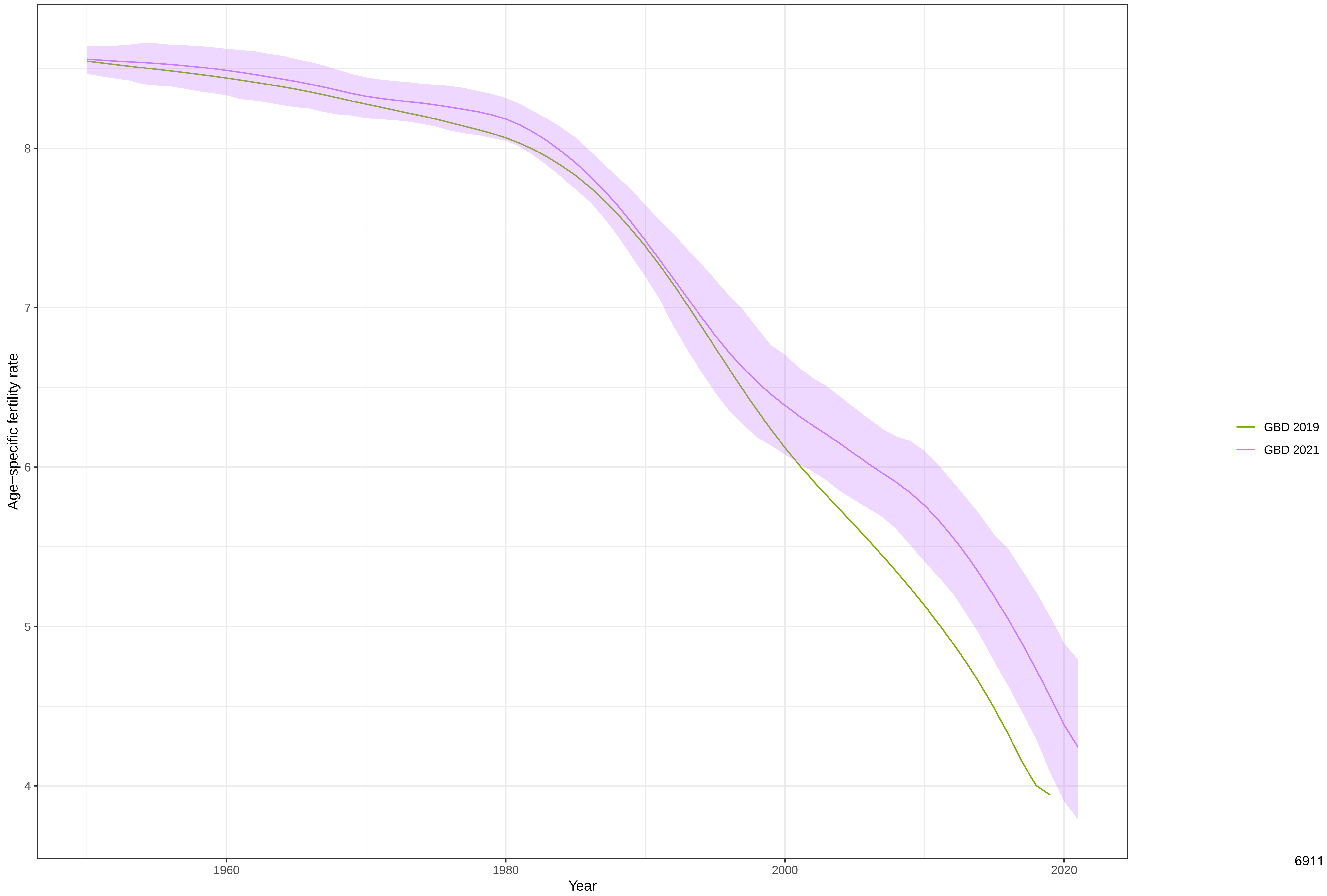

Age−specific fertility fitted trend lines, 1950−2021: Busia, Ages: 10−14
Data points enclosed with a diamond are reference sources

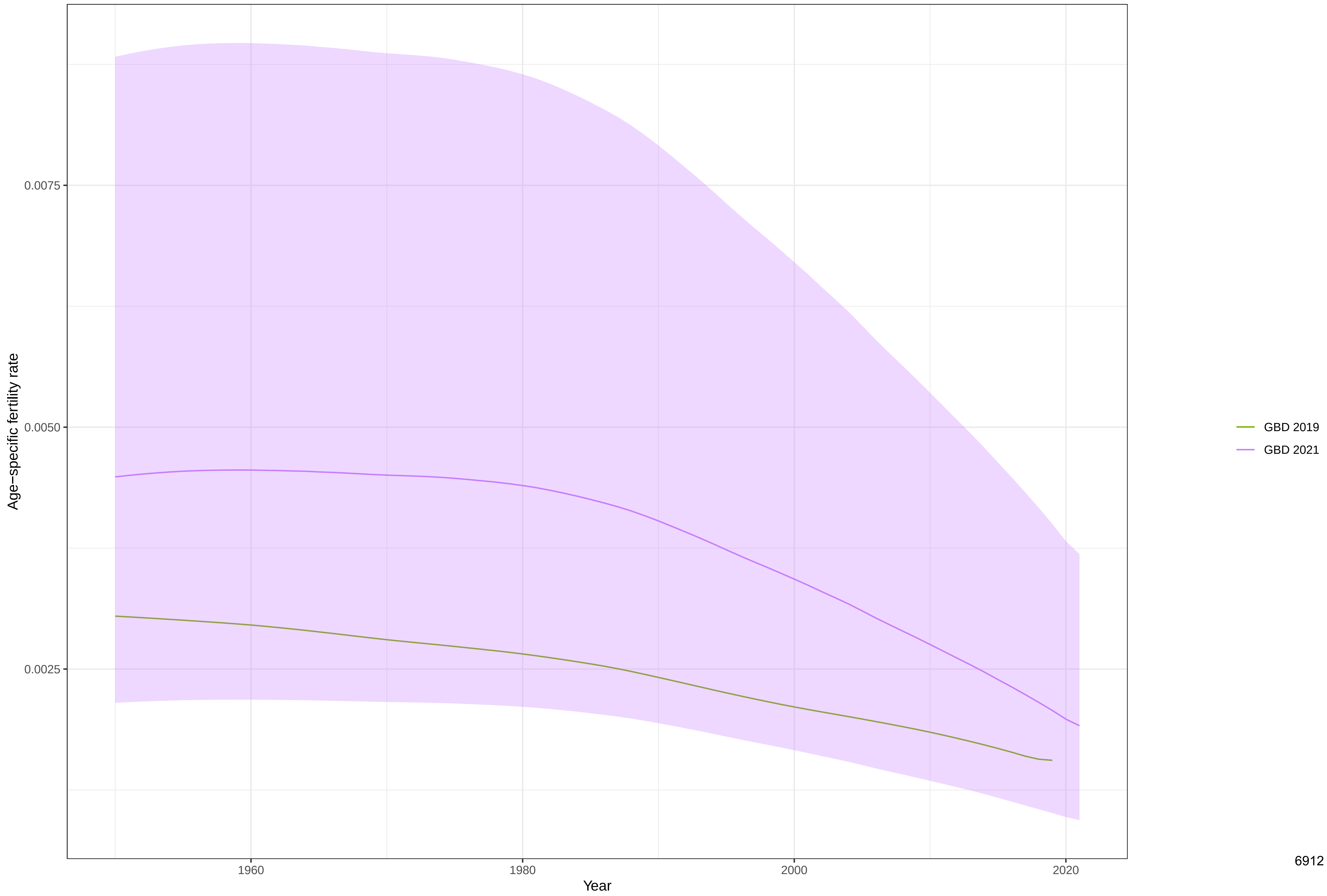

Age−specific fertility fitted trend lines, 1950−2021: Busia, Ages: 15−19
Data points enclosed with a diamond are reference sources

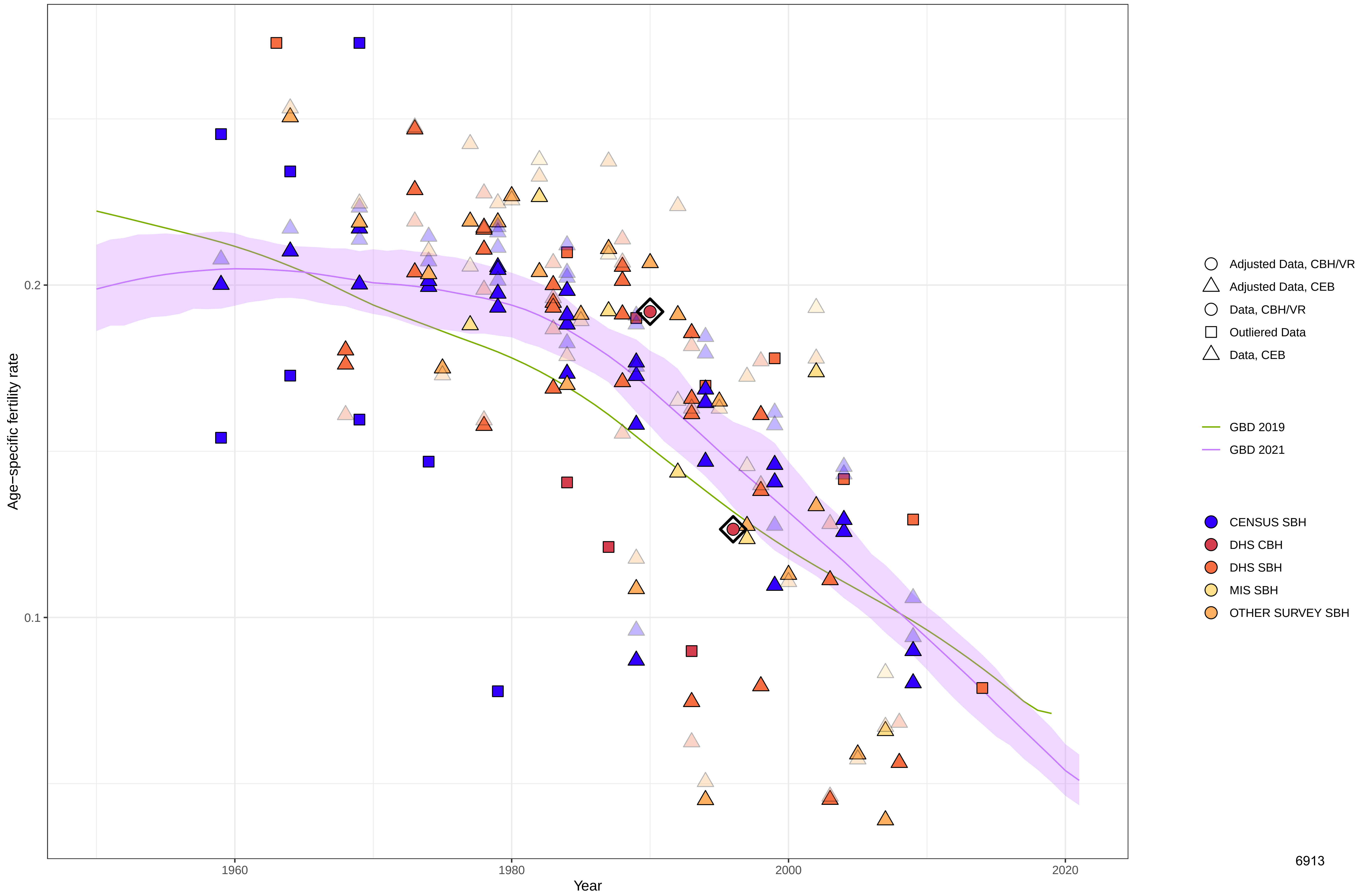

Age−specific fertility fitted trend lines, 1950−2021: Busia, Ages: 20−24
Data points enclosed with a diamond are reference sources

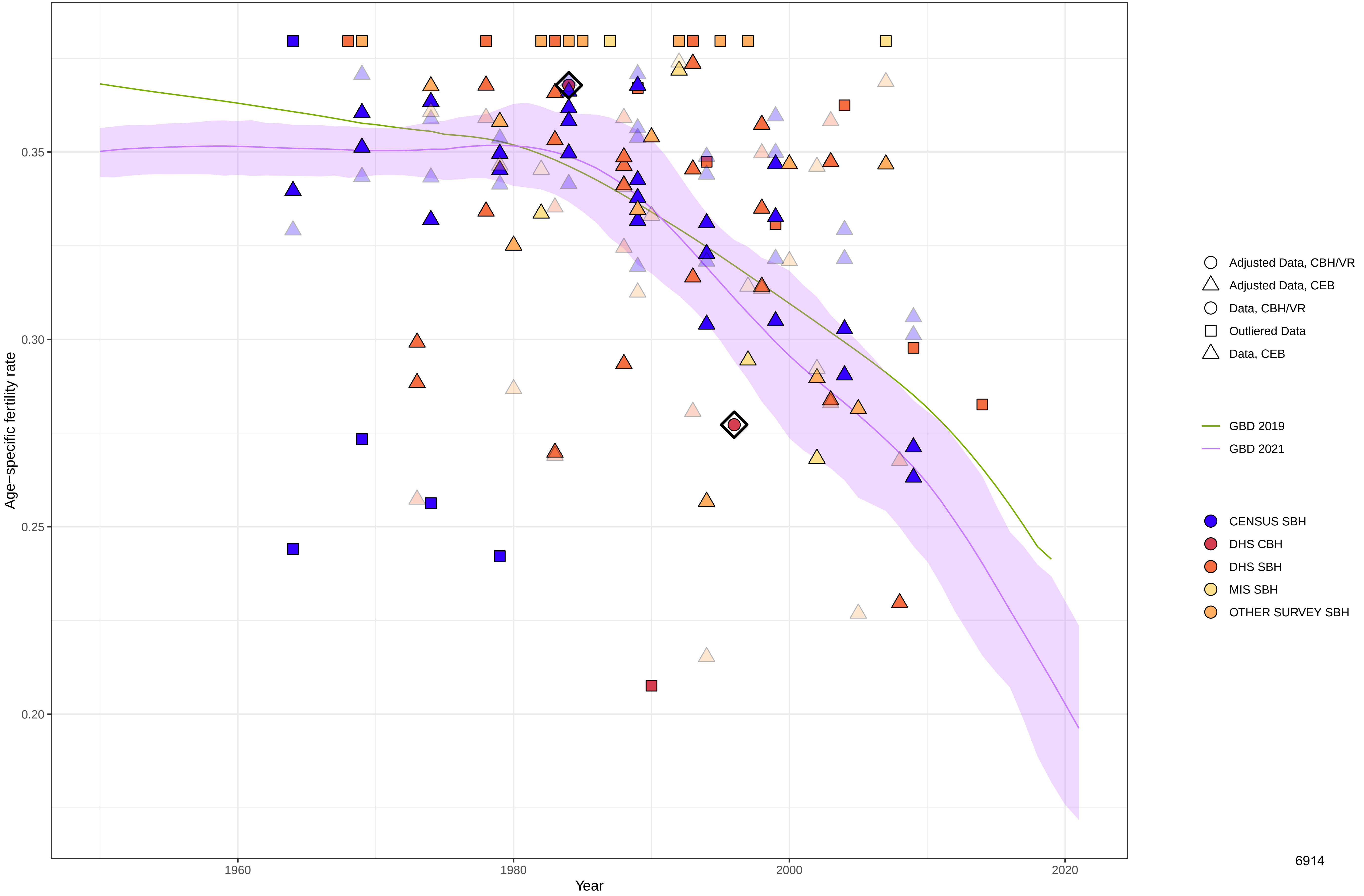

Age−specific fertility fitted trend lines, 1950−2021: Busia, Ages: 25−29
Data points enclosed with a diamond are reference sources

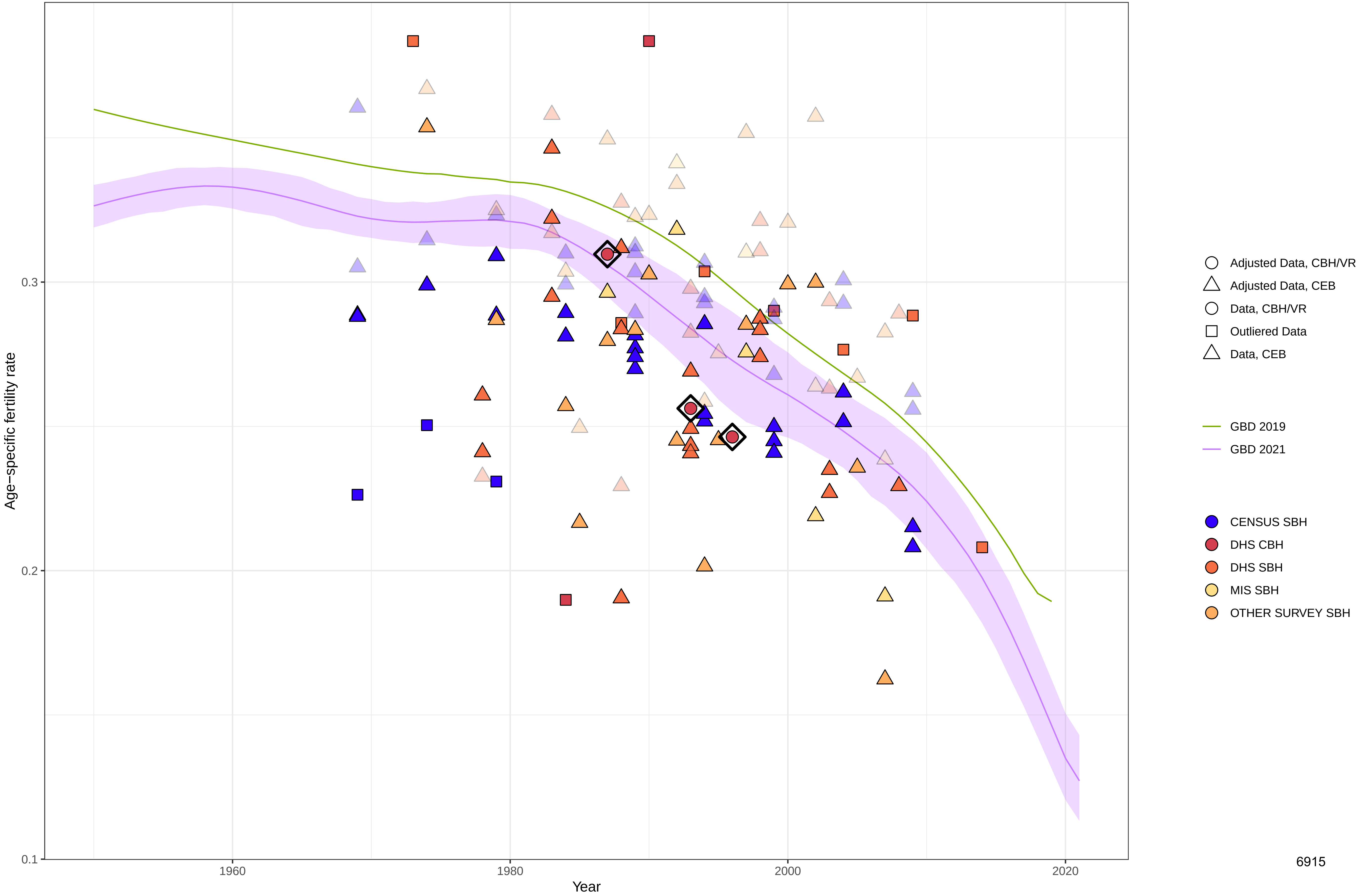

Age−specific fertility fitted trend lines, 1950−2021: Busia, Ages: 30−34
Data points enclosed with a diamond are reference sources

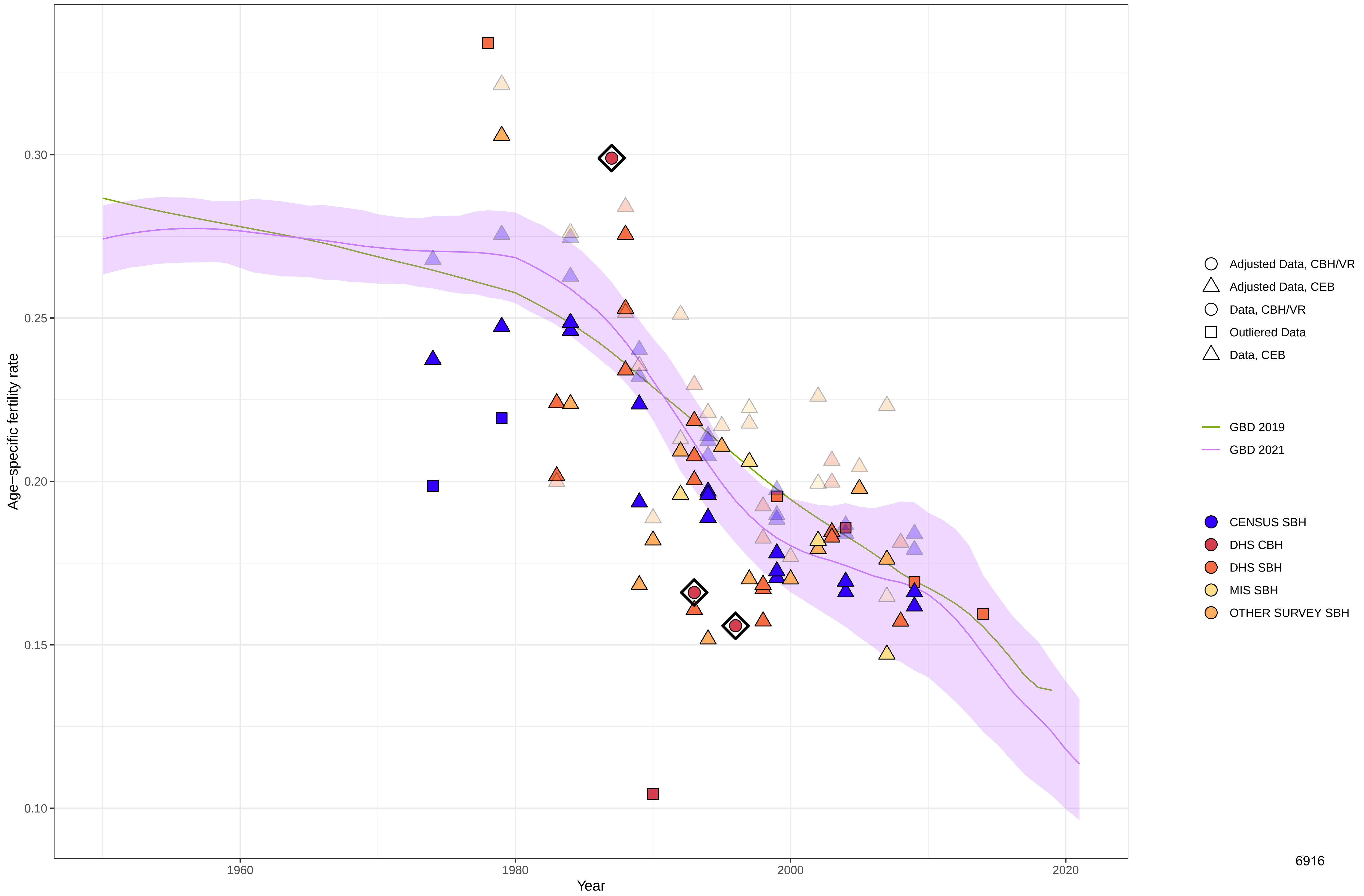

Age−specific fertility fitted trend lines, 1950−2021: Busia, Ages: 35−39
Data points enclosed with a diamond are reference sources

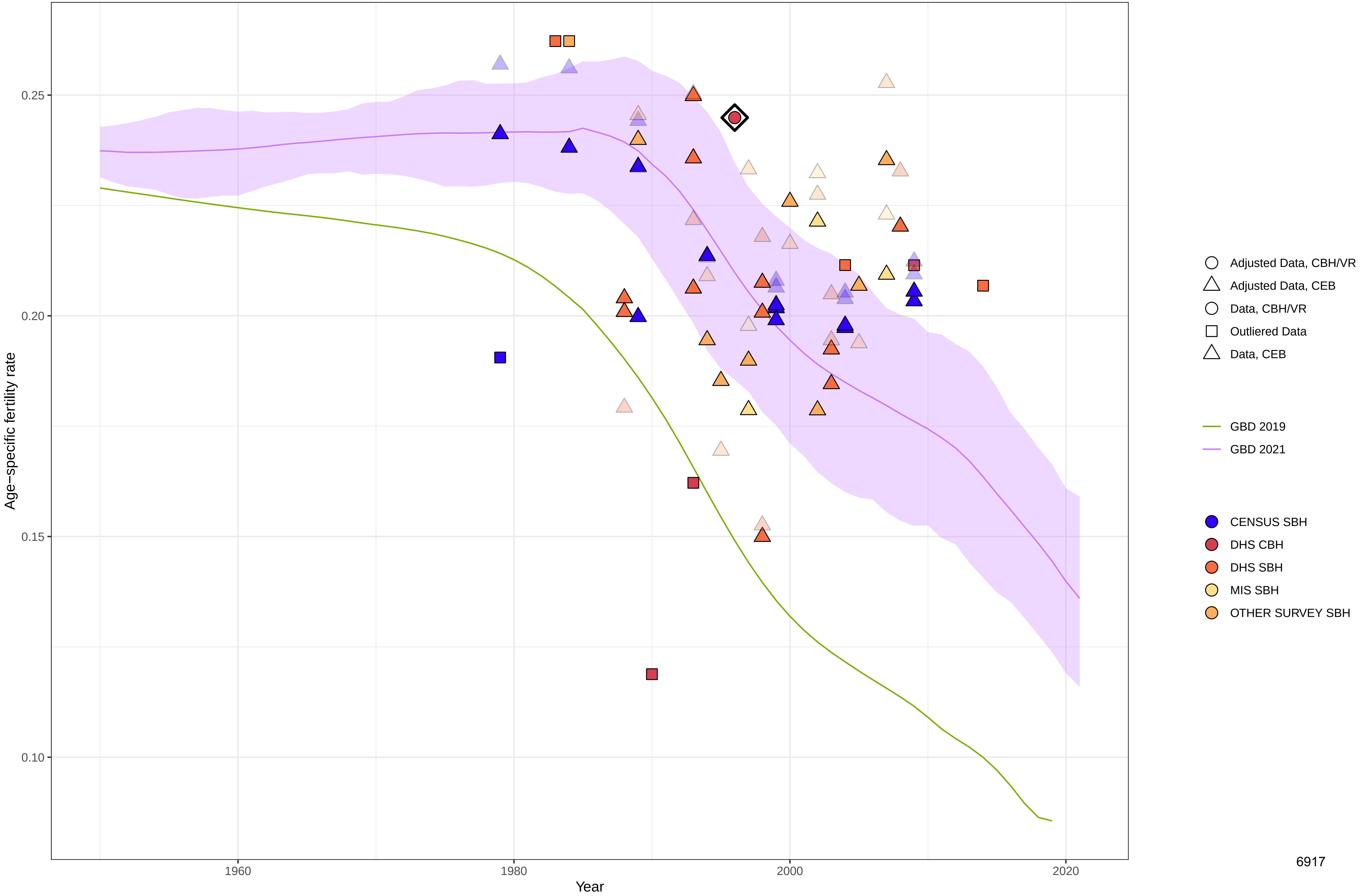

Age−specific fertility fitted trend lines, 1950−2021: Busia, Ages: 40−44
Data points enclosed with a diamond are reference sources

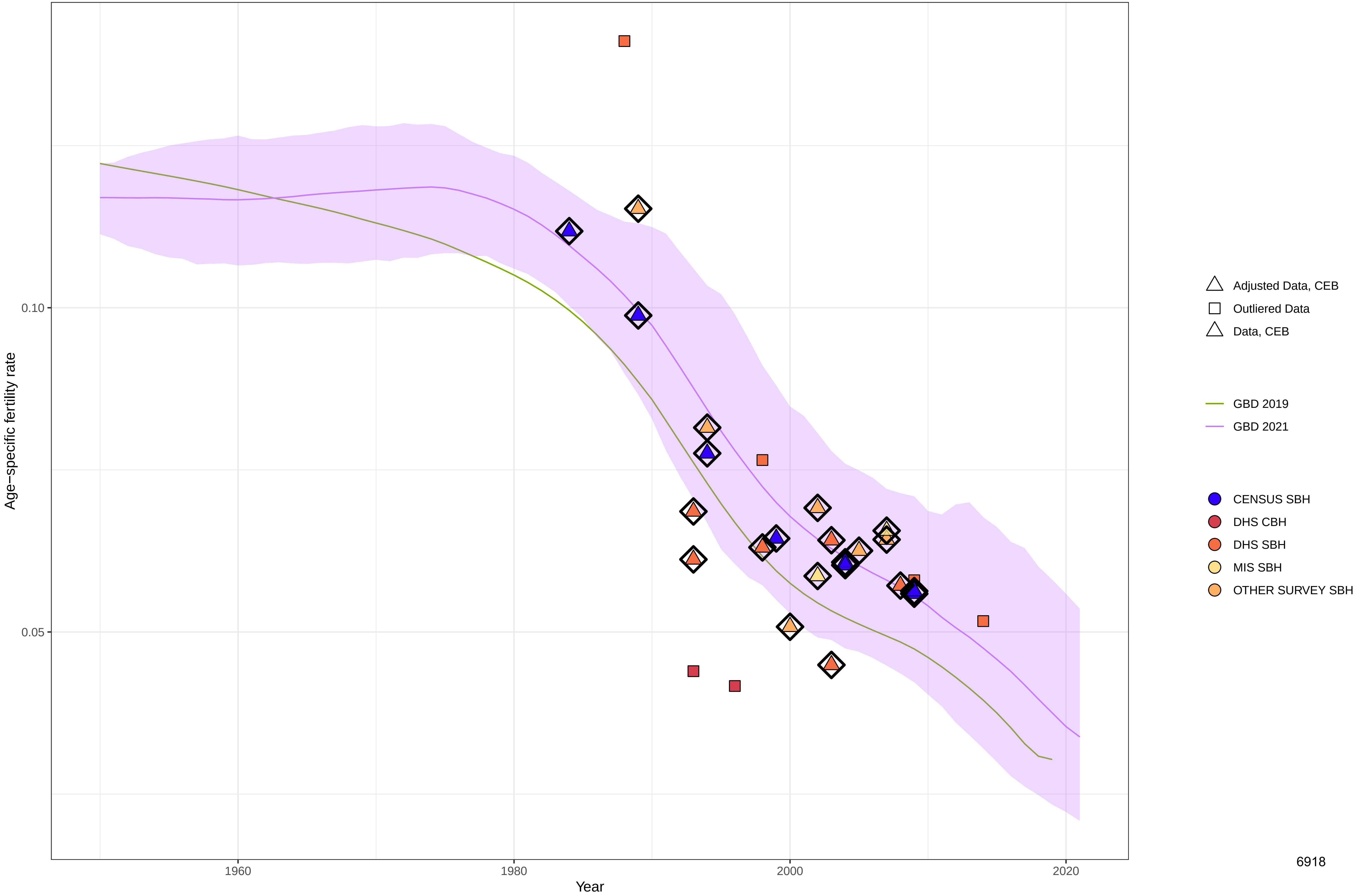

Age−specific fertility fitted trend lines, 1950−2021: Busia, Ages: 45−49
Data points enclosed with a diamond are reference sources

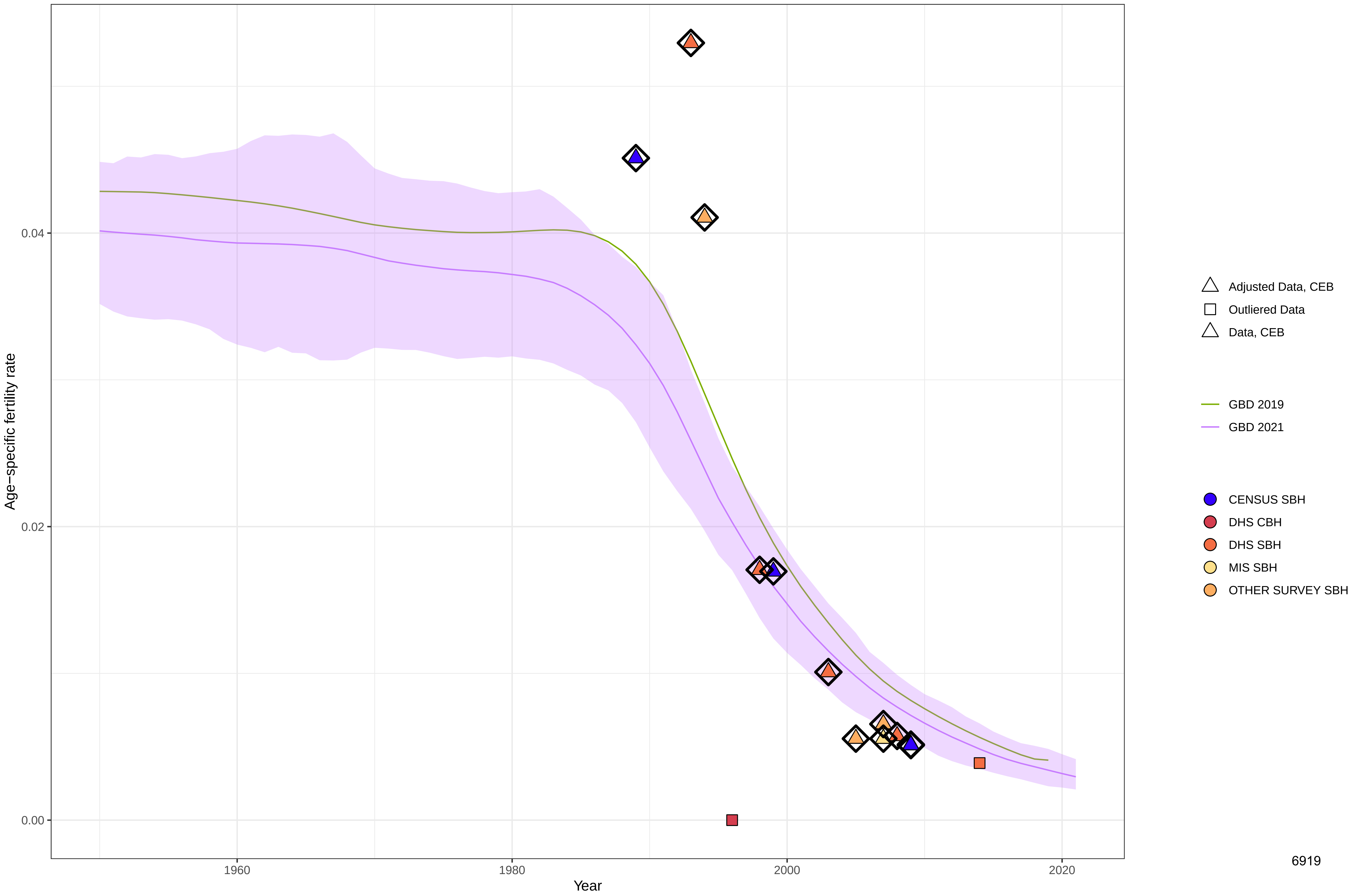

6919

Age−specific fertility fitted trend lines, 1950−2021: Busia, Ages: 50−54
Data points enclosed with a diamond are reference sources

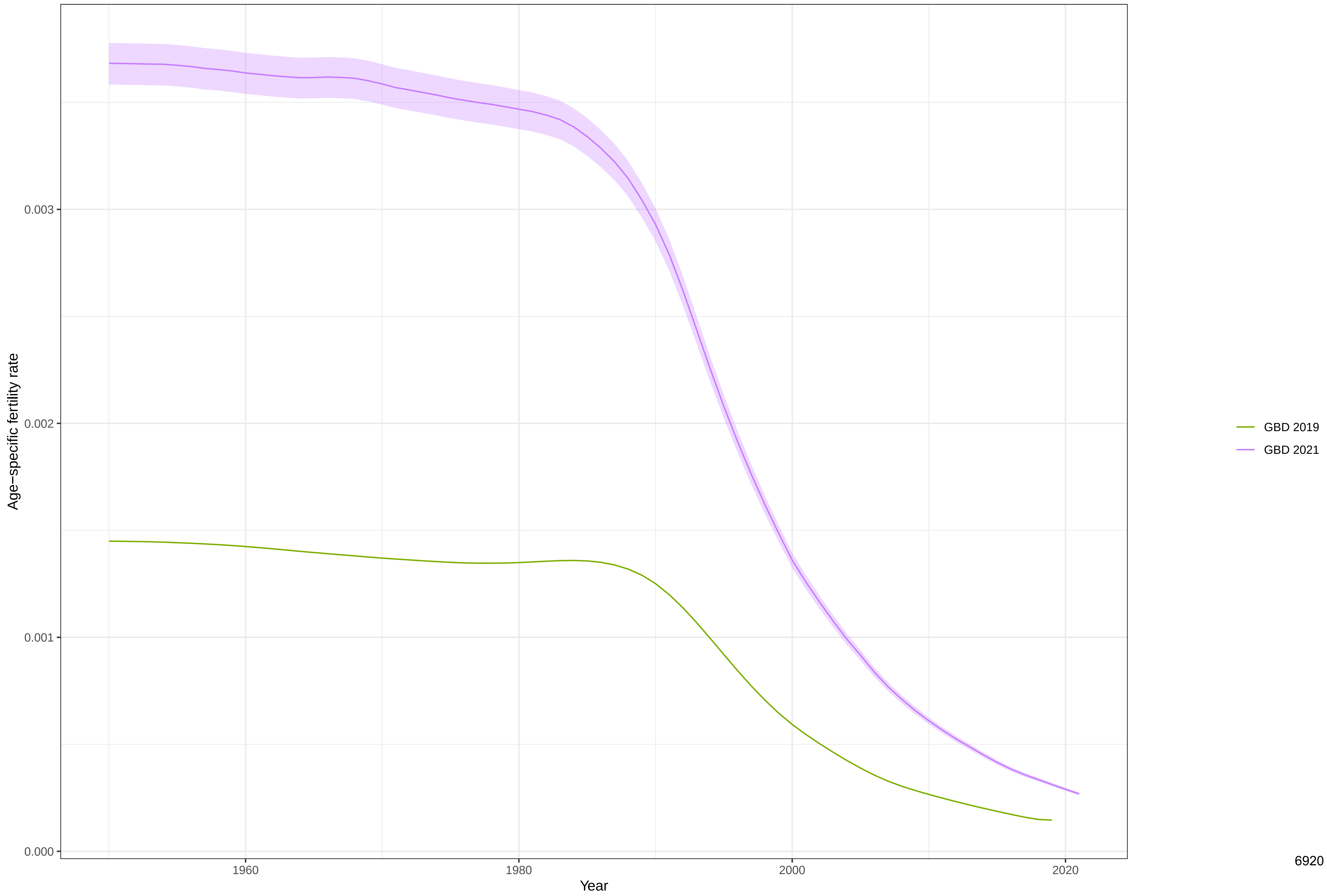

Total fertility rate fitted trend lines, 1950−2021: Busia TFR
Data points enclosed with a diamond are reference sources

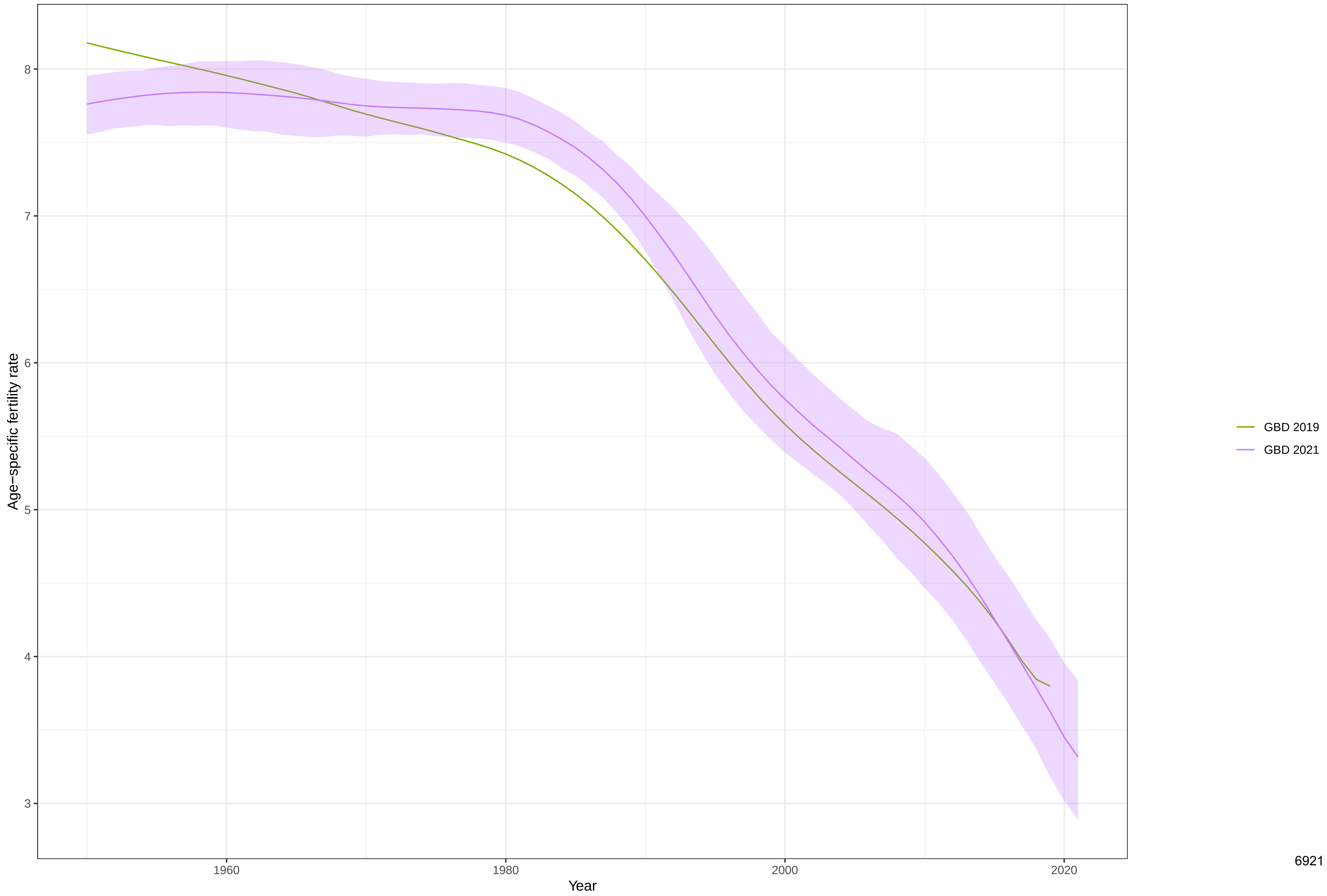

6921

Age−specific fertility fitted trend lines, 1950−2021: Elgeyo Marakwet, Ages: 10−14
Data points enclosed with a diamond are reference sources

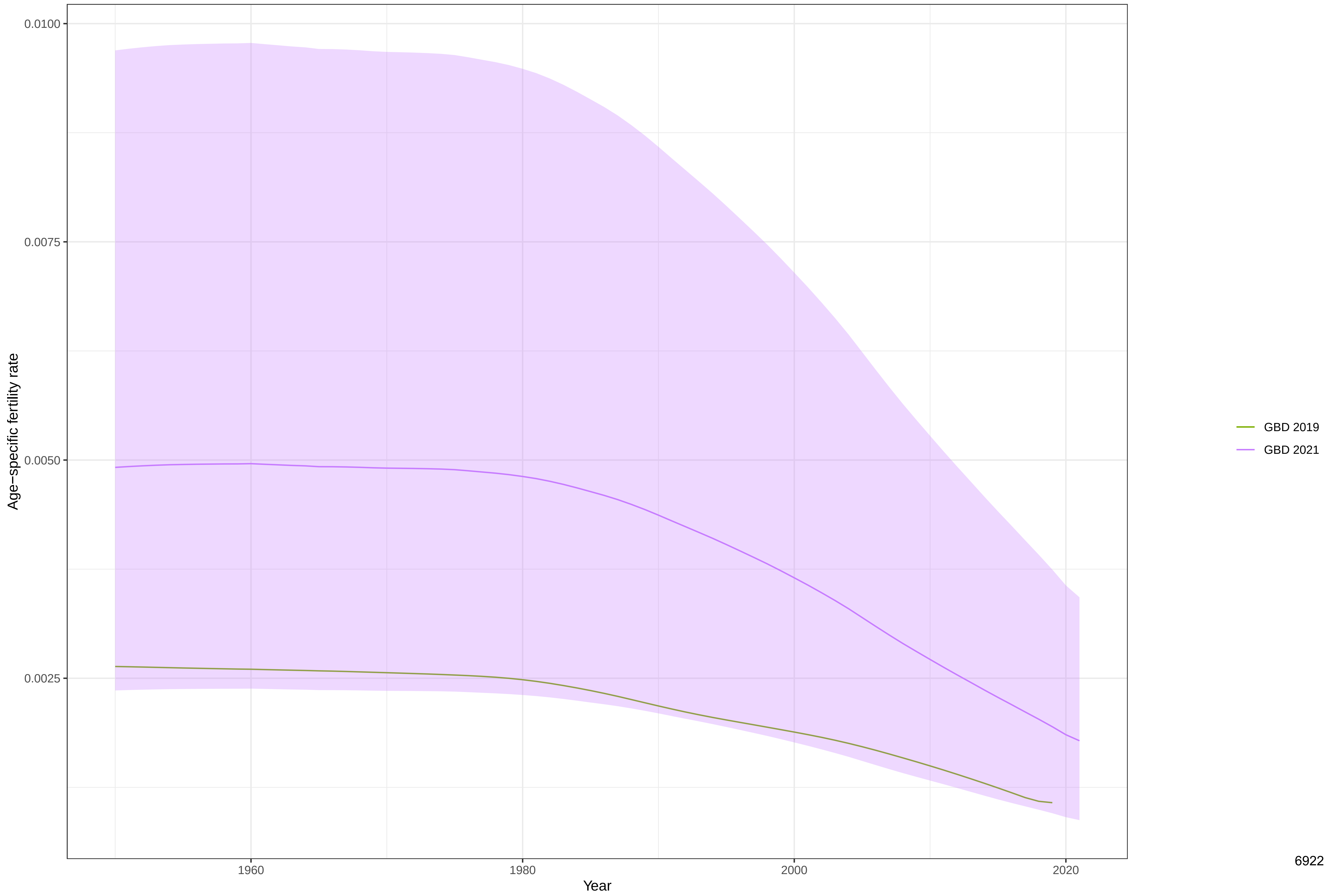

Age−specific fertility fitted trend lines, 1950−2021: Elgeyo Marakwet, Ages: 15−19
Data points enclosed with a diamond are reference sources

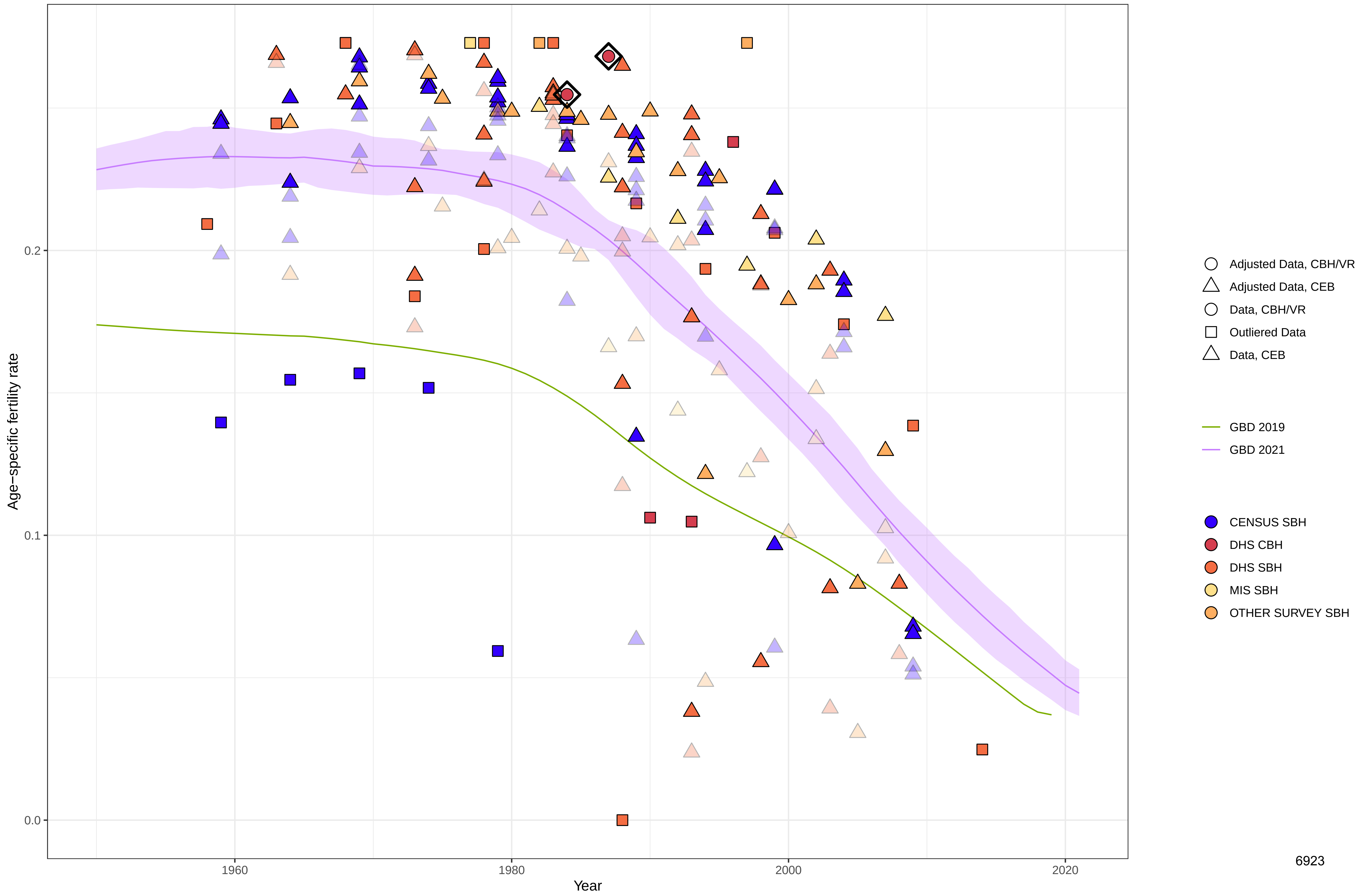

Age−specific fertility fitted trend lines, 1950−2021: Elgeyo Marakwet, Ages: 20−24
Data points enclosed with a diamond are reference sources

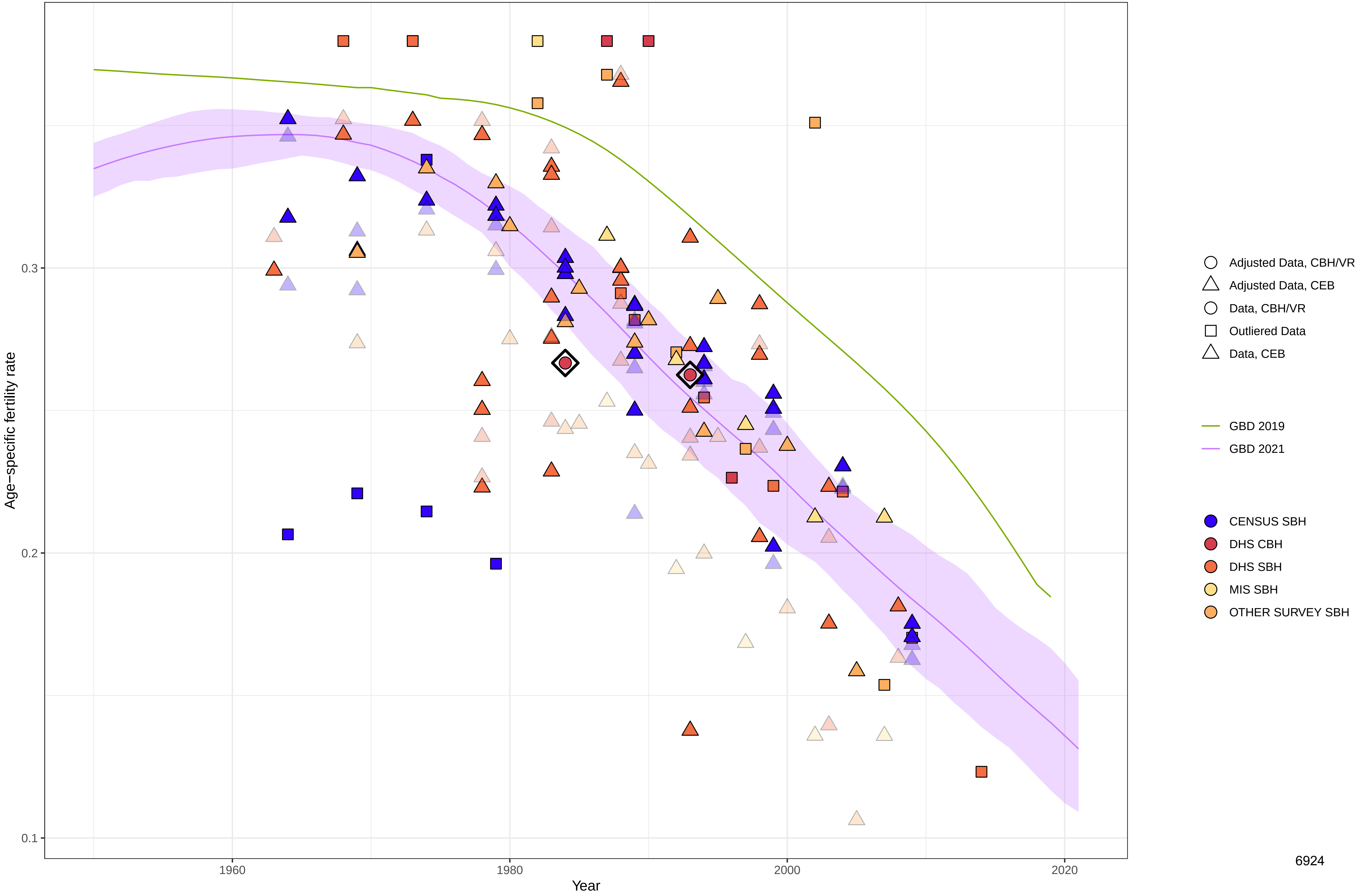

Age−specific fertility fitted trend lines, 1950−2021: Elgeyo Marakwet, Ages: 25−29
Data points enclosed with a diamond are reference sources

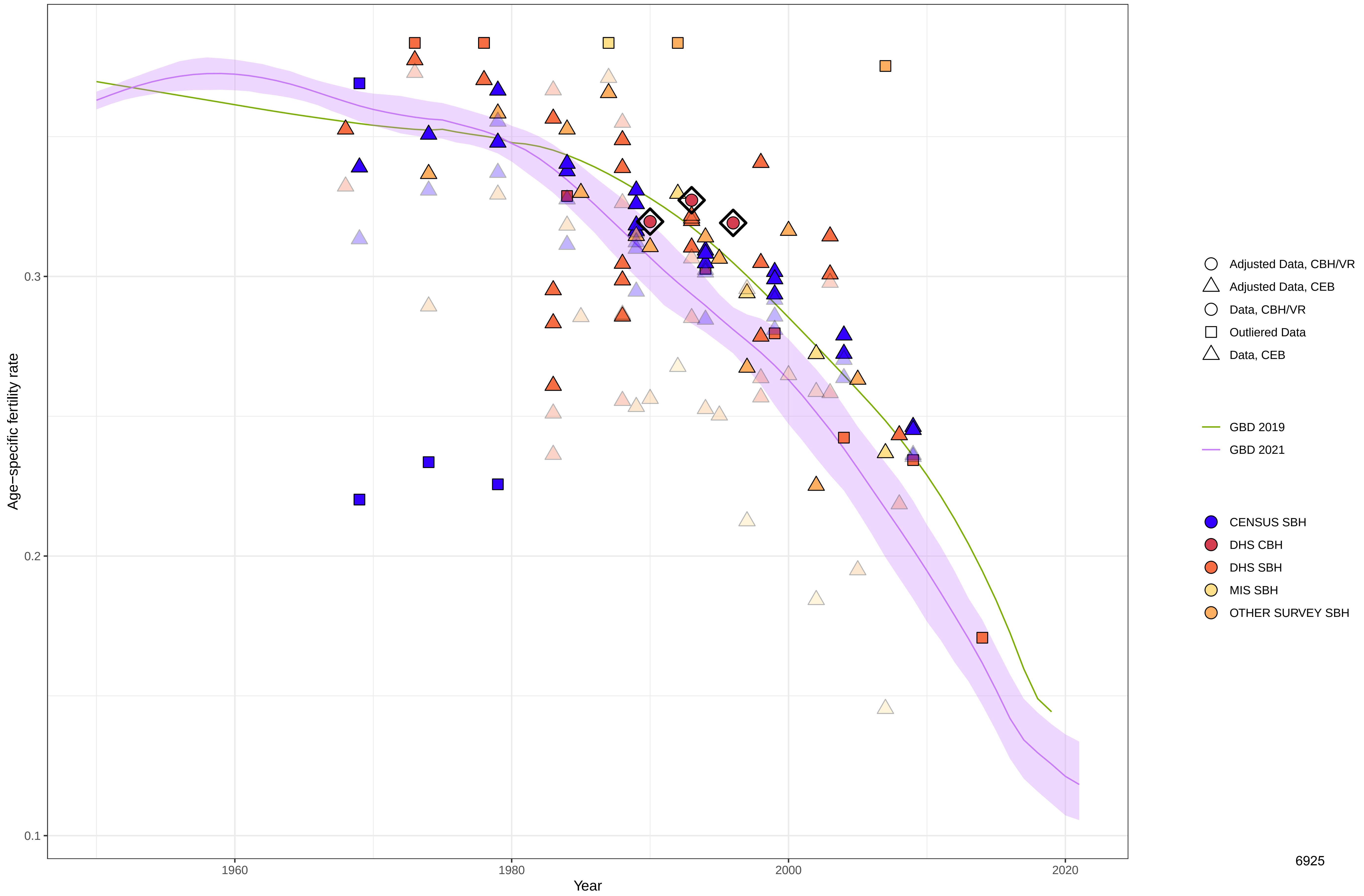

Age−specific fertility fitted trend lines, 1950−2021: Elgeyo Marakwet, Ages: 30−34
Data points enclosed with a diamond are reference sources

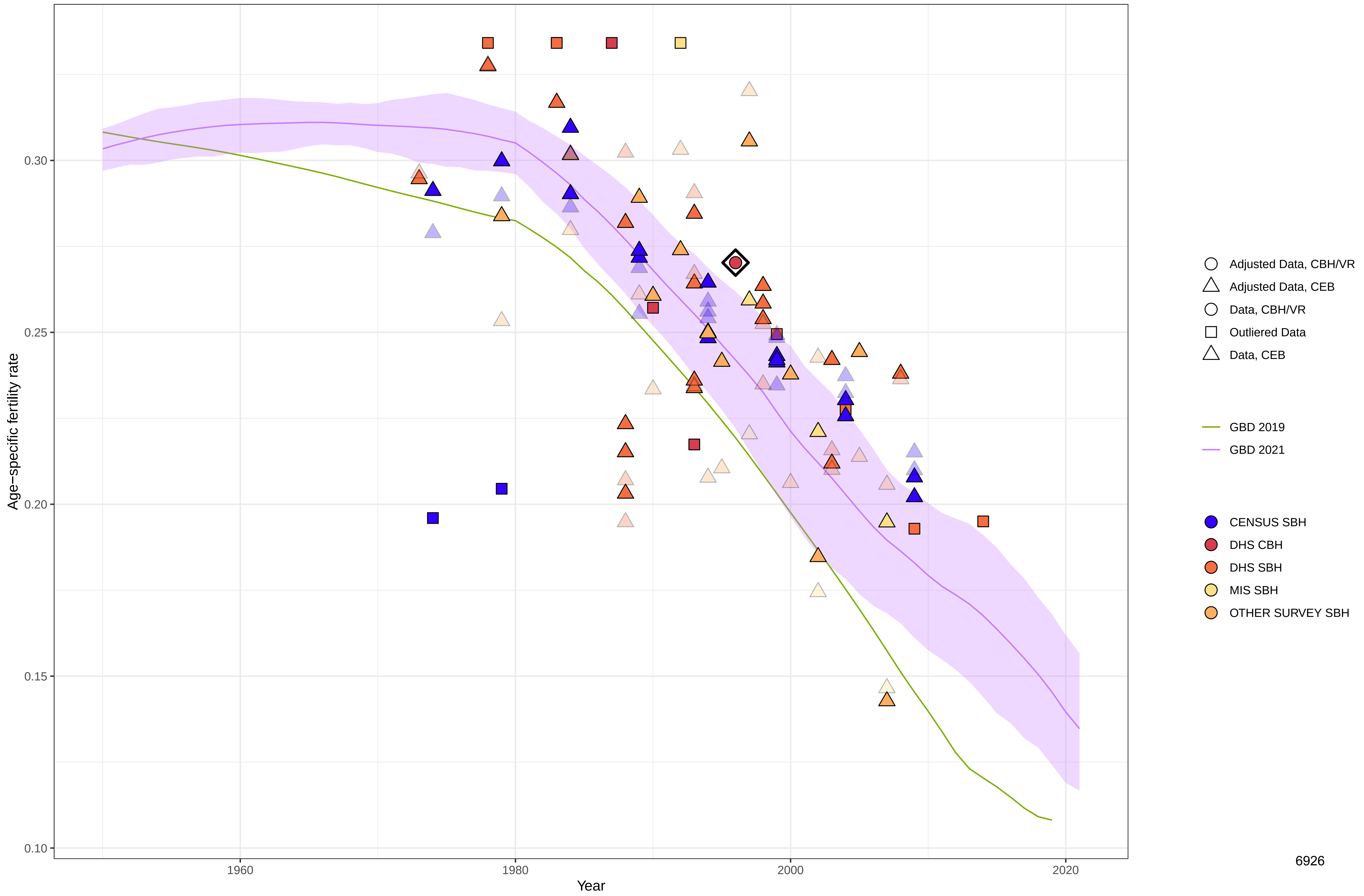

6926

Age−specific fertility fitted trend lines, 1950−2021: Elgeyo Marakwet, Ages: 35−39
Data points enclosed with a diamond are reference sources

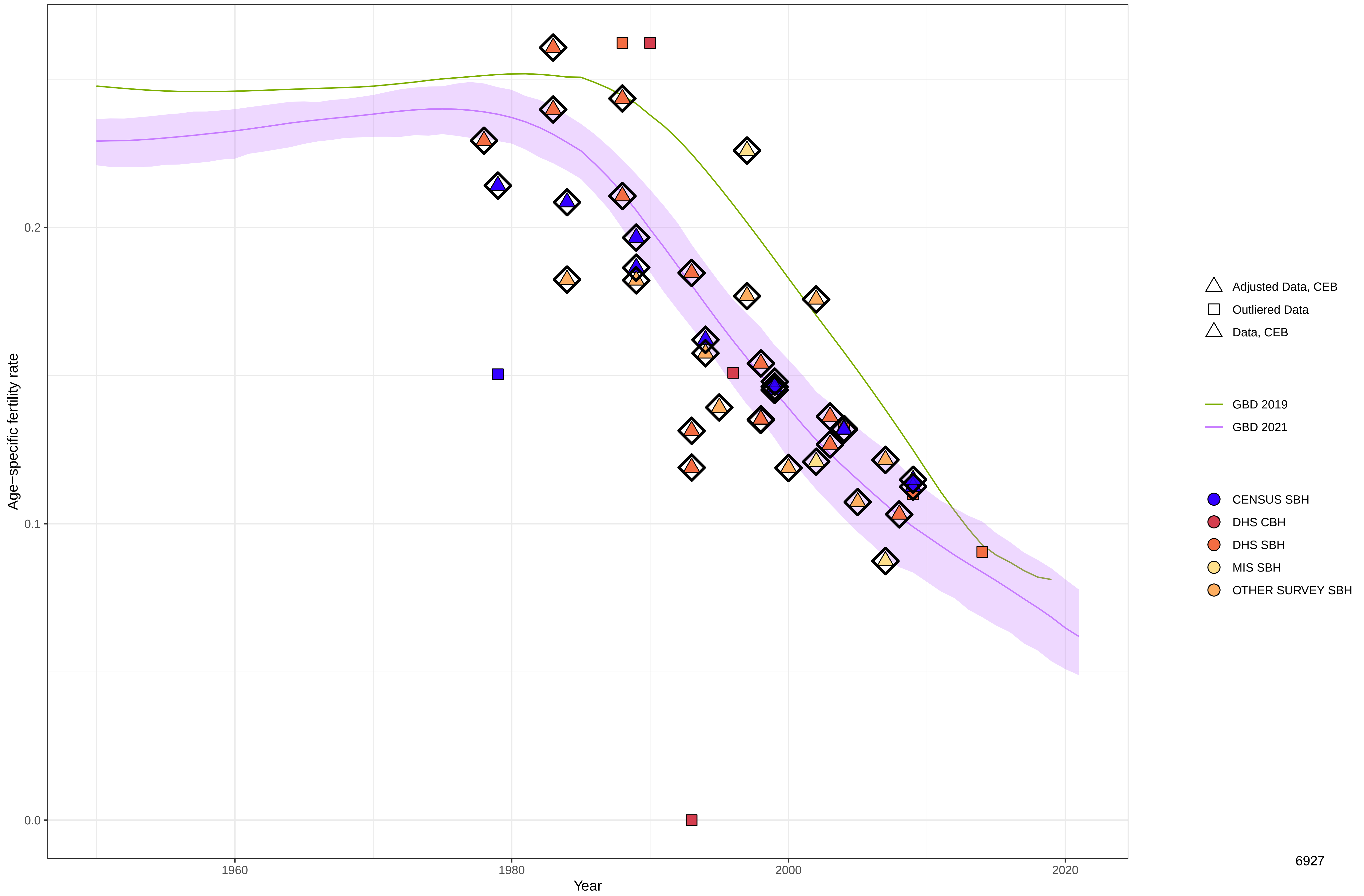

Age−specific fertility fitted trend lines, 1950−2021: Elgeyo Marakwet, Ages: 40−44
Data points enclosed with a diamond are reference sources

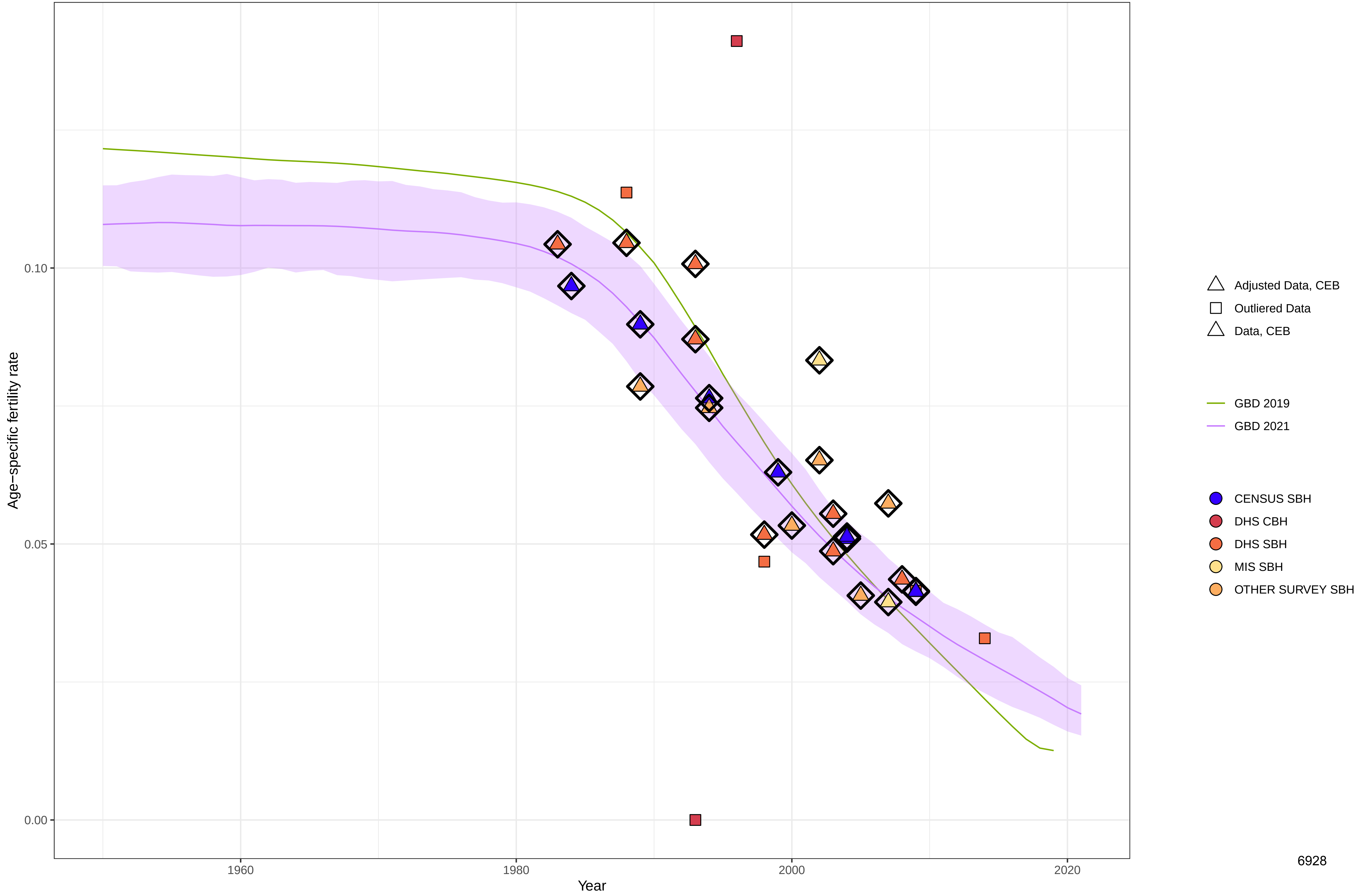

Age−specific fertility fitted trend lines, 1950−2021: Elgeyo Marakwet, Ages: 45−49
Data points enclosed with a diamond are reference sources

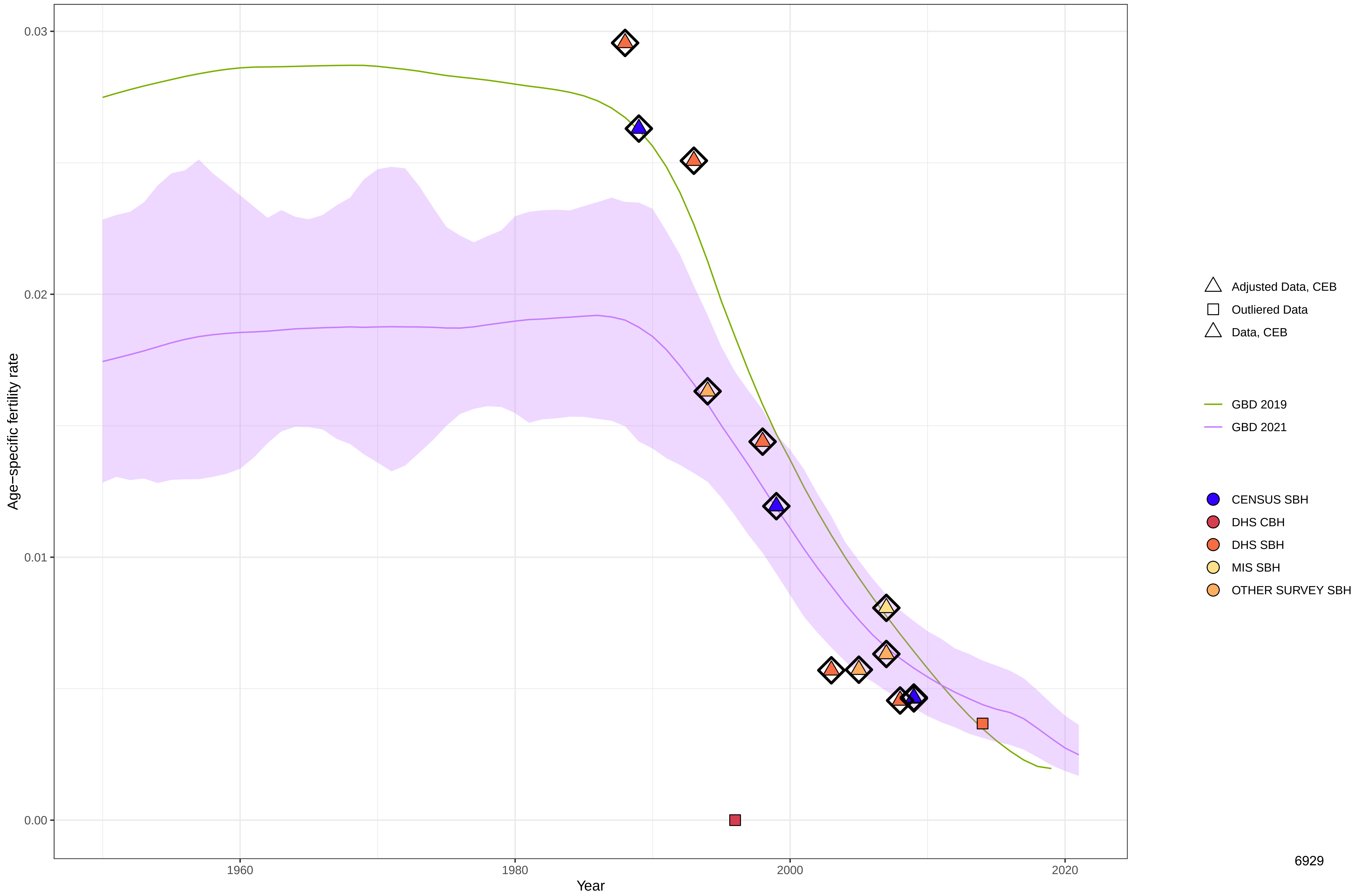

Age−specific fertility fitted trend lines, 1950−2021: Elgeyo Marakwet, Ages: 50−54
Data points enclosed with a diamond are reference sources

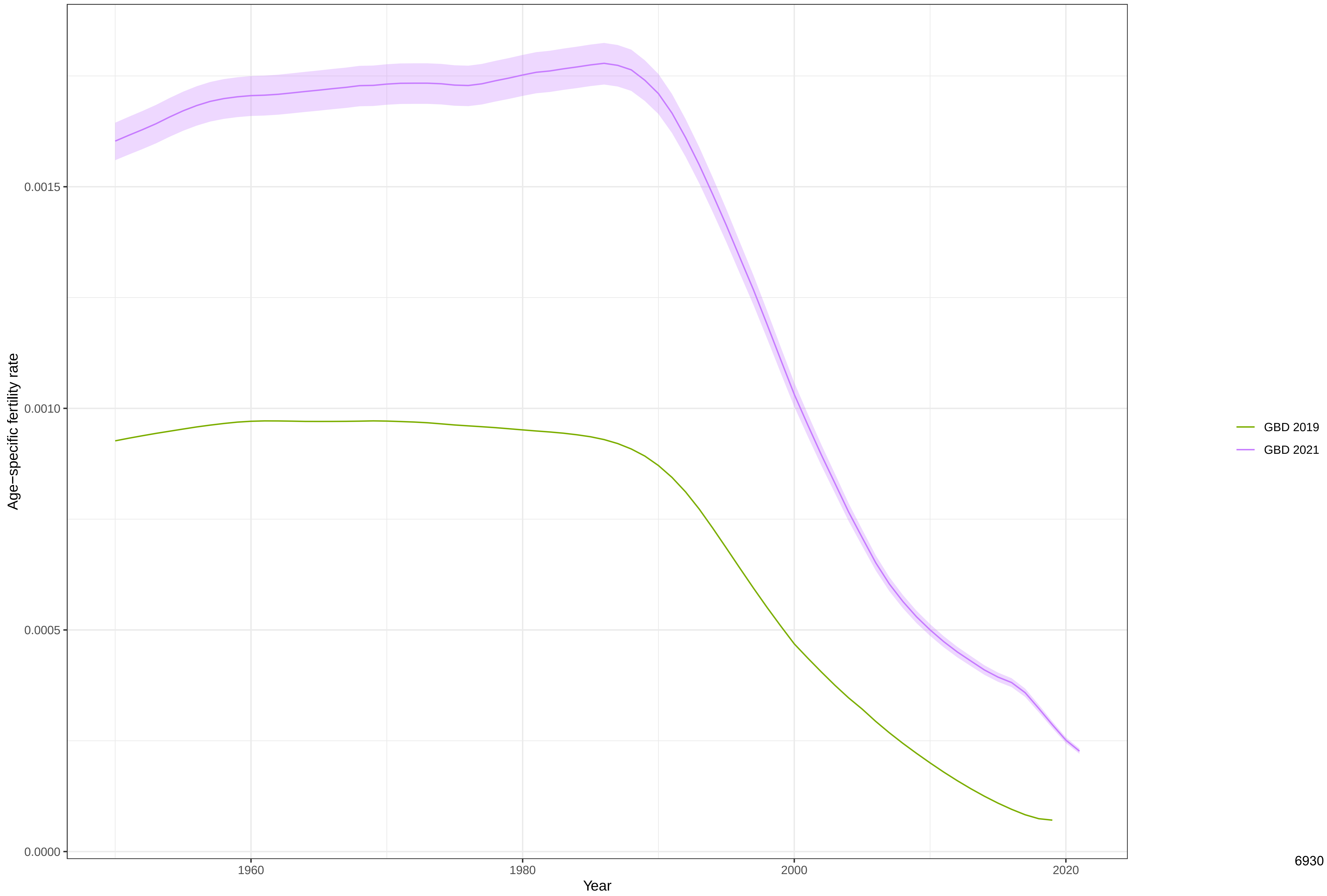

Total fertility rate fitted trend lines, 1950−2021: Elgeyo Marakwet TFR
Data points enclosed with a diamond are reference sources

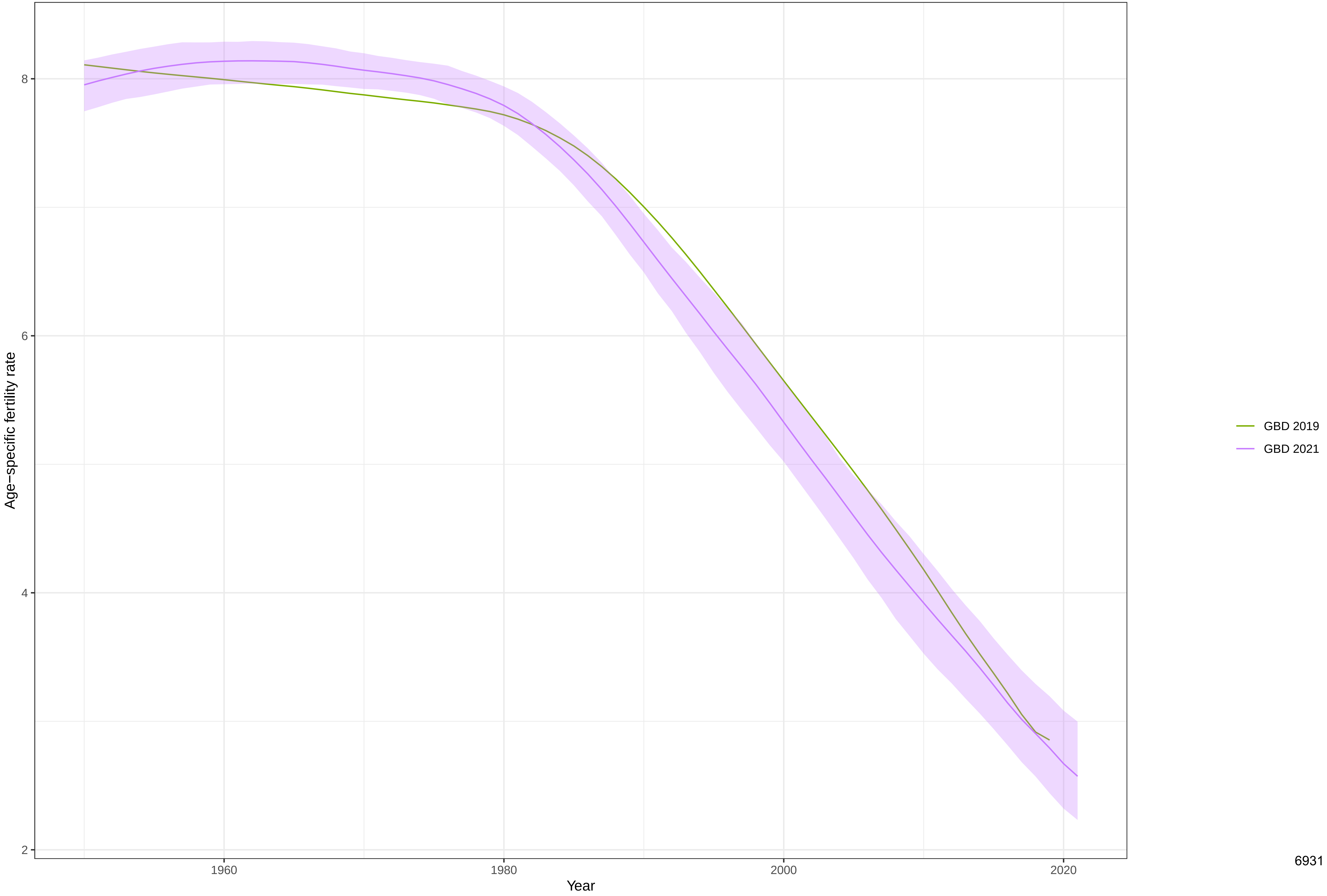

6931

Age−specific fertility fitted trend lines, 1950−2021: Embu, Ages: 10−14
Data points enclosed with a diamond are reference sources

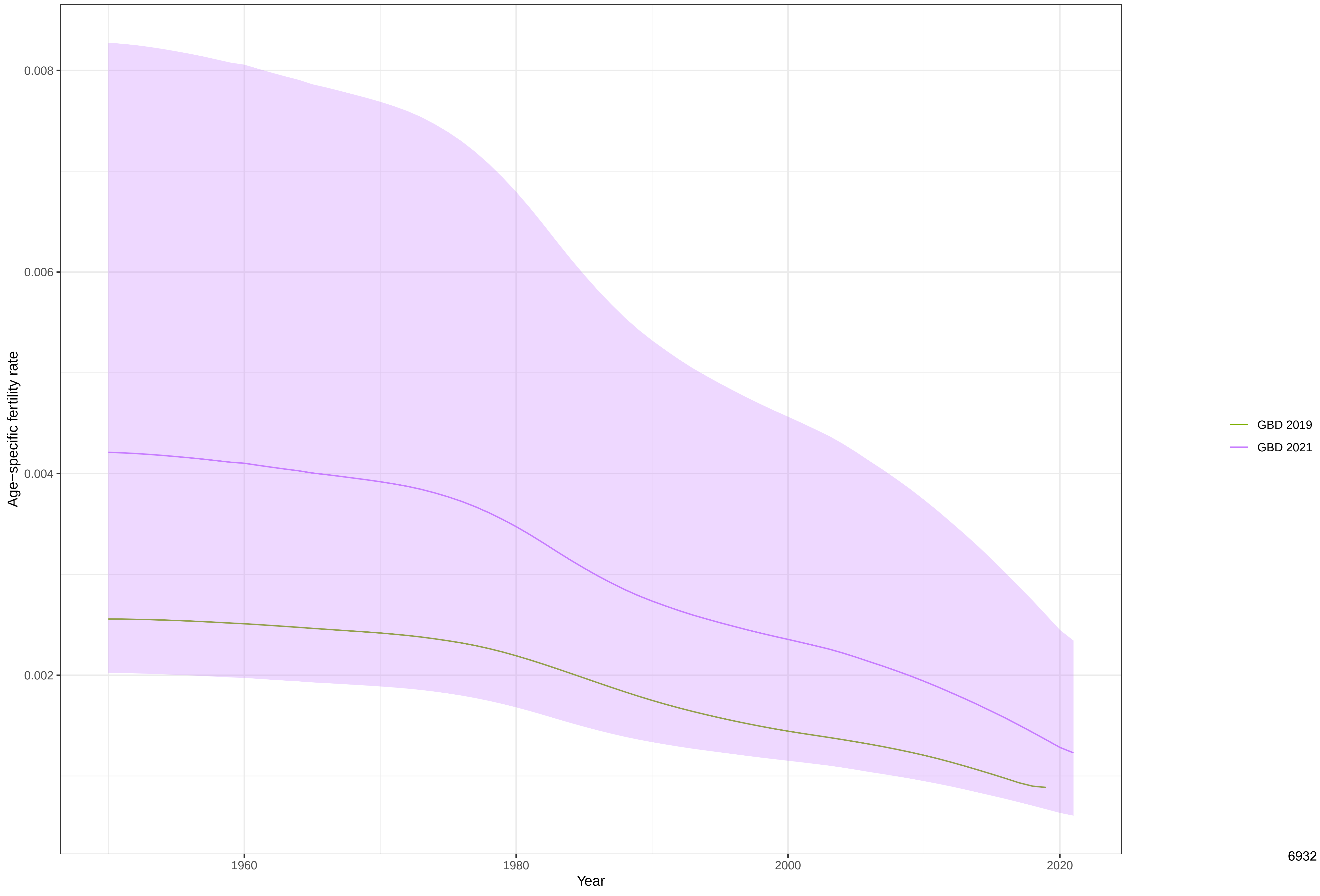

Age−specific fertility fitted trend lines, 1950−2021: Embu, Ages: 15−19
Data points enclosed with a diamond are reference sources

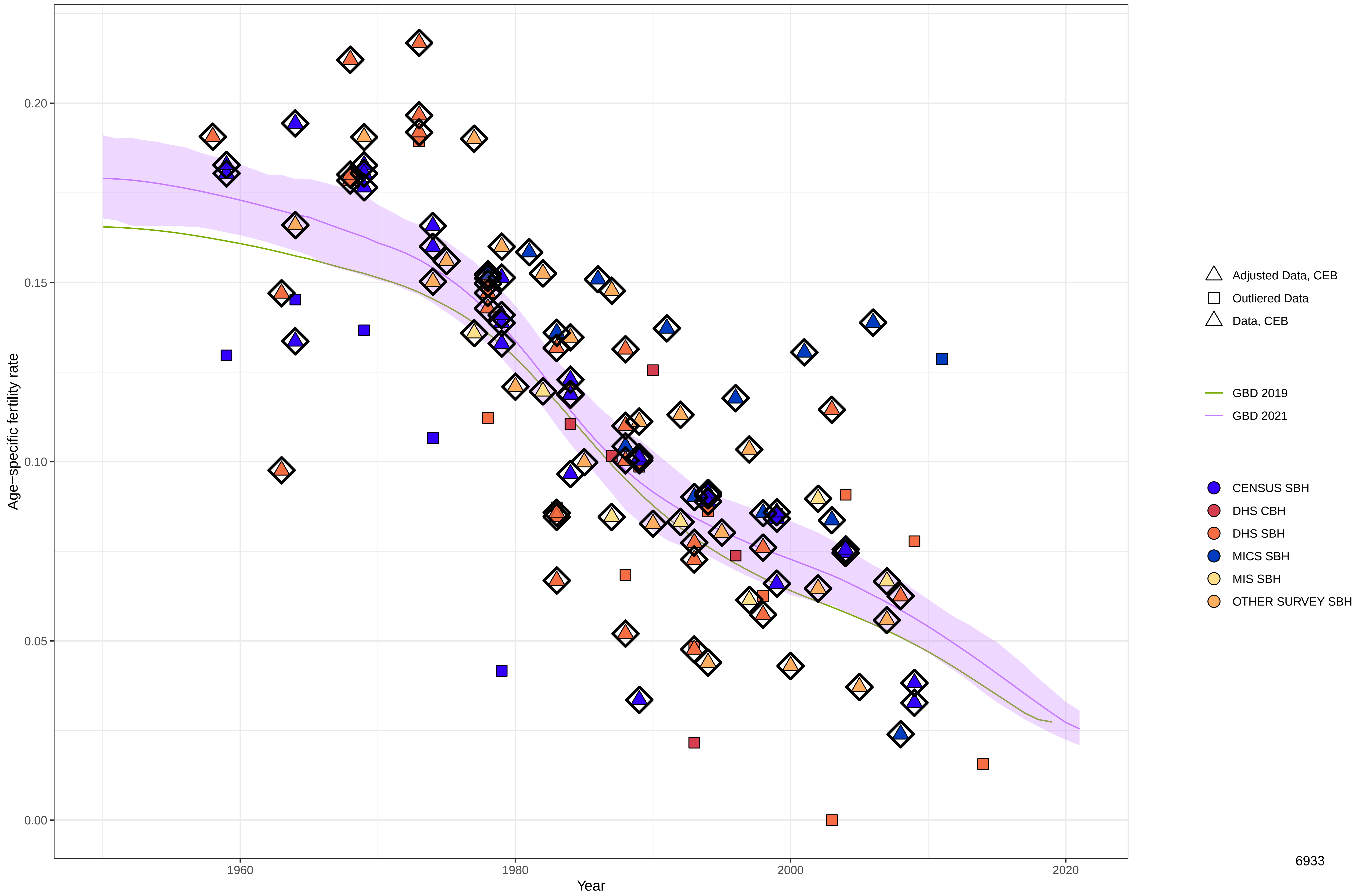

Age−specific fertility fitted trend lines, 1950−2021: Embu, Ages: 20−24
Data points enclosed with a diamond are reference sources

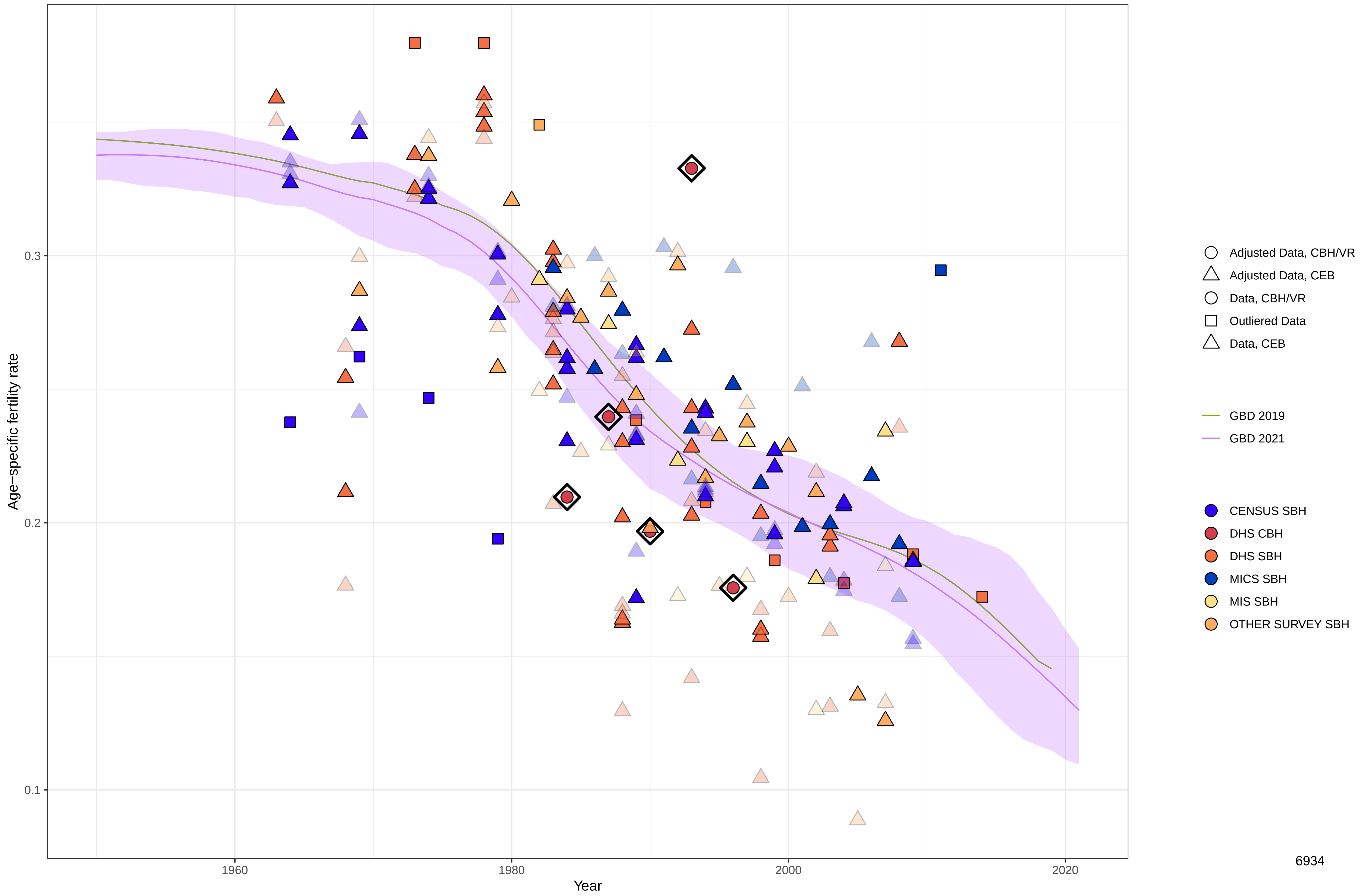

Age−specific fertility fitted trend lines, 1950−2021: Embu, Ages: 25−29
Data points enclosed with a diamond are reference sources

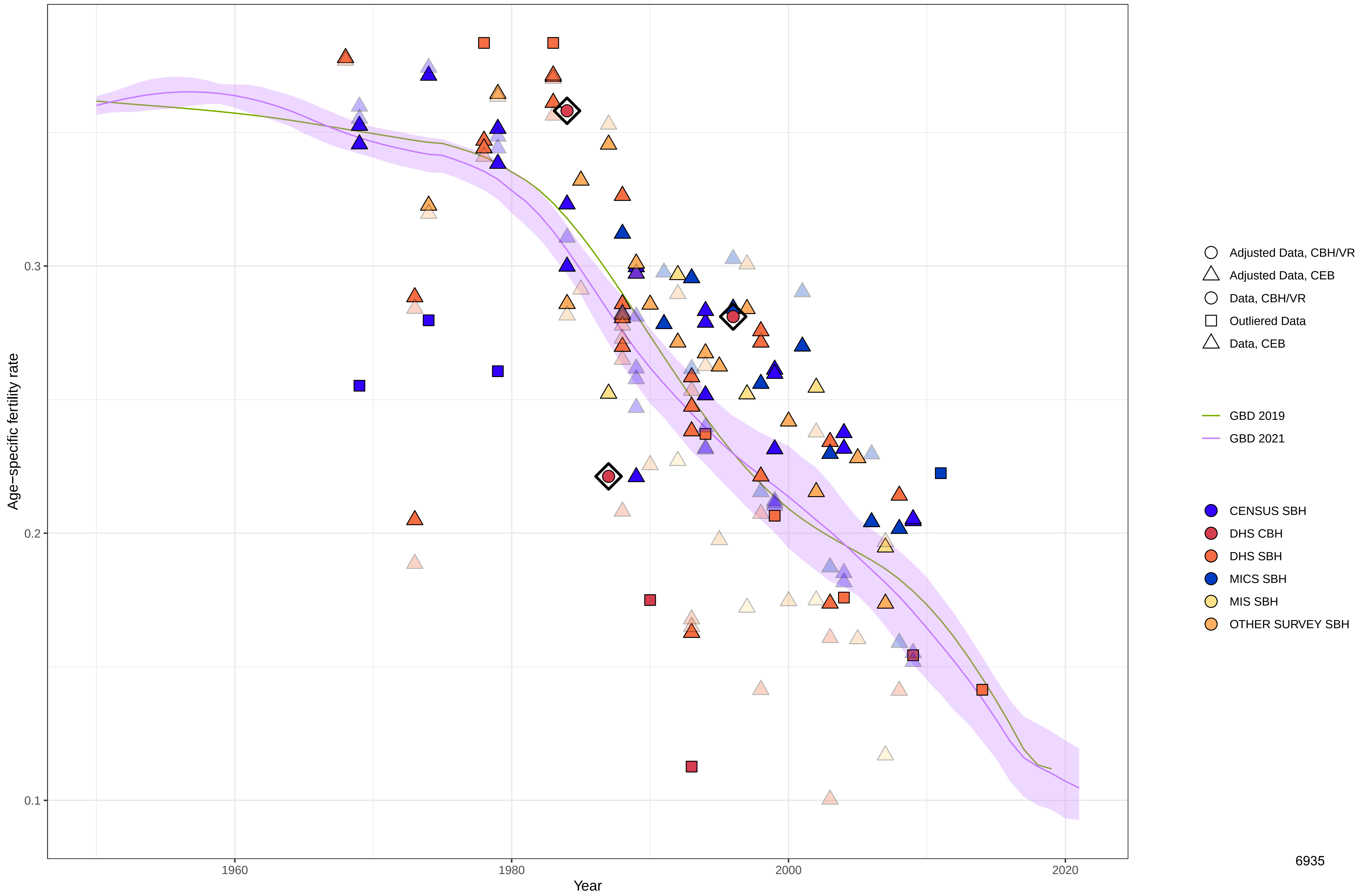

Age−specific fertility fitted trend lines, 1950−2021: Embu, Ages: 30−34
Data points enclosed with a diamond are reference sources

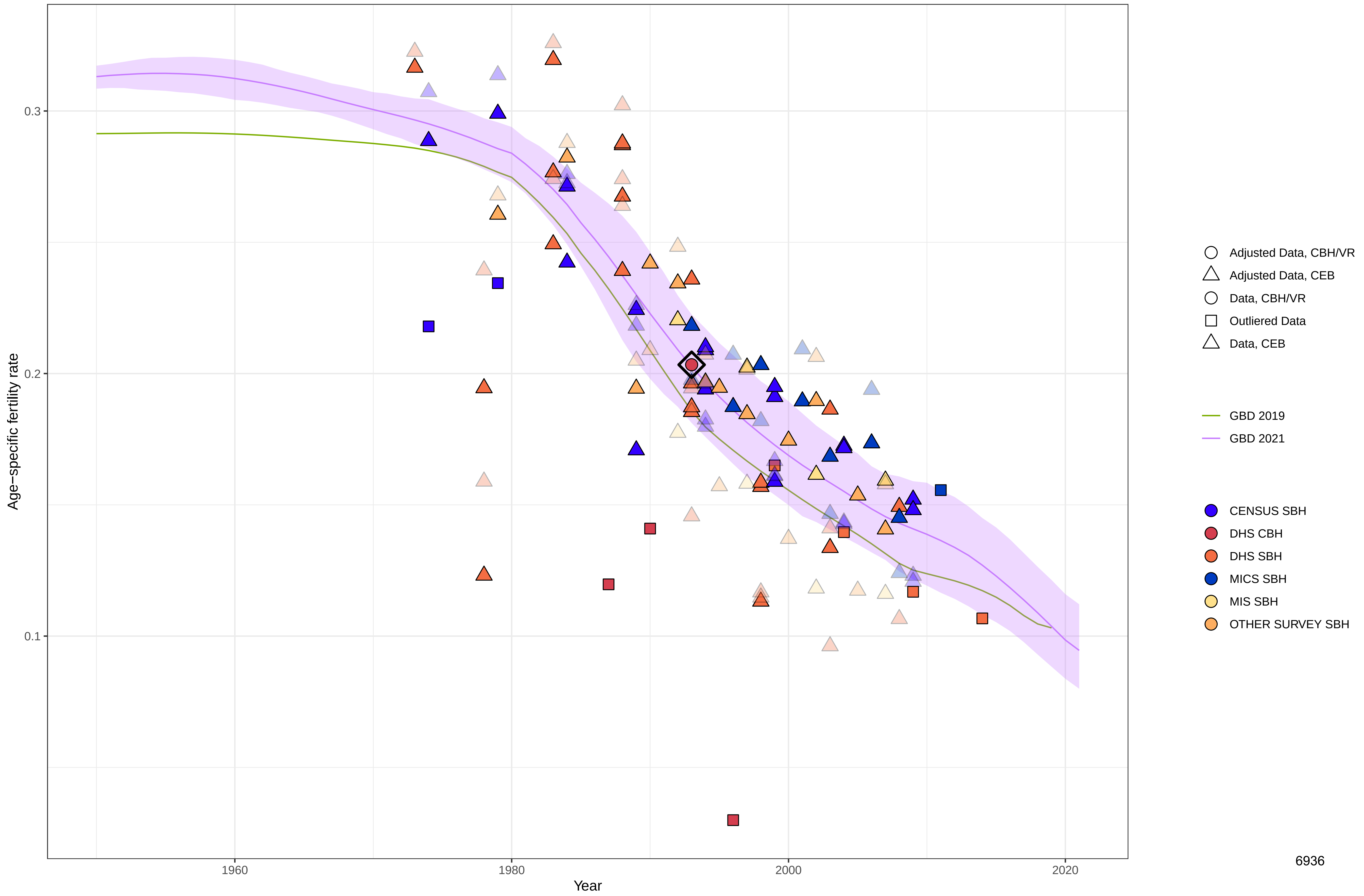

Age−specific fertility fitted trend lines, 1950−2021: Embu, Ages: 35−39
Data points enclosed with a diamond are reference sources

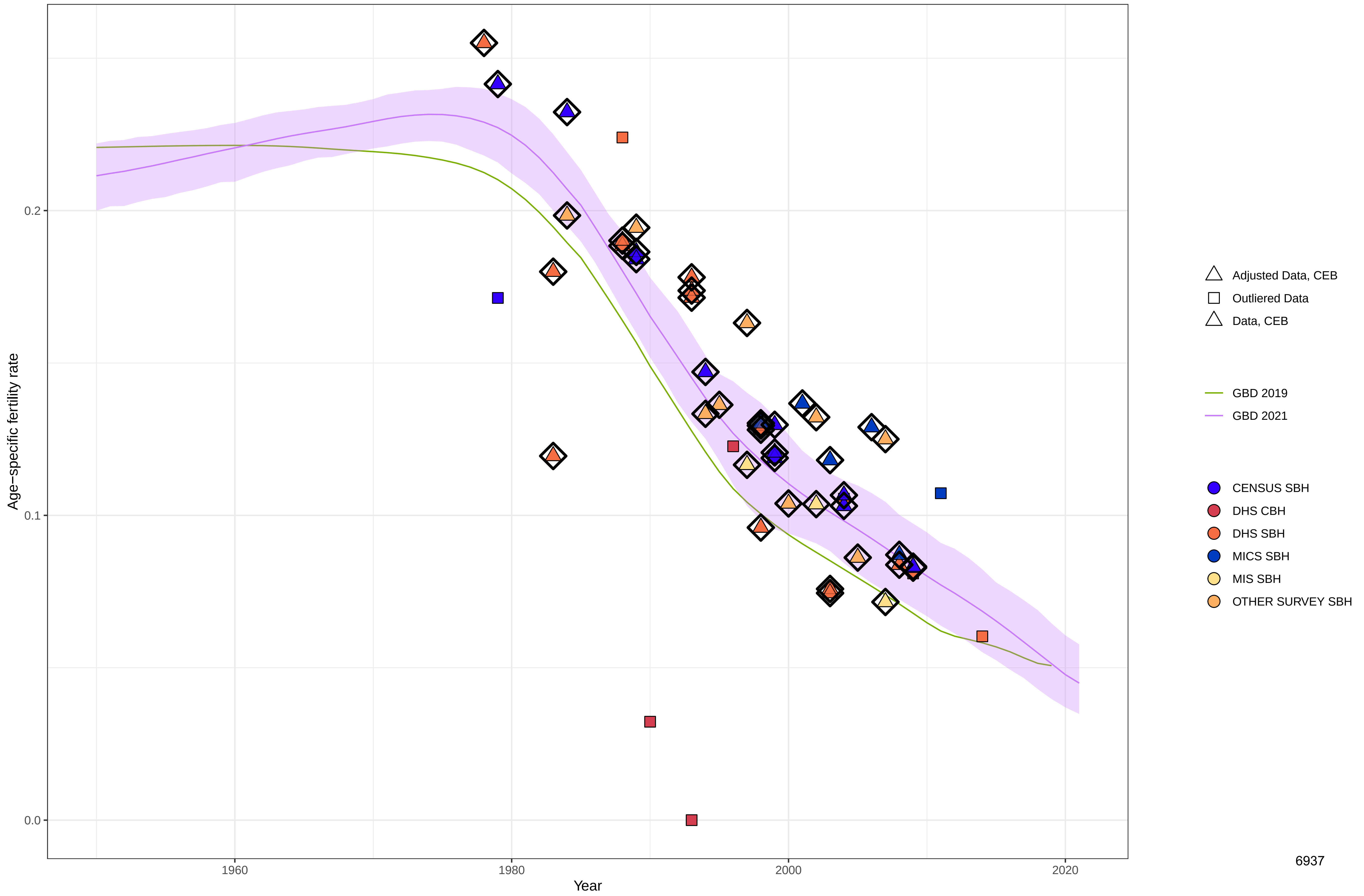

Age−specific fertility fitted trend lines, 1950−2021: Embu, Ages: 40−44
Data points enclosed with a diamond are reference sources

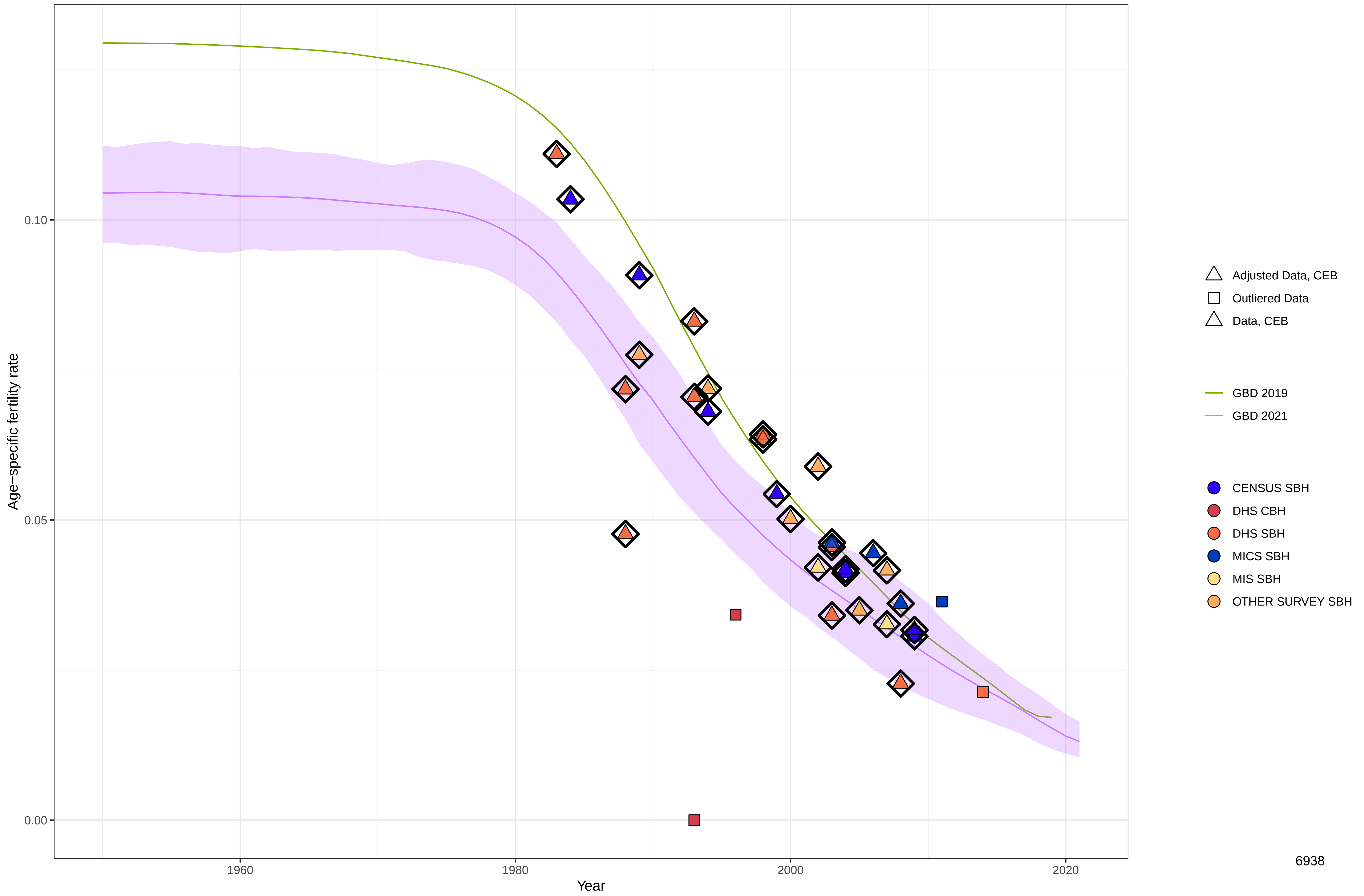

Age−specific fertility fitted trend lines, 1950−2021: Embu, Ages: 45−49
Data points enclosed with a diamond are reference sources

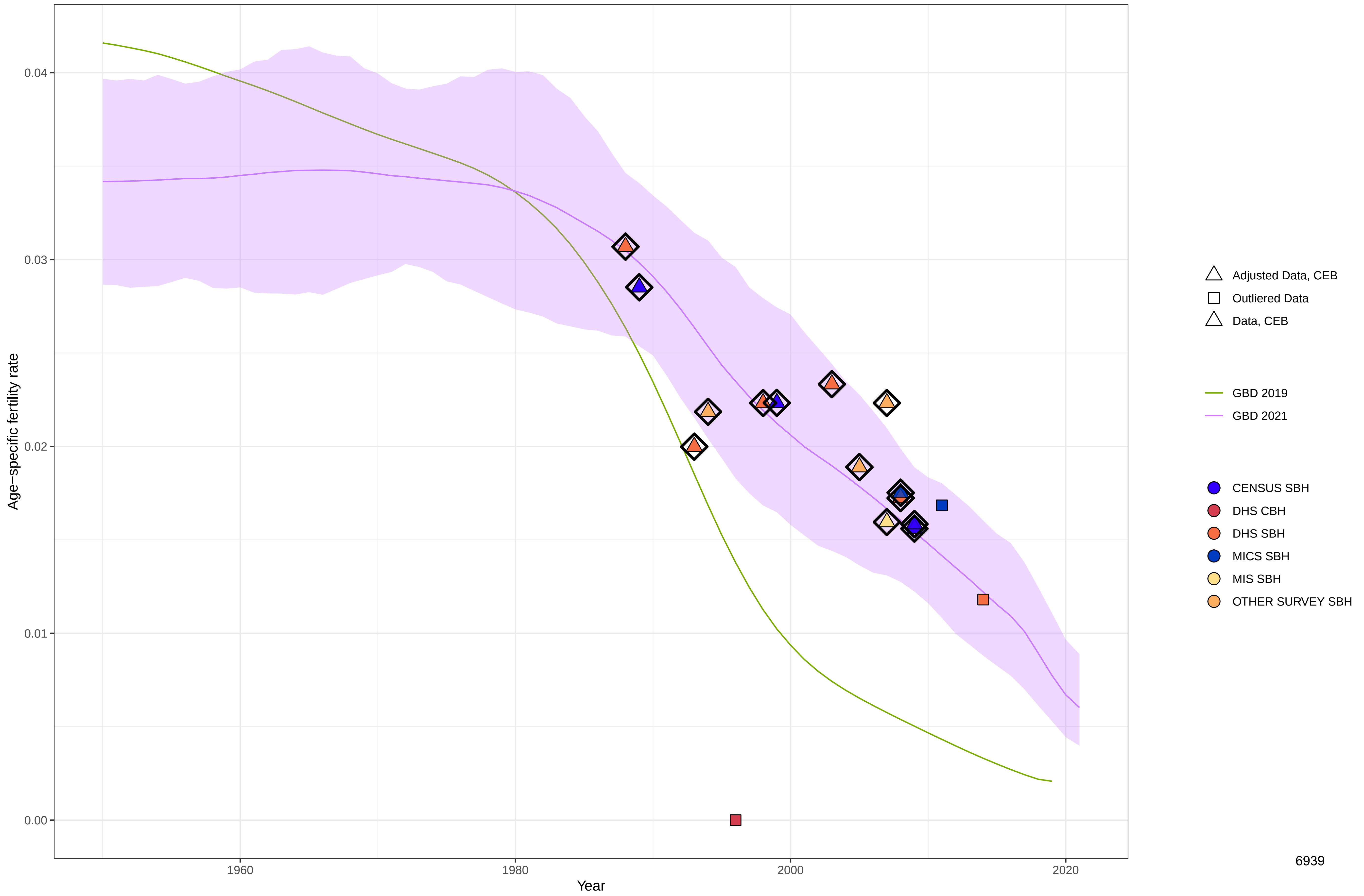

Age−specific fertility fitted trend lines, 1950−2021: Embu, Ages: 50−54
Data points enclosed with a diamond are reference sources

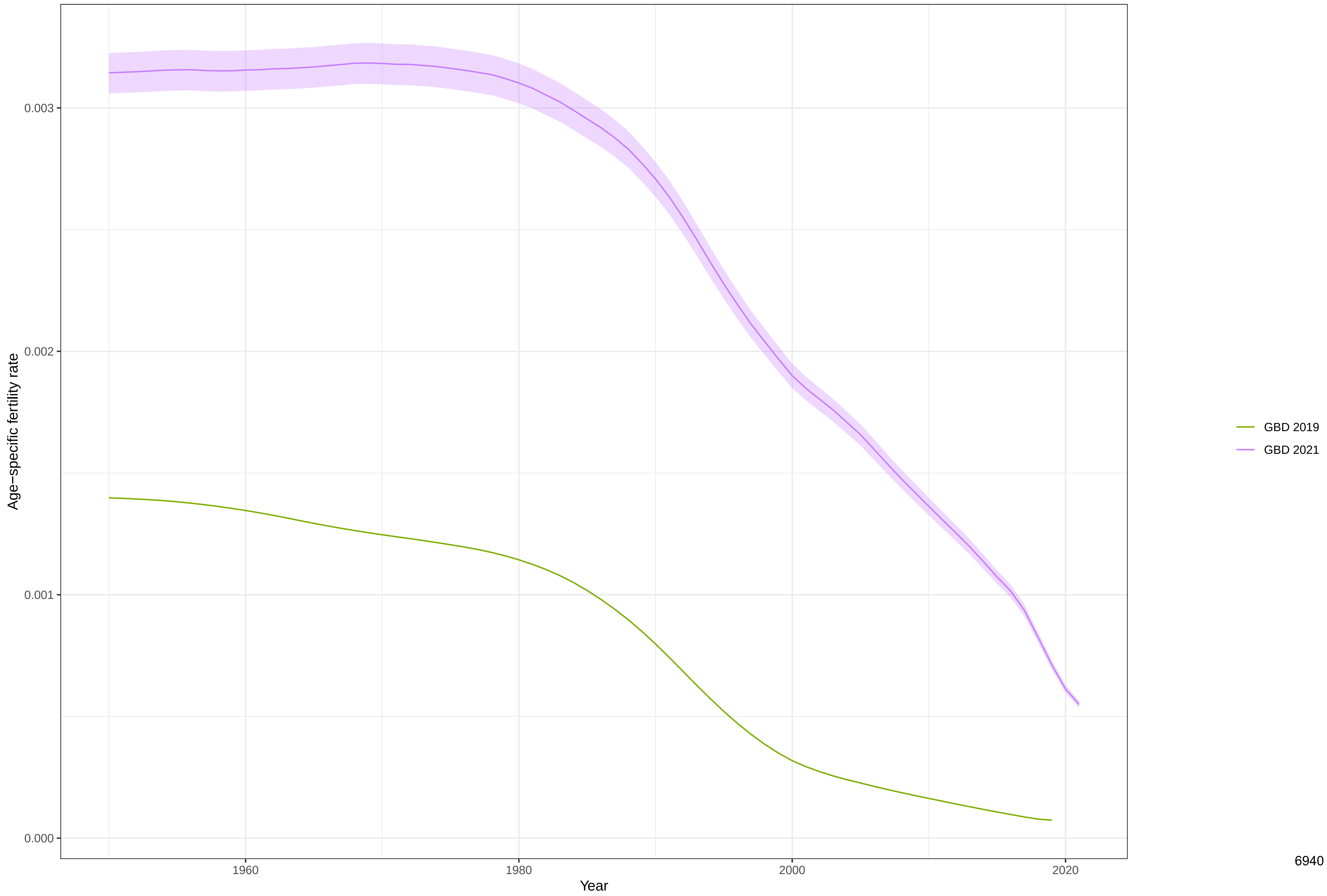

Total fertility rate fitted trend lines, 1950−2021: Embu TFR
Data points enclosed with a diamond are reference sources

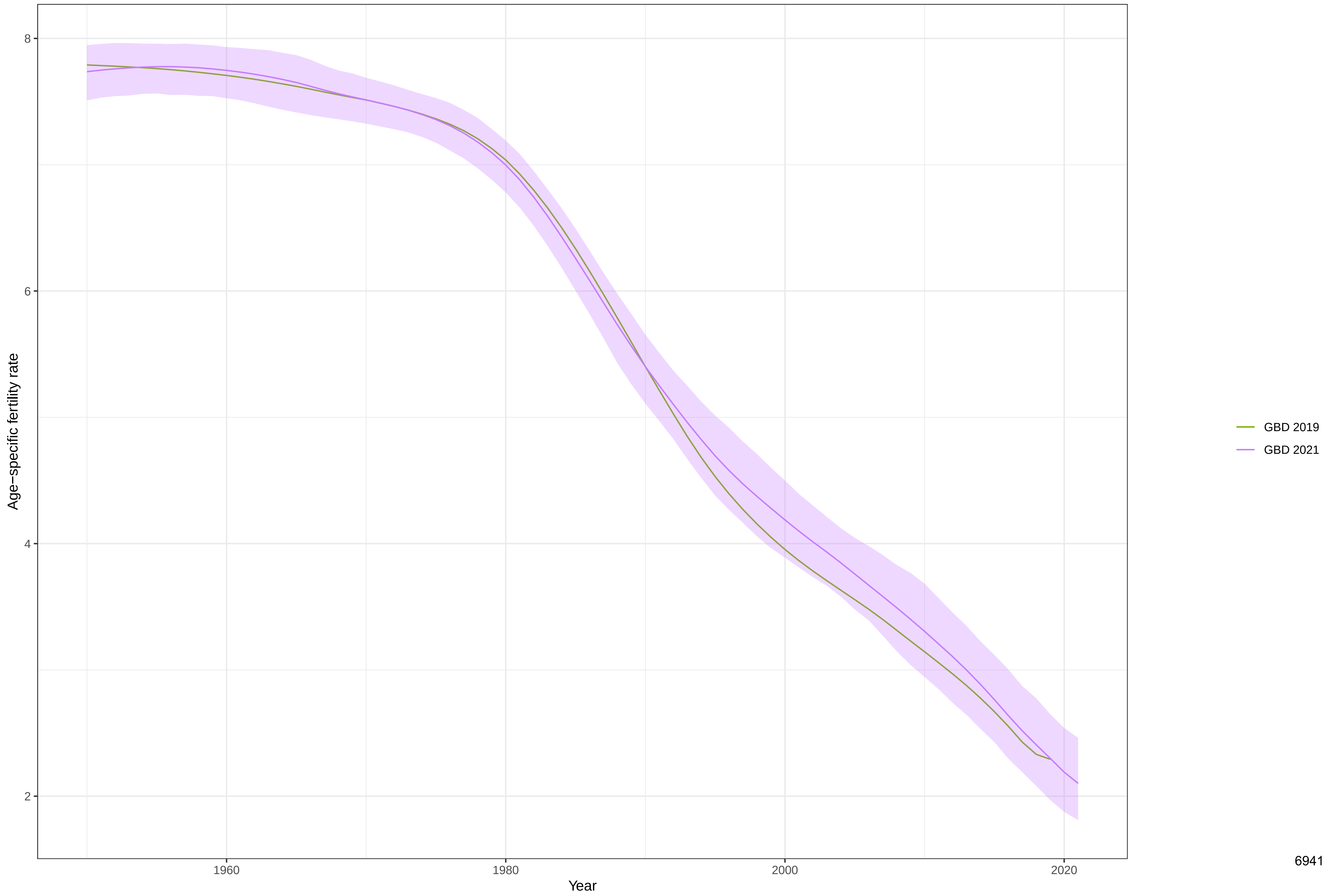

Age−specific fertility fitted trend lines, 1950−2021: Garissa, Ages: 10−14
Data points enclosed with a diamond are reference sources

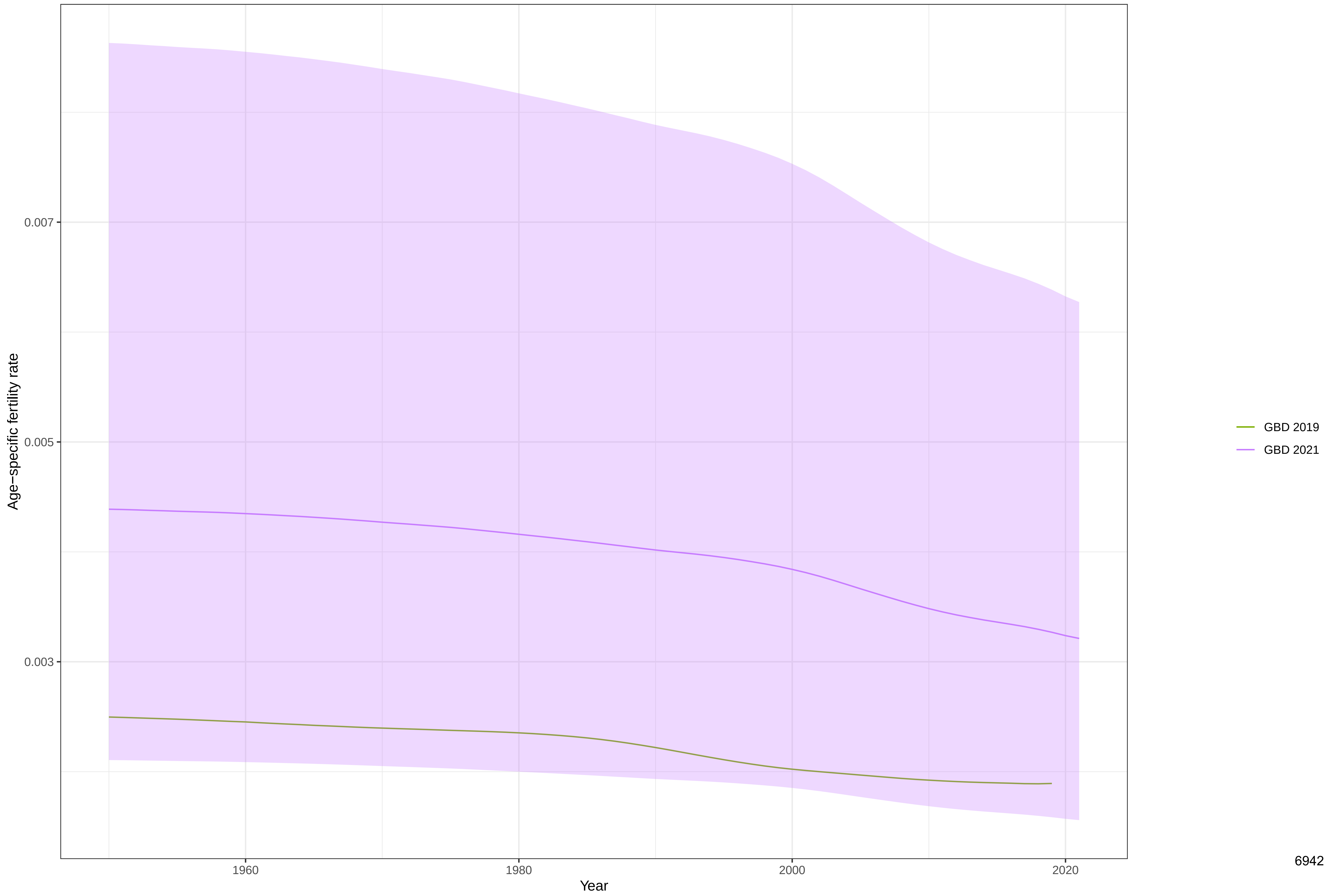

Age−specific fertility fitted trend lines, 1950−2021: Garissa, Ages: 15−19
Data points enclosed with a diamond are reference sources

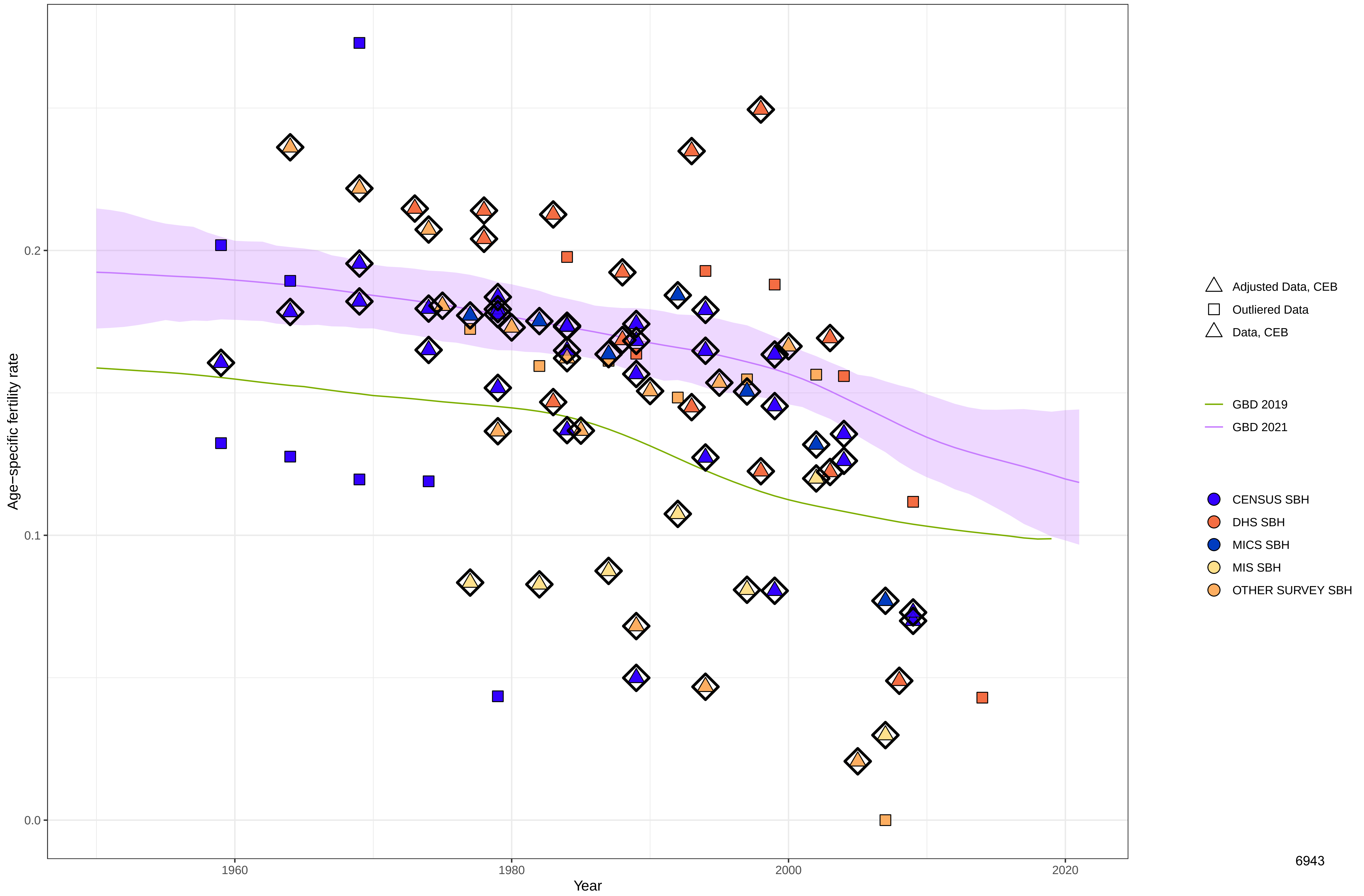

Age−specific fertility fitted trend lines, 1950−2021: Garissa, Ages: 20−24
Data points enclosed with a diamond are reference sources

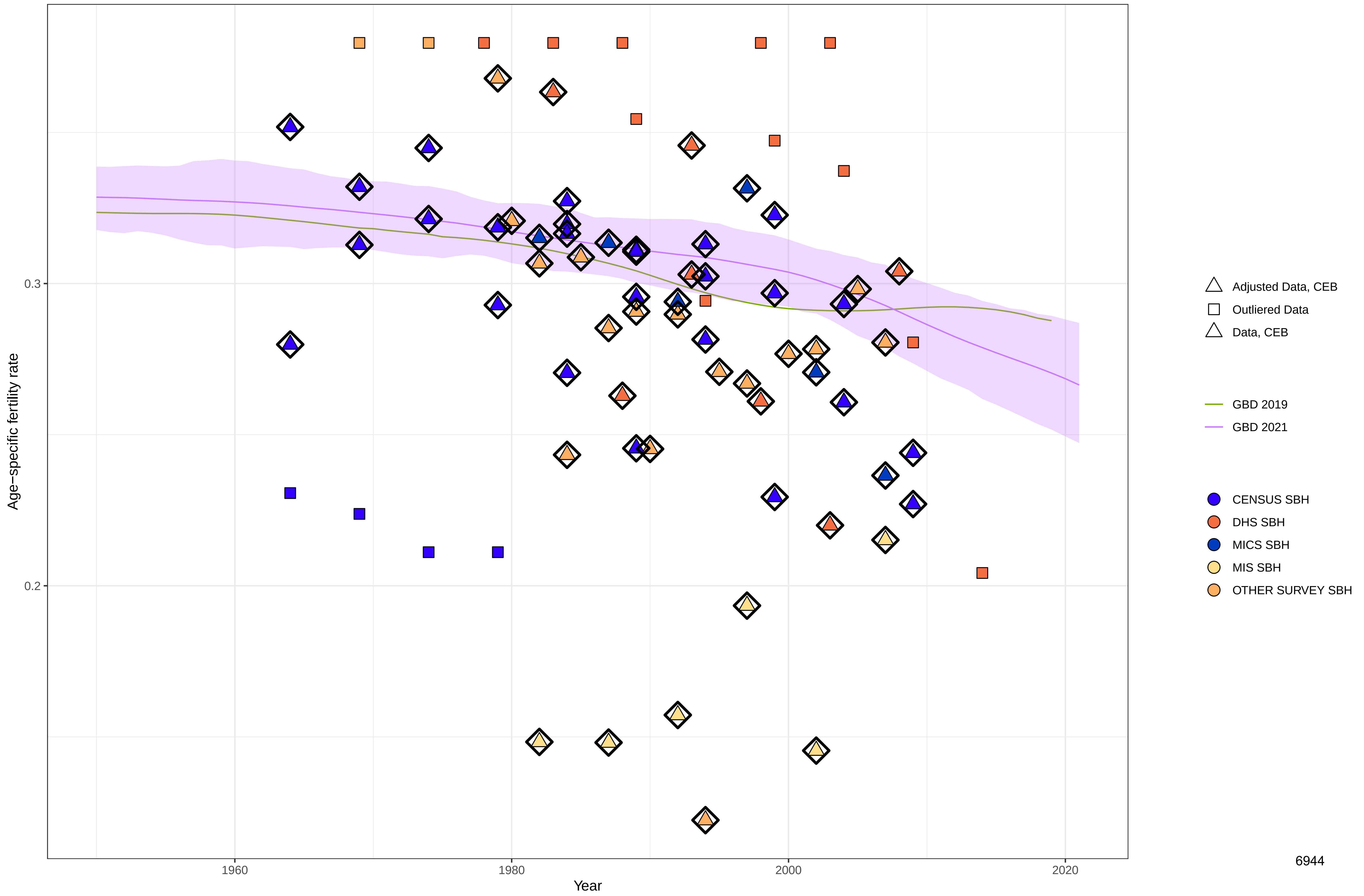

6944

Age−specific fertility fitted trend lines, 1950−2021: Garissa, Ages: 25−29
Data points enclosed with a diamond are reference sources

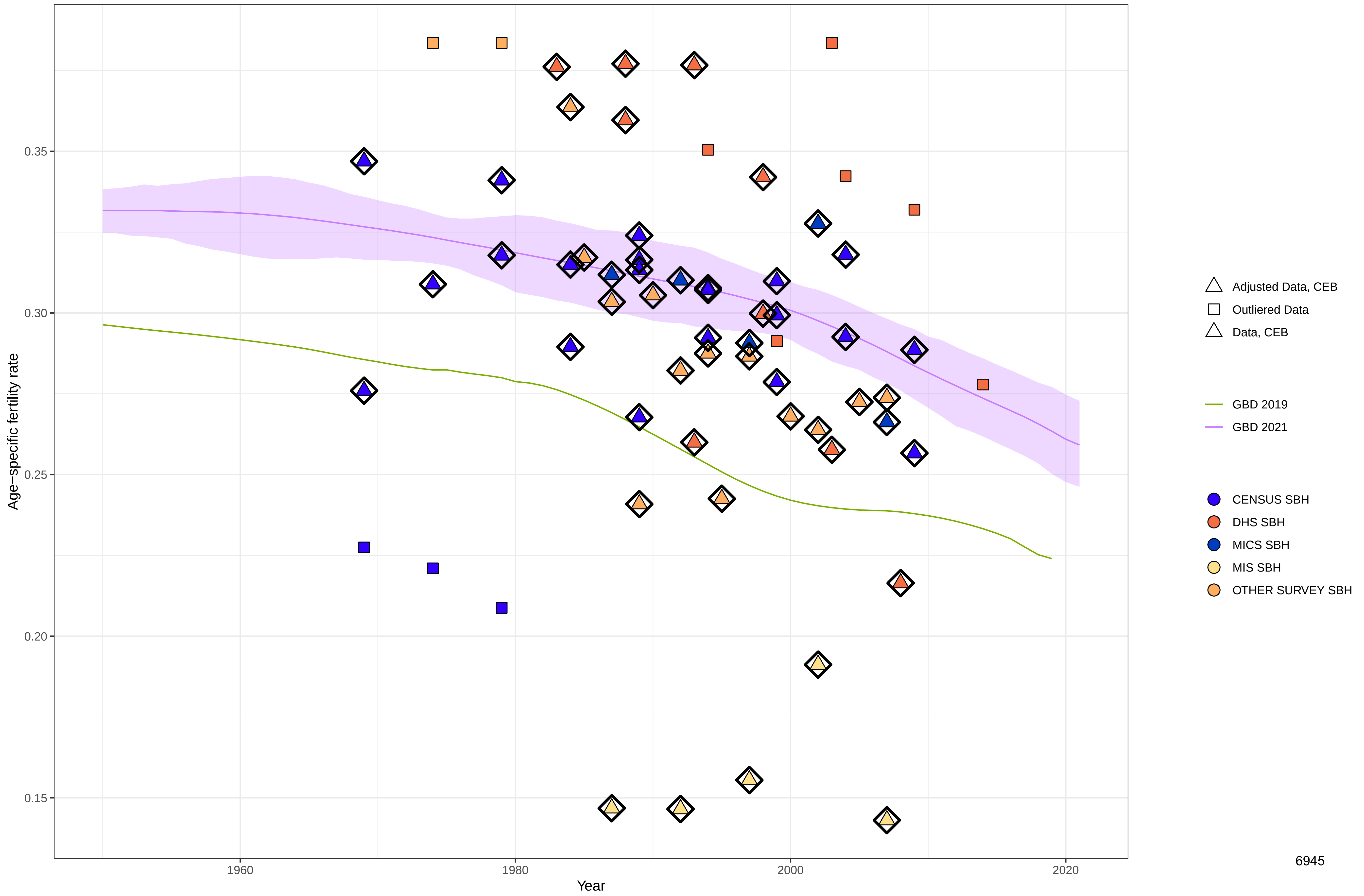

Age−specific fertility fitted trend lines, 1950−2021: Garissa, Ages: 30−34
Data points enclosed with a diamond are reference sources

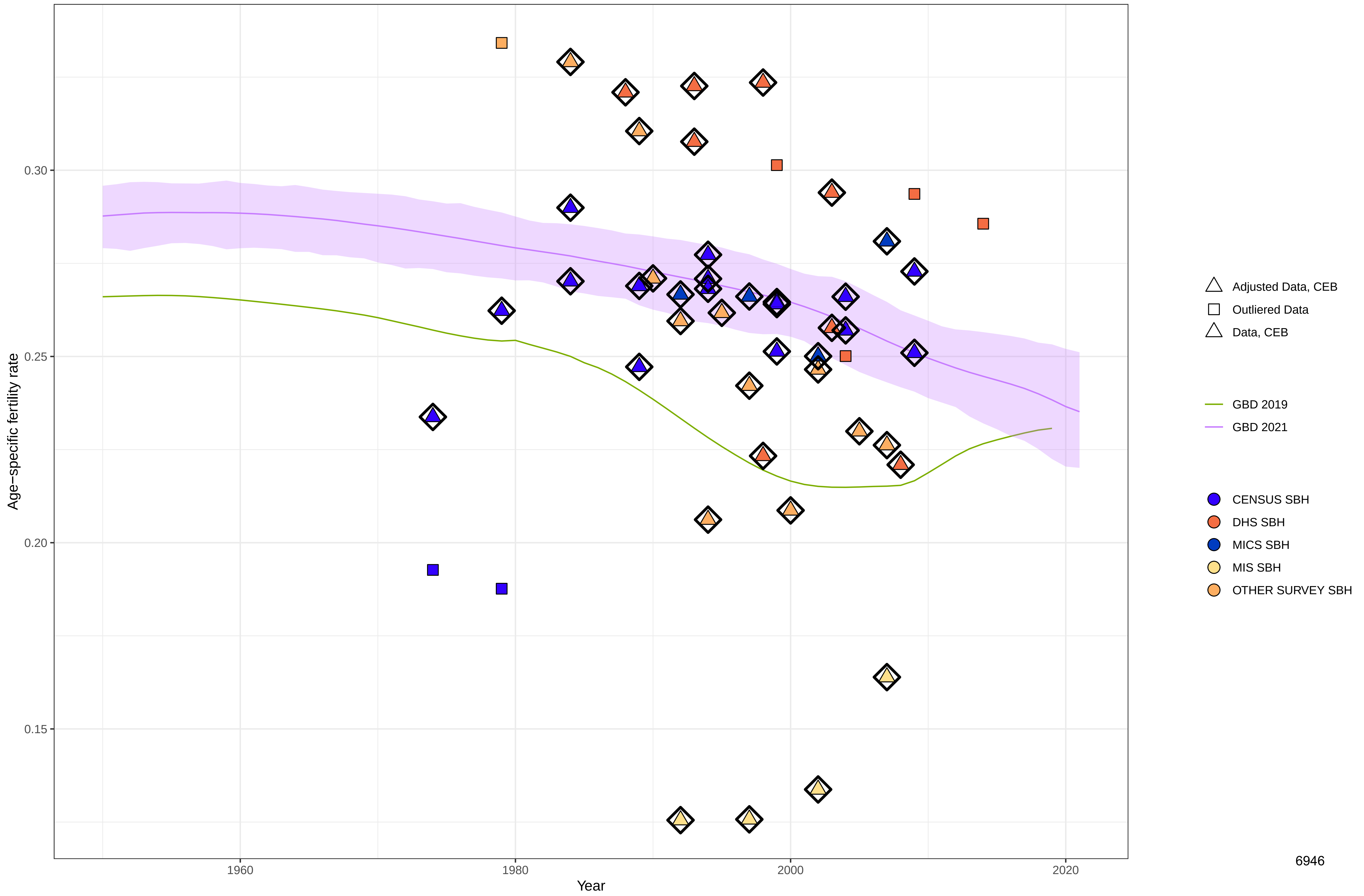

Age−specific fertility fitted trend lines, 1950−2021: Garissa, Ages: 35−39
Data points enclosed with a diamond are reference sources

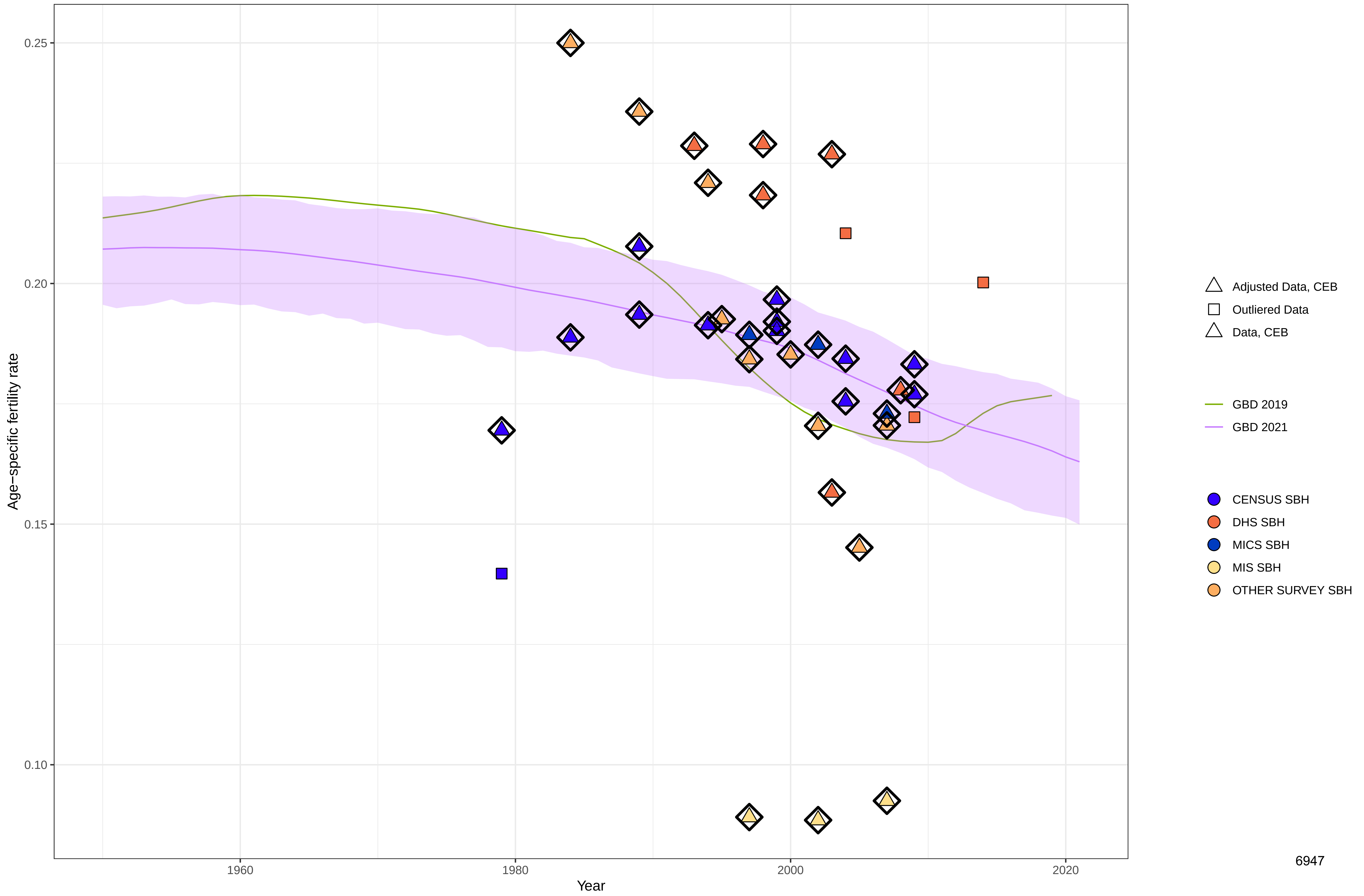

Age−specific fertility fitted trend lines, 1950−2021: Garissa, Ages: 40−44
Data points enclosed with a diamond are reference sources

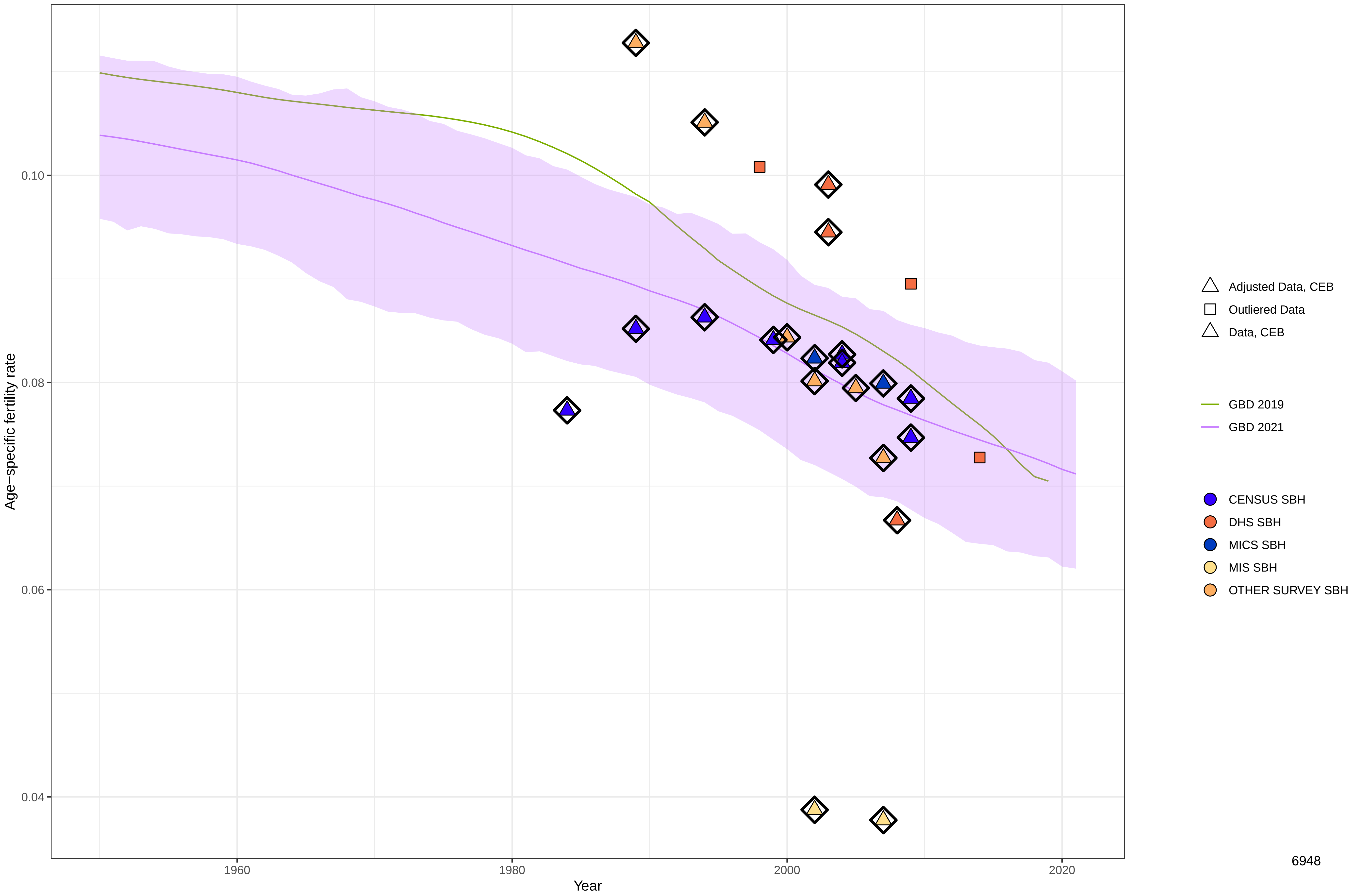

Age−specific fertility fitted trend lines, 1950−2021: Garissa, Ages: 45−49
Data points enclosed with a diamond are reference sources

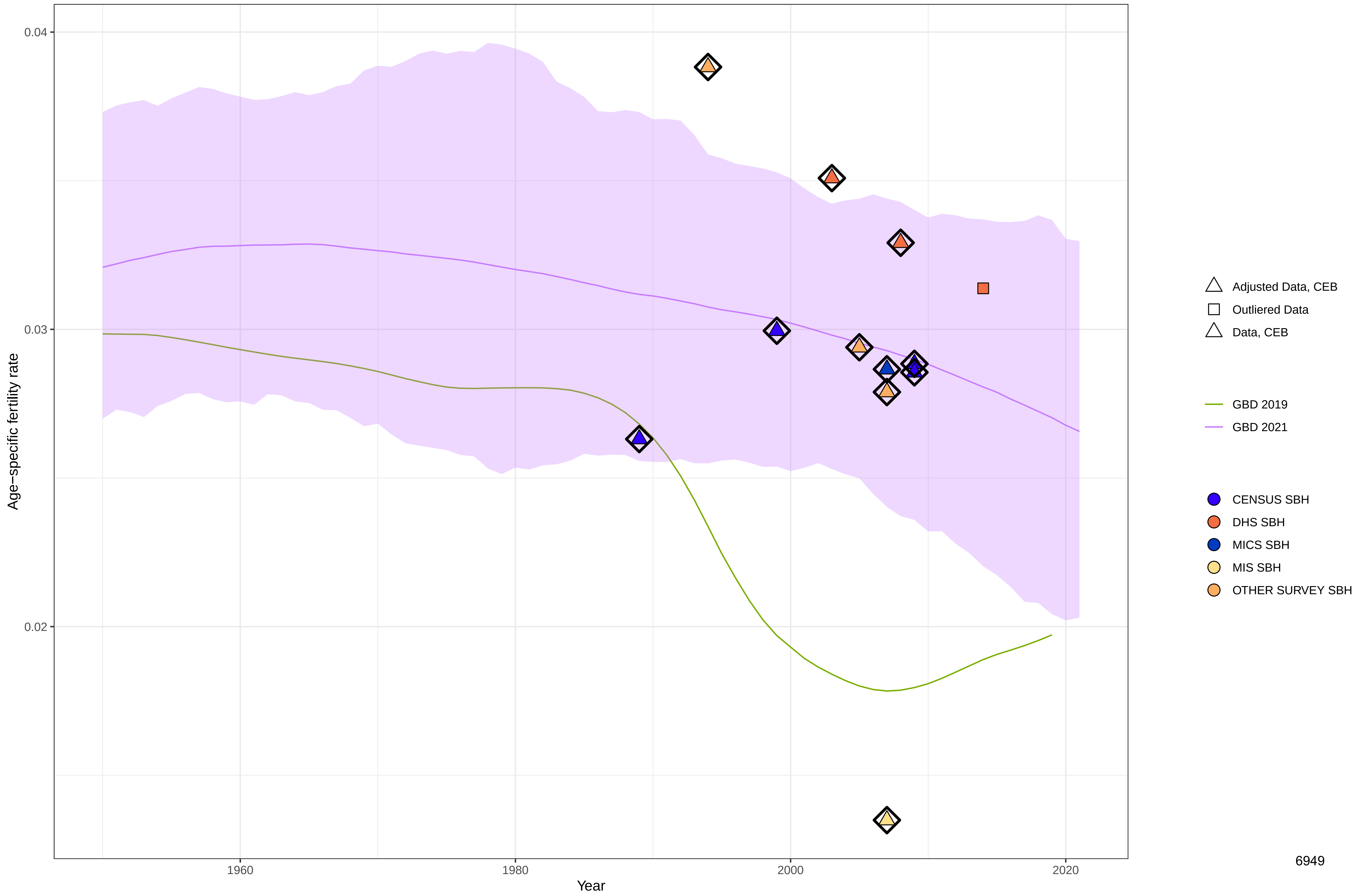

6949

Age−specific fertility fitted trend lines, 1950−2021: Garissa, Ages: 50−54
Data points enclosed with a diamond are reference sources

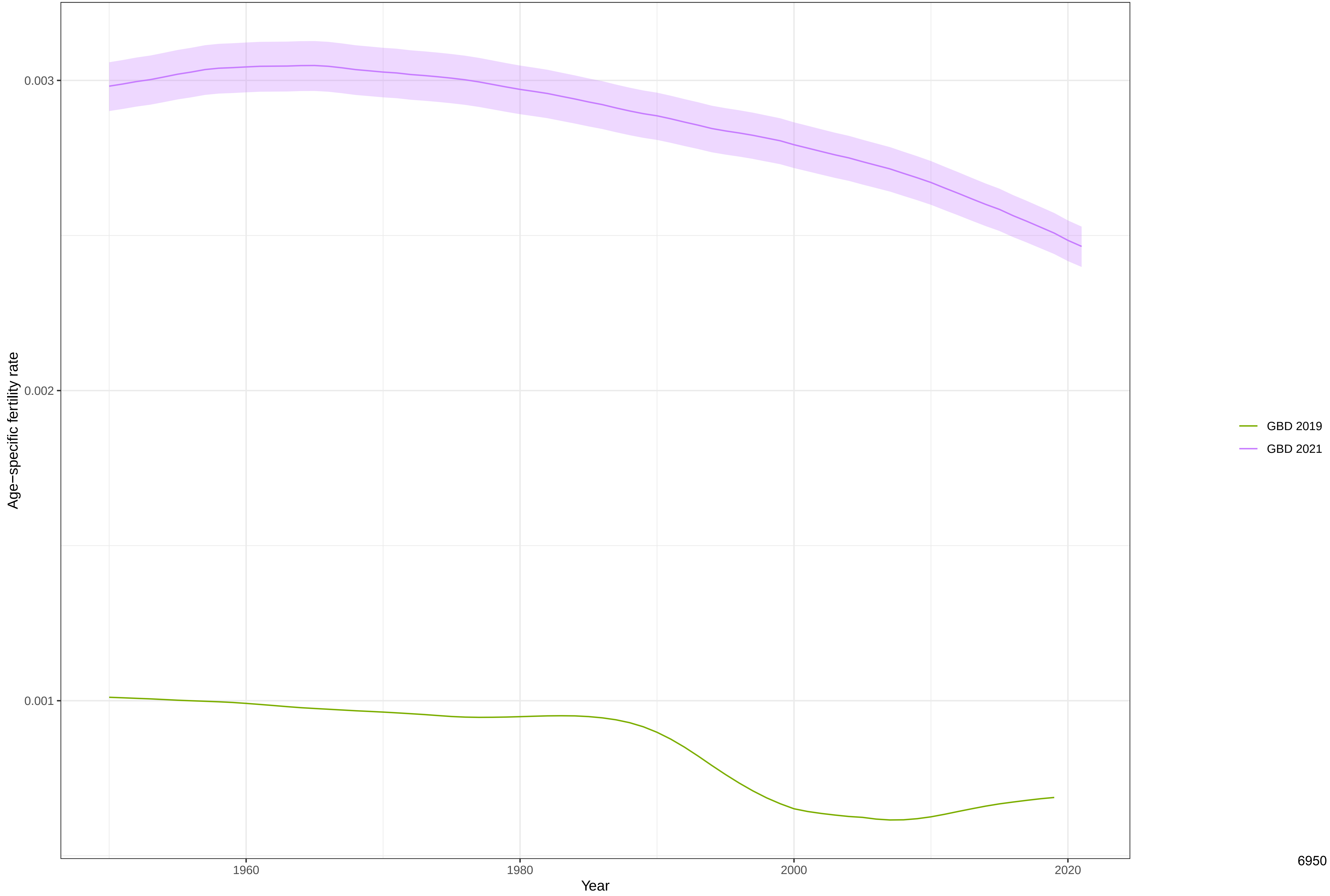

Total fertility rate fitted trend lines, 1950−2021: Garissa TFR
Data points enclosed with a diamond are reference sources

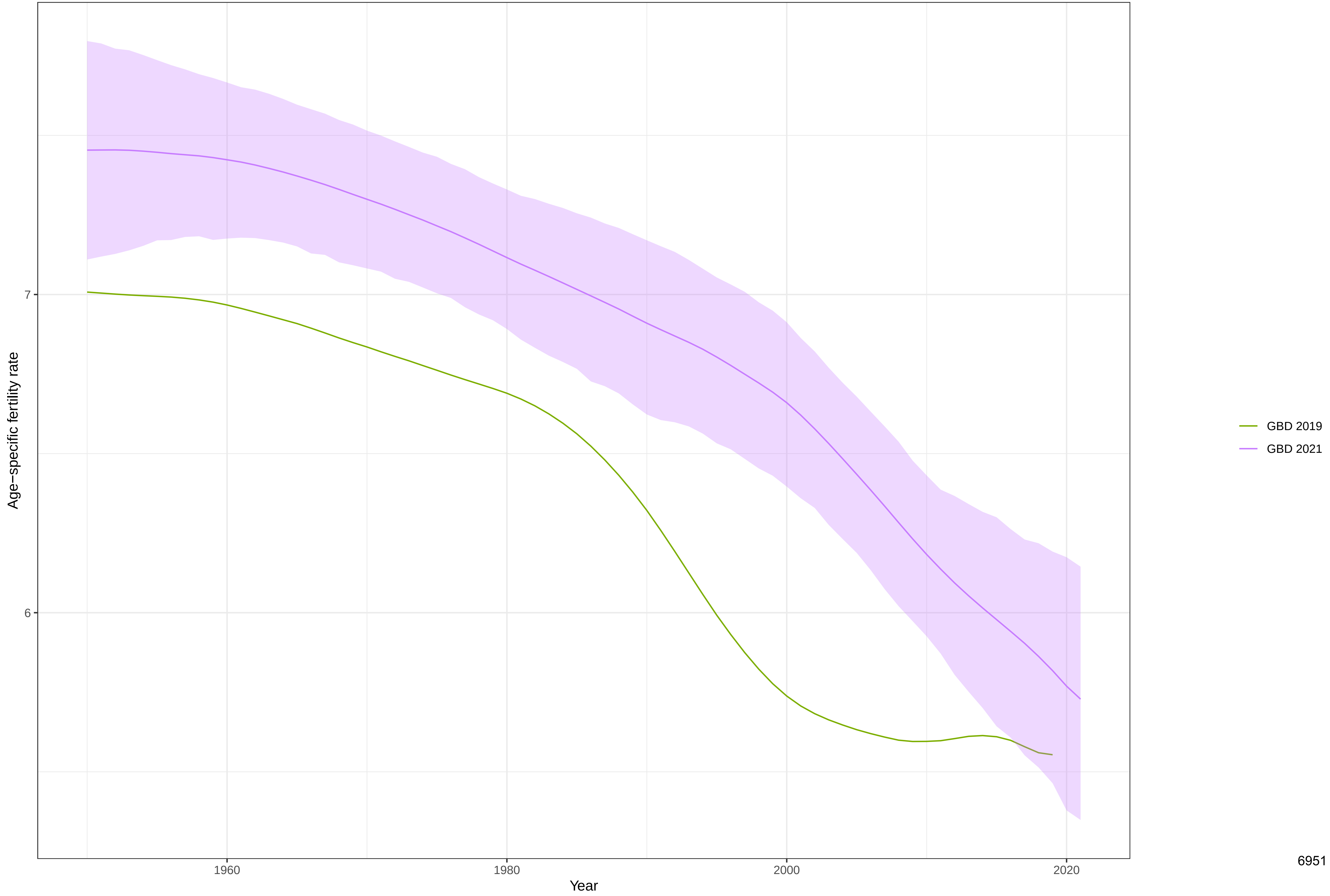

Age−specific fertility fitted trend lines, 1950−2021: Homa Bay, Ages: 10−14
Data points enclosed with a diamond are reference sources

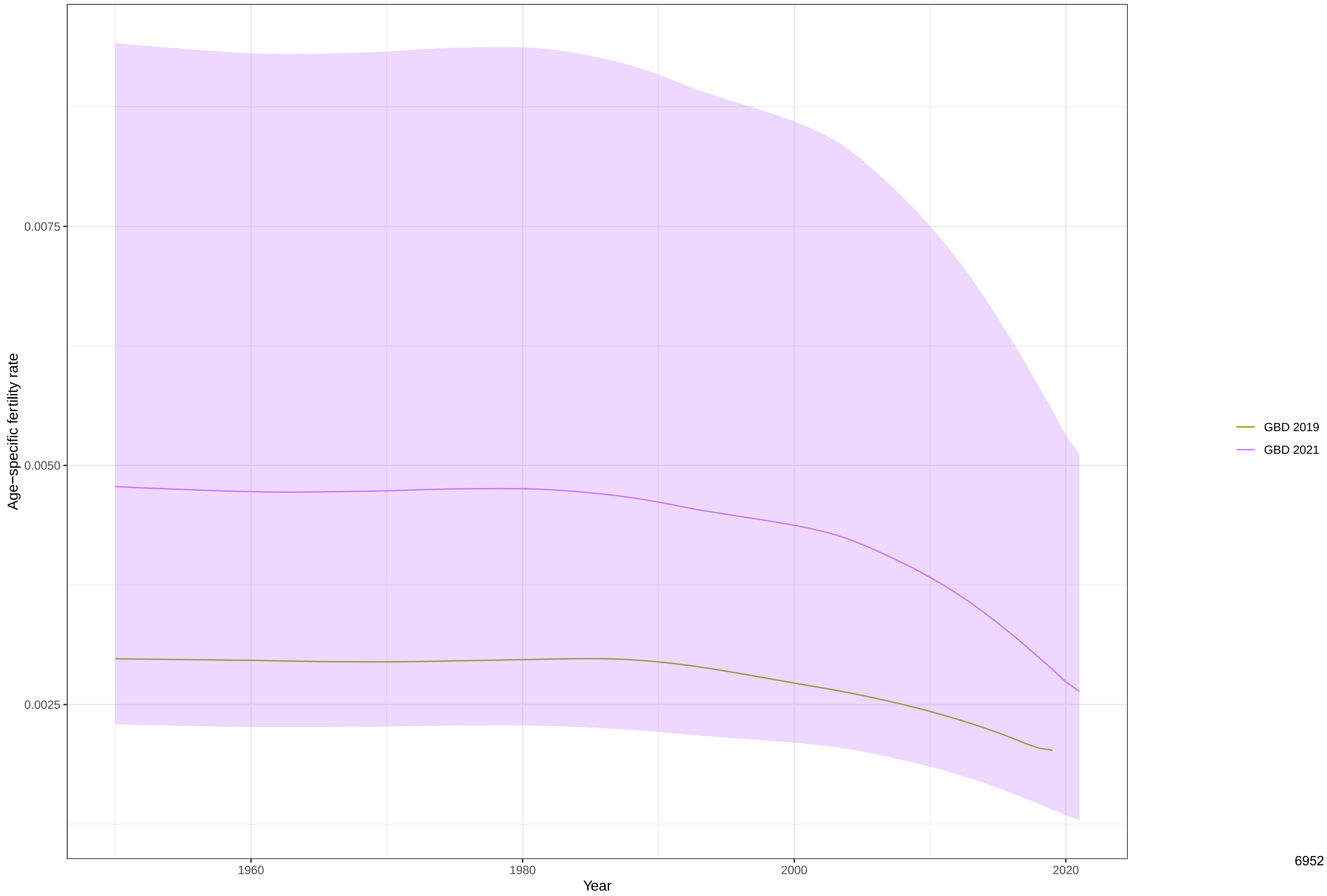

Age−specific fertility fitted trend lines, 1950−2021: Homa Bay, Ages: 15−19
Data points enclosed with a diamond are reference sources

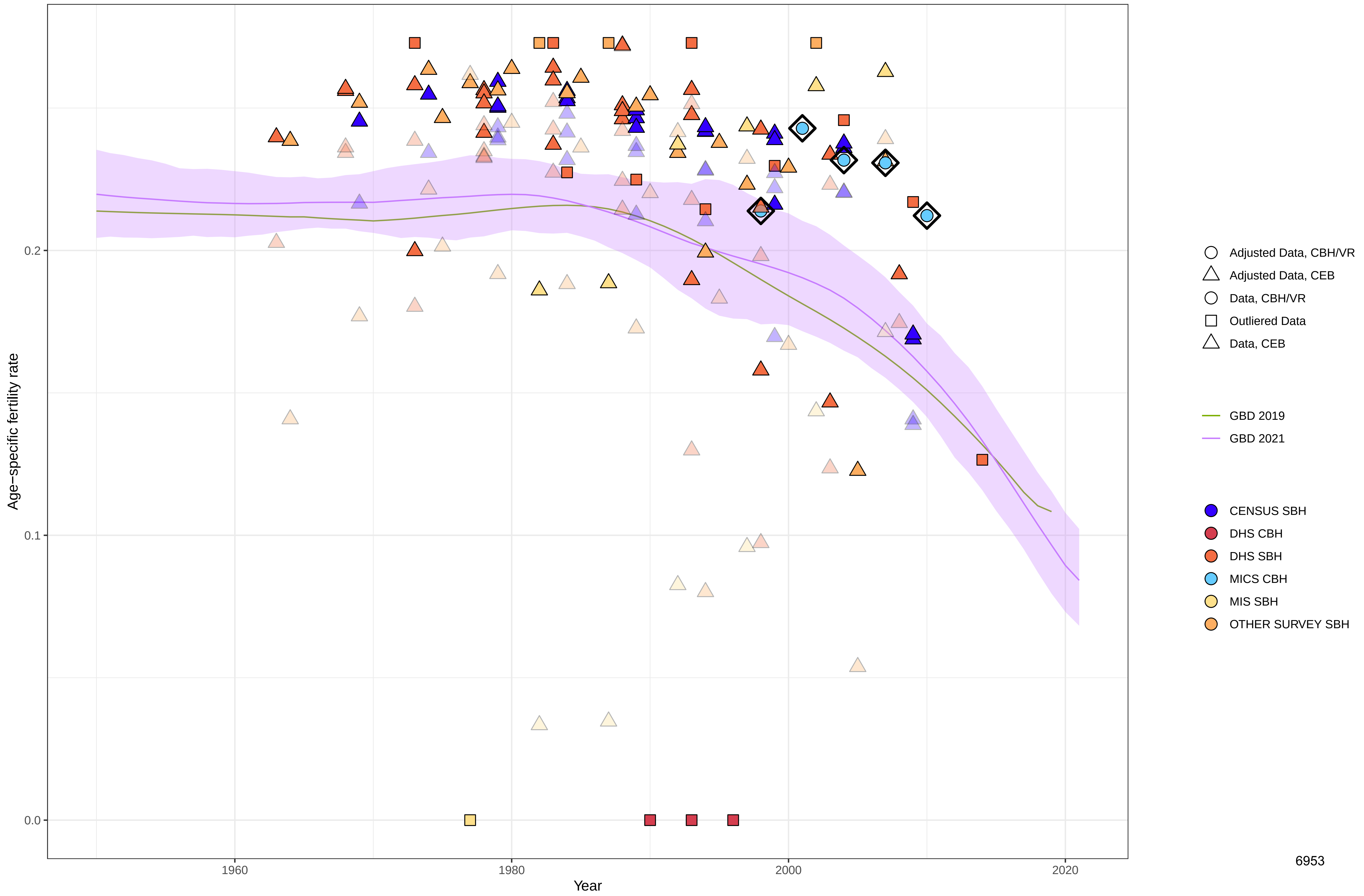

Age−specific fertility fitted trend lines, 1950−2021: Homa Bay, Ages: 20−24
Data points enclosed with a diamond are reference sources

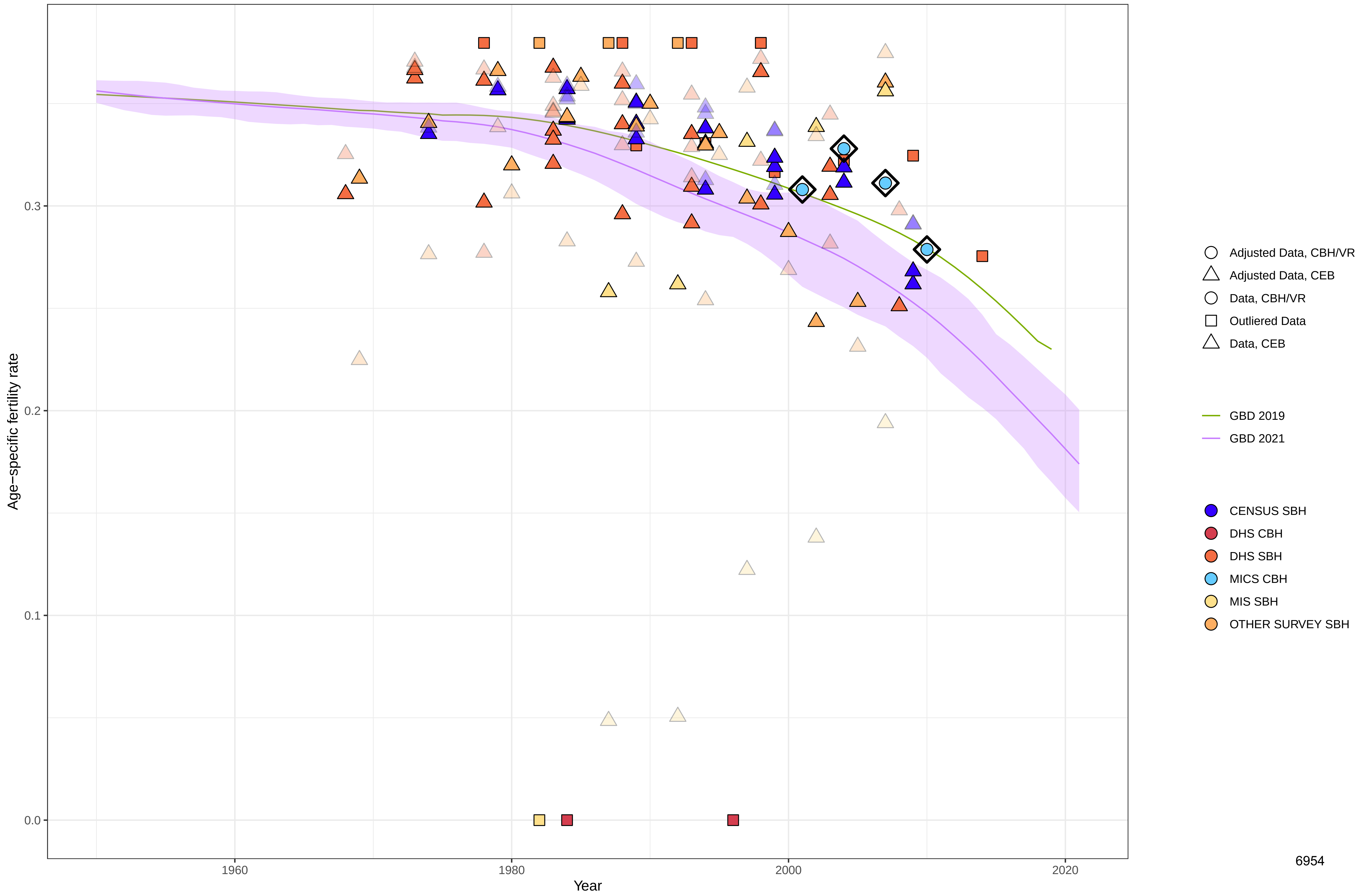

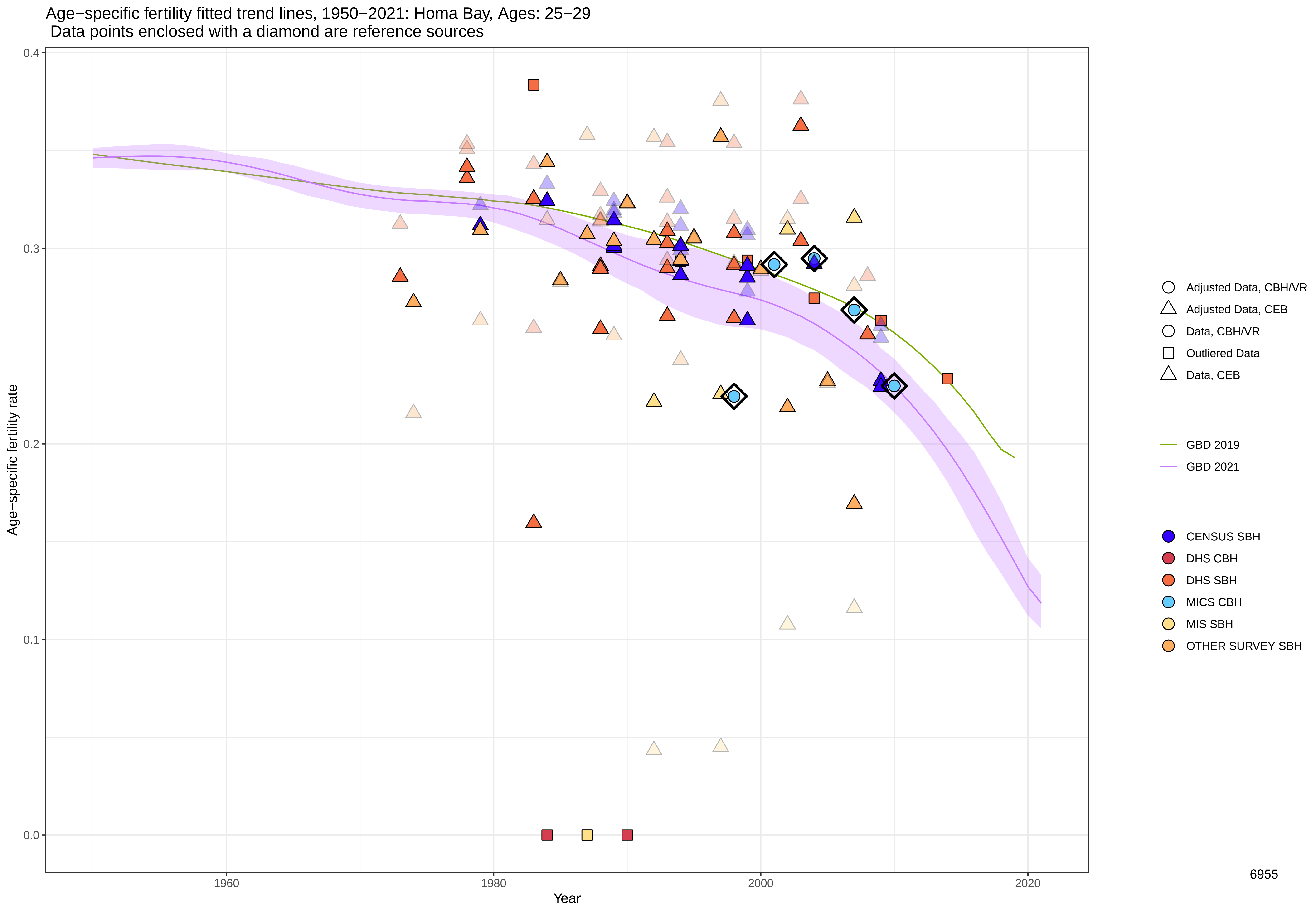

Age−specific fertility fitted trend lines, 1950−2021: Homa Bay, Ages: 25−29
Data points enclosed with a diamond are reference sources
Age−specific fertility rate
Year
0.0
0.1
0.2
0.3
0.4
1960
1980
2000
2020
Adjusted Data, CBH/VR
Adjusted Data, CEB
Data, CBH/VR
Outliered Data
Data, CEB
GBD 2019
GBD 2021
CENSUS SBH
DHS CBH
DHS SBH
MICS CBH
MIS SBH
OTHER SURVEY SBH

Age−specific fertility fitted trend lines, 1950−2021: Homa Bay, Ages: 30−34
Data points enclosed with a diamond are reference sources

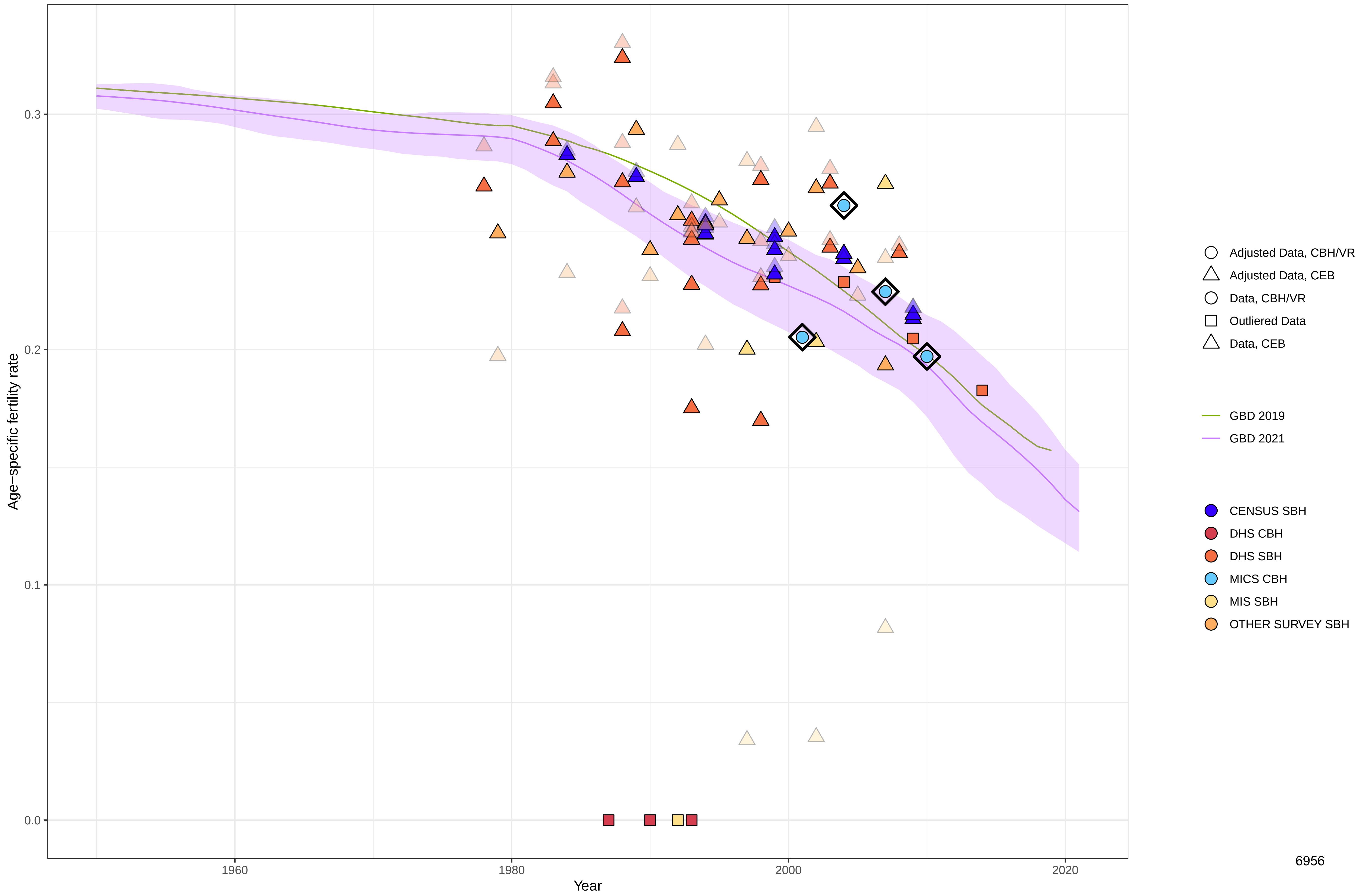

Age−specific fertility fitted trend lines, 1950−2021: Homa Bay, Ages: 35−39
Data points enclosed with a diamond are reference sources

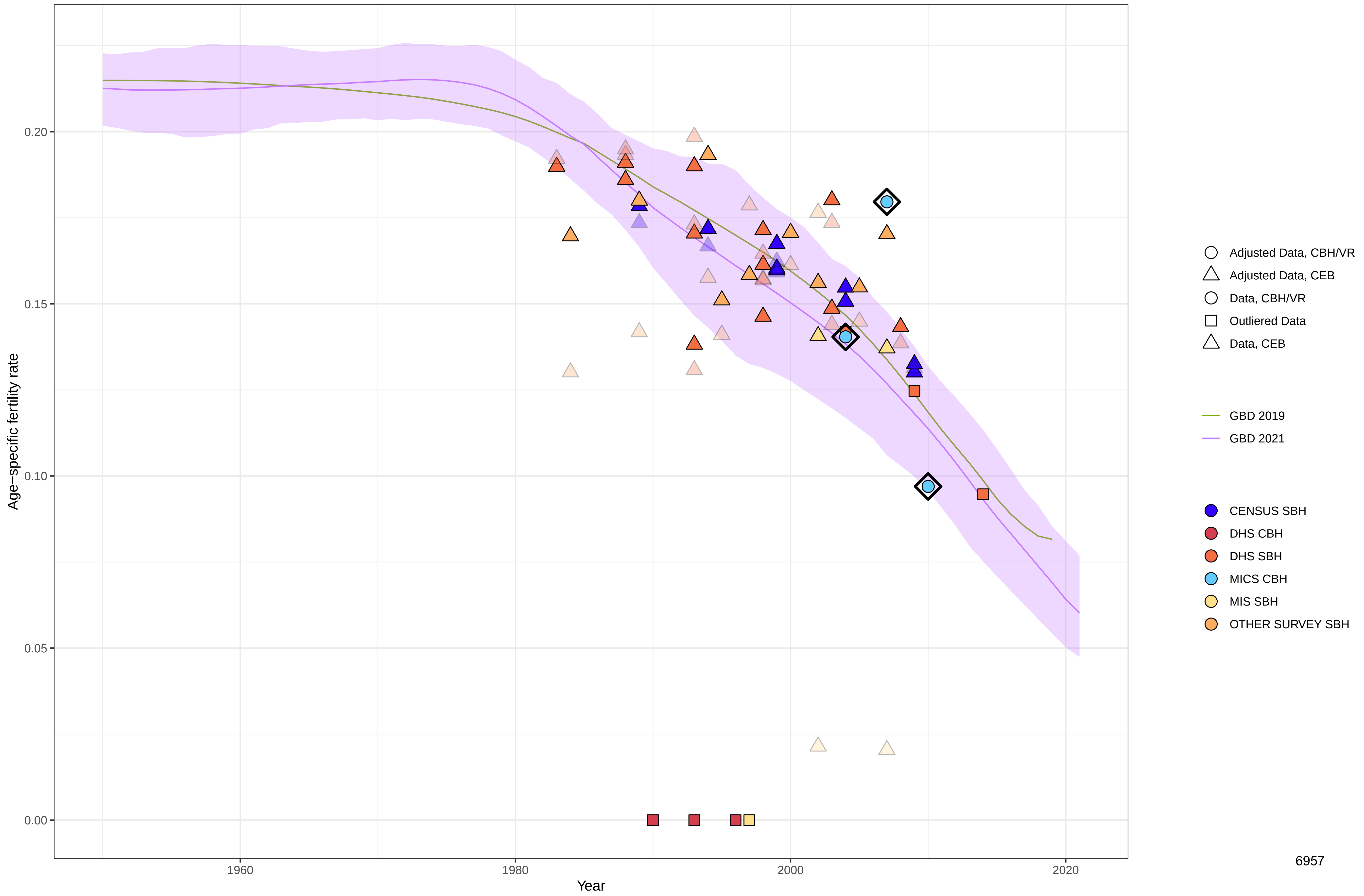

Age−specific fertility fitted trend lines, 1950−2021: Homa Bay, Ages: 40−44
Data points enclosed with a diamond are reference sources

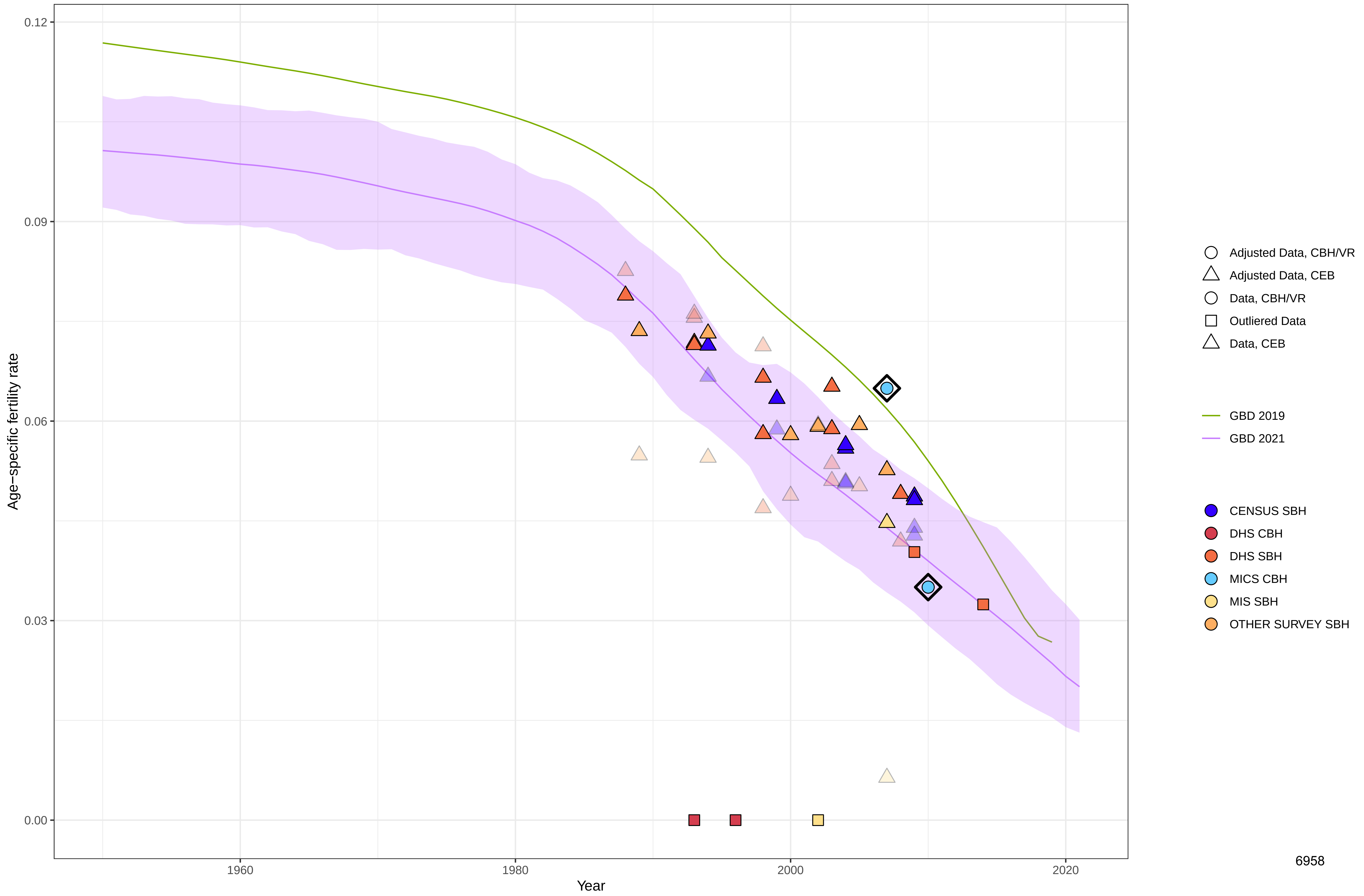

Age−specific fertility fitted trend lines, 1950−2021: Homa Bay, Ages: 45−49
Data points enclosed with a diamond are reference sources

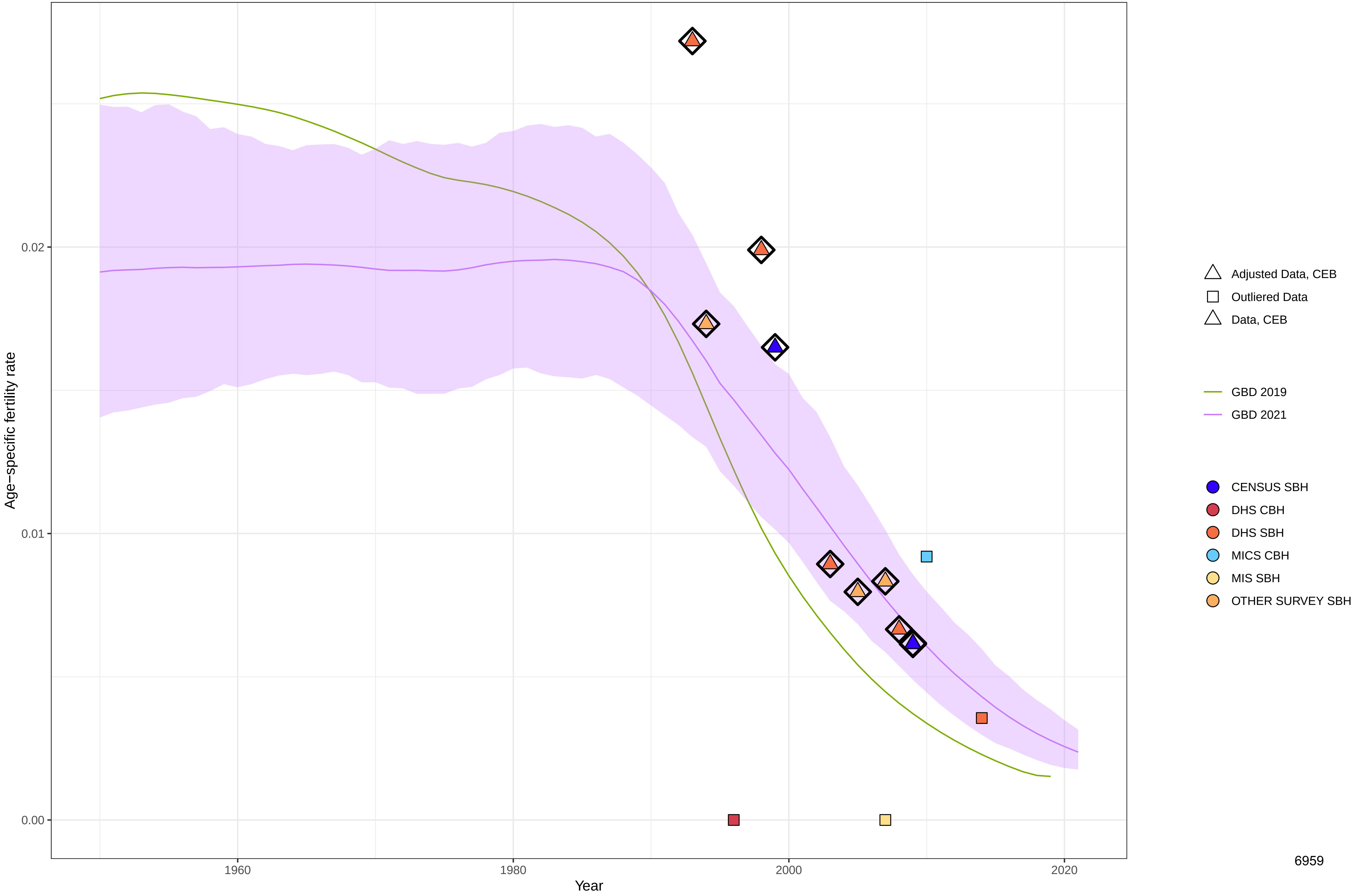

Age−specific fertility fitted trend lines, 1950−2021: Homa Bay, Ages: 50−54
Data points enclosed with a diamond are reference sources

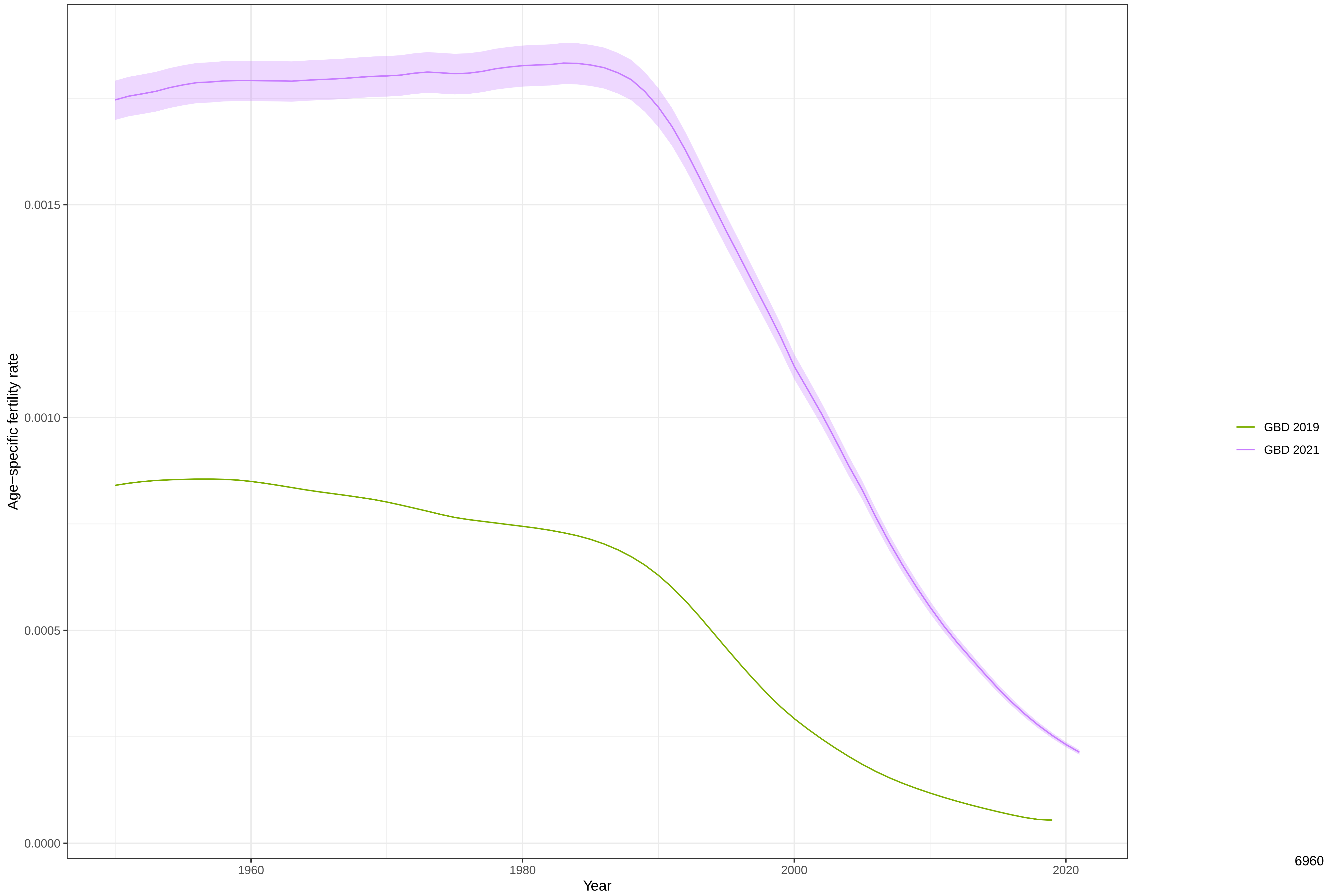

Total fertility rate fitted trend lines, 1950−2021: Homa Bay TFR
Data points enclosed with a diamond are reference sources

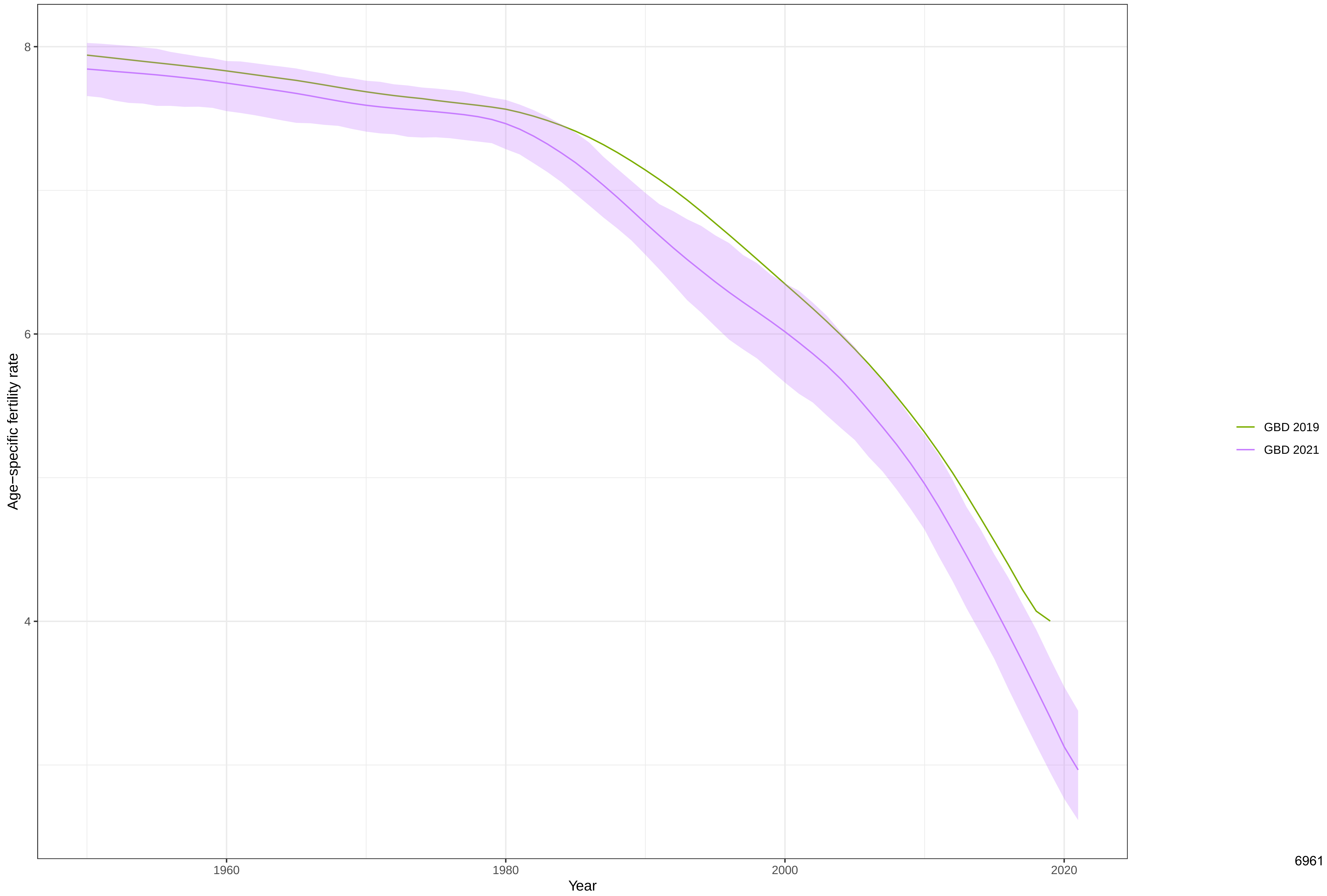

Age−specific fertility fitted trend lines, 1950−2021: Isiolo, Ages: 10−14
Data points enclosed with a diamond are reference sources

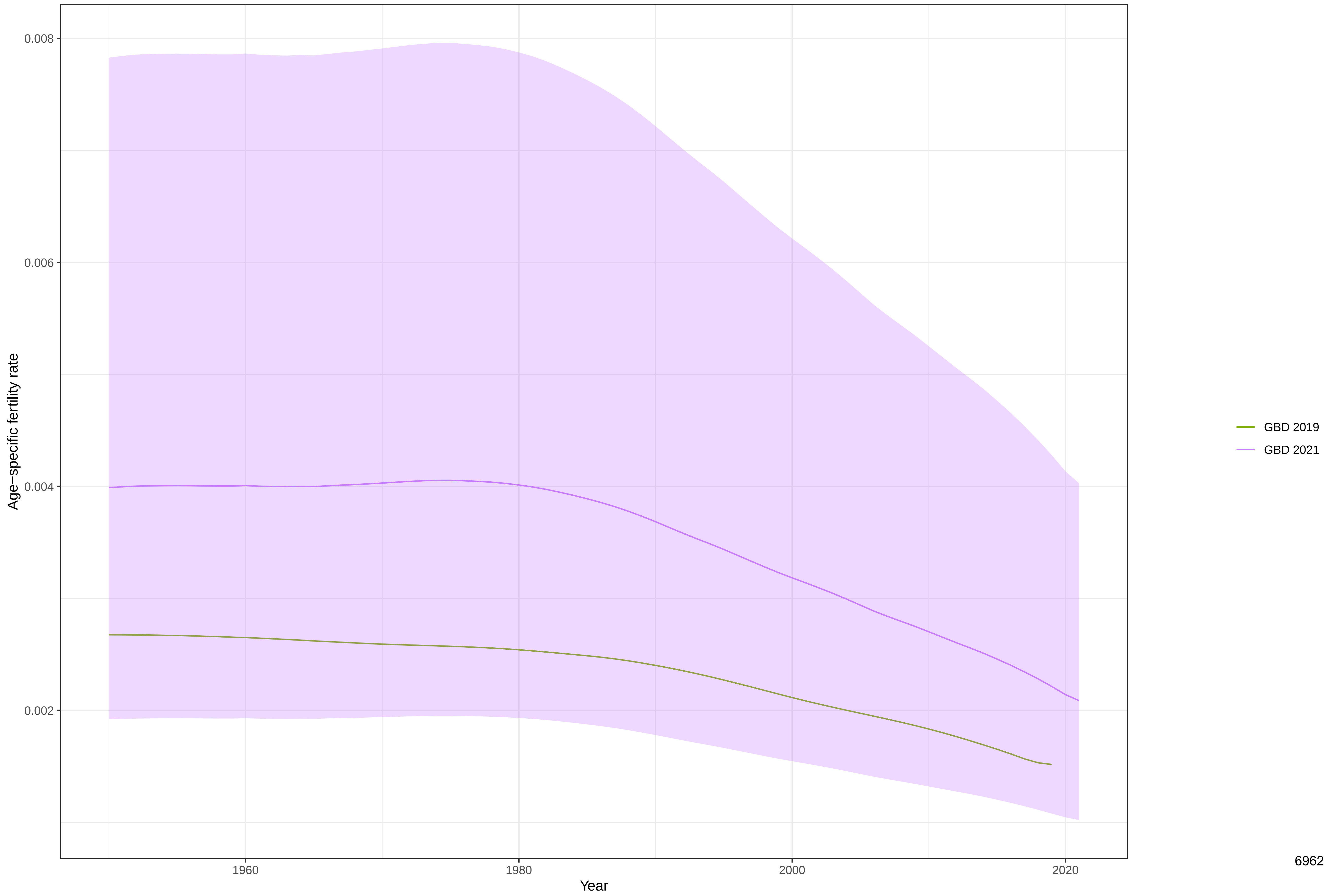

Age−specific fertility fitted trend lines, 1950−2021: Isiolo, Ages: 15−19
Data points enclosed with a diamond are reference sources

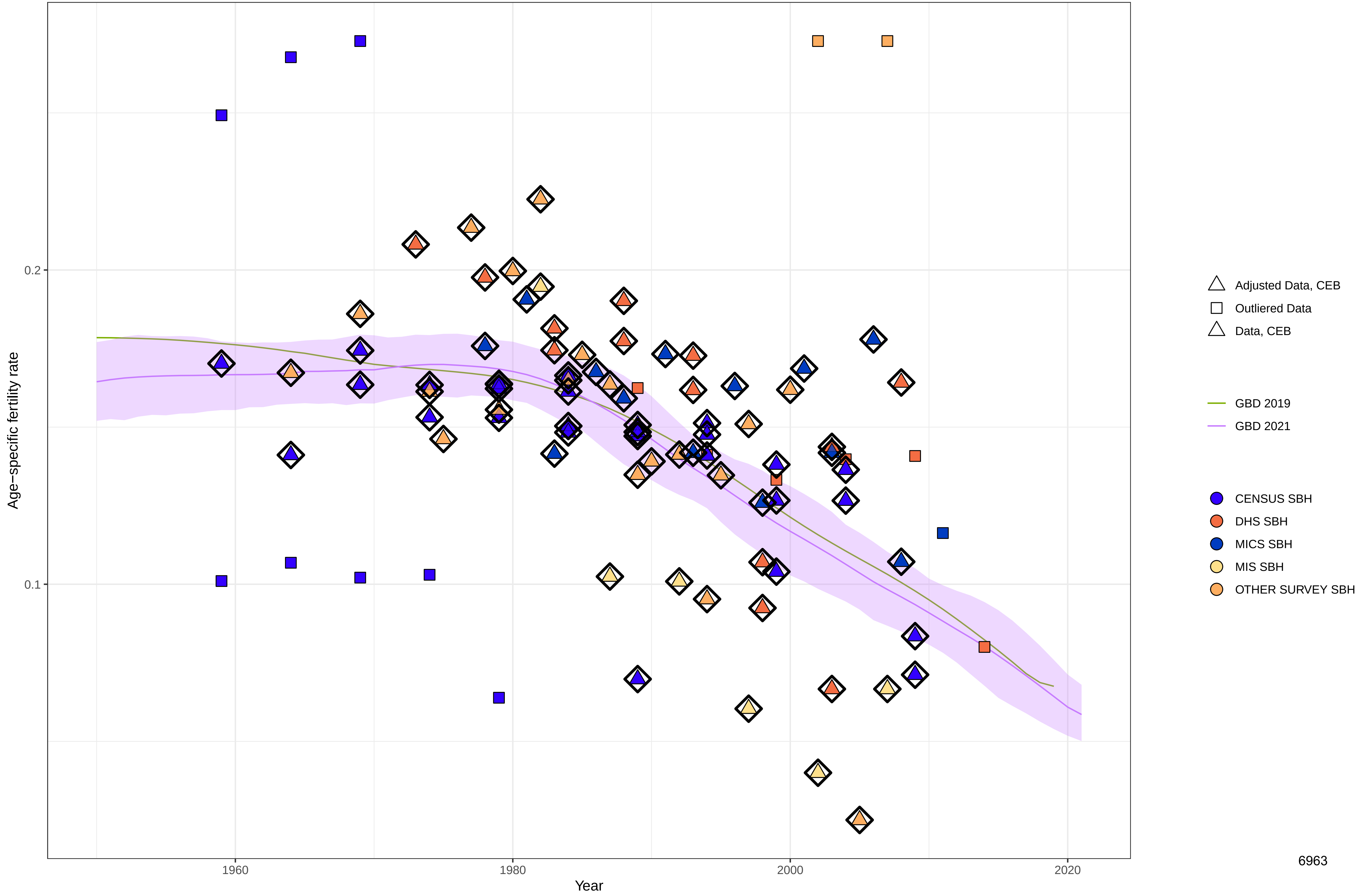

Age−specific fertility fitted trend lines, 1950−2021: Isiolo, Ages: 20−24
Data points enclosed with a diamond are reference sources

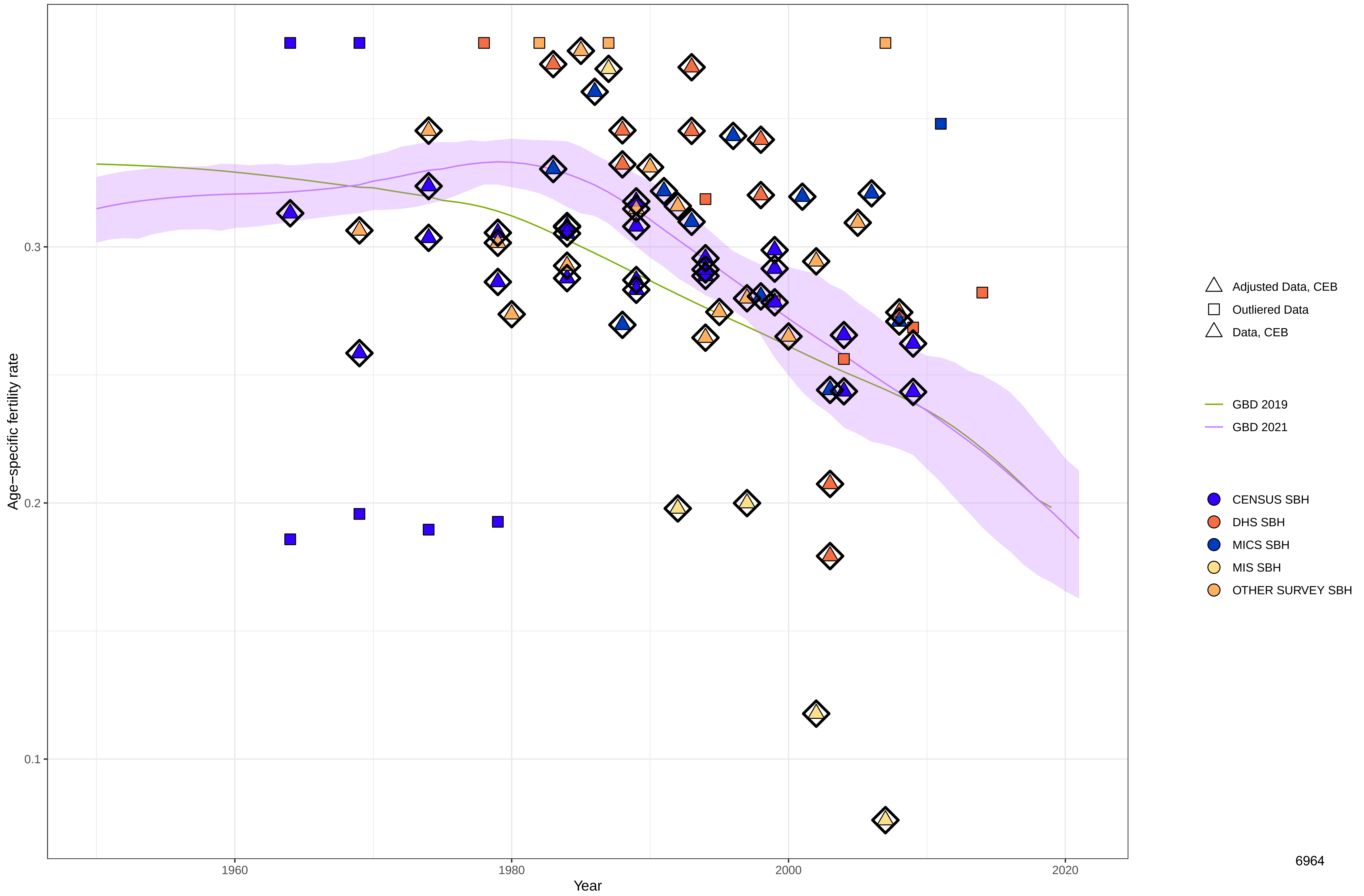

Age−specific fertility fitted trend lines, 1950−2021: Isiolo, Ages: 25−29
Data points enclosed with a diamond are reference sources

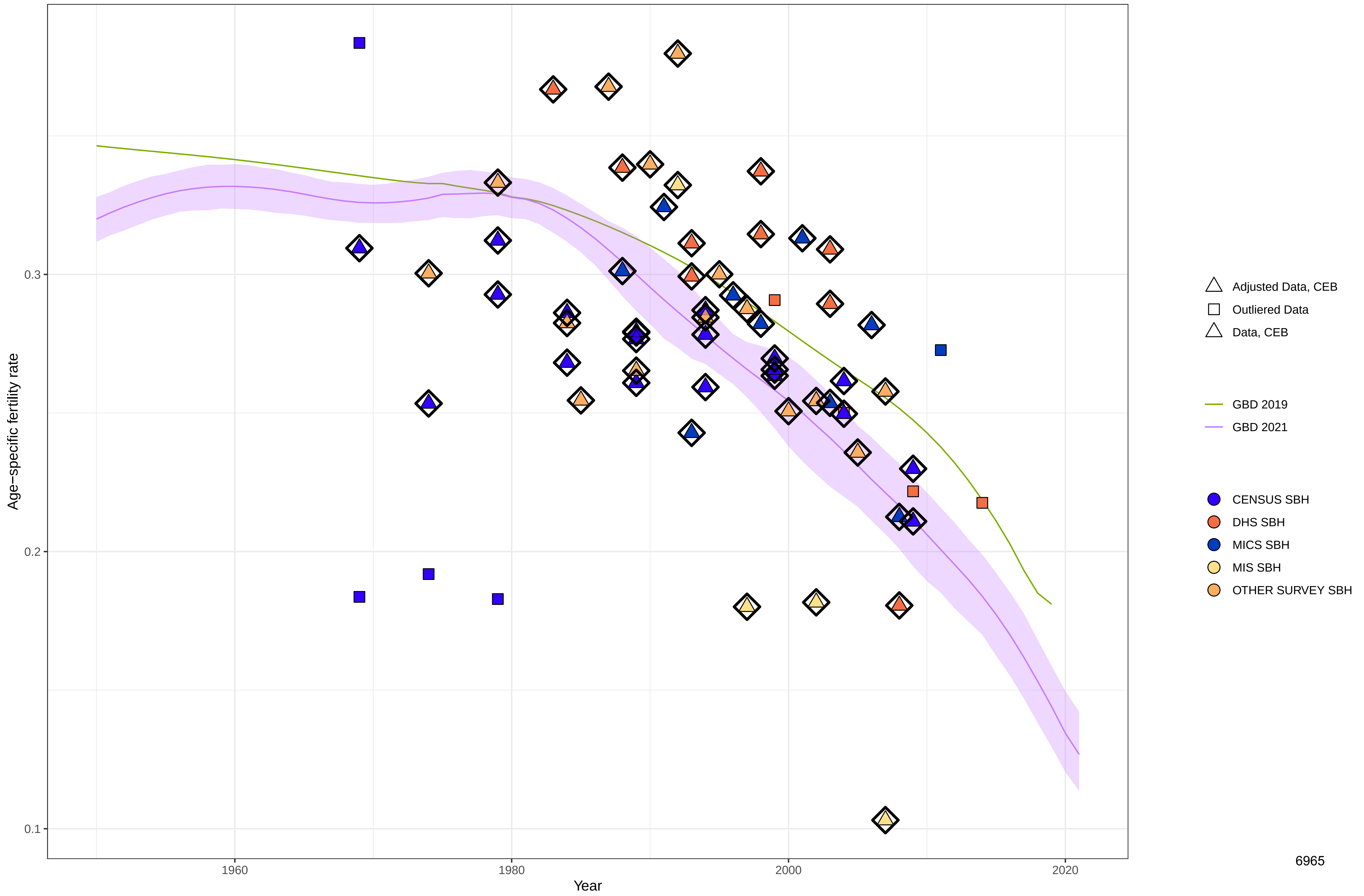

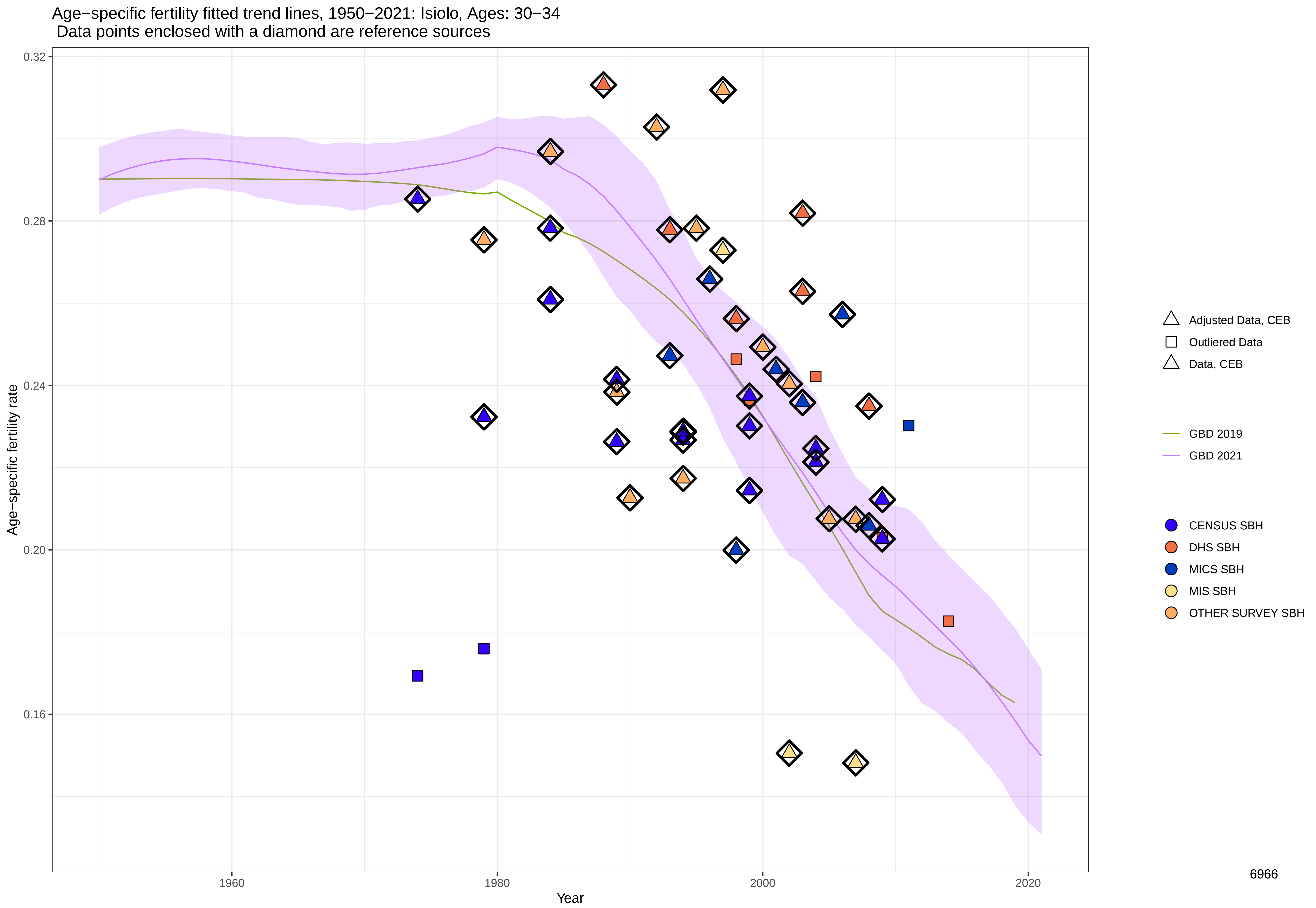
Age−specific fertility fitted trend lines, 1950−2021: Isiolo, Ages: 30−34
Data points enclosed with a diamond are reference sources
Age−specific fertility rate
Year
0.32
0.28
0.24
0.20
0.16
1960
1980
2000
2020
Adjusted Data, CEB
Outliered Data
Data, CEB
GBD 2019
GBD 2021
CENSUS SBH
DHS SBH
MICS SBH
MIS SBH
OTHER SURVEY SBH

Age−specific fertility fitted trend lines, 1950−2021: Isiolo, Ages: 35−39
Data points enclosed with a diamond are reference sources

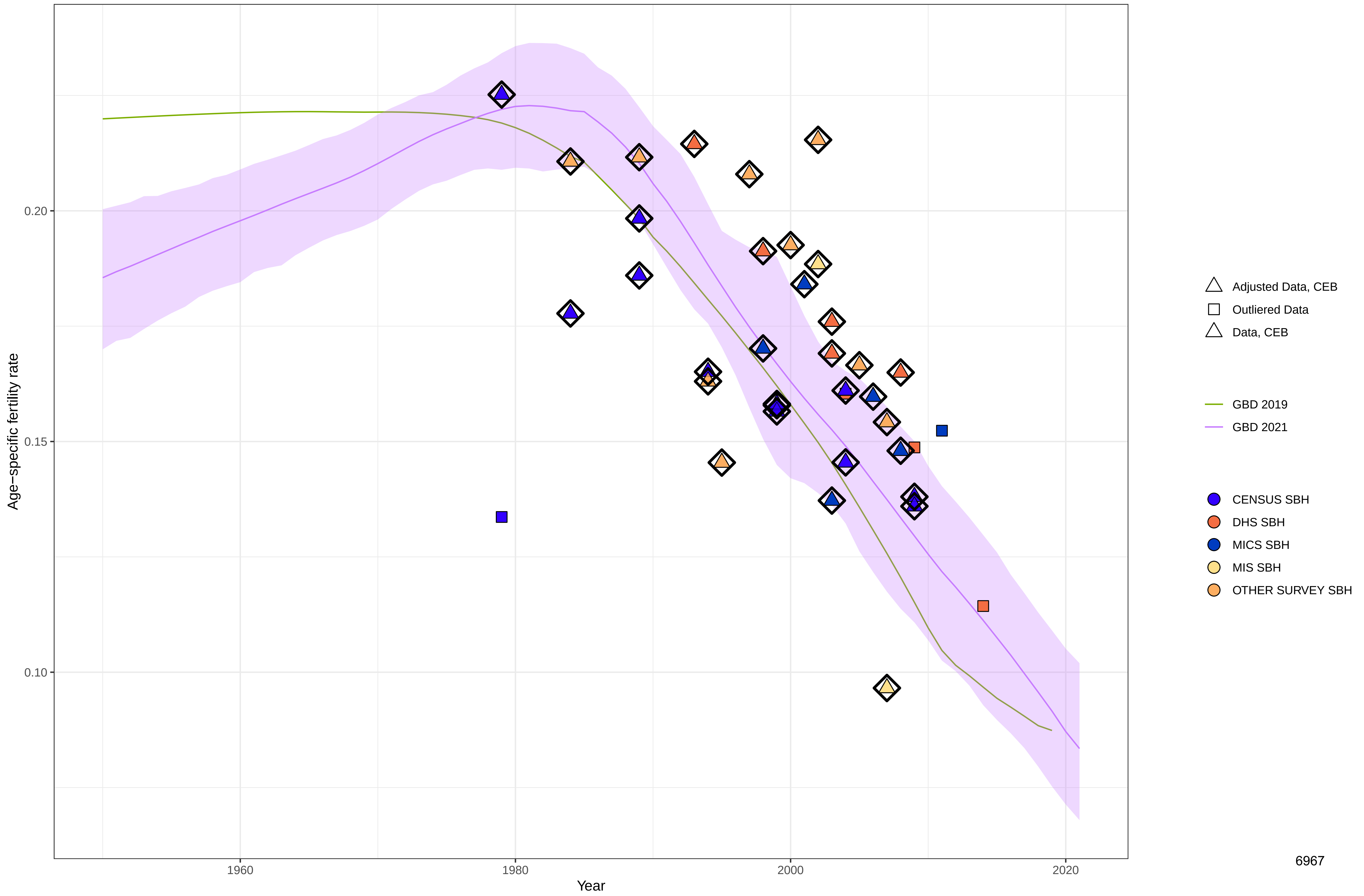

6967

Age−specific fertility fitted trend lines, 1950−2021: Isiolo, Ages: 40−44
Data points enclosed with a diamond are reference sources

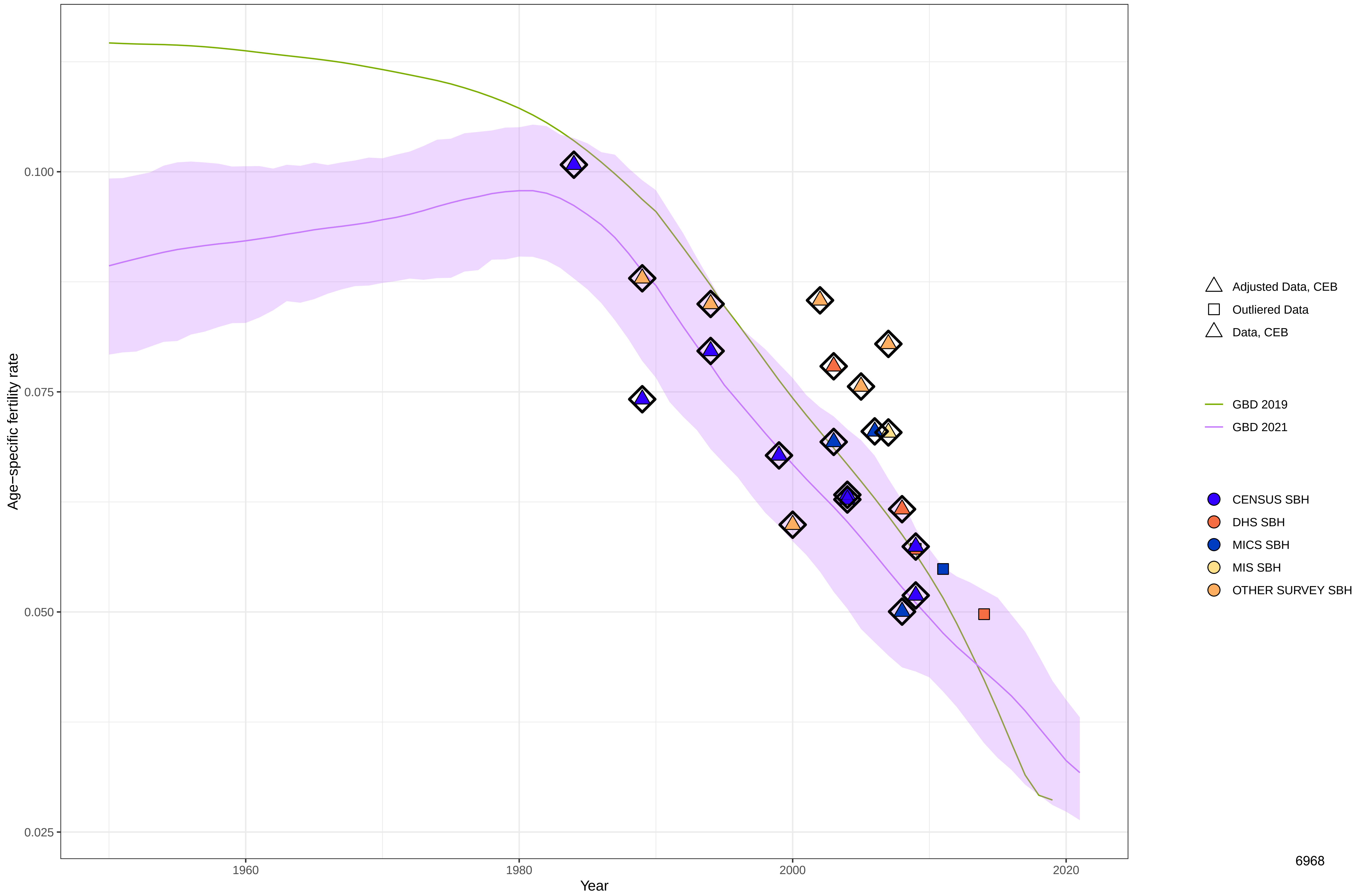

Age−specific fertility fitted trend lines, 1950−2021: Isiolo, Ages: 45−49
Data points enclosed with a diamond are reference sources

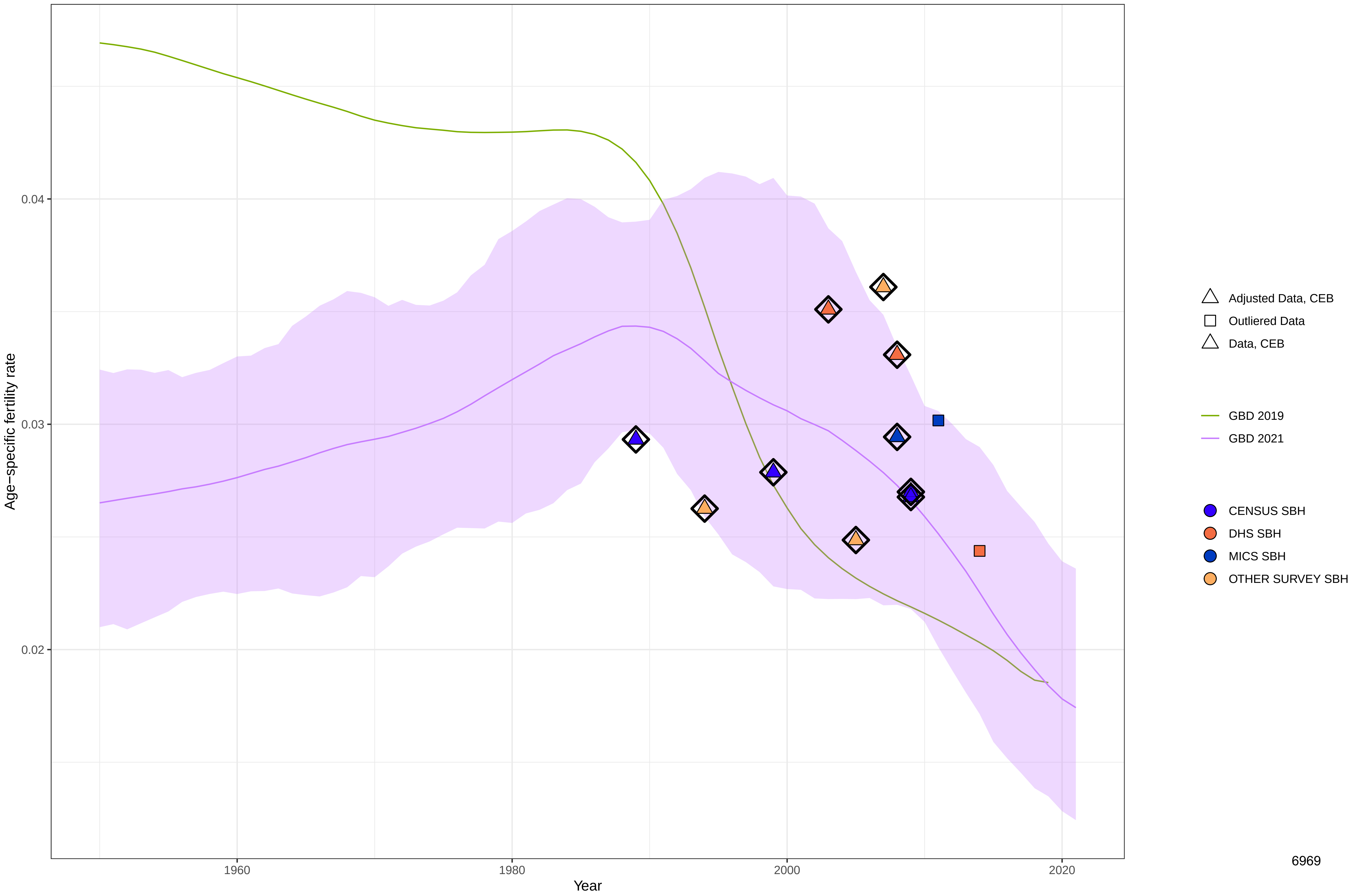

Age−specific fertility fitted trend lines, 1950−2021: Isiolo, Ages: 50−54
Data points enclosed with a diamond are reference sources

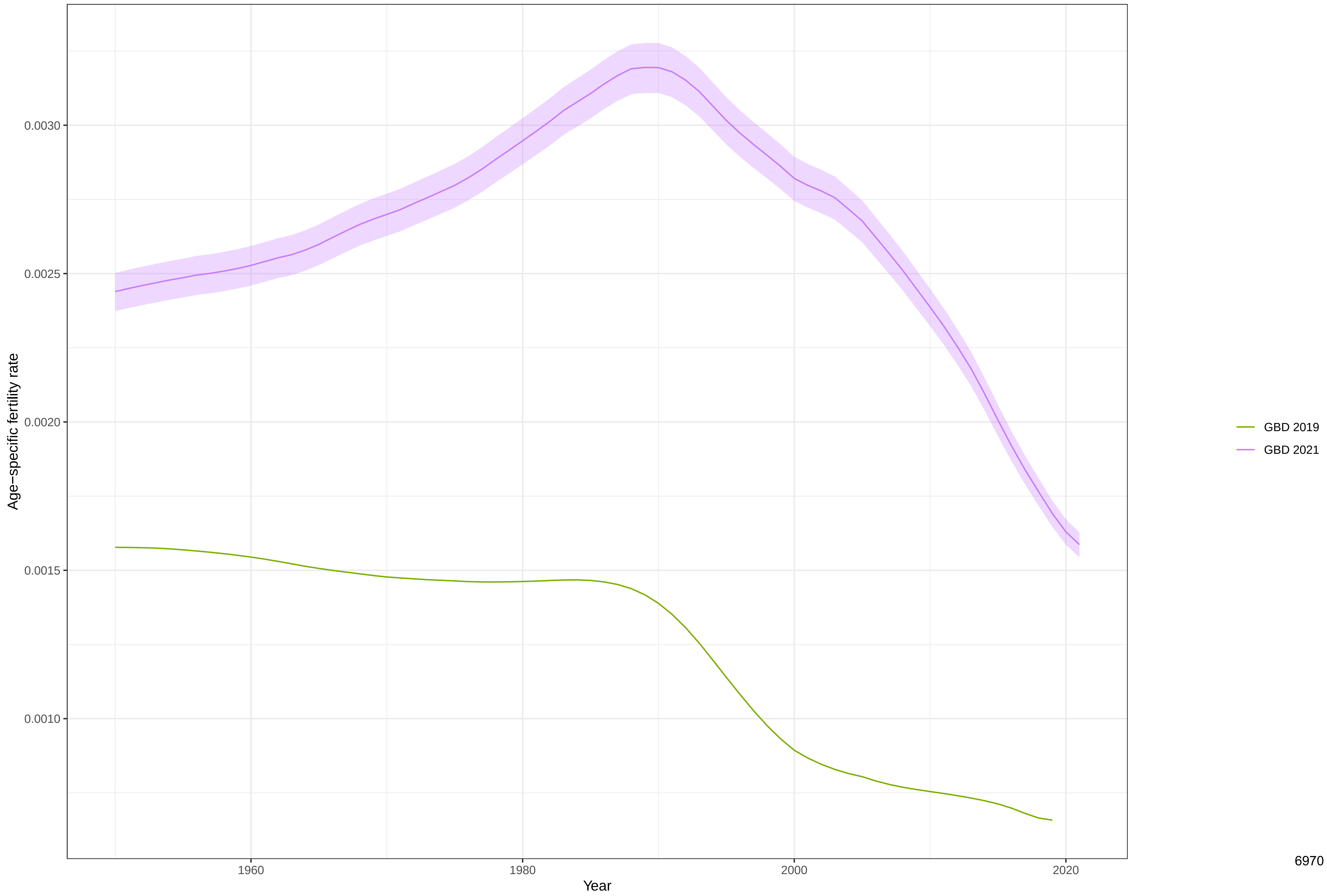

Total fertility rate fitted trend lines, 1950−2021: Isiolo TFR
Data points enclosed with a diamond are reference sources

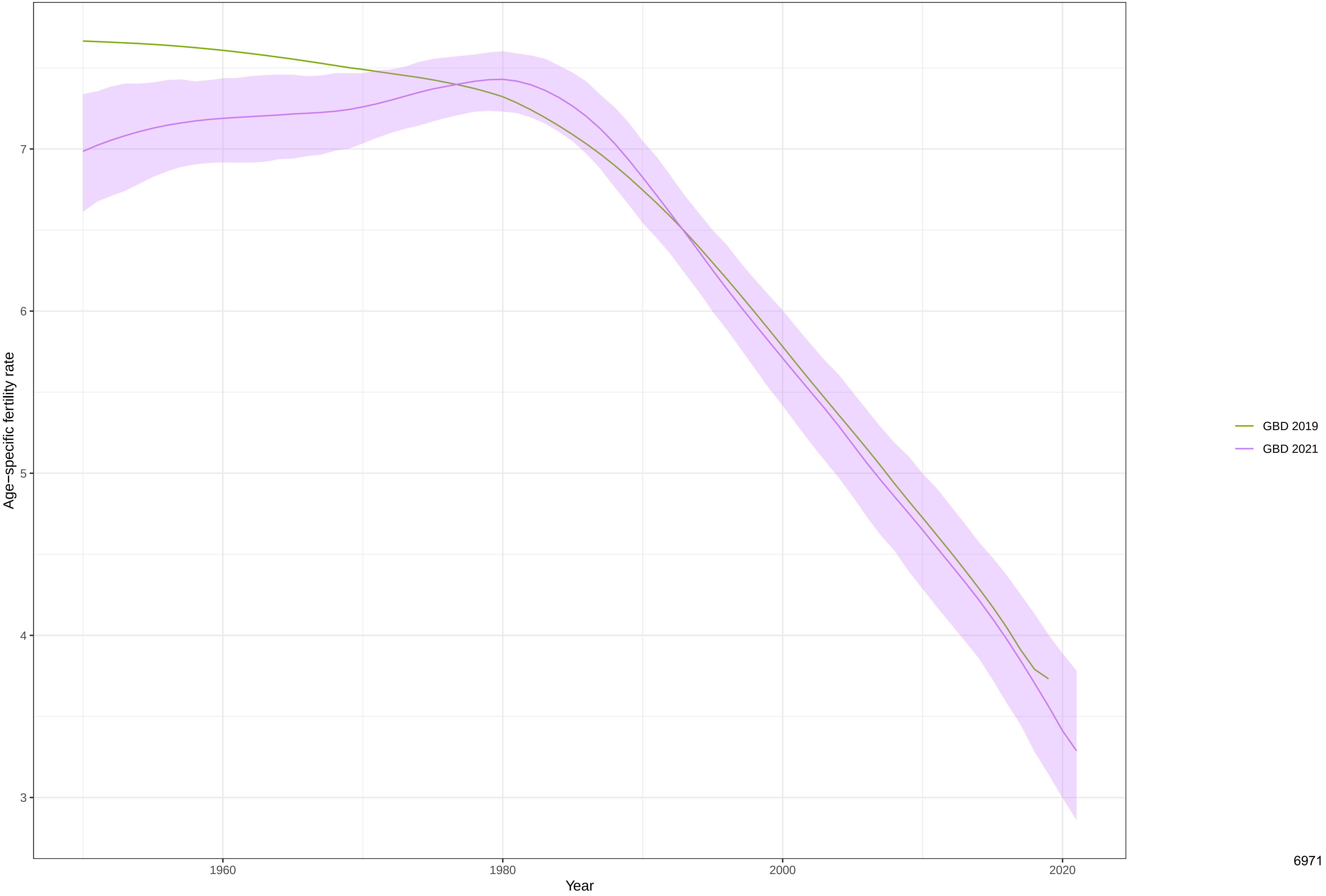

Age−specific fertility fitted trend lines, 1950−2021: Kajiado, Ages: 10−14
Data points enclosed with a diamond are reference sources

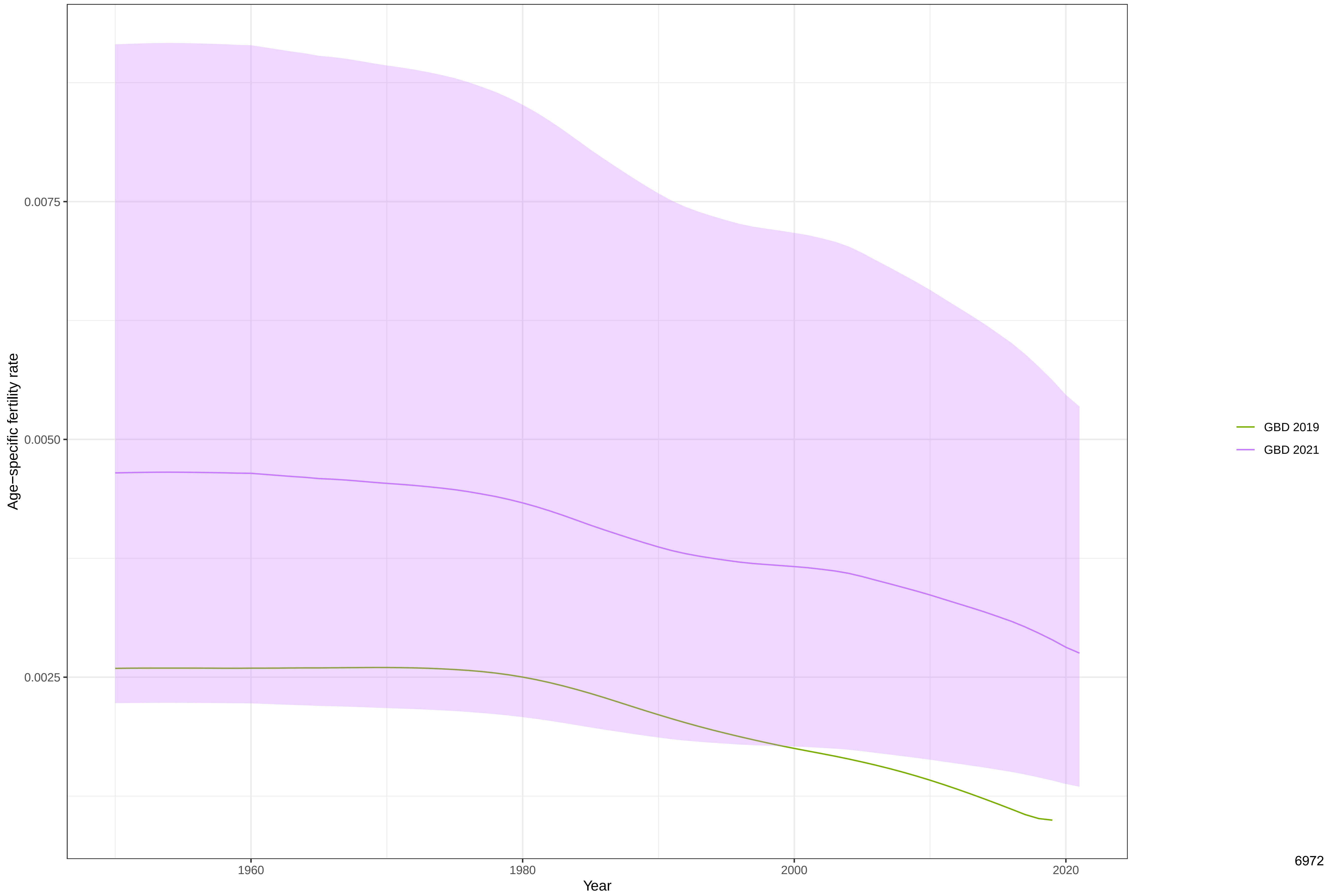

Age−specific fertility fitted trend lines, 1950−2021: Kajiado, Ages: 15−19
Data points enclosed with a diamond are reference sources

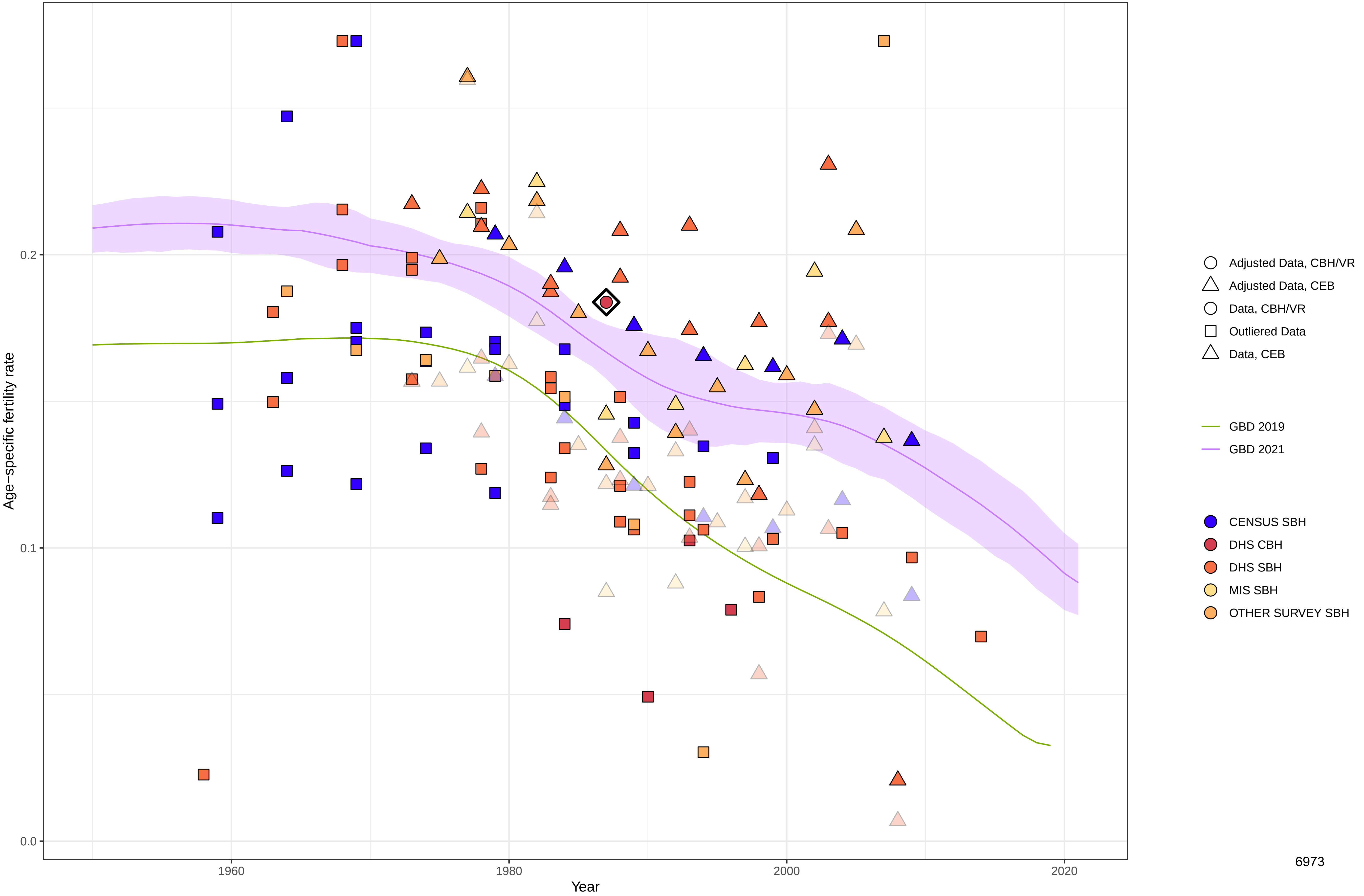

Age−specific fertility fitted trend lines, 1950−2021: Kajiado, Ages: 20−24
Data points enclosed with a diamond are reference sources

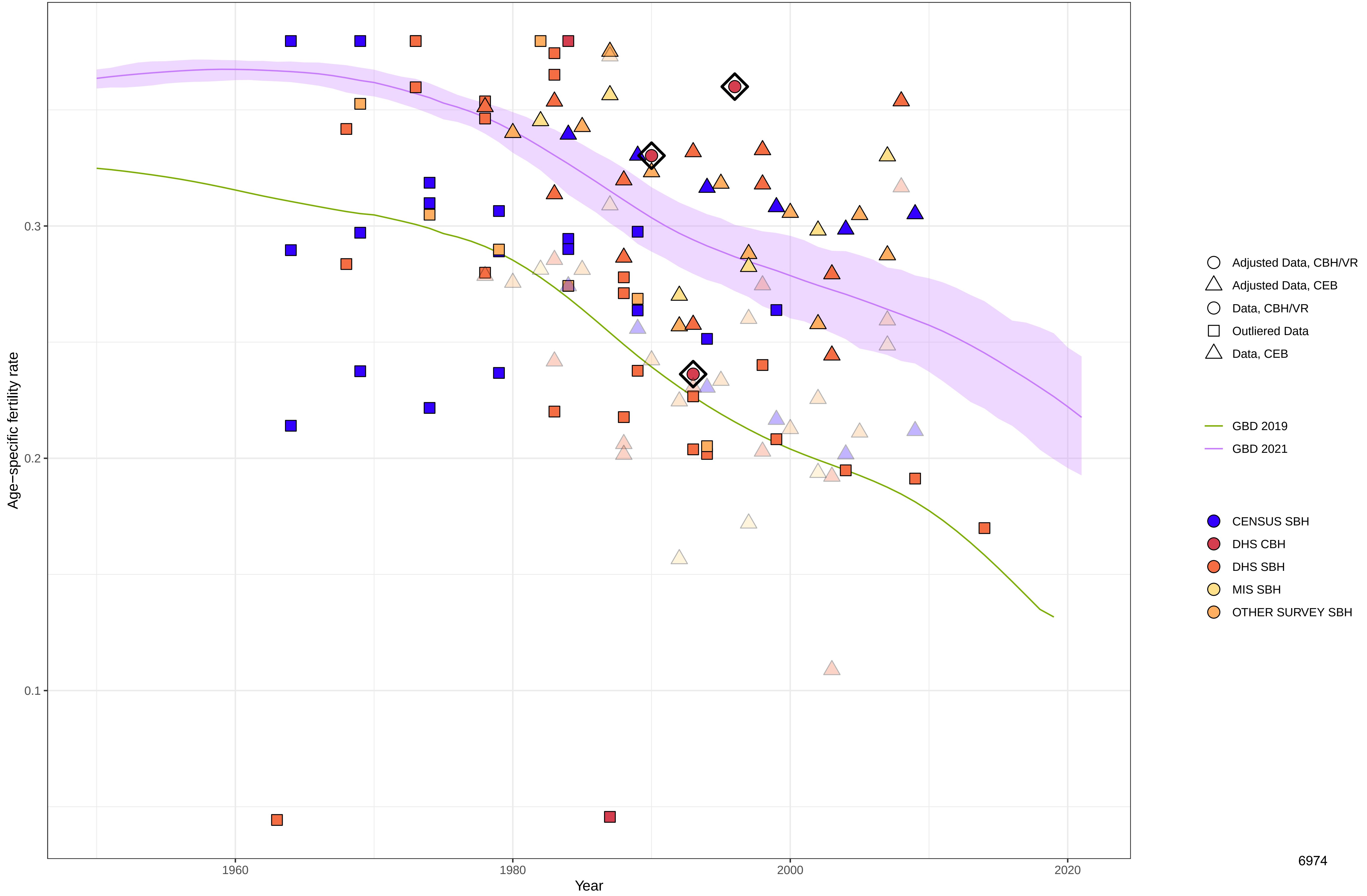

Age−specific fertility fitted trend lines, 1950−2021: Kajiado, Ages: 25−29
Data points enclosed with a diamond are reference sources

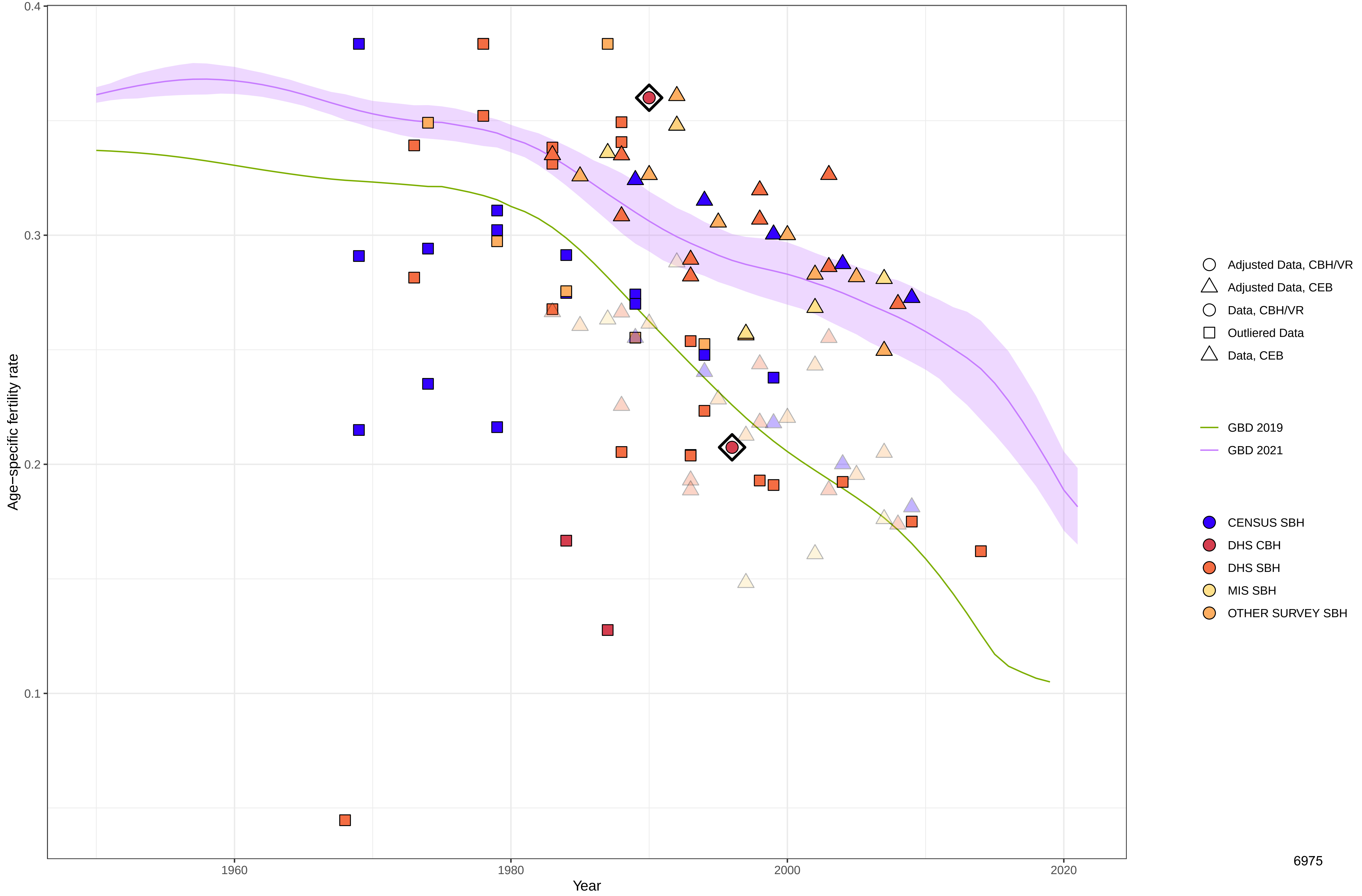

Age−specific fertility fitted trend lines, 1950−2021: Kajiado, Ages: 30−34
Data points enclosed with a diamond are reference sources

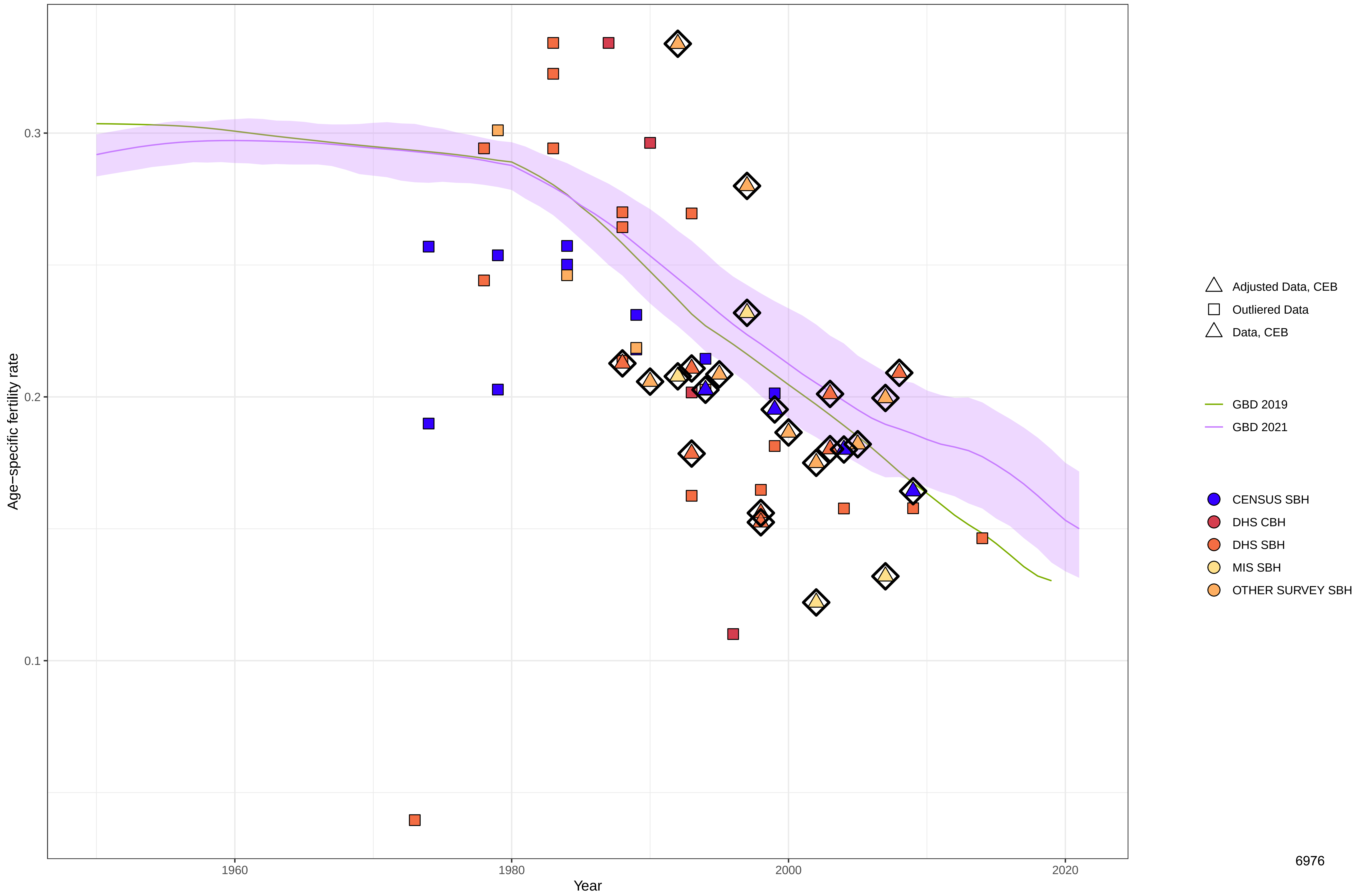

Age−specific fertility fitted trend lines, 1950−2021: Kajiado, Ages: 35−39
Data points enclosed with a diamond are reference sources

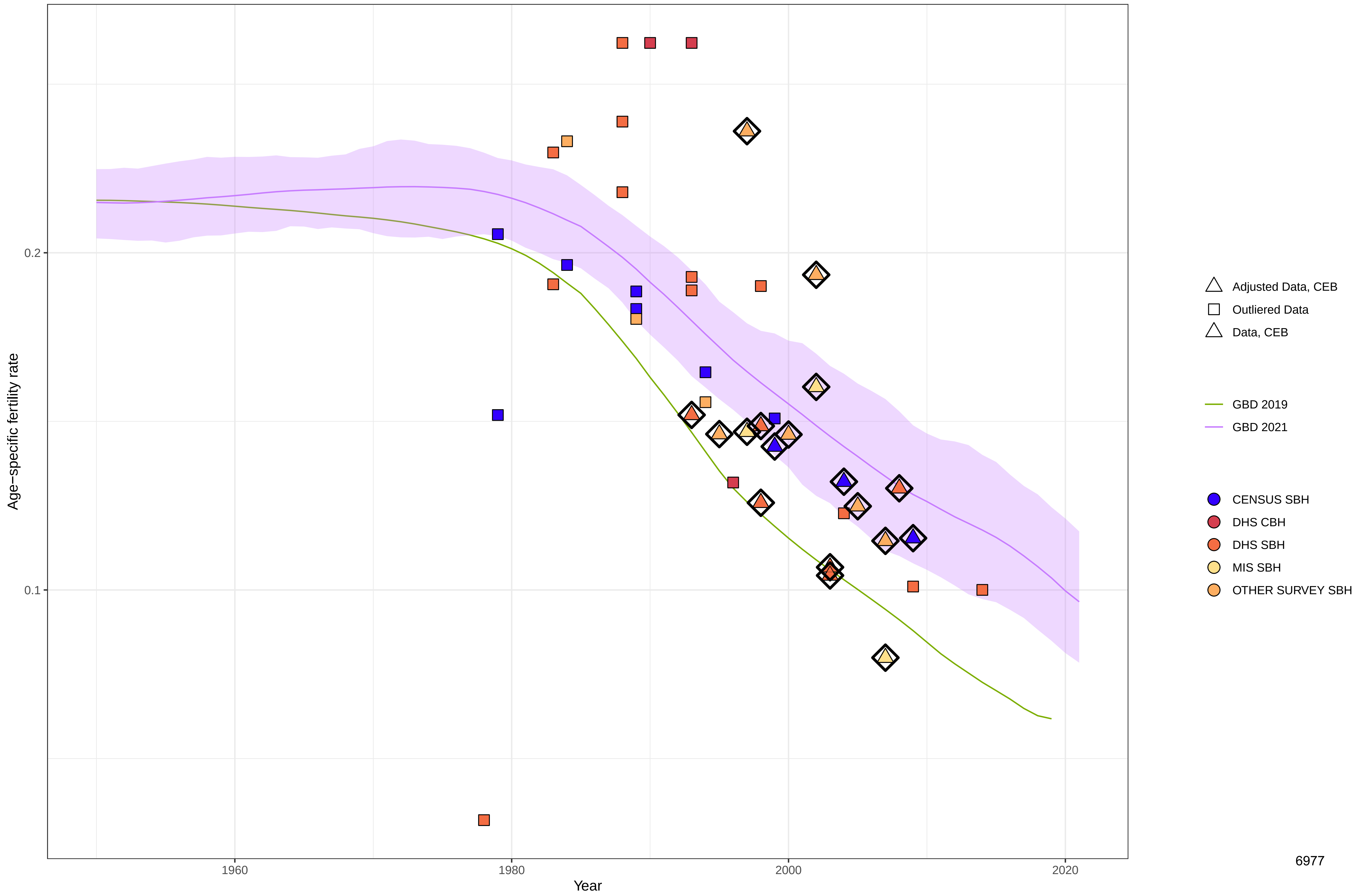

Age−specific fertility fitted trend lines, 1950−2021: Kajiado, Ages: 40−44
Data points enclosed with a diamond are reference sources

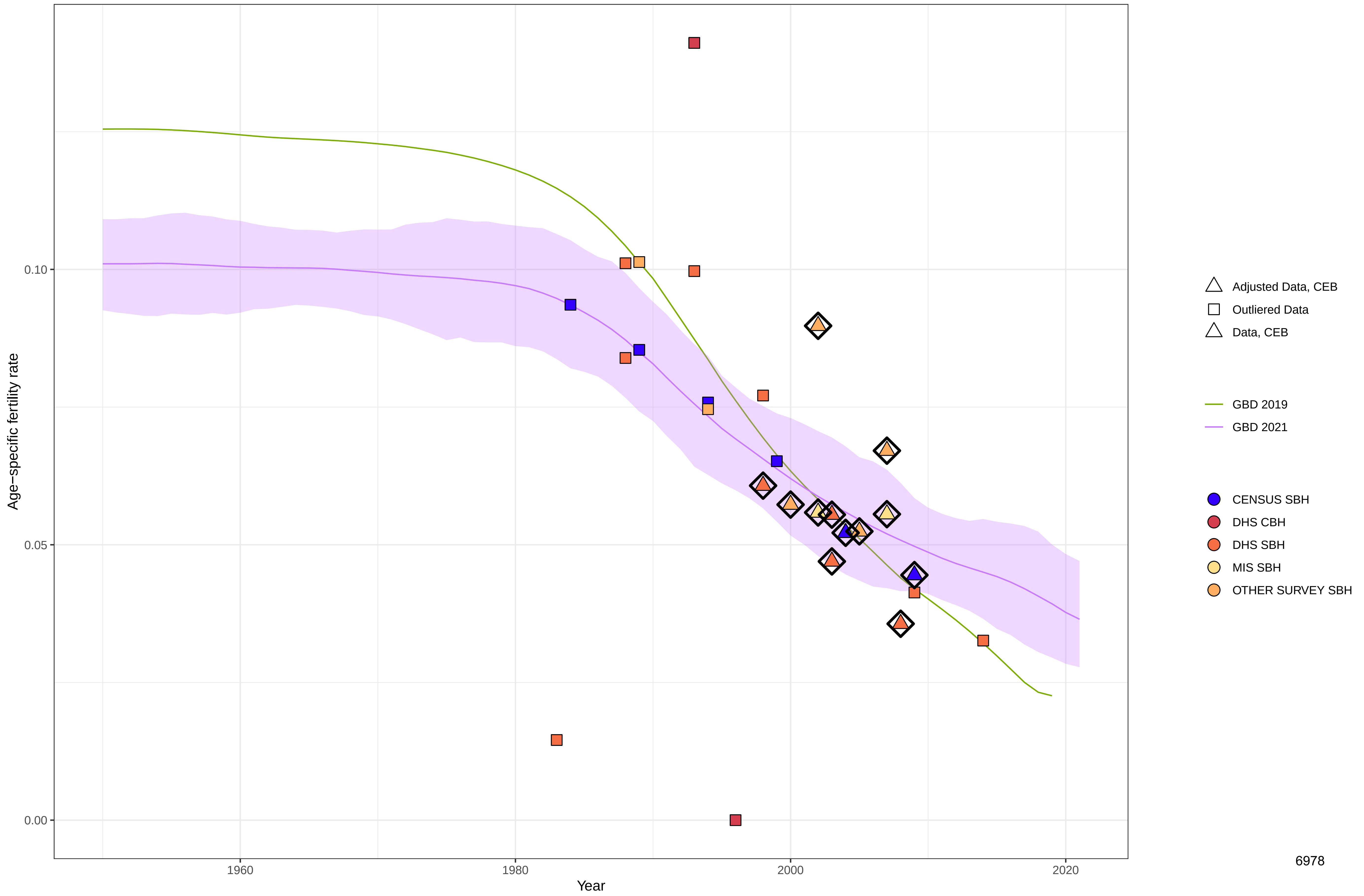

Age−specific fertility fitted trend lines, 1950−2021: Kajiado, Ages: 45−49
Data points enclosed with a diamond are reference sources

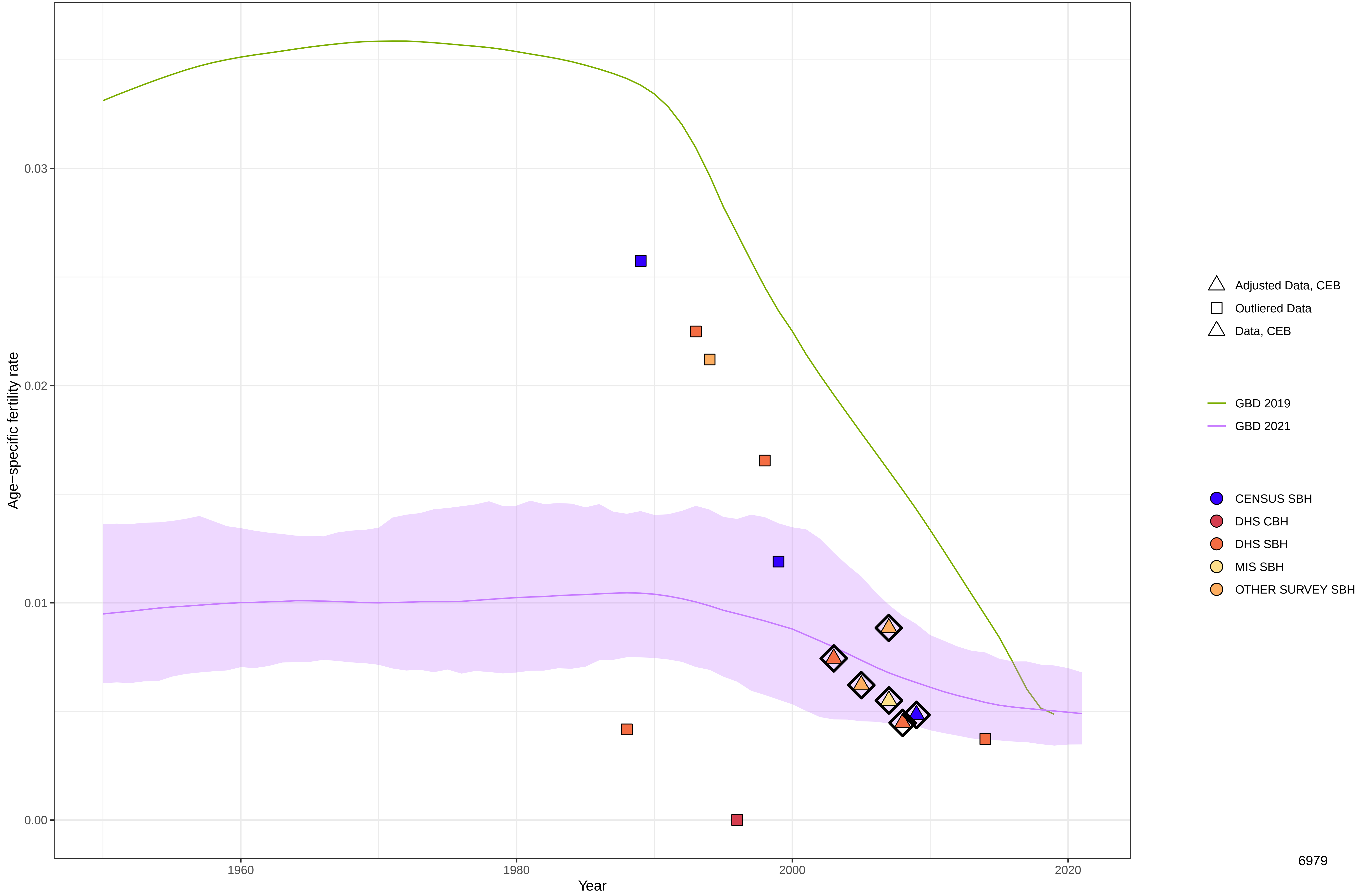

Age−specific fertility fitted trend lines, 1950−2021: Kajiado, Ages: 50−54
Data points enclosed with a diamond are reference sources

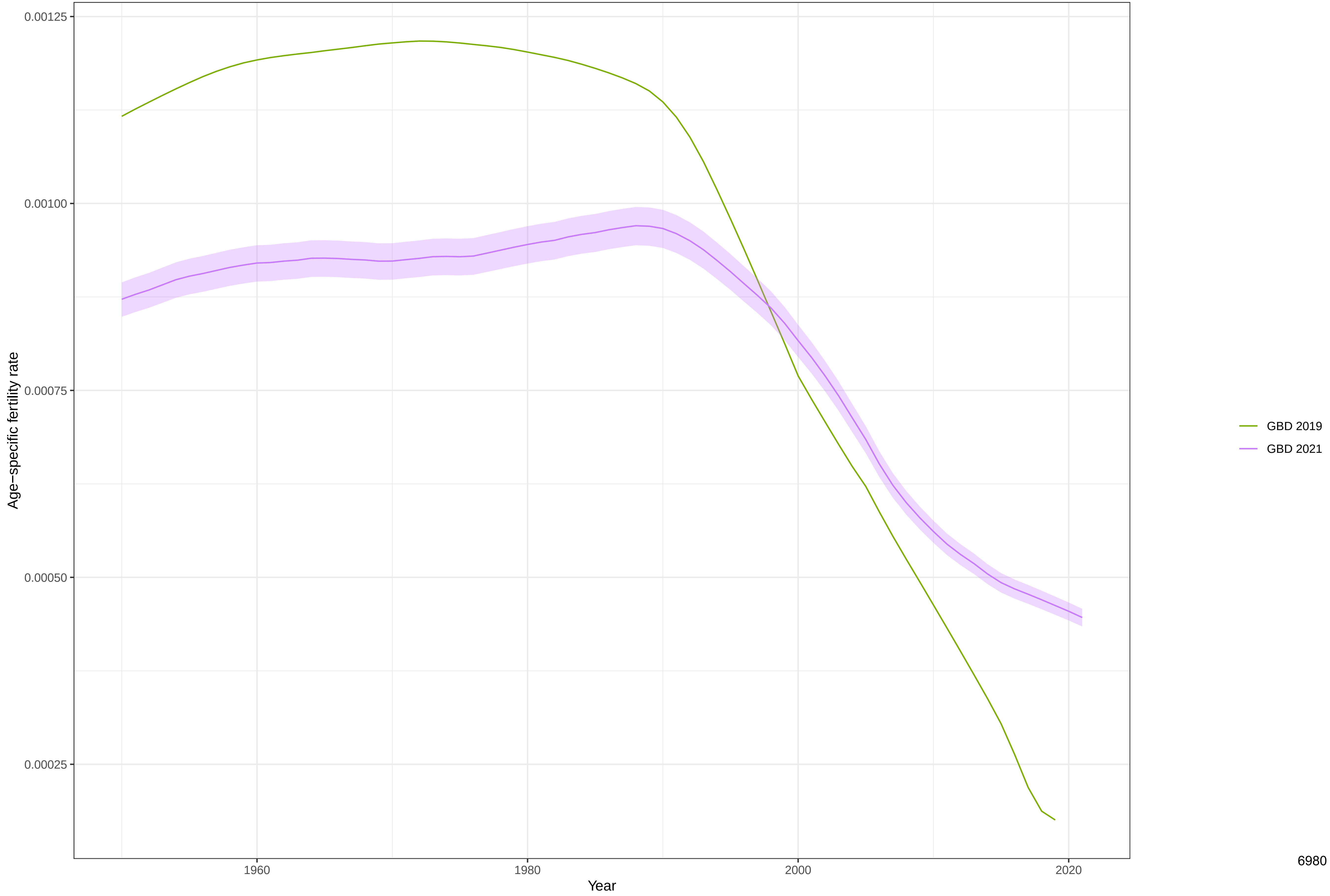

Total fertility rate fitted trend lines, 1950−2021: Kajiado TFR
Data points enclosed with a diamond are reference sources

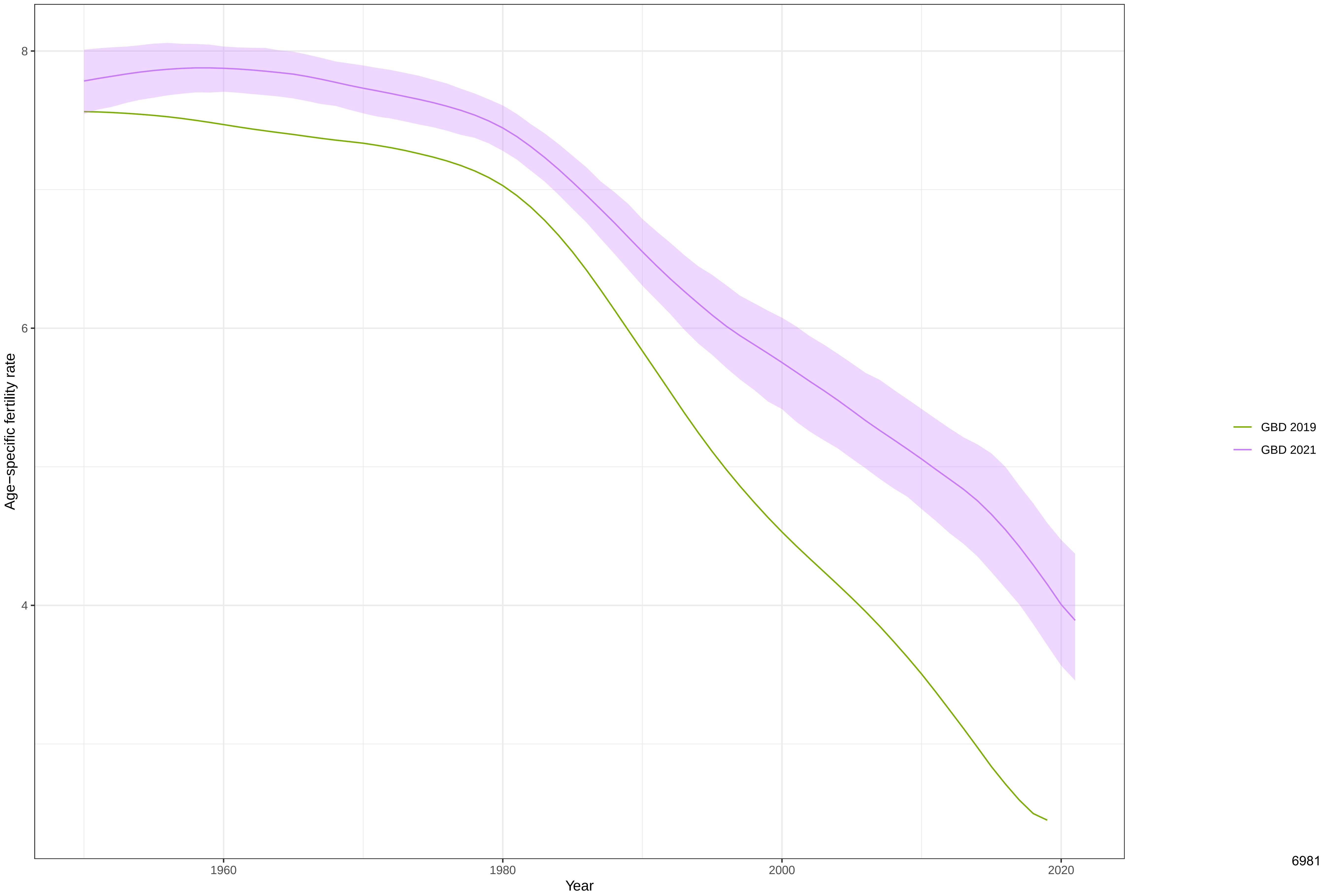

Age−specific fertility fitted trend lines, 1950−2021: Kakamega, Ages: 10−14
Data points enclosed with a diamond are reference sources

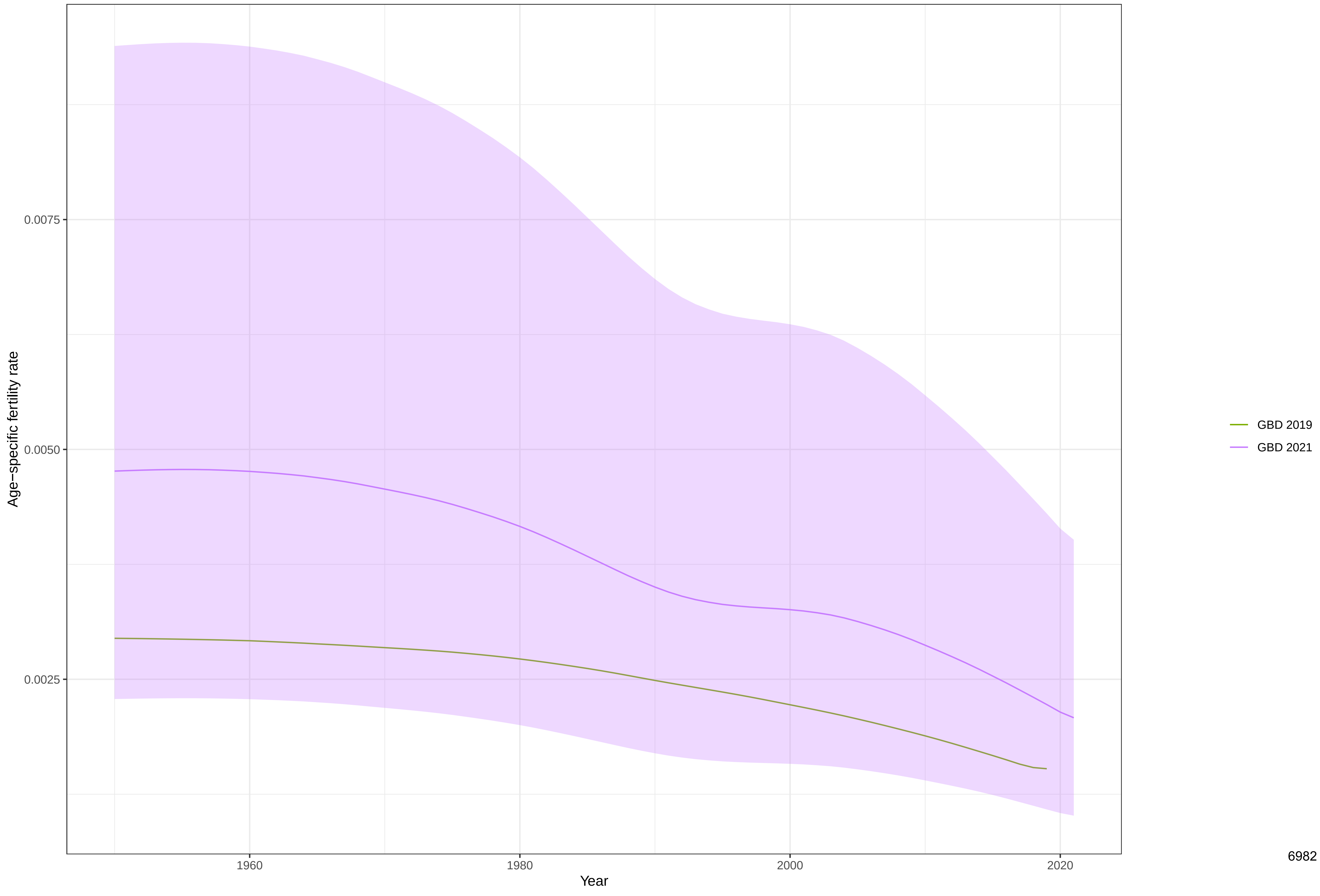

Age−specific fertility fitted trend lines, 1950−2021: Kakamega, Ages: 15−19
Data points enclosed with a diamond are reference sources

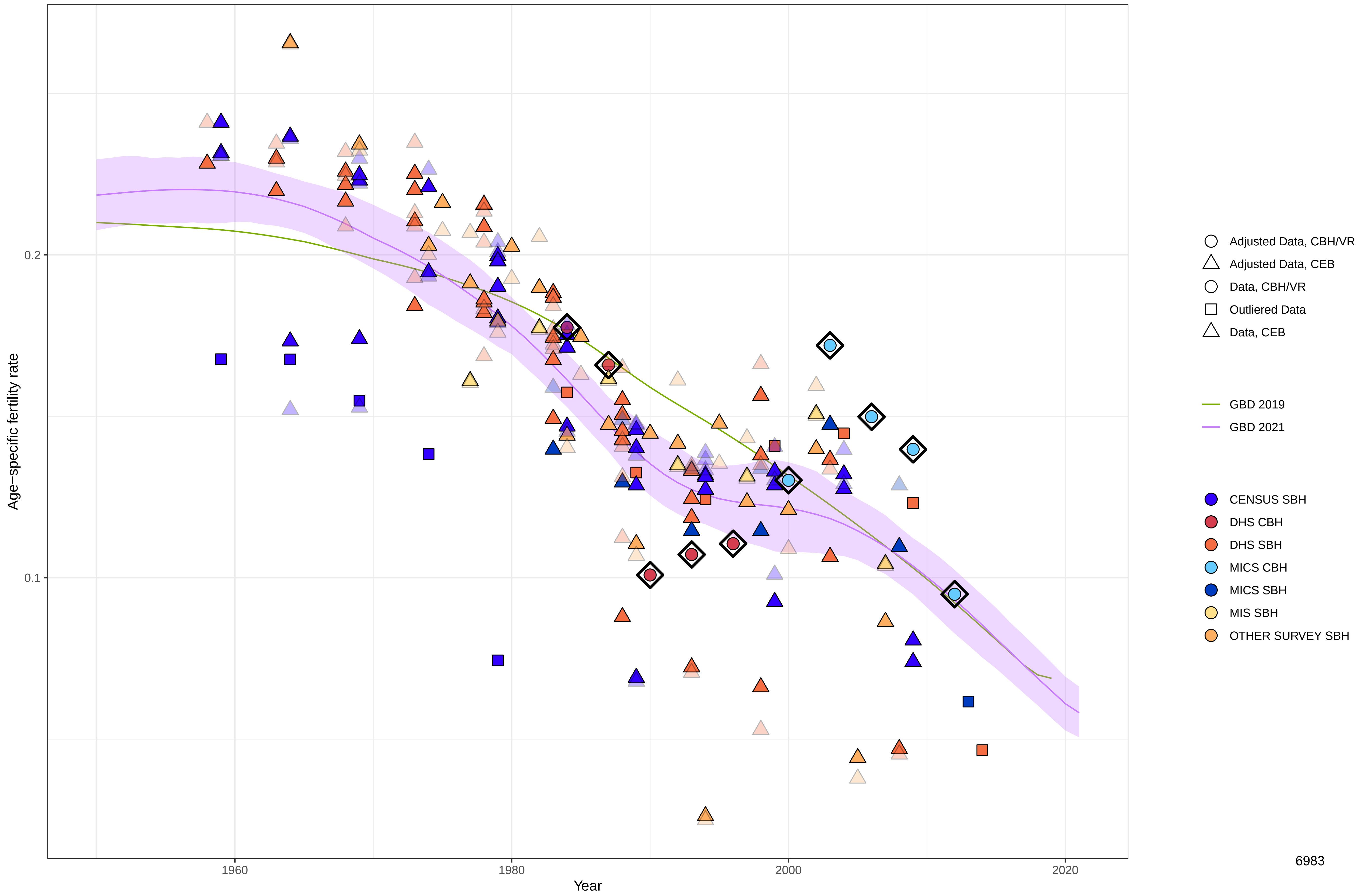

Age−specific fertility fitted trend lines, 1950−2021: Kakamega, Ages: 20−24
Data points enclosed with a diamond are reference sources

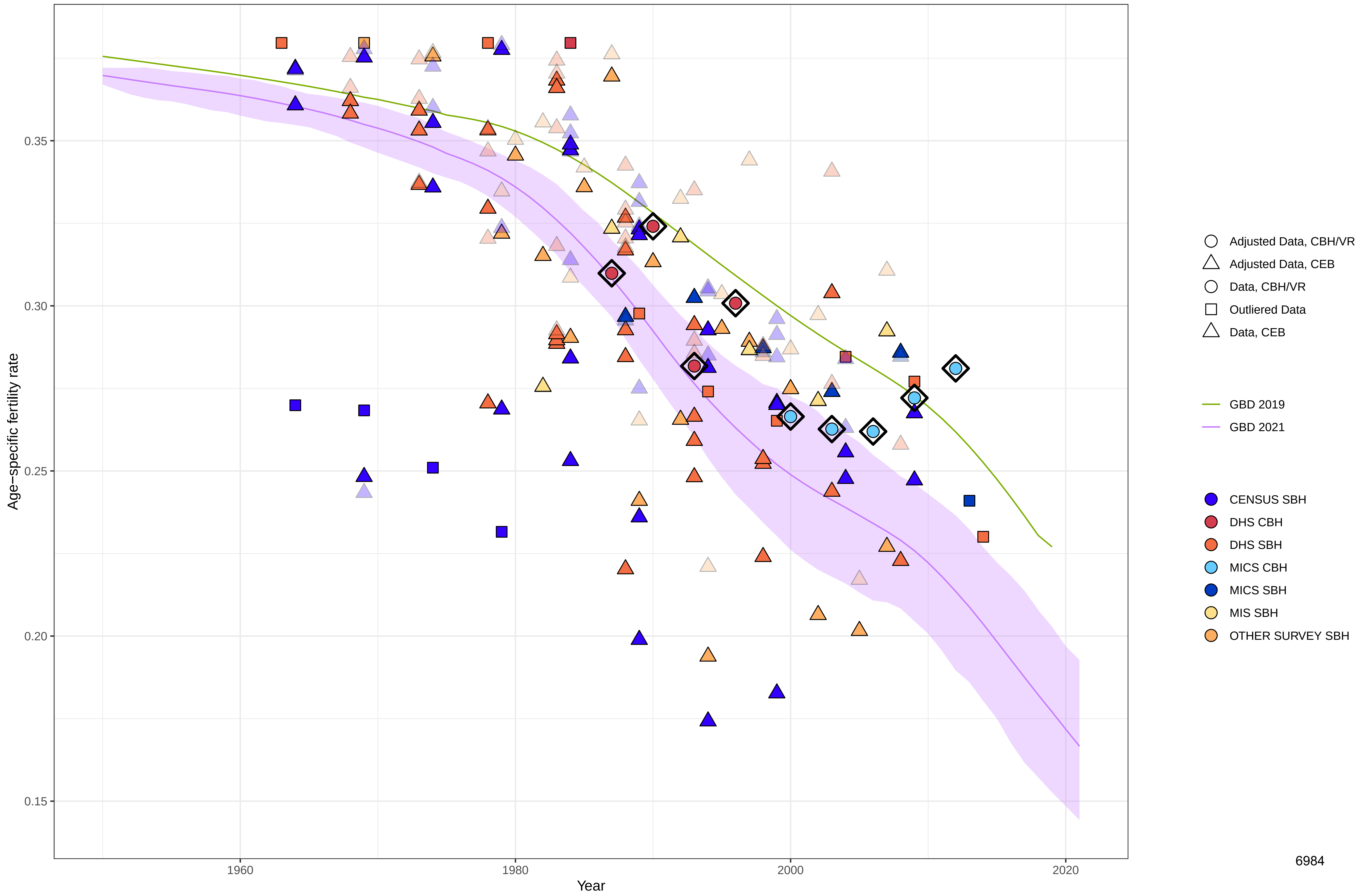

Age−specific fertility fitted trend lines, 1950−2021: Kakamega, Ages: 25−29
Data points enclosed with a diamond are reference sources

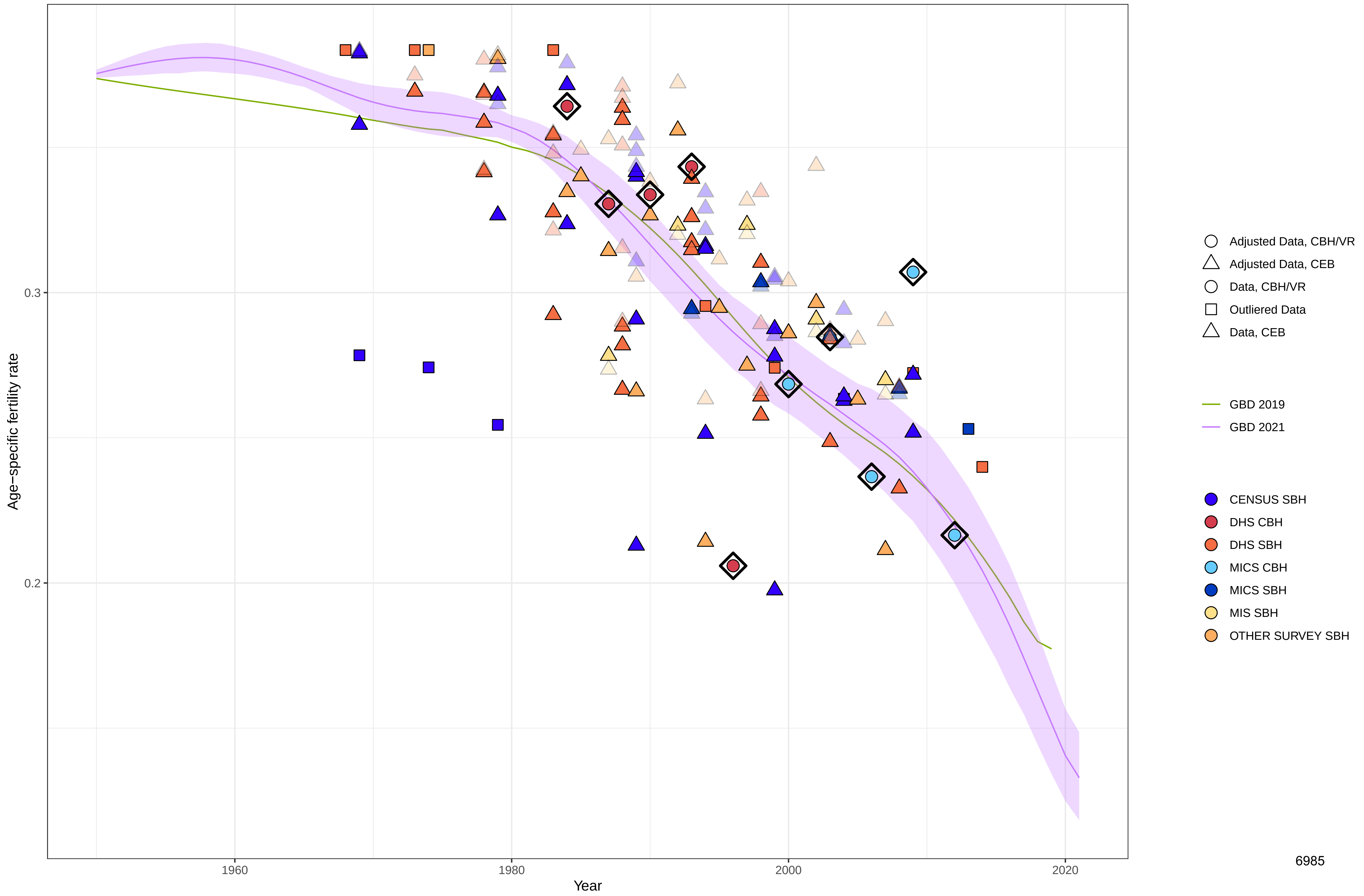

Age−specific fertility fitted trend lines, 1950−2021: Kakamega, Ages: 30−34
Data points enclosed with a diamond are reference sources

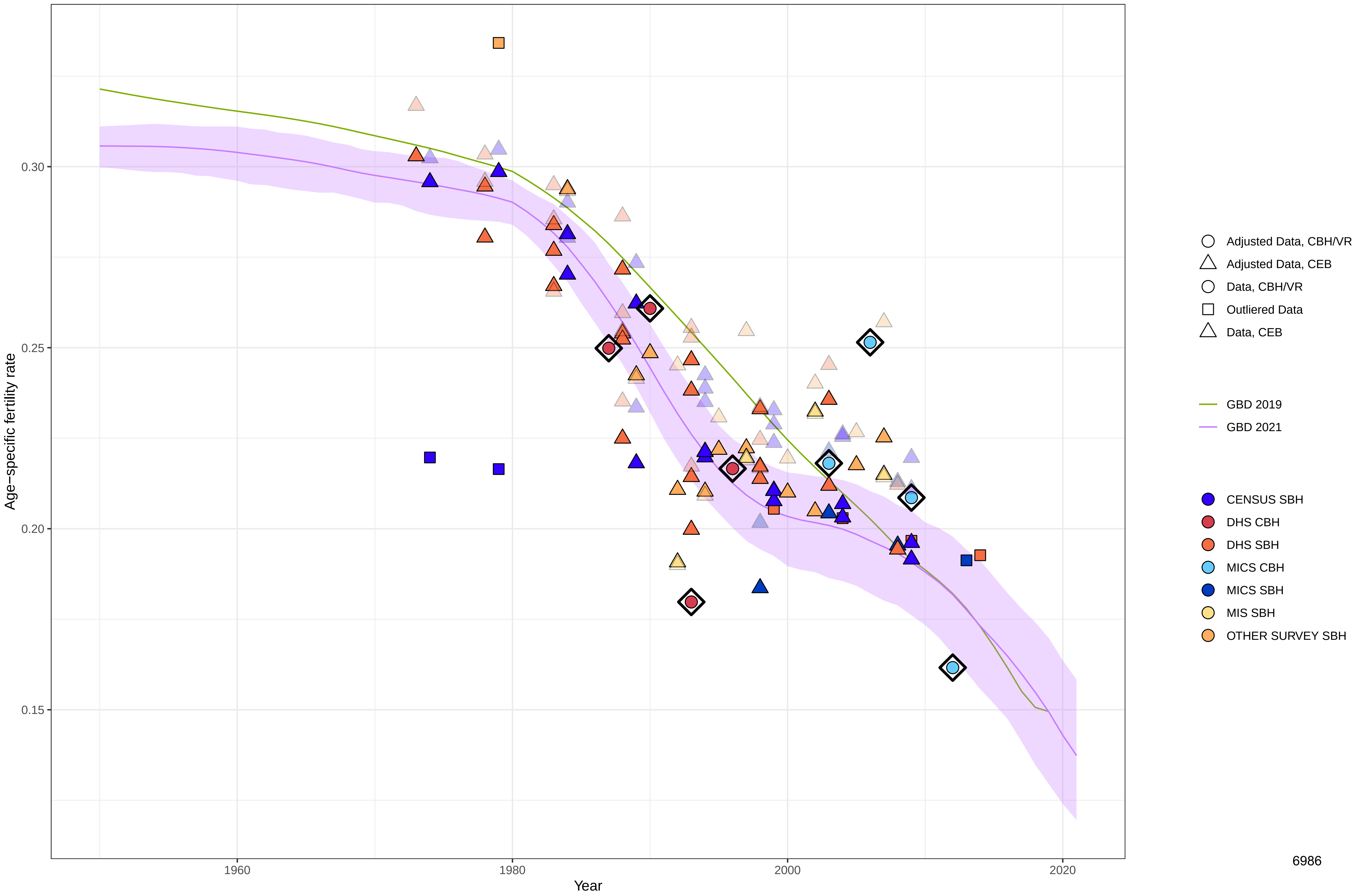

6986

Age−specific fertility fitted trend lines, 1950−2021: Kakamega, Ages: 35−39
Data points enclosed with a diamond are reference sources

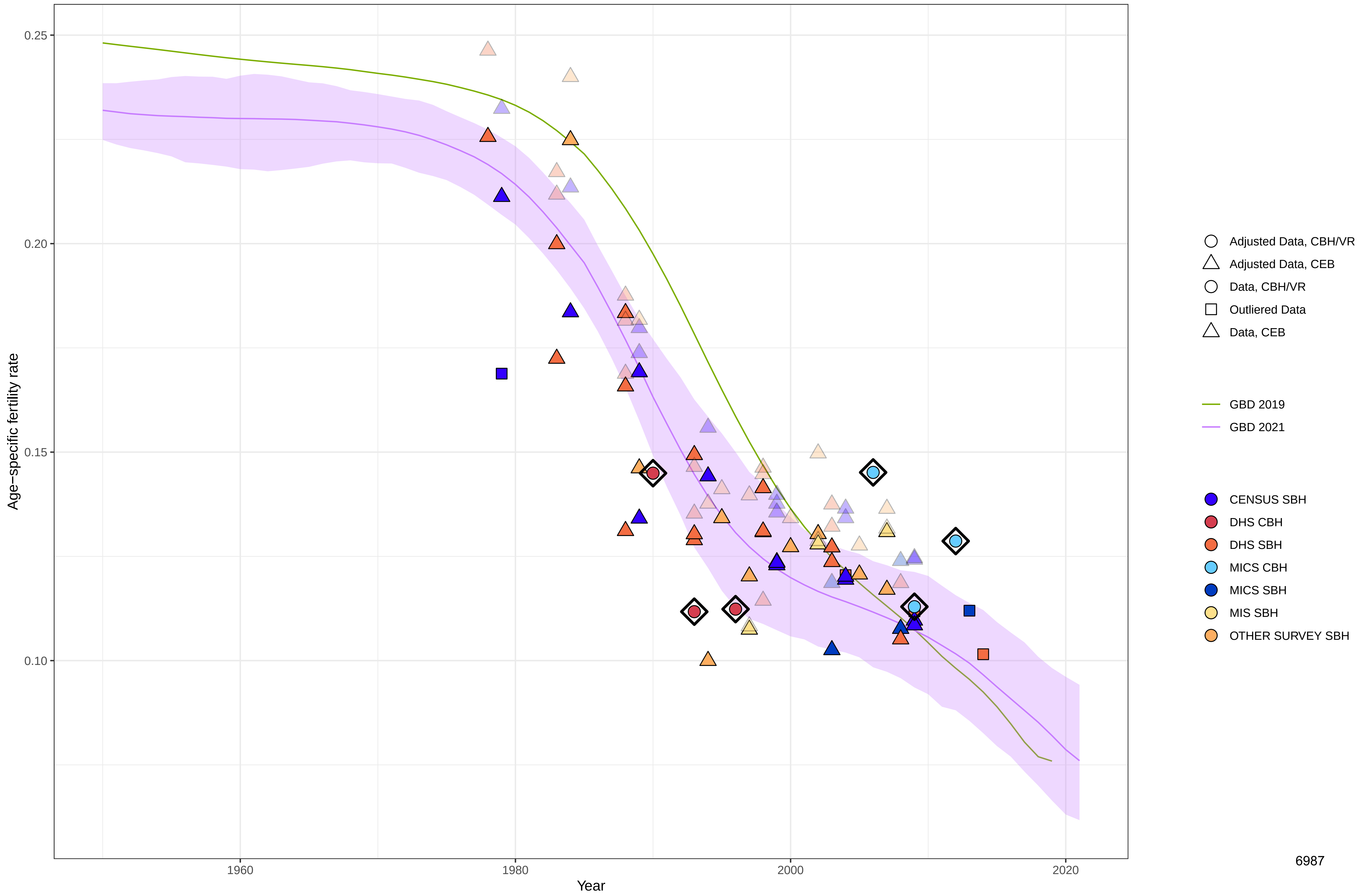

6987

Age−specific fertility fitted trend lines, 1950−2021: Kakamega, Ages: 40−44
Data points enclosed with a diamond are reference sources

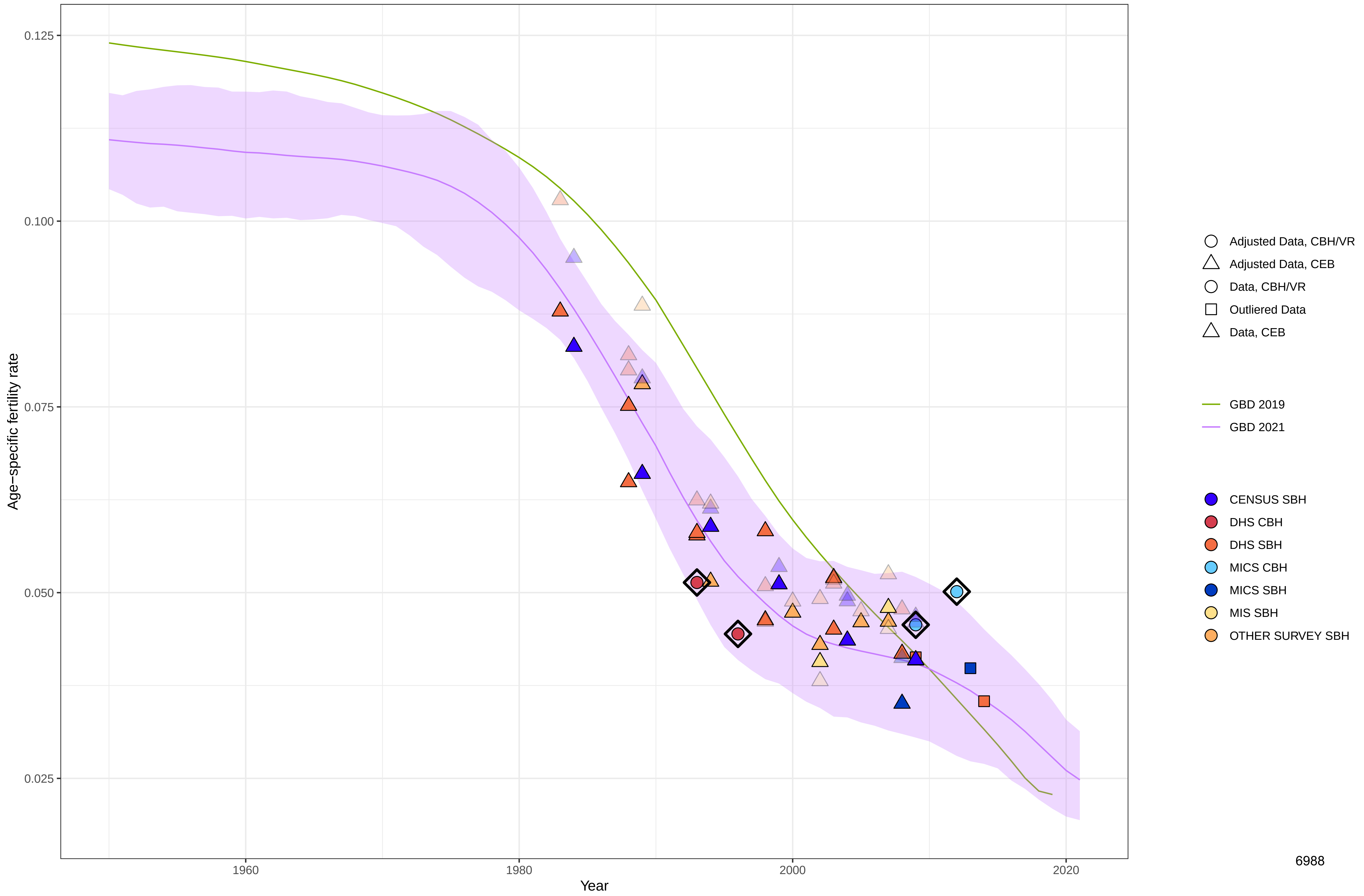

Age−specific fertility fitted trend lines, 1950−2021: Kakamega, Ages: 45−49
Data points enclosed with a diamond are reference sources

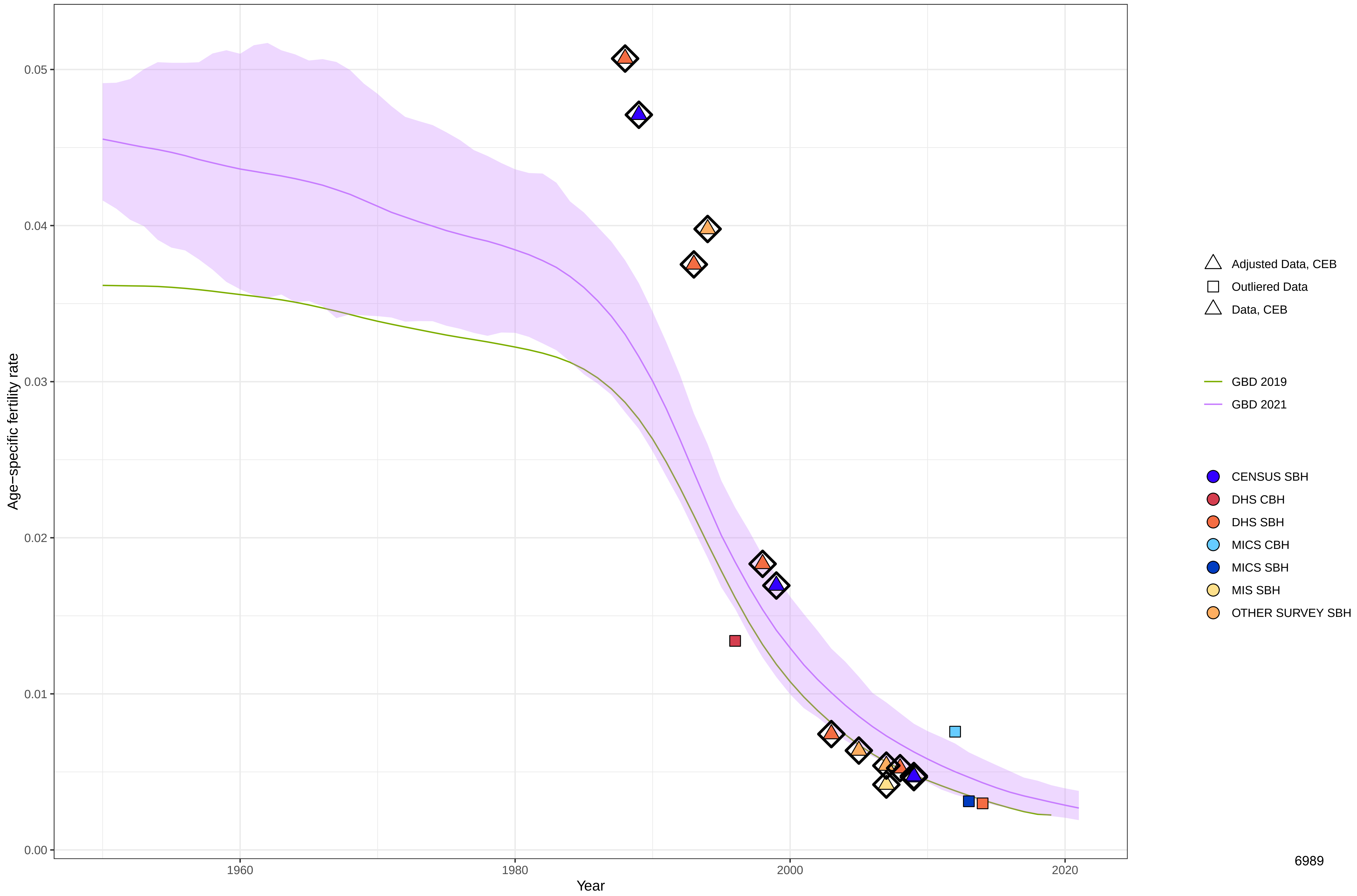

Age−specific fertility fitted trend lines, 1950−2021: Kakamega, Ages: 50−54
Data points enclosed with a diamond are reference sources

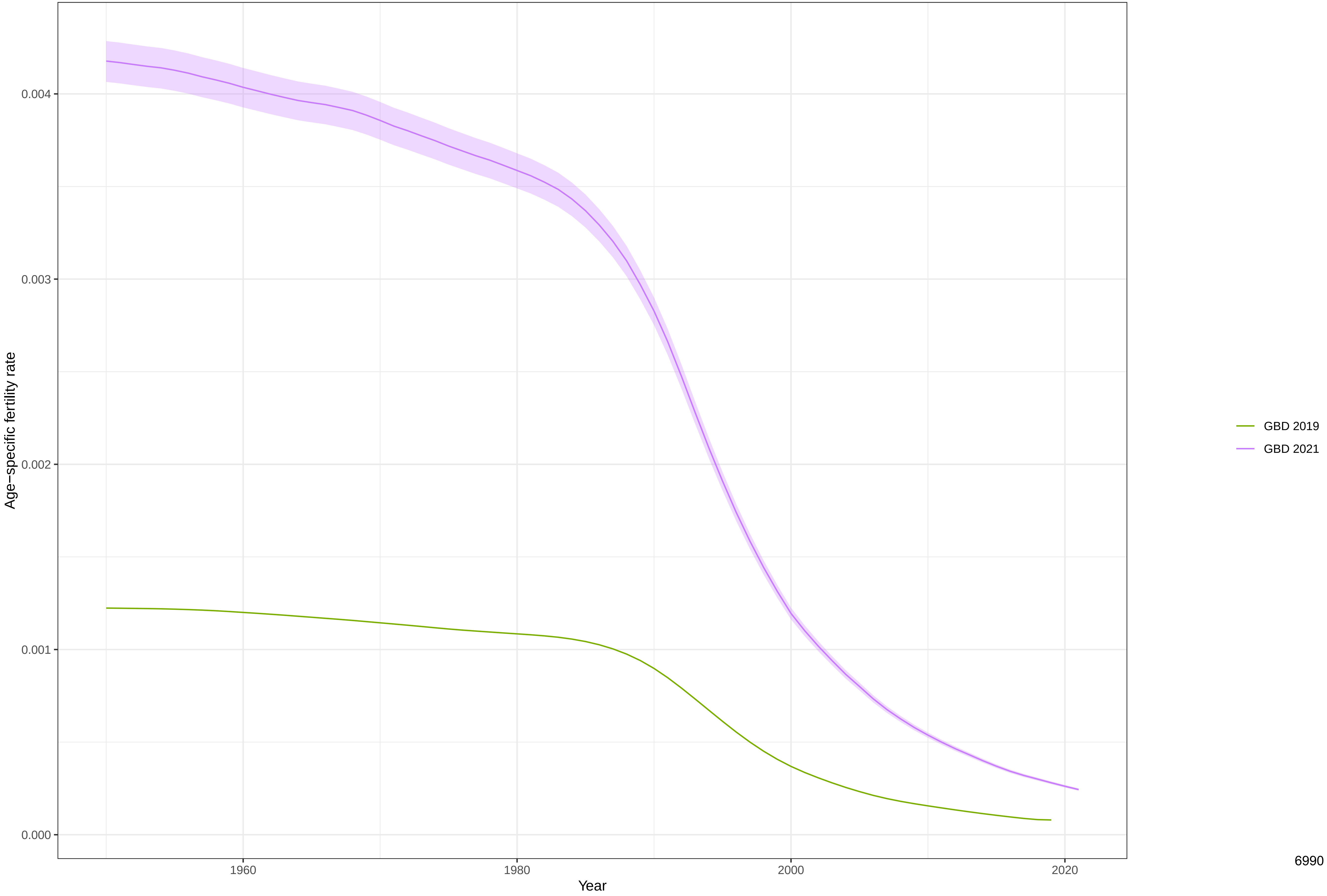

Total fertility rate fitted trend lines, 1950−2021: Kakamega TFR
Data points enclosed with a diamond are reference sources

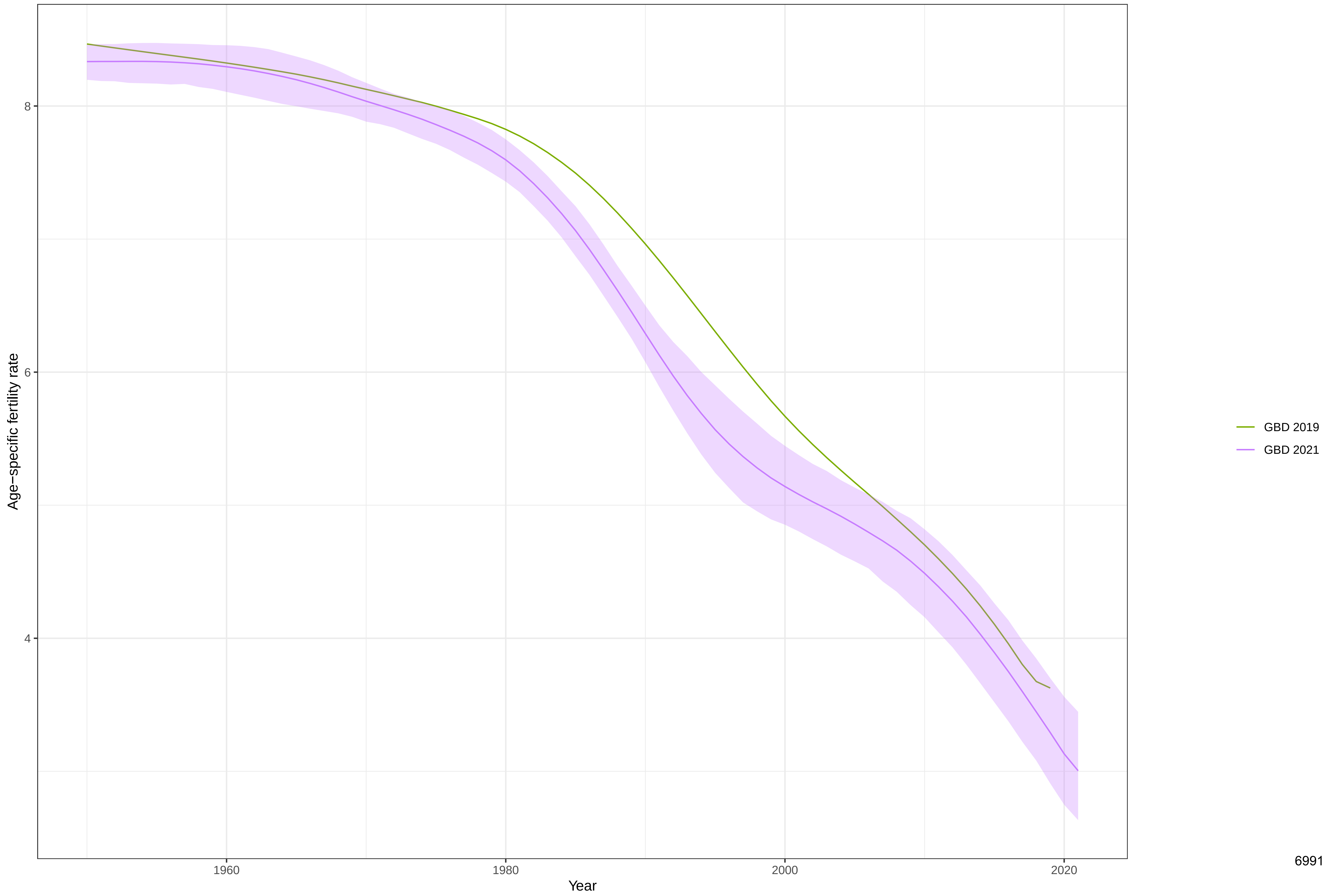

Age−specific fertility fitted trend lines, 1950−2021: Kericho, Ages: 10−14
Data points enclosed with a diamond are reference sources

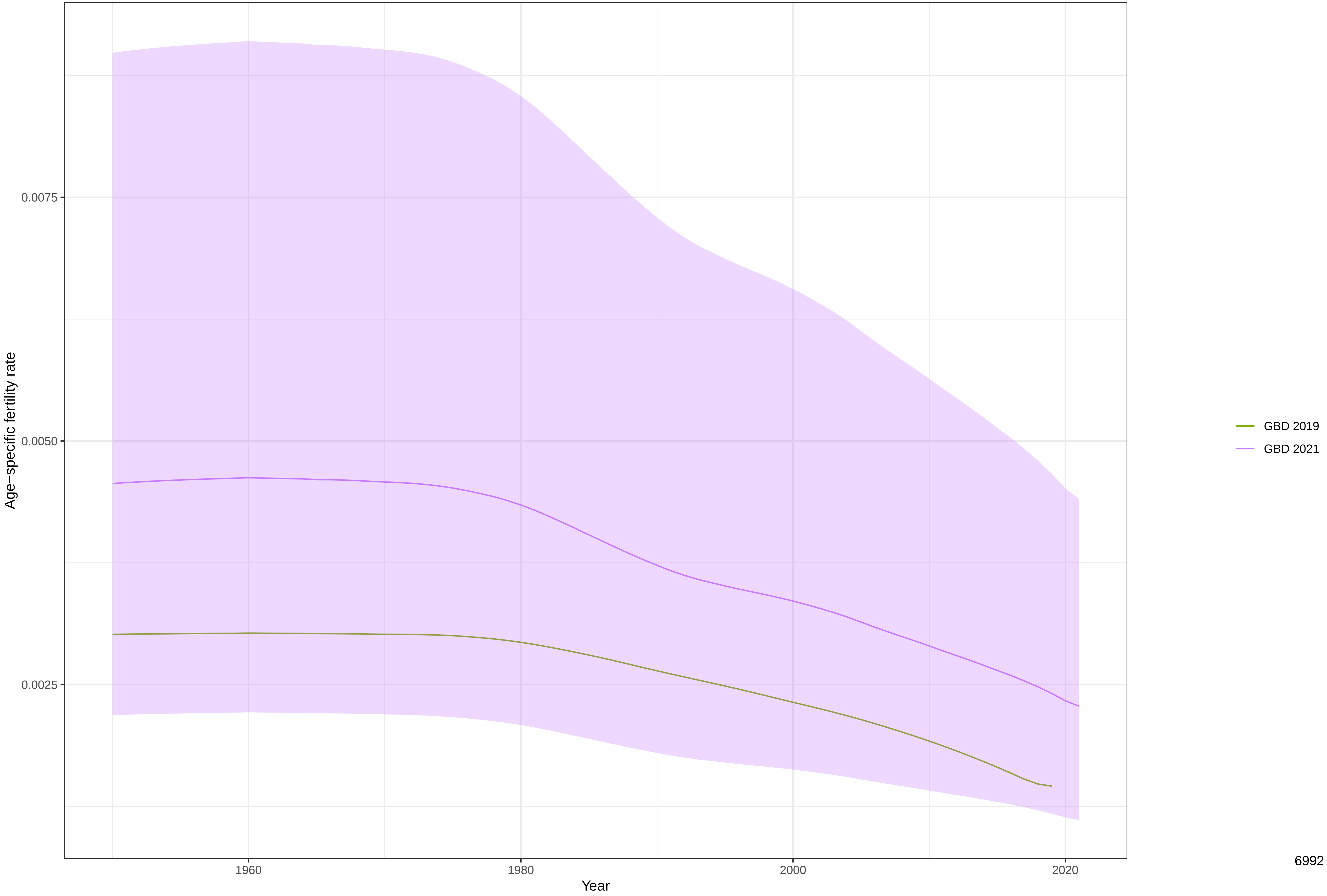

Age−specific fertility fitted trend lines, 1950−2021: Kericho, Ages: 15−19
Data points enclosed with a diamond are reference sources

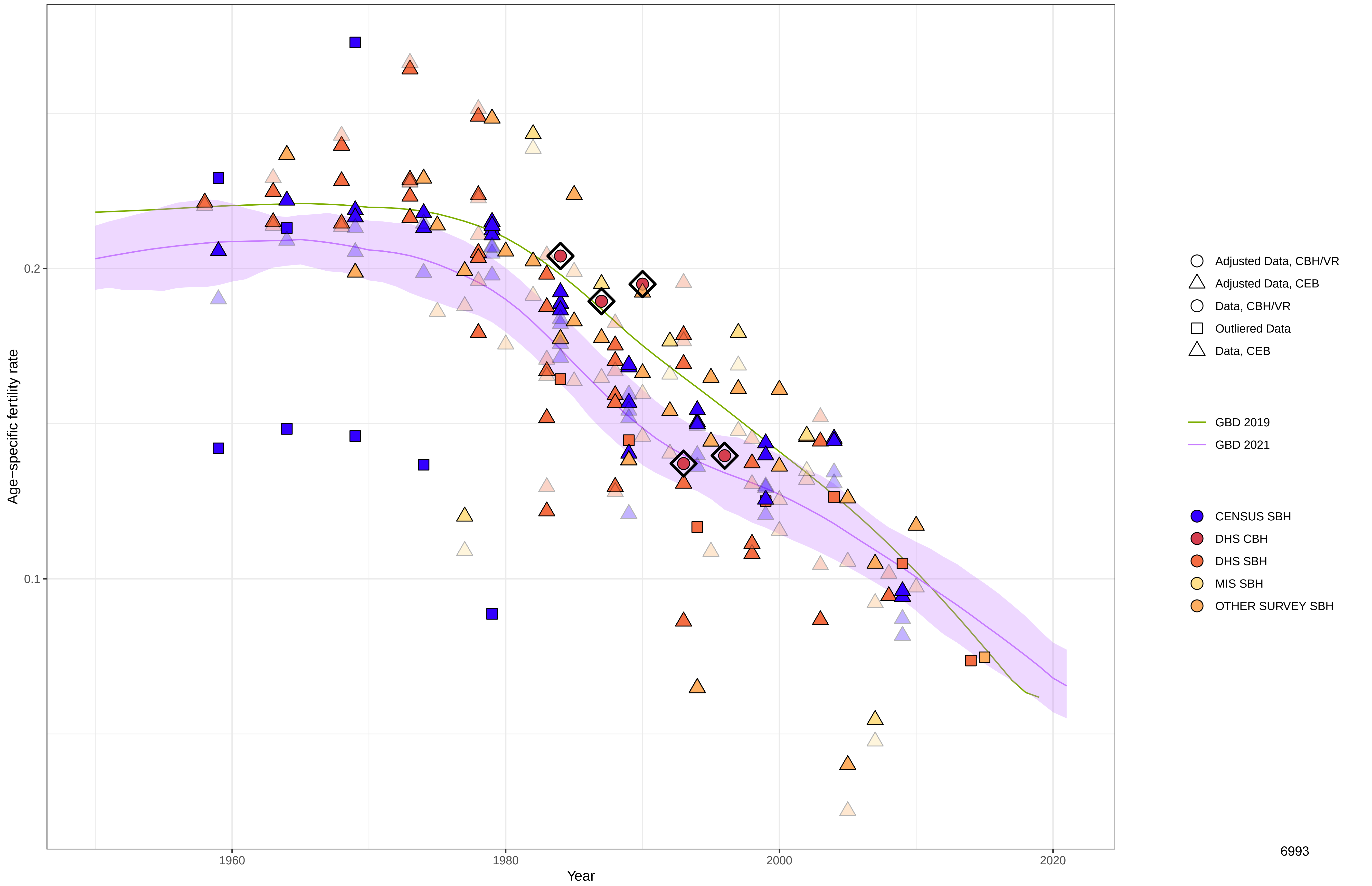

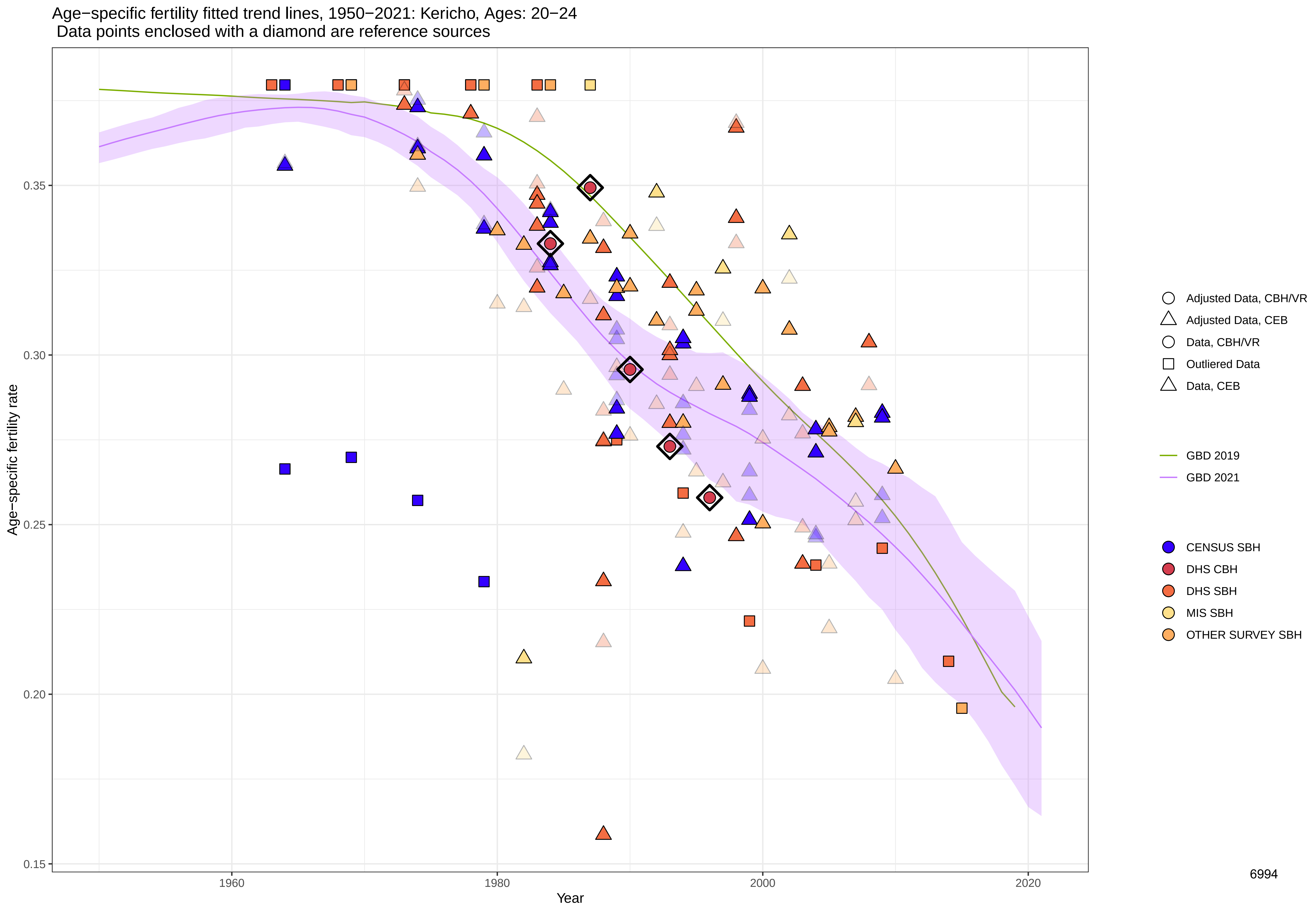

Age−specific fertility fitted trend lines, 1950−2021: Kericho, Ages: 20−24
Data points enclosed with a diamond are reference sources
Age−specific fertility rate
0.15
0.20
0.25
0.30
0.35
1960
1980
2000
2020
Year
Adjusted Data, CBH/VR
Adjusted Data, CEB
Data, CBH/VR
Outliered Data
Data, CEB
GBD 2019
GBD 2021
CENSUS SBH
DHS CBH
DHS SBH
MIS SBH
OTHER SURVEY SBH

Age−specific fertility fitted trend lines, 1950−2021: Kericho, Ages: 25−29
Data points enclosed with a diamond are reference sources

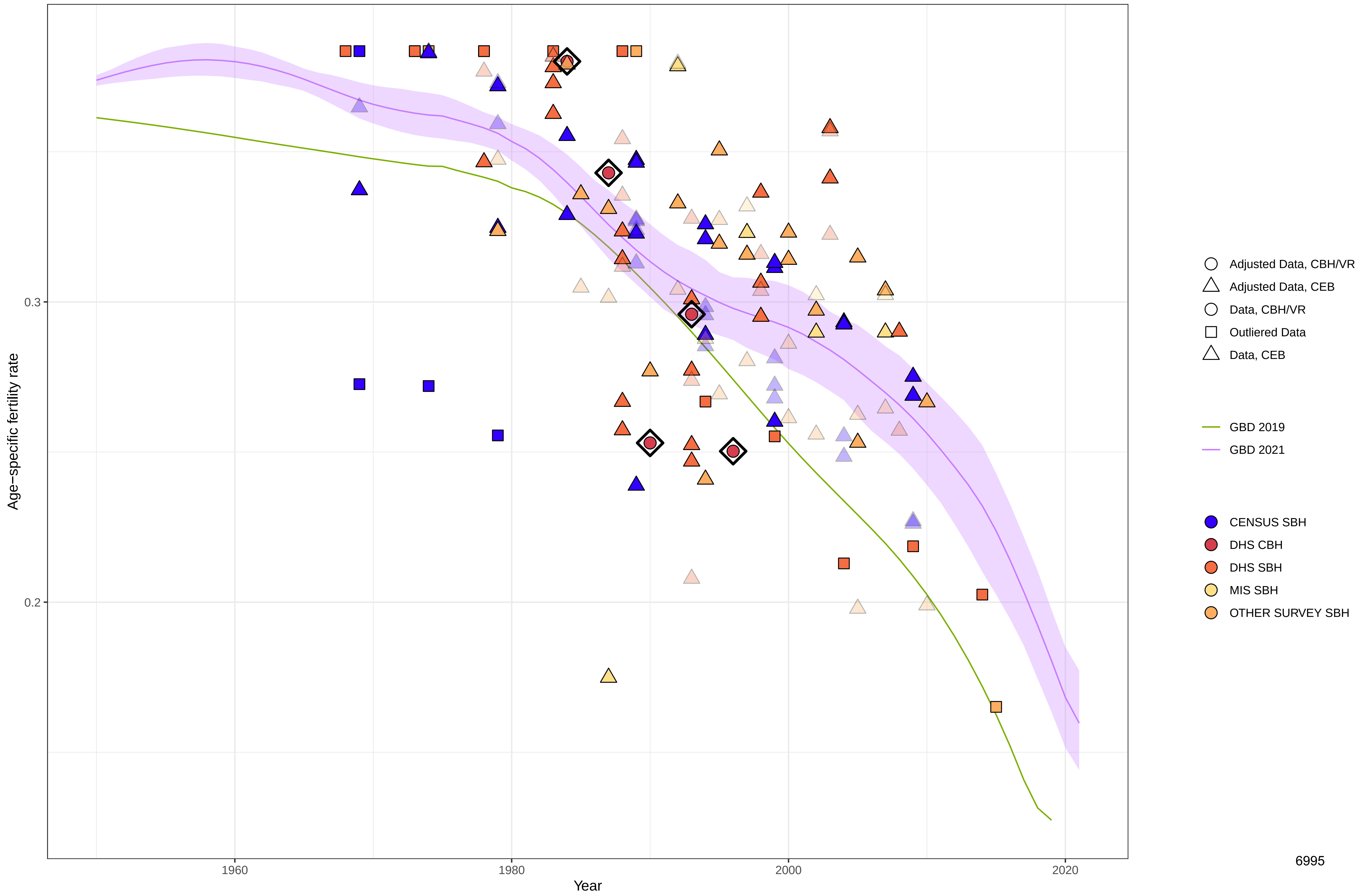

Age−specific fertility fitted trend lines, 1950−2021: Kericho, Ages: 30−34
Data points enclosed with a diamond are reference sources

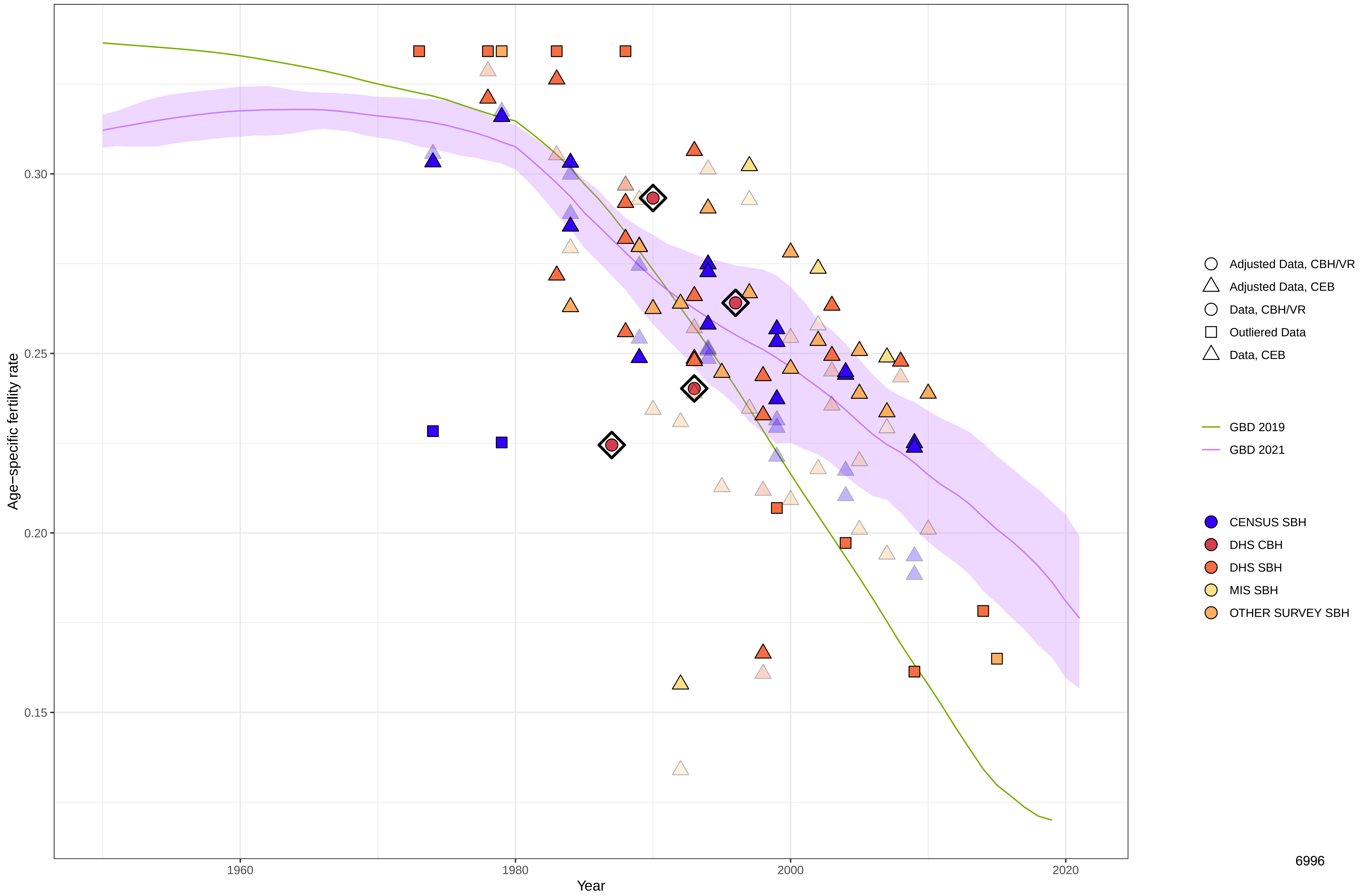

6996

Age−specific fertility fitted trend lines, 1950−2021: Kericho, Ages: 35−39
Data points enclosed with a diamond are reference sources

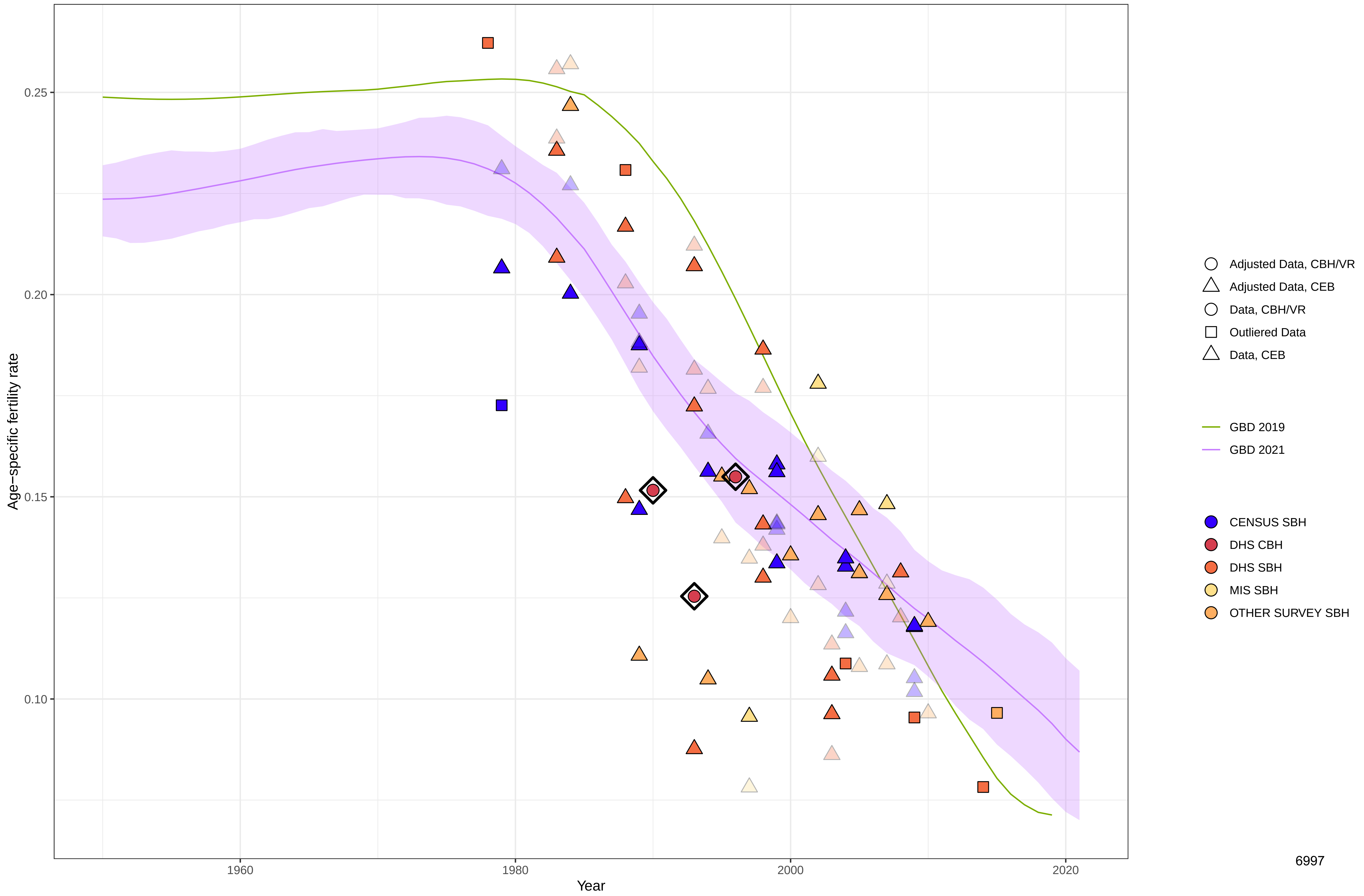

Age−specific fertility fitted trend lines, 1950−2021: Kericho, Ages: 40−44
Data points enclosed with a diamond are reference sources

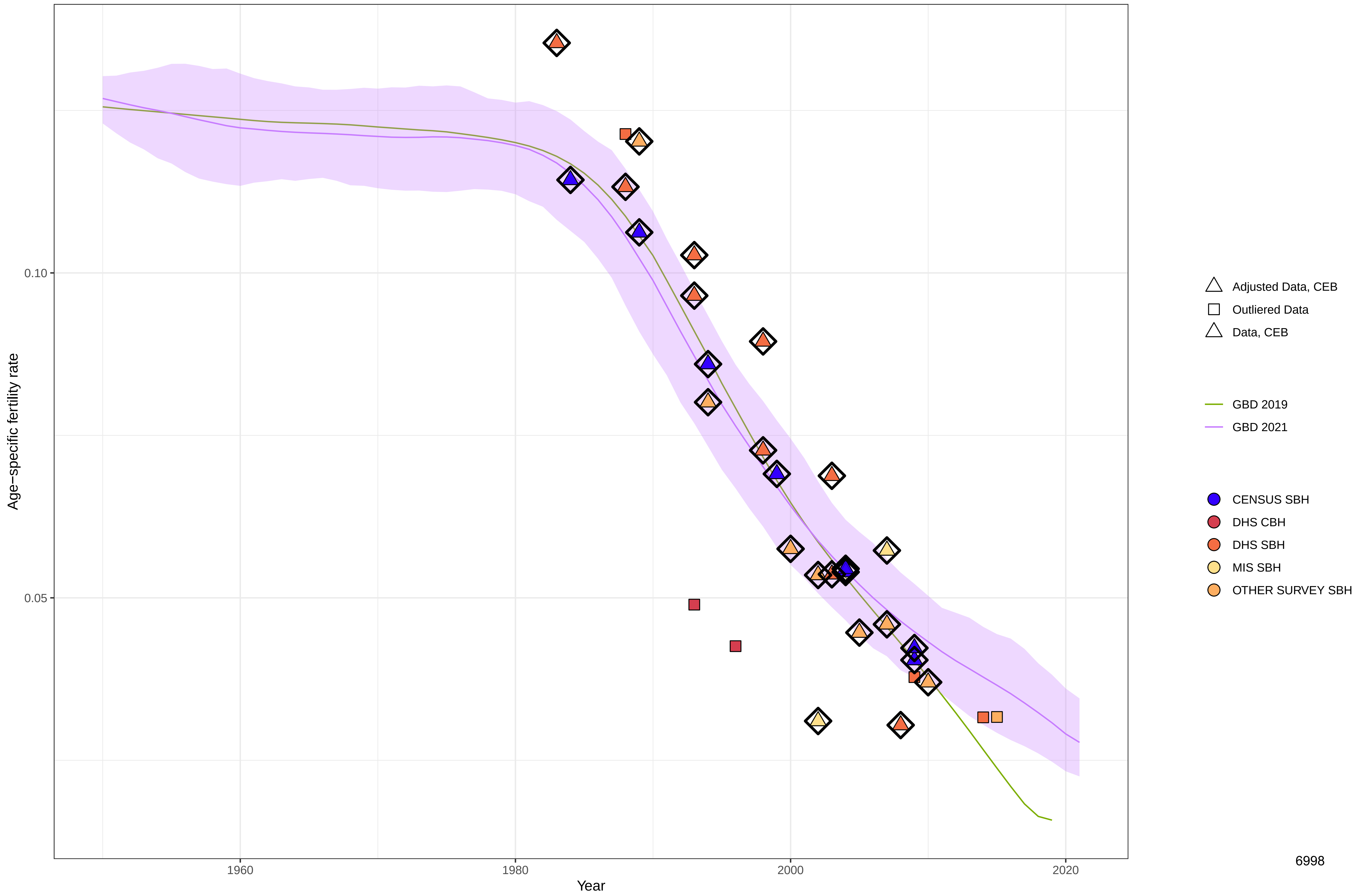

Age−specific fertility fitted trend lines, 1950−2021: Kericho, Ages: 45−49
Data points enclosed with a diamond are reference sources

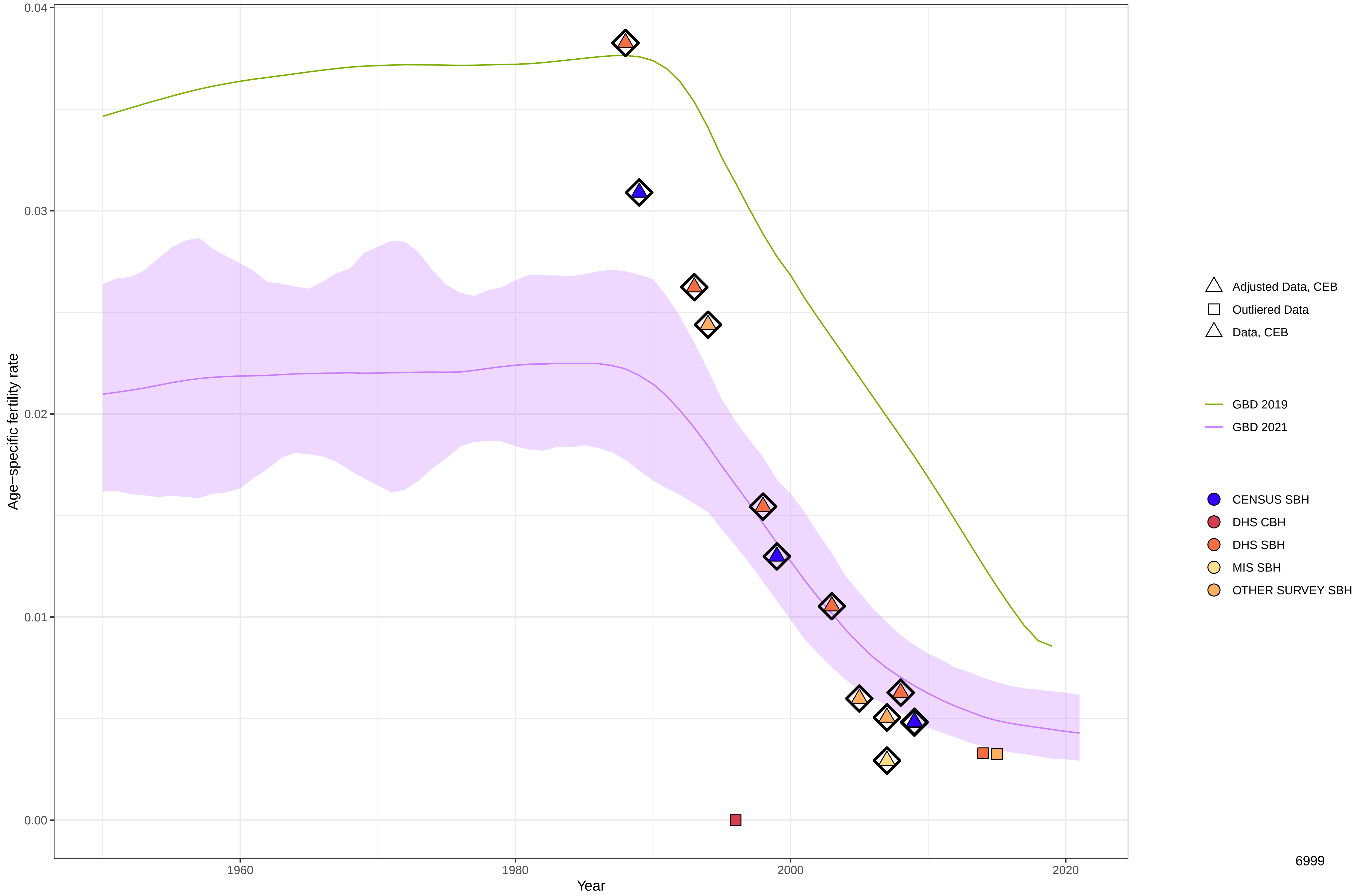

Age−specific fertility fitted trend lines, 1950−2021: Kericho, Ages: 50−54
Data points enclosed with a diamond are reference sources

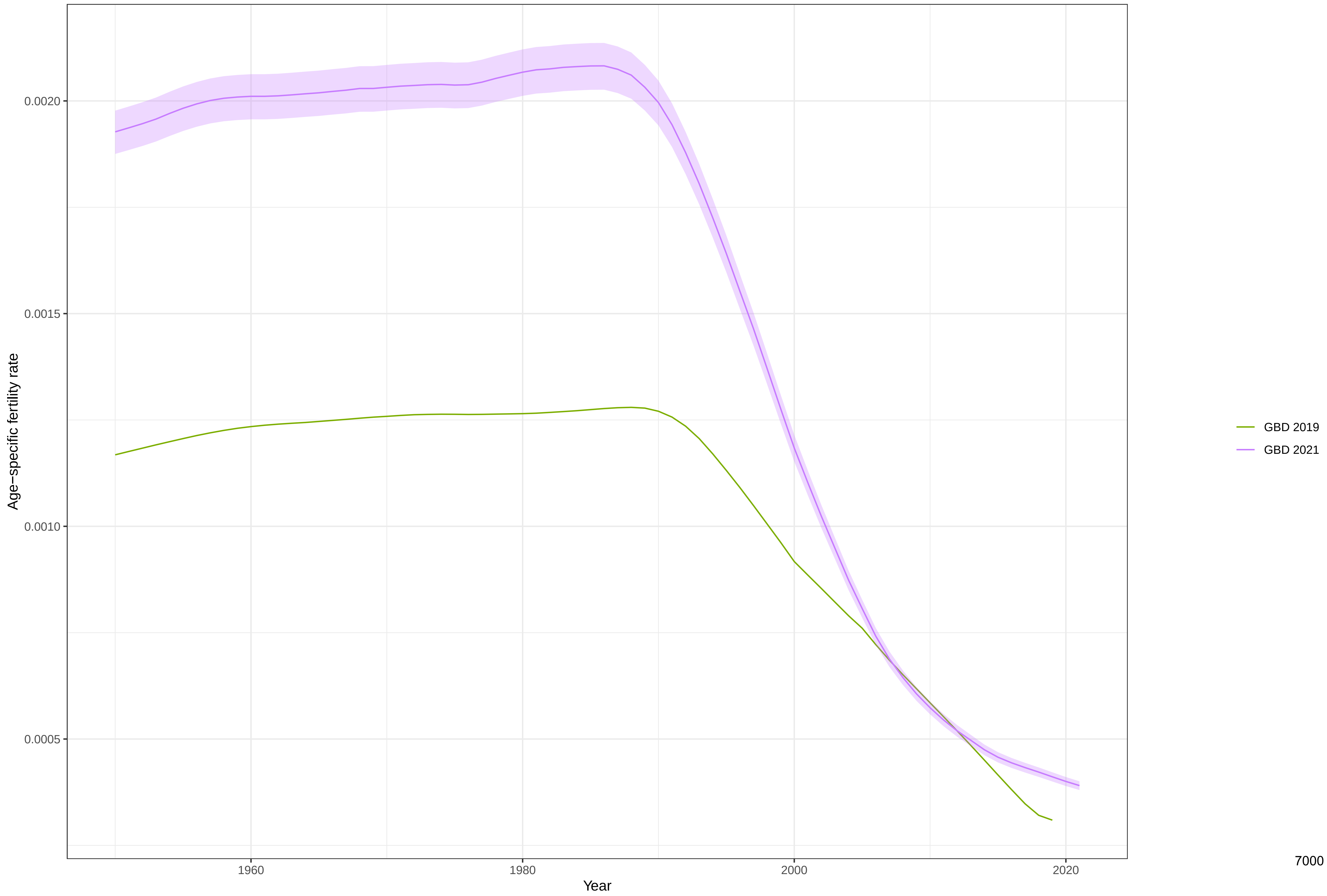

Total fertility rate fitted trend lines, 1950−2021: Kericho TFR
Data points enclosed with a diamond are reference sources

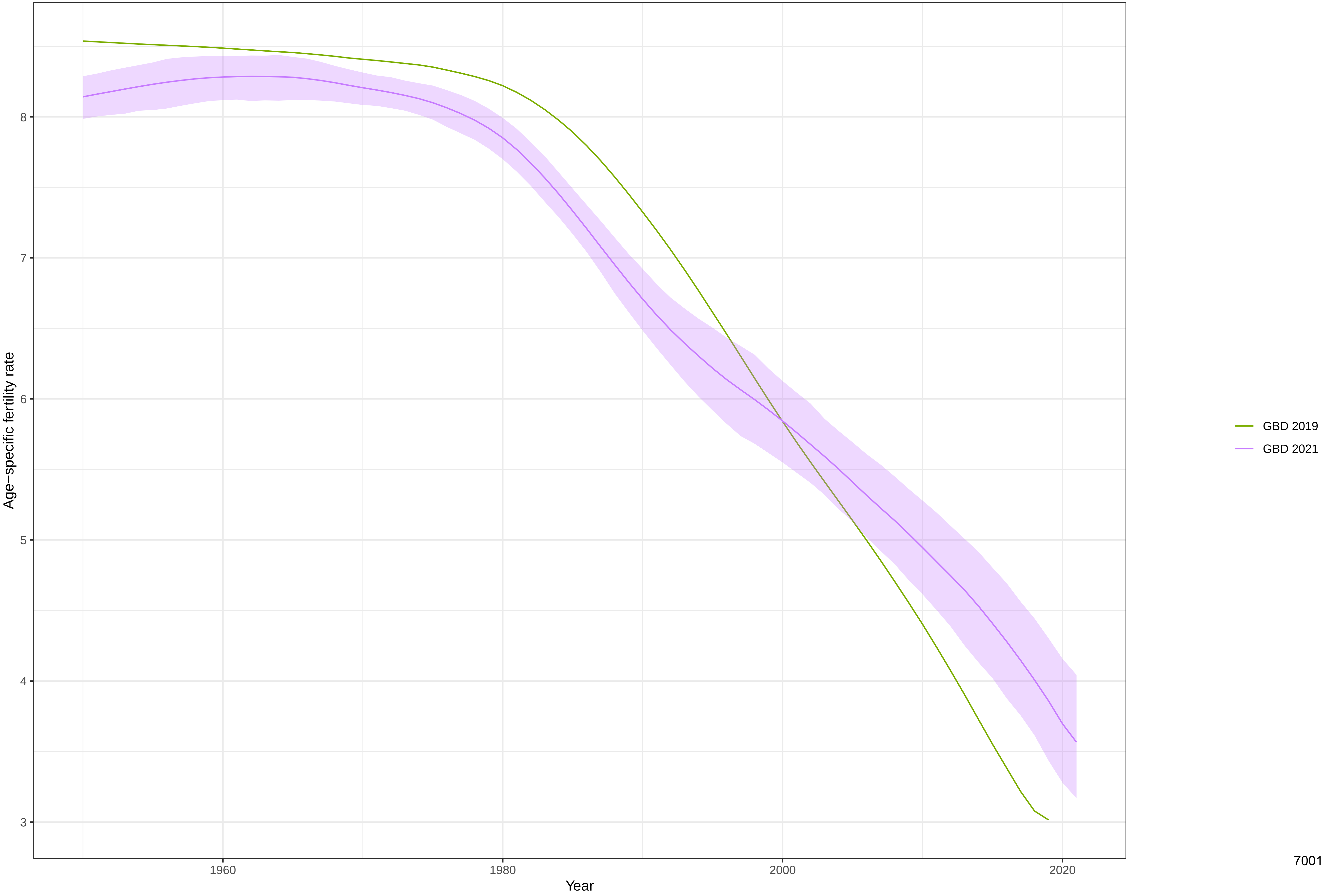

Age−specific fertility fitted trend lines, 1950−2021: Kiambu, Ages: 10−14
Data points enclosed with a diamond are reference sources

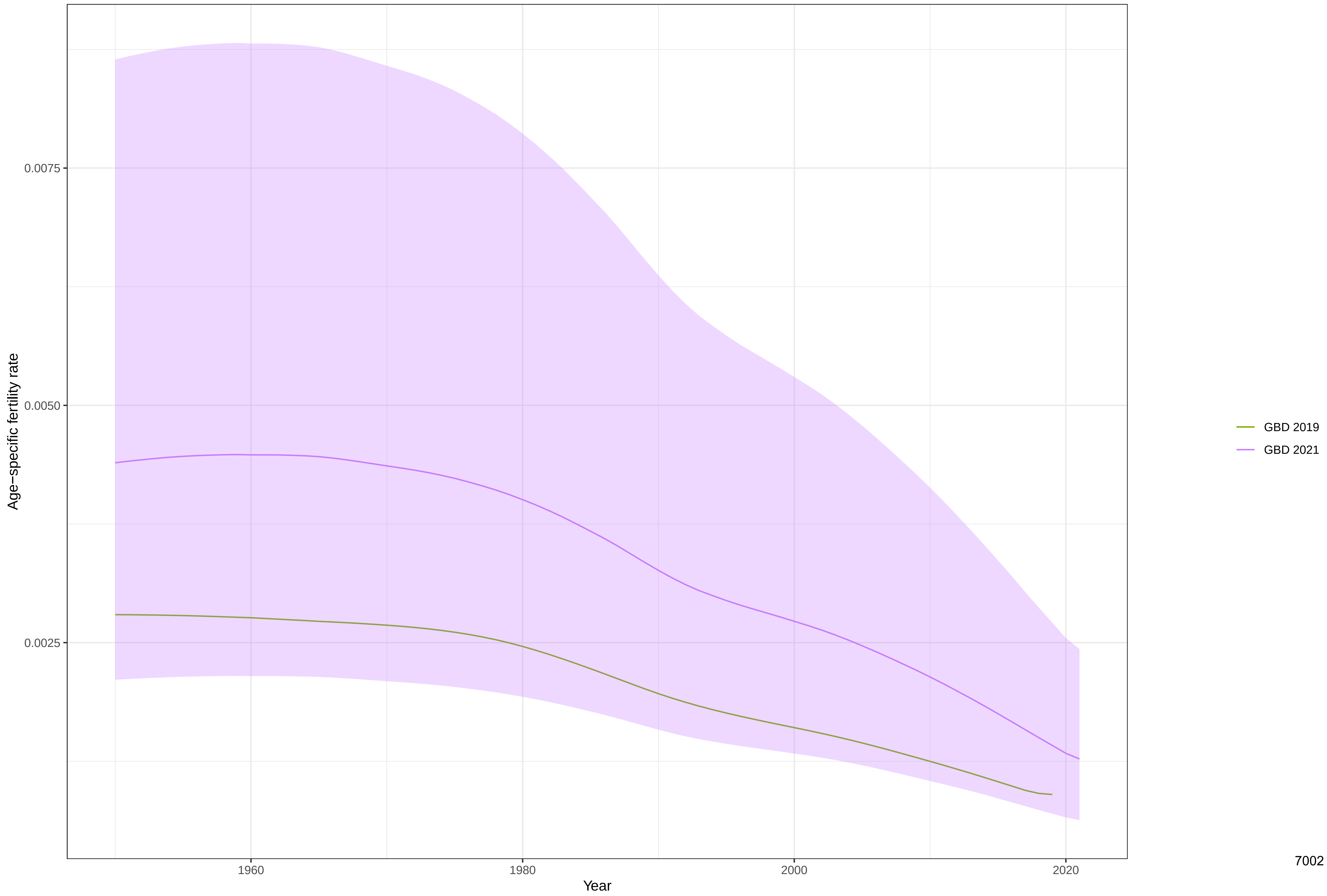

Age−specific fertility fitted trend lines, 1950−2021: Kiambu, Ages: 15−19
Data points enclosed with a diamond are reference sources

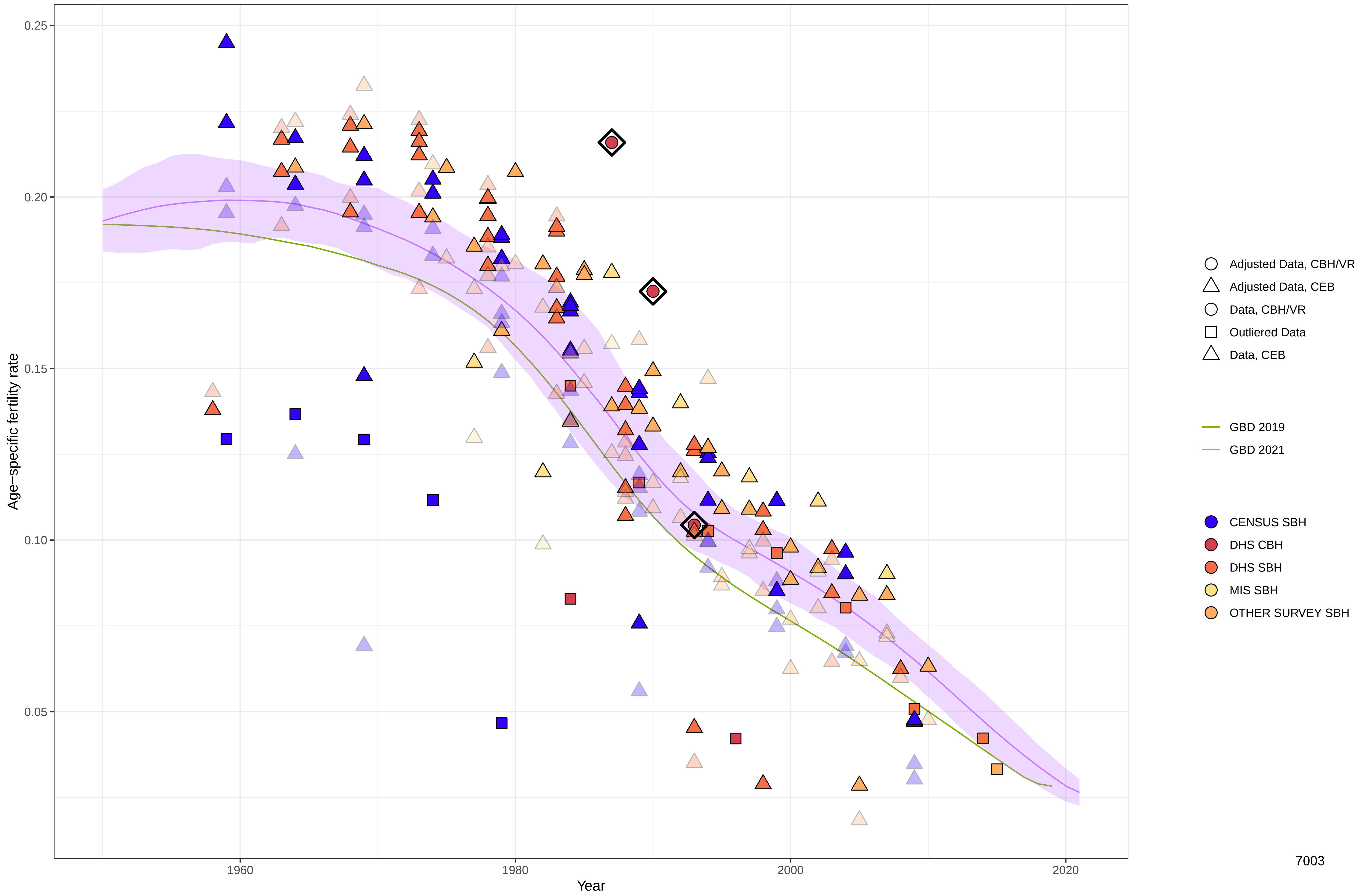

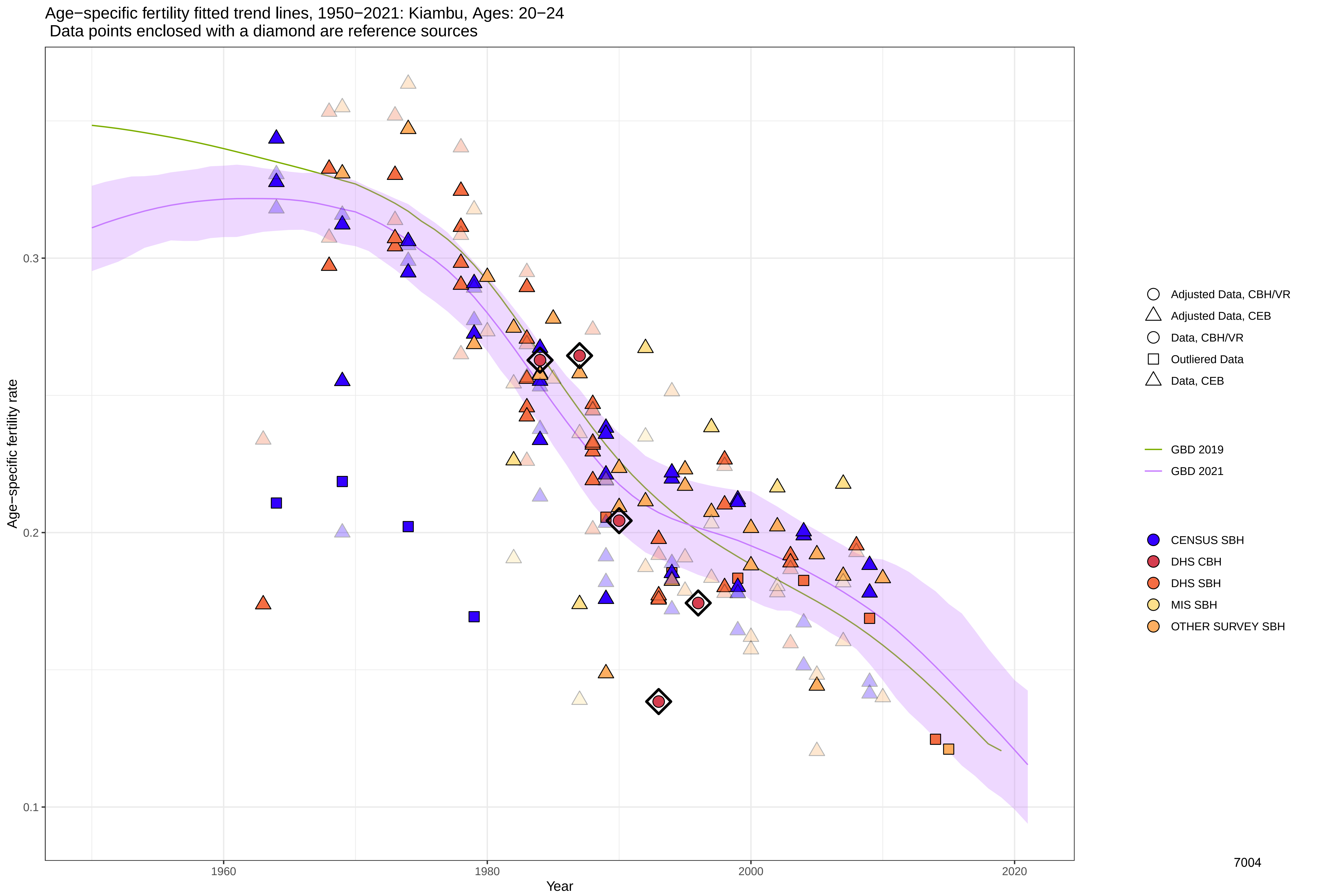

Age−specific fertility fitted trend lines, 1950−2021: Kiambu, Ages: 20−24
Data points enclosed with a diamond are reference sources
Age−specific fertility rate
Year
0.1
0.2
0.3
1960
1980
2000
2020
Adjusted Data, CBH/VR
Adjusted Data, CEB
Data, CBH/VR
Outliered Data
Data, CEB
GBD 2019
GBD 2021
CENSUS SBH
DHS CBH
DHS SBH
MIS SBH
OTHER SURVEY SBH

Age−specific fertility fitted trend lines, 1950−2021: Kiambu, Ages: 25−29
Data points enclosed with a diamond are reference sources

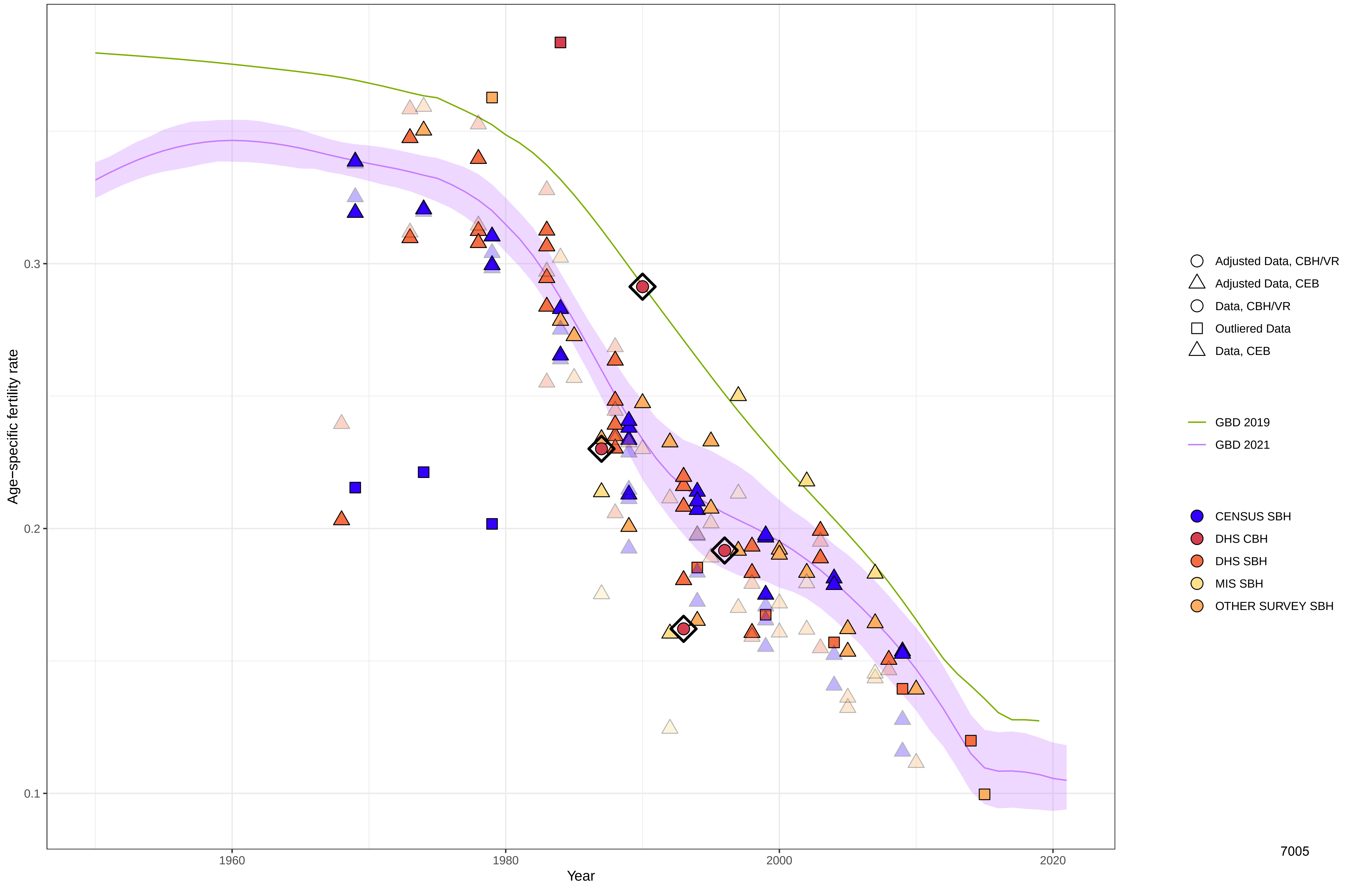

Age−specific fertility fitted trend lines, 1950−2021: Kiambu, Ages: 30−34
Data points enclosed with a diamond are reference sources

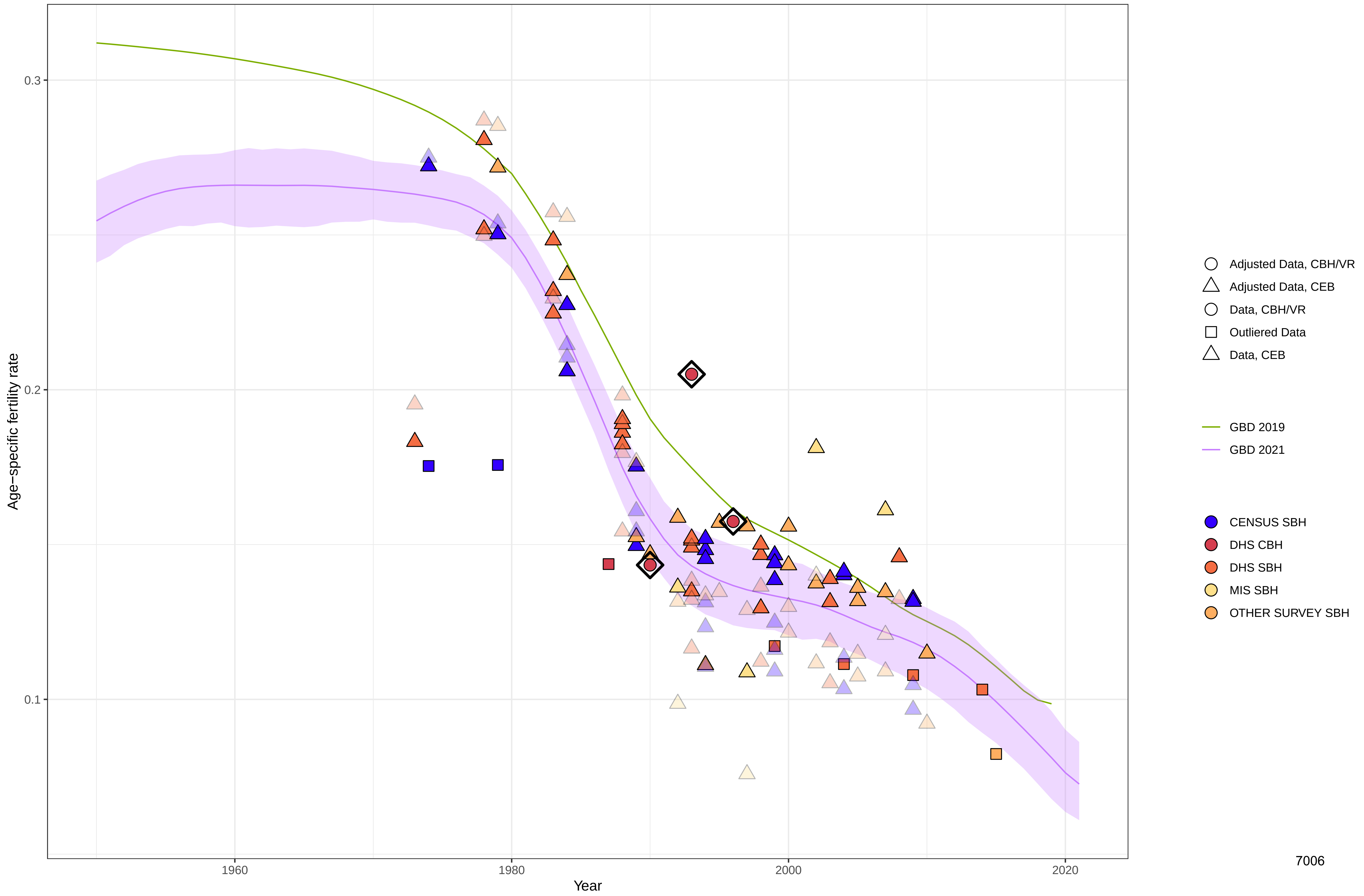

Age−specific fertility fitted trend lines, 1950−2021: Kiambu, Ages: 35−39
Data points enclosed with a diamond are reference sources

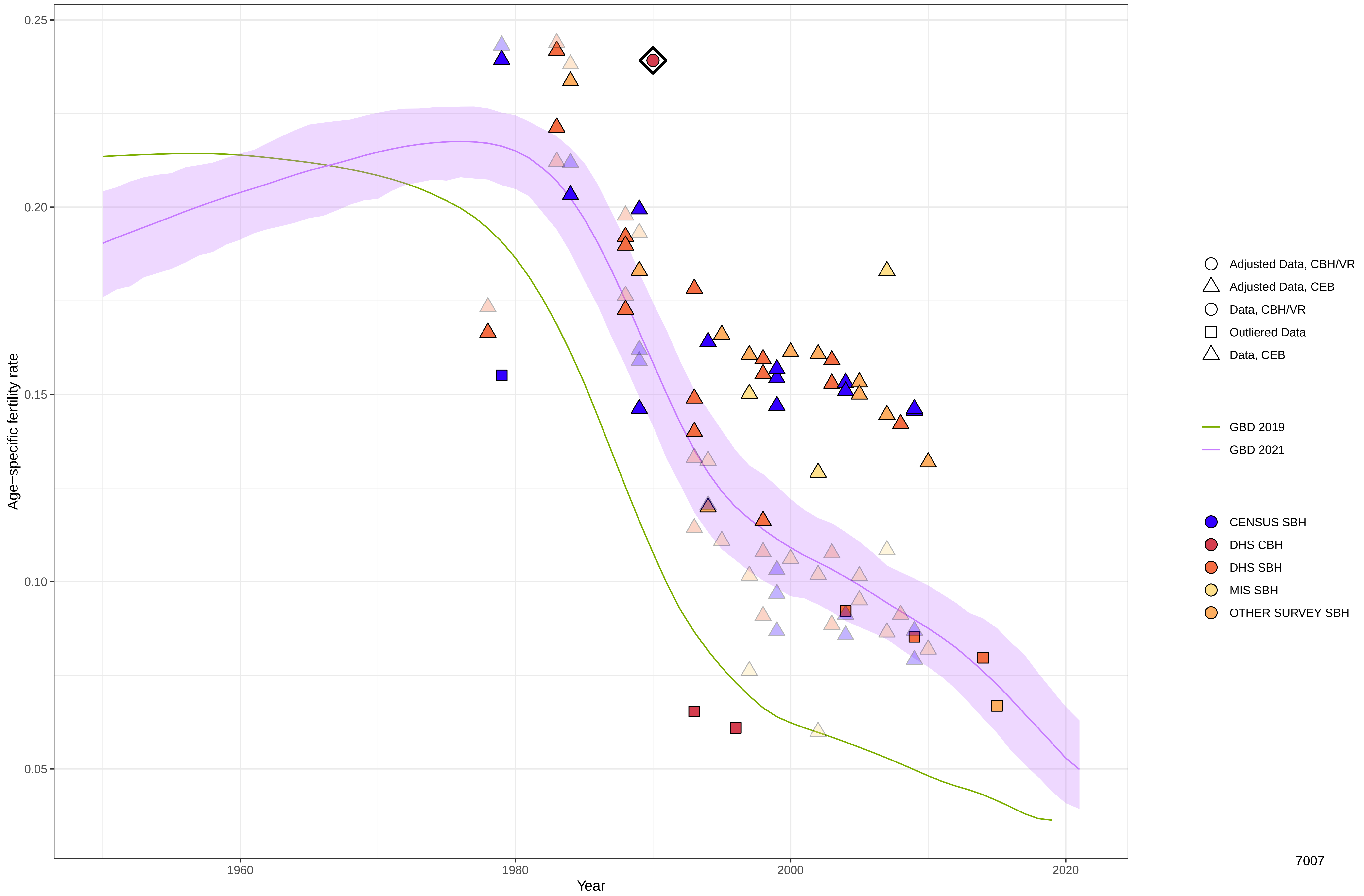

Age−specific fertility fitted trend lines, 1950−2021: Kiambu, Ages: 40−44
Data points enclosed with a diamond are reference sources

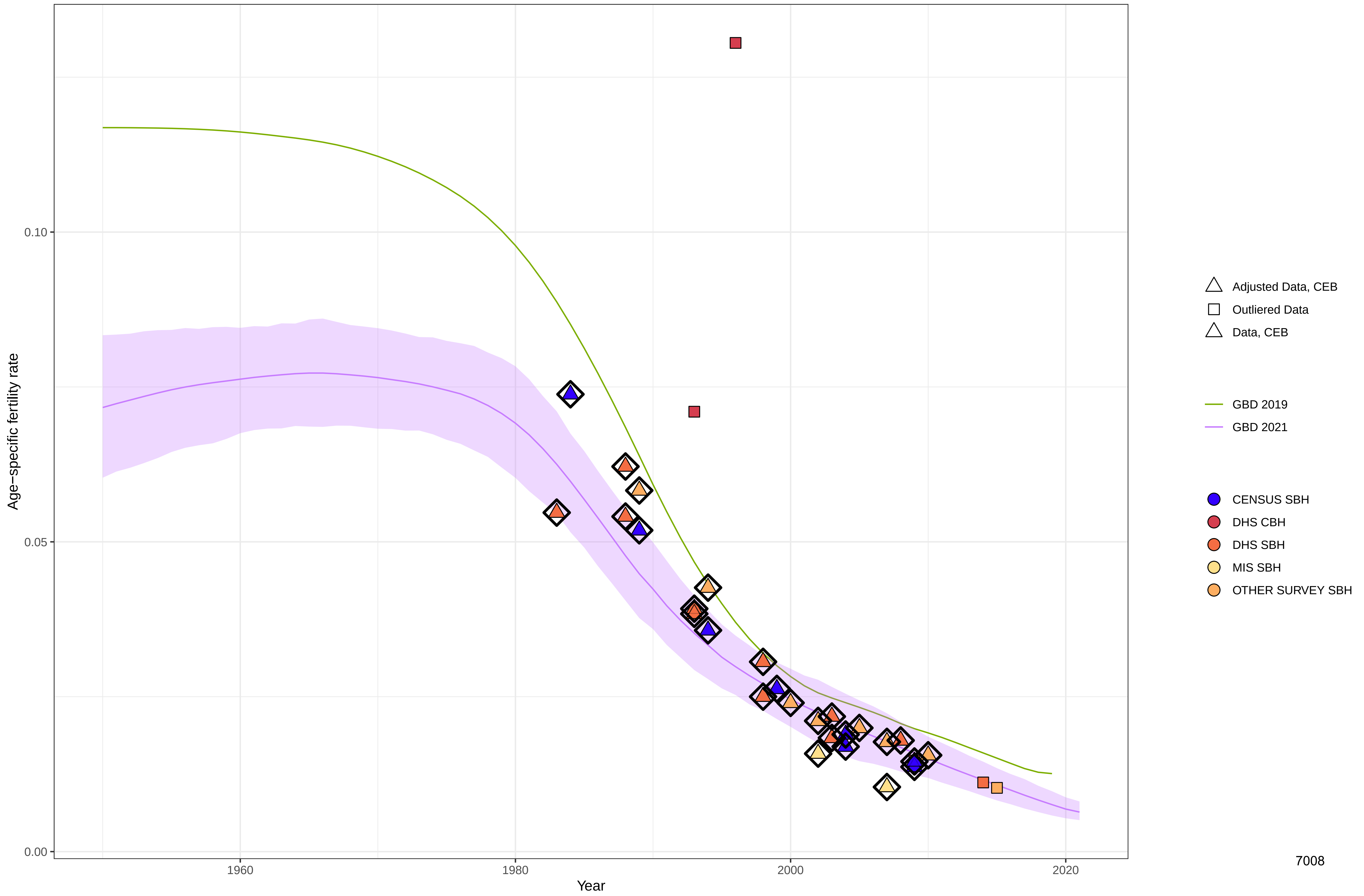

Age−specific fertility fitted trend lines, 1950−2021: Kiambu, Ages: 45−49
Data points enclosed with a diamond are reference sources

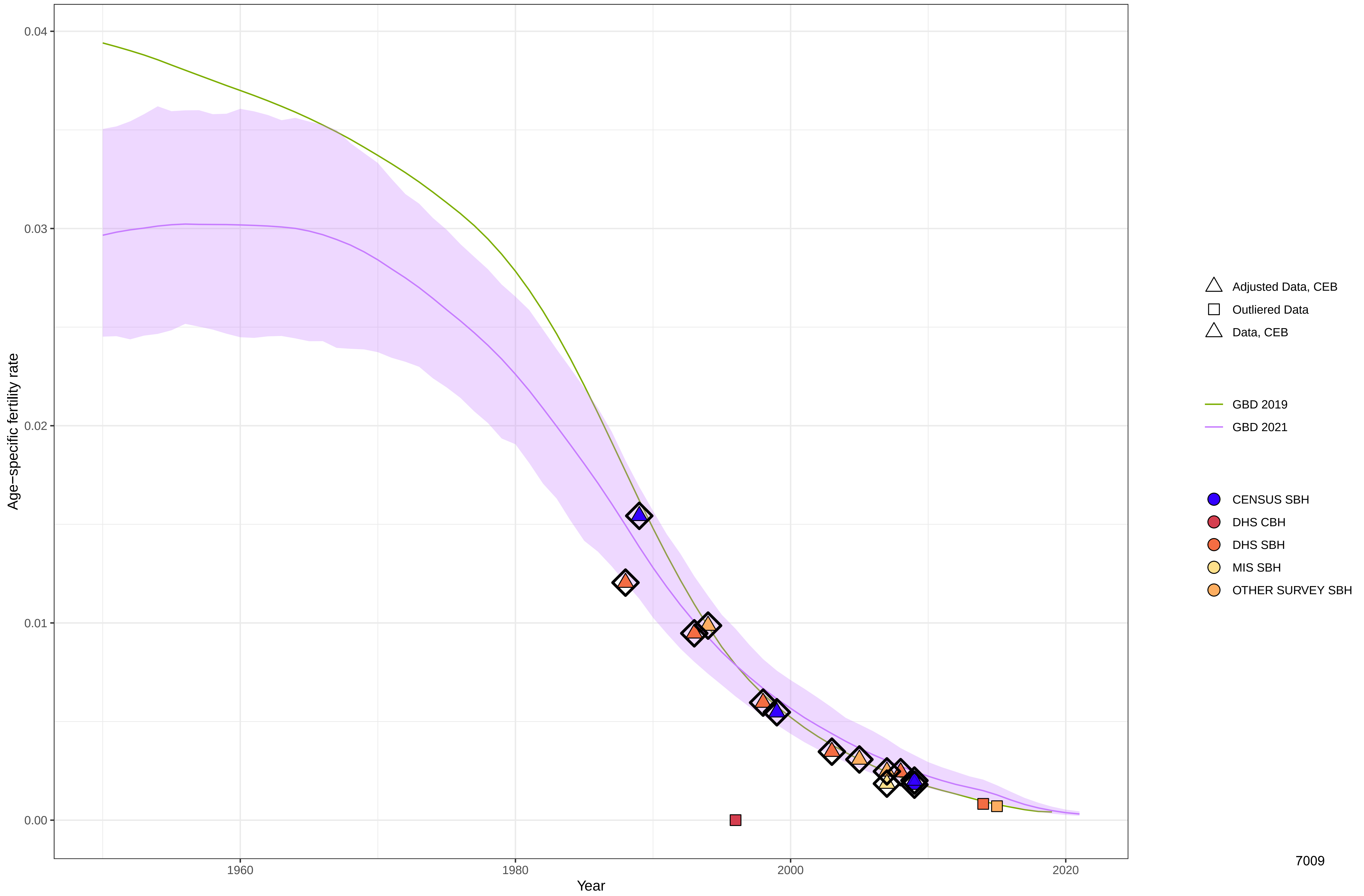

Age−specific fertility fitted trend lines, 1950−2021: Kiambu, Ages: 50−54
Data points enclosed with a diamond are reference sources

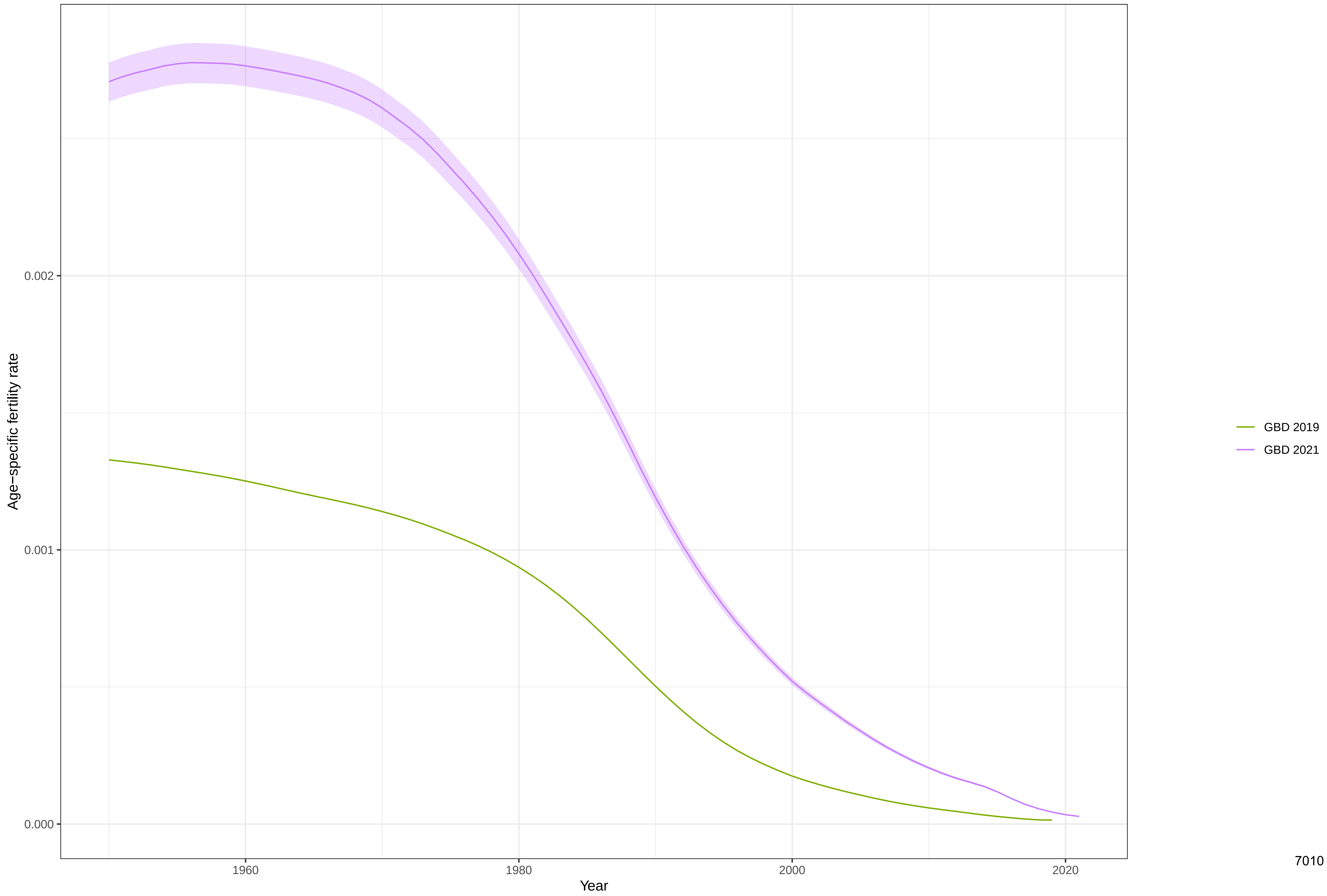

Total fertility rate fitted trend lines, 1950−2021: Kiambu TFR
Data points enclosed with a diamond are reference sources

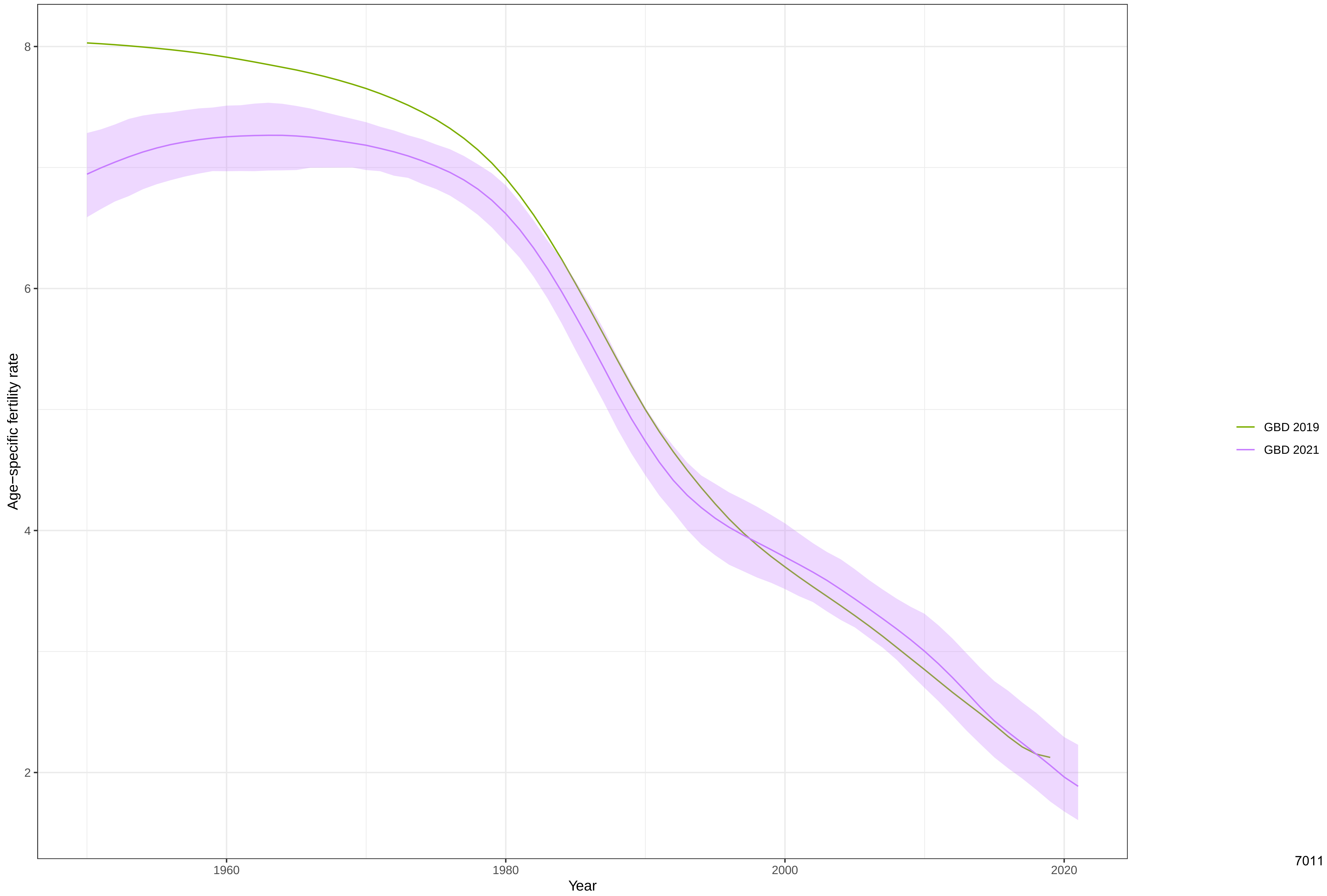

Age−specific fertility fitted trend lines, 1950−2021: Kilifi, Ages: 10−14
Data points enclosed with a diamond are reference sources

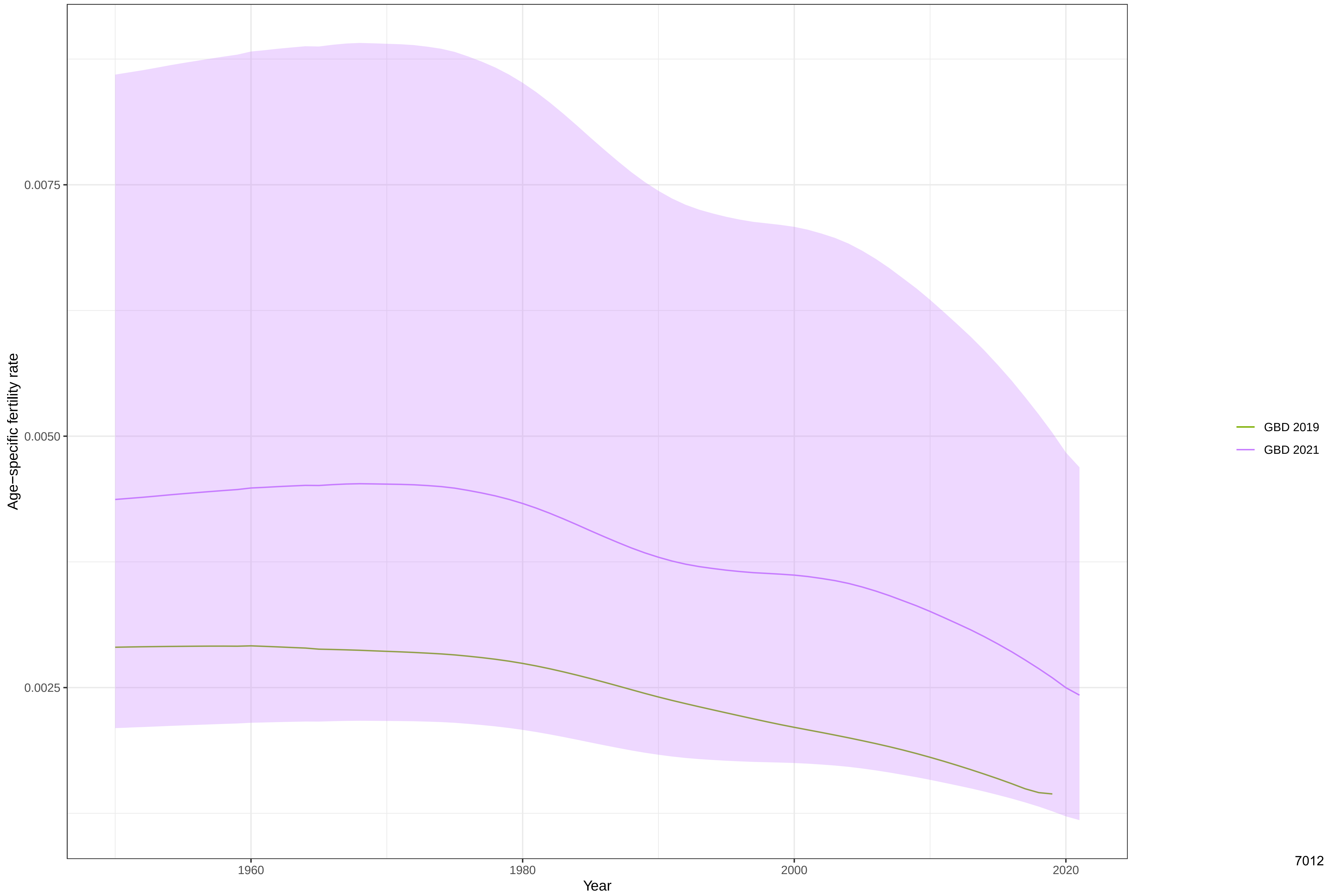

Age−specific fertility fitted trend lines, 1950−2021: Kilifi, Ages: 15−19
Data points enclosed with a diamond are reference sources

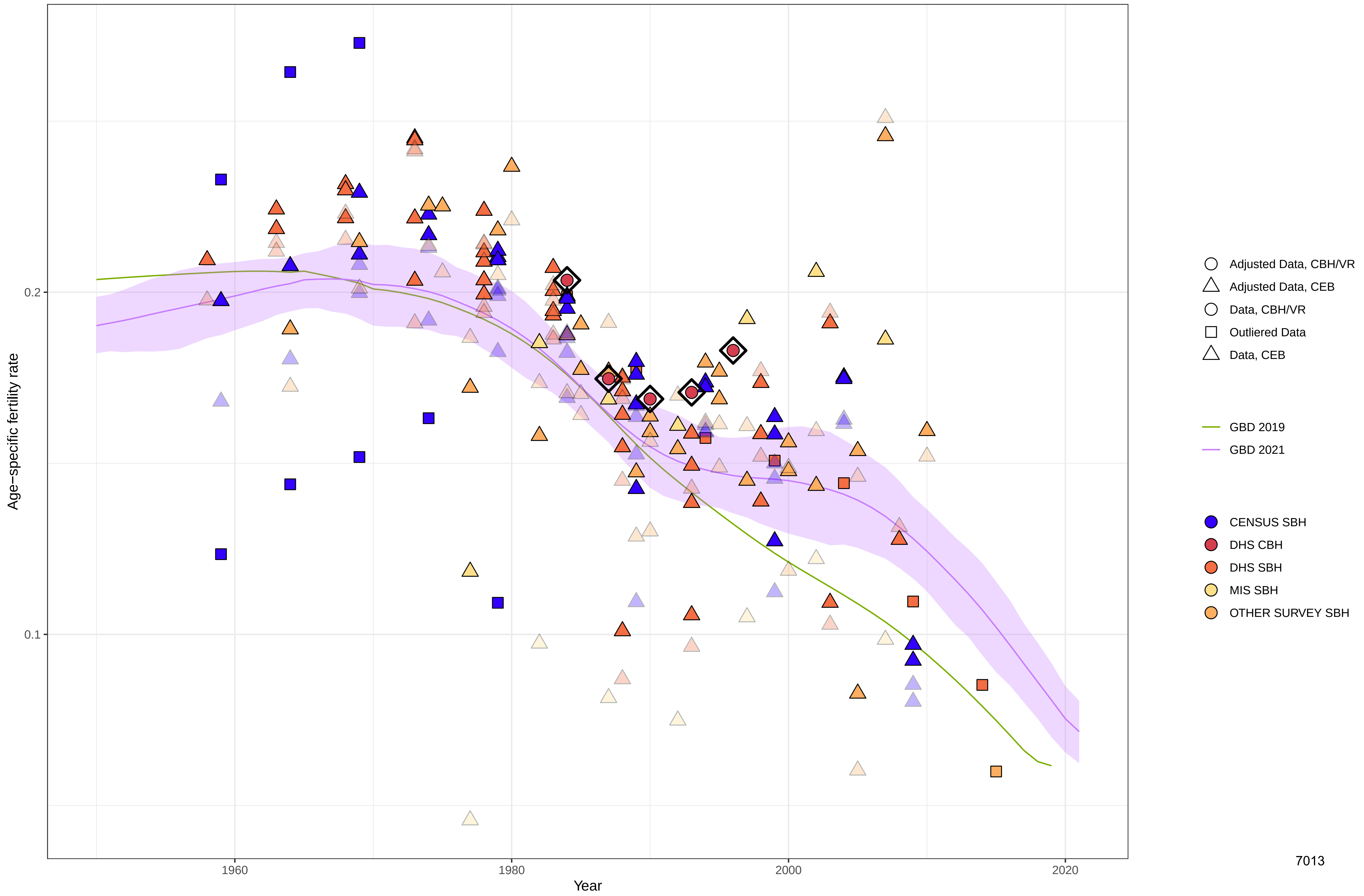

Age−specific fertility fitted trend lines, 1950−2021: Kilifi, Ages: 20−24
Data points enclosed with a diamond are reference sources

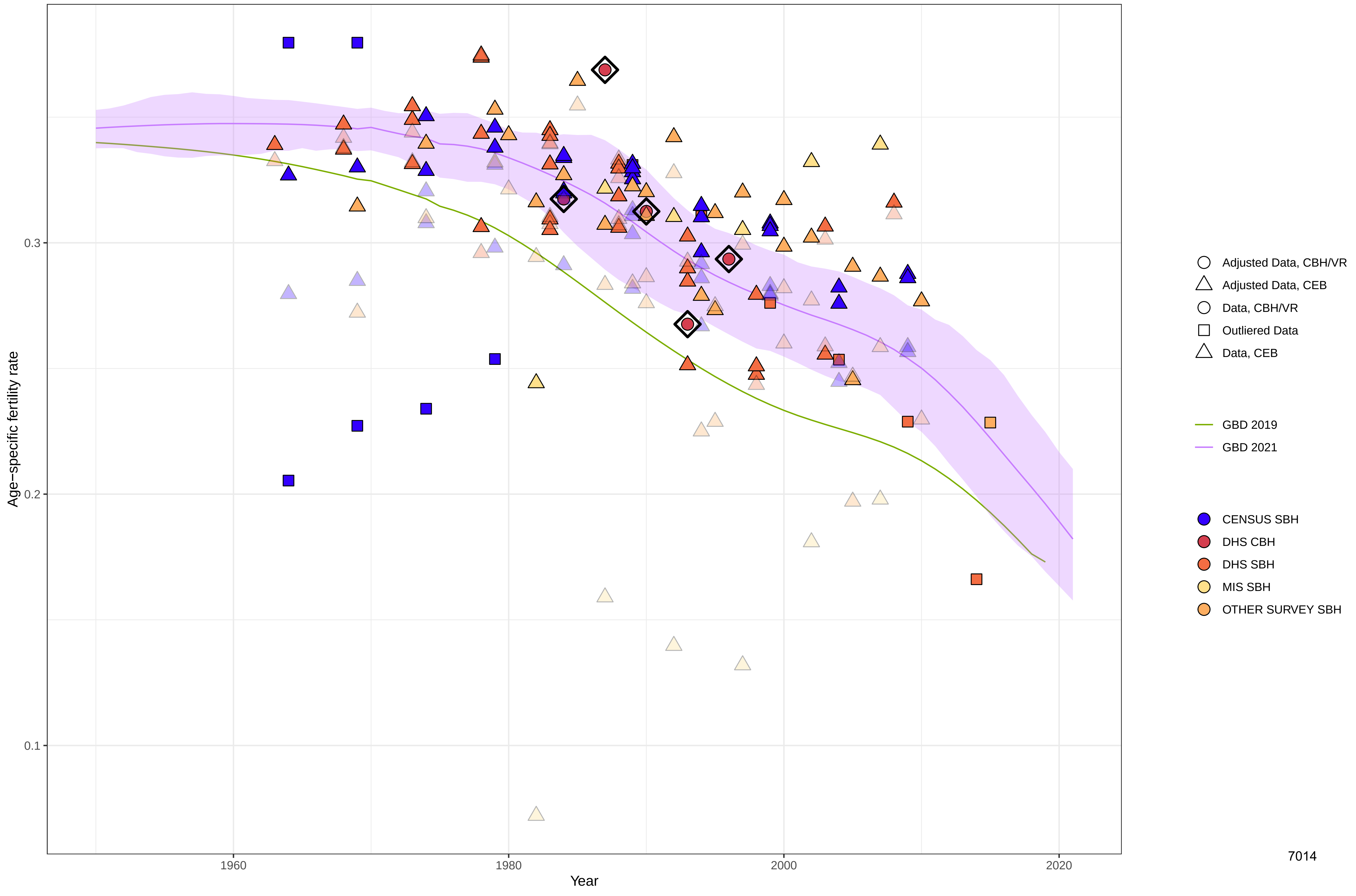

7014

Age−specific fertility fitted trend lines, 1950−2021: Kilifi, Ages: 25−29
Data points enclosed with a diamond are reference sources

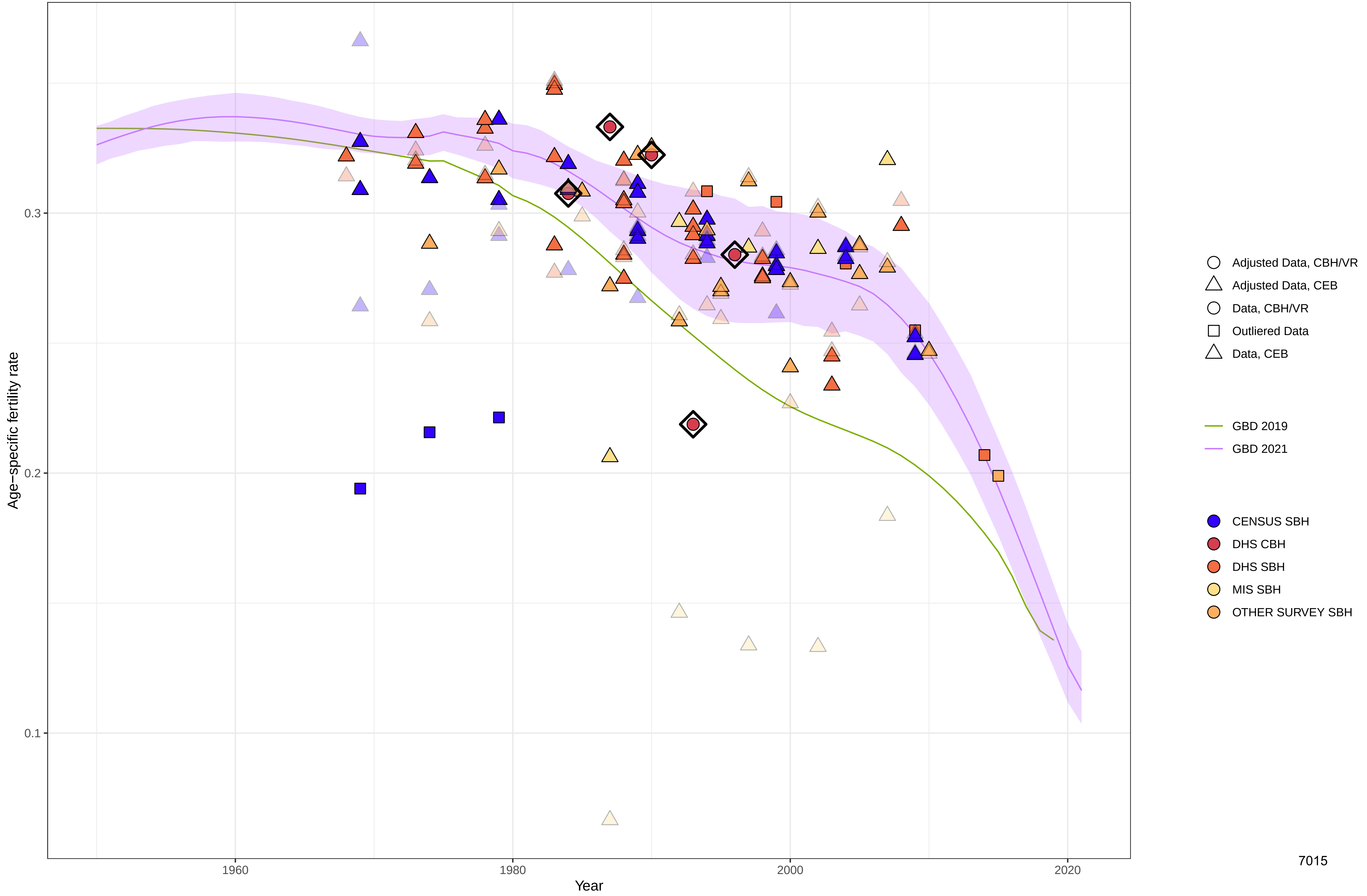

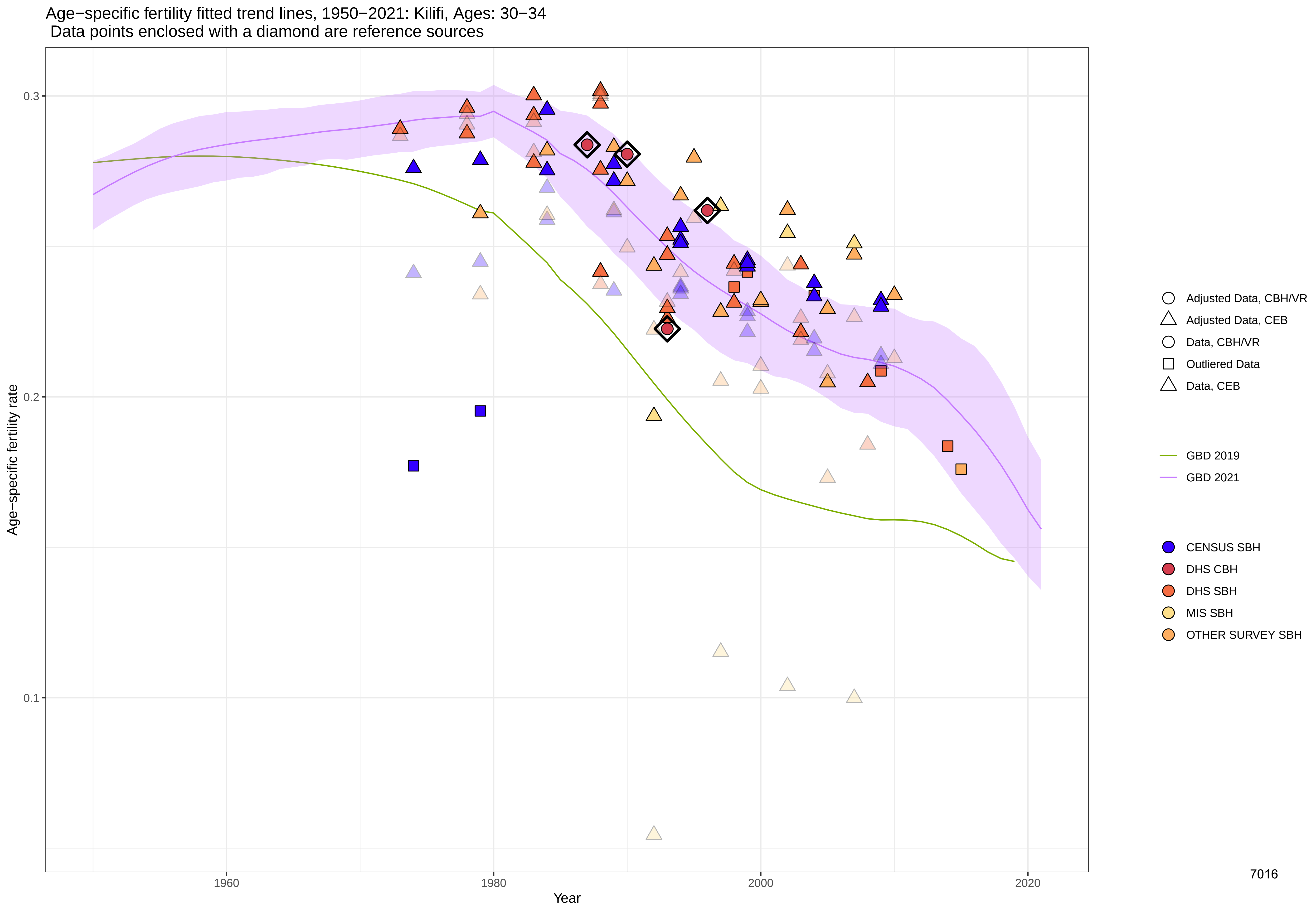

Age−specific fertility fitted trend lines, 1950−2021: Kilifi, Ages: 30−34
Data points enclosed with a diamond are reference sources
Age−specific fertility rate
0.3
0.2
0.1
Year
1960
1980
2000
2020
Adjusted Data, CBH/VR
Adjusted Data, CEB
Data, CBH/VR
Outliered Data
Data, CEB
GBD 2019
GBD 2021
CENSUS SBH
DHS CBH
DHS SBH
MIS SBH
OTHER SURVEY SBH

Age−specific fertility fitted trend lines, 1950−2021: Kilifi, Ages: 35−39
Data points enclosed with a diamond are reference sources

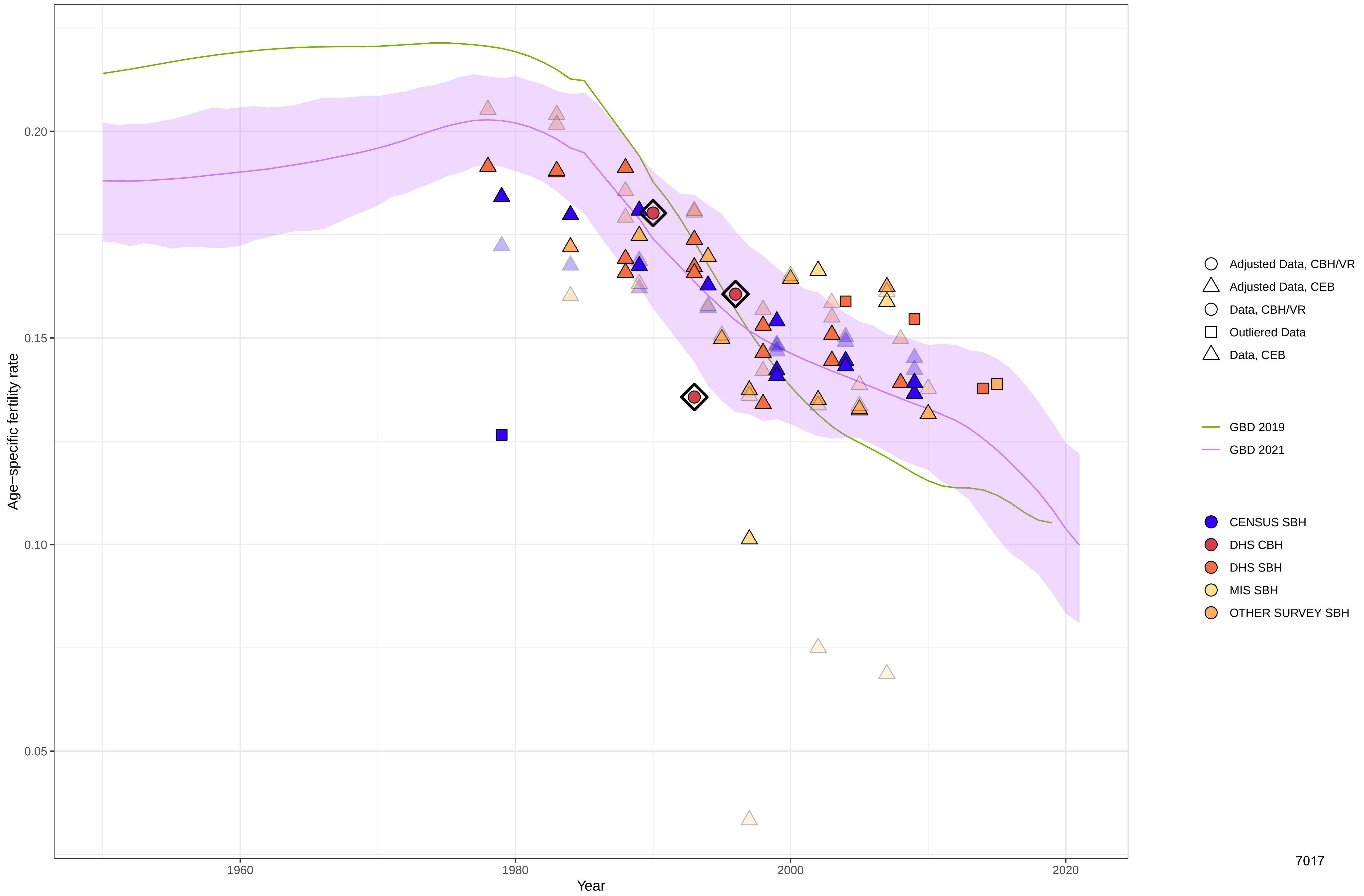

7017

Age−specific fertility fitted trend lines, 1950−2021: Kilifi, Ages: 40−44
Data points enclosed with a diamond are reference sources

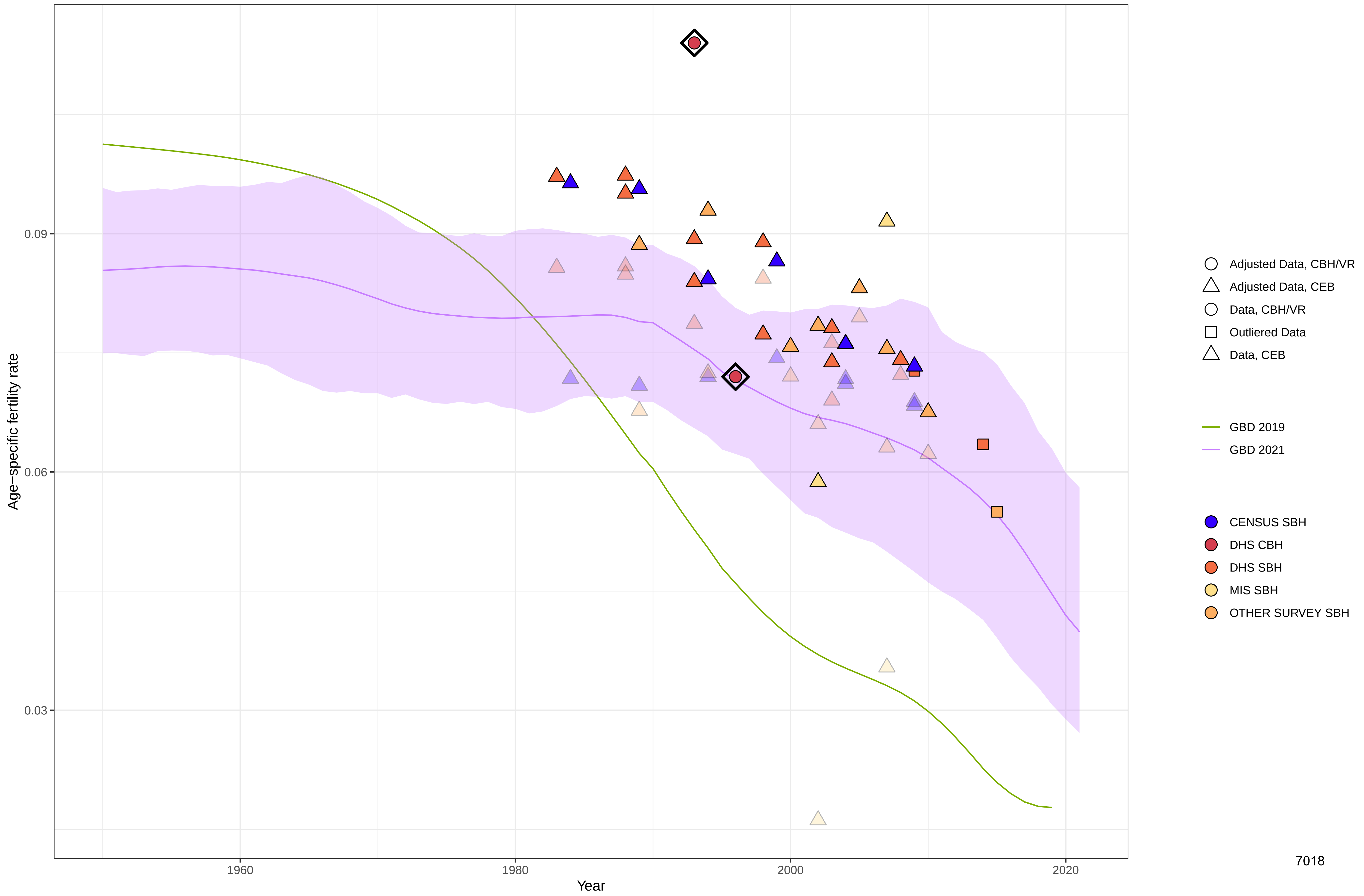

7018

Age−specific fertility fitted trend lines, 1950−2021: Kilifi, Ages: 45−49
Data points enclosed with a diamond are reference sources

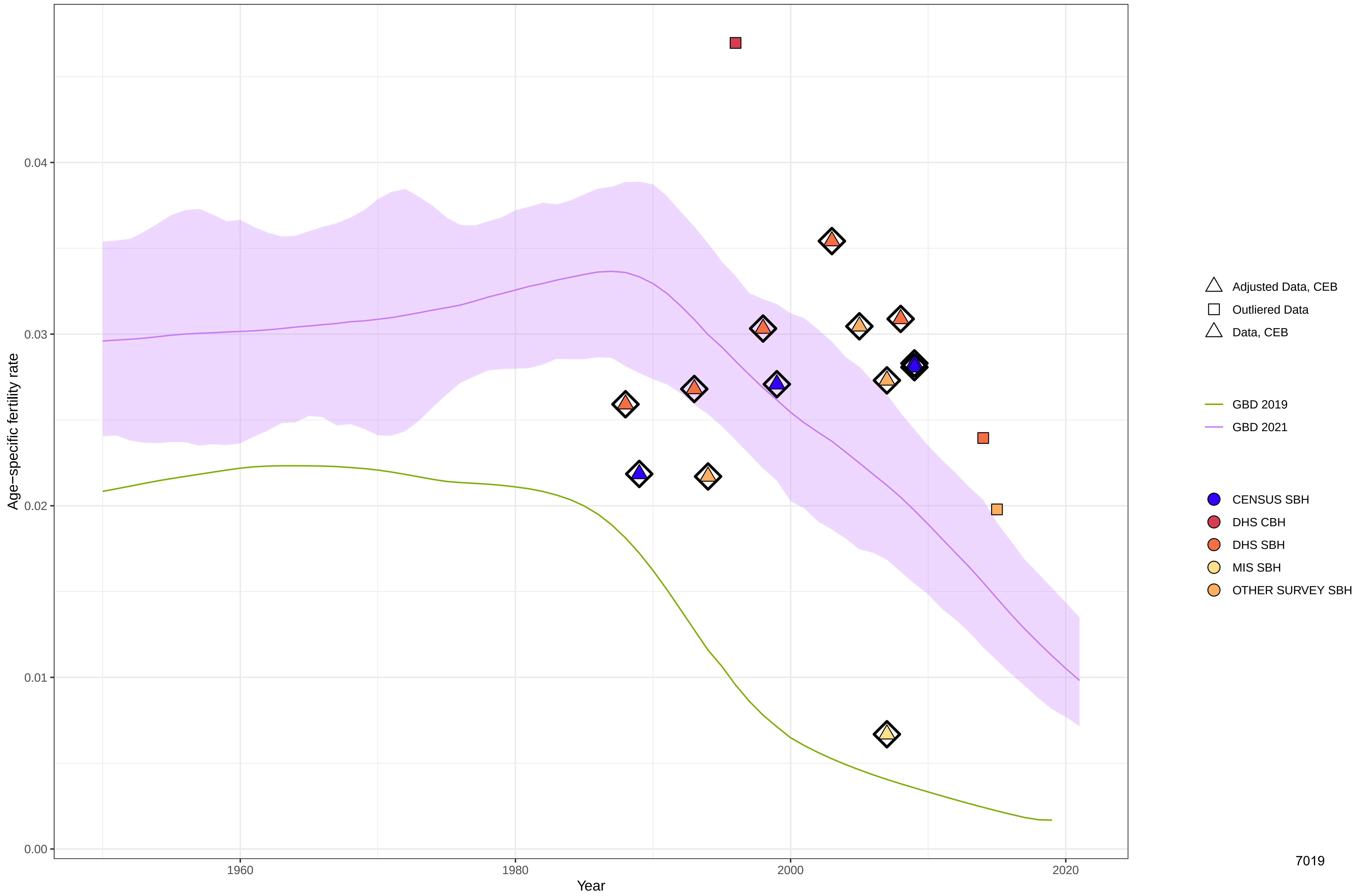

Age−specific fertility fitted trend lines, 1950−2021: Kilifi, Ages: 50−54
Data points enclosed with a diamond are reference sources

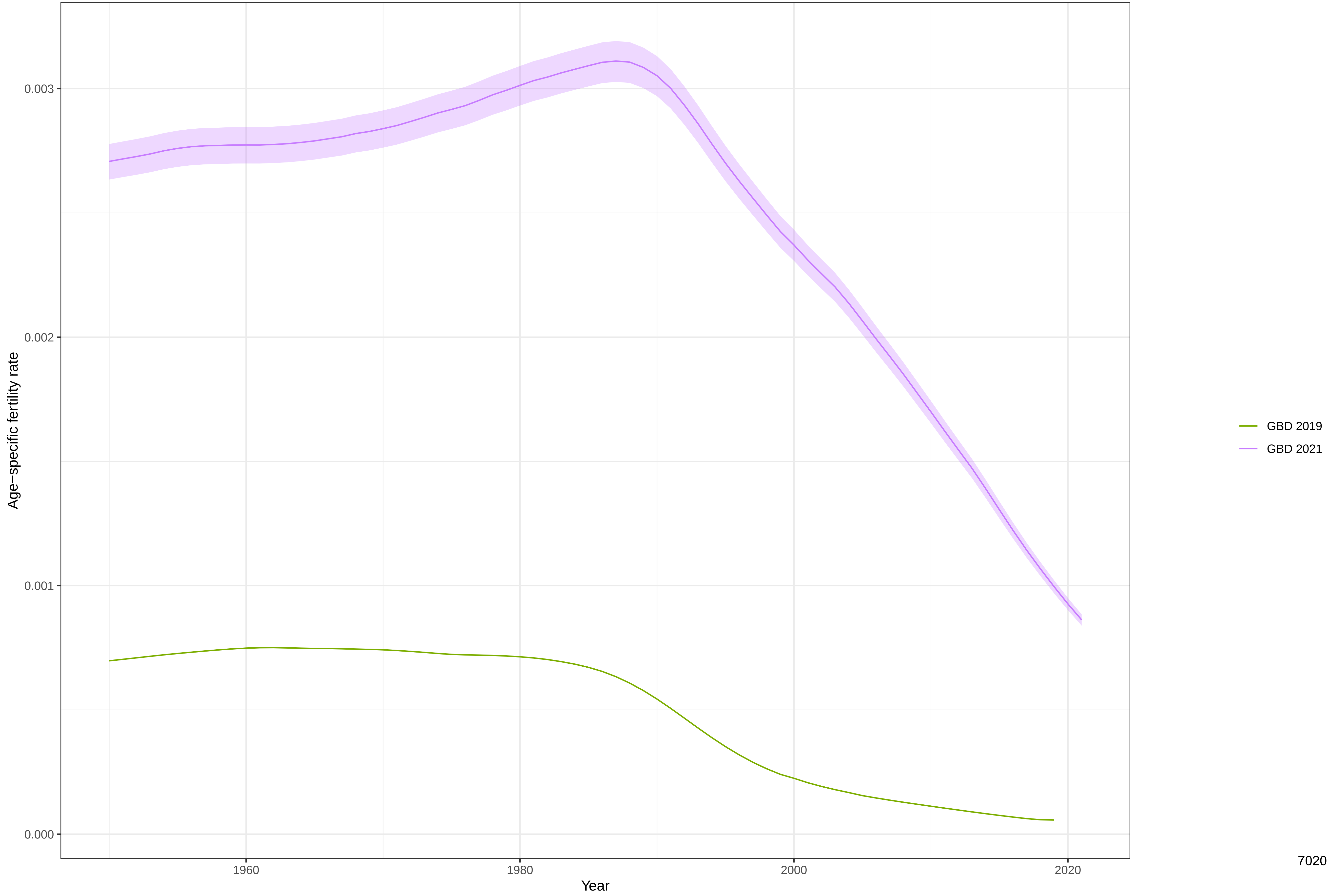

Total fertility rate fitted trend lines, 1950−2021: Kilifi TFR
Data points enclosed with a diamond are reference sources

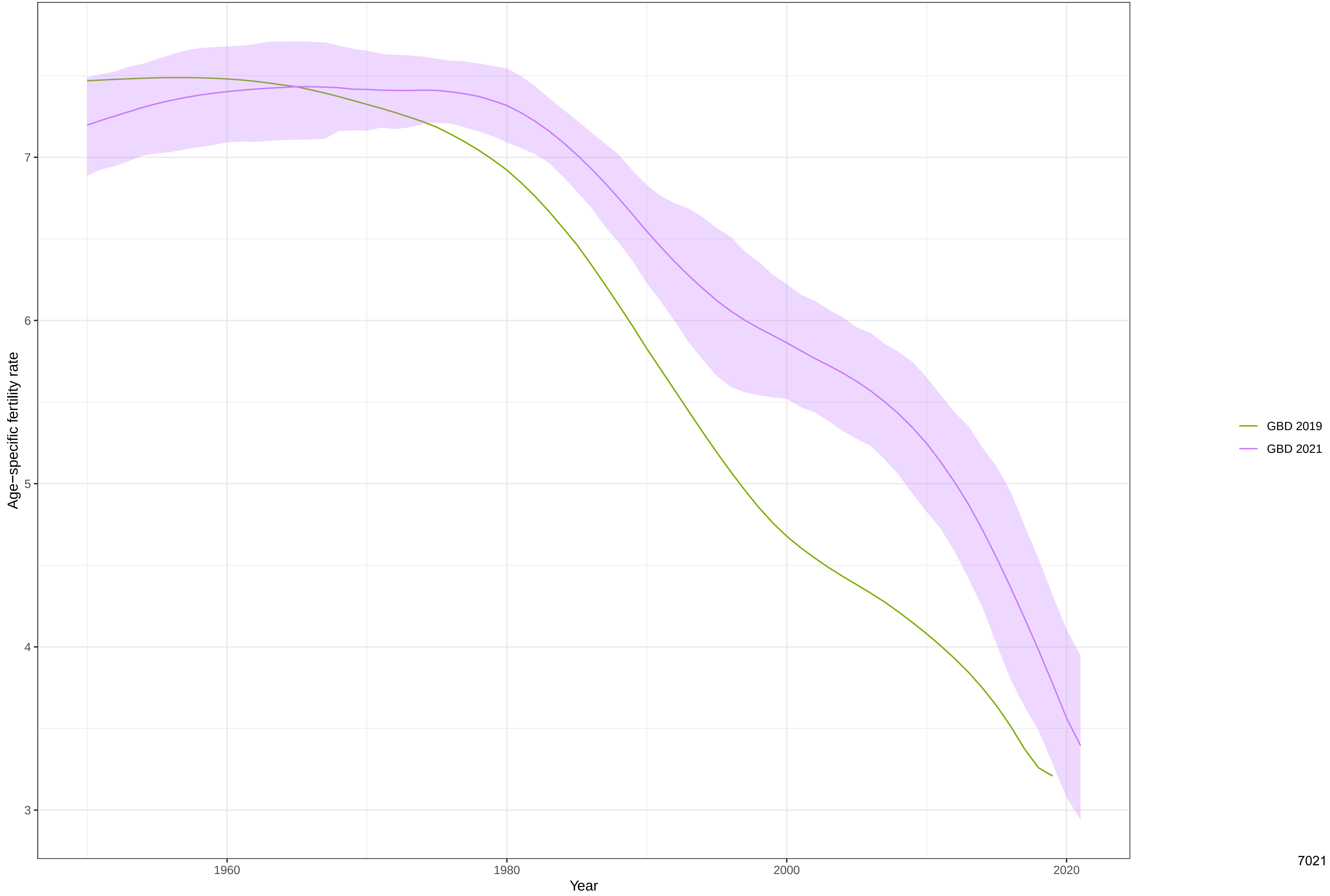

Age−specific fertility fitted trend lines, 1950−2021: Kirinyaga, Ages: 10−14
Data points enclosed with a diamond are reference sources

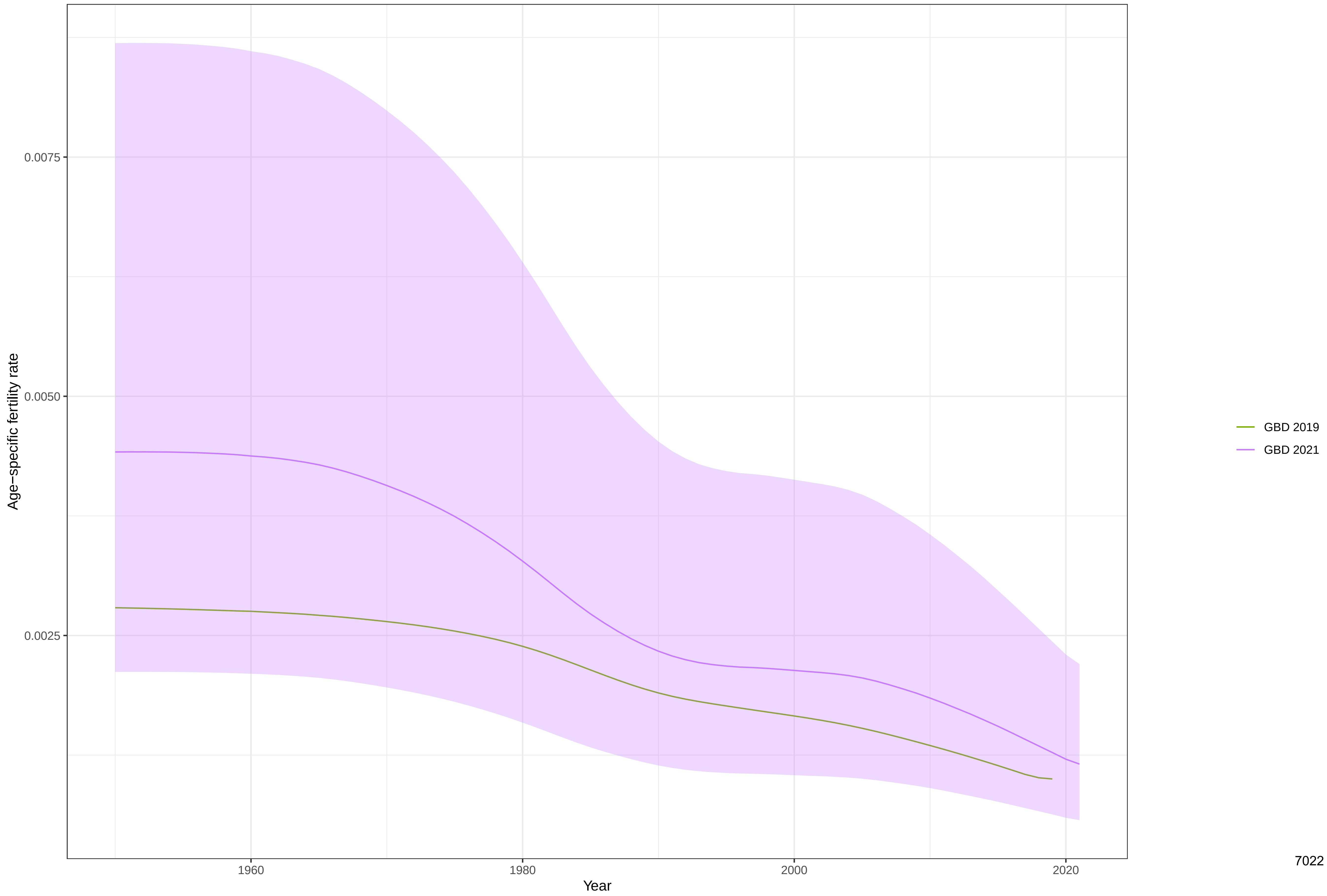

Age−specific fertility fitted trend lines, 1950−2021: Kirinyaga, Ages: 15−19
Data points enclosed with a diamond are reference sources

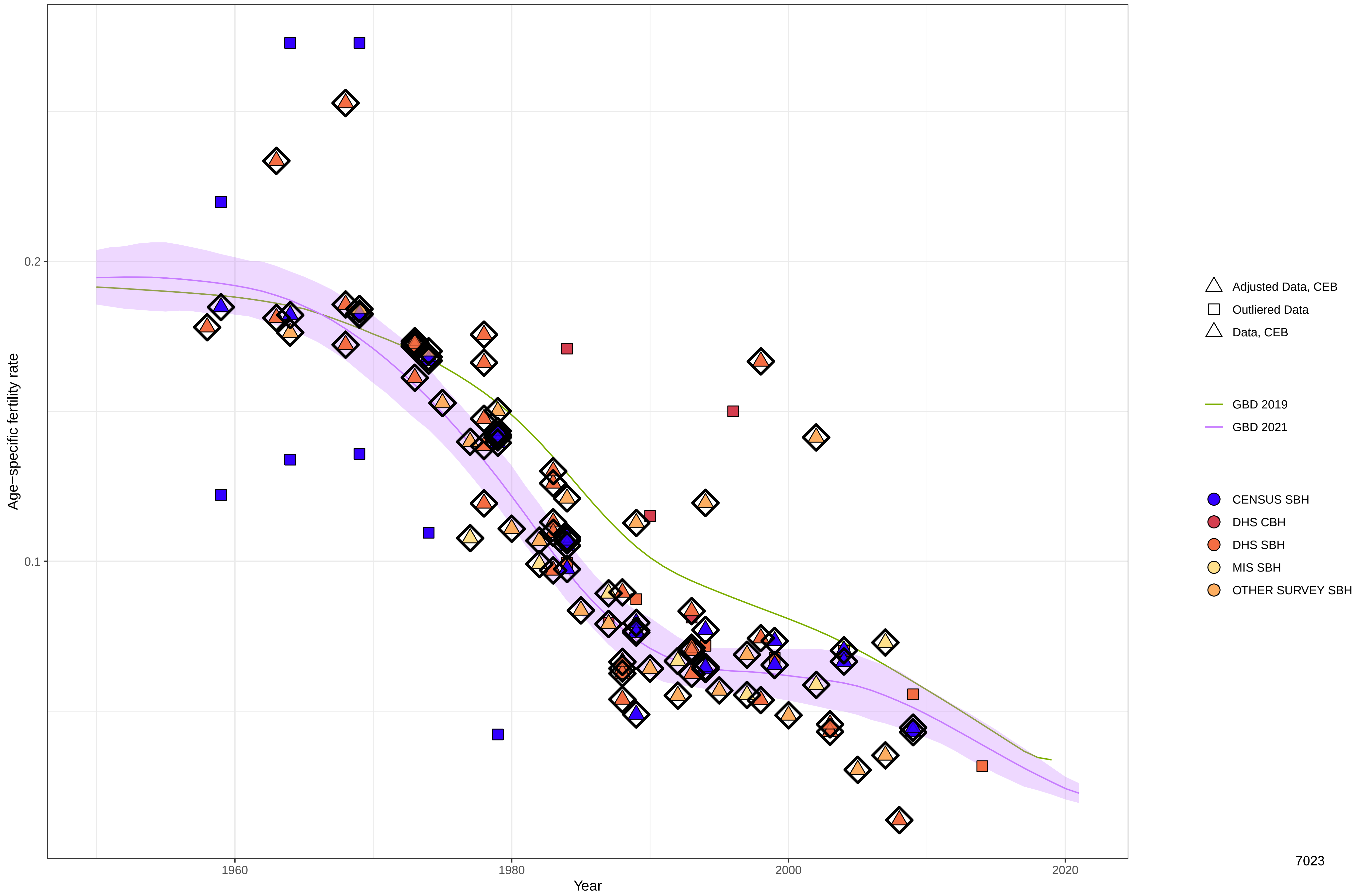

Age−specific fertility fitted trend lines, 1950−2021: Kirinyaga, Ages: 20−24
Data points enclosed with a diamond are reference sources

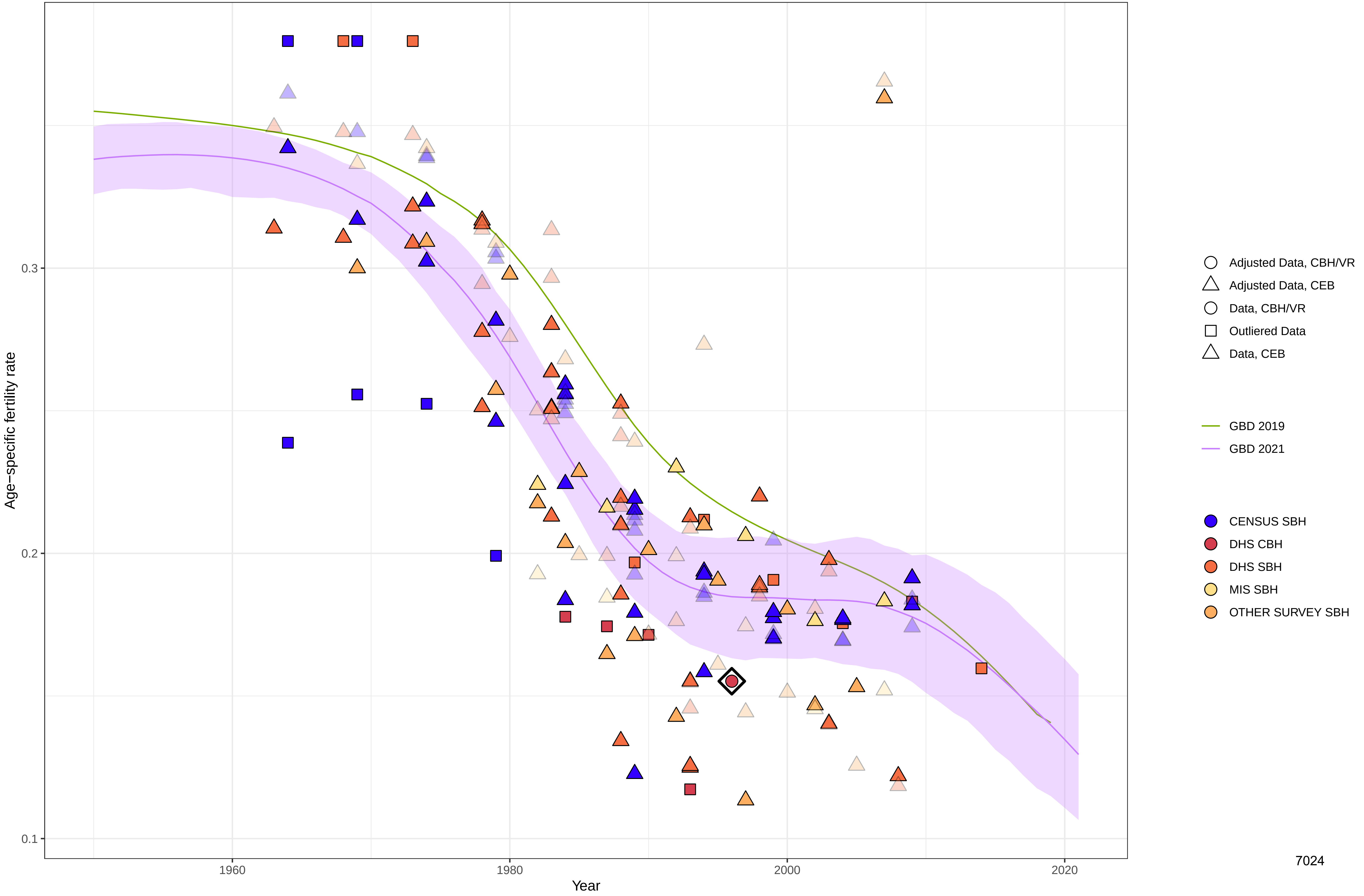

Age−specific fertility fitted trend lines, 1950−2021: Kirinyaga, Ages: 25−29
Data points enclosed with a diamond are reference sources

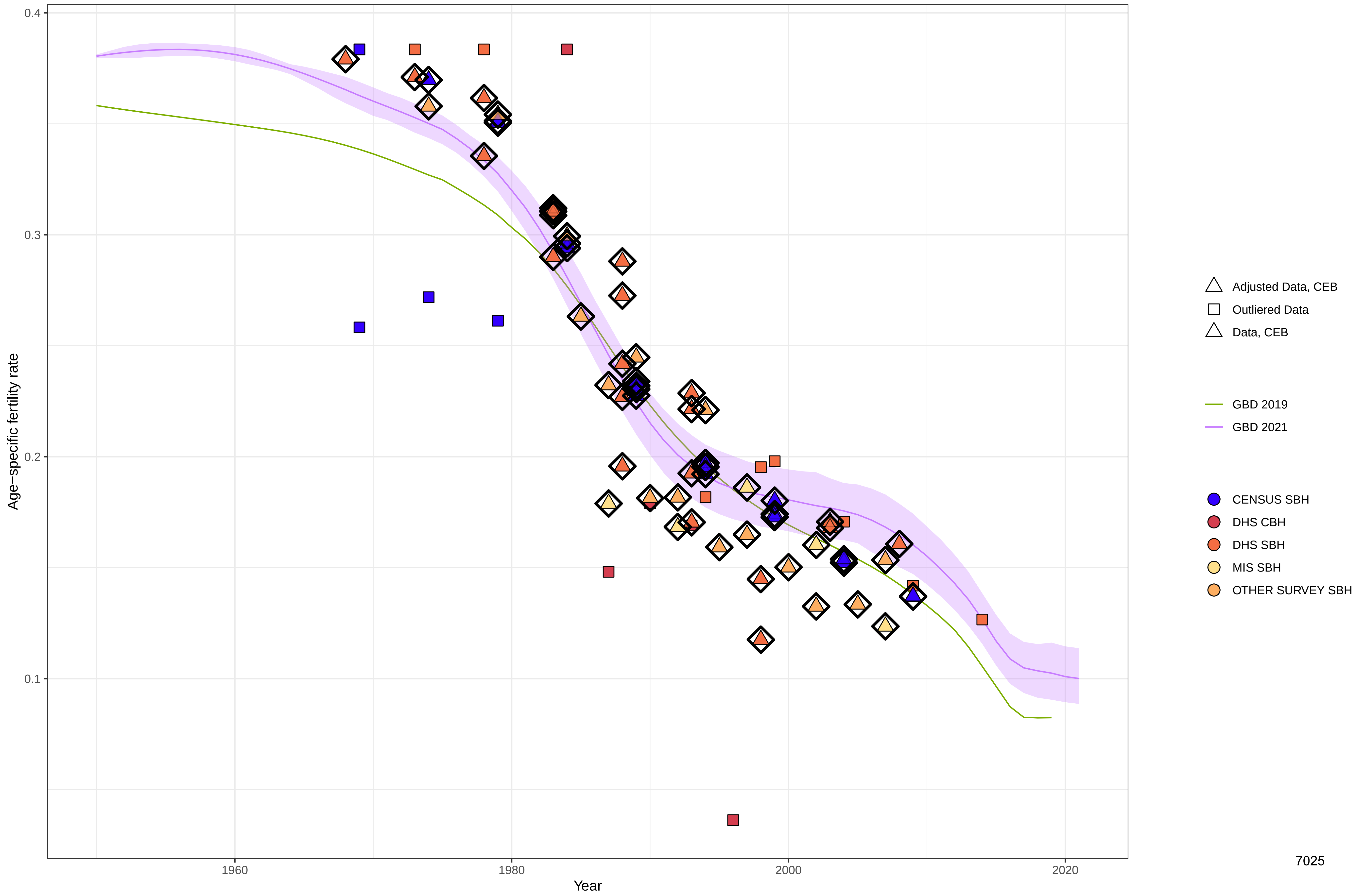

Age−specific fertility fitted trend lines, 1950−2021: Kirinyaga, Ages: 30−34
Data points enclosed with a diamond are reference sources

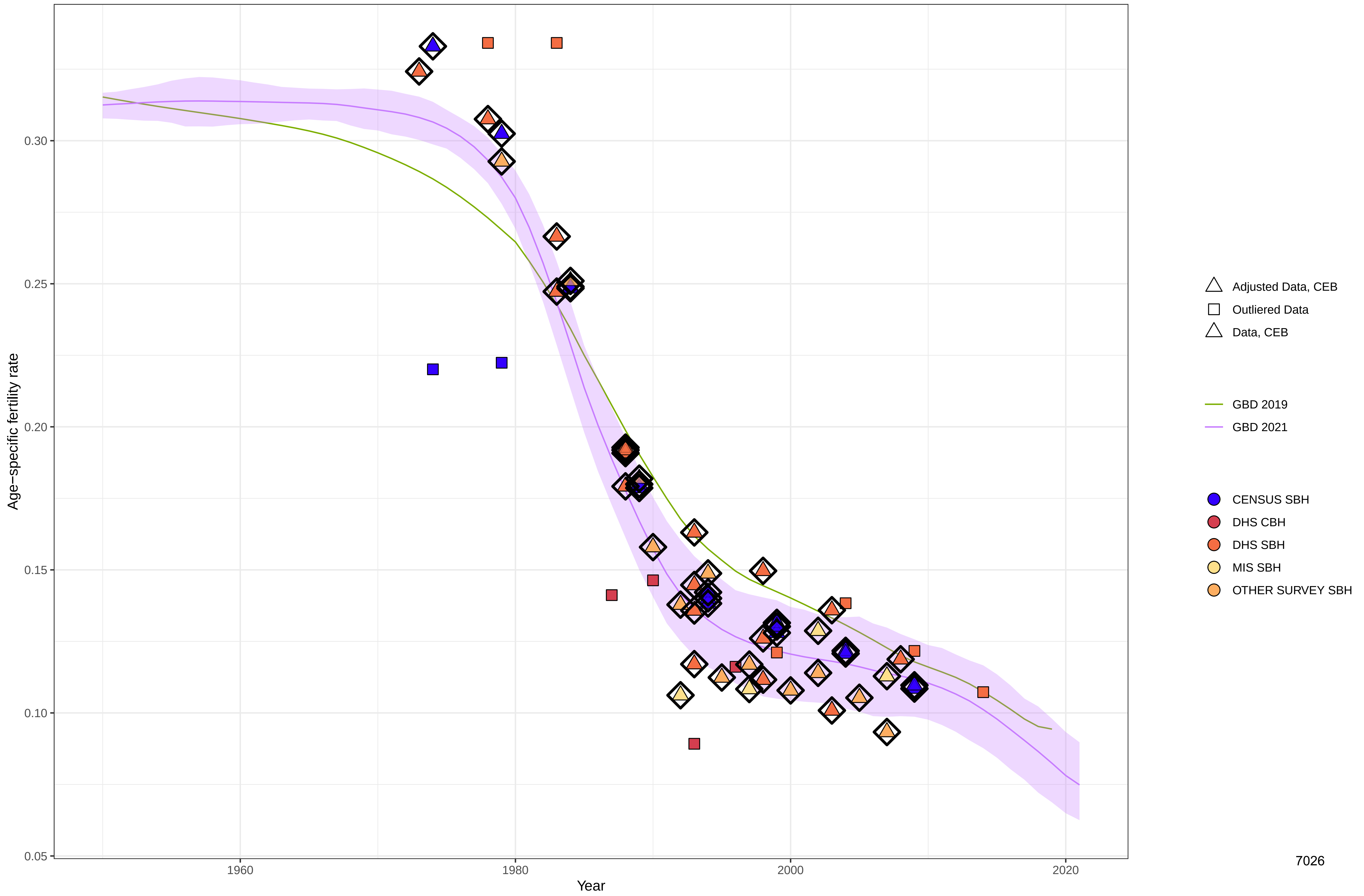

Age−specific fertility fitted trend lines, 1950−2021: Kirinyaga, Ages: 35−39
Data points enclosed with a diamond are reference sources

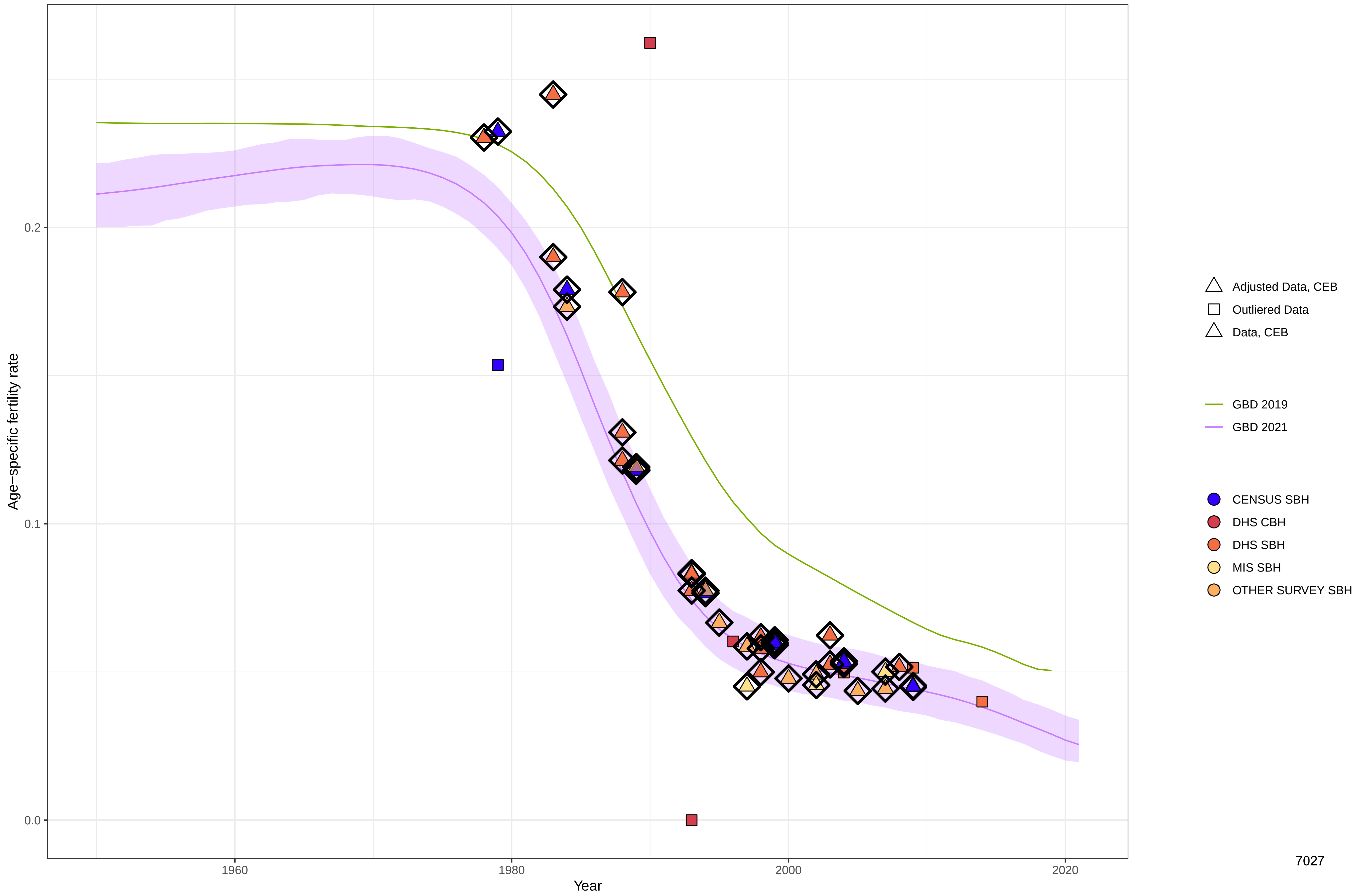

Age−specific fertility fitted trend lines, 1950−2021: Kirinyaga, Ages: 40−44
Data points enclosed with a diamond are reference sources

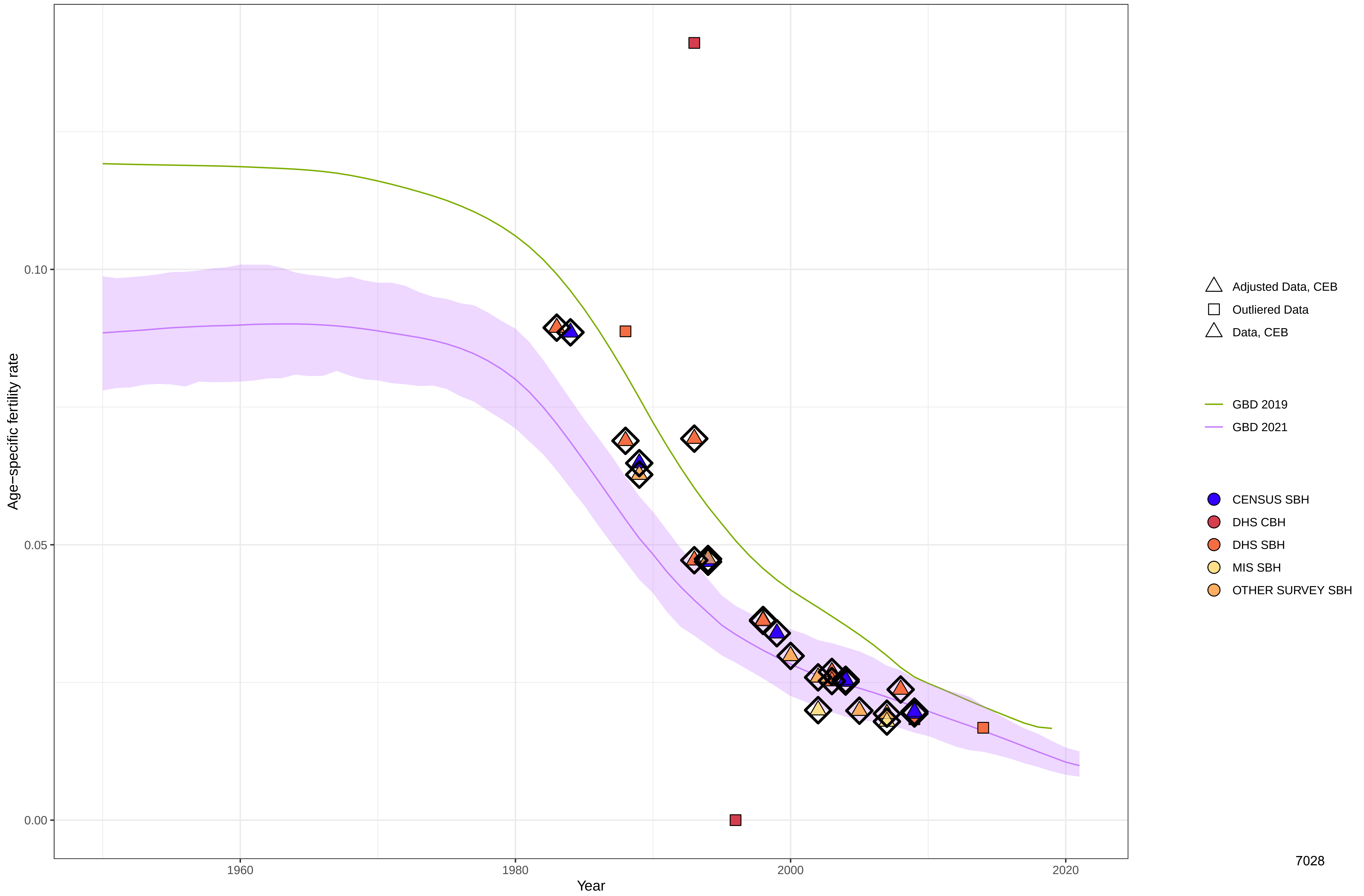

Age−specific fertility fitted trend lines, 1950−2021: Kirinyaga, Ages: 45−49
Data points enclosed with a diamond are reference sources

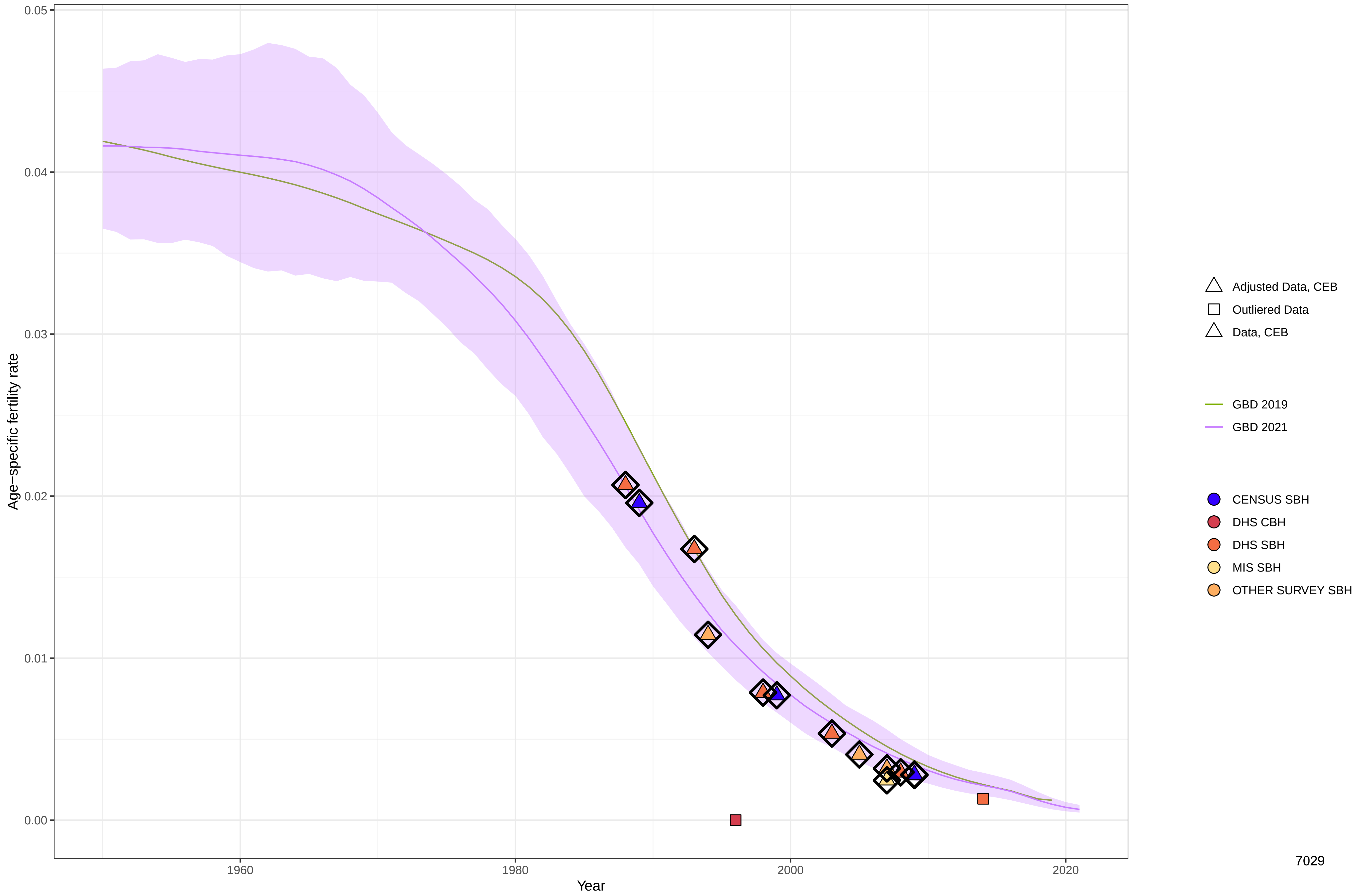

Age−specific fertility fitted trend lines, 1950−2021: Kirinyaga, Ages: 50−54
Data points enclosed with a diamond are reference sources

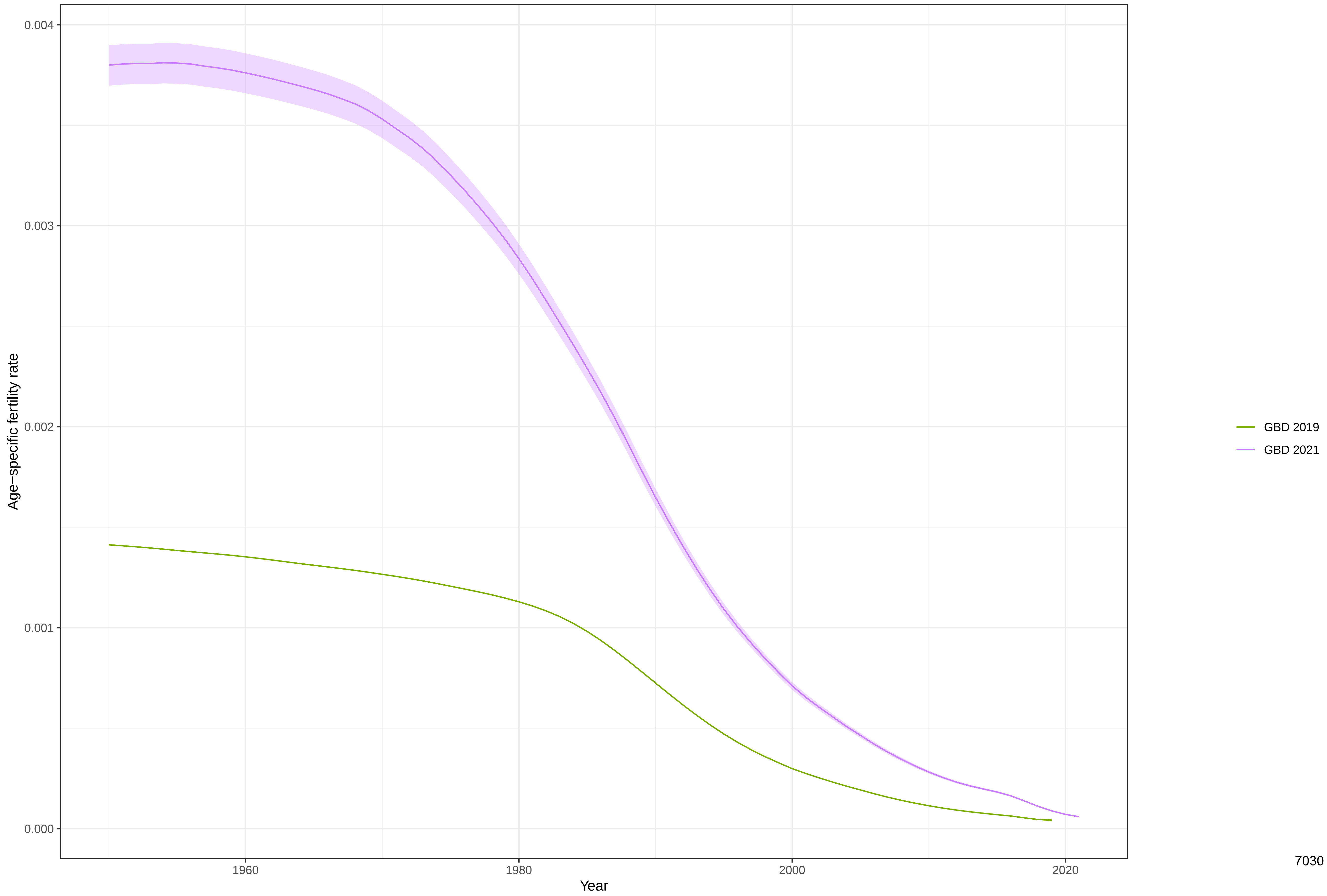

Total fertility rate fitted trend lines, 1950−2021: Kirinyaga TFR
Data points enclosed with a diamond are reference sources

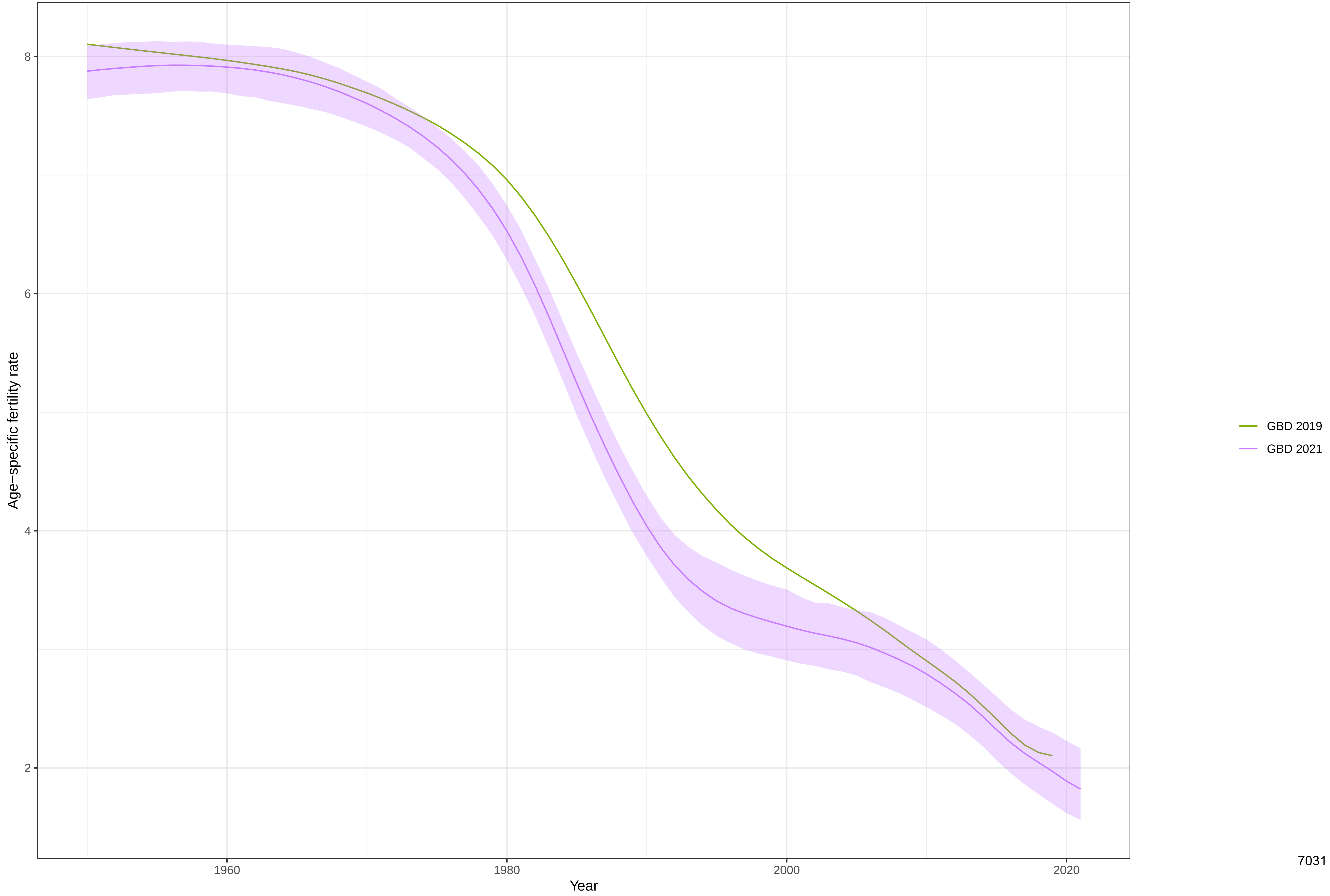

Age−specific fertility fitted trend lines, 1950−2021: Kisii, Ages: 10−14
Data points enclosed with a diamond are reference sources

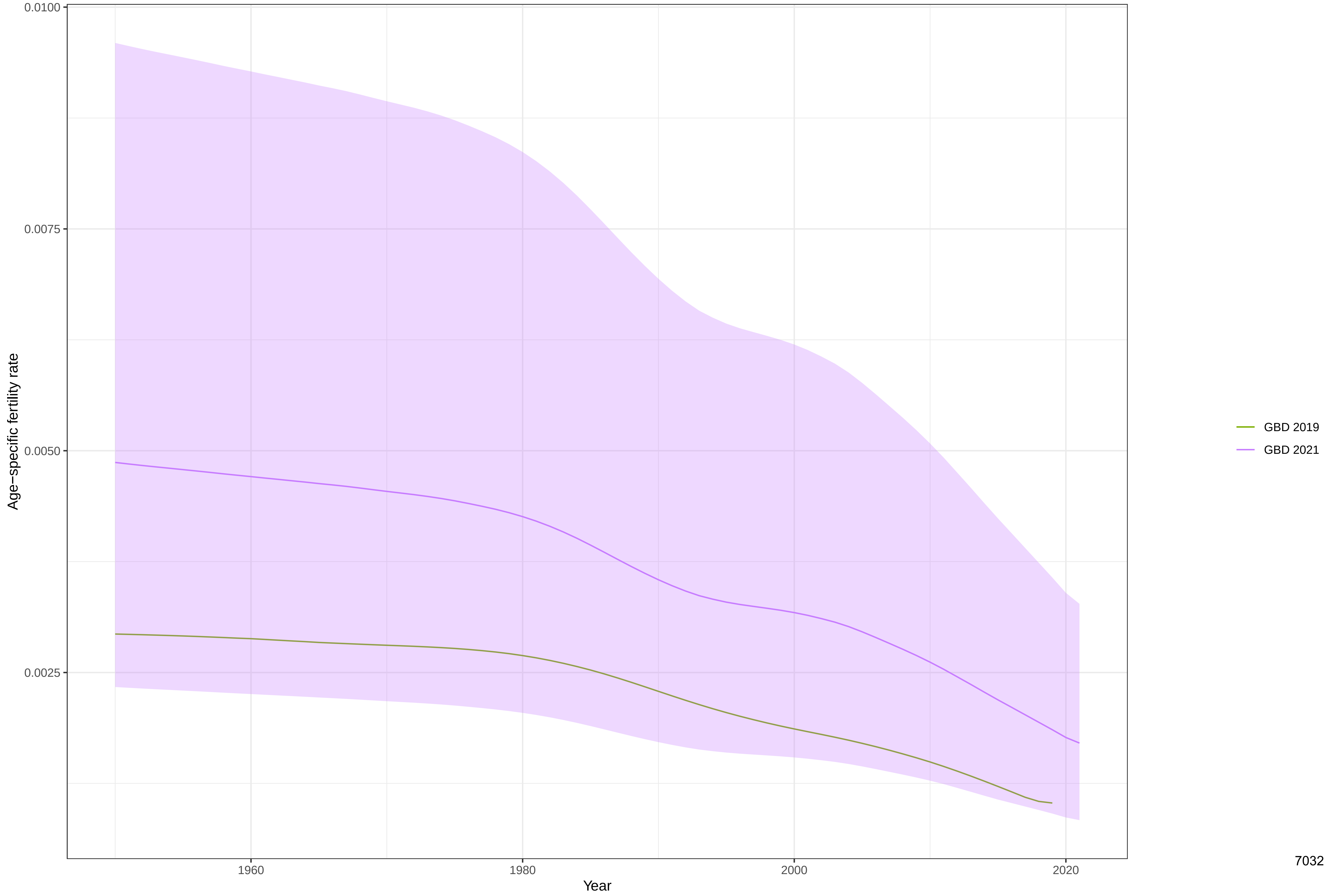

Age−specific fertility fitted trend lines, 1950−2021: Kisii, Ages: 15−19
Data points enclosed with a diamond are reference sources

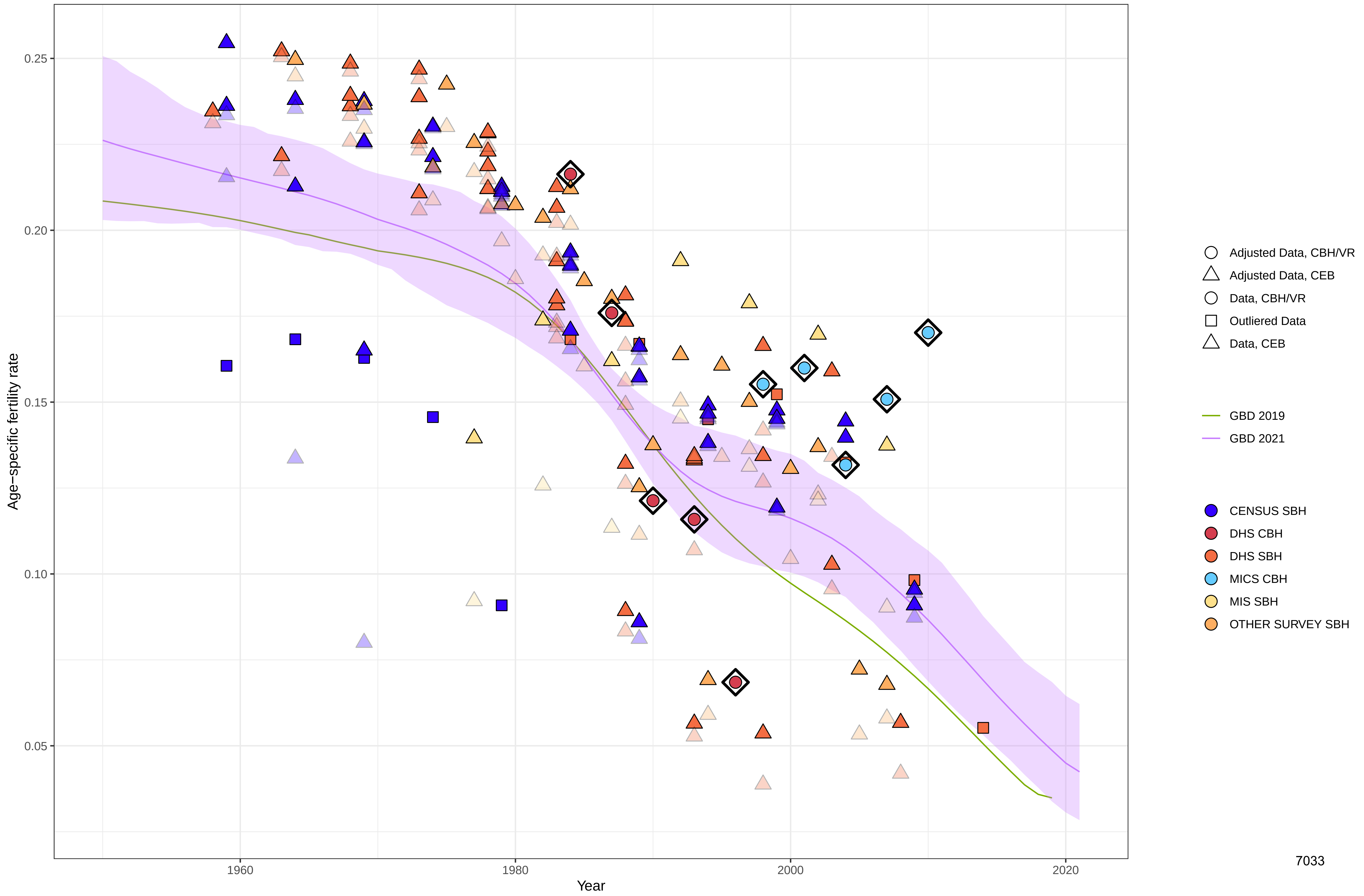

Age−specific fertility fitted trend lines, 1950−2021: Kisii, Ages: 20−24
Data points enclosed with a diamond are reference sources

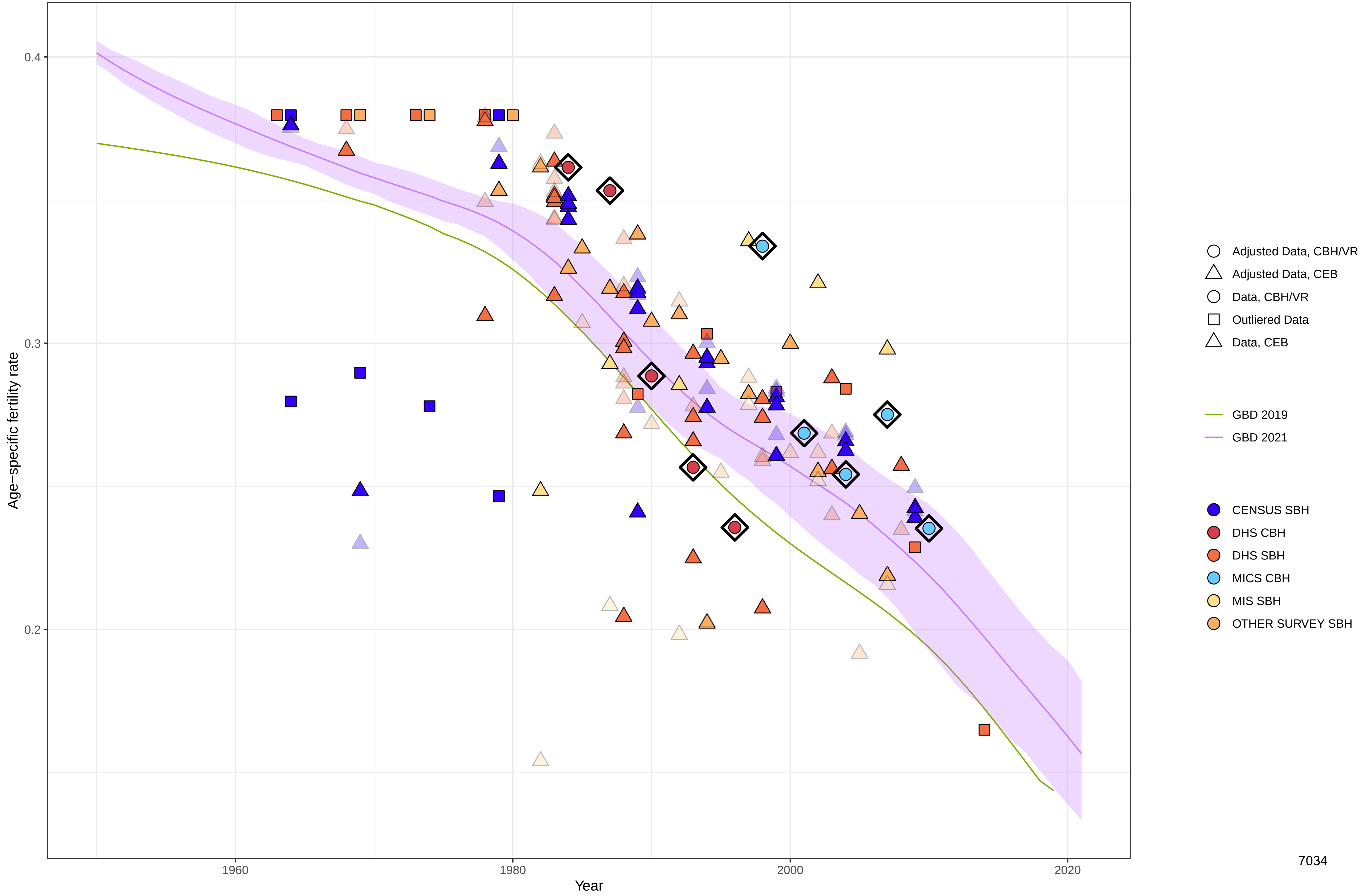

Age−specific fertility fitted trend lines, 1950−2021: Kisii, Ages: 25−29
Data points enclosed with a diamond are reference sources

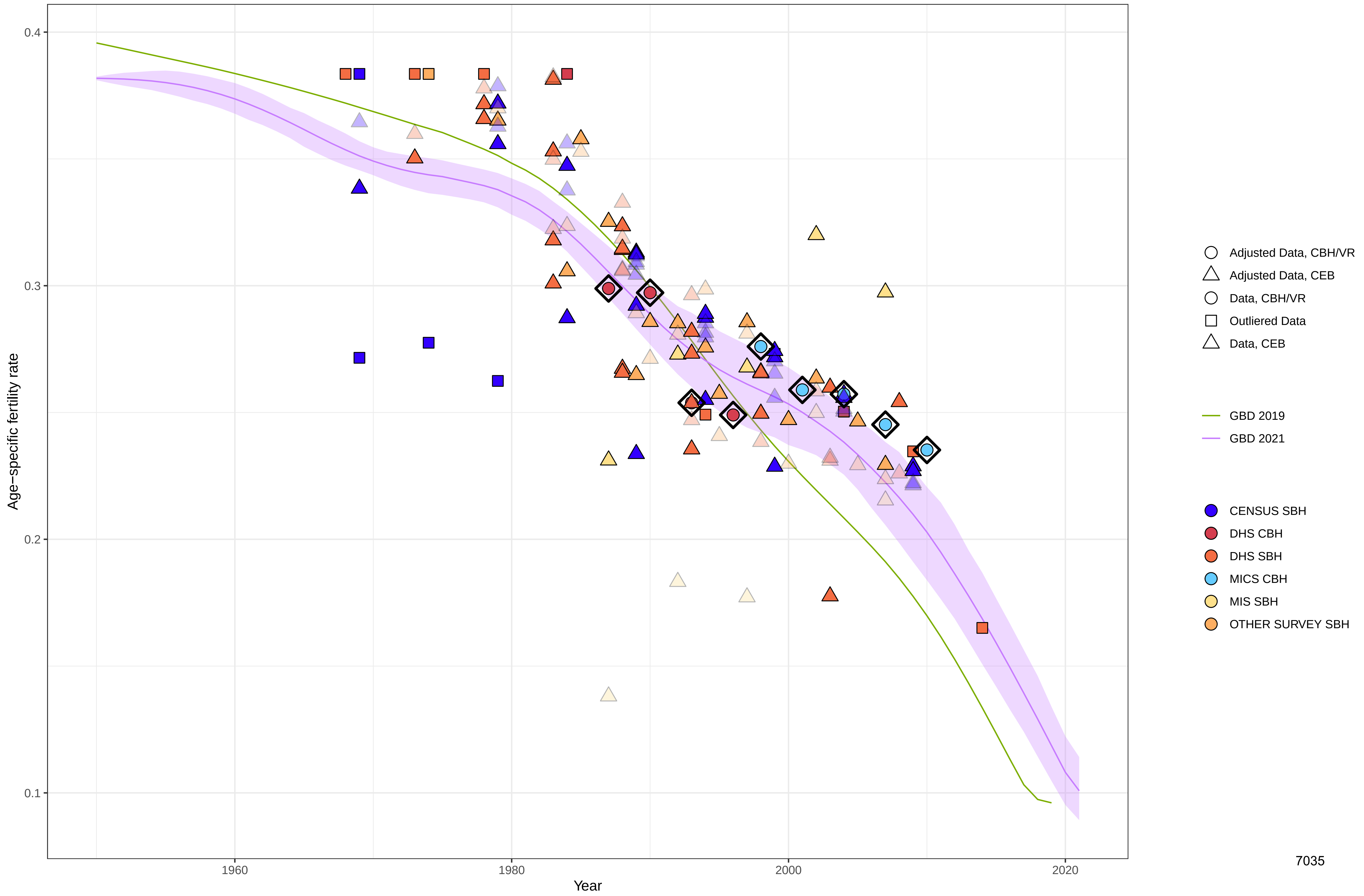

7035

Age−specific fertility fitted trend lines, 1950−2021: Kisii, Ages: 30−34
Data points enclosed with a diamond are reference sources

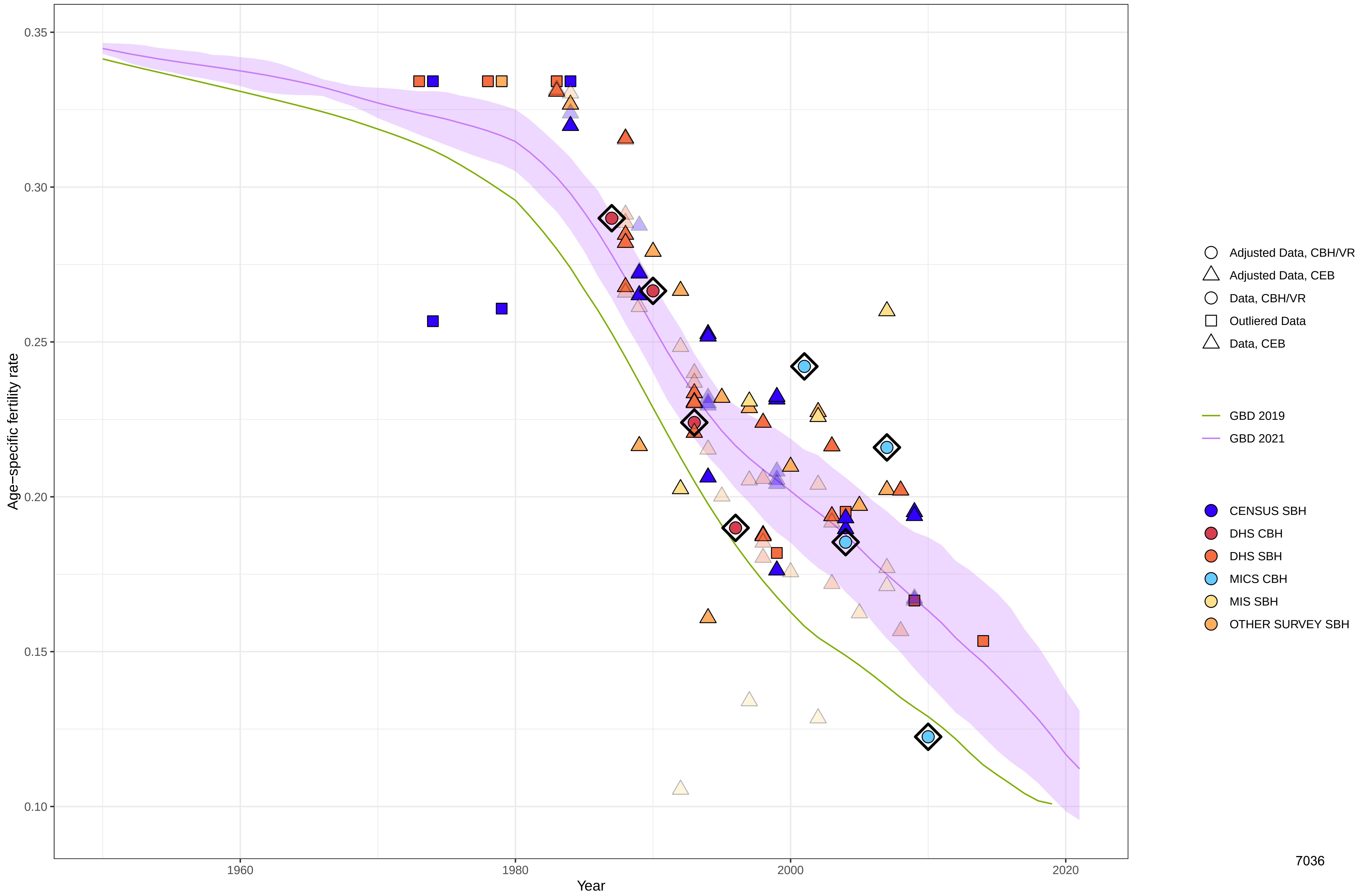

Age−specific fertility fitted trend lines, 1950−2021: Kisii, Ages: 35−39
Data points enclosed with a diamond are reference sources

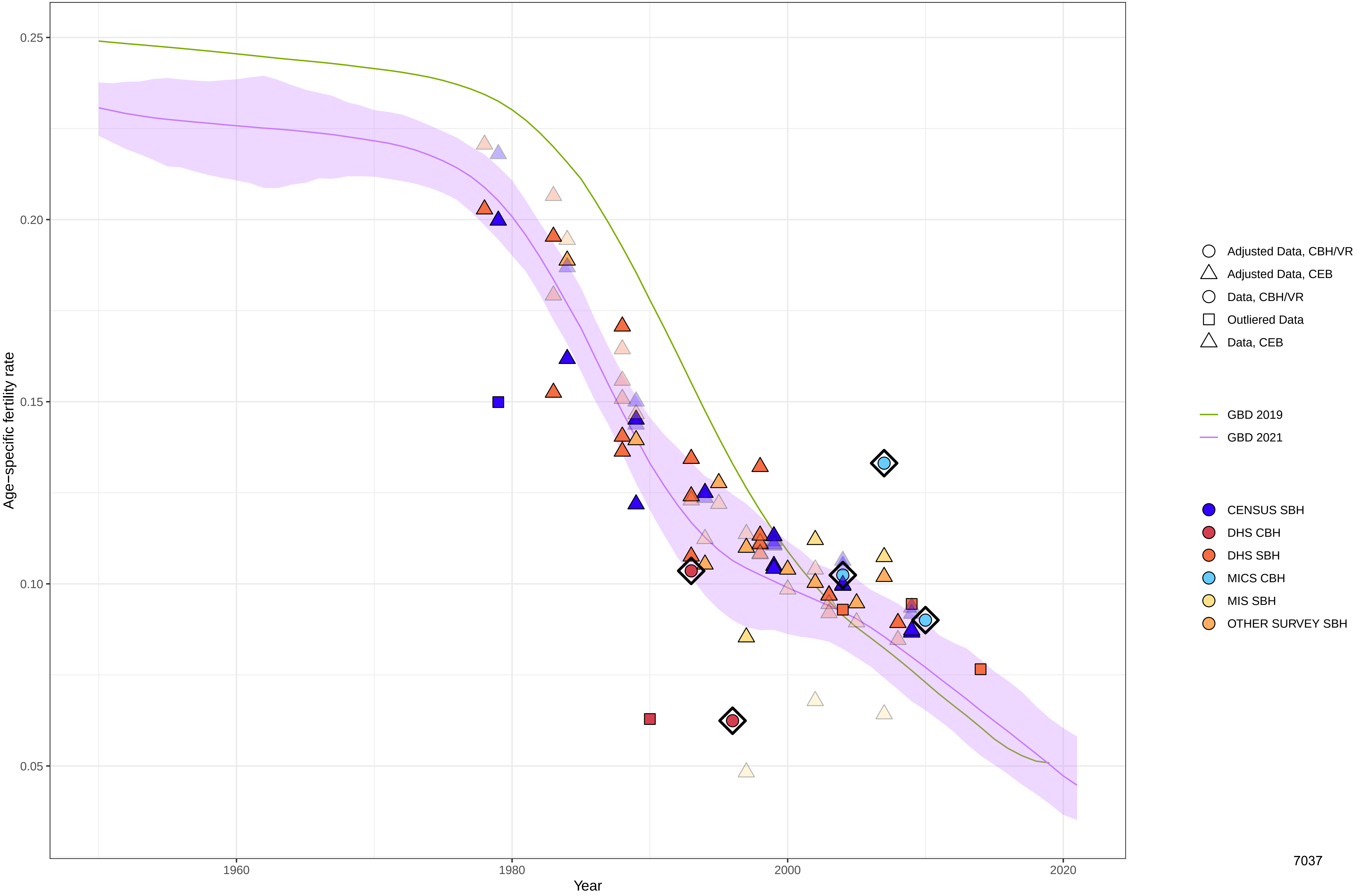

Age−specific fertility fitted trend lines, 1950−2021: Kisii, Ages: 40−44
Data points enclosed with a diamond are reference sources

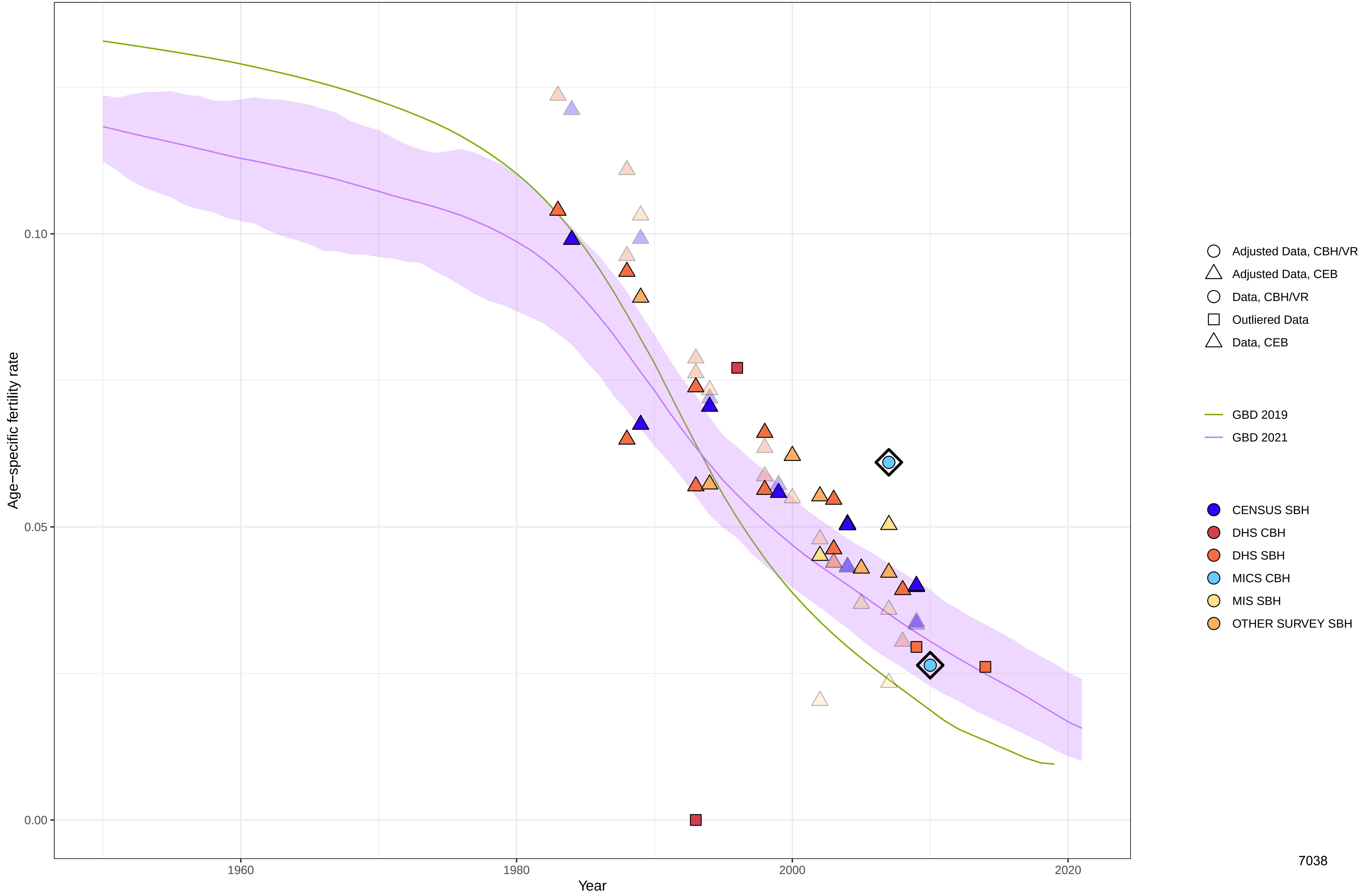

7038

Age−specific fertility fitted trend lines, 1950−2021: Kisii, Ages: 45−49
Data points enclosed with a diamond are reference sources

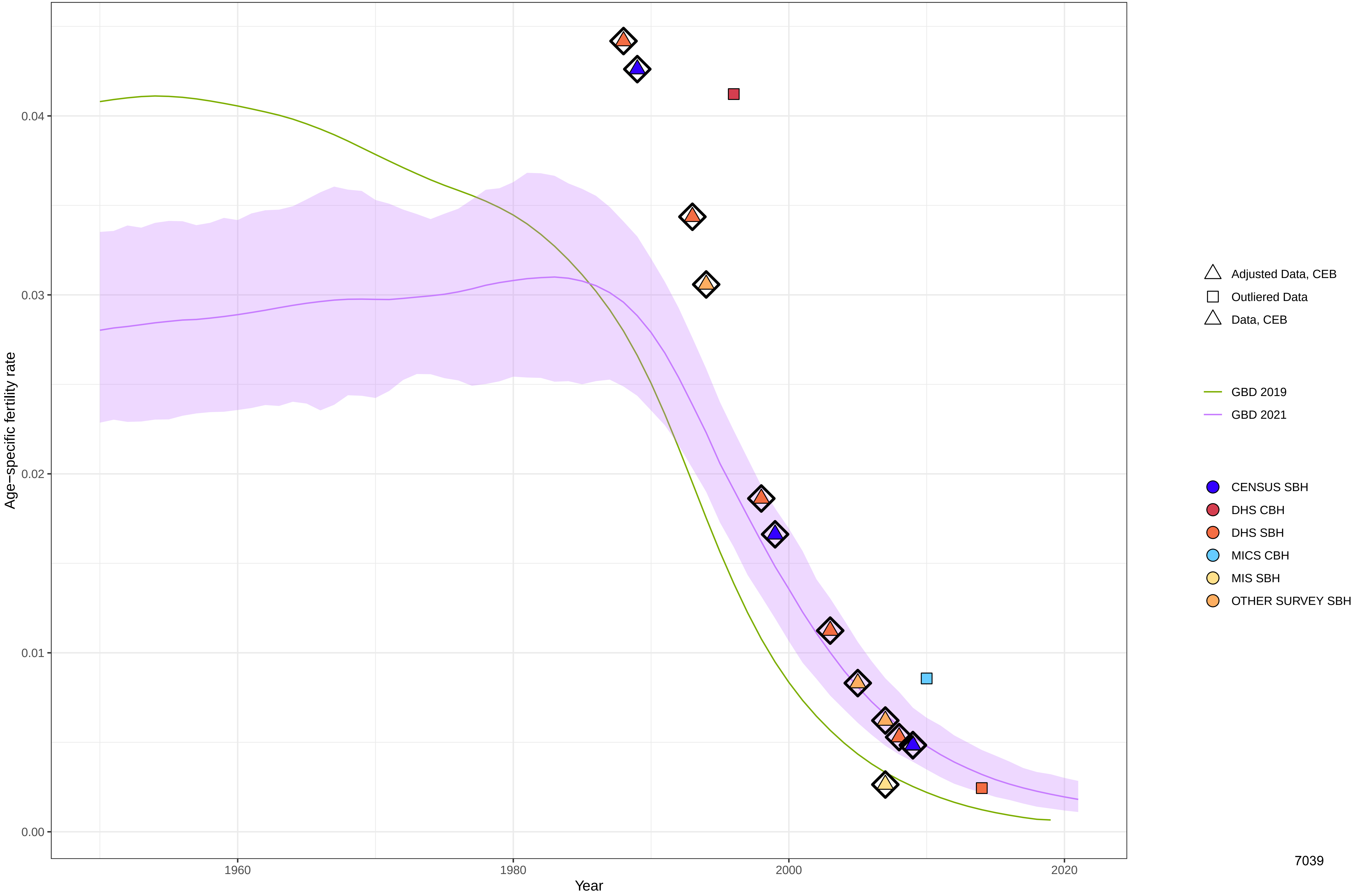

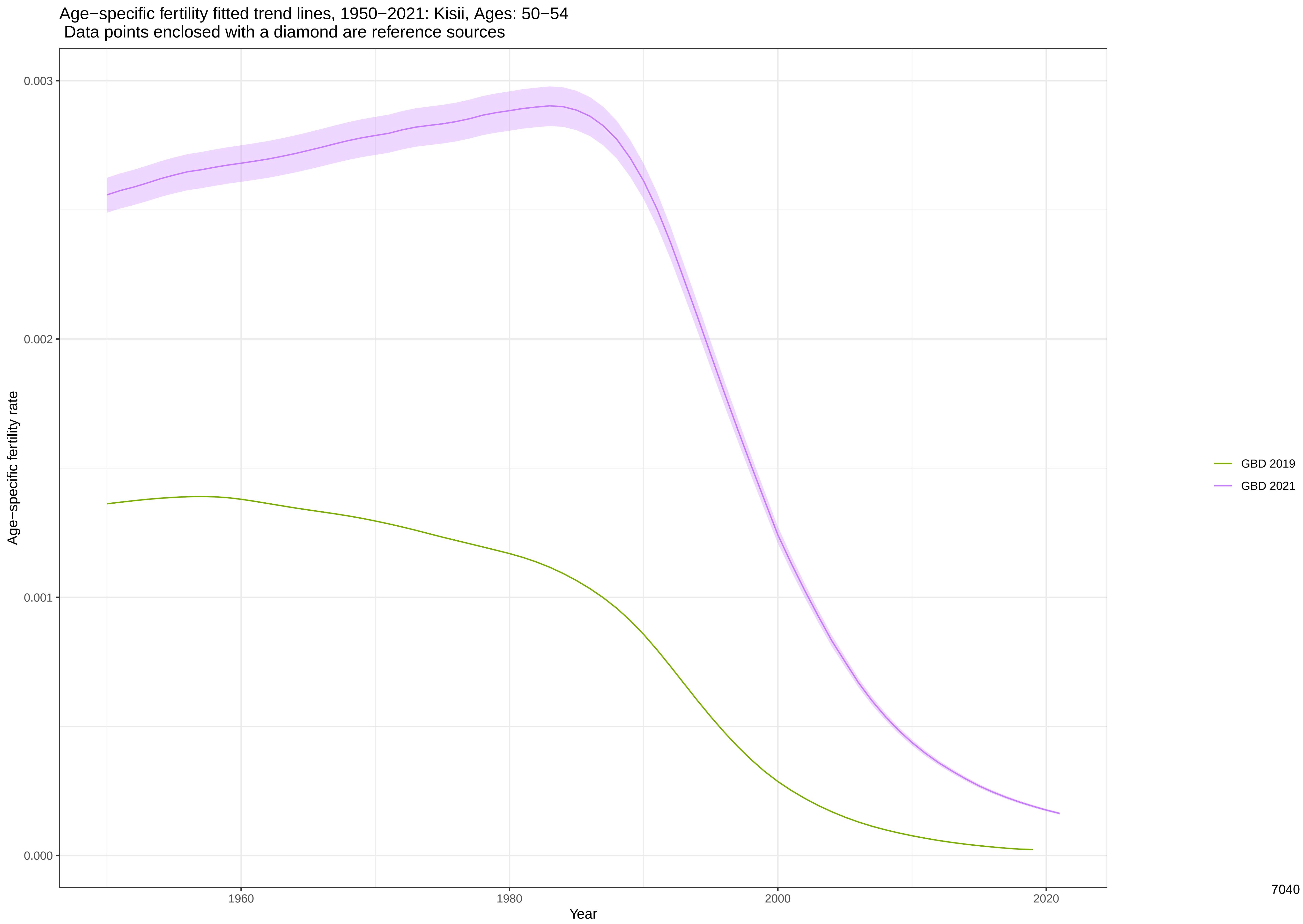

Age−specific fertility fitted trend lines, 1950−2021: Kisii, Ages: 50−54
Data points enclosed with a diamond are reference sources
0.003
0.002
0.001
0.000
Age−specific fertility rate
1960
1980
2000
2020
Year
GBD 2019
GBD 2021

Total fertility rate fitted trend lines, 1950−2021: Kisii TFR
Data points enclosed with a diamond are reference sources

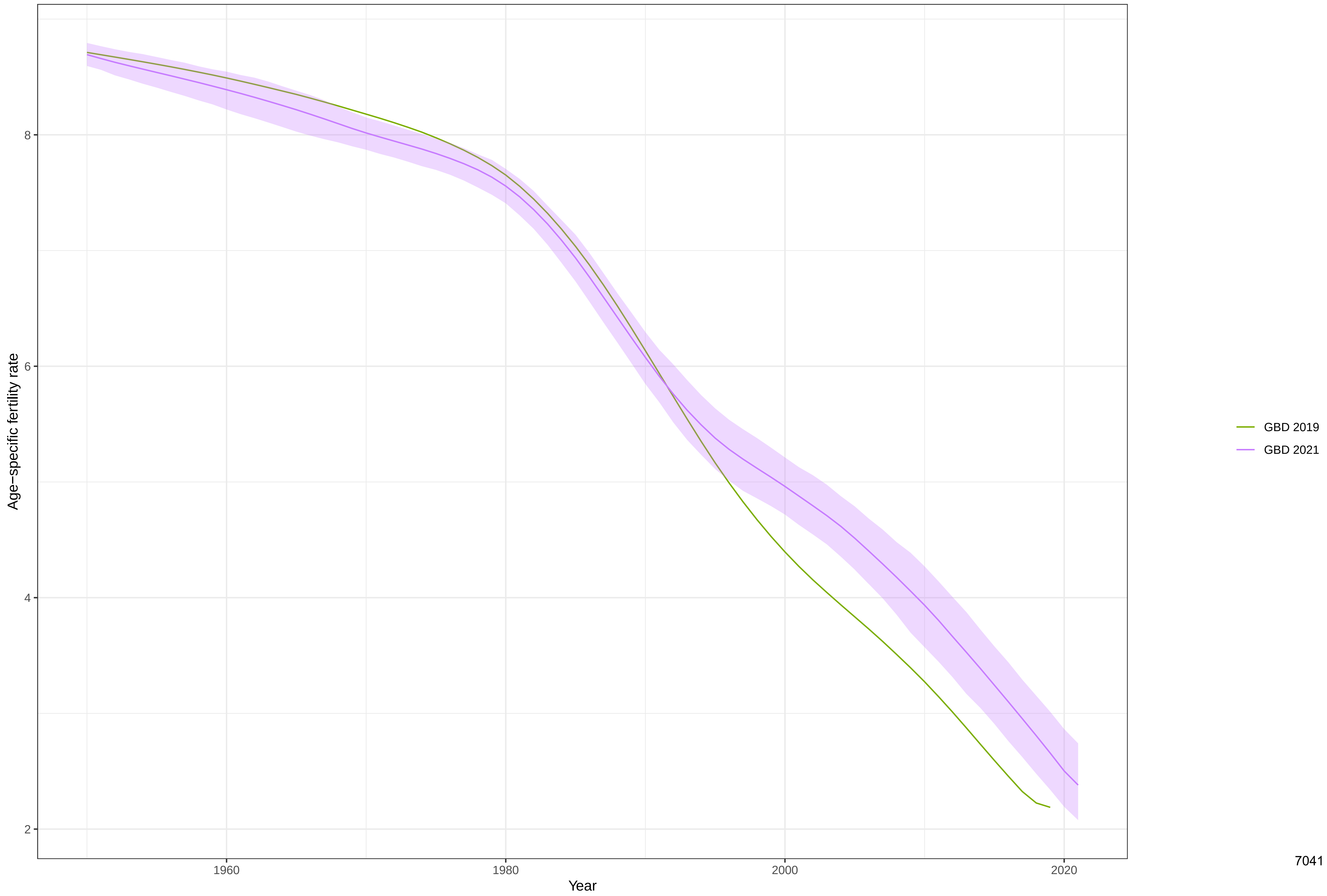

Age−specific fertility fitted trend lines, 1950−2021: Kisumu, Ages: 10−14
Data points enclosed with a diamond are reference sources

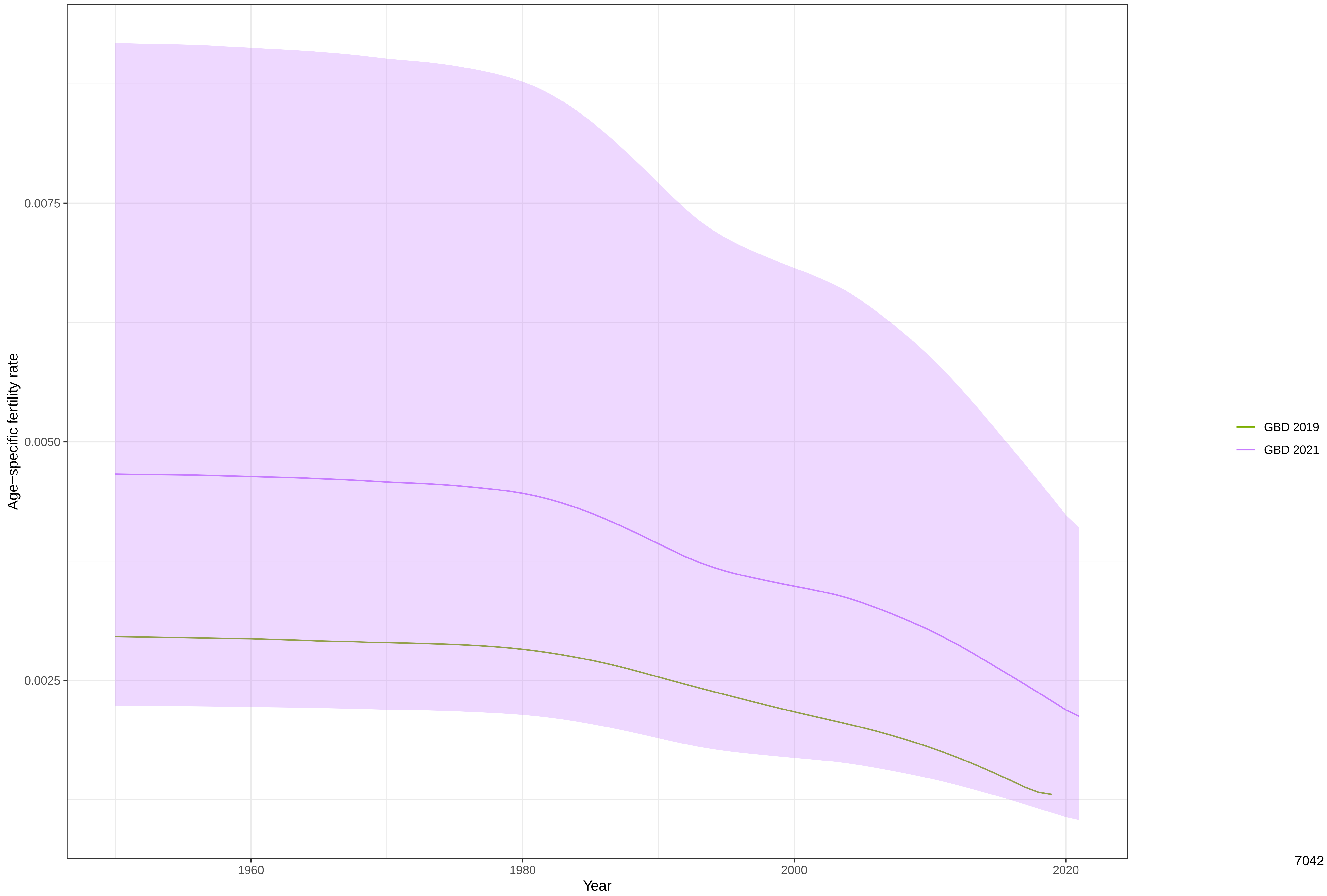

Age−specific fertility fitted trend lines, 1950−2021: Kisumu, Ages: 15−19
Data points enclosed with a diamond are reference sources

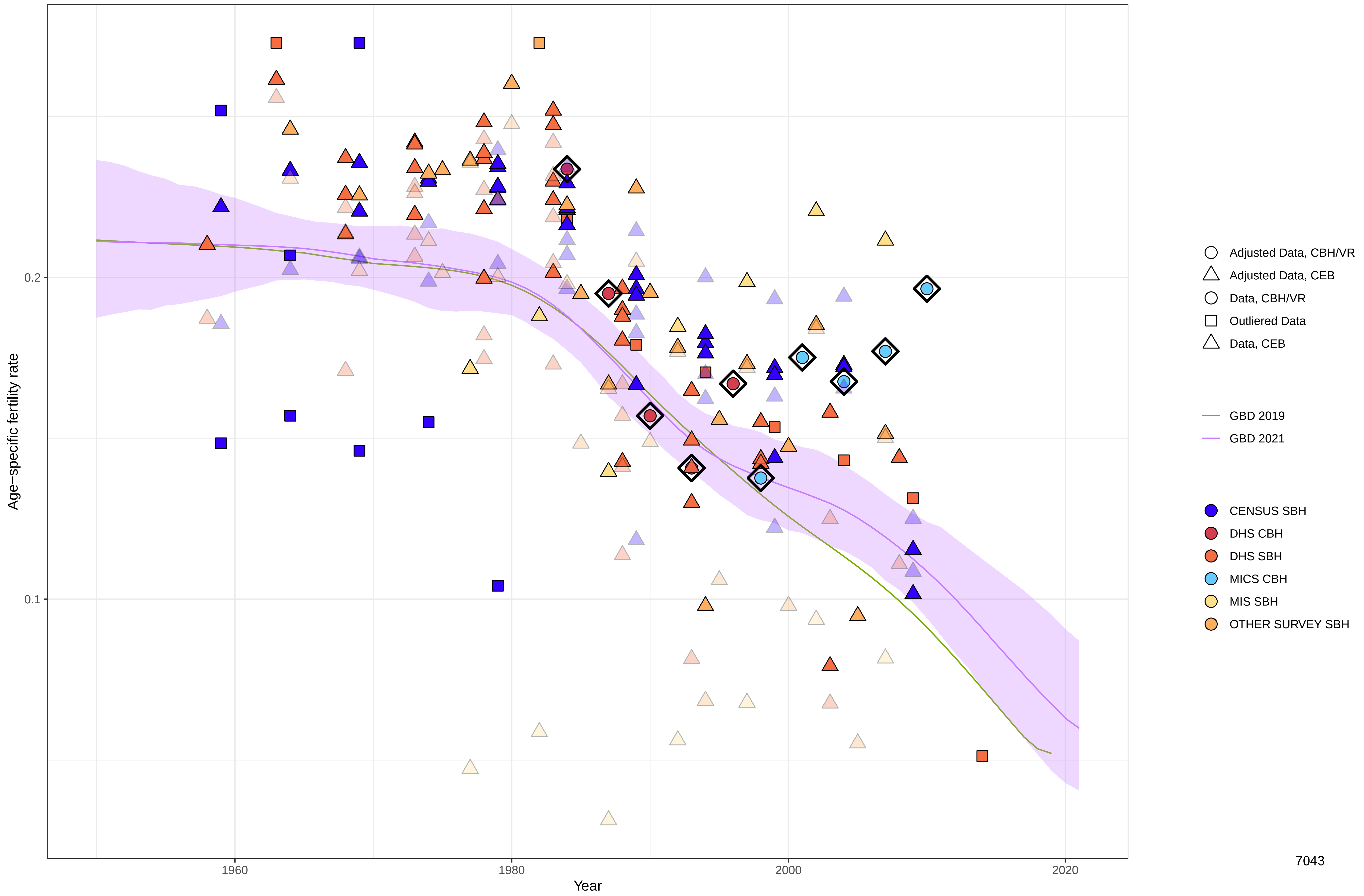

Age−specific fertility fitted trend lines, 1950−2021: Kisumu, Ages: 20−24
Data points enclosed with a diamond are reference sources

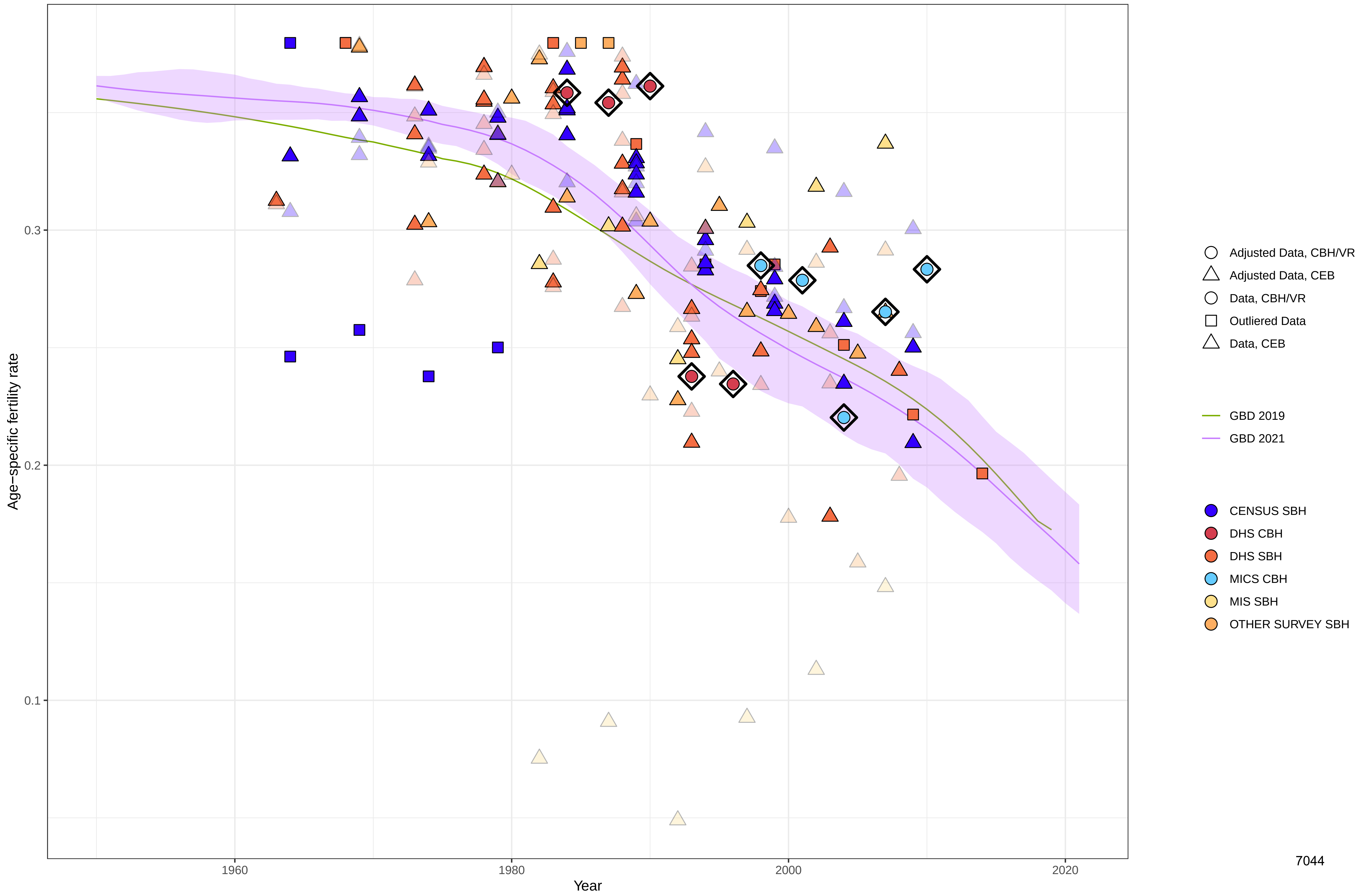

Age−specific fertility fitted trend lines, 1950−2021: Kisumu, Ages: 25−29
Data points enclosed with a diamond are reference sources

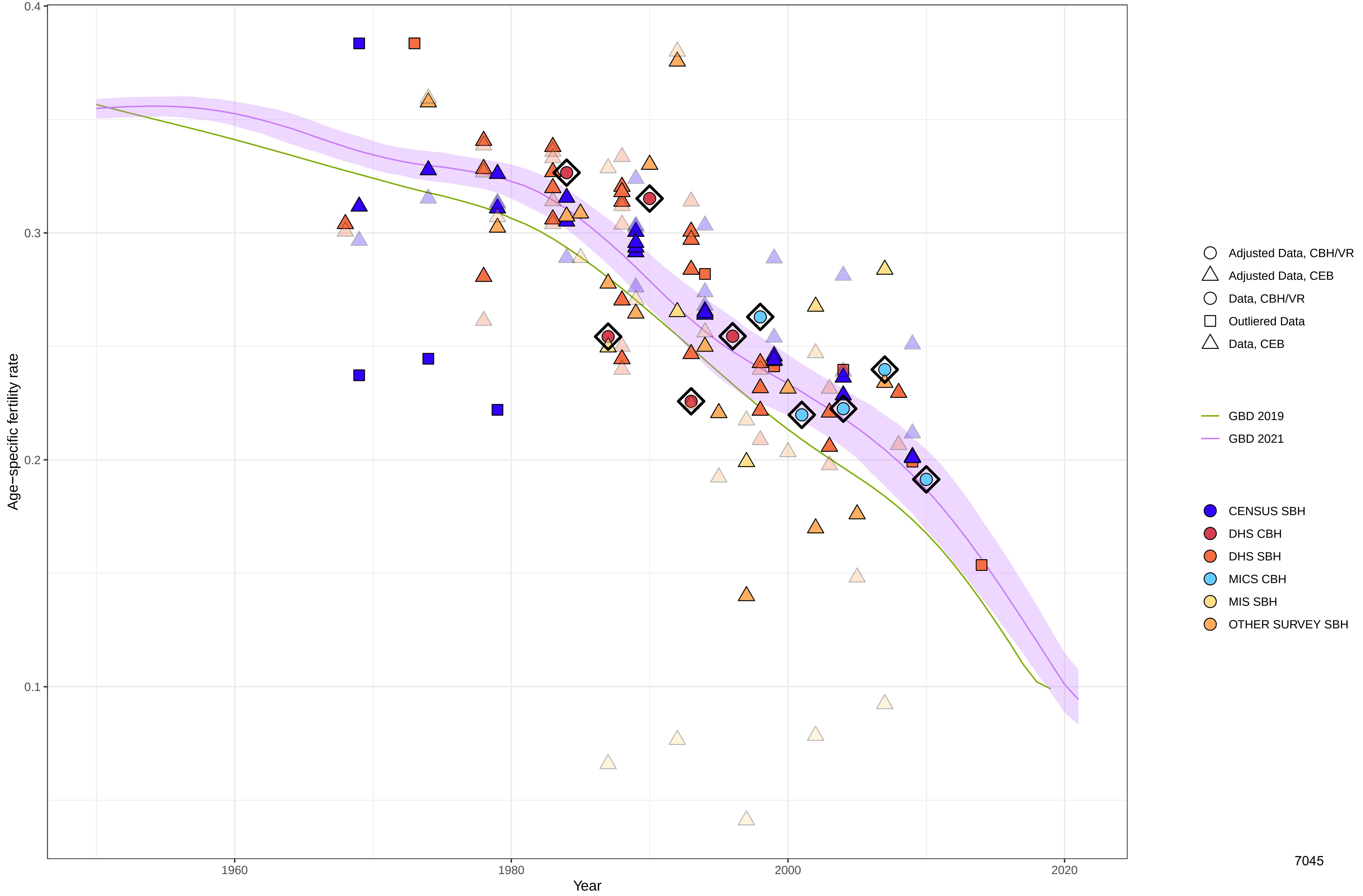

Age−specific fertility fitted trend lines, 1950−2021: Kisumu, Ages: 30−34
Data points enclosed with a diamond are reference sources

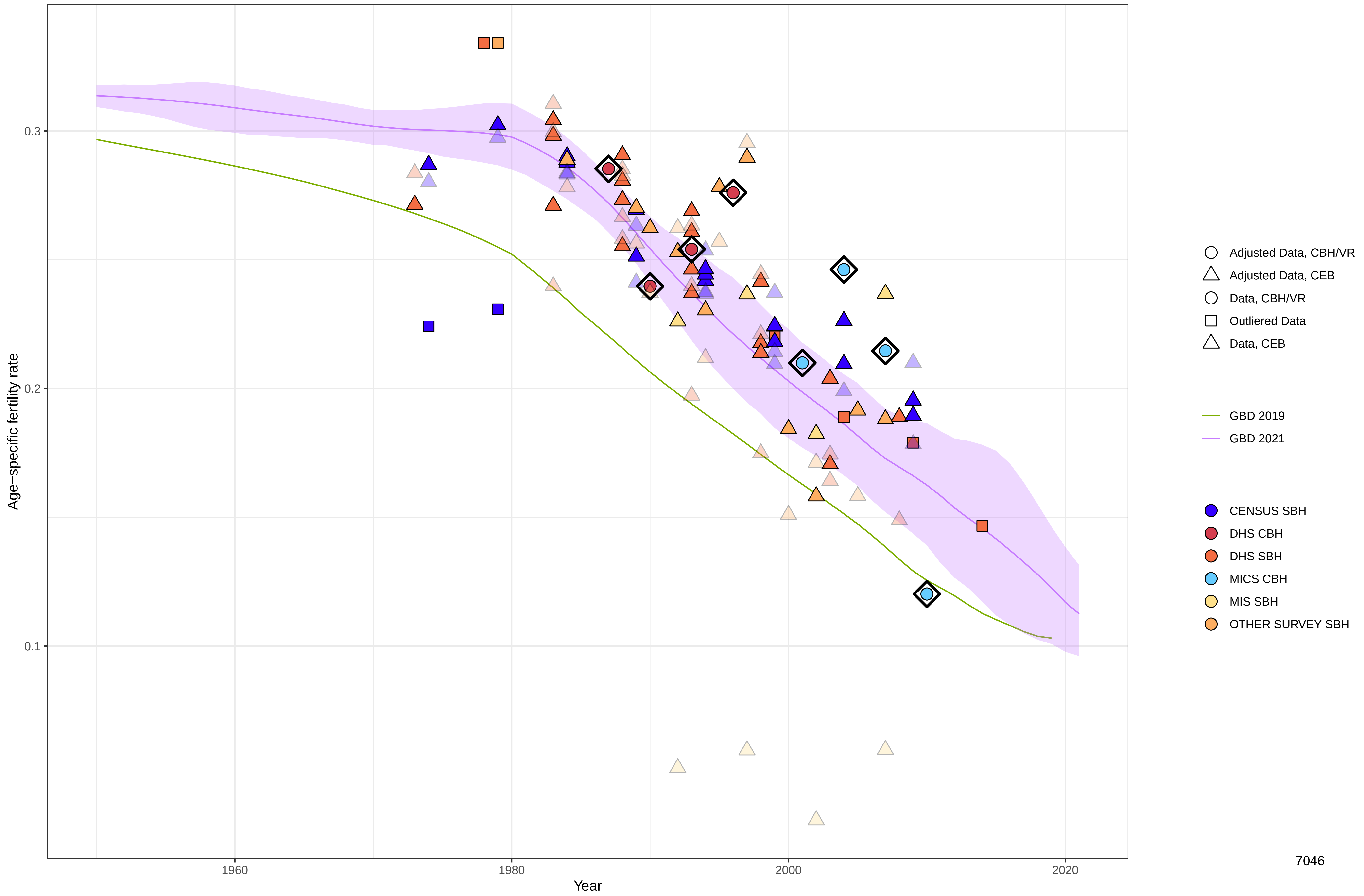

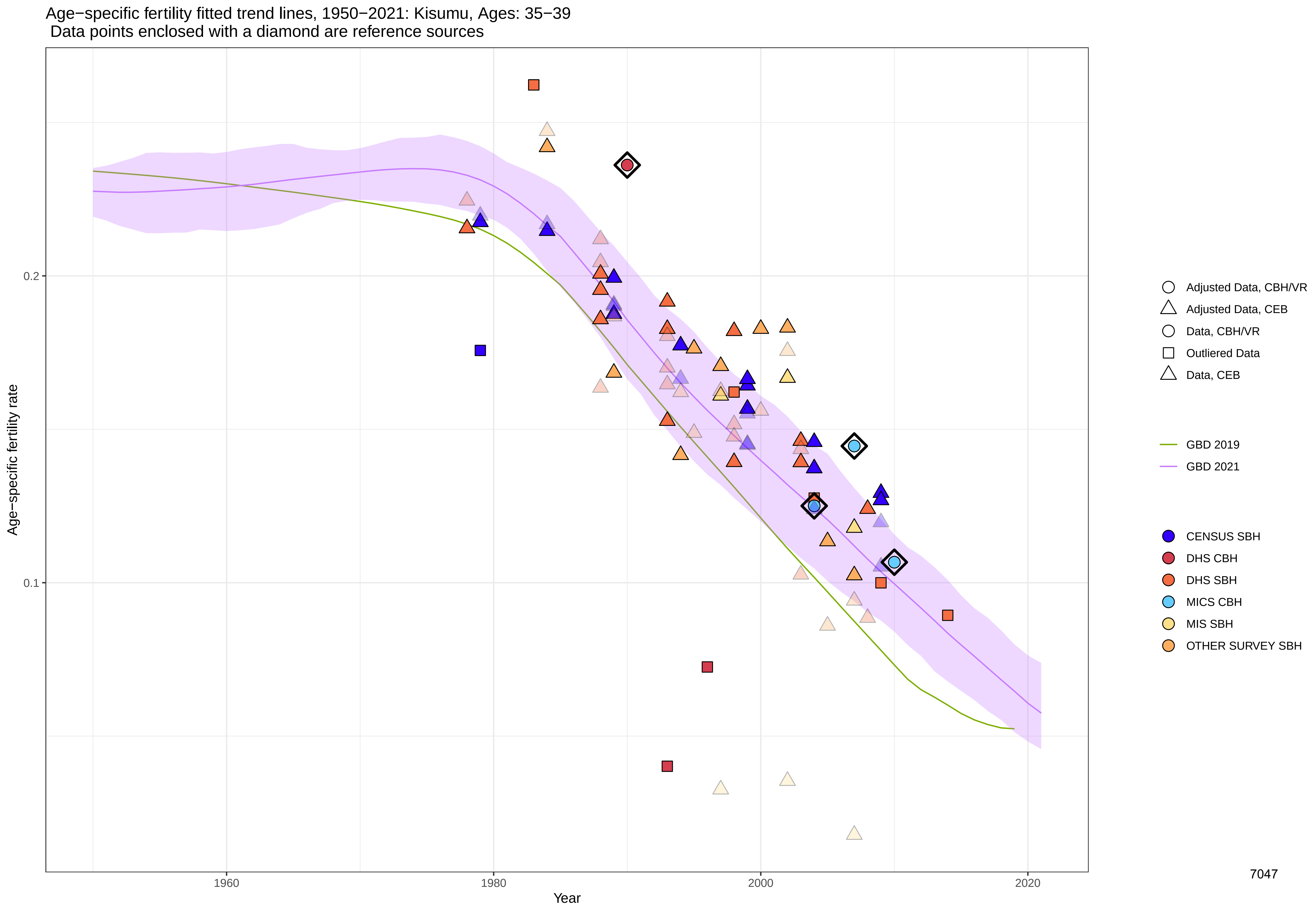
Age−specific fertility fitted trend lines, 1950−2021: Kisumu, Ages: 35−39
Data points enclosed with a diamond are reference sources
Age−specific fertility rate
0.2
0.1
1960
1980
2000
2020
Year
Adjusted Data, CBH/VR
Adjusted Data, CEB
Data, CBH/VR
Outliered Data
Data, CEB
GBD 2019
GBD 2021
CENSUS SBH
DHS CBH
DHS SBH
MICS CBH
MIS SBH
OTHER SURVEY SBH

7047

Age−specific fertility fitted trend lines, 1950−2021: Kisumu, Ages: 40−44
Data points enclosed with a diamond are reference sources

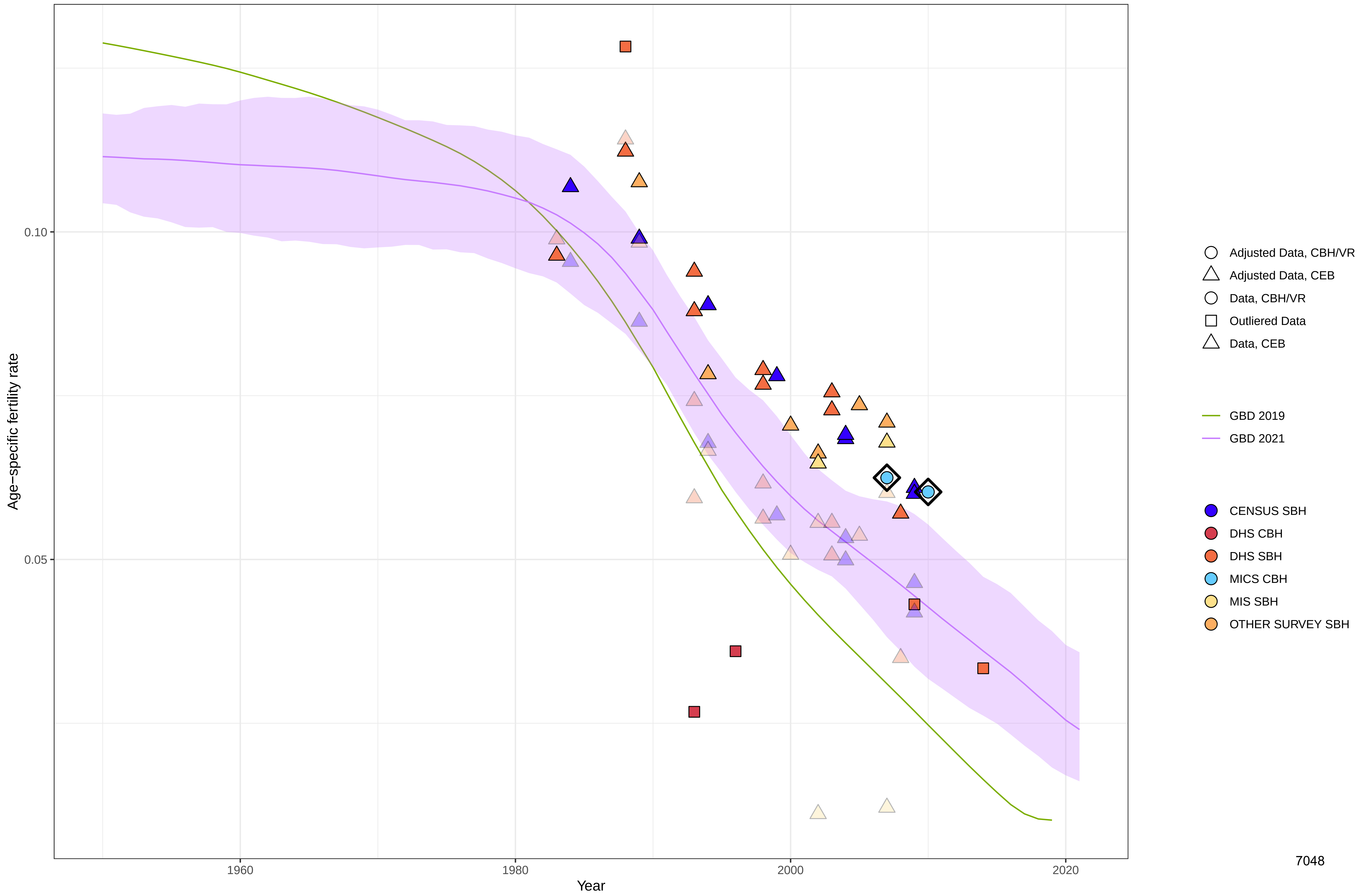

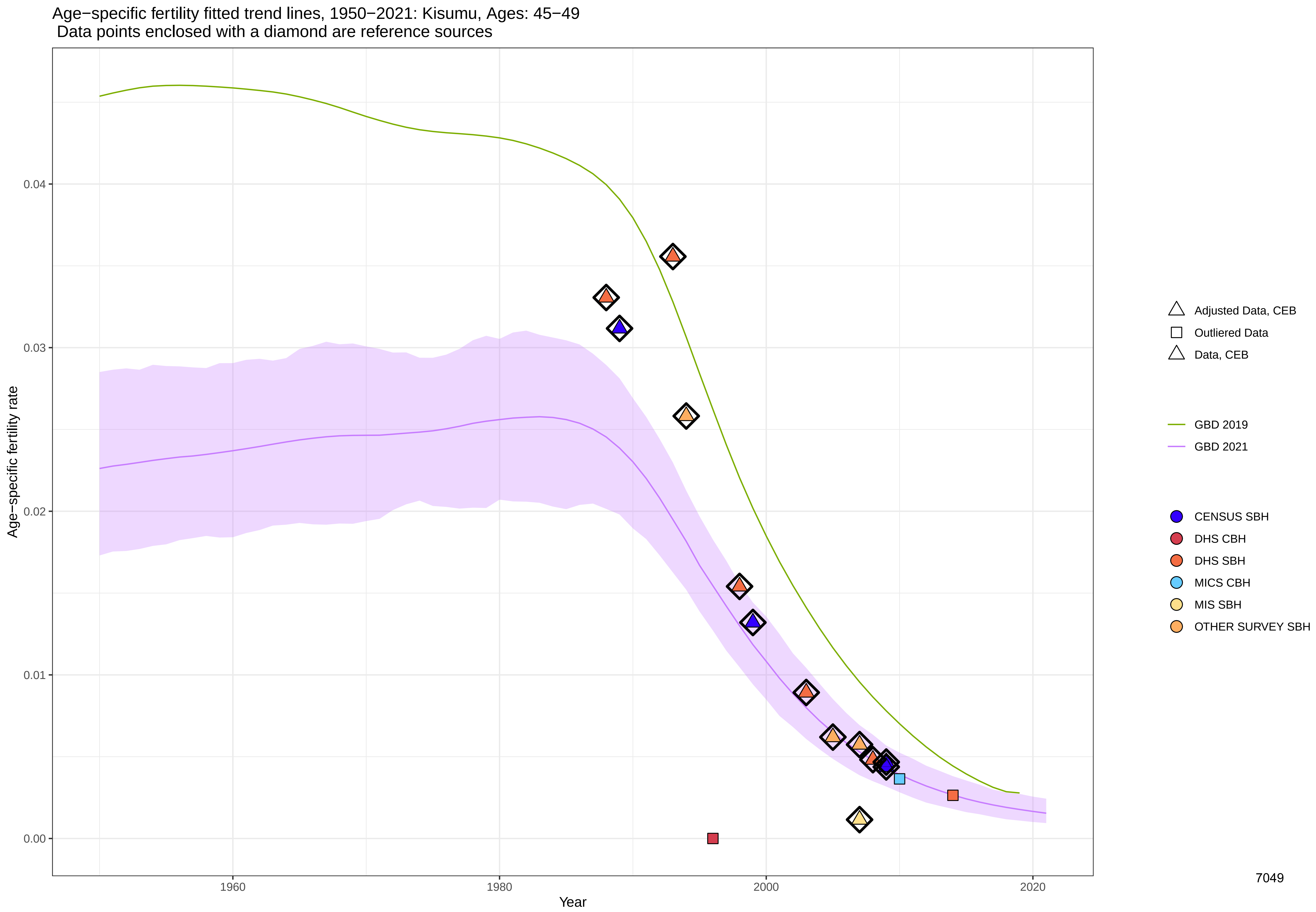

Age−specific fertility fitted trend lines, 1950−2021: Kisumu, Ages: 45−49
Data points enclosed with a diamond are reference sources
Age−specific fertility rate
0.00
0.01
0.02
0.03
0.04
1960
1980
2000
2020
Year
Adjusted Data, CEB
Outliered Data
Data, CEB
GBD 2019
GBD 2021
CENSUS SBH
DHS CBH
DHS SBH
MICS CBH
MIS SBH
OTHER SURVEY SBH

Age−specific fertility fitted trend lines, 1950−2021: Kisumu, Ages: 50−54
Data points enclosed with a diamond are reference sources

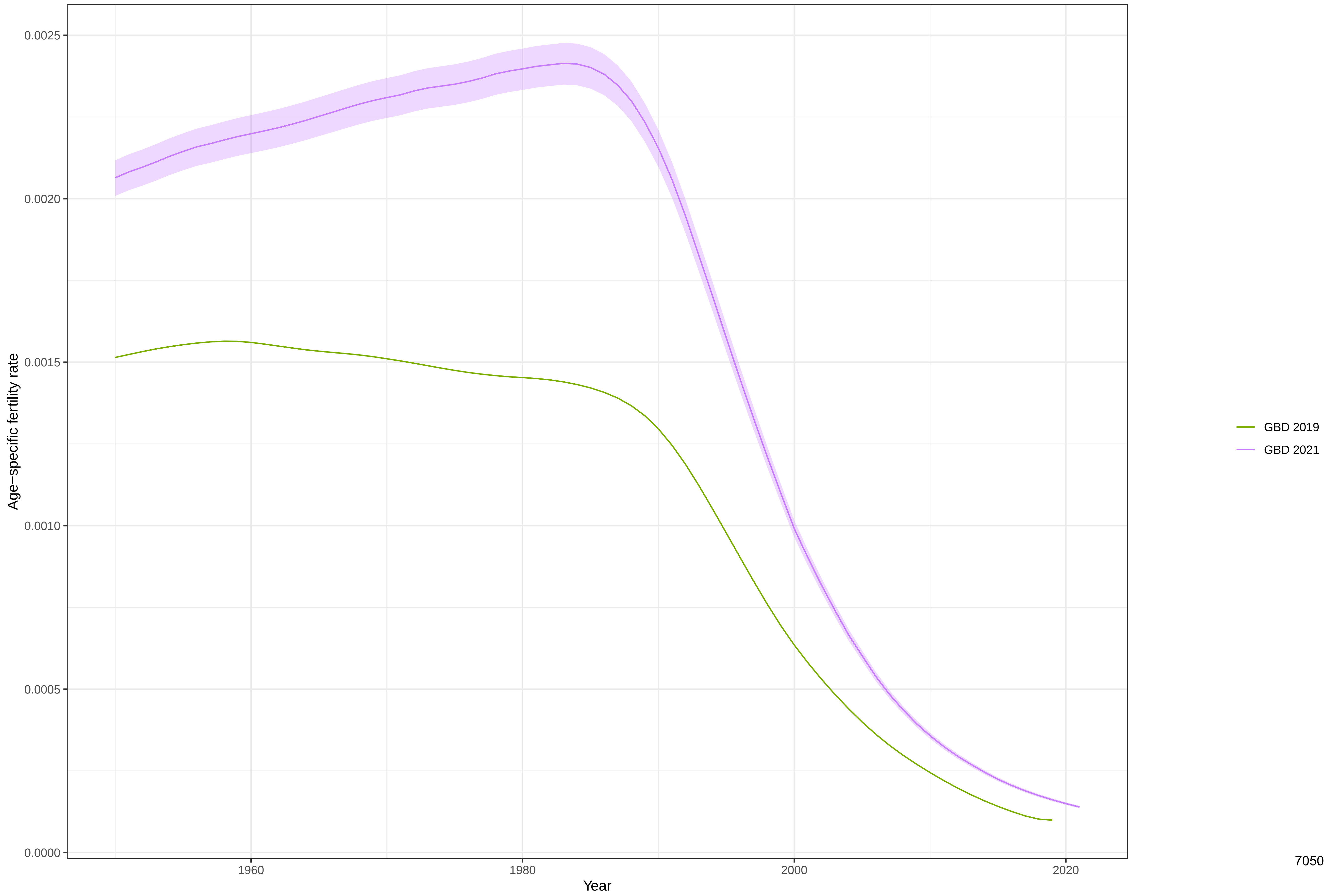

Total fertility rate fitted trend lines, 1950−2021: Kisumu TFR
Data points enclosed with a diamond are reference sources

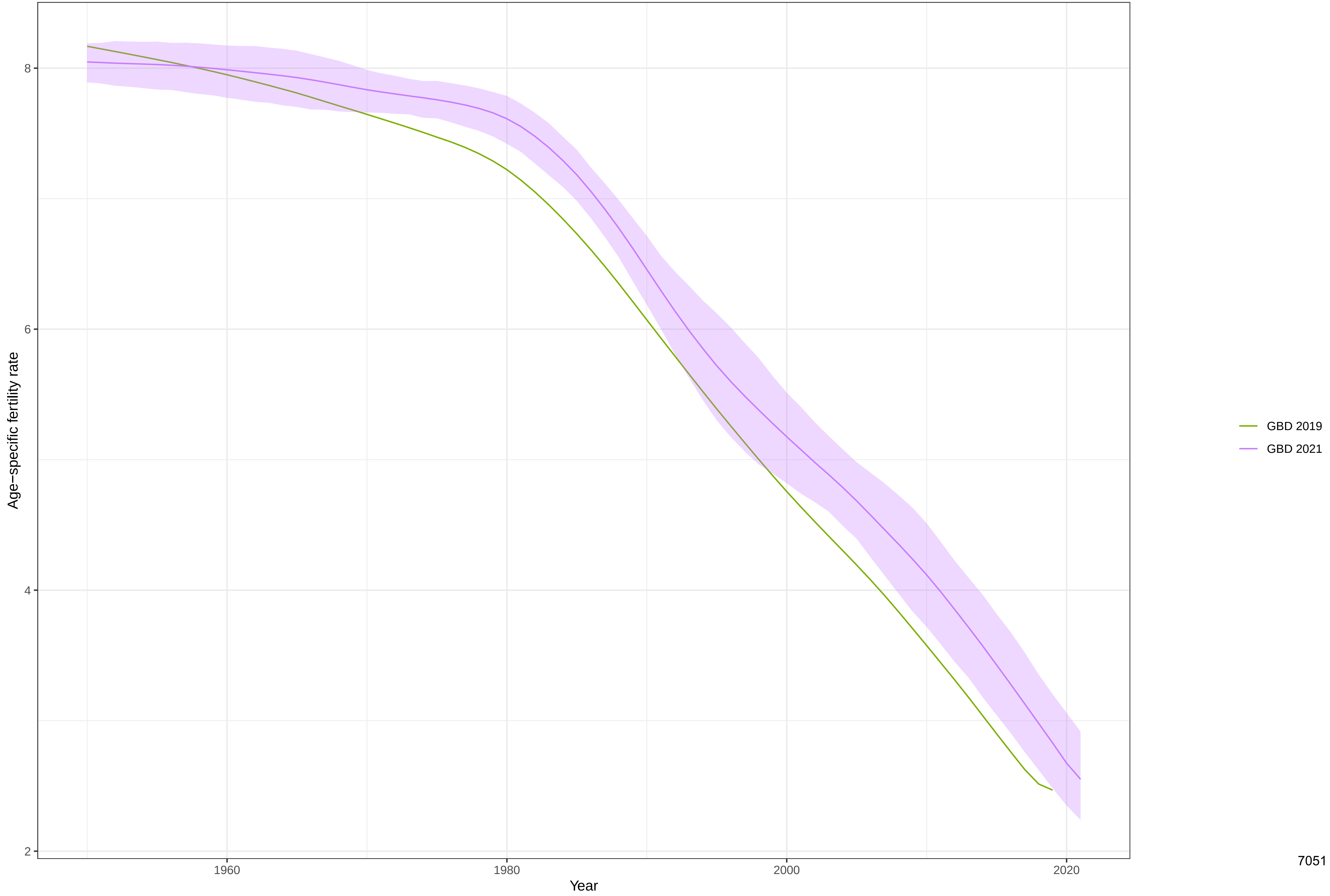

Age−specific fertility fitted trend lines, 1950−2021: Kitui, Ages: 10−14
Data points enclosed with a diamond are reference sources

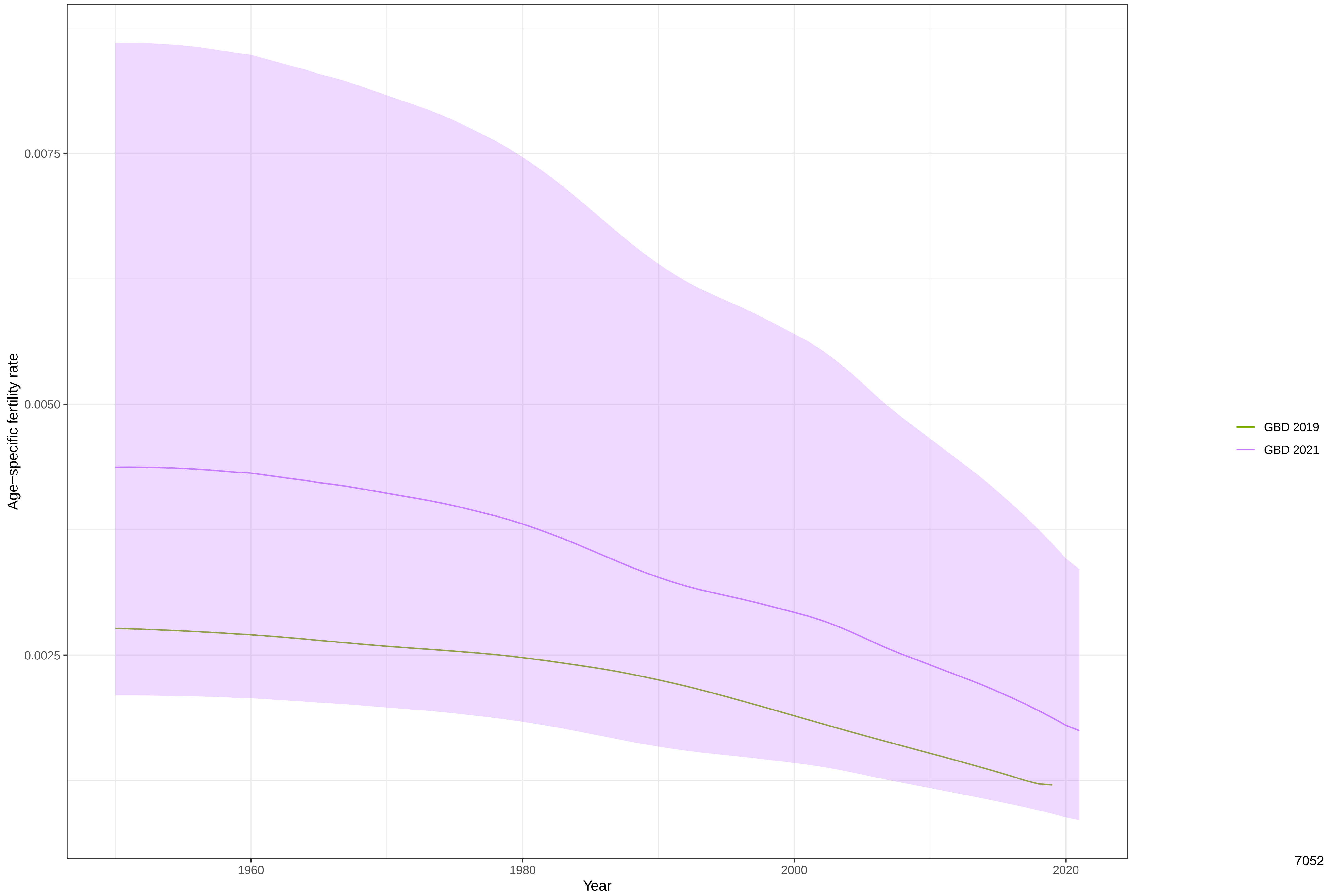

Age−specific fertility fitted trend lines, 1950−2021: Kitui, Ages: 15−19
Data points enclosed with a diamond are reference sources

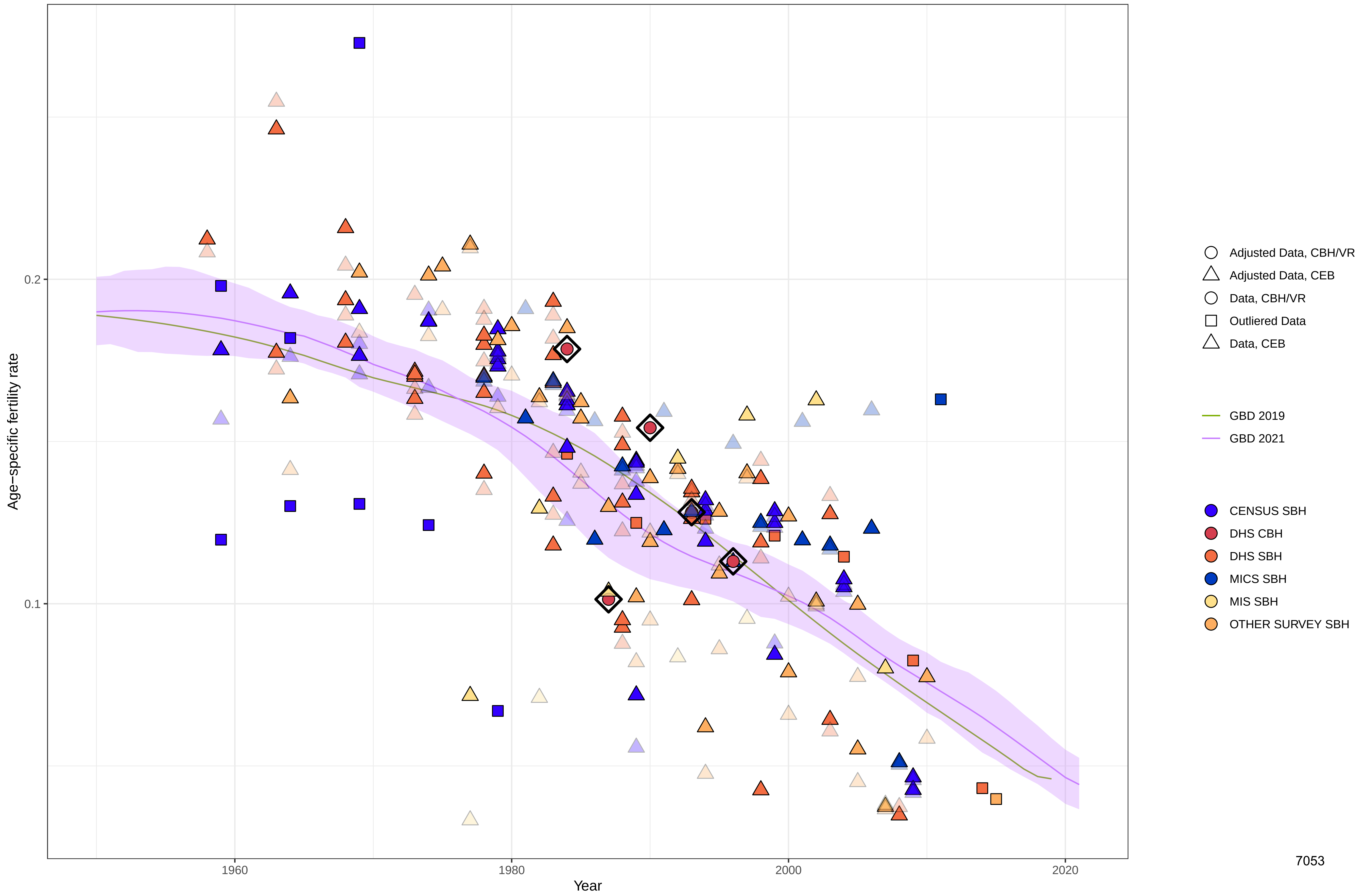

Age−specific fertility fitted trend lines, 1950−2021: Kitui, Ages: 20−24
Data points enclosed with a diamond are reference sources

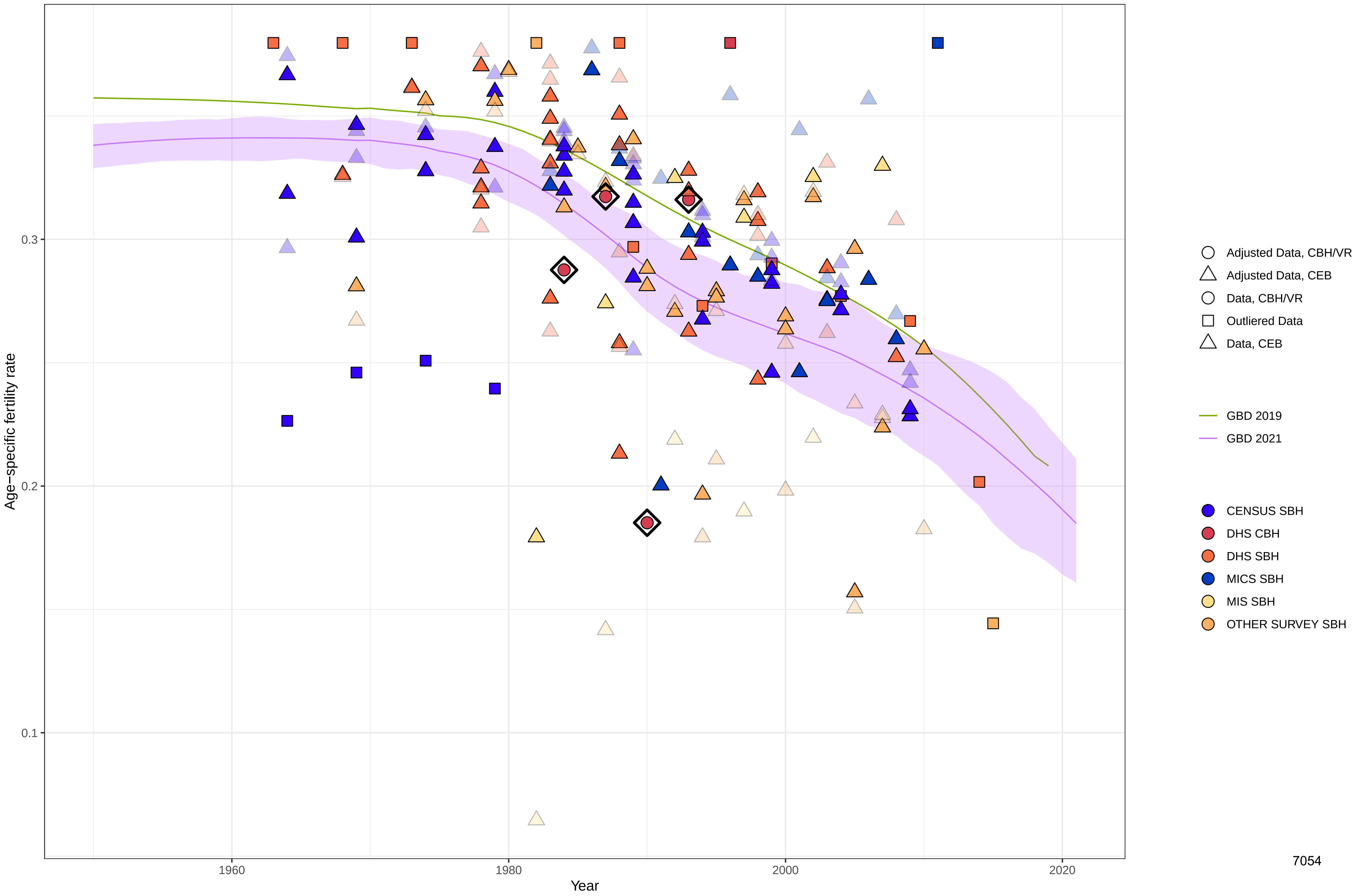

Age−specific fertility fitted trend lines, 1950−2021: Kitui, Ages: 25−29
Data points enclosed with a diamond are reference sources

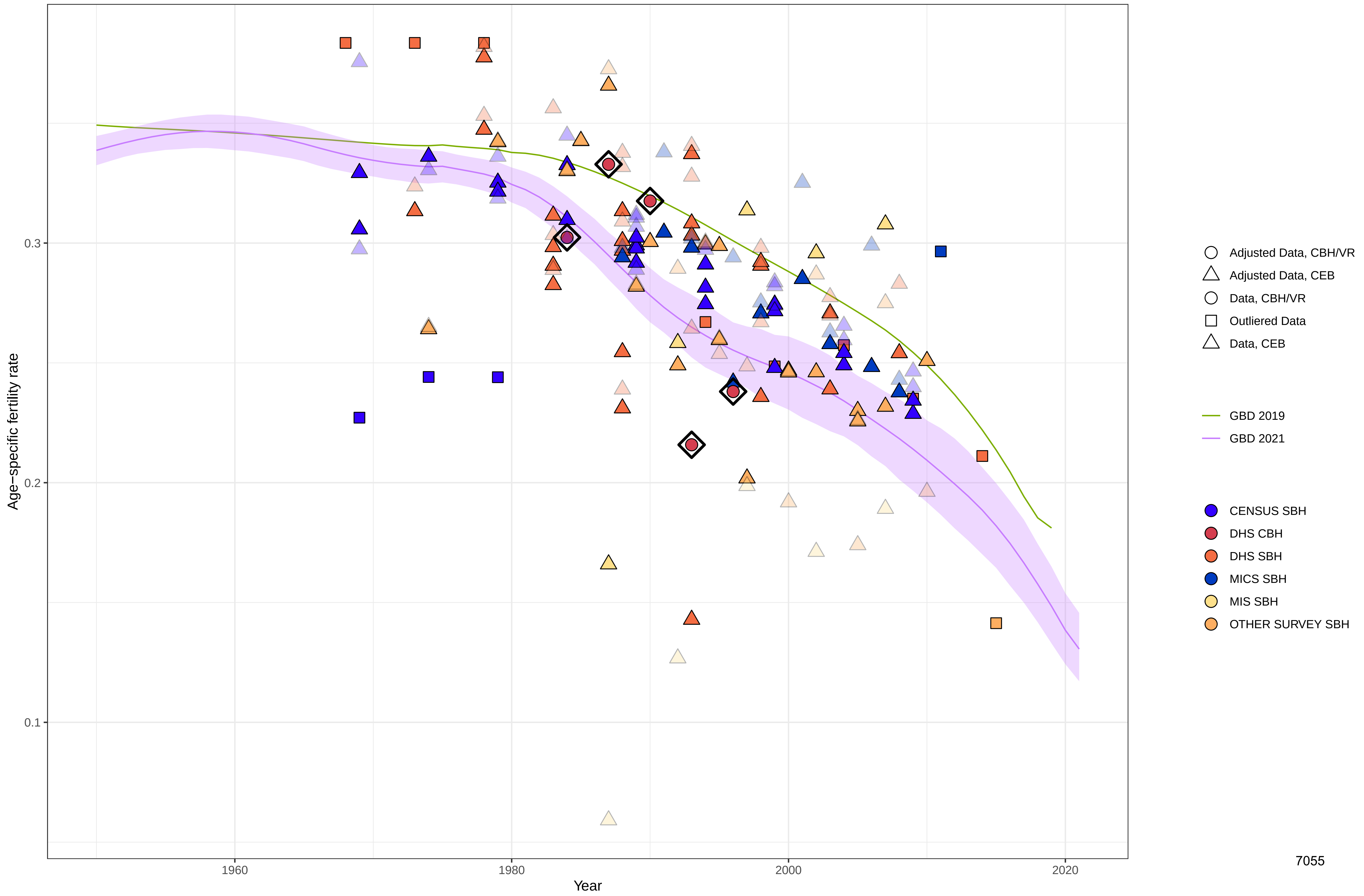

Age−specific fertility fitted trend lines, 1950−2021: Kitui, Ages: 30−34
Data points enclosed with a diamond are reference sources

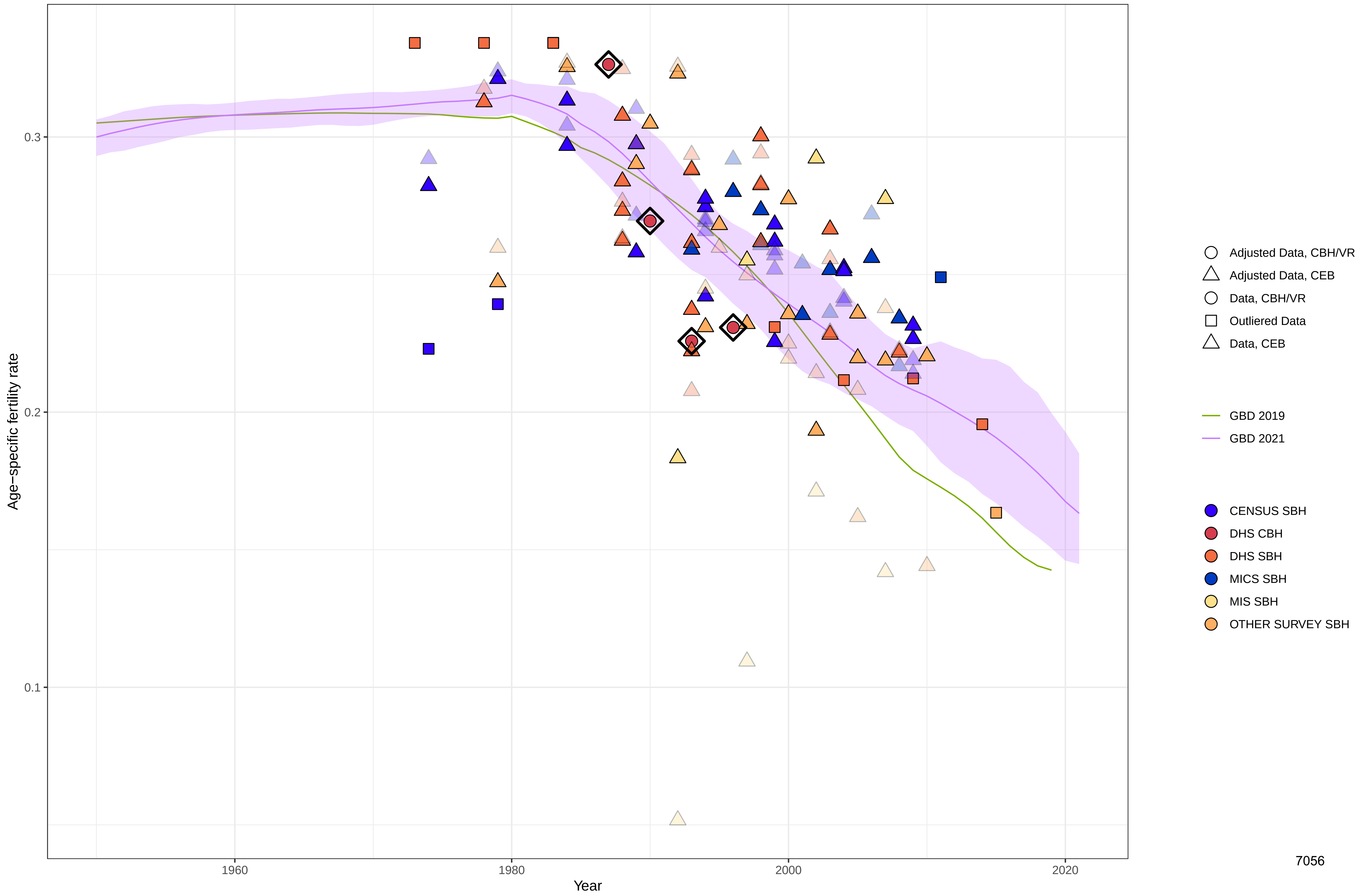

Age−specific fertility fitted trend lines, 1950−2021: Kitui, Ages: 35−39
Data points enclosed with a diamond are reference sources

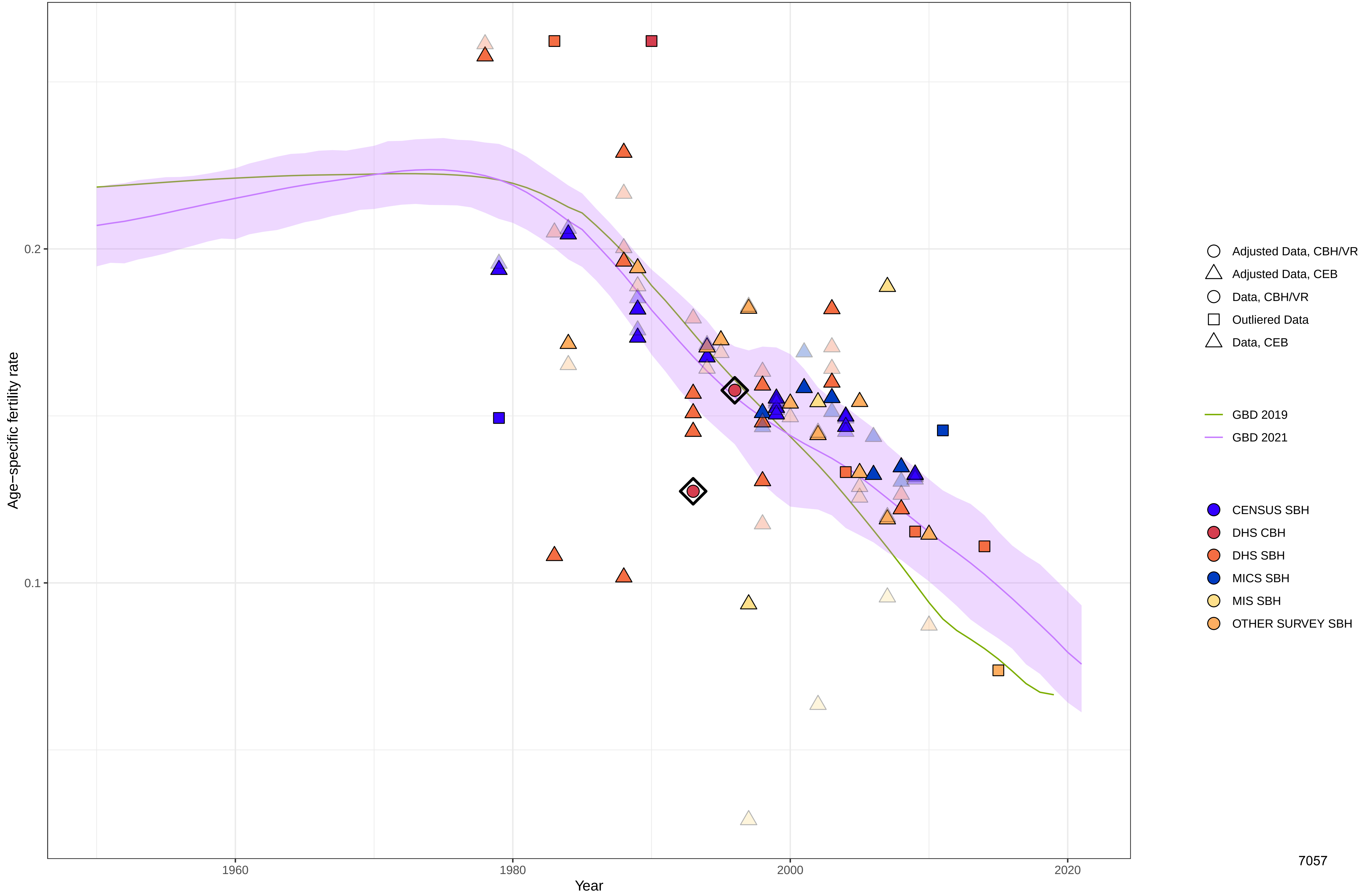

Age−specific fertility fitted trend lines, 1950−2021: Kitui, Ages: 40−44
Data points enclosed with a diamond are reference sources

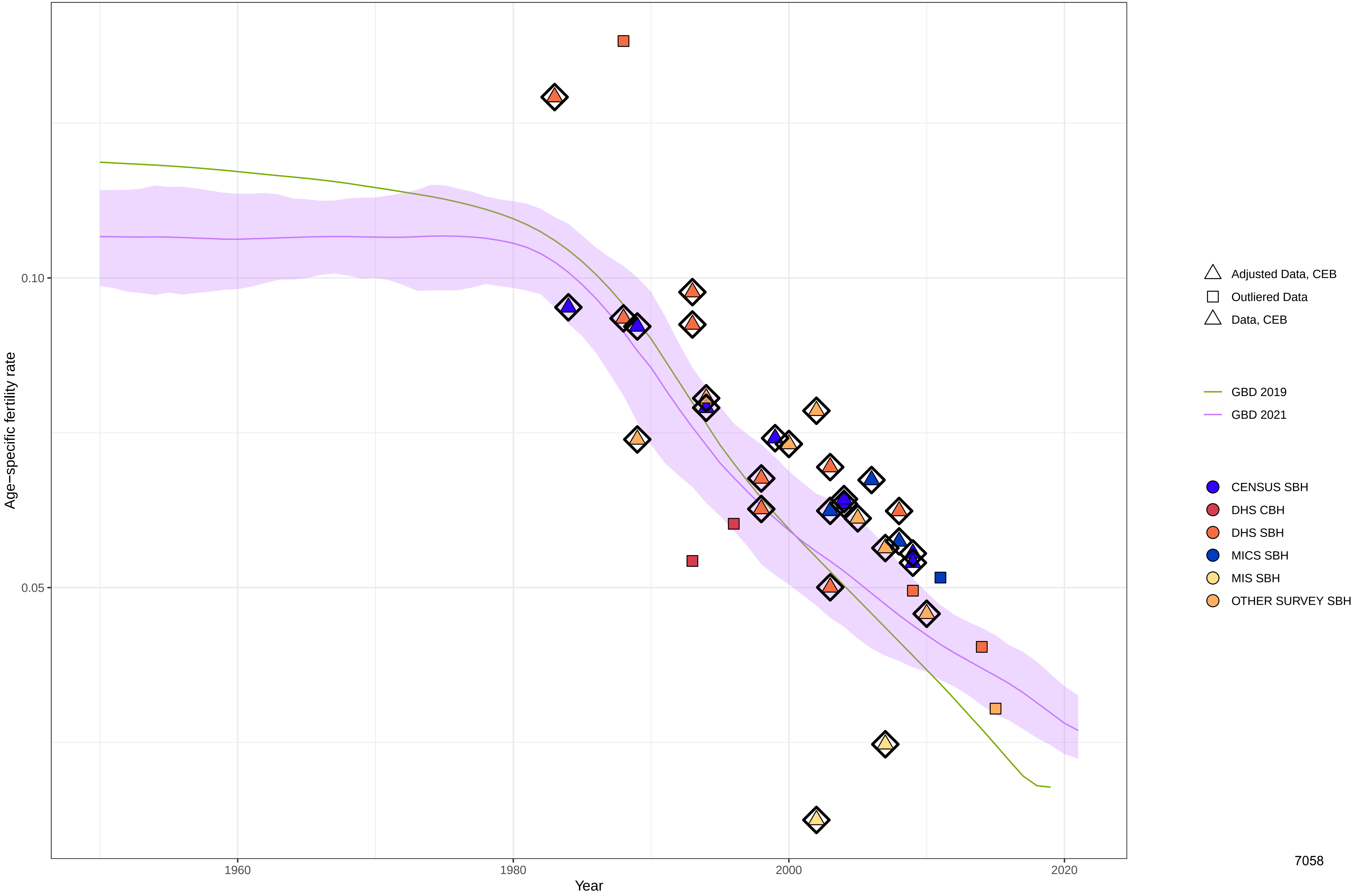

7058

Age−specific fertility fitted trend lines, 1950−2021: Kitui, Ages: 45−49
Data points enclosed with a diamond are reference sources

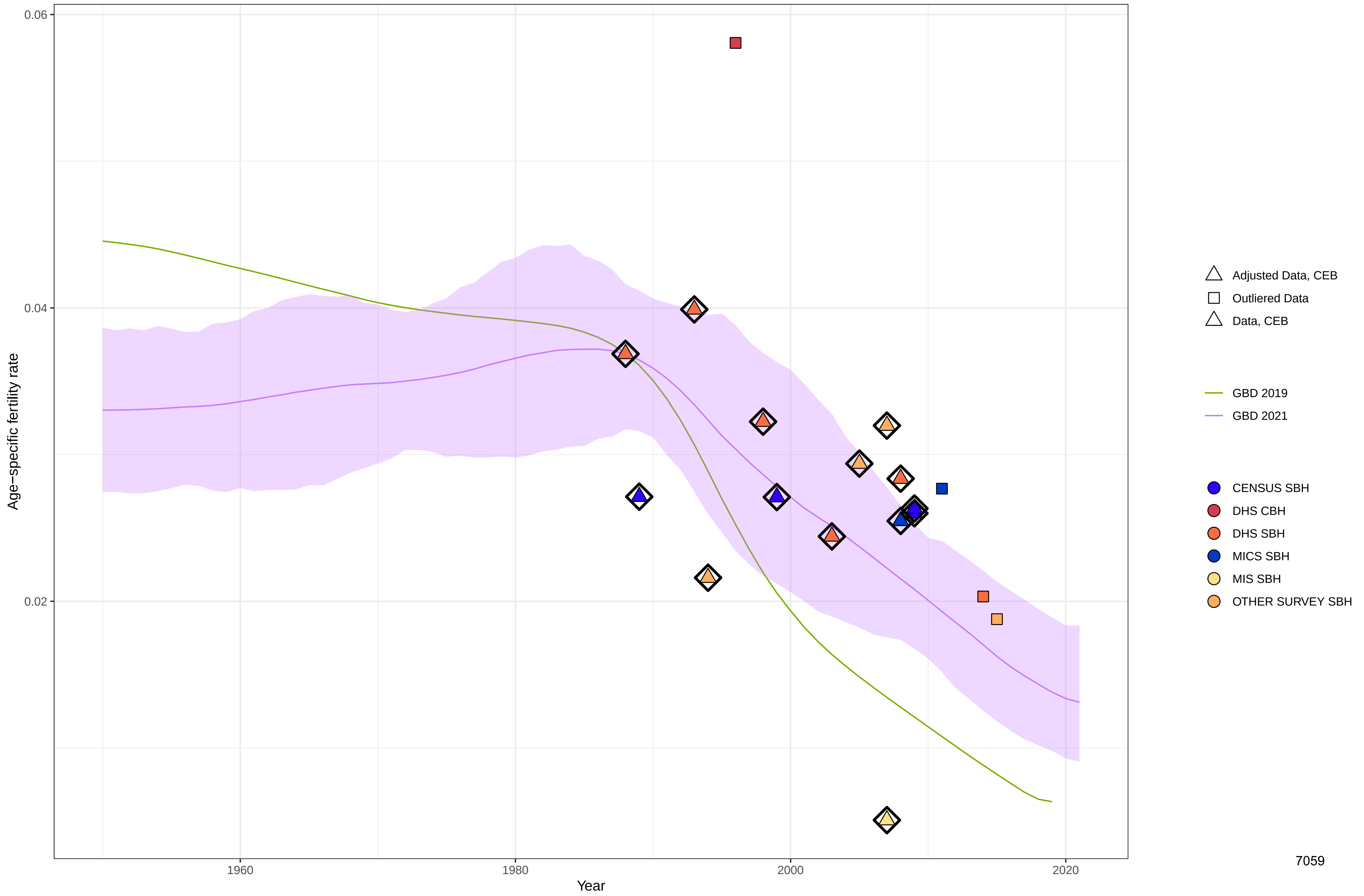

Age−specific fertility fitted trend lines, 1950−2021: Kitui, Ages: 50−54
Data points enclosed with a diamond are reference sources

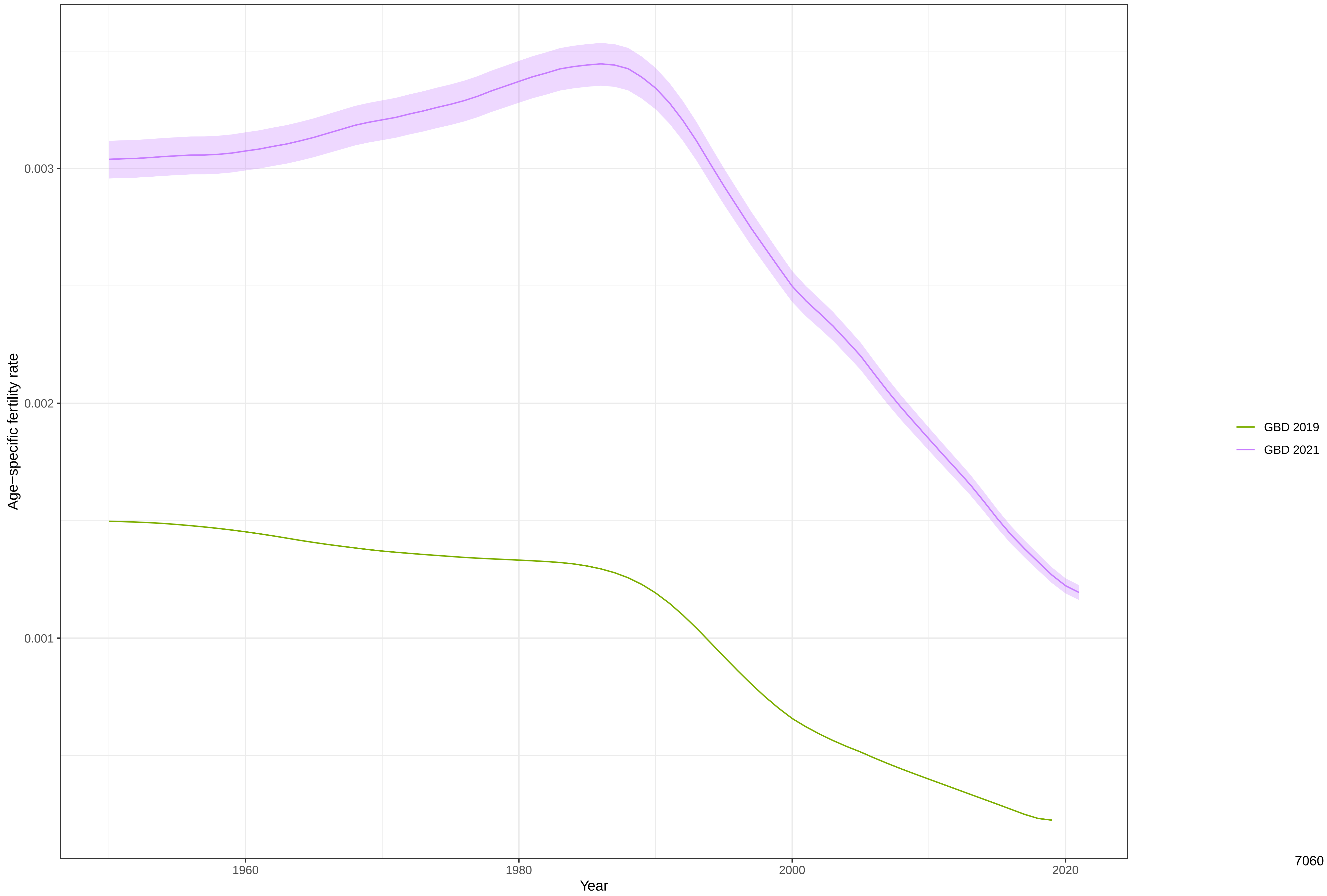

Total fertility rate fitted trend lines, 1950−2021: Kitui TFR
Data points enclosed with a diamond are reference sources

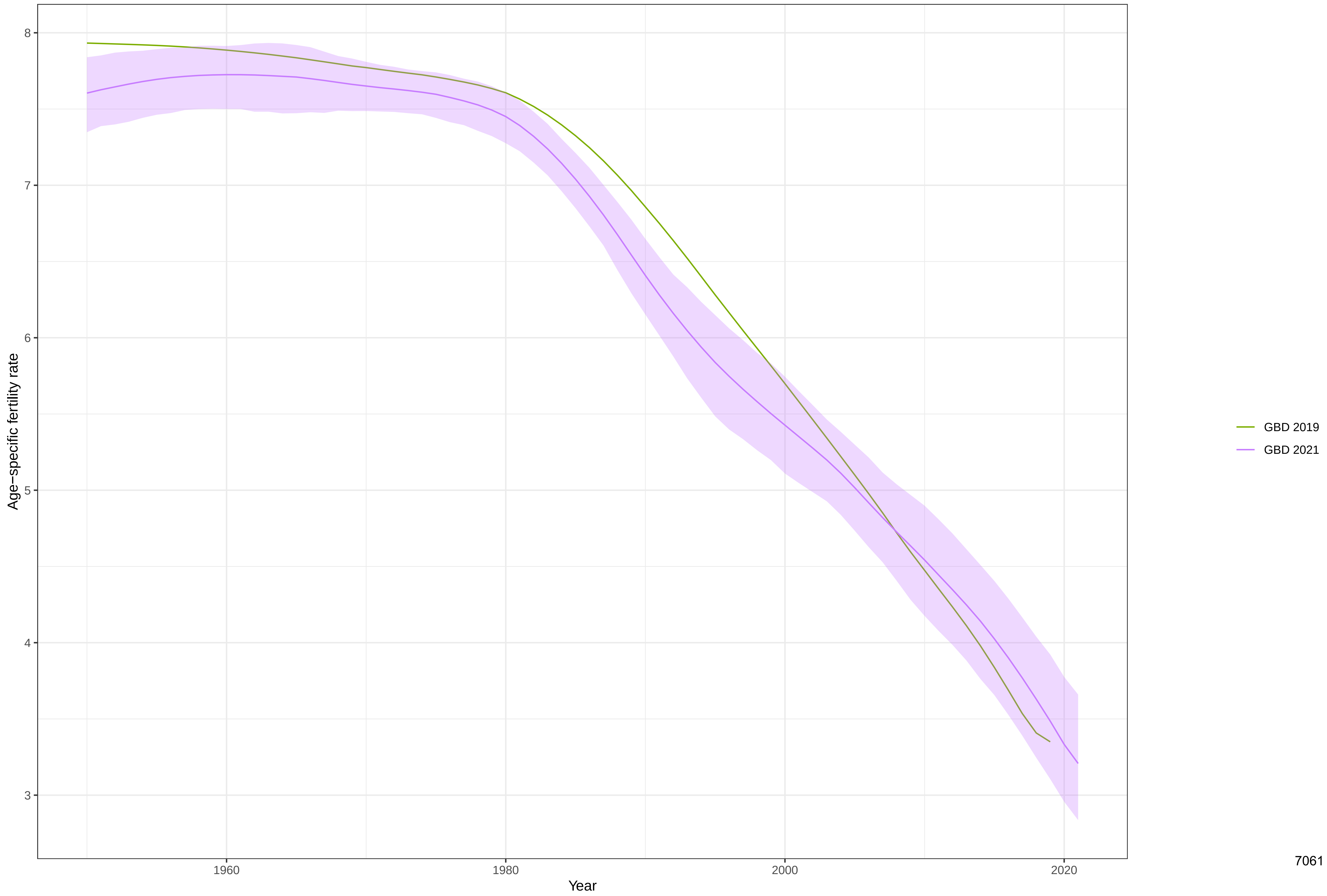

Age−specific fertility fitted trend lines, 1950−2021: Kwale, Ages: 10−14
Data points enclosed with a diamond are reference sources

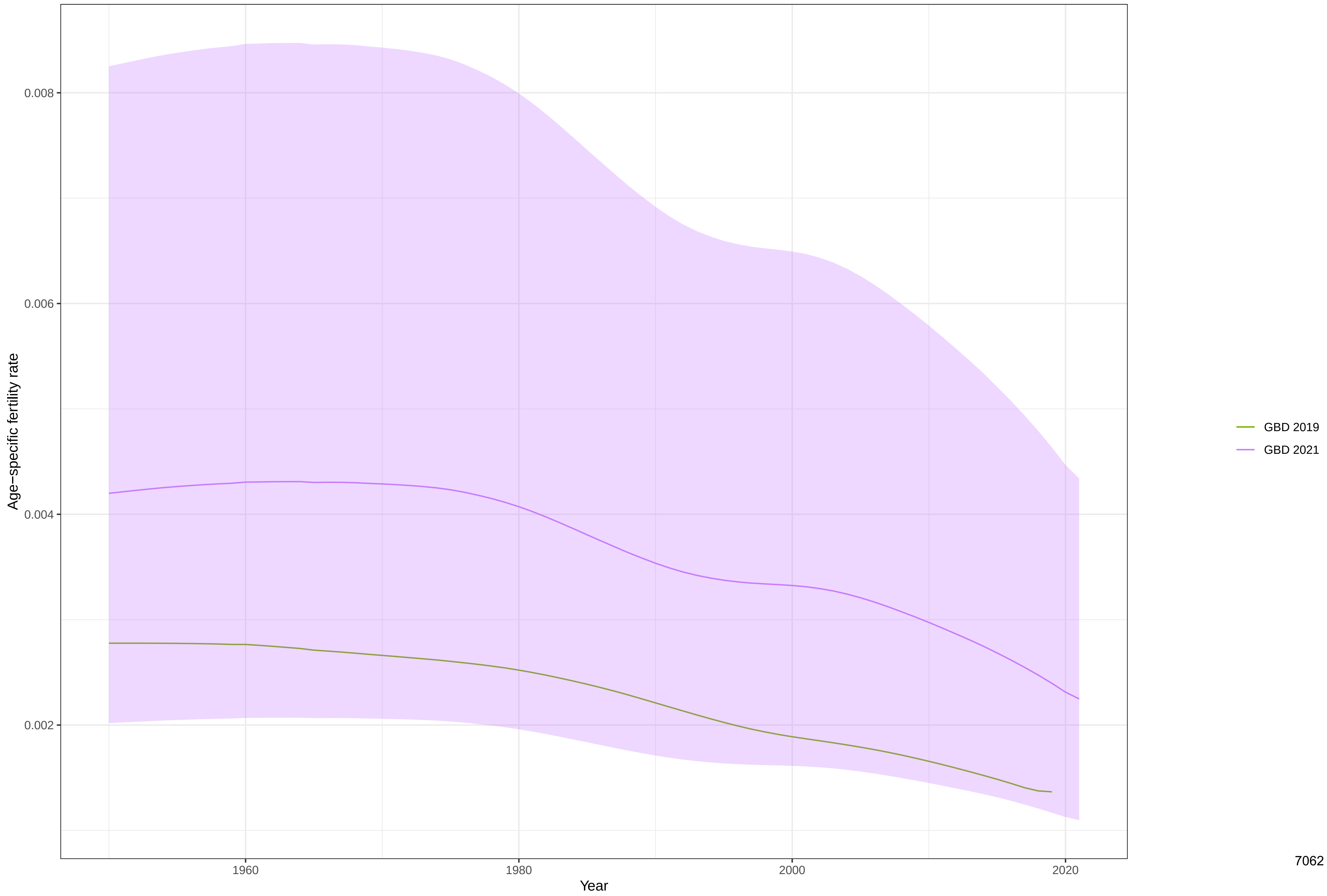

Age−specific fertility fitted trend lines, 1950−2021: Kwale, Ages: 15−19
Data points enclosed with a diamond are reference sources

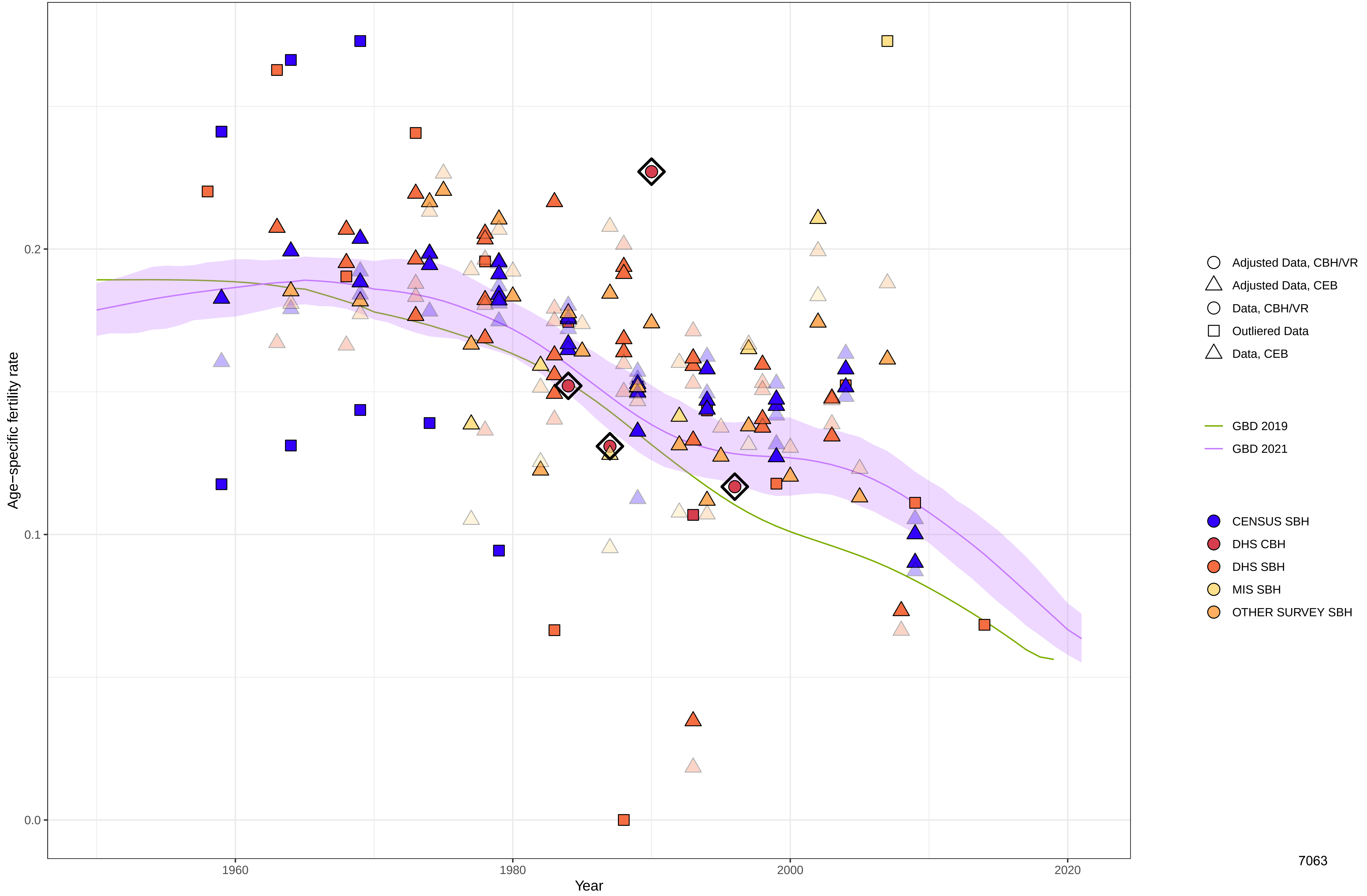

Age−specific fertility fitted trend lines, 1950−2021: Kwale, Ages: 20−24
Data points enclosed with a diamond are reference sources

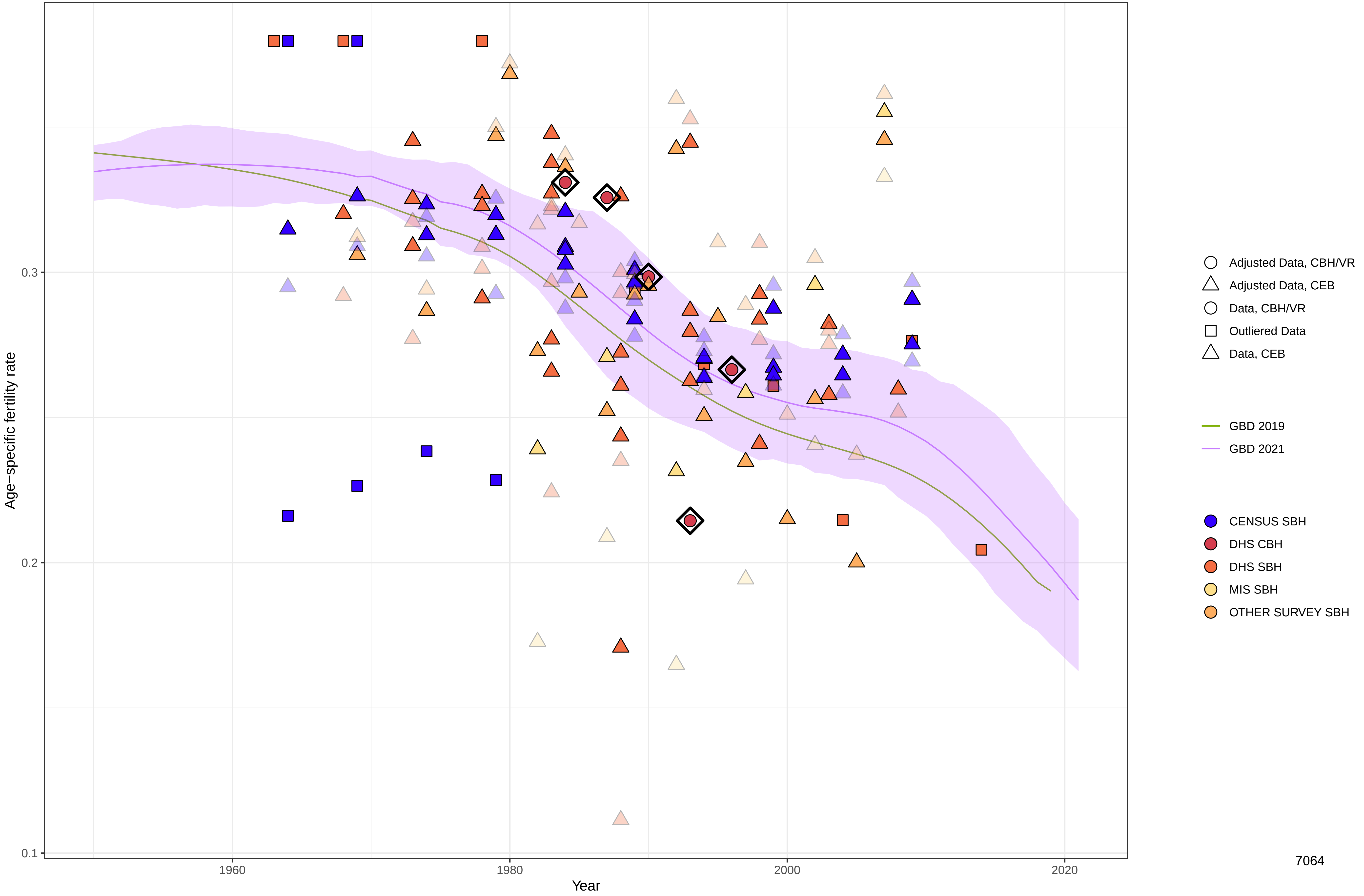

Age−specific fertility fitted trend lines, 1950−2021: Kwale, Ages: 25−29
Data points enclosed with a diamond are reference sources

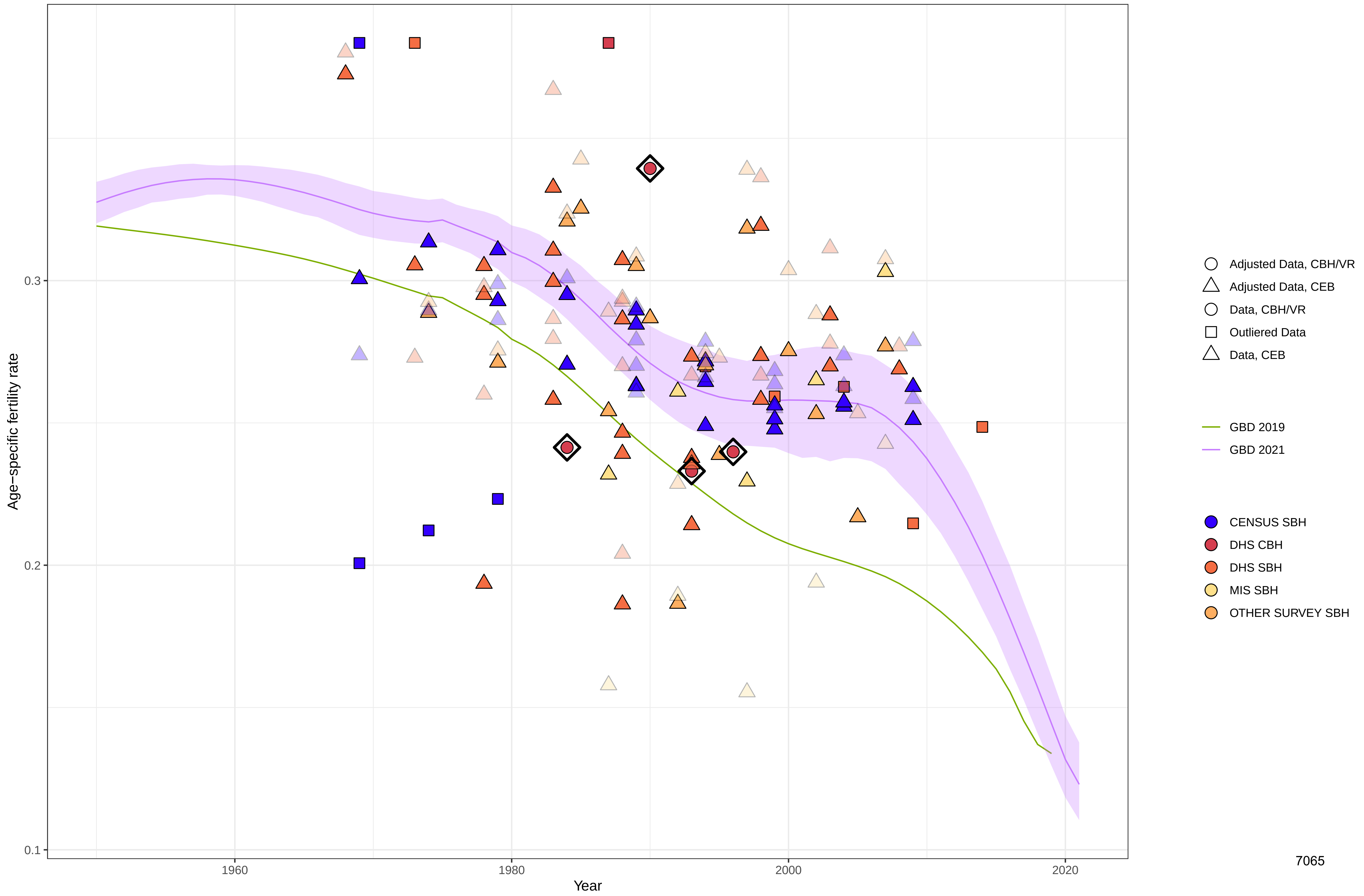

Age−specific fertility fitted trend lines, 1950−2021: Kwale, Ages: 30−34
Data points enclosed with a diamond are reference sources

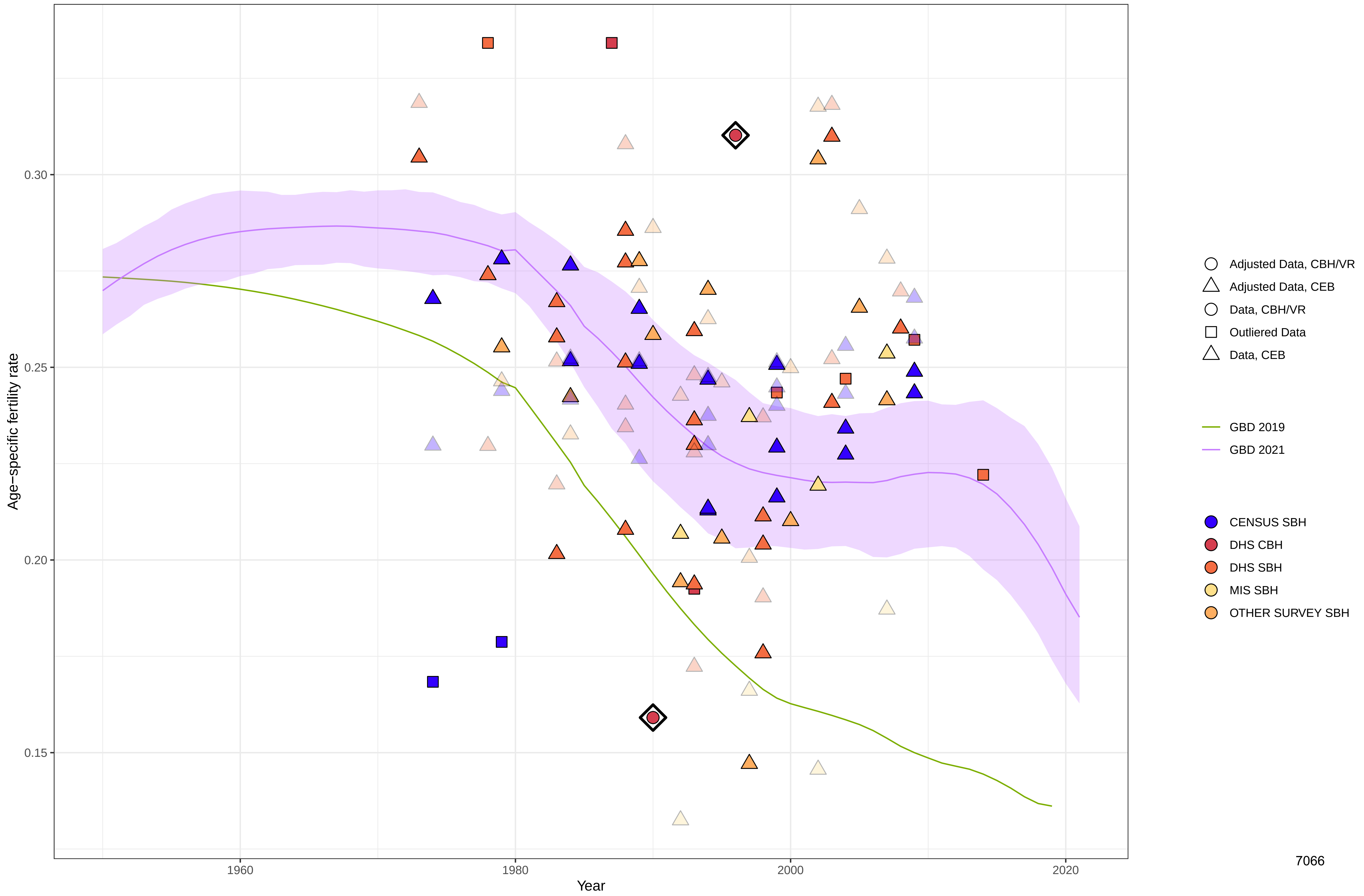

Age−specific fertility fitted trend lines, 1950−2021: Kwale, Ages: 35−39
Data points enclosed with a diamond are reference sources

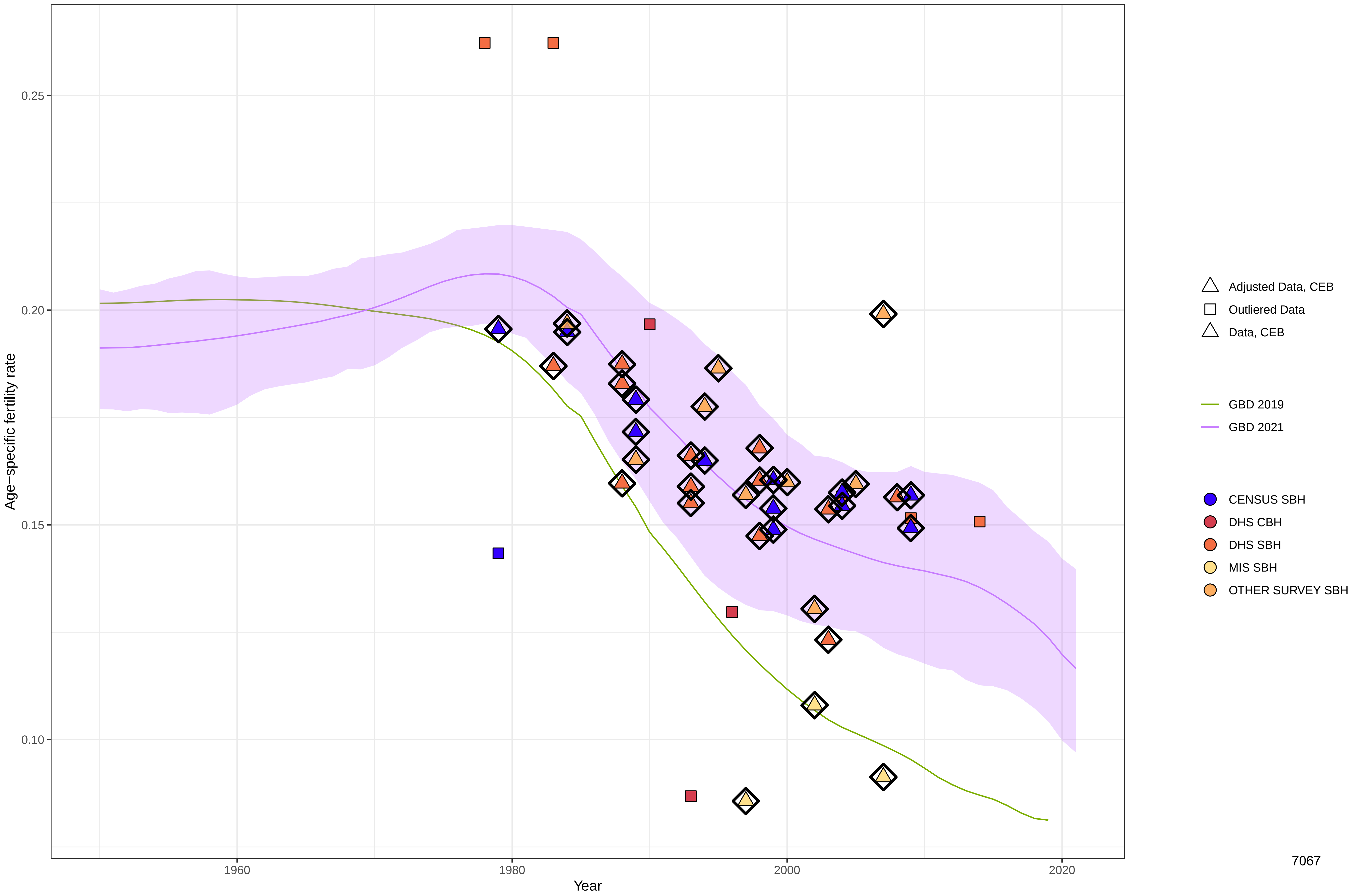

7067

Age−specific fertility fitted trend lines, 1950−2021: Kwale, Ages: 40−44
Data points enclosed with a diamond are reference sources

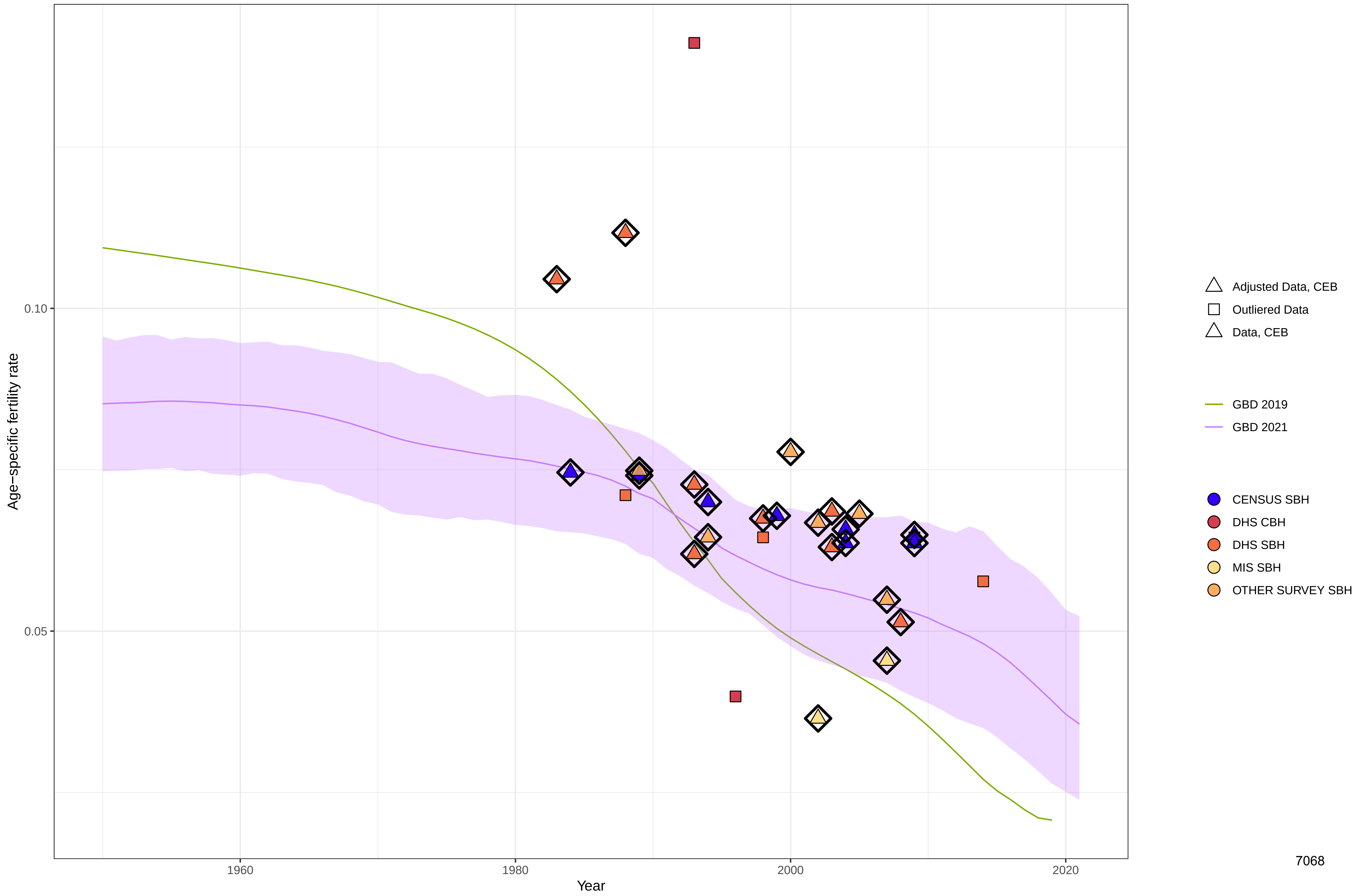

Age−specific fertility fitted trend lines, 1950−2021: Kwale, Ages: 45−49
Data points enclosed with a diamond are reference sources

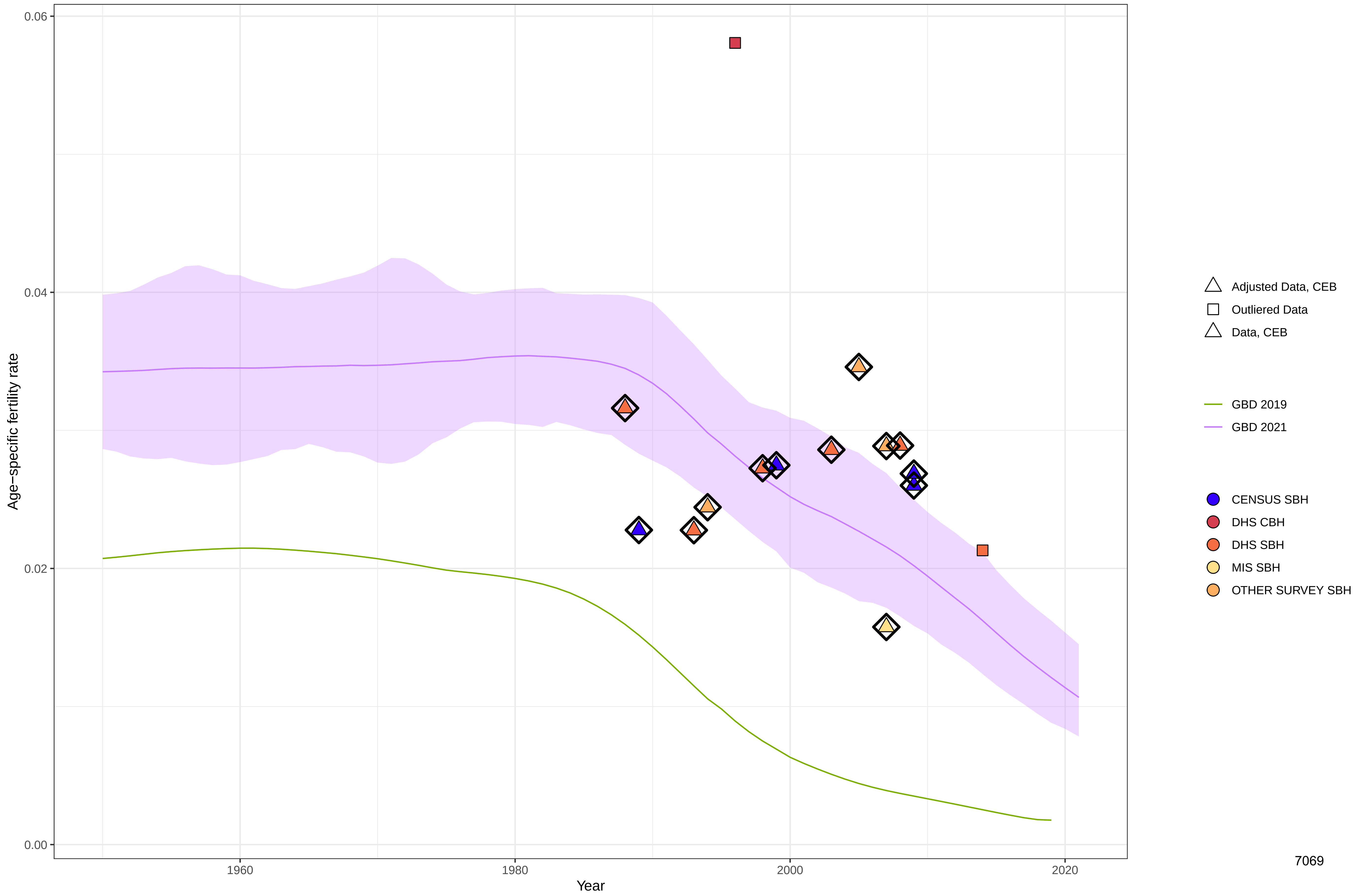

Age−specific fertility fitted trend lines, 1950−2021: Kwale, Ages: 50−54
Data points enclosed with a diamond are reference sources

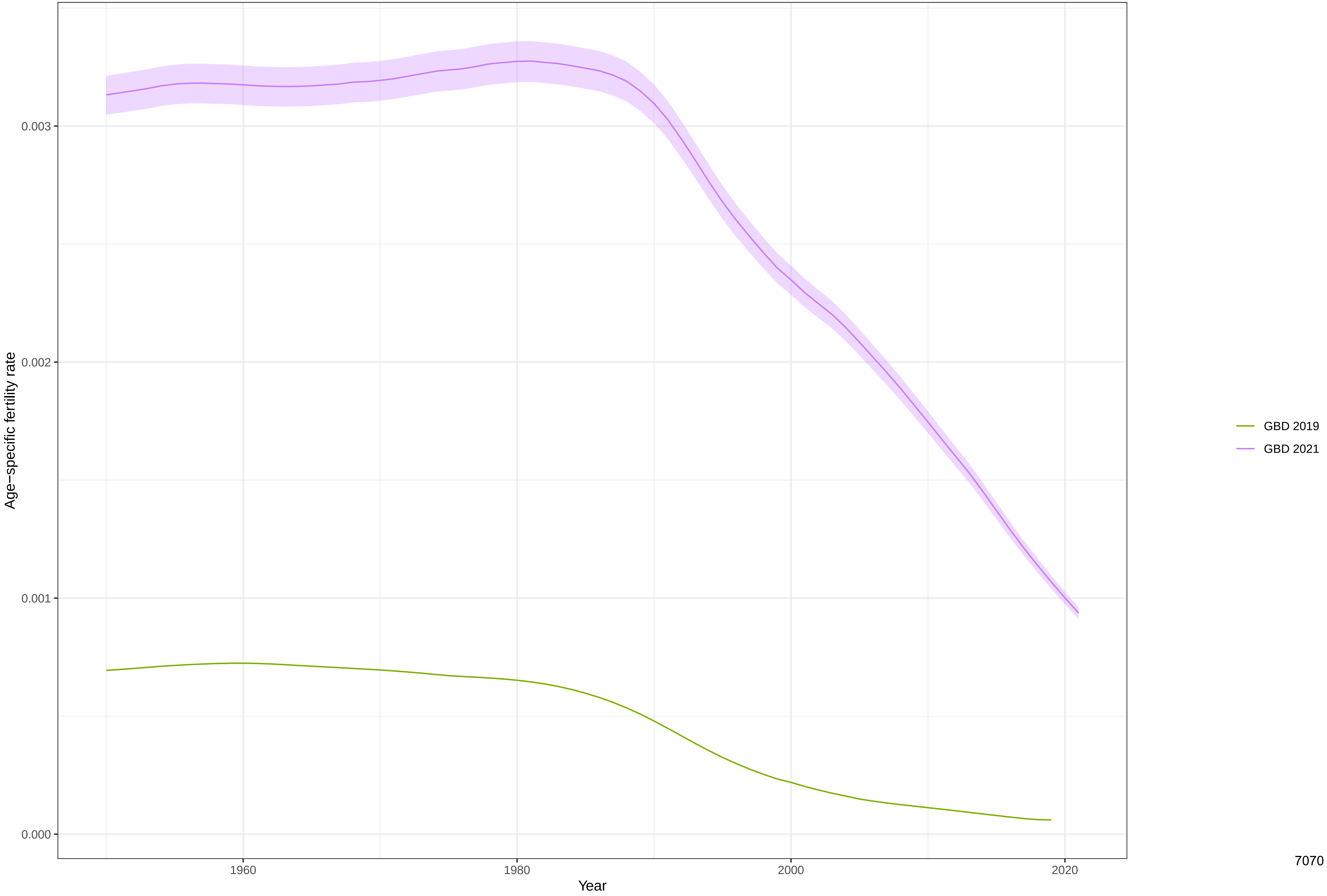

Total fertility rate fitted trend lines, 1950−2021: Kwale TFR
Data points enclosed with a diamond are reference sources

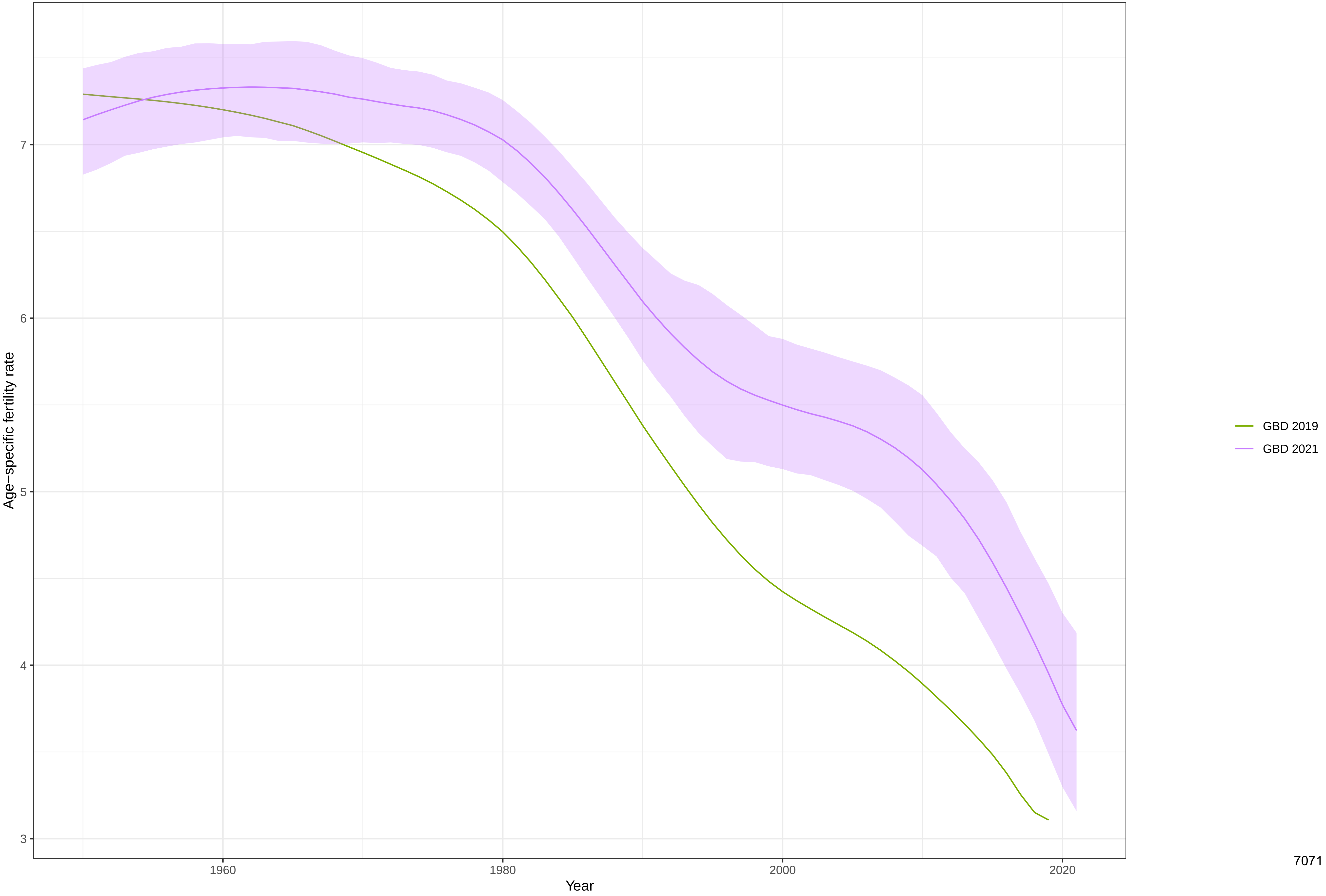

Age−specific fertility fitted trend lines, 1950−2021: Laikipia, Ages: 10−14
Data points enclosed with a diamond are reference sources

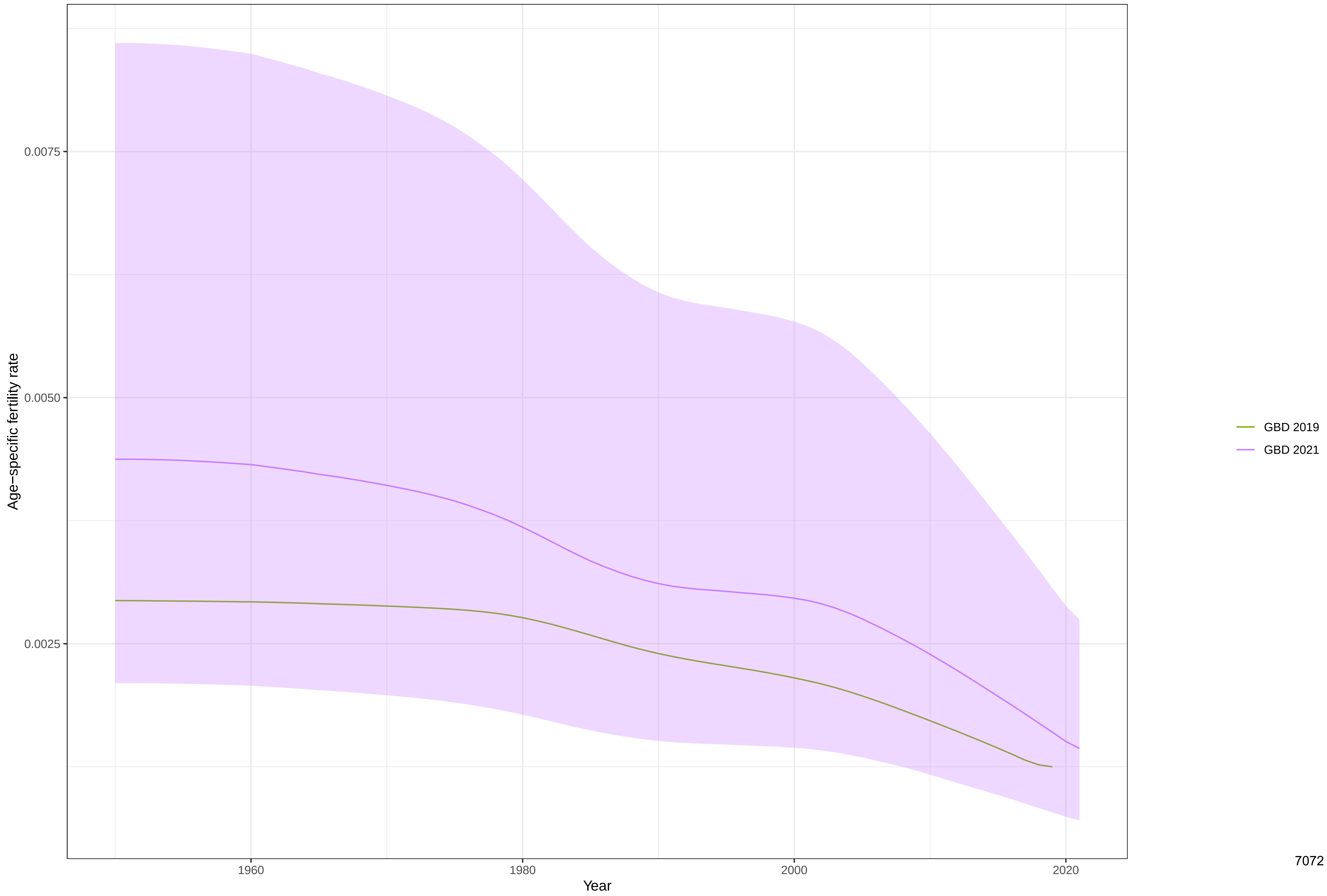

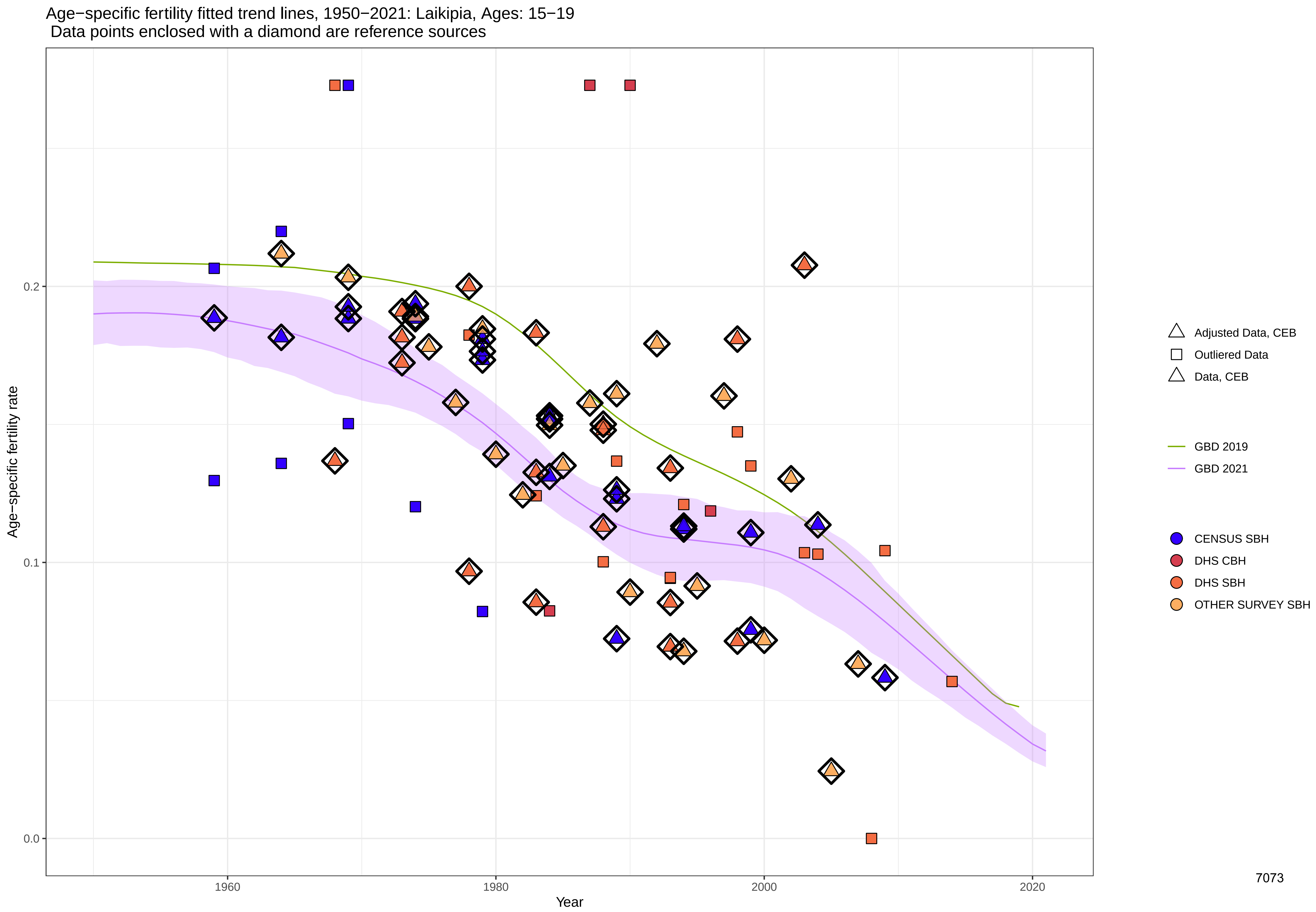

Age−specific fertility fitted trend lines, 1950−2021: Laikipia, Ages: 15−19
Data points enclosed with a diamond are reference sources
Age−specific fertility rate
0.0
0.1
0.2
Year
1960
1980
2000
2020
Adjusted Data, CEB
Outliered Data
Data, CEB
GBD 2019
GBD 2021
CENSUS SBH
DHS CBH
DHS SBH
OTHER SURVEY SBH

Age−specific fertility fitted trend lines, 1950−2021: Laikipia, Ages: 20−24
Data points enclosed with a diamond are reference sources

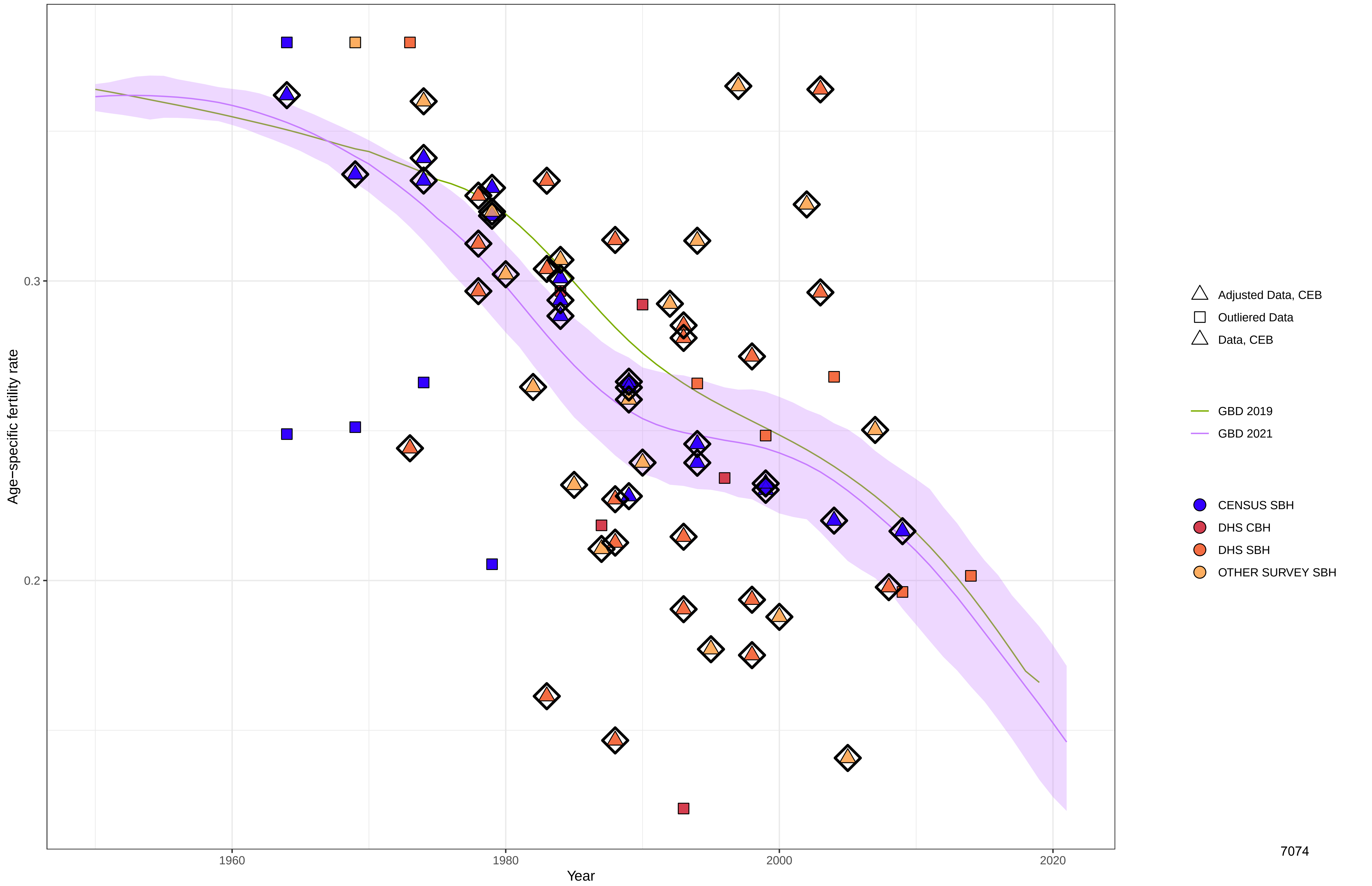

Age−specific fertility fitted trend lines, 1950−2021: Laikipia, Ages: 25−29
Data points enclosed with a diamond are reference sources

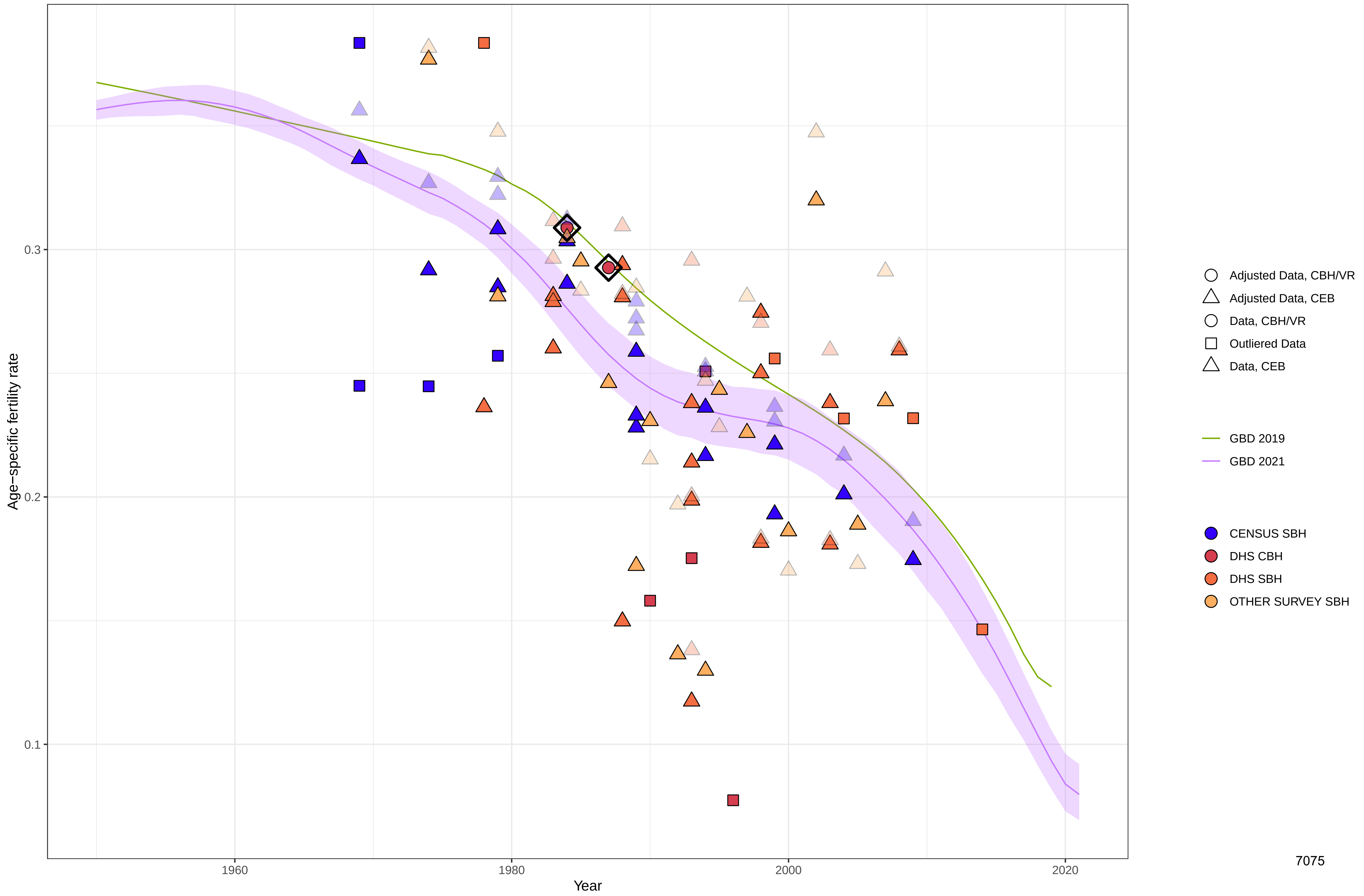

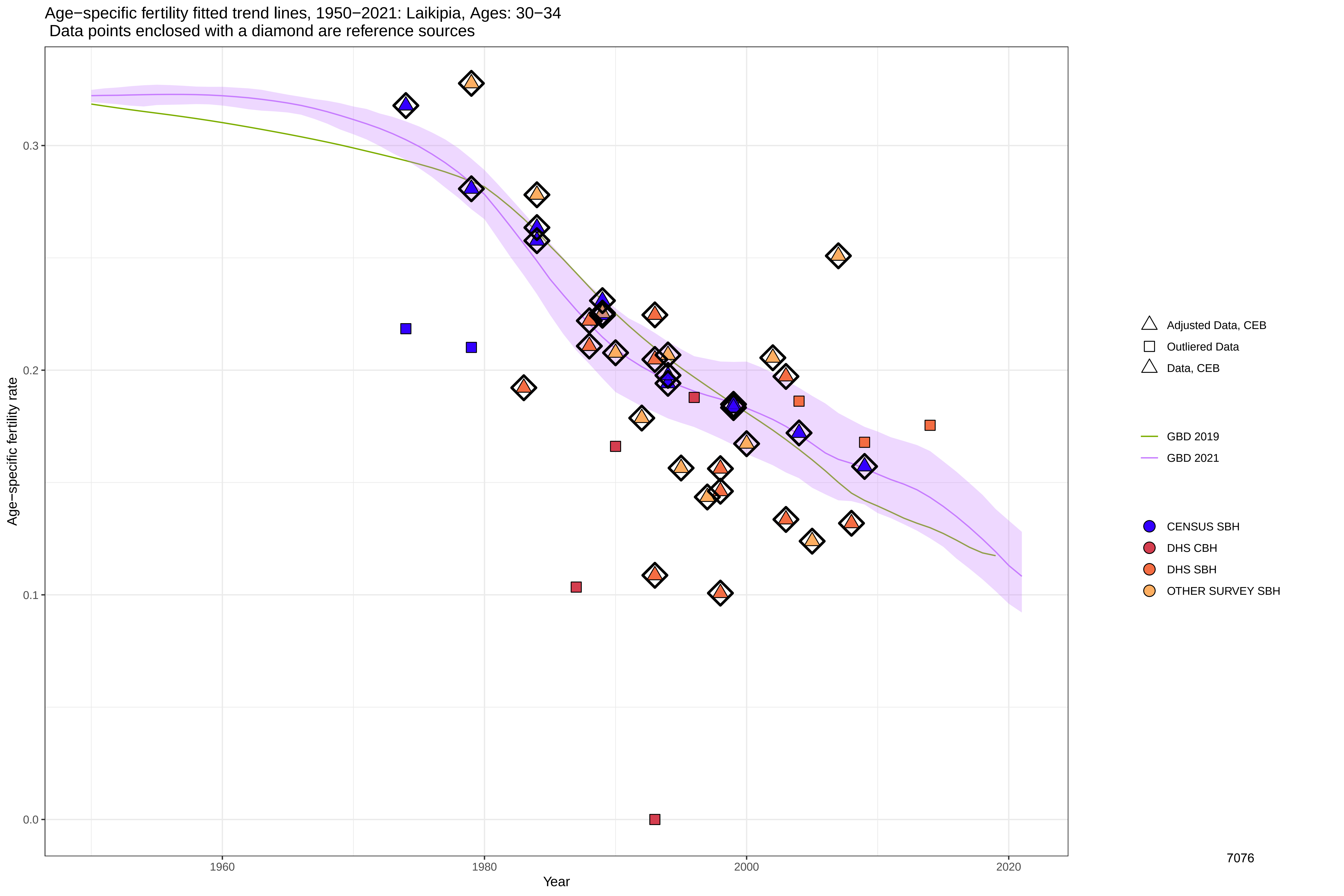

Age−specific fertility fitted trend lines, 1950−2021: Laikipia, Ages: 30−34
Data points enclosed with a diamond are reference sources
Age−specific fertility rate
Year
0.0
0.1
0.2
0.3
1960
1980
2000
2020
Adjusted Data, CEB
Outliered Data
Data, CEB
GBD 2019
GBD 2021
CENSUS SBH
DHS CBH
DHS SBH
OTHER SURVEY SBH
7076

Age−specific fertility fitted trend lines, 1950−2021: Laikipia, Ages: 35−39
Data points enclosed with a diamond are reference sources

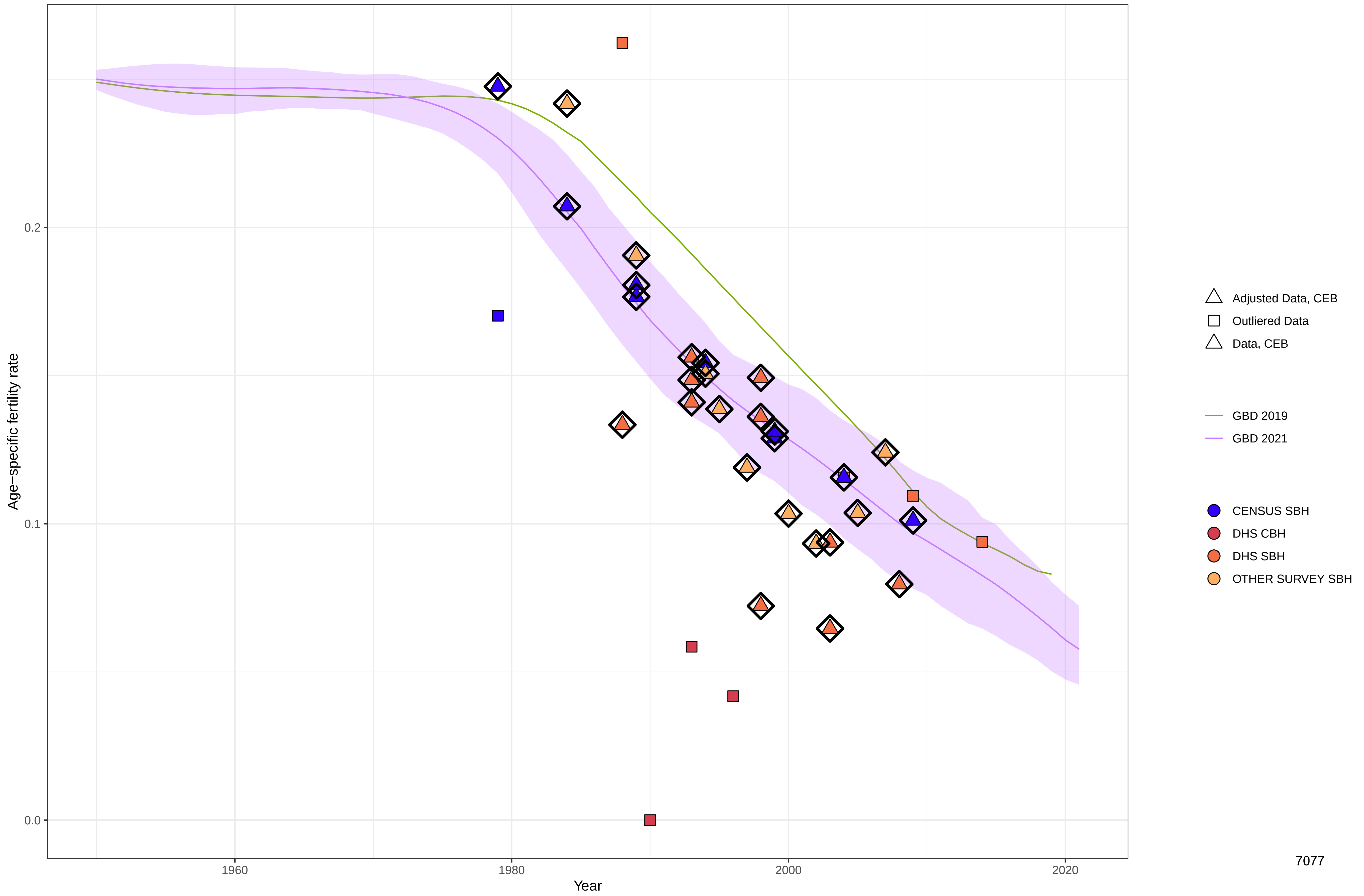

7077

Age−specific fertility fitted trend lines, 1950−2021: Laikipia, Ages: 40−44
Data points enclosed with a diamond are reference sources

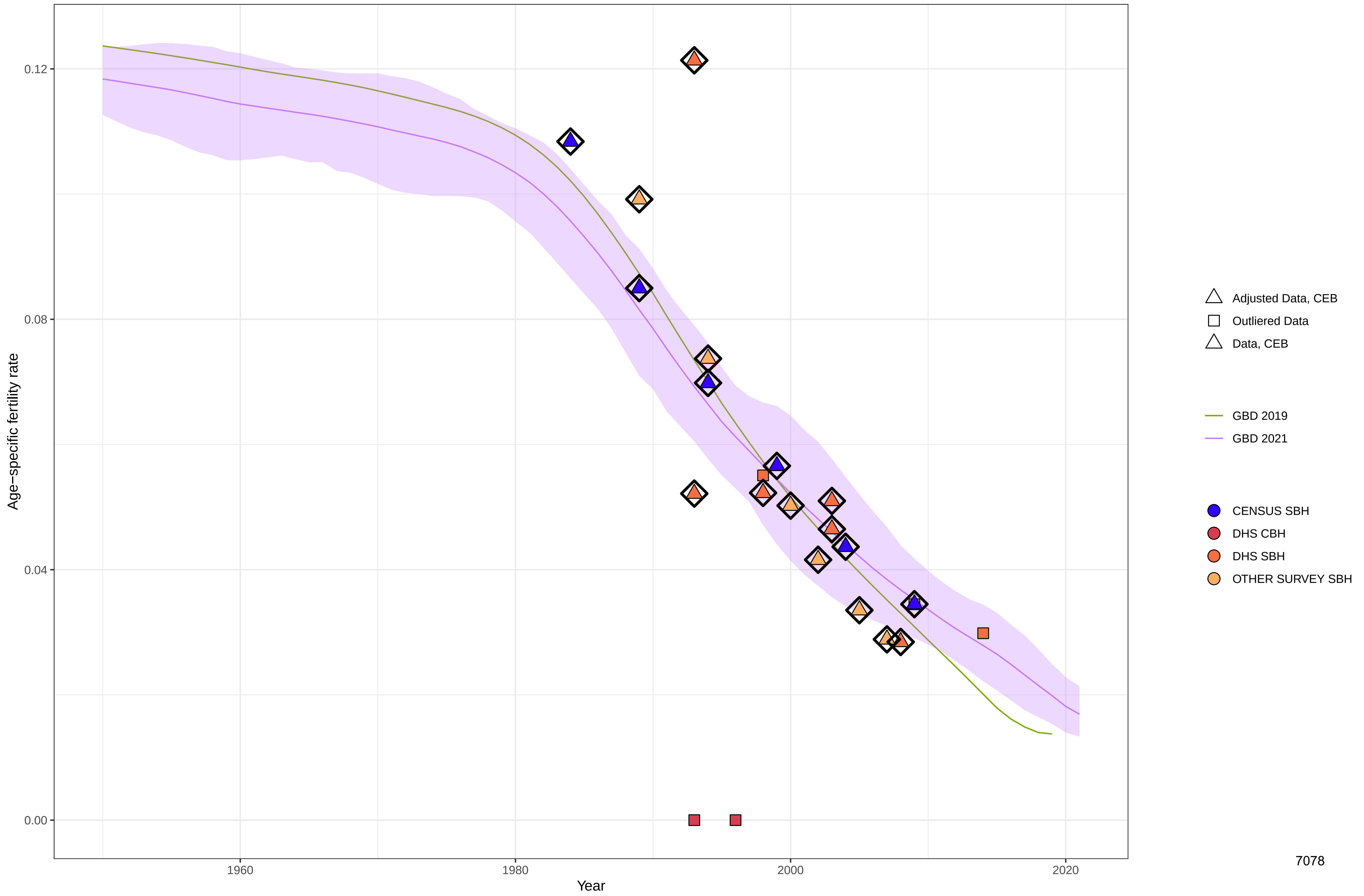

Age−specific fertility fitted trend lines, 1950−2021: Laikipia, Ages: 45−49
Data points enclosed with a diamond are reference sources

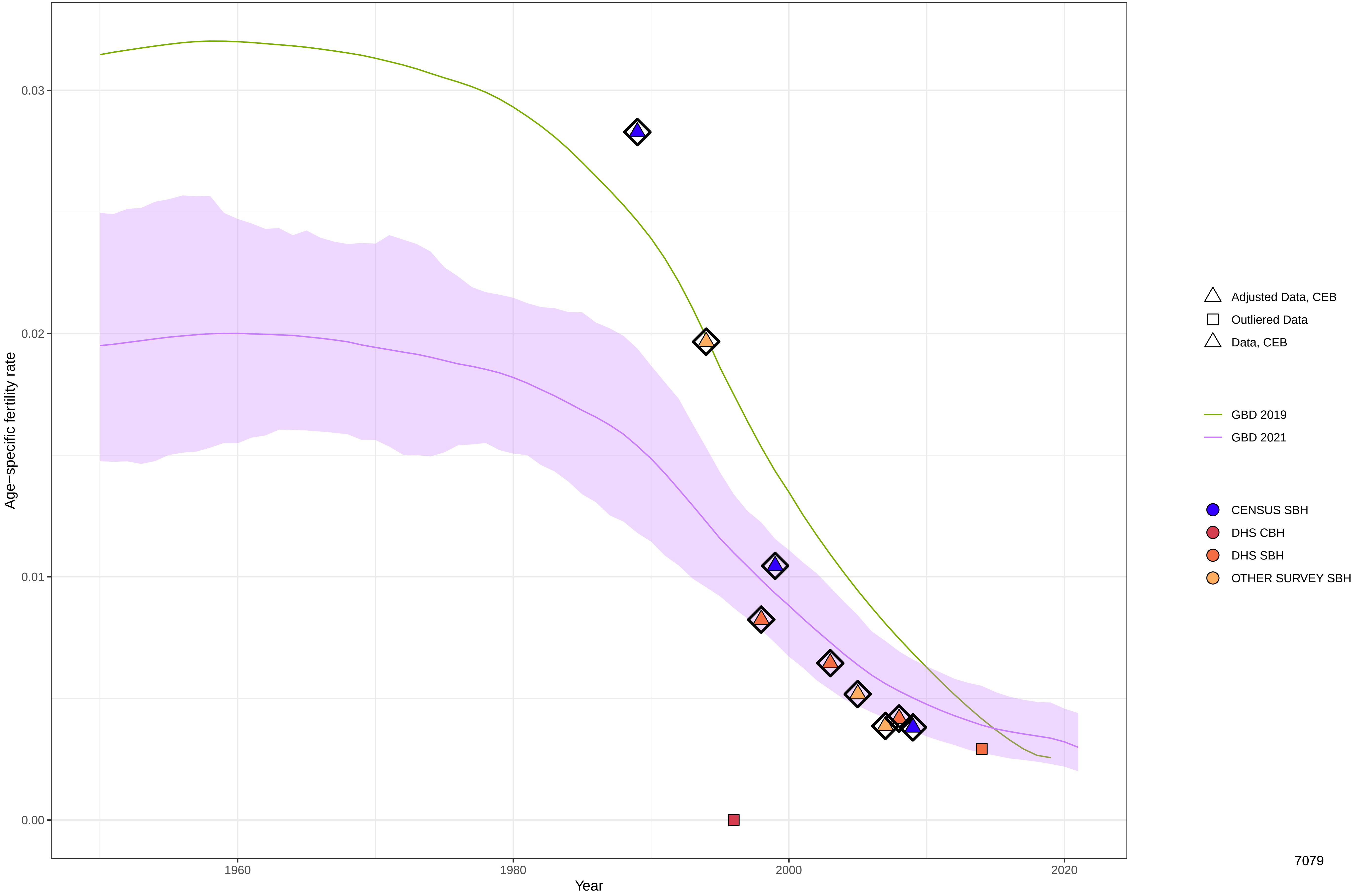

Age−specific fertility fitted trend lines, 1950−2021: Laikipia, Ages: 50−54
Data points enclosed with a diamond are reference sources

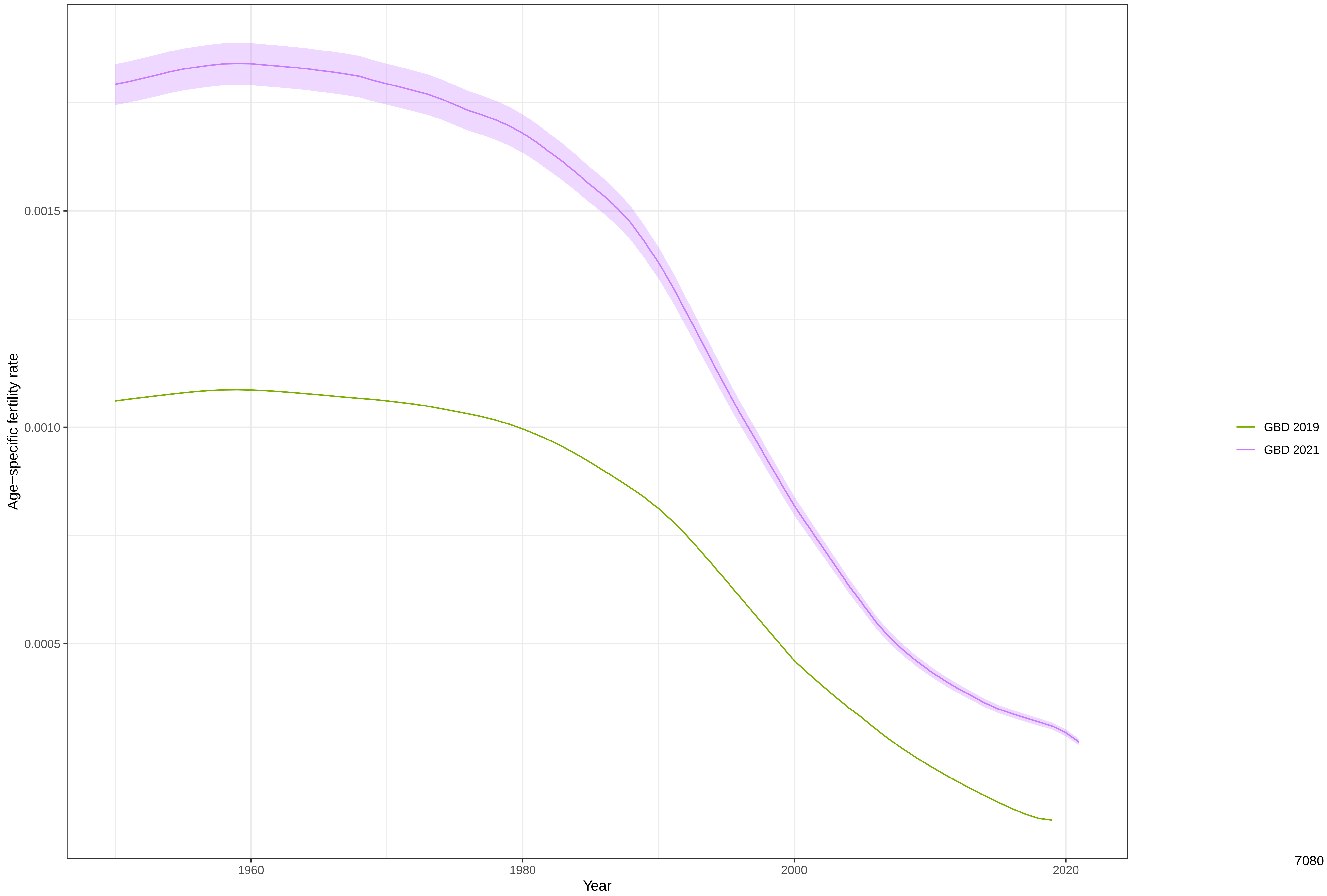

Total fertility rate fitted trend lines, 1950−2021: Laikipia TFR
Data points enclosed with a diamond are reference sources

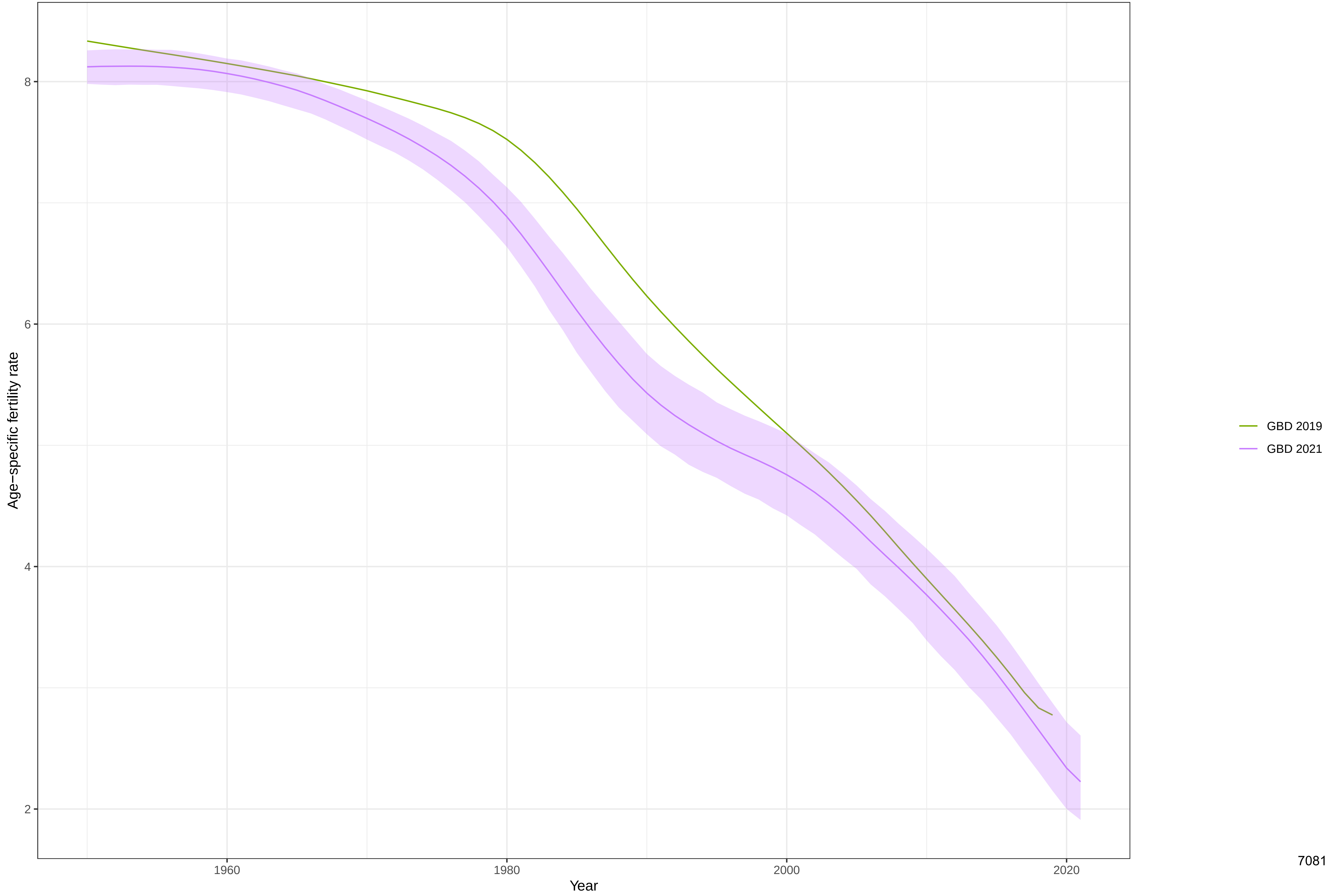

Age−specific fertility fitted trend lines, 1950−2021: Lamu, Ages: 10−14
Data points enclosed with a diamond are reference sources

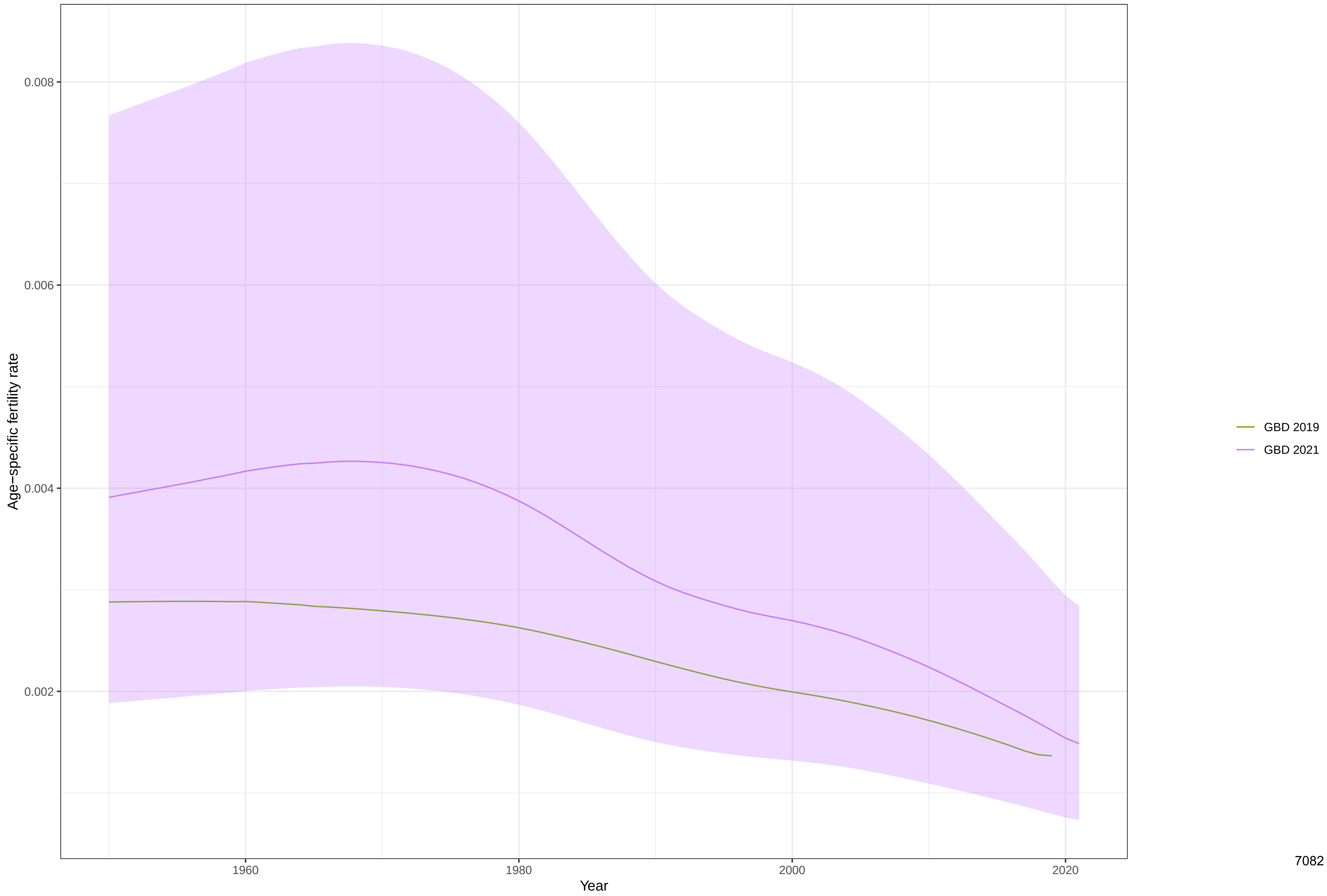

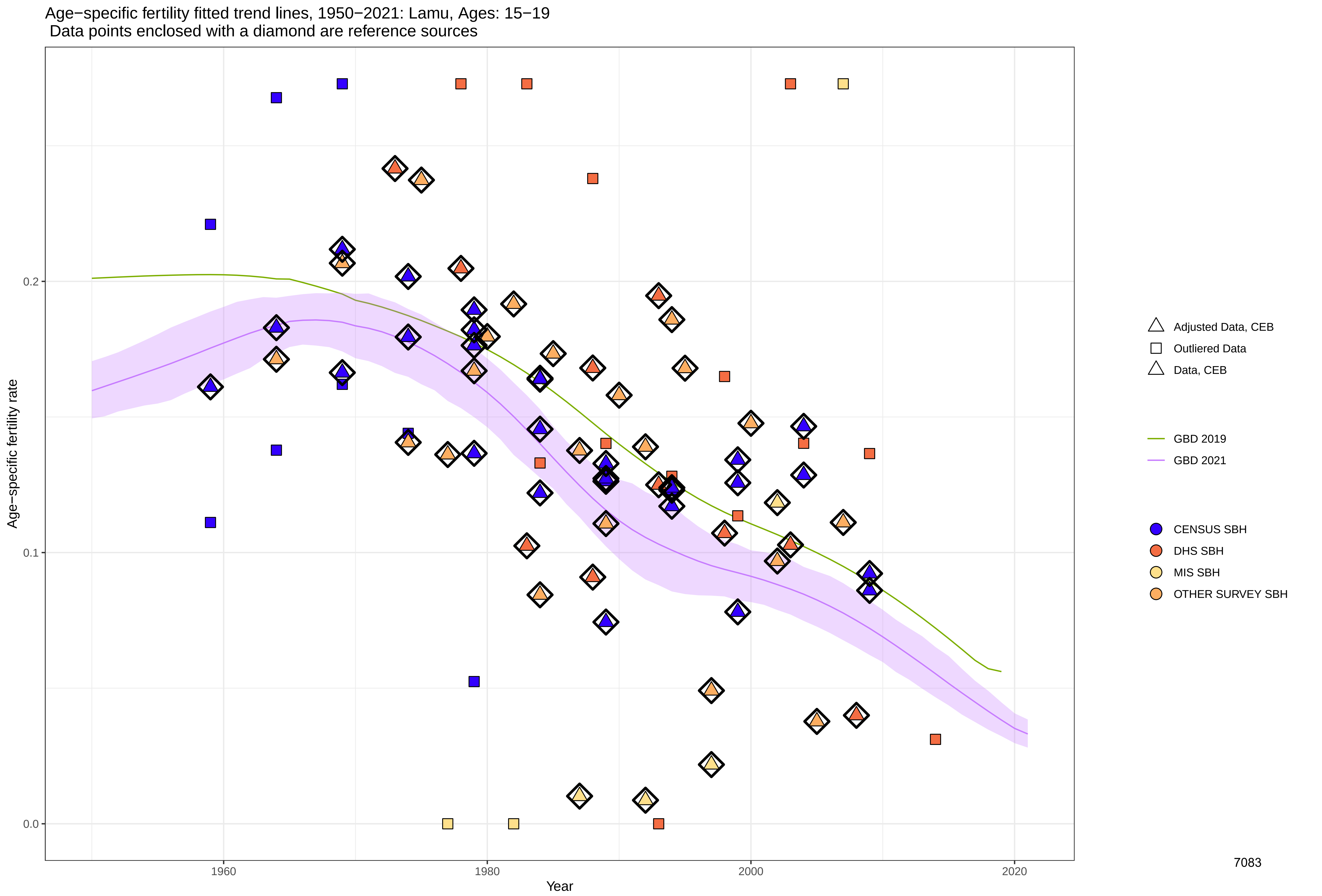

Age−specific fertility fitted trend lines, 1950−2021: Lamu, Ages: 15−19
Data points enclosed with a diamond are reference sources
Age−specific fertility rate
0.0
0.1
0.2
Year
1960
1980
2000
2020
Adjusted Data, CEB
Outliered Data
Data, CEB
GBD 2019
GBD 2021
CENSUS SBH
DHS SBH
MIS SBH
OTHER SURVEY SBH

Age−specific fertility fitted trend lines, 1950−2021: Lamu, Ages: 20−24
Data points enclosed with a diamond are reference sources

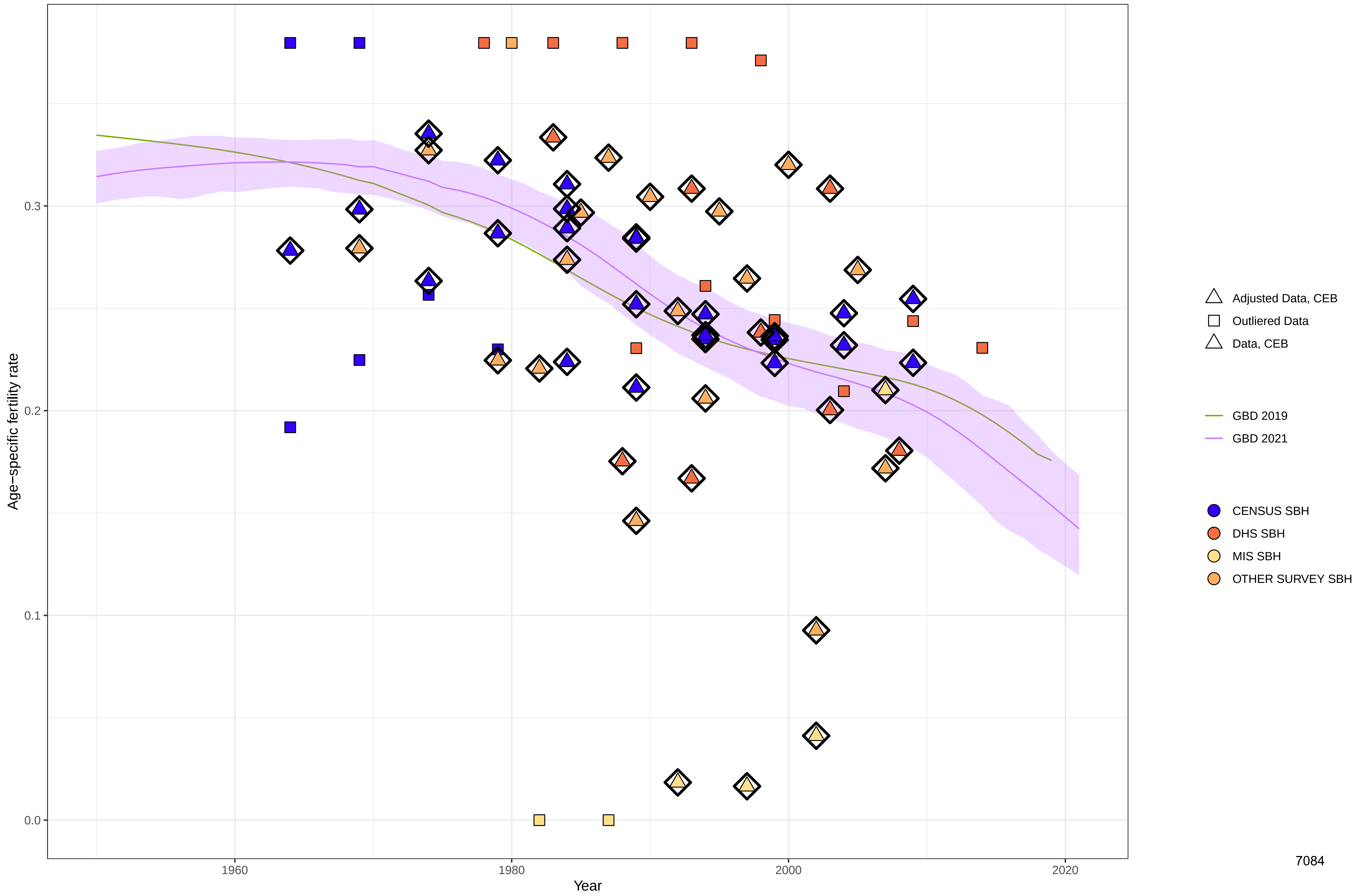

Age−specific fertility fitted trend lines, 1950−2021: Lamu, Ages: 25−29
Data points enclosed with a diamond are reference sources

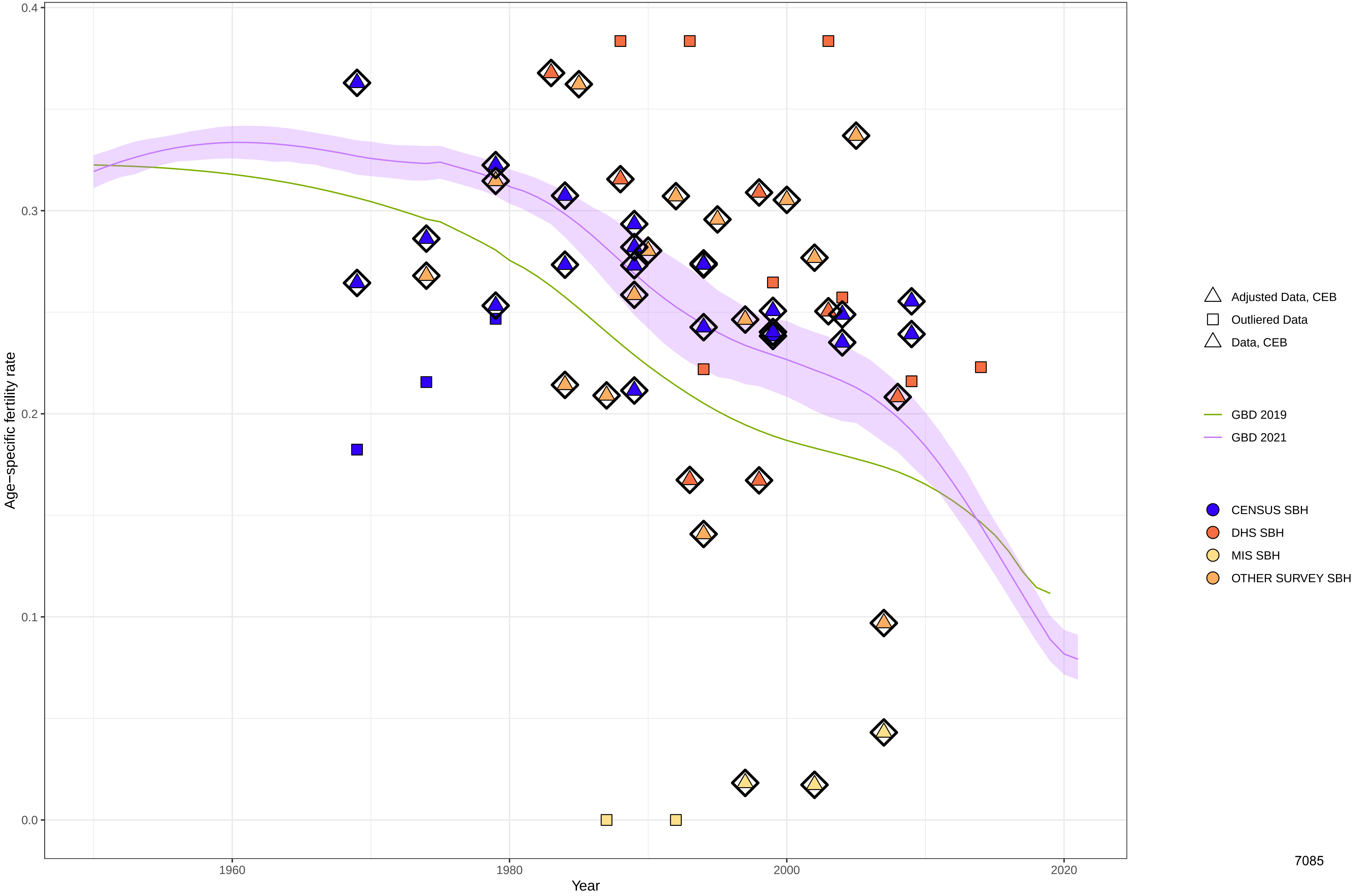

Age−specific fertility fitted trend lines, 1950−2021: Lamu, Ages: 30−34
Data points enclosed with a diamond are reference sources

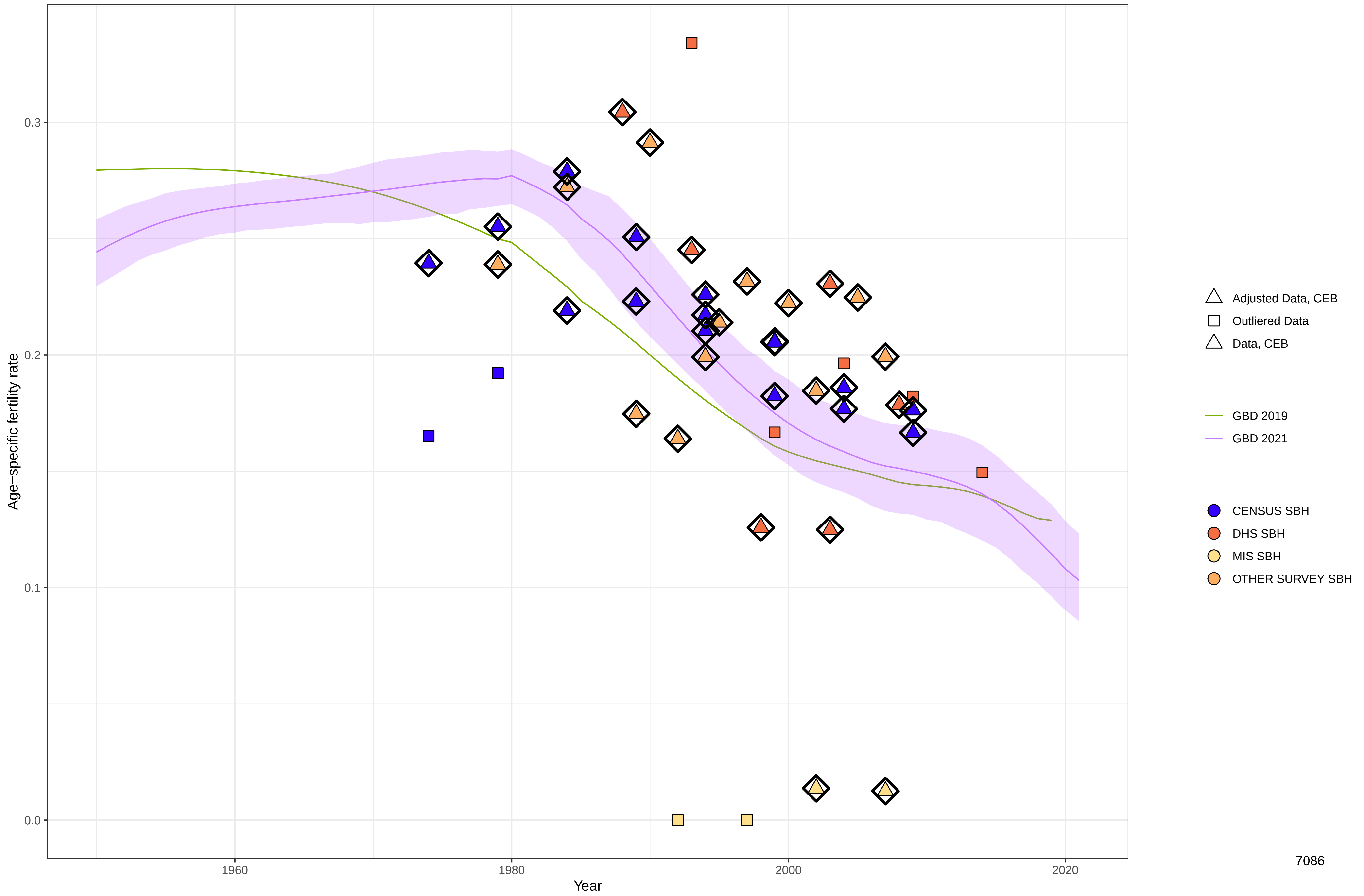

Age−specific fertility fitted trend lines, 1950−2021: Lamu, Ages: 35−39
Data points enclosed with a diamond are reference sources

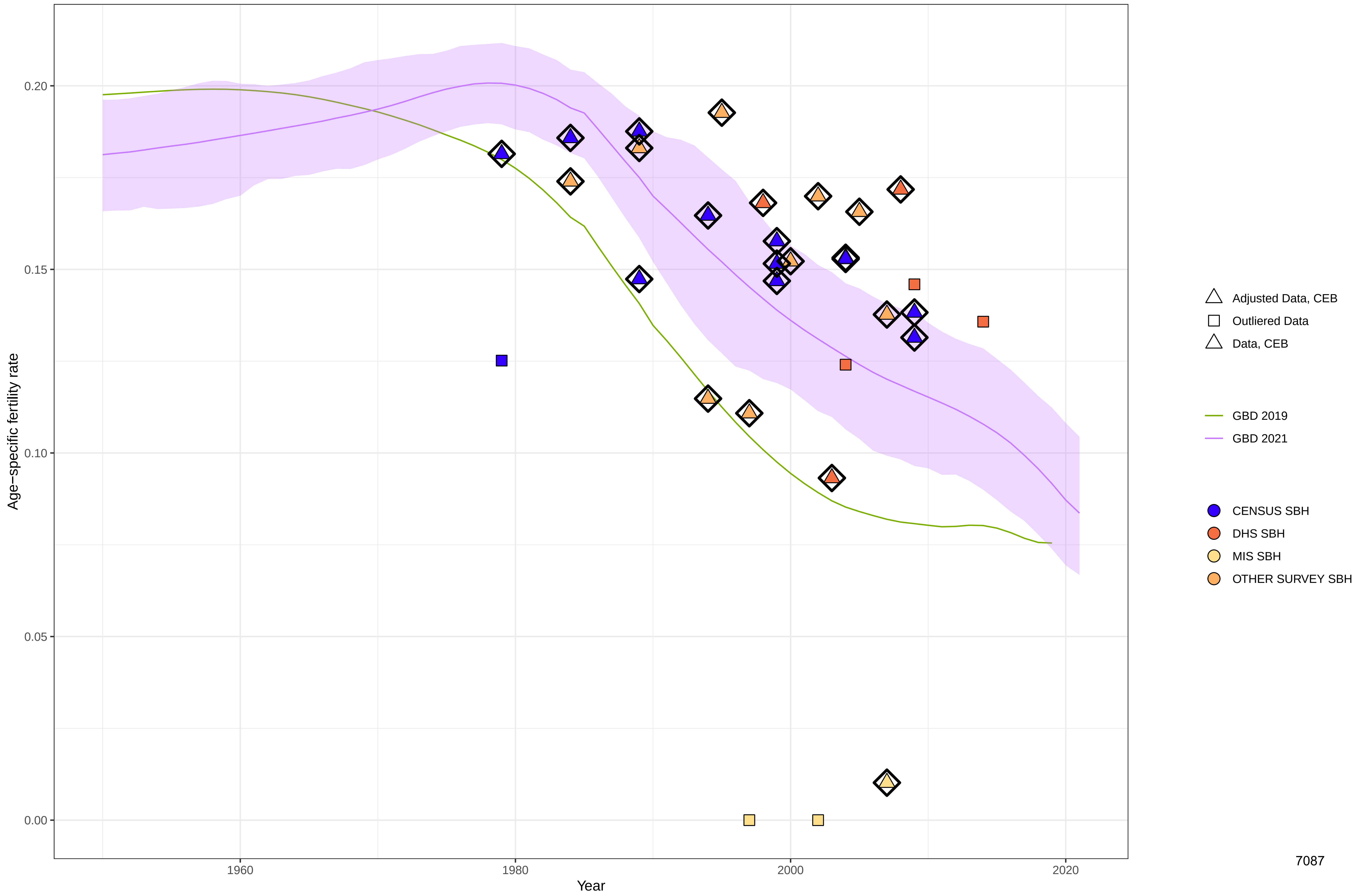

Age−specific fertility fitted trend lines, 1950−2021: Lamu, Ages: 40−44
Data points enclosed with a diamond are reference sources

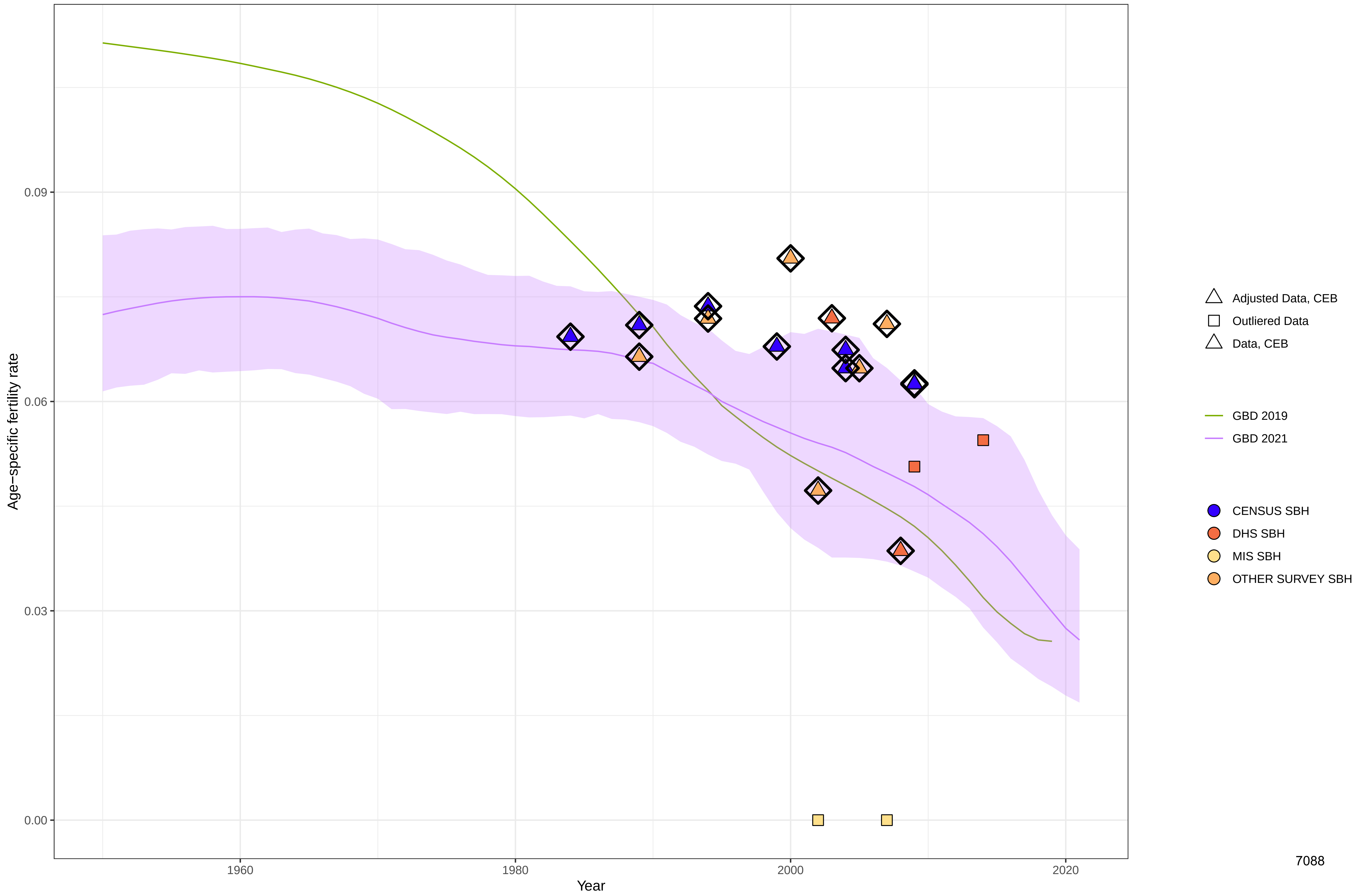

Age−specific fertility fitted trend lines, 1950−2021: Lamu, Ages: 45−49
Data points enclosed with a diamond are reference sources

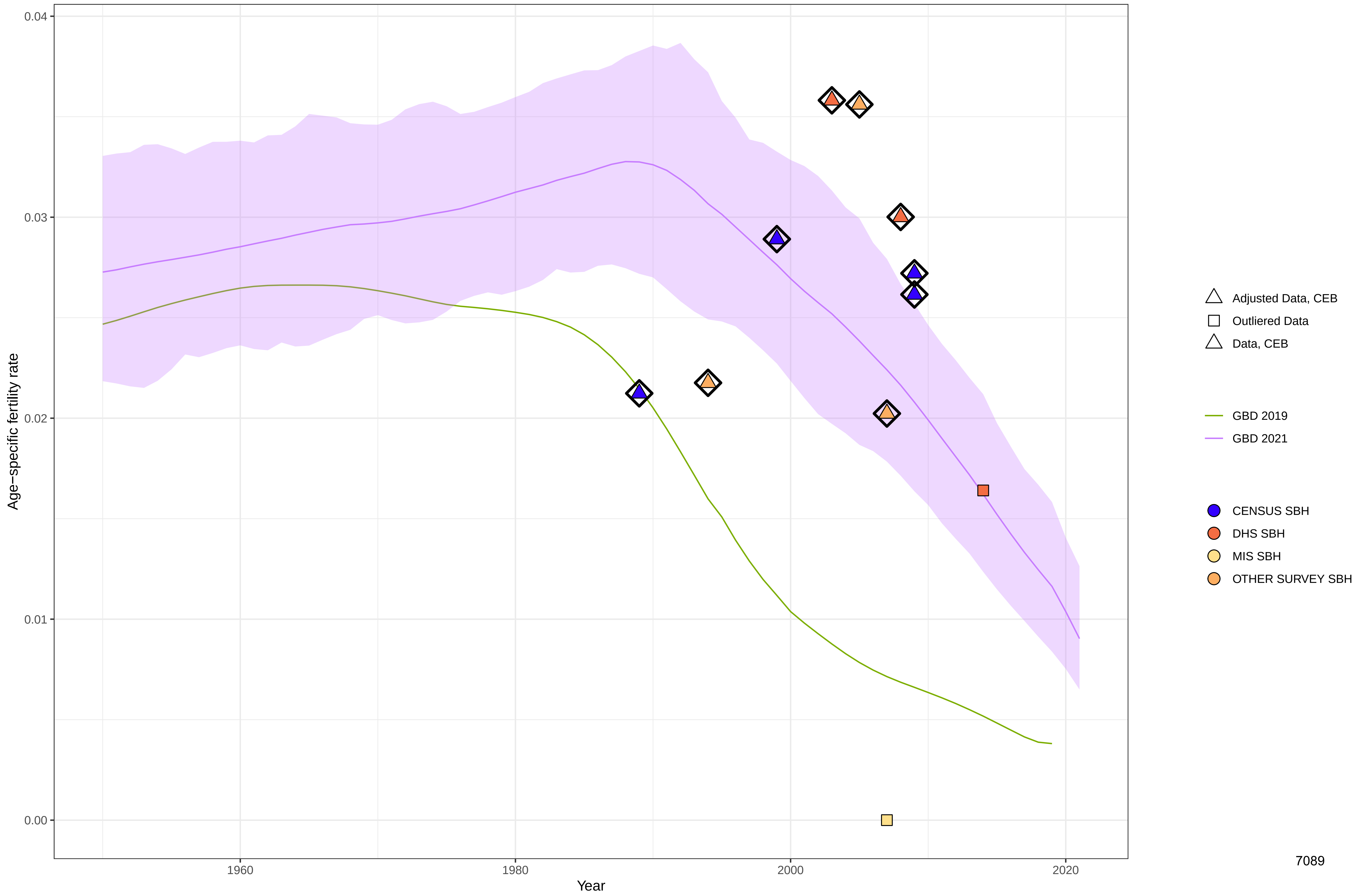

7089

Age−specific fertility fitted trend lines, 1950−2021: Lamu, Ages: 50−54
Data points enclosed with a diamond are reference sources

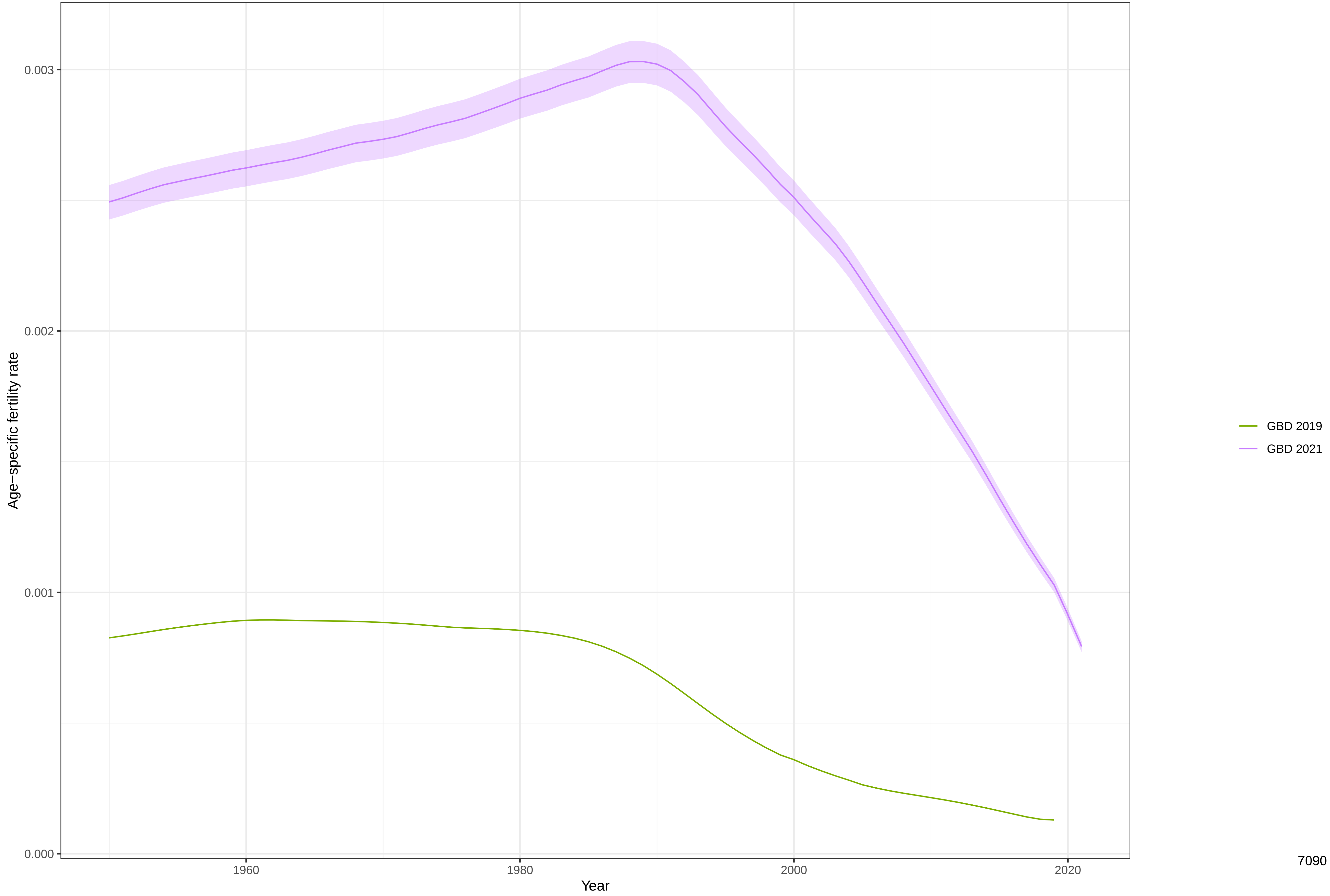

Total fertility rate fitted trend lines, 1950−2021: Lamu TFR
Data points enclosed with a diamond are reference sources

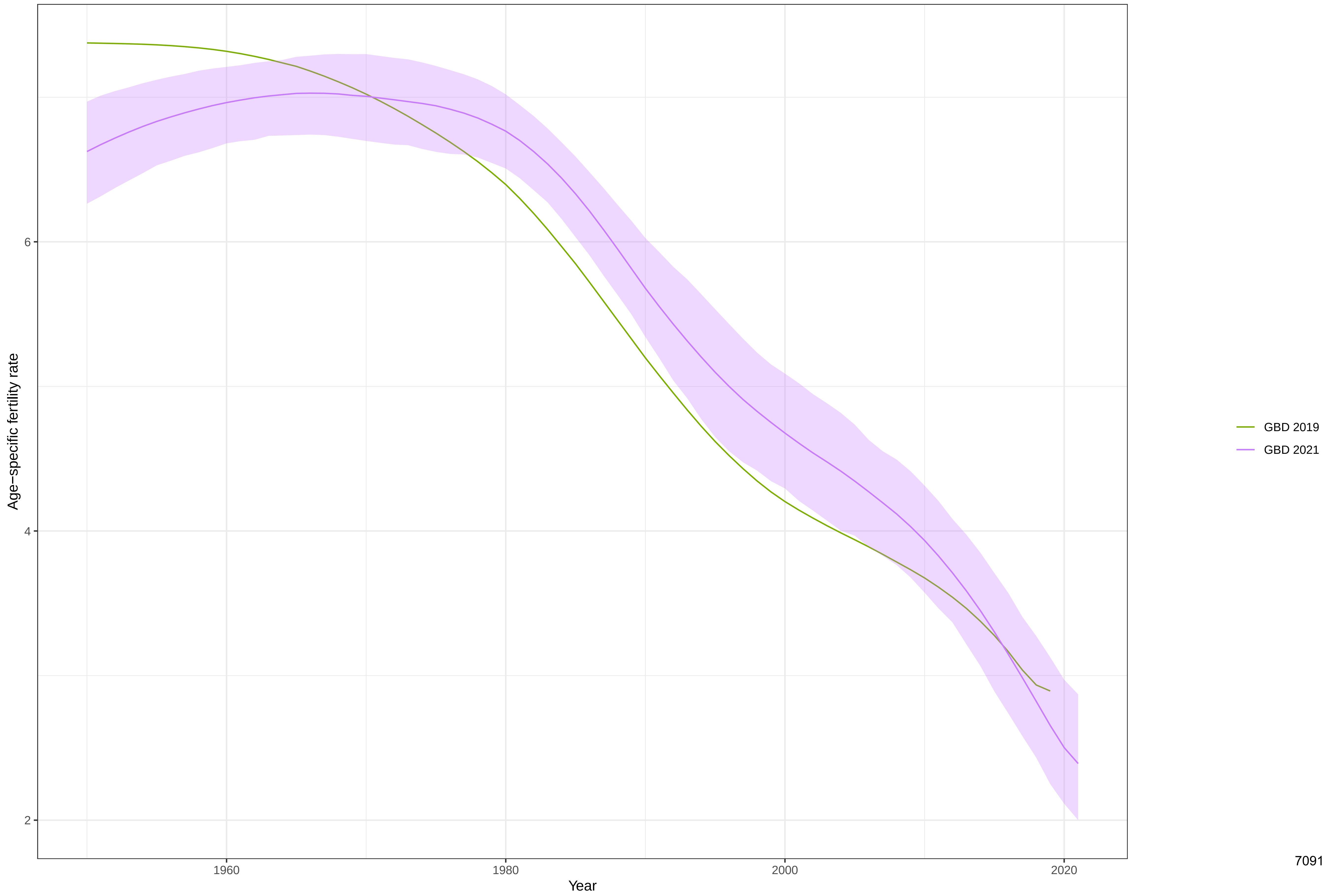

7091

Age−specific fertility fitted trend lines, 1950−2021: Machakos, Ages: 10−14
Data points enclosed with a diamond are reference sources

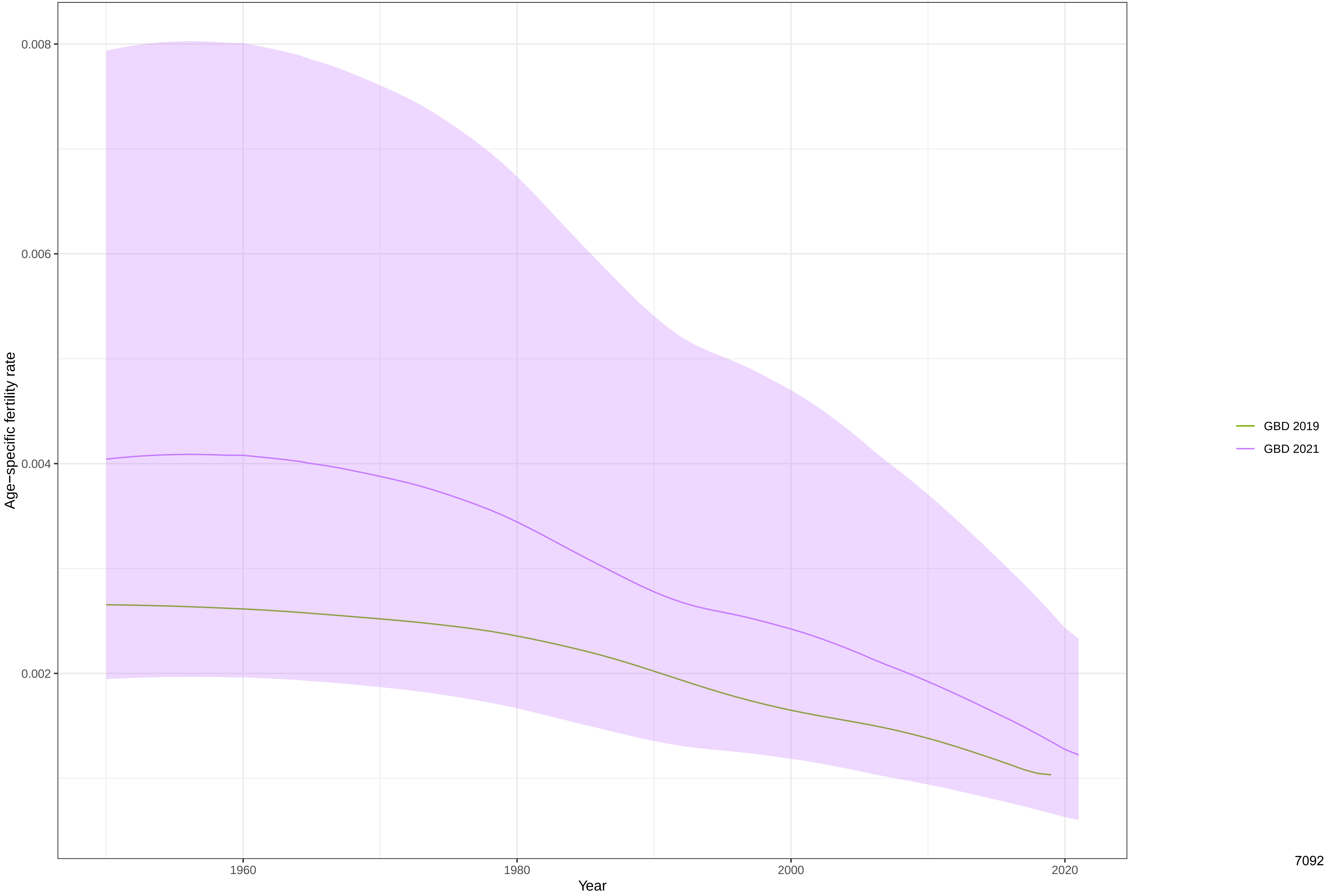

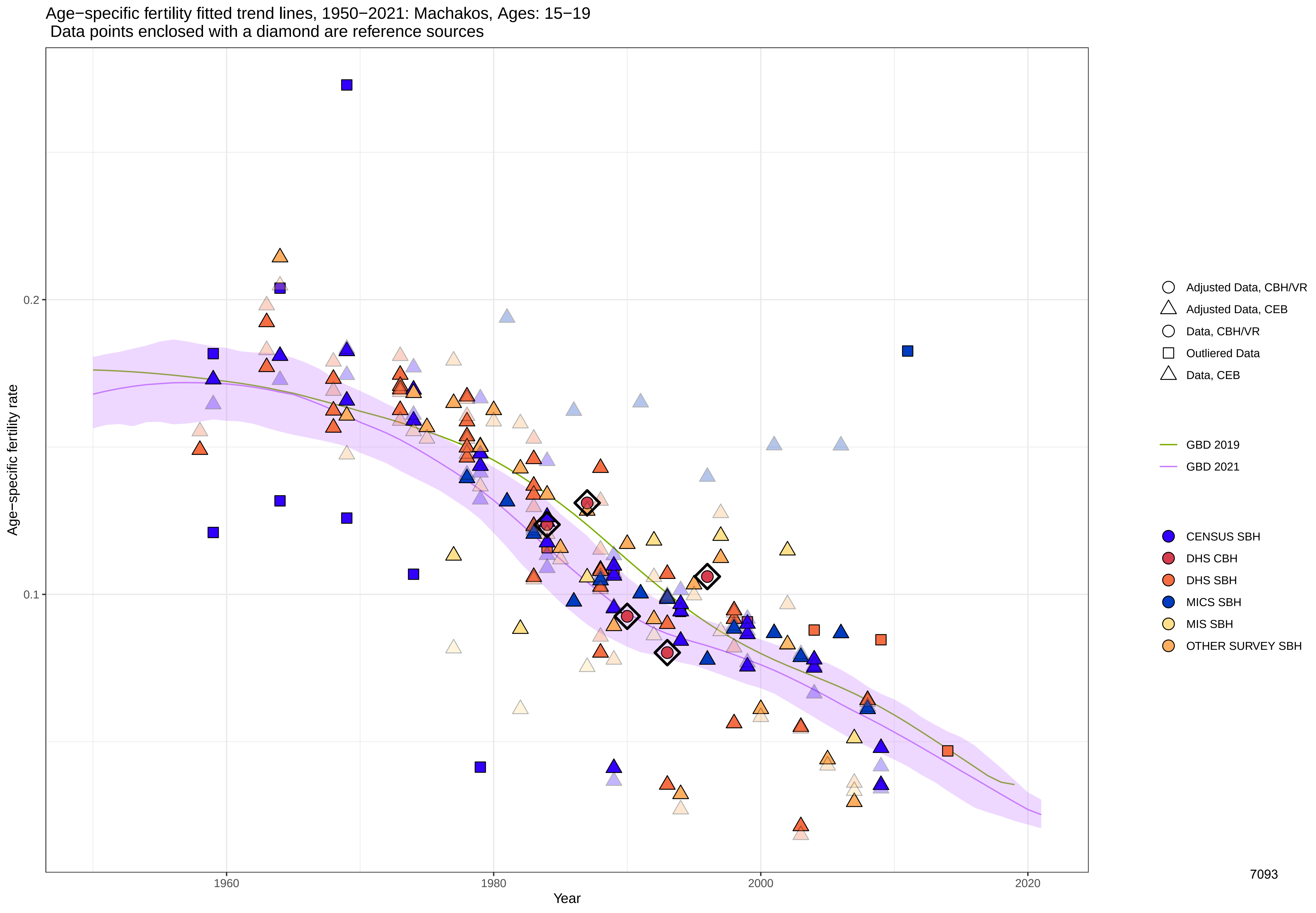

Age−specific fertility fitted trend lines, 1950−2021: Machakos, Ages: 15−19
Data points enclosed with a diamond are reference sources
Age−specific fertility rate
0.2
0.1
1960
1980
2000
2020
Year
Adjusted Data, CBH/VR
Adjusted Data, CEB
Data, CBH/VR
Outliered Data
Data, CEB
GBD 2019
GBD 2021
CENSUS SBH
DHS CBH
DHS SBH
MICS SBH
MIS SBH
OTHER SURVEY SBH

Age−specific fertility fitted trend lines, 1950−2021: Machakos, Ages: 20−24
Data points enclosed with a diamond are reference sources

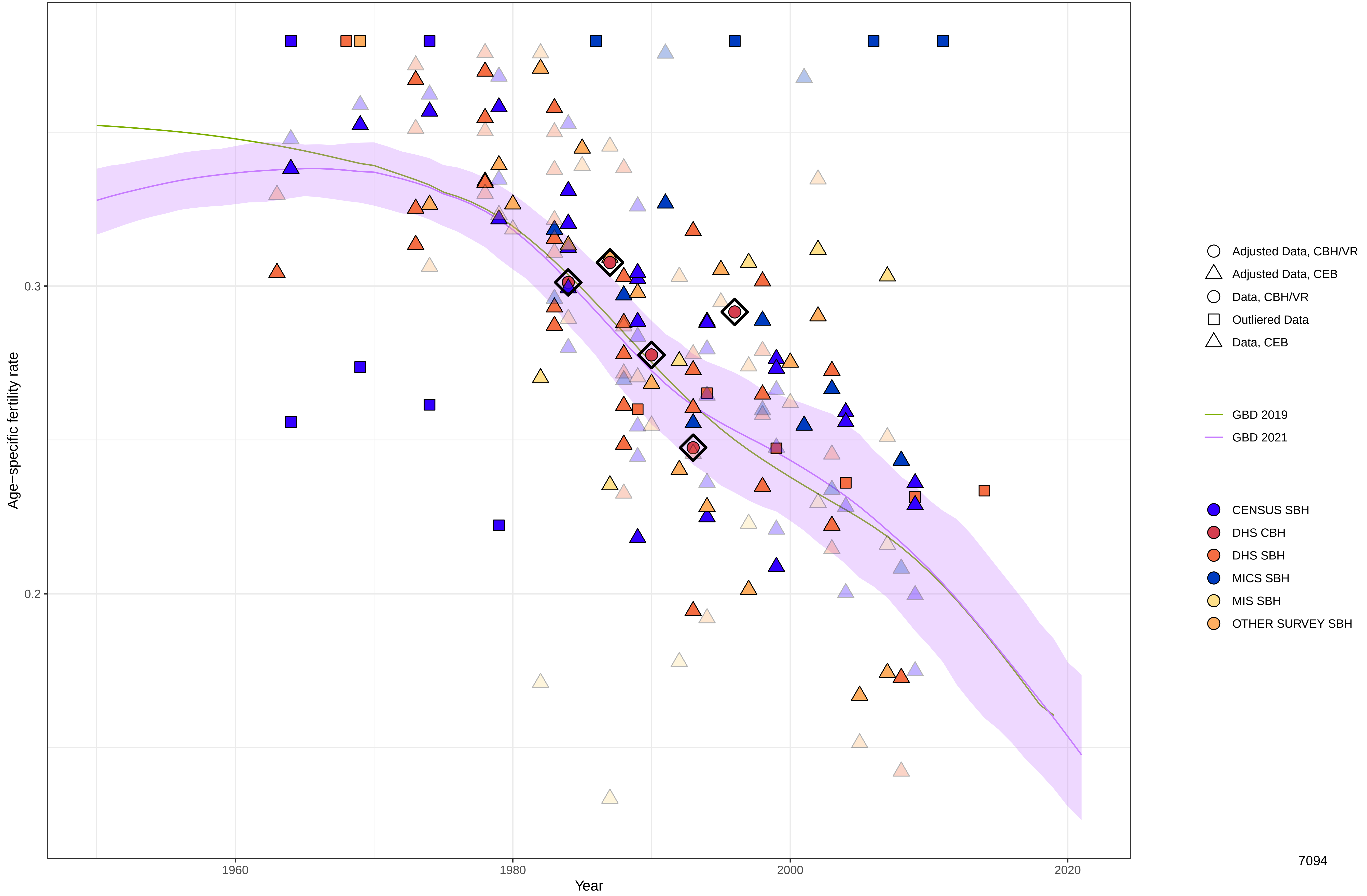

Age−specific fertility fitted trend lines, 1950−2021: Machakos, Ages: 25−29
Data points enclosed with a diamond are reference sources

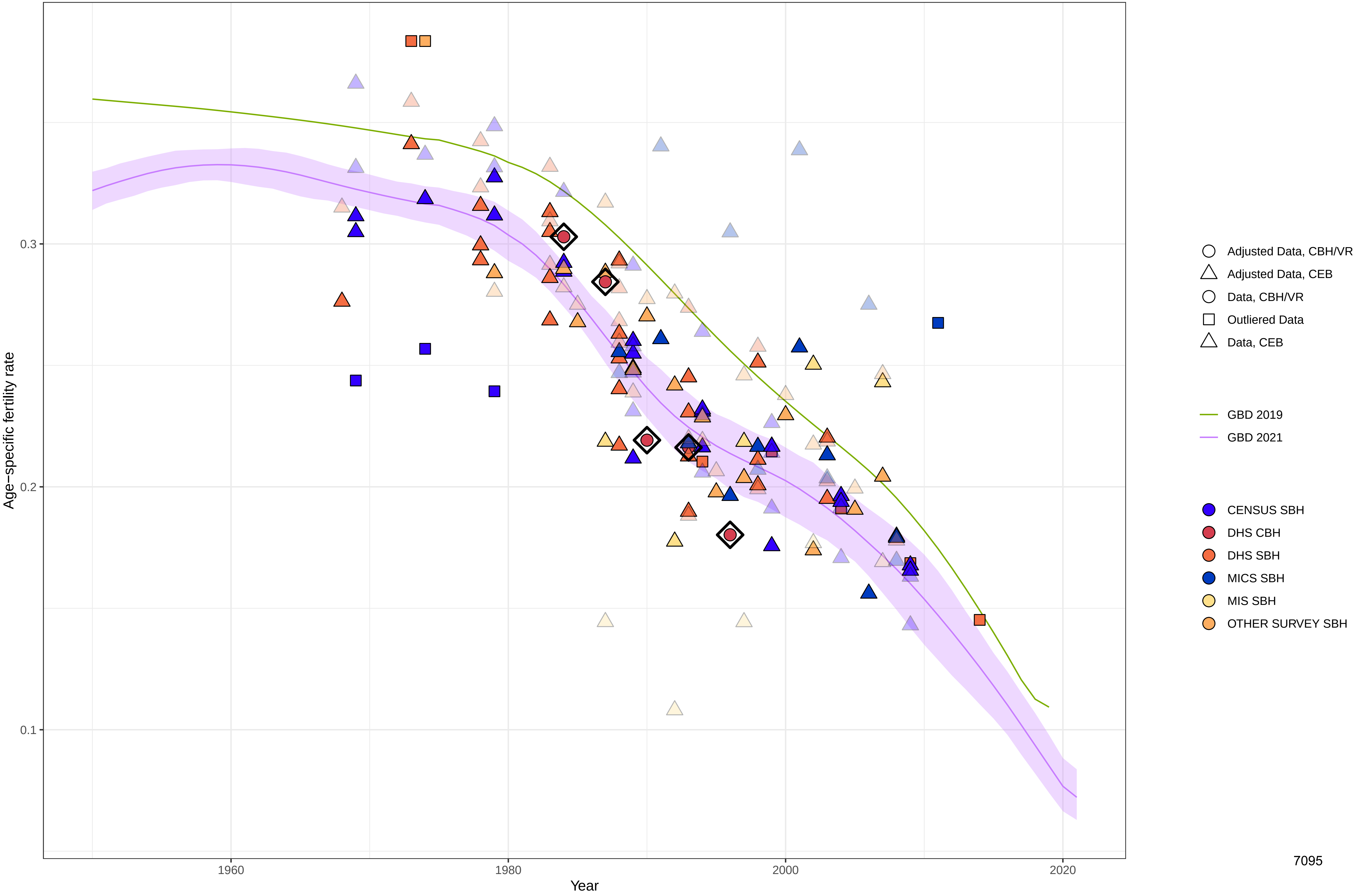

Age−specific fertility fitted trend lines, 1950−2021: Machakos, Ages: 30−34
Data points enclosed with a diamond are reference sources

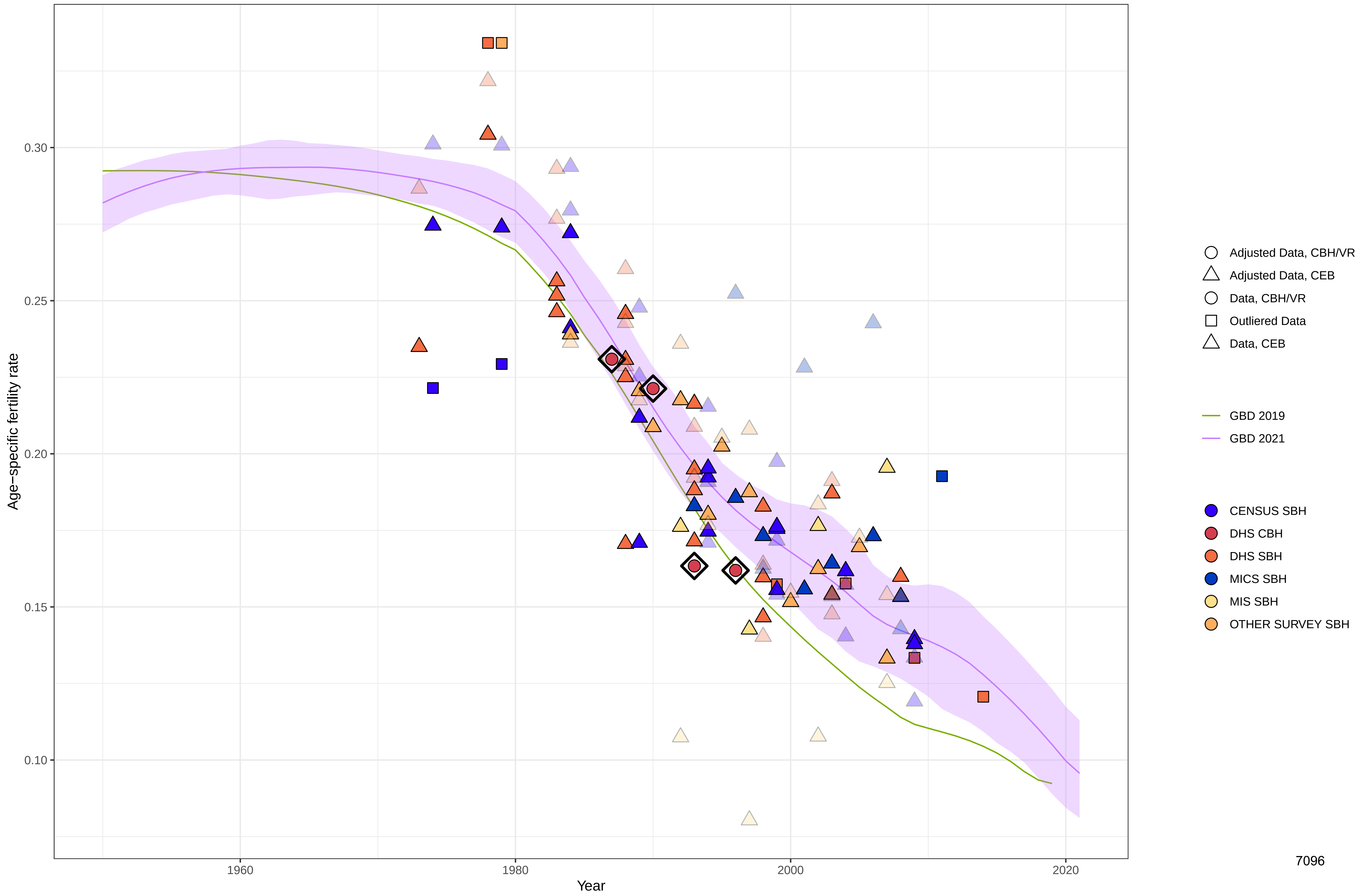

Age−specific fertility fitted trend lines, 1950−2021: Machakos, Ages: 35−39
Data points enclosed with a diamond are reference sources

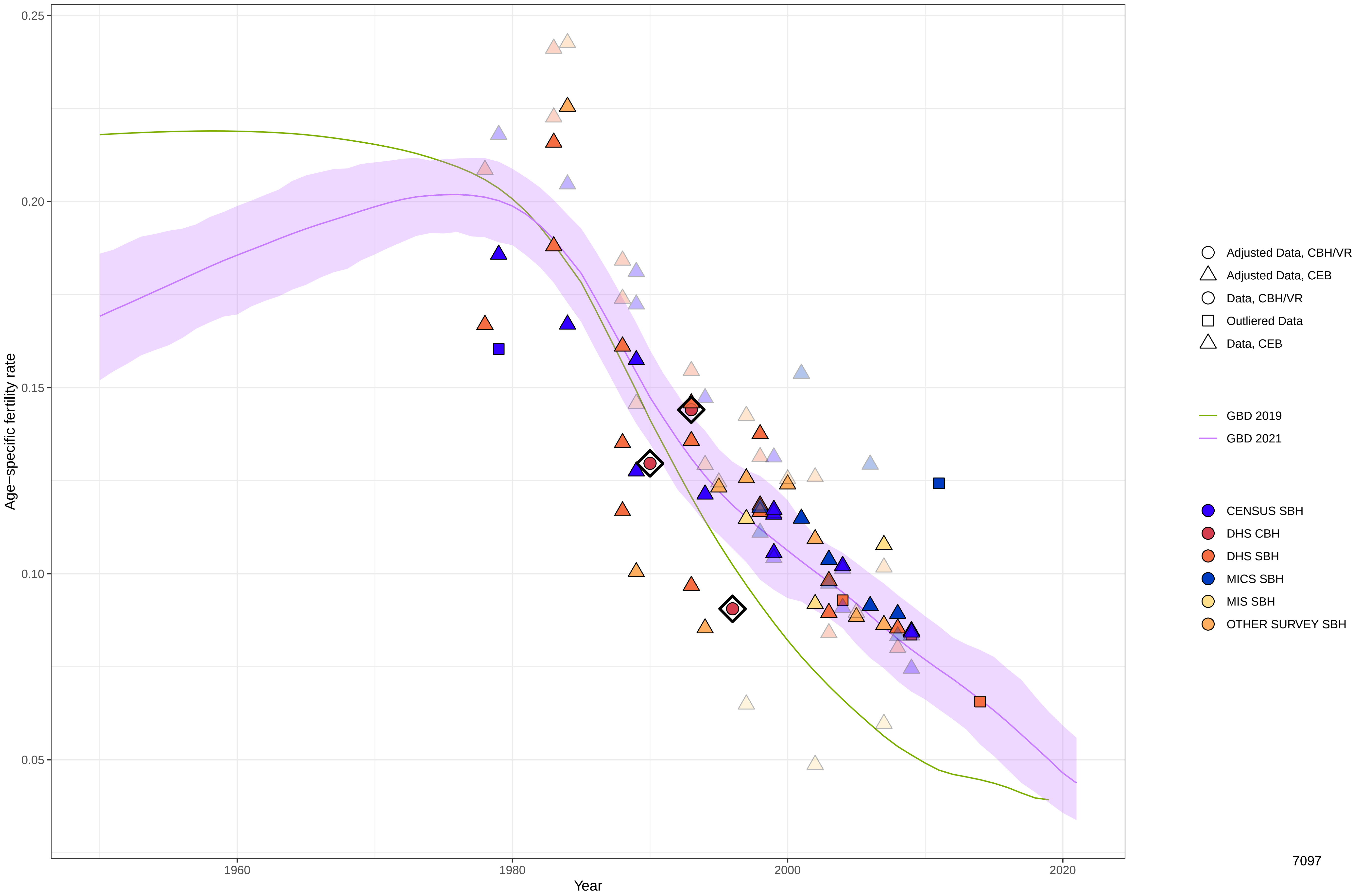

Age−specific fertility fitted trend lines, 1950−2021: Machakos, Ages: 40−44
Data points enclosed with a diamond are reference sources

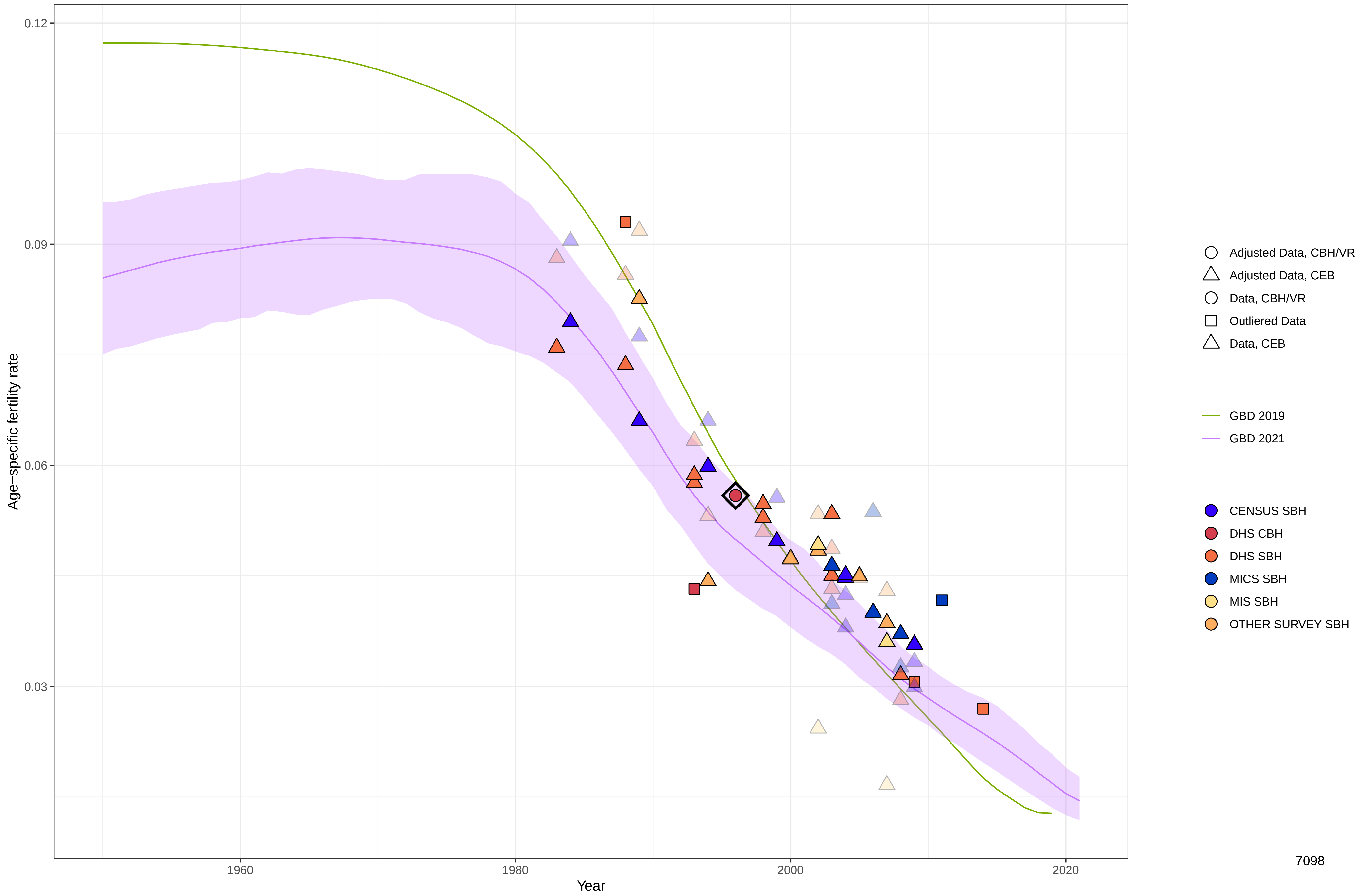

Age−specific fertility fitted trend lines, 1950−2021: Machakos, Ages: 45−49
Data points enclosed with a diamond are reference sources

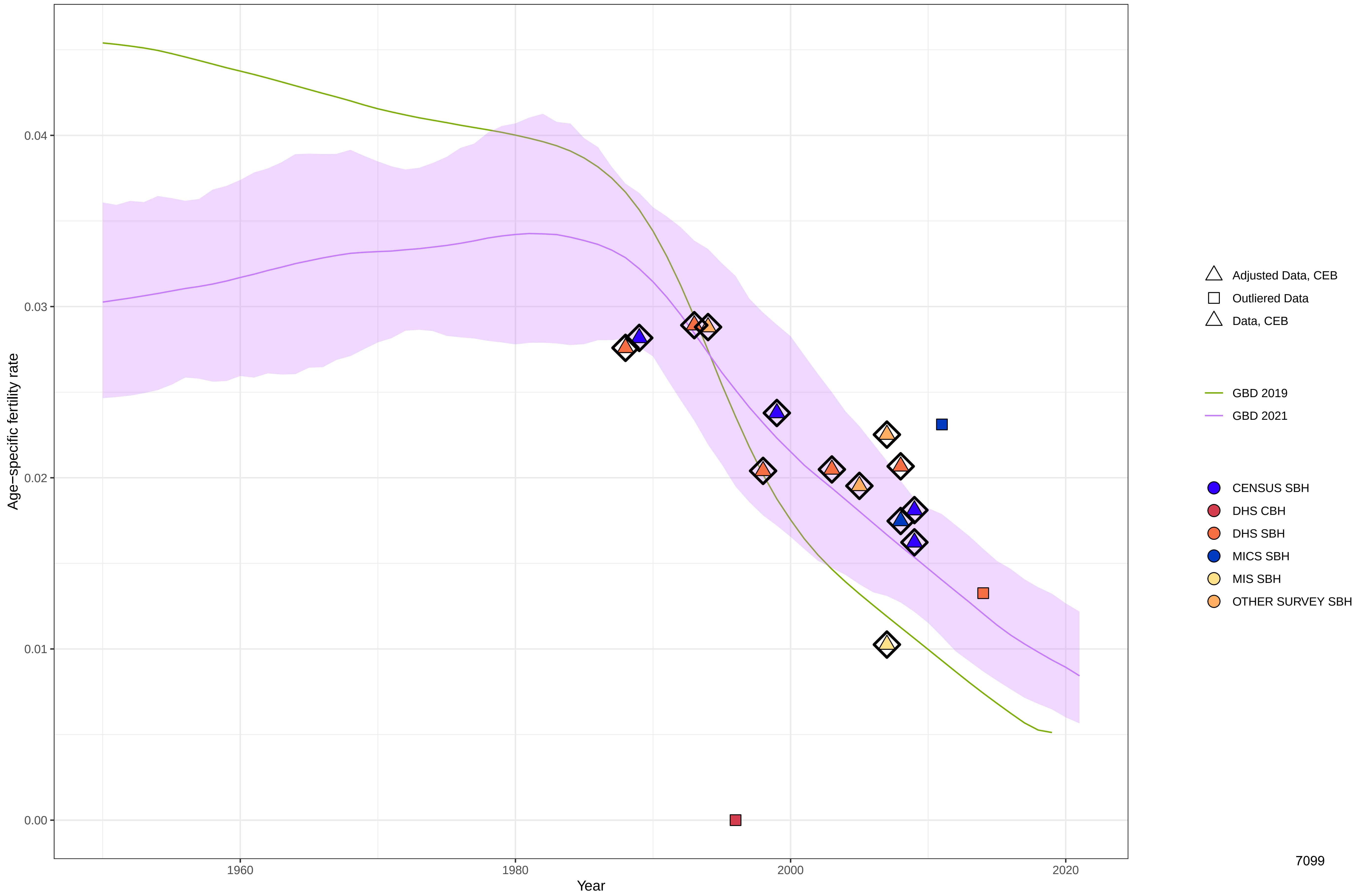

Age−specific fertility fitted trend lines, 1950−2021: Machakos, Ages: 50−54
Data points enclosed with a diamond are reference sources

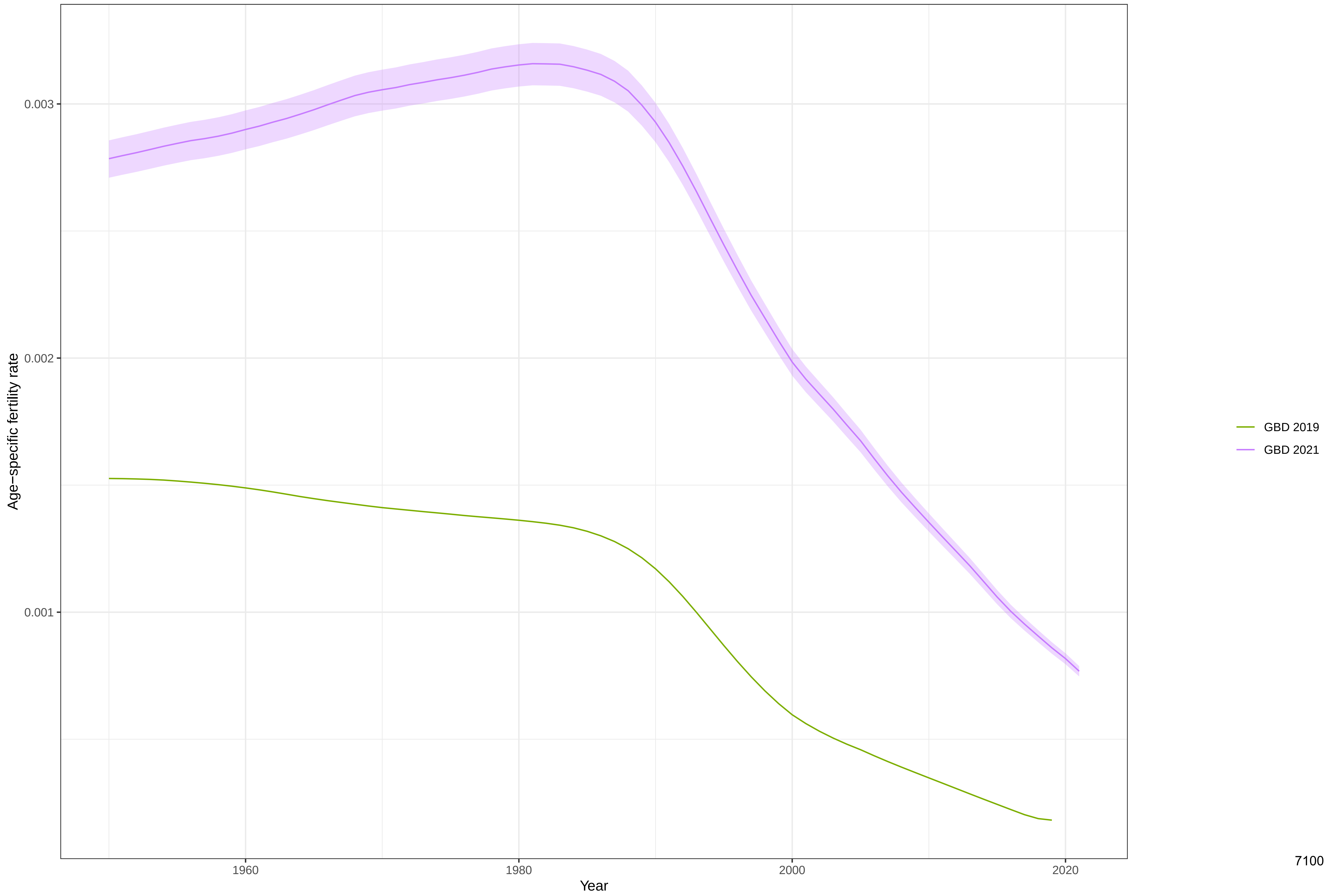

Total fertility rate fitted trend lines, 1950−2021: Machakos TFR
Data points enclosed with a diamond are reference sources

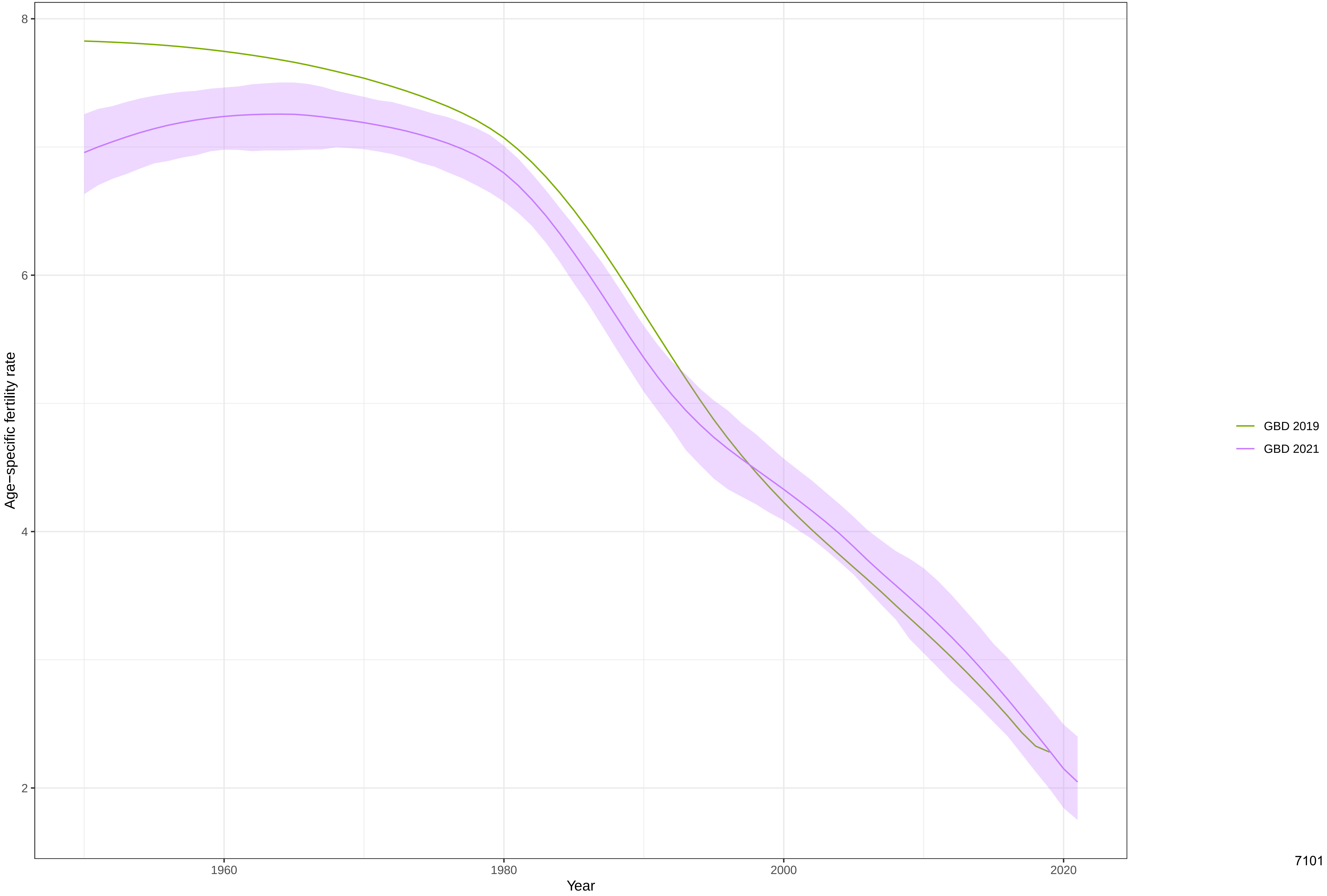

Age−specific fertility fitted trend lines, 1950−2021: Makueni, Ages: 10−14
Data points enclosed with a diamond are reference sources

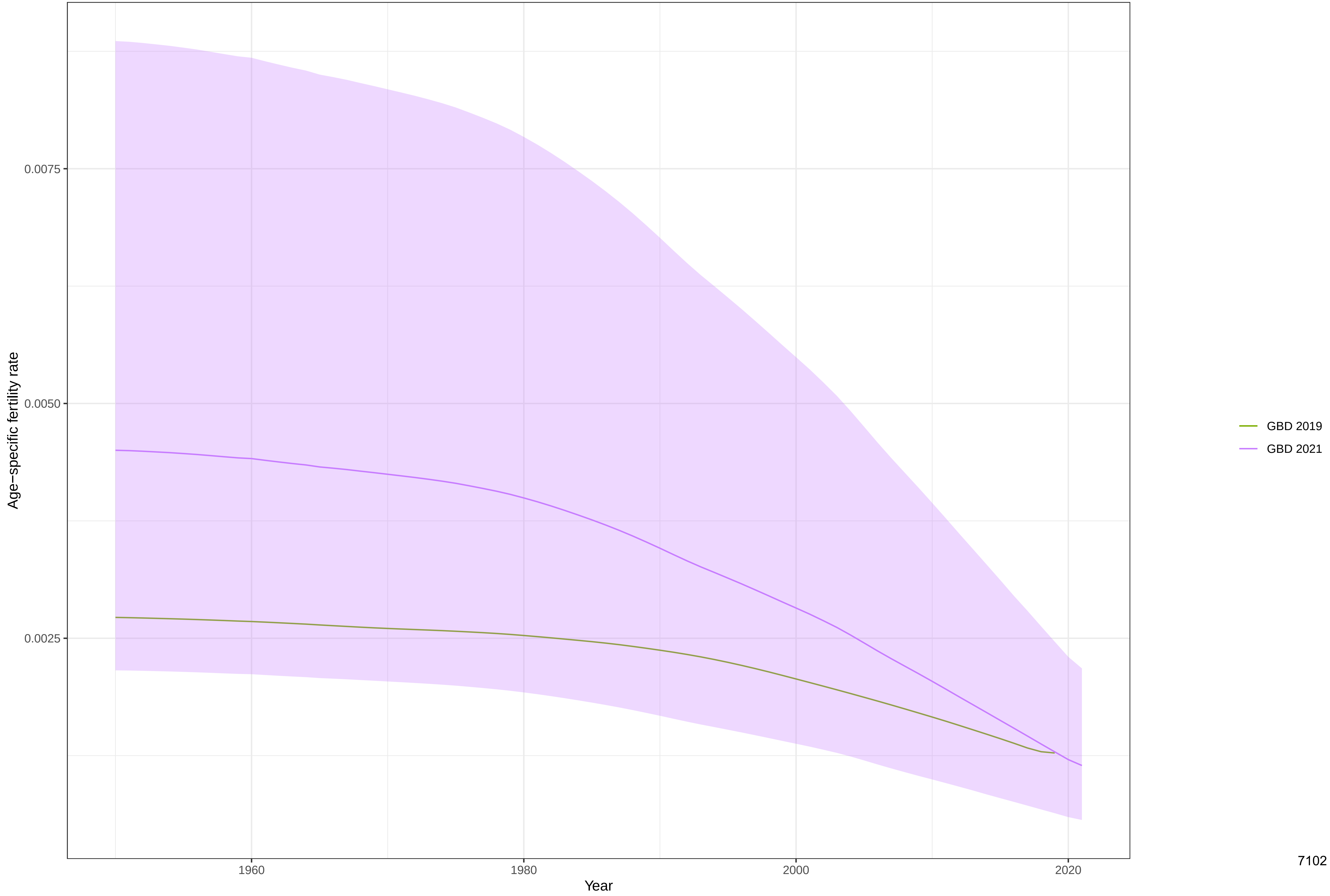

Age−specific fertility fitted trend lines, 1950−2021: Makueni, Ages: 15−19
Data points enclosed with a diamond are reference sources

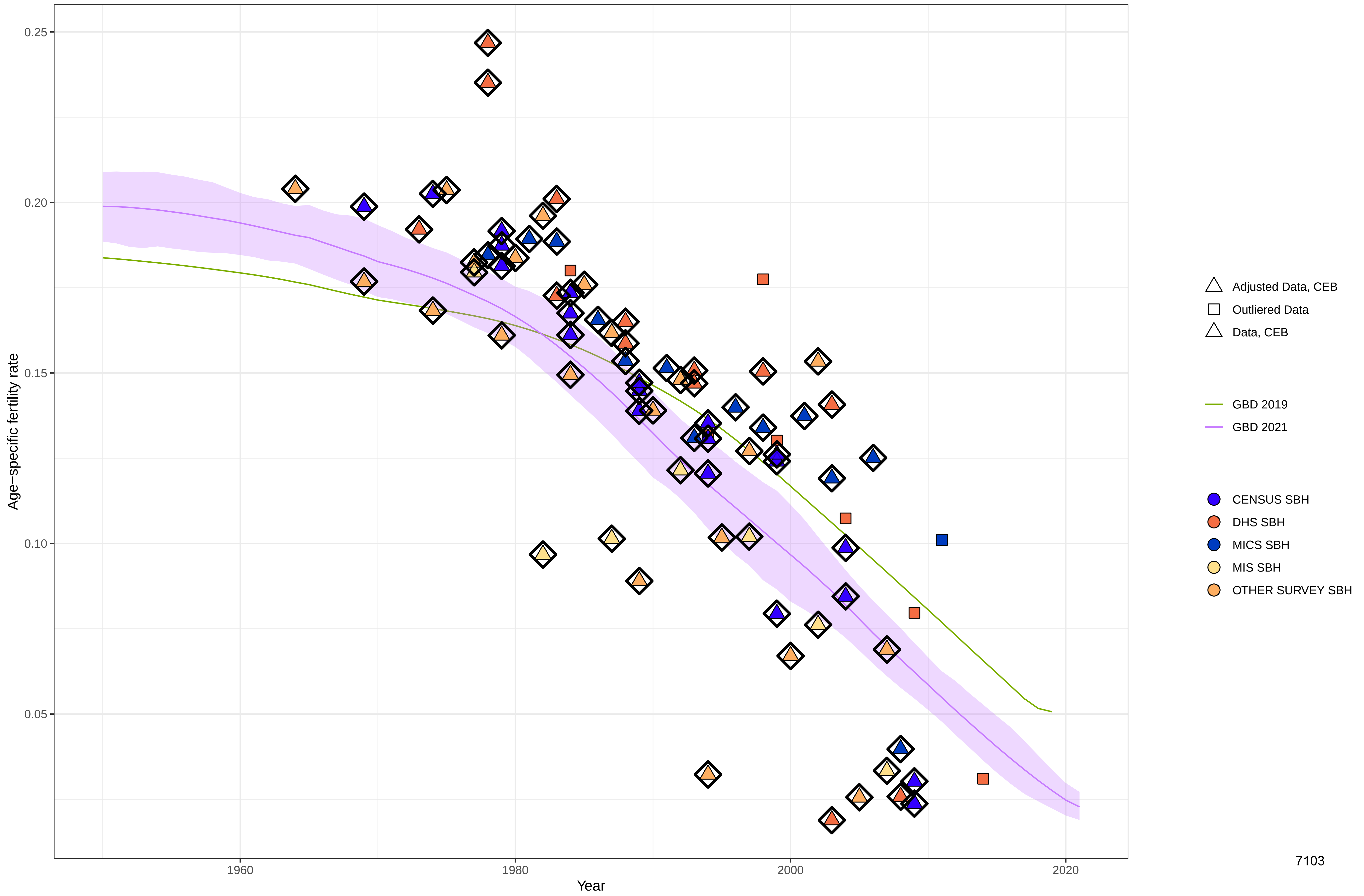

Age−specific fertility fitted trend lines, 1950−2021: Makueni, Ages: 20−24
Data points enclosed with a diamond are reference sources

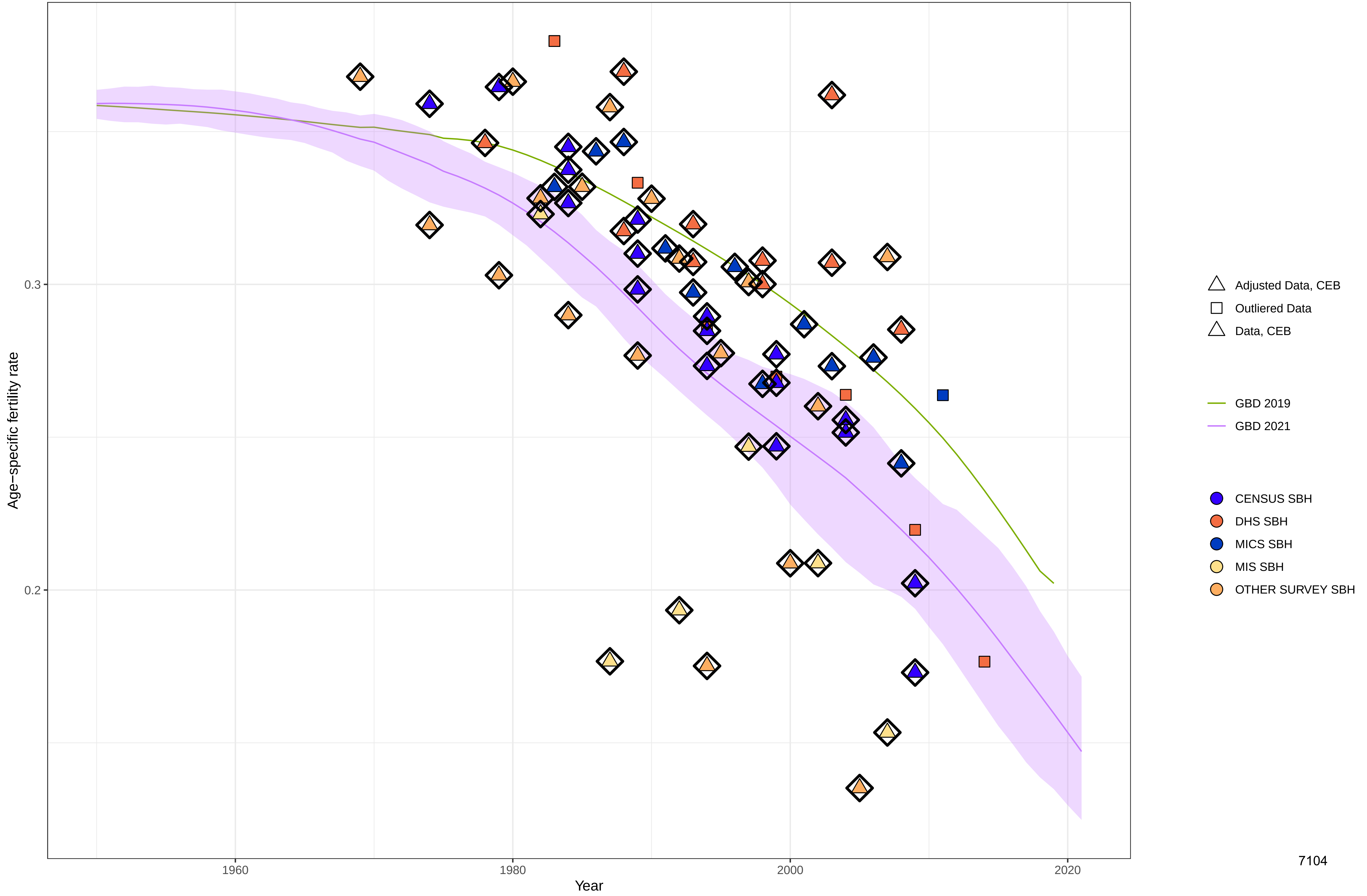

Age−specific fertility fitted trend lines, 1950−2021: Makueni, Ages: 25−29
Data points enclosed with a diamond are reference sources

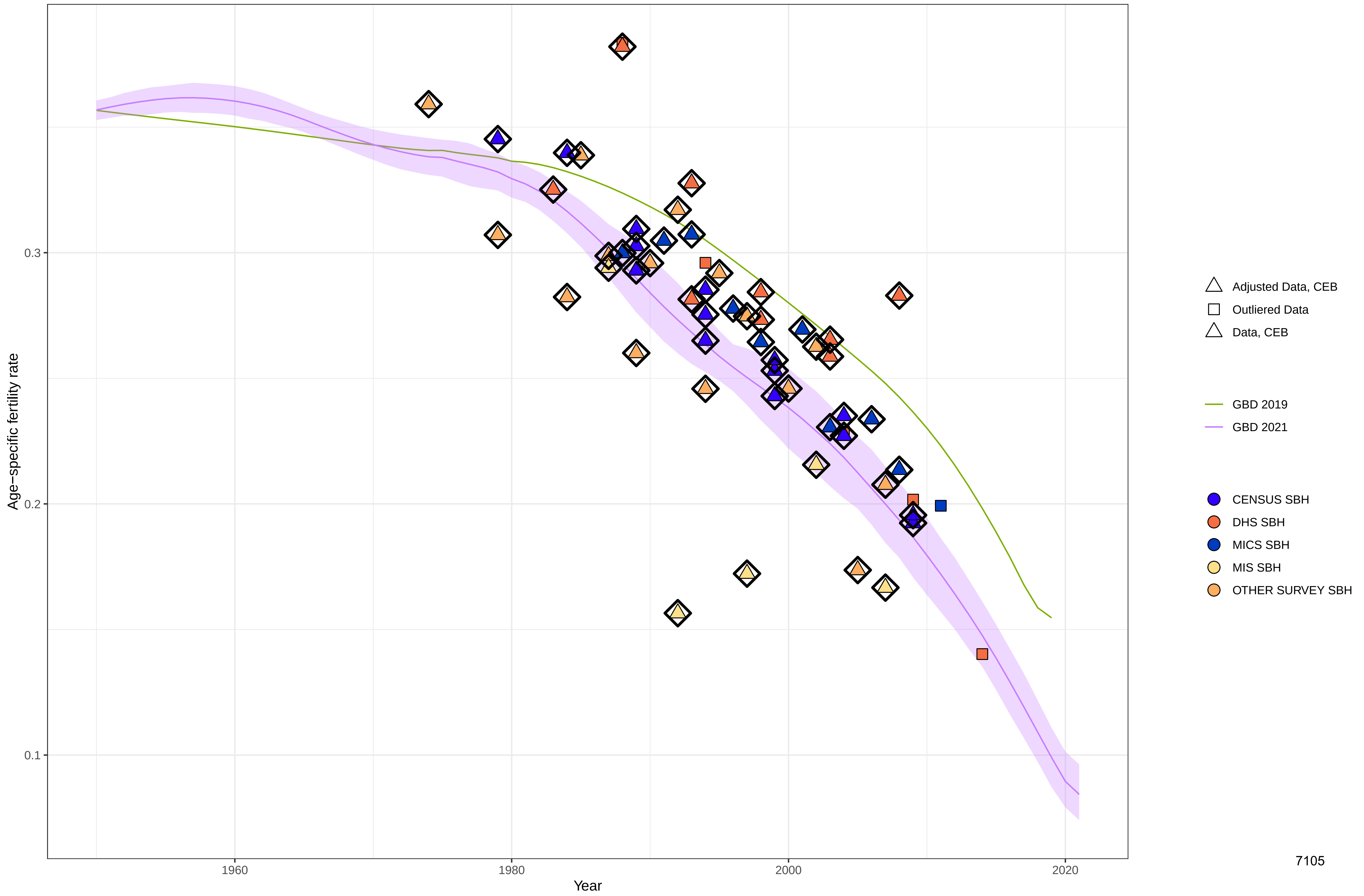

Age−specific fertility fitted trend lines, 1950−2021: Makueni, Ages: 30−34
Data points enclosed with a diamond are reference sources

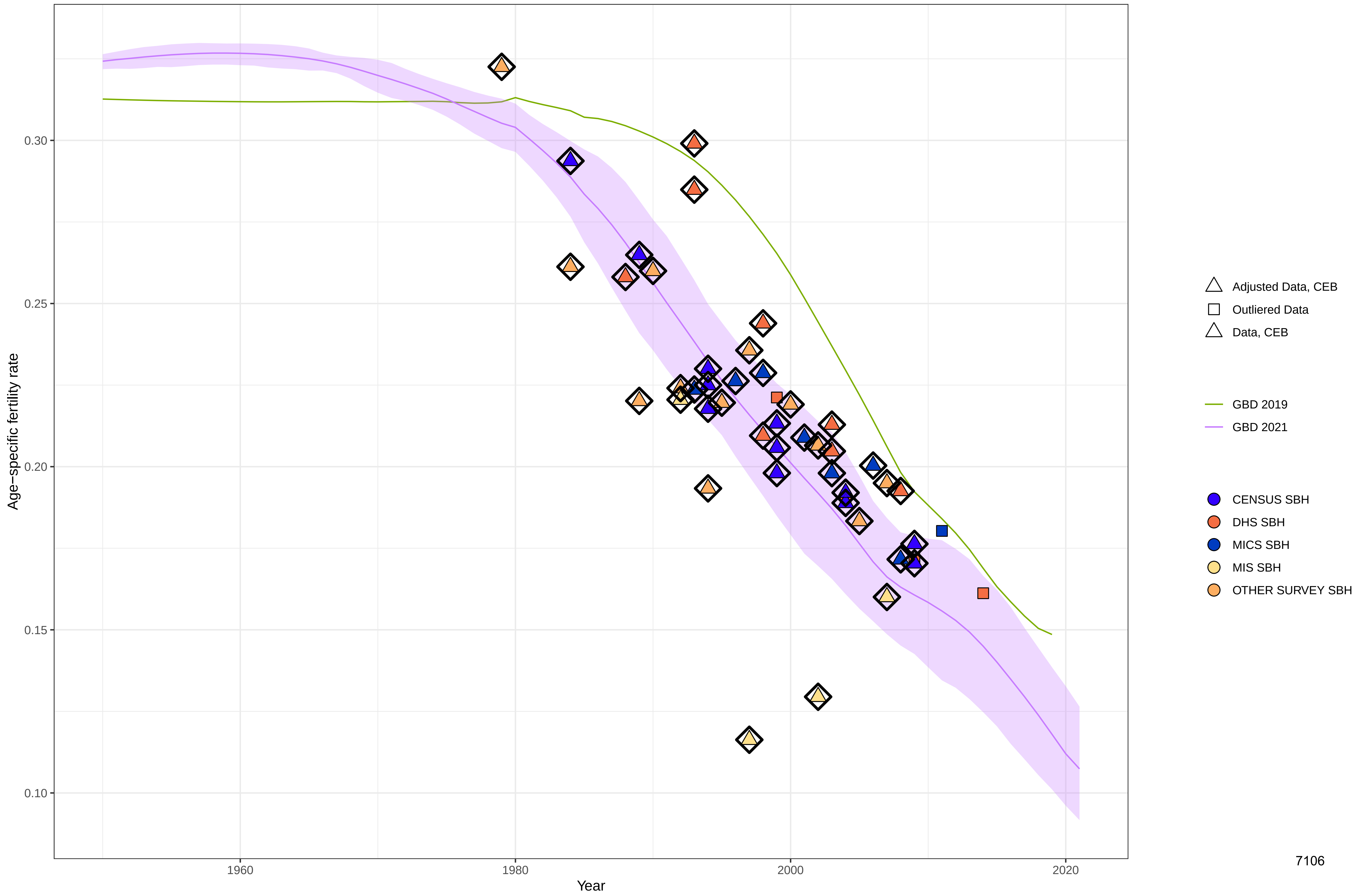

7106

Age−specific fertility fitted trend lines, 1950−2021: Makueni, Ages: 35−39
Data points enclosed with a diamond are reference sources

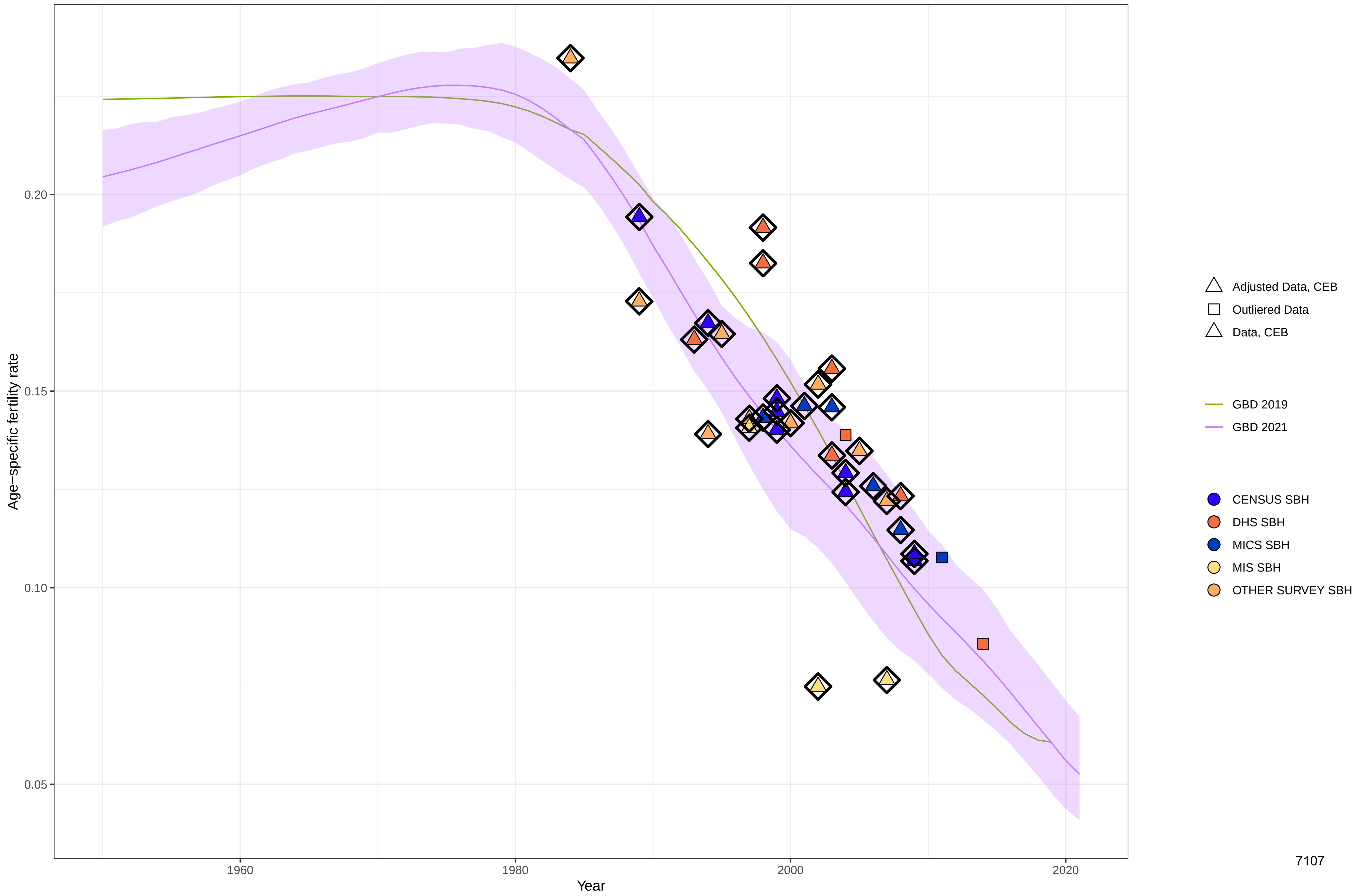

Age−specific fertility fitted trend lines, 1950−2021: Makueni, Ages: 40−44
Data points enclosed with a diamond are reference sources

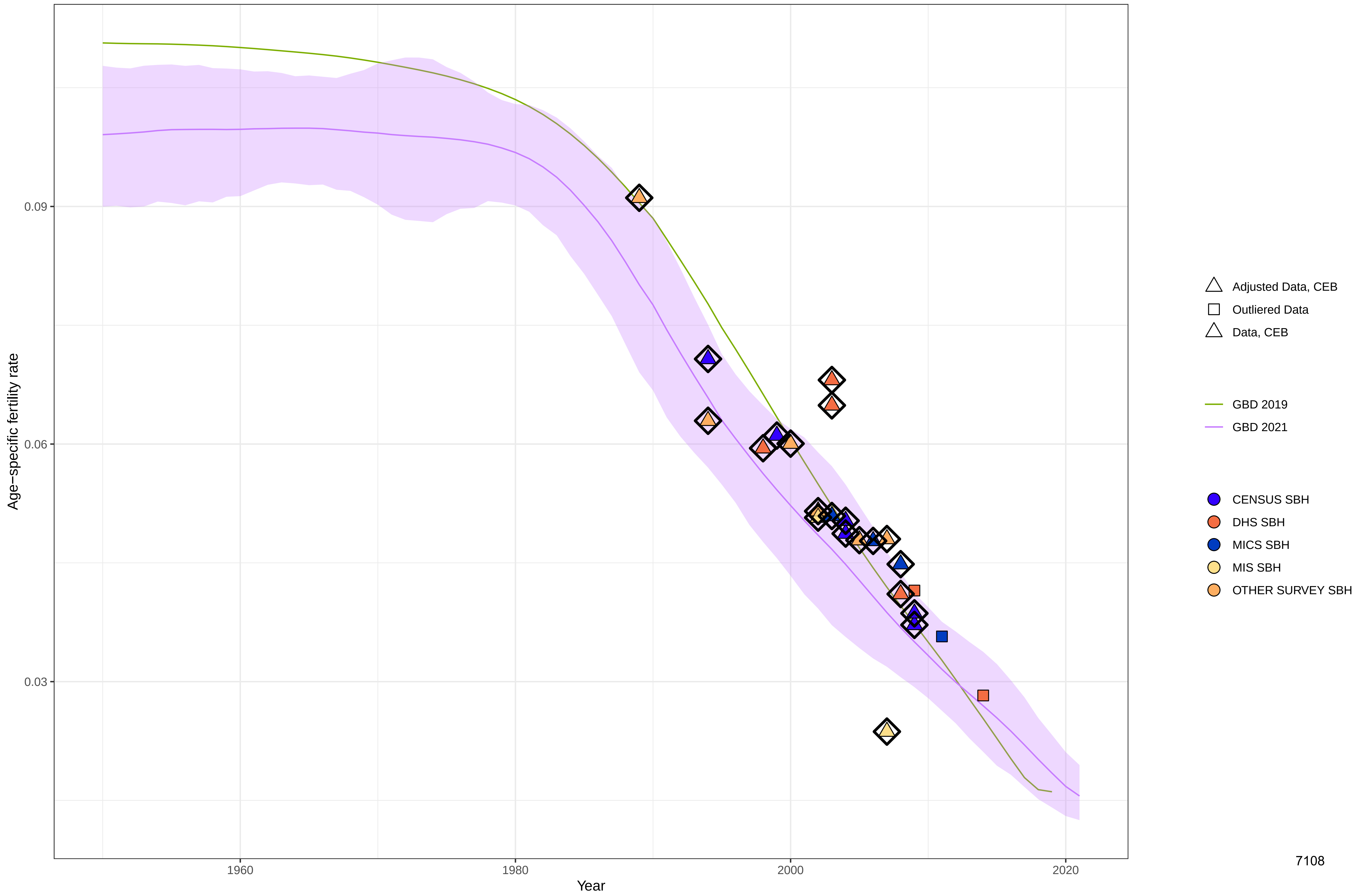

Age−specific fertility fitted trend lines, 1950−2021: Makueni, Ages: 45−49
Data points enclosed with a diamond are reference sources

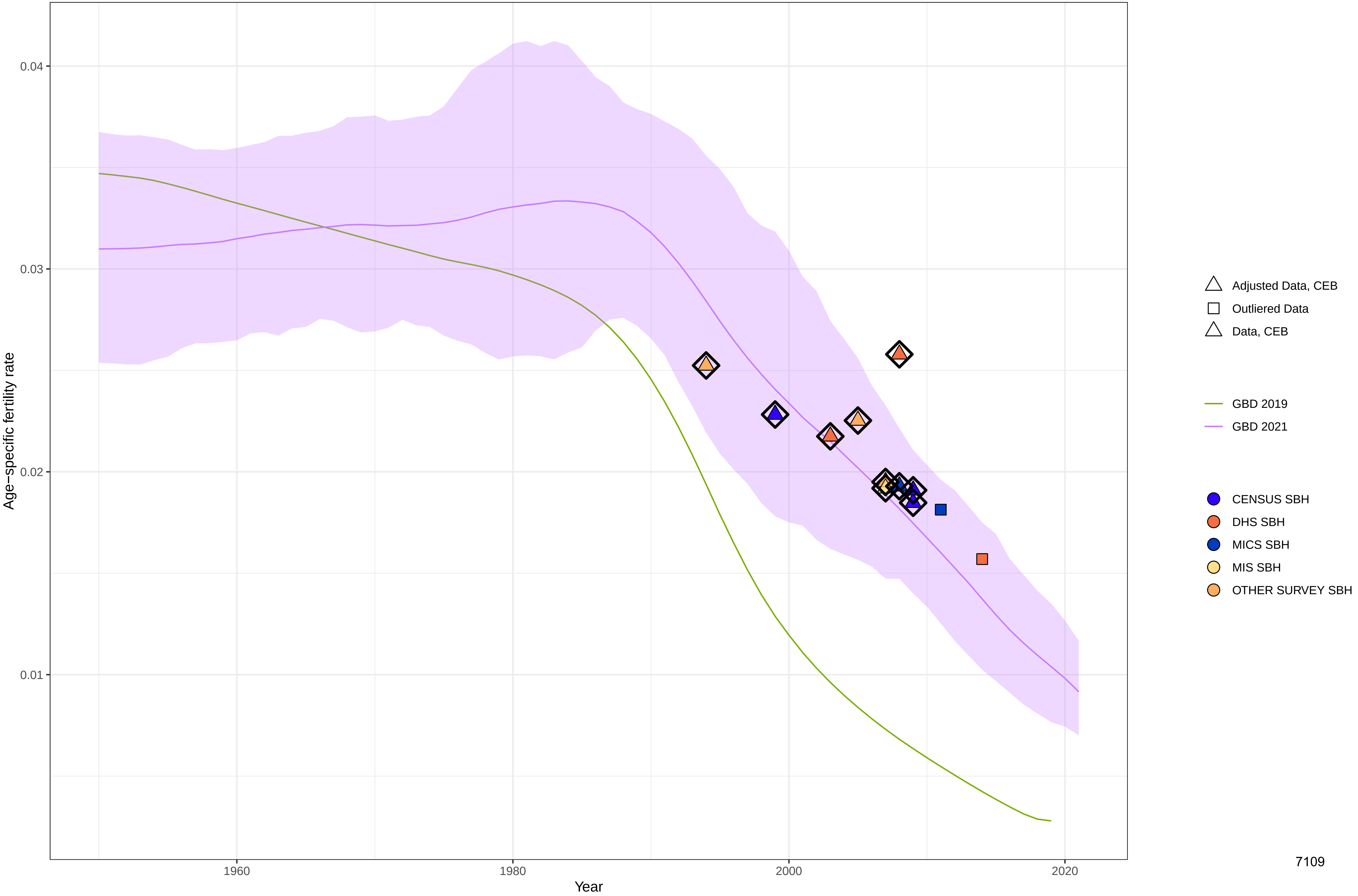

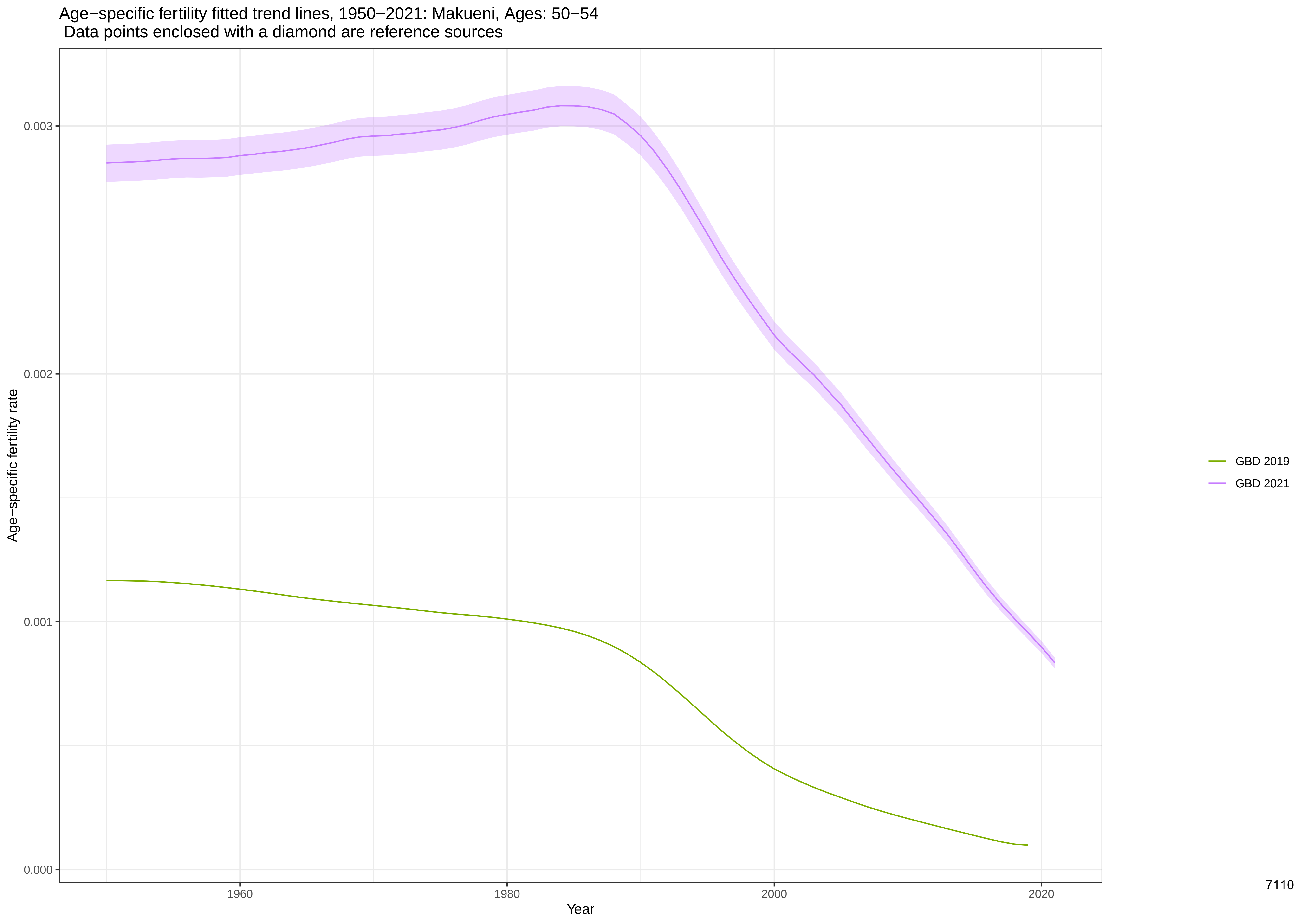

Age−specific fertility fitted trend lines, 1950−2021: Makueni, Ages: 50−54
Data points enclosed with a diamond are reference sources
Age−specific fertility rate
0.000
0.001
0.002
0.003
1960
1980
2000
2020
Year
GBD 2019
GBD 2021

Total fertility rate fitted trend lines, 1950−2021: Makueni TFR
Data points enclosed with a diamond are reference sources

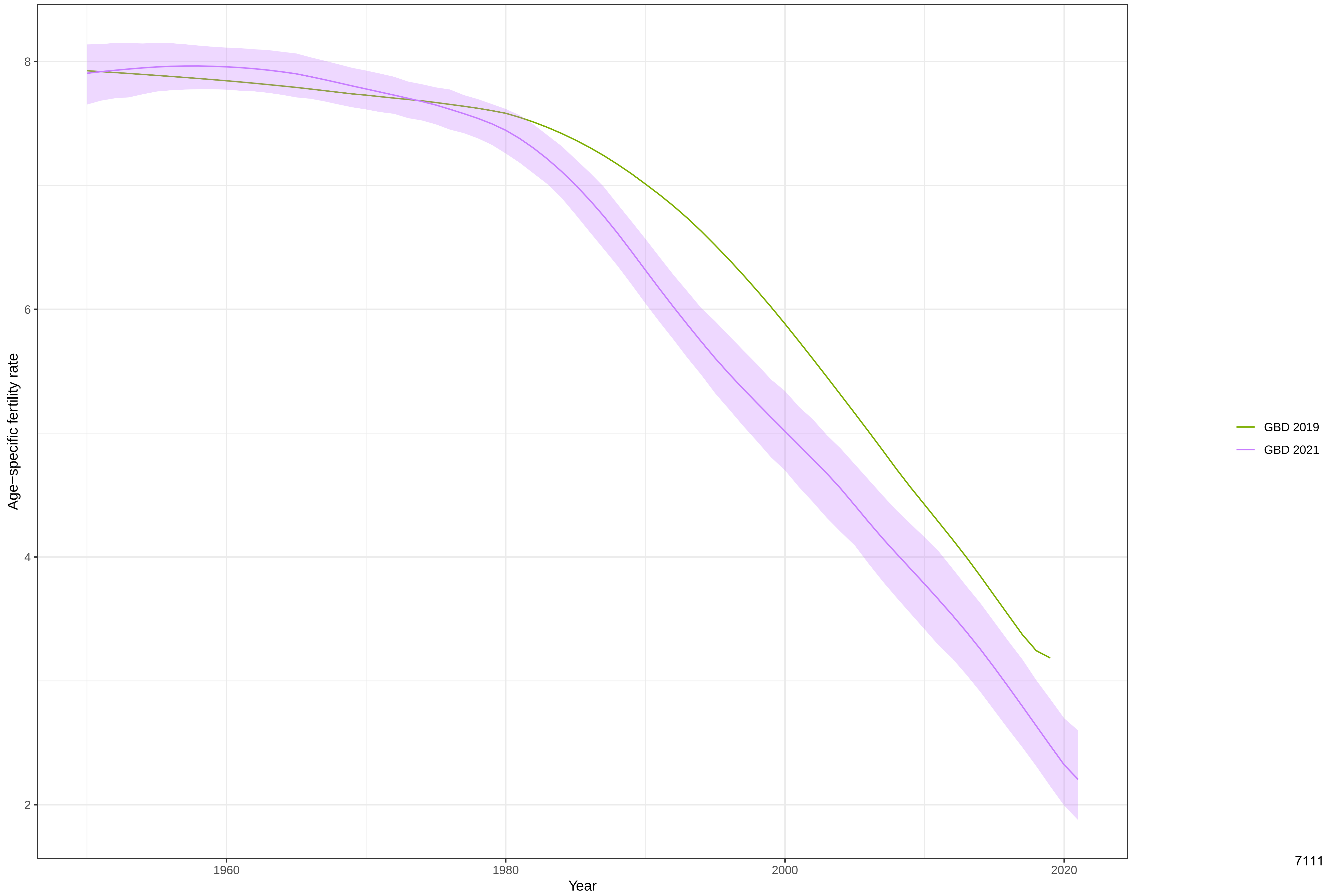

Age−specific fertility fitted trend lines, 1950−2021: Mandera, Ages: 10−14
Data points enclosed with a diamond are reference sources

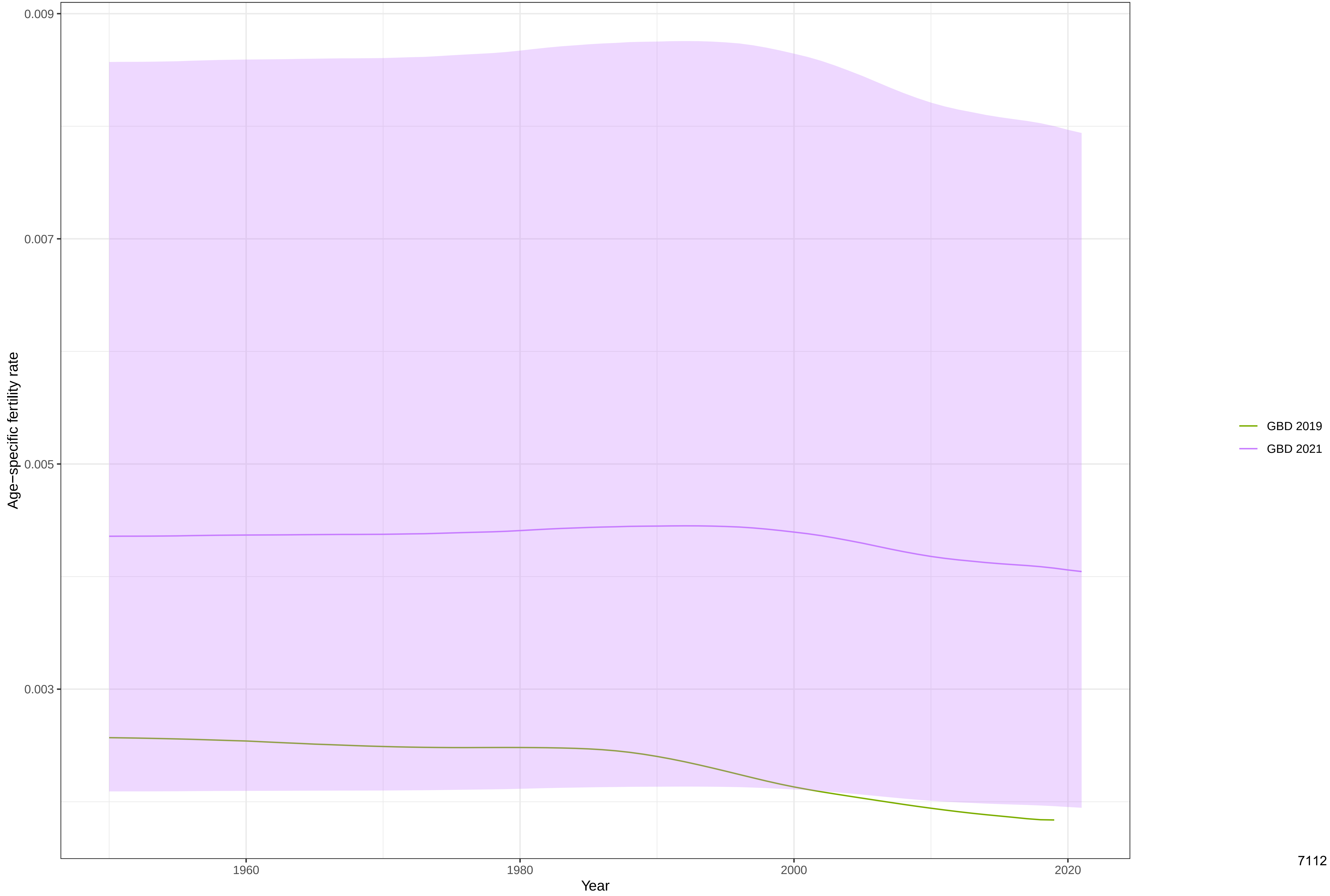

Age−specific fertility fitted trend lines, 1950−2021: Mandera, Ages: 15−19
Data points enclosed with a diamond are reference sources

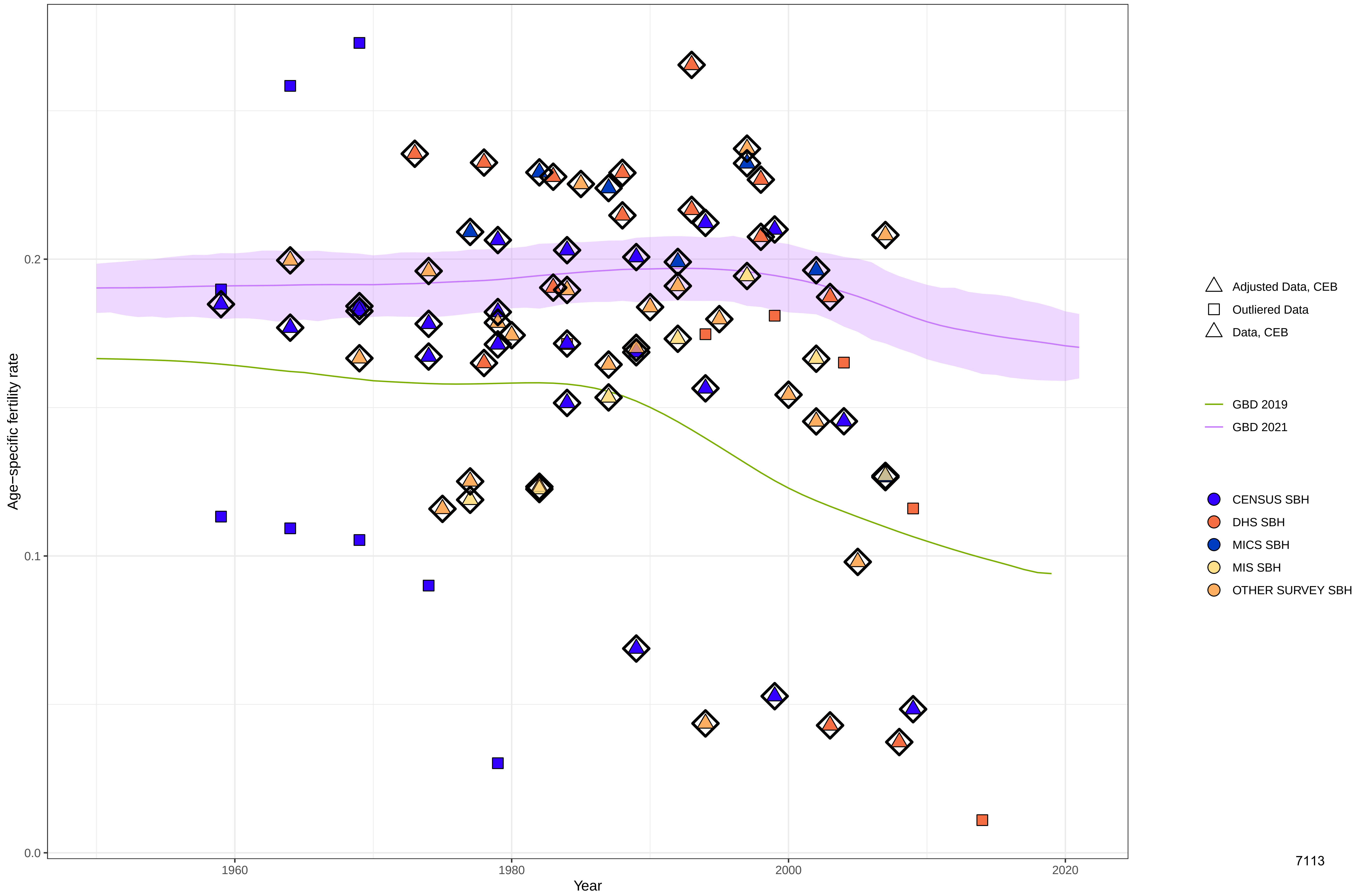

Age−specific fertility fitted trend lines, 1950−2021: Mandera, Ages: 20−24
Data points enclosed with a diamond are reference sources

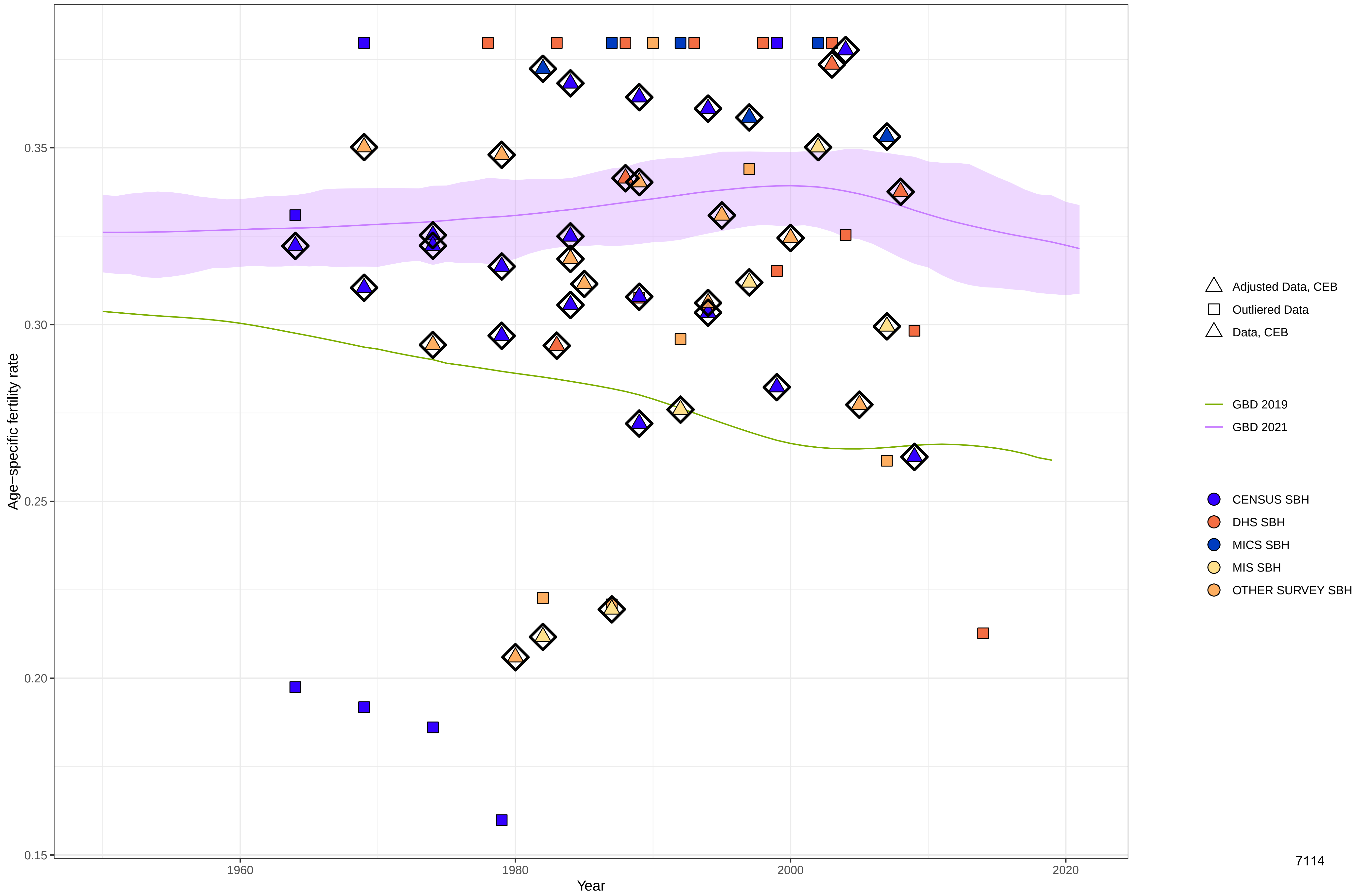

7114

Age−specific fertility fitted trend lines, 1950−2021: Mandera, Ages: 25−29
Data points enclosed with a diamond are reference sources

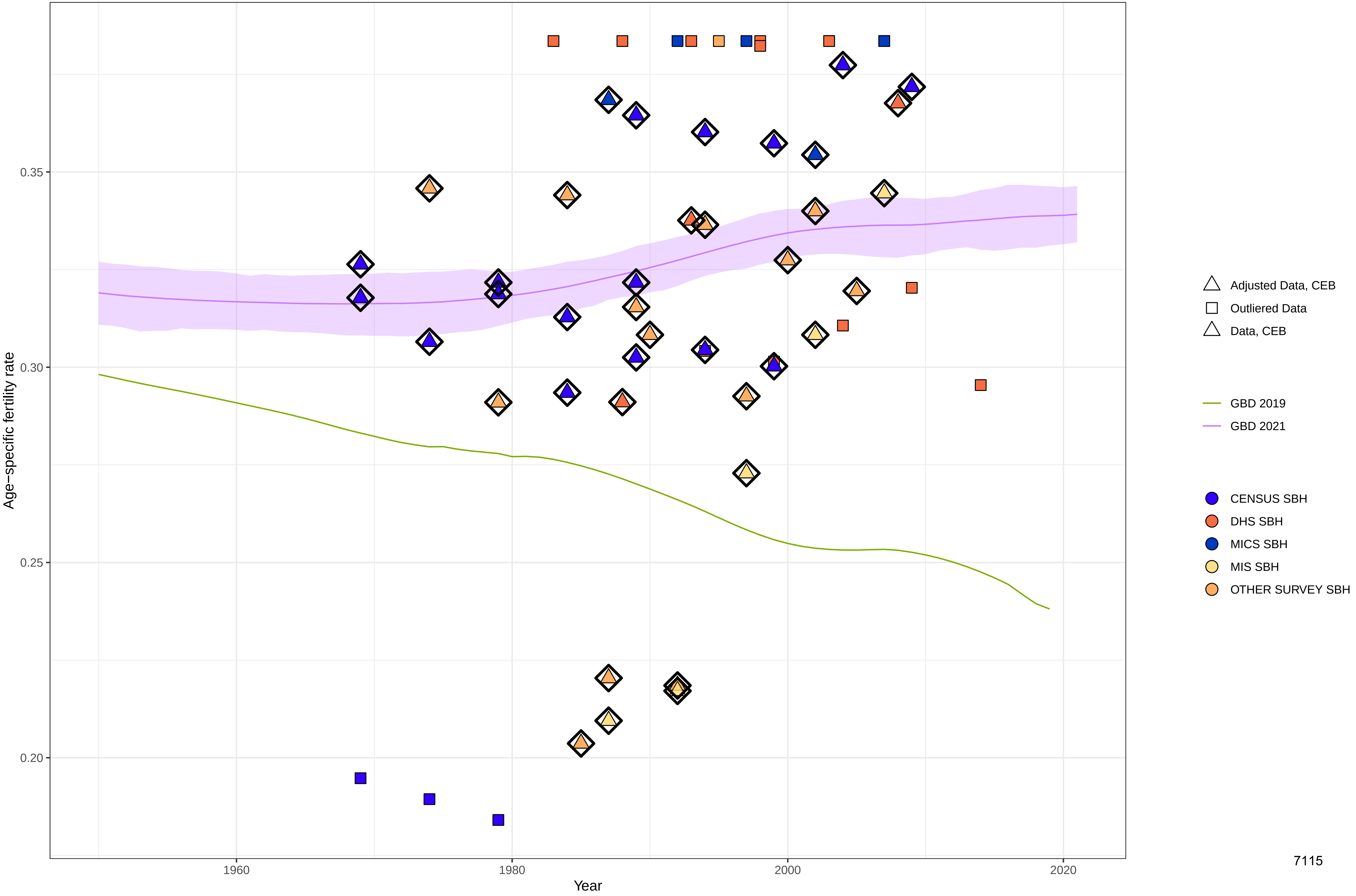

Age−specific fertility fitted trend lines, 1950−2021: Mandera, Ages: 30−34
Data points enclosed with a diamond are reference sources

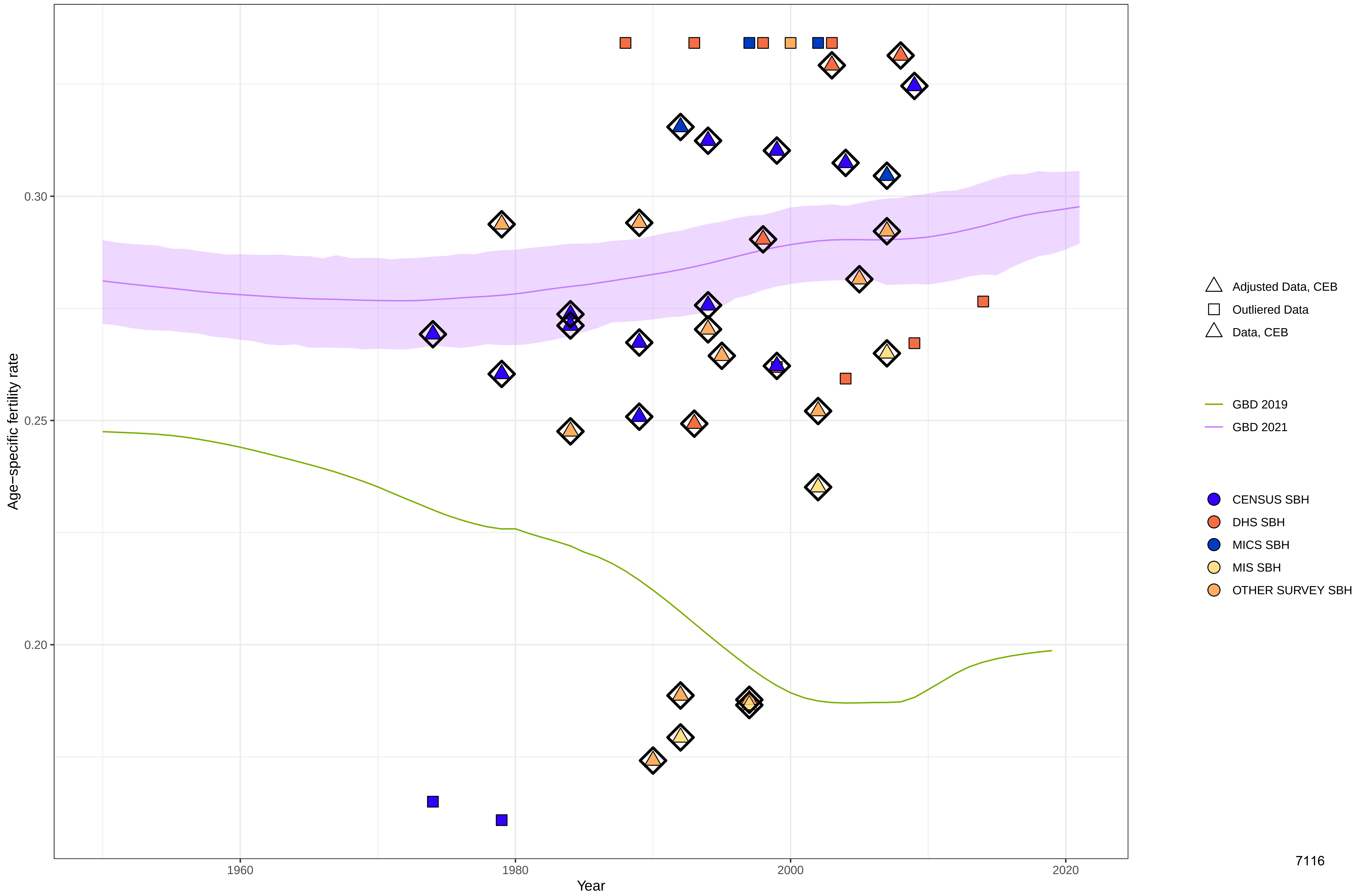

Age−specific fertility fitted trend lines, 1950−2021: Mandera, Ages: 35−39
Data points enclosed with a diamond are reference sources

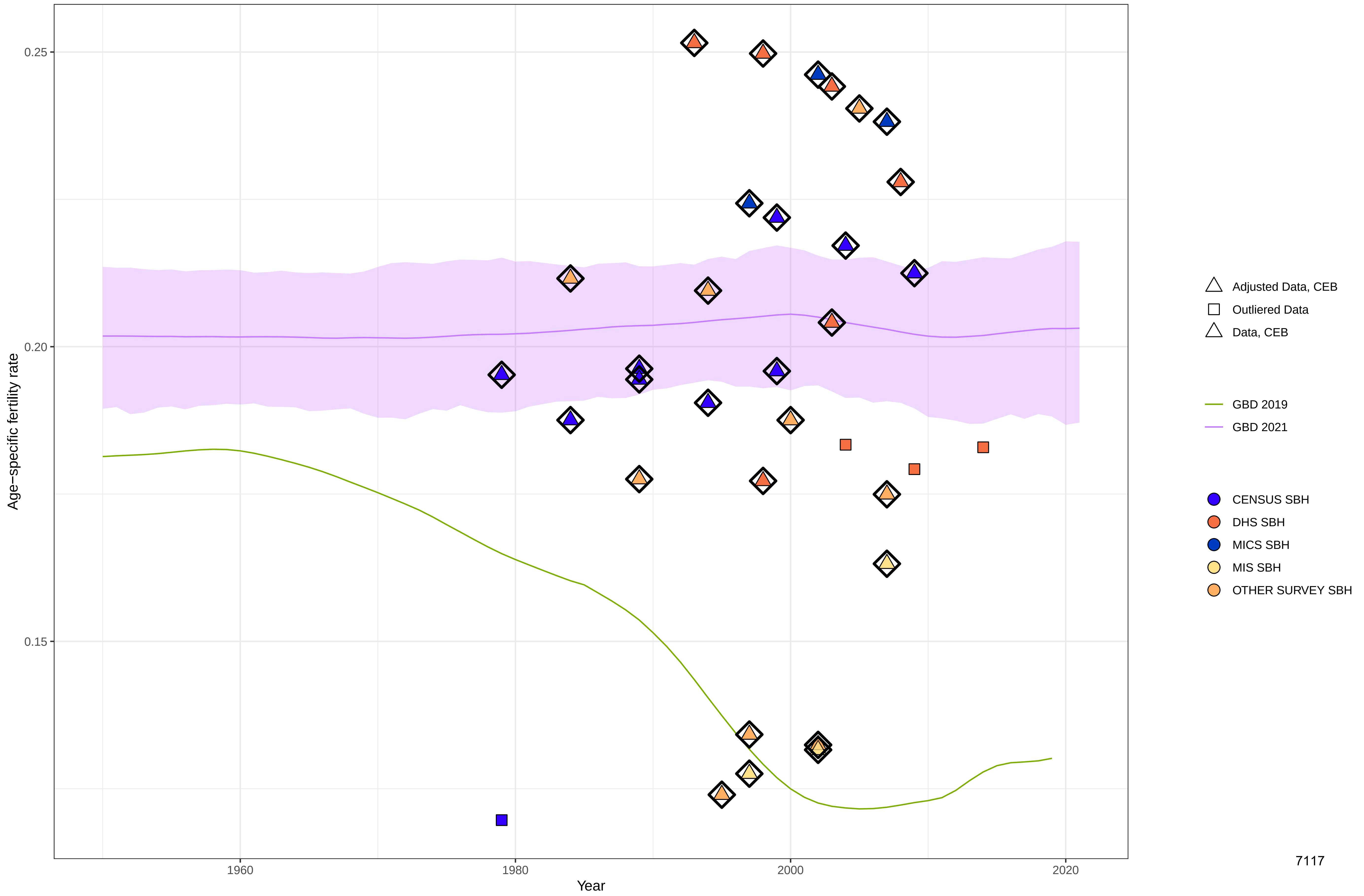

Age−specific fertility fitted trend lines, 1950−2021: Mandera, Ages: 40−44
Data points enclosed with a diamond are reference sources

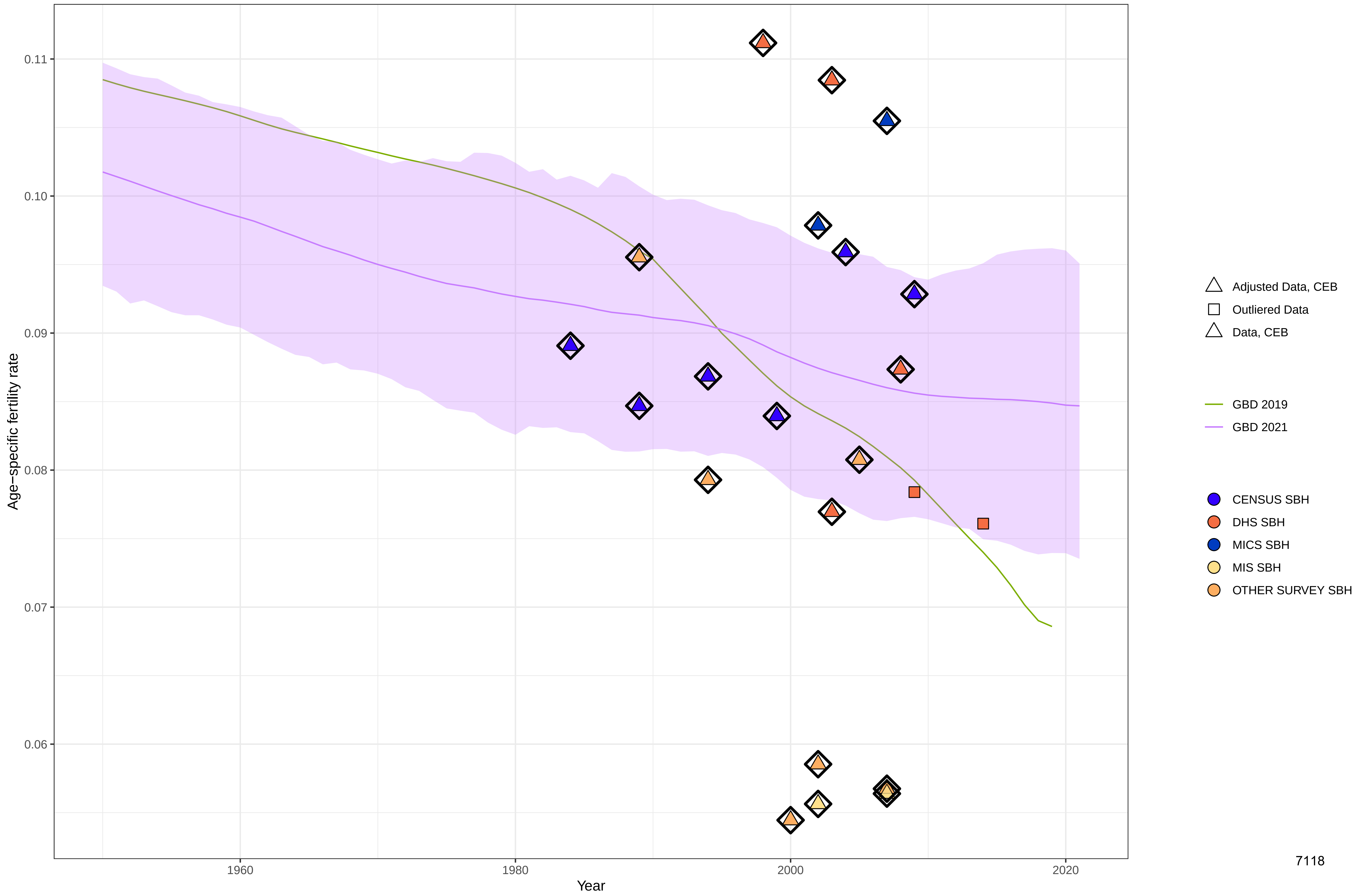

Age−specific fertility fitted trend lines, 1950−2021: Mandera, Ages: 45−49
Data points enclosed with a diamond are reference sources

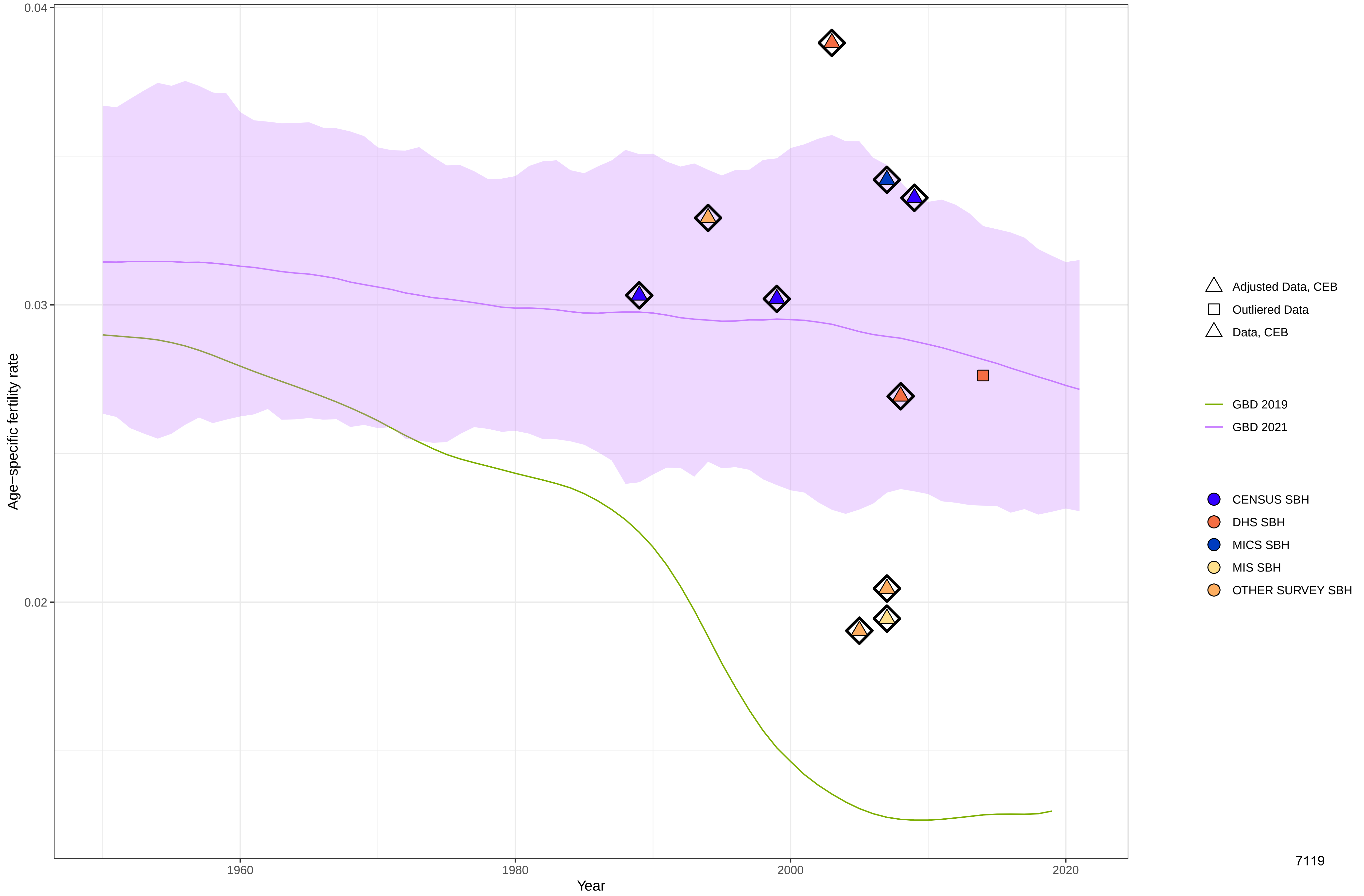

Age−specific fertility fitted trend lines, 1950−2021: Mandera, Ages: 50−54
Data points enclosed with a diamond are reference sources

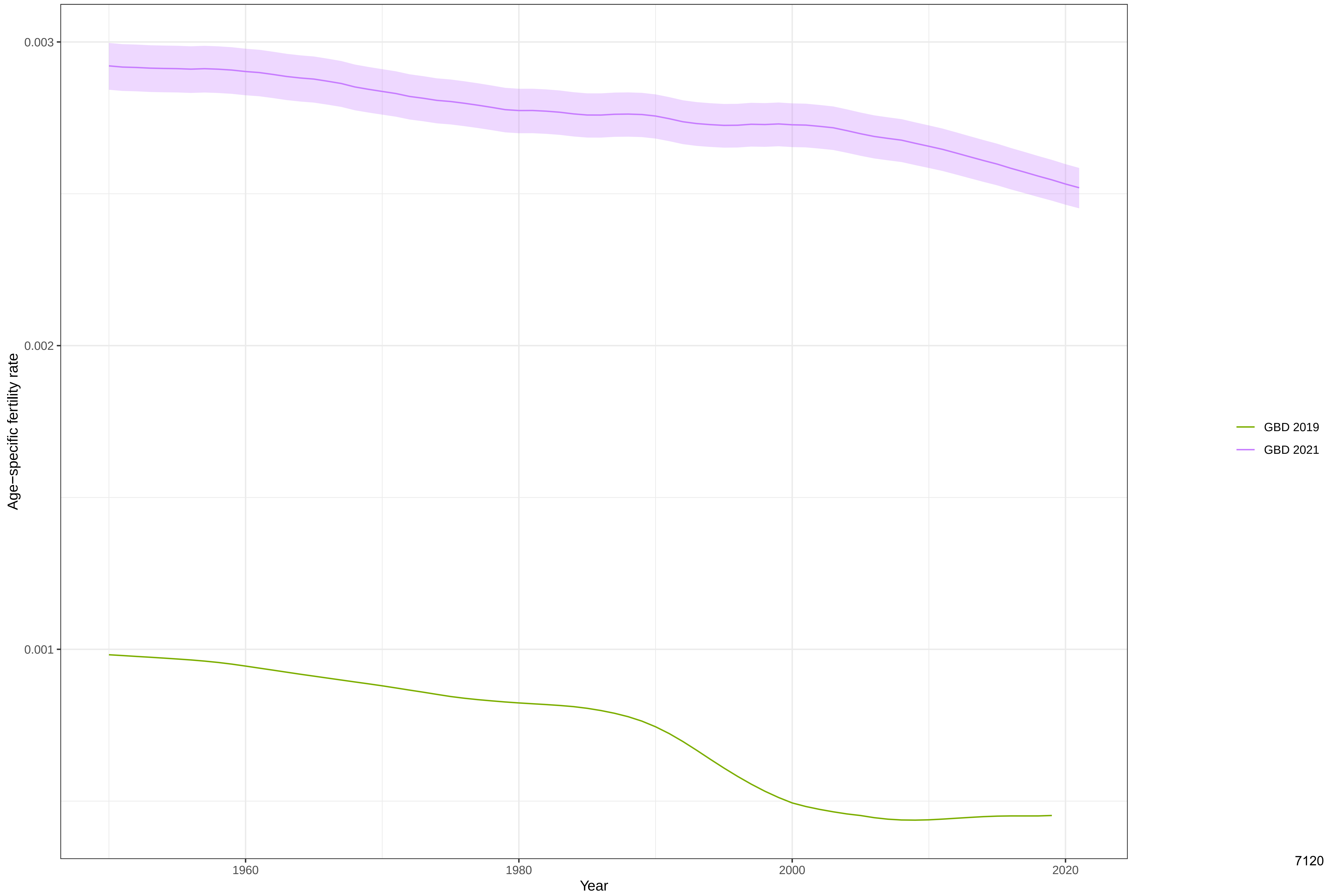

Total fertility rate fitted trend lines, 1950−2021: Mandera TFR
Data points enclosed with a diamond are reference sources

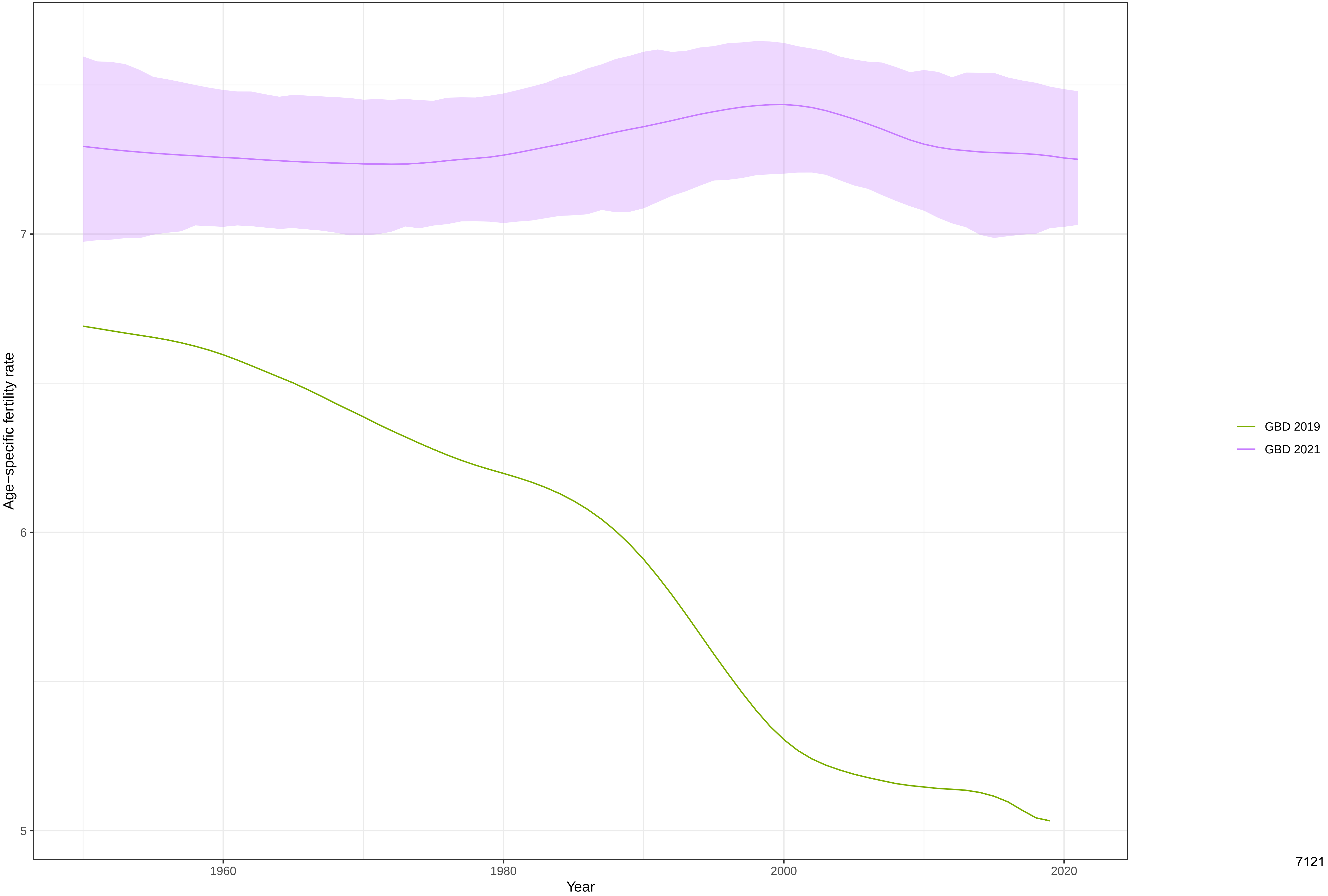

Age−specific fertility fitted trend lines, 1950−2021: Marsabit, Ages: 10−14
Data points enclosed with a diamond are reference sources

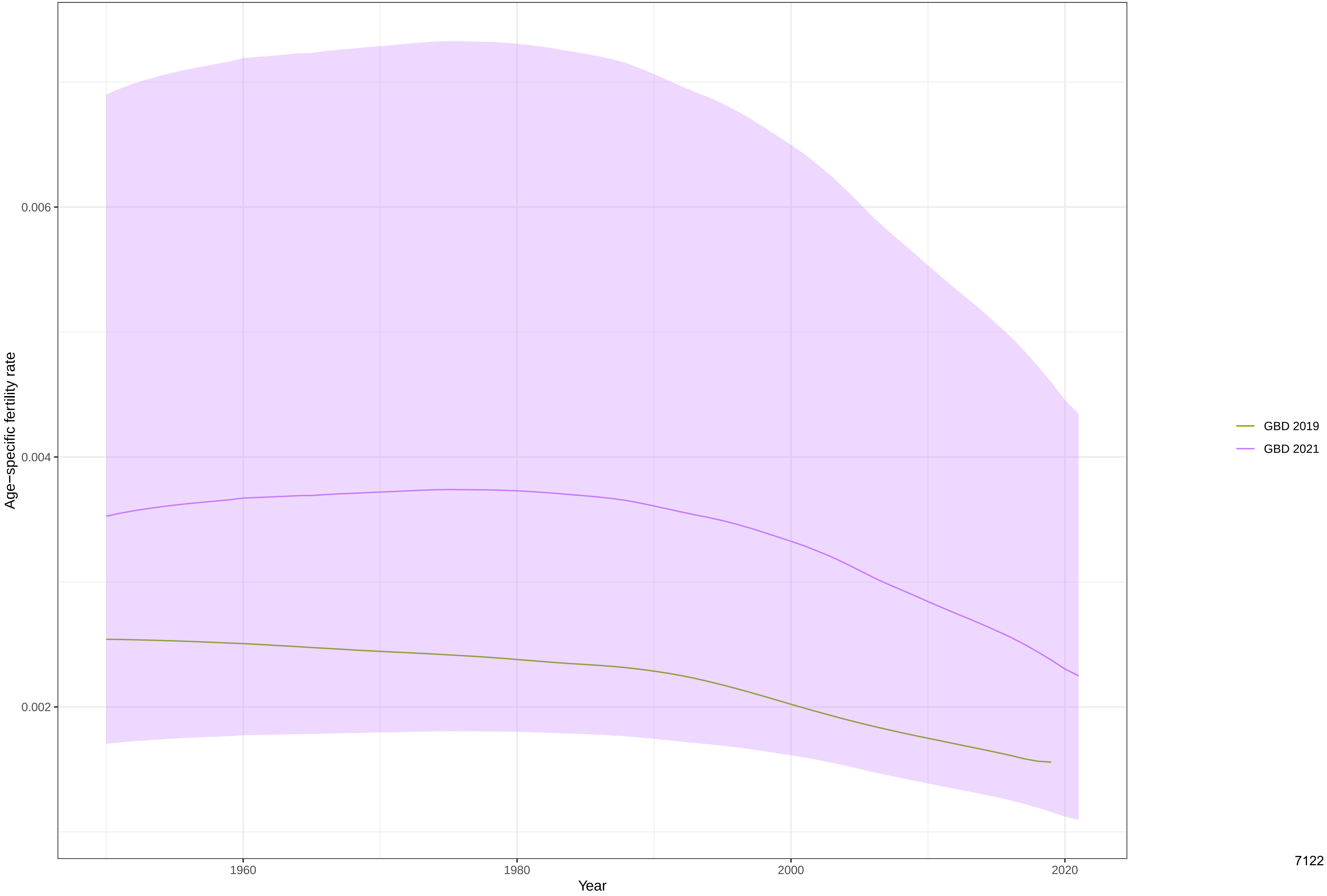

Age−specific fertility fitted trend lines, 1950−2021: Marsabit, Ages: 15−19
Data points enclosed with a diamond are reference sources

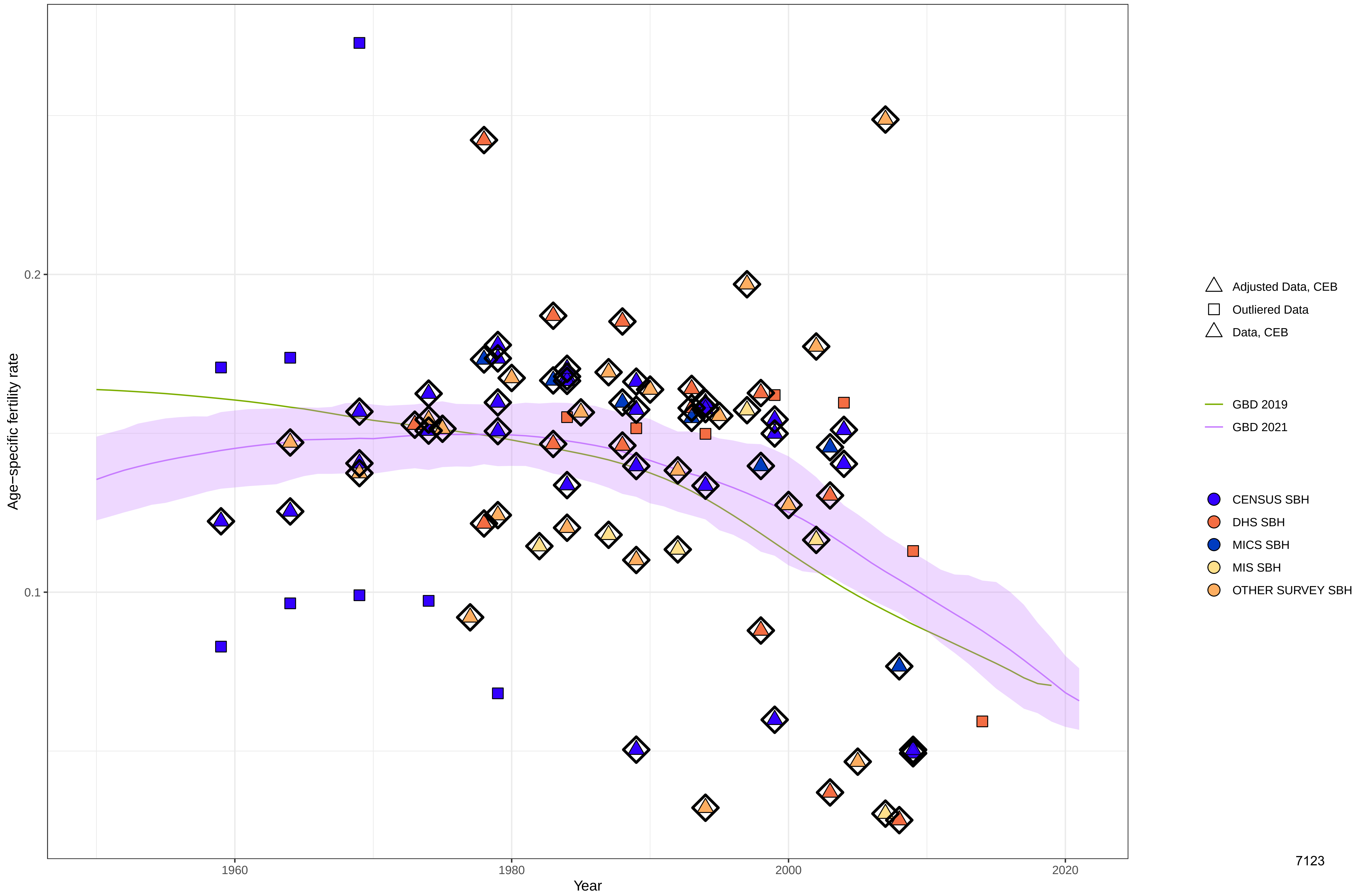

Age−specific fertility fitted trend lines, 1950−2021: Marsabit, Ages: 20−24
Data points enclosed with a diamond are reference sources

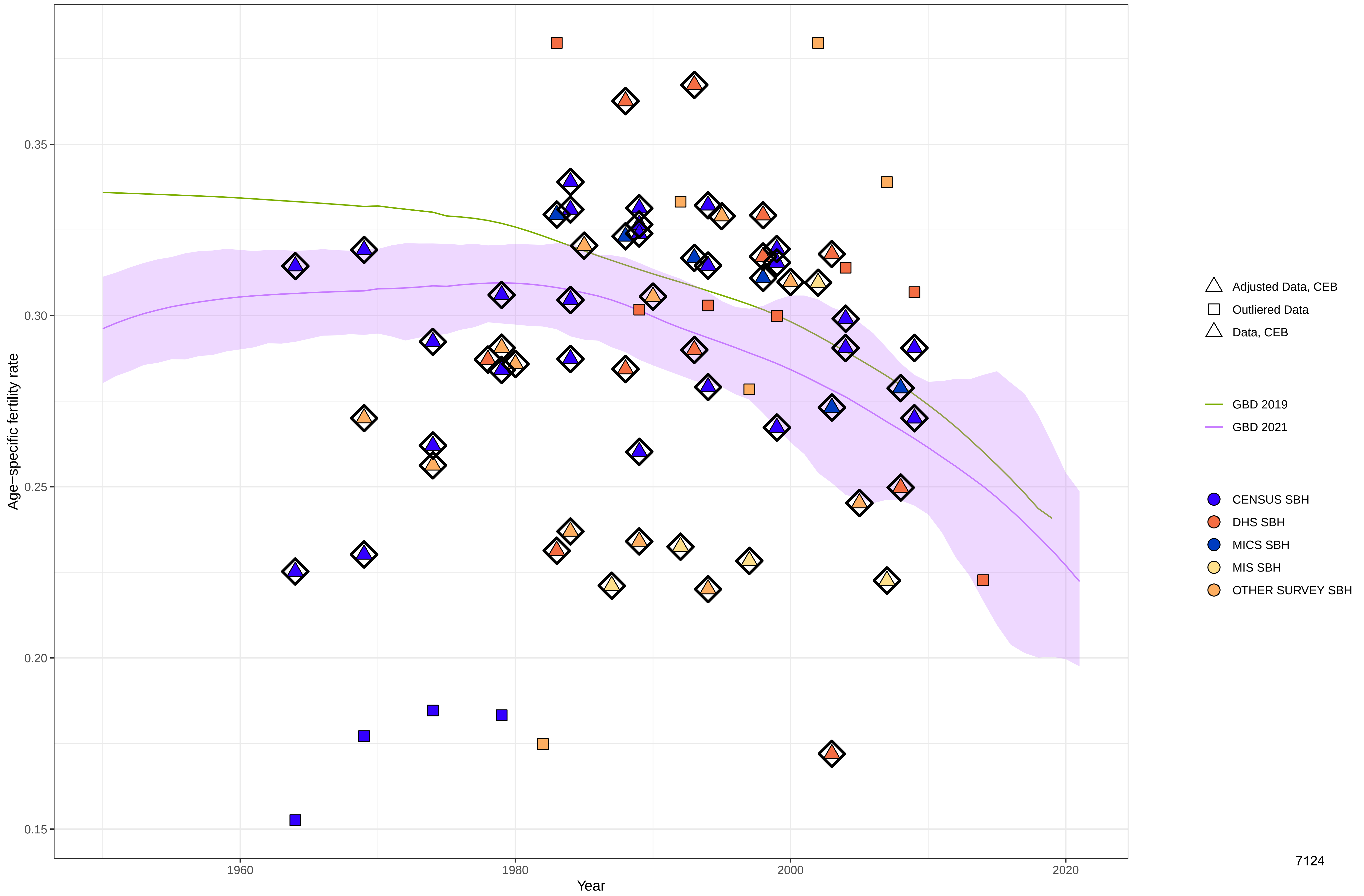

Age−specific fertility fitted trend lines, 1950−2021: Marsabit, Ages: 25−29
Data points enclosed with a diamond are reference sources

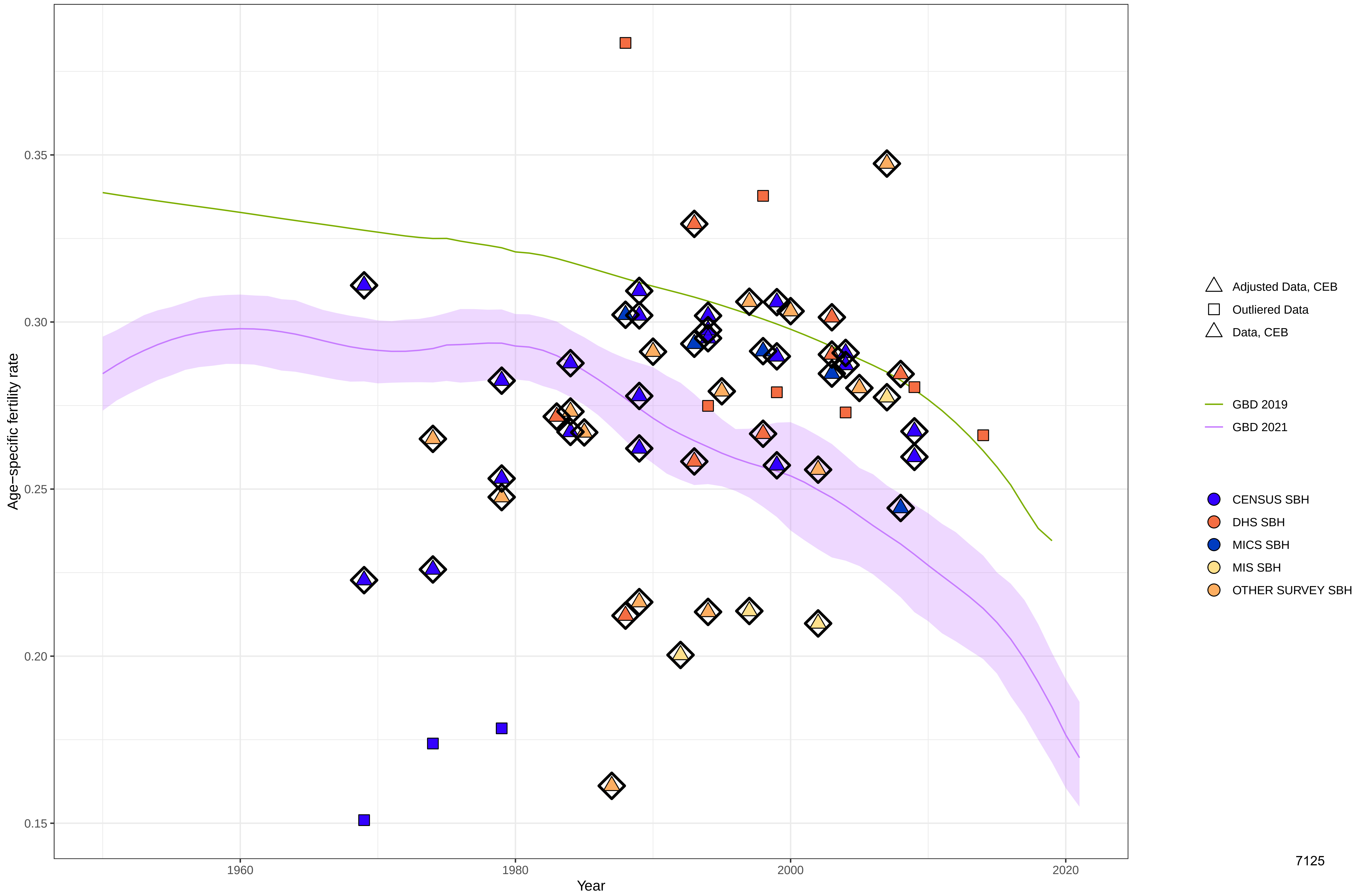

Age−specific fertility fitted trend lines, 1950−2021: Marsabit, Ages: 30−34
Data points enclosed with a diamond are reference sources

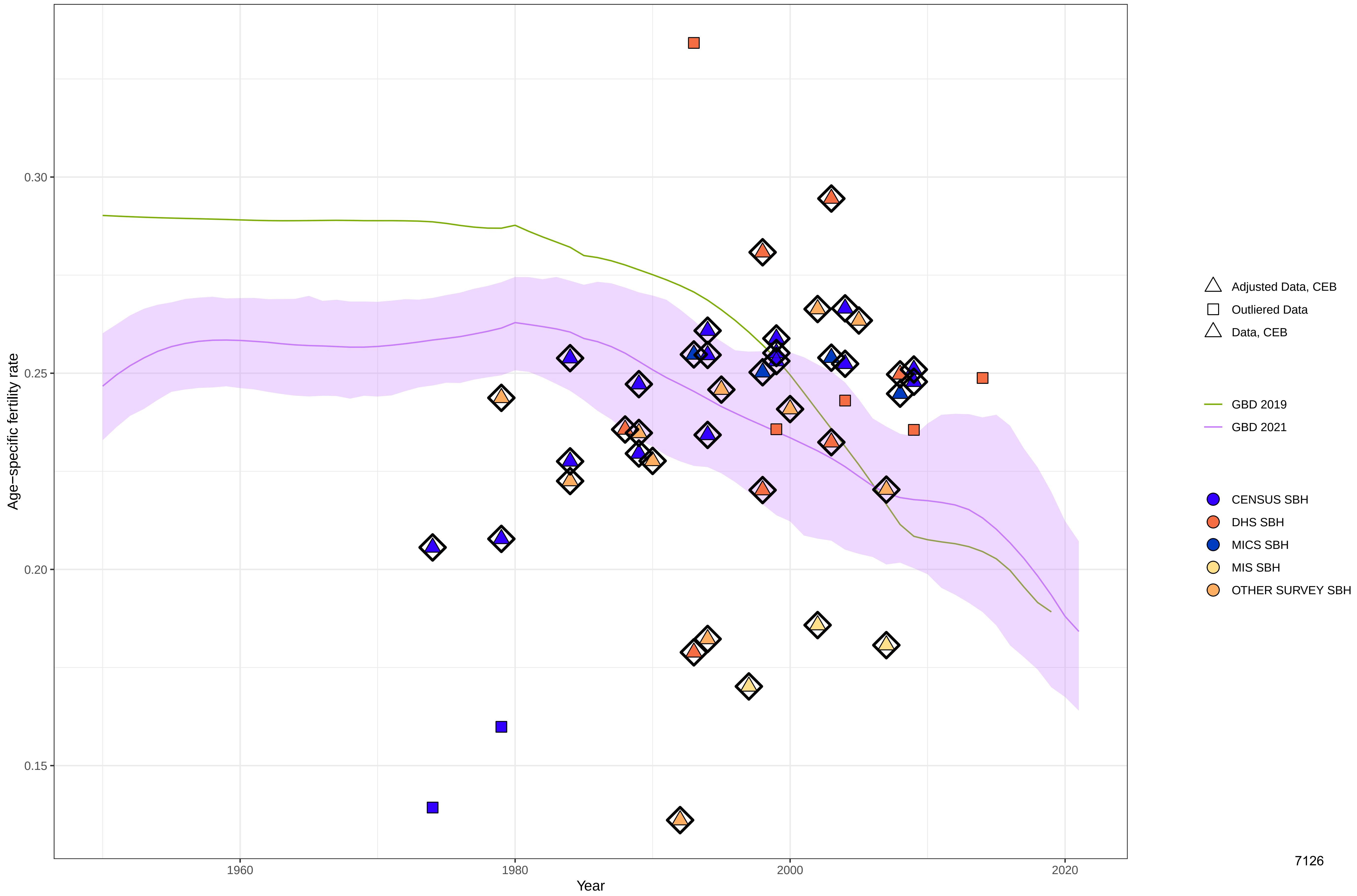

Age−specific fertility fitted trend lines, 1950−2021: Marsabit, Ages: 35−39
Data points enclosed with a diamond are reference sources

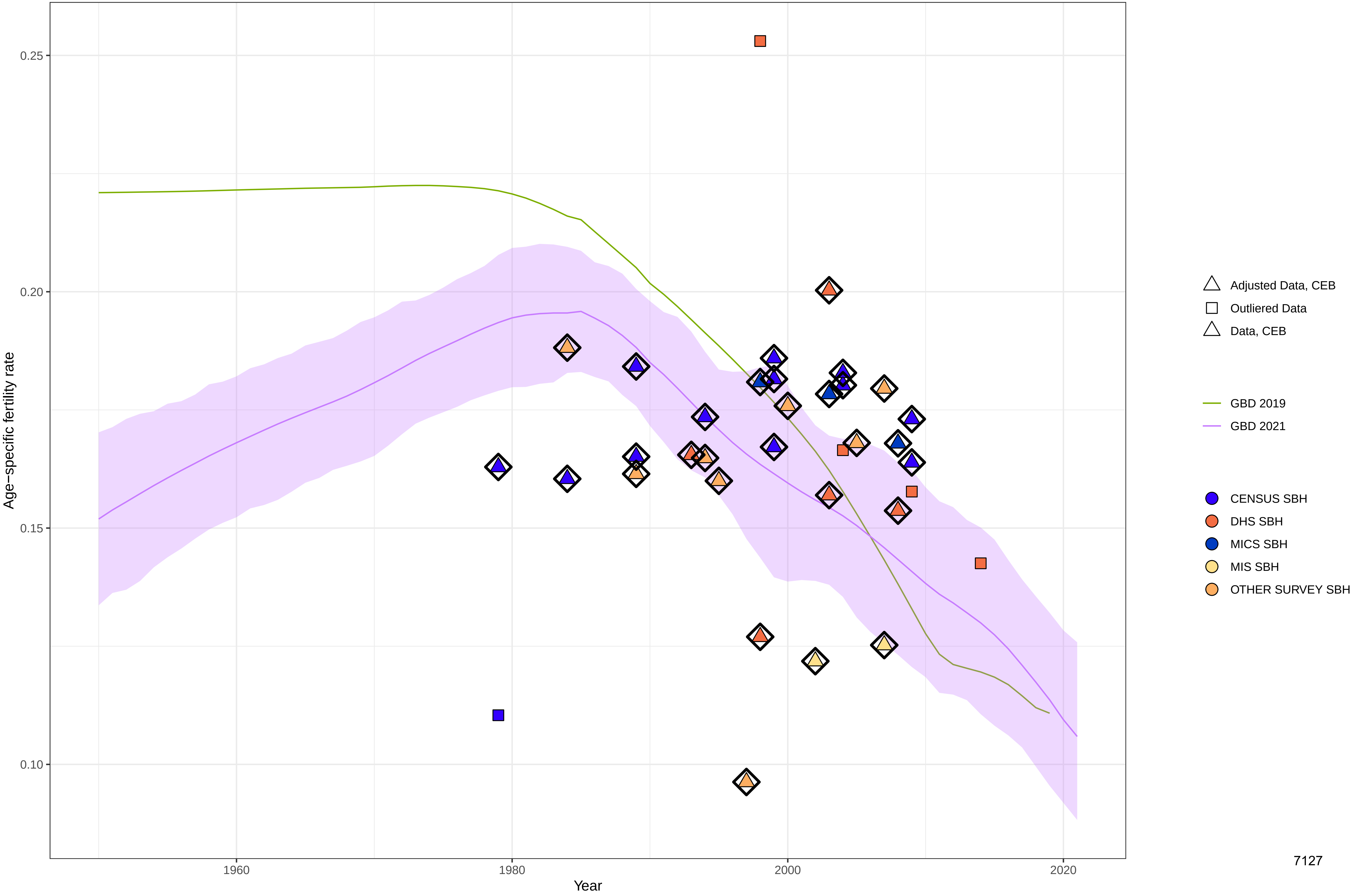

Age−specific fertility fitted trend lines, 1950−2021: Marsabit, Ages: 40−44
Data points enclosed with a diamond are reference sources

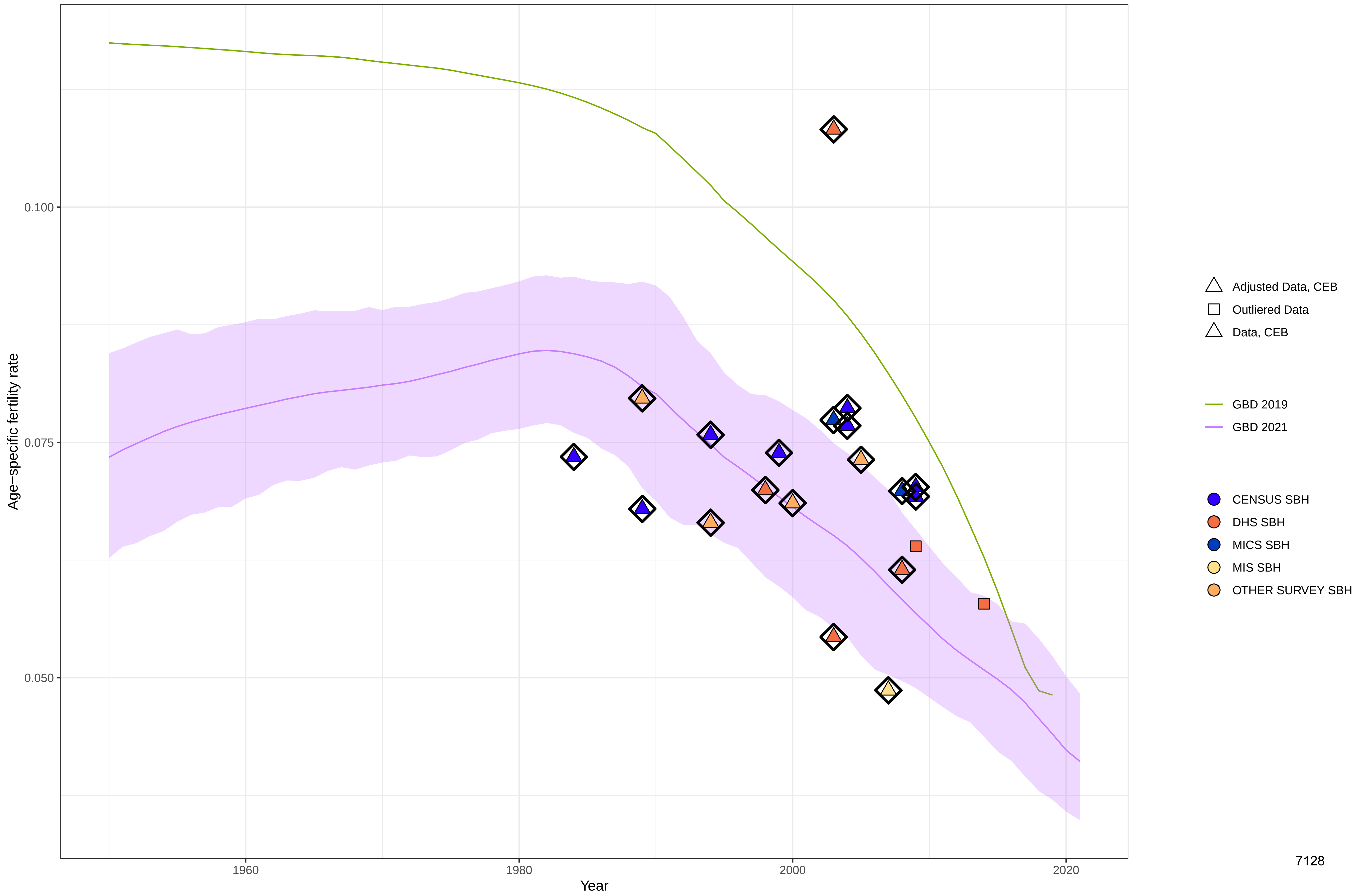

Age−specific fertility fitted trend lines, 1950−2021: Marsabit, Ages: 45−49
Data points enclosed with a diamond are reference sources

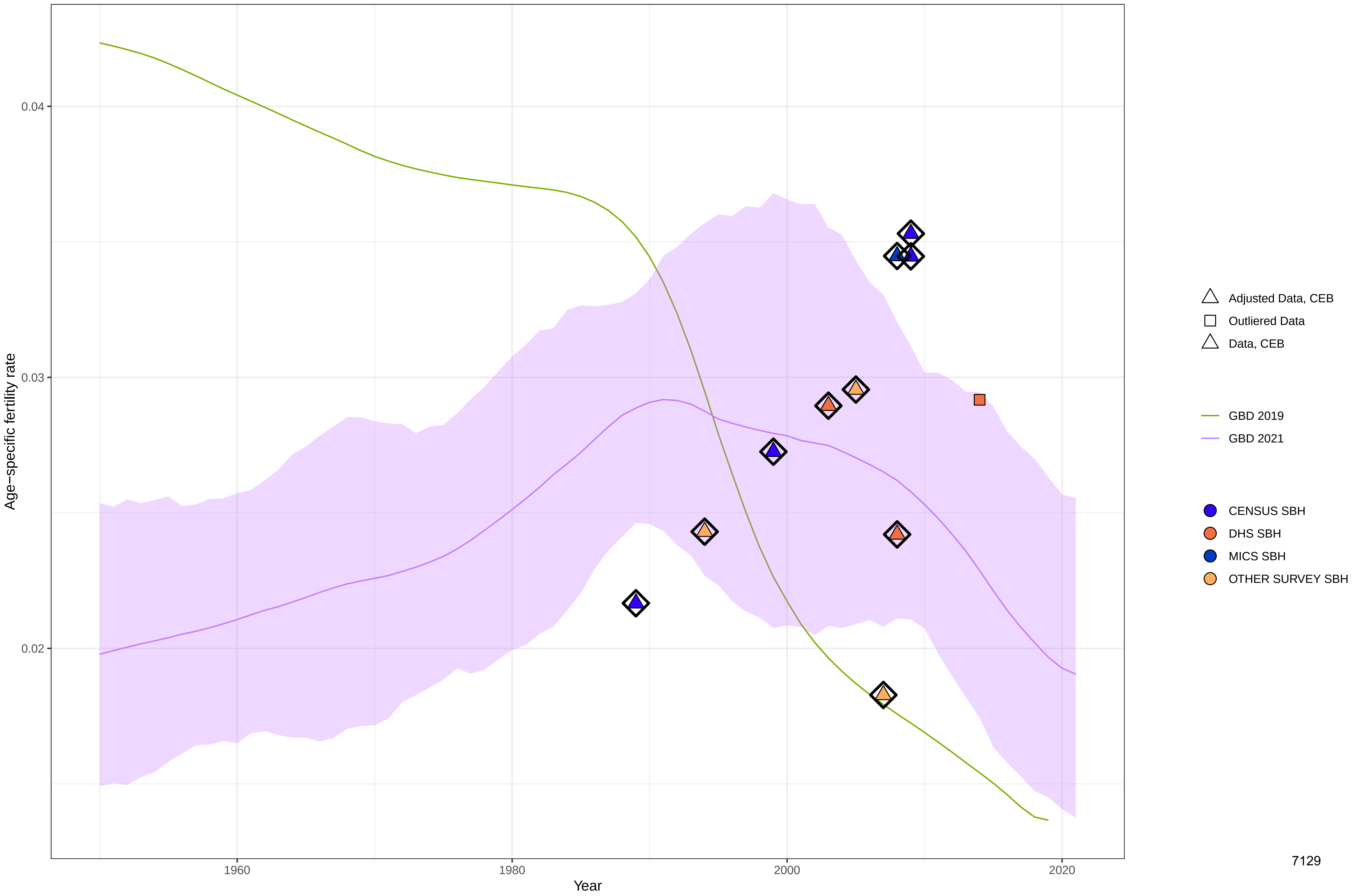

Age−specific fertility fitted trend lines, 1950−2021: Marsabit, Ages: 50−54
Data points enclosed with a diamond are reference sources

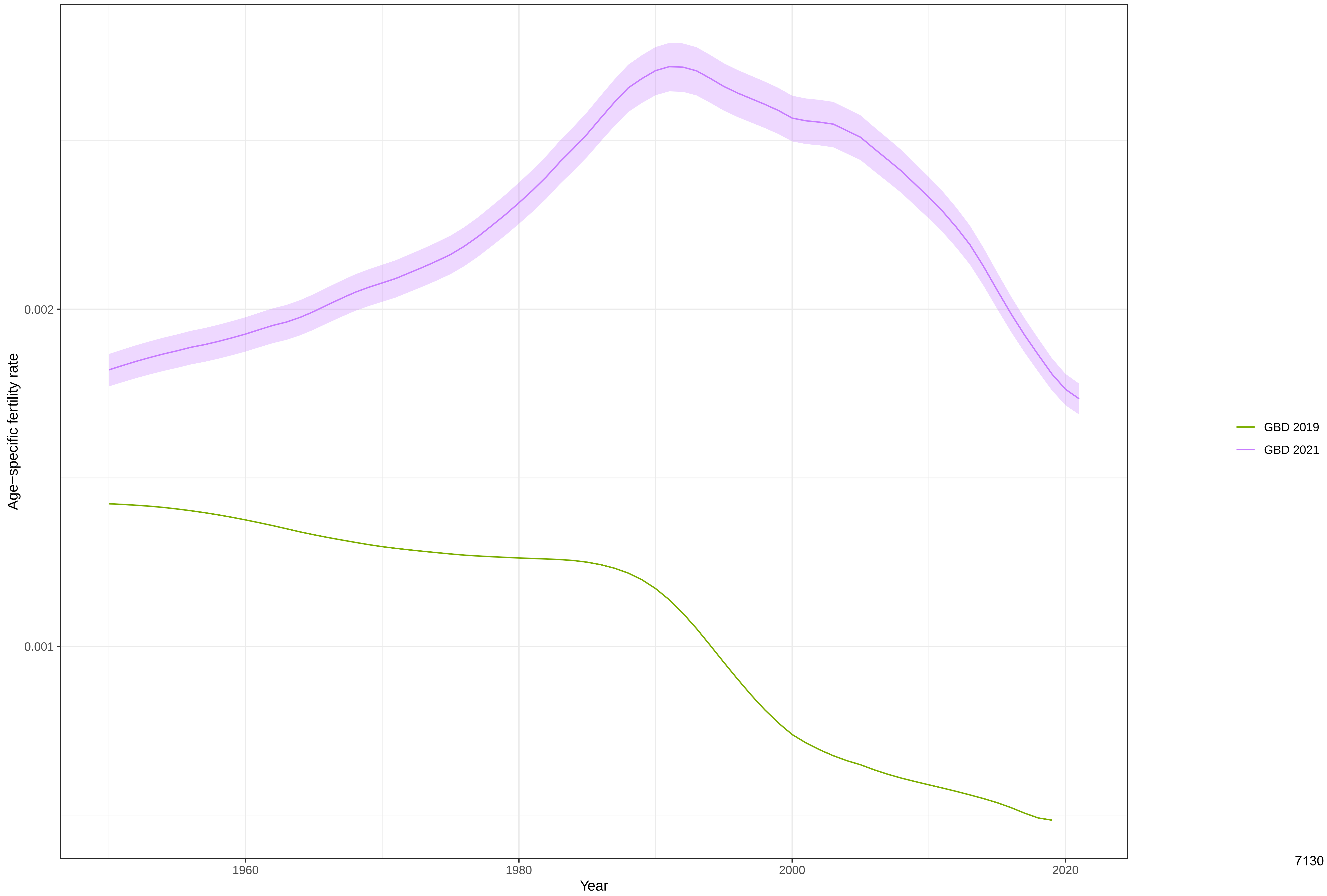

Total fertility rate fitted trend lines, 1950−2021: Marsabit TFR
Data points enclosed with a diamond are reference sources

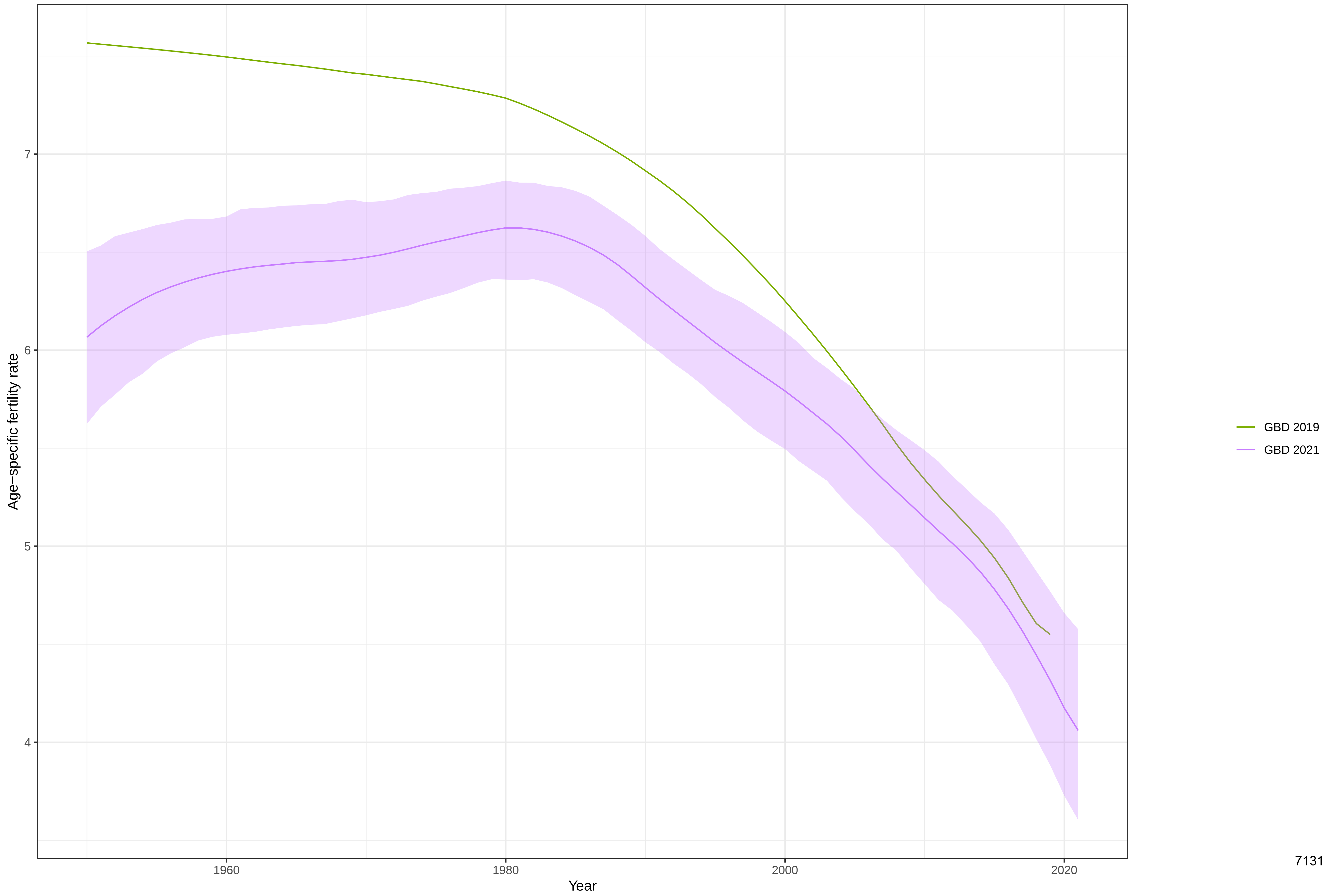

Age−specific fertility fitted trend lines, 1950−2021: Meru, Ages: 10−14
Data points enclosed with a diamond are reference sources

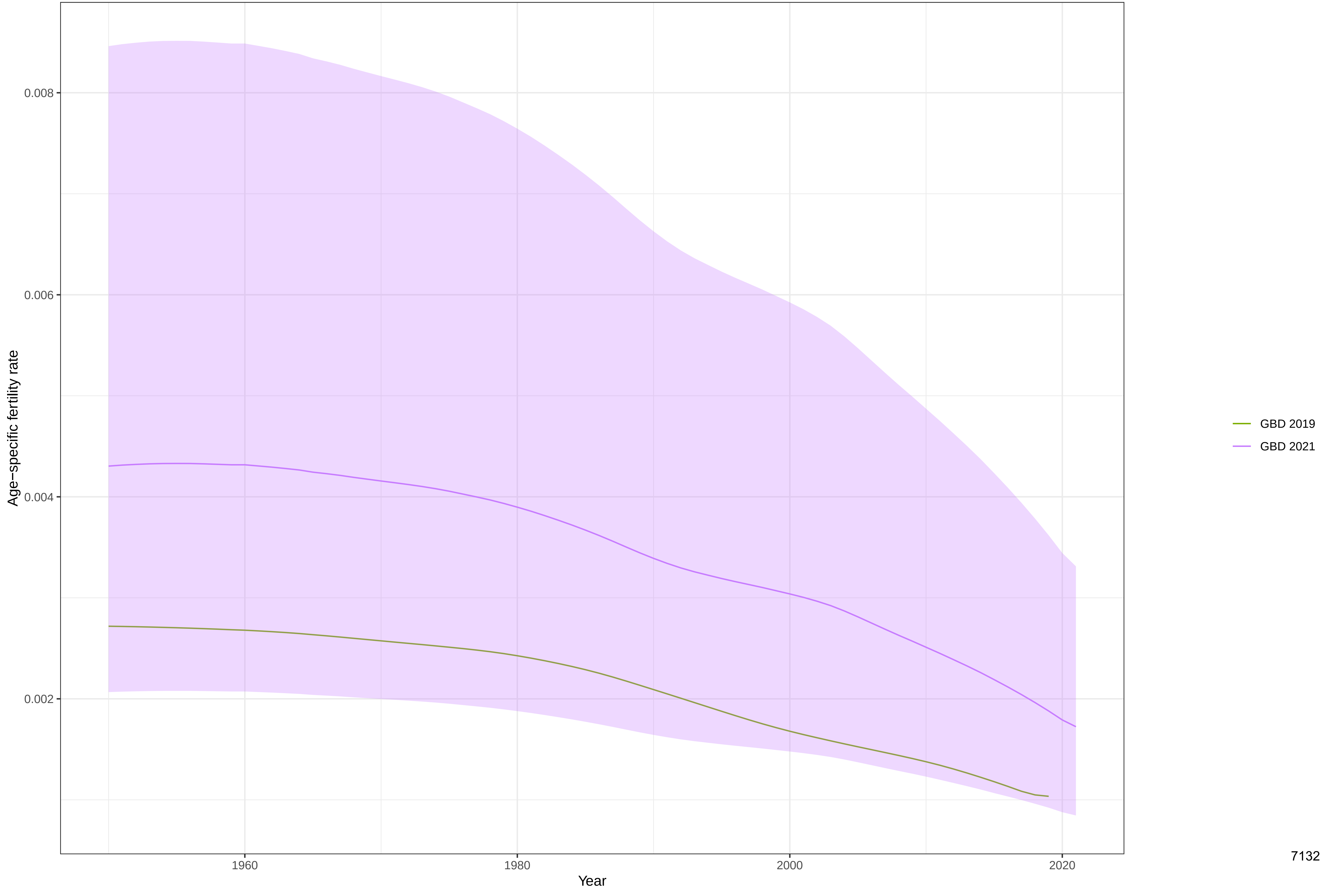

Age−specific fertility fitted trend lines, 1950−2021: Meru, Ages: 15−19
Data points enclosed with a diamond are reference sources

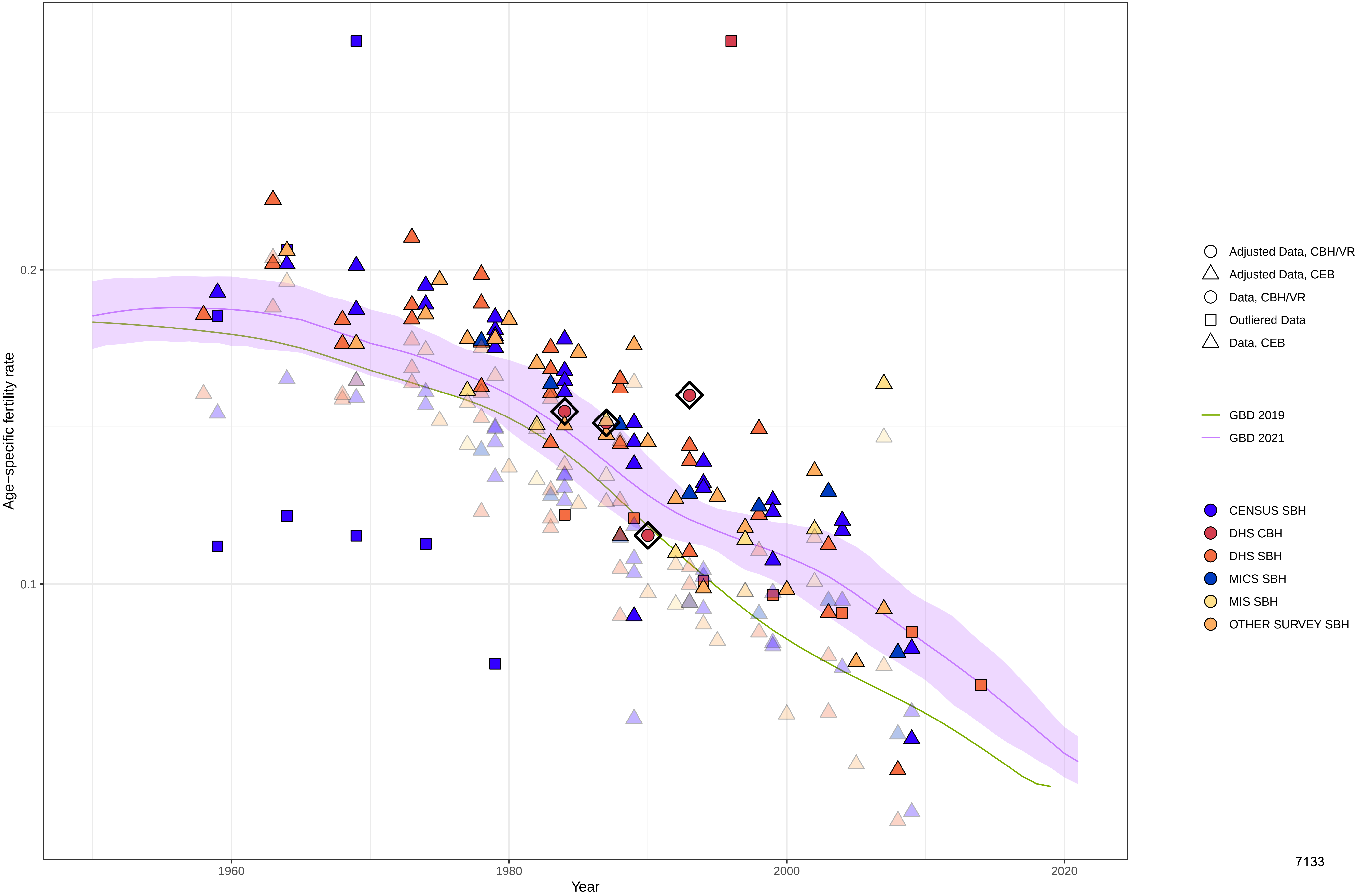

Age−specific fertility fitted trend lines, 1950−2021: Meru, Ages: 20−24
Data points enclosed with a diamond are reference sources

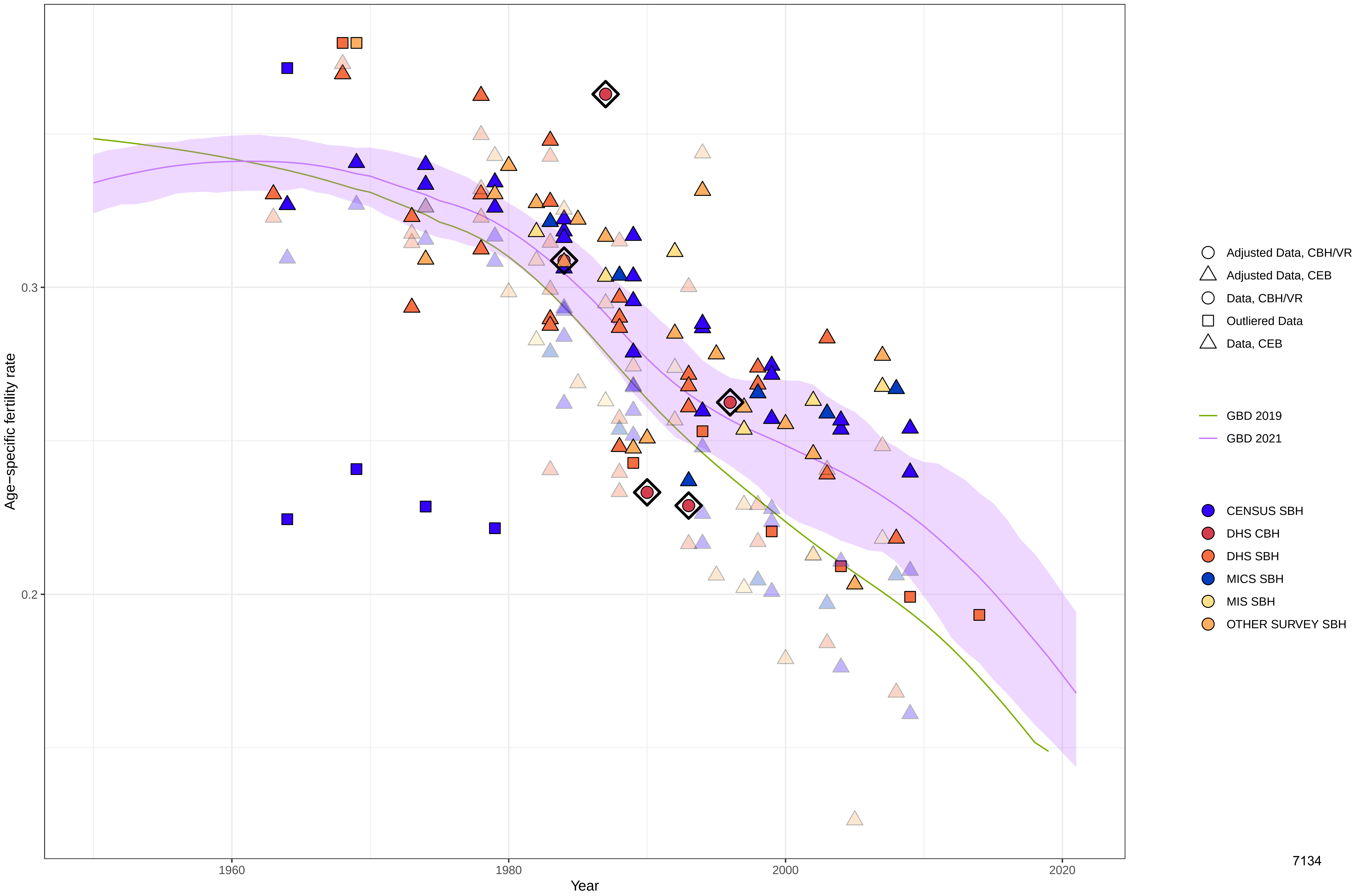

Age−specific fertility fitted trend lines, 1950−2021: Meru, Ages: 25−29
Data points enclosed with a diamond are reference sources

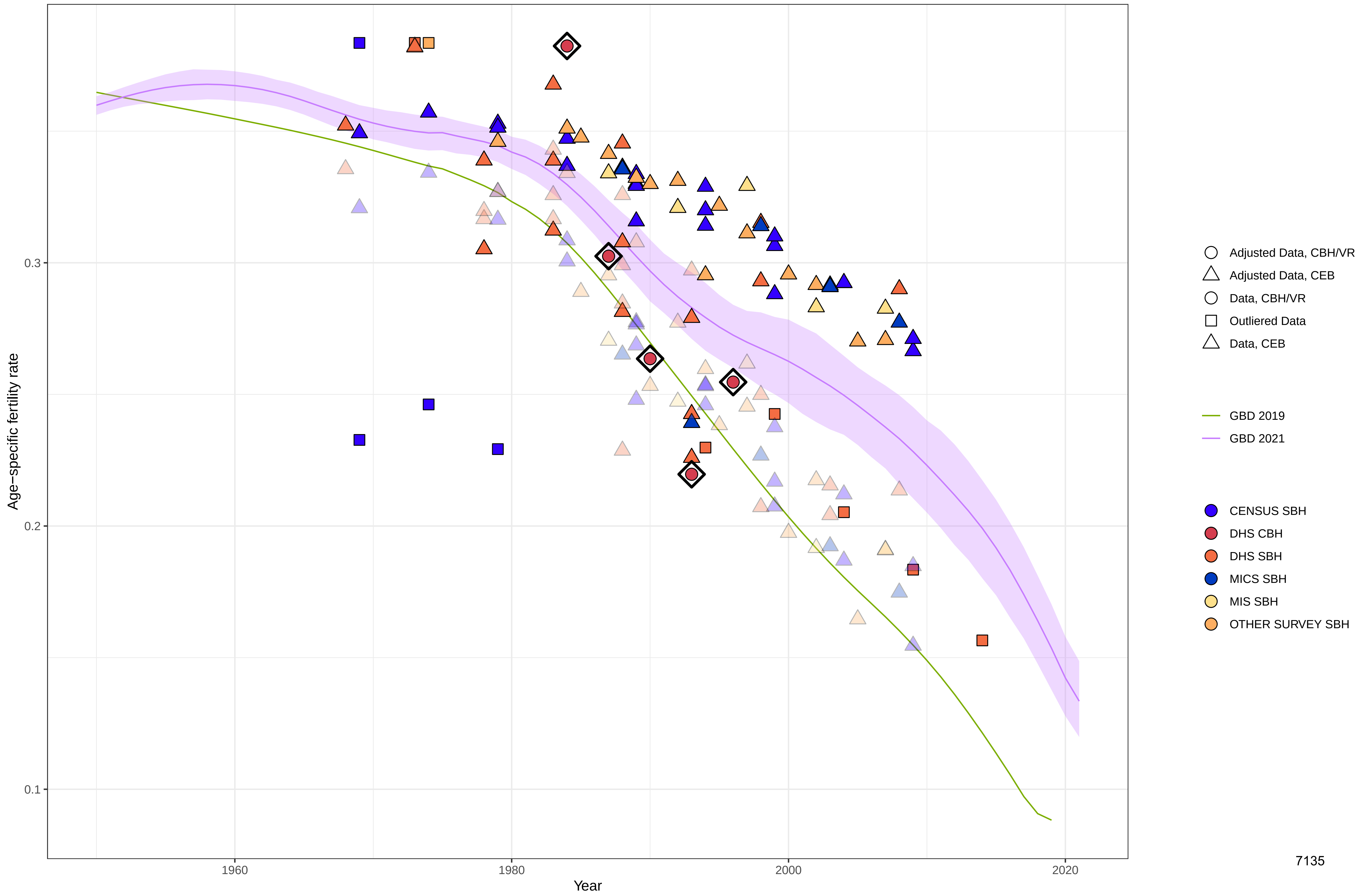

Age−specific fertility fitted trend lines, 1950−2021: Meru, Ages: 30−34
Data points enclosed with a diamond are reference sources

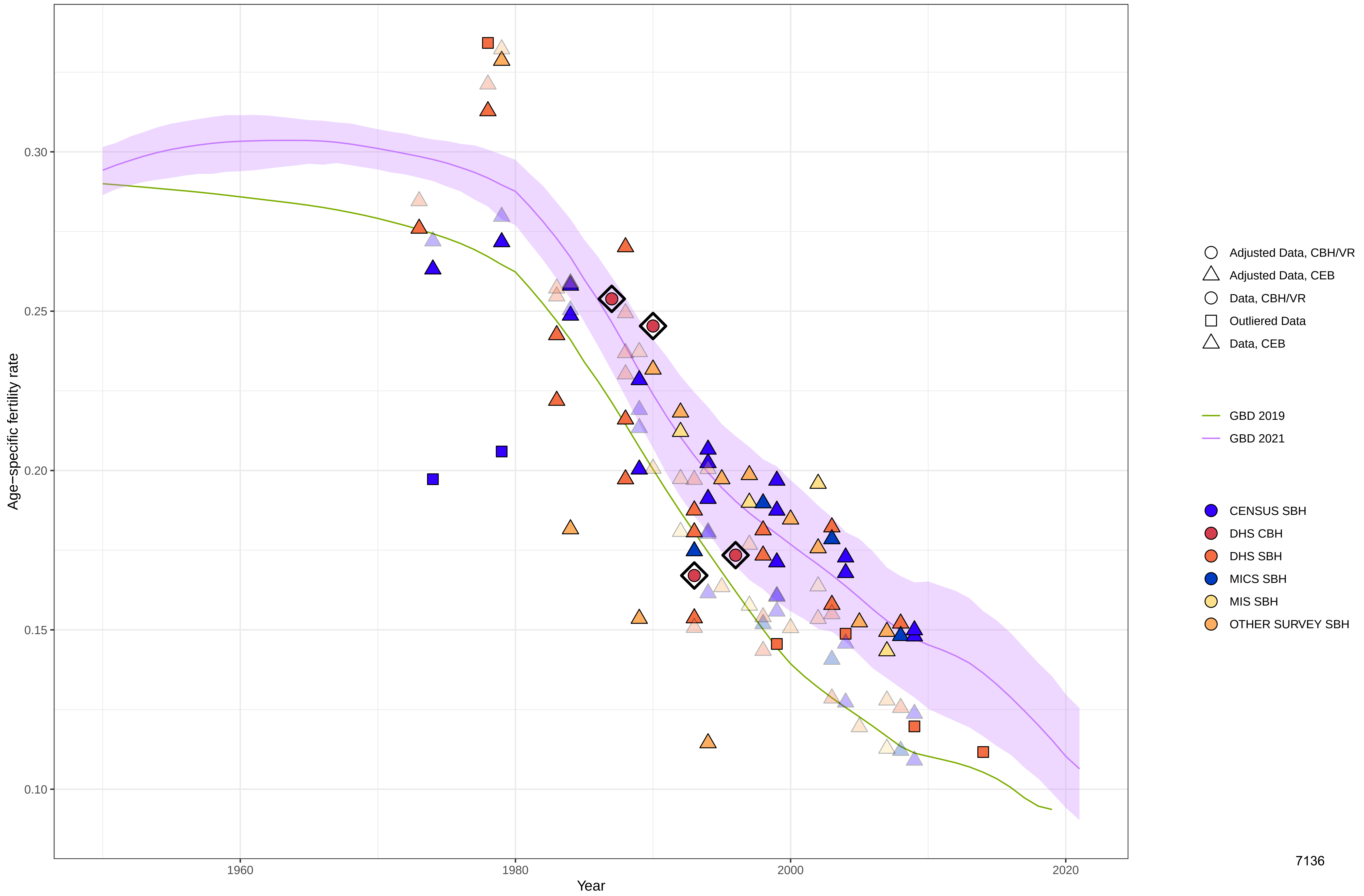

Age−specific fertility fitted trend lines, 1950−2021: Meru, Ages: 35−39
Data points enclosed with a diamond are reference sources

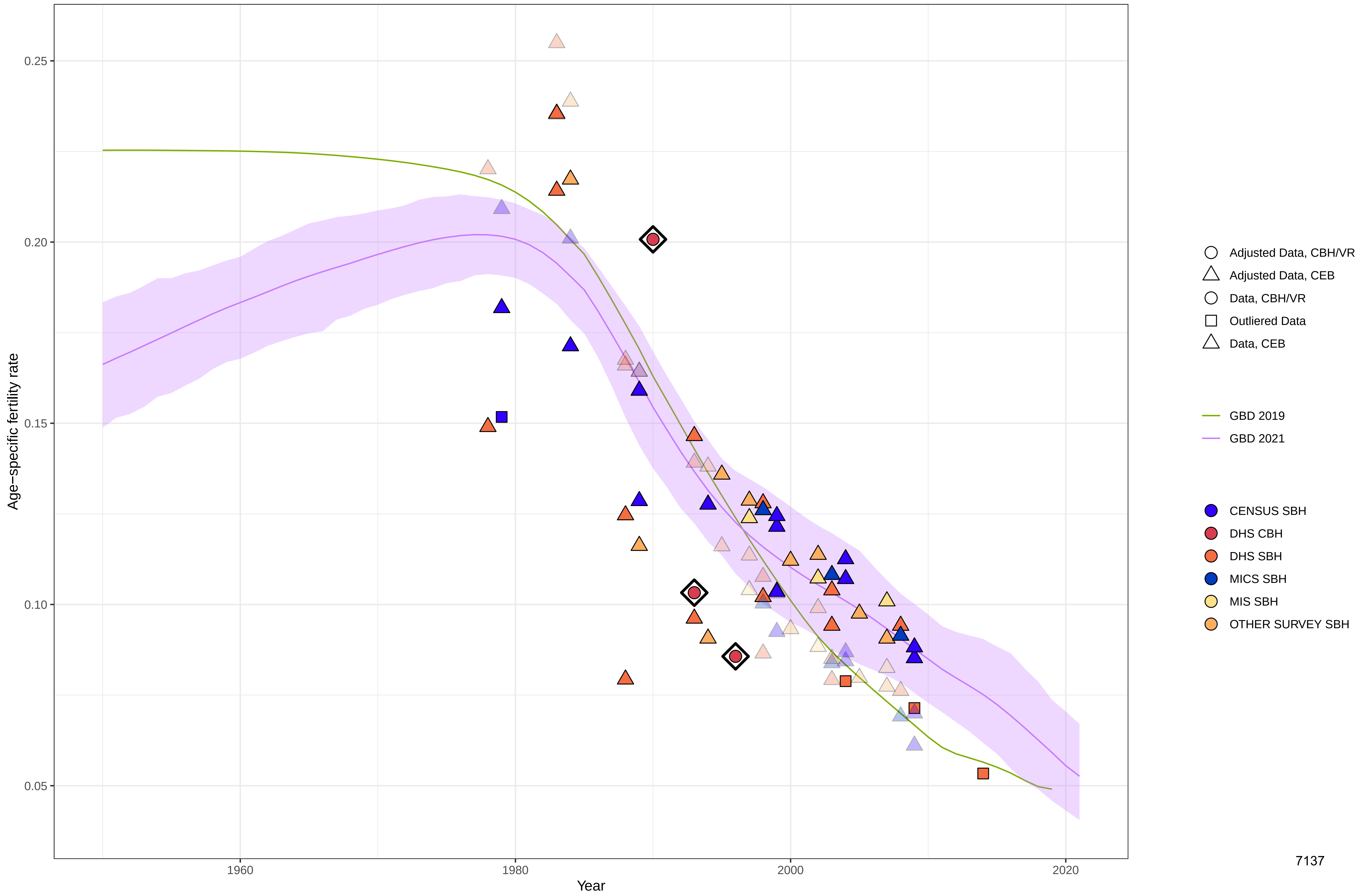

Age−specific fertility fitted trend lines, 1950−2021: Meru, Ages: 40−44
Data points enclosed with a diamond are reference sources

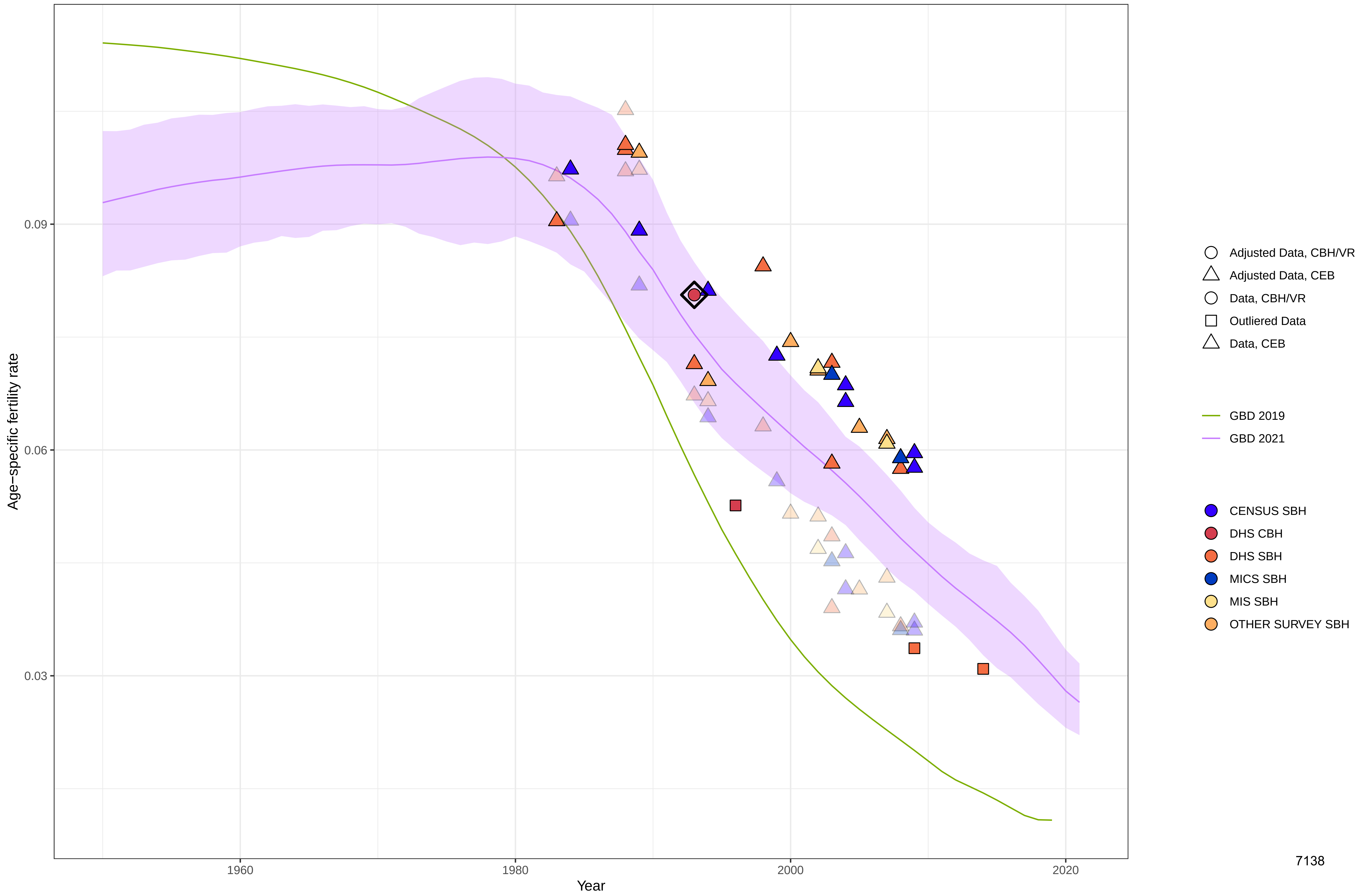

Age−specific fertility fitted trend lines, 1950−2021: Meru, Ages: 45−49
Data points enclosed with a diamond are reference sources

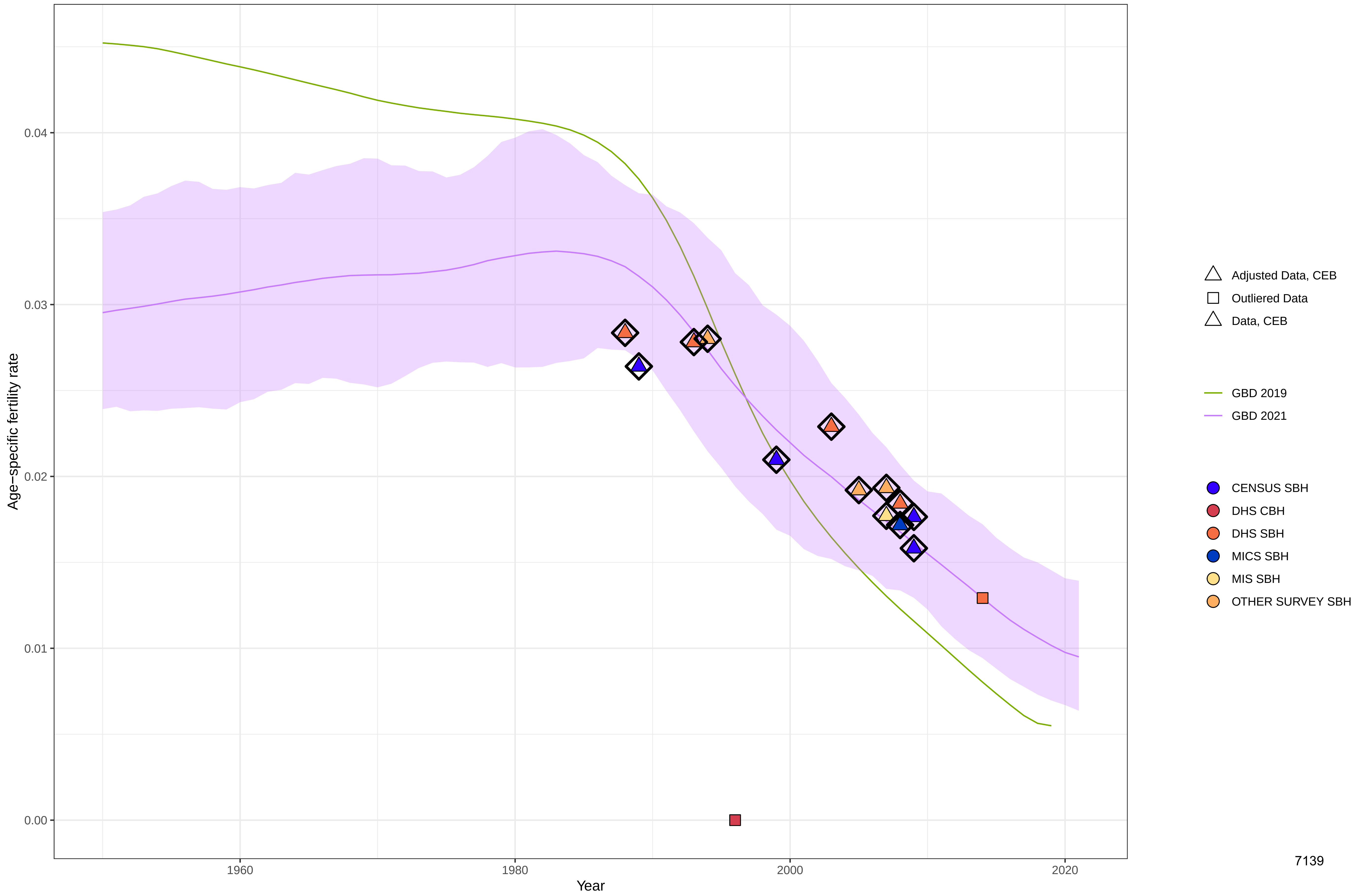

Age−specific fertility fitted trend lines, 1950−2021: Meru, Ages: 50−54
Data points enclosed with a diamond are reference sources

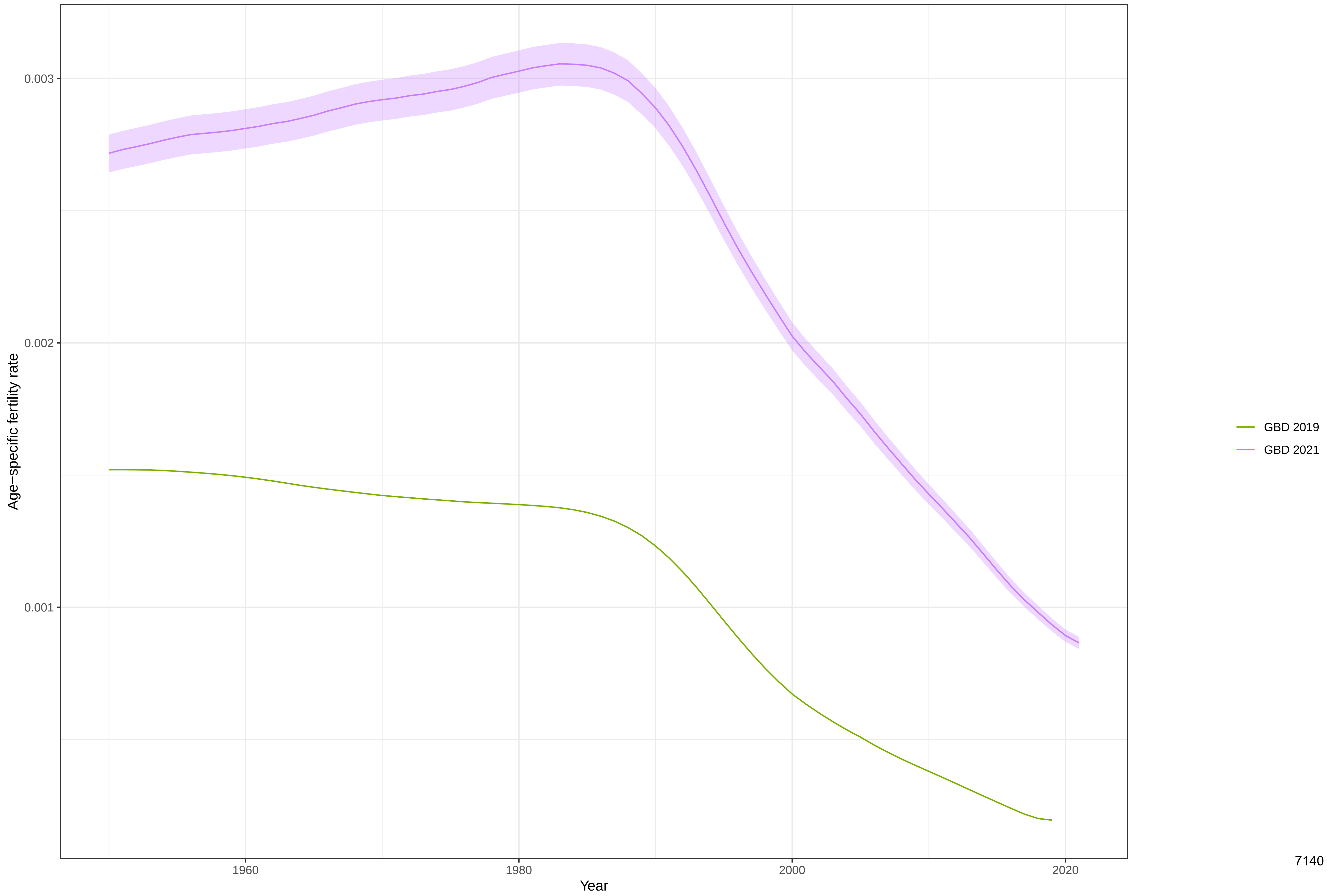

Total fertility rate fitted trend lines, 1950−2021: Meru TFR
Data points enclosed with a diamond are reference sources

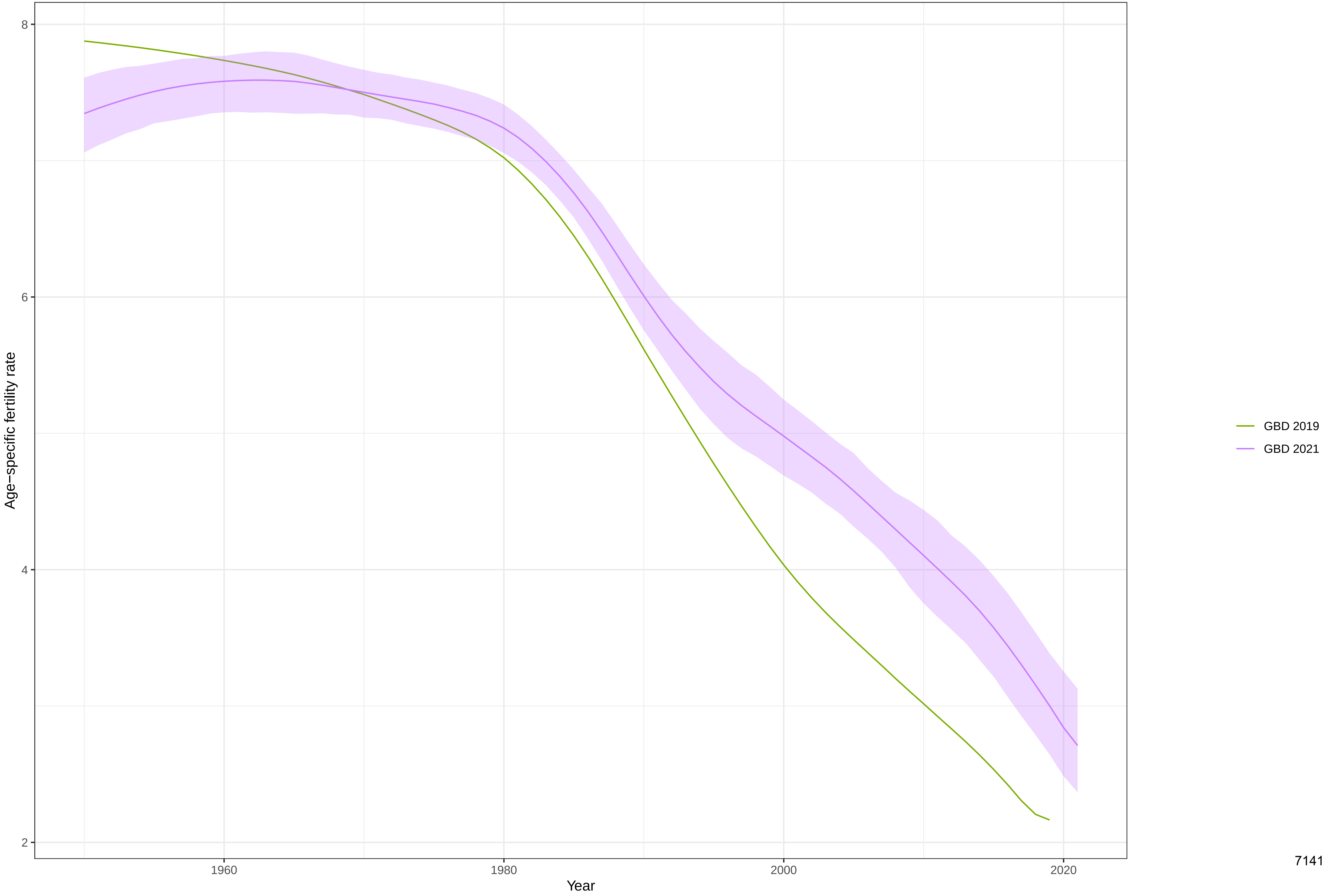

Age−specific fertility fitted trend lines, 1950−2021: Migori, Ages: 10−14
Data points enclosed with a diamond are reference sources

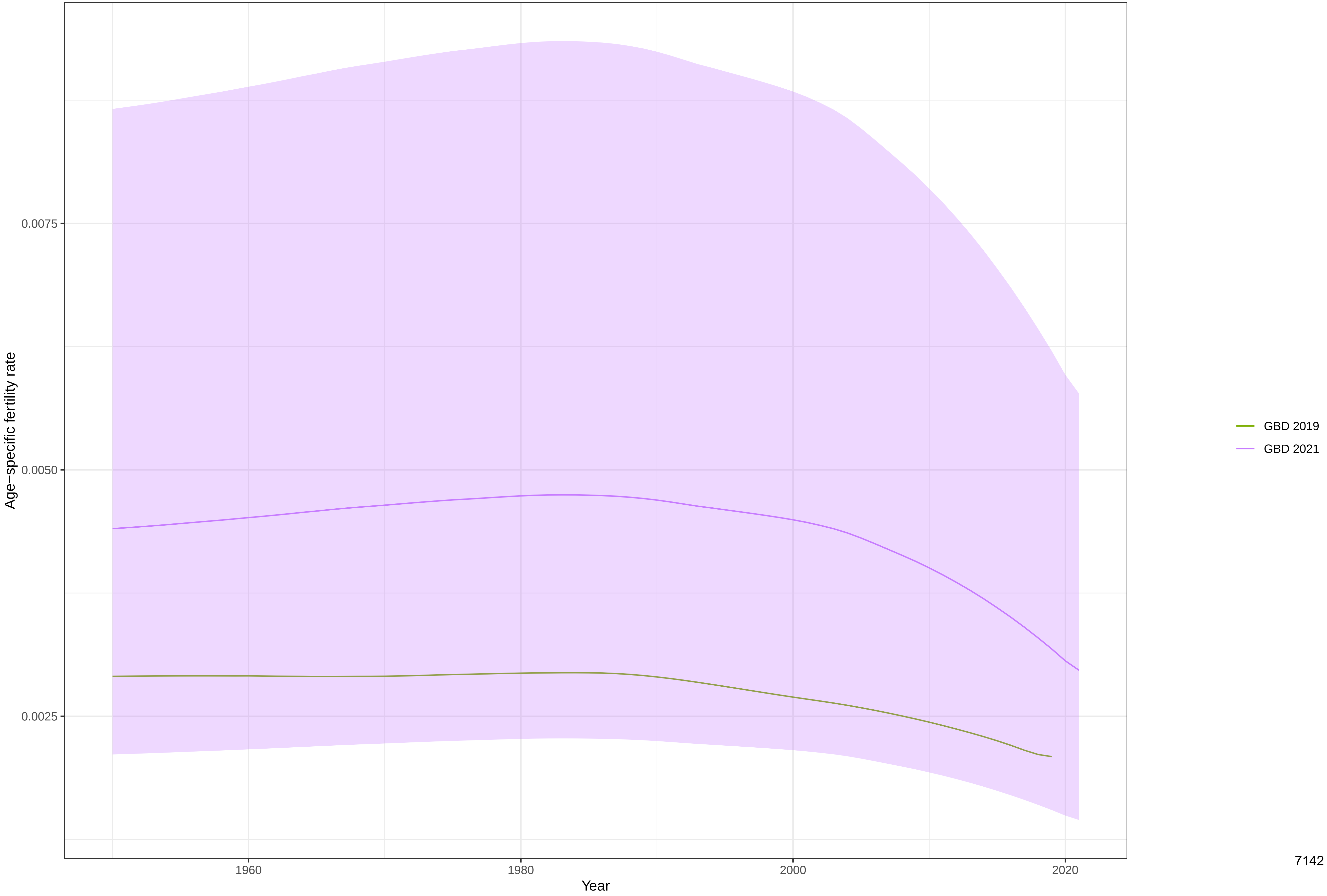

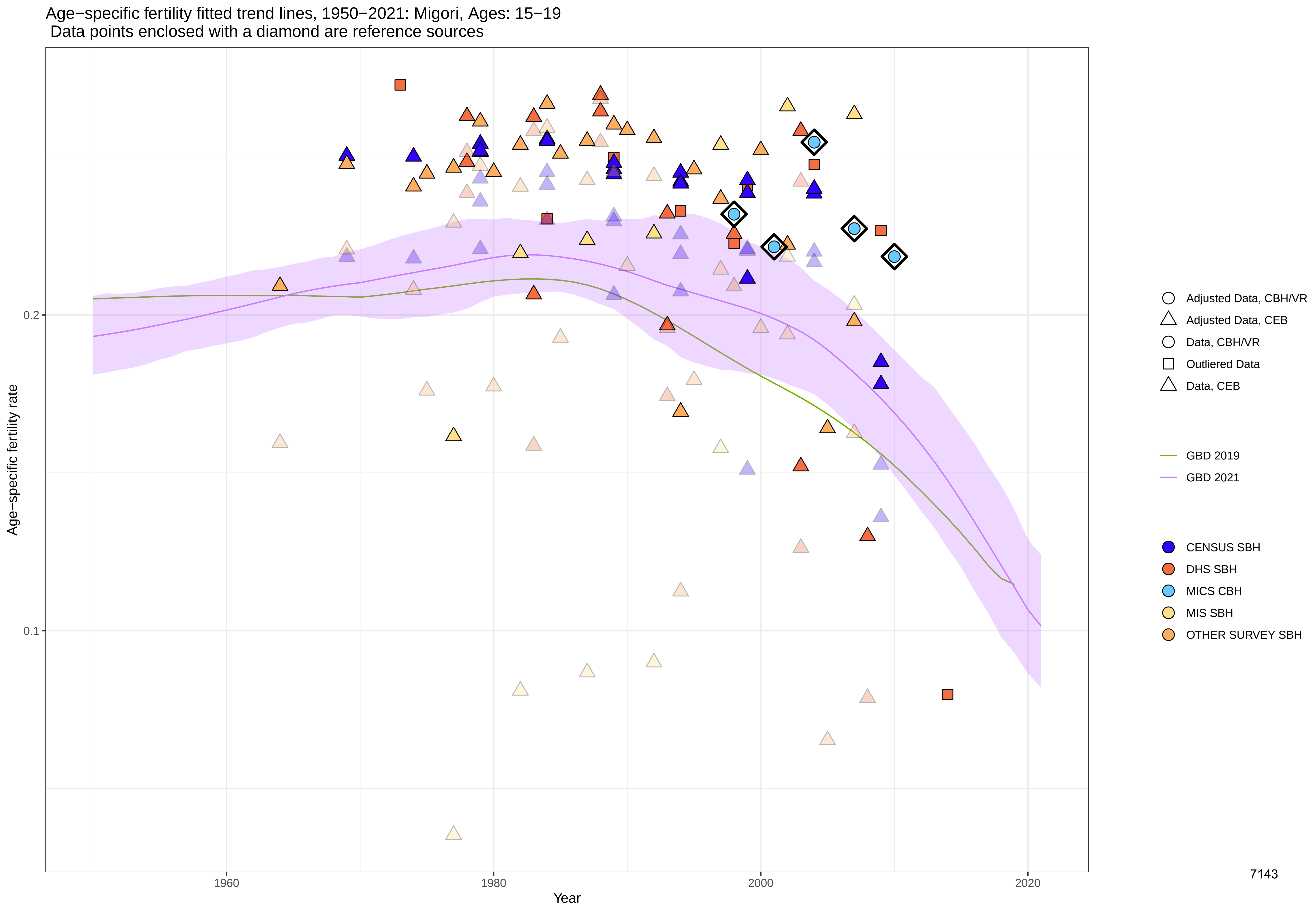

Age−specific fertility fitted trend lines, 1950−2021: Migori, Ages: 15−19
Data points enclosed with a diamond are reference sources
Age−specific fertility rate
Year
0.2
0.1
1960
1980
2000
2020
Adjusted Data, CBH/VR
Adjusted Data, CEB
Data, CBH/VR
Outliered Data
Data, CEB
GBD 2019
GBD 2021
CENSUS SBH
DHS SBH
MICS CBH
MIS SBH
OTHER SURVEY SBH

Age−specific fertility fitted trend lines, 1950−2021: Migori, Ages: 20−24
Data points enclosed with a diamond are reference sources

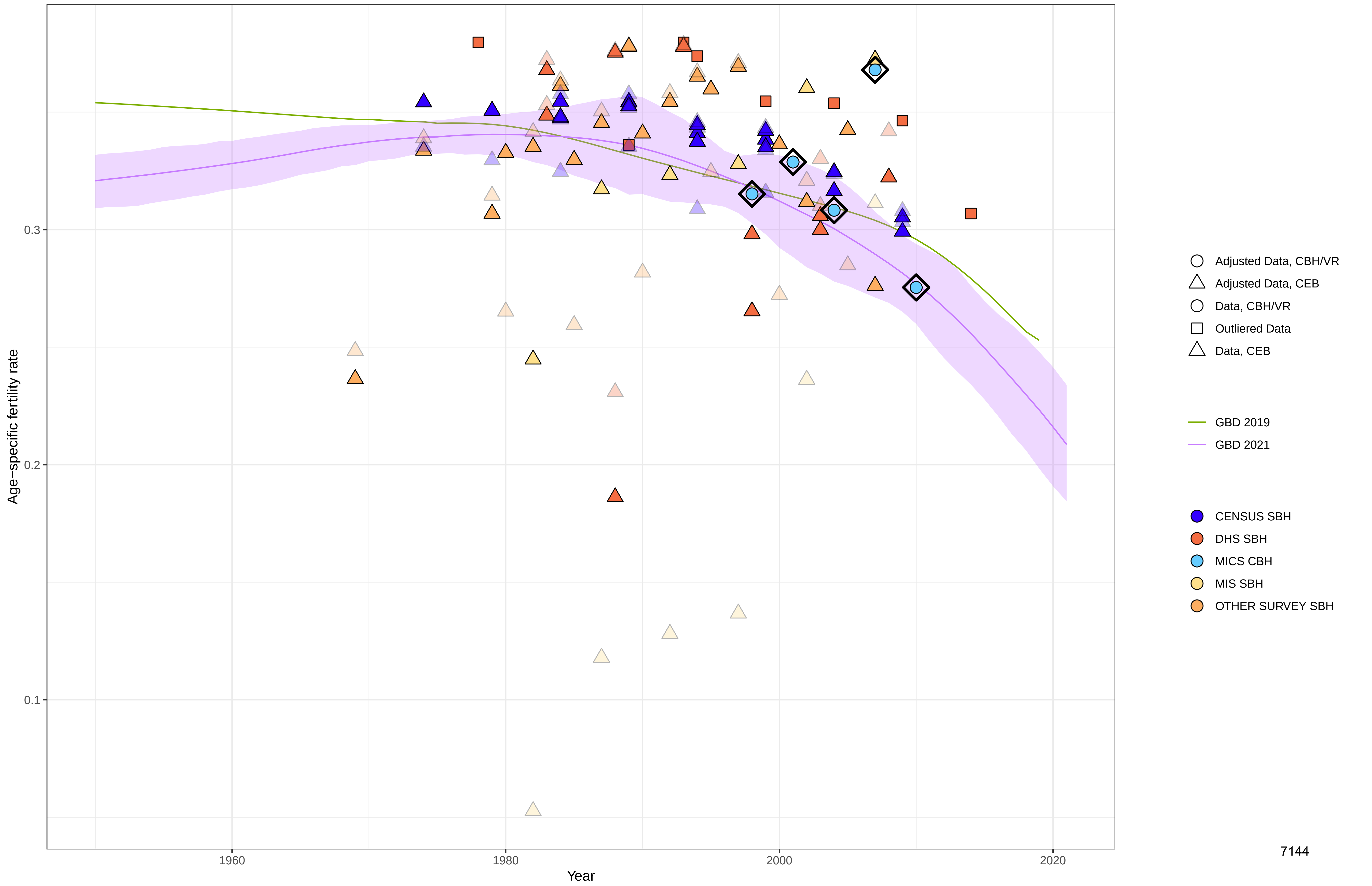

Age−specific fertility fitted trend lines, 1950−2021: Migori, Ages: 25−29
Data points enclosed with a diamond are reference sources

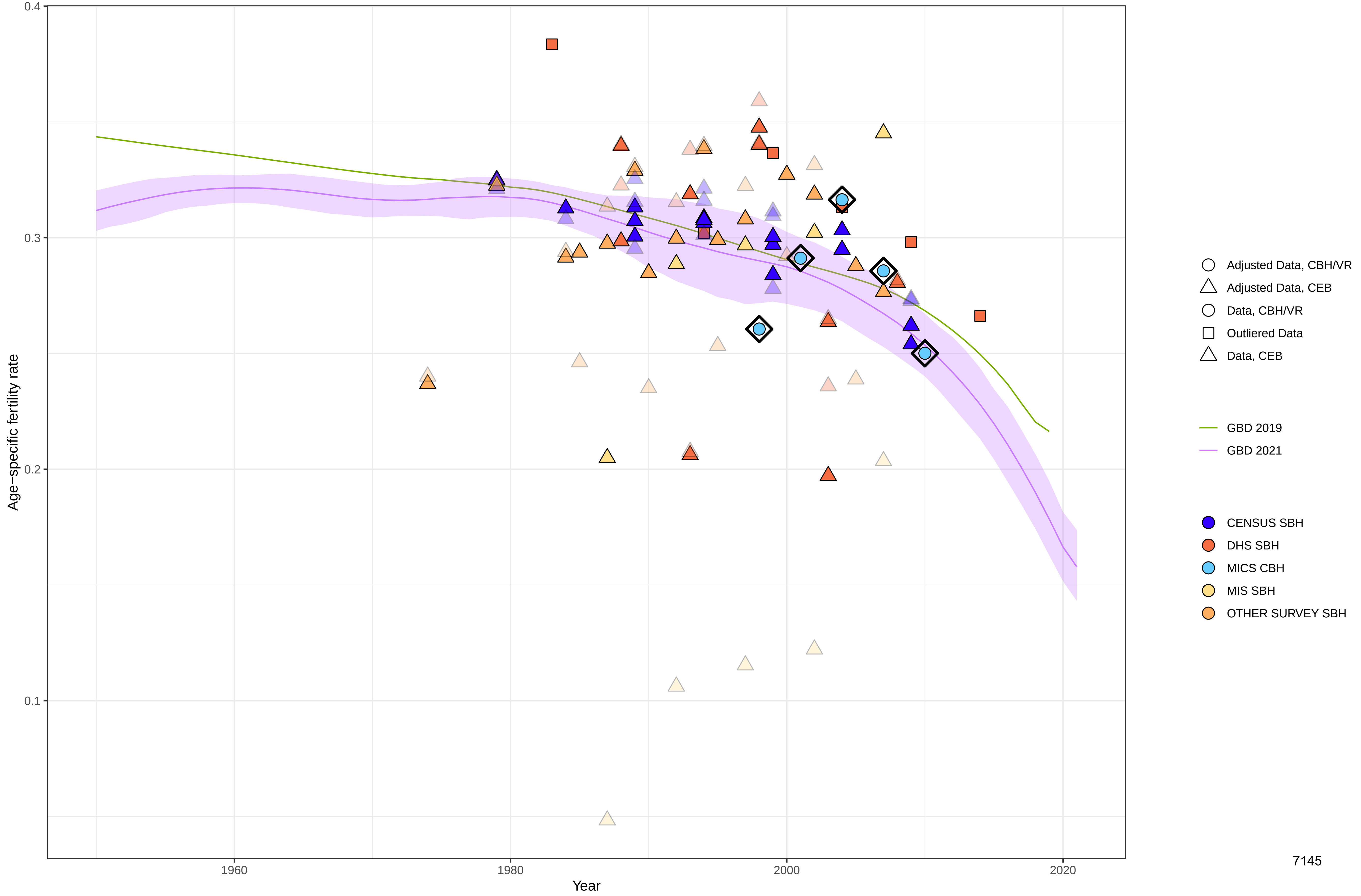

Age−specific fertility fitted trend lines, 1950−2021: Migori, Ages: 30−34
Data points enclosed with a diamond are reference sources

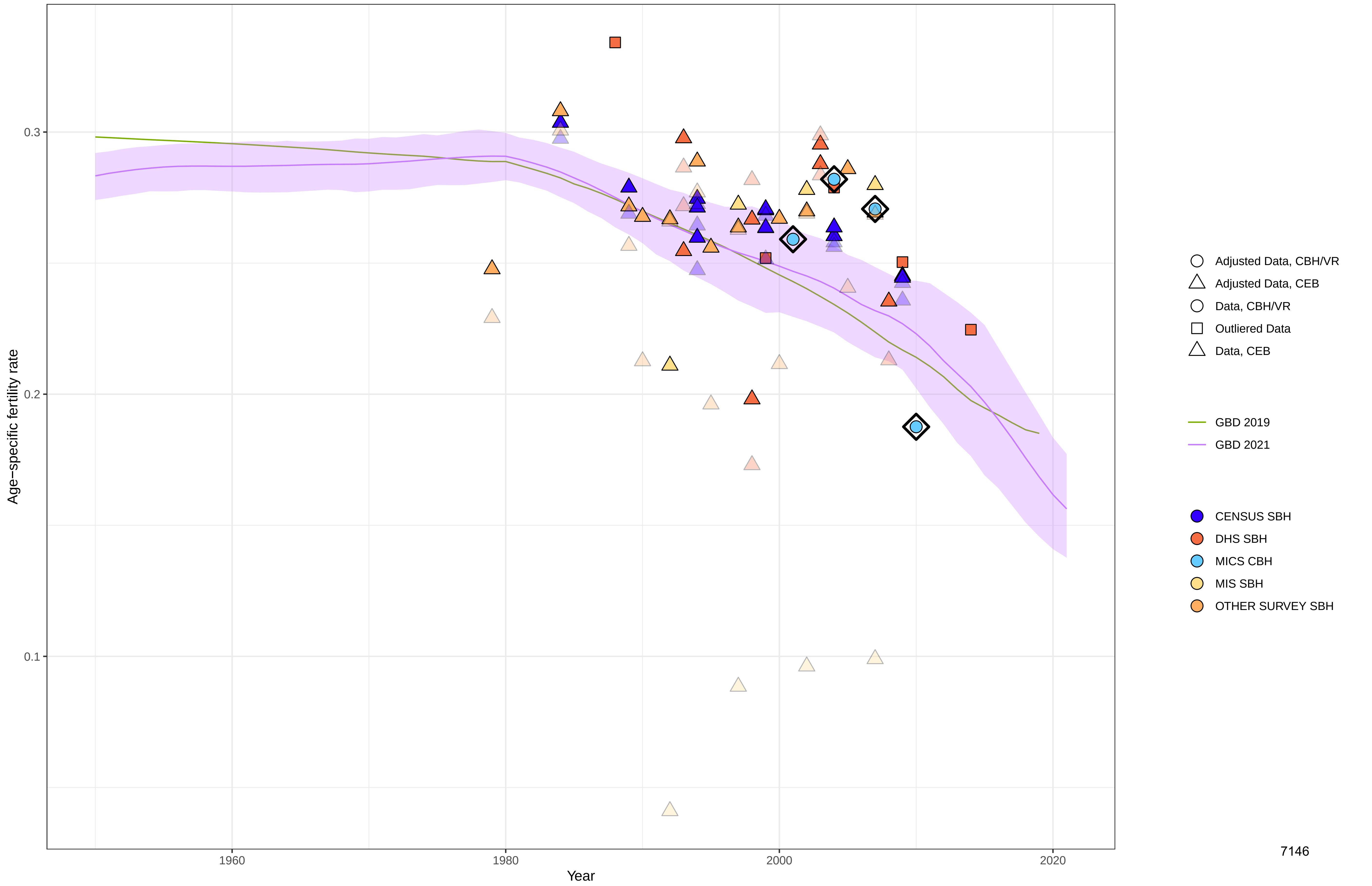

Age−specific fertility fitted trend lines, 1950−2021: Migori, Ages: 35−39
Data points enclosed with a diamond are reference sources

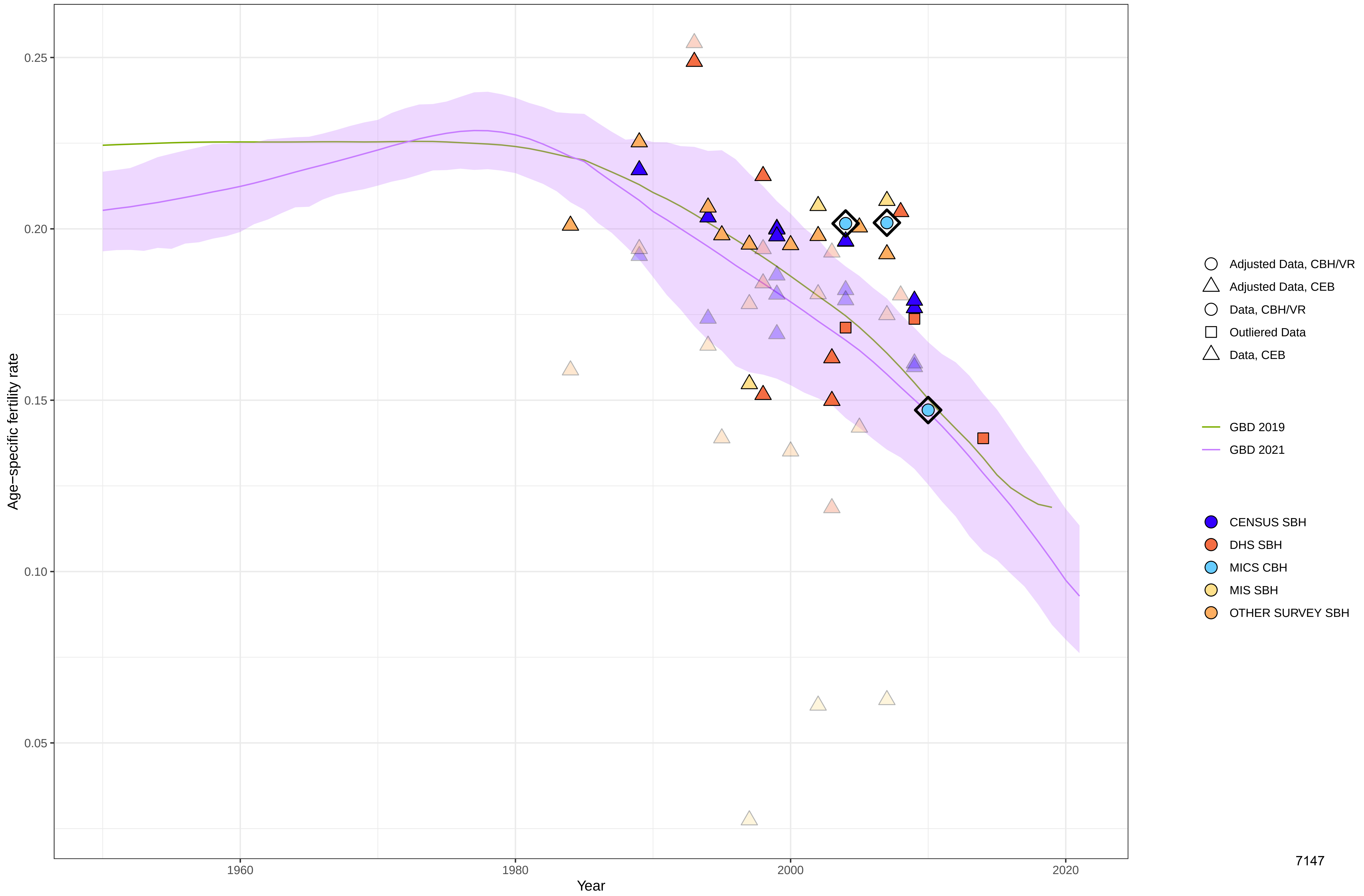

7147

Age−specific fertility fitted trend lines, 1950−2021: Migori, Ages: 40−44
Data points enclosed with a diamond are reference sources

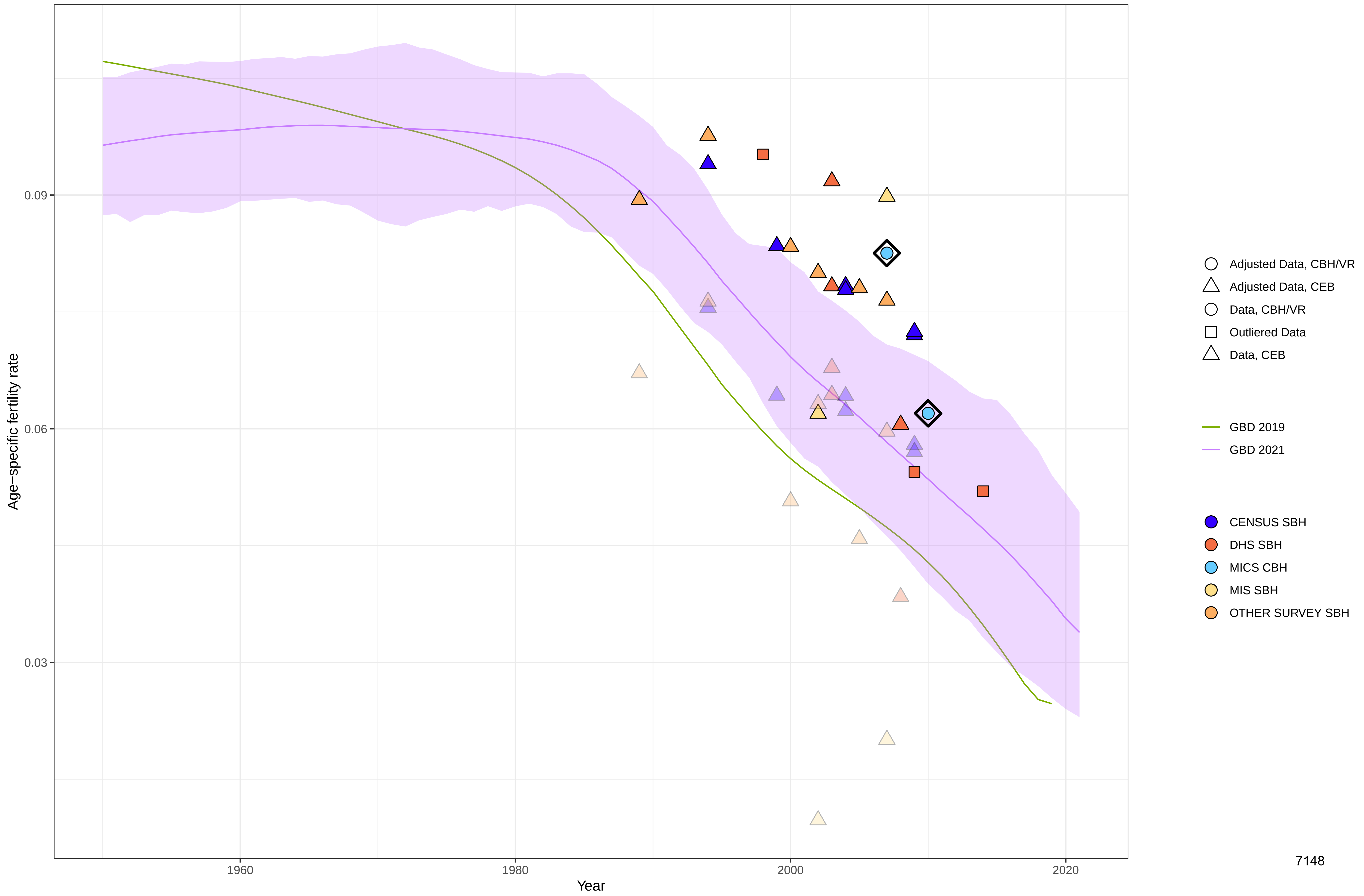

Age−specific fertility fitted trend lines, 1950−2021: Migori, Ages: 45−49
Data points enclosed with a diamond are reference sources

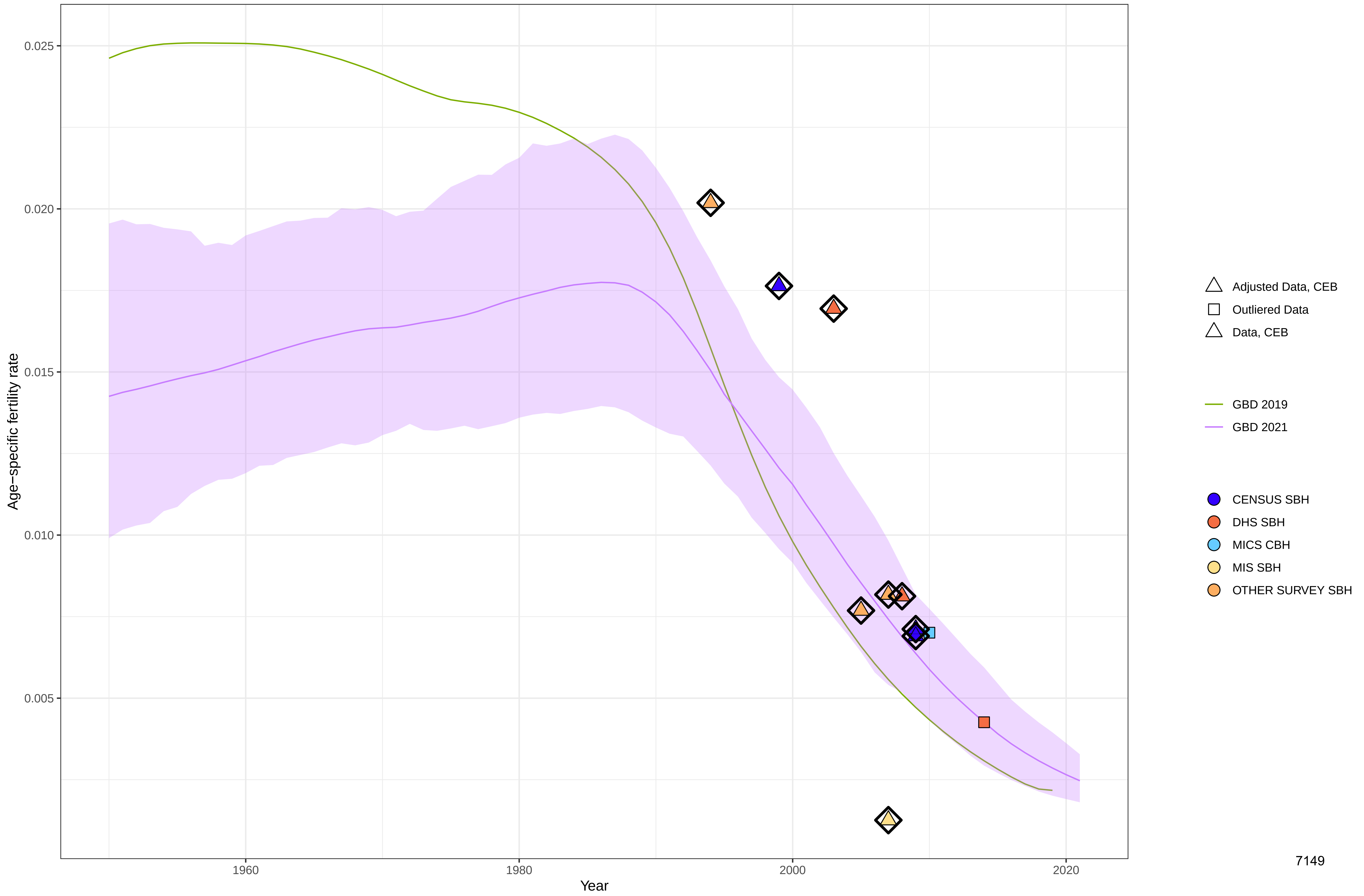

Age−specific fertility fitted trend lines, 1950−2021: Migori, Ages: 50−54
Data points enclosed with a diamond are reference sources

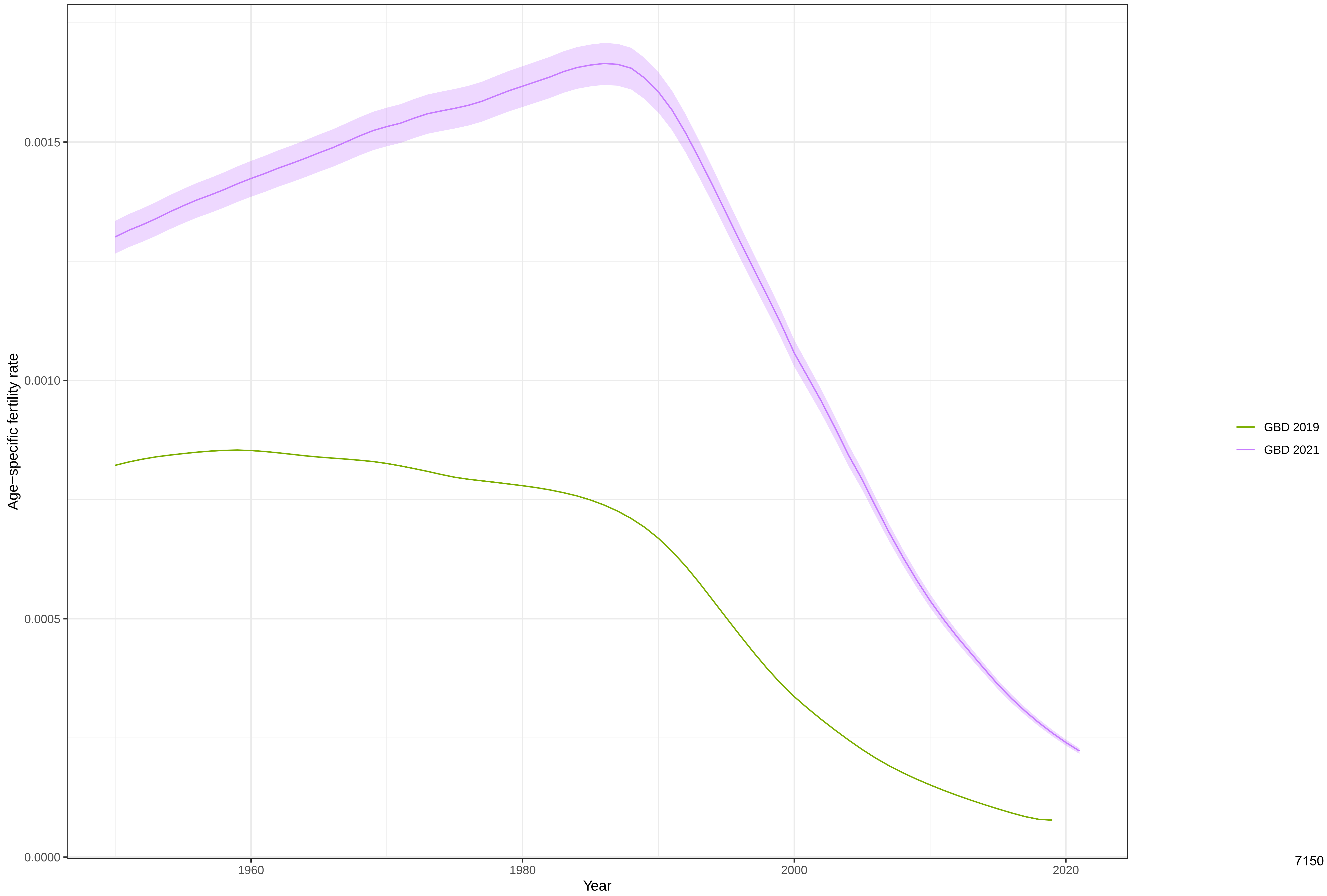

Total fertility rate fitted trend lines, 1950−2021: Migori TFR
Data points enclosed with a diamond are reference sources

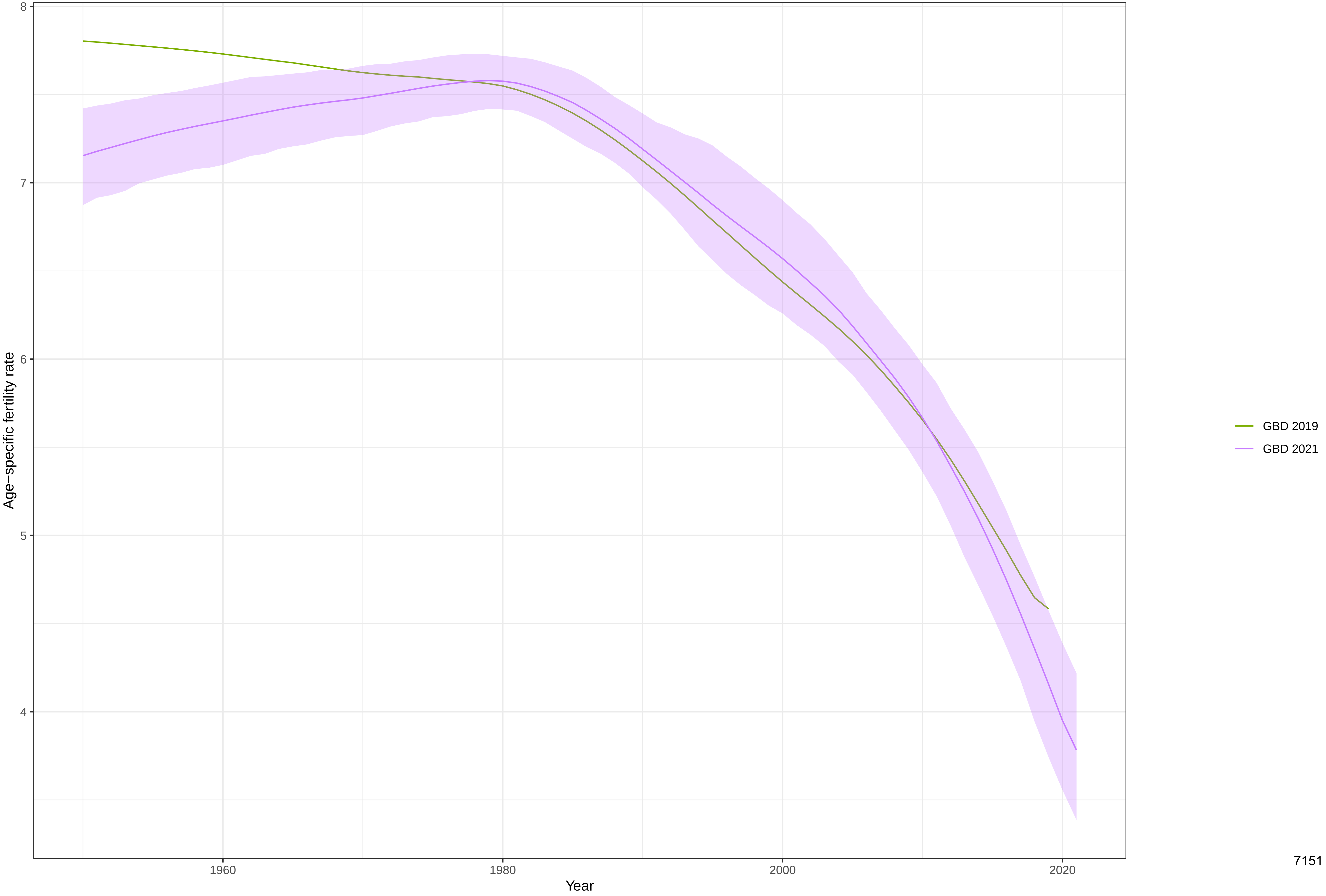

Age−specific fertility fitted trend lines, 1950−2021: Mombasa, Ages: 10−14
Data points enclosed with a diamond are reference sources

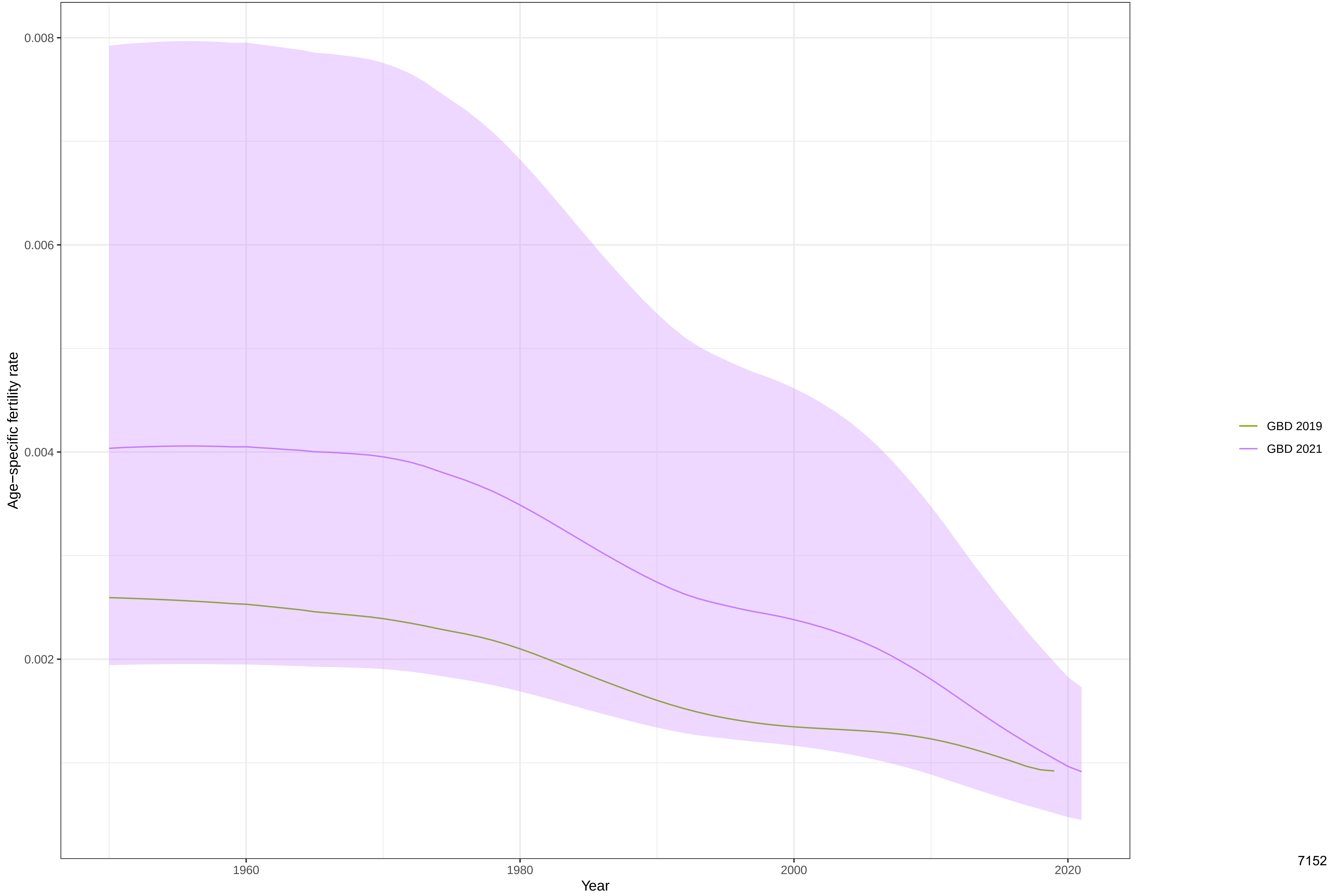

Age−specific fertility fitted trend lines, 1950−2021: Mombasa, Ages: 15−19
Data points enclosed with a diamond are reference sources

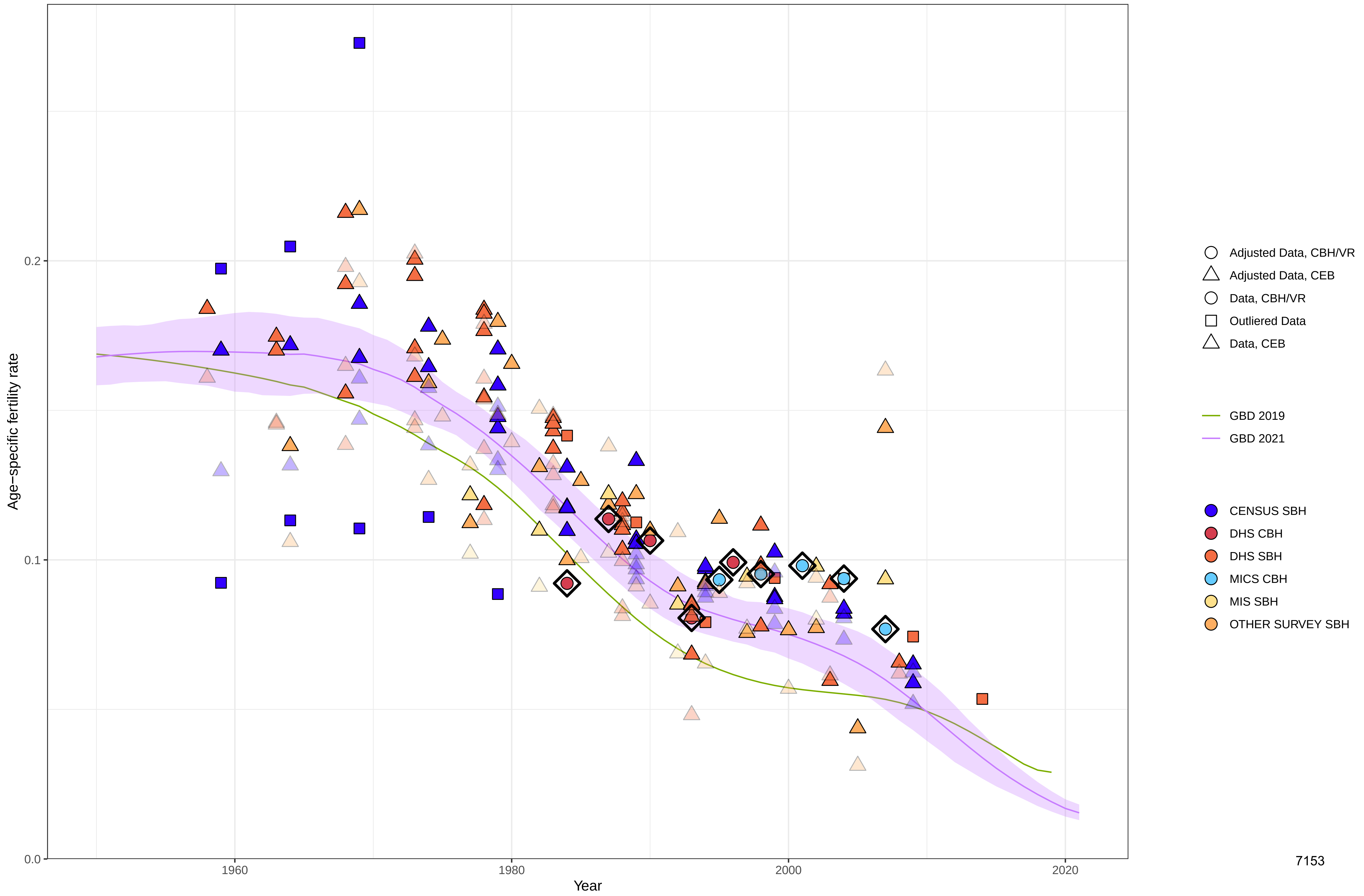

Age−specific fertility fitted trend lines, 1950−2021: Mombasa, Ages: 20−24
Data points enclosed with a diamond are reference sources

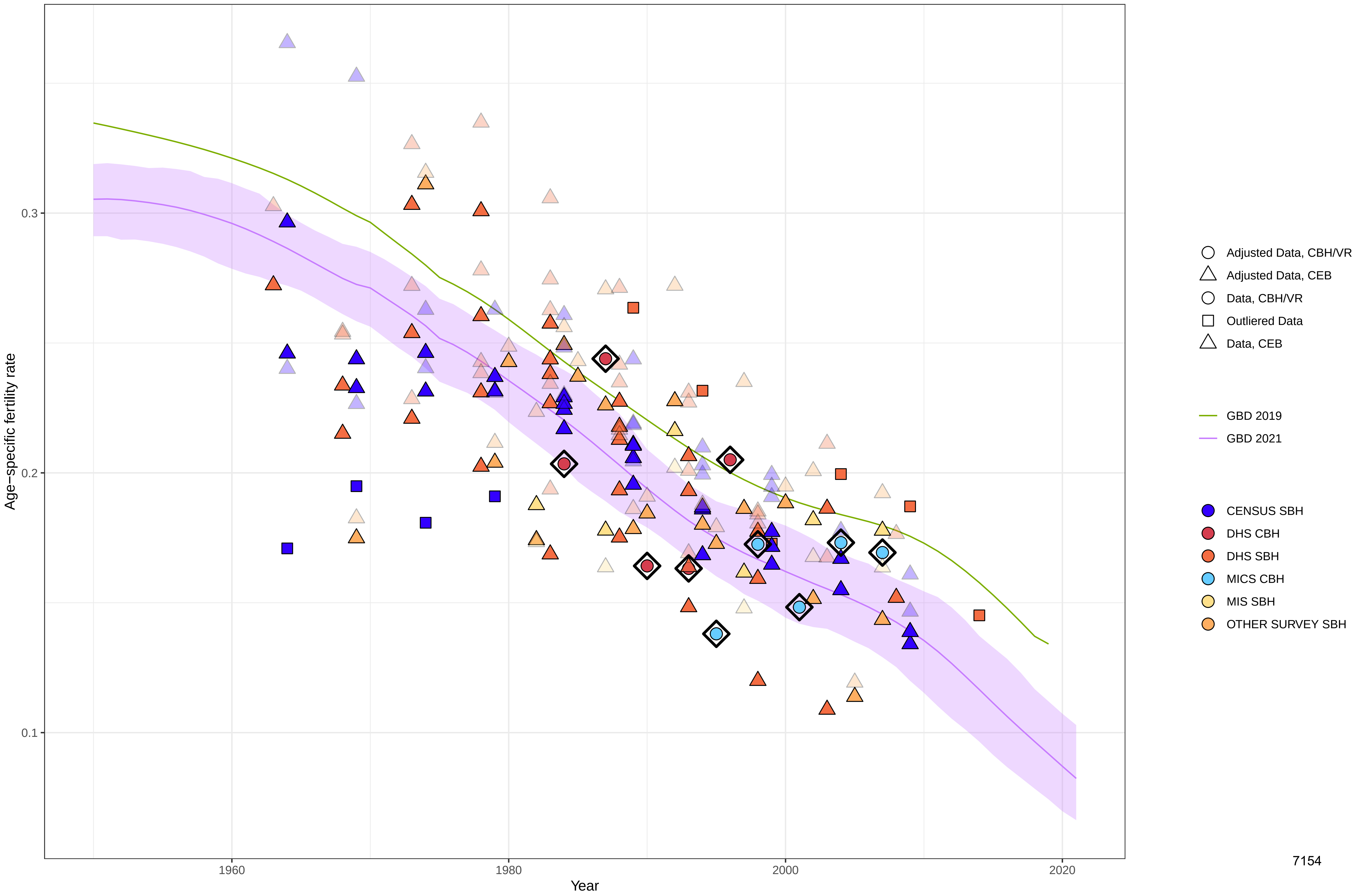

7154

Age−specific fertility fitted trend lines, 1950−2021: Mombasa, Ages: 25−29
Data points enclosed with a diamond are reference sources

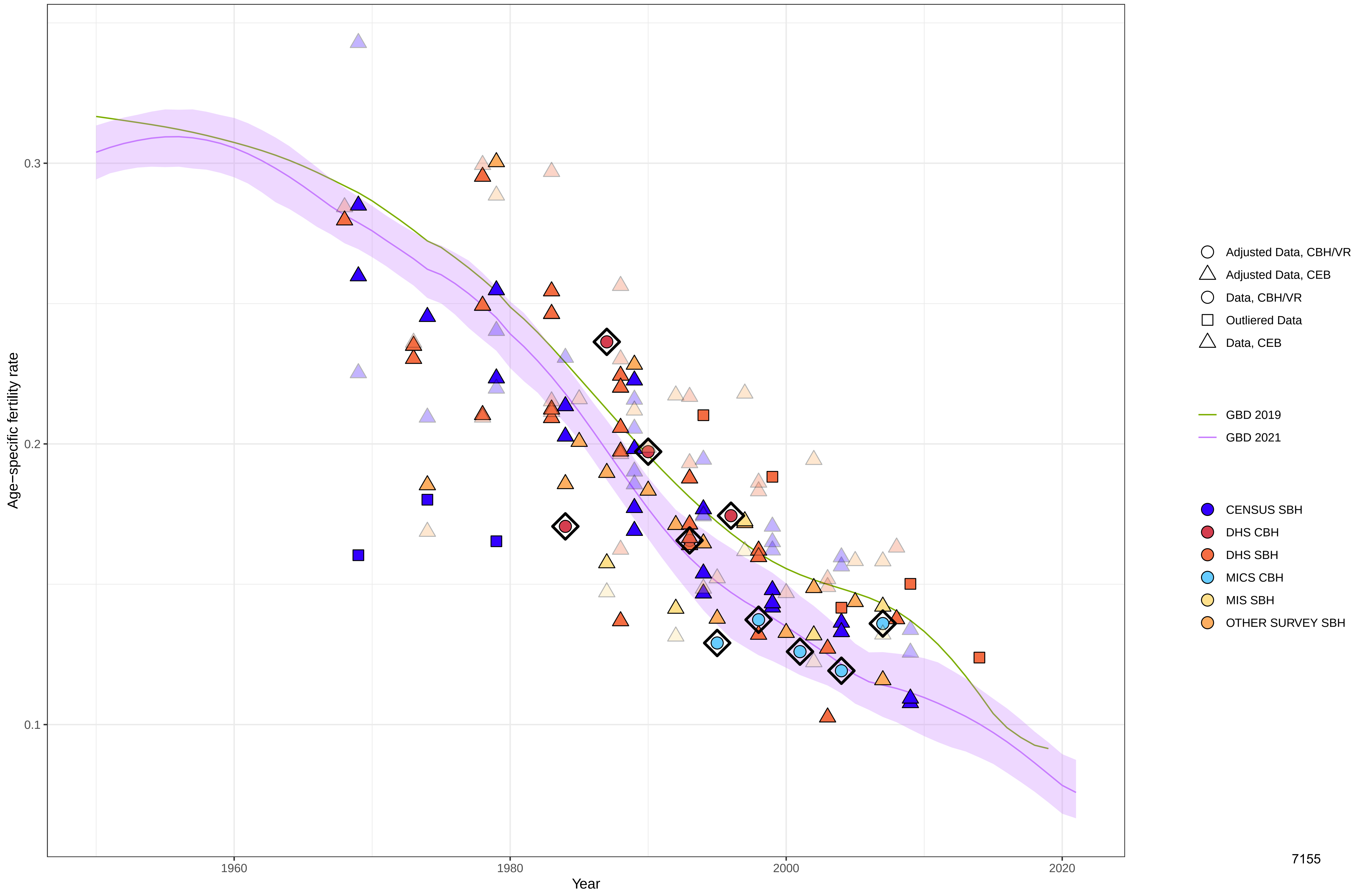

7155

Age−specific fertility fitted trend lines, 1950−2021: Mombasa, Ages: 30−34
Data points enclosed with a diamond are reference sources

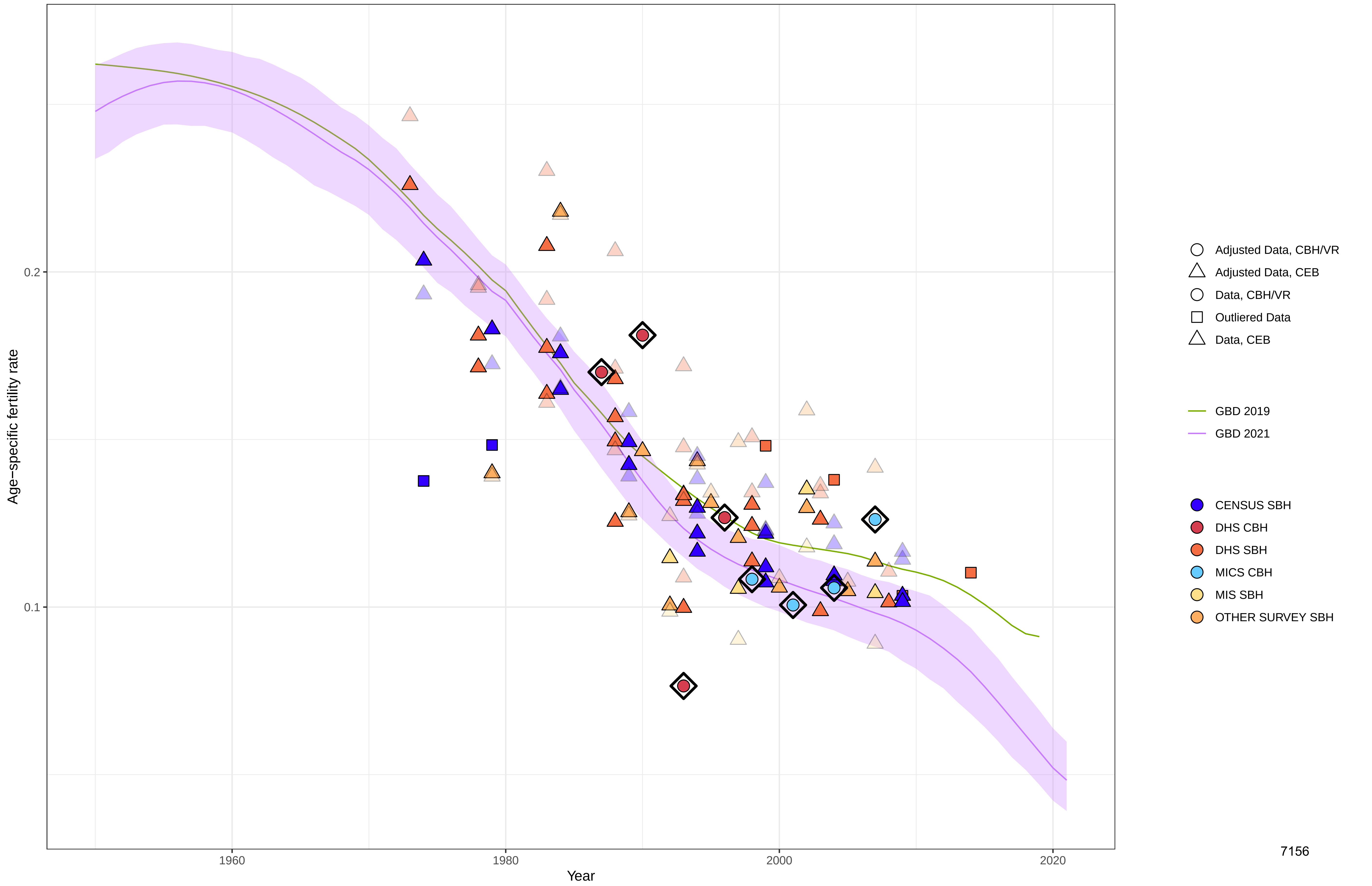

Age−specific fertility fitted trend lines, 1950−2021: Mombasa, Ages: 35−39
Data points enclosed with a diamond are reference sources

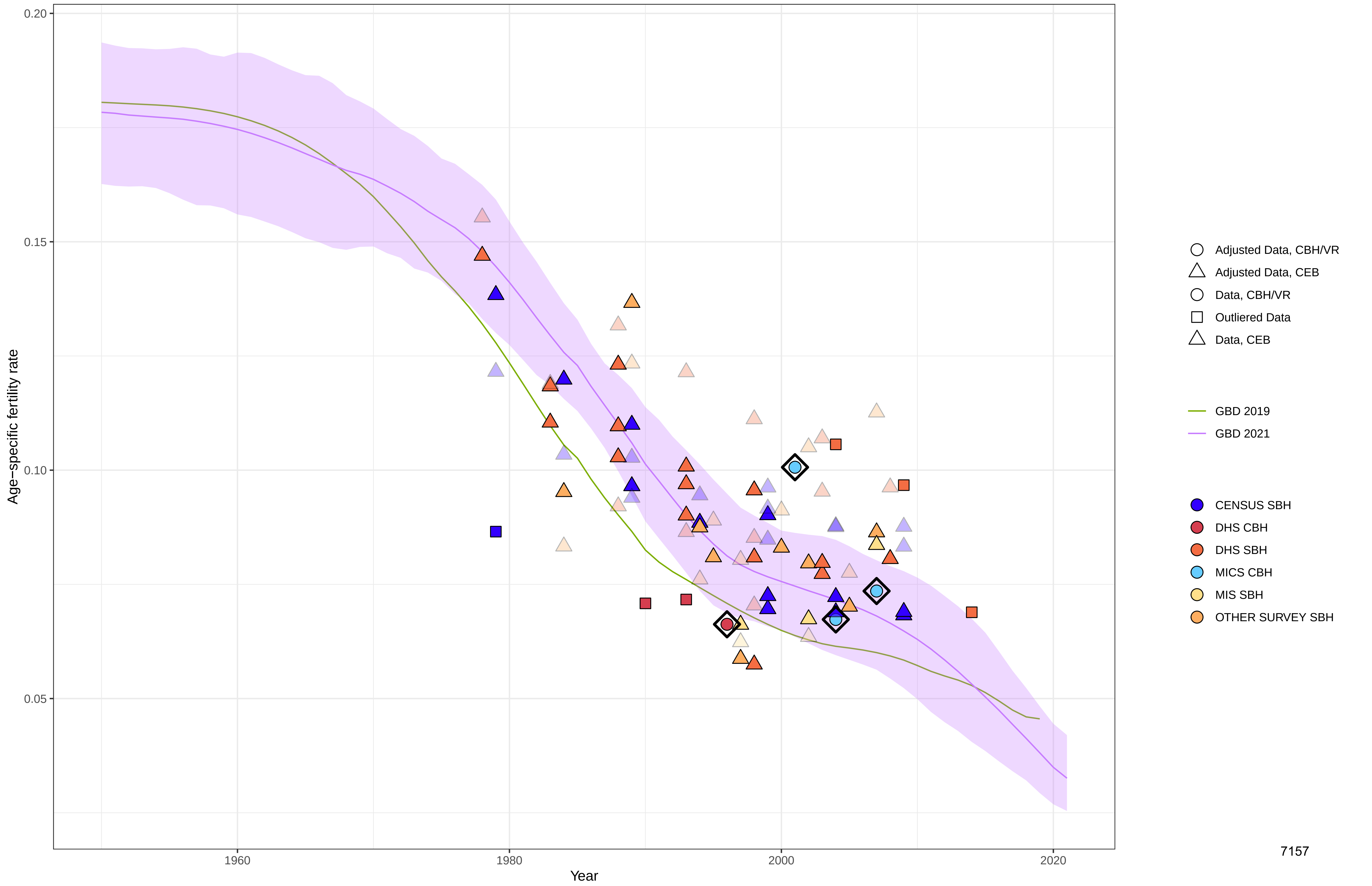

Age−specific fertility fitted trend lines, 1950−2021: Mombasa, Ages: 40−44
Data points enclosed with a diamond are reference sources

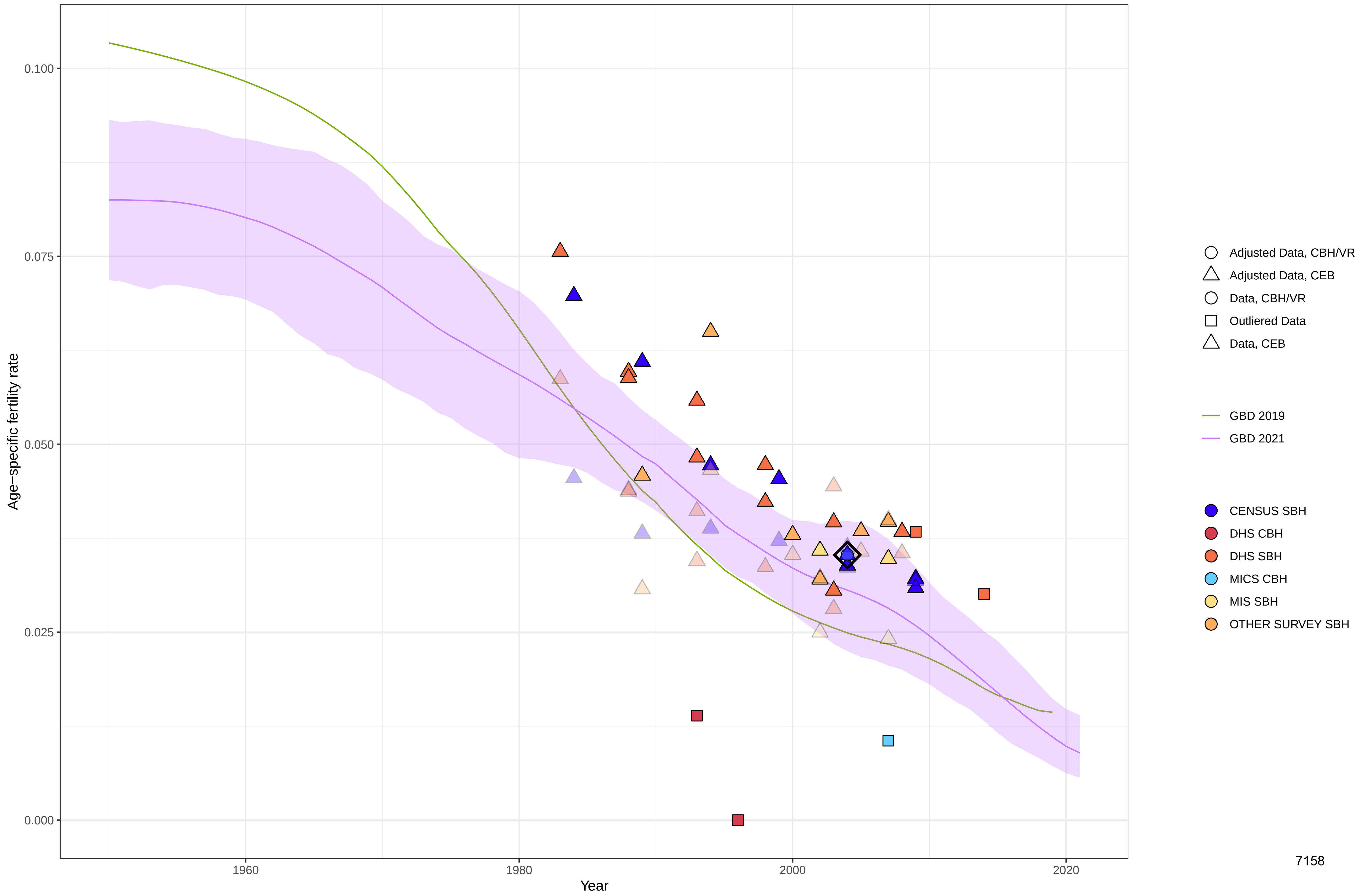

Age−specific fertility fitted trend lines, 1950−2021: Mombasa, Ages: 45−49
Data points enclosed with a diamond are reference sources

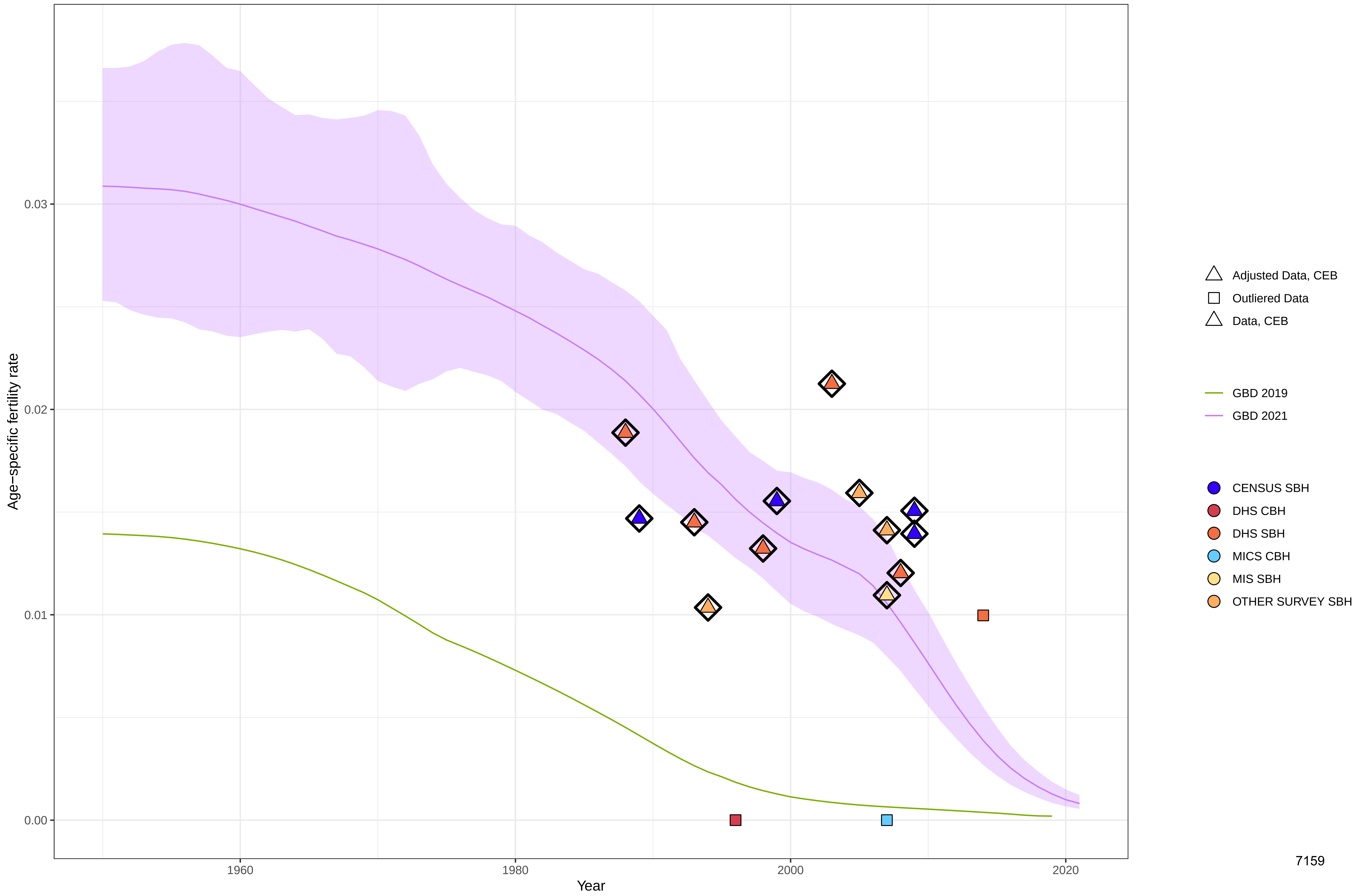

Age−specific fertility fitted trend lines, 1950−2021: Mombasa, Ages: 50−54
Data points enclosed with a diamond are reference sources

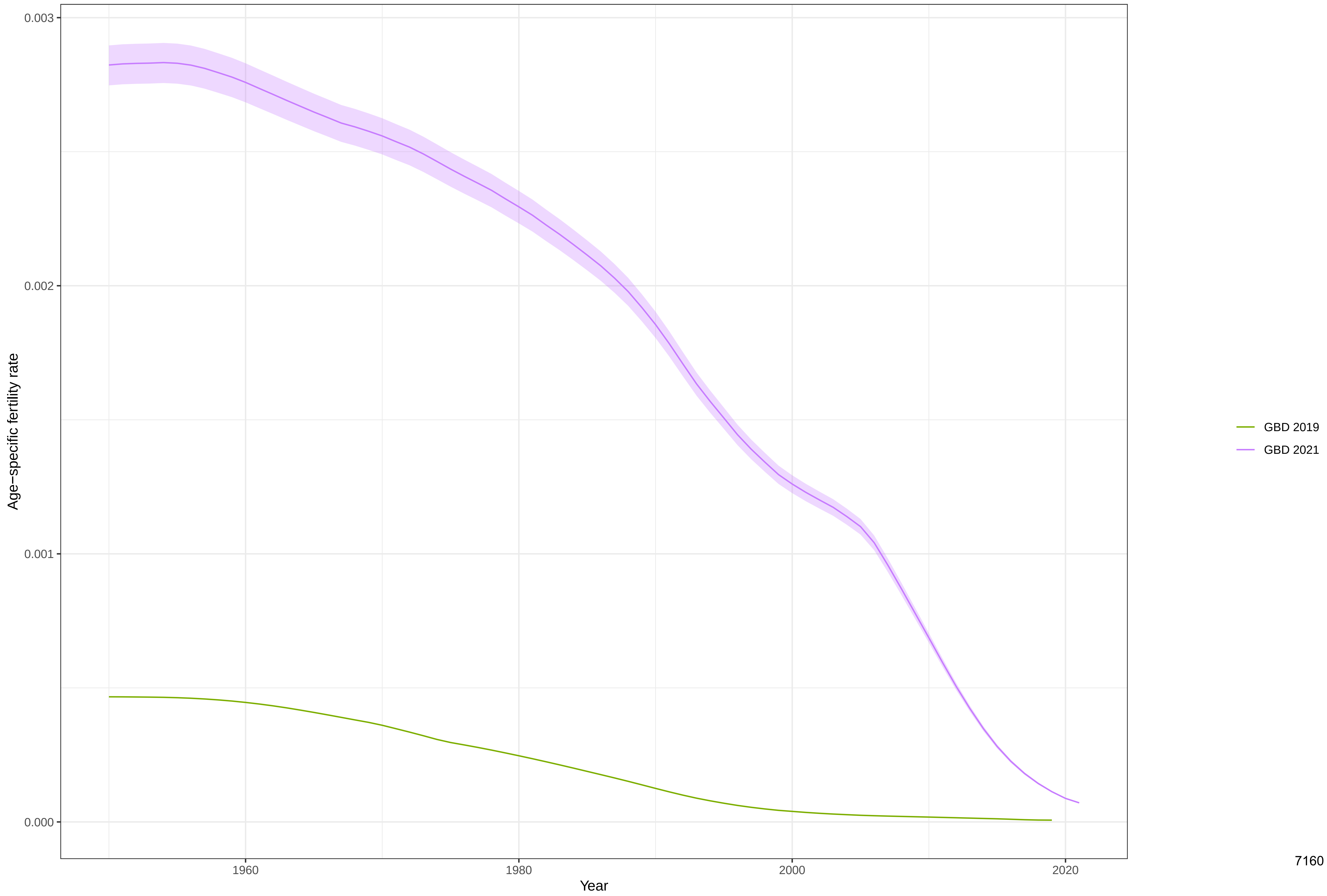

Total fertility rate fitted trend lines, 1950−2021: Mombasa TFR
Data points enclosed with a diamond are reference sources

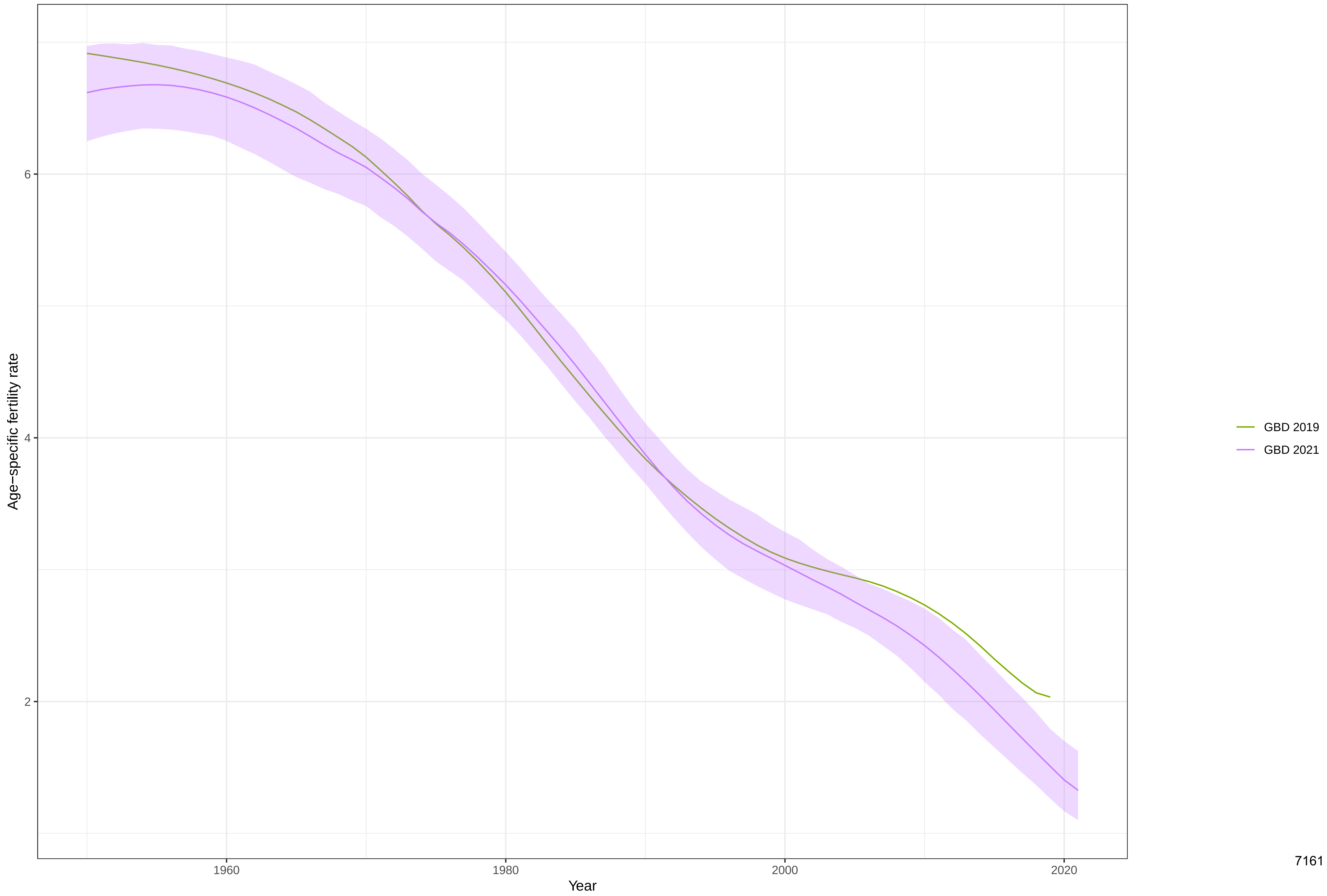

Age−specific fertility fitted trend lines, 1950−2021: Murang'a, Ages: 10−14
Data points enclosed with a diamond are reference sources

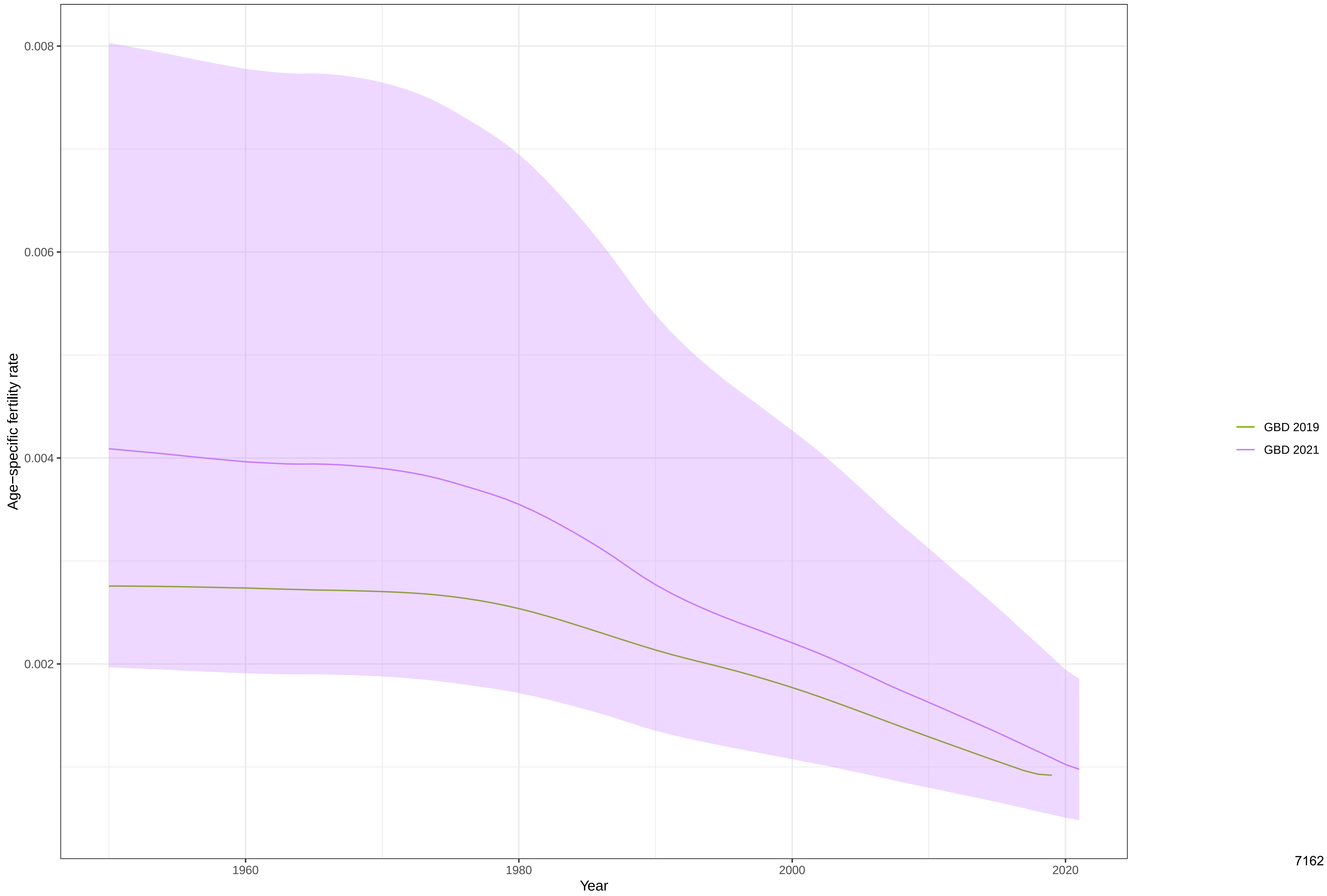

Age−specific fertility fitted trend lines, 1950−2021: Murang'a, Ages: 15−19
Data points enclosed with a diamond are reference sources

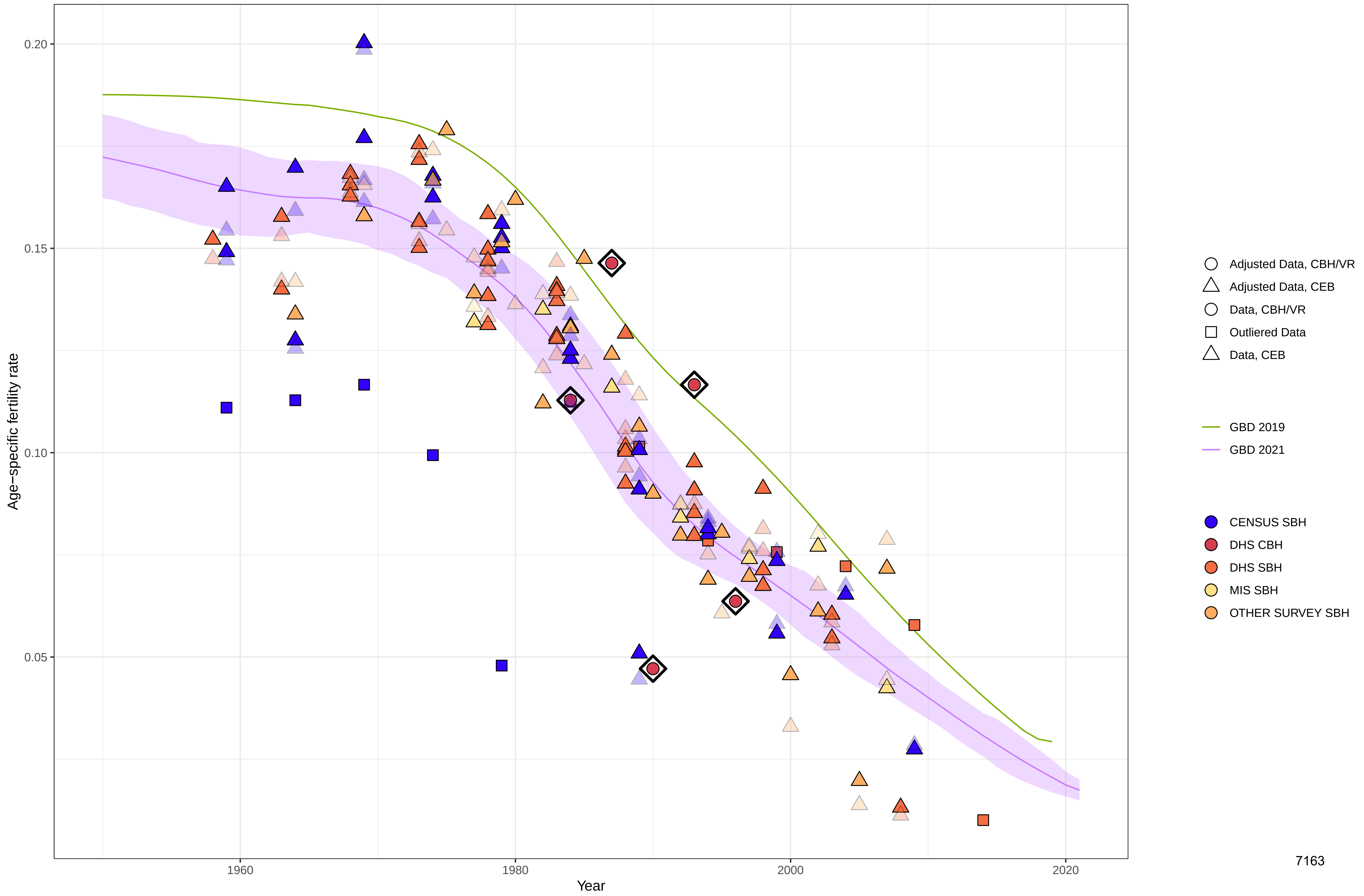

Age−specific fertility fitted trend lines, 1950−2021: Murang'a, Ages: 20−24
Data points enclosed with a diamond are reference sources

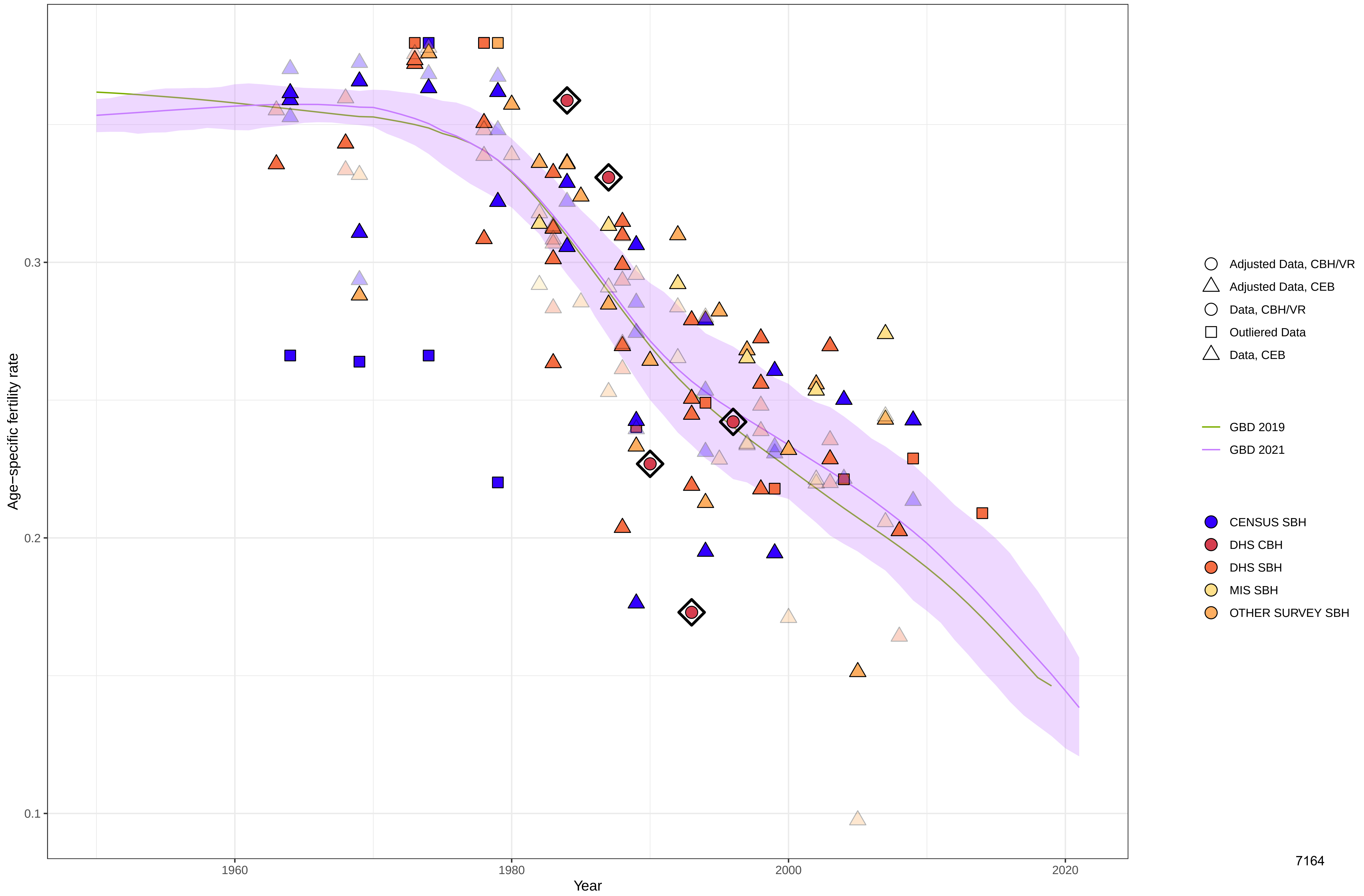

Age−specific fertility fitted trend lines, 1950−2021: Murang'a, Ages: 25−29
Data points enclosed with a diamond are reference sources

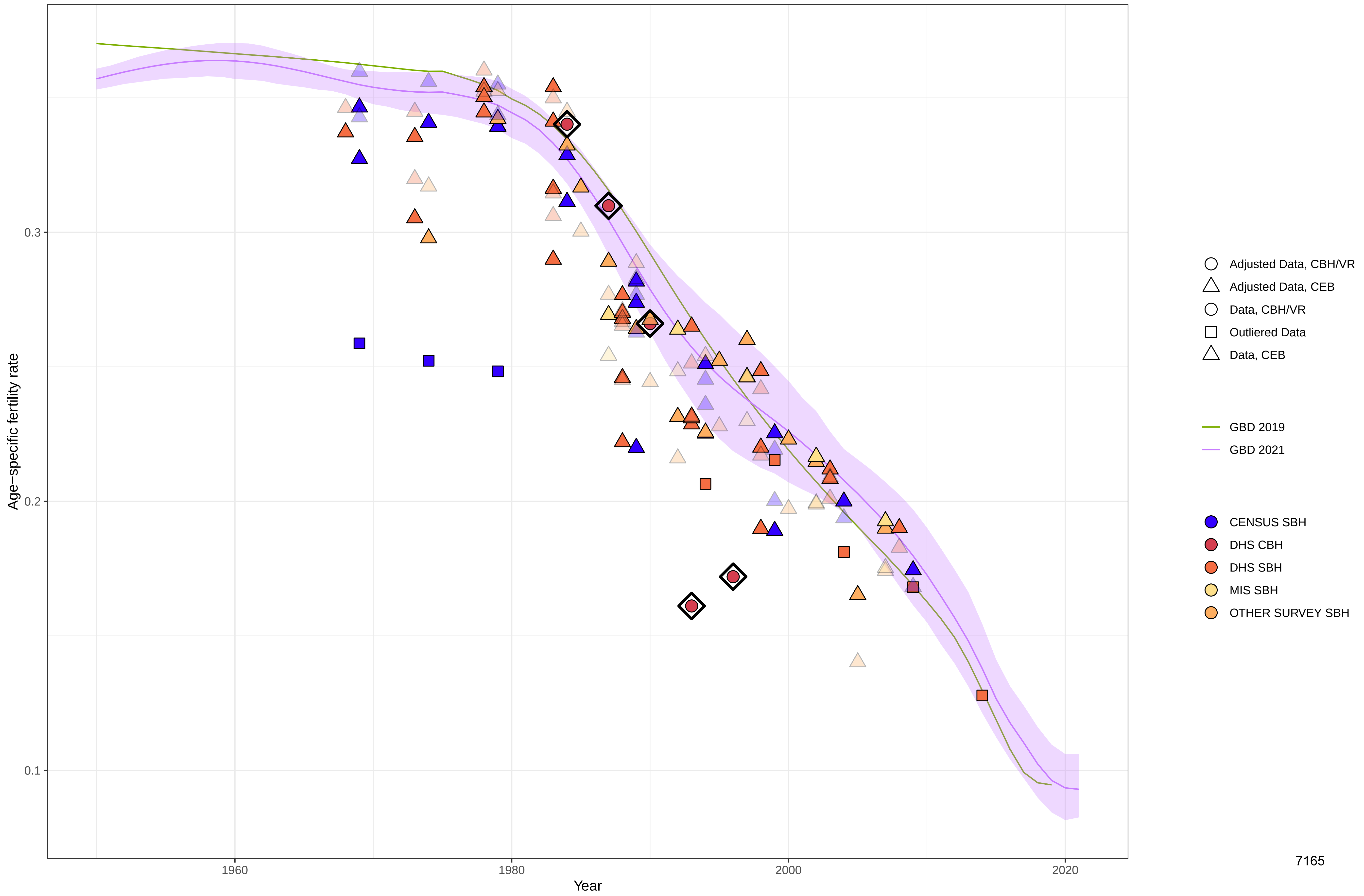

Age−specific fertility fitted trend lines, 1950−2021: Murang'a, Ages: 30−34
Data points enclosed with a diamond are reference sources

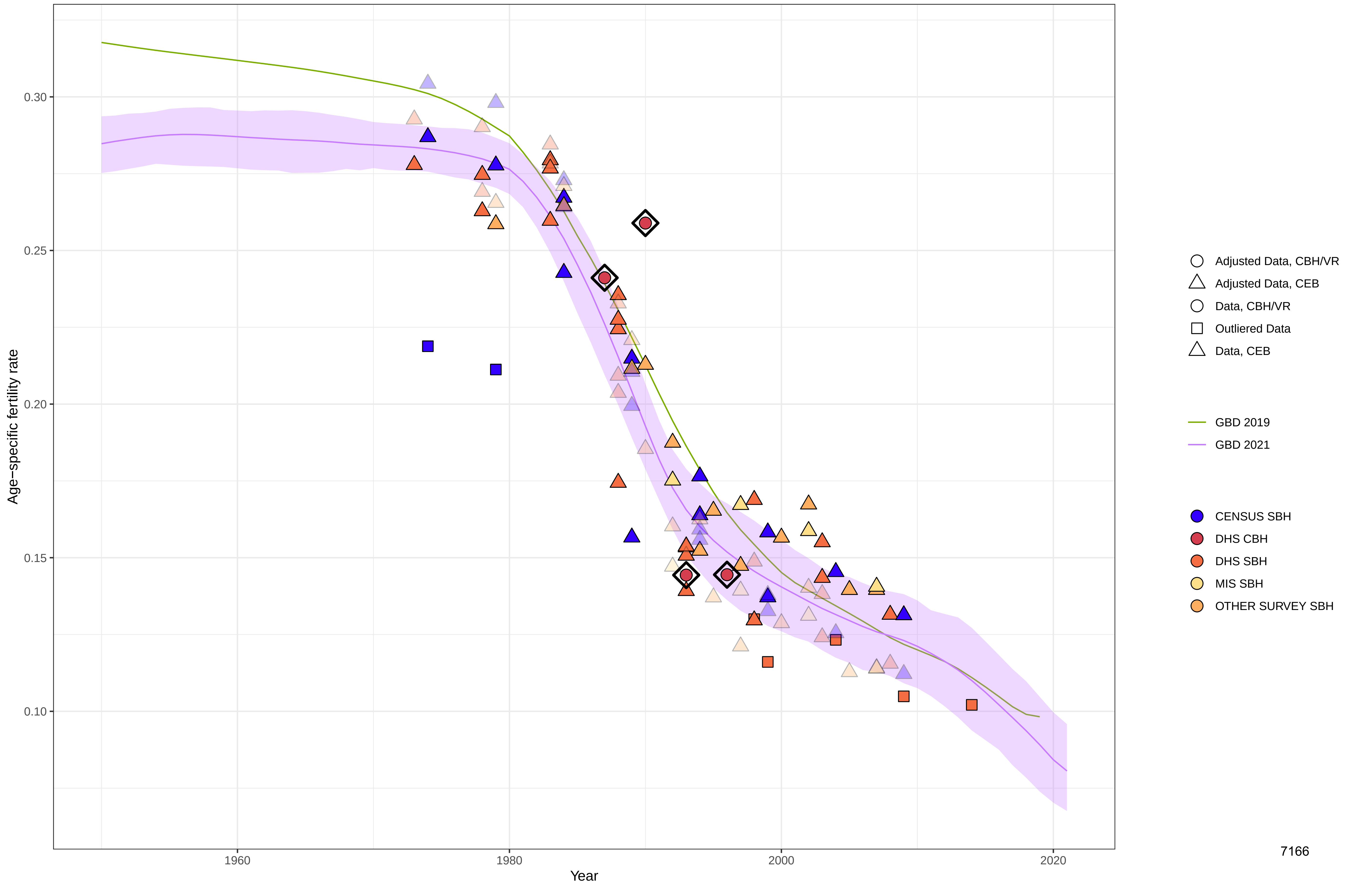

7166

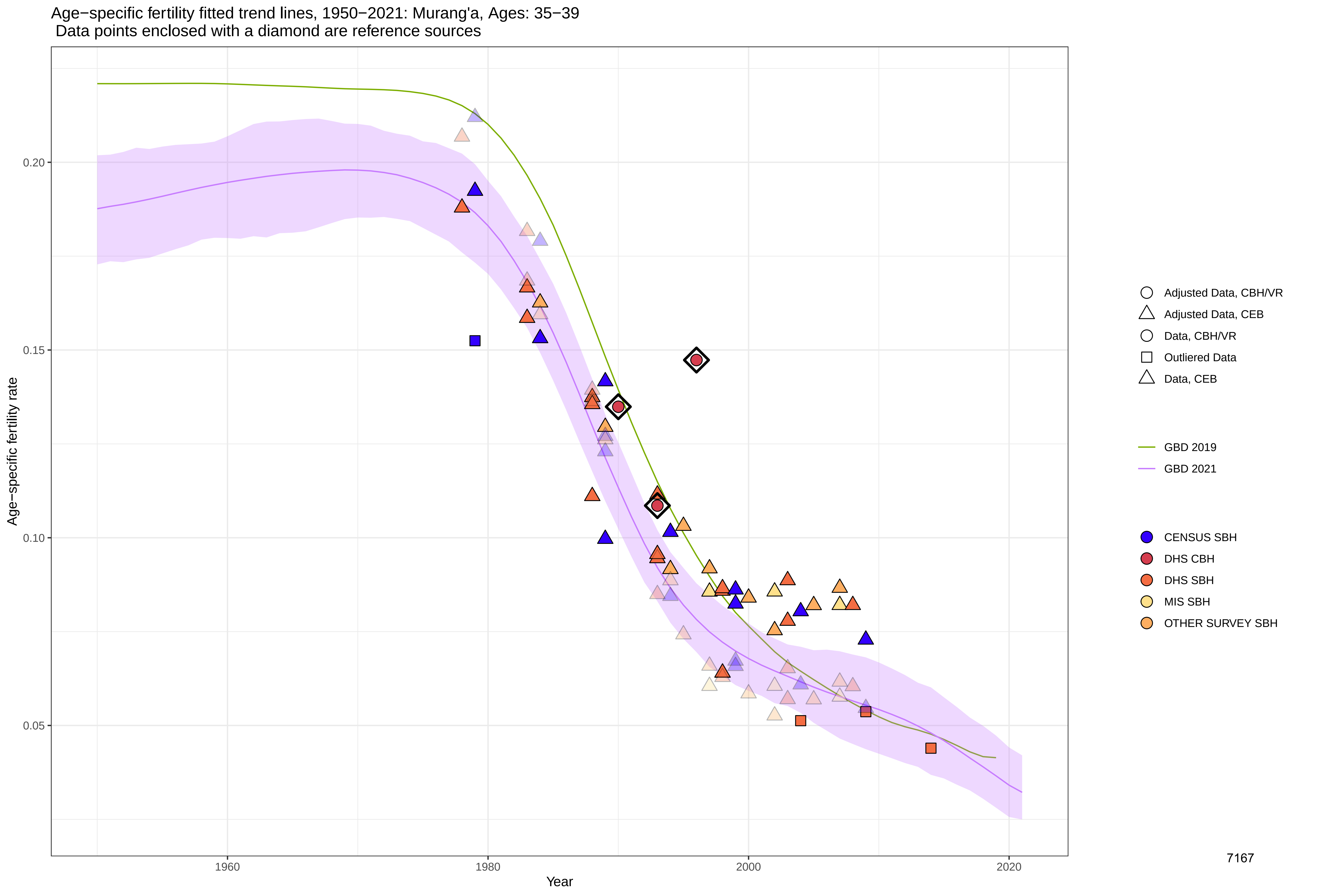

Age−specific fertility fitted trend lines, 1950−2021: Murang'a, Ages: 35−39
Data points enclosed with a diamond are reference sources
Age−specific fertility rate
0.20
0.15
0.10
0.05
1960
1980
2000
2020
Year
Adjusted Data, CBH/VR
Adjusted Data, CEB
Data, CBH/VR
Outliered Data
Data, CEB
GBD 2019
GBD 2021
CENSUS SBH
DHS CBH
DHS SBH
MIS SBH
OTHER SURVEY SBH
7167

Age−specific fertility fitted trend lines, 1950−2021: Murang'a, Ages: 40−44
Data points enclosed with a diamond are reference sources

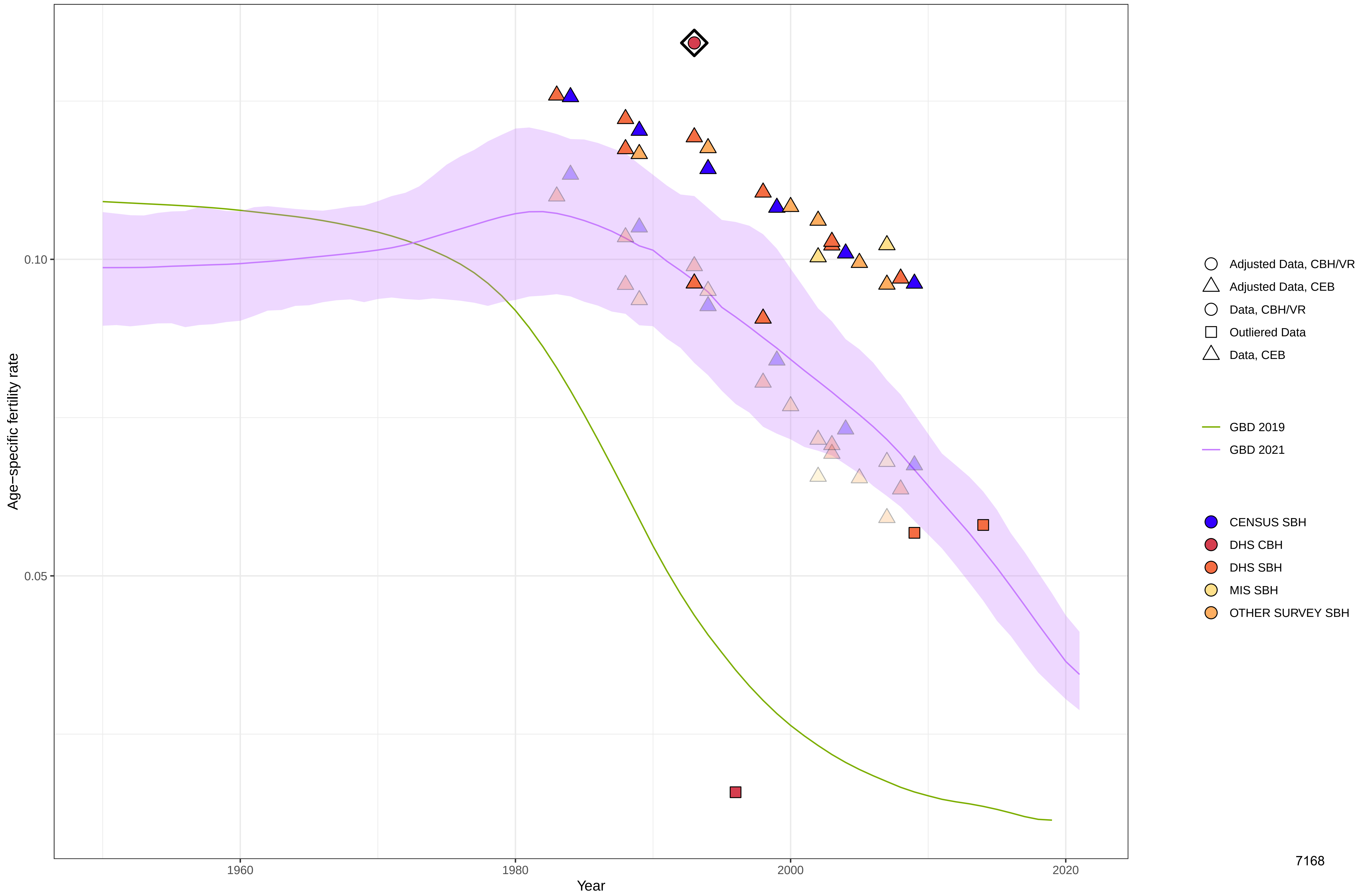

7168

Age−specific fertility fitted trend lines, 1950−2021: Murang'a, Ages: 45−49
Data points enclosed with a diamond are reference sources

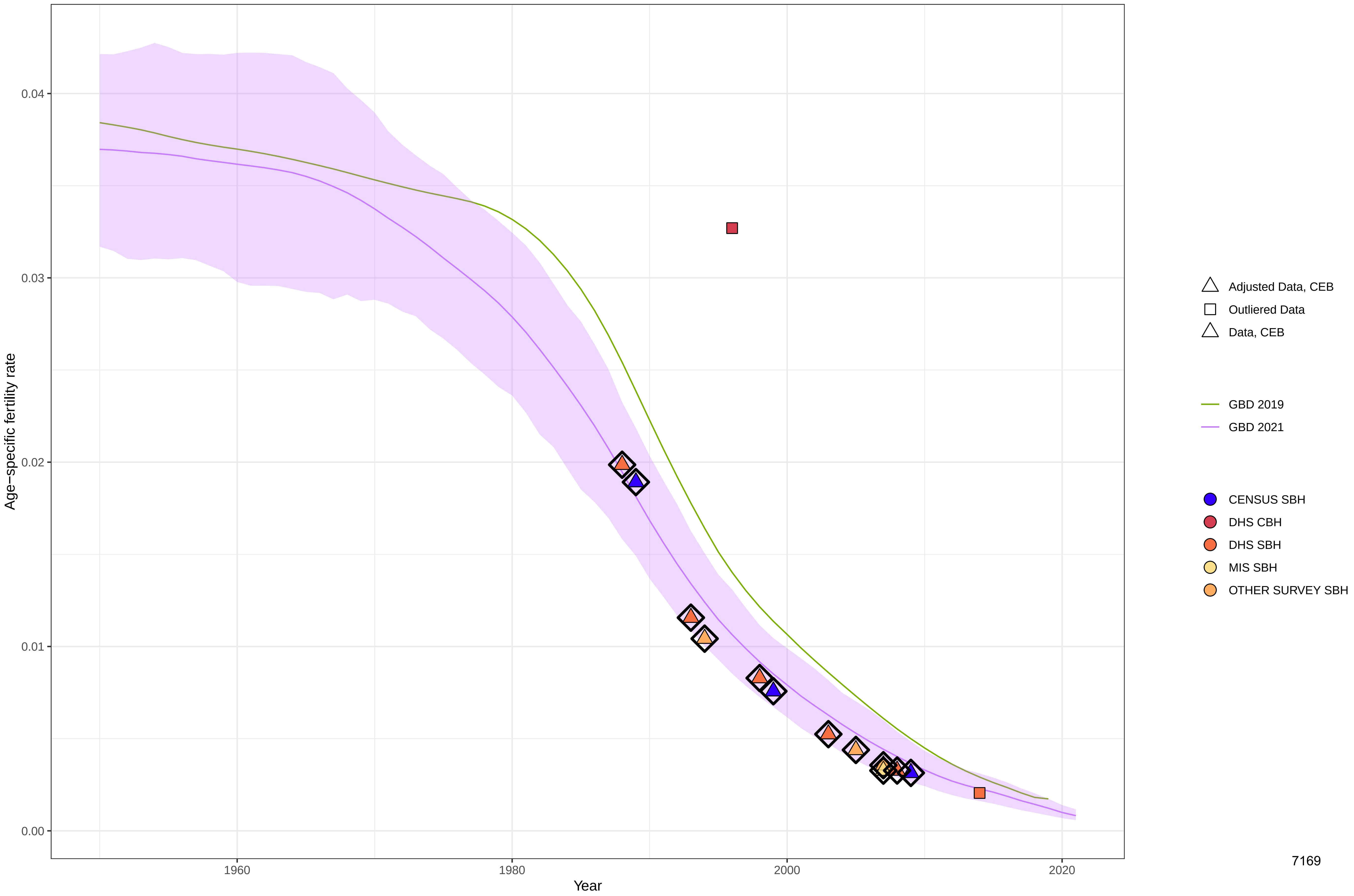

Age−specific fertility fitted trend lines, 1950−2021: Murang'a, Ages: 50−54
Data points enclosed with a diamond are reference sources

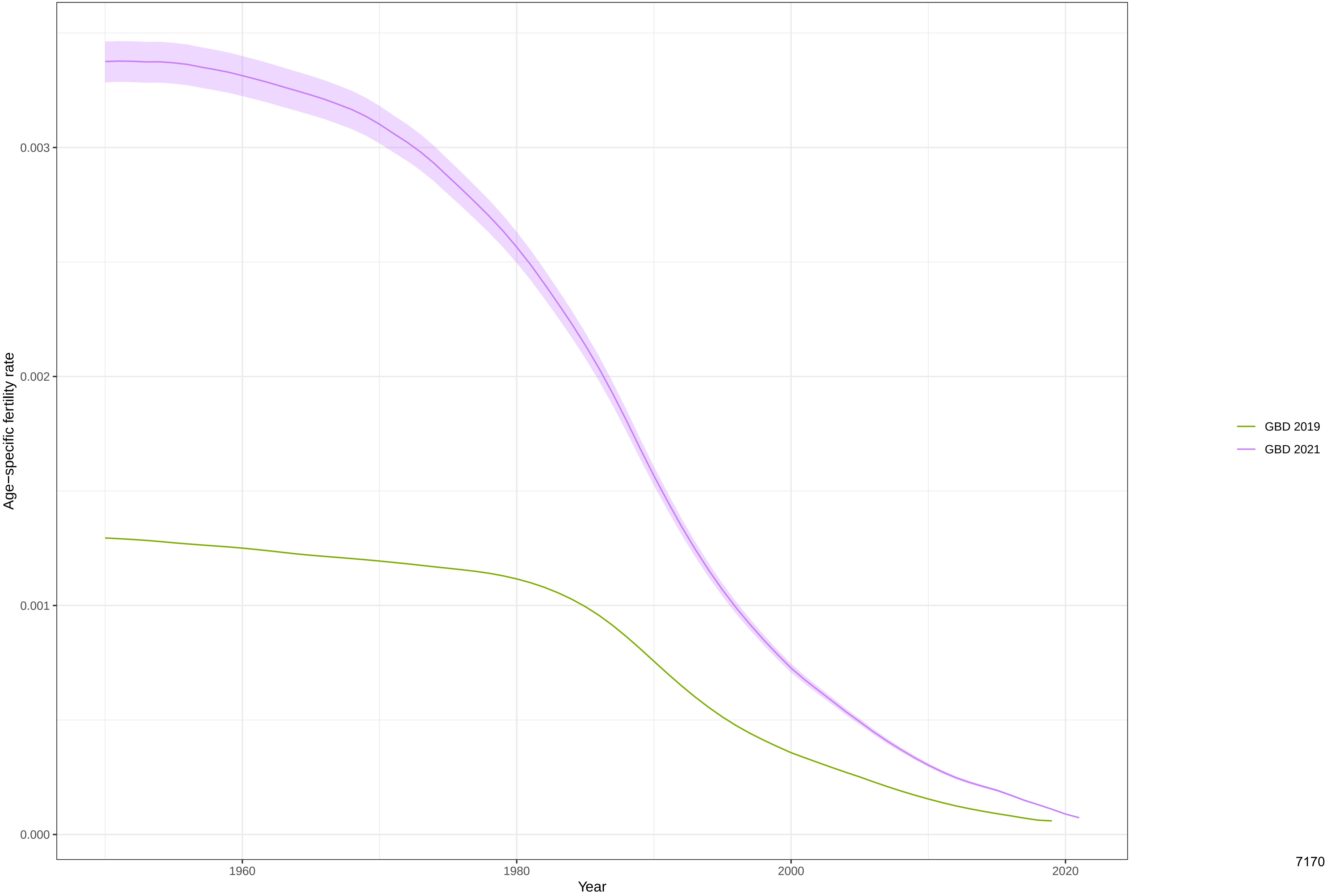

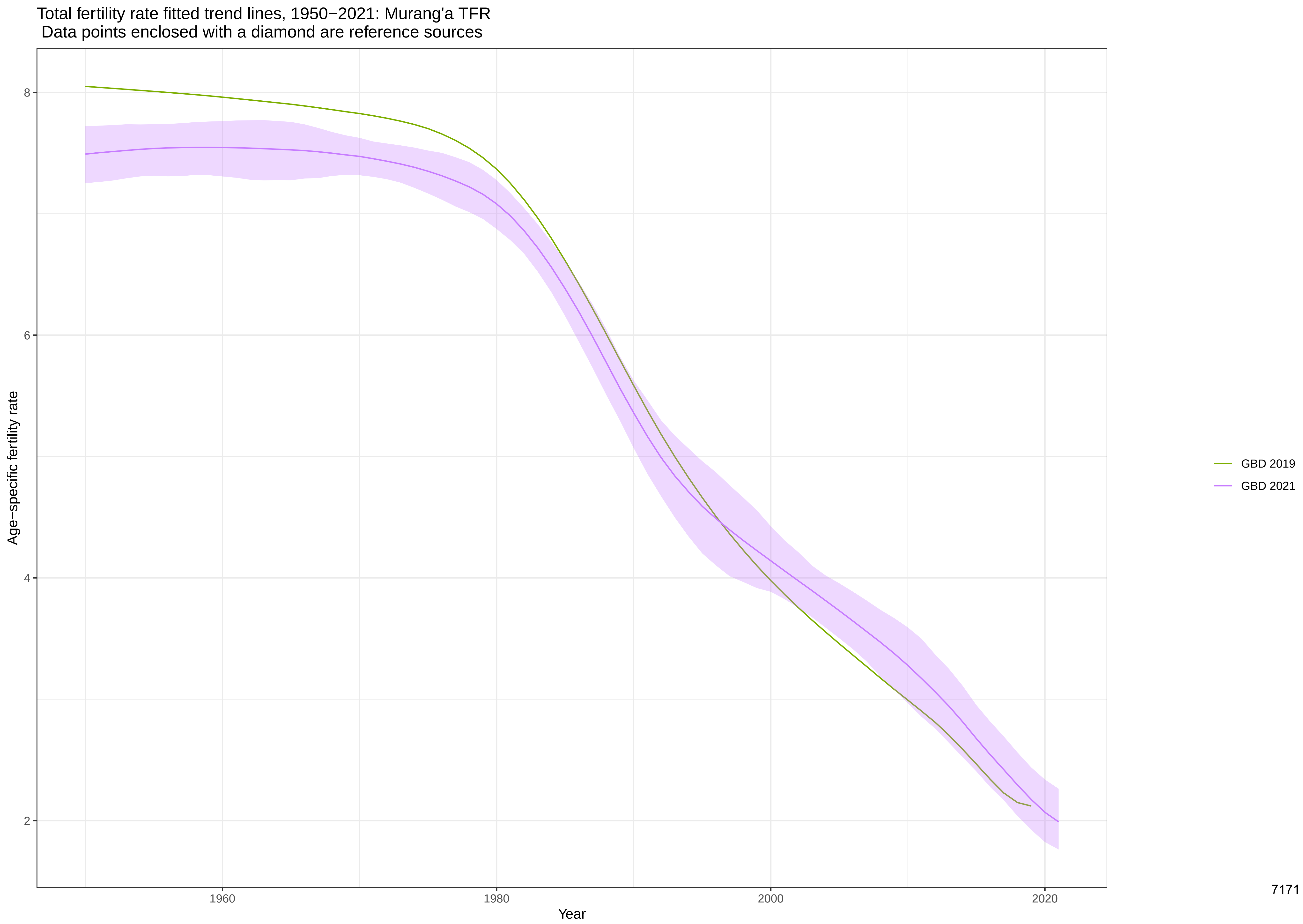
Total fertility rate fitted trend lines, 1950−2021: Murang'a TFR
Data points enclosed with a diamond are reference sources
Age−specific fertility rate
8
6
4
2
1960
1980
2000
2020
Year
GBD 2019
GBD 2021

Age−specific fertility fitted trend lines, 1950−2021: Nairobi, Ages: 10−14
Data points enclosed with a diamond are reference sources

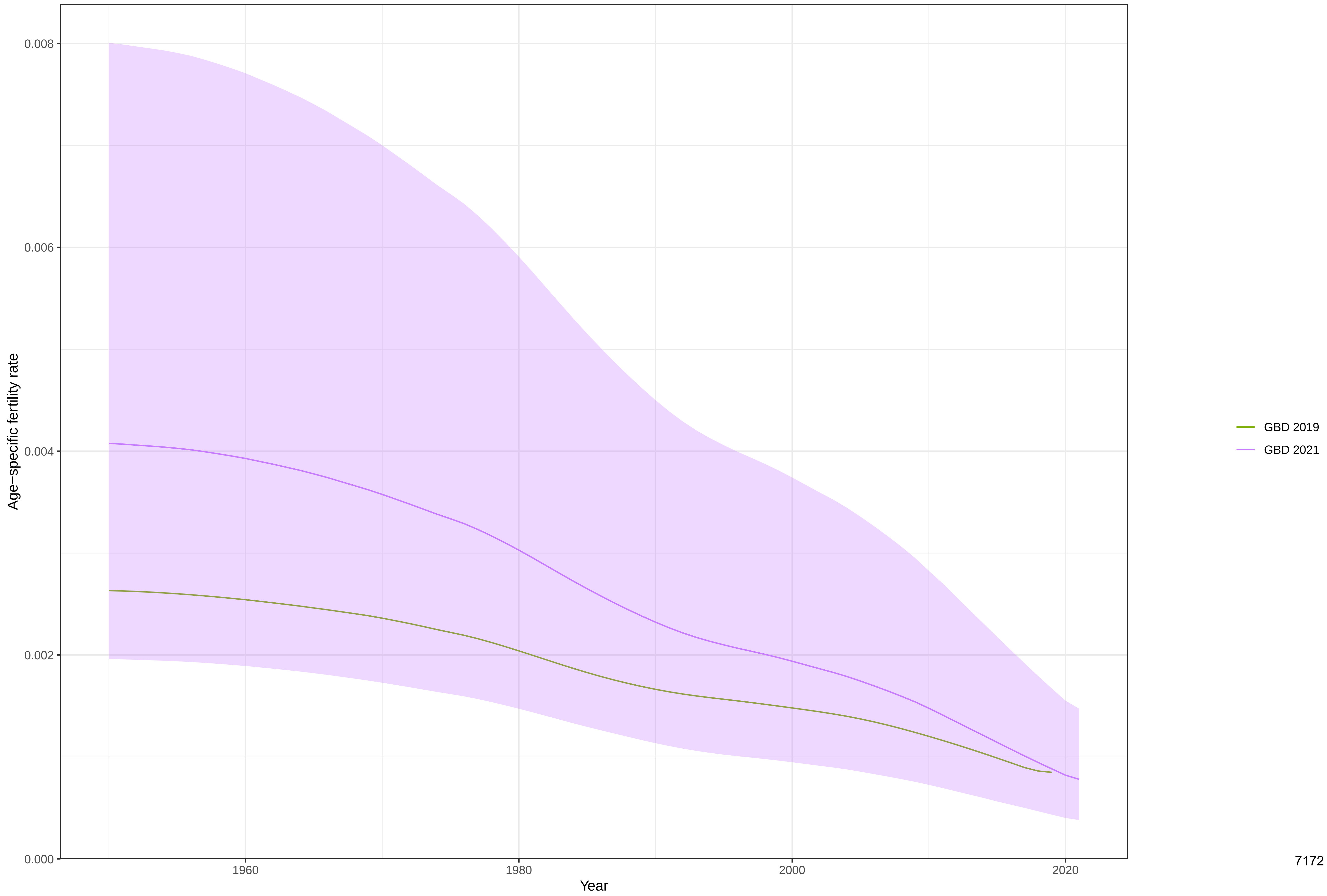

Age−specific fertility fitted trend lines, 1950−2021: Nairobi, Ages: 15−19
Data points enclosed with a diamond are reference sources

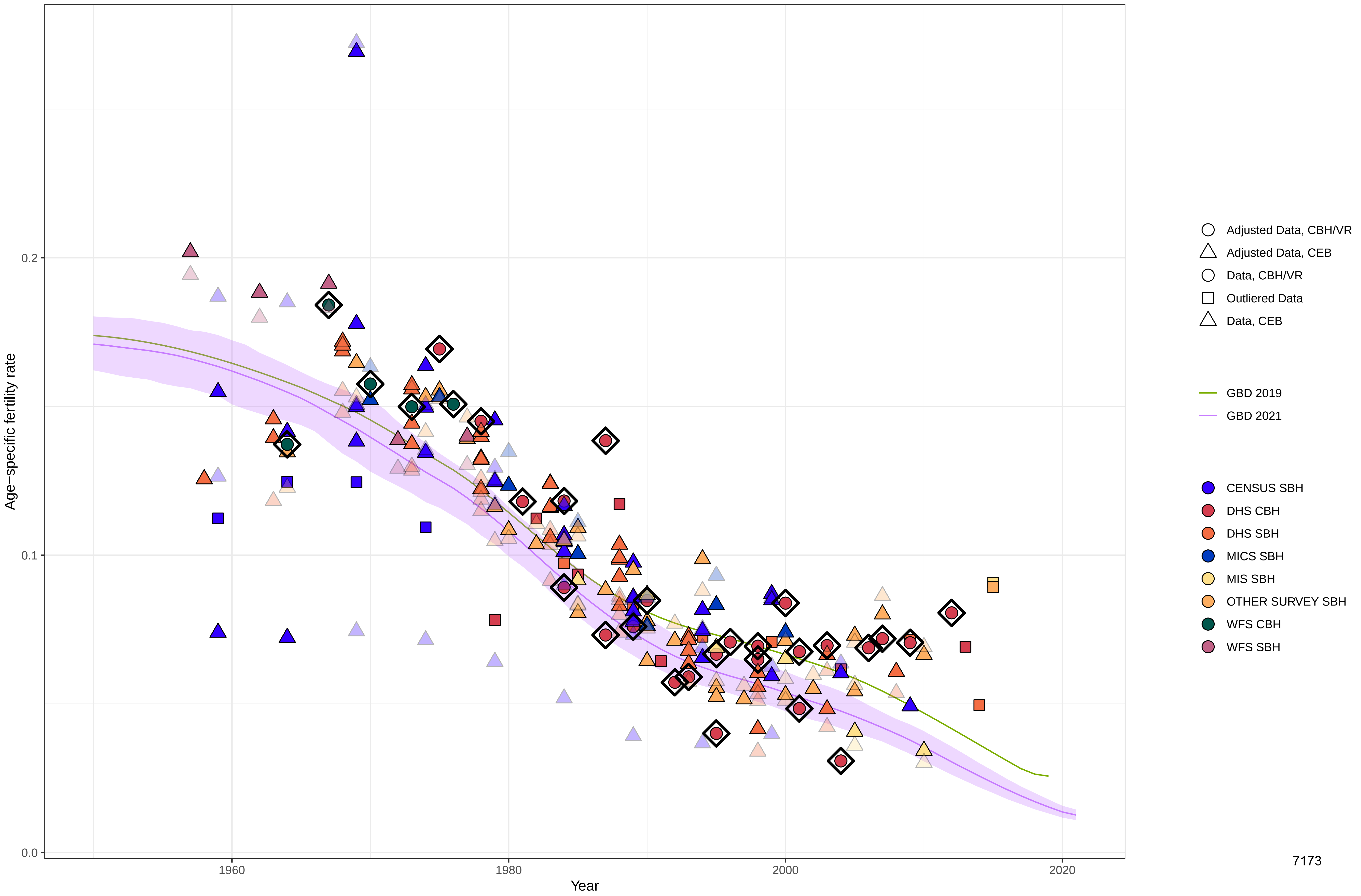

Age−specific fertility fitted trend lines, 1950−2021: Nairobi, Ages: 20−24
Data points enclosed with a diamond are reference sources

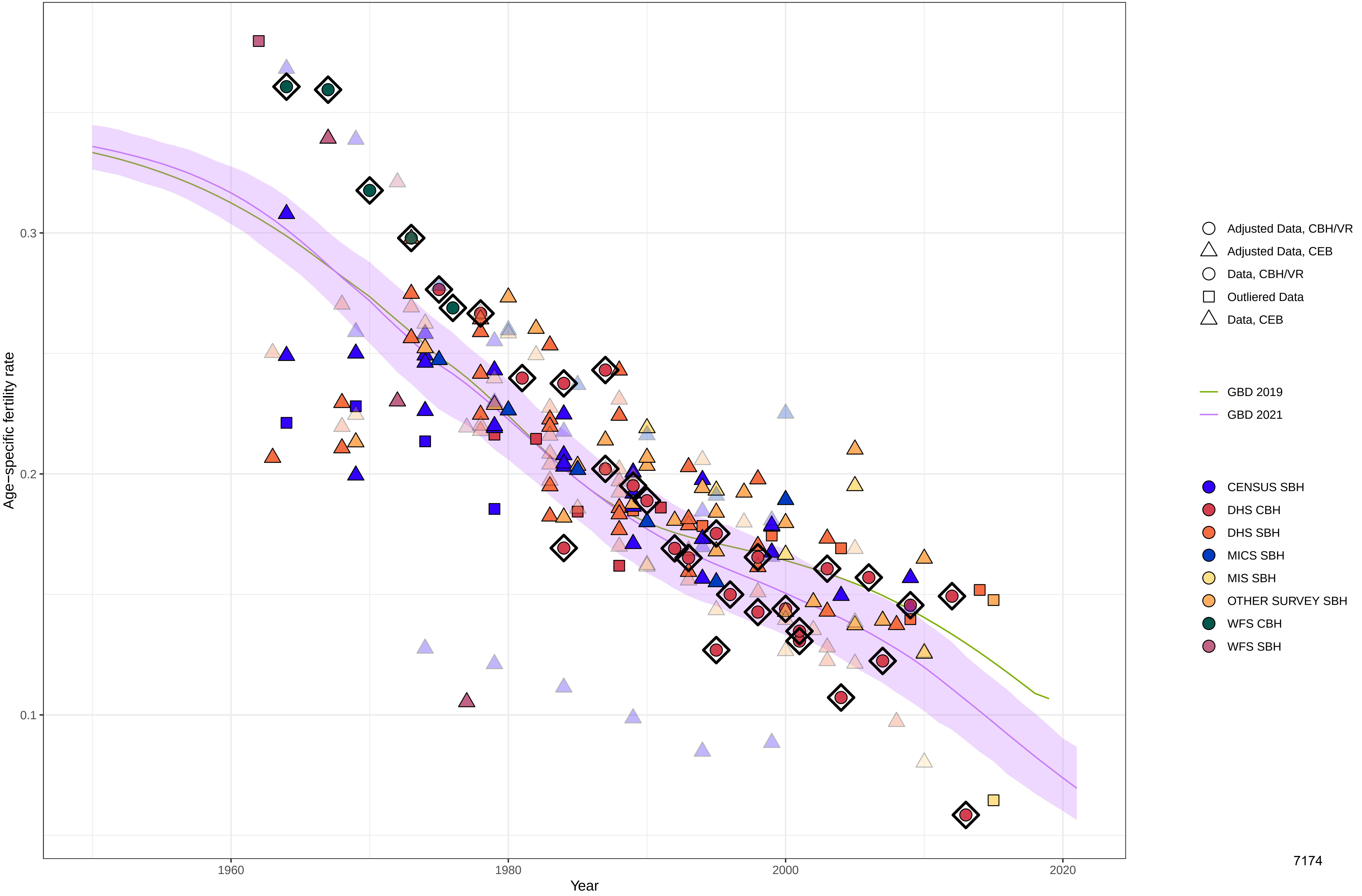

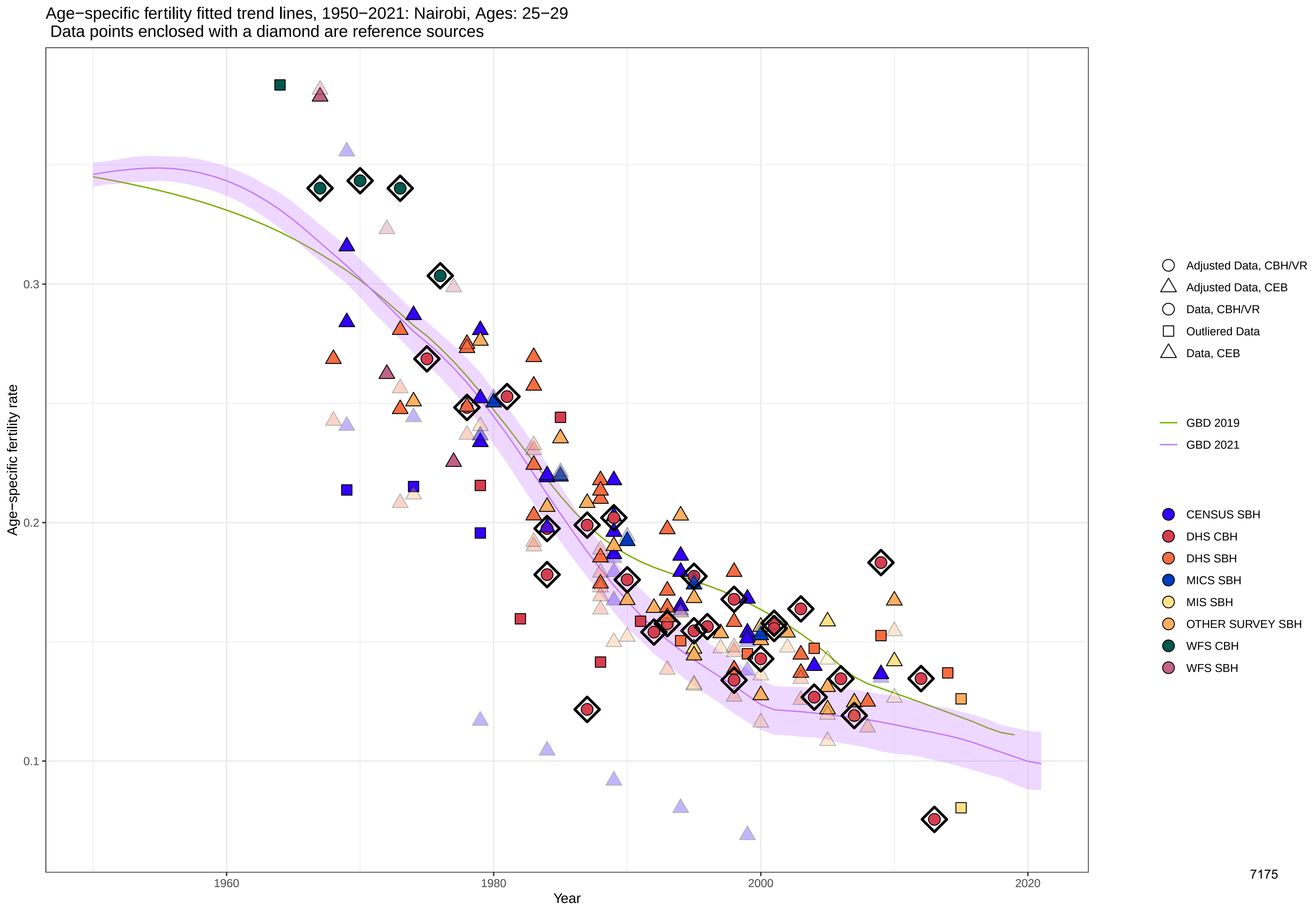

Age−specific fertility fitted trend lines, 1950−2021: Nairobi, Ages: 25−29
Data points enclosed with a diamond are reference sources
Age−specific fertility rate
Year
0.1
0.2
0.3
1960
1980
2000
2020
Adjusted Data, CBH/VR
Adjusted Data, CEB
Data, CBH/VR
Outliered Data
Data, CEB
GBD 2019
GBD 2021
CENSUS SBH
DHS CBH
DHS SBH
MICS SBH
MIS SBH
OTHER SURVEY SBH
WFS CBH
WFS SBH

Age−specific fertility fitted trend lines, 1950−2021: Nairobi, Ages: 30−34
Data points enclosed with a diamond are reference sources

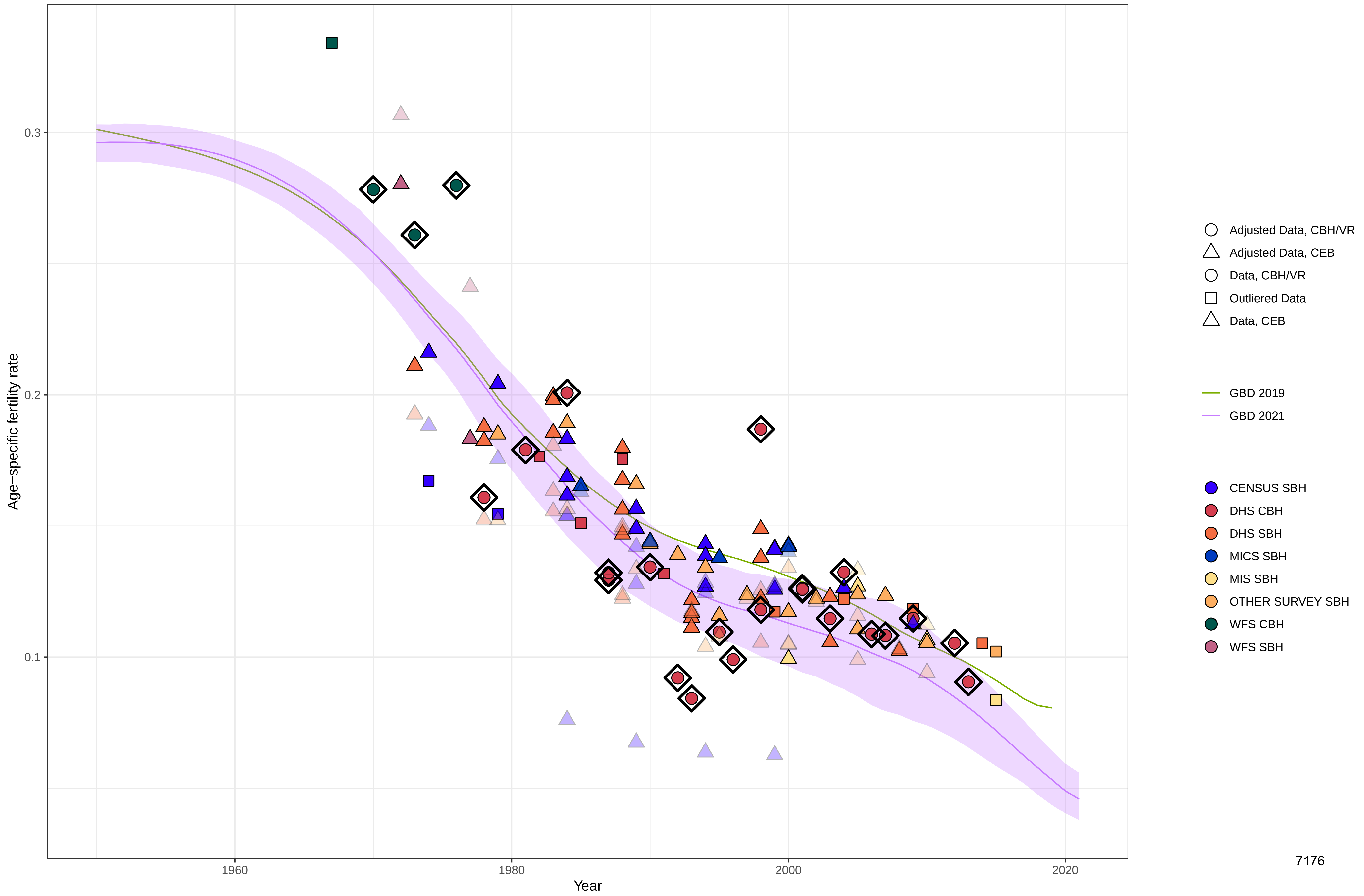

7176

Age−specific fertility fitted trend lines, 1950−2021: Nairobi, Ages: 35−39
Data points enclosed with a diamond are reference sources

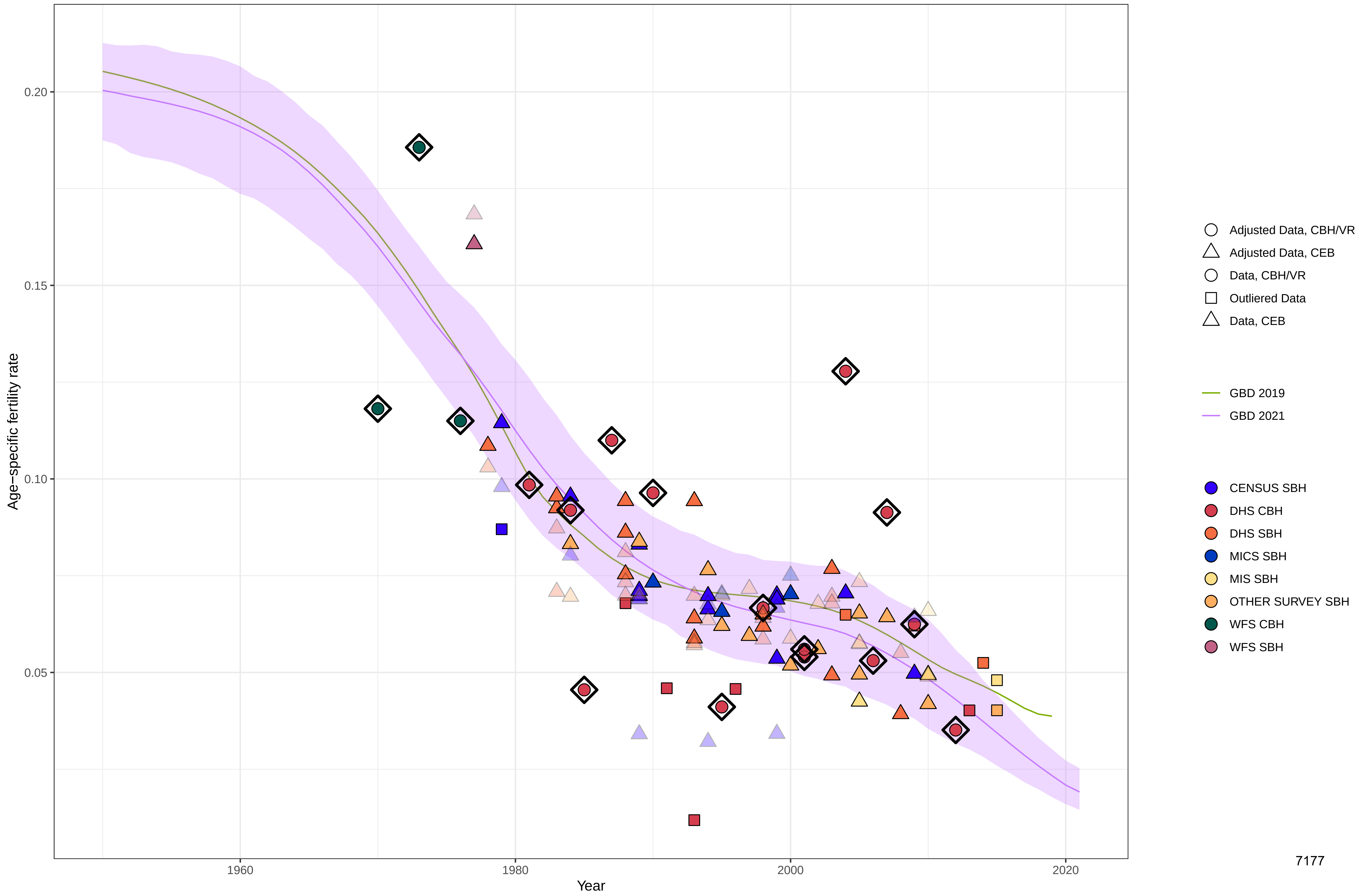

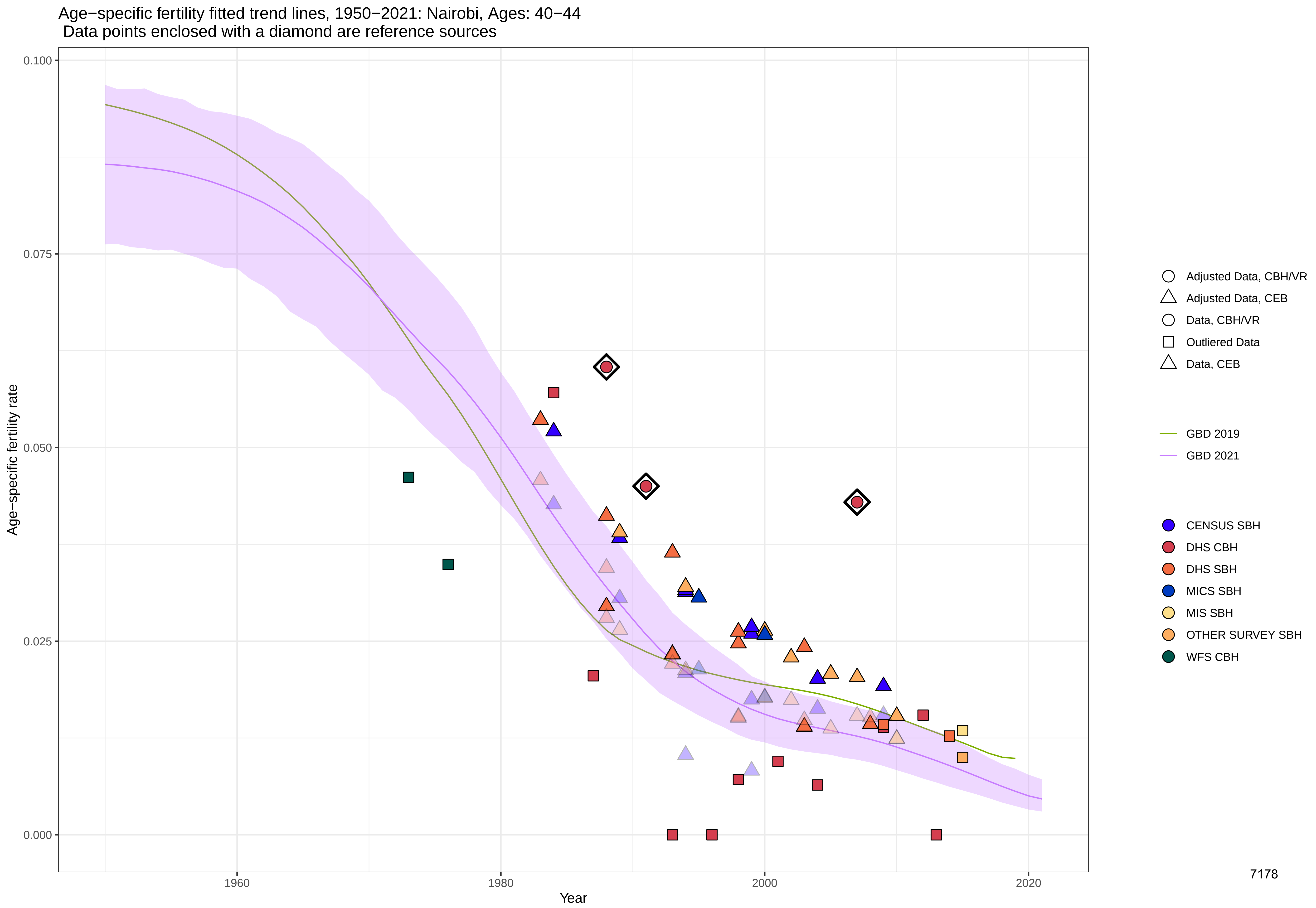

Age−specific fertility fitted trend lines, 1950−2021: Nairobi, Ages: 40−44
Data points enclosed with a diamond are reference sources
Age−specific fertility rate
Year
0.100
0.075
0.050
0.025
0.000
1960
1980
2000
2020
Adjusted Data, CBH/VR
Adjusted Data, CEB
Data, CBH/VR
Outliered Data
Data, CEB
GBD 2019
GBD 2021
CENSUS SBH
DHS CBH
DHS SBH
MICS SBH
MIS SBH
OTHER SURVEY SBH
WFS CBH

Age−specific fertility fitted trend lines, 1950−2021: Nairobi, Ages: 45−49
Data points enclosed with a diamond are reference sources

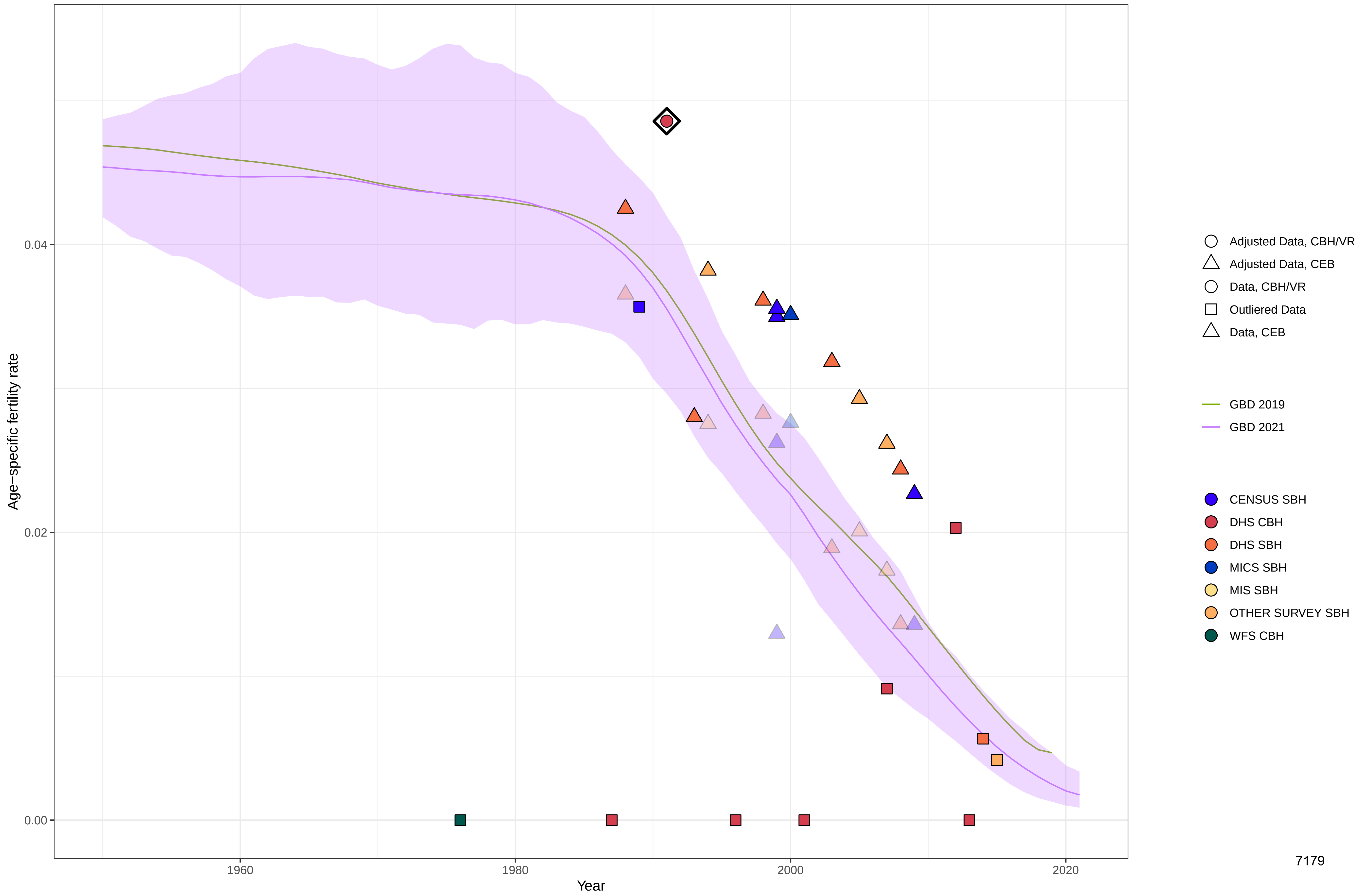

7179

Age−specific fertility fitted trend lines, 1950−2021: Nairobi, Ages: 50−54
Data points enclosed with a diamond are reference sources

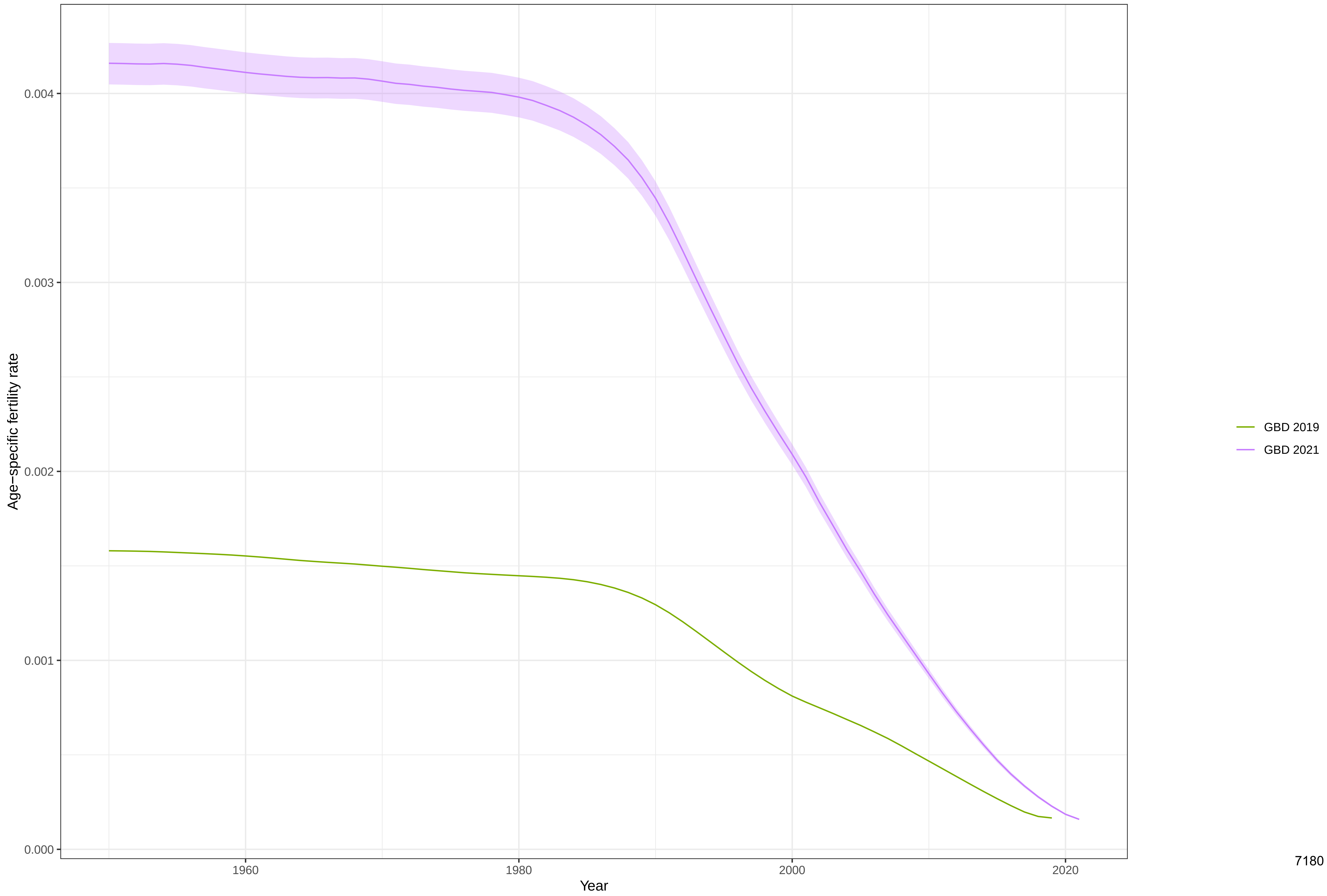

Total fertility rate fitted trend lines, 1950−2021: Nairobi TFR
Data points enclosed with a diamond are reference sources

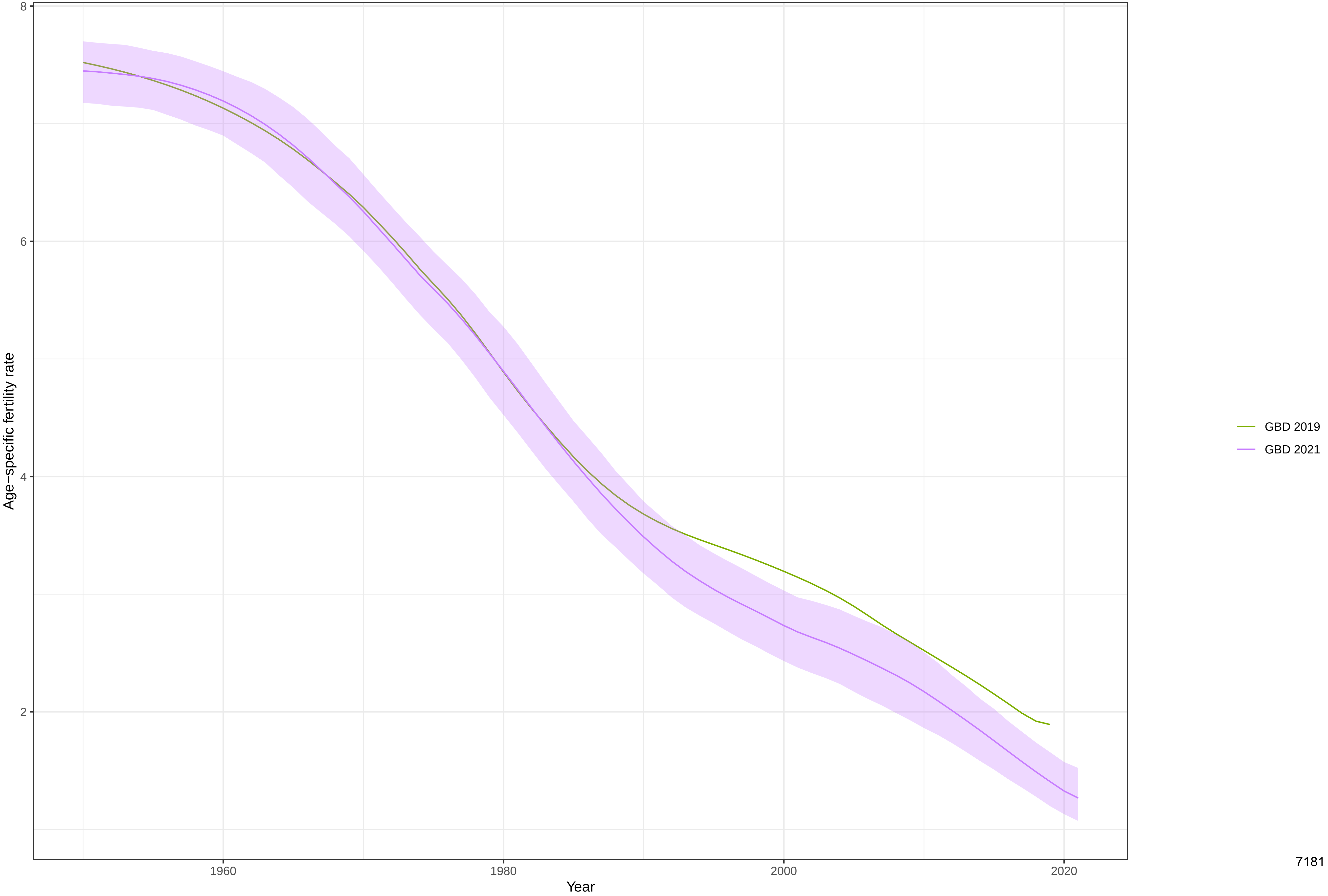

Age−specific fertility fitted trend lines, 1950−2021: Nakuru, Ages: 10−14
Data points enclosed with a diamond are reference sources

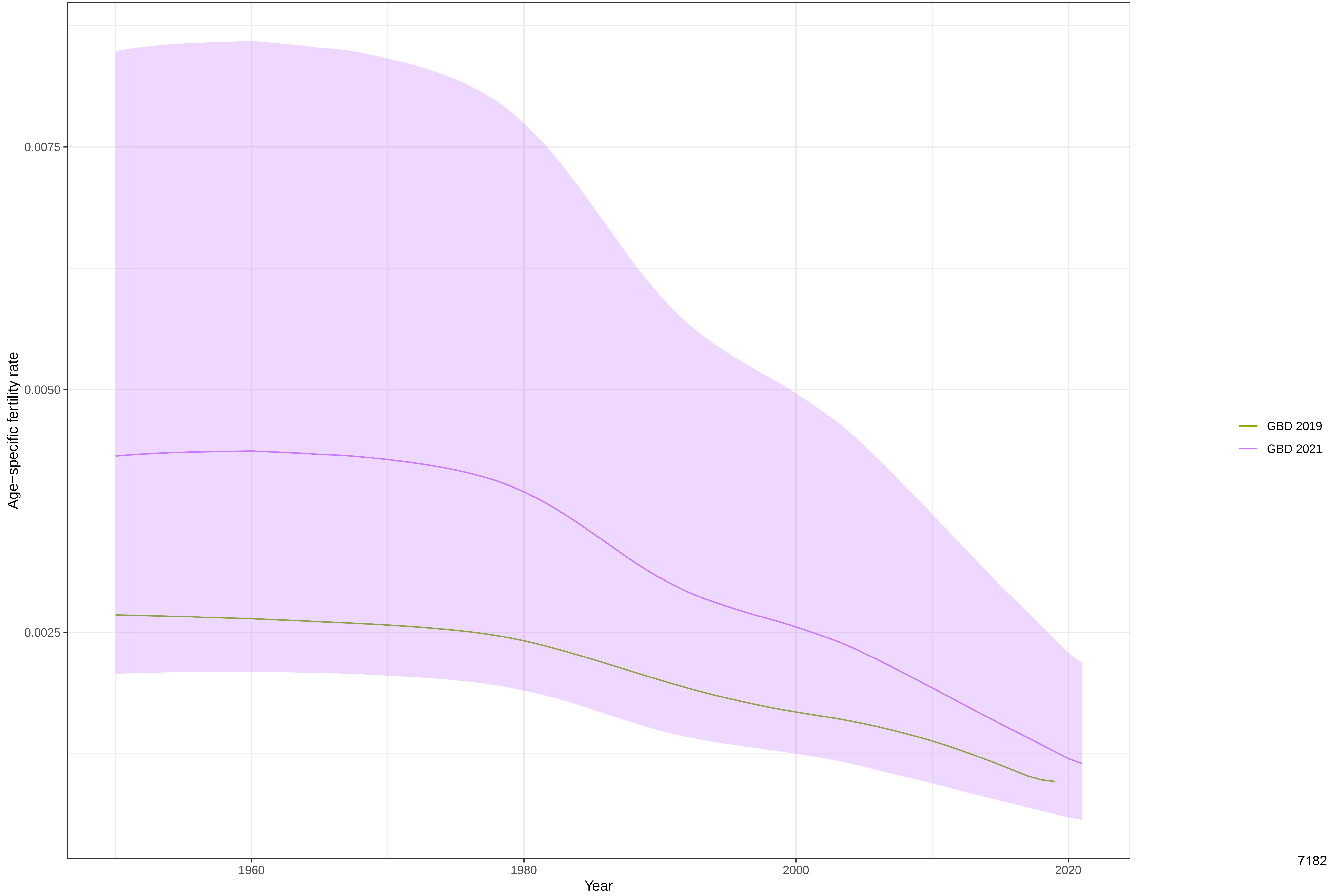

Age−specific fertility fitted trend lines, 1950−2021: Nakuru, Ages: 15−19
Data points enclosed with a diamond are reference sources

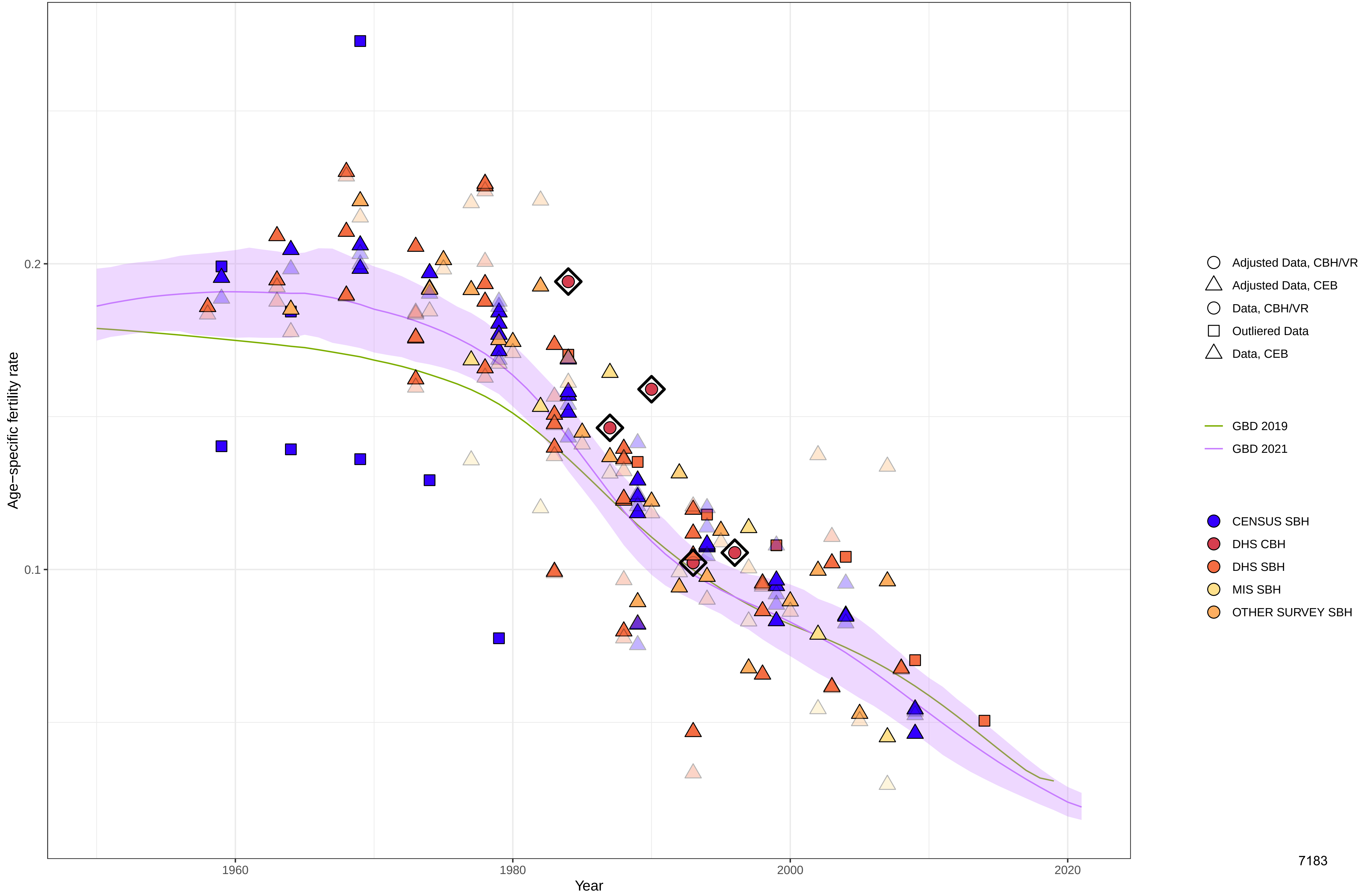

Age−specific fertility fitted trend lines, 1950−2021: Nakuru, Ages: 20−24
Data points enclosed with a diamond are reference sources

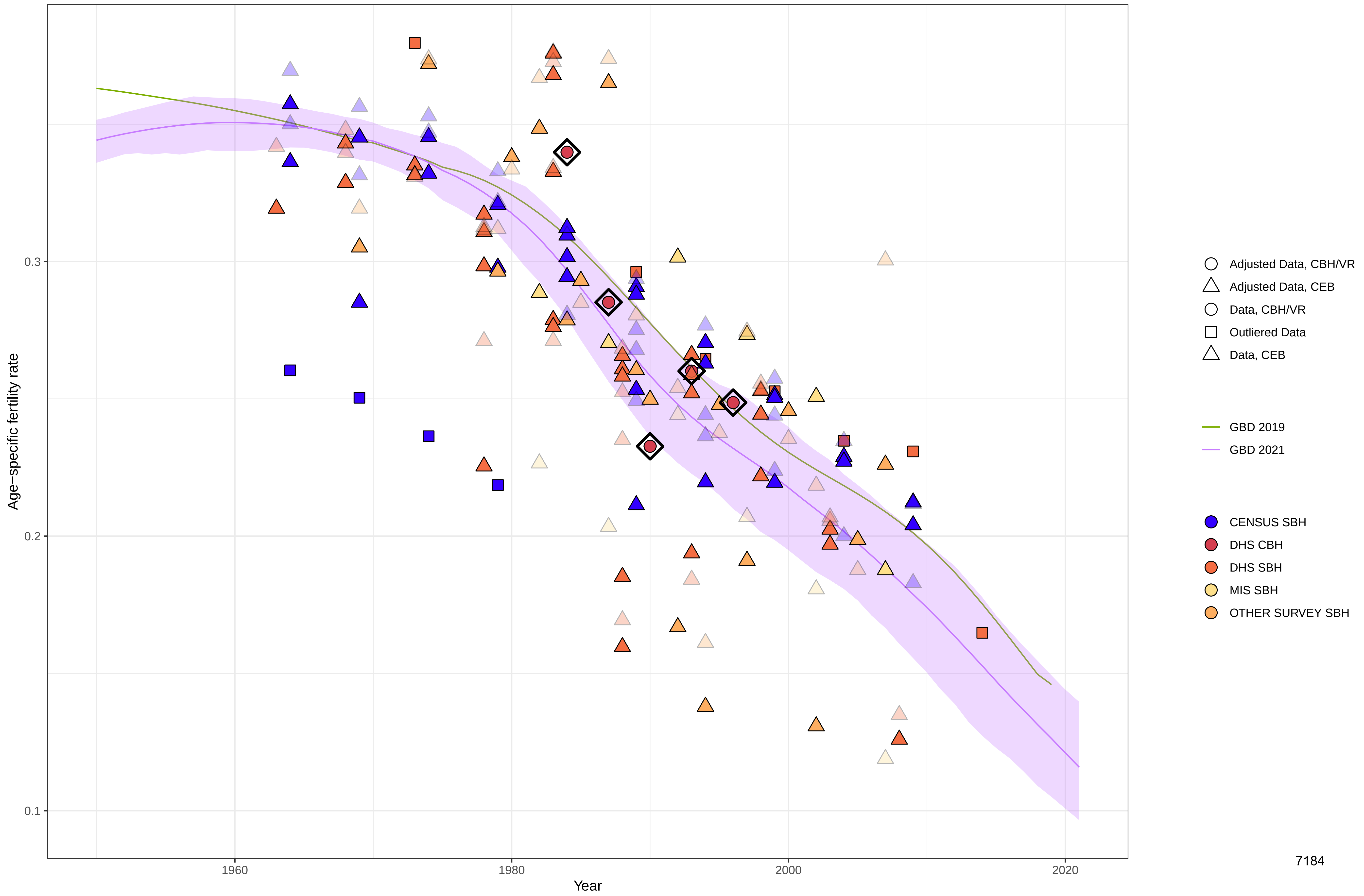

Age−specific fertility fitted trend lines, 1950−2021: Nakuru, Ages: 25−29
Data points enclosed with a diamond are reference sources

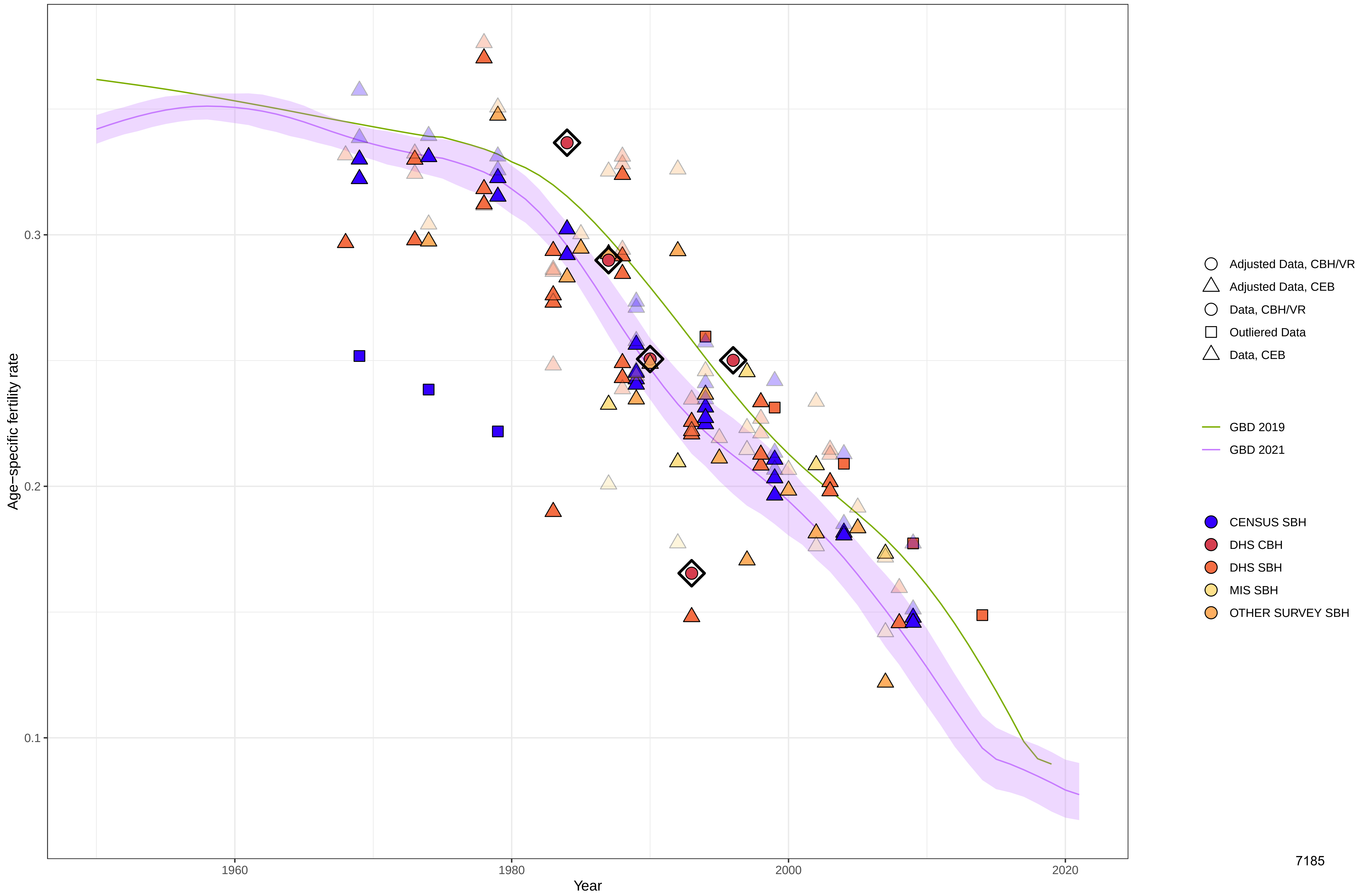

Age−specific fertility fitted trend lines, 1950−2021: Nakuru, Ages: 30−34
Data points enclosed with a diamond are reference sources

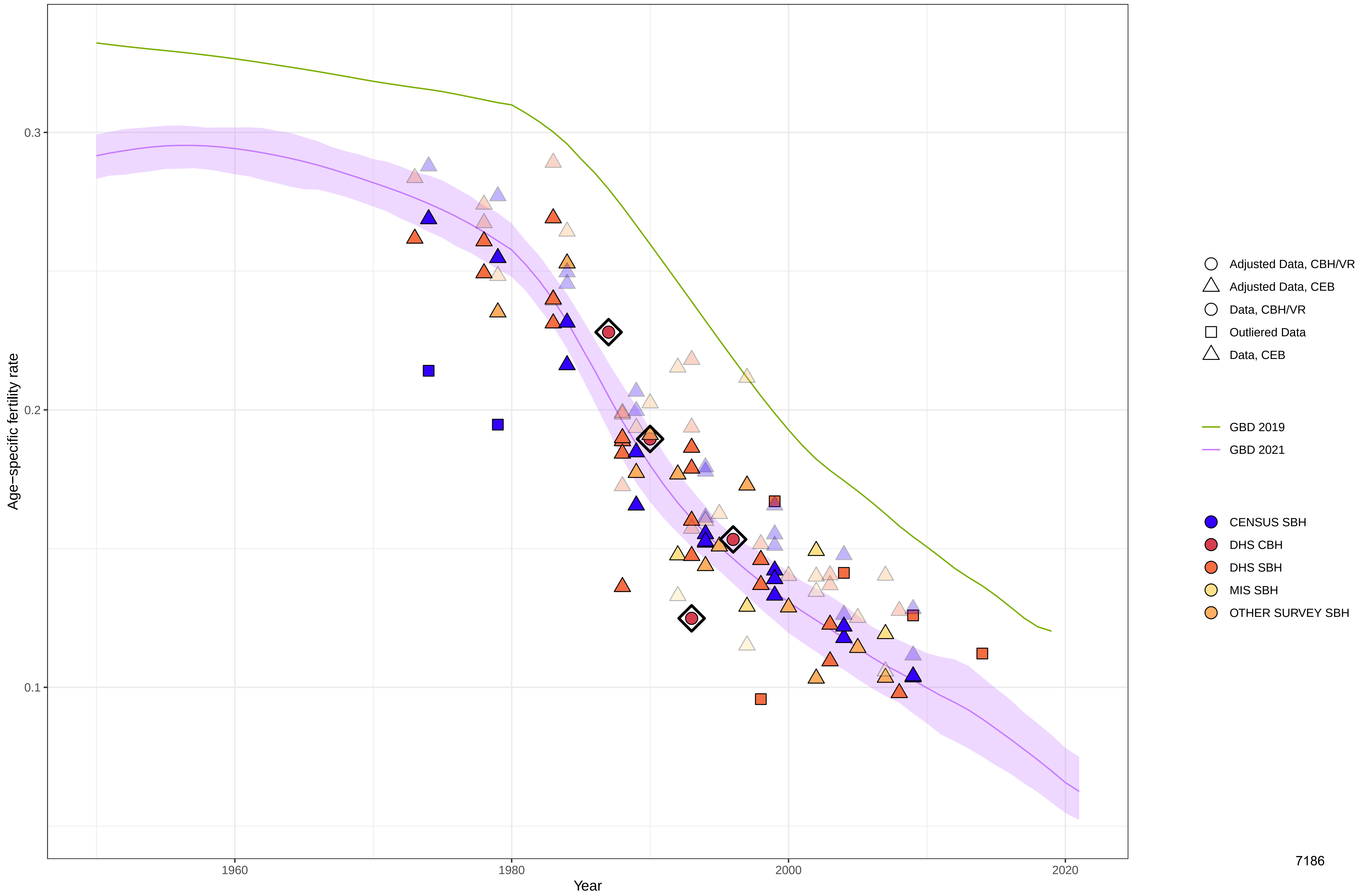

7186

Age−specific fertility fitted trend lines, 1950−2021: Nakuru, Ages: 35−39
Data points enclosed with a diamond are reference sources

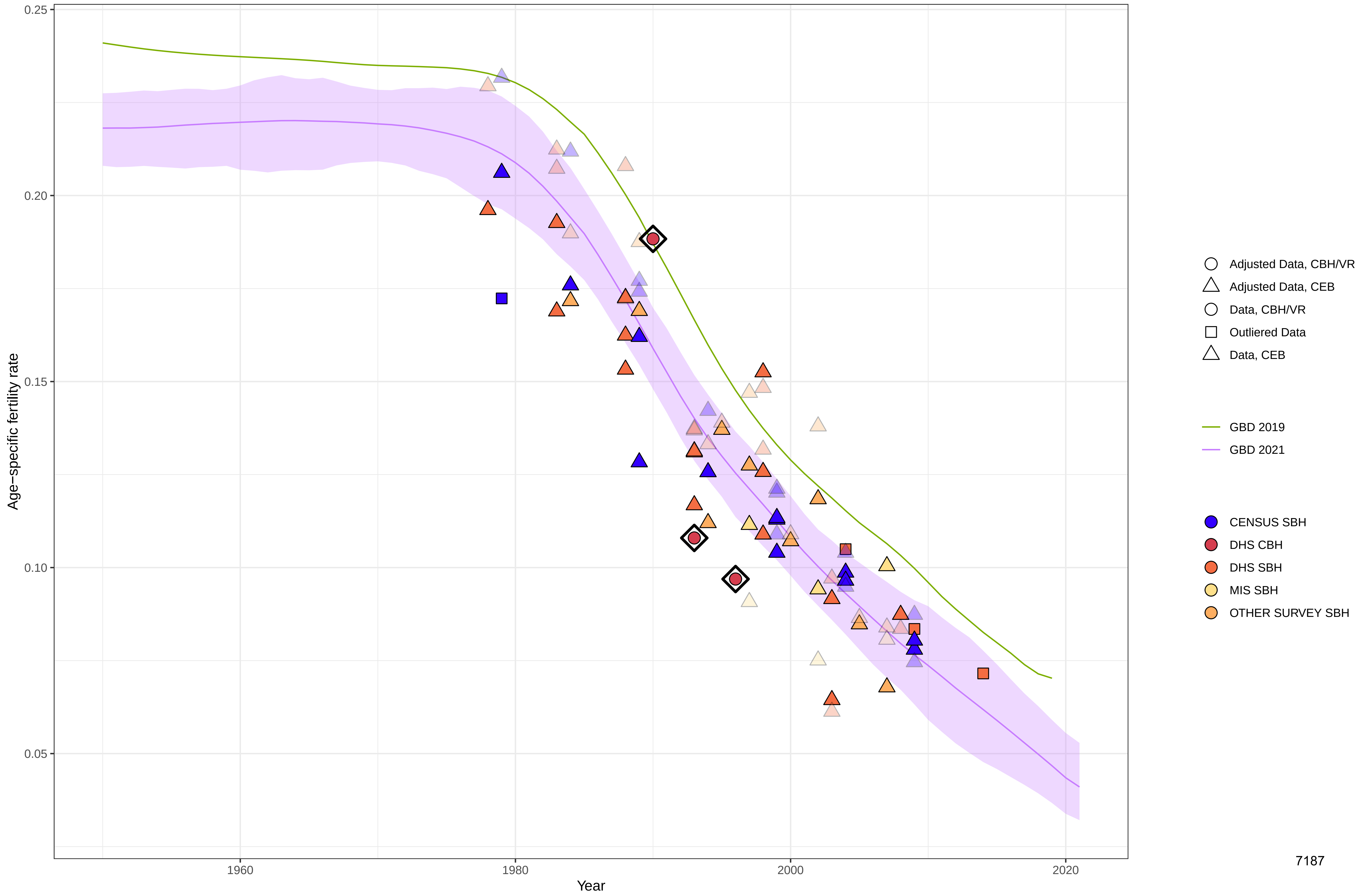

Age−specific fertility fitted trend lines, 1950−2021: Nakuru, Ages: 40−44
Data points enclosed with a diamond are reference sources

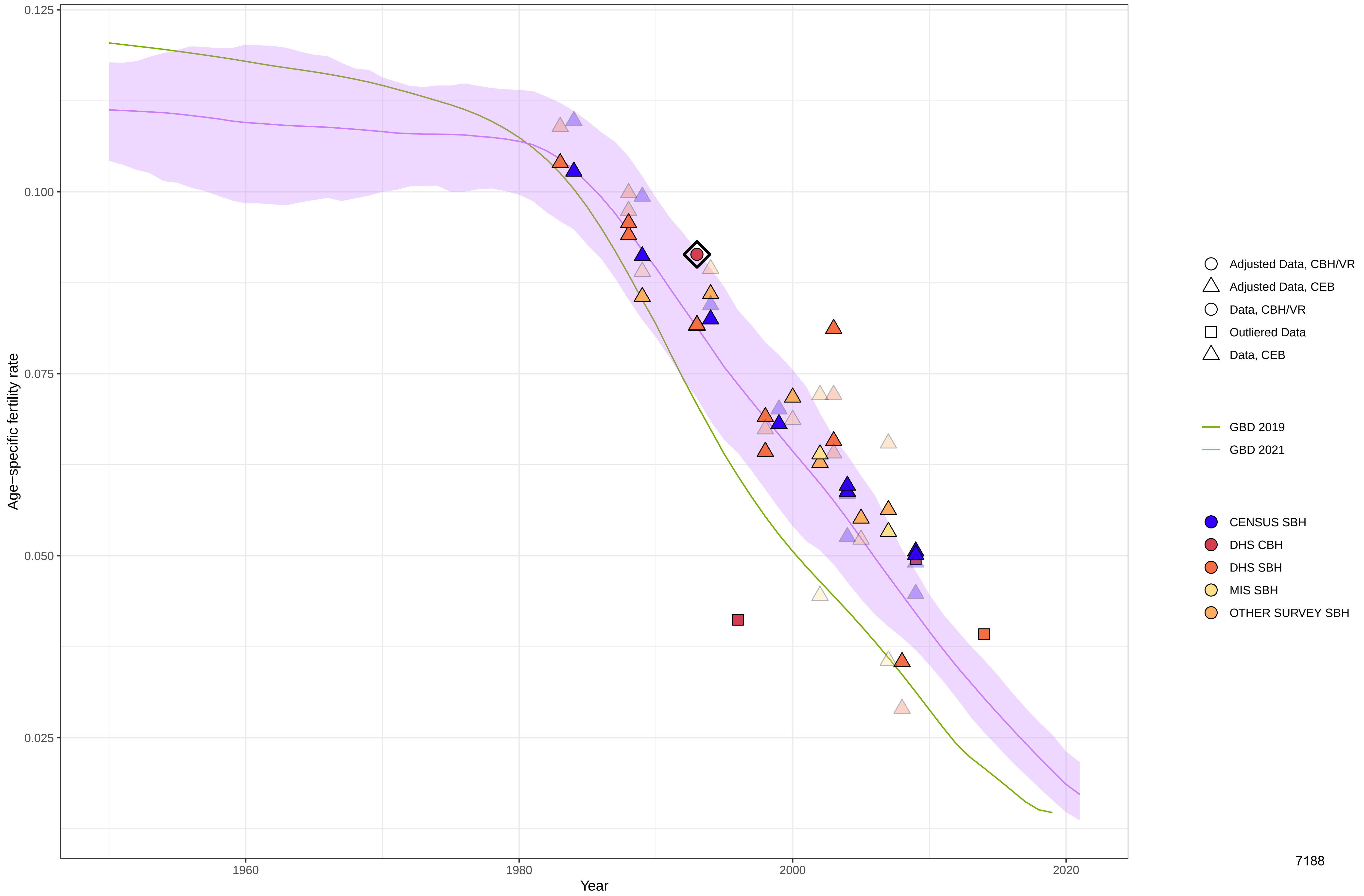

Age−specific fertility fitted trend lines, 1950−2021: Nakuru, Ages: 45−49
Data points enclosed with a diamond are reference sources

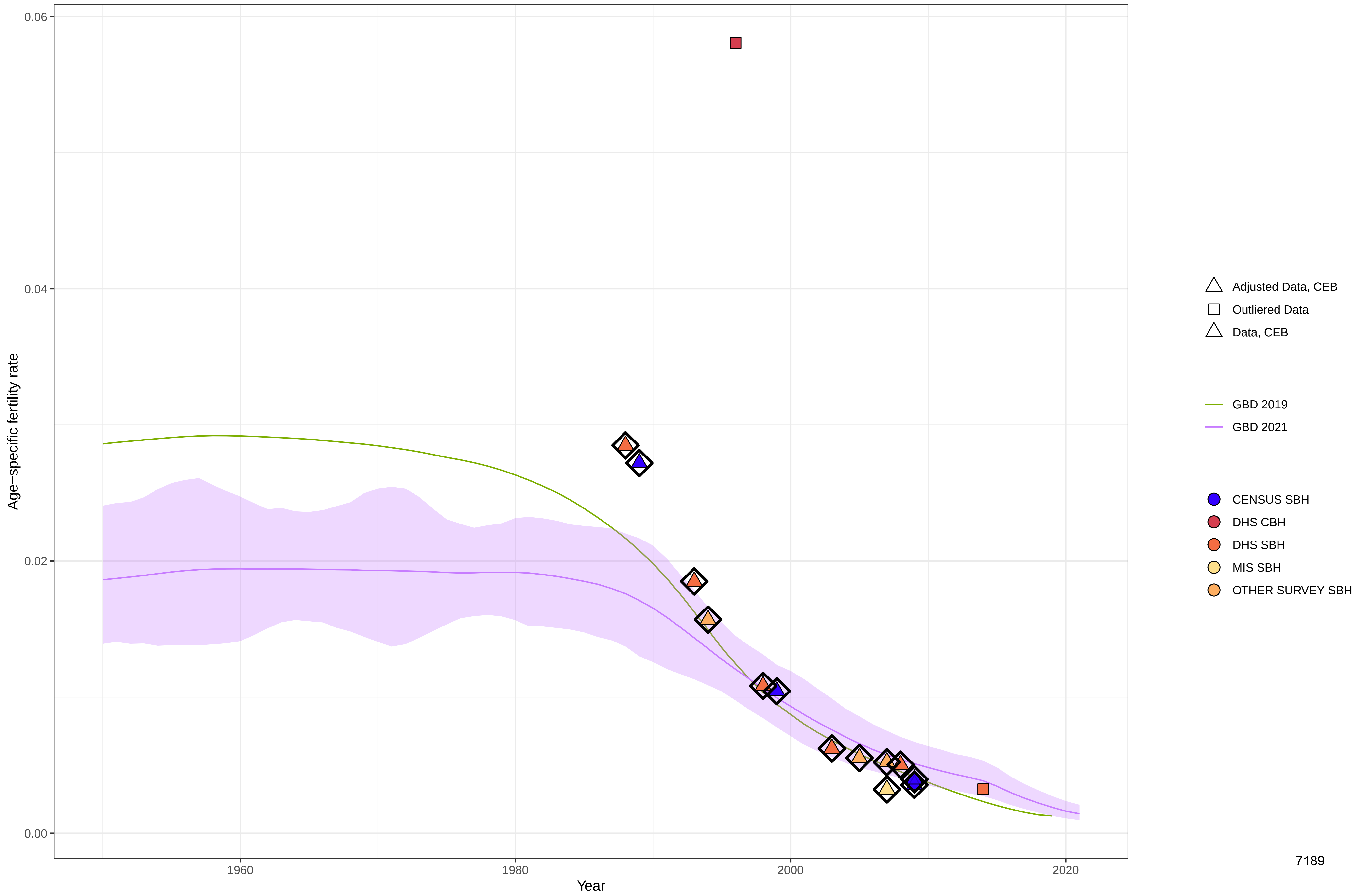

Age−specific fertility fitted trend lines, 1950−2021: Nakuru, Ages: 50−54
Data points enclosed with a diamond are reference sources

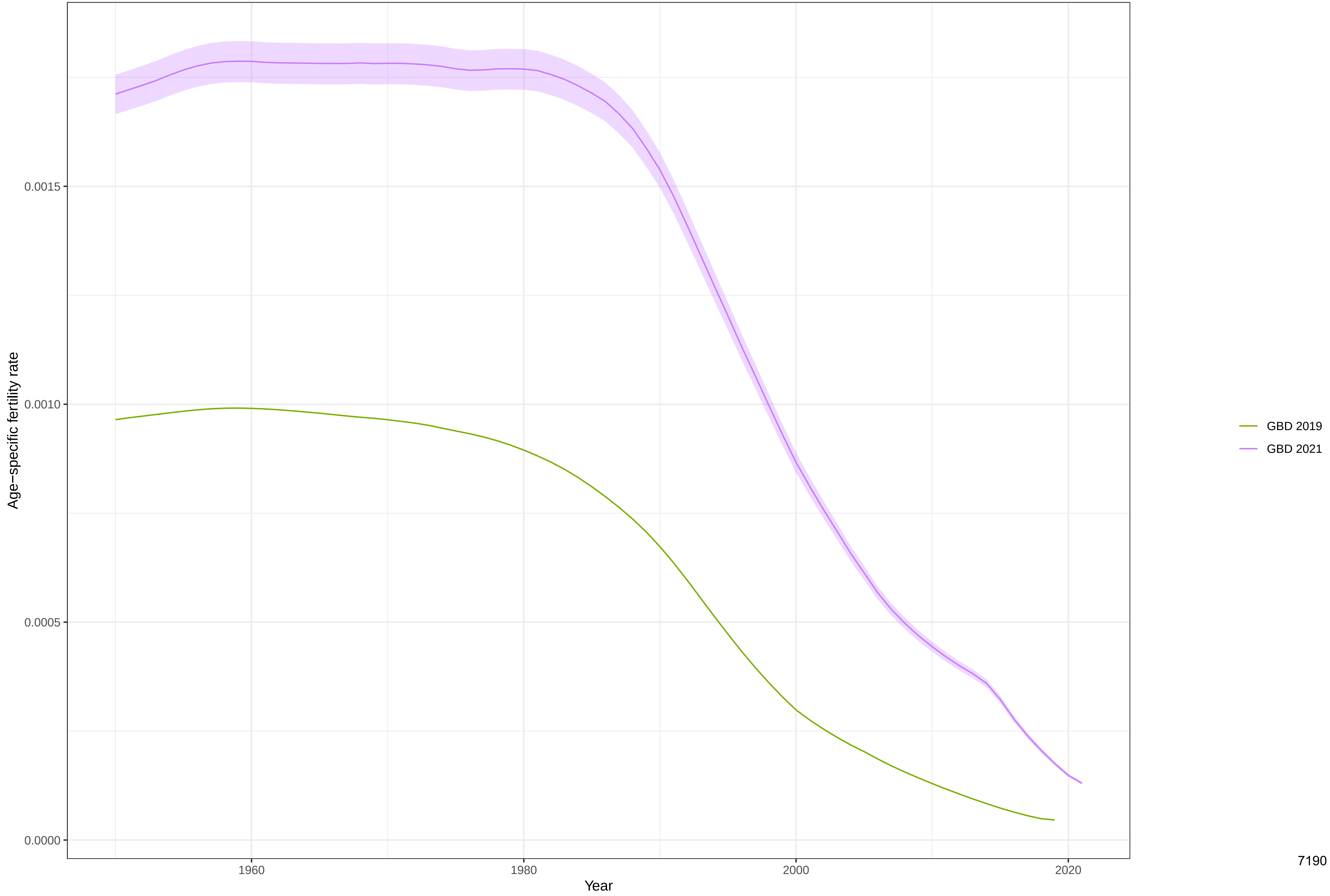

Total fertility rate fitted trend lines, 1950−2021: Nakuru TFR
Data points enclosed with a diamond are reference sources

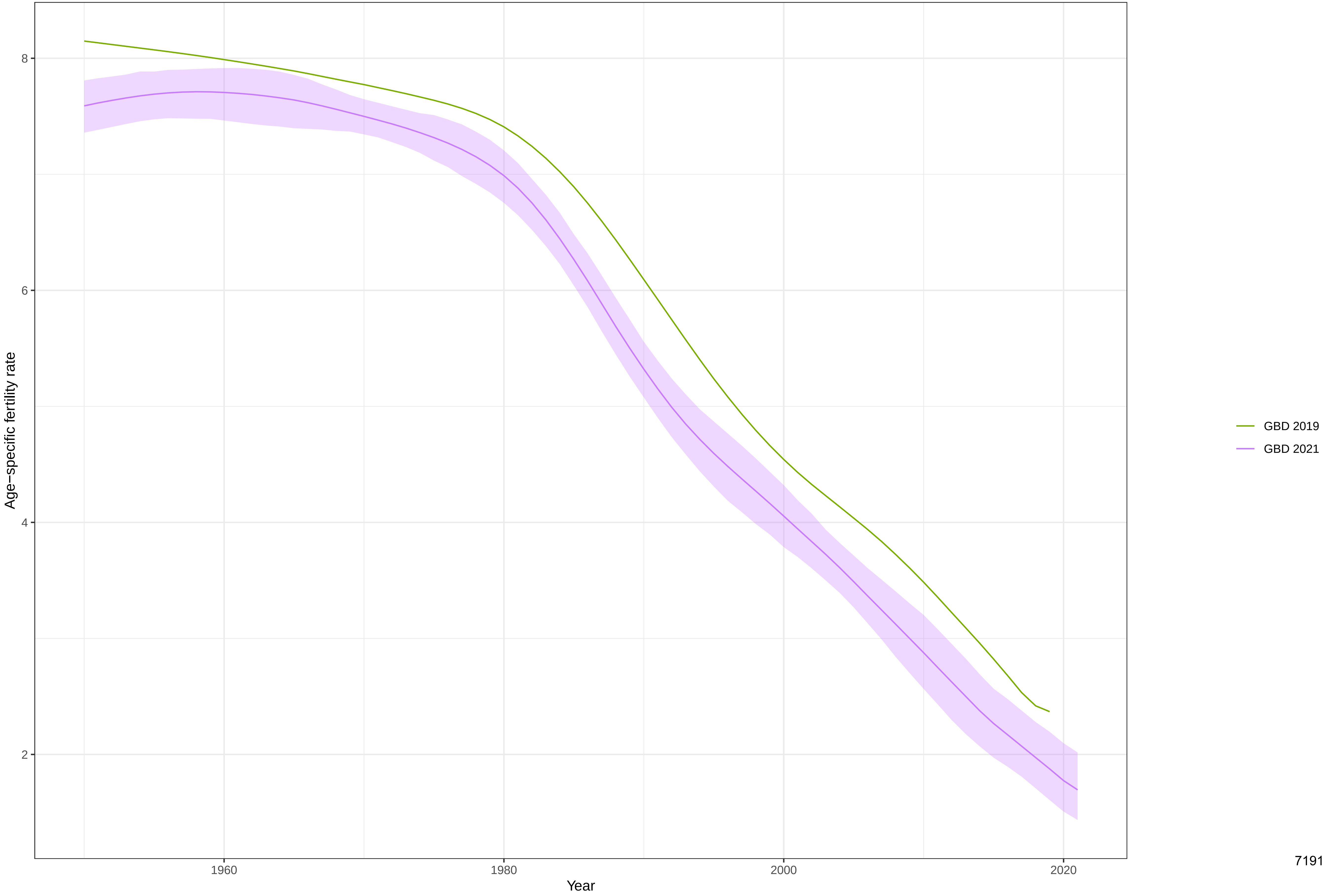

Age−specific fertility fitted trend lines, 1950−2021: Nandi, Ages: 10−14
Data points enclosed with a diamond are reference sources

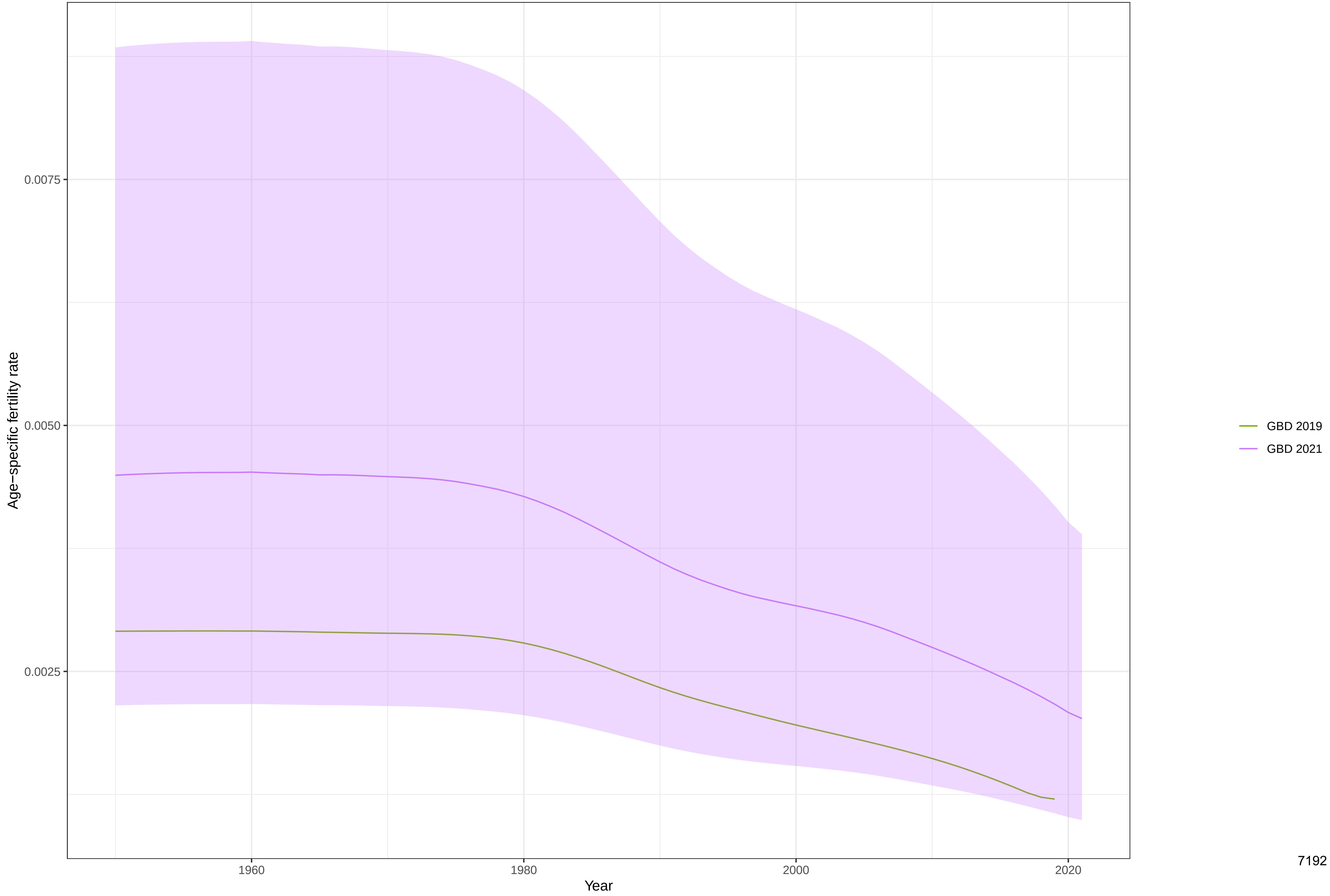

Age−specific fertility fitted trend lines, 1950−2021: Nandi, Ages: 15−19
Data points enclosed with a diamond are reference sources

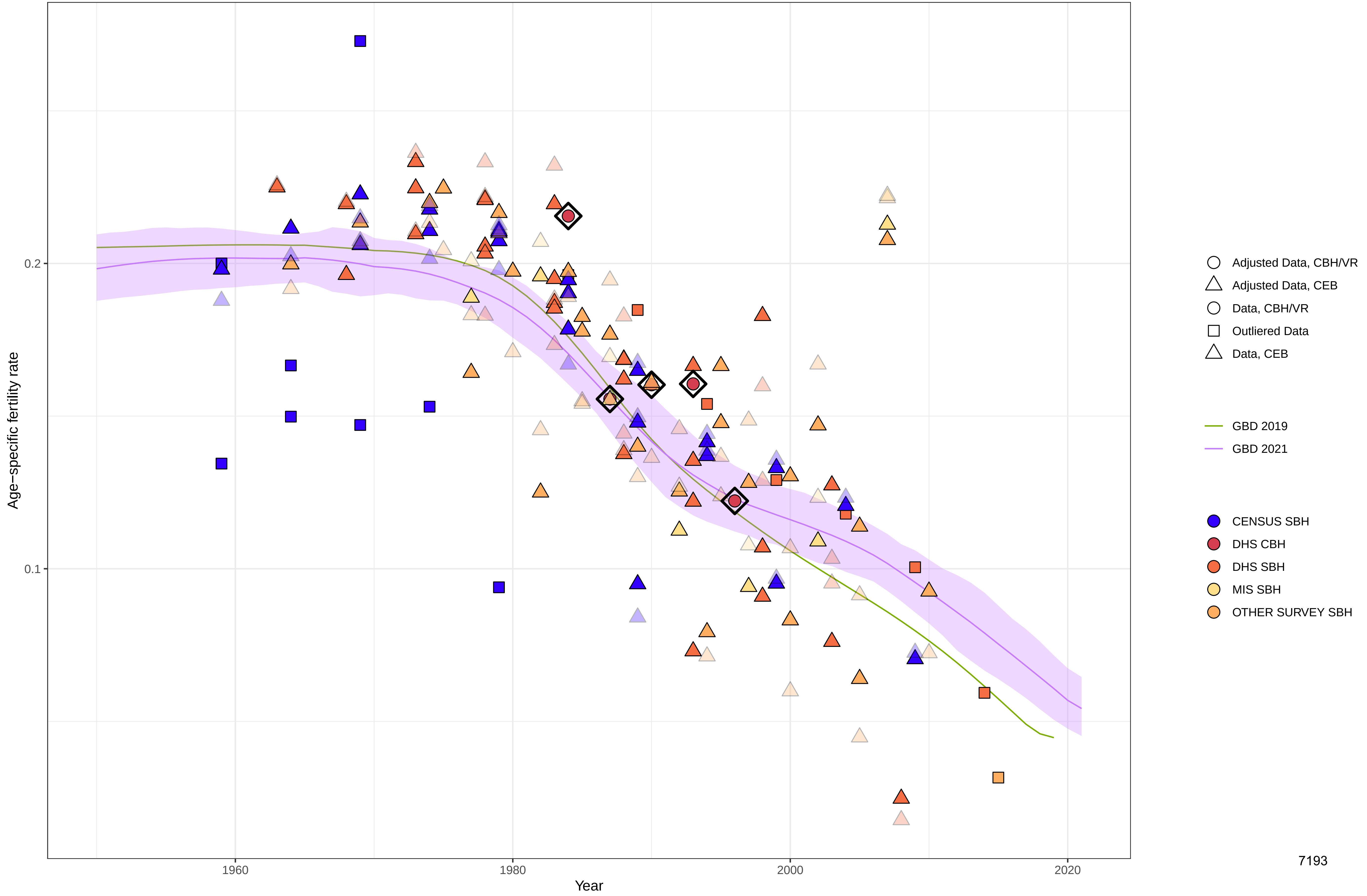

Age−specific fertility fitted trend lines, 1950−2021: Nandi, Ages: 20−24
Data points enclosed with a diamond are reference sources

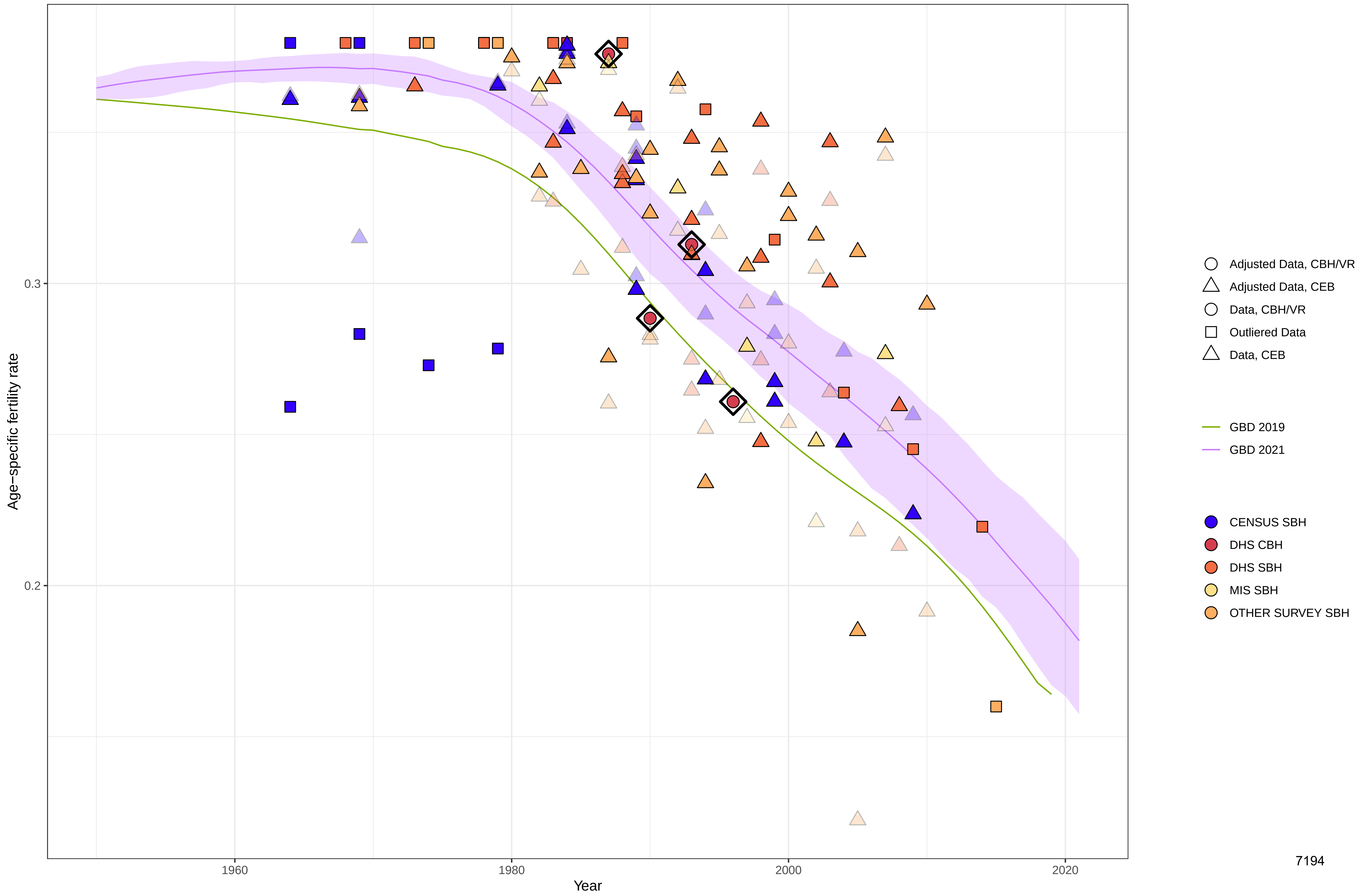

Age−specific fertility fitted trend lines, 1950−2021: Nandi, Ages: 25−29
Data points enclosed with a diamond are reference sources

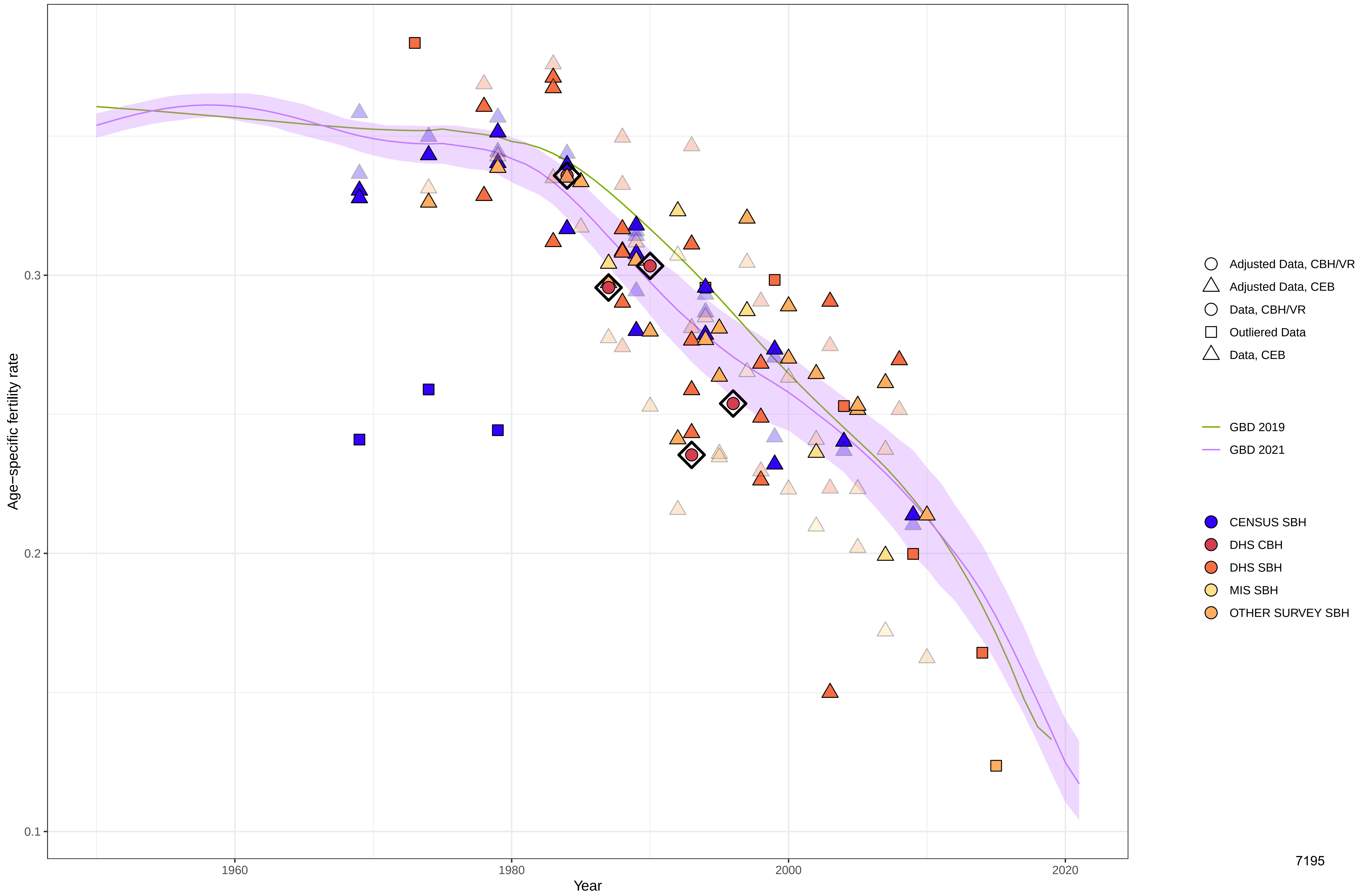

Age−specific fertility fitted trend lines, 1950−2021: Nandi, Ages: 30−34
Data points enclosed with a diamond are reference sources

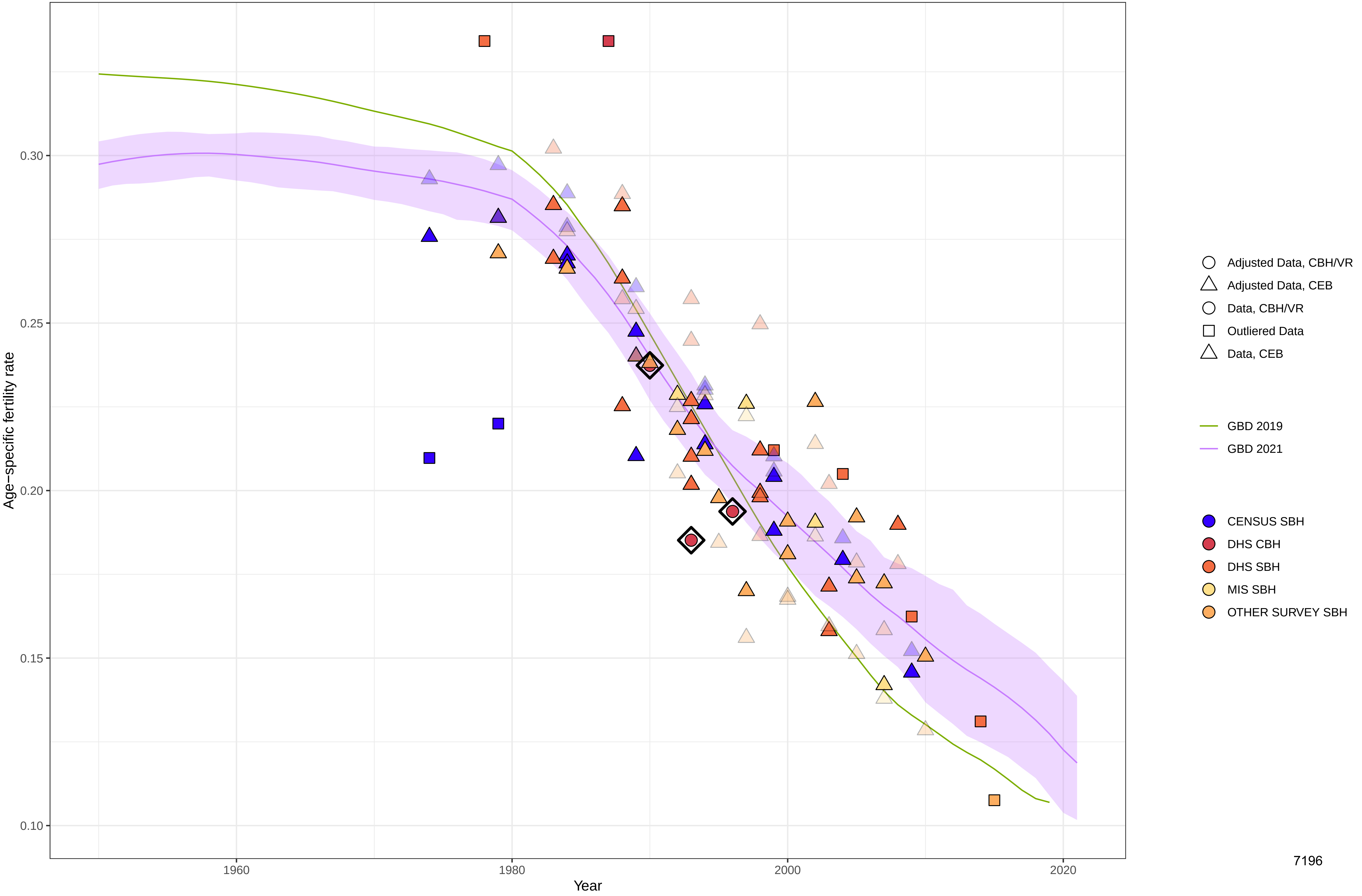

Age−specific fertility fitted trend lines, 1950−2021: Nandi, Ages: 35−39
Data points enclosed with a diamond are reference sources

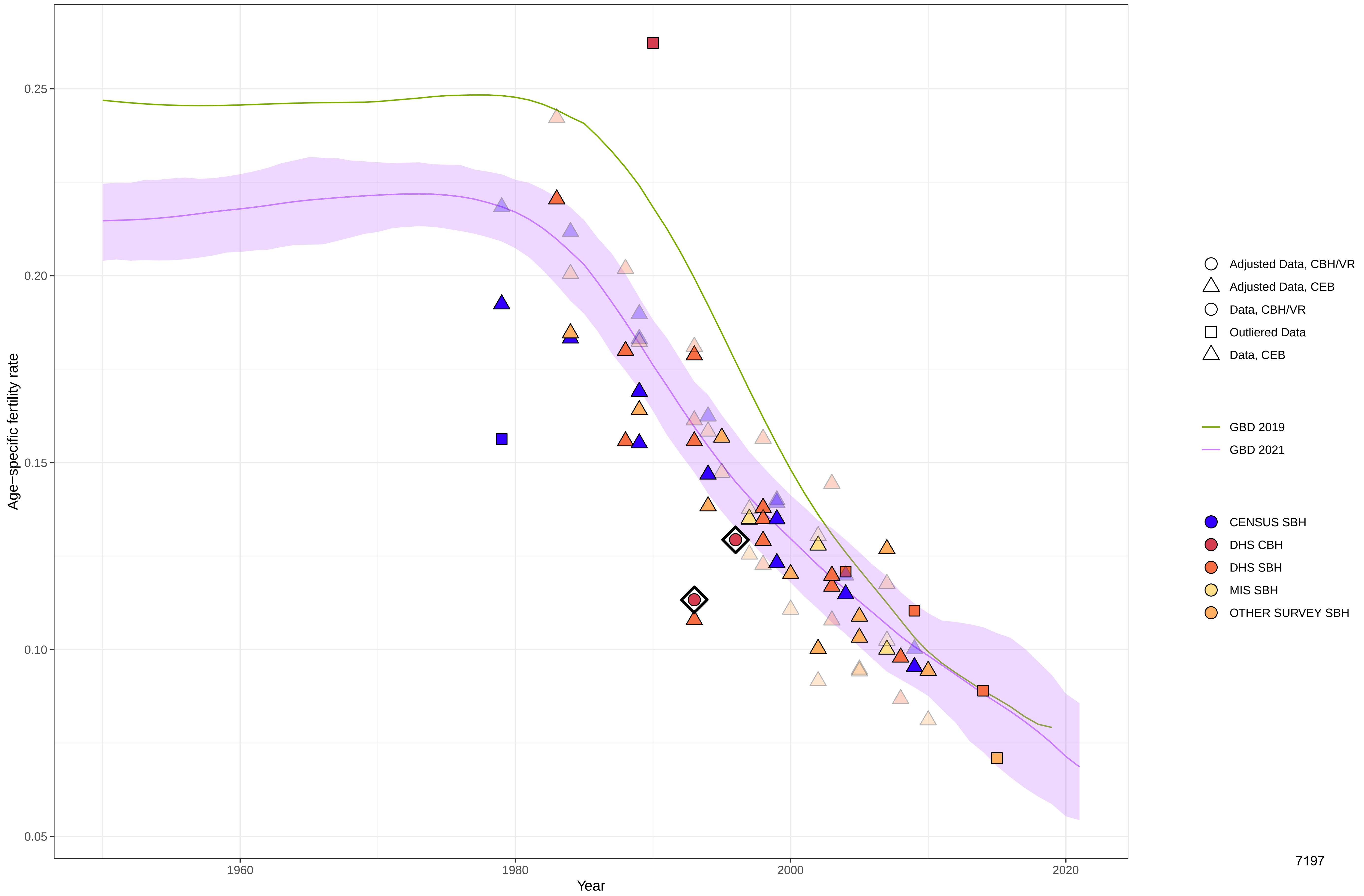

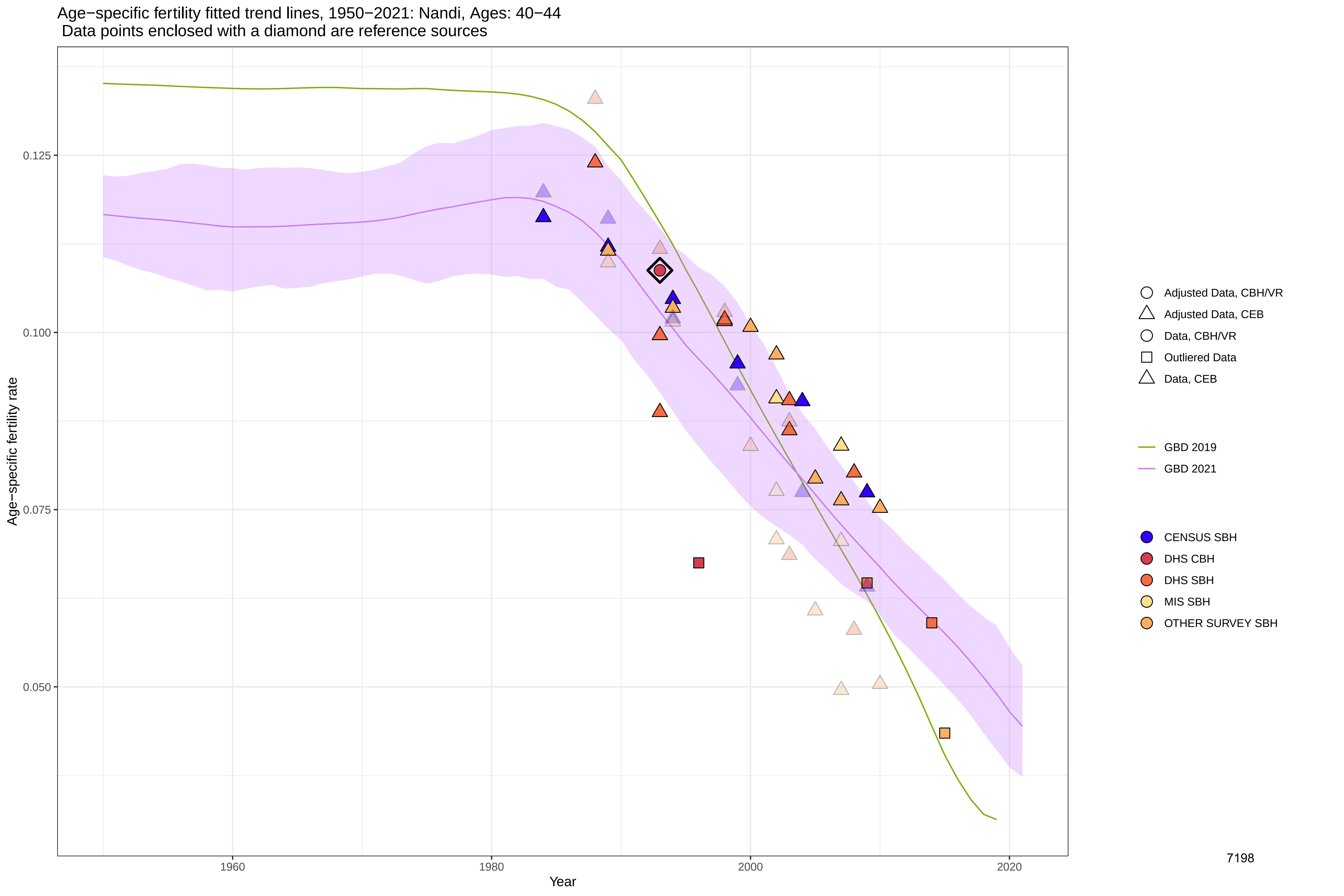

Age−specific fertility fitted trend lines, 1950−2021: Nandi, Ages: 40−44
Data points enclosed with a diamond are reference sources
Age−specific fertility rate
0.125
0.100
0.075
0.050
1960
1980
2000
2020
Year
Adjusted Data, CBH/VR
Adjusted Data, CEB
Data, CBH/VR
Outliered Data
Data, CEB
GBD 2019
GBD 2021
CENSUS SBH
DHS CBH
DHS SBH
MIS SBH
OTHER SURVEY SBH

Age−specific fertility fitted trend lines, 1950−2021: Nandi, Ages: 45−49
Data points enclosed with a diamond are reference sources

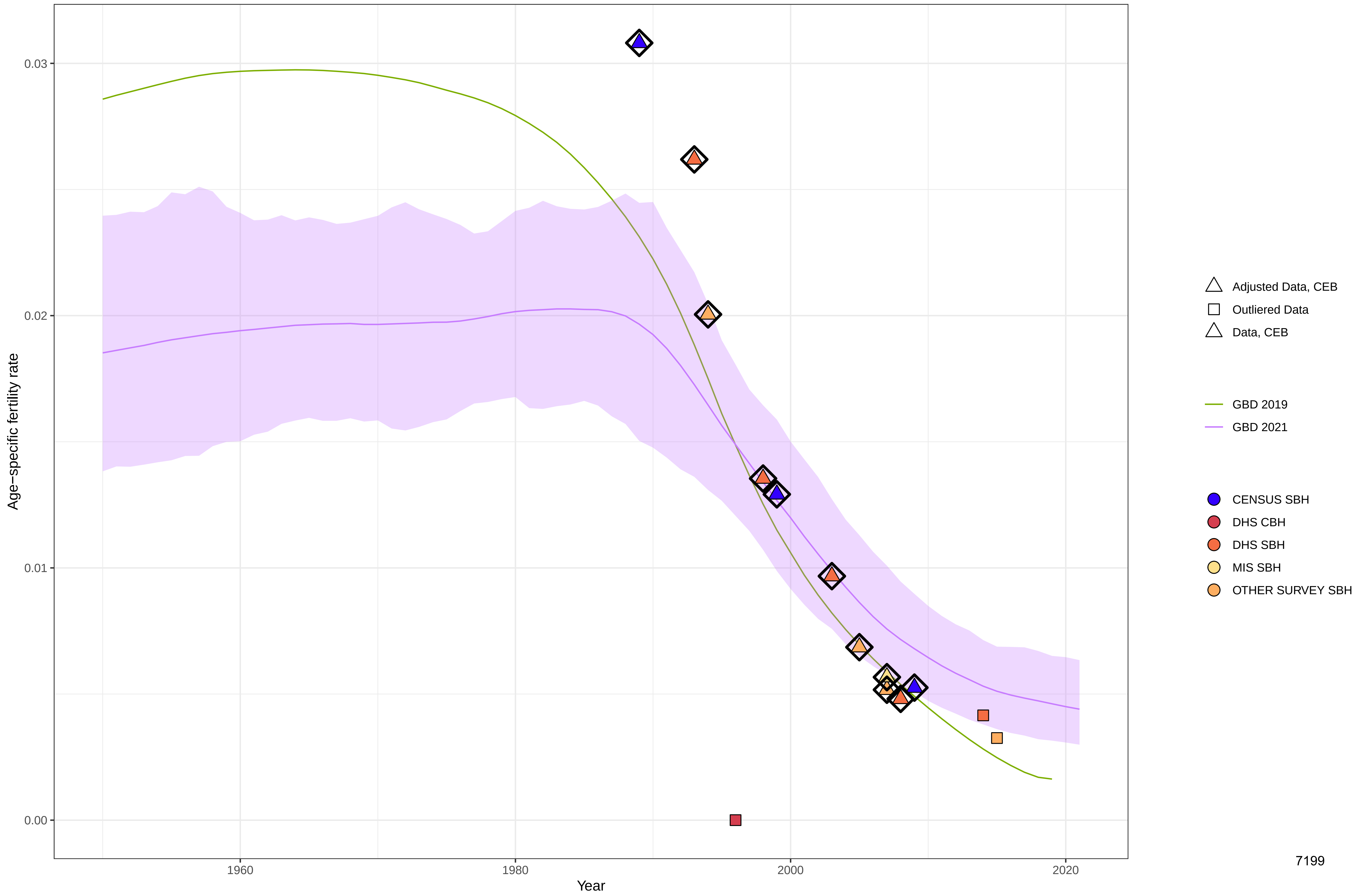

Age−specific fertility fitted trend lines, 1950−2021: Nandi, Ages: 50−54
Data points enclosed with a diamond are reference sources

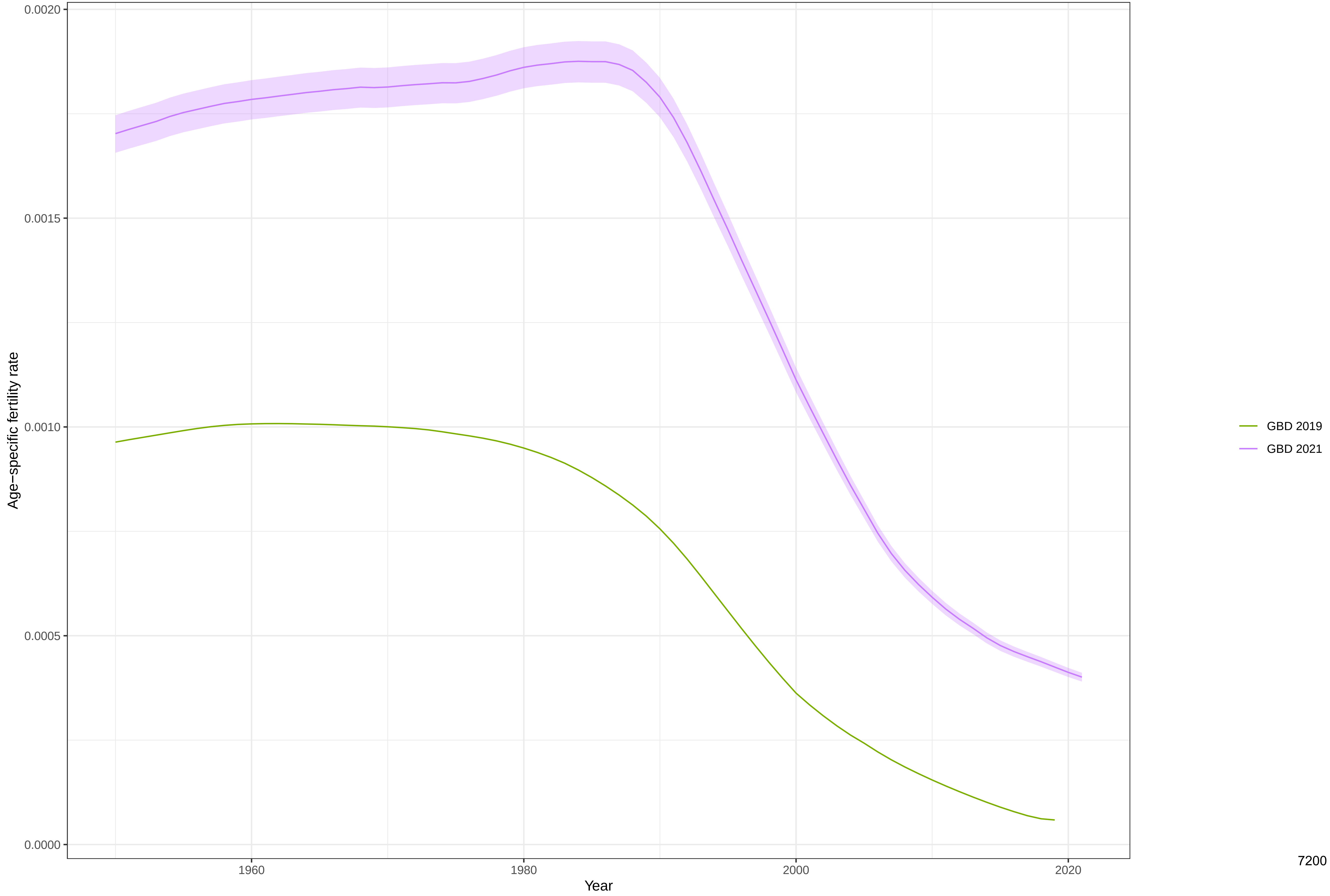

Total fertility rate fitted trend lines, 1950−2021: Nandi TFR
Data points enclosed with a diamond are reference sources

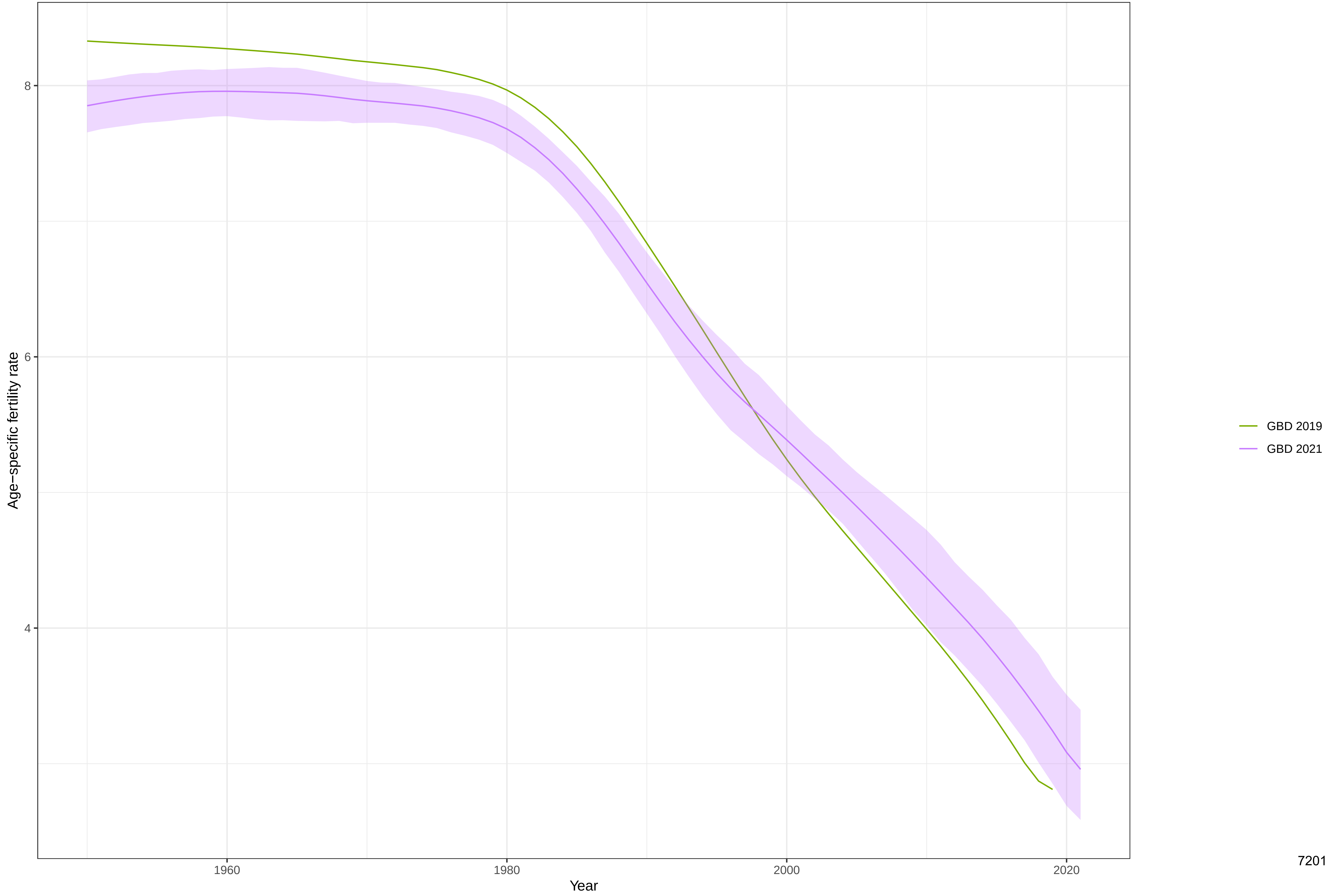

Age−specific fertility fitted trend lines, 1950−2021: Narok, Ages: 10−14
Data points enclosed with a diamond are reference sources

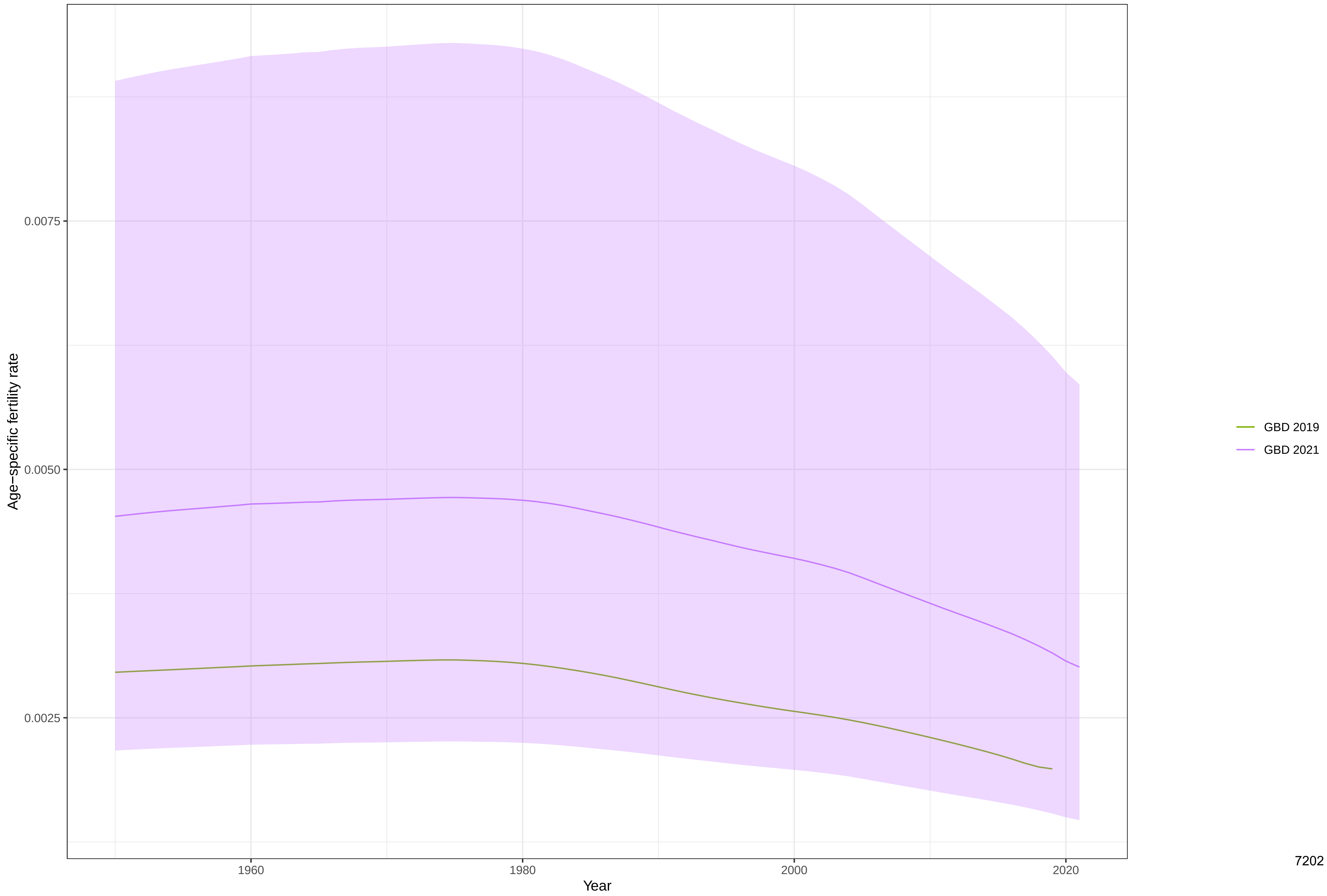

Age−specific fertility fitted trend lines, 1950−2021: Narok, Ages: 15−19
Data points enclosed with a diamond are reference sources

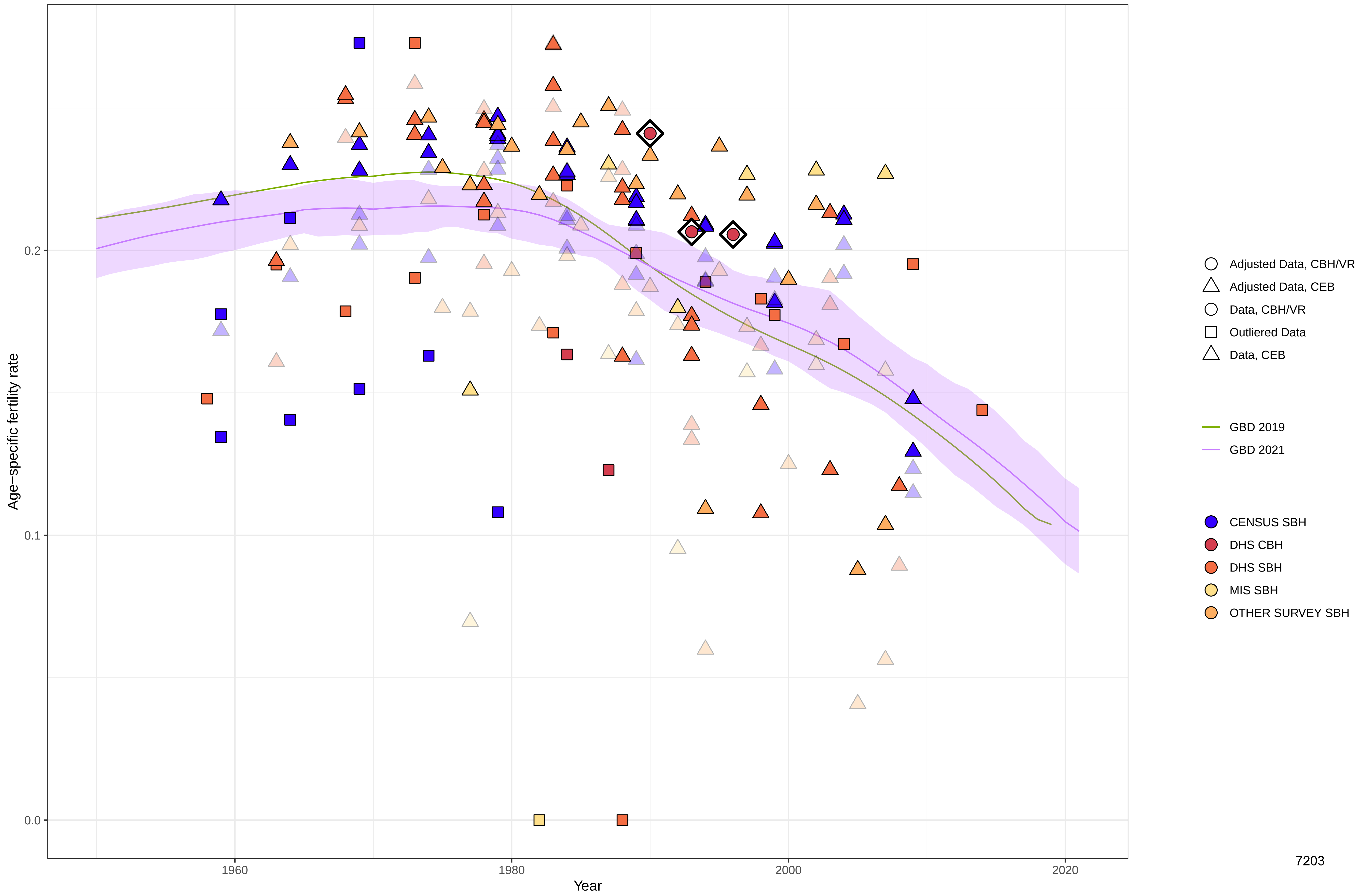

Age−specific fertility fitted trend lines, 1950−2021: Narok, Ages: 20−24
Data points enclosed with a diamond are reference sources

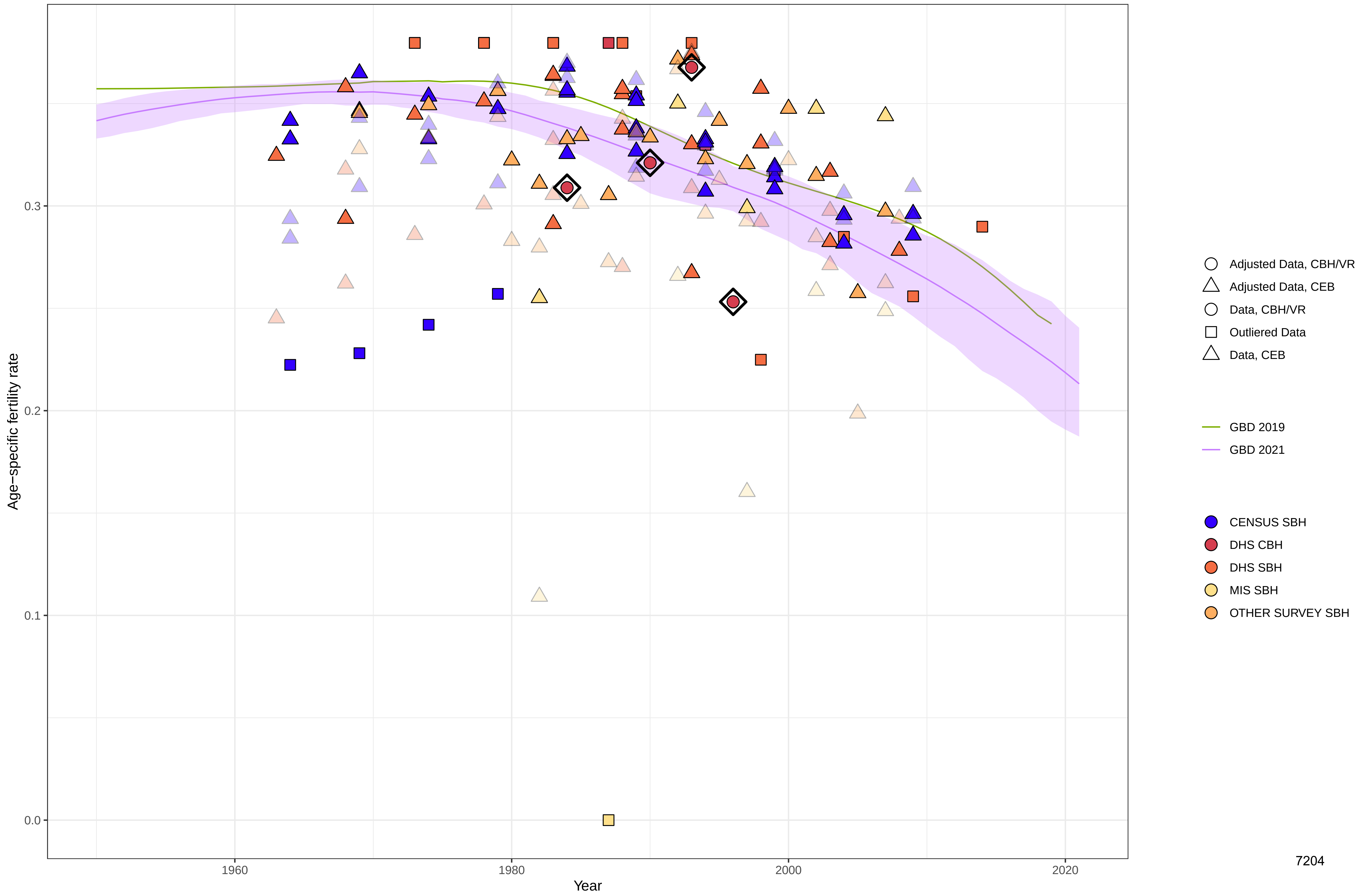

Age−specific fertility fitted trend lines, 1950−2021: Narok, Ages: 25−29
Data points enclosed with a diamond are reference sources

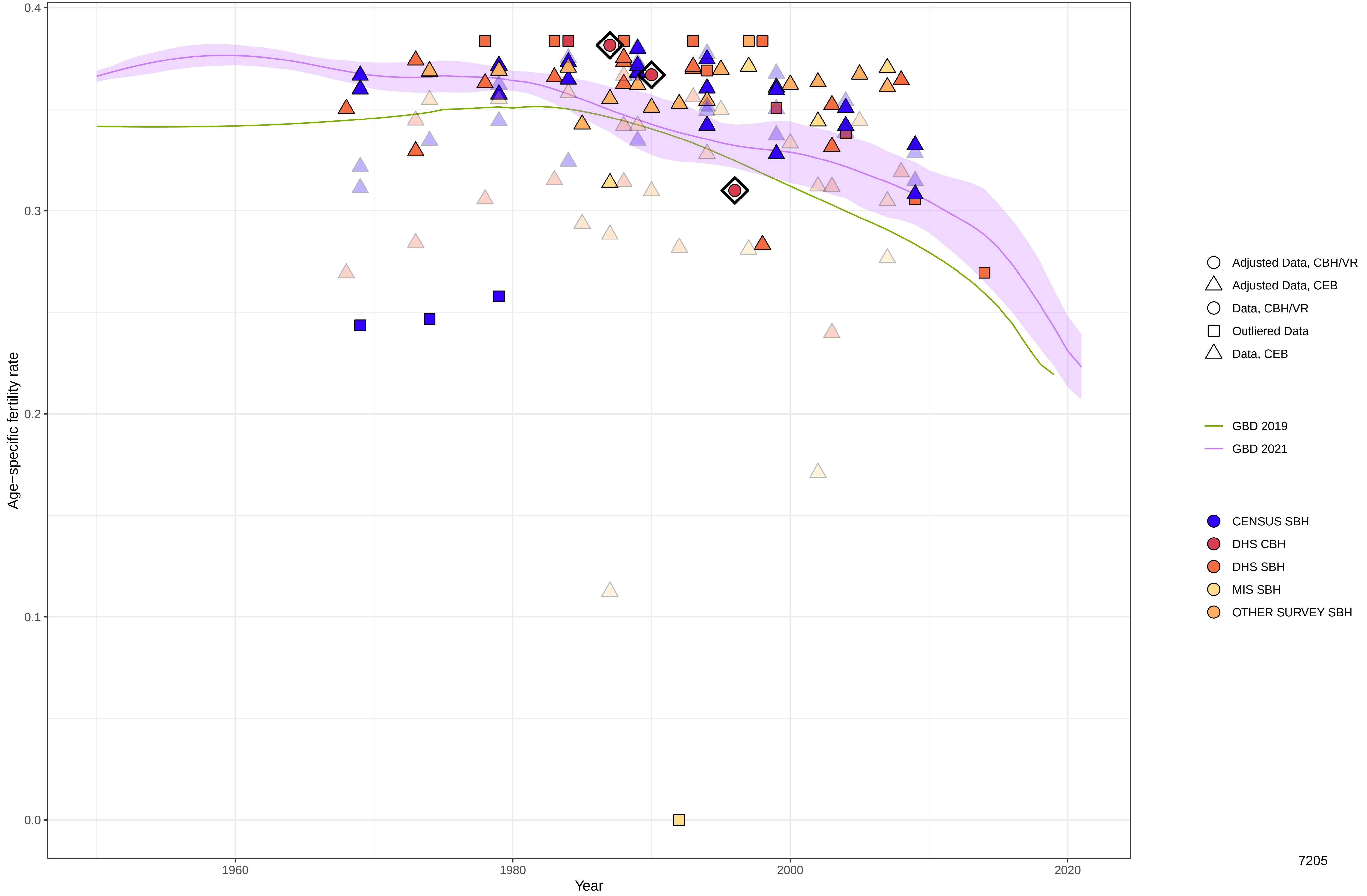

Age−specific fertility fitted trend lines, 1950−2021: Narok, Ages: 30−34
Data points enclosed with a diamond are reference sources

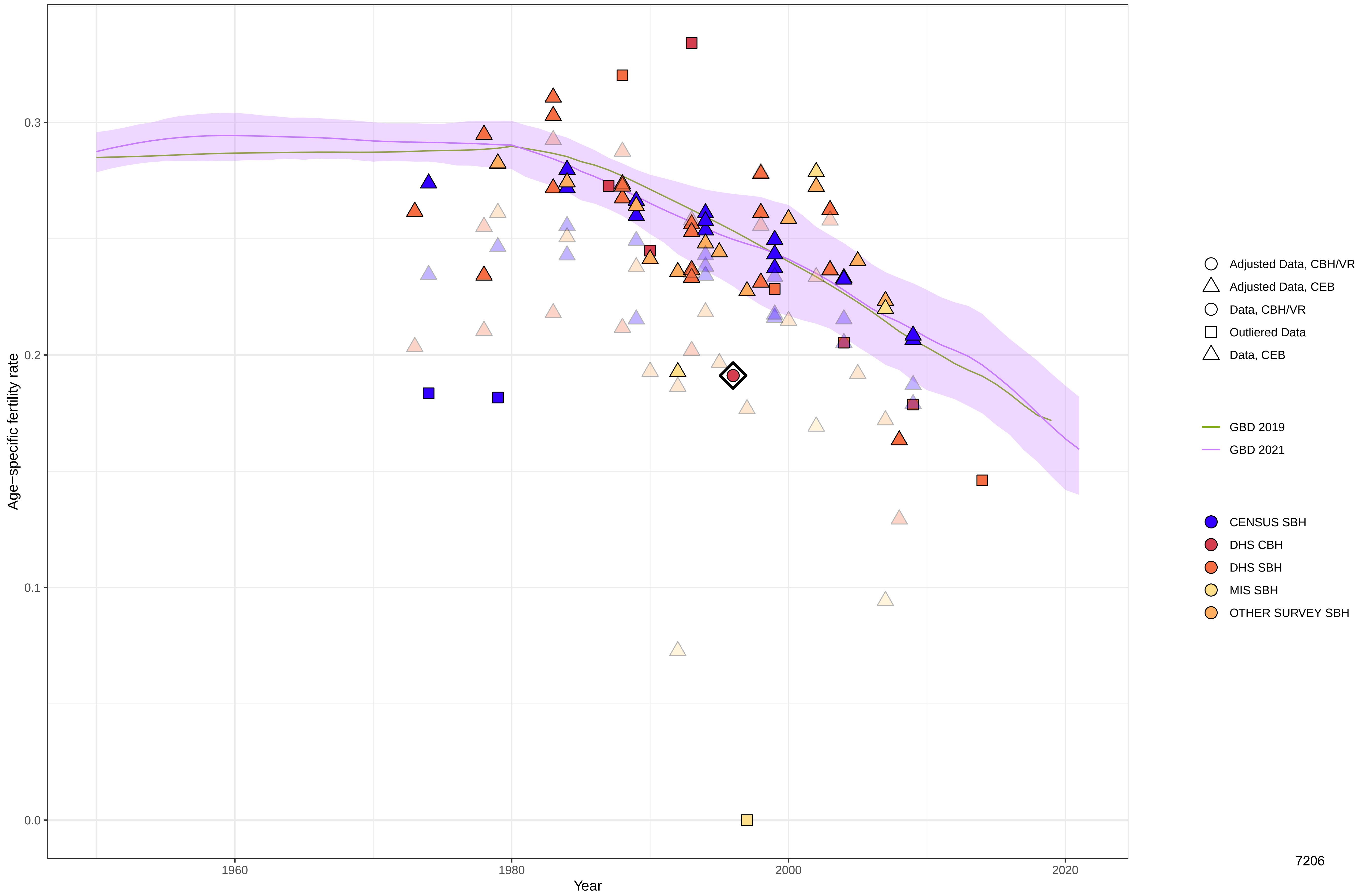

Age−specific fertility fitted trend lines, 1950−2021: Narok, Ages: 35−39
Data points enclosed with a diamond are reference sources

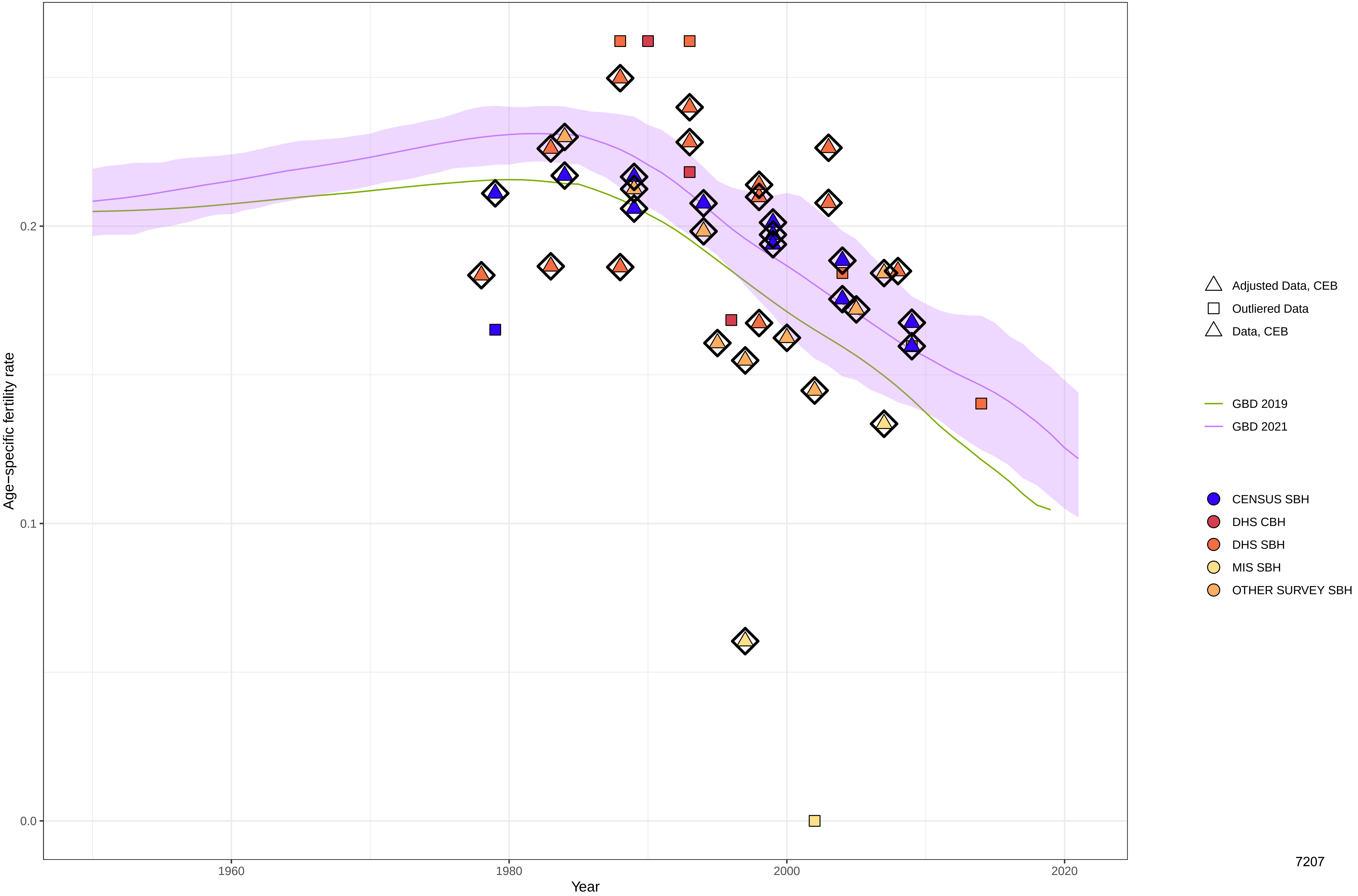

Age−specific fertility fitted trend lines, 1950−2021: Narok, Ages: 40−44
Data points enclosed with a diamond are reference sources

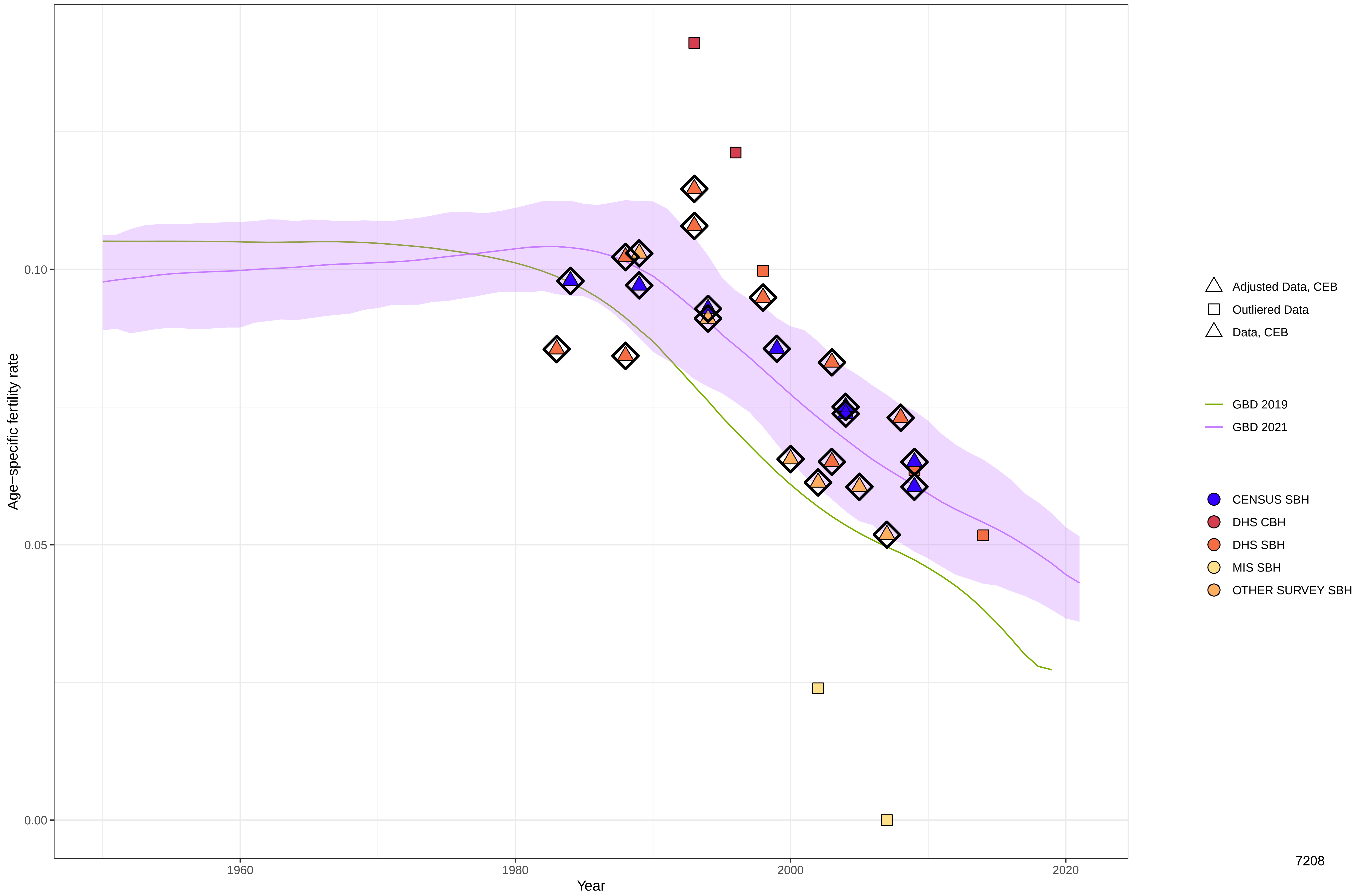

7208

Age−specific fertility fitted trend lines, 1950−2021: Narok, Ages: 45−49
Data points enclosed with a diamond are reference sources

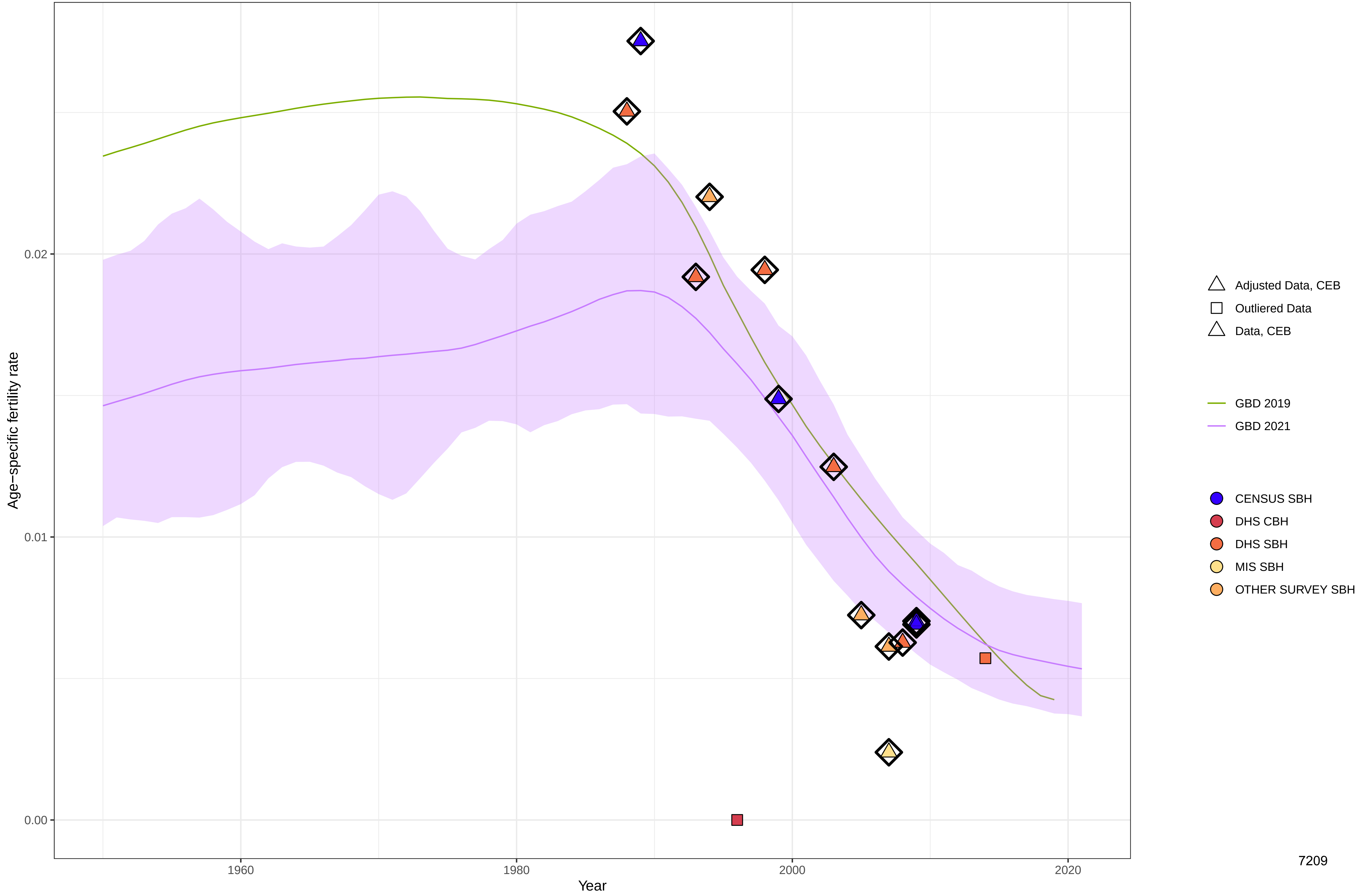

Age−specific fertility fitted trend lines, 1950−2021: Narok, Ages: 50−54
Data points enclosed with a diamond are reference sources

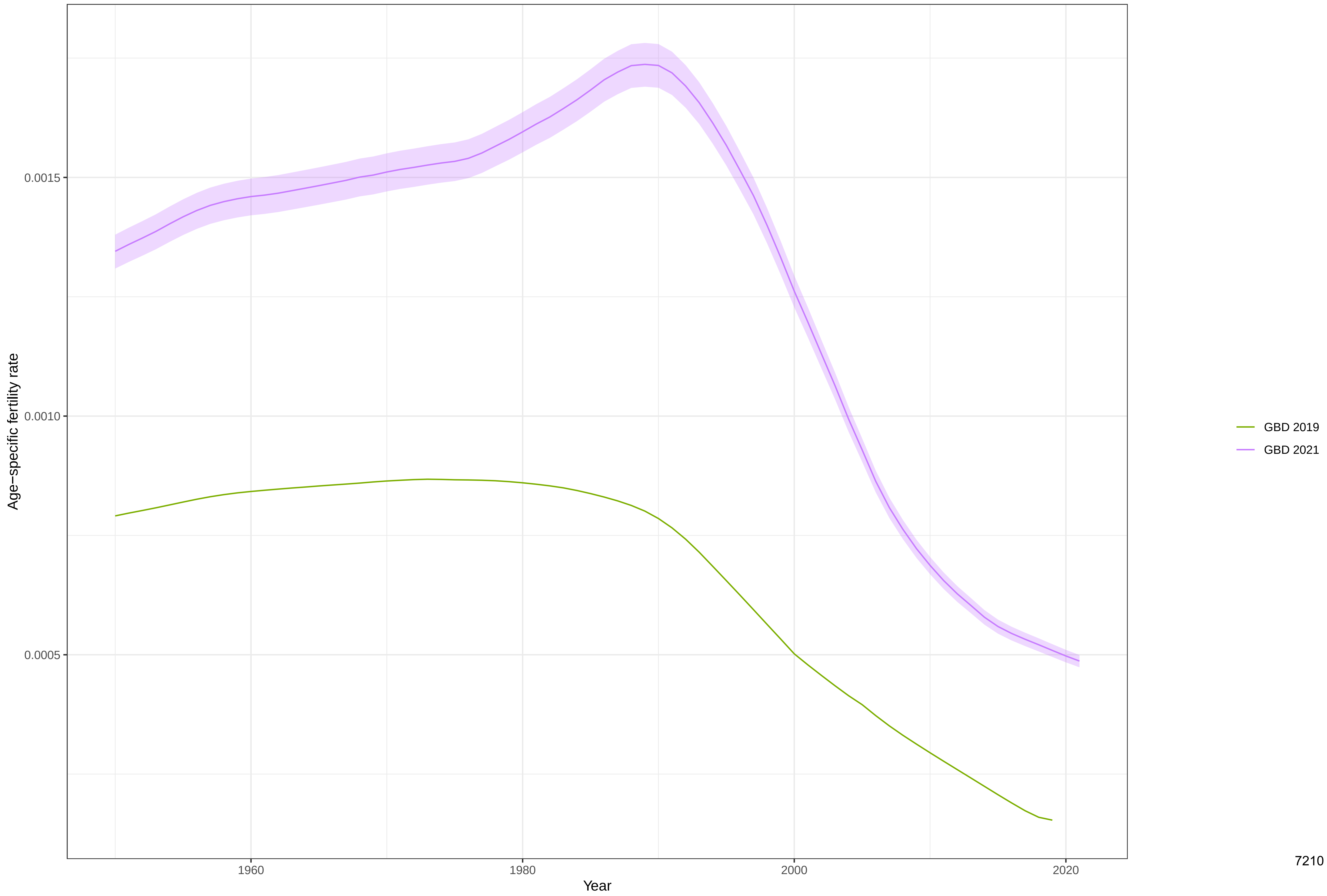

Total fertility rate fitted trend lines, 1950−2021: Narok TFR
Data points enclosed with a diamond are reference sources

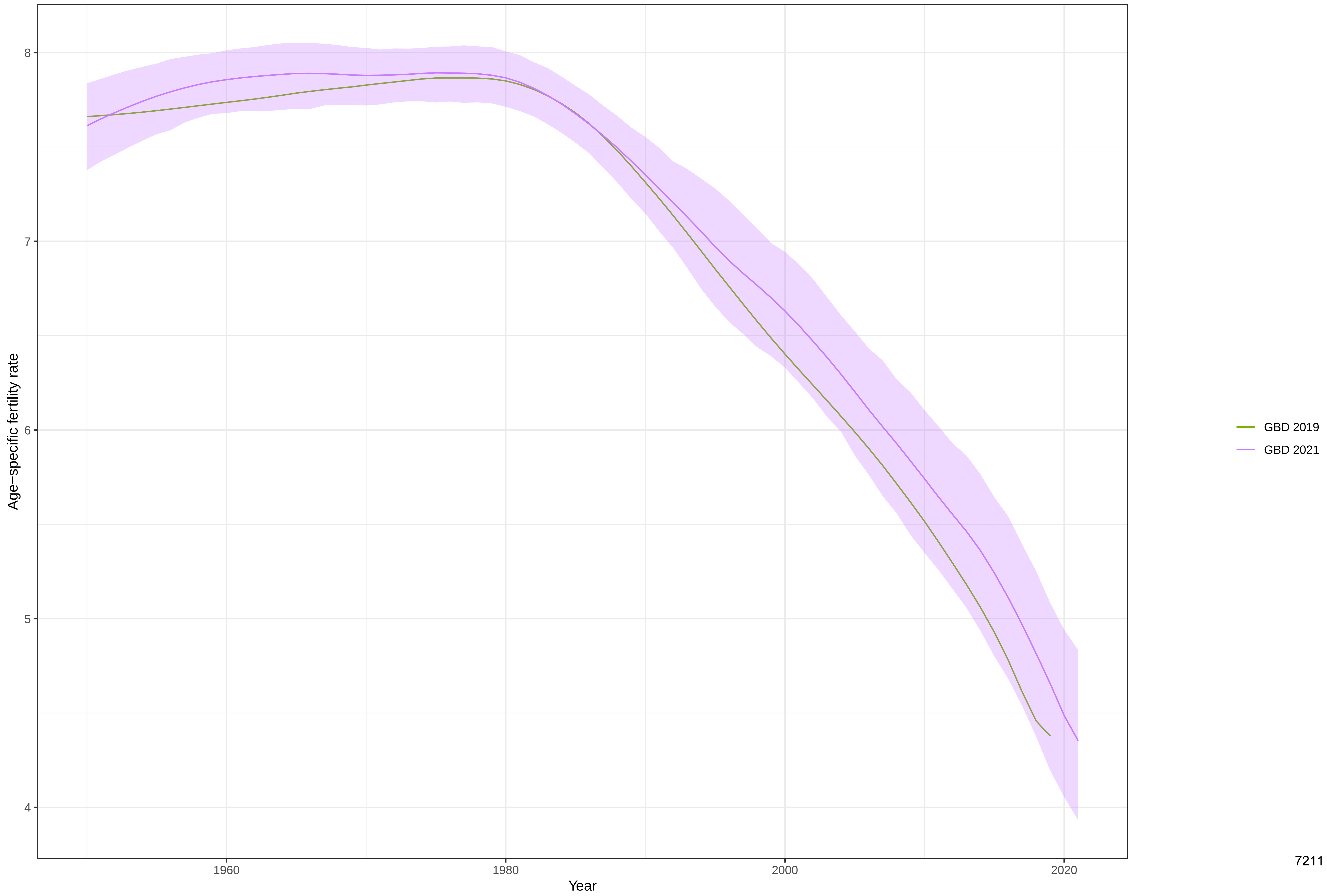

Age−specific fertility fitted trend lines, 1950−2021: Nyamira, Ages: 10−14
Data points enclosed with a diamond are reference sources

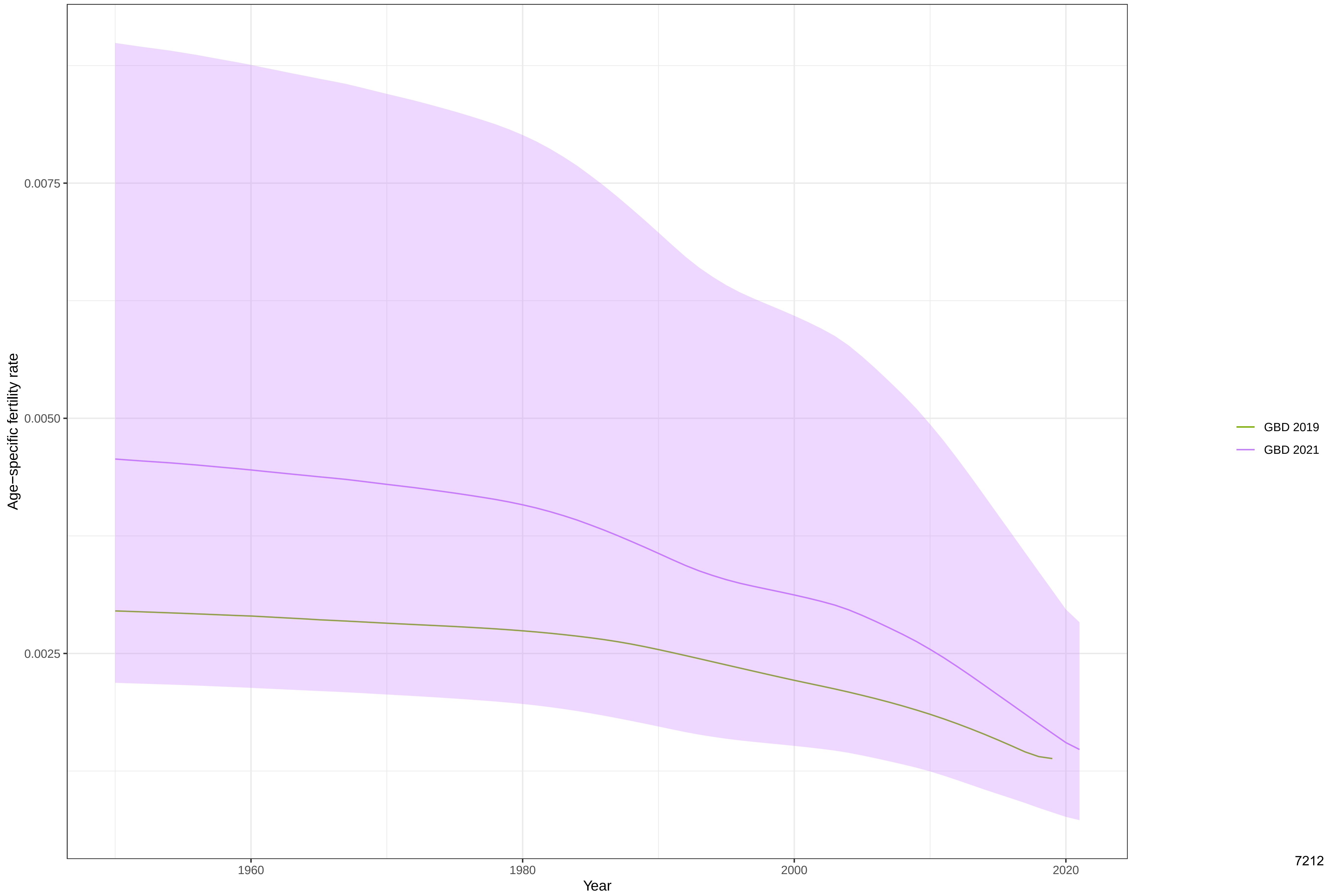

7212

Age−specific fertility fitted trend lines, 1950−2021: Nyamira, Ages: 15−19
Data points enclosed with a diamond are reference sources

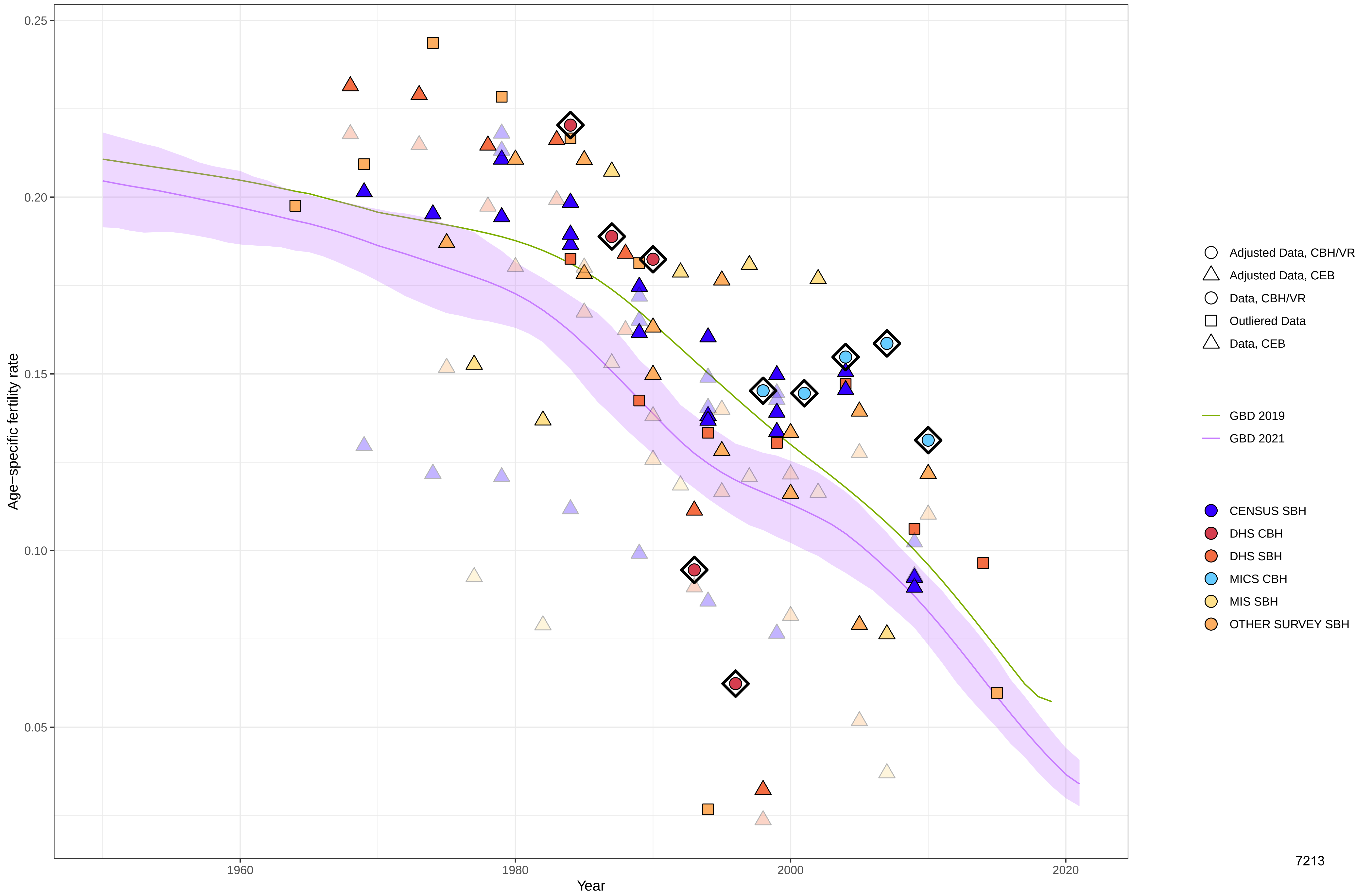

7213

Age−specific fertility fitted trend lines, 1950−2021: Nyamira, Ages: 20−24
Data points enclosed with a diamond are reference sources

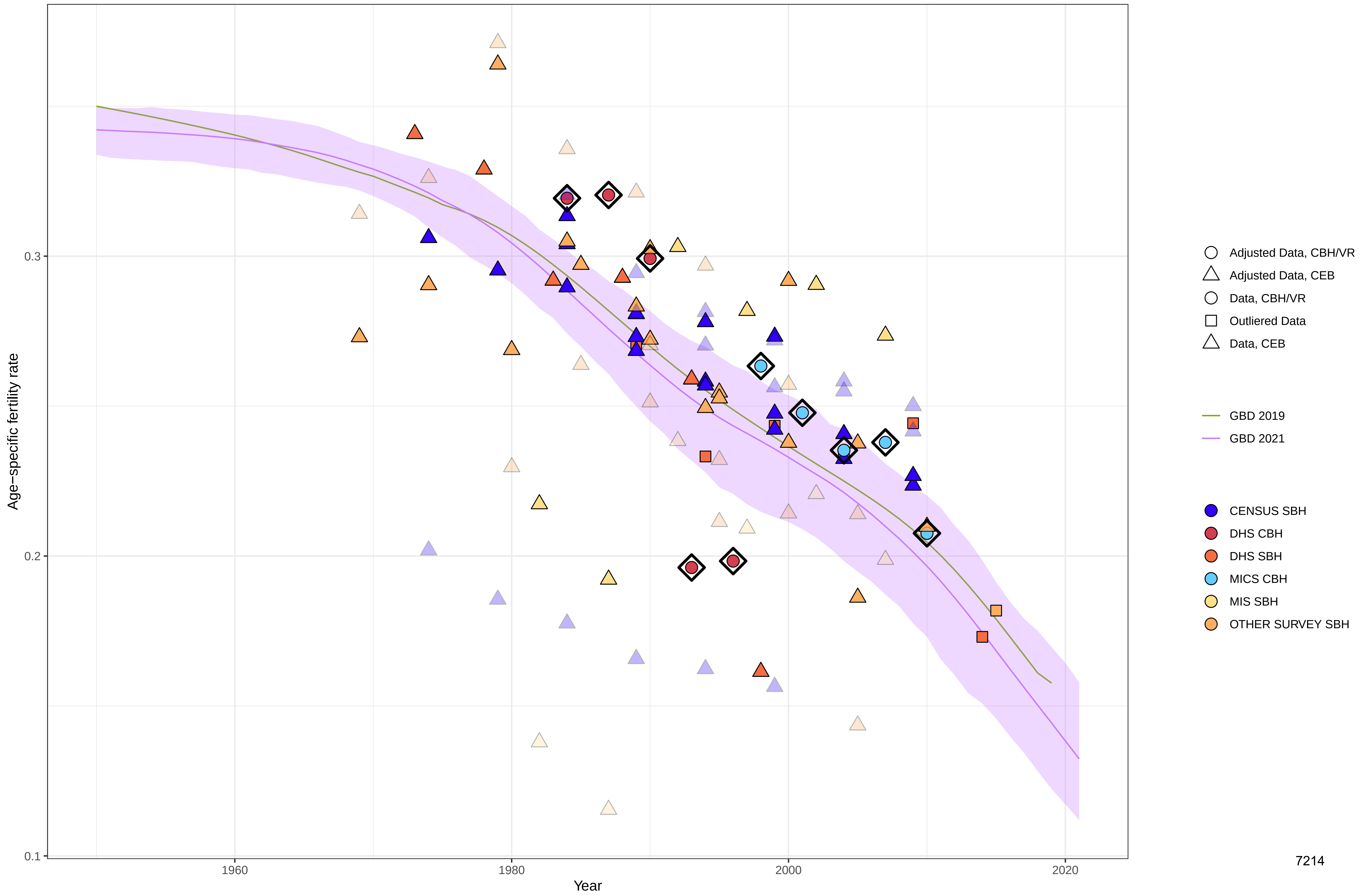

7214

Age−specific fertility fitted trend lines, 1950−2021: Nyamira, Ages: 25−29
Data points enclosed with a diamond are reference sources

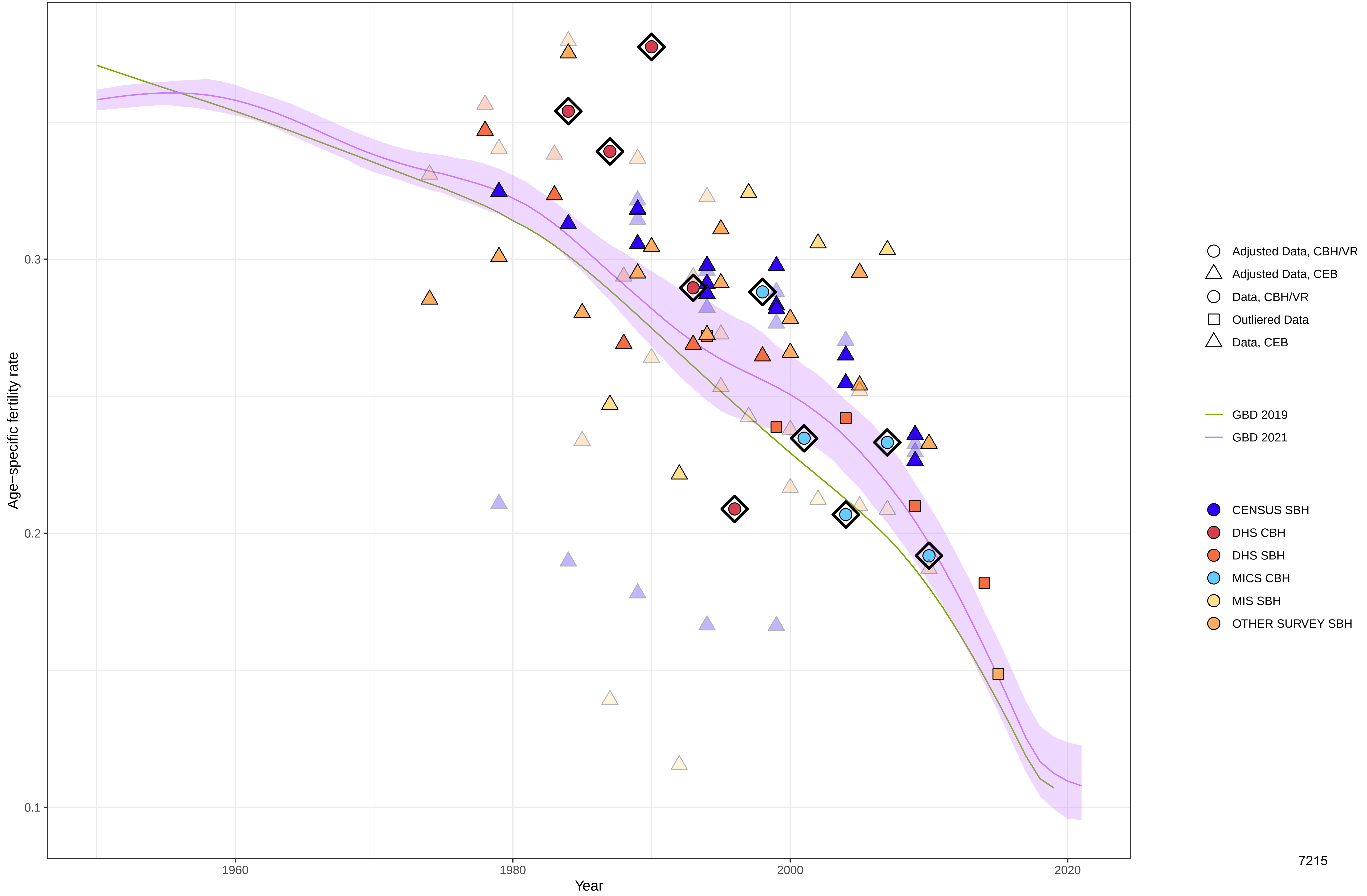

Age−specific fertility fitted trend lines, 1950−2021: Nyamira, Ages: 30−34
Data points enclosed with a diamond are reference sources

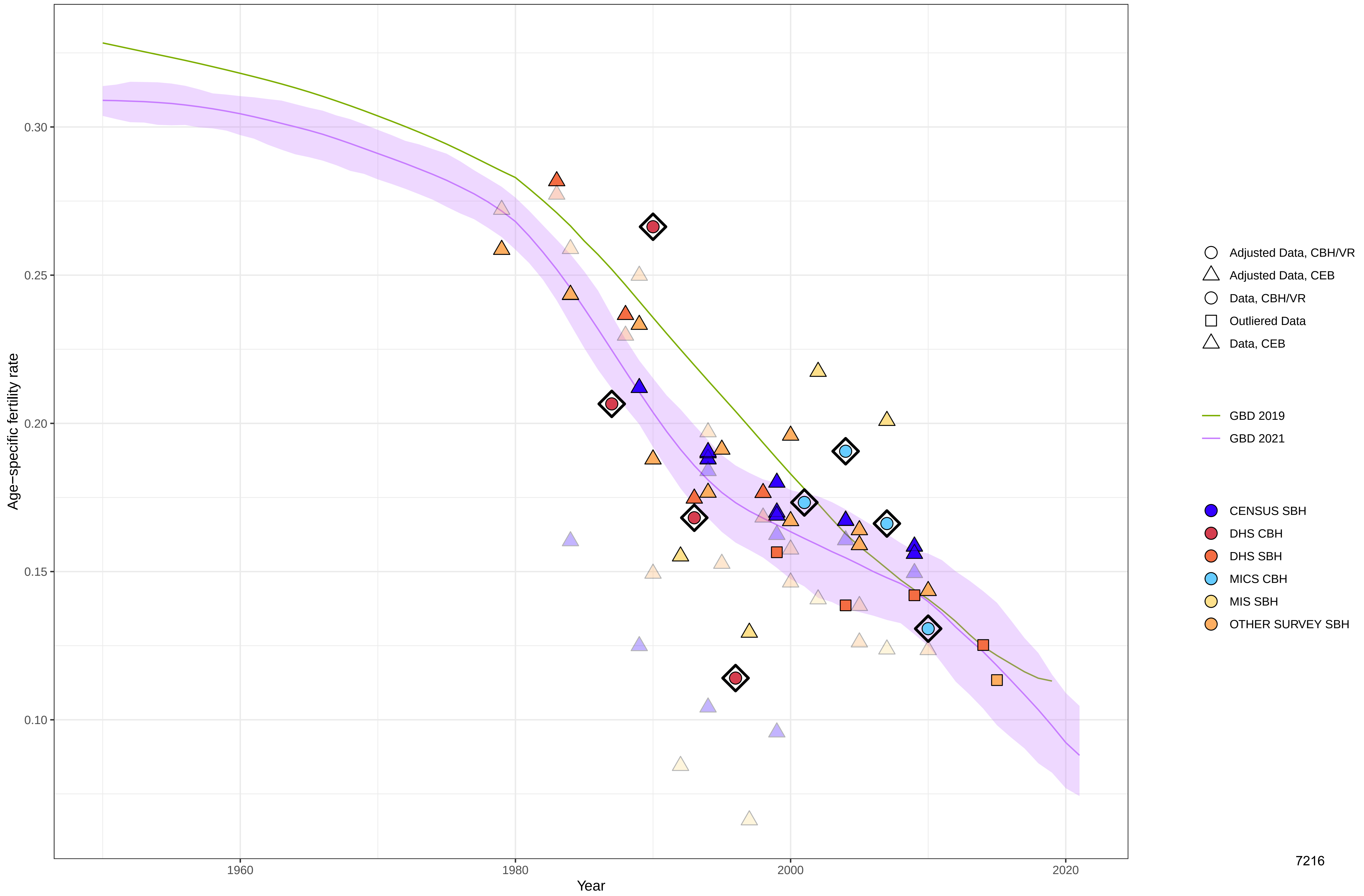

Age−specific fertility fitted trend lines, 1950−2021: Nyamira, Ages: 35−39
Data points enclosed with a diamond are reference sources

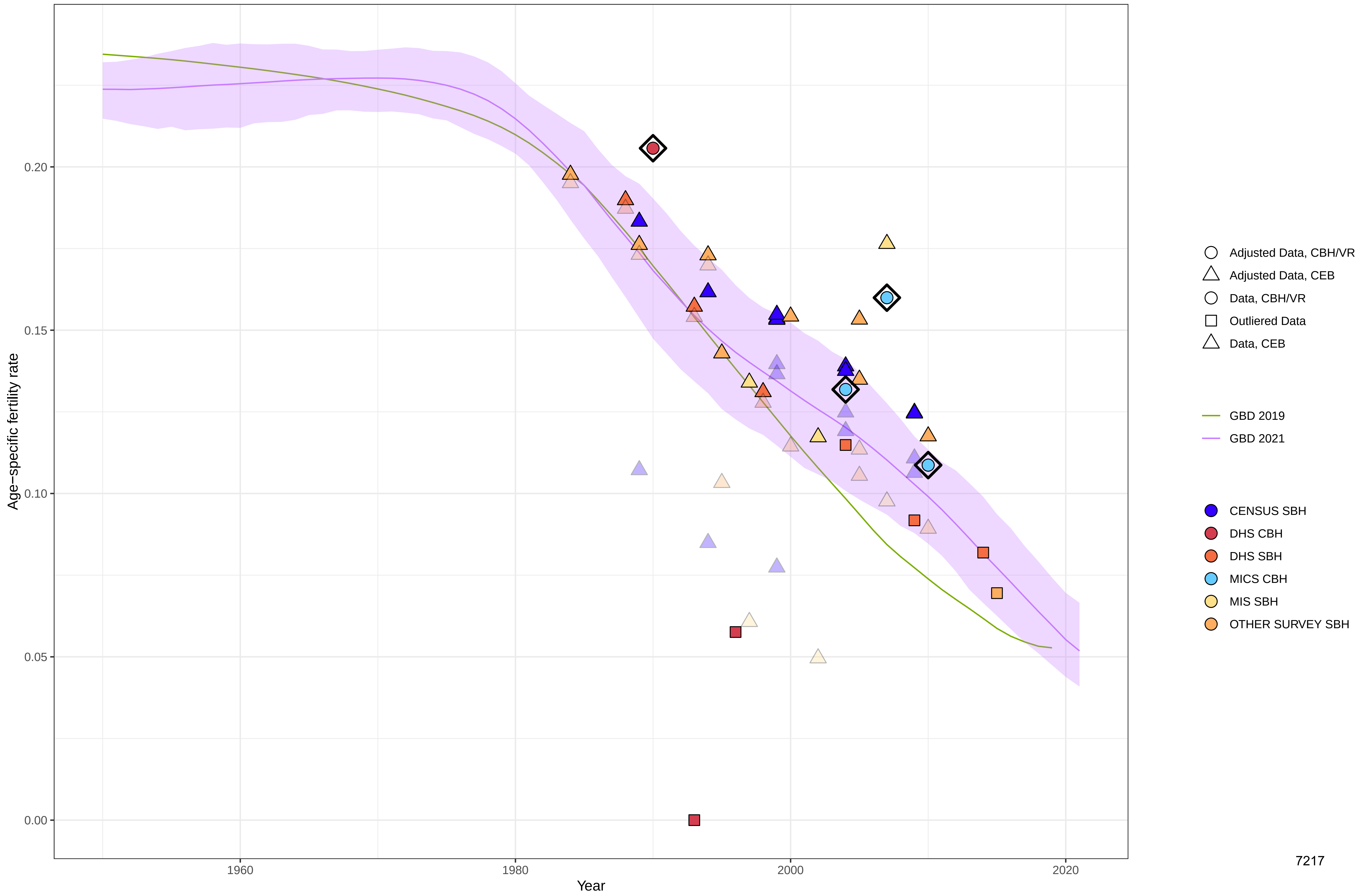

Age−specific fertility fitted trend lines, 1950−2021: Nyamira, Ages: 40−44
Data points enclosed with a diamond are reference sources

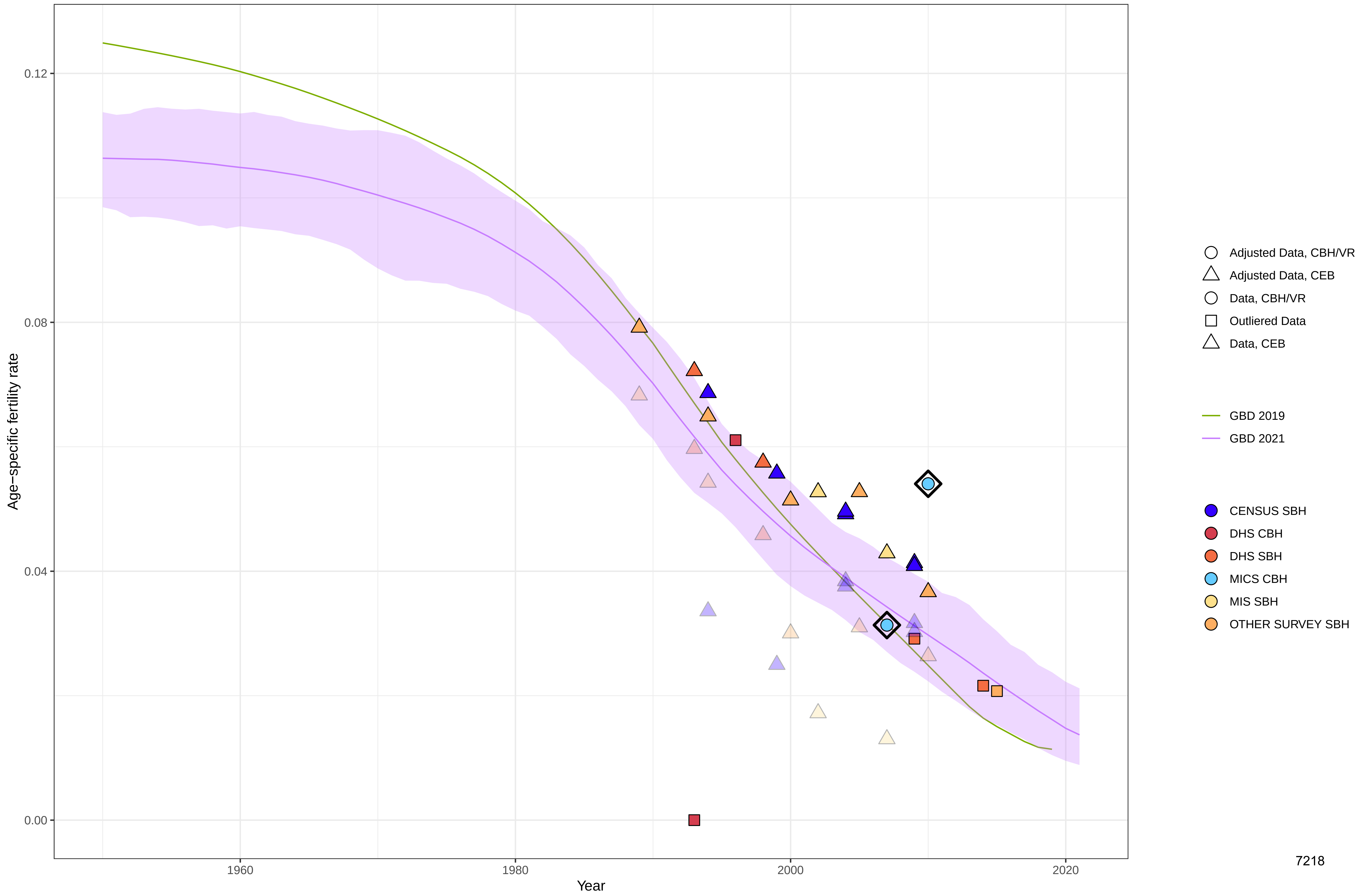

Age−specific fertility fitted trend lines, 1950−2021: Nyamira, Ages: 45−49
Data points enclosed with a diamond are reference sources

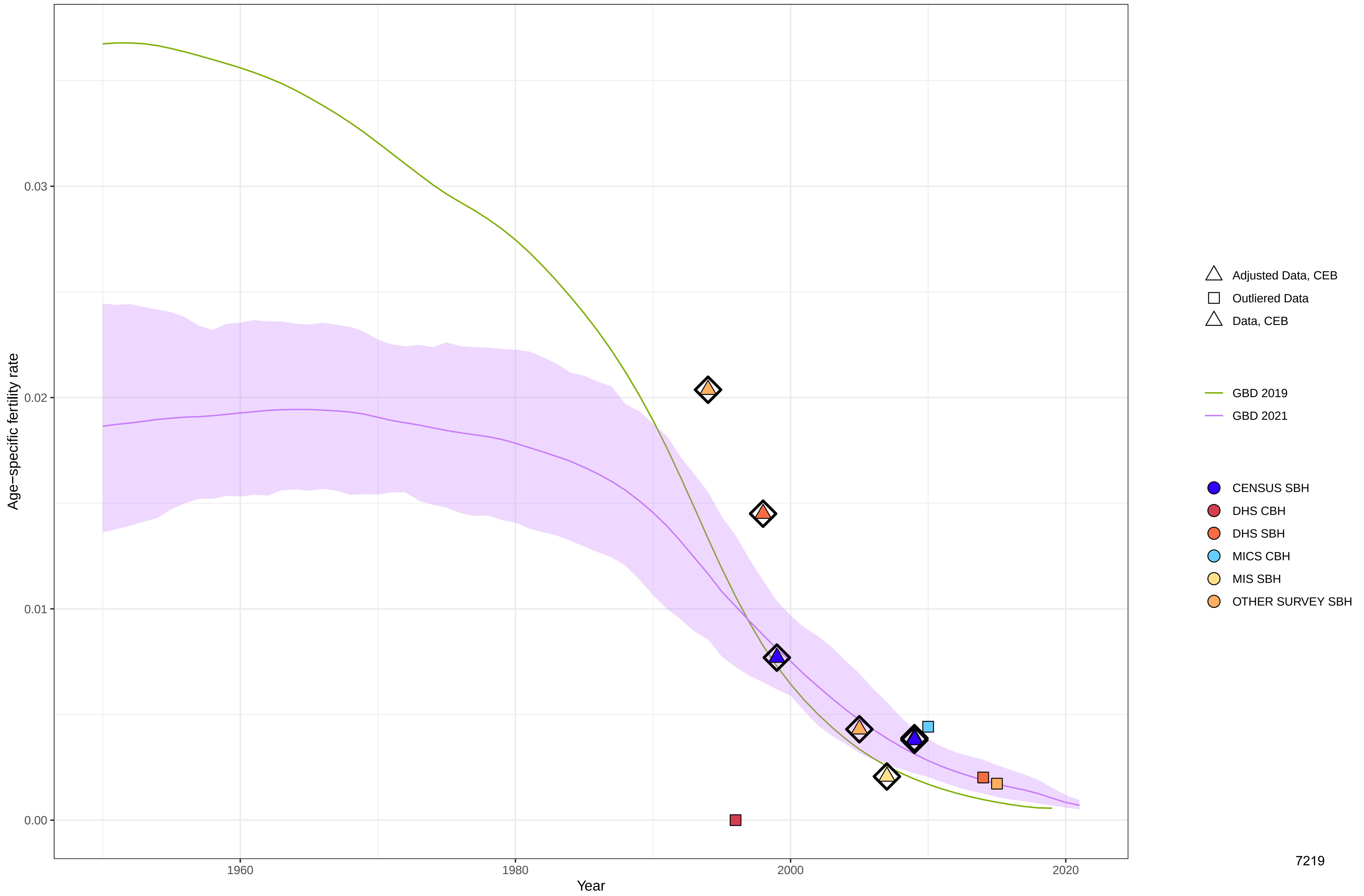

Age−specific fertility fitted trend lines, 1950−2021: Nyamira, Ages: 50−54
Data points enclosed with a diamond are reference sources

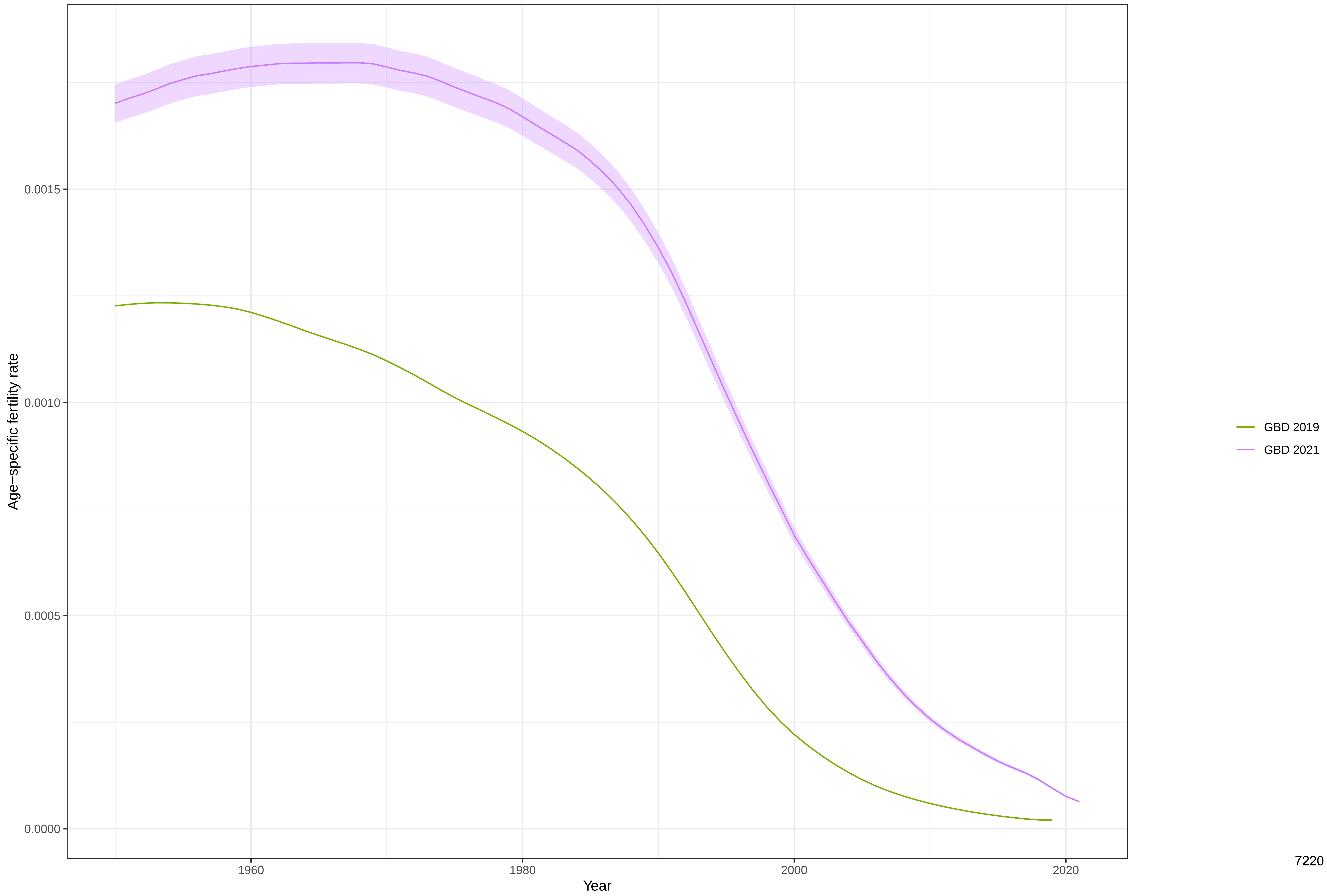

Total fertility rate fitted trend lines, 1950−2021: Nyamira TFR
Data points enclosed with a diamond are reference sources

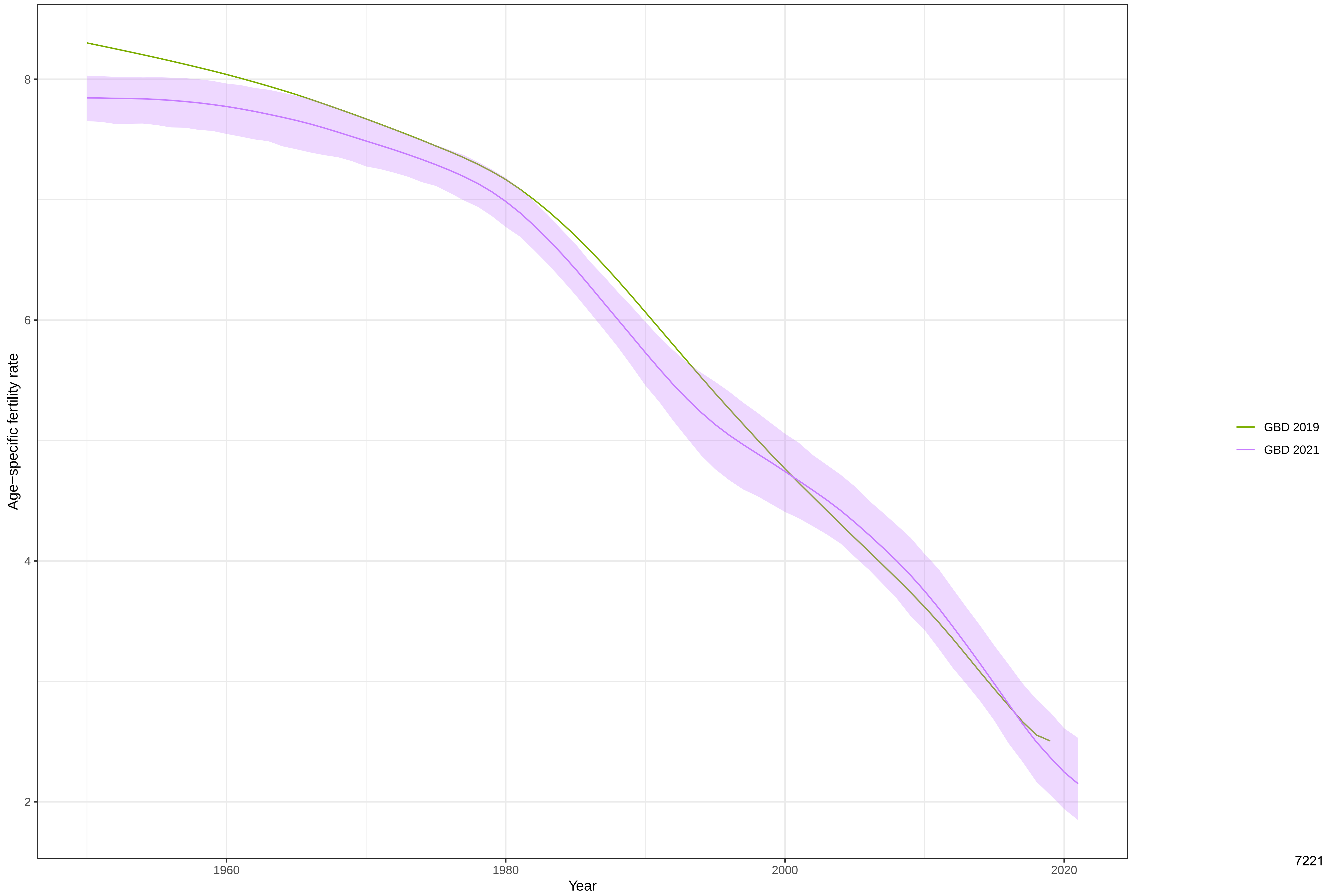

Age−specific fertility fitted trend lines, 1950−2021: Nyandarua, Ages: 10−14
Data points enclosed with a diamond are reference sources

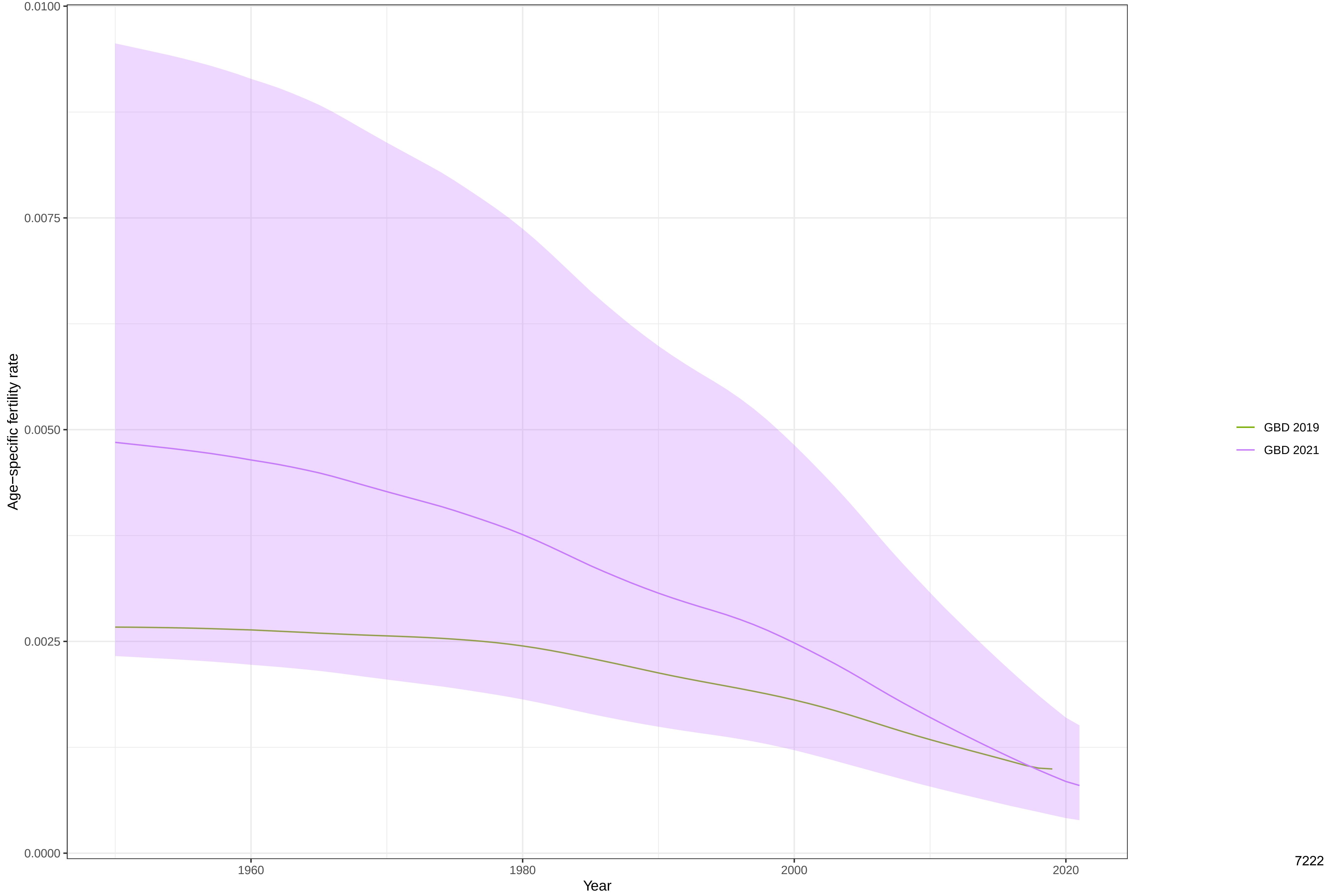

Age−specific fertility fitted trend lines, 1950−2021: Nyandarua, Ages: 15−19
Data points enclosed with a diamond are reference sources

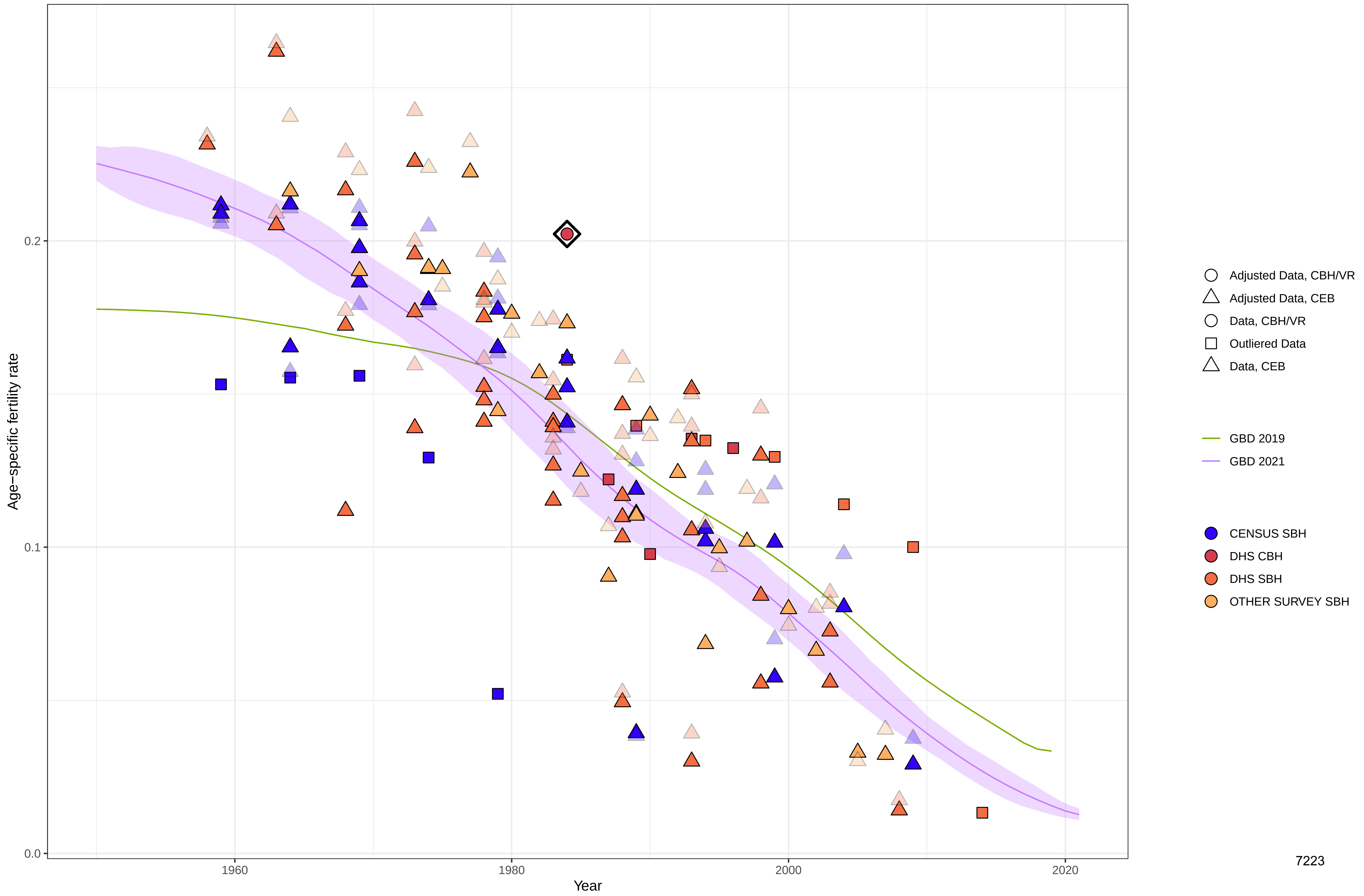

Age−specific fertility fitted trend lines, 1950−2021: Nyandarua, Ages: 20−24
Data points enclosed with a diamond are reference sources

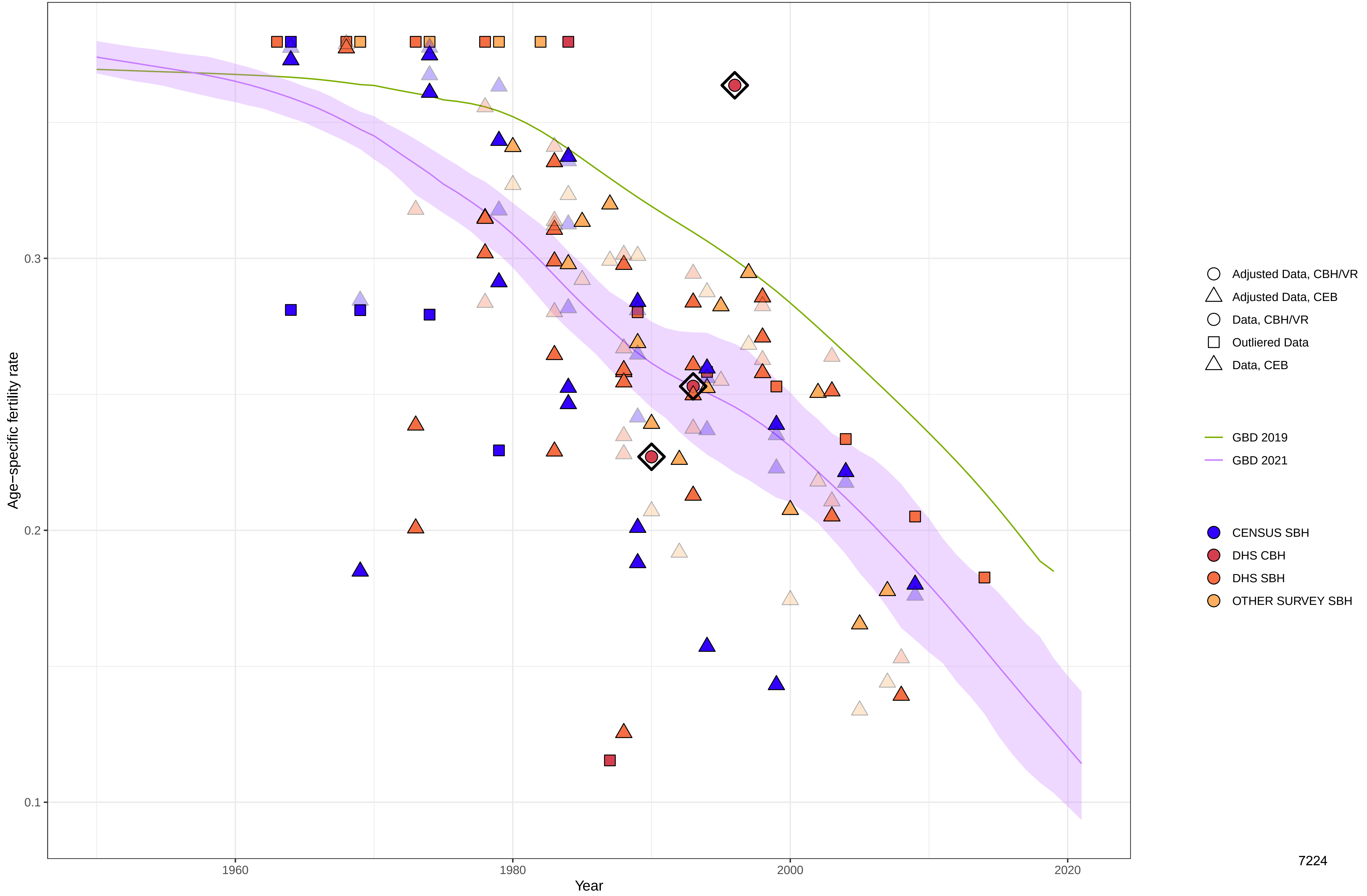

Age−specific fertility fitted trend lines, 1950−2021: Nyandarua, Ages: 25−29
Data points enclosed with a diamond are reference sources

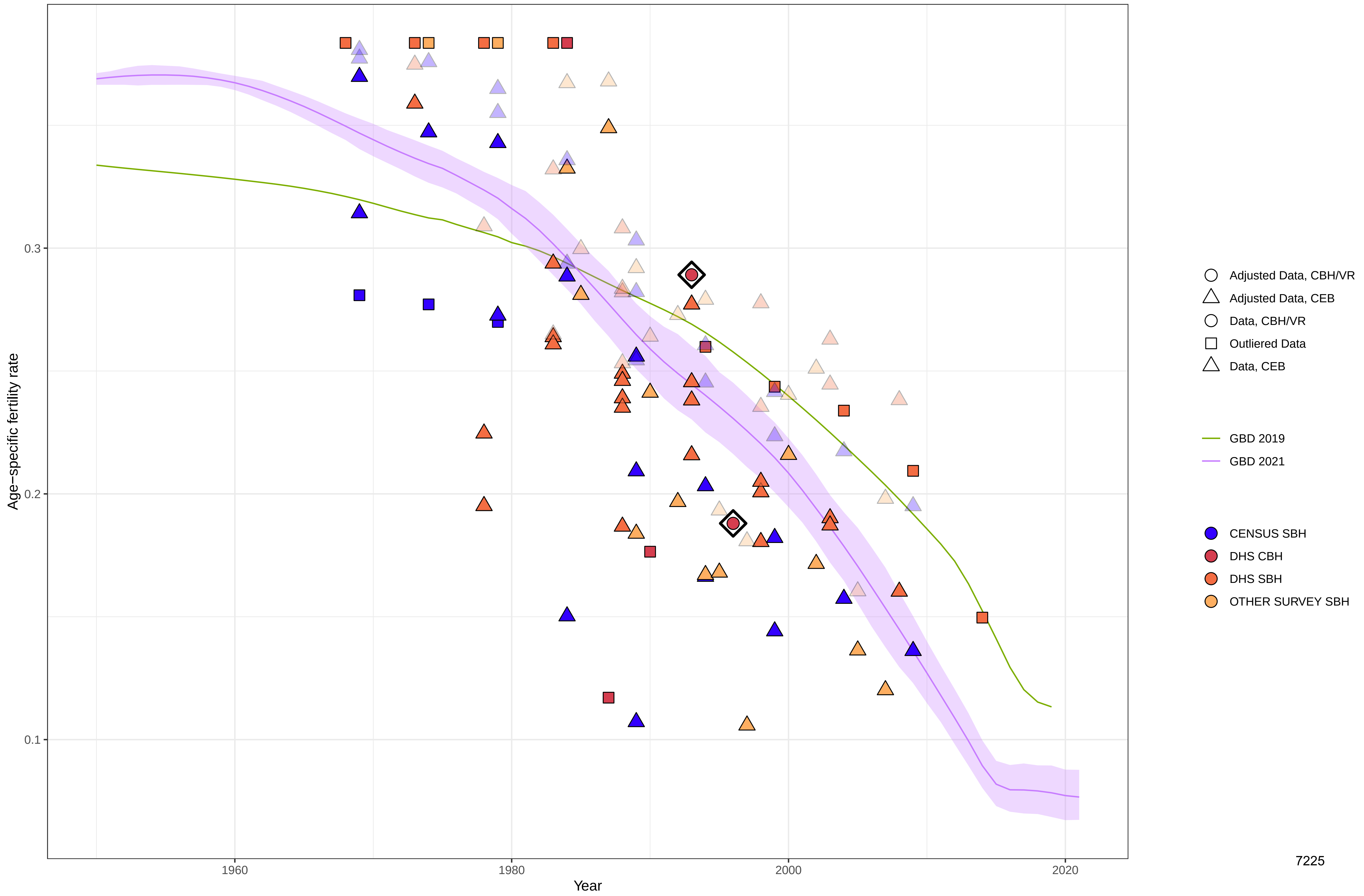

Age−specific fertility fitted trend lines, 1950−2021: Nyandarua, Ages: 30−34
Data points enclosed with a diamond are reference sources

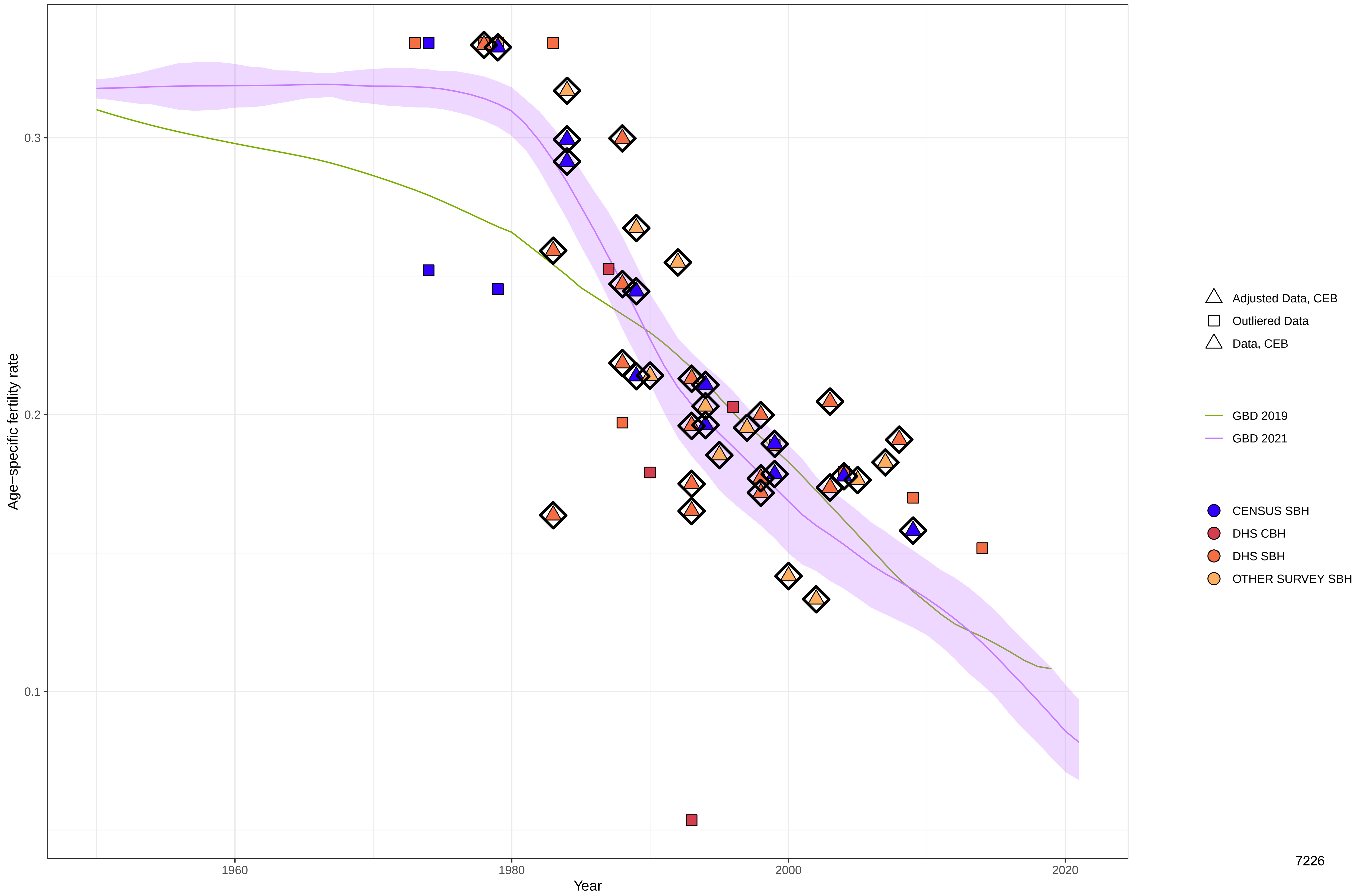

Age−specific fertility fitted trend lines, 1950−2021: Nyandarua, Ages: 35−39
Data points enclosed with a diamond are reference sources

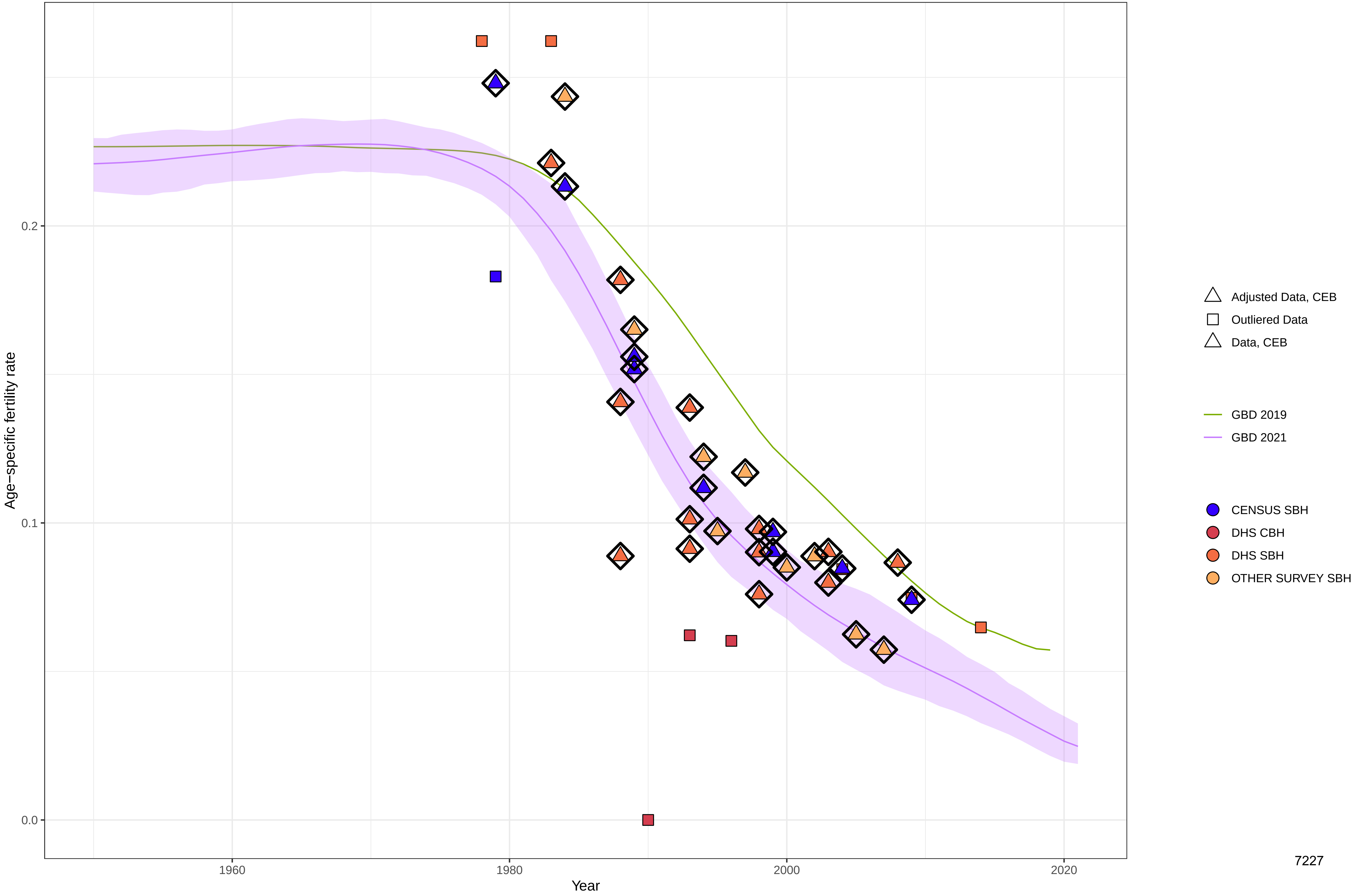

Age−specific fertility fitted trend lines, 1950−2021: Nyandarua, Ages: 40−44
Data points enclosed with a diamond are reference sources

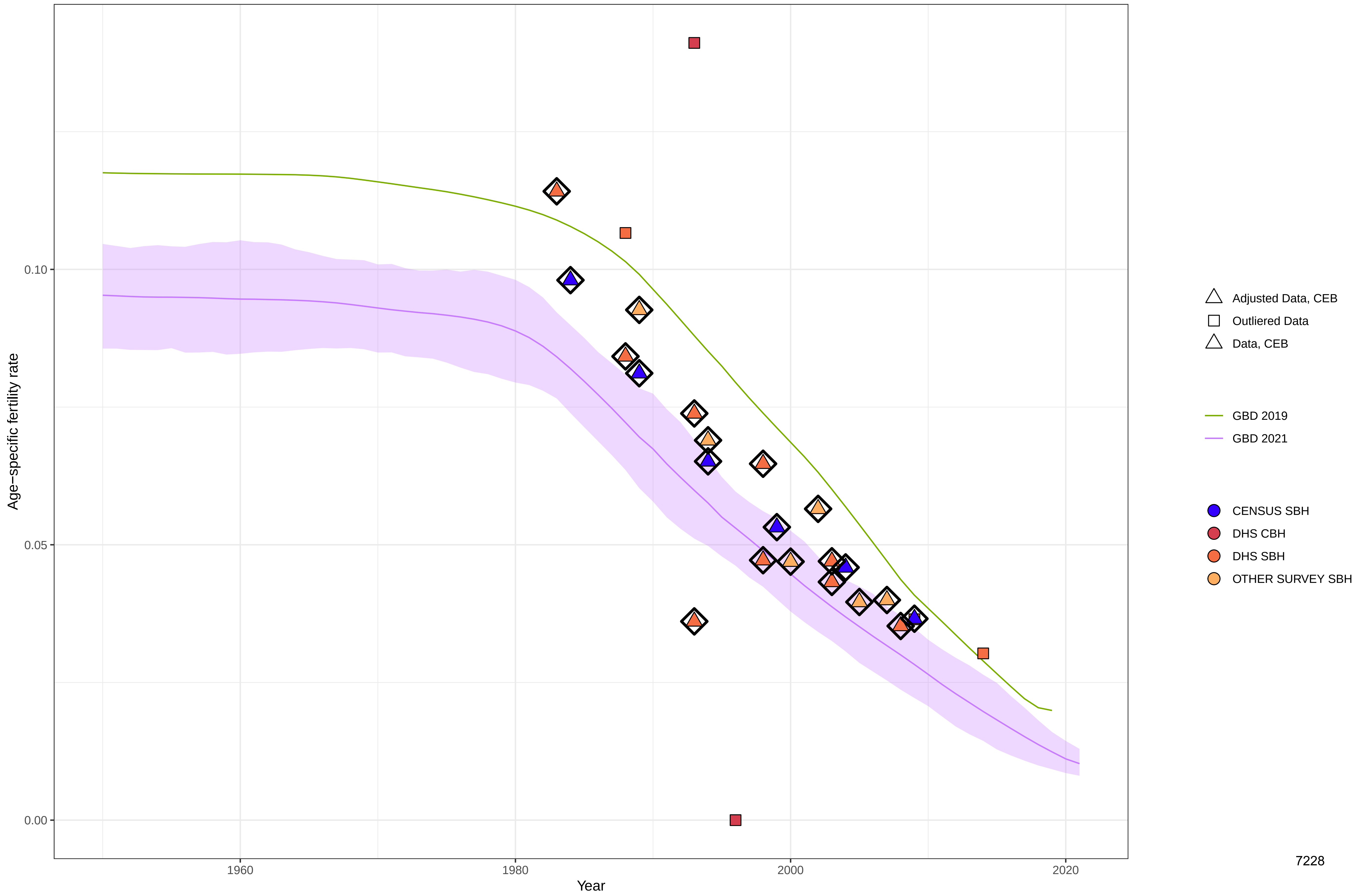

7228

Age−specific fertility fitted trend lines, 1950−2021: Nyandarua, Ages: 45−49
Data points enclosed with a diamond are reference sources

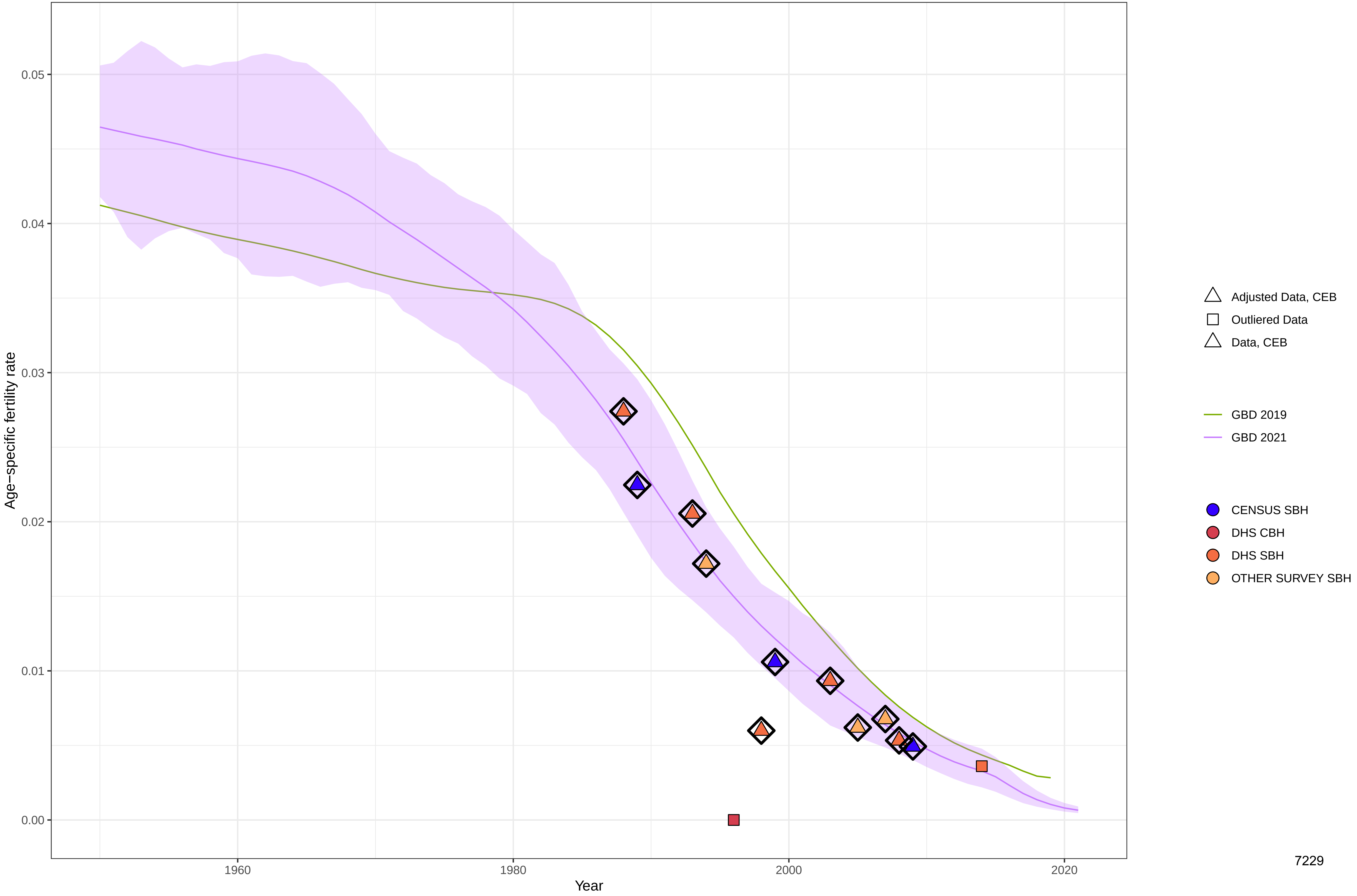

Age−specific fertility fitted trend lines, 1950−2021: Nyandarua, Ages: 50−54
Data points enclosed with a diamond are reference sources

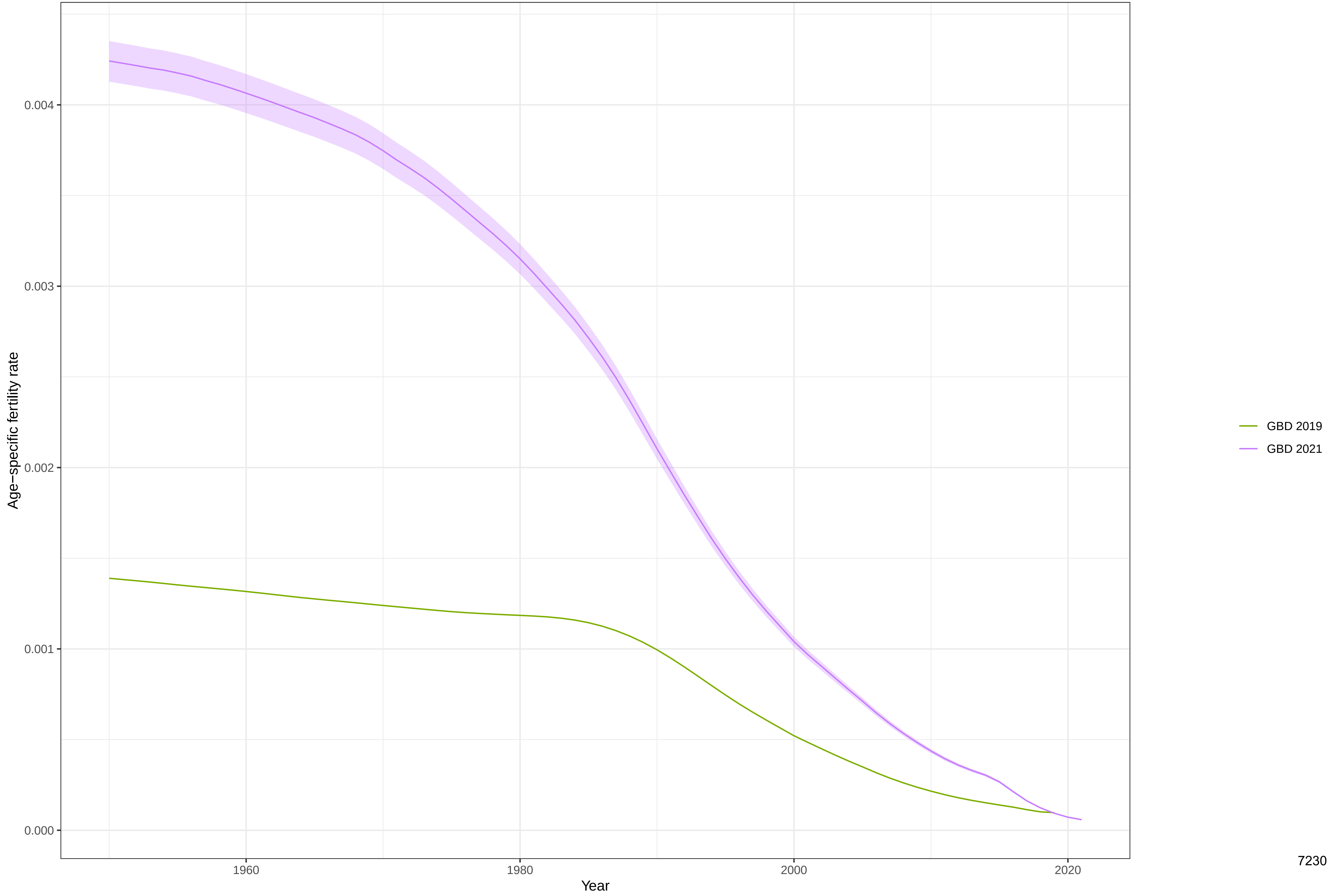

Total fertility rate fitted trend lines, 1950−2021: Nyandarua TFR
Data points enclosed with a diamond are reference sources

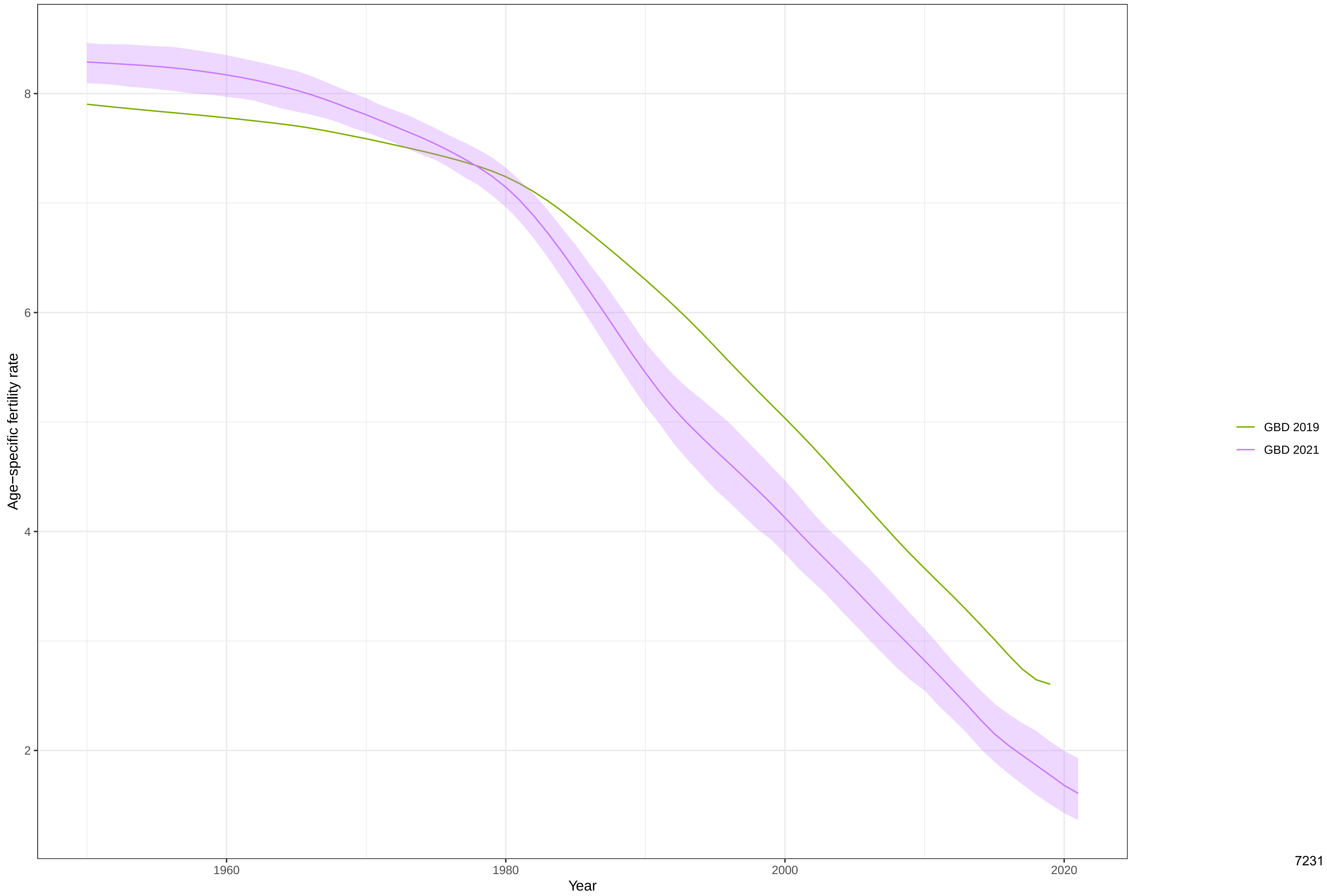

Age−specific fertility fitted trend lines, 1950−2021: Nyeri, Ages: 10−14
Data points enclosed with a diamond are reference sources

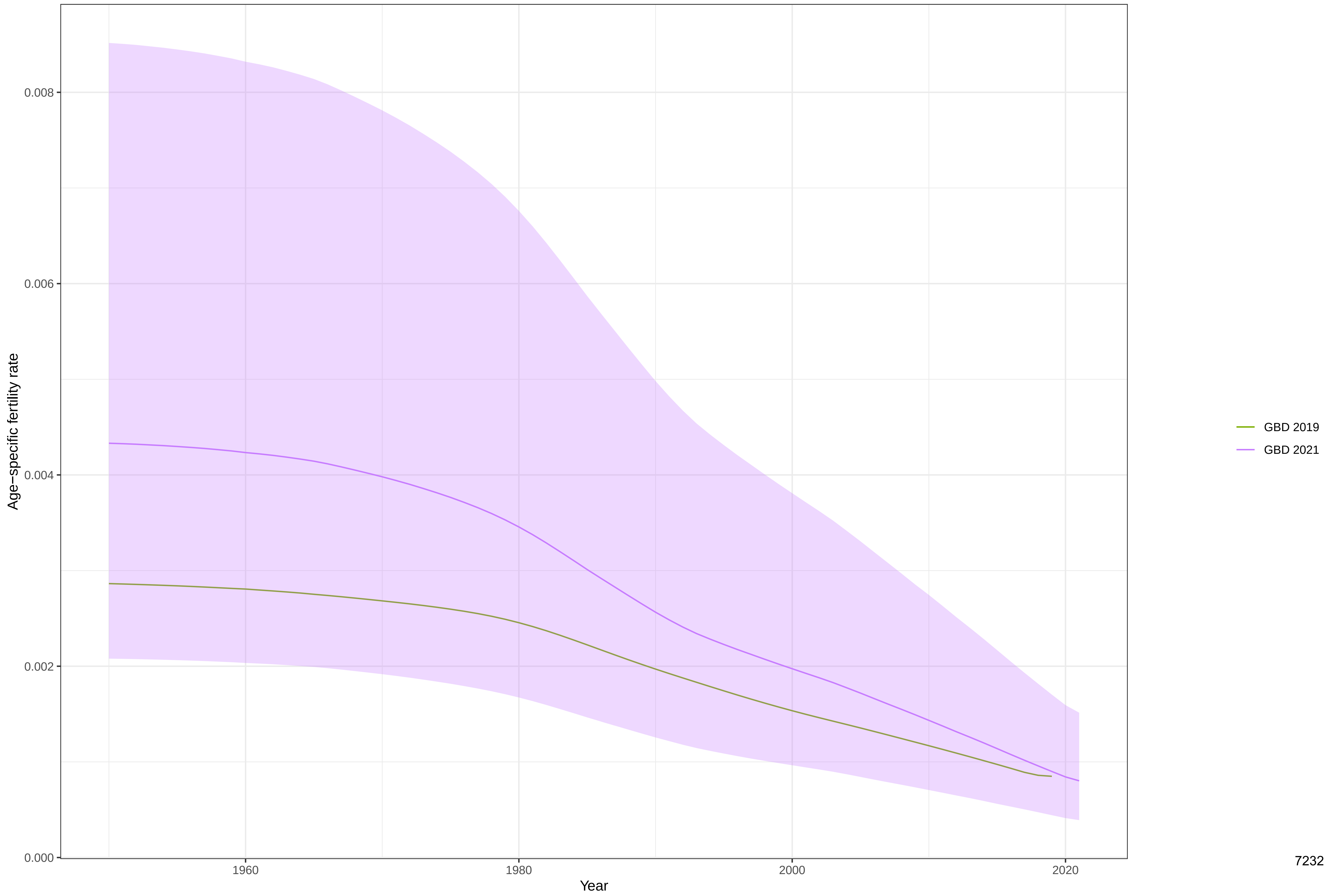

Age−specific fertility fitted trend lines, 1950−2021: Nyeri, Ages: 15−19
Data points enclosed with a diamond are reference sources

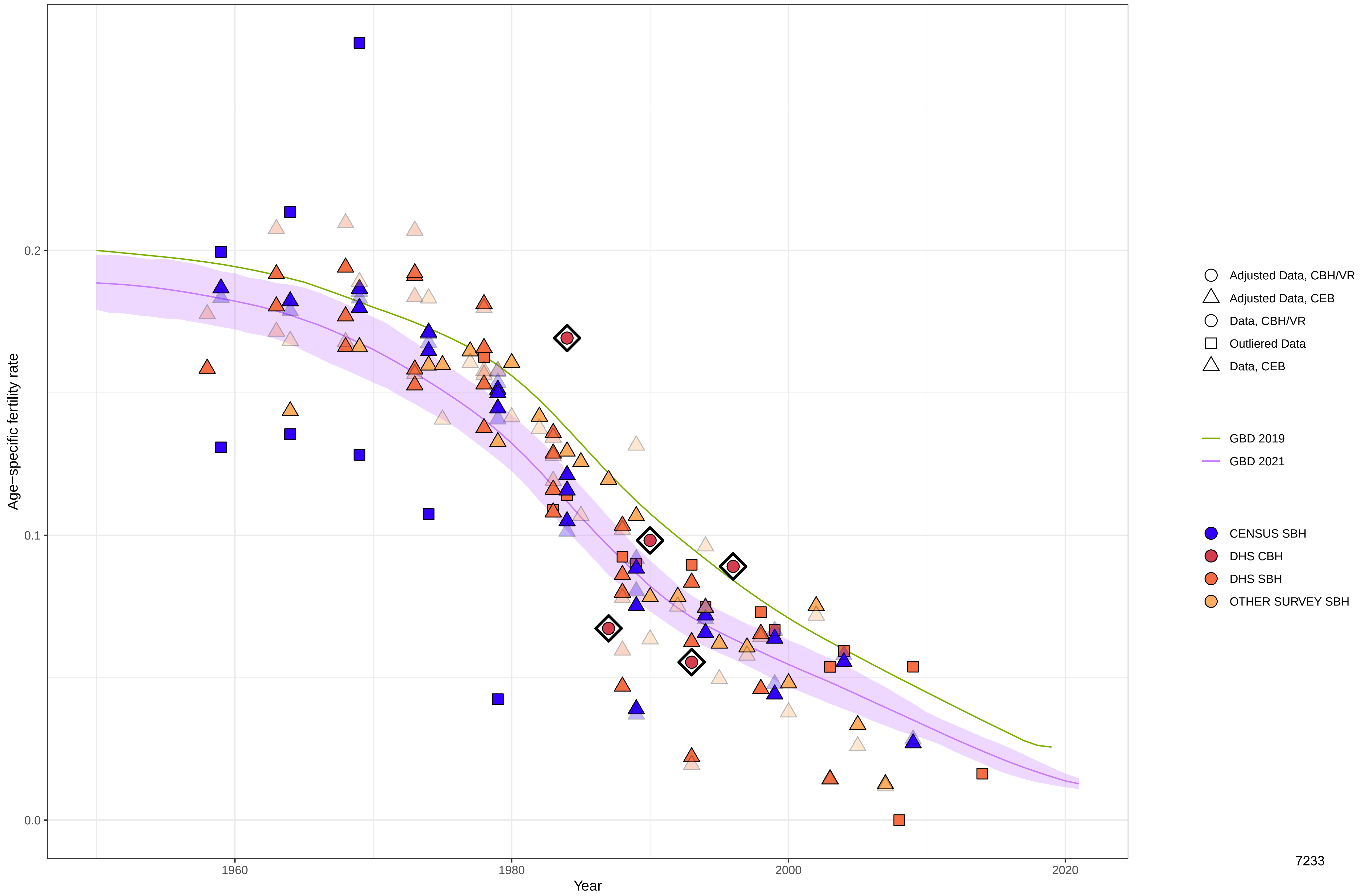

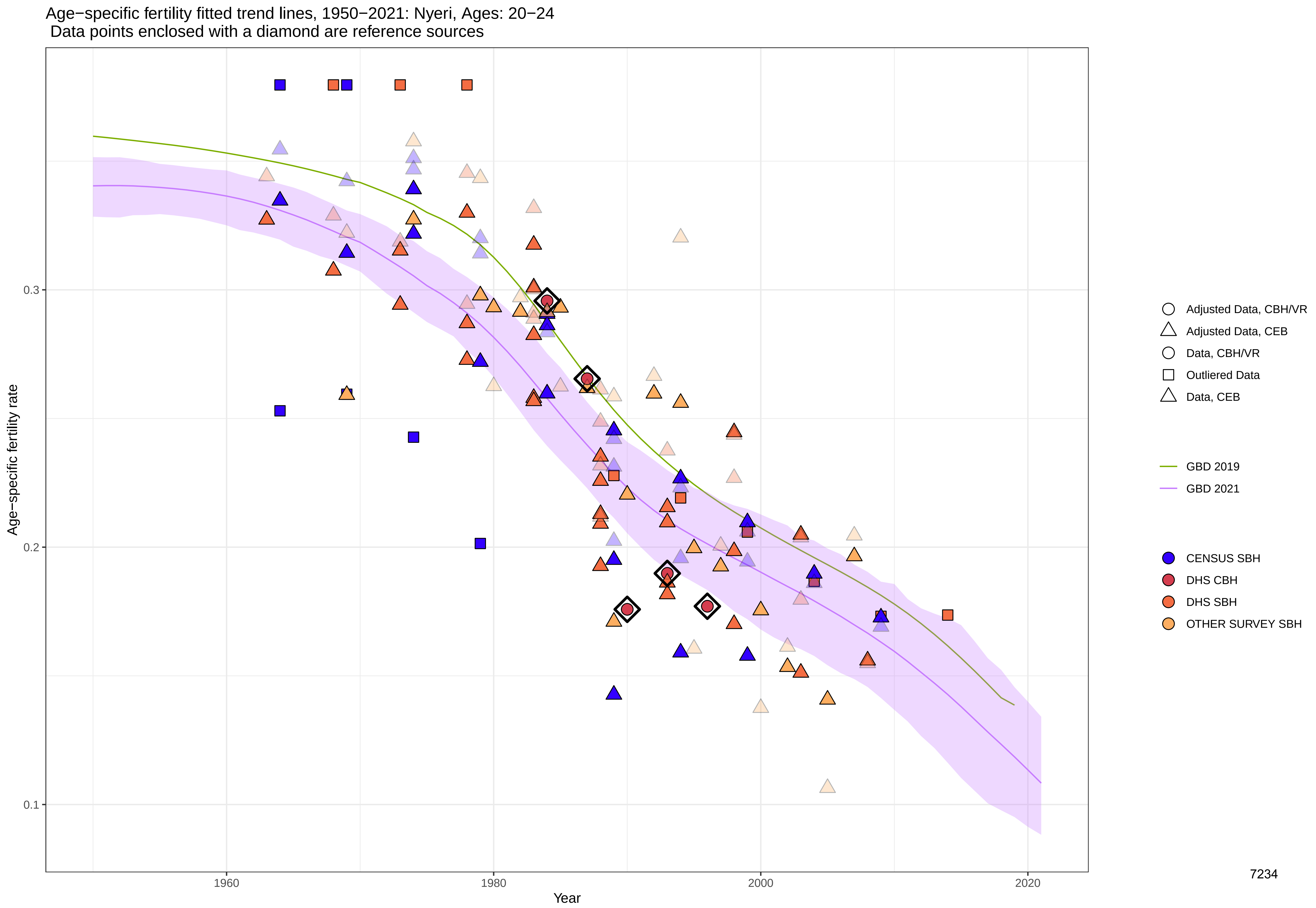
Age−specific fertility fitted trend lines, 1950−2021: Nyeri, Ages: 20−24
Data points enclosed with a diamond are reference sources
Age−specific fertility rate
Year
0.1
0.2
0.3
1960
1980
2000
2020
Adjusted Data, CBH/VR
Adjusted Data, CEB
Data, CBH/VR
Outliered Data
Data, CEB
GBD 2019
GBD 2021
CENSUS SBH
DHS CBH
DHS SBH
OTHER SURVEY SBH

Age−specific fertility fitted trend lines, 1950−2021: Nyeri, Ages: 25−29
Data points enclosed with a diamond are reference sources

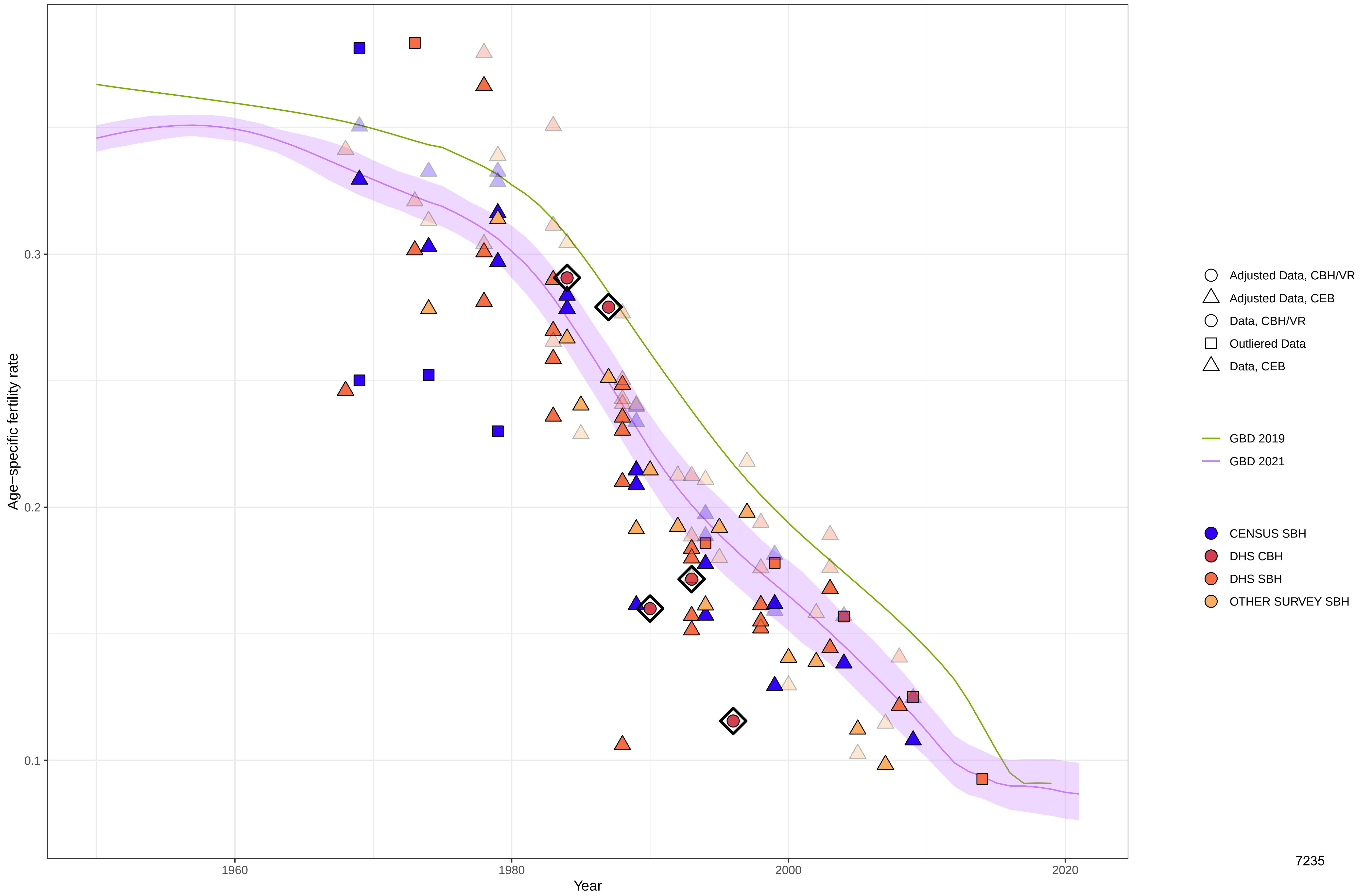

Age−specific fertility fitted trend lines, 1950−2021: Nyeri, Ages: 30−34
Data points enclosed with a diamond are reference sources

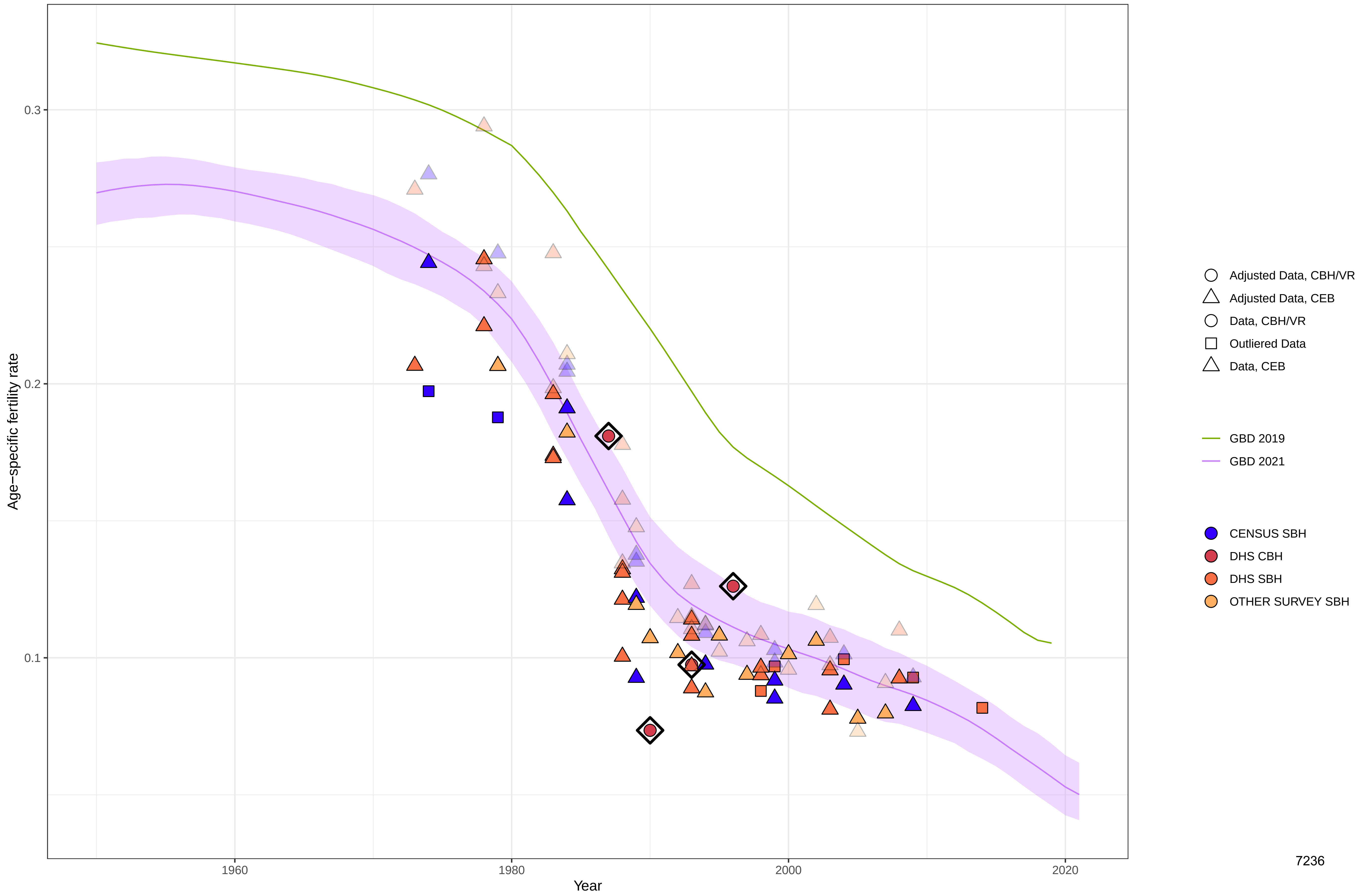

Age−specific fertility fitted trend lines, 1950−2021: Nyeri, Ages: 35−39
Data points enclosed with a diamond are reference sources

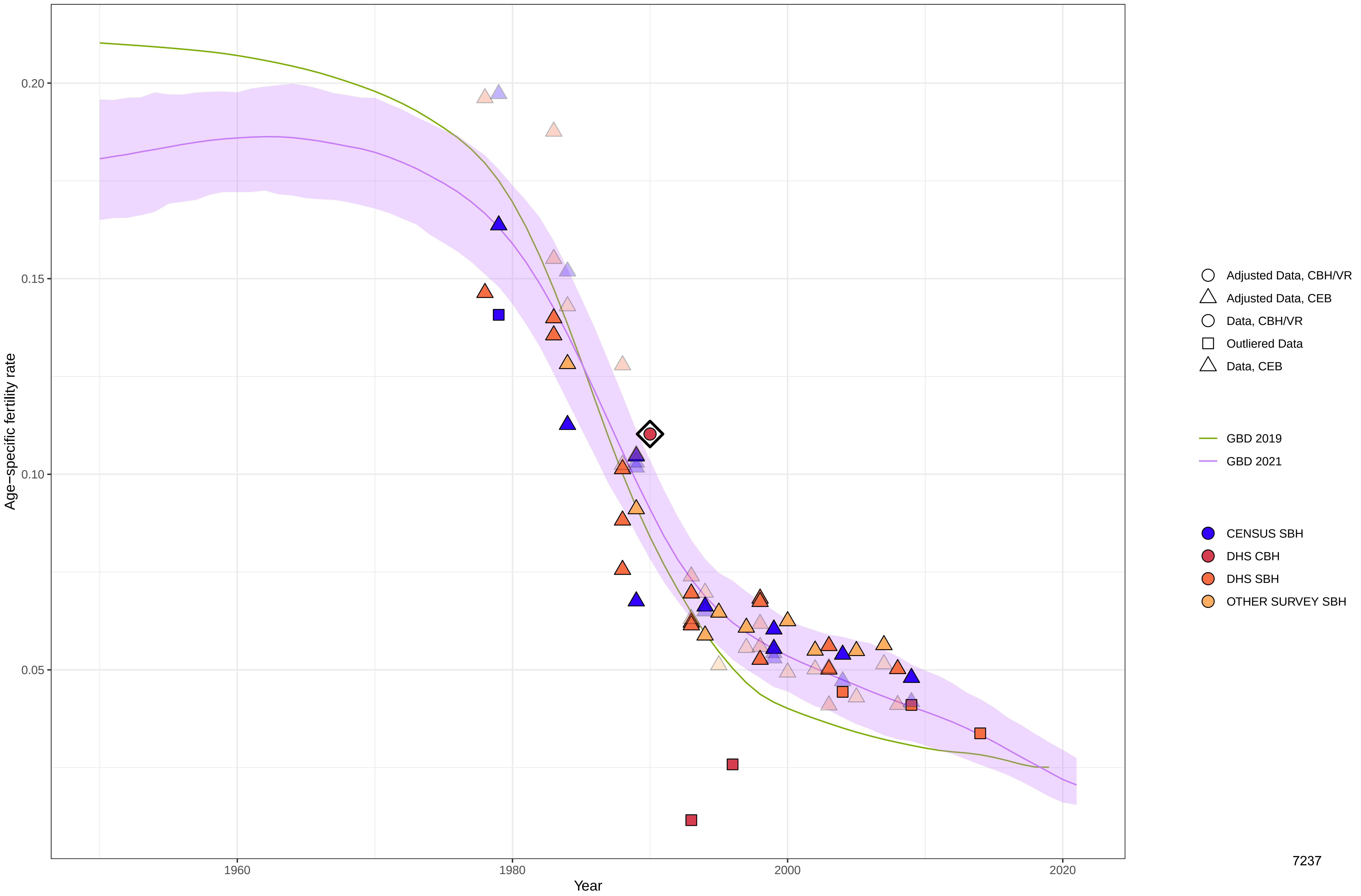

Age−specific fertility fitted trend lines, 1950−2021: Nyeri, Ages: 40−44
Data points enclosed with a diamond are reference sources

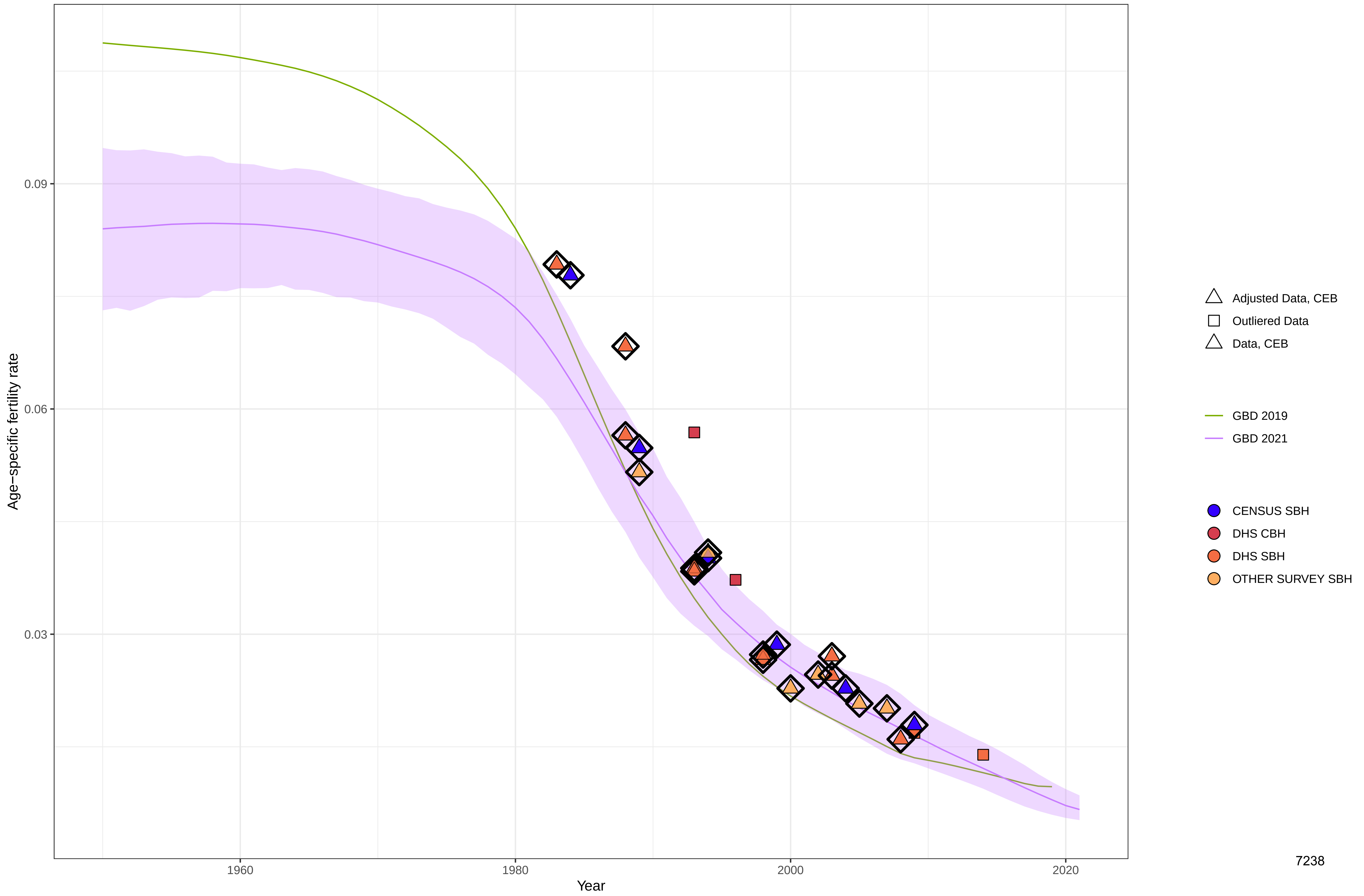

Age−specific fertility fitted trend lines, 1950−2021: Nyeri, Ages: 45−49
Data points enclosed with a diamond are reference sources

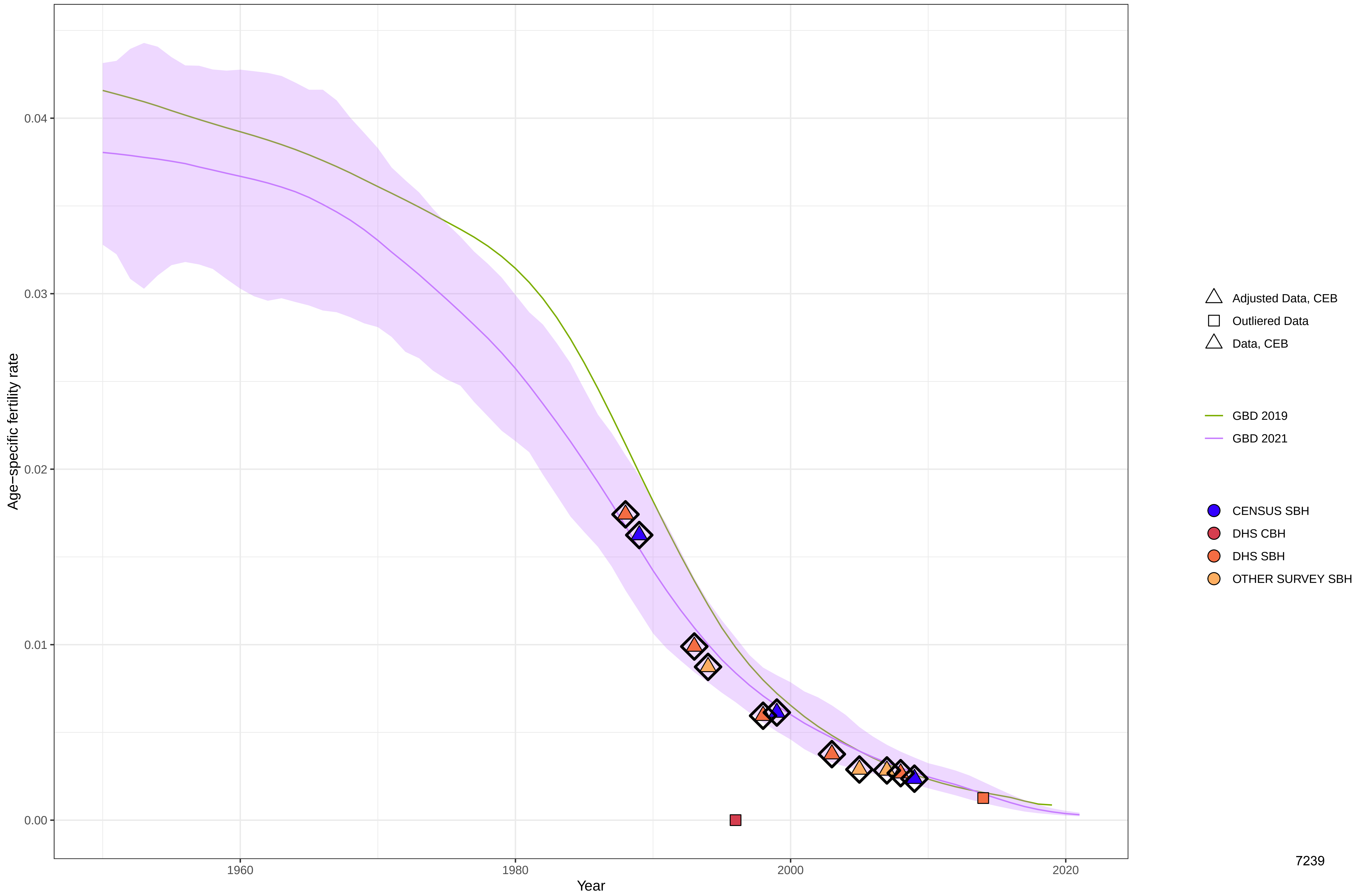

Age−specific fertility fitted trend lines, 1950−2021: Nyeri, Ages: 50−54
Data points enclosed with a diamond are reference sources

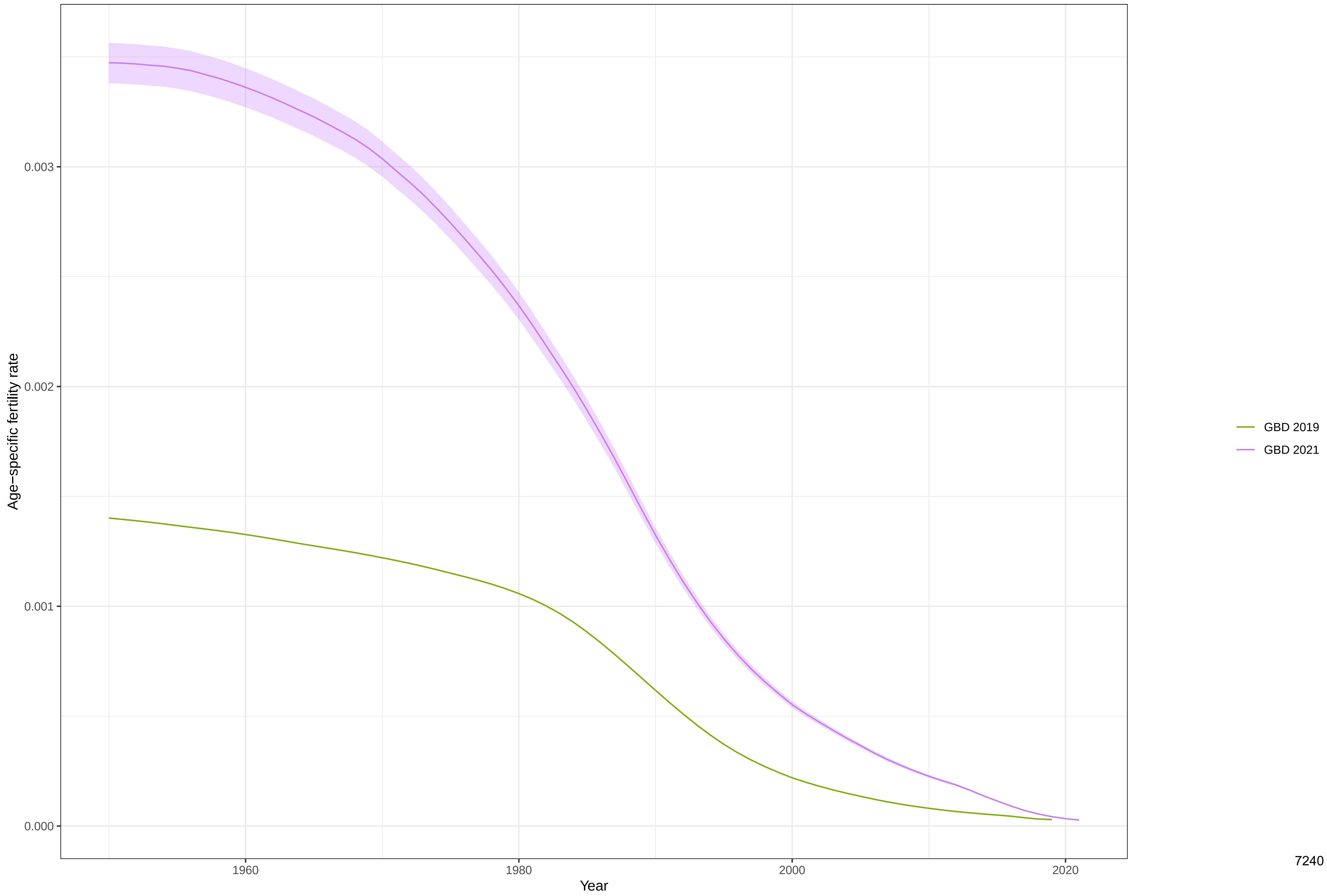

Total fertility rate fitted trend lines, 1950−2021: Nyeri TFR
Data points enclosed with a diamond are reference sources

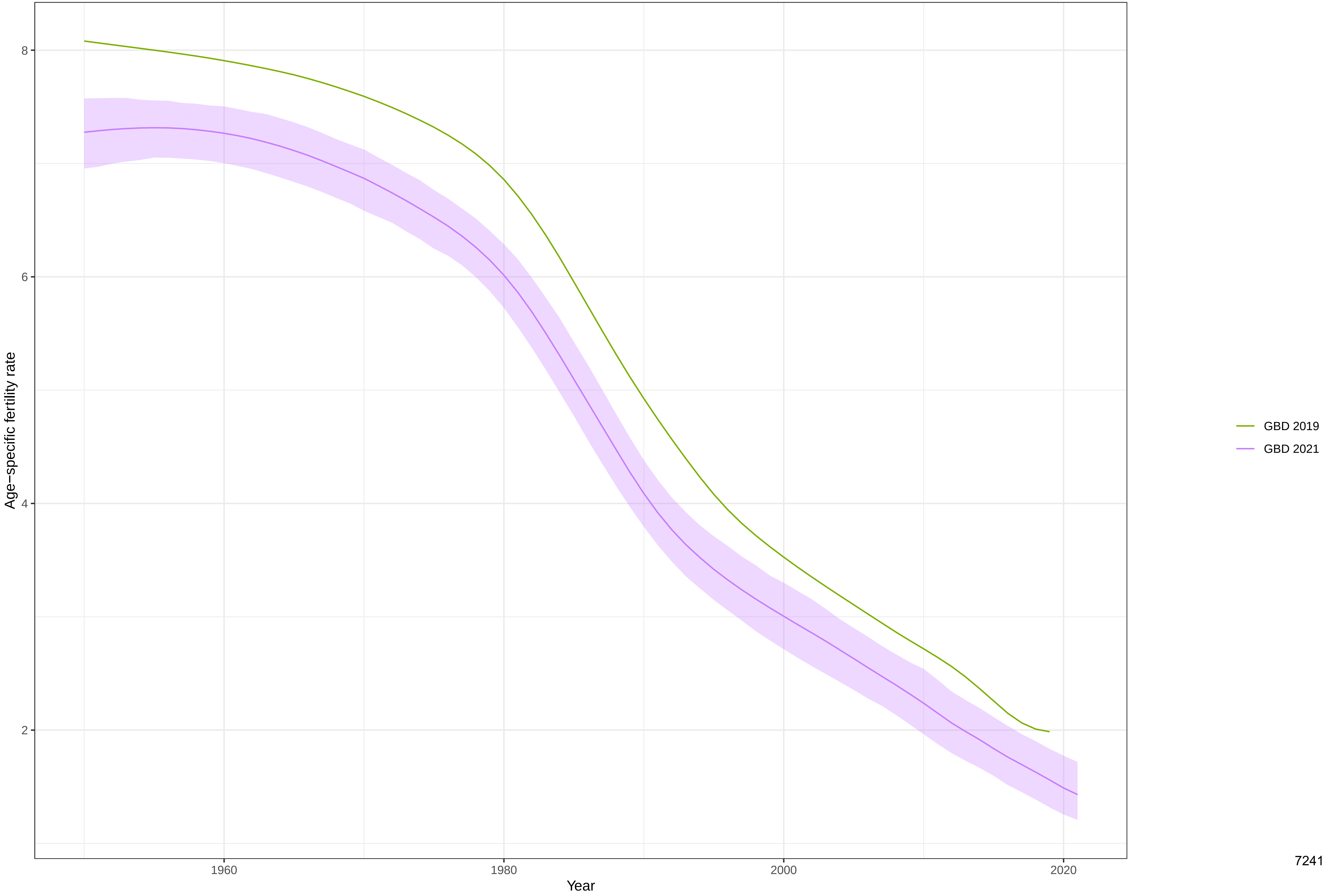

Age−specific fertility fitted trend lines, 1950−2021: Samburu, Ages: 10−14
Data points enclosed with a diamond are reference sources

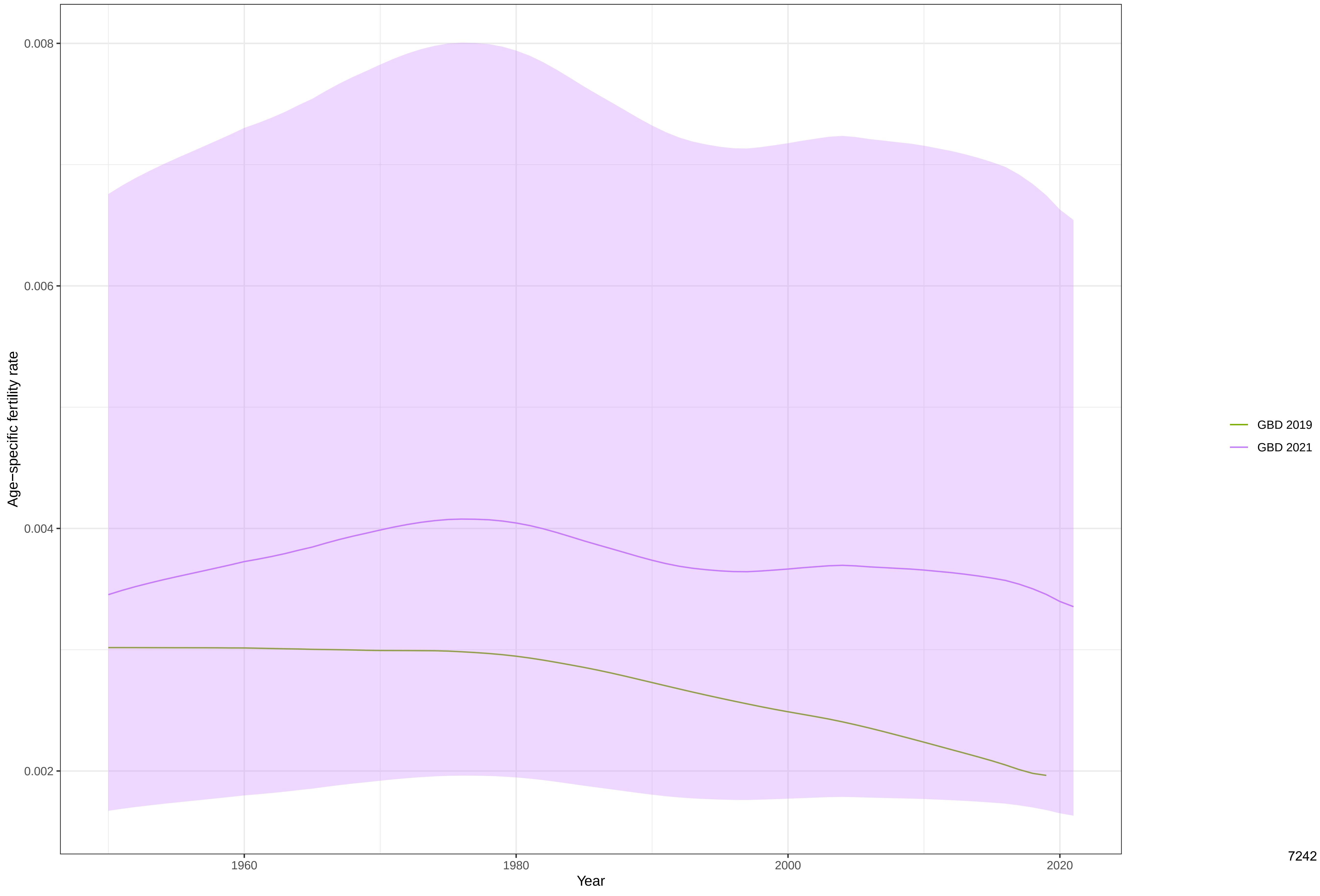

Age−specific fertility fitted trend lines, 1950−2021: Samburu, Ages: 15−19
Data points enclosed with a diamond are reference sources

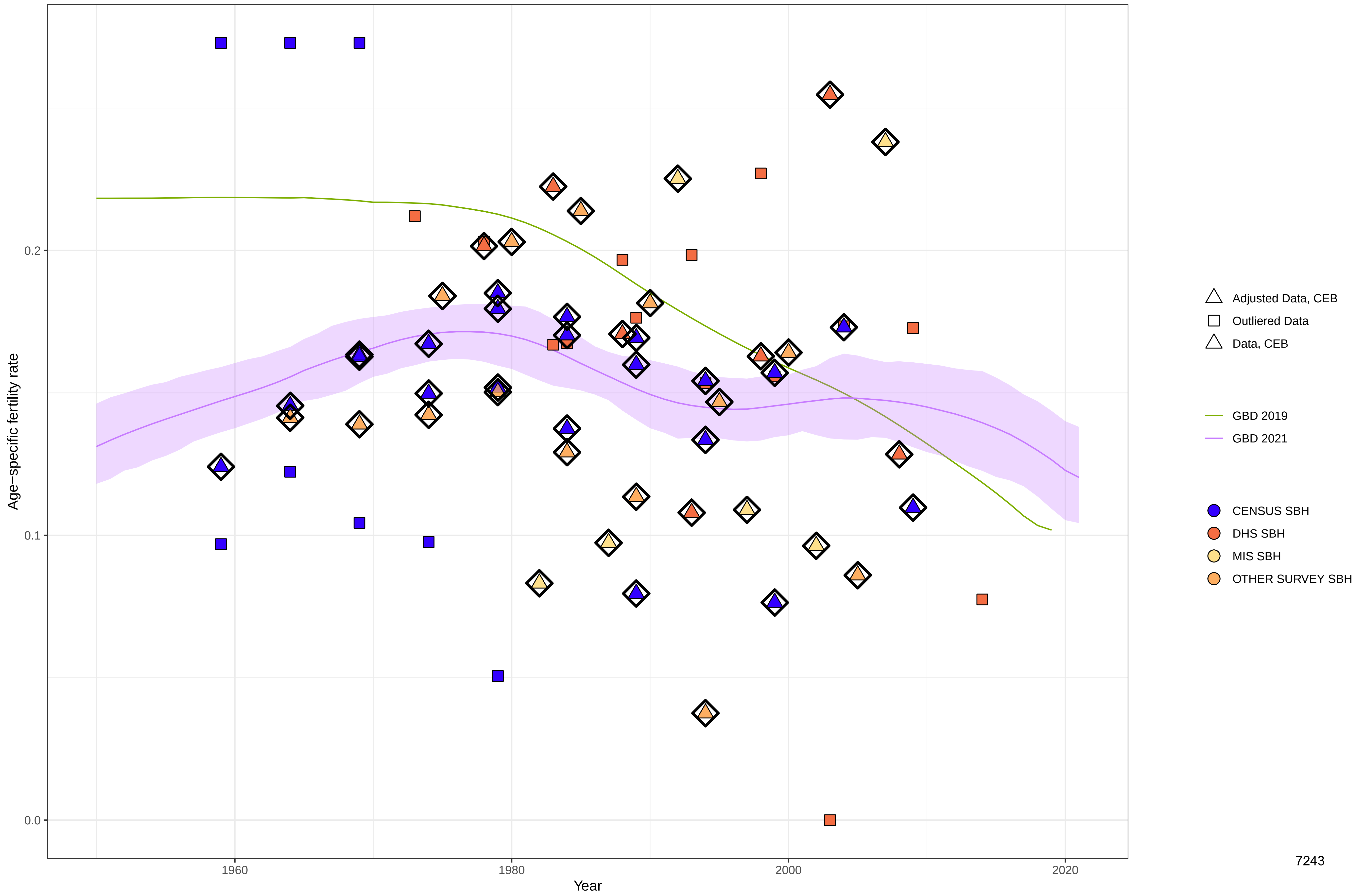

Age−specific fertility fitted trend lines, 1950−2021: Samburu, Ages: 20−24
Data points enclosed with a diamond are reference sources

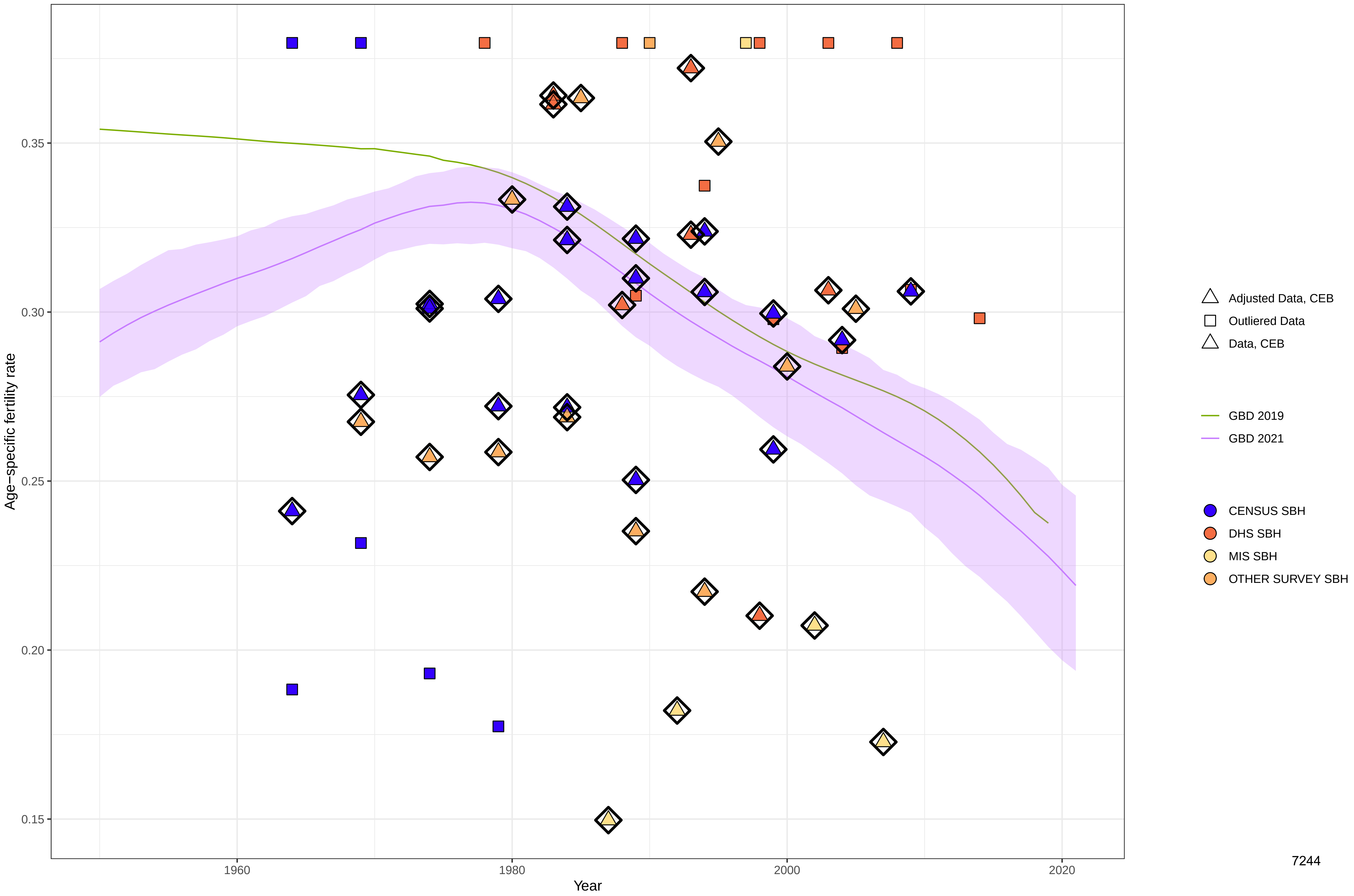

Age−specific fertility fitted trend lines, 1950−2021: Samburu, Ages: 25−29
Data points enclosed with a diamond are reference sources

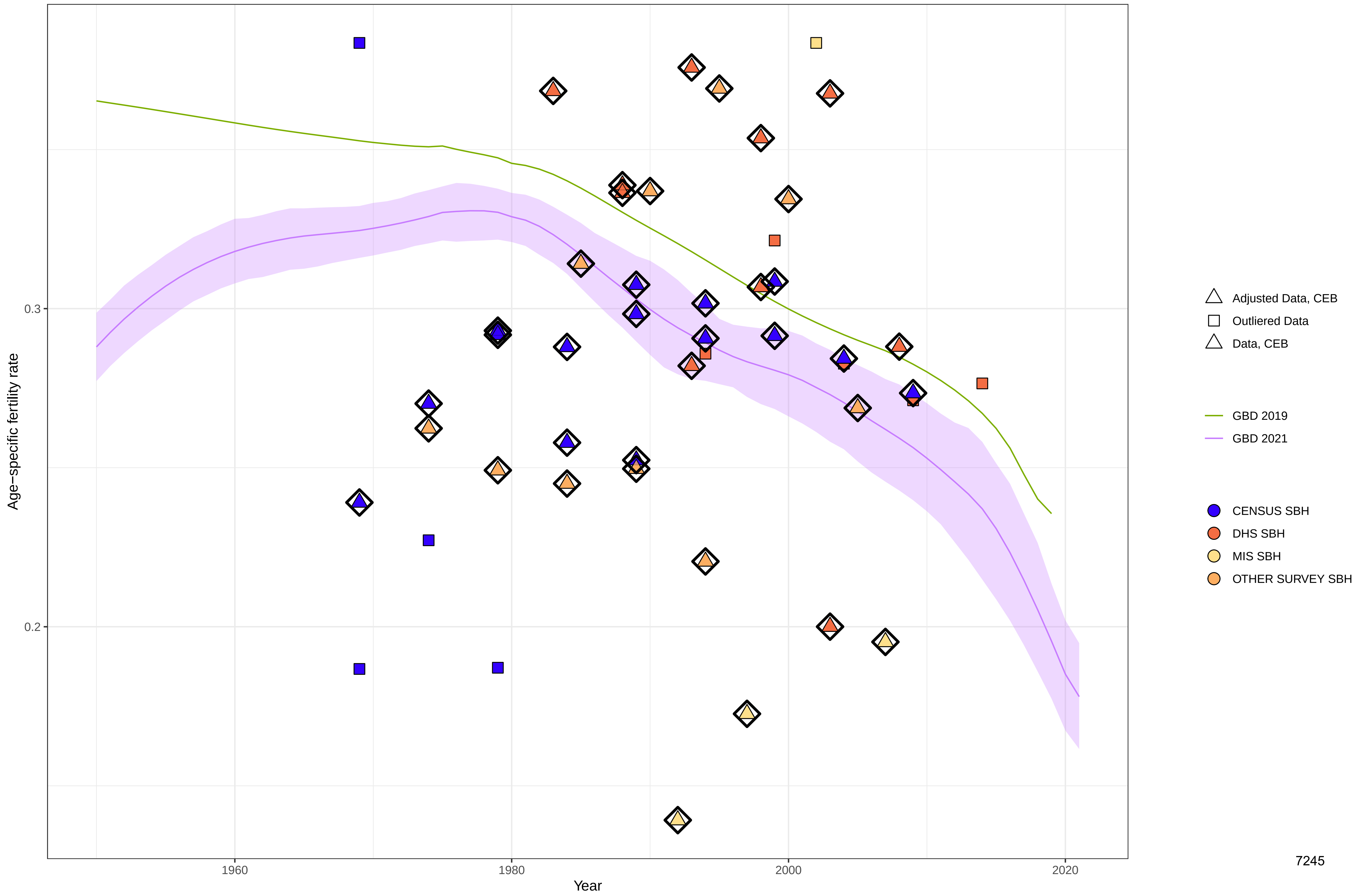

Age−specific fertility fitted trend lines, 1950−2021: Samburu, Ages: 30−34
Data points enclosed with a diamond are reference sources

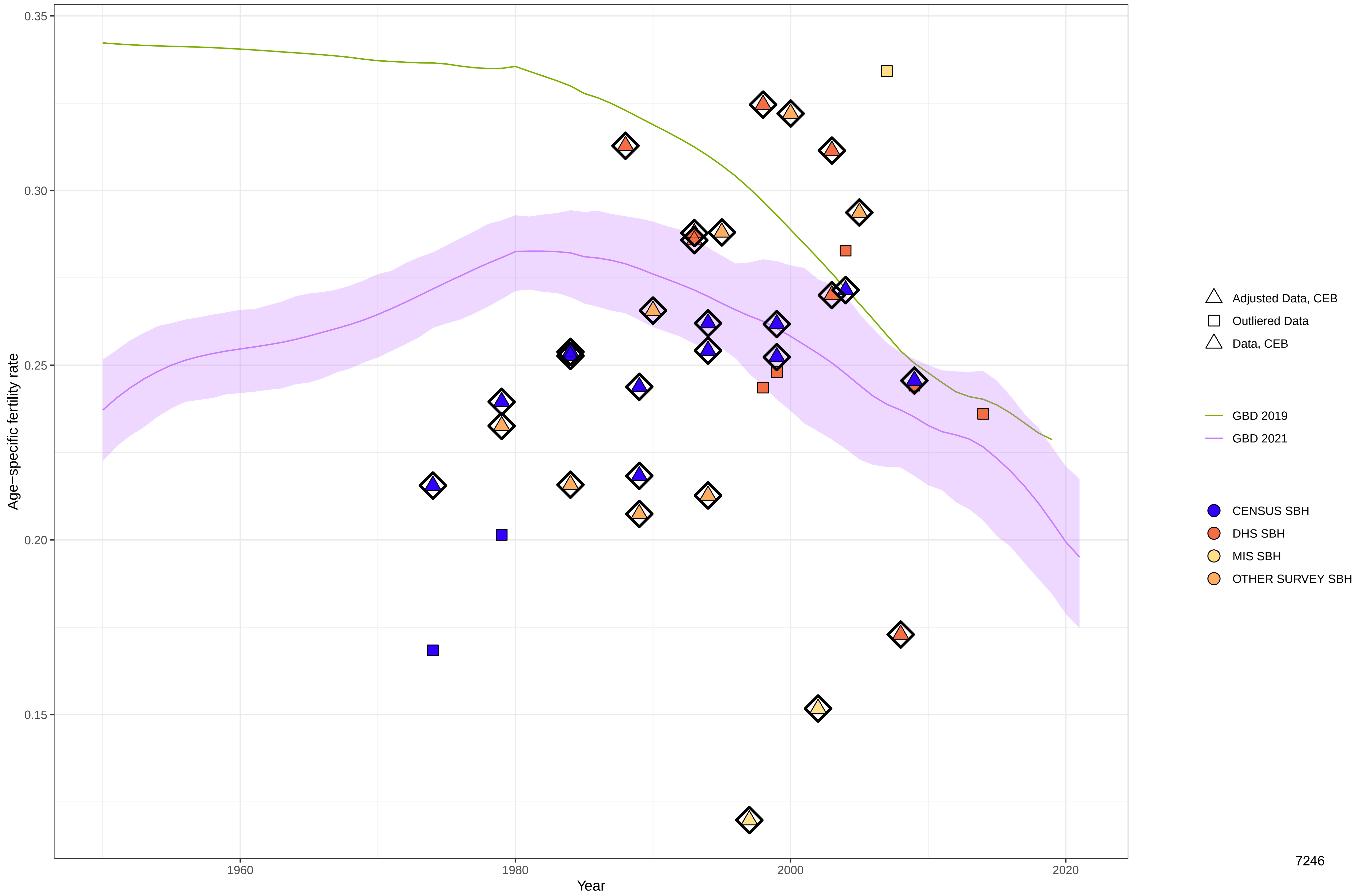

Age−specific fertility fitted trend lines, 1950−2021: Samburu, Ages: 35−39
Data points enclosed with a diamond are reference sources

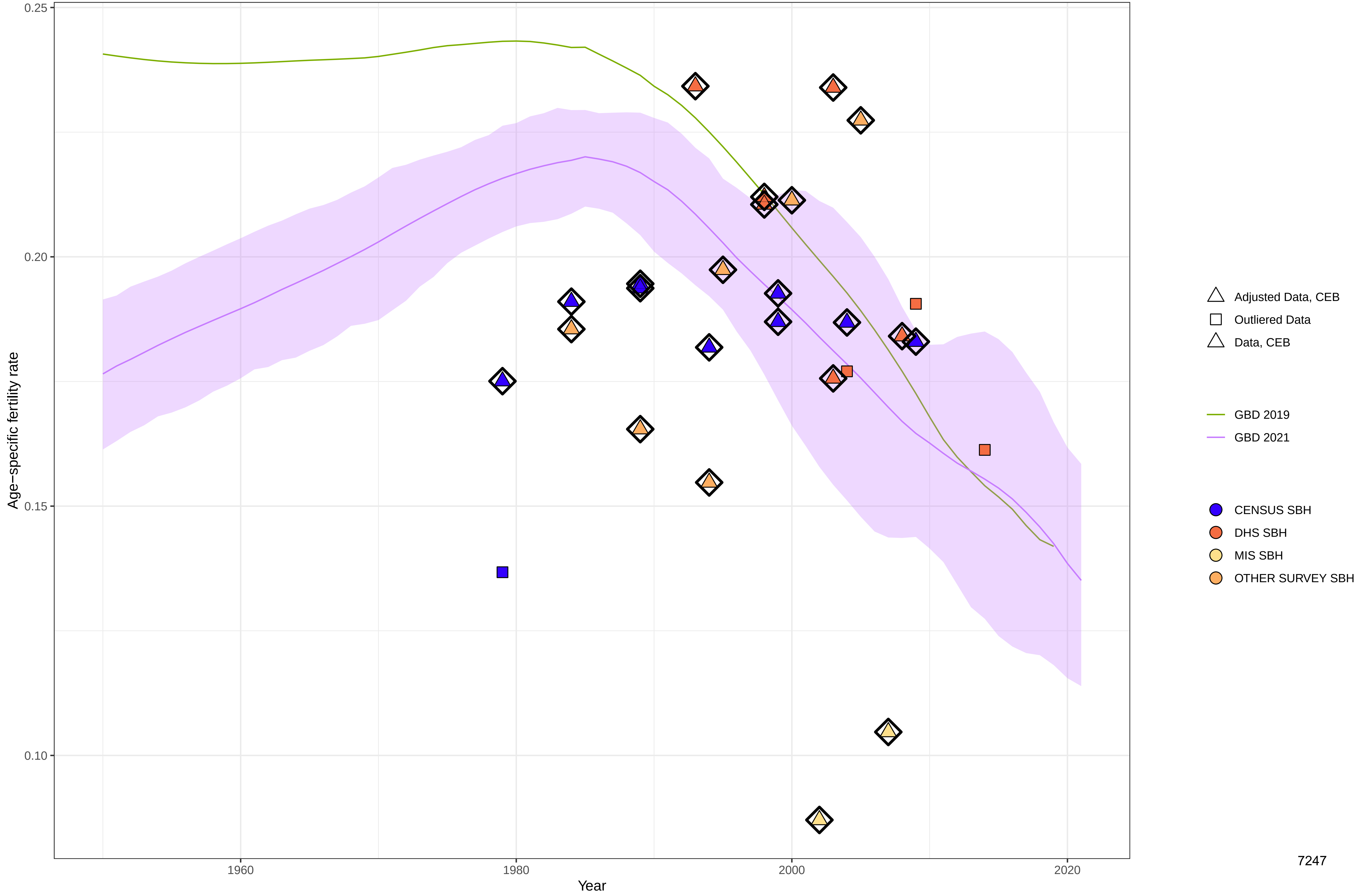

7247

Age−specific fertility fitted trend lines, 1950−2021: Samburu, Ages: 40−44
Data points enclosed with a diamond are reference sources

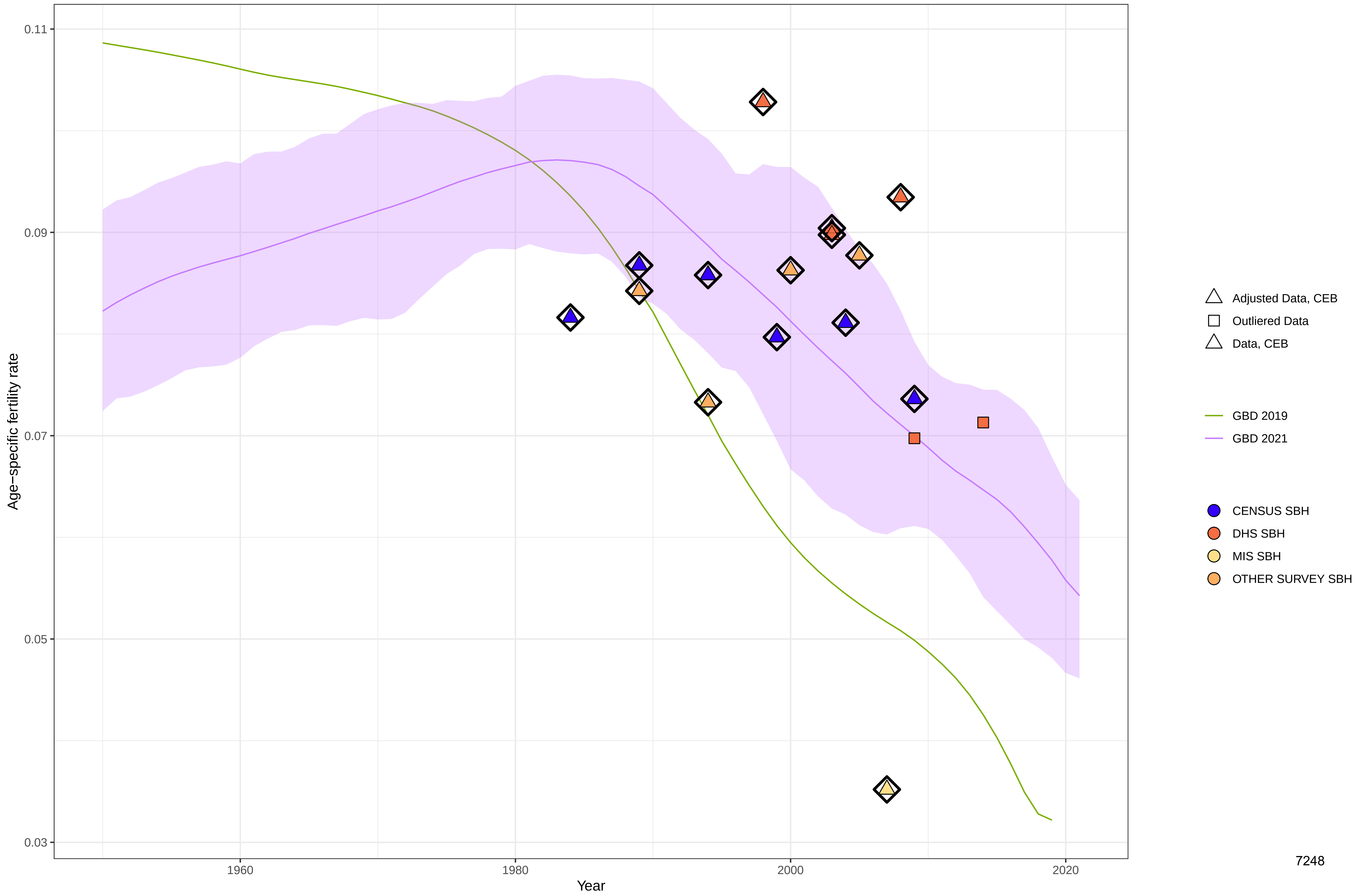

Age−specific fertility fitted trend lines, 1950−2021: Samburu, Ages: 45−49
Data points enclosed with a diamond are reference sources

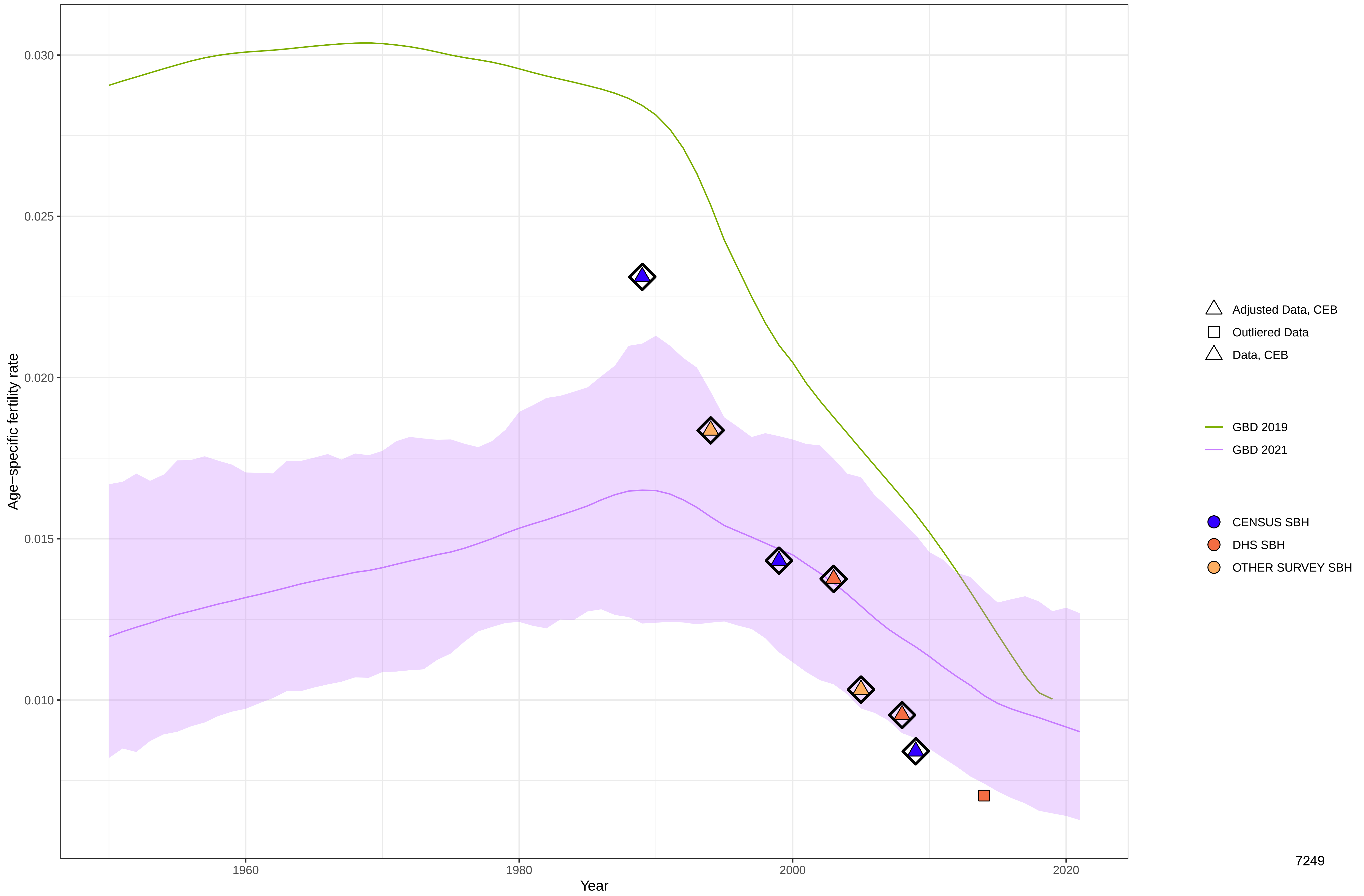

Age−specific fertility fitted trend lines, 1950−2021: Samburu, Ages: 50−54
Data points enclosed with a diamond are reference sources

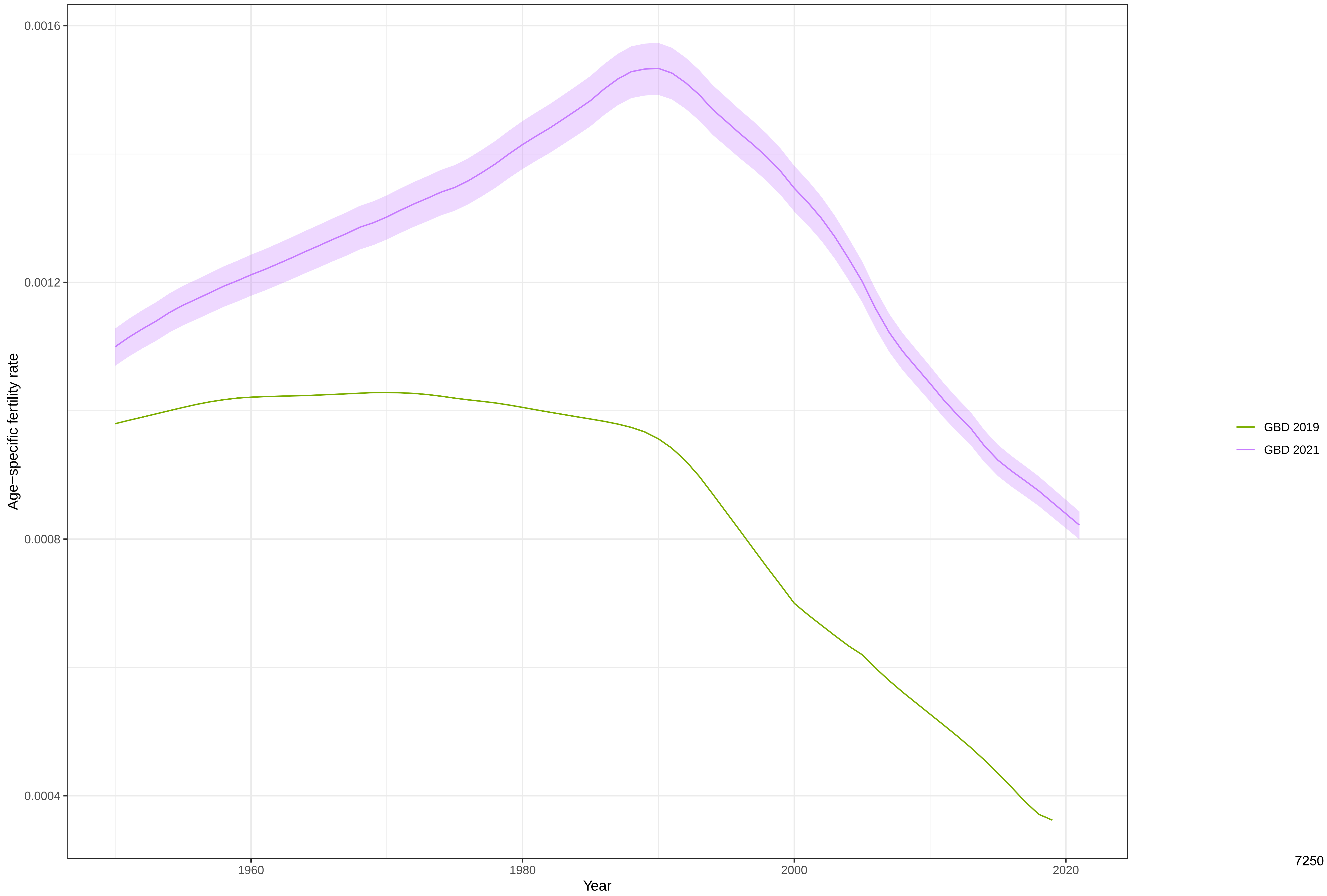

Total fertility rate fitted trend lines, 1950−2021: Samburu TFR
Data points enclosed with a diamond are reference sources

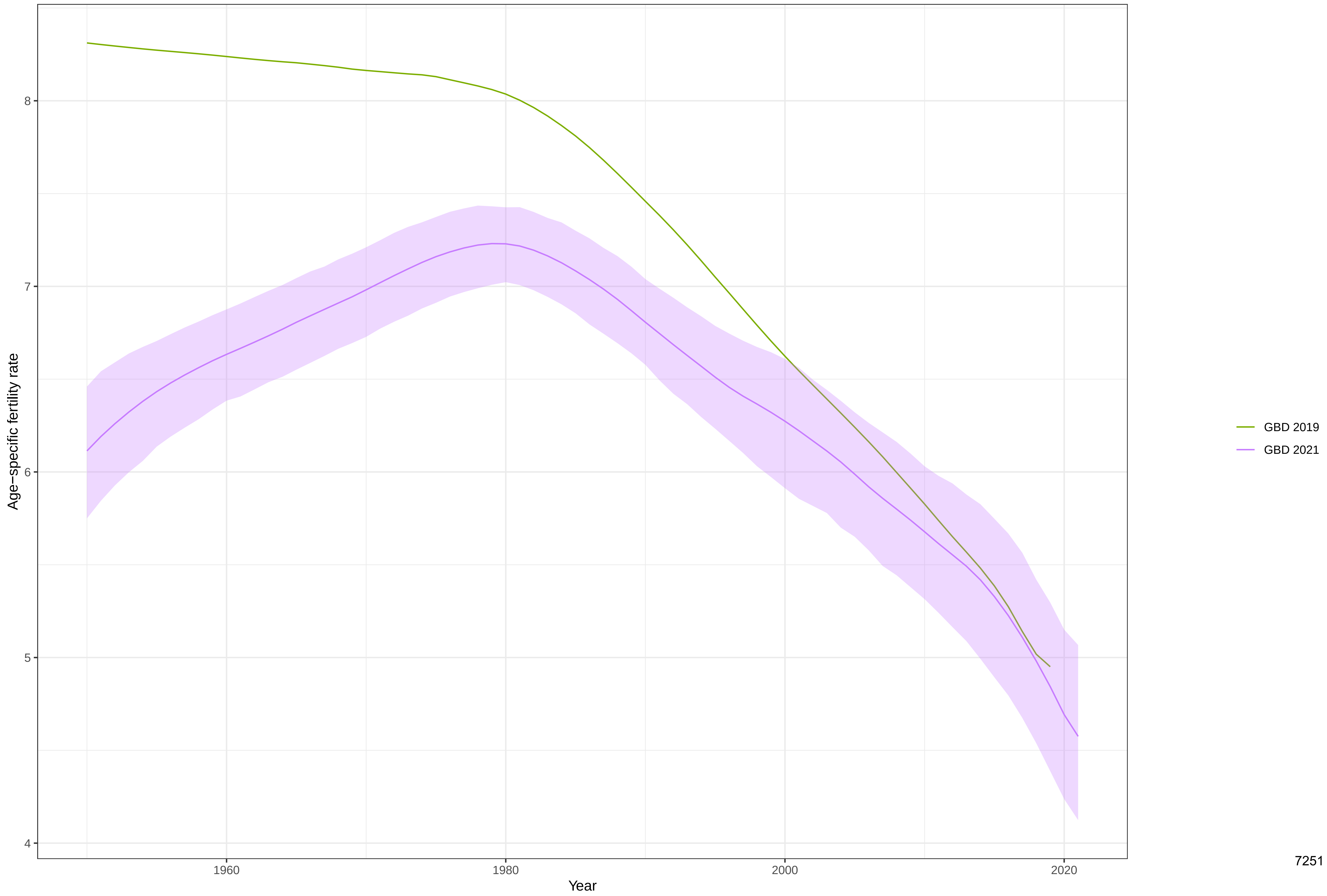

Age−specific fertility fitted trend lines, 1950−2021: Siaya, Ages: 10−14
Data points enclosed with a diamond are reference sources

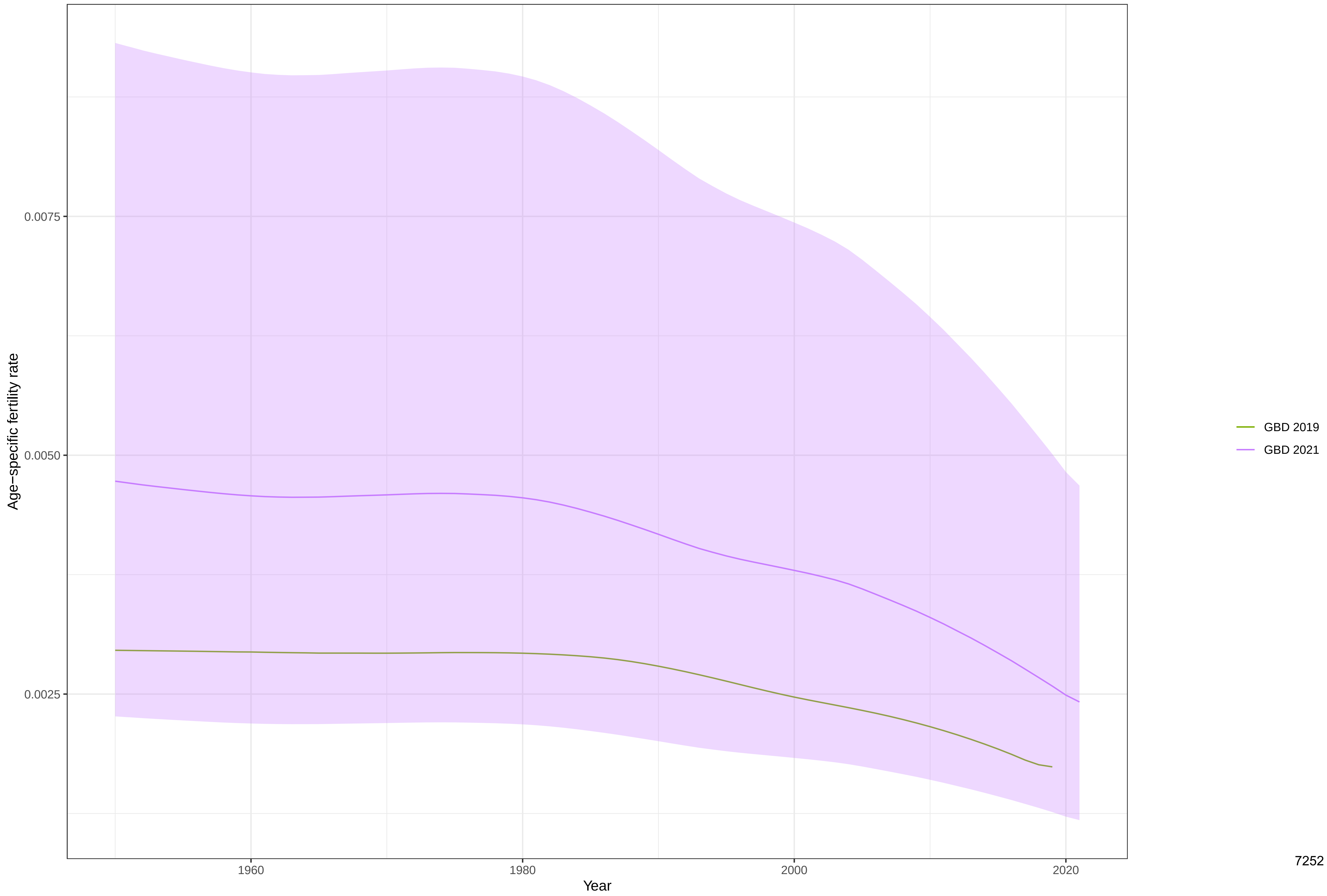

Age−specific fertility fitted trend lines, 1950−2021: Siaya, Ages: 15−19
Data points enclosed with a diamond are reference sources

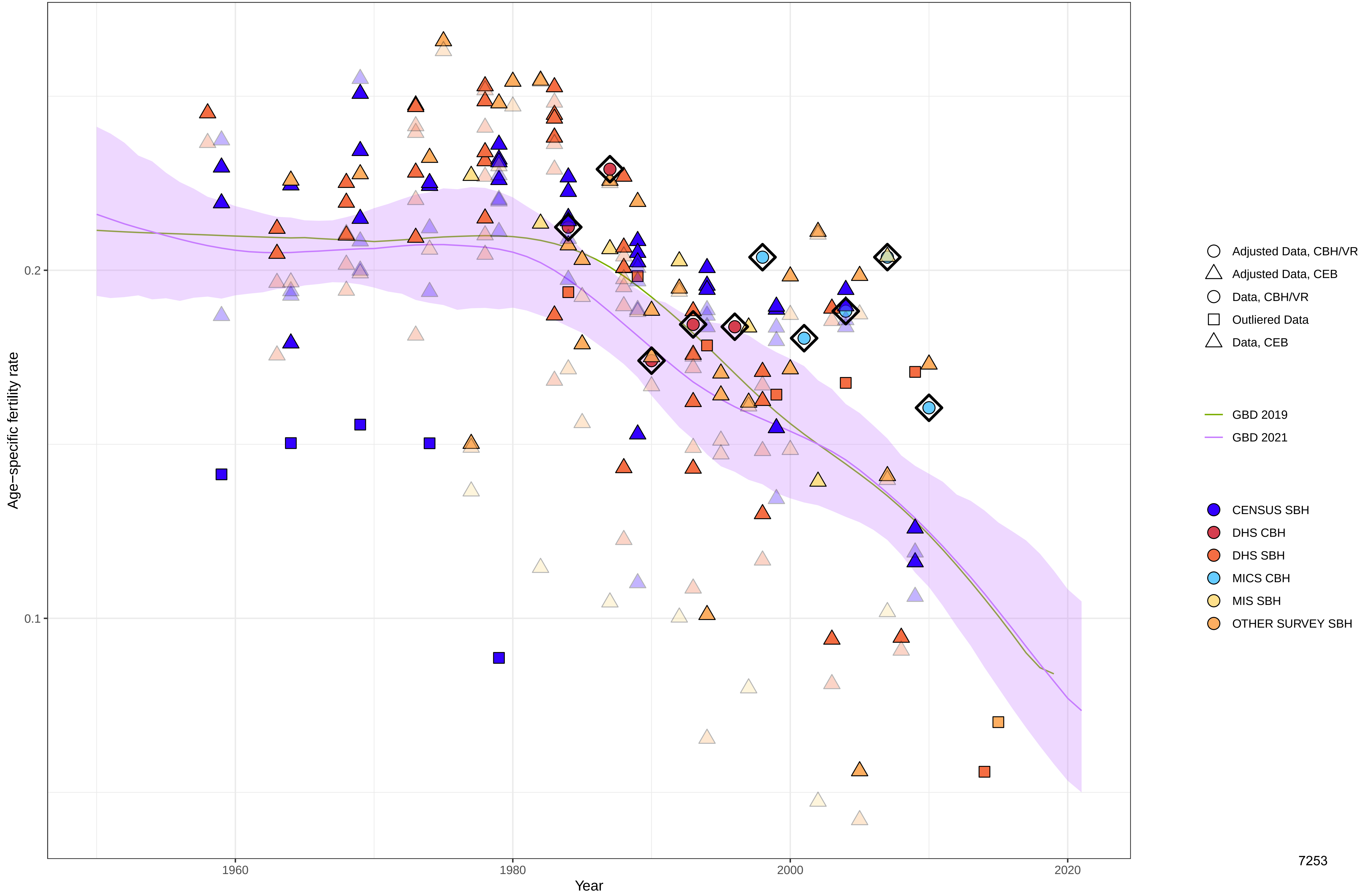

Age−specific fertility fitted trend lines, 1950−2021: Siaya, Ages: 20−24
Data points enclosed with a diamond are reference sources

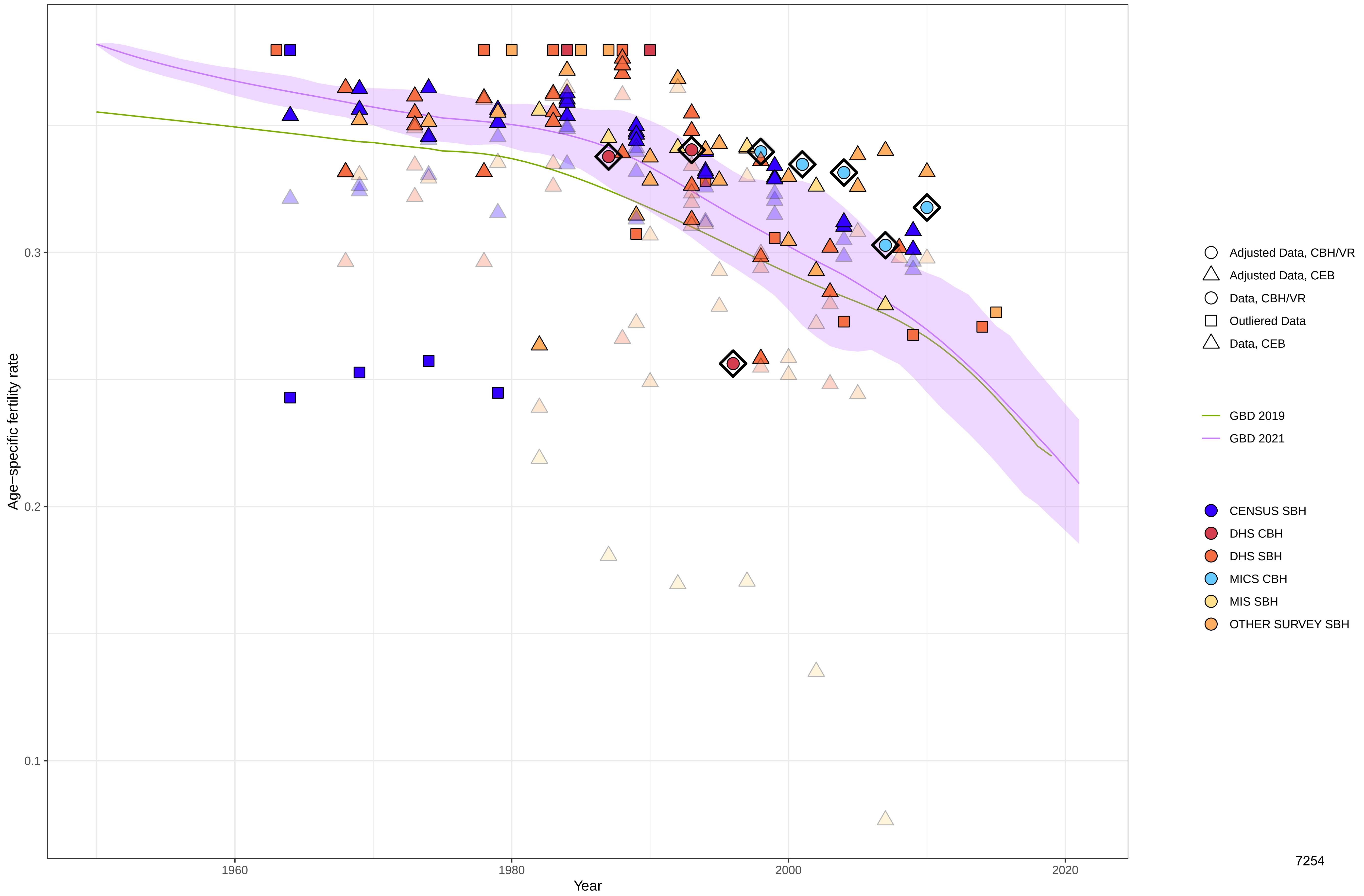

Age−specific fertility fitted trend lines, 1950−2021: Siaya, Ages: 25−29
Data points enclosed with a diamond are reference sources

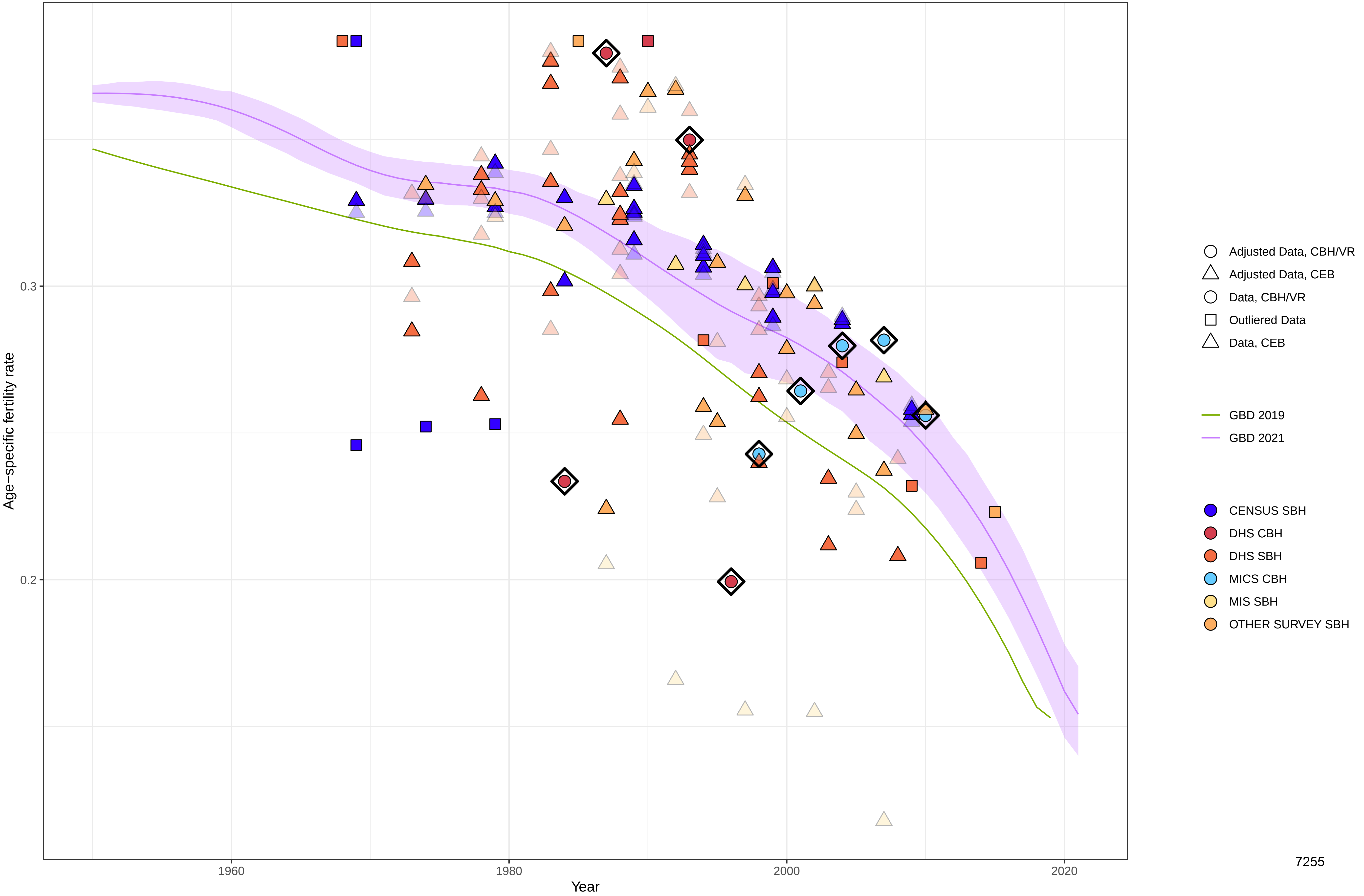

Age−specific fertility fitted trend lines, 1950−2021: Siaya, Ages: 30−34
Data points enclosed with a diamond are reference sources

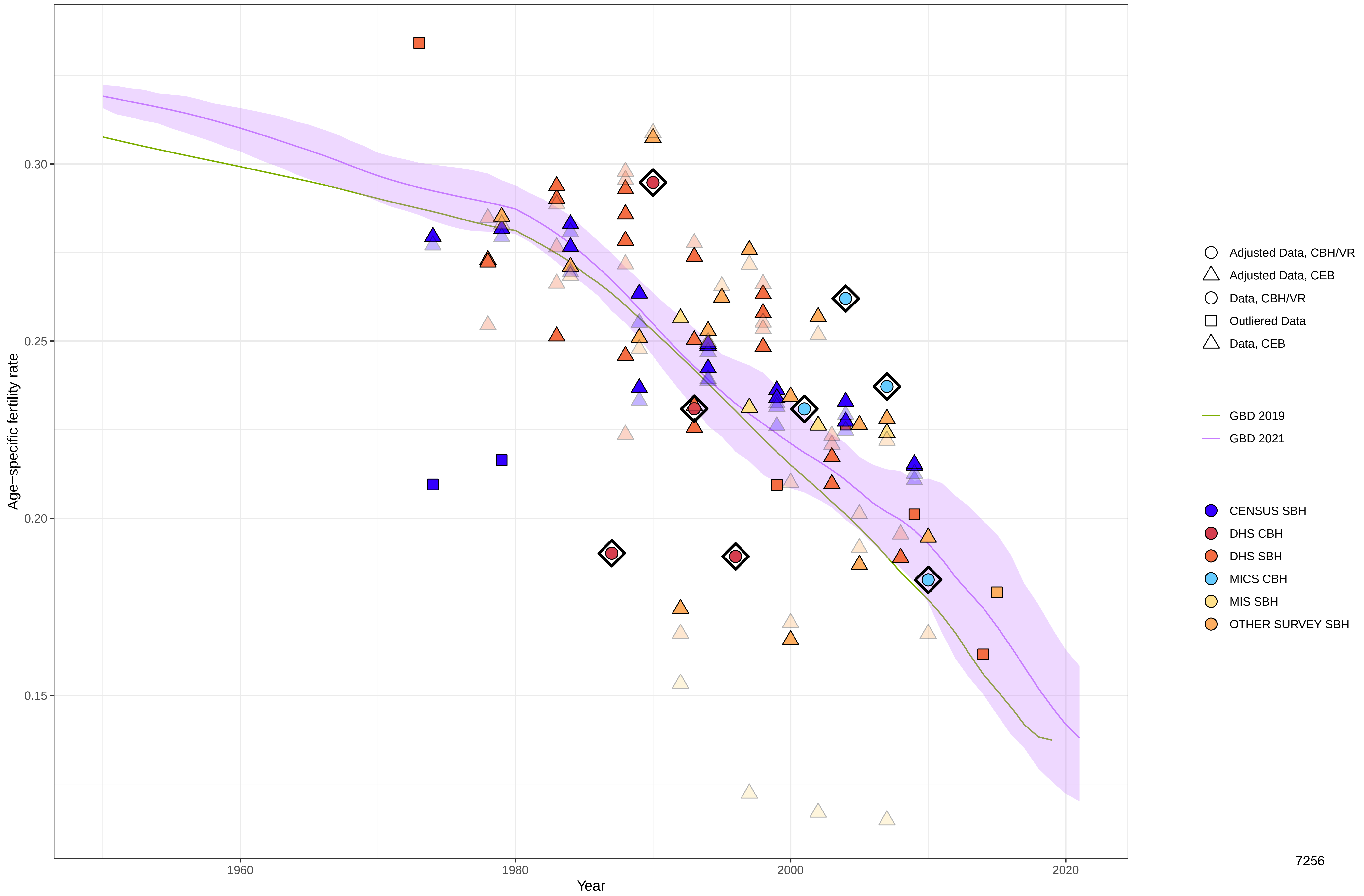

Age−specific fertility fitted trend lines, 1950−2021: Siaya, Ages: 35−39
Data points enclosed with a diamond are reference sources

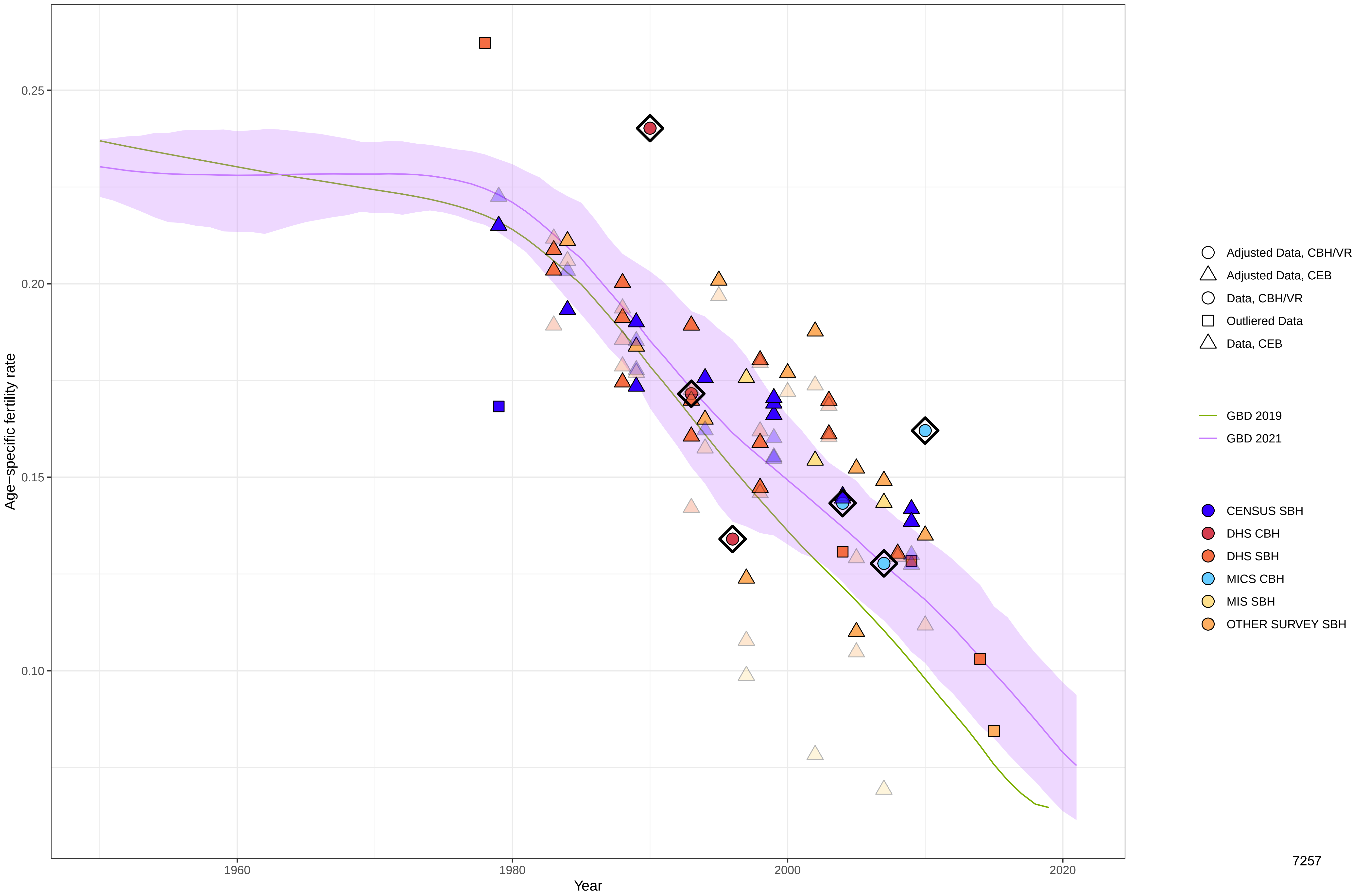

Age−specific fertility fitted trend lines, 1950−2021: Siaya, Ages: 40−44
Data points enclosed with a diamond are reference sources

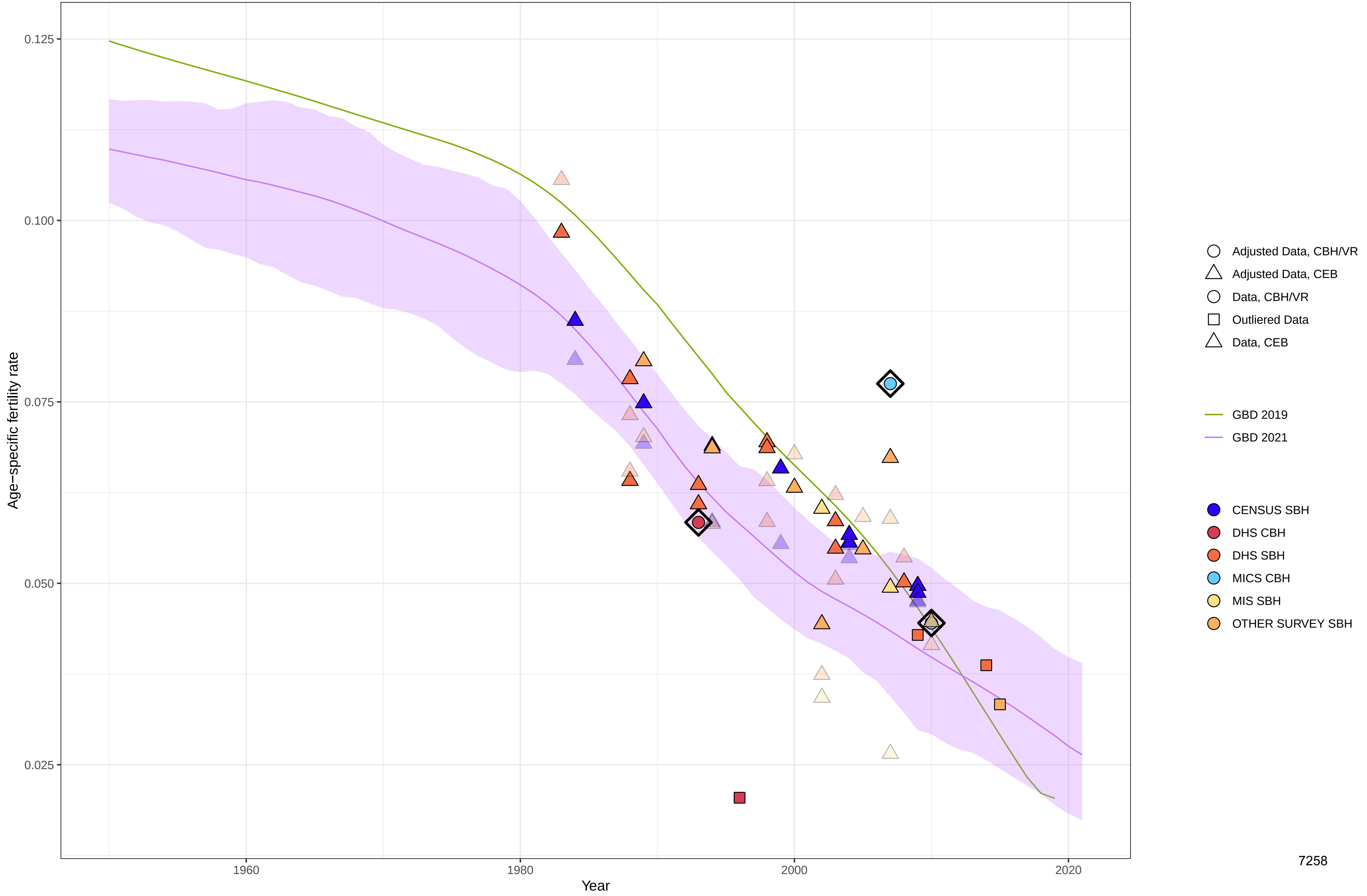

Age−specific fertility fitted trend lines, 1950−2021: Siaya, Ages: 45−49
Data points enclosed with a diamond are reference sources

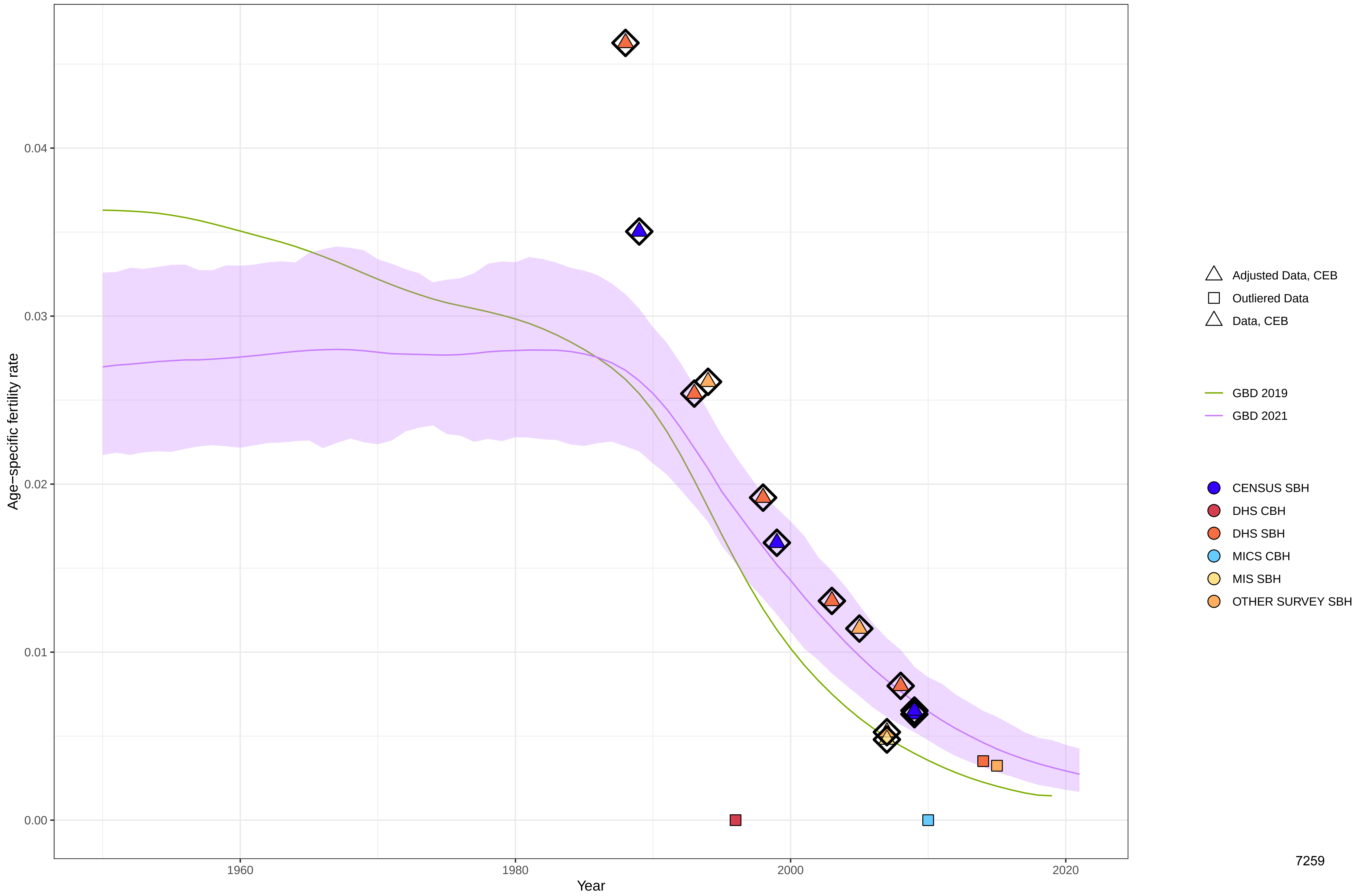

Age−specific fertility fitted trend lines, 1950−2021: Siaya, Ages: 50−54
Data points enclosed with a diamond are reference sources

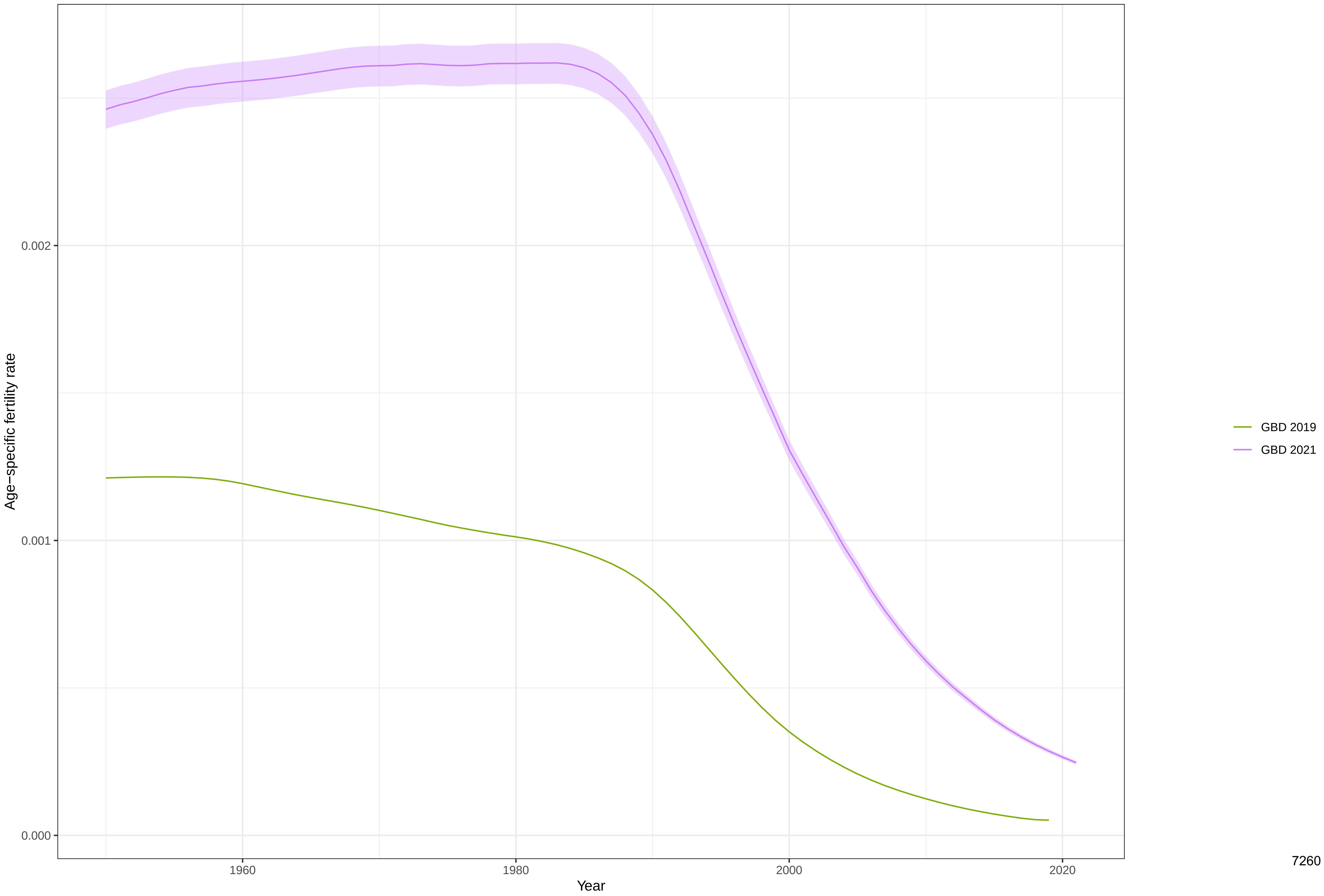

Total fertility rate fitted trend lines, 1950−2021: Siaya TFR
Data points enclosed with a diamond are reference sources

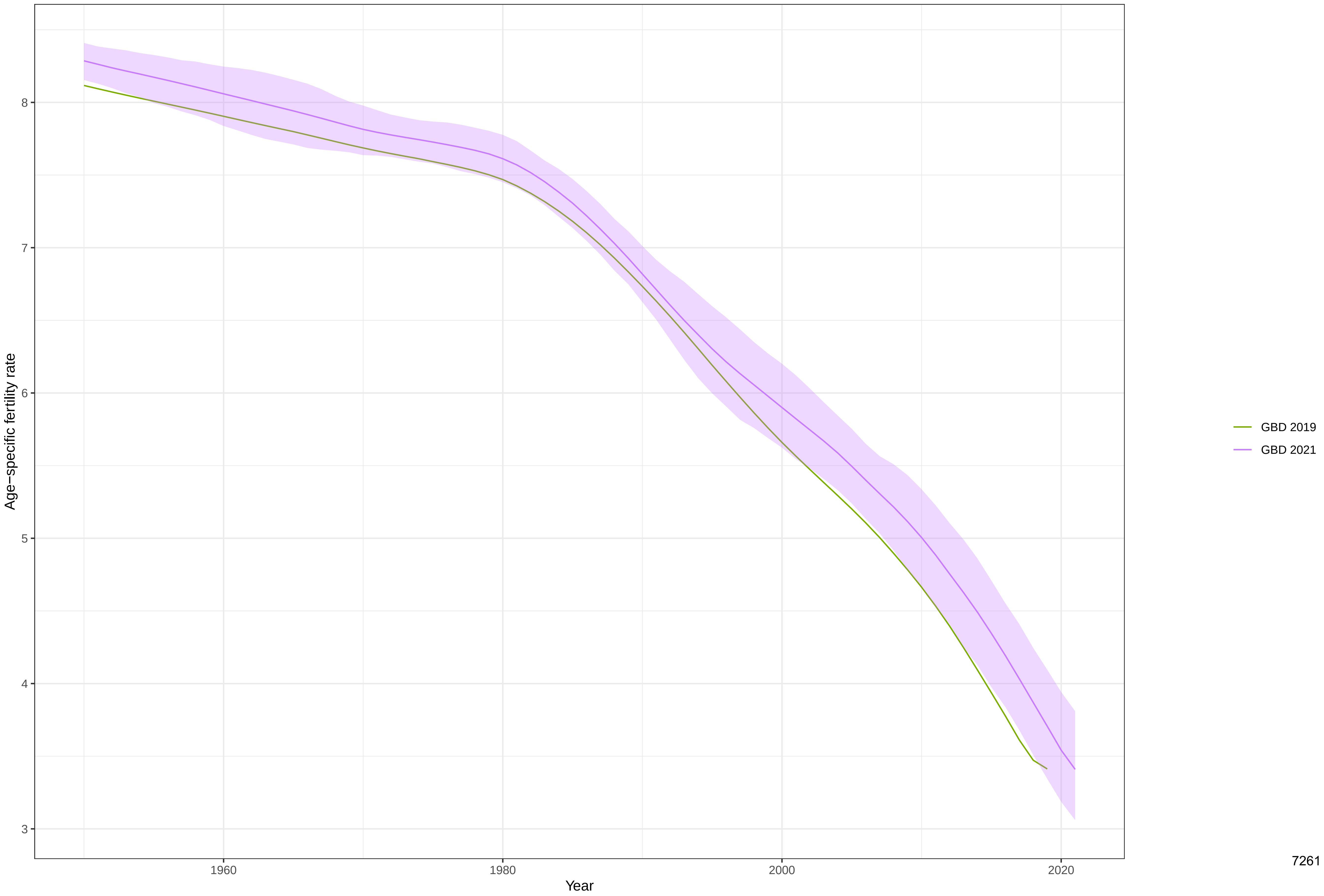

Age−specific fertility fitted trend lines, 1950−2021: Taita Taveta, Ages: 10−14
Data points enclosed with a diamond are reference sources

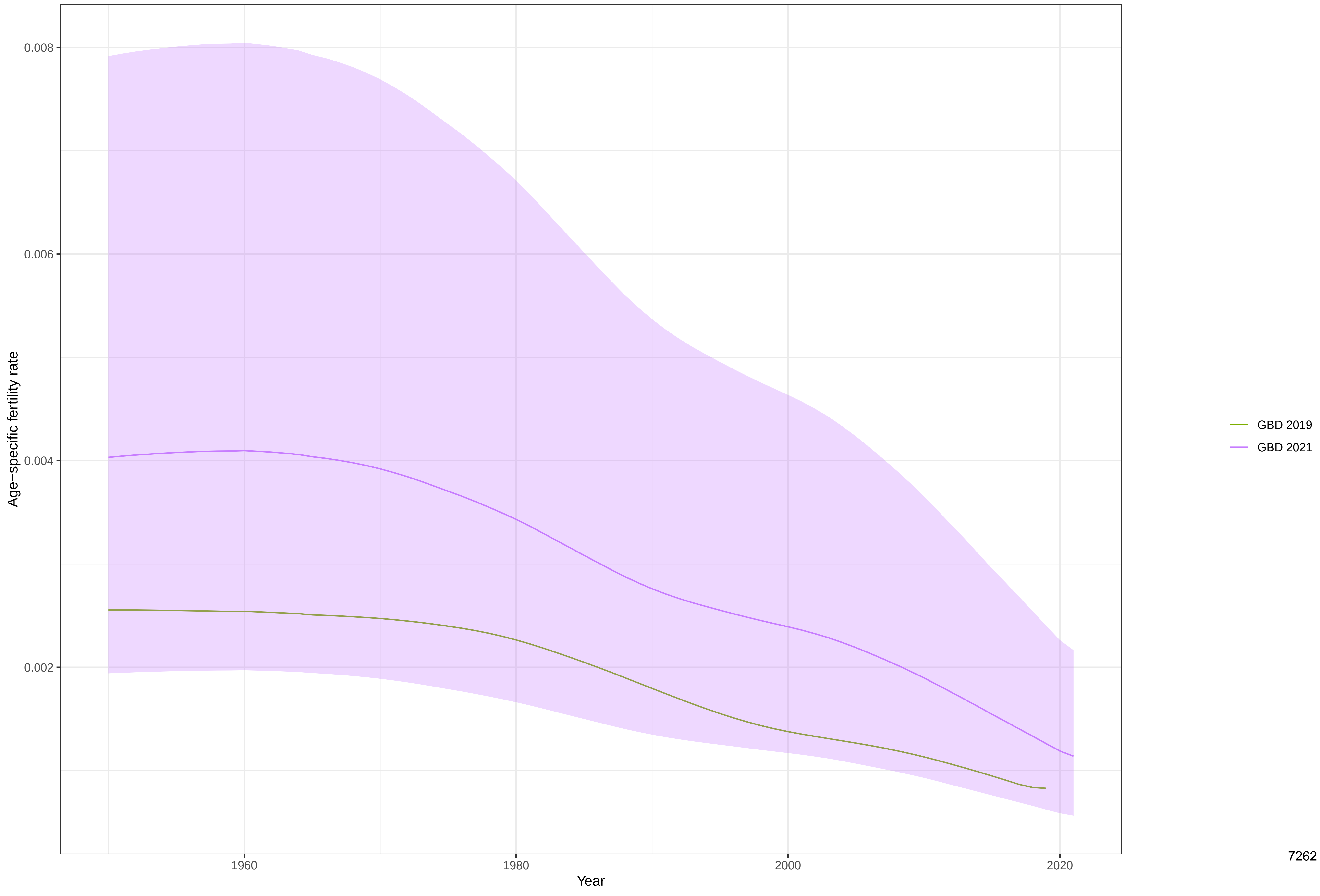

Age−specific fertility fitted trend lines, 1950−2021: Taita Taveta, Ages: 15−19
Data points enclosed with a diamond are reference sources

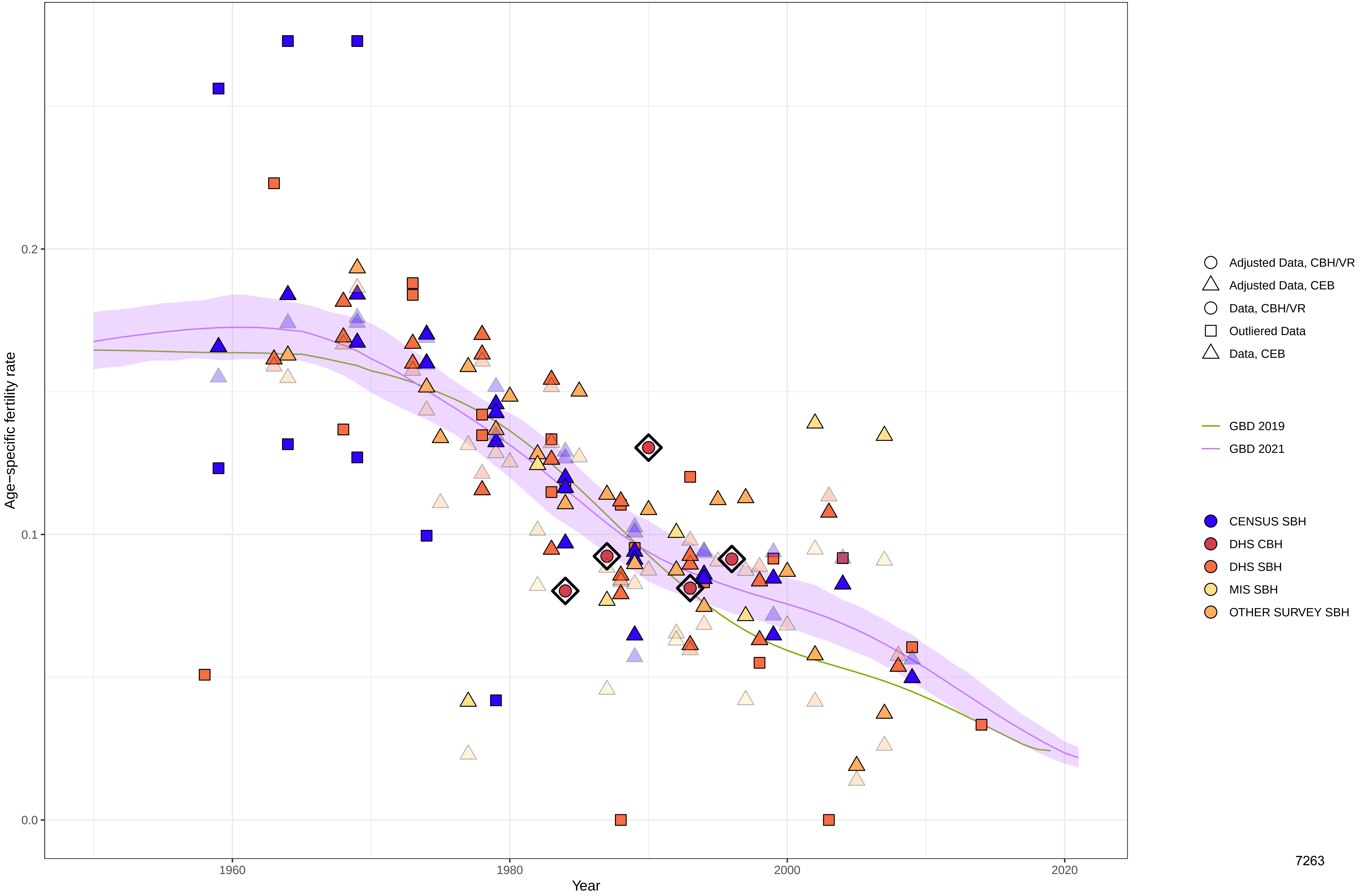

Age−specific fertility fitted trend lines, 1950−2021: Taita Taveta, Ages: 20−24
Data points enclosed with a diamond are reference sources

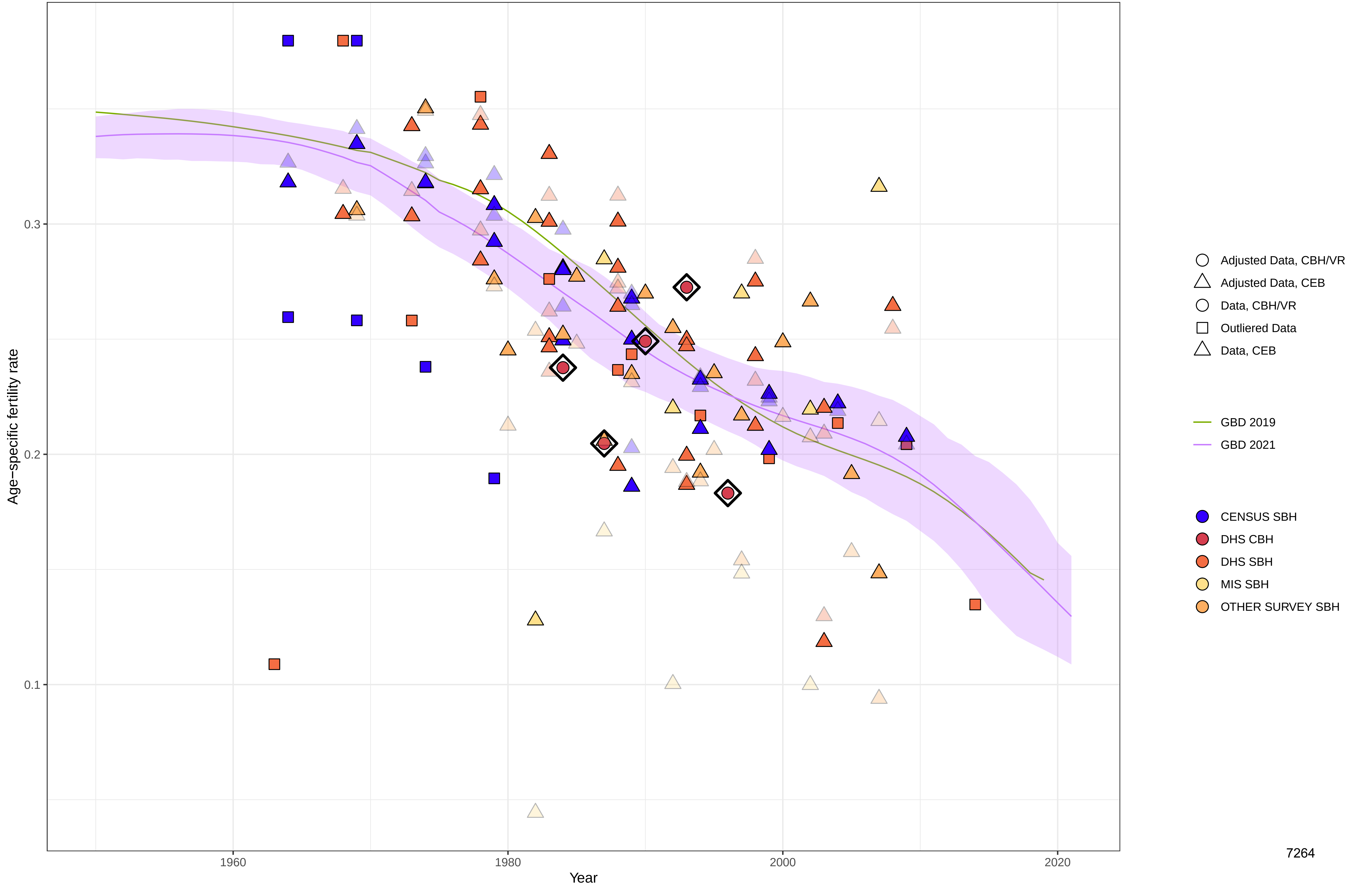

Age−specific fertility fitted trend lines, 1950−2021: Taita Taveta, Ages: 25−29
Data points enclosed with a diamond are reference sources

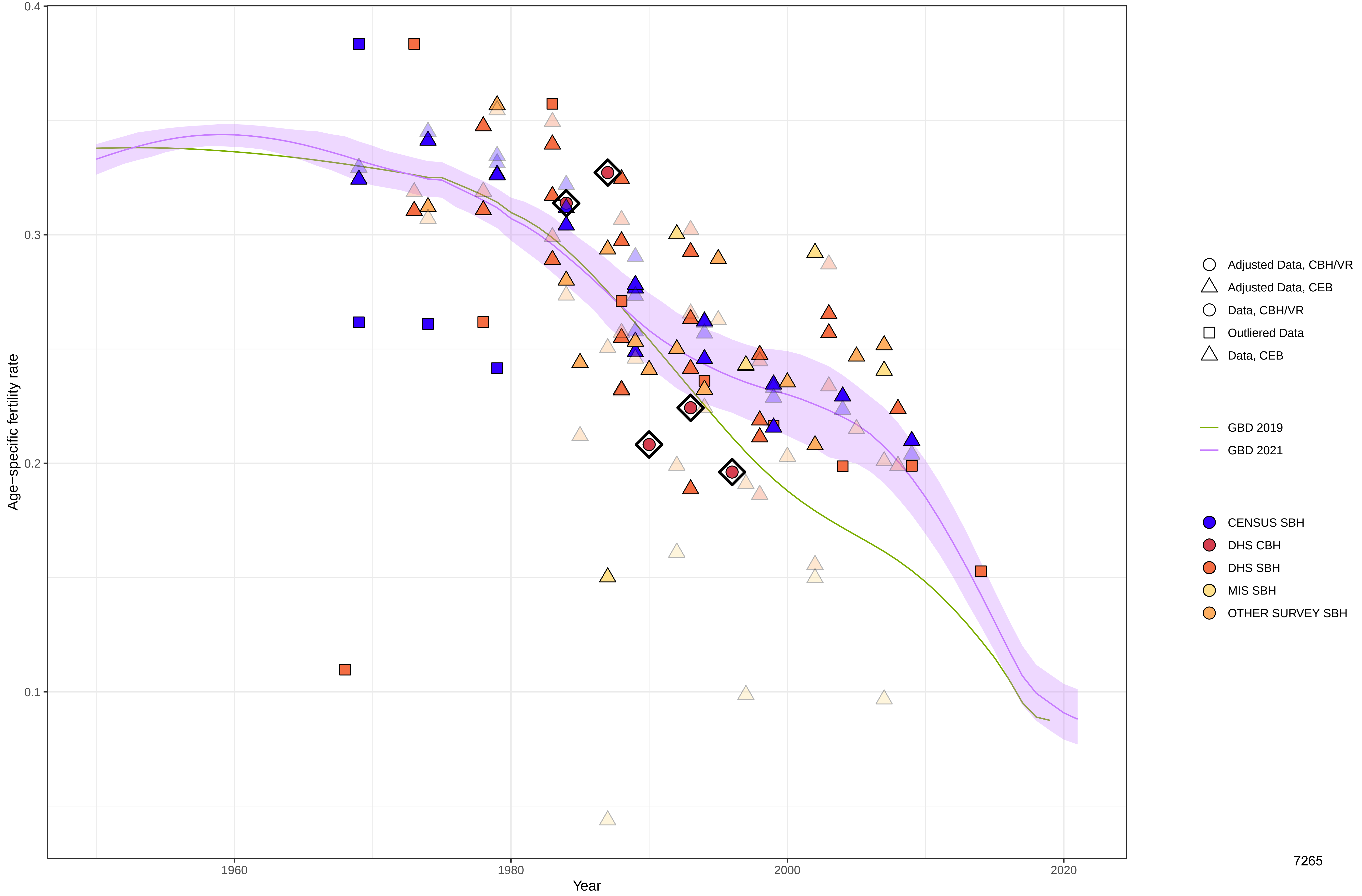

Age−specific fertility fitted trend lines, 1950−2021: Taita Taveta, Ages: 30−34
Data points enclosed with a diamond are reference sources

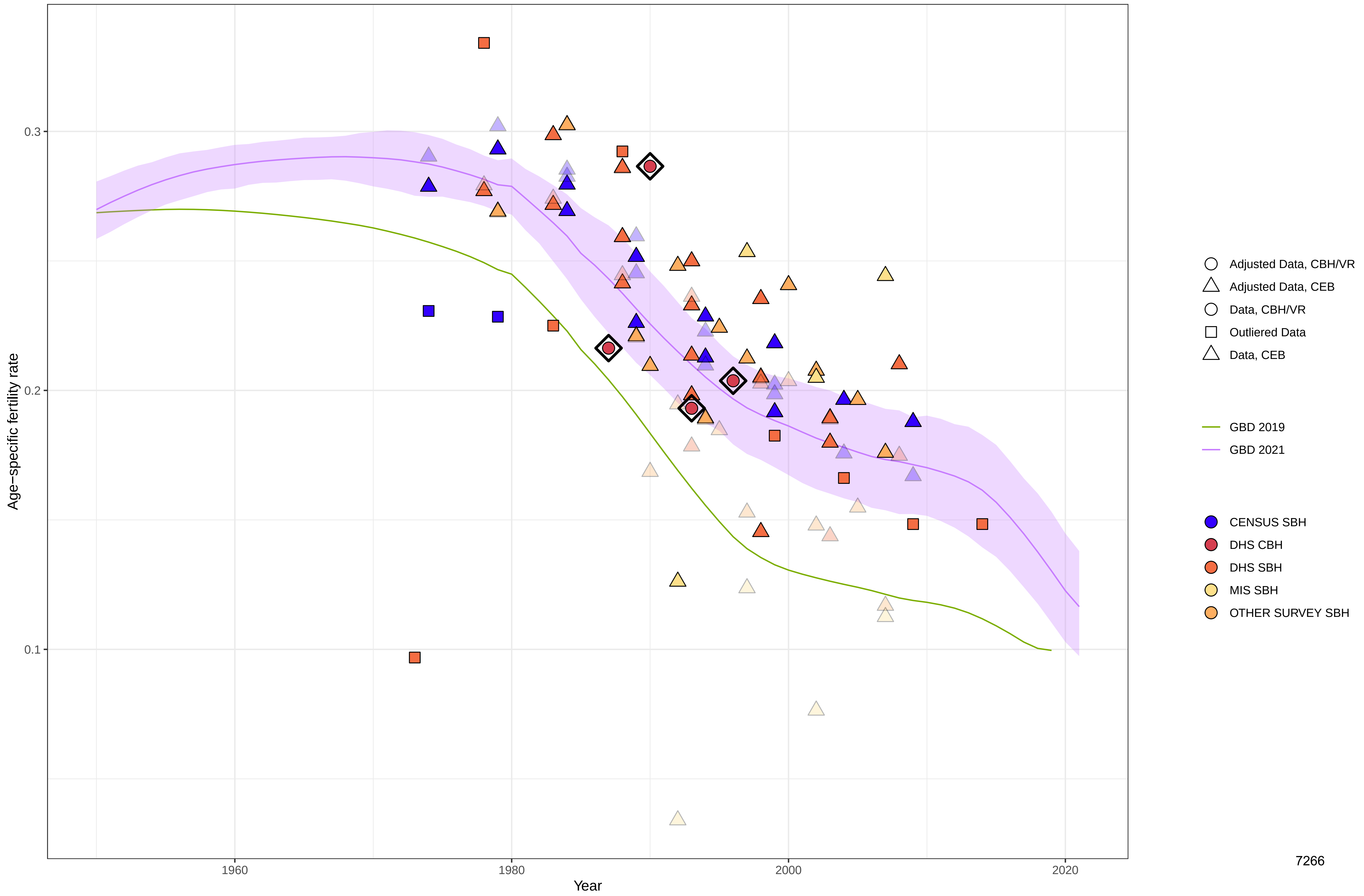

Age−specific fertility fitted trend lines, 1950−2021: Taita Taveta, Ages: 35−39
Data points enclosed with a diamond are reference sources

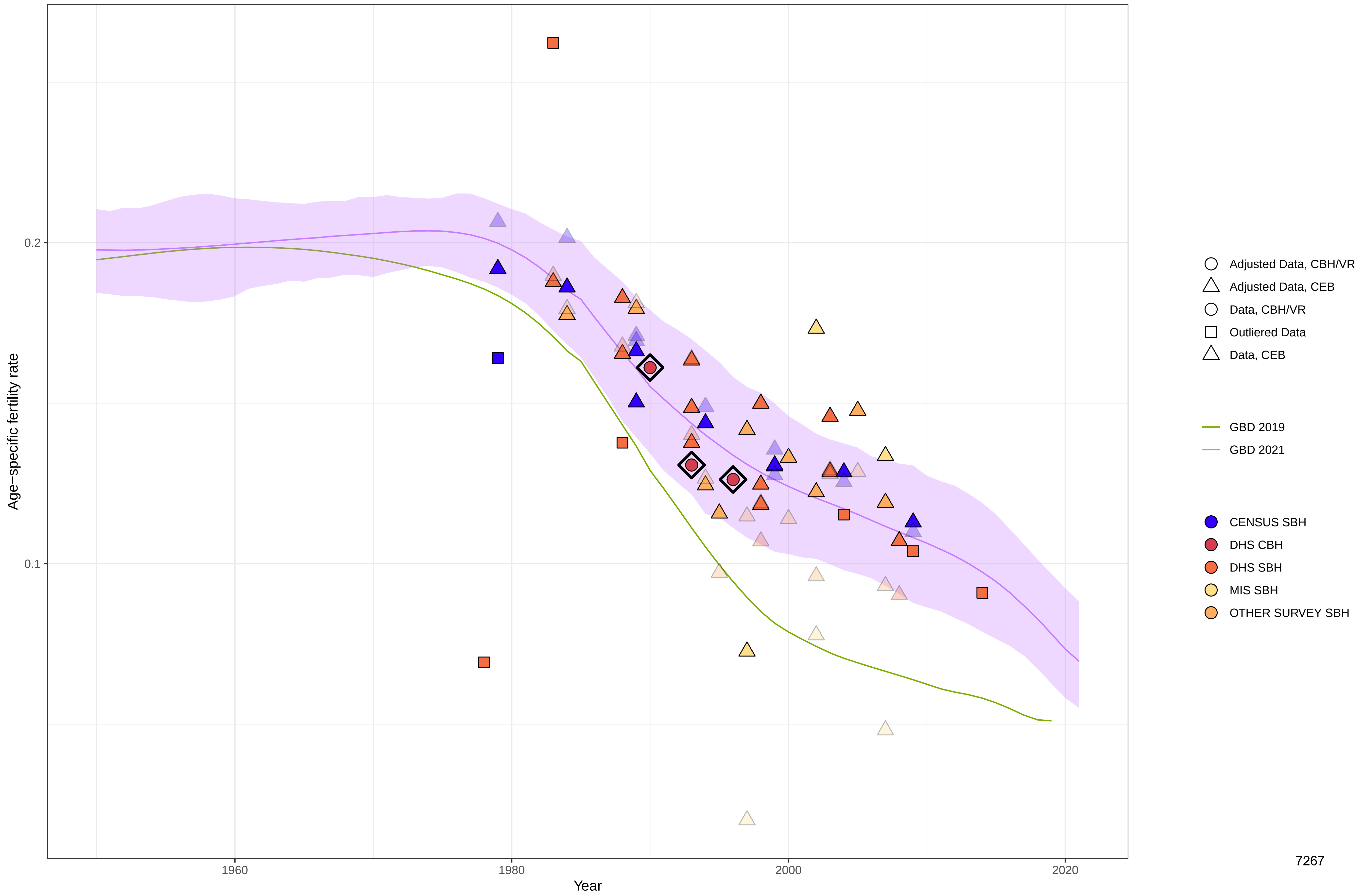

Age−specific fertility fitted trend lines, 1950−2021: Taita Taveta, Ages: 40−44
Data points enclosed with a diamond are reference sources

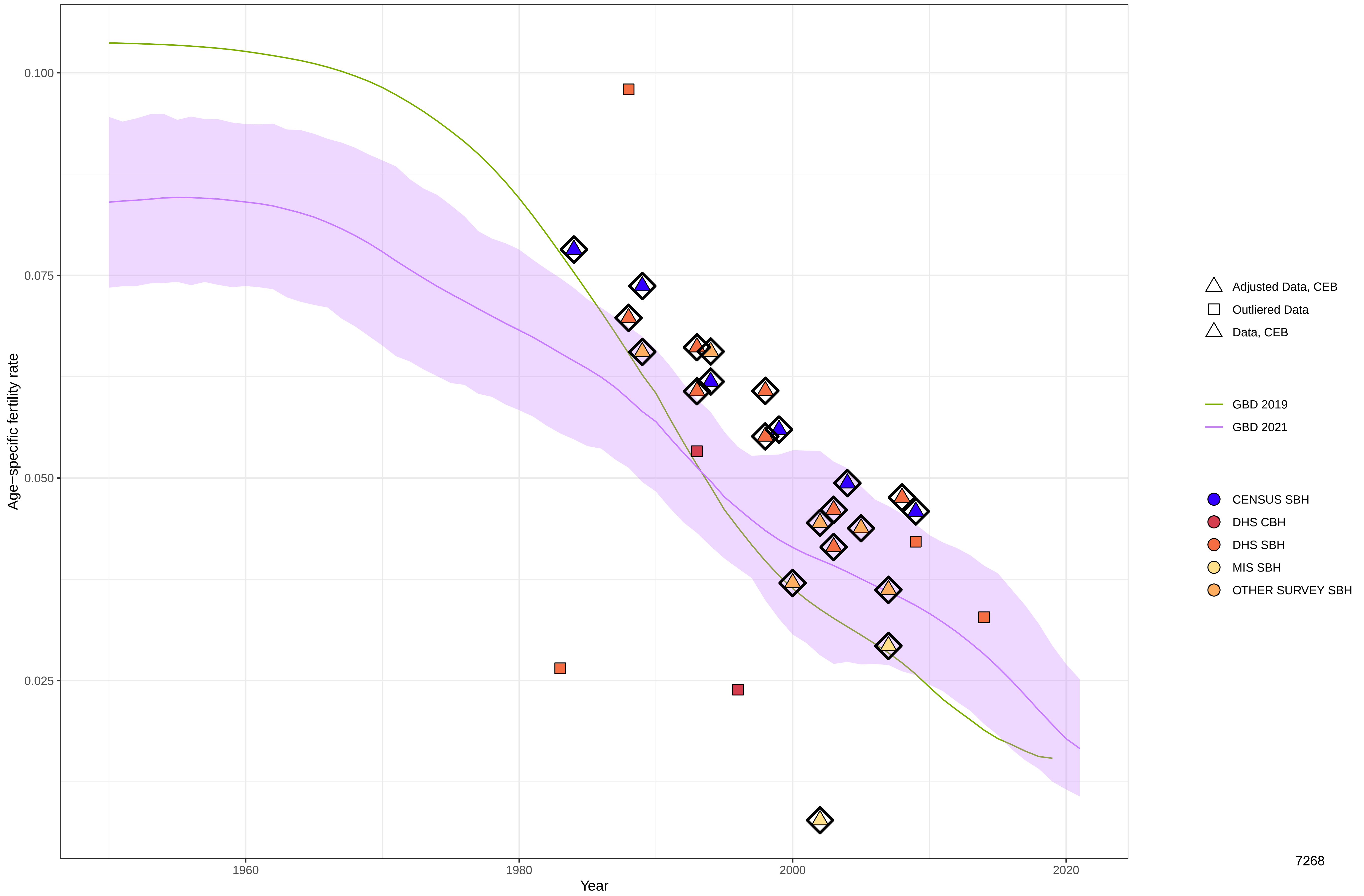

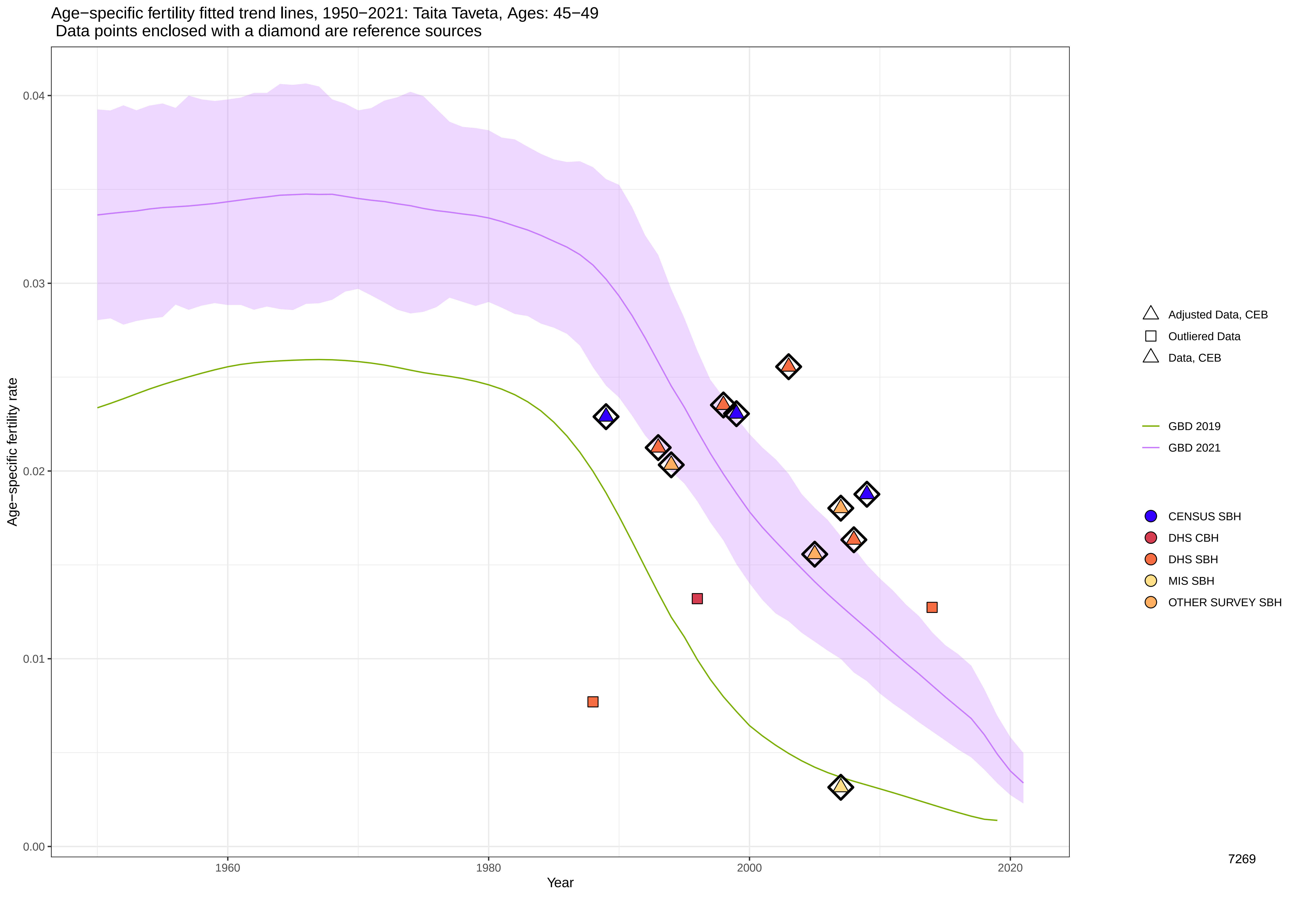

Age−specific fertility fitted trend lines, 1950−2021: Taita Taveta, Ages: 45−49
Data points enclosed with a diamond are reference sources
Age−specific fertility rate
0.00
0.01
0.02
0.03
0.04
Year
1960
1980
2000
2020
Adjusted Data, CEB
Outliered Data
Data, CEB
GBD 2019
GBD 2021
CENSUS SBH
DHS CBH
DHS SBH
MIS SBH
OTHER SURVEY SBH

7269

Age−specific fertility fitted trend lines, 1950−2021: Taita Taveta, Ages: 50−54
Data points enclosed with a diamond are reference sources

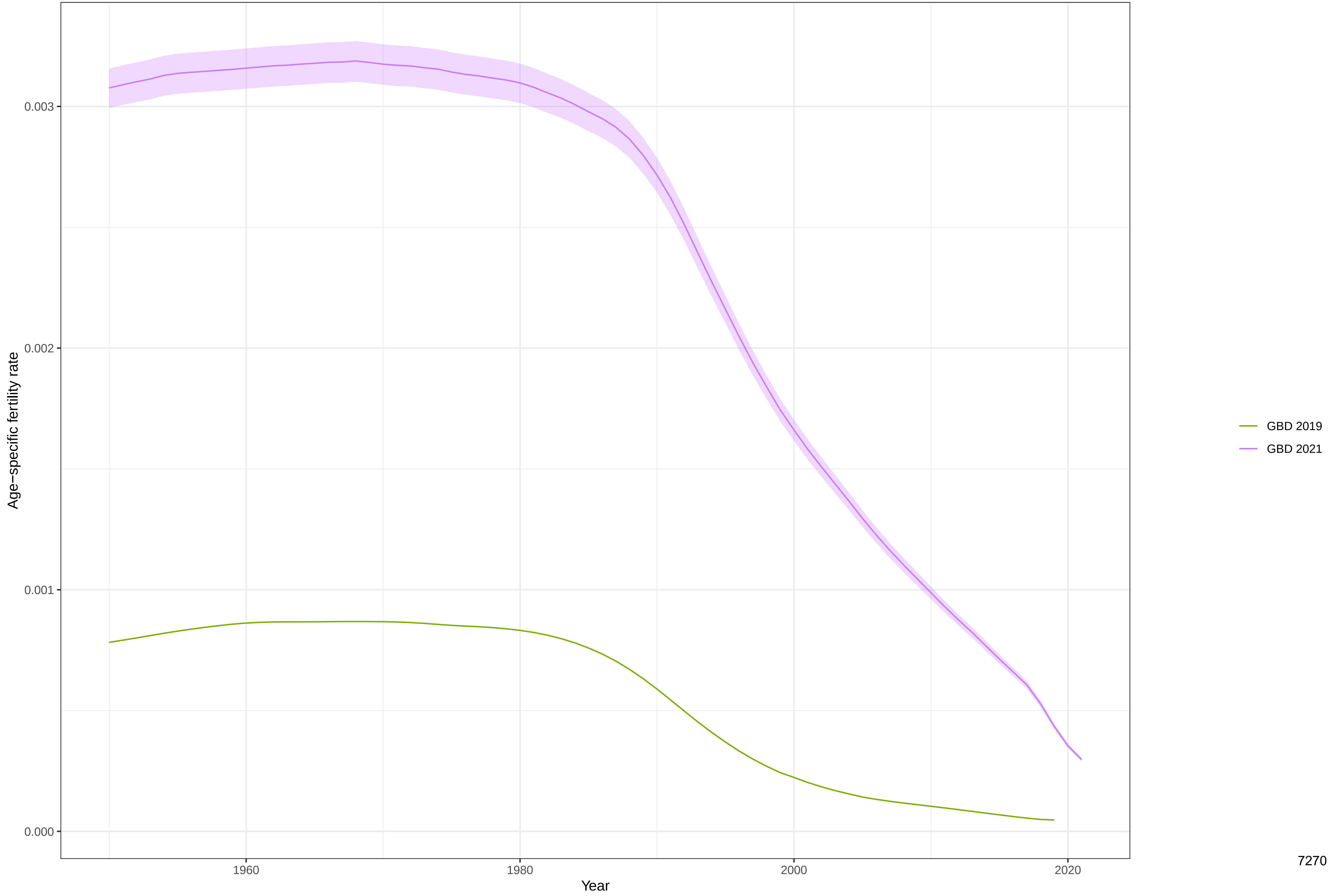

Total fertility rate fitted trend lines, 1950−2021: Taita Taveta TFR
Data points enclosed with a diamond are reference sources

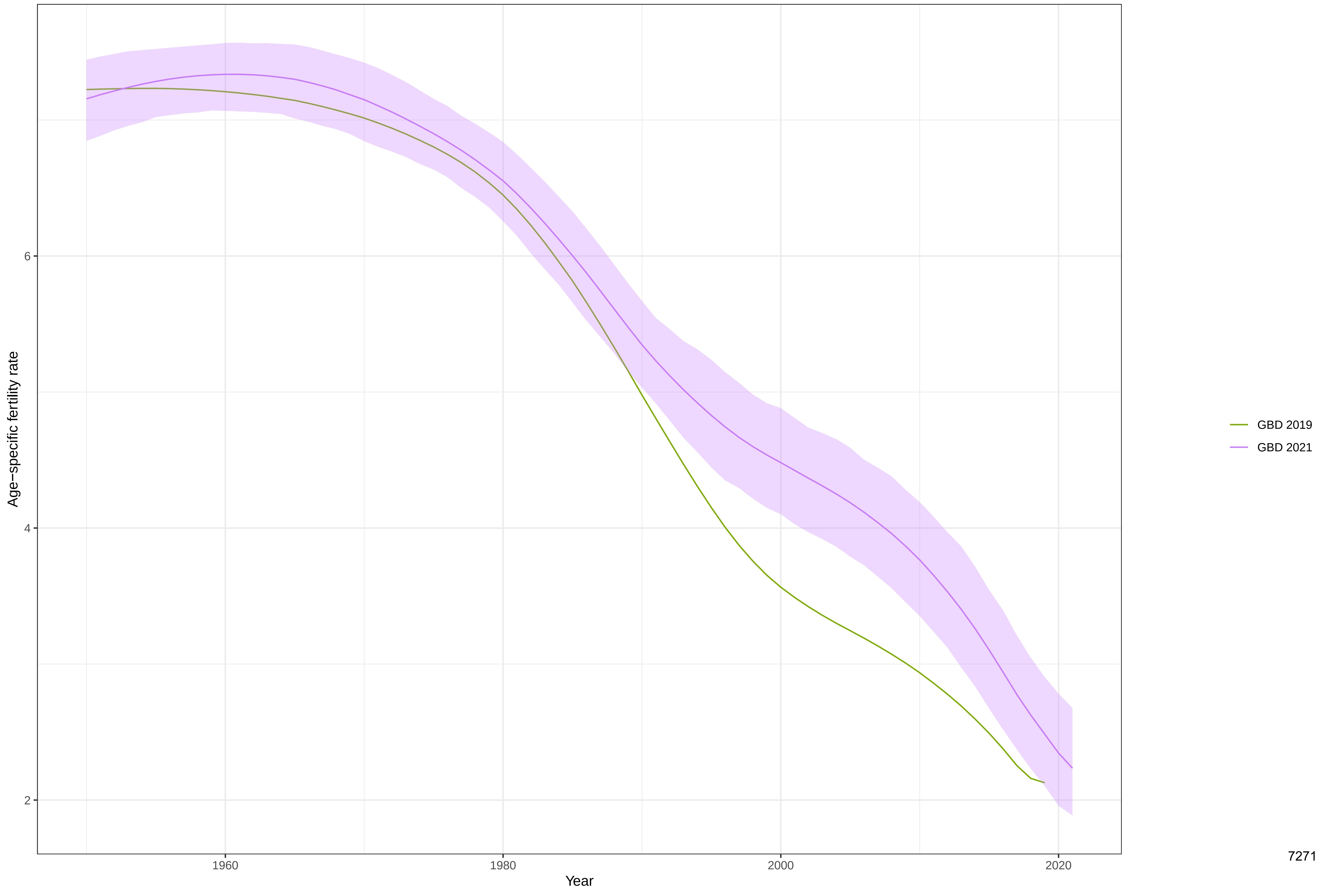

Age−specific fertility fitted trend lines, 1950−2021: Tana River, Ages: 10−14
Data points enclosed with a diamond are reference sources

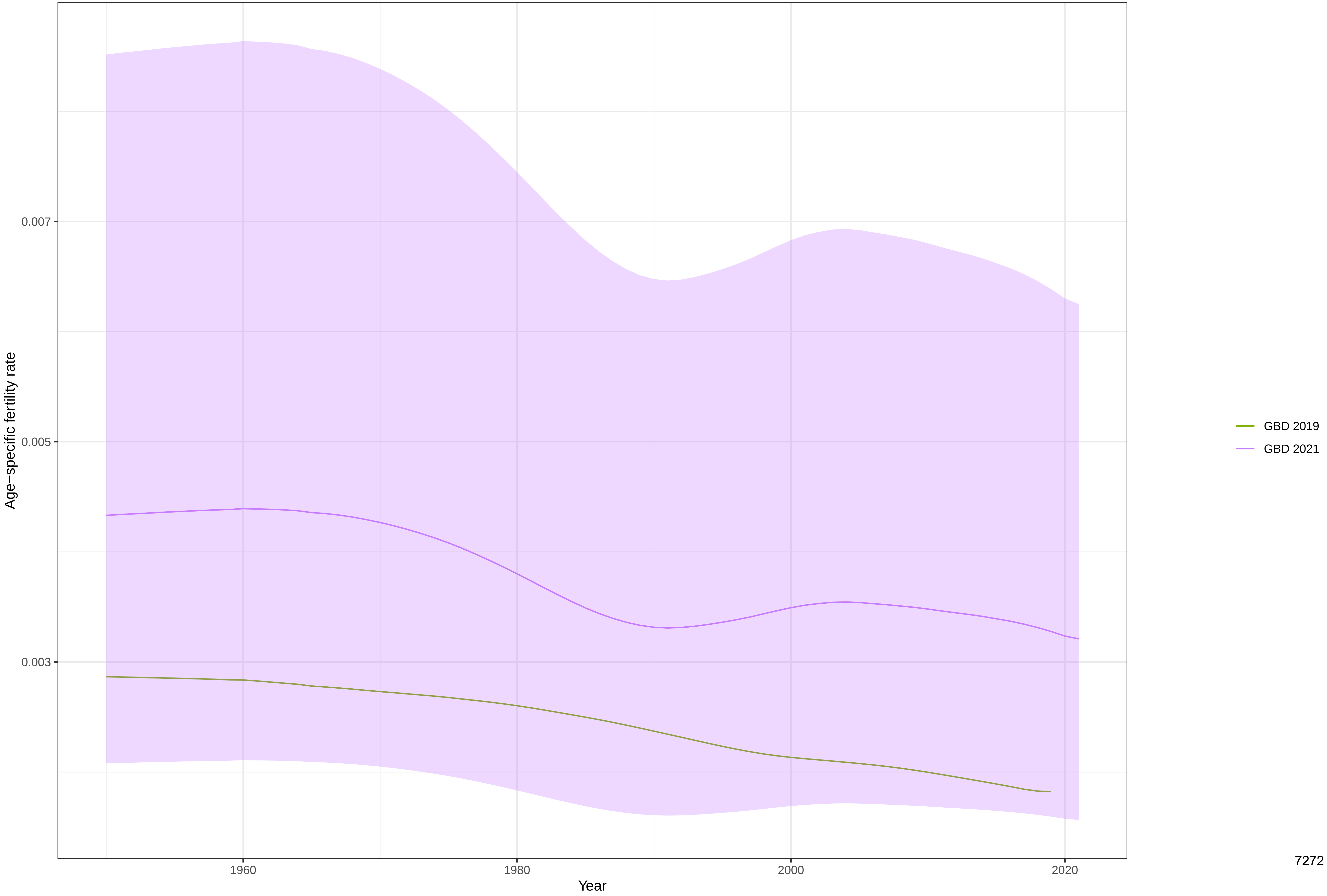

Age−specific fertility fitted trend lines, 1950−2021: Tana River, Ages: 15−19
Data points enclosed with a diamond are reference sources

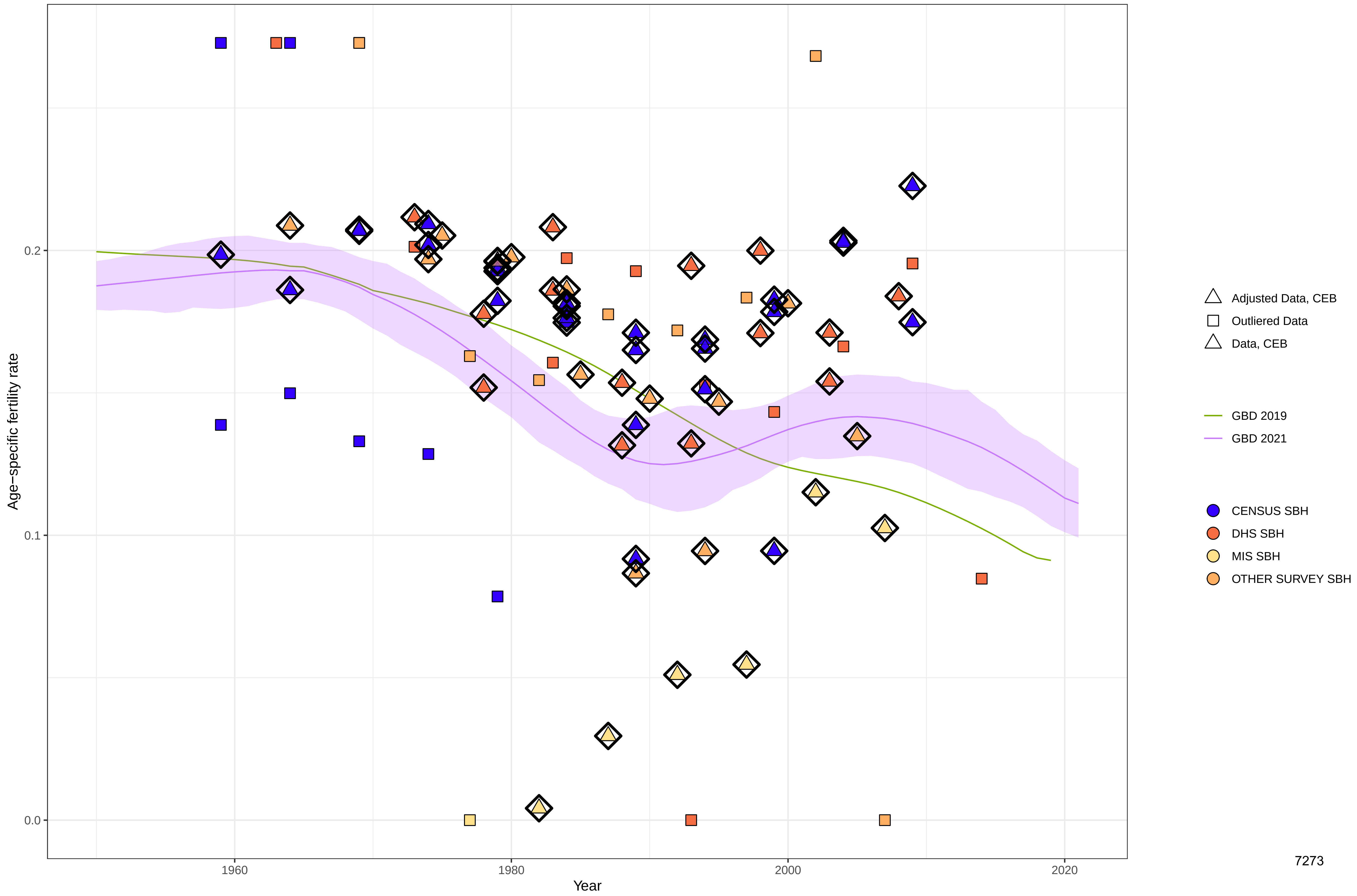

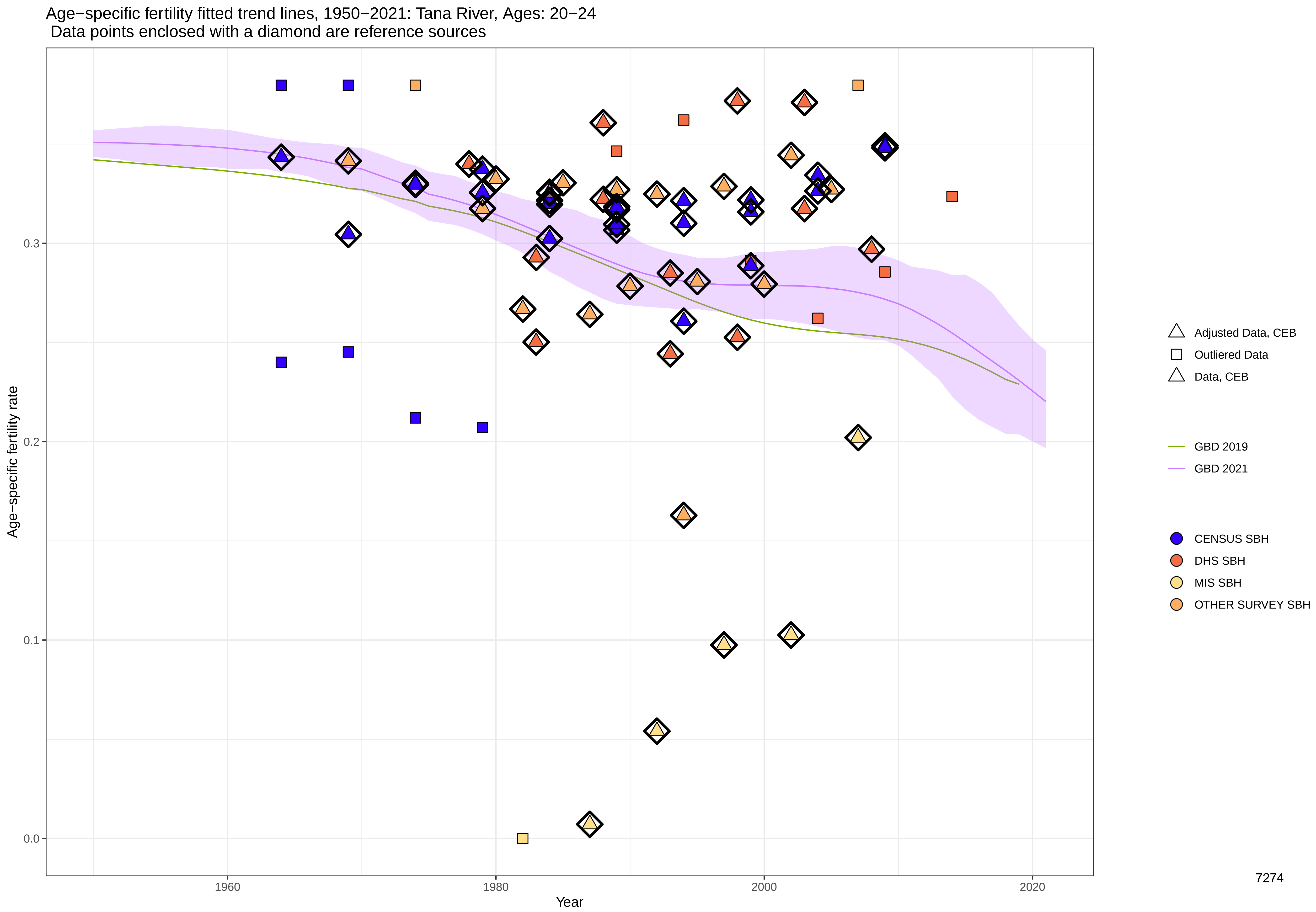
Age−specific fertility fitted trend lines, 1950−2021: Tana River, Ages: 20−24
Data points enclosed with a diamond are reference sources
Age−specific fertility rate
Year
0.0
0.1
0.2
0.3
1960
1980
2000
2020
Adjusted Data, CEB
Outliered Data
Data, CEB
GBD 2019
GBD 2021
CENSUS SBH
DHS SBH
MIS SBH
OTHER SURVEY SBH

Age−specific fertility fitted trend lines, 1950−2021: Tana River, Ages: 25−29
Data points enclosed with a diamond are reference sources

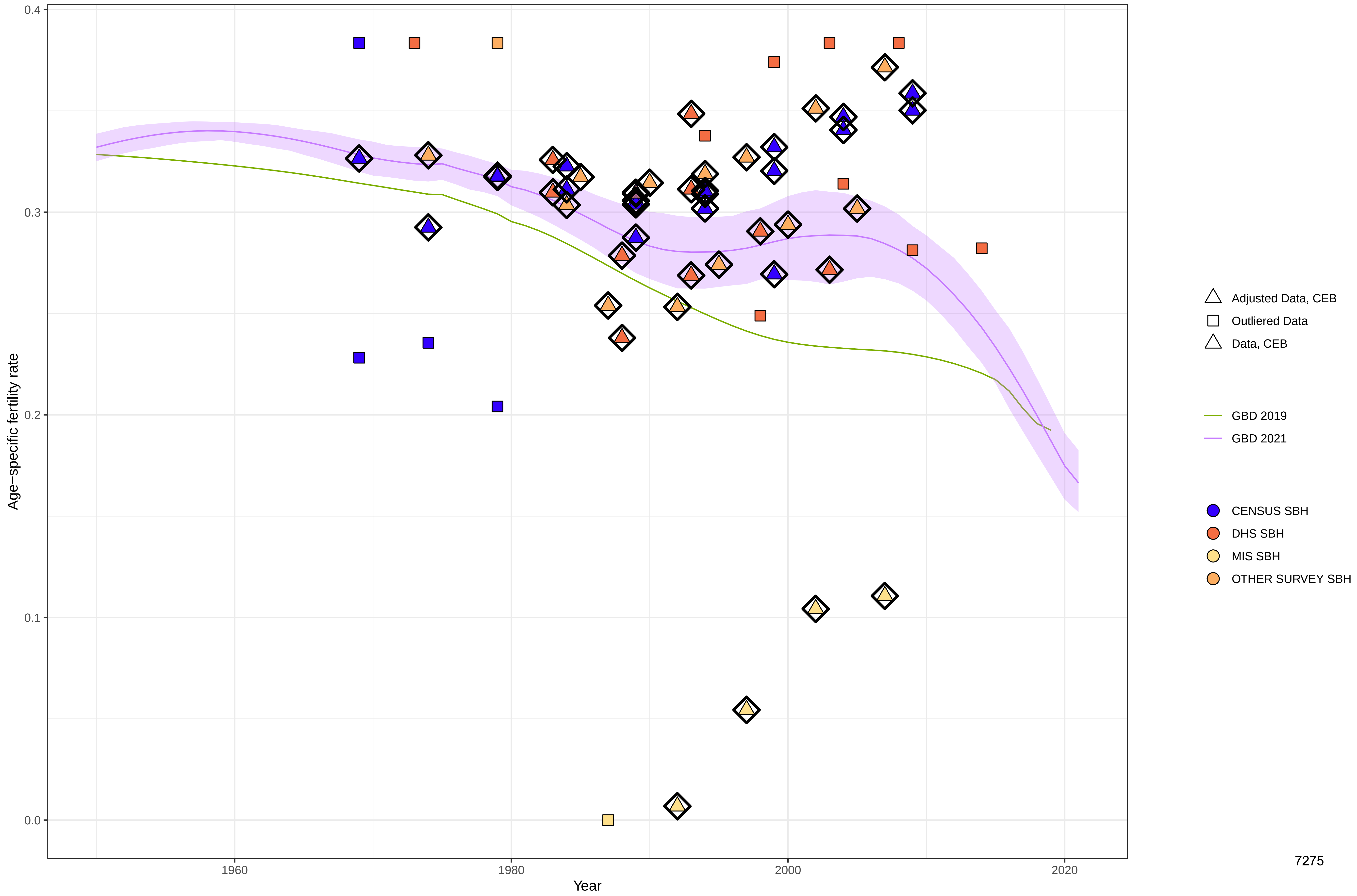

Age−specific fertility fitted trend lines, 1950−2021: Tana River, Ages: 30−34
Data points enclosed with a diamond are reference sources

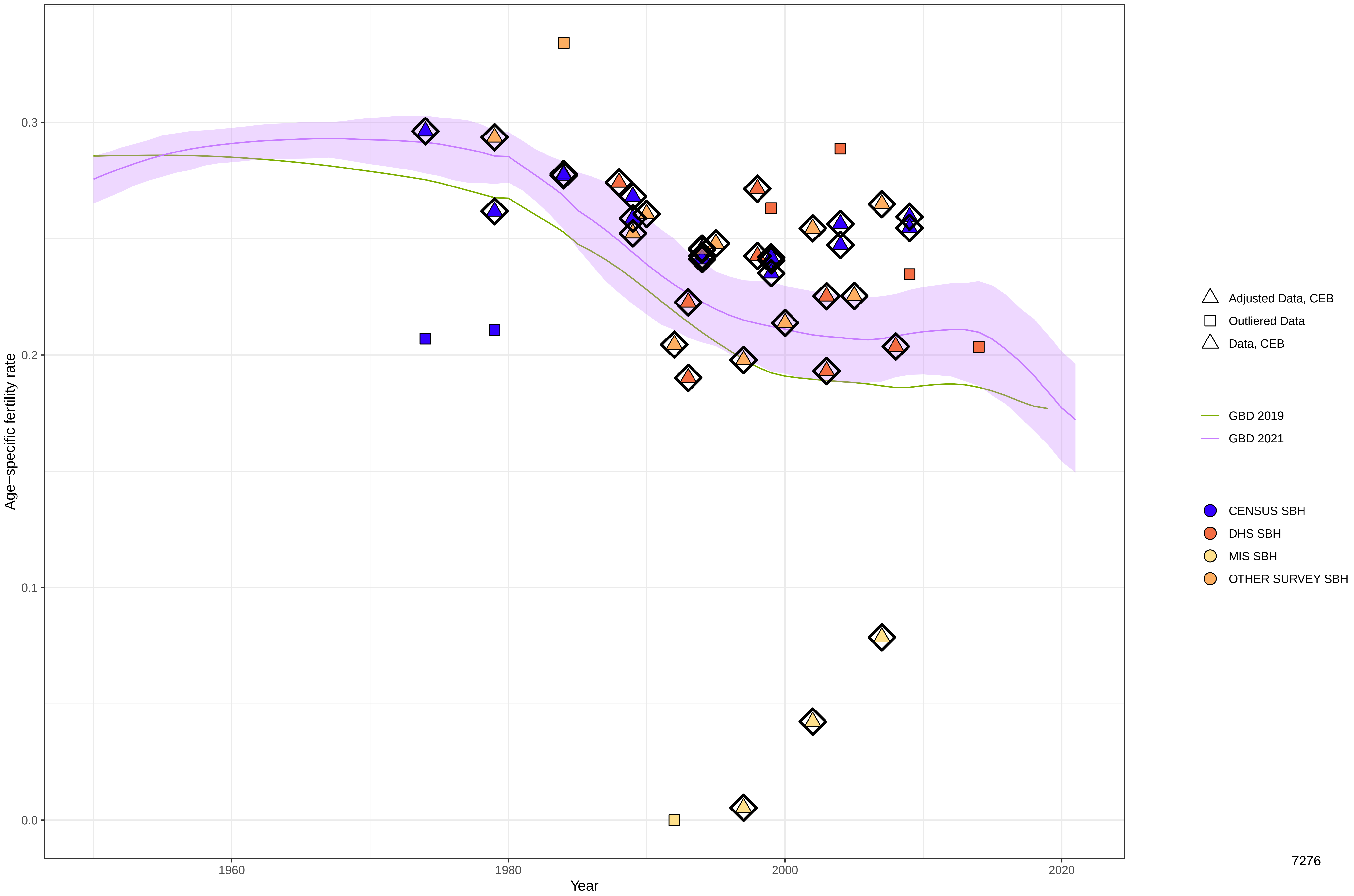

Age−specific fertility fitted trend lines, 1950−2021: Tana River, Ages: 35−39
Data points enclosed with a diamond are reference sources

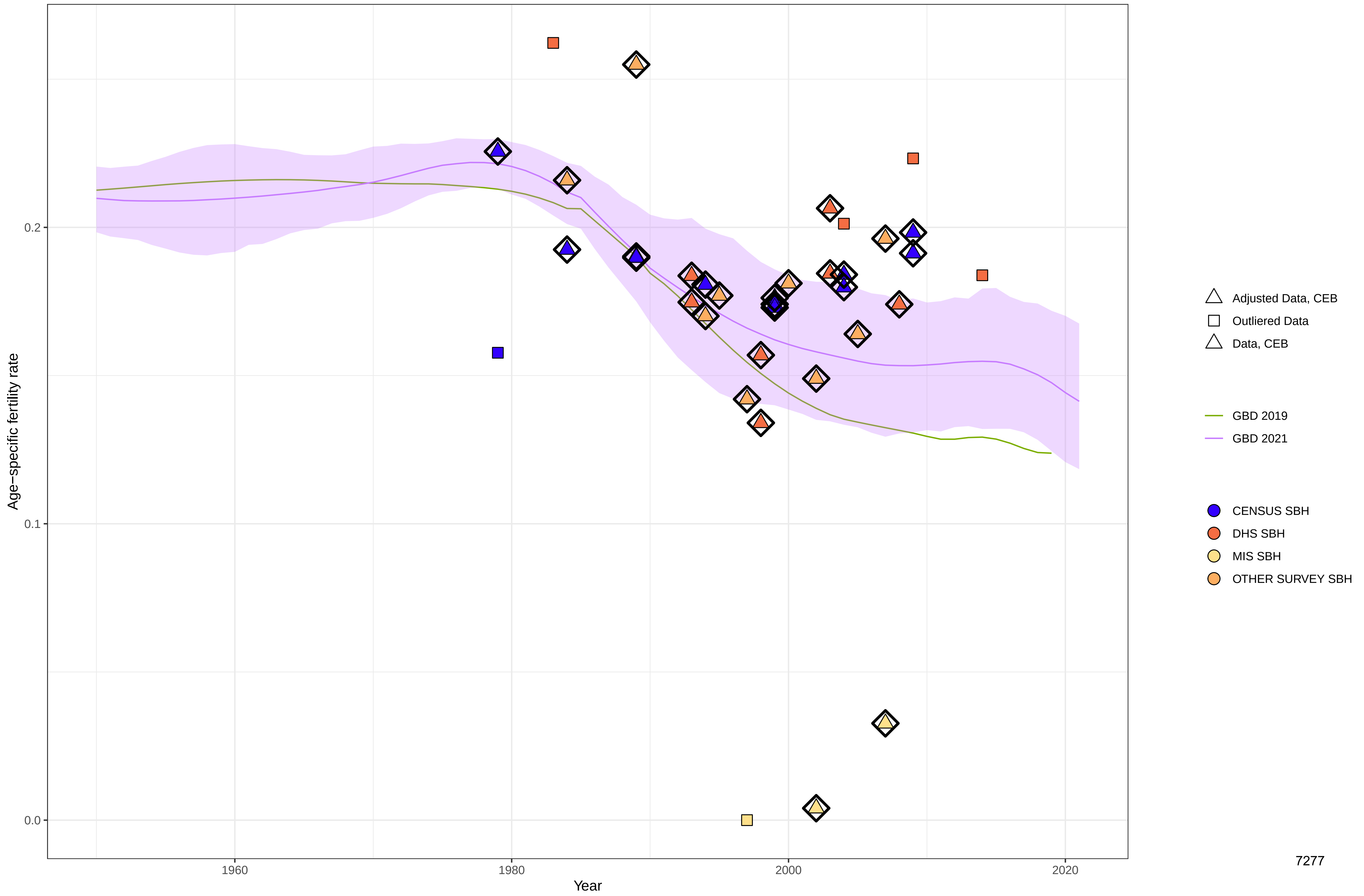

Age−specific fertility fitted trend lines, 1950−2021: Tana River, Ages: 40−44
Data points enclosed with a diamond are reference sources

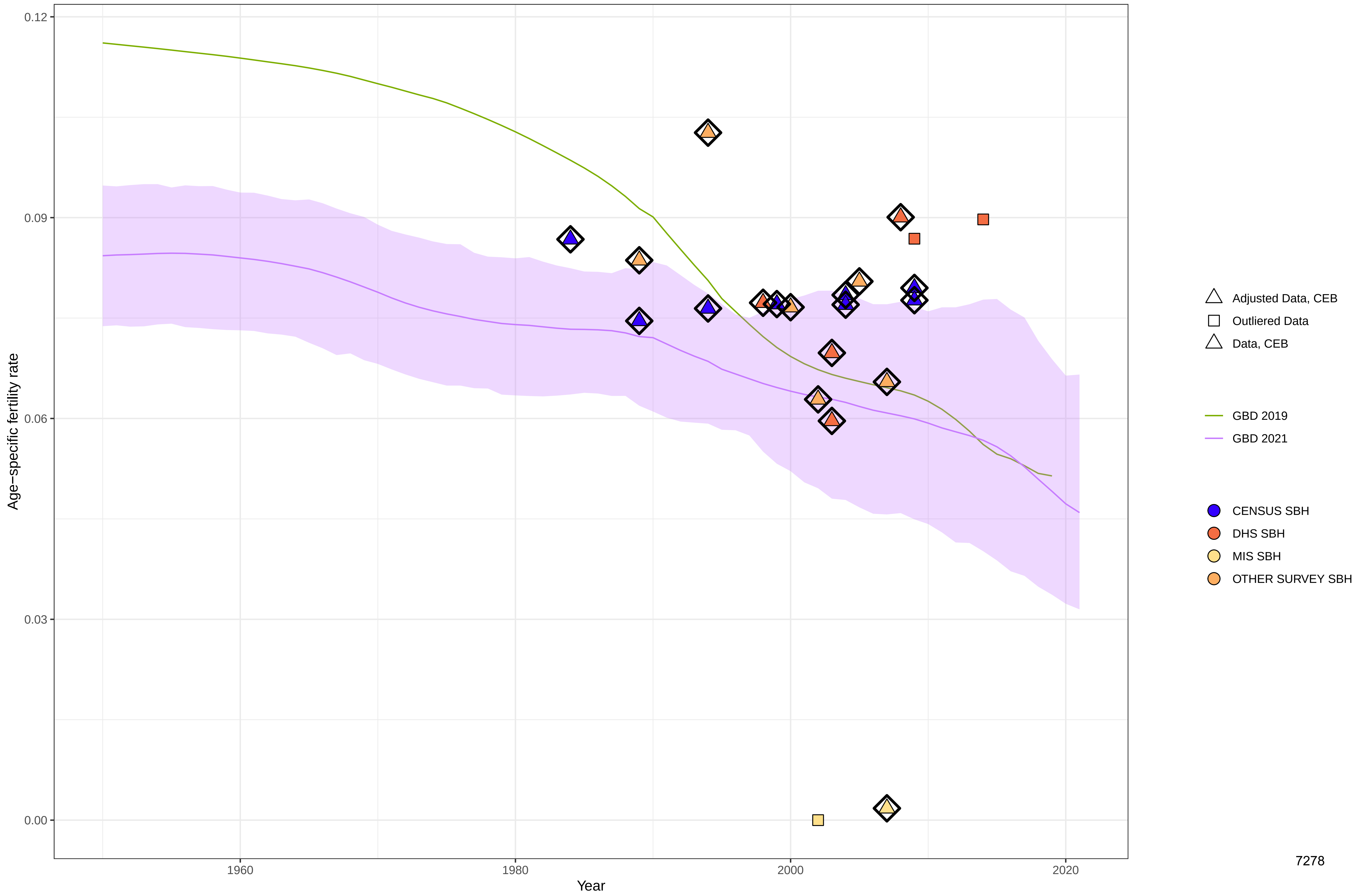

Age−specific fertility fitted trend lines, 1950−2021: Tana River, Ages: 45−49
Data points enclosed with a diamond are reference sources

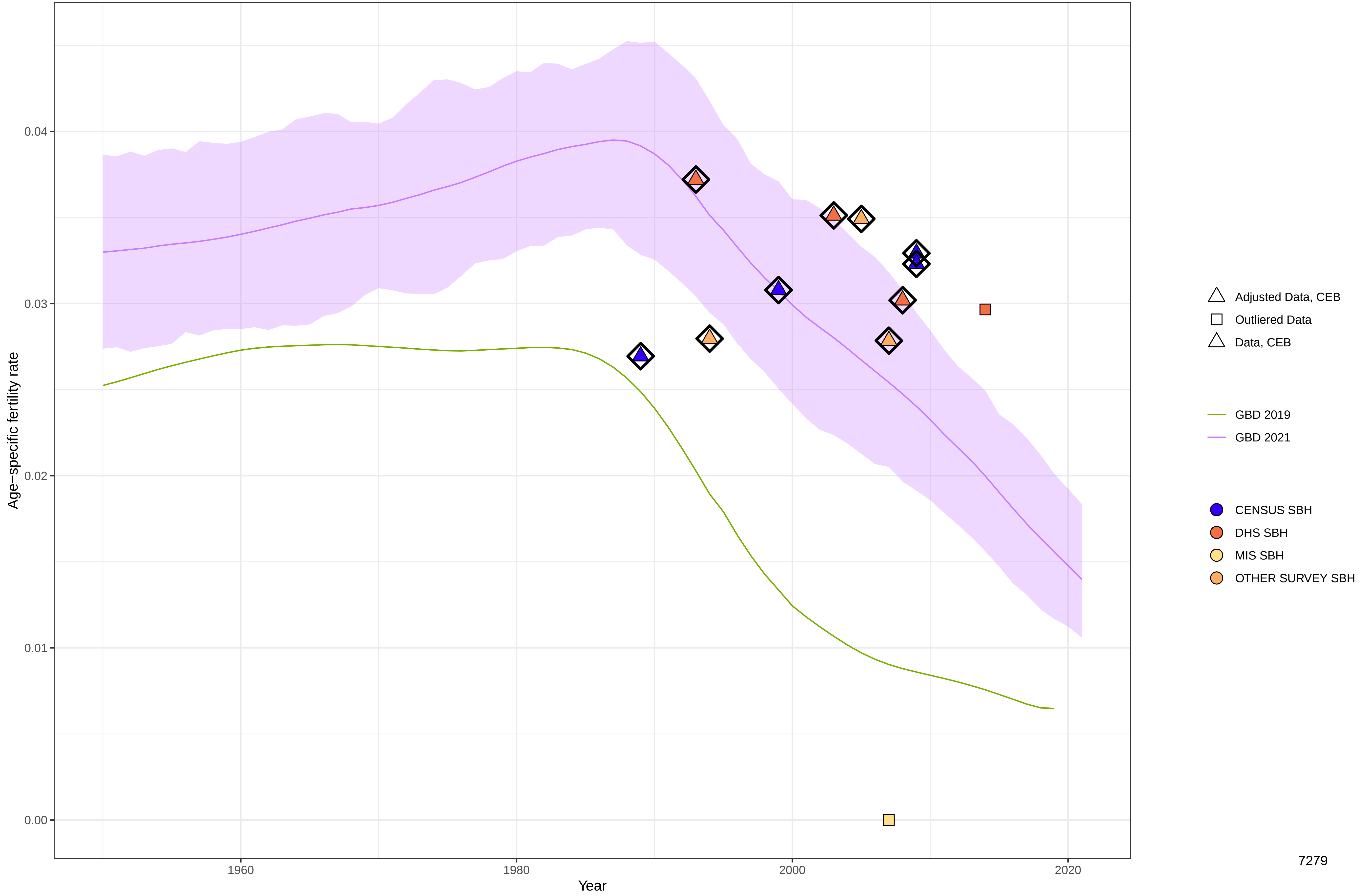

Age−specific fertility fitted trend lines, 1950−2021: Tana River, Ages: 50−54
Data points enclosed with a diamond are reference sources

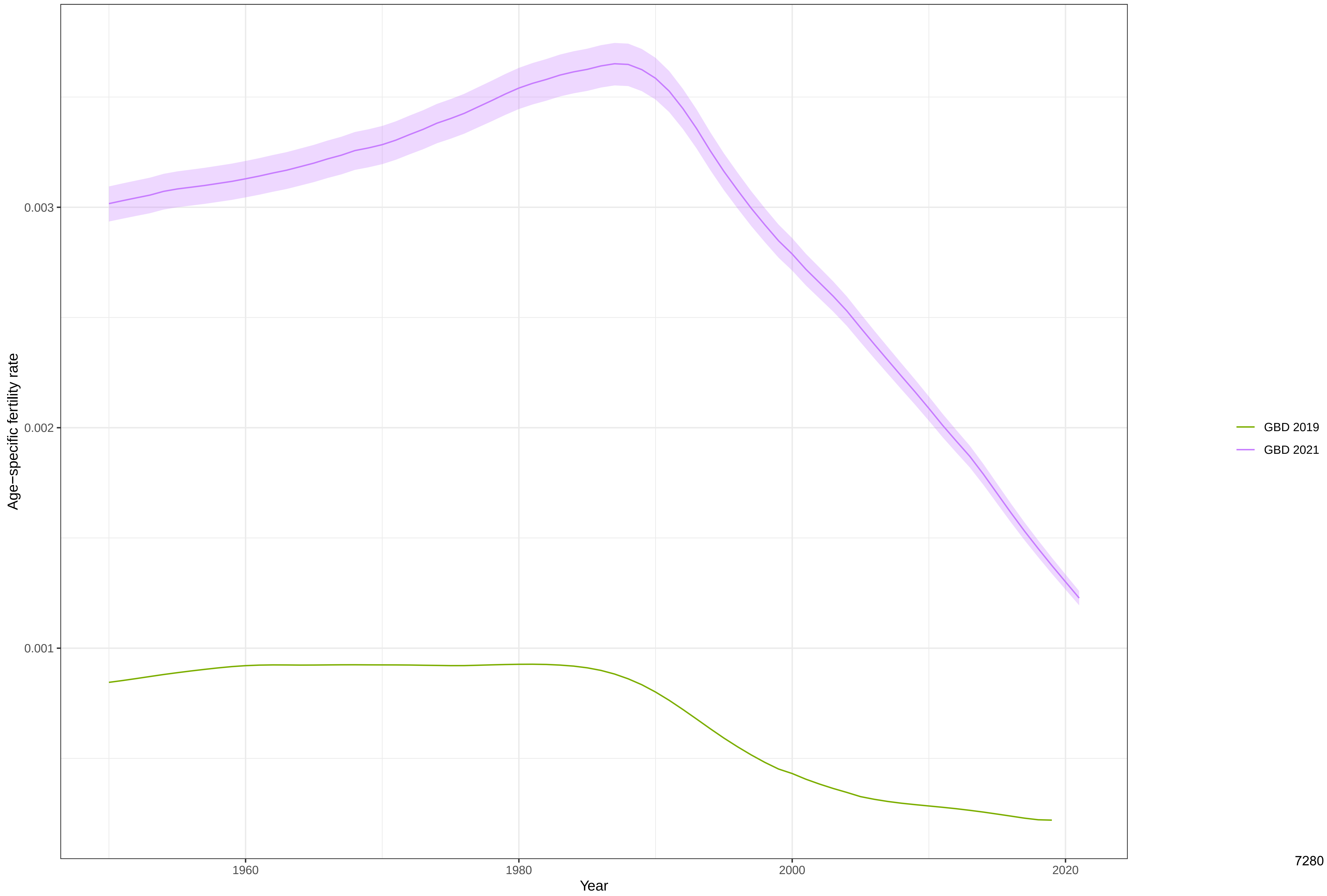

Total fertility rate fitted trend lines, 1950−2021: Tana River TFR
Data points enclosed with a diamond are reference sources

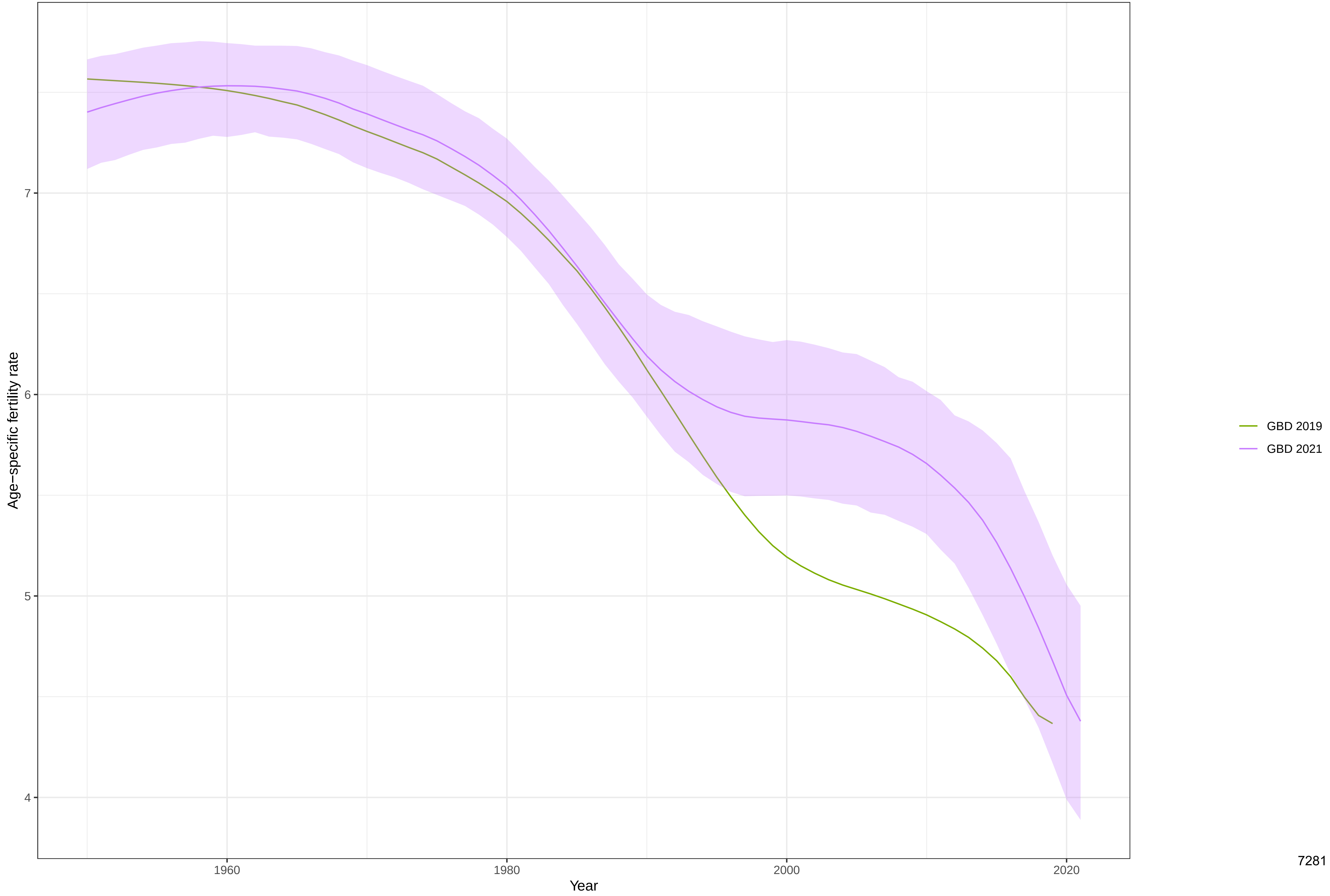

Age−specific fertility fitted trend lines, 1950−2021: Tharaka Nithi, Ages: 10−14
Data points enclosed with a diamond are reference sources

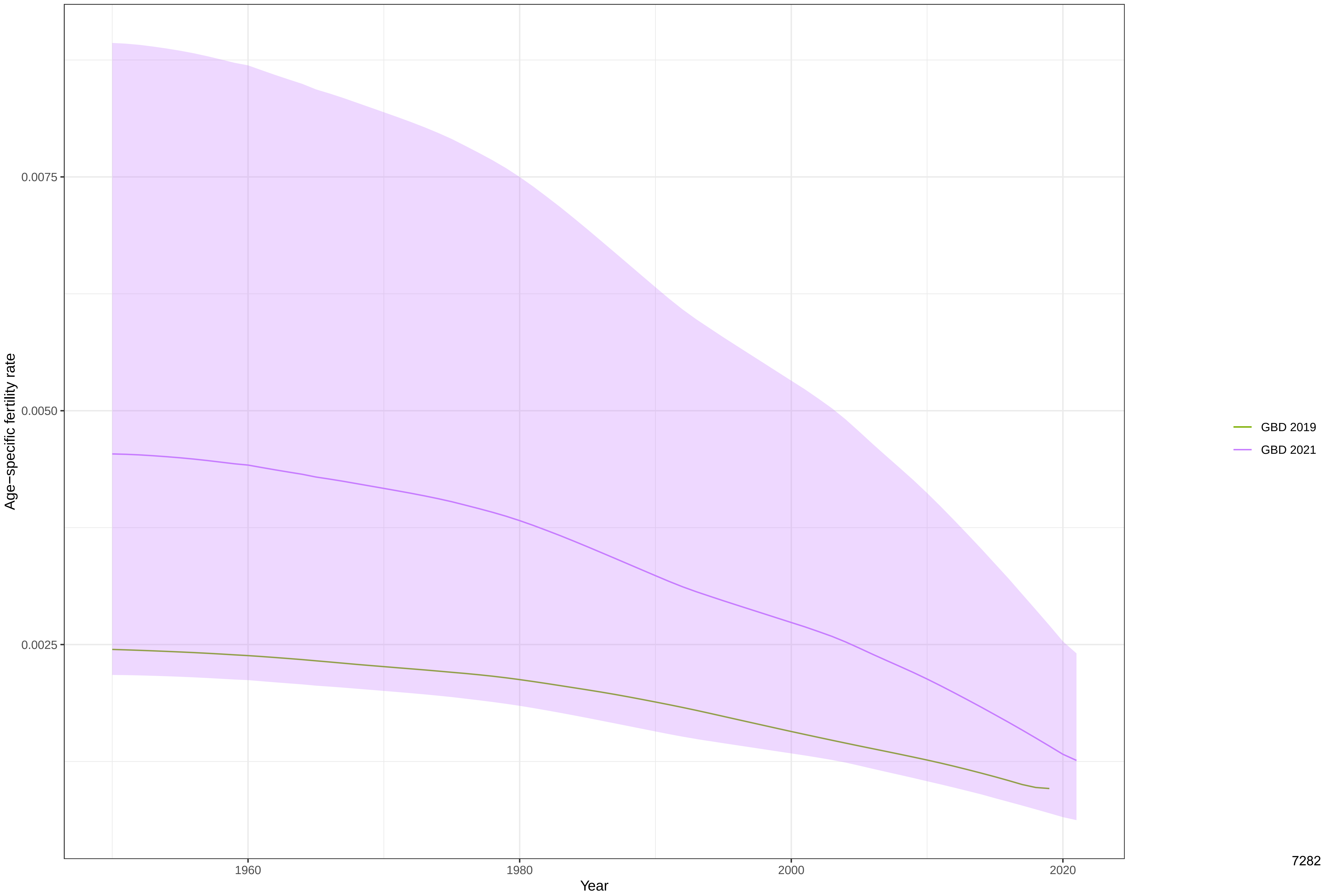

Age−specific fertility fitted trend lines, 1950−2021: Tharaka Nithi, Ages: 15−19
Data points enclosed with a diamond are reference sources

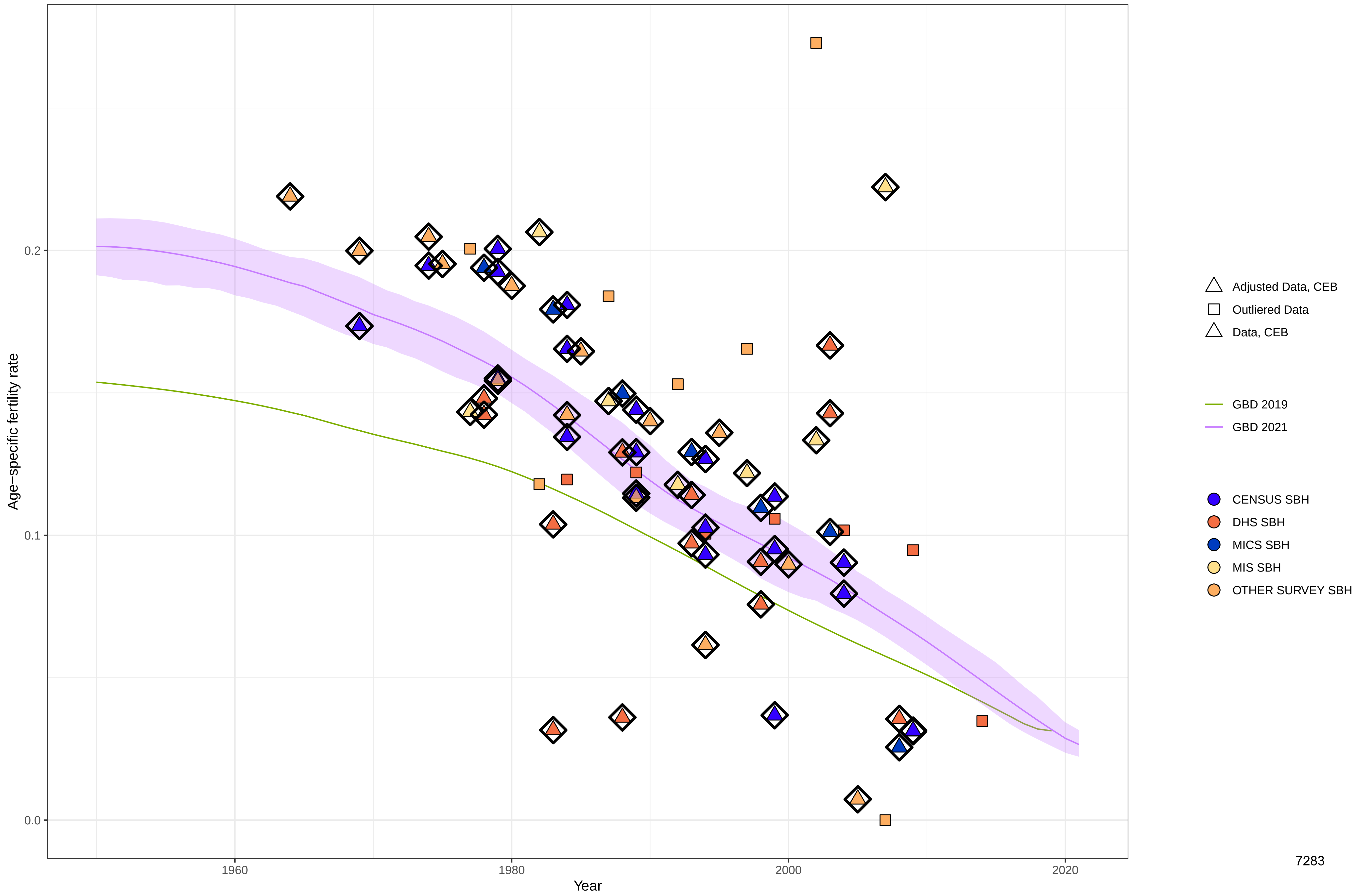

Age−specific fertility fitted trend lines, 1950−2021: Tharaka Nithi, Ages: 20−24
Data points enclosed with a diamond are reference sources

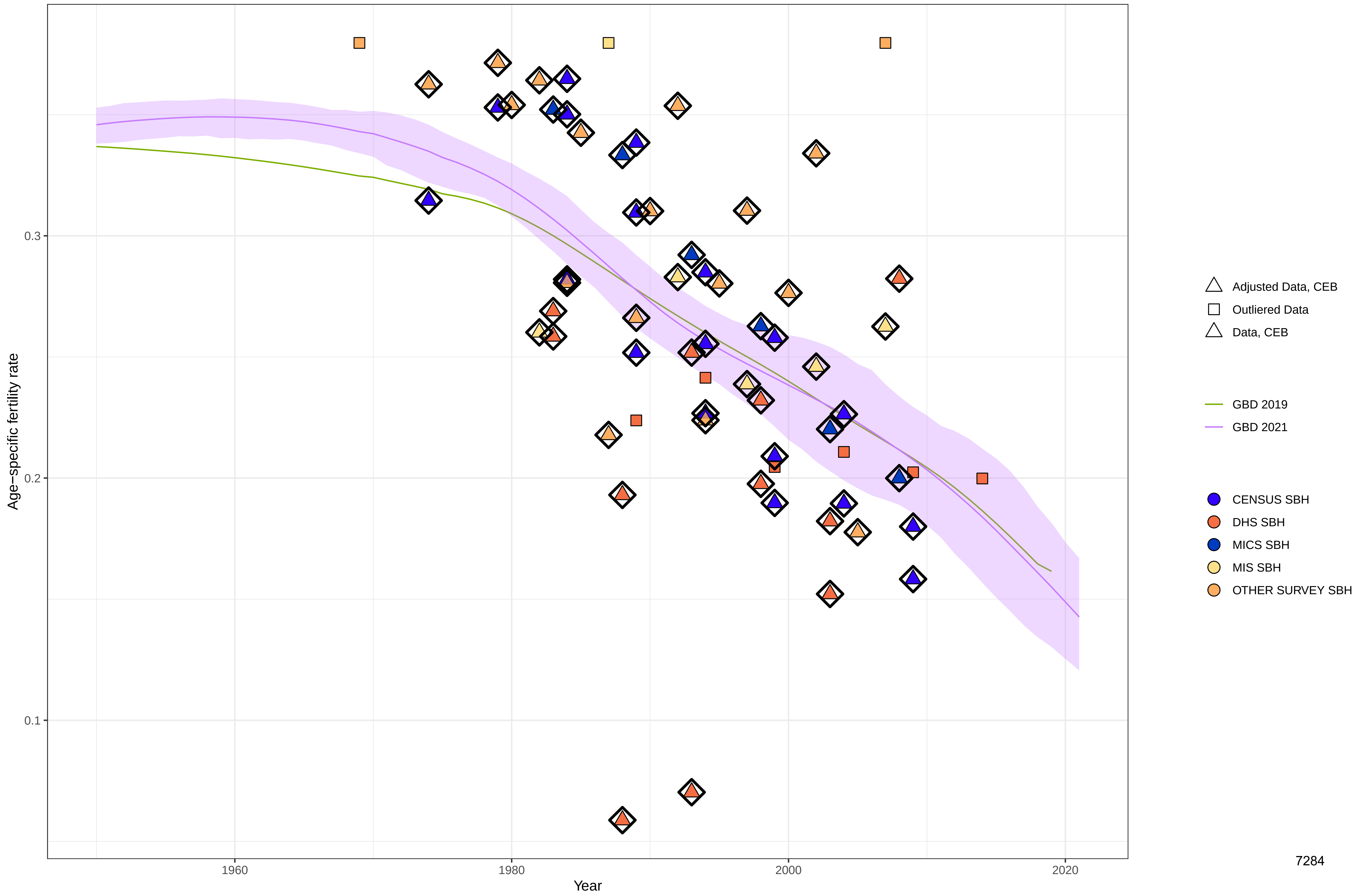

Age−specific fertility fitted trend lines, 1950−2021: Tharaka Nithi, Ages: 25−29
Data points enclosed with a diamond are reference sources

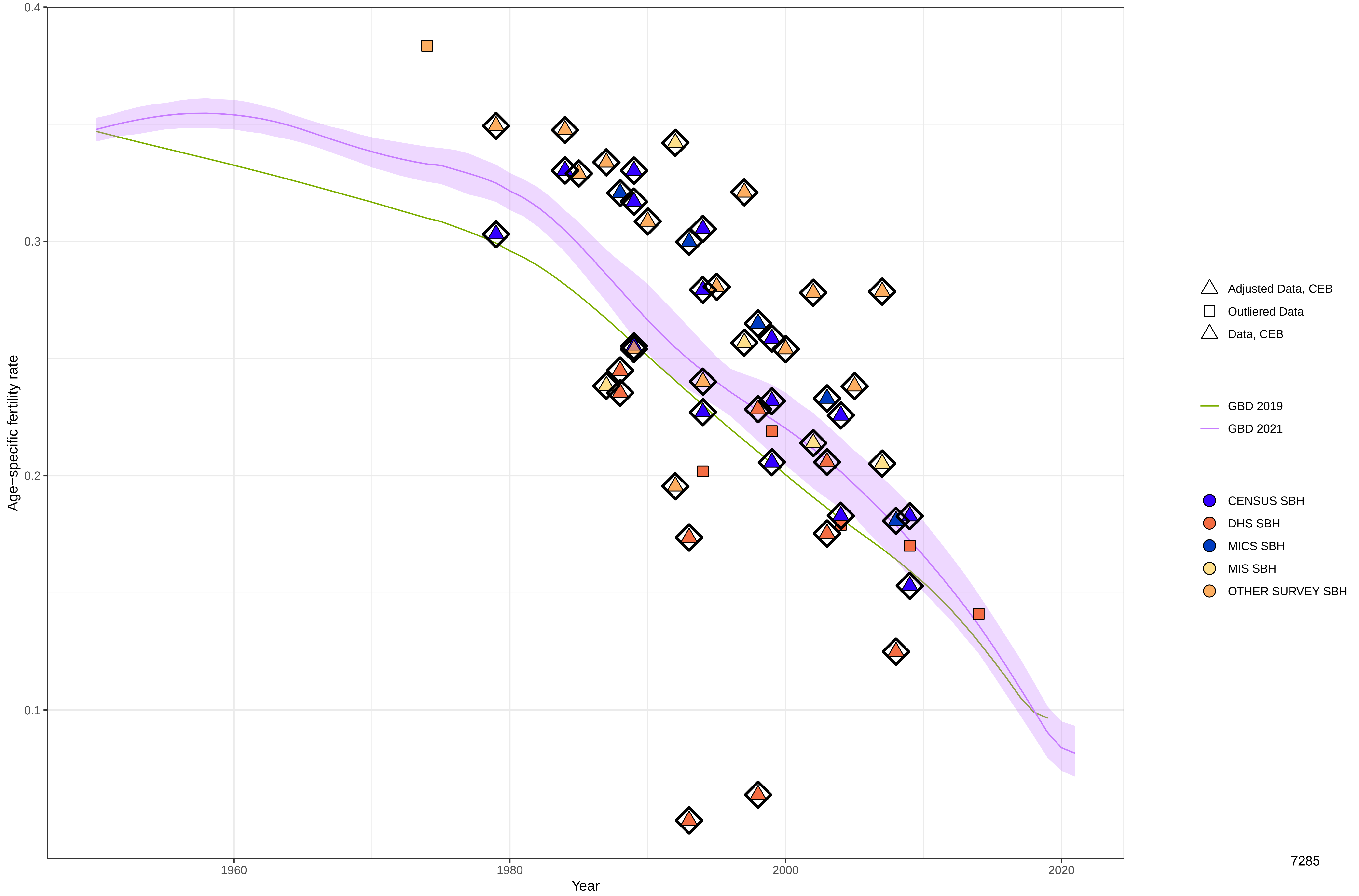

Age−specific fertility fitted trend lines, 1950−2021: Tharaka Nithi, Ages: 30−34
Data points enclosed with a diamond are reference sources

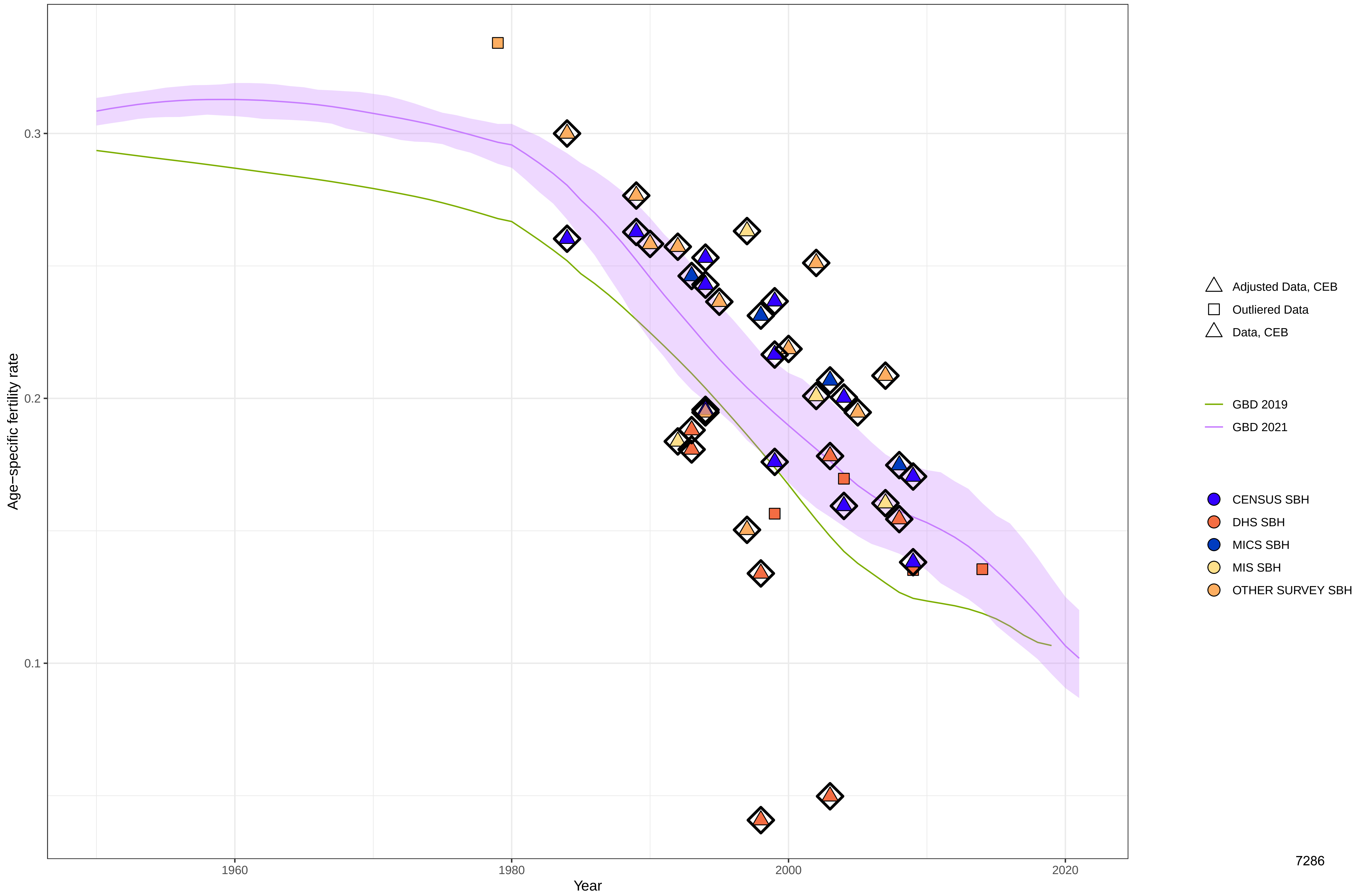

Age−specific fertility fitted trend lines, 1950−2021: Tharaka Nithi, Ages: 35−39
Data points enclosed with a diamond are reference sources

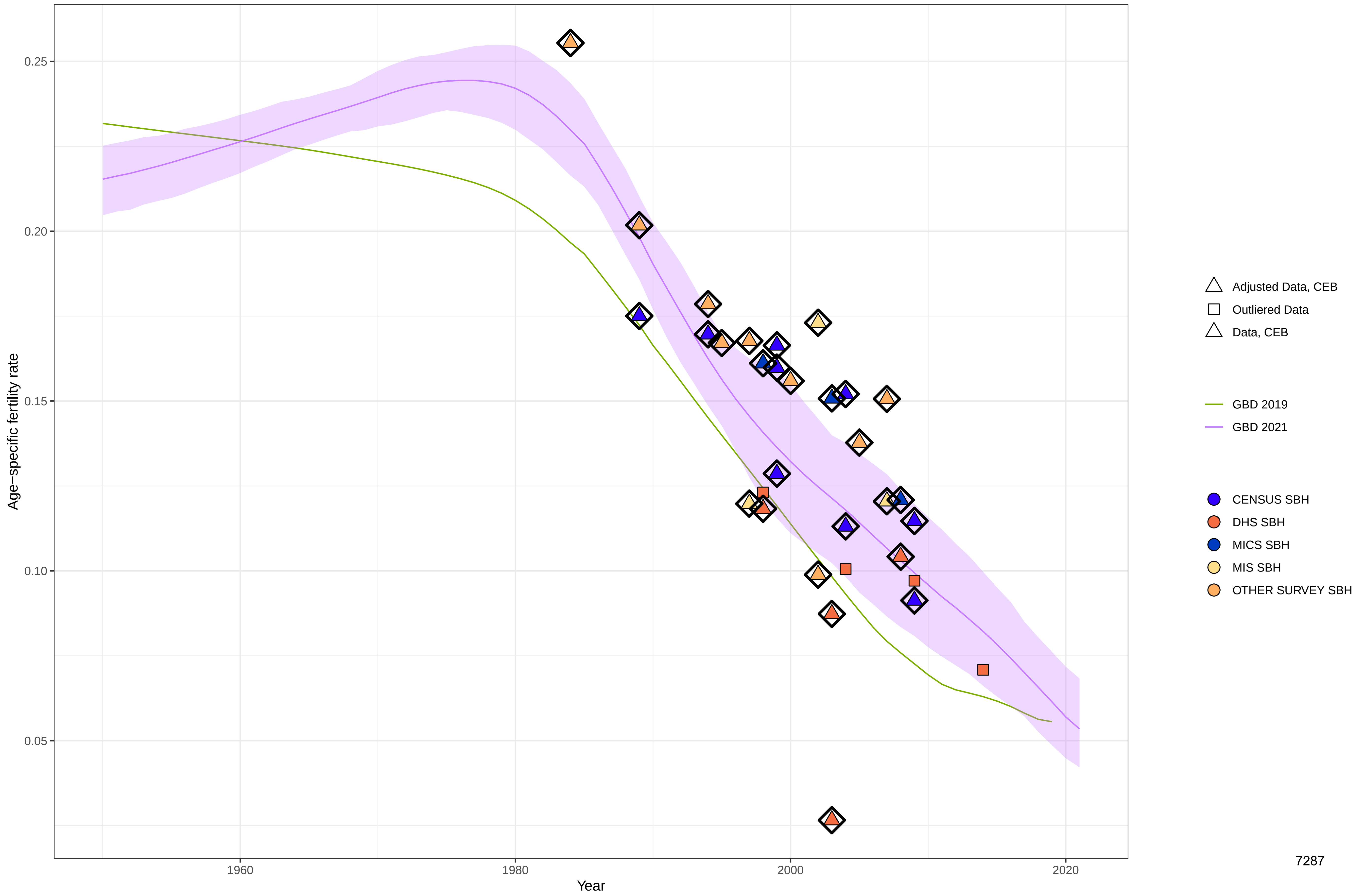

Age−specific fertility fitted trend lines, 1950−2021: Tharaka Nithi, Ages: 40−44
Data points enclosed with a diamond are reference sources

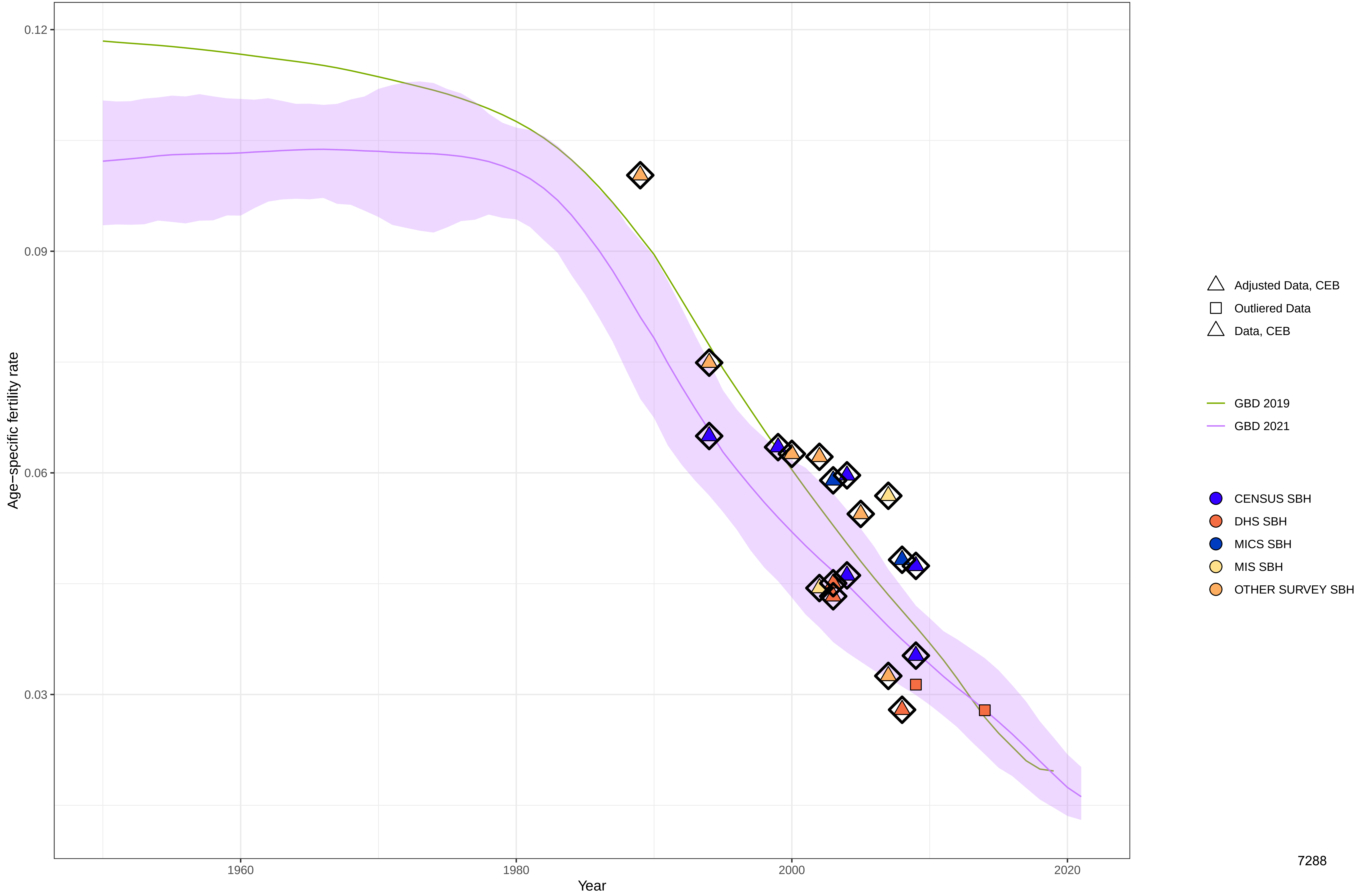

Age−specific fertility fitted trend lines, 1950−2021: Tharaka Nithi, Ages: 45−49
Data points enclosed with a diamond are reference sources

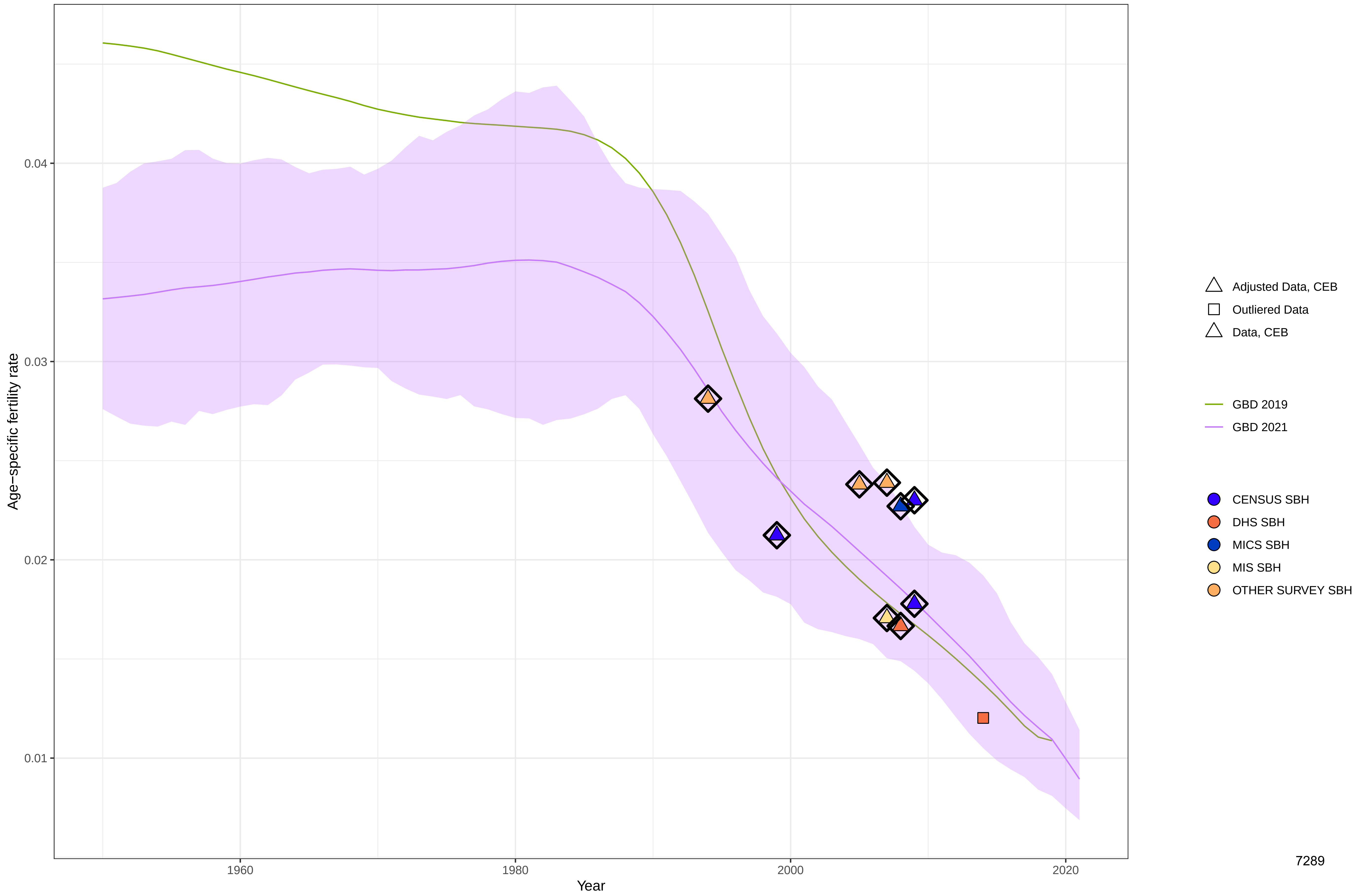

Age−specific fertility fitted trend lines, 1950−2021: Tharaka Nithi, Ages: 50−54
Data points enclosed with a diamond are reference sources

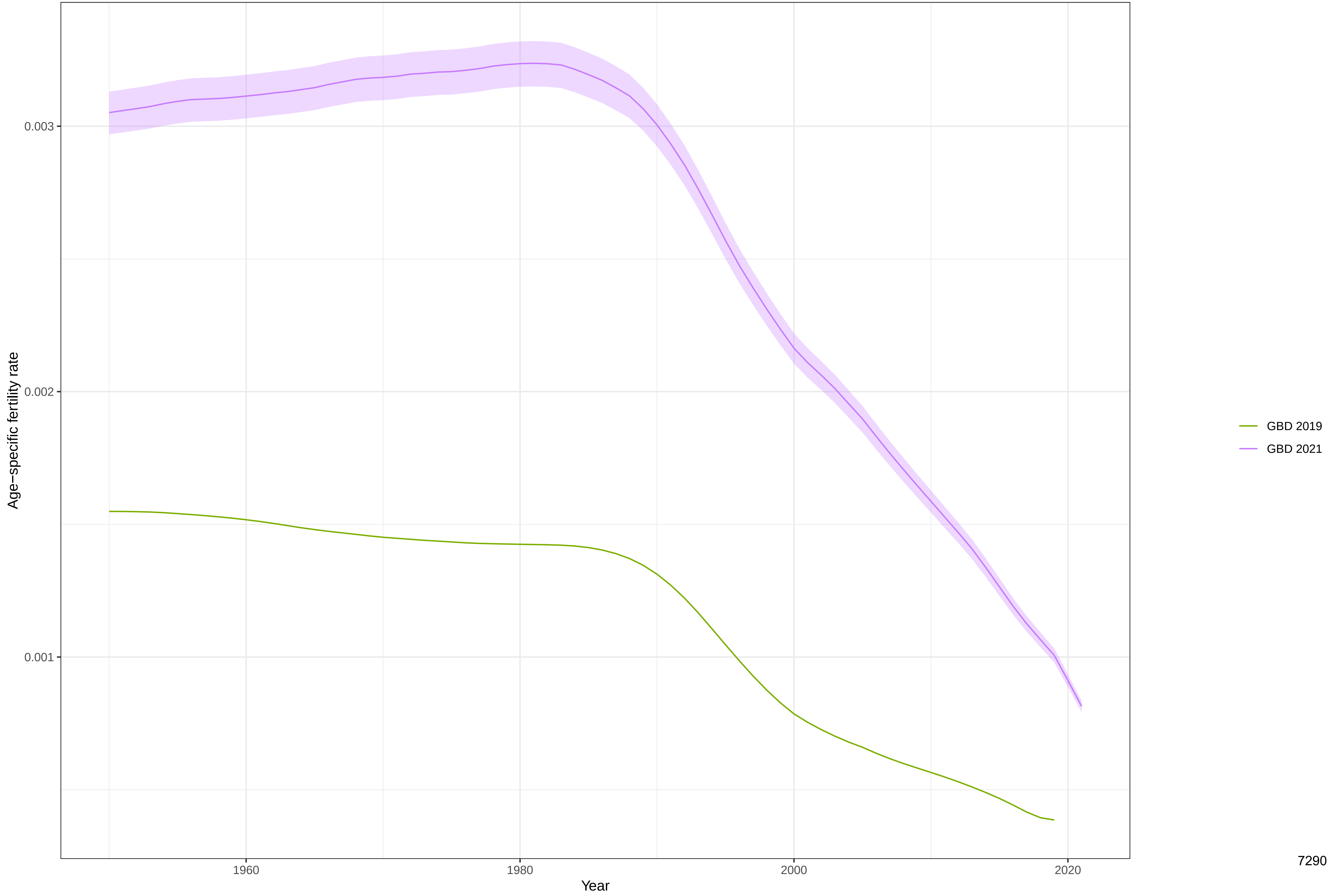

Total fertility rate fitted trend lines, 1950−2021: Tharaka Nithi TFR
Data points enclosed with a diamond are reference sources

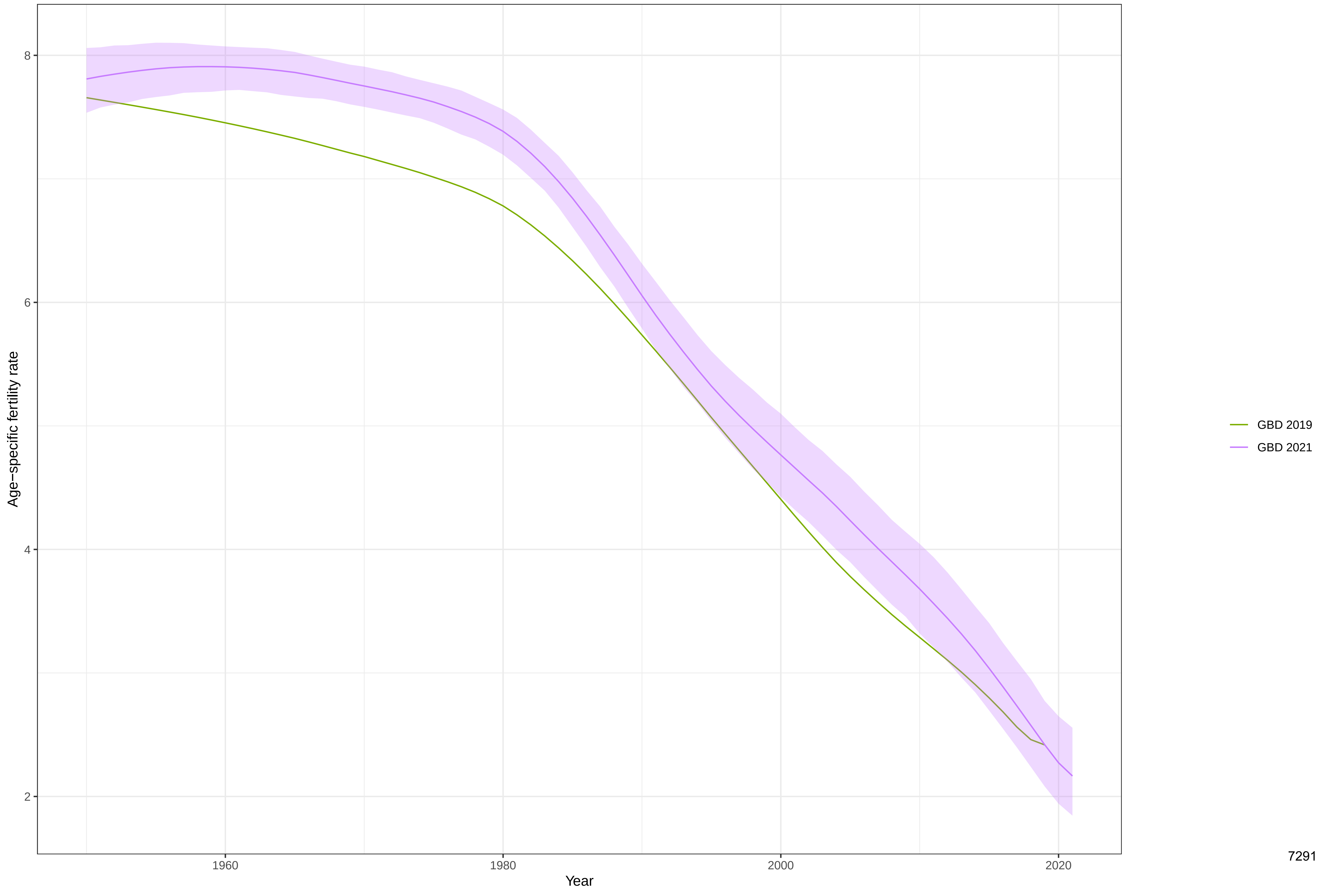

Age−specific fertility fitted trend lines, 1950−2021: Trans Nzoia, Ages: 10−14
Data points enclosed with a diamond are reference sources

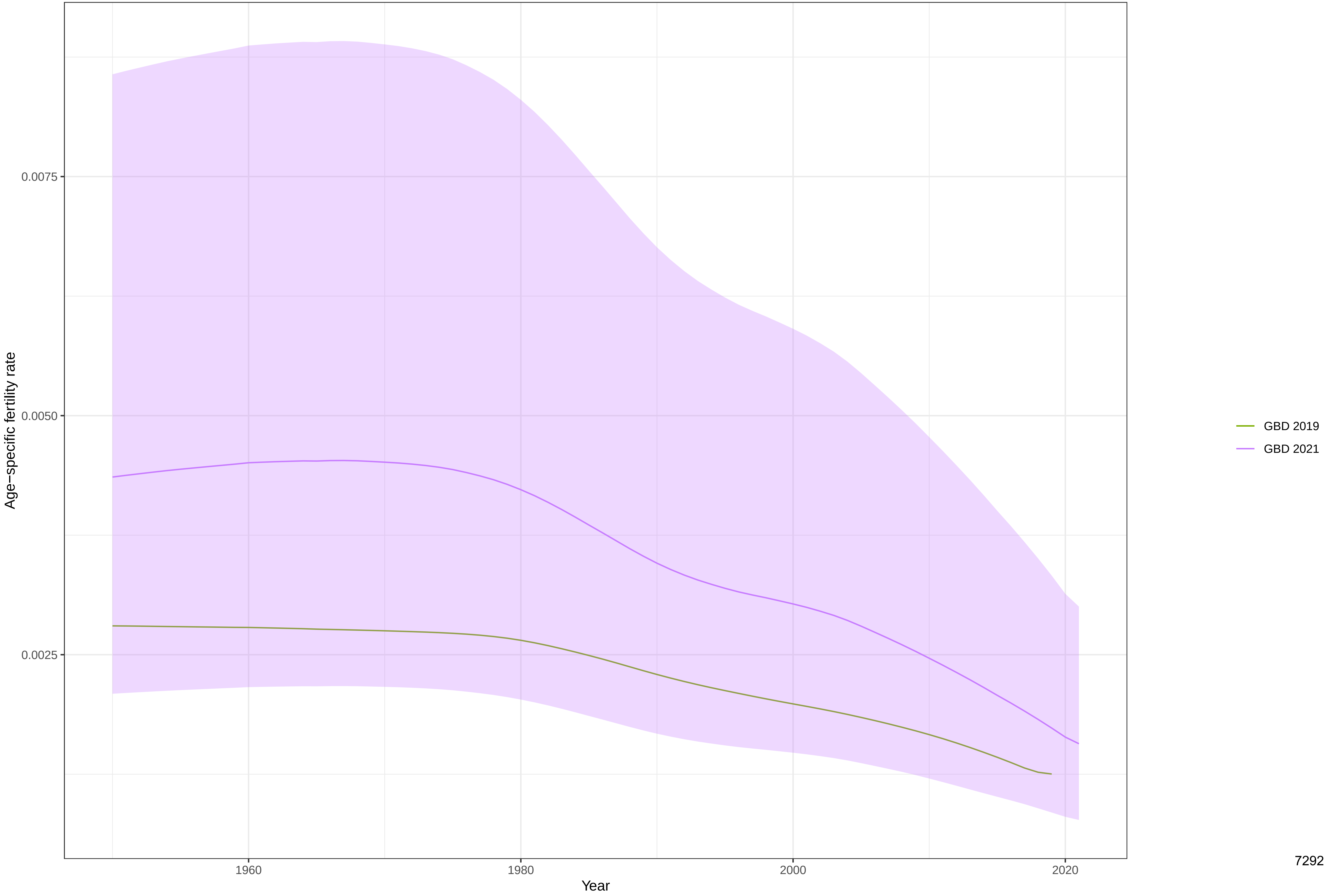

Age−specific fertility fitted trend lines, 1950−2021: Trans Nzoia, Ages: 15−19
Data points enclosed with a diamond are reference sources

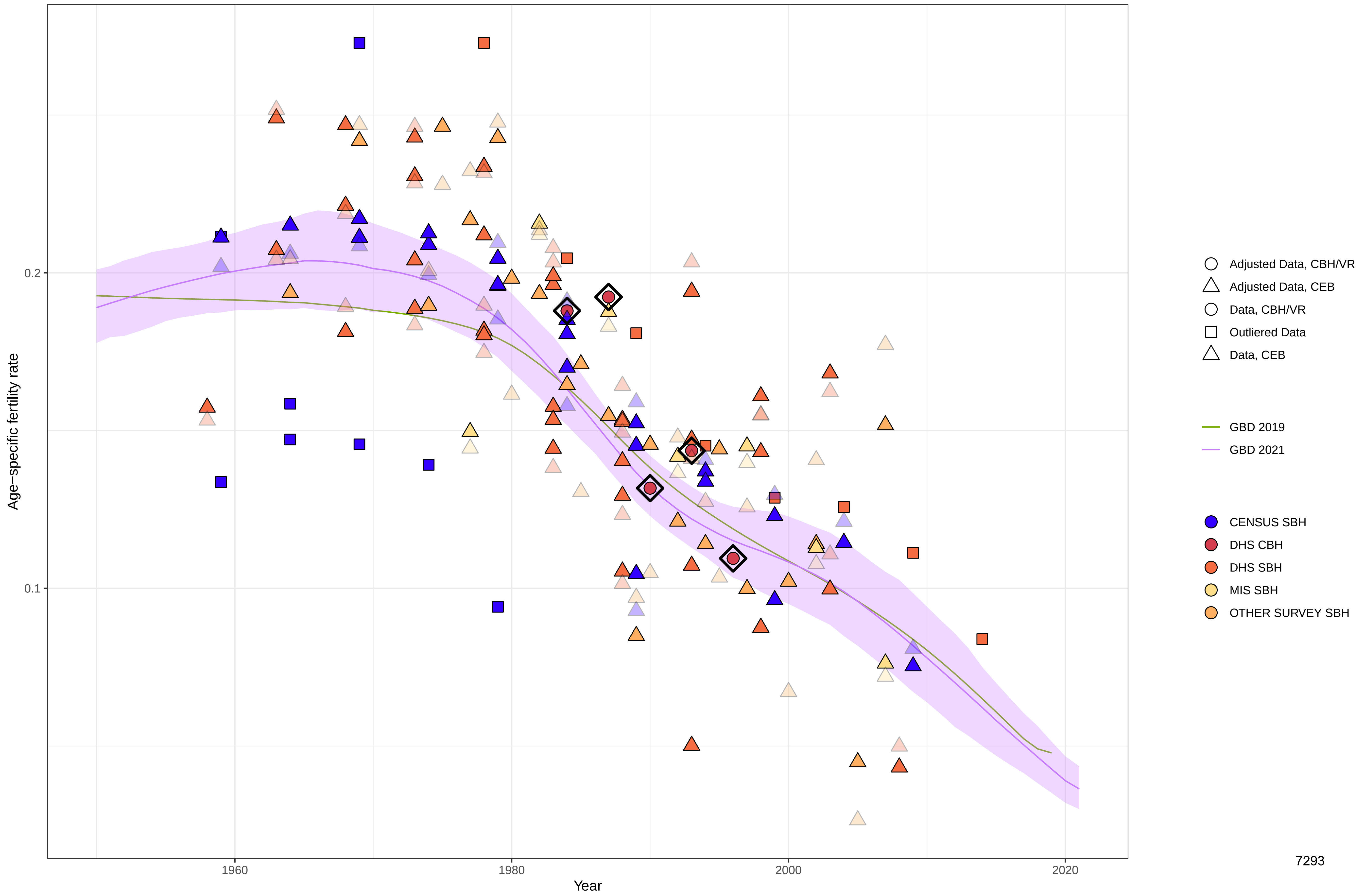

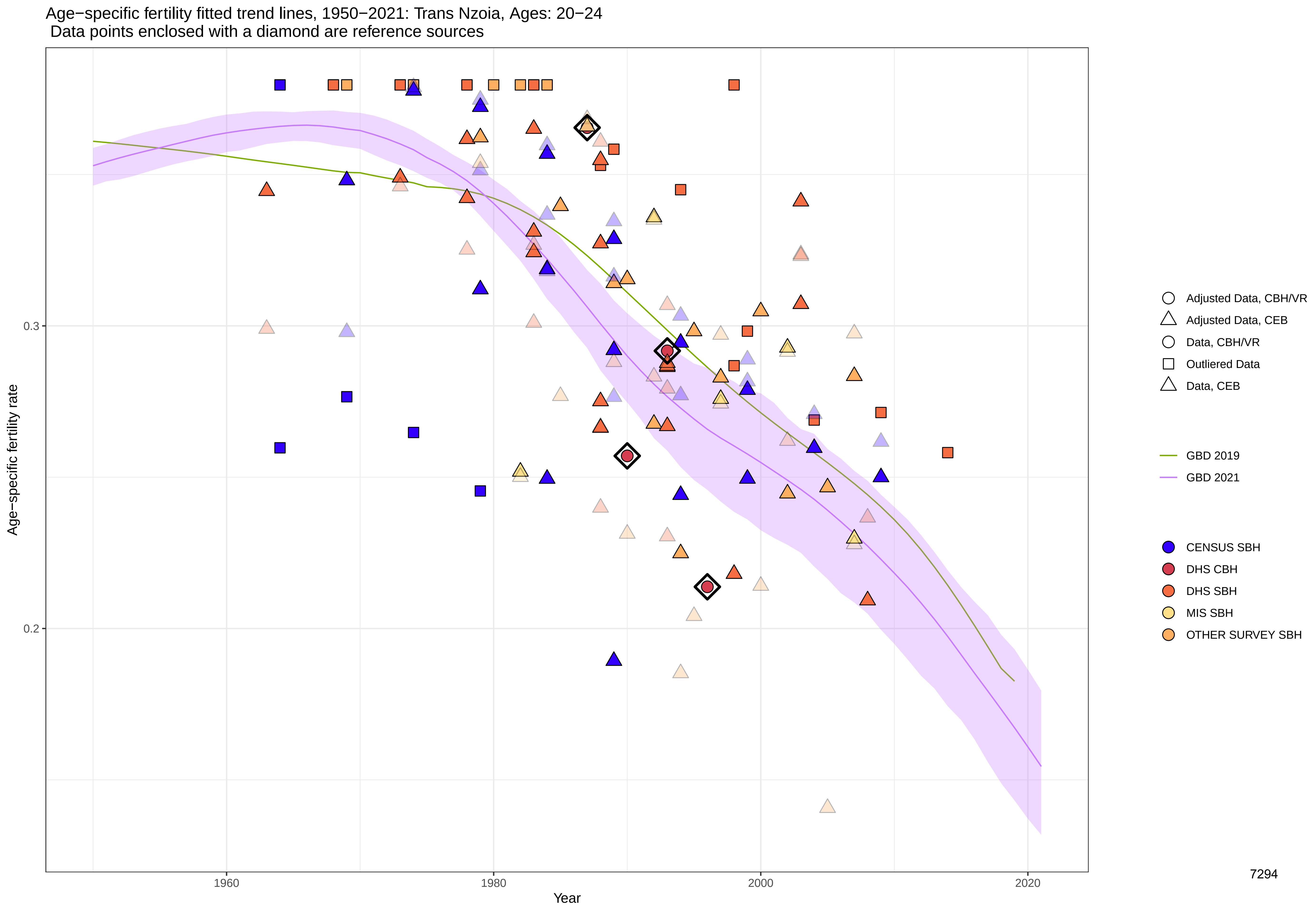

Age−specific fertility fitted trend lines, 1950−2021: Trans Nzoia, Ages: 20−24
Data points enclosed with a diamond are reference sources
Age−specific fertility rate
Year
0.3
0.2
1960
1980
2000
2020
Adjusted Data, CBH/VR
Adjusted Data, CEB
Data, CBH/VR
Outliered Data
Data, CEB
GBD 2019
GBD 2021
CENSUS SBH
DHS CBH
DHS SBH
MIS SBH
OTHER SURVEY SBH

Age−specific fertility fitted trend lines, 1950−2021: Trans Nzoia, Ages: 25−29
Data points enclosed with a diamond are reference sources

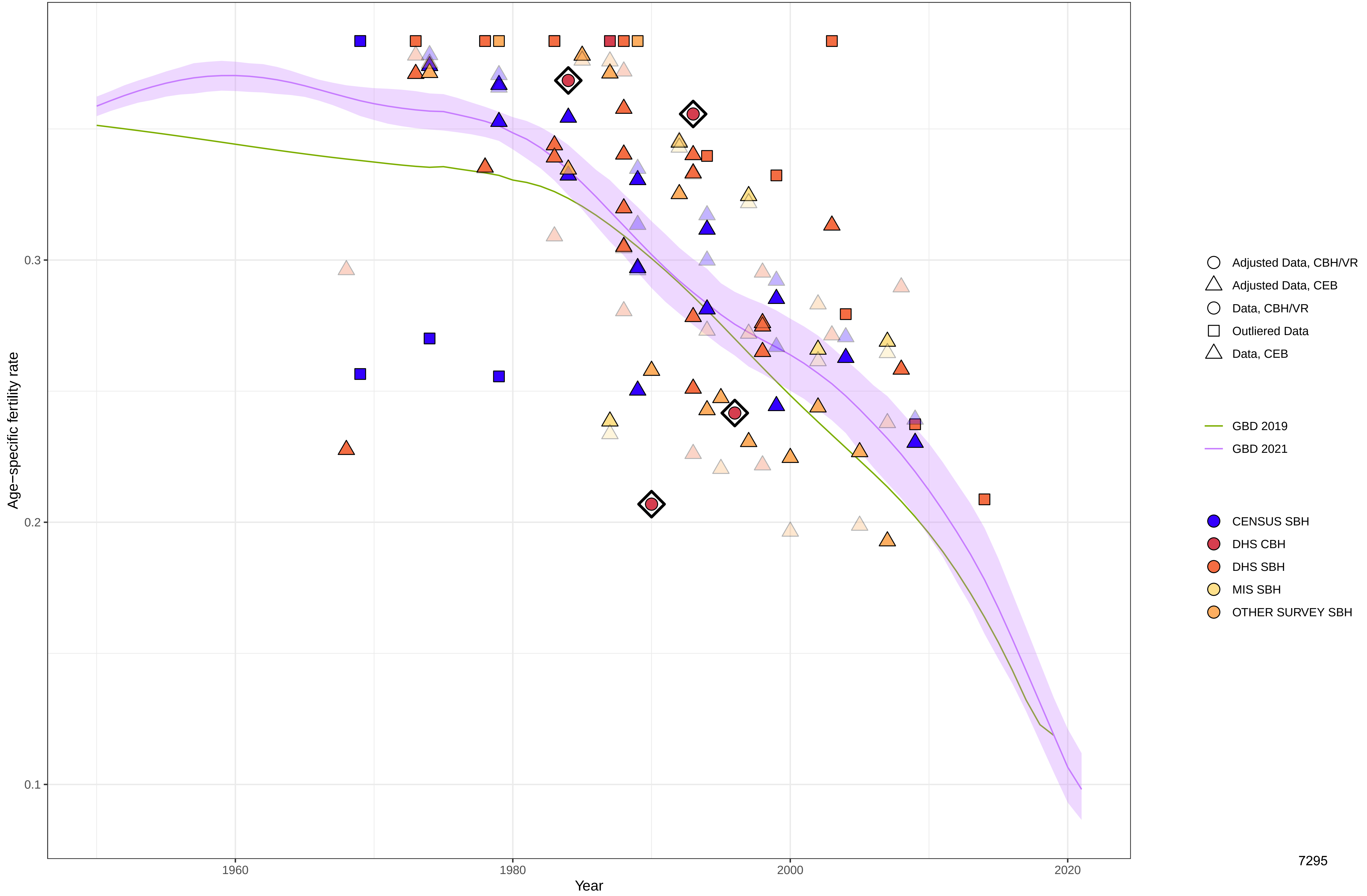

Age−specific fertility fitted trend lines, 1950−2021: Trans Nzoia, Ages: 30−34
Data points enclosed with a diamond are reference sources

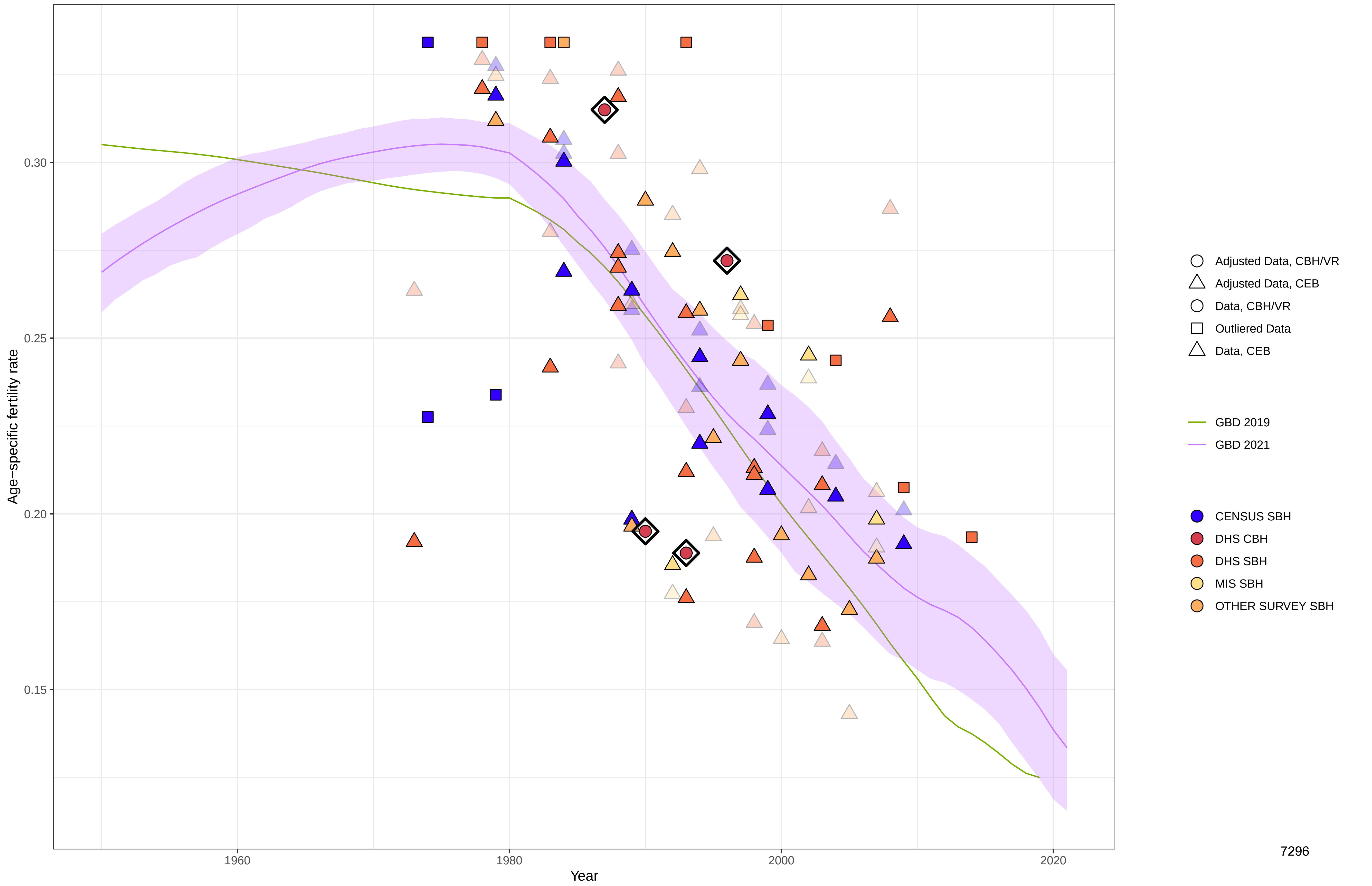

Age−specific fertility fitted trend lines, 1950−2021: Trans Nzoia, Ages: 35−39
Data points enclosed with a diamond are reference sources

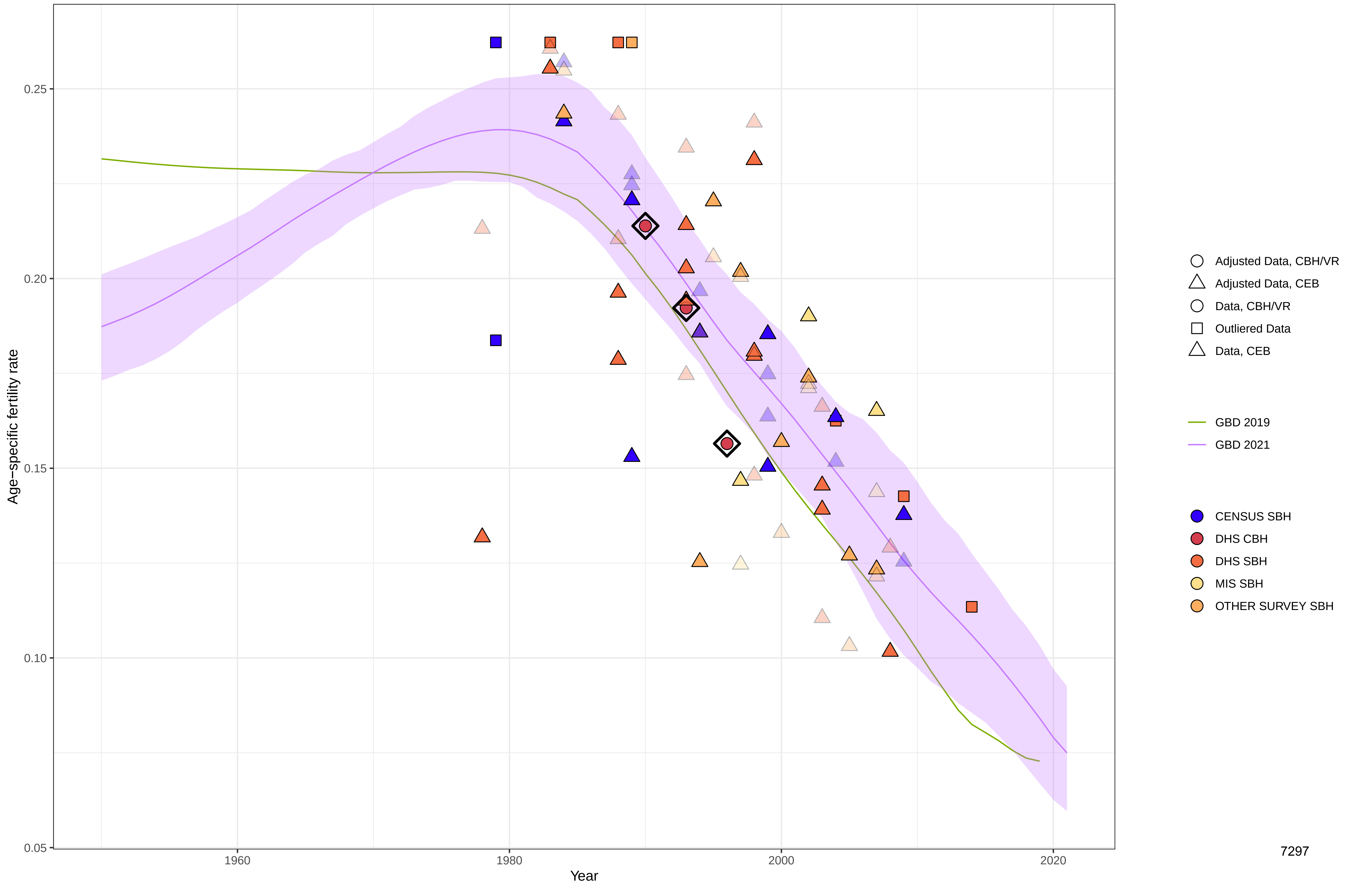

Age−specific fertility fitted trend lines, 1950−2021: Trans Nzoia, Ages: 40−44
Data points enclosed with a diamond are reference sources

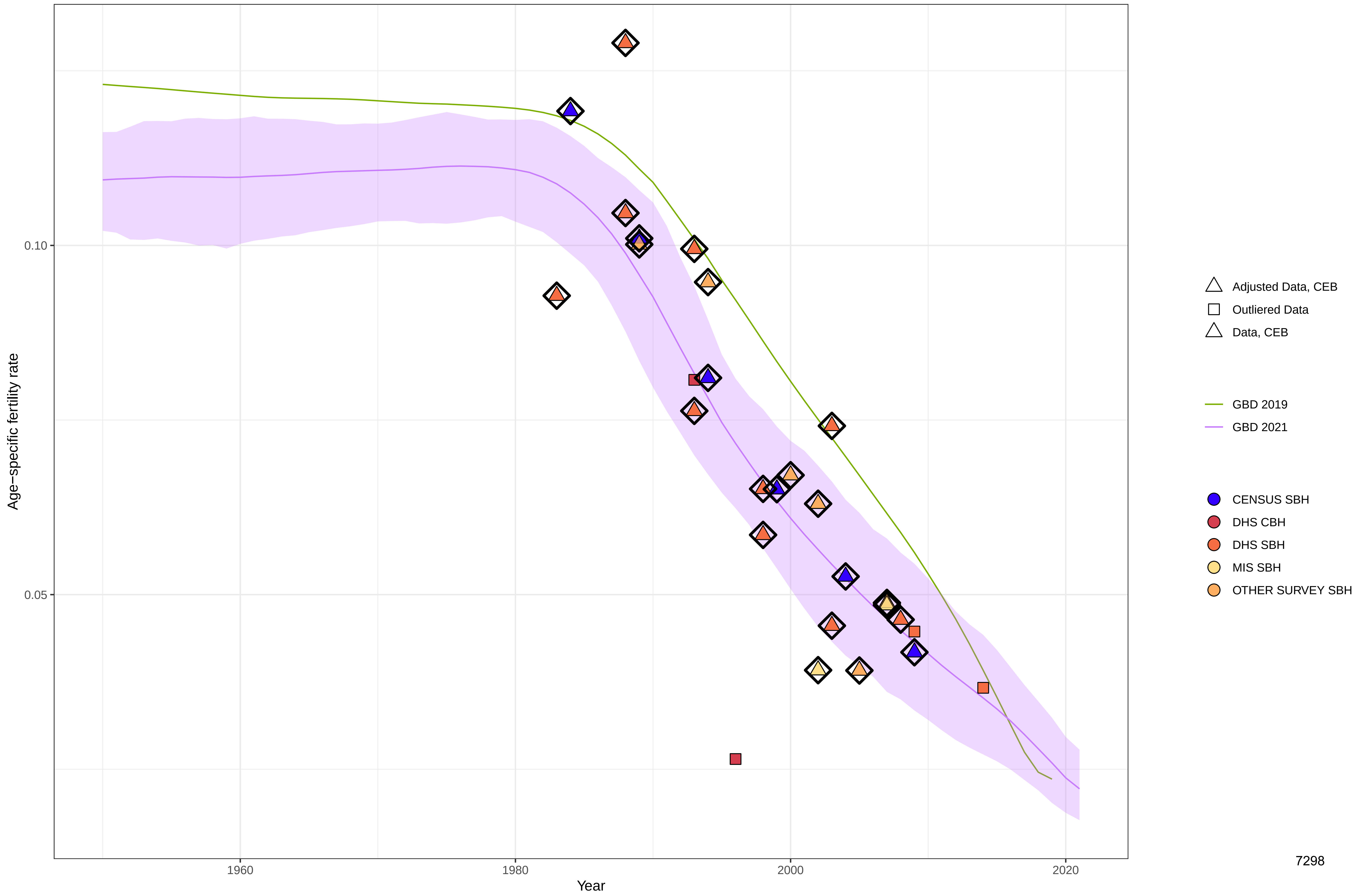

Age−specific fertility fitted trend lines, 1950−2021: Trans Nzoia, Ages: 45−49
Data points enclosed with a diamond are reference sources

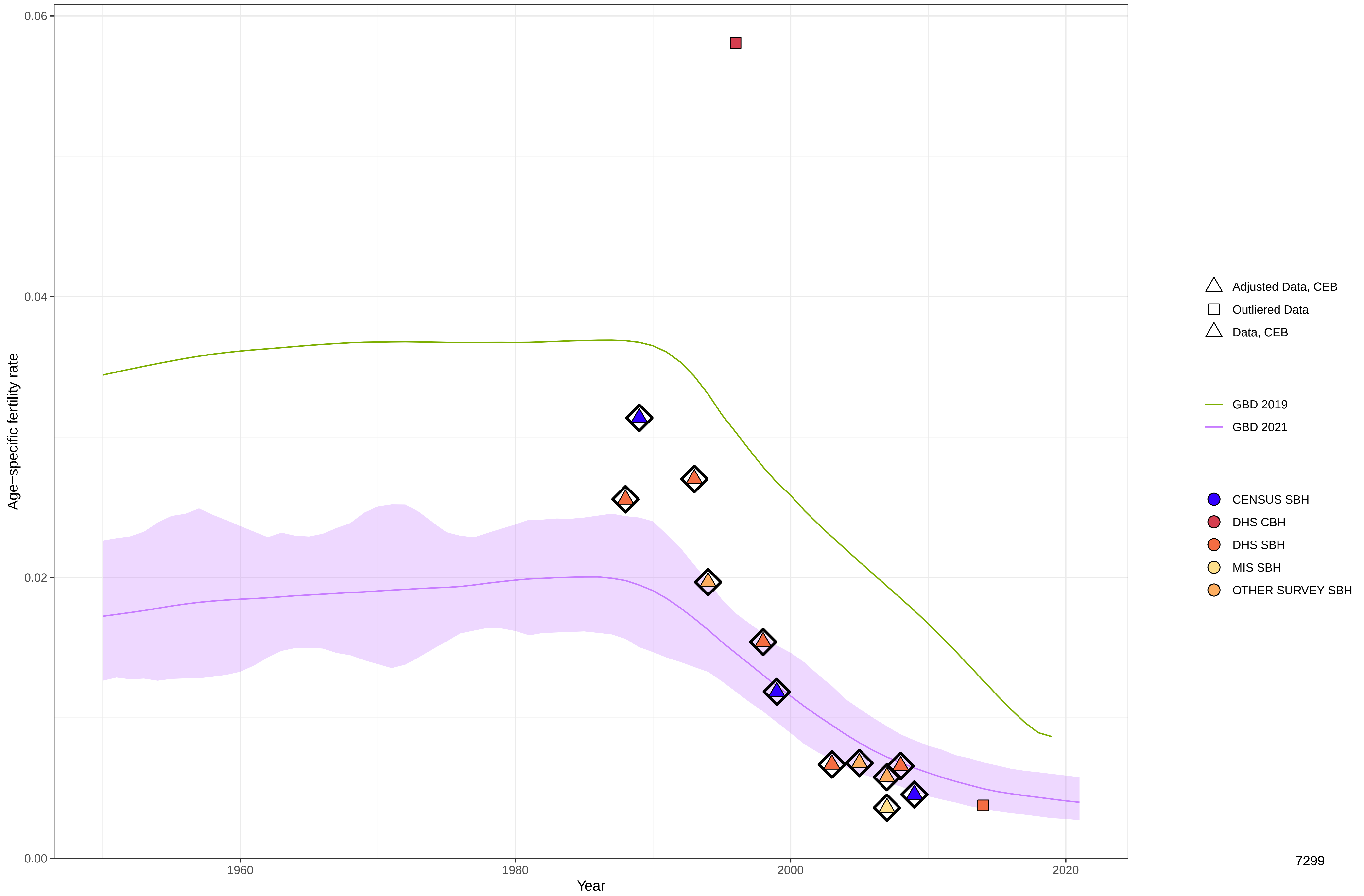

Age−specific fertility fitted trend lines, 1950−2021: Trans Nzoia, Ages: 50−54
Data points enclosed with a diamond are reference sources

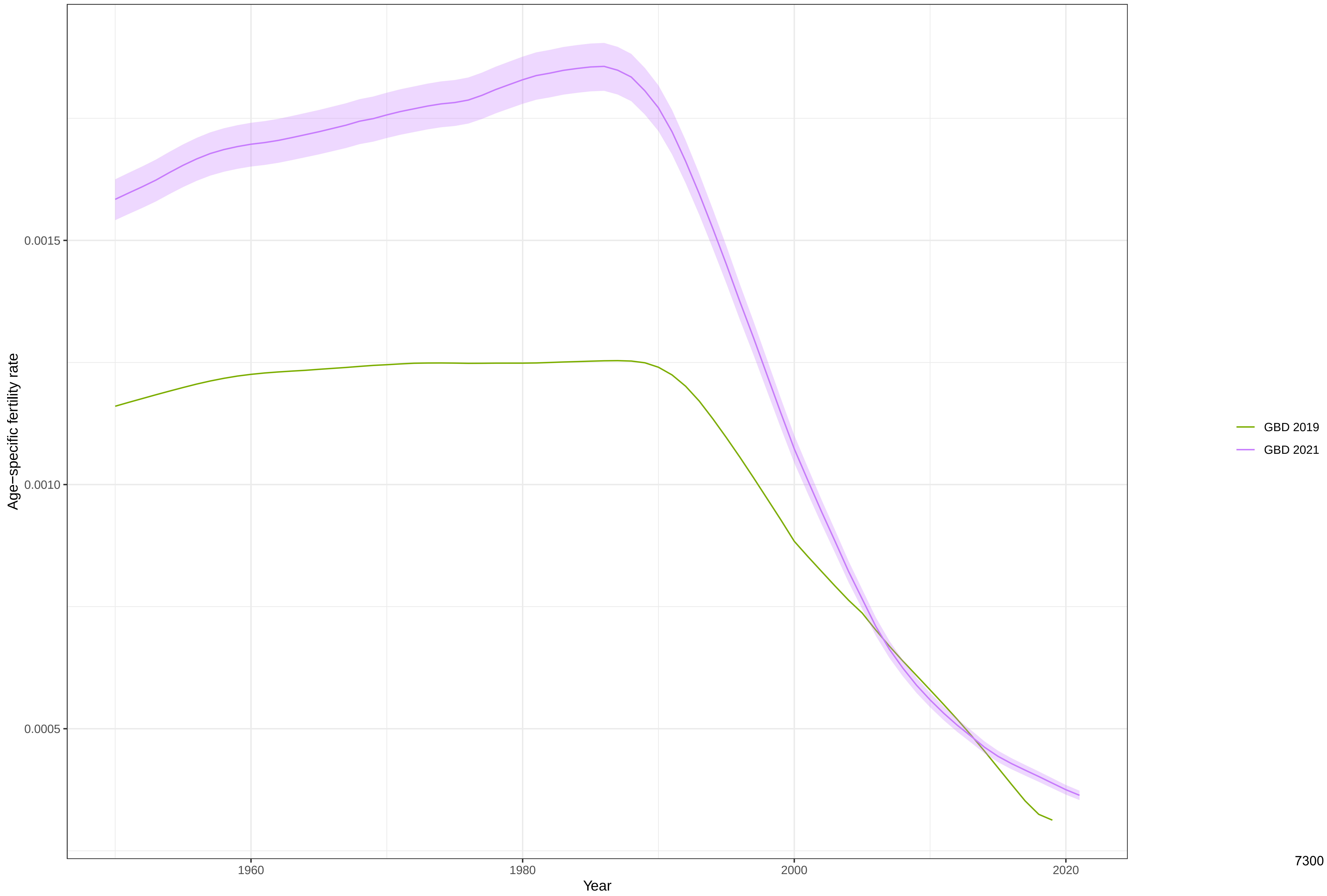

Total fertility rate fitted trend lines, 1950−2021: Trans Nzoia TFR
Data points enclosed with a diamond are reference sources

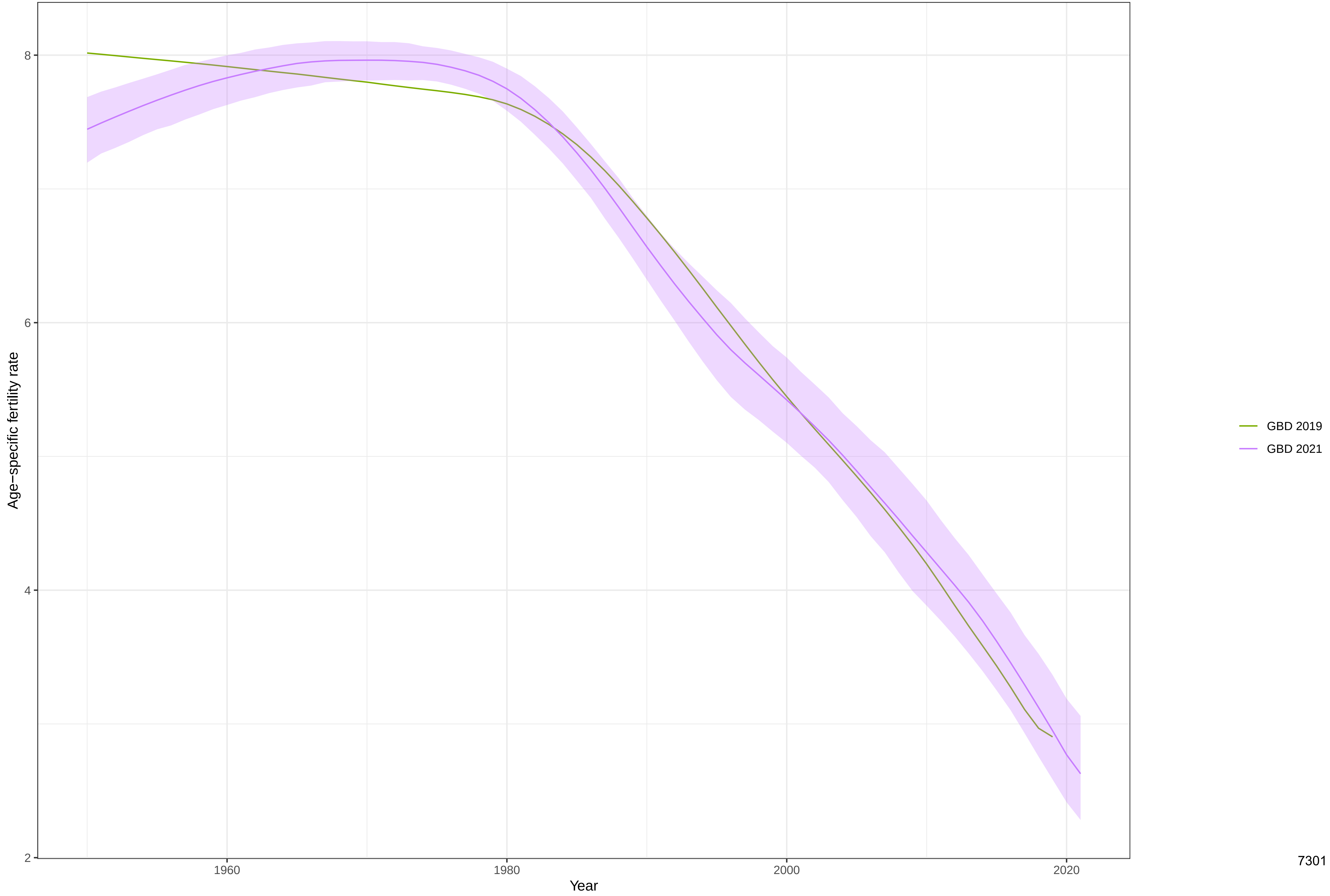

Age−specific fertility fitted trend lines, 1950−2021: Turkana, Ages: 10−14
Data points enclosed with a diamond are reference sources

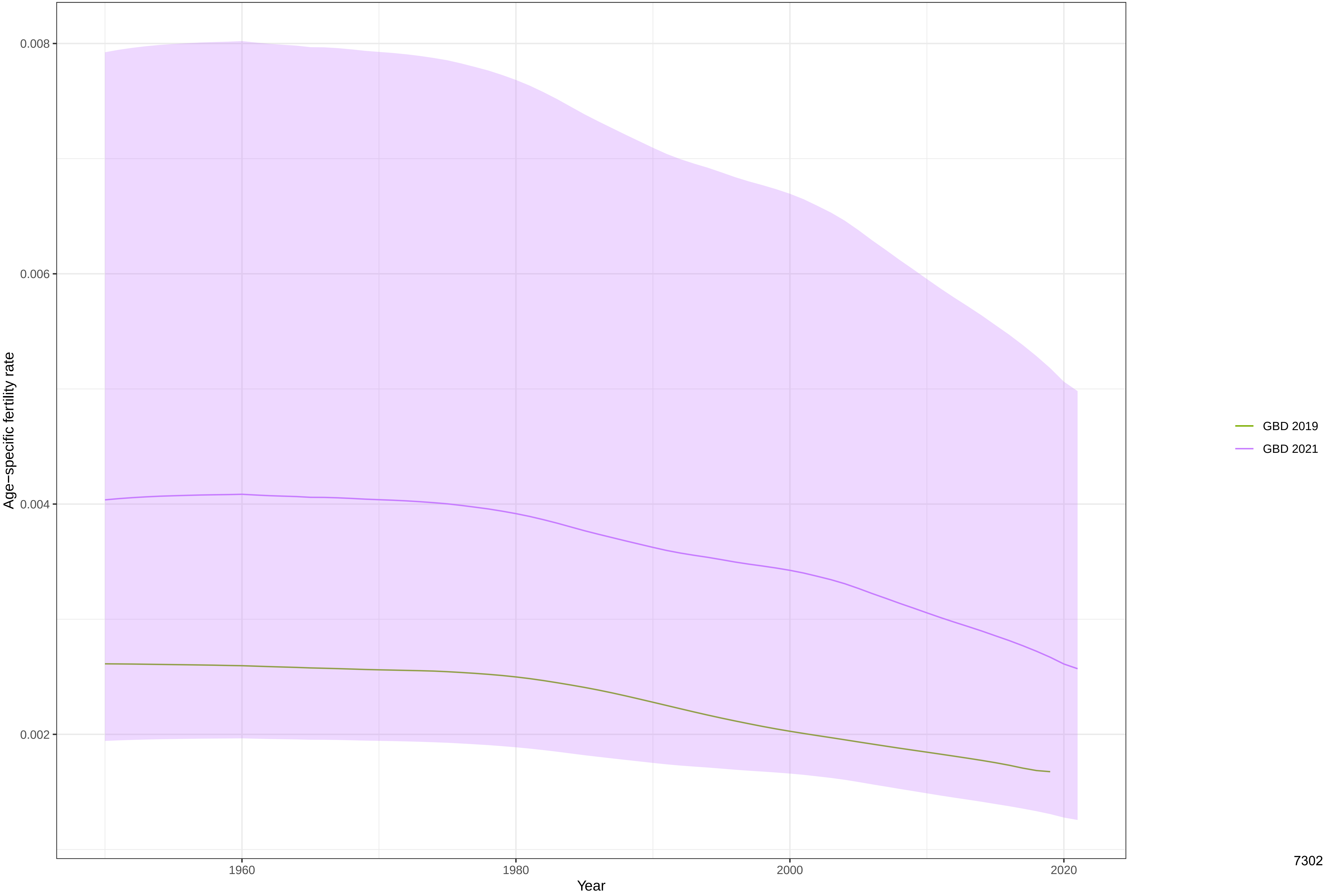

Age−specific fertility fitted trend lines, 1950−2021: Turkana, Ages: 15−19
Data points enclosed with a diamond are reference sources

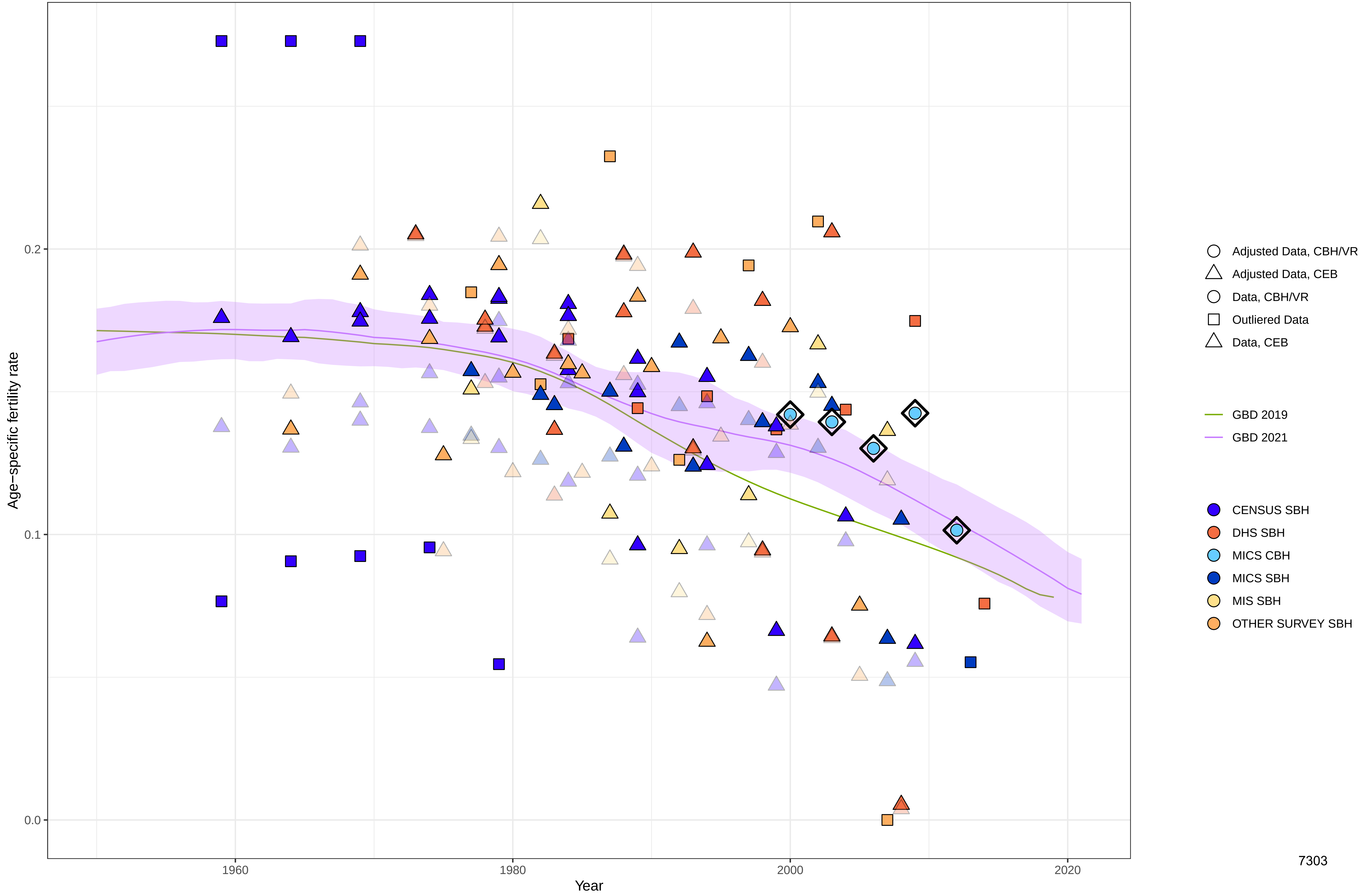

Age−specific fertility fitted trend lines, 1950−2021: Turkana, Ages: 20−24
Data points enclosed with a diamond are reference sources

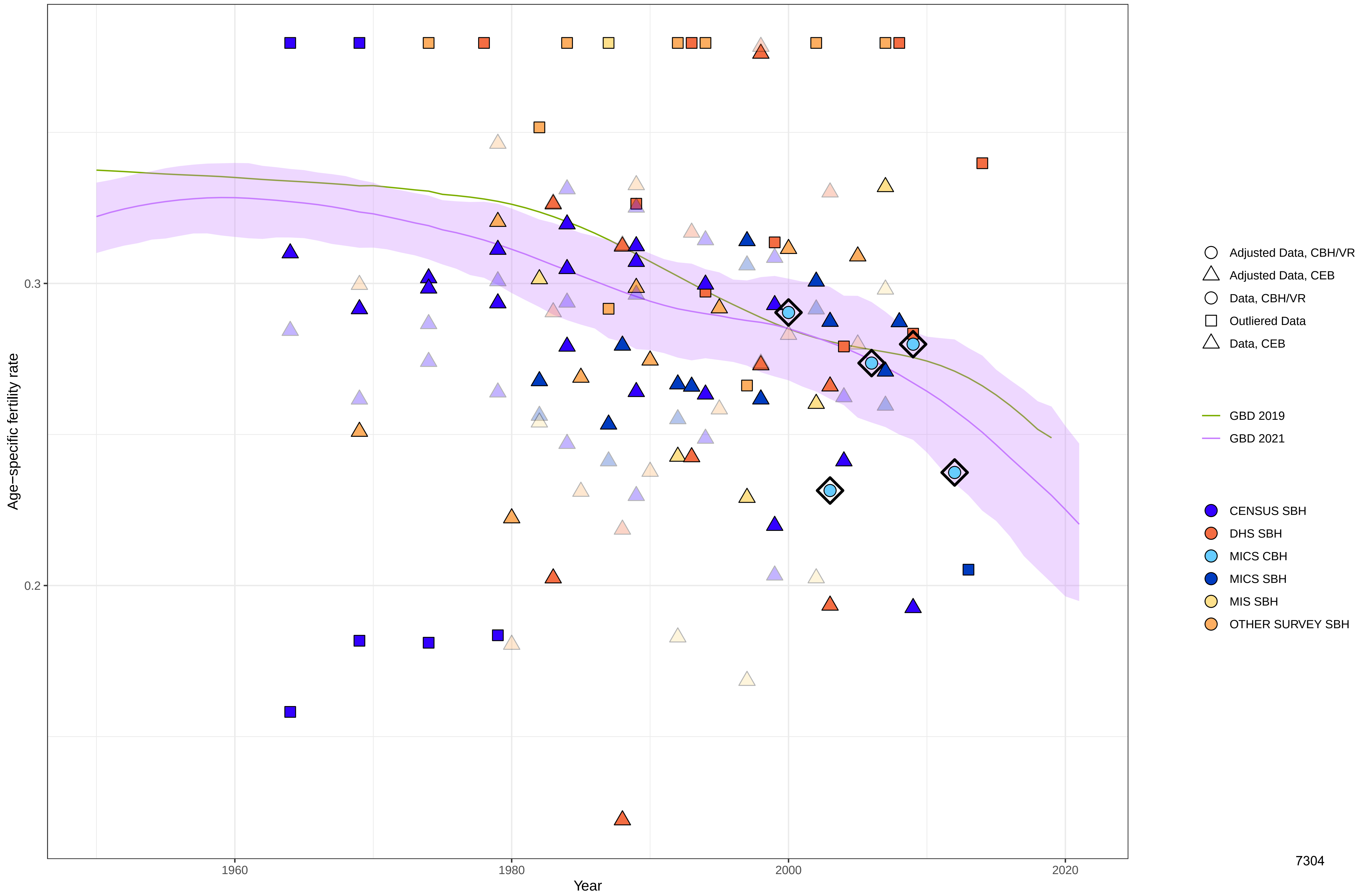

Age−specific fertility fitted trend lines, 1950−2021: Turkana, Ages: 25−29
Data points enclosed with a diamond are reference sources

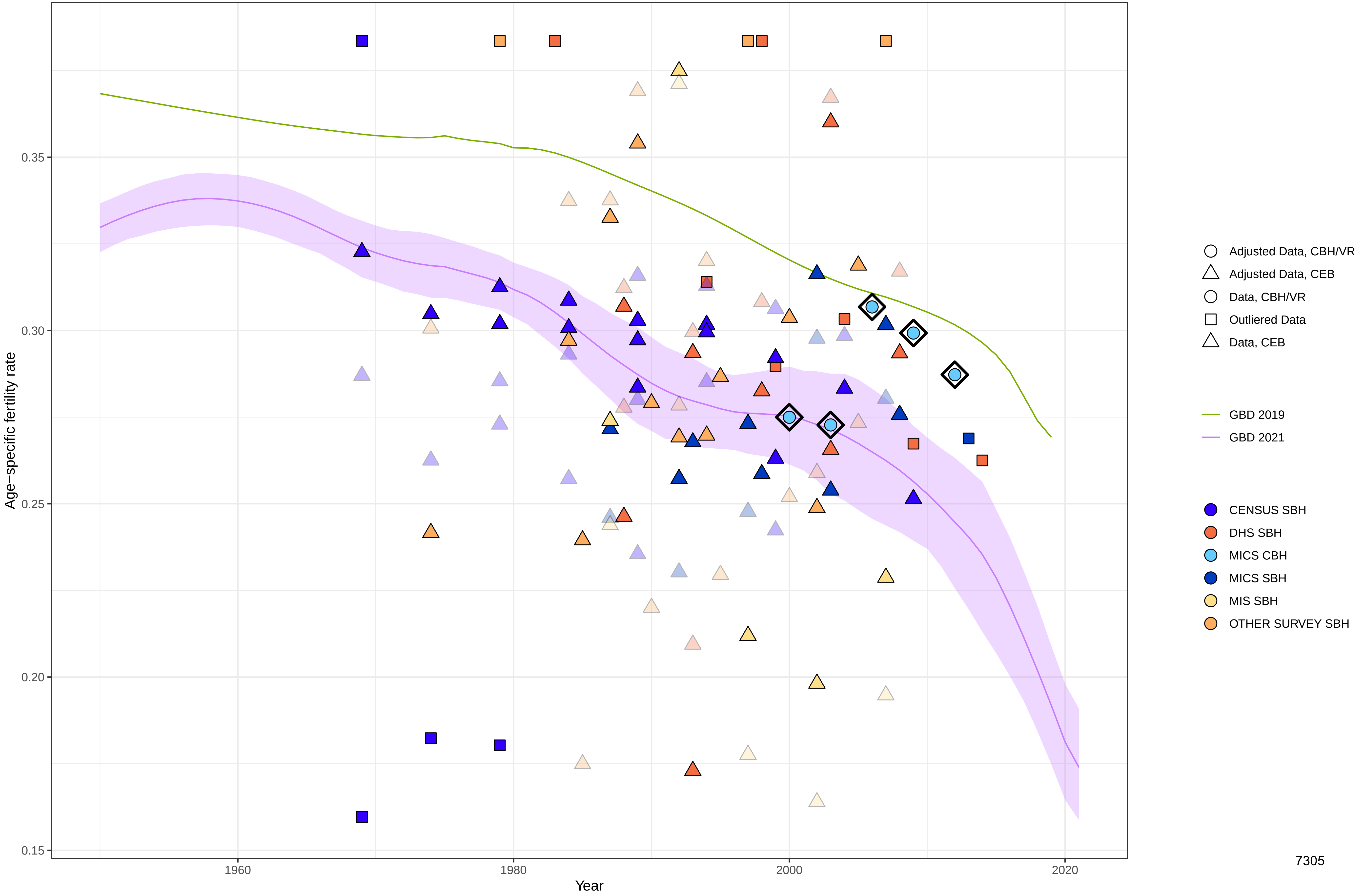

Age−specific fertility fitted trend lines, 1950−2021: Turkana, Ages: 30−34
Data points enclosed with a diamond are reference sources

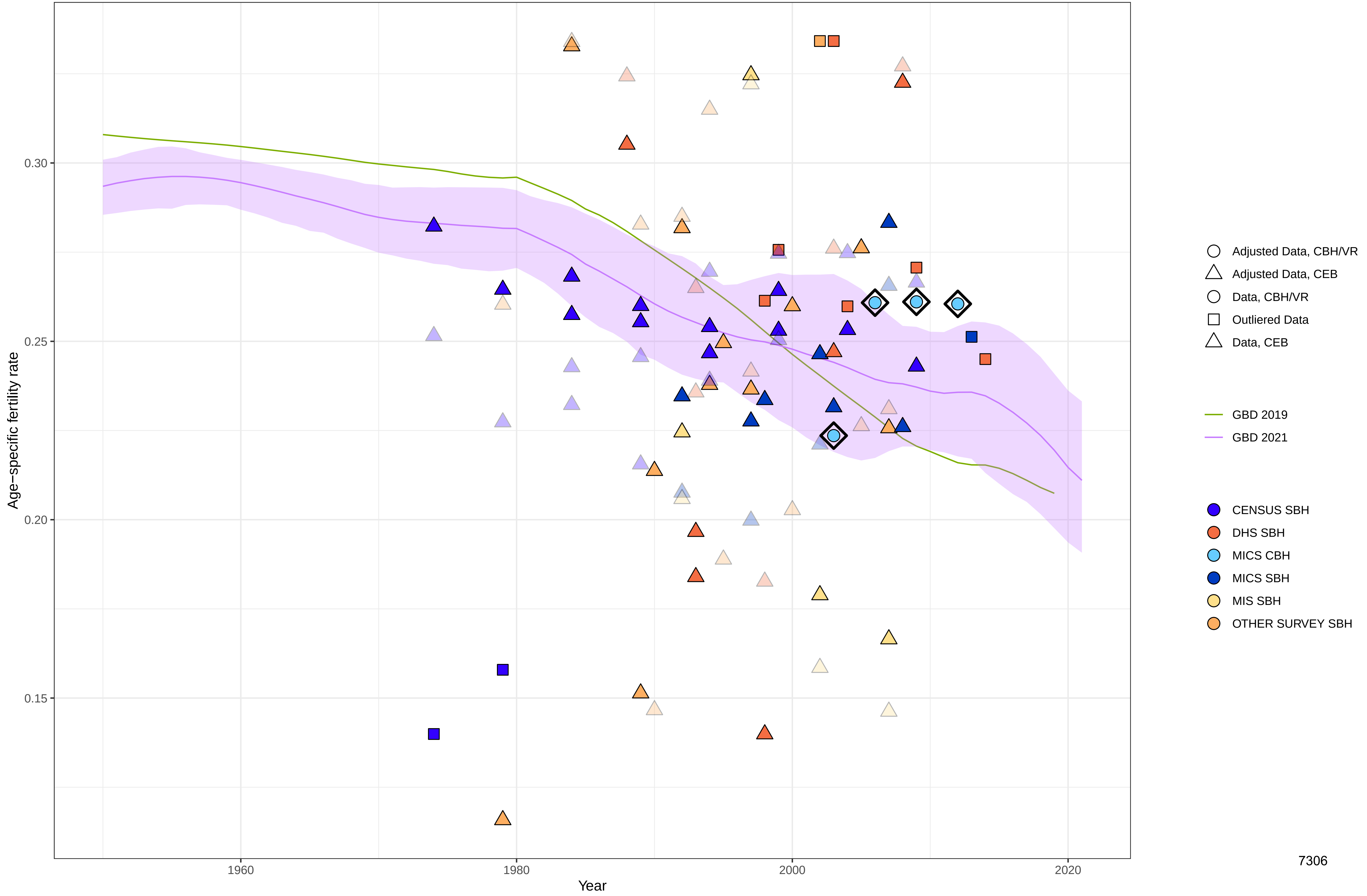

Age−specific fertility fitted trend lines, 1950−2021: Turkana, Ages: 35−39
Data points enclosed with a diamond are reference sources

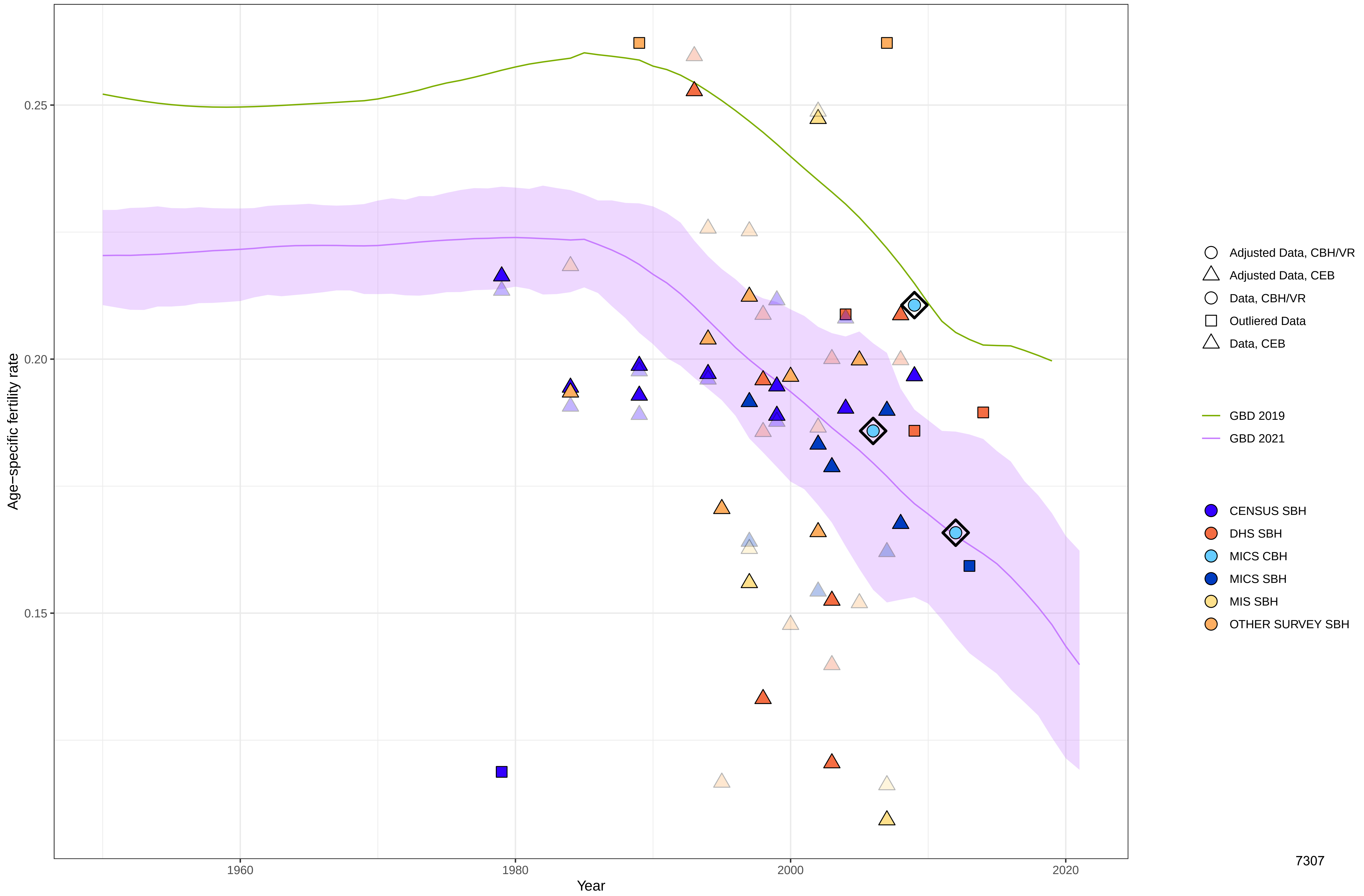

Age−specific fertility fitted trend lines, 1950−2021: Turkana, Ages: 40−44
Data points enclosed with a diamond are reference sources

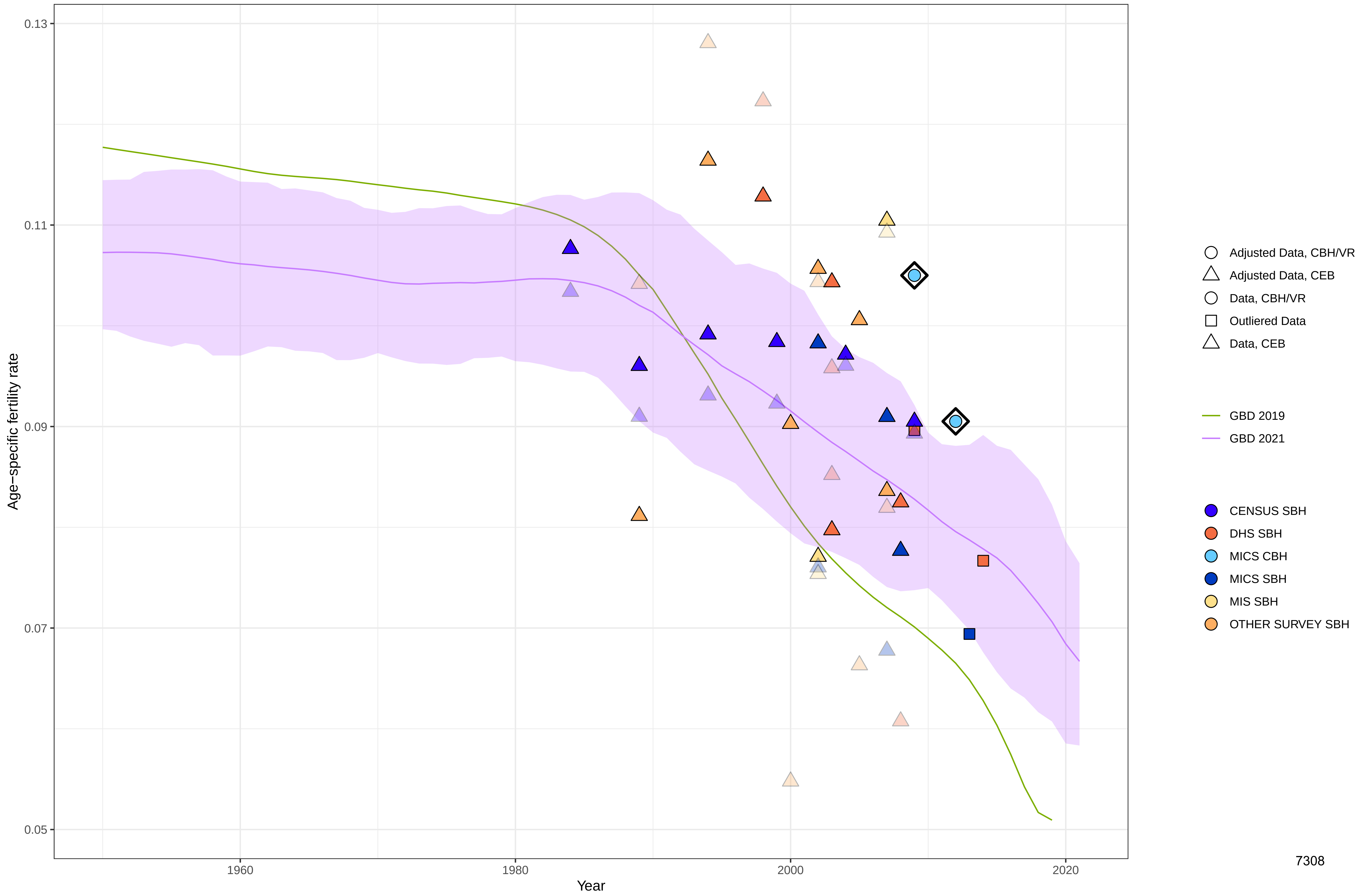

Age−specific fertility fitted trend lines, 1950−2021: Turkana, Ages: 45−49
Data points enclosed with a diamond are reference sources

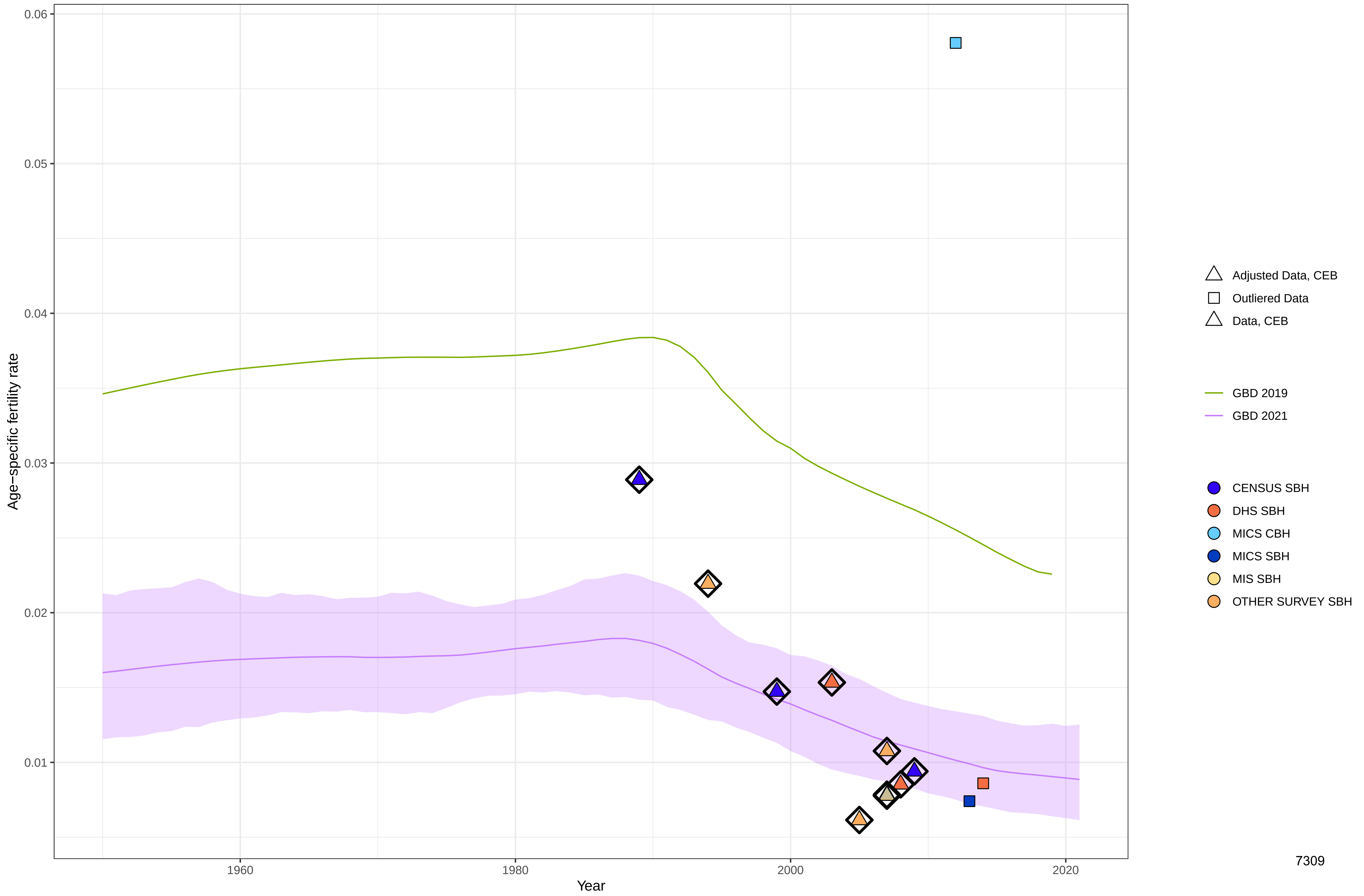

7309

Age−specific fertility fitted trend lines, 1950−2021: Turkana, Ages: 50−54
Data points enclosed with a diamond are reference sources

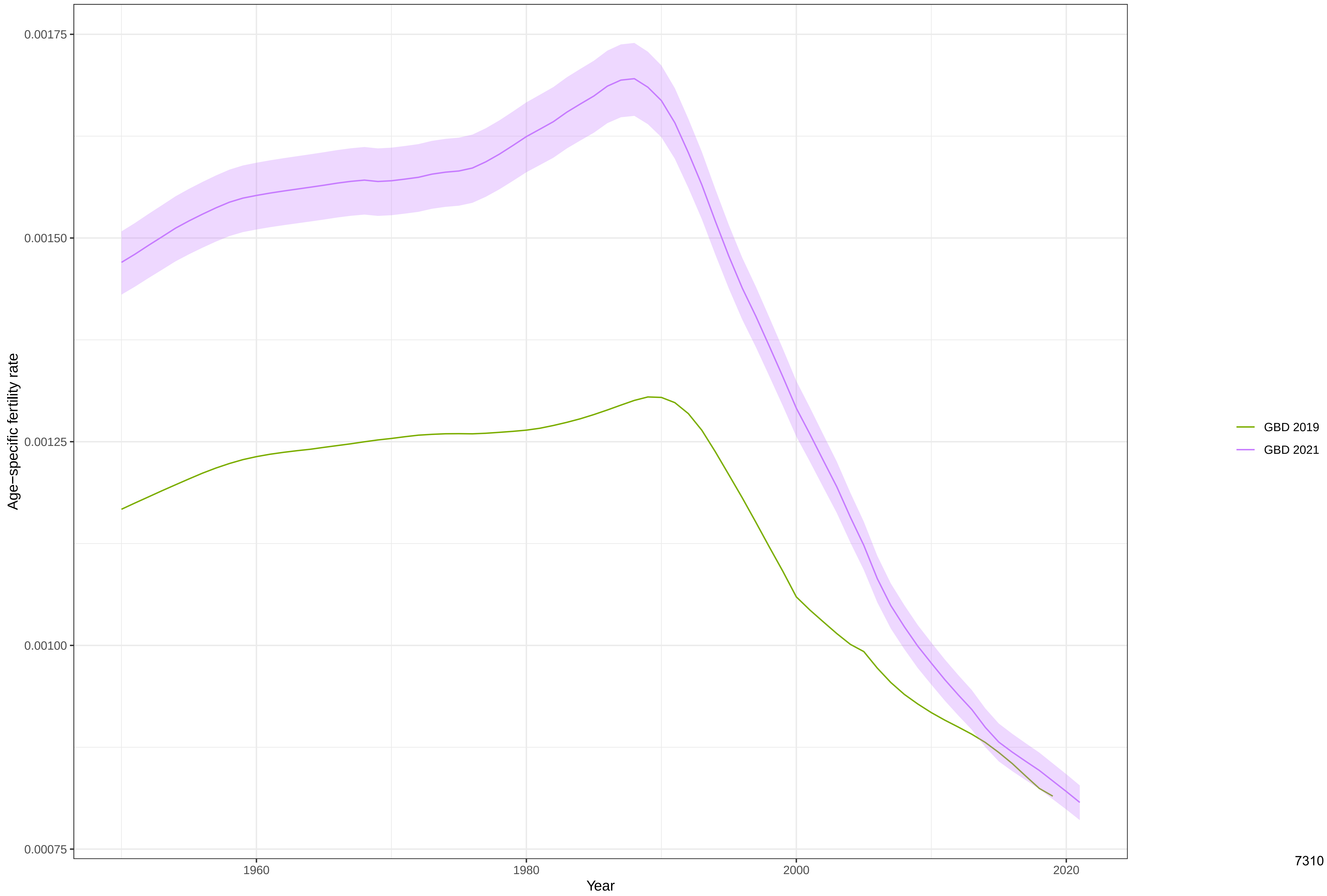

7310

Total fertility rate fitted trend lines, 1950−2021: Turkana TFR
Data points enclosed with a diamond are reference sources

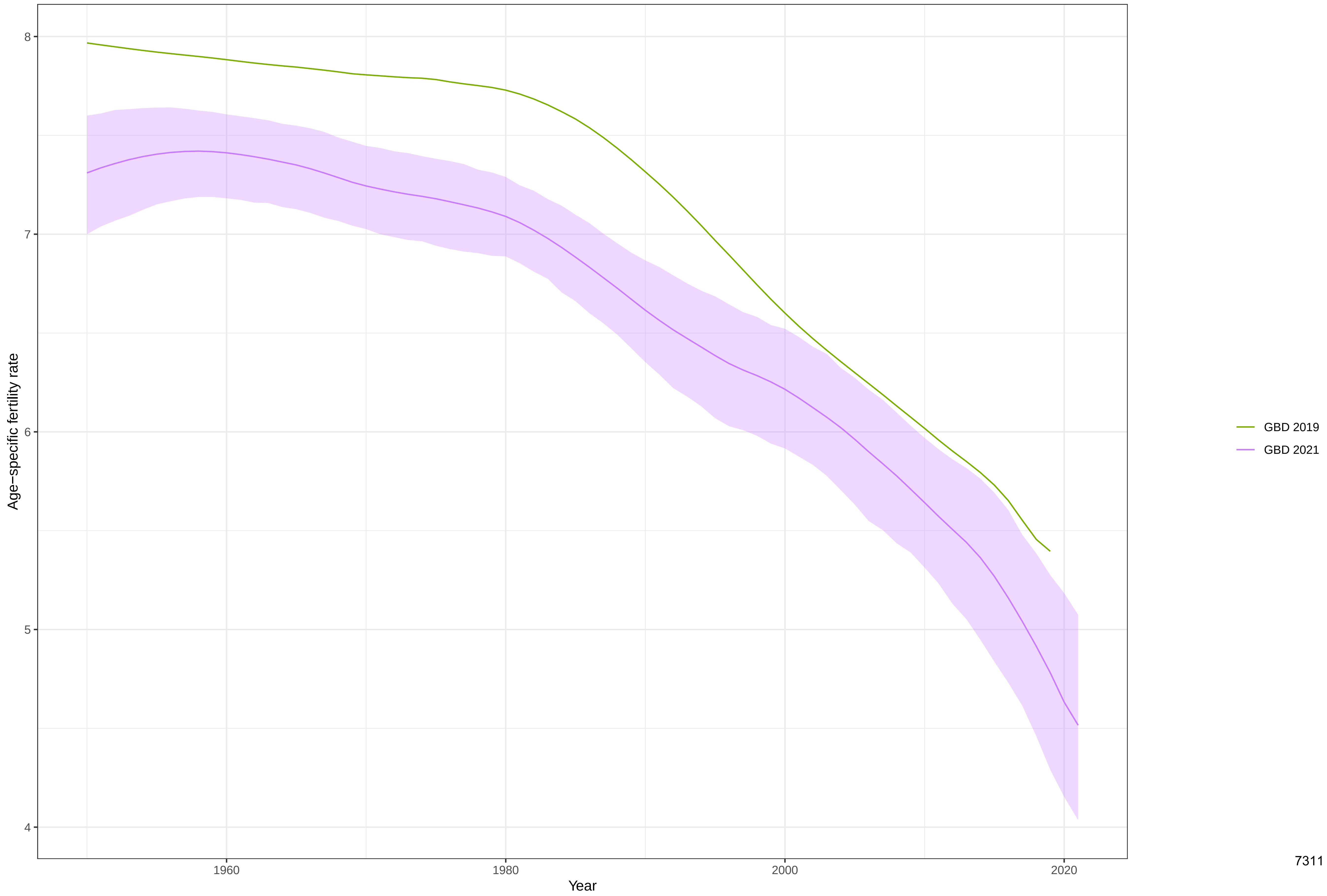

Age−specific fertility fitted trend lines, 1950−2021: Uasin Gishu, Ages: 10−14
Data points enclosed with a diamond are reference sources

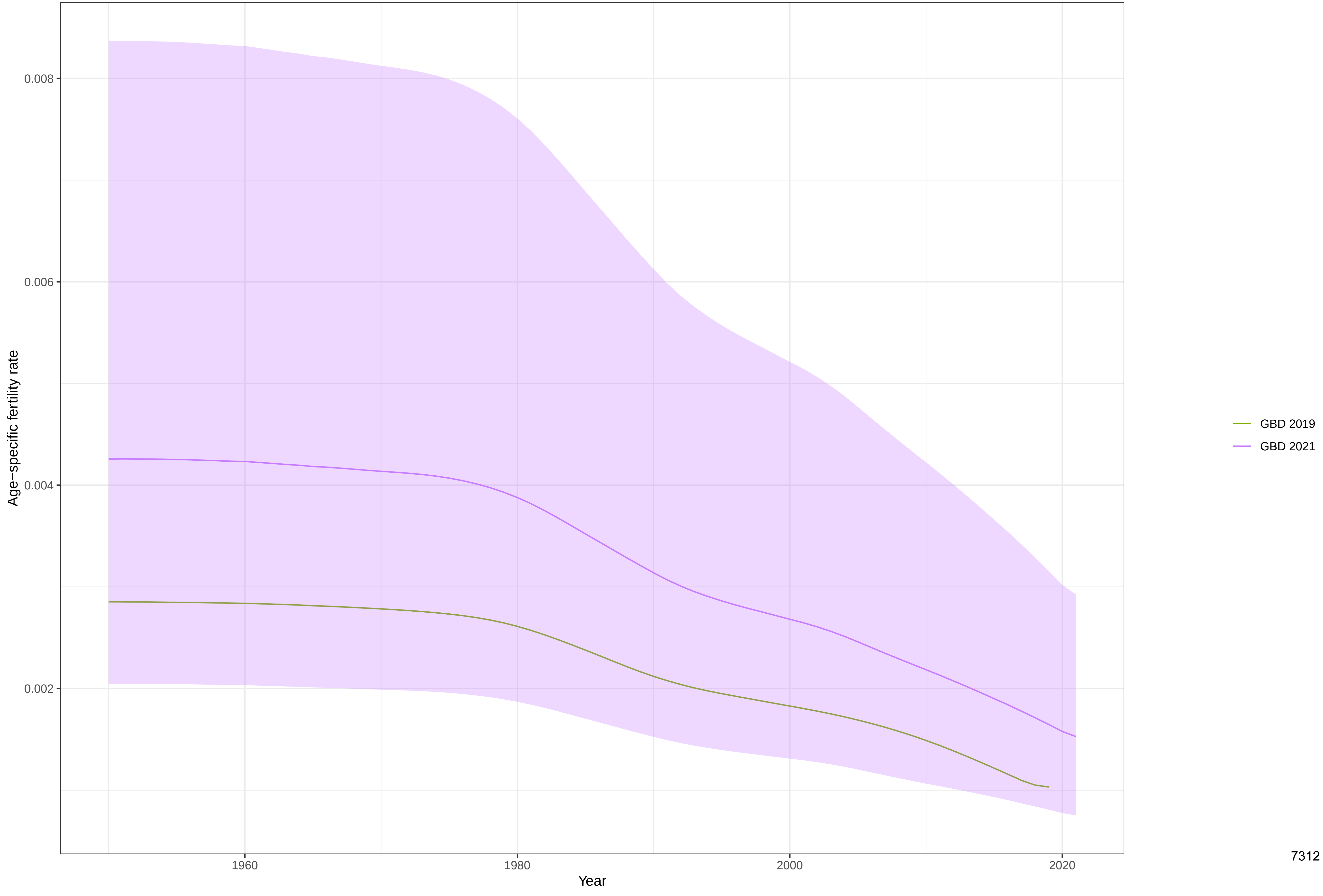

Age−specific fertility fitted trend lines, 1950−2021: Uasin Gishu, Ages: 15−19
Data points enclosed with a diamond are reference sources

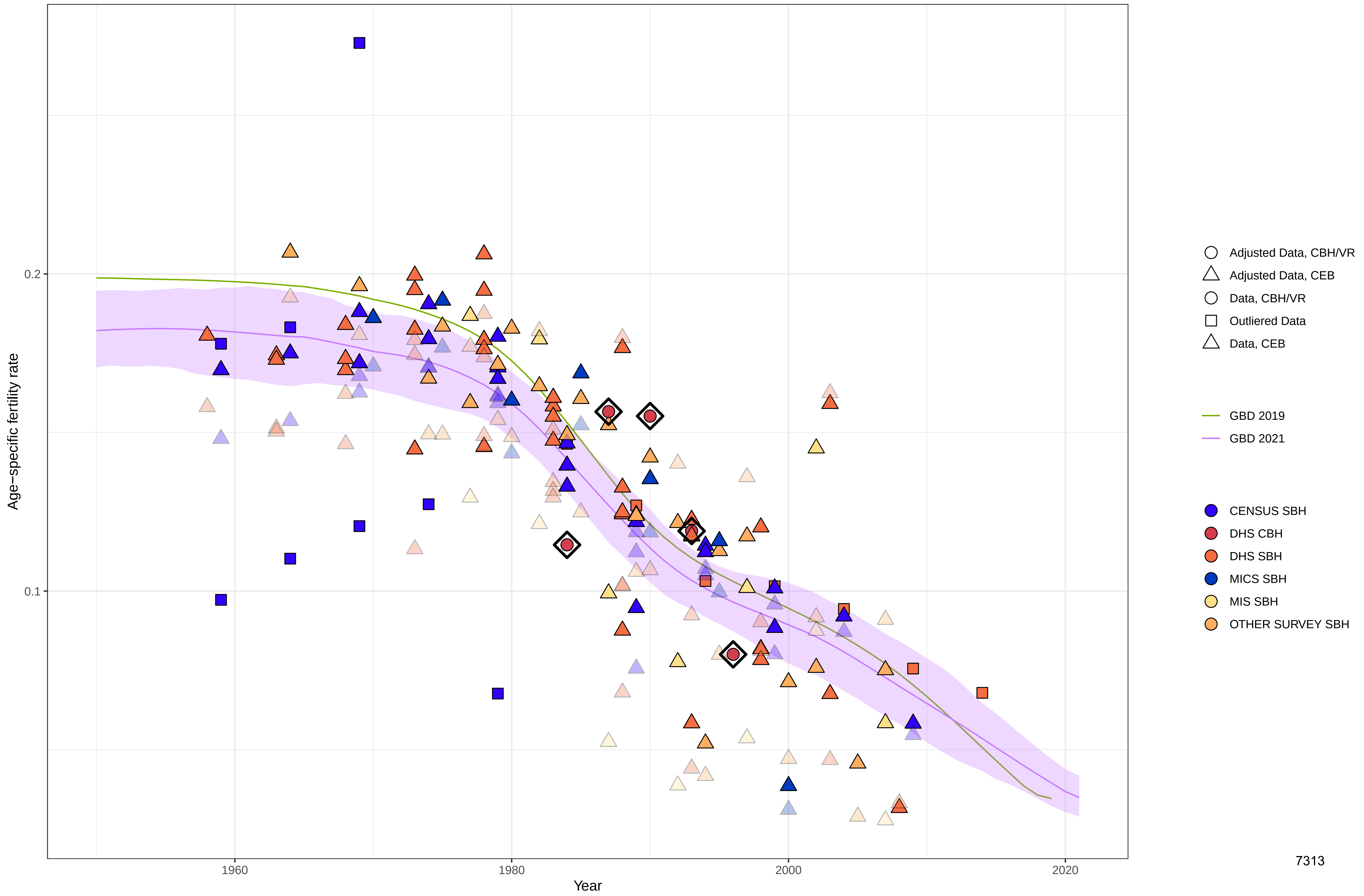

Age−specific fertility fitted trend lines, 1950−2021: Uasin Gishu, Ages: 20−24
Data points enclosed with a diamond are reference sources

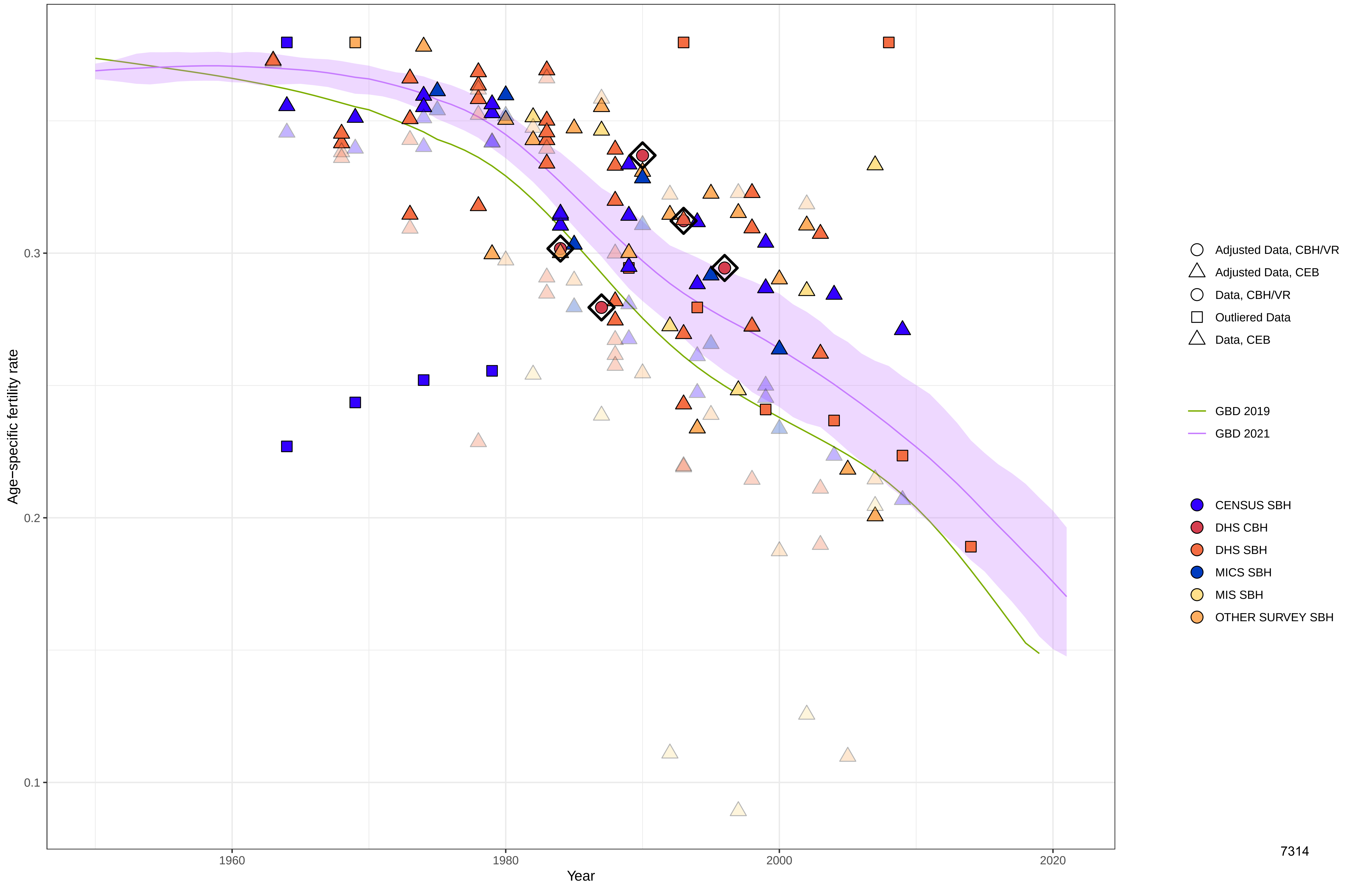

Age−specific fertility fitted trend lines, 1950−2021: Uasin Gishu, Ages: 25−29
Data points enclosed with a diamond are reference sources

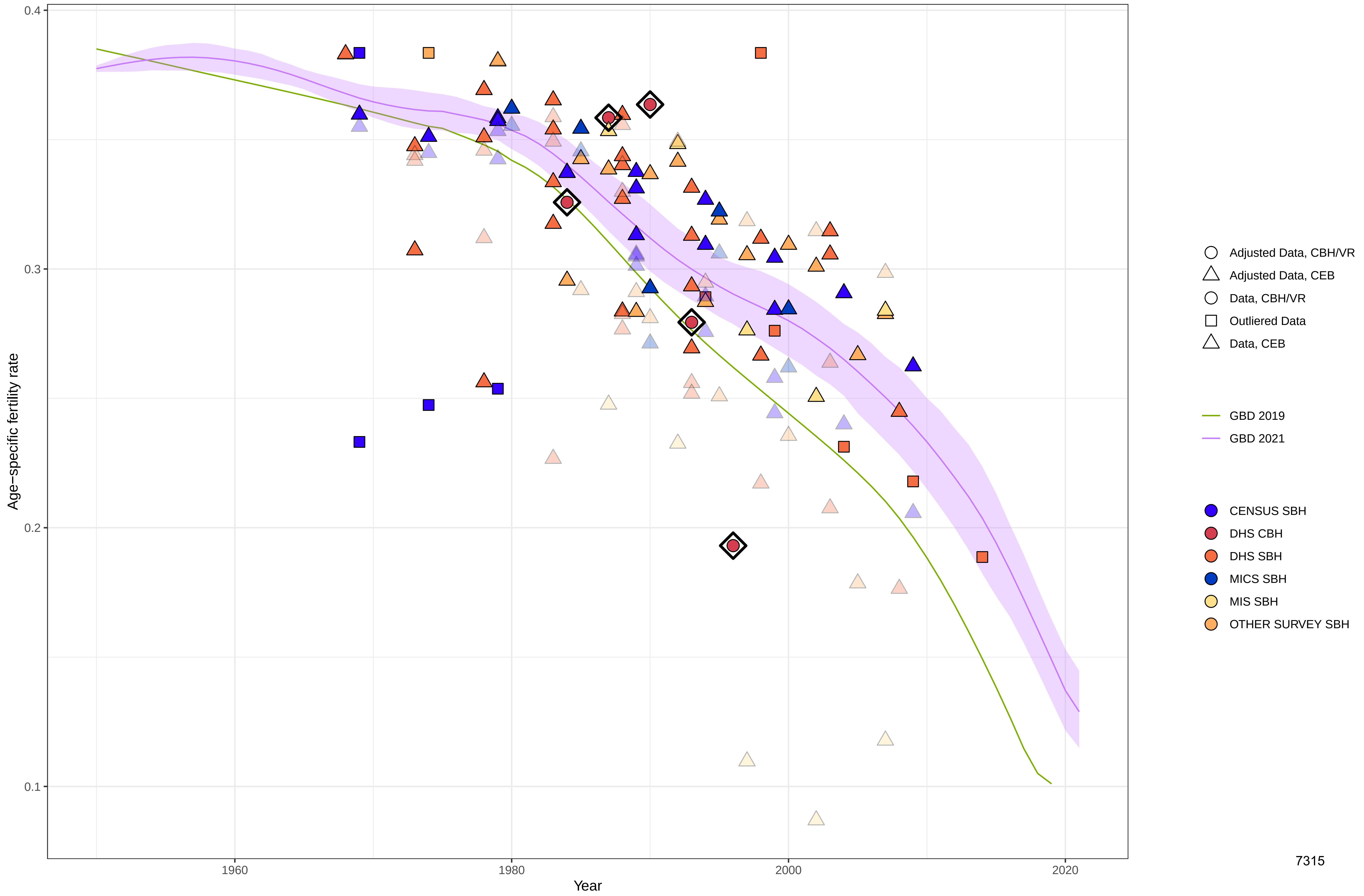

Age−specific fertility fitted trend lines, 1950−2021: Uasin Gishu, Ages: 30−34
Data points enclosed with a diamond are reference sources

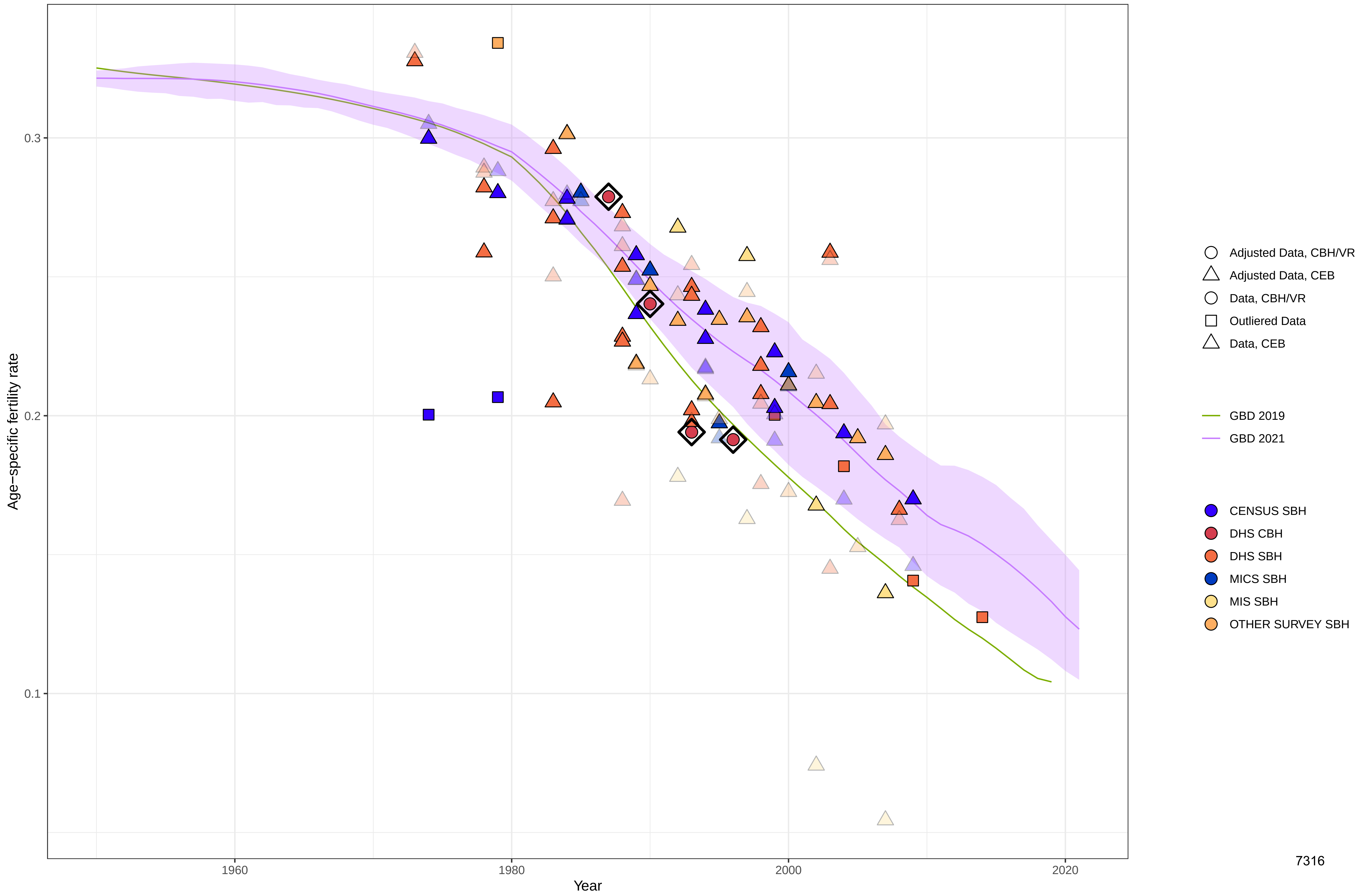

Age−specific fertility fitted trend lines, 1950−2021: Uasin Gishu, Ages: 35−39
Data points enclosed with a diamond are reference sources

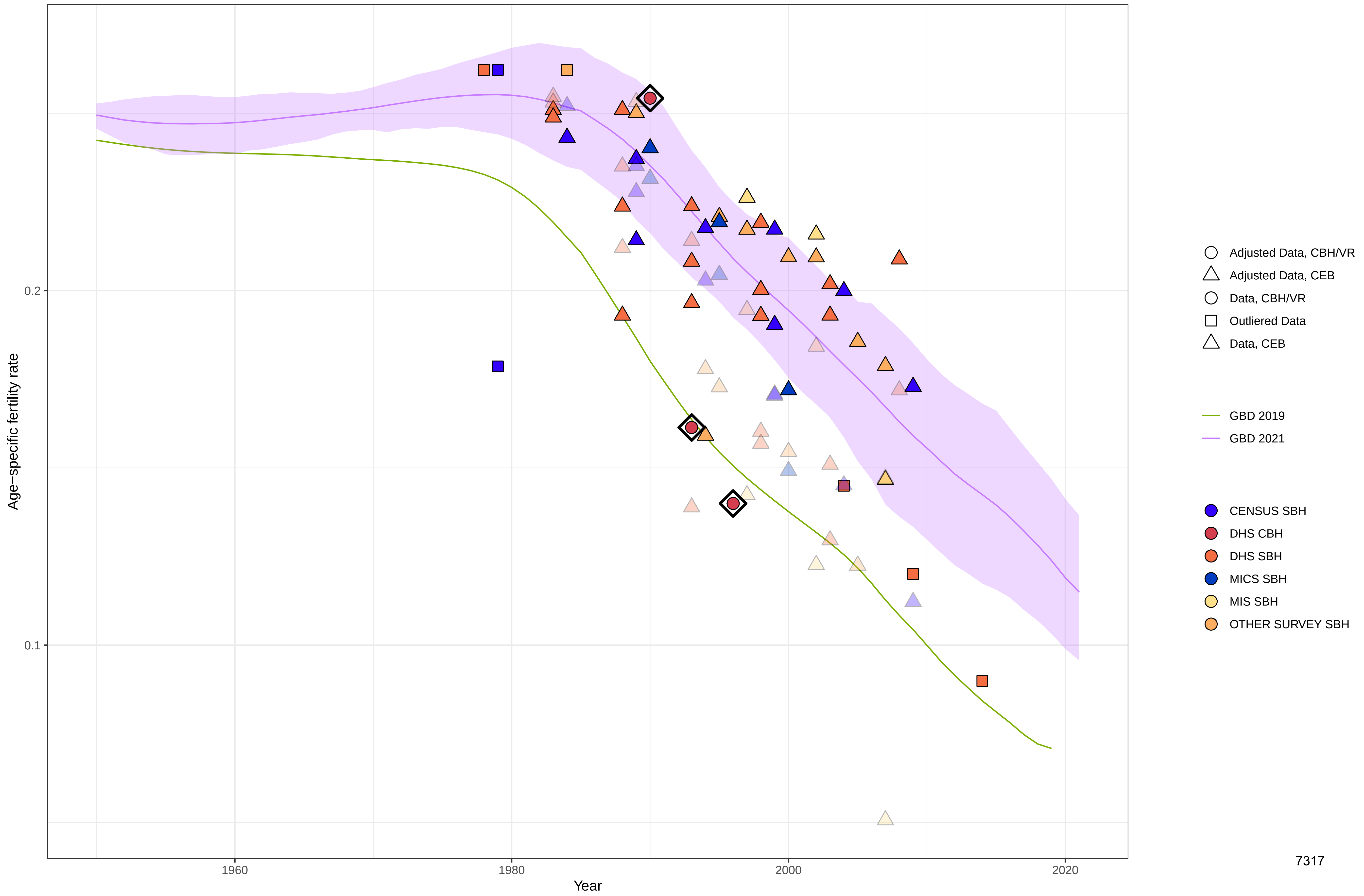

Age−specific fertility fitted trend lines, 1950−2021: Uasin Gishu, Ages: 40−44
Data points enclosed with a diamond are reference sources

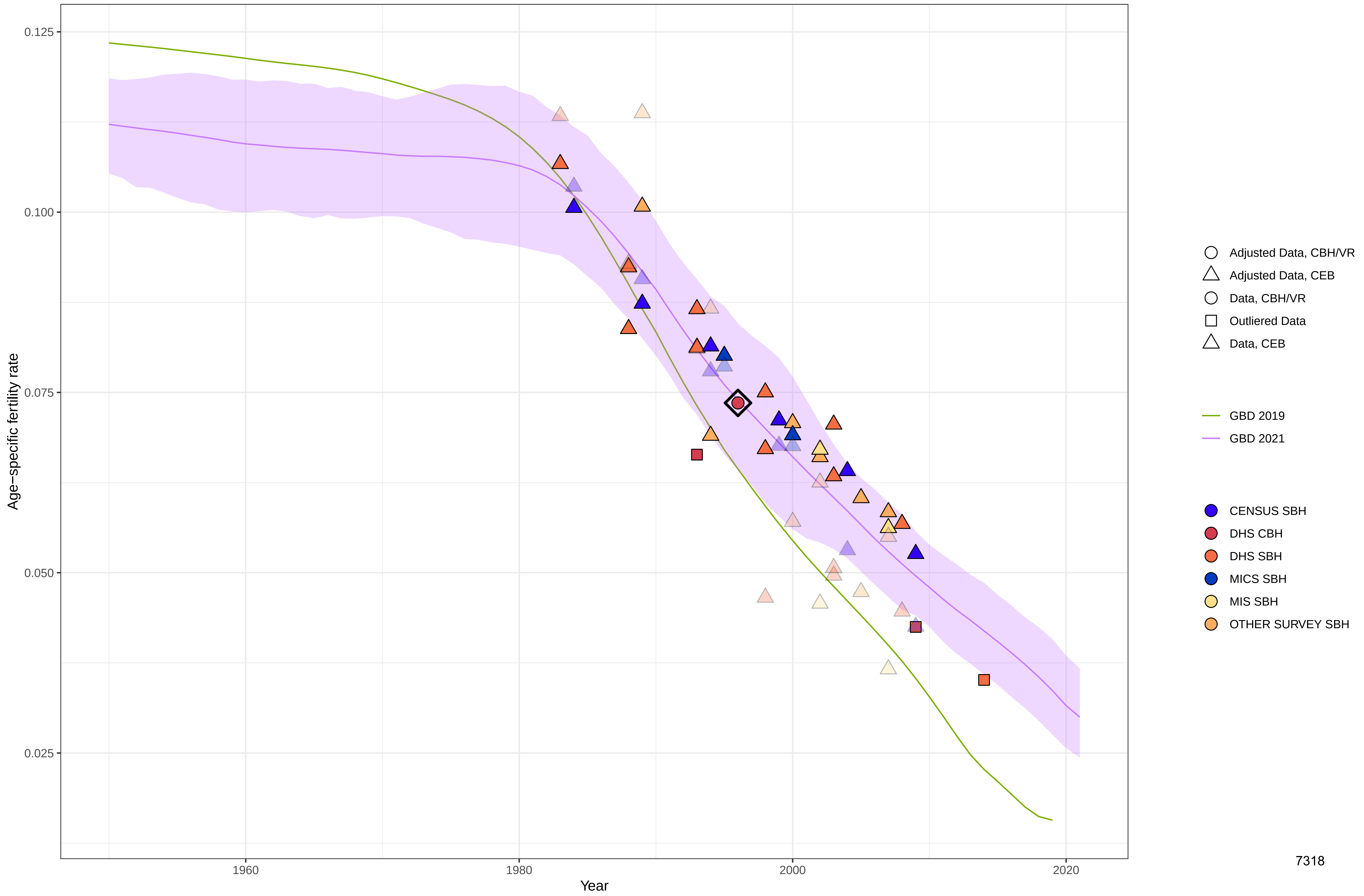

Age−specific fertility fitted trend lines, 1950−2021: Uasin Gishu, Ages: 45−49
Data points enclosed with a diamond are reference sources

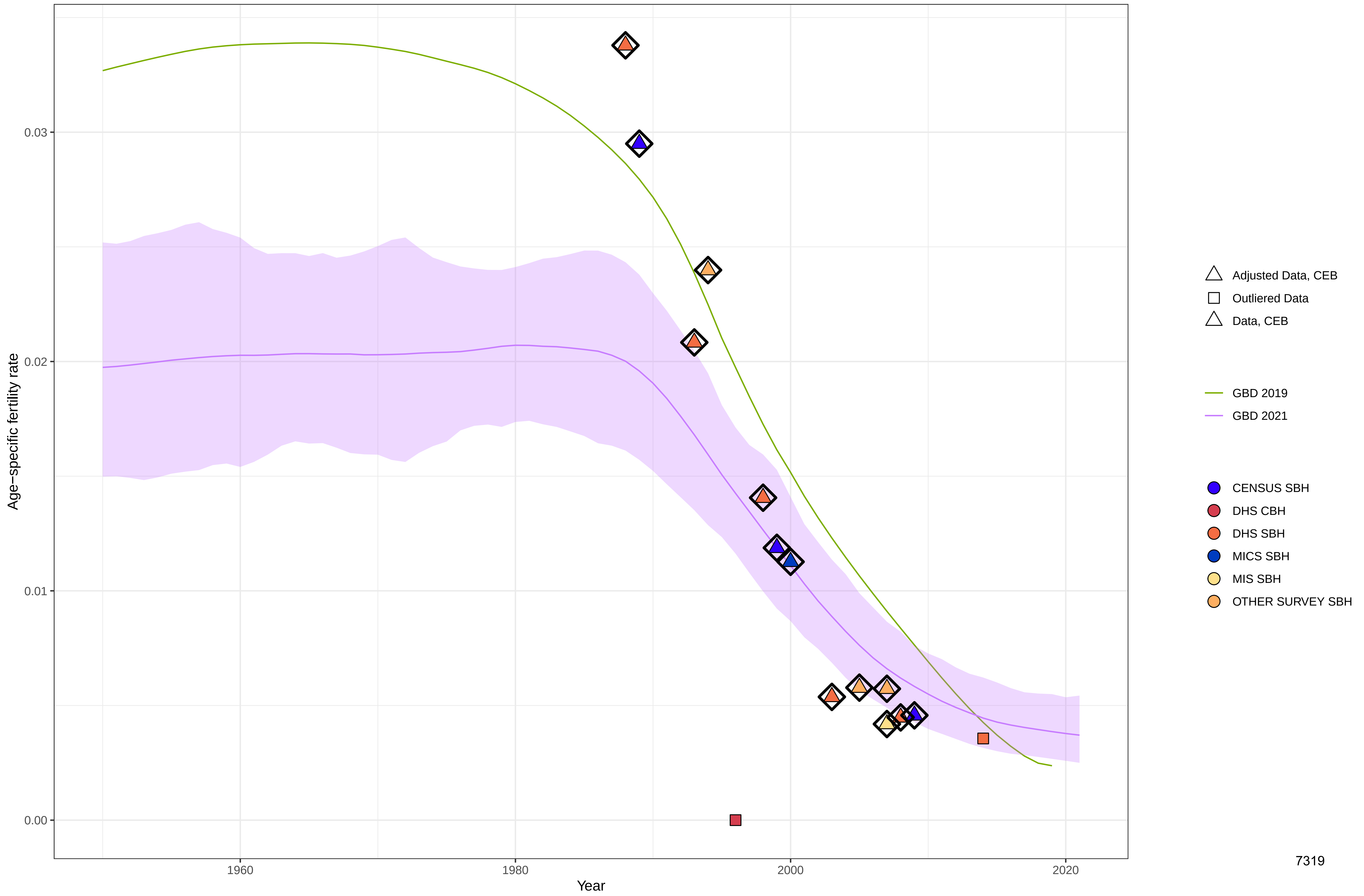

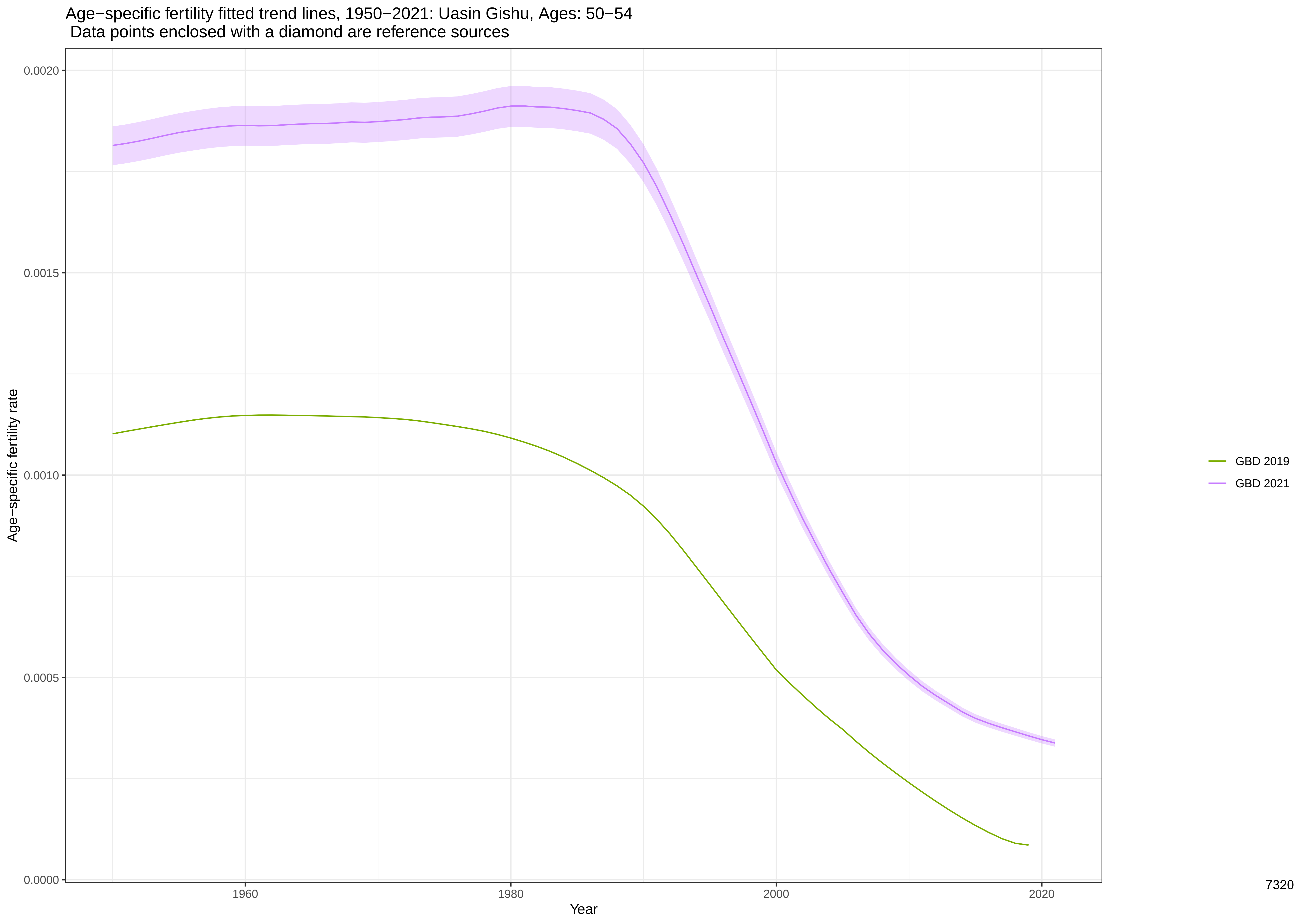

Age−specific fertility fitted trend lines, 1950−2021: Uasin Gishu, Ages: 50−54
Data points enclosed with a diamond are reference sources
0.0020
0.0015
0.0010
0.0005
0.0000
Age−specific fertility rate
1960
1980
2000
2020
Year
GBD 2019
GBD 2021

Total fertility rate fitted trend lines, 1950−2021: Uasin Gishu TFR
Data points enclosed with a diamond are reference sources

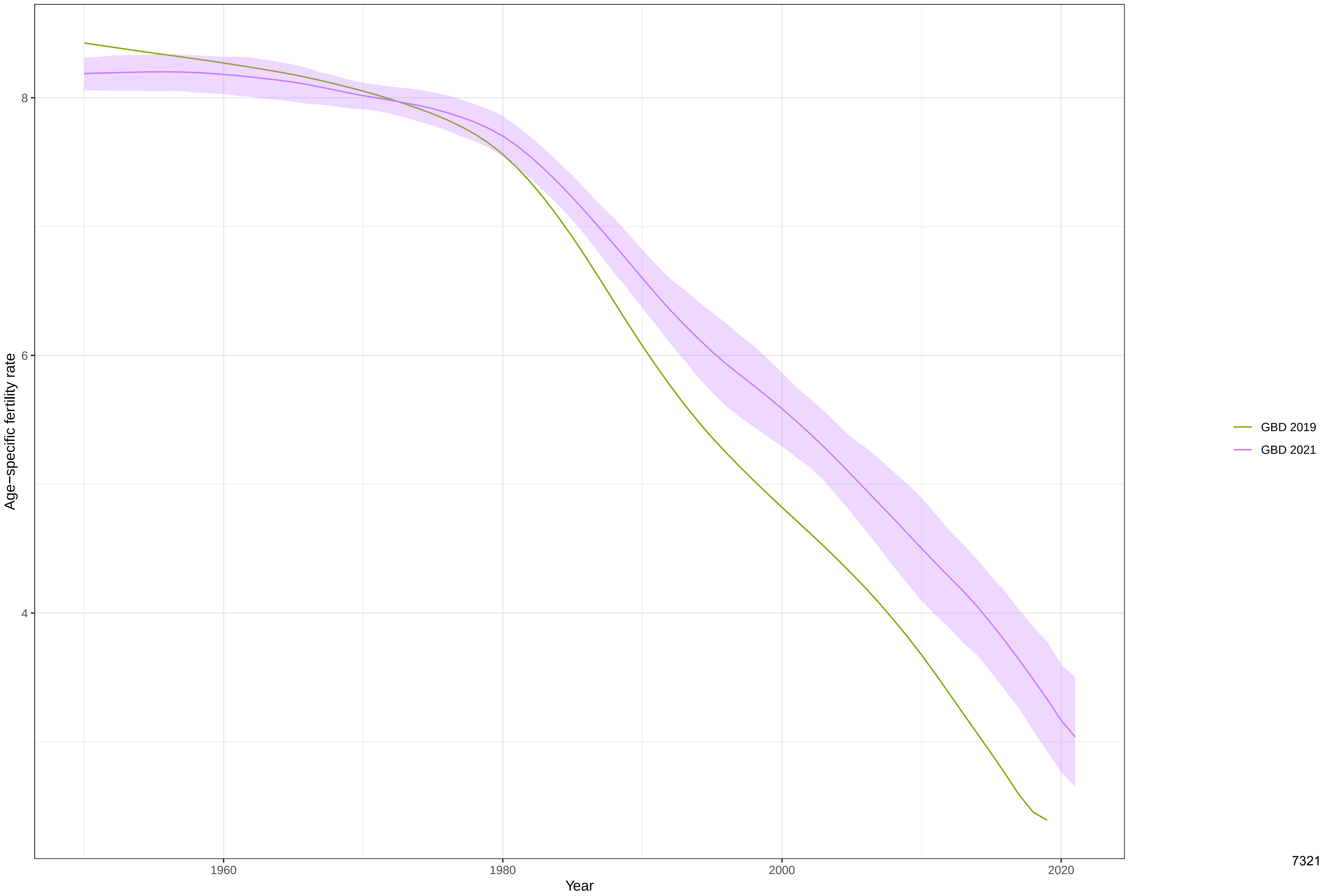

Age−specific fertility fitted trend lines, 1950−2021: Vihiga, Ages: 10−14
Data points enclosed with a diamond are reference sources

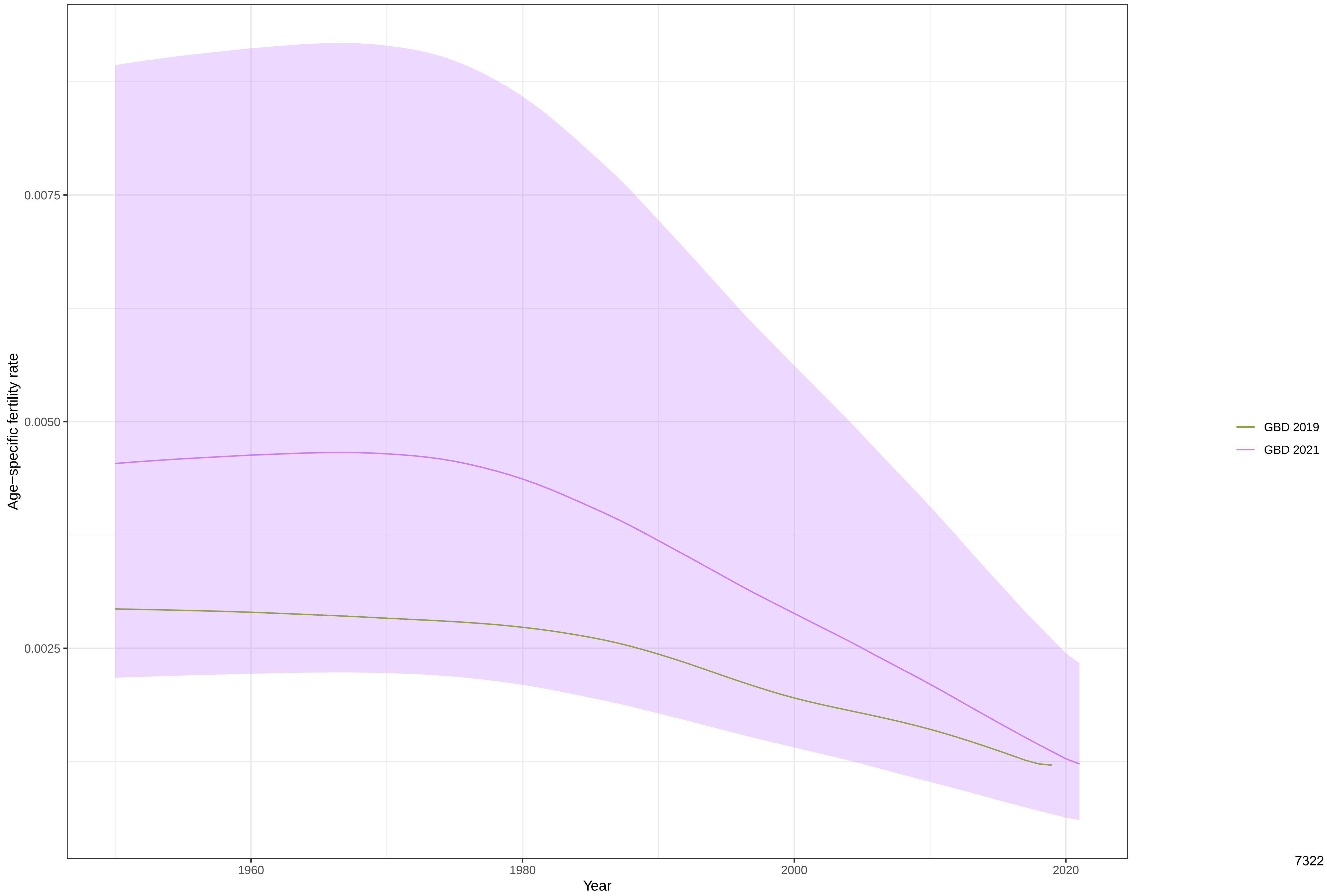

Age−specific fertility fitted trend lines, 1950−2021: Vihiga, Ages: 15−19
Data points enclosed with a diamond are reference sources

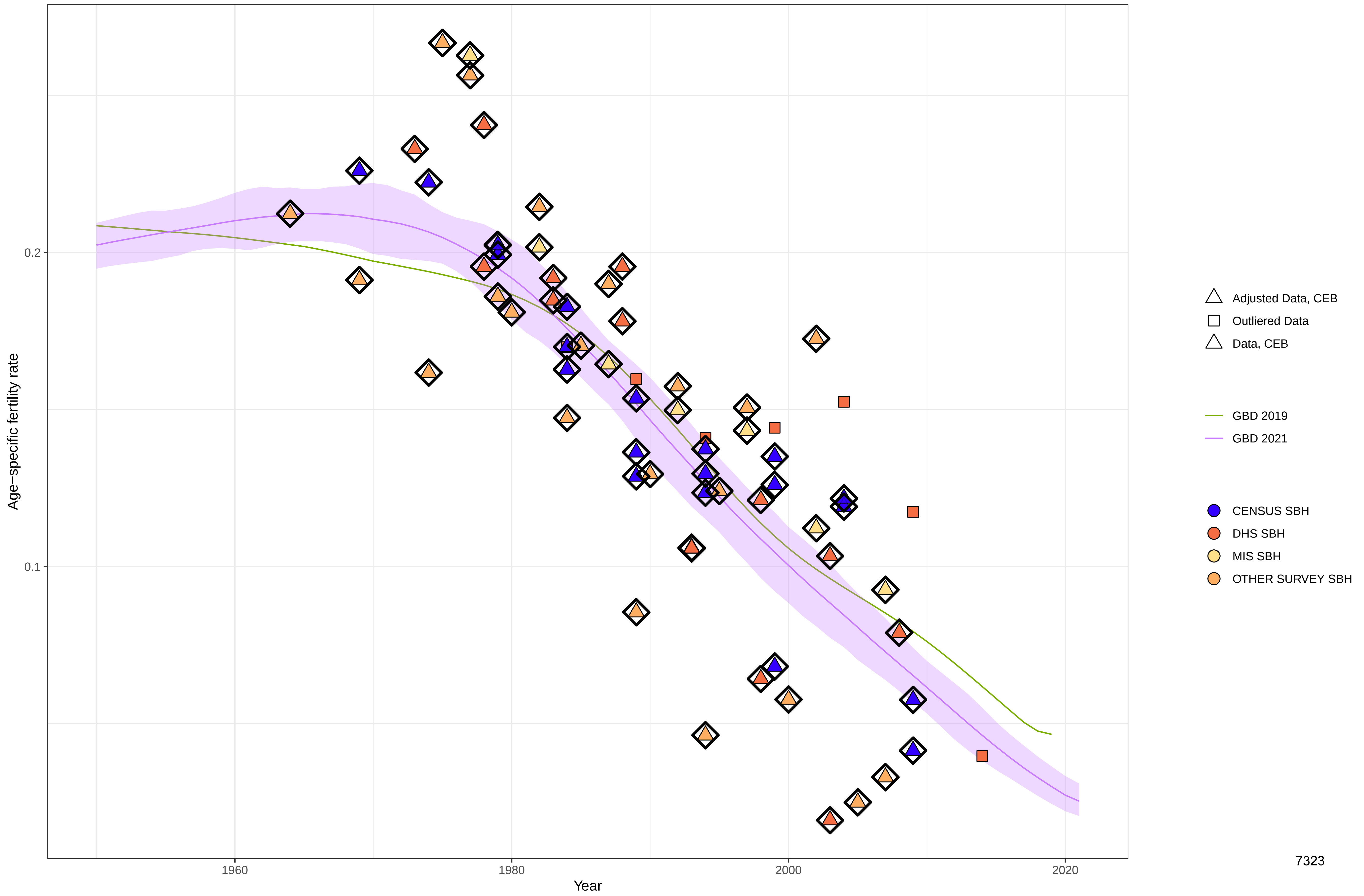

Age−specific fertility fitted trend lines, 1950−2021: Vihiga, Ages: 20−24
Data points enclosed with a diamond are reference sources

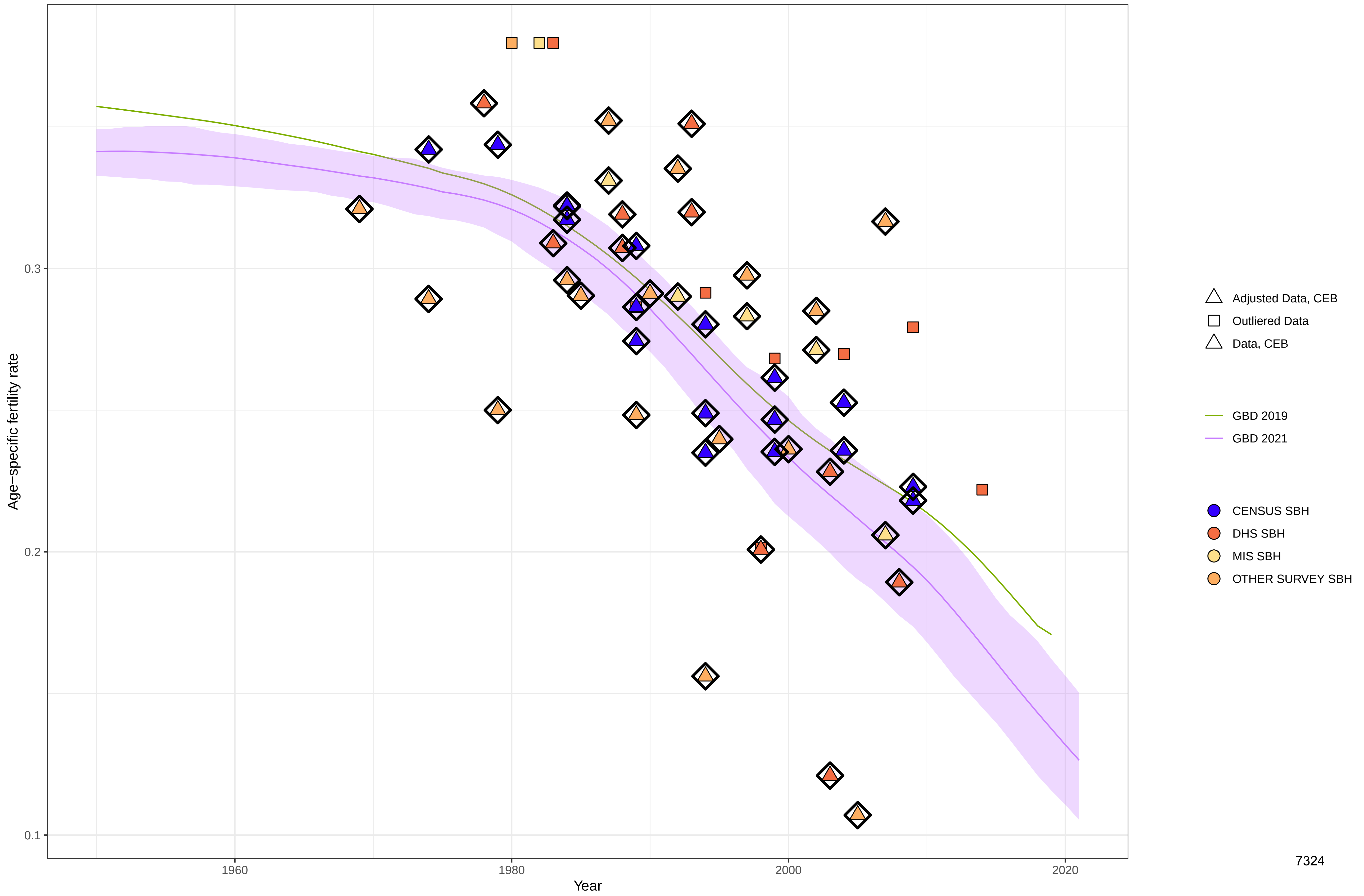

Age−specific fertility fitted trend lines, 1950−2021: Vihiga, Ages: 25−29
Data points enclosed with a diamond are reference sources

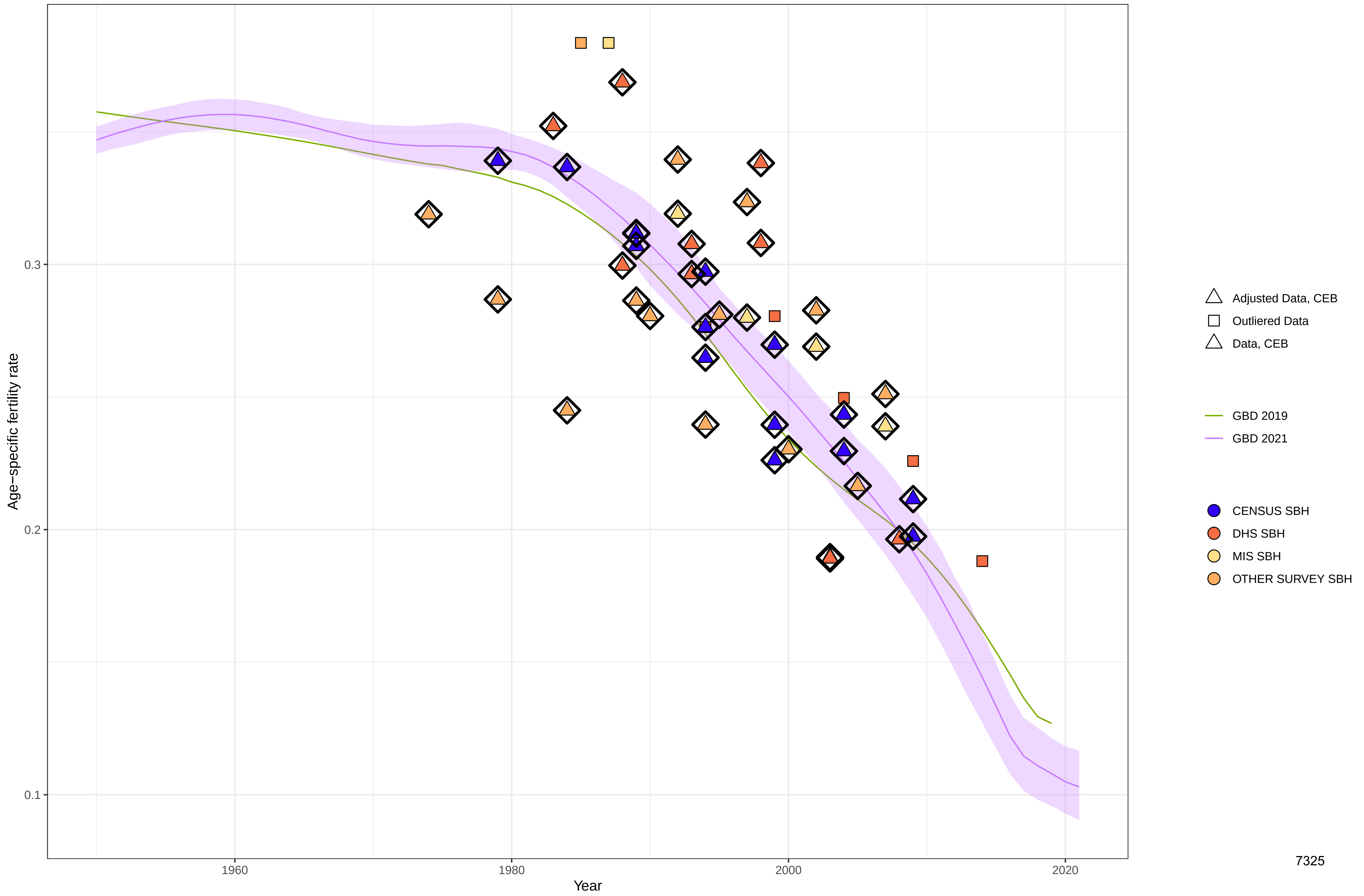

Age−specific fertility fitted trend lines, 1950−2021: Vihiga, Ages: 30−34
Data points enclosed with a diamond are reference sources

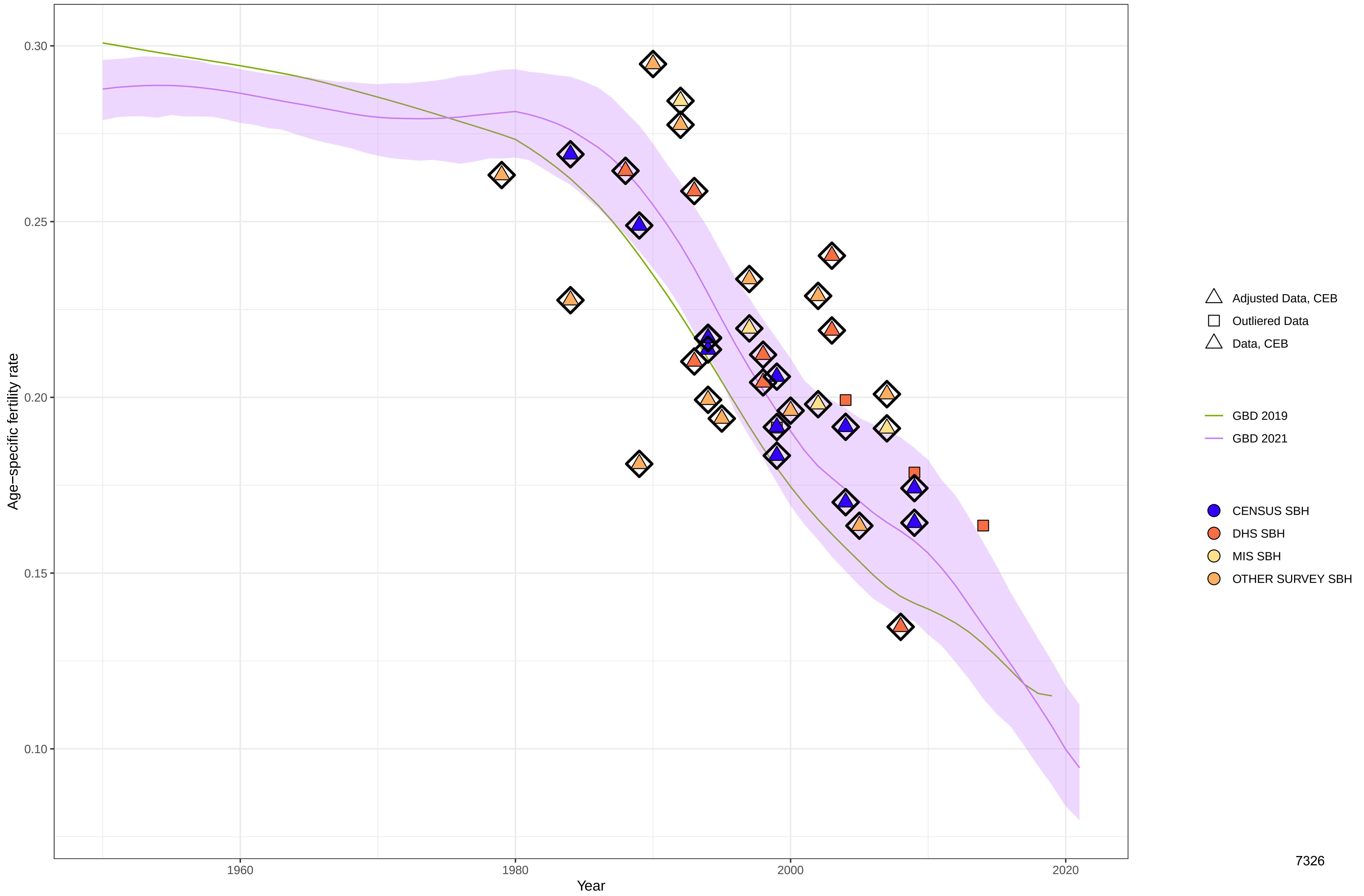

Age−specific fertility fitted trend lines, 1950−2021: Vihiga, Ages: 35−39
Data points enclosed with a diamond are reference sources

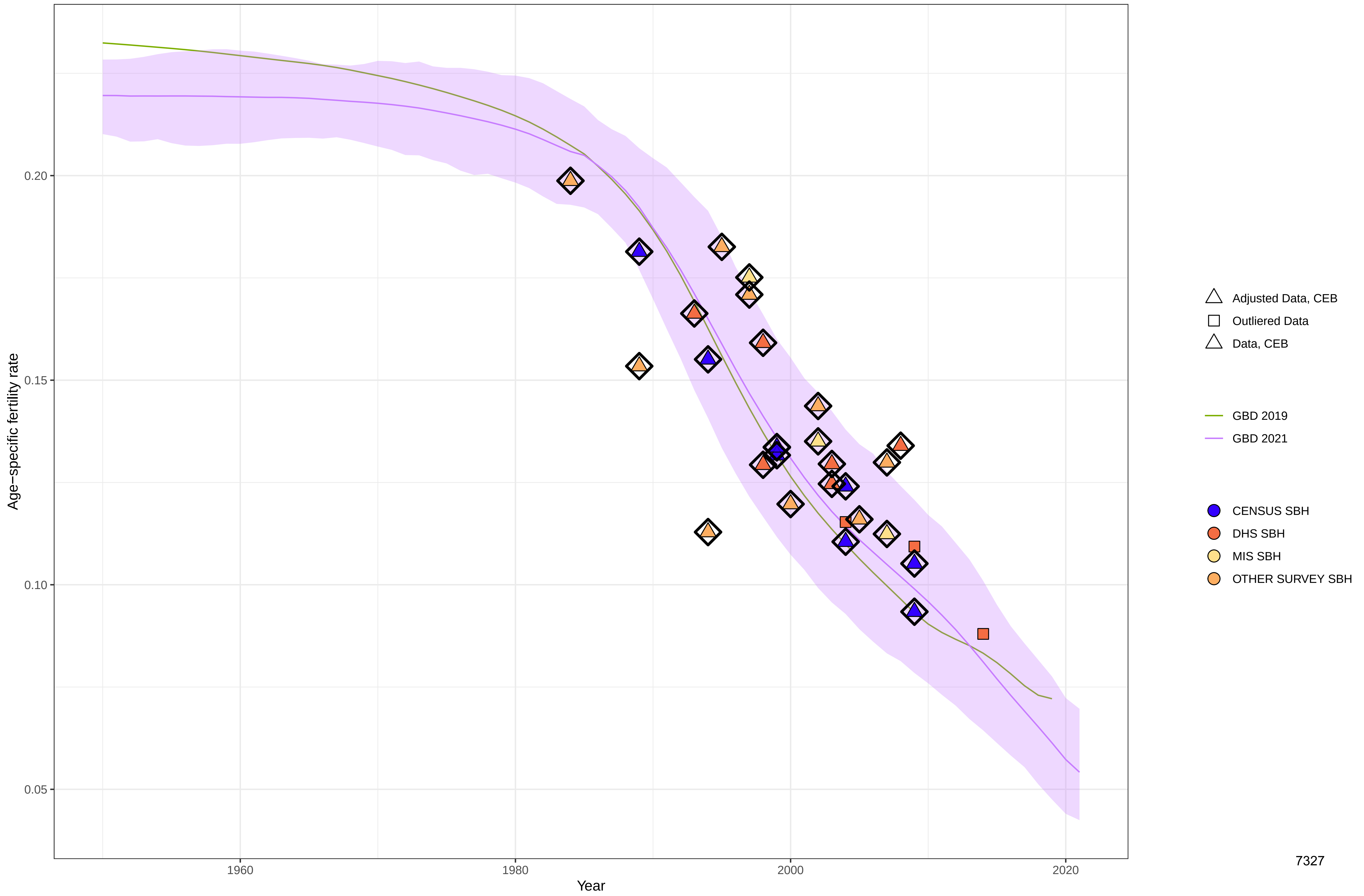

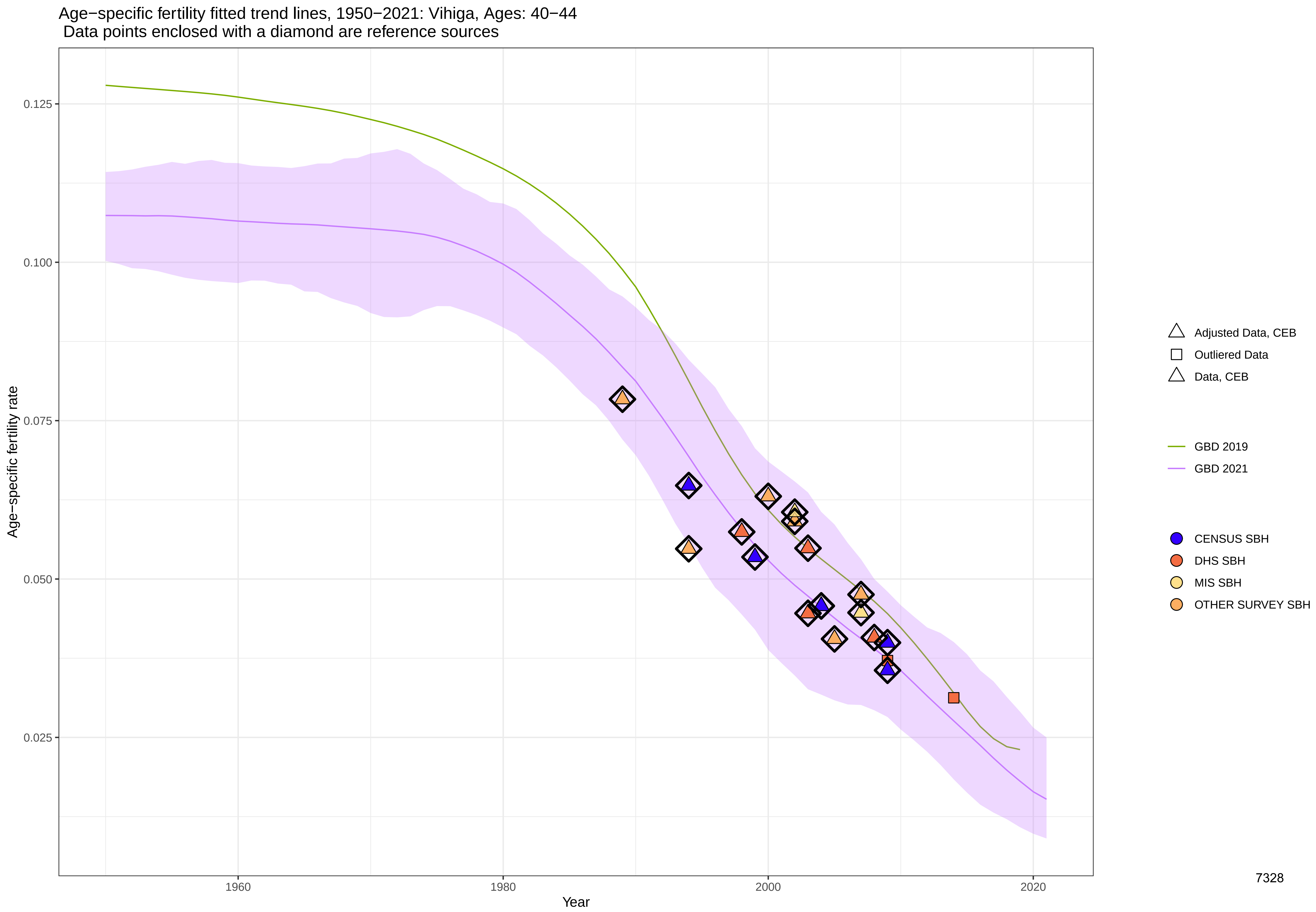

Age−specific fertility fitted trend lines, 1950−2021: Vihiga, Ages: 40−44
Data points enclosed with a diamond are reference sources
Age−specific fertility rate
0.125
0.100
0.075
0.050
0.025
1960
1980
2000
2020
Year
Adjusted Data, CEB
Outliered Data
Data, CEB
GBD 2019
GBD 2021
CENSUS SBH
DHS SBH
MIS SBH
OTHER SURVEY SBH

Age−specific fertility fitted trend lines, 1950−2021: Vihiga, Ages: 45−49
Data points enclosed with a diamond are reference sources

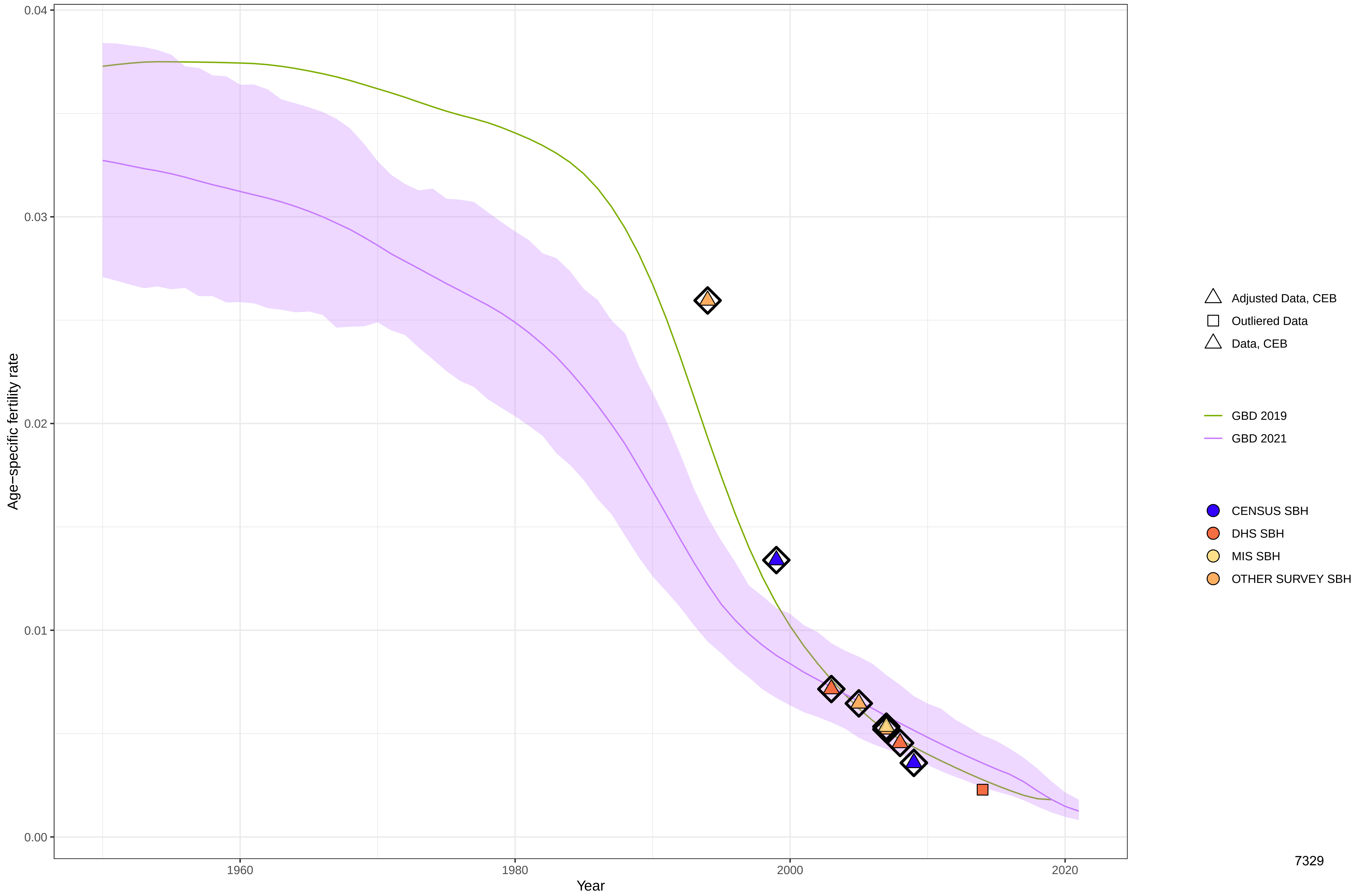

Age−specific fertility fitted trend lines, 1950−2021: Vihiga, Ages: 50−54
Data points enclosed with a diamond are reference sources

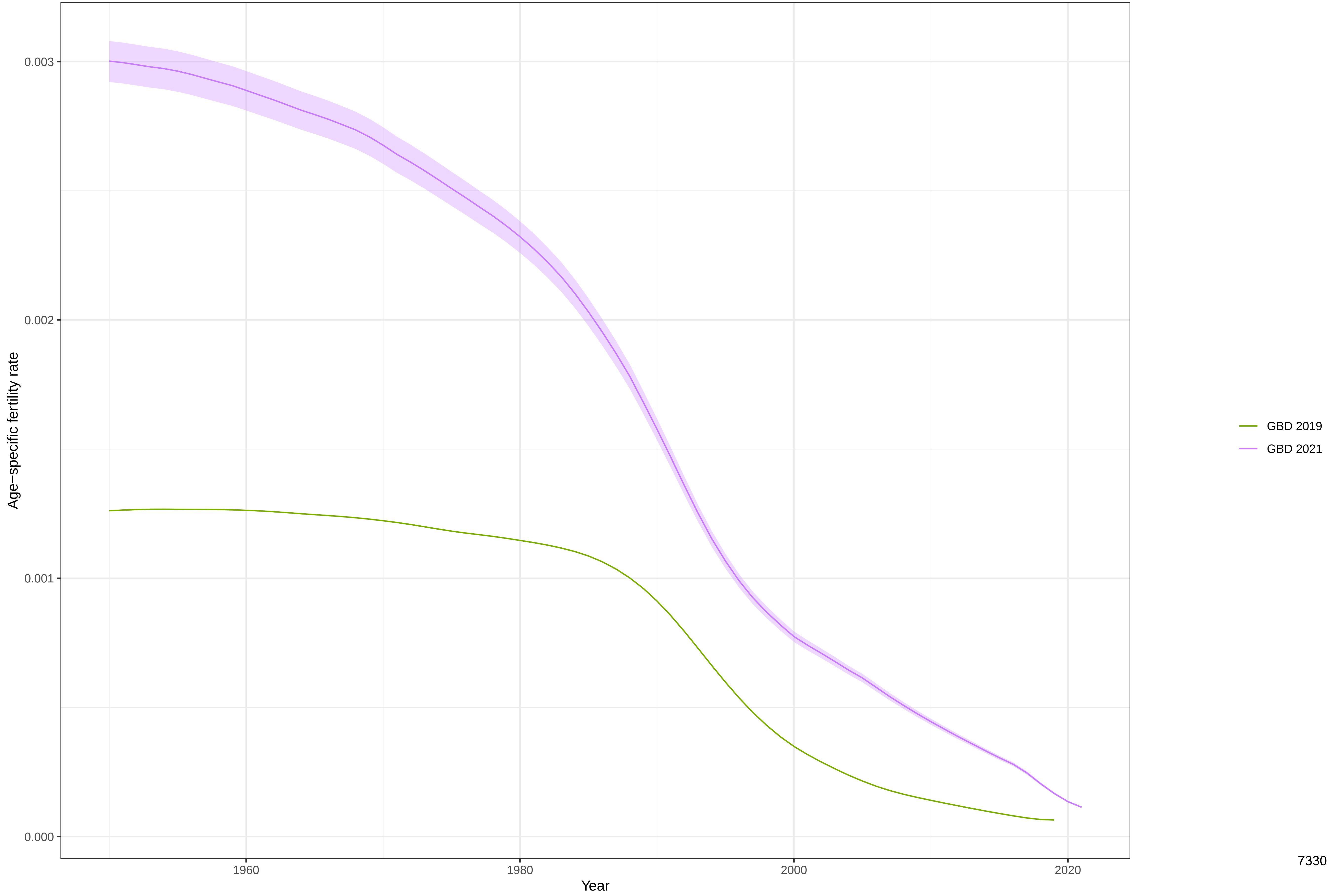

Total fertility rate fitted trend lines, 1950−2021: Vihiga TFR
Data points enclosed with a diamond are reference sources

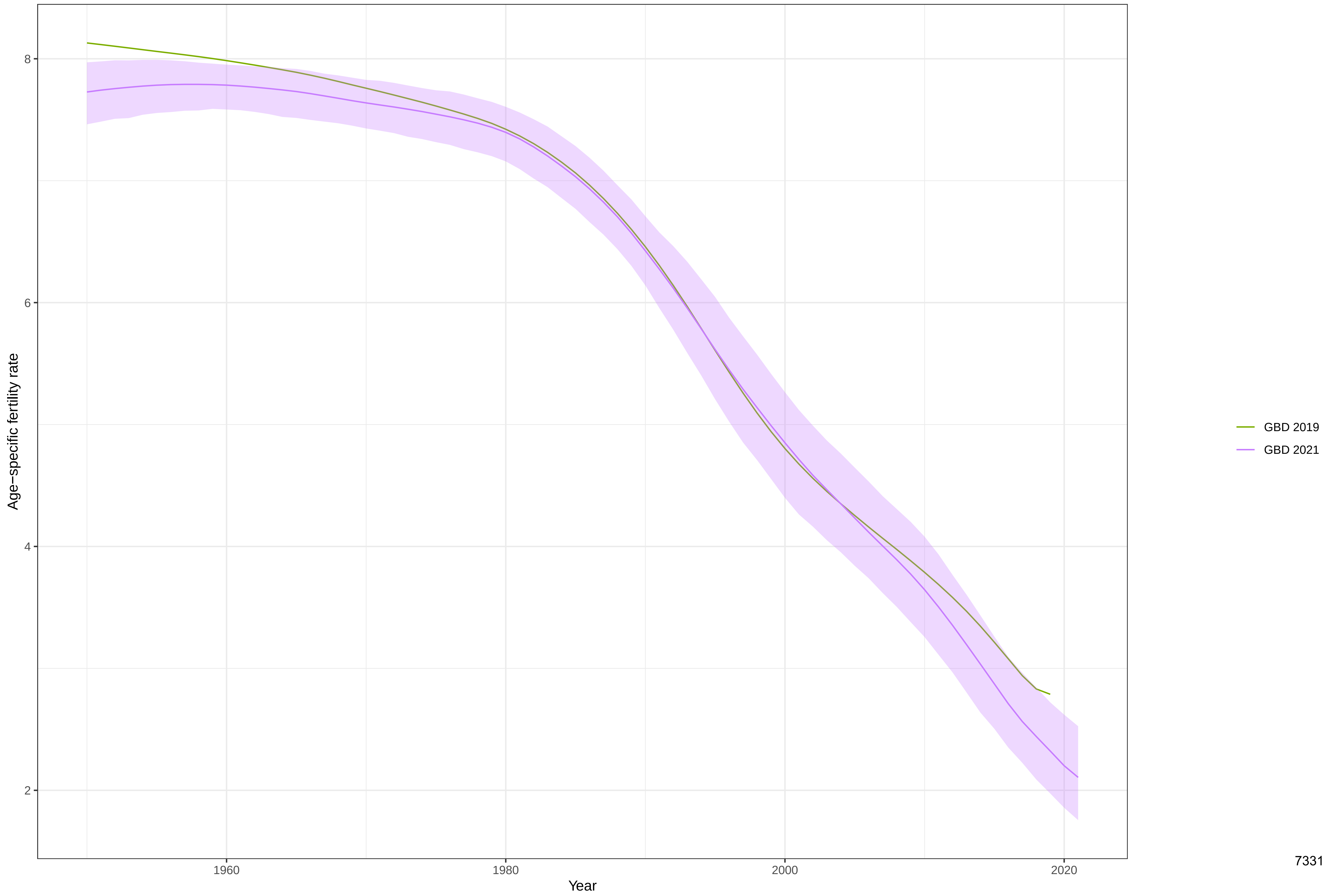

Age−specific fertility fitted trend lines, 1950−2021: Wajir, Ages: 10−14
Data points enclosed with a diamond are reference sources

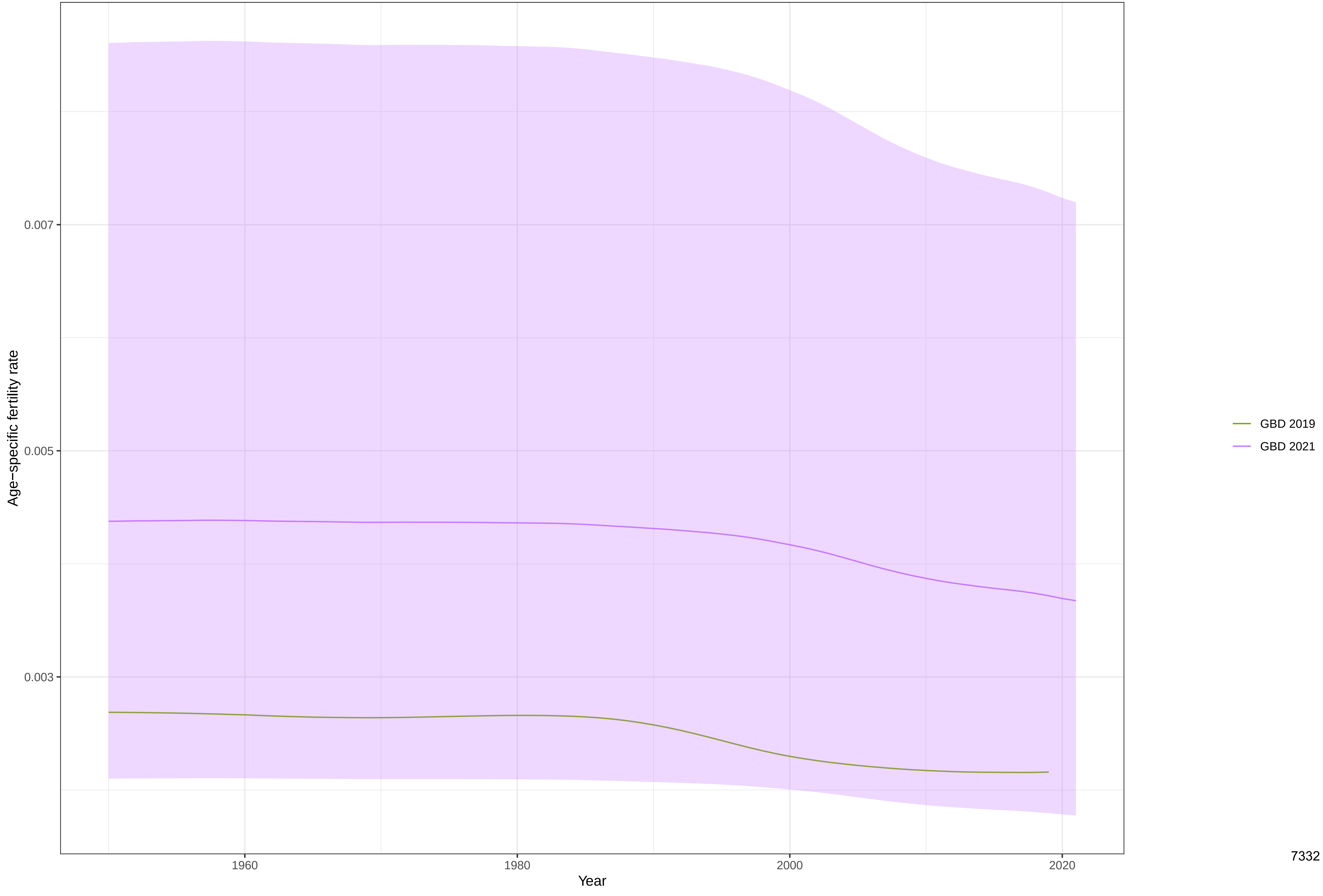

Age−specific fertility fitted trend lines, 1950−2021: Wajir, Ages: 15−19
Data points enclosed with a diamond are reference sources

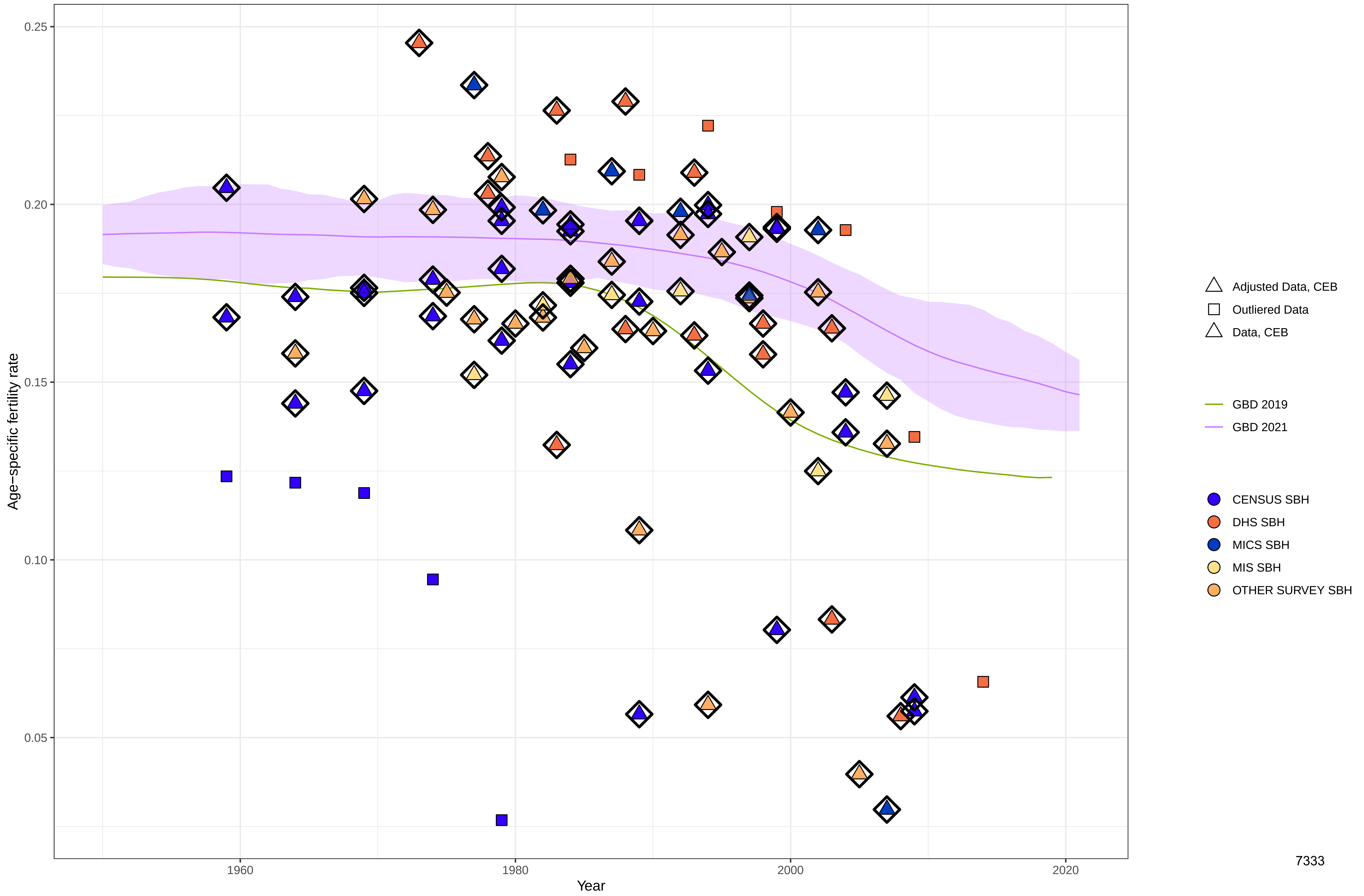

7333

Age−specific fertility fitted trend lines, 1950−2021: Wajir, Ages: 20−24
Data points enclosed with a diamond are reference sources

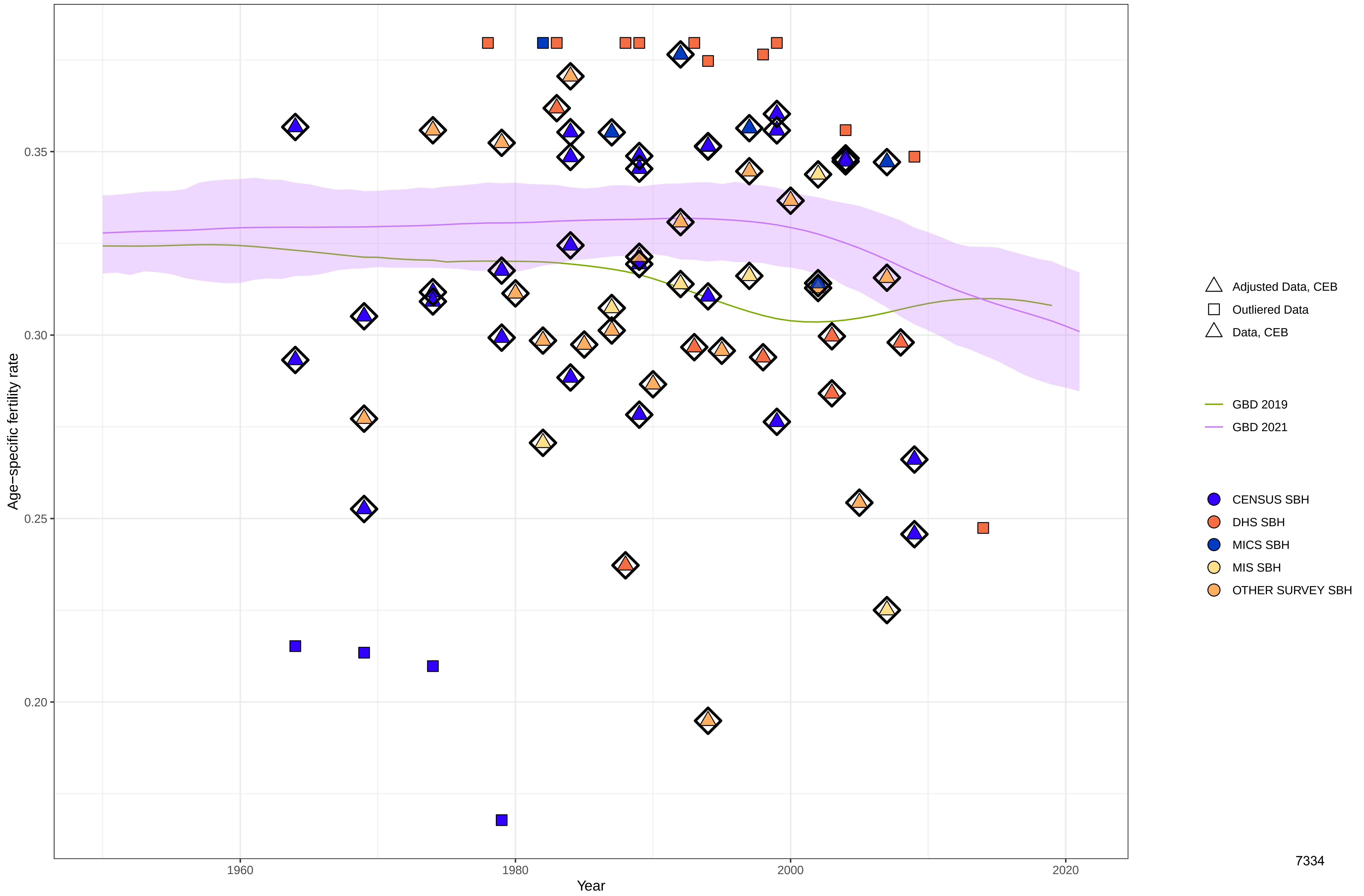

Age−specific fertility fitted trend lines, 1950−2021: Wajir, Ages: 25−29
Data points enclosed with a diamond are reference sources

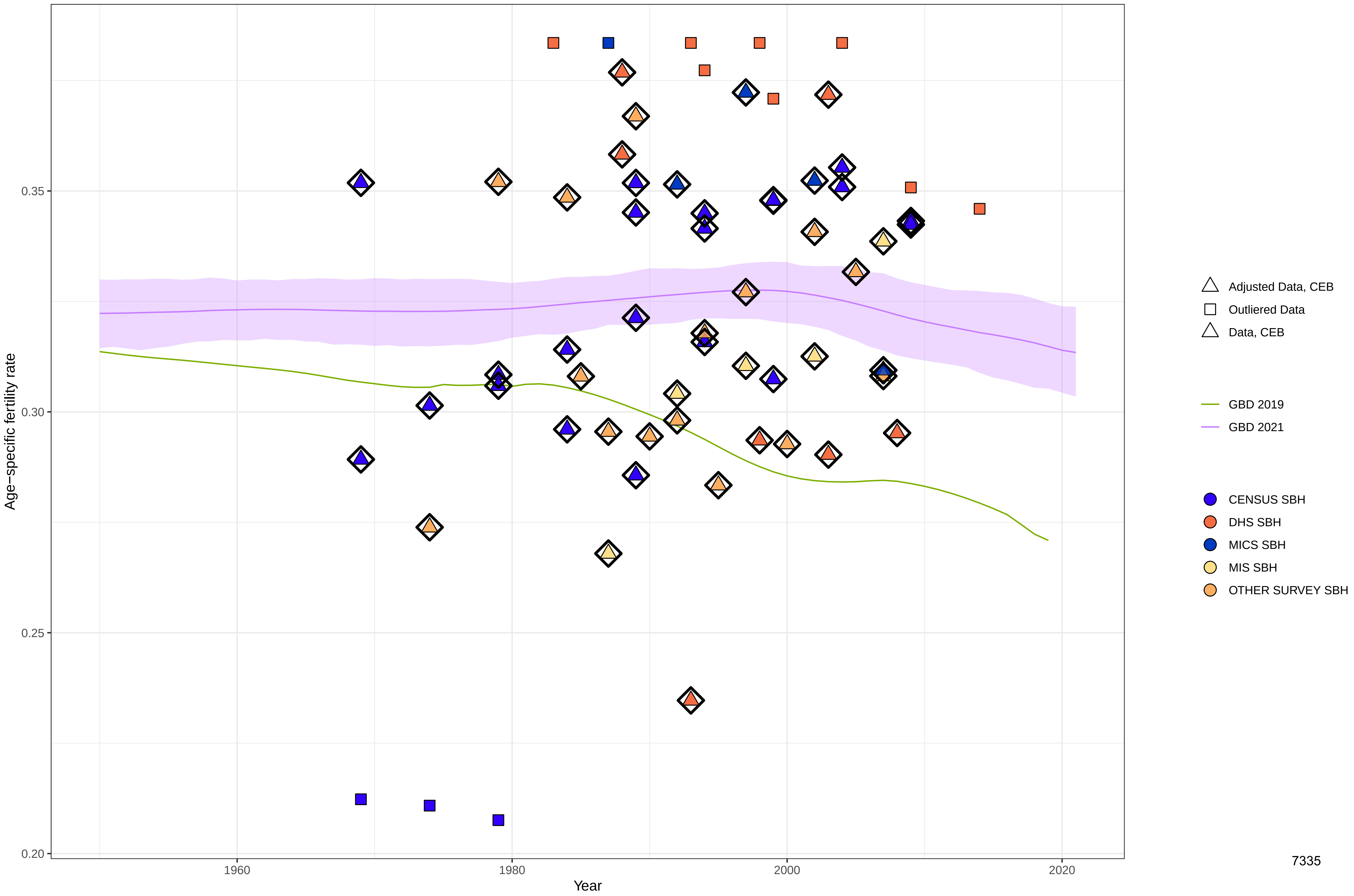

Age−specific fertility fitted trend lines, 1950−2021: Wajir, Ages: 30−34
Data points enclosed with a diamond are reference sources

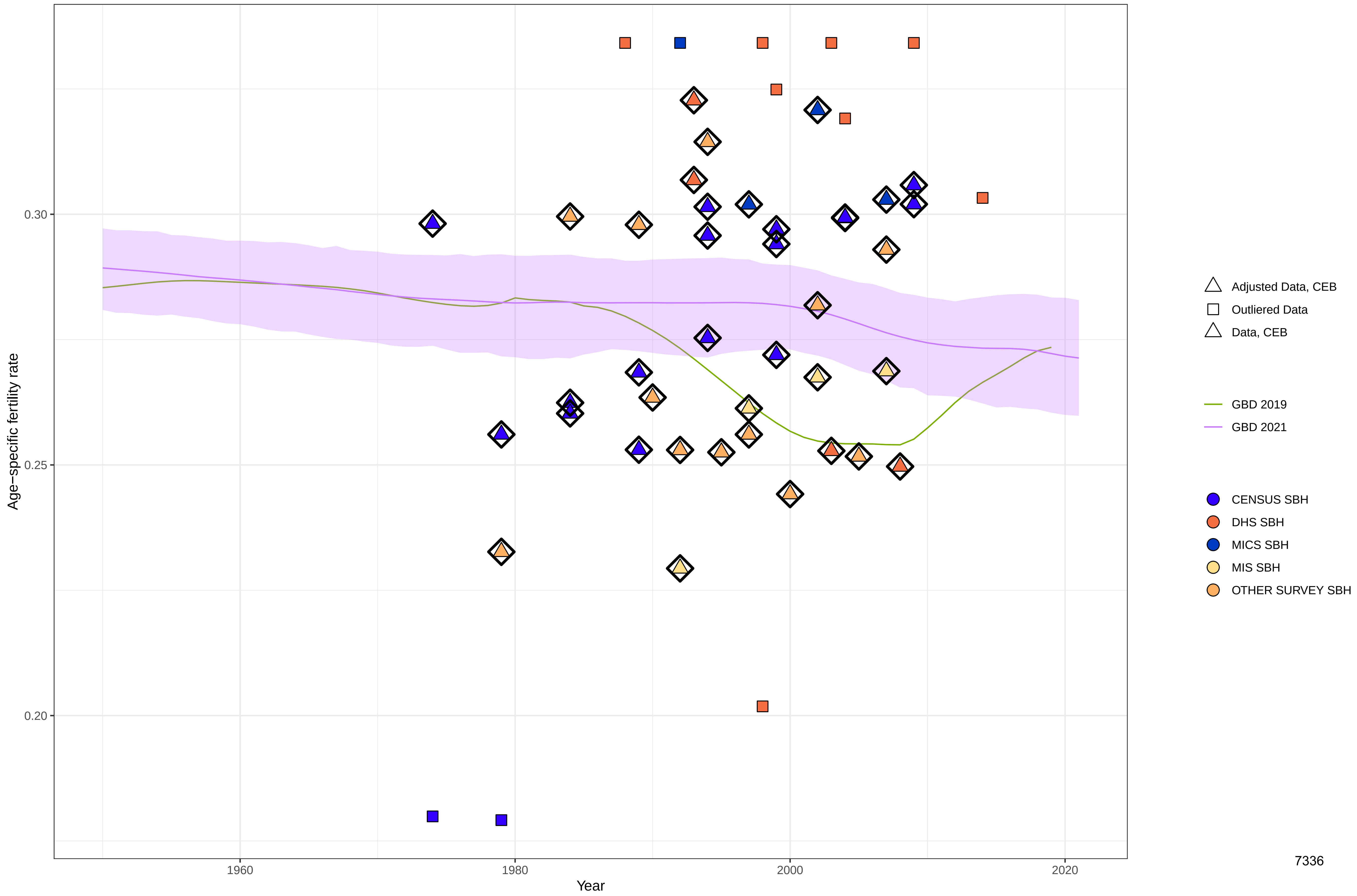

Age−specific fertility fitted trend lines, 1950−2021: Wajir, Ages: 35−39
Data points enclosed with a diamond are reference sources

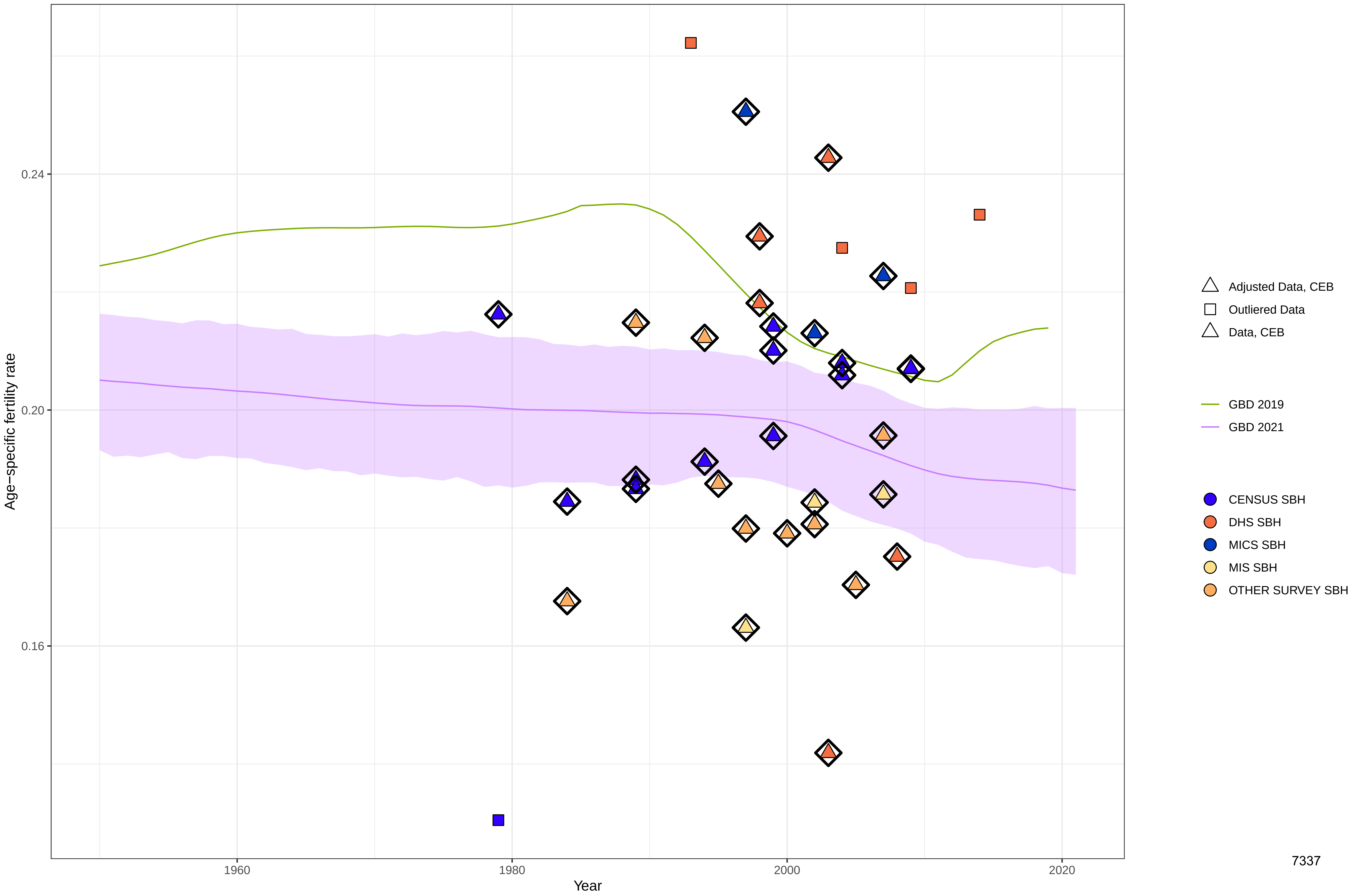

Age−specific fertility fitted trend lines, 1950−2021: Wajir, Ages: 40−44
Data points enclosed with a diamond are reference sources

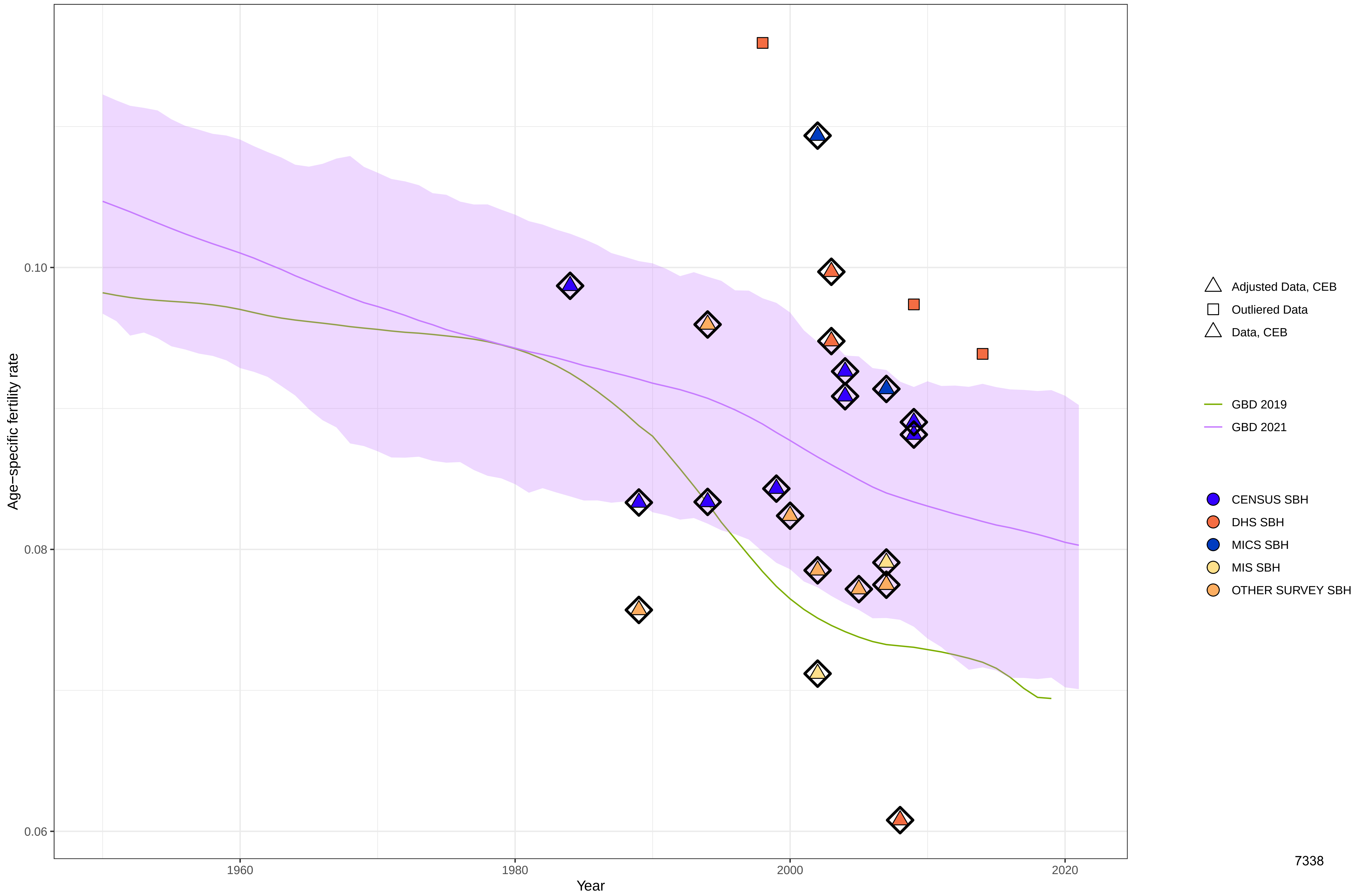

Age−specific fertility fitted trend lines, 1950−2021: Wajir, Ages: 45−49
Data points enclosed with a diamond are reference sources

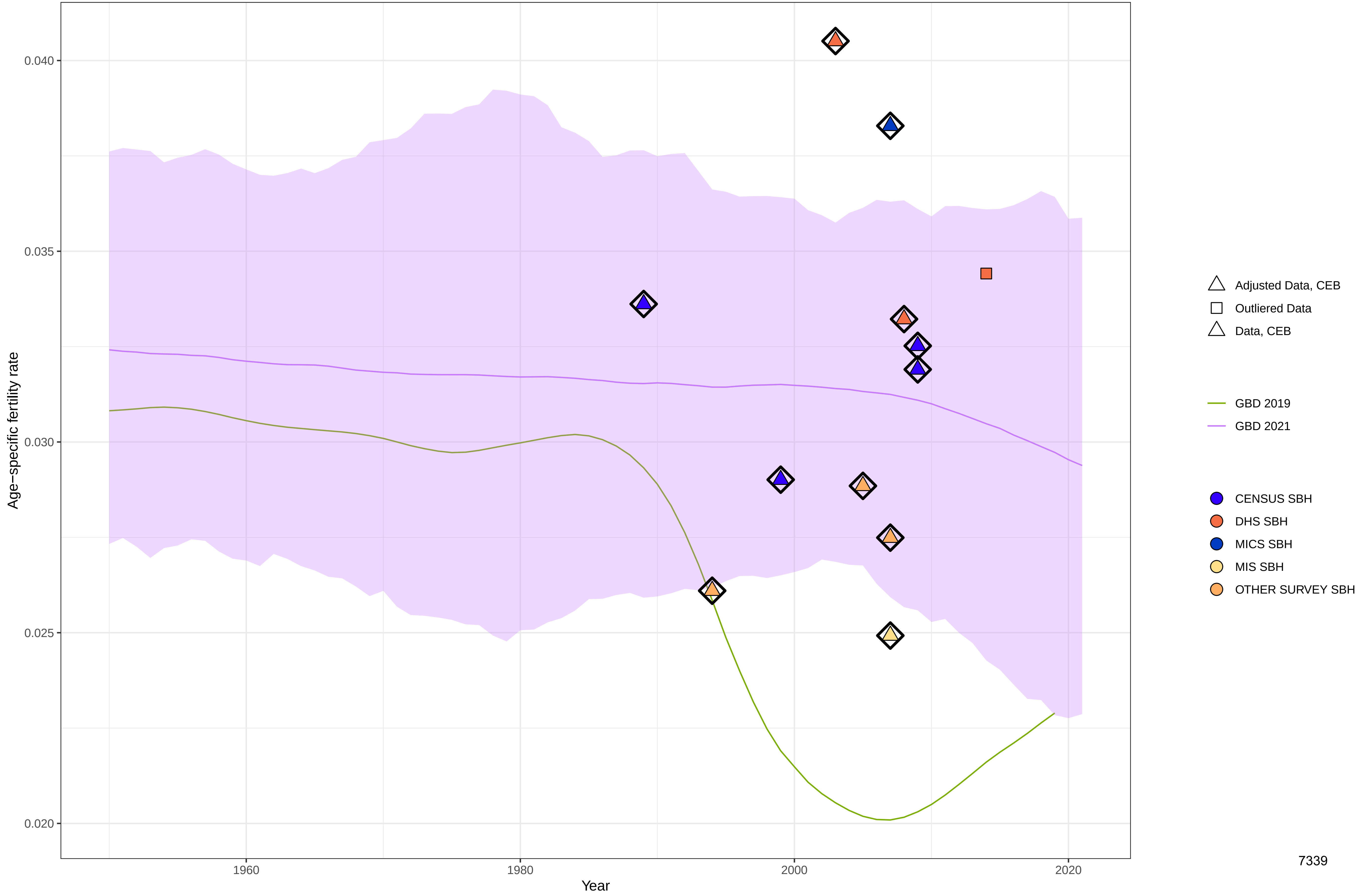

Age−specific fertility fitted trend lines, 1950−2021: Wajir, Ages: 50−54
Data points enclosed with a diamond are reference sources

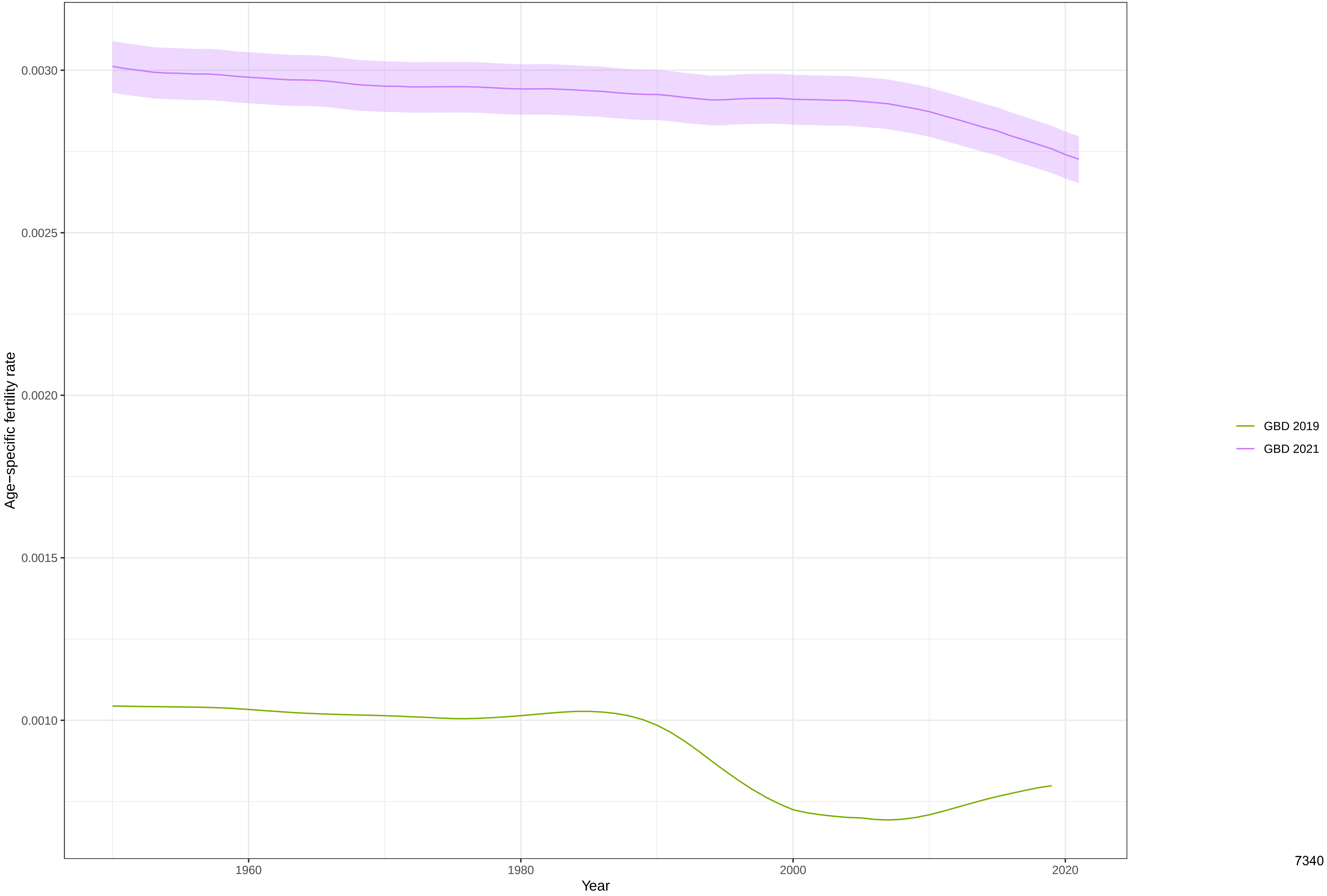

Total fertility rate fitted trend lines, 1950−2021: Wajir TFR
Data points enclosed with a diamond are reference sources

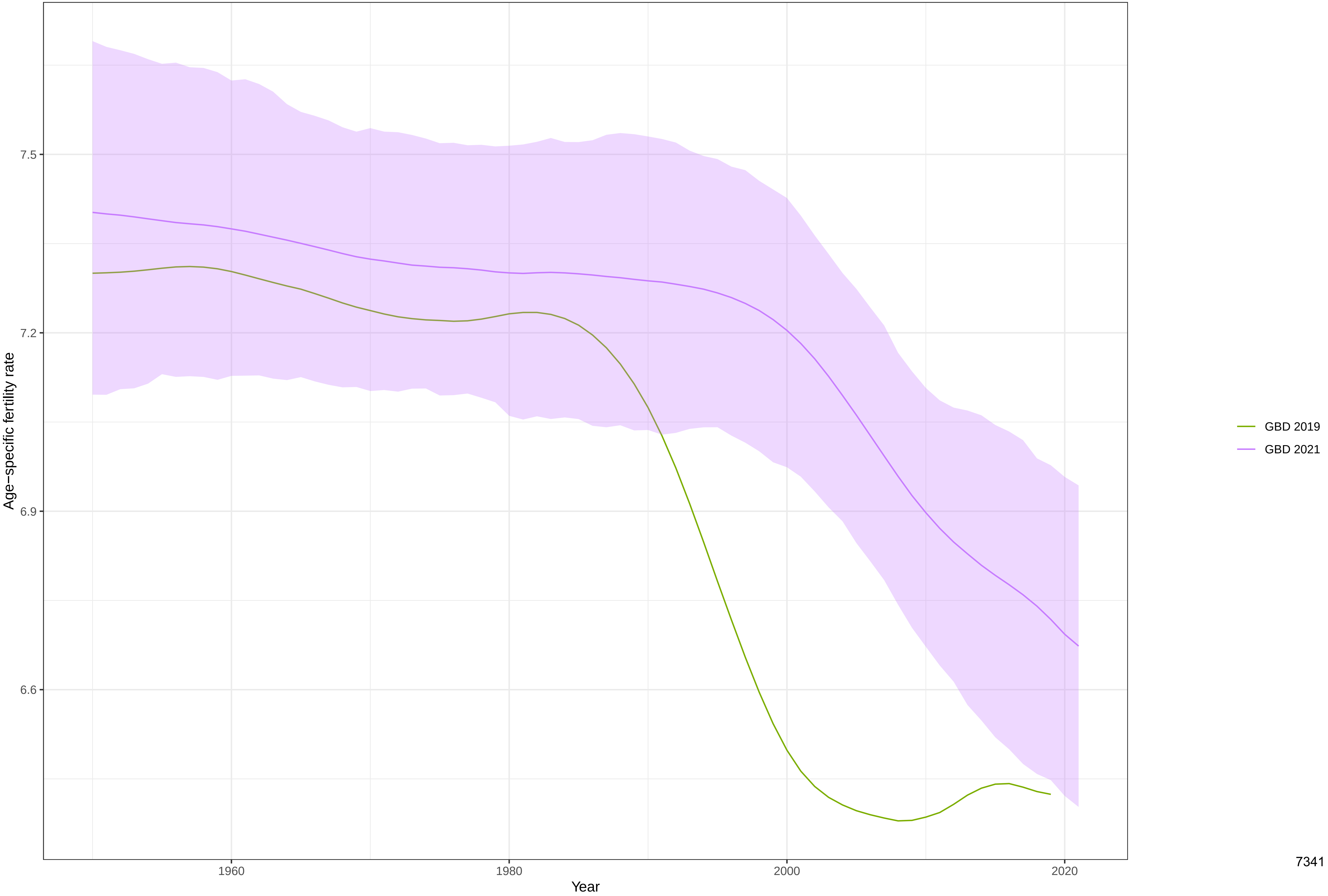

Age−specific fertility fitted trend lines, 1950−2021: West Pokot, Ages: 10−14
Data points enclosed with a diamond are reference sources

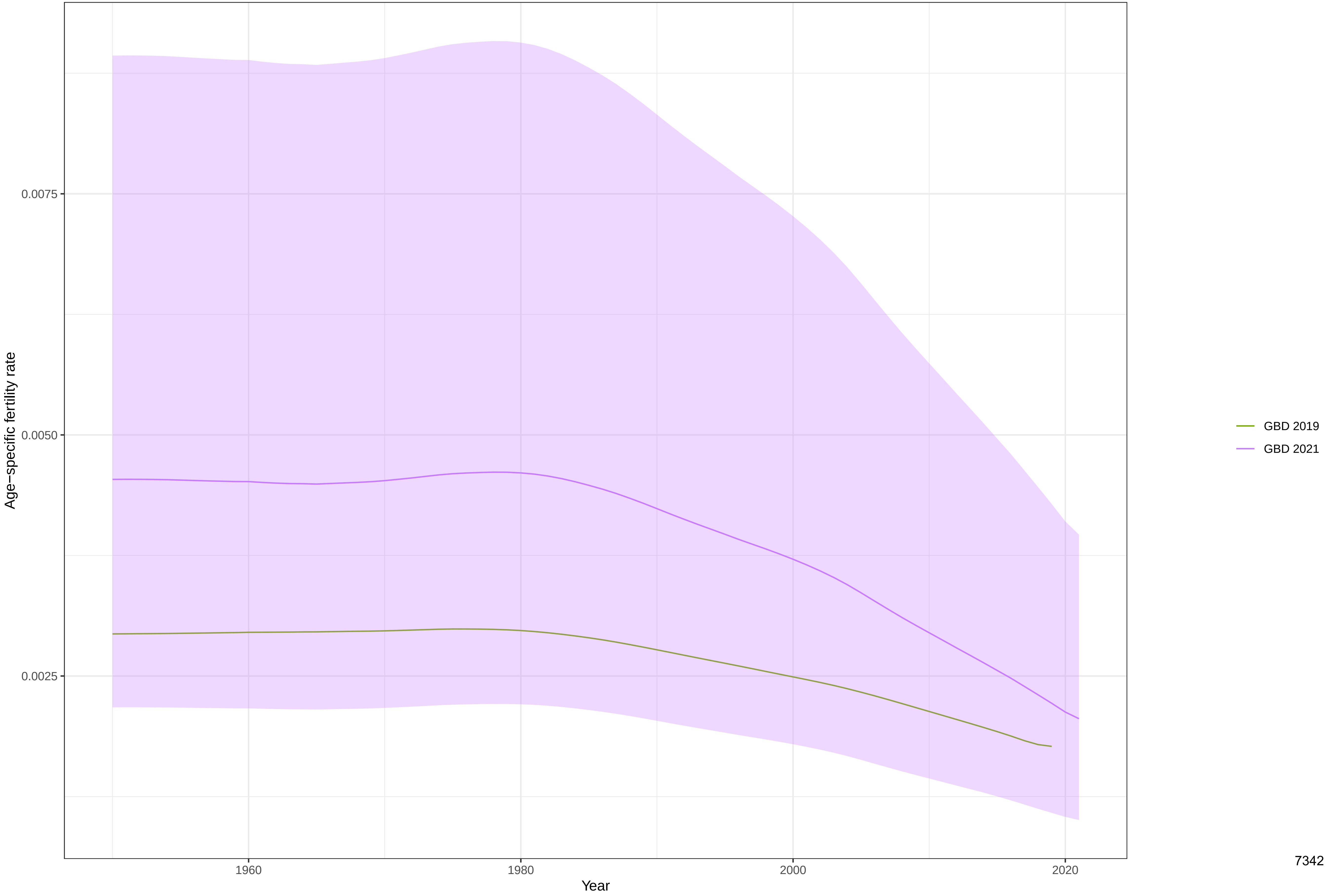

Age−specific fertility fitted trend lines, 1950−2021: West Pokot, Ages: 15−19
Data points enclosed with a diamond are reference sources

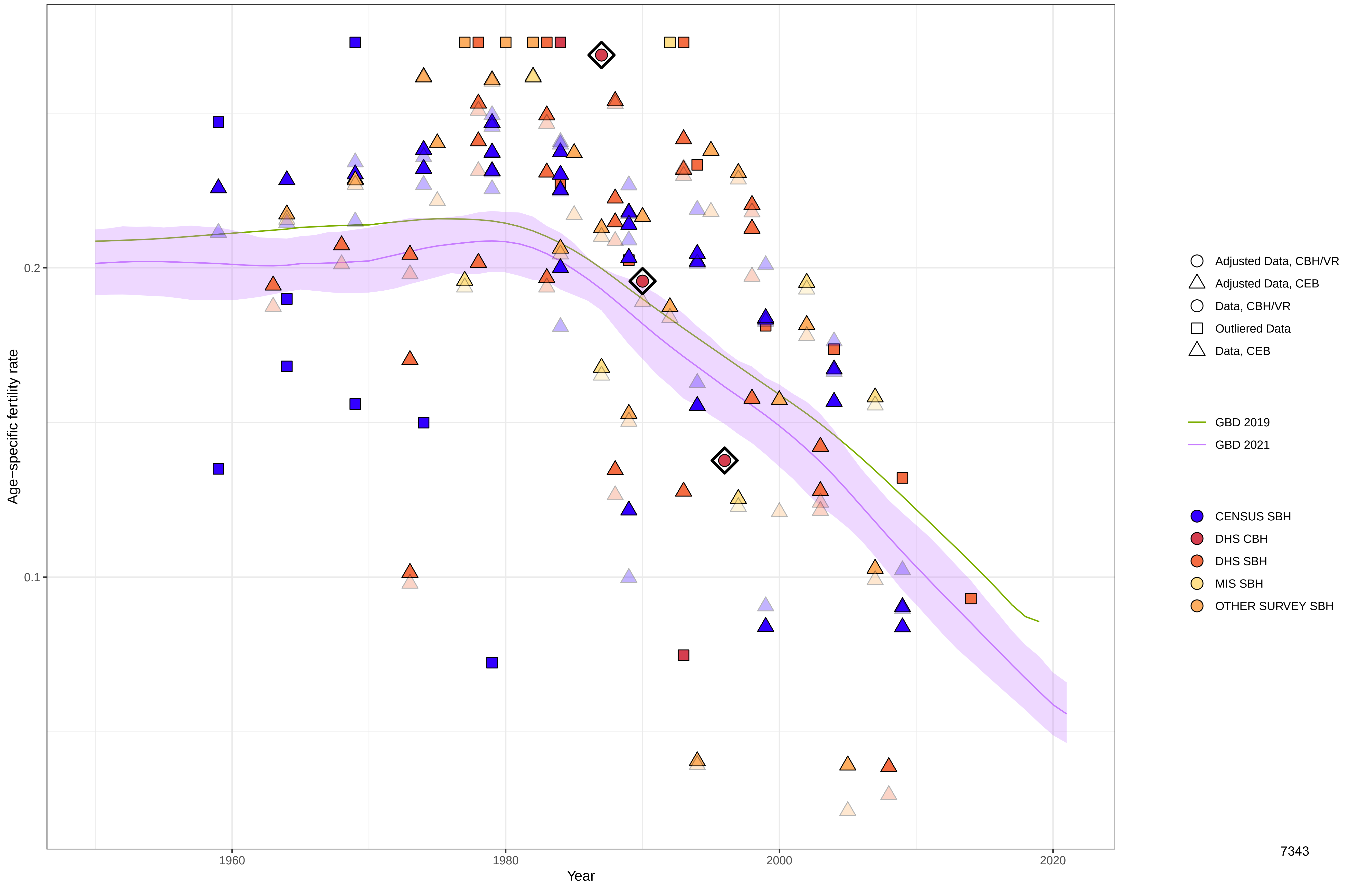

Age−specific fertility fitted trend lines, 1950−2021: West Pokot, Ages: 20−24
Data points enclosed with a diamond are reference sources

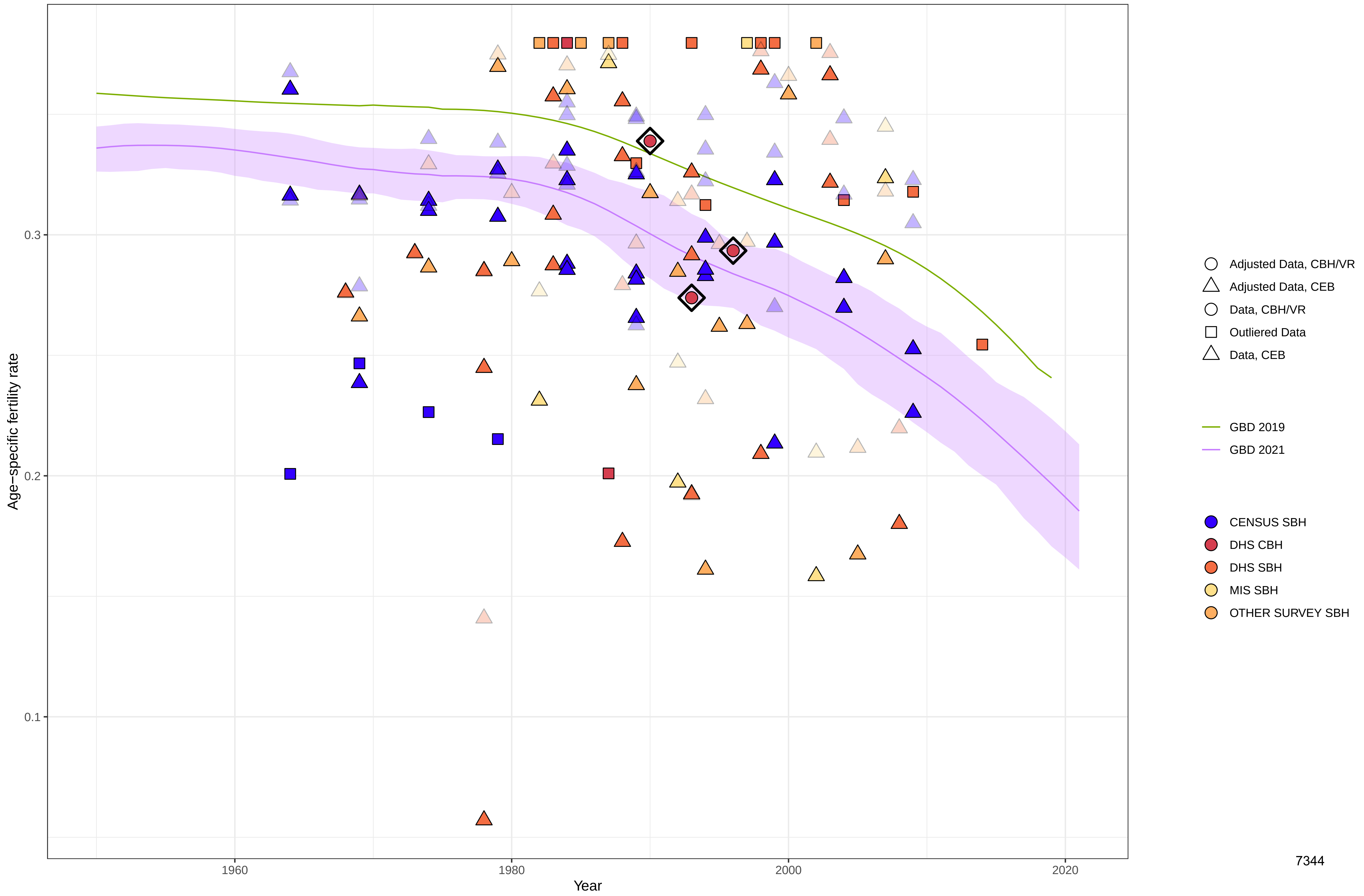

Age−specific fertility fitted trend lines, 1950−2021: West Pokot, Ages: 25−29
Data points enclosed with a diamond are reference sources

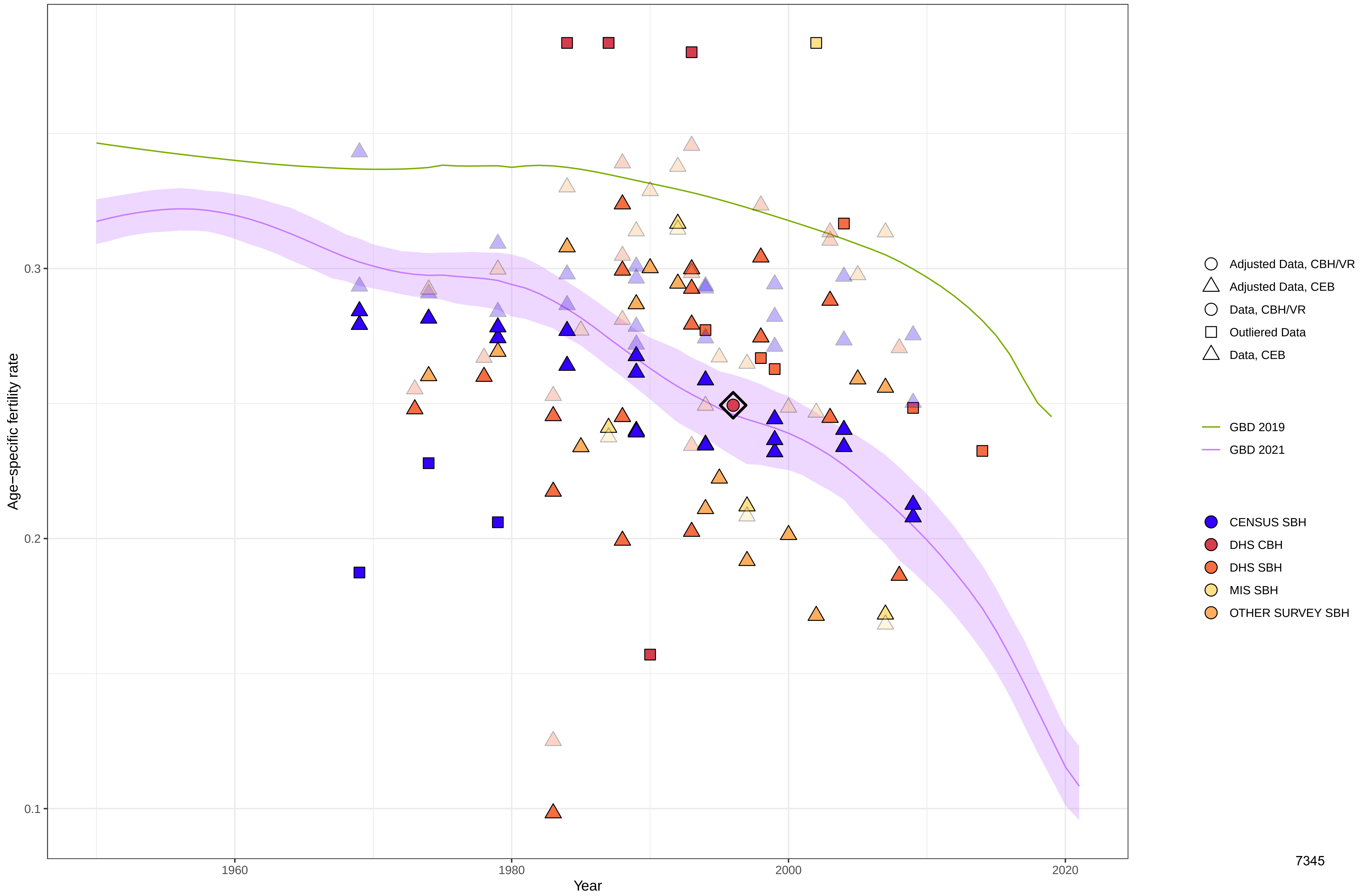

Age−specific fertility fitted trend lines, 1950−2021: West Pokot, Ages: 30−34
Data points enclosed with a diamond are reference sources

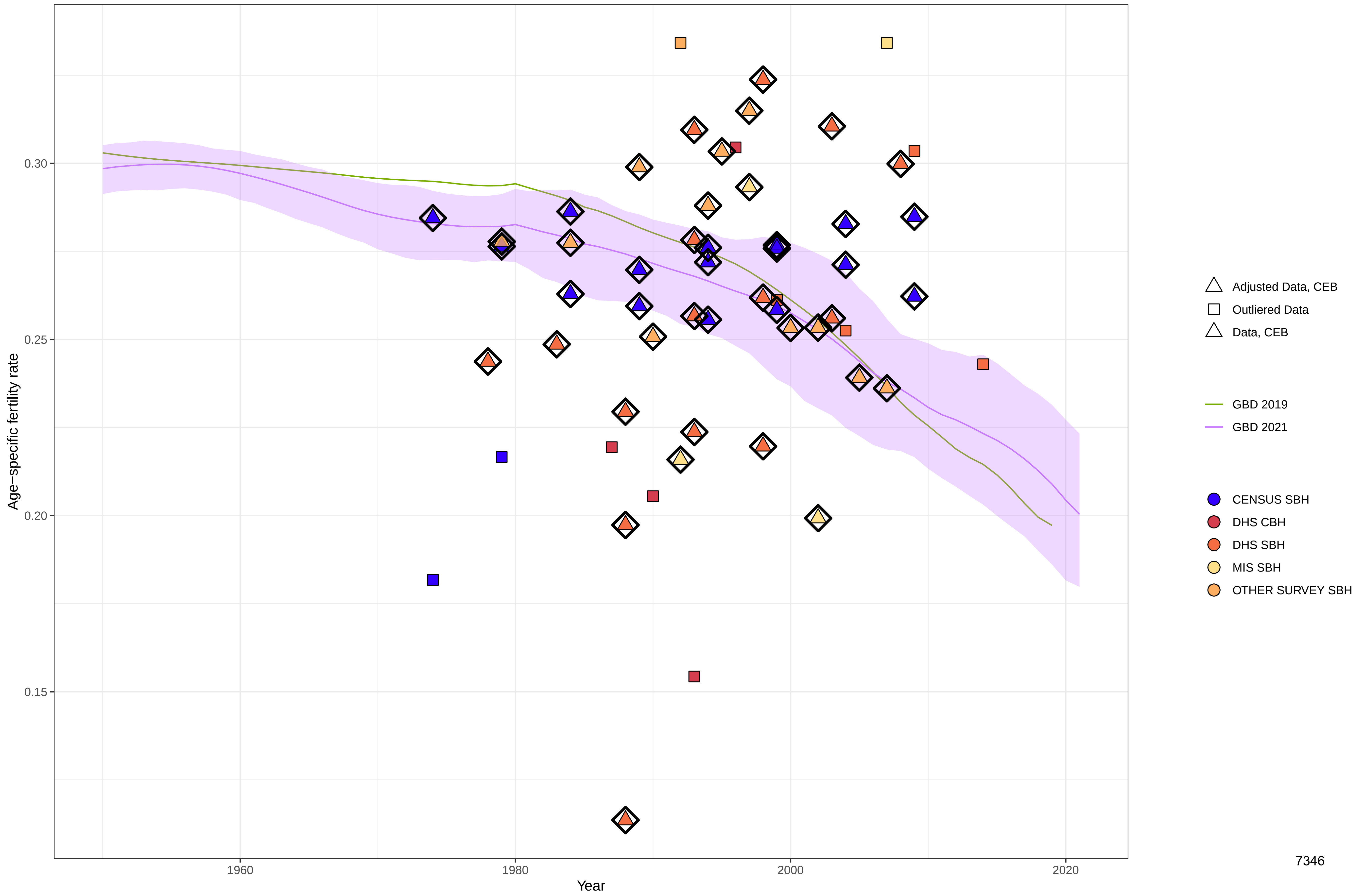

Age−specific fertility fitted trend lines, 1950−2021: West Pokot, Ages: 35−39
Data points enclosed with a diamond are reference sources

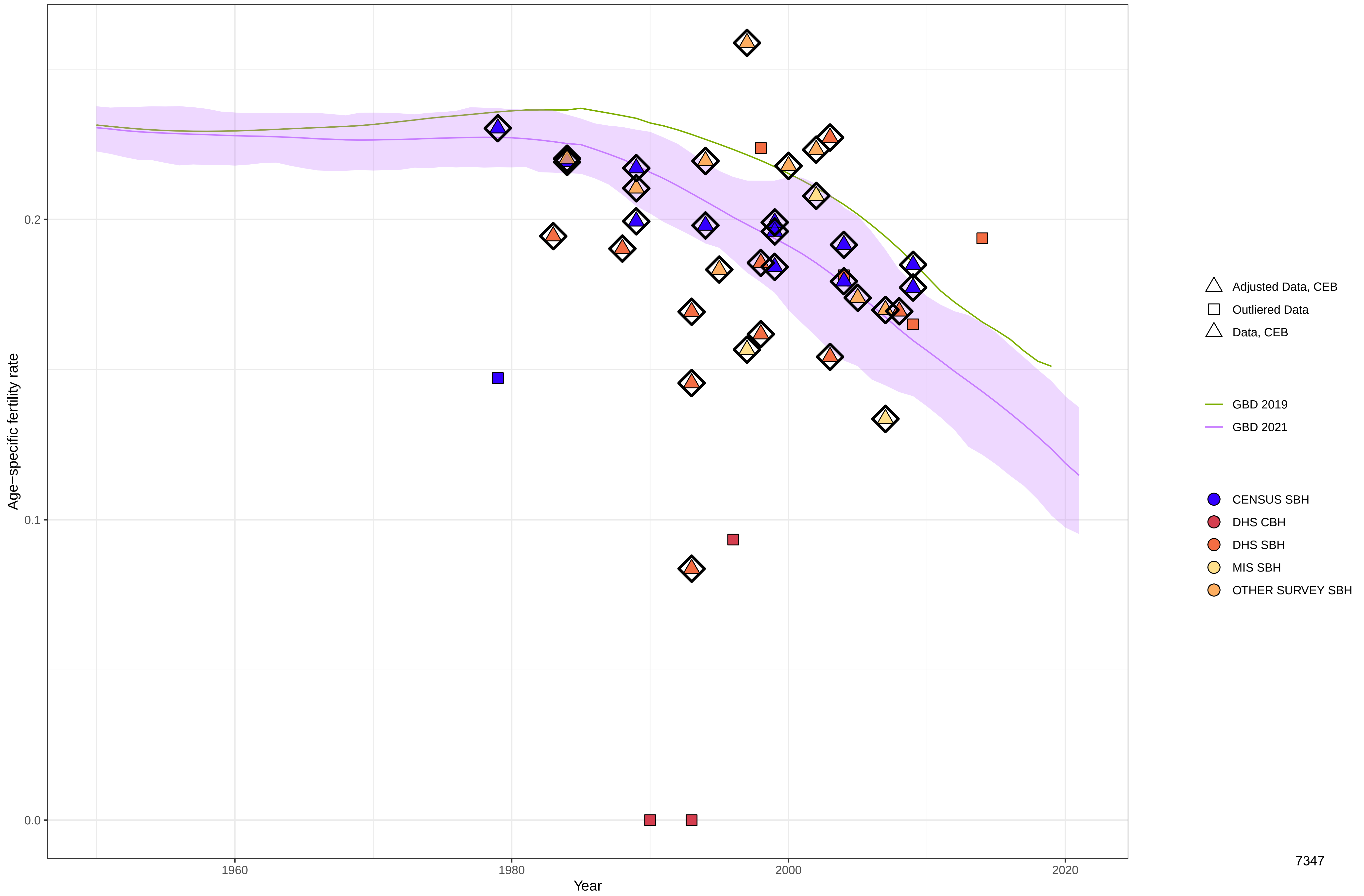

Age−specific fertility fitted trend lines, 1950−2021: West Pokot, Ages: 40−44
Data points enclosed with a diamond are reference sources

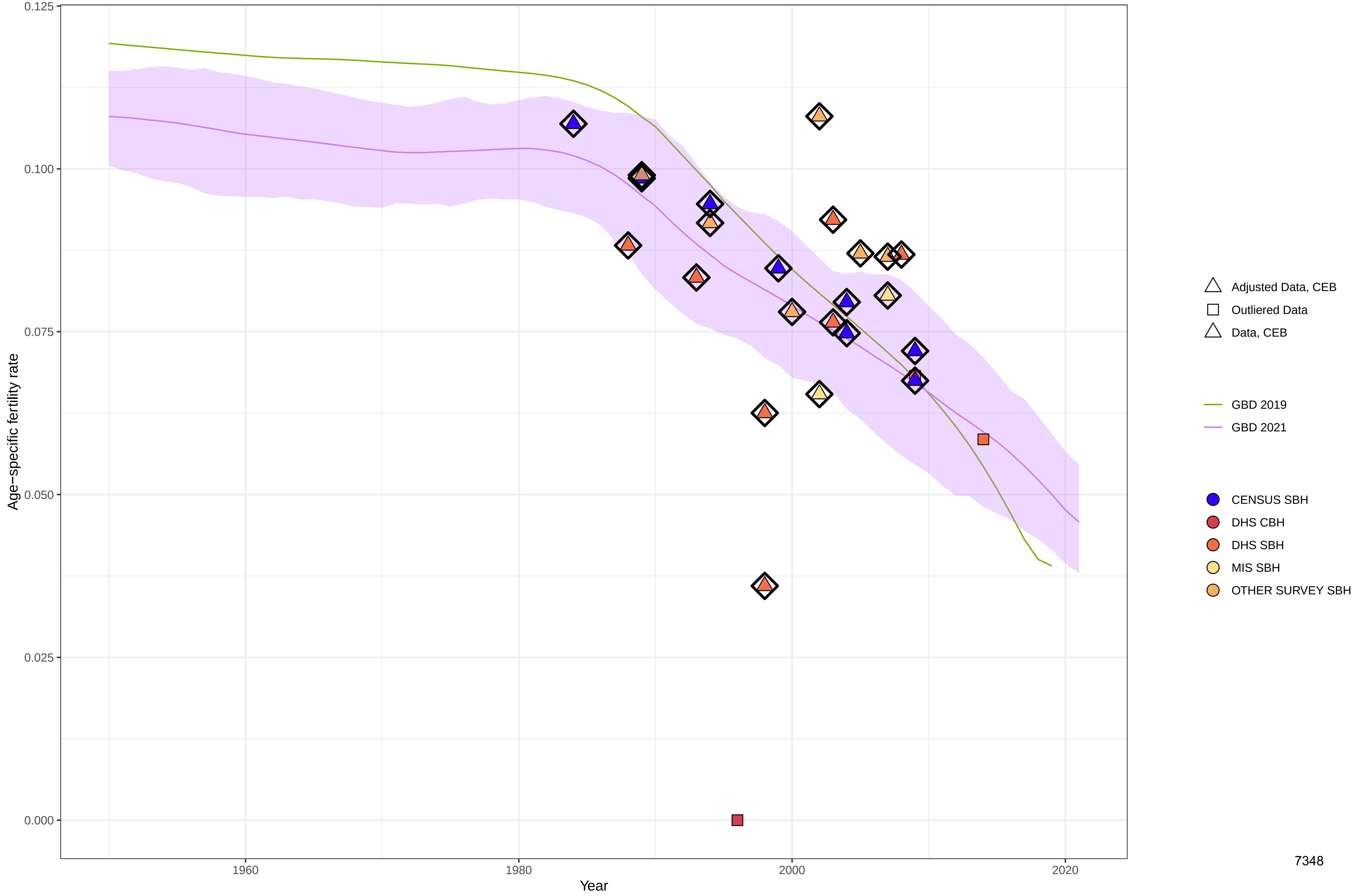

Age−specific fertility fitted trend lines, 1950−2021: West Pokot, Ages: 45−49
Data points enclosed with a diamond are reference sources

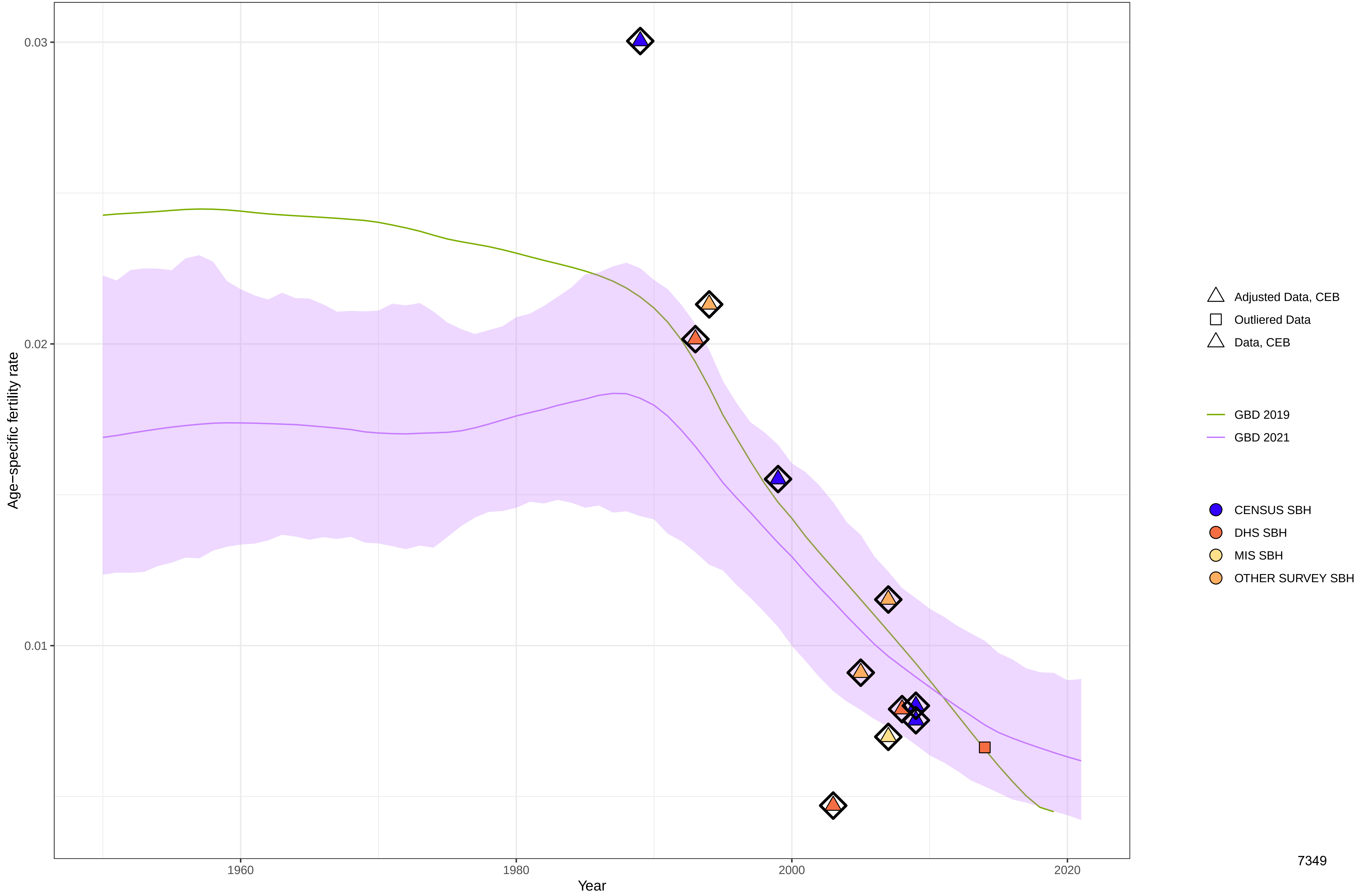

Age−specific fertility fitted trend lines, 1950−2021: West Pokot, Ages: 50−54
Data points enclosed with a diamond are reference sources

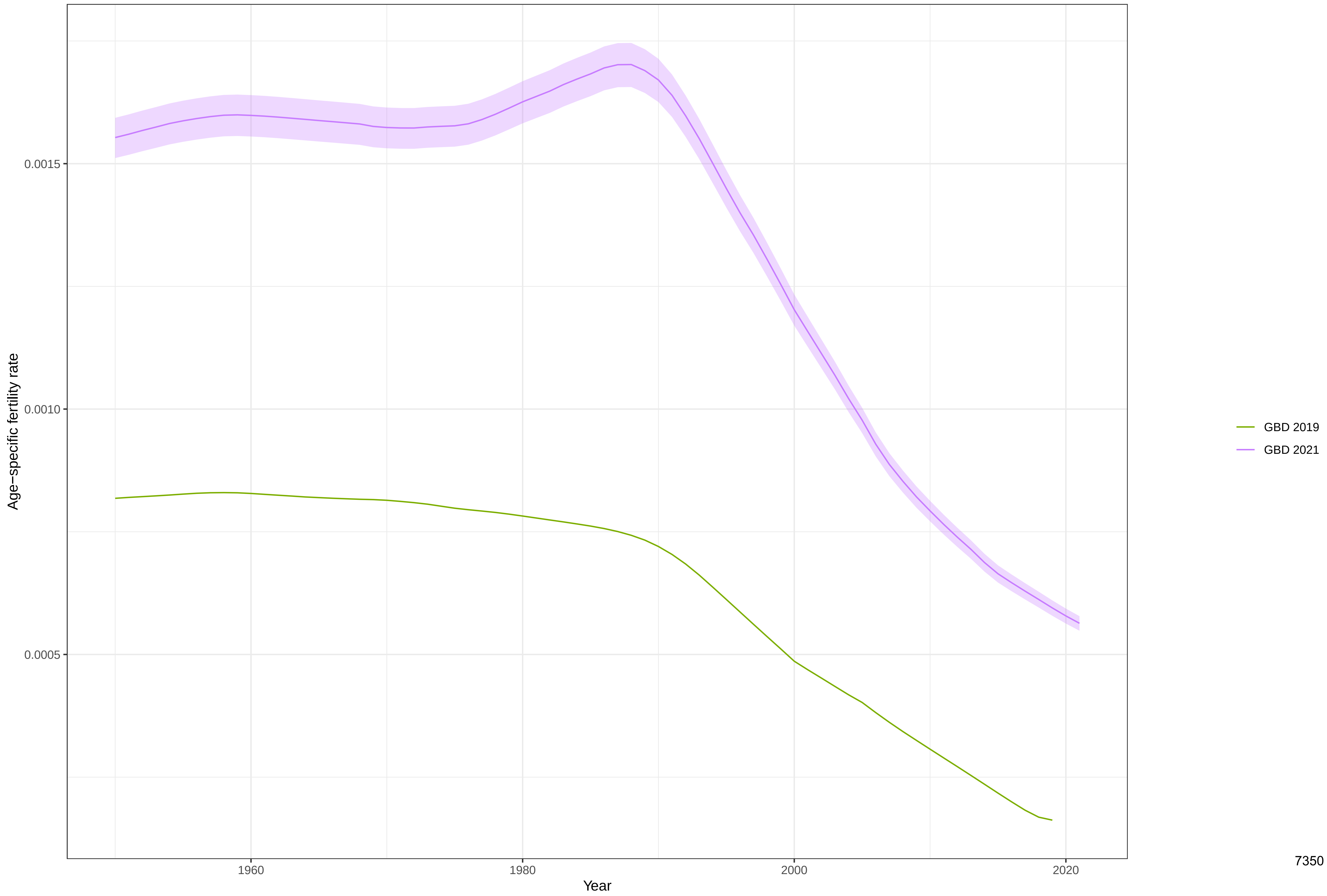

Total fertility rate fitted trend lines, 1950−2021: West Pokot TFR
Data points enclosed with a diamond are reference sources

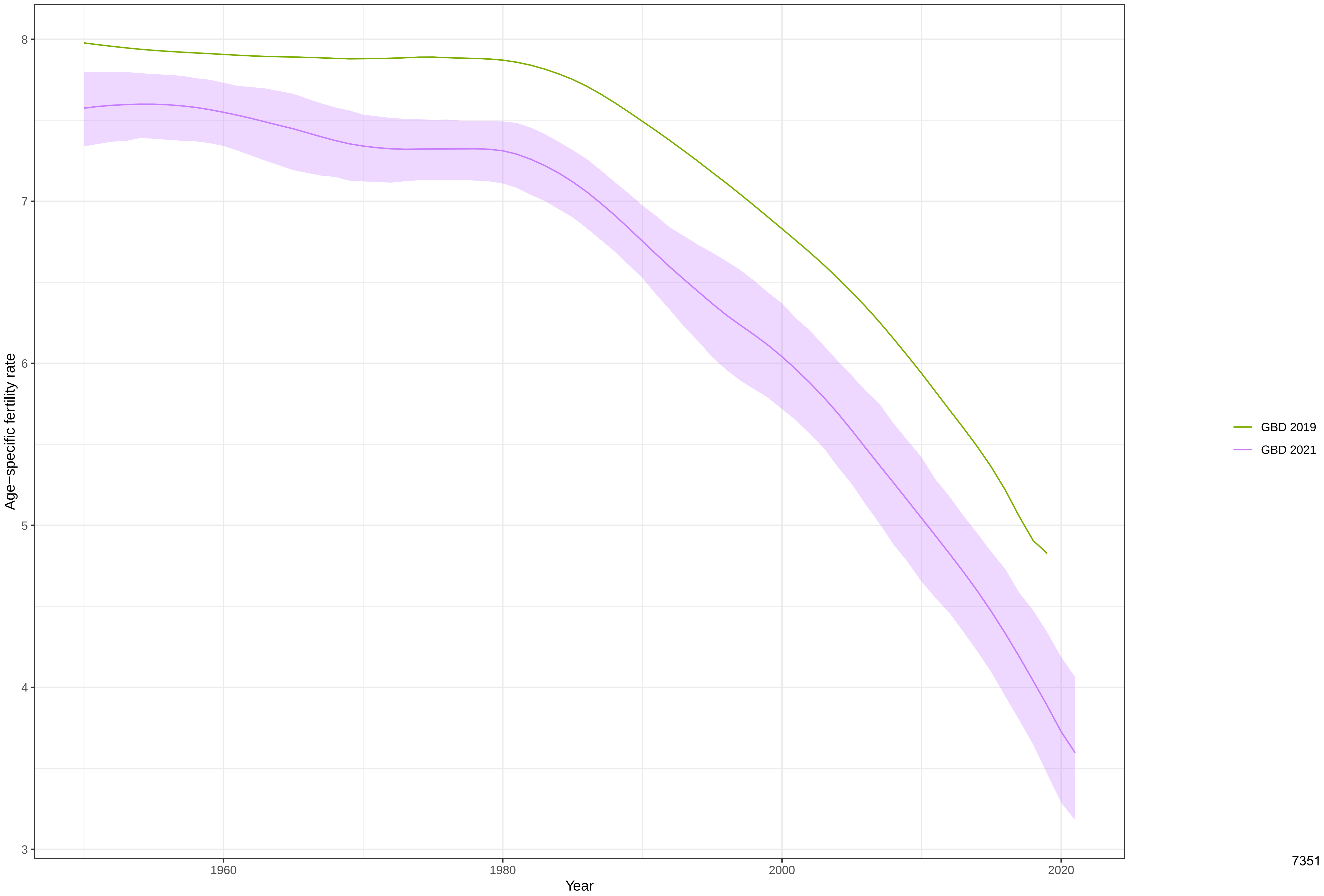

Age−specific fertility fitted trend lines, 1950−2021: Madagascar, Ages: 10−14
Data points enclosed with a diamond are reference sources

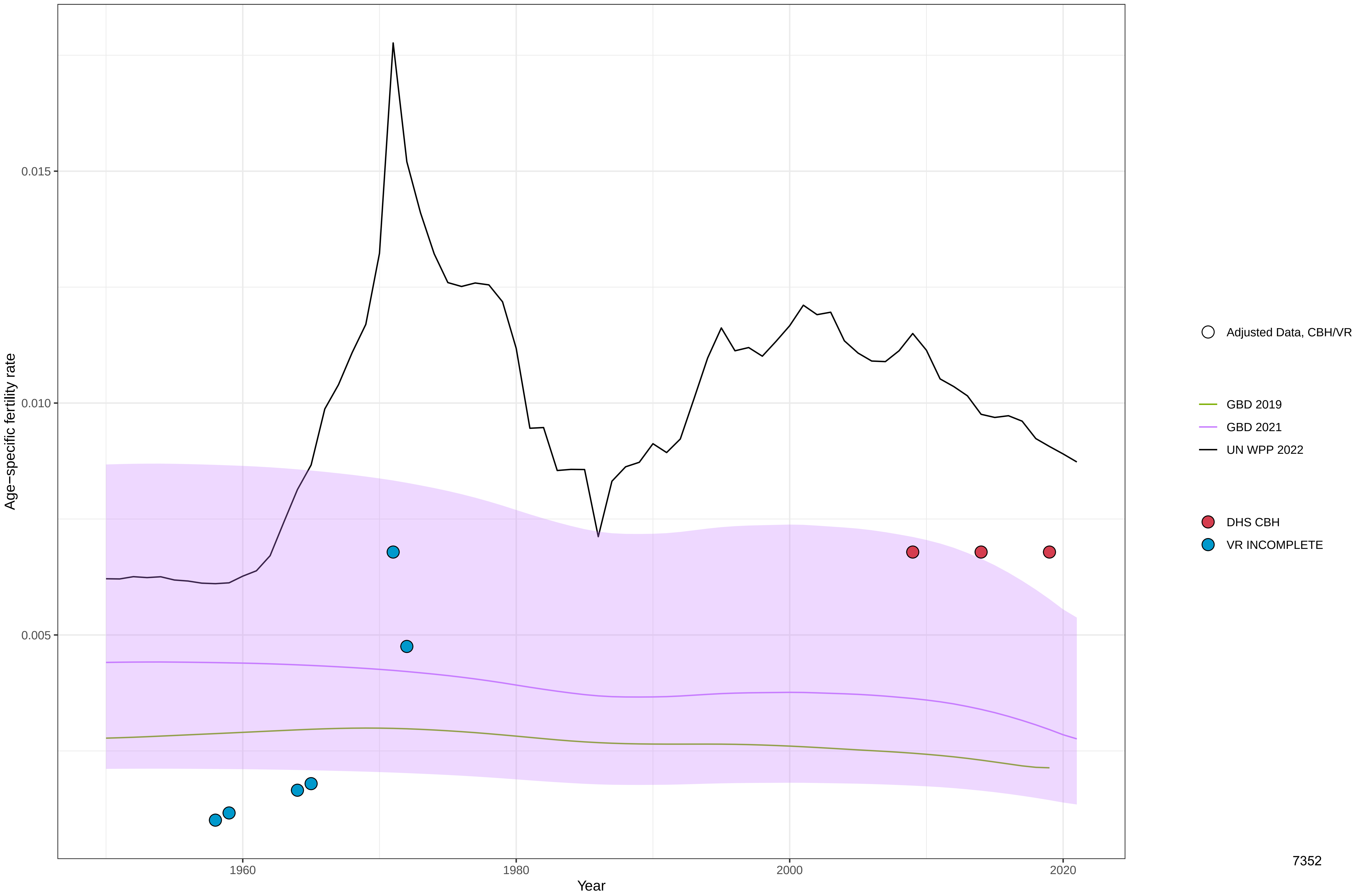

Age−specific fertility fitted trend lines, 1950−2021: Madagascar, Ages: 15−19
Data points enclosed with a diamond are reference sources

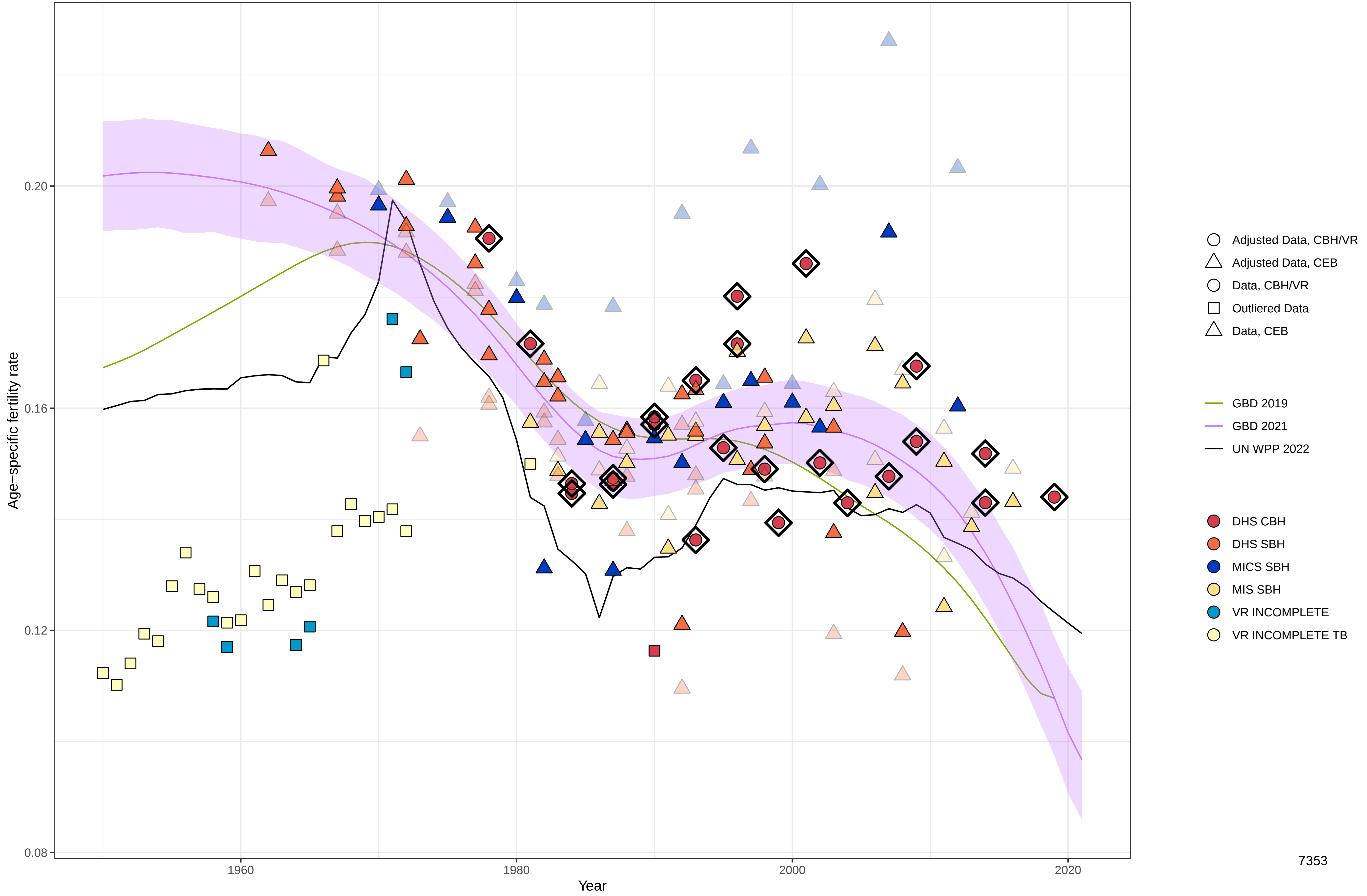

7353

Age−specific fertility fitted trend lines, 1950−2021: Madagascar, Ages: 20−24
Data points enclosed with a diamond are reference sources

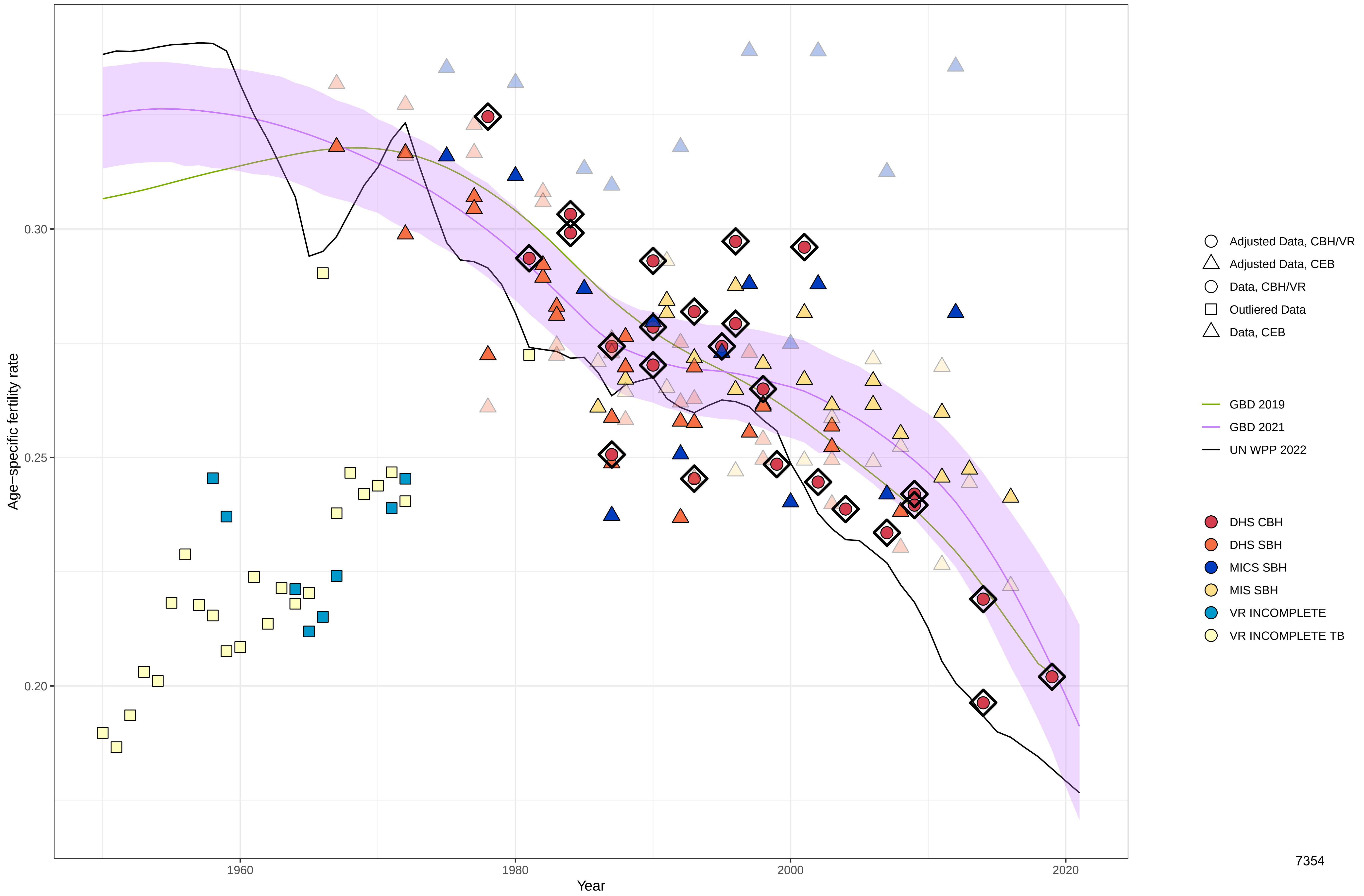

Age−specific fertility fitted trend lines, 1950−2021: Madagascar, Ages: 25−29
Data points enclosed with a diamond are reference sources

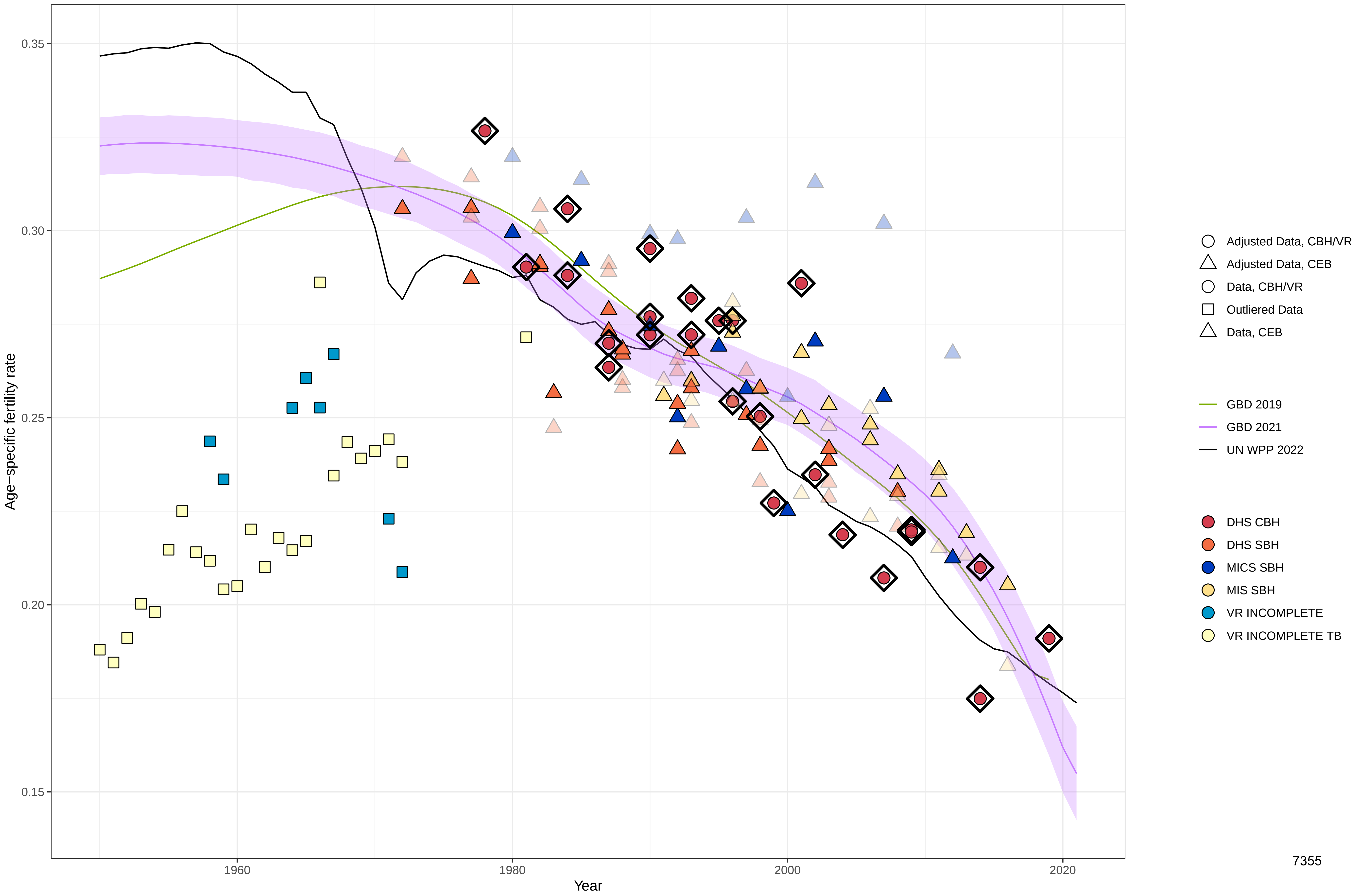

Age−specific fertility fitted trend lines, 1950−2021: Madagascar, Ages: 30−34
Data points enclosed with a diamond are reference sources

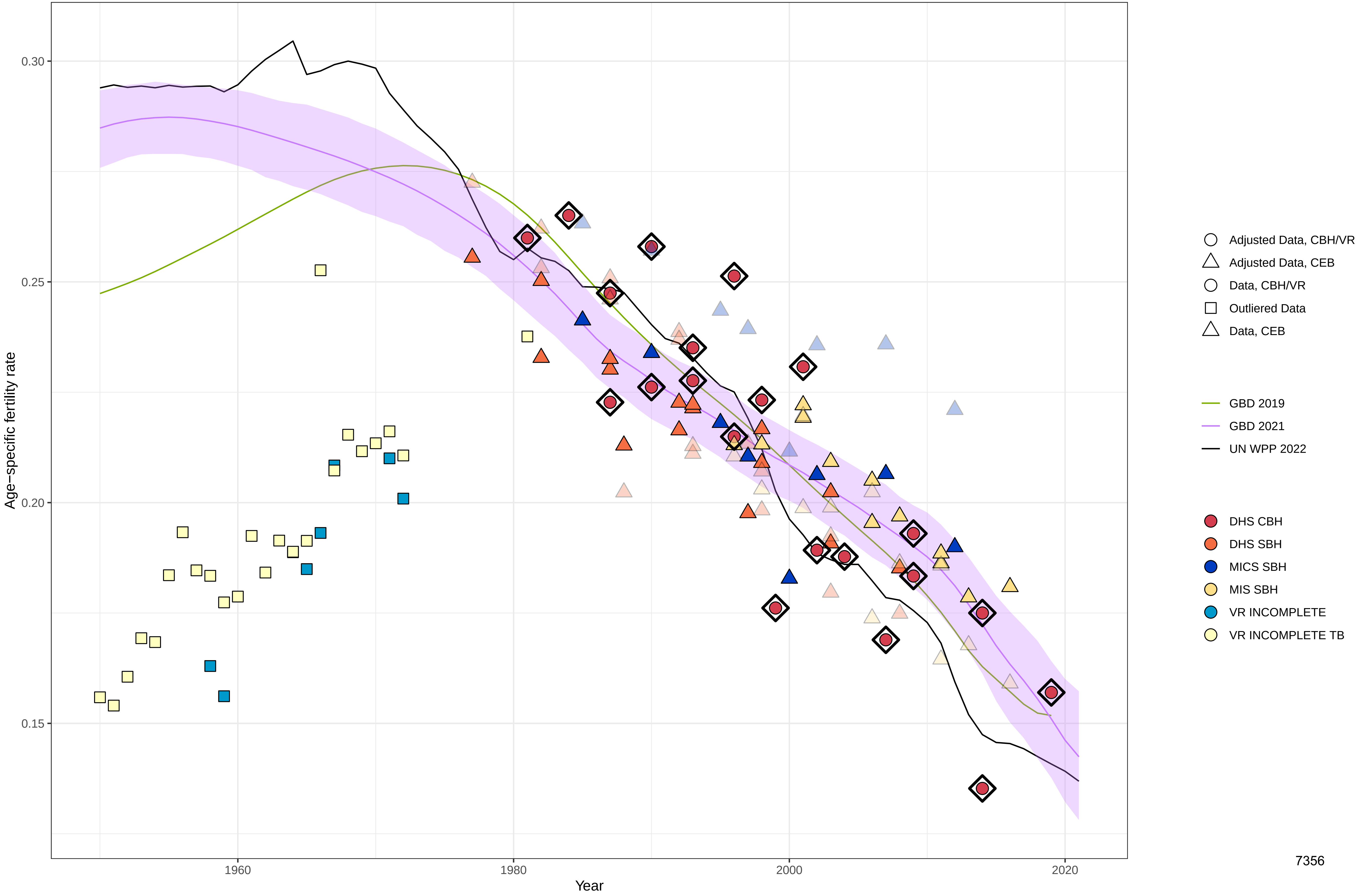

Age−specific fertility fitted trend lines, 1950−2021: Madagascar, Ages: 35−39
Data points enclosed with a diamond are reference sources

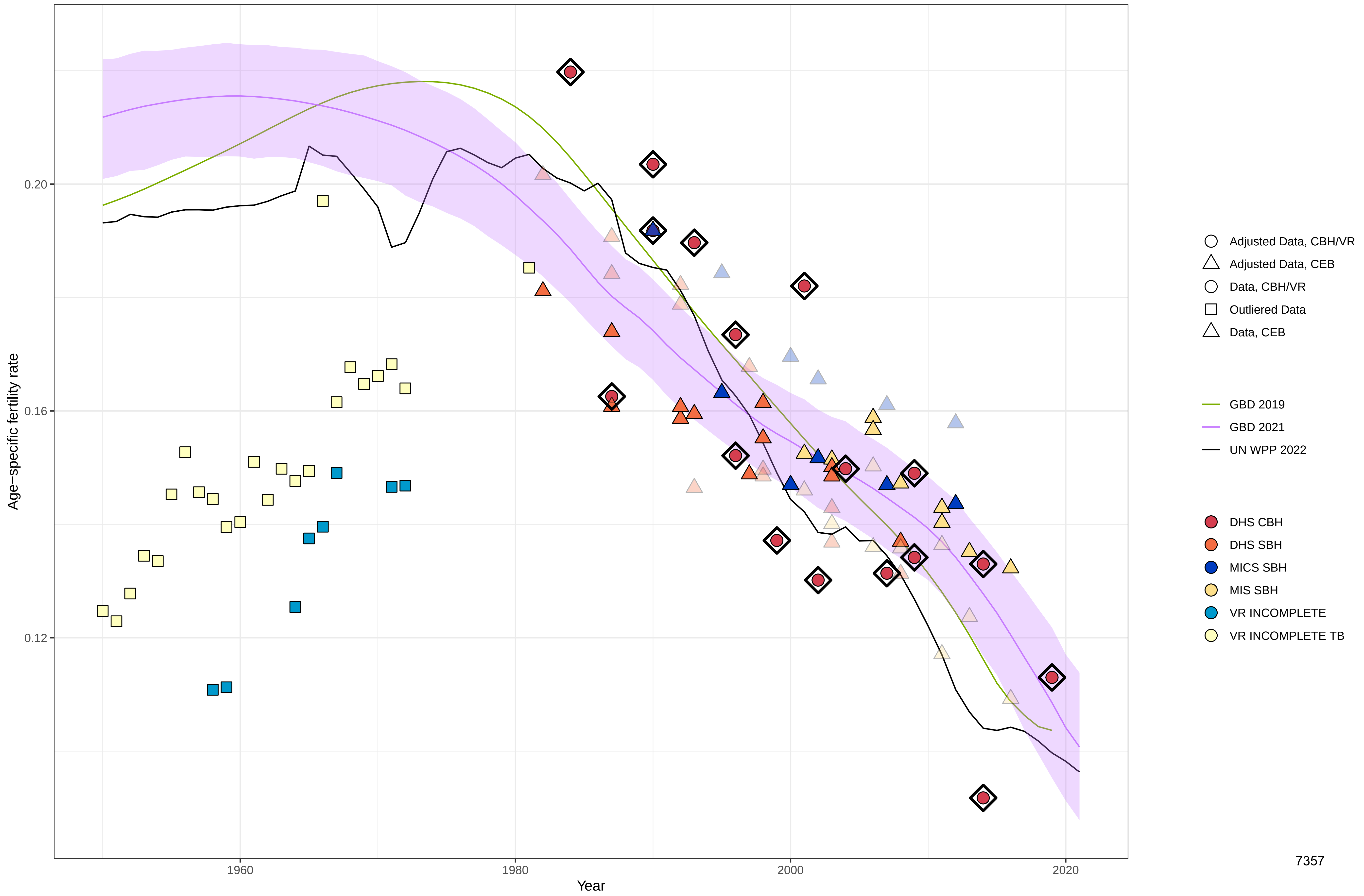

Age−specific fertility fitted trend lines, 1950−2021: Madagascar, Ages: 40−44
Data points enclosed with a diamond are reference sources

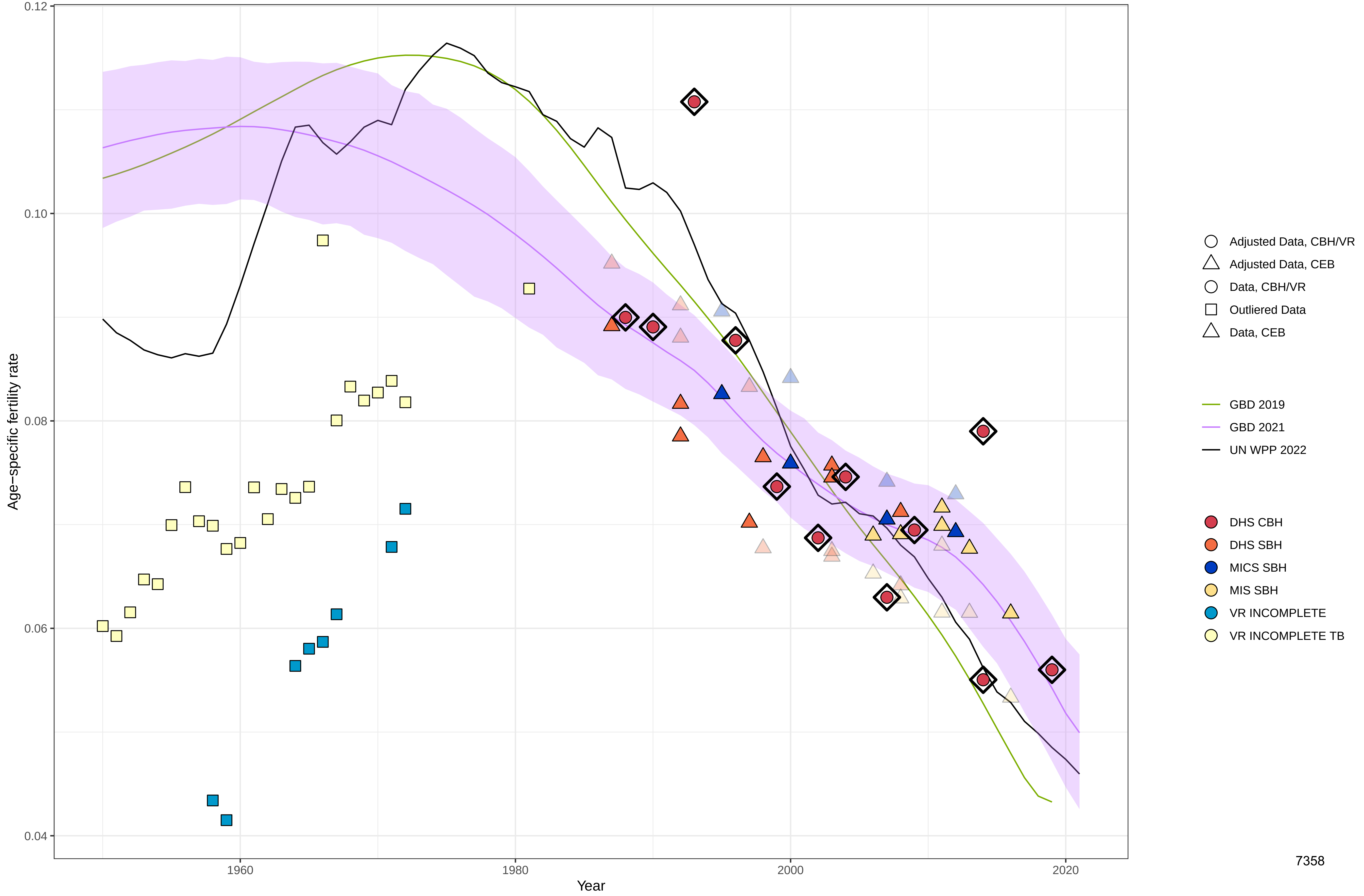

Age−specific fertility fitted trend lines, 1950−2021: Madagascar, Ages: 45−49
Data points enclosed with a diamond are reference sources

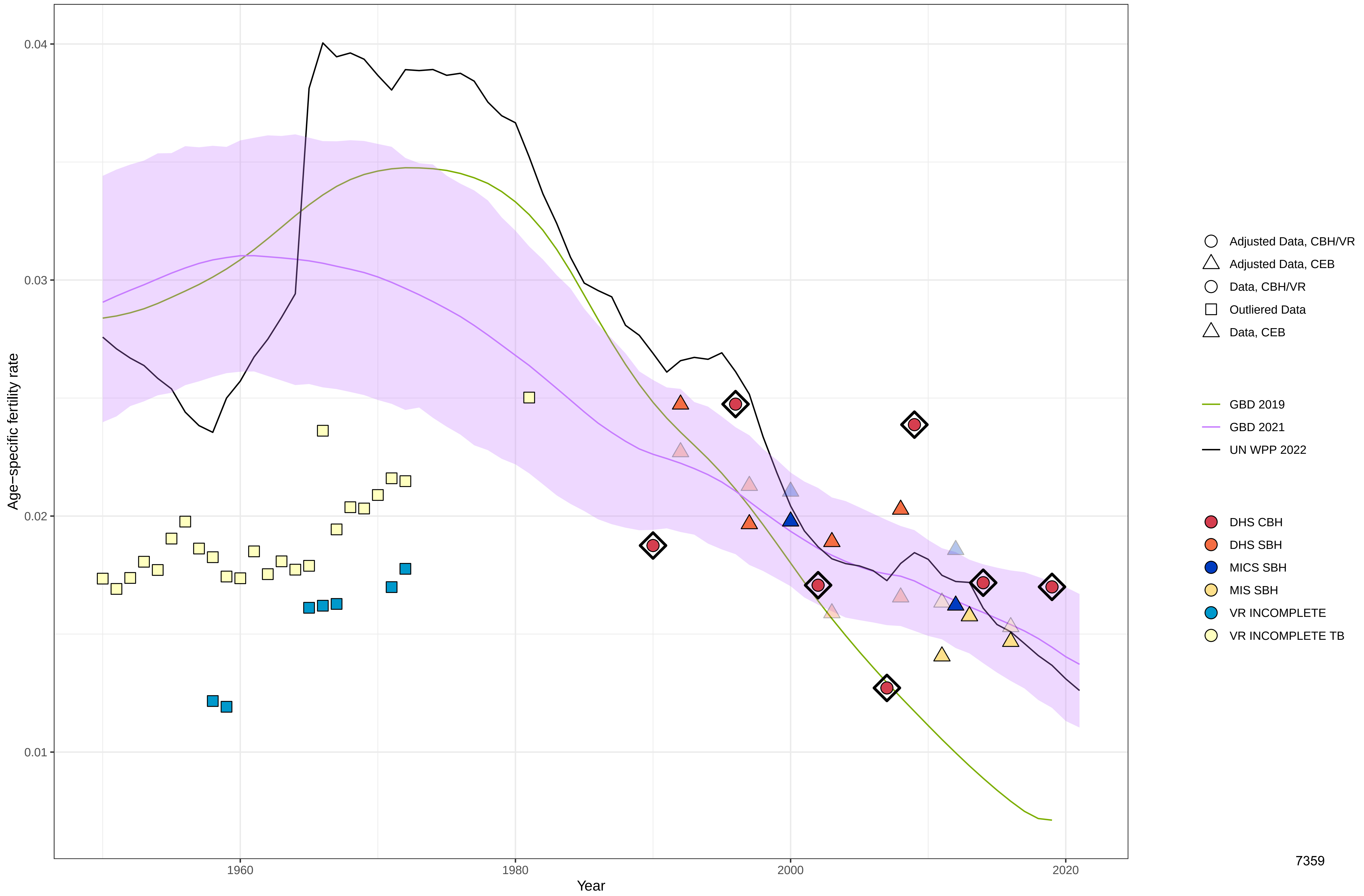

Age−specific fertility fitted trend lines, 1950−2021: Madagascar, Ages: 50−54
Data points enclosed with a diamond are reference sources

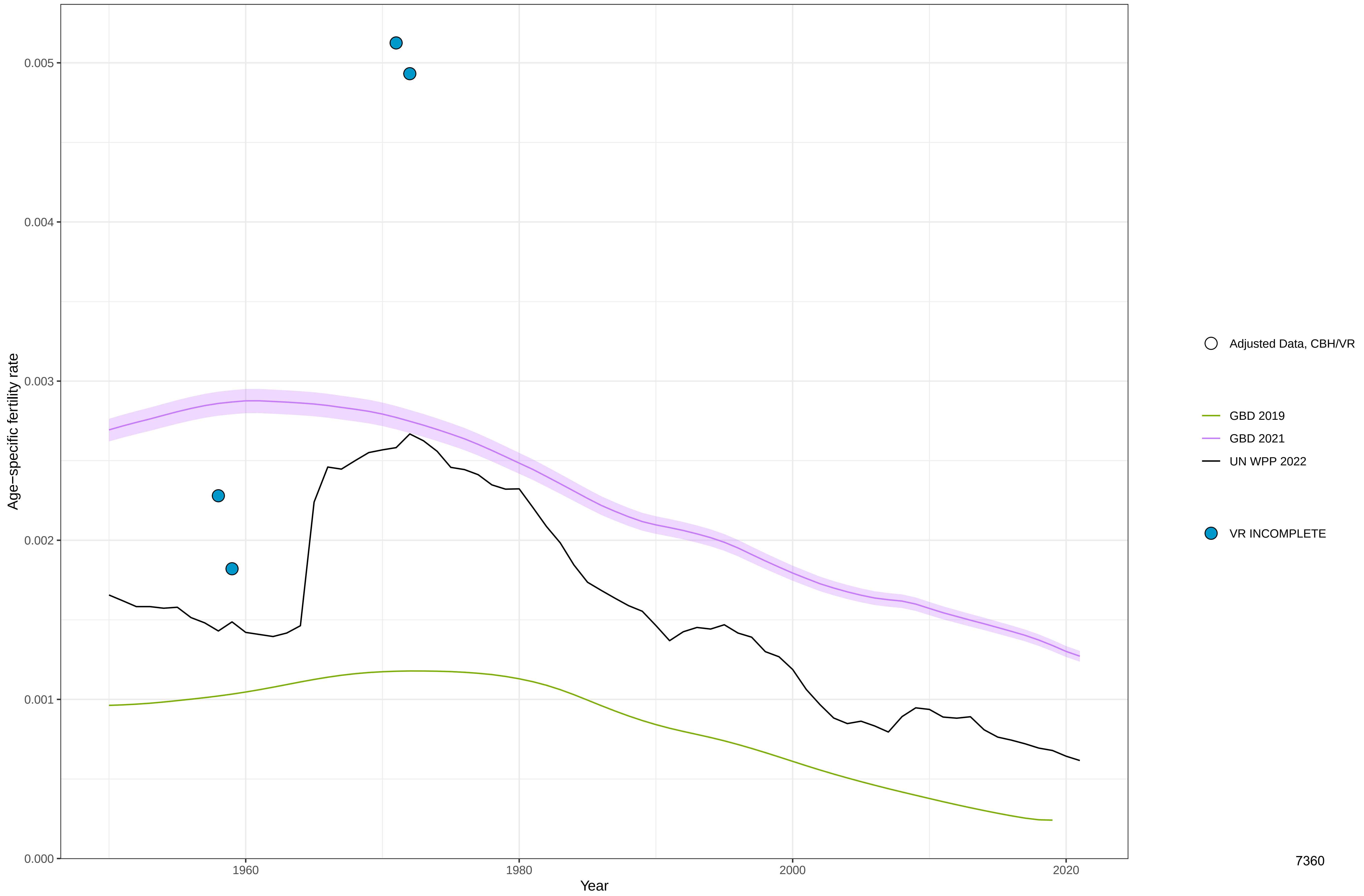

Total fertility rate fitted trend lines, 1950−2021: Madagascar TFR
Data points enclosed with a diamond are reference sources

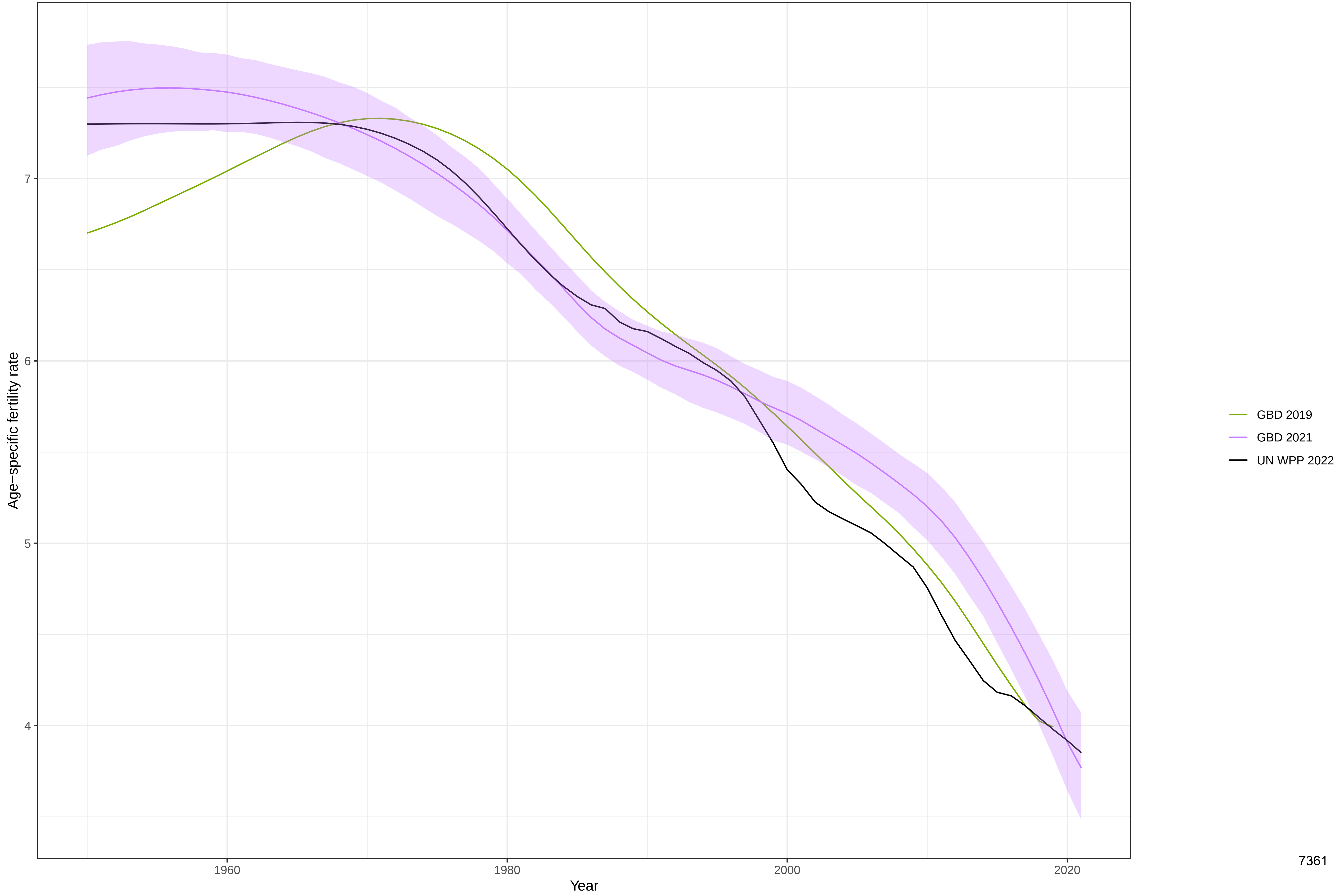

Age−specific fertility fitted trend lines, 1950−2021: Malawi, Ages: 10−14
Data points enclosed with a diamond are reference sources

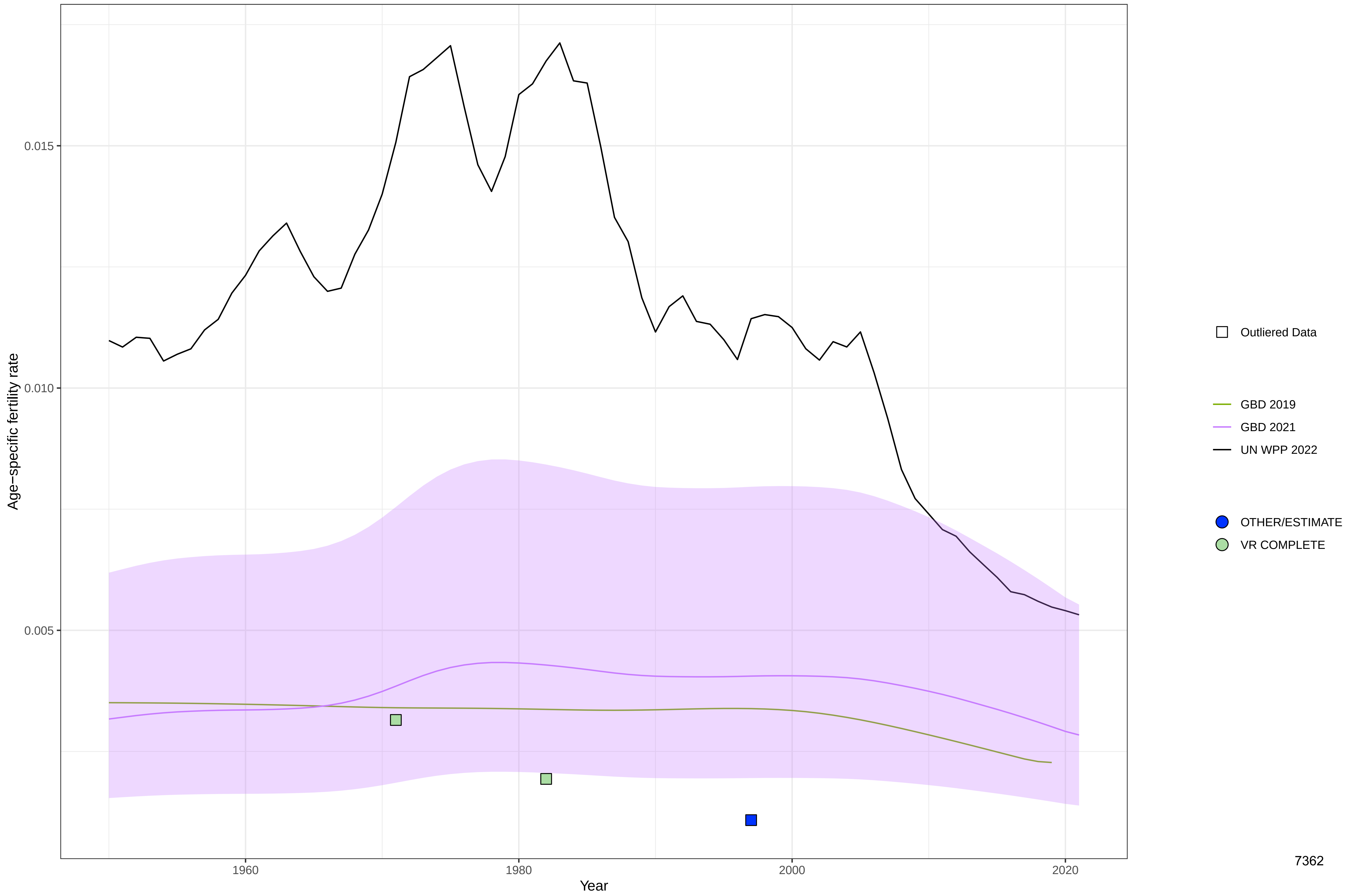

Age−specific fertility fitted trend lines, 1950−2021: Malawi, Ages: 15−19
Data points enclosed with a diamond are reference sources

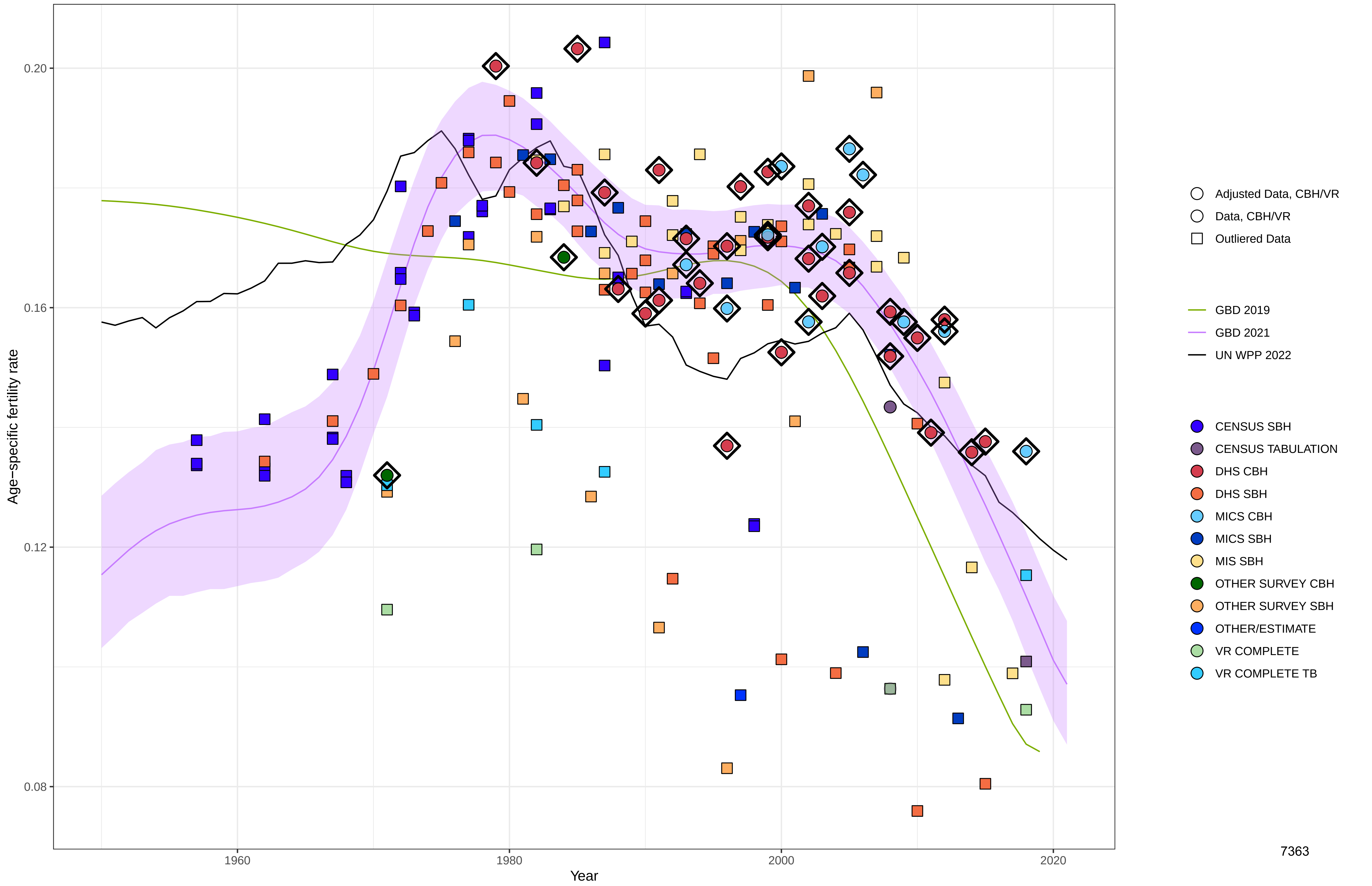

Age−specific fertility fitted trend lines, 1950−2021: Malawi, Ages: 20−24
Data points enclosed with a diamond are reference sources

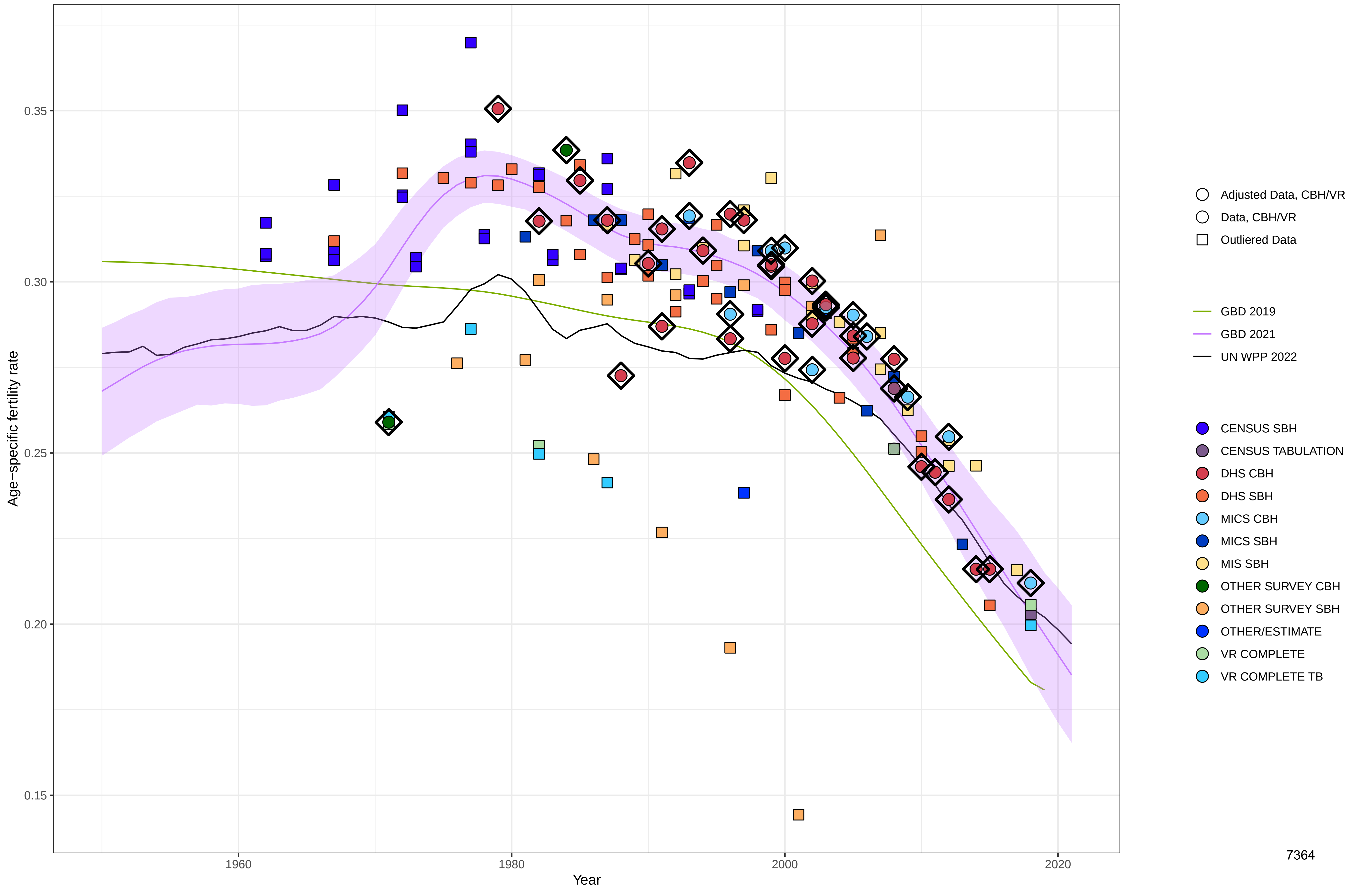

Age−specific fertility fitted trend lines, 1950−2021: Malawi, Ages: 25−29
Data points enclosed with a diamond are reference sources

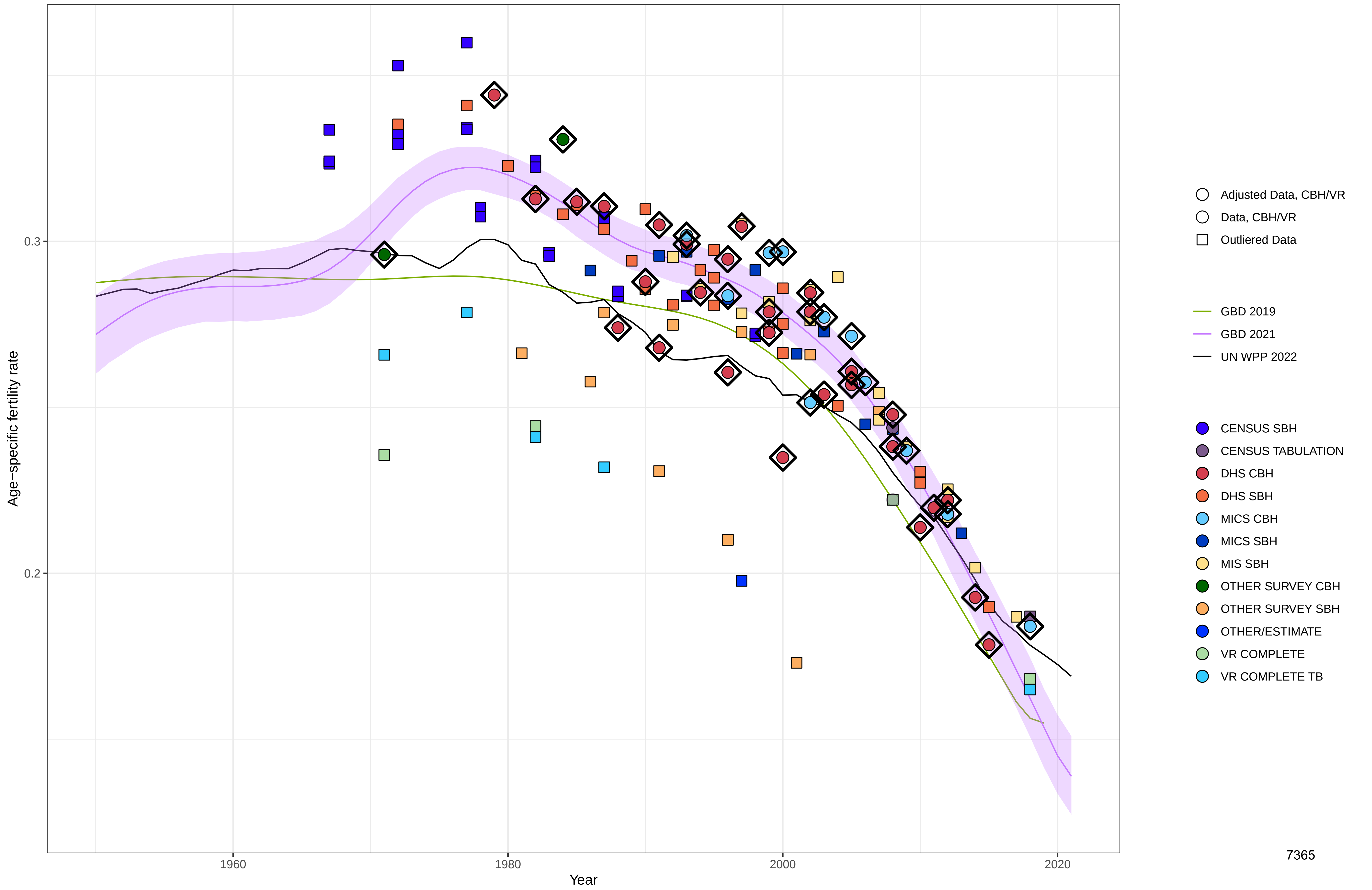

Age−specific fertility fitted trend lines, 1950−2021: Malawi, Ages: 30−34
Data points enclosed with a diamond are reference sources

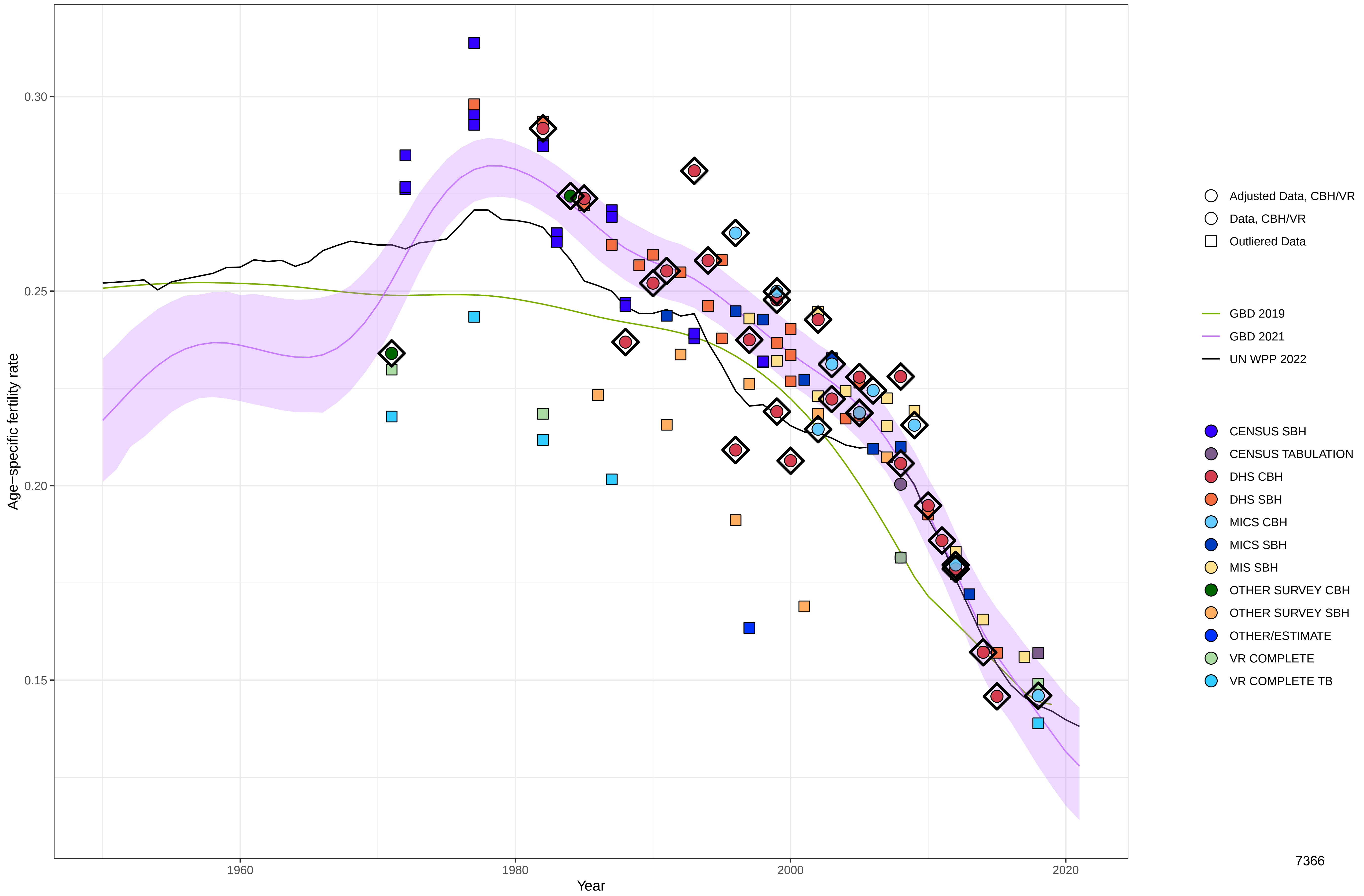

Age−specific fertility fitted trend lines, 1950−2021: Malawi, Ages: 35−39
Data points enclosed with a diamond are reference sources

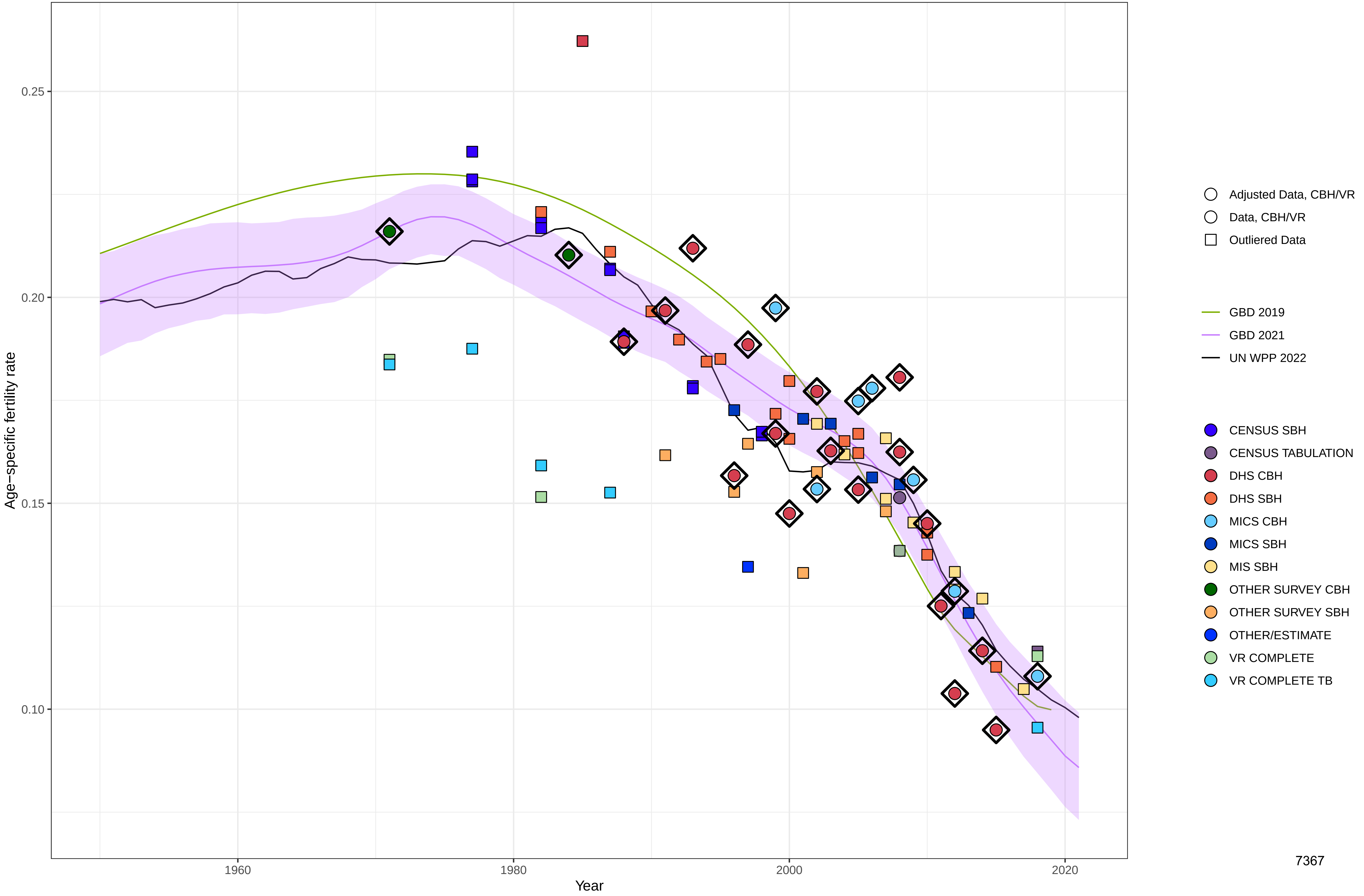

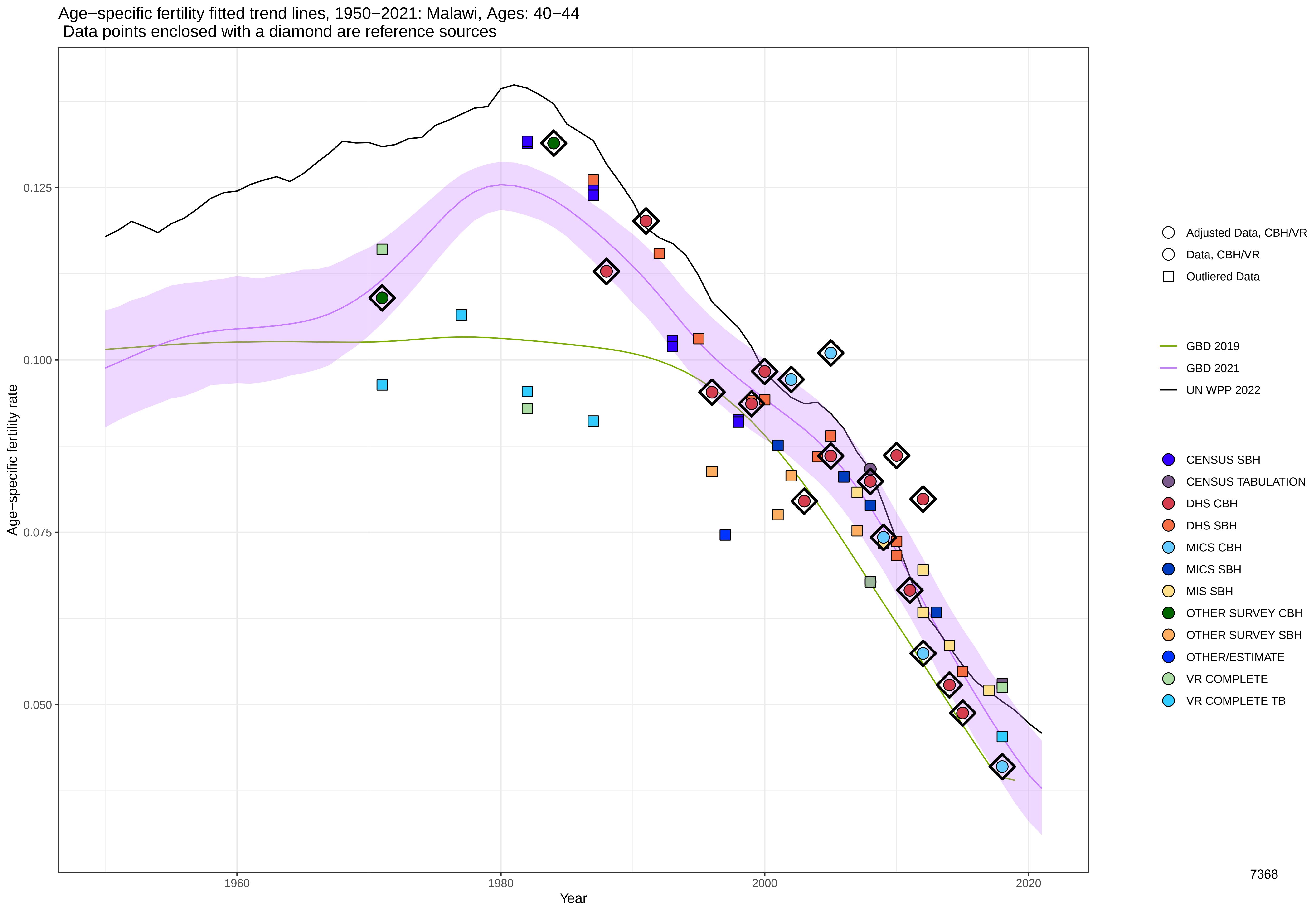

Age−specific fertility fitted trend lines, 1950−2021: Malawi, Ages: 40−44
Data points enclosed with a diamond are reference sources
Age−specific fertility rate
0.125
0.100
0.075
0.050
Year
1960
1980
2000
2020
Adjusted Data, CBH/VR
Data, CBH/VR
Outliered Data
GBD 2019
GBD 2021
UN WPP 2022
CENSUS SBH
CENSUS TABULATION
DHS CBH
DHS SBH
MICS CBH
MICS SBH
MIS SBH
OTHER SURVEY CBH
OTHER SURVEY SBH
OTHER/ESTIMATE
VR COMPLETE
VR COMPLETE TB

Age−specific fertility fitted trend lines, 1950−2021: Malawi, Ages: 45−49
Data points enclosed with a diamond are reference sources

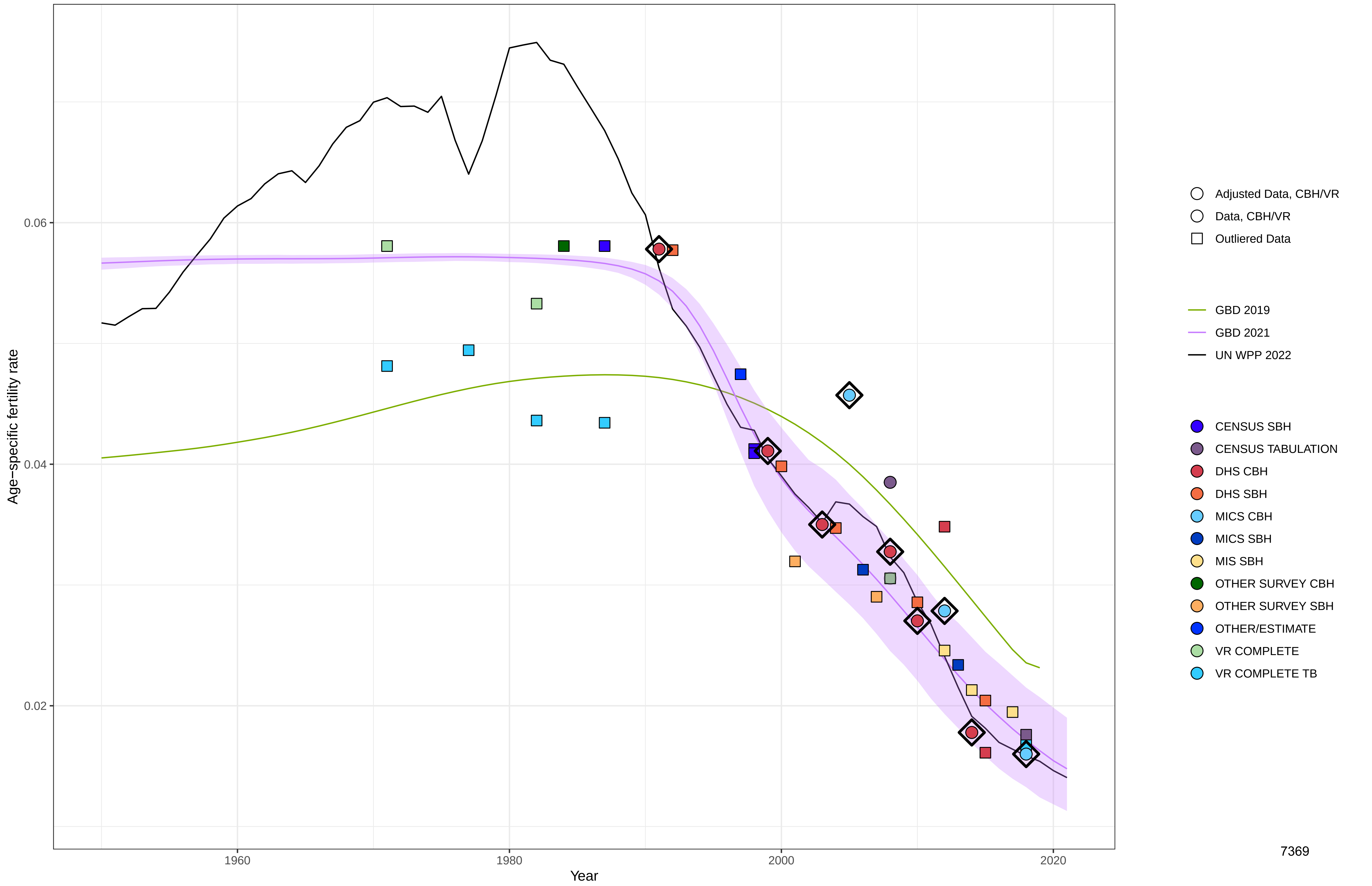

Age−specific fertility fitted trend lines, 1950−2021: Malawi, Ages: 50−54
Data points enclosed with a diamond are reference sources

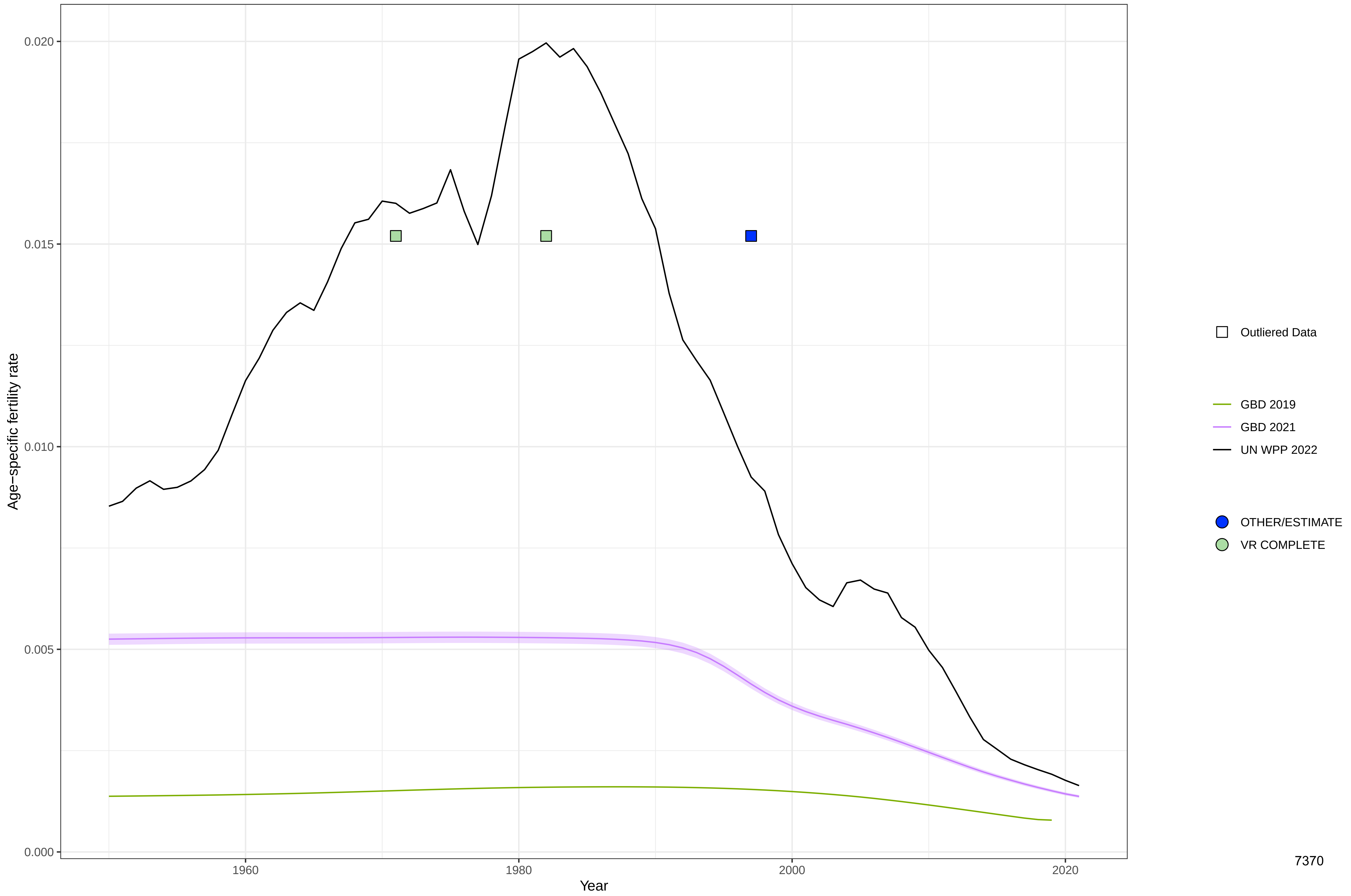

Total fertility rate fitted trend lines, 1950−2021: Malawi TFR
Data points enclosed with a diamond are reference sources

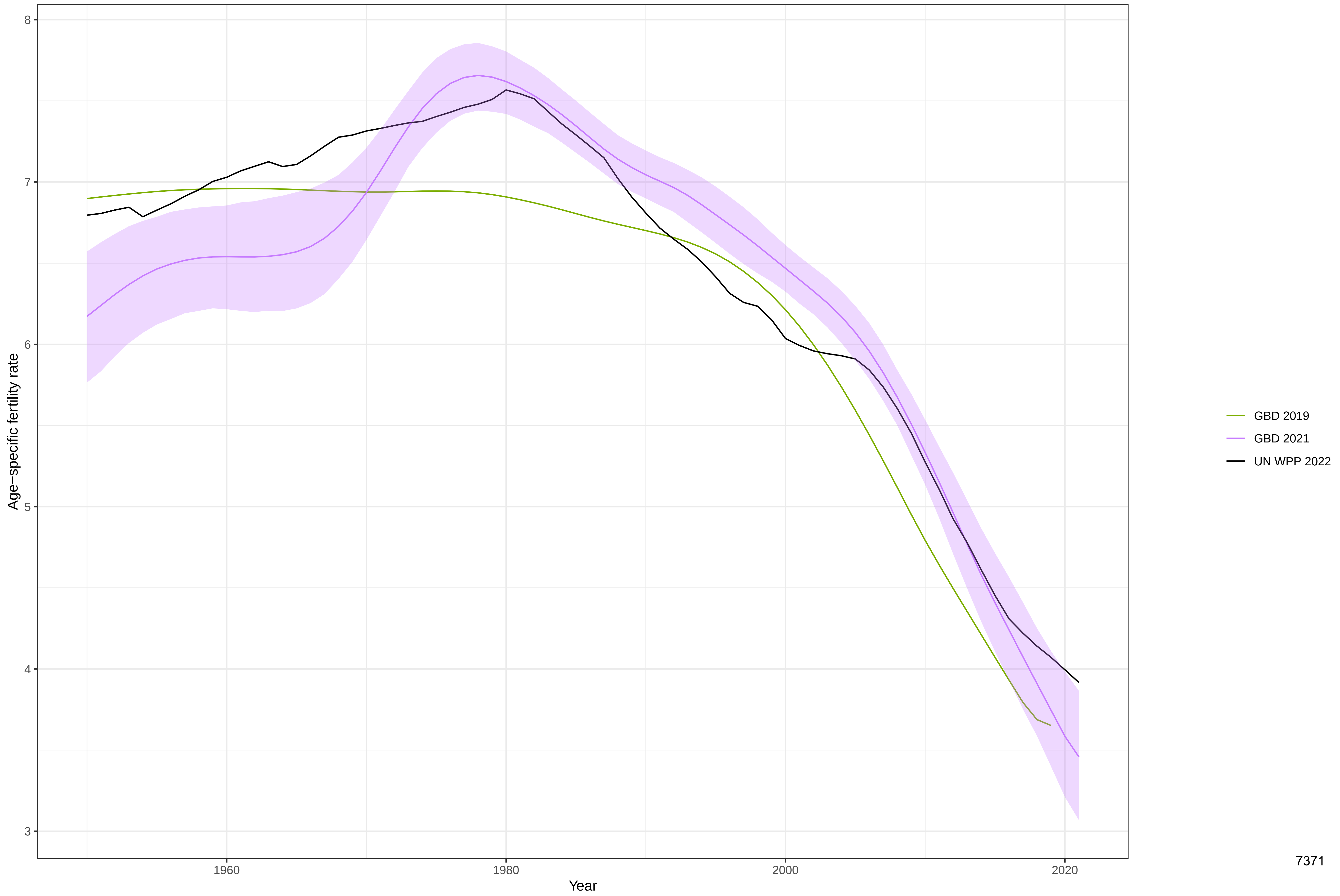

Age−specific fertility fitted trend lines, 1950−2021: Mozambique, Ages: 10−14
Data points enclosed with a diamond are reference sources

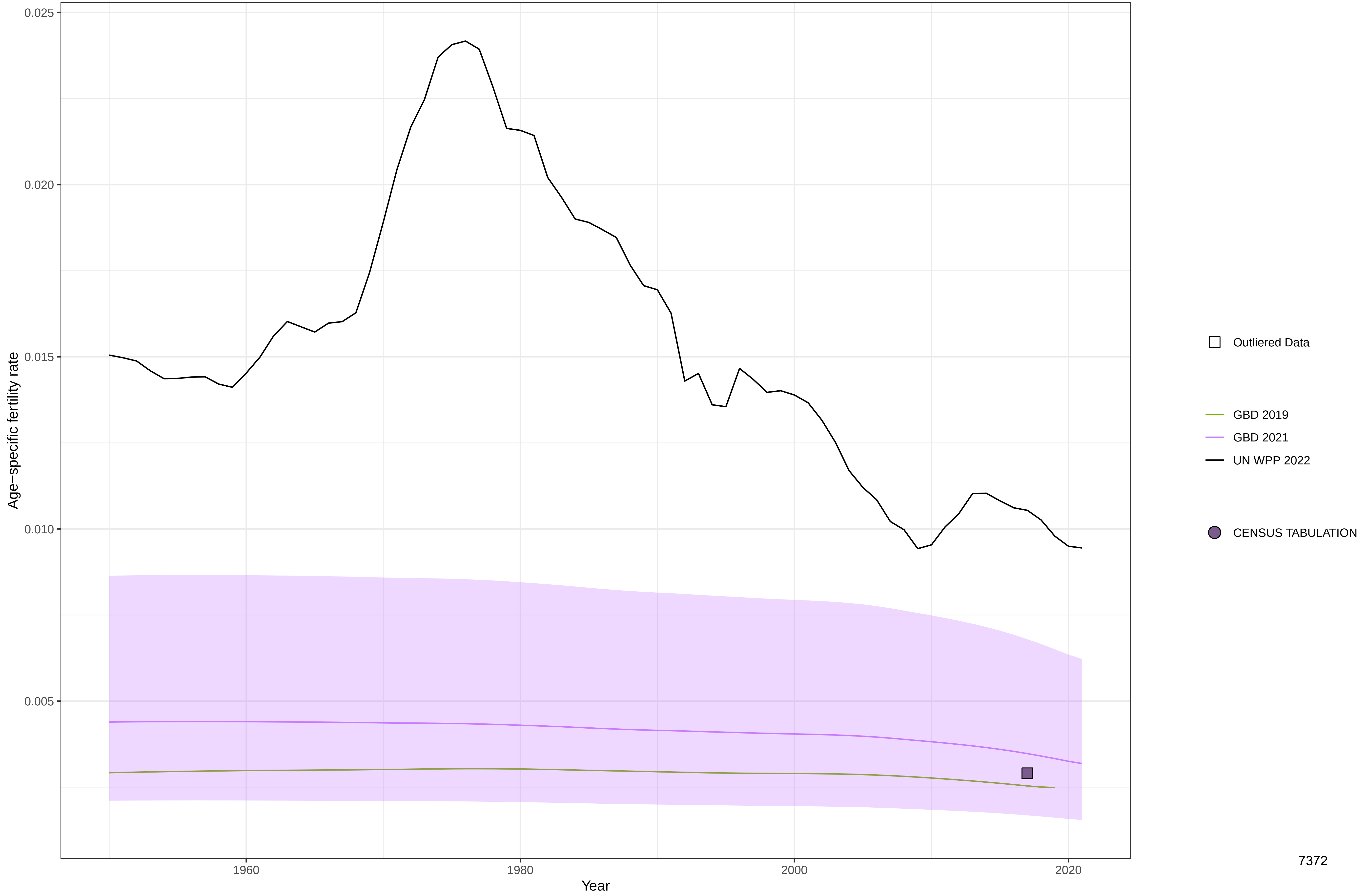

Age−specific fertility fitted trend lines, 1950−2021: Mozambique, Ages: 15−19
Data points enclosed with a diamond are reference sources

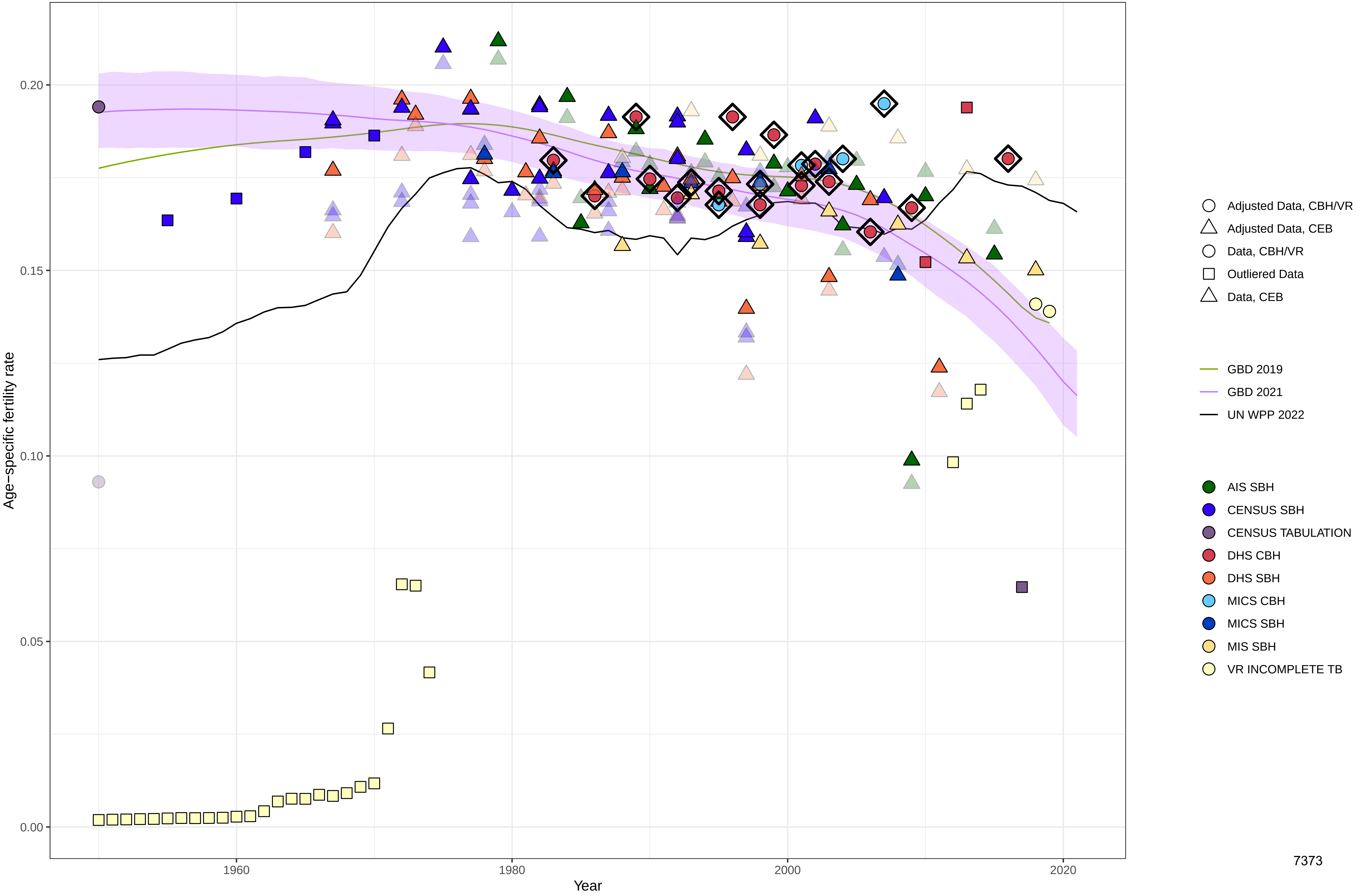

Age−specific fertility fitted trend lines, 1950−2021: Mozambique, Ages: 20−24
Data points enclosed with a diamond are reference sources

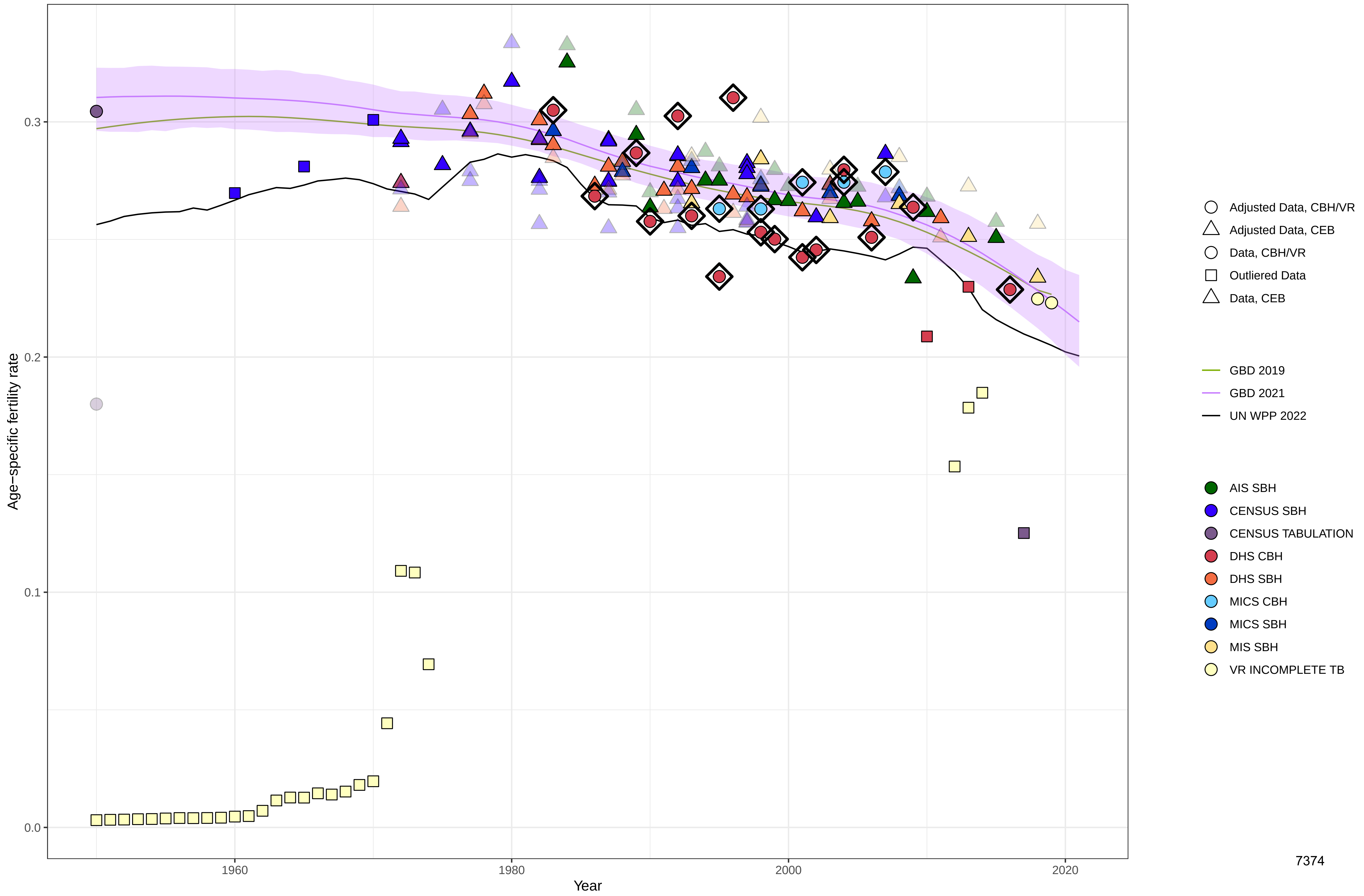

Age−specific fertility fitted trend lines, 1950−2021: Mozambique, Ages: 25−29
Data points enclosed with a diamond are reference sources

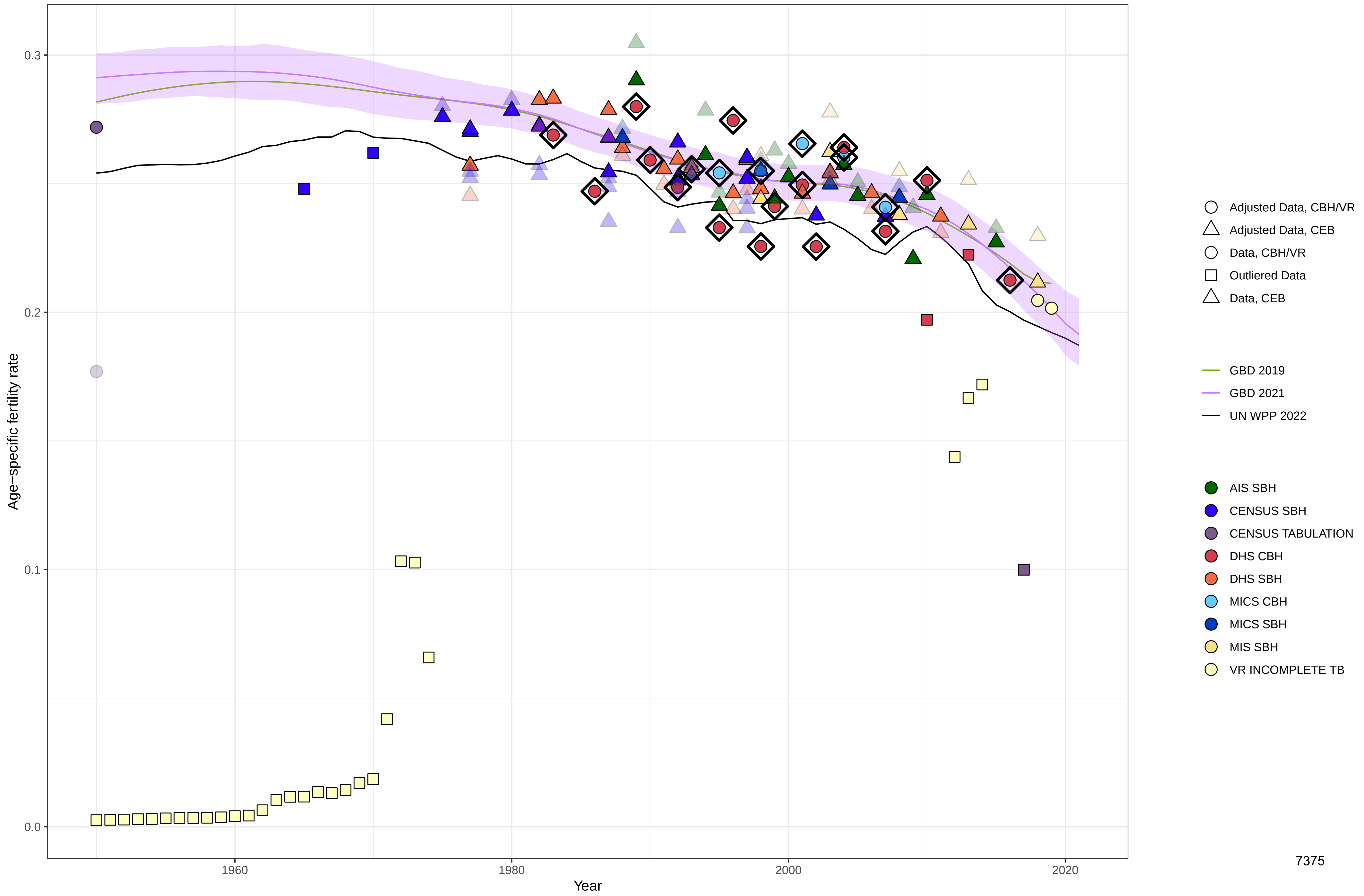

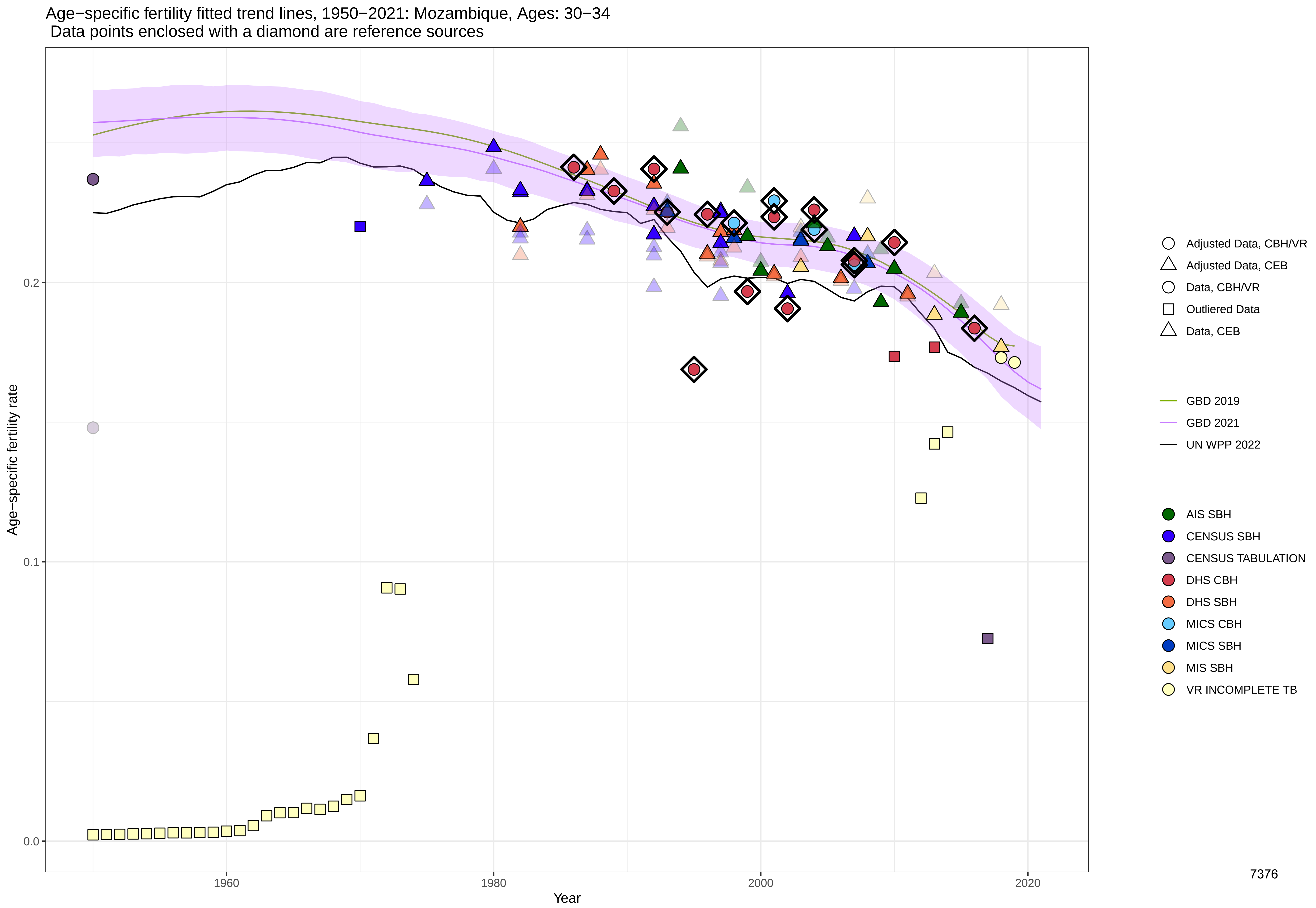

Age−specific fertility fitted trend lines, 1950−2021: Mozambique, Ages: 30−34
Data points enclosed with a diamond are reference sources
Age−specific fertility rate
Year
0.0
0.1
0.2
1960
1980
2000
2020
Adjusted Data, CBH/VR
Adjusted Data, CEB
Data, CBH/VR
Outliered Data
Data, CEB
GBD 2019
GBD 2021
UN WPP 2022
AIS SBH
CENSUS SBH
CENSUS TABULATION
DHS CBH
DHS SBH
MICS CBH
MICS SBH
MIS SBH
VR INCOMPLETE TB

Age−specific fertility fitted trend lines, 1950−2021: Mozambique, Ages: 35−39
Data points enclosed with a diamond are reference sources

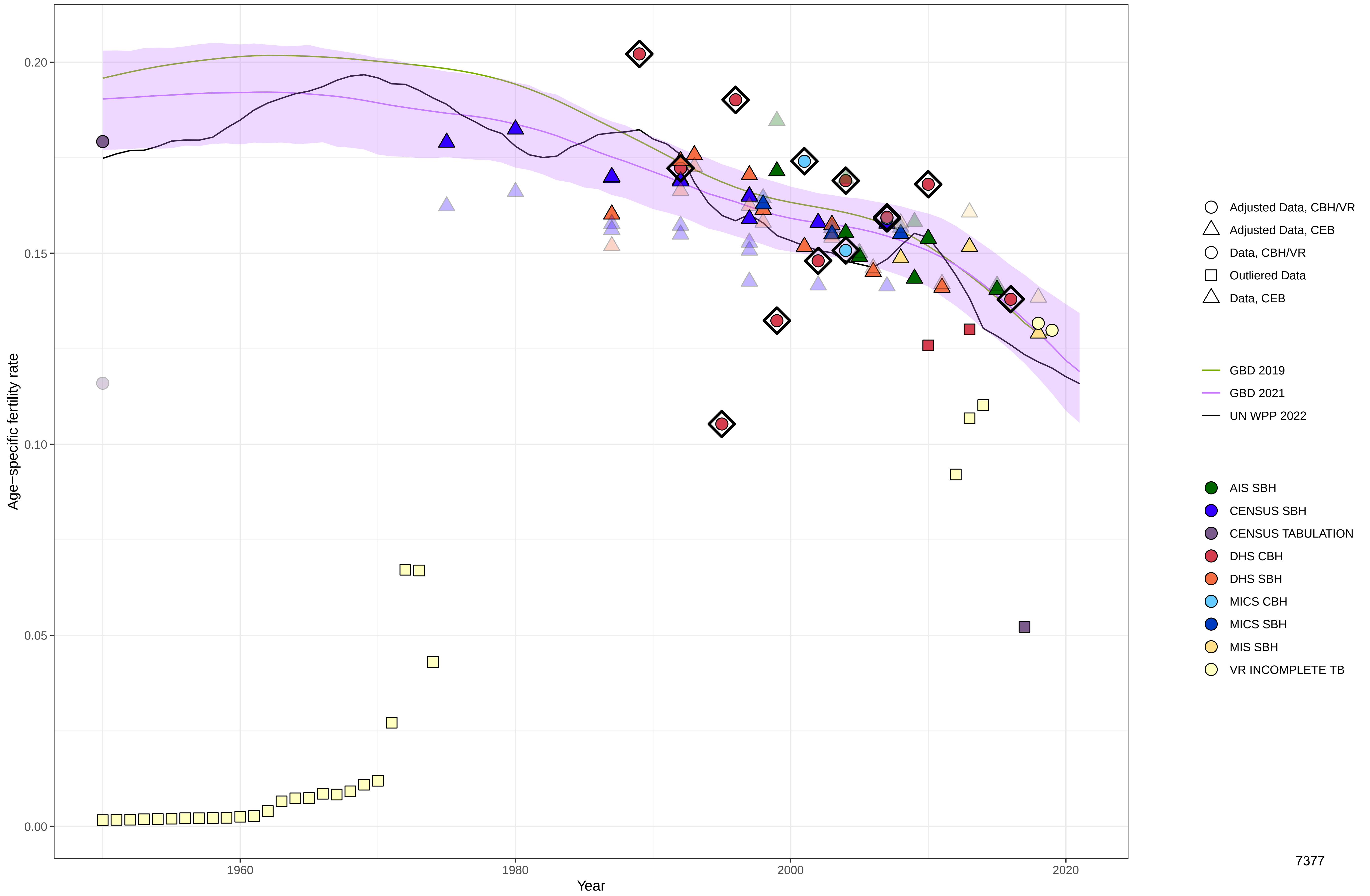

Age−specific fertility fitted trend lines, 1950−2021: Mozambique, Ages: 40−44
Data points enclosed with a diamond are reference sources

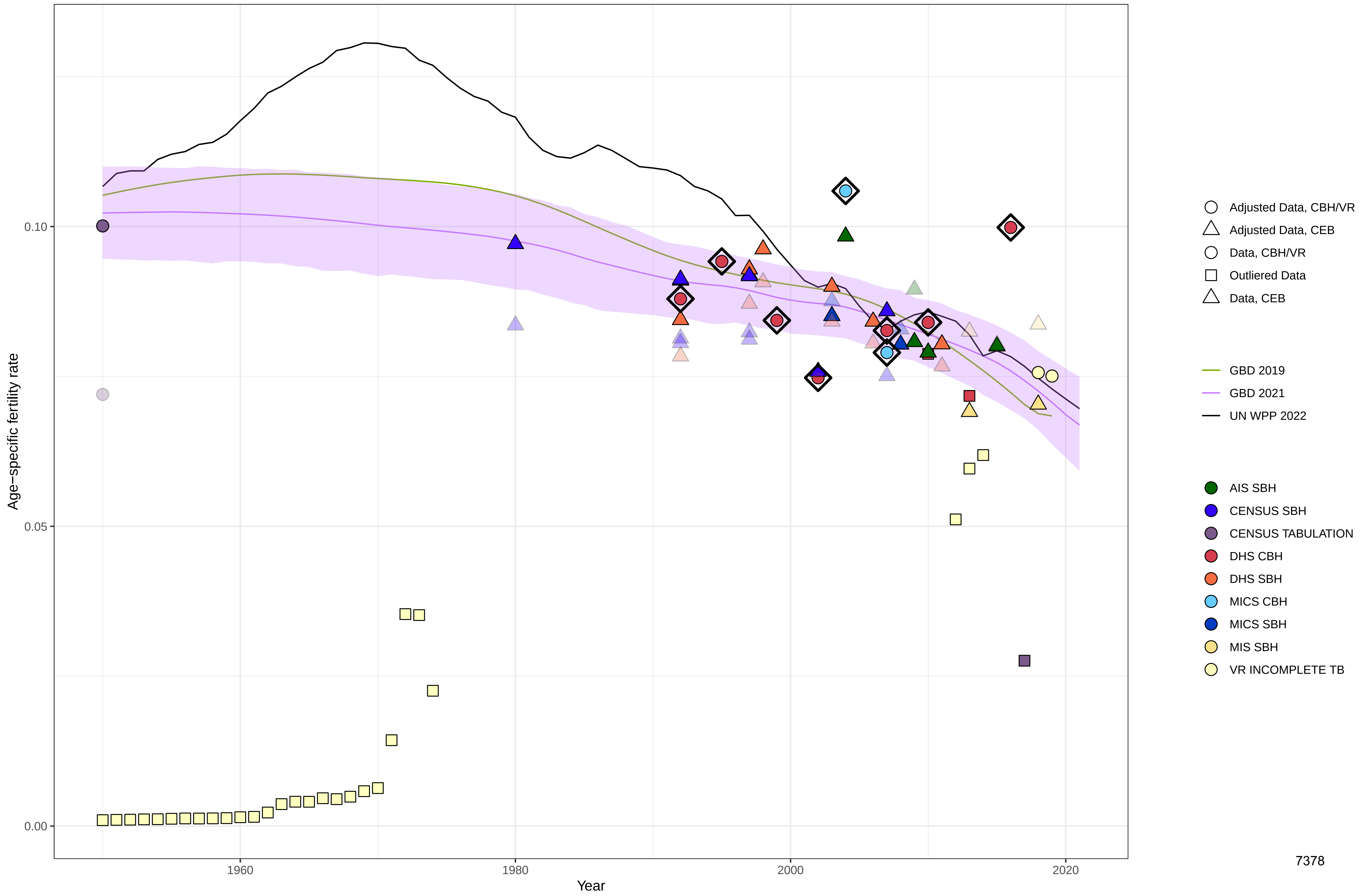

Age−specific fertility fitted trend lines, 1950−2021: Mozambique, Ages: 45−49
Data points enclosed with a diamond are reference sources

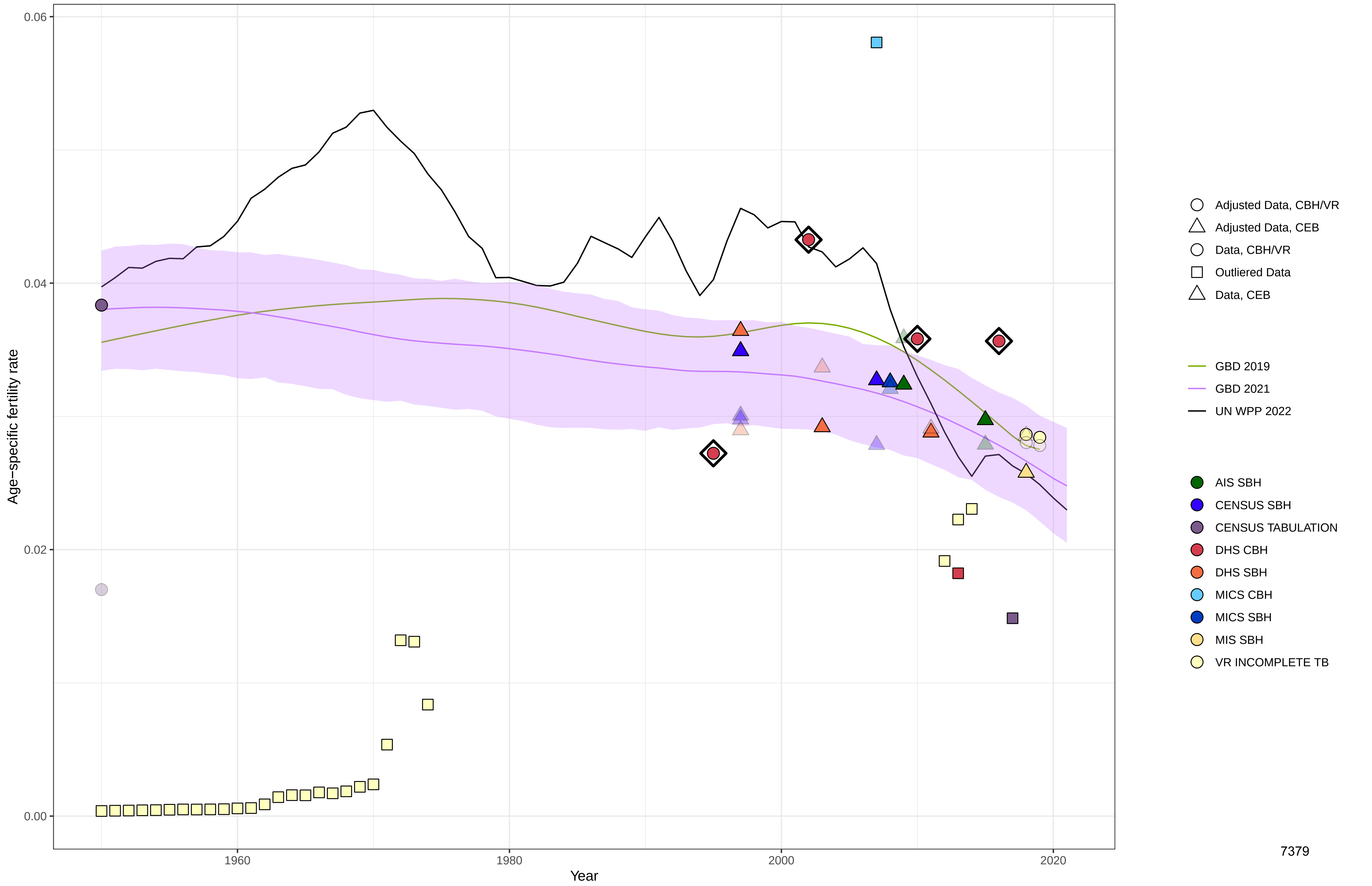

Age−specific fertility fitted trend lines, 1950−2021: Mozambique, Ages: 50−54
Data points enclosed with a diamond are reference sources

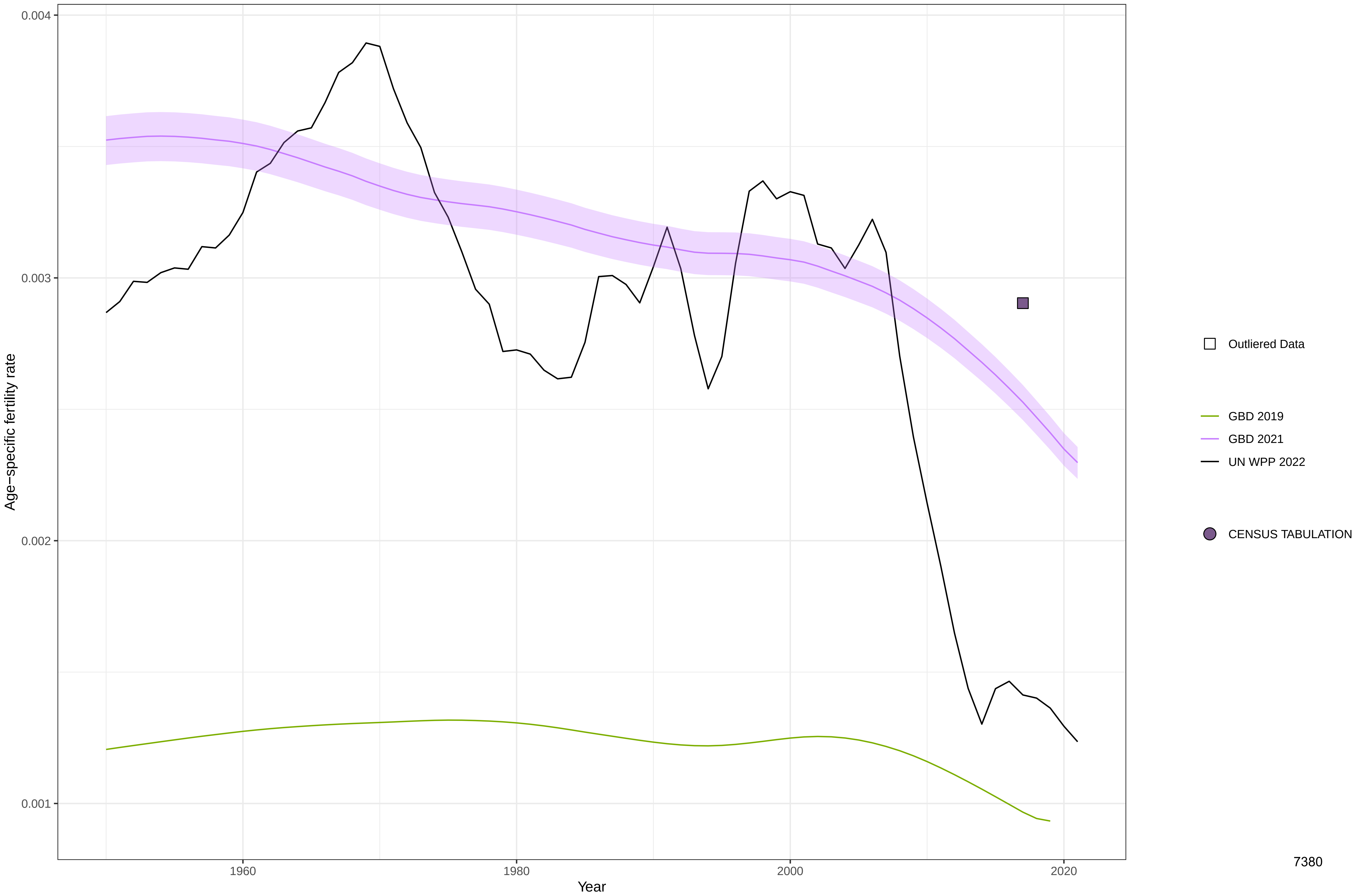

Total fertility rate fitted trend lines, 1950−2021: Mozambique TFR
Data points enclosed with a diamond are reference sources

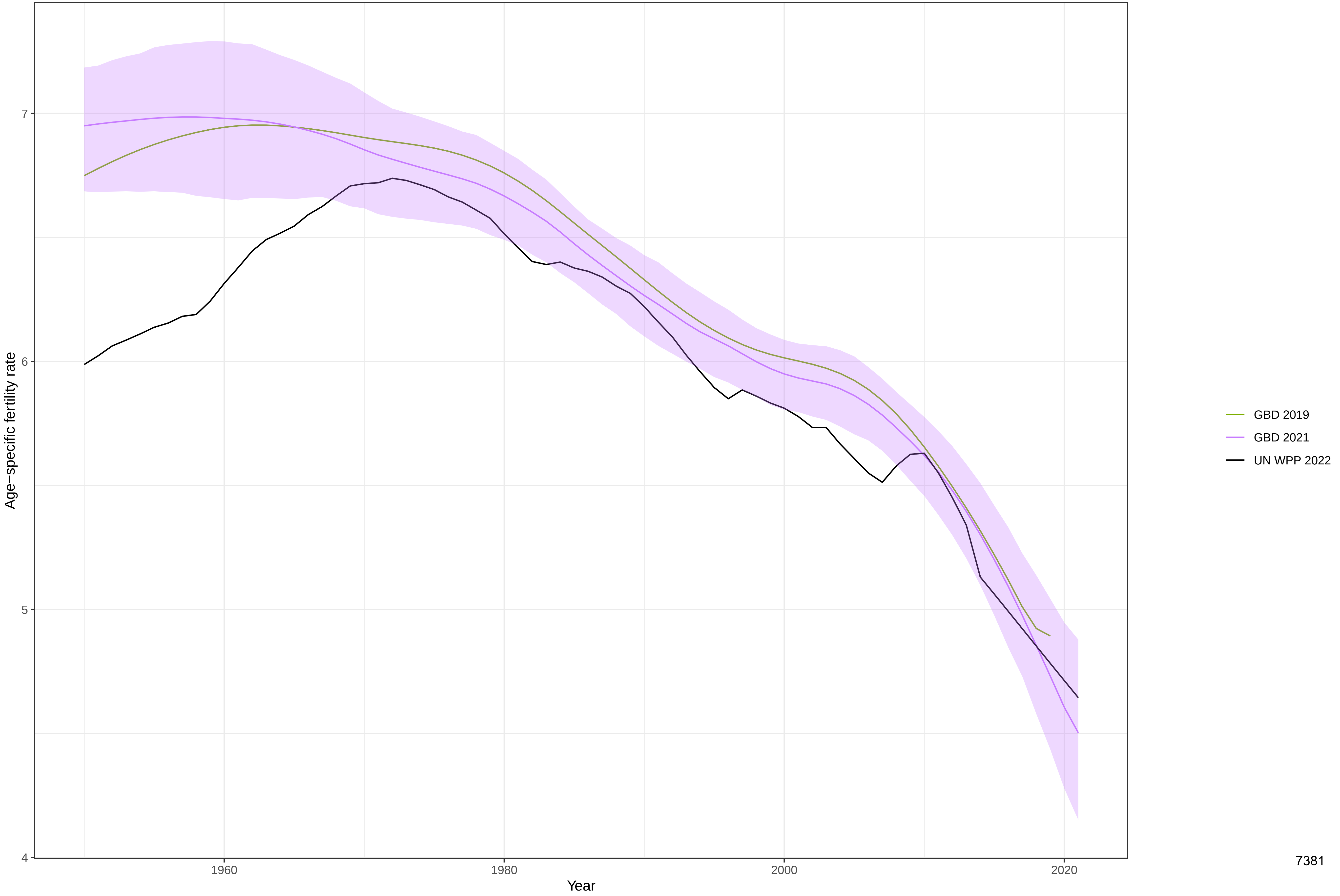

Age−specific fertility fitted trend lines, 1950−2021: Rwanda, Ages: 10−14
Data points enclosed with a diamond are reference sources

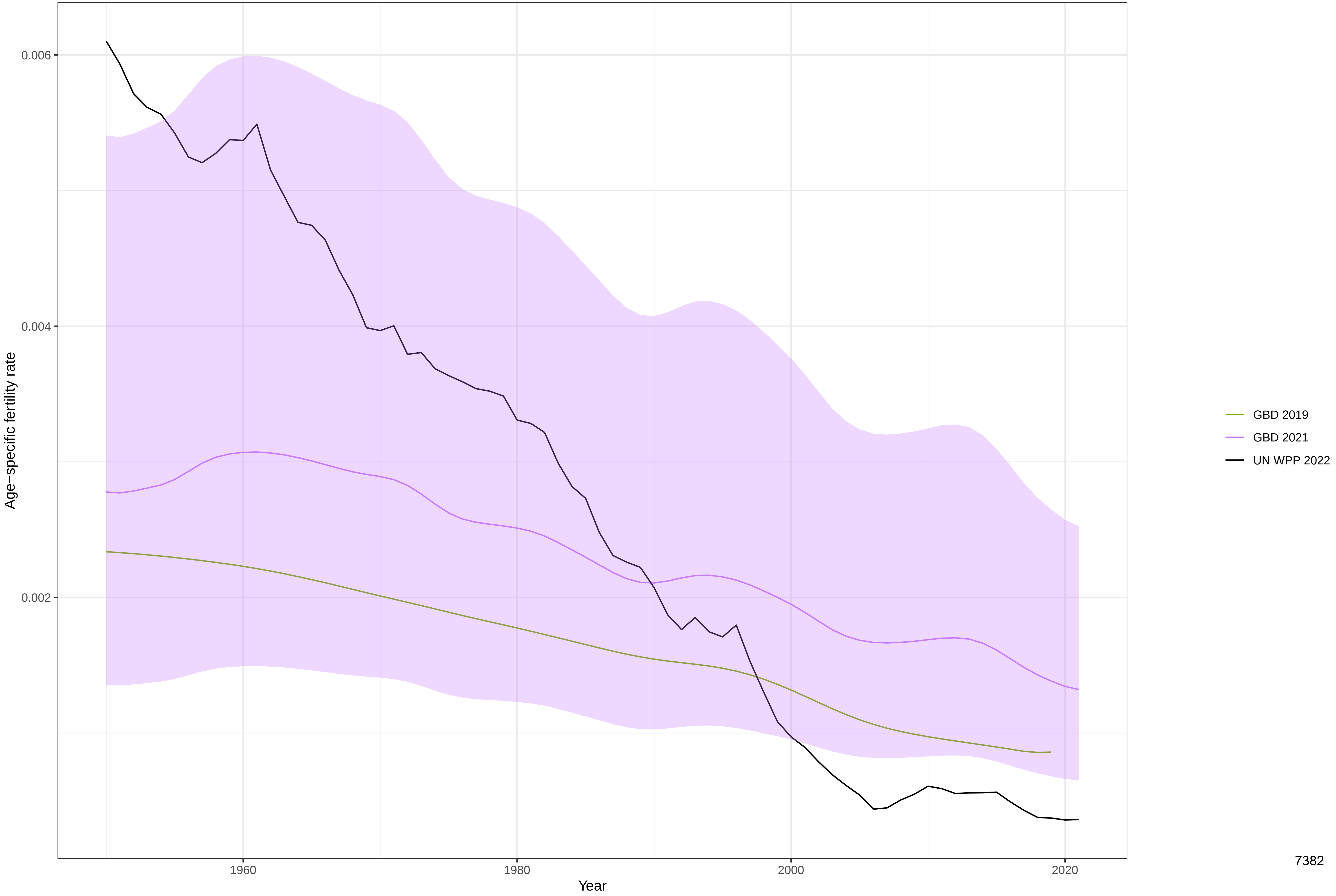

Age−specific fertility fitted trend lines, 1950−2021: Rwanda, Ages: 15−19
Data points enclosed with a diamond are reference sources

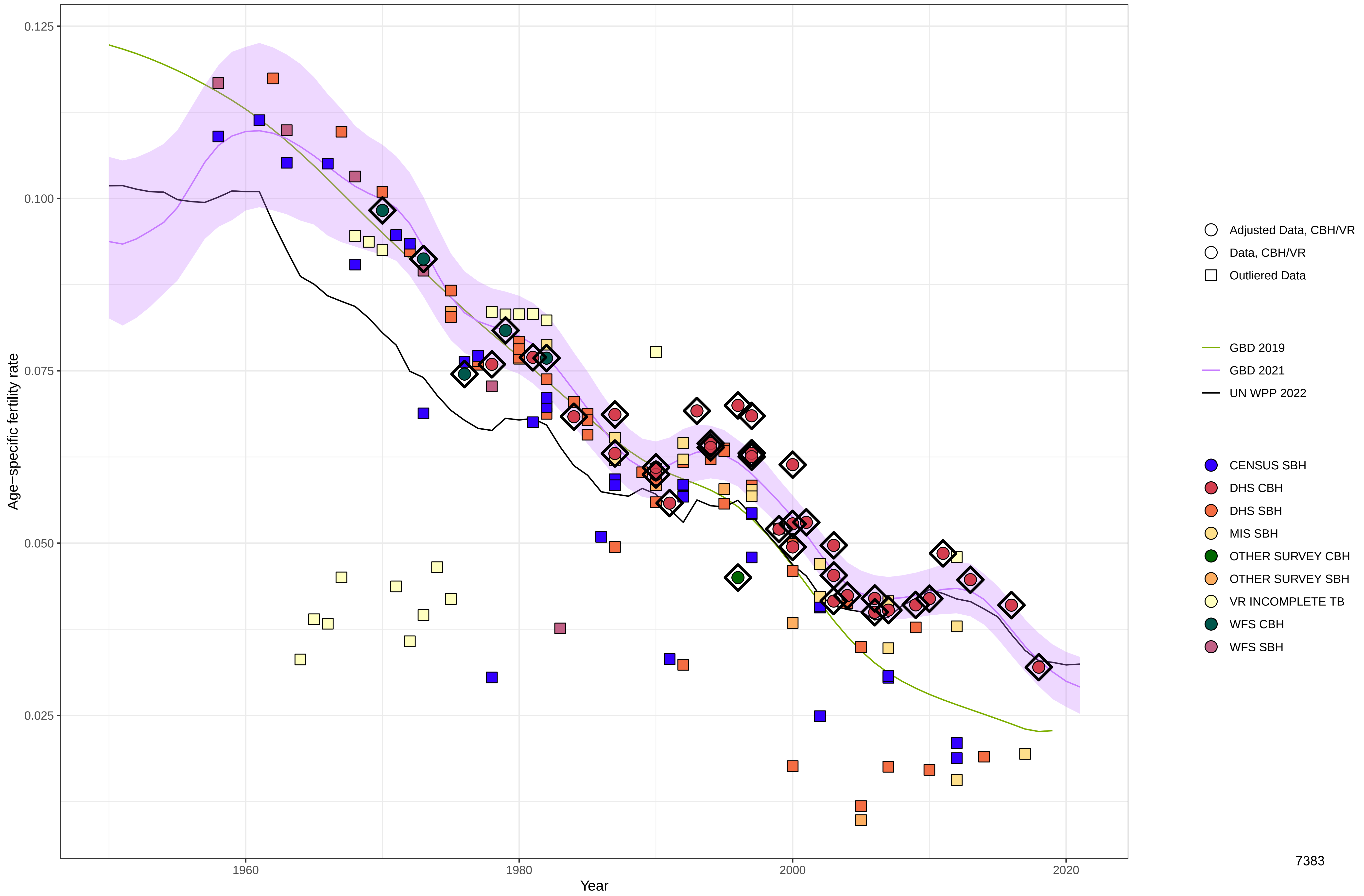

7383

Age−specific fertility fitted trend lines, 1950−2021: Rwanda, Ages: 20−24
Data points enclosed with a diamond are reference sources

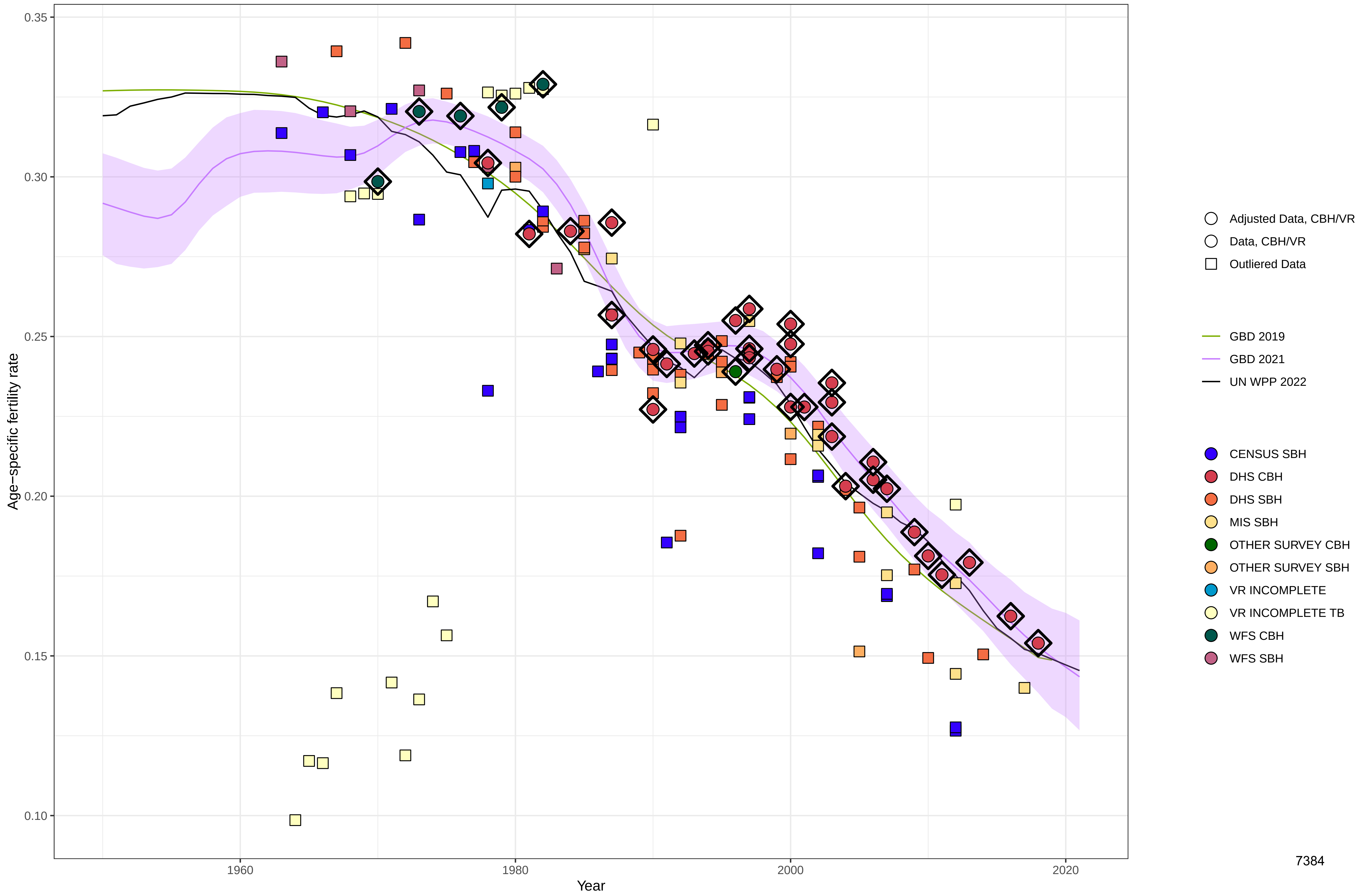

Age−specific fertility fitted trend lines, 1950−2021: Rwanda, Ages: 25−29
Data points enclosed with a diamond are reference sources

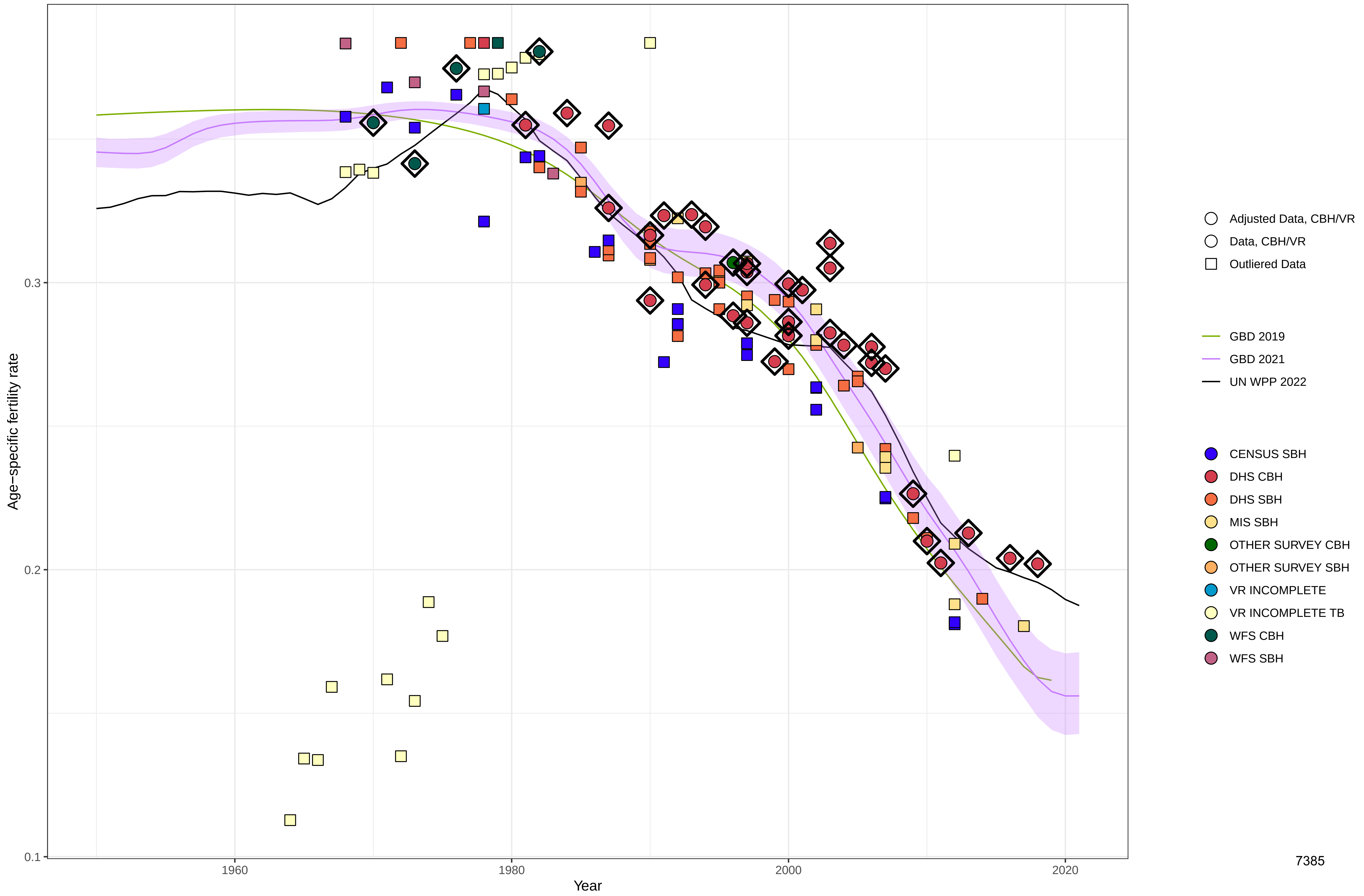

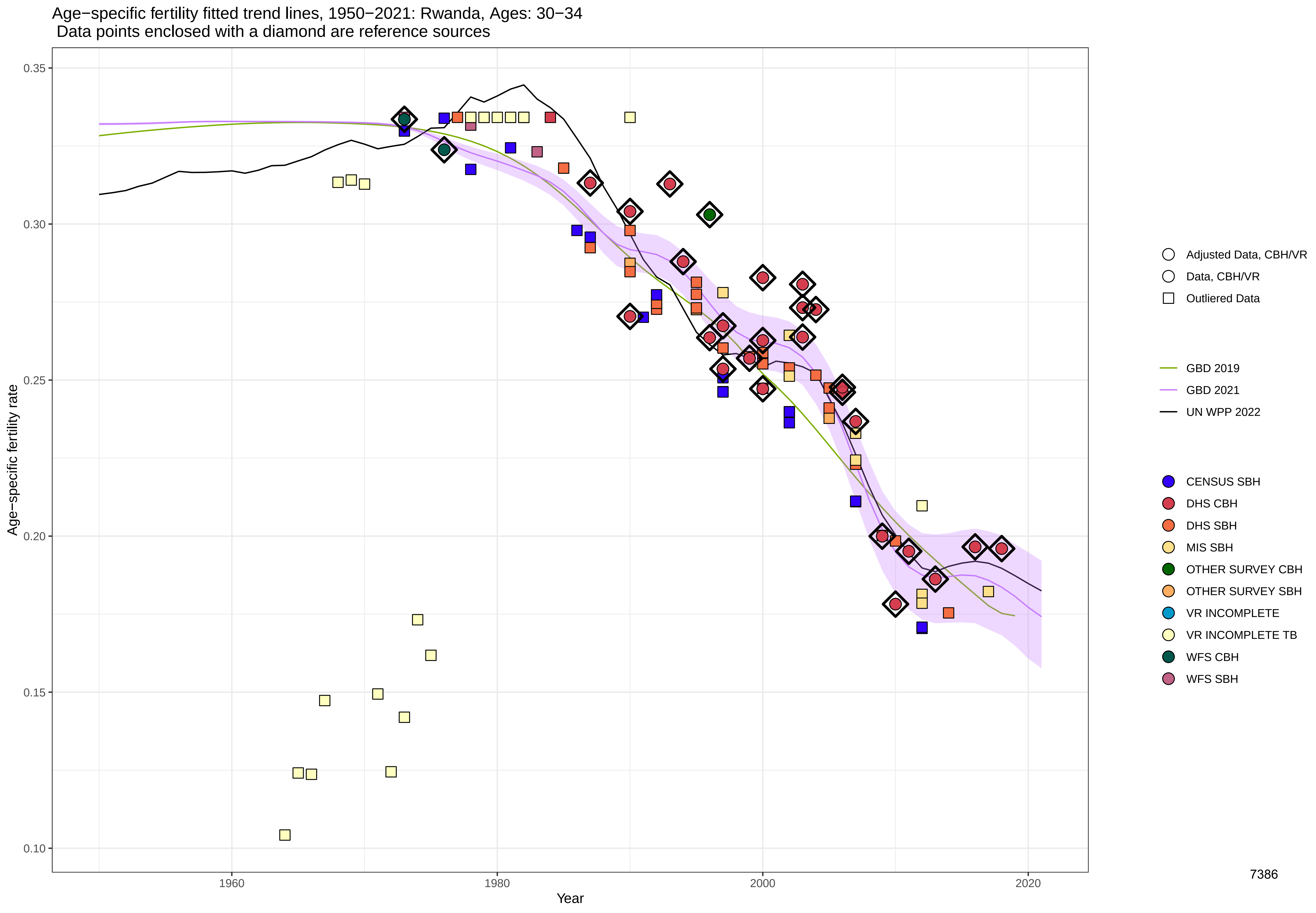

Age−specific fertility fitted trend lines, 1950−2021: Rwanda, Ages: 30−34
Data points enclosed with a diamond are reference sources
Age−specific fertility rate
Year
0.35
0.30
0.25
0.20
0.15
0.10
1960
1980
2000
2020
Adjusted Data, CBH/VR
Data, CBH/VR
Outliered Data
GBD 2019
GBD 2021
UN WPP 2022
CENSUS SBH
DHS CBH
DHS SBH
MIS SBH
OTHER SURVEY CBH
OTHER SURVEY SBH
VR INCOMPLETE
VR INCOMPLETE TB
WFS CBH
WFS SBH

Age−specific fertility fitted trend lines, 1950−2021: Rwanda, Ages: 35−39
Data points enclosed with a diamond are reference sources

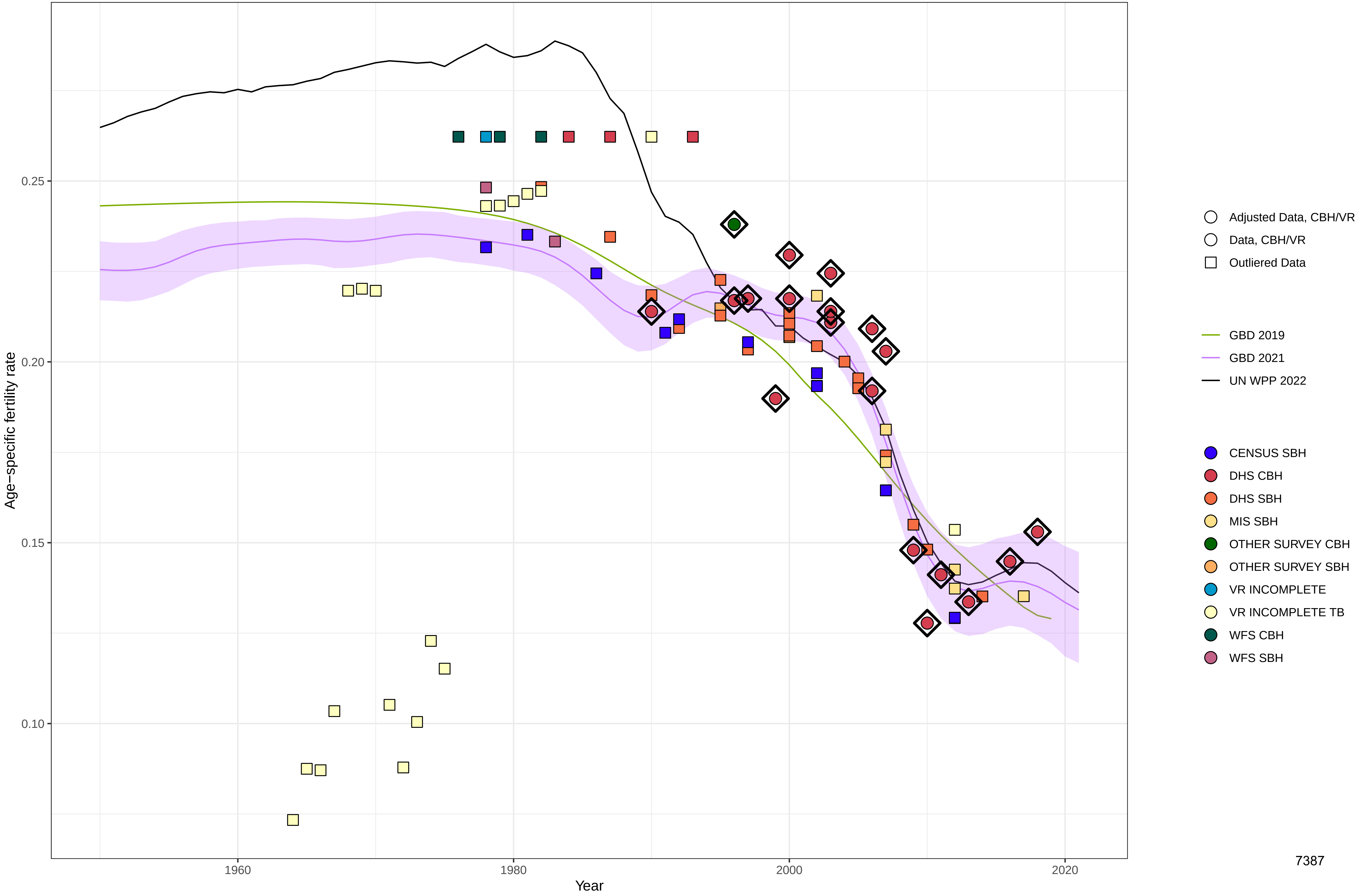

Age−specific fertility fitted trend lines, 1950−2021: Rwanda, Ages: 40−44
Data points enclosed with a diamond are reference sources

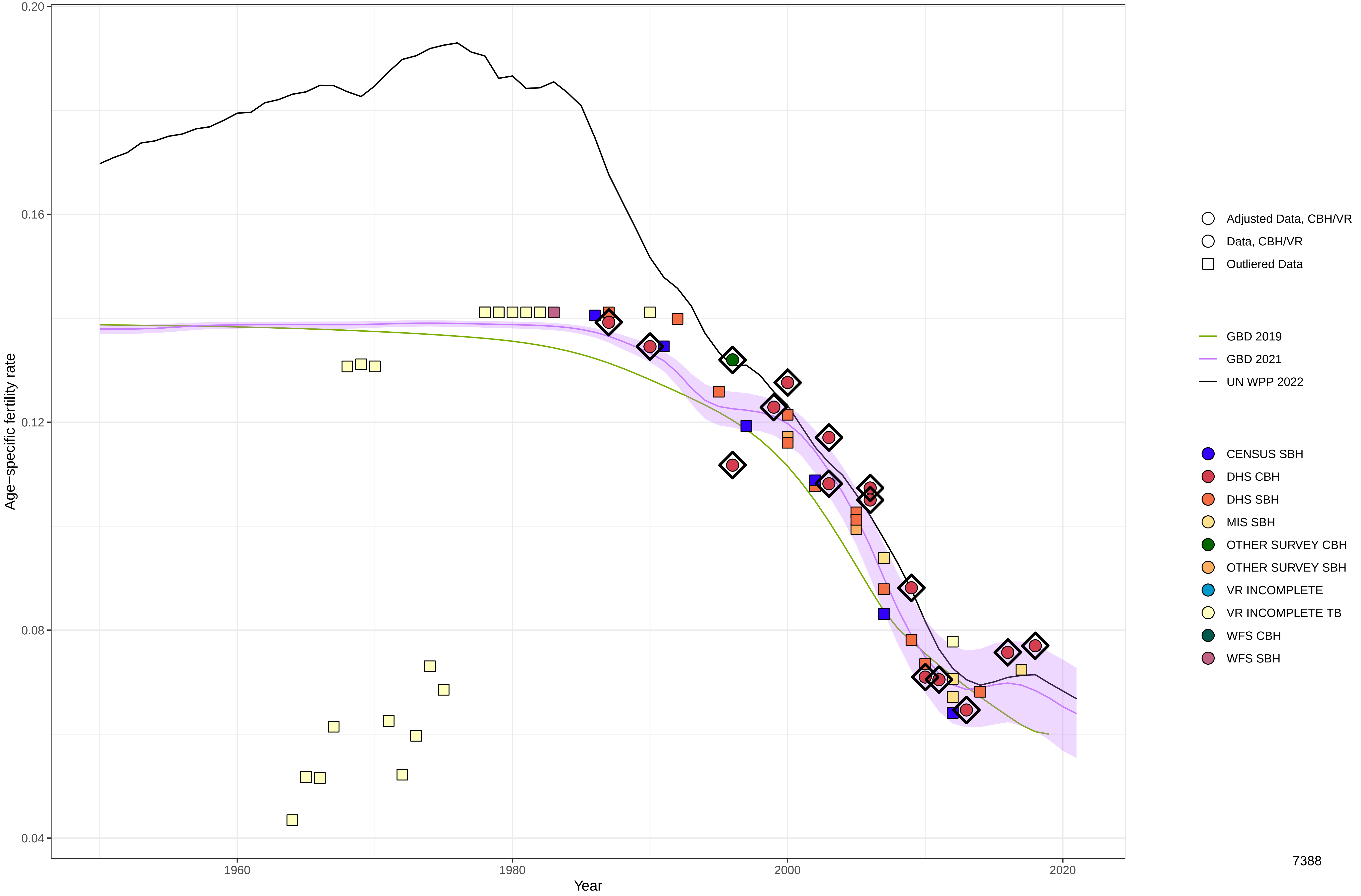

Age−specific fertility fitted trend lines, 1950−2021: Rwanda, Ages: 45−49
Data points enclosed with a diamond are reference sources

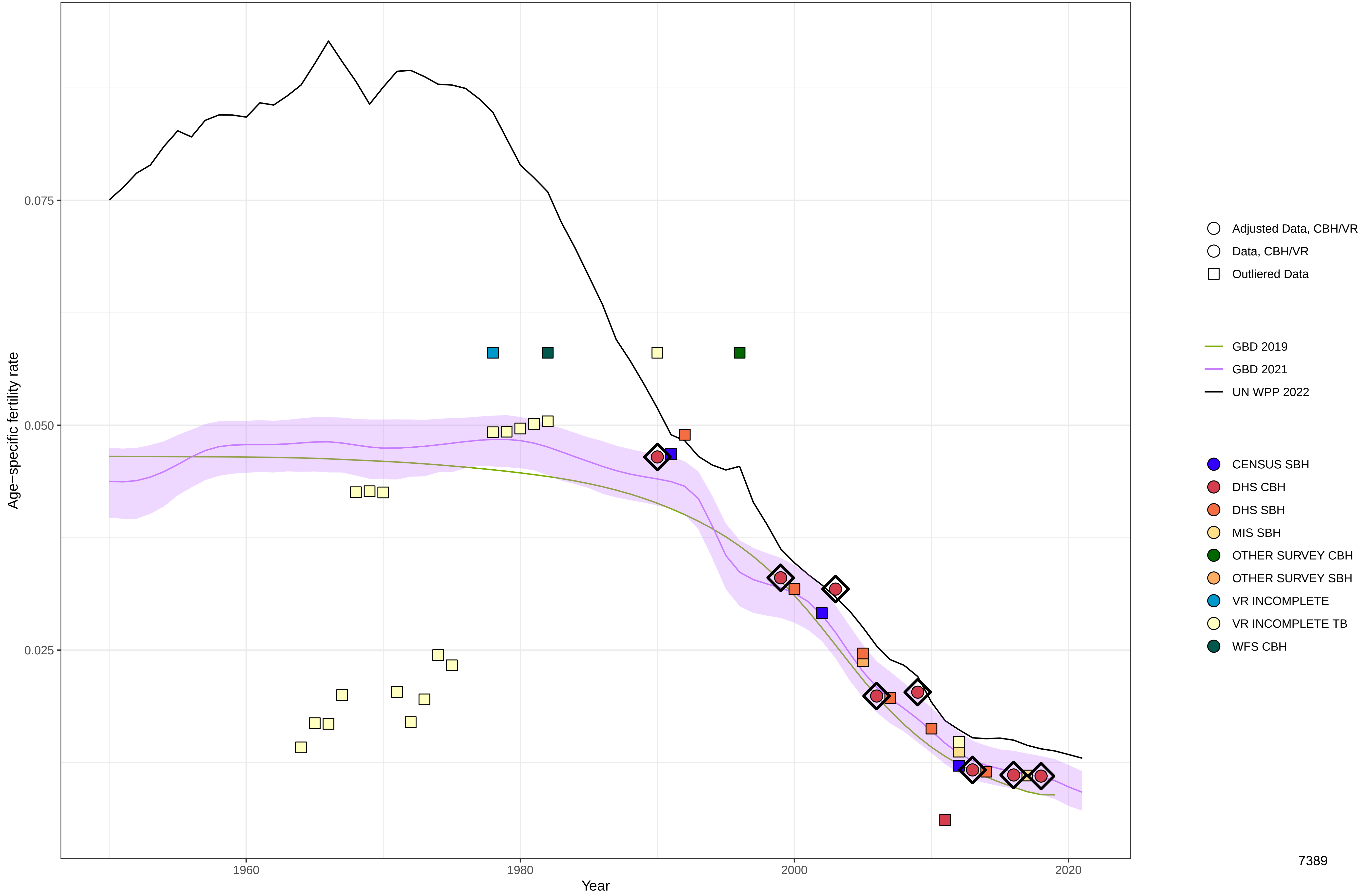

Age−specific fertility fitted trend lines, 1950−2021: Rwanda, Ages: 50−54
Data points enclosed with a diamond are reference sources

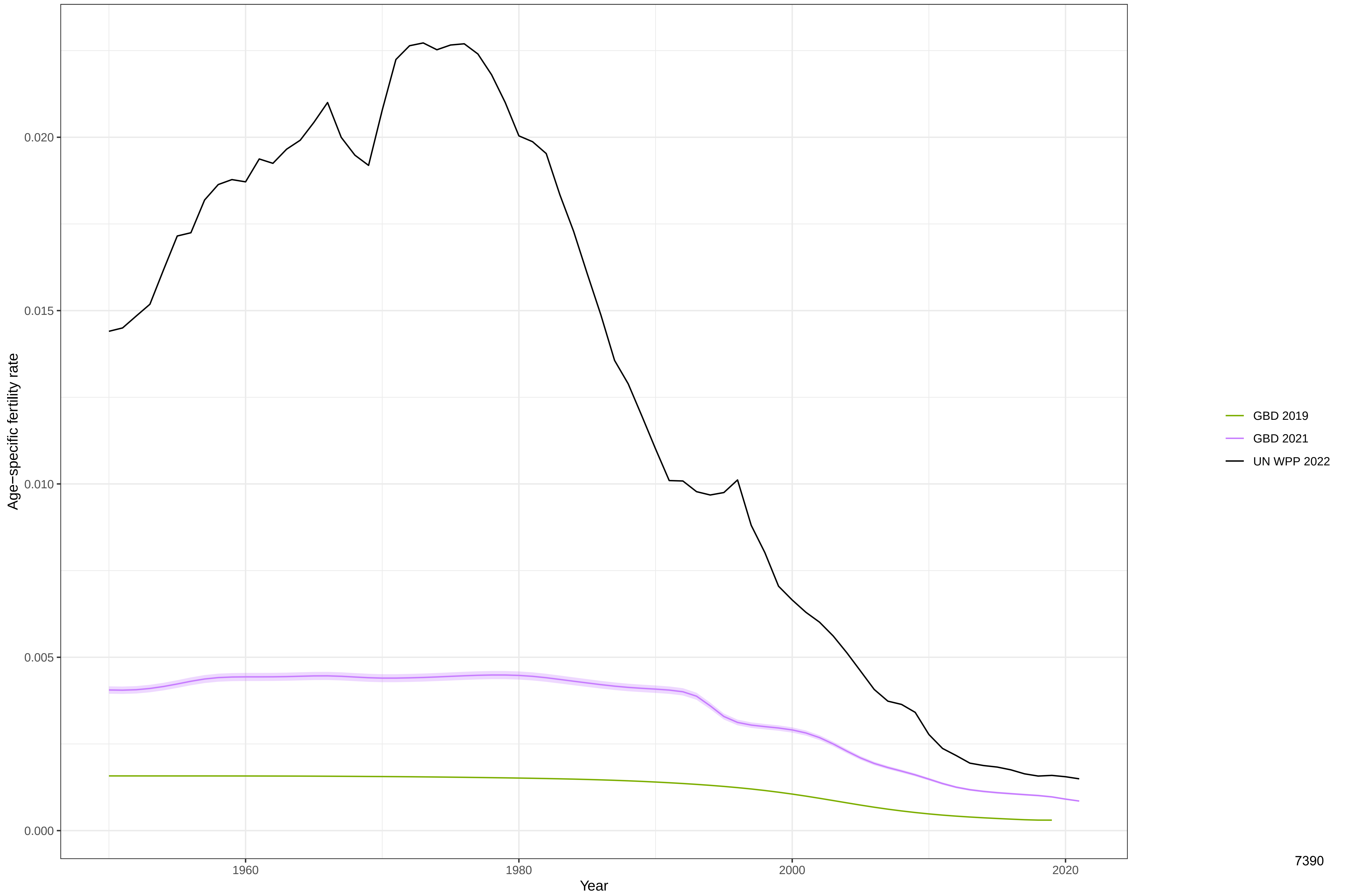

Total fertility rate fitted trend lines, 1950−2021: Rwanda TFR
Data points enclosed with a diamond are reference sources

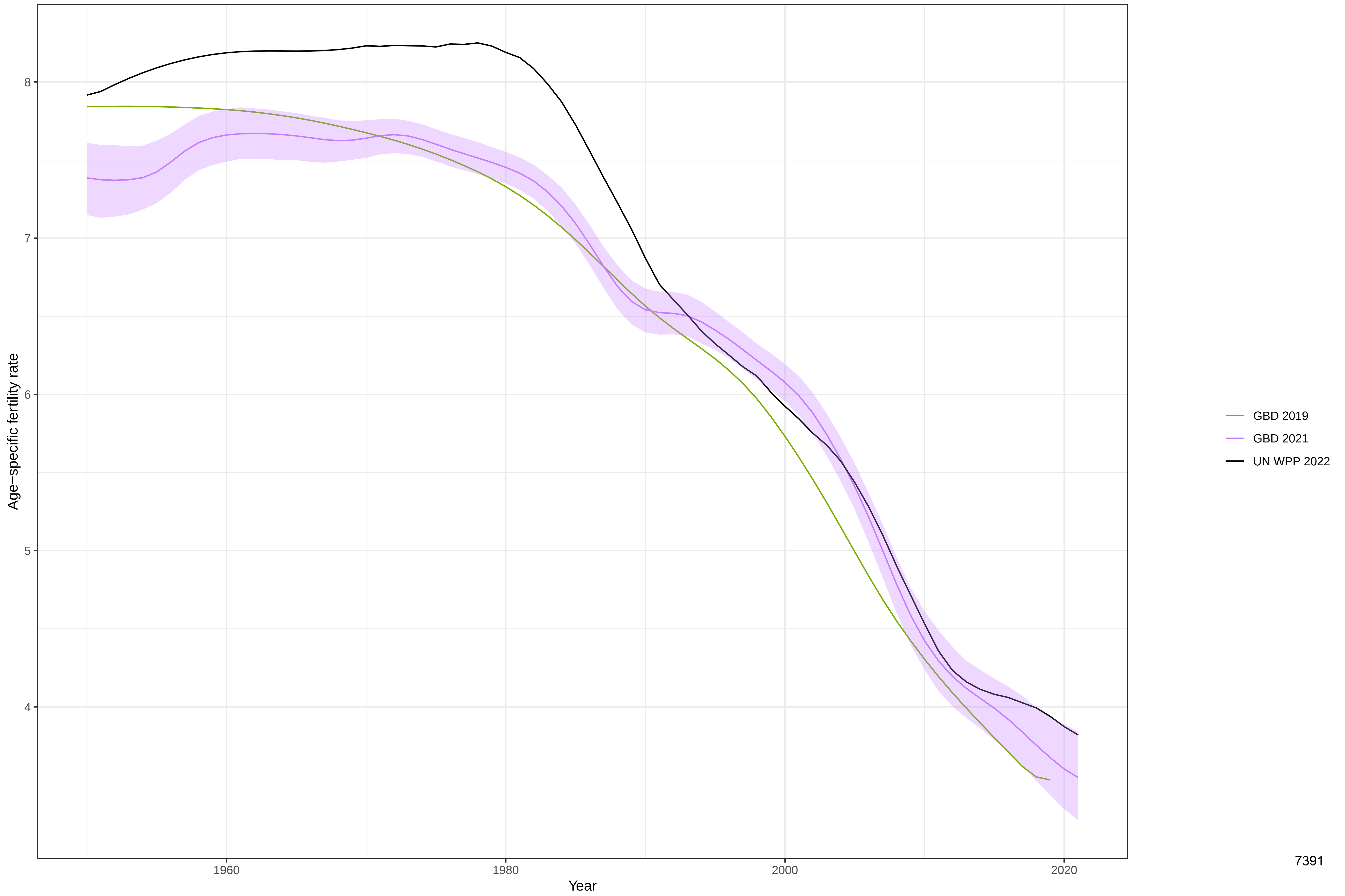

Age−specific fertility fitted trend lines, 1950−2021: Somalia, Ages: 10−14
Data points enclosed with a diamond are reference sources

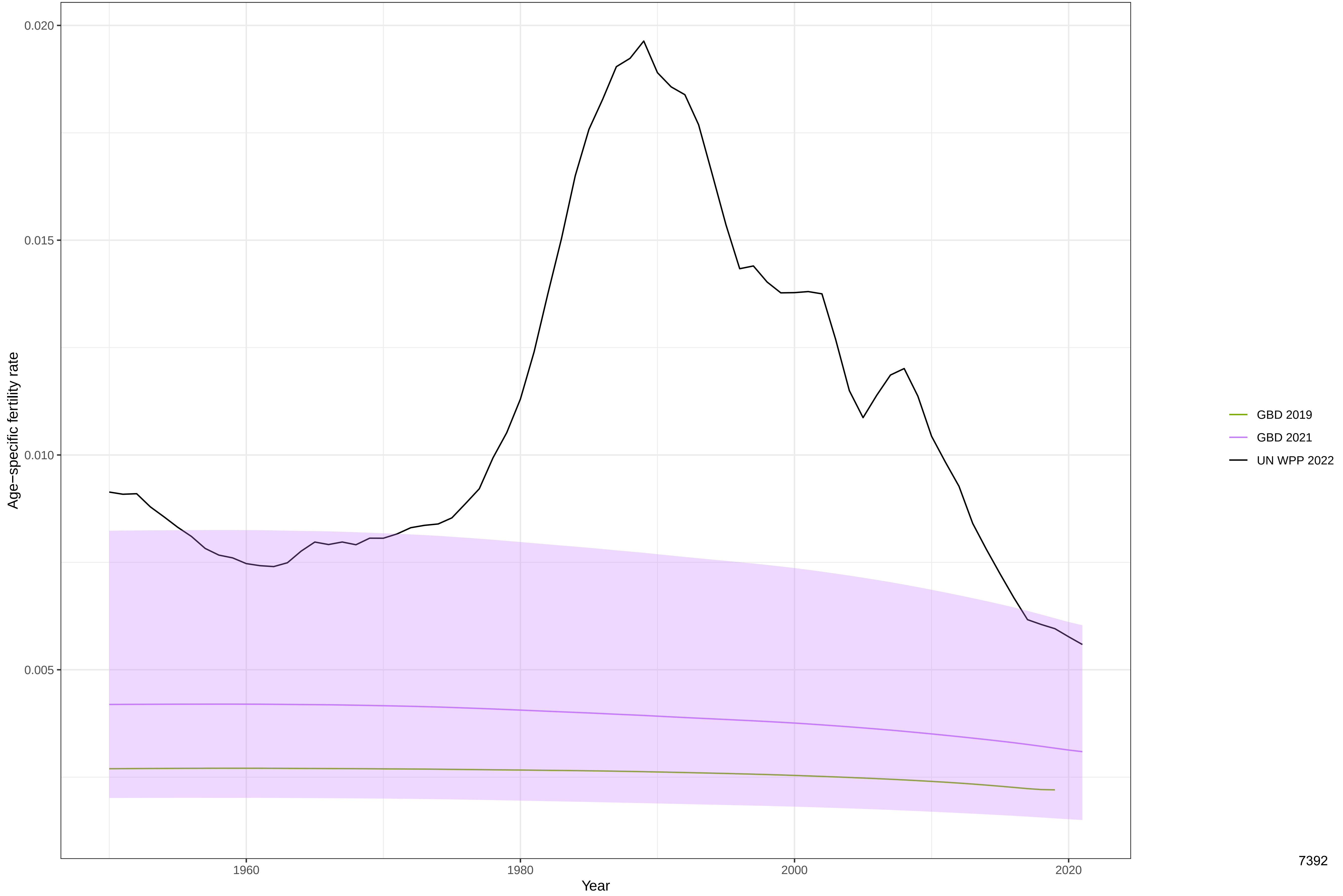

Age−specific fertility fitted trend lines, 1950−2021: Somalia, Ages: 15−19
Data points enclosed with a diamond are reference sources

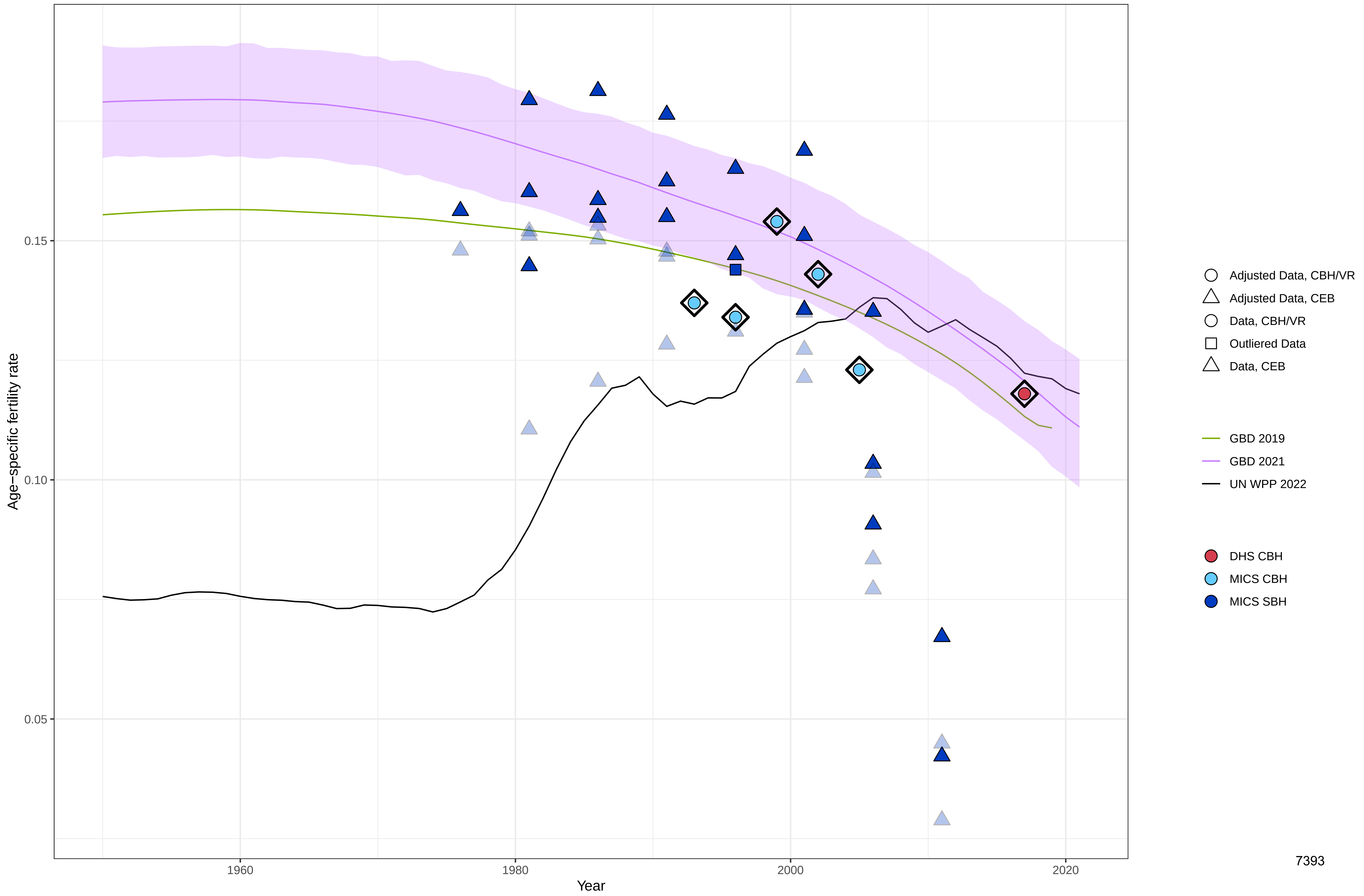

Age−specific fertility fitted trend lines, 1950−2021: Somalia, Ages: 20−24
Data points enclosed with a diamond are reference sources

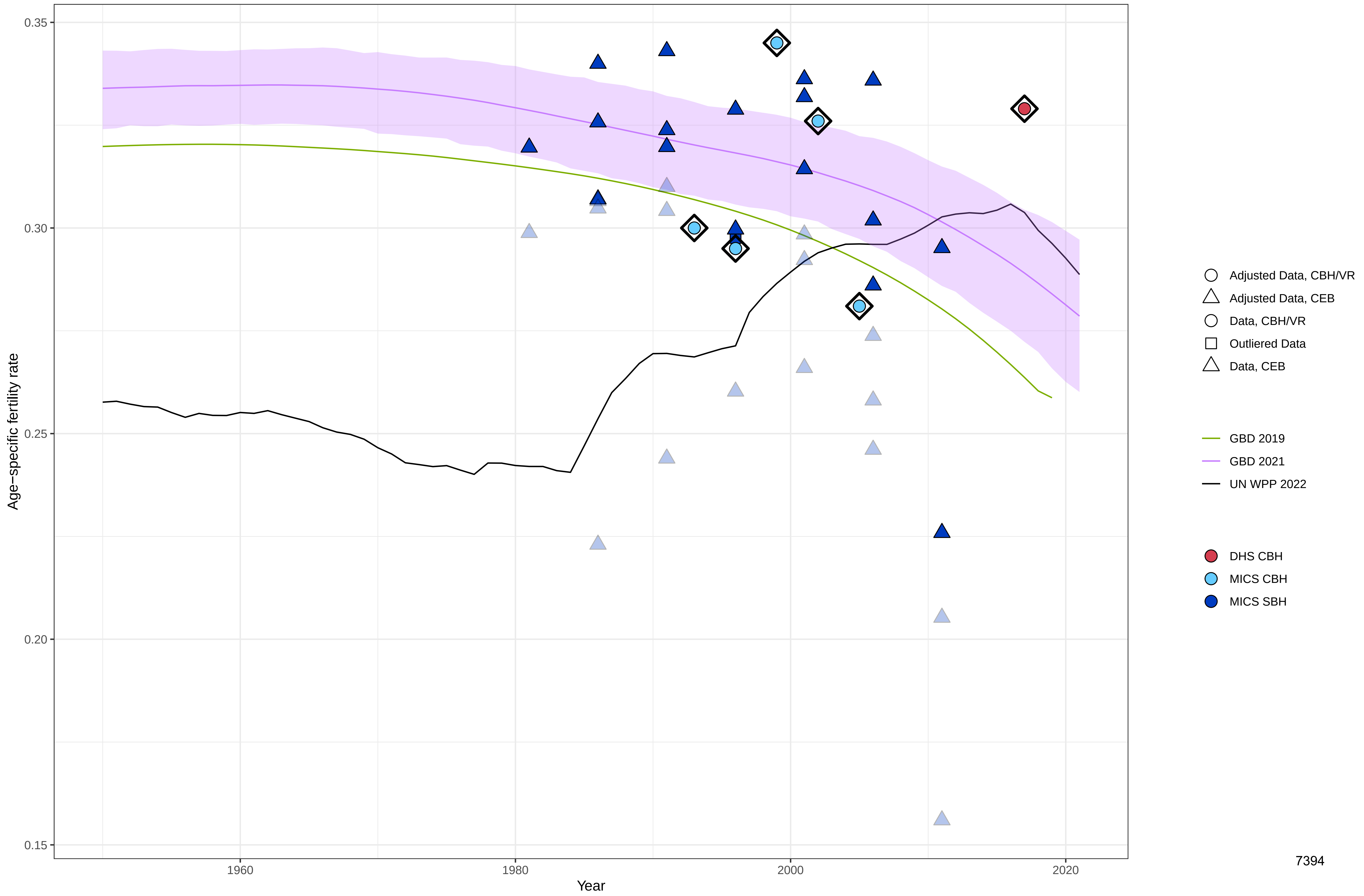

Age−specific fertility fitted trend lines, 1950−2021: Somalia, Ages: 25−29
Data points enclosed with a diamond are reference sources

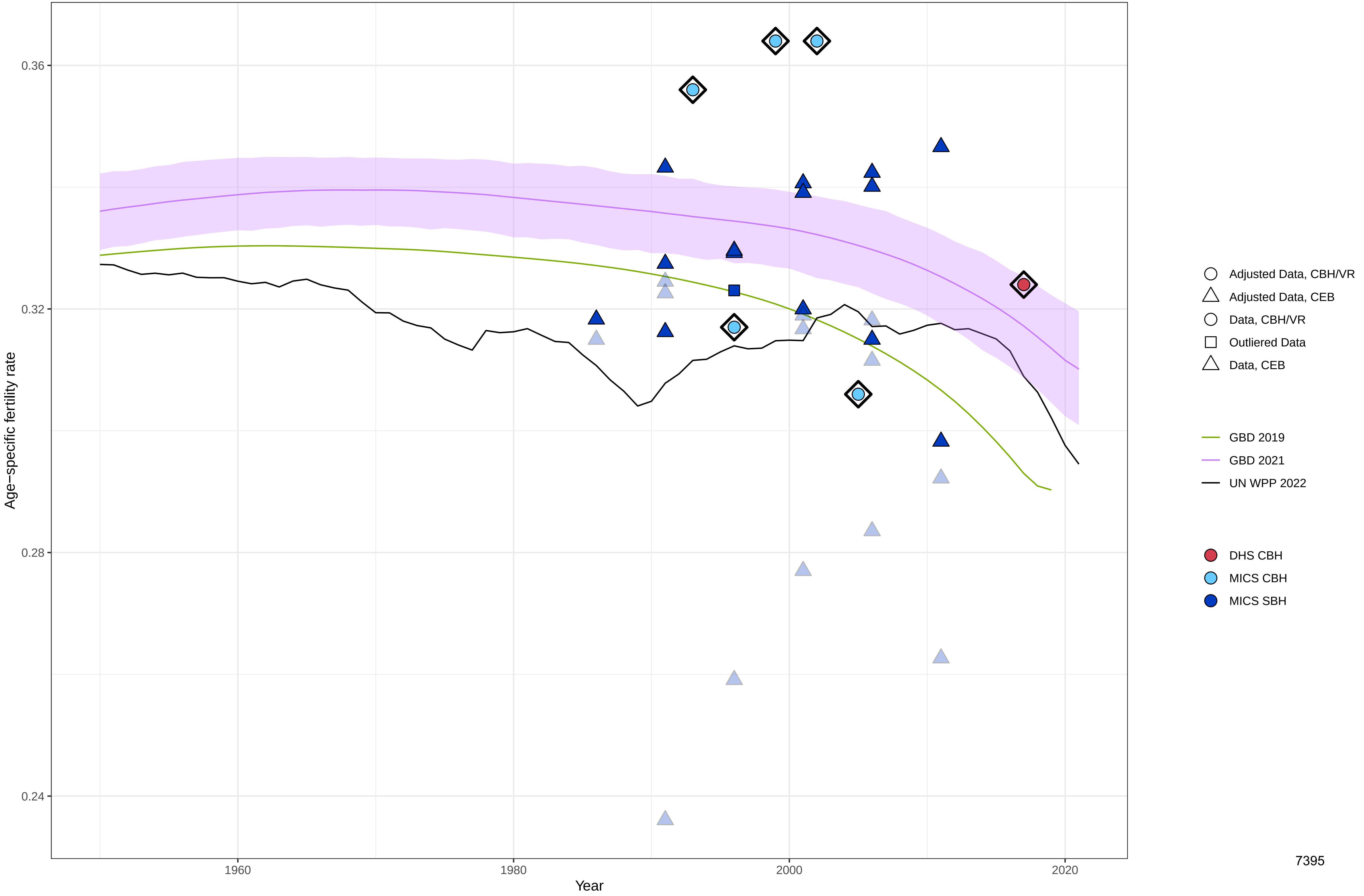

Age−specific fertility fitted trend lines, 1950−2021: Somalia, Ages: 30−34
Data points enclosed with a diamond are reference sources

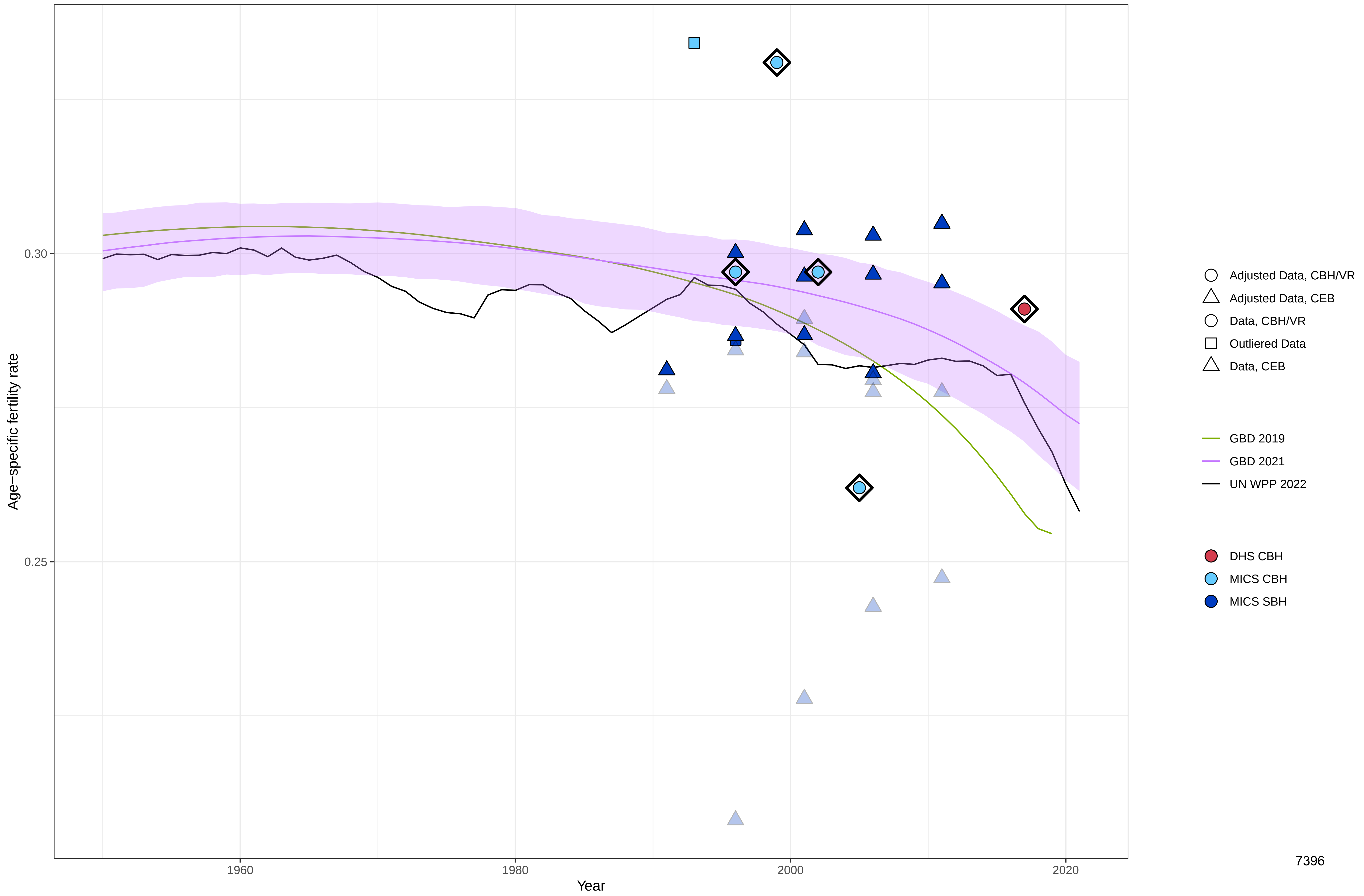

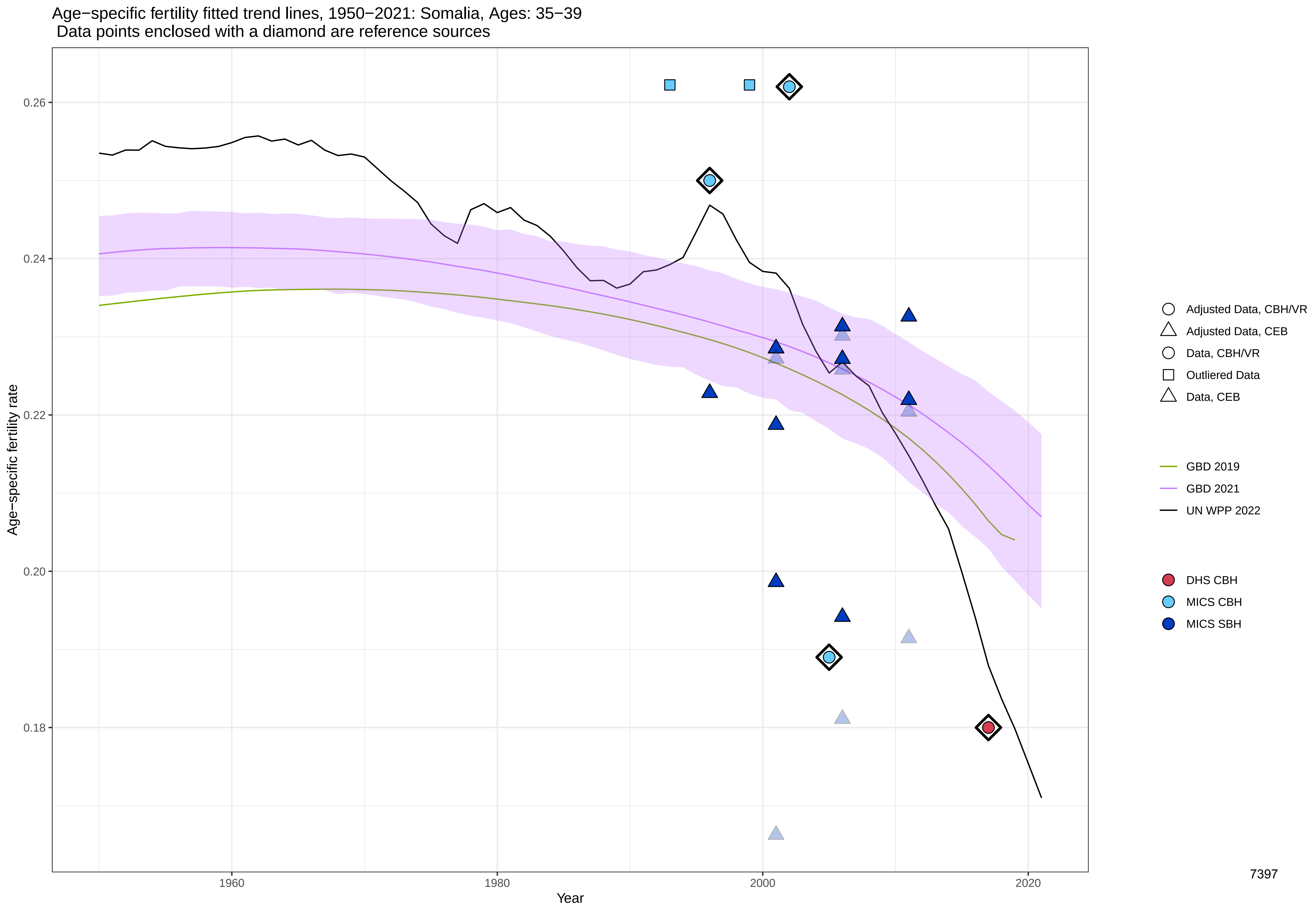

Age−specific fertility fitted trend lines, 1950−2021: Somalia, Ages: 35−39
Data points enclosed with a diamond are reference sources
Age−specific fertility rate
0.26
0.24
0.22
0.20
0.18
1960
1980
2000
2020
Year
Adjusted Data, CBH/VR
Adjusted Data, CEB
Data, CBH/VR
Outliered Data
Data, CEB
GBD 2019
GBD 2021
UN WPP 2022
DHS CBH
MICS CBH
MICS SBH

Age−specific fertility fitted trend lines, 1950−2021: Somalia, Ages: 40−44
Data points enclosed with a diamond are reference sources

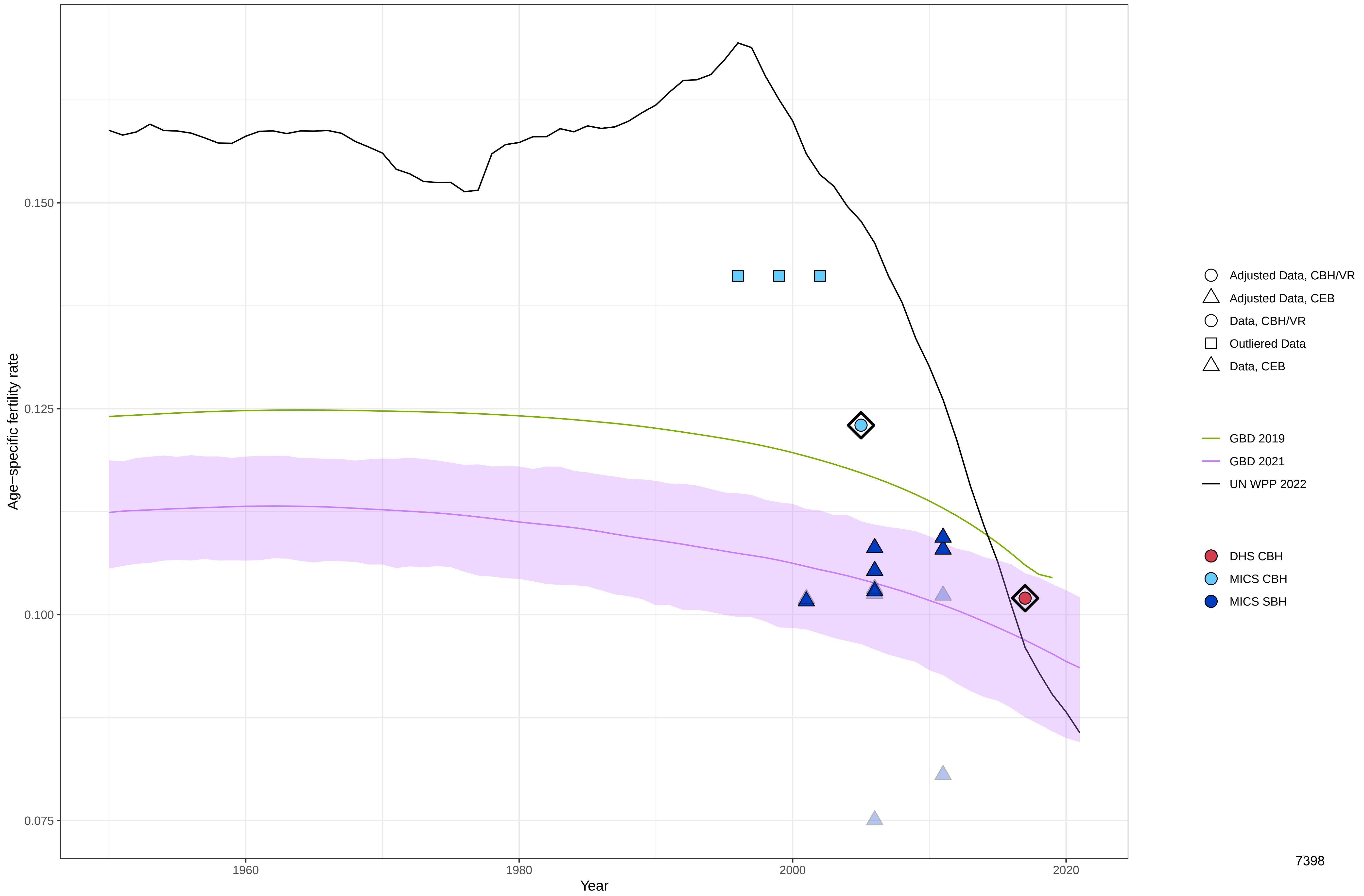

Age−specific fertility fitted trend lines, 1950−2021: Somalia, Ages: 45−49
Data points enclosed with a diamond are reference sources

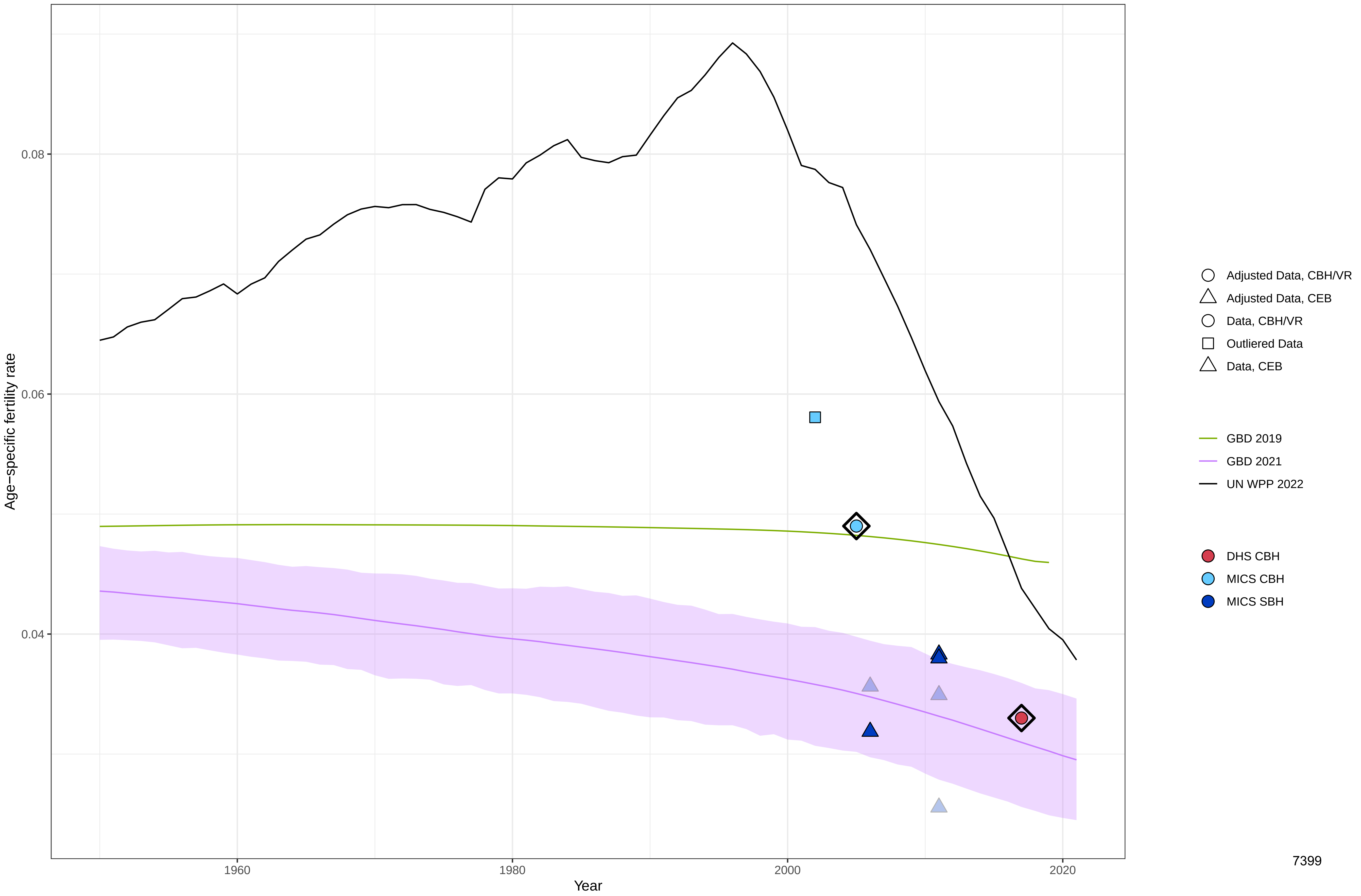

Age−specific fertility fitted trend lines, 1950−2021: Somalia, Ages: 50−54
Data points enclosed with a diamond are reference sources

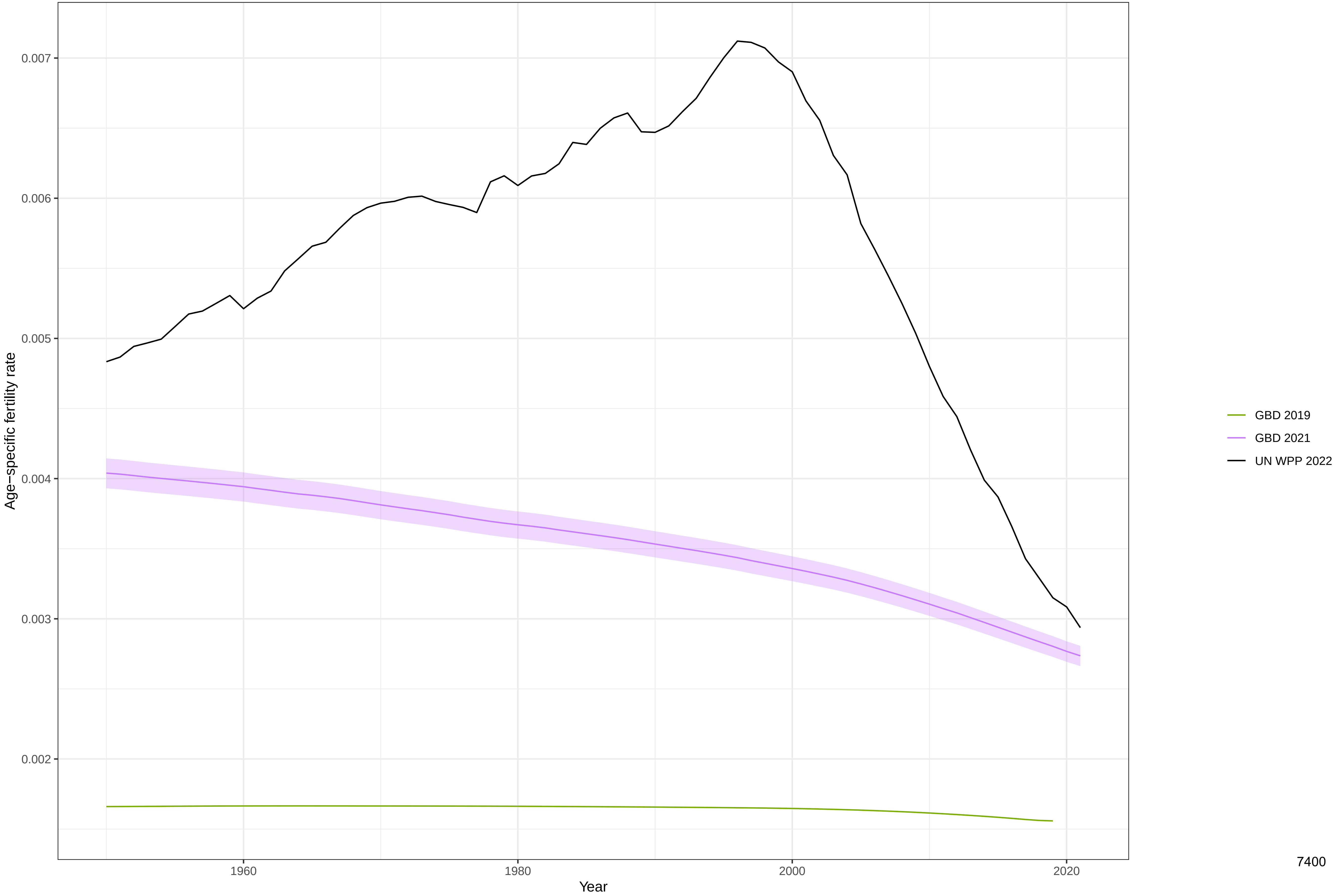

Total fertility rate fitted trend lines, 1950−2021: Somalia TFR
Data points enclosed with a diamond are reference sources

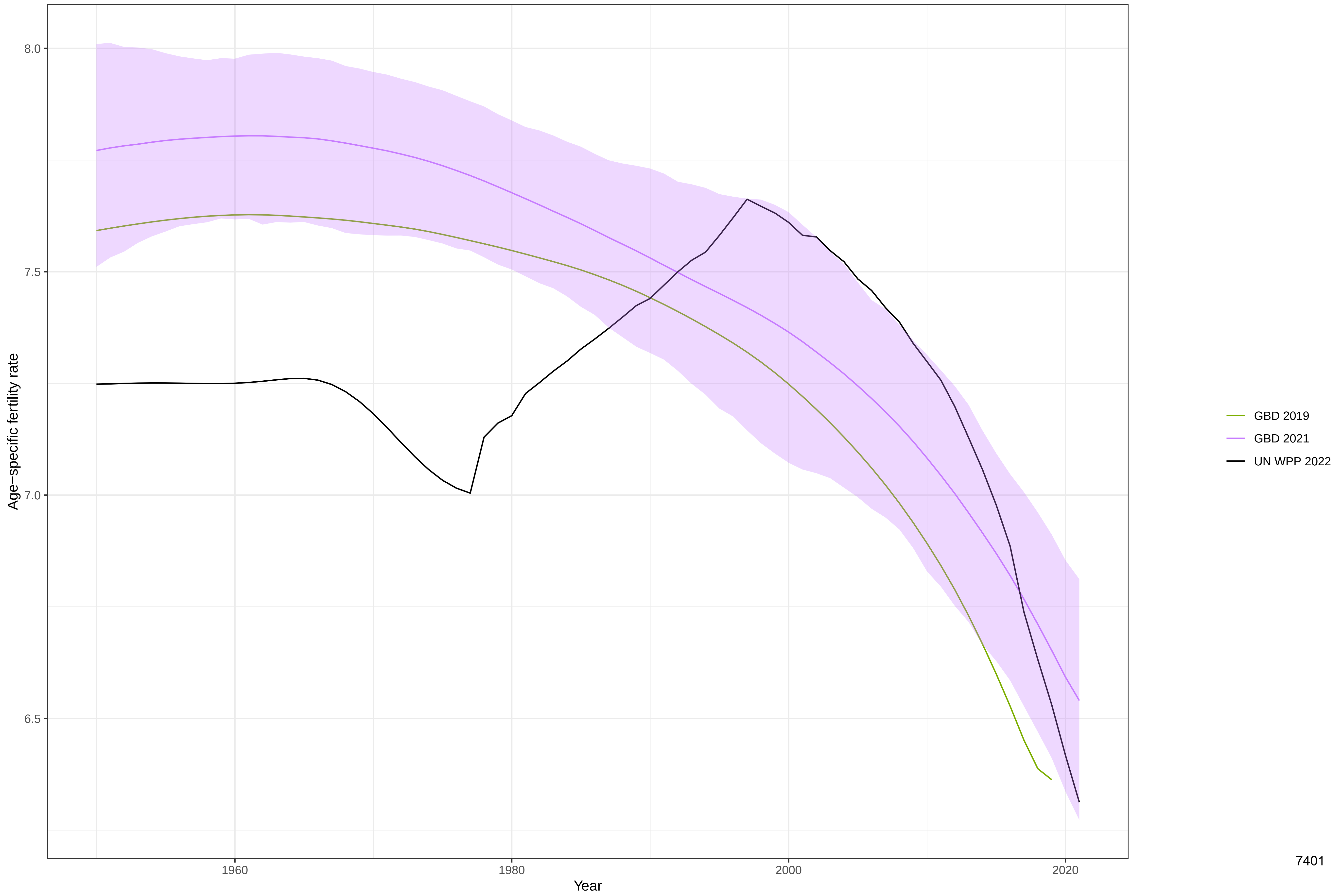

Age−specific fertility fitted trend lines, 1950−2021: South Sudan, Ages: 10−14
Data points enclosed with a diamond are reference sources

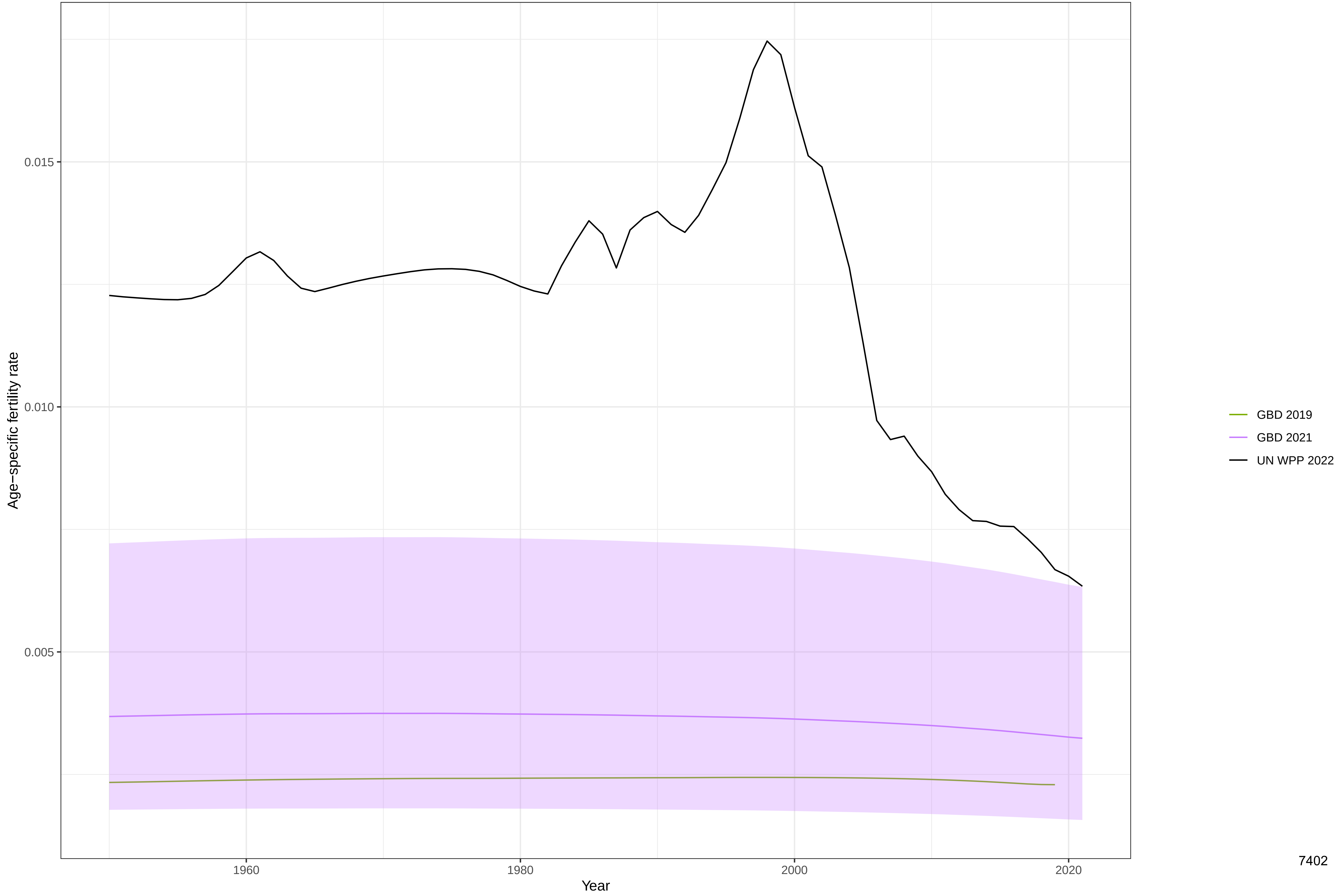

7402

Age−specific fertility fitted trend lines, 1950−2021: South Sudan, Ages: 15−19
Data points enclosed with a diamond are reference sources

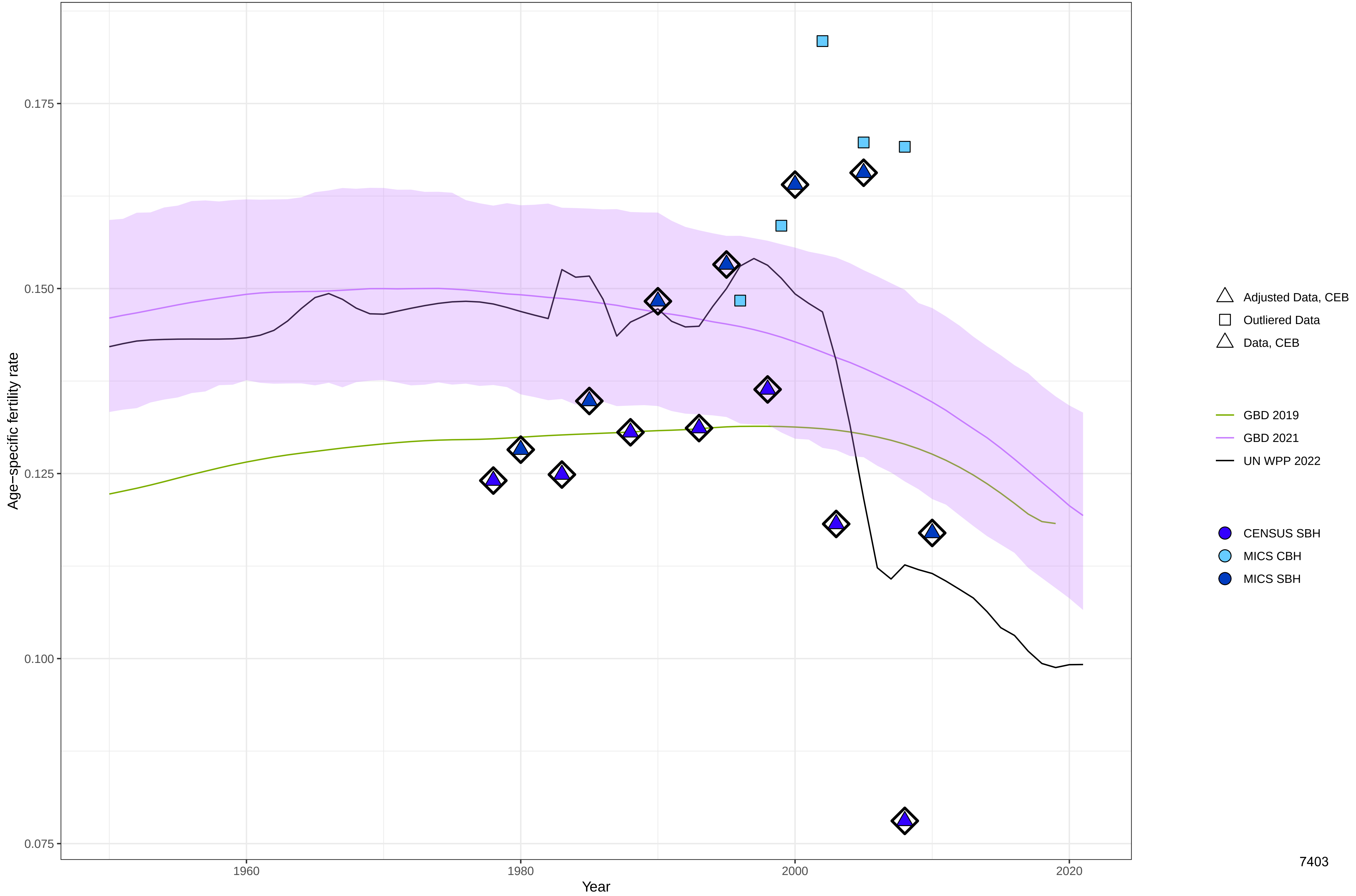

Age−specific fertility fitted trend lines, 1950−2021: South Sudan, Ages: 20−24
Data points enclosed with a diamond are reference sources

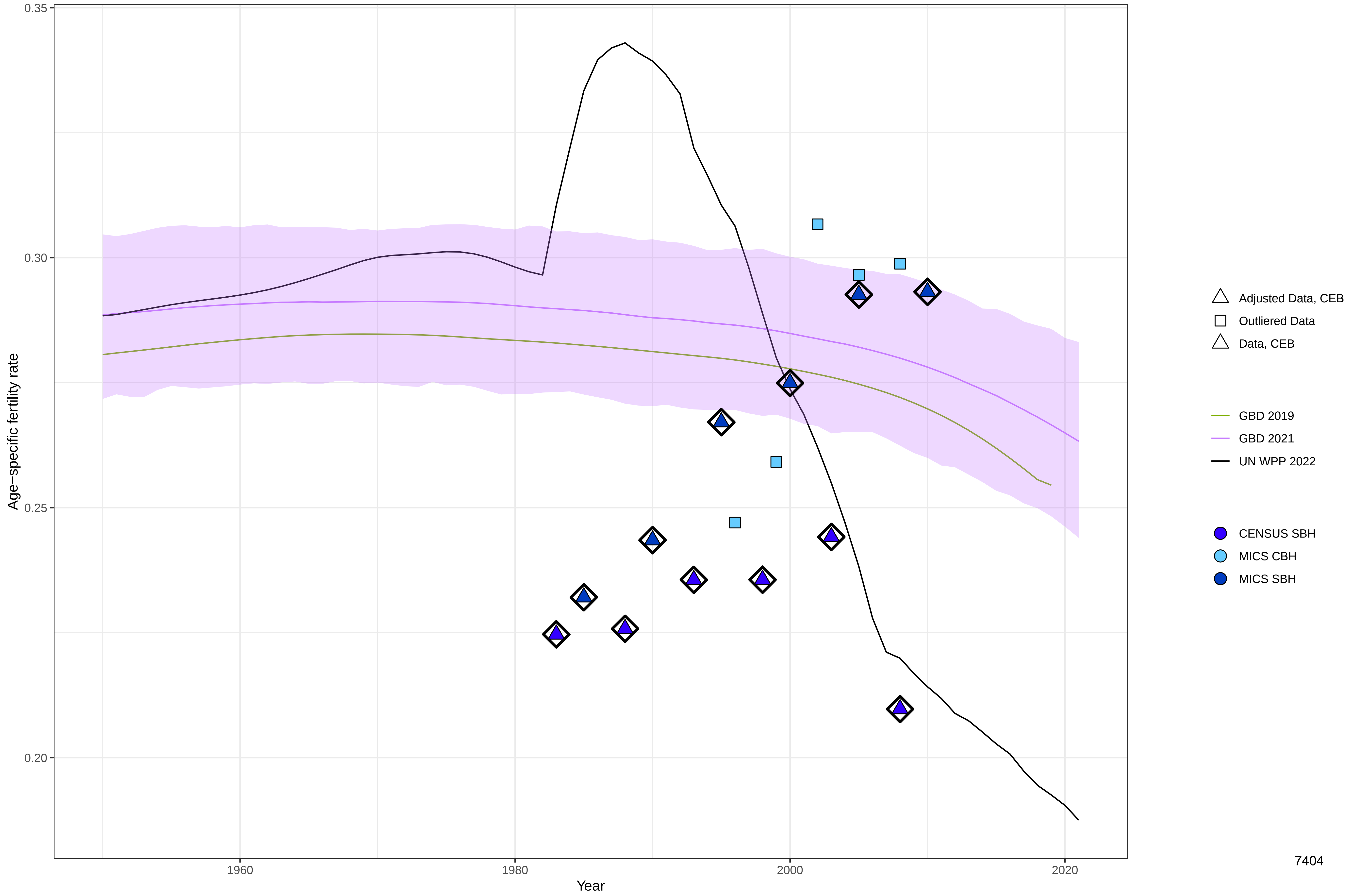

Age−specific fertility fitted trend lines, 1950−2021: South Sudan, Ages: 25−29
Data points enclosed with a diamond are reference sources

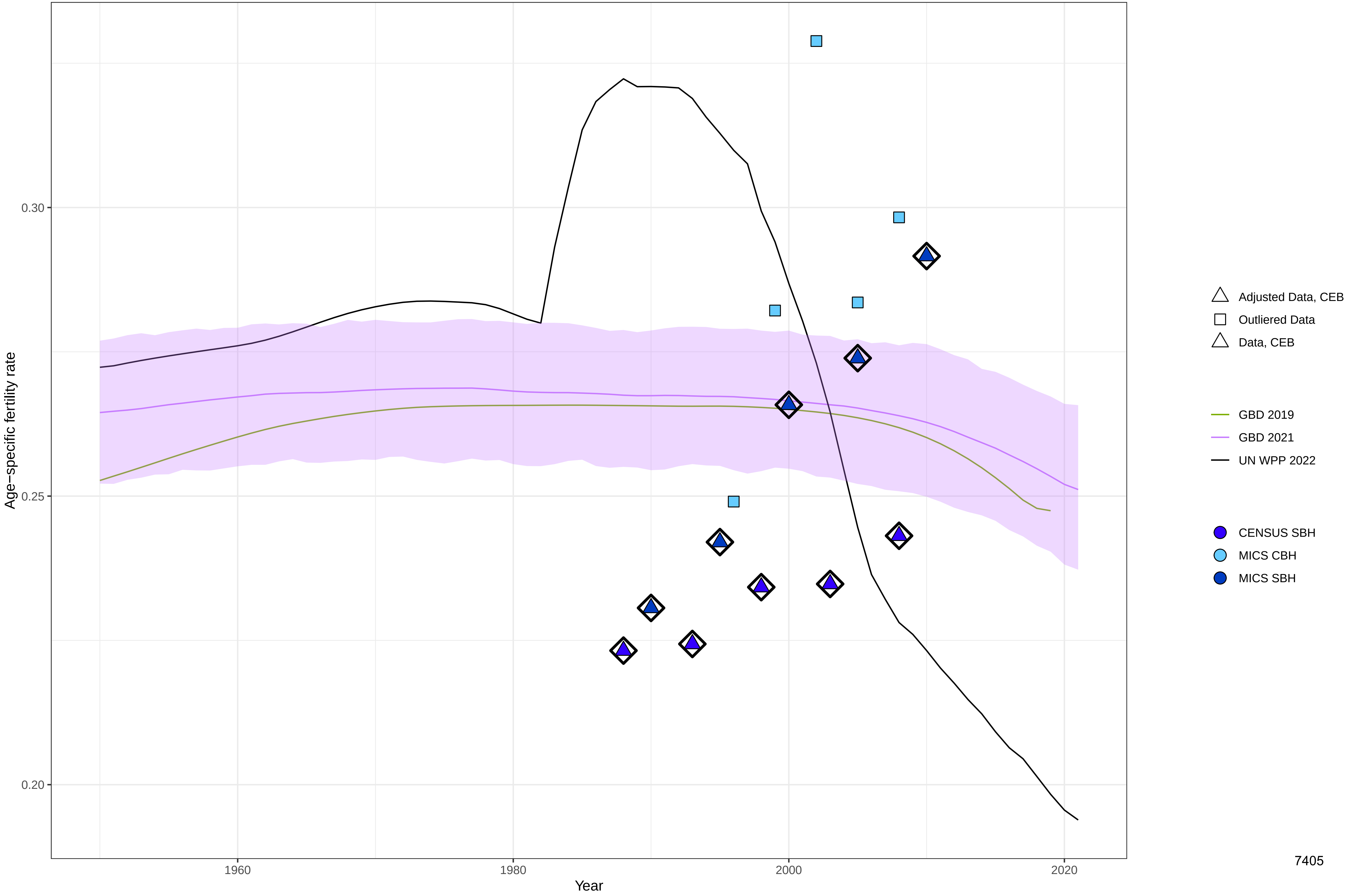

Age−specific fertility fitted trend lines, 1950−2021: South Sudan, Ages: 30−34
Data points enclosed with a diamond are reference sources

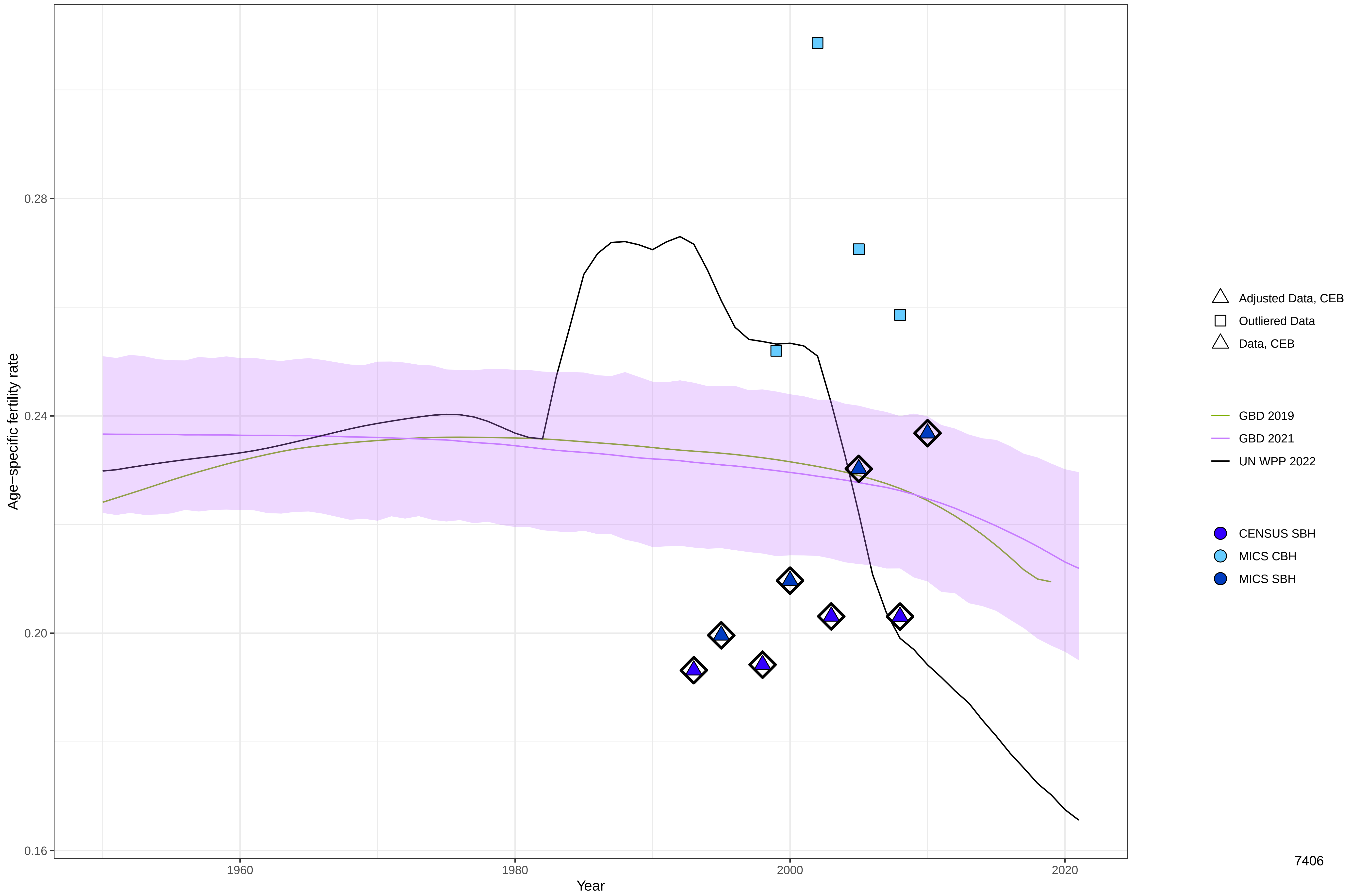

Age−specific fertility fitted trend lines, 1950−2021: South Sudan, Ages: 35−39
Data points enclosed with a diamond are reference sources

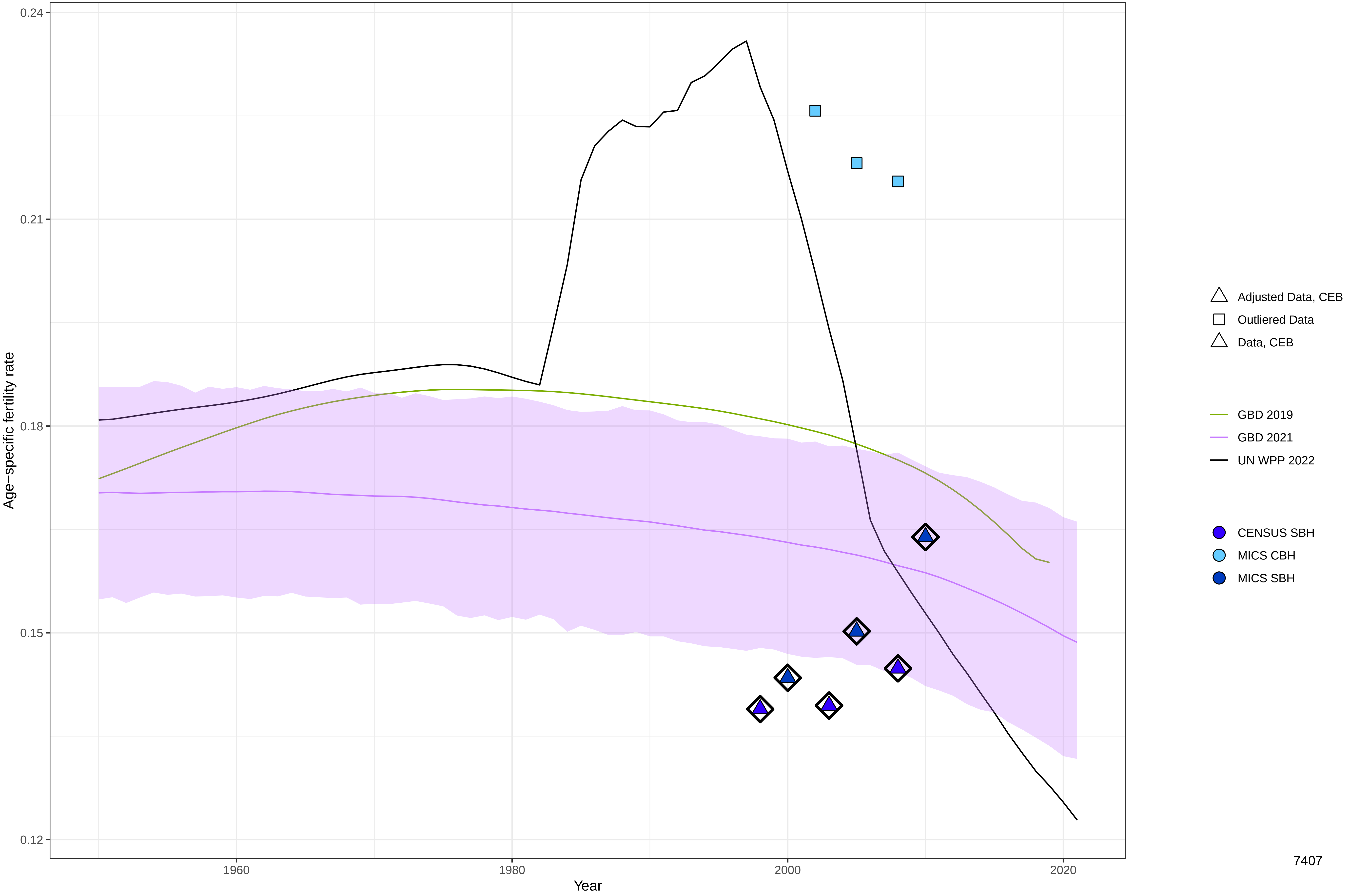

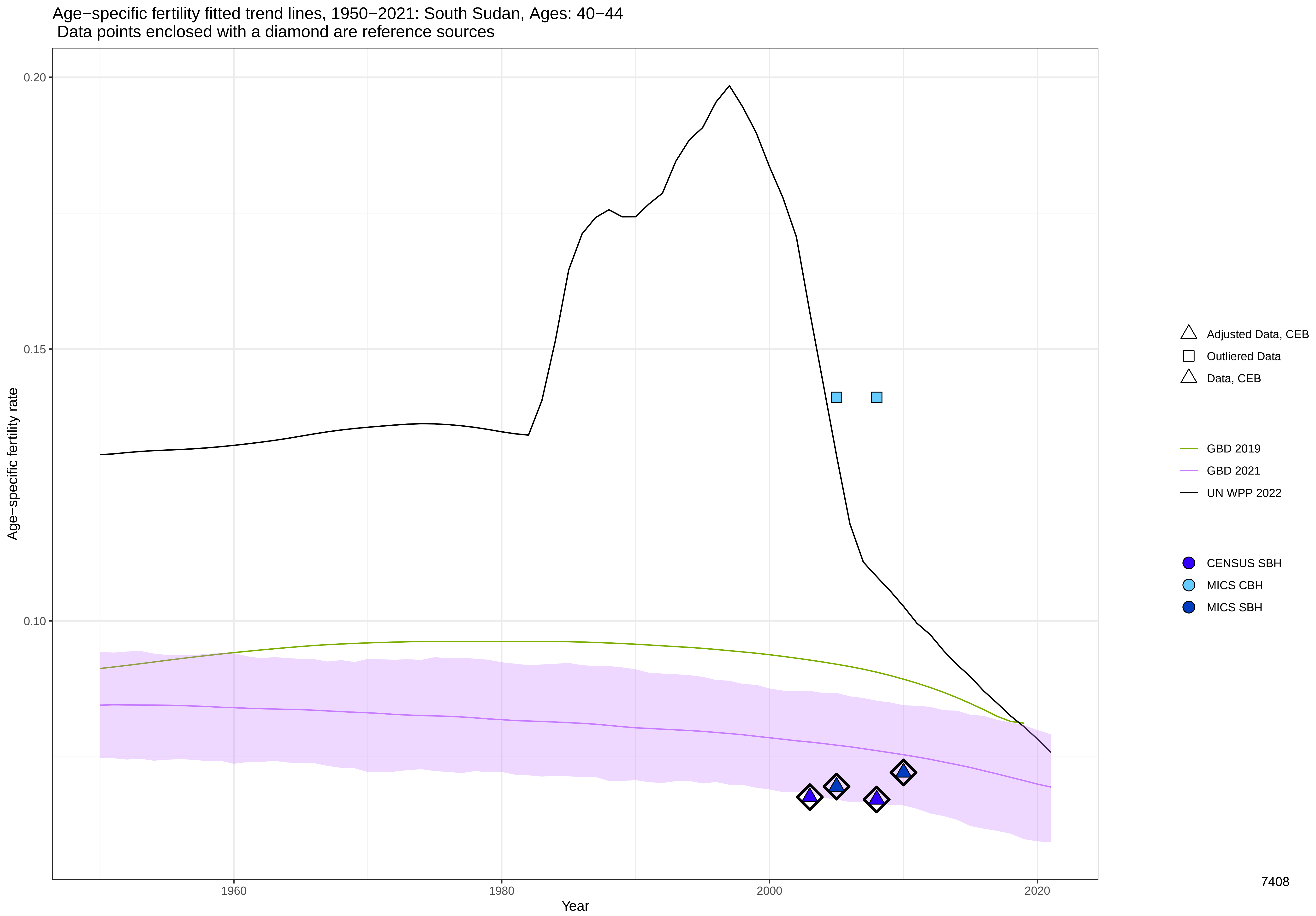

Age−specific fertility fitted trend lines, 1950−2021: South Sudan, Ages: 40−44
Data points enclosed with a diamond are reference sources
Age−specific fertility rate
0.20
0.15
0.10
Year
1960
1980
2000
2020
Adjusted Data, CEB
Outliered Data
Data, CEB
GBD 2019
GBD 2021
UN WPP 2022
CENSUS SBH
MICS CBH
MICS SBH

Age−specific fertility fitted trend lines, 1950−2021: South Sudan, Ages: 45−49
Data points enclosed with a diamond are reference sources

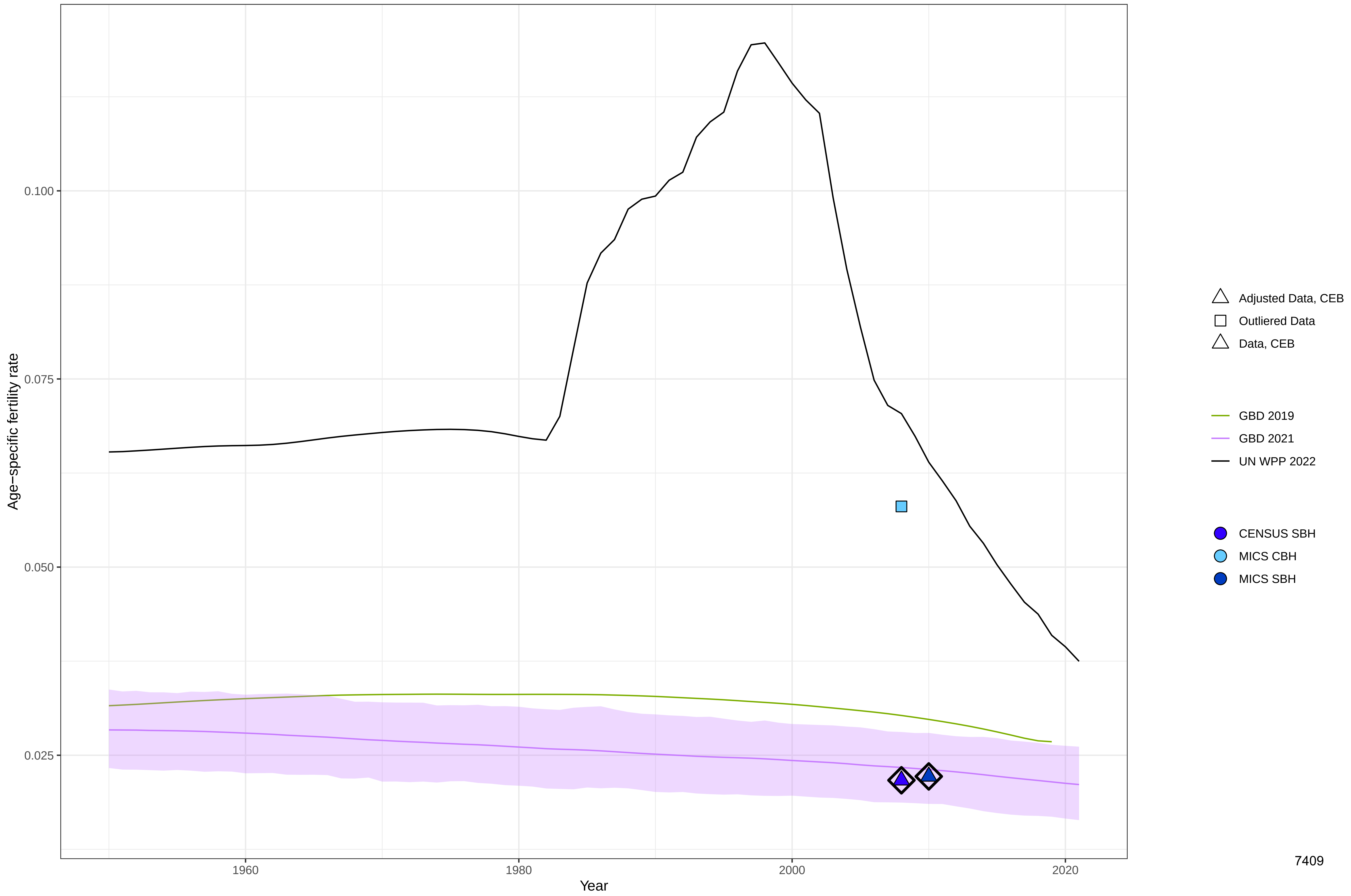

Age−specific fertility fitted trend lines, 1950−2021: South Sudan, Ages: 50−54
Data points enclosed with a diamond are reference sources

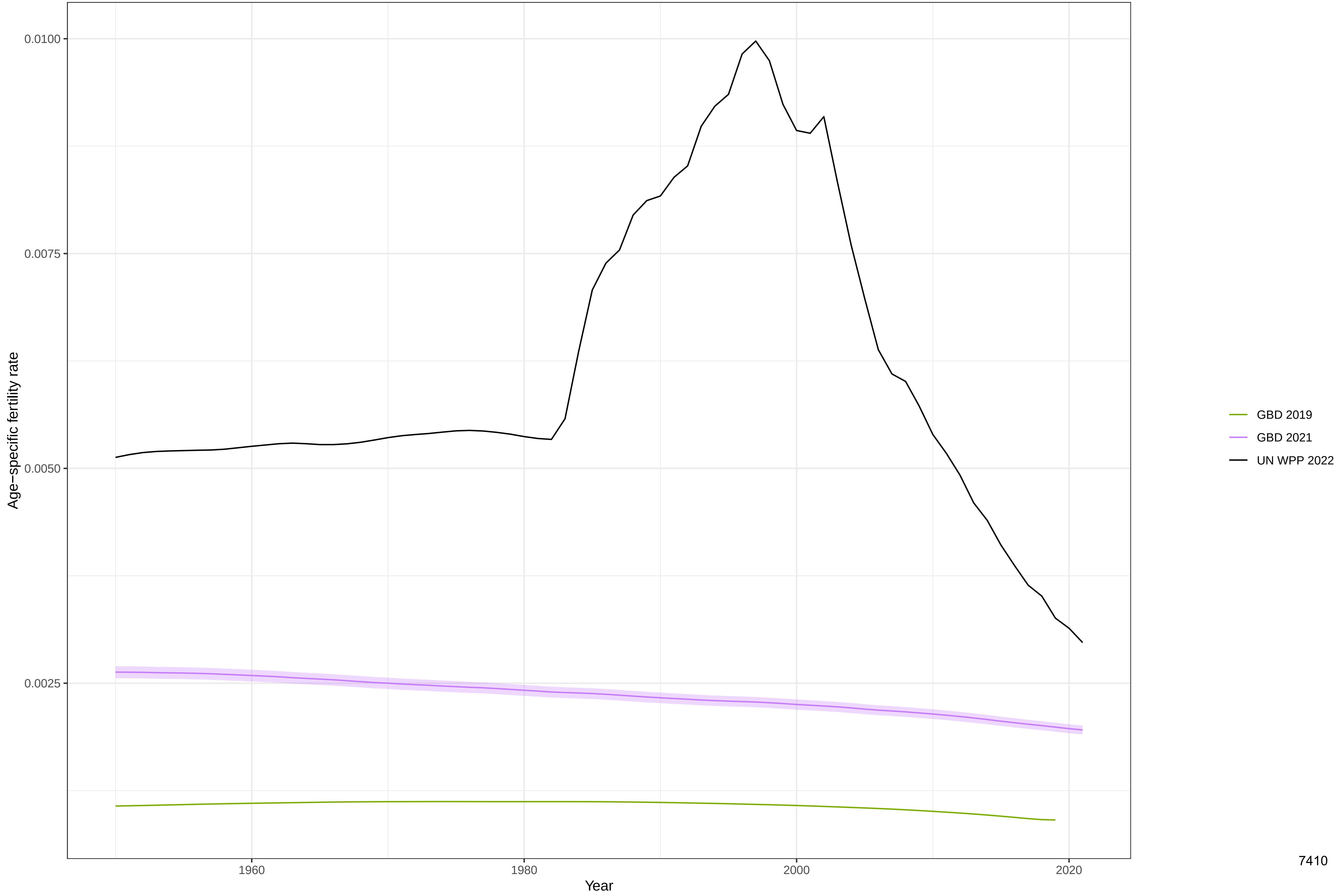

Total fertility rate fitted trend lines, 1950−2021: South Sudan TFR
Data points enclosed with a diamond are reference sources

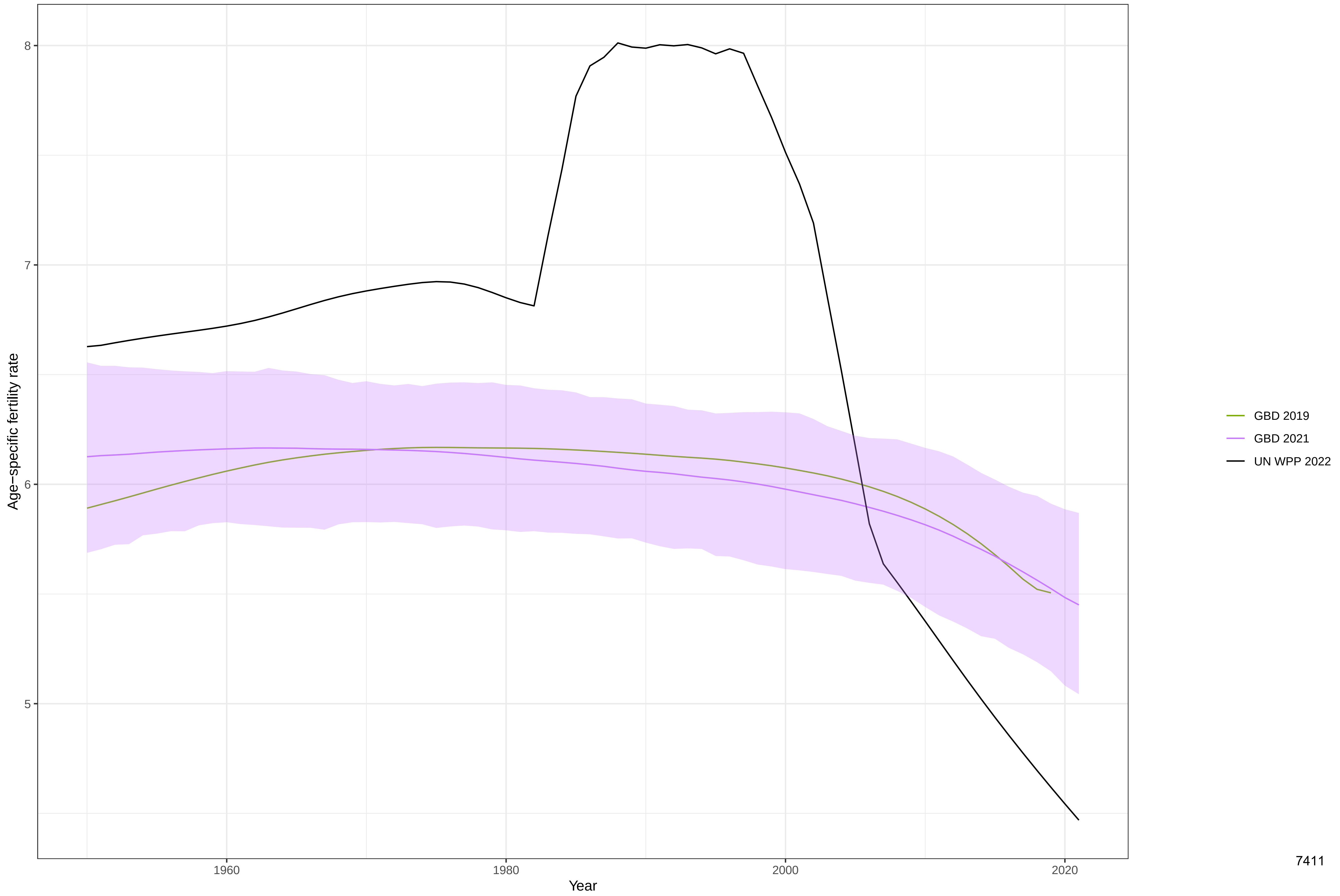

Age−specific fertility fitted trend lines, 1950−2021: Uganda, Ages: 10−14
Data points enclosed with a diamond are reference sources

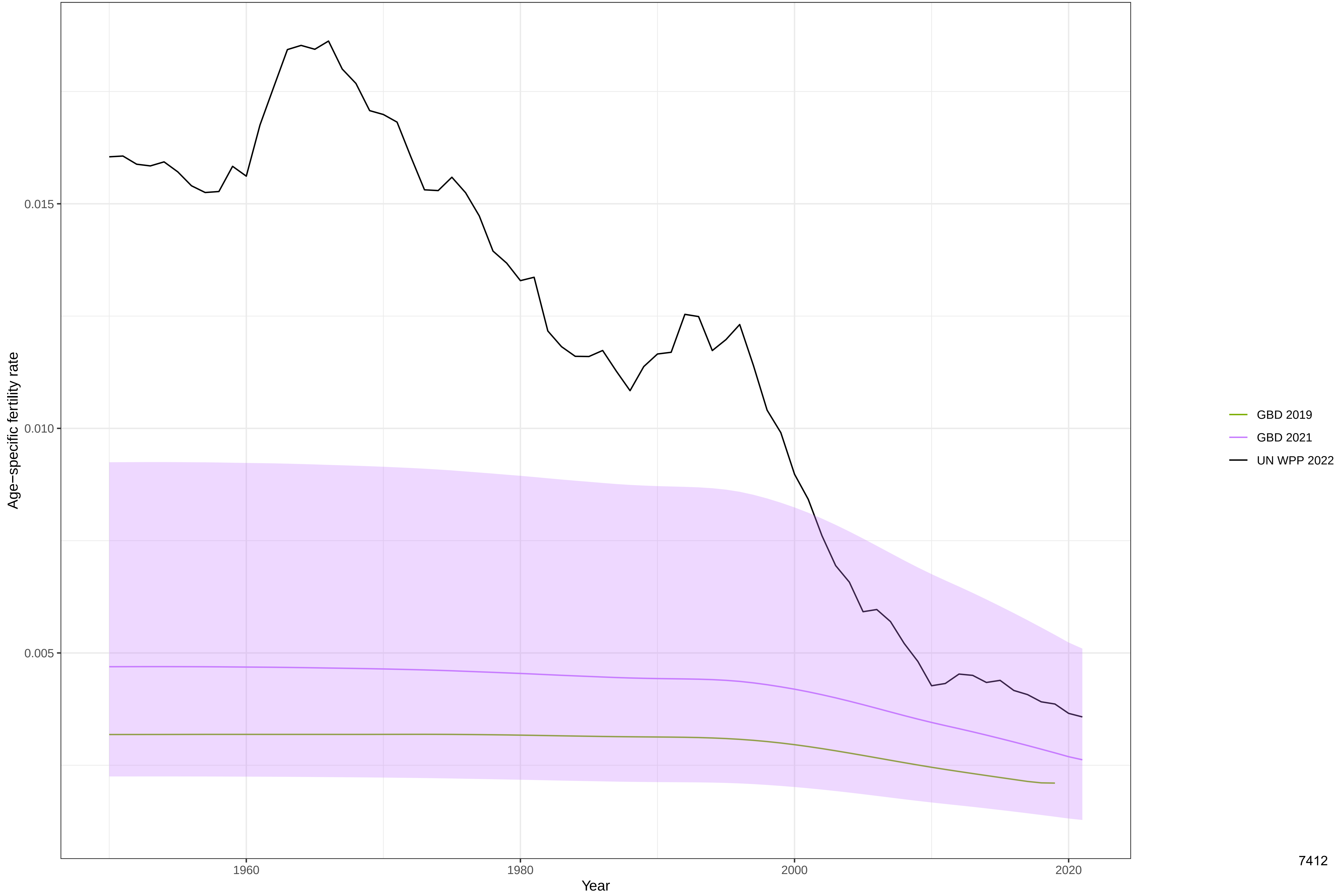

Age−specific fertility fitted trend lines, 1950−2021: Uganda, Ages: 15−19
Data points enclosed with a diamond are reference sources

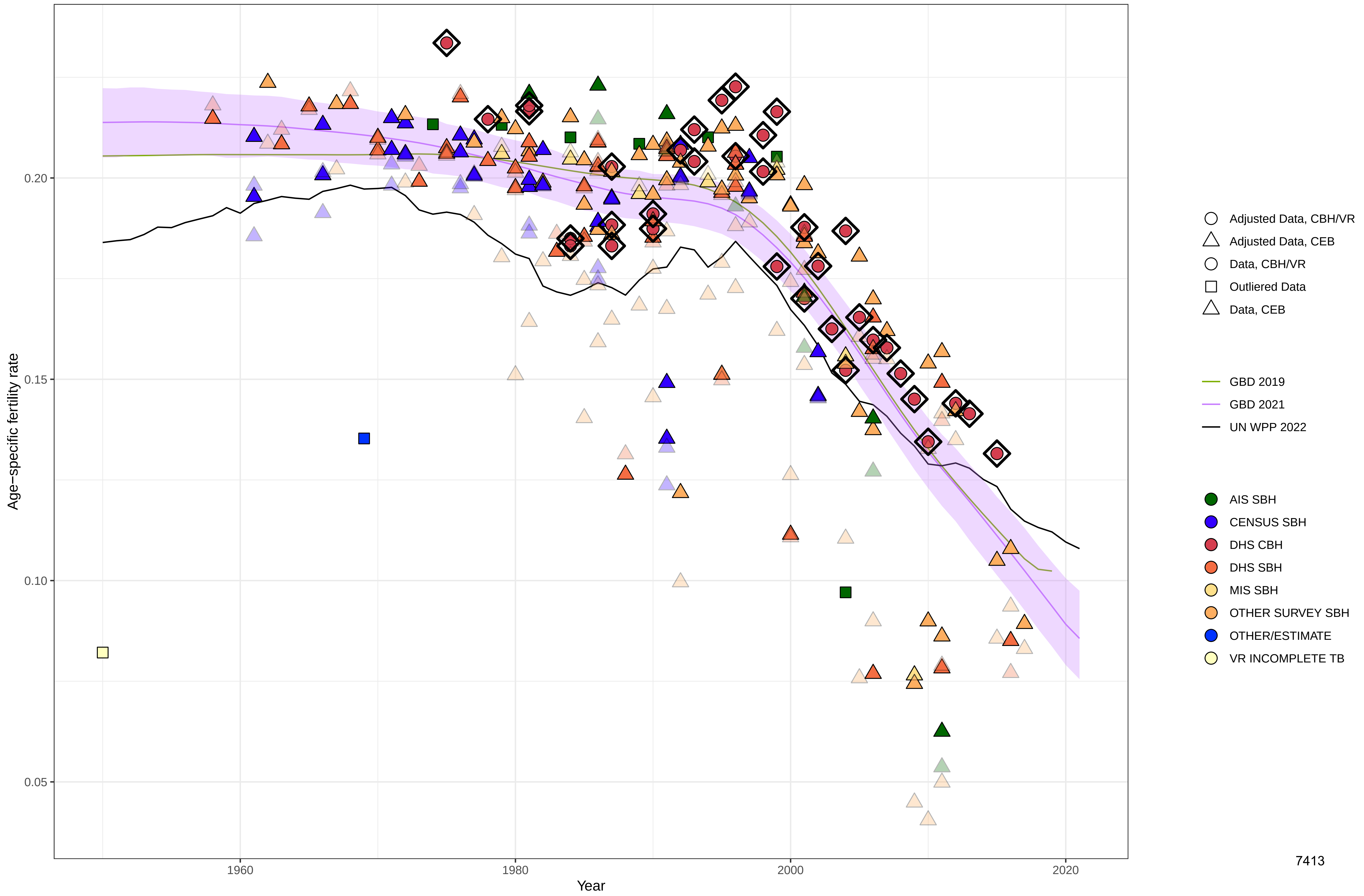

7413

Age−specific fertility fitted trend lines, 1950−2021: Uganda, Ages: 20−24
Data points enclosed with a diamond are reference sources

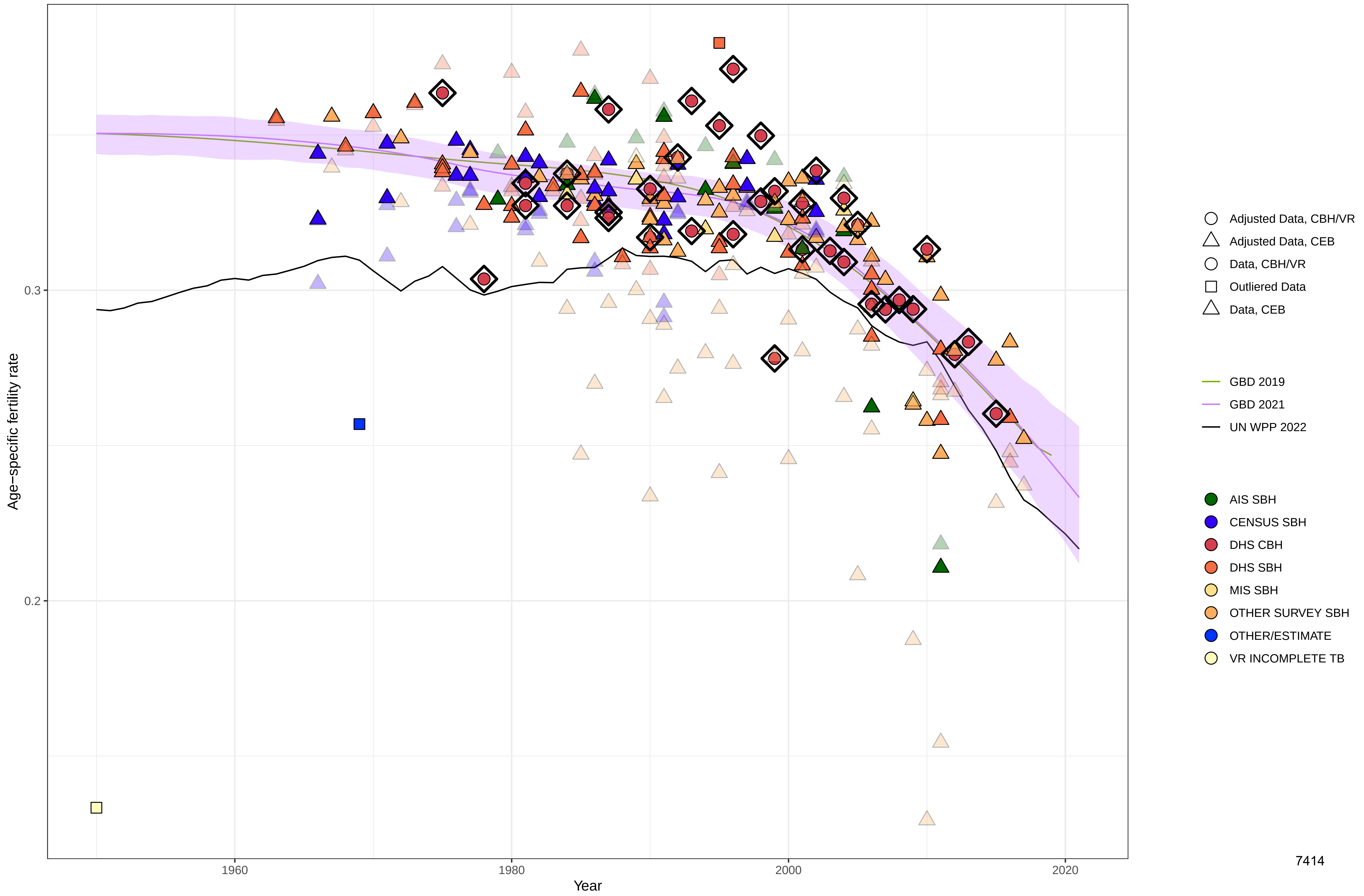

Age−specific fertility fitted trend lines, 1950−2021: Uganda, Ages: 25−29
Data points enclosed with a diamond are reference sources

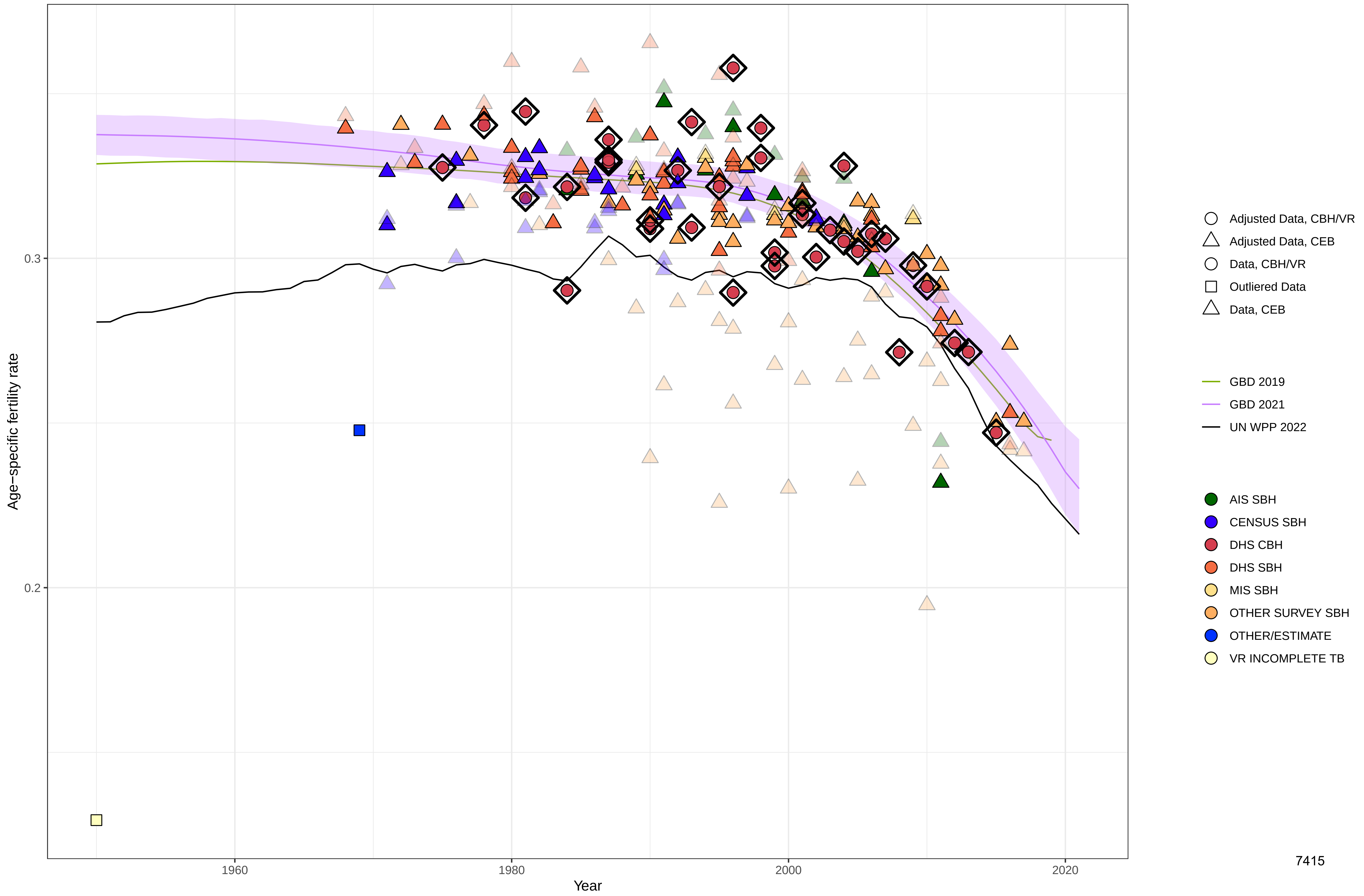

Age−specific fertility fitted trend lines, 1950−2021: Uganda, Ages: 30−34
Data points enclosed with a diamond are reference sources

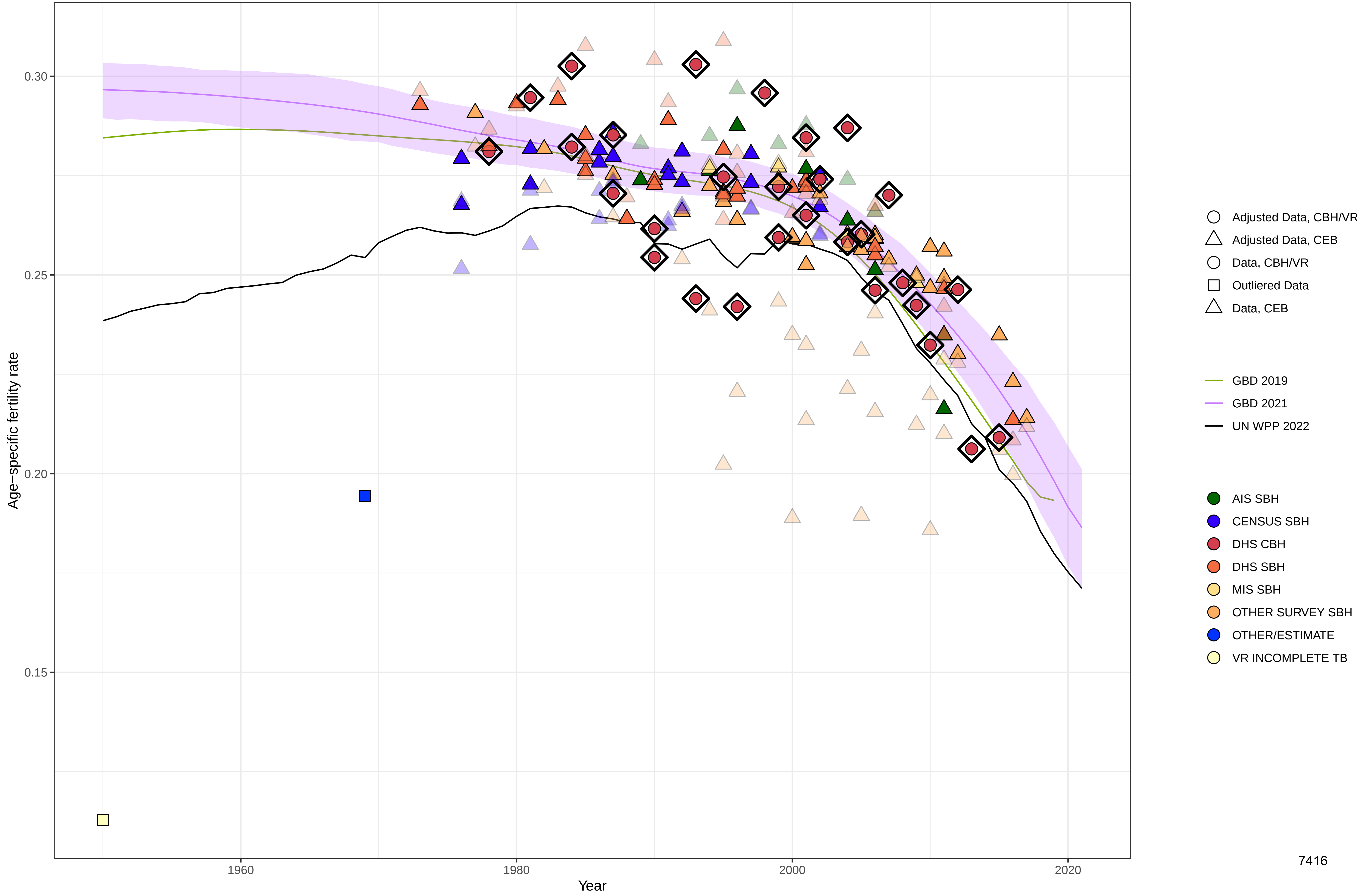

Age−specific fertility fitted trend lines, 1950−2021: Uganda, Ages: 35−39
Data points enclosed with a diamond are reference sources

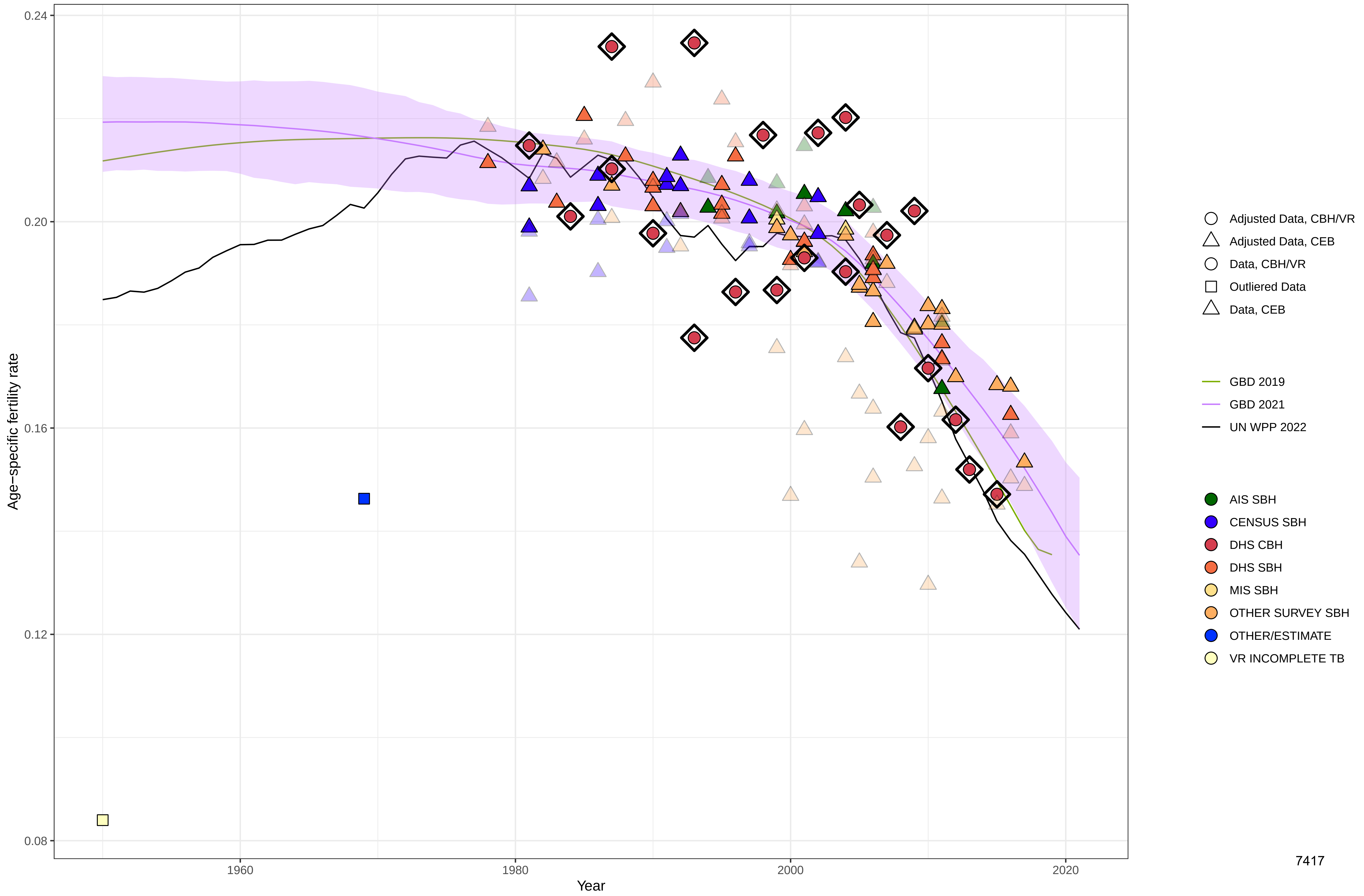

Age−specific fertility fitted trend lines, 1950−2021: Uganda, Ages: 40−44
Data points enclosed with a diamond are reference sources

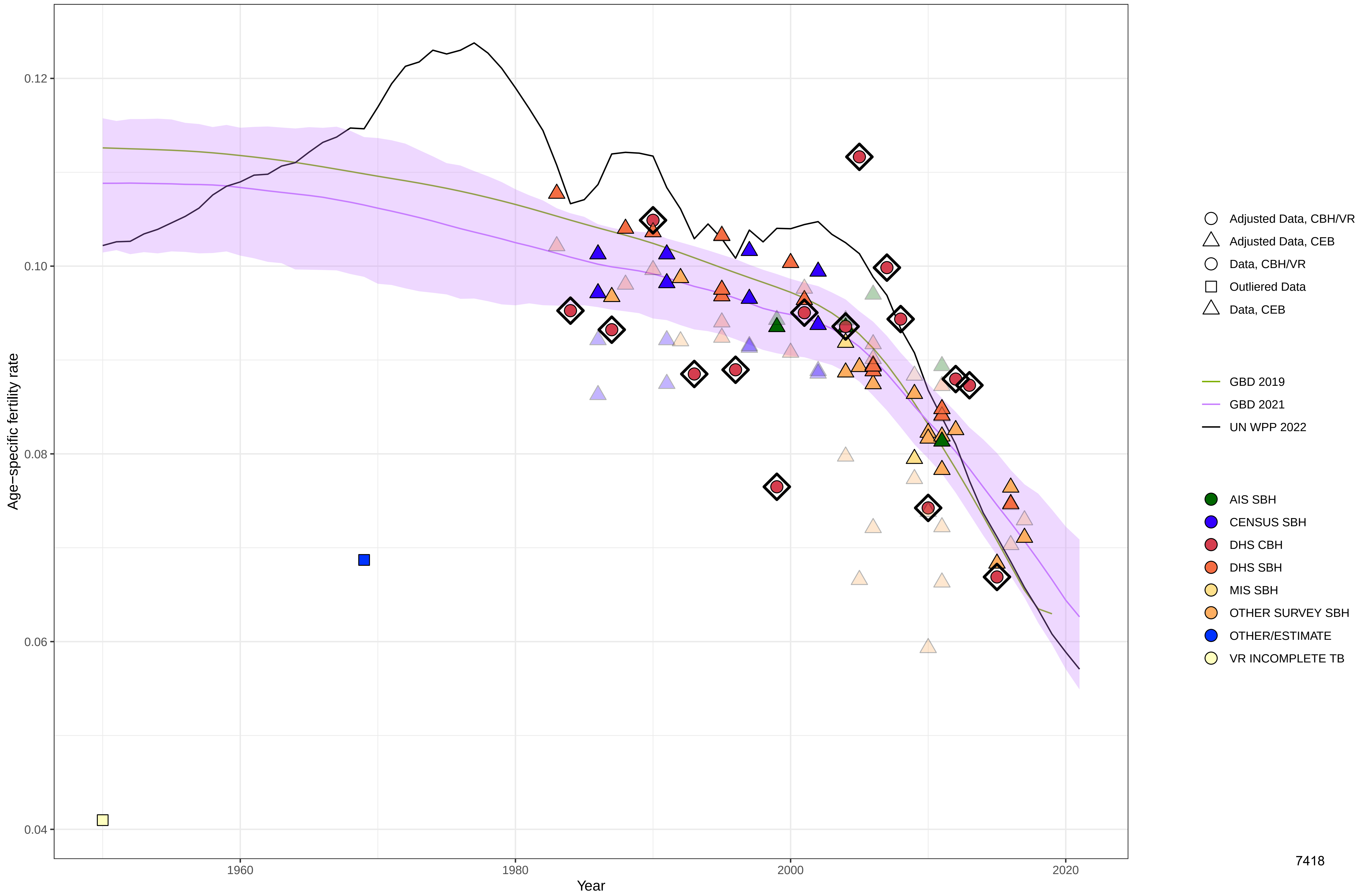

Age−specific fertility fitted trend lines, 1950−2021: Uganda, Ages: 45−49
Data points enclosed with a diamond are reference sources

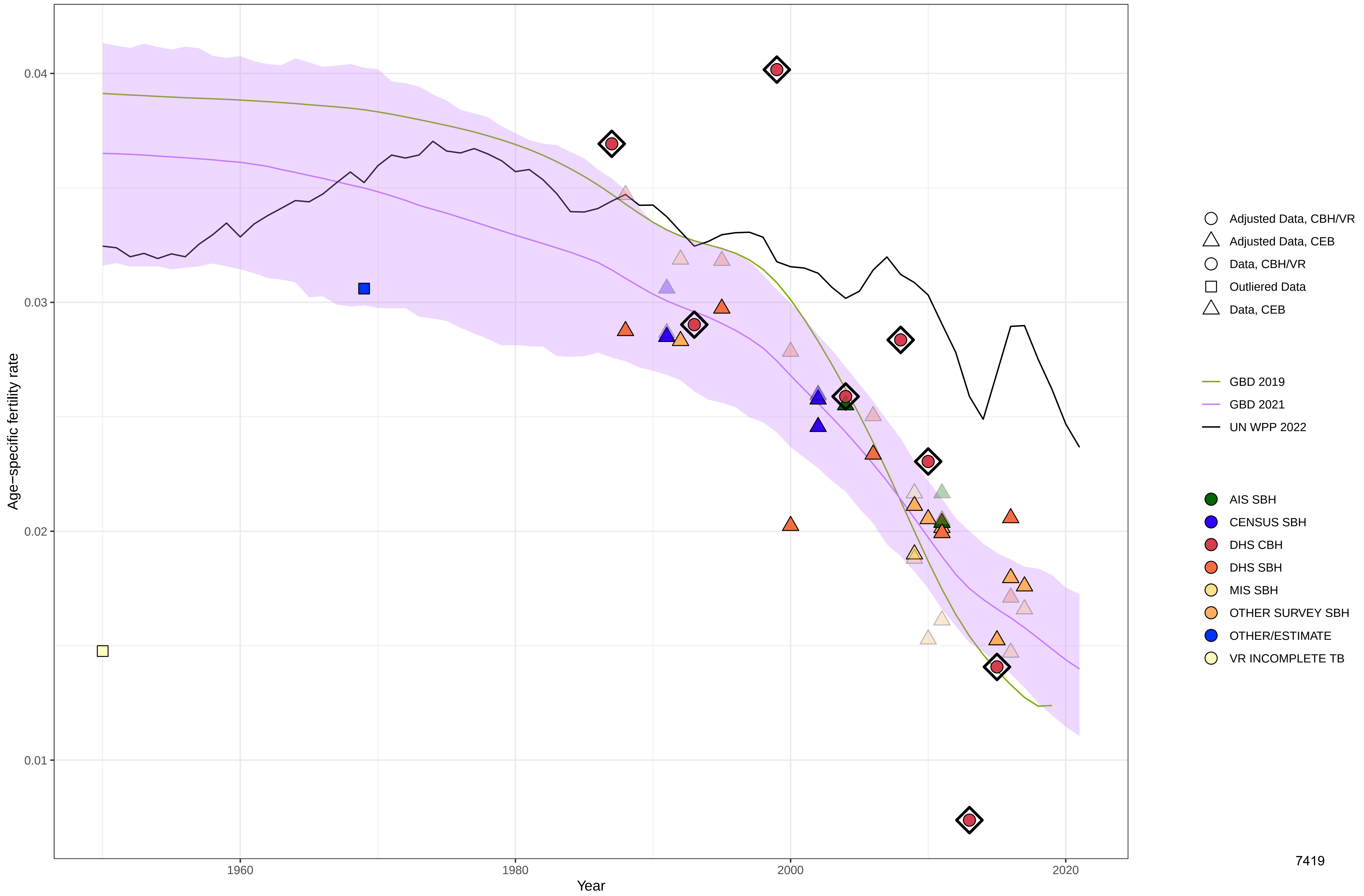

Age−specific fertility fitted trend lines, 1950−2021: Uganda, Ages: 50−54
Data points enclosed with a diamond are reference sources

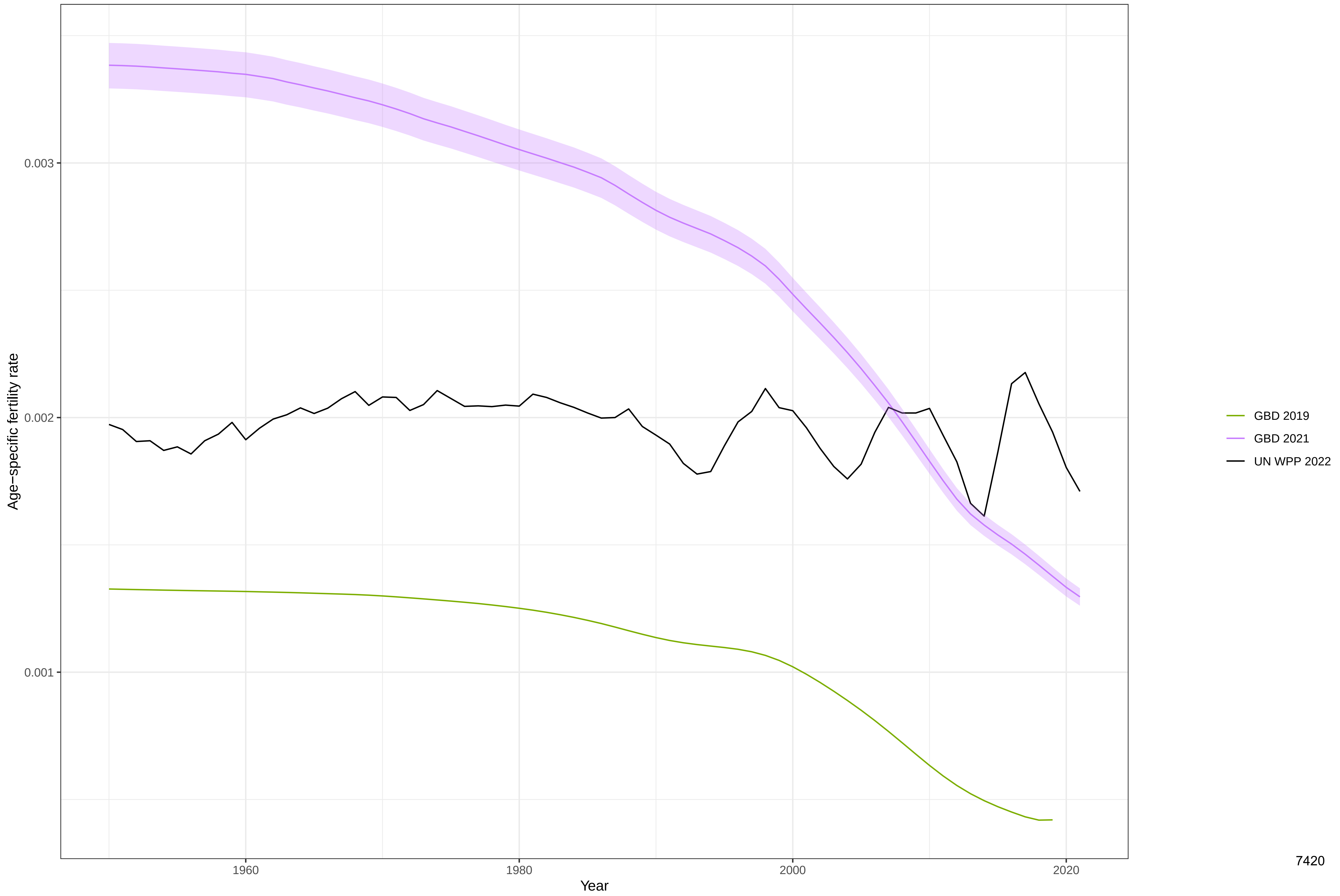

Total fertility rate fitted trend lines, 1950−2021: Uganda TFR
Data points enclosed with a diamond are reference sources

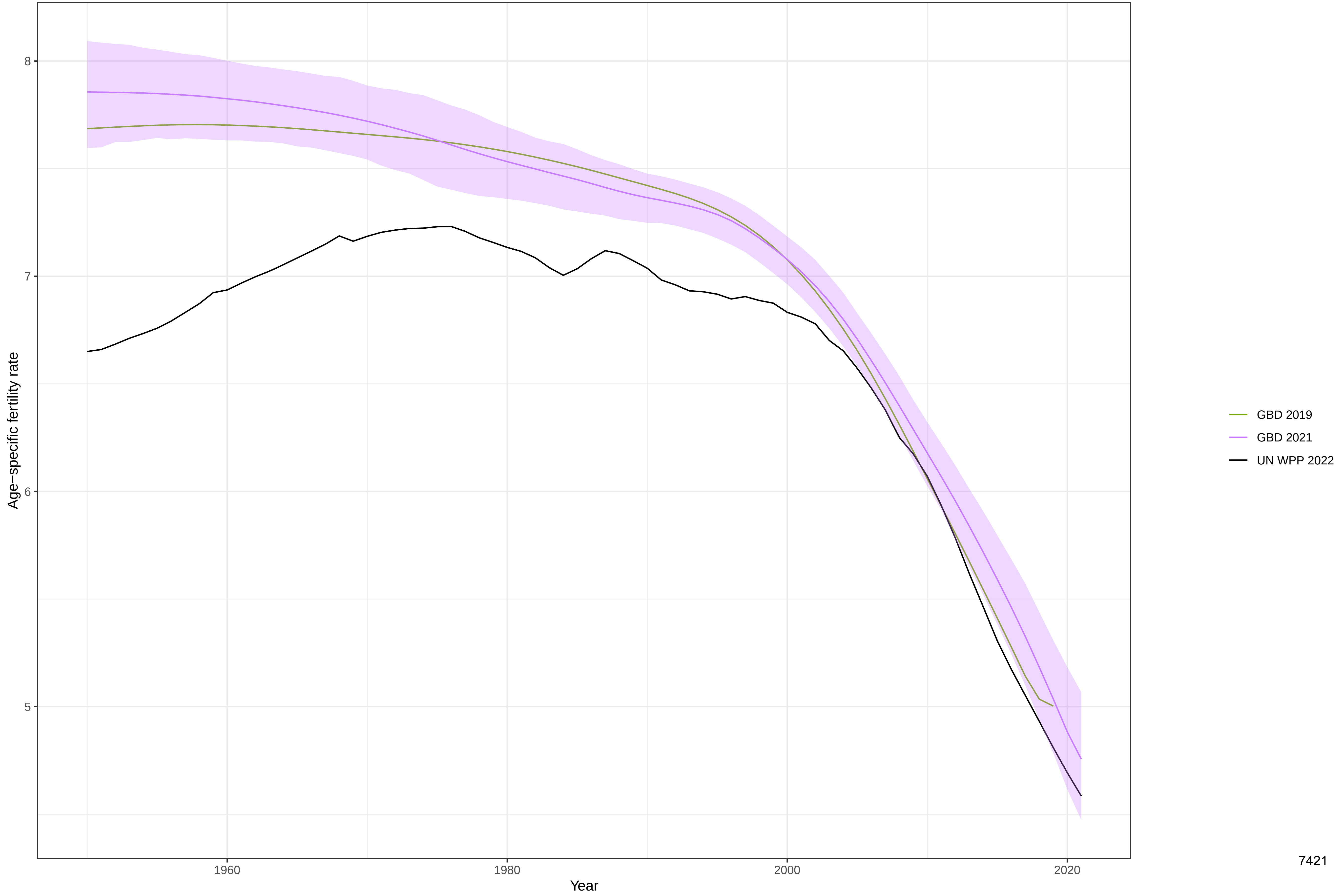

Age−specific fertility fitted trend lines, 1950−2021: Tanzania, Ages: 10−14
Data points enclosed with a diamond are reference sources

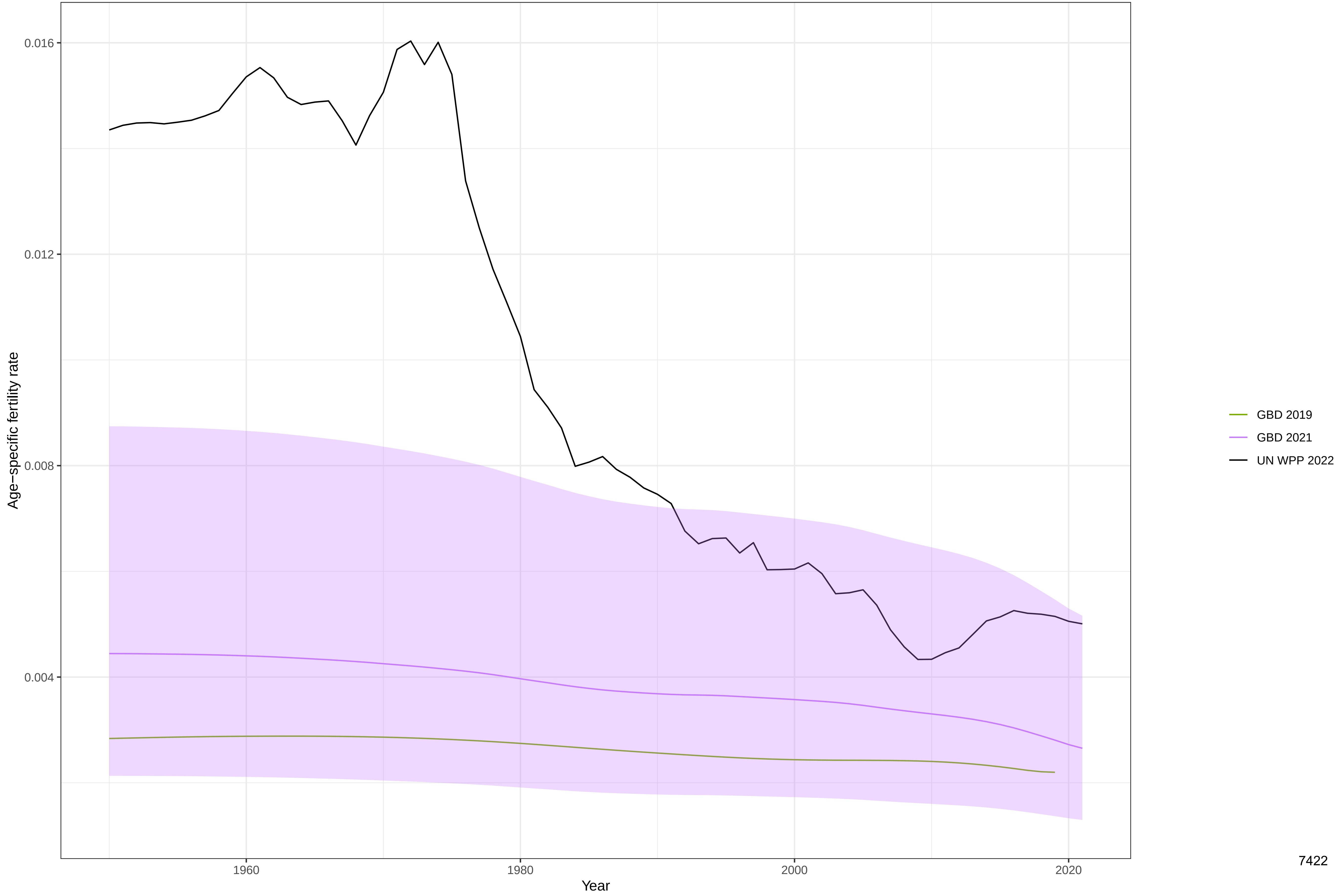

Age−specific fertility fitted trend lines, 1950−2021: Tanzania, Ages: 15−19
Data points enclosed with a diamond are reference sources

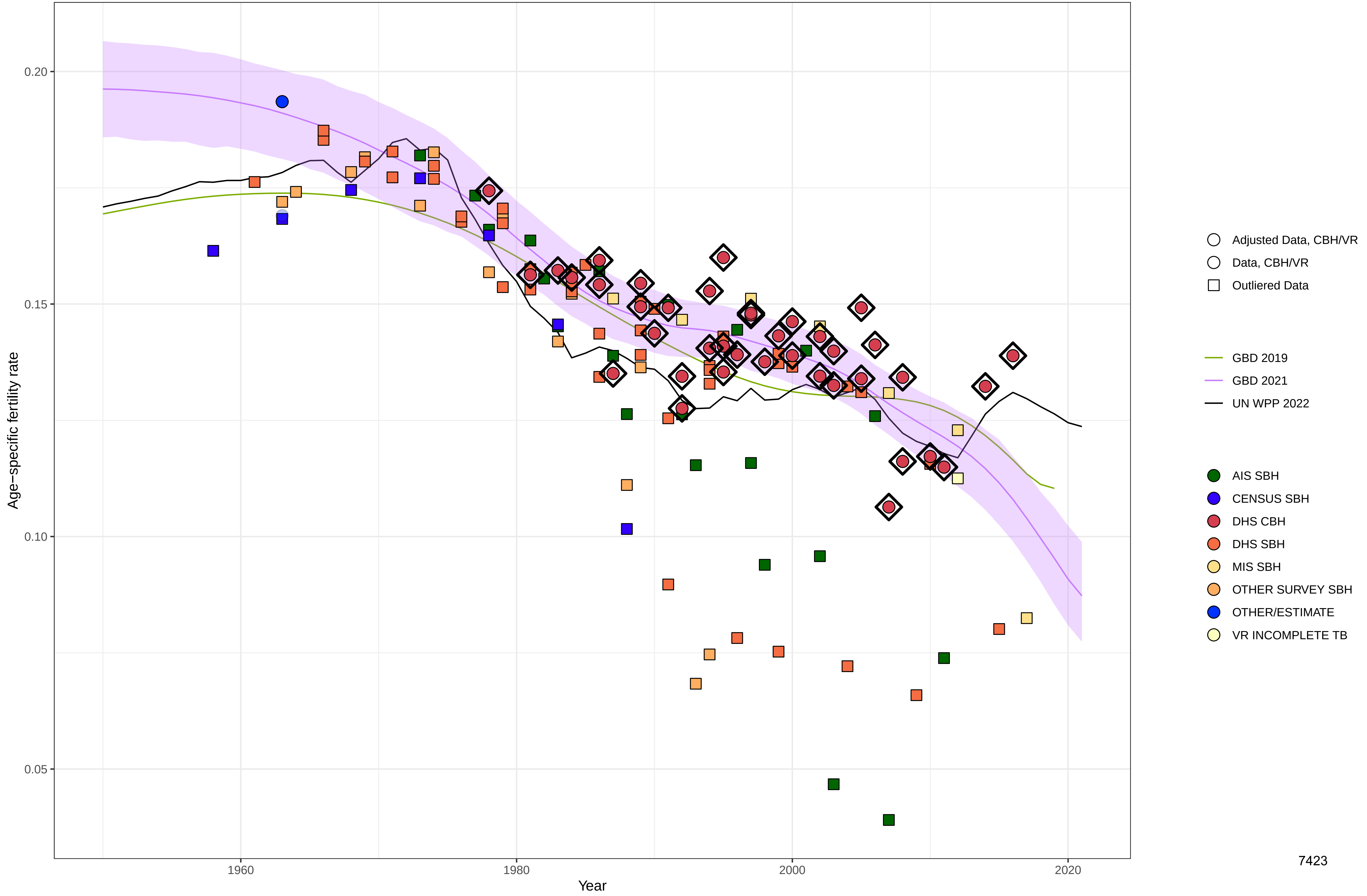

Age−specific fertility fitted trend lines, 1950−2021: Tanzania, Ages: 20−24
Data points enclosed with a diamond are reference sources

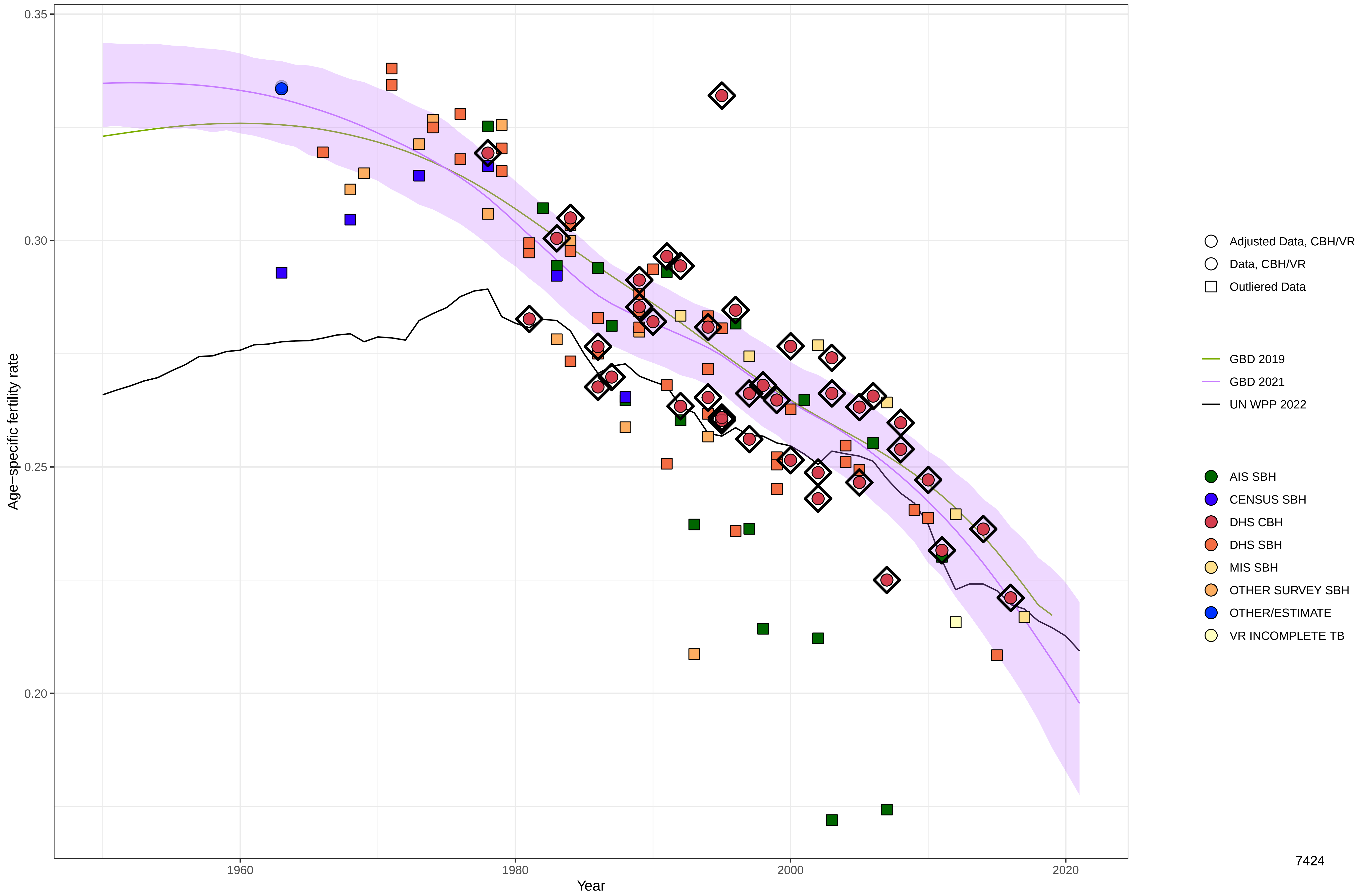

Age−specific fertility fitted trend lines, 1950−2021: Tanzania, Ages: 25−29
Data points enclosed with a diamond are reference sources

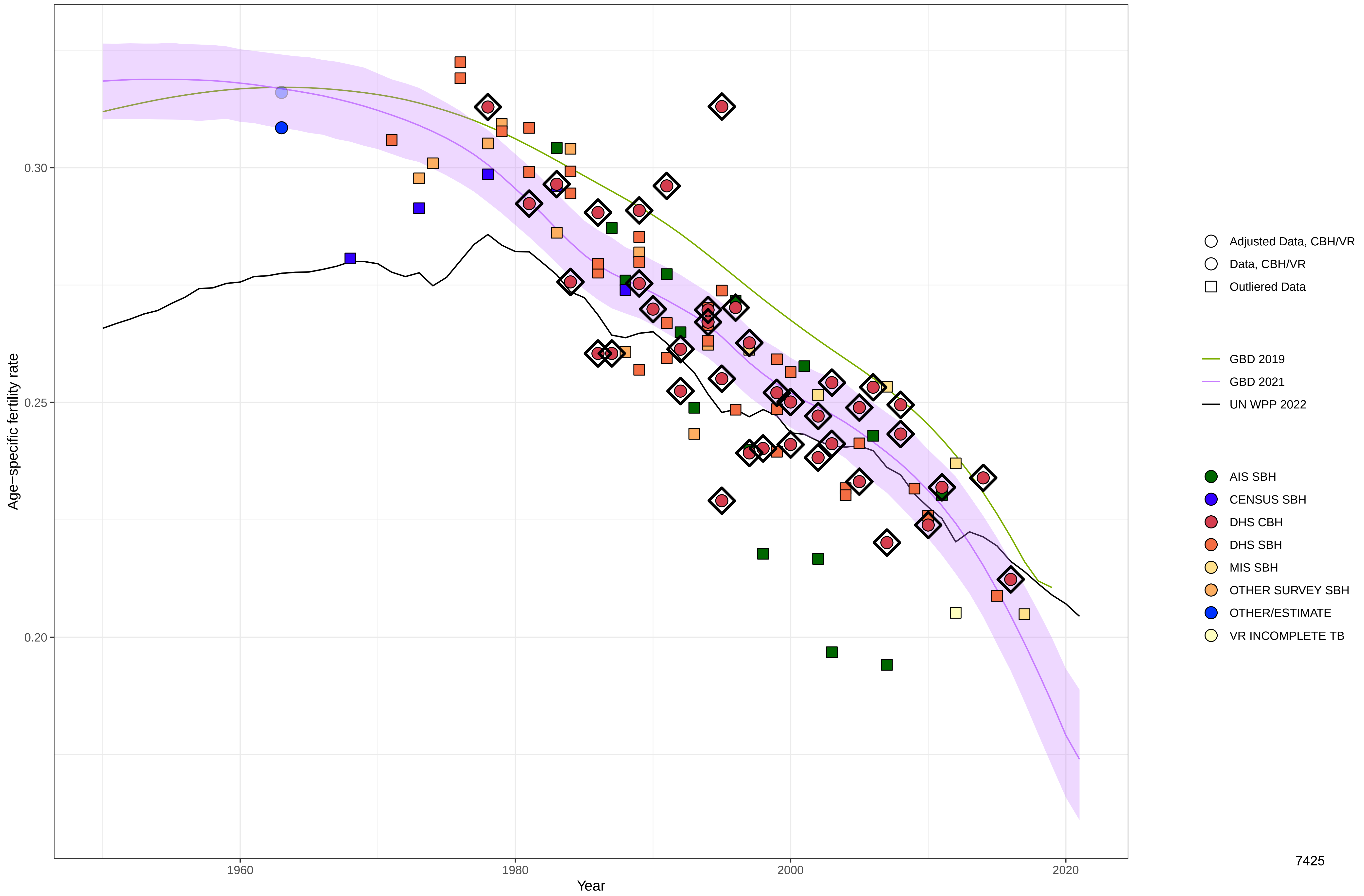

Age−specific fertility fitted trend lines, 1950−2021: Tanzania, Ages: 30−34
Data points enclosed with a diamond are reference sources

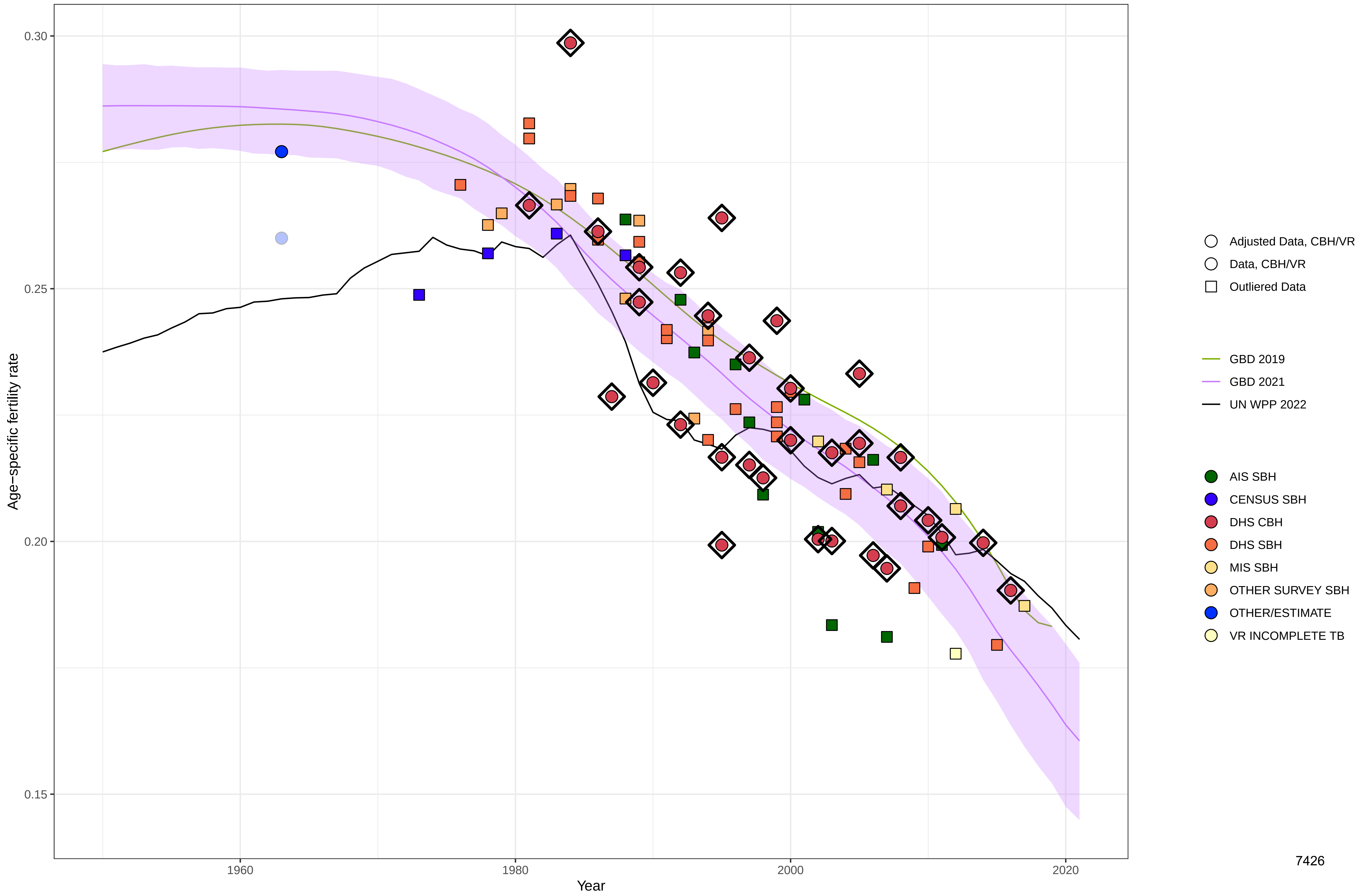

Age−specific fertility fitted trend lines, 1950−2021: Tanzania, Ages: 35−39
Data points enclosed with a diamond are reference sources

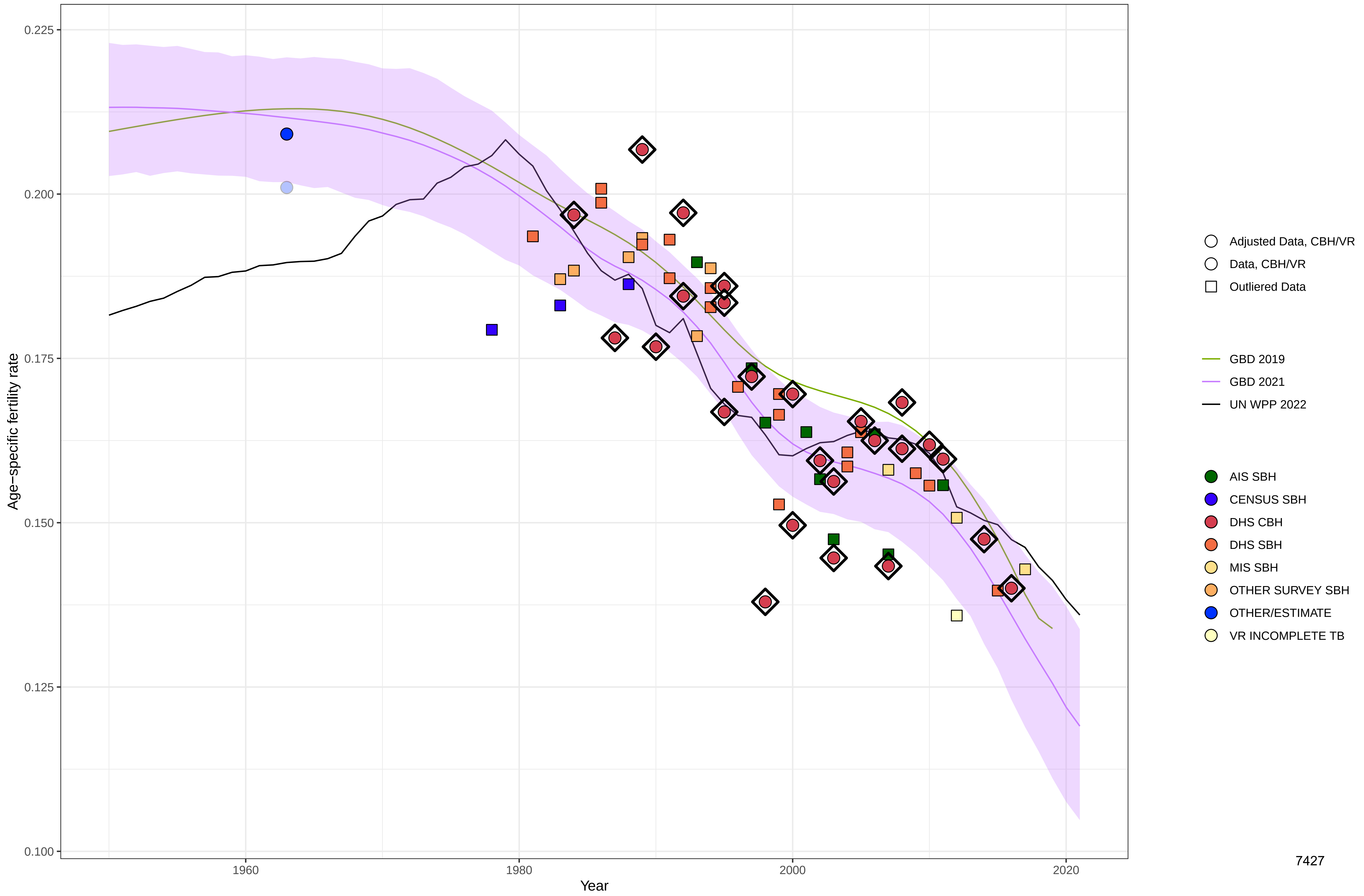

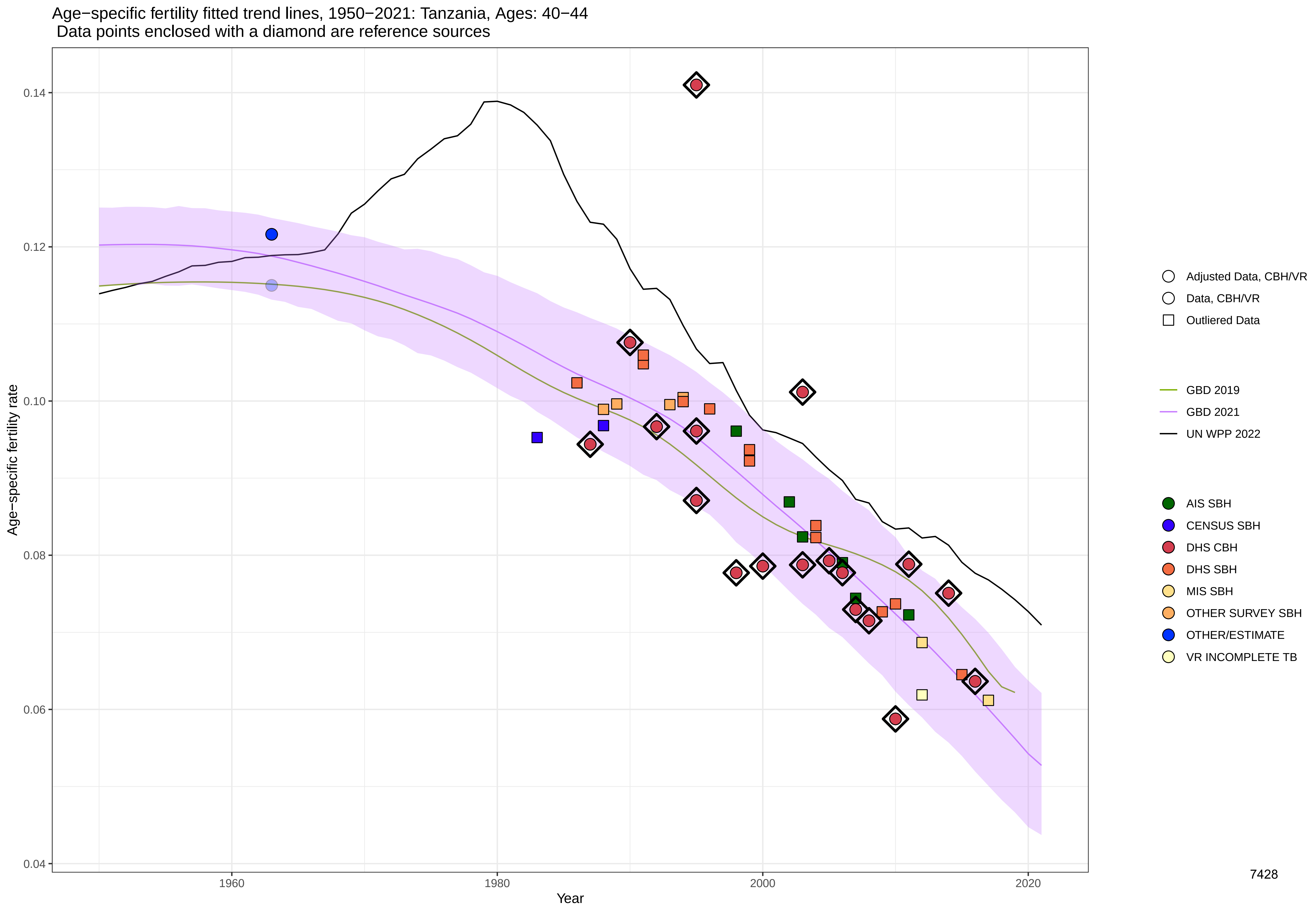
Age−specific fertility fitted trend lines, 1950−2021: Tanzania, Ages: 40−44
Data points enclosed with a diamond are reference sources
Age−specific fertility rate
0.04
0.06
0.08
0.10
0.12
0.14
Year
1960
1980
2000
2020
Adjusted Data, CBH/VR
Data, CBH/VR
Outliered Data
GBD 2019
GBD 2021
UN WPP 2022
AIS SBH
CENSUS SBH
DHS CBH
DHS SBH
MIS SBH
OTHER SURVEY SBH
OTHER/ESTIMATE
VR INCOMPLETE TB

Age−specific fertility fitted trend lines, 1950−2021: Tanzania, Ages: 45−49
Data points enclosed with a diamond are reference sources

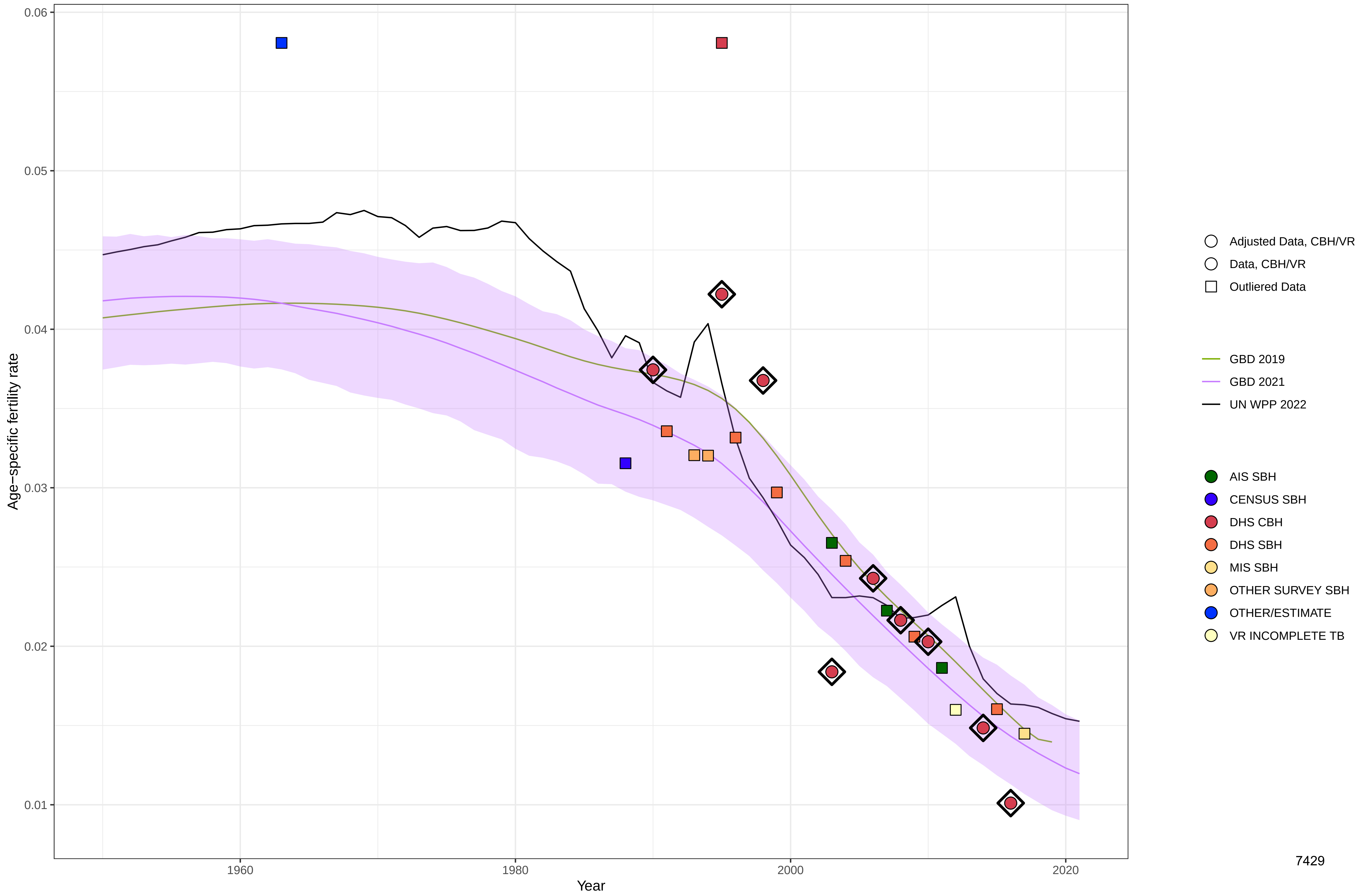

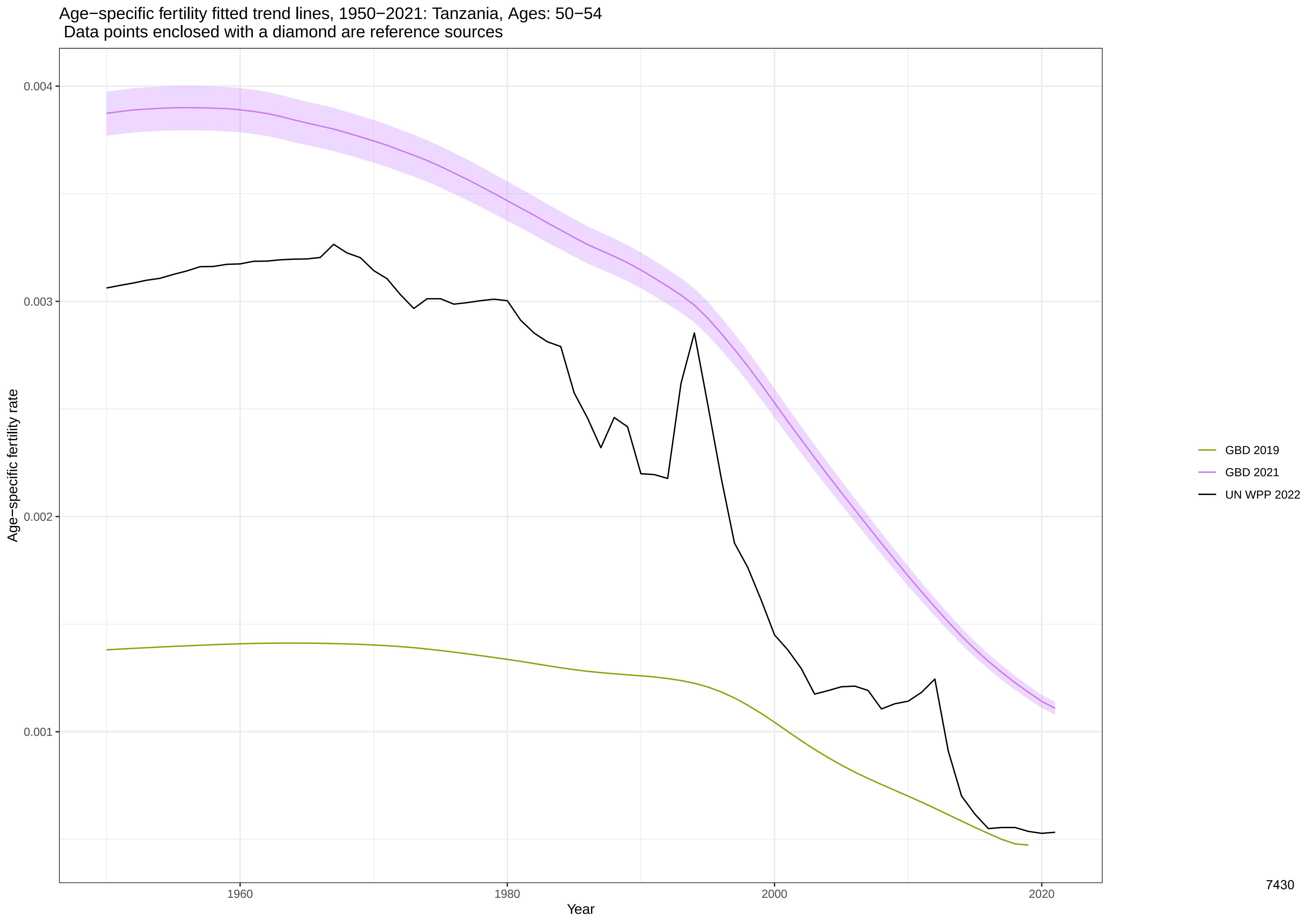

Age−specific fertility fitted trend lines, 1950−2021: Tanzania, Ages: 50−54
Data points enclosed with a diamond are reference sources
0.004
0.003
0.002
0.001
Age−specific fertility rate
1960
1980
2000
2020
Year
GBD 2019
GBD 2021
UN WPP 2022

Total fertility rate fitted trend lines, 1950−2021: Tanzania TFR
Data points enclosed with a diamond are reference sources

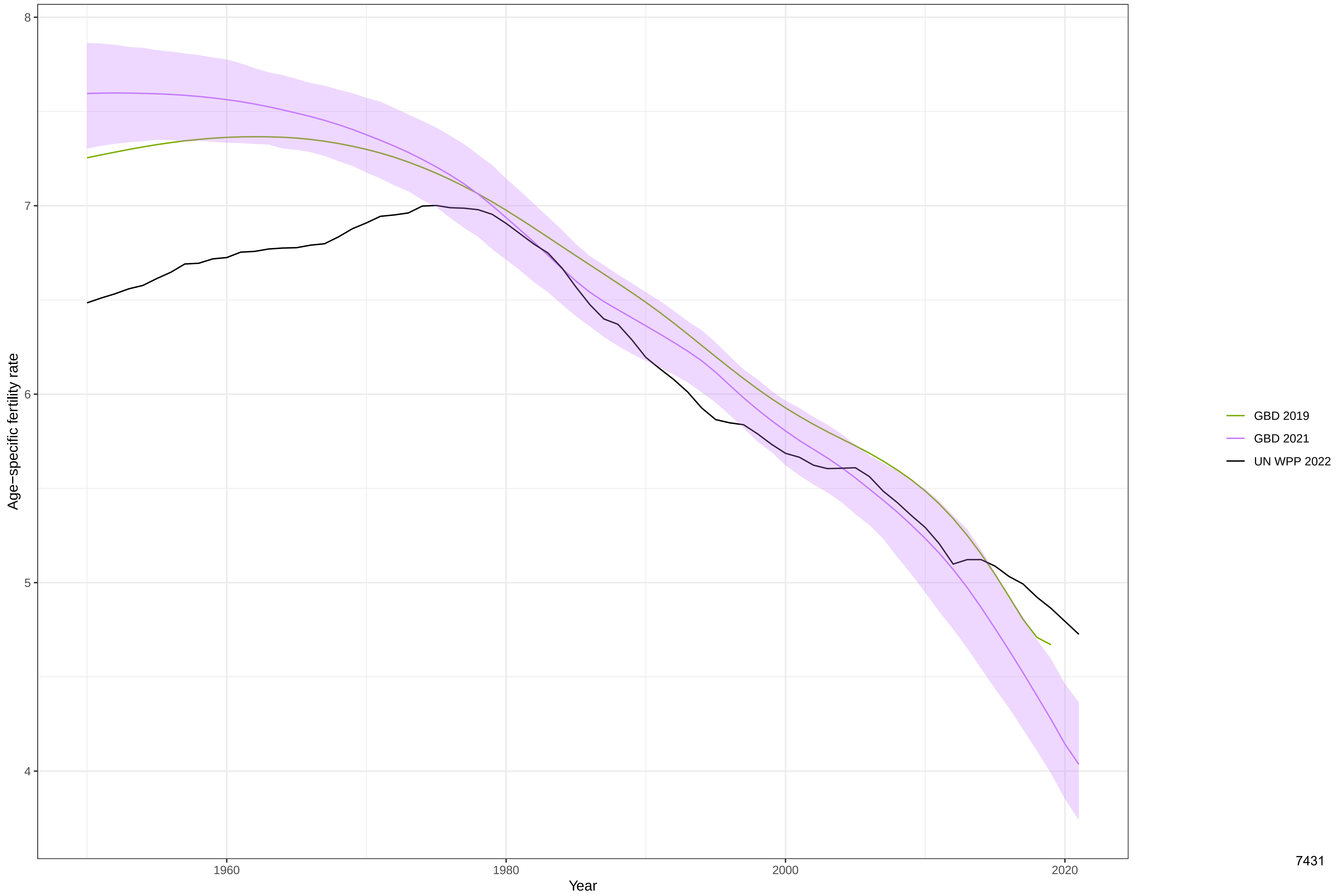

Age−specific fertility fitted trend lines, 1950−2021: Zambia, Ages: 10−14
Data points enclosed with a diamond are reference sources

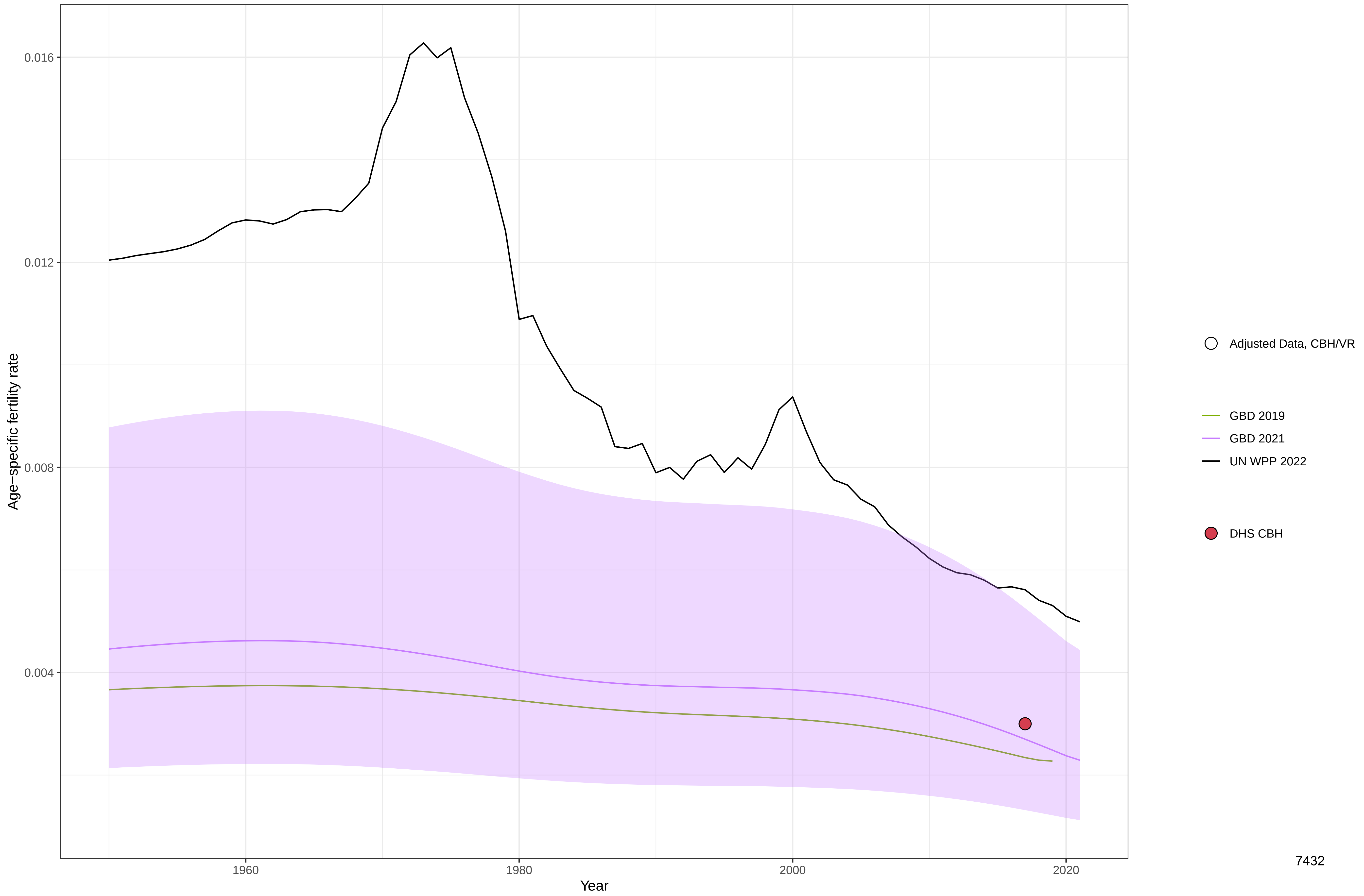

Age−specific fertility fitted trend lines, 1950−2021: Zambia, Ages: 15−19
Data points enclosed with a diamond are reference sources

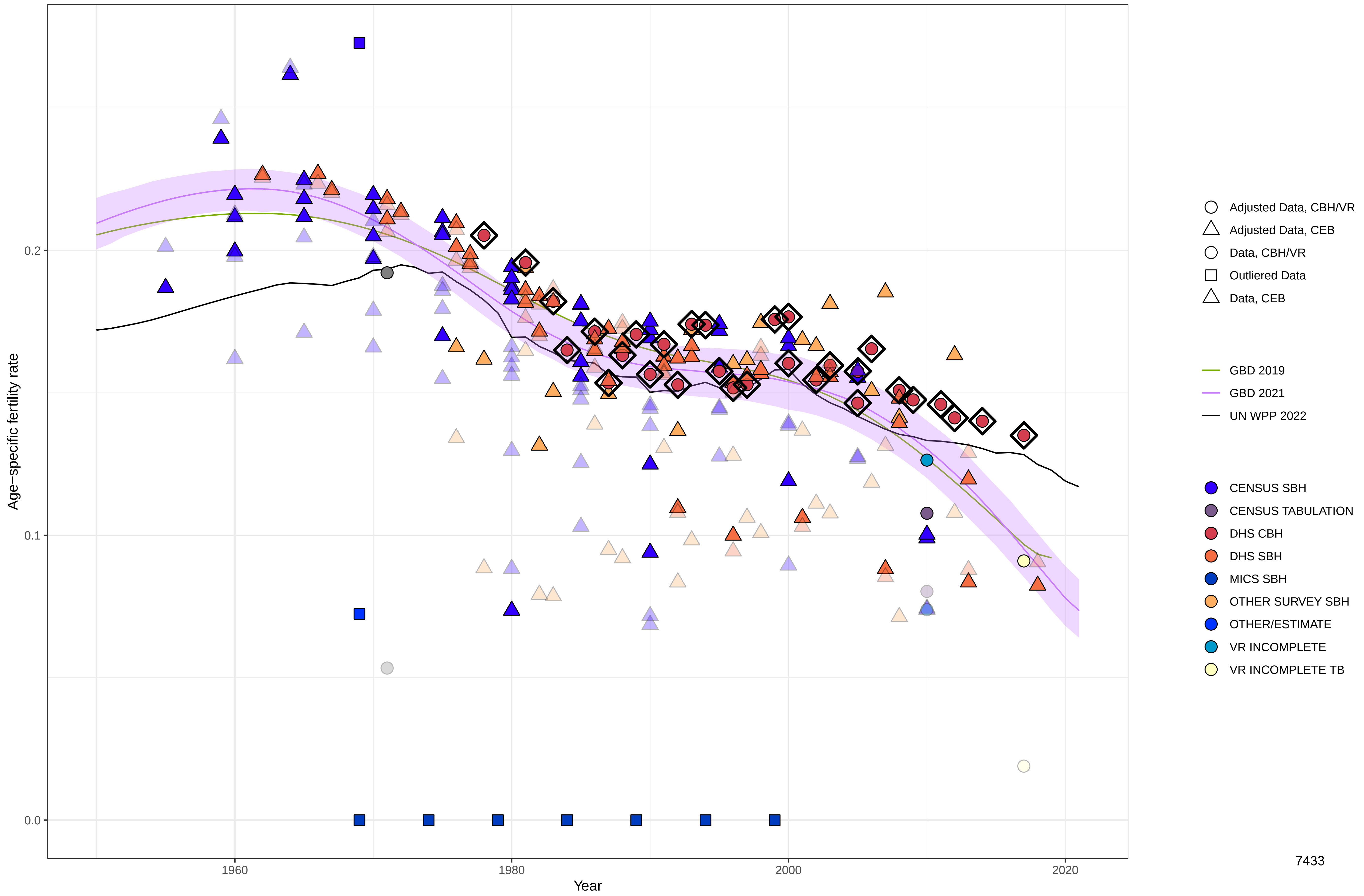

Age−specific fertility fitted trend lines, 1950−2021: Zambia, Ages: 20−24
Data points enclosed with a diamond are reference sources

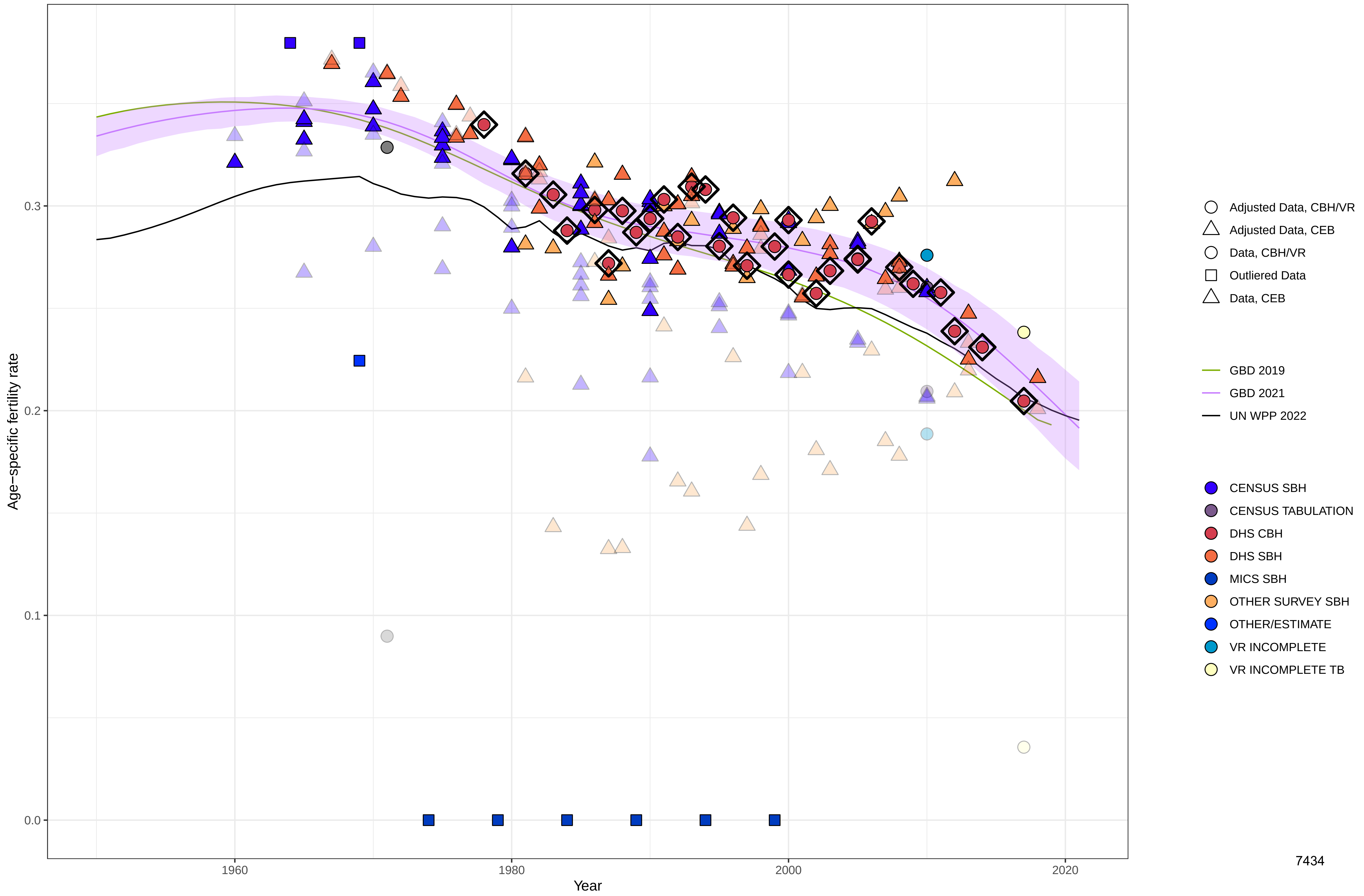

Age−specific fertility fitted trend lines, 1950−2021: Zambia, Ages: 25−29
Data points enclosed with a diamond are reference sources

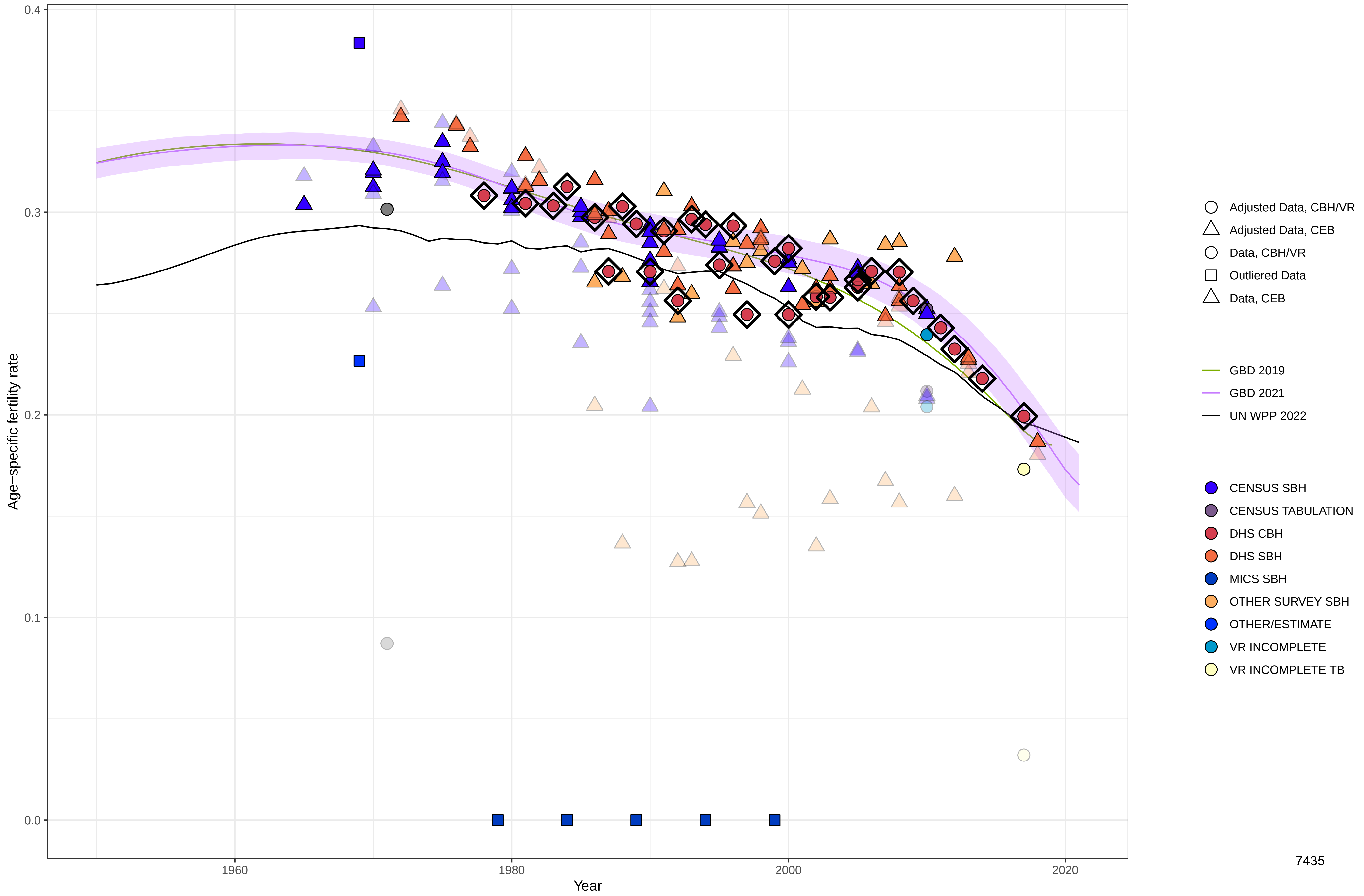

Age−specific fertility fitted trend lines, 1950−2021: Zambia, Ages: 30−34
Data points enclosed with a diamond are reference sources

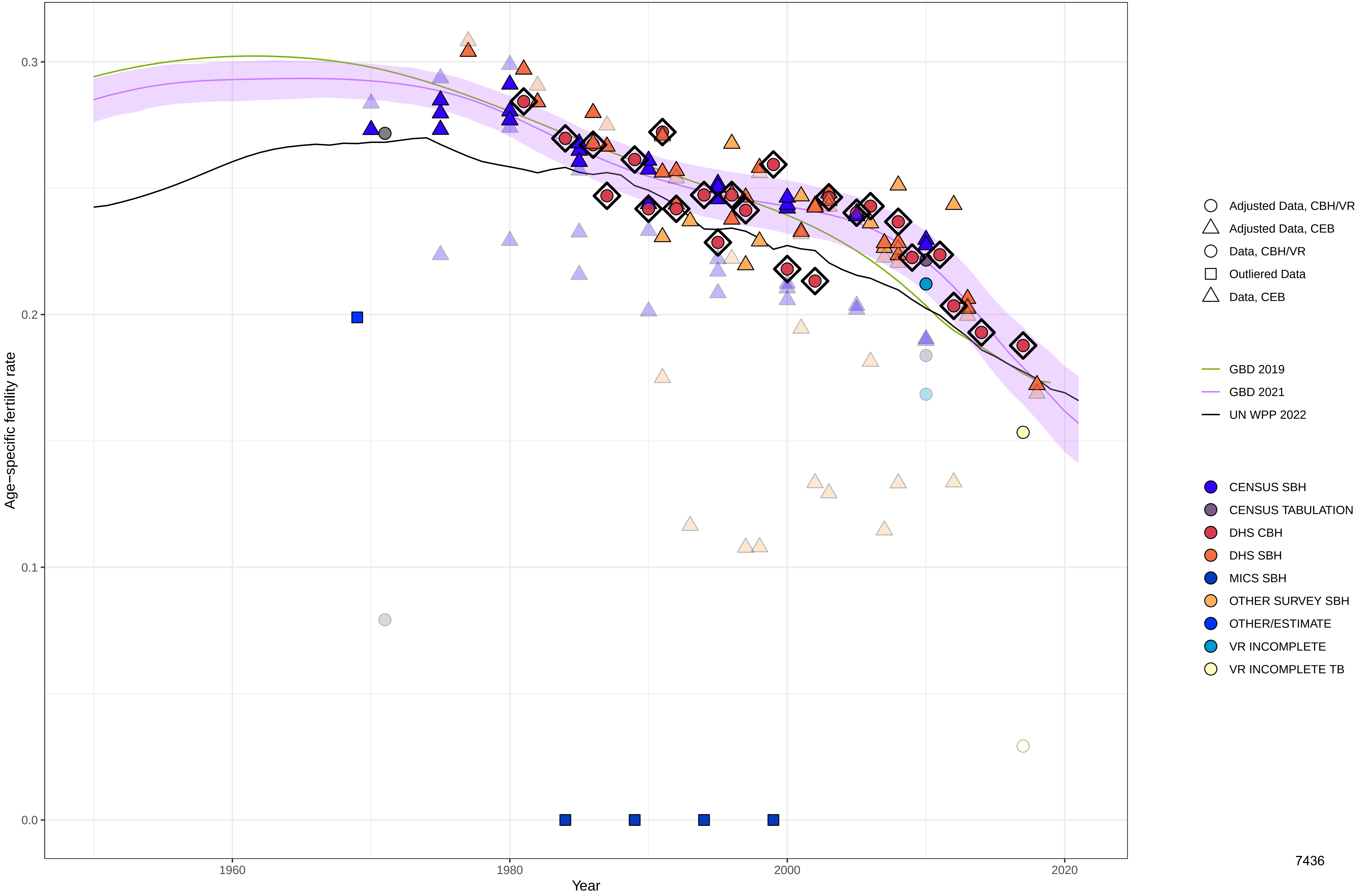

Age−specific fertility fitted trend lines, 1950−2021: Zambia, Ages: 35−39
Data points enclosed with a diamond are reference sources

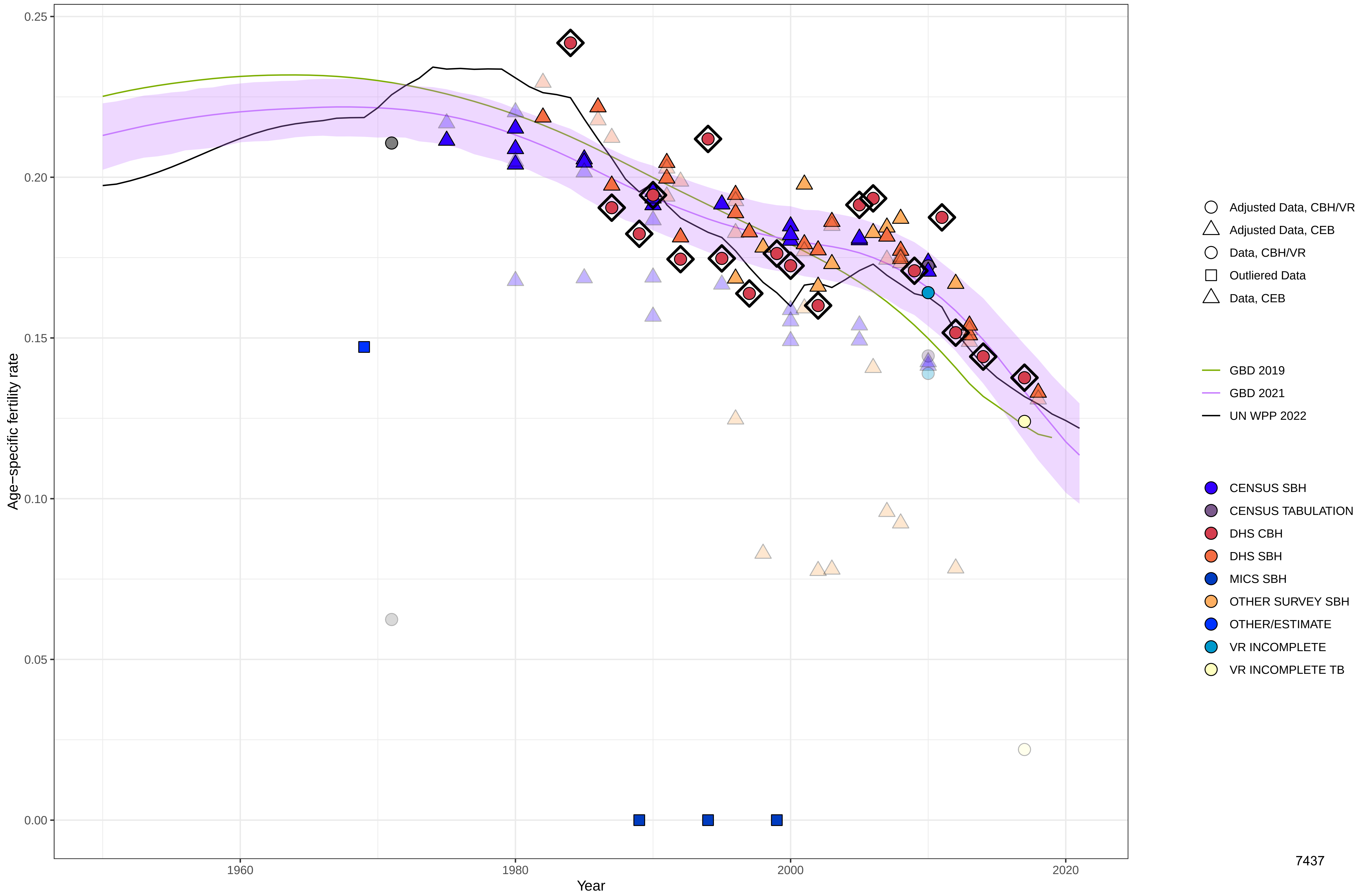

Age−specific fertility fitted trend lines, 1950−2021: Zambia, Ages: 40−44
Data points enclosed with a diamond are reference sources

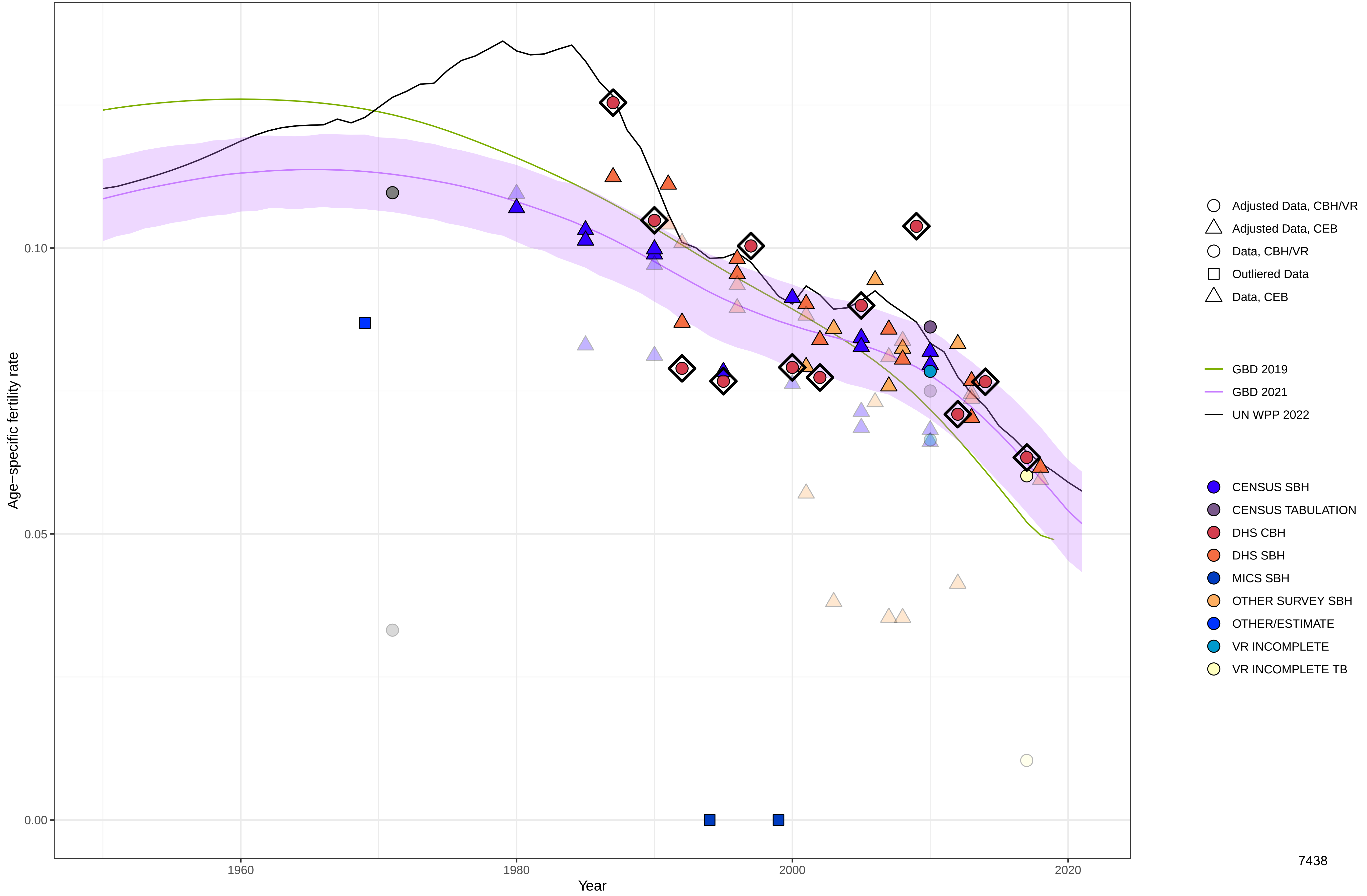

Age−specific fertility fitted trend lines, 1950−2021: Zambia, Ages: 45−49
Data points enclosed with a diamond are reference sources

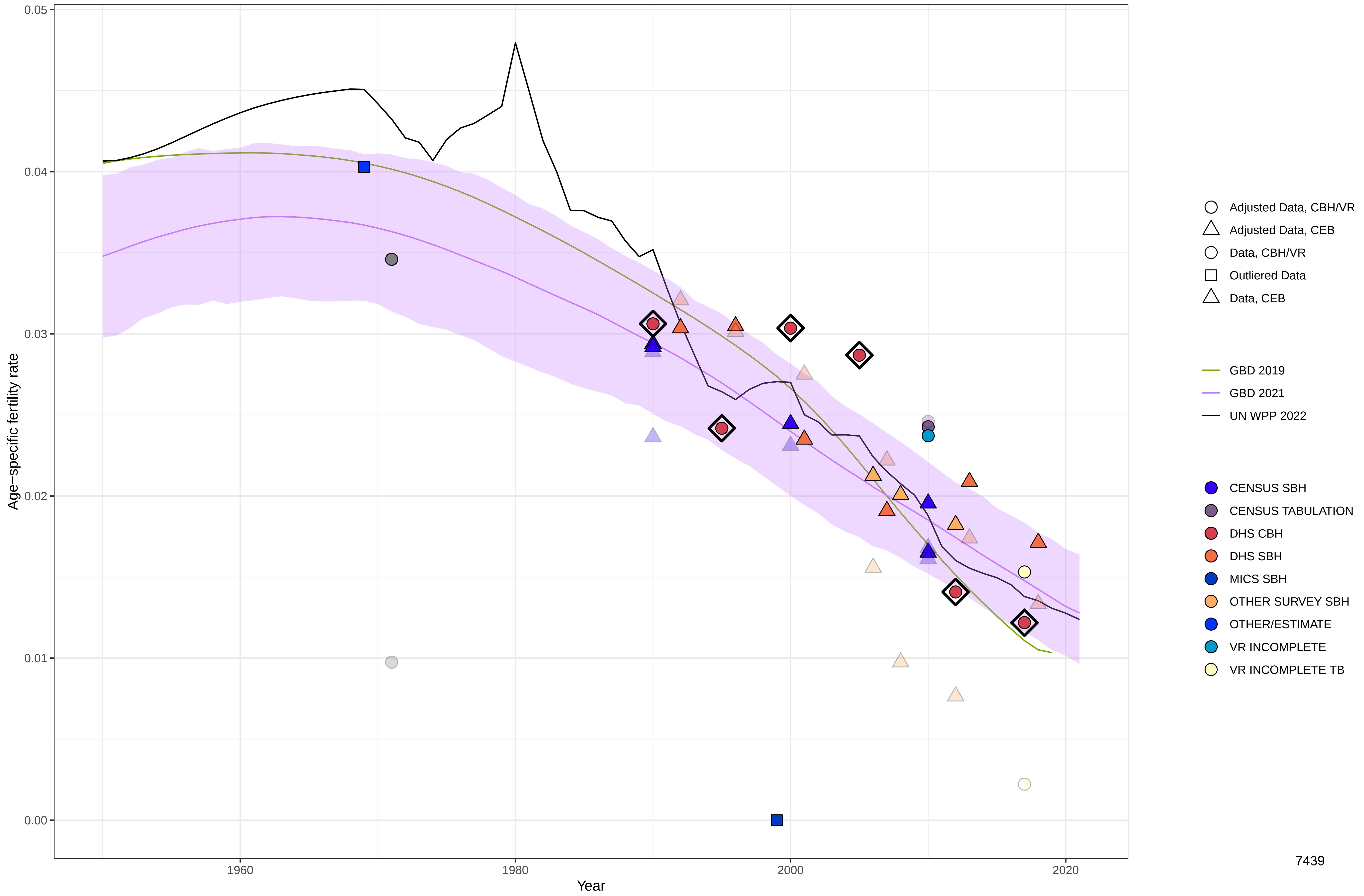

Age−specific fertility fitted trend lines, 1950−2021: Zambia, Ages: 50−54
Data points enclosed with a diamond are reference sources

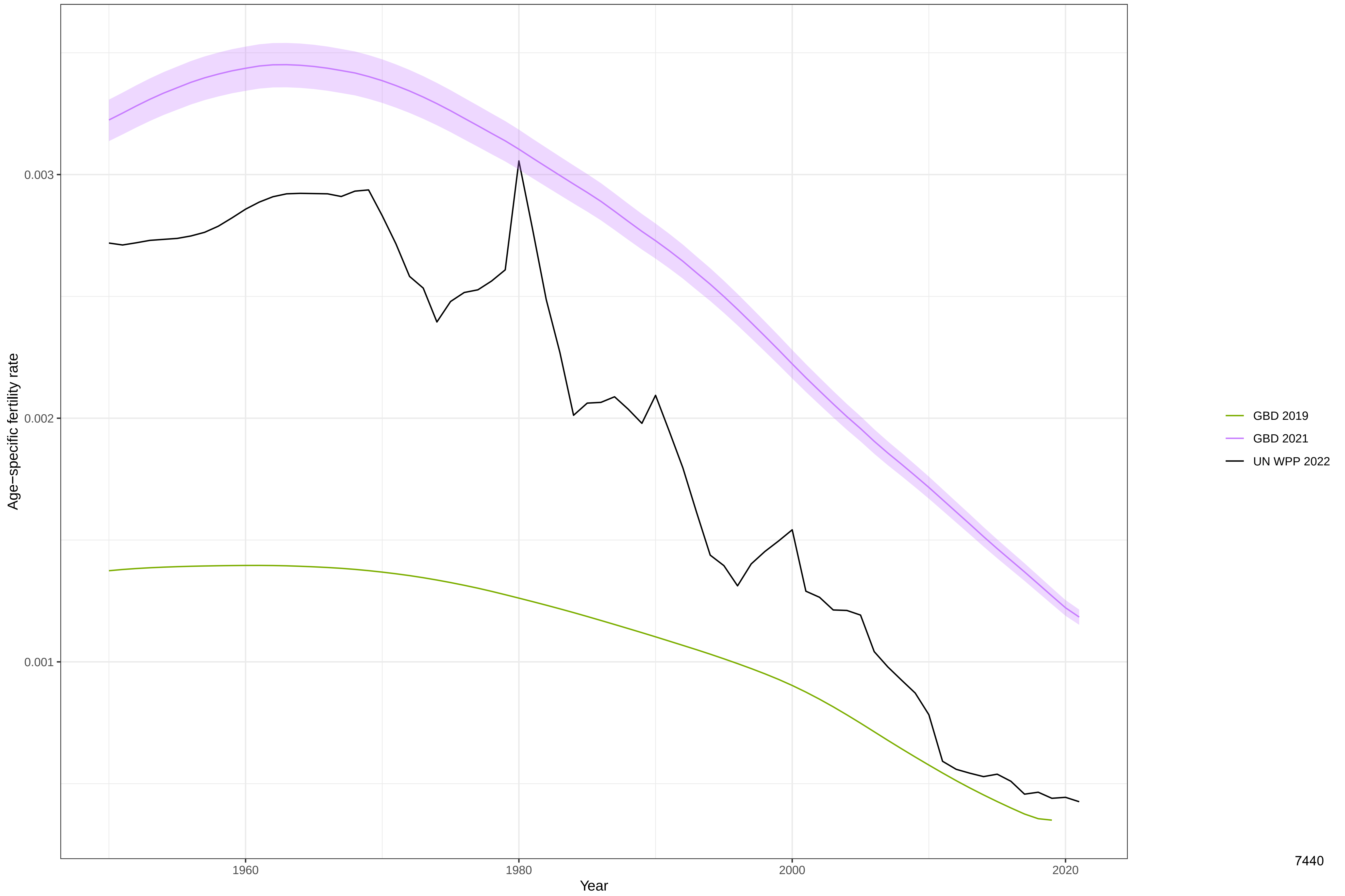

Total fertility rate fitted trend lines, 1950−2021: Zambia TFR
Data points enclosed with a diamond are reference sources

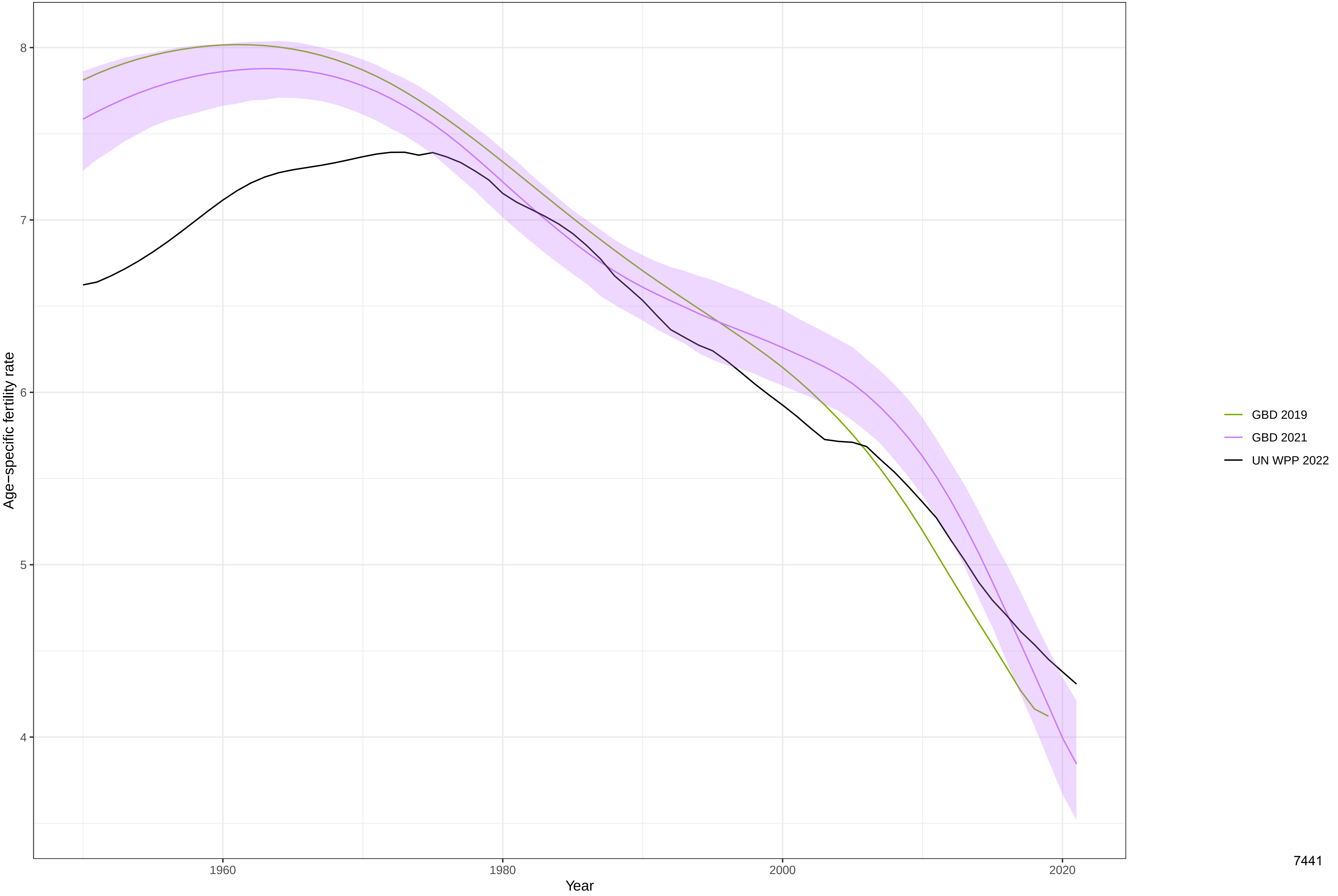

Age−specific fertility fitted trend lines, 1950−2021: Botswana, Ages: 10−14
Data points enclosed with a diamond are reference sources

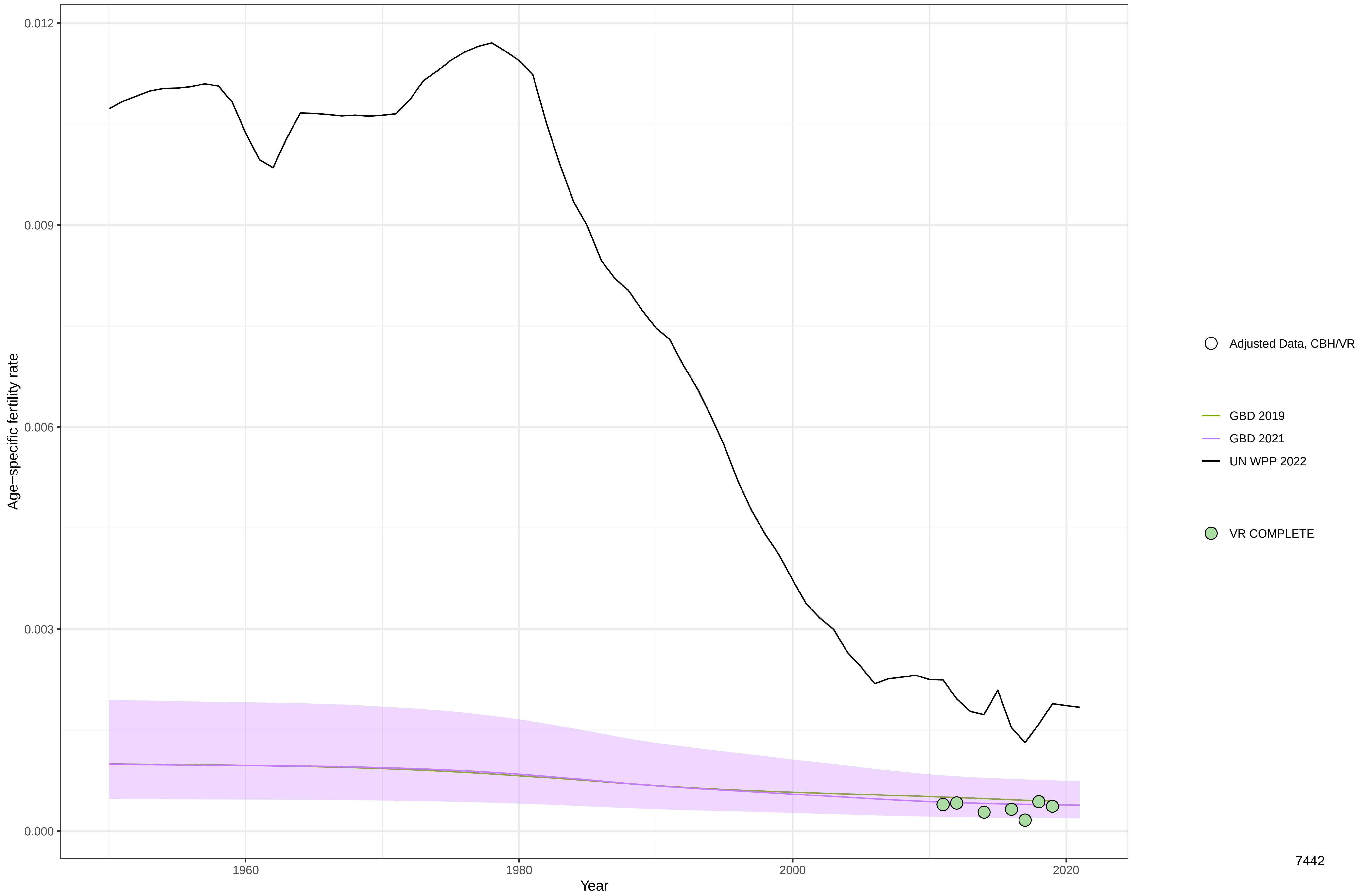

Age−specific fertility fitted trend lines, 1950−2021: Botswana, Ages: 15−19
Data points enclosed with a diamond are reference sources

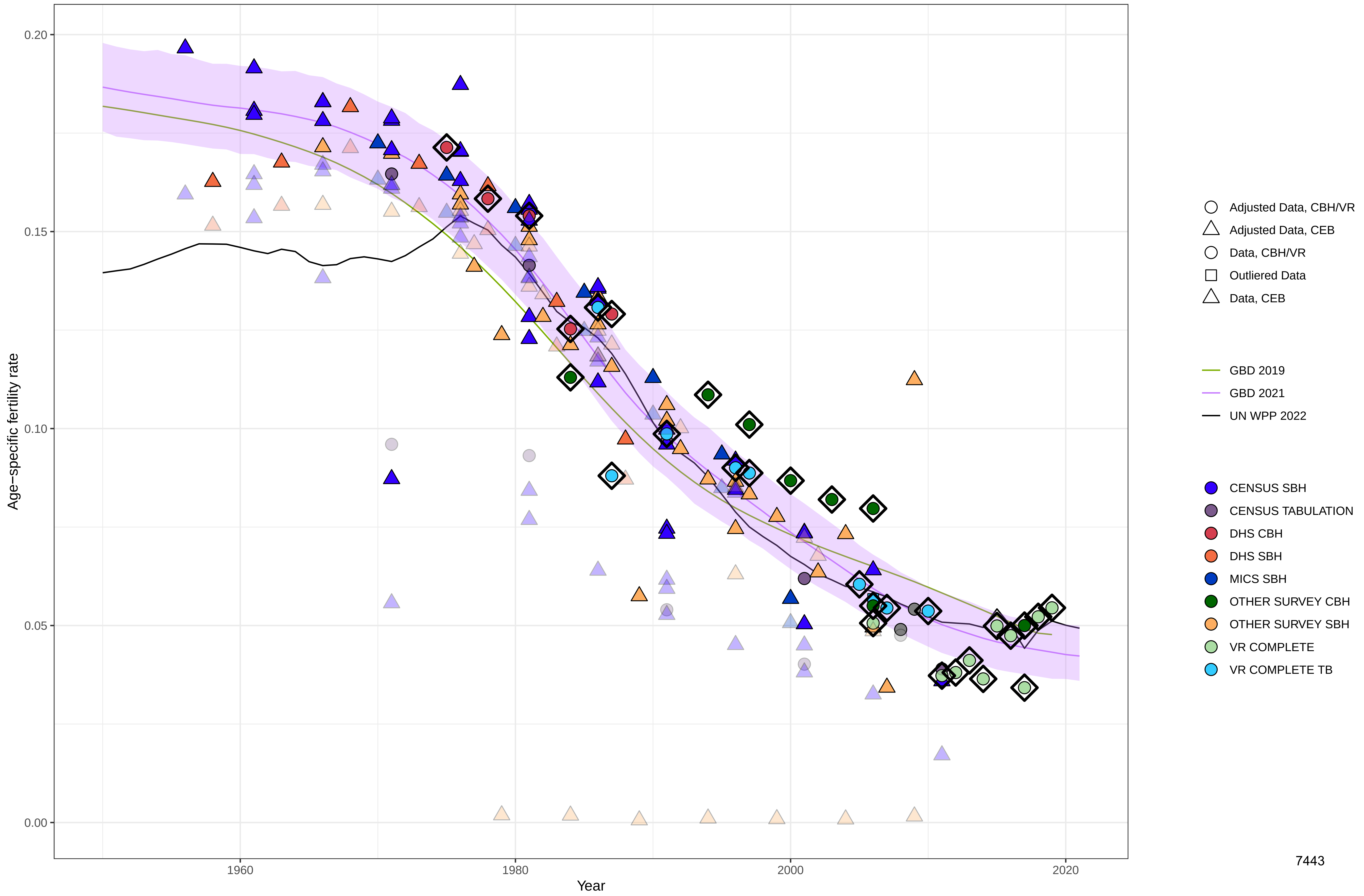

Age−specific fertility fitted trend lines, 1950−2021: Botswana, Ages: 20−24
Data points enclosed with a diamond are reference sources

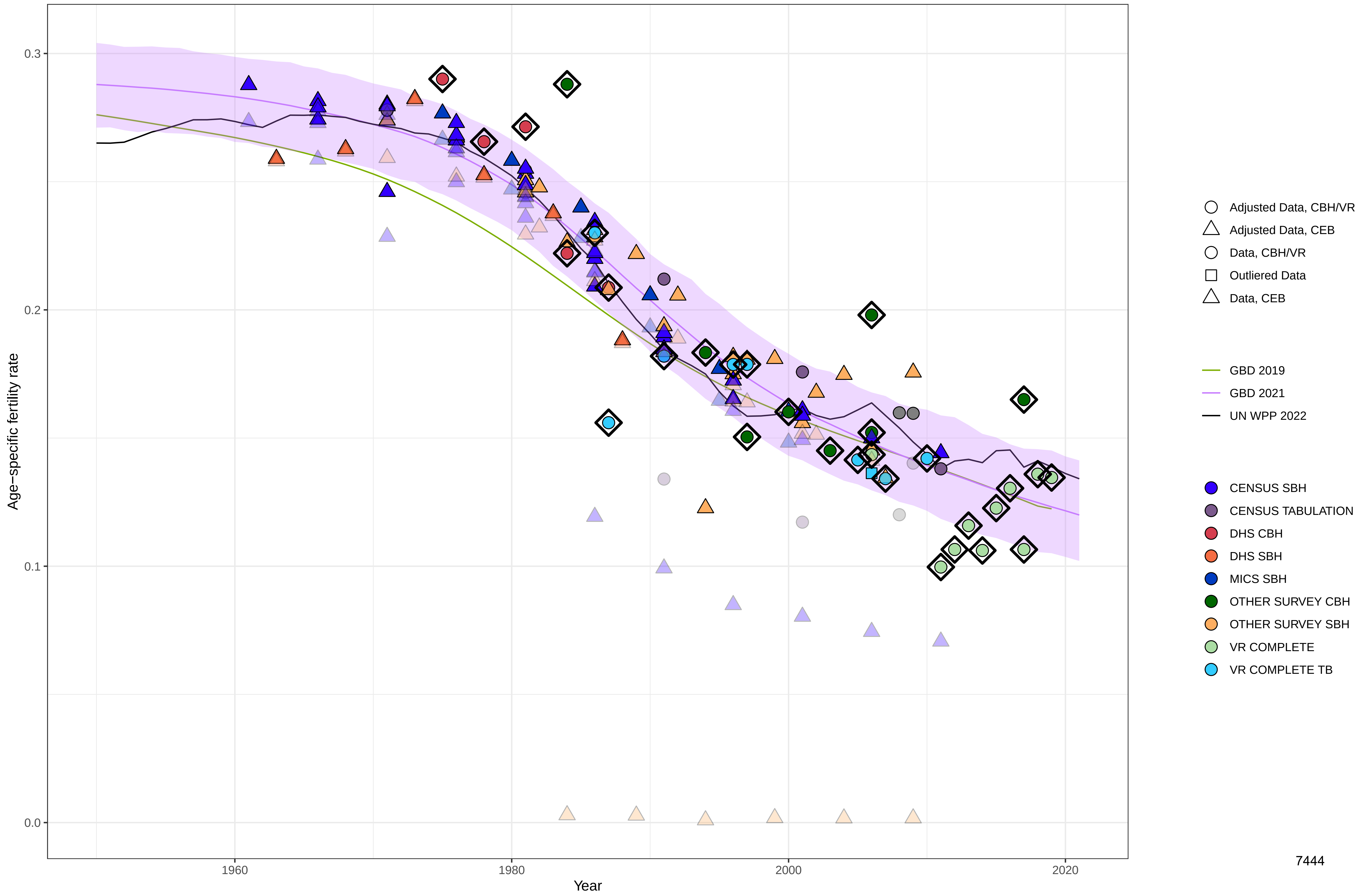

Age−specific fertility fitted trend lines, 1950−2021: Botswana, Ages: 25−29
Data points enclosed with a diamond are reference sources

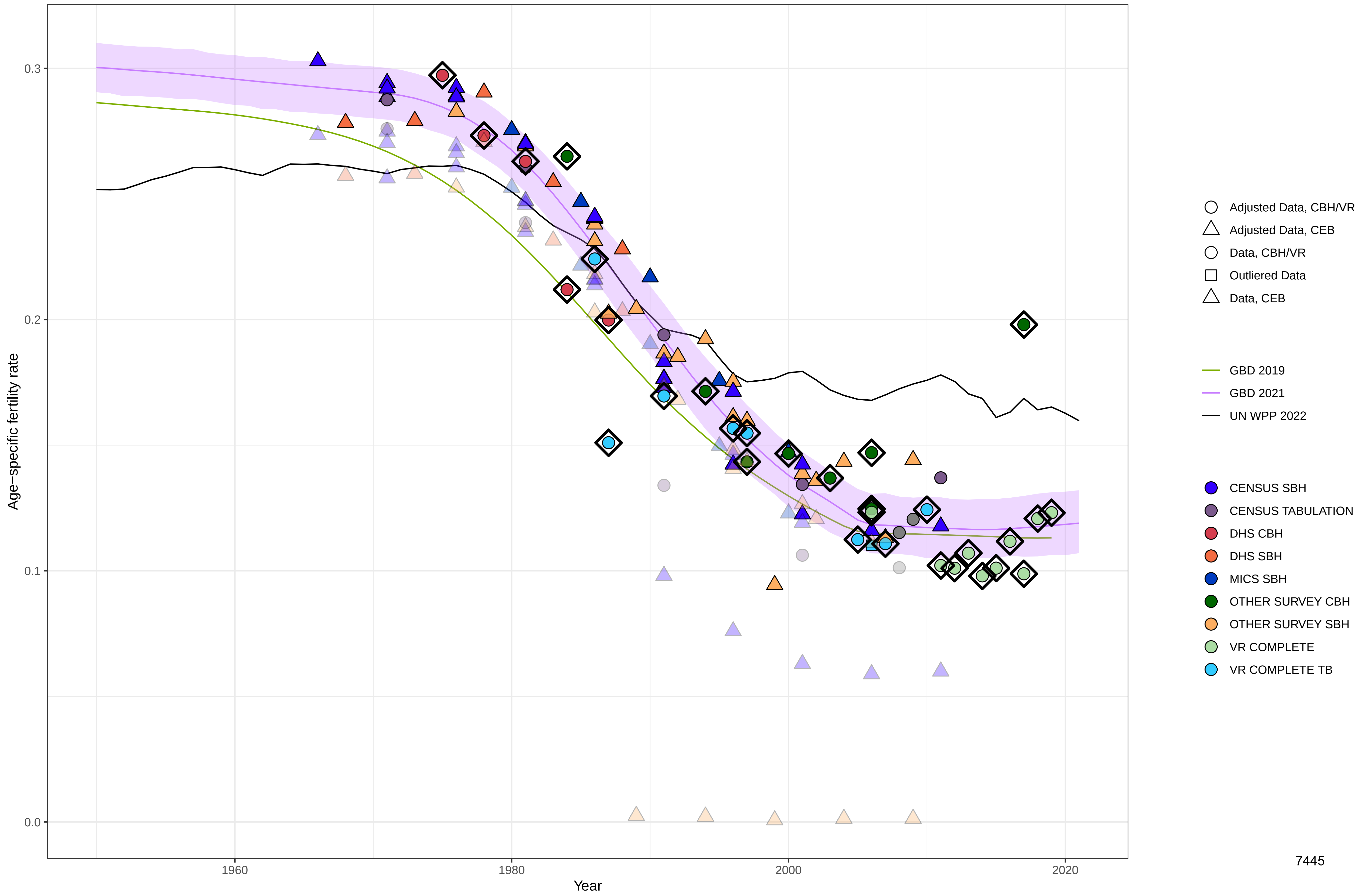

Age−specific fertility fitted trend lines, 1950−2021: Botswana, Ages: 30−34
Data points enclosed with a diamond are reference sources

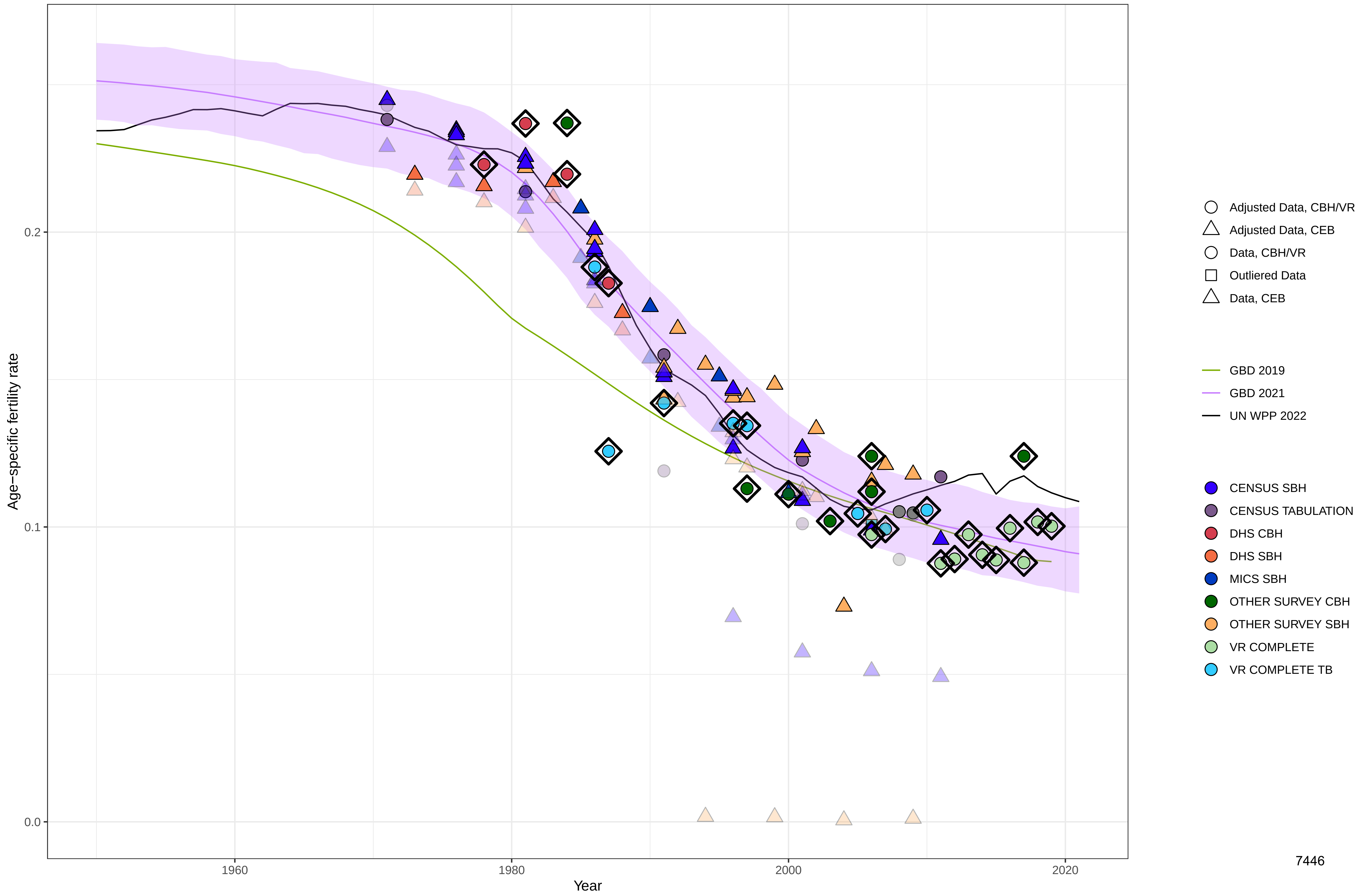

Age−specific fertility fitted trend lines, 1950−2021: Botswana, Ages: 35−39
Data points enclosed with a diamond are reference sources

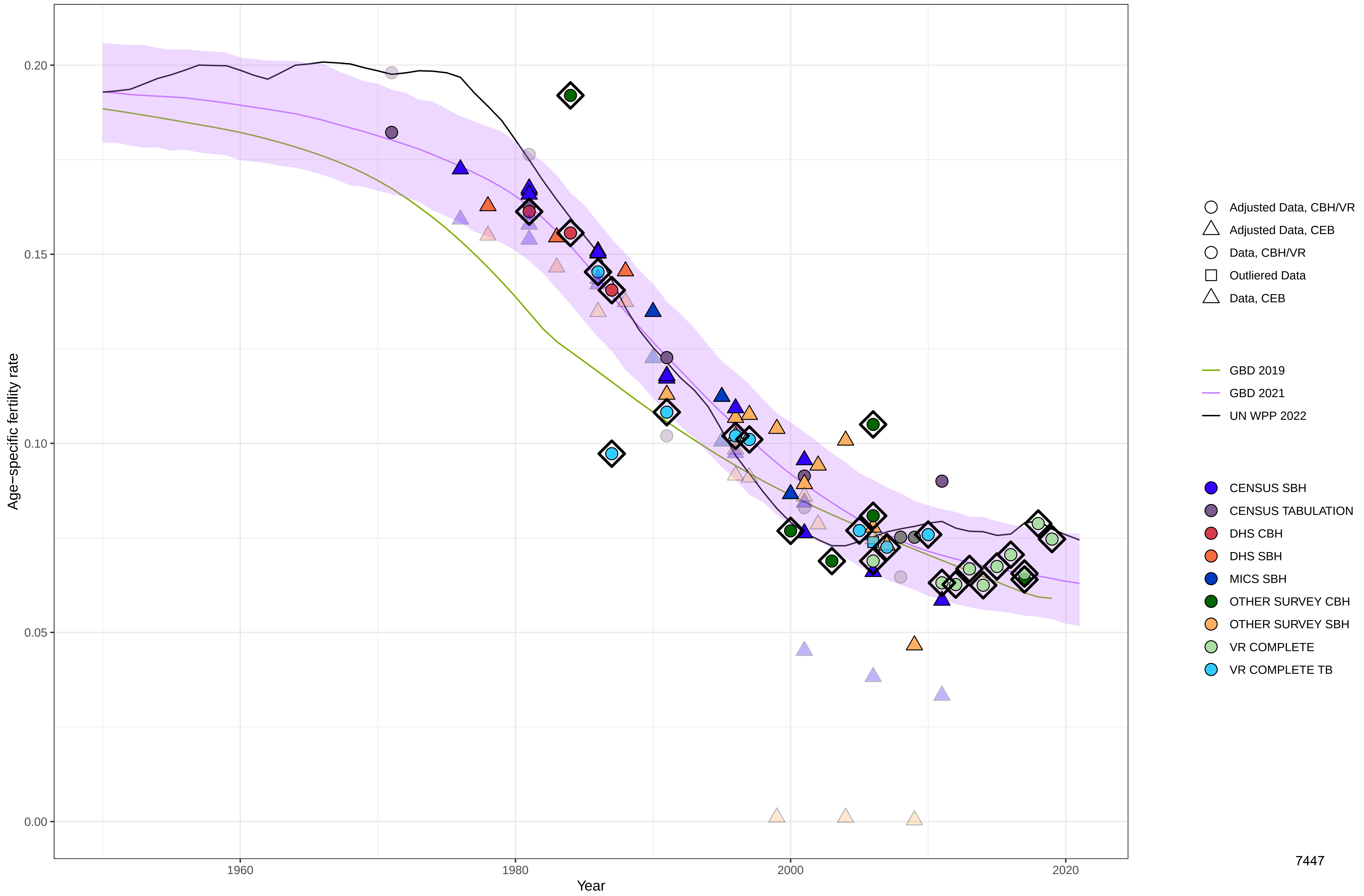

Age−specific fertility fitted trend lines, 1950−2021: Botswana, Ages: 40−44
Data points enclosed with a diamond are reference sources

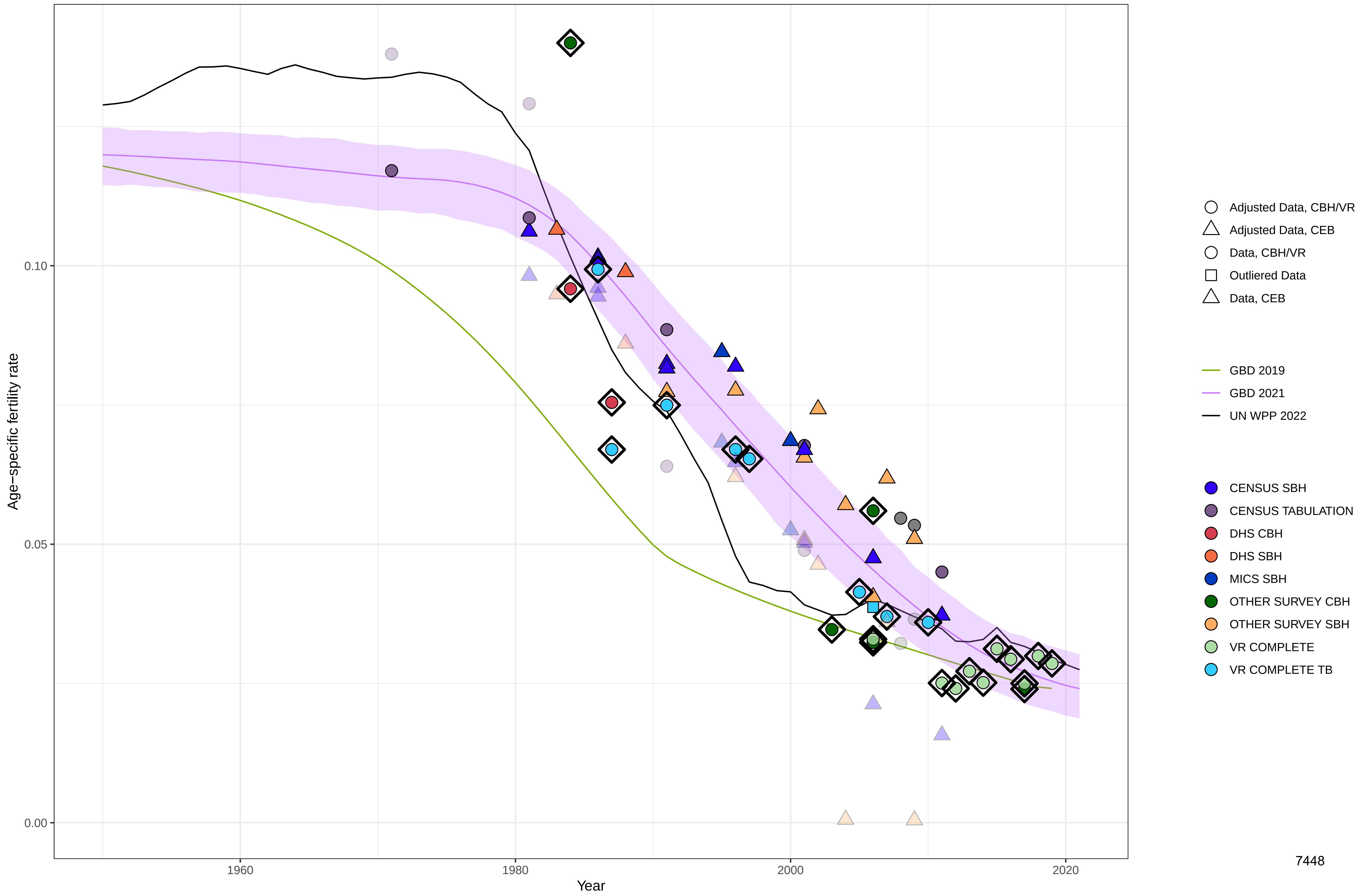

Age−specific fertility fitted trend lines, 1950−2021: Botswana, Ages: 45−49
Data points enclosed with a diamond are reference sources

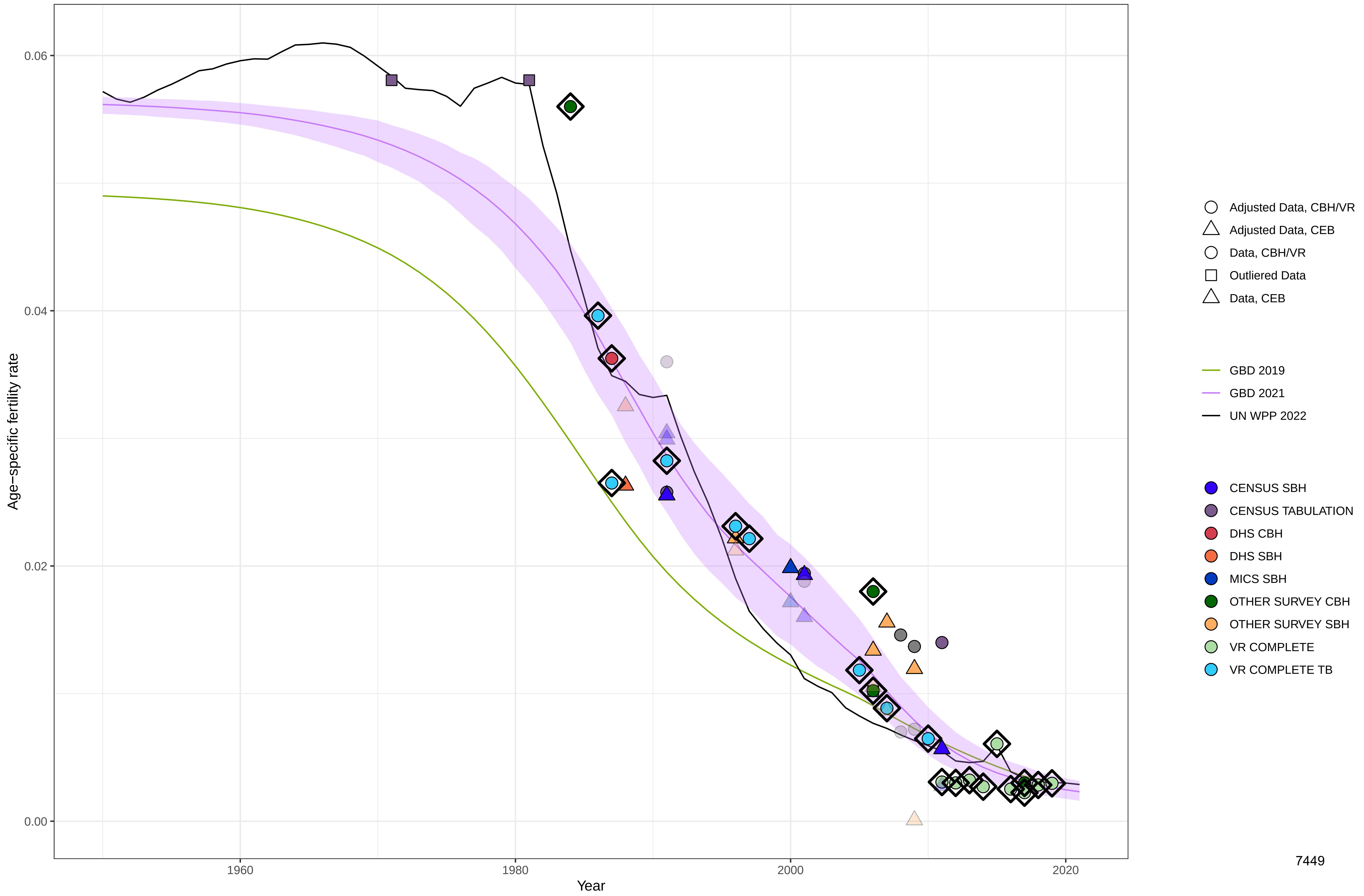

Age−specific fertility fitted trend lines, 1950−2021: Botswana, Ages: 50−54
Data points enclosed with a diamond are reference sources

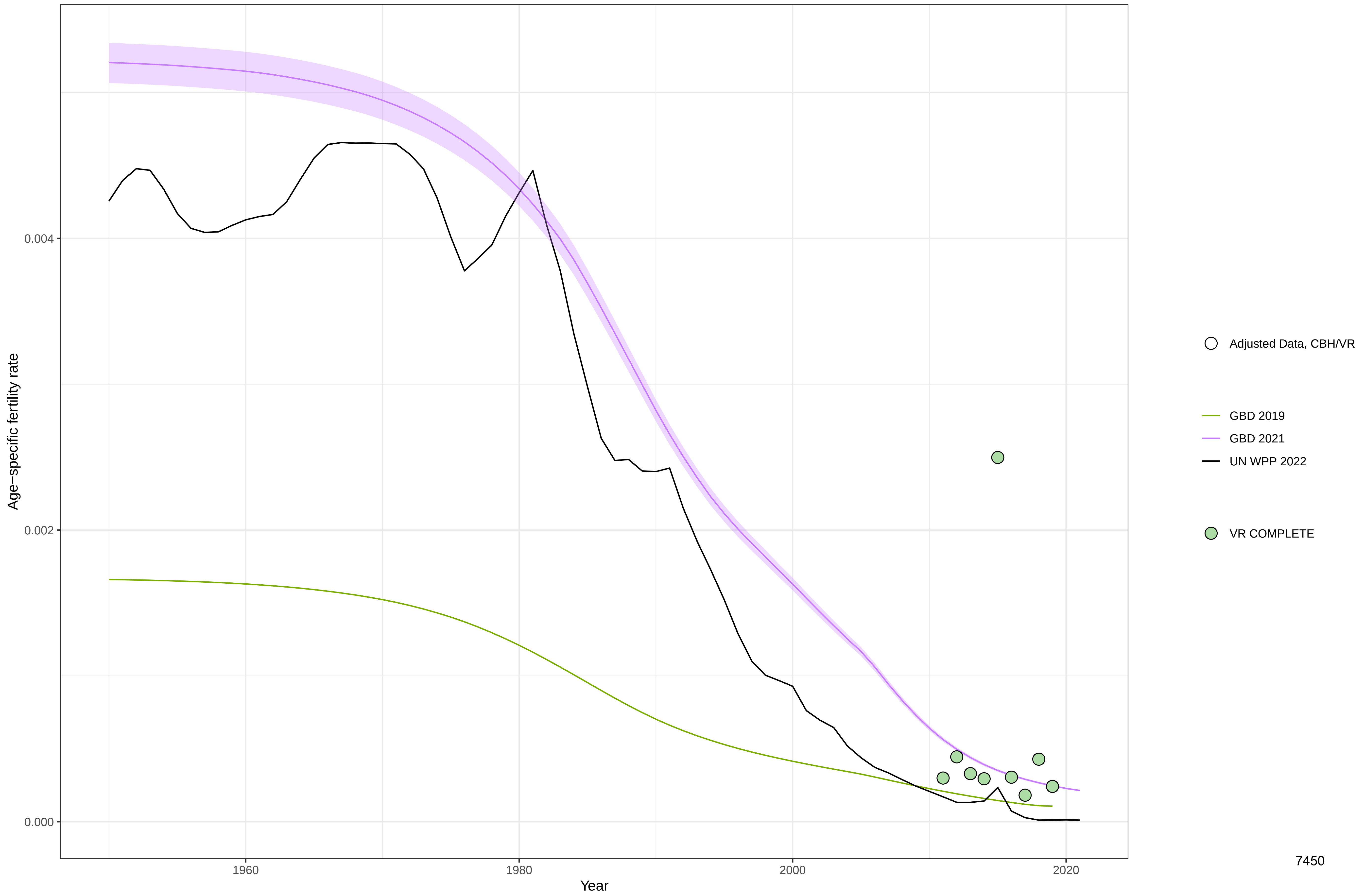

Total fertility rate fitted trend lines, 1950−2021: Botswana TFR
Data points enclosed with a diamond are reference sources

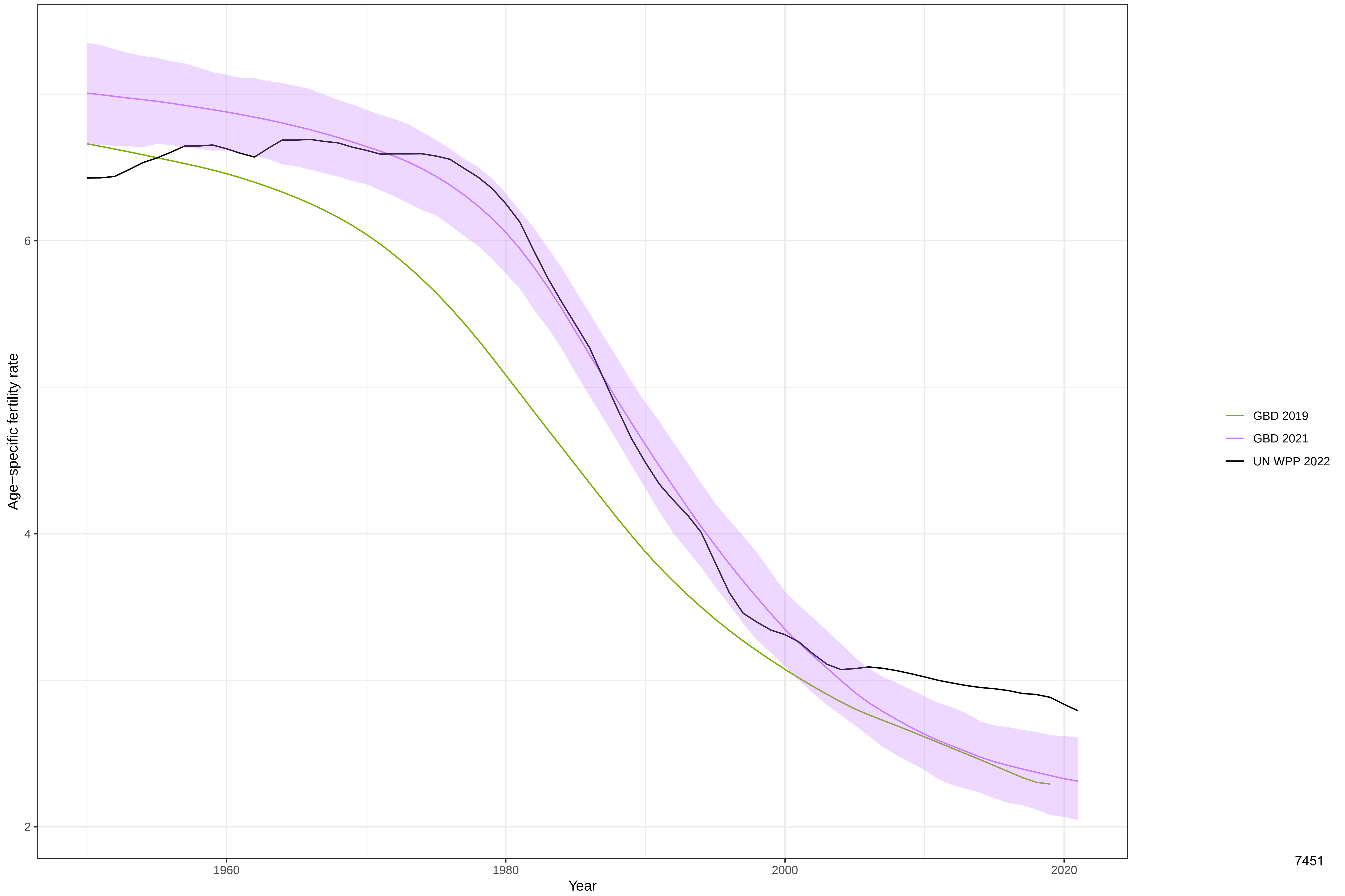

Age−specific fertility fitted trend lines, 1950−2021: Eswatini, Ages: 10−14
Data points enclosed with a diamond are reference sources

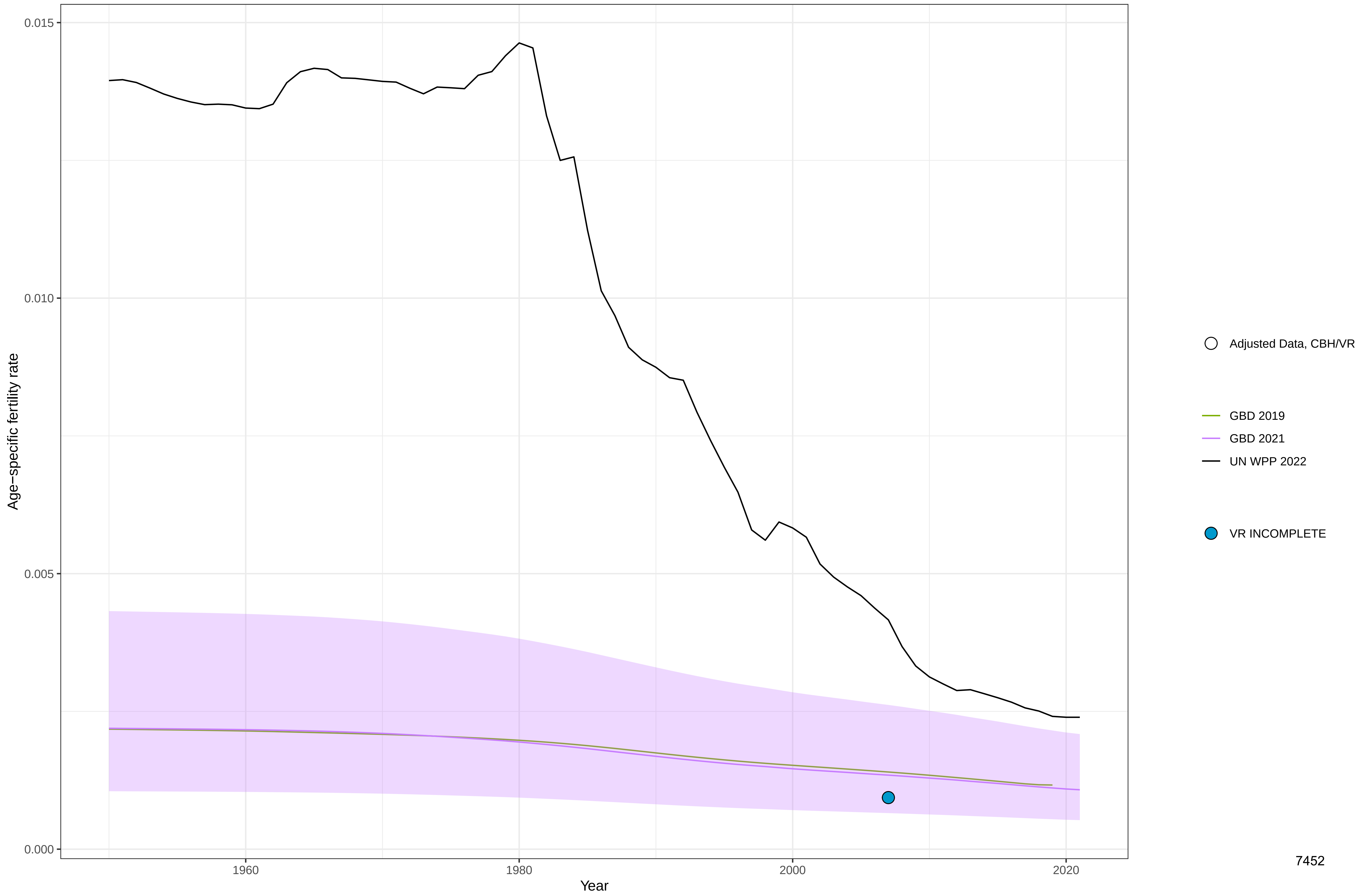

Age−specific fertility fitted trend lines, 1950−2021: Eswatini, Ages: 15−19
Data points enclosed with a diamond are reference sources

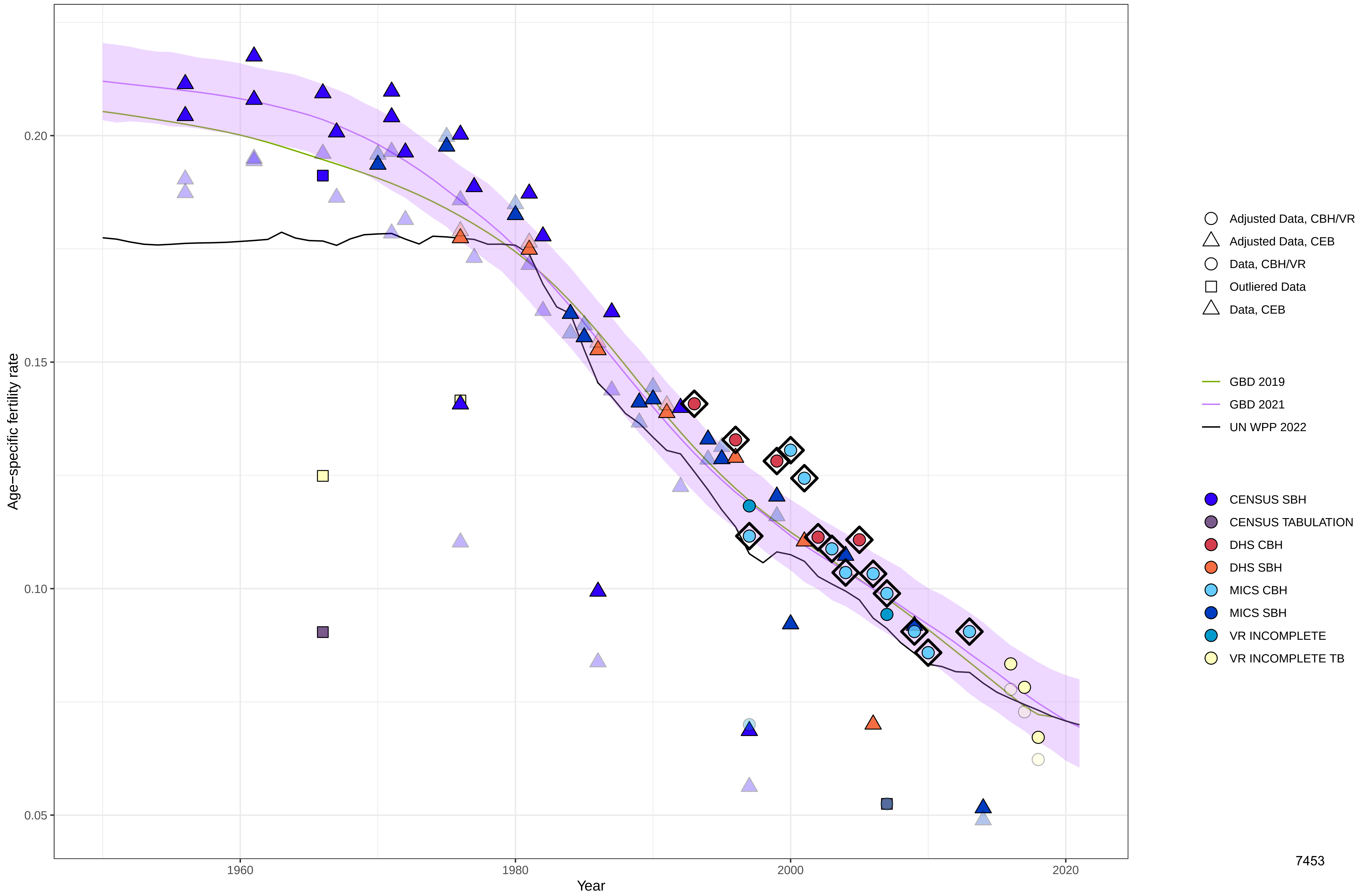

Age−specific fertility fitted trend lines, 1950−2021: Eswatini, Ages: 20−24
Data points enclosed with a diamond are reference sources

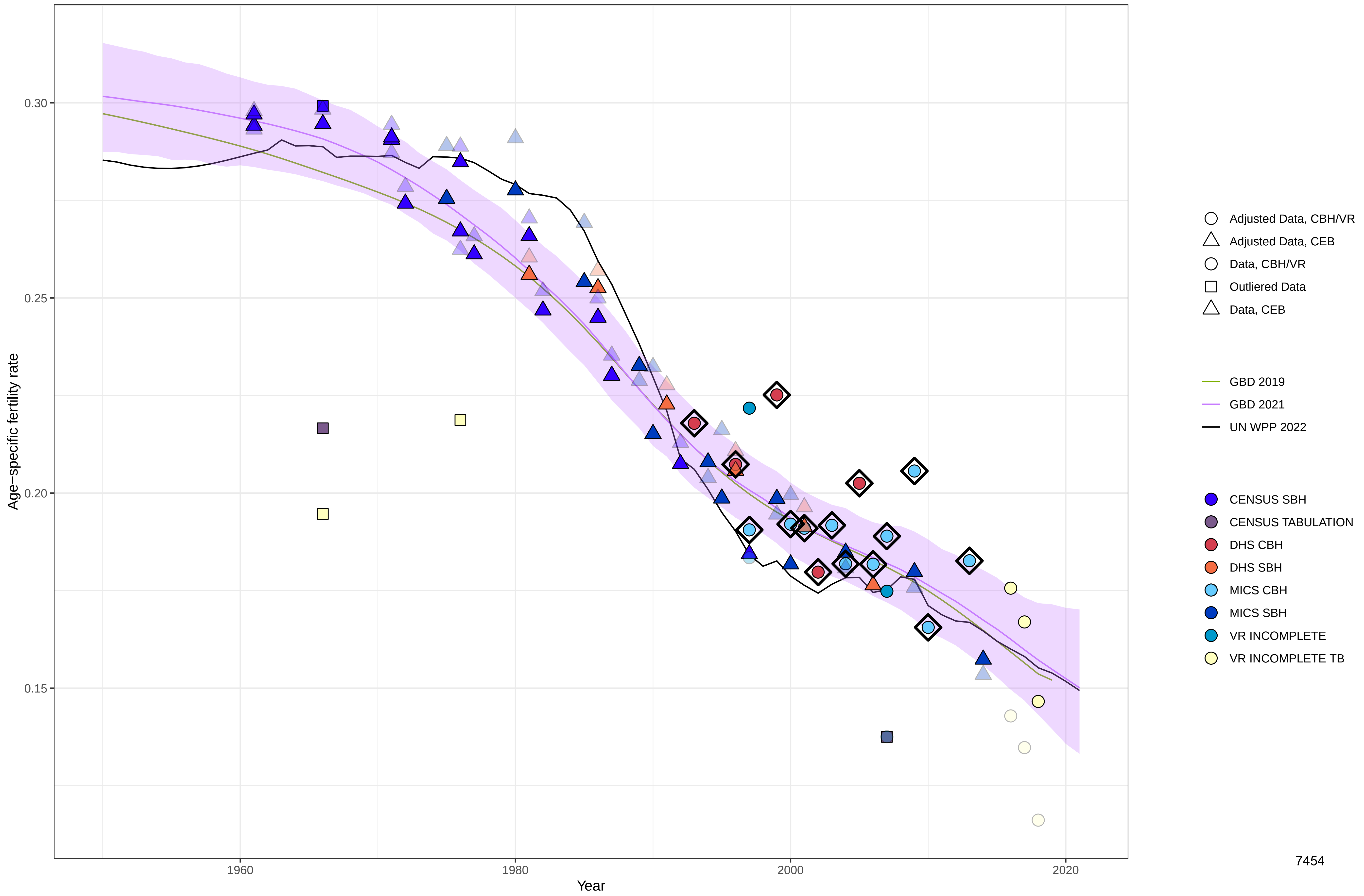

Age−specific fertility fitted trend lines, 1950−2021: Eswatini, Ages: 25−29
Data points enclosed with a diamond are reference sources

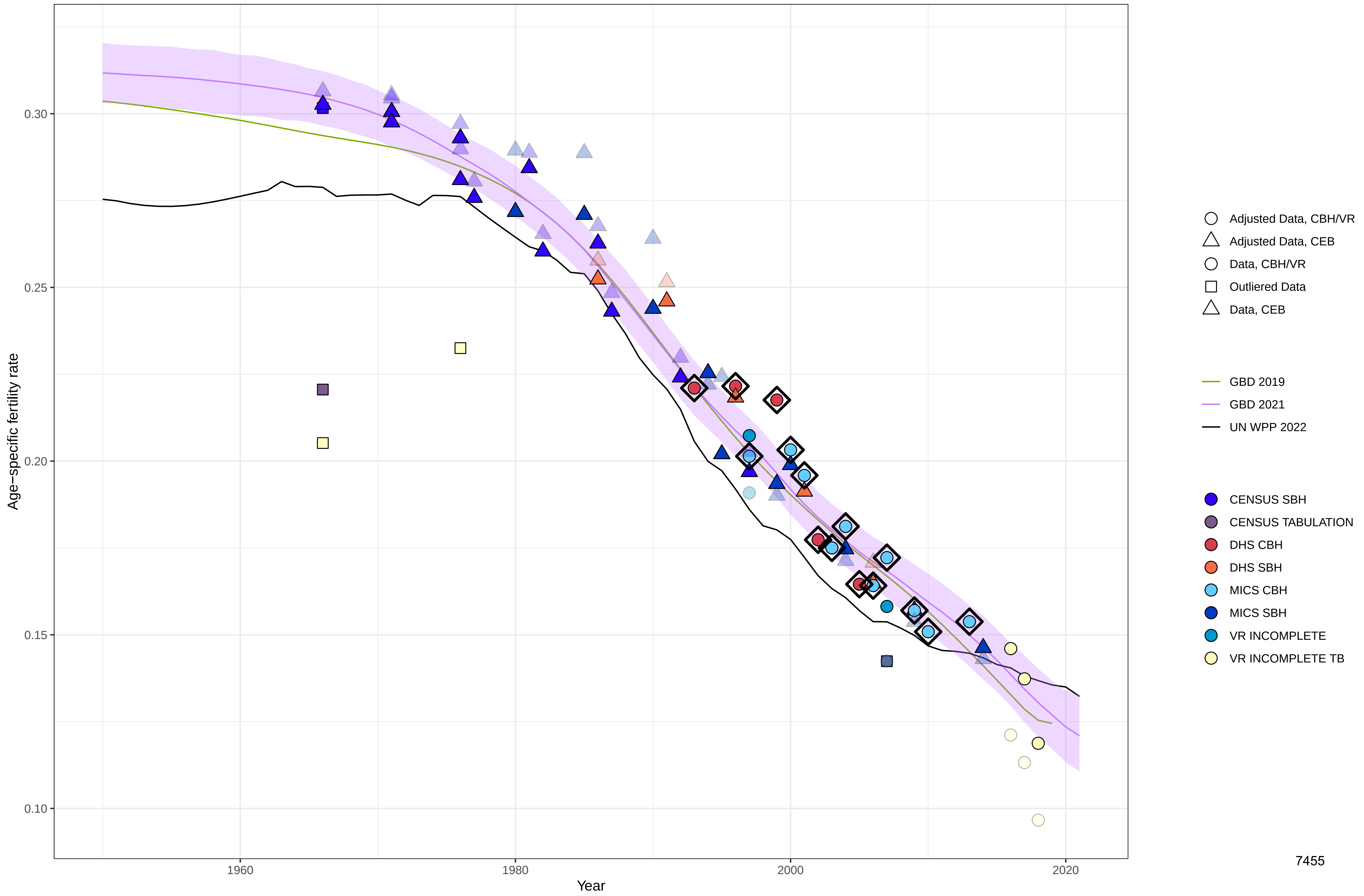

Age−specific fertility fitted trend lines, 1950−2021: Eswatini, Ages: 30−34
Data points enclosed with a diamond are reference sources

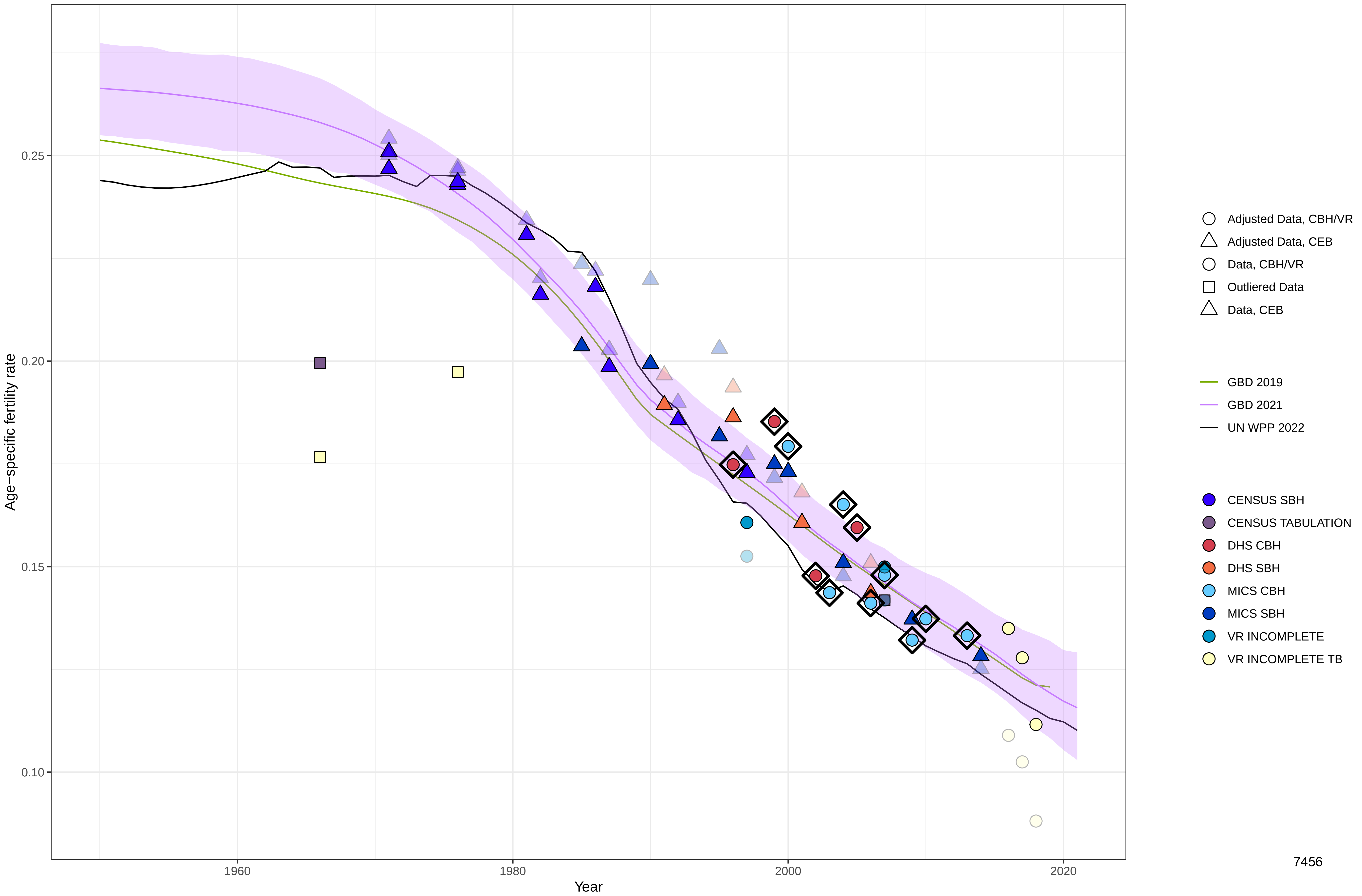

Age−specific fertility fitted trend lines, 1950−2021: Eswatini, Ages: 35−39
Data points enclosed with a diamond are reference sources

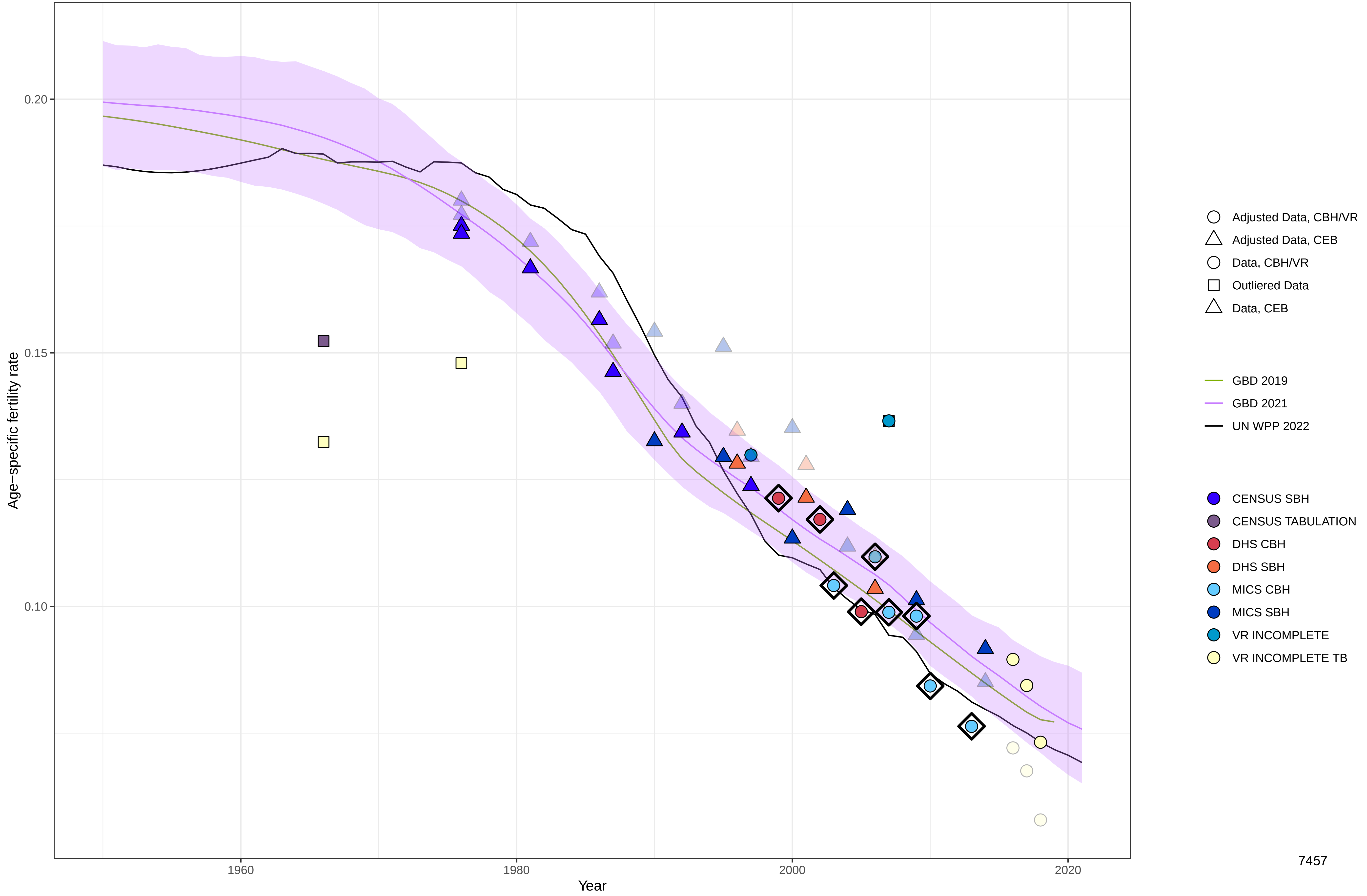

Age−specific fertility fitted trend lines, 1950−2021: Eswatini, Ages: 40−44
Data points enclosed with a diamond are reference sources

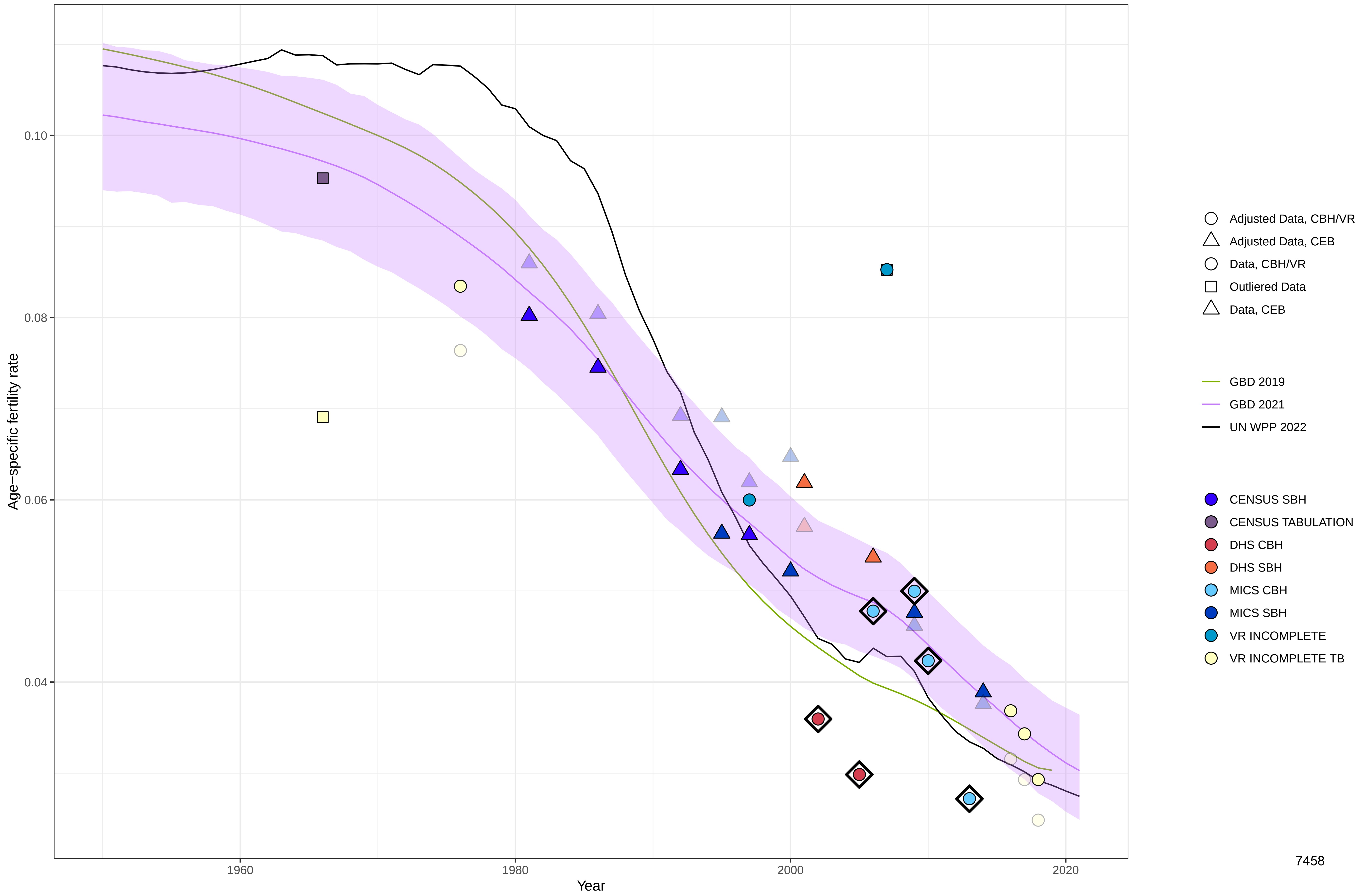

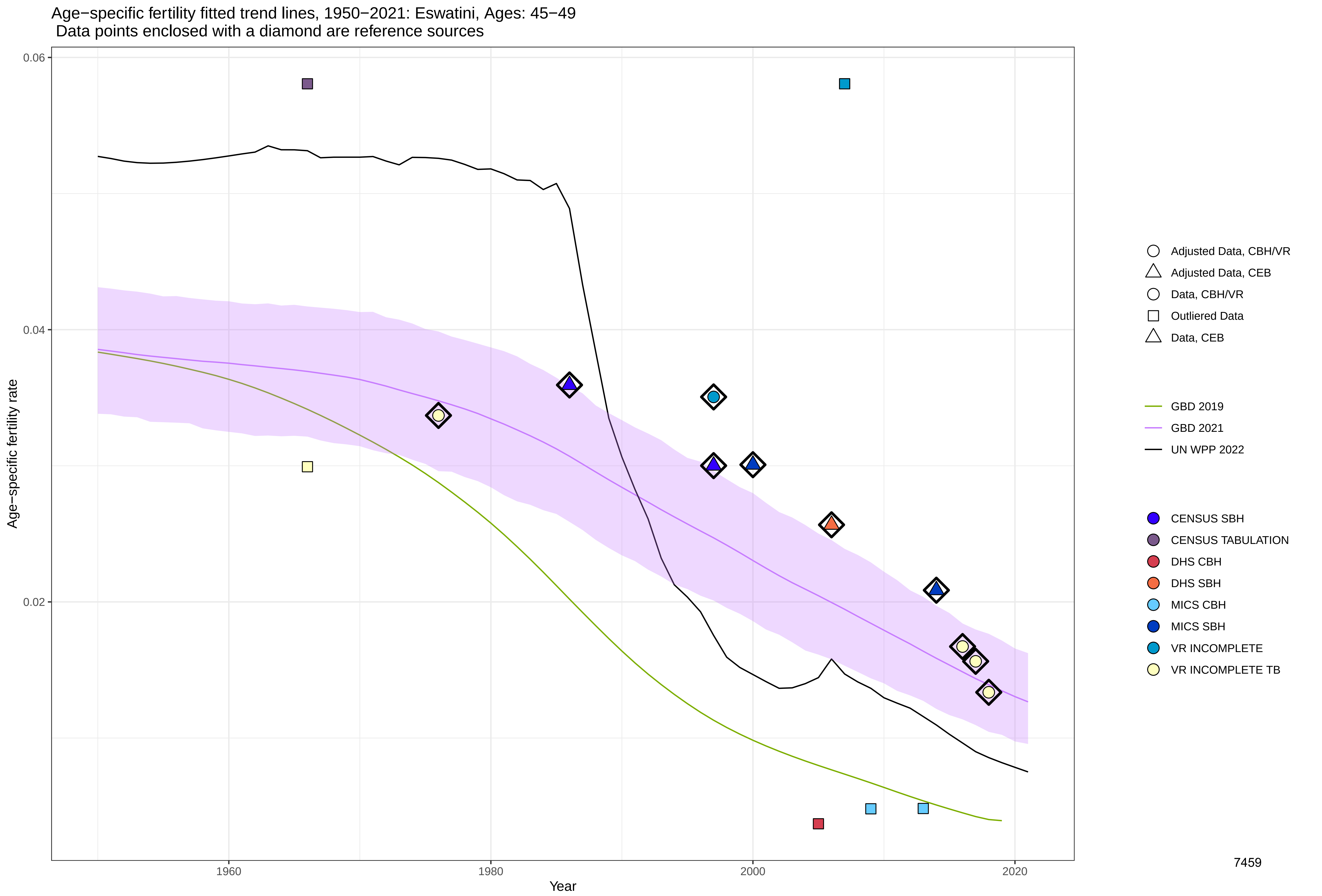
Age−specific fertility fitted trend lines, 1950−2021: Eswatini, Ages: 45−49
Data points enclosed with a diamond are reference sources
0.06
0.04
0.02
Age−specific fertility rate
1960
1980
2000
2020
Year
Adjusted Data, CBH/VR
Adjusted Data, CEB
Data, CBH/VR
Outliered Data
Data, CEB
GBD 2019
GBD 2021
UN WPP 2022
CENSUS SBH
CENSUS TABULATION
DHS CBH
DHS SBH
MICS CBH
MICS SBH
VR INCOMPLETE
VR INCOMPLETE TB

Age−specific fertility fitted trend lines, 1950−2021: Eswatini, Ages: 50−54
Data points enclosed with a diamond are reference sources

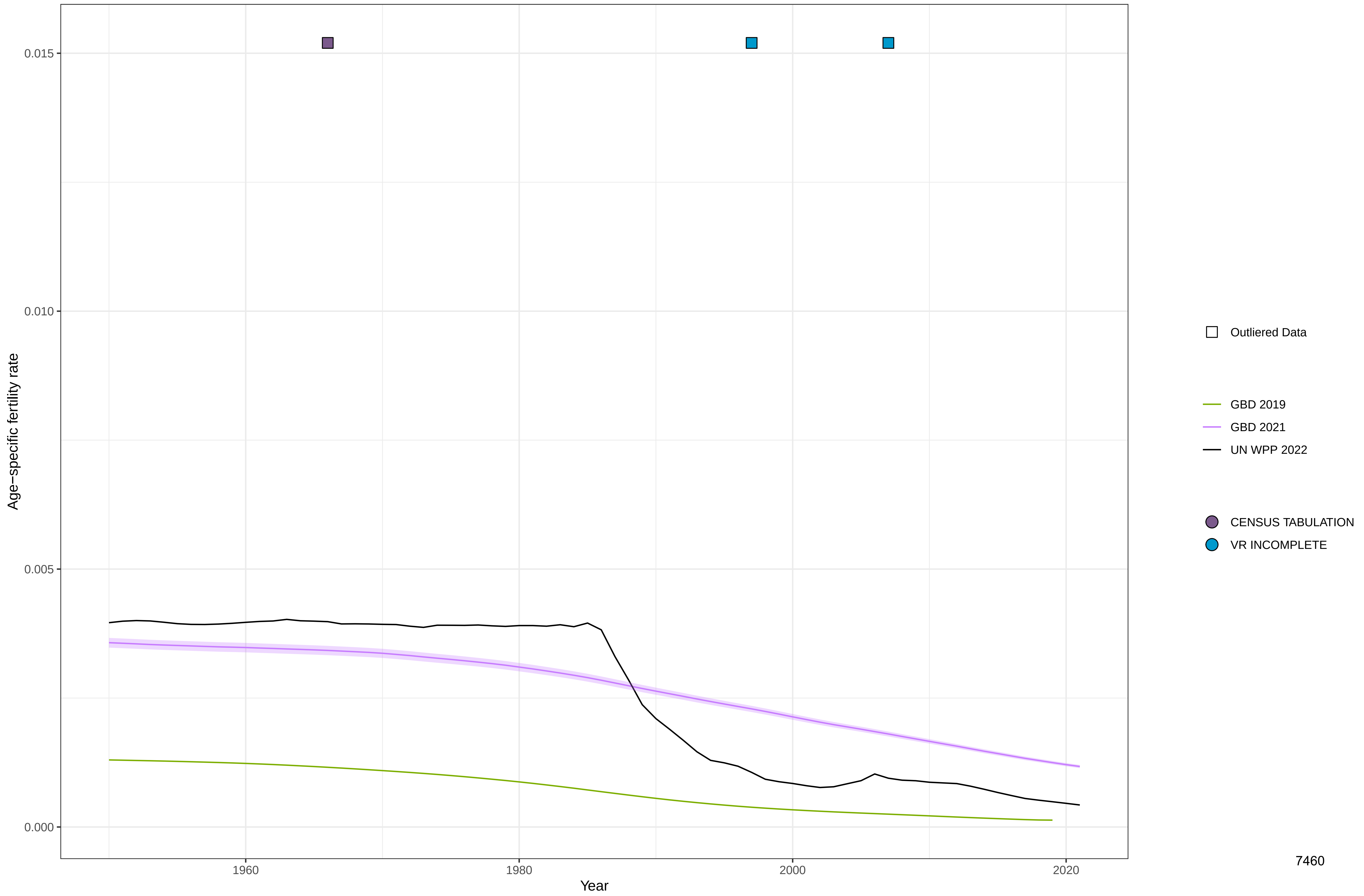

Total fertility rate fitted trend lines, 1950−2021: Eswatini TFR
Data points enclosed with a diamond are reference sources

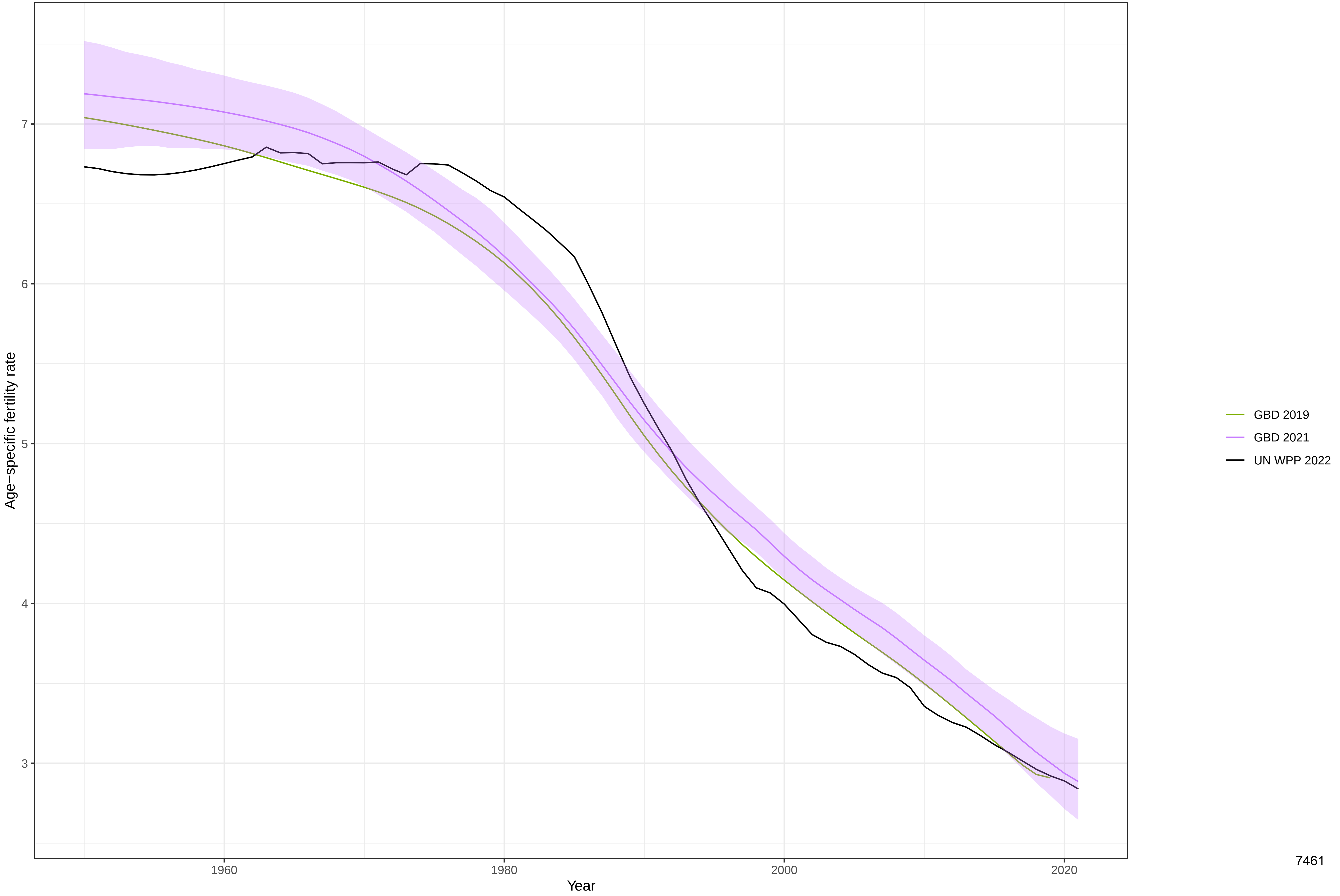

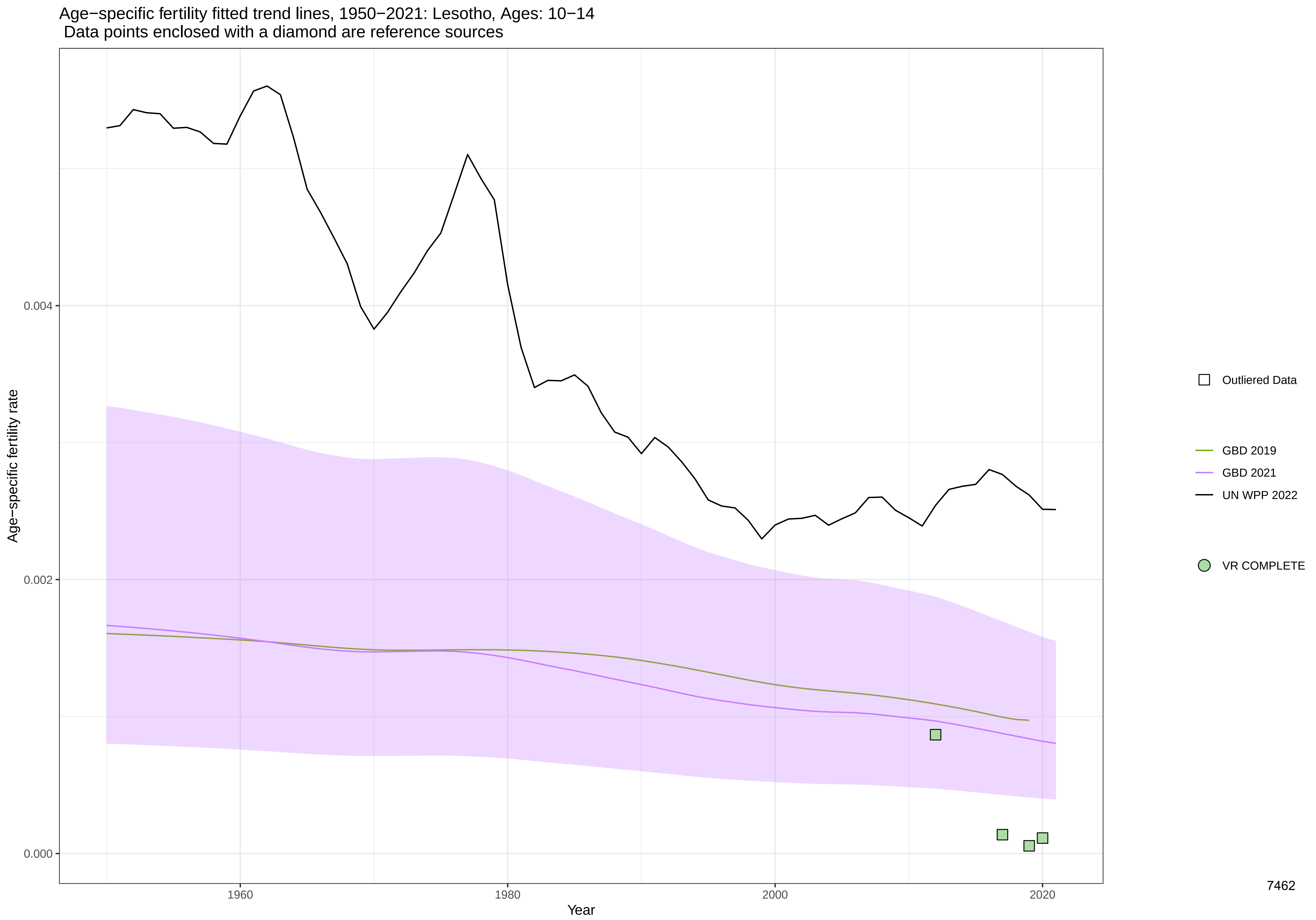

Age−specific fertility fitted trend lines, 1950−2021: Lesotho, Ages: 10−14
Data points enclosed with a diamond are reference sources
Age−specific fertility rate
0.000
0.002
0.004
1960
1980
2000
2020
Year
Outliered Data
GBD 2019
GBD 2021
UN WPP 2022
VR COMPLETE

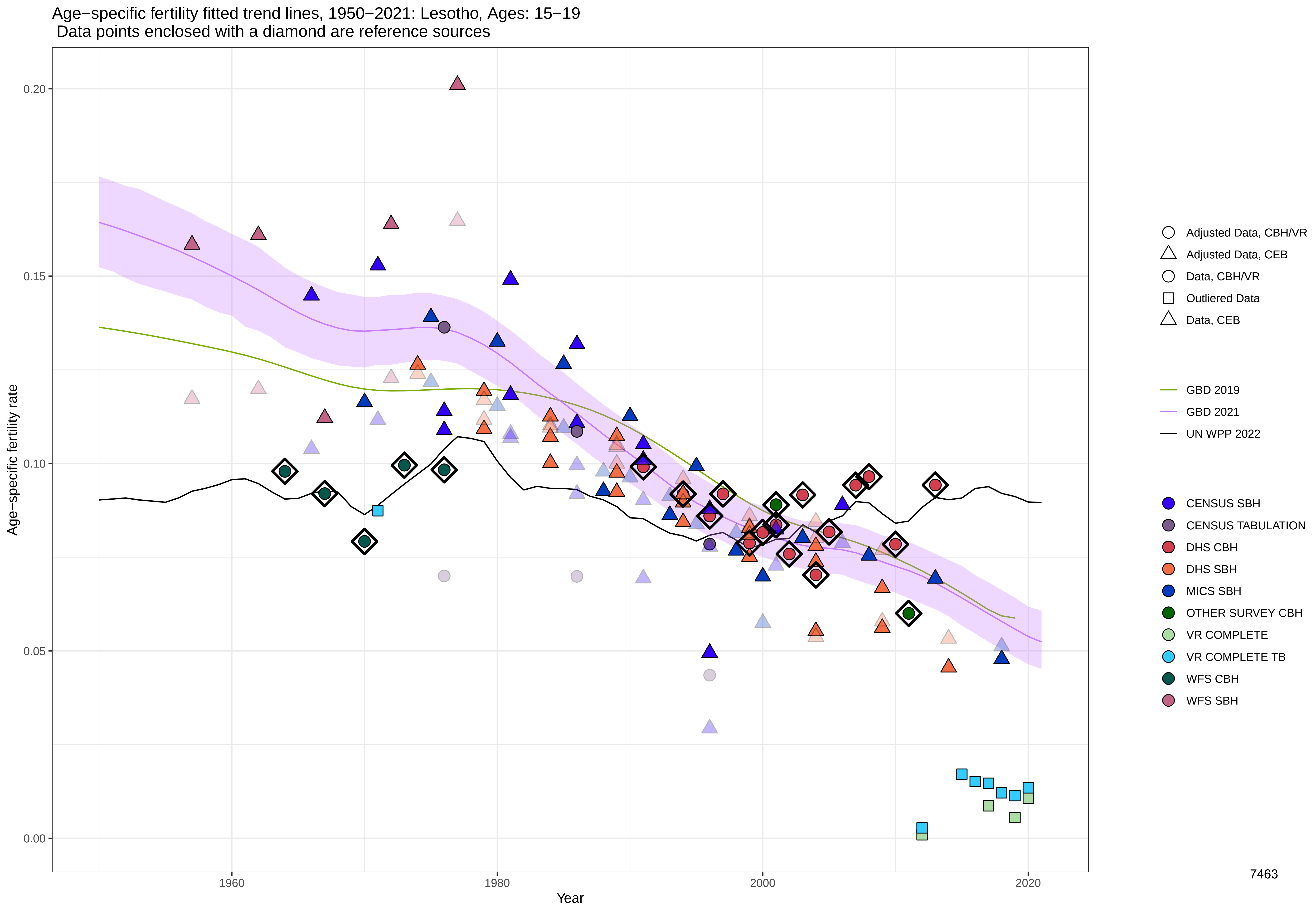

Age−specific fertility fitted trend lines, 1950−2021: Lesotho, Ages: 15−19
Data points enclosed with a diamond are reference sources
Age−specific fertility rate
Year
0.00
0.05
0.10
0.15
0.20
1960
1980
2000
2020
Adjusted Data, CBH/VR
Adjusted Data, CEB
Data, CBH/VR
Outliered Data
Data, CEB
GBD 2019
GBD 2021
UN WPP 2022
CENSUS SBH
CENSUS TABULATION
DHS CBH
DHS SBH
MICS SBH
OTHER SURVEY CBH
VR COMPLETE
VR COMPLETE TB
WFS CBH
WFS SBH

Age−specific fertility fitted trend lines, 1950−2021: Lesotho, Ages: 20−24
Data points enclosed with a diamond are reference sources

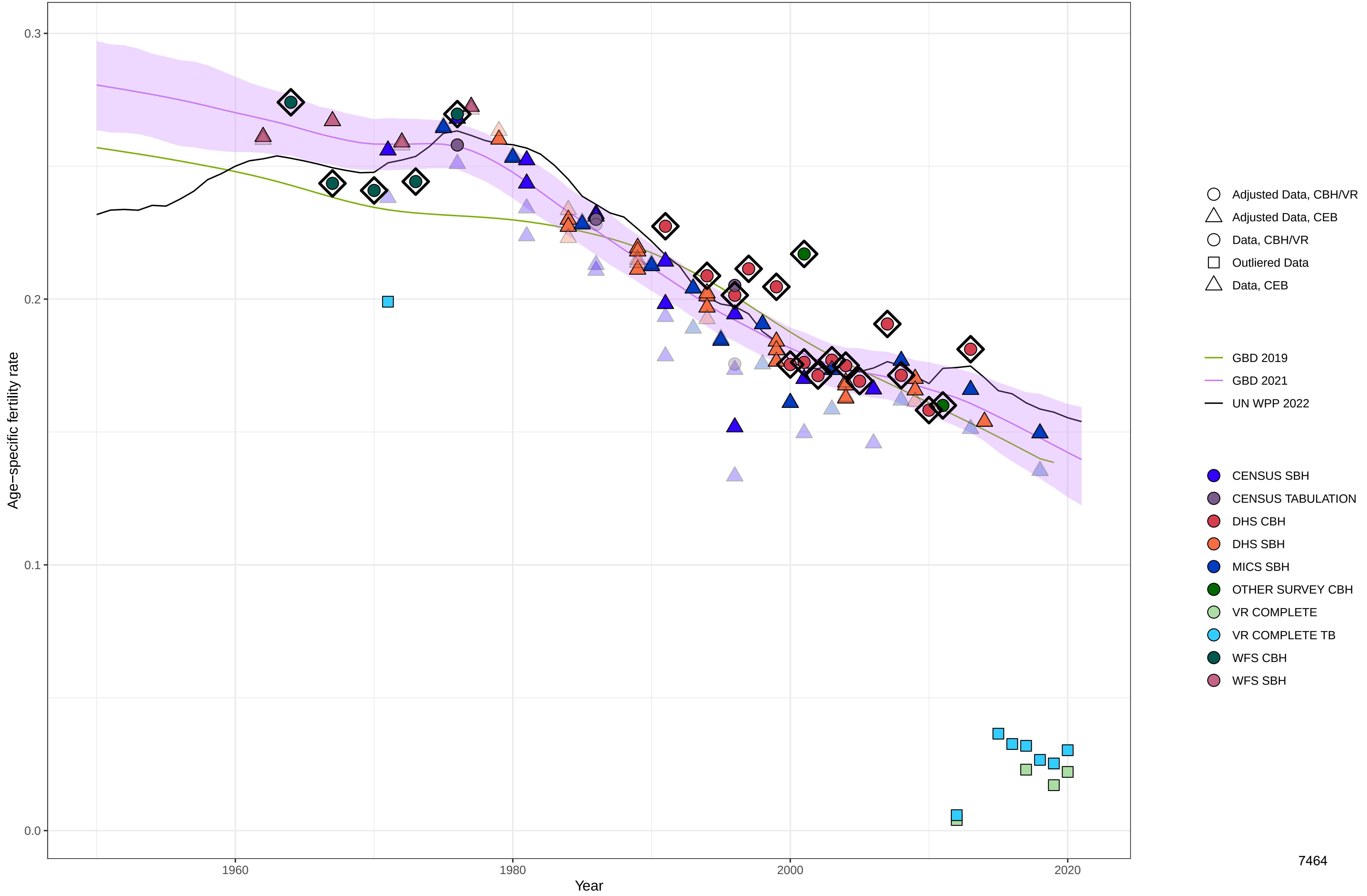

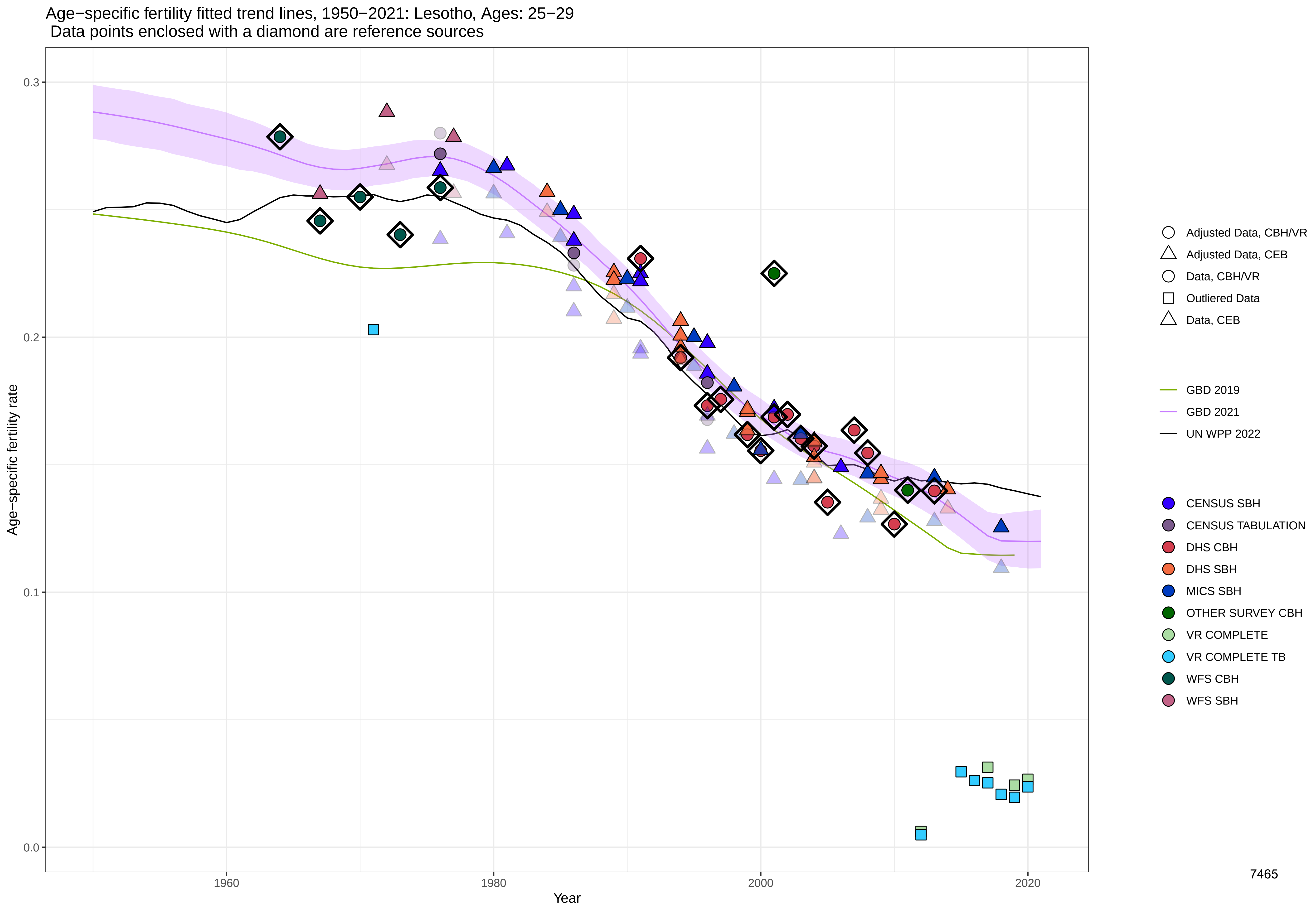
Age−specific fertility fitted trend lines, 1950−2021: Lesotho, Ages: 25−29
Data points enclosed with a diamond are reference sources
Age−specific fertility rate
0.0
0.1
0.2
0.3
Year
1960
1980
2000
2020
Adjusted Data, CBH/VR
Adjusted Data, CEB
Data, CBH/VR
Outliered Data
Data, CEB
GBD 2019
GBD 2021
UN WPP 2022
CENSUS SBH
CENSUS TABULATION
DHS CBH
DHS SBH
MICS SBH
OTHER SURVEY CBH
VR COMPLETE
VR COMPLETE TB
WFS CBH
WFS SBH

Age−specific fertility fitted trend lines, 1950−2021: Lesotho, Ages: 30−34
Data points enclosed with a diamond are reference sources

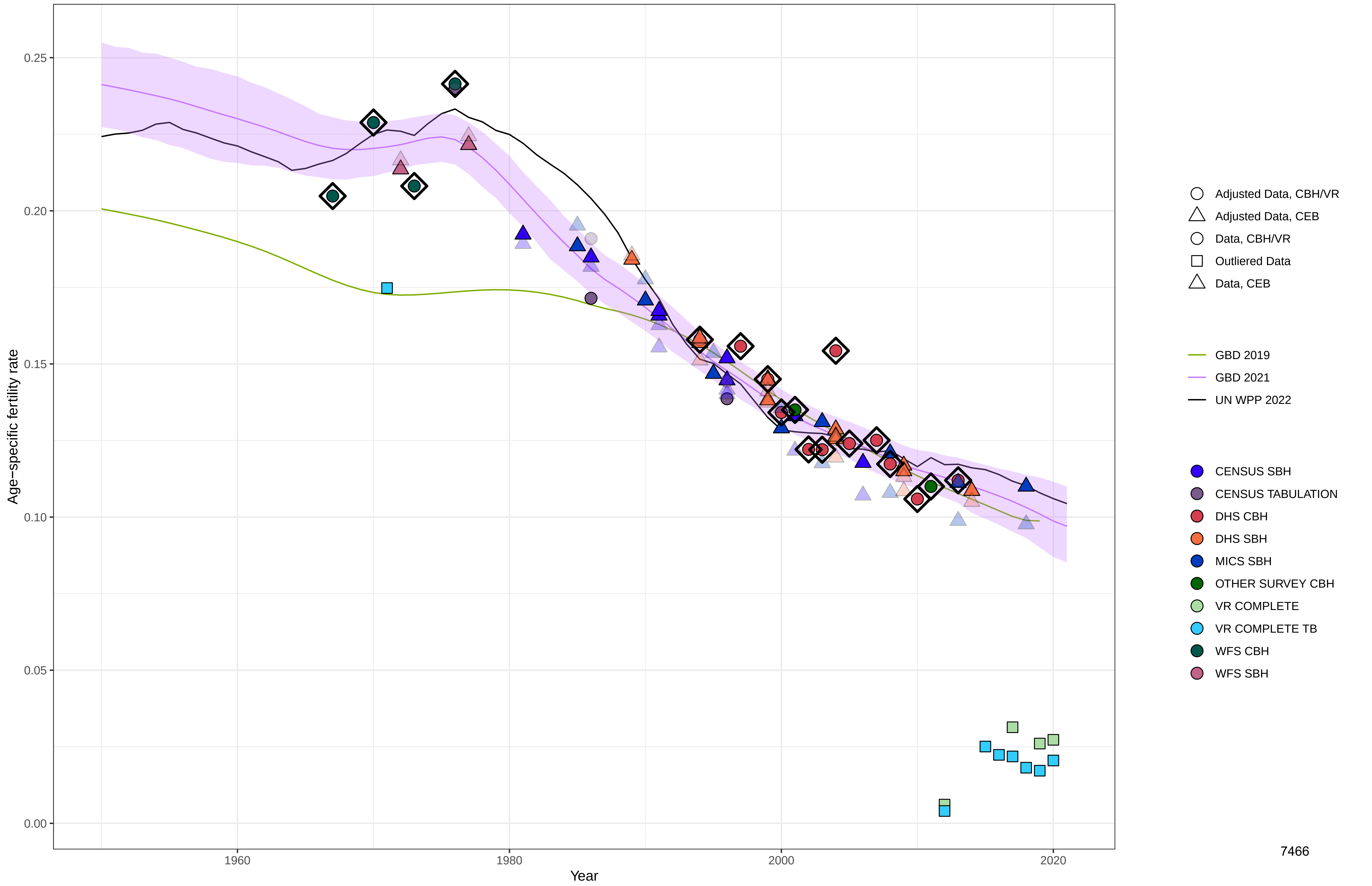

Age−specific fertility fitted trend lines, 1950−2021: Lesotho, Ages: 35−39
Data points enclosed with a diamond are reference sources

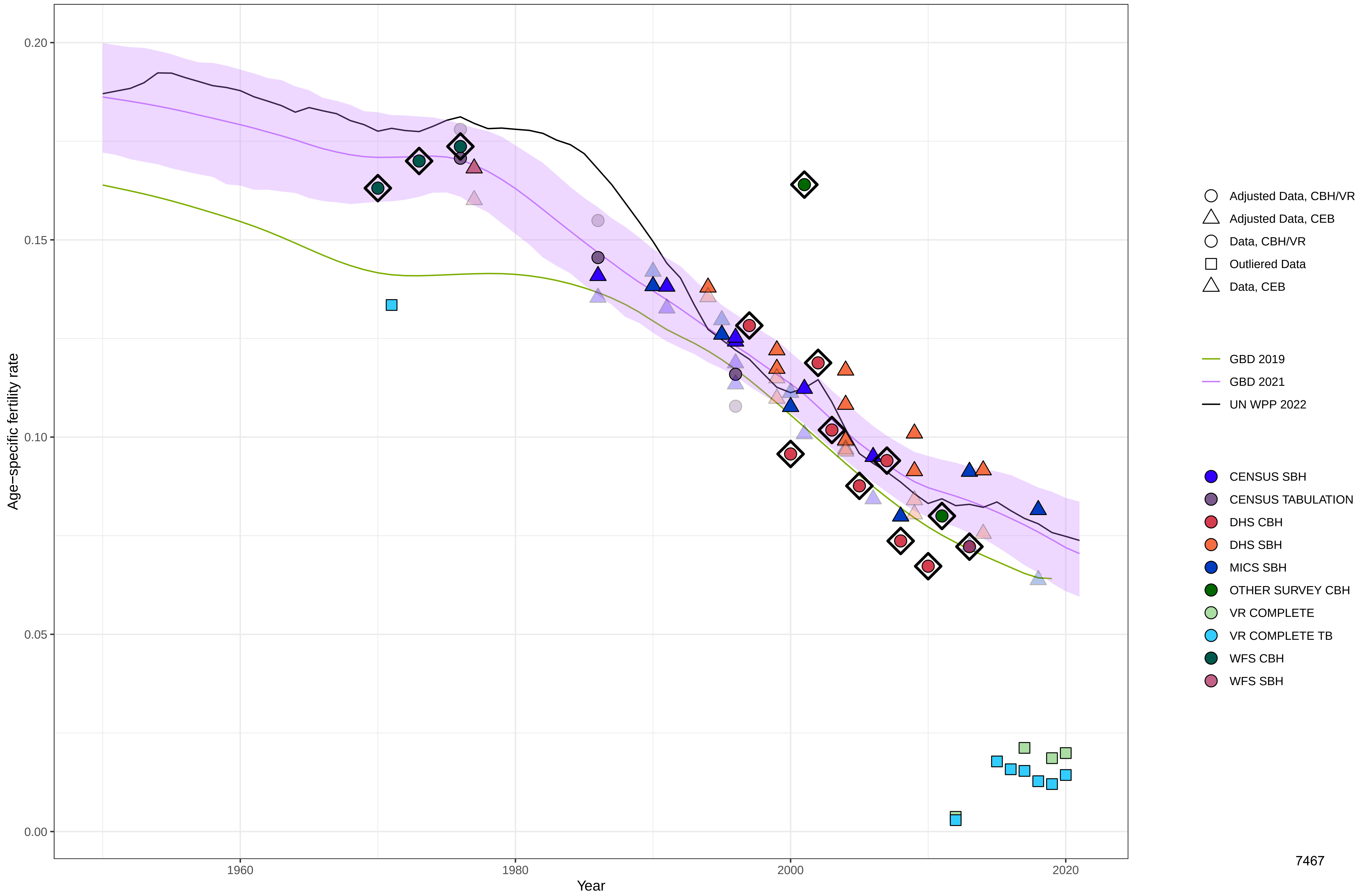

7467

Age−specific fertility fitted trend lines, 1950−2021: Lesotho, Ages: 40−44
Data points enclosed with a diamond are reference sources

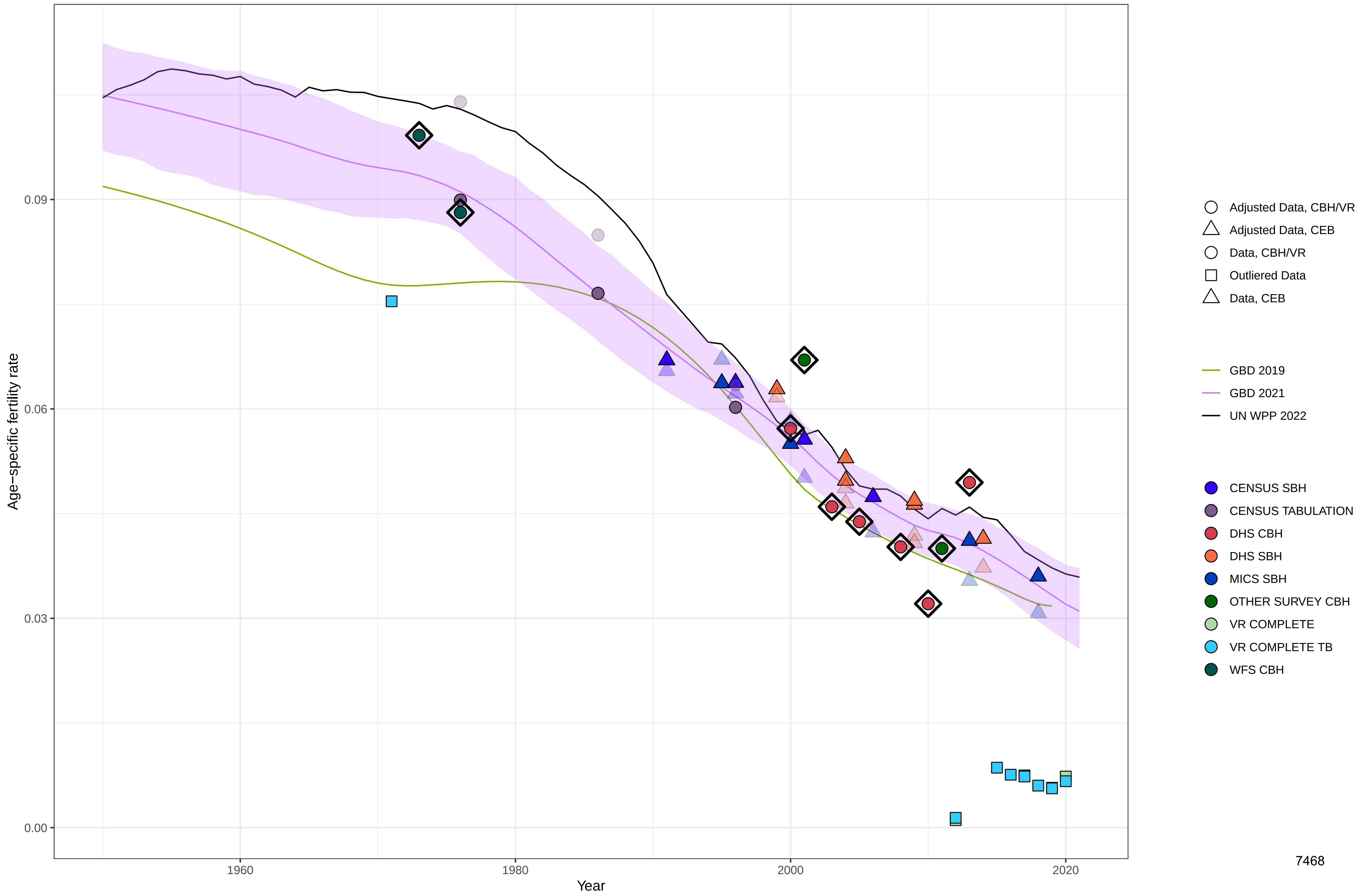

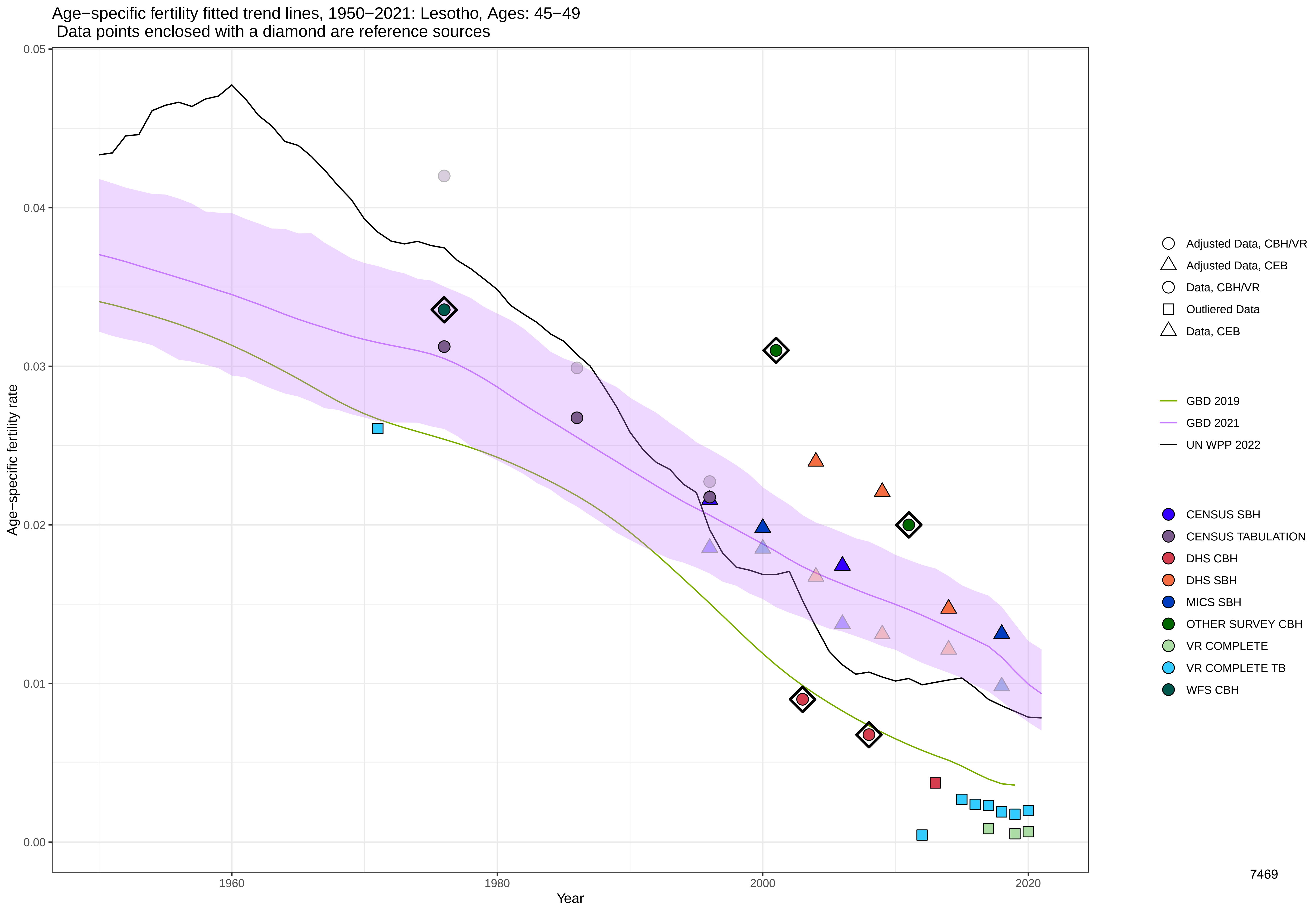

Age−specific fertility fitted trend lines, 1950−2021: Lesotho, Ages: 45−49
Data points enclosed with a diamond are reference sources
Age−specific fertility rate
Year
0.00
0.01
0.02
0.03
0.04
0.05
1960
1980
2000
2020
Adjusted Data, CBH/VR
Adjusted Data, CEB
Data, CBH/VR
Outliered Data
Data, CEB
GBD 2019
GBD 2021
UN WPP 2022
CENSUS SBH
CENSUS TABULATION
DHS CBH
DHS SBH
MICS SBH
OTHER SURVEY CBH
VR COMPLETE
VR COMPLETE TB
WFS CBH

Age−specific fertility fitted trend lines, 1950−2021: Lesotho, Ages: 50−54
Data points enclosed with a diamond are reference sources

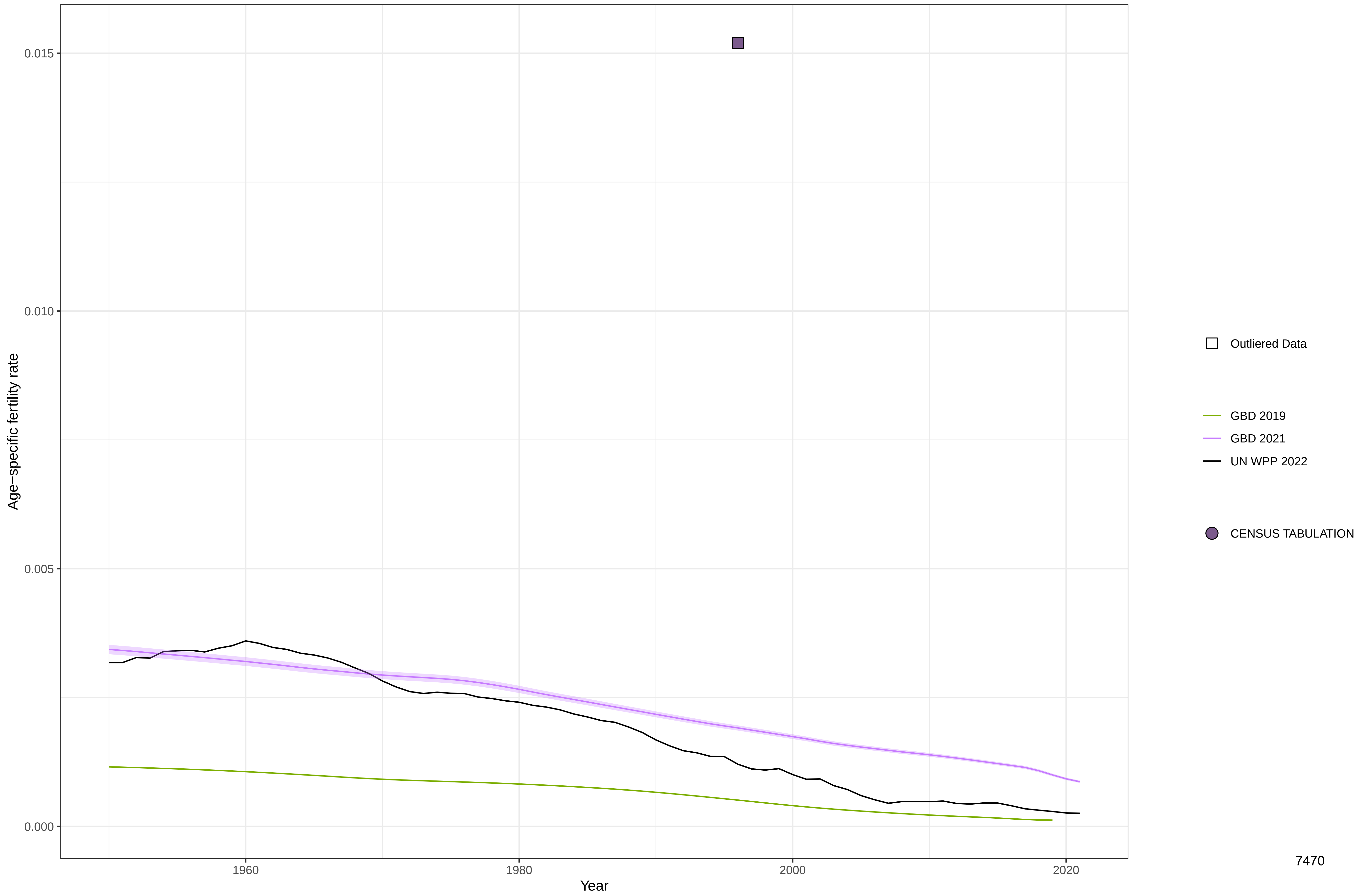

Total fertility rate fitted trend lines, 1950−2021: Lesotho TFR
Data points enclosed with a diamond are reference sources

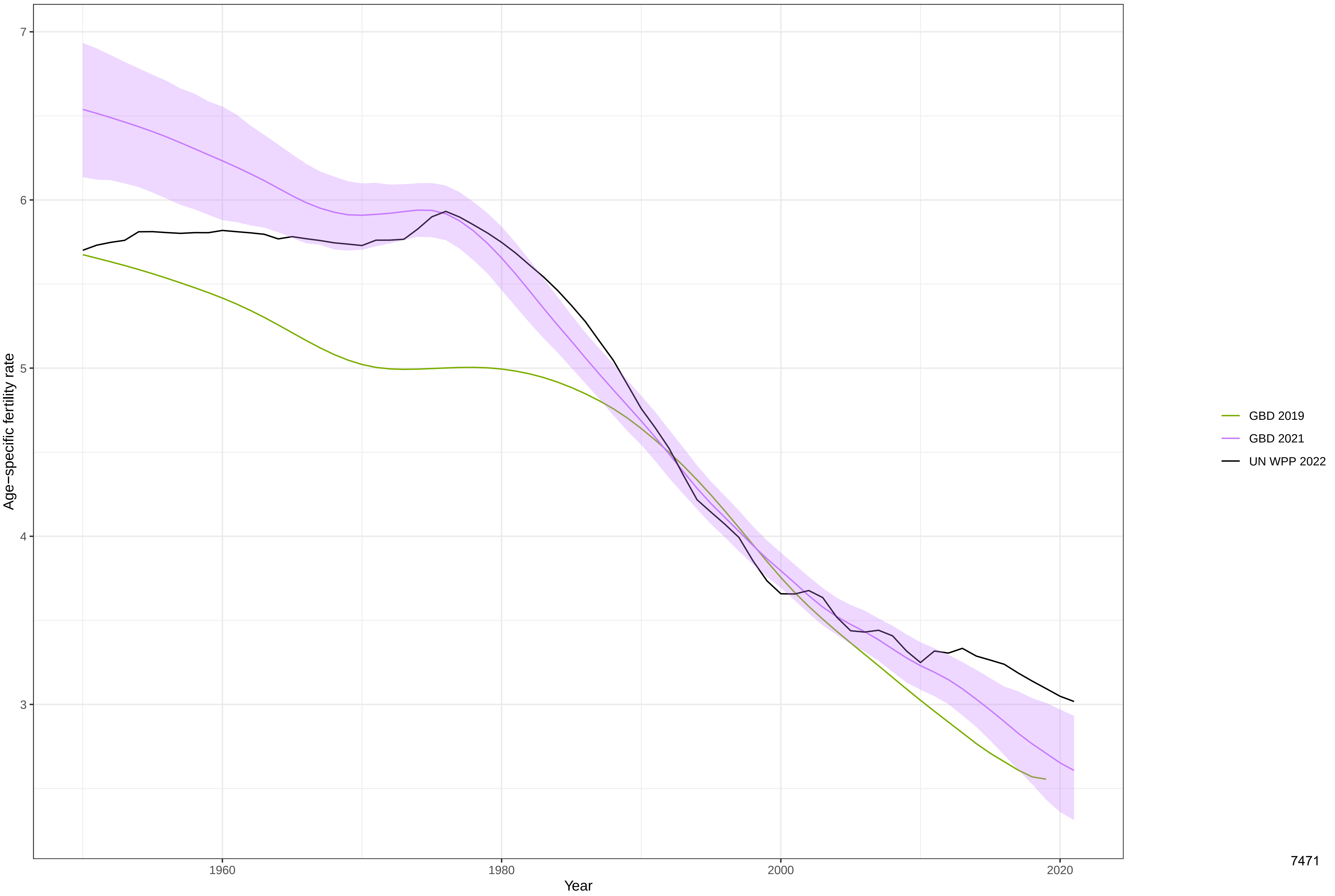

Age−specific fertility fitted trend lines, 1950−2021: Namibia, Ages: 10−14
Data points enclosed with a diamond are reference sources

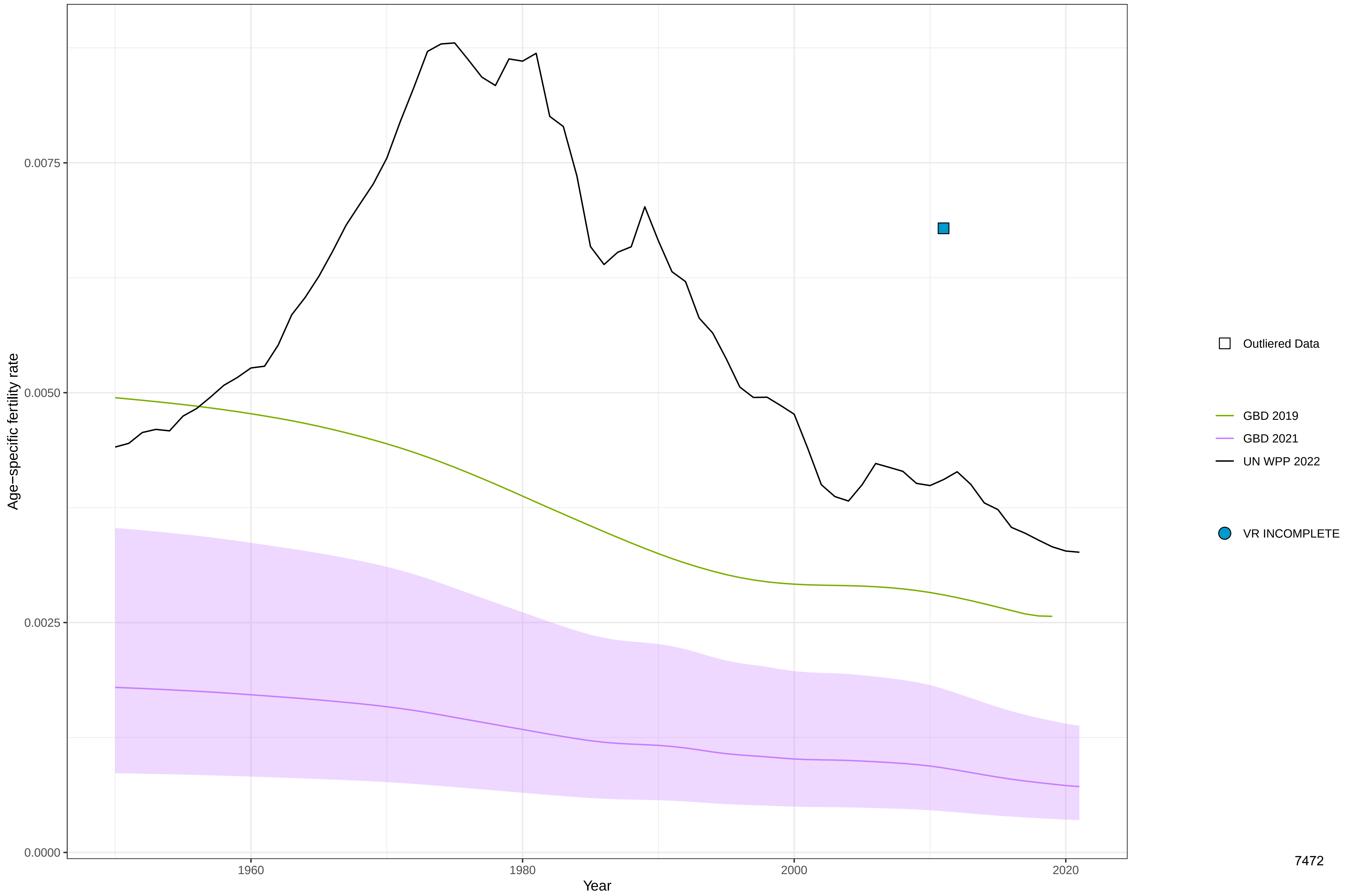

Age−specific fertility fitted trend lines, 1950−2021: Namibia, Ages: 15−19
Data points enclosed with a diamond are reference sources

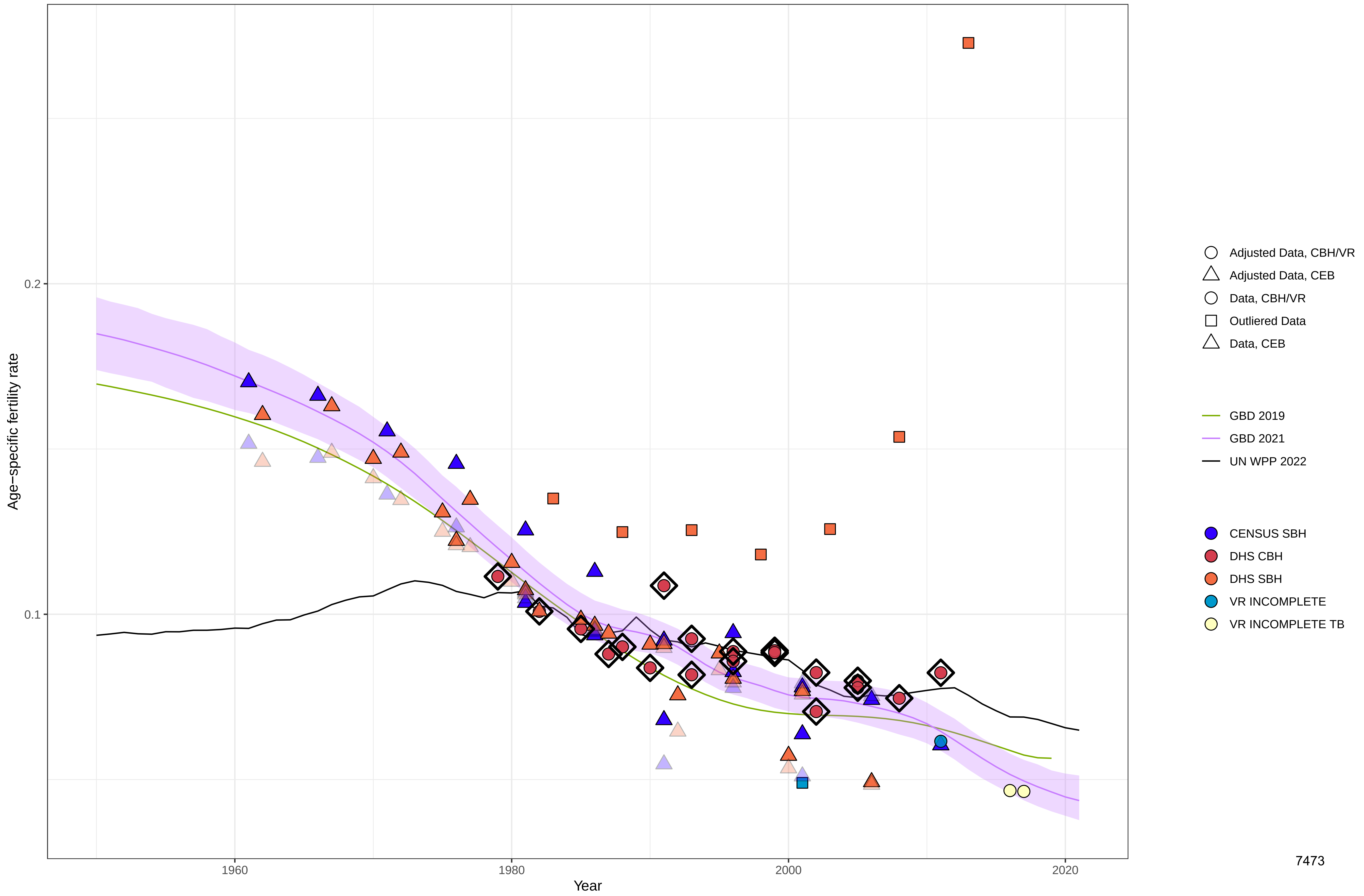

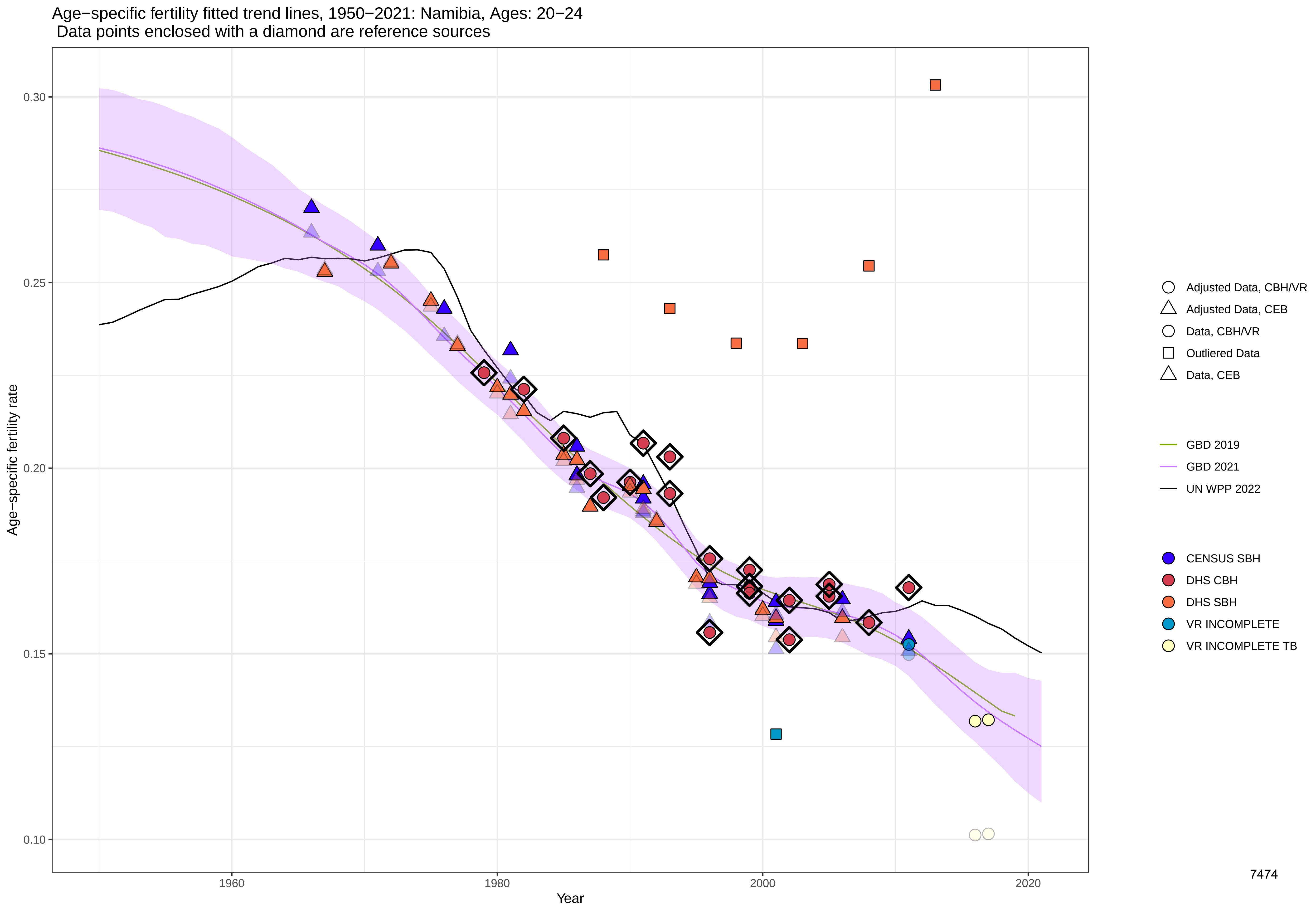

Age−specific fertility fitted trend lines, 1950−2021: Namibia, Ages: 20−24
Data points enclosed with a diamond are reference sources
Age−specific fertility rate
Year
0.10
0.15
0.20
0.25
0.30
1960
1980
2000
2020
Adjusted Data, CBH/VR
Adjusted Data, CEB
Data, CBH/VR
Outliered Data
Data, CEB
GBD 2019
GBD 2021
UN WPP 2022
CENSUS SBH
DHS CBH
DHS SBH
VR INCOMPLETE
VR INCOMPLETE TB

Age−specific fertility fitted trend lines, 1950−2021: Namibia, Ages: 25−29
Data points enclosed with a diamond are reference sources

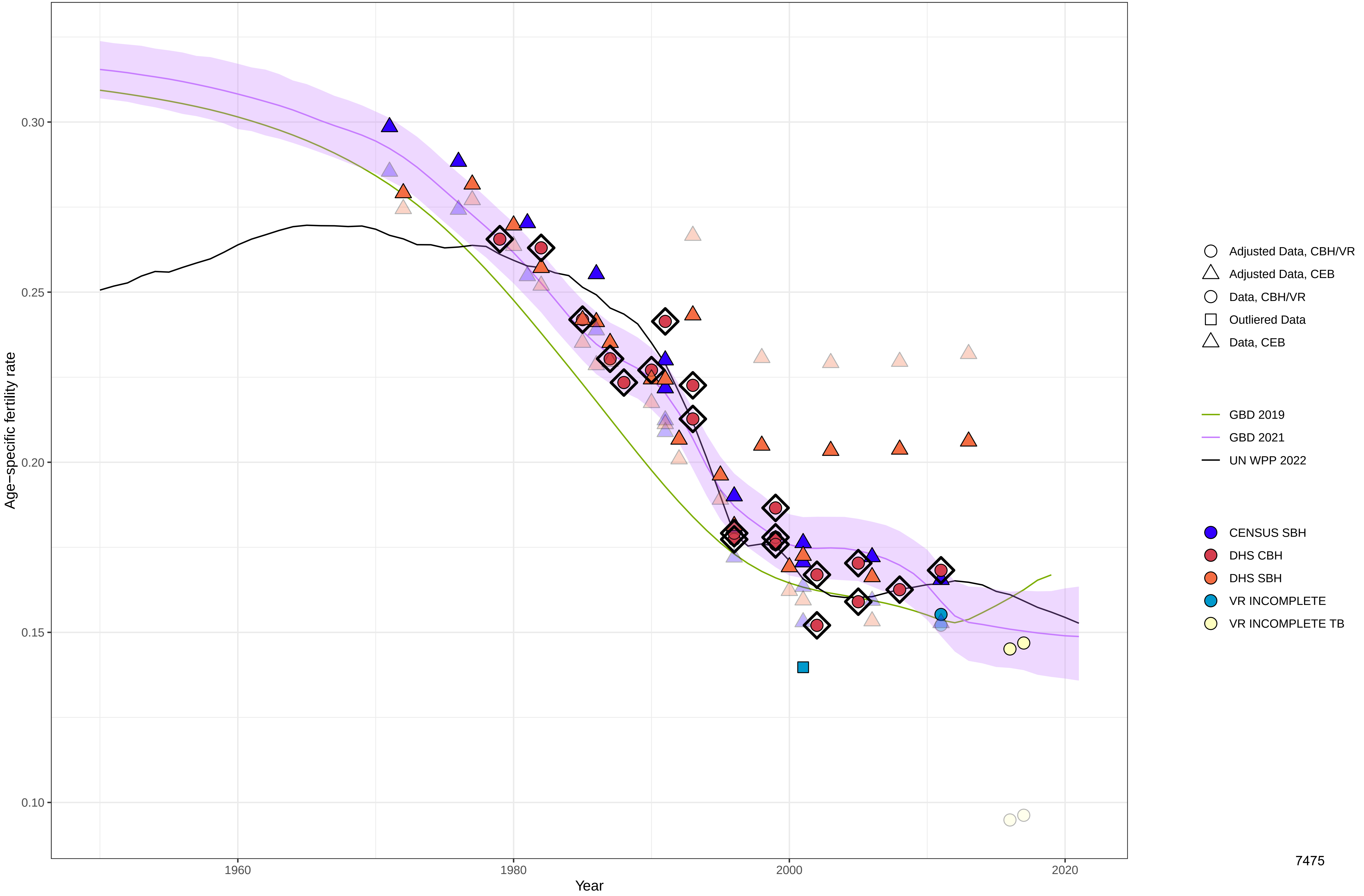

Age−specific fertility fitted trend lines, 1950−2021: Namibia, Ages: 30−34
Data points enclosed with a diamond are reference sources

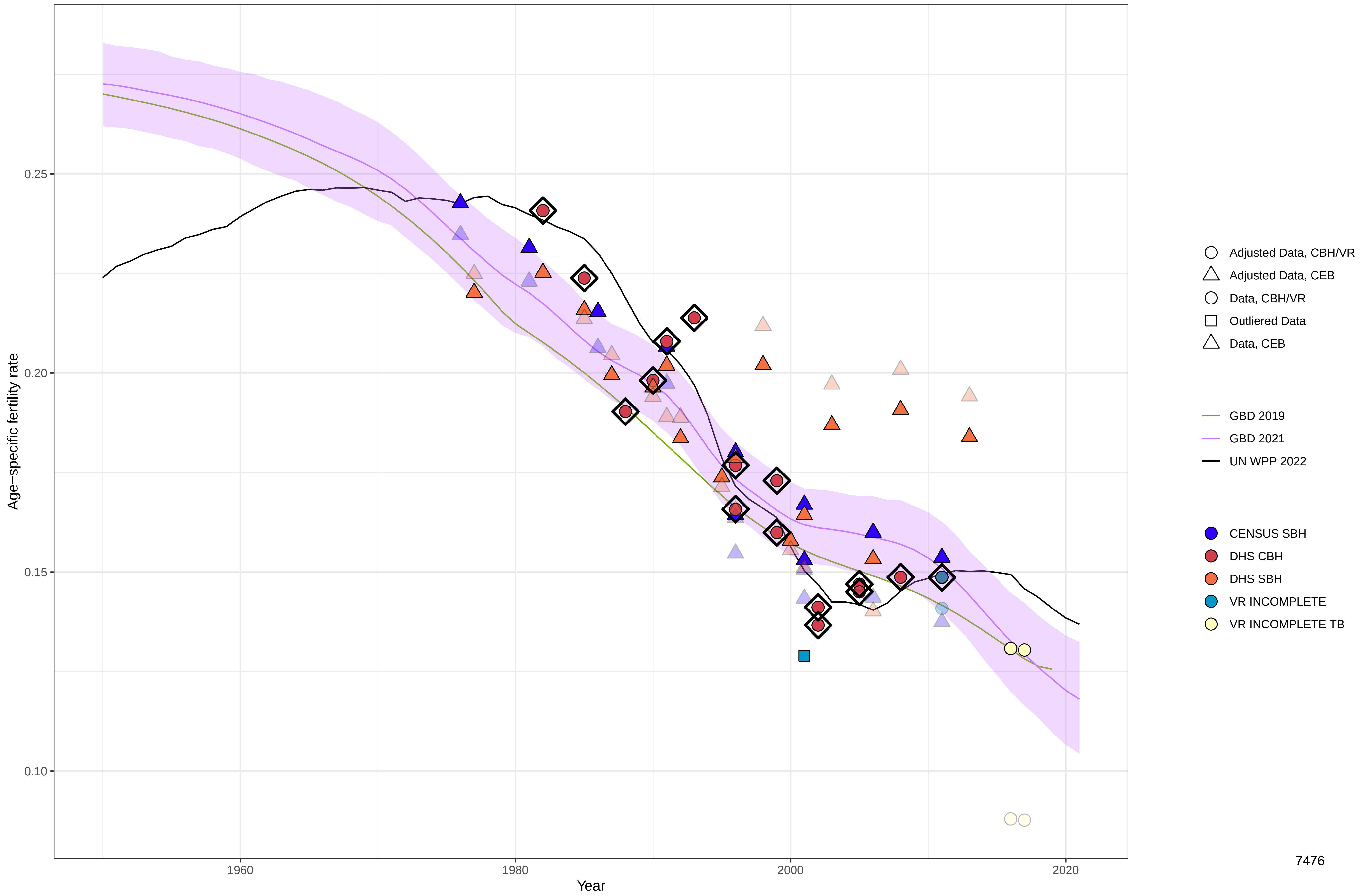

Age−specific fertility fitted trend lines, 1950−2021: Namibia, Ages: 35−39
Data points enclosed with a diamond are reference sources

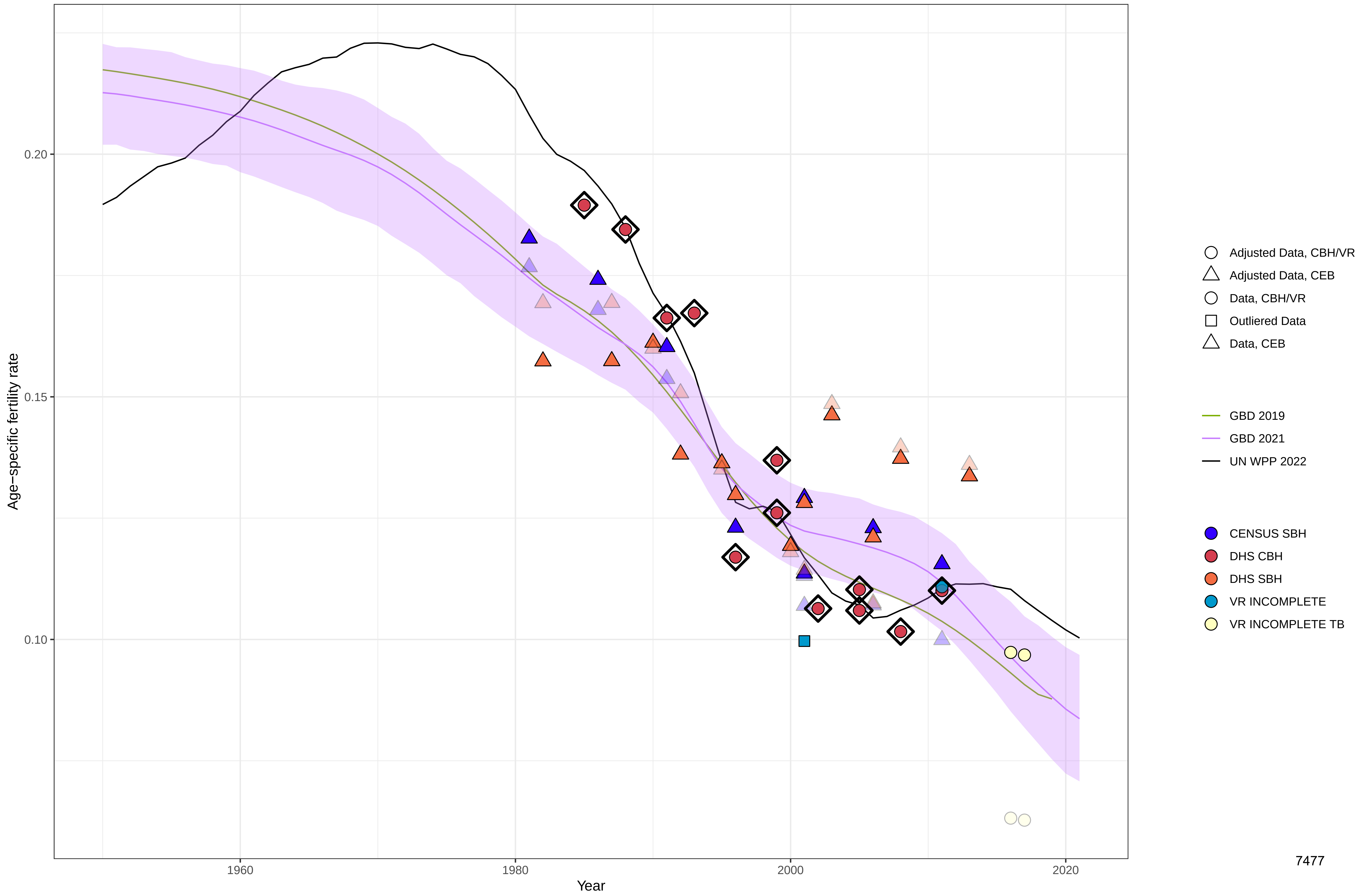

Age−specific fertility fitted trend lines, 1950−2021: Namibia, Ages: 40−44
Data points enclosed with a diamond are reference sources

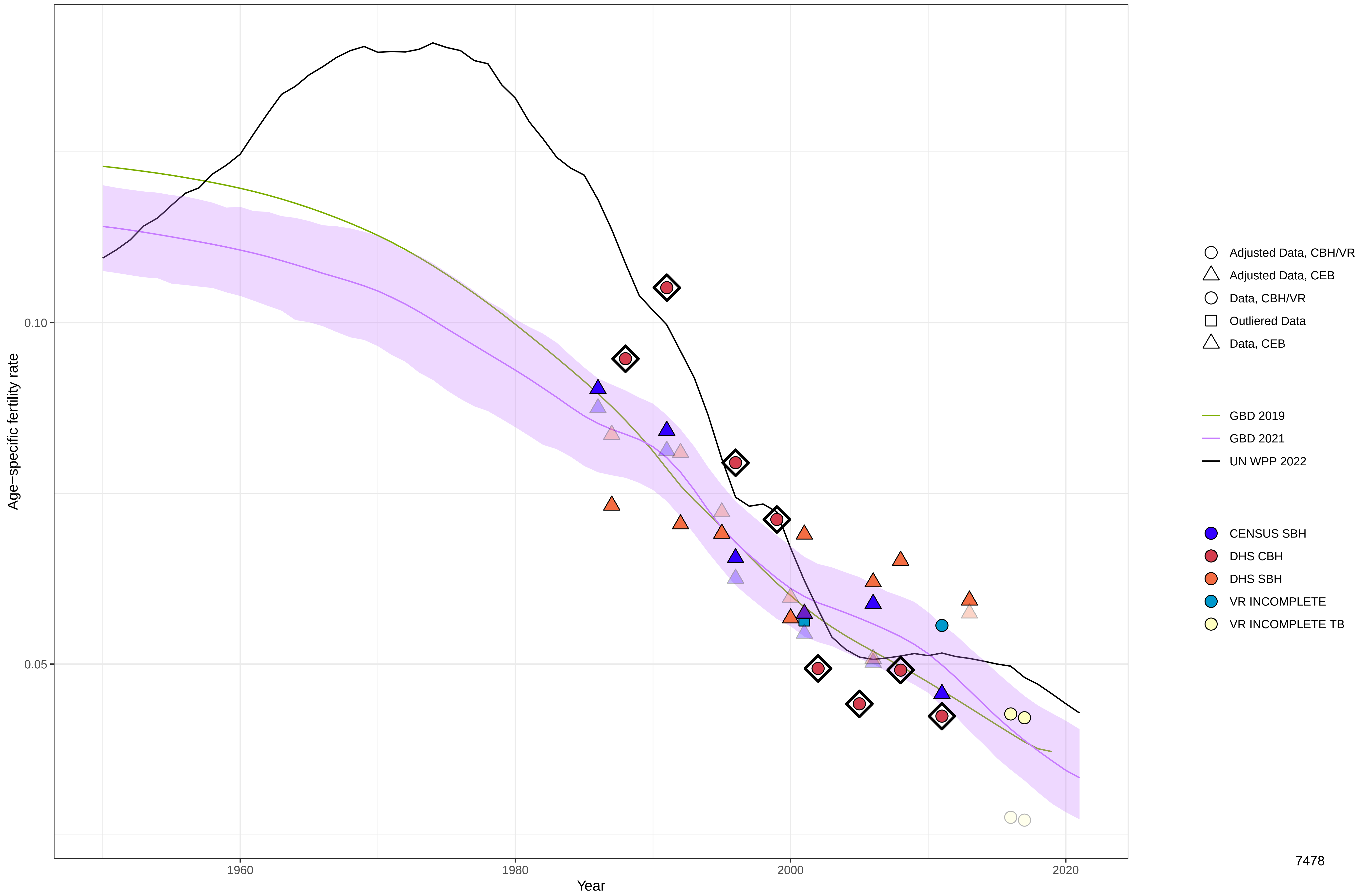

Age−specific fertility fitted trend lines, 1950−2021: Namibia, Ages: 45−49
Data points enclosed with a diamond are reference sources

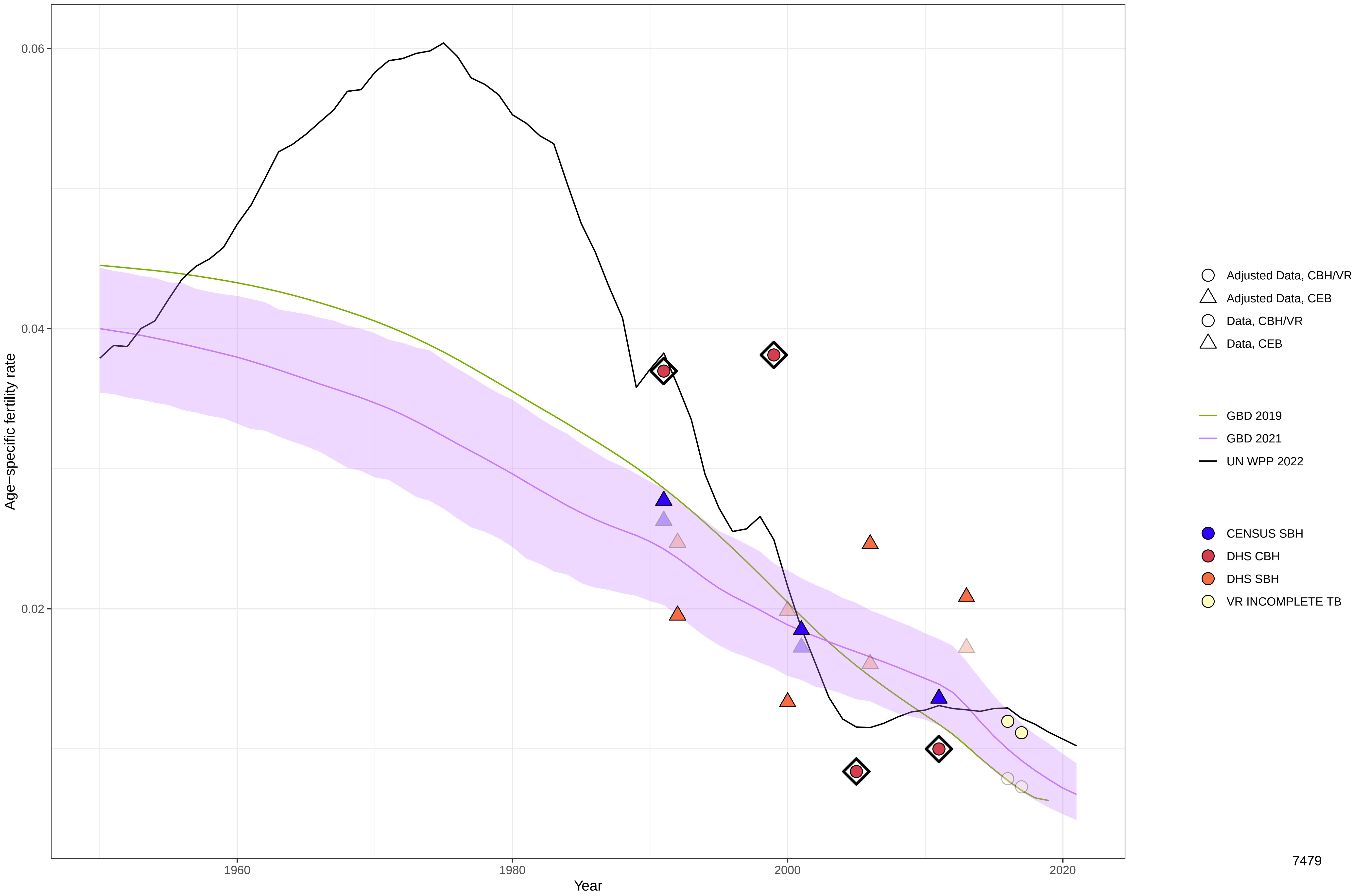

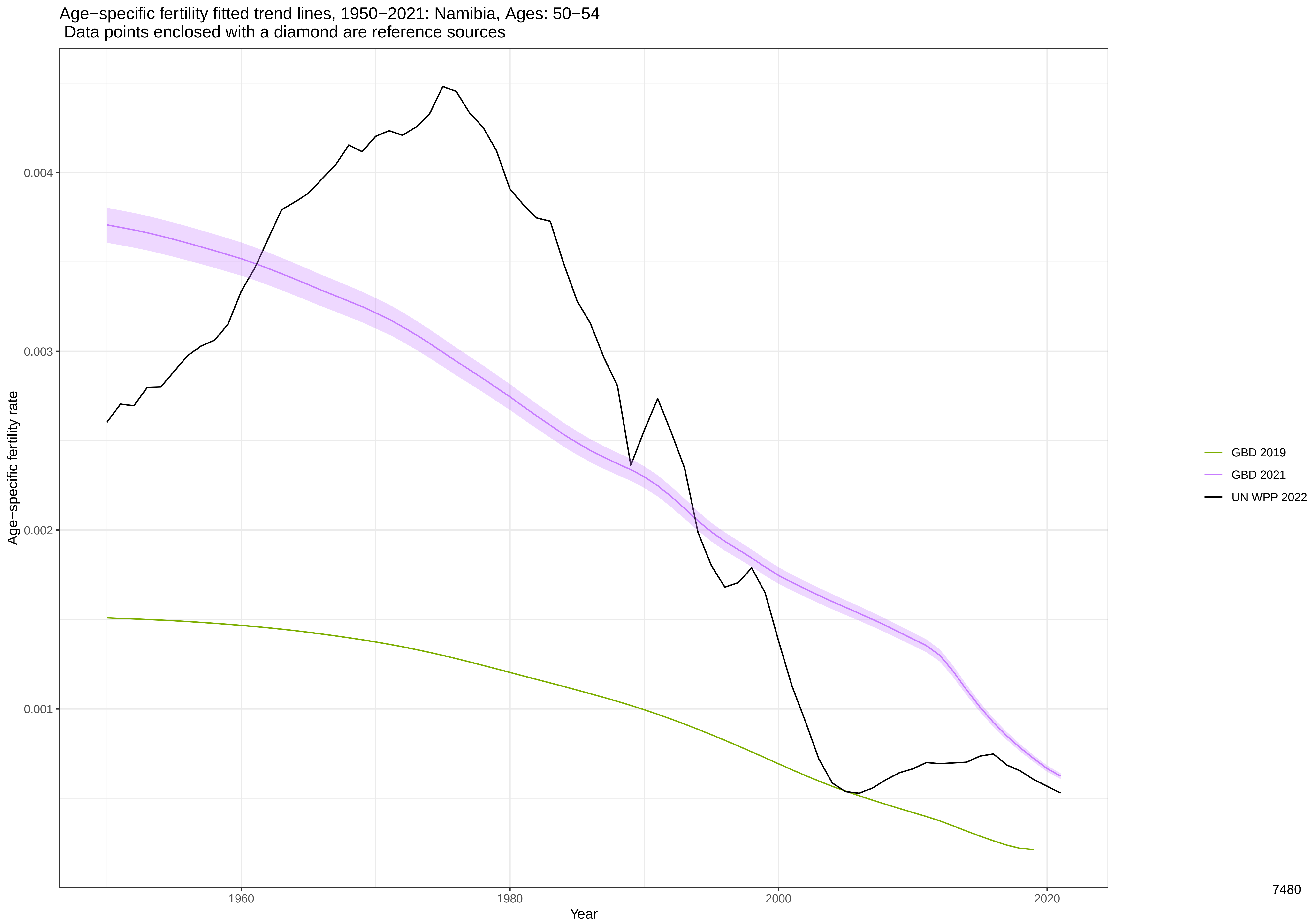
Age−specific fertility fitted trend lines, 1950−2021: Namibia, Ages: 50−54
Data points enclosed with a diamond are reference sources
Age−specific fertility rate
0.004
0.003
0.002
0.001
1960
1980
2000
2020
Year
GBD 2019
GBD 2021
UN WPP 2022

Total fertility rate fitted trend lines, 1950−2021: Namibia TFR
Data points enclosed with a diamond are reference sources

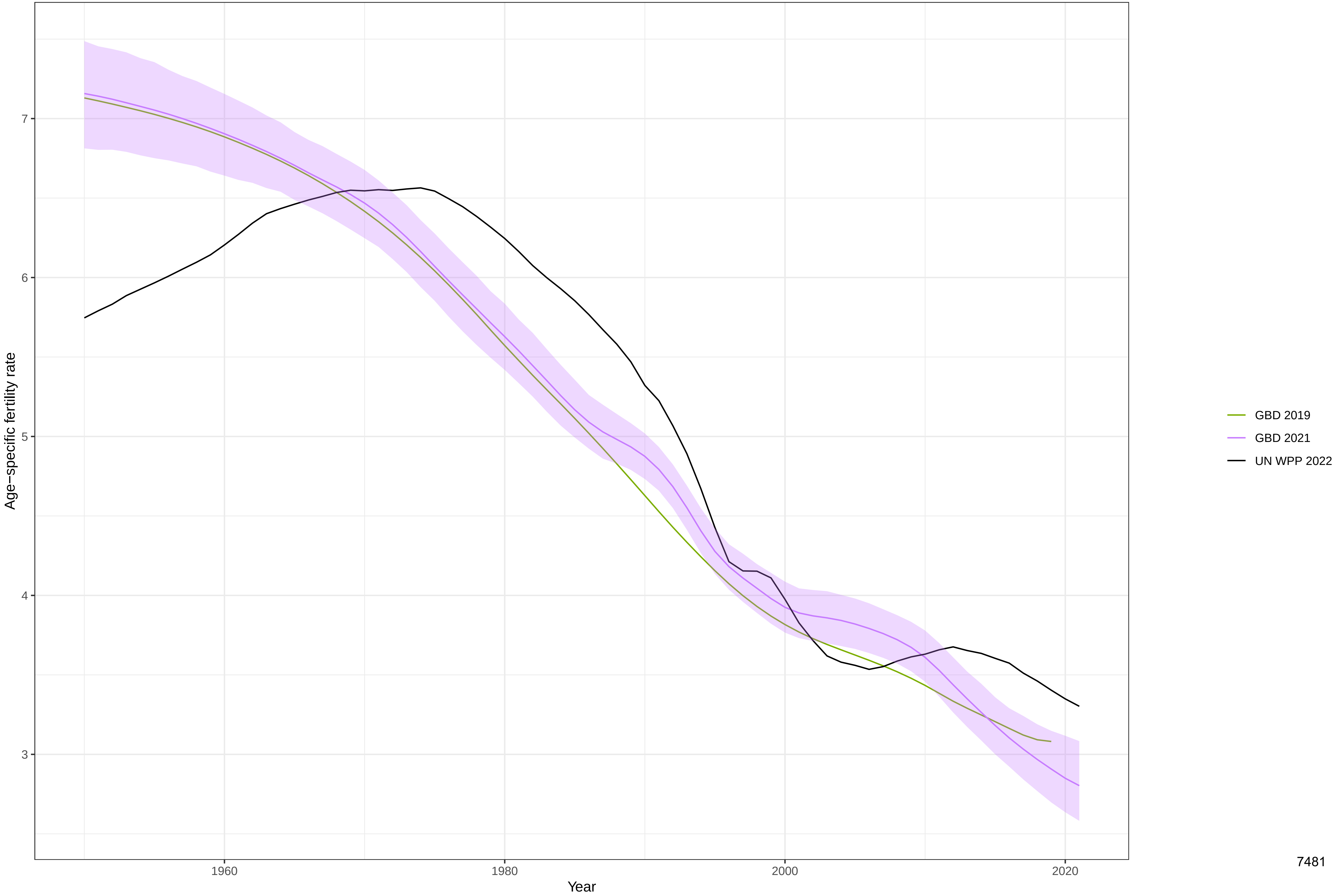

Age−specific fertility fitted trend lines, 1950−2021: South Africa, Ages: 10−14
Data points enclosed with a diamond are reference sources

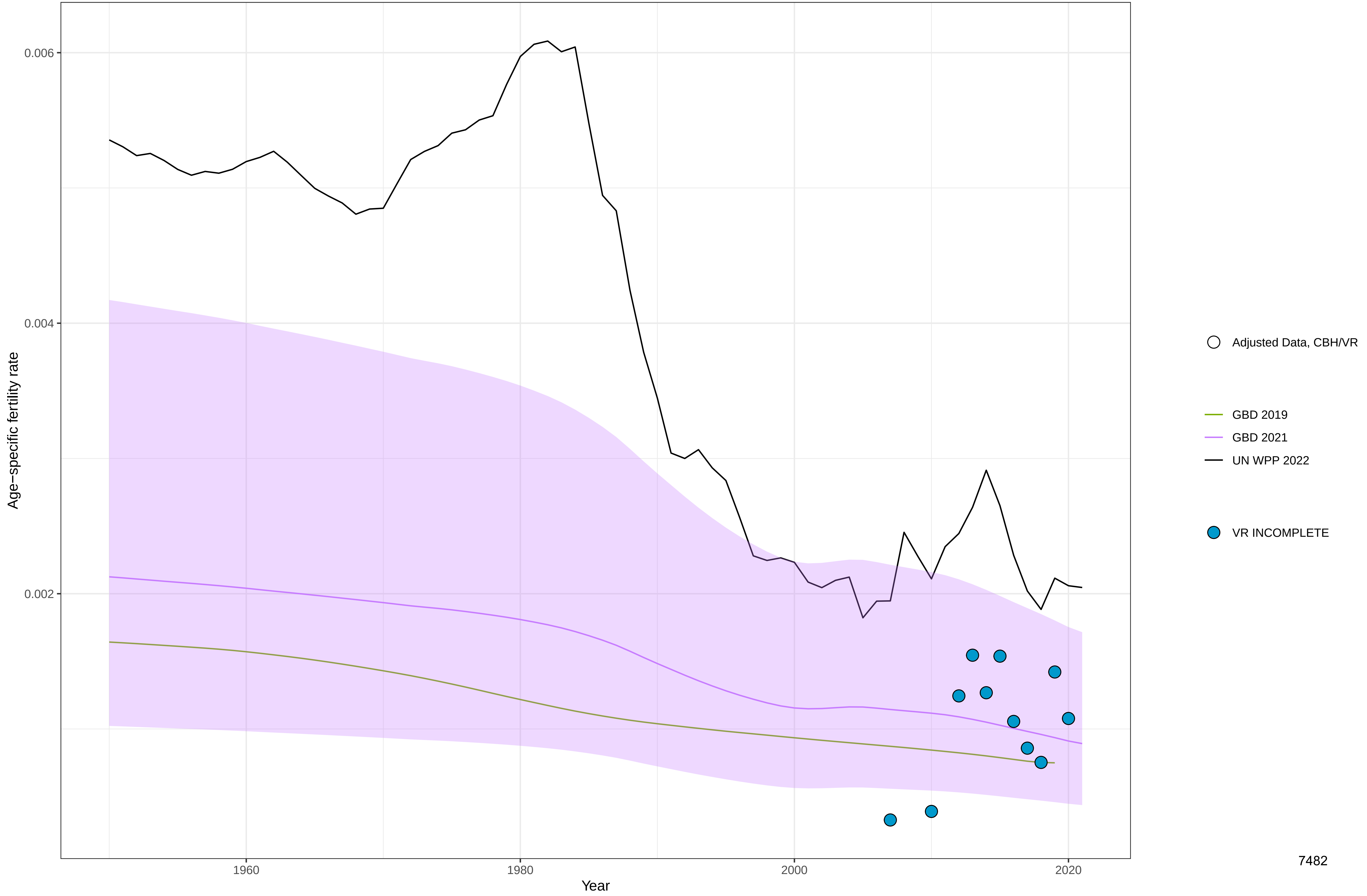

Age−specific fertility fitted trend lines, 1950−2021: South Africa, Ages: 15−19
Data points enclosed with a diamond are reference sources

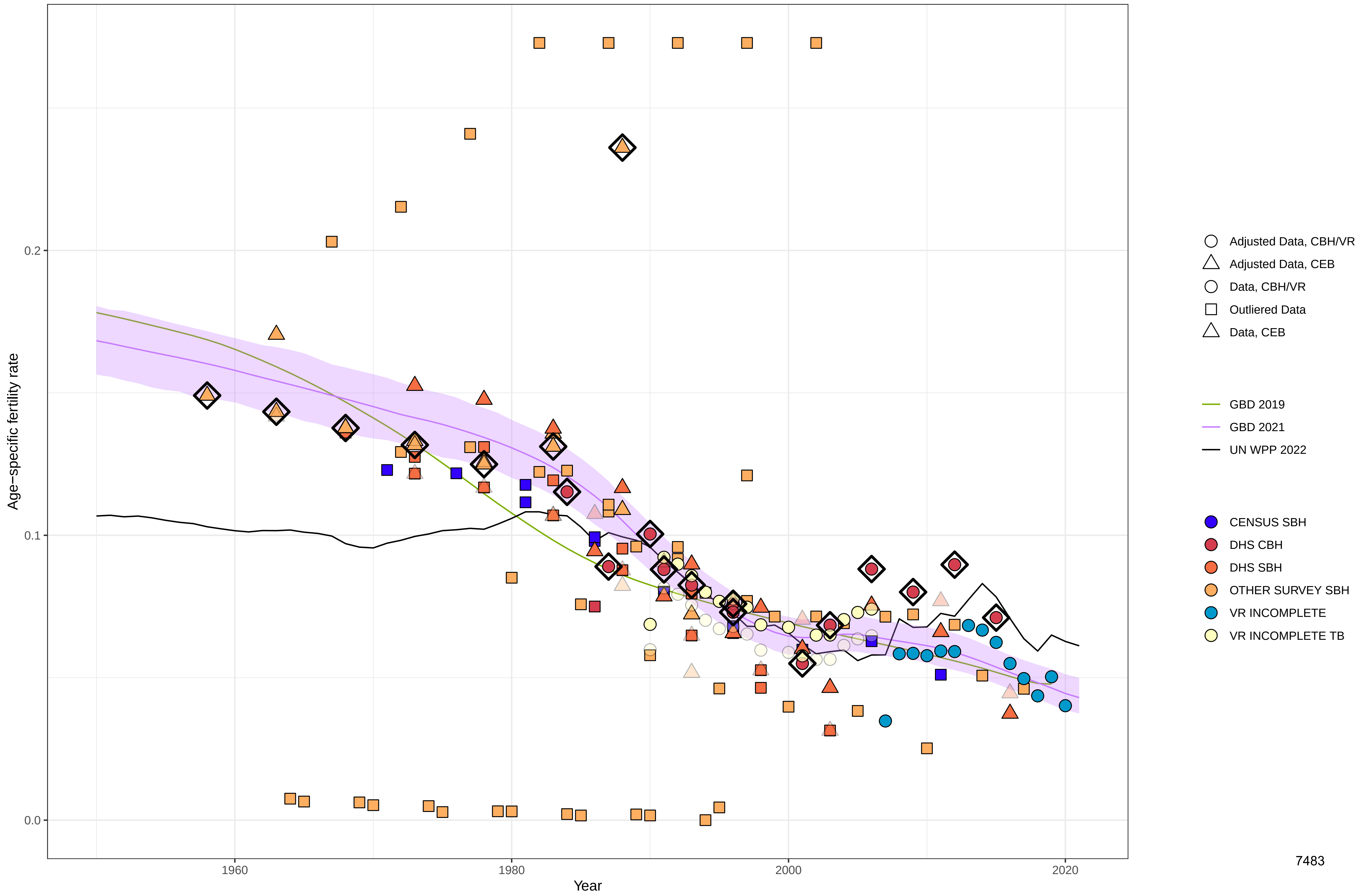

Age−specific fertility fitted trend lines, 1950−2021: South Africa, Ages: 20−24
Data points enclosed with a diamond are reference sources

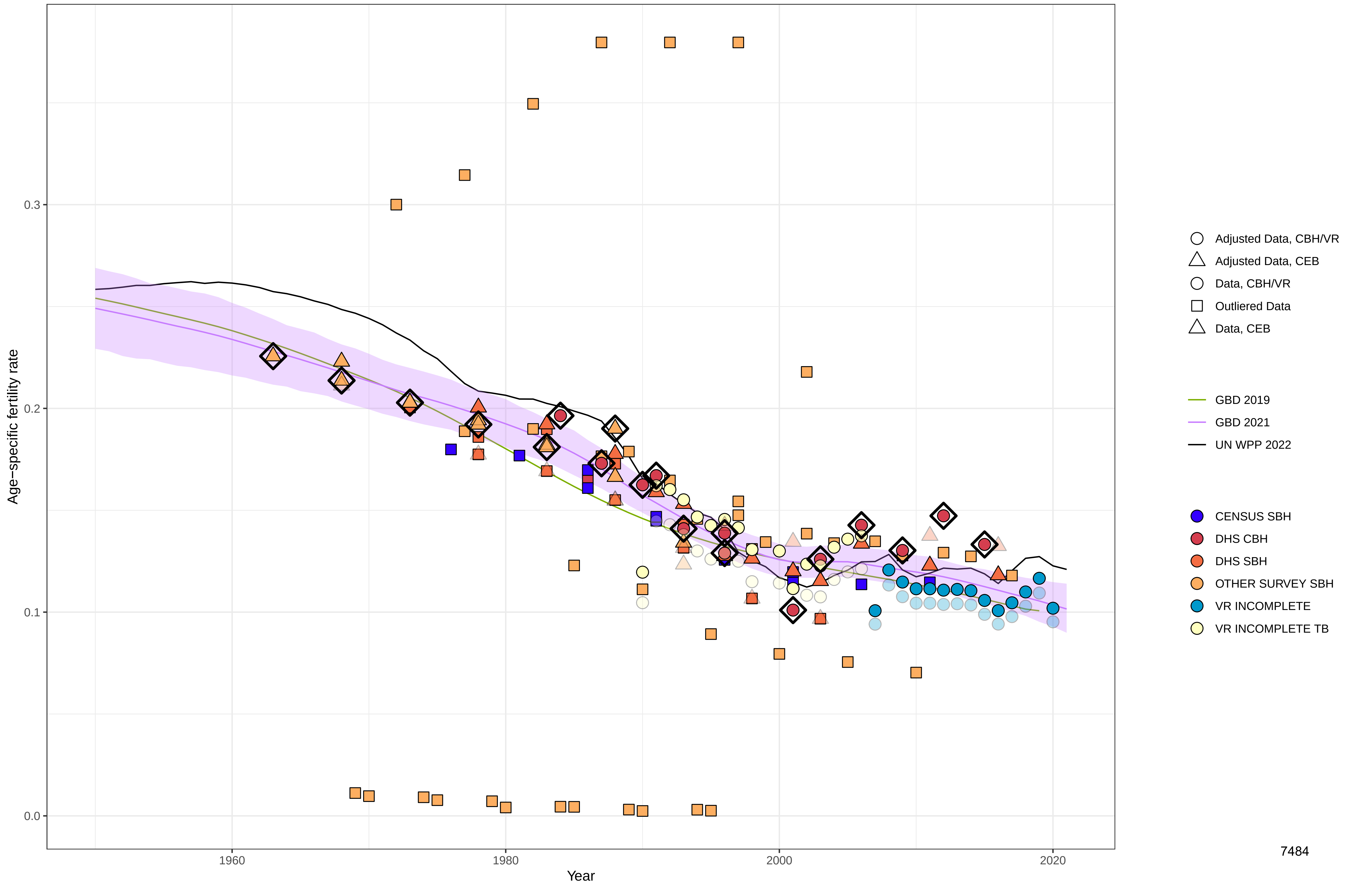

Age−specific fertility fitted trend lines, 1950−2021: South Africa, Ages: 25−29
Data points enclosed with a diamond are reference sources

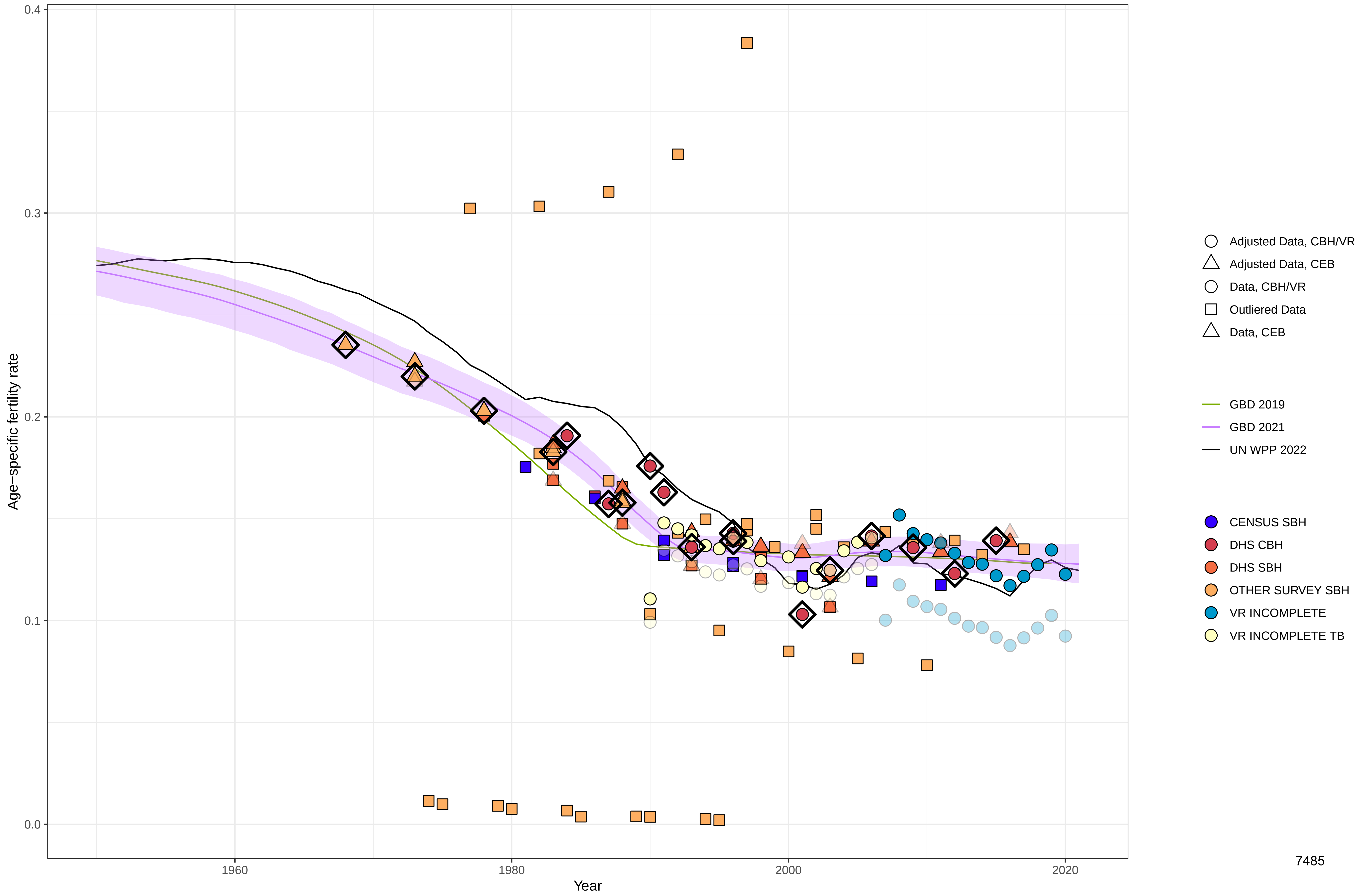

Age−specific fertility fitted trend lines, 1950−2021: South Africa, Ages: 30−34
Data points enclosed with a diamond are reference sources

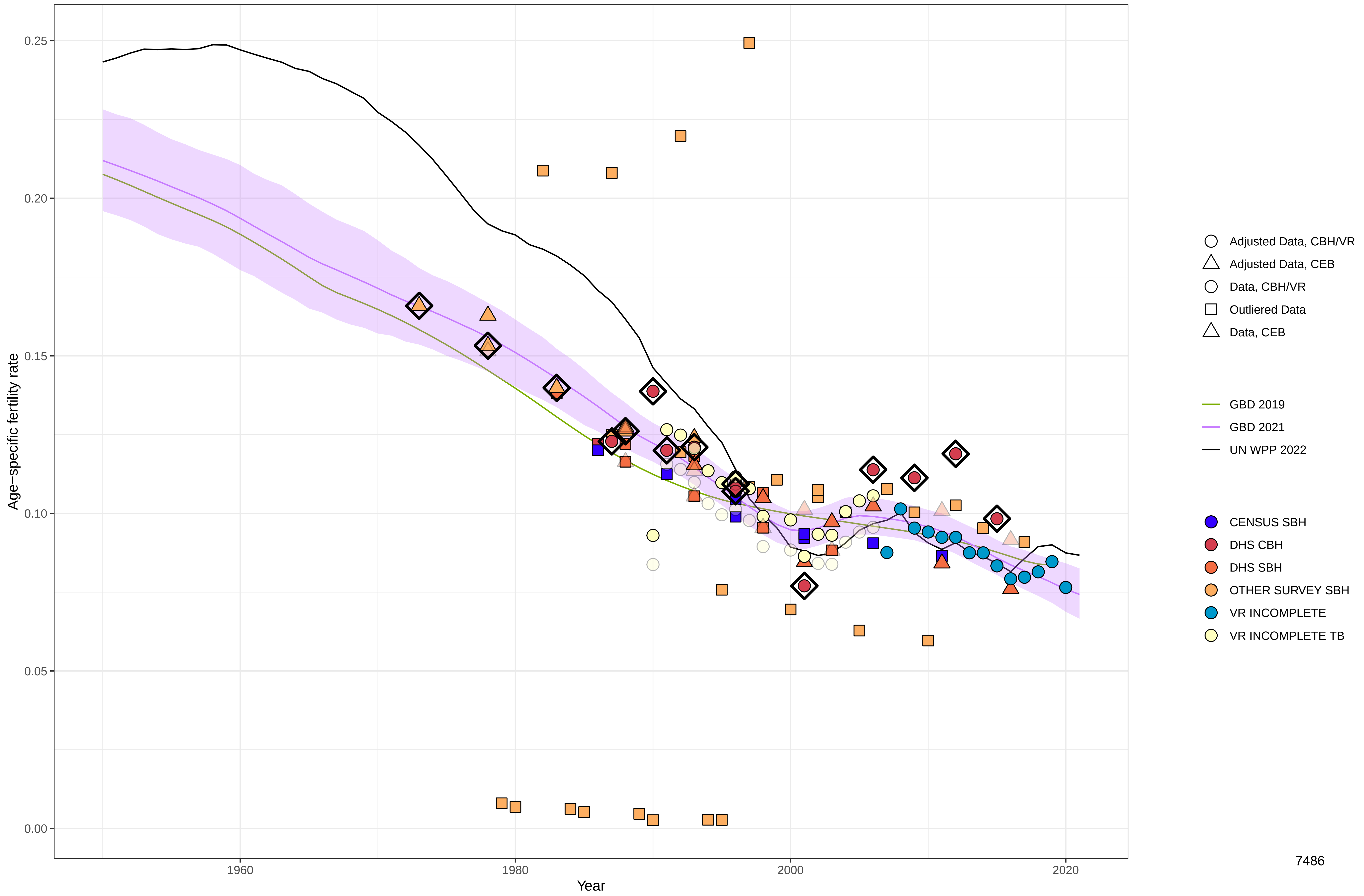

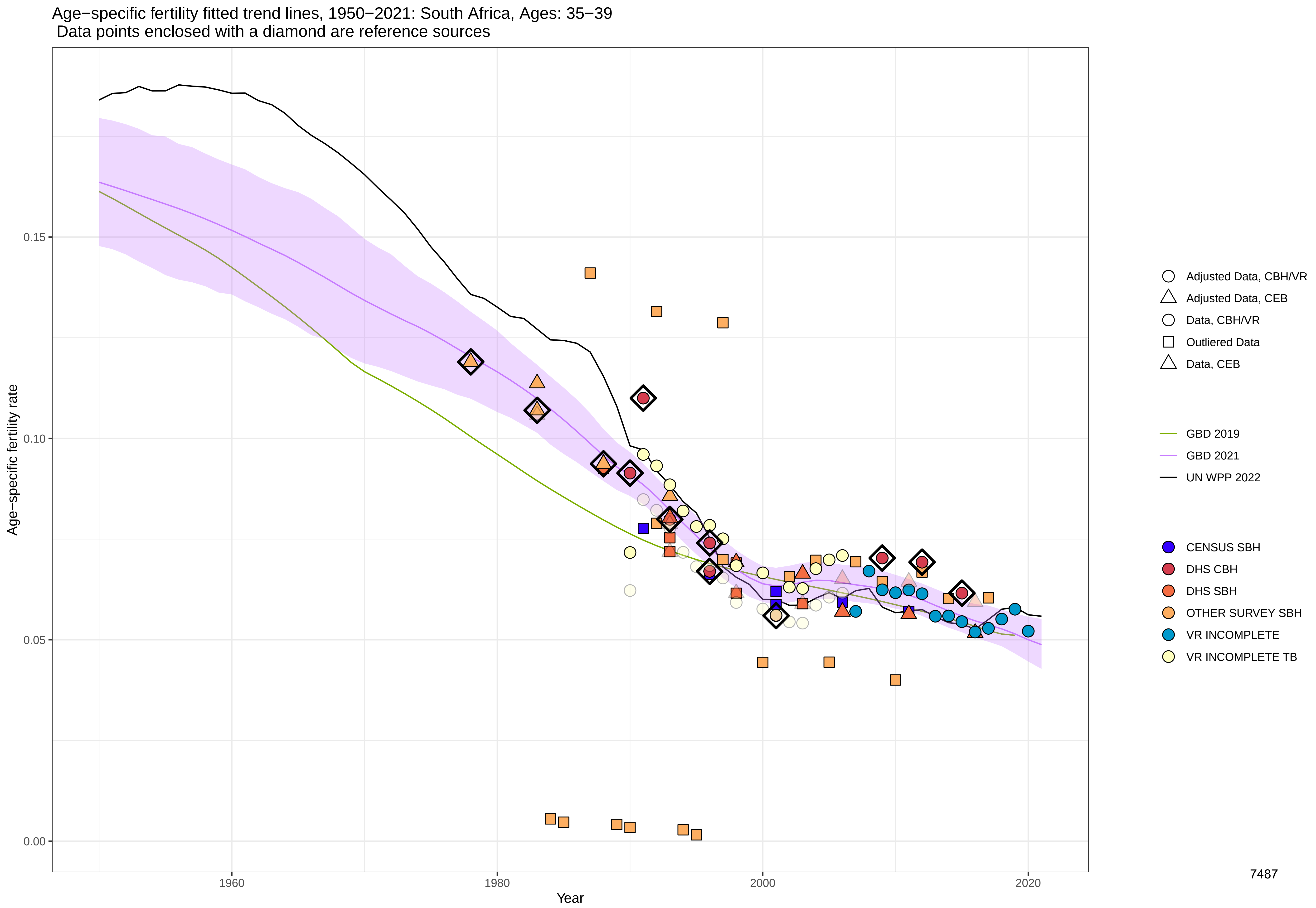

Age−specific fertility fitted trend lines, 1950−2021: South Africa, Ages: 35−39
Data points enclosed with a diamond are reference sources
Age−specific fertility rate
0.00
0.05
0.10
0.15
Year
1960
1980
2000
2020
Adjusted Data, CBH/VR
Adjusted Data, CEB
Data, CBH/VR
Outliered Data
Data, CEB
GBD 2019
GBD 2021
UN WPP 2022
CENSUS SBH
DHS CBH
DHS SBH
OTHER SURVEY SBH
VR INCOMPLETE
VR INCOMPLETE TB

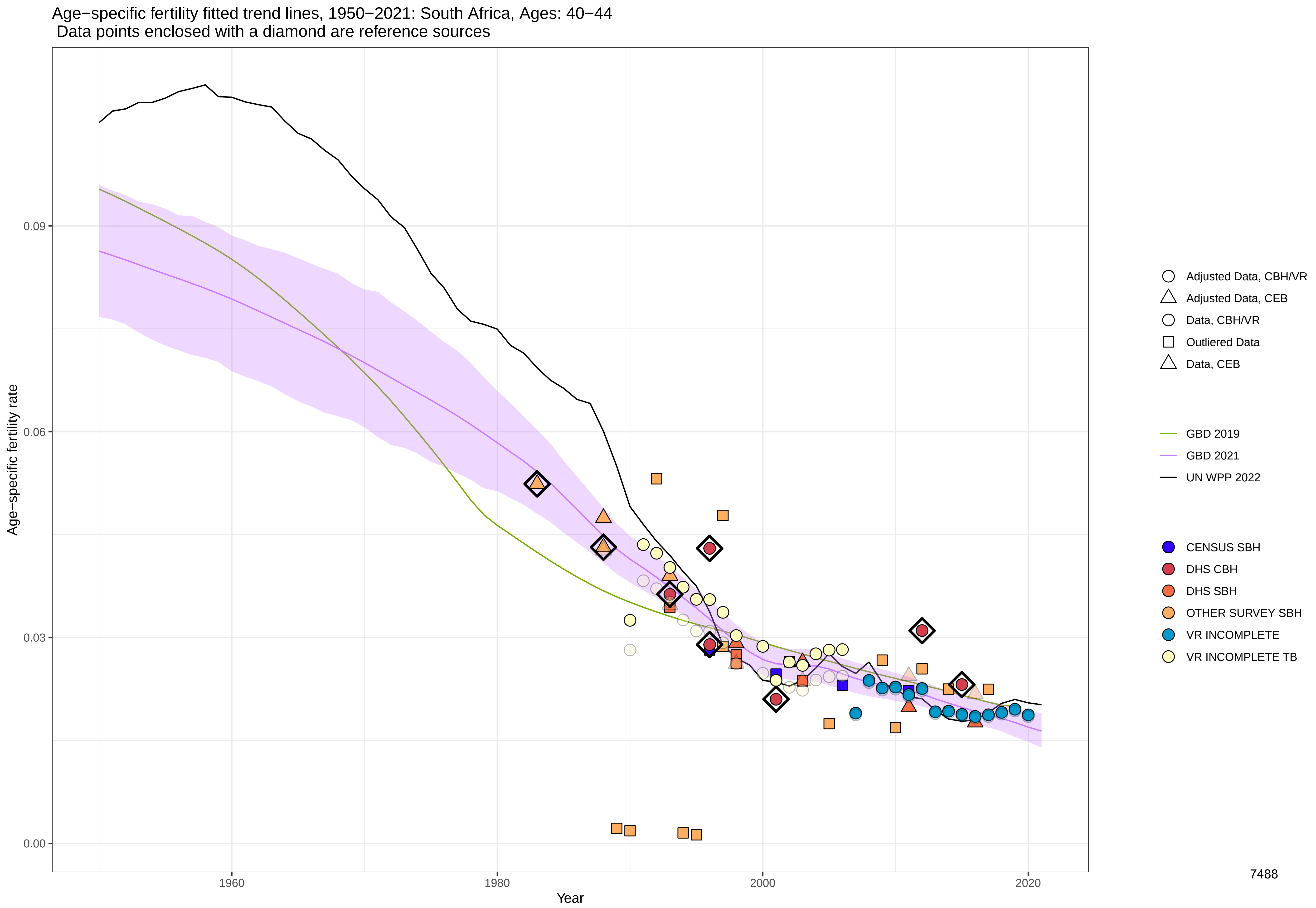

Age−specific fertility fitted trend lines, 1950−2021: South Africa, Ages: 40−44
Data points enclosed with a diamond are reference sources
Age−specific fertility rate
0.00
0.03
0.06
0.09
Year
1960
1980
2000
2020
Adjusted Data, CBH/VR
Adjusted Data, CEB
Data, CBH/VR
Outliered Data
Data, CEB
GBD 2019
GBD 2021
UN WPP 2022
CENSUS SBH
DHS CBH
DHS SBH
OTHER SURVEY SBH
VR INCOMPLETE
VR INCOMPLETE TB

Age−specific fertility fitted trend lines, 1950−2021: South Africa, Ages: 45−49
Data points enclosed with a diamond are reference sources

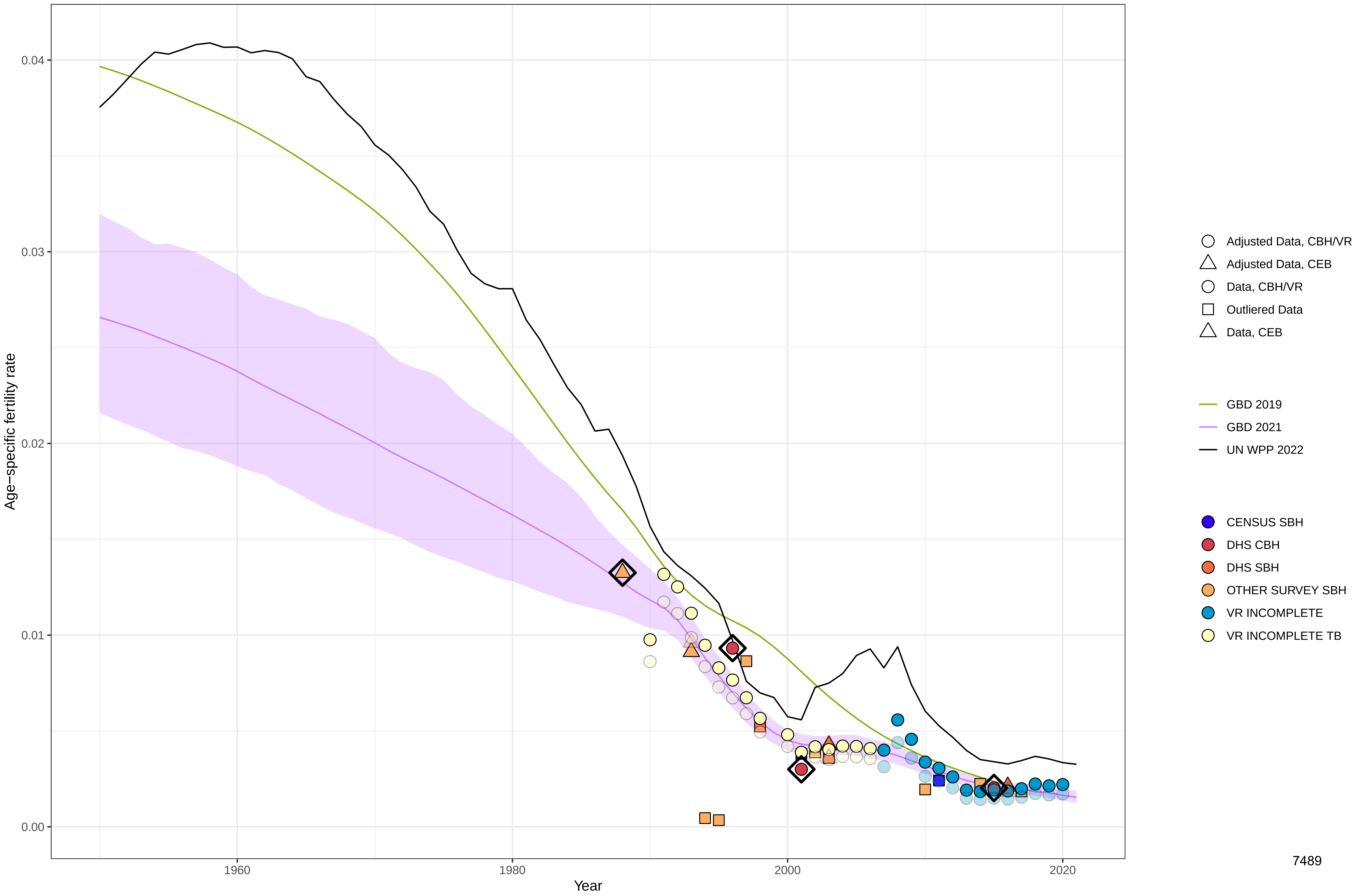

Age−specific fertility fitted trend lines, 1950−2021: South Africa, Ages: 50−54
Data points enclosed with a diamond are reference sources

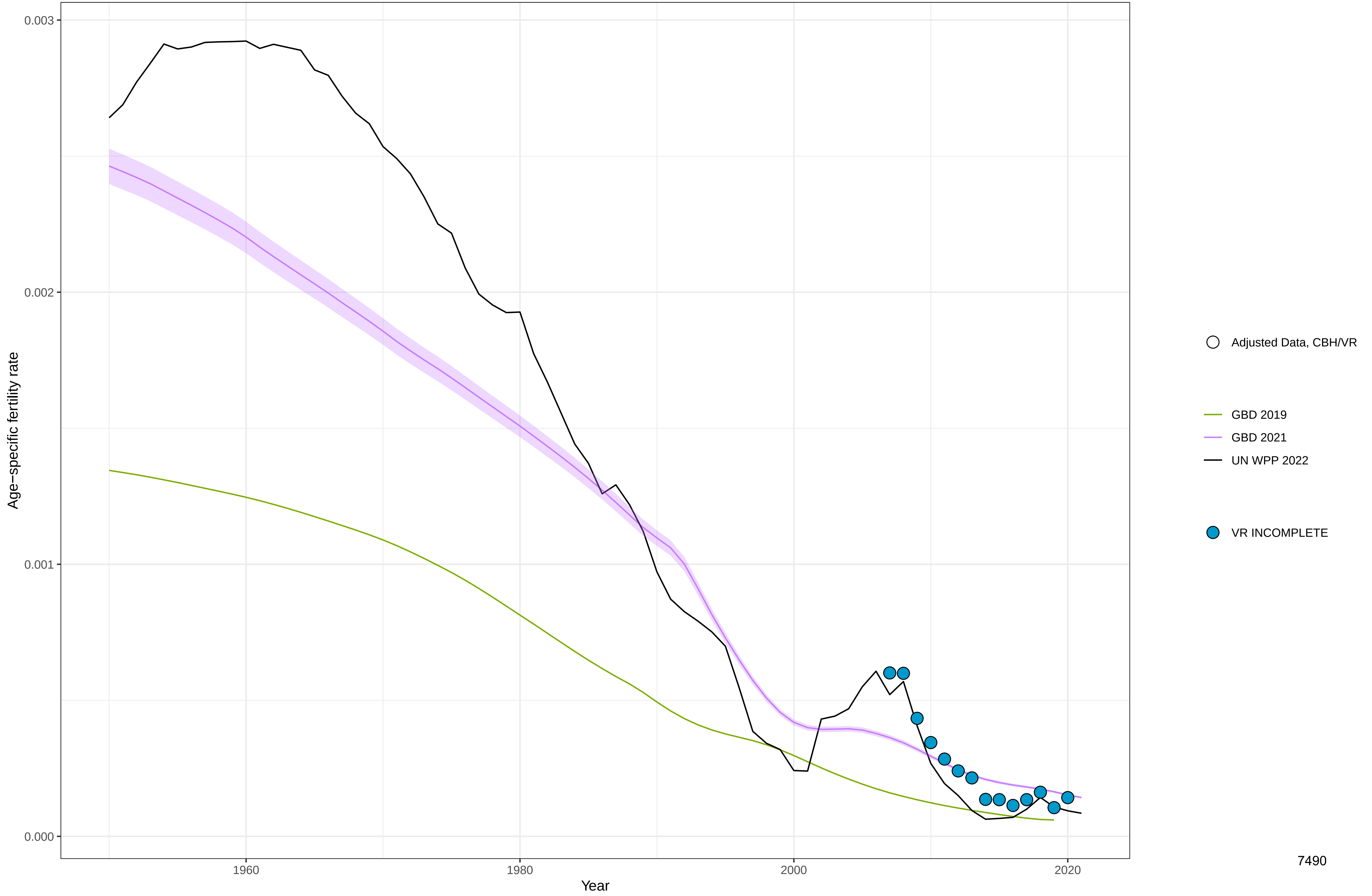

Total fertility rate fitted trend lines, 1950−2021: South Africa TFR
Data points enclosed with a diamond are reference sources

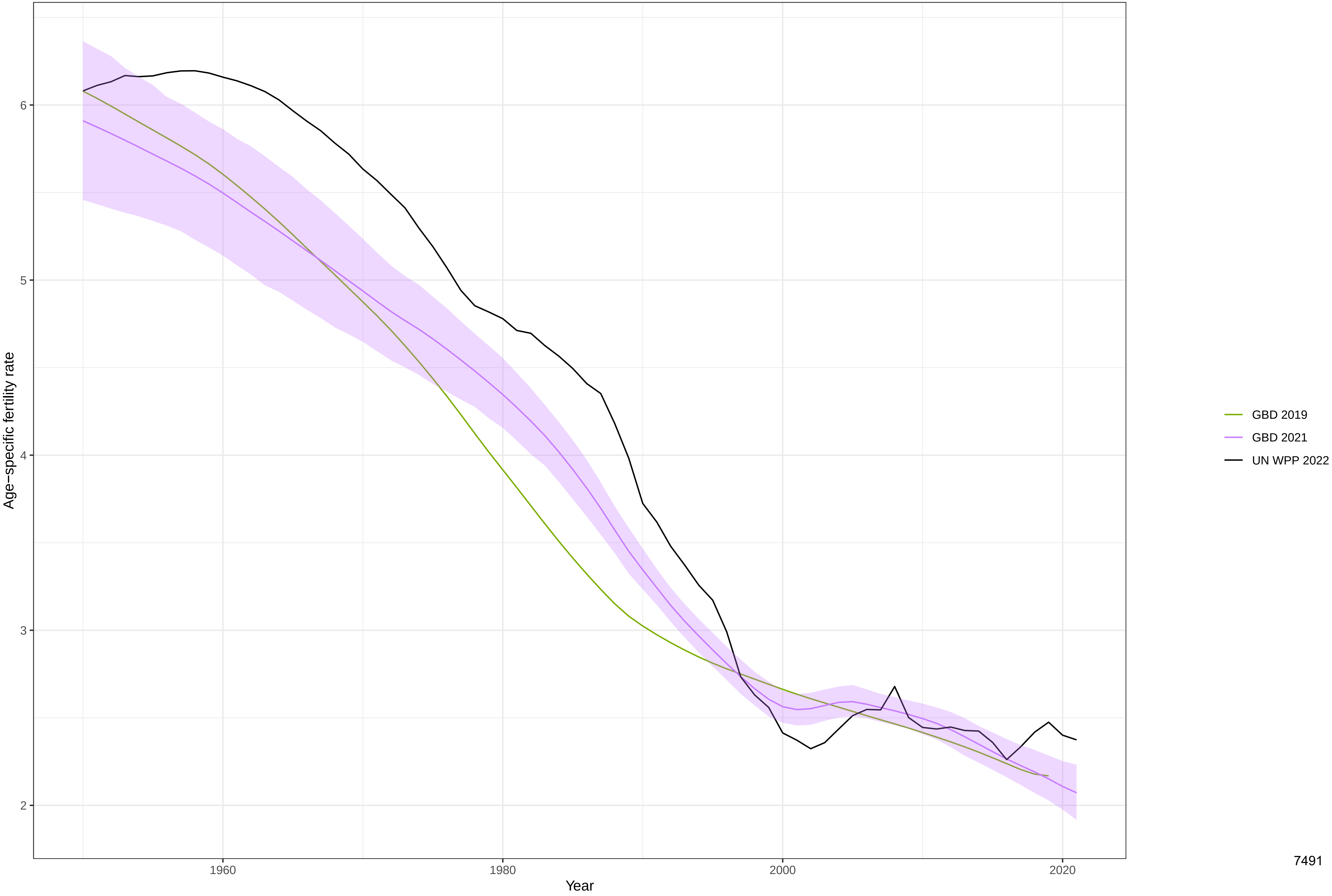

Age−specific fertility fitted trend lines, 1950−2021: Eastern Cape, Ages: 10−14
Data points enclosed with a diamond are reference sources

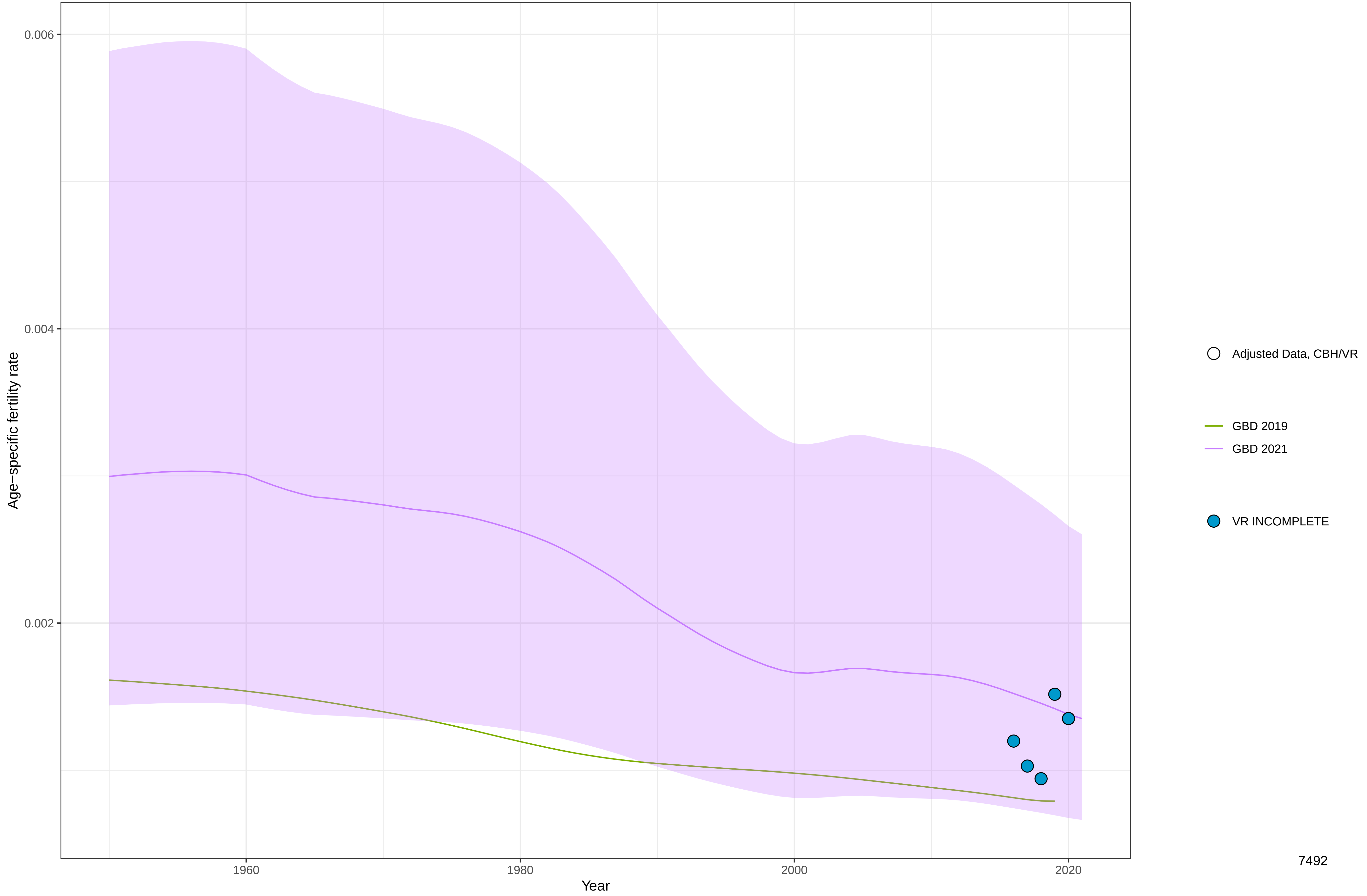

Age−specific fertility fitted trend lines, 1950−2021: Eastern Cape, Ages: 15−19
Data points enclosed with a diamond are reference sources

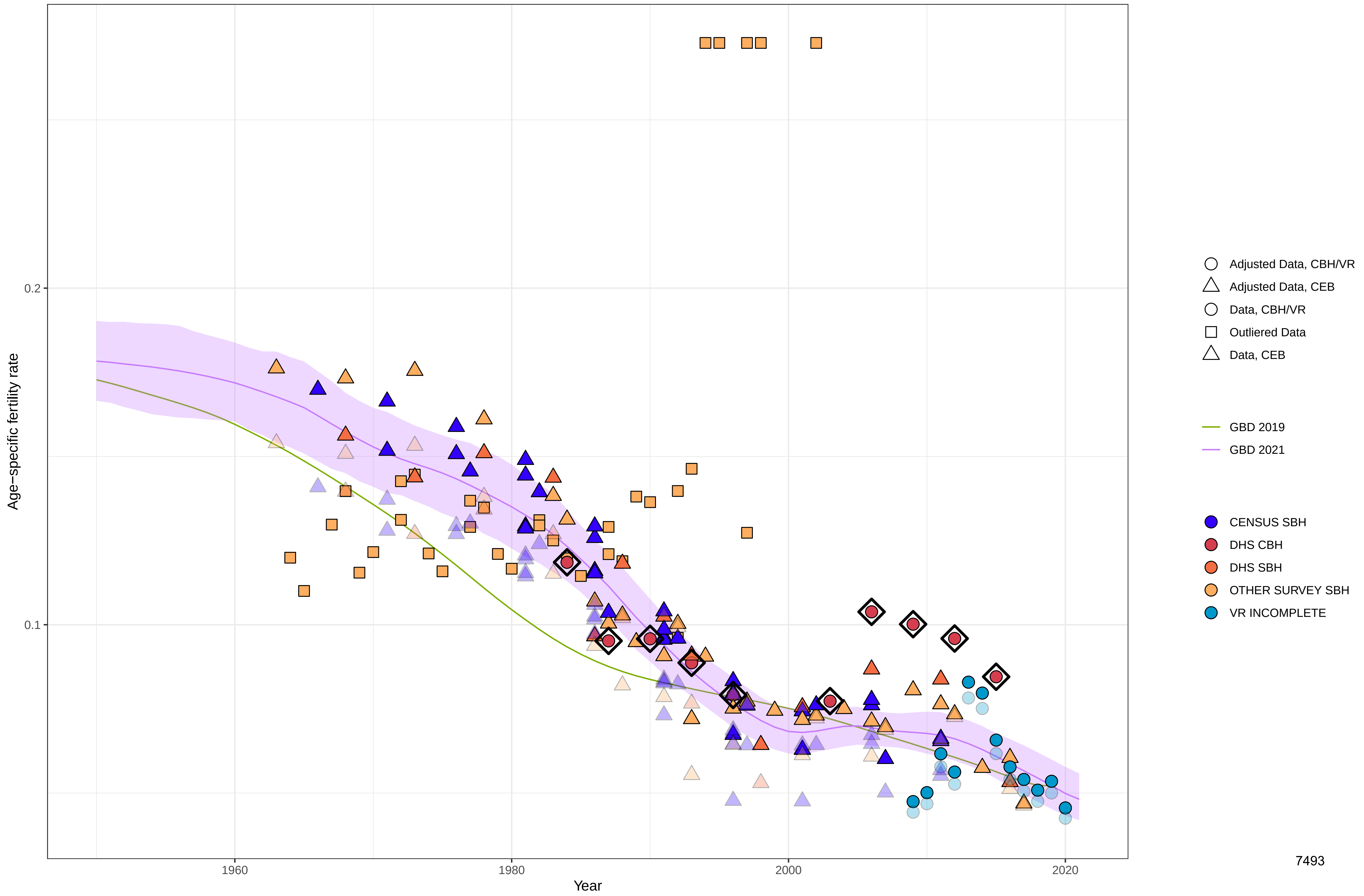

Age−specific fertility fitted trend lines, 1950−2021: Eastern Cape, Ages: 20−24
Data points enclosed with a diamond are reference sources

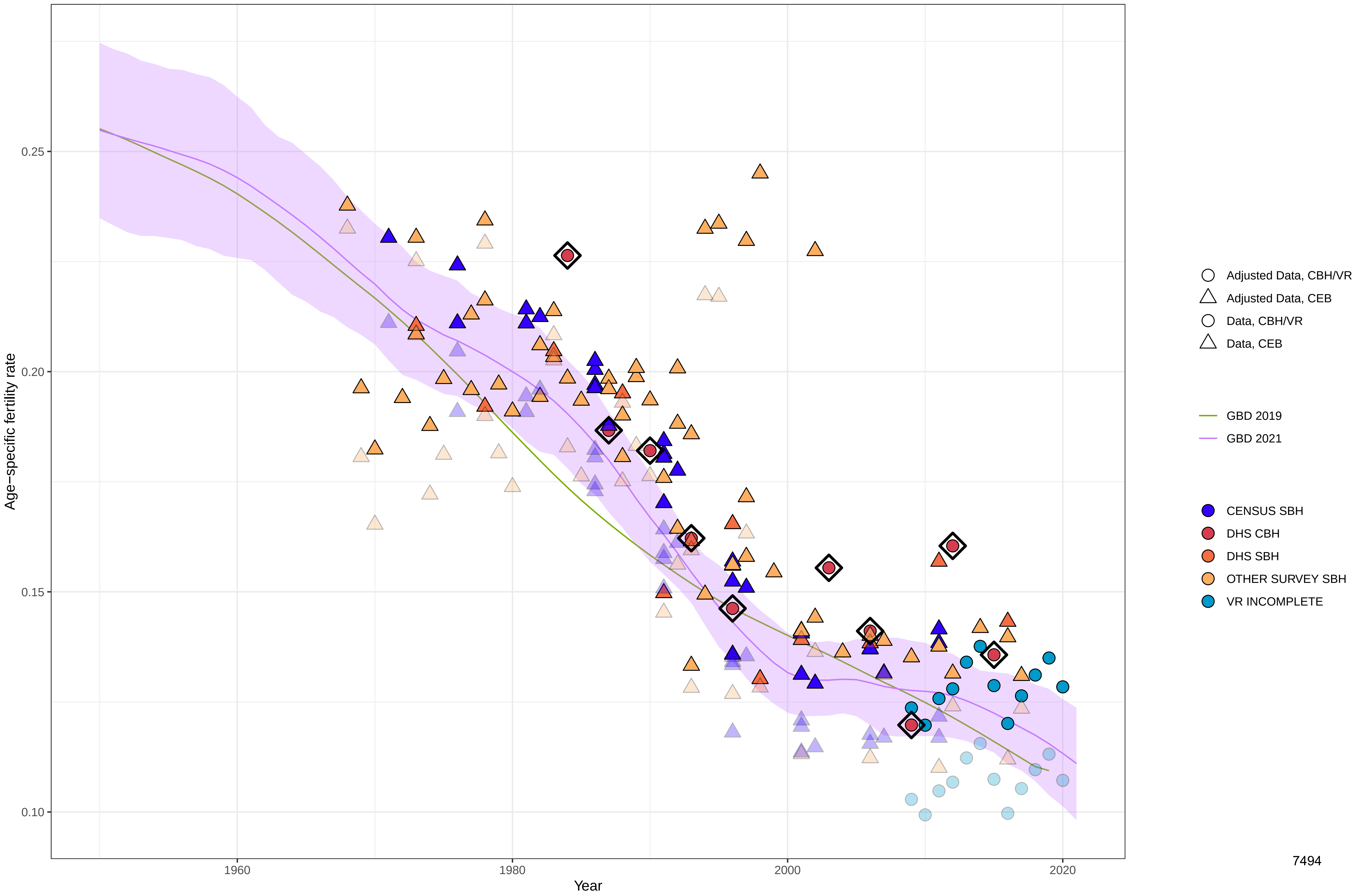

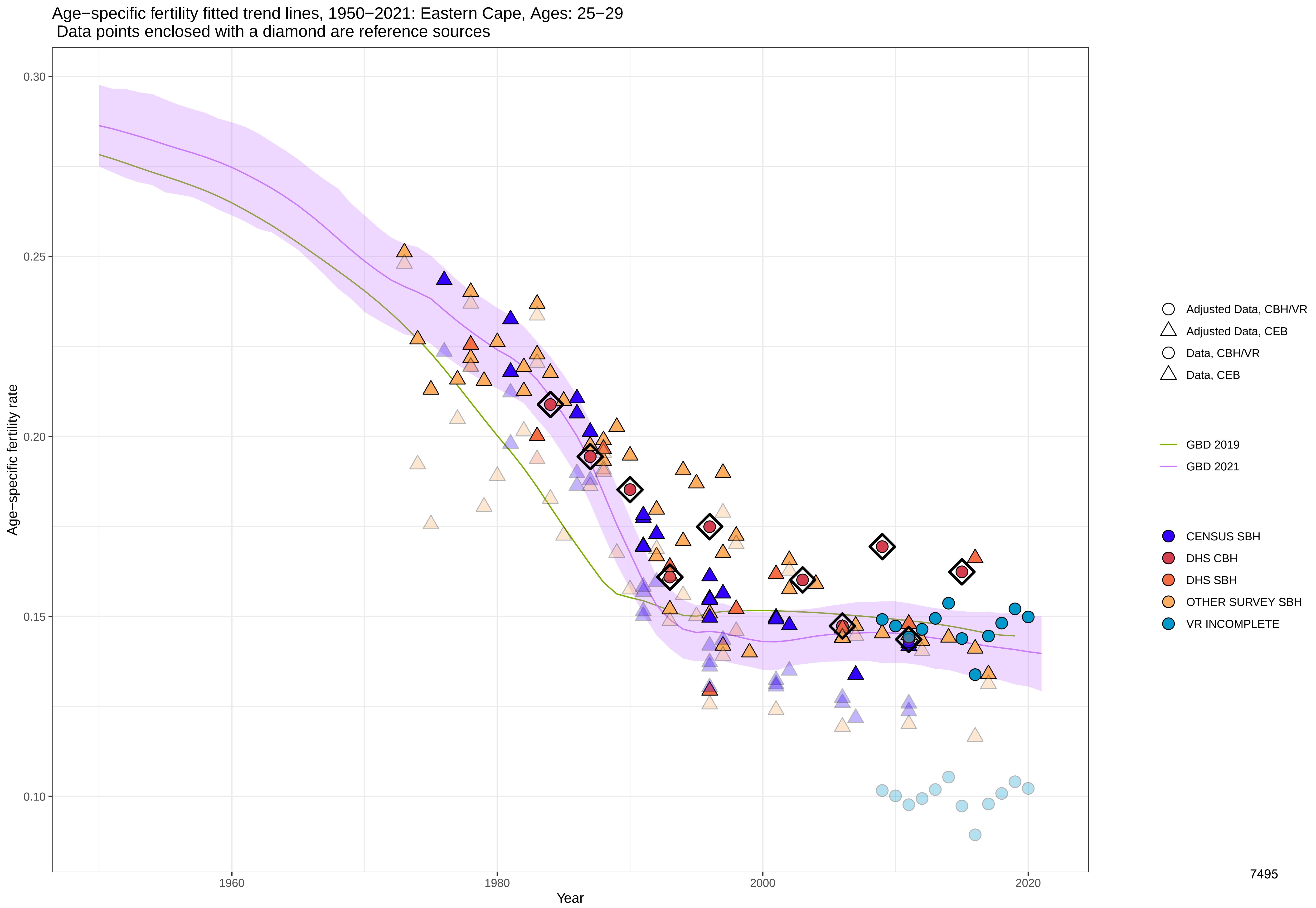

Age−specific fertility fitted trend lines, 1950−2021: Eastern Cape, Ages: 25−29
Data points enclosed with a diamond are reference sources
Age−specific fertility rate
Year
0.30
0.25
0.20
0.15
0.10
1960
1980
2000
2020
Adjusted Data, CBH/VR
Adjusted Data, CEB
Data, CBH/VR
Data, CEB
GBD 2019
GBD 2021
CENSUS SBH
DHS CBH
DHS SBH
OTHER SURVEY SBH
VR INCOMPLETE

Age−specific fertility fitted trend lines, 1950−2021: Eastern Cape, Ages: 30−34
Data points enclosed with a diamond are reference sources

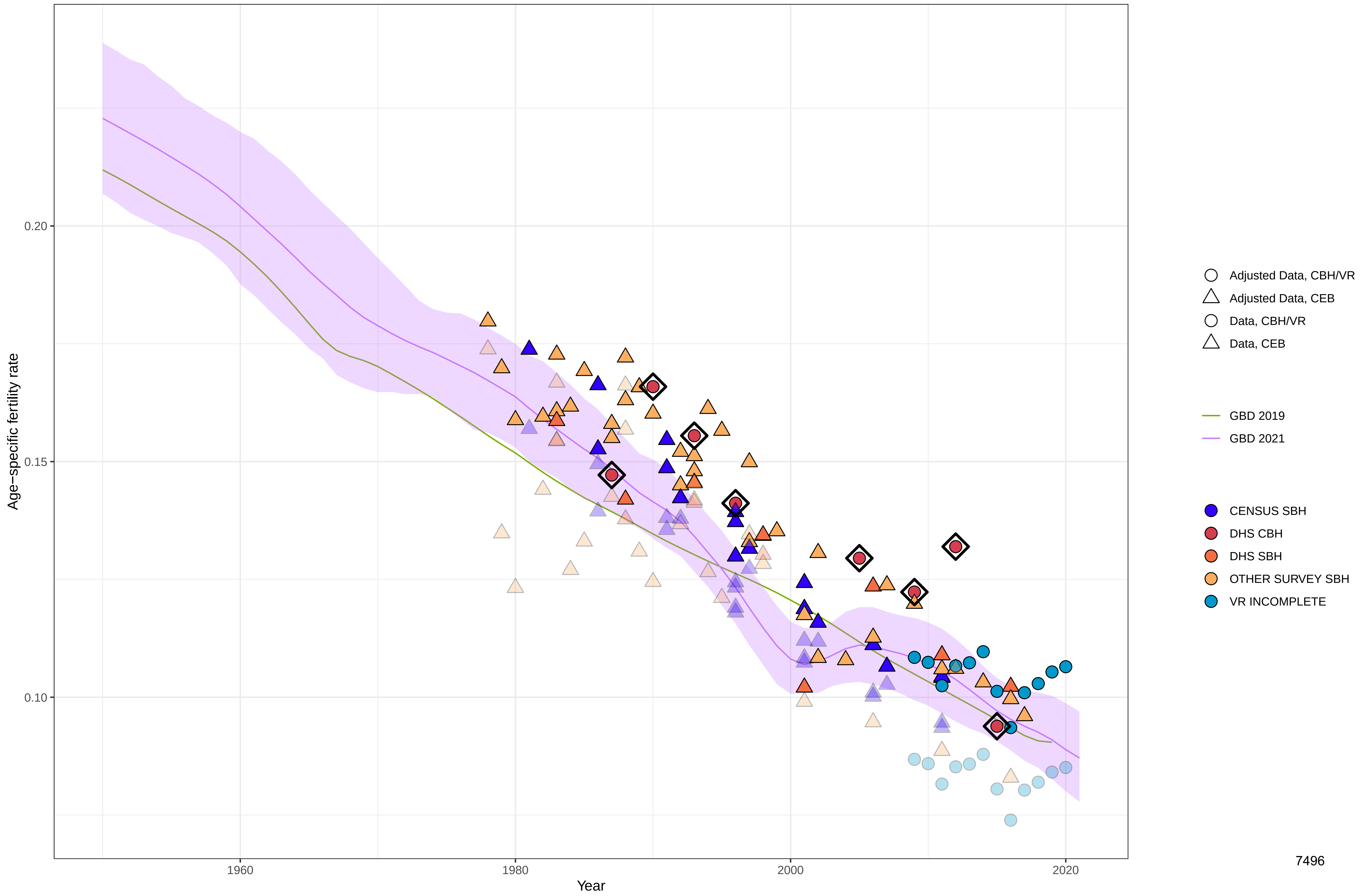

Age−specific fertility fitted trend lines, 1950−2021: Eastern Cape, Ages: 35−39
Data points enclosed with a diamond are reference sources

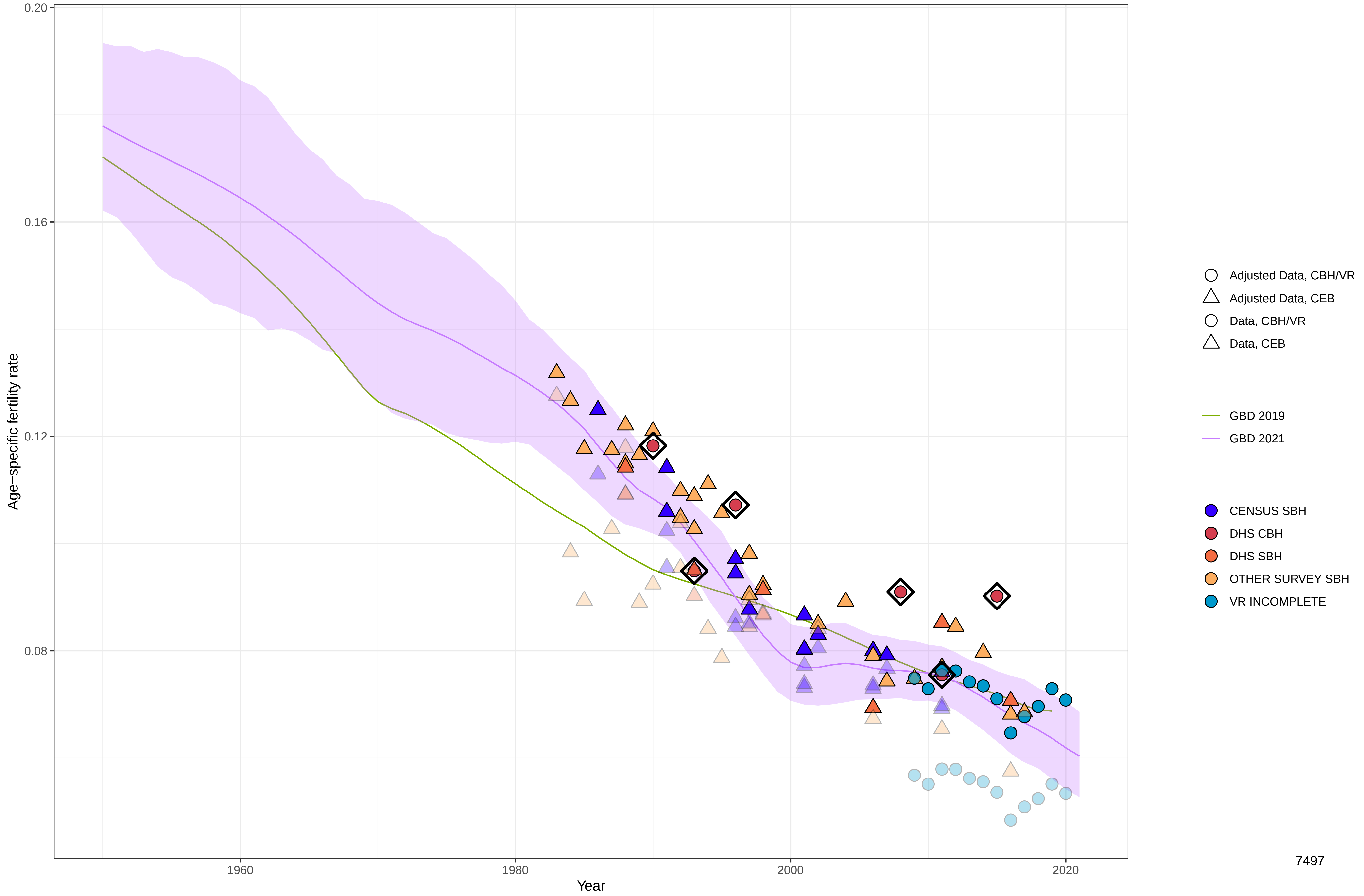

Age−specific fertility fitted trend lines, 1950−2021: Eastern Cape, Ages: 40−44
Data points enclosed with a diamond are reference sources

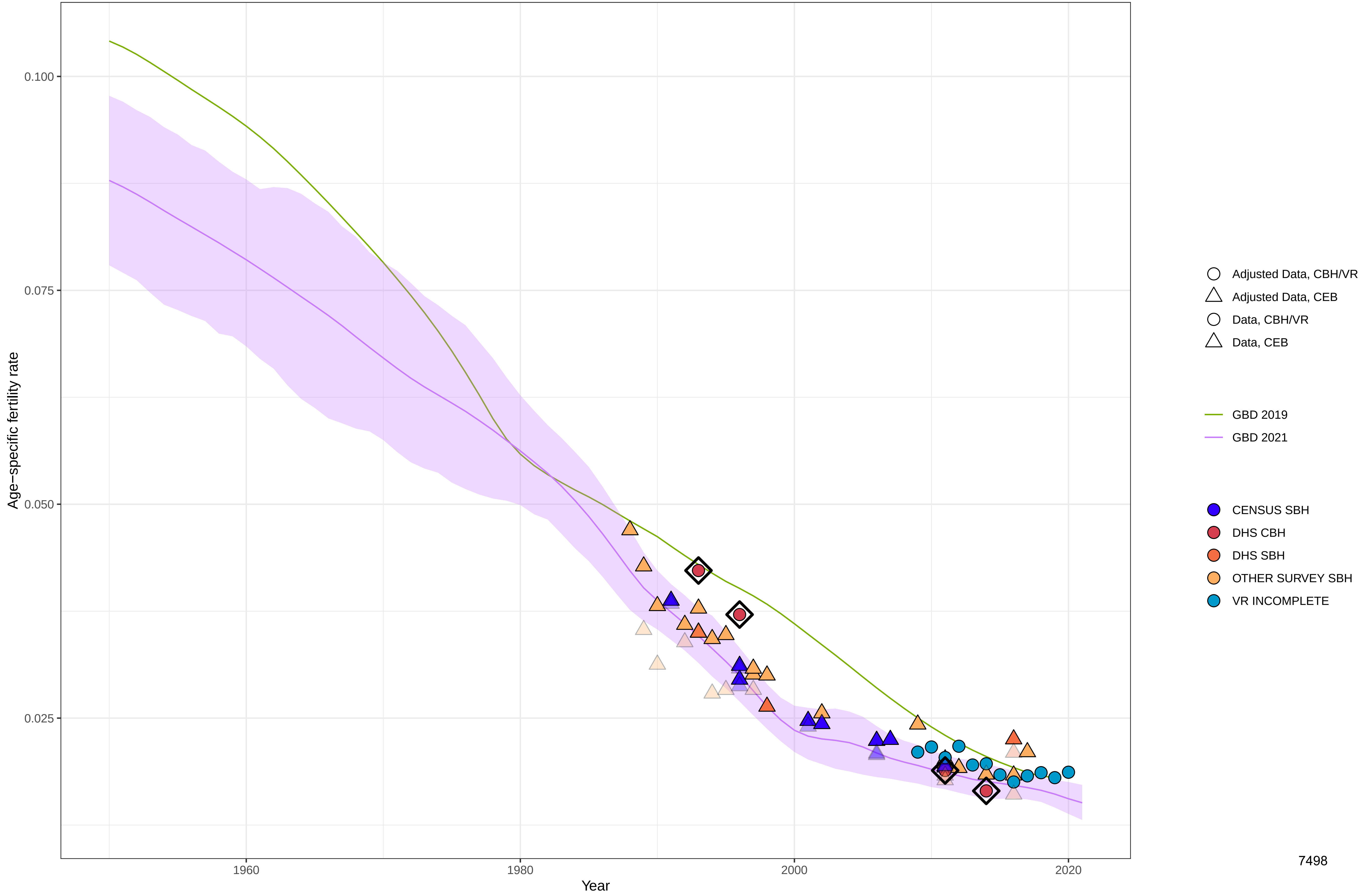

Age−specific fertility fitted trend lines, 1950−2021: Eastern Cape, Ages: 45−49
Data points enclosed with a diamond are reference sources

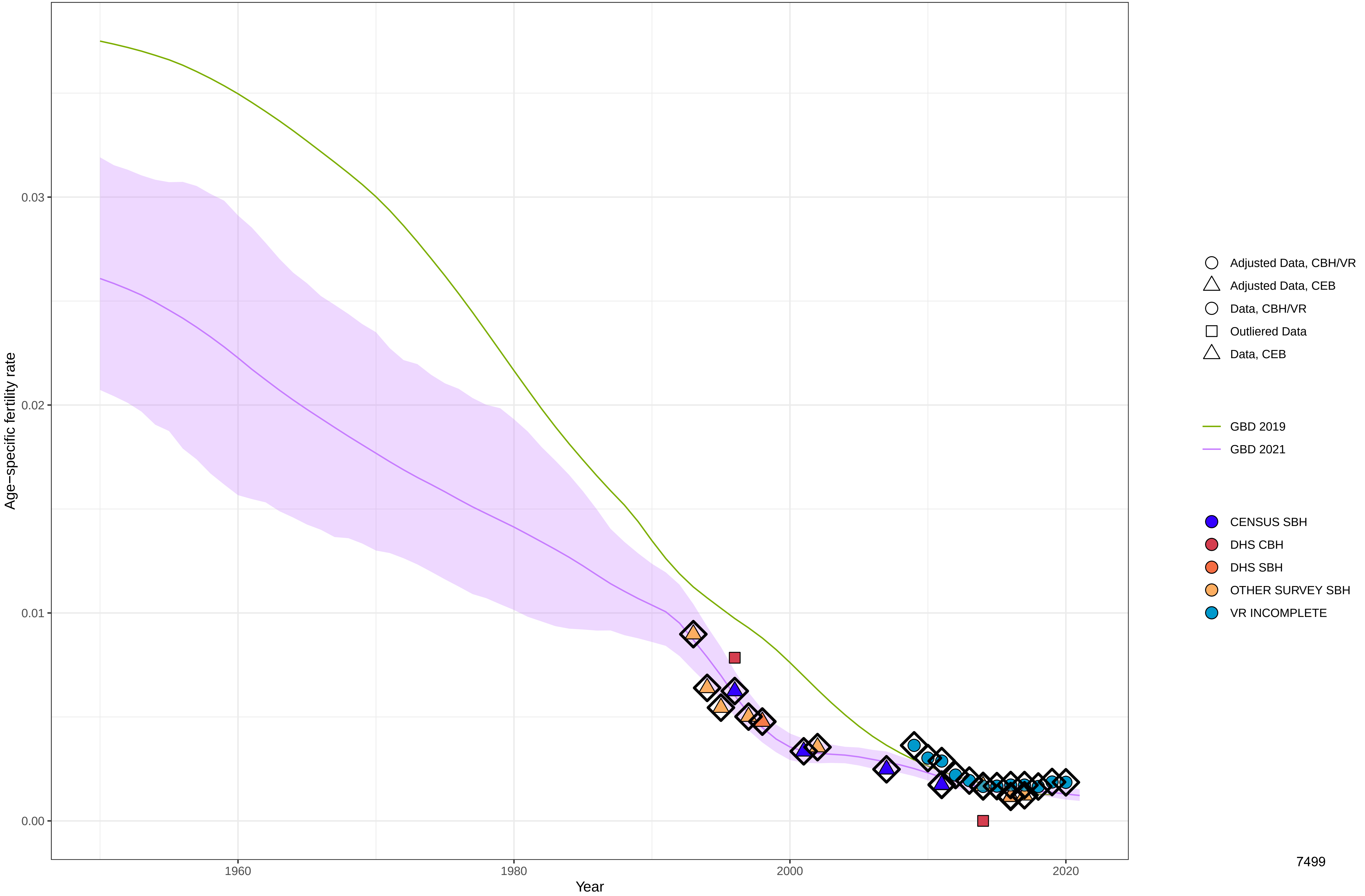

Age−specific fertility fitted trend lines, 1950−2021: Eastern Cape, Ages: 50−54
Data points enclosed with a diamond are reference sources

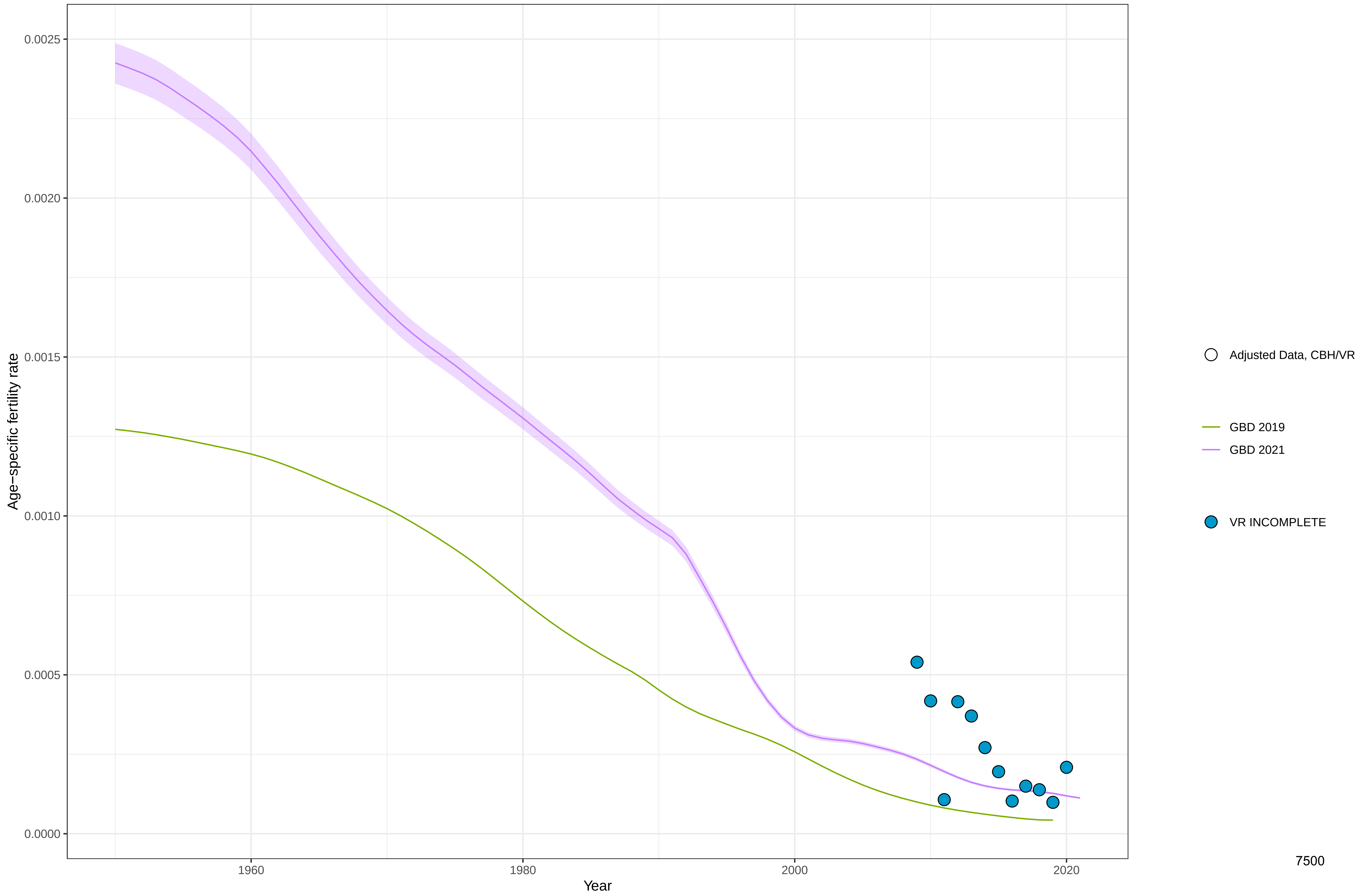

Total fertility rate fitted trend lines, 1950−2021: Eastern Cape TFR
Data points enclosed with a diamond are reference sources

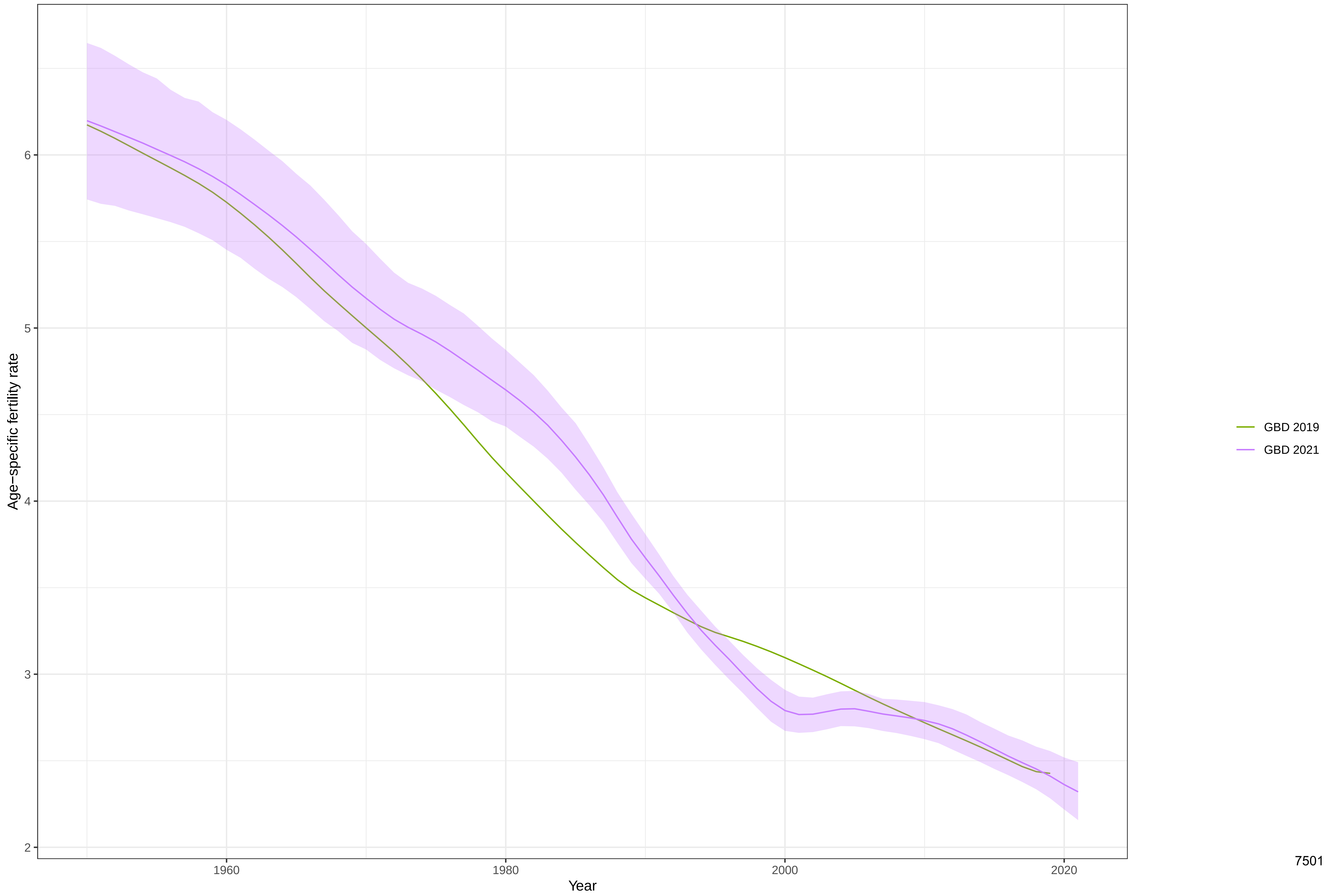

Age−specific fertility fitted trend lines, 1950−2021: Free State, Ages: 10−14
Data points enclosed with a diamond are reference sources

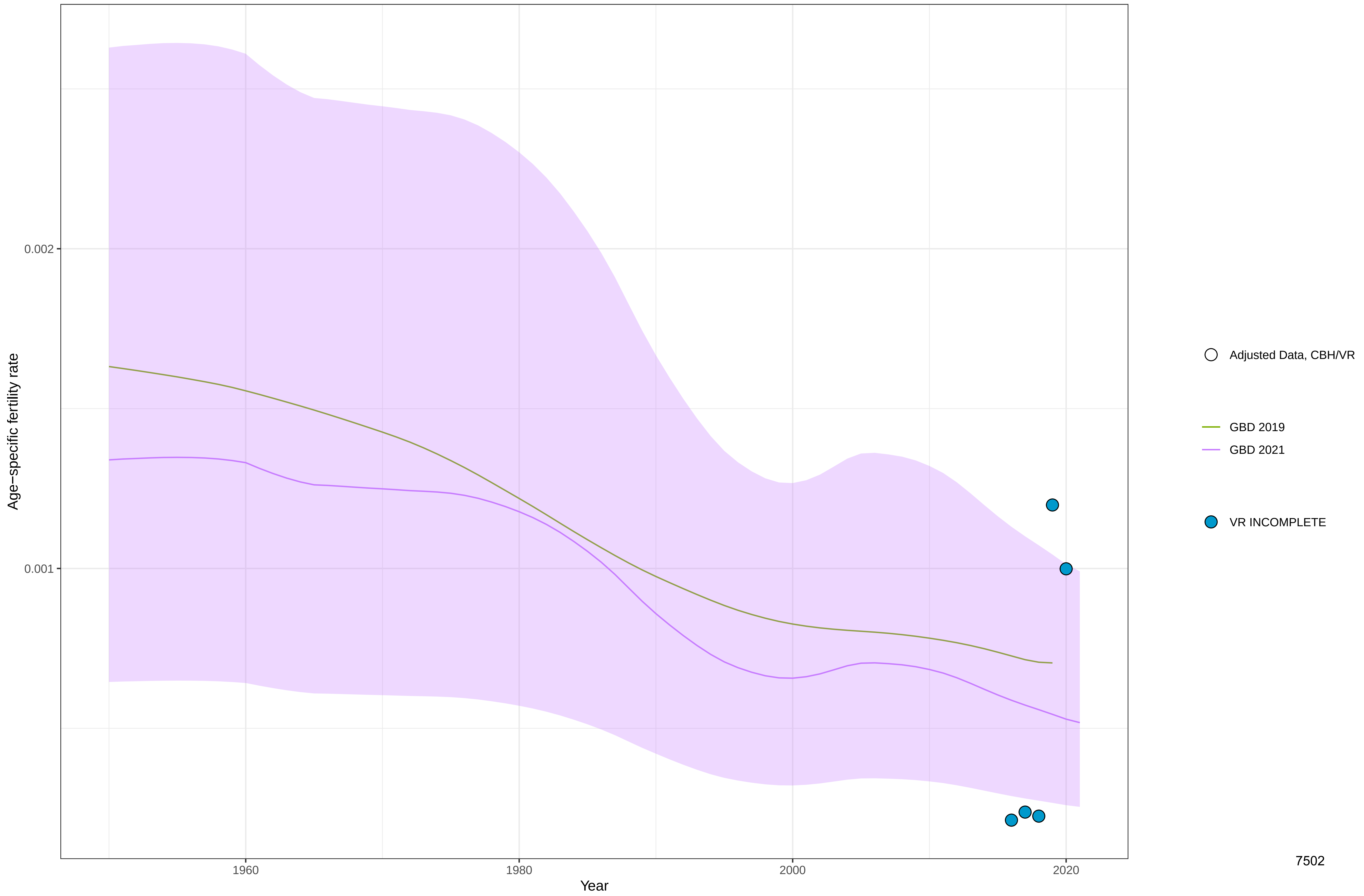

Age−specific fertility fitted trend lines, 1950−2021: Free State, Ages: 15−19
Data points enclosed with a diamond are reference sources

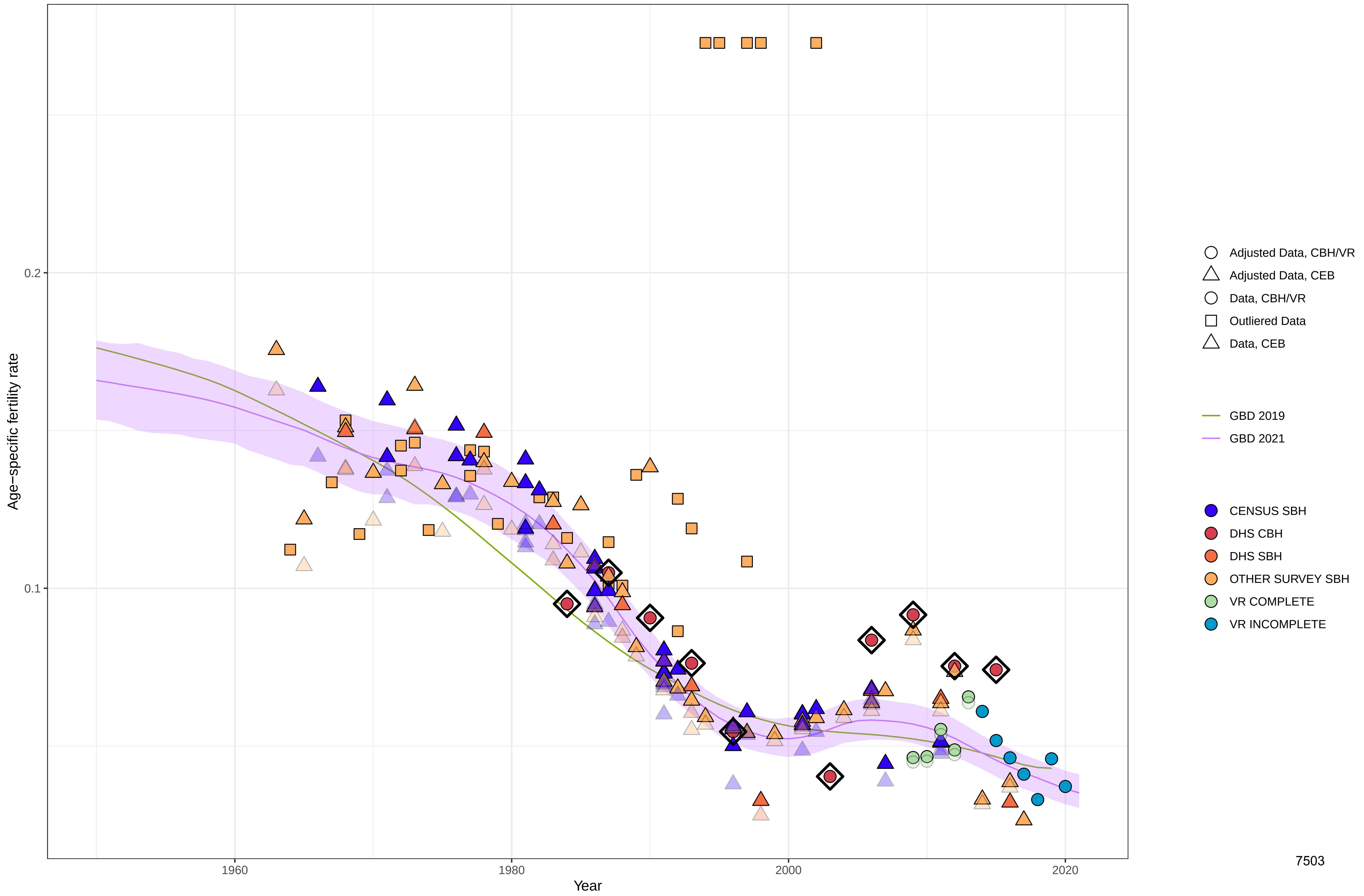

Age−specific fertility fitted trend lines, 1950−2021: Free State, Ages: 20−24
Data points enclosed with a diamond are reference sources

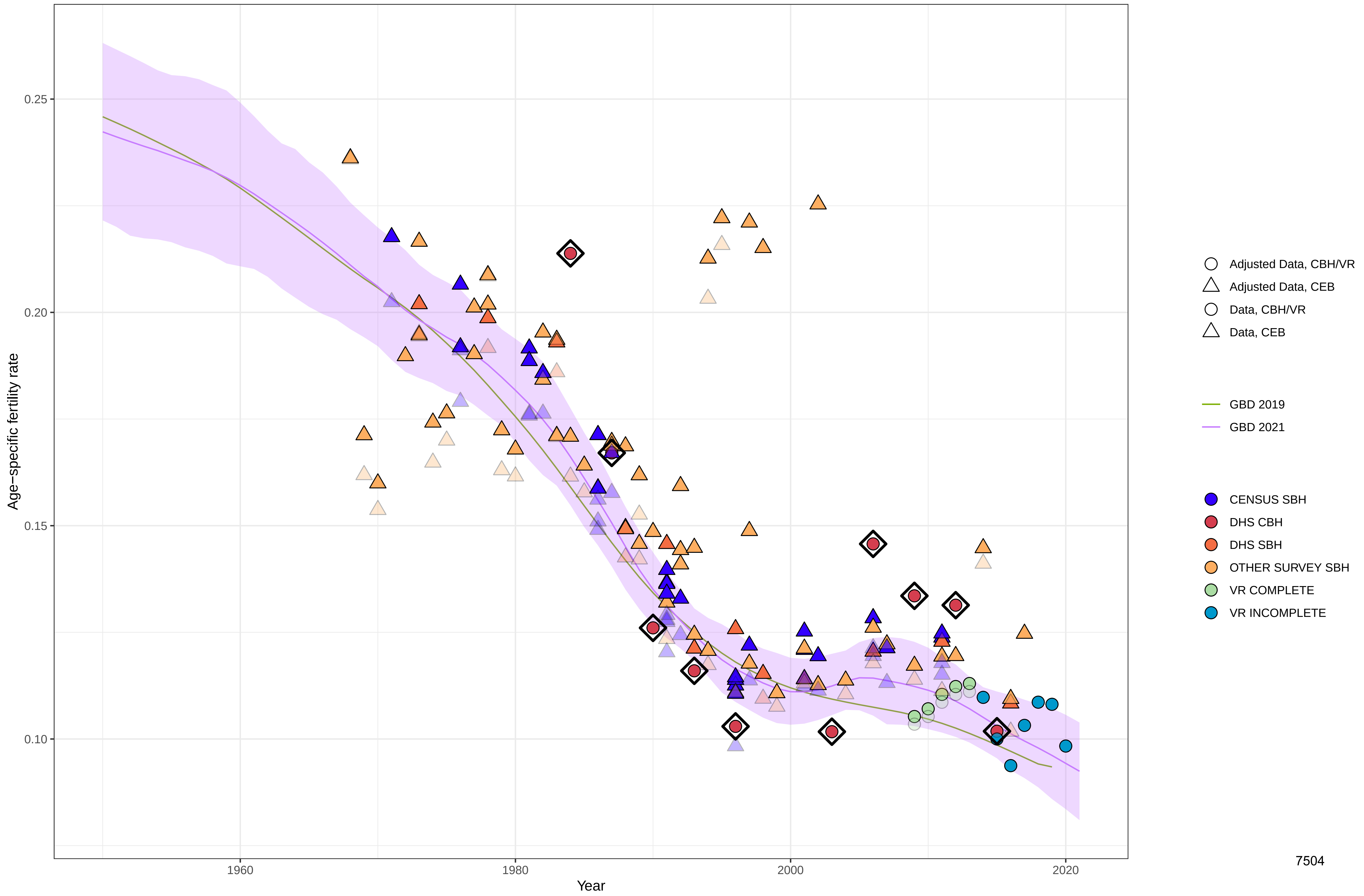

Age−specific fertility fitted trend lines, 1950−2021: Free State, Ages: 25−29
Data points enclosed with a diamond are reference sources

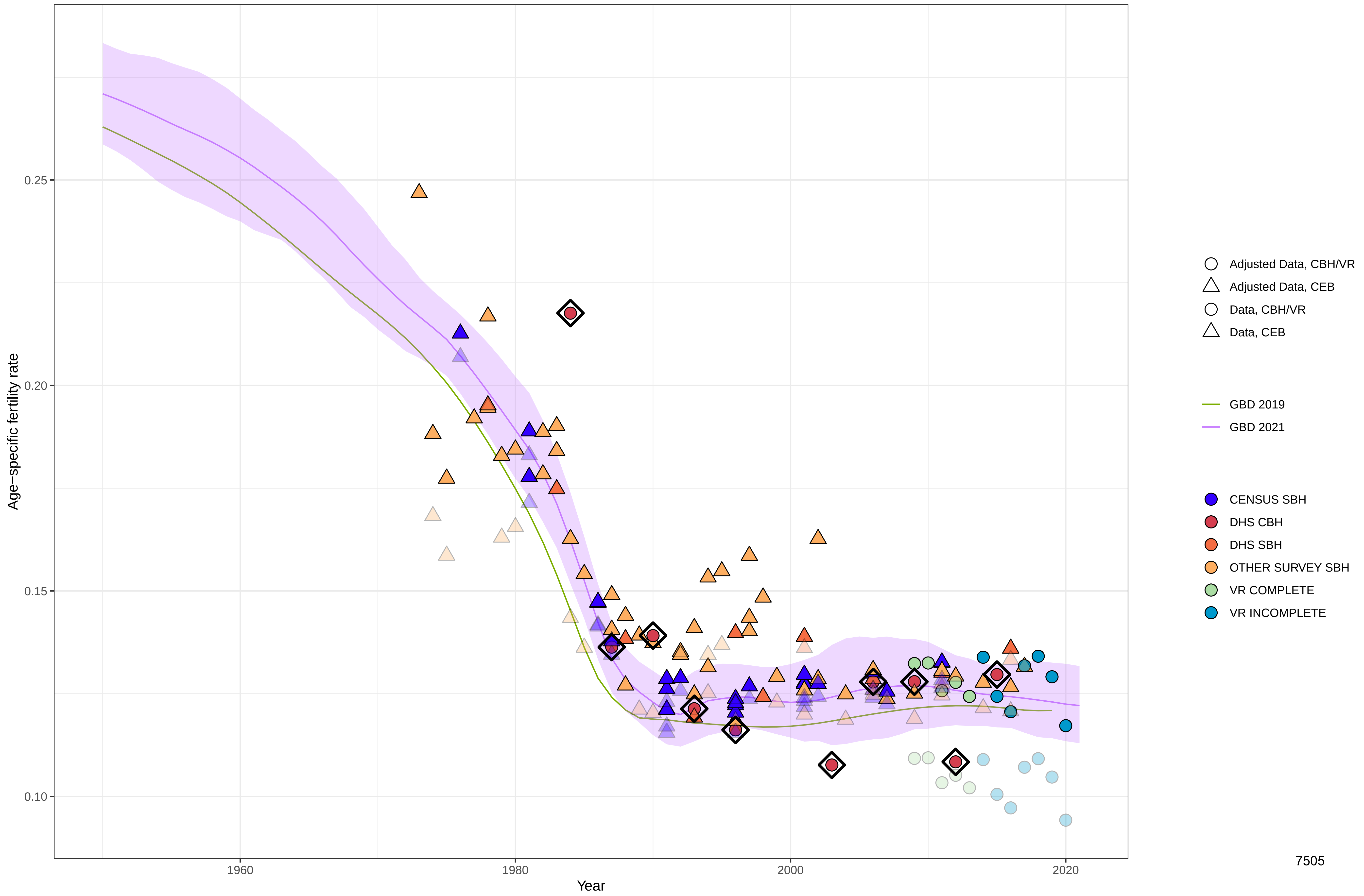

Age−specific fertility fitted trend lines, 1950−2021: Free State, Ages: 30−34
Data points enclosed with a diamond are reference sources

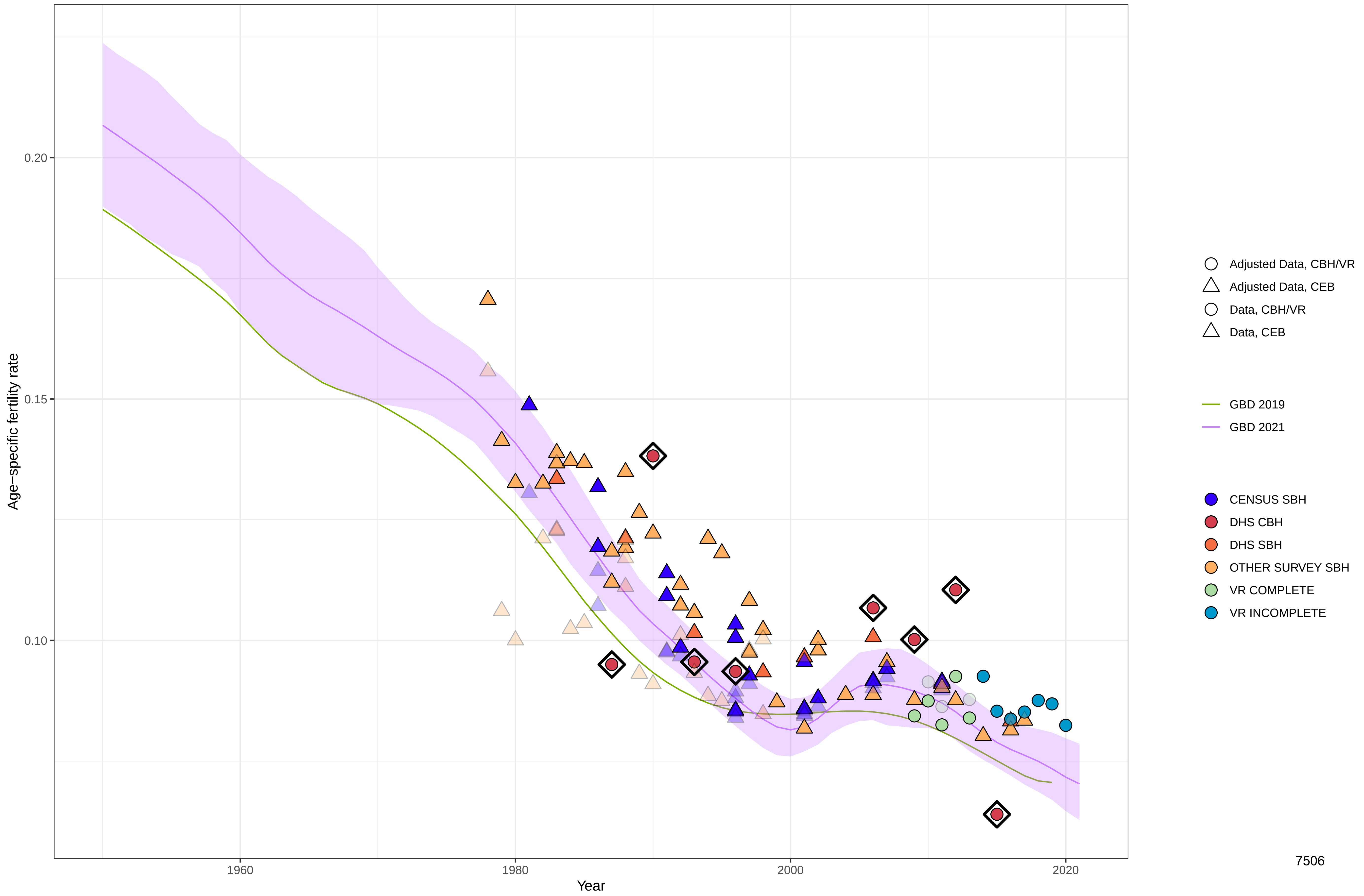

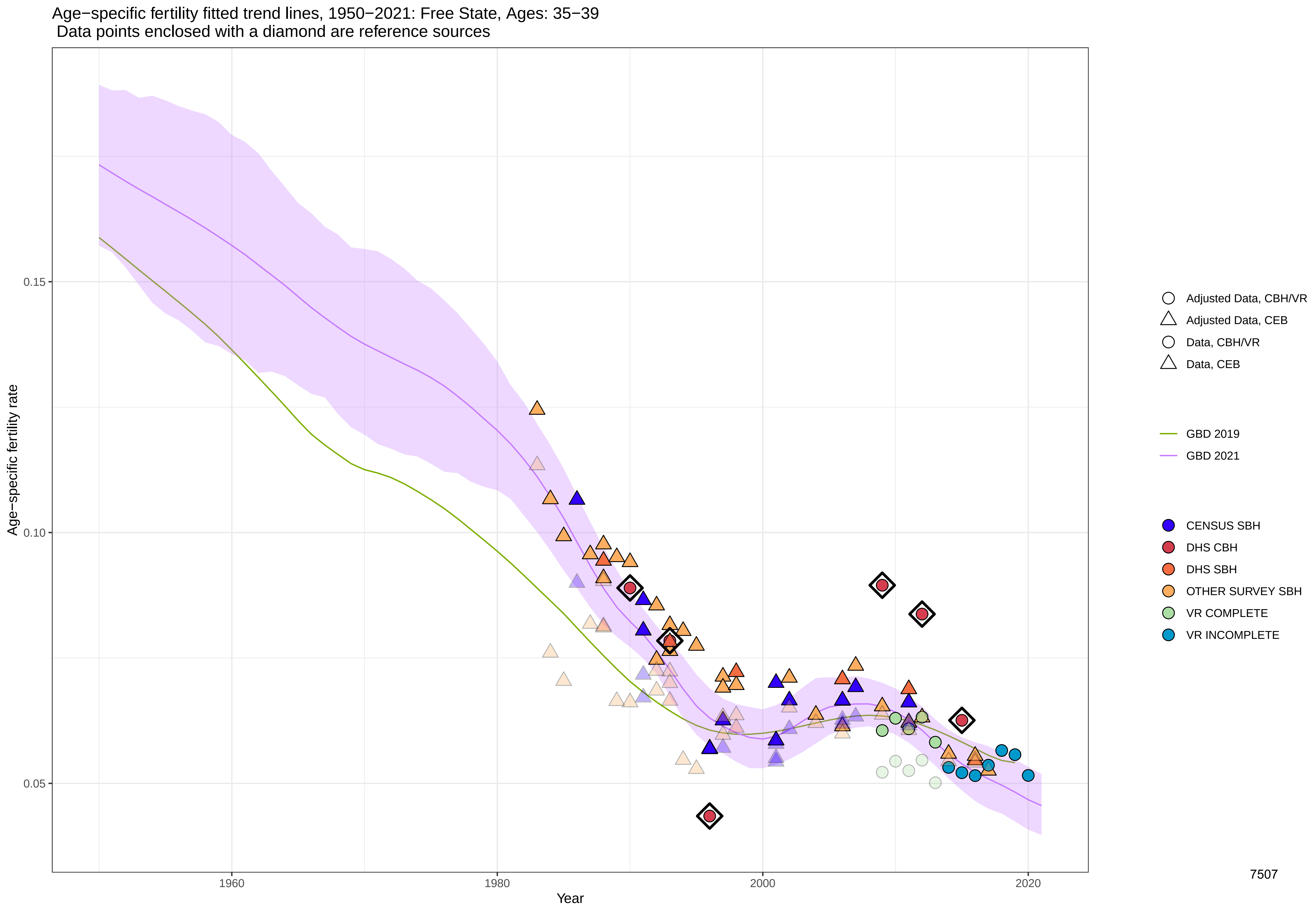

Age−specific fertility fitted trend lines, 1950−2021: Free State, Ages: 35−39
Data points enclosed with a diamond are reference sources
Age−specific fertility rate
0.15
0.10
0.05
1960
1980
2000
2020
Year
Adjusted Data, CBH/VR
Adjusted Data, CEB
Data, CBH/VR
Data, CEB
GBD 2019
GBD 2021
CENSUS SBH
DHS CBH
DHS SBH
OTHER SURVEY SBH
VR COMPLETE
VR INCOMPLETE

Age−specific fertility fitted trend lines, 1950−2021: Free State, Ages: 40−44
Data points enclosed with a diamond are reference sources

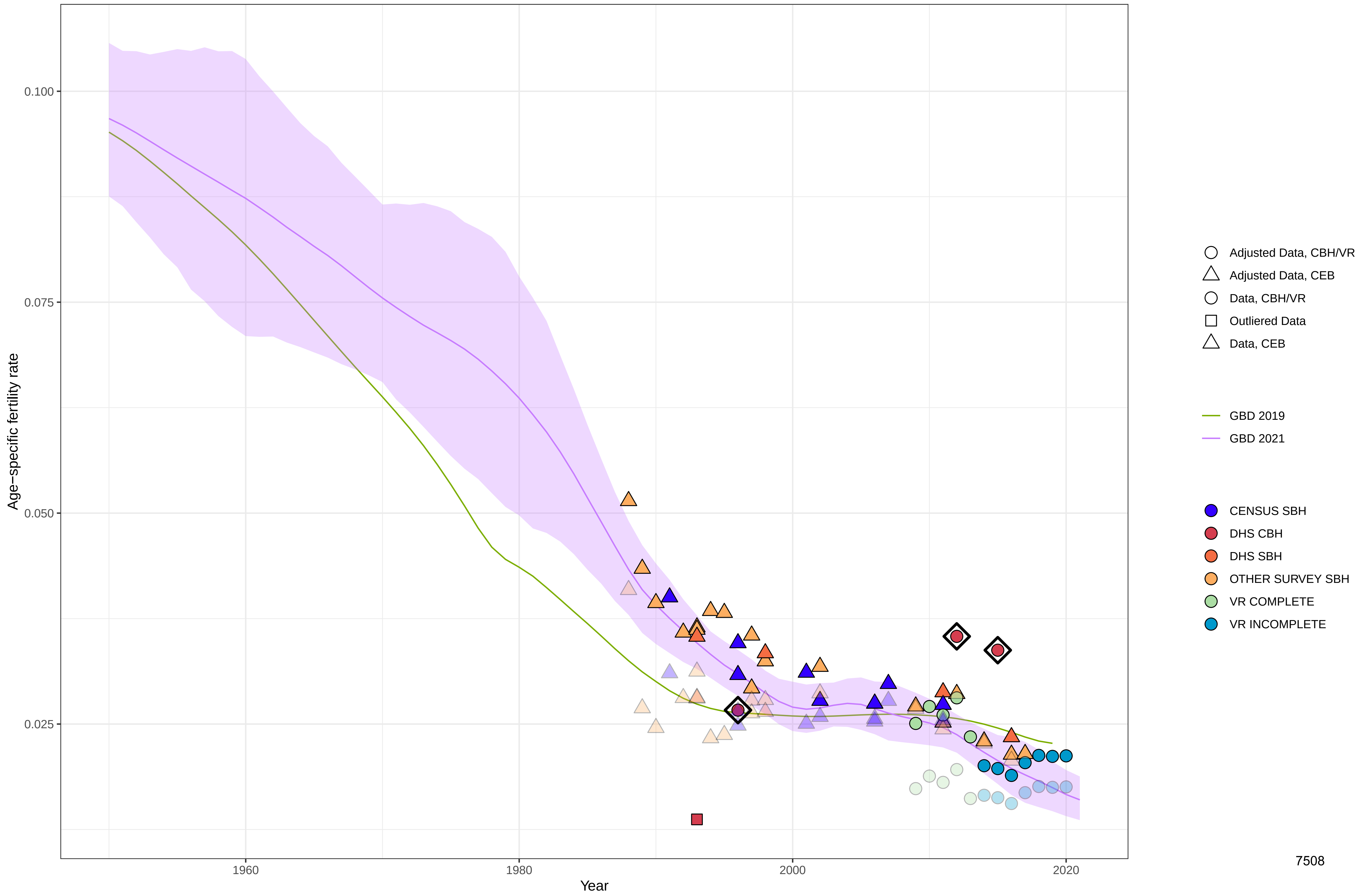

Age−specific fertility fitted trend lines, 1950−2021: Free State, Ages: 45−49
Data points enclosed with a diamond are reference sources

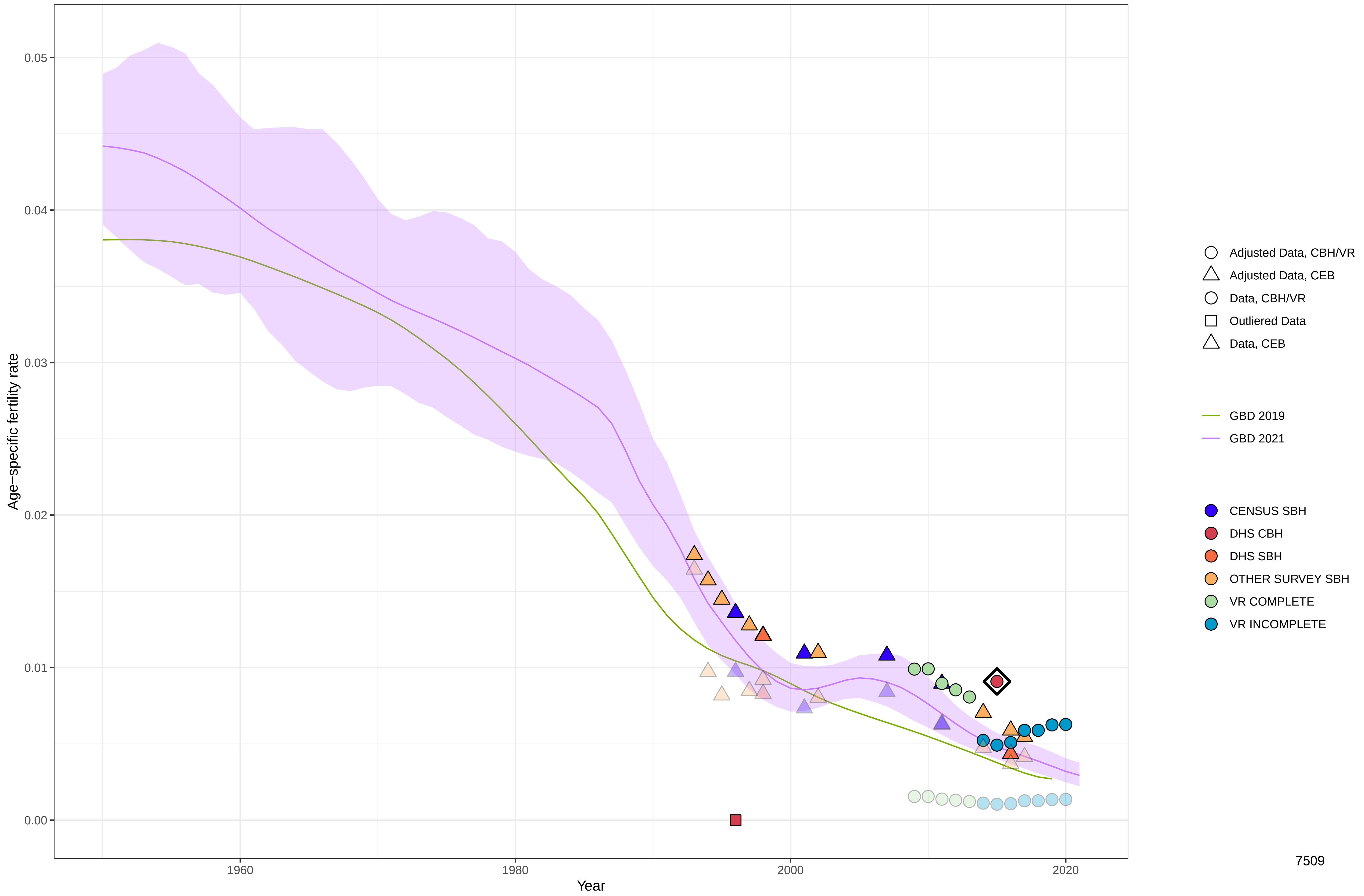

Age−specific fertility fitted trend lines, 1950−2021: Free State, Ages: 50−54
Data points enclosed with a diamond are reference sources

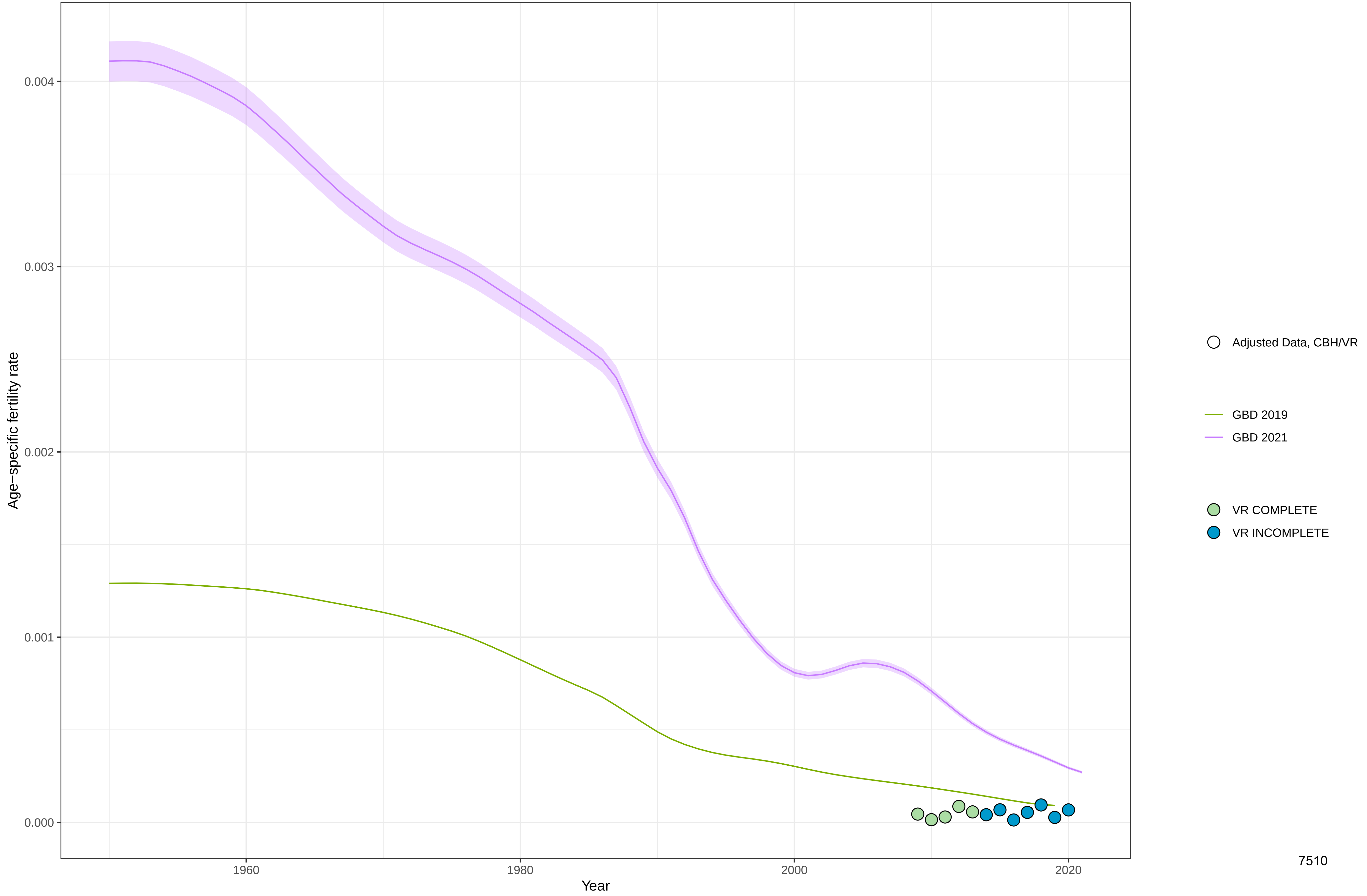

Total fertility rate fitted trend lines, 1950−2021: Free State TFR
Data points enclosed with a diamond are reference sources

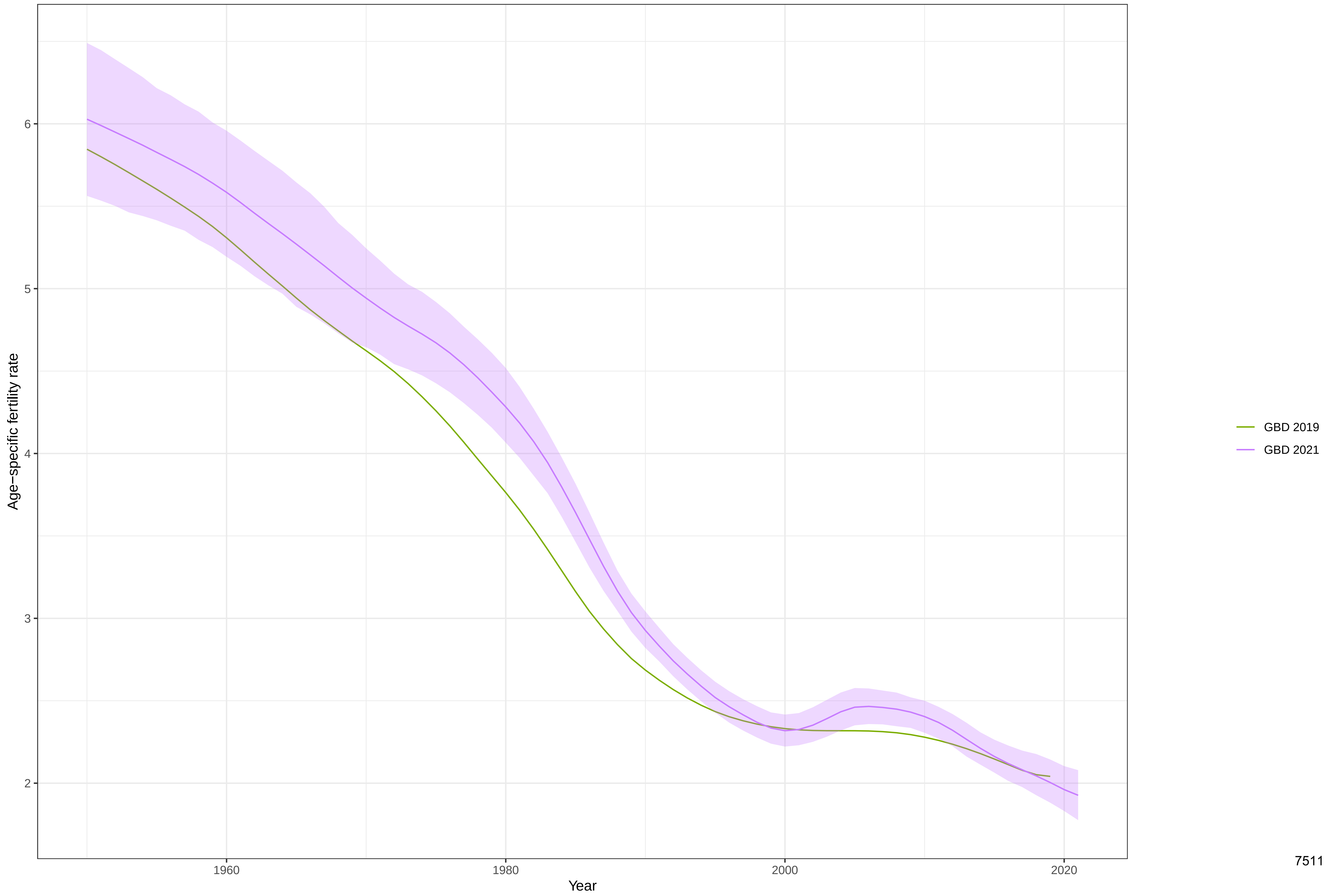

Age−specific fertility fitted trend lines, 1950−2021: Gauteng, Ages: 10−14
Data points enclosed with a diamond are reference sources

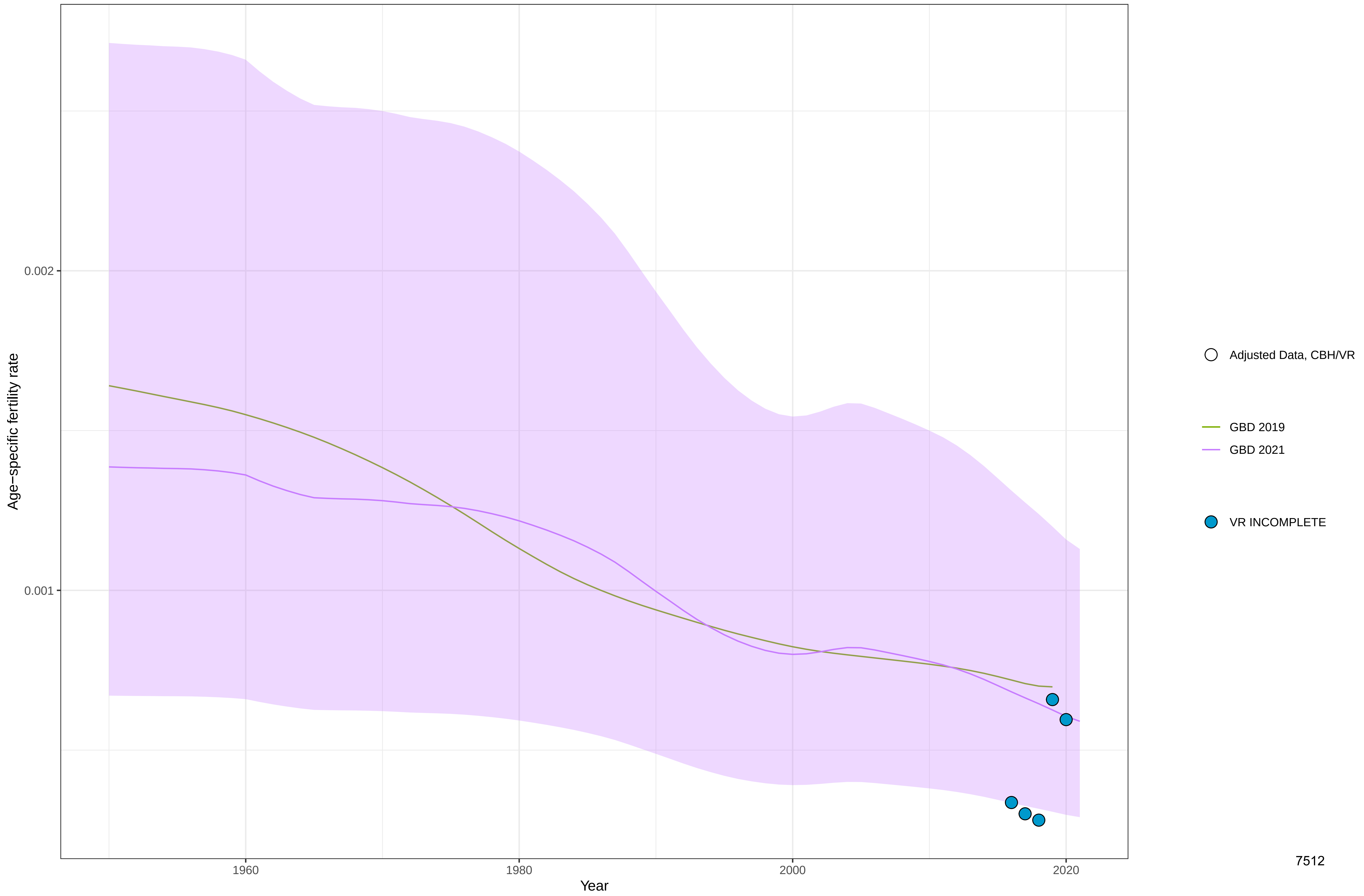

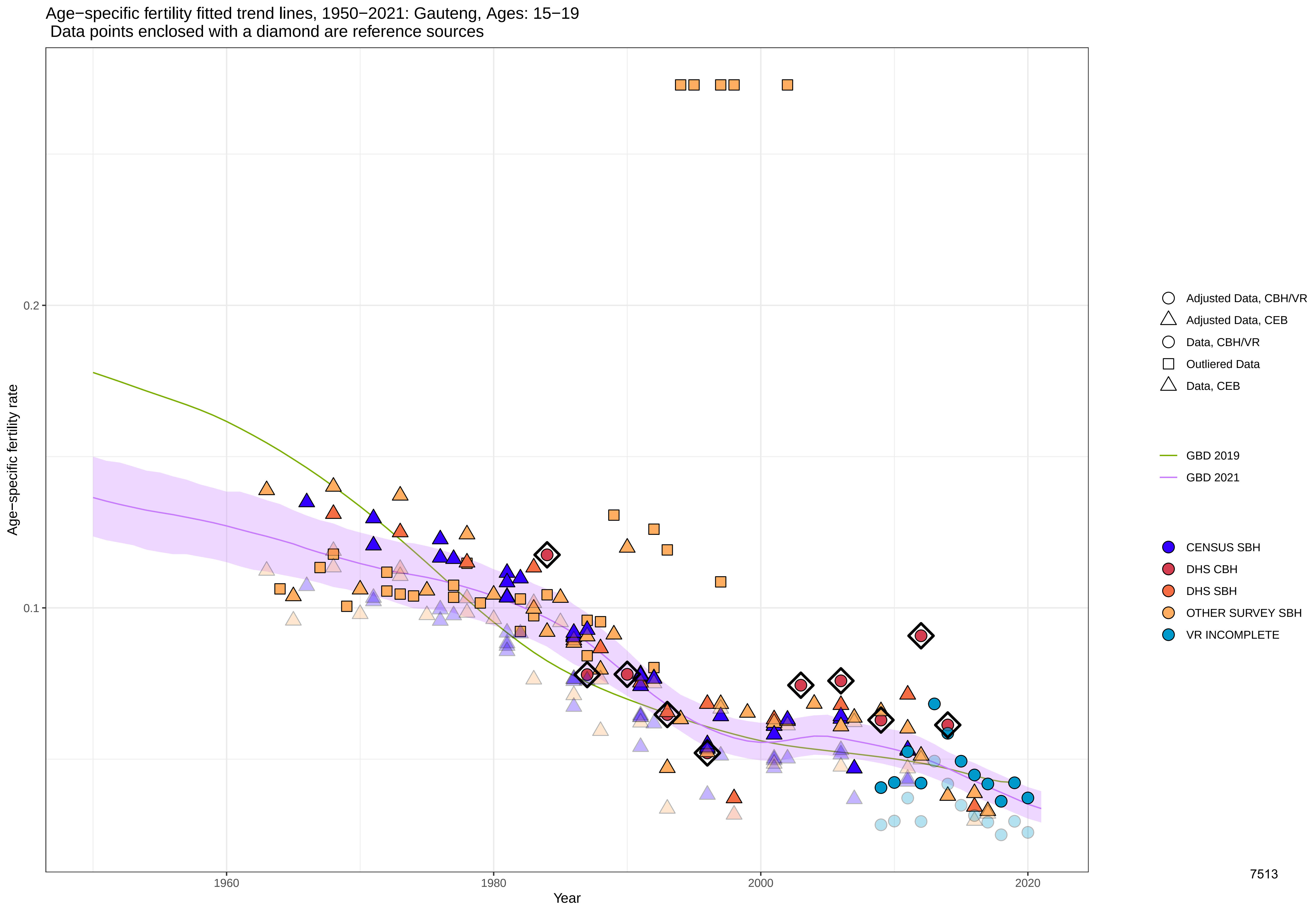
Age−specific fertility fitted trend lines, 1950−2021: Gauteng, Ages: 15−19
Data points enclosed with a diamond are reference sources
Age−specific fertility rate
0.2
0.1
Year
1960
1980
2000
2020
Adjusted Data, CBH/VR
Adjusted Data, CEB
Data, CBH/VR
Outliered Data
Data, CEB
GBD 2019
GBD 2021
CENSUS SBH
DHS CBH
DHS SBH
OTHER SURVEY SBH
VR INCOMPLETE

7513

Age−specific fertility fitted trend lines, 1950−2021: Gauteng, Ages: 20−24
Data points enclosed with a diamond are reference sources

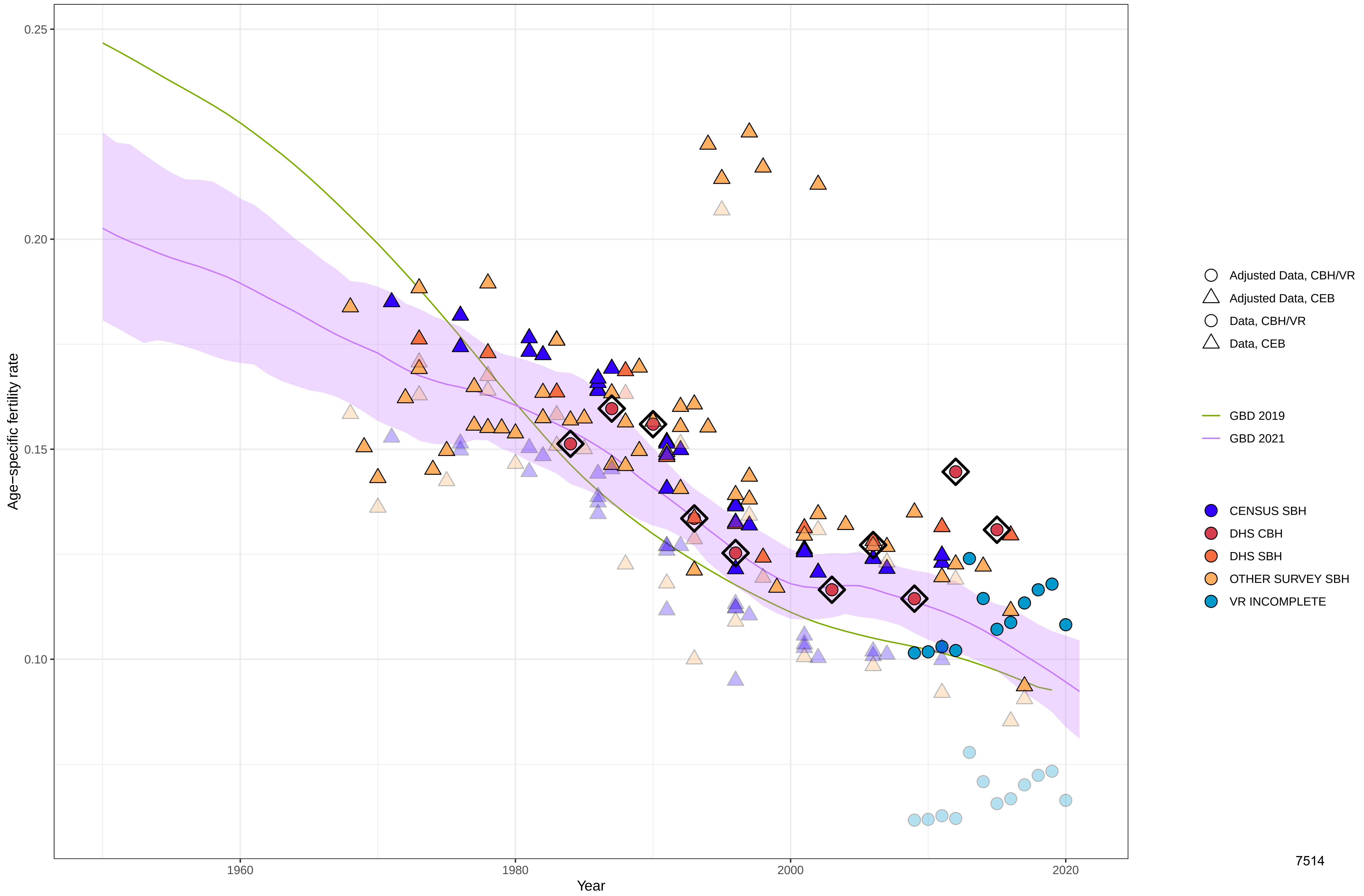

Age−specific fertility fitted trend lines, 1950−2021: Gauteng, Ages: 25−29
Data points enclosed with a diamond are reference sources

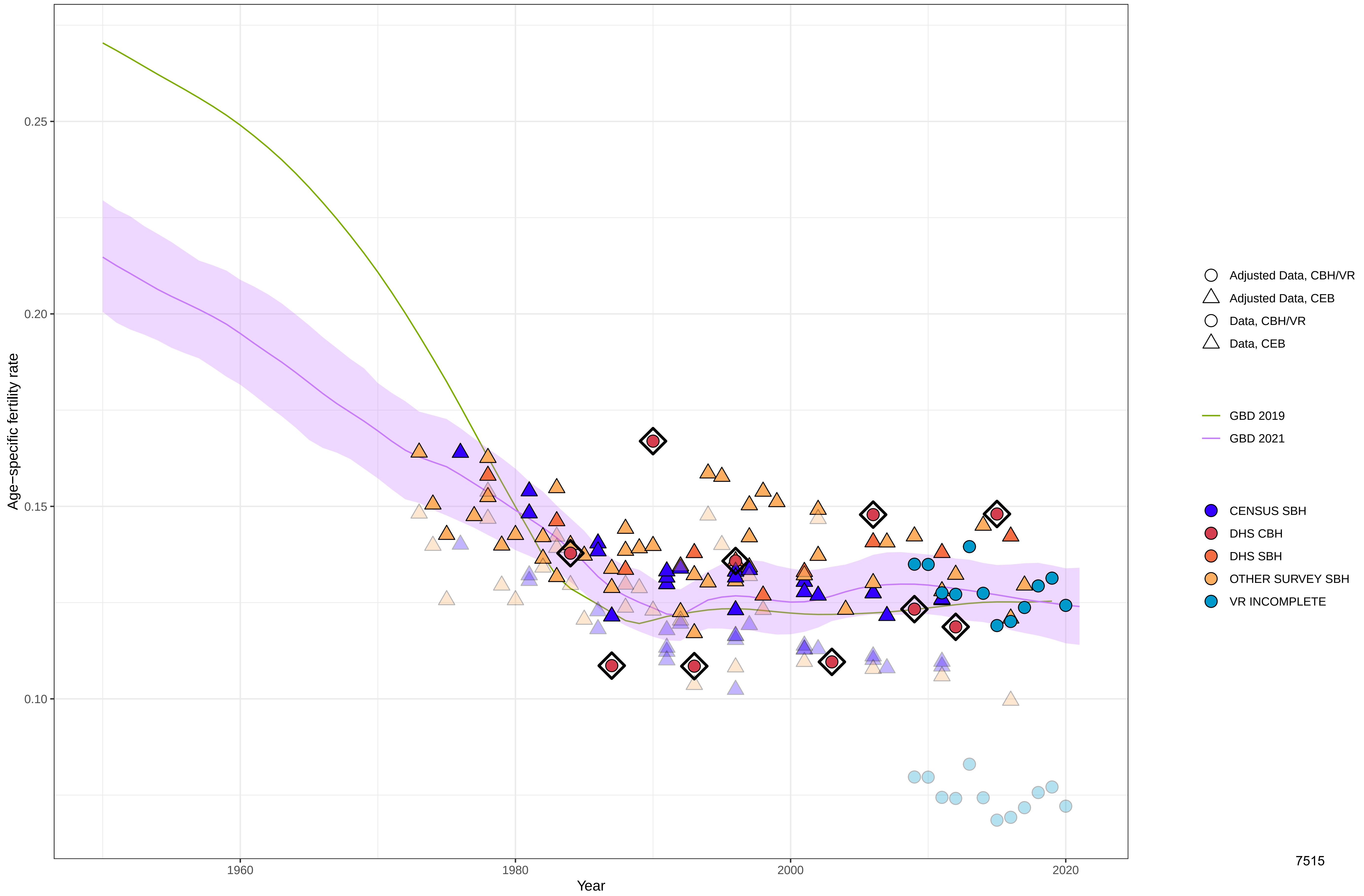

Age−specific fertility fitted trend lines, 1950−2021: Gauteng, Ages: 30−34
Data points enclosed with a diamond are reference sources

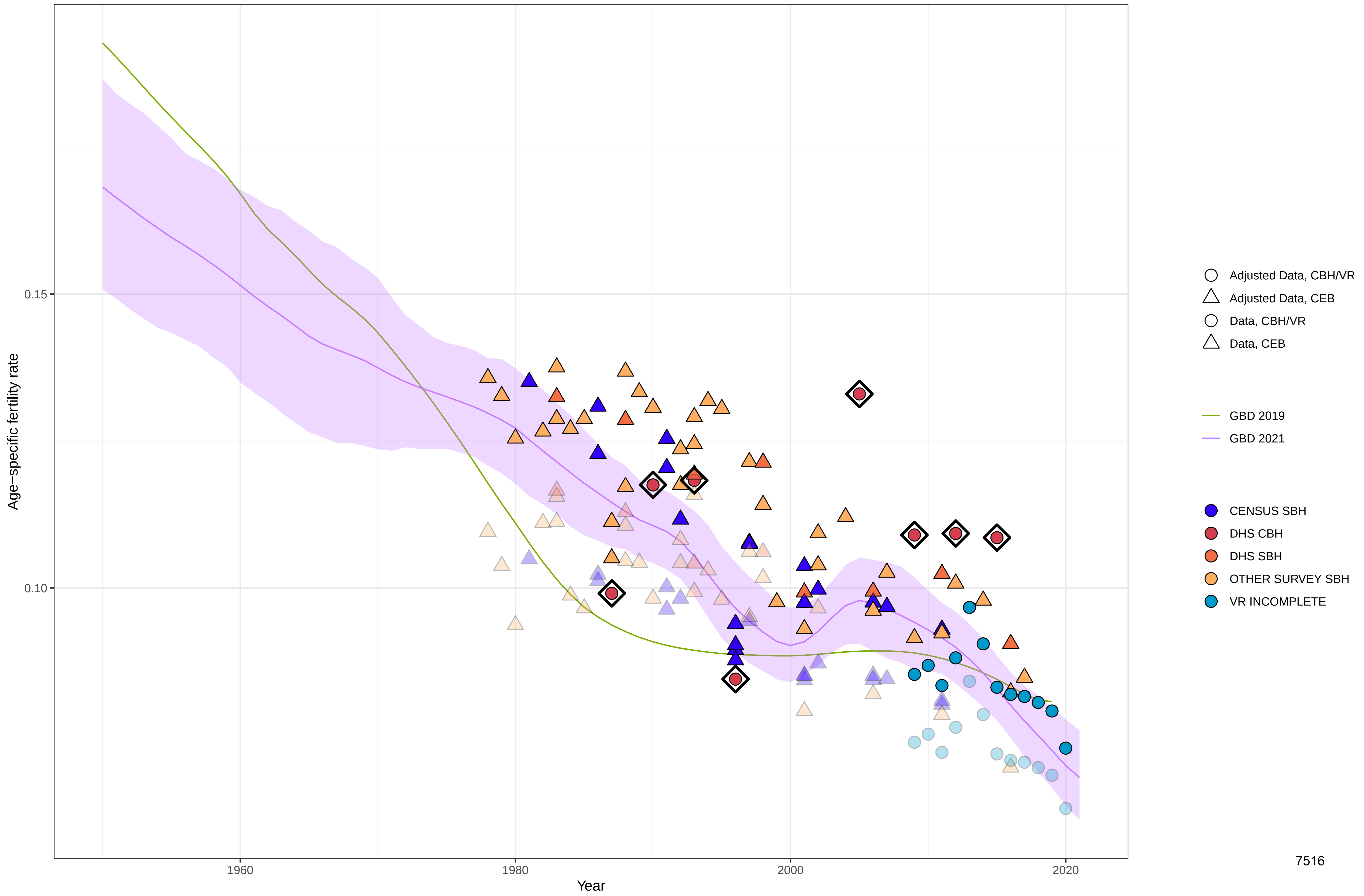

Age−specific fertility fitted trend lines, 1950−2021: Gauteng, Ages: 35−39
Data points enclosed with a diamond are reference sources

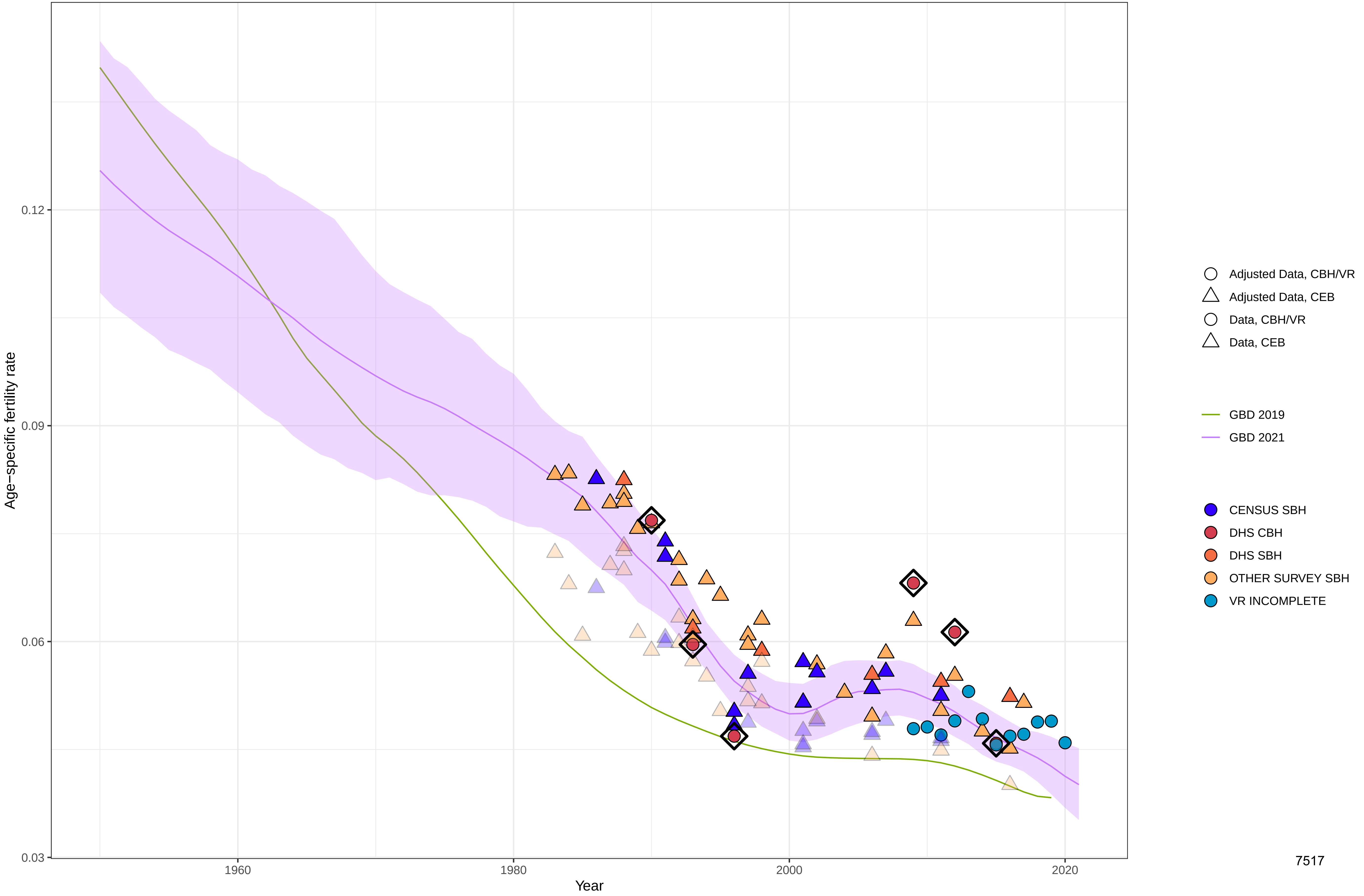

Age−specific fertility fitted trend lines, 1950−2021: Gauteng, Ages: 40−44
Data points enclosed with a diamond are reference sources

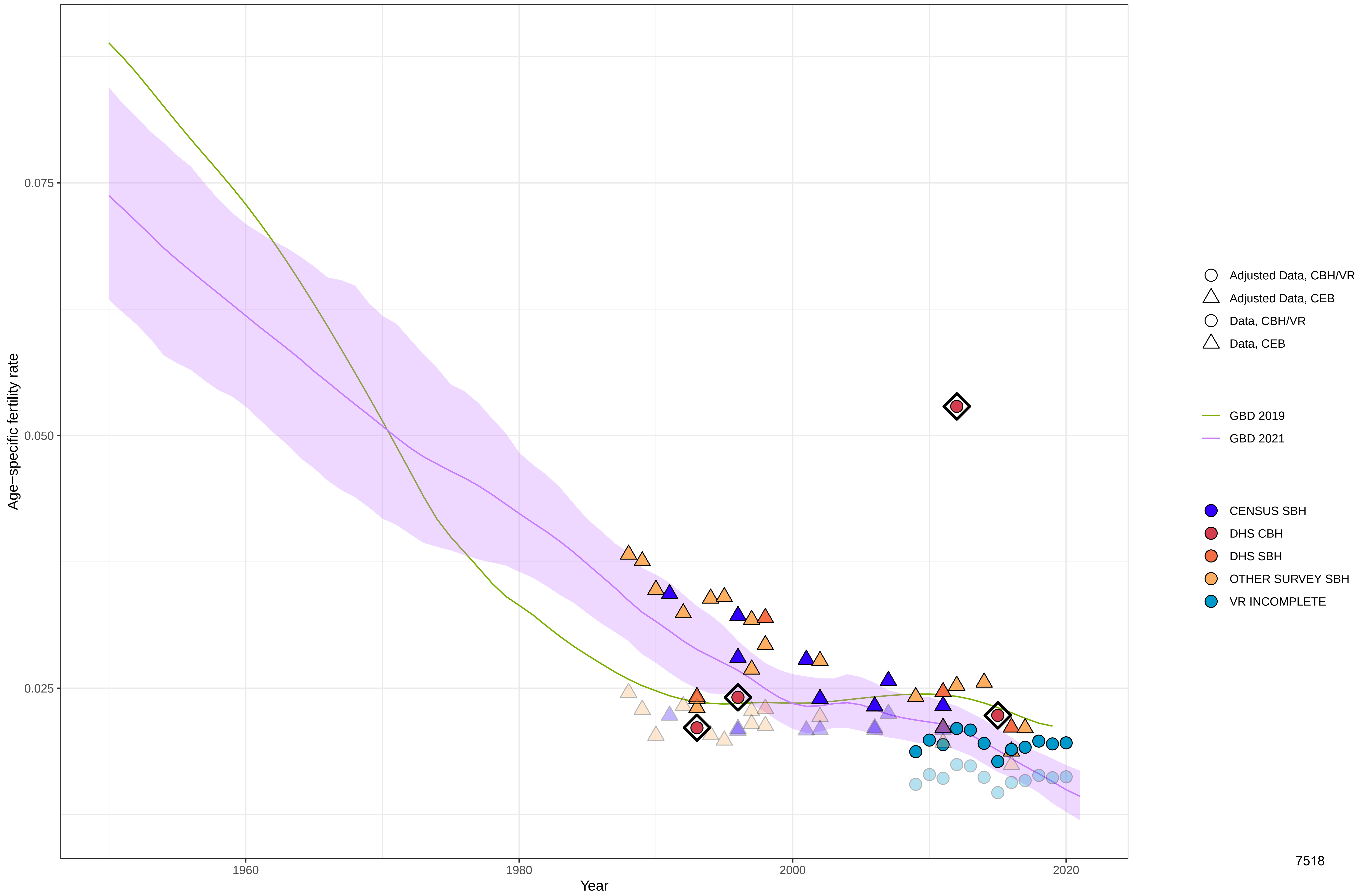

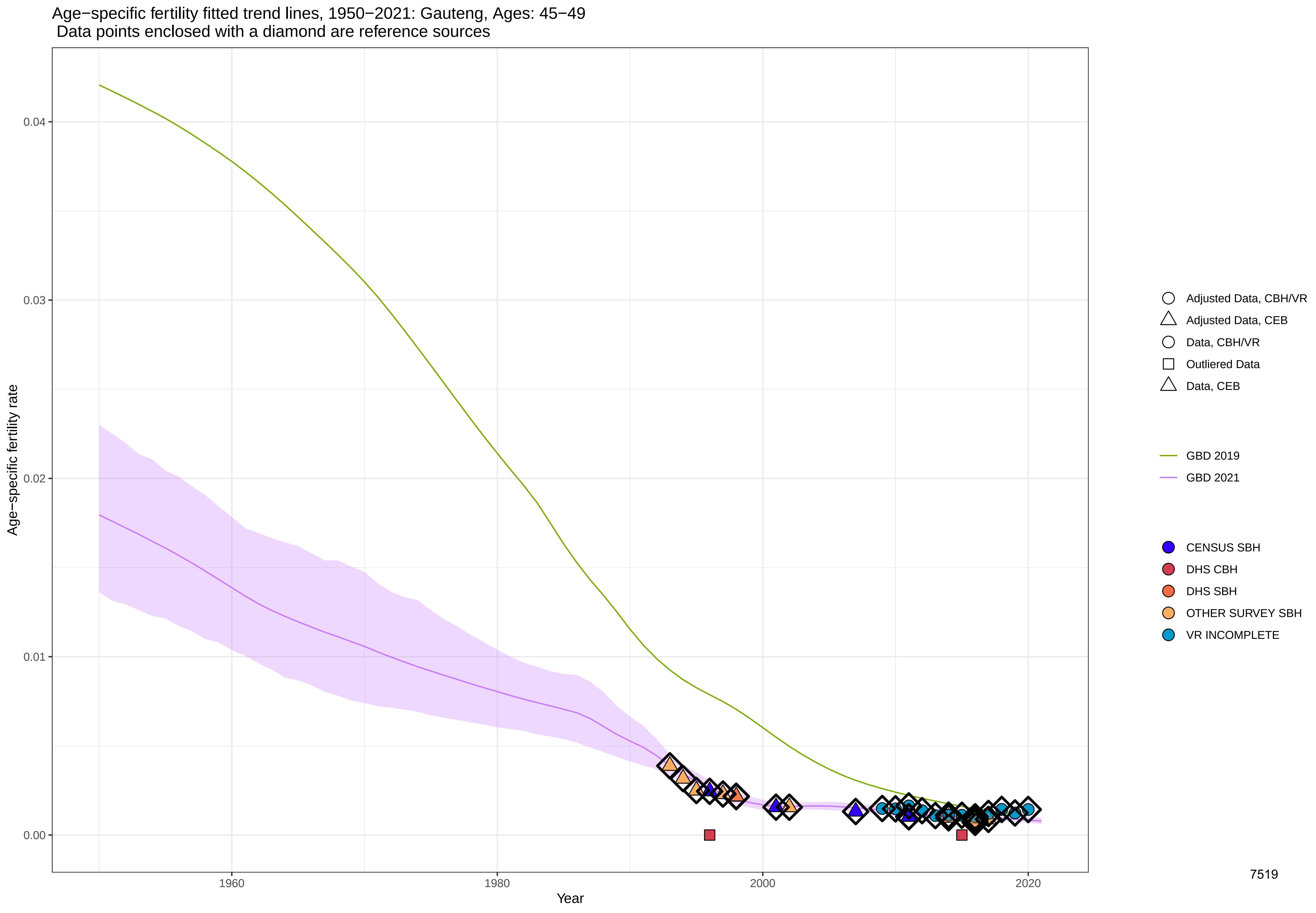

Age−specific fertility fitted trend lines, 1950−2021: Gauteng, Ages: 45−49
Data points enclosed with a diamond are reference sources
Age−specific fertility rate
0.04
0.03
0.02
0.01
0.00
1960
1980
2000
2020
Year
Adjusted Data, CBH/VR
Adjusted Data, CEB
Data, CBH/VR
Outliered Data
Data, CEB
GBD 2019
GBD 2021
CENSUS SBH
DHS CBH
DHS SBH
OTHER SURVEY SBH
VR INCOMPLETE

Age−specific fertility fitted trend lines, 1950−2021: Gauteng, Ages: 50−54
Data points enclosed with a diamond are reference sources

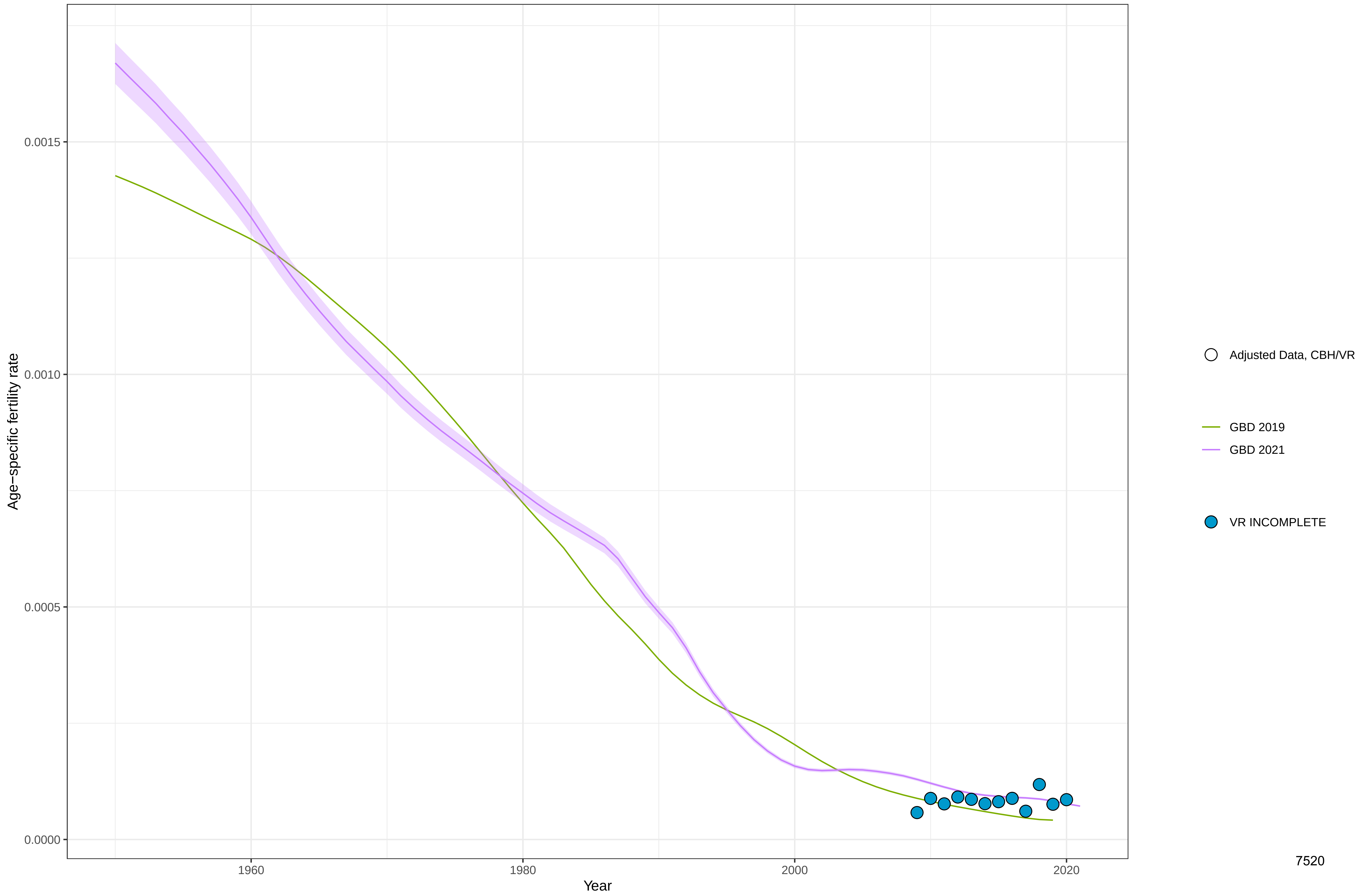

Total fertility rate fitted trend lines, 1950−2021: Gauteng TFR
Data points enclosed with a diamond are reference sources

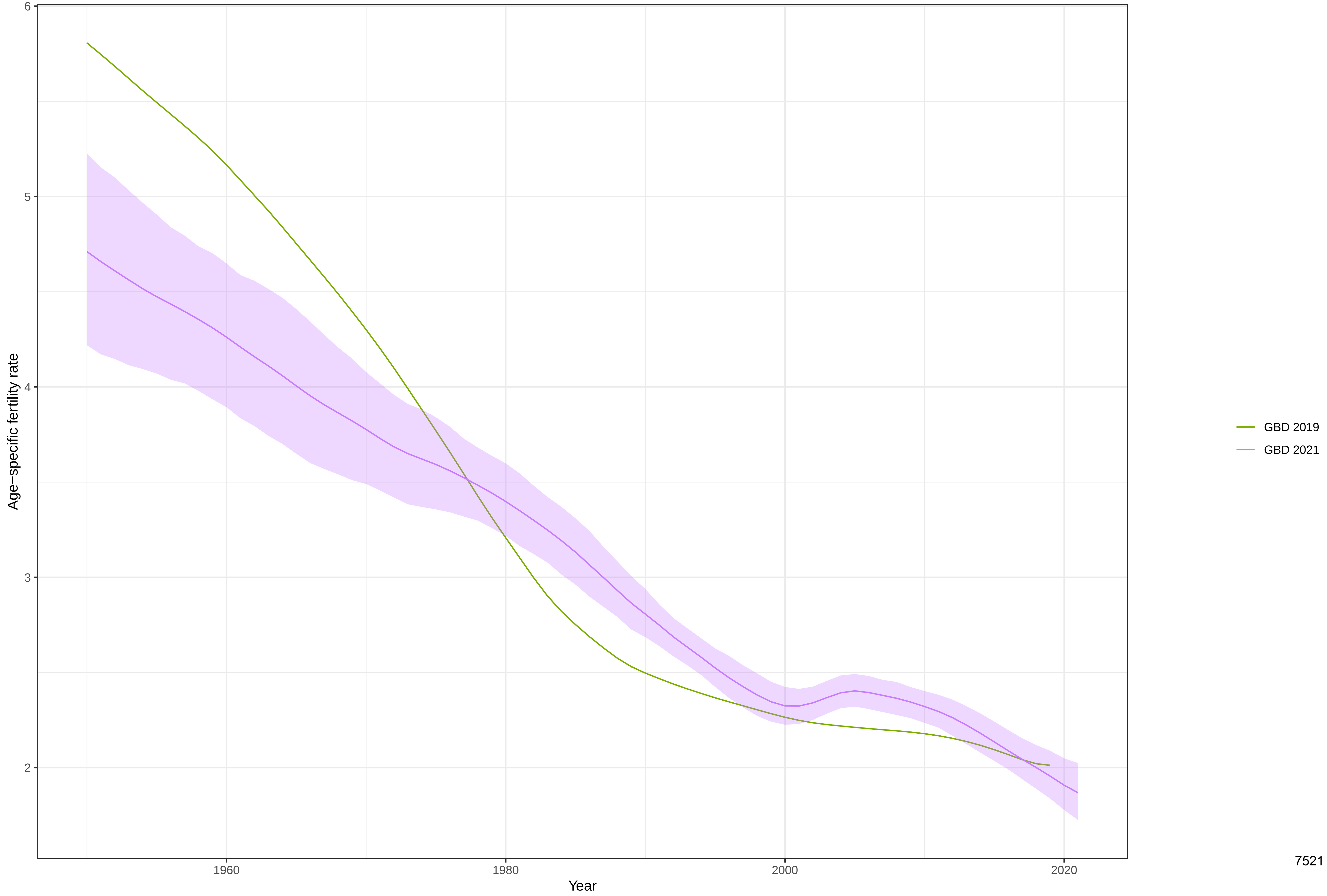

Age−specific fertility fitted trend lines, 1950−2021: KwaZulu−Natal, Ages: 10−14
Data points enclosed with a diamond are reference sources

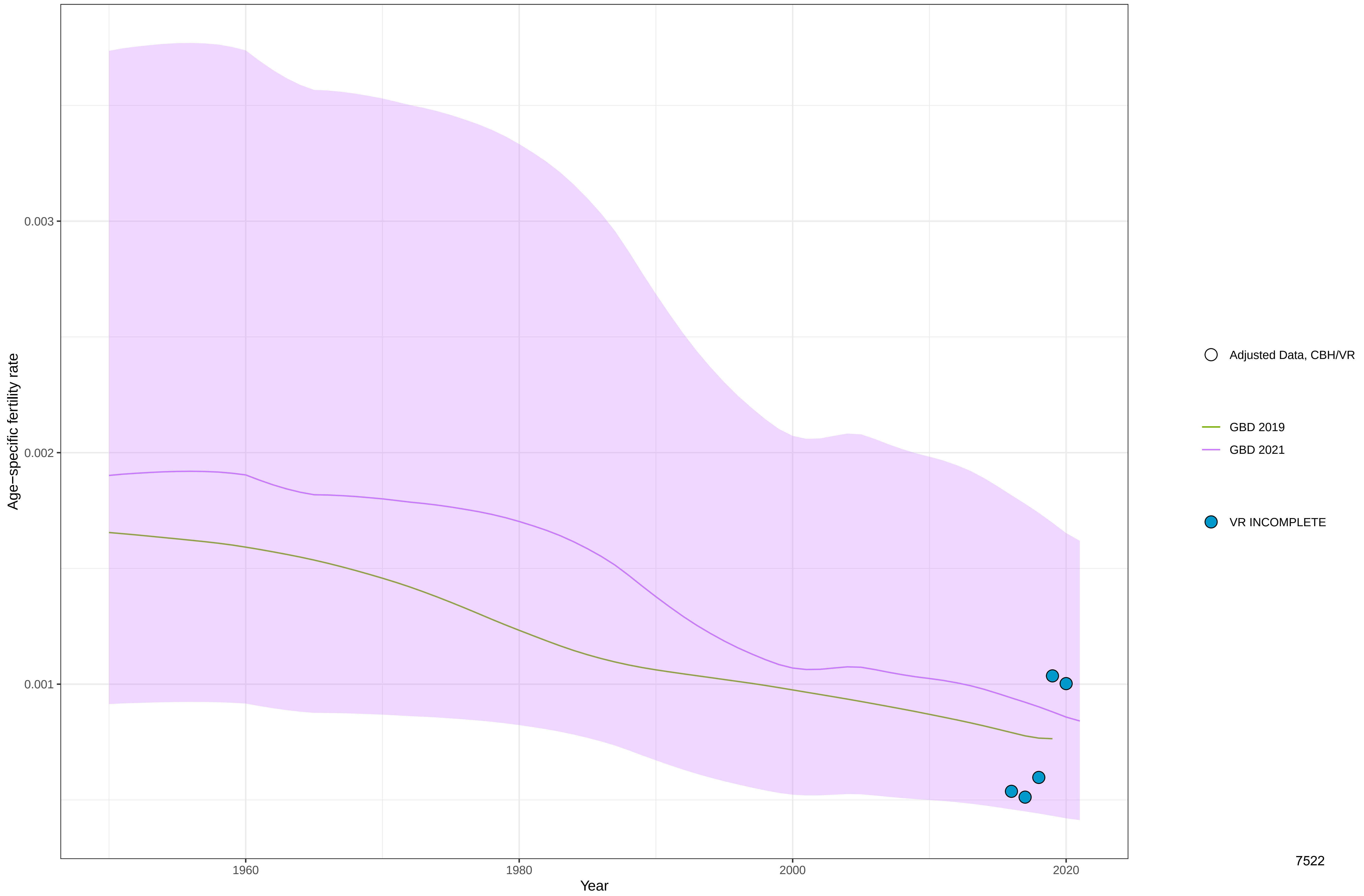

Age−specific fertility fitted trend lines, 1950−2021: KwaZulu−Natal, Ages: 15−19
Data points enclosed with a diamond are reference sources

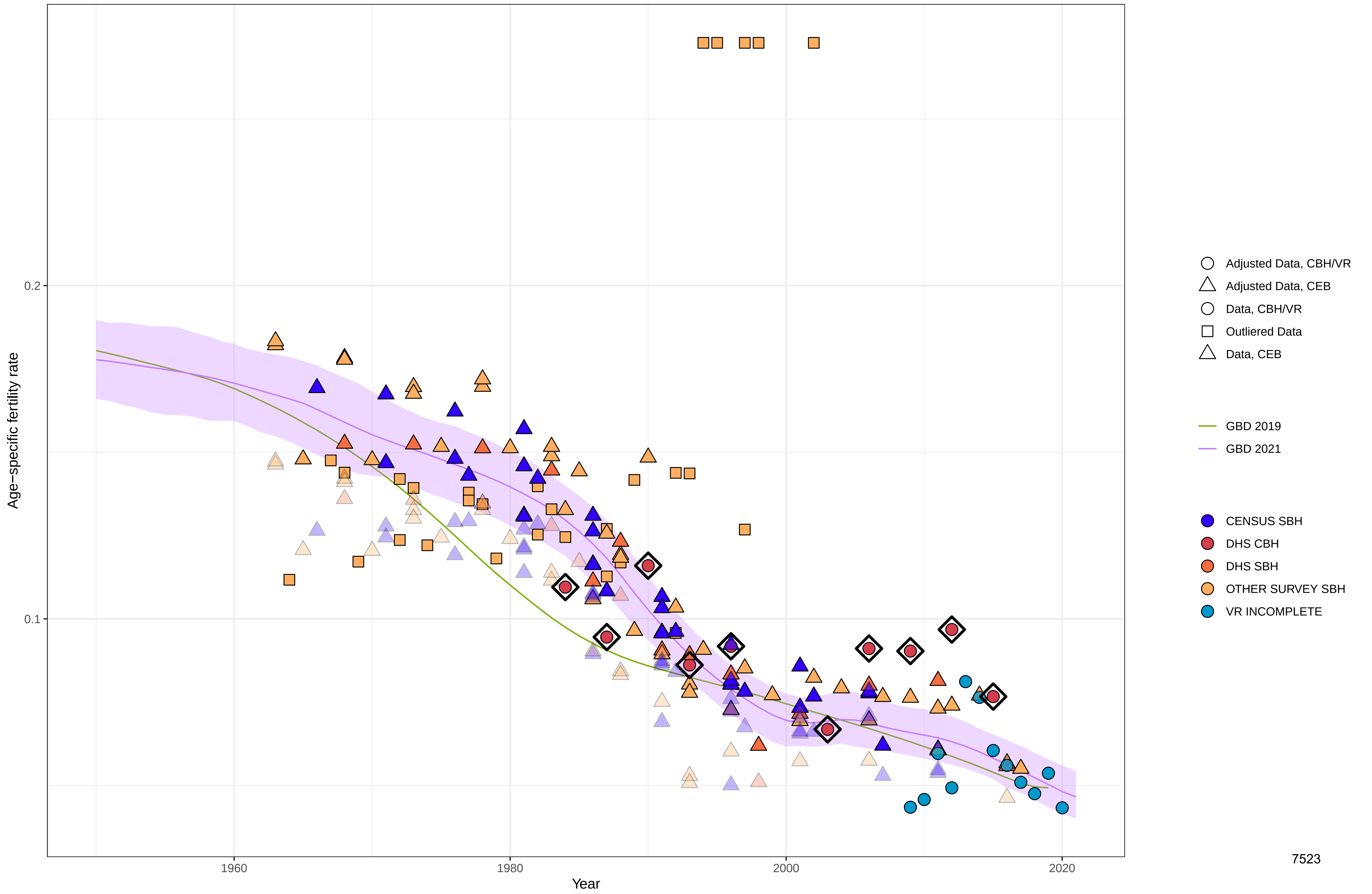

Age−specific fertility fitted trend lines, 1950−2021: KwaZulu−Natal, Ages: 20−24
Data points enclosed with a diamond are reference sources

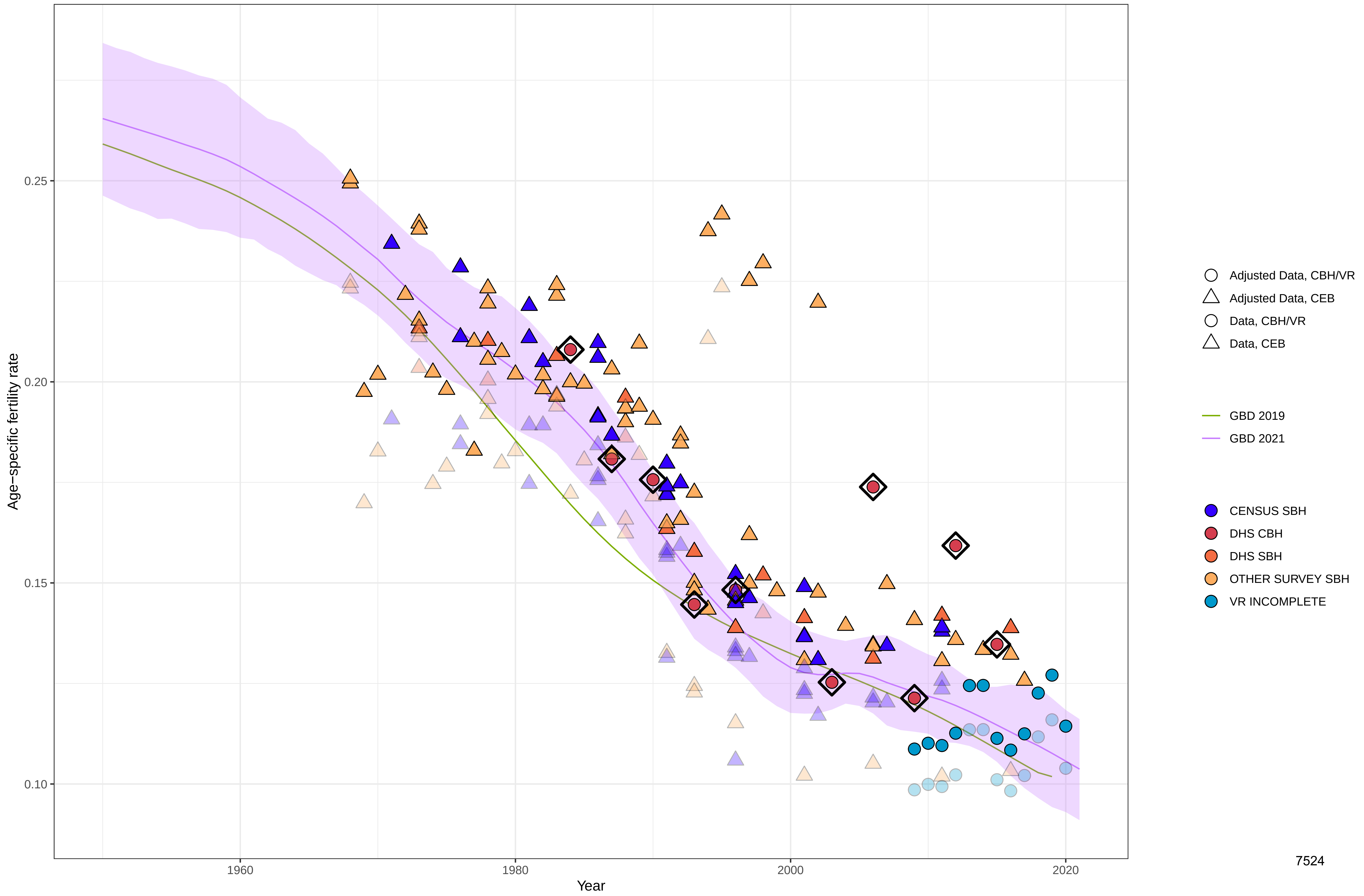

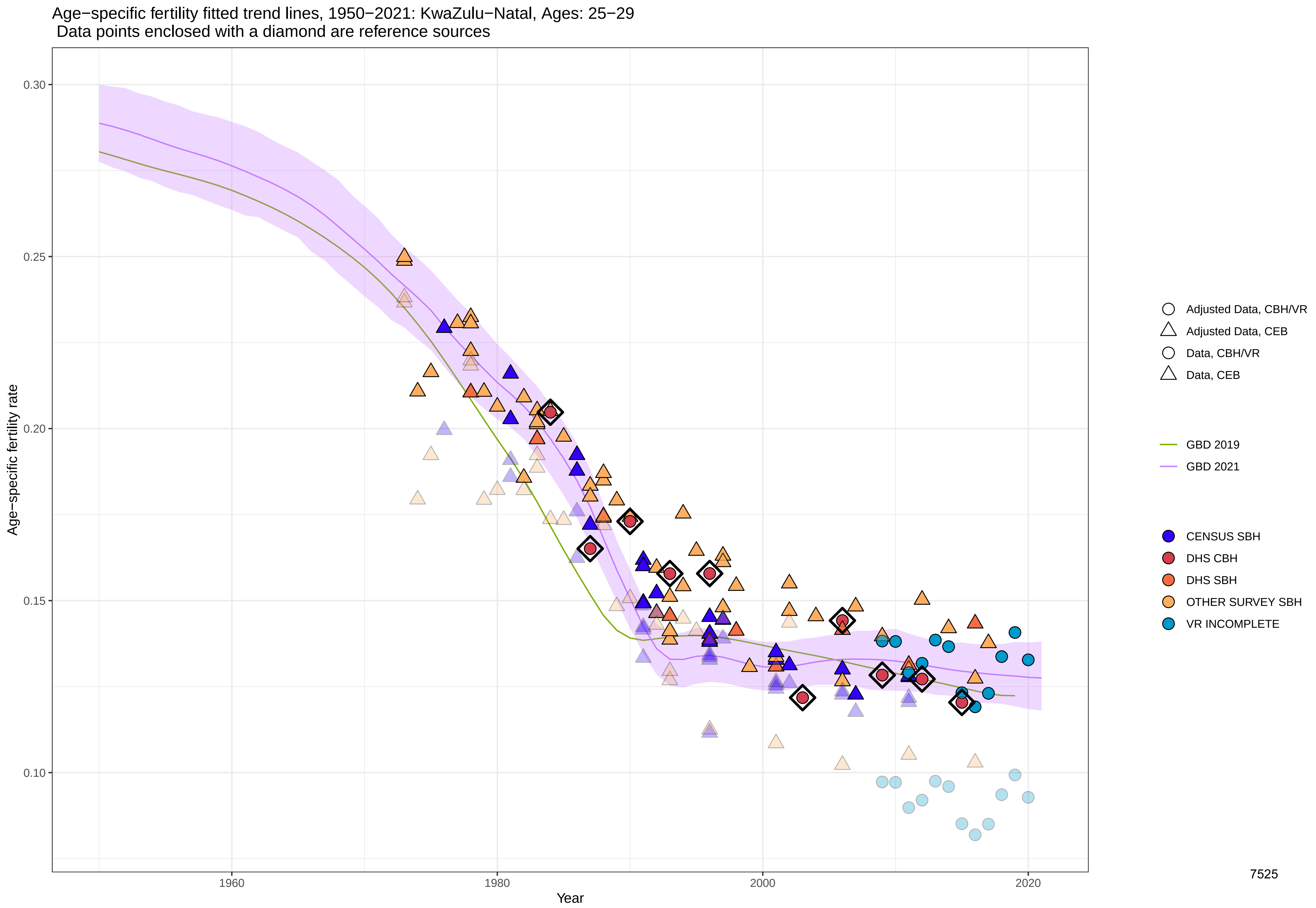

Age−specific fertility fitted trend lines, 1950−2021: KwaZulu−Natal, Ages: 25−29
Data points enclosed with a diamond are reference sources
Age−specific fertility rate
Year
0.30
0.25
0.20
0.15
0.10
1960
1980
2000
2020
Adjusted Data, CBH/VR
Adjusted Data, CEB
Data, CBH/VR
Data, CEB
GBD 2019
GBD 2021
CENSUS SBH
DHS CBH
DHS SBH
OTHER SURVEY SBH
VR INCOMPLETE

Age−specific fertility fitted trend lines, 1950−2021: KwaZulu−Natal, Ages: 30−34
Data points enclosed with a diamond are reference sources

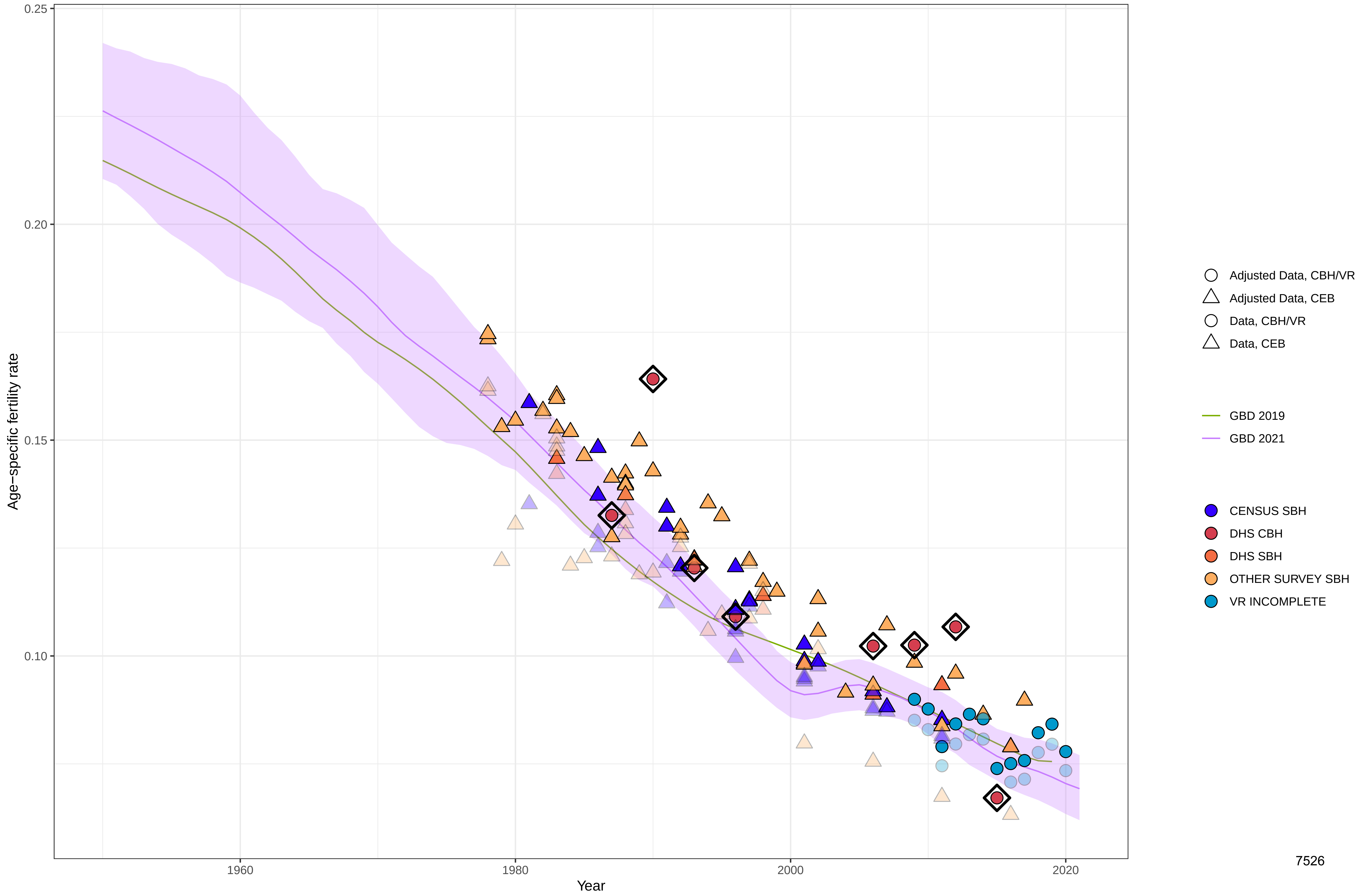

Age−specific fertility fitted trend lines, 1950−2021: KwaZulu−Natal, Ages: 35−39
Data points enclosed with a diamond are reference sources

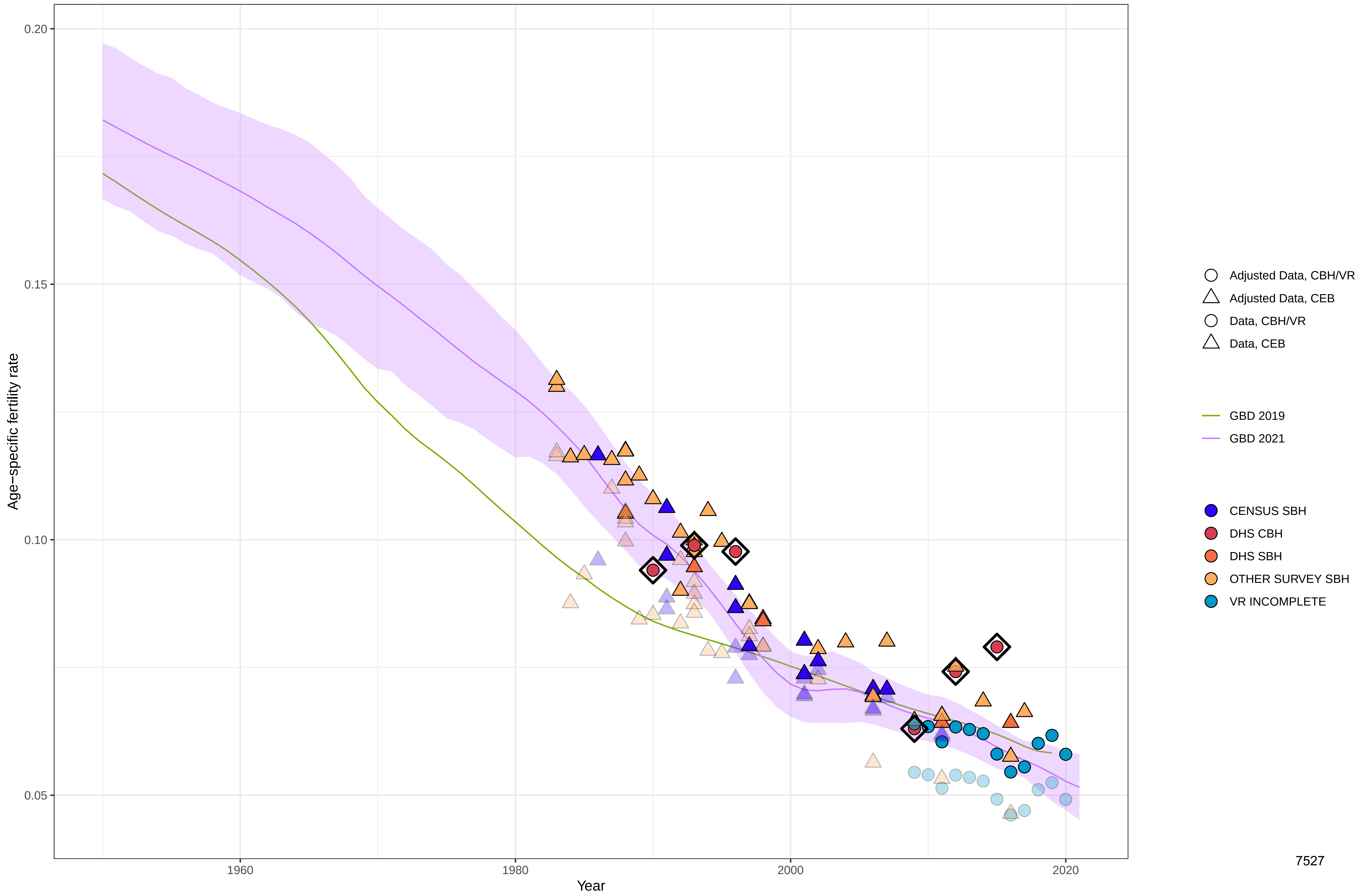

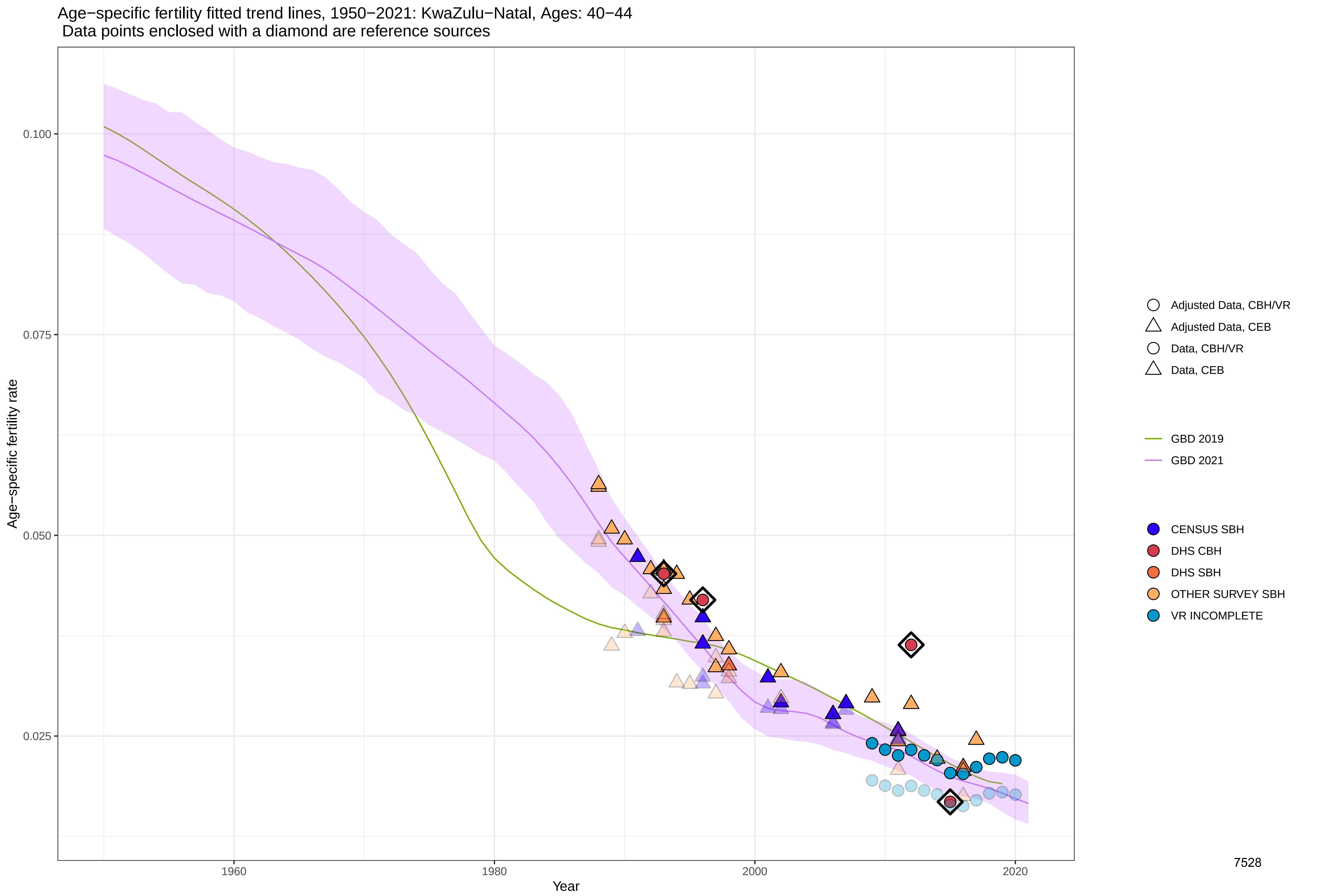

Age−specific fertility fitted trend lines, 1950−2021: KwaZulu−Natal, Ages: 40−44
Data points enclosed with a diamond are reference sources
Age−specific fertility rate
0.100
0.075
0.050
0.025
1960
1980
2000
2020
Year
Adjusted Data, CBH/VR
Adjusted Data, CEB
Data, CBH/VR
Data, CEB
GBD 2019
GBD 2021
CENSUS SBH
DHS CBH
DHS SBH
OTHER SURVEY SBH
VR INCOMPLETE

7528

Age−specific fertility fitted trend lines, 1950−2021: KwaZulu−Natal, Ages: 45−49
Data points enclosed with a diamond are reference sources

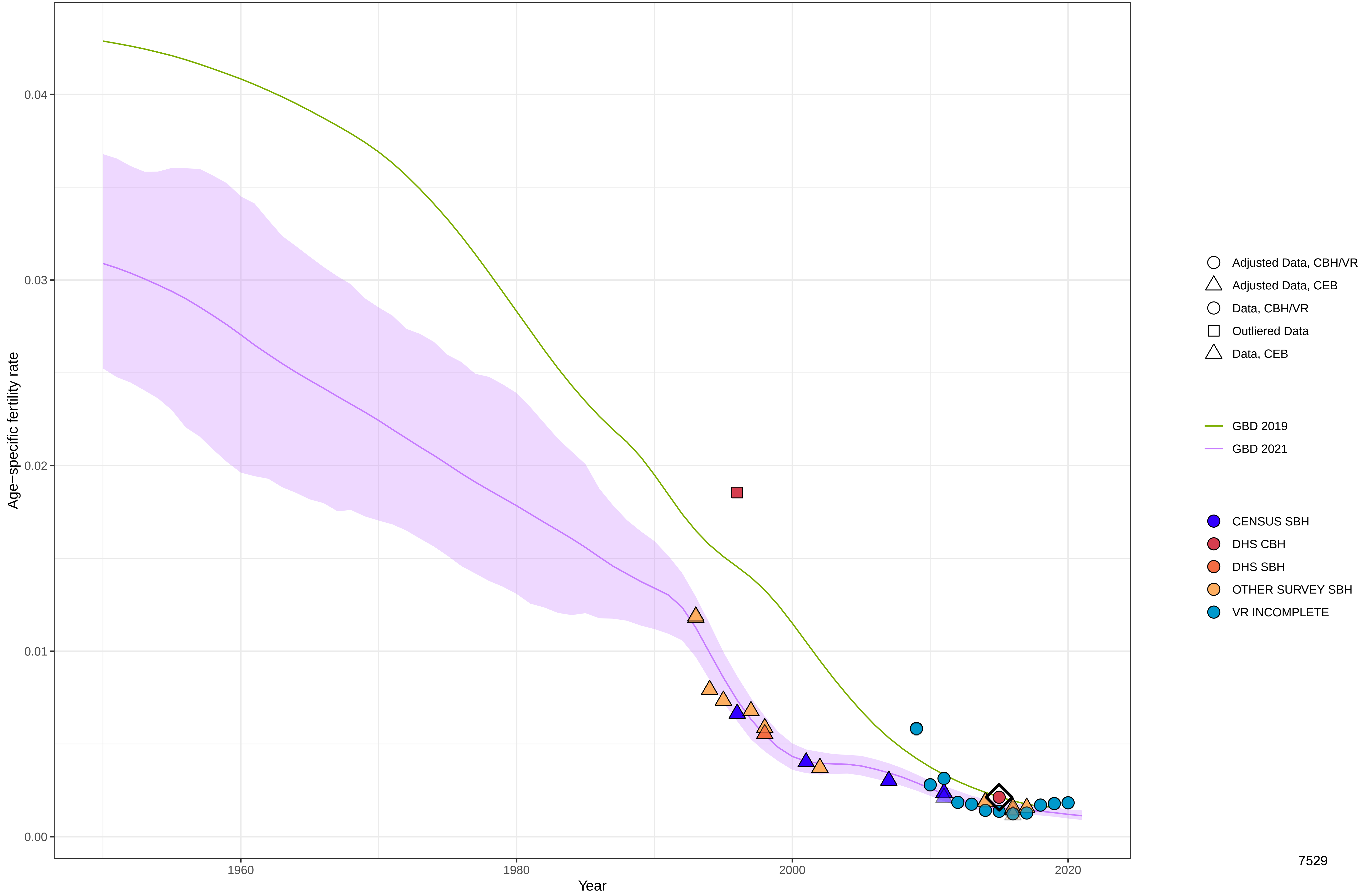

Age−specific fertility fitted trend lines, 1950−2021: KwaZulu−Natal, Ages: 50−54
Data points enclosed with a diamond are reference sources

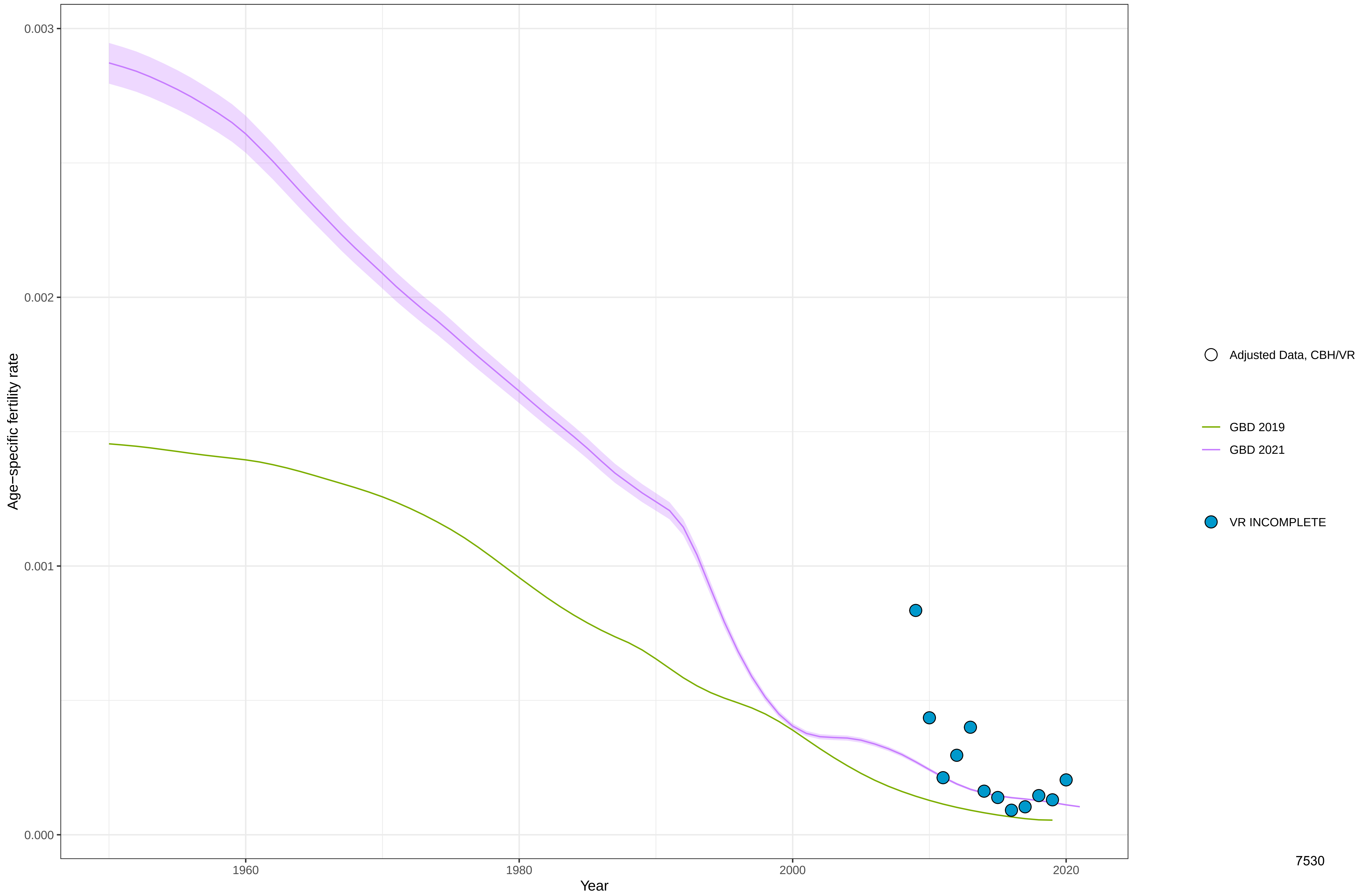

Total fertility rate fitted trend lines, 1950−2021: KwaZulu−Natal TFR
Data points enclosed with a diamond are reference sources

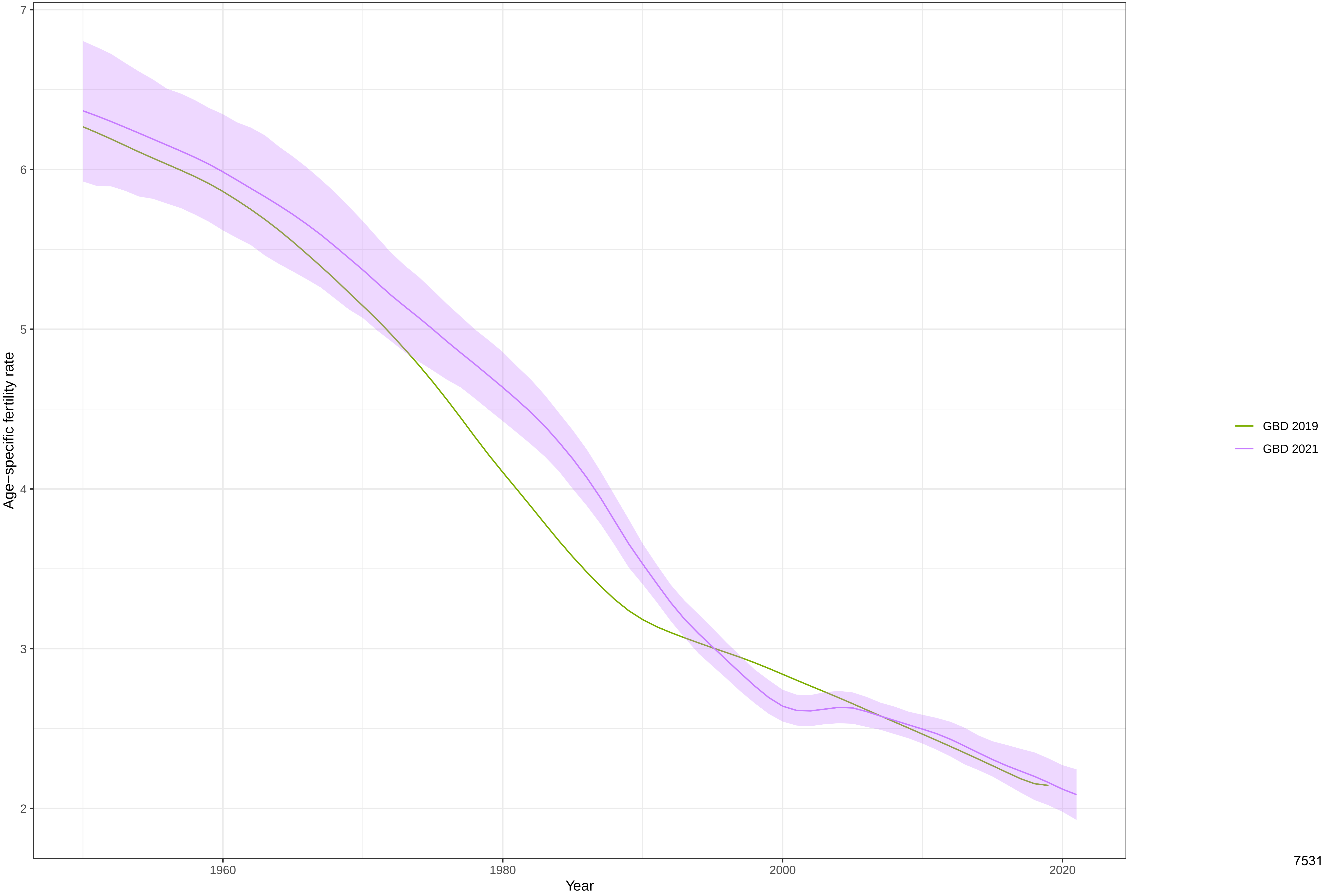

Age−specific fertility fitted trend lines, 1950−2021: Limpopo, Ages: 10−14
Data points enclosed with a diamond are reference sources

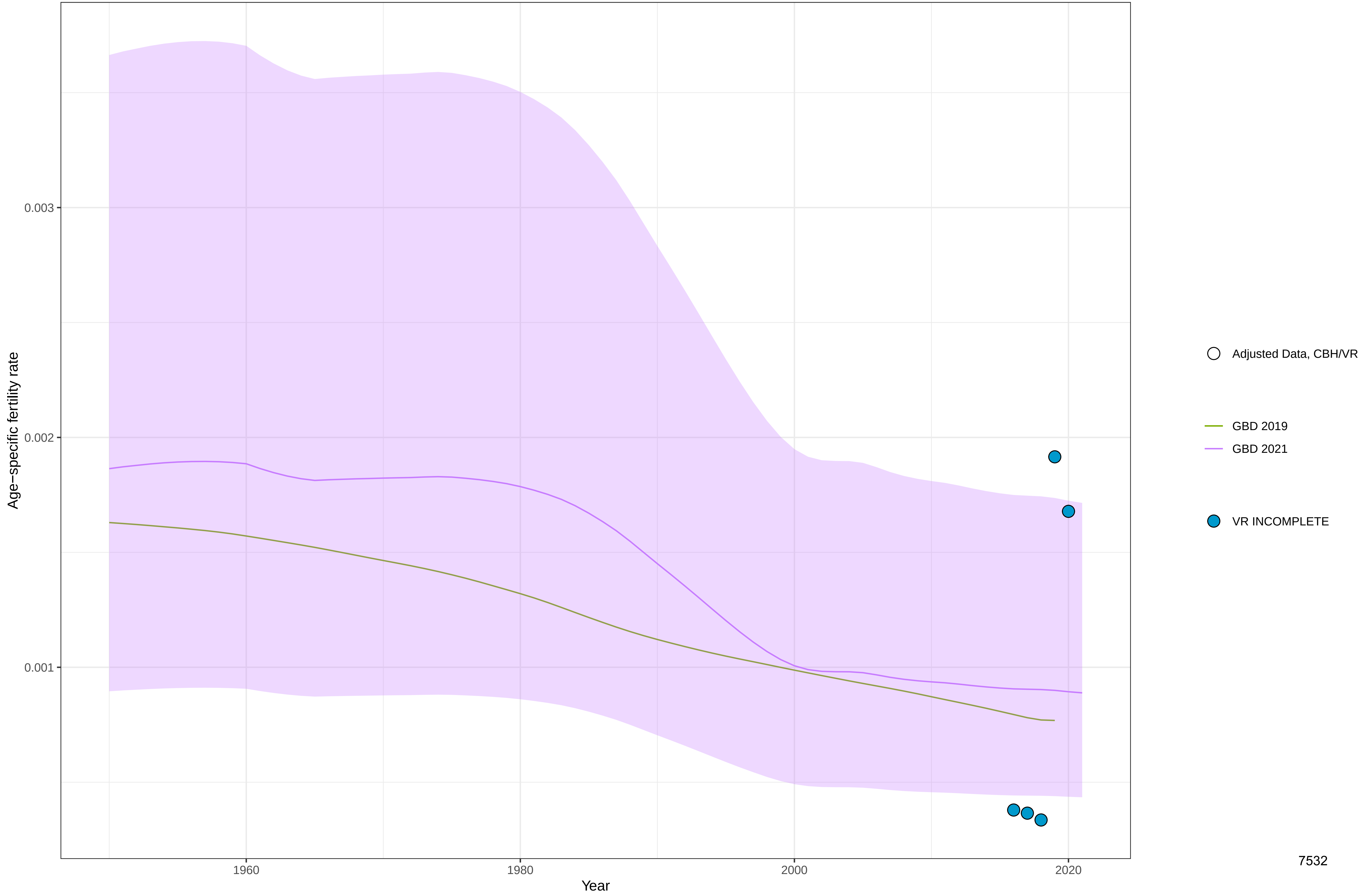

Age−specific fertility fitted trend lines, 1950−2021: Limpopo, Ages: 15−19
Data points enclosed with a diamond are reference sources

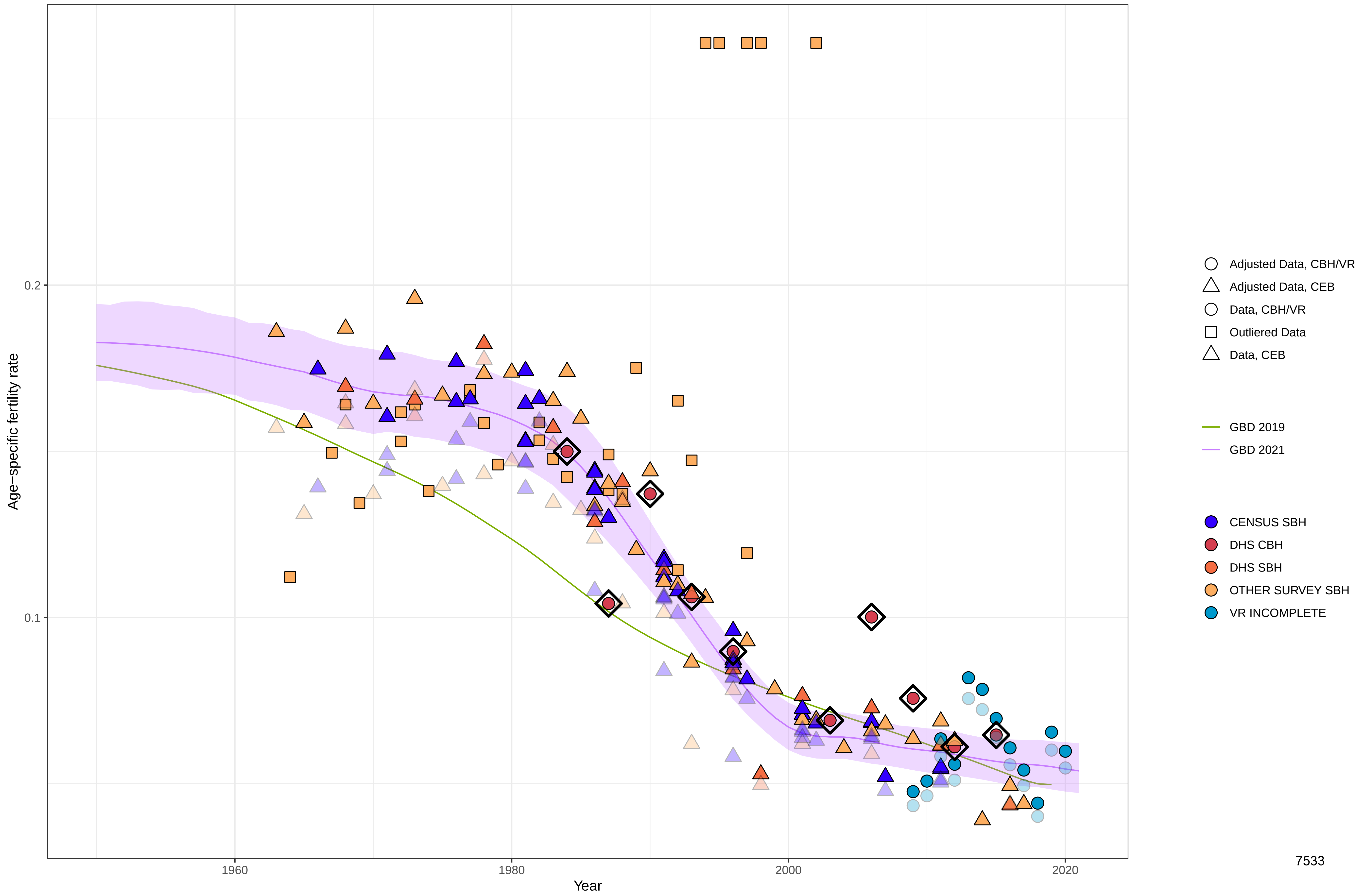

Age−specific fertility fitted trend lines, 1950−2021: Limpopo, Ages: 20−24
Data points enclosed with a diamond are reference sources

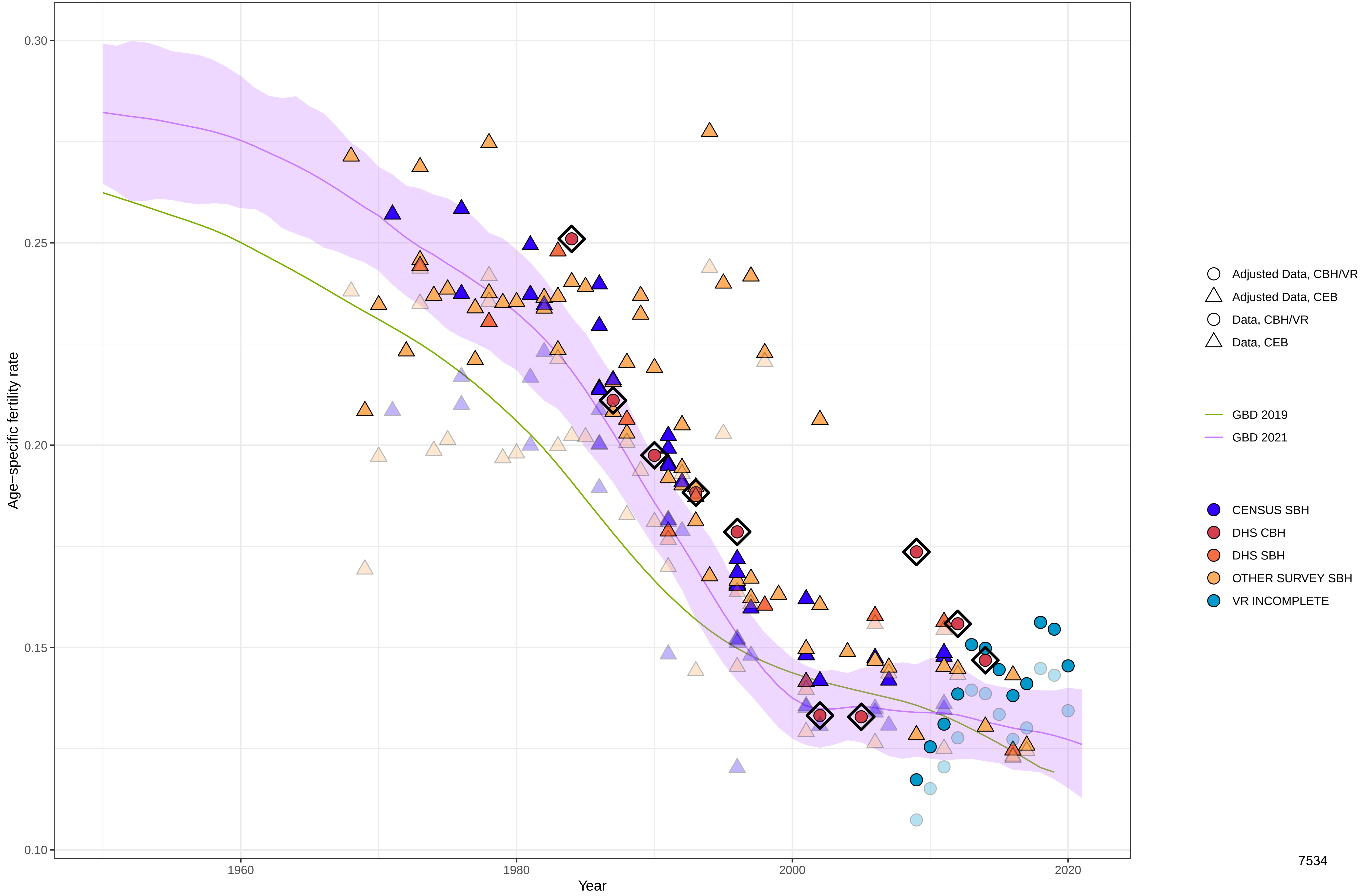

Age−specific fertility fitted trend lines, 1950−2021: Limpopo, Ages: 25−29
Data points enclosed with a diamond are reference sources

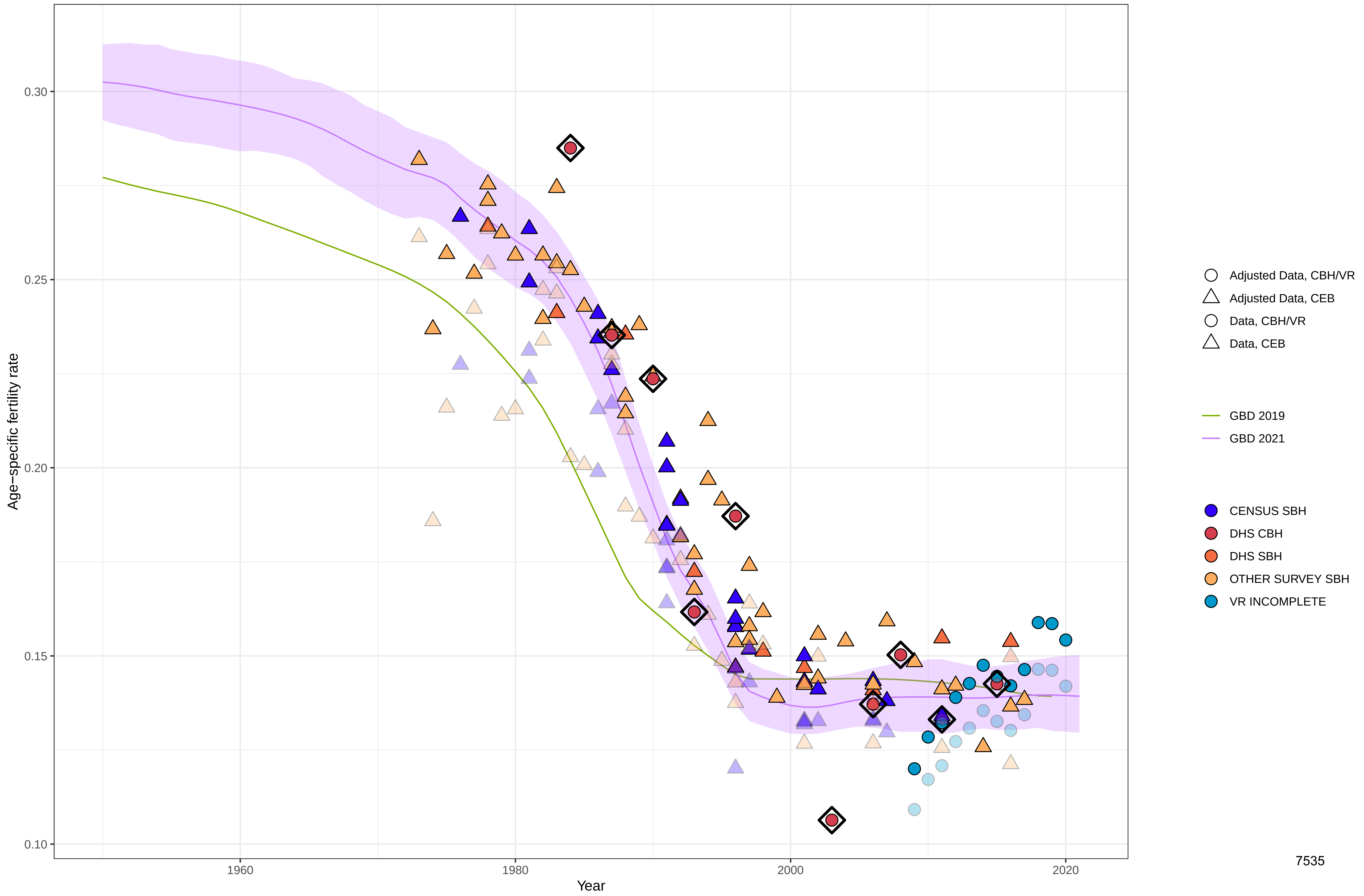

Age−specific fertility fitted trend lines, 1950−2021: Limpopo, Ages: 30−34
Data points enclosed with a diamond are reference sources

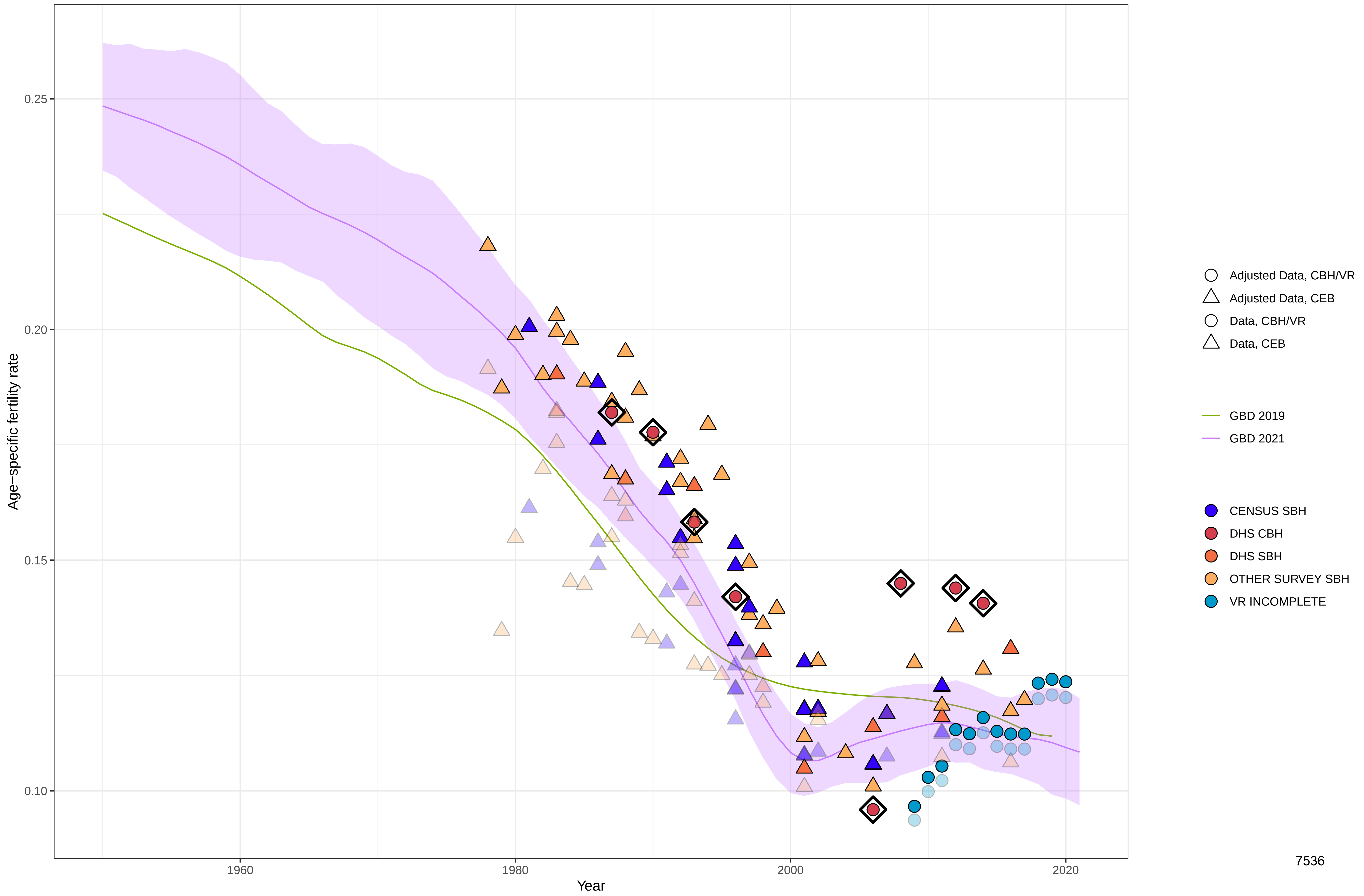

Age−specific fertility fitted trend lines, 1950−2021: Limpopo, Ages: 35−39
Data points enclosed with a diamond are reference sources

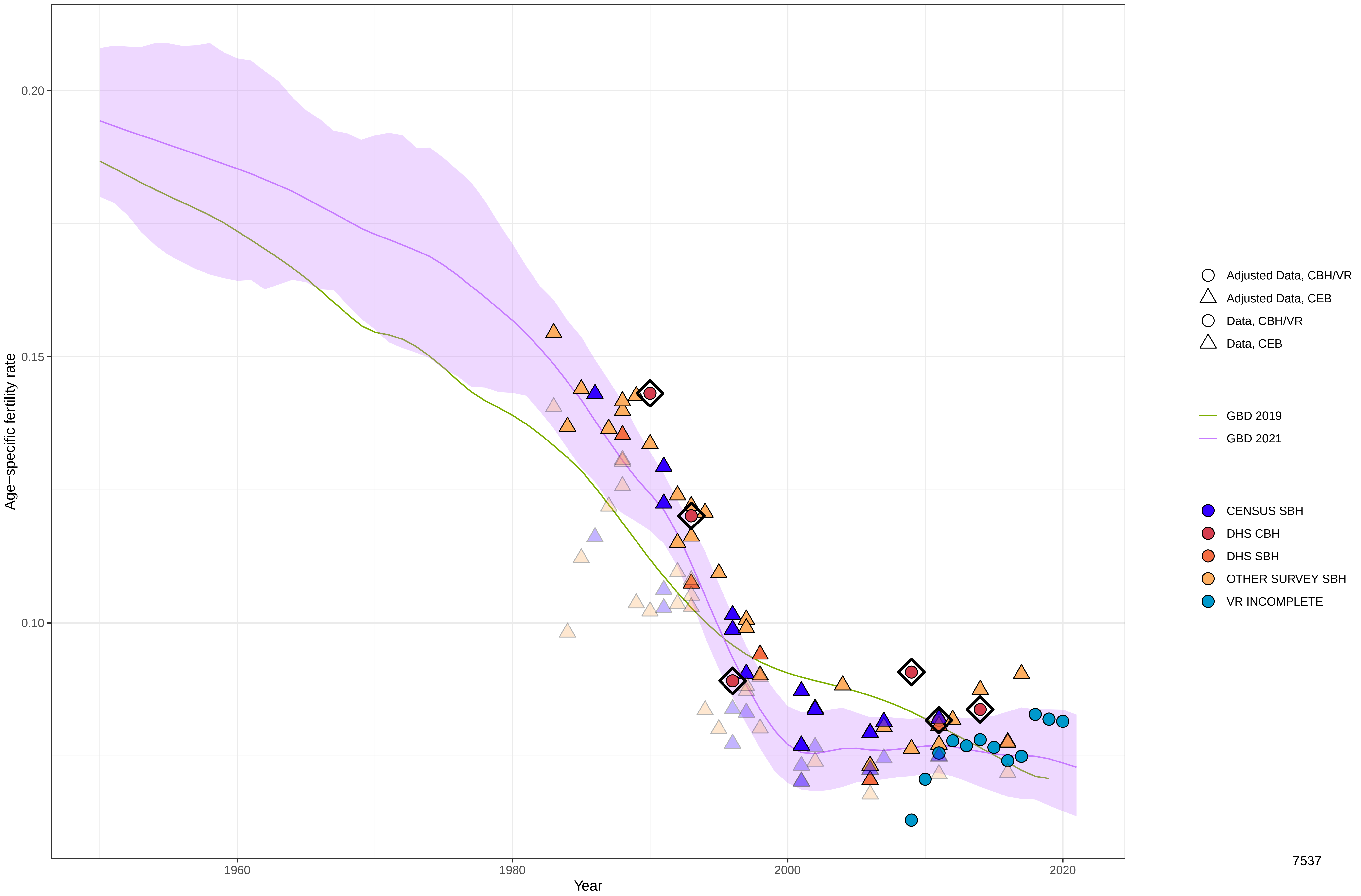

Age−specific fertility fitted trend lines, 1950−2021: Limpopo, Ages: 40−44
Data points enclosed with a diamond are reference sources

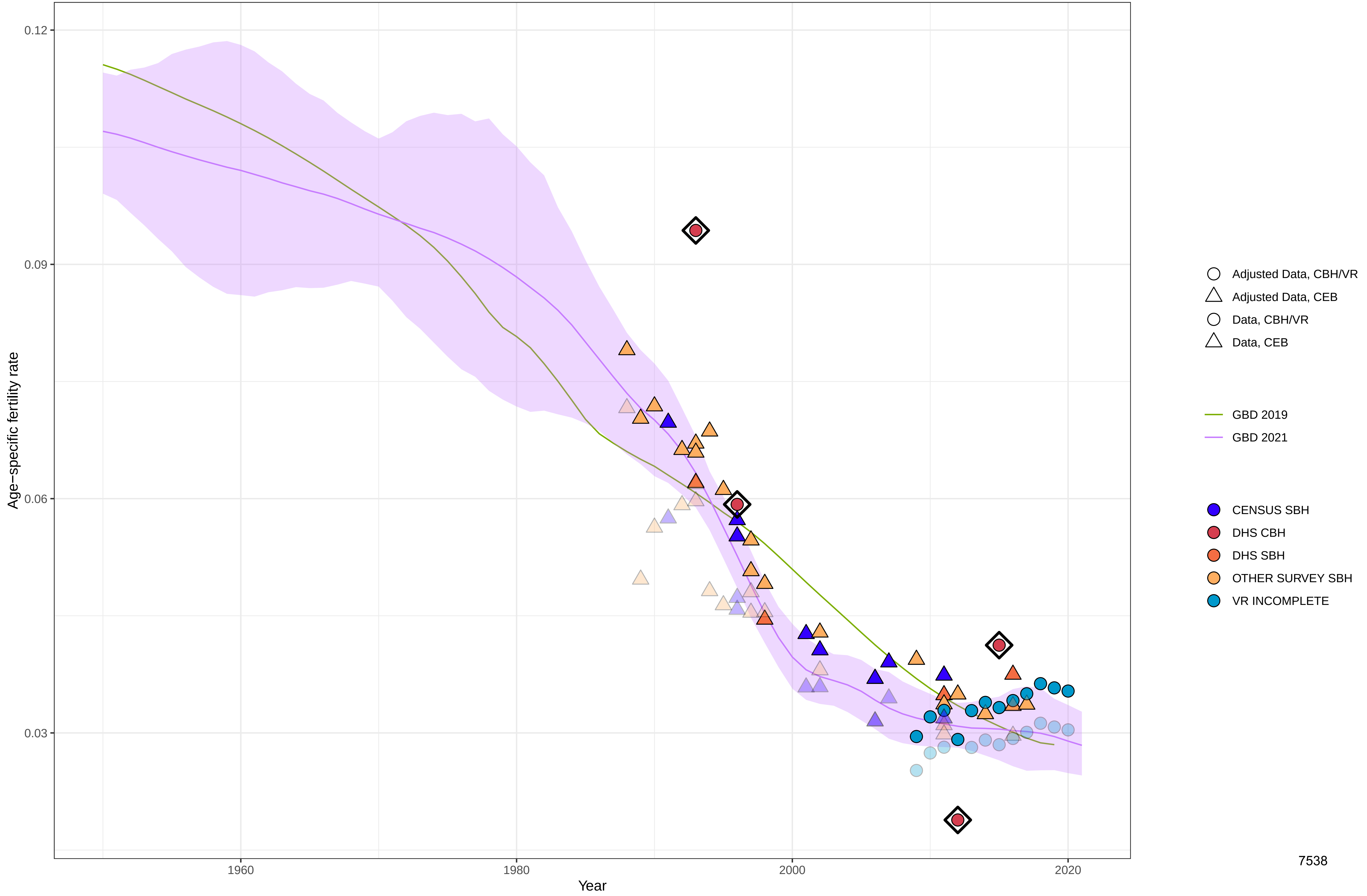

Age−specific fertility fitted trend lines, 1950−2021: Limpopo, Ages: 45−49
Data points enclosed with a diamond are reference sources

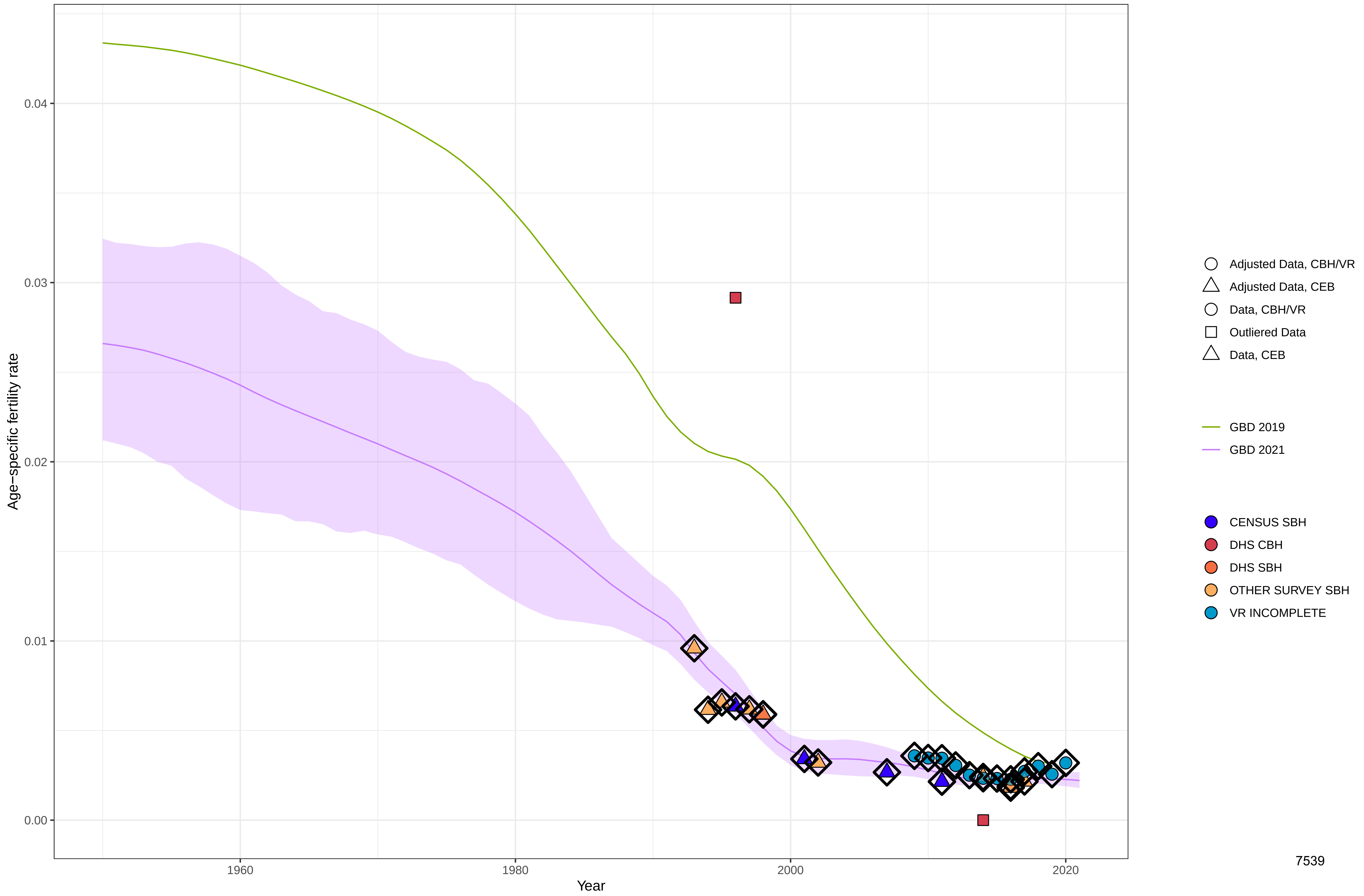

Age−specific fertility fitted trend lines, 1950−2021: Limpopo, Ages: 50−54
Data points enclosed with a diamond are reference sources

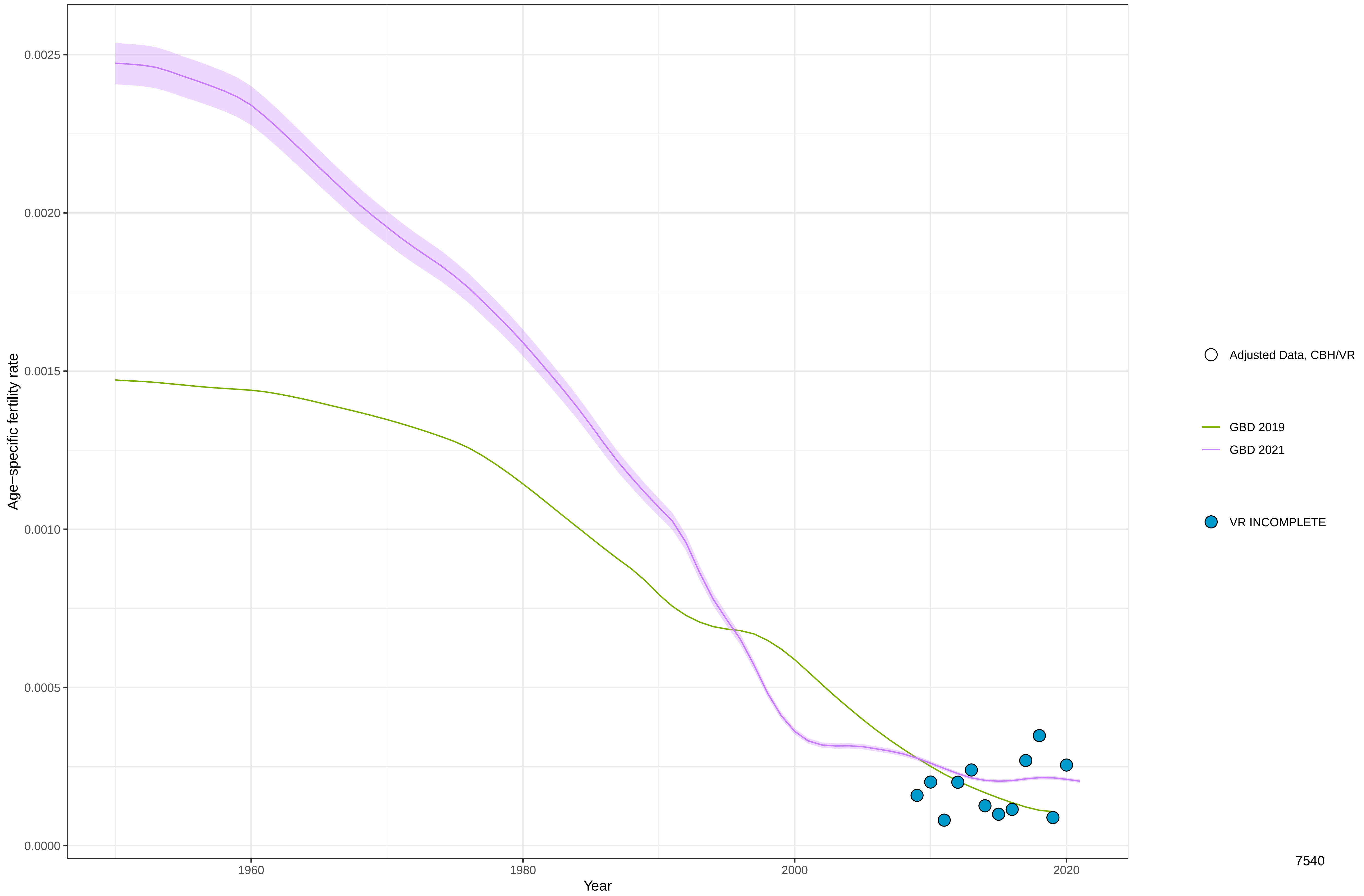

Total fertility rate fitted trend lines, 1950−2021: Limpopo TFR
Data points enclosed with a diamond are reference sources

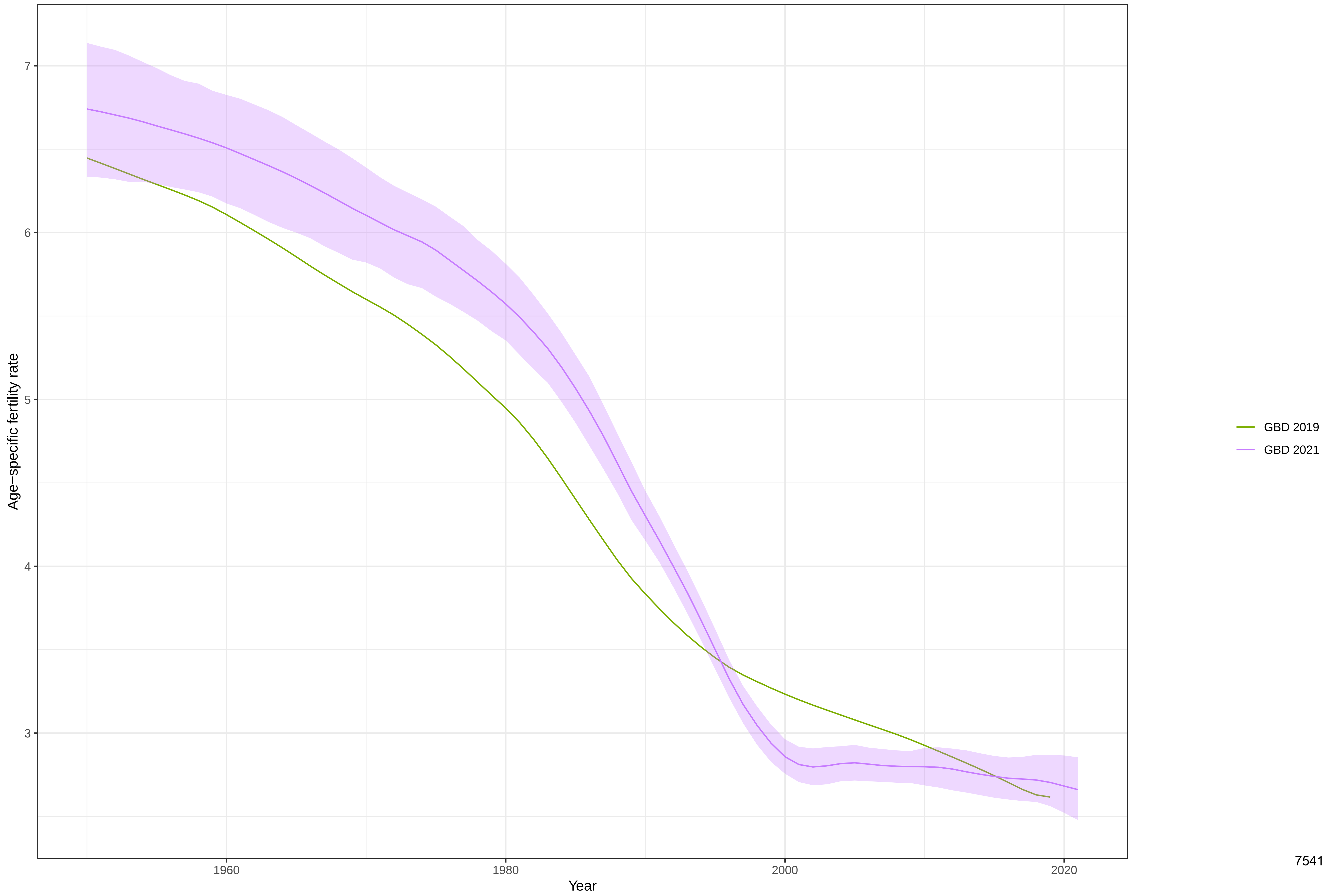

Age−specific fertility fitted trend lines, 1950−2021: Mpumalanga, Ages: 10−14
Data points enclosed with a diamond are reference sources

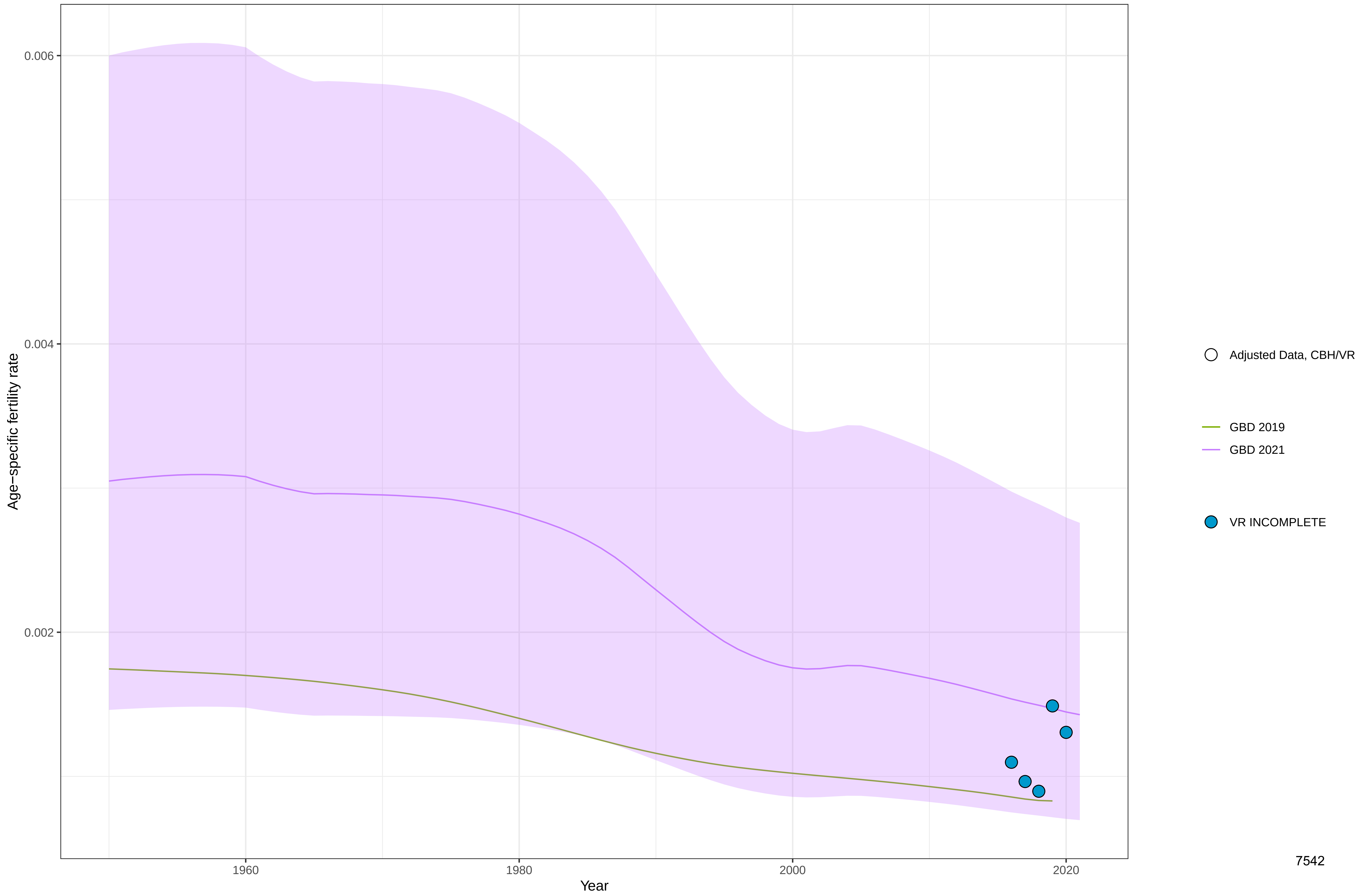

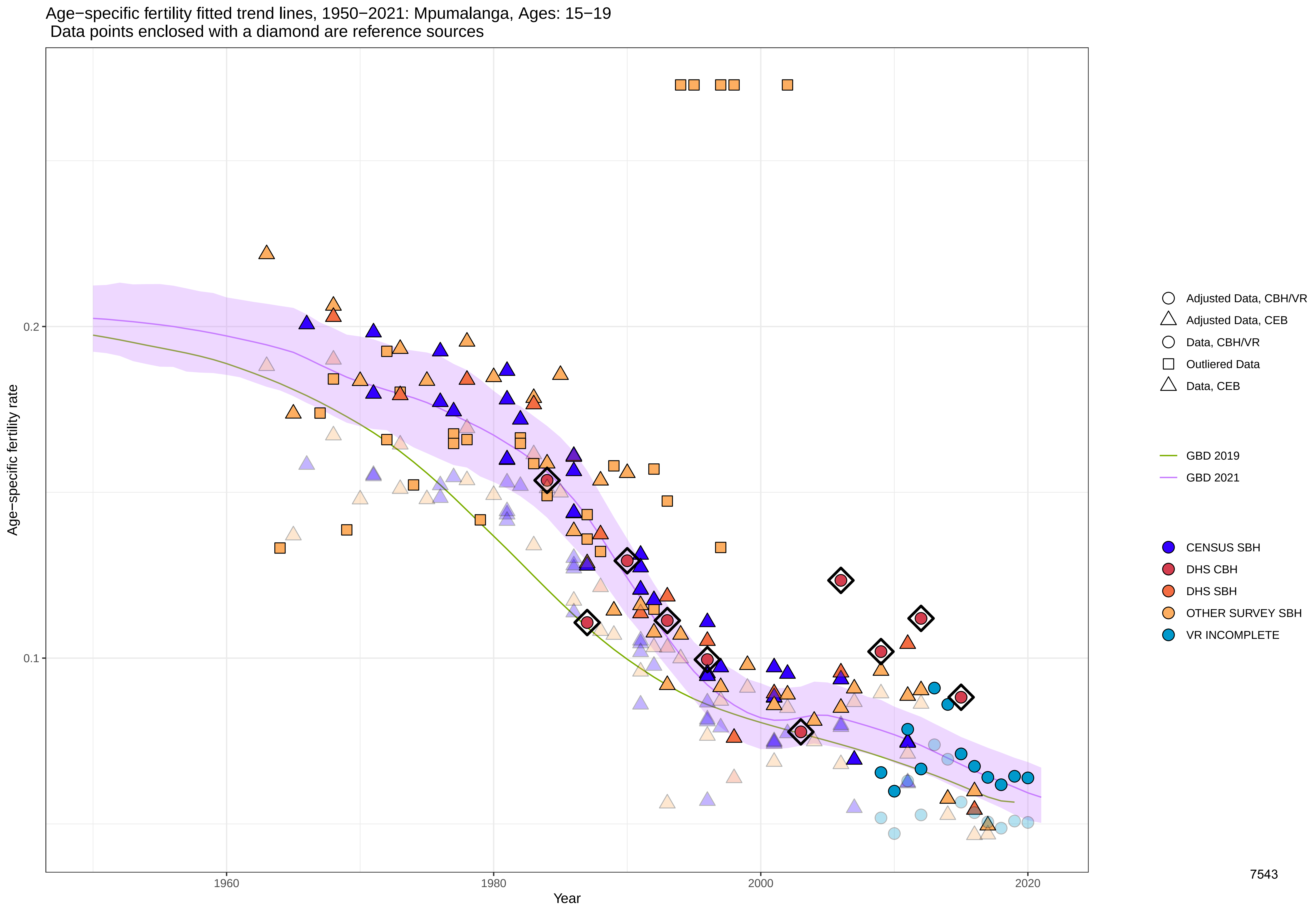

Age−specific fertility fitted trend lines, 1950−2021: Mpumalanga, Ages: 15−19
Data points enclosed with a diamond are reference sources
Age−specific fertility rate
0.2
0.1
1960
1980
2000
2020
Year
Adjusted Data, CBH/VR
Adjusted Data, CEB
Data, CBH/VR
Outliered Data
Data, CEB
GBD 2019
GBD 2021
CENSUS SBH
DHS CBH
DHS SBH
OTHER SURVEY SBH
VR INCOMPLETE

7543

Age−specific fertility fitted trend lines, 1950−2021: Mpumalanga, Ages: 20−24
Data points enclosed with a diamond are reference sources

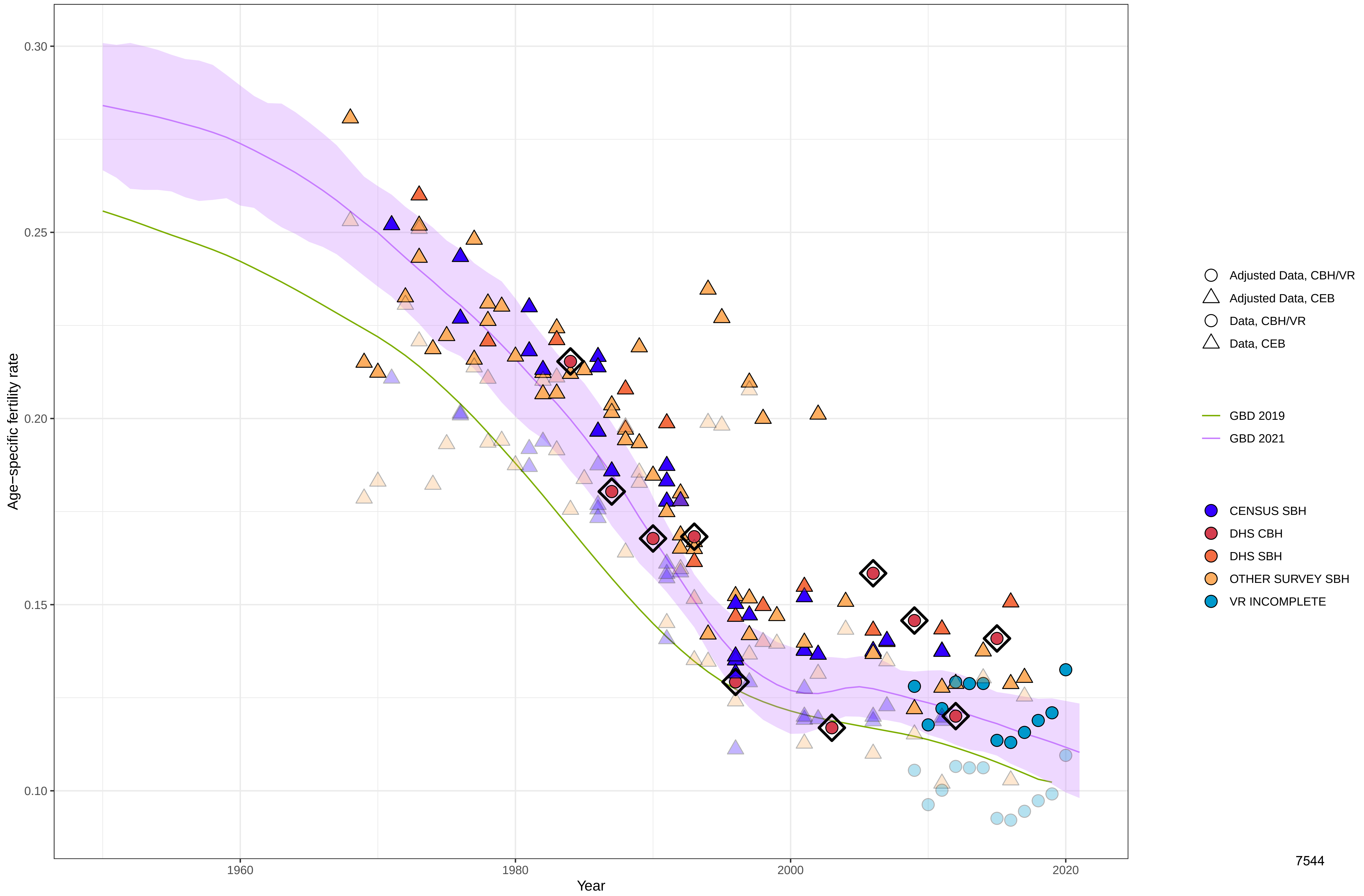

Age−specific fertility fitted trend lines, 1950−2021: Mpumalanga, Ages: 25−29
Data points enclosed with a diamond are reference sources

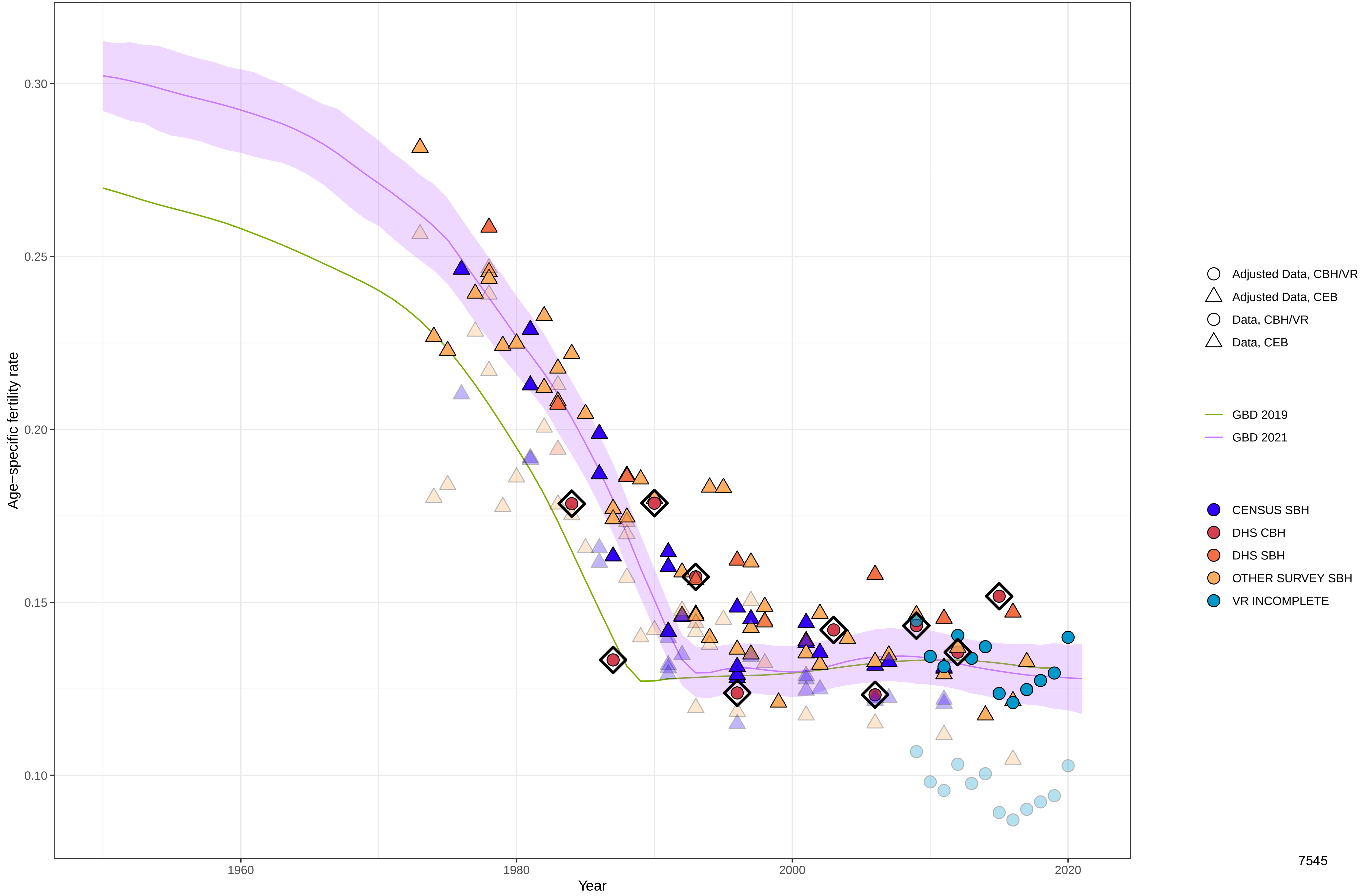

Age−specific fertility fitted trend lines, 1950−2021: Mpumalanga, Ages: 30−34
Data points enclosed with a diamond are reference sources

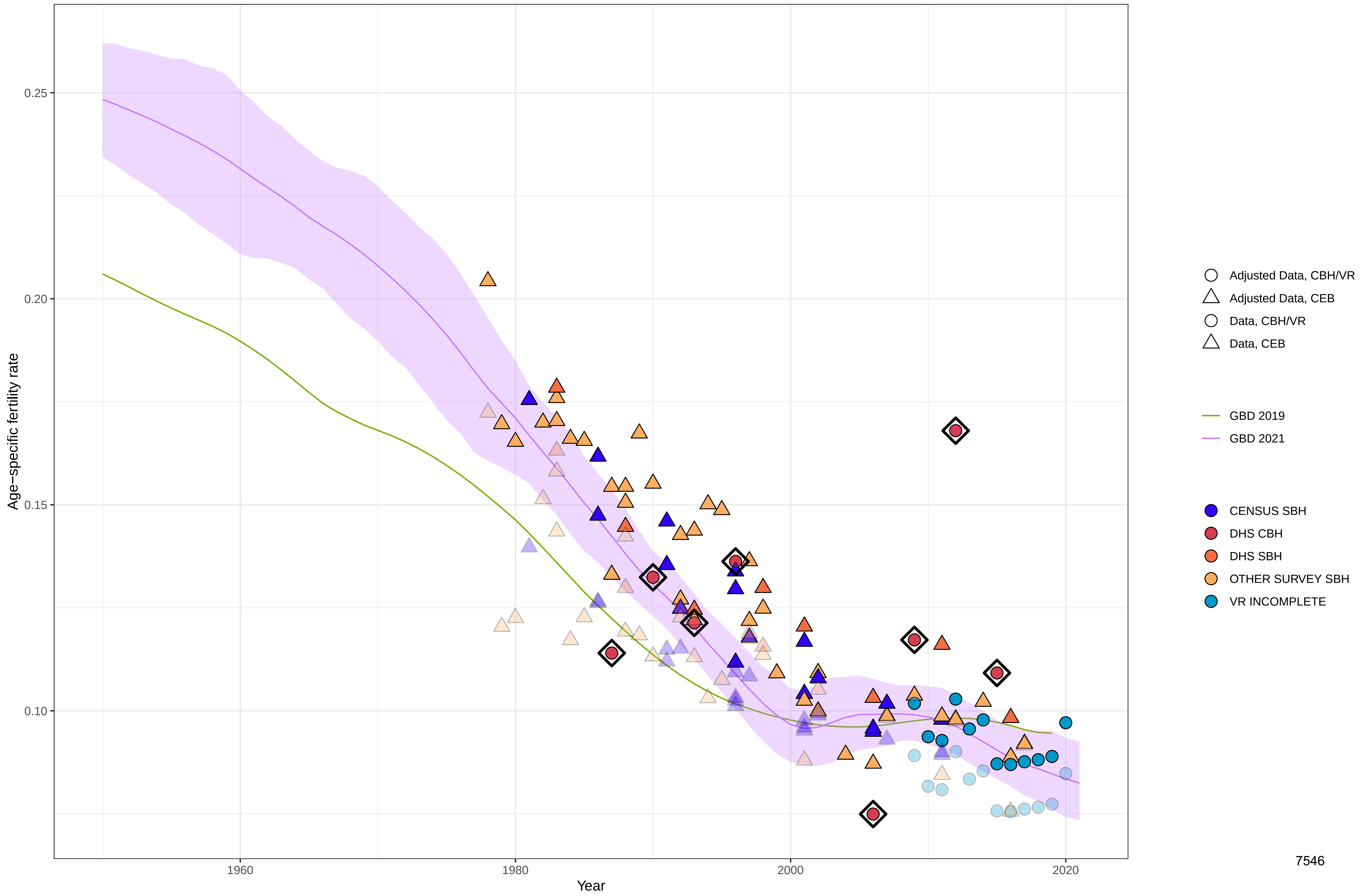

Age−specific fertility fitted trend lines, 1950−2021: Mpumalanga, Ages: 35−39
Data points enclosed with a diamond are reference sources

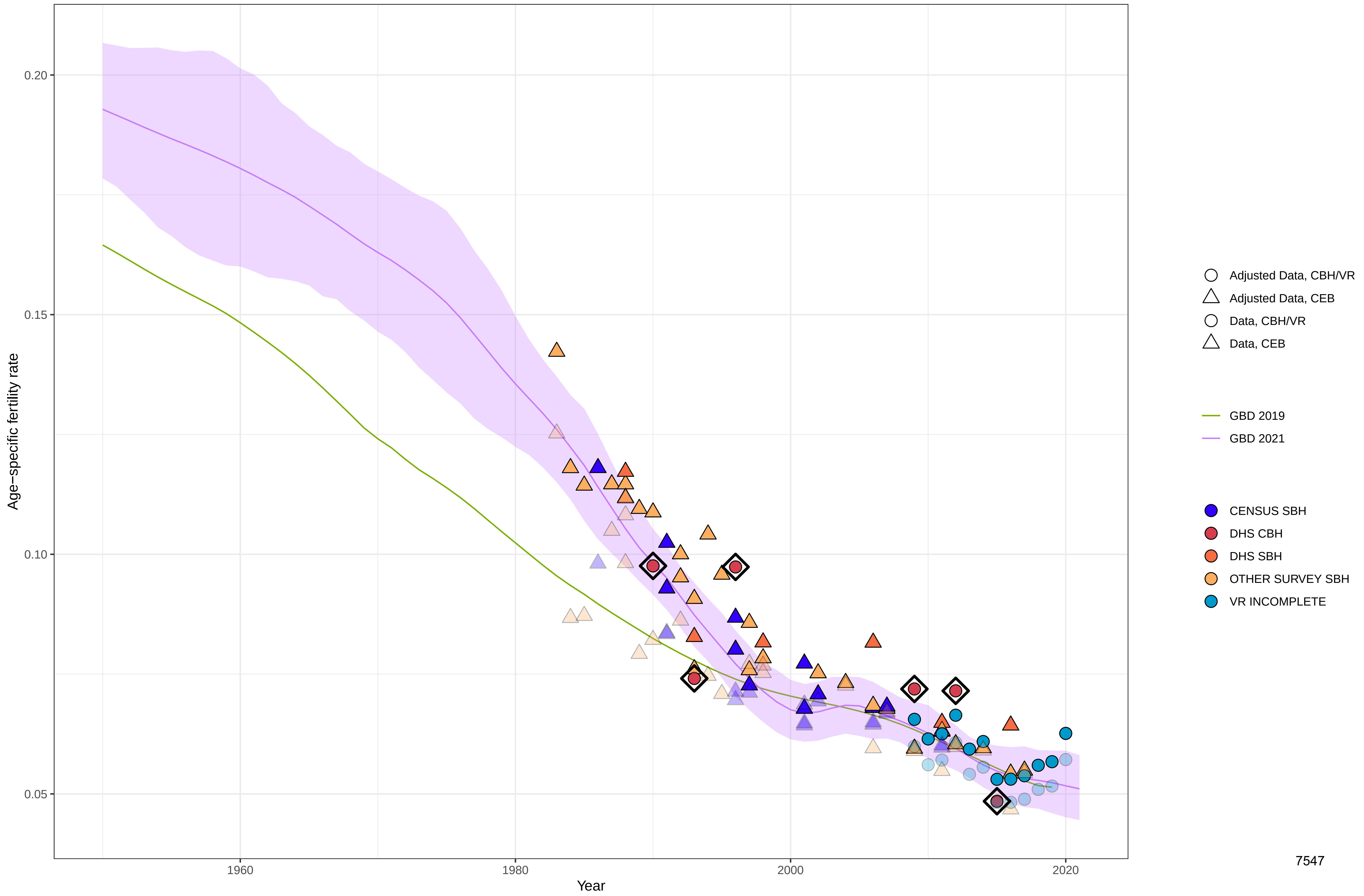

Age−specific fertility fitted trend lines, 1950−2021: Mpumalanga, Ages: 40−44
Data points enclosed with a diamond are reference sources

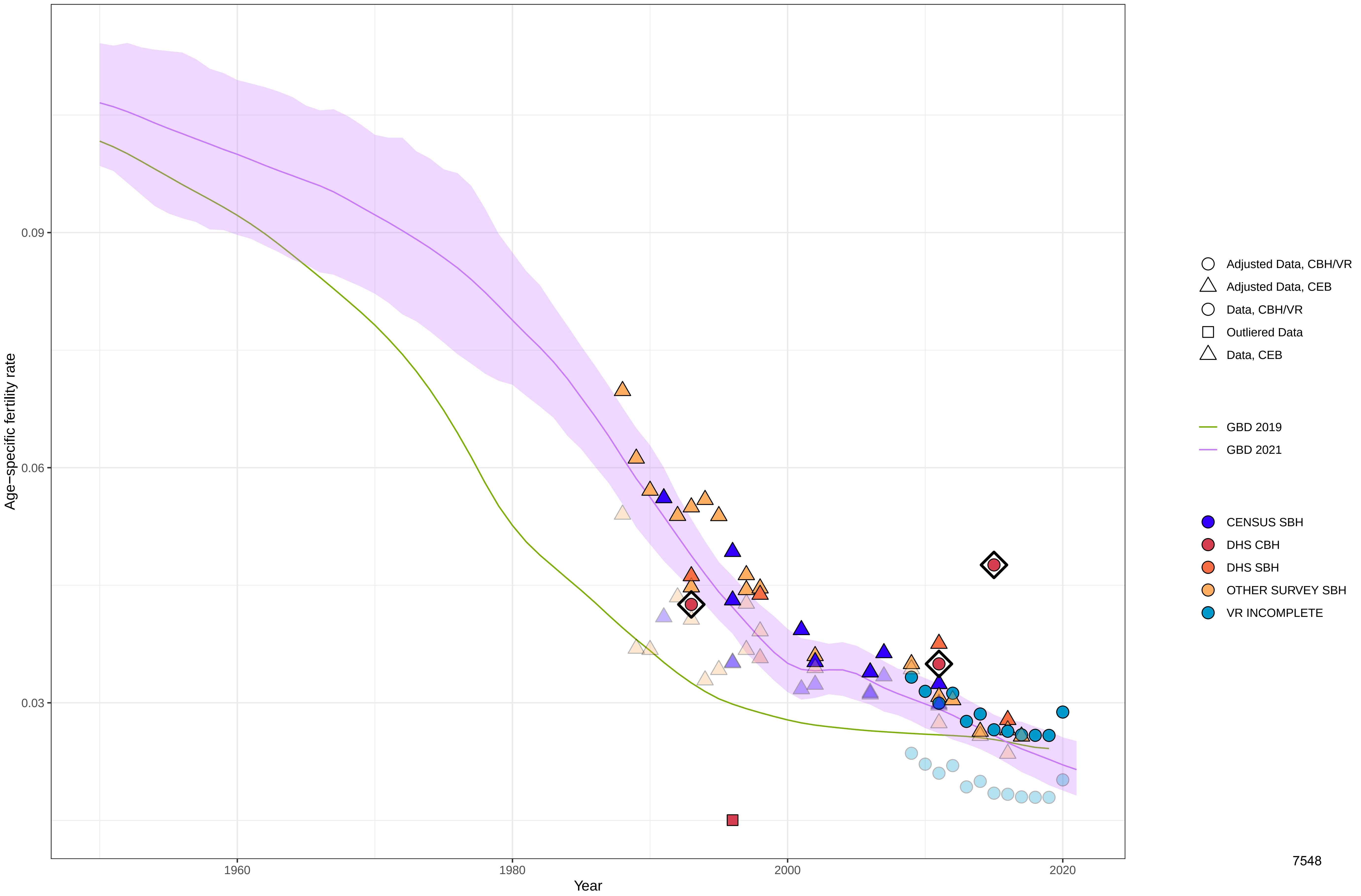

Age−specific fertility fitted trend lines, 1950−2021: Mpumalanga, Ages: 45−49
Data points enclosed with a diamond are reference sources

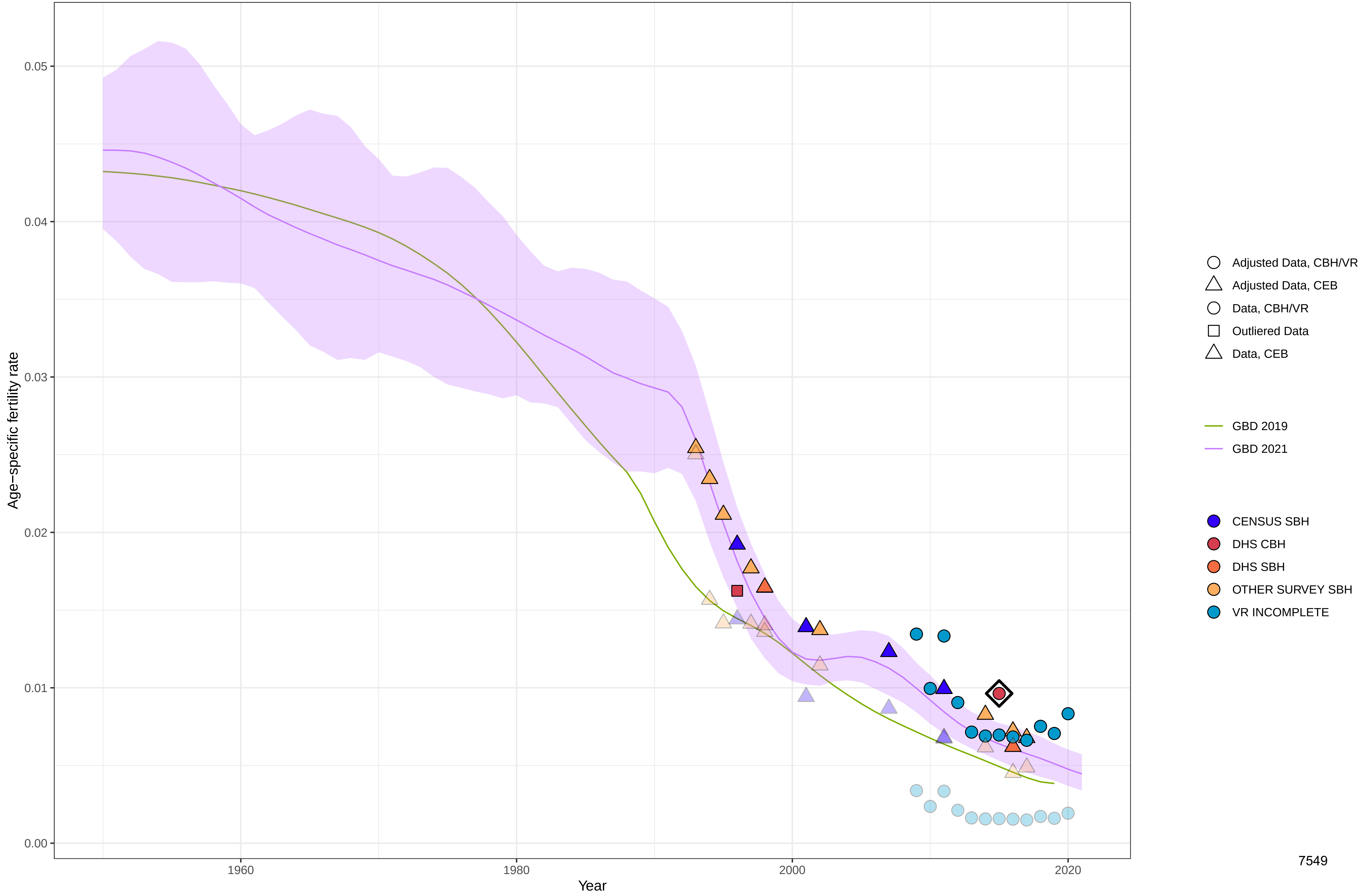

Age−specific fertility fitted trend lines, 1950−2021: Mpumalanga, Ages: 50−54
Data points enclosed with a diamond are reference sources

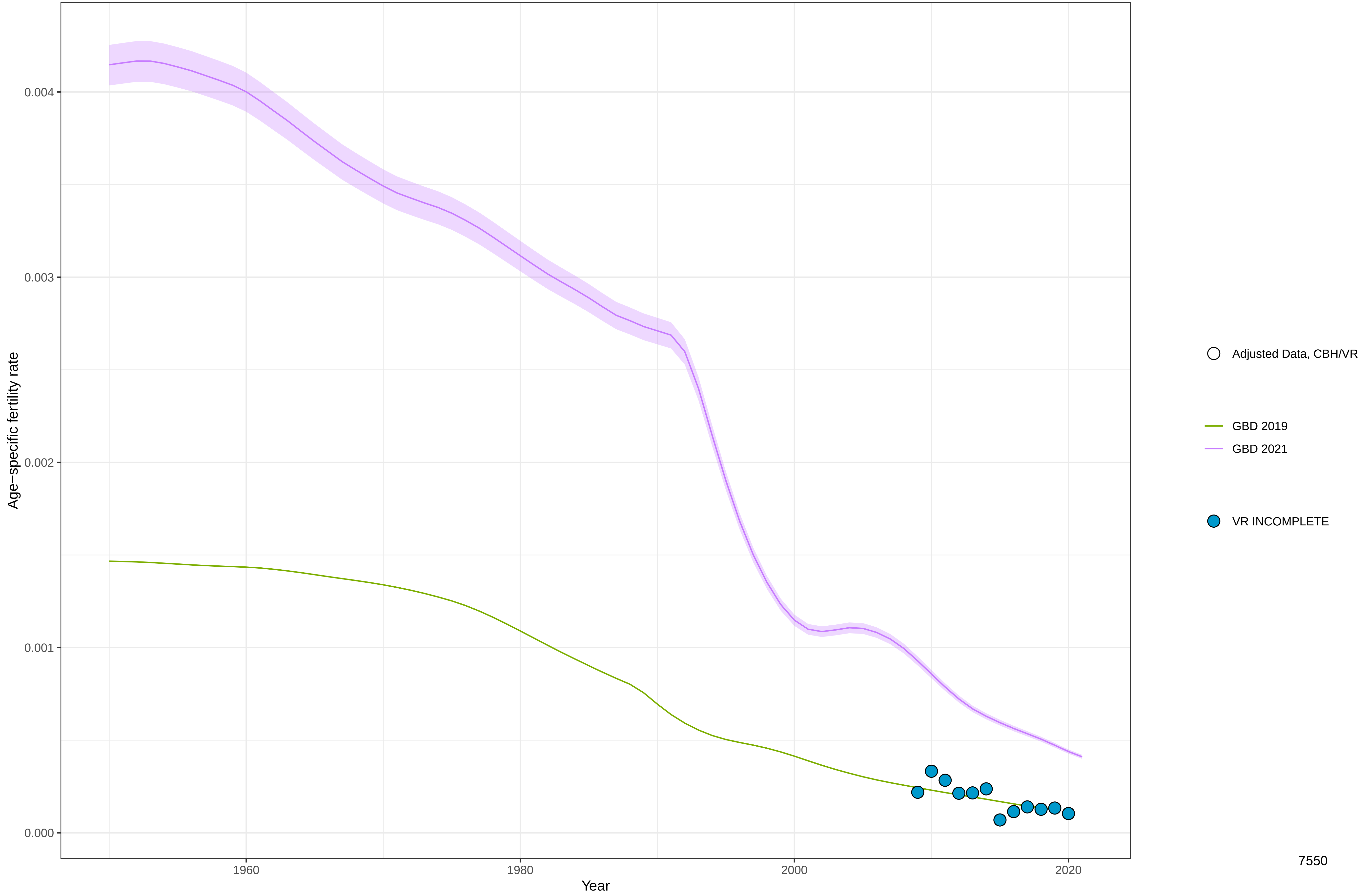

Total fertility rate fitted trend lines, 1950−2021: Mpumalanga TFR
Data points enclosed with a diamond are reference sources

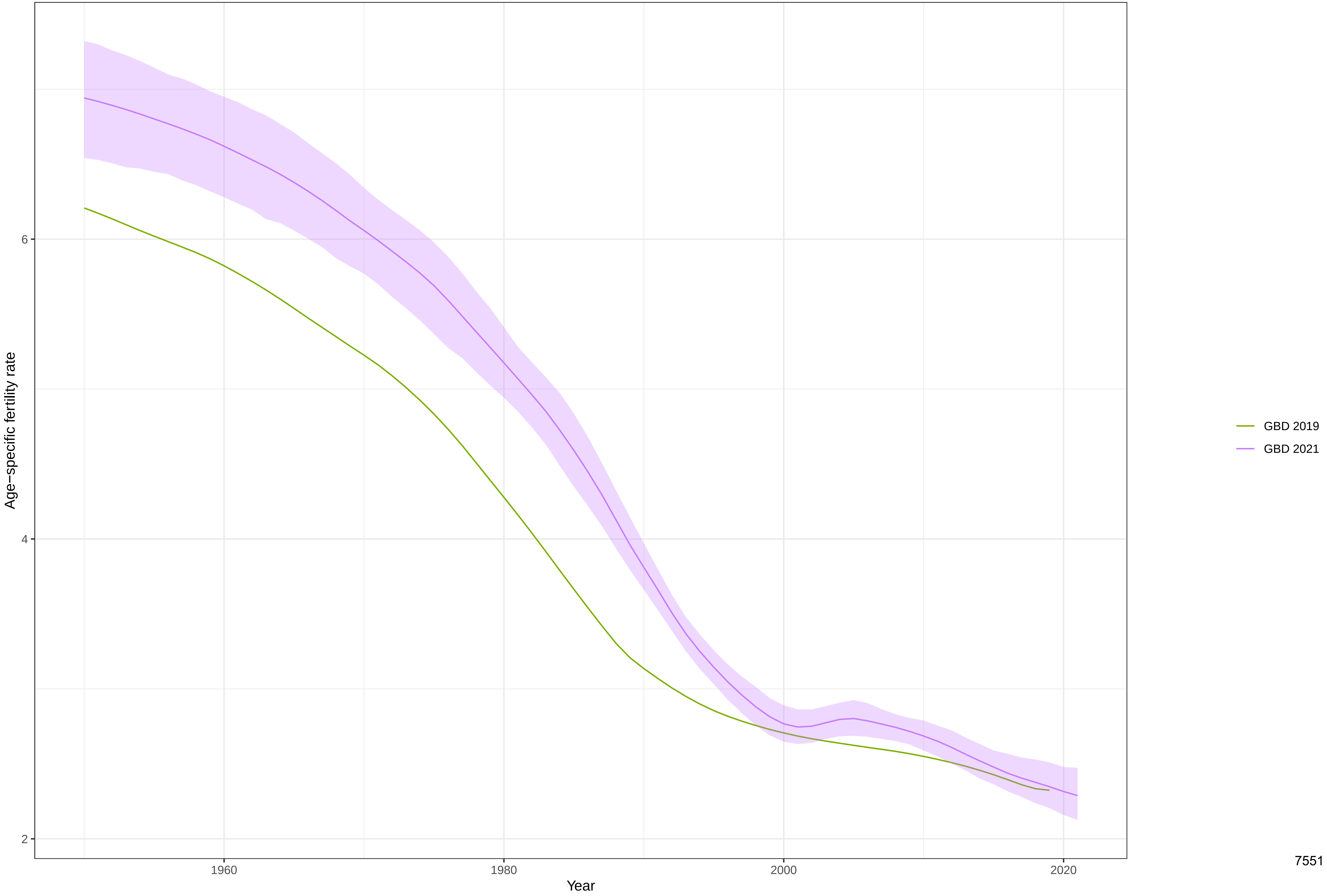

7551

Age−specific fertility fitted trend lines, 1950−2021: North West, Ages: 10−14
Data points enclosed with a diamond are reference sources

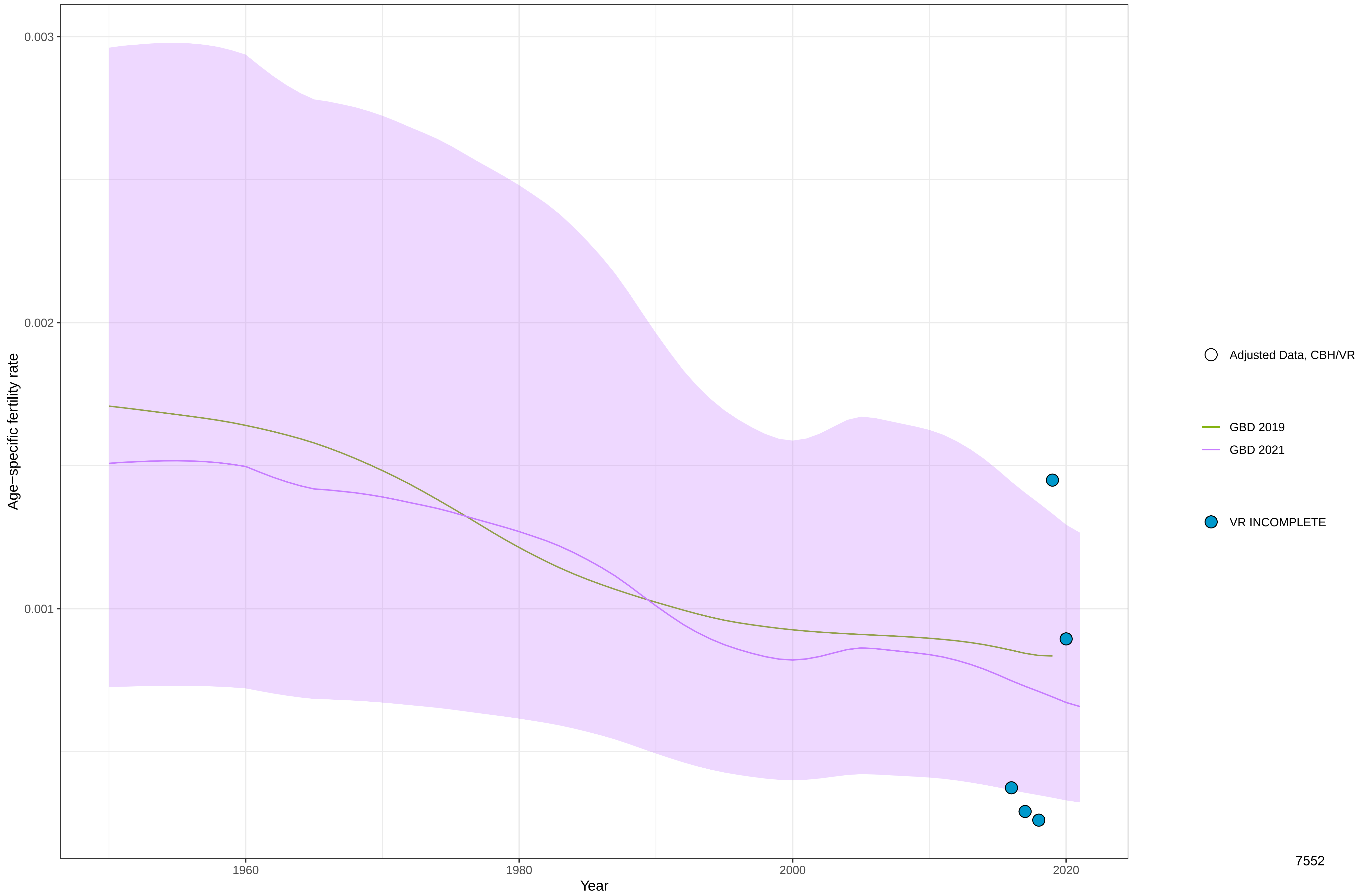

Age−specific fertility fitted trend lines, 1950−2021: North West, Ages: 15−19
Data points enclosed with a diamond are reference sources

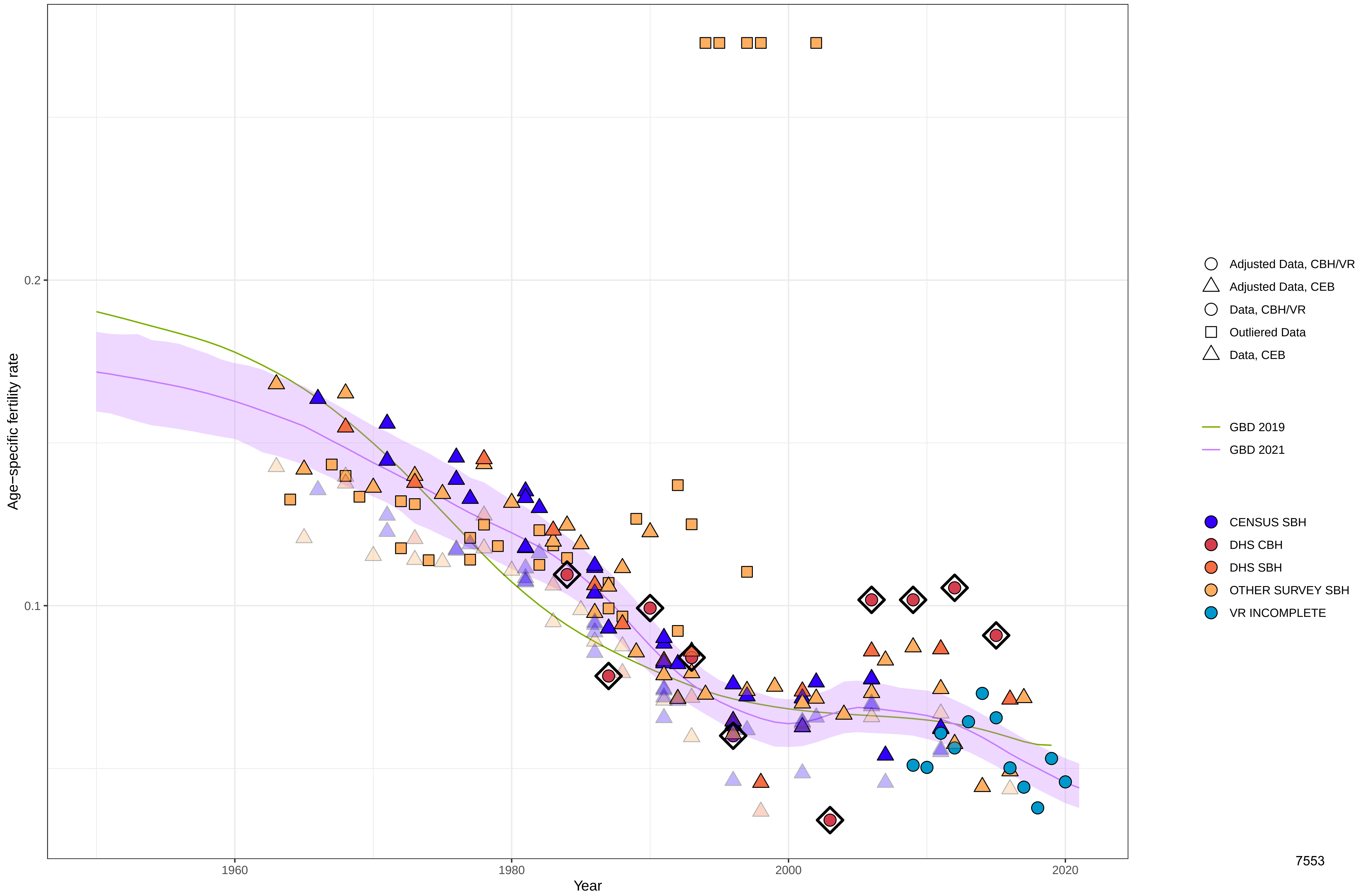

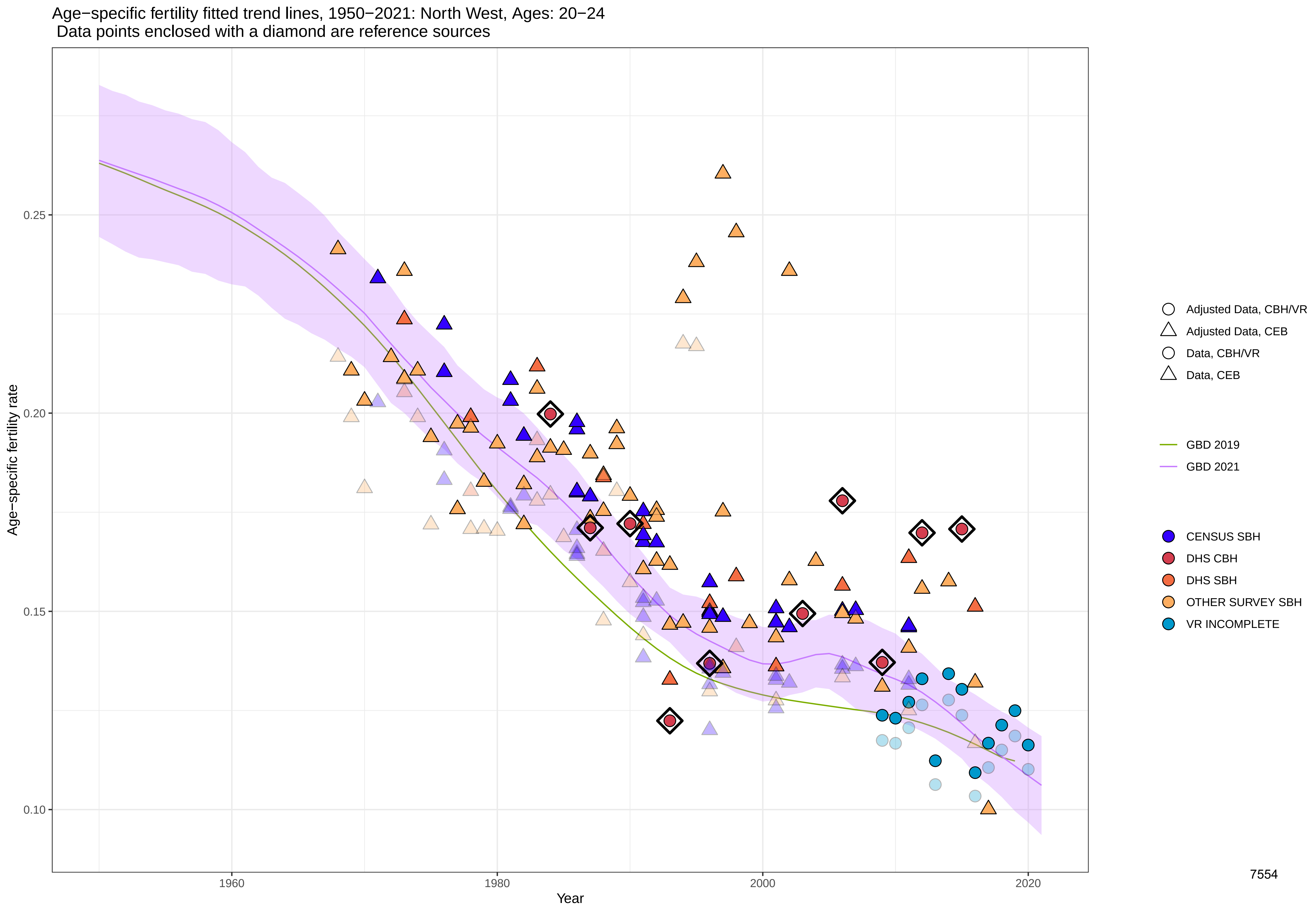

Age−specific fertility fitted trend lines, 1950−2021: North West, Ages: 20−24
Data points enclosed with a diamond are reference sources
Age−specific fertility rate
0.25
0.20
0.15
0.10
Year
1960
1980
2000
2020
Adjusted Data, CBH/VR
Adjusted Data, CEB
Data, CBH/VR
Data, CEB
GBD 2019
GBD 2021
CENSUS SBH
DHS CBH
DHS SBH
OTHER SURVEY SBH
VR INCOMPLETE

Age−specific fertility fitted trend lines, 1950−2021: North West, Ages: 25−29
Data points enclosed with a diamond are reference sources

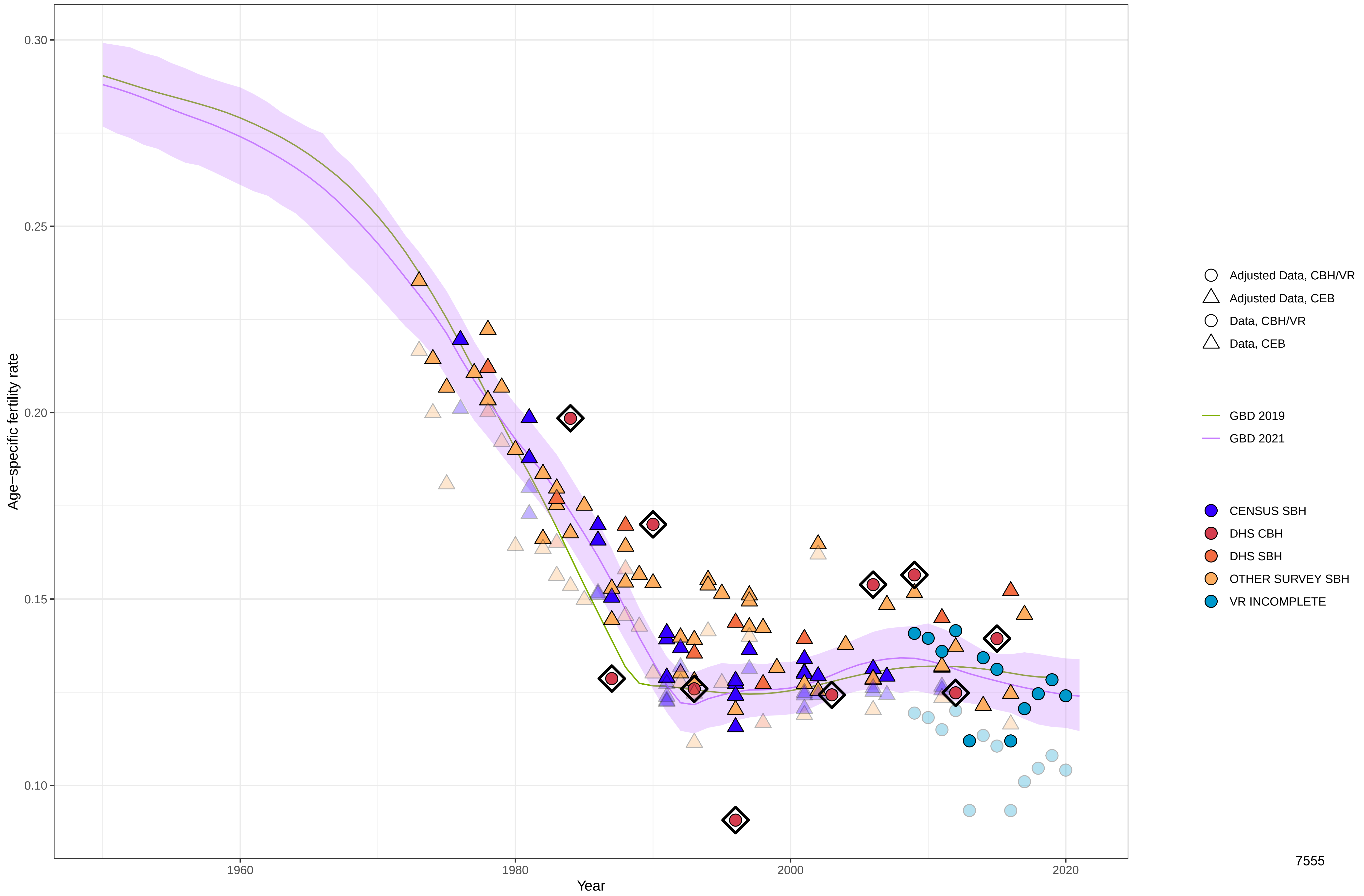

Age−specific fertility fitted trend lines, 1950−2021: North West, Ages: 30−34
Data points enclosed with a diamond are reference sources

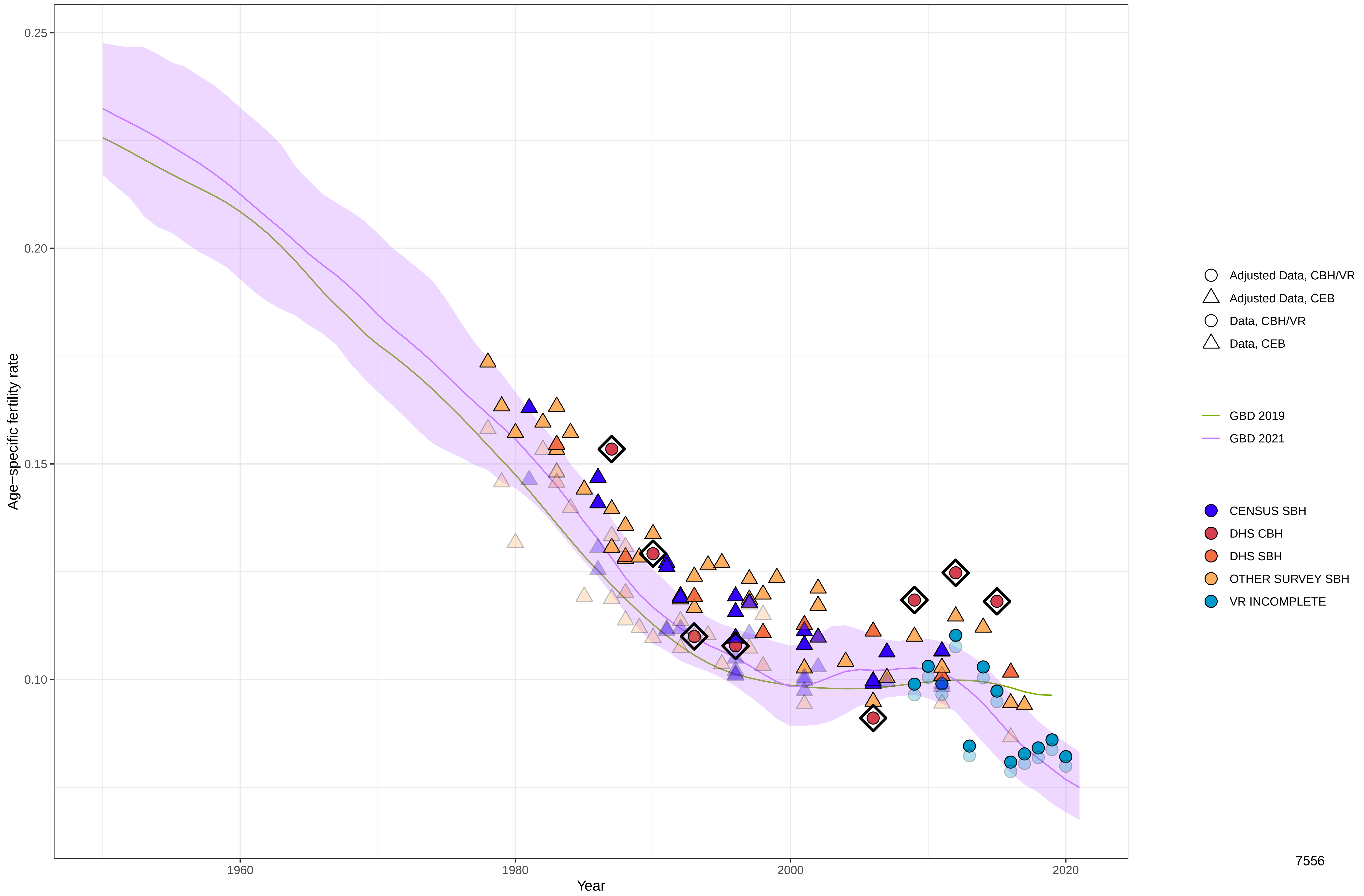

7556

Age−specific fertility fitted trend lines, 1950−2021: North West, Ages: 35−39
Data points enclosed with a diamond are reference sources

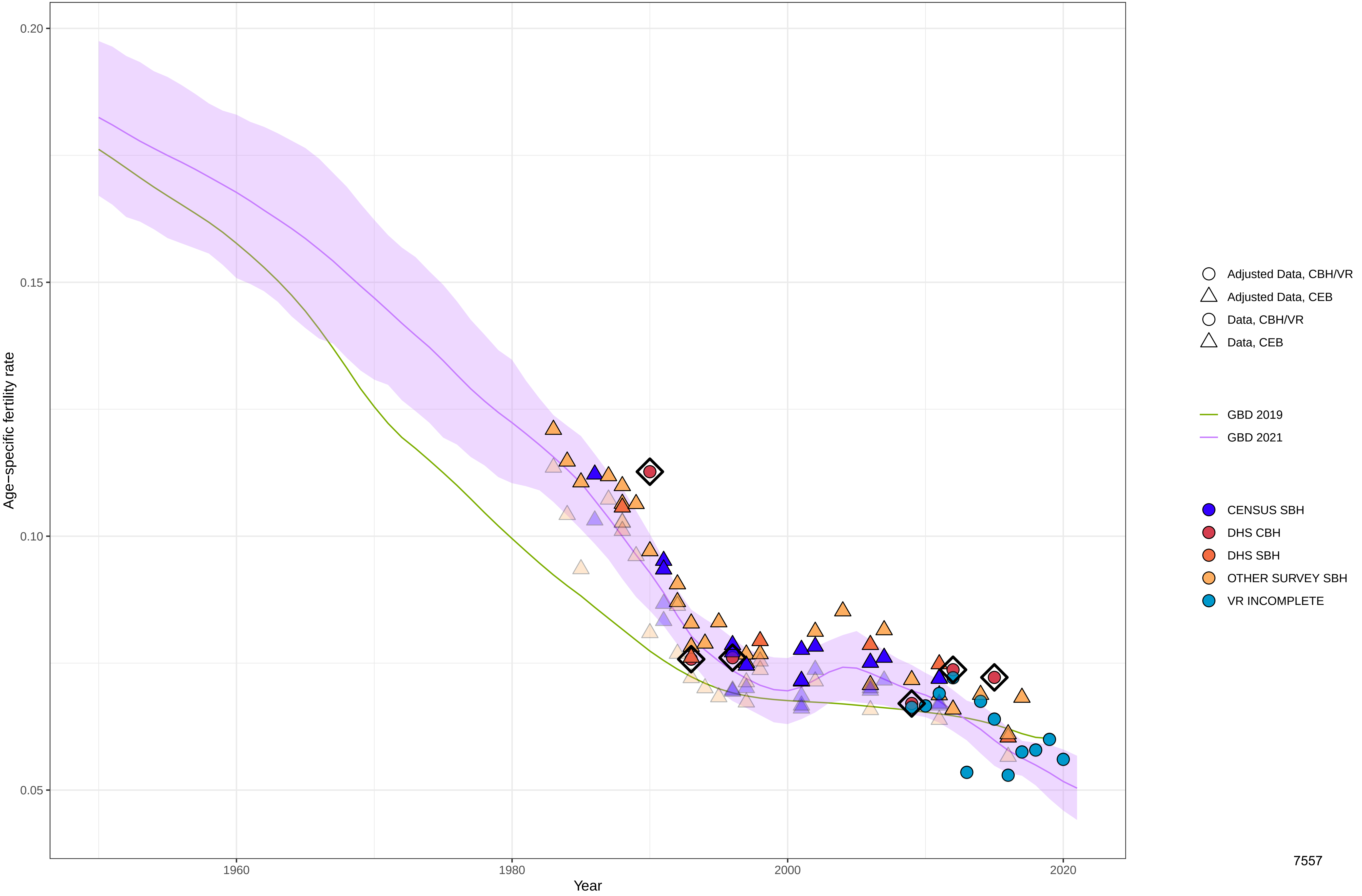

Age−specific fertility fitted trend lines, 1950−2021: North West, Ages: 40−44
Data points enclosed with a diamond are reference sources

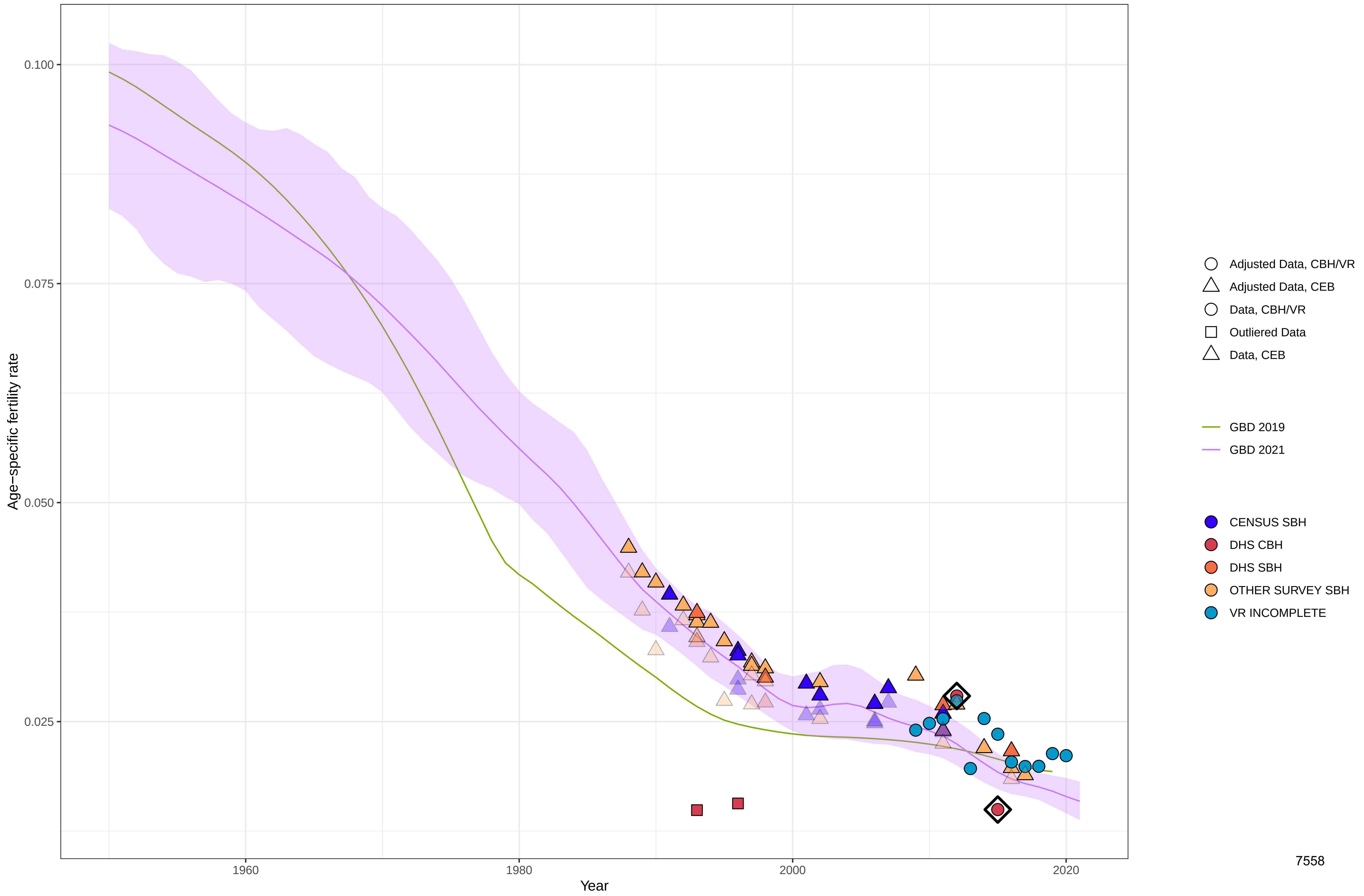

Age−specific fertility fitted trend lines, 1950−2021: North West, Ages: 45−49
Data points enclosed with a diamond are reference sources

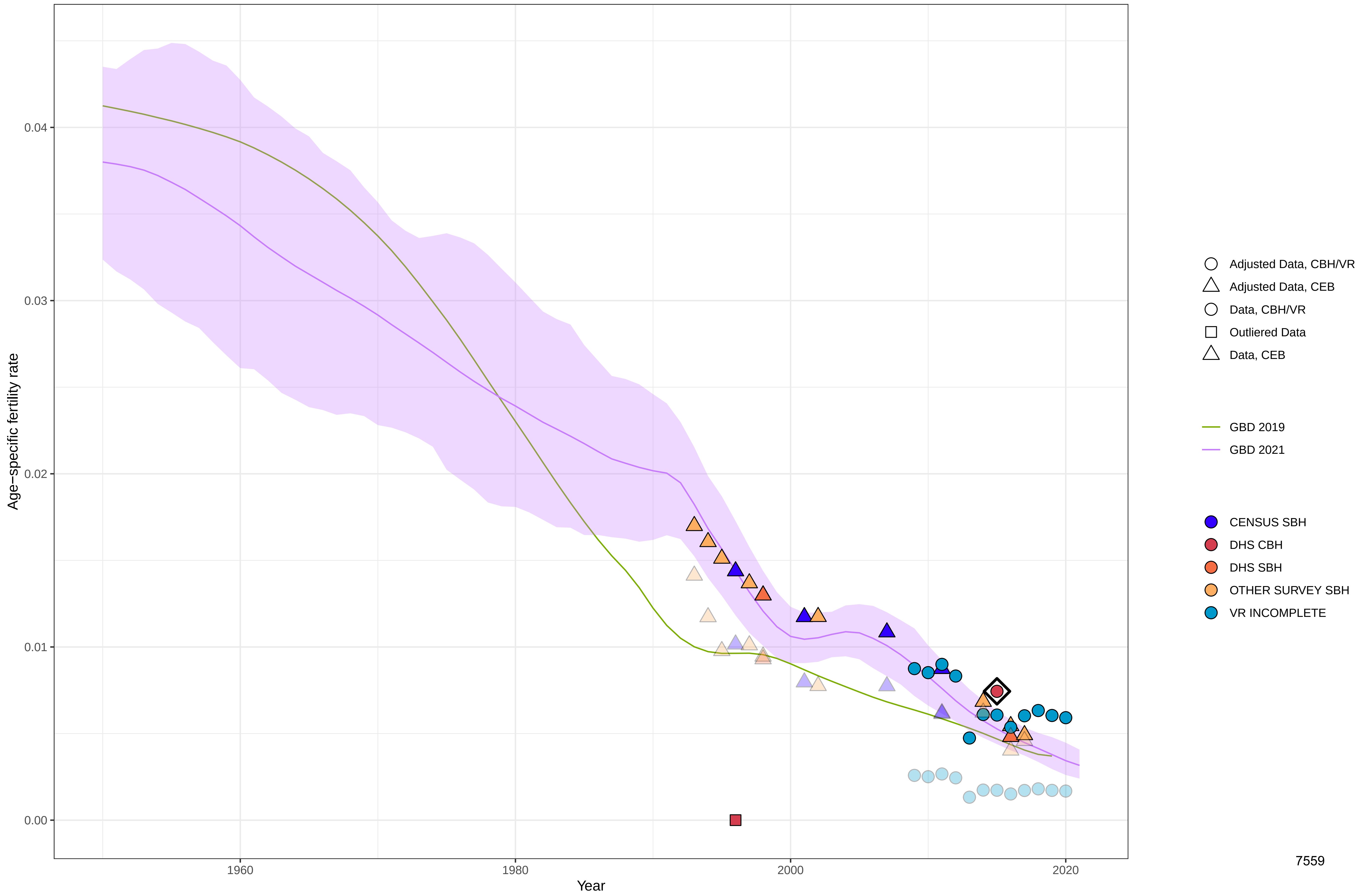

Age−specific fertility fitted trend lines, 1950−2021: North West, Ages: 50−54
Data points enclosed with a diamond are reference sources

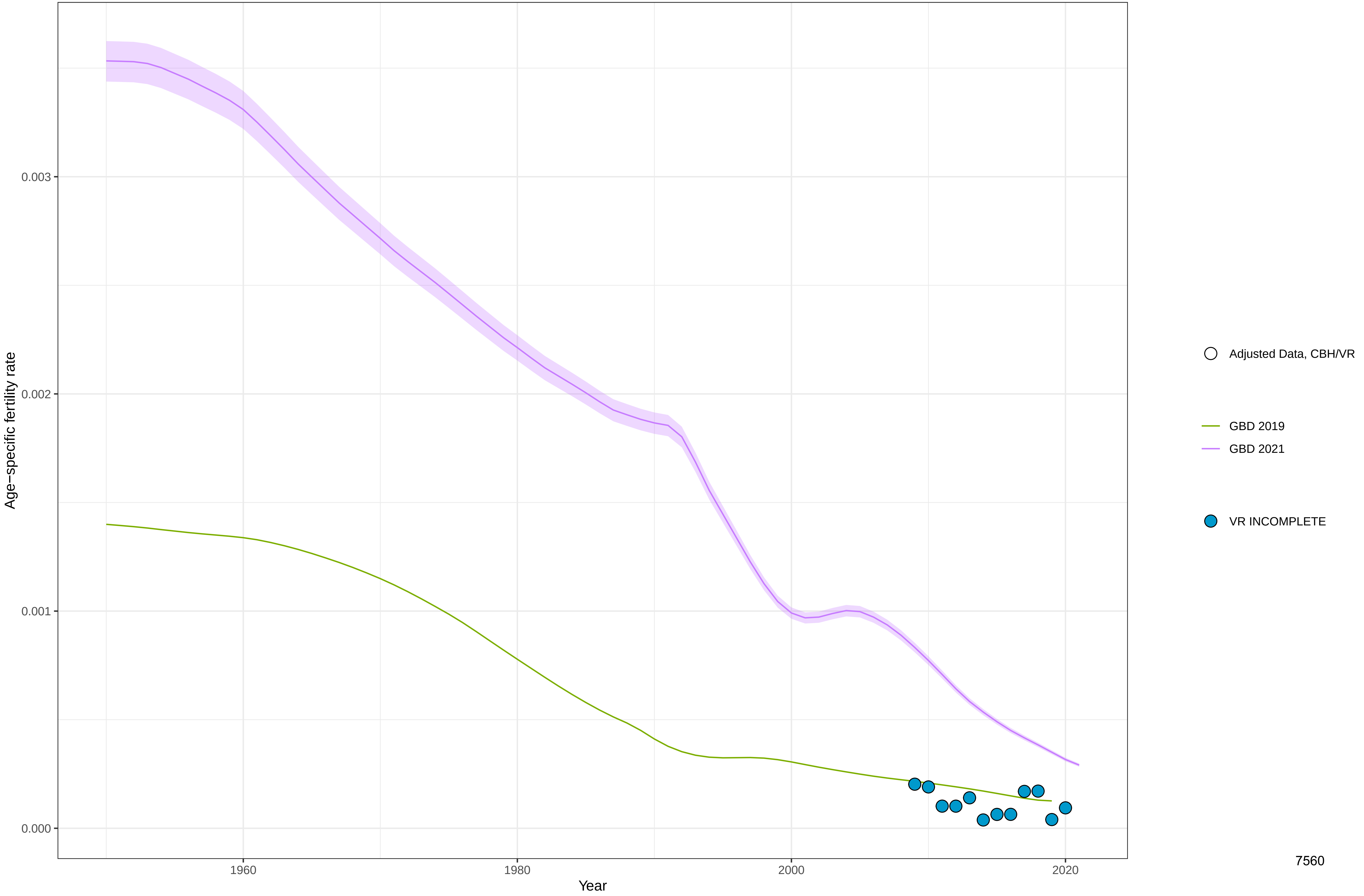

Total fertility rate fitted trend lines, 1950−2021: North West TFR
Data points enclosed with a diamond are reference sources

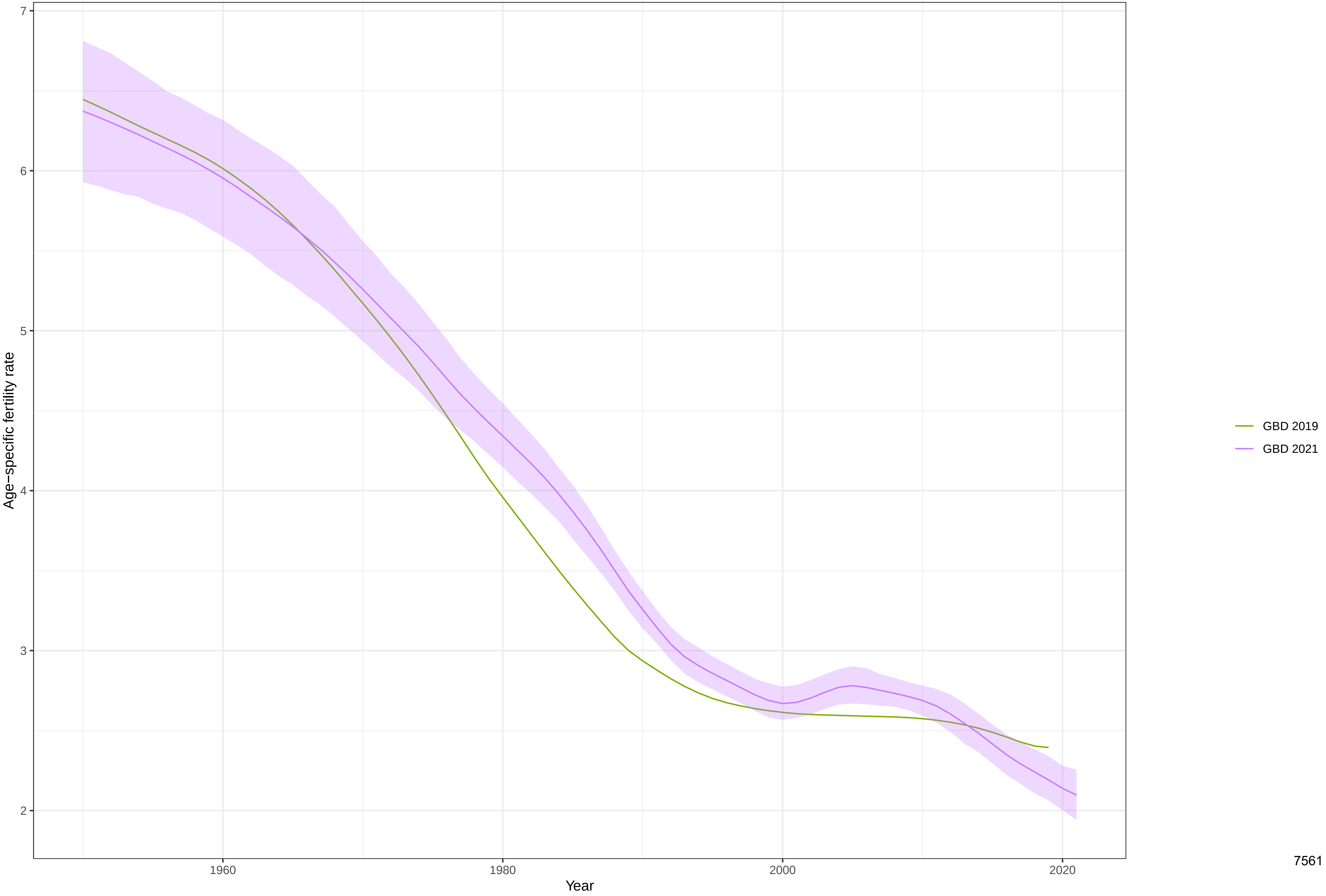

Age−specific fertility fitted trend lines, 1950−2021: Northern Cape, Ages: 10−14
Data points enclosed with a diamond are reference sources

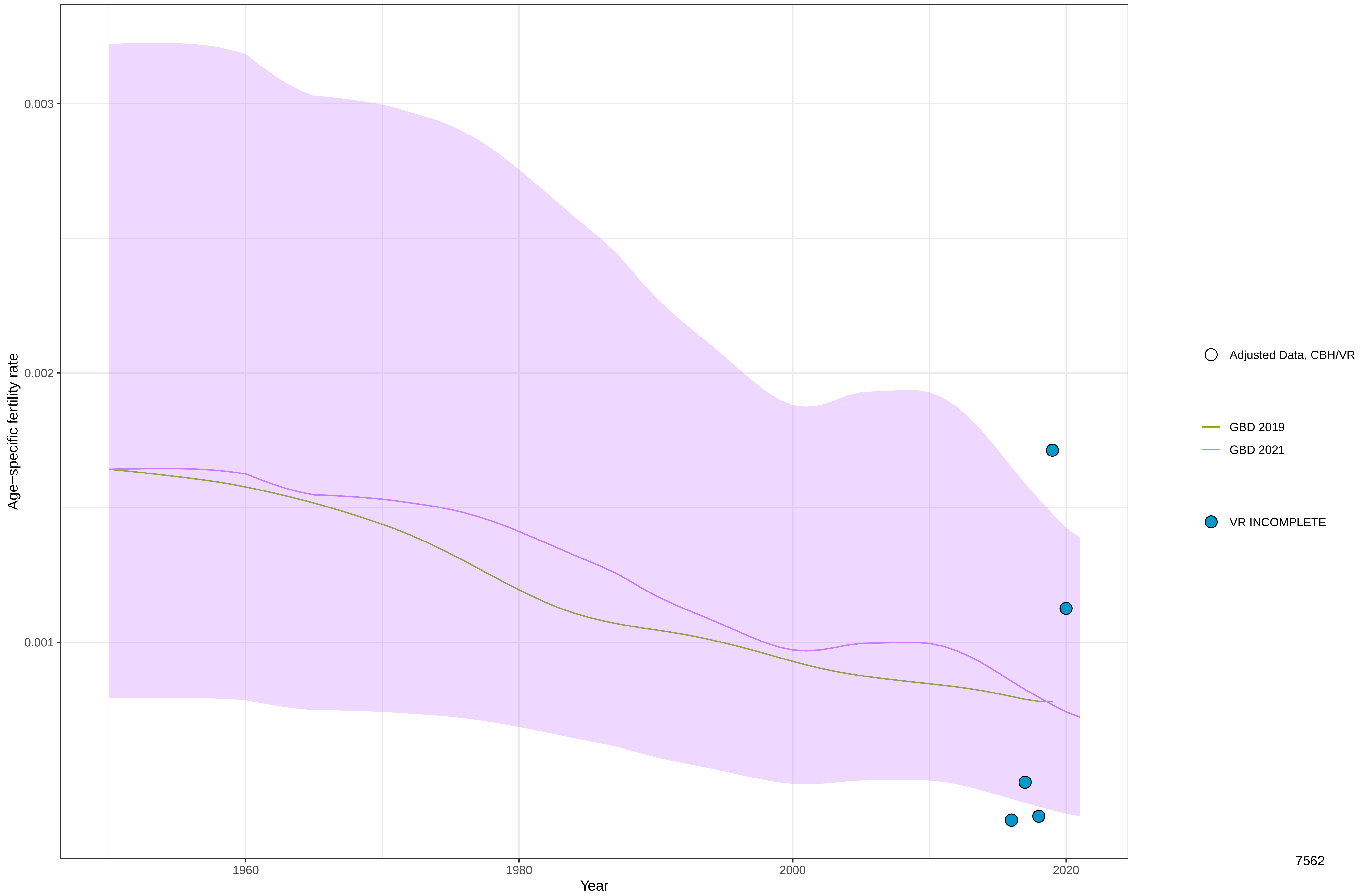

7562

Age−specific fertility fitted trend lines, 1950−2021: Northern Cape, Ages: 15−19
Data points enclosed with a diamond are reference sources

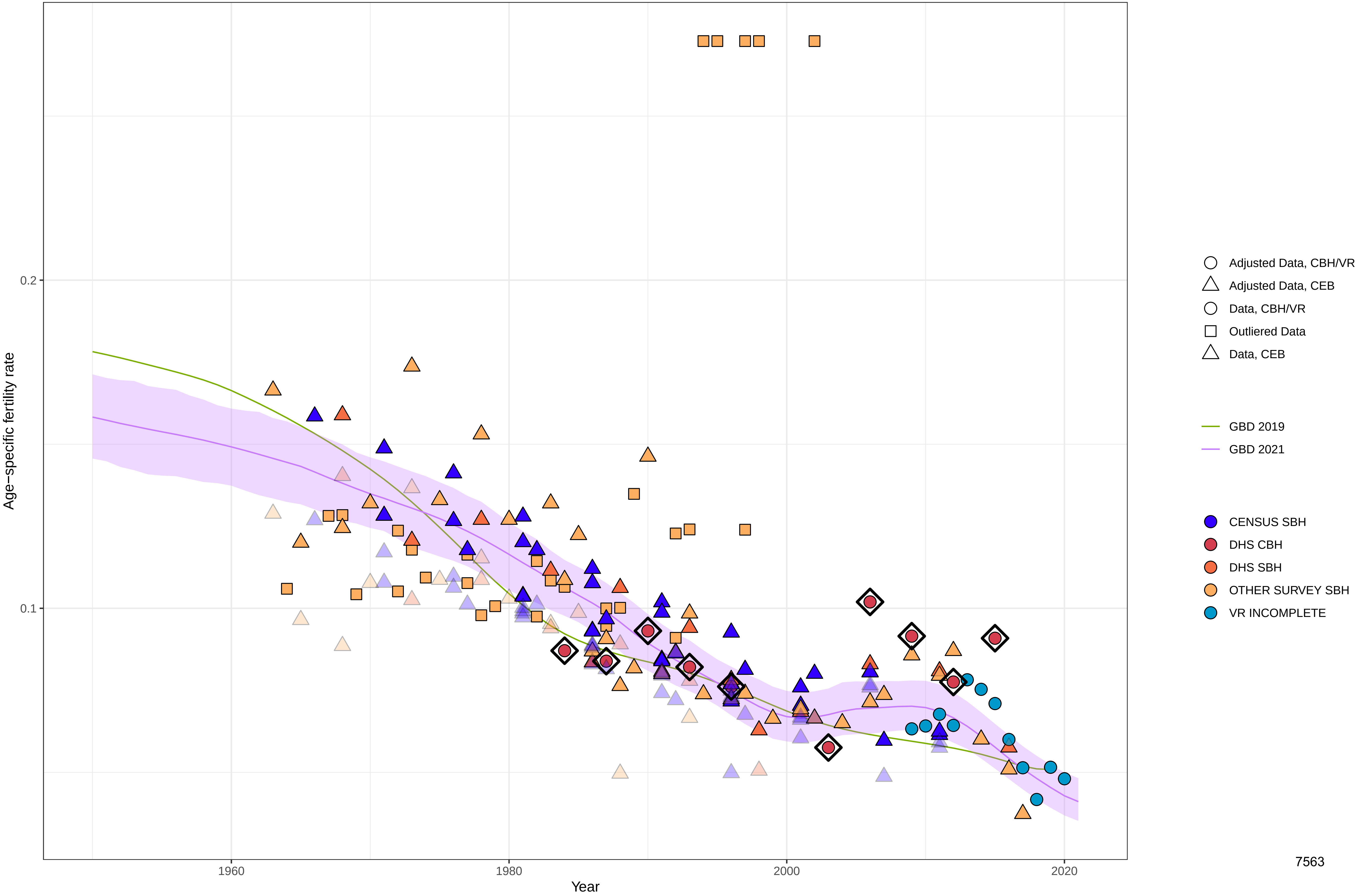

Age−specific fertility fitted trend lines, 1950−2021: Northern Cape, Ages: 20−24
Data points enclosed with a diamond are reference sources

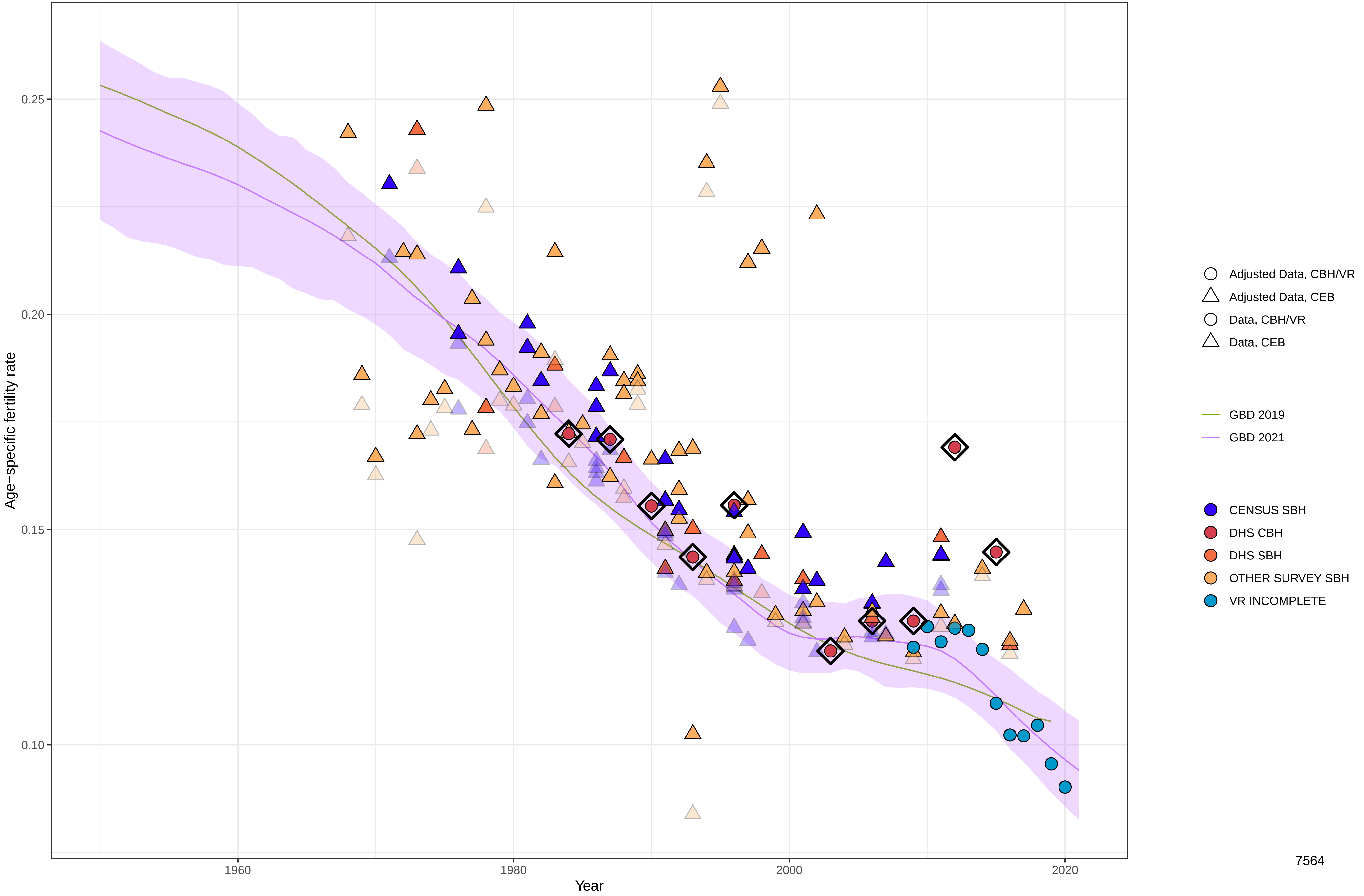

Age−specific fertility fitted trend lines, 1950−2021: Northern Cape, Ages: 25−29
Data points enclosed with a diamond are reference sources

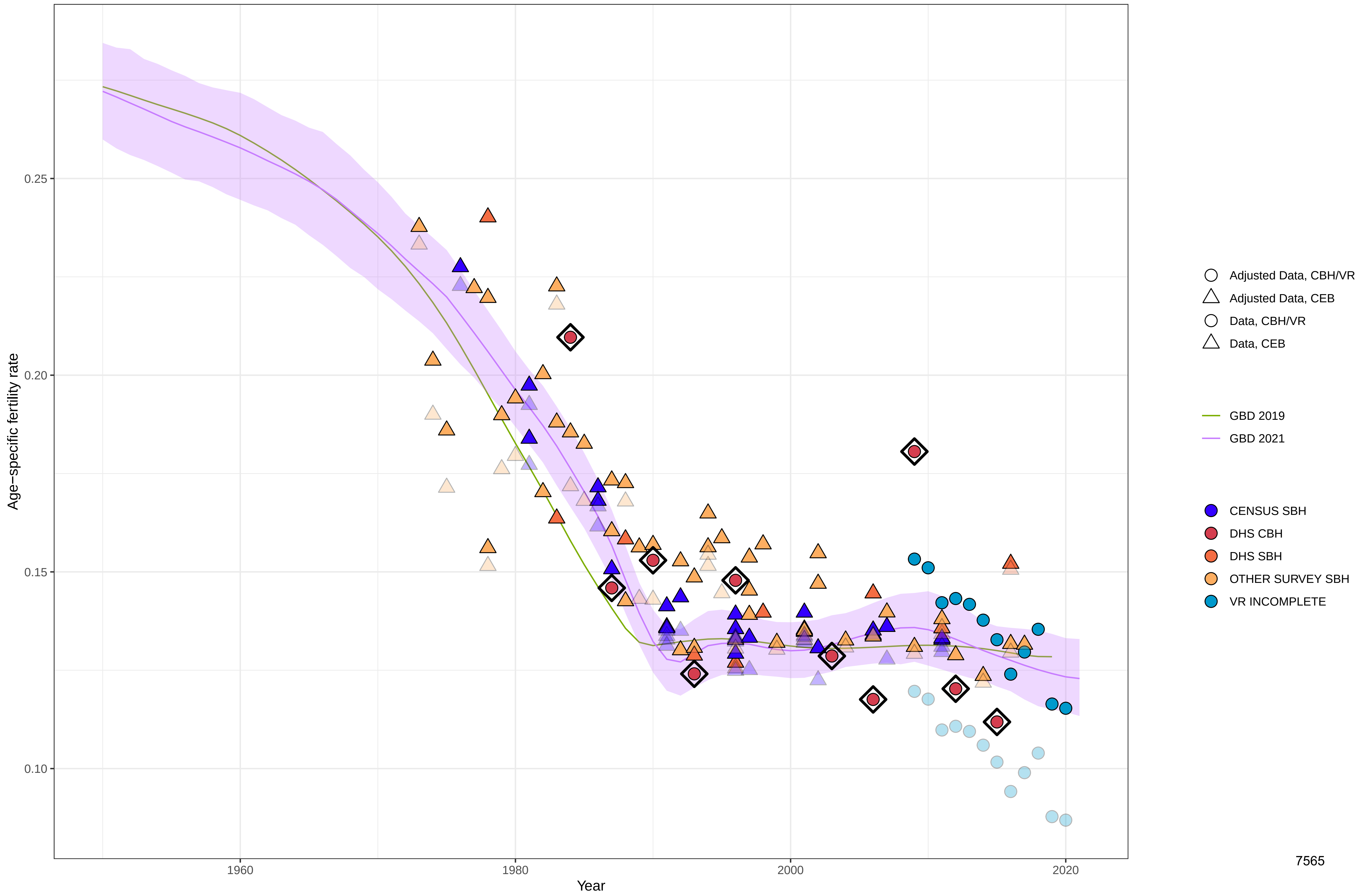

Age−specific fertility fitted trend lines, 1950−2021: Northern Cape, Ages: 30−34
Data points enclosed with a diamond are reference sources

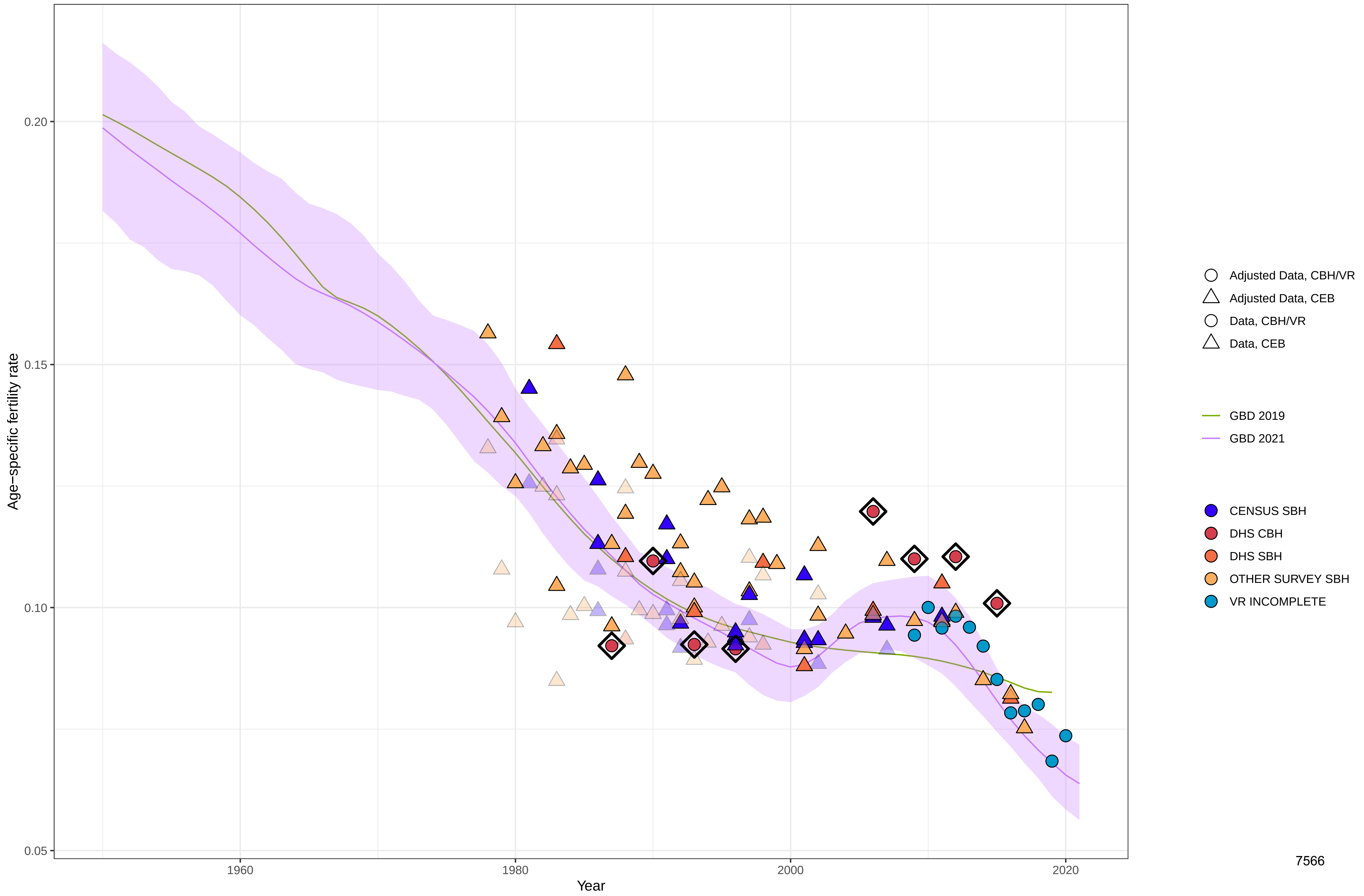

Age−specific fertility fitted trend lines, 1950−2021: Northern Cape, Ages: 35−39
Data points enclosed with a diamond are reference sources

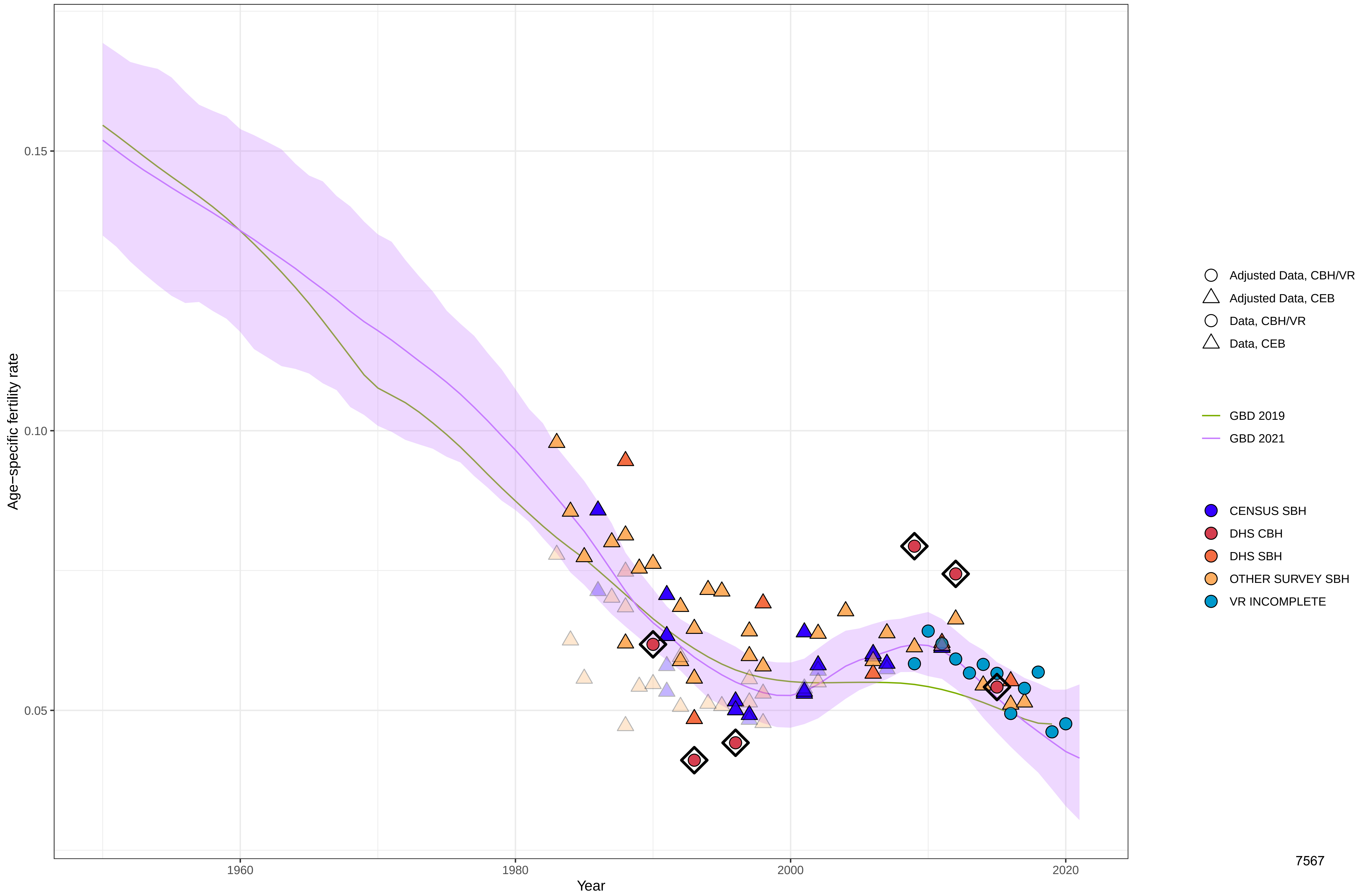

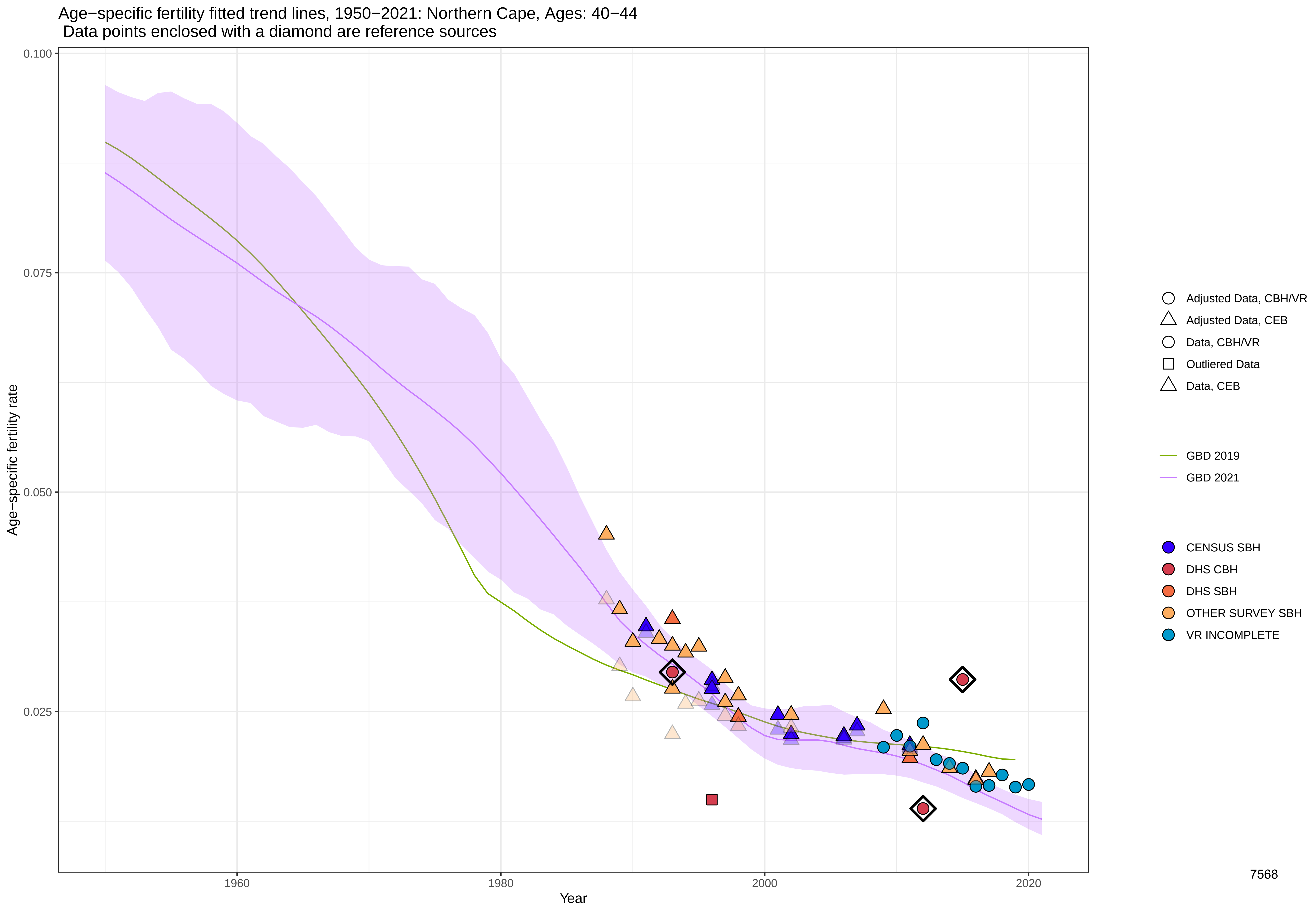

Age−specific fertility fitted trend lines, 1950−2021: Northern Cape, Ages: 40−44
Data points enclosed with a diamond are reference sources
Age−specific fertility rate
0.100
0.075
0.050
0.025
1960
1980
2000
2020
Year
Adjusted Data, CBH/VR
Adjusted Data, CEB
Data, CBH/VR
Outliered Data
Data, CEB
GBD 2019
GBD 2021
CENSUS SBH
DHS CBH
DHS SBH
OTHER SURVEY SBH
VR INCOMPLETE

7568

Age−specific fertility fitted trend lines, 1950−2021: Northern Cape, Ages: 45−49
Data points enclosed with a diamond are reference sources

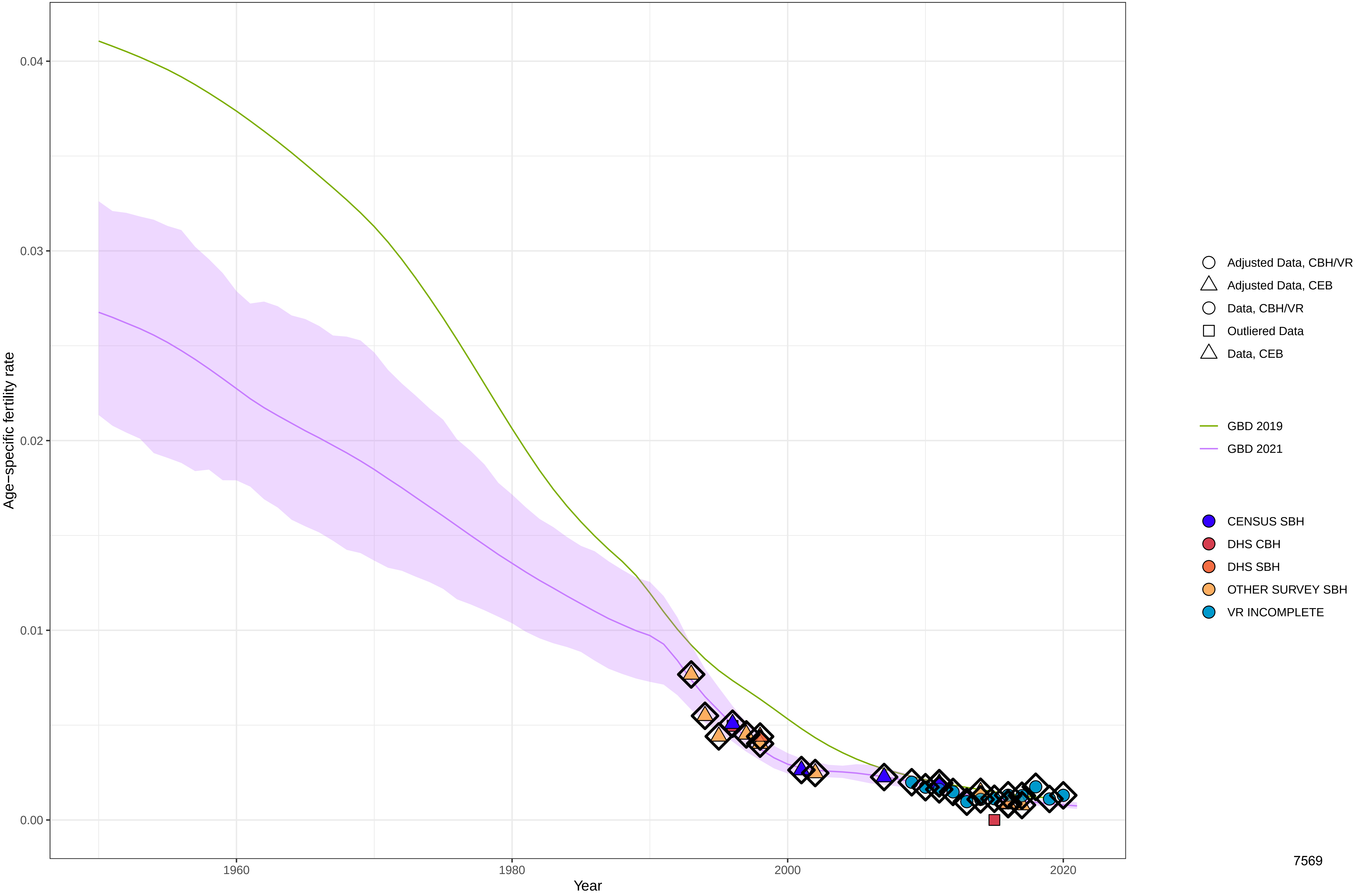

Age−specific fertility fitted trend lines, 1950−2021: Northern Cape, Ages: 50−54
Data points enclosed with a diamond are reference sources

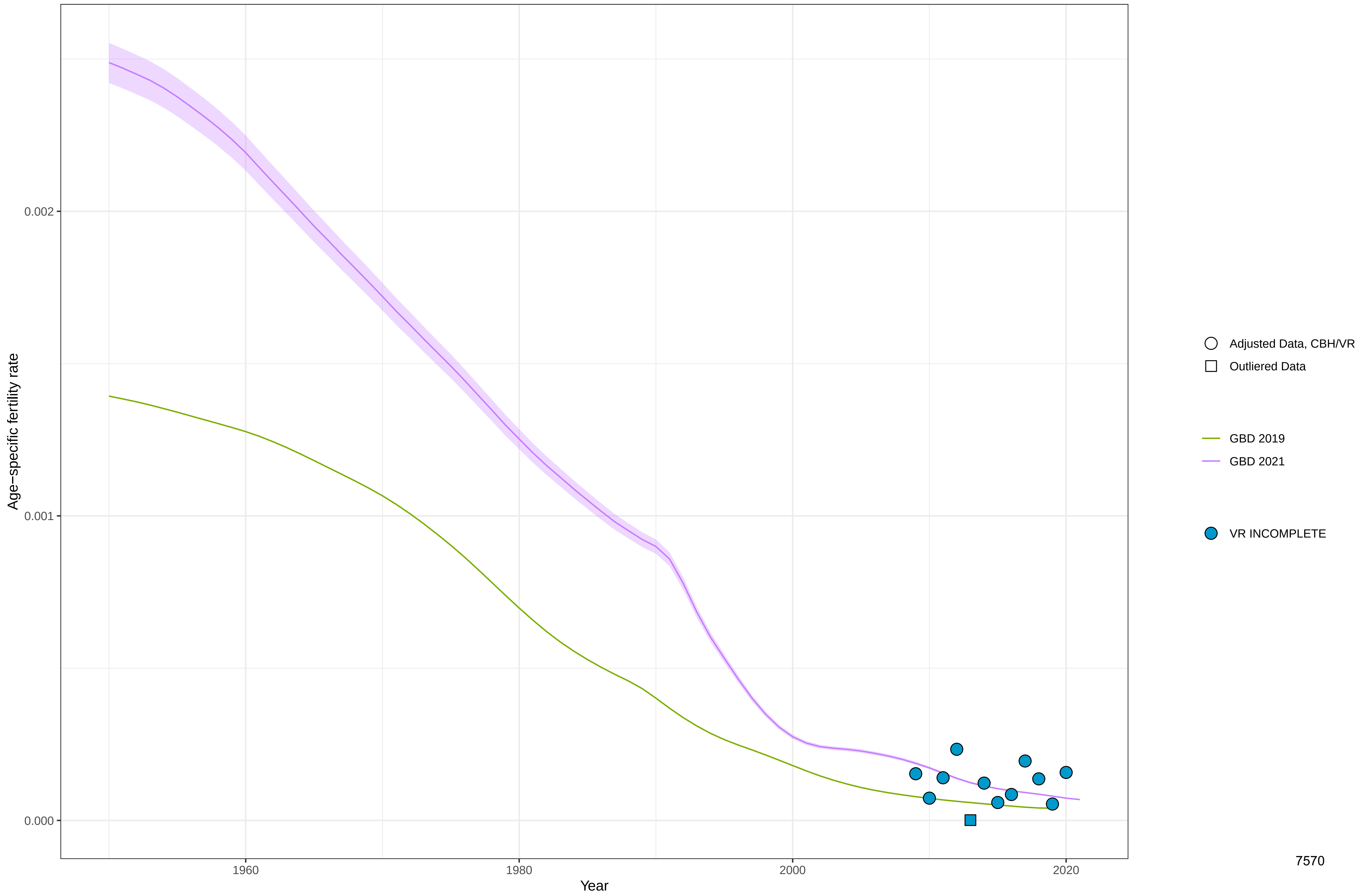

Total fertility rate fitted trend lines, 1950−2021: Northern Cape TFR
Data points enclosed with a diamond are reference sources

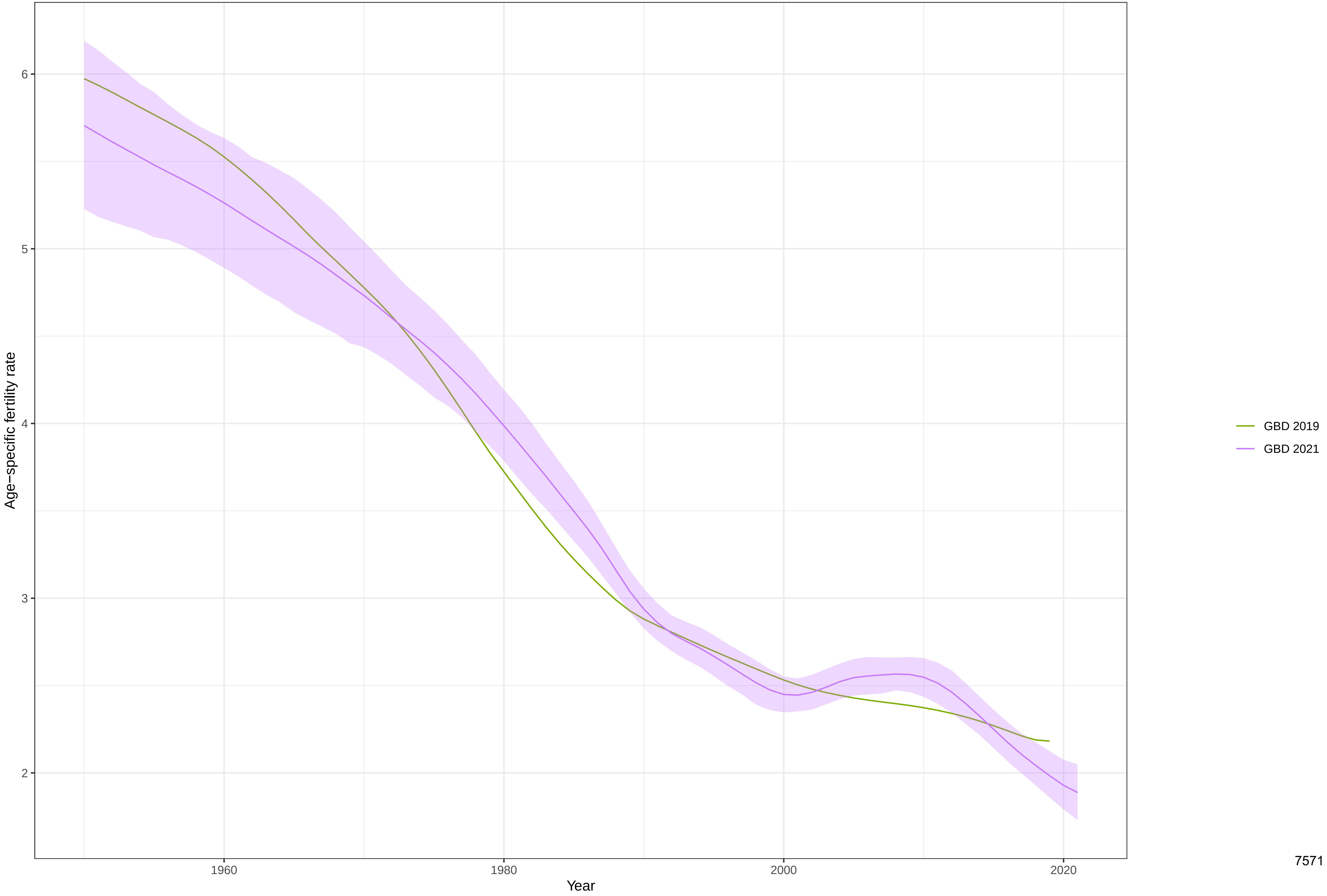

Age−specific fertility fitted trend lines, 1950−2021: Western Cape, Ages: 10−14
Data points enclosed with a diamond are reference sources

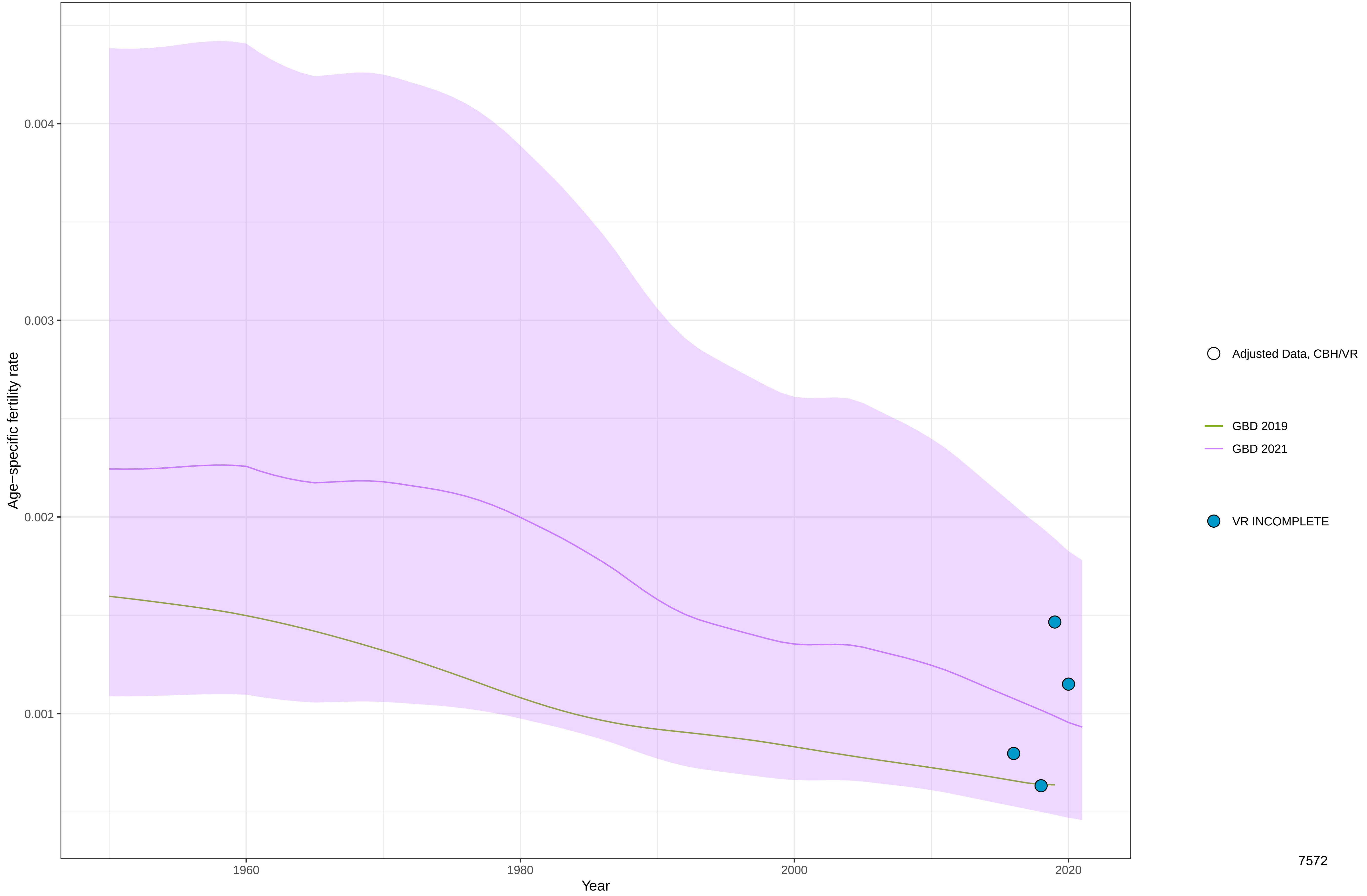

Age−specific fertility fitted trend lines, 1950−2021: Western Cape, Ages: 15−19
Data points enclosed with a diamond are reference sources

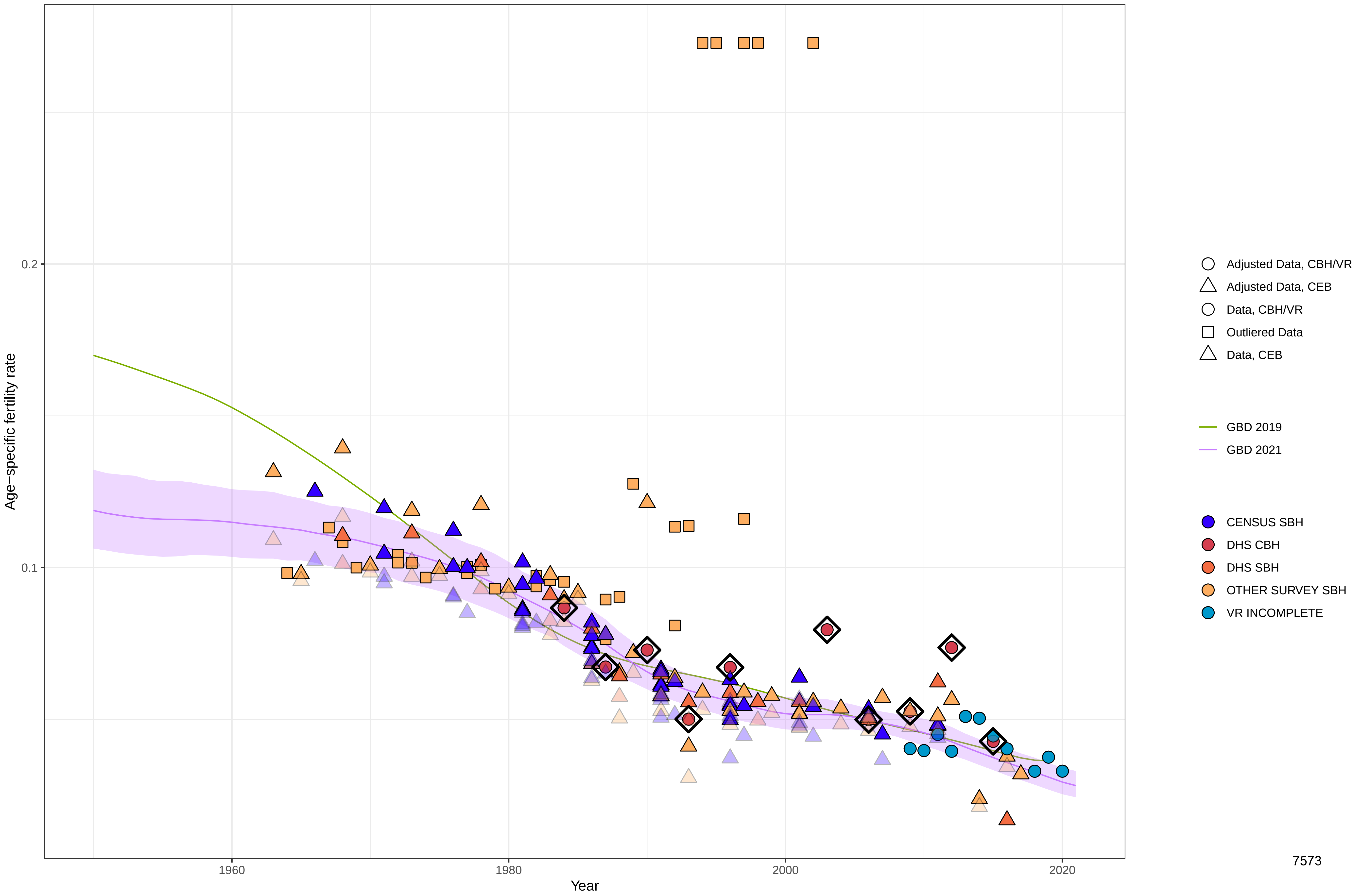

Age−specific fertility fitted trend lines, 1950−2021: Western Cape, Ages: 20−24
Data points enclosed with a diamond are reference sources

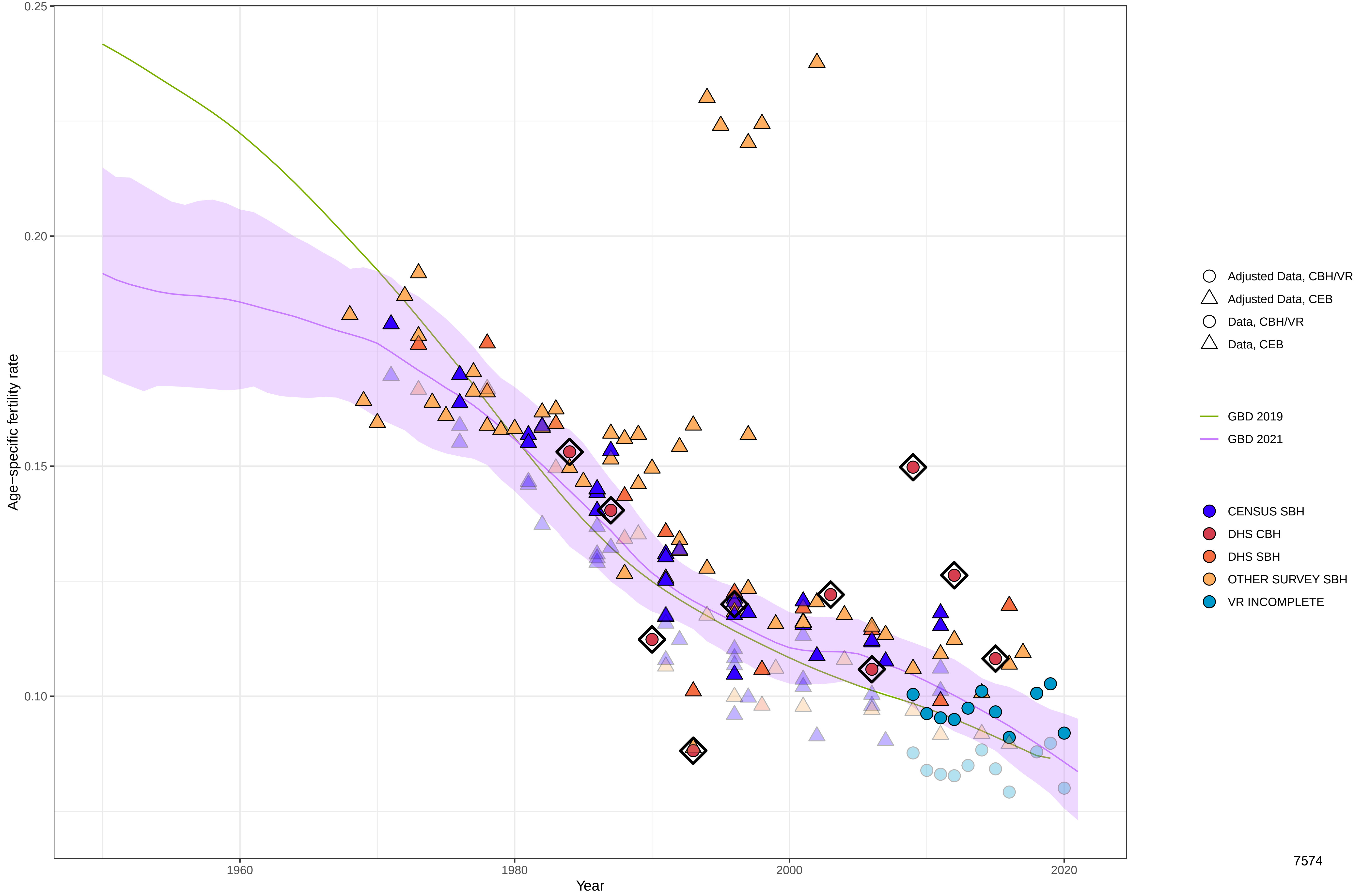

Age−specific fertility fitted trend lines, 1950−2021: Western Cape, Ages: 25−29
Data points enclosed with a diamond are reference sources

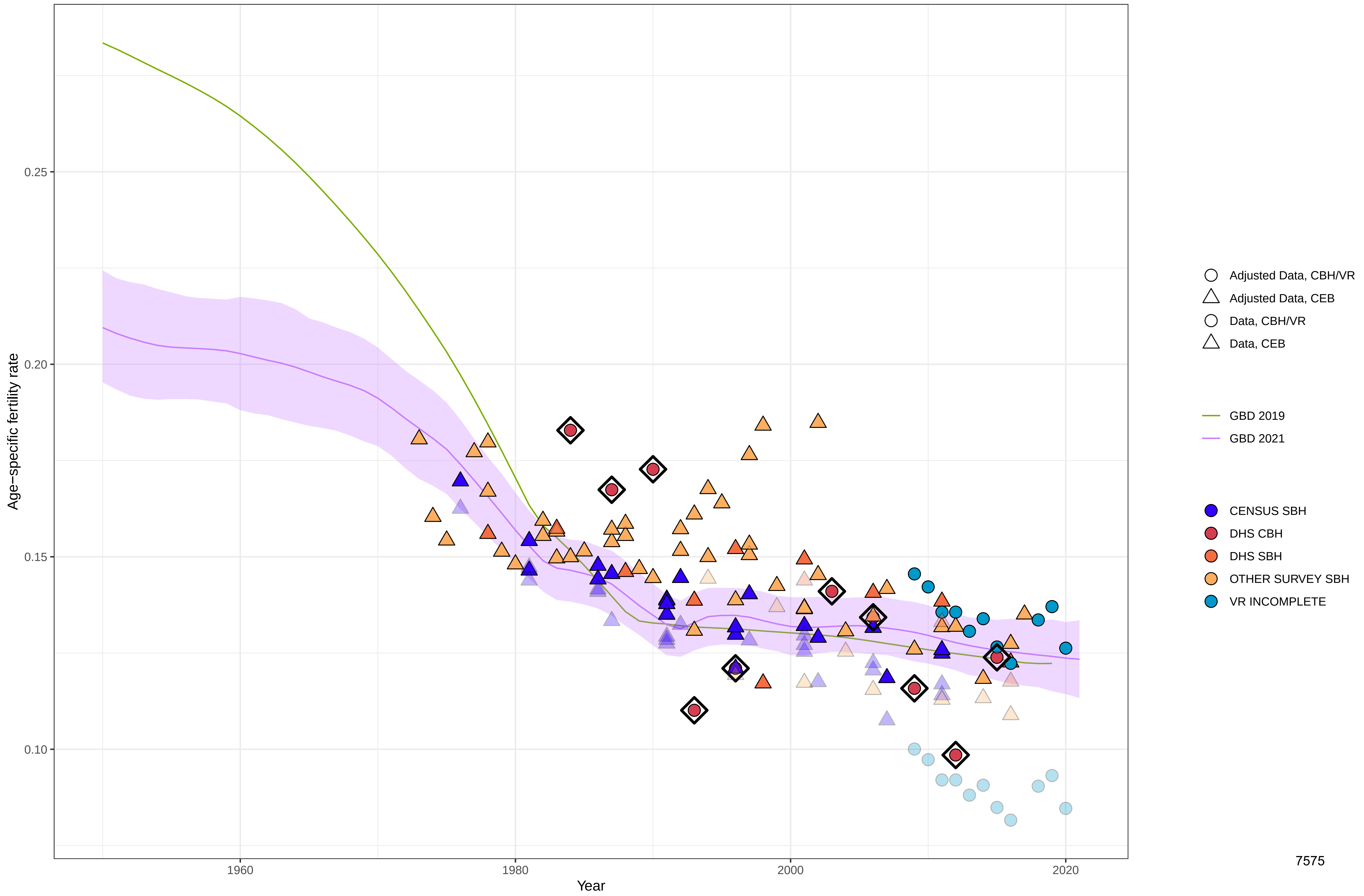

Age−specific fertility fitted trend lines, 1950−2021: Western Cape, Ages: 30−34
Data points enclosed with a diamond are reference sources

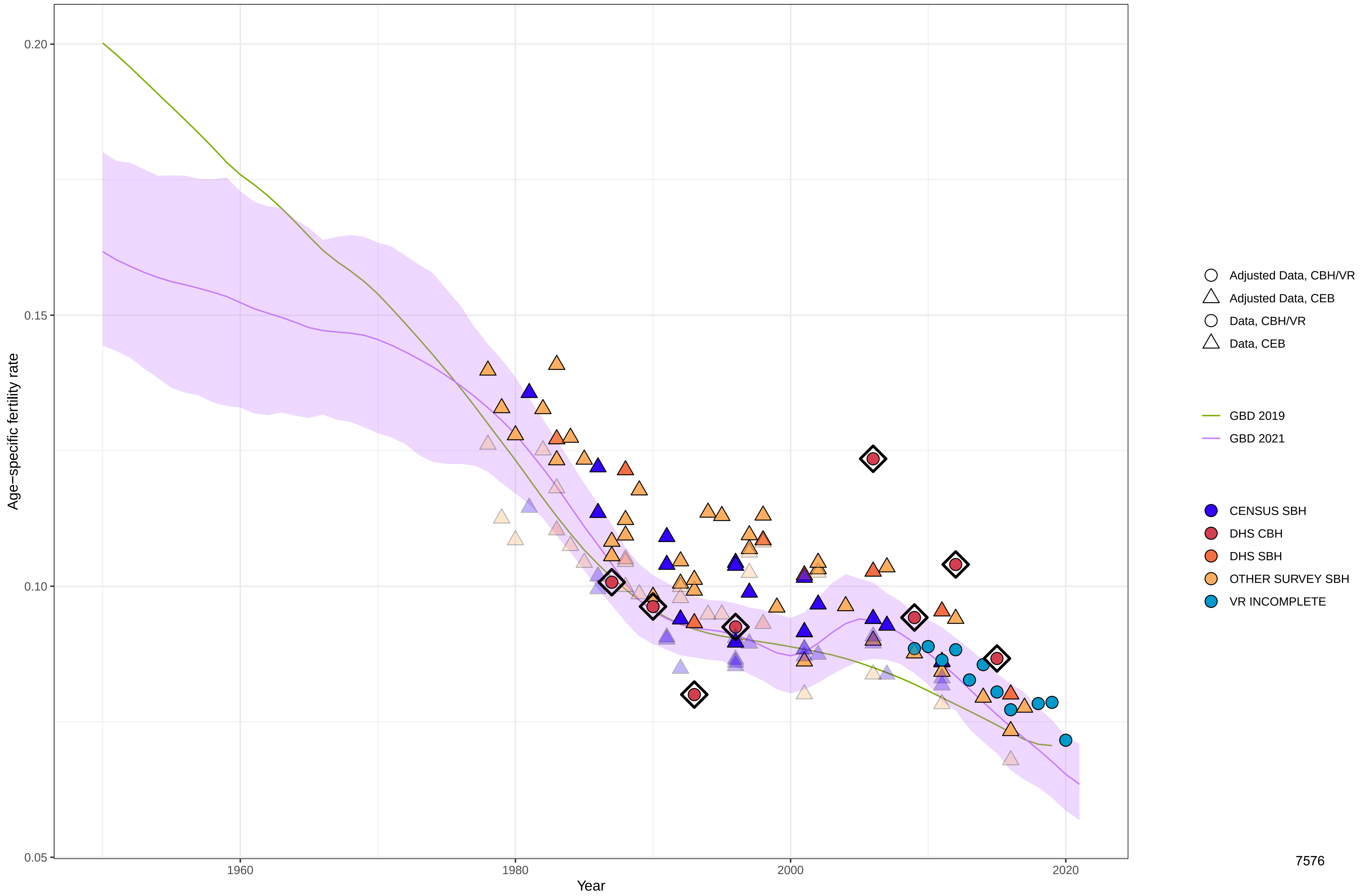

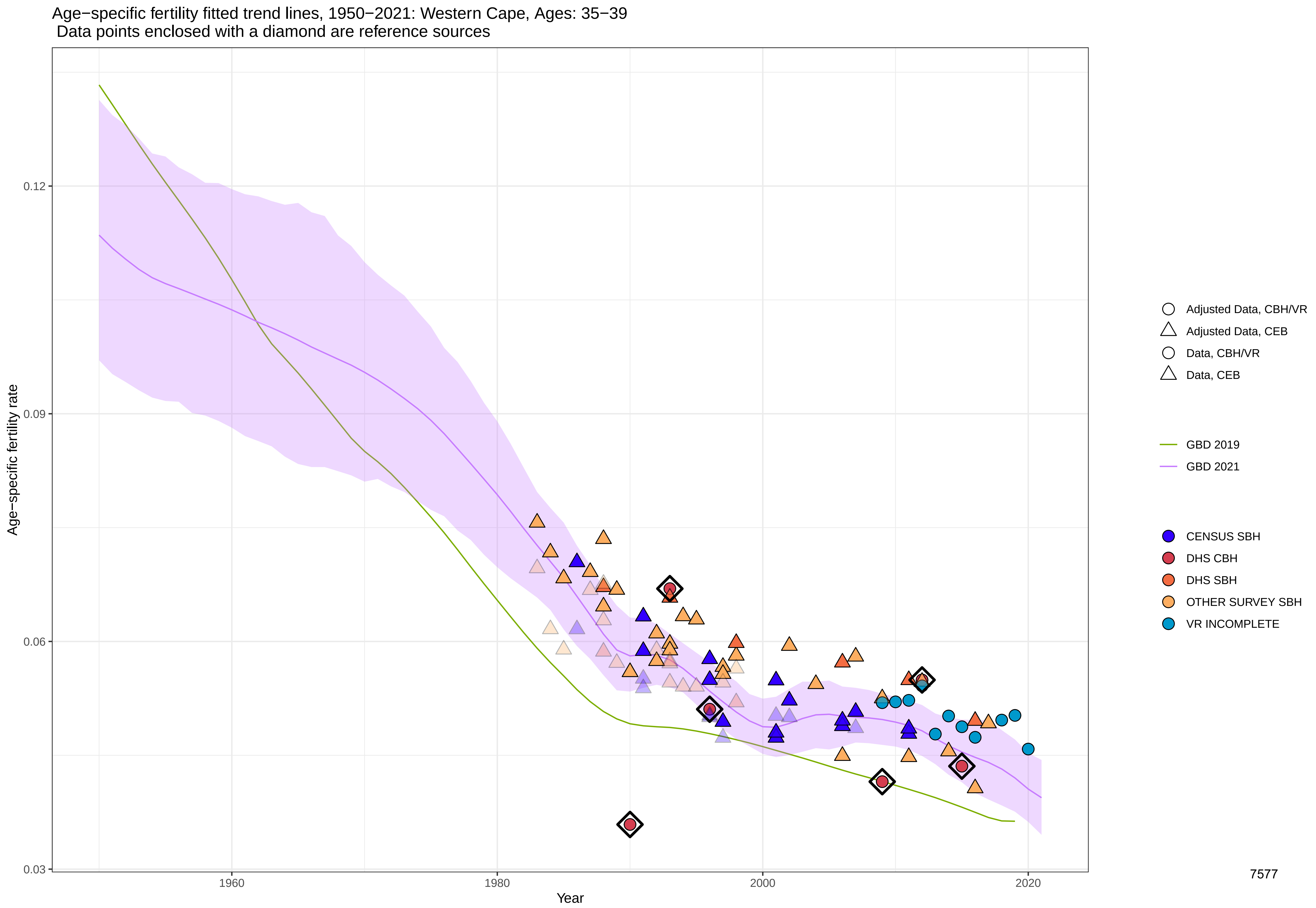

Age−specific fertility fitted trend lines, 1950−2021: Western Cape, Ages: 35−39
Data points enclosed with a diamond are reference sources
Age−specific fertility rate
0.12
0.09
0.06
0.03
1960
1980
2000
2020
Year
Adjusted Data, CBH/VR
Adjusted Data, CEB
Data, CBH/VR
Data, CEB
GBD 2019
GBD 2021
CENSUS SBH
DHS CBH
DHS SBH
OTHER SURVEY SBH
VR INCOMPLETE

Age−specific fertility fitted trend lines, 1950−2021: Western Cape, Ages: 40−44
Data points enclosed with a diamond are reference sources

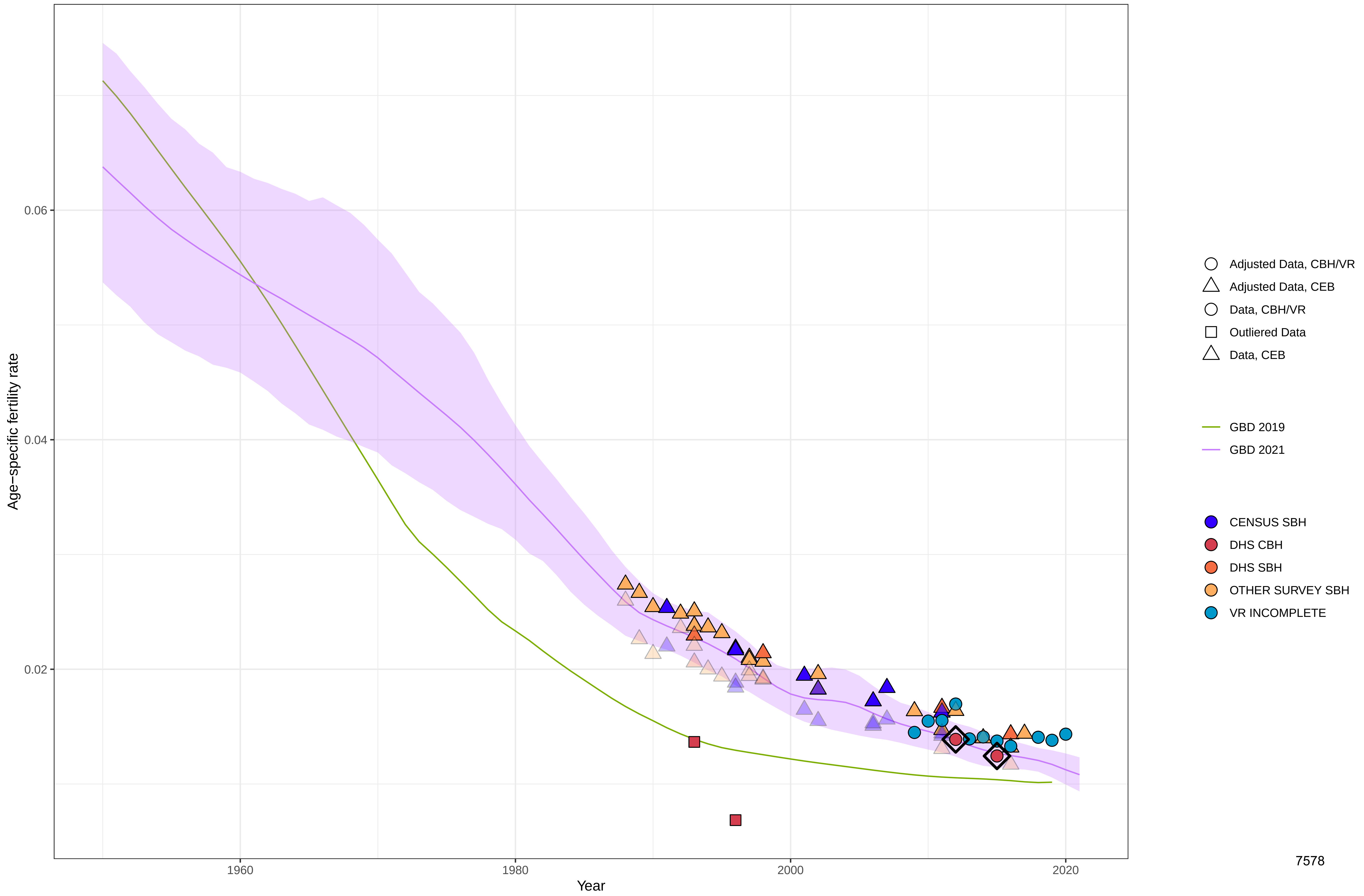

Age−specific fertility fitted trend lines, 1950−2021: Western Cape, Ages: 45−49
Data points enclosed with a diamond are reference sources

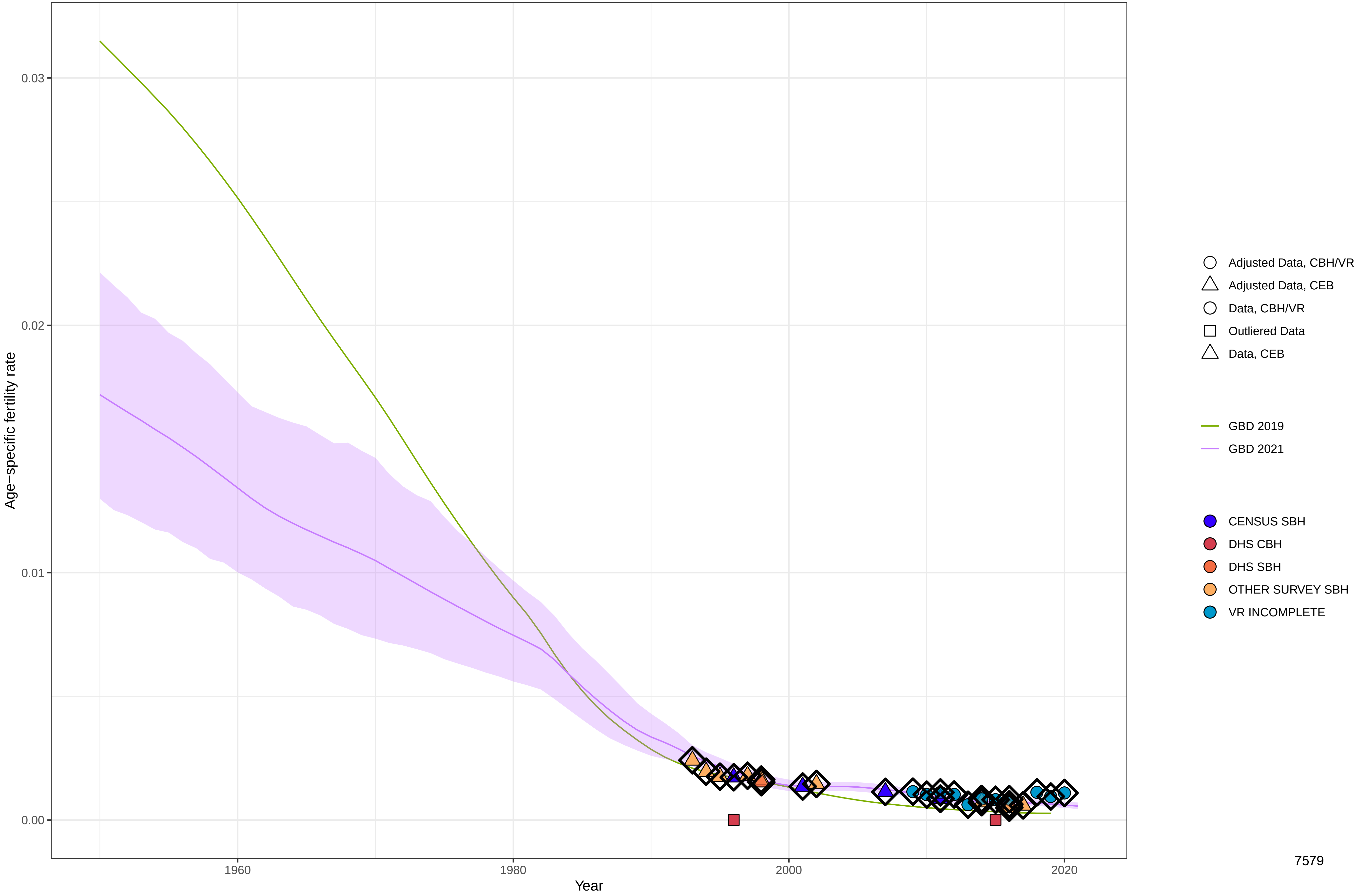

Age−specific fertility fitted trend lines, 1950−2021: Western Cape, Ages: 50−54
Data points enclosed with a diamond are reference sources

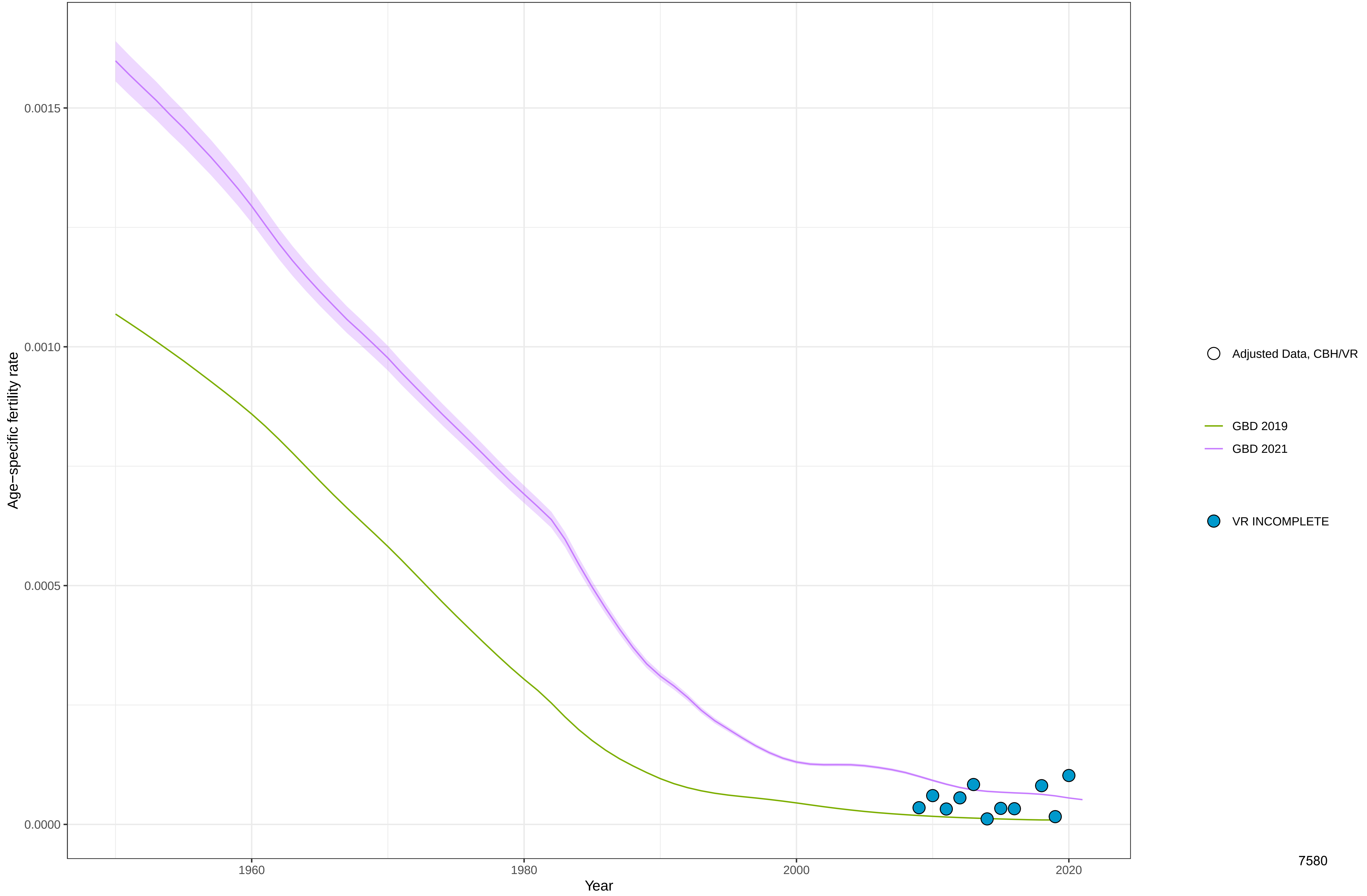

Total fertility rate fitted trend lines, 1950−2021: Western Cape TFR
Data points enclosed with a diamond are reference sources

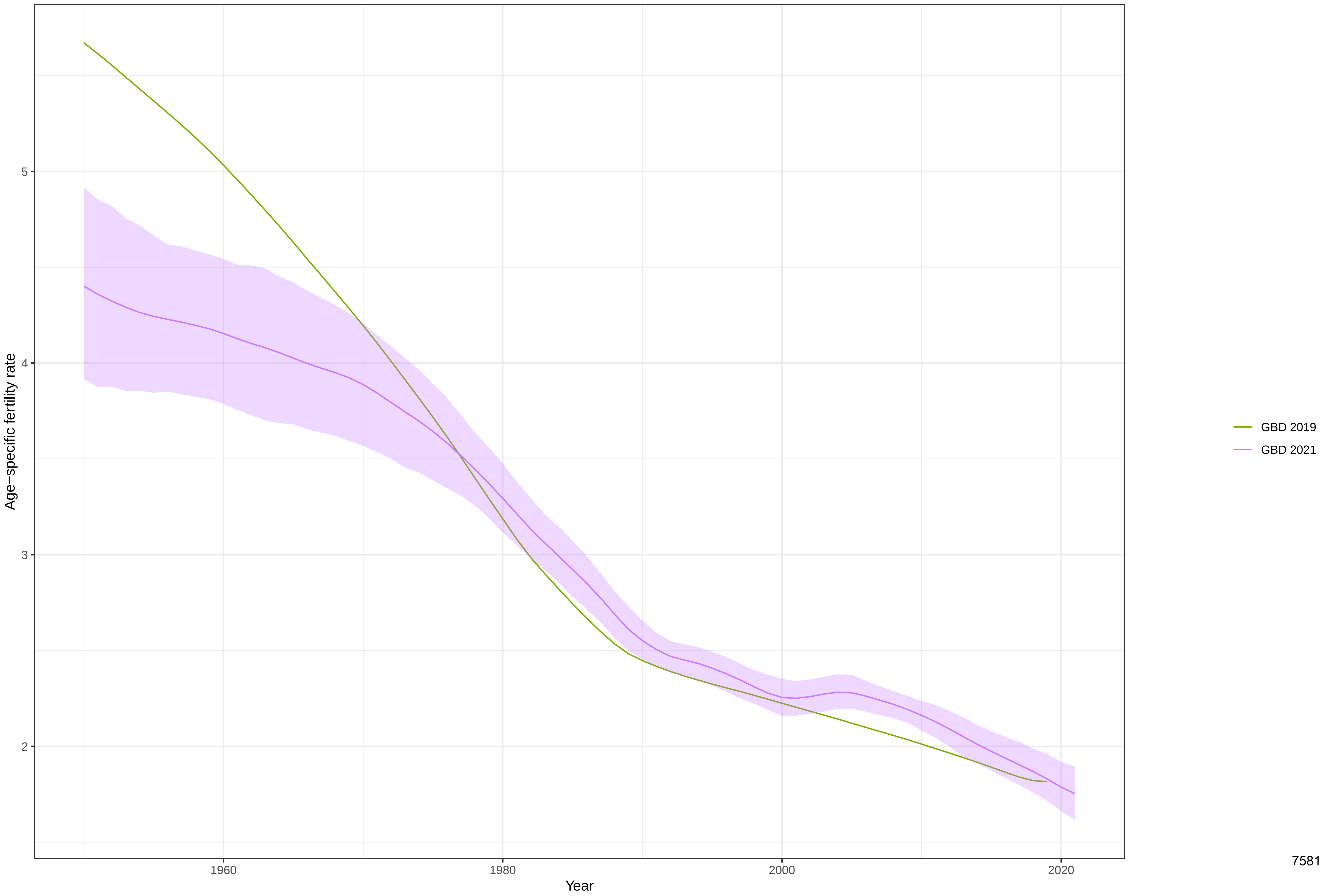

Age−specific fertility fitted trend lines, 1950−2021: Zimbabwe, Ages: 10−14
Data points enclosed with a diamond are reference sources

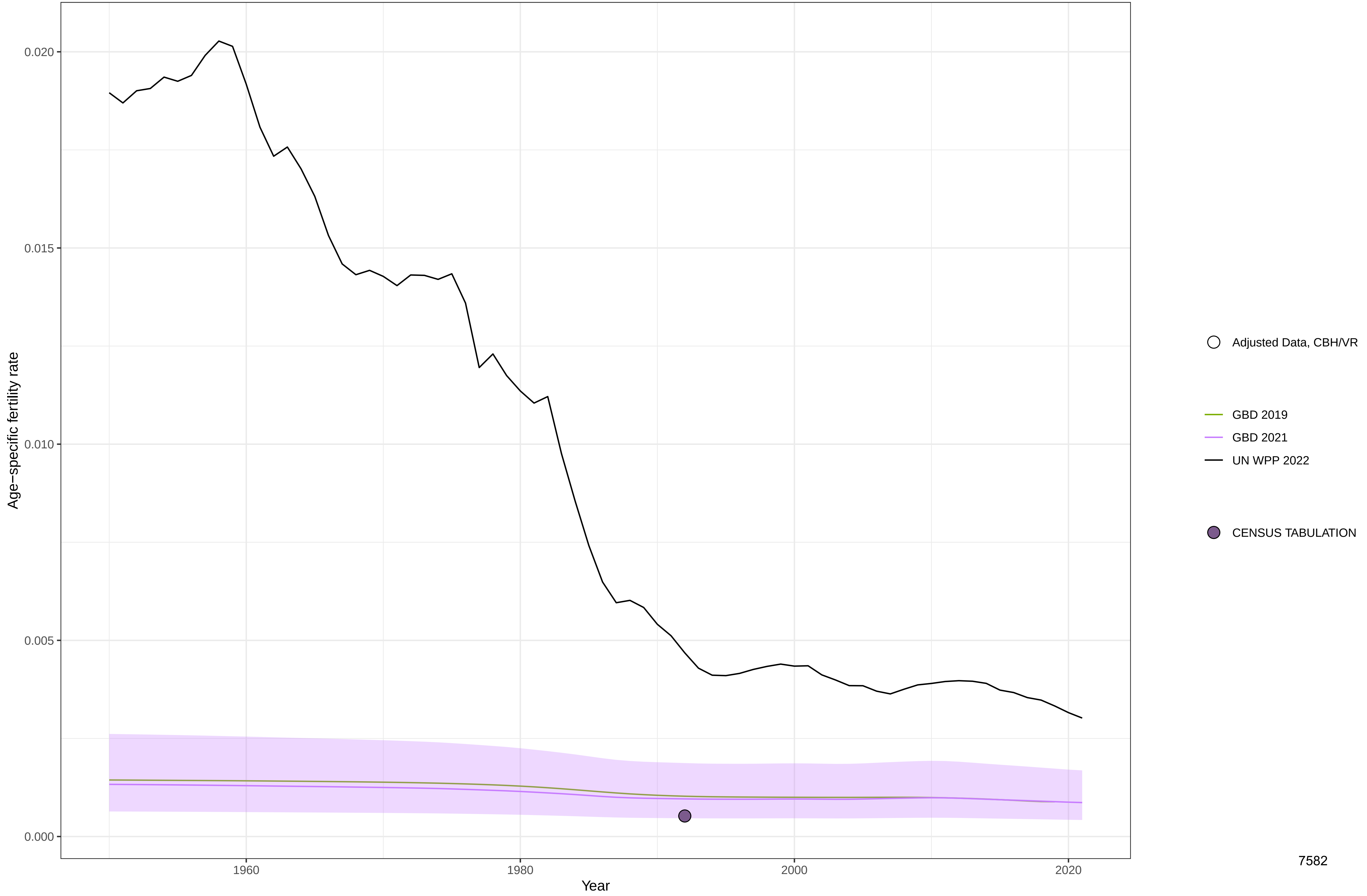

Age−specific fertility fitted trend lines, 1950−2021: Zimbabwe, Ages: 15−19
Data points enclosed with a diamond are reference sources

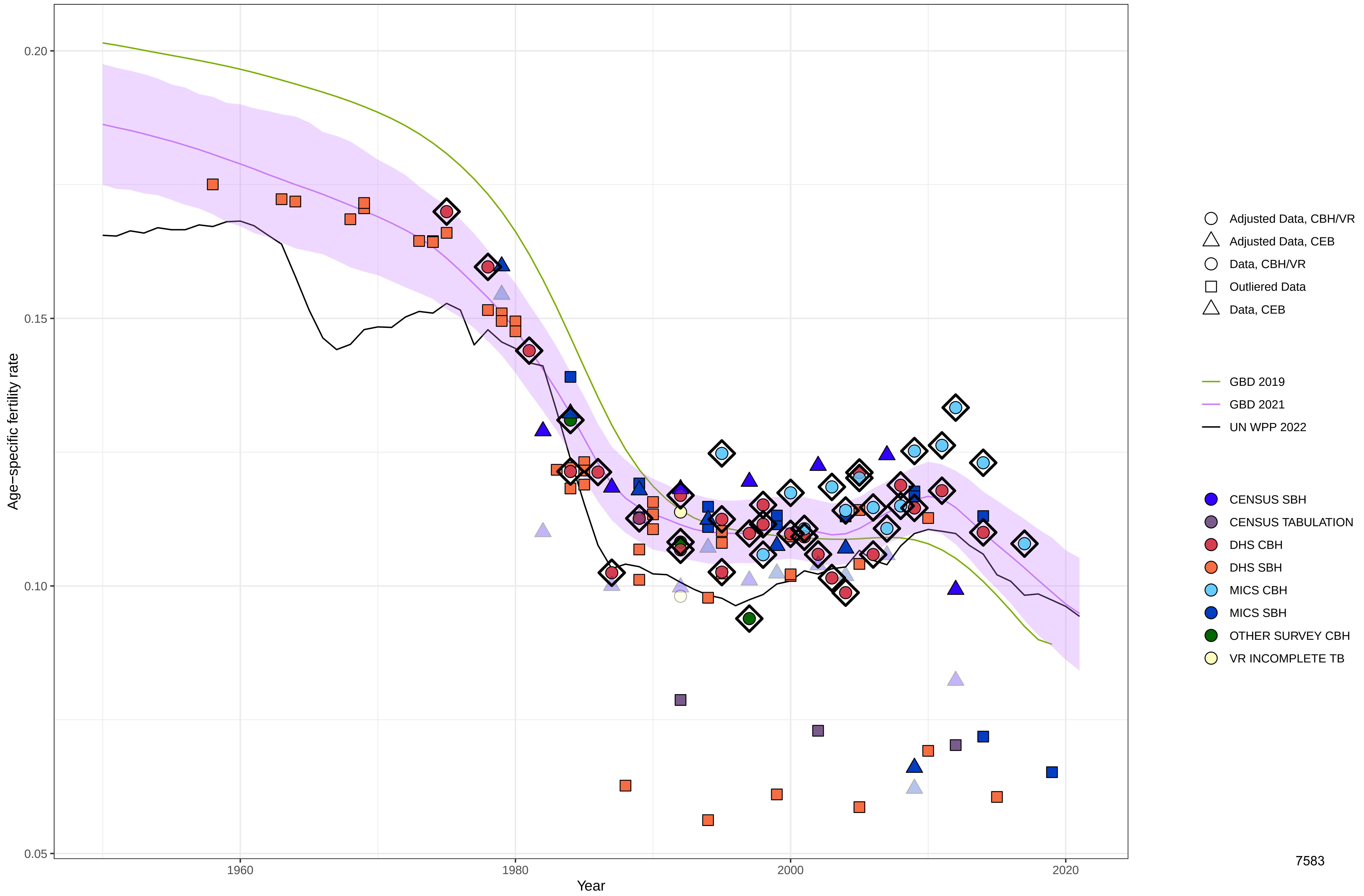

Age−specific fertility fitted trend lines, 1950−2021: Zimbabwe, Ages: 20−24
Data points enclosed with a diamond are reference sources

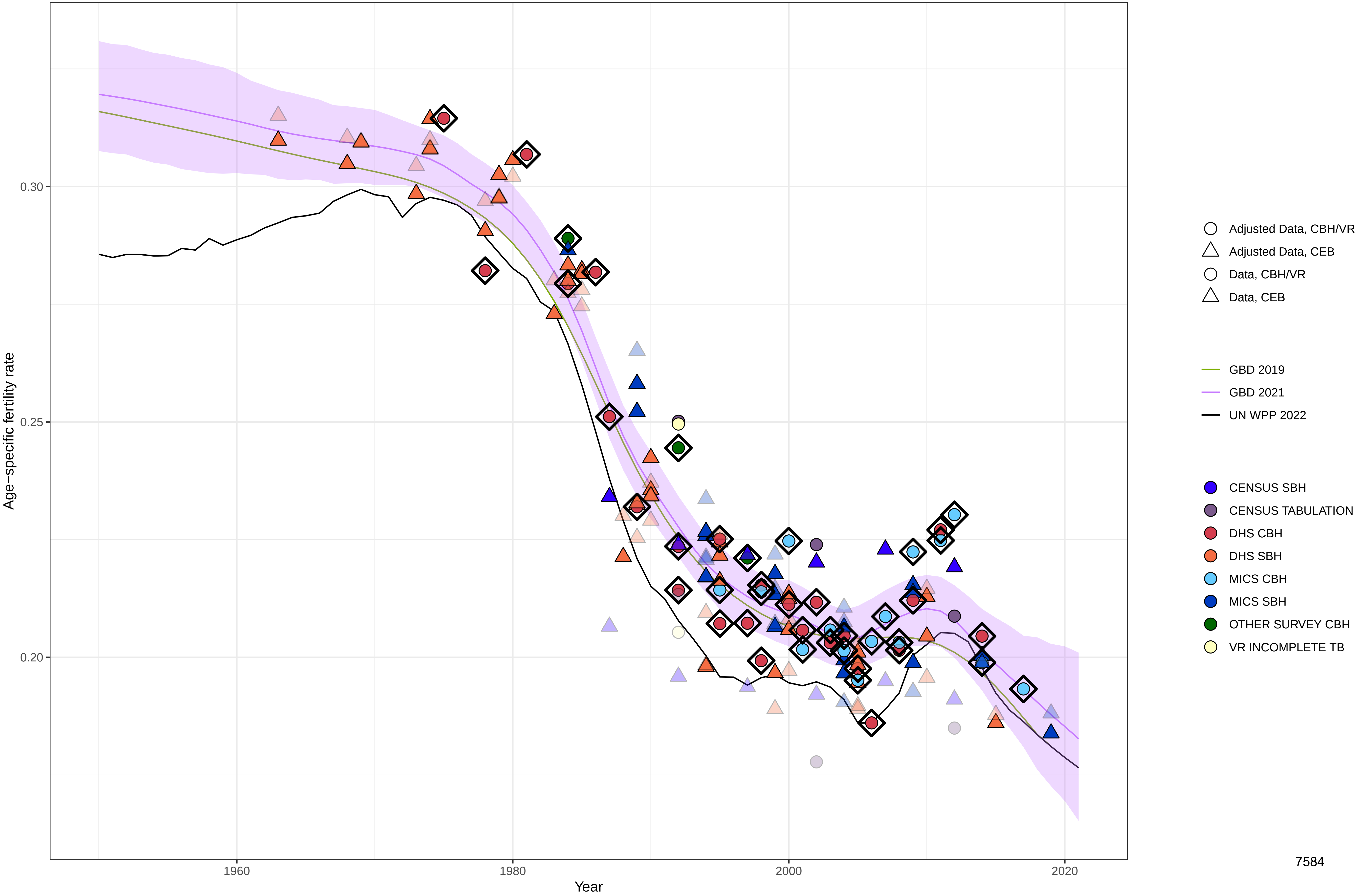

Age−specific fertility fitted trend lines, 1950−2021: Zimbabwe, Ages: 25−29
Data points enclosed with a diamond are reference sources

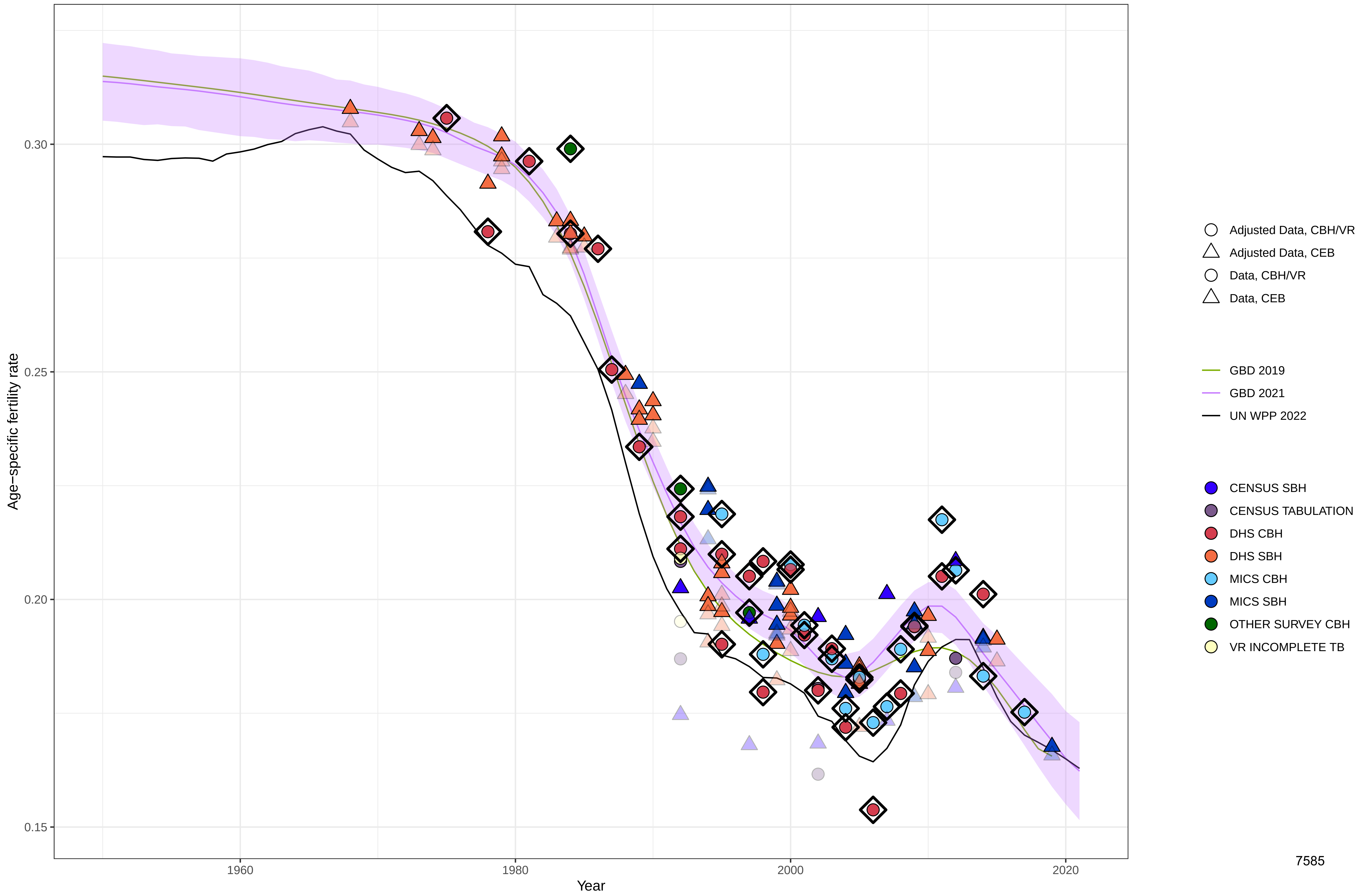

Age−specific fertility fitted trend lines, 1950−2021: Zimbabwe, Ages: 30−34
Data points enclosed with a diamond are reference sources

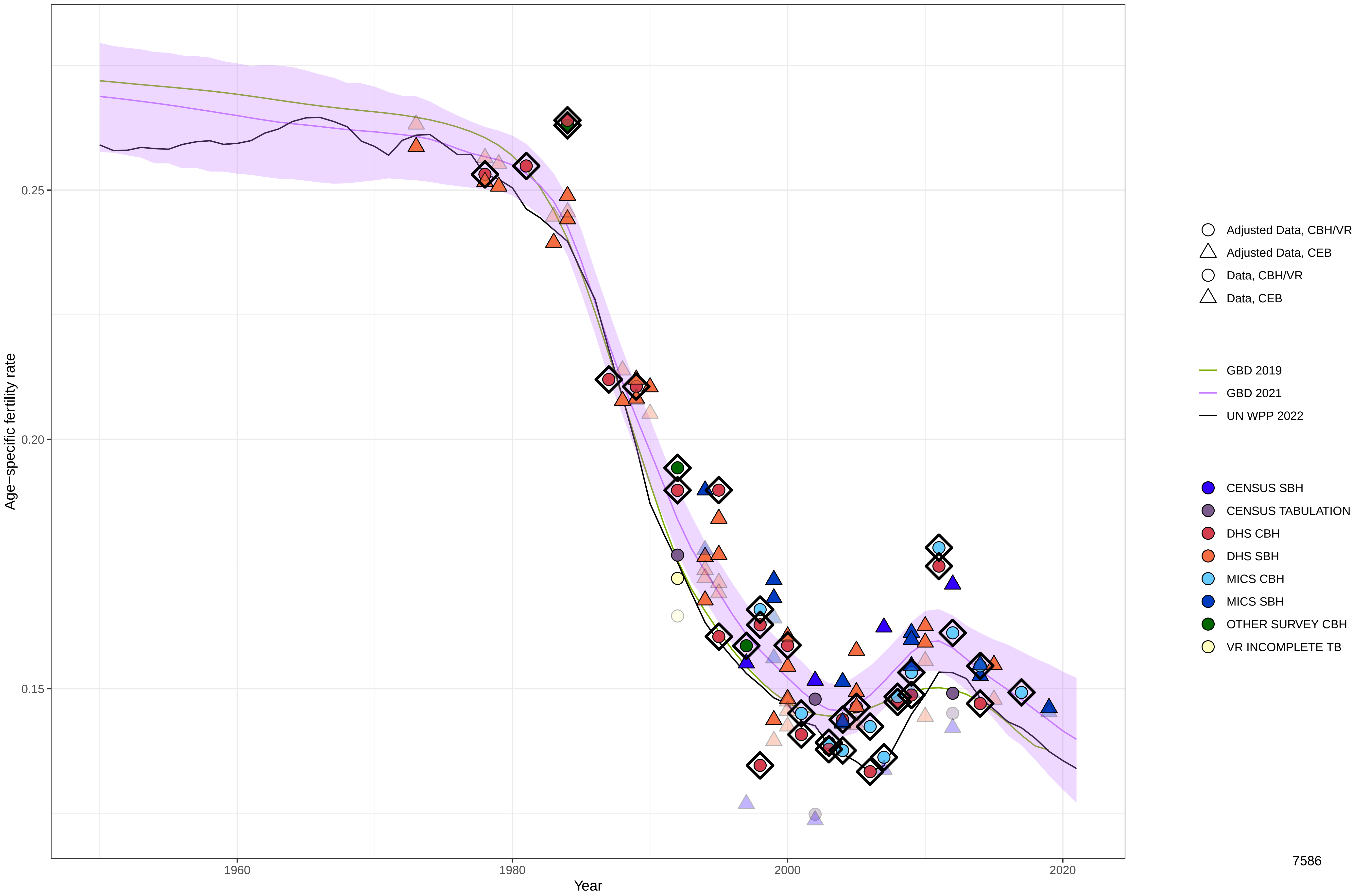

Age−specific fertility fitted trend lines, 1950−2021: Zimbabwe, Ages: 35−39
Data points enclosed with a diamond are reference sources

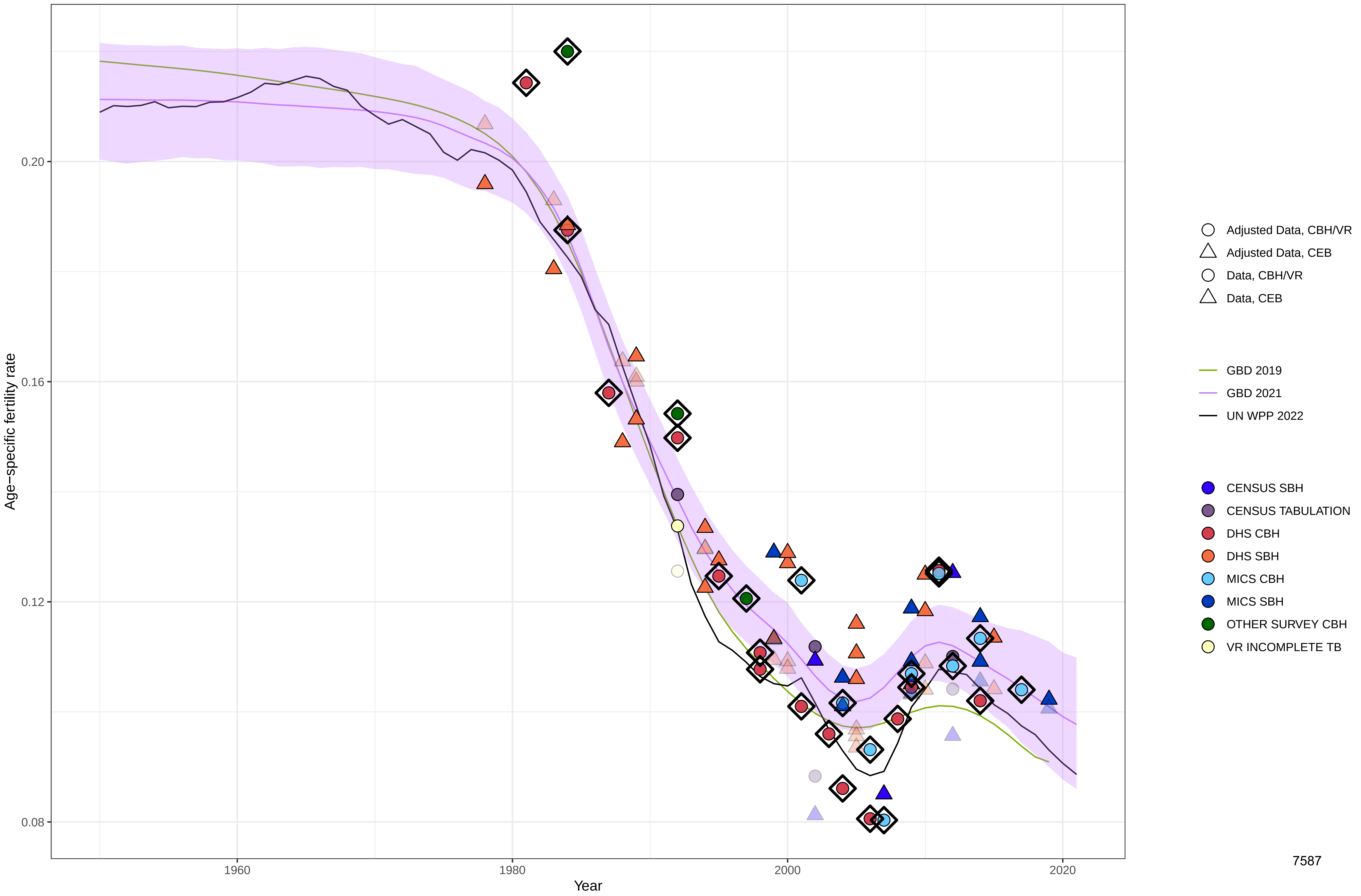

Age−specific fertility fitted trend lines, 1950−2021: Zimbabwe, Ages: 40−44
Data points enclosed with a diamond are reference sources

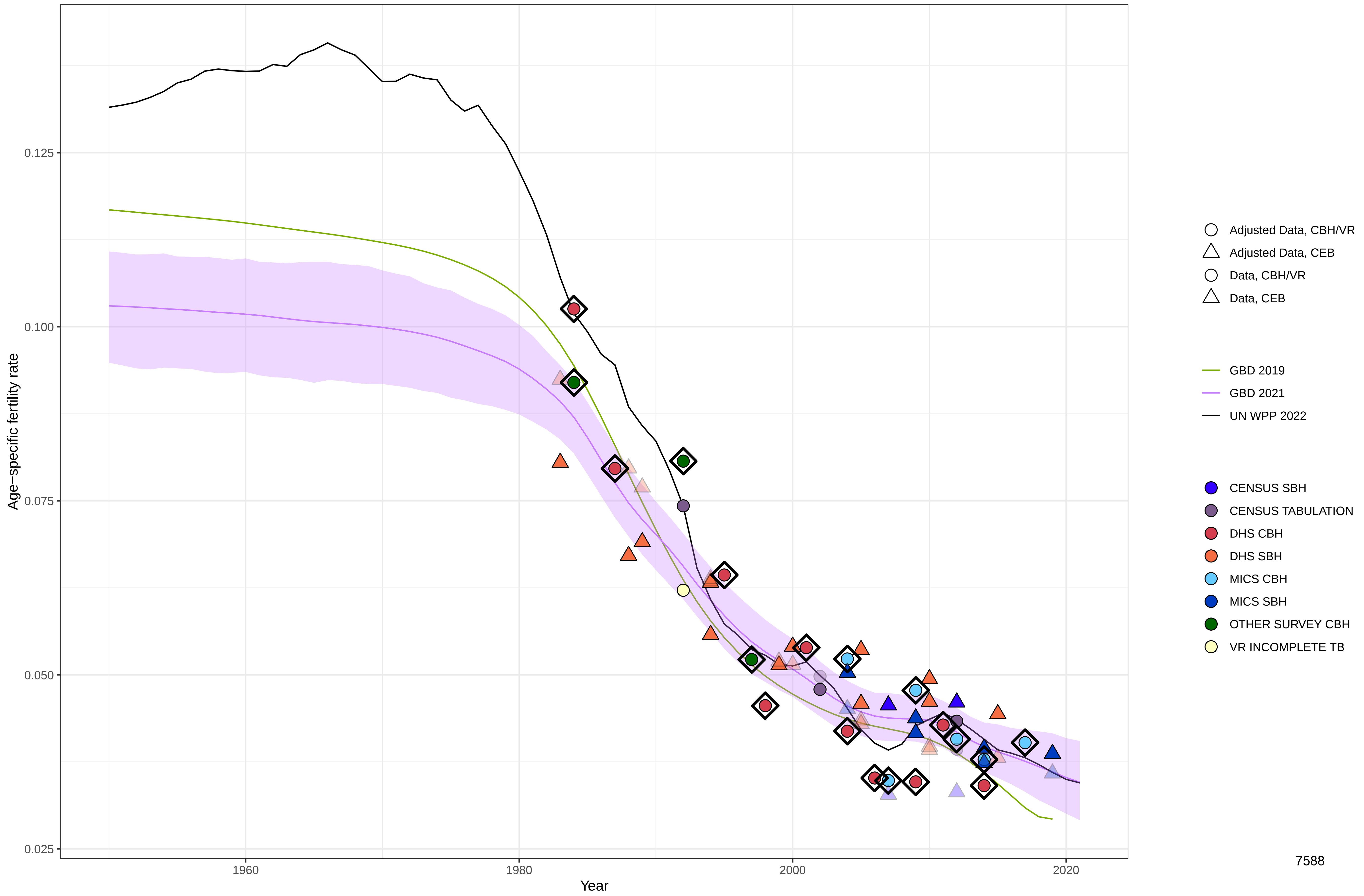

Age−specific fertility fitted trend lines, 1950−2021: Zimbabwe, Ages: 45−49
Data points enclosed with a diamond are reference sources

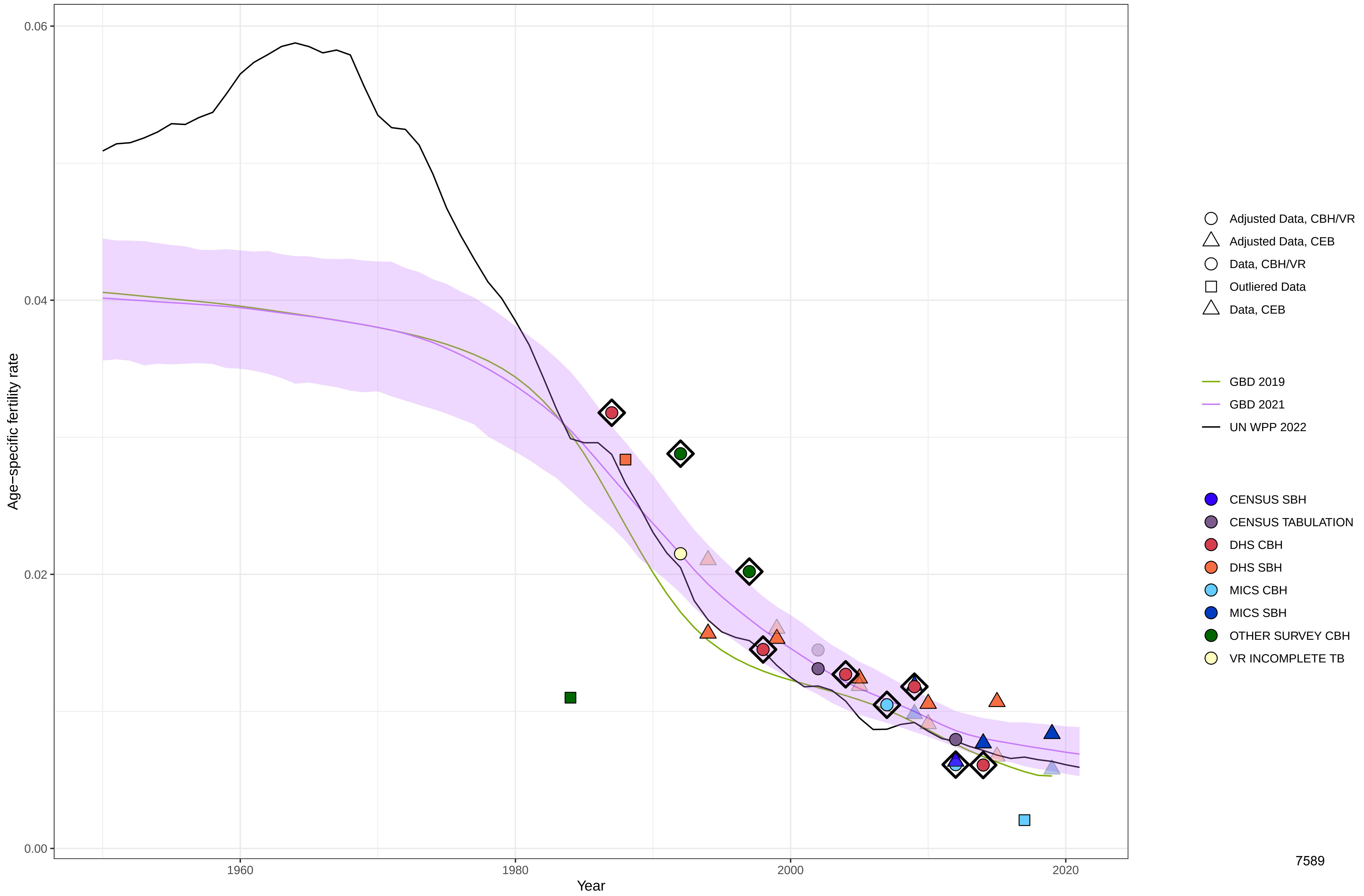

Age−specific fertility fitted trend lines, 1950−2021: Zimbabwe, Ages: 50−54
Data points enclosed with a diamond are reference sources

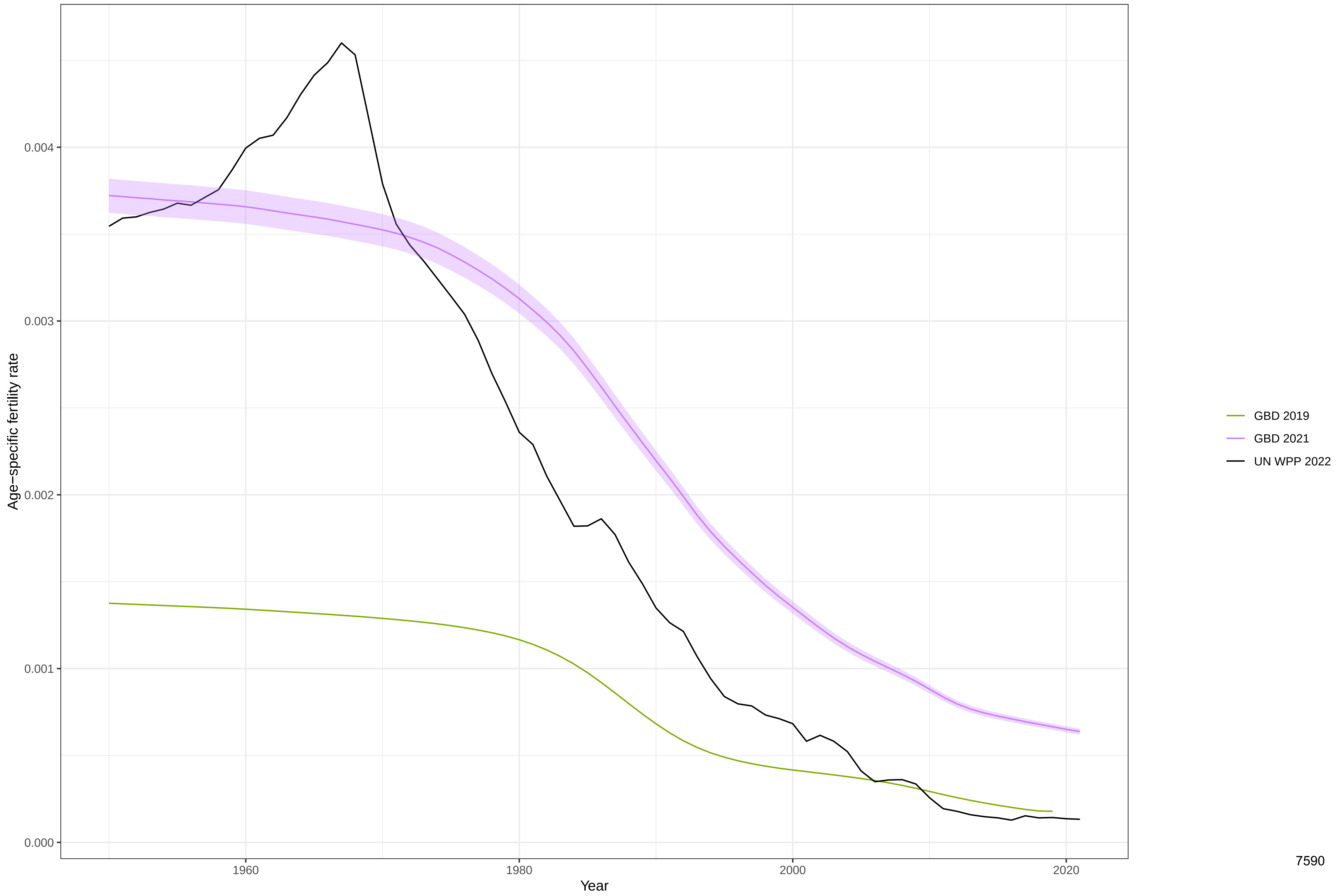

Total fertility rate fitted trend lines, 1950−2021: Zimbabwe TFR
Data points enclosed with a diamond are reference sources

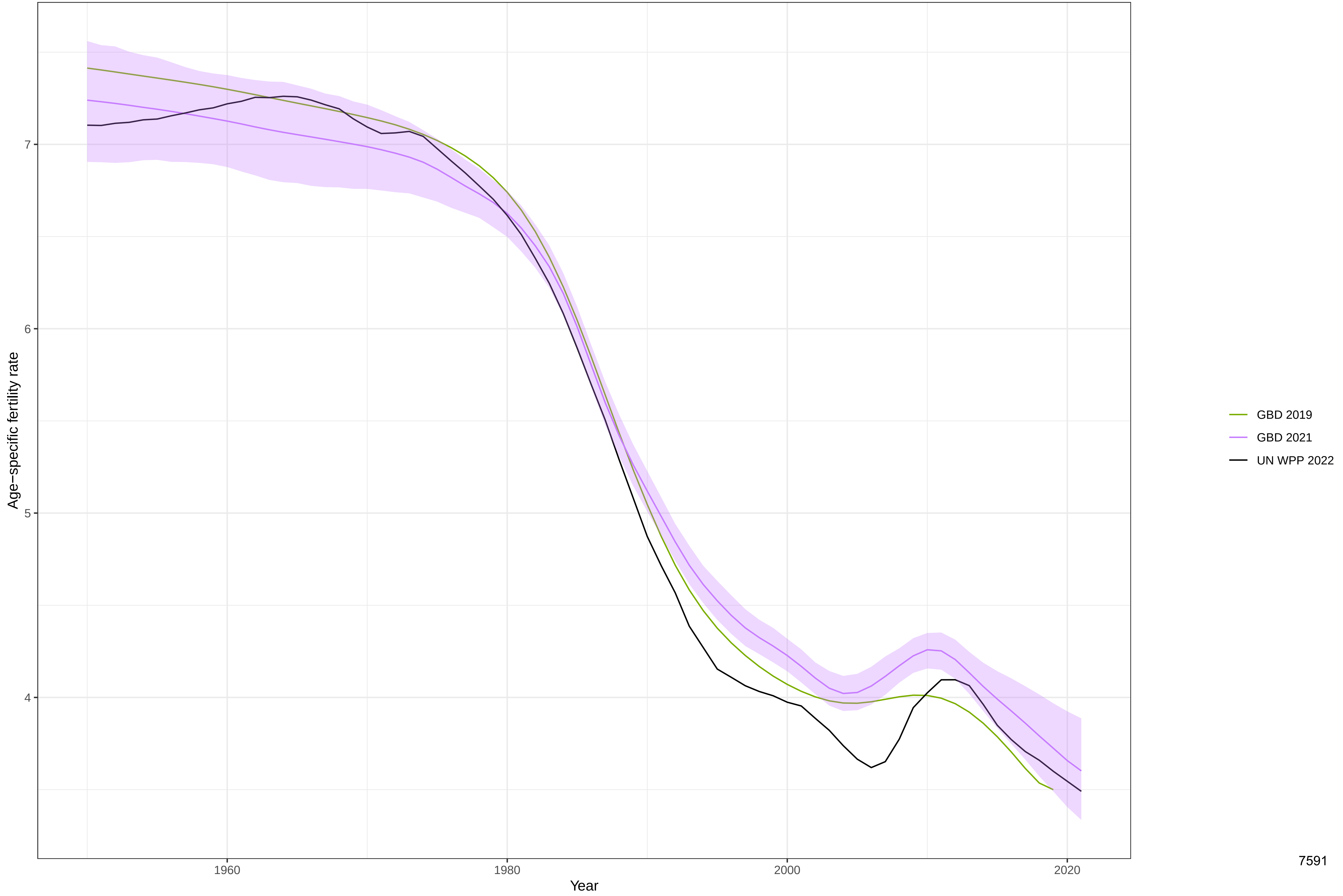

Age−specific fertility fitted trend lines, 1950−2021: Benin, Ages: 10−14
Data points enclosed with a diamond are reference sources

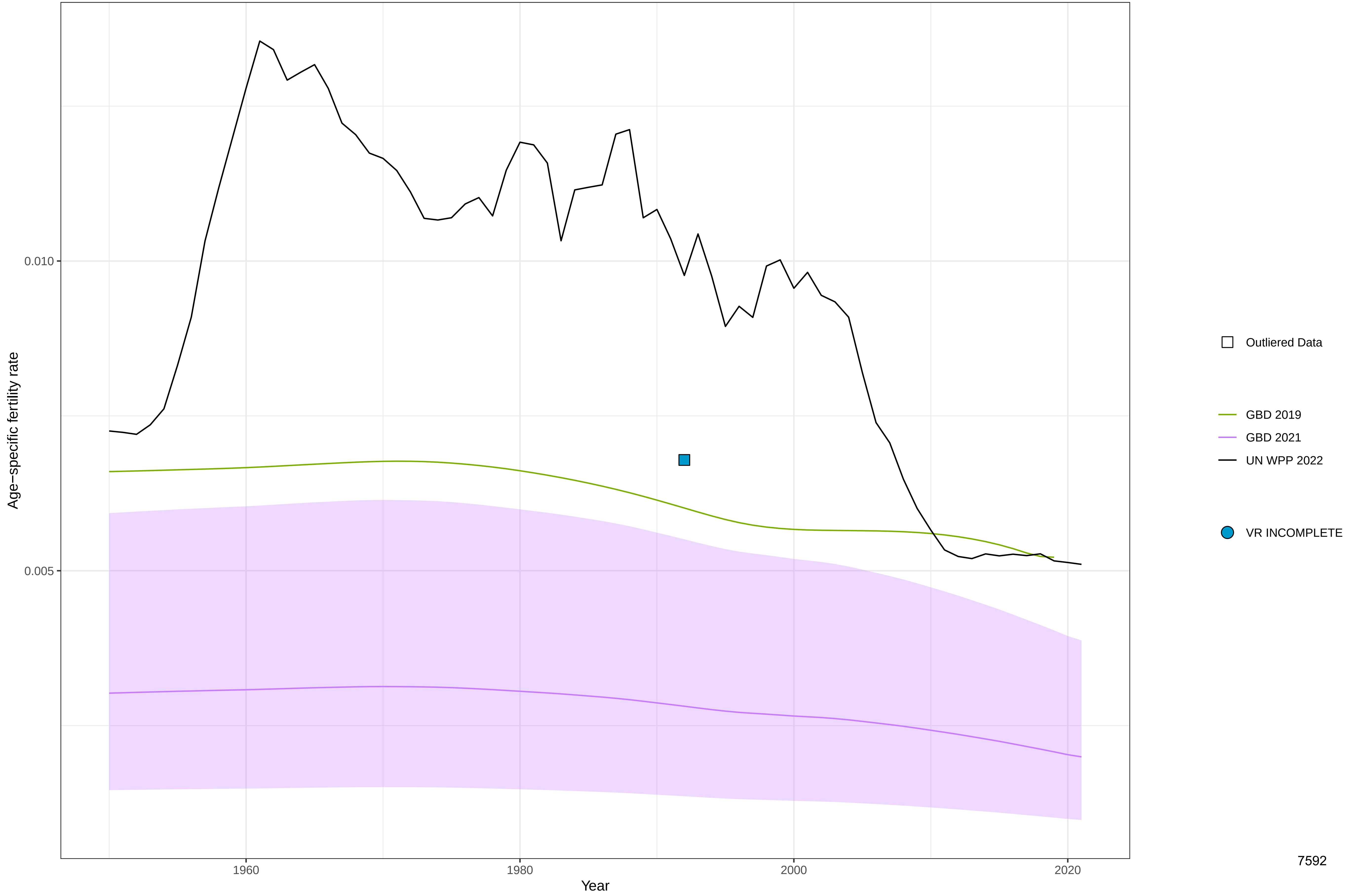

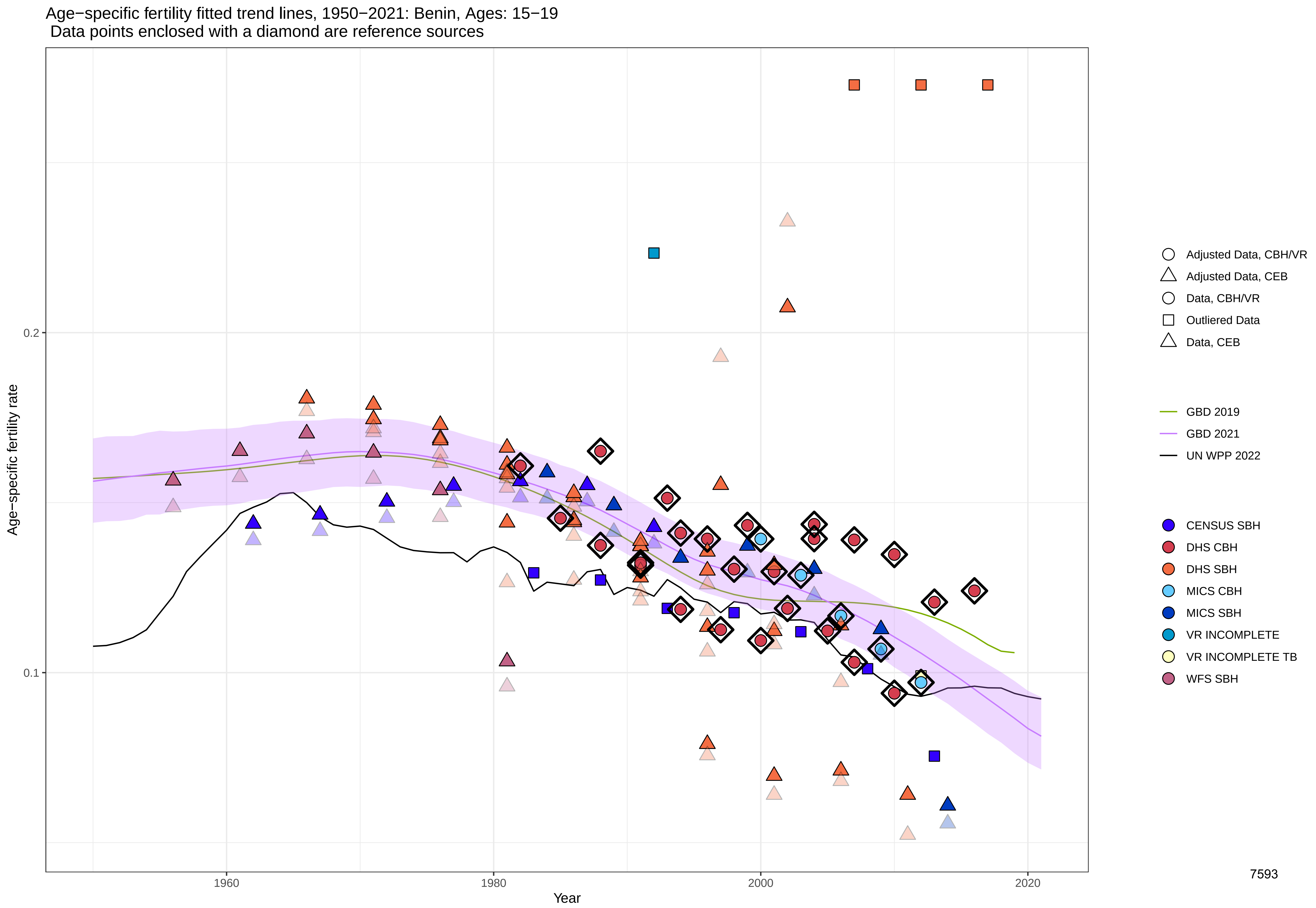

Age−specific fertility fitted trend lines, 1950−2021: Benin, Ages: 15−19
Data points enclosed with a diamond are reference sources
Age−specific fertility rate
0.2
0.1
1960
1980
2000
2020
Year
Adjusted Data, CBH/VR
Adjusted Data, CEB
Data, CBH/VR
Outliered Data
Data, CEB
GBD 2019
GBD 2021
UN WPP 2022
CENSUS SBH
DHS CBH
DHS SBH
MICS CBH
MICS SBH
VR INCOMPLETE
VR INCOMPLETE TB
WFS SBH

Age−specific fertility fitted trend lines, 1950−2021: Benin, Ages: 20−24
Data points enclosed with a diamond are reference sources

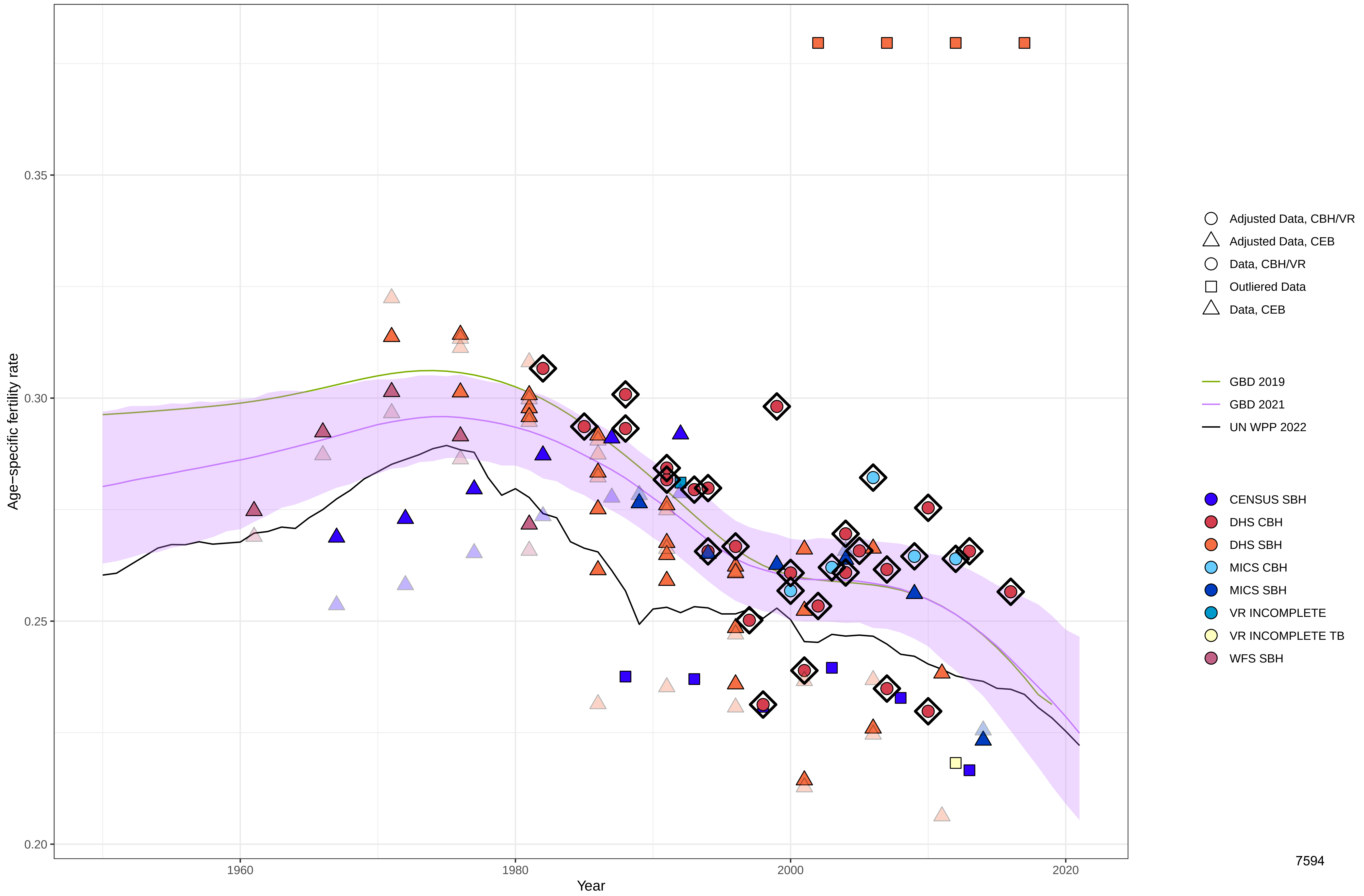

Age−specific fertility fitted trend lines, 1950−2021: Benin, Ages: 25−29
Data points enclosed with a diamond are reference sources

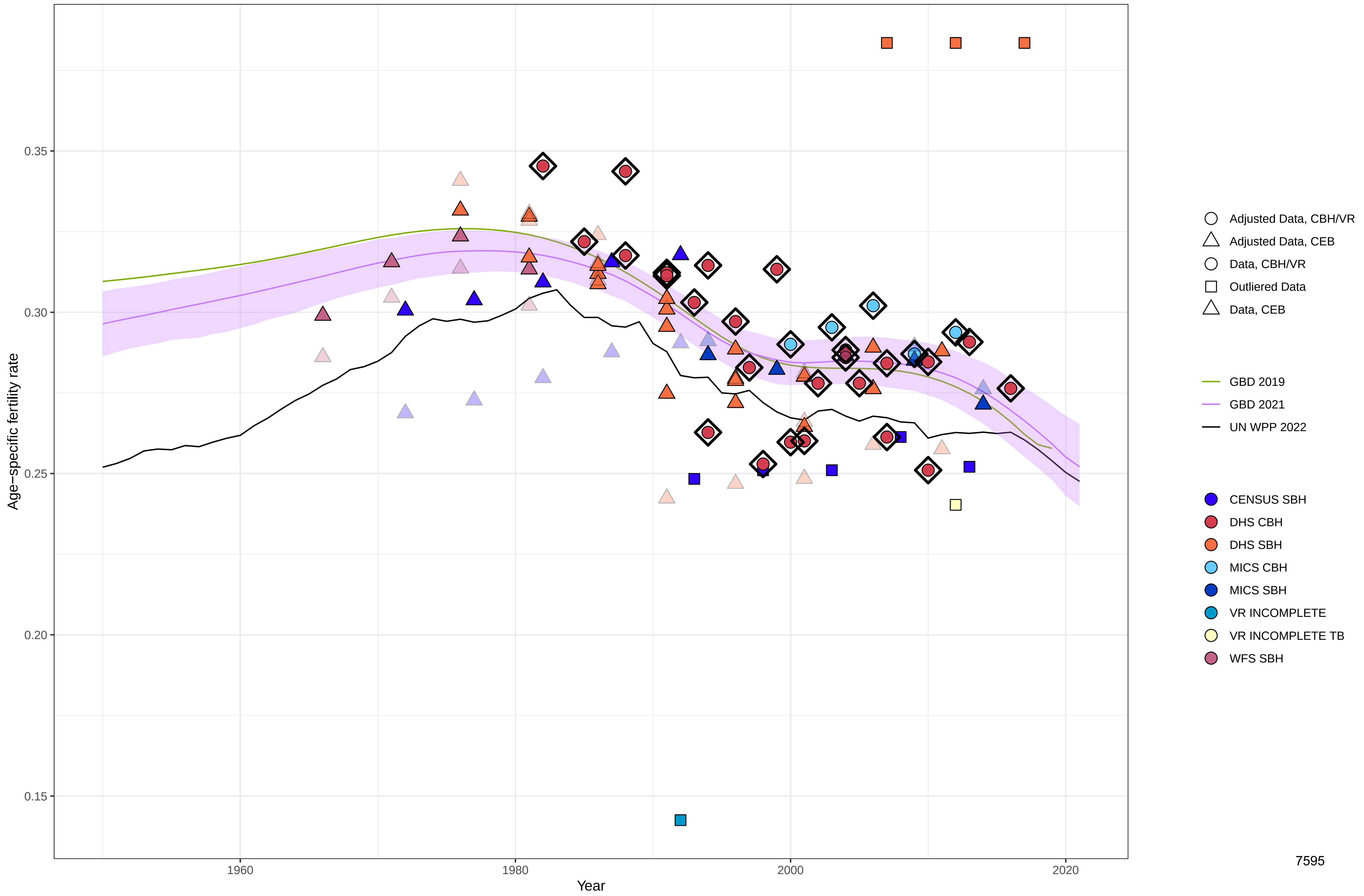

Age−specific fertility fitted trend lines, 1950−2021: Benin, Ages: 30−34
Data points enclosed with a diamond are reference sources

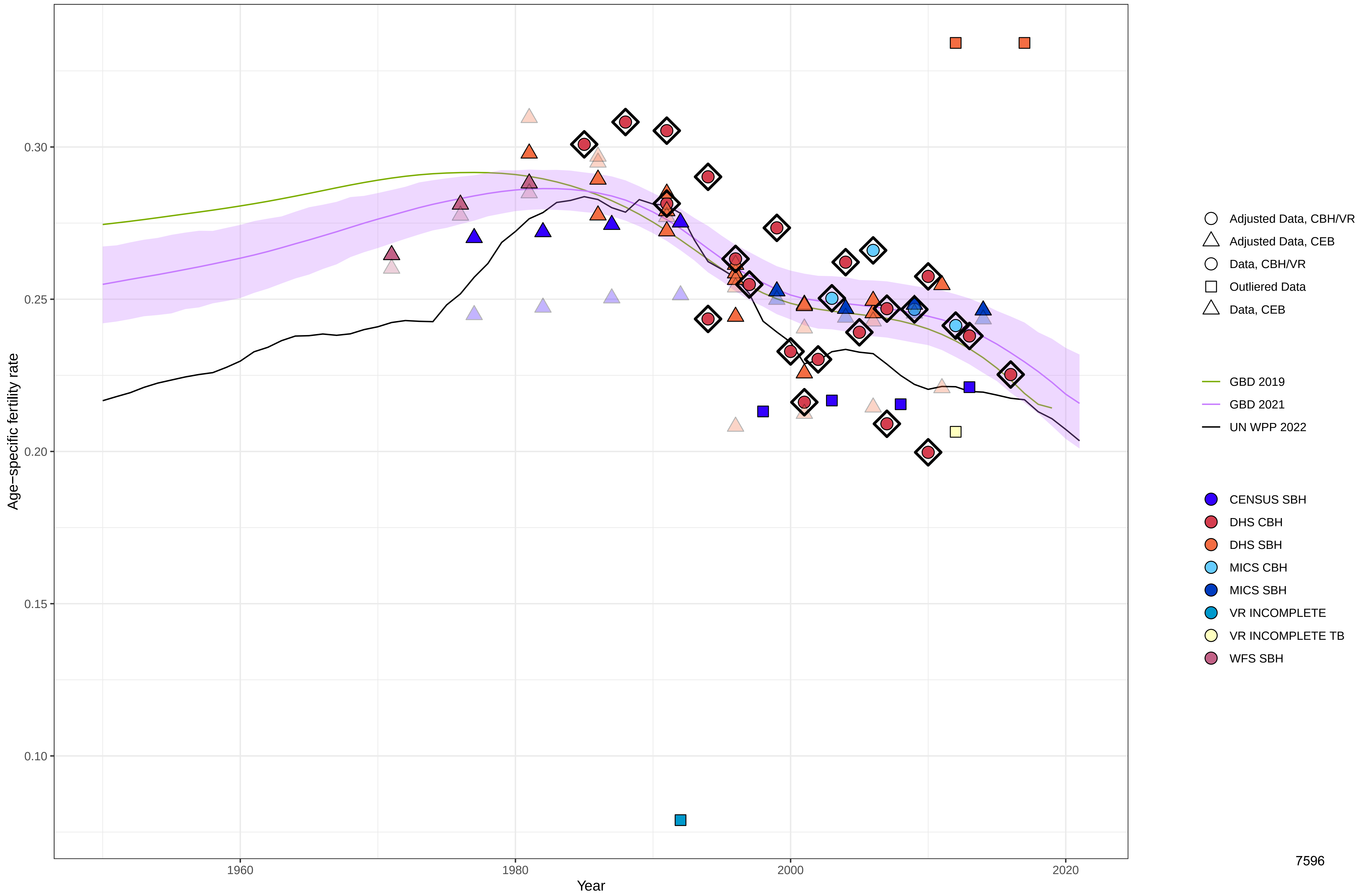

Age−specific fertility fitted trend lines, 1950−2021: Benin, Ages: 35−39
Data points enclosed with a diamond are reference sources

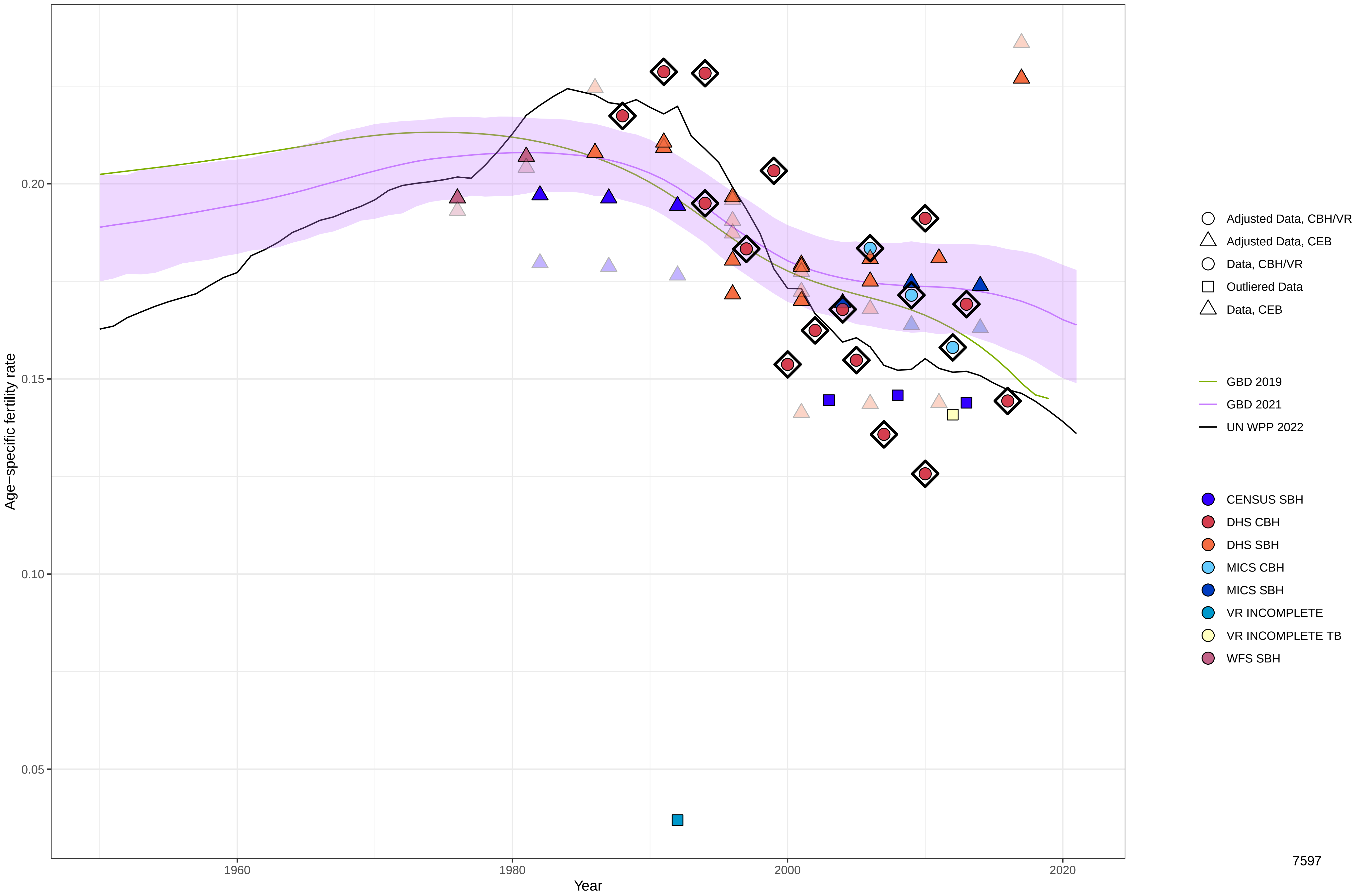

Age−specific fertility fitted trend lines, 1950−2021: Benin, Ages: 40−44
Data points enclosed with a diamond are reference sources

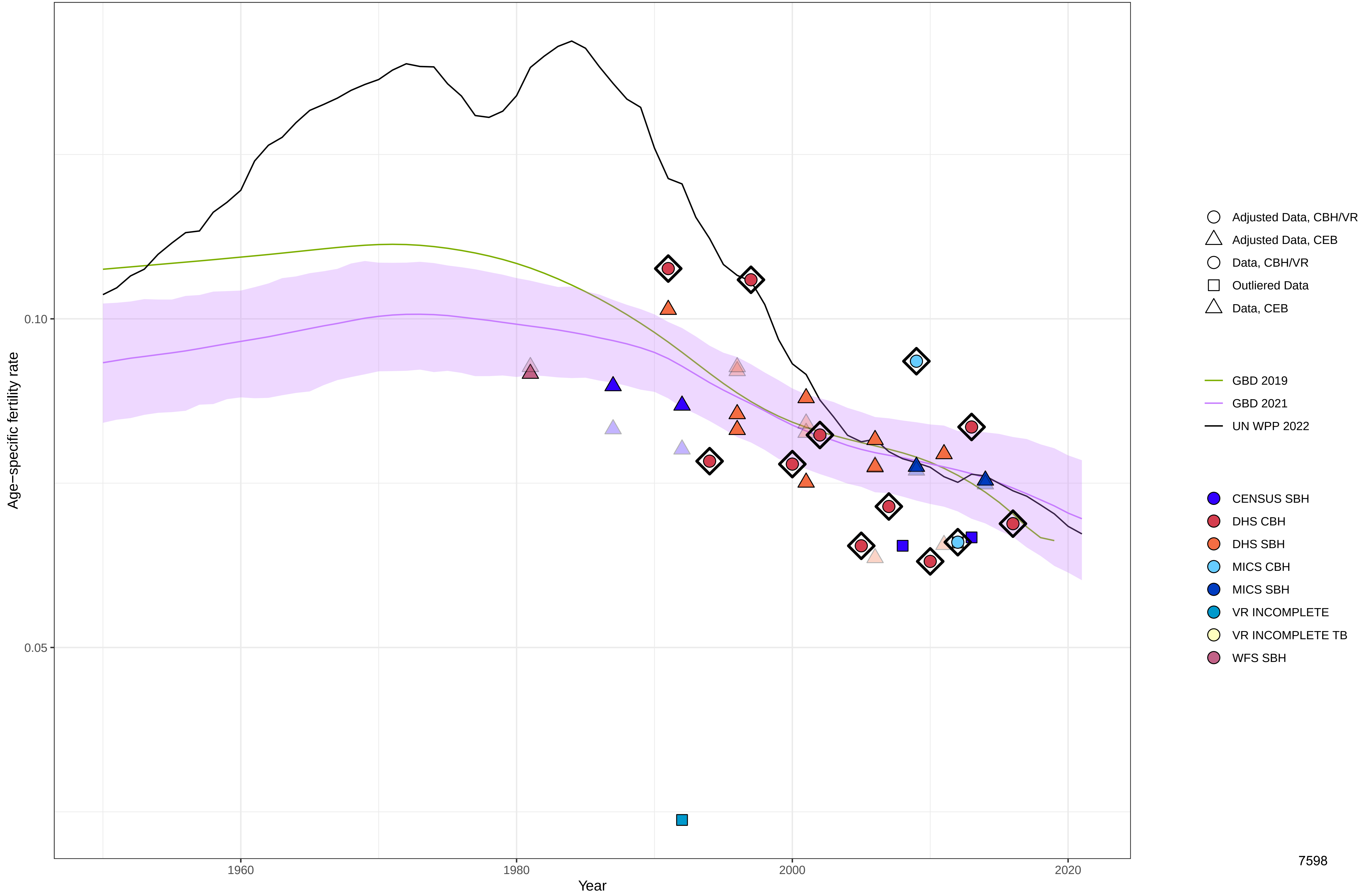

Age−specific fertility fitted trend lines, 1950−2021: Benin, Ages: 45−49
Data points enclosed with a diamond are reference sources

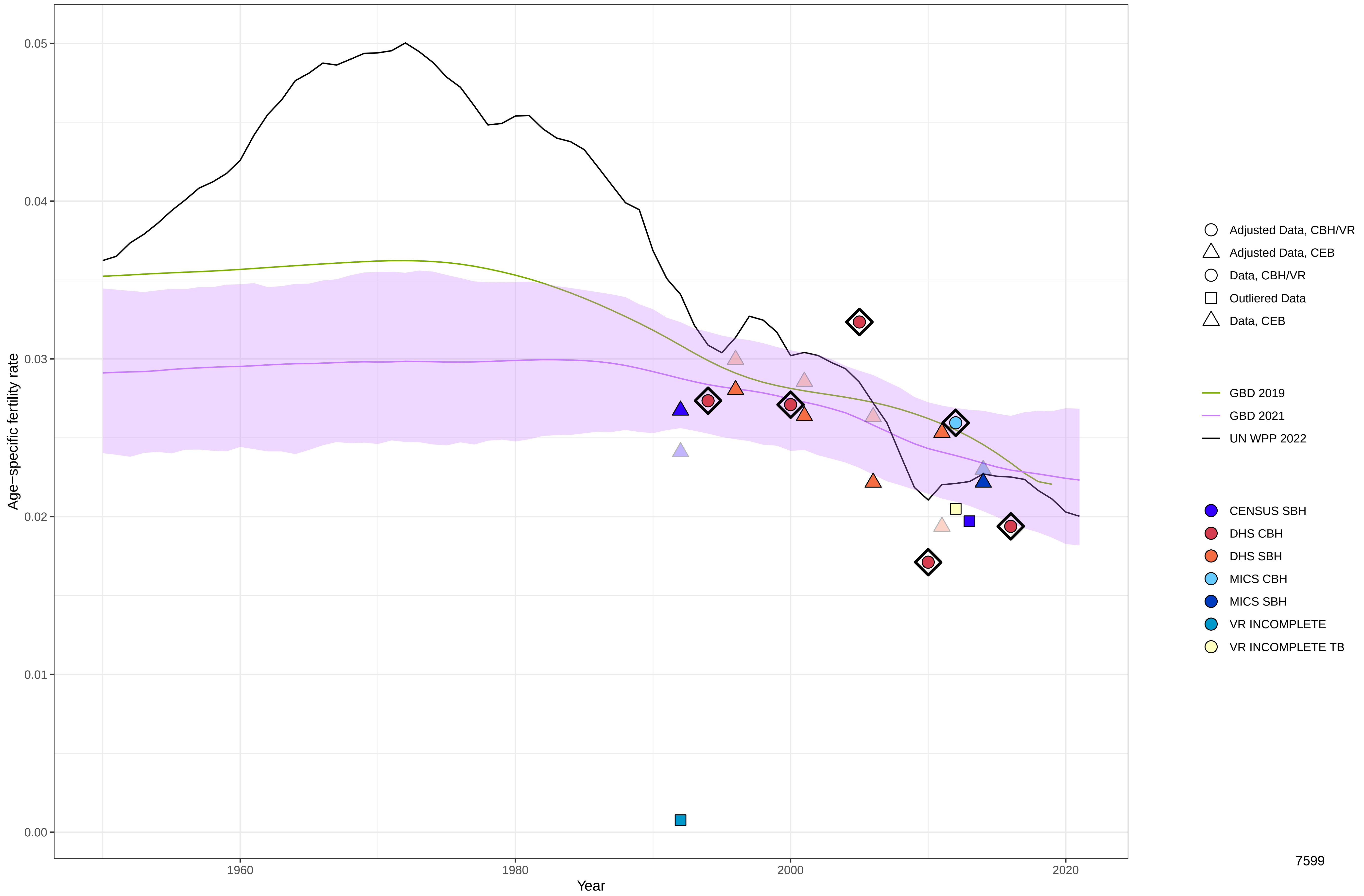

Age−specific fertility fitted trend lines, 1950−2021: Benin, Ages: 50−54
Data points enclosed with a diamond are reference sources

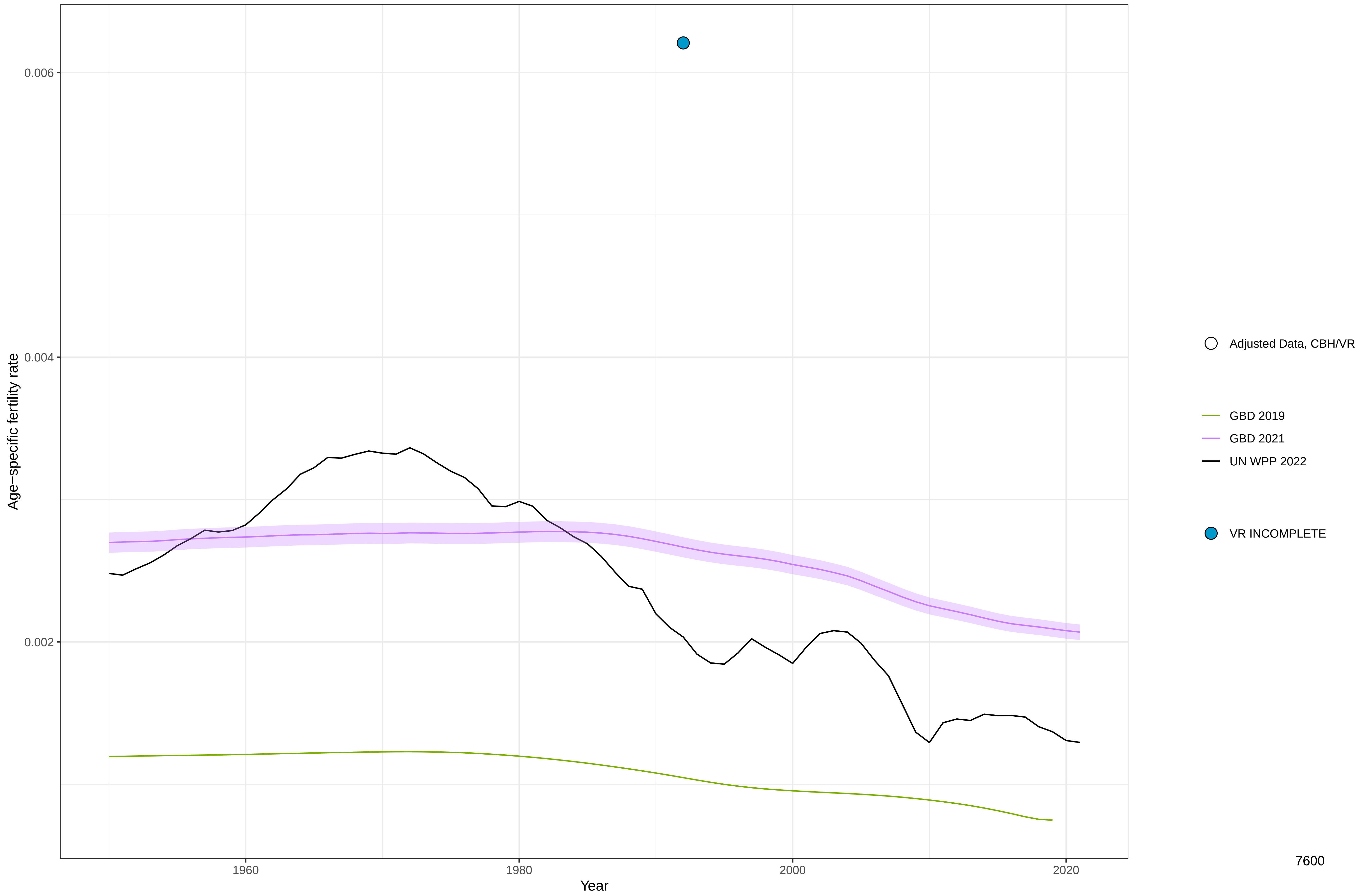

Total fertility rate fitted trend lines, 1950−2021: Benin TFR
Data points enclosed with a diamond are reference sources

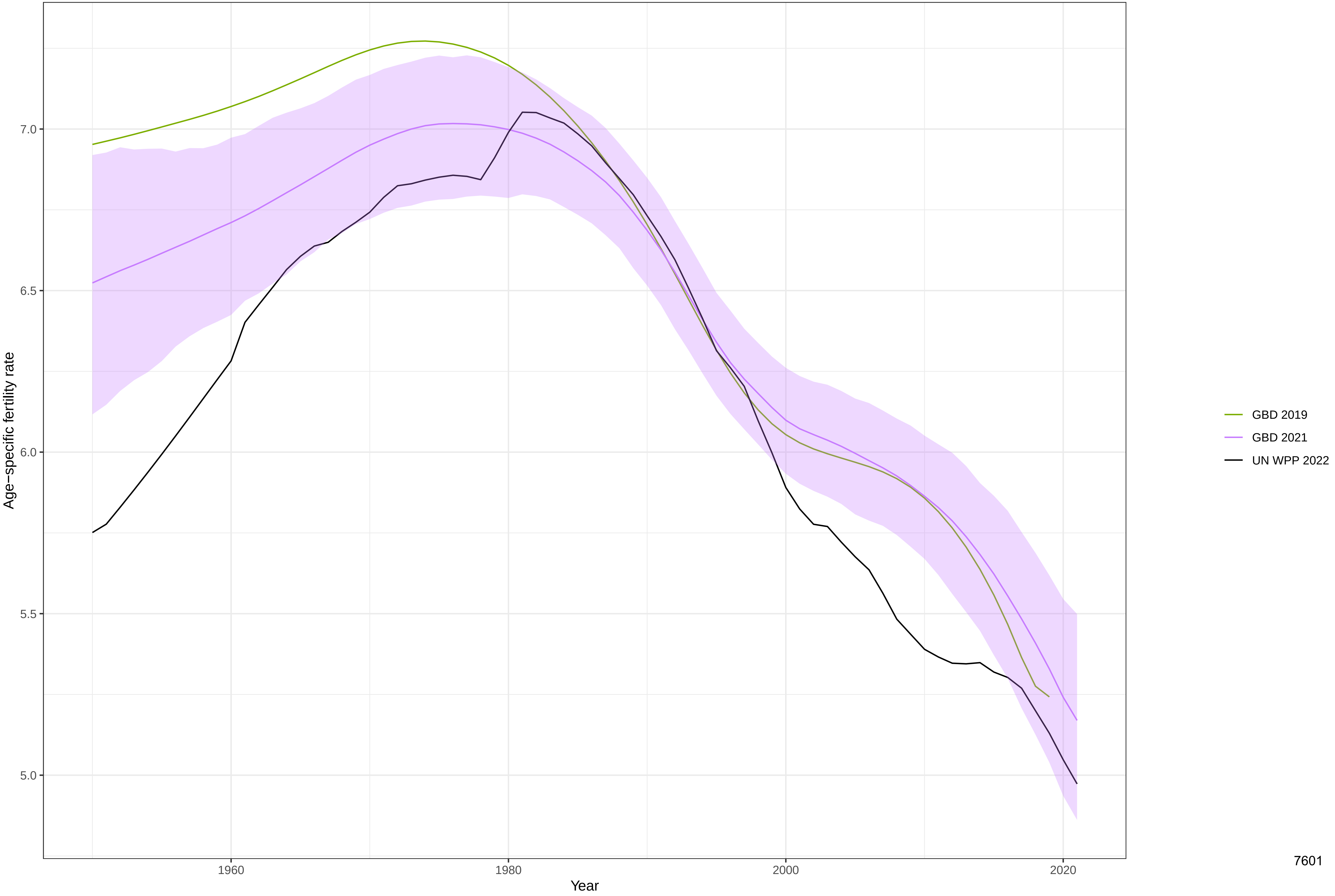

7601

Age−specific fertility fitted trend lines, 1950−2021: Burkina Faso, Ages: 10−14
Data points enclosed with a diamond are reference sources

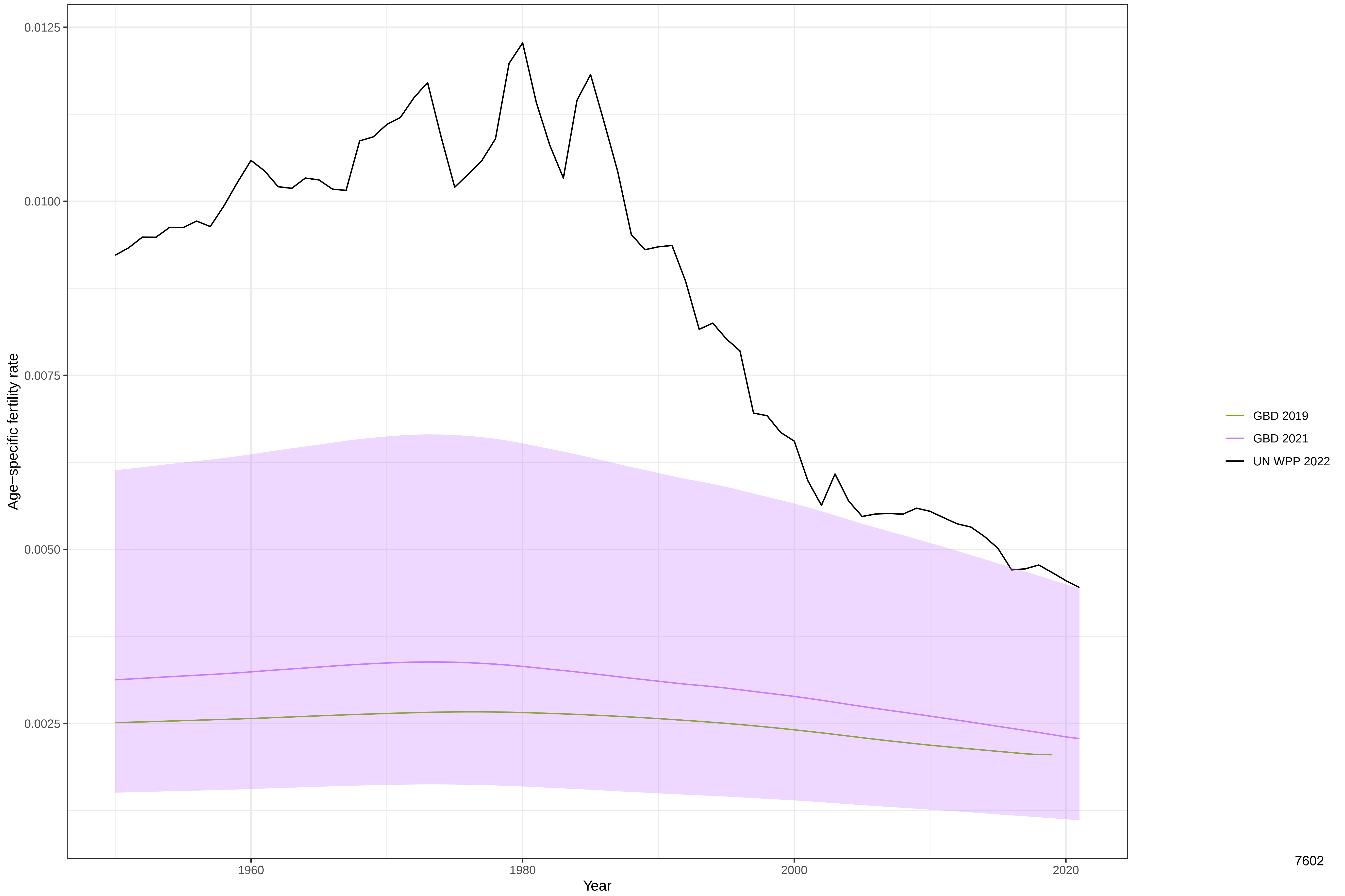

Age−specific fertility fitted trend lines, 1950−2021: Burkina Faso, Ages: 15−19
Data points enclosed with a diamond are reference sources

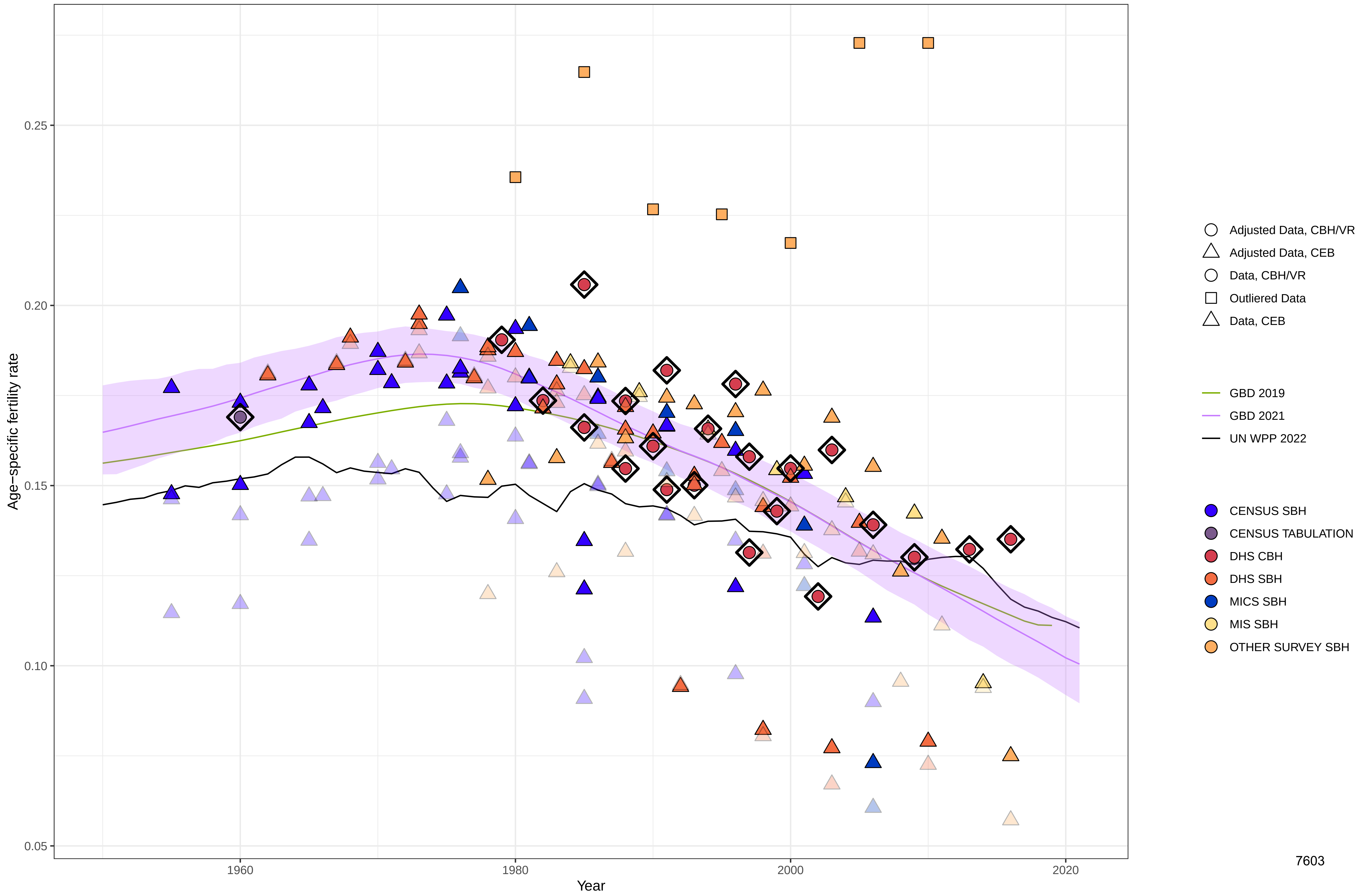

Age−specific fertility fitted trend lines, 1950−2021: Burkina Faso, Ages: 20−24
Data points enclosed with a diamond are reference sources

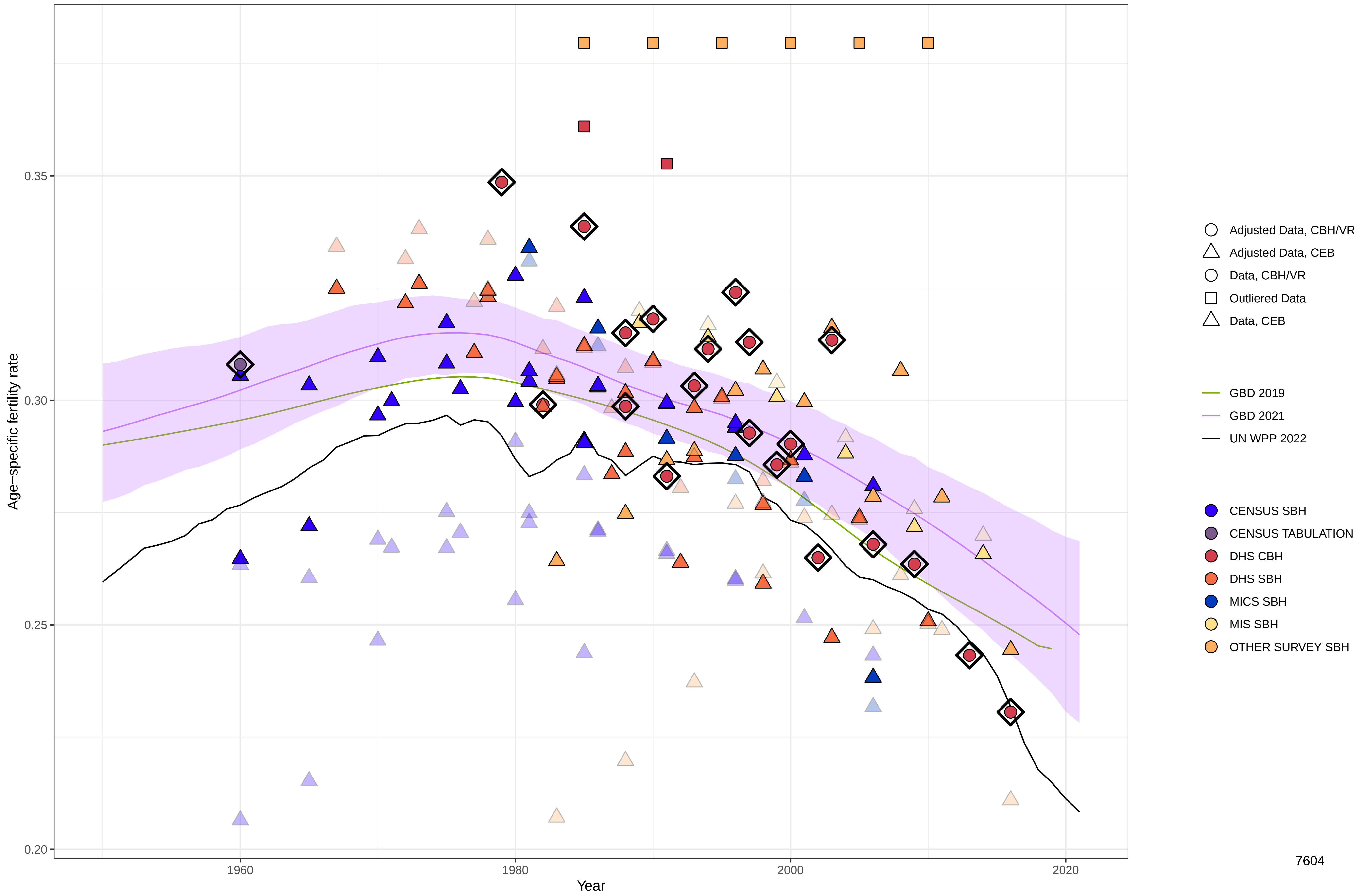

Age−specific fertility fitted trend lines, 1950−2021: Burkina Faso, Ages: 25−29
Data points enclosed with a diamond are reference sources

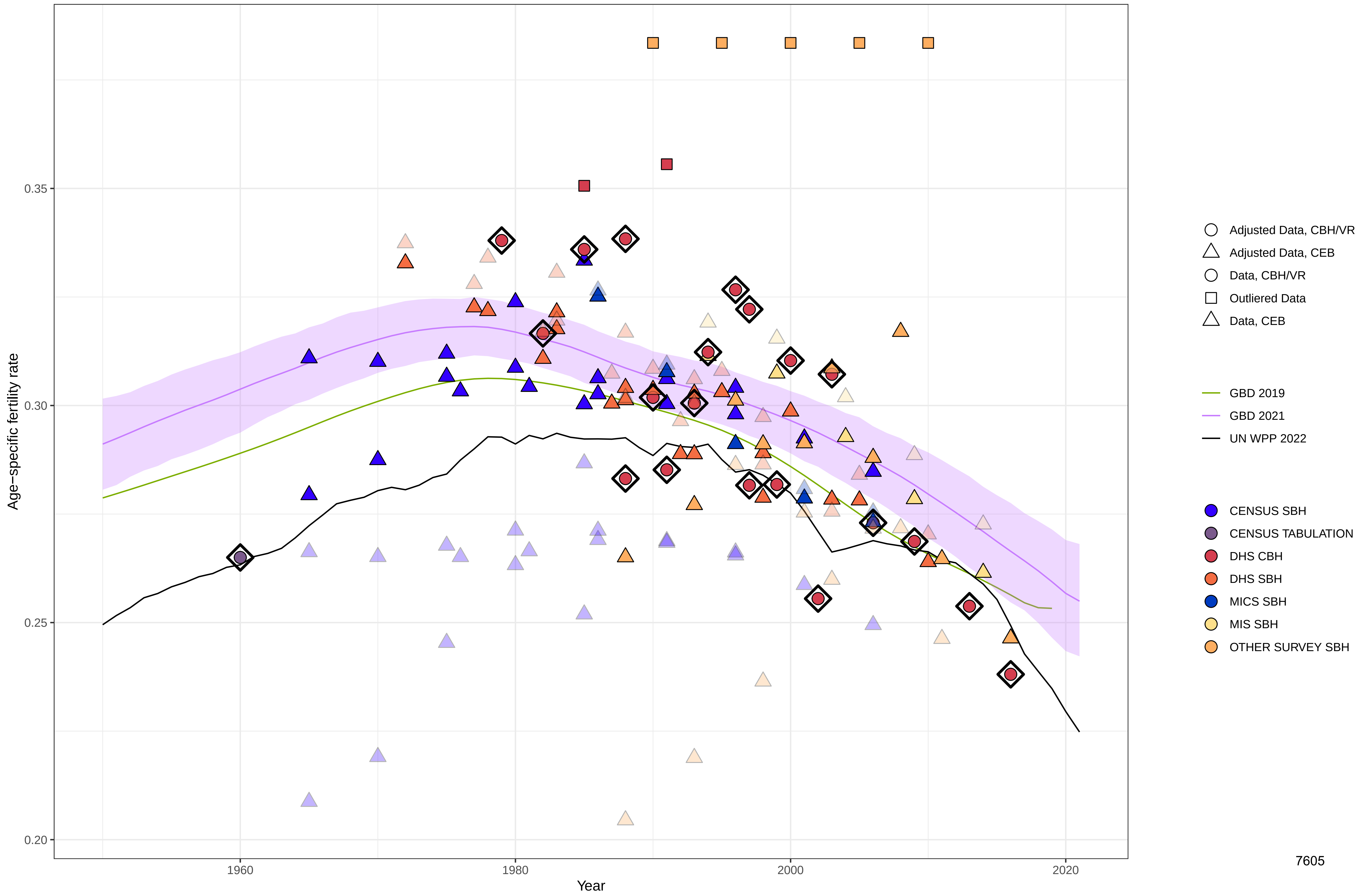

Age−specific fertility fitted trend lines, 1950−2021: Burkina Faso, Ages: 30−34
Data points enclosed with a diamond are reference sources

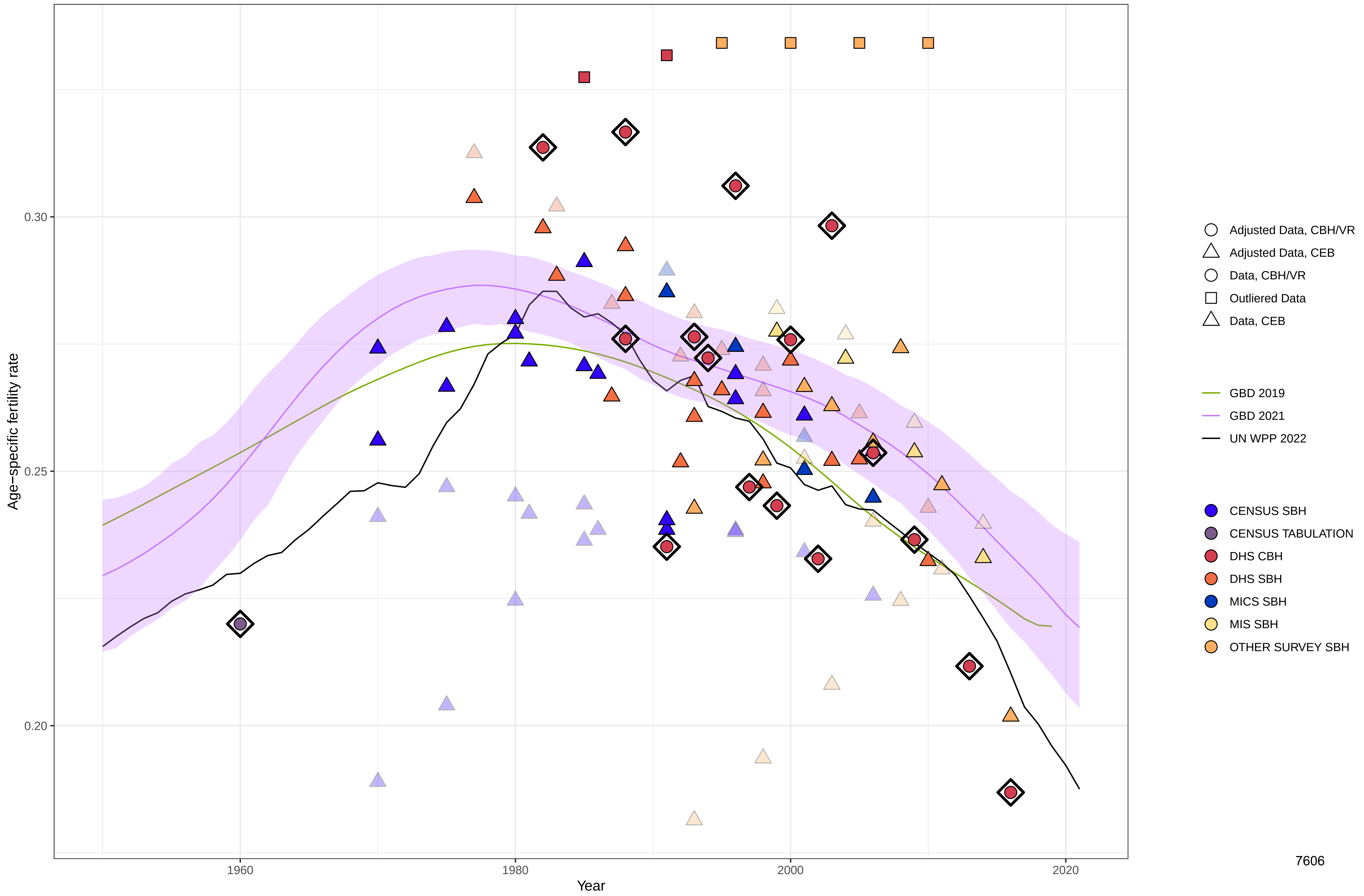

Age−specific fertility fitted trend lines, 1950−2021: Burkina Faso, Ages: 35−39
Data points enclosed with a diamond are reference sources

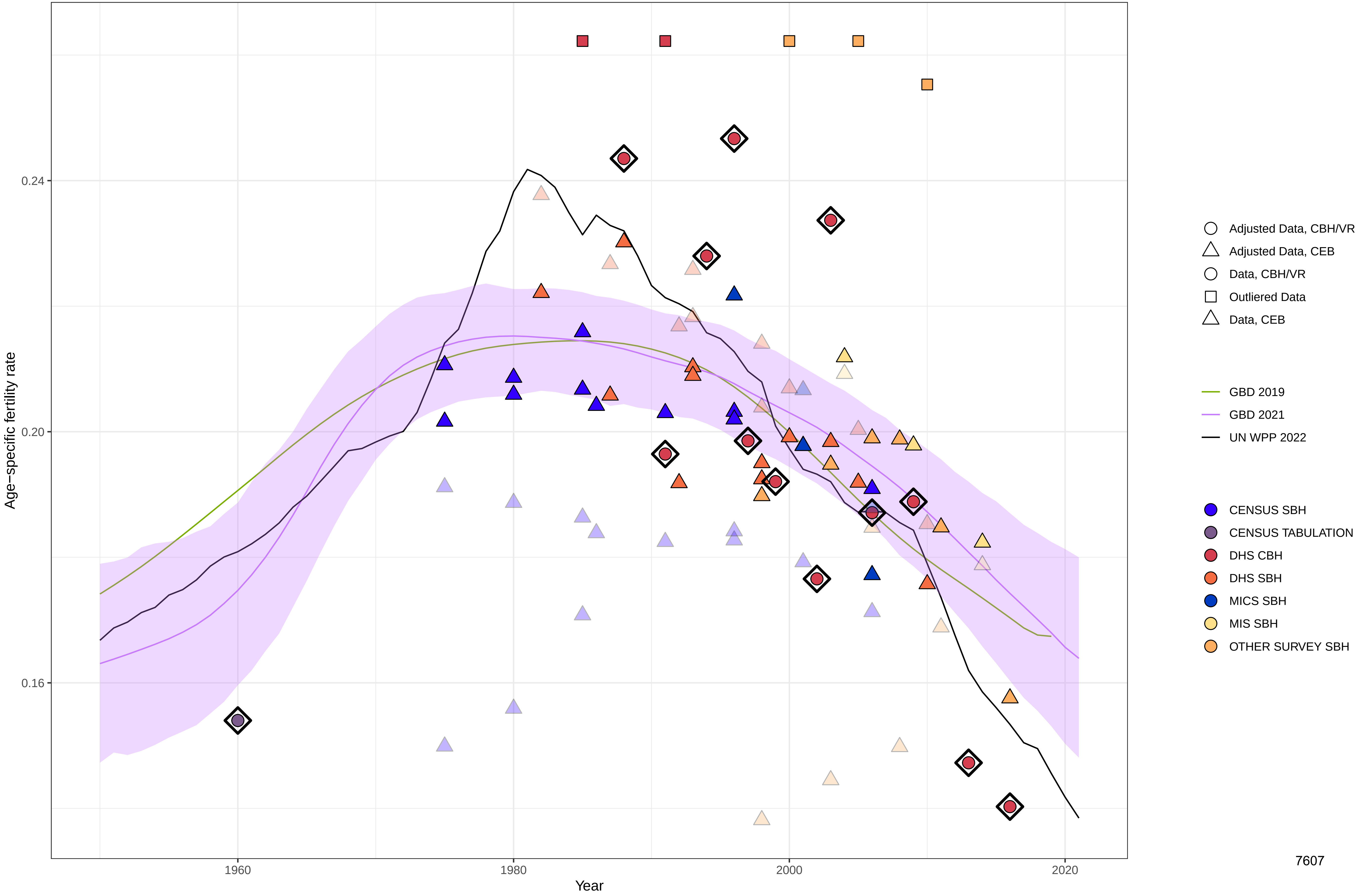

Age−specific fertility fitted trend lines, 1950−2021: Burkina Faso, Ages: 40−44
Data points enclosed with a diamond are reference sources

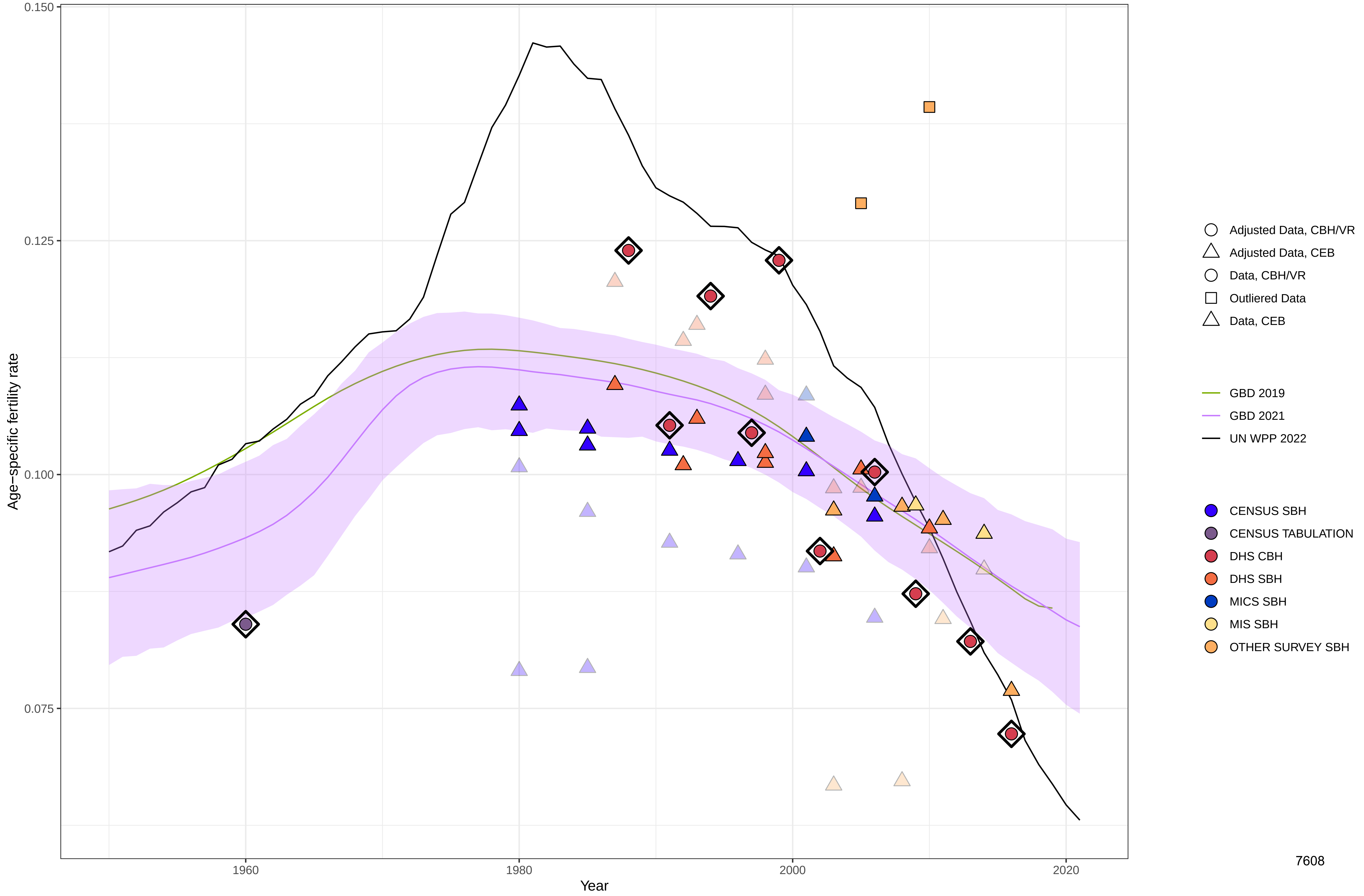

Age−specific fertility fitted trend lines, 1950−2021: Burkina Faso, Ages: 45−49
Data points enclosed with a diamond are reference sources

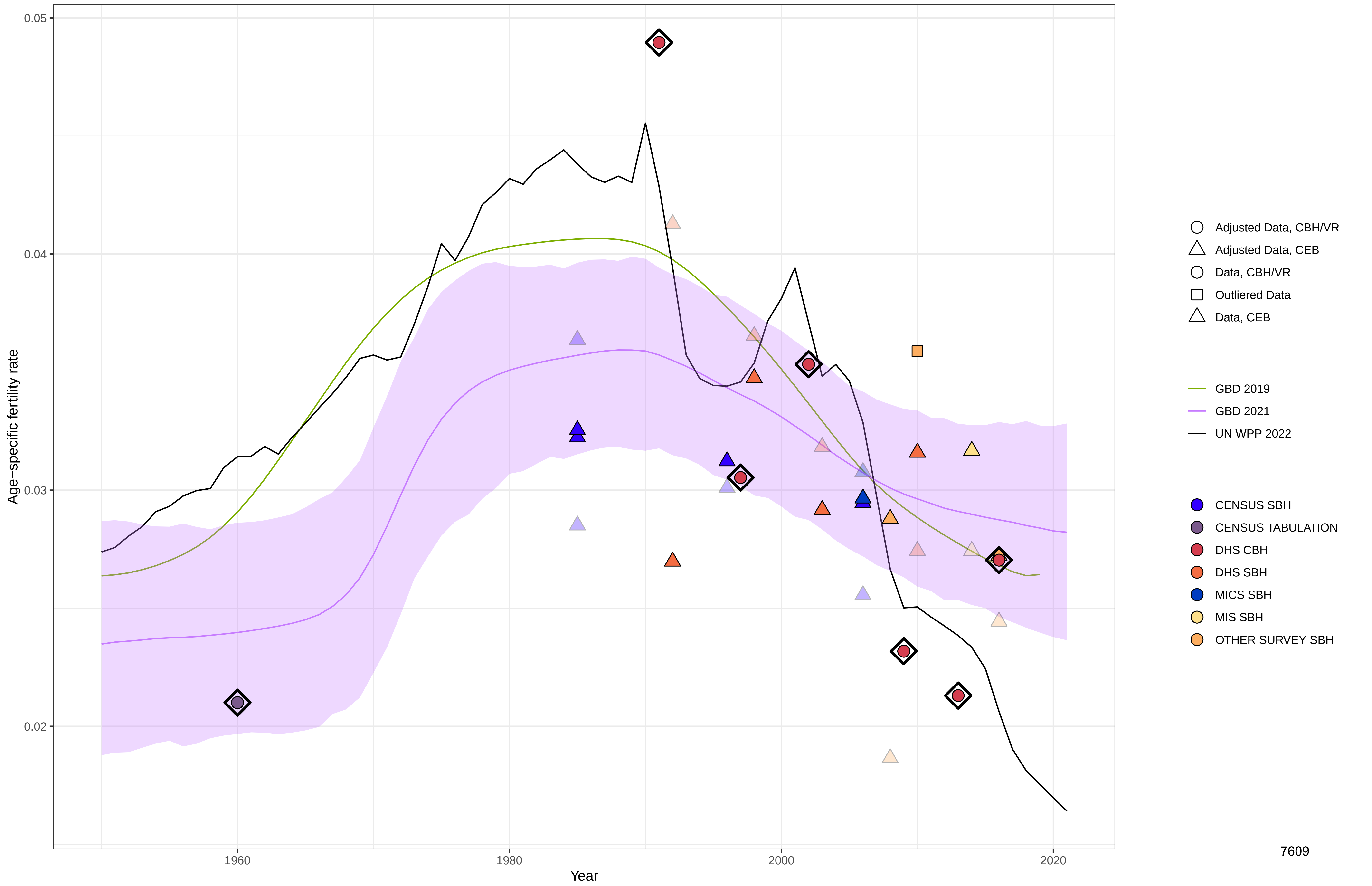

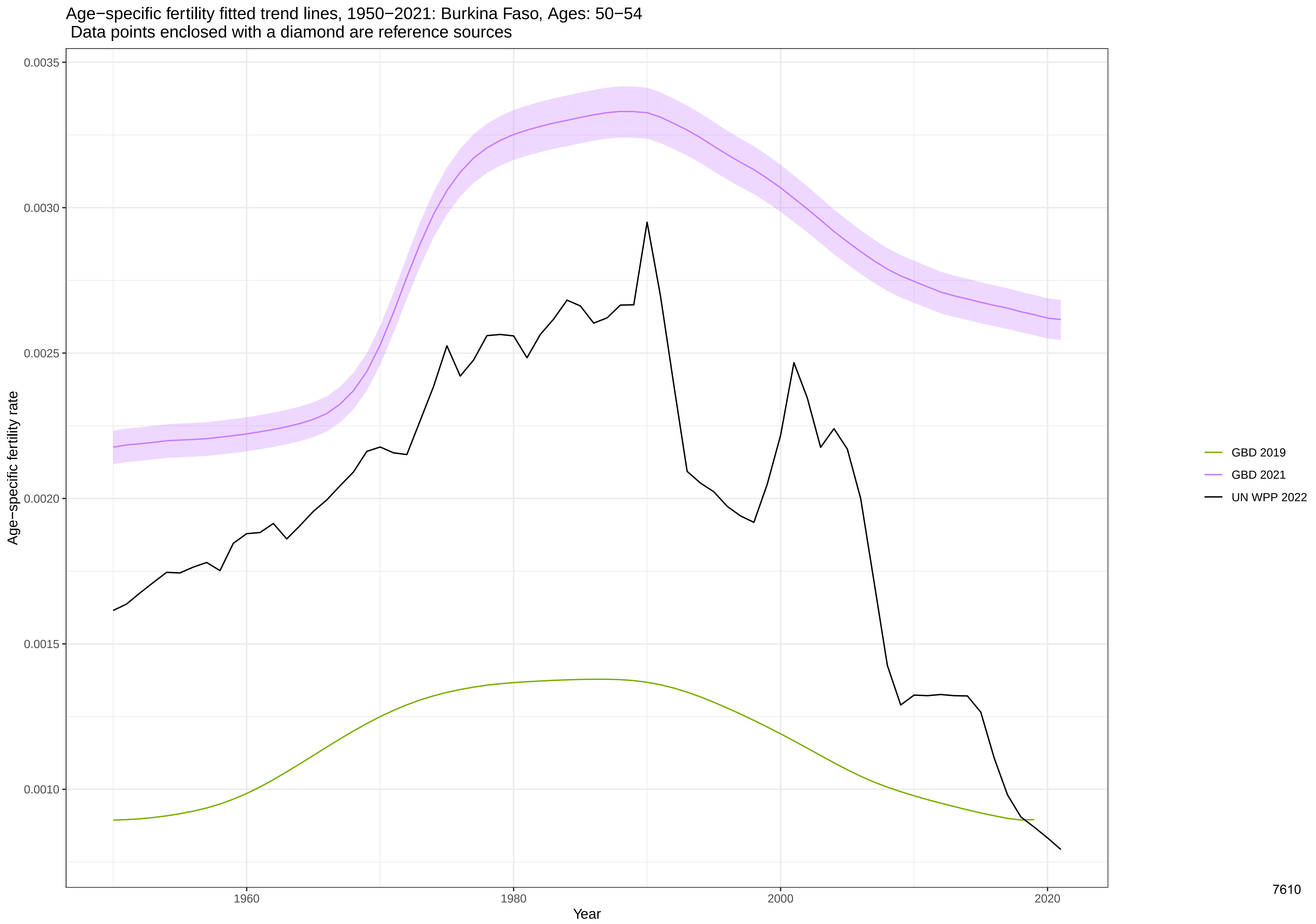

Age−specific fertility fitted trend lines, 1950−2021: Burkina Faso, Ages: 50−54
Data points enclosed with a diamond are reference sources
0.0035
0.0030
0.0025
0.0020
0.0015
0.0010
Age−specific fertility rate
1960
1980
2000
2020
Year
GBD 2019
GBD 2021
UN WPP 2022

7610

Total fertility rate fitted trend lines, 1950−2021: Burkina Faso TFR
Data points enclosed with a diamond are reference sources

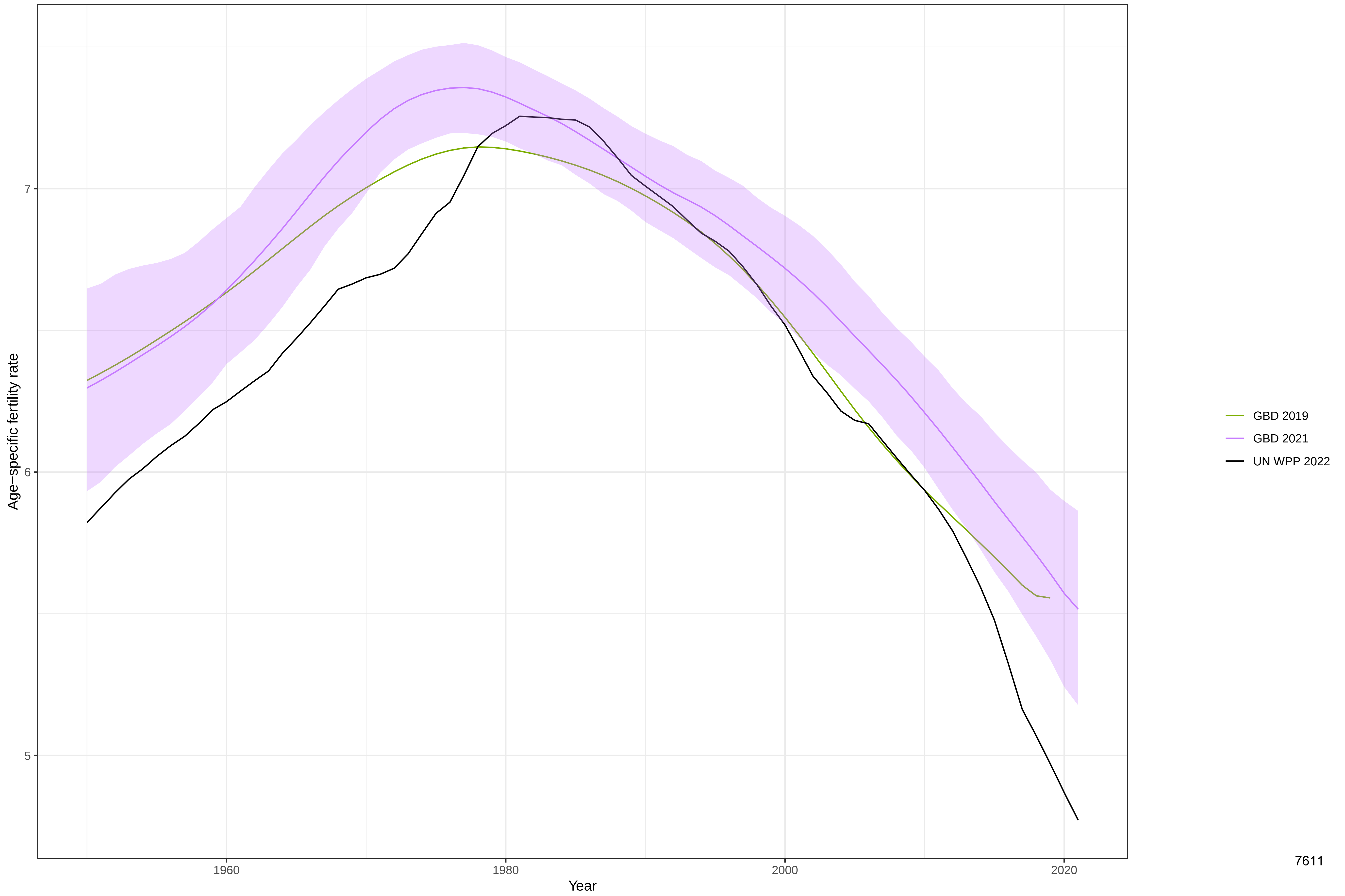

Age−specific fertility fitted trend lines, 1950−2021: Cabo Verde, Ages: 10−14
Data points enclosed with a diamond are reference sources

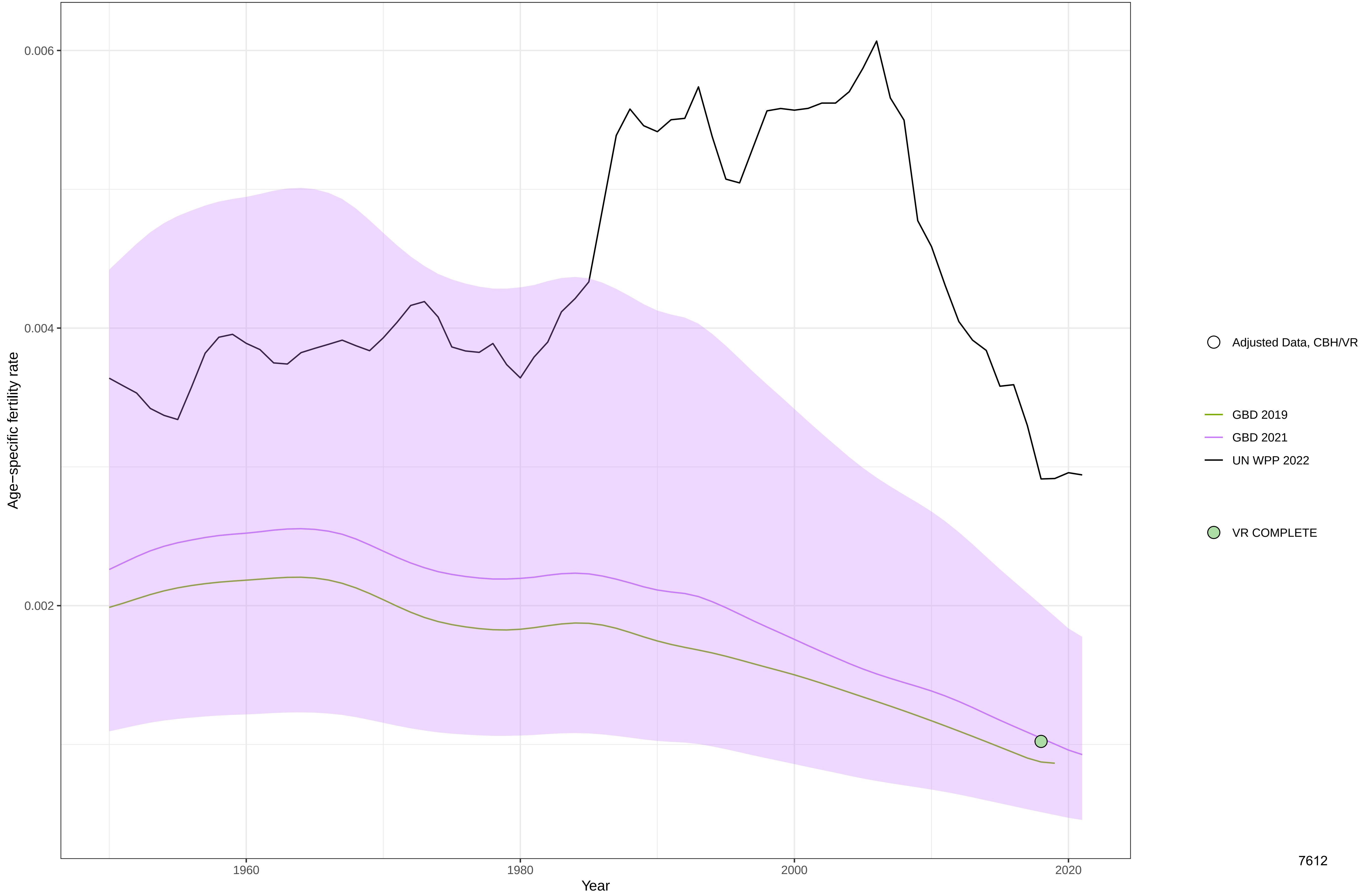

Age−specific fertility fitted trend lines, 1950−2021: Cabo Verde, Ages: 15−19
Data points enclosed with a diamond are reference sources

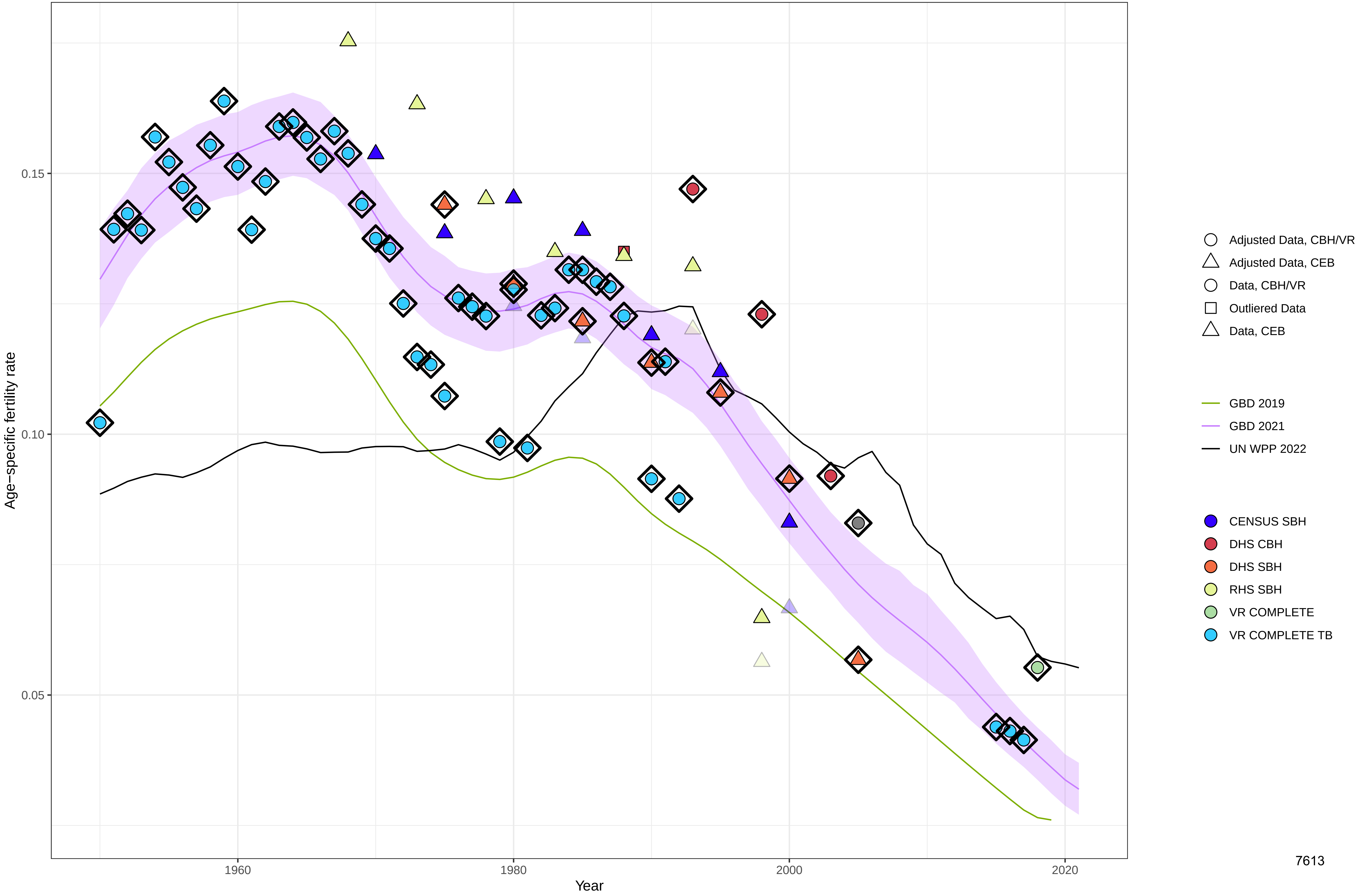

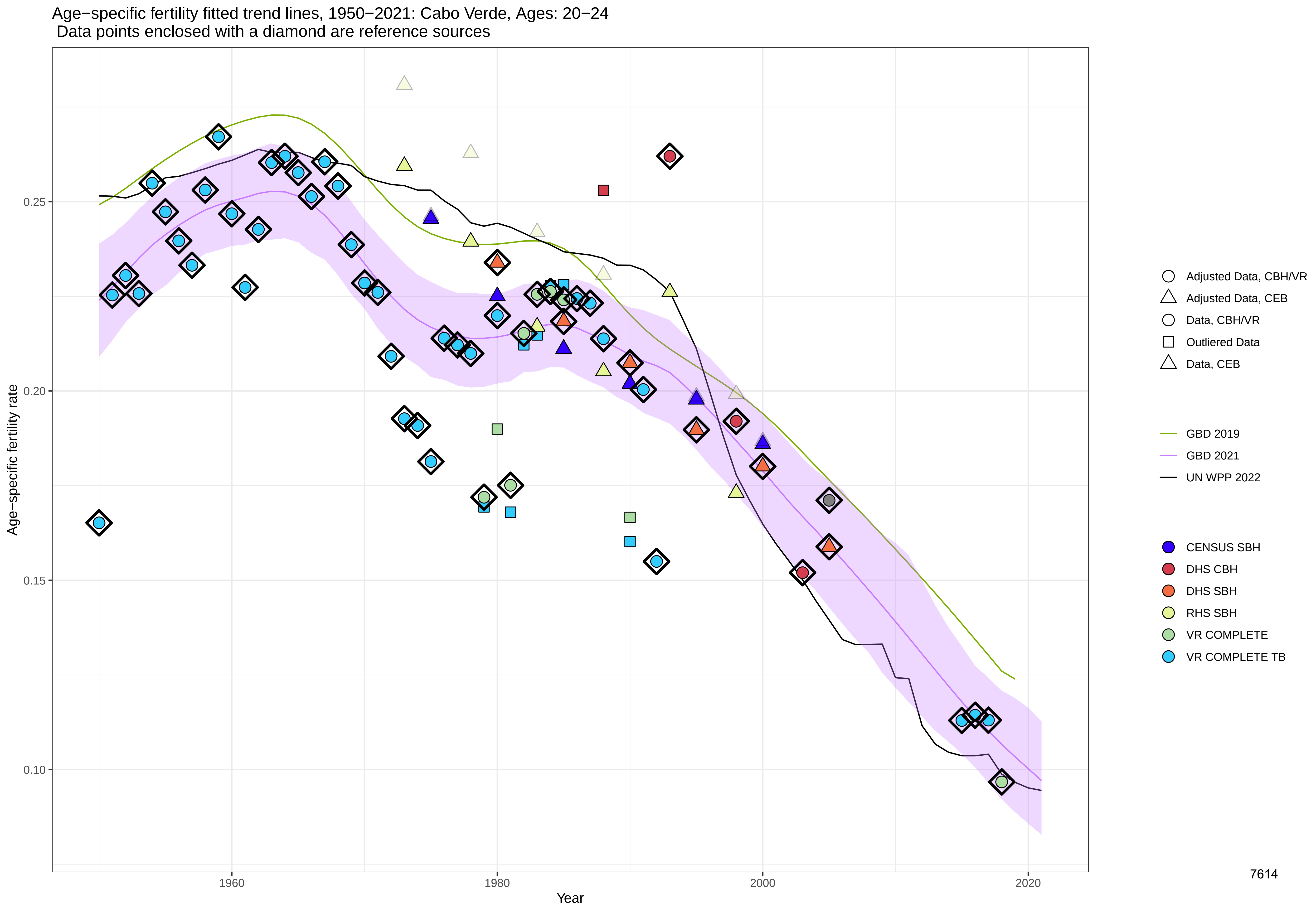
Age−specific fertility fitted trend lines, 1950−2021: Cabo Verde, Ages: 20−24
Data points enclosed with a diamond are reference sources
Age−specific fertility rate
0.25
0.20
0.15
0.10
1960
1980
2000
2020
Year
Adjusted Data, CBH/VR
Adjusted Data, CEB
Data, CBH/VR
Outliered Data
Data, CEB
GBD 2019
GBD 2021
UN WPP 2022
CENSUS SBH
DHS CBH
DHS SBH
RHS SBH
VR COMPLETE
VR COMPLETE TB

Age−specific fertility fitted trend lines, 1950−2021: Cabo Verde, Ages: 25−29
Data points enclosed with a diamond are reference sources

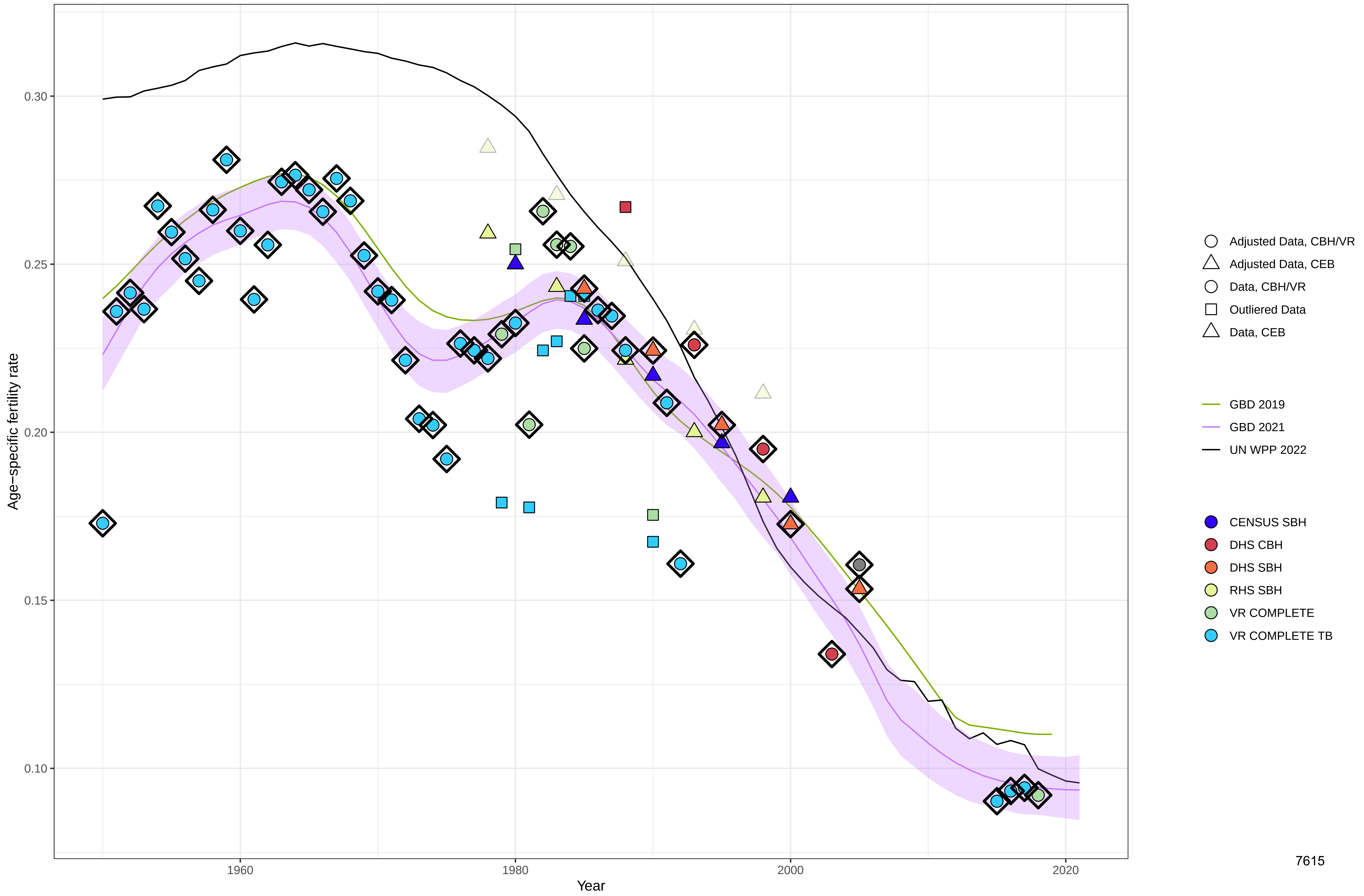

Age−specific fertility fitted trend lines, 1950−2021: Cabo Verde, Ages: 30−34
Data points enclosed with a diamond are reference sources

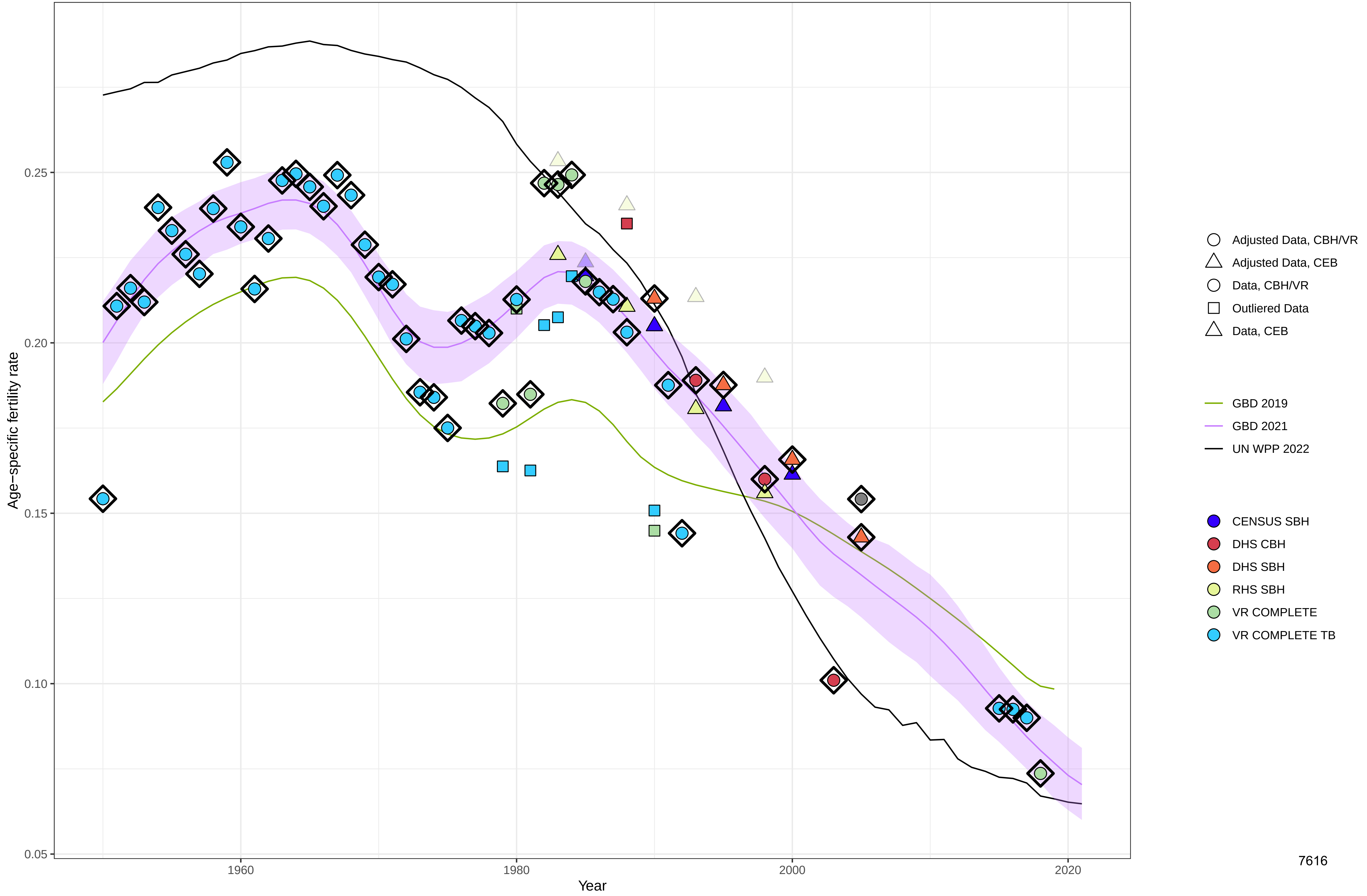

Age−specific fertility fitted trend lines, 1950−2021: Cabo Verde, Ages: 35−39
Data points enclosed with a diamond are reference sources

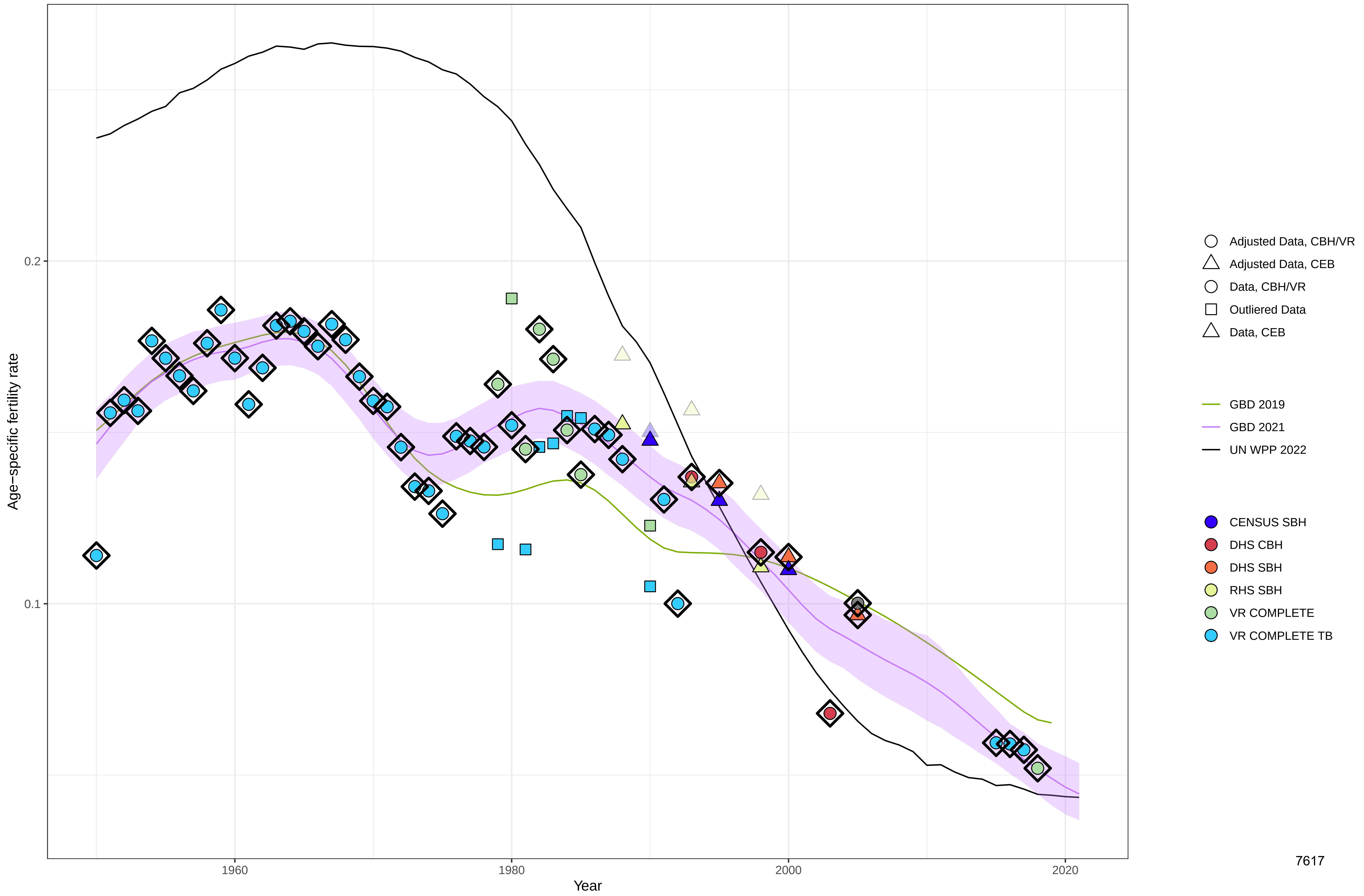

Age−specific fertility fitted trend lines, 1950−2021: Cabo Verde, Ages: 40−44
Data points enclosed with a diamond are reference sources

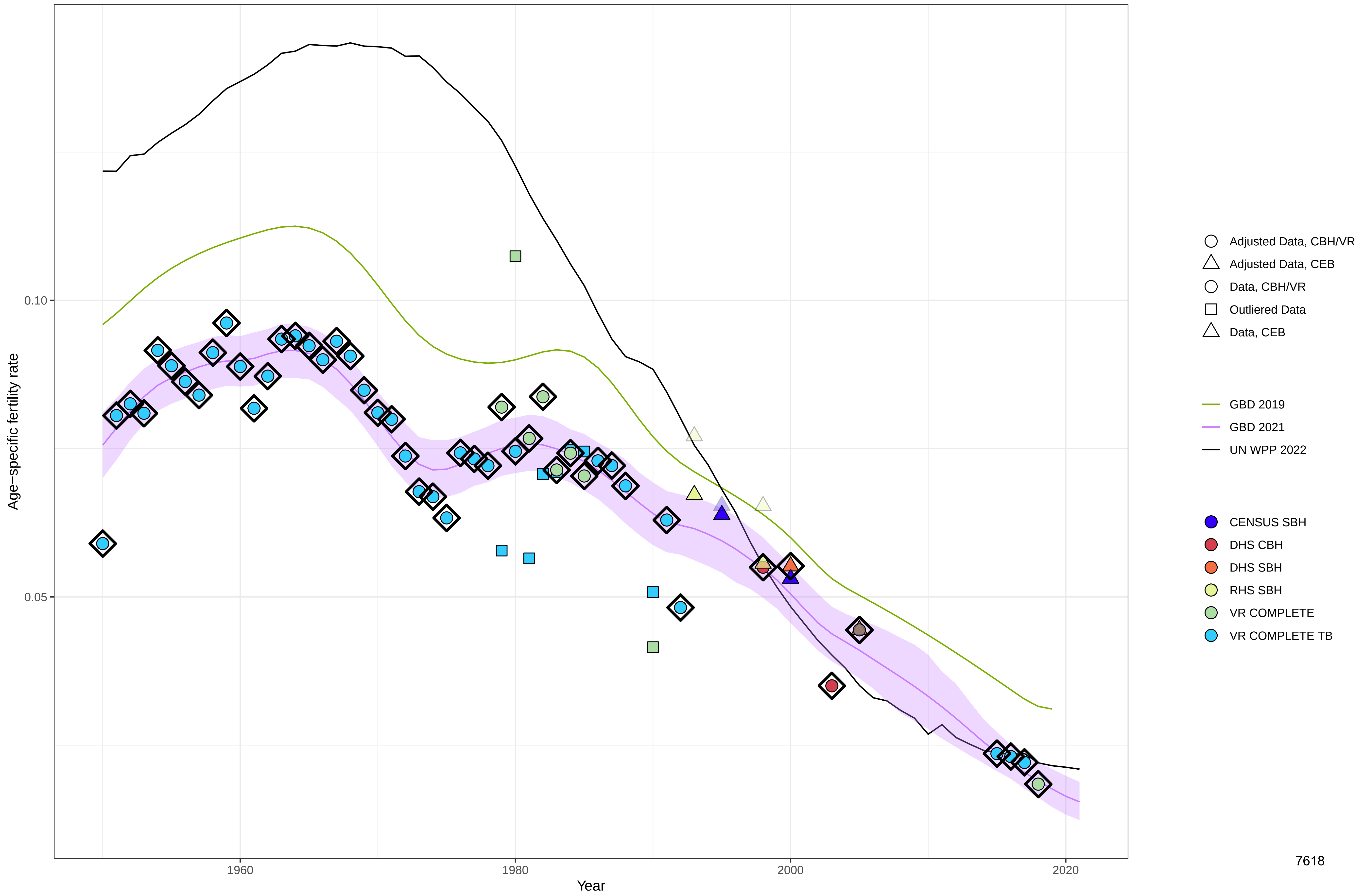

Age−specific fertility fitted trend lines, 1950−2021: Cabo Verde, Ages: 45−49
Data points enclosed with a diamond are reference sources

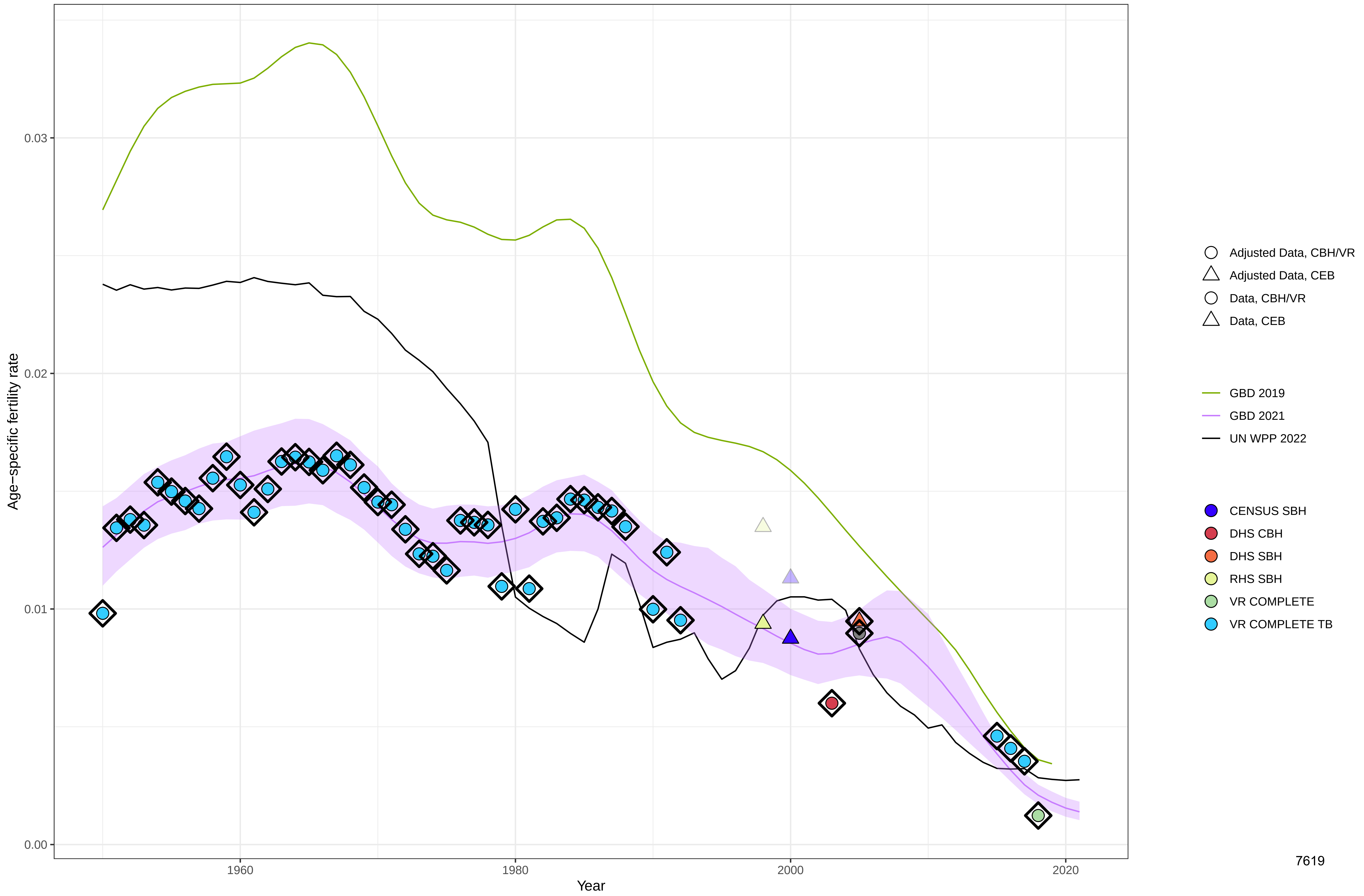

Age−specific fertility fitted trend lines, 1950−2021: Cabo Verde, Ages: 50−54
Data points enclosed with a diamond are reference sources

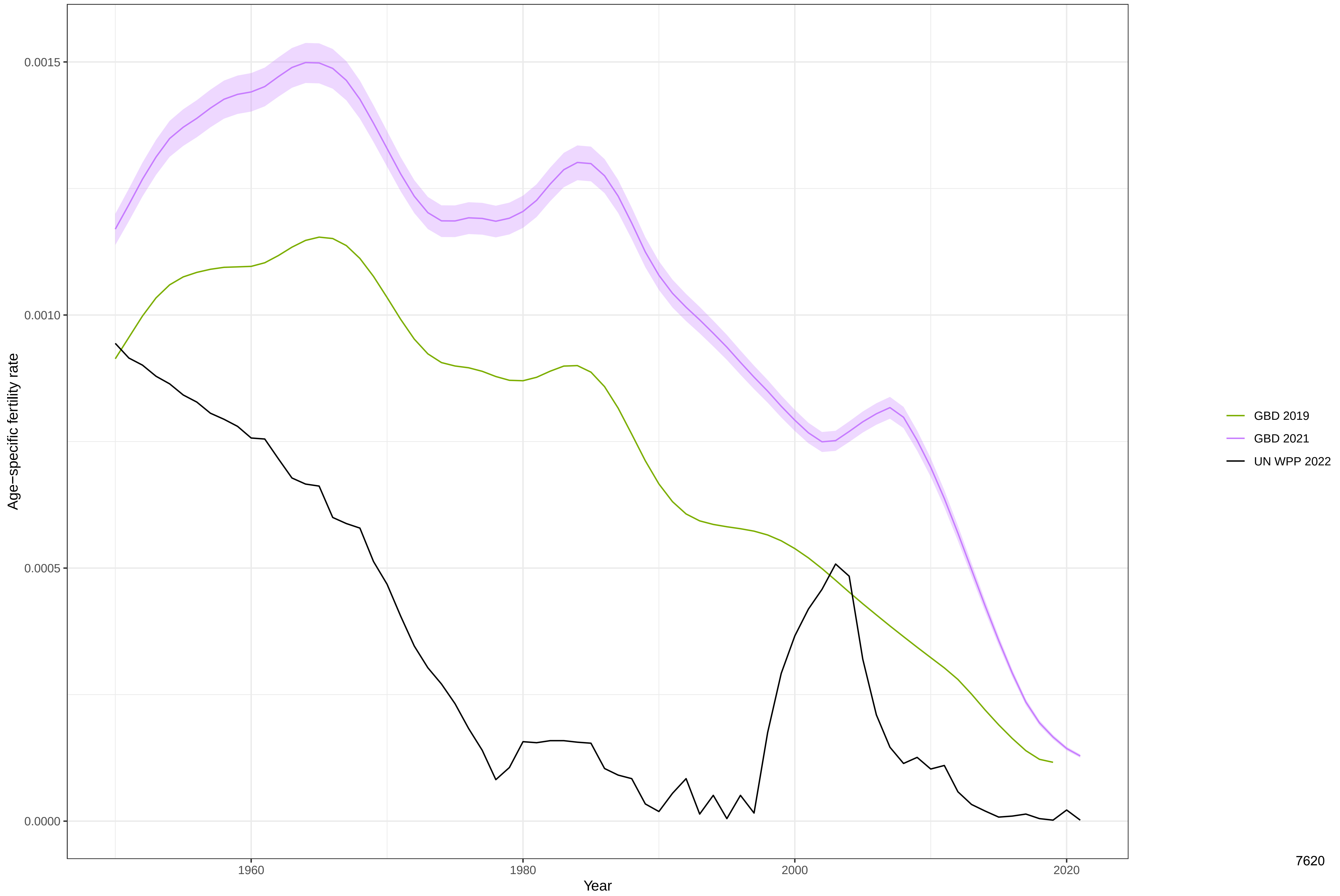

Total fertility rate fitted trend lines, 1950−2021: Cabo Verde TFR
Data points enclosed with a diamond are reference sources

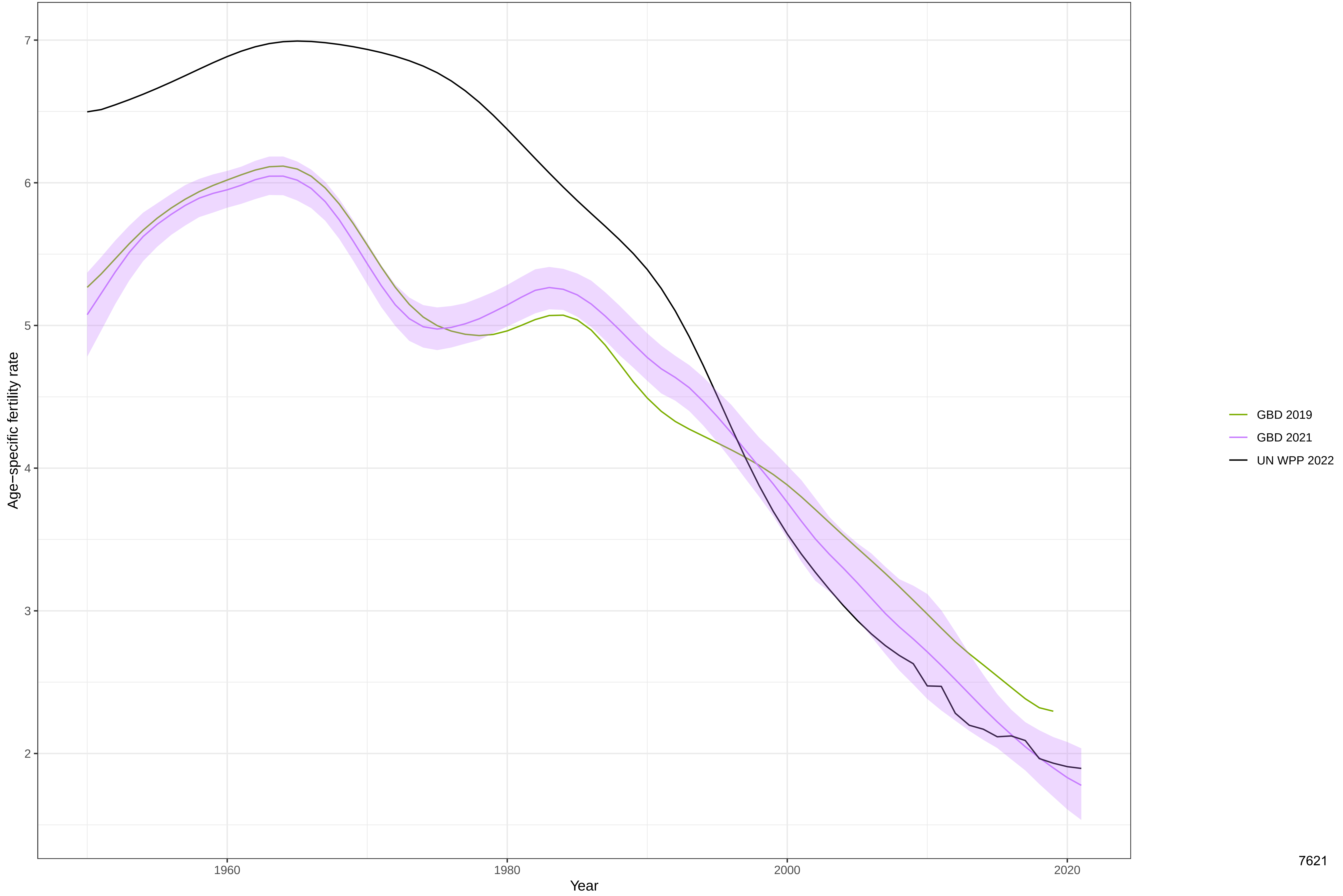

Age−specific fertility fitted trend lines, 1950−2021: Cameroon, Ages: 10−14
Data points enclosed with a diamond are reference sources

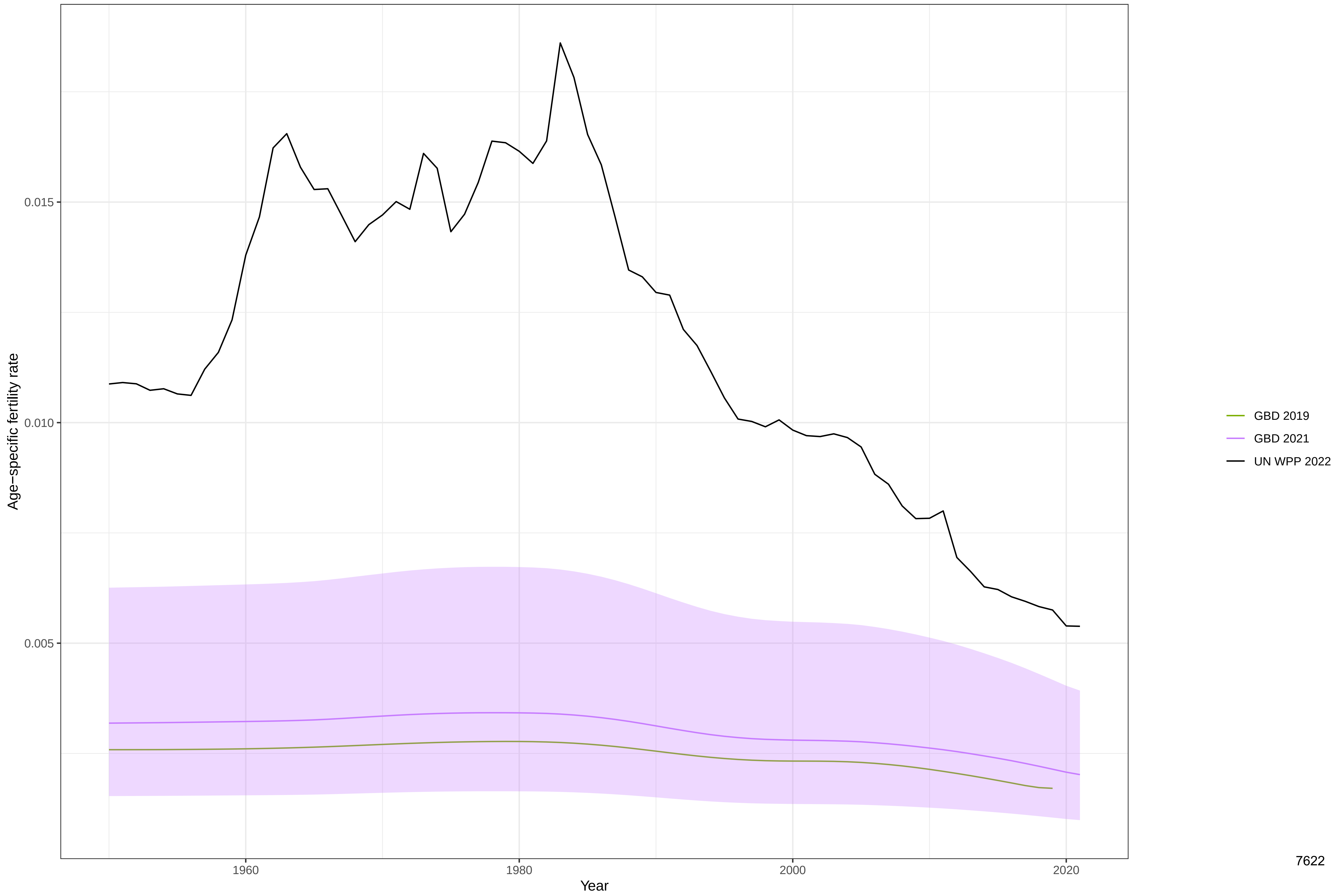

Age−specific fertility fitted trend lines, 1950−2021: Cameroon, Ages: 15−19
Data points enclosed with a diamond are reference sources

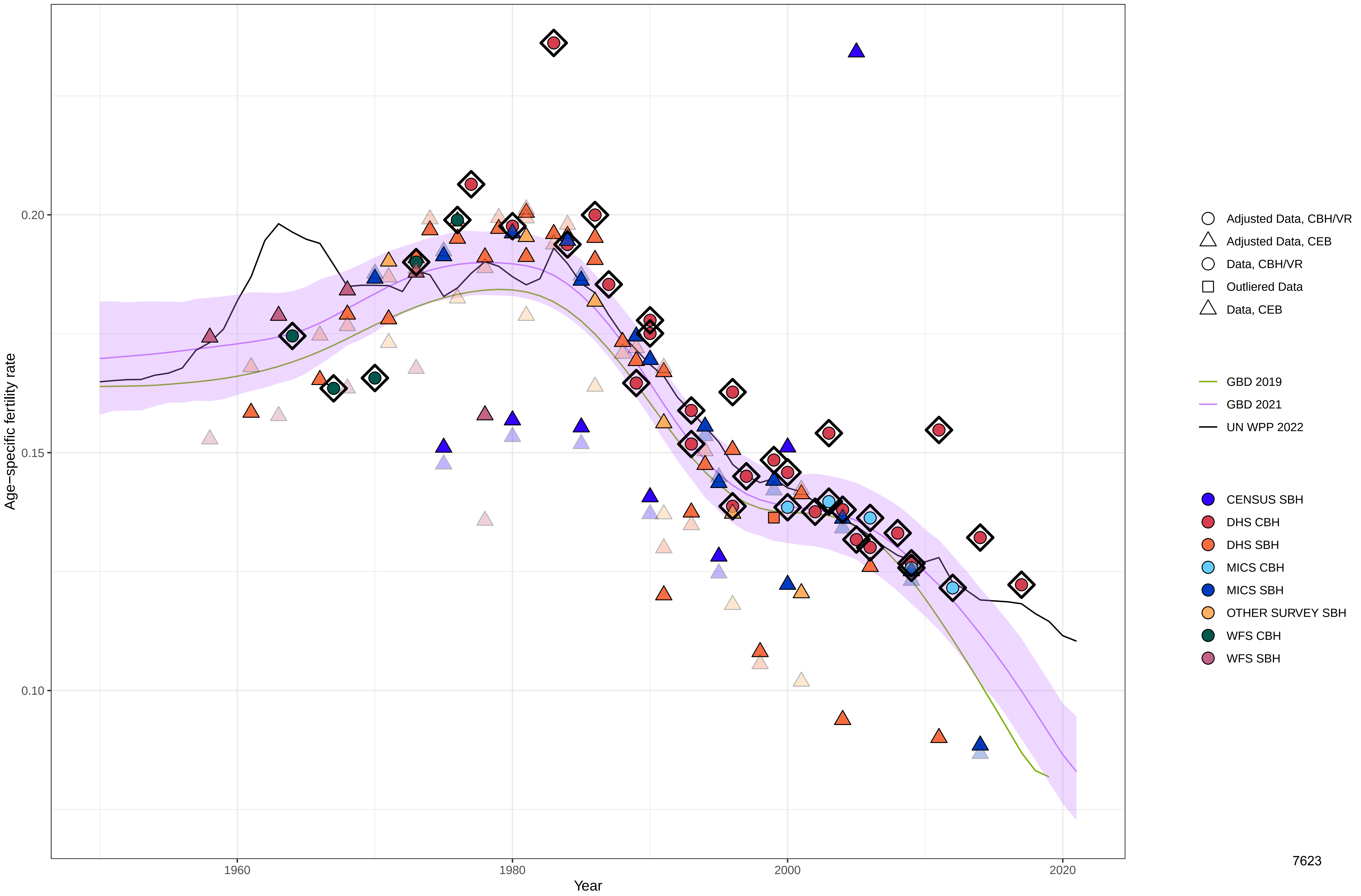

Age−specific fertility fitted trend lines, 1950−2021: Cameroon, Ages: 20−24
Data points enclosed with a diamond are reference sources

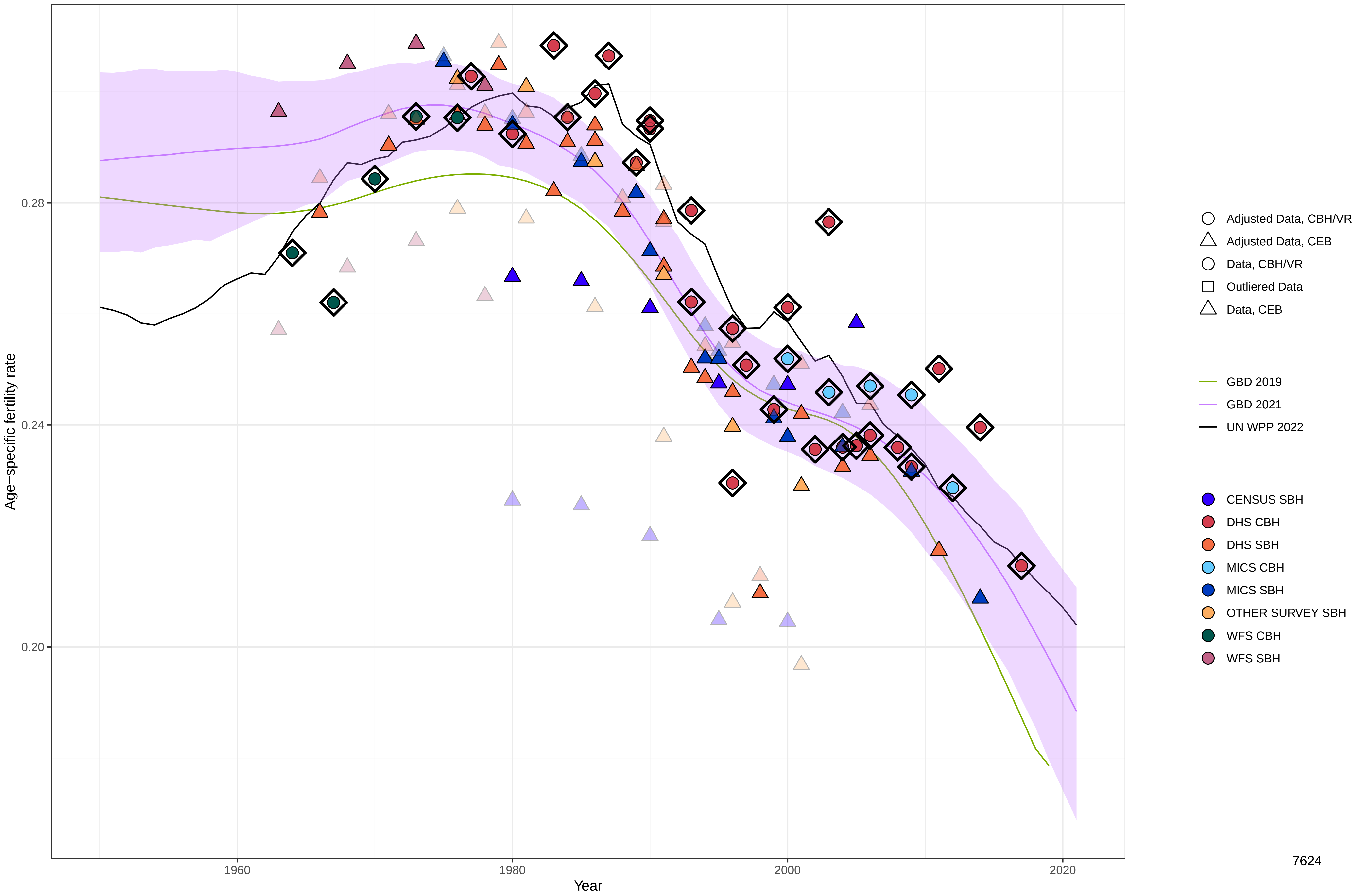

Age−specific fertility fitted trend lines, 1950−2021: Cameroon, Ages: 25−29
Data points enclosed with a diamond are reference sources

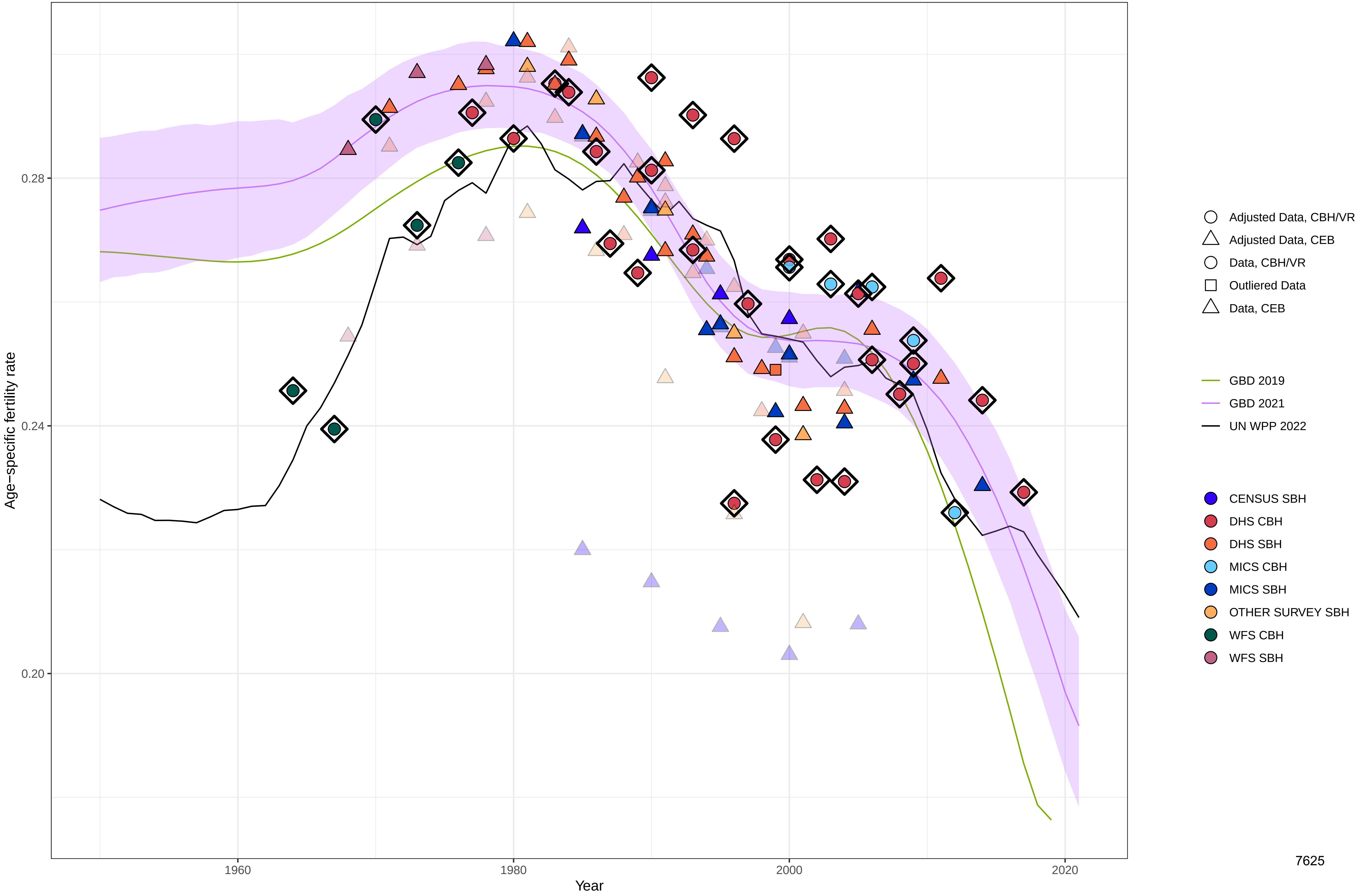

Age−specific fertility fitted trend lines, 1950−2021: Cameroon, Ages: 30−34
Data points enclosed with a diamond are reference sources

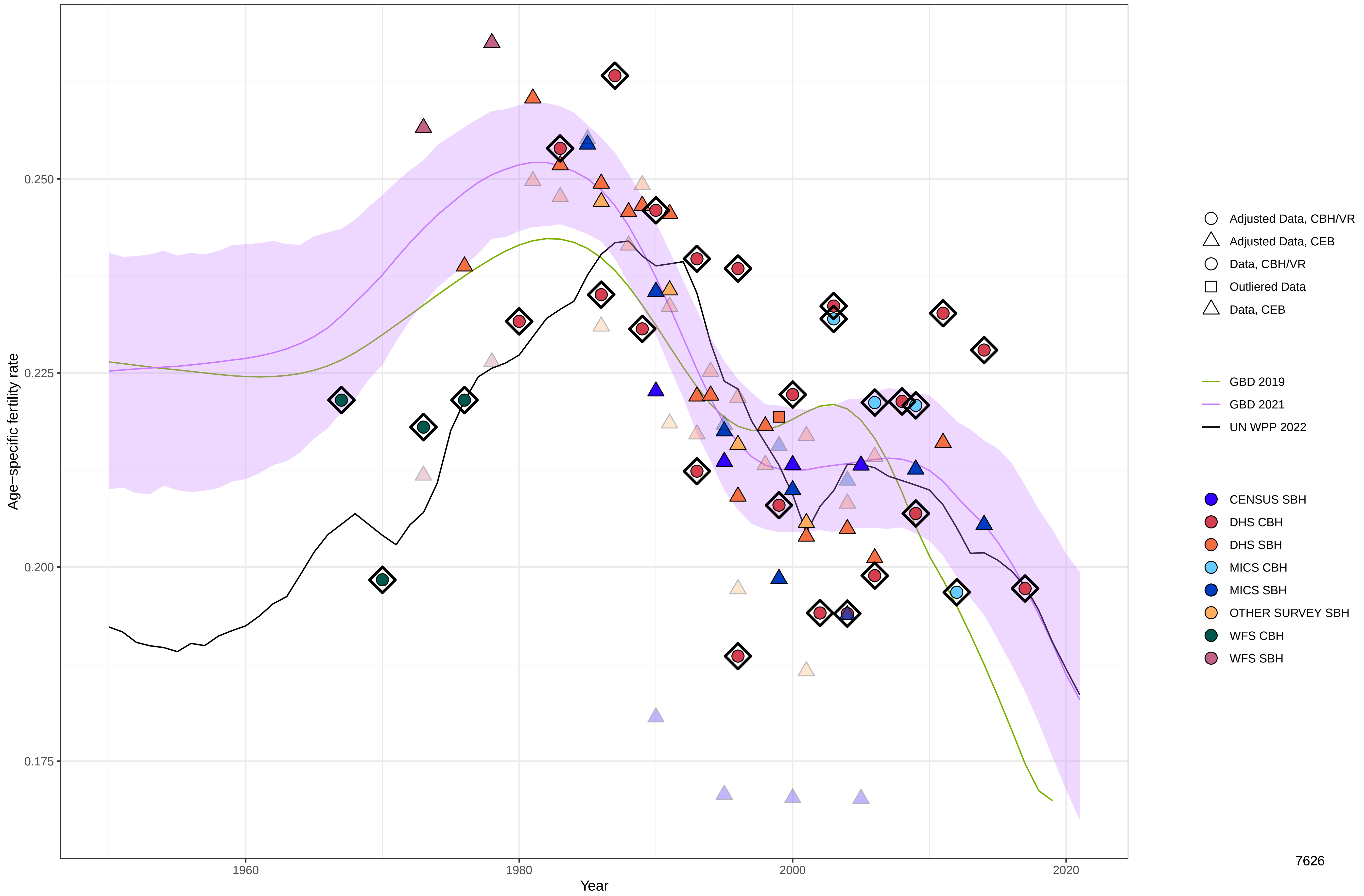

Age−specific fertility fitted trend lines, 1950−2021: Cameroon, Ages: 35−39
Data points enclosed with a diamond are reference sources

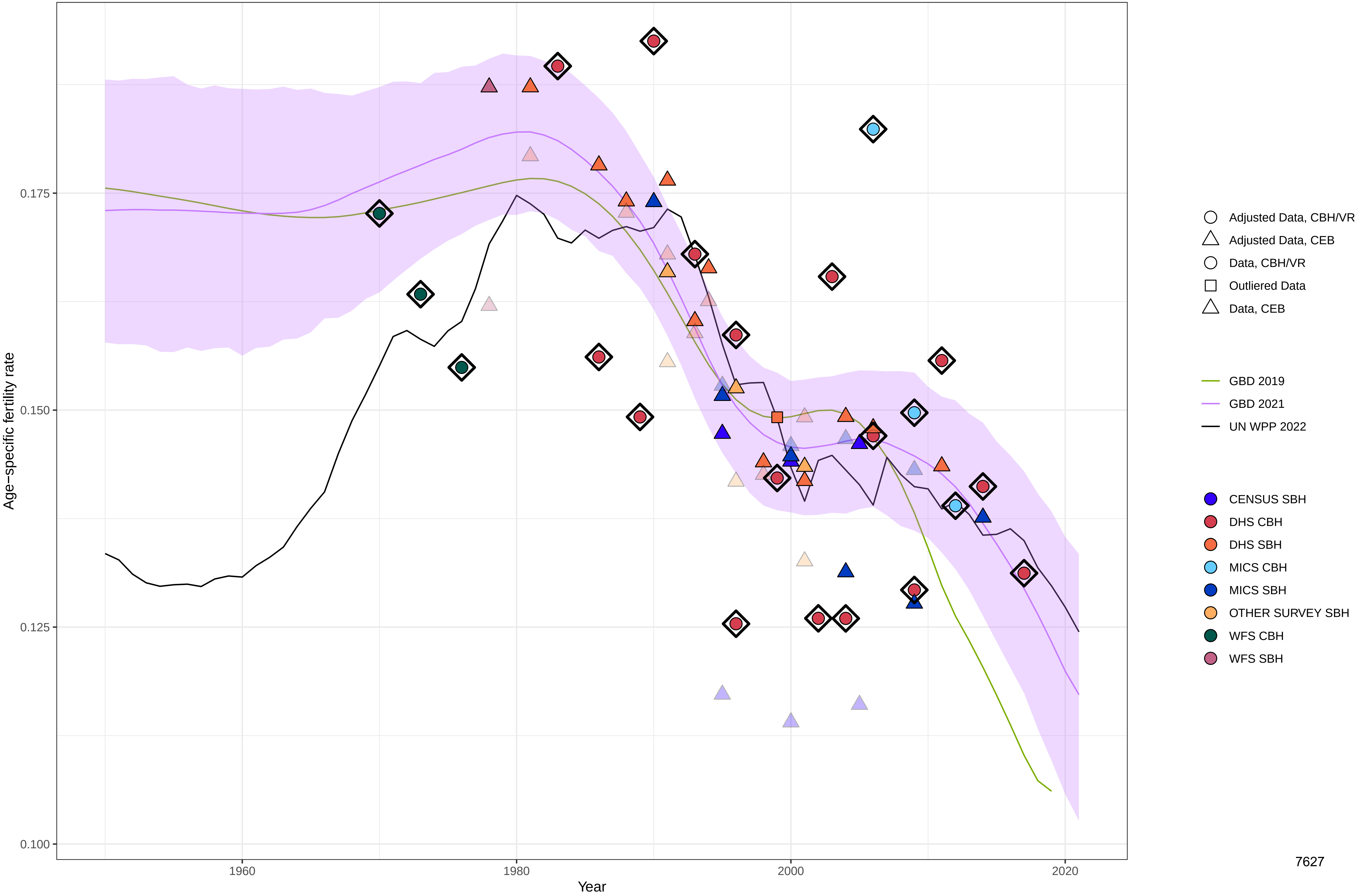

Age−specific fertility fitted trend lines, 1950−2021: Cameroon, Ages: 40−44
Data points enclosed with a diamond are reference sources

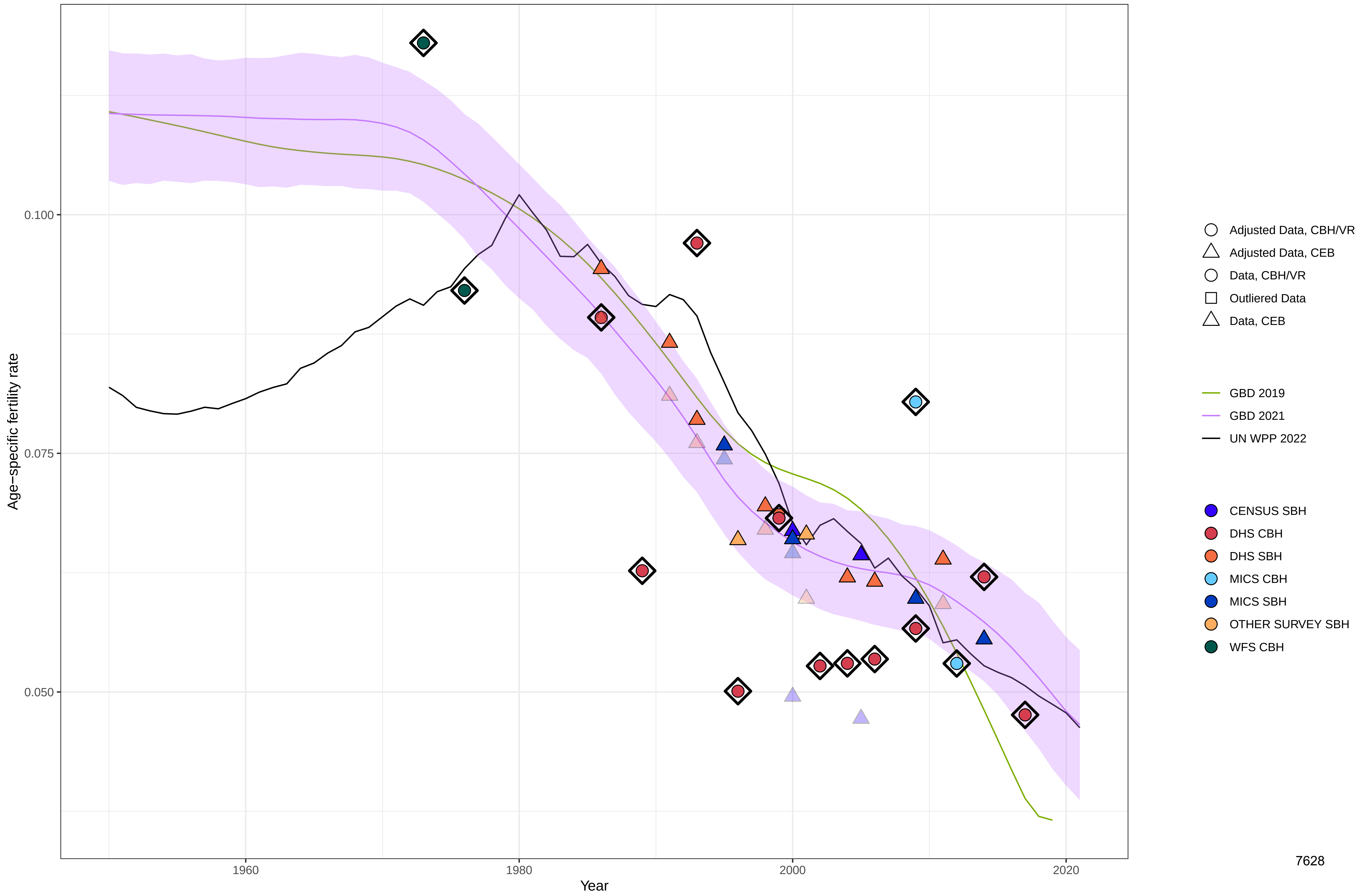

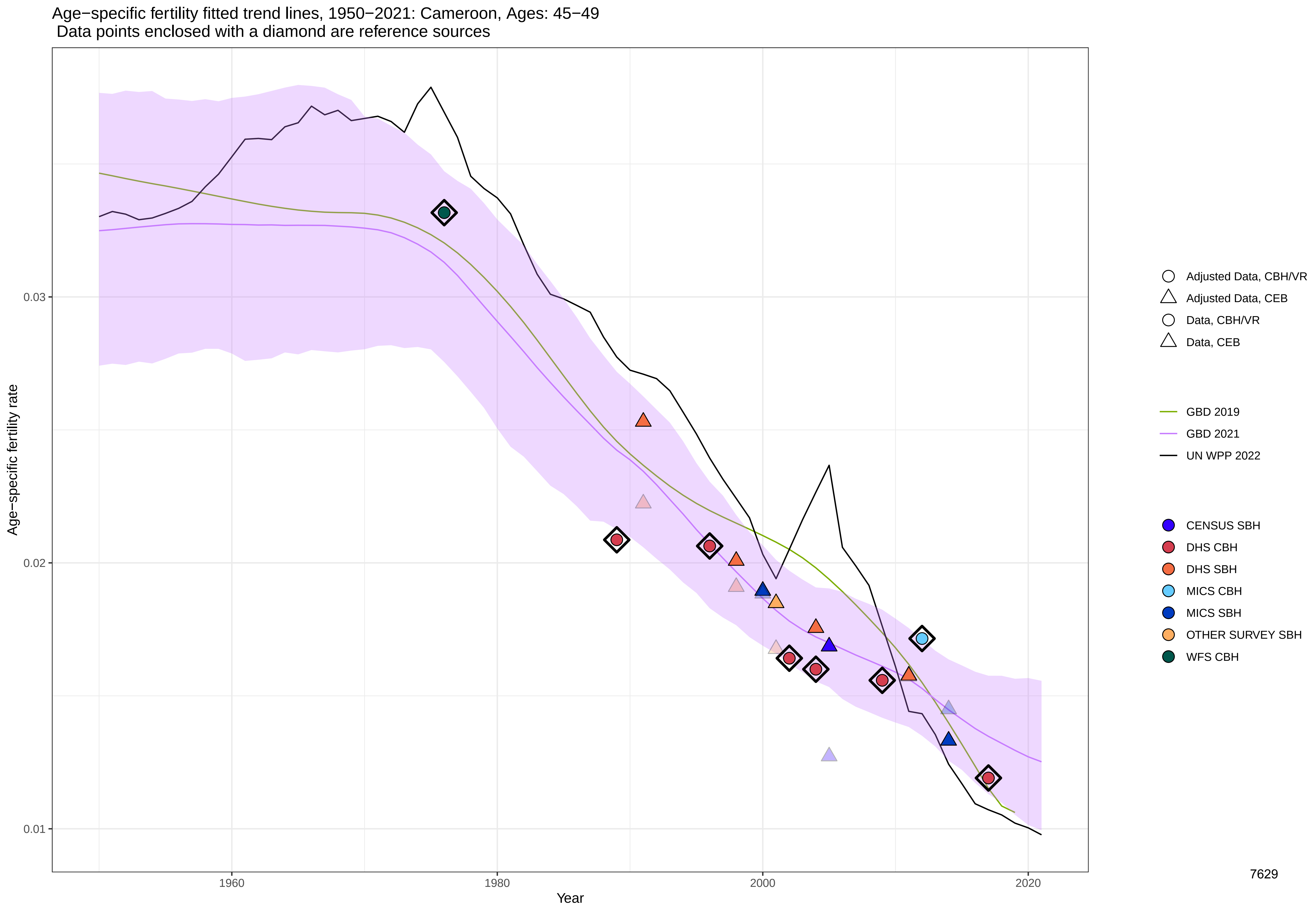

Age−specific fertility fitted trend lines, 1950−2021: Cameroon, Ages: 45−49
Data points enclosed with a diamond are reference sources
Age−specific fertility rate
0.03
0.02
0.01
1960
1980
2000
2020
Year
Adjusted Data, CBH/VR
Adjusted Data, CEB
Data, CBH/VR
Data, CEB
GBD 2019
GBD 2021
UN WPP 2022
CENSUS SBH
DHS CBH
DHS SBH
MICS CBH
MICS SBH
OTHER SURVEY SBH
WFS CBH

Age−specific fertility fitted trend lines, 1950−2021: Cameroon, Ages: 50−54
Data points enclosed with a diamond are reference sources

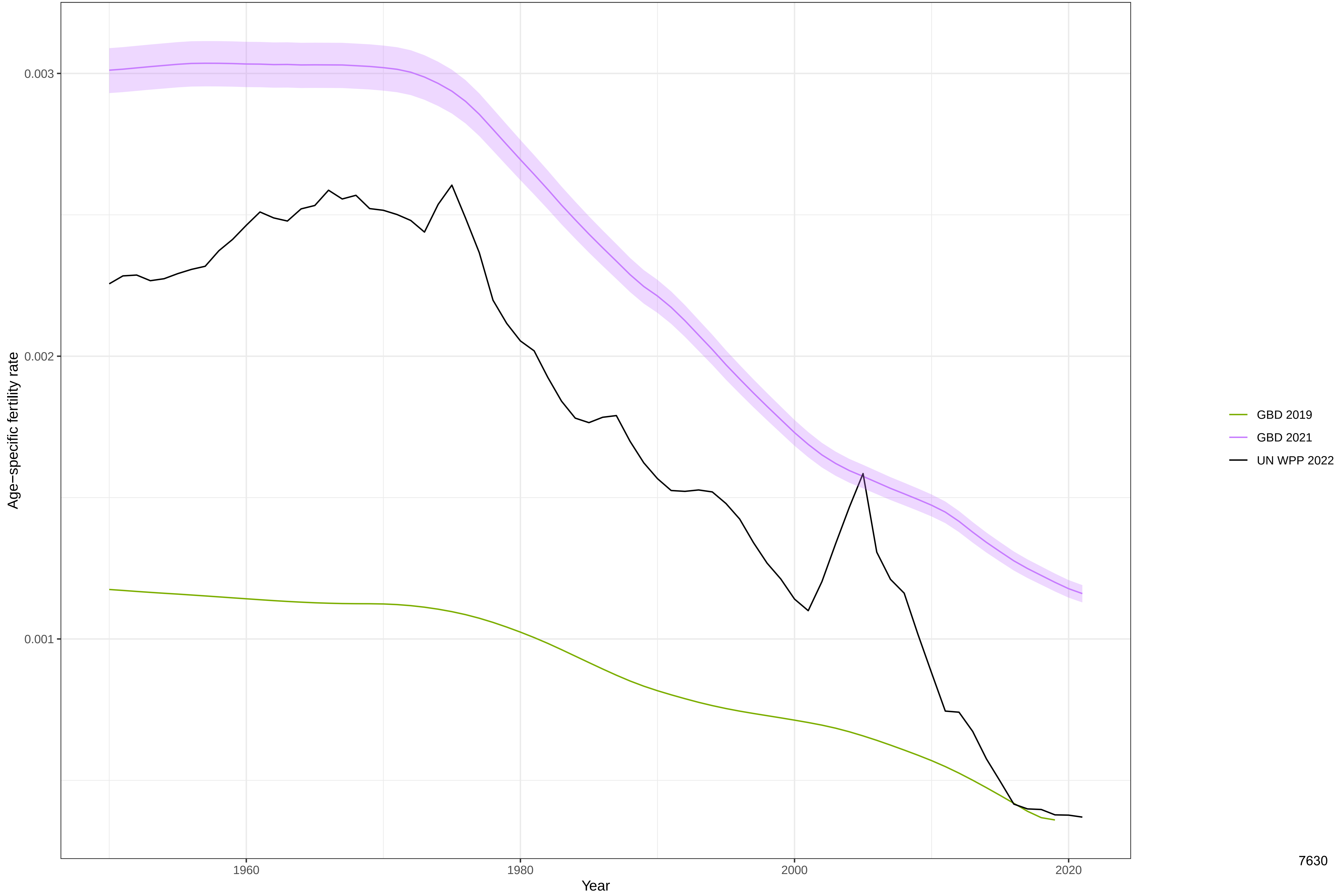

Total fertility rate fitted trend lines, 1950−2021: Cameroon TFR
Data points enclosed with a diamond are reference sources

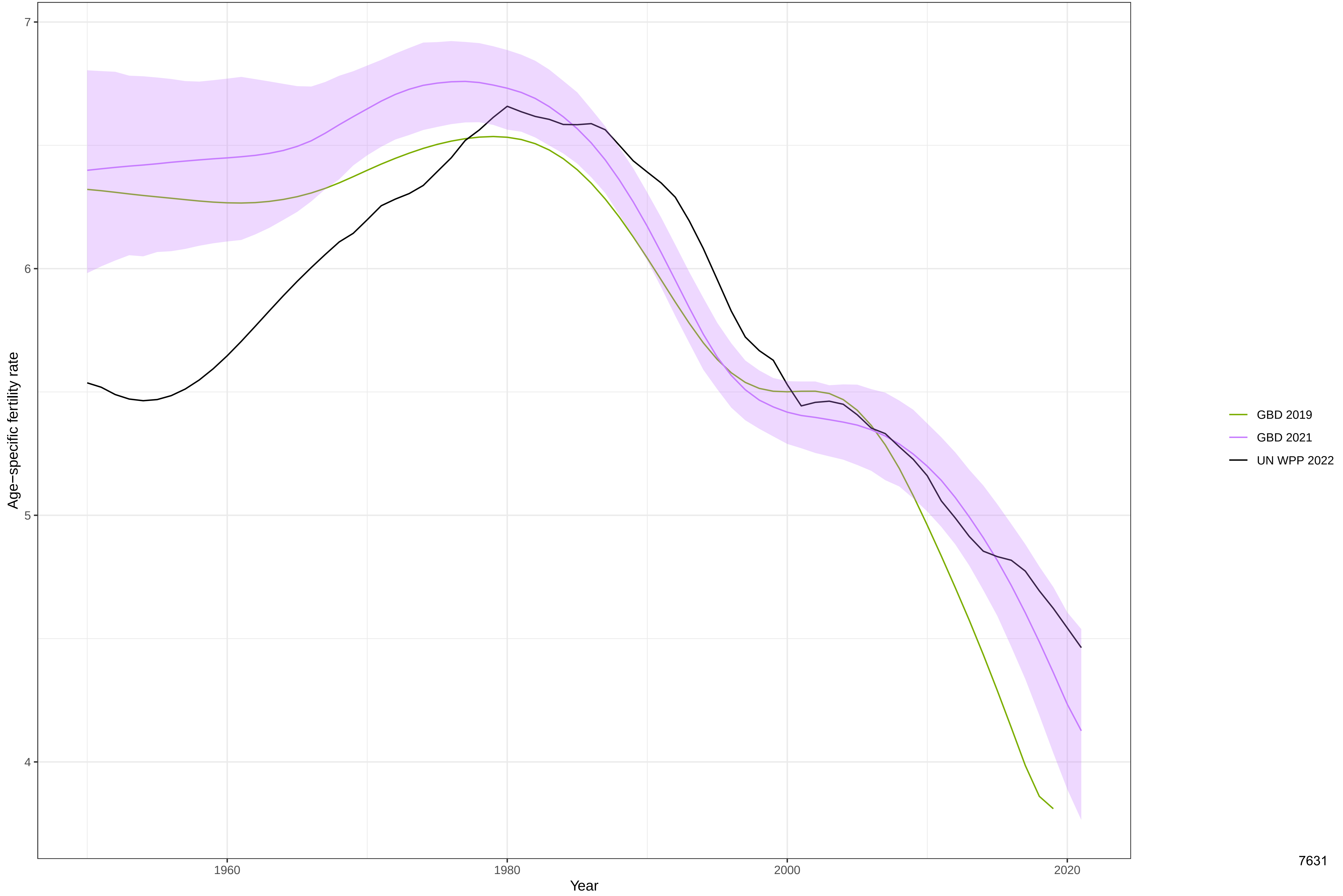

Age−specific fertility fitted trend lines, 1950−2021: Chad, Ages: 10−14
Data points enclosed with a diamond are reference sources

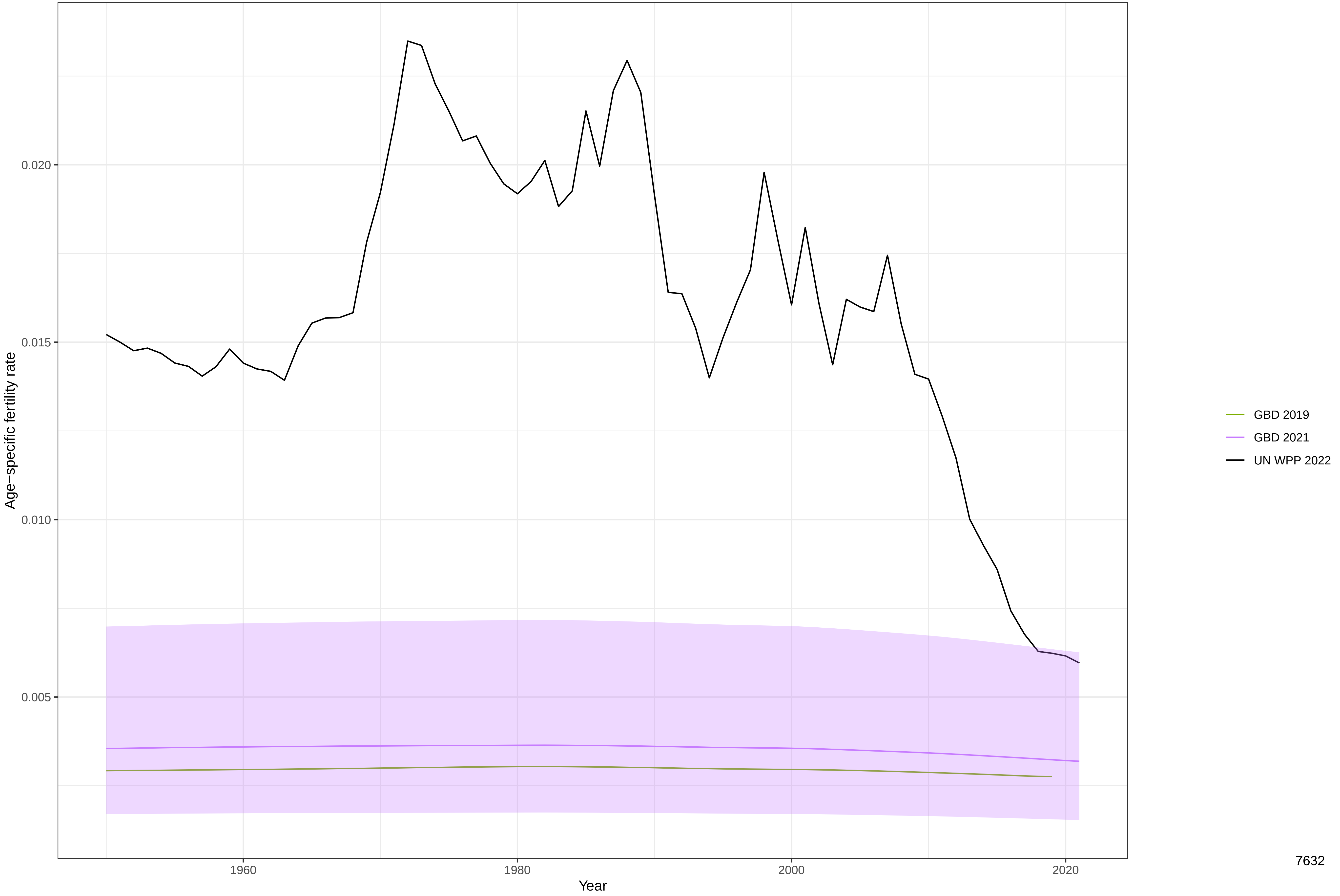

Age−specific fertility fitted trend lines, 1950−2021: Chad, Ages: 15−19
Data points enclosed with a diamond are reference sources

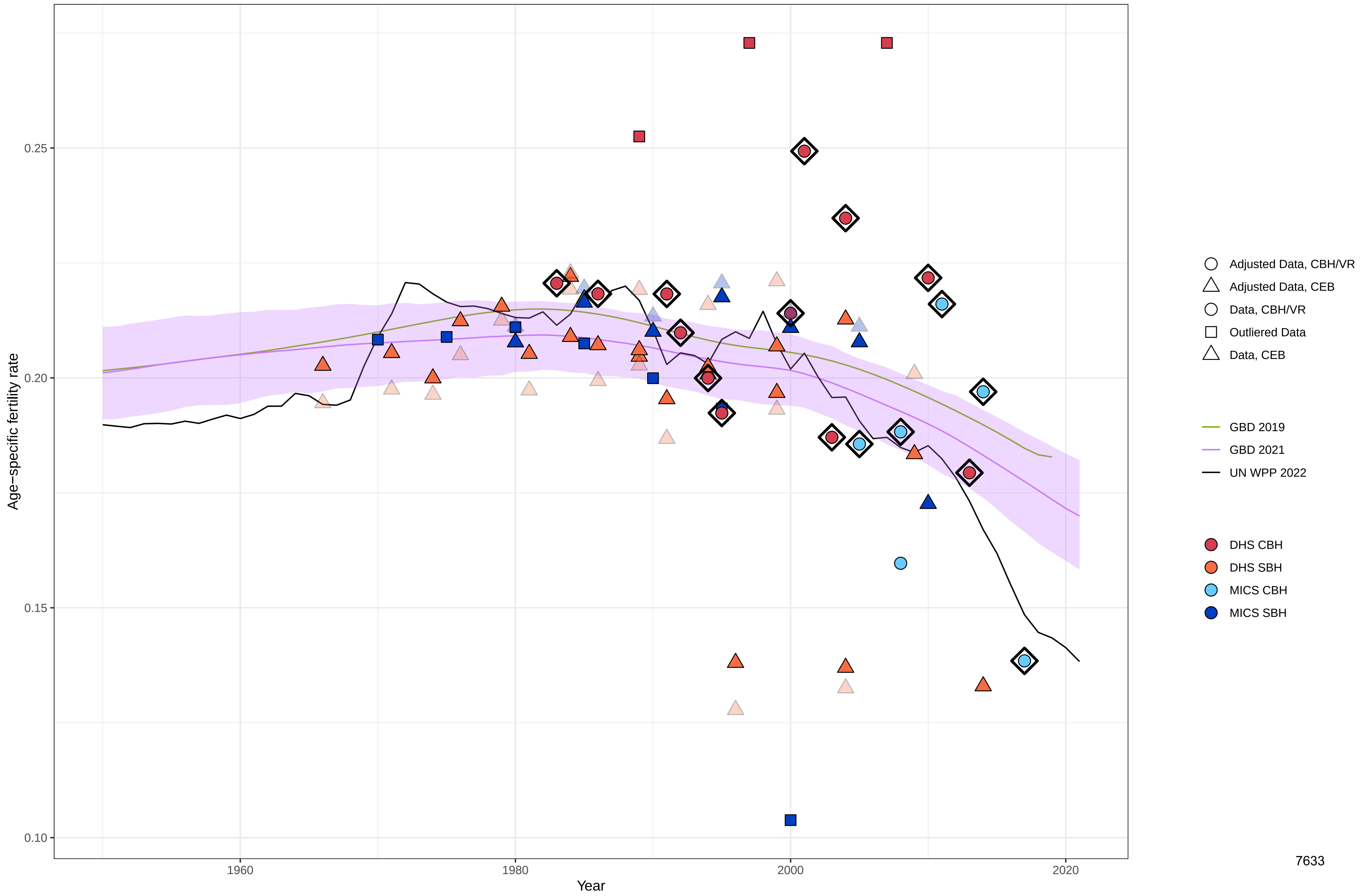

Age−specific fertility fitted trend lines, 1950−2021: Chad, Ages: 20−24
Data points enclosed with a diamond are reference sources

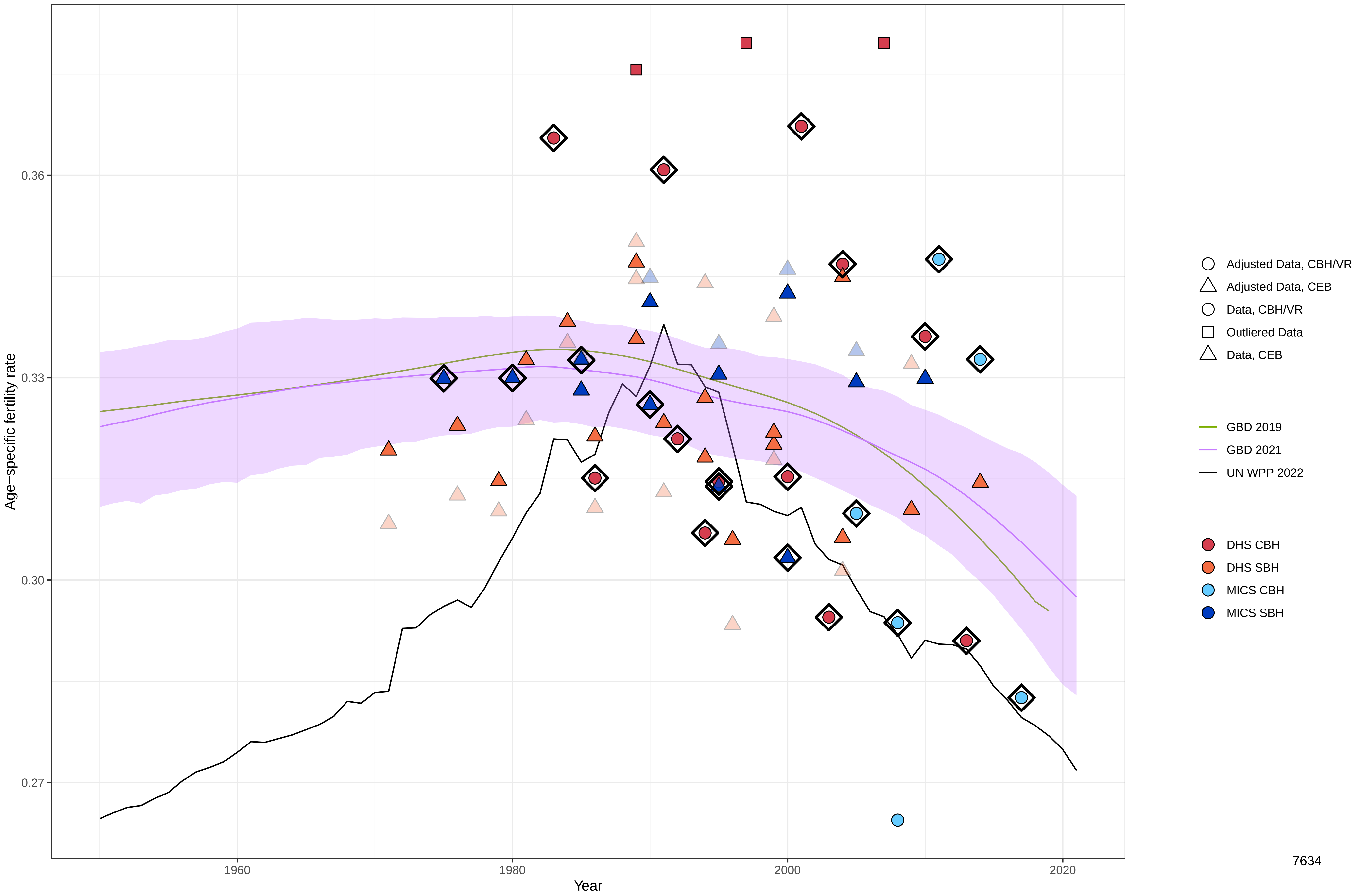

Age−specific fertility fitted trend lines, 1950−2021: Chad, Ages: 25−29
Data points enclosed with a diamond are reference sources

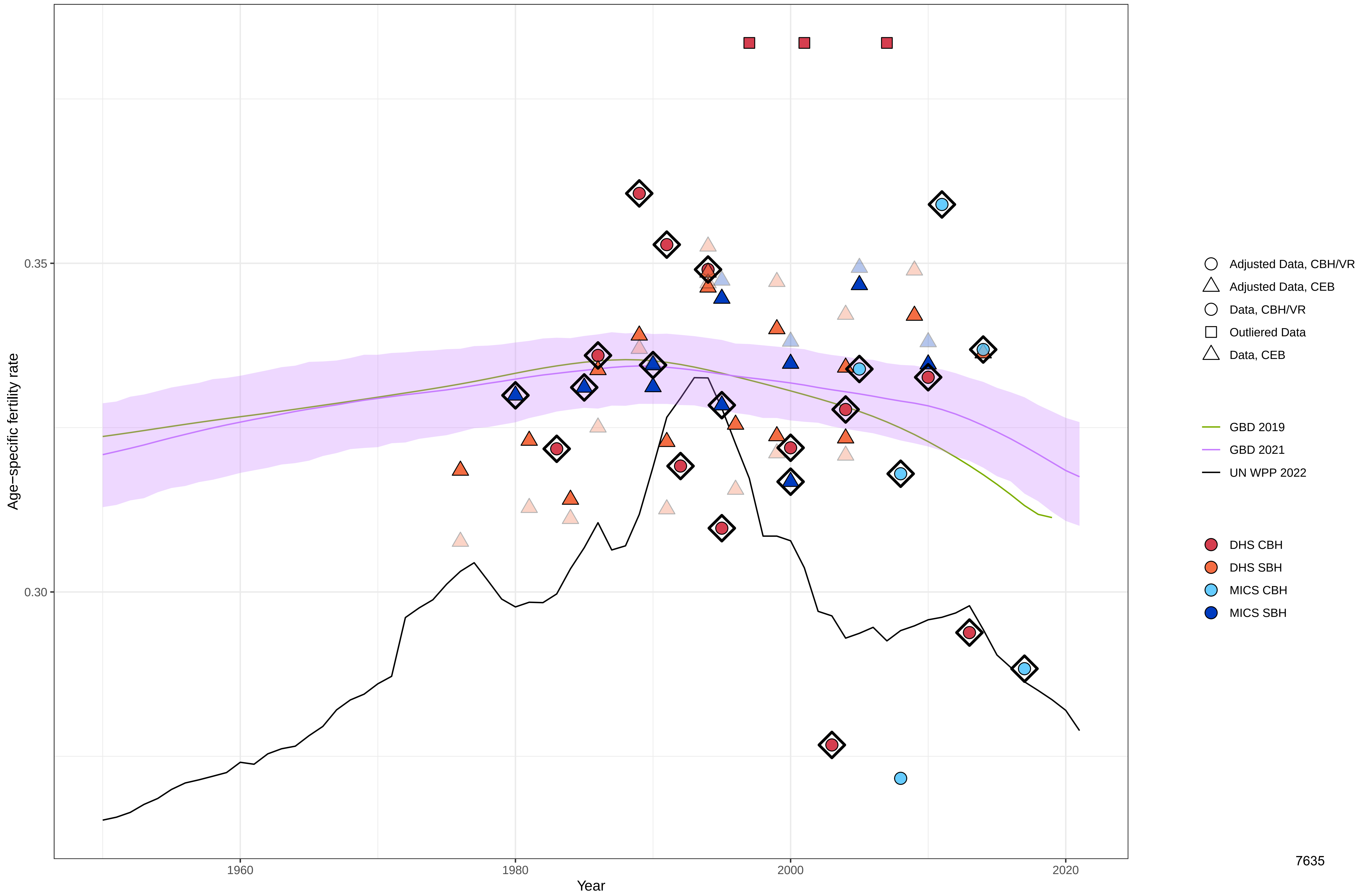

Age−specific fertility fitted trend lines, 1950−2021: Chad, Ages: 30−34
Data points enclosed with a diamond are reference sources

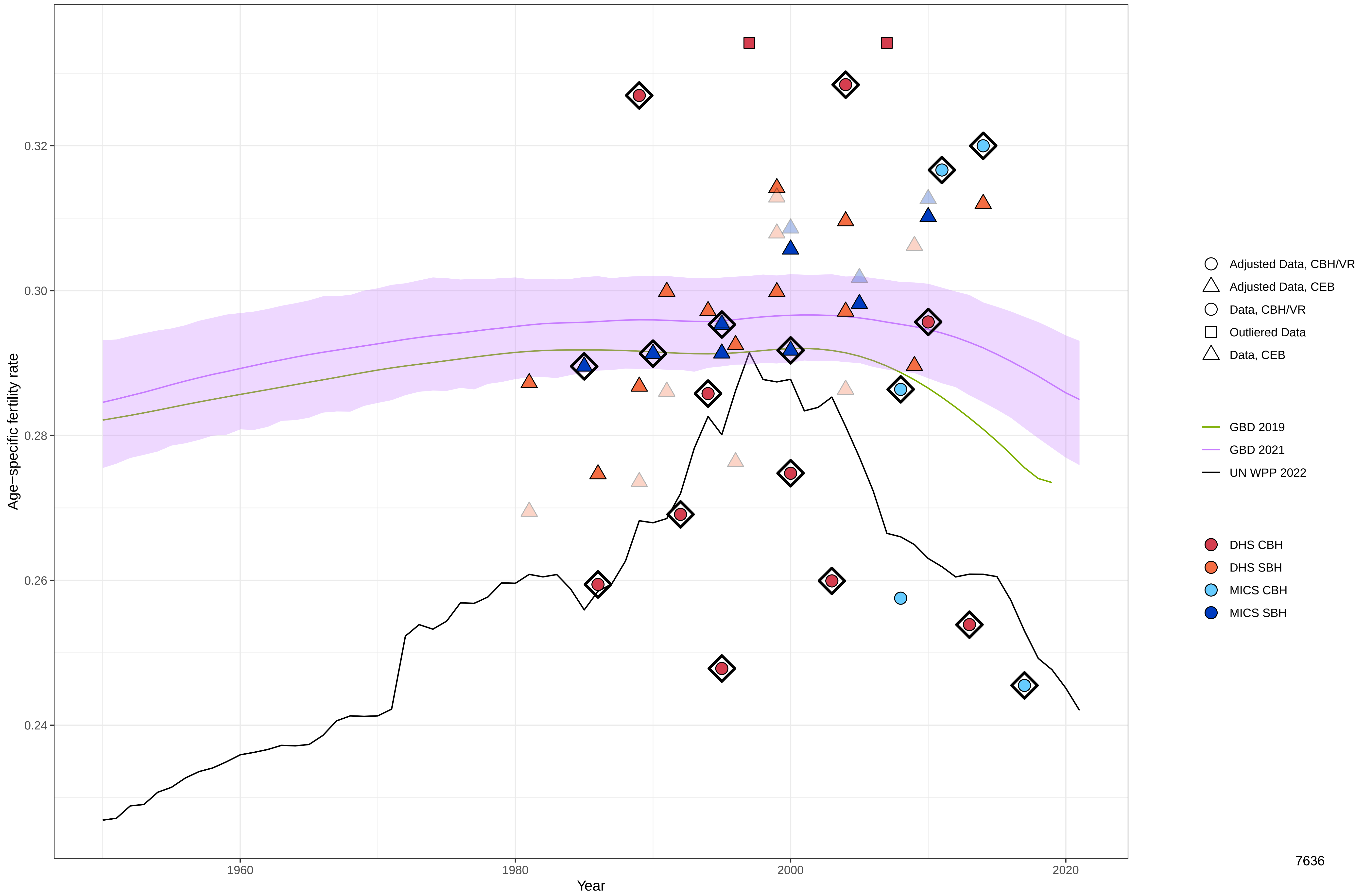

Age−specific fertility fitted trend lines, 1950−2021: Chad, Ages: 35−39
Data points enclosed with a diamond are reference sources

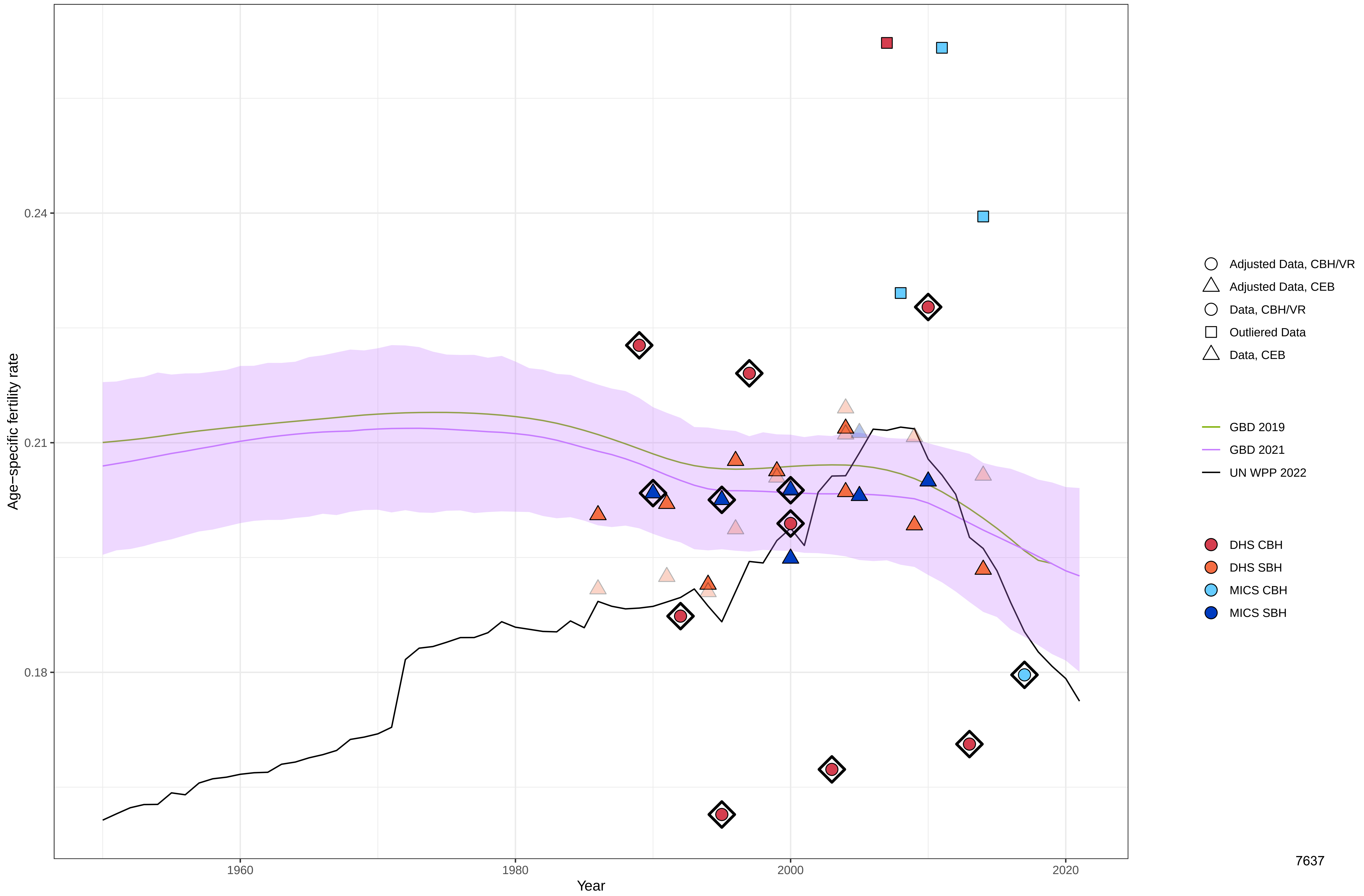

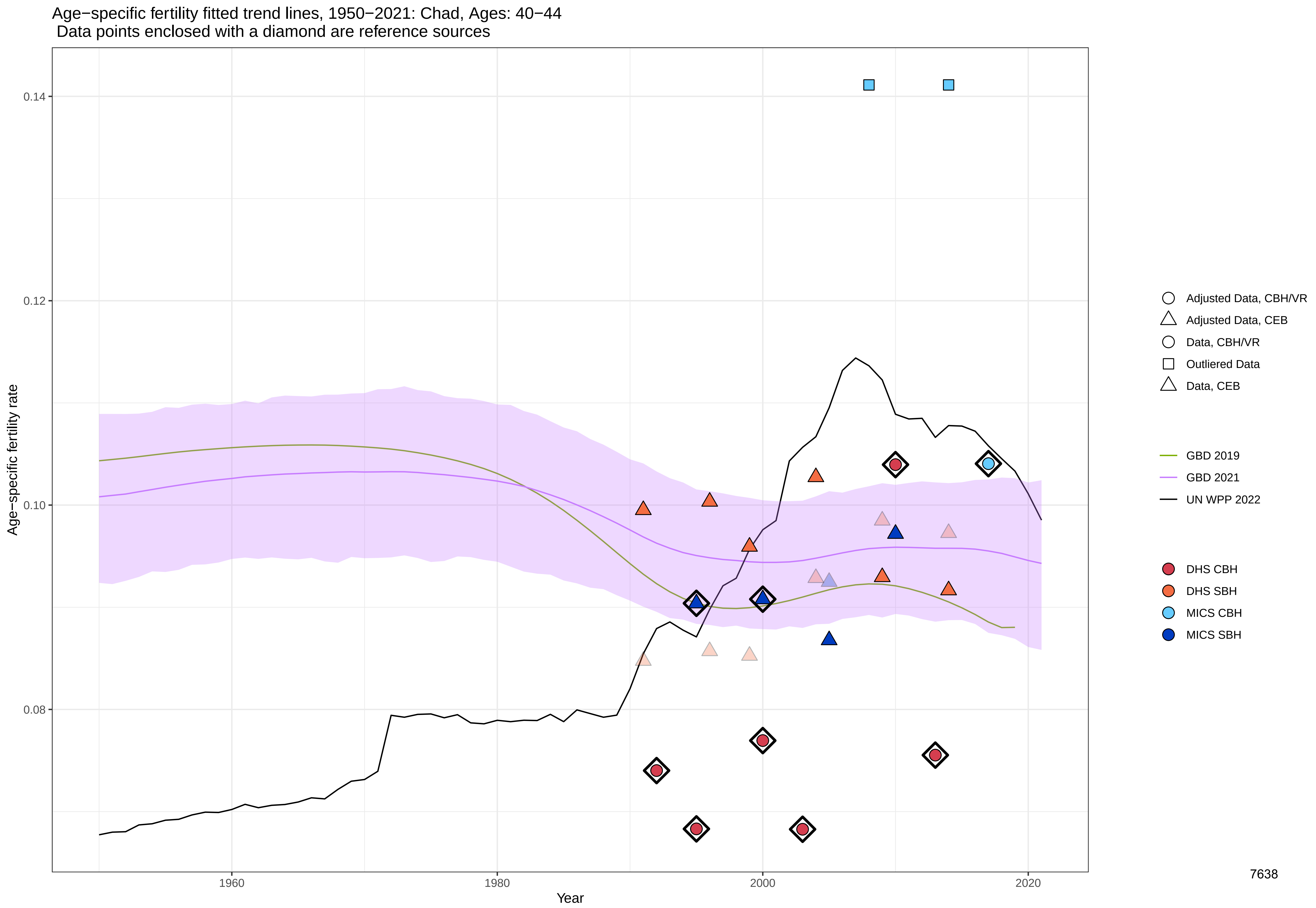

Age−specific fertility fitted trend lines, 1950−2021: Chad, Ages: 40−44
Data points enclosed with a diamond are reference sources
Age−specific fertility rate
0.14
0.12
0.10
0.08
1960
1980
2000
2020
Year
Adjusted Data, CBH/VR
Adjusted Data, CEB
Data, CBH/VR
Outliered Data
Data, CEB
GBD 2019
GBD 2021
UN WPP 2022
DHS CBH
DHS SBH
MICS CBH
MICS SBH

7638

Age−specific fertility fitted trend lines, 1950−2021: Chad, Ages: 45−49
Data points enclosed with a diamond are reference sources

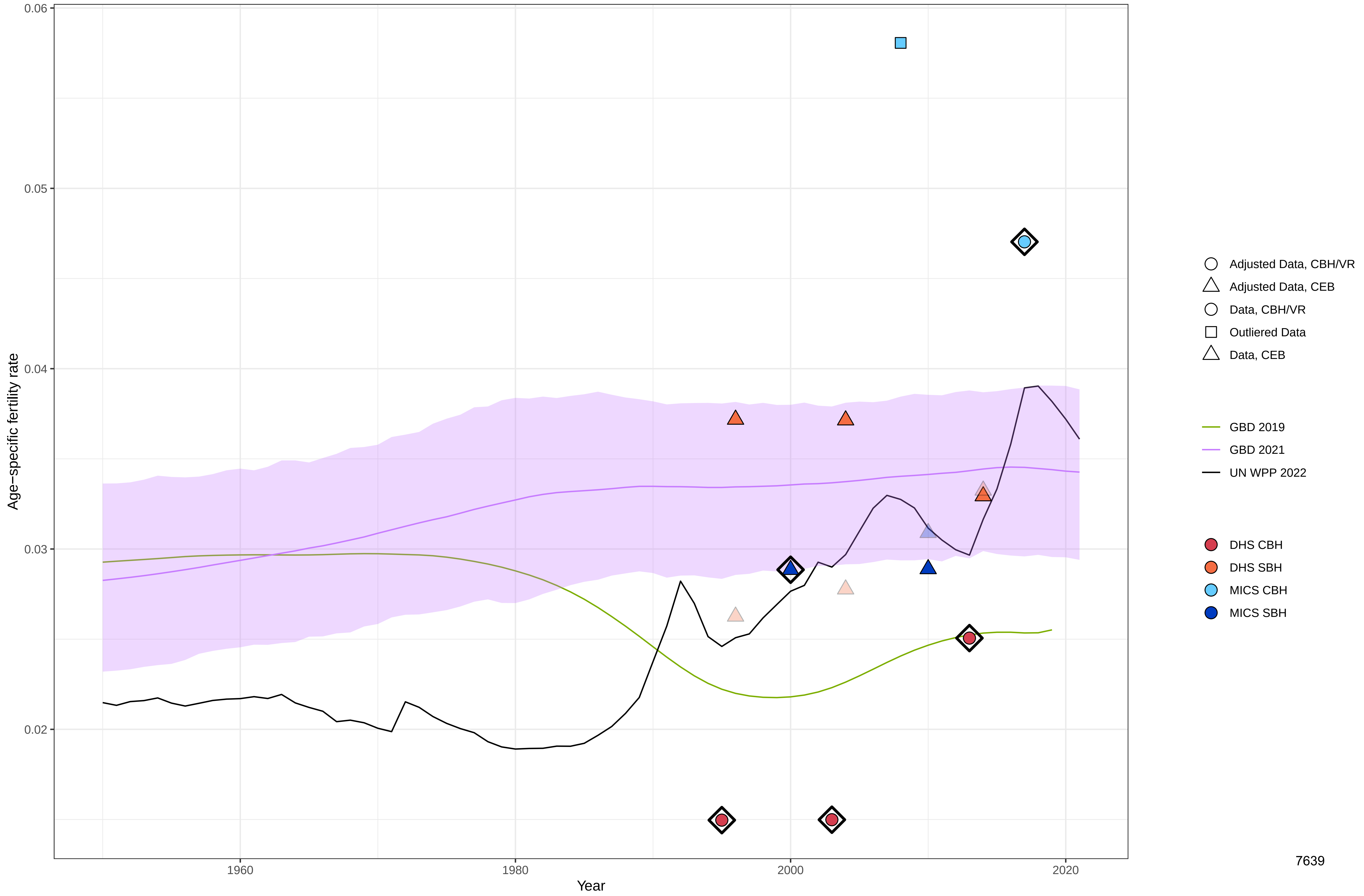

Age−specific fertility fitted trend lines, 1950−2021: Chad, Ages: 50−54
Data points enclosed with a diamond are reference sources

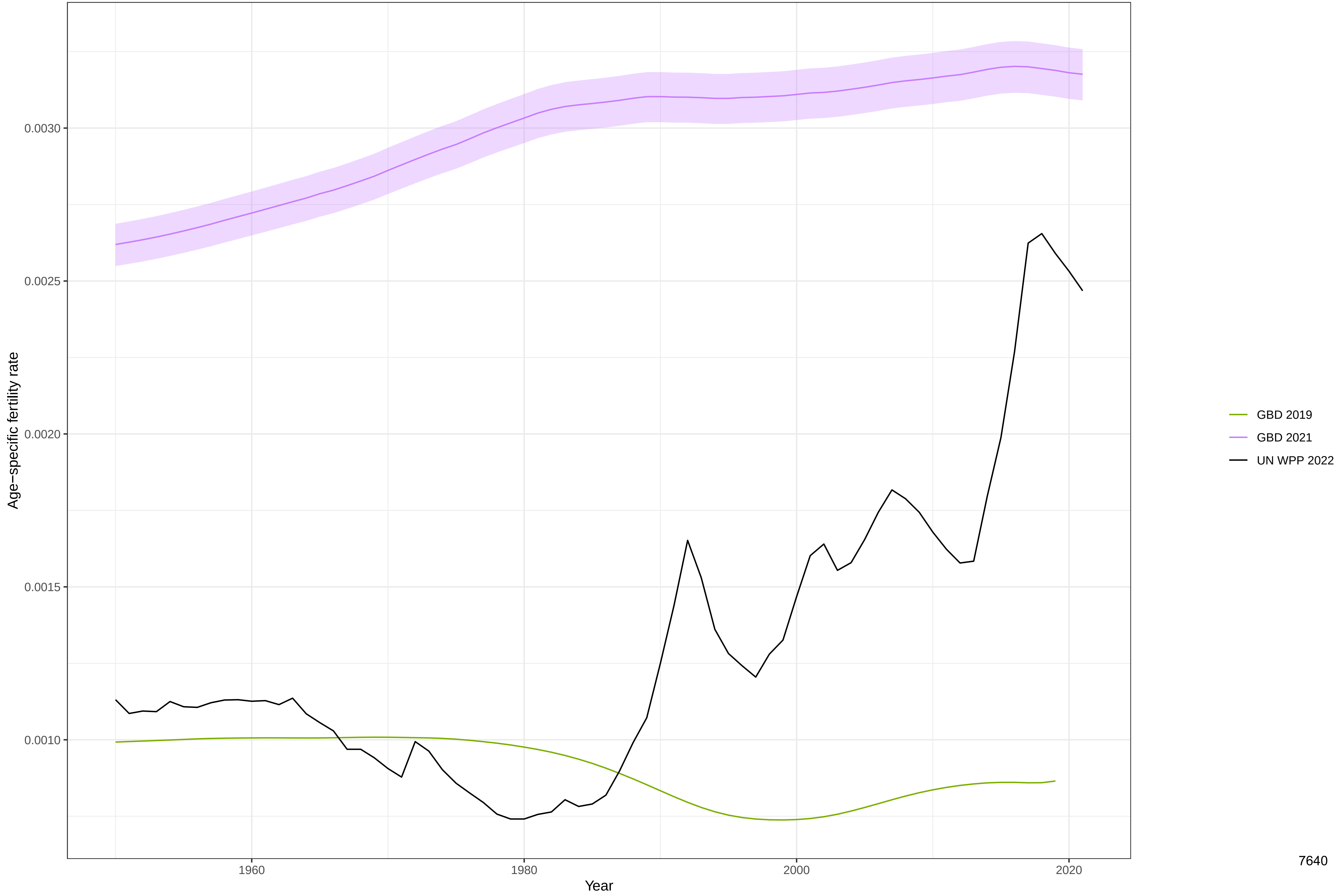

Total fertility rate fitted trend lines, 1950−2021: Chad TFR
Data points enclosed with a diamond are reference sources

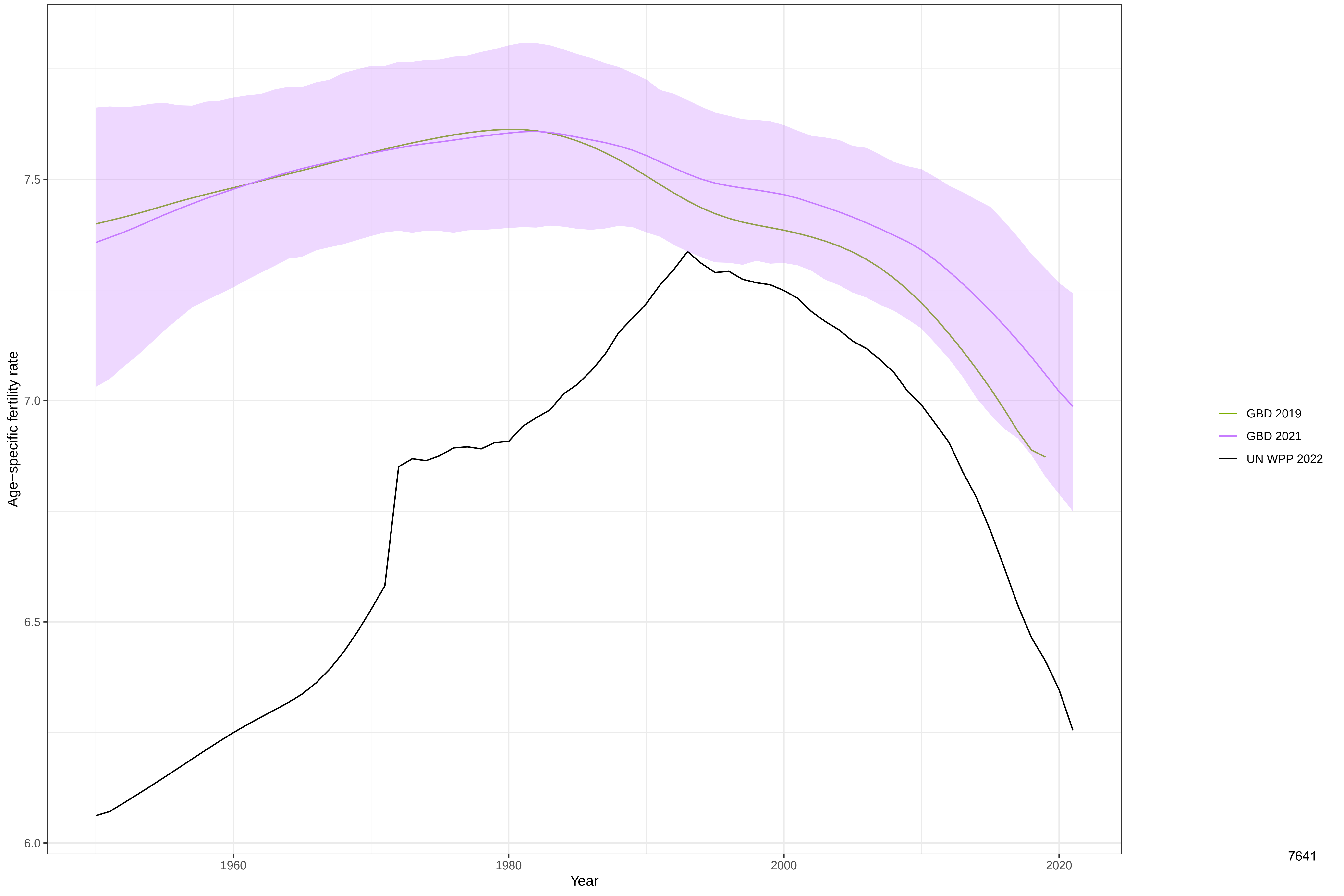

Age−specific fertility fitted trend lines, 1950−2021: Côte d'Ivoire, Ages: 10−14
Data points enclosed with a diamond are reference sources

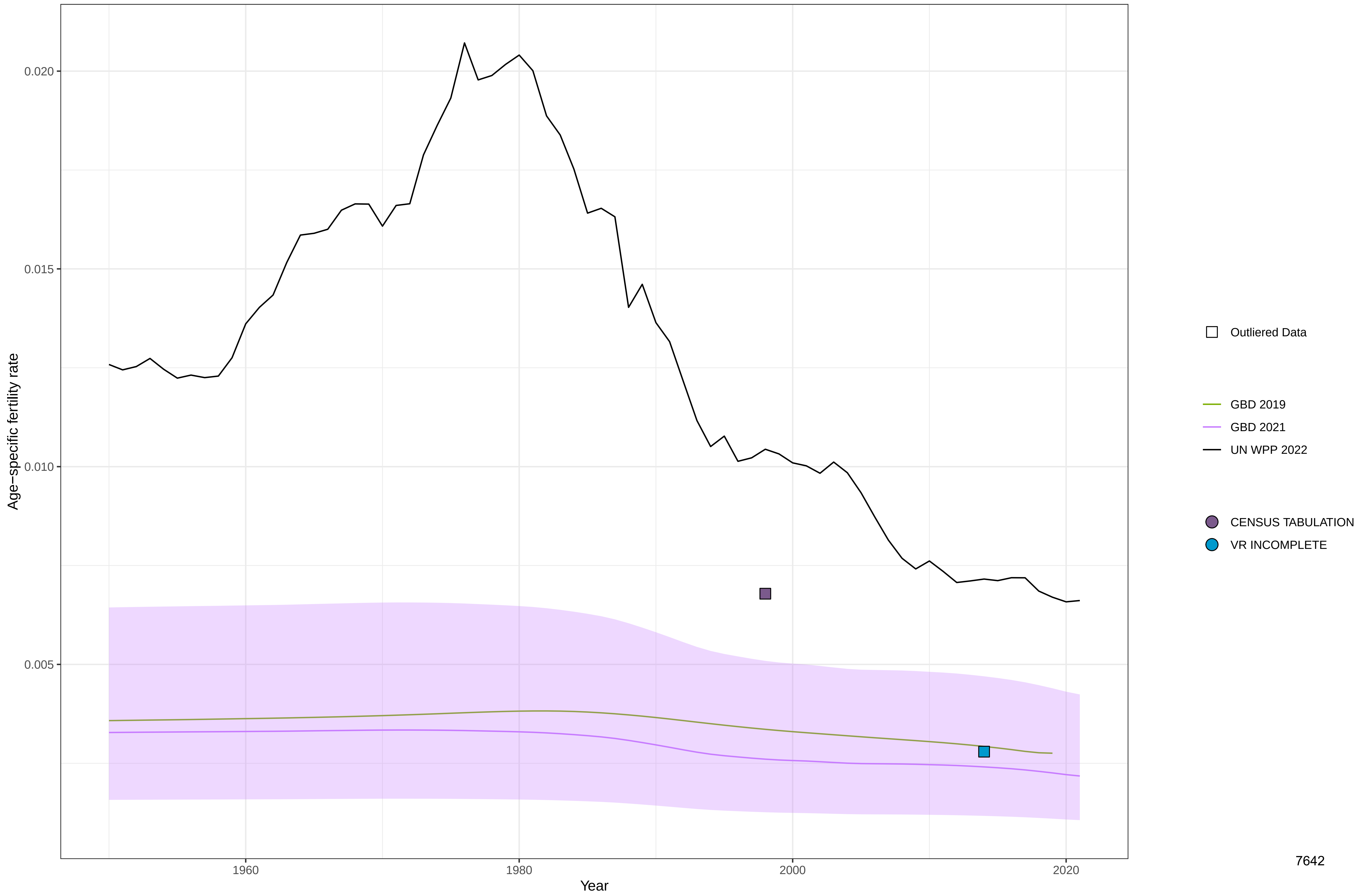

Age−specific fertility fitted trend lines, 1950−2021: Côte d'Ivoire, Ages: 15−19
Data points enclosed with a diamond are reference sources

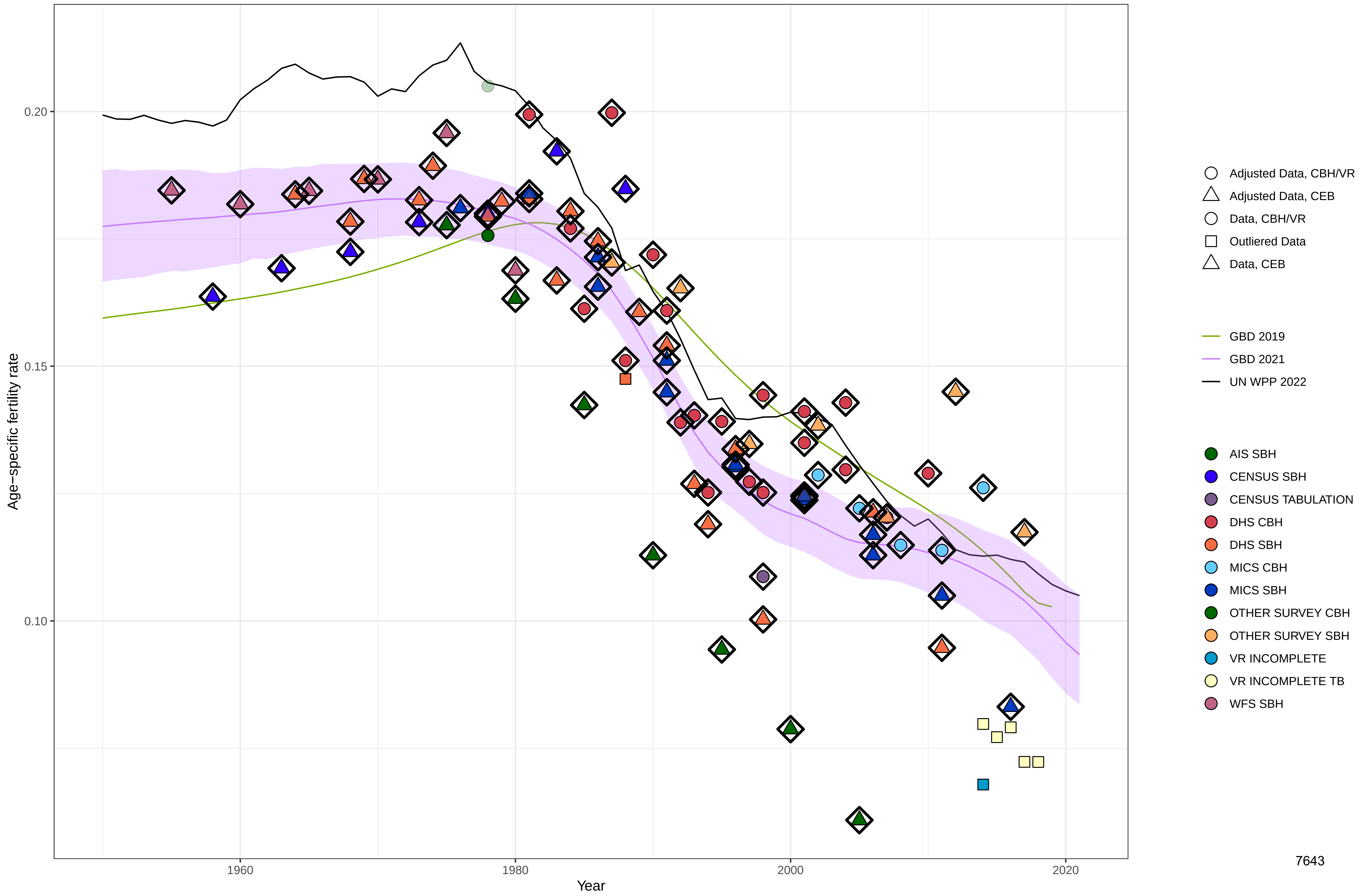

Age−specific fertility fitted trend lines, 1950−2021: Côte d'Ivoire, Ages: 20−24
Data points enclosed with a diamond are reference sources

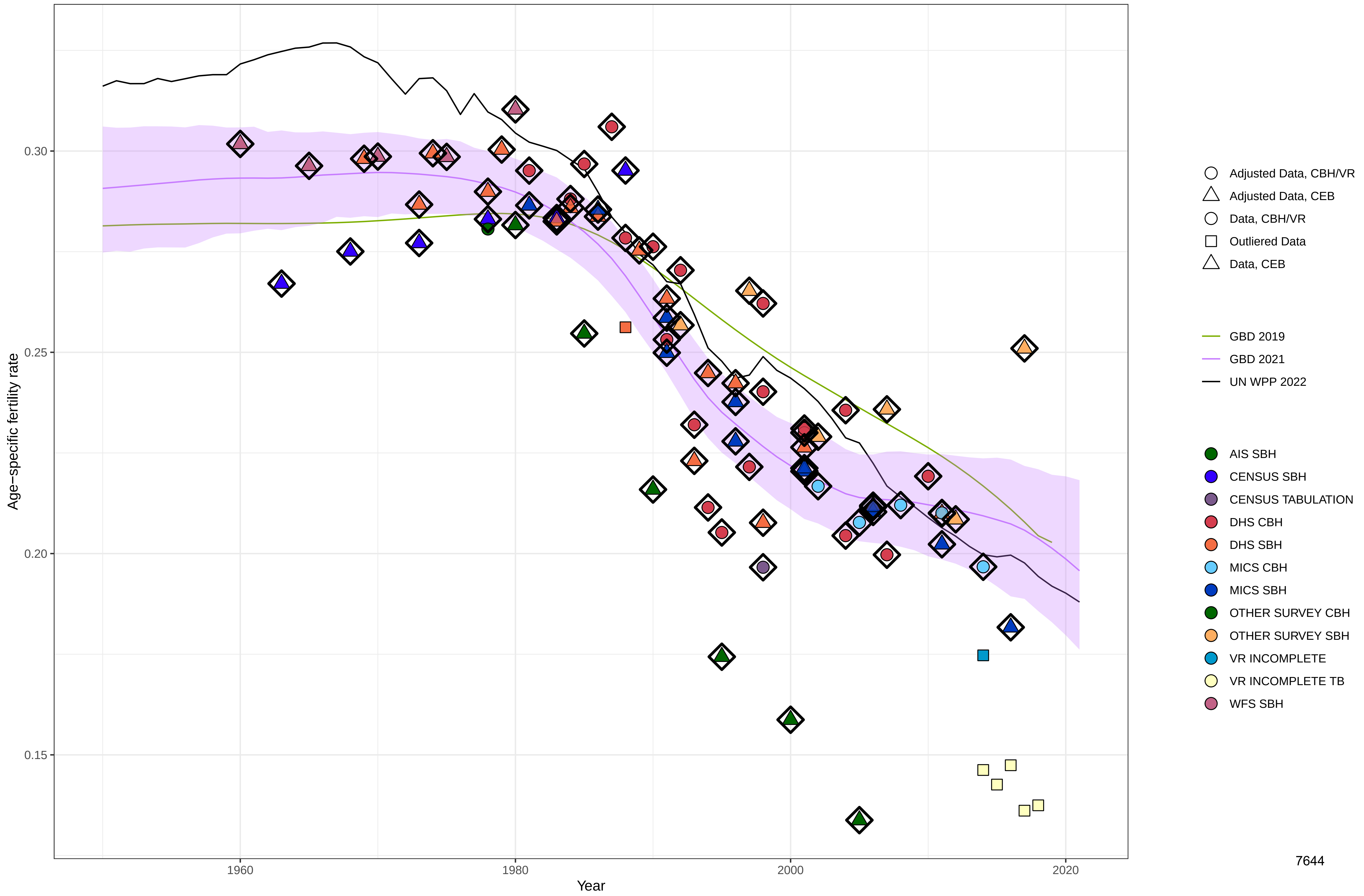

Age−specific fertility fitted trend lines, 1950−2021: Côte d'Ivoire, Ages: 25−29
Data points enclosed with a diamond are reference sources

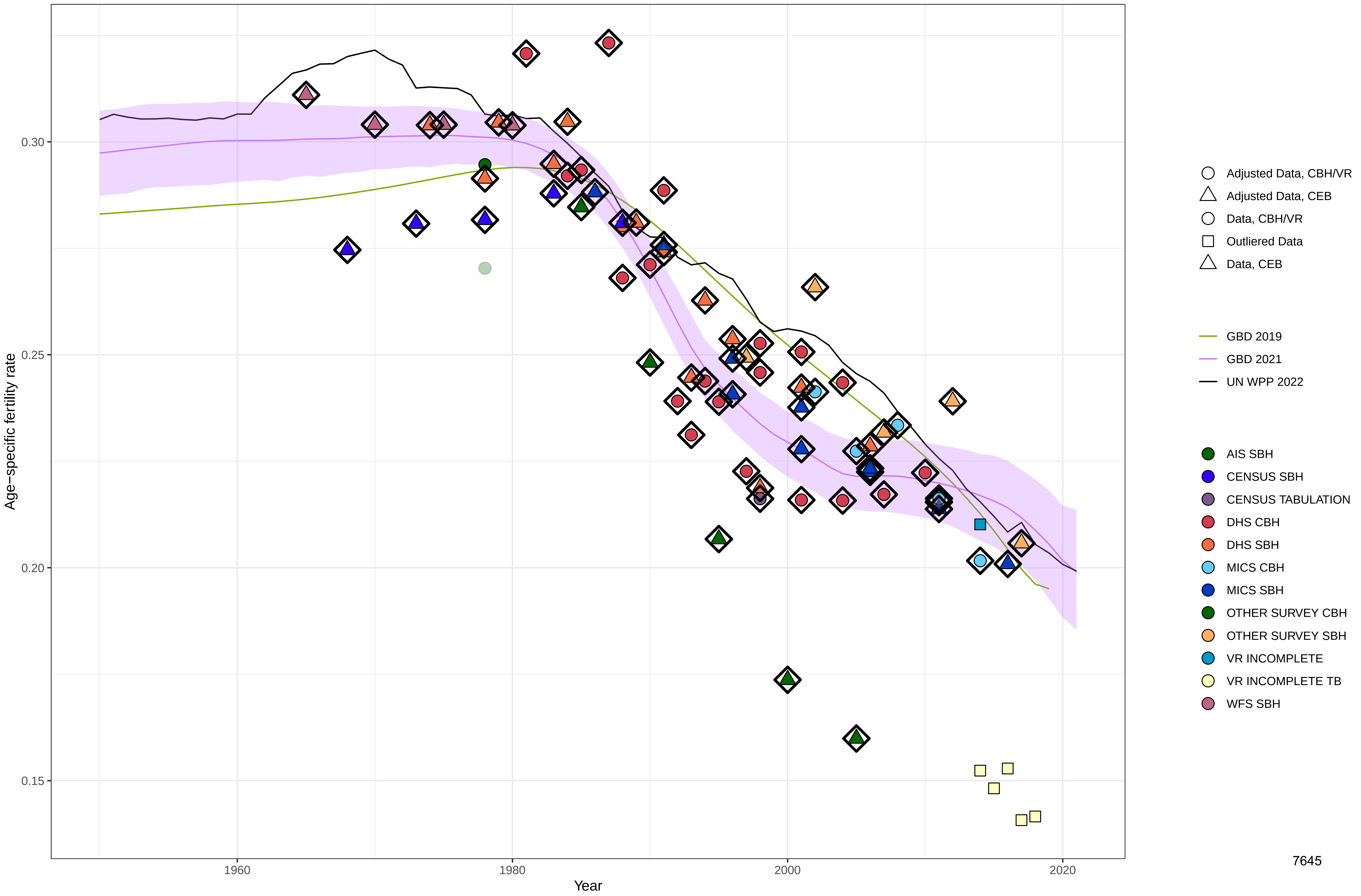

Age−specific fertility fitted trend lines, 1950−2021: Côte d'Ivoire, Ages: 30−34
Data points enclosed with a diamond are reference sources

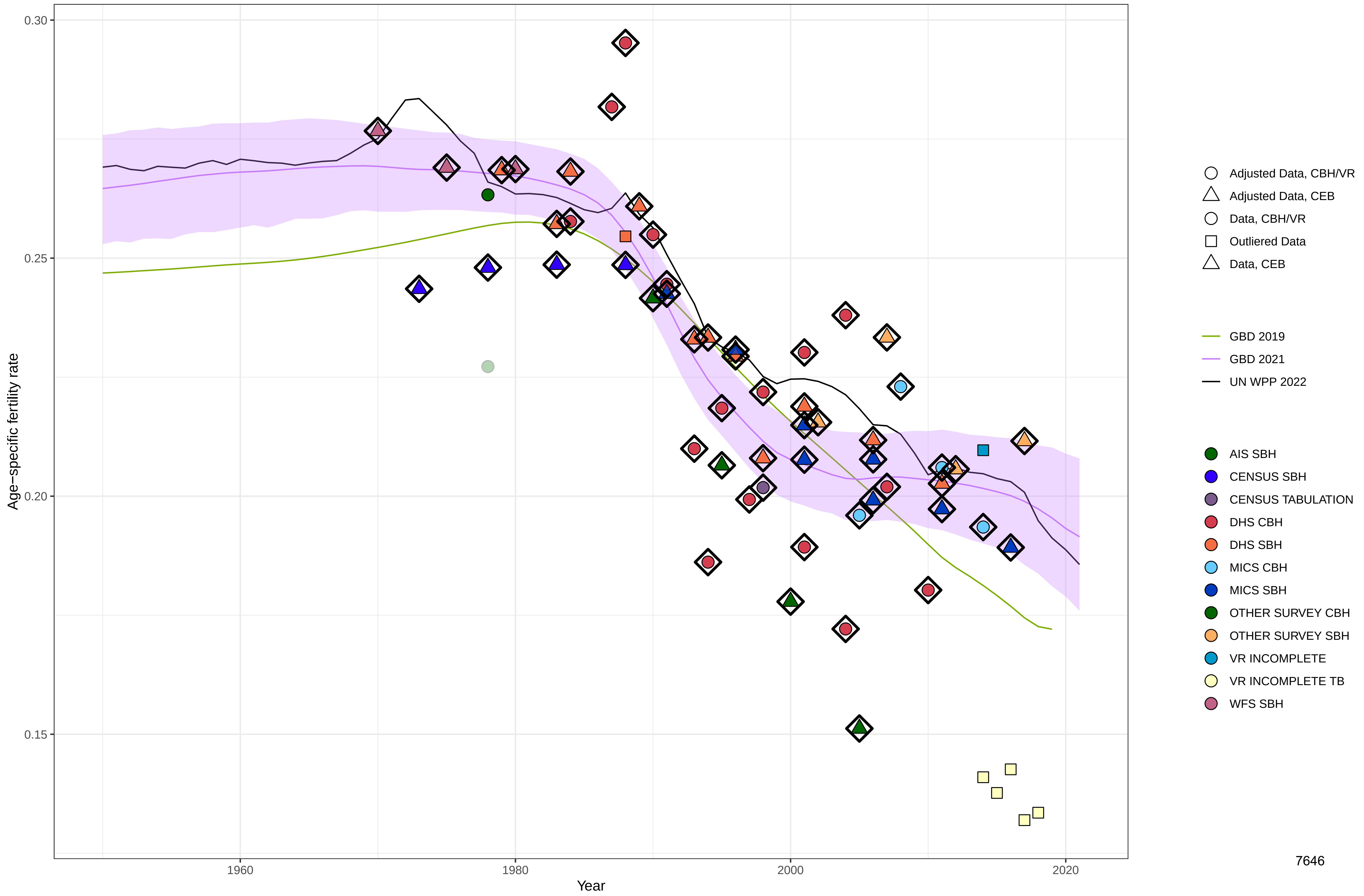

Age−specific fertility fitted trend lines, 1950−2021: Côte d'Ivoire, Ages: 35−39
Data points enclosed with a diamond are reference sources

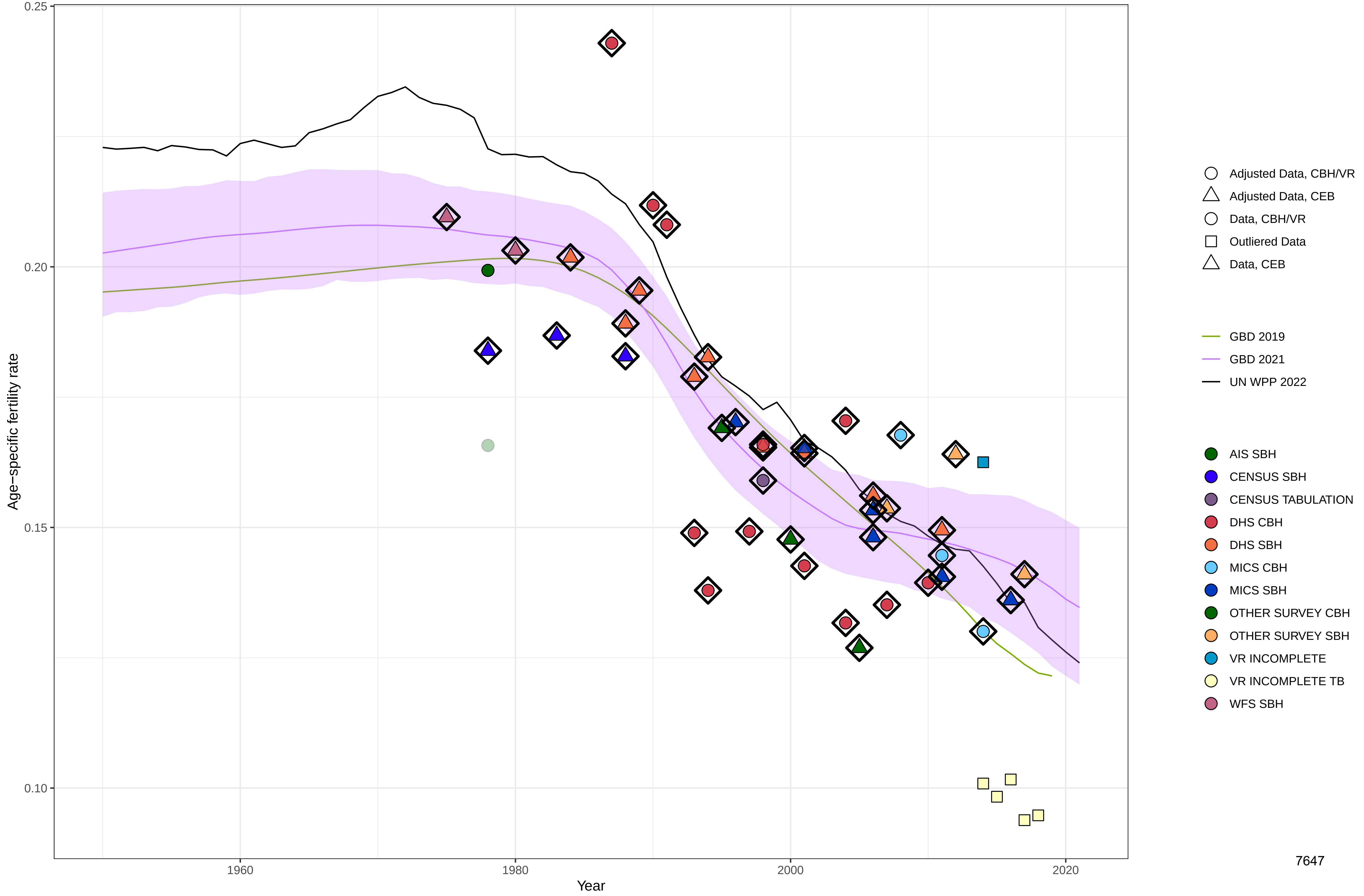

Age−specific fertility fitted trend lines, 1950−2021: Côte d'Ivoire, Ages: 40−44
Data points enclosed with a diamond are reference sources

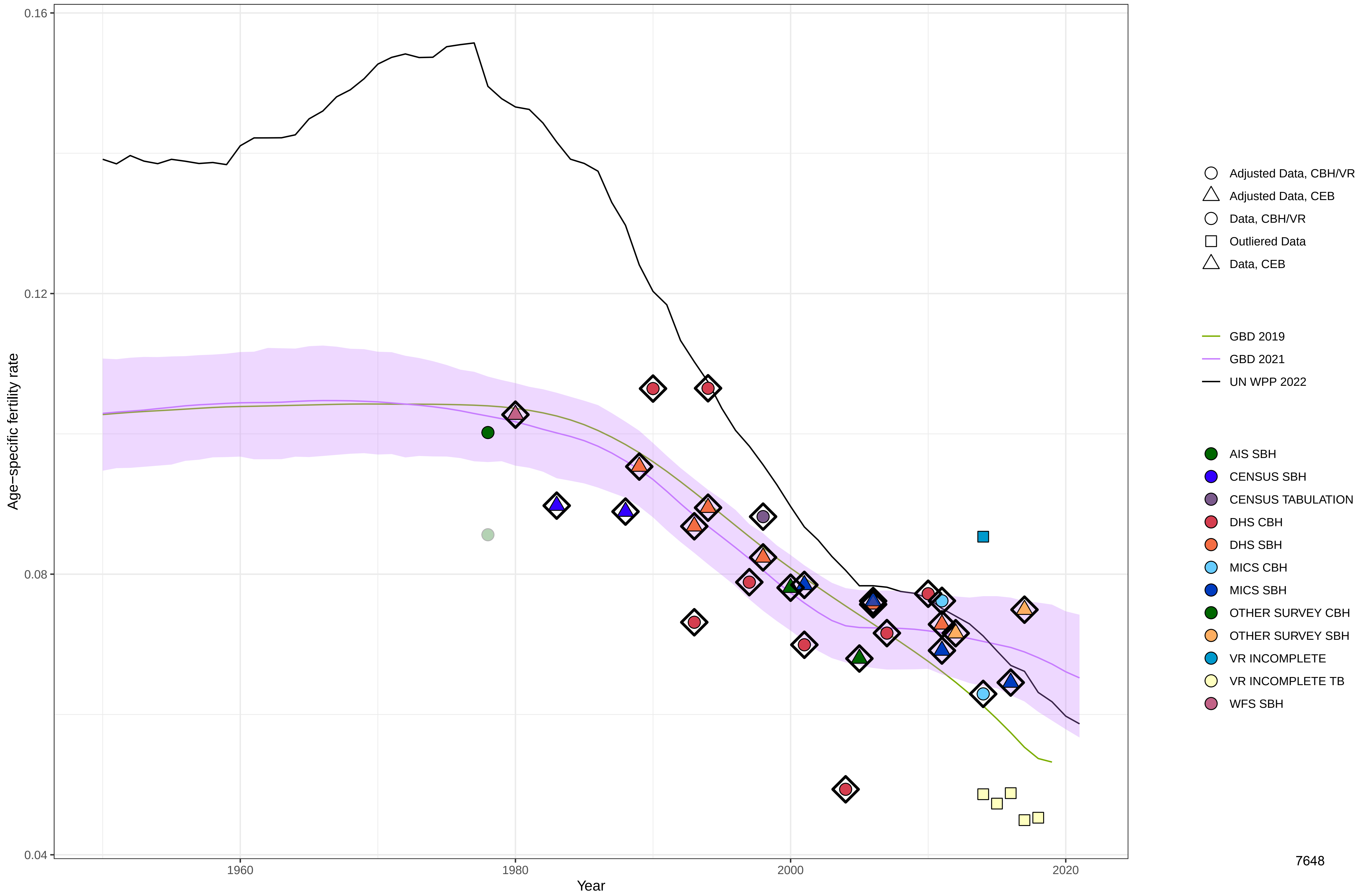

Age−specific fertility fitted trend lines, 1950−2021: Côte d'Ivoire, Ages: 45−49
Data points enclosed with a diamond are reference sources

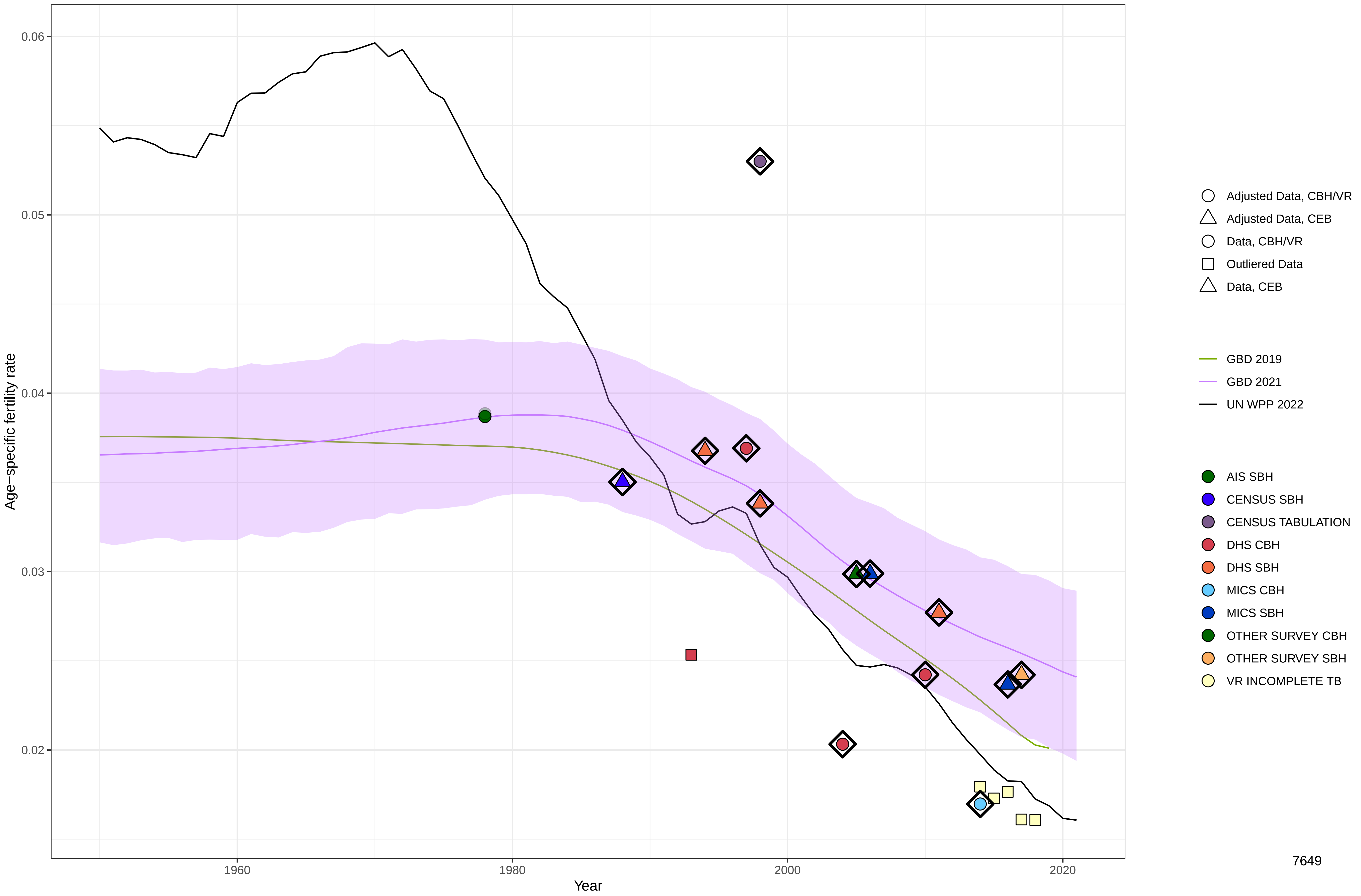

Age−specific fertility fitted trend lines, 1950−2021: Côte d'Ivoire, Ages: 50−54
Data points enclosed with a diamond are reference sources

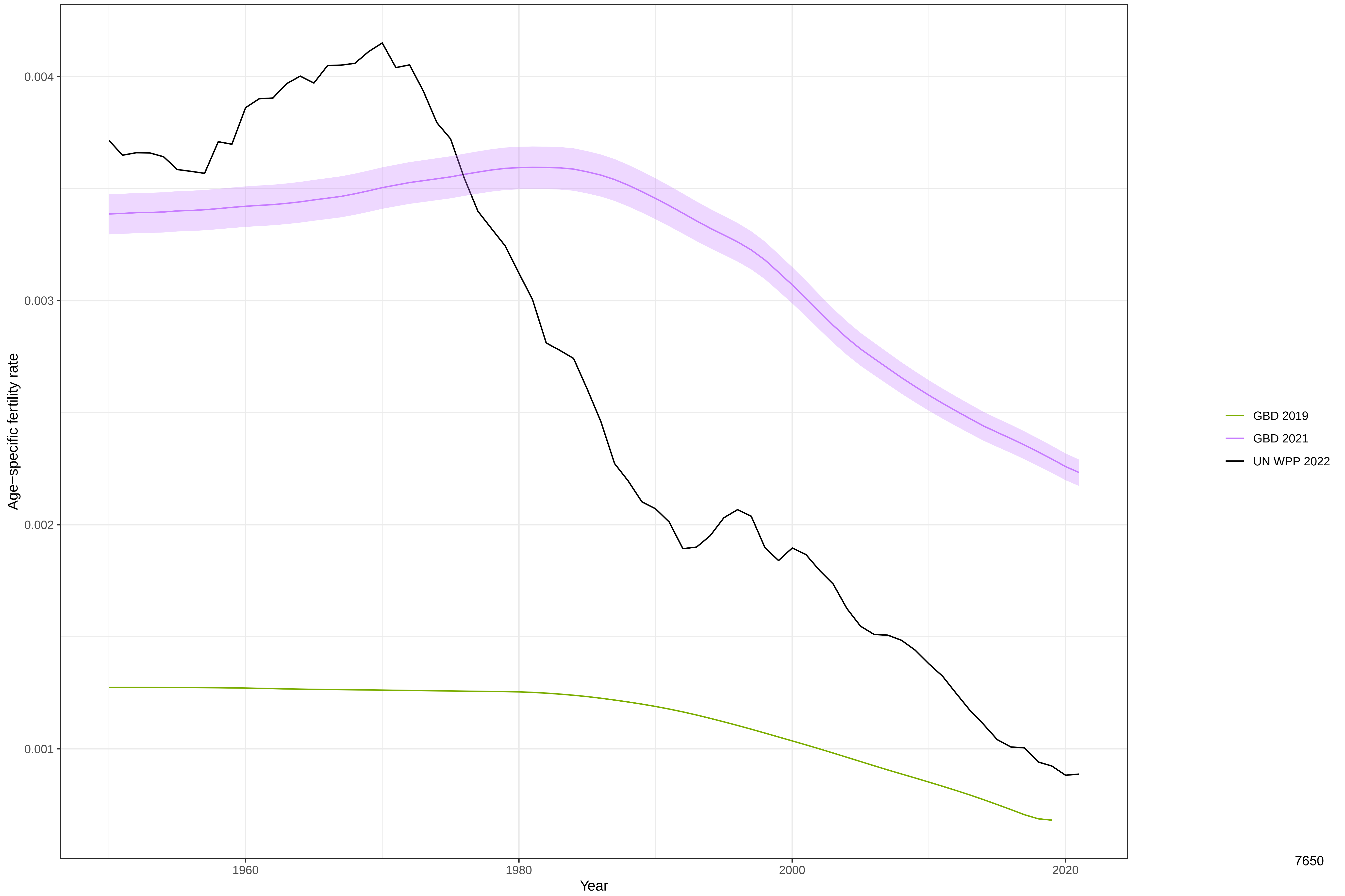

Total fertility rate fitted trend lines, 1950−2021: Côte d'Ivoire TFR
Data points enclosed with a diamond are reference sources

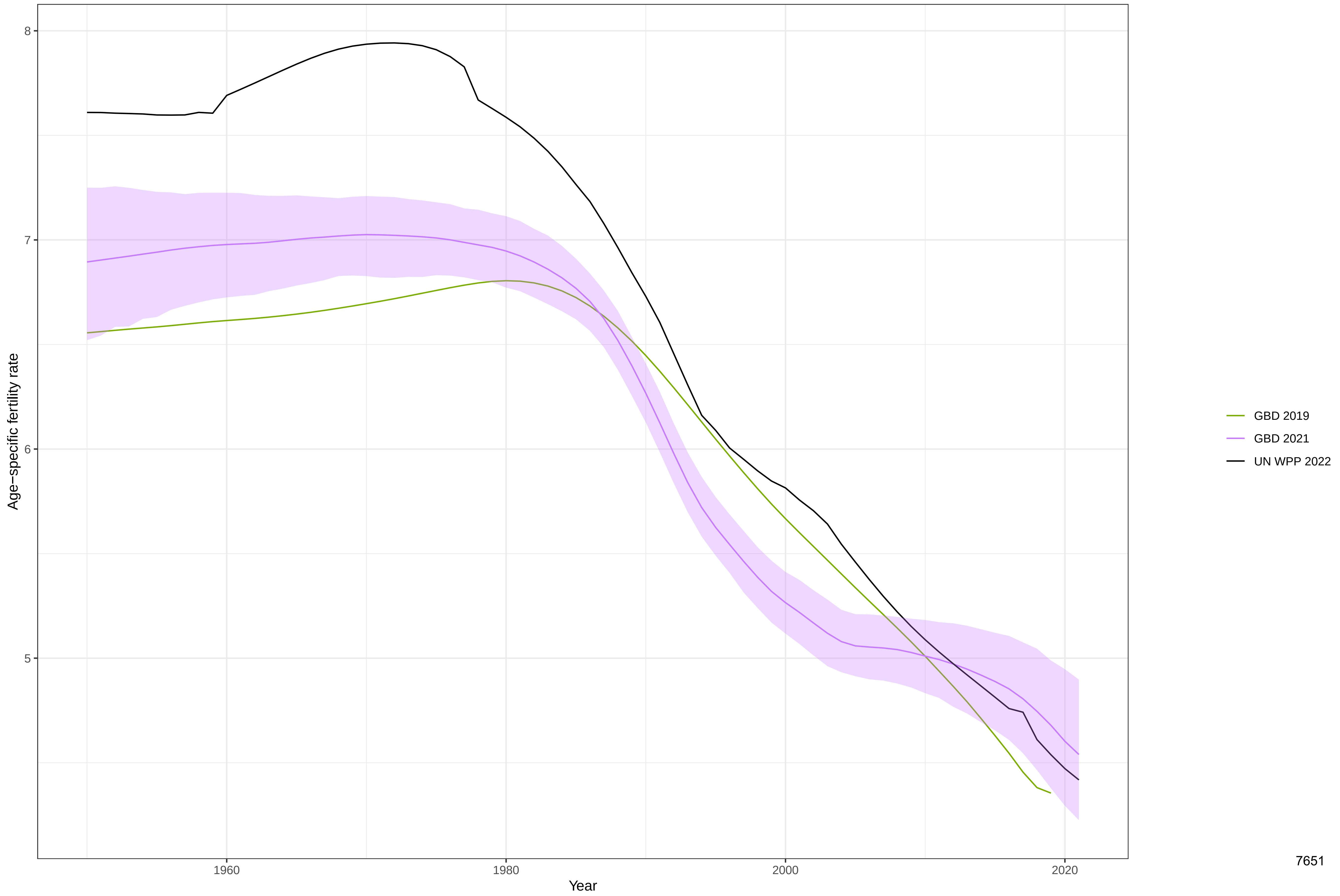

Age−specific fertility fitted trend lines, 1950−2021: The Gambia, Ages: 10−14
Data points enclosed with a diamond are reference sources

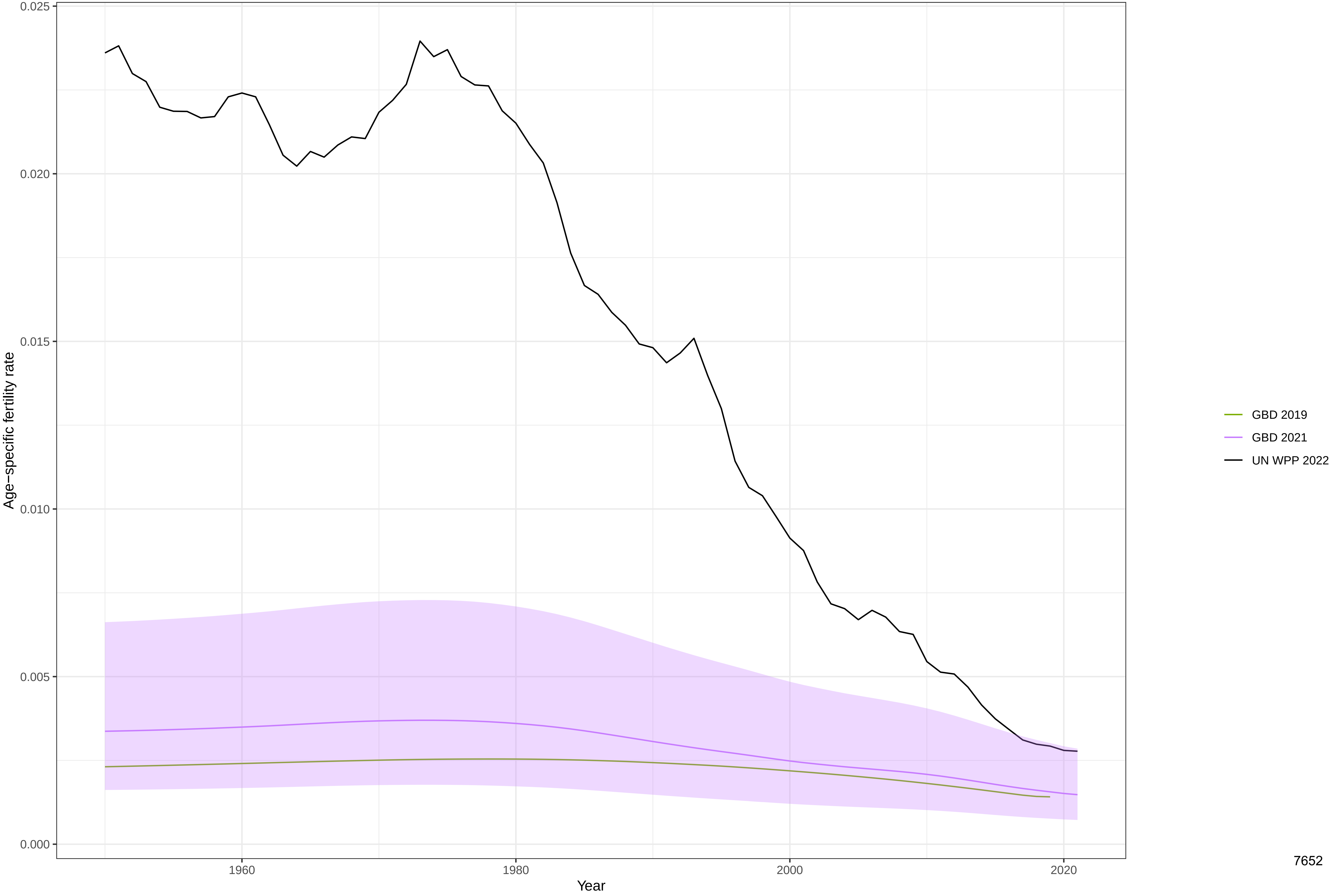

Age−specific fertility fitted trend lines, 1950−2021: The Gambia, Ages: 15−19
Data points enclosed with a diamond are reference sources

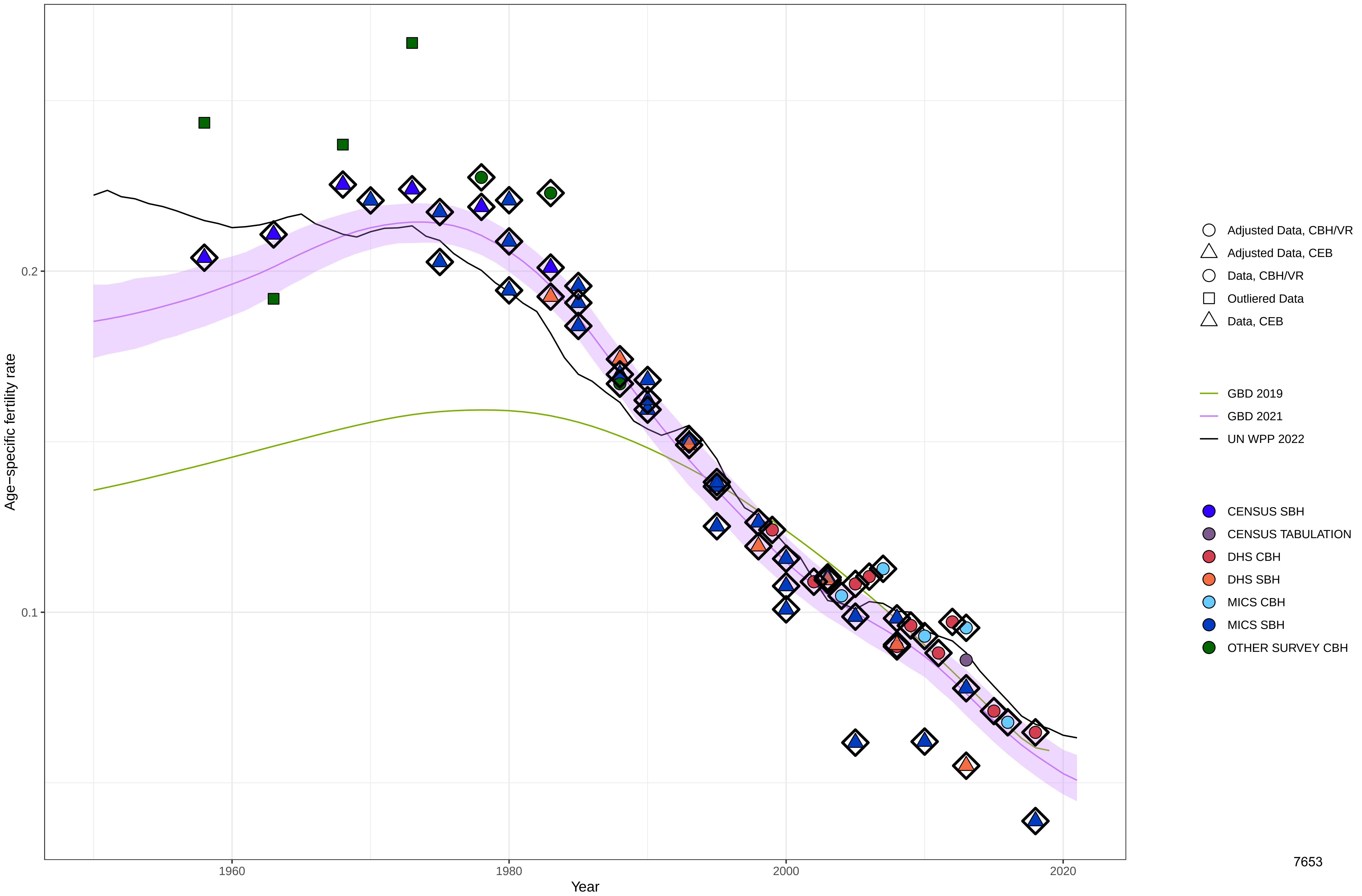

Age−specific fertility fitted trend lines, 1950−2021: The Gambia, Ages: 20−24
Data points enclosed with a diamond are reference sources

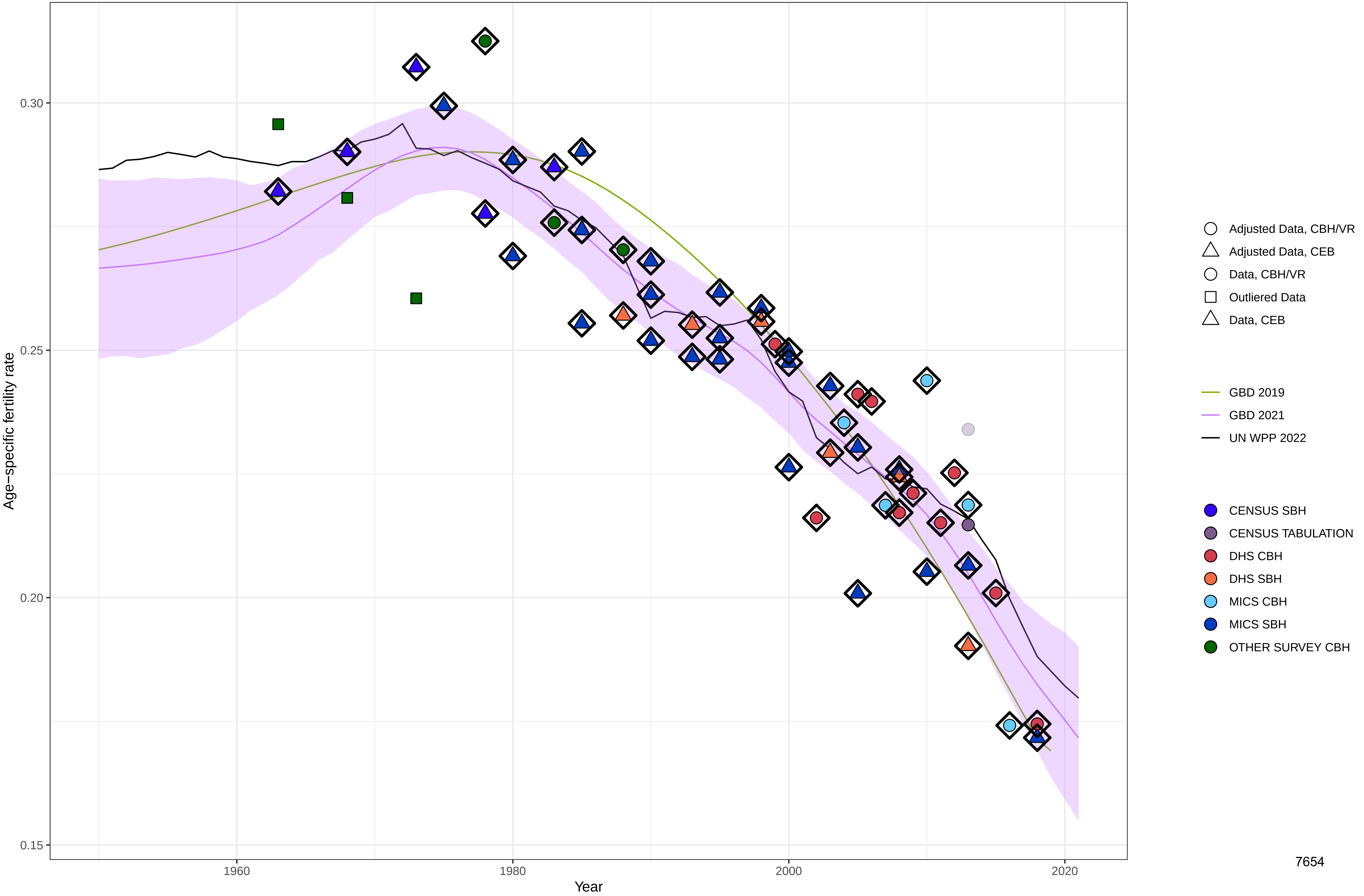

Age−specific fertility fitted trend lines, 1950−2021: The Gambia, Ages: 25−29
Data points enclosed with a diamond are reference sources

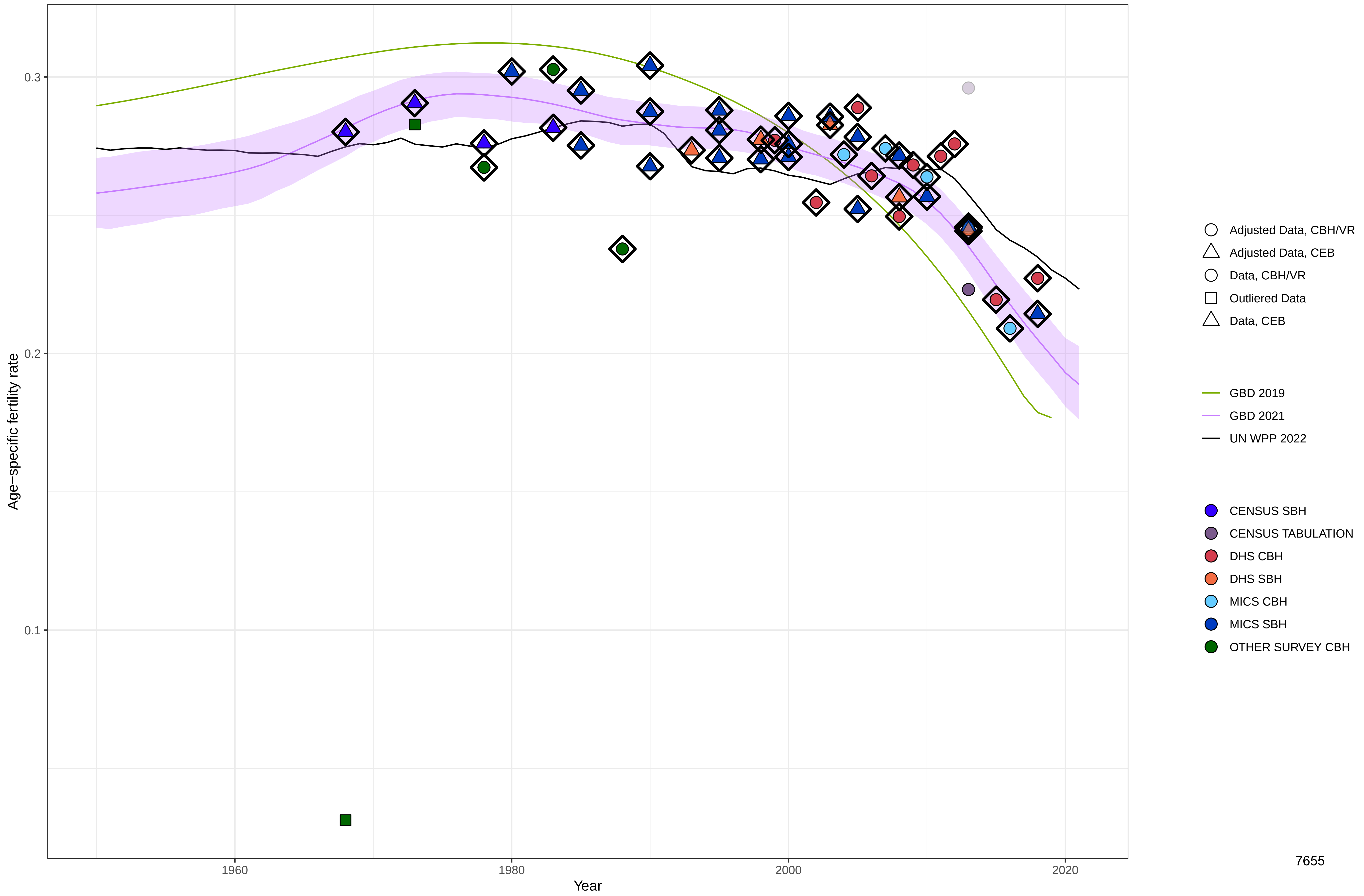

Age−specific fertility fitted trend lines, 1950−2021: The Gambia, Ages: 30−34
Data points enclosed with a diamond are reference sources

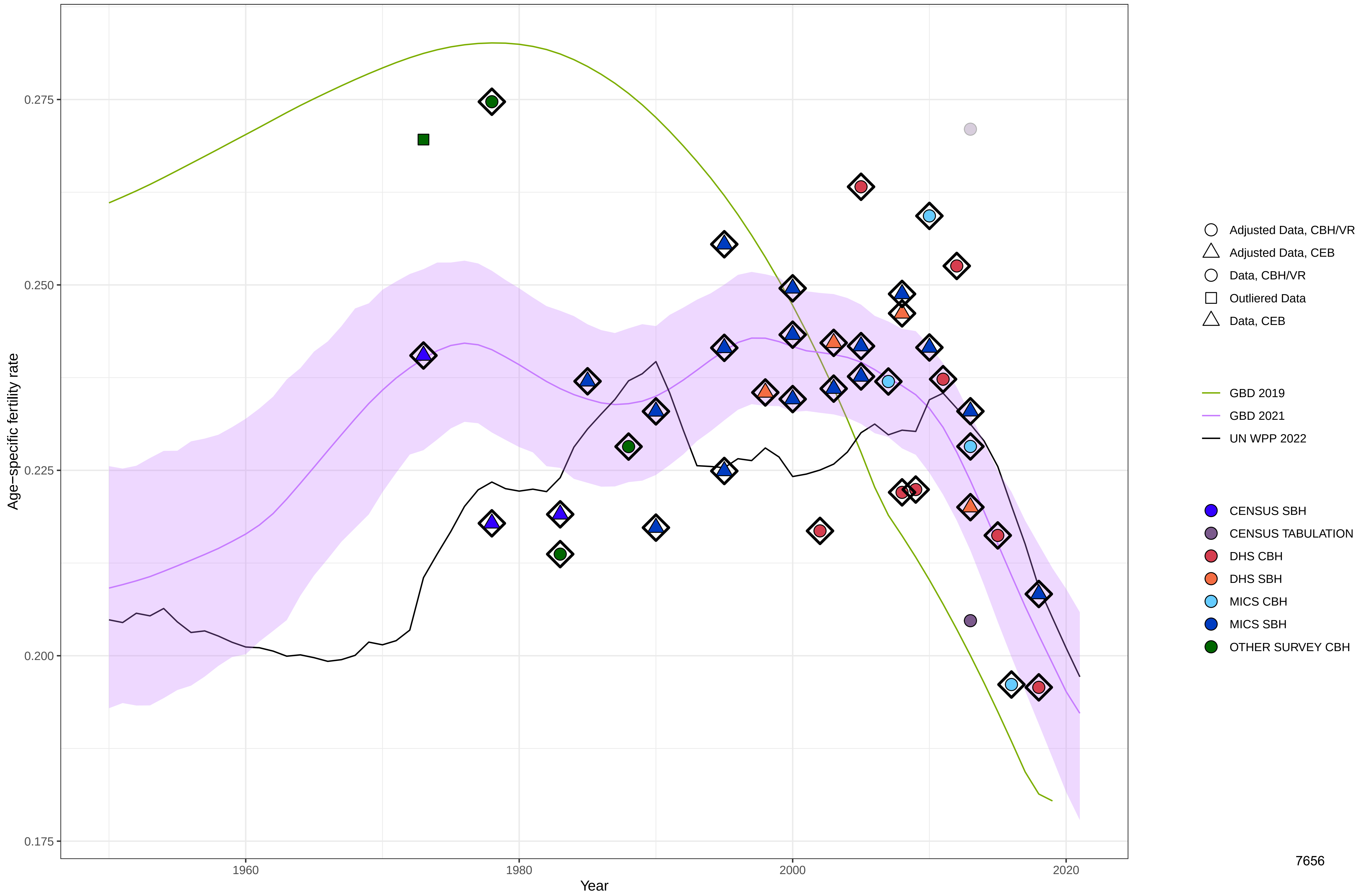

Age−specific fertility fitted trend lines, 1950−2021: The Gambia, Ages: 35−39
Data points enclosed with a diamond are reference sources

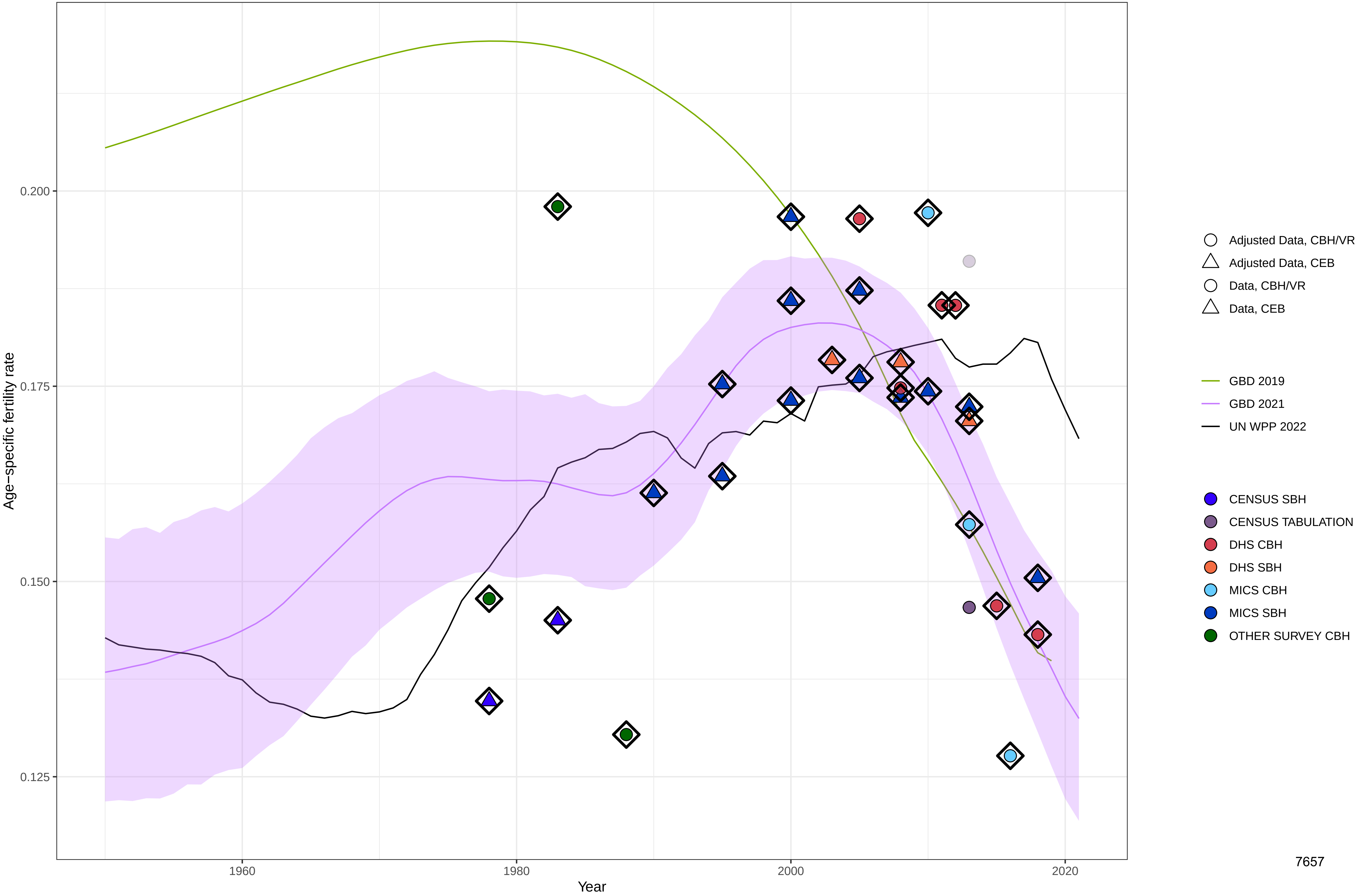

Age−specific fertility fitted trend lines, 1950−2021: The Gambia, Ages: 40−44
Data points enclosed with a diamond are reference sources

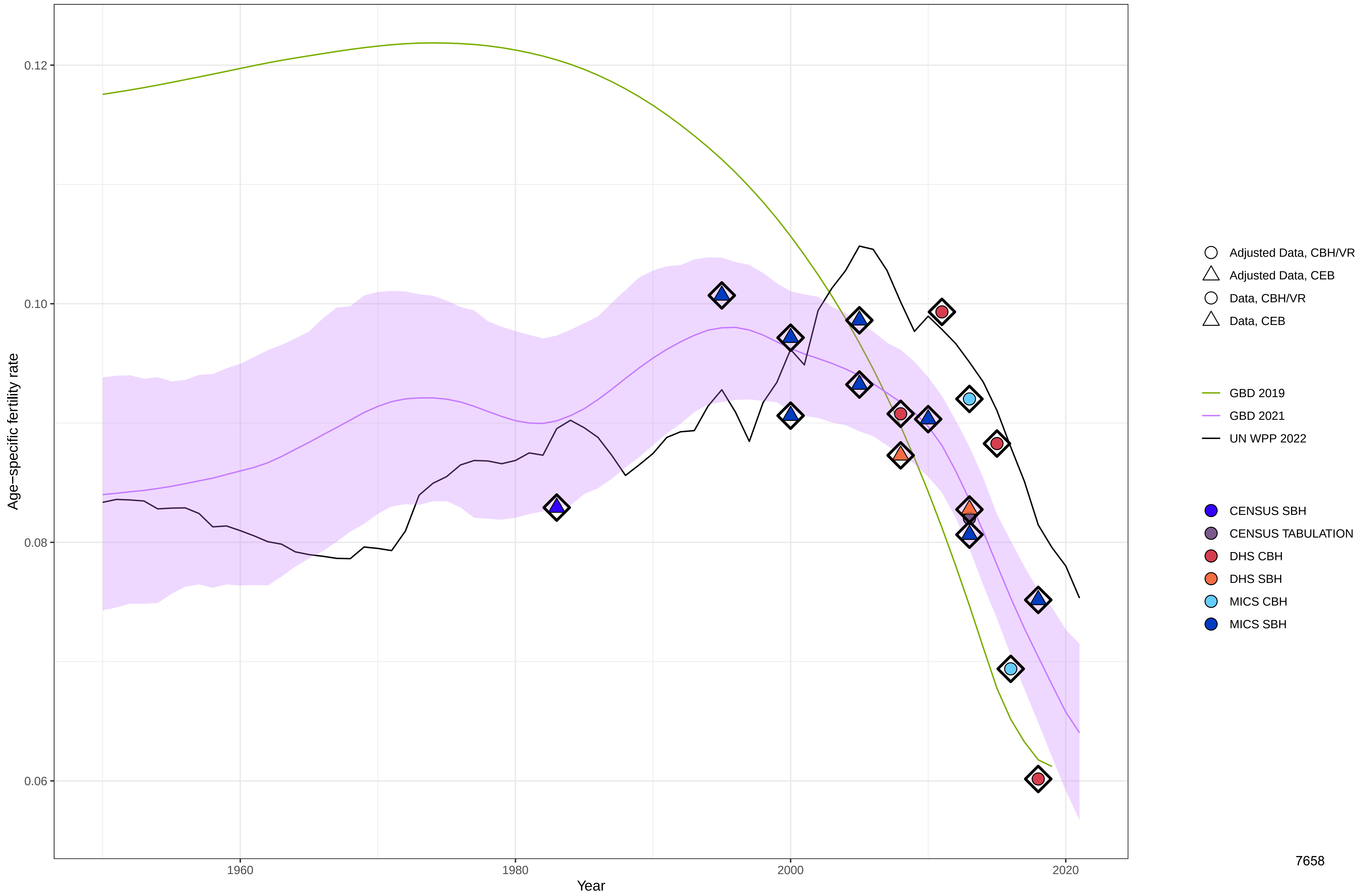

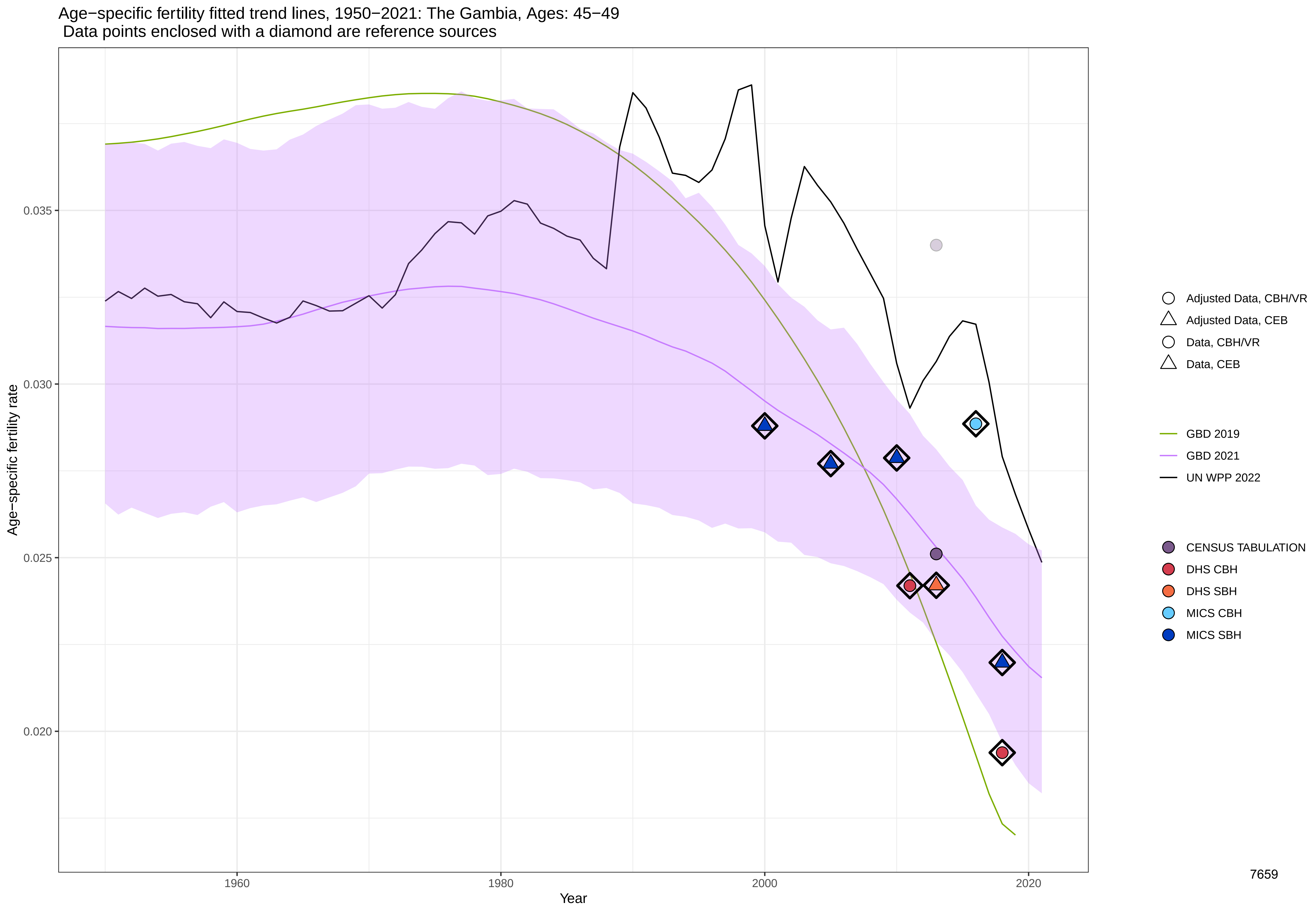

Age−specific fertility fitted trend lines, 1950−2021: The Gambia, Ages: 45−49
Data points enclosed with a diamond are reference sources
Age−specific fertility rate
0.035
0.030
0.025
0.020
Year
1960
1980
2000
2020
Adjusted Data, CBH/VR
Adjusted Data, CEB
Data, CBH/VR
Data, CEB
GBD 2019
GBD 2021
UN WPP 2022
CENSUS TABULATION
DHS CBH
DHS SBH
MICS CBH
MICS SBH

7659

Age−specific fertility fitted trend lines, 1950−2021: The Gambia, Ages: 50−54
Data points enclosed with a diamond are reference sources

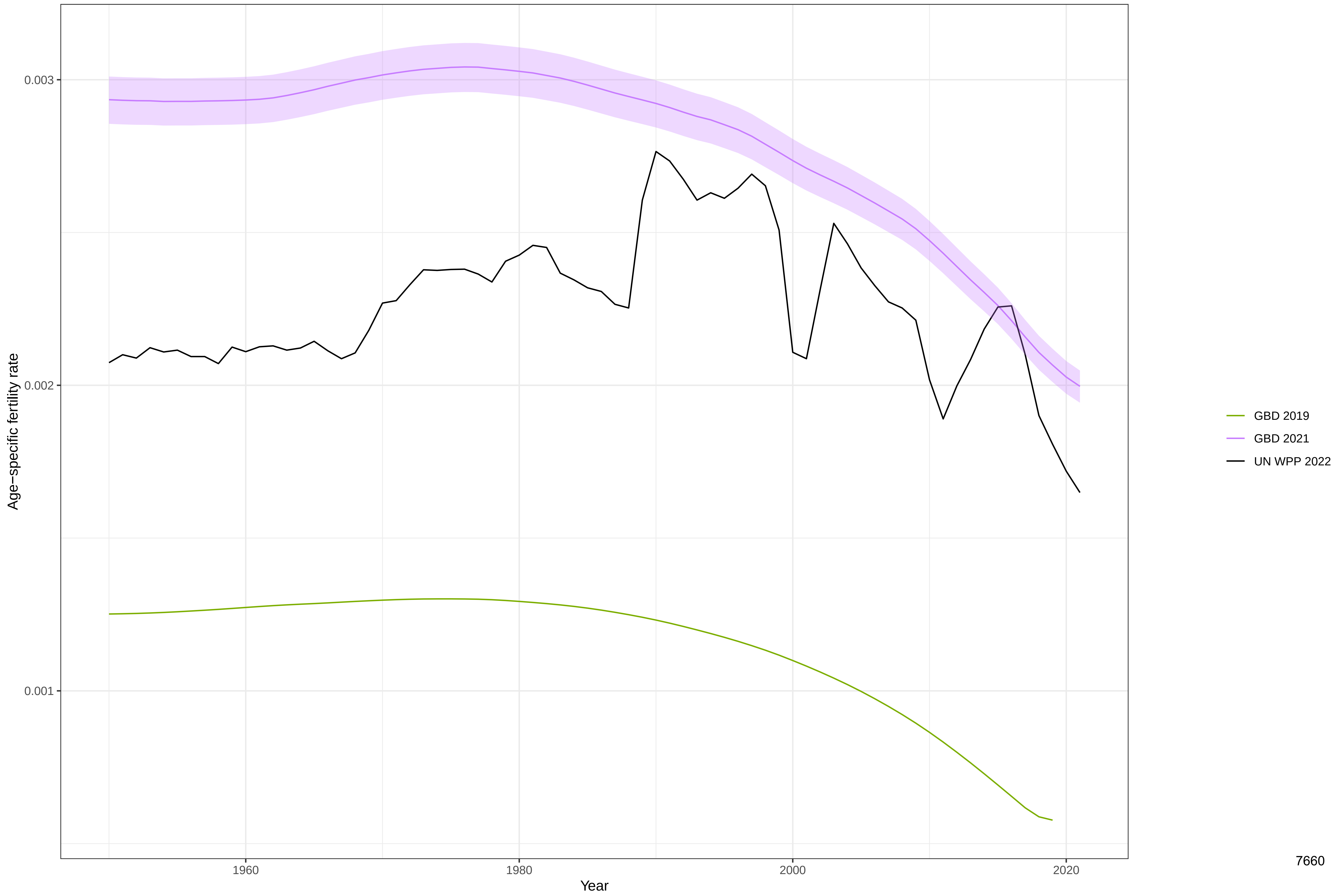

Total fertility rate fitted trend lines, 1950−2021: The Gambia TFR
Data points enclosed with a diamond are reference sources

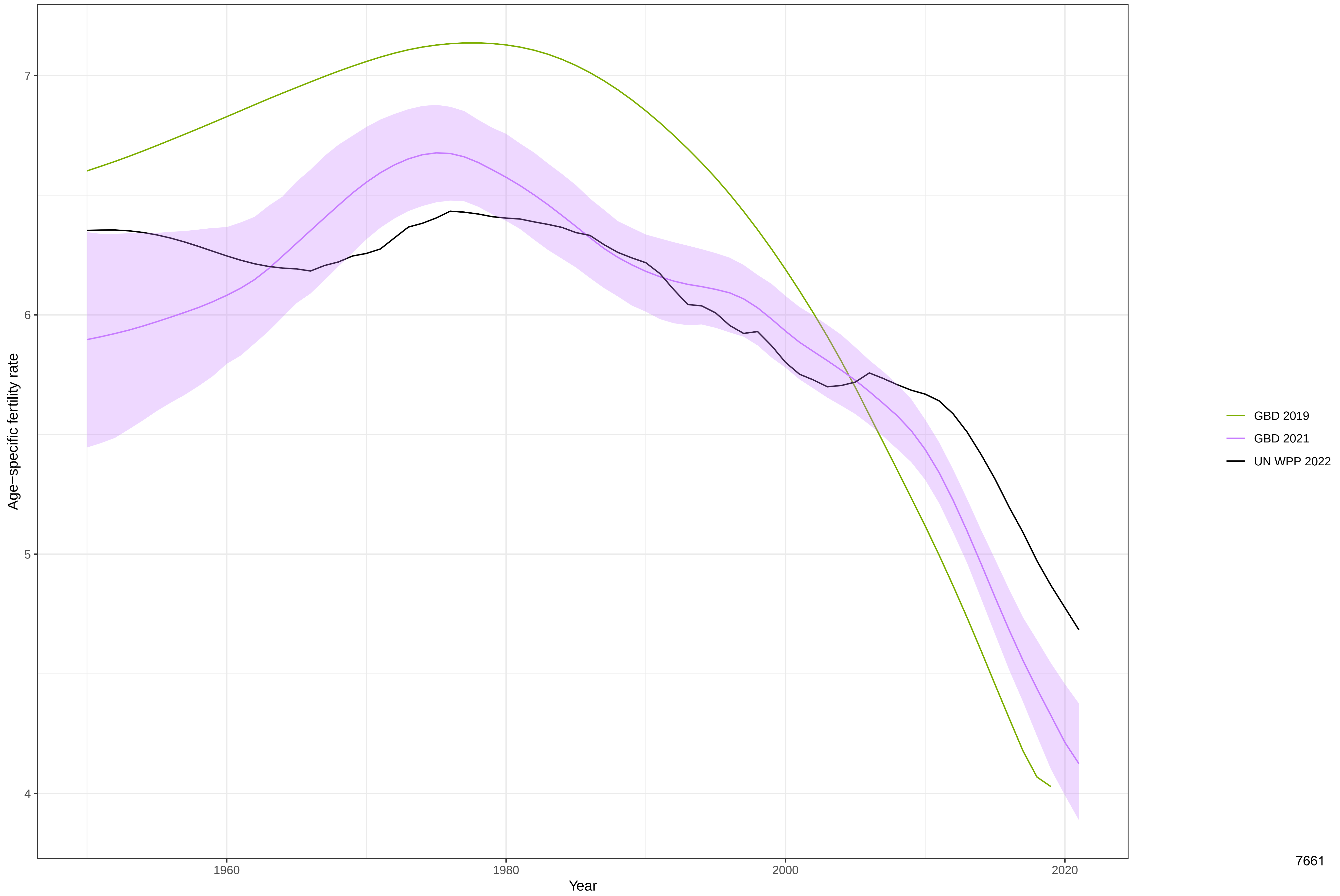

Age−specific fertility fitted trend lines, 1950−2021: Ghana, Ages: 10−14
Data points enclosed with a diamond are reference sources

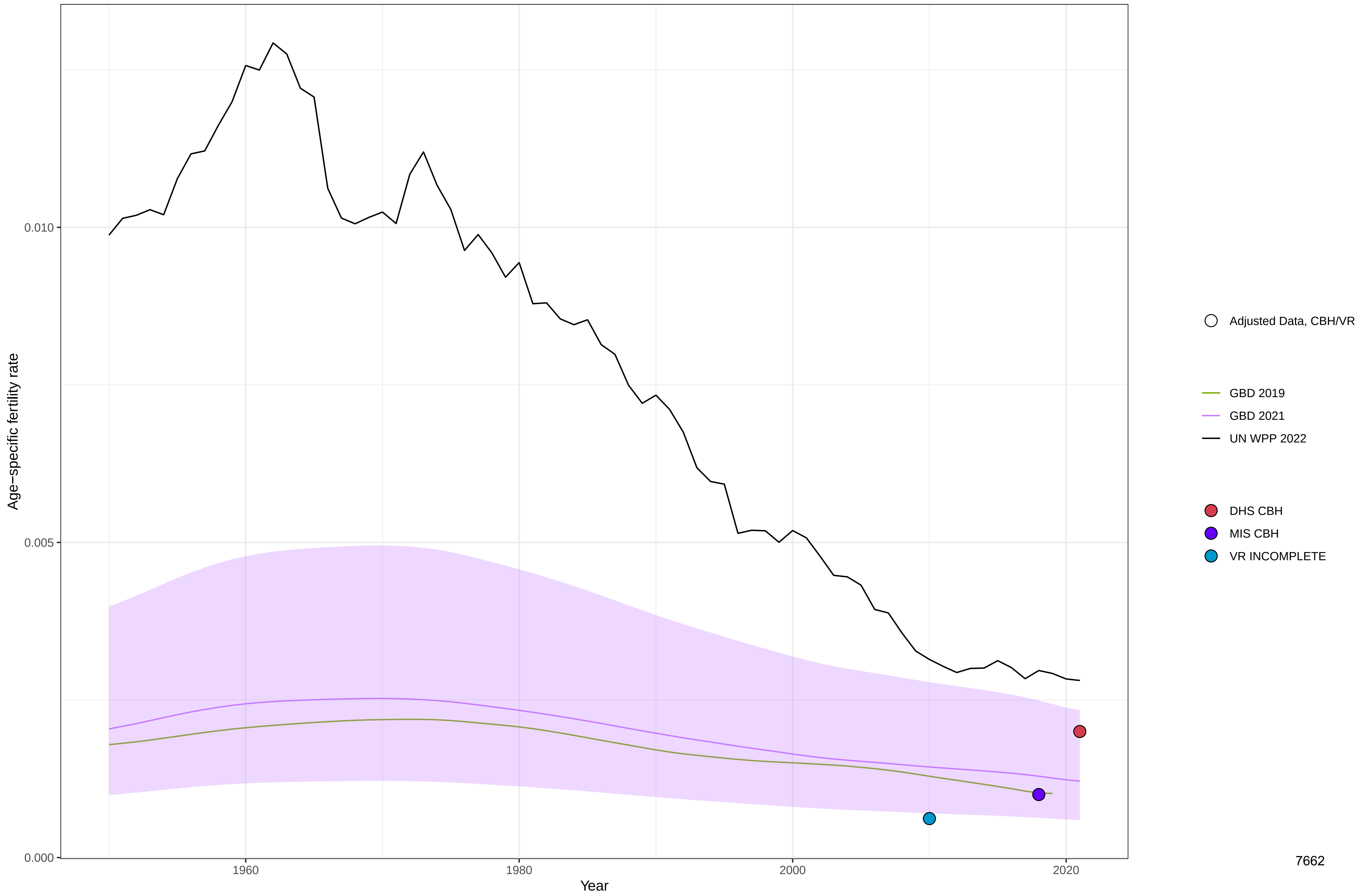

Age−specific fertility fitted trend lines, 1950−2021: Ghana, Ages: 15−19
Data points enclosed with a diamond are reference sources

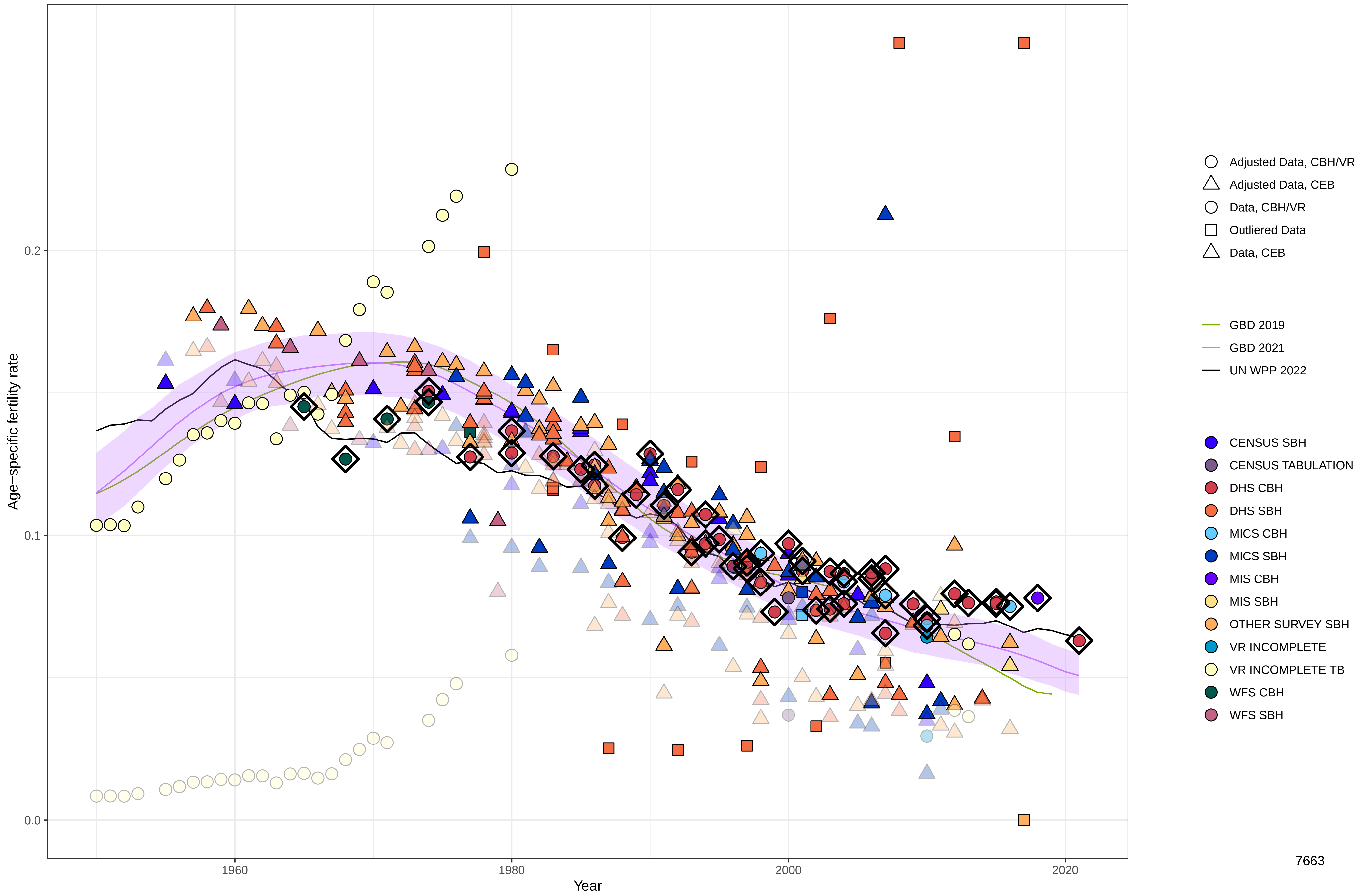

Age−specific fertility fitted trend lines, 1950−2021: Ghana, Ages: 20−24
Data points enclosed with a diamond are reference sources

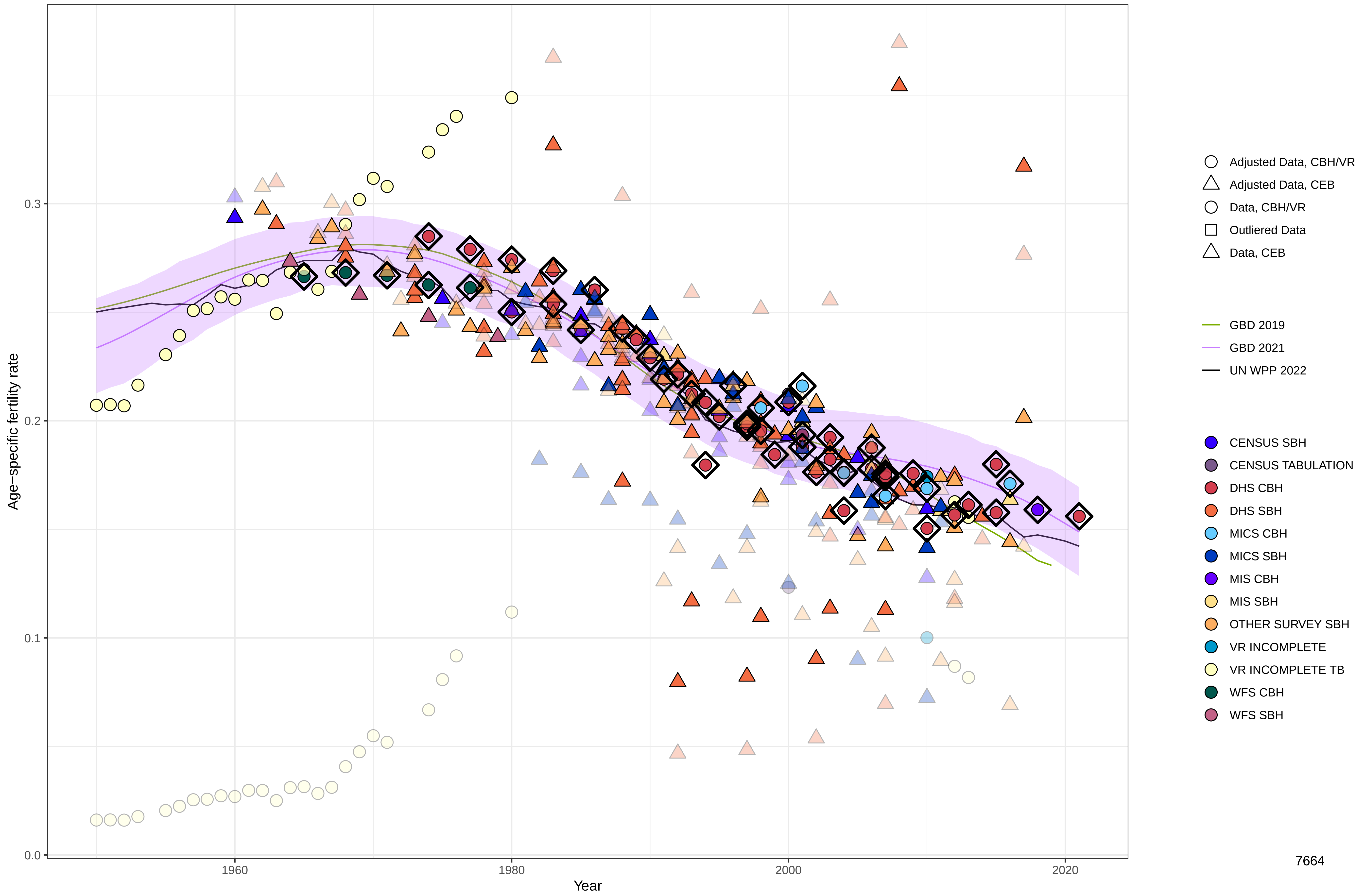

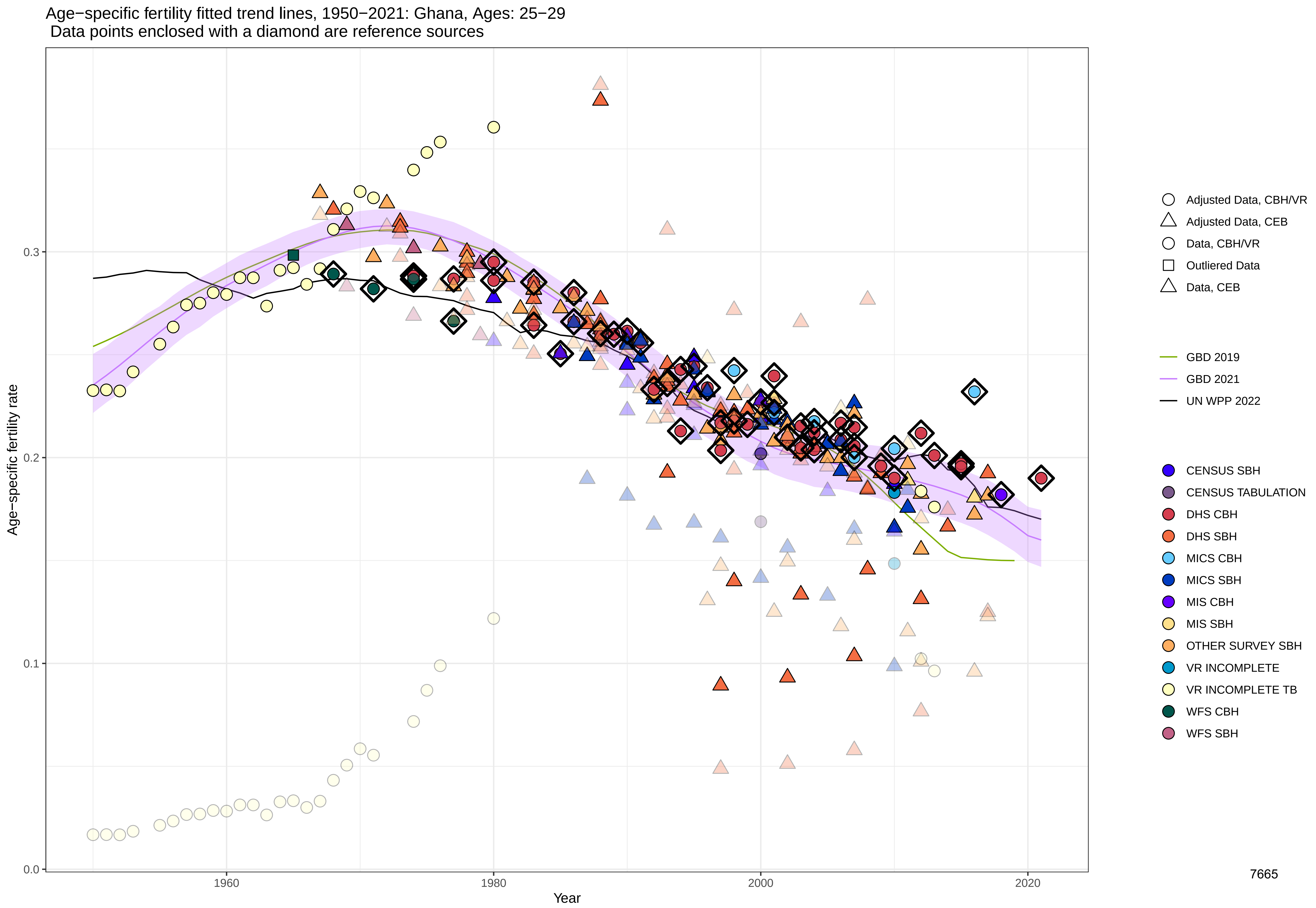

Age−specific fertility fitted trend lines, 1950−2021: Ghana, Ages: 25−29
Data points enclosed with a diamond are reference sources
Age−specific fertility rate
Year
0.0
0.1
0.2
0.3
1960
1980
2000
2020
Adjusted Data, CBH/VR
Adjusted Data, CEB
Data, CBH/VR
Outliered Data
Data, CEB
GBD 2019
GBD 2021
UN WPP 2022
CENSUS SBH
CENSUS TABULATION
DHS CBH
DHS SBH
MICS CBH
MICS SBH
MIS CBH
MIS SBH
OTHER SURVEY SBH
VR INCOMPLETE
VR INCOMPLETE TB
WFS CBH
WFS SBH

Age−specific fertility fitted trend lines, 1950−2021: Ghana, Ages: 30−34
Data points enclosed with a diamond are reference sources

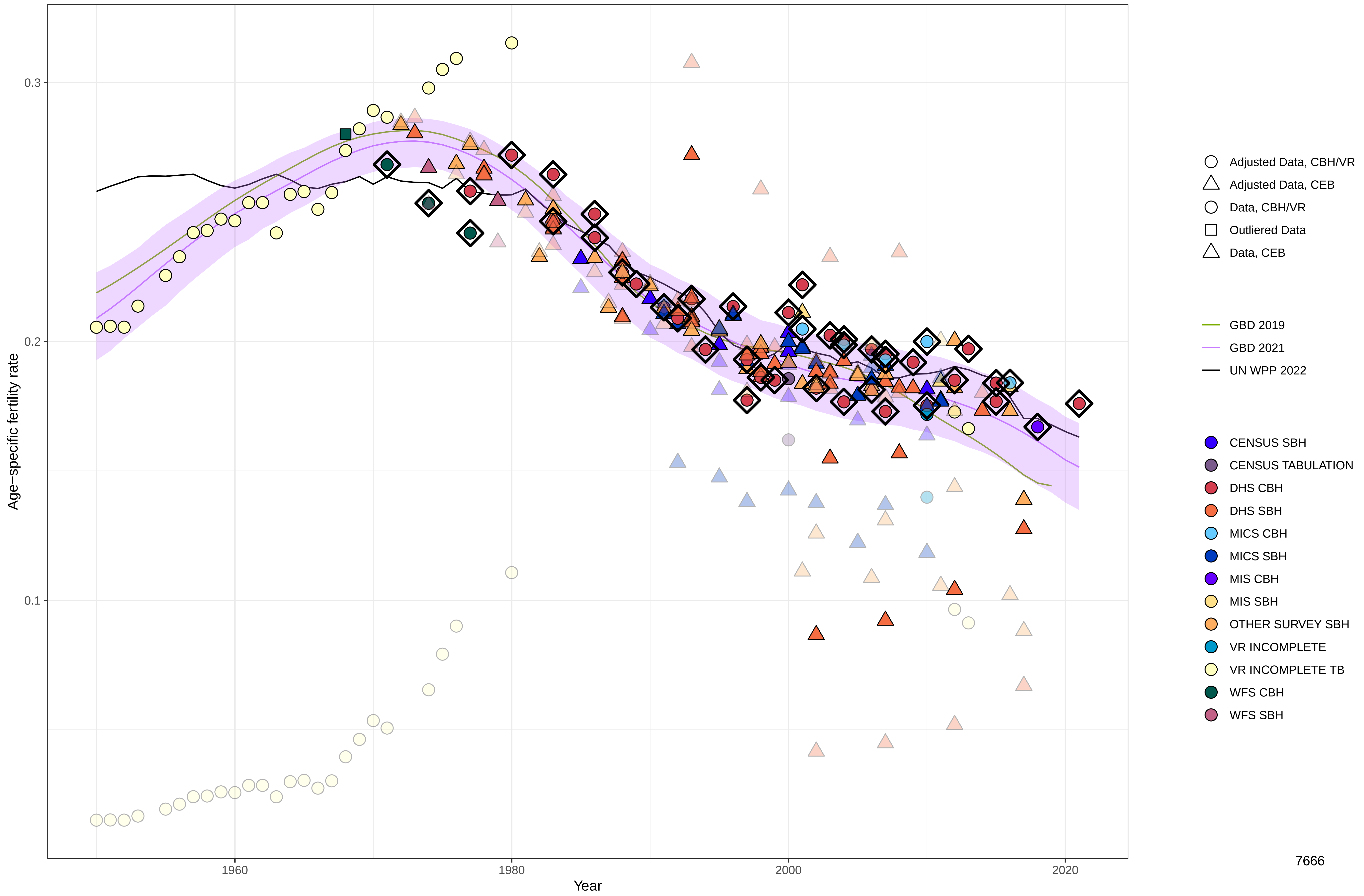

Age−specific fertility fitted trend lines, 1950−2021: Ghana, Ages: 35−39
Data points enclosed with a diamond are reference sources

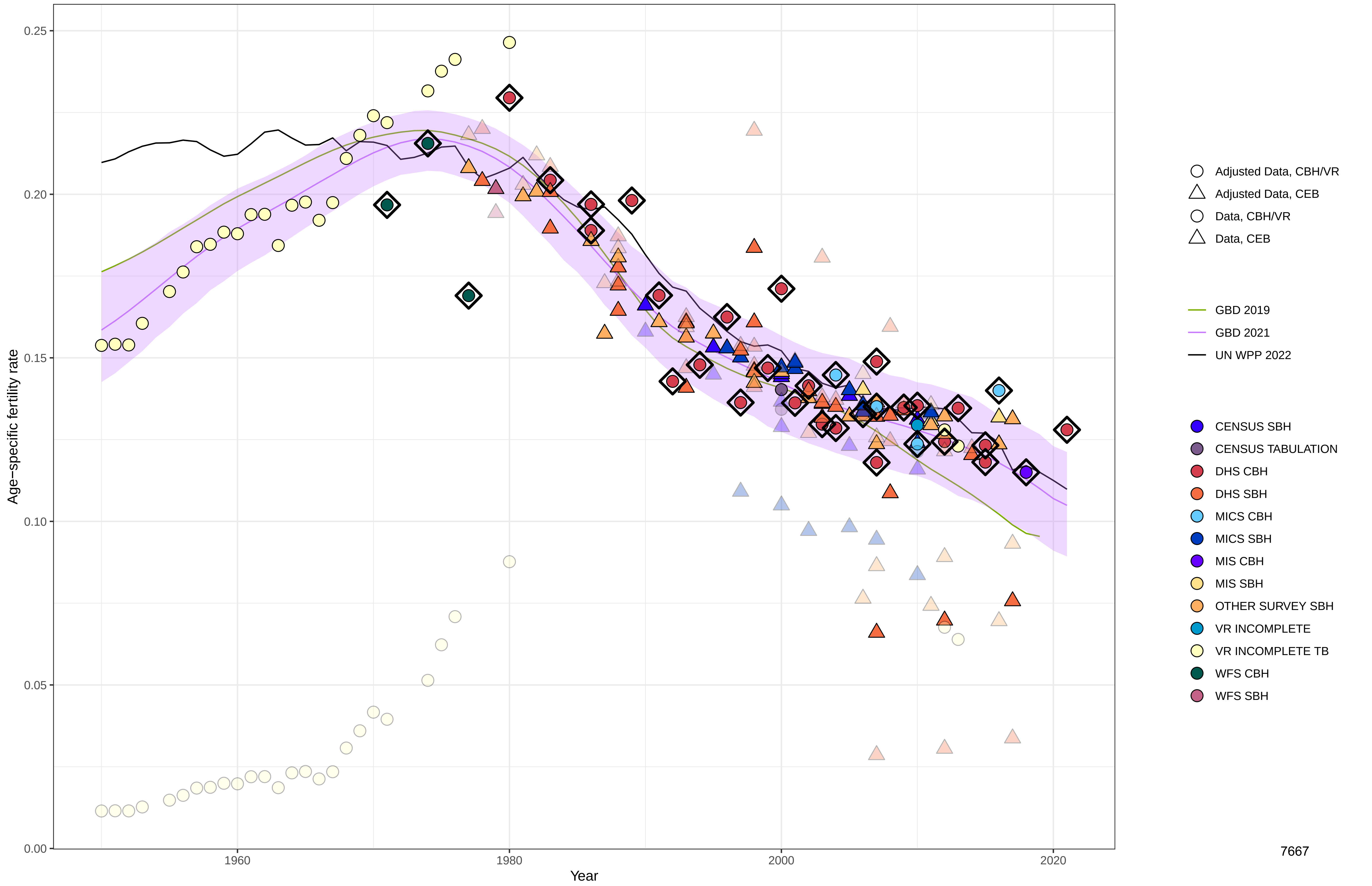

Age−specific fertility fitted trend lines, 1950−2021: Ghana, Ages: 40−44
Data points enclosed with a diamond are reference sources

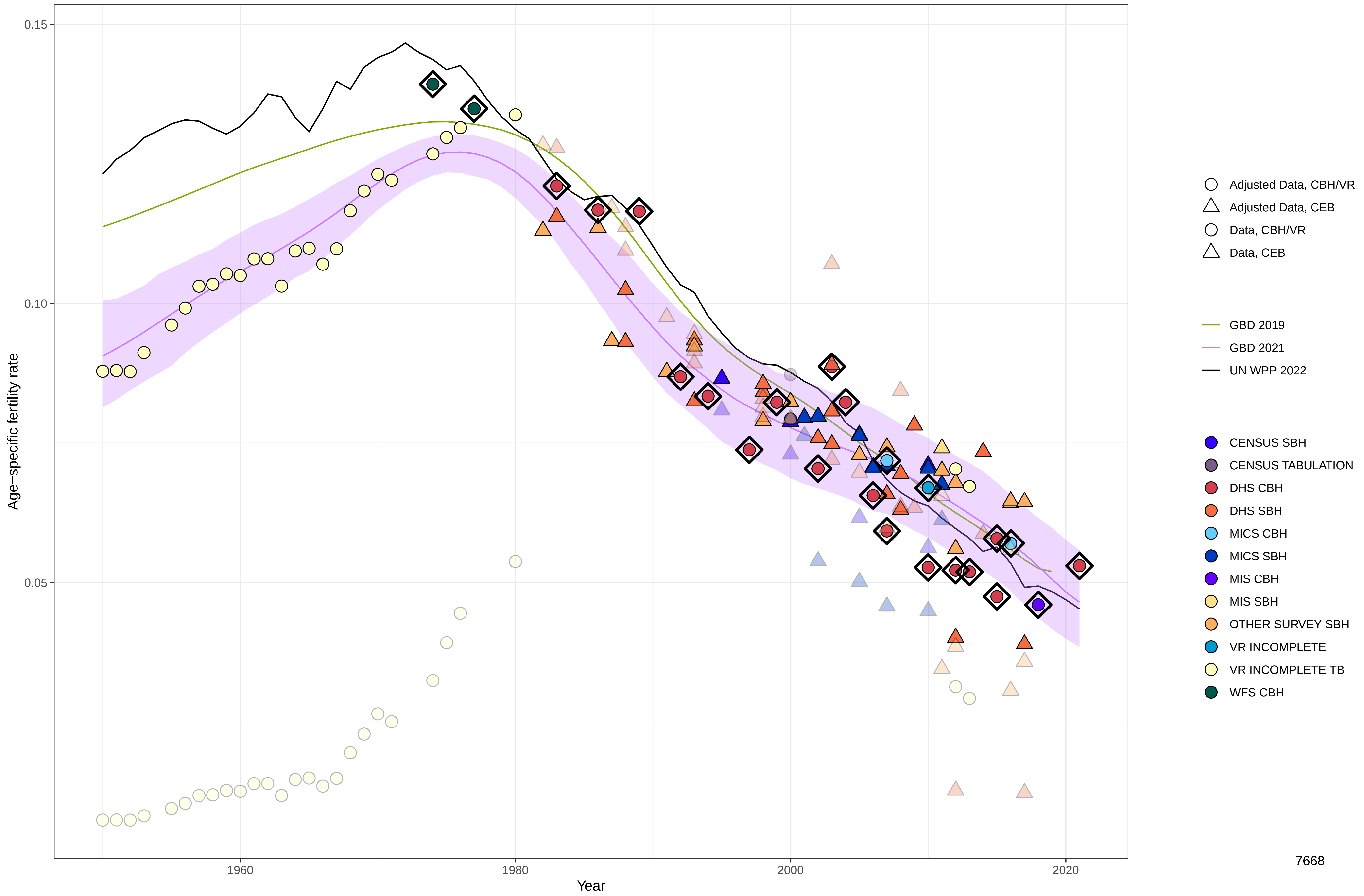

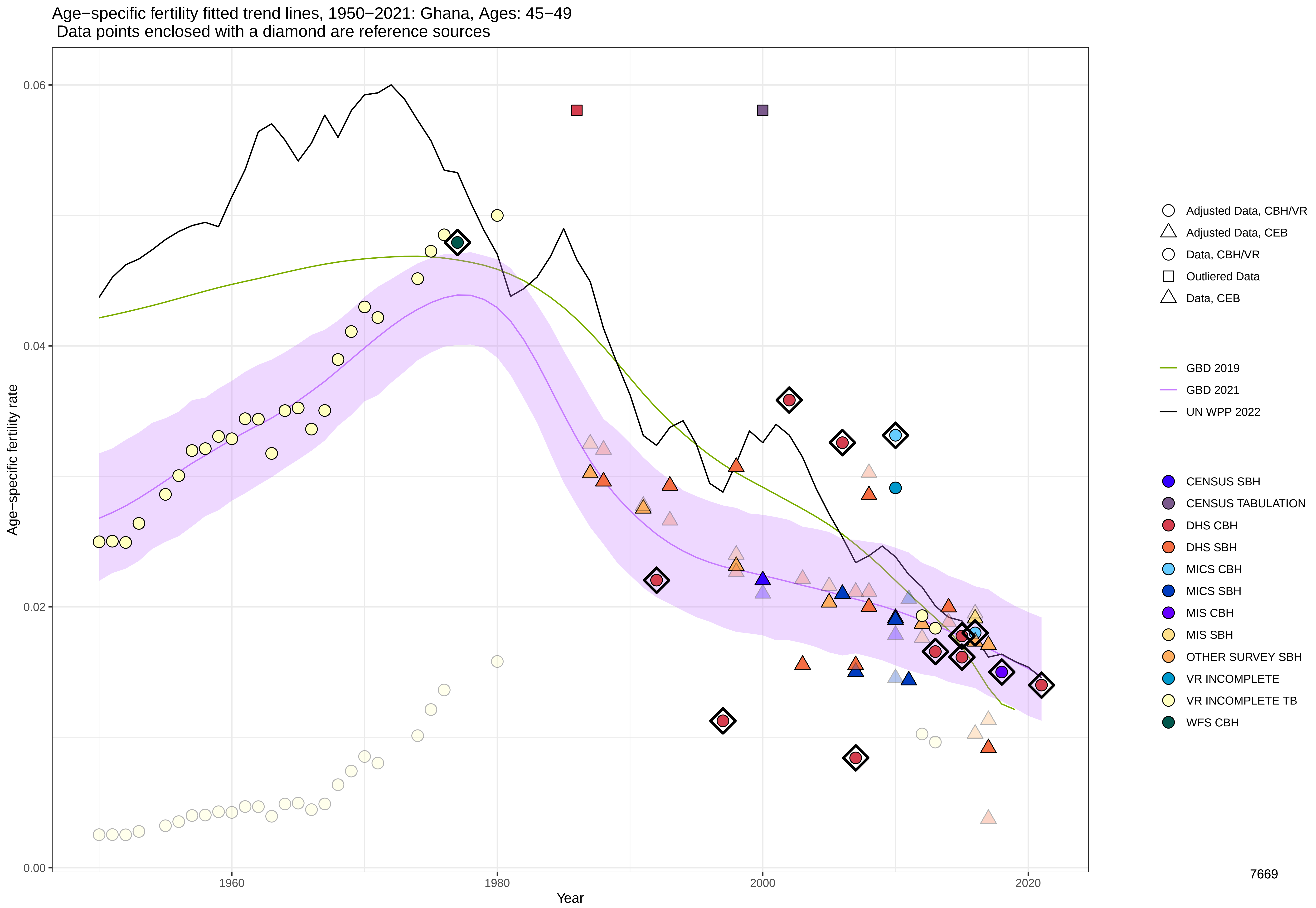

Age−specific fertility fitted trend lines, 1950−2021: Ghana, Ages: 45−49
Data points enclosed with a diamond are reference sources
Age−specific fertility rate
Year
0.00
0.02
0.04
0.06
1960
1980
2000
2020
Adjusted Data, CBH/VR
Adjusted Data, CEB
Data, CBH/VR
Outliered Data
Data, CEB
GBD 2019
GBD 2021
UN WPP 2022
CENSUS SBH
CENSUS TABULATION
DHS CBH
DHS SBH
MICS CBH
MICS SBH
MIS CBH
MIS SBH
OTHER SURVEY SBH
VR INCOMPLETE
VR INCOMPLETE TB
WFS CBH

Age−specific fertility fitted trend lines, 1950−2021: Ghana, Ages: 50−54
Data points enclosed with a diamond are reference sources

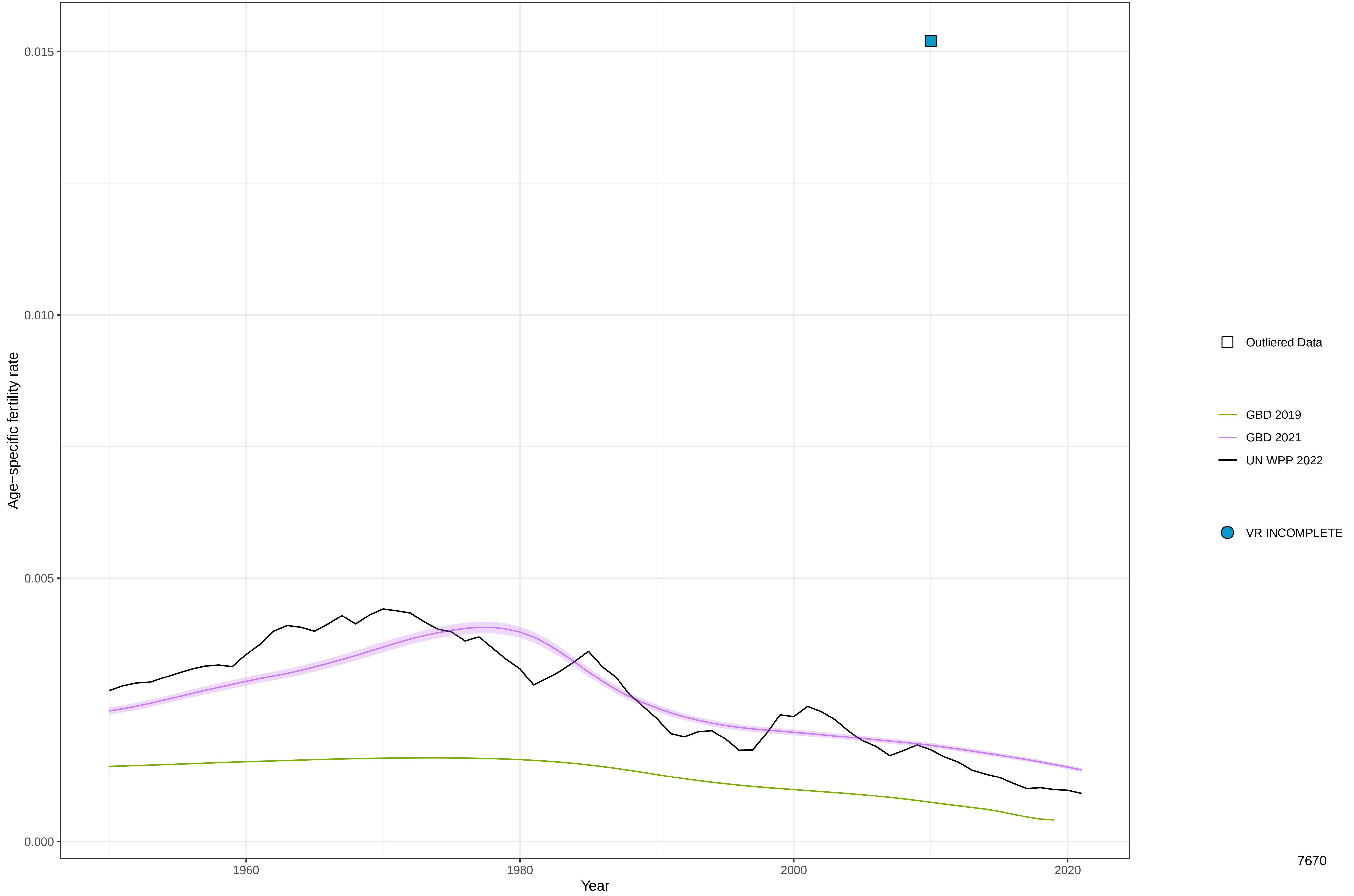

Total fertility rate fitted trend lines, 1950−2021: Ghana TFR
Data points enclosed with a diamond are reference sources

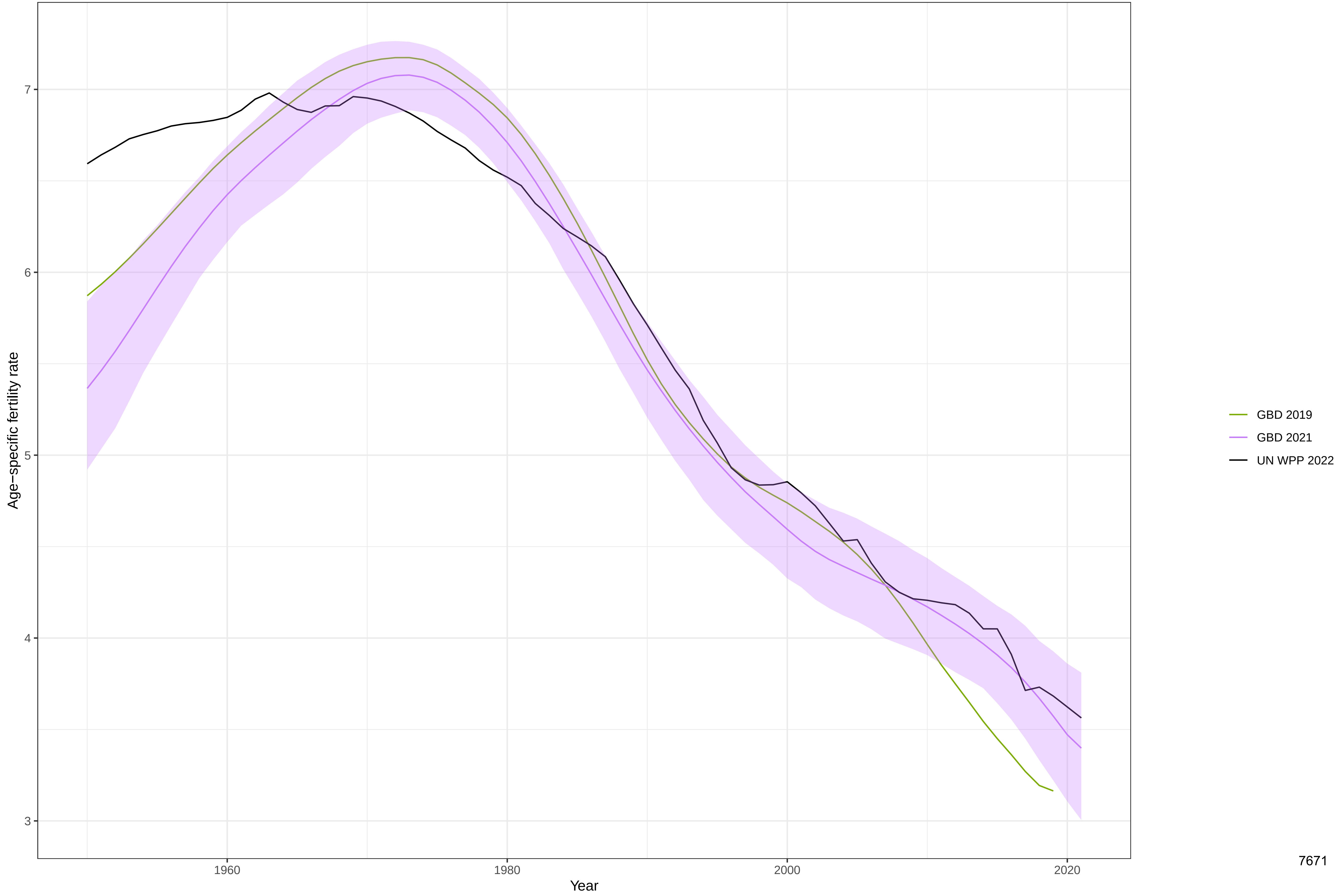

Age−specific fertility fitted trend lines, 1950−2021: Guinea, Ages: 10−14
Data points enclosed with a diamond are reference sources

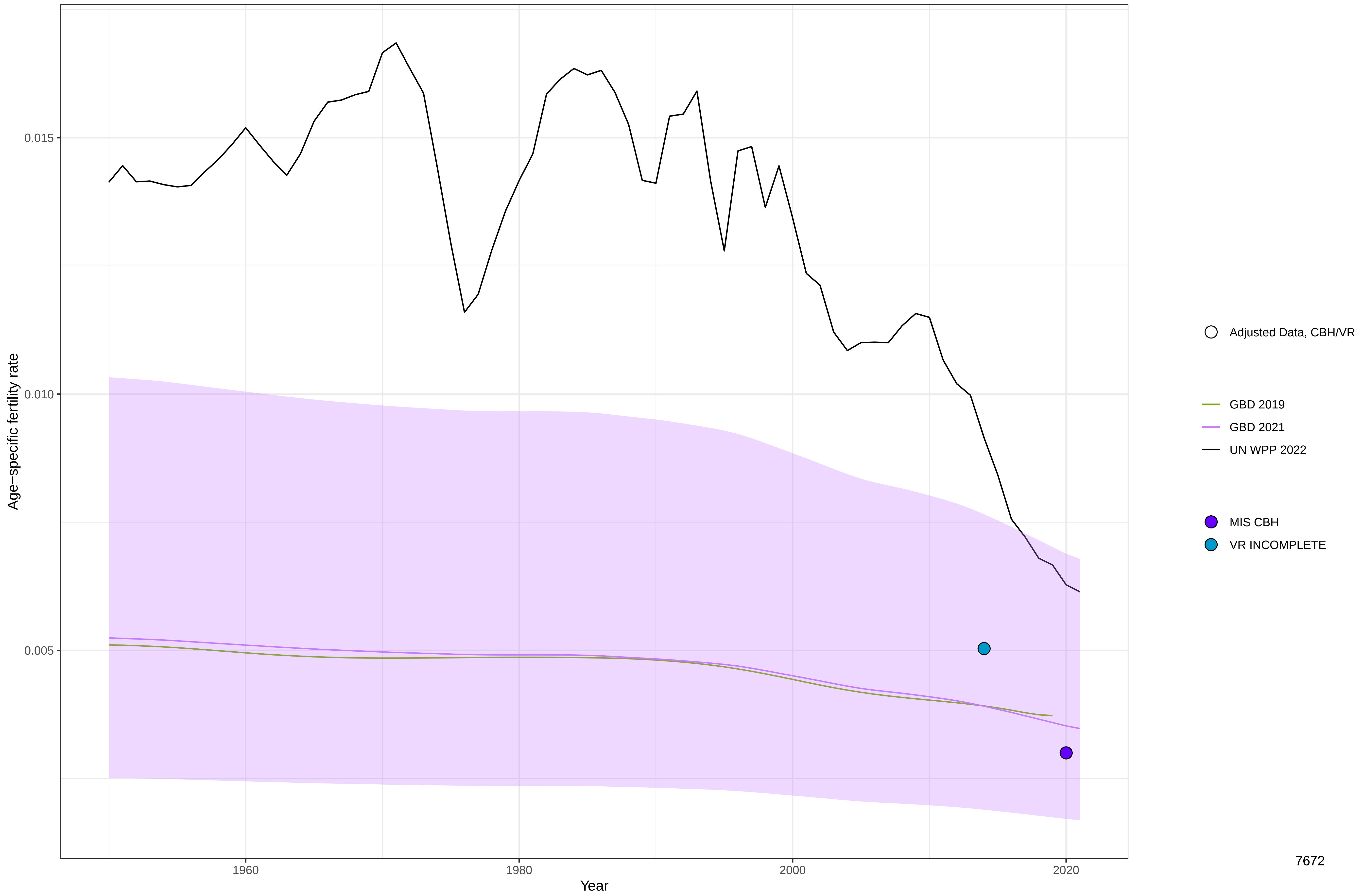

Age−specific fertility fitted trend lines, 1950−2021: Guinea, Ages: 15−19
Data points enclosed with a diamond are reference sources

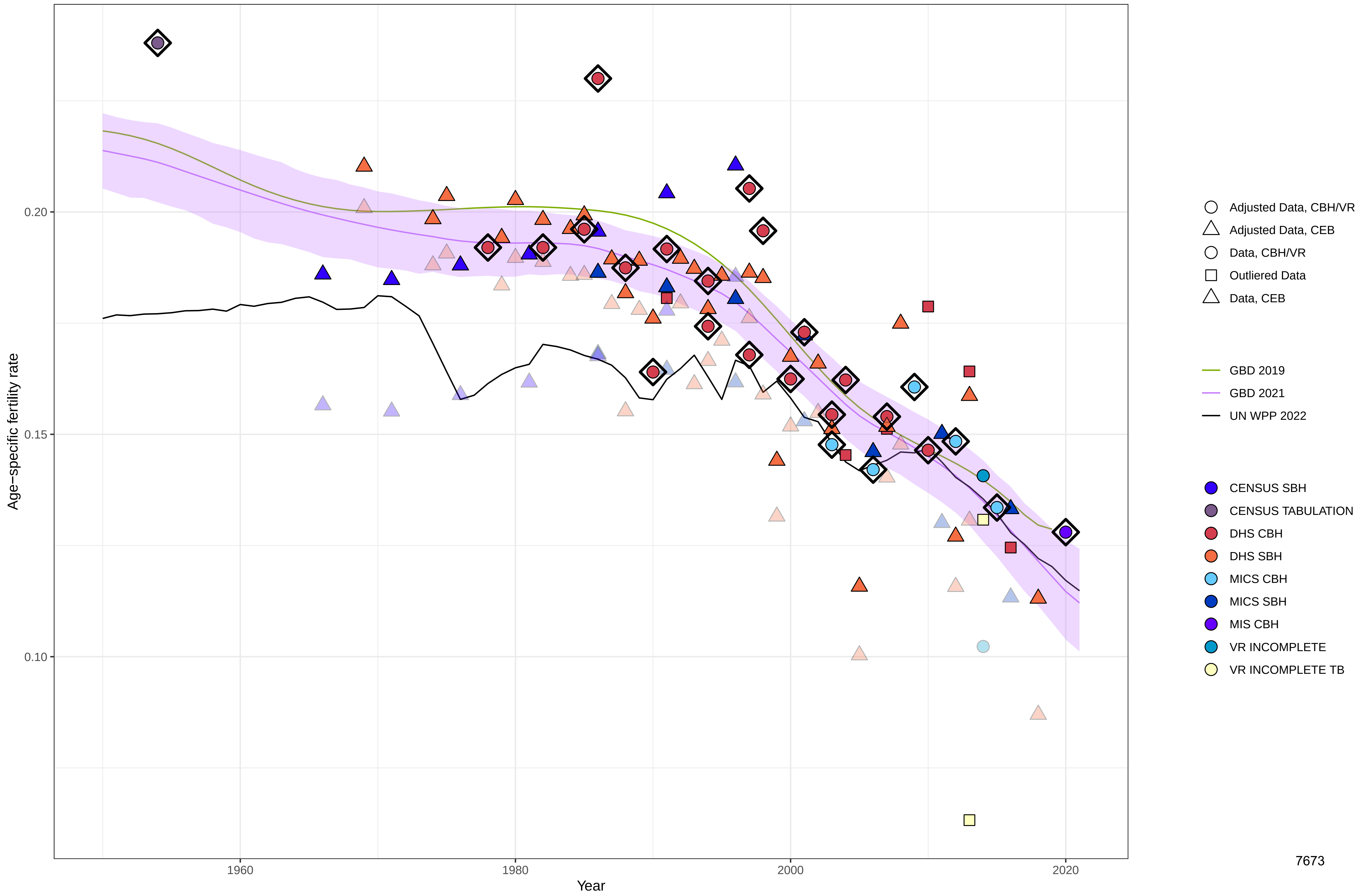

Age−specific fertility fitted trend lines, 1950−2021: Guinea, Ages: 20−24
Data points enclosed with a diamond are reference sources

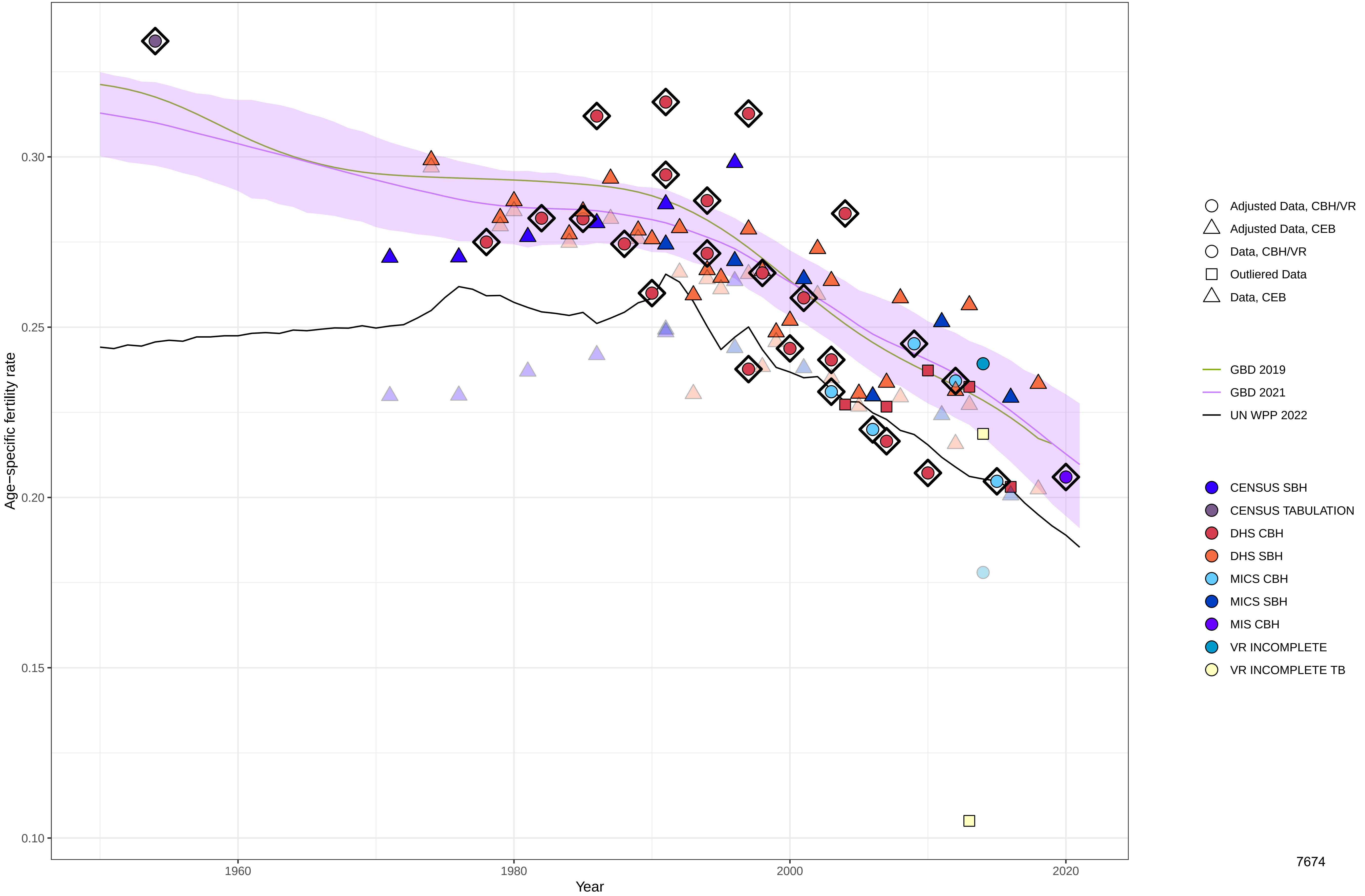

Age−specific fertility fitted trend lines, 1950−2021: Guinea, Ages: 25−29
Data points enclosed with a diamond are reference sources

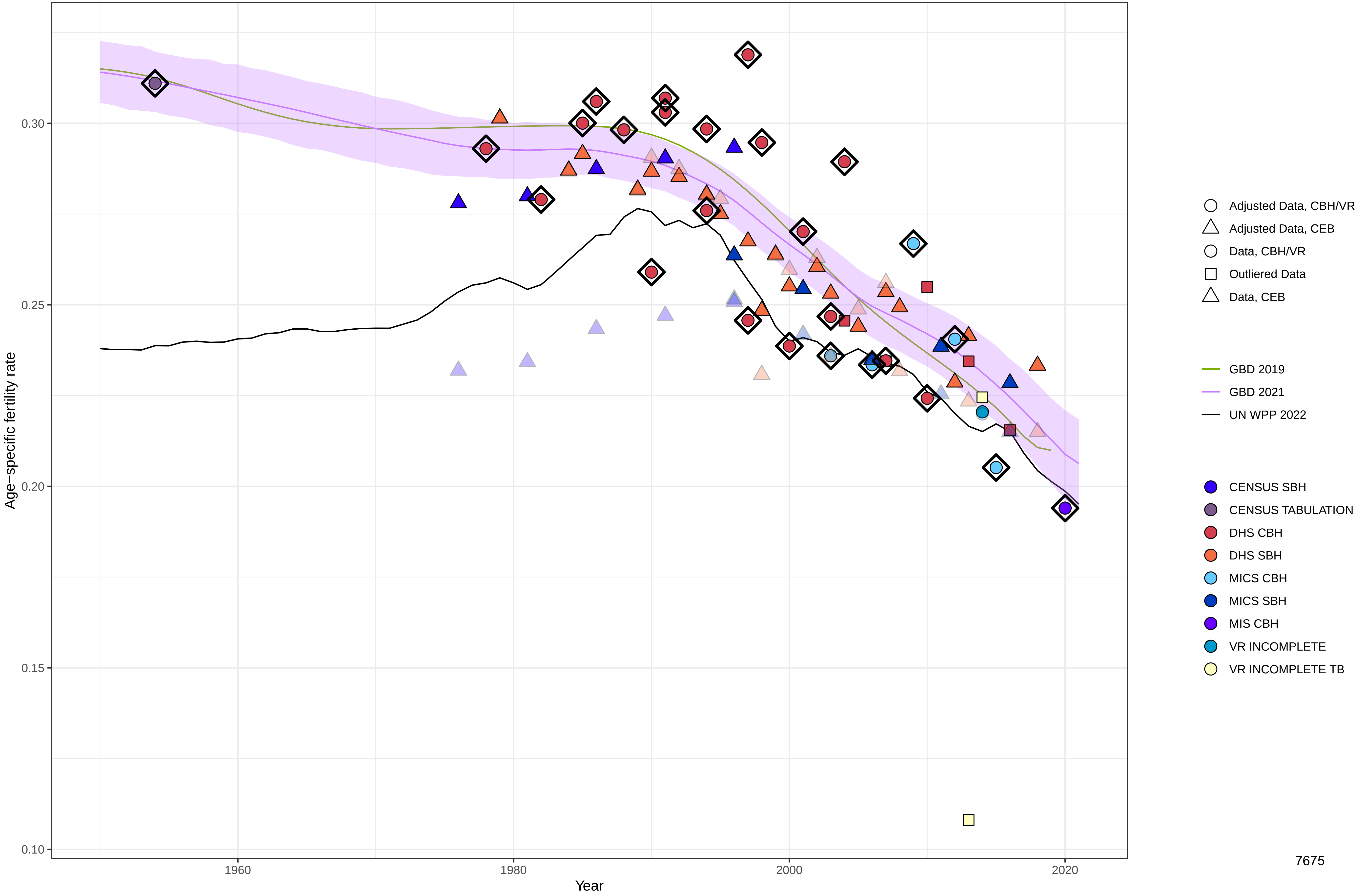

Age−specific fertility fitted trend lines, 1950−2021: Guinea, Ages: 30−34
Data points enclosed with a diamond are reference sources

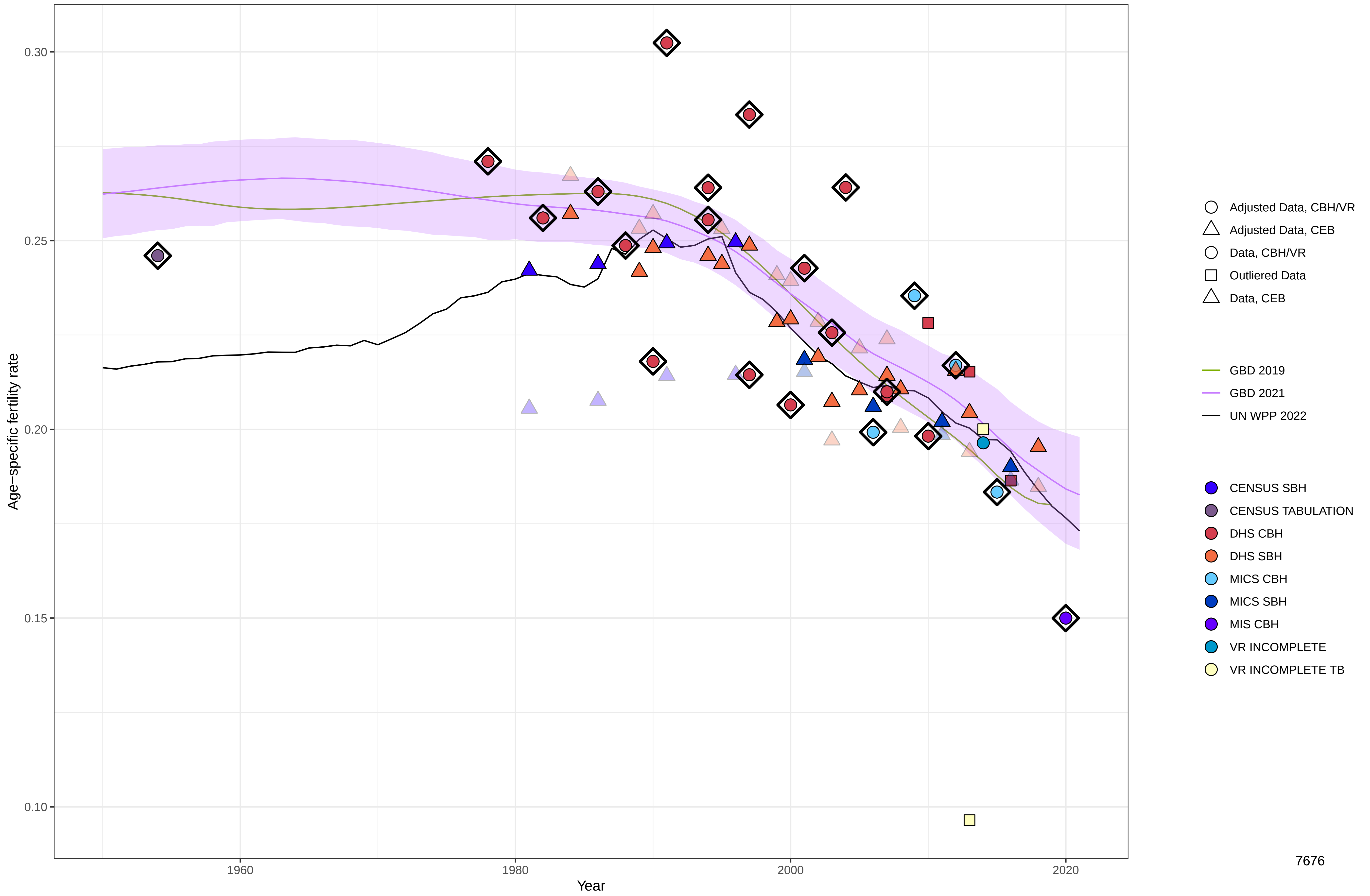

7676

Age−specific fertility fitted trend lines, 1950−2021: Guinea, Ages: 35−39
Data points enclosed with a diamond are reference sources

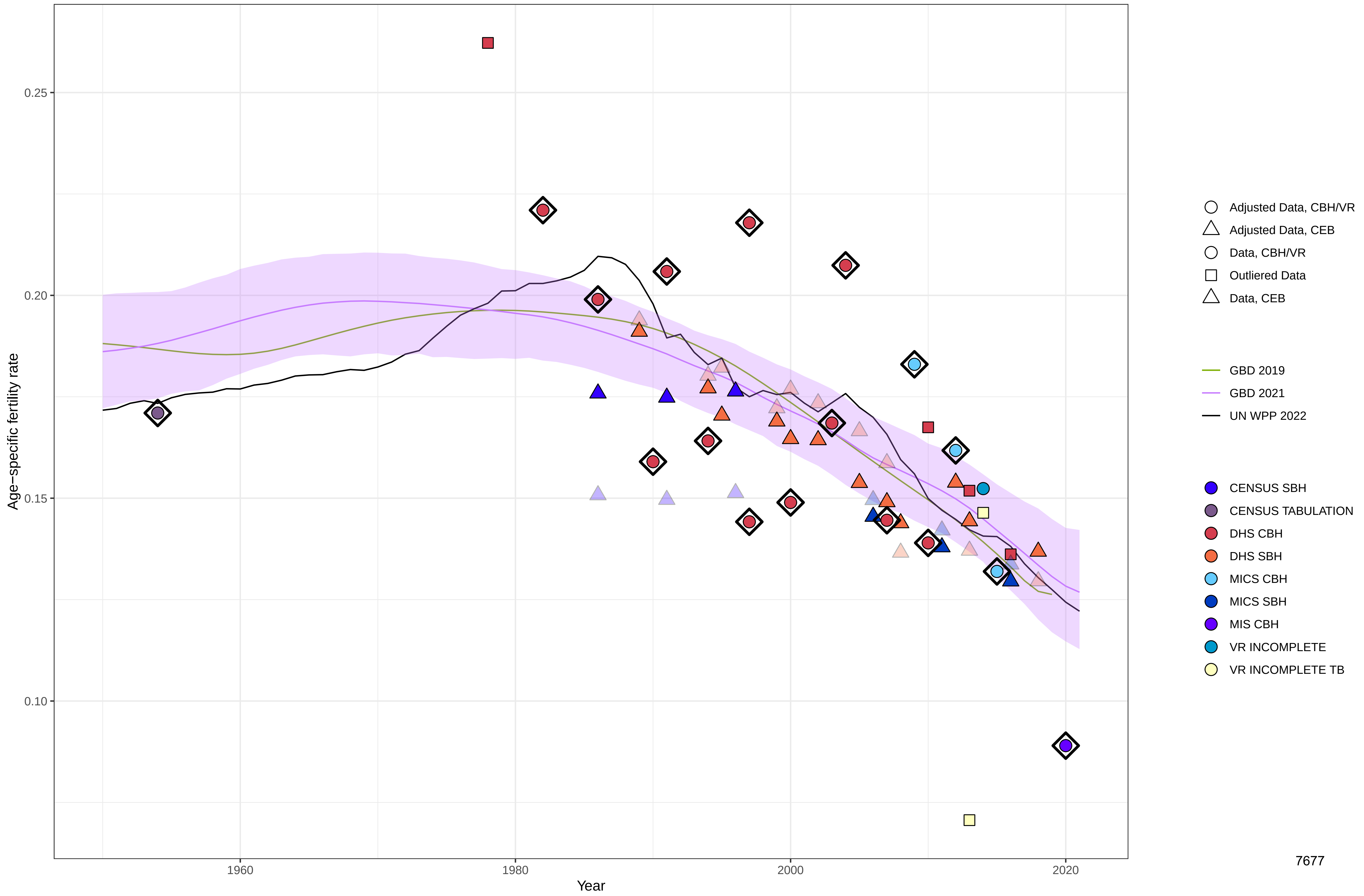

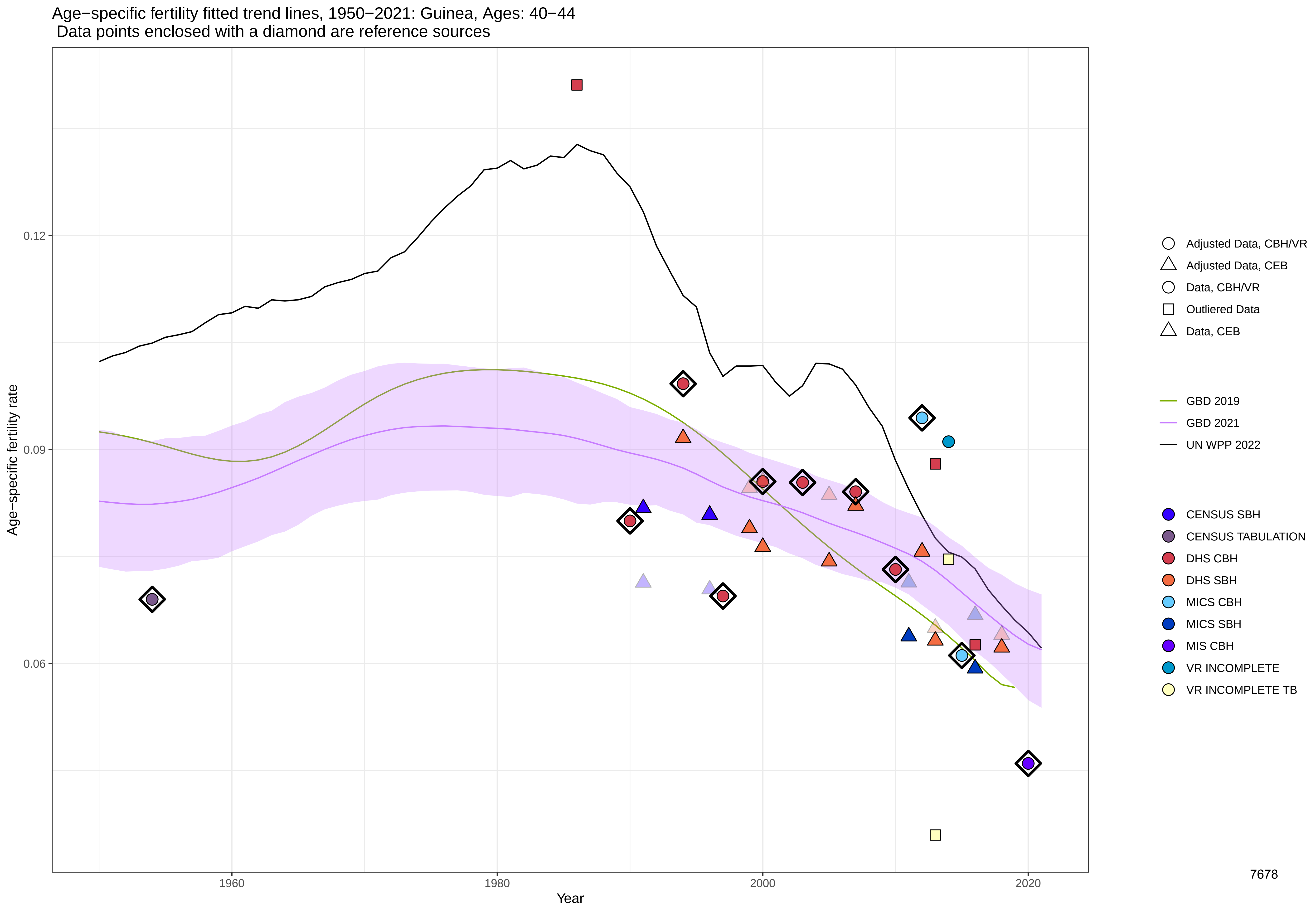

Age−specific fertility fitted trend lines, 1950−2021: Guinea, Ages: 40−44
Data points enclosed with a diamond are reference sources
Age−specific fertility rate
0.12
0.09
0.06
1960
1980
2000
2020
Year
Adjusted Data, CBH/VR
Adjusted Data, CEB
Data, CBH/VR
Outliered Data
Data, CEB
GBD 2019
GBD 2021
UN WPP 2022
CENSUS SBH
CENSUS TABULATION
DHS CBH
DHS SBH
MICS CBH
MICS SBH
MIS CBH
VR INCOMPLETE
VR INCOMPLETE TB

Age−specific fertility fitted trend lines, 1950−2021: Guinea, Ages: 45−49
Data points enclosed with a diamond are reference sources

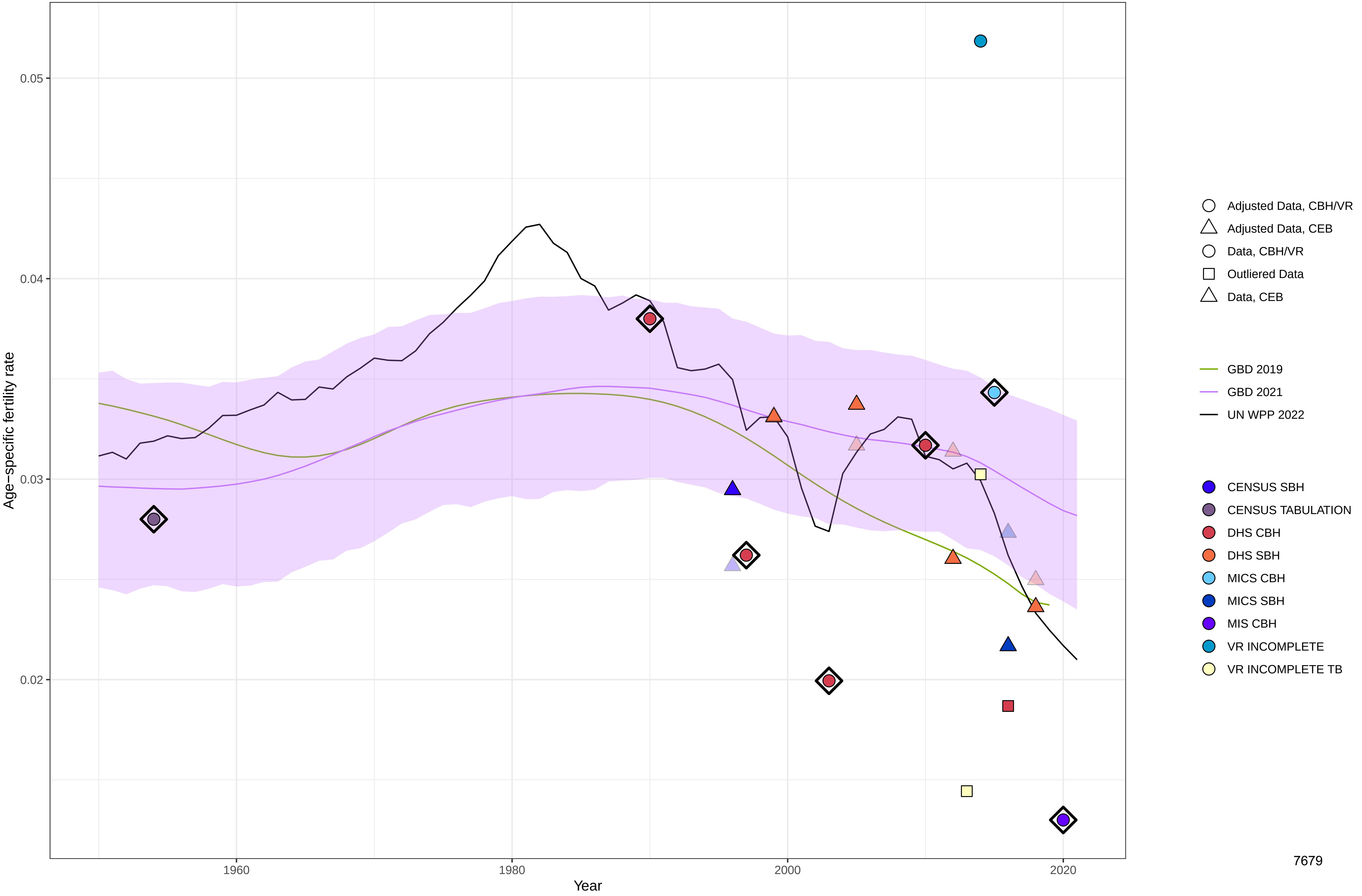

7679

Age−specific fertility fitted trend lines, 1950−2021: Guinea, Ages: 50−54
Data points enclosed with a diamond are reference sources

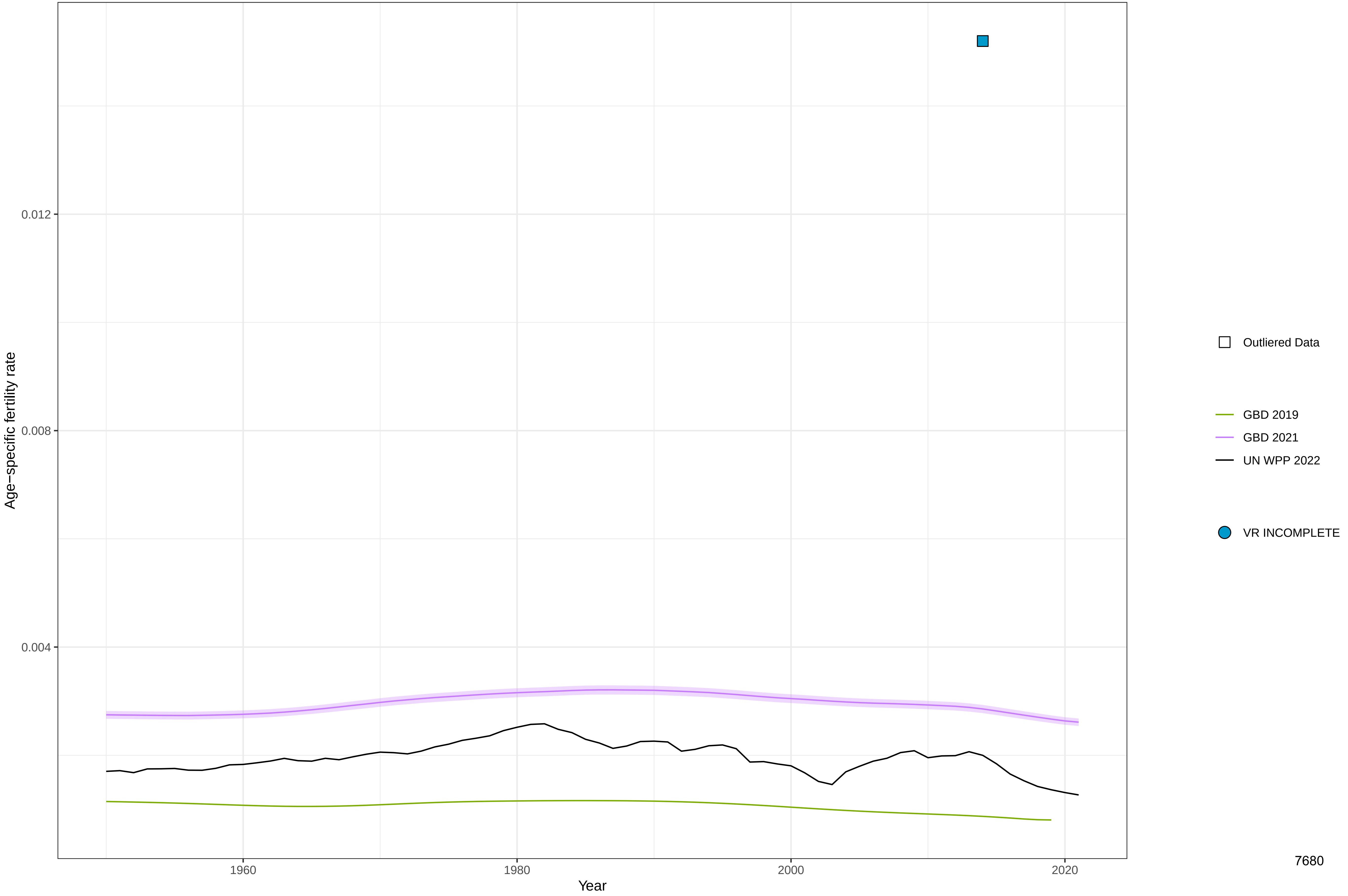

Total fertility rate fitted trend lines, 1950−2021: Guinea TFR
Data points enclosed with a diamond are reference sources

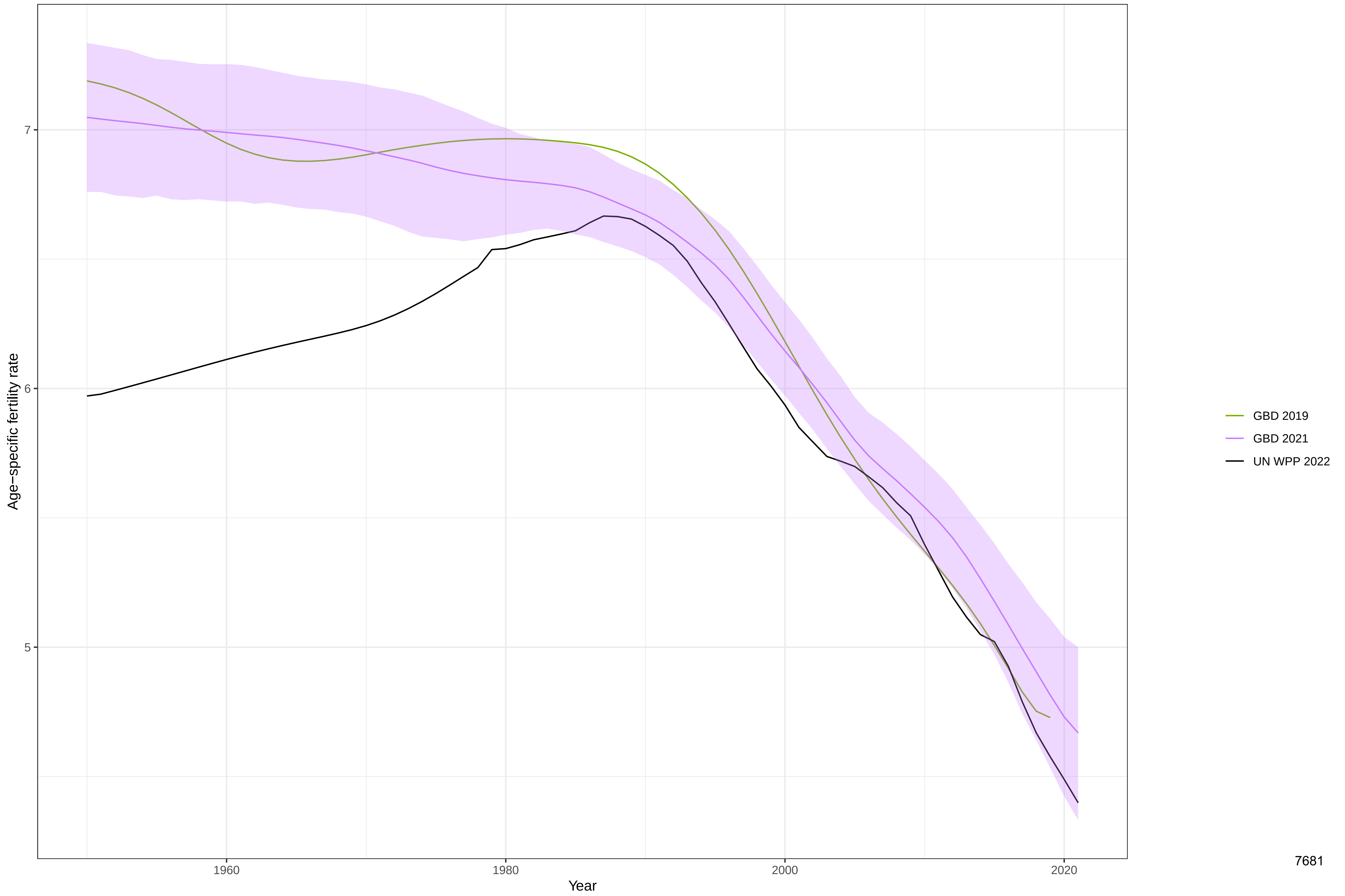

Age−specific fertility fitted trend lines, 1950−2021: Guinea−Bissau, Ages: 10−14
Data points enclosed with a diamond are reference sources

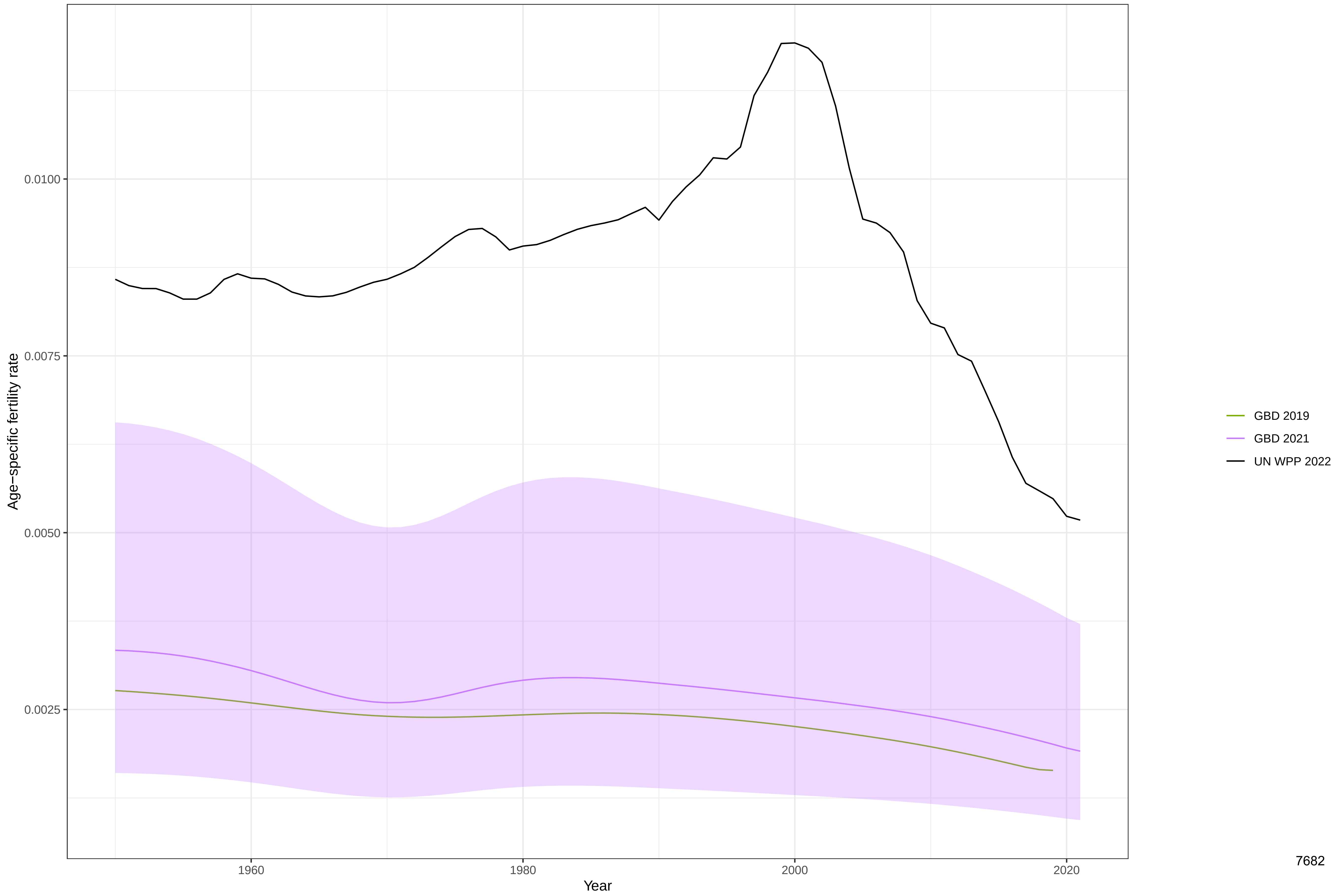

Age−specific fertility fitted trend lines, 1950−2021: Guinea−Bissau, Ages: 15−19
Data points enclosed with a diamond are reference sources

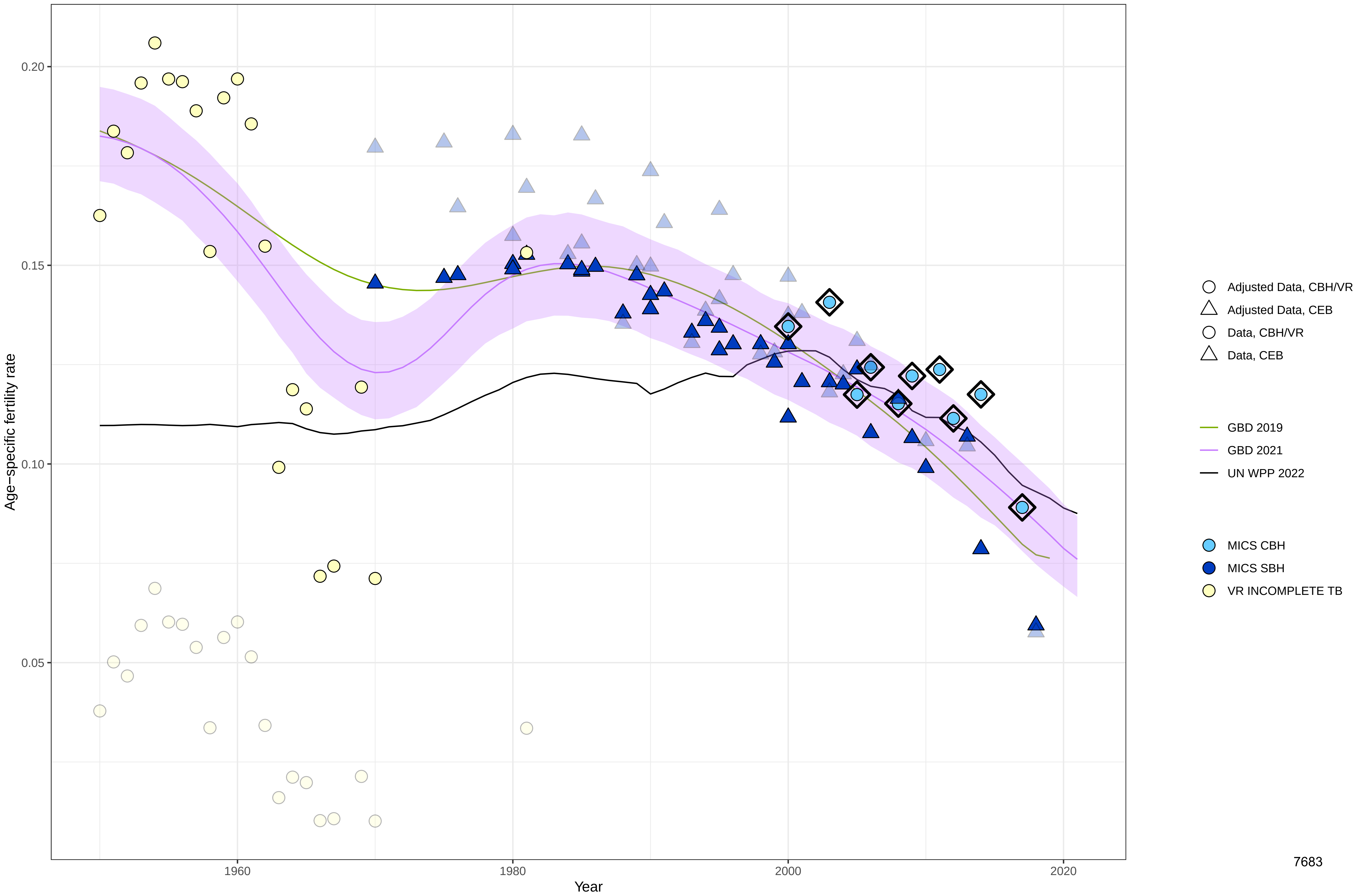

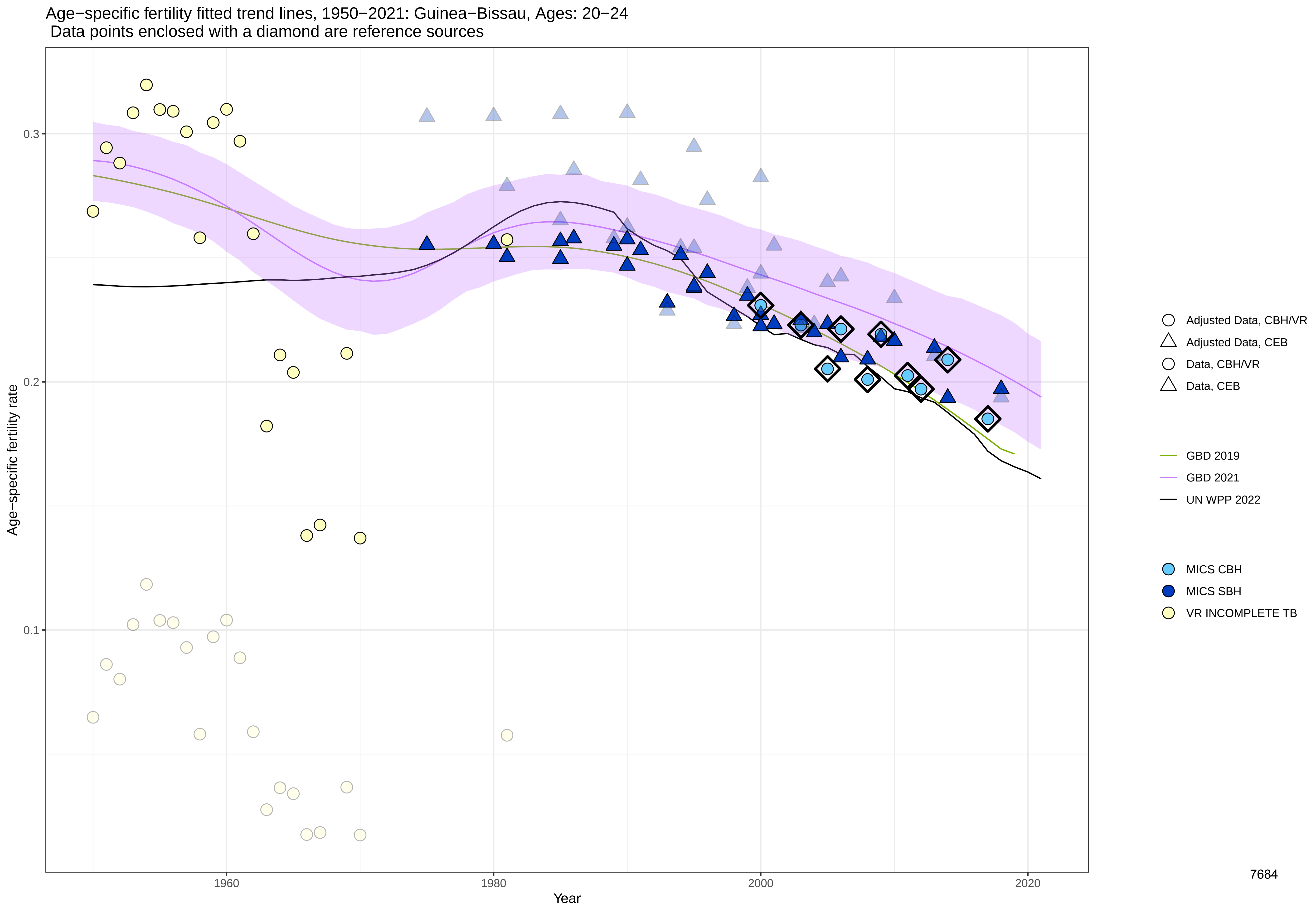

Age−specific fertility fitted trend lines, 1950−2021: Guinea−Bissau, Ages: 20−24
Data points enclosed with a diamond are reference sources
Age−specific fertility rate
0.1
0.2
0.3
Year
1960
1980
2000
2020
Adjusted Data, CBH/VR
Adjusted Data, CEB
Data, CBH/VR
Data, CEB
GBD 2019
GBD 2021
UN WPP 2022
MICS CBH
MICS SBH
VR INCOMPLETE TB

Age−specific fertility fitted trend lines, 1950−2021: Guinea−Bissau, Ages: 25−29
Data points enclosed with a diamond are reference sources

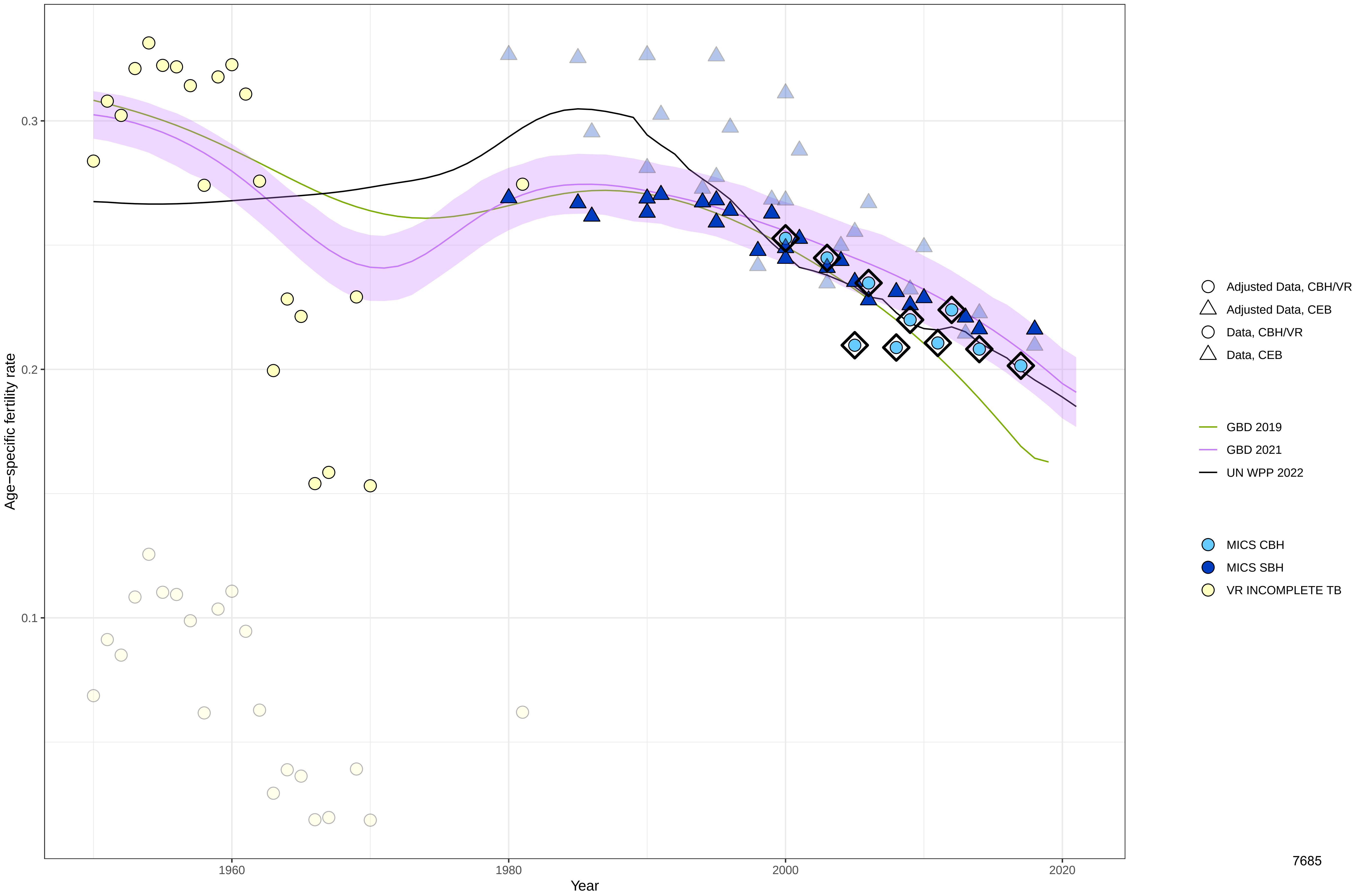

Age−specific fertility fitted trend lines, 1950−2021: Guinea−Bissau, Ages: 30−34
Data points enclosed with a diamond are reference sources

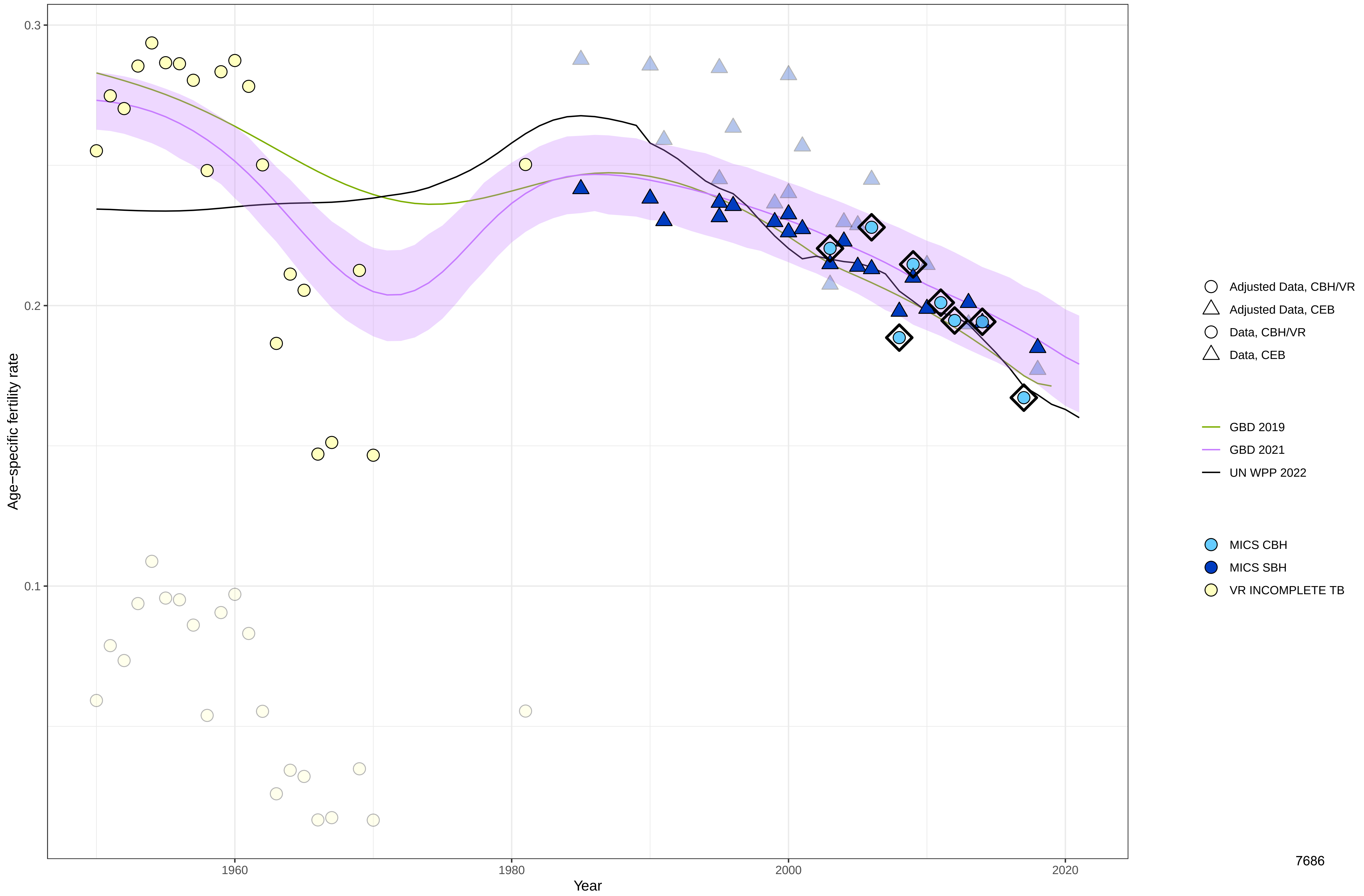

Age−specific fertility fitted trend lines, 1950−2021: Guinea−Bissau, Ages: 35−39
Data points enclosed with a diamond are reference sources

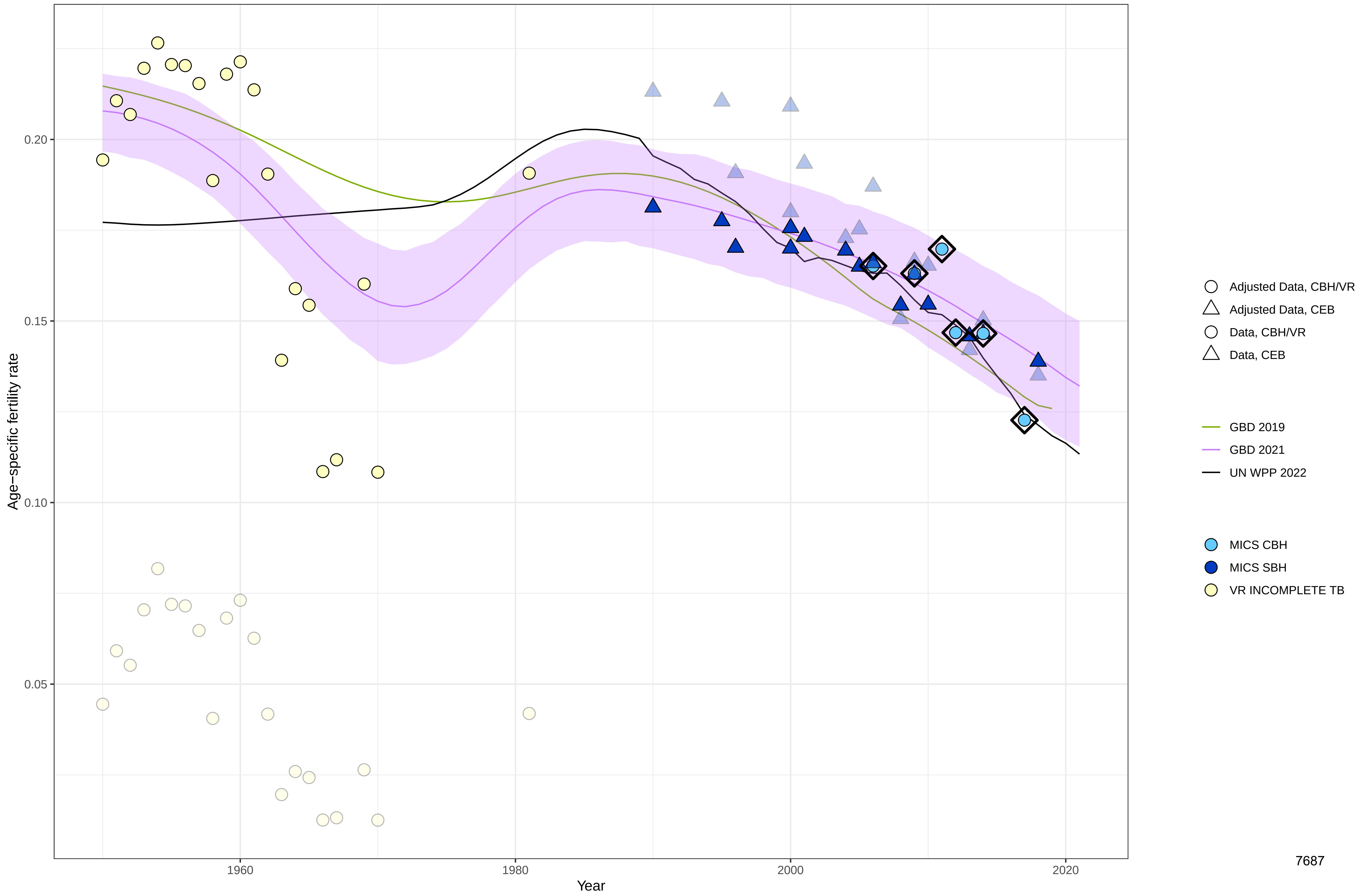

Age−specific fertility fitted trend lines, 1950−2021: Guinea−Bissau, Ages: 40−44
Data points enclosed with a diamond are reference sources

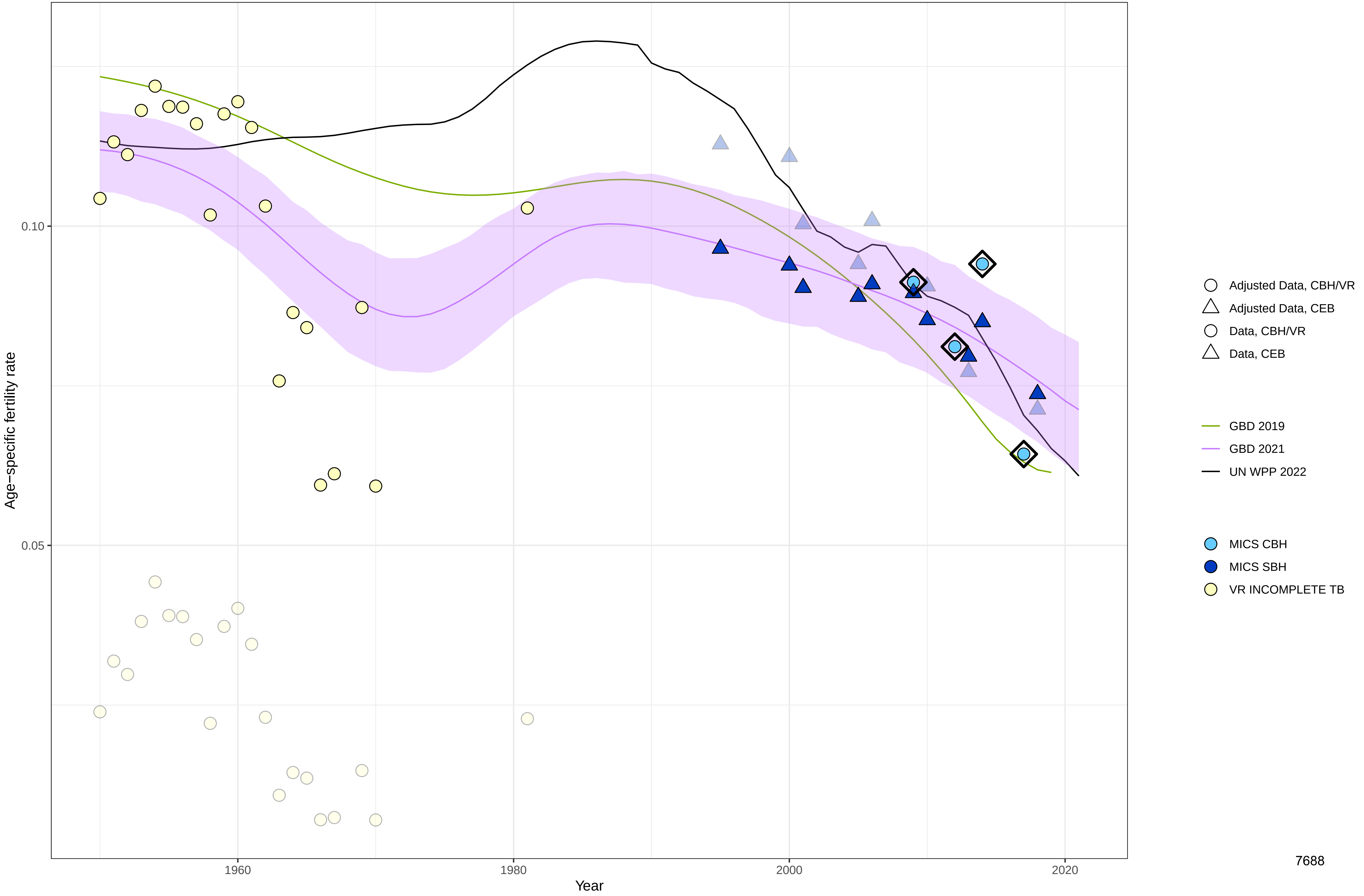

Age−specific fertility fitted trend lines, 1950−2021: Guinea−Bissau, Ages: 45−49
Data points enclosed with a diamond are reference sources

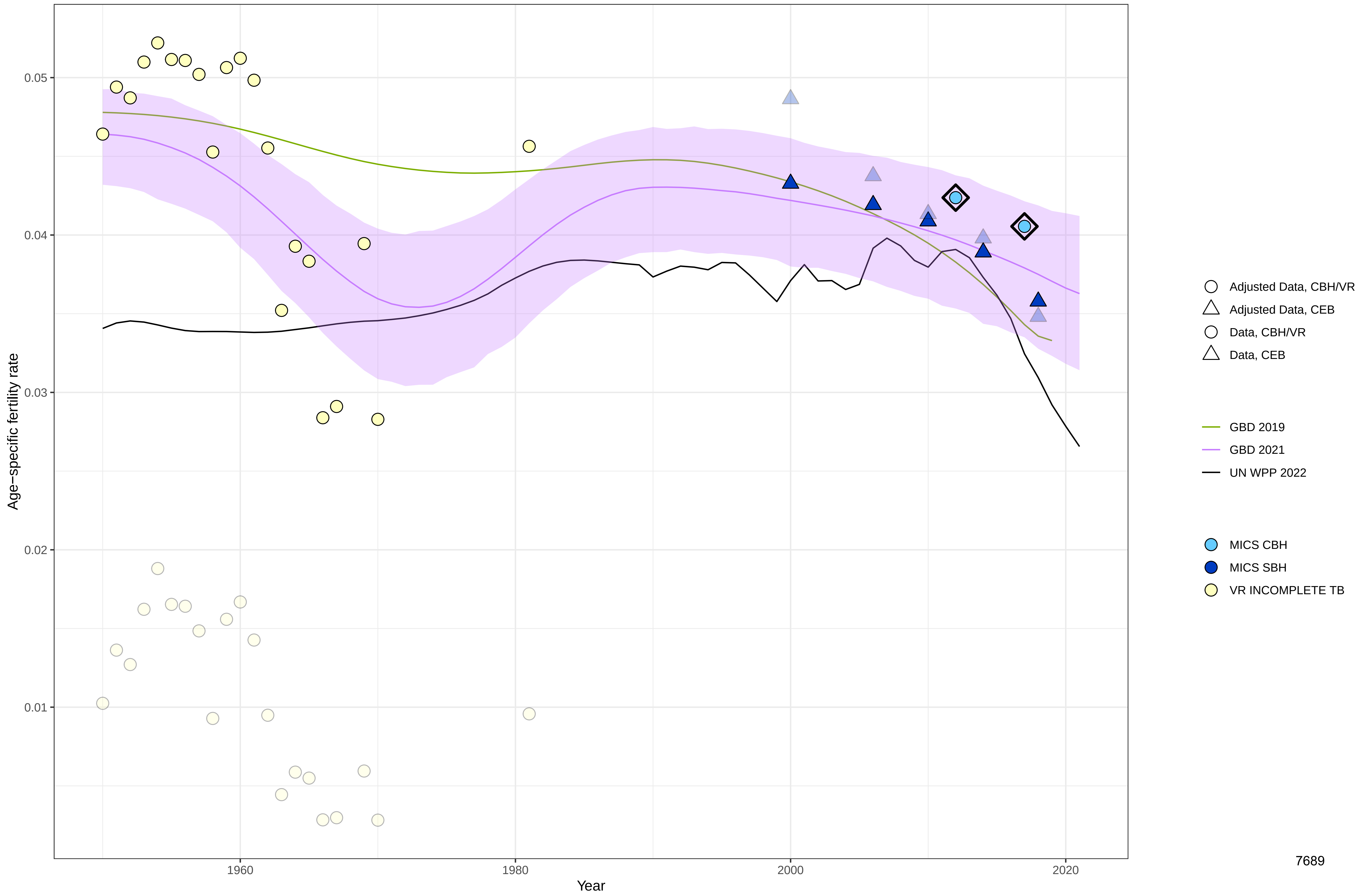

Age−specific fertility fitted trend lines, 1950−2021: Guinea−Bissau, Ages: 50−54
Data points enclosed with a diamond are reference sources

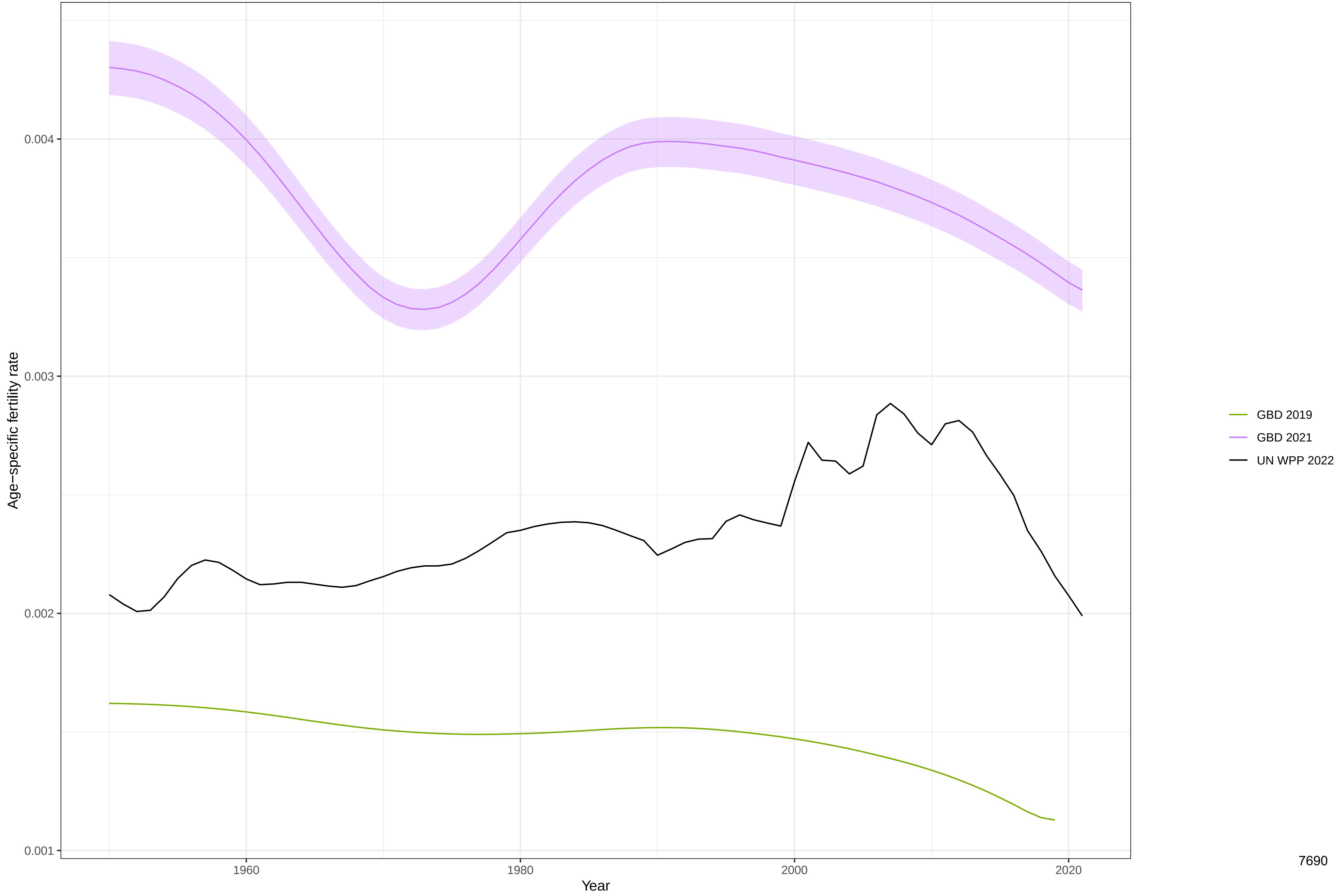

Total fertility rate fitted trend lines, 1950−2021: Guinea−Bissau TFR
Data points enclosed with a diamond are reference sources

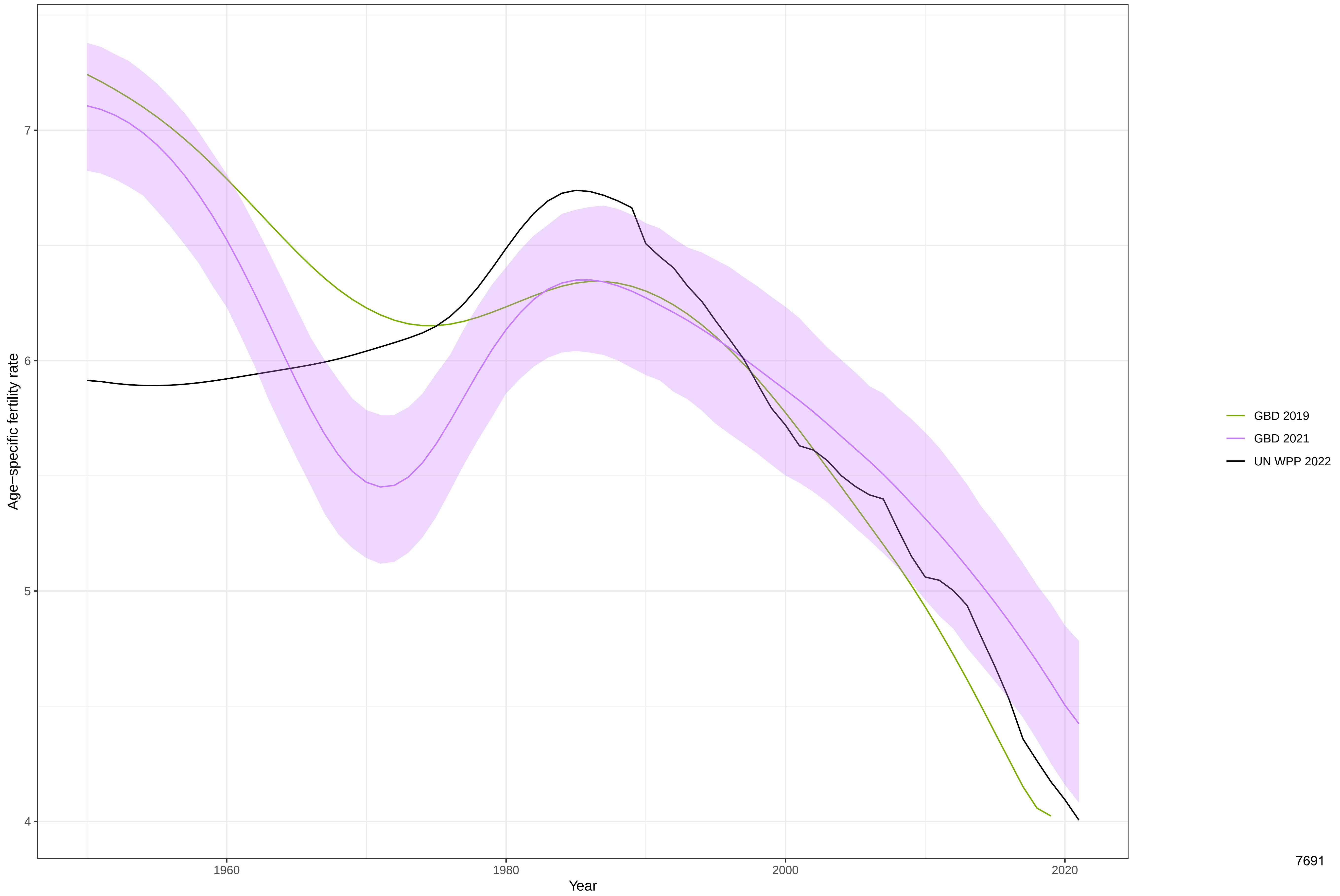

Age−specific fertility fitted trend lines, 1950−2021: Liberia, Ages: 10−14
Data points enclosed with a diamond are reference sources

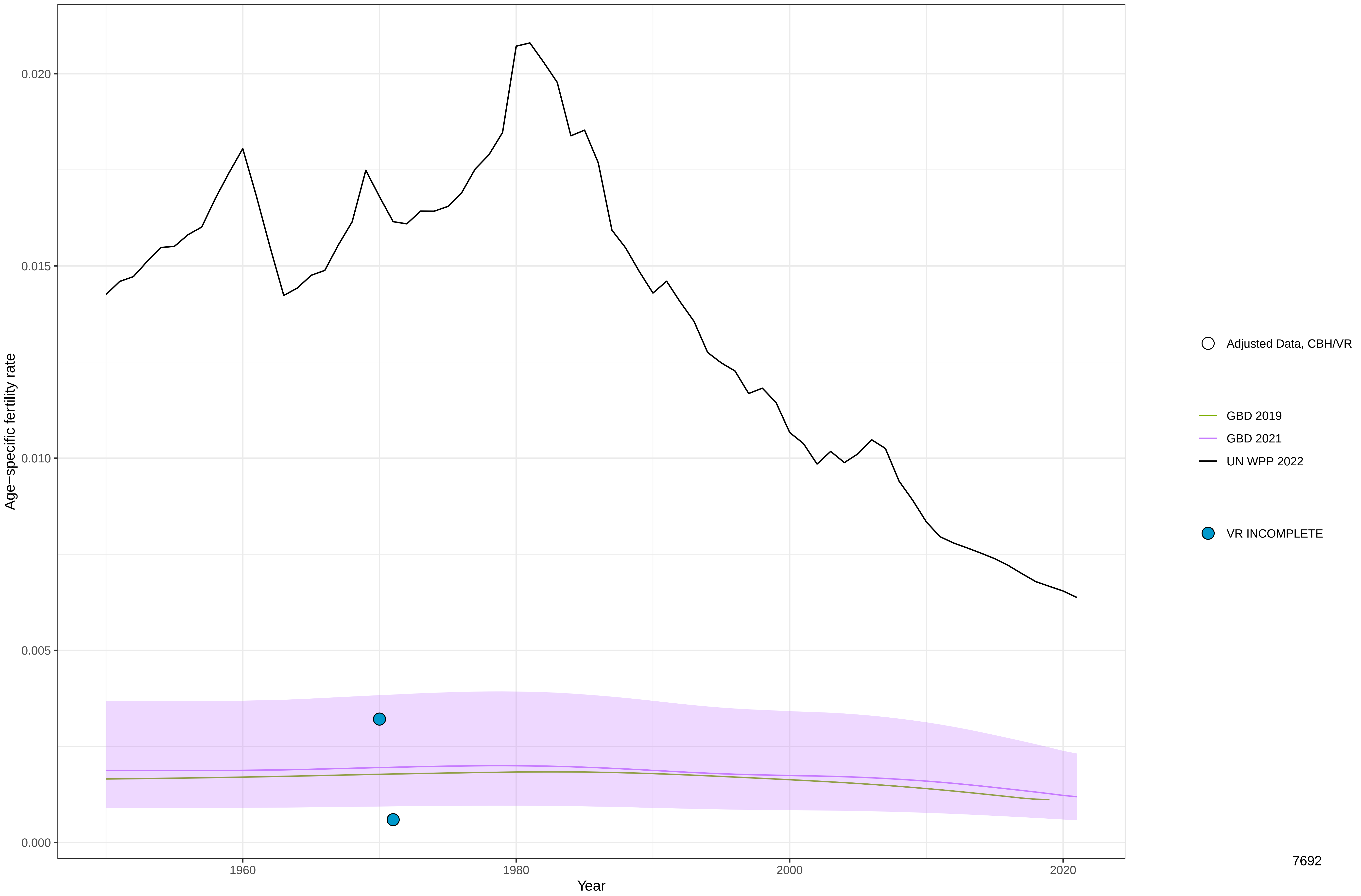

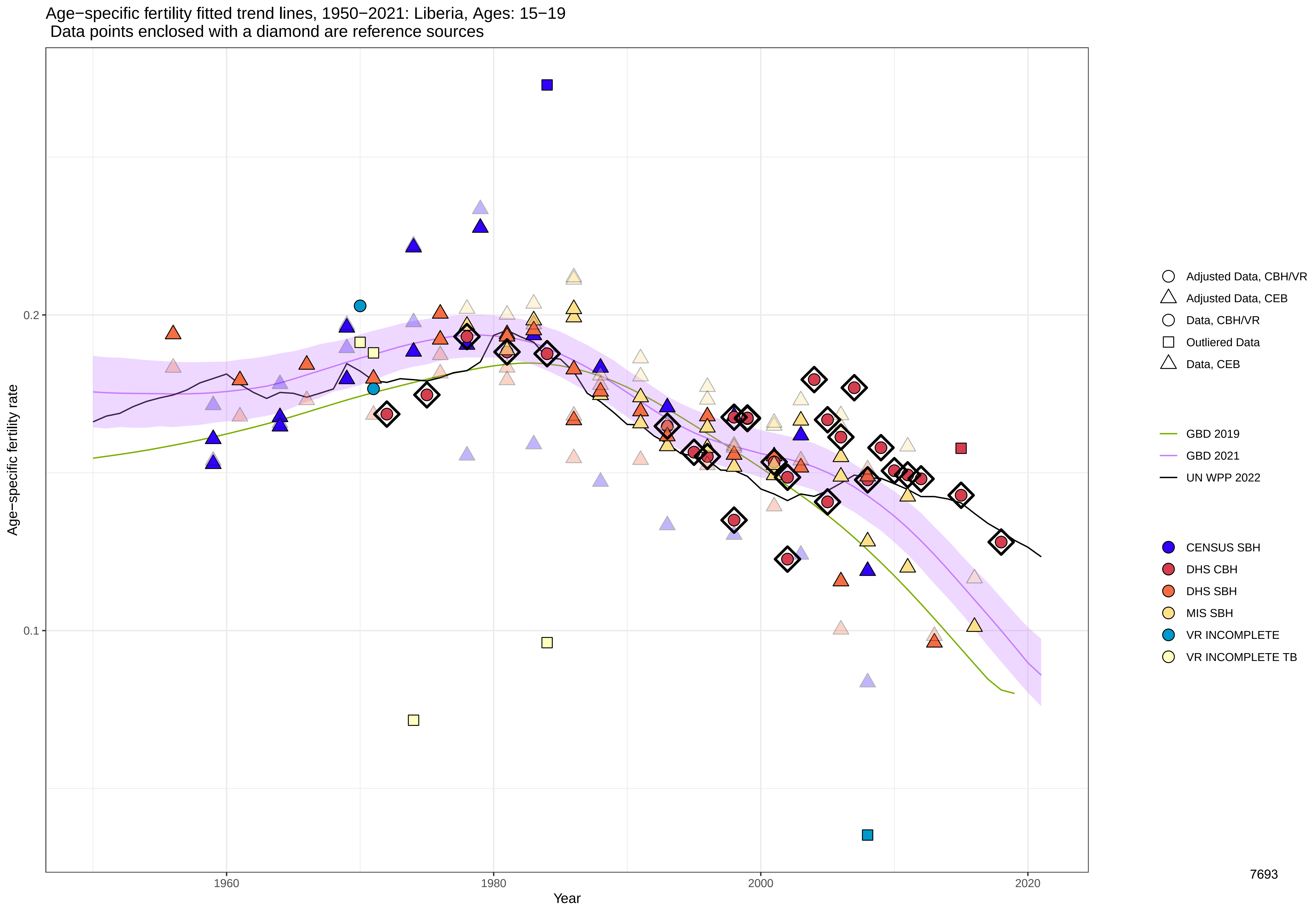
Age−specific fertility fitted trend lines, 1950−2021: Liberia, Ages: 15−19
Data points enclosed with a diamond are reference sources
Age−specific fertility rate
0.2
0.1
Year
1960
1980
2000
2020
Adjusted Data, CBH/VR
Adjusted Data, CEB
Data, CBH/VR
Outliered Data
Data, CEB
GBD 2019
GBD 2021
UN WPP 2022
CENSUS SBH
DHS CBH
DHS SBH
MIS SBH
VR INCOMPLETE
VR INCOMPLETE TB

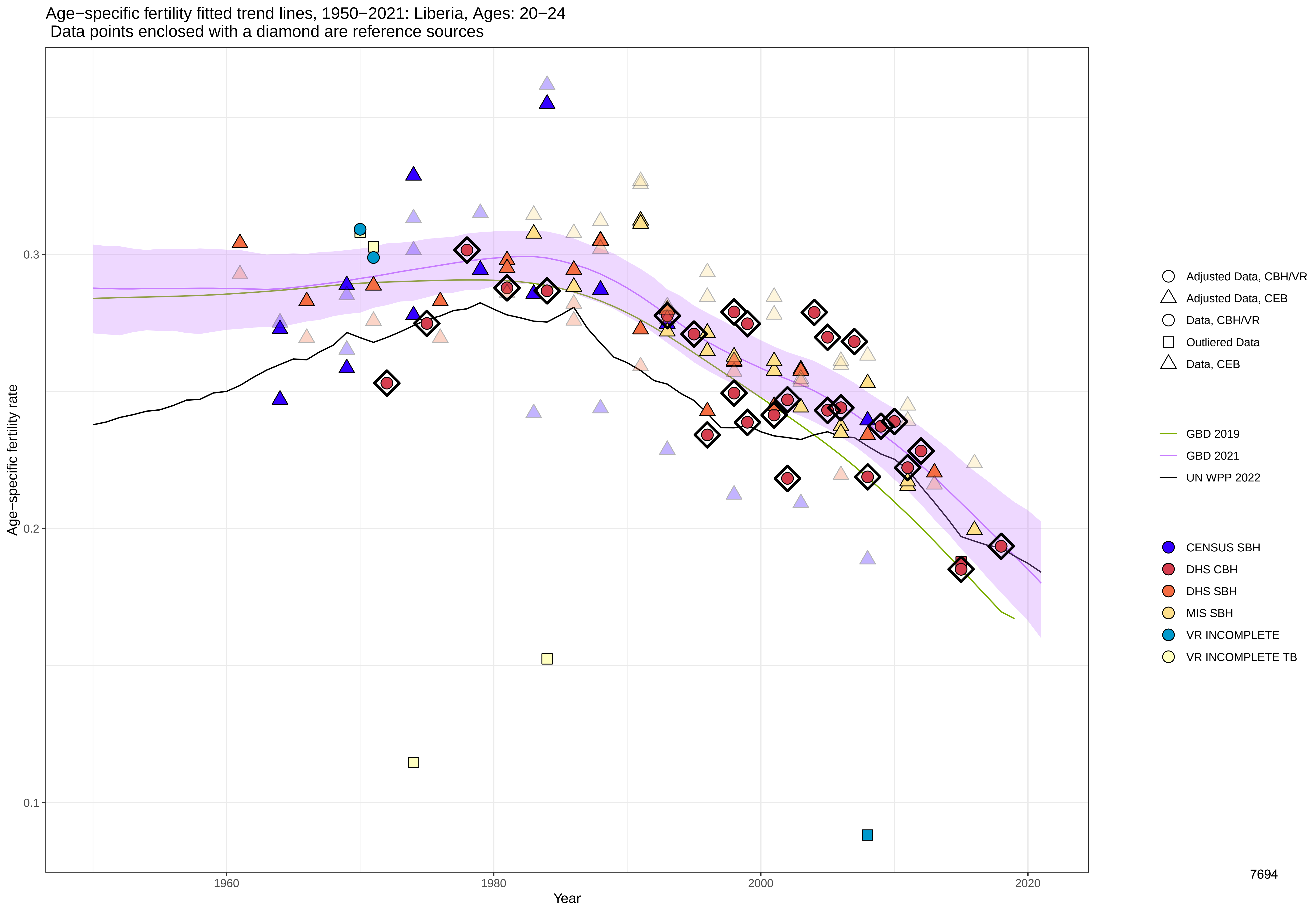

Age−specific fertility fitted trend lines, 1950−2021: Liberia, Ages: 20−24
Data points enclosed with a diamond are reference sources
Age−specific fertility rate
Year
0.1
0.2
0.3
1960
1980
2000
2020
Adjusted Data, CBH/VR
Adjusted Data, CEB
Data, CBH/VR
Outliered Data
Data, CEB
GBD 2019
GBD 2021
UN WPP 2022
CENSUS SBH
DHS CBH
DHS SBH
MIS SBH
VR INCOMPLETE
VR INCOMPLETE TB

7694

Age−specific fertility fitted trend lines, 1950−2021: Liberia, Ages: 25−29
Data points enclosed with a diamond are reference sources

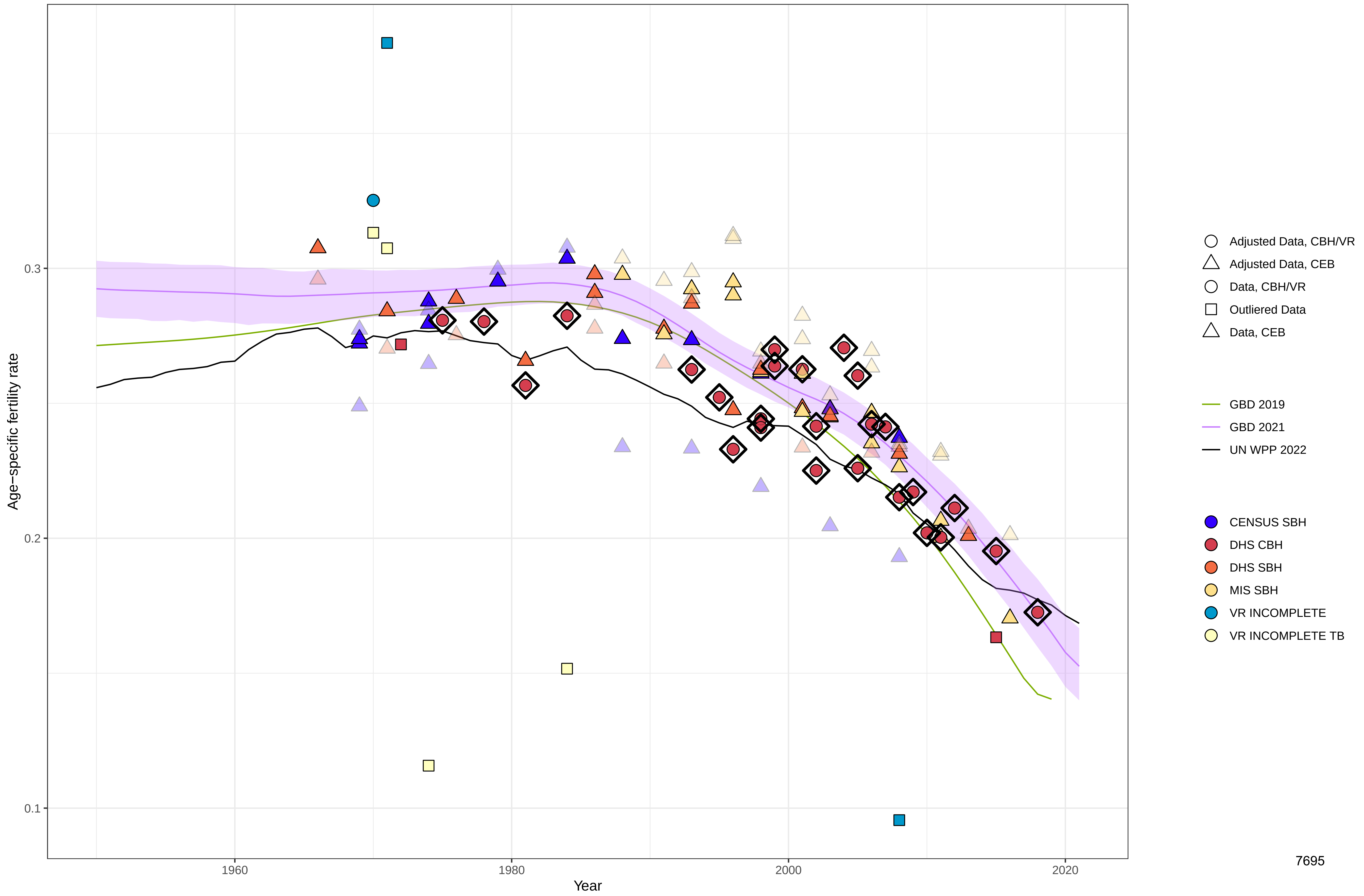

Age−specific fertility fitted trend lines, 1950−2021: Liberia, Ages: 30−34
Data points enclosed with a diamond are reference sources

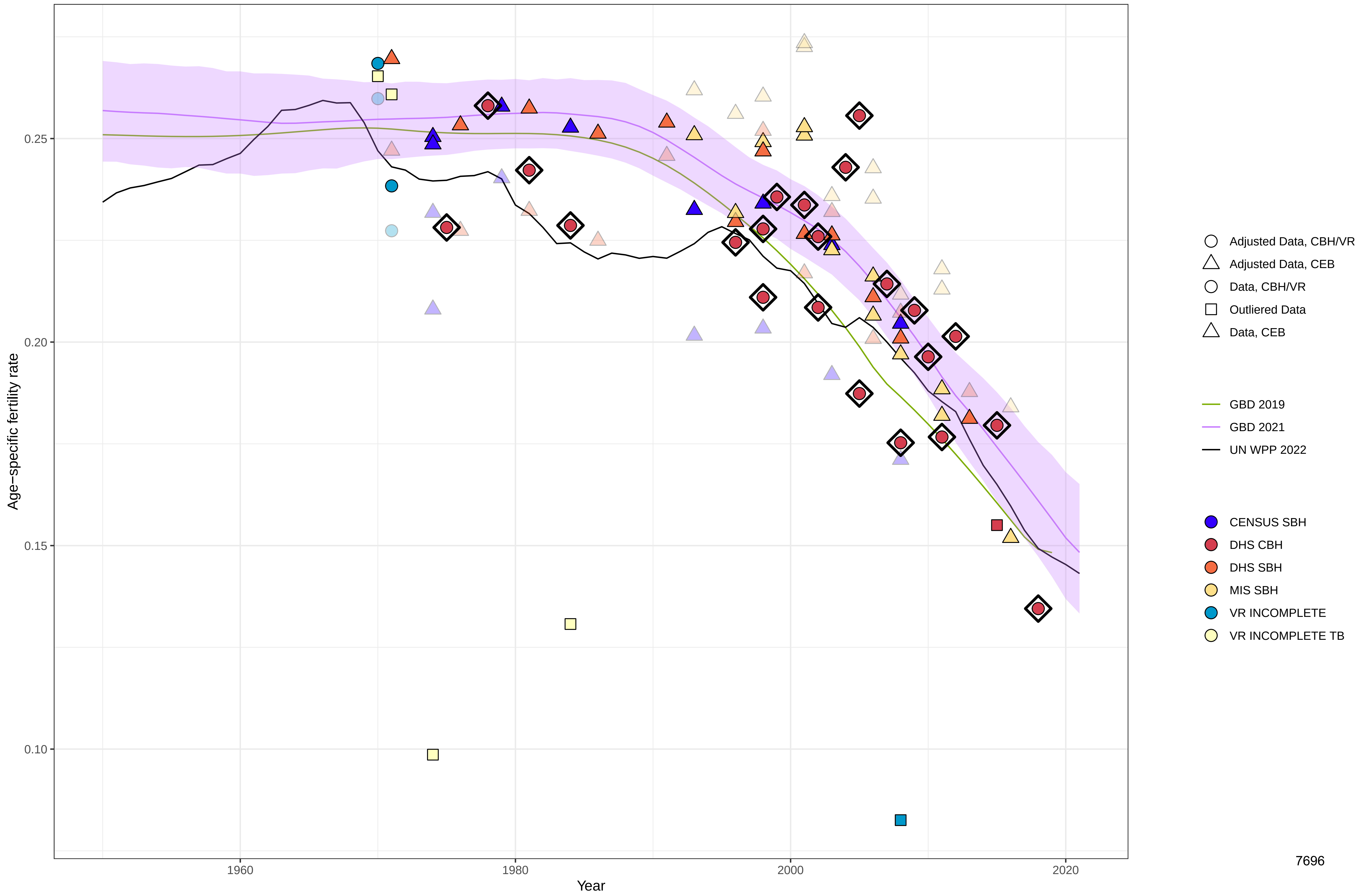

Age−specific fertility fitted trend lines, 1950−2021: Liberia, Ages: 35−39
Data points enclosed with a diamond are reference sources

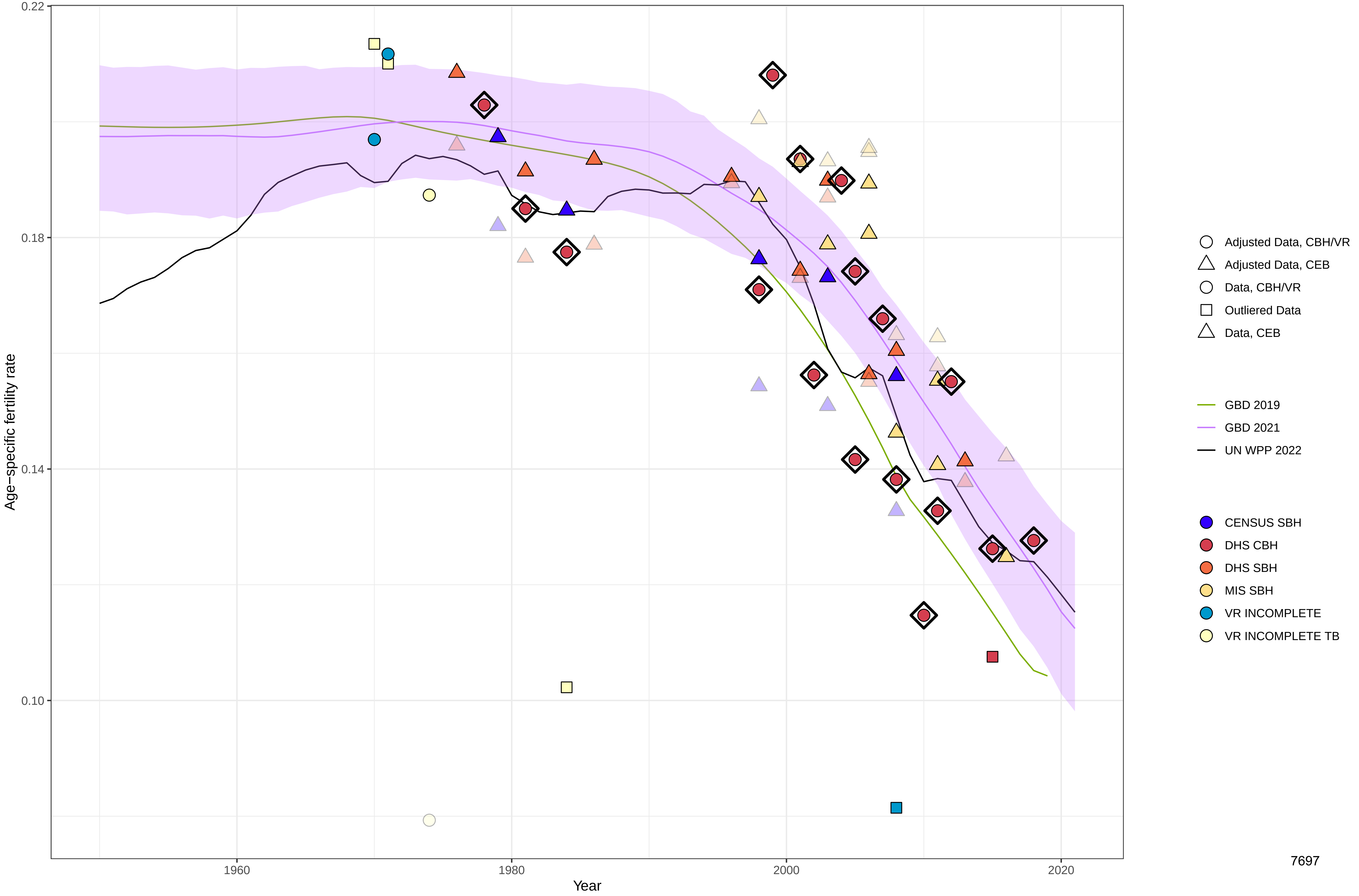

Age−specific fertility fitted trend lines, 1950−2021: Liberia, Ages: 40−44
Data points enclosed with a diamond are reference sources

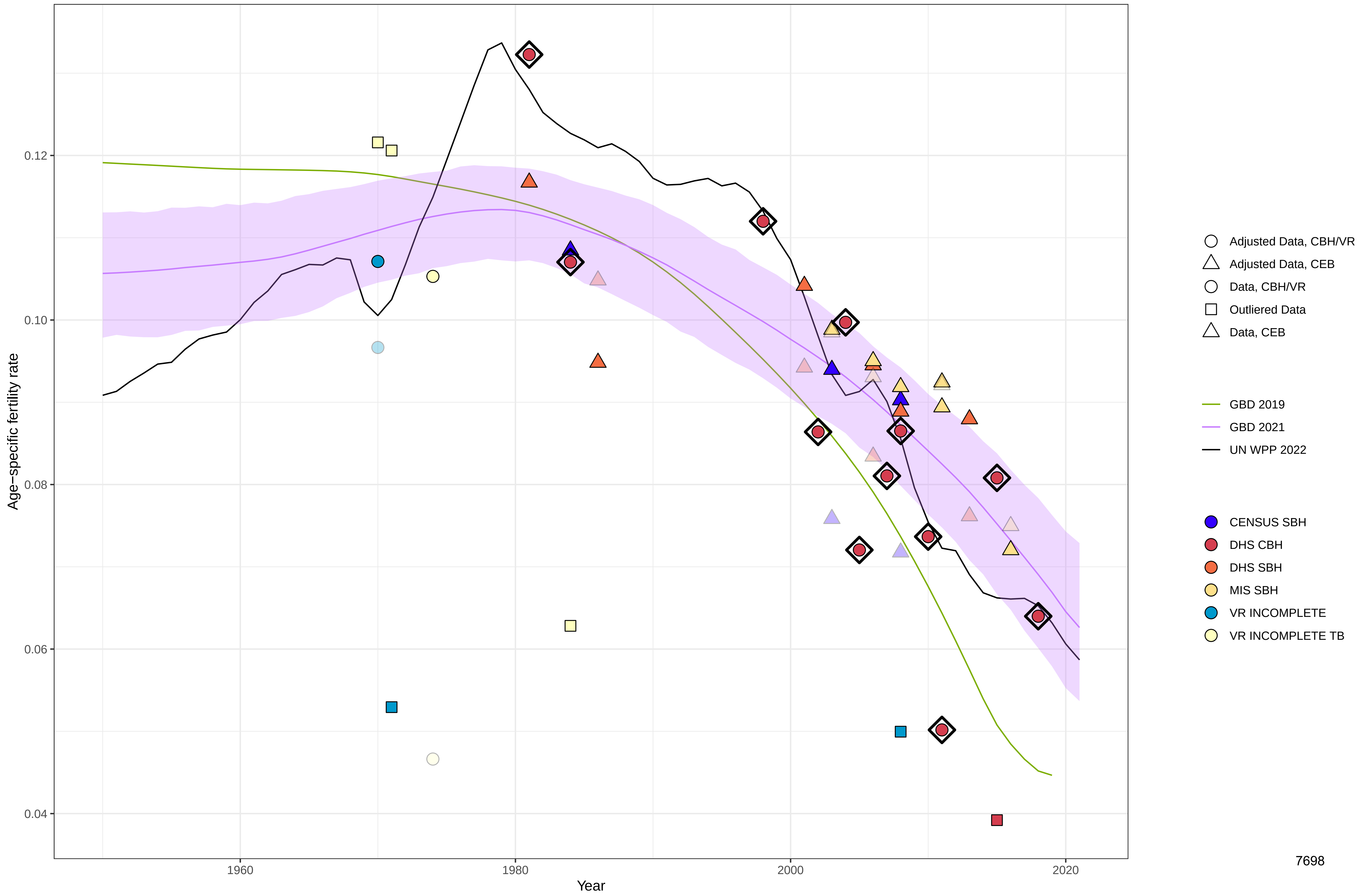

Age−specific fertility fitted trend lines, 1950−2021: Liberia, Ages: 45−49
Data points enclosed with a diamond are reference sources

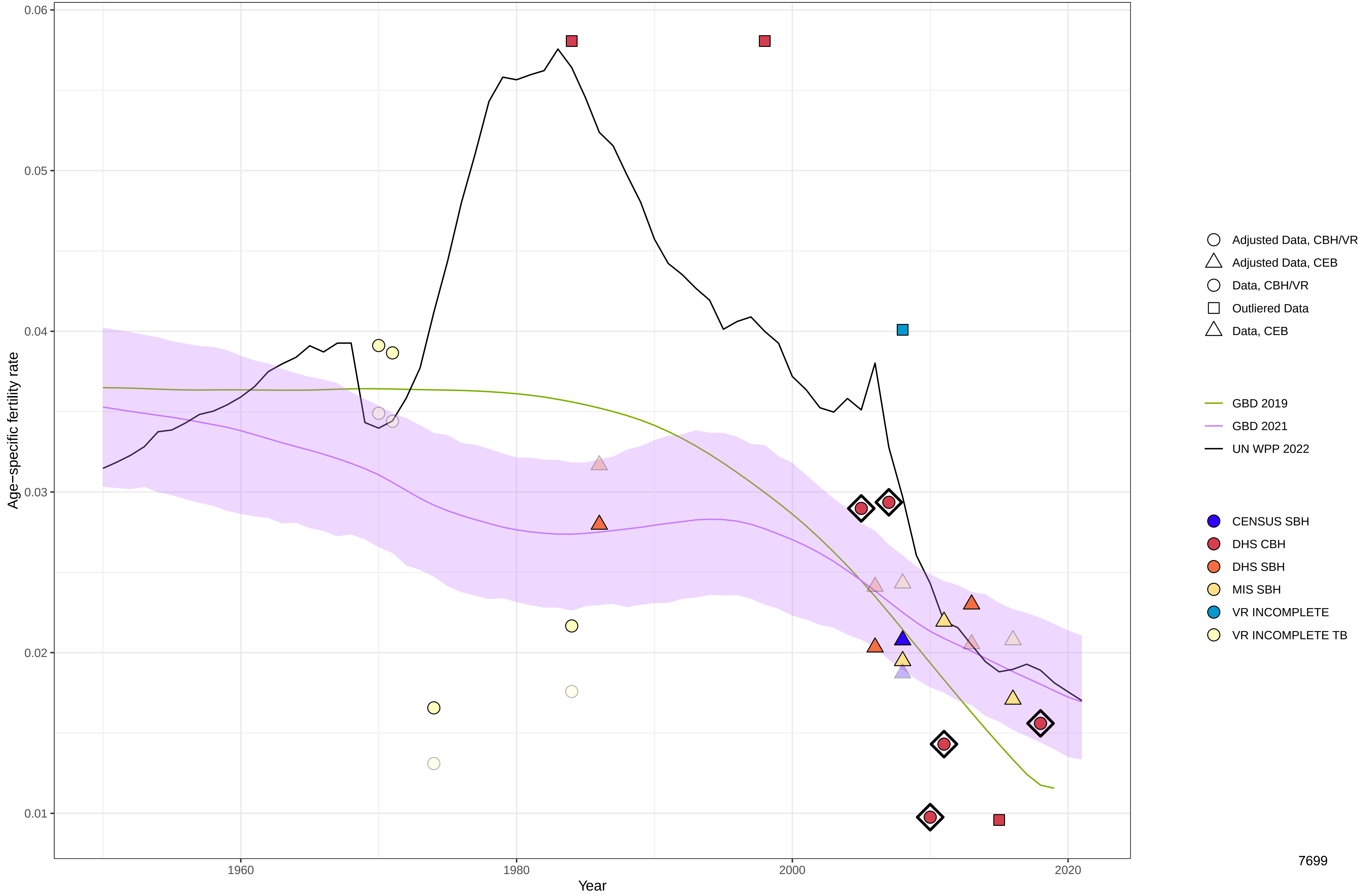

Age−specific fertility fitted trend lines, 1950−2021: Liberia, Ages: 50−54
Data points enclosed with a diamond are reference sources

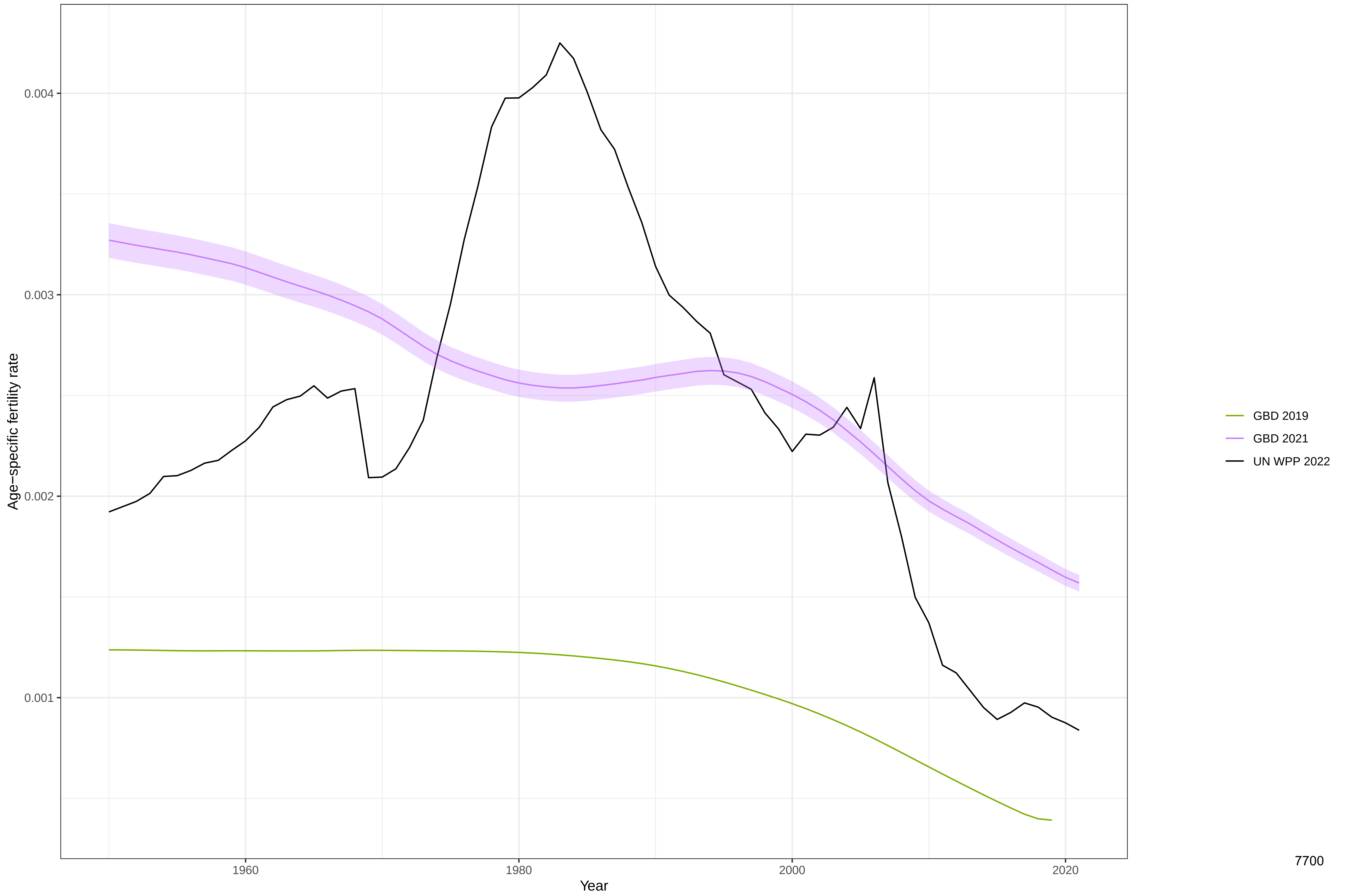

Total fertility rate fitted trend lines, 1950−2021: Liberia TFR
Data points enclosed with a diamond are reference sources

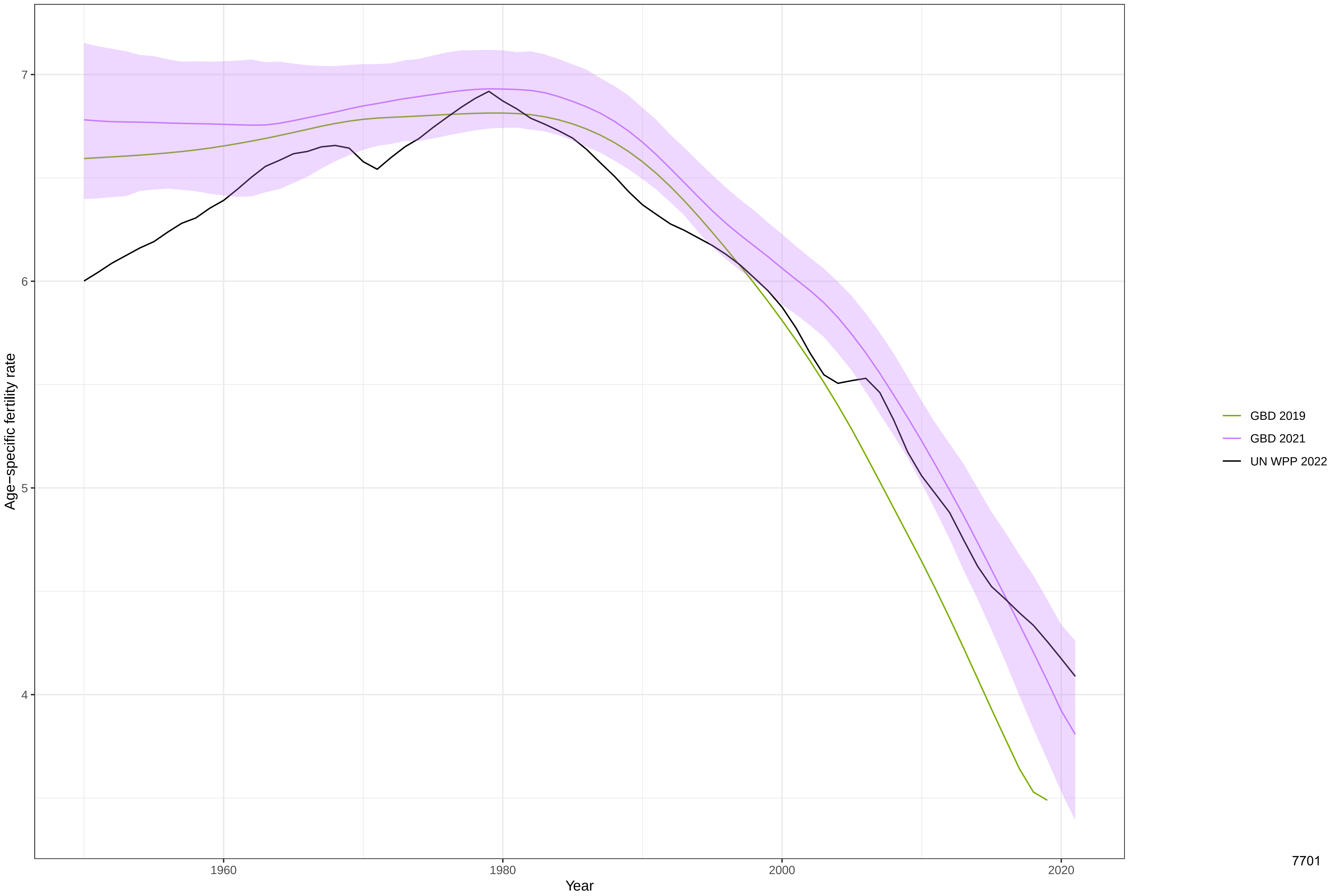

Age−specific fertility fitted trend lines, 1950−2021: Mali, Ages: 10−14
Data points enclosed with a diamond are reference sources

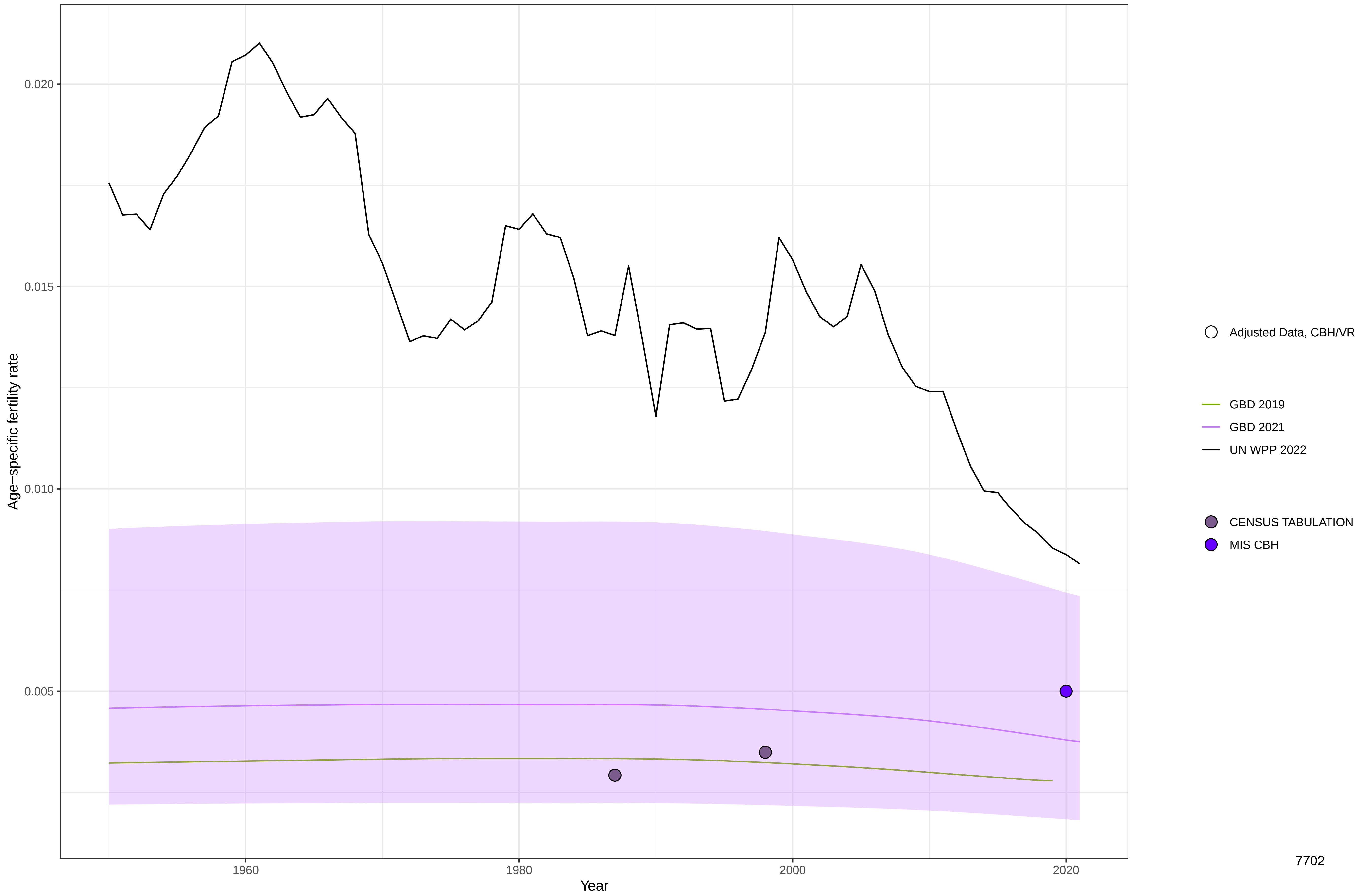

Age−specific fertility fitted trend lines, 1950−2021: Mali, Ages: 15−19
Data points enclosed with a diamond are reference sources

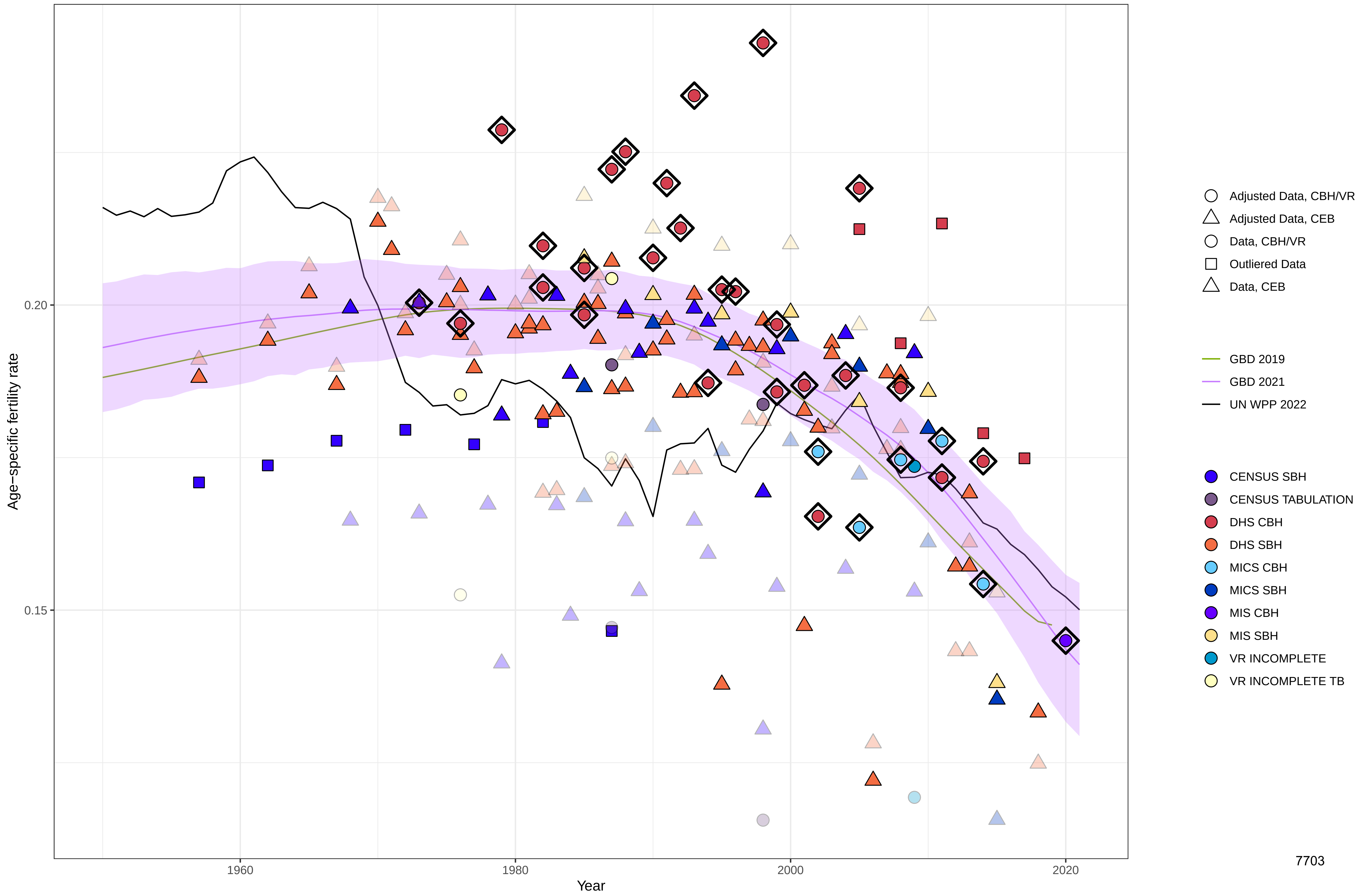

Age−specific fertility fitted trend lines, 1950−2021: Mali, Ages: 20−24
Data points enclosed with a diamond are reference sources

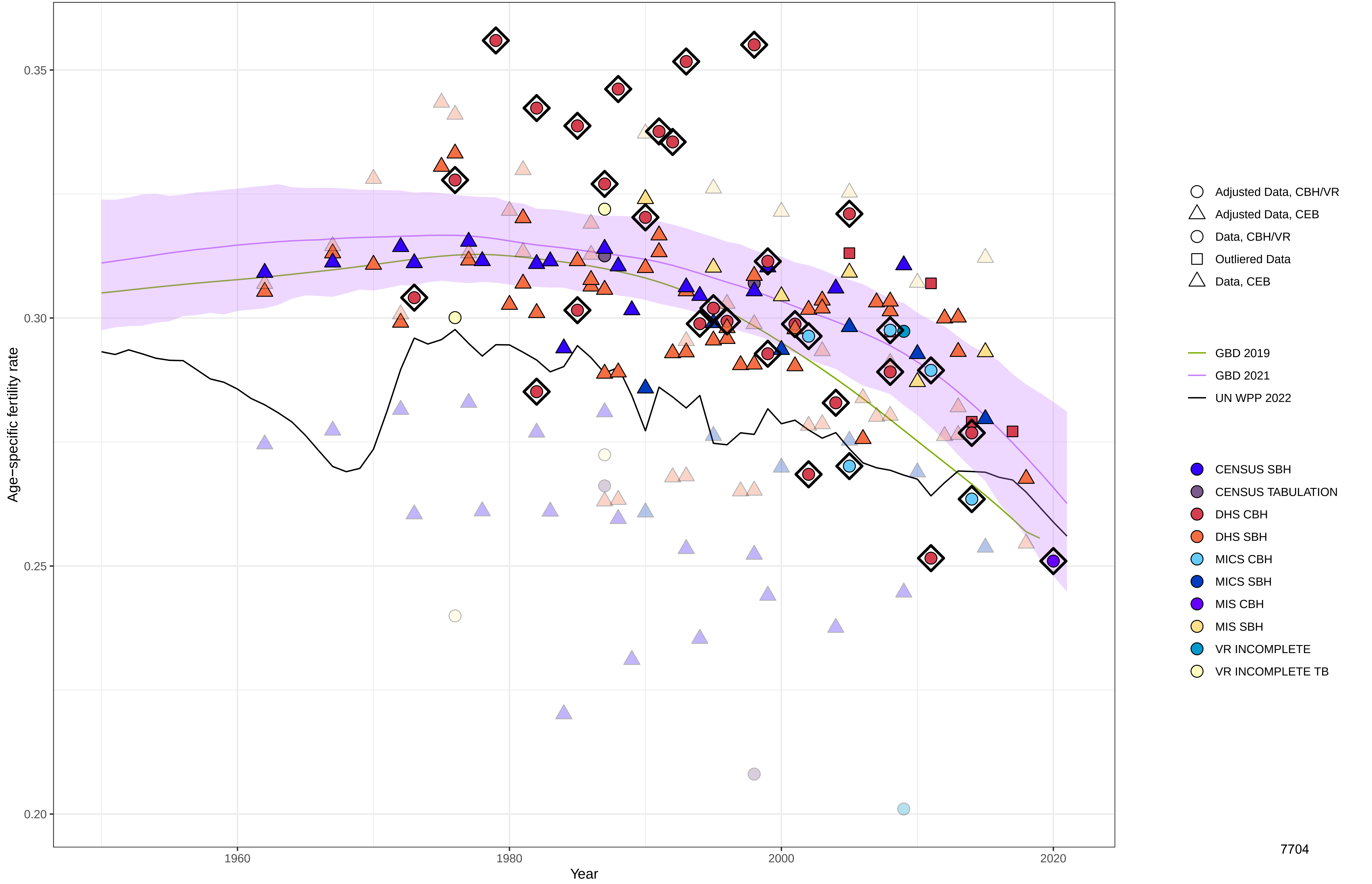

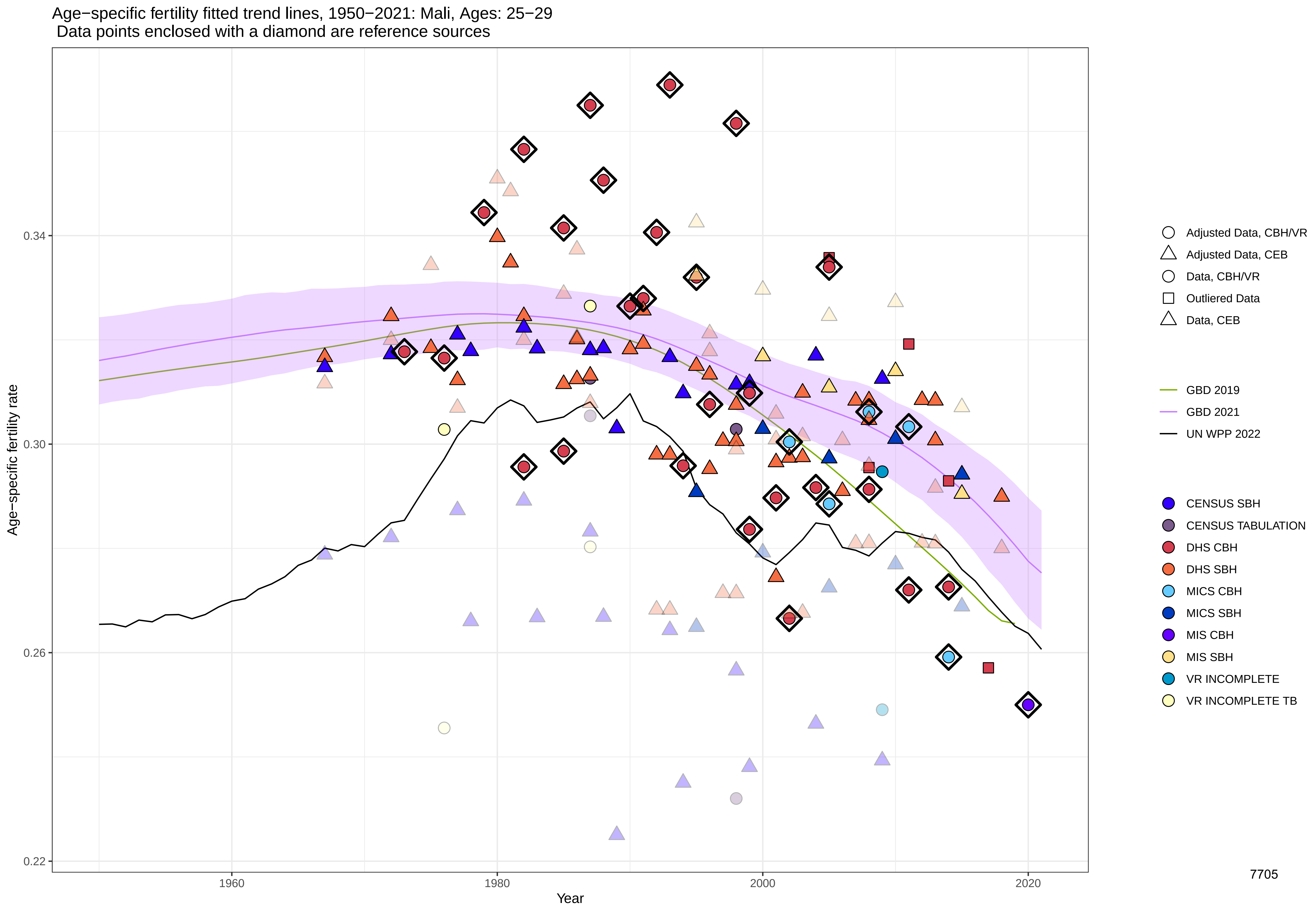

Age−specific fertility fitted trend lines, 1950−2021: Mali, Ages: 25−29
Data points enclosed with a diamond are reference sources
Age−specific fertility rate
Year
0.22
0.26
0.30
0.34
1960
1980
2000
2020
Adjusted Data, CBH/VR
Adjusted Data, CEB
Data, CBH/VR
Outliered Data
Data, CEB
GBD 2019
GBD 2021
UN WPP 2022
CENSUS SBH
CENSUS TABULATION
DHS CBH
DHS SBH
MICS CBH
MICS SBH
MIS CBH
MIS SBH
VR INCOMPLETE
VR INCOMPLETE TB

Age−specific fertility fitted trend lines, 1950−2021: Mali, Ages: 30−34
Data points enclosed with a diamond are reference sources

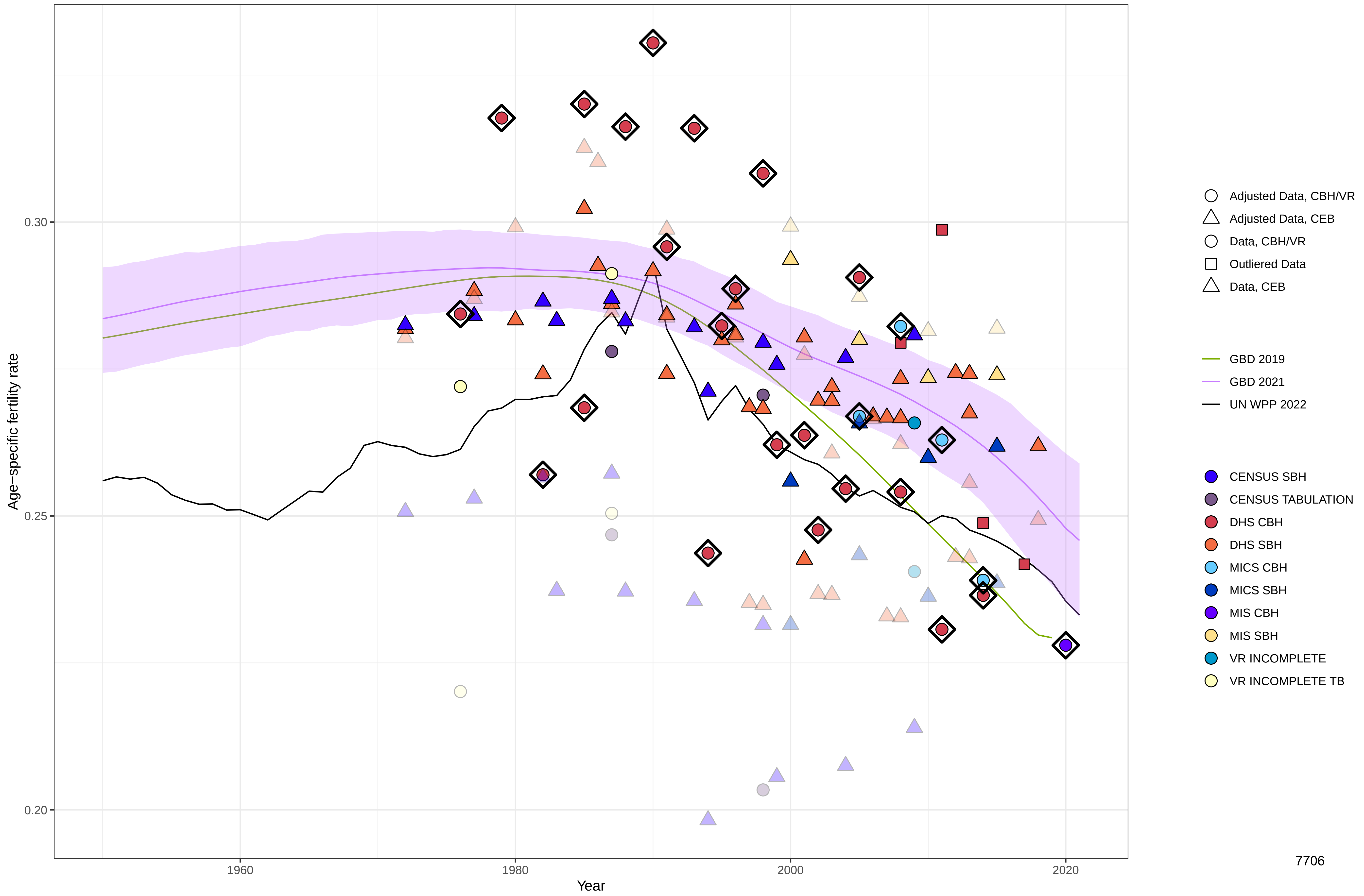

Age−specific fertility fitted trend lines, 1950−2021: Mali, Ages: 35−39
Data points enclosed with a diamond are reference sources

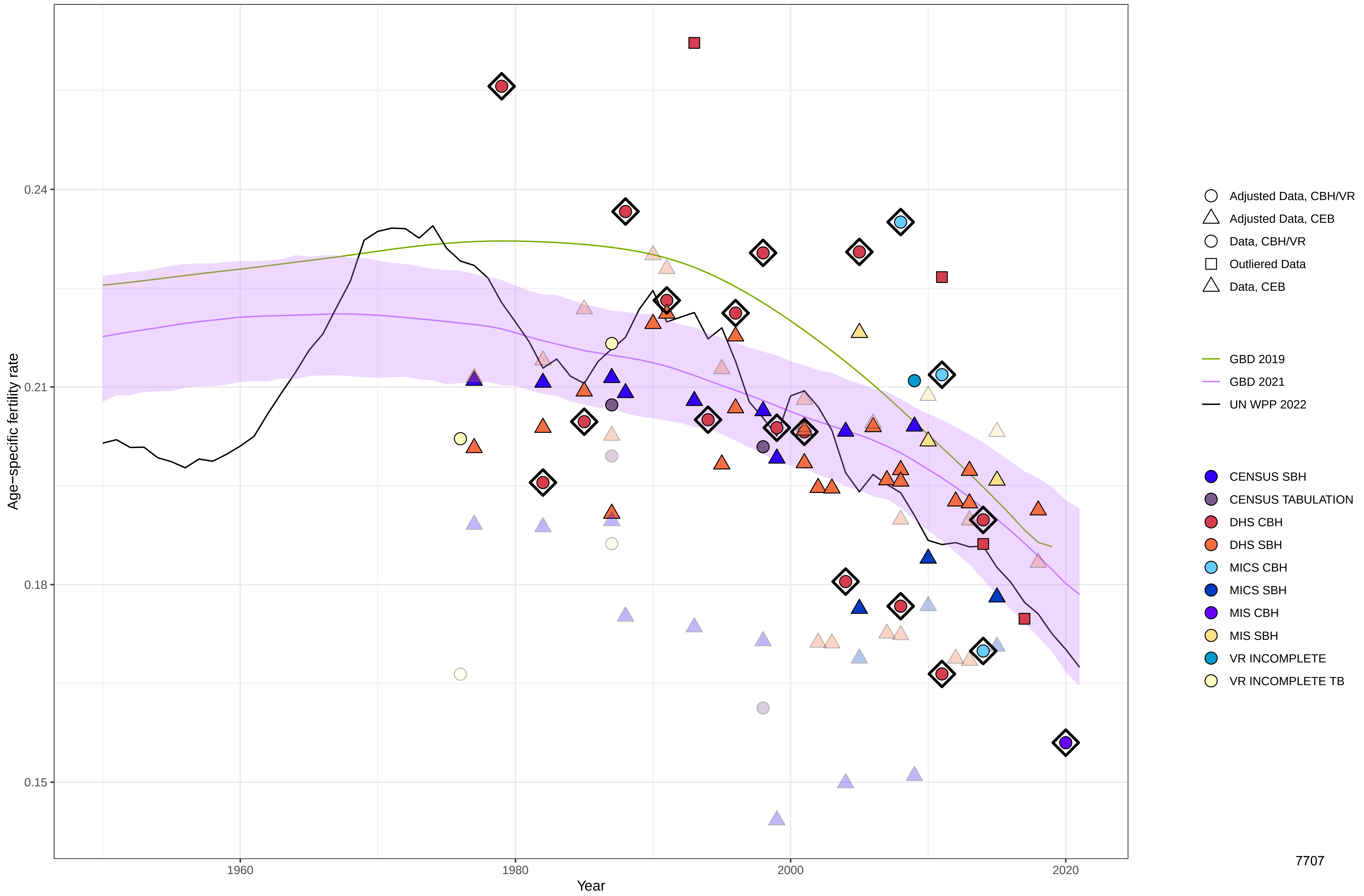

Age−specific fertility fitted trend lines, 1950−2021: Mali, Ages: 40−44
Data points enclosed with a diamond are reference sources

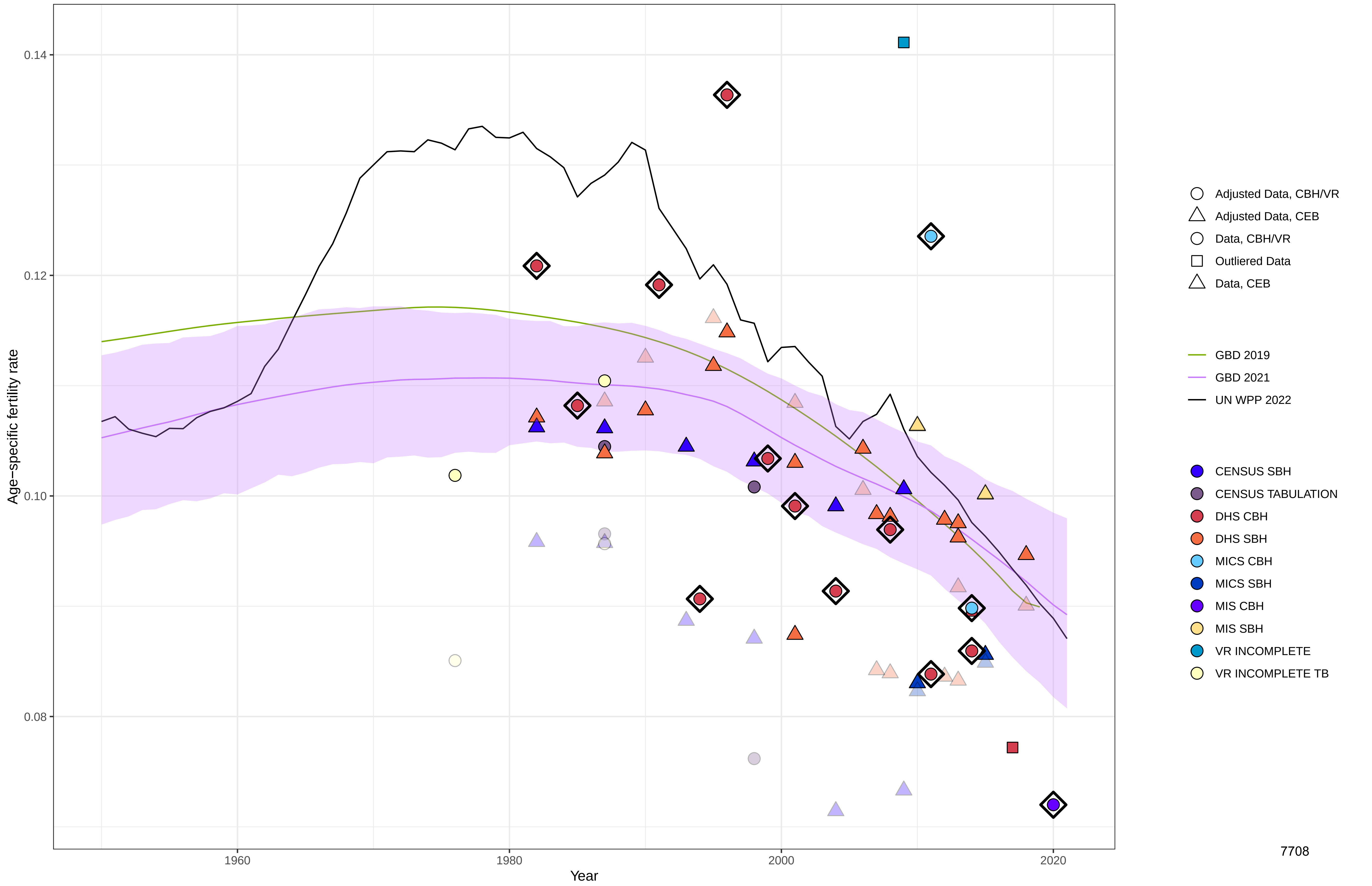

7708

Age−specific fertility fitted trend lines, 1950−2021: Mali, Ages: 45−49
Data points enclosed with a diamond are reference sources

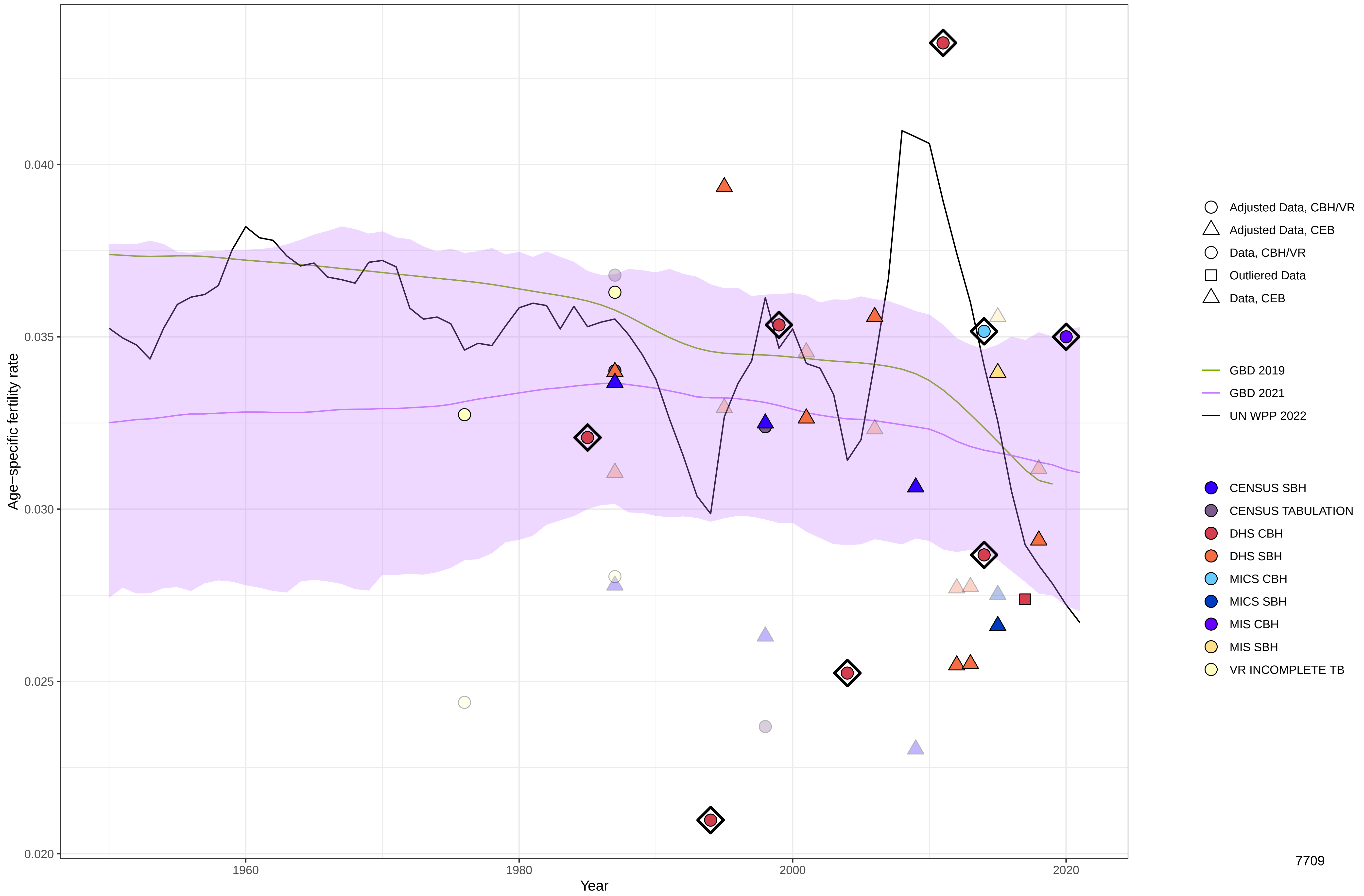

Age−specific fertility fitted trend lines, 1950−2021: Mali, Ages: 50−54
Data points enclosed with a diamond are reference sources

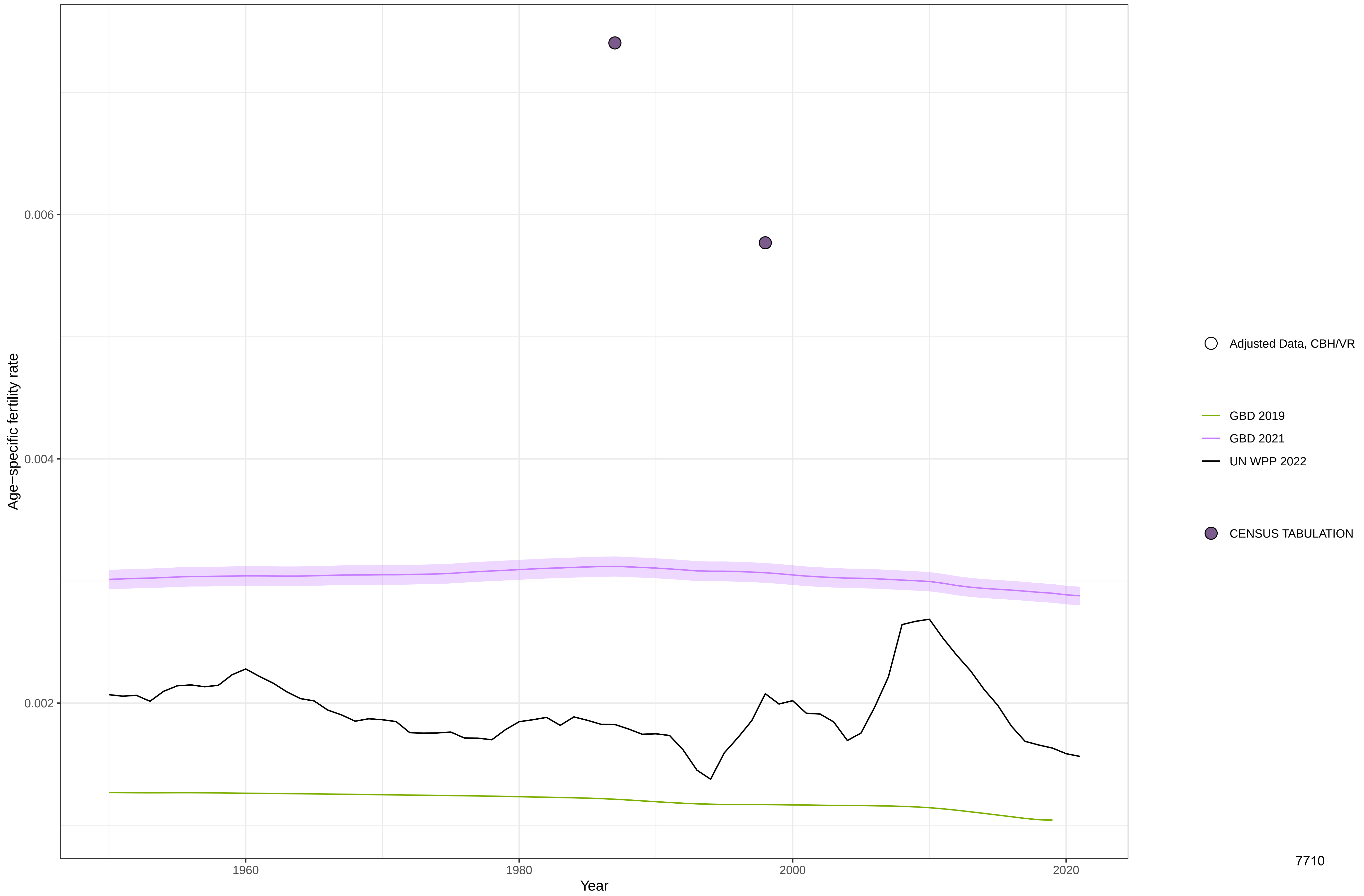

Total fertility rate fitted trend lines, 1950−2021: Mali TFR
Data points enclosed with a diamond are reference sources

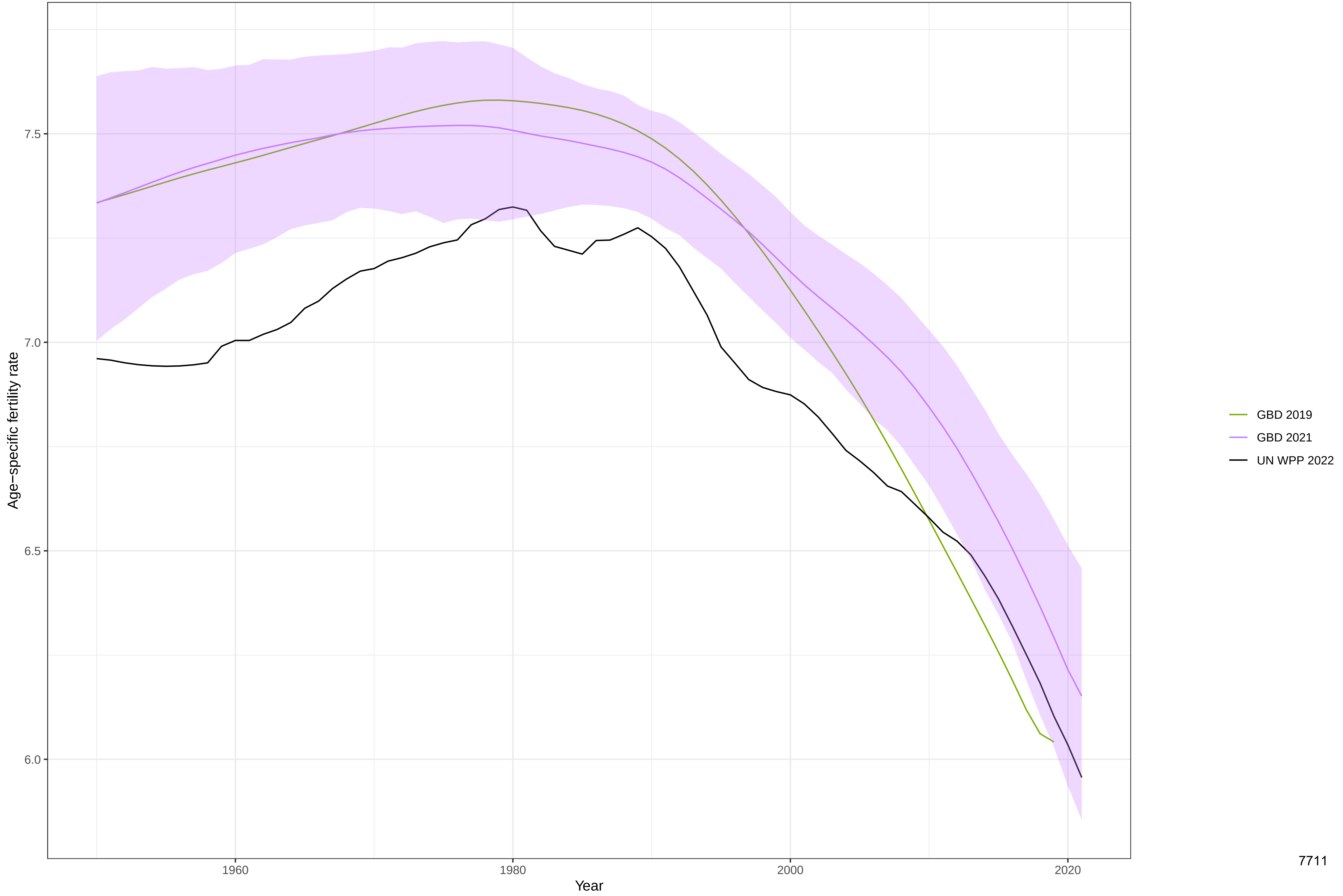

Age−specific fertility fitted trend lines, 1950−2021: Mauritania, Ages: 10−14
Data points enclosed with a diamond are reference sources

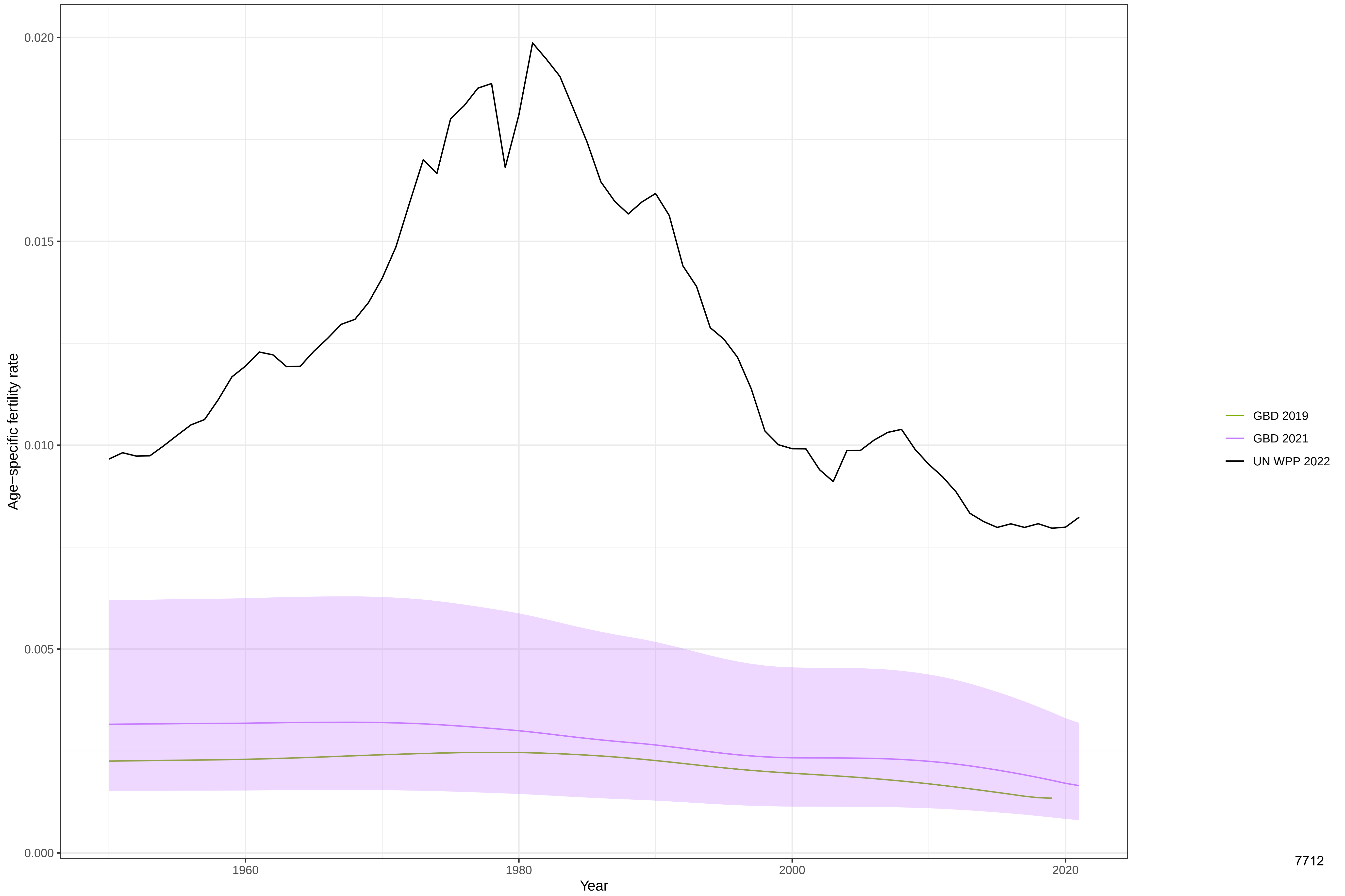

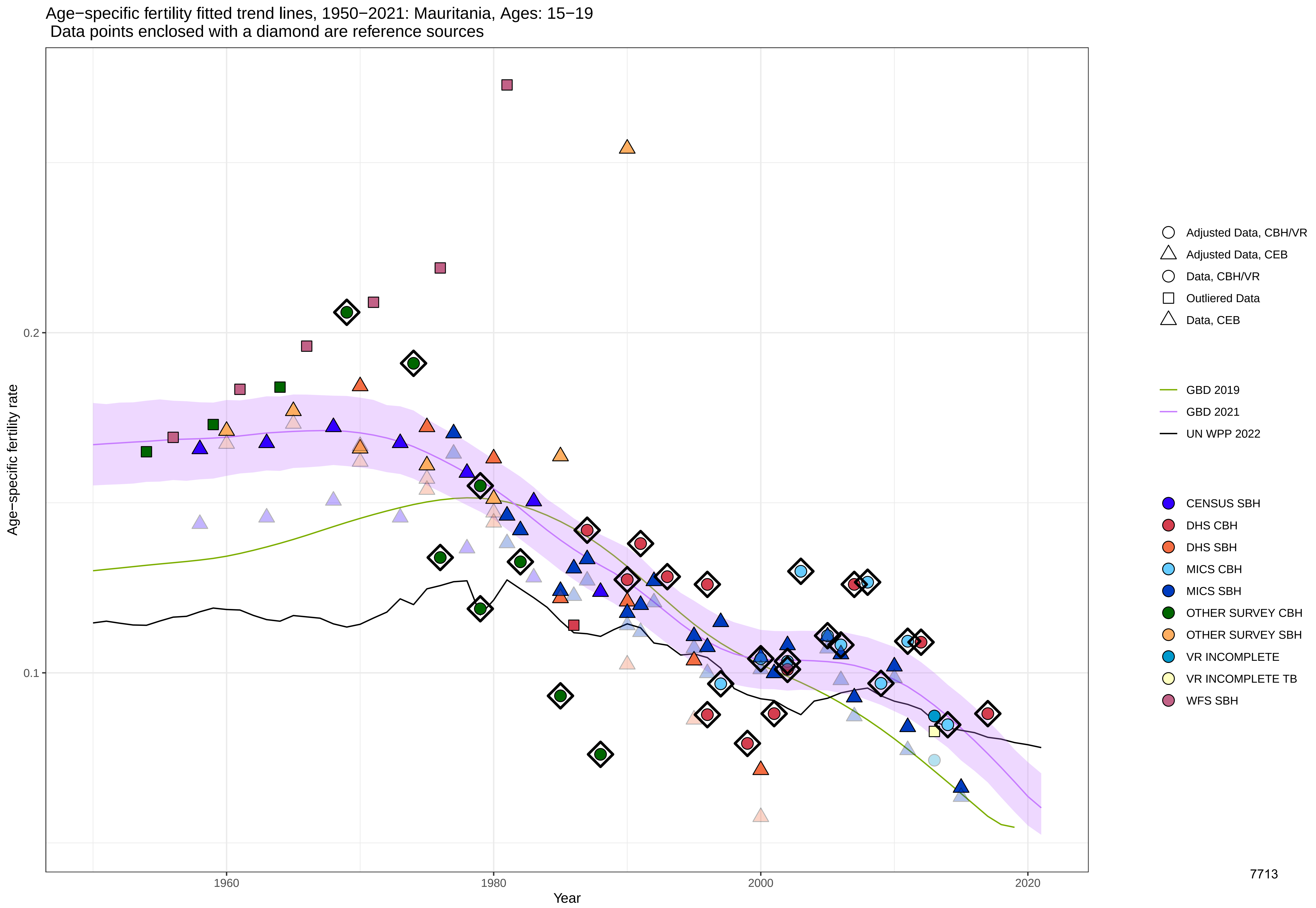

Age−specific fertility fitted trend lines, 1950−2021: Mauritania, Ages: 15−19
Data points enclosed with a diamond are reference sources
Age−specific fertility rate
0.2
0.1
1960
1980
2000
2020
Year
Adjusted Data, CBH/VR
Adjusted Data, CEB
Data, CBH/VR
Outliered Data
Data, CEB
GBD 2019
GBD 2021
UN WPP 2022
CENSUS SBH
DHS CBH
DHS SBH
MICS CBH
MICS SBH
OTHER SURVEY CBH
OTHER SURVEY SBH
VR INCOMPLETE
VR INCOMPLETE TB
WFS SBH

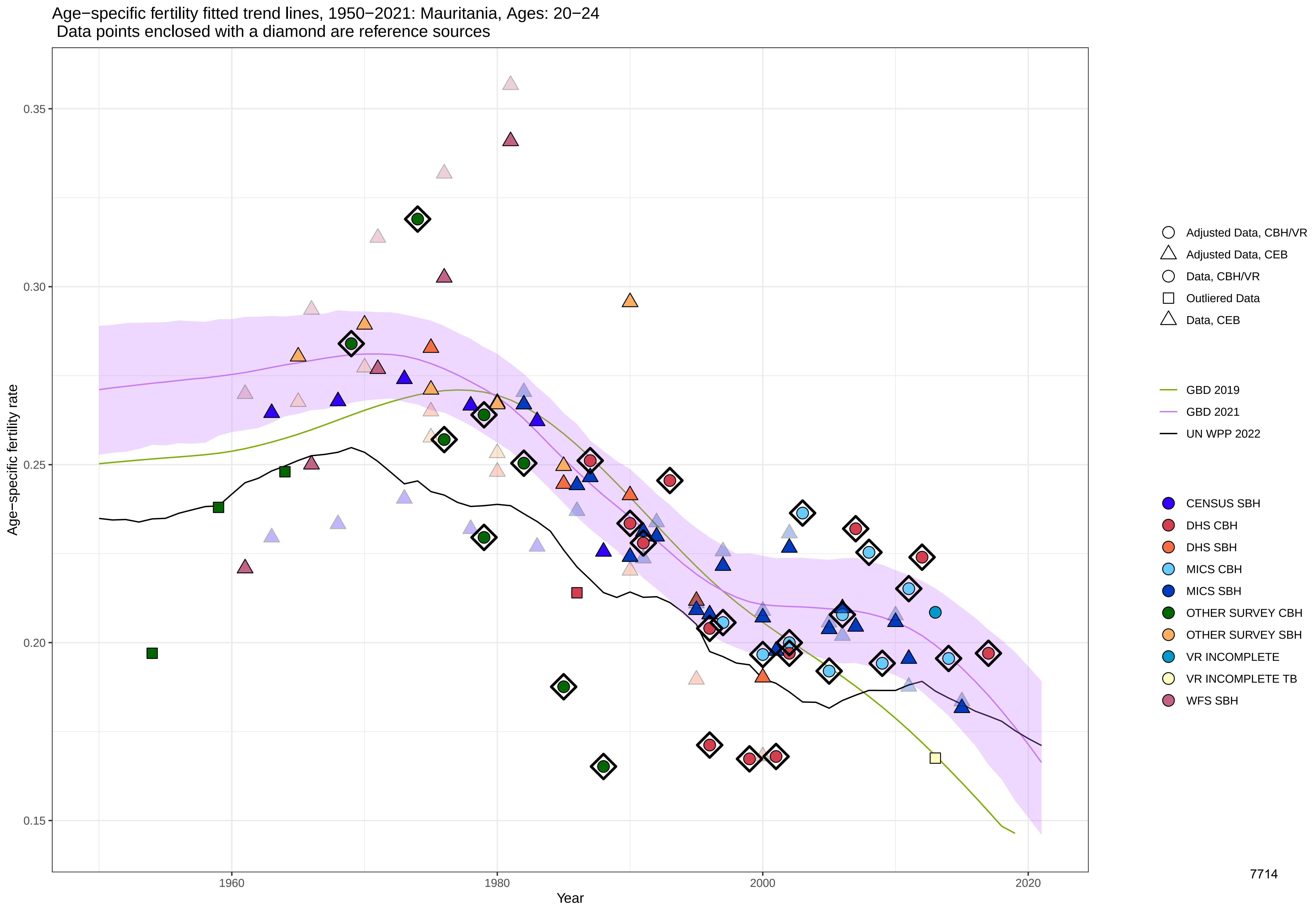
Age−specific fertility fitted trend lines, 1950−2021: Mauritania, Ages: 20−24
Data points enclosed with a diamond are reference sources
Age−specific fertility rate
0.35
0.30
0.25
0.20
0.15
1960
1980
2000
2020
Year
Adjusted Data, CBH/VR
Adjusted Data, CEB
Data, CBH/VR
Outliered Data
Data, CEB
GBD 2019
GBD 2021
UN WPP 2022
CENSUS SBH
DHS CBH
DHS SBH
MICS CBH
MICS SBH
OTHER SURVEY CBH
OTHER SURVEY SBH
VR INCOMPLETE
VR INCOMPLETE TB
WFS SBH

7714

Age−specific fertility fitted trend lines, 1950−2021: Mauritania, Ages: 25−29
Data points enclosed with a diamond are reference sources

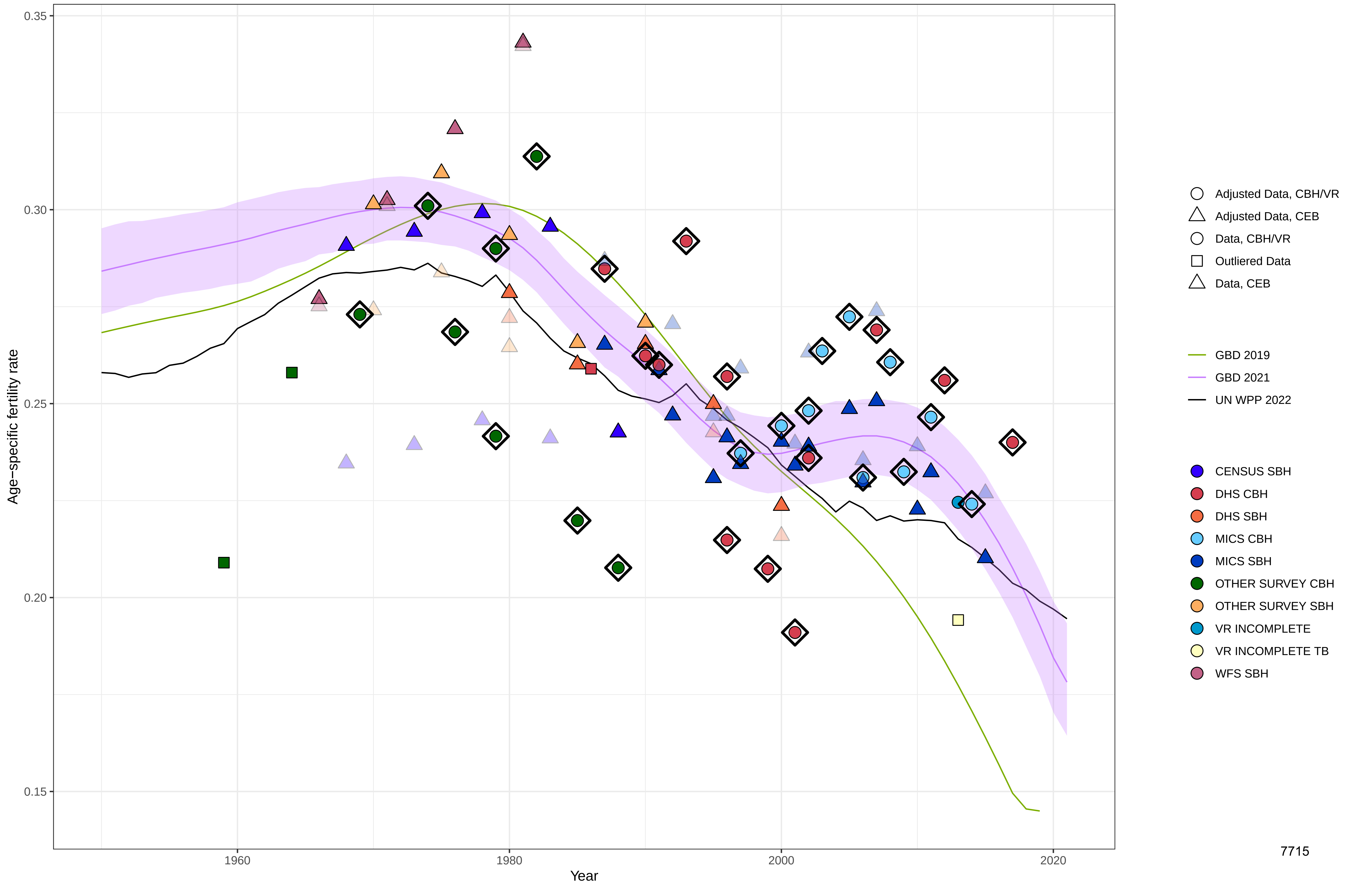

Age−specific fertility fitted trend lines, 1950−2021: Mauritania, Ages: 30−34
Data points enclosed with a diamond are reference sources

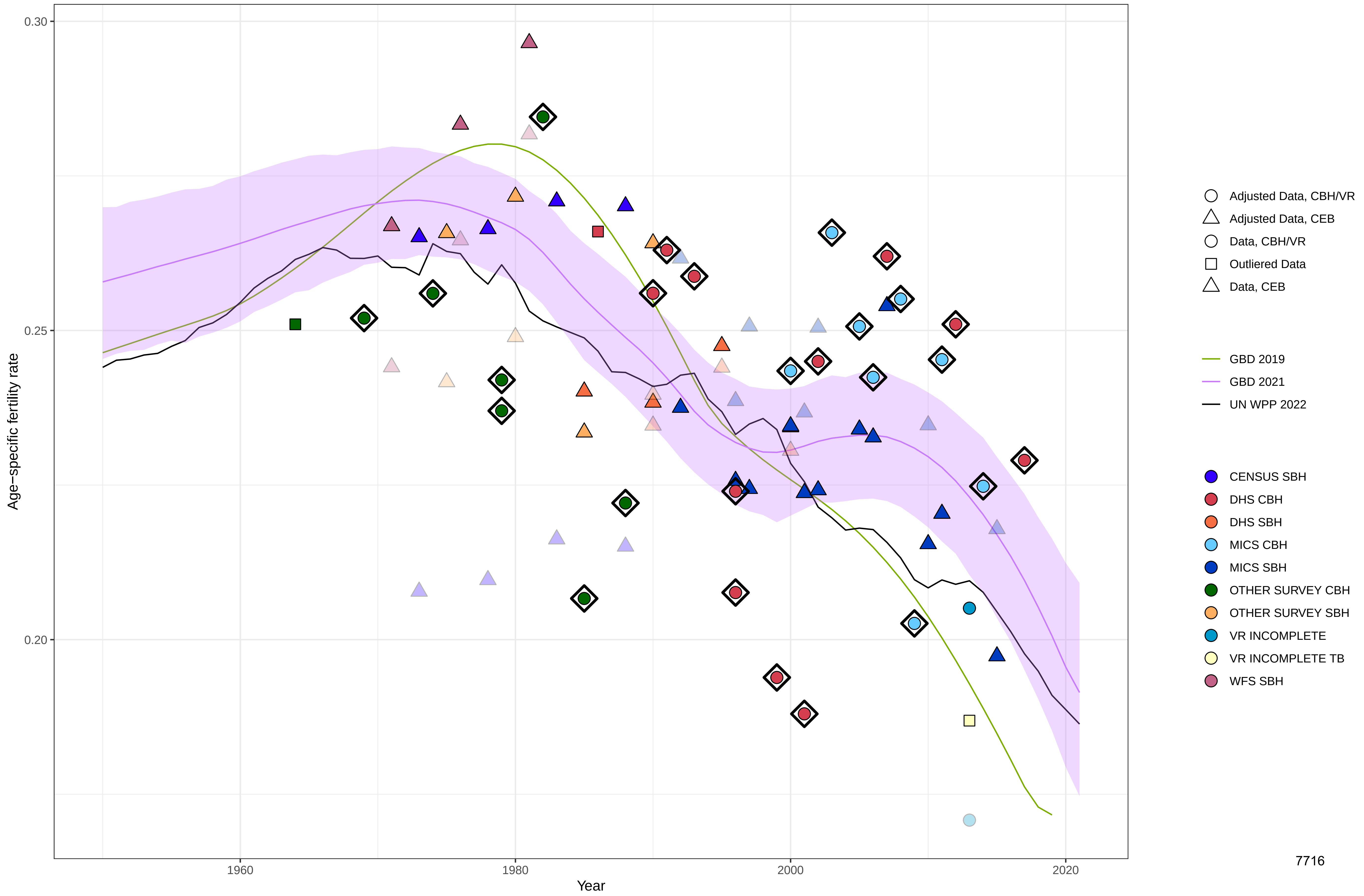

Age−specific fertility fitted trend lines, 1950−2021: Mauritania, Ages: 35−39
Data points enclosed with a diamond are reference sources

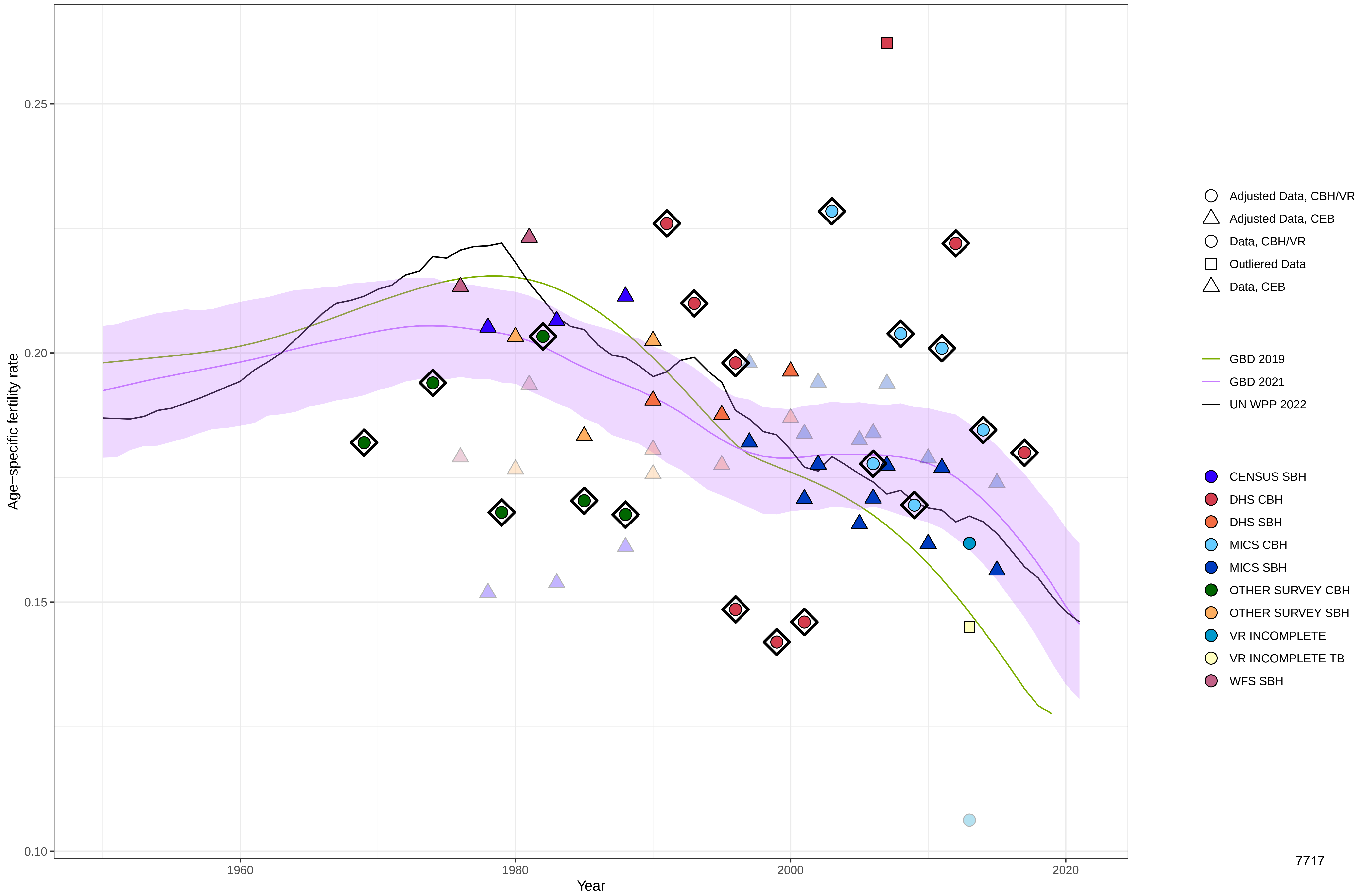

Age−specific fertility fitted trend lines, 1950−2021: Mauritania, Ages: 40−44
Data points enclosed with a diamond are reference sources

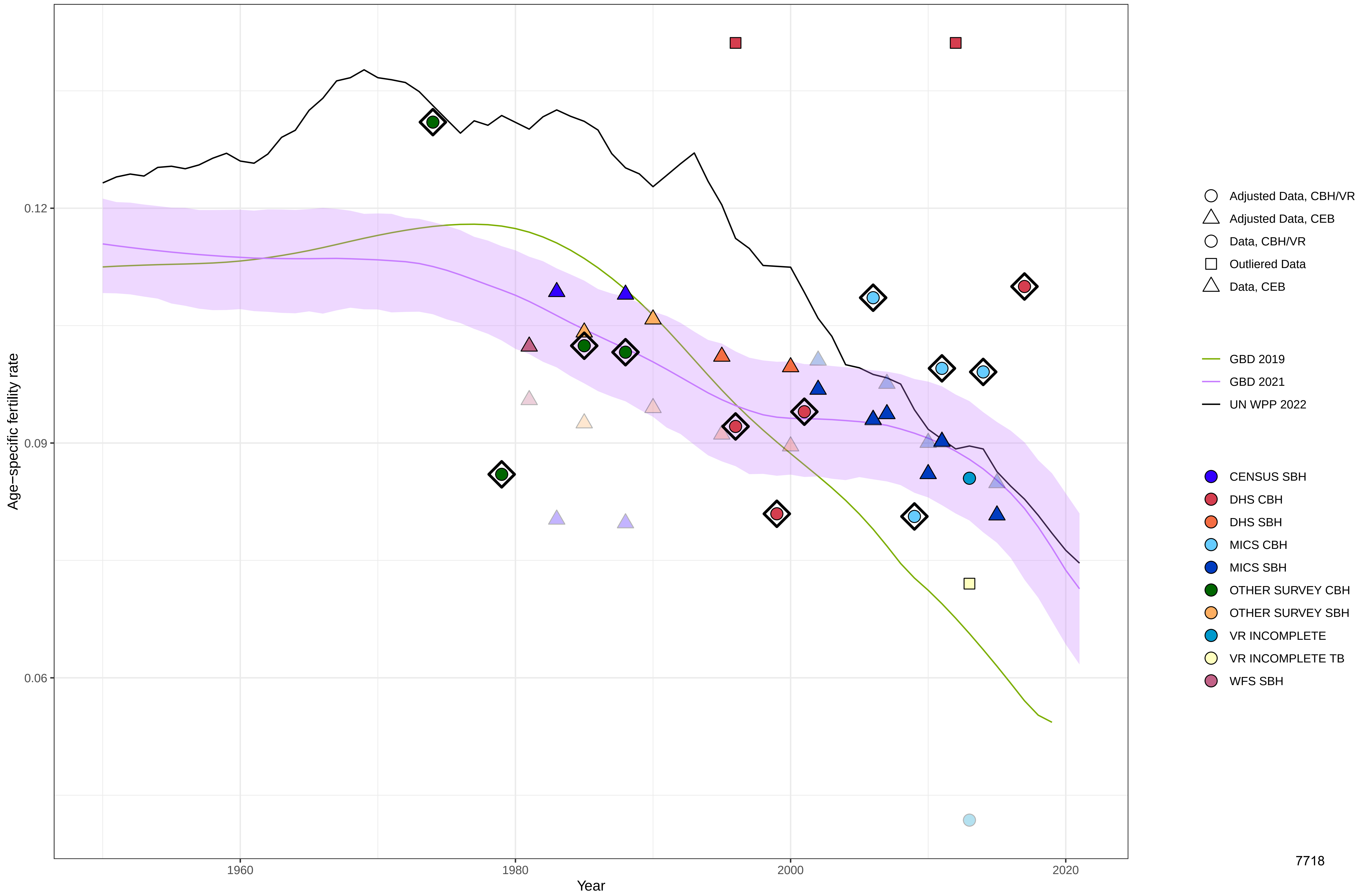

Age−specific fertility fitted trend lines, 1950−2021: Mauritania, Ages: 45−49
Data points enclosed with a diamond are reference sources

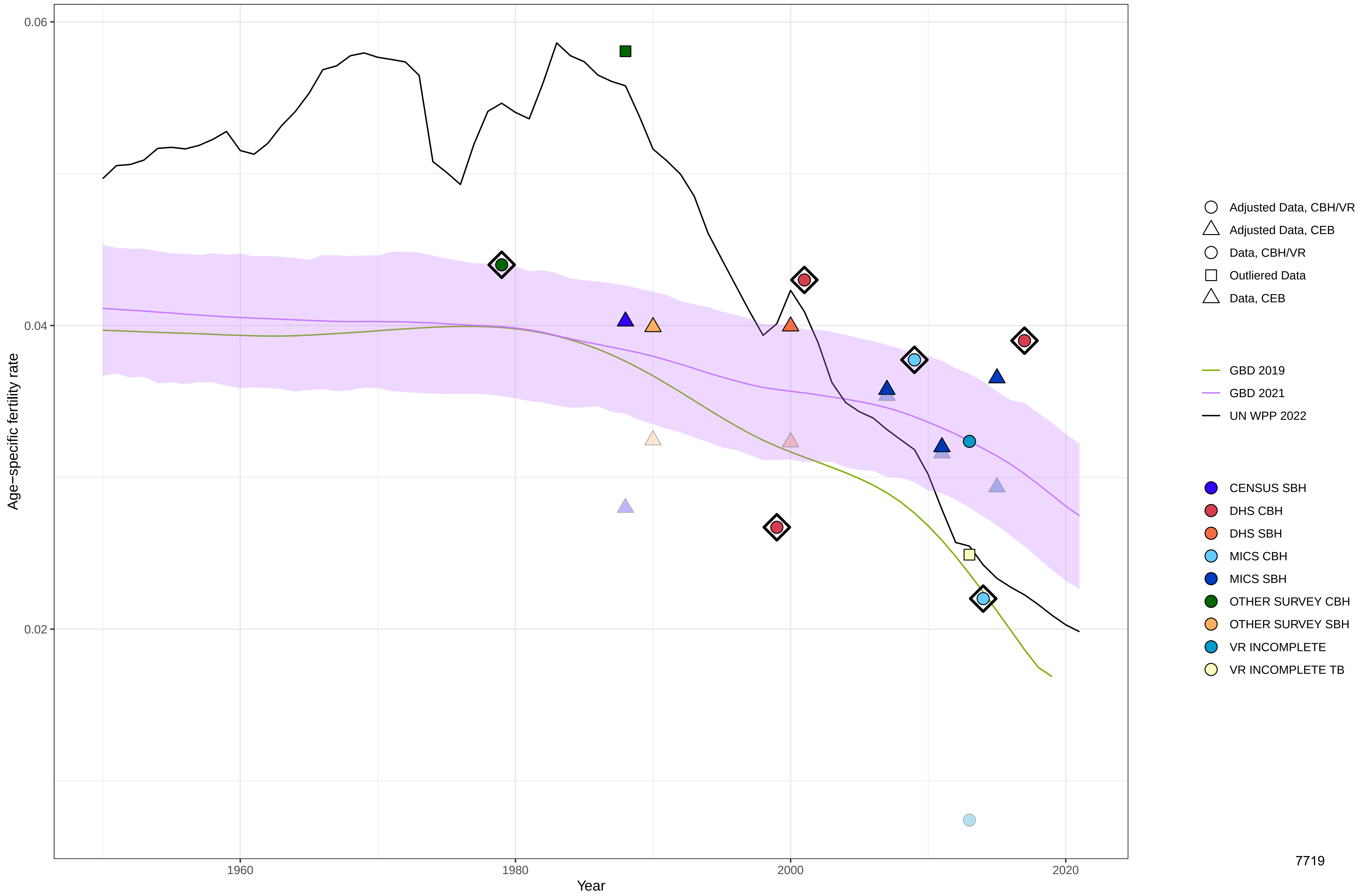

Age−specific fertility fitted trend lines, 1950−2021: Mauritania, Ages: 50−54
Data points enclosed with a diamond are reference sources

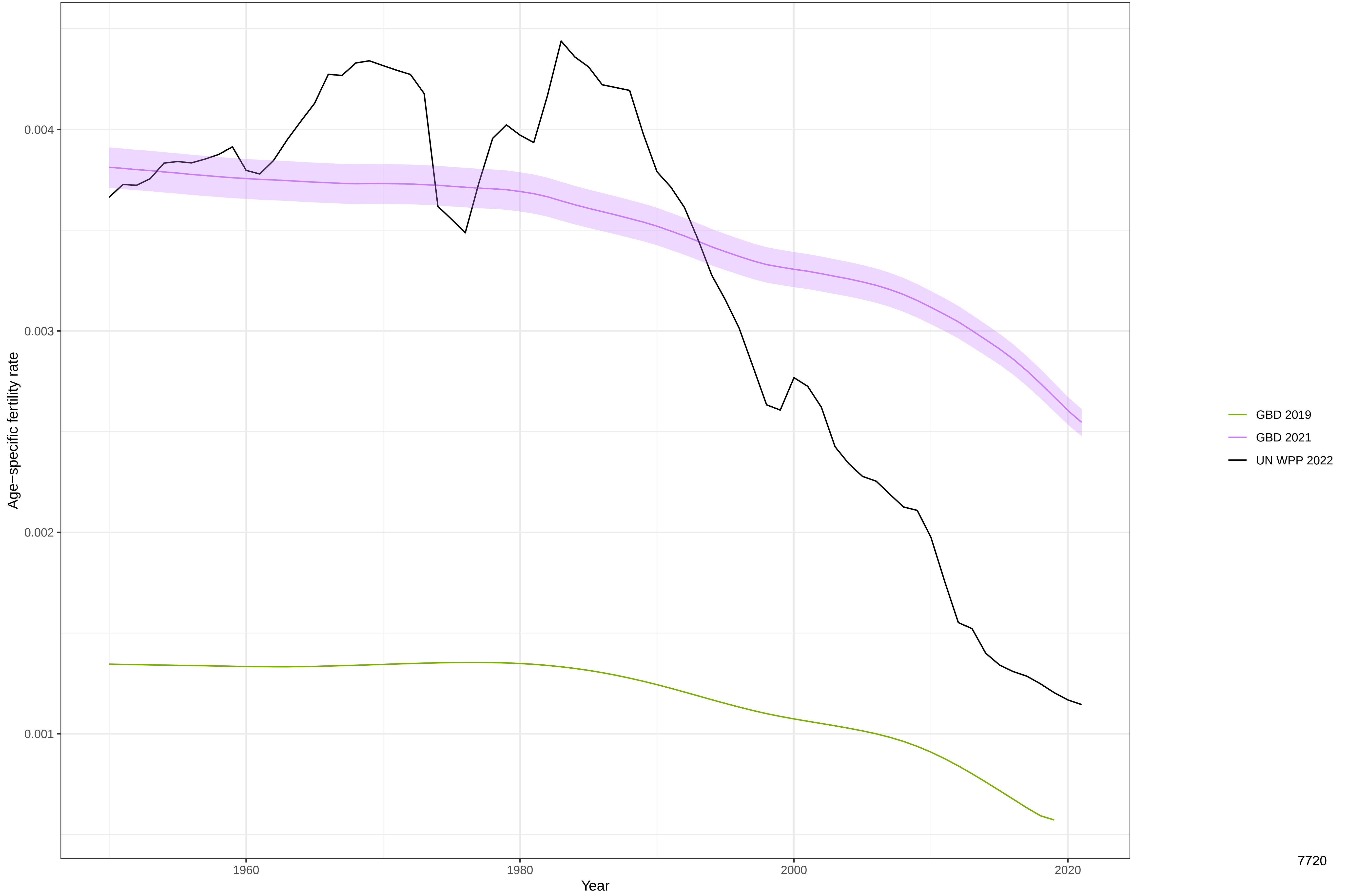

Total fertility rate fitted trend lines, 1950−2021: Mauritania TFR
Data points enclosed with a diamond are reference sources

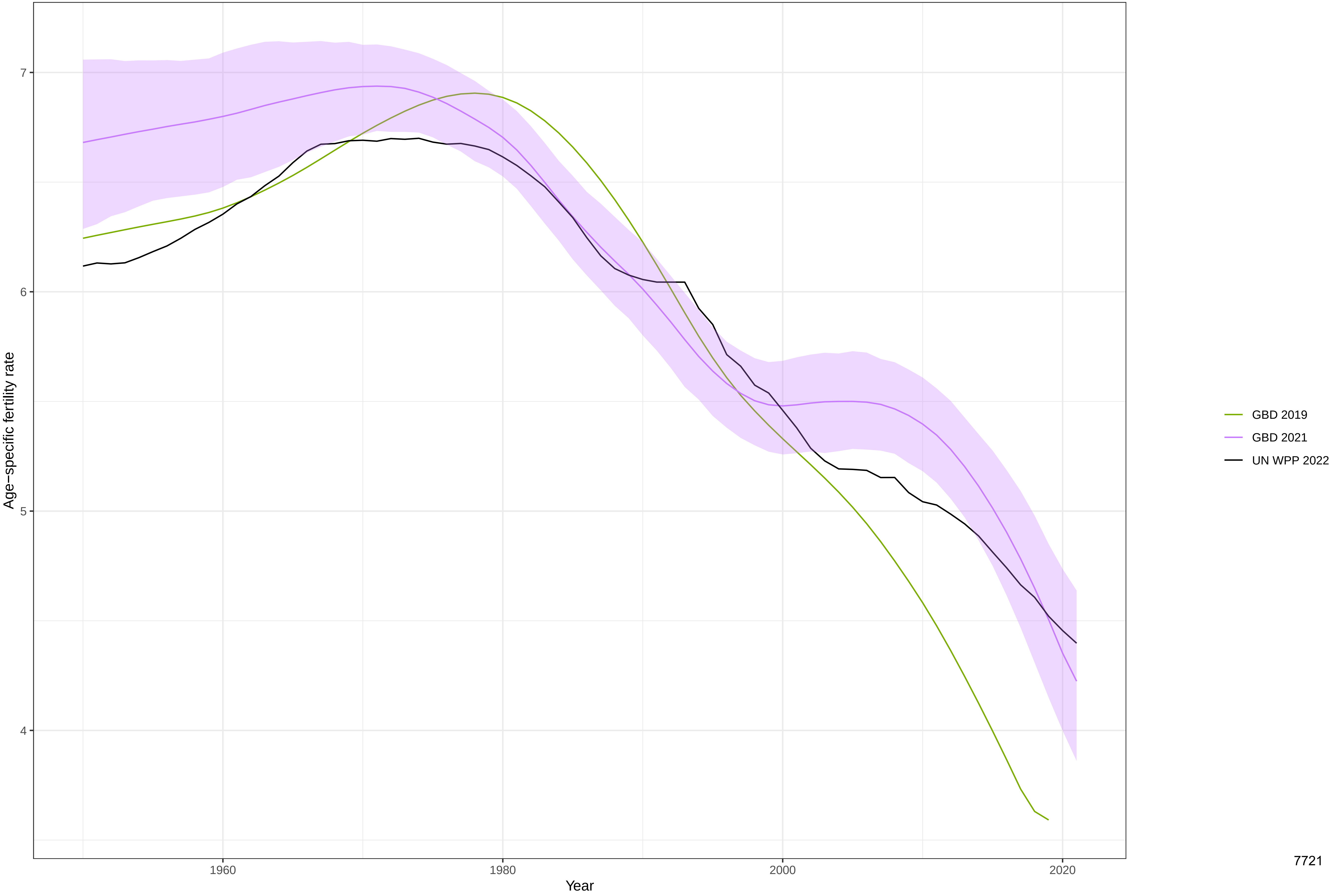

7721

Age−specific fertility fitted trend lines, 1950−2021: Niger, Ages: 10−14
Data points enclosed with a diamond are reference sources

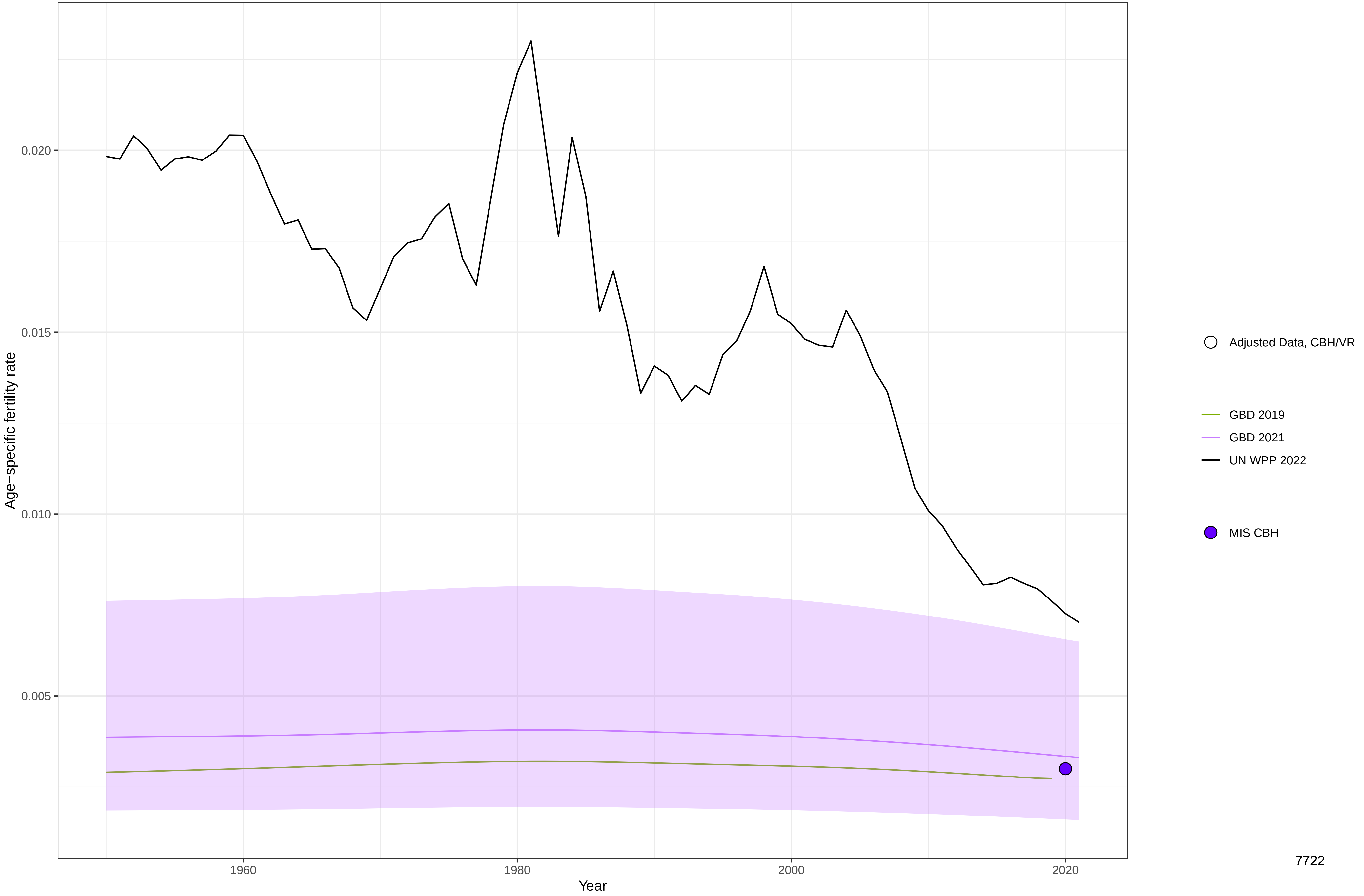

Age−specific fertility fitted trend lines, 1950−2021: Niger, Ages: 15−19
Data points enclosed with a diamond are reference sources

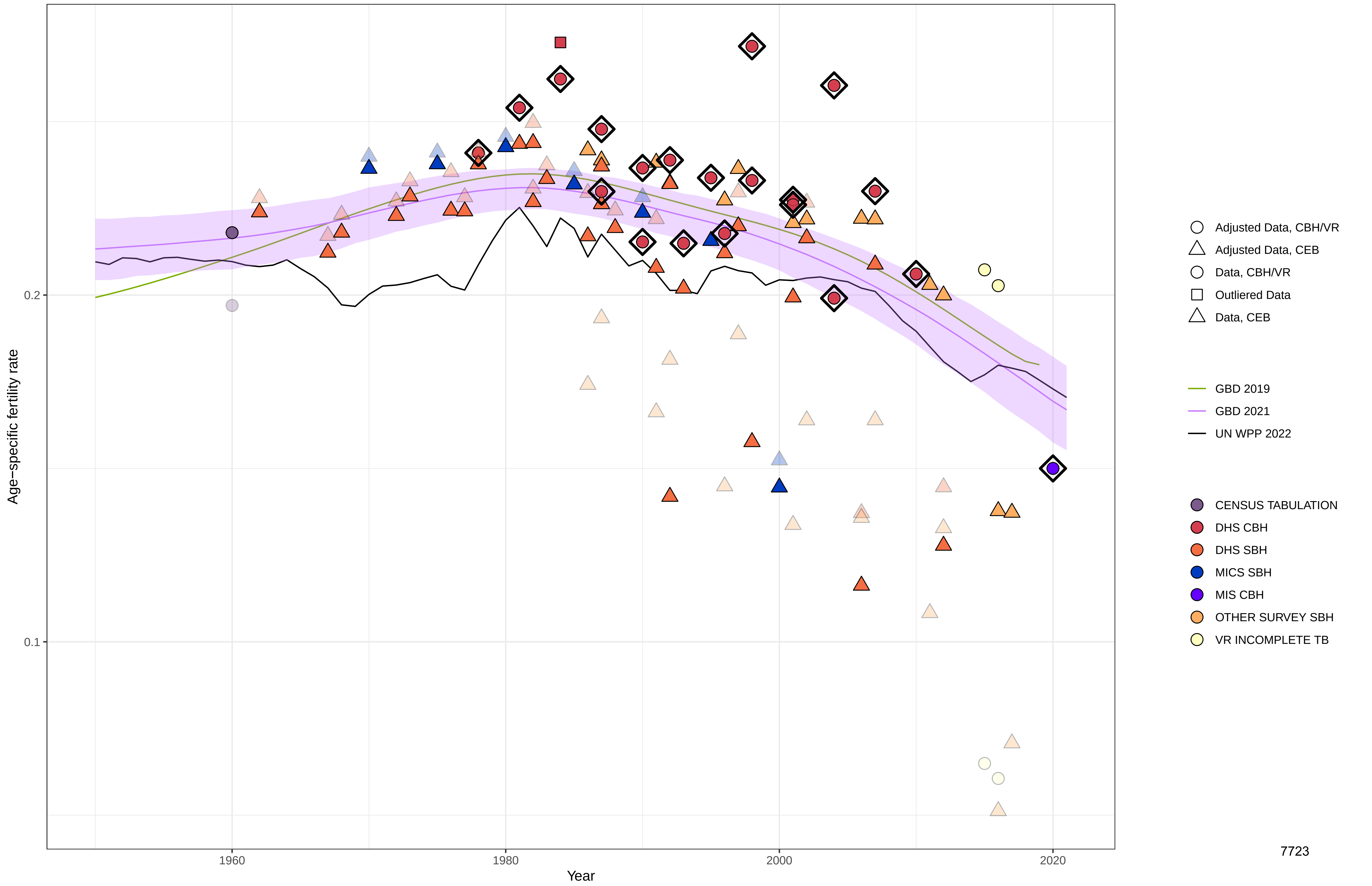

Age−specific fertility fitted trend lines, 1950−2021: Niger, Ages: 20−24
Data points enclosed with a diamond are reference sources

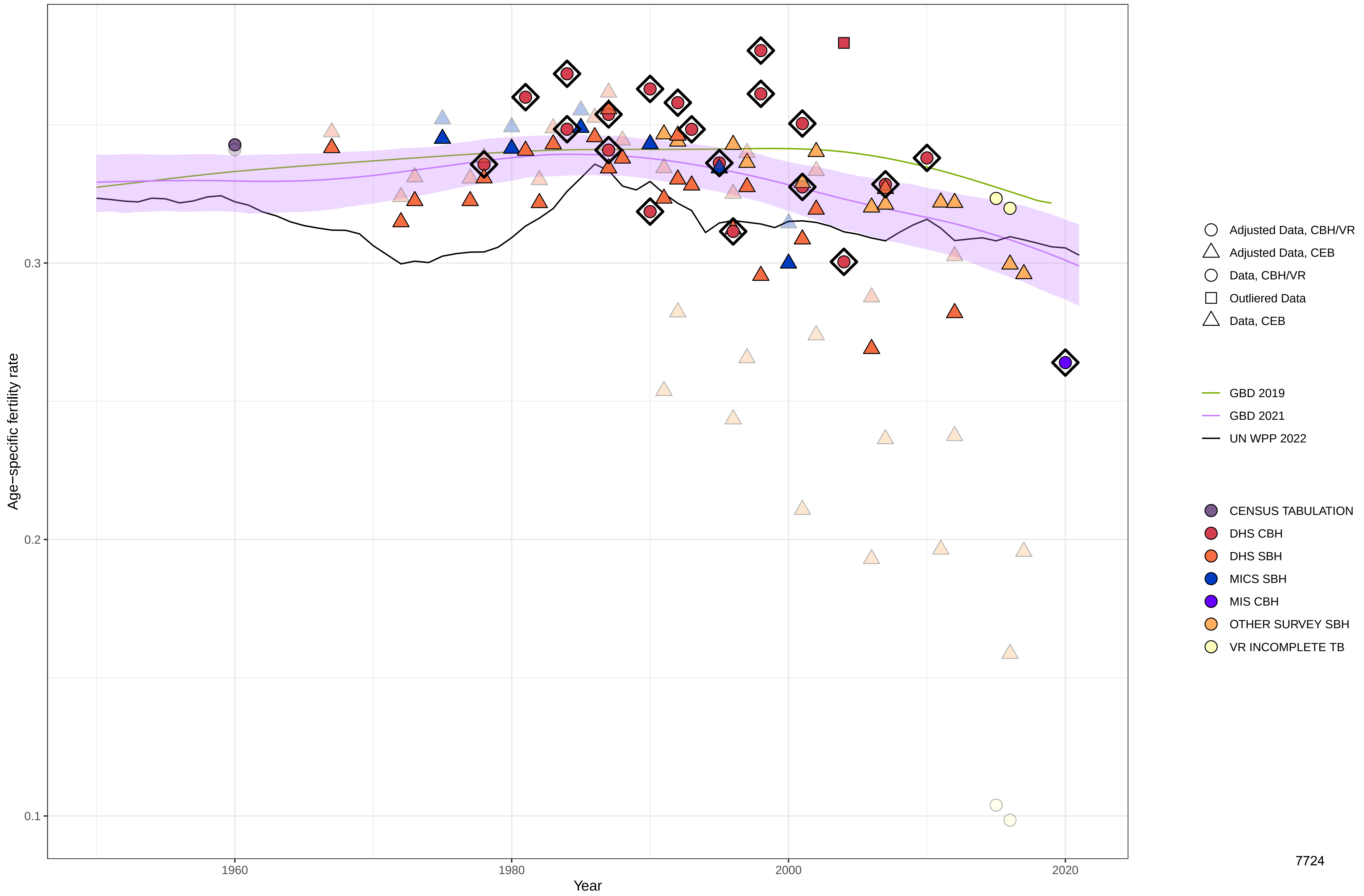

Age−specific fertility fitted trend lines, 1950−2021: Niger, Ages: 25−29
Data points enclosed with a diamond are reference sources

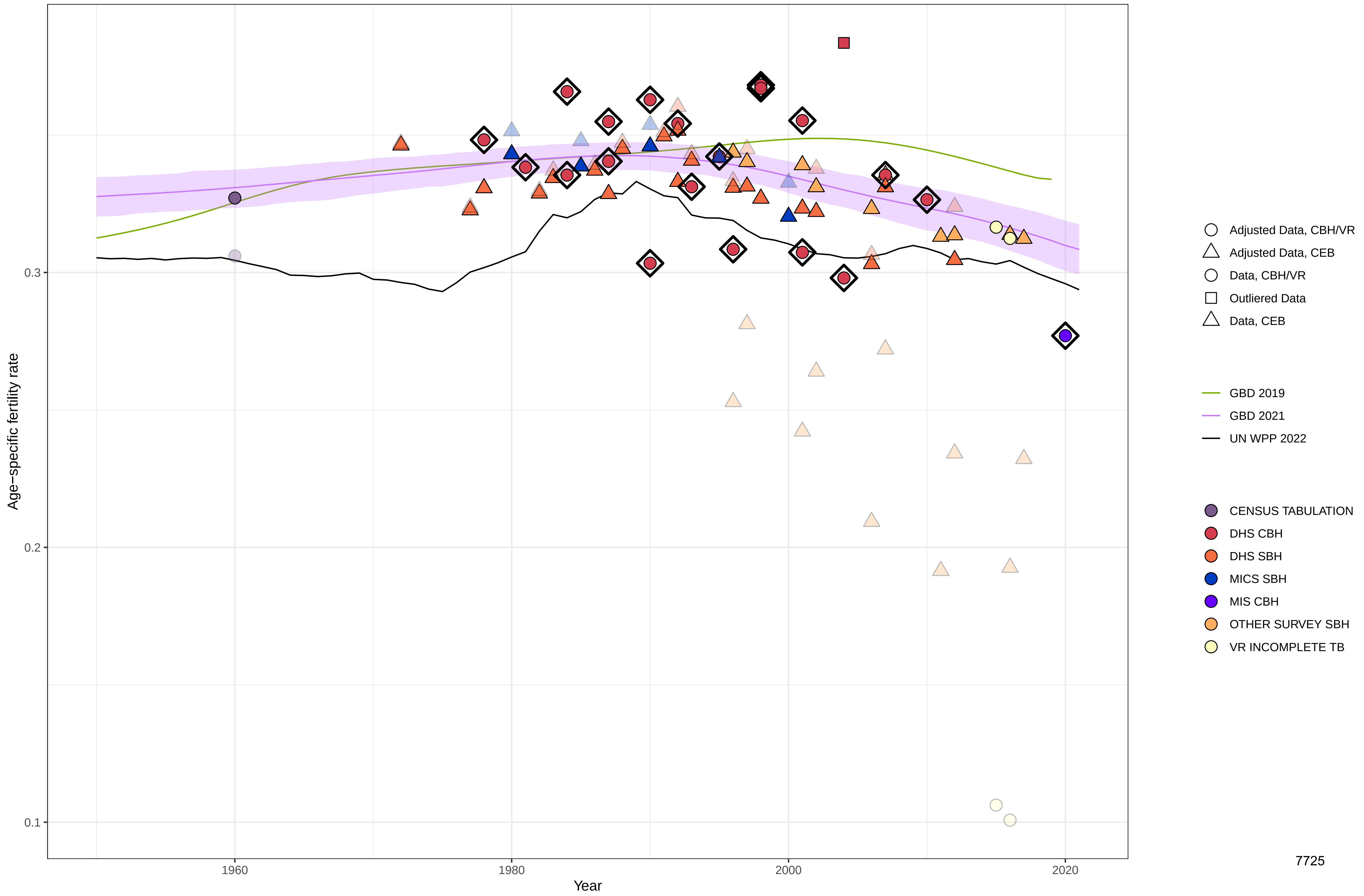

Age−specific fertility fitted trend lines, 1950−2021: Niger, Ages: 30−34
Data points enclosed with a diamond are reference sources

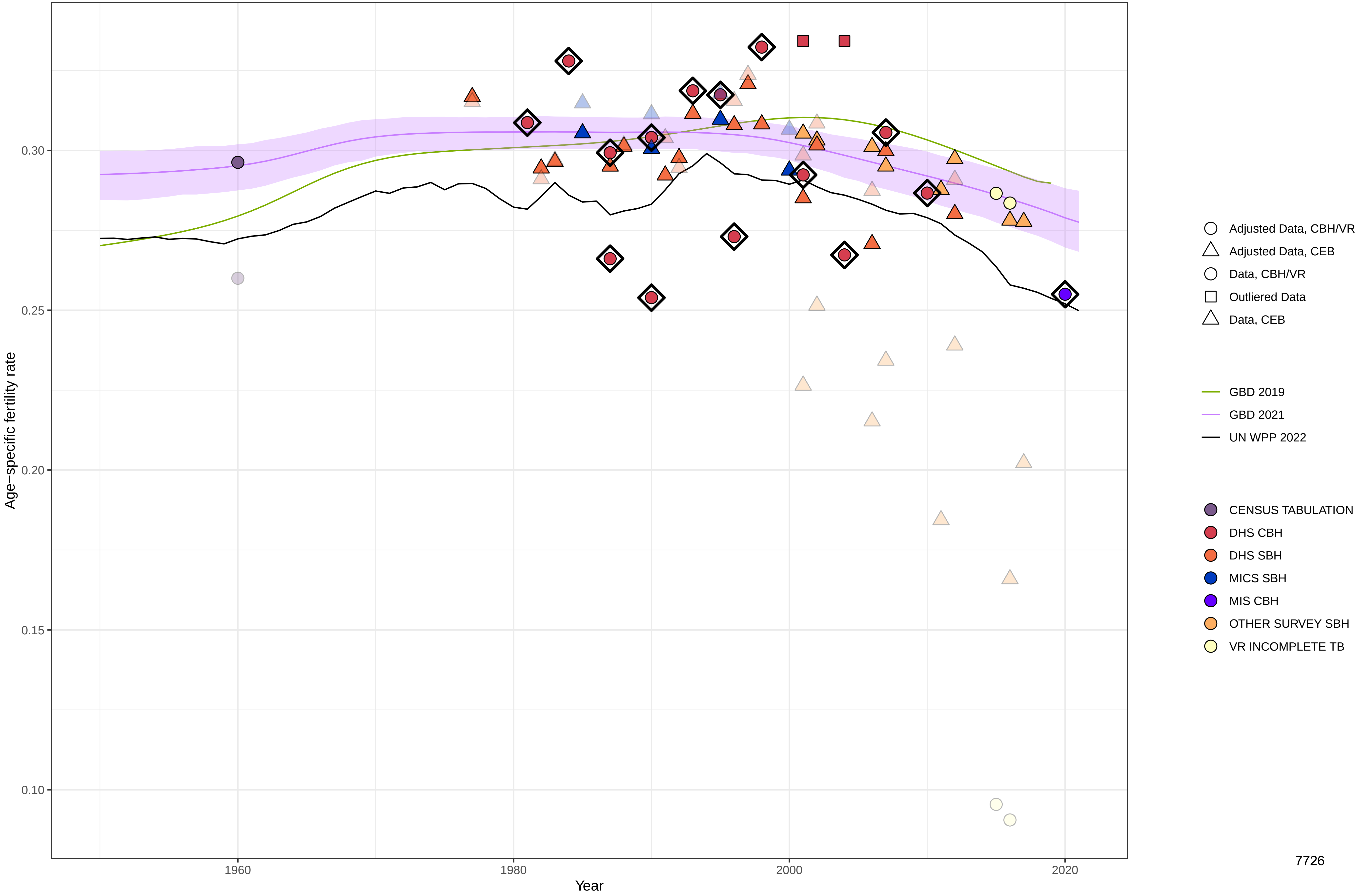

Age−specific fertility fitted trend lines, 1950−2021: Niger, Ages: 35−39
Data points enclosed with a diamond are reference sources

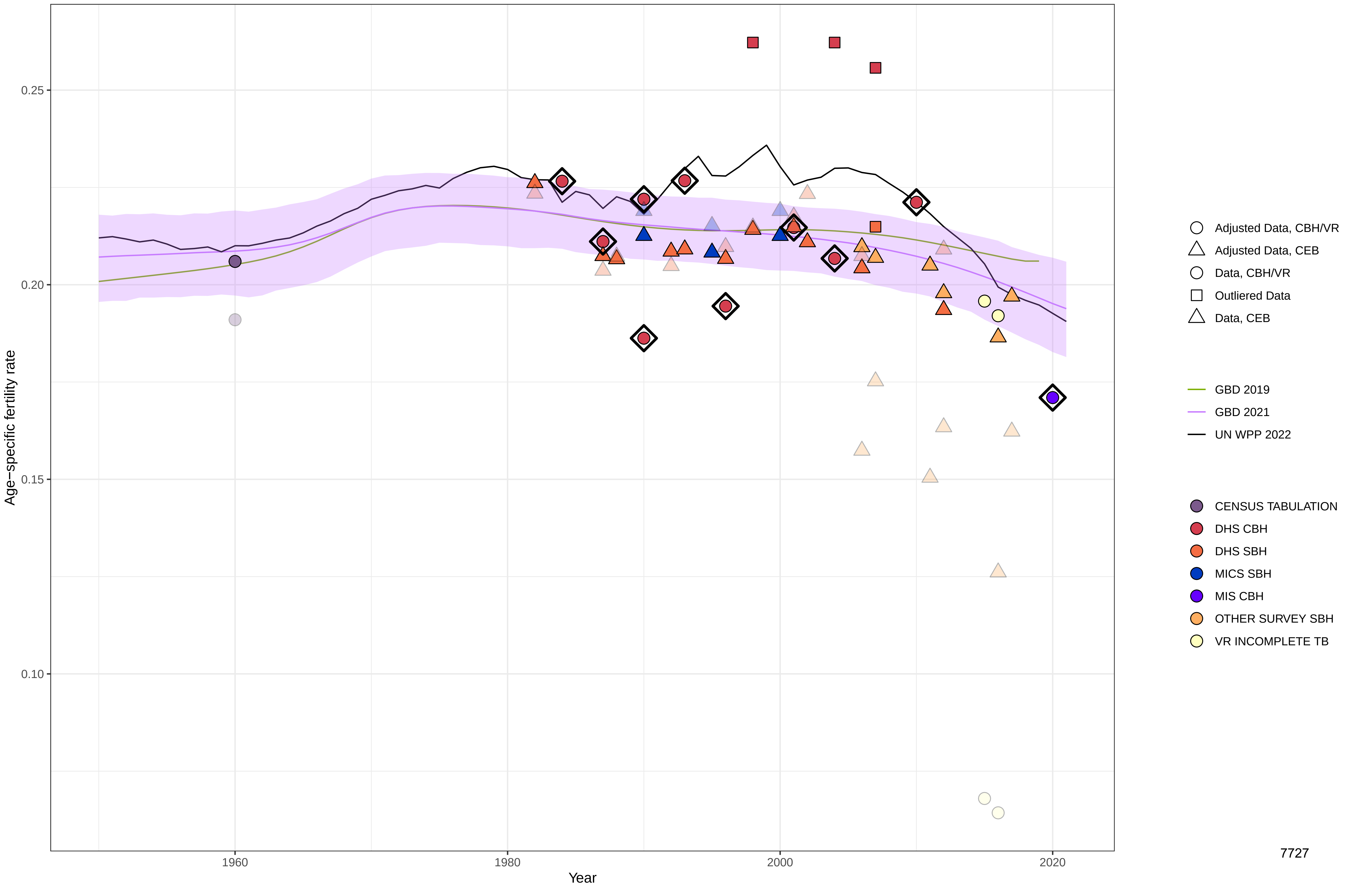

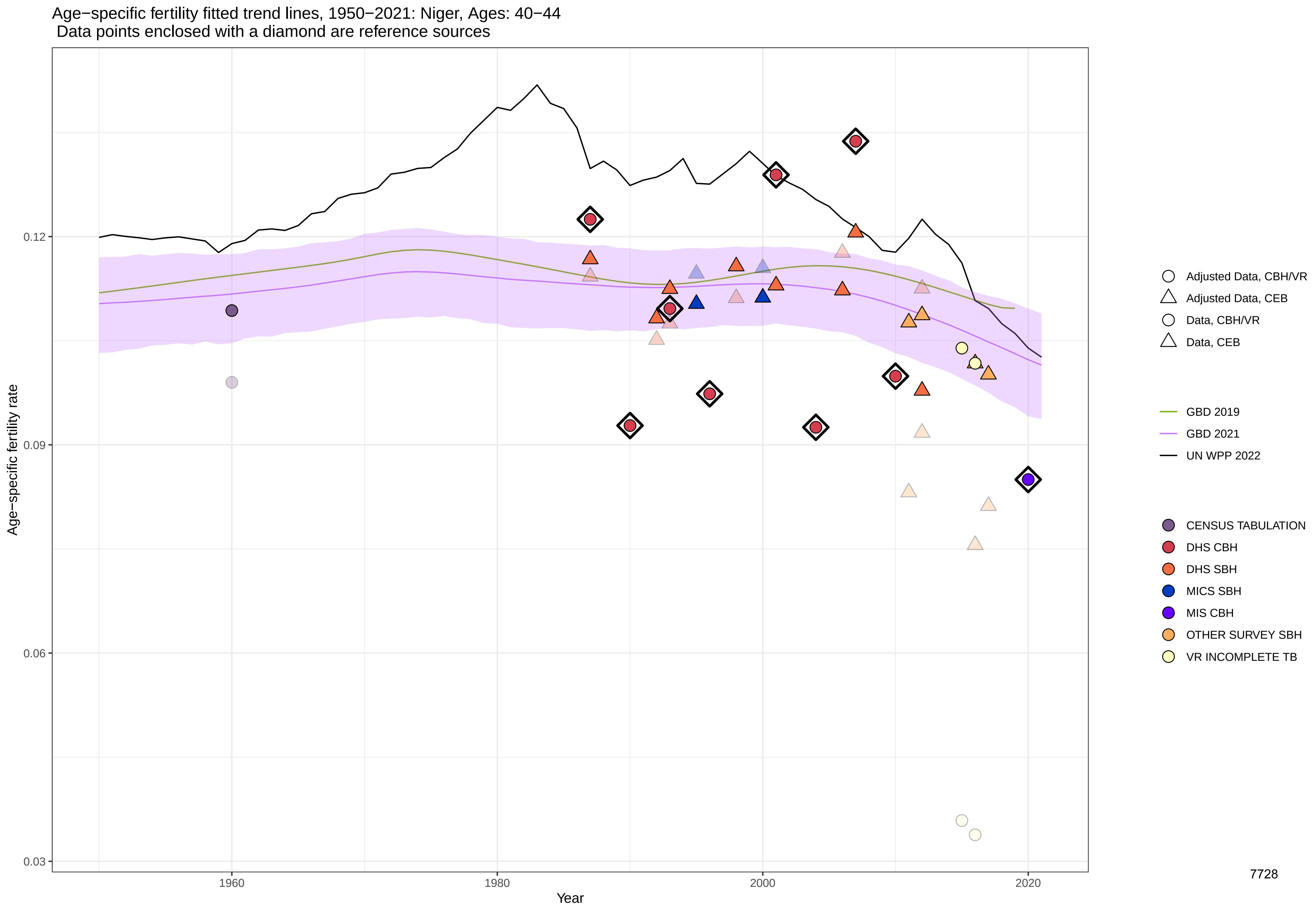

Age−specific fertility fitted trend lines, 1950−2021: Niger, Ages: 40−44
Data points enclosed with a diamond are reference sources
Age−specific fertility rate
0.12
0.09
0.06
0.03
Year
1960
1980
2000
2020
Adjusted Data, CBH/VR
Adjusted Data, CEB
Data, CBH/VR
Data, CEB
GBD 2019
GBD 2021
UN WPP 2022
CENSUS TABULATION
DHS CBH
DHS SBH
MICS SBH
MIS CBH
OTHER SURVEY SBH
VR INCOMPLETE TB

Age−specific fertility fitted trend lines, 1950−2021: Niger, Ages: 45−49
Data points enclosed with a diamond are reference sources

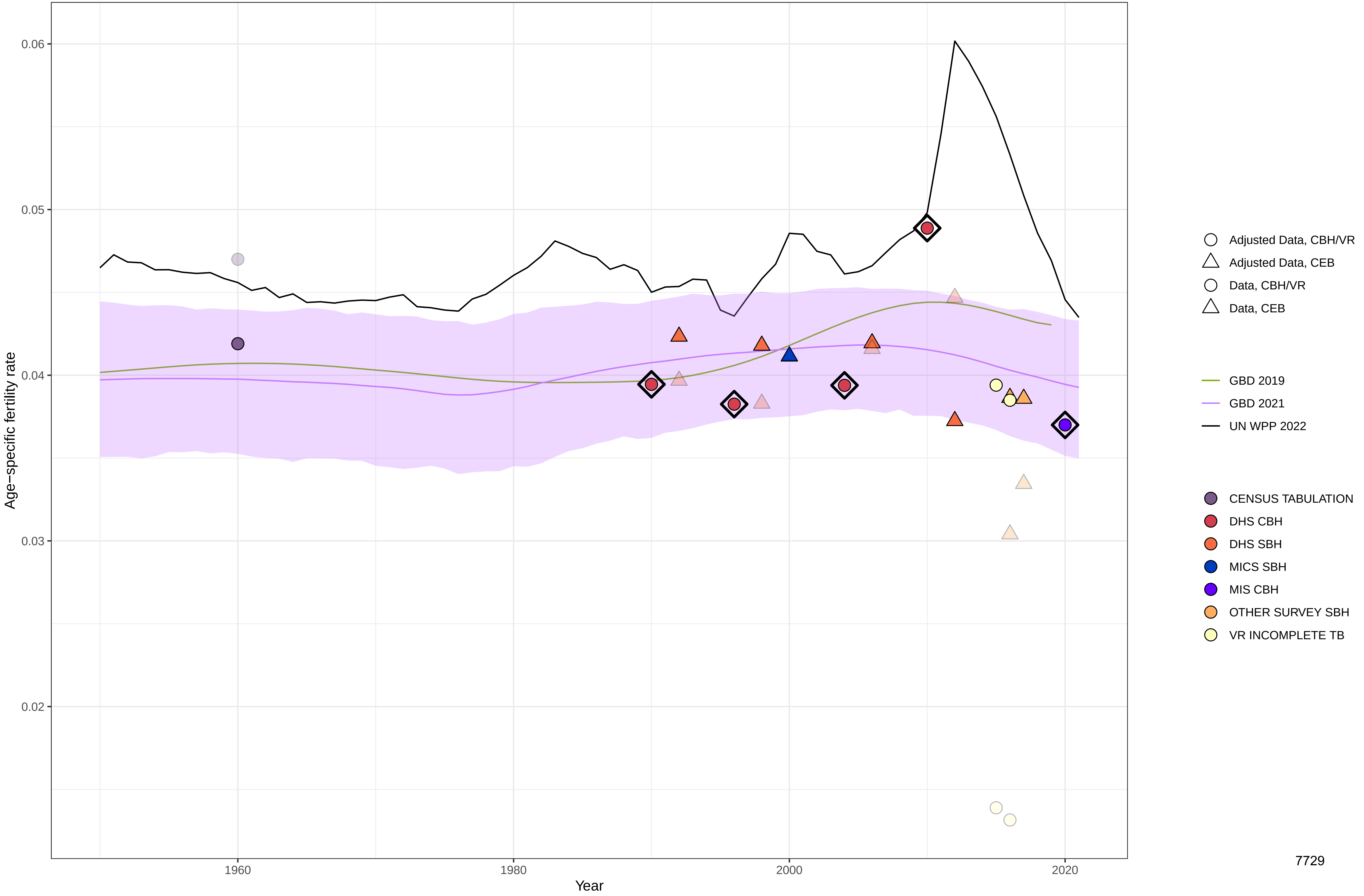

Age−specific fertility fitted trend lines, 1950−2021: Niger, Ages: 50−54
Data points enclosed with a diamond are reference sources

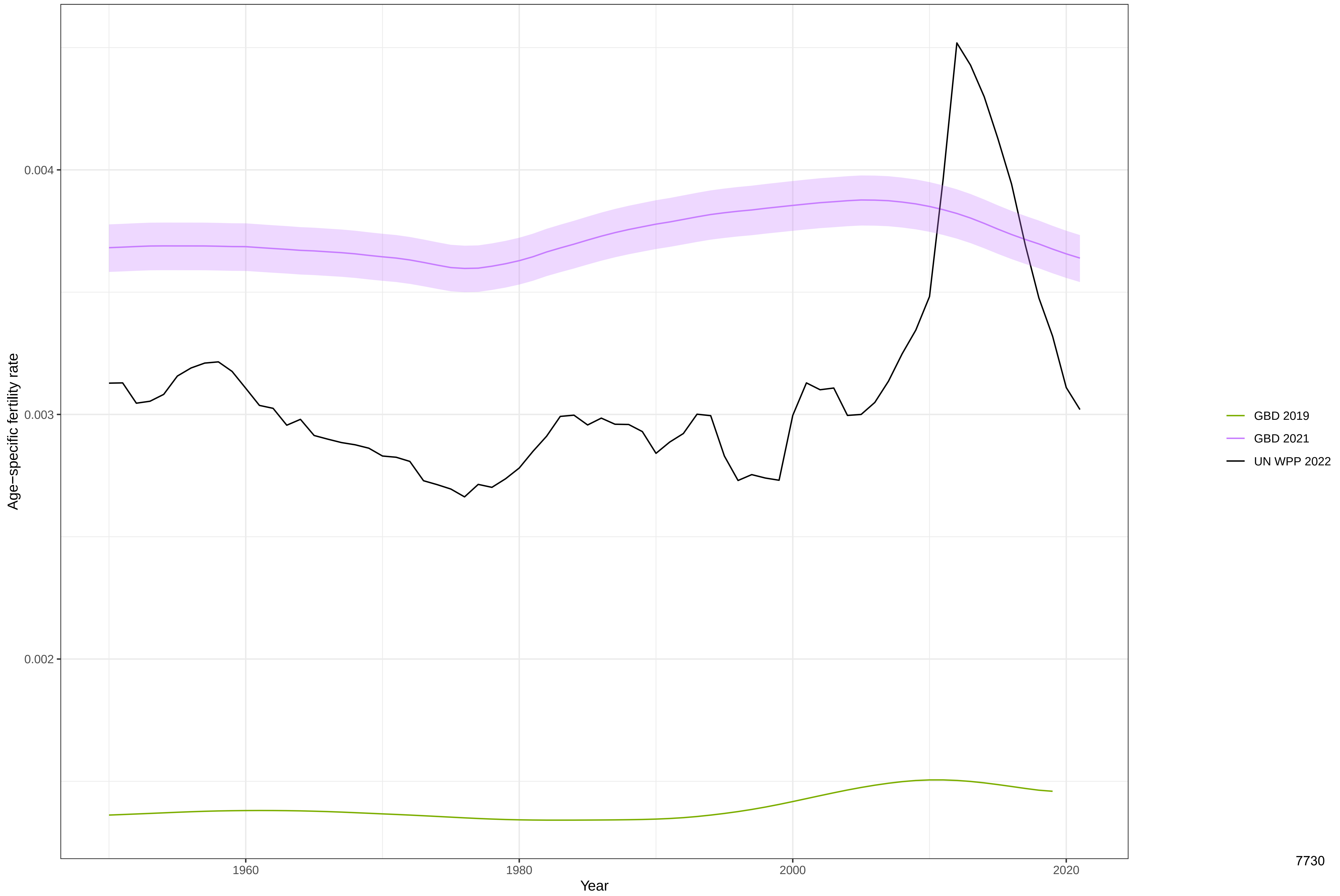

Total fertility rate fitted trend lines, 1950−2021: Niger TFR
Data points enclosed with a diamond are reference sources

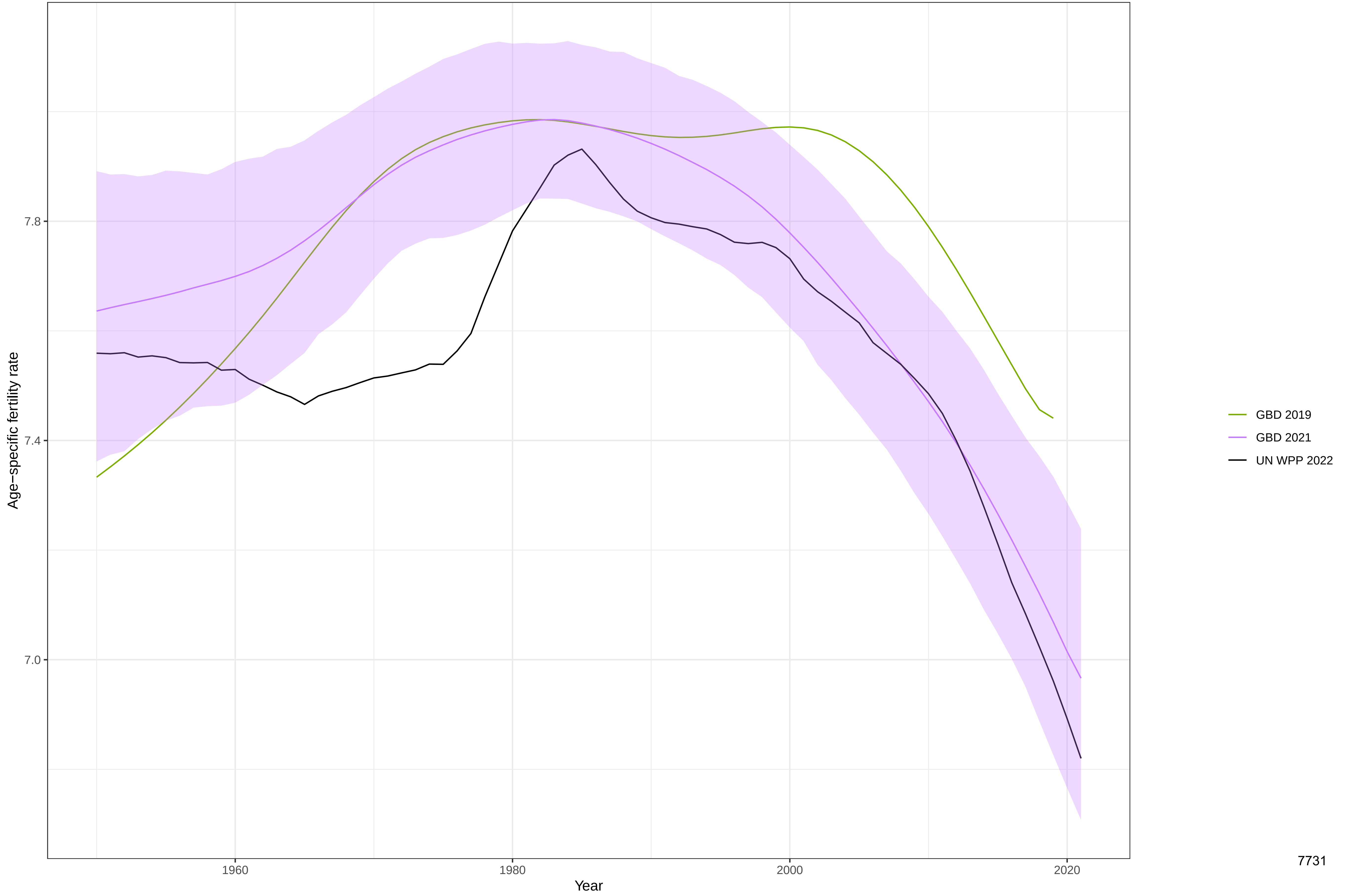

7731

Age−specific fertility fitted trend lines, 1950−2021: Nigeria, Ages: 10−14
Data points enclosed with a diamond are reference sources

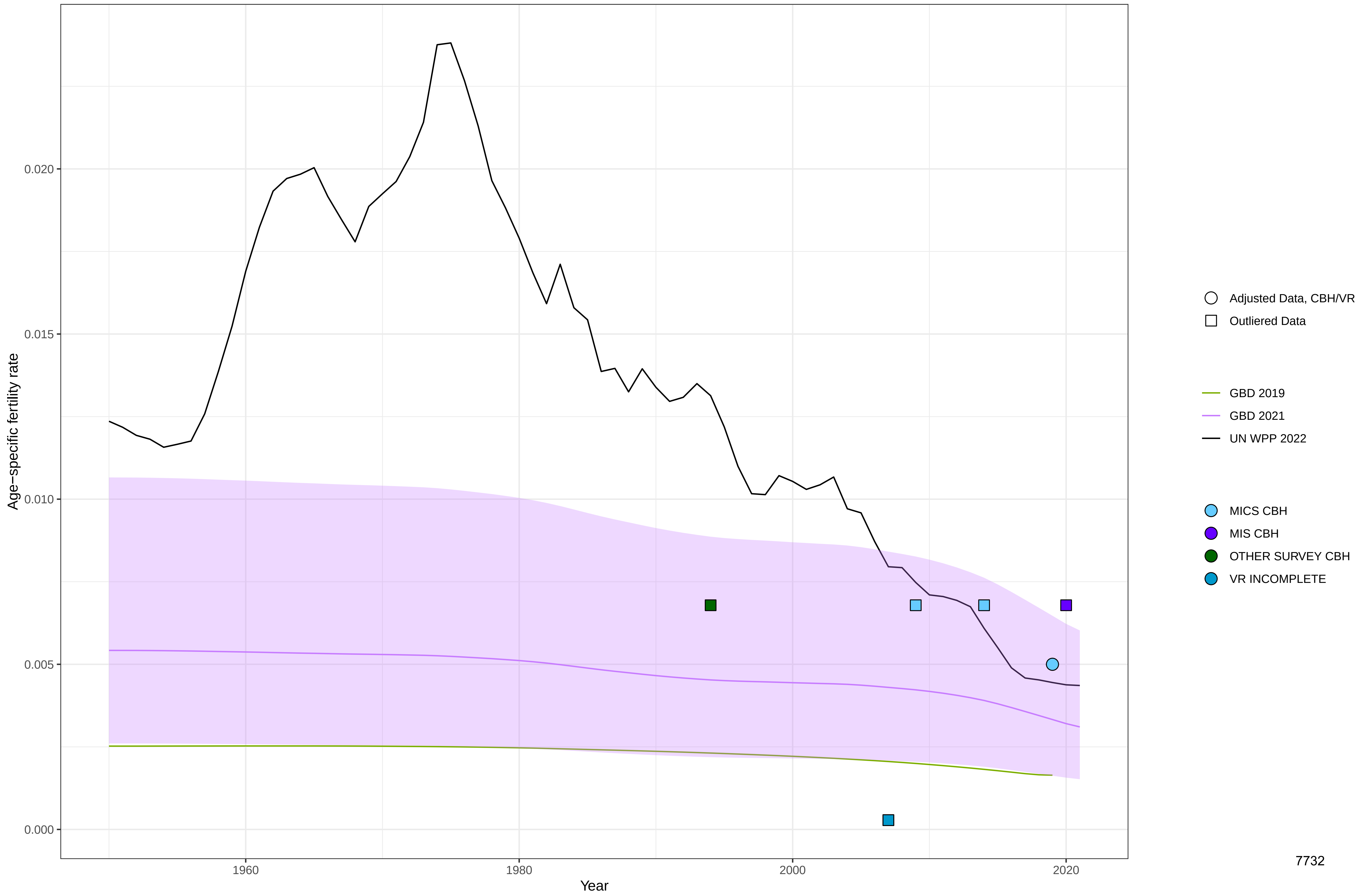

Age−specific fertility fitted trend lines, 1950−2021: Nigeria, Ages: 15−19
Data points enclosed with a diamond are reference sources

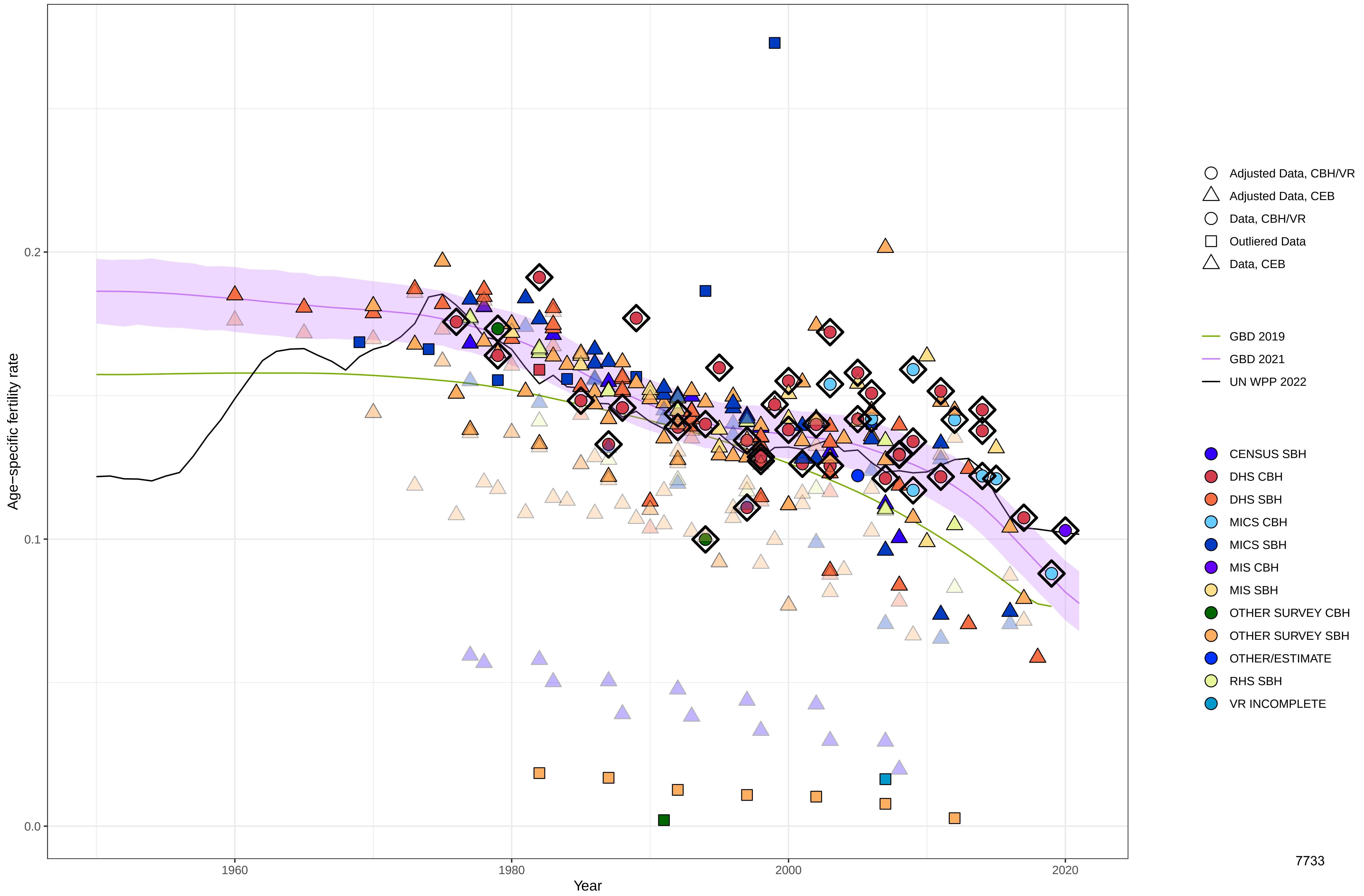

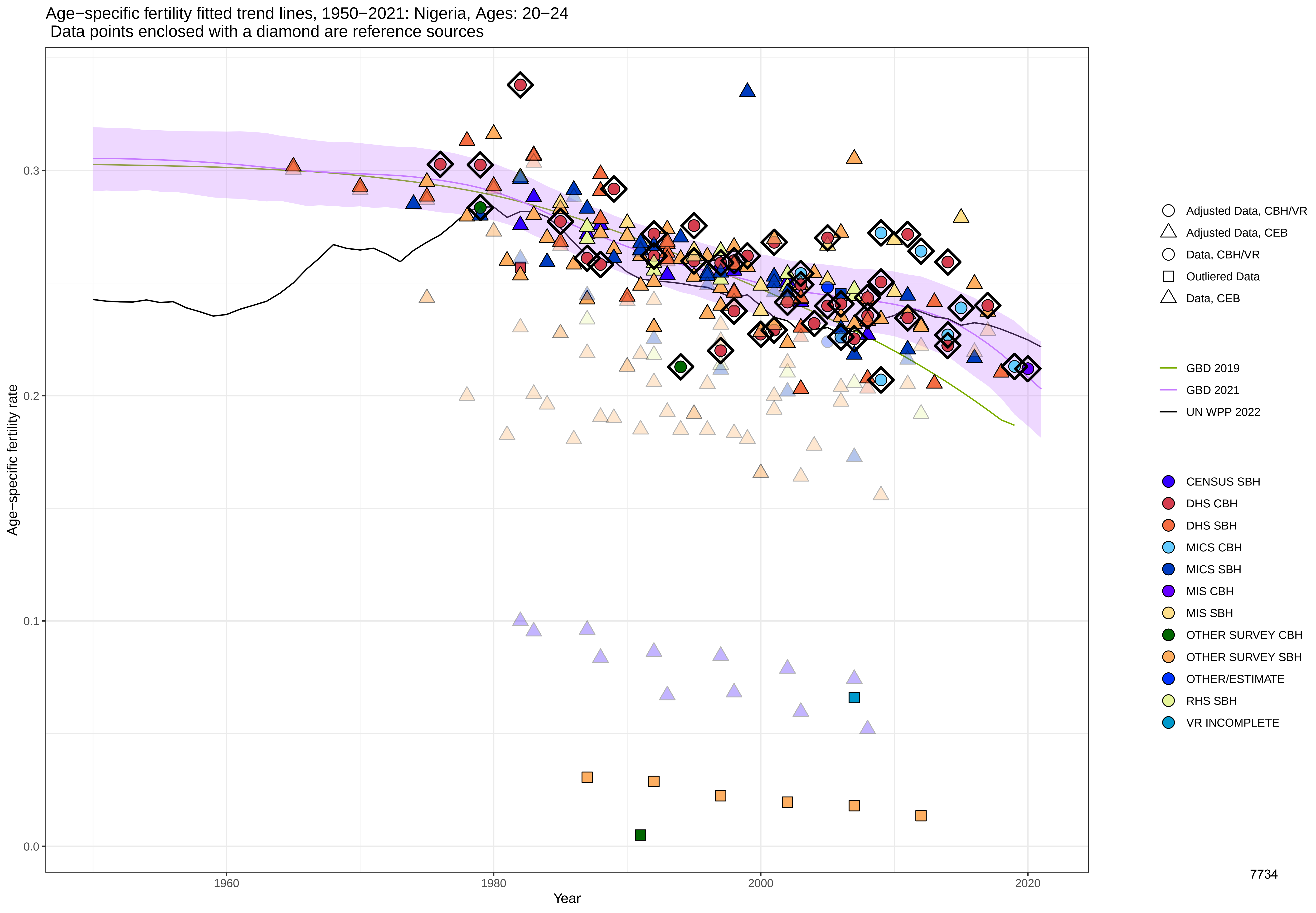

Age−specific fertility fitted trend lines, 1950−2021: Nigeria, Ages: 20−24
Data points enclosed with a diamond are reference sources
Age−specific fertility rate
0.0
0.1
0.2
0.3
Year
1960
1980
2000
2020
Adjusted Data, CBH/VR
Adjusted Data, CEB
Data, CBH/VR
Outliered Data
Data, CEB
GBD 2019
GBD 2021
UN WPP 2022
CENSUS SBH
DHS CBH
DHS SBH
MICS CBH
MICS SBH
MIS CBH
MIS SBH
OTHER SURVEY CBH
OTHER SURVEY SBH
OTHER/ESTIMATE
RHS SBH
VR INCOMPLETE

Age−specific fertility fitted trend lines, 1950−2021: Nigeria, Ages: 25−29
Data points enclosed with a diamond are reference sources

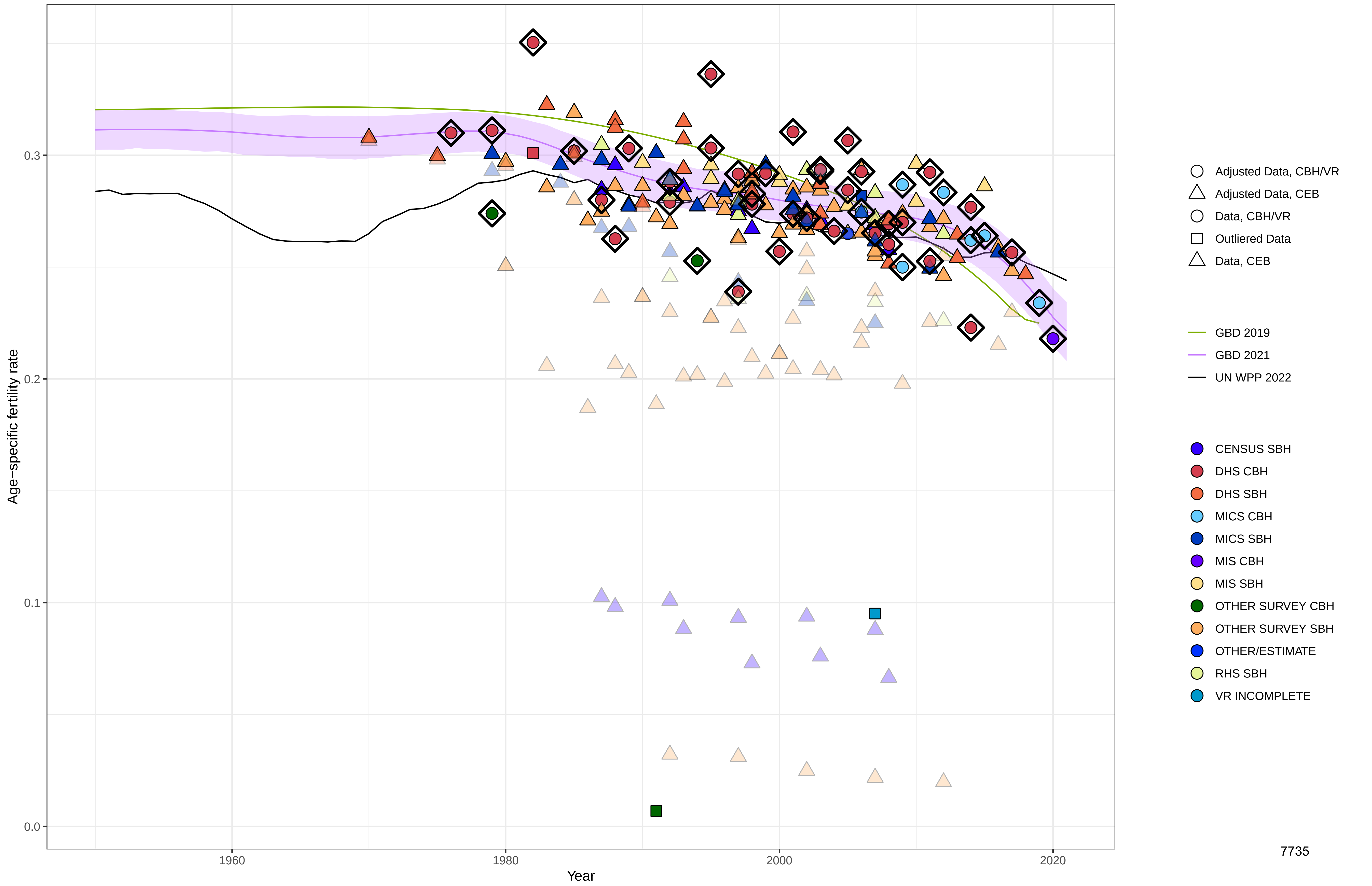

Age−specific fertility fitted trend lines, 1950−2021: Nigeria, Ages: 30−34
Data points enclosed with a diamond are reference sources

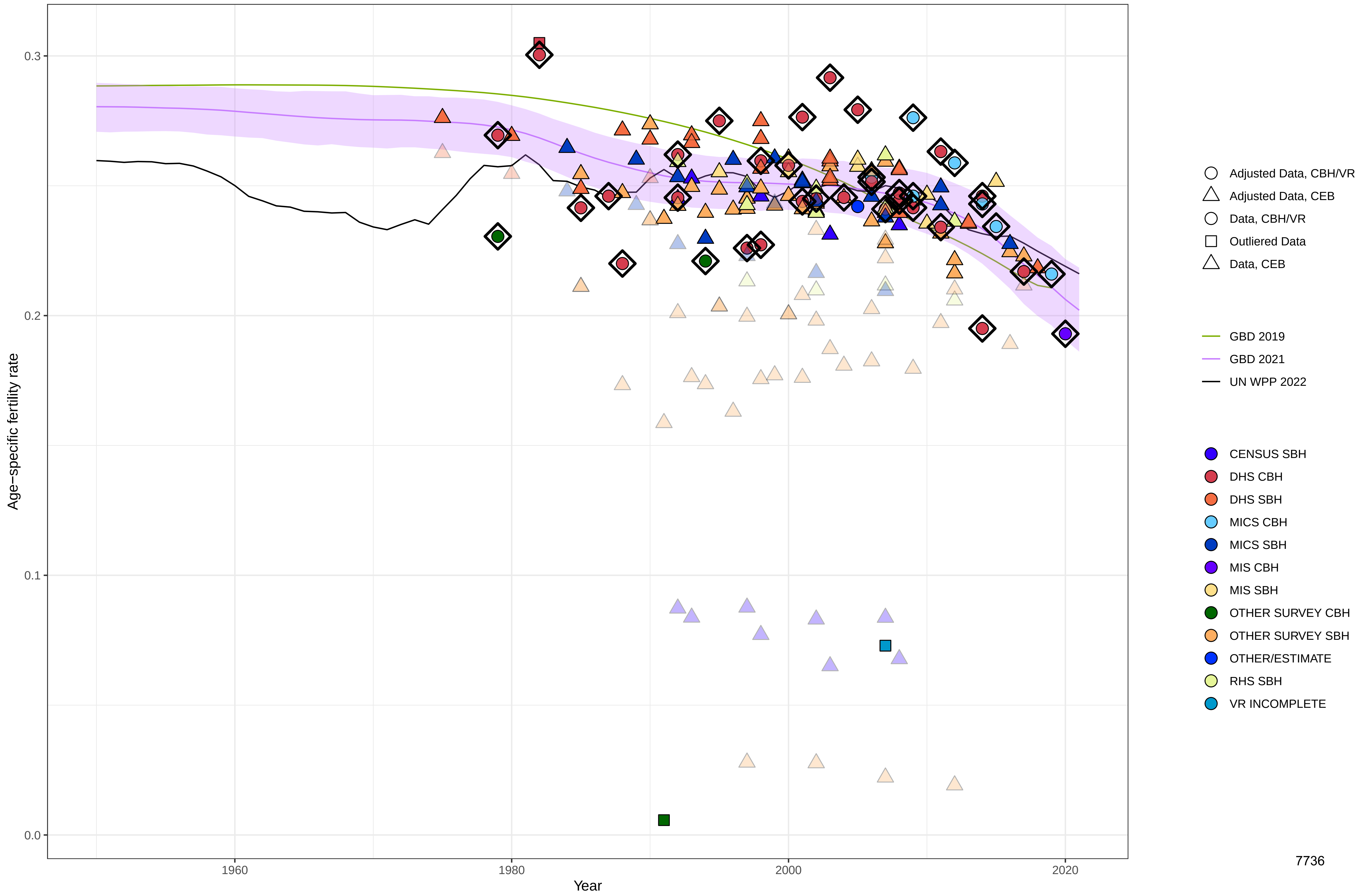

Age−specific fertility fitted trend lines, 1950−2021: Nigeria, Ages: 35−39
Data points enclosed with a diamond are reference sources

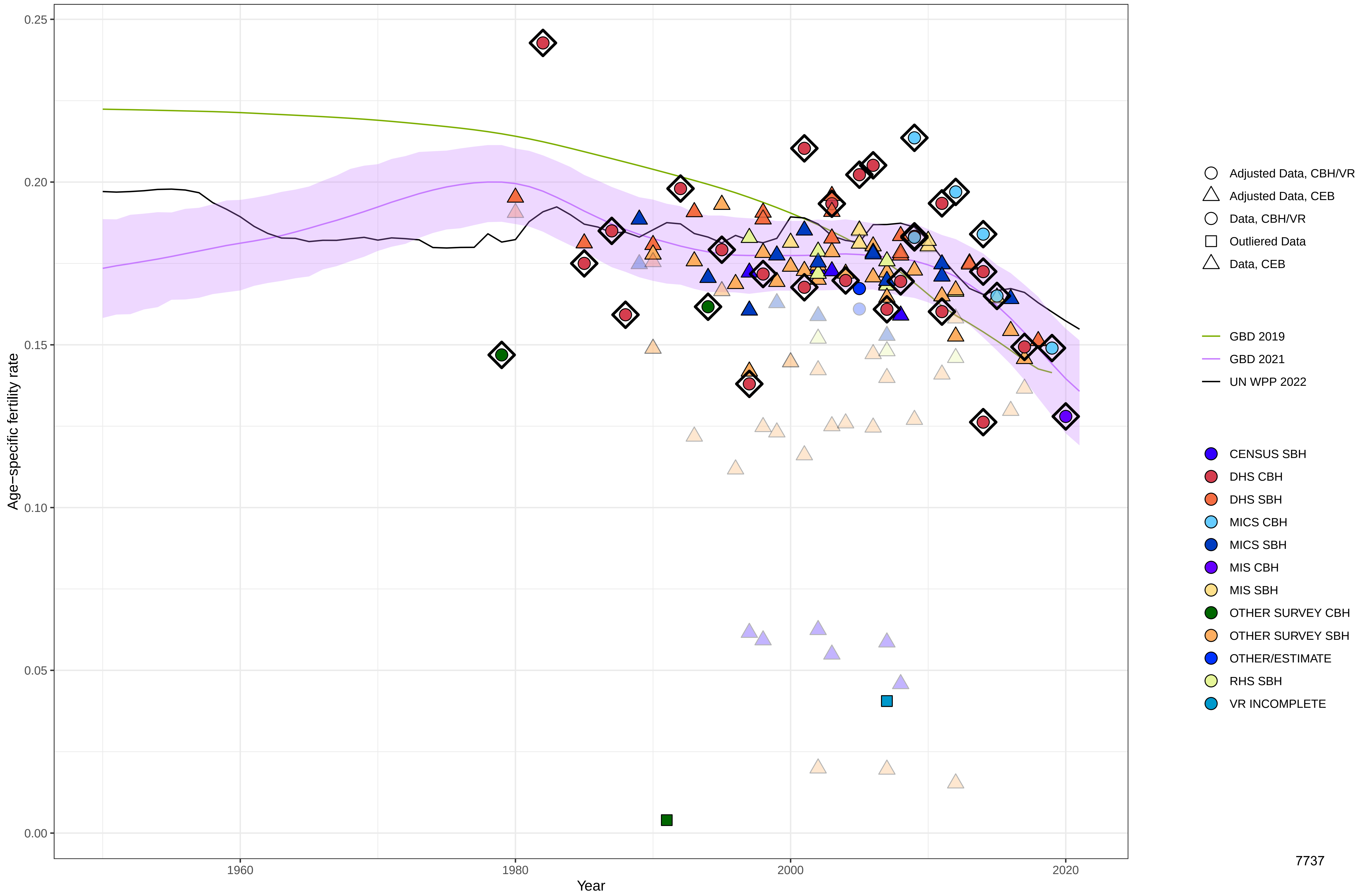

Age−specific fertility fitted trend lines, 1950−2021: Nigeria, Ages: 40−44
Data points enclosed with a diamond are reference sources

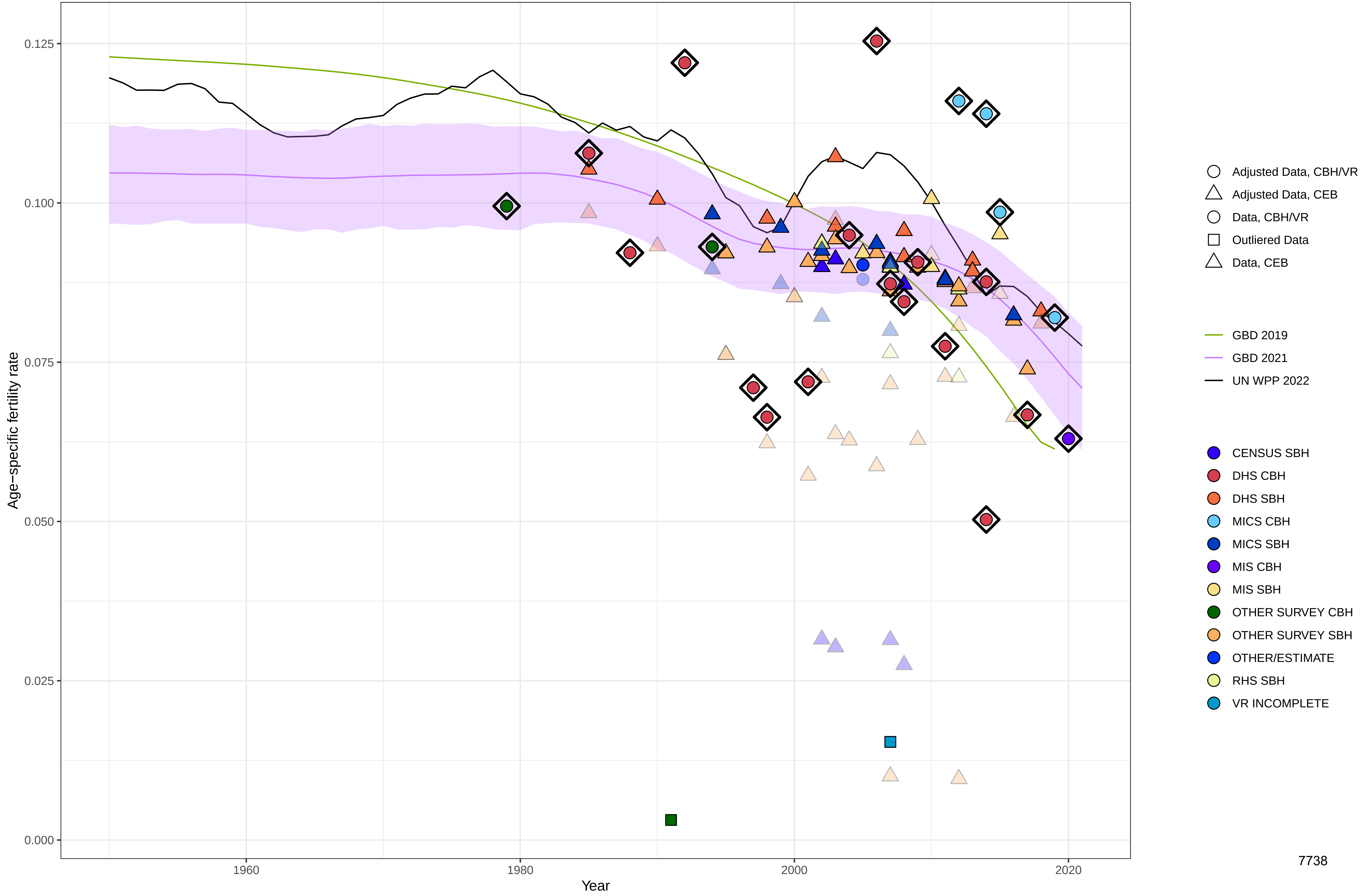

Age−specific fertility fitted trend lines, 1950−2021: Nigeria, Ages: 45−49
Data points enclosed with a diamond are reference sources

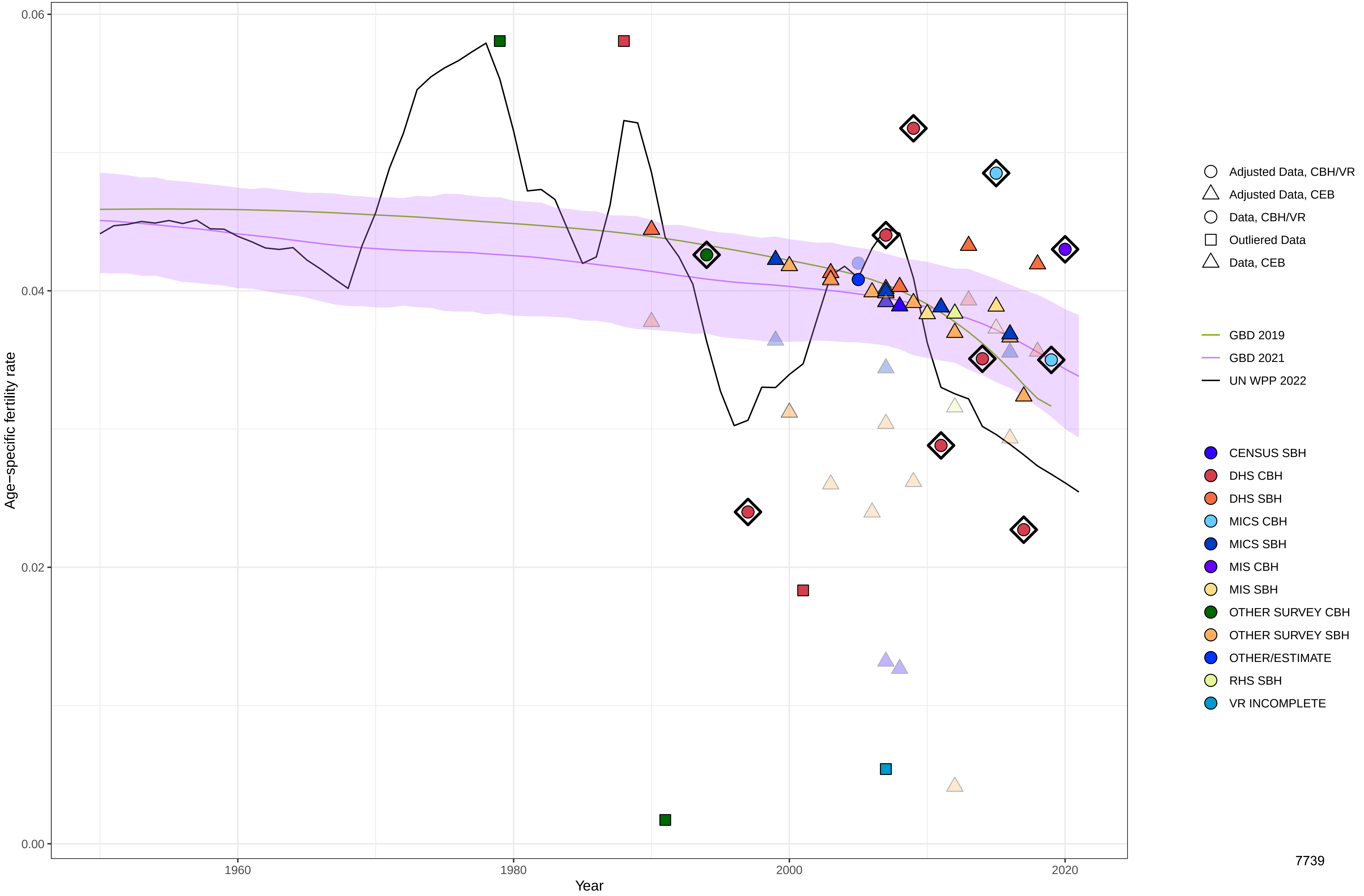

Age−specific fertility fitted trend lines, 1950−2021: Nigeria, Ages: 50−54
Data points enclosed with a diamond are reference sources

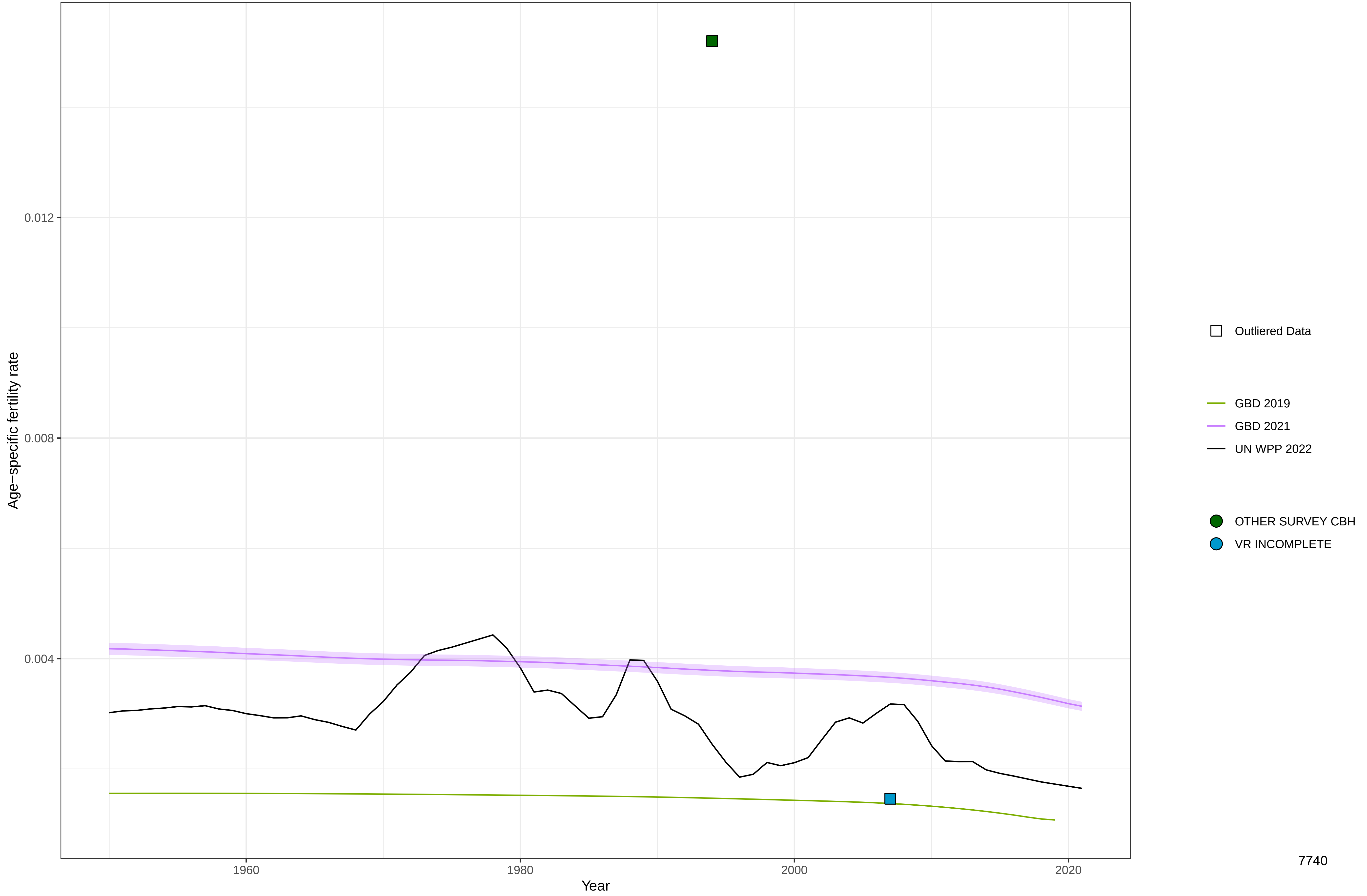

Total fertility rate fitted trend lines, 1950−2021: Nigeria TFR
Data points enclosed with a diamond are reference sources

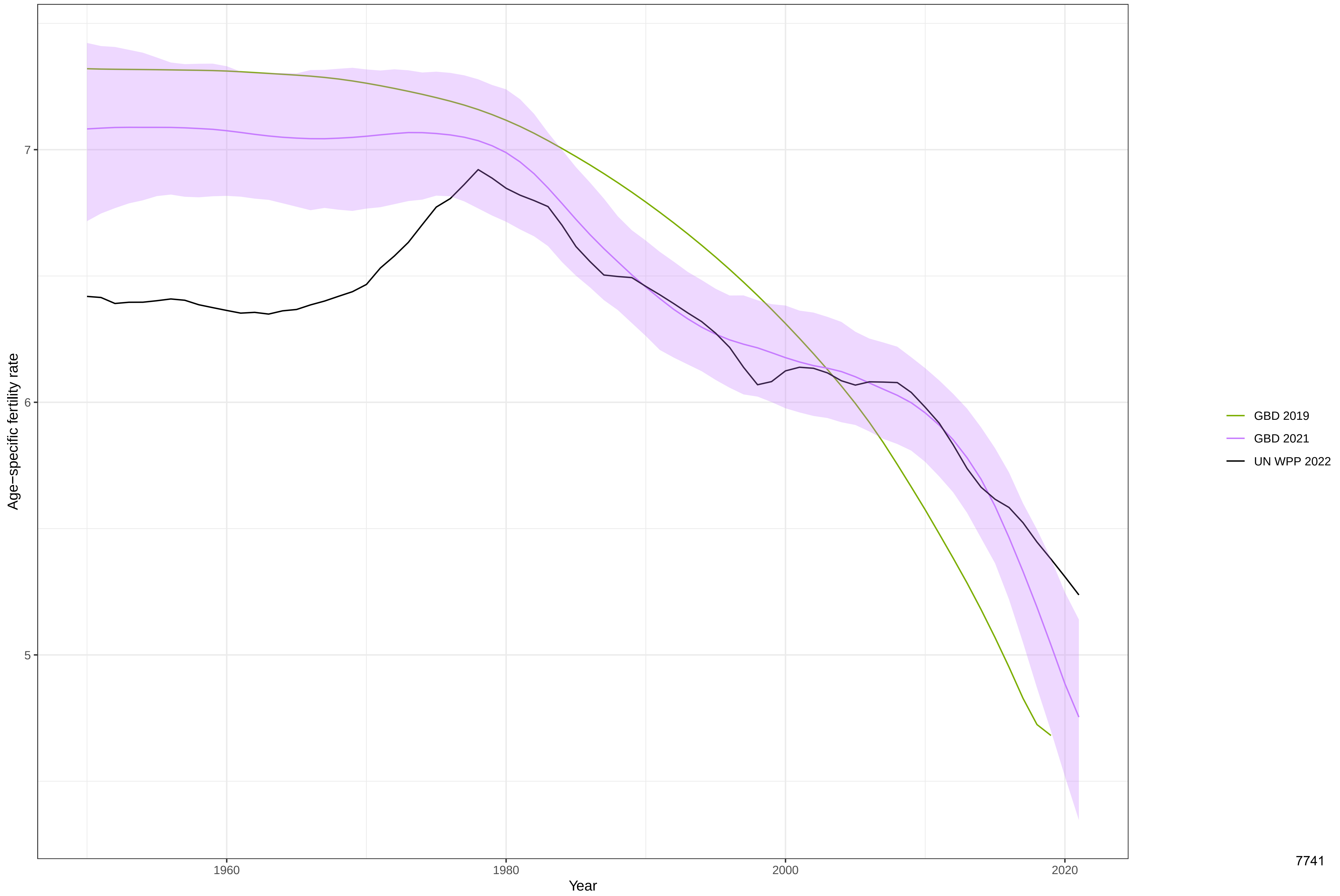

7741

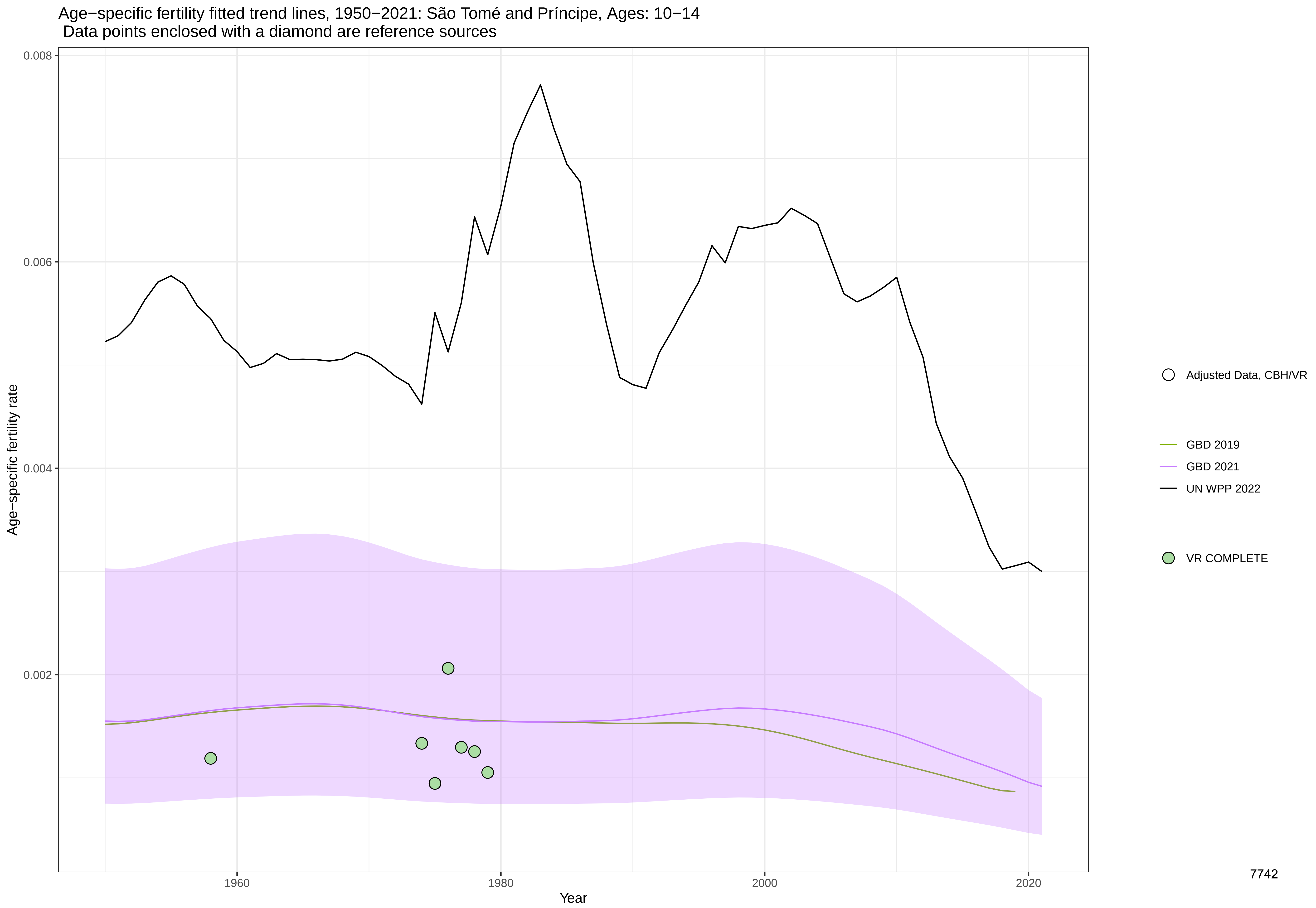

Age−specific fertility fitted trend lines, 1950−2021: São Tomé and Príncipe, Ages: 10−14
Data points enclosed with a diamond are reference sources
0.008
0.006
0.004
0.002
1960
1980
2000
2020
Year
Age−specific fertility rate
Adjusted Data, CBH/VR
GBD 2019
GBD 2021
UN WPP 2022
VR COMPLETE

Age−specific fertility fitted trend lines, 1950−2021: São Tomé and Príncipe, Ages: 15−19
Data points enclosed with a diamond are reference sources

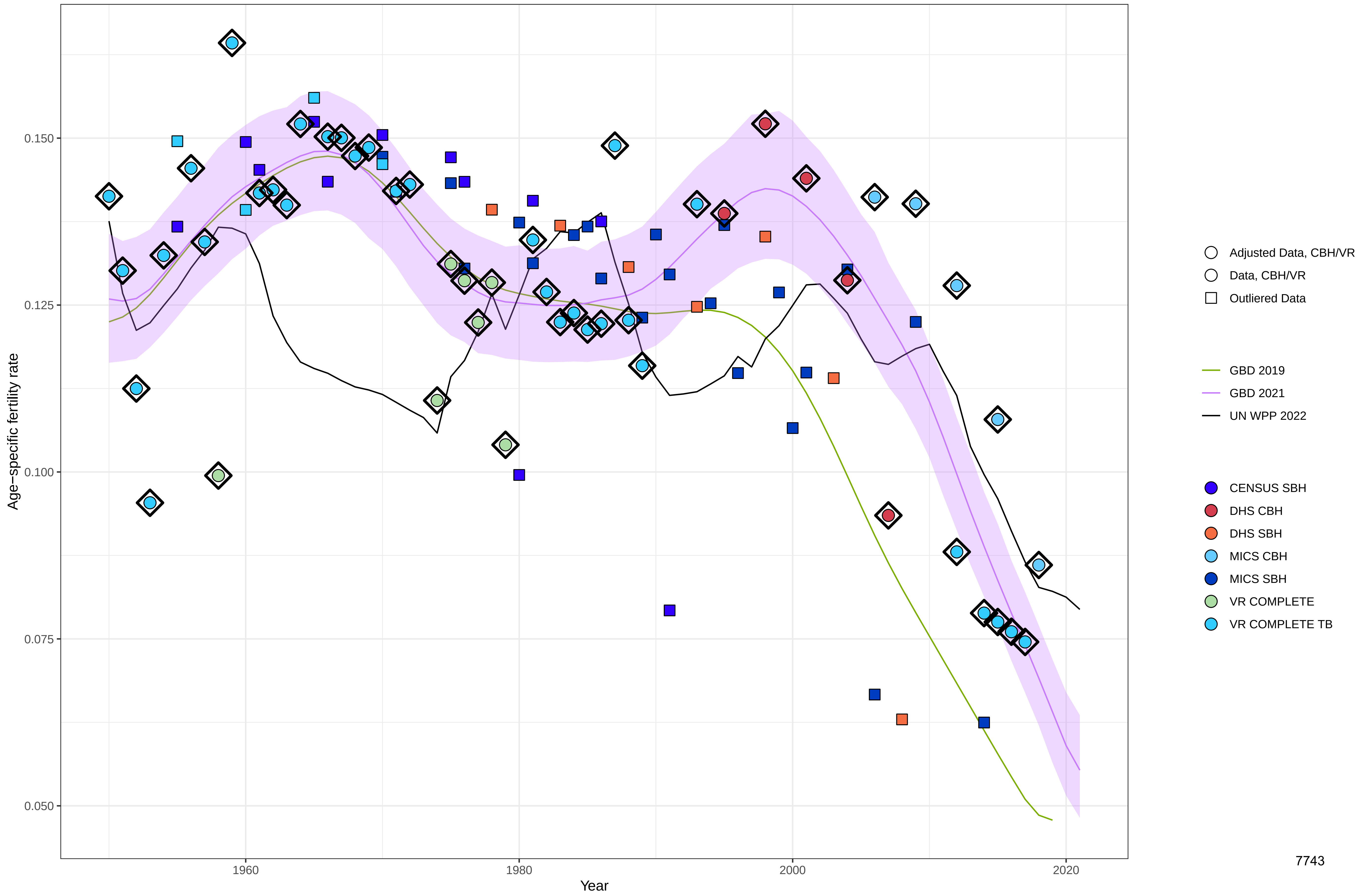

Age−specific fertility fitted trend lines, 1950−2021: São Tomé and Príncipe, Ages: 20−24
Data points enclosed with a diamond are reference sources

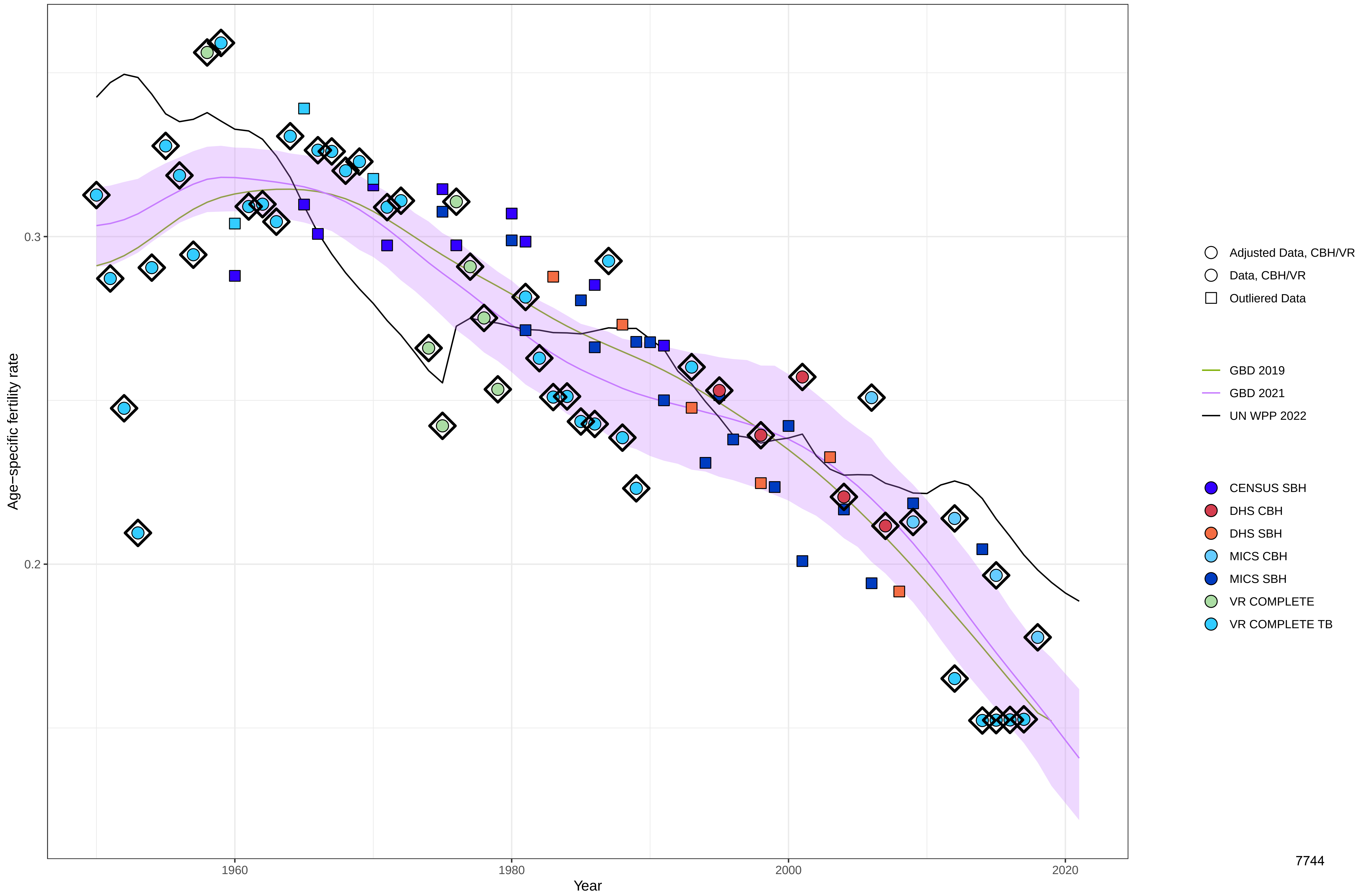

Age−specific fertility fitted trend lines, 1950−2021: São Tomé and Príncipe, Ages: 25−29
Data points enclosed with a diamond are reference sources

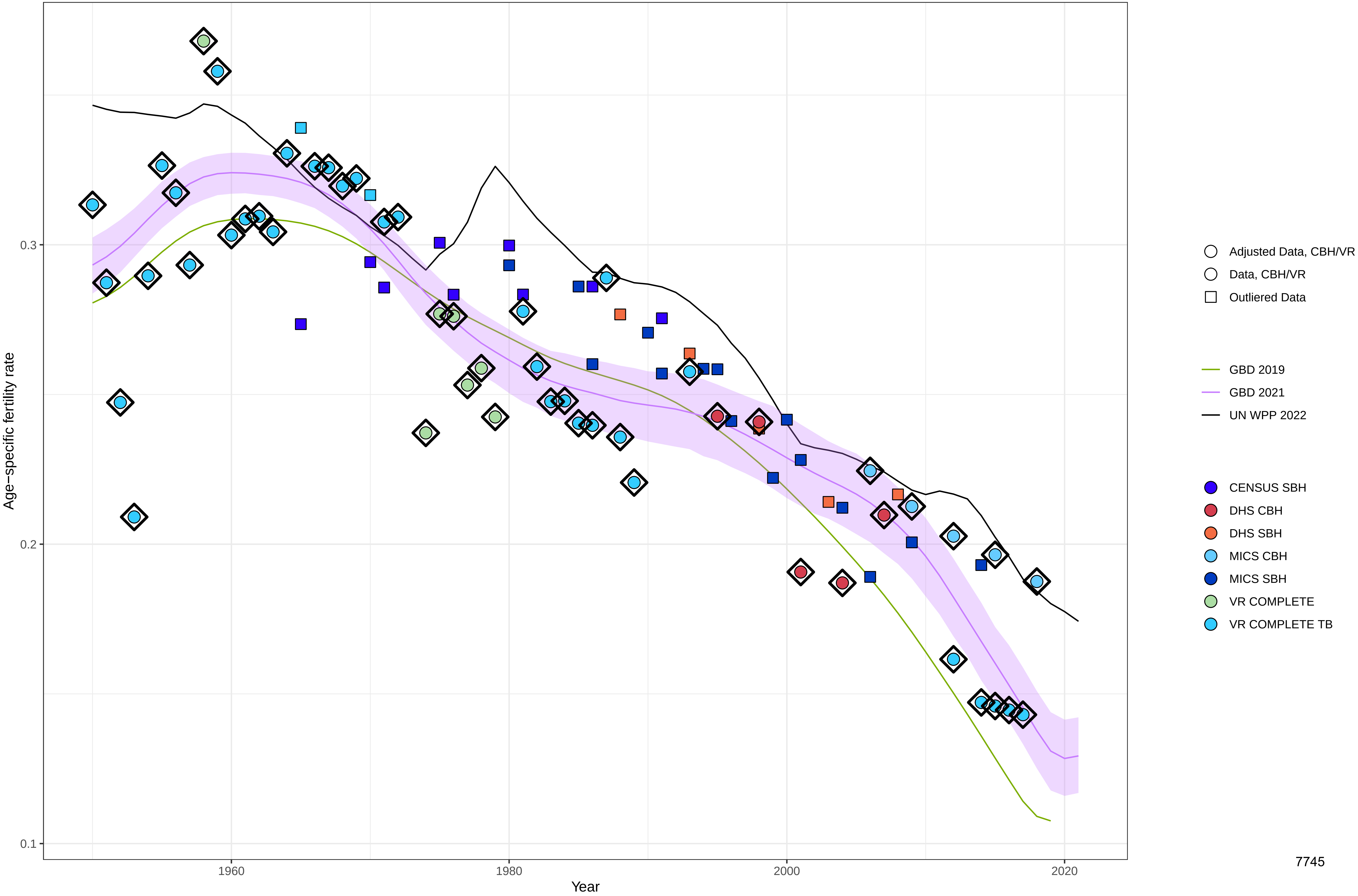

Age−specific fertility fitted trend lines, 1950−2021: São Tomé and Príncipe, Ages: 30−34
Data points enclosed with a diamond are reference sources

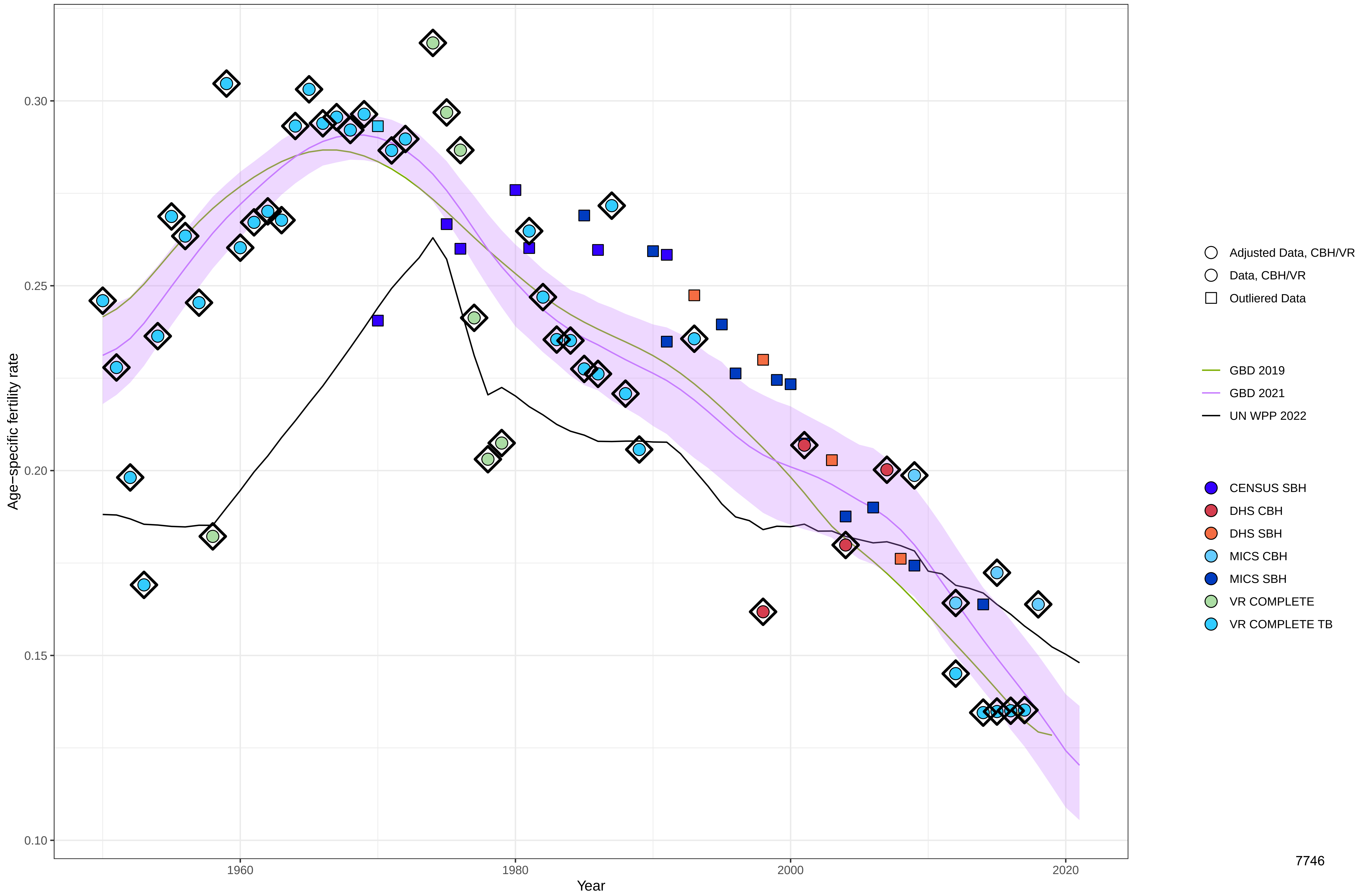

Age−specific fertility fitted trend lines, 1950−2021: São Tomé and Príncipe, Ages: 35−39
Data points enclosed with a diamond are reference sources

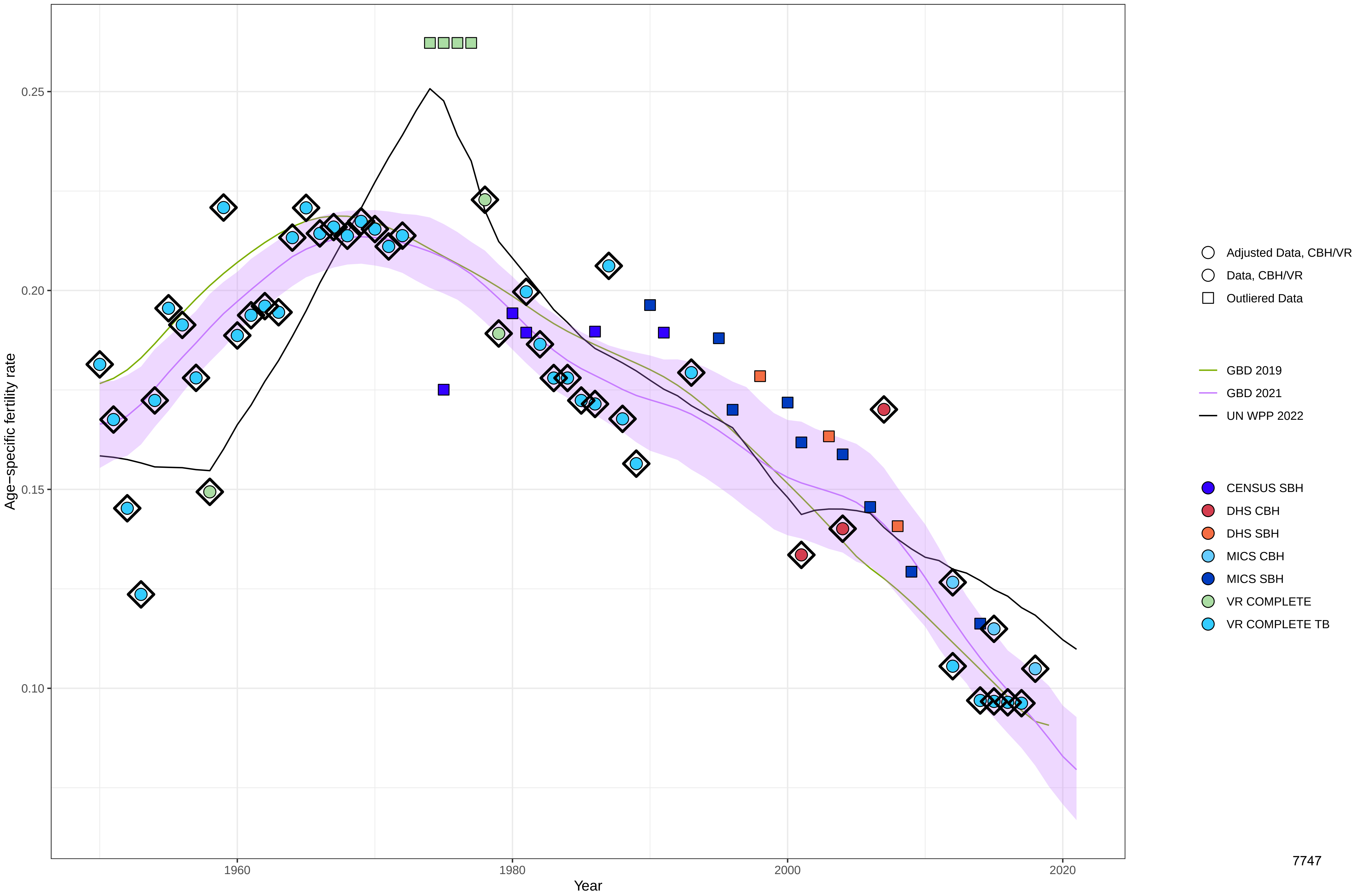

Age−specific fertility fitted trend lines, 1950−2021: São Tomé and Príncipe, Ages: 40−44
Data points enclosed with a diamond are reference sources

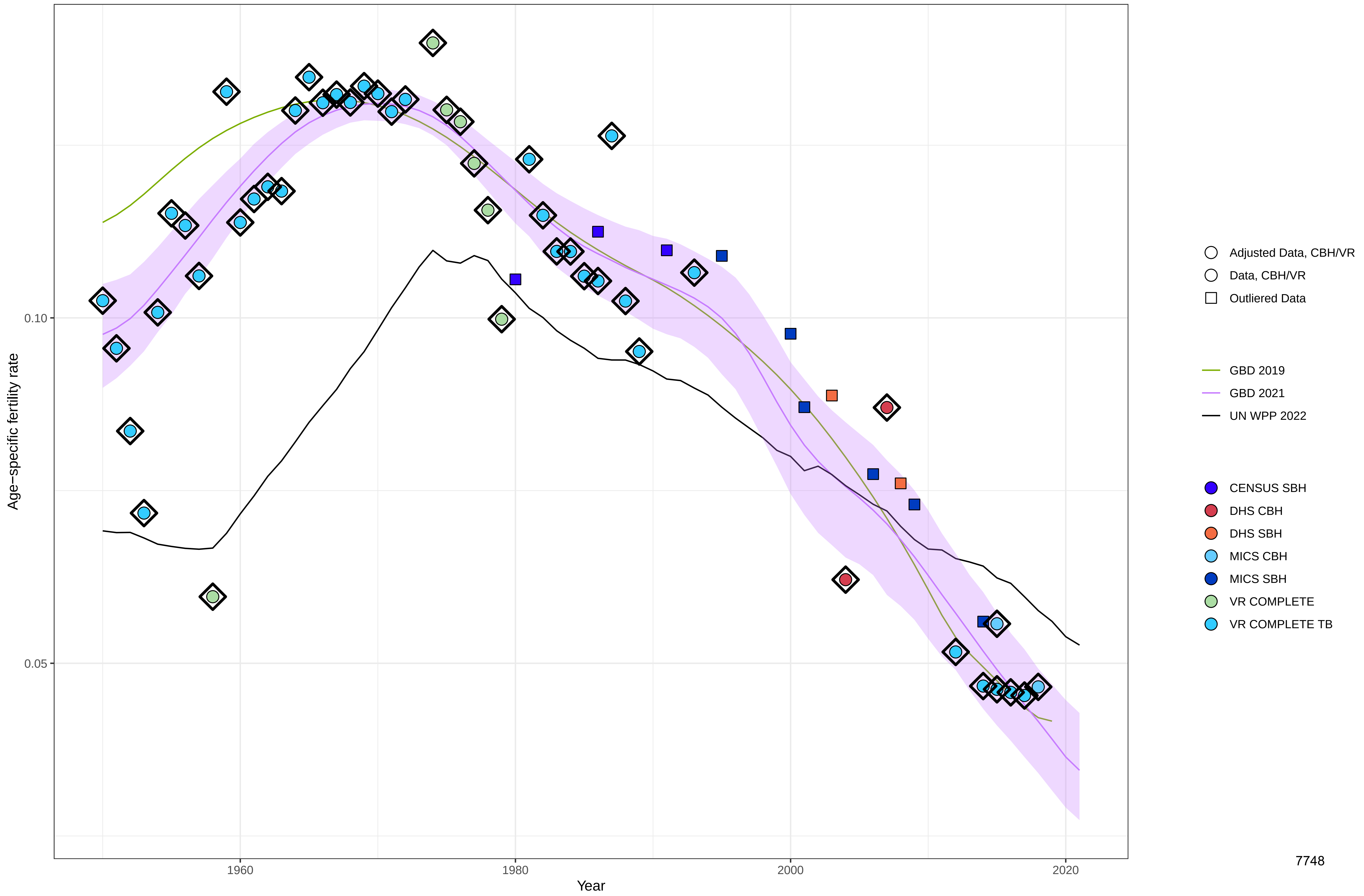

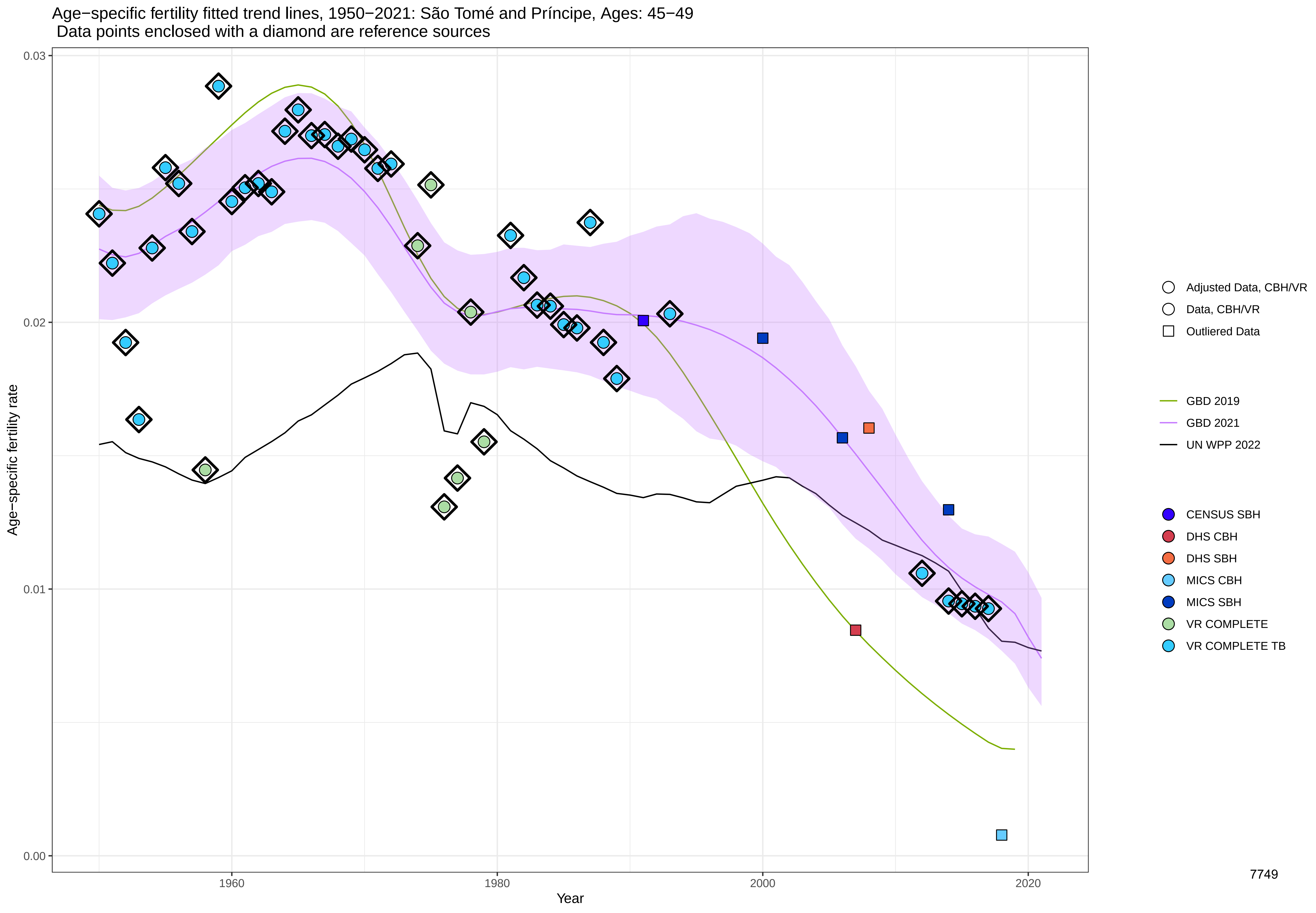

Age−specific fertility fitted trend lines, 1950−2021: São Tomé and Príncipe, Ages: 45−49
Data points enclosed with a diamond are reference sources
0.03
0.02
0.01
0.00
Age−specific fertility rate
1960
1980
2000
2020
Year
Adjusted Data, CBH/VR
Data, CBH/VR
Outliered Data
GBD 2019
GBD 2021
UN WPP 2022
CENSUS SBH
DHS CBH
DHS SBH
MICS CBH
MICS SBH
VR COMPLETE
VR COMPLETE TB

Age−specific fertility fitted trend lines, 1950−2021: São Tomé and Príncipe, Ages: 50−54
Data points enclosed with a diamond are reference sources

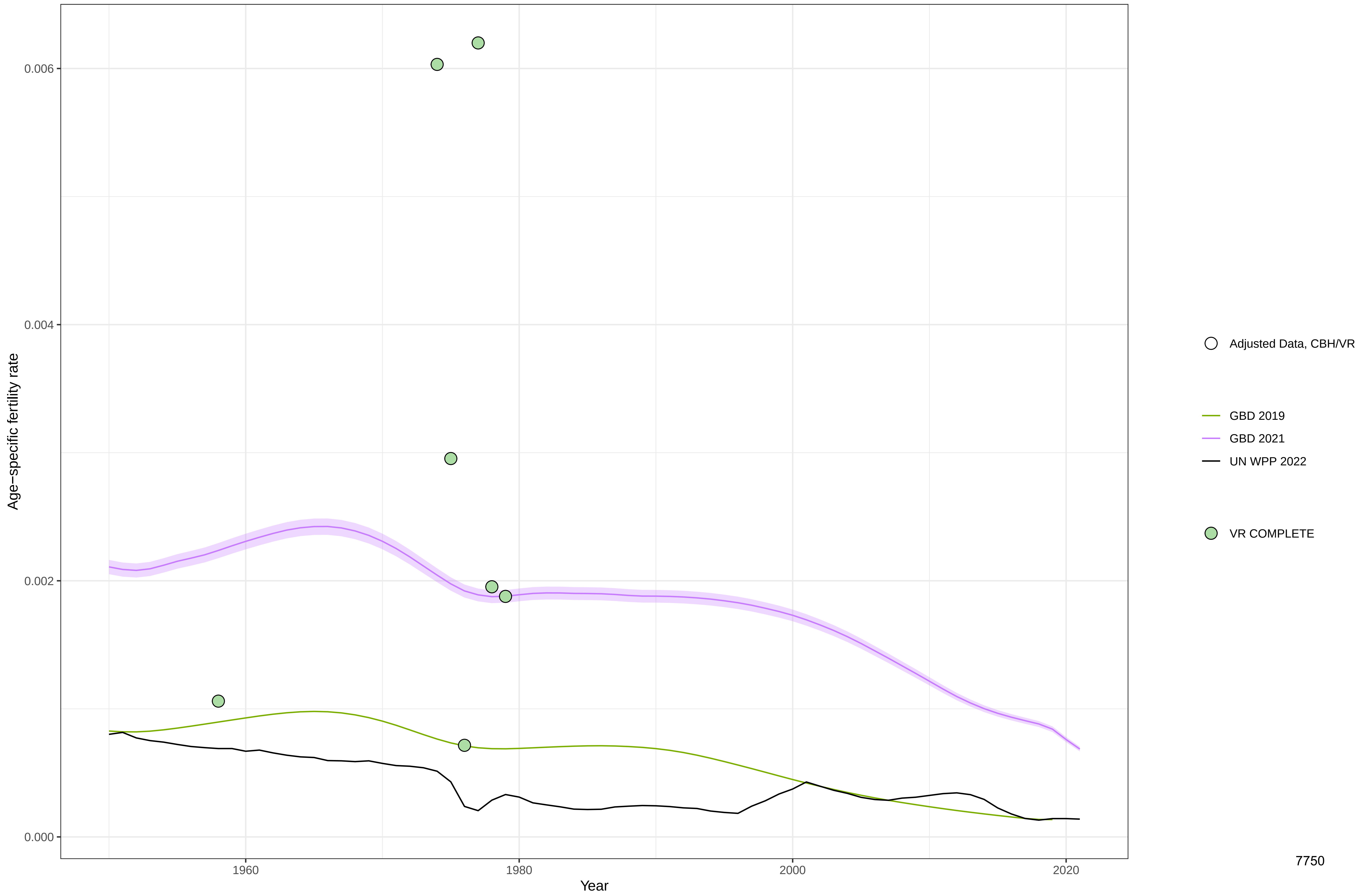

Total fertility rate fitted trend lines, 1950−2021: São Tomé and Príncipe TFR
Data points enclosed with a diamond are reference sources

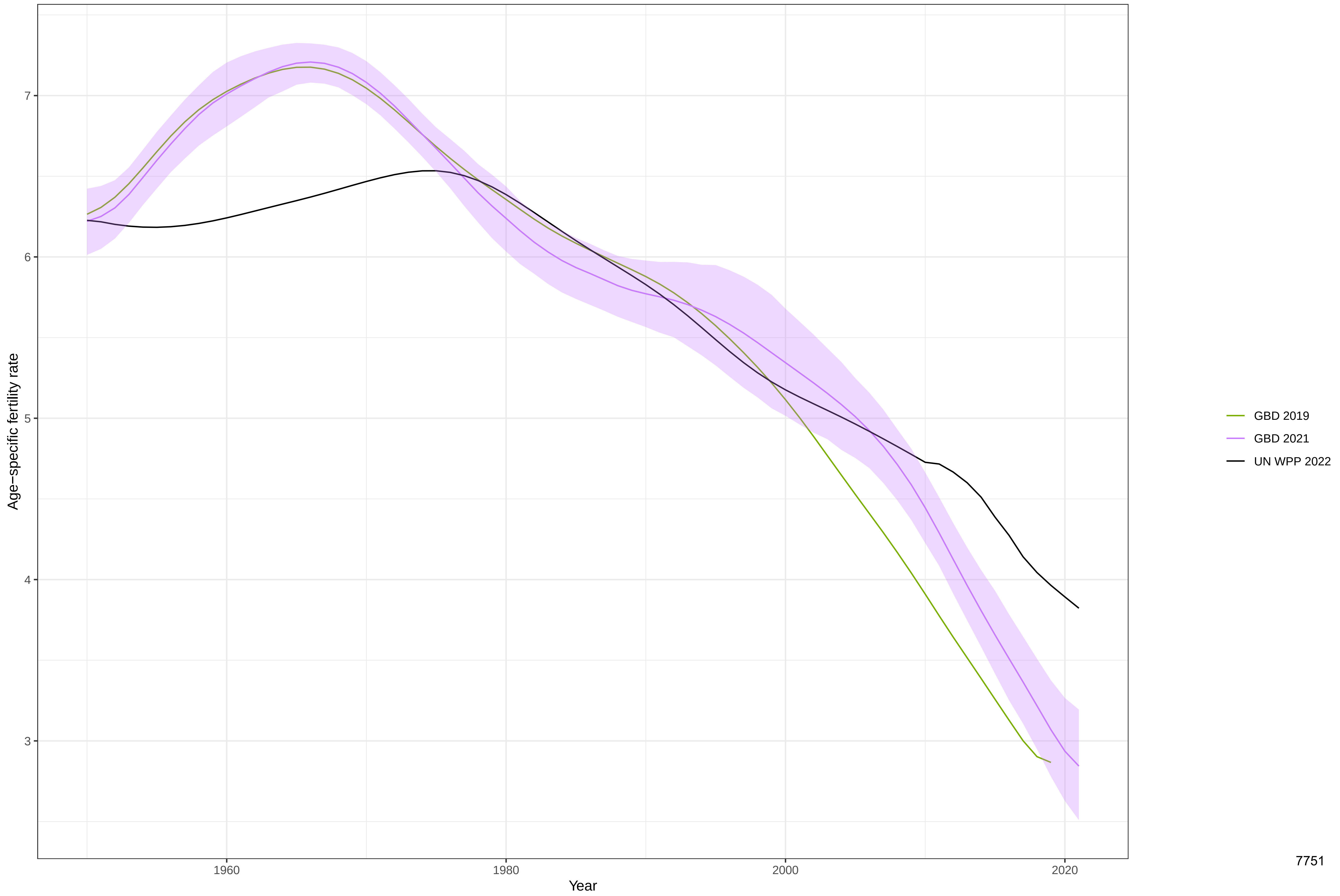

Age−specific fertility fitted trend lines, 1950−2021: Senegal, Ages: 10−14
Data points enclosed with a diamond are reference sources

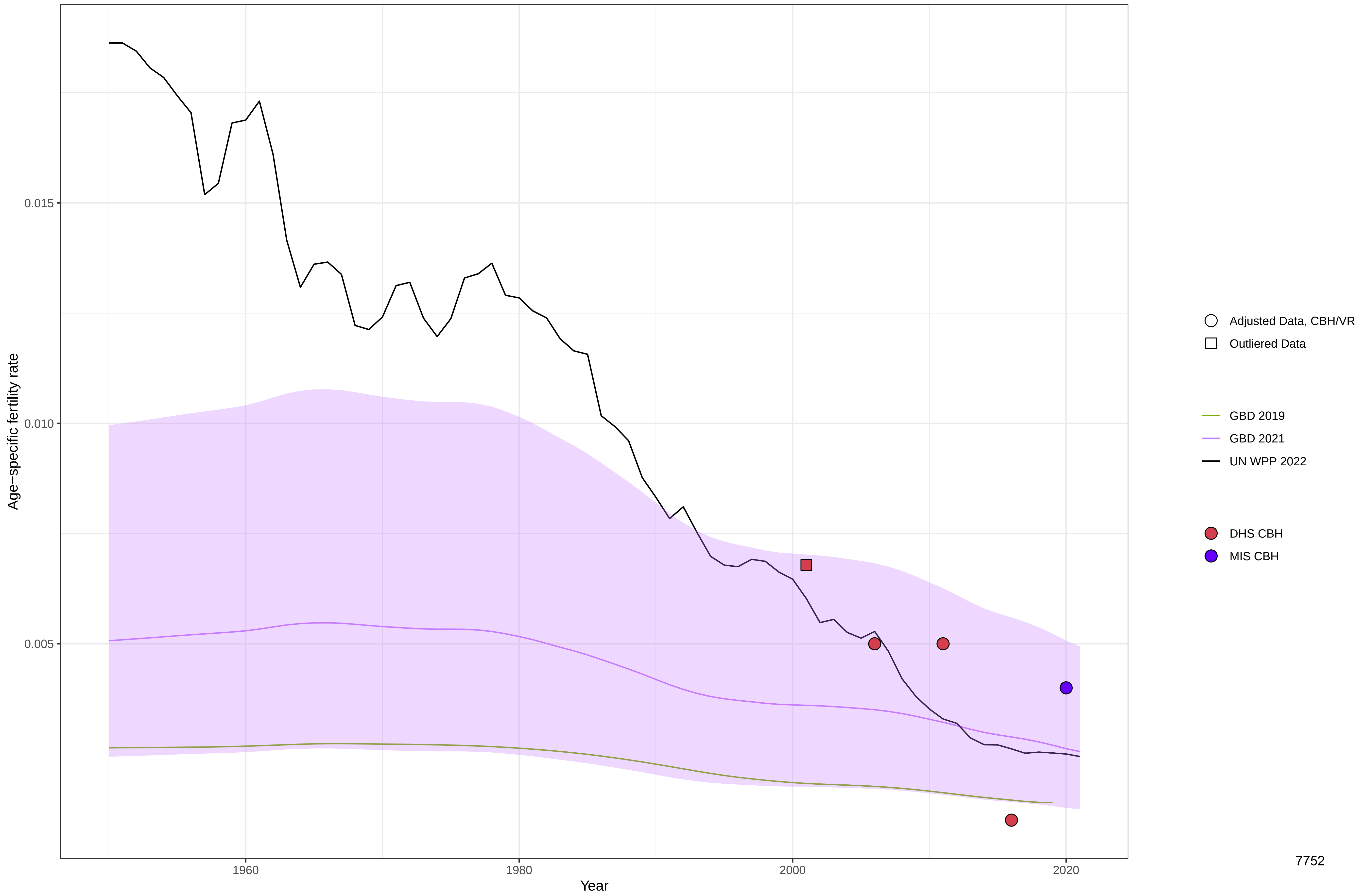

Age−specific fertility fitted trend lines, 1950−2021: Senegal, Ages: 15−19
Data points enclosed with a diamond are reference sources

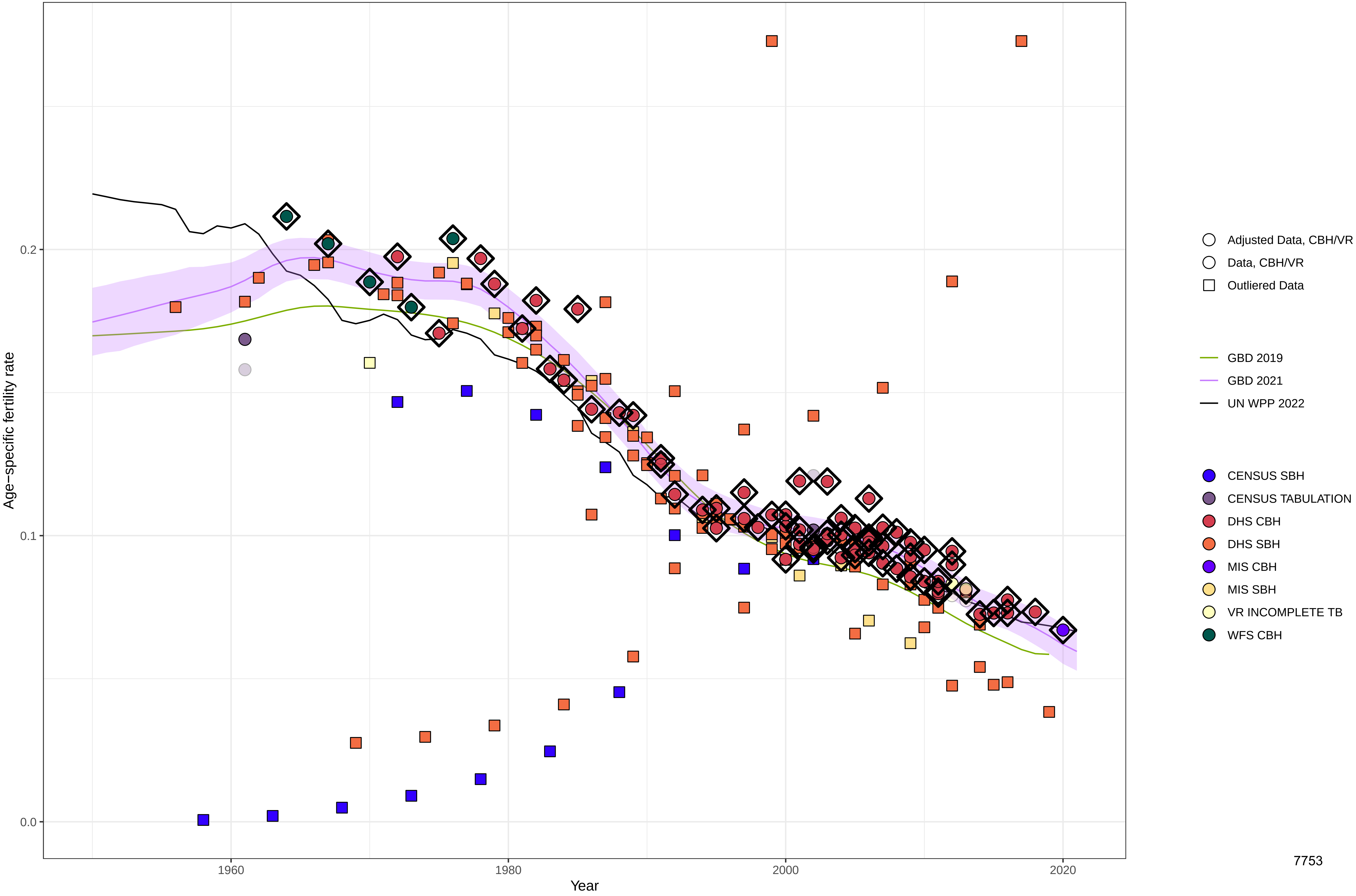

Age−specific fertility fitted trend lines, 1950−2021: Senegal, Ages: 20−24
Data points enclosed with a diamond are reference sources

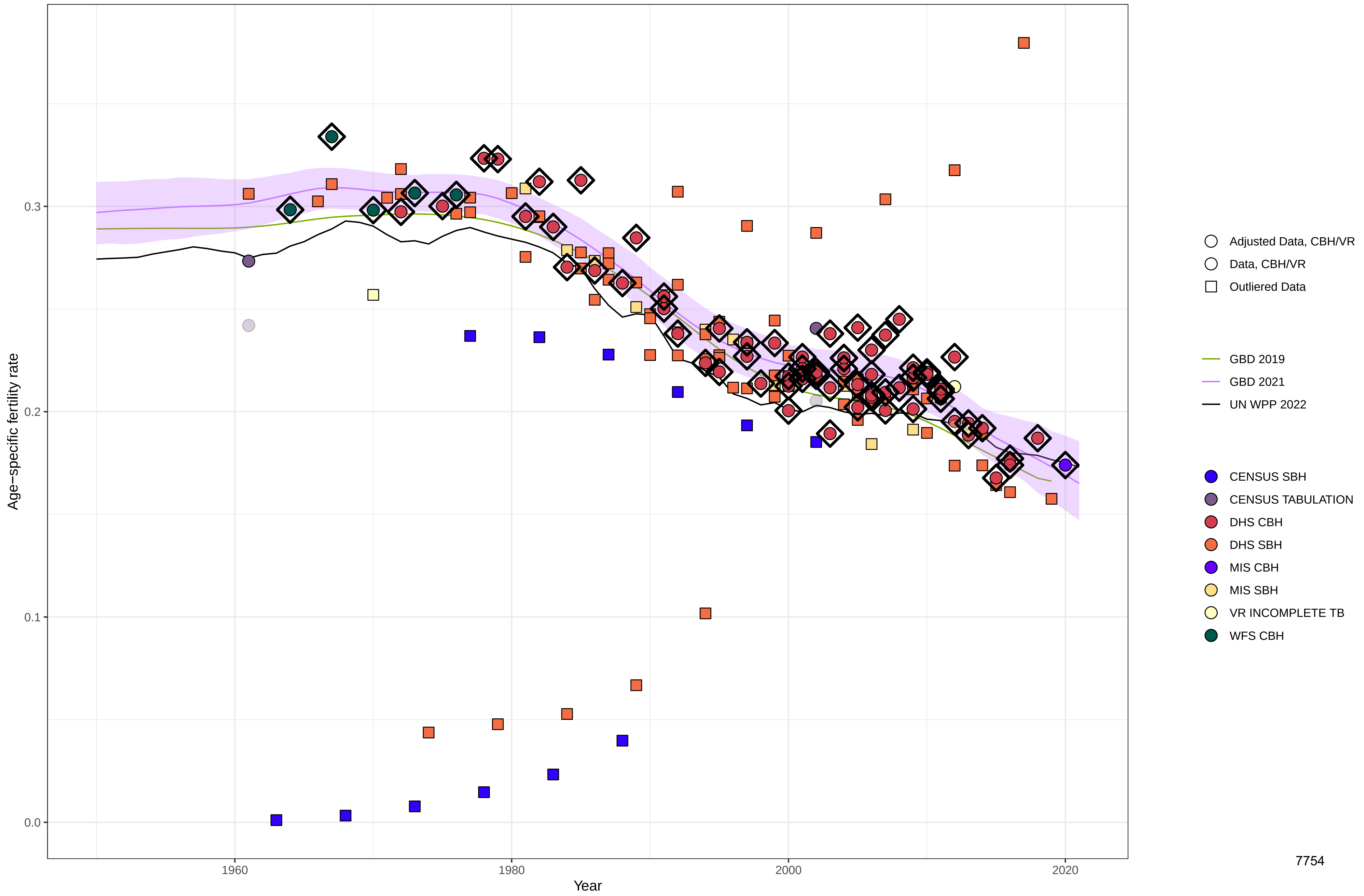

Age−specific fertility fitted trend lines, 1950−2021: Senegal, Ages: 25−29
Data points enclosed with a diamond are reference sources

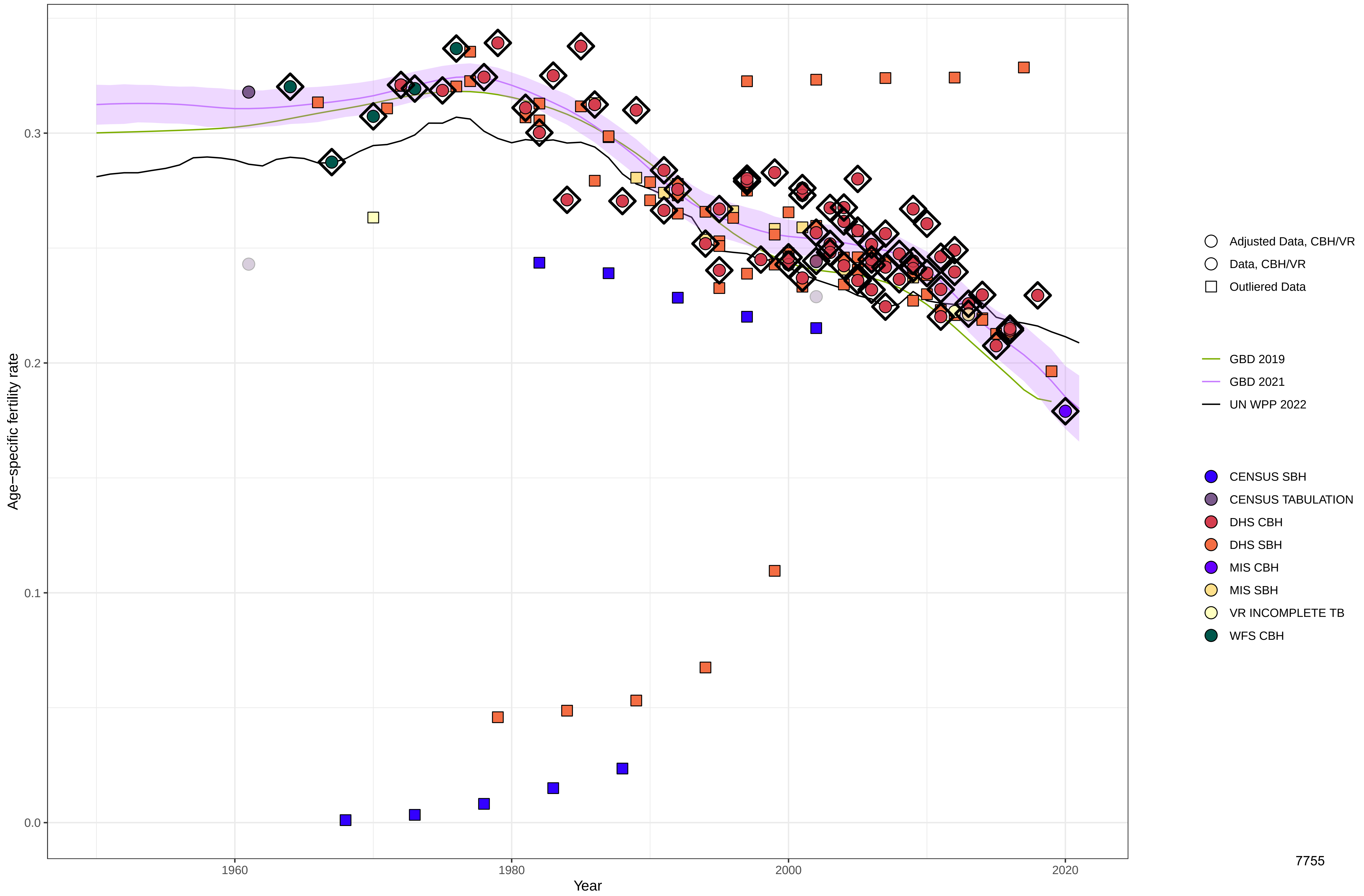

Age−specific fertility fitted trend lines, 1950−2021: Senegal, Ages: 30−34
Data points enclosed with a diamond are reference sources

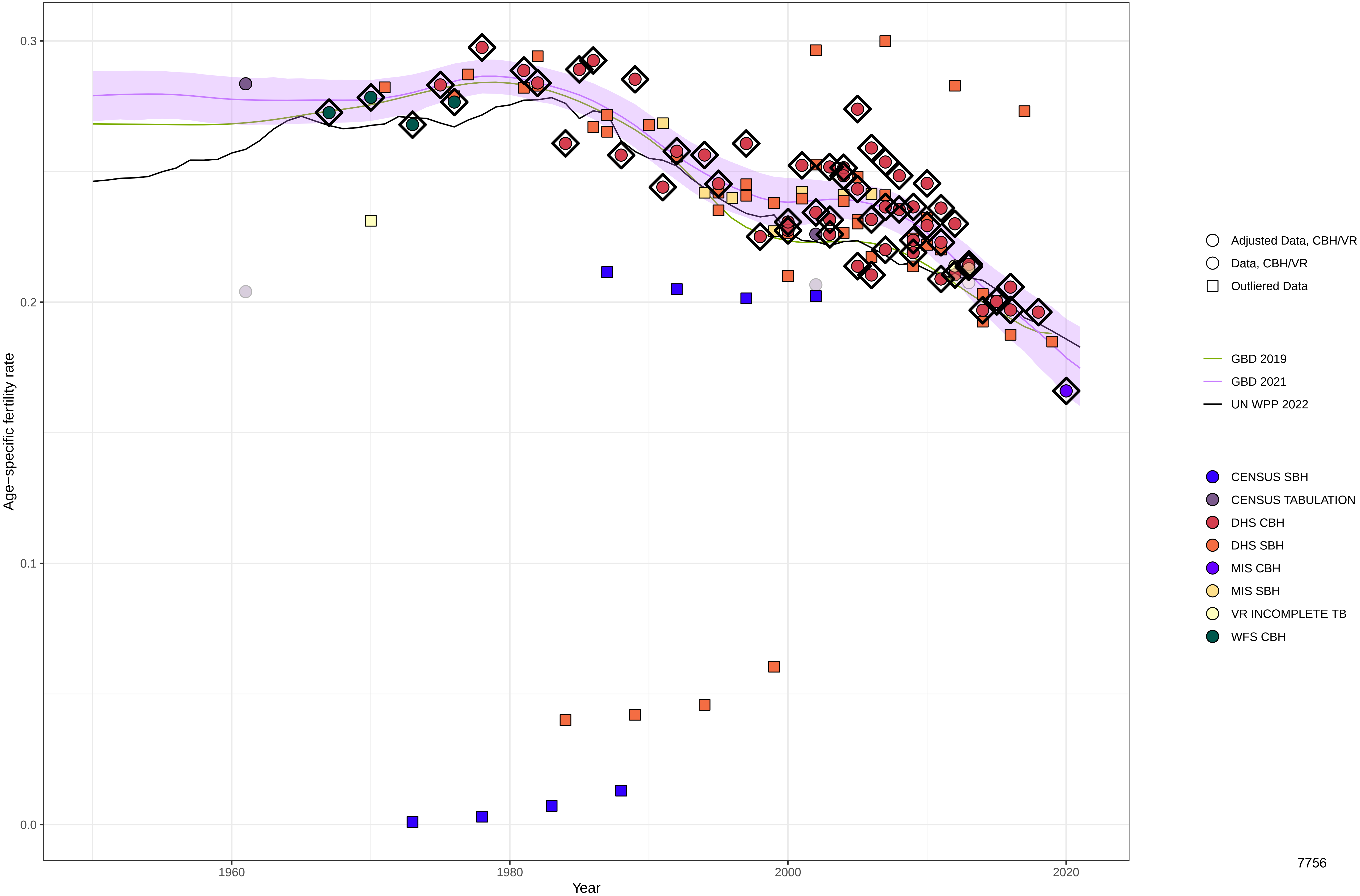

Age−specific fertility fitted trend lines, 1950−2021: Senegal, Ages: 35−39
Data points enclosed with a diamond are reference sources

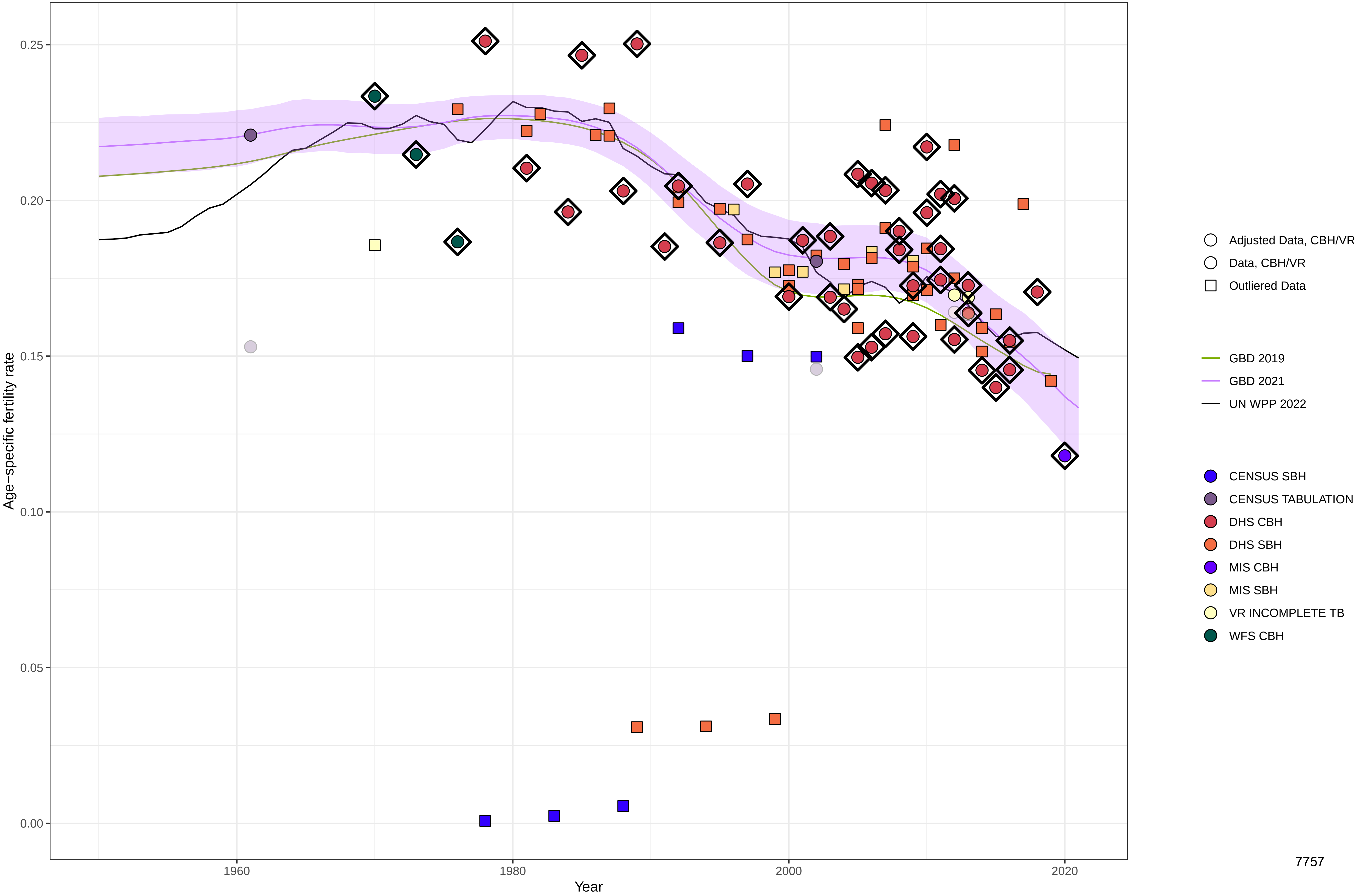

Age−specific fertility fitted trend lines, 1950−2021: Senegal, Ages: 40−44
Data points enclosed with a diamond are reference sources

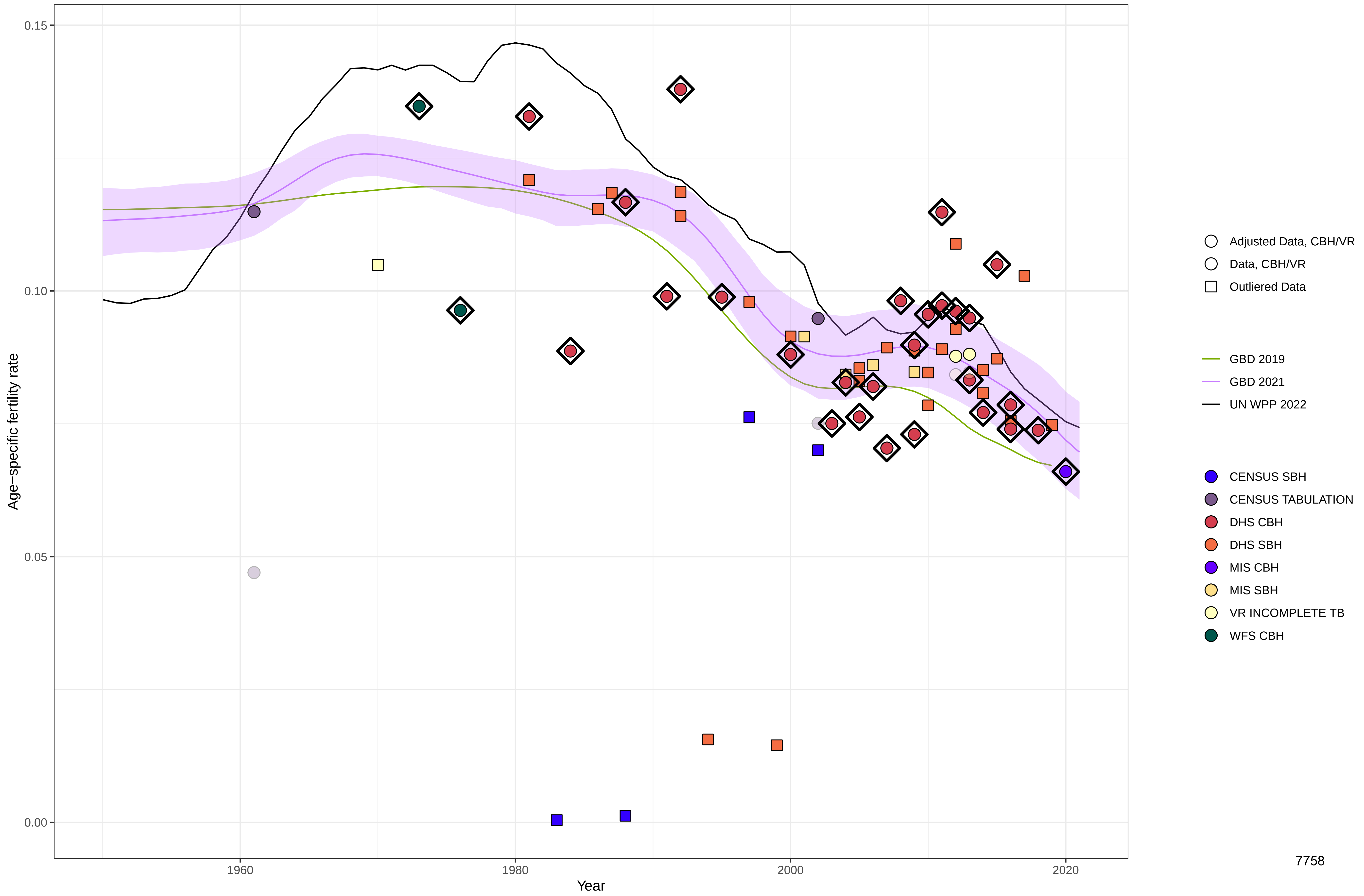

Age−specific fertility fitted trend lines, 1950−2021: Senegal, Ages: 45−49
Data points enclosed with a diamond are reference sources

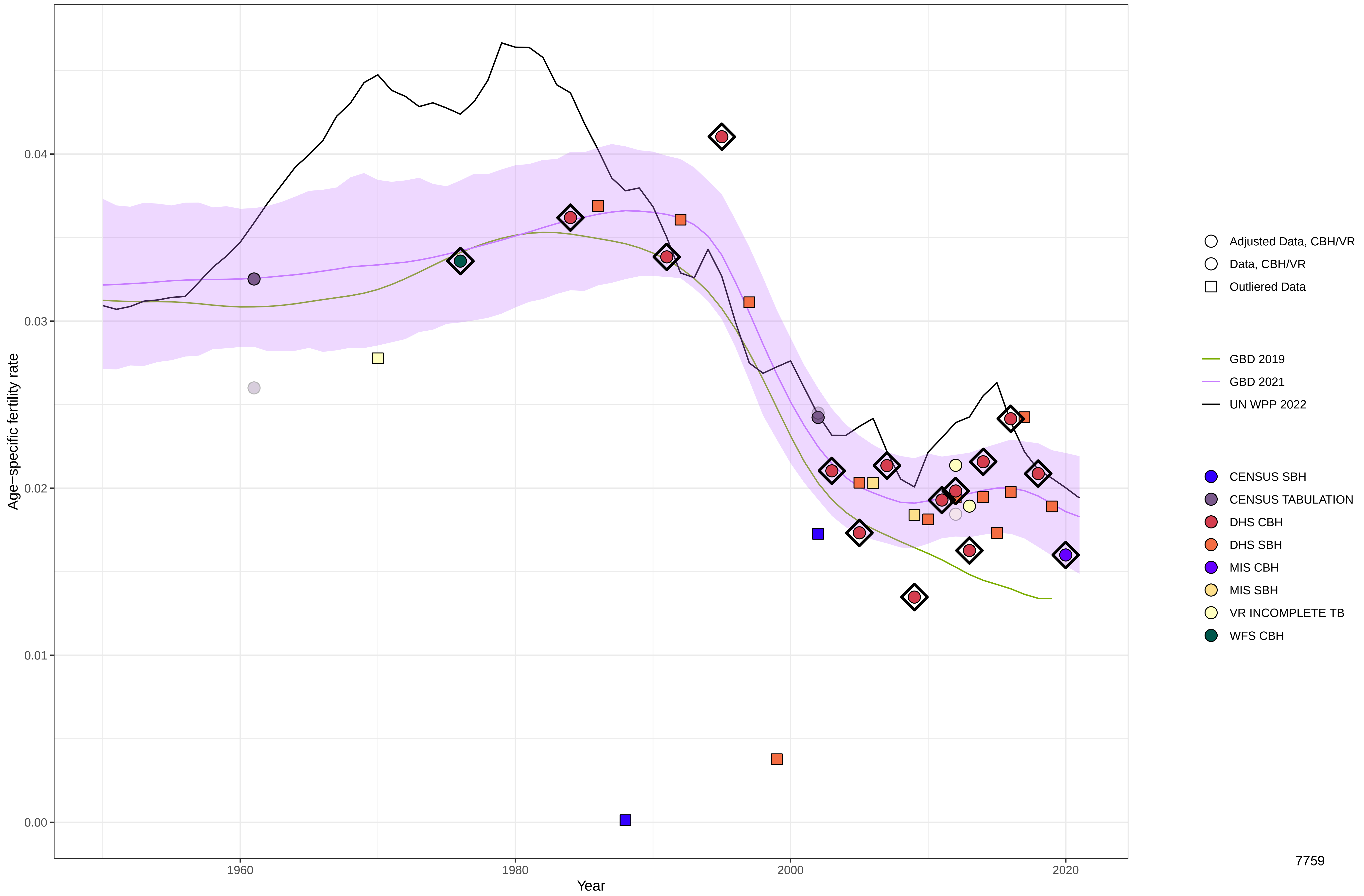

Age−specific fertility fitted trend lines, 1950−2021: Senegal, Ages: 50−54
Data points enclosed with a diamond are reference sources

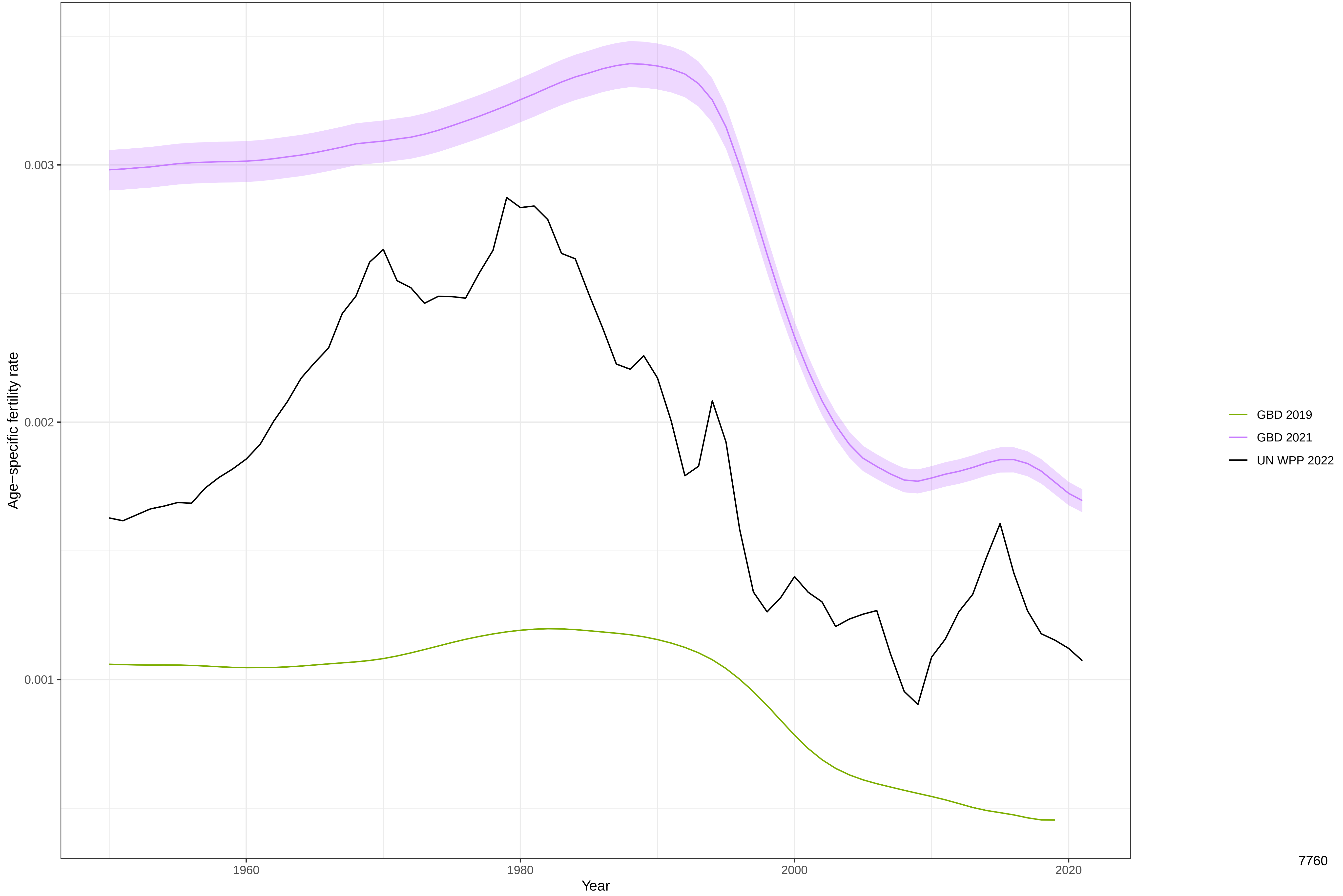

Total fertility rate fitted trend lines, 1950−2021: Senegal TFR
Data points enclosed with a diamond are reference sources

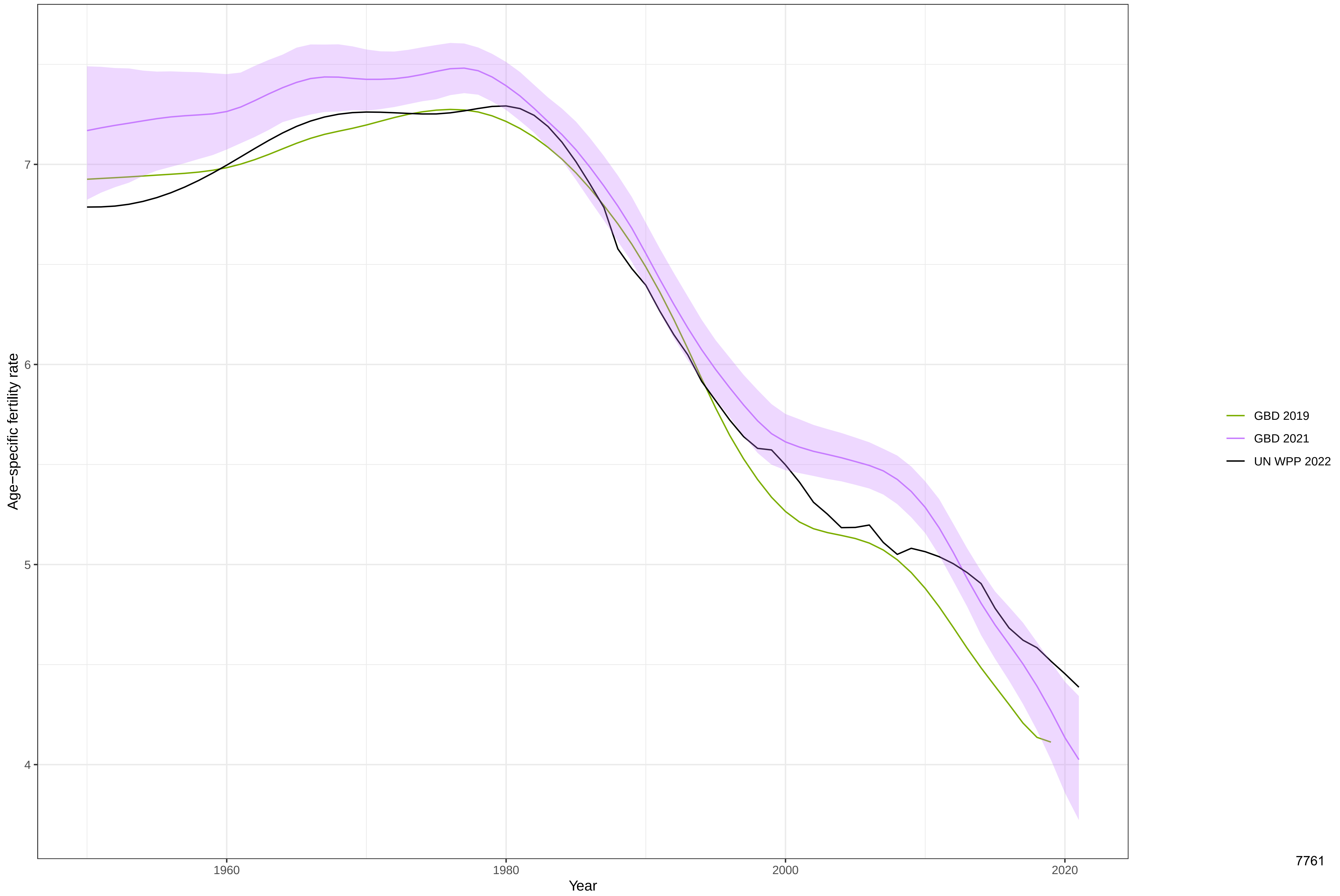

Age−specific fertility fitted trend lines, 1950−2021: Sierra Leone, Ages: 10−14
Data points enclosed with a diamond are reference sources

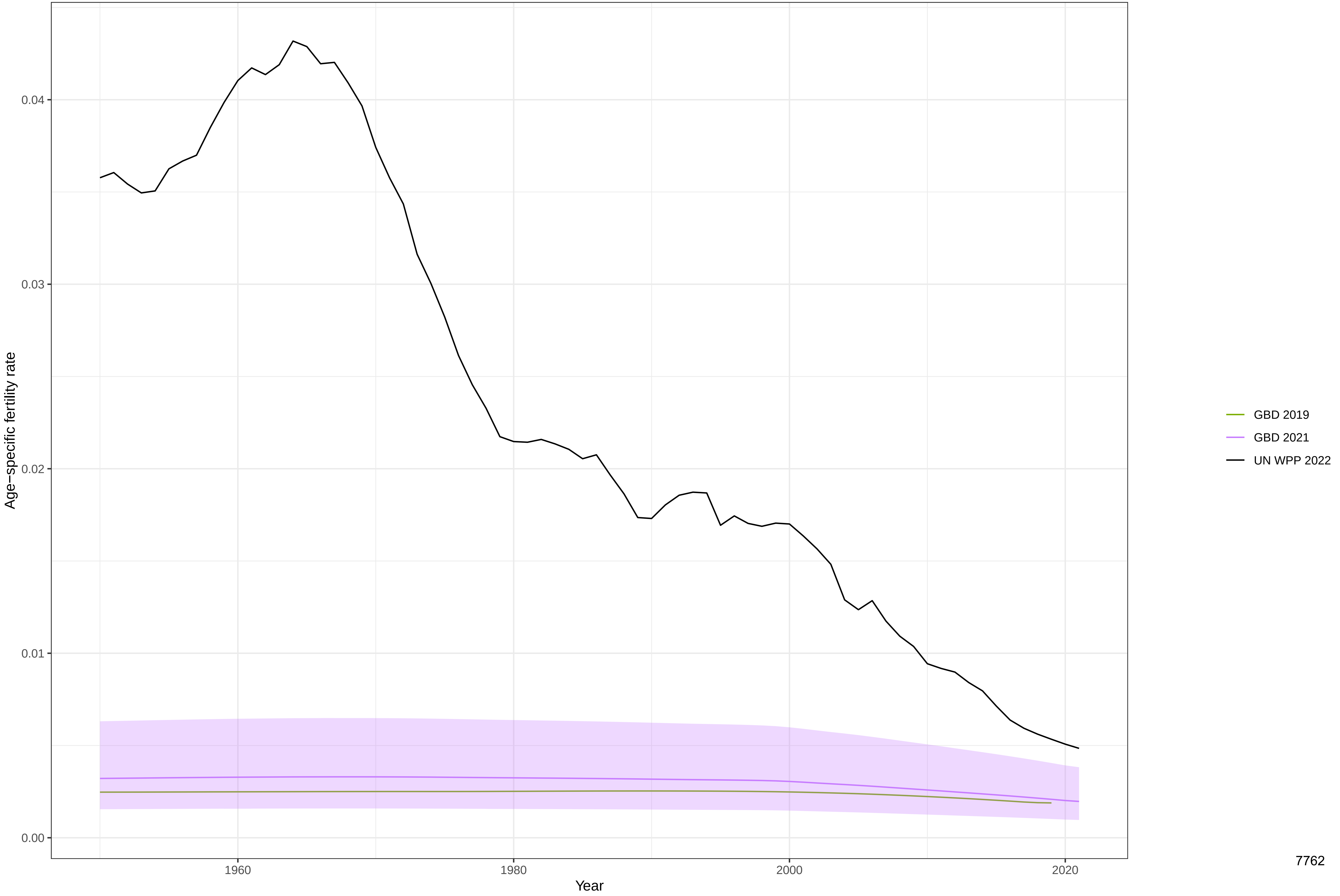

Age−specific fertility fitted trend lines, 1950−2021: Sierra Leone, Ages: 15−19
Data points enclosed with a diamond are reference sources

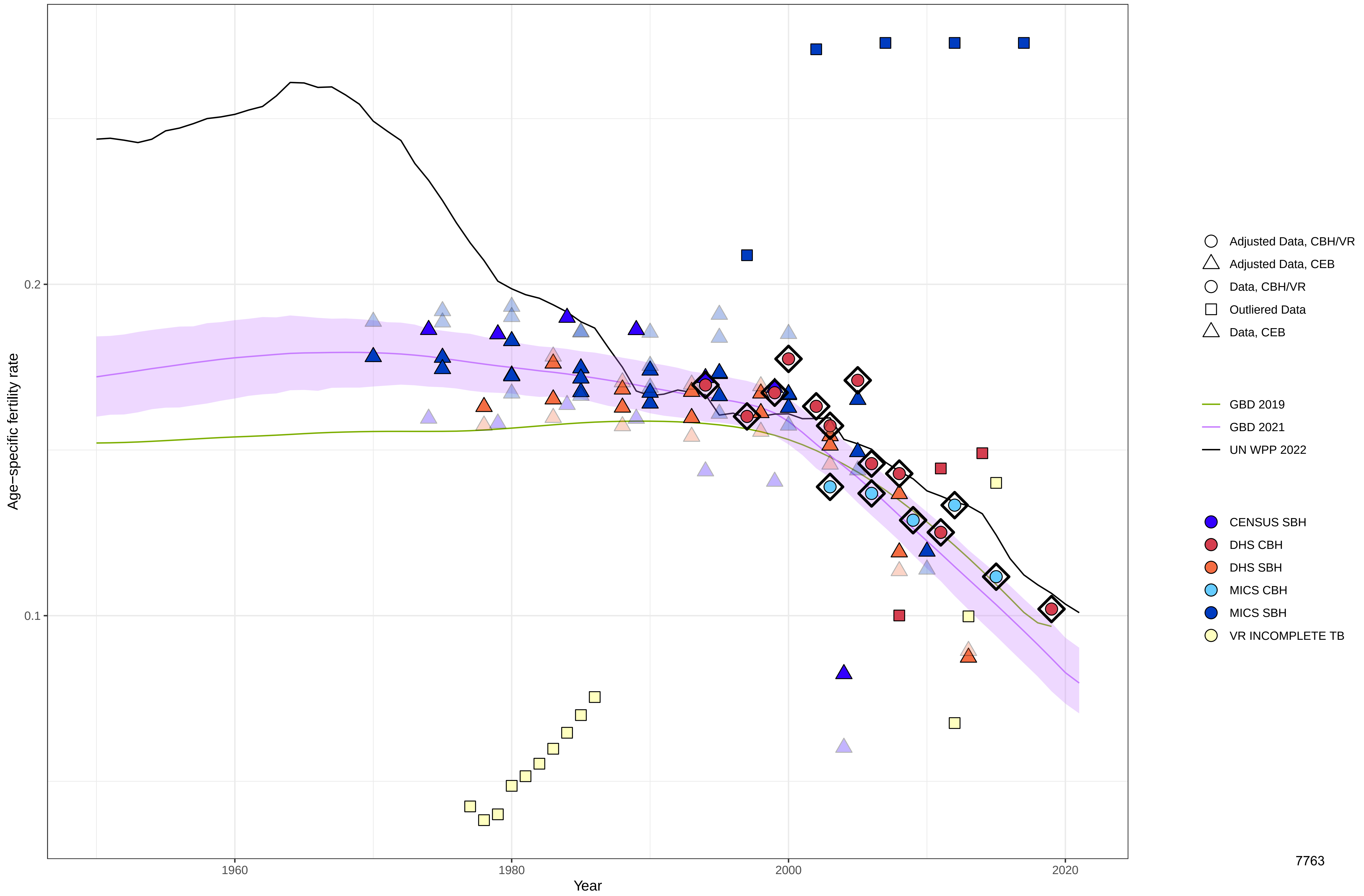

Age−specific fertility fitted trend lines, 1950−2021: Sierra Leone, Ages: 20−24
Data points enclosed with a diamond are reference sources

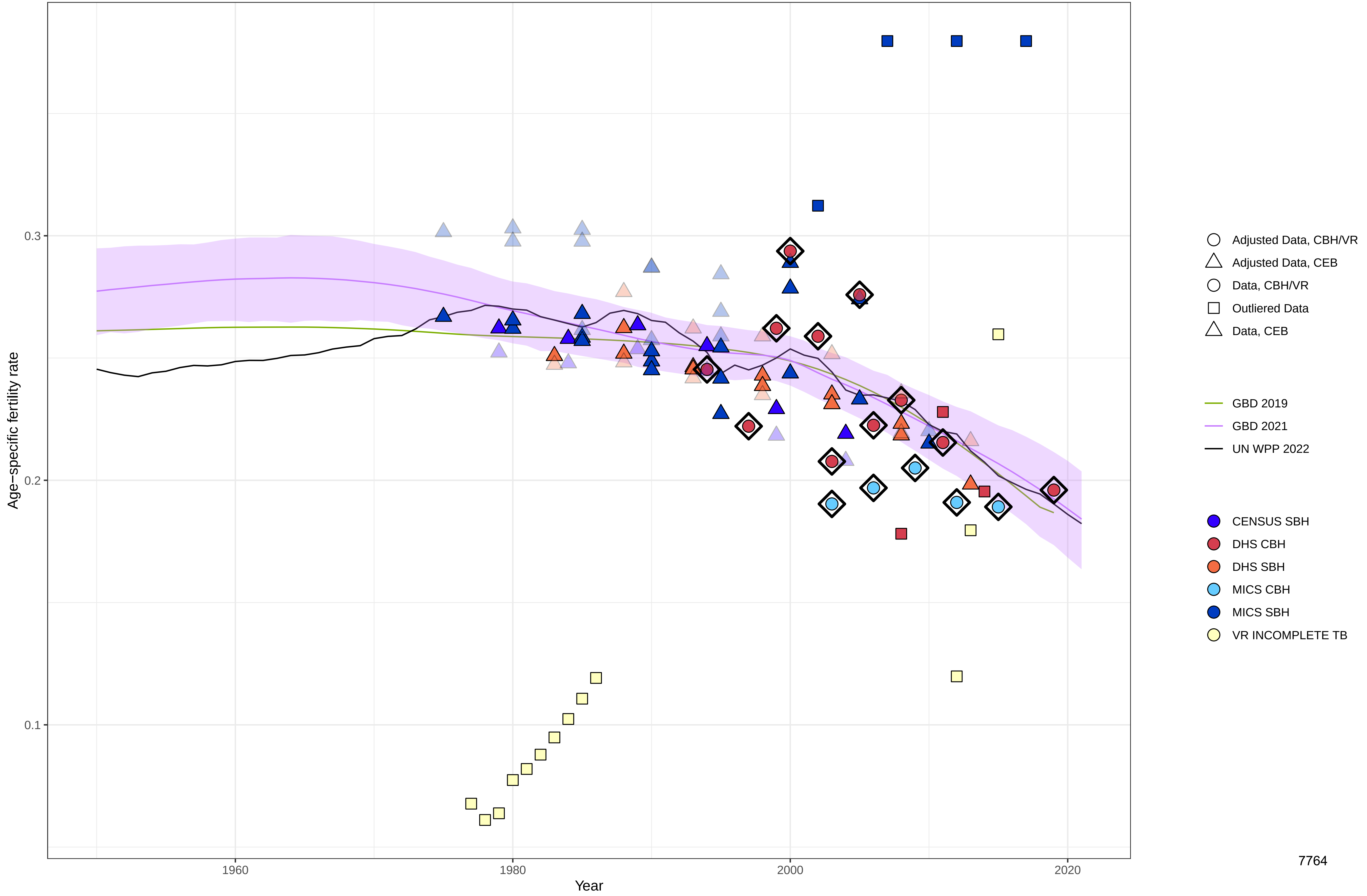

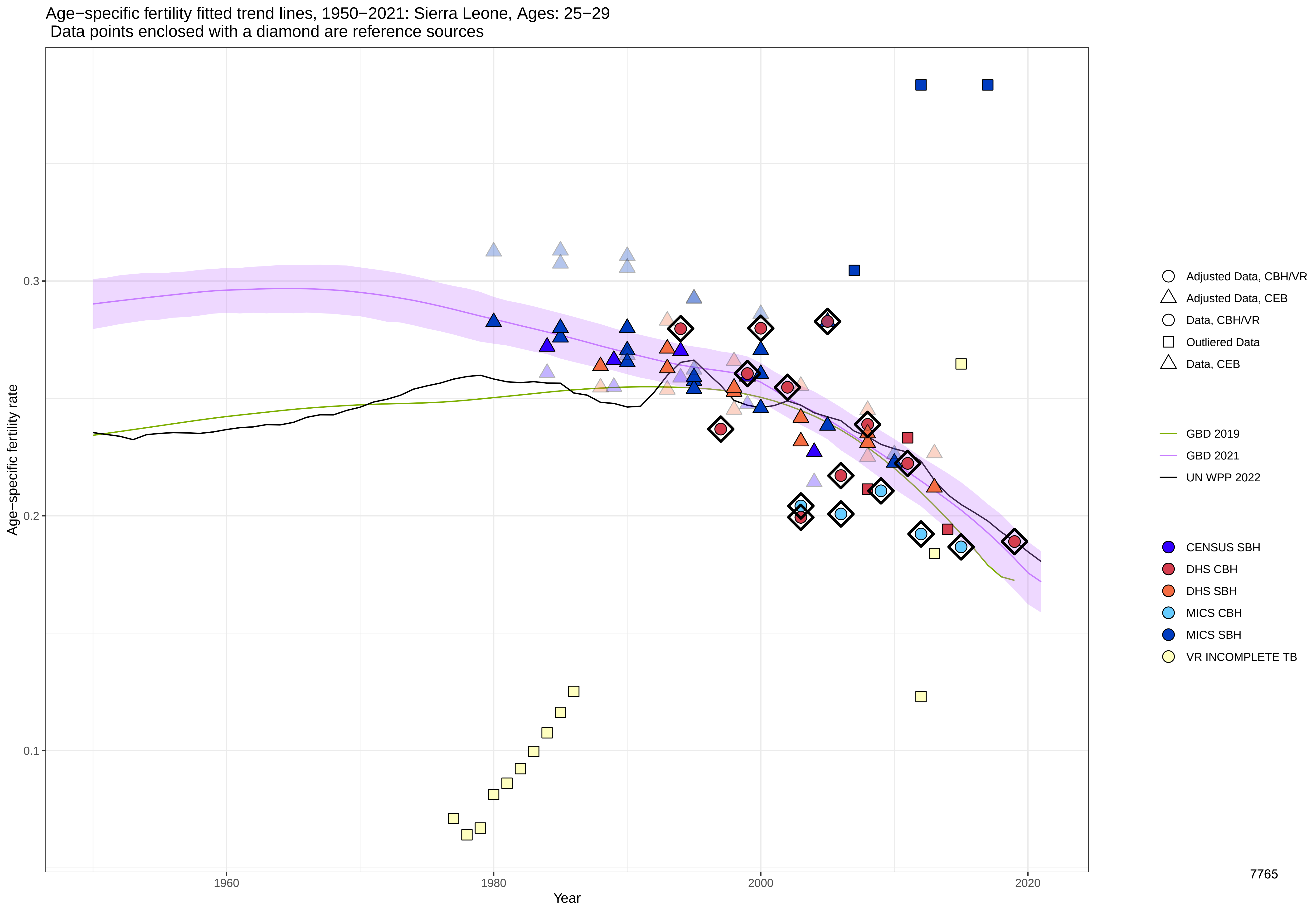

Age−specific fertility fitted trend lines, 1950−2021: Sierra Leone, Ages: 25−29
Data points enclosed with a diamond are reference sources
Age−specific fertility rate
0.1
0.2
0.3
Year
1960
1980
2000
2020
Adjusted Data, CBH/VR
Adjusted Data, CEB
Data, CBH/VR
Outliered Data
Data, CEB
GBD 2019
GBD 2021
UN WPP 2022
CENSUS SBH
DHS CBH
DHS SBH
MICS CBH
MICS SBH
VR INCOMPLETE TB

Age−specific fertility fitted trend lines, 1950−2021: Sierra Leone, Ages: 30−34
Data points enclosed with a diamond are reference sources

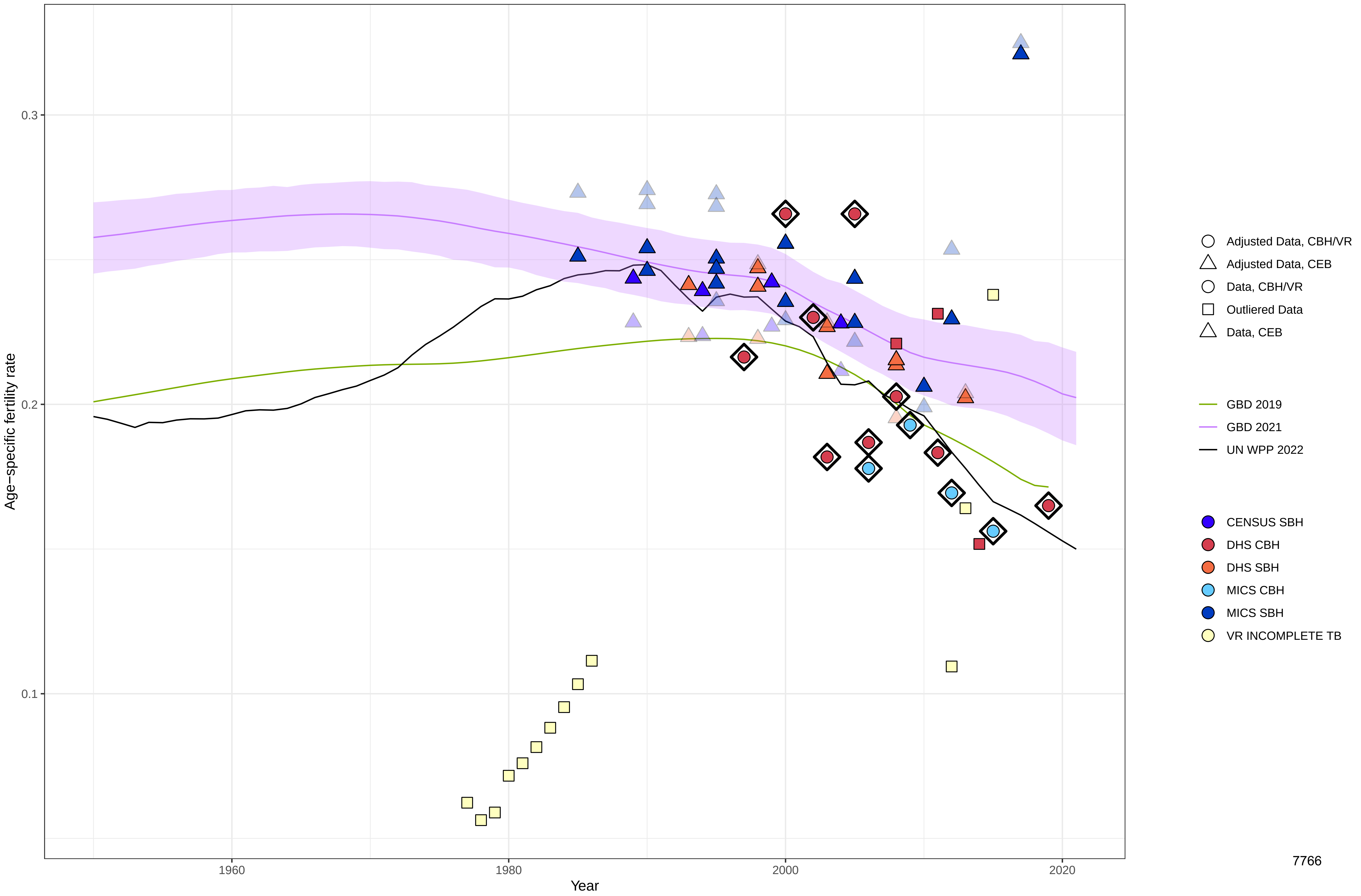

Age−specific fertility fitted trend lines, 1950−2021: Sierra Leone, Ages: 35−39
Data points enclosed with a diamond are reference sources

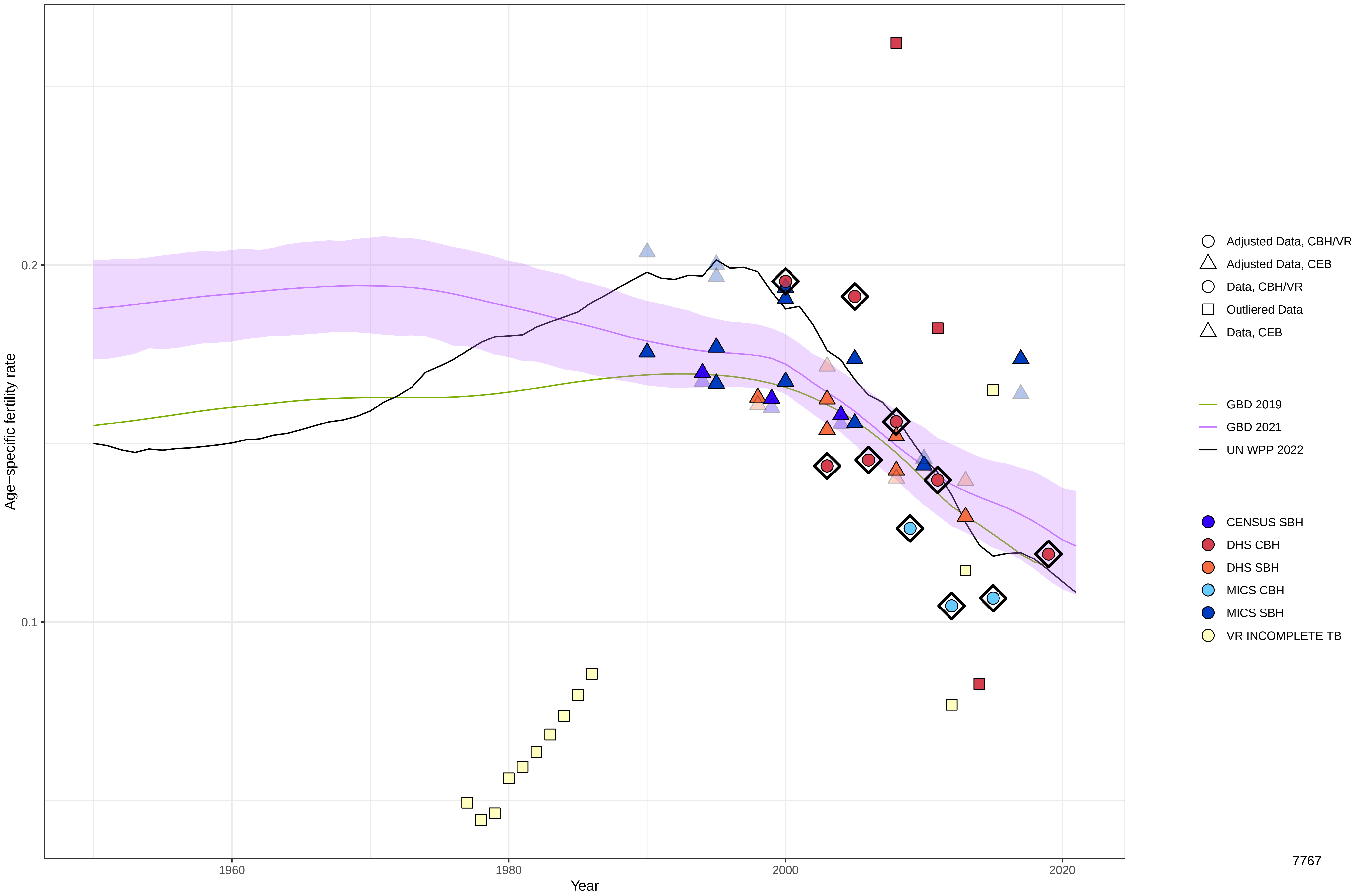

Age−specific fertility fitted trend lines, 1950−2021: Sierra Leone, Ages: 40−44
Data points enclosed with a diamond are reference sources

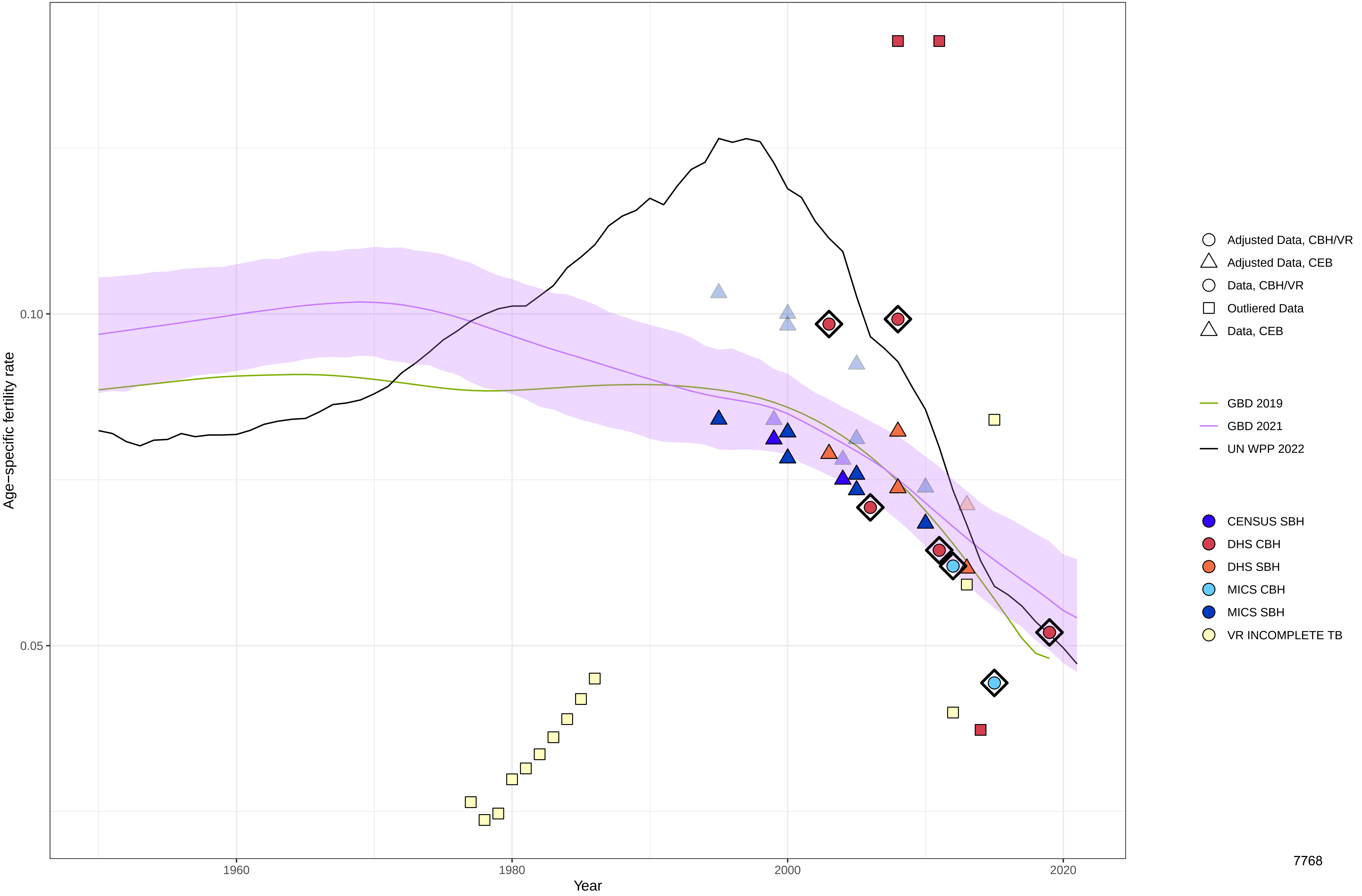

Age−specific fertility fitted trend lines, 1950−2021: Sierra Leone, Ages: 45−49
Data points enclosed with a diamond are reference sources

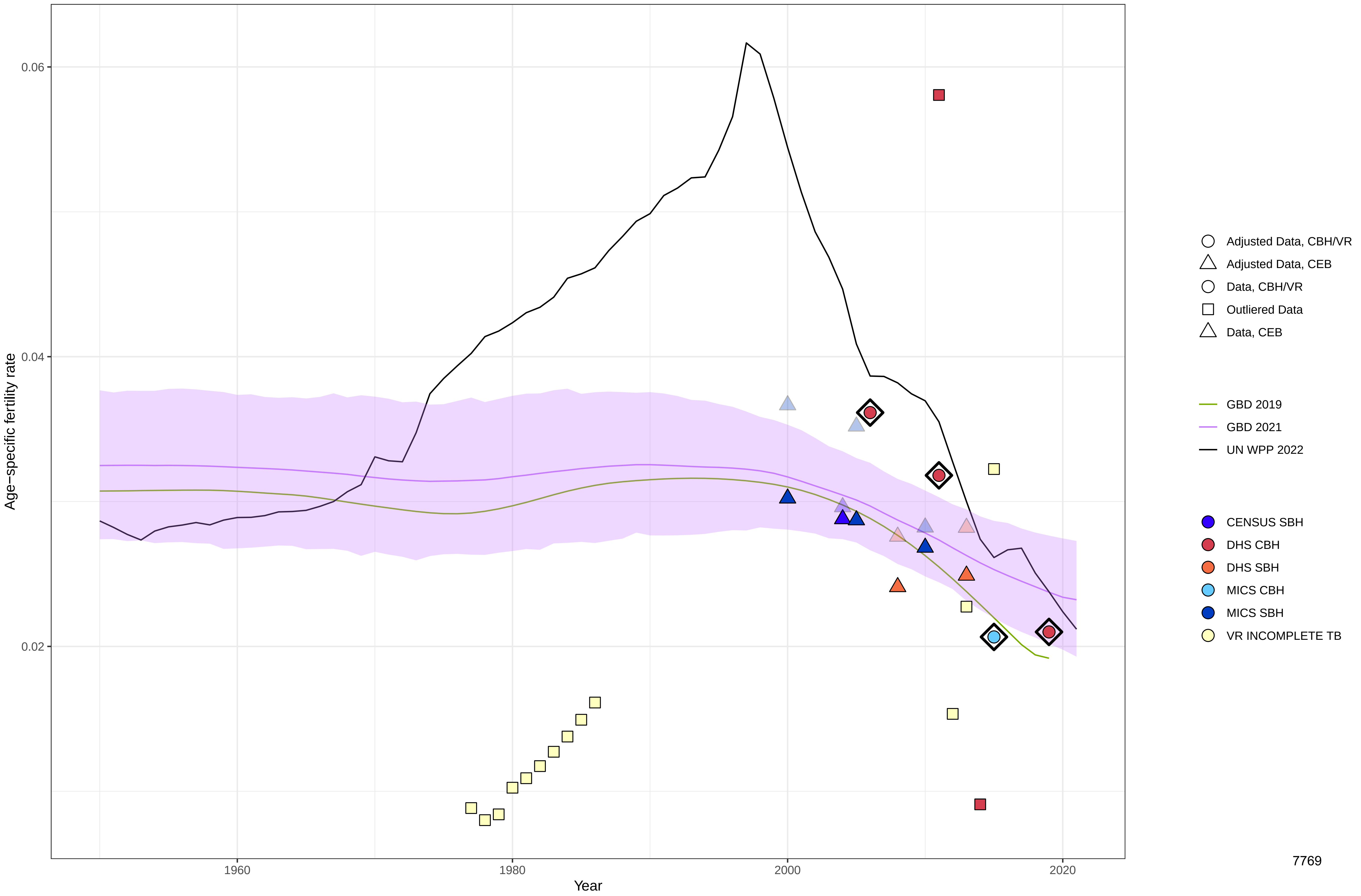

Age−specific fertility fitted trend lines, 1950−2021: Sierra Leone, Ages: 50−54
Data points enclosed with a diamond are reference sources

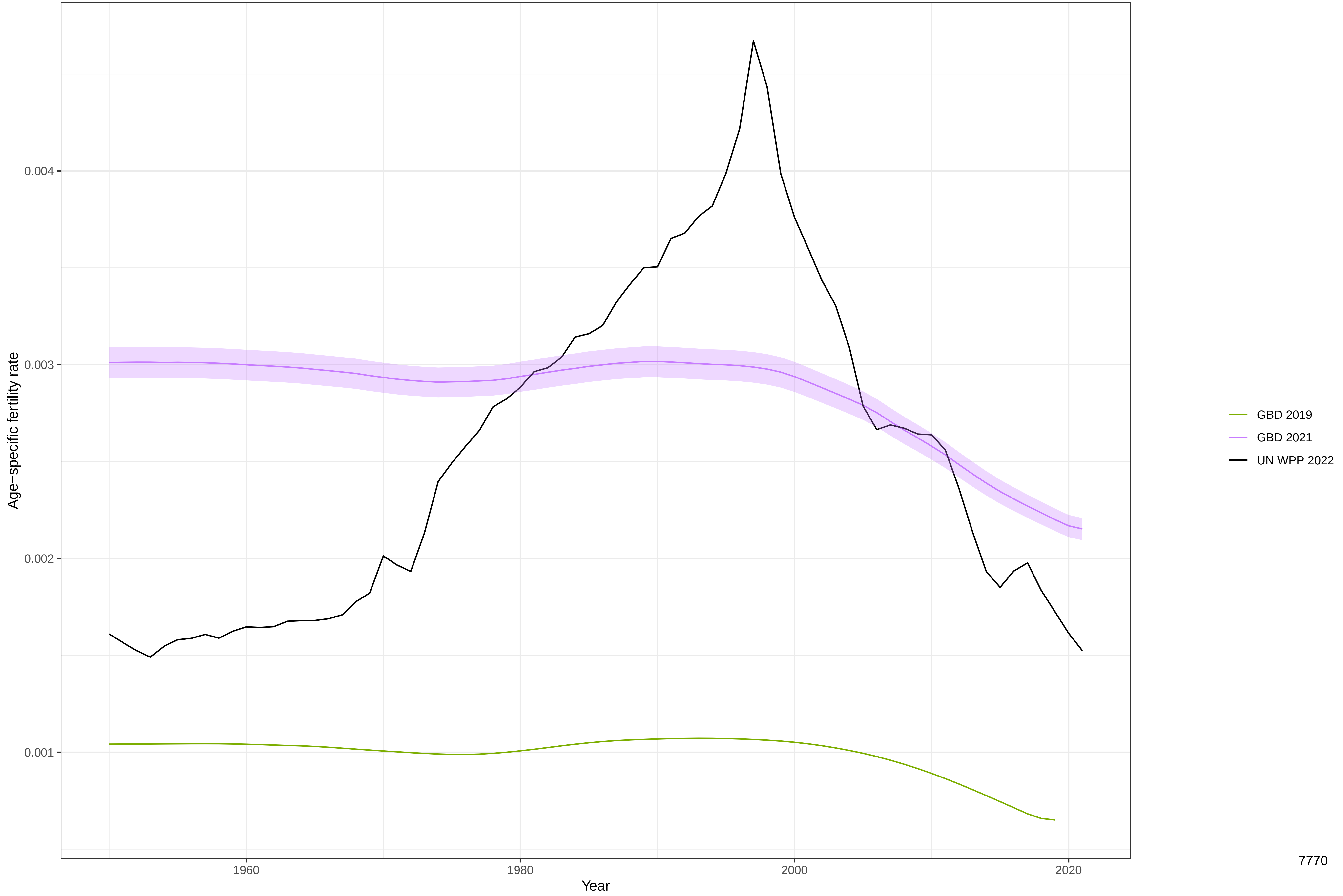

Total fertility rate fitted trend lines, 1950−2021: Sierra Leone TFR
Data points enclosed with a diamond are reference sources

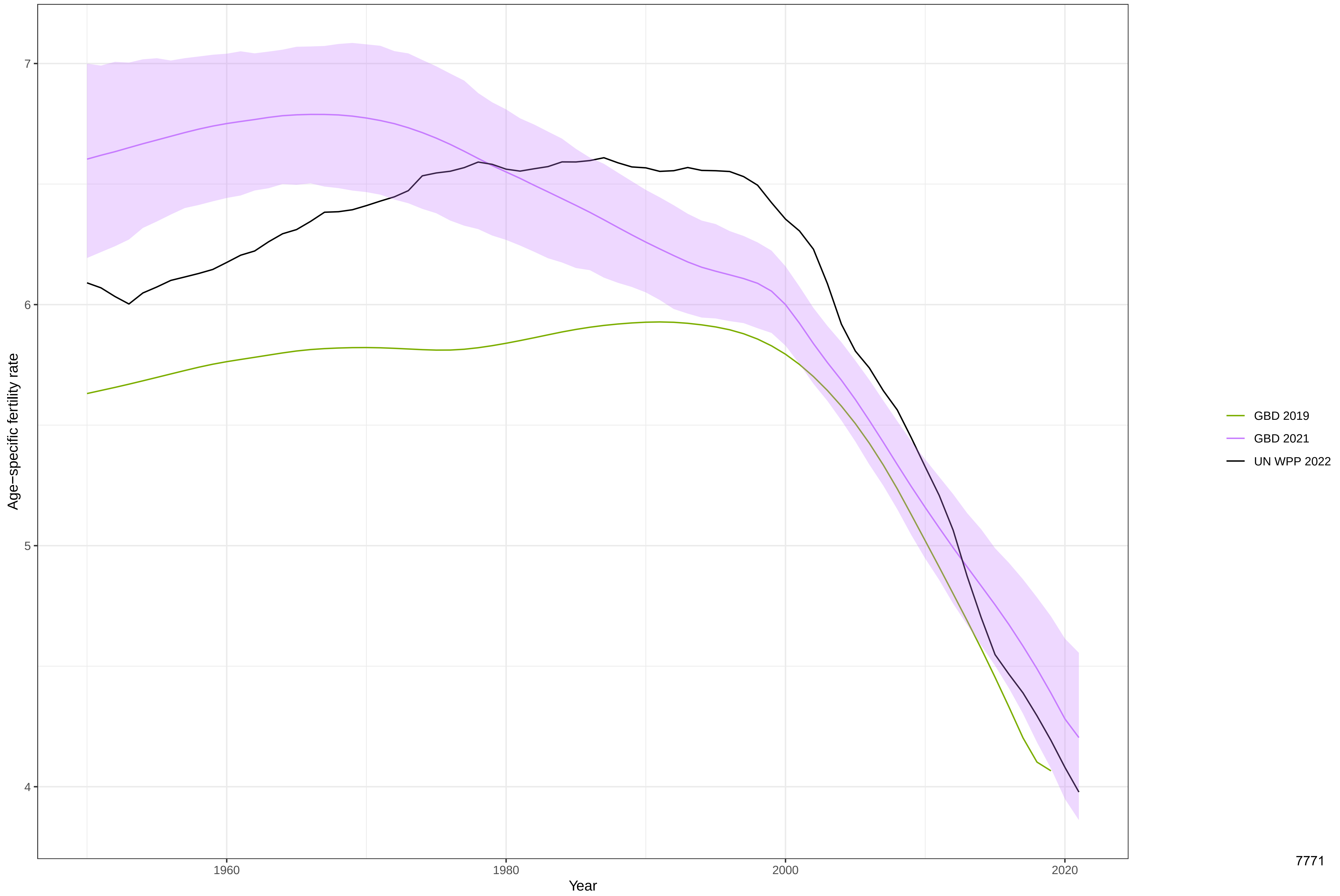

Age−specific fertility fitted trend lines, 1950−2021: Togo, Ages: 10−14
Data points enclosed with a diamond are reference sources

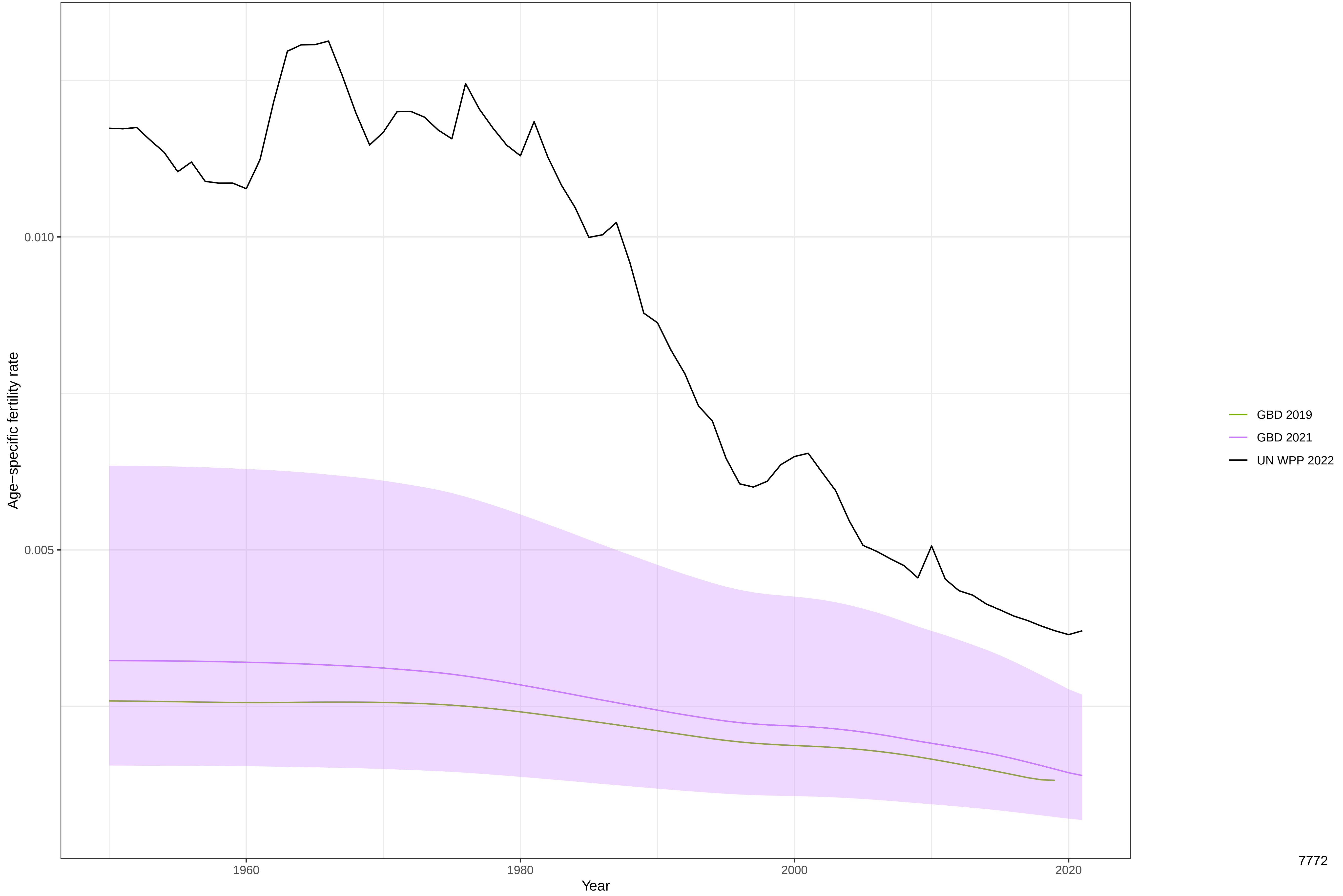

Age−specific fertility fitted trend lines, 1950−2021: Togo, Ages: 15−19
Data points enclosed with a diamond are reference sources

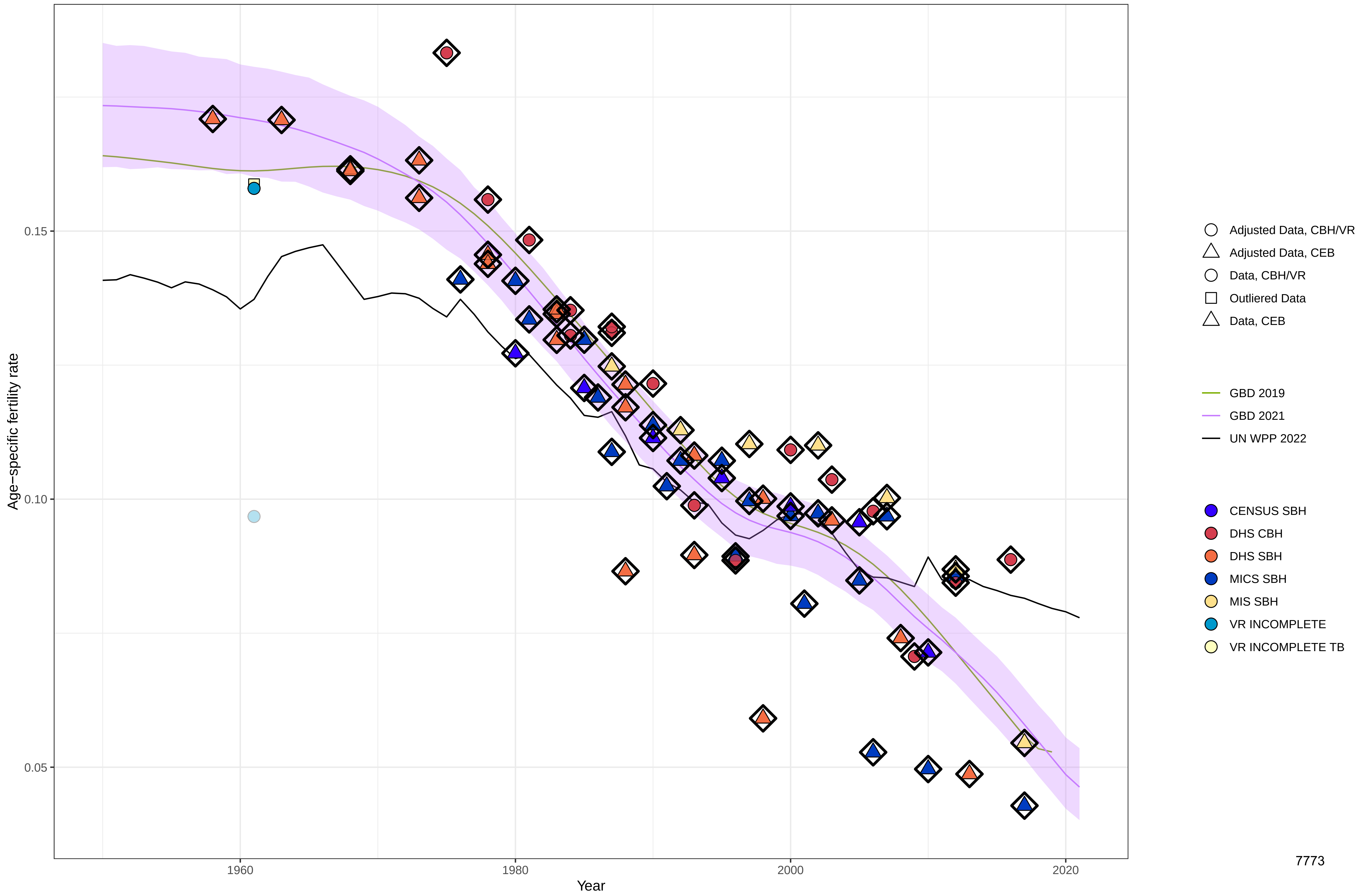

Age−specific fertility fitted trend lines, 1950−2021: Togo, Ages: 20−24
Data points enclosed with a diamond are reference sources

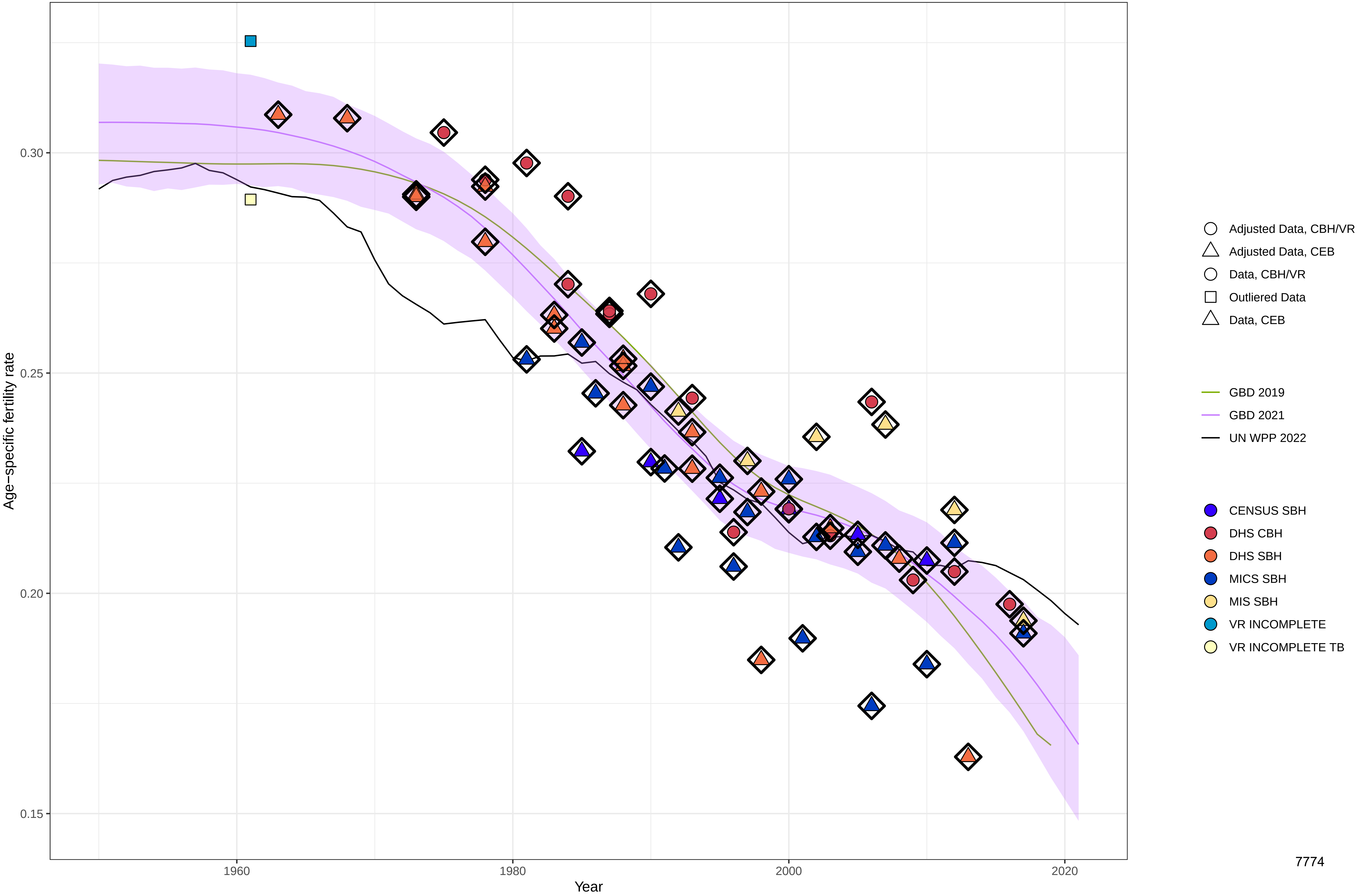

Age−specific fertility fitted trend lines, 1950−2021: Togo, Ages: 25−29
Data points enclosed with a diamond are reference sources

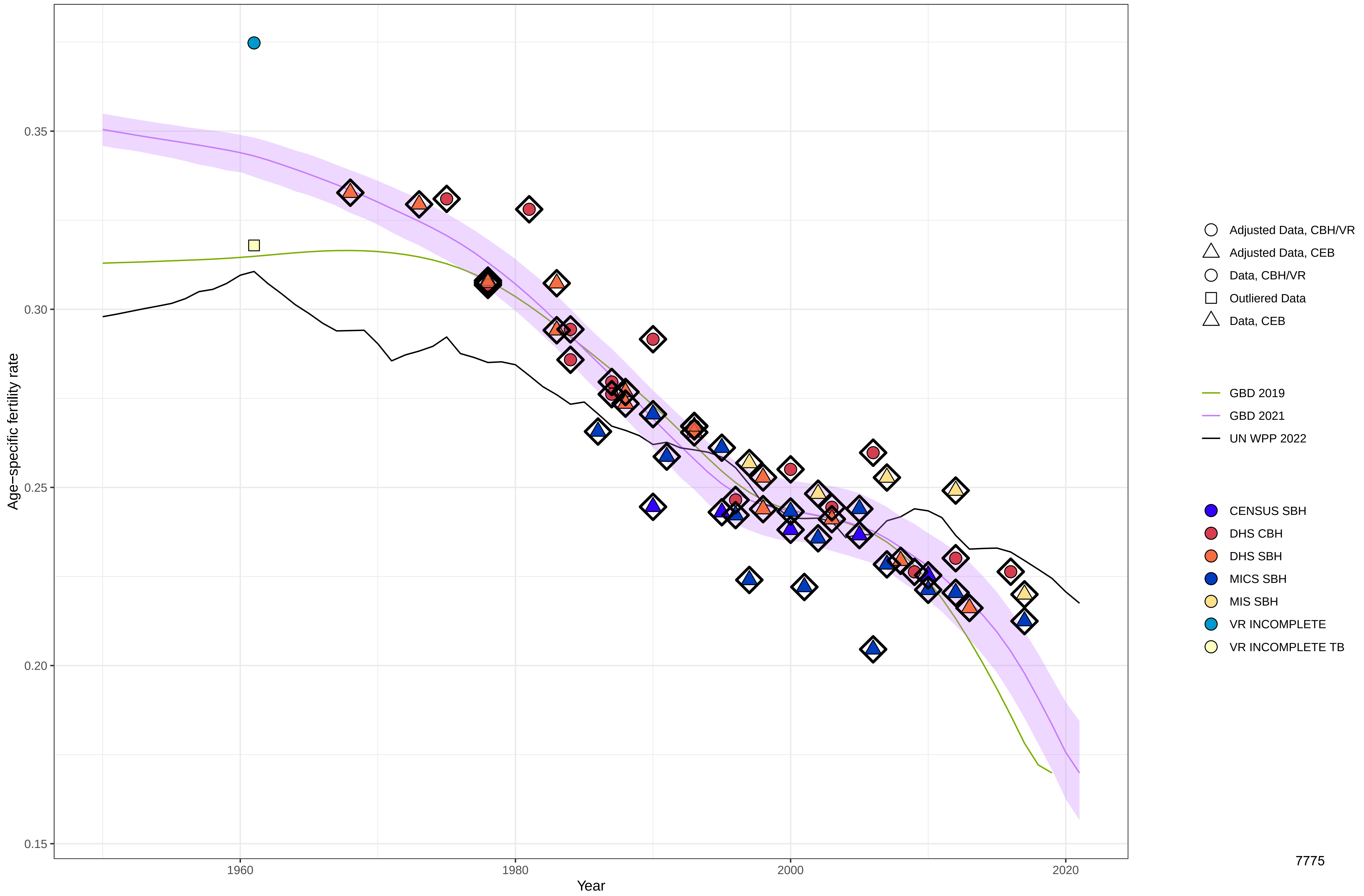

Age−specific fertility fitted trend lines, 1950−2021: Togo, Ages: 30−34
Data points enclosed with a diamond are reference sources

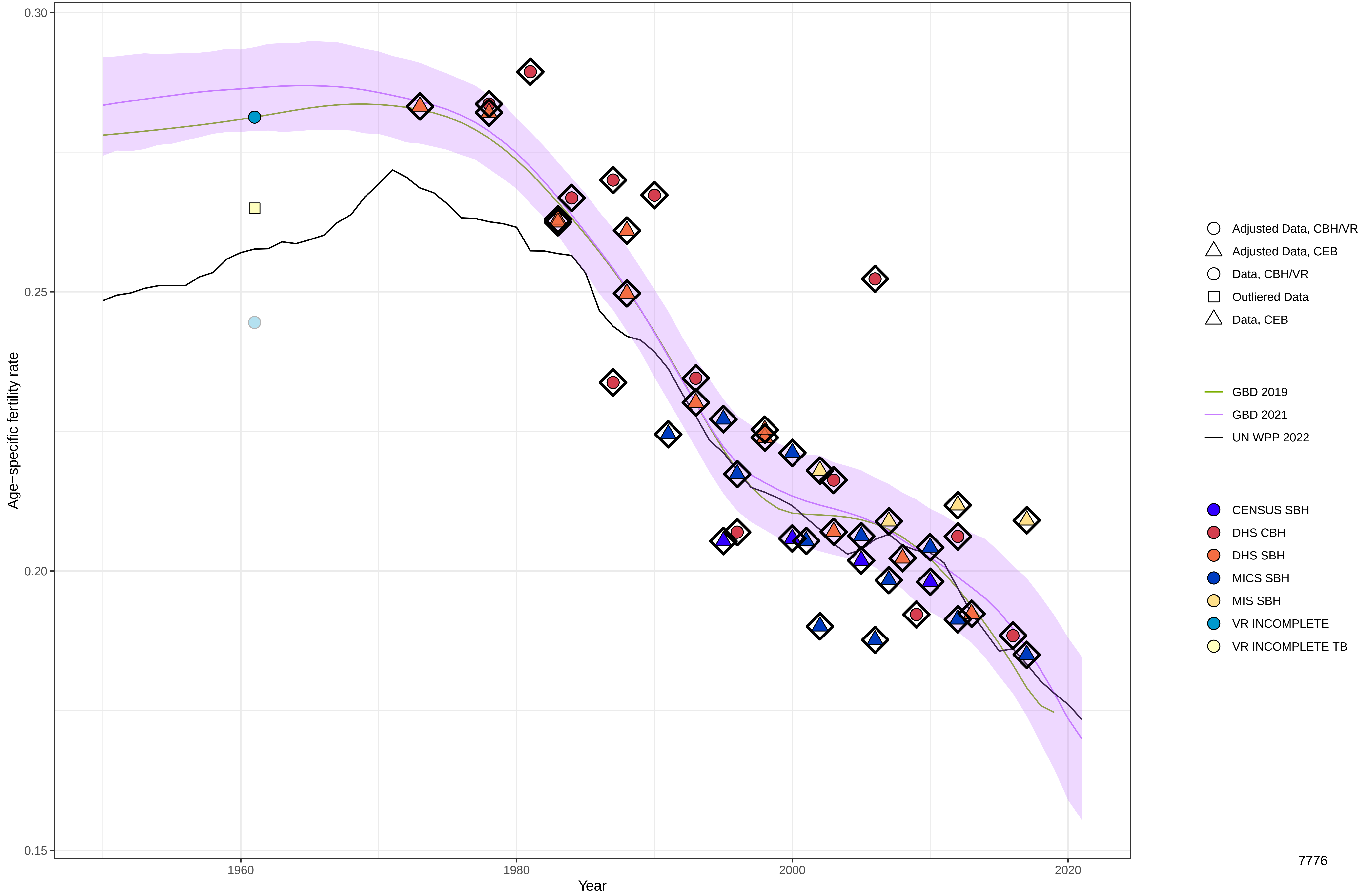

Age−specific fertility fitted trend lines, 1950−2021: Togo, Ages: 35−39
Data points enclosed with a diamond are reference sources

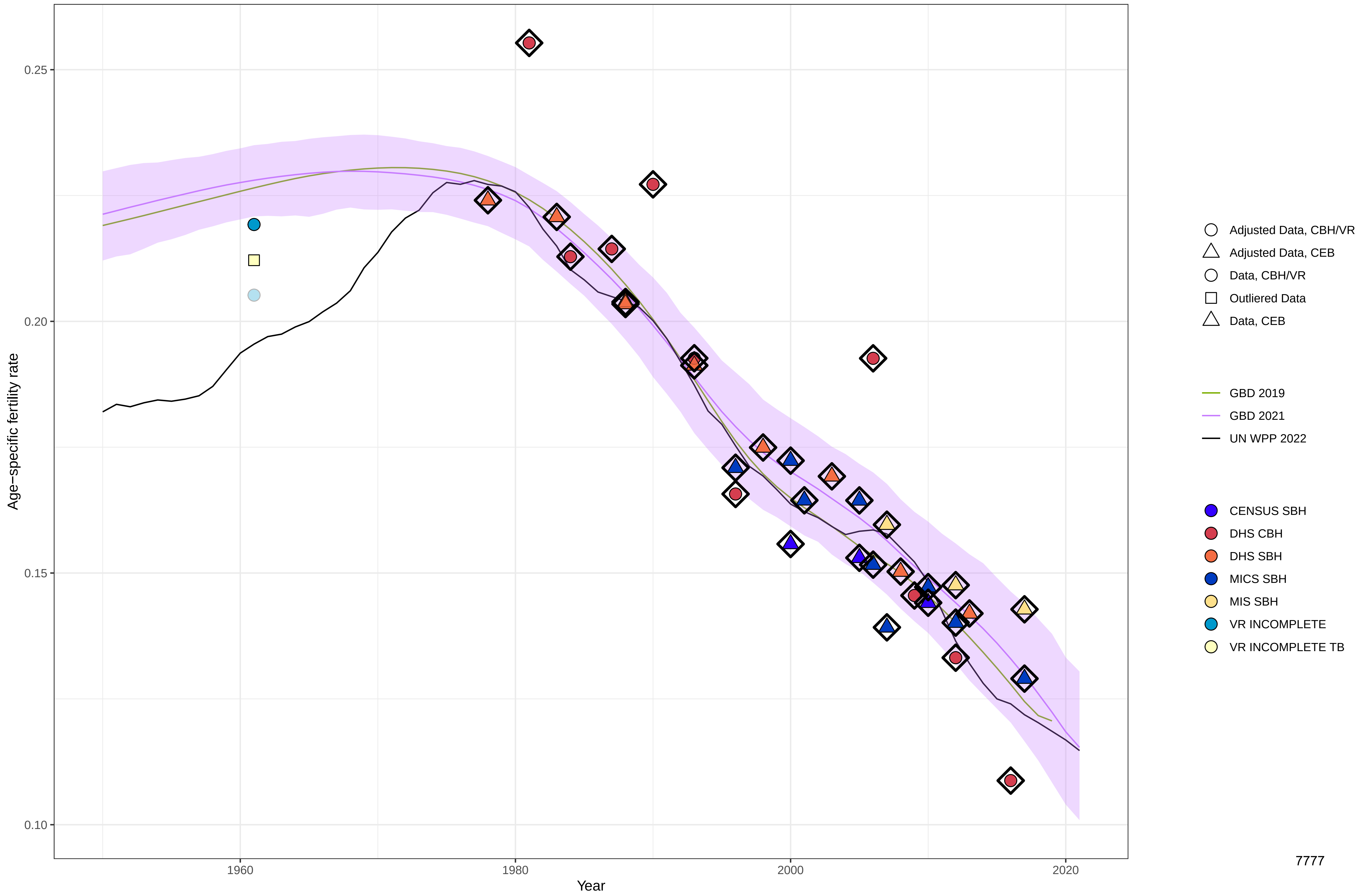

Age−specific fertility fitted trend lines, 1950−2021: Togo, Ages: 40−44
Data points enclosed with a diamond are reference sources

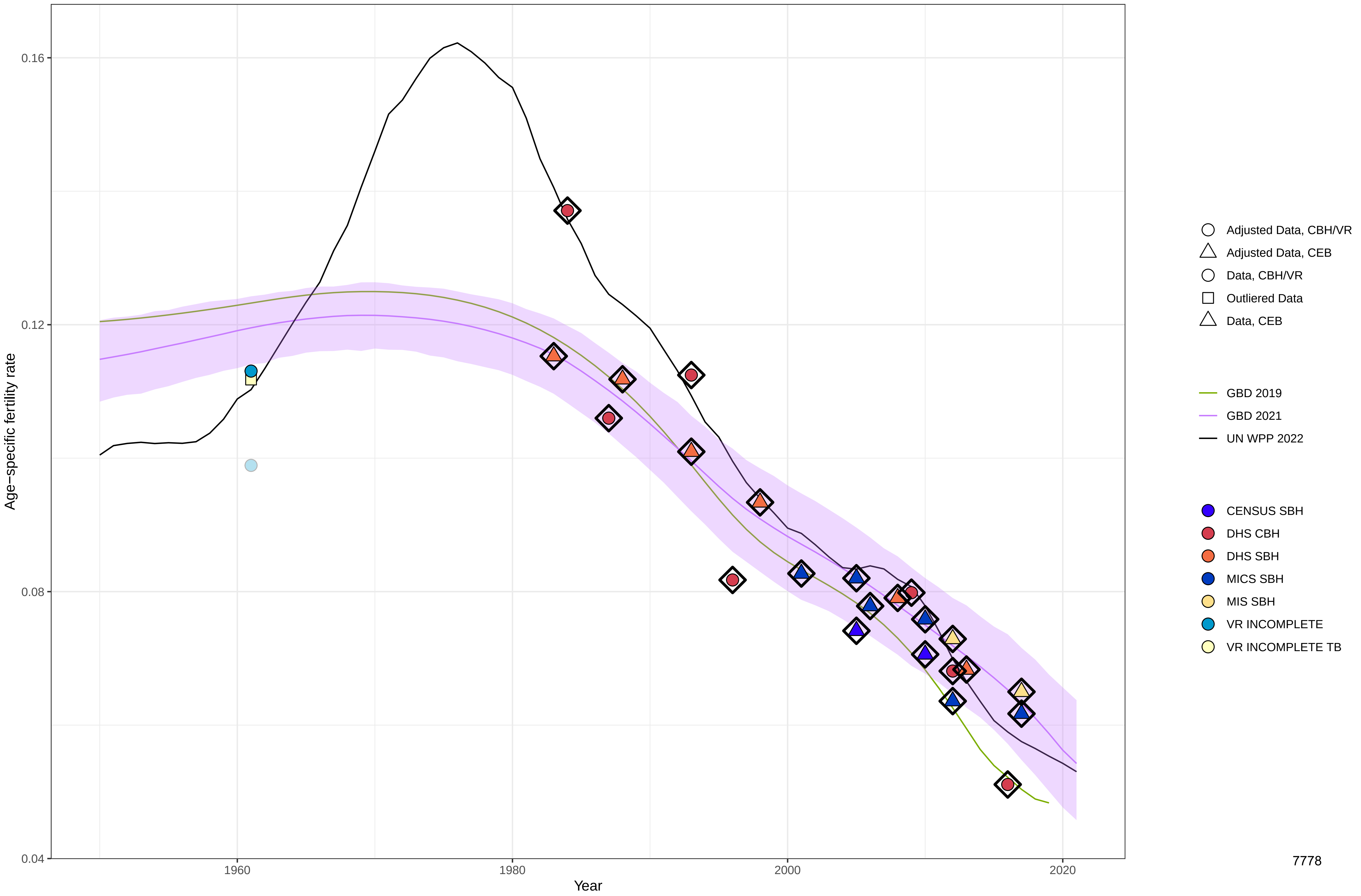

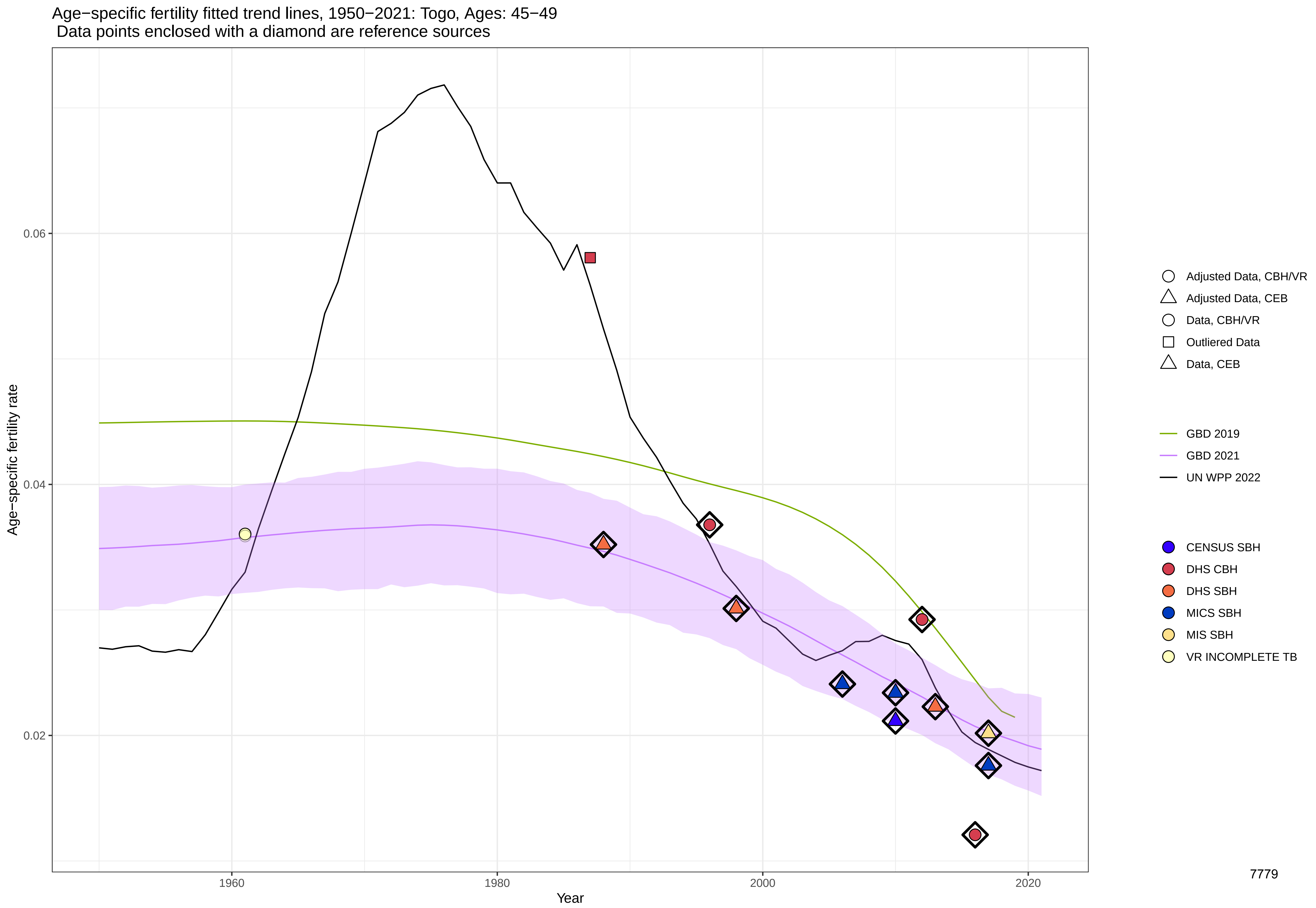

Age−specific fertility fitted trend lines, 1950−2021: Togo, Ages: 45−49
Data points enclosed with a diamond are reference sources
Age−specific fertility rate
0.06
0.04
0.02
1960
1980
2000
2020
Year
Adjusted Data, CBH/VR
Adjusted Data, CEB
Data, CBH/VR
Outliered Data
Data, CEB
GBD 2019
GBD 2021
UN WPP 2022
CENSUS SBH
DHS CBH
DHS SBH
MICS SBH
MIS SBH
VR INCOMPLETE TB

Age−specific fertility fitted trend lines, 1950−2021: Togo, Ages: 50−54
Data points enclosed with a diamond are reference sources

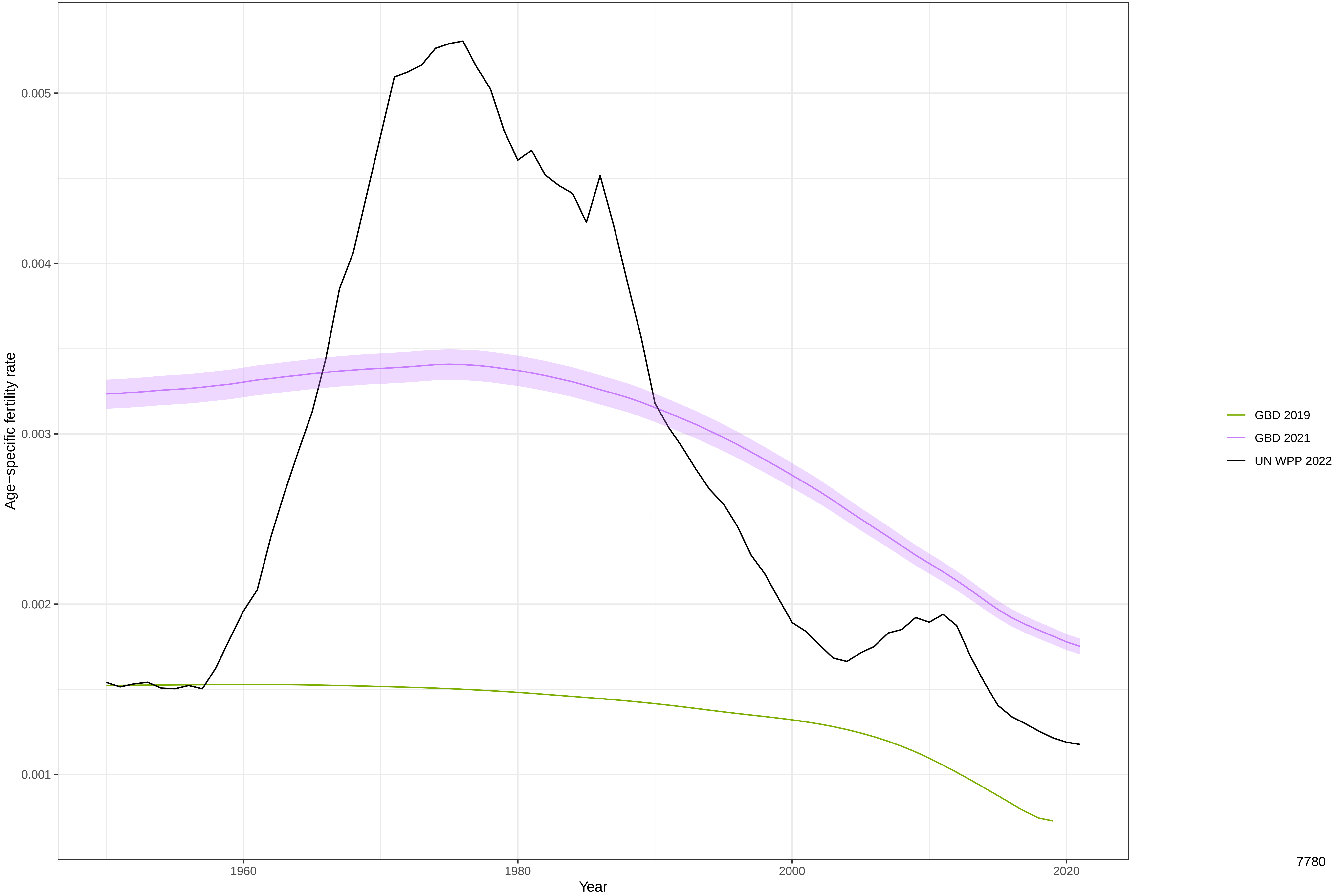

Total fertility rate fitted trend lines, 1950−2021: Togo TFR
Data points enclosed with a diamond are reference sources

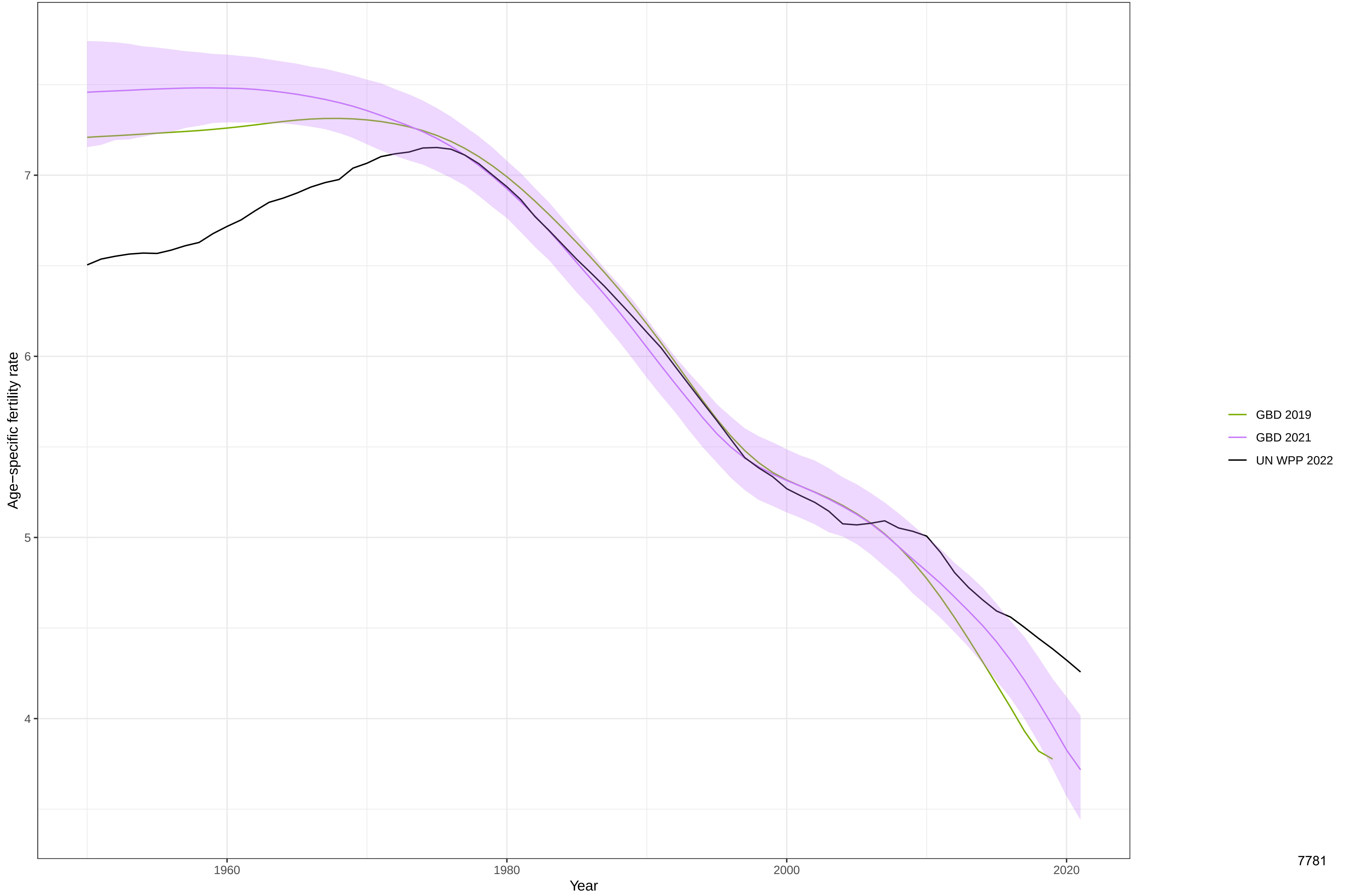

Armenia

Supplemental Results Figure S2: Covariates used in fertility forecasts by location, 1970-2135

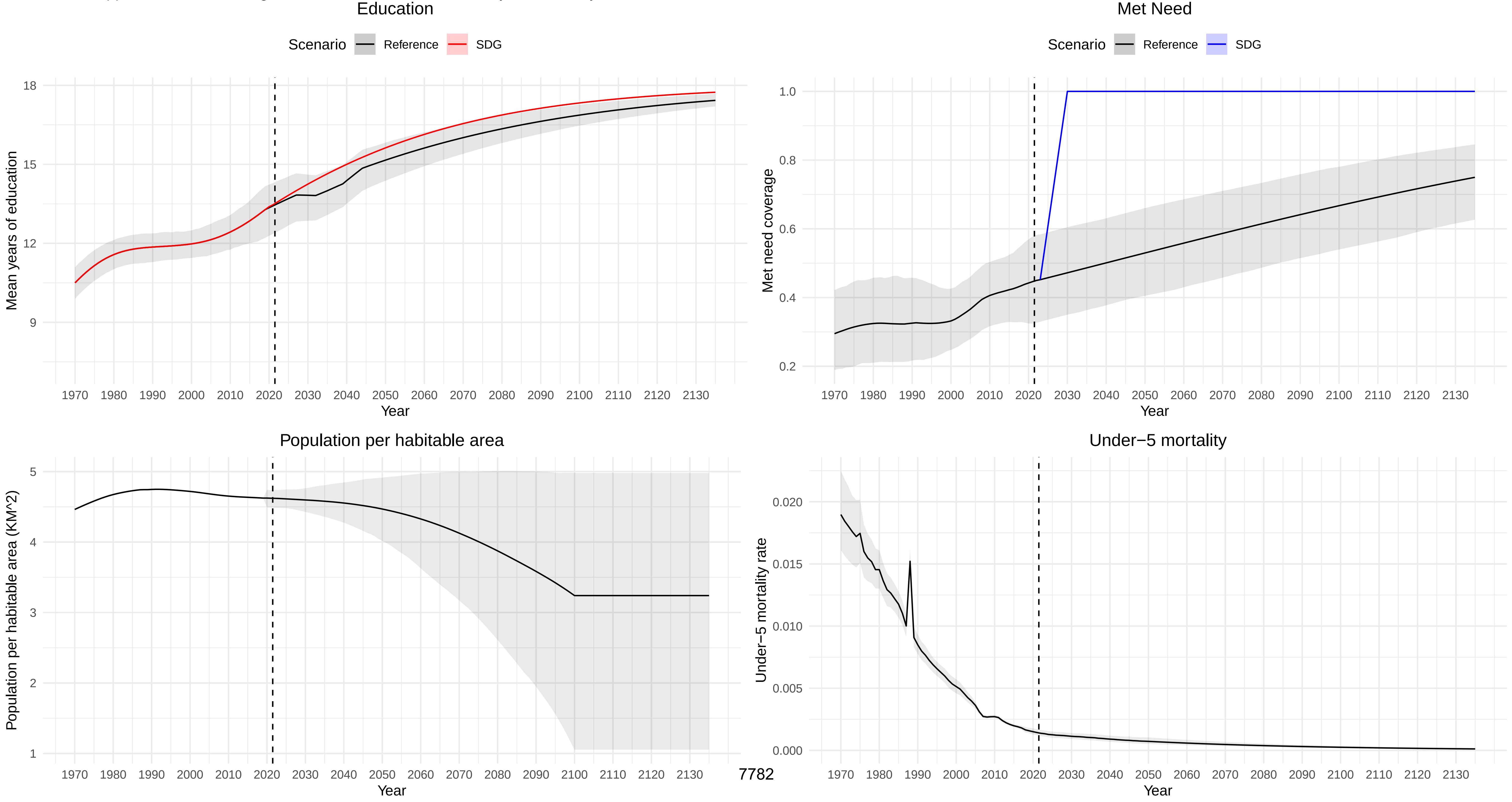

Azerbaijan

Supplemental Results Figure S2: Covariates used in fertility forecasts by location, 1970-2135

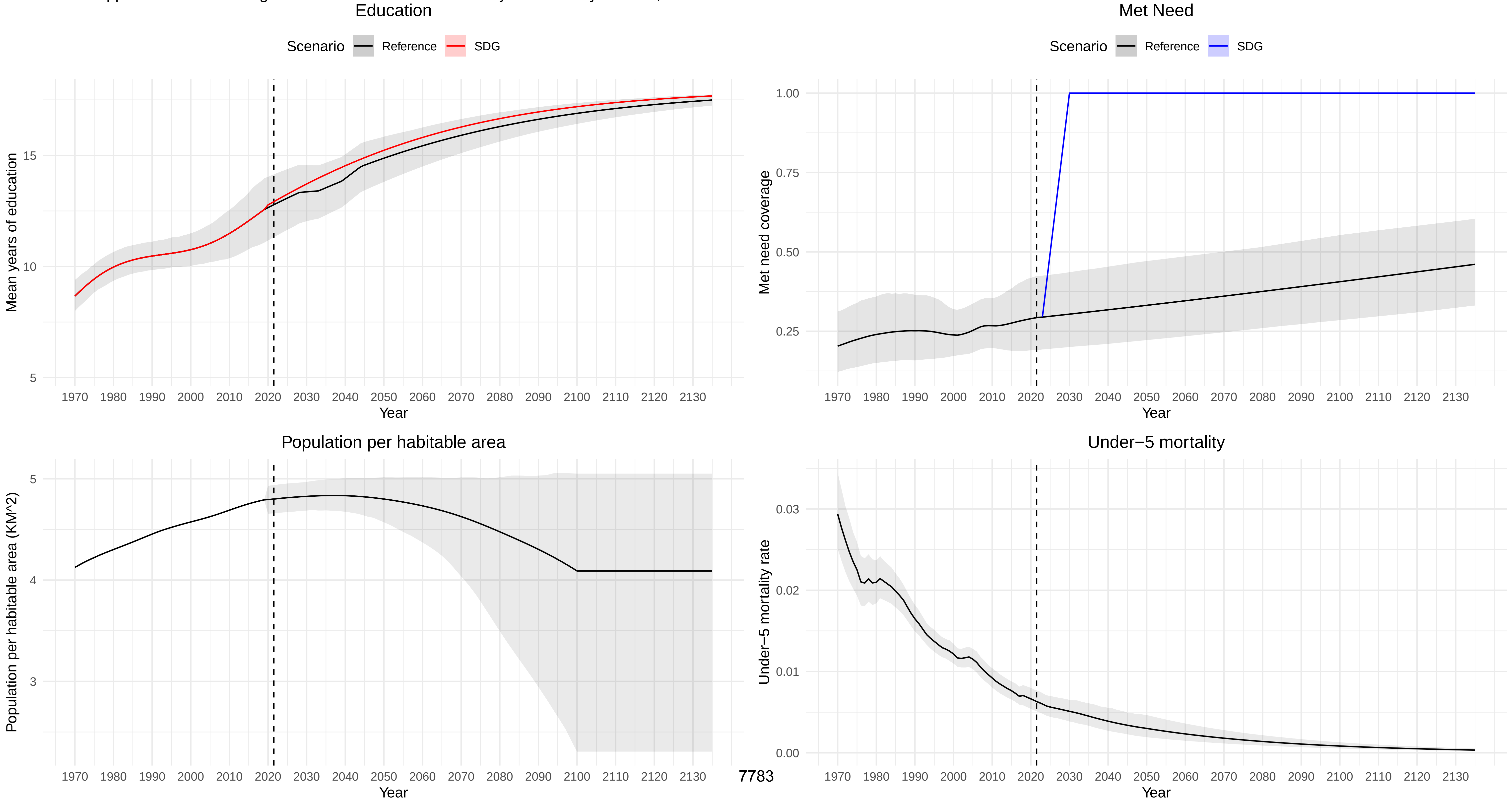

Georgia

Supplemental Results Figure S2: Covariates used in fertility forecasts by location, 1970-2135

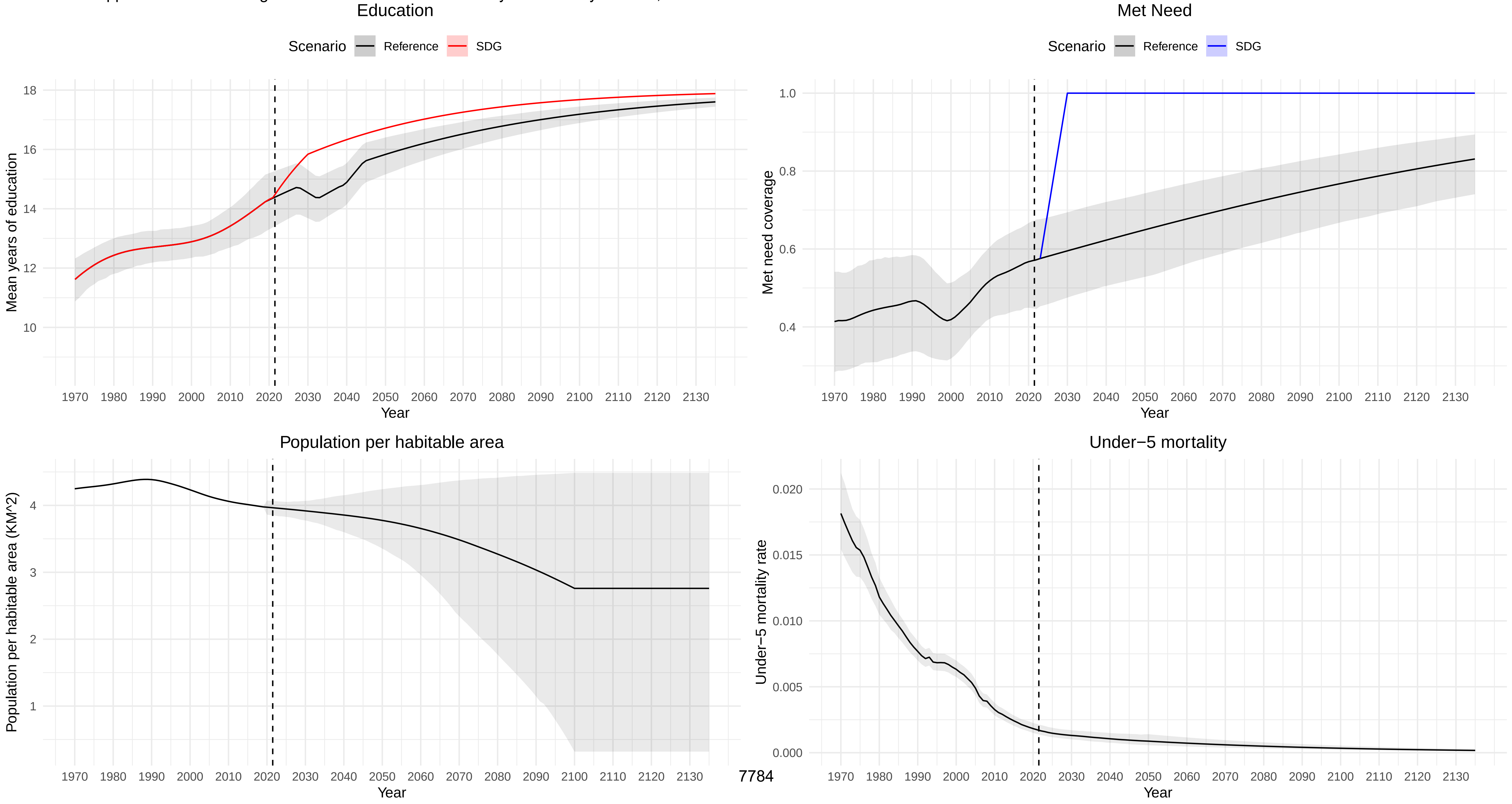

Kazakhstan

Supplemental Results Figure S2: Covariates used in fertility forecasts by location, 1970-2135

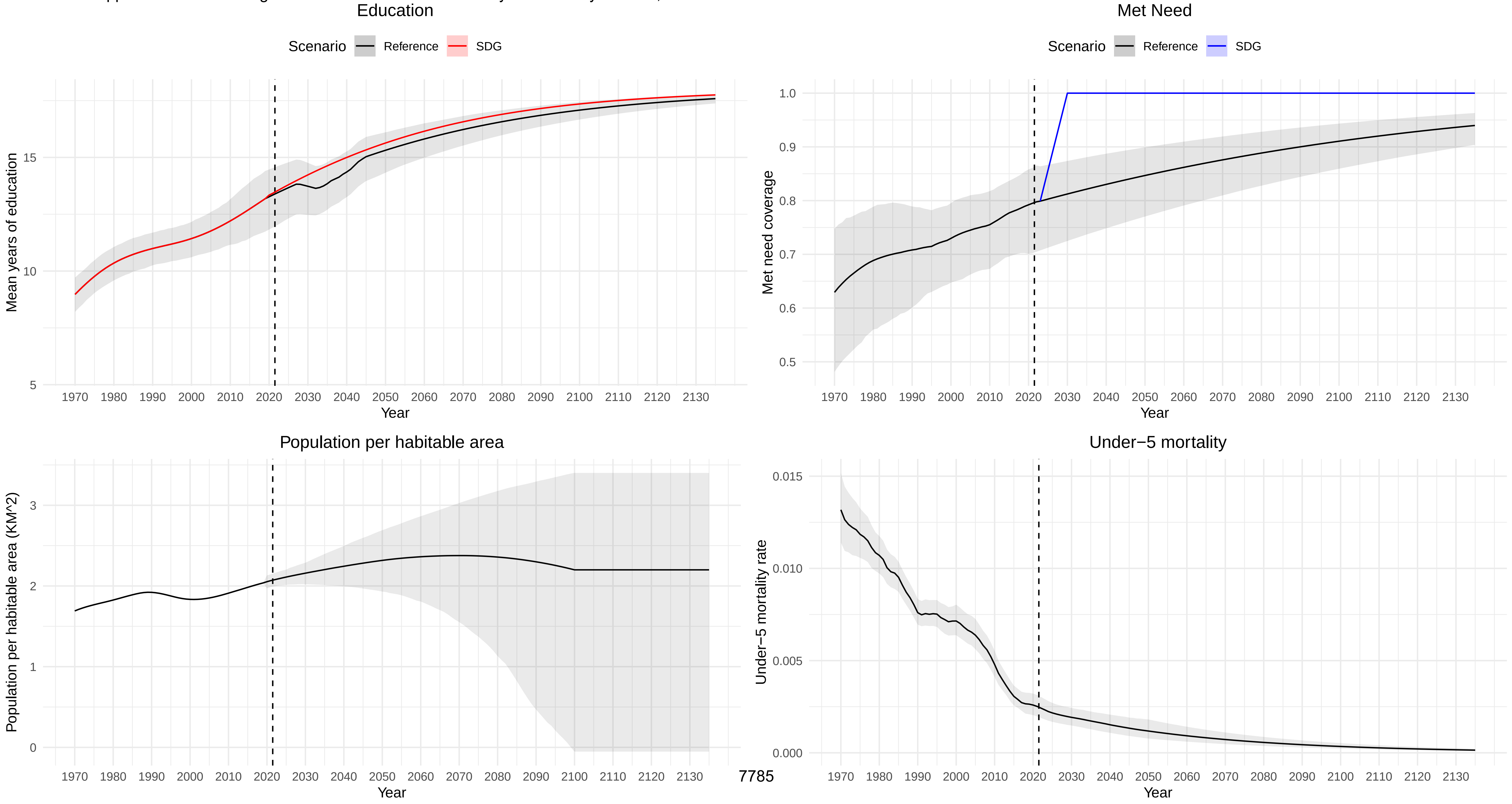

Kyrgyzstan

Supplemental Results Figure S2: Covariates used in fertility forecasts by location, 1970-2135

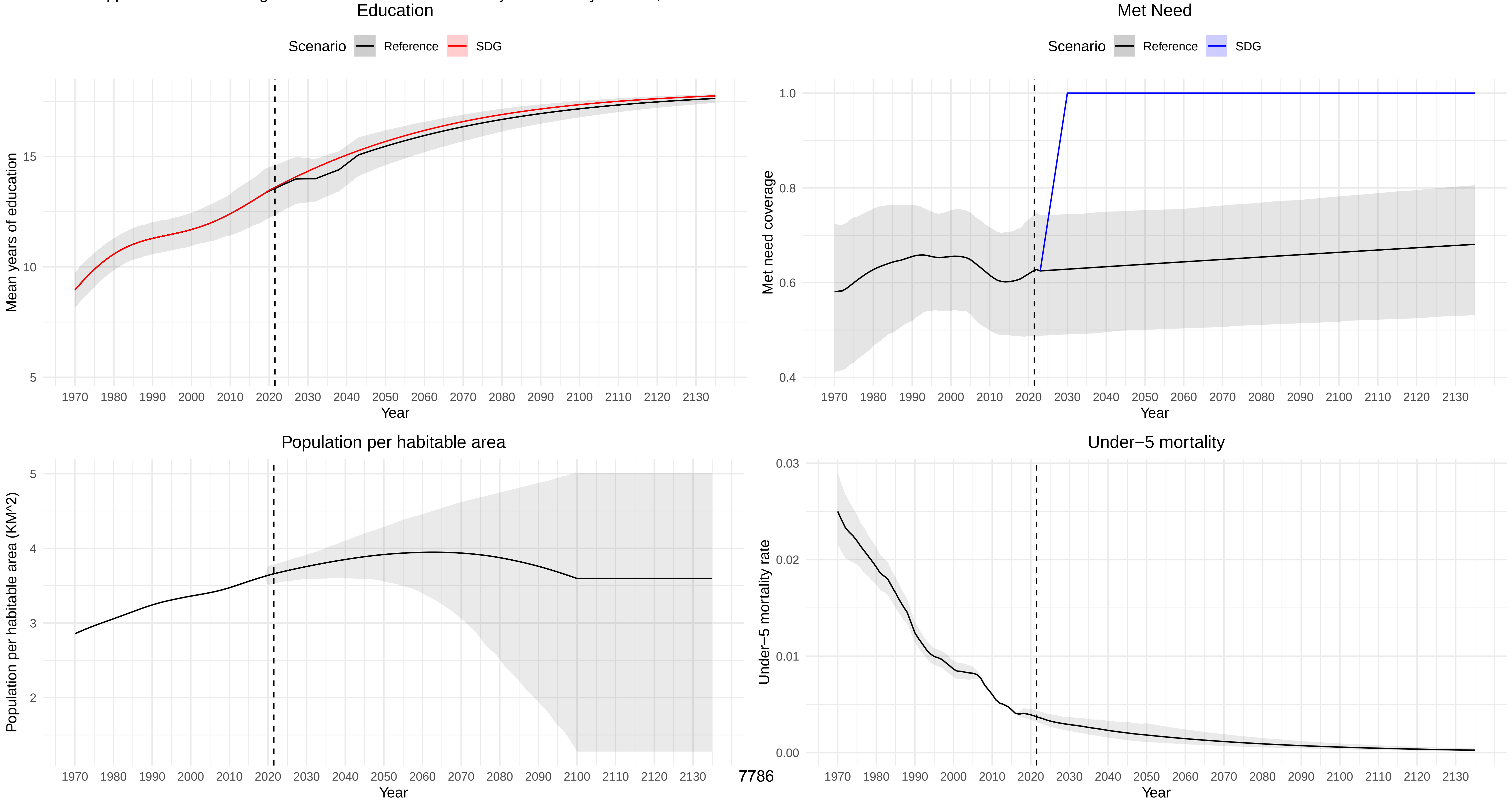

Mongolia

Supplemental Results Figure S2: Covariates used in fertility forecasts by location, 1970-2135

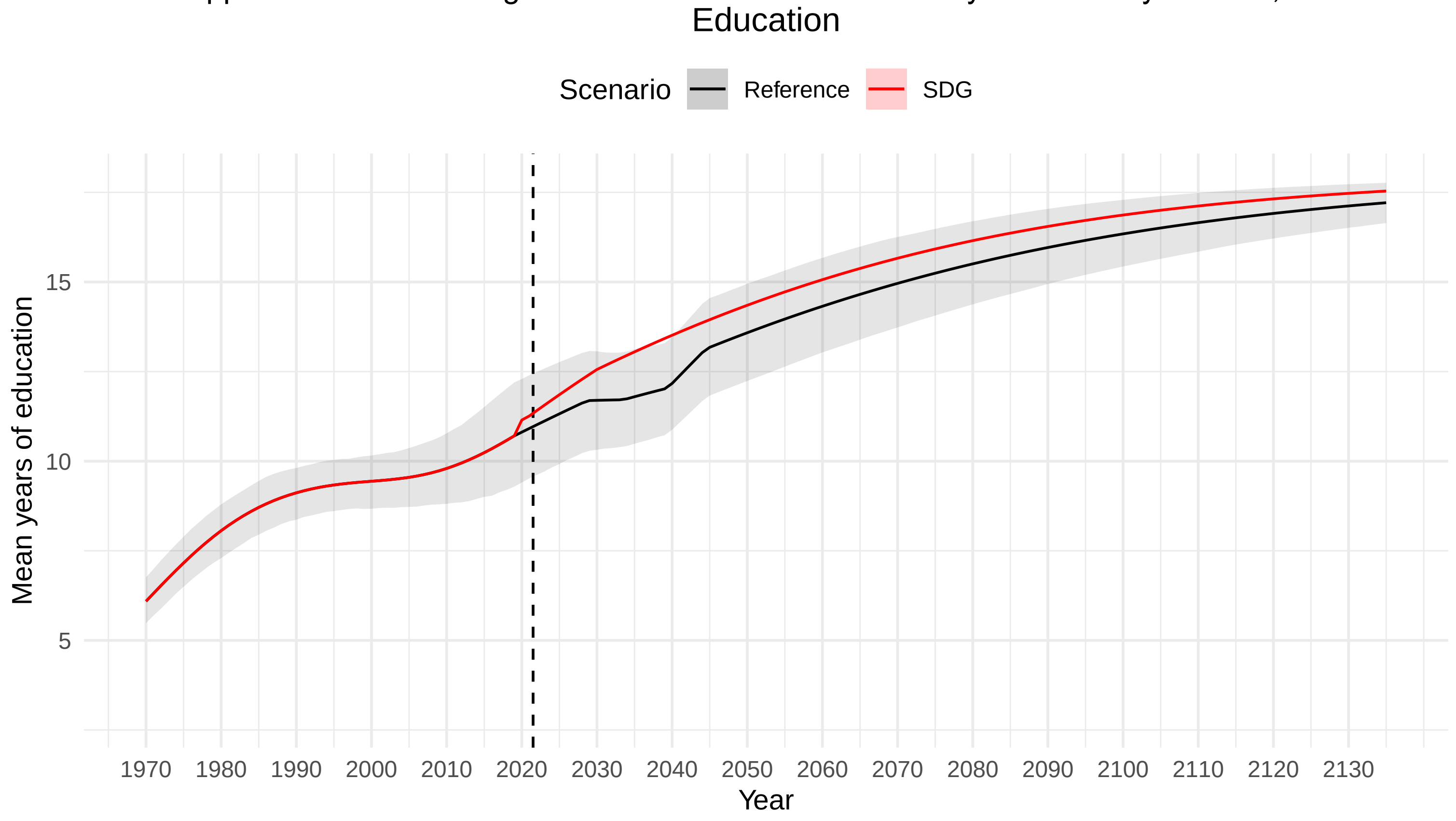

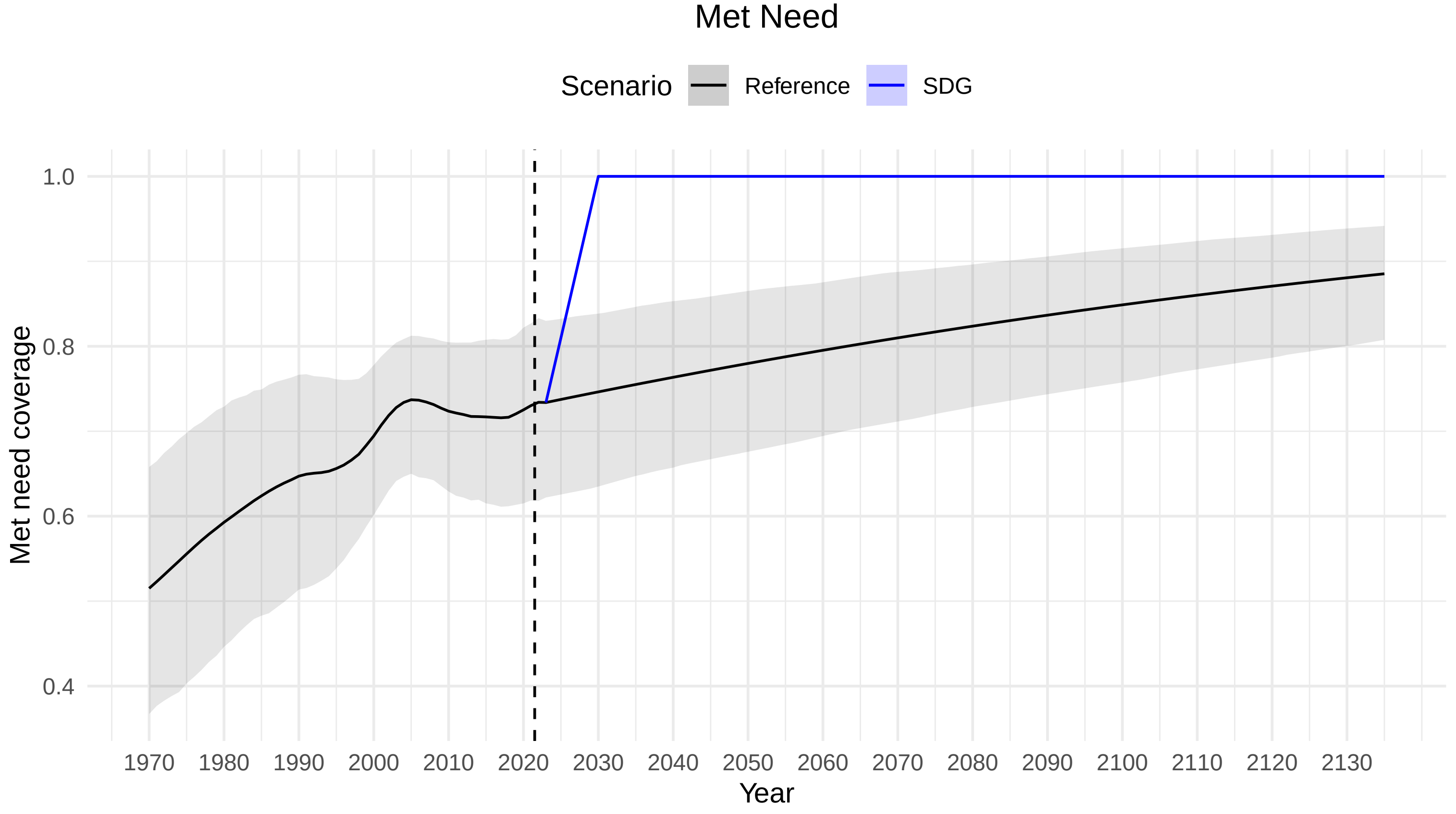

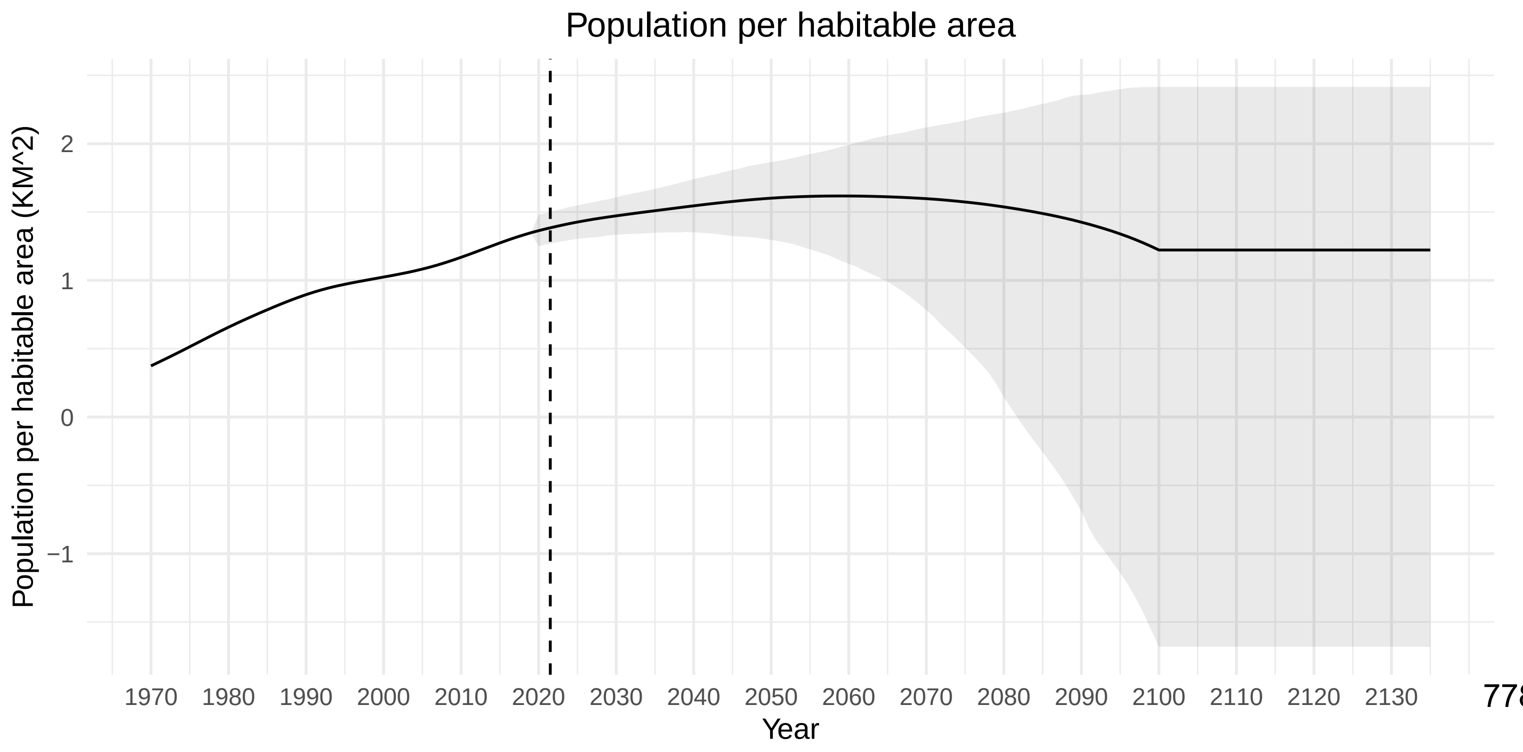

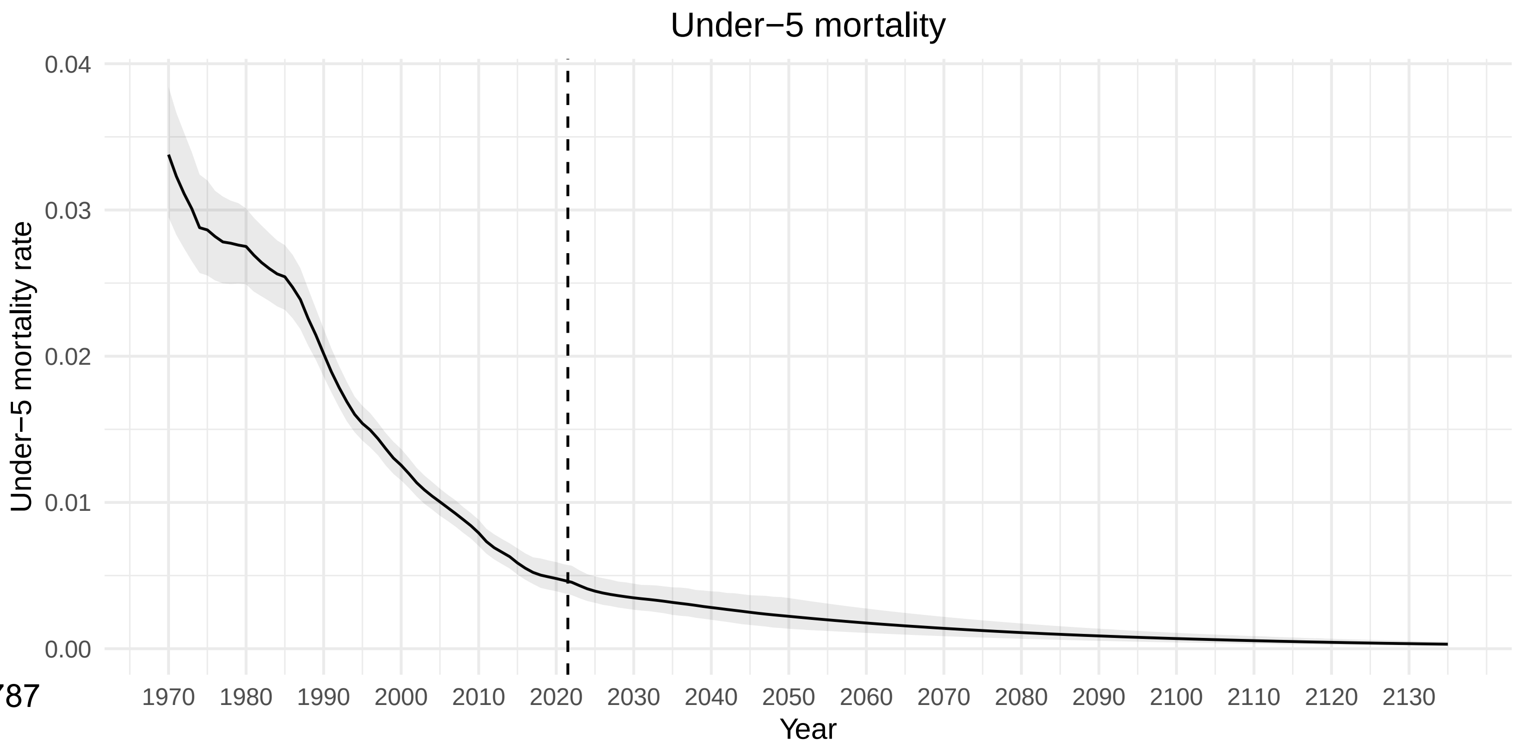

Tajikistan

Supplemental Results Figure S2: Covariates used in fertility forecasts by location, 1970-2135

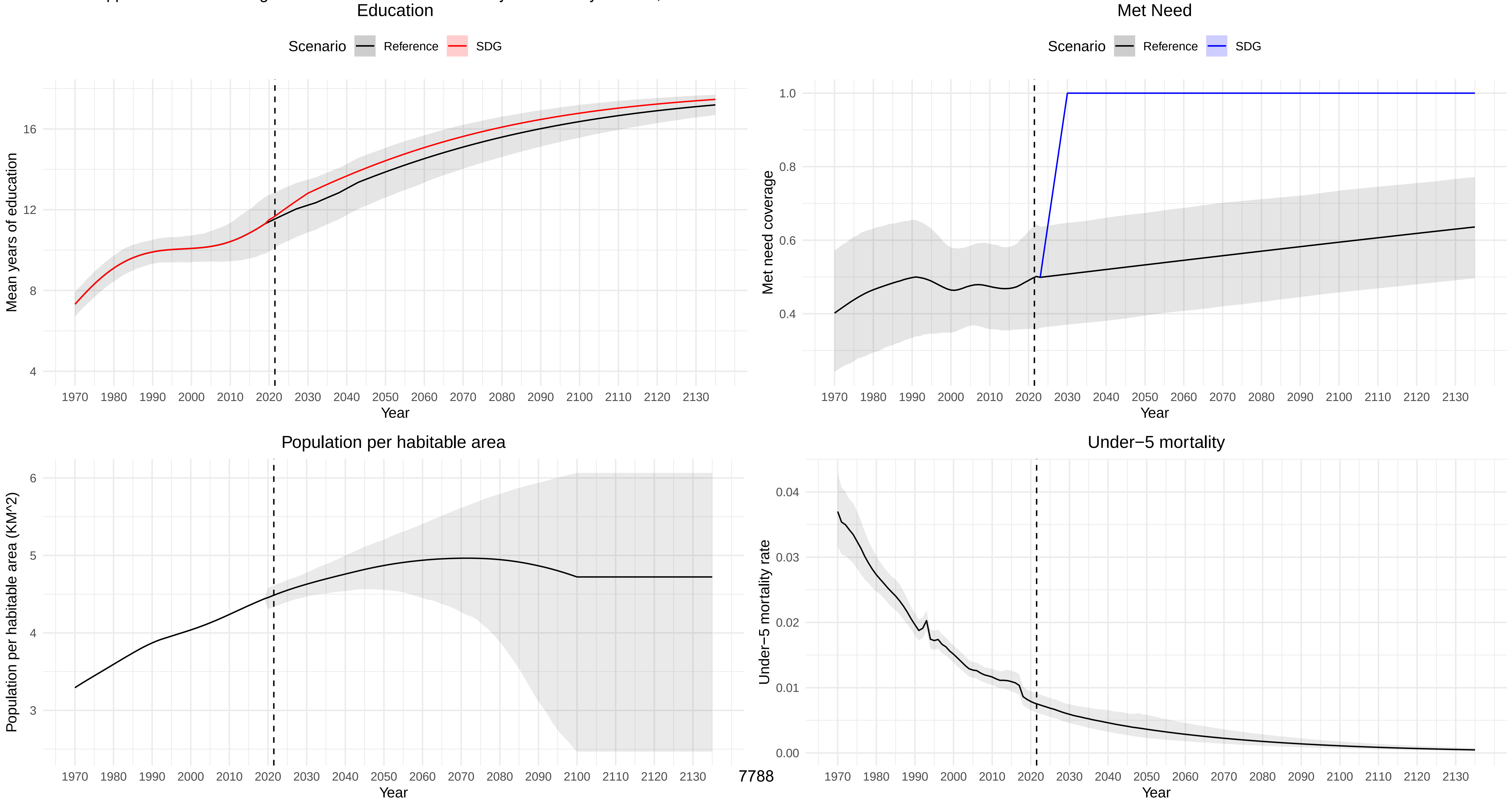

Turkmenistan

Supplemental Results Figure S2: Covariates used in fertility forecasts by location, 1970-2135

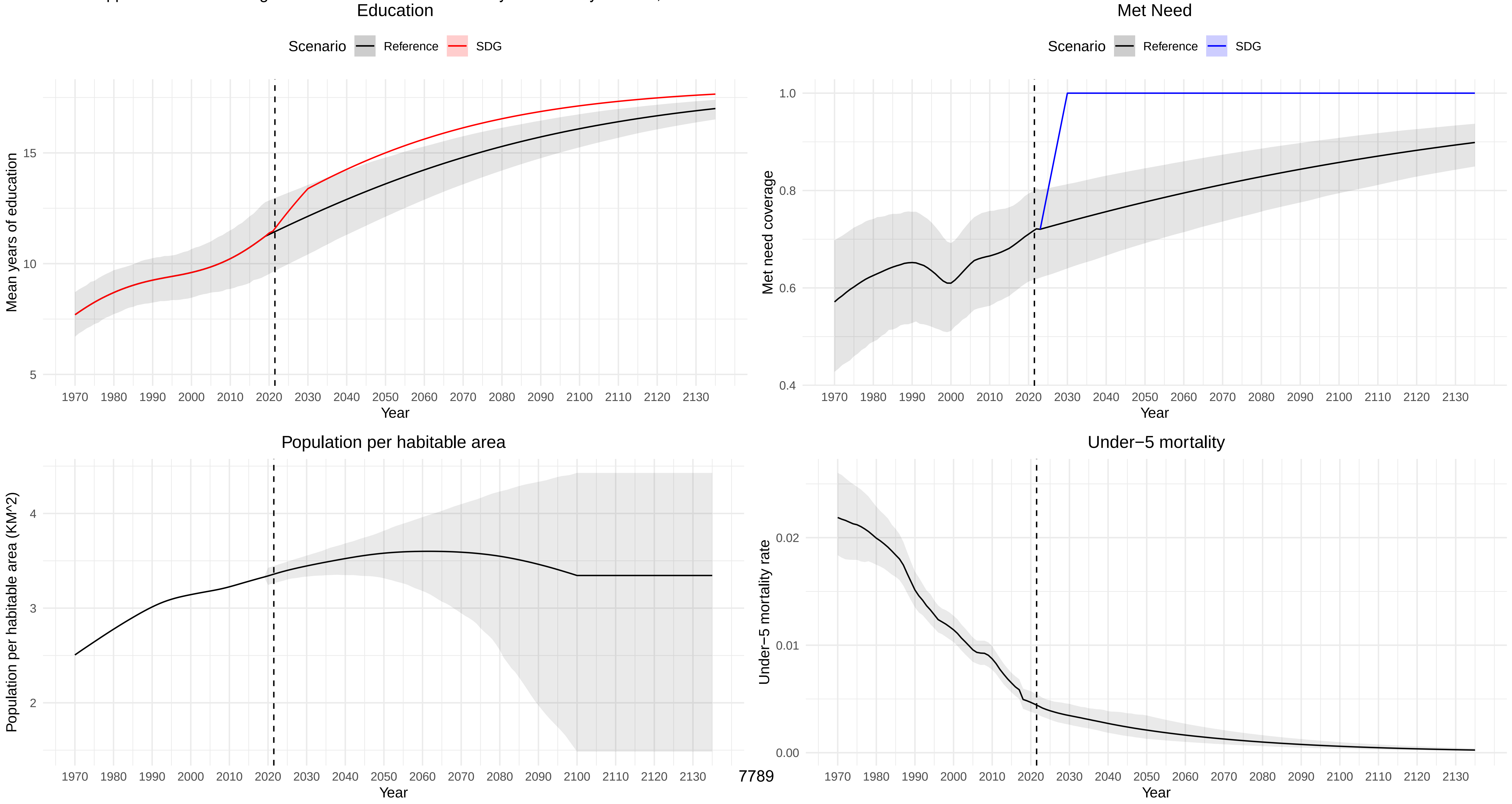

Uzbekistan

Supplemental Results Figure S2: Covariates used in fertility forecasts by location, 1970-2135

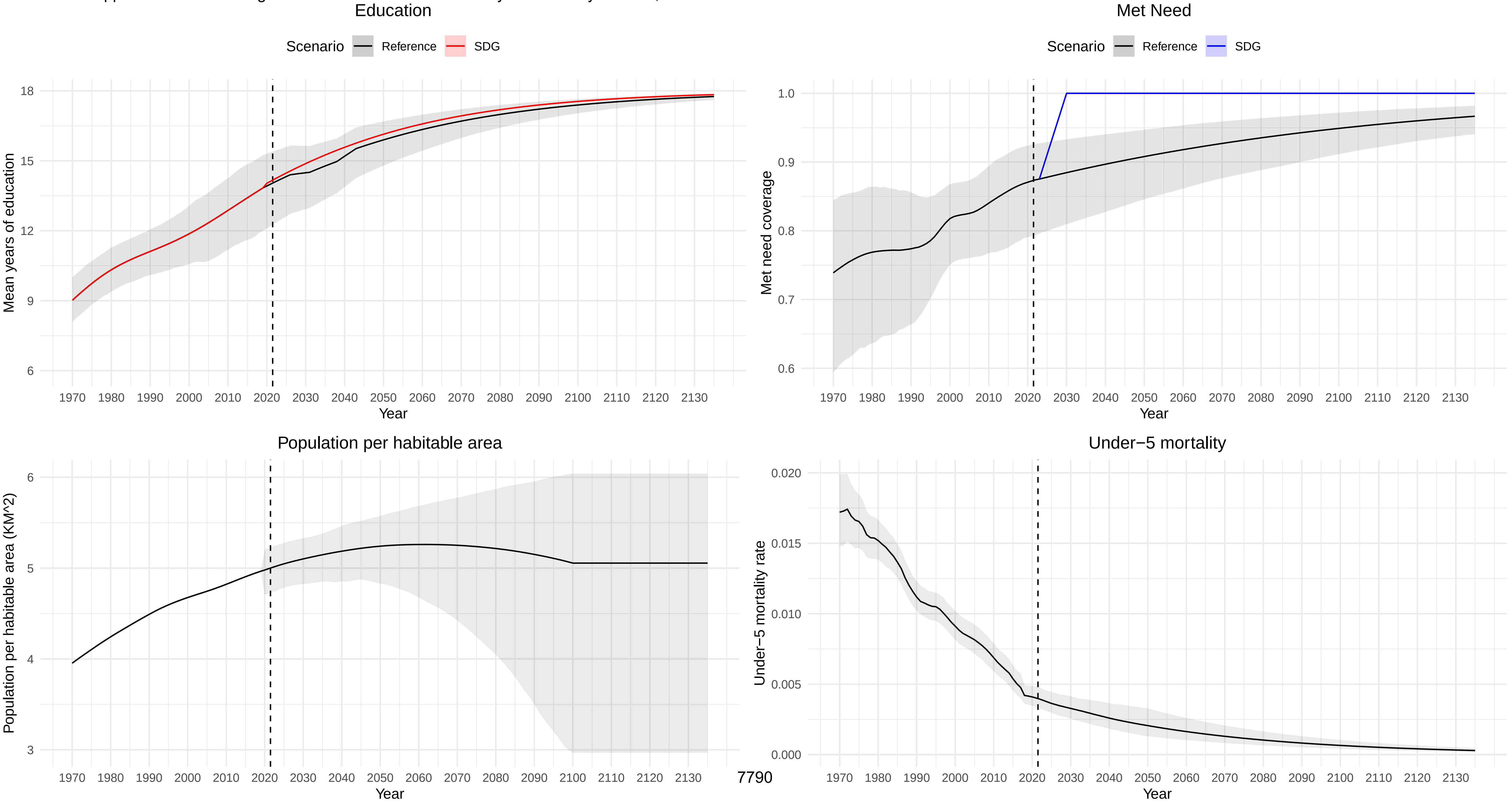

Albania

Supplemental Results Figure S2: Covariates used in fertility forecasts by location, 1970-2135

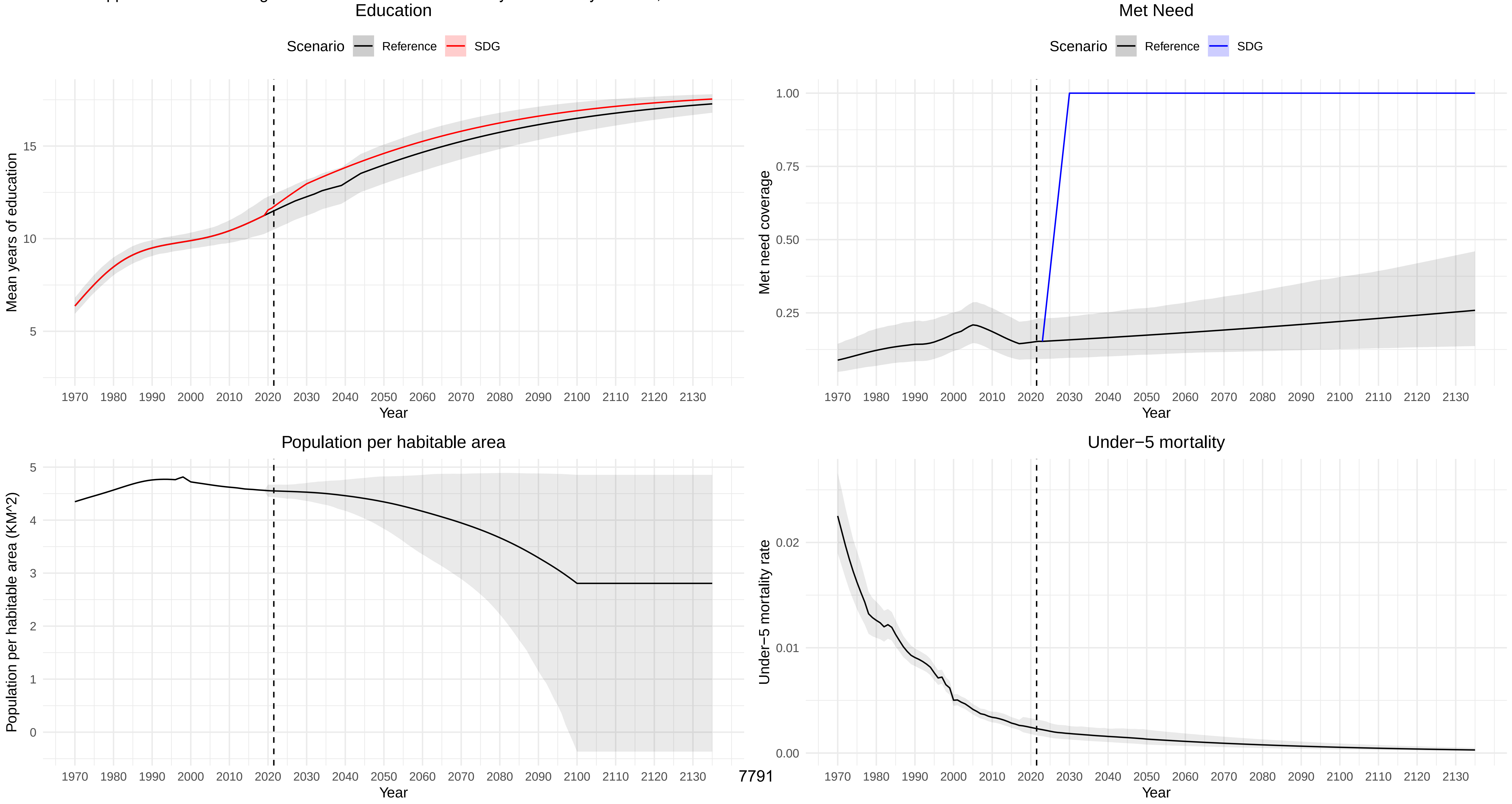

Bosnia and Herzegovina

Supplemental Results Figure S2: Covariates used in fertility forecasts by location, 1970-2135

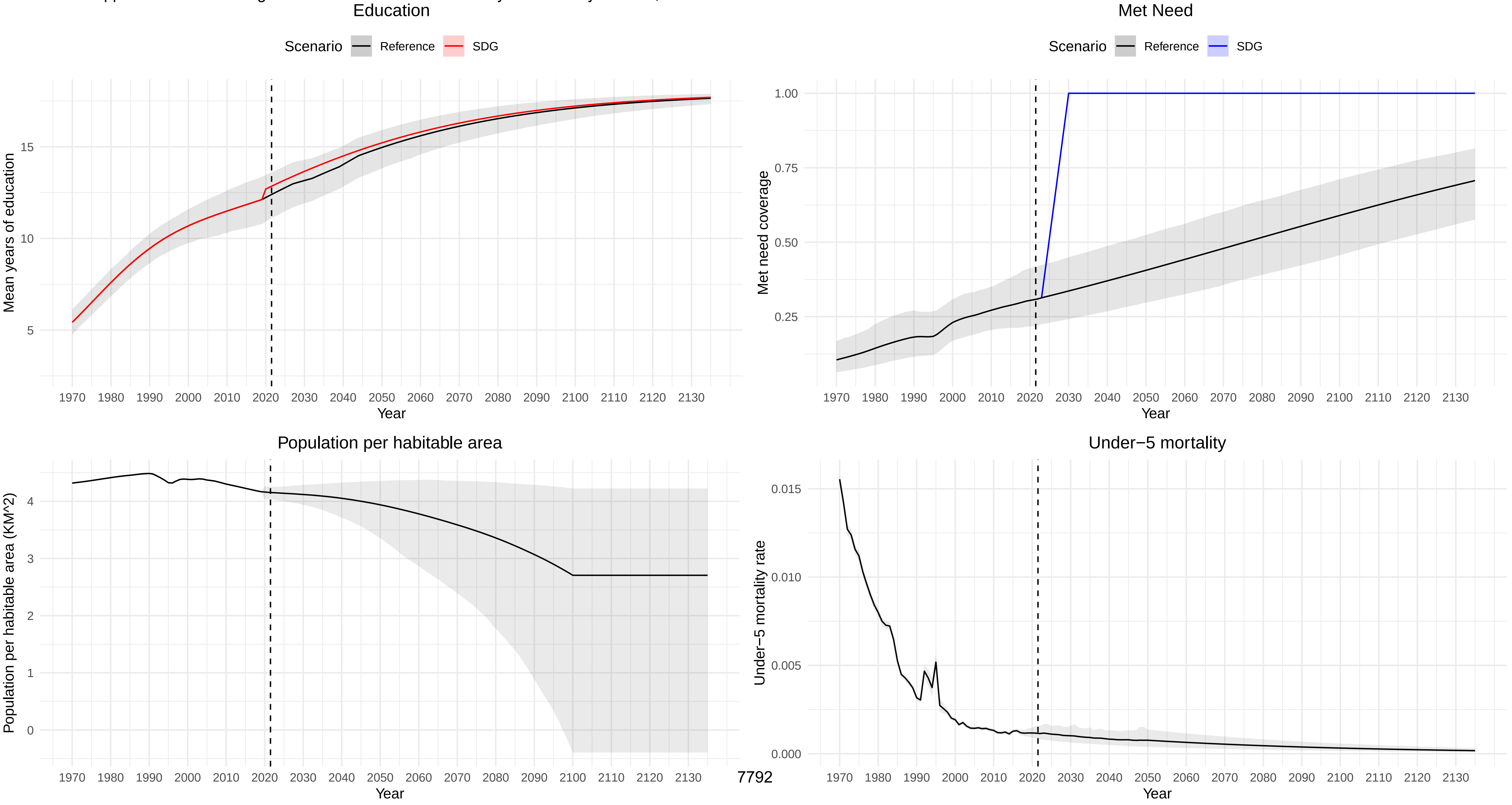

Bulgaria

Supplemental Results Figure S2: Covariates used in fertility forecasts by location, 1970-2135

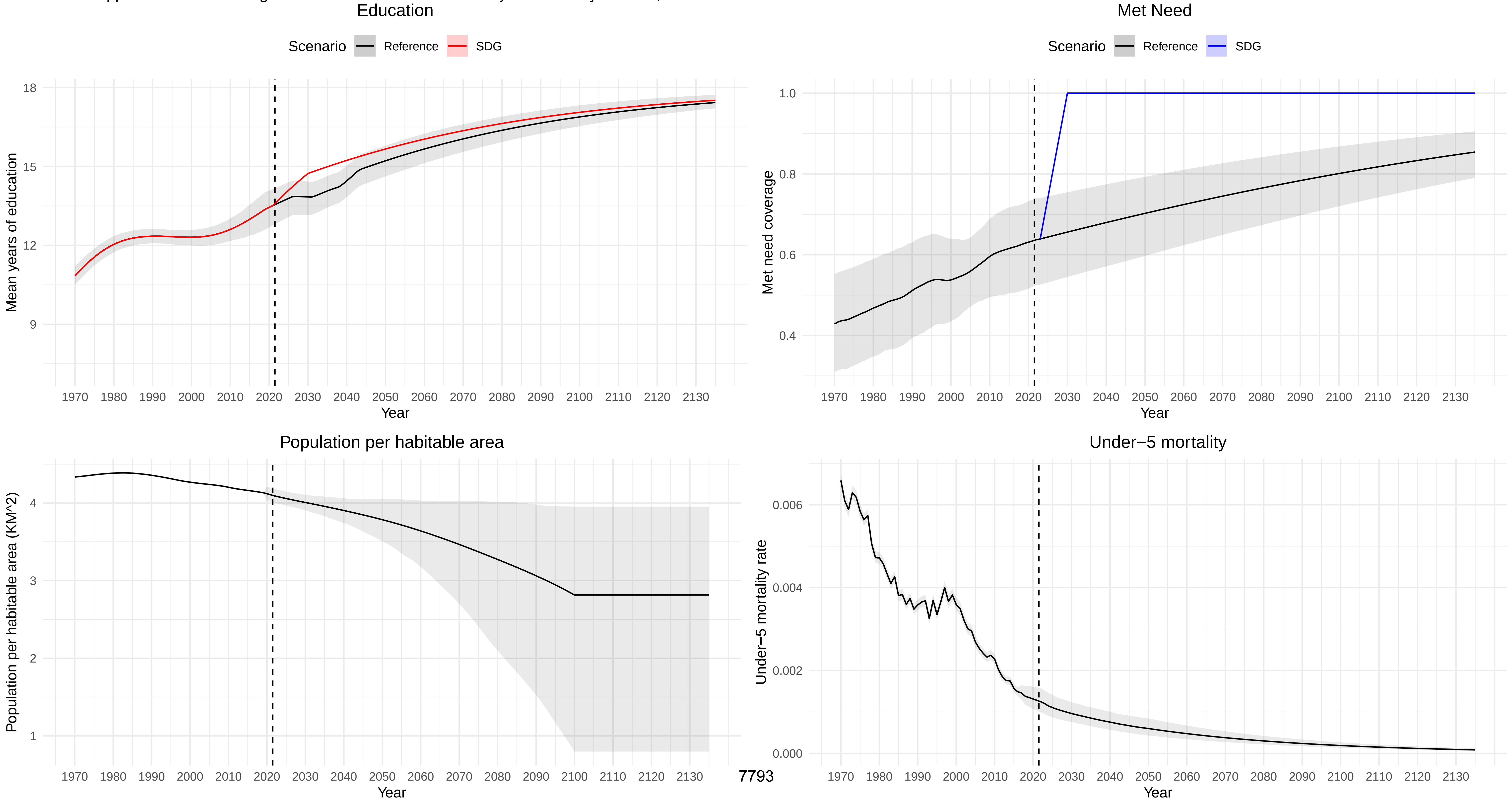

Croatia

Supplemental Results Figure S2: Covariates used in fertility forecasts by location, 1970-2135

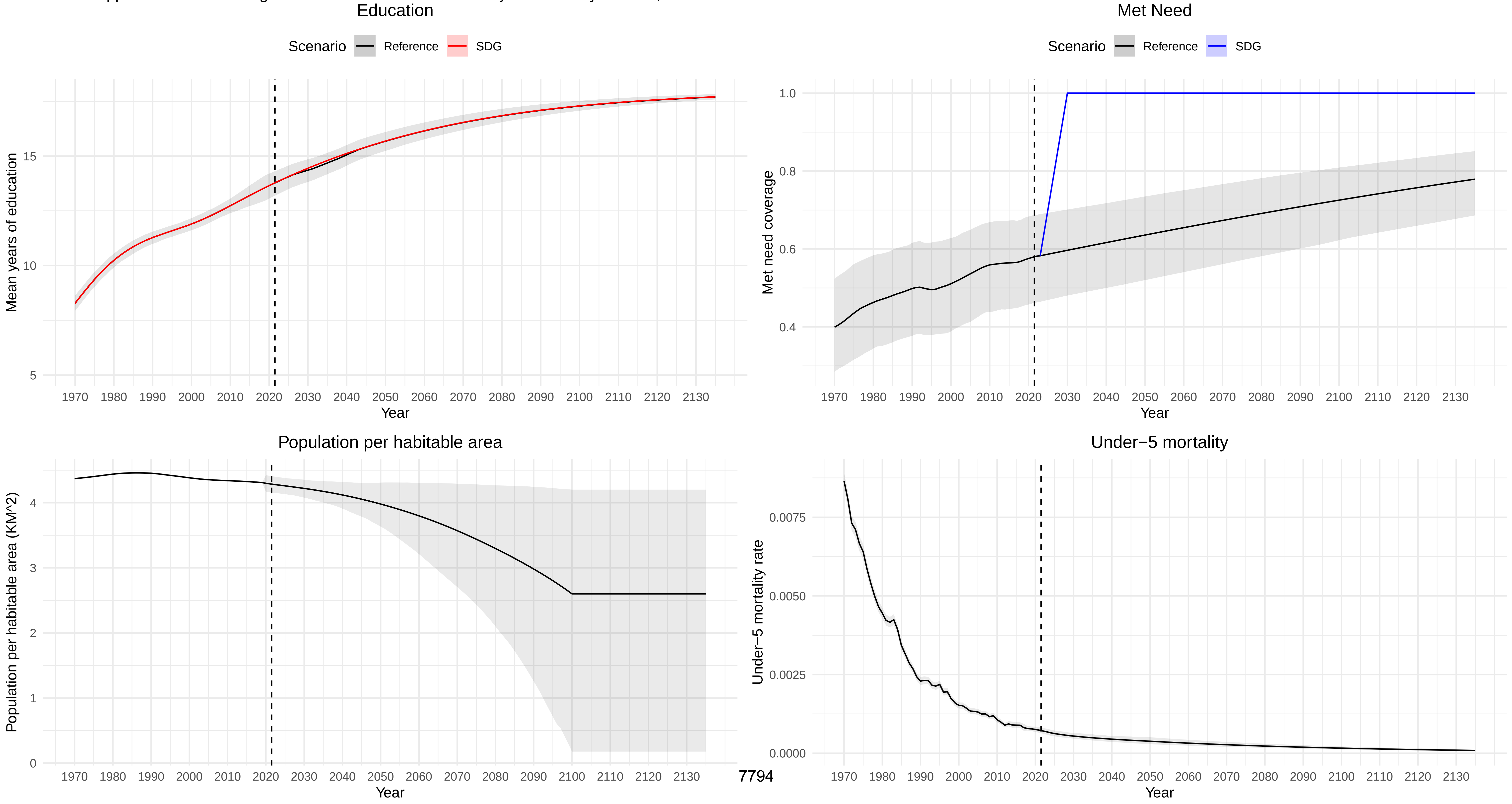

Czechia

Supplemental Results Figure S2: Covariates used in fertility forecasts by location, 1970-2135

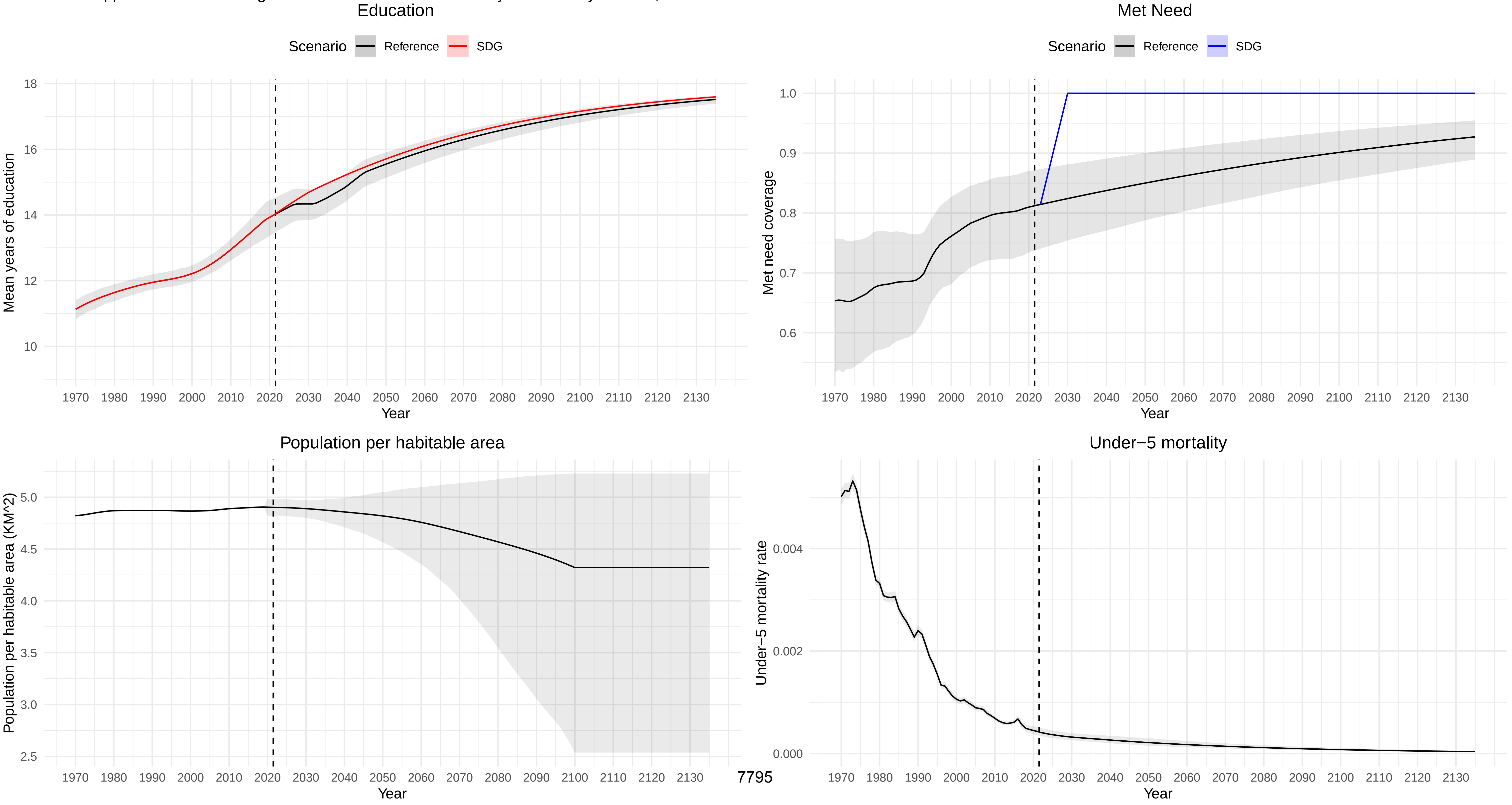

Hungary

Supplemental Results Figure S2: Covariates used in fertility forecasts by location, 1970-2135

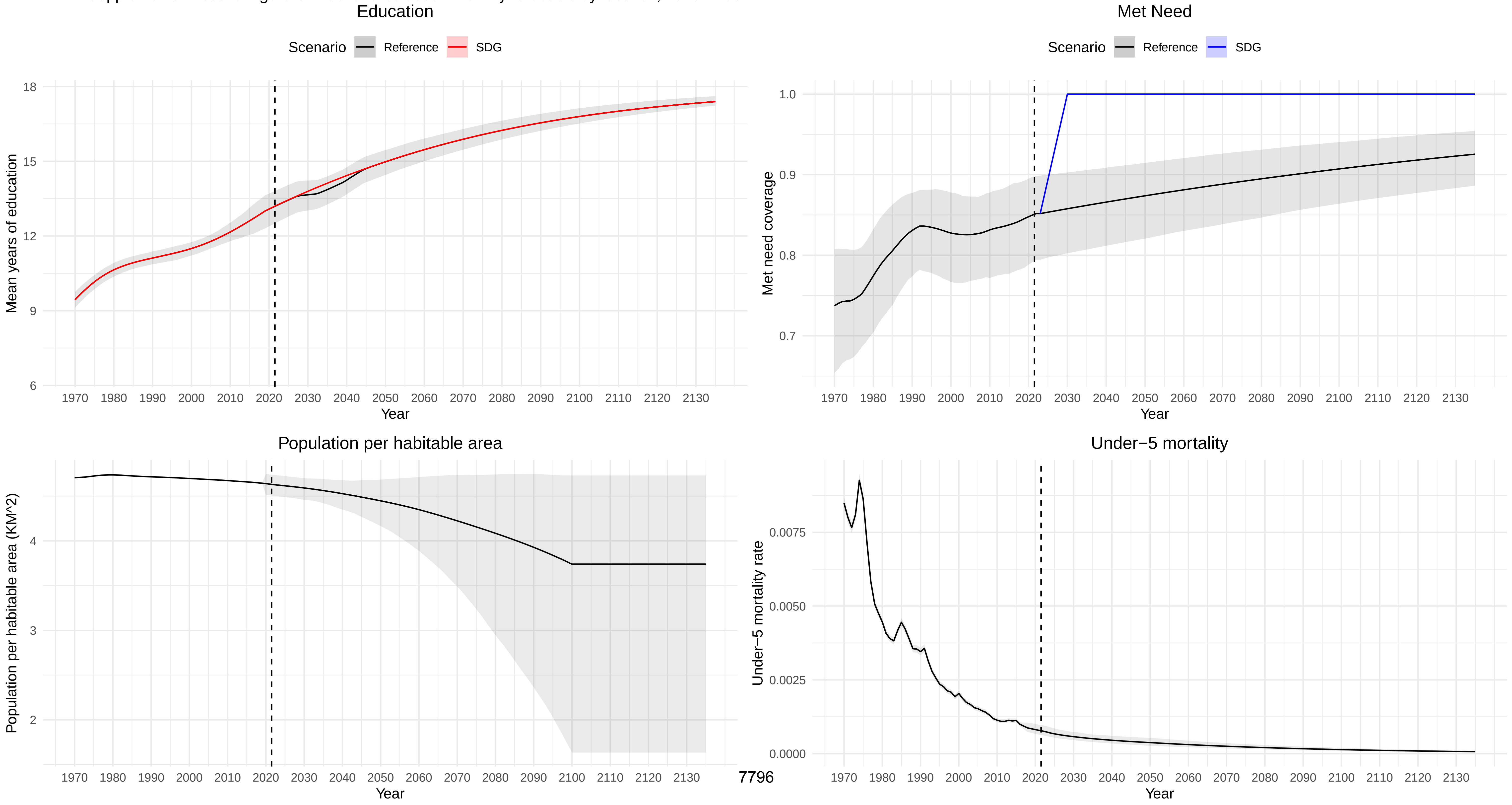

Montenegro

Supplemental Results Figure S2: Covariates used in fertility forecasts by location, 1970-2135

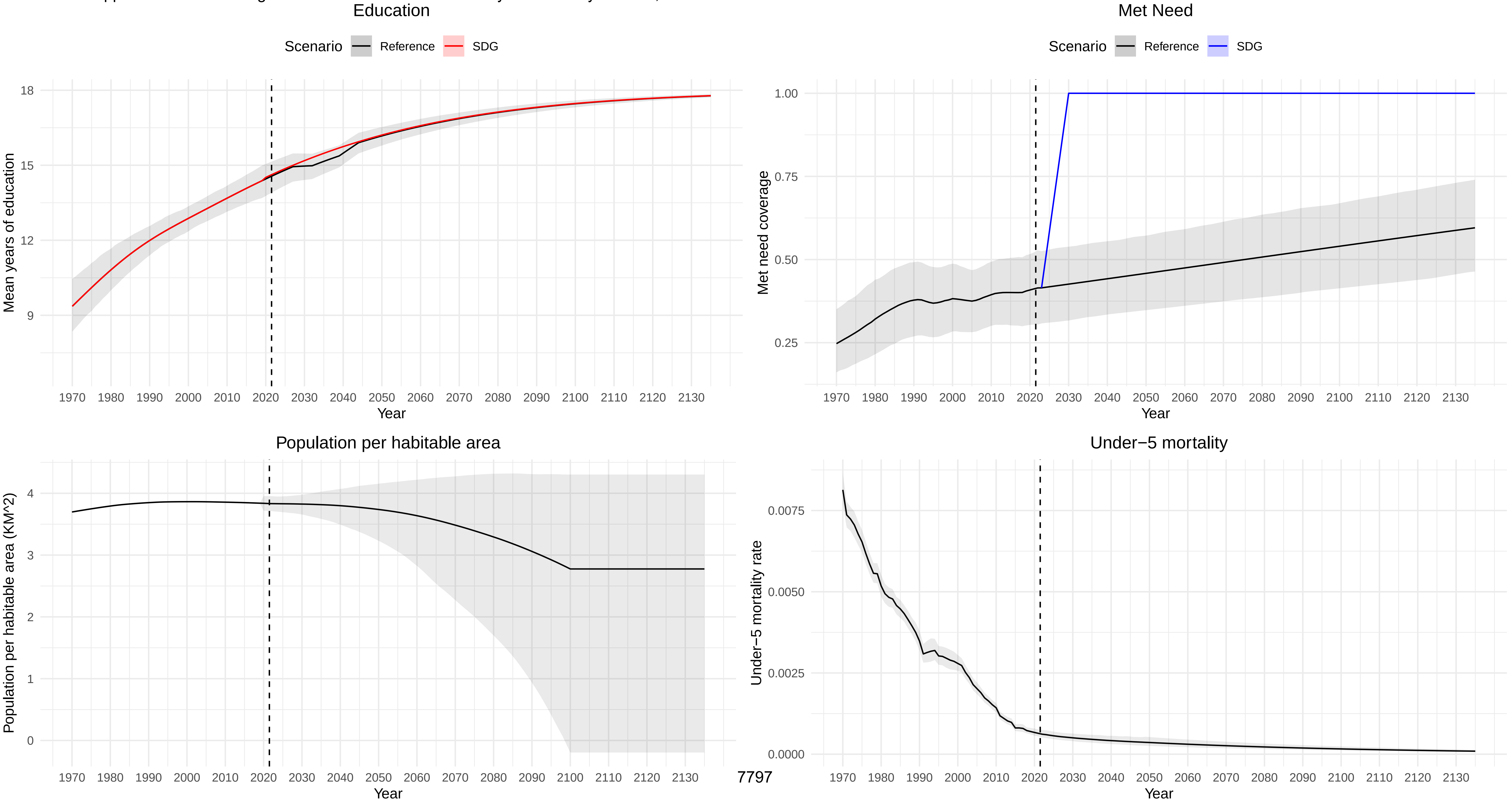

North Macedonia

Supplemental Results Figure S2: Covariates used in fertility forecasts by location, 1970-2135

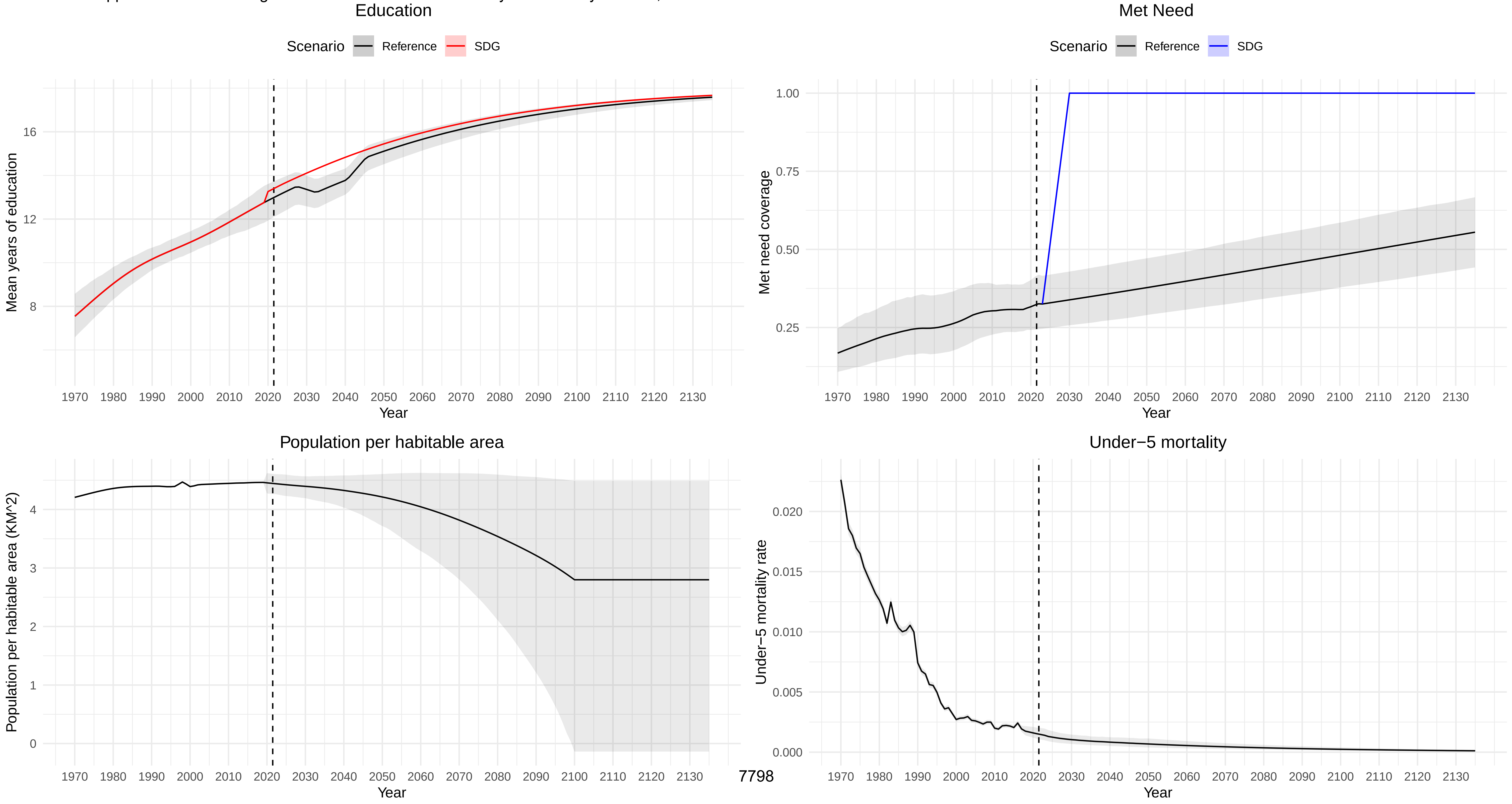

Poland

Supplemental Results Figure S2: Covariates used in fertility forecasts by location, 1970-2135

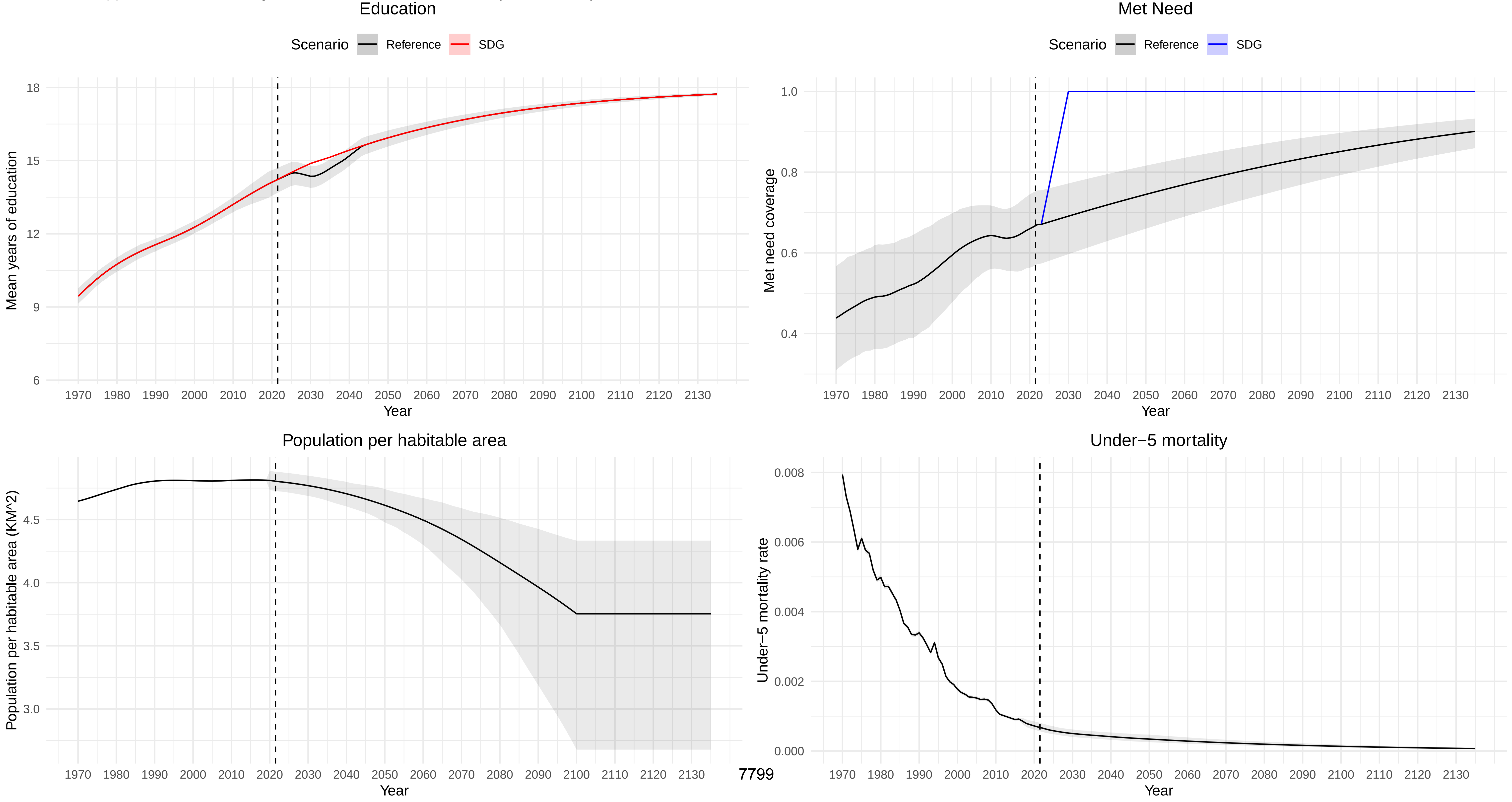

Romania

Supplemental Results Figure S2: Covariates used in fertility forecasts by location, 1970-2135

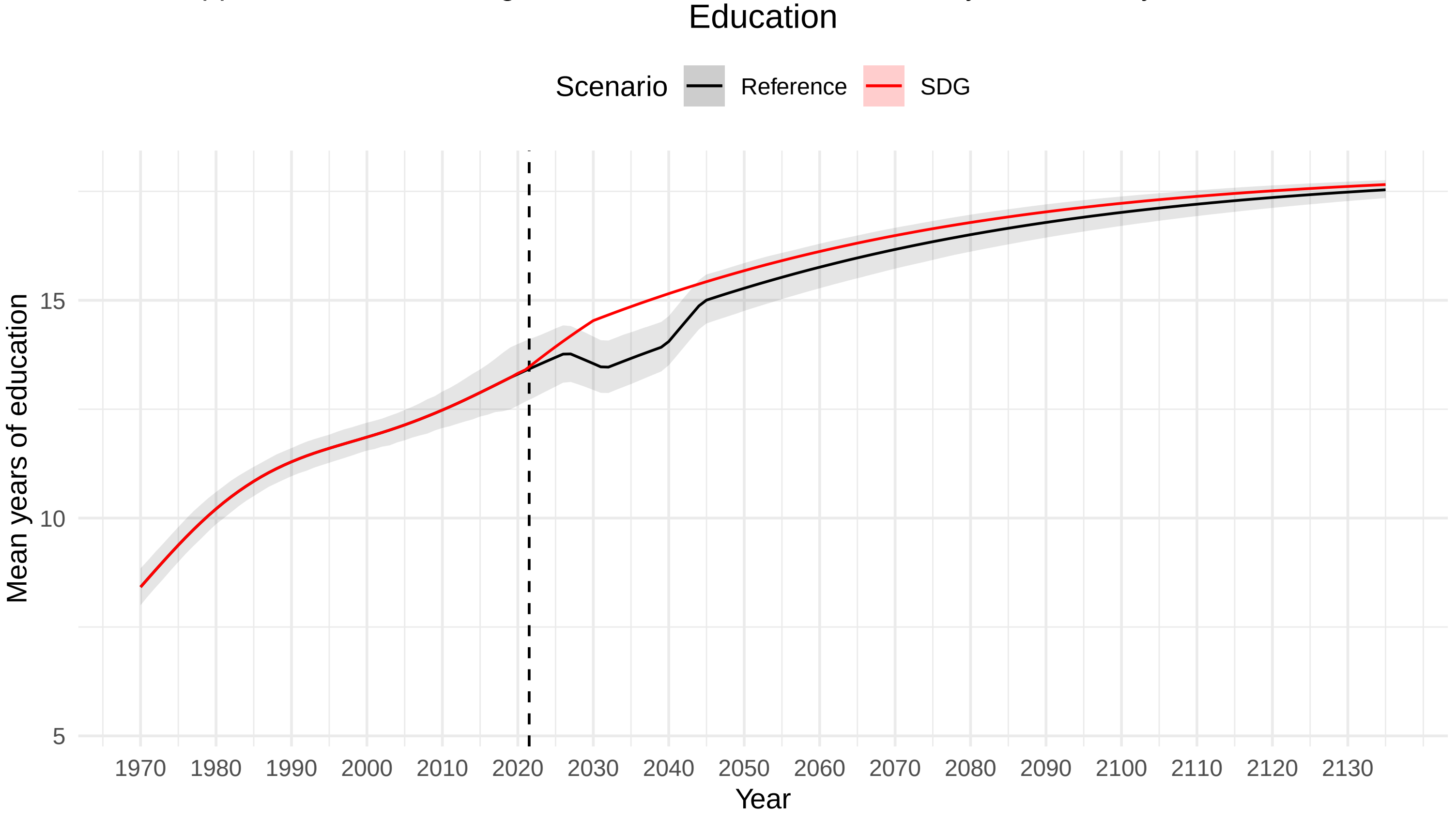

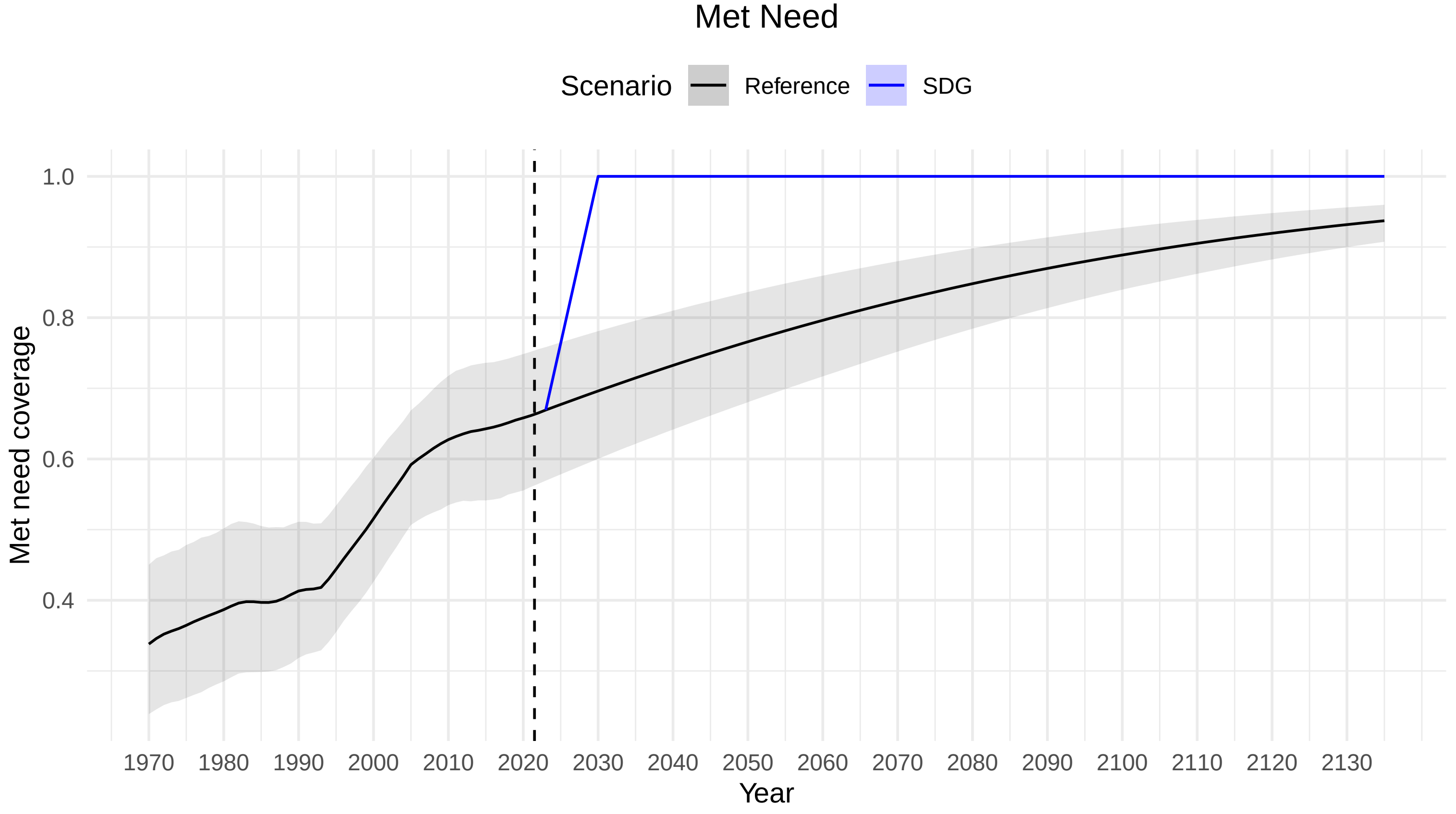

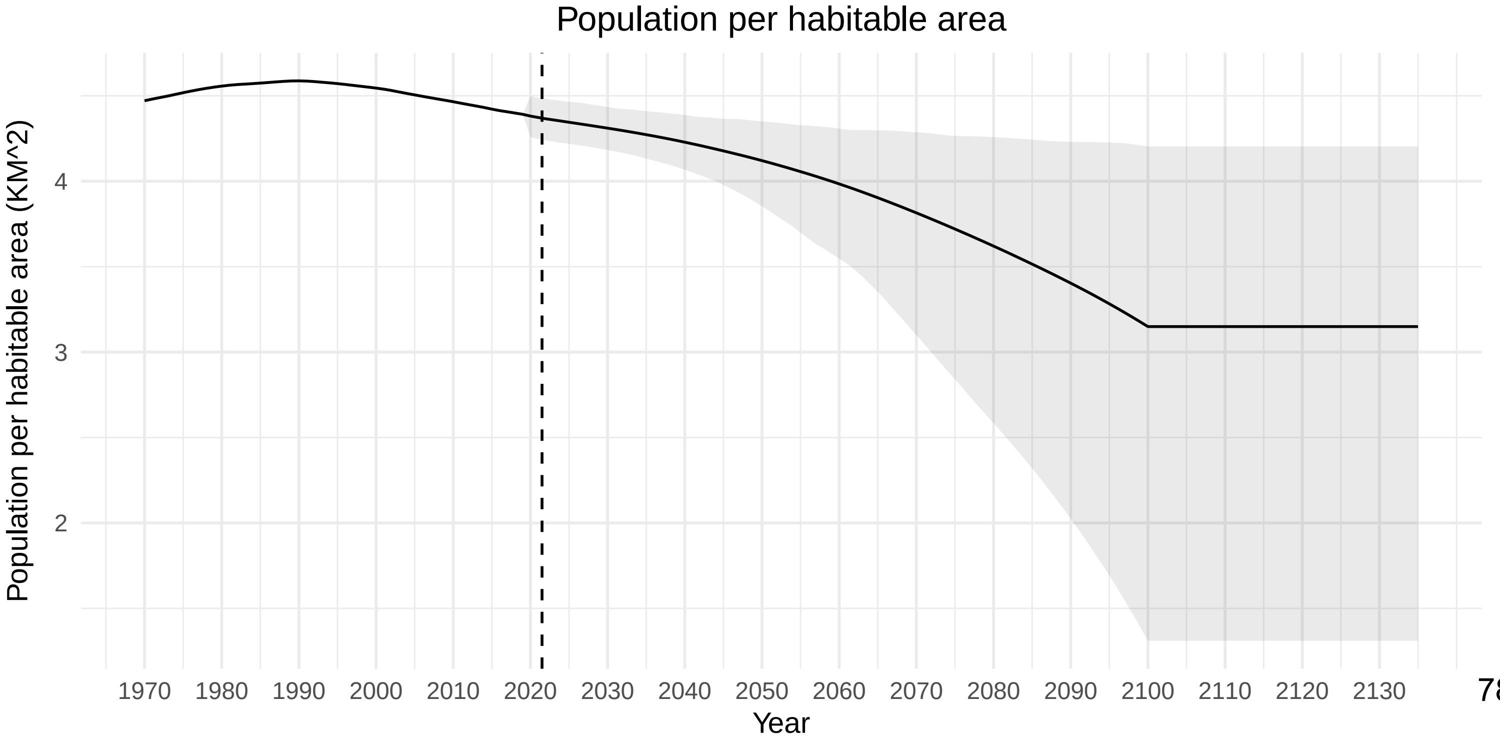

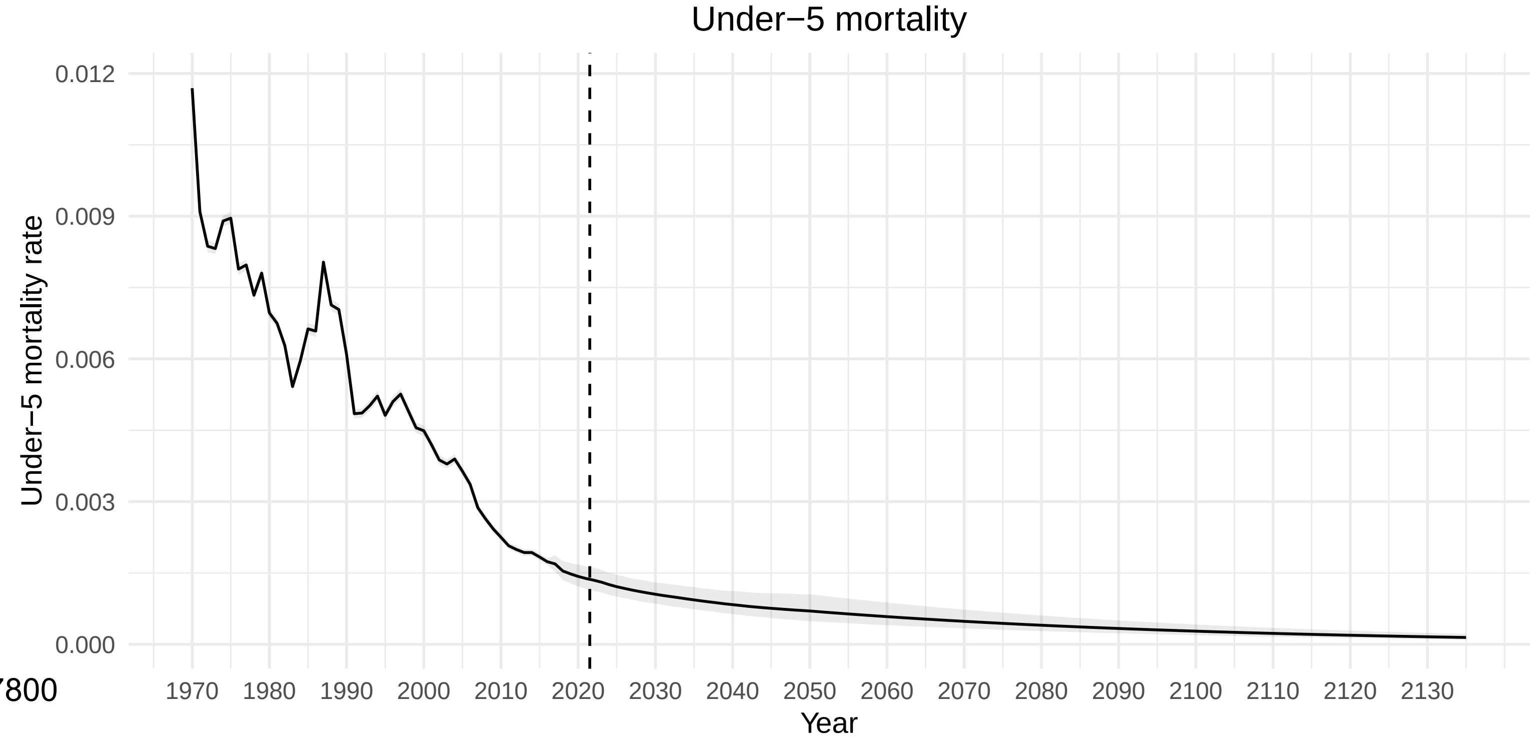

Serbia

Supplemental Results Figure S2: Covariates used in fertility forecasts by location, 1970-2135

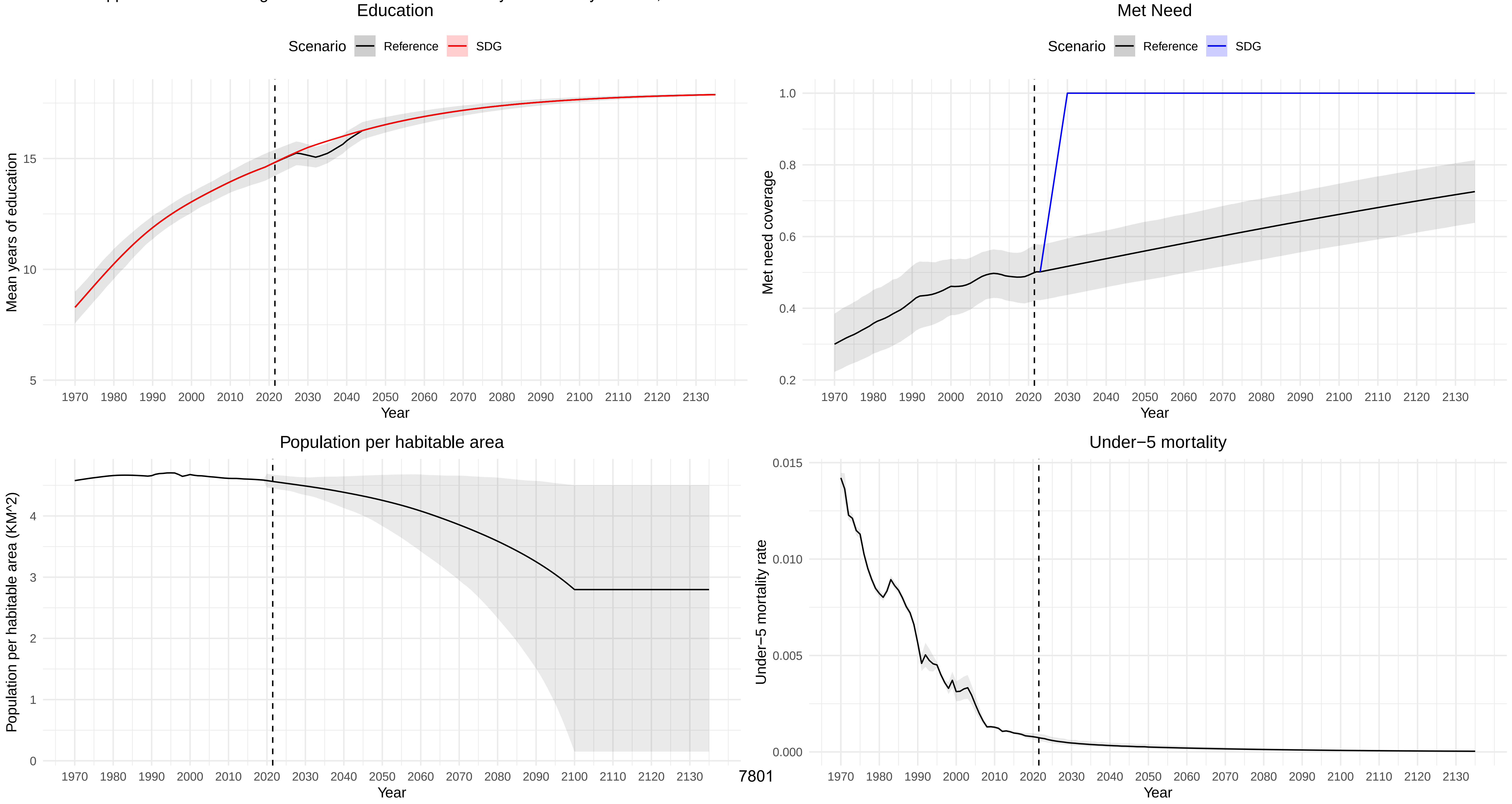

Slovakia

Supplemental Results Figure S2: Covariates used in fertility forecasts by location, 1970-2135

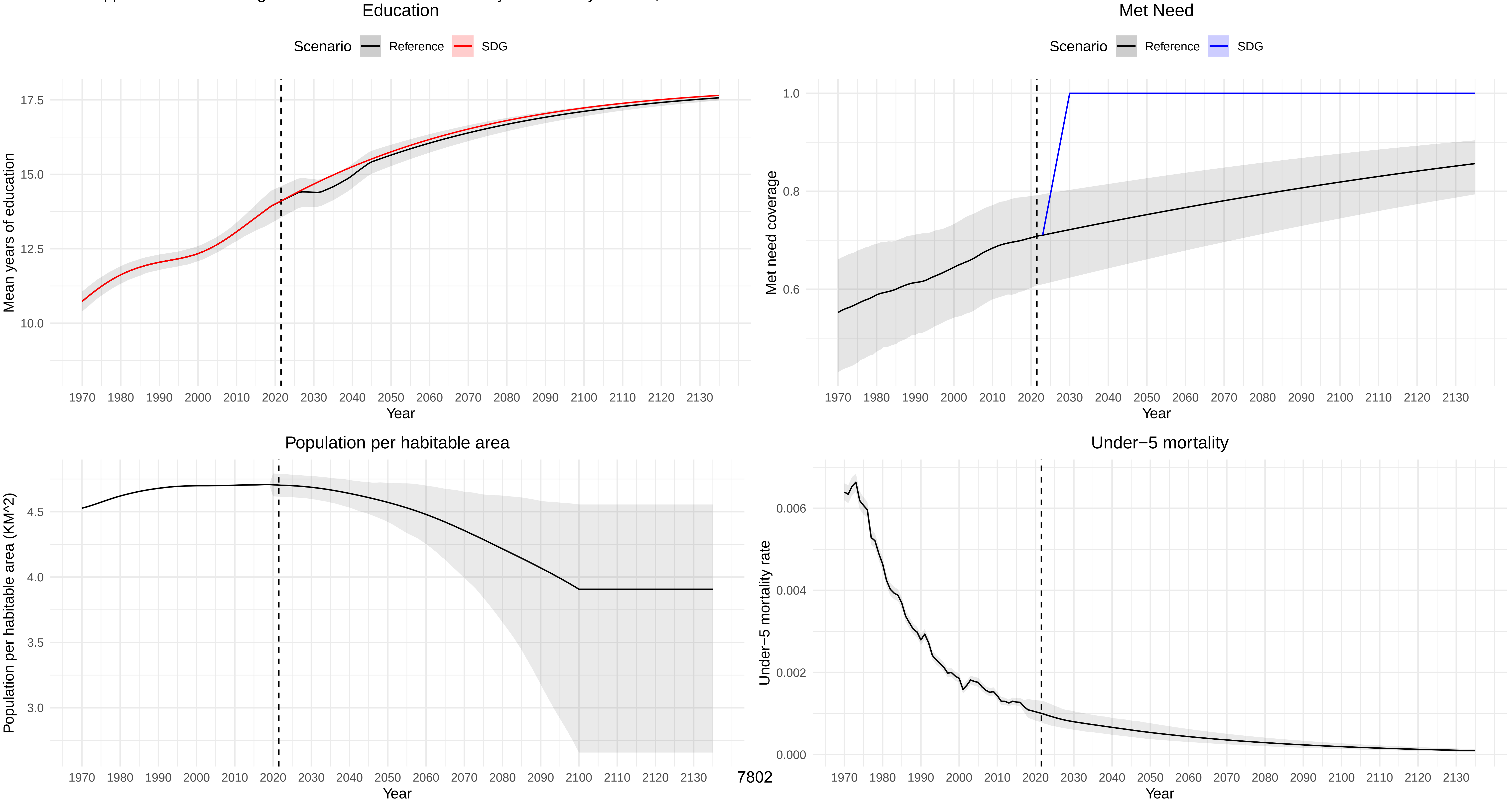

Slovenia

Supplemental Results Figure S2: Covariates used in fertility forecasts by location, 1970-2135

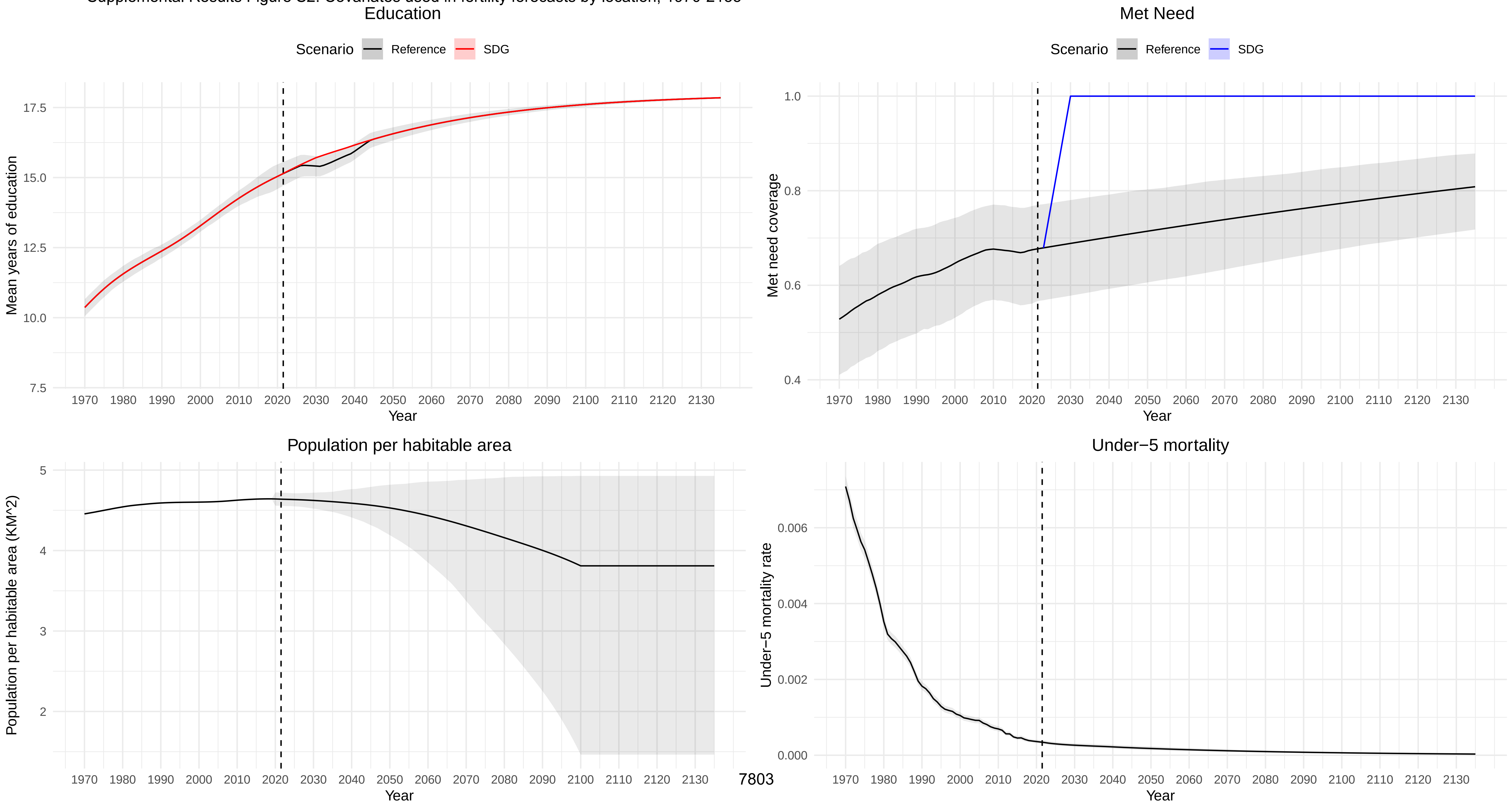

Belarus

Supplemental Results Figure S2: Covariates used in fertility forecasts by location, 1970-2135

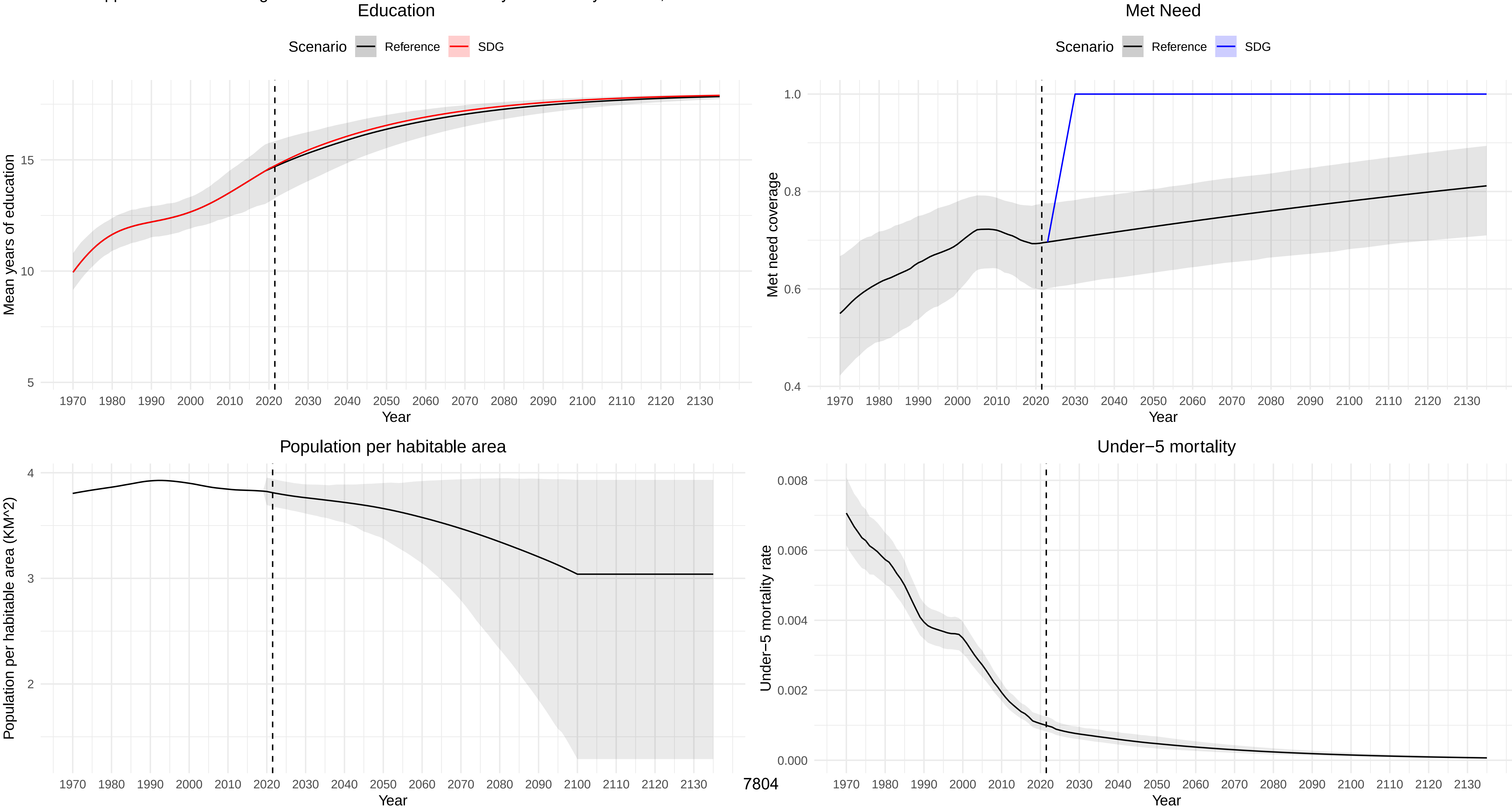

Estonia

Supplemental Results Figure S2: Covariates used in fertility forecasts by location, 1970-2135

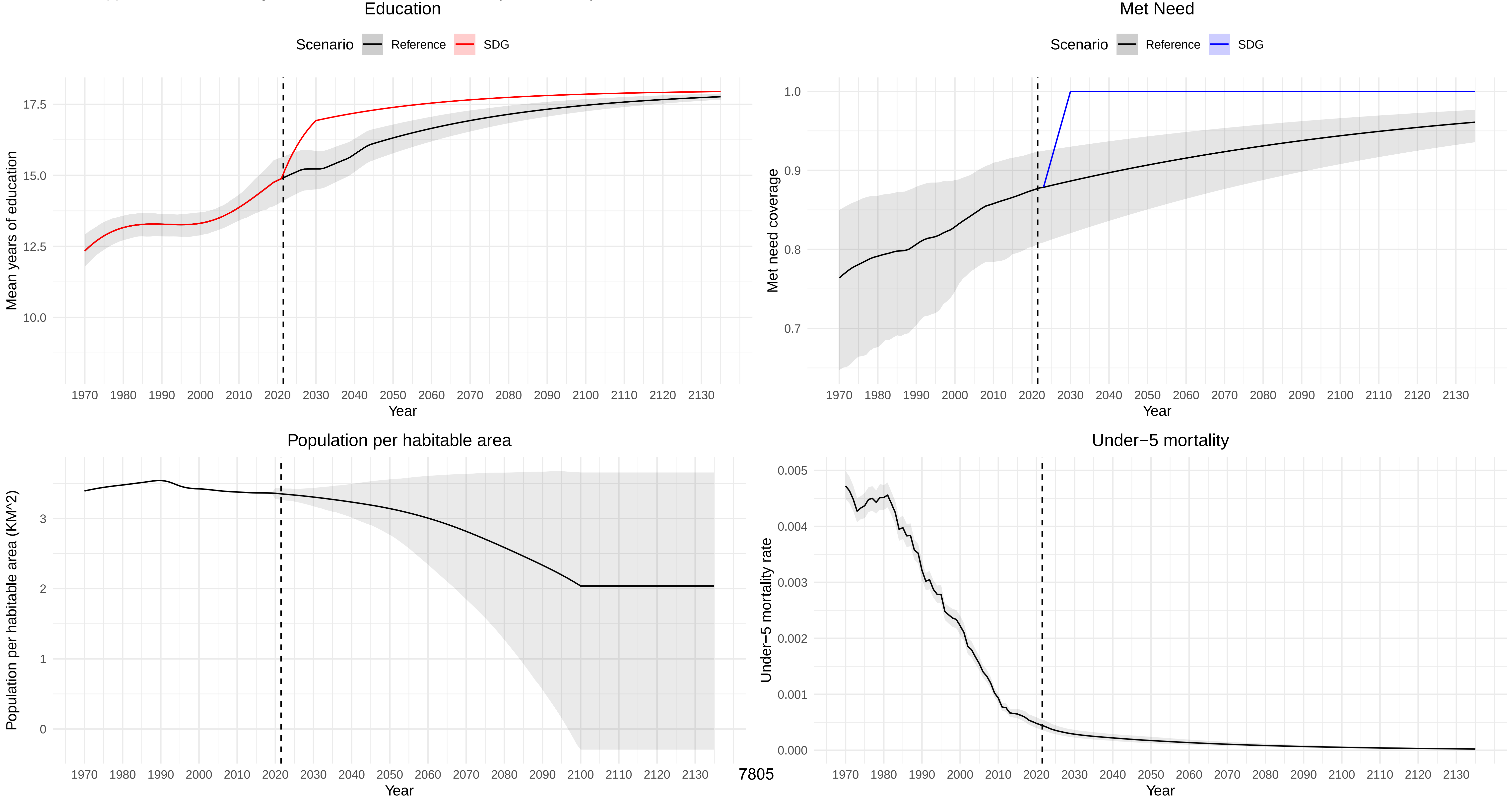

Latvia

Supplemental Results Figure S2: Covariates used in fertility forecasts by location, 1970-2135

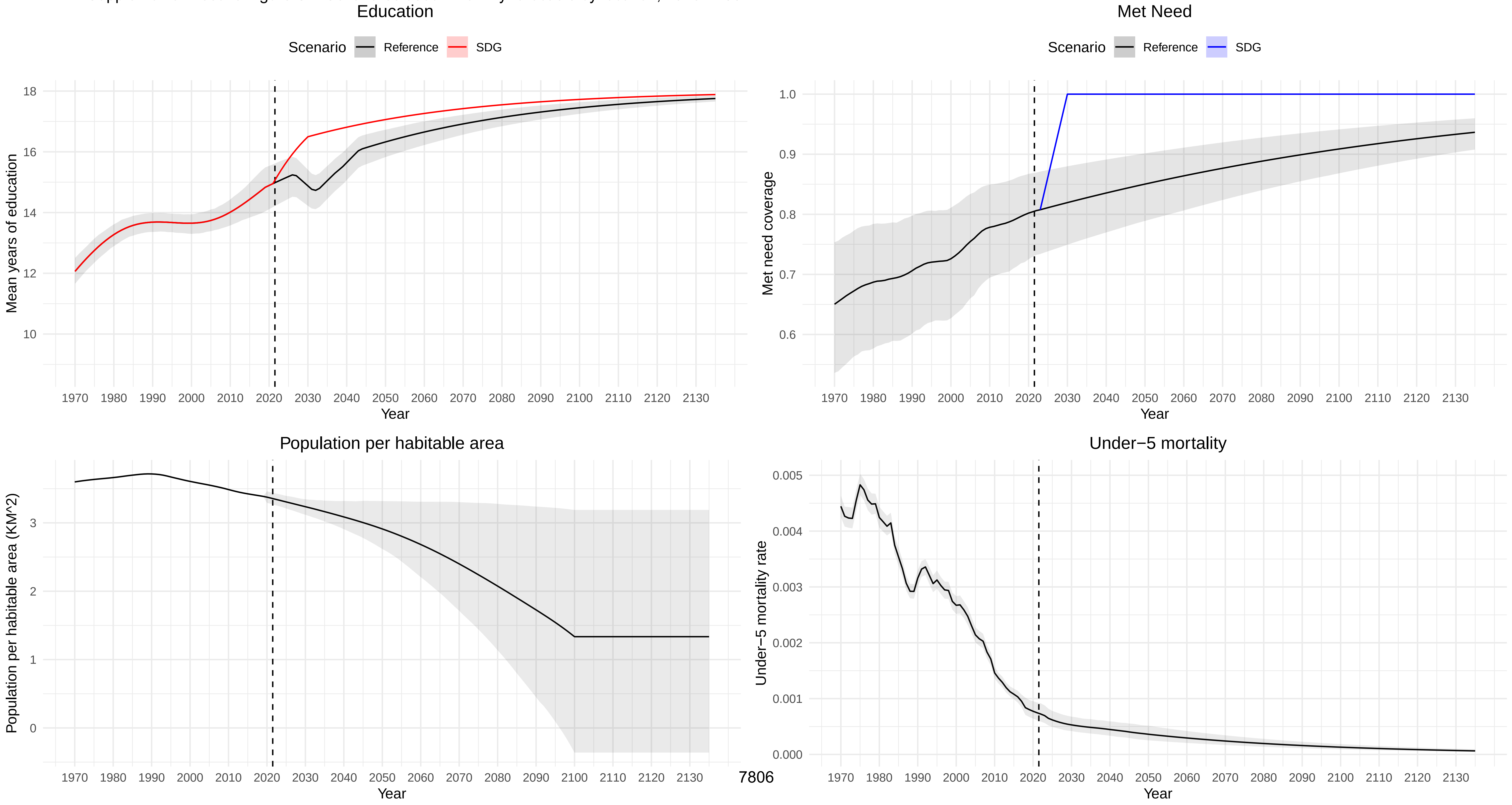

Lithuania

Supplemental Results Figure S2: Covariates used in fertility forecasts by location, 1970-2135

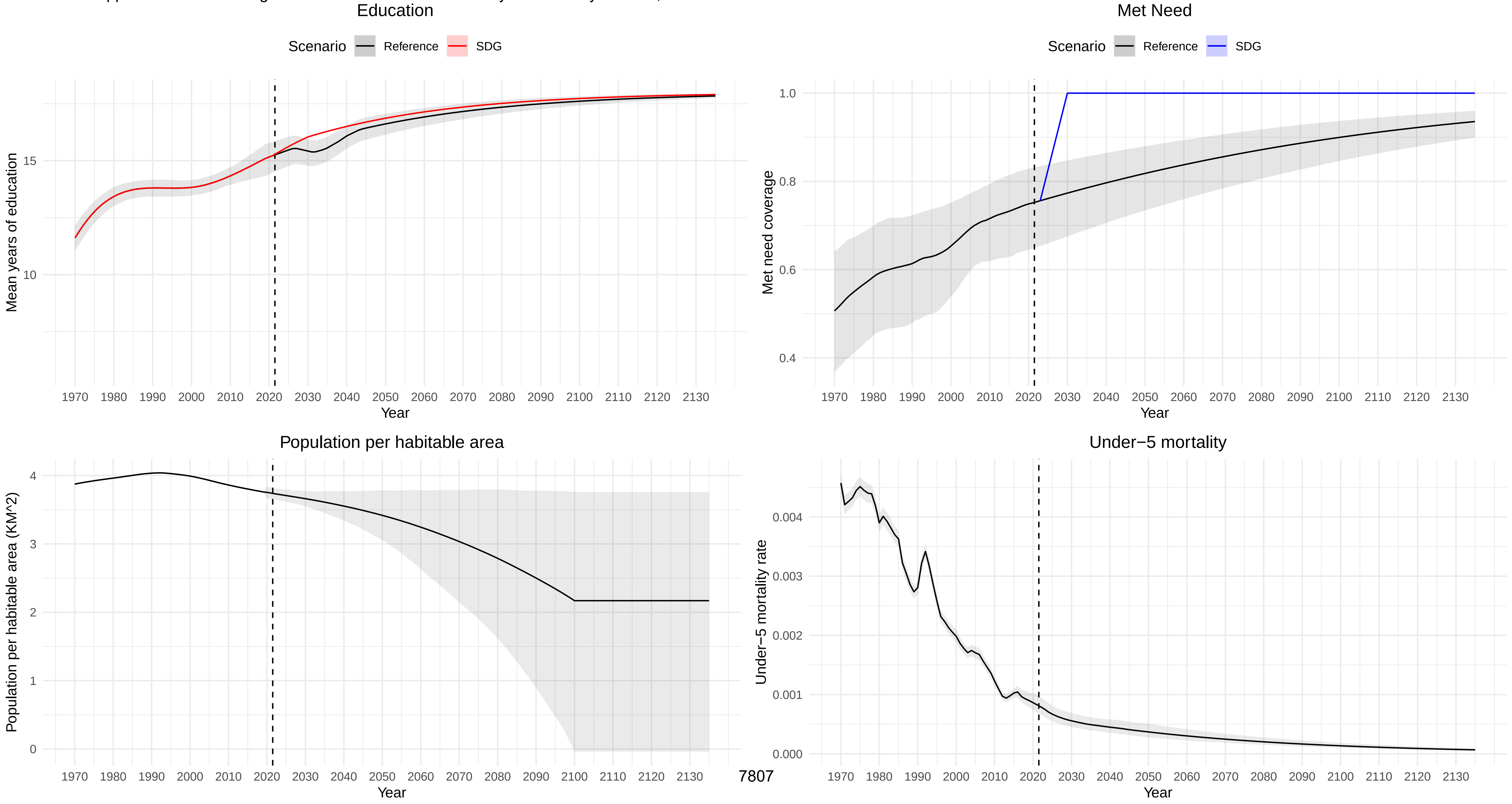

Republic of Moldova

Supplemental Results Figure S2: Covariates used in fertility forecasts by location, 1970-2135

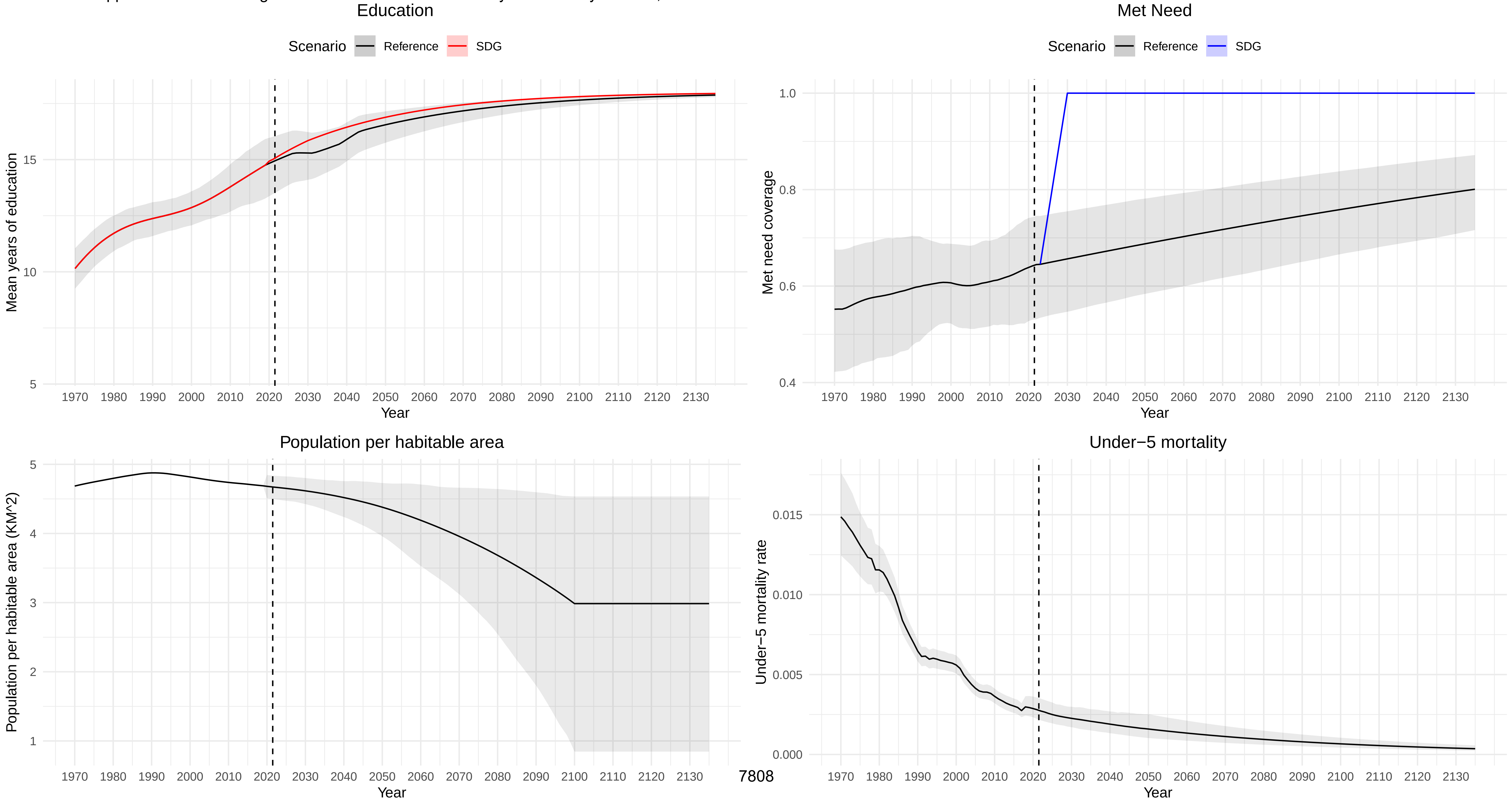

Russia

Supplemental Results Figure S2: Covariates used in fertility forecasts by location, 1970-2135

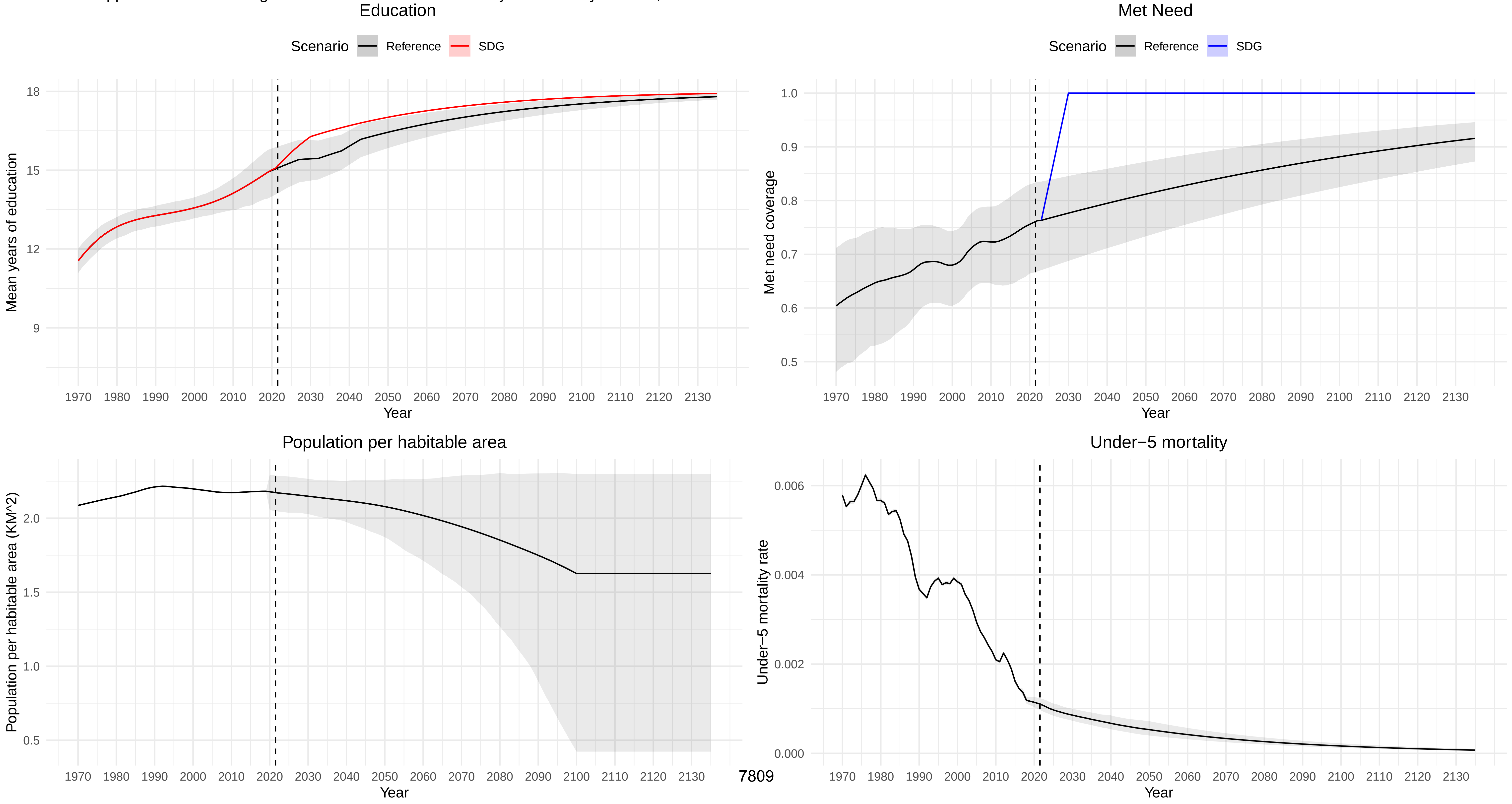

Ukraine

Supplemental Results Figure S2: Covariates used in fertility forecasts by location, 1970-2135

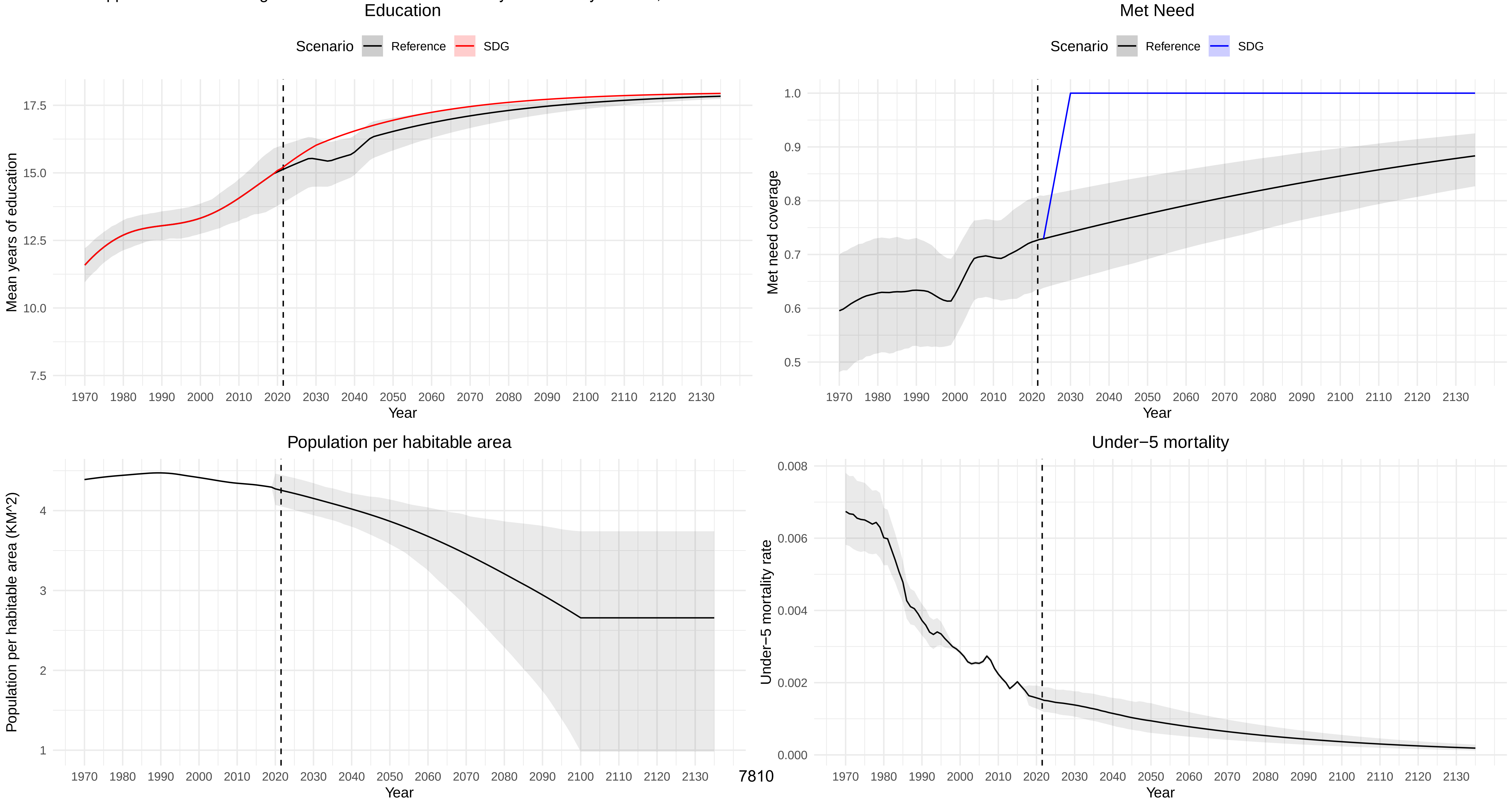

Australia

Supplemental Results Figure S2: Covariates used in fertility forecasts by location, 1970-2135

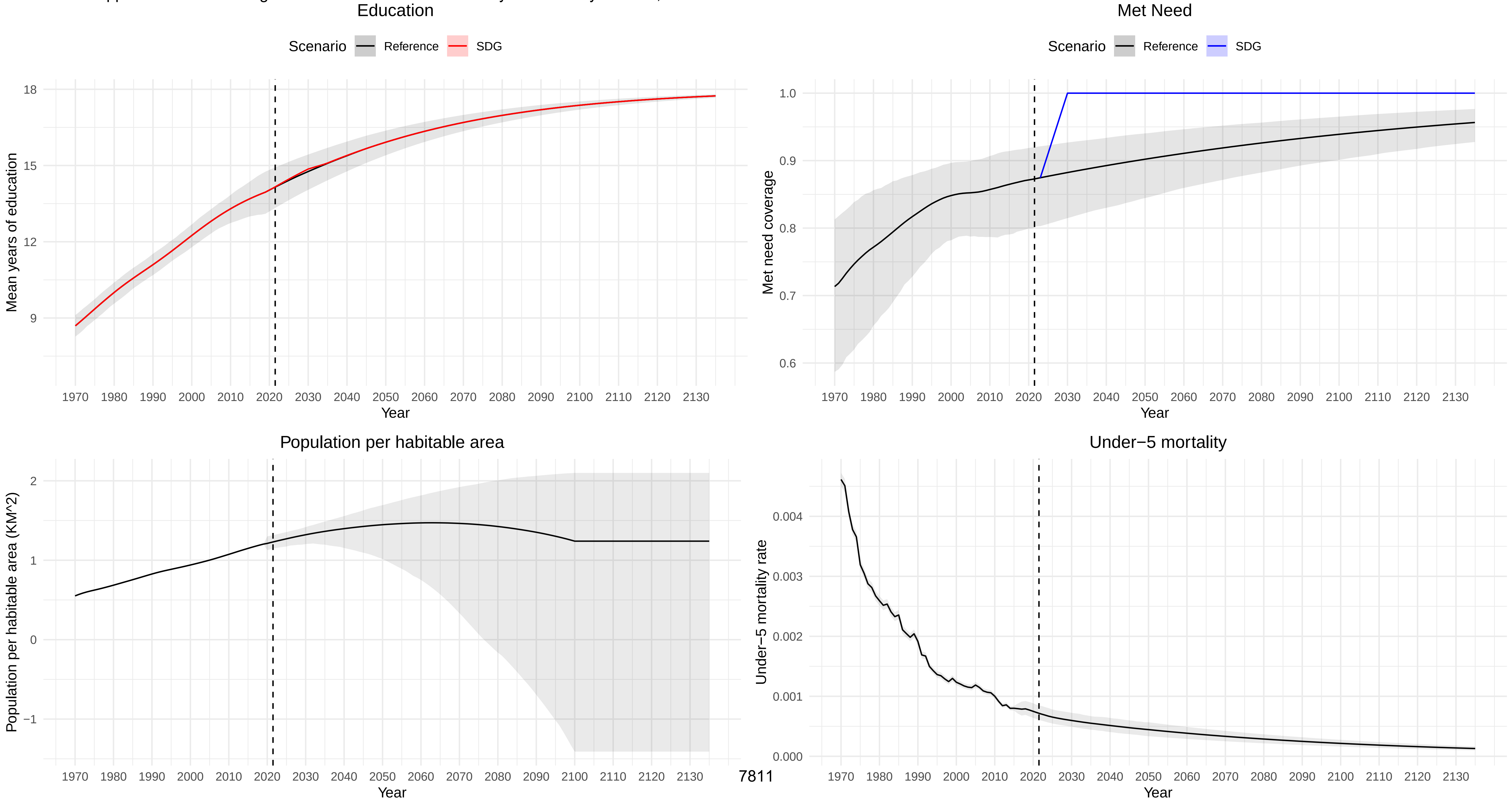

New Zealand

Supplemental Results Figure S2: Covariates used in fertility forecasts by location, 1970-2135

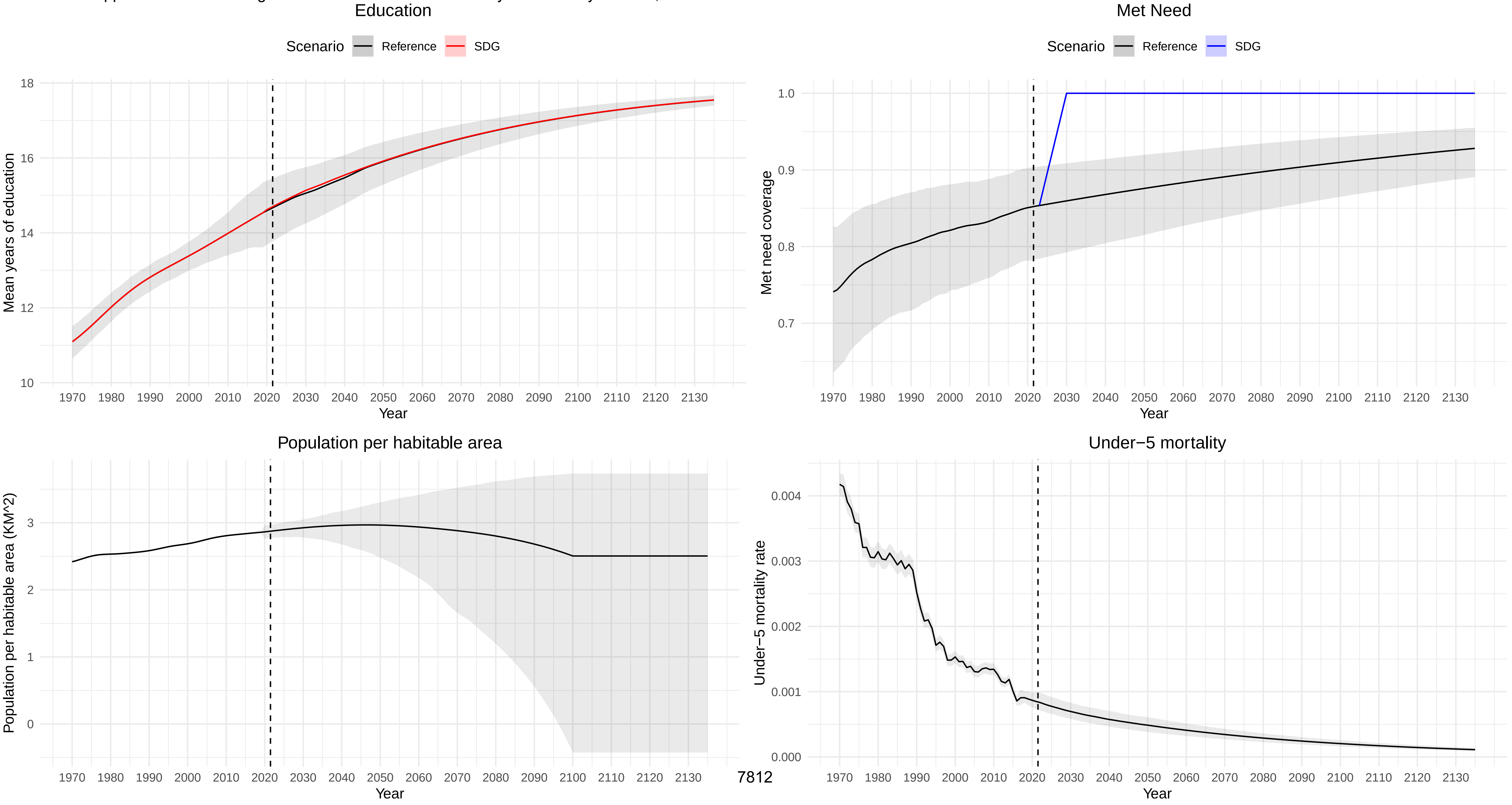

Brunei Darussalam

Supplemental Results Figure S2: Covariates used in fertility forecasts by location, 1970-2135

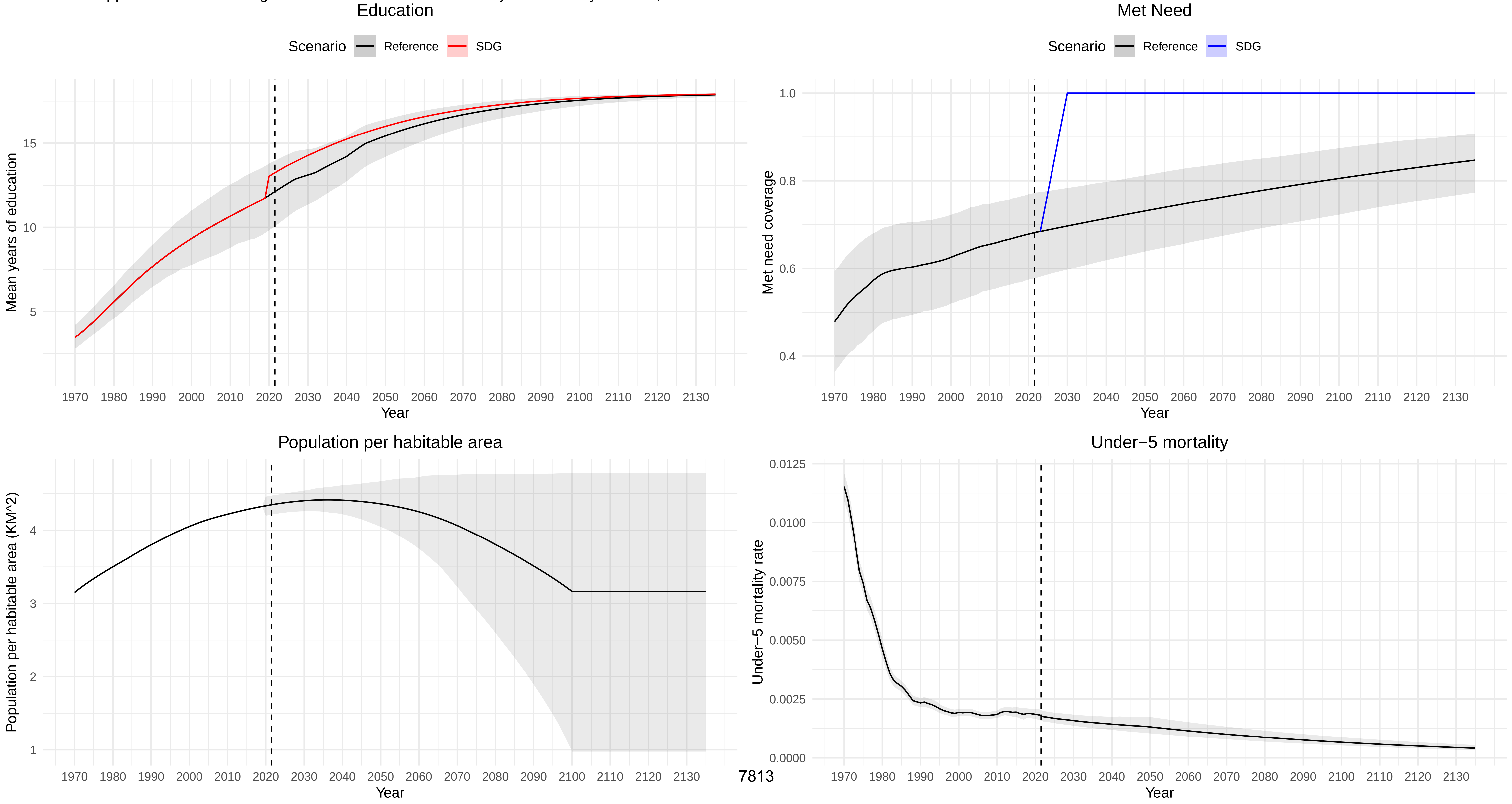

Japan

Supplemental Results Figure S2: Covariates used in fertility forecasts by location, 1970-2135

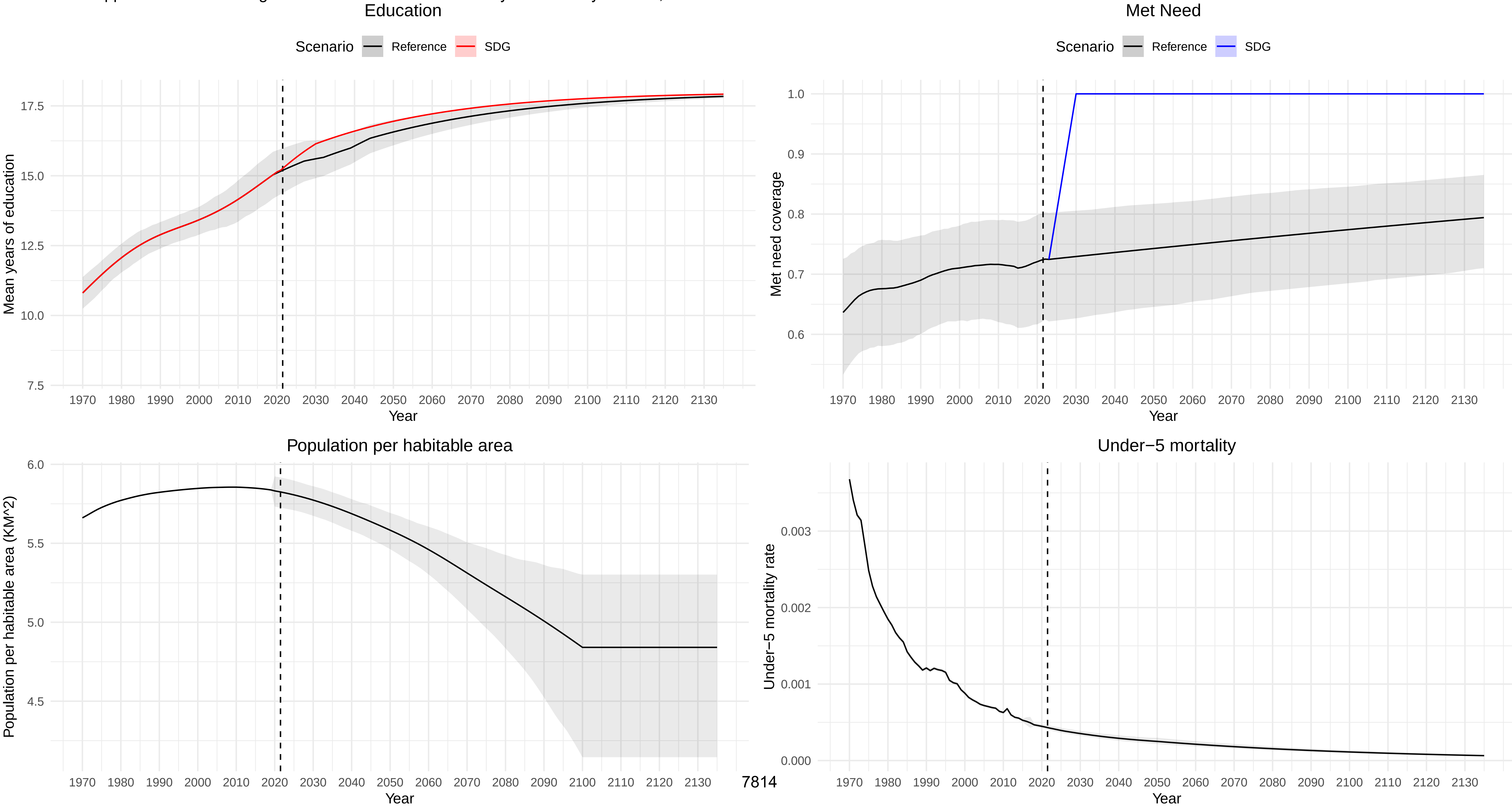

South Korea

Supplemental Results Figure S2: Covariates used in fertility forecasts by location, 1970-2135

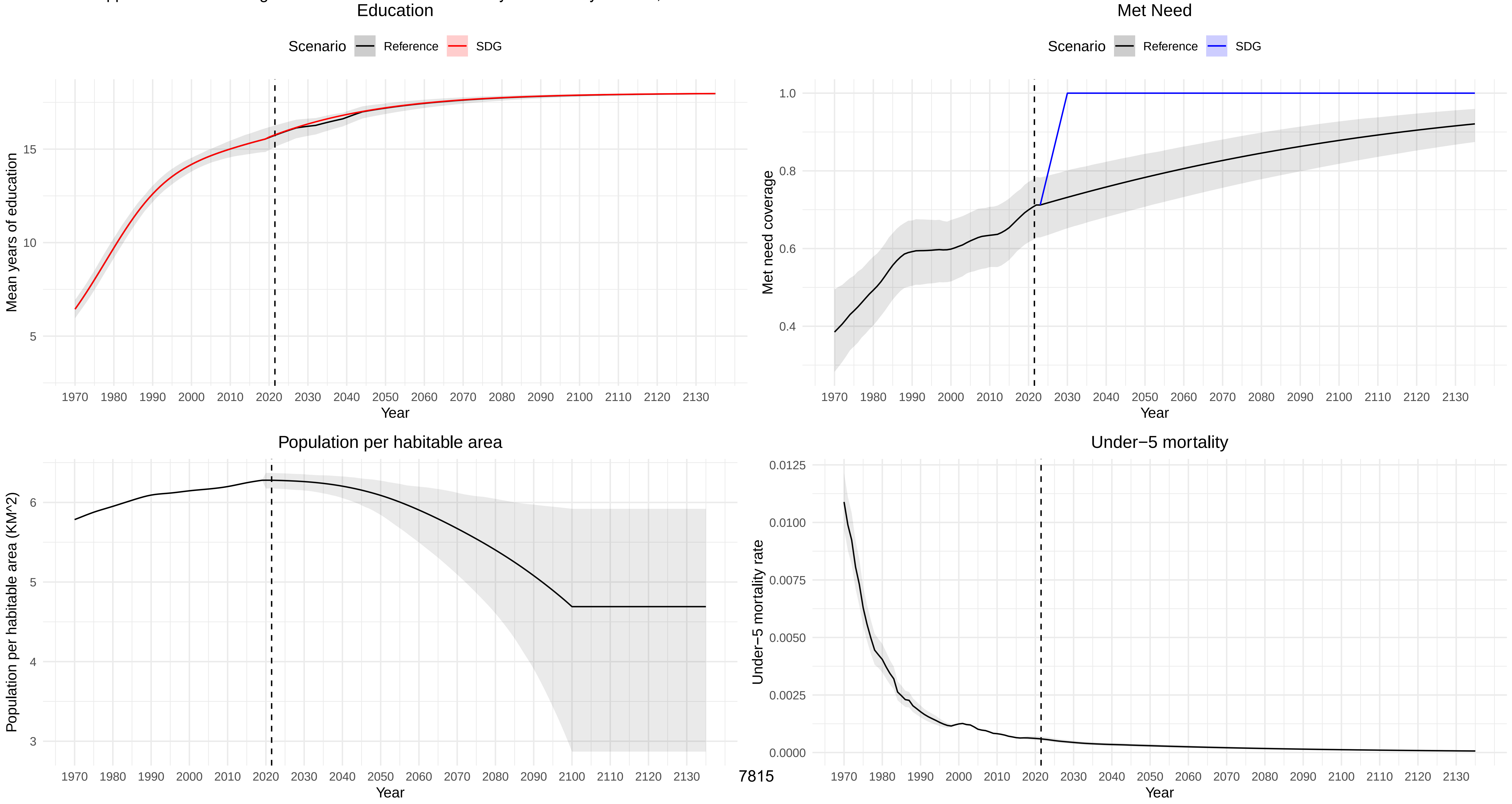

Singapore

Supplemental Results Figure S2: Covariates used in fertility forecasts by location, 1970-2135

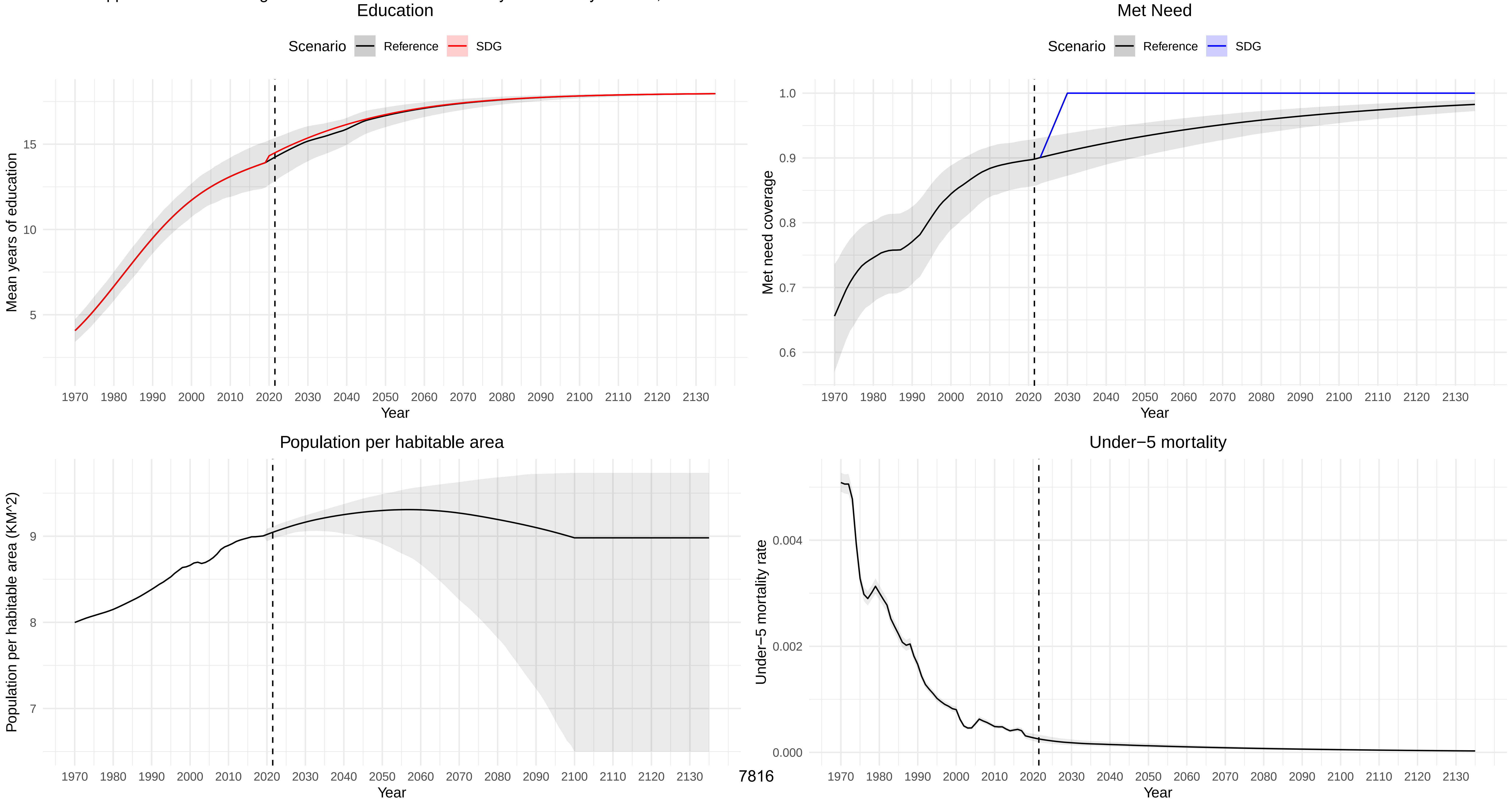

Canada

Supplemental Results Figure S2: Covariates used in fertility forecasts by location, 1970-2135

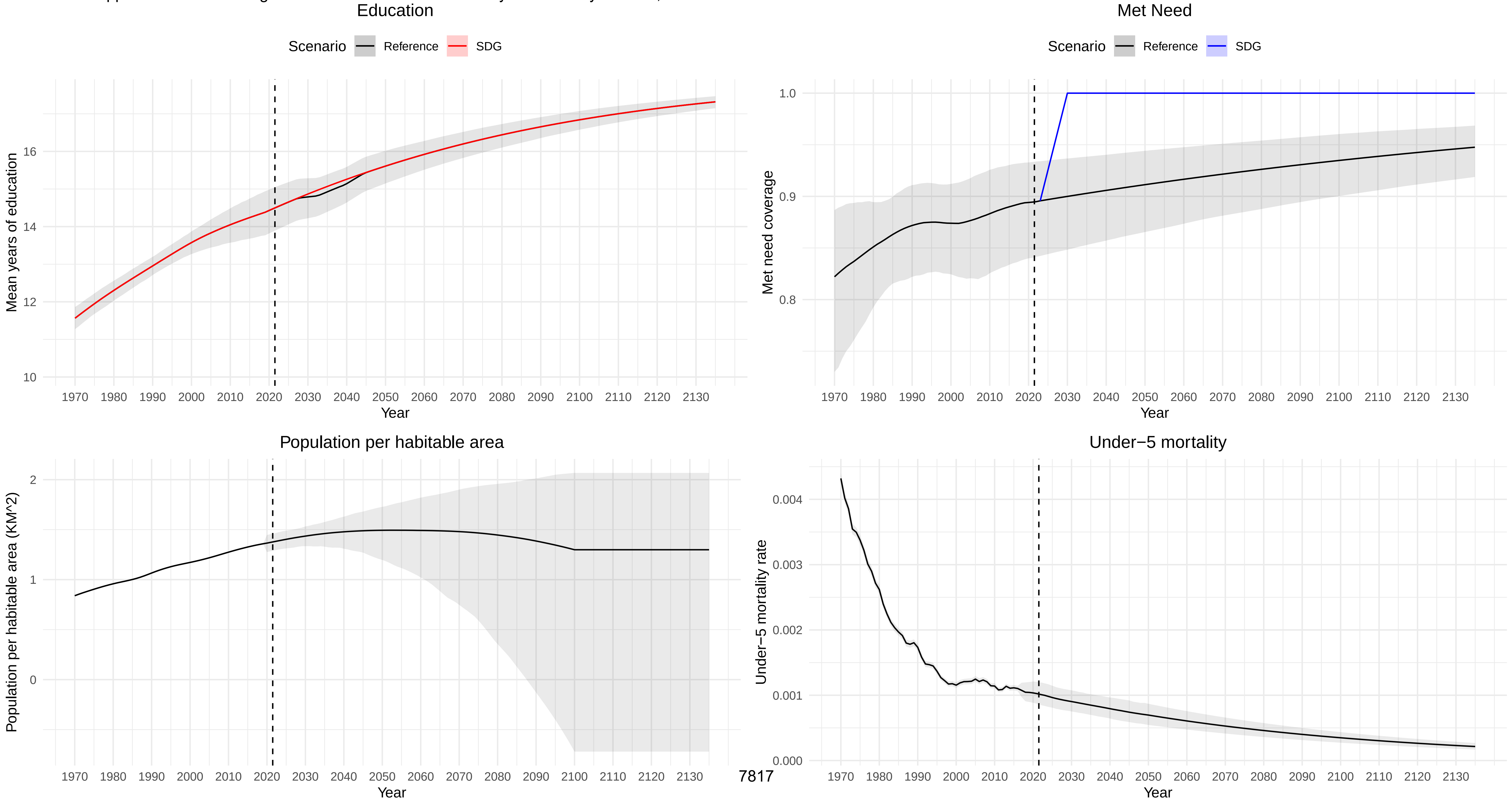

Greenland

Supplemental Results Figure S2: Covariates used in fertility forecasts by location, 1970-2135

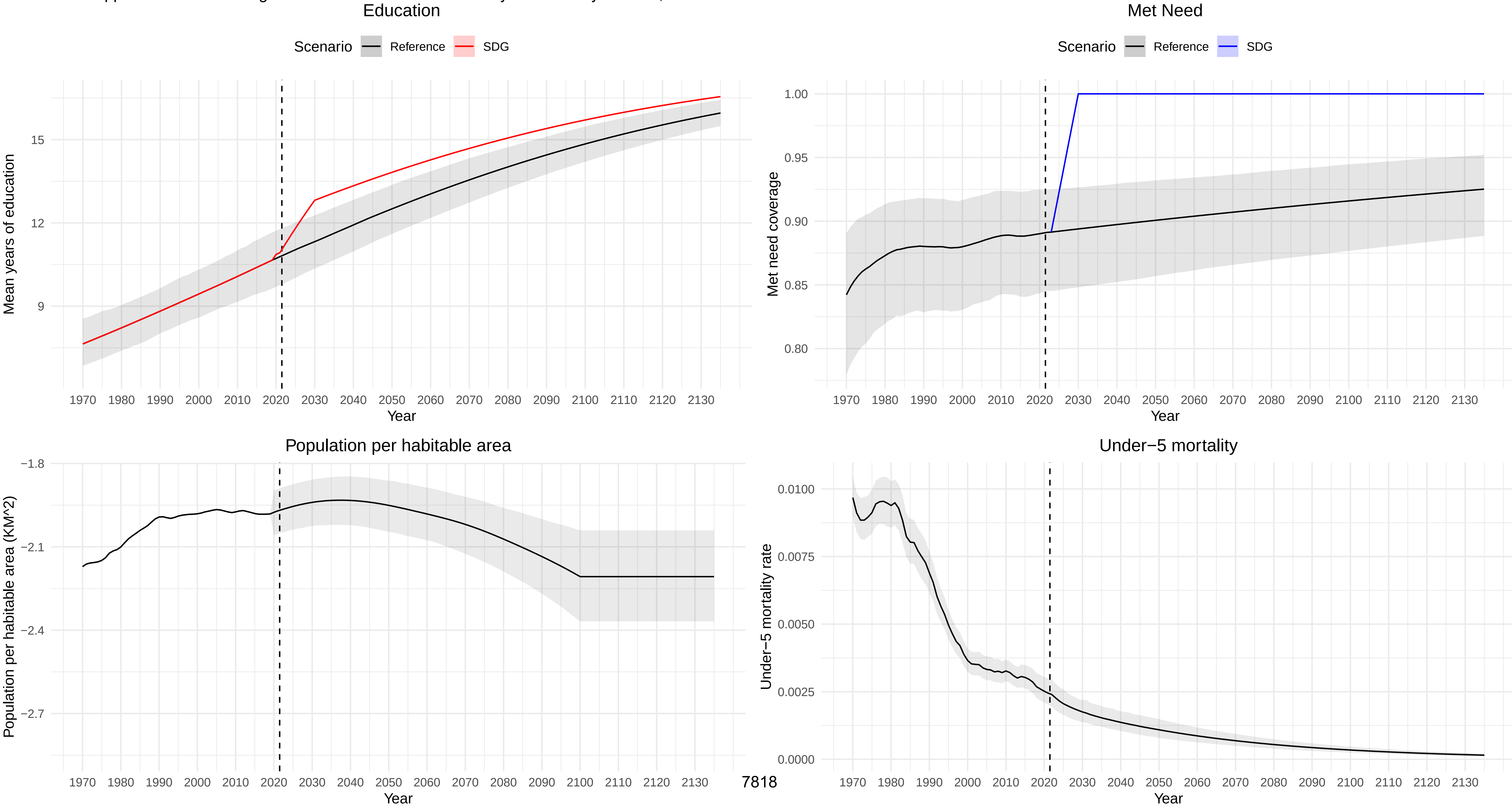

USA

Supplemental Results Figure S2: Covariates used in fertility forecasts by location, 1970-2135

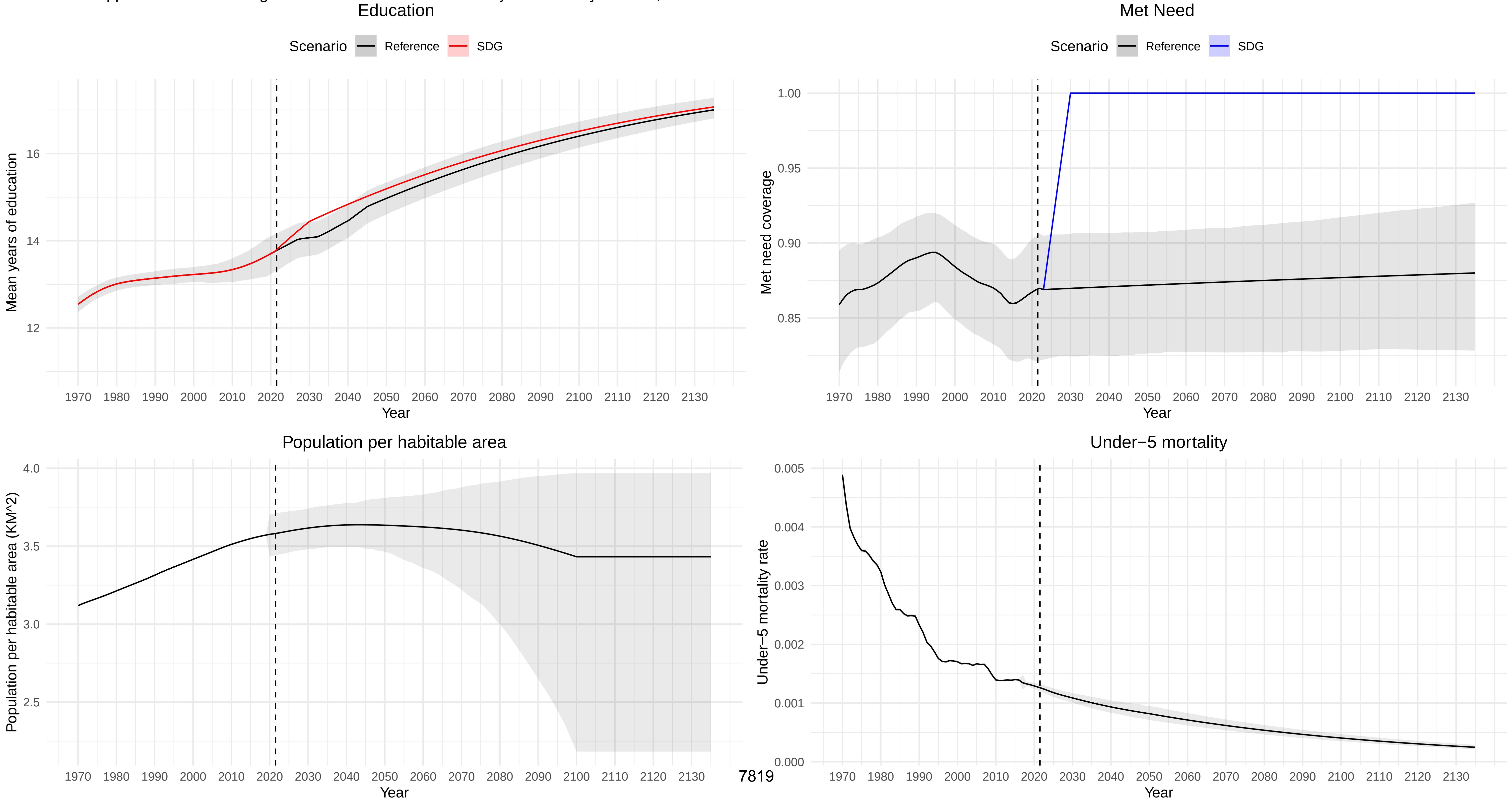

Argentina

Supplemental Results Figure S2: Covariates used in fertility forecasts by location, 1970-2135

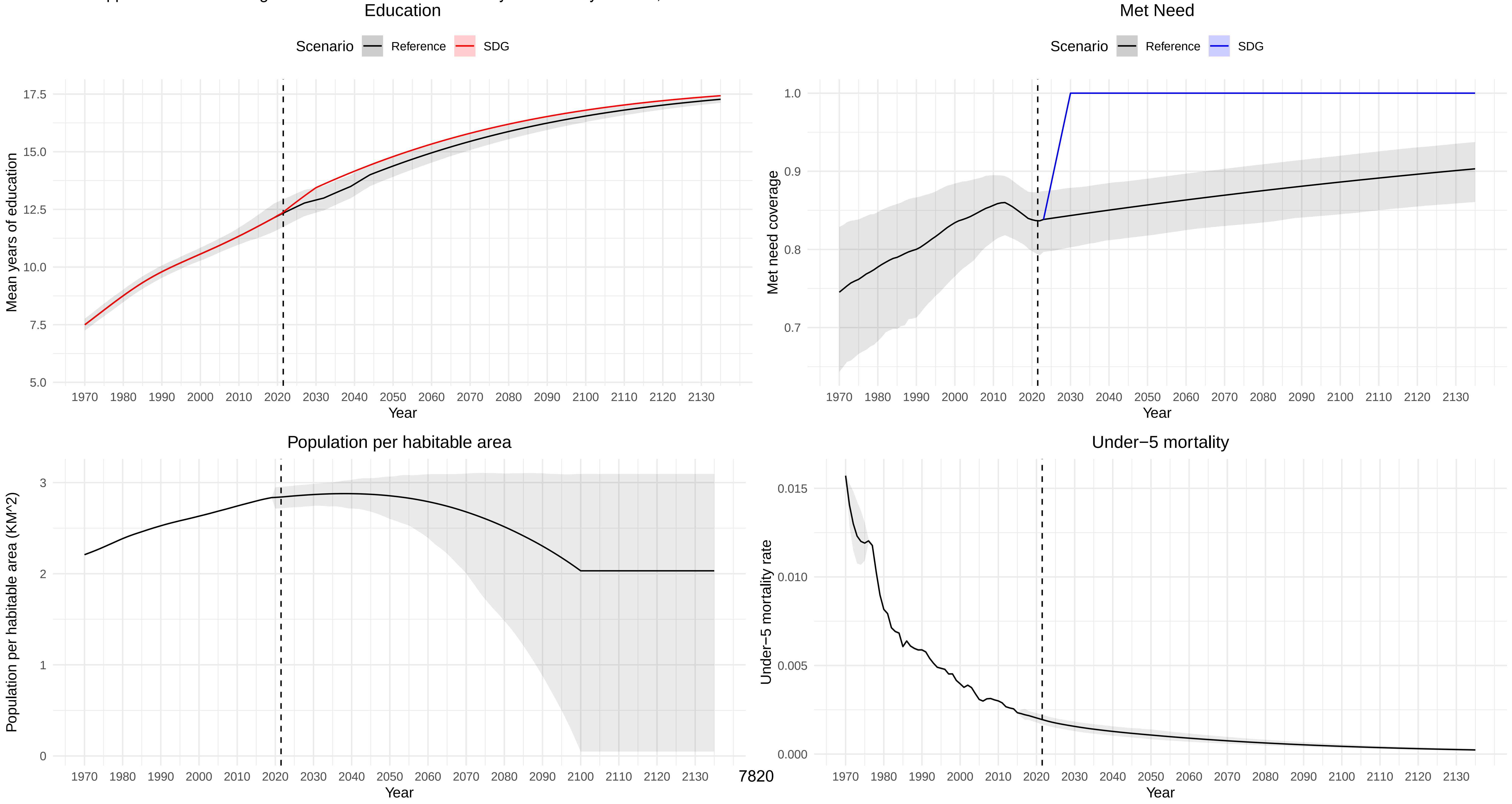

Chile

Supplemental Results Figure S2: Covariates used in fertility forecasts by location, 1970-2135

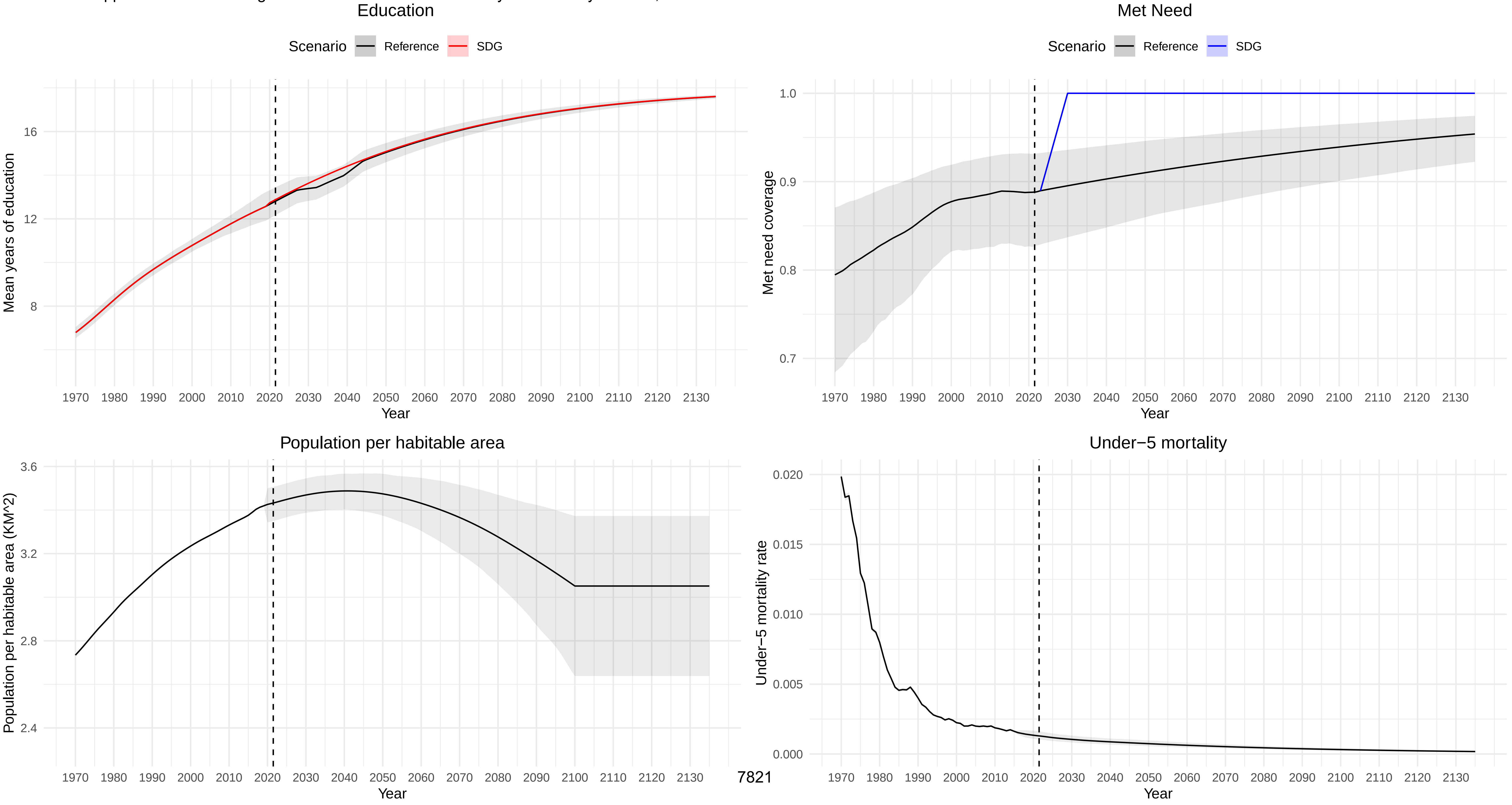

Uruguay

Supplemental Results Figure S2: Covariates used in fertility forecasts by location, 1970-2135

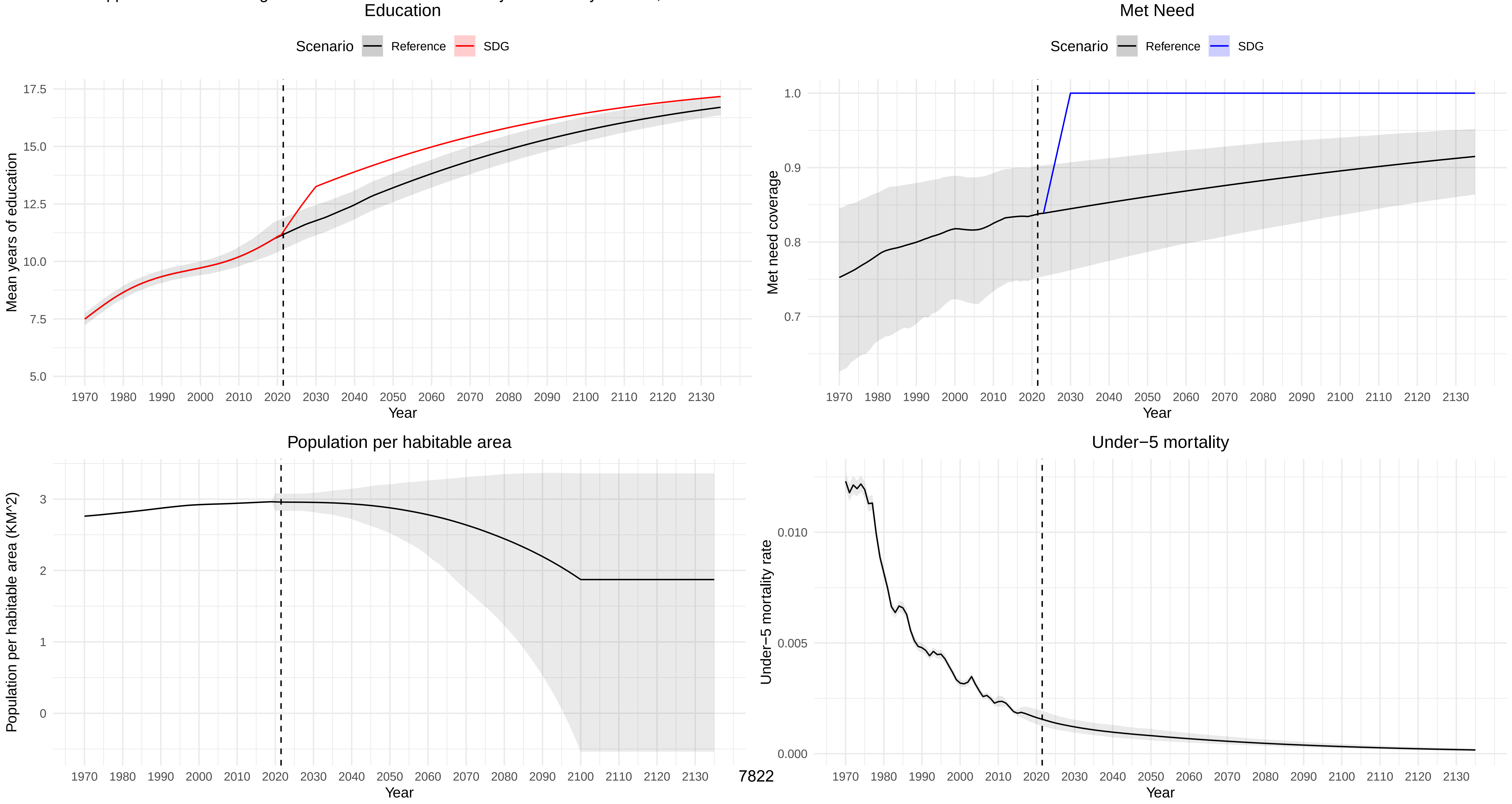

Andorra

Supplemental Results Figure S2: Covariates used in fertility forecasts by location, 1970-2135

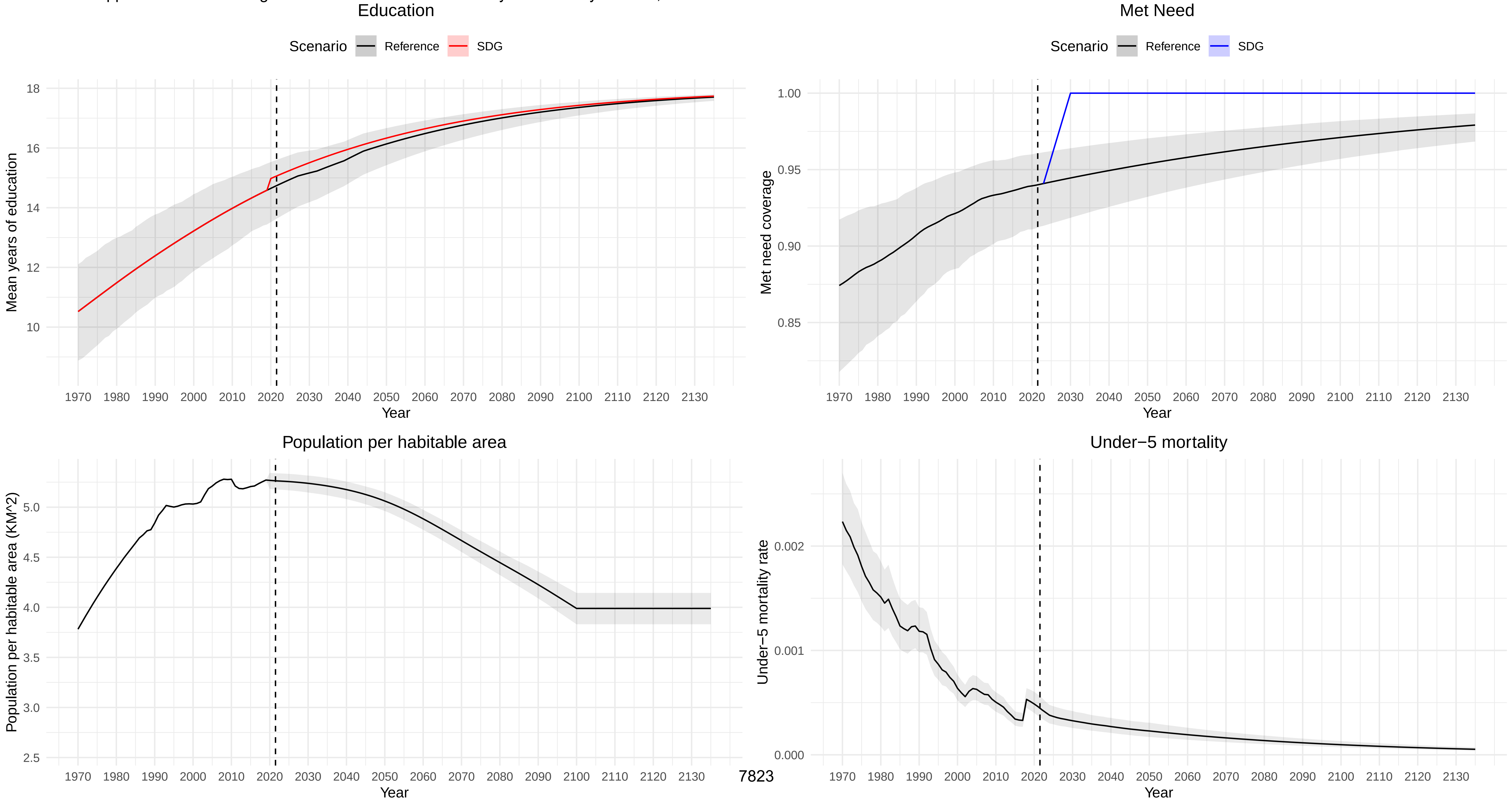

Austria

Supplemental Results Figure S2: Covariates used in fertility forecasts by location, 1970-2135

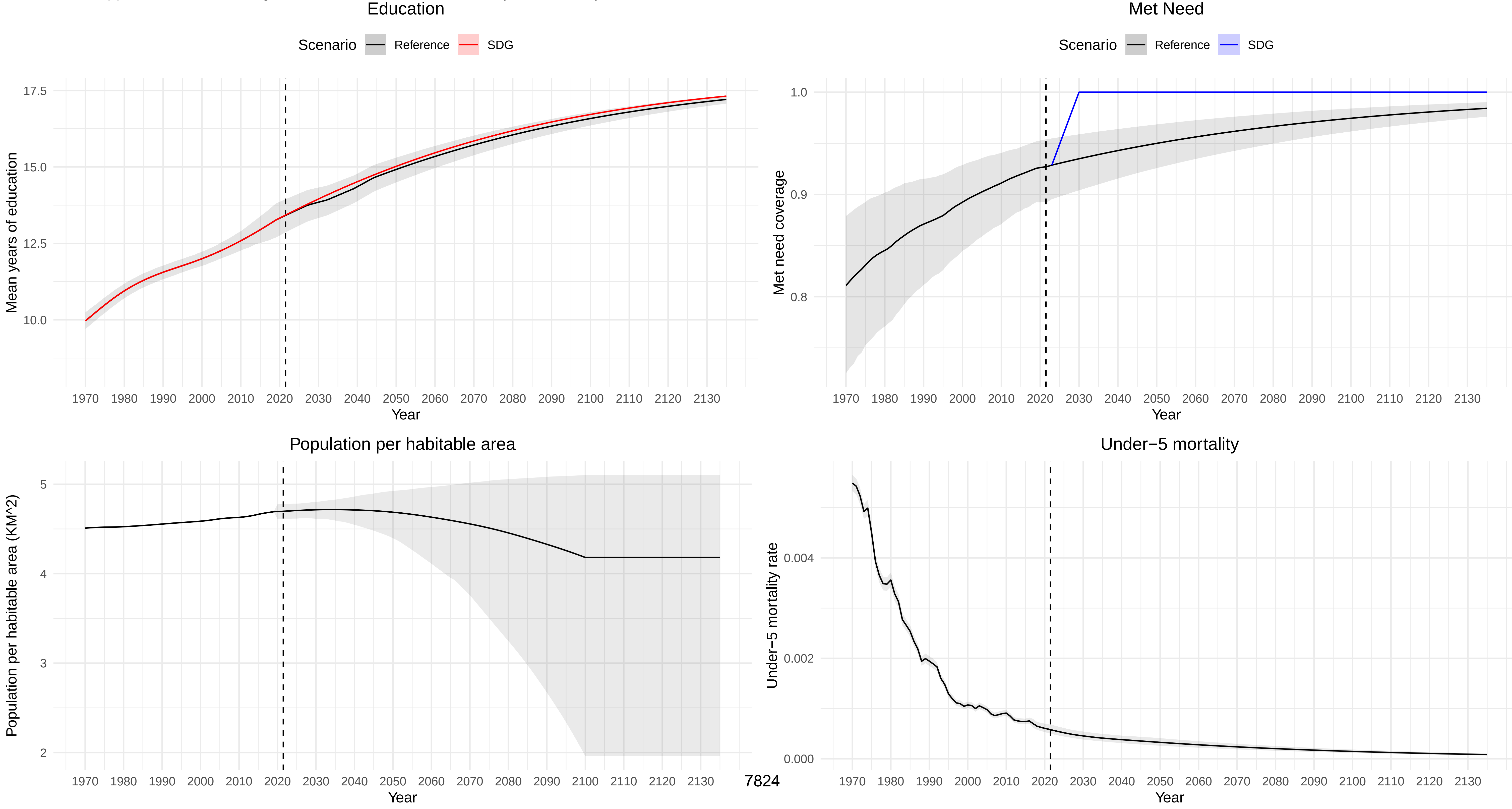

Belgium

Supplemental Results Figure S2: Covariates used in fertility forecasts by location, 1970-2135

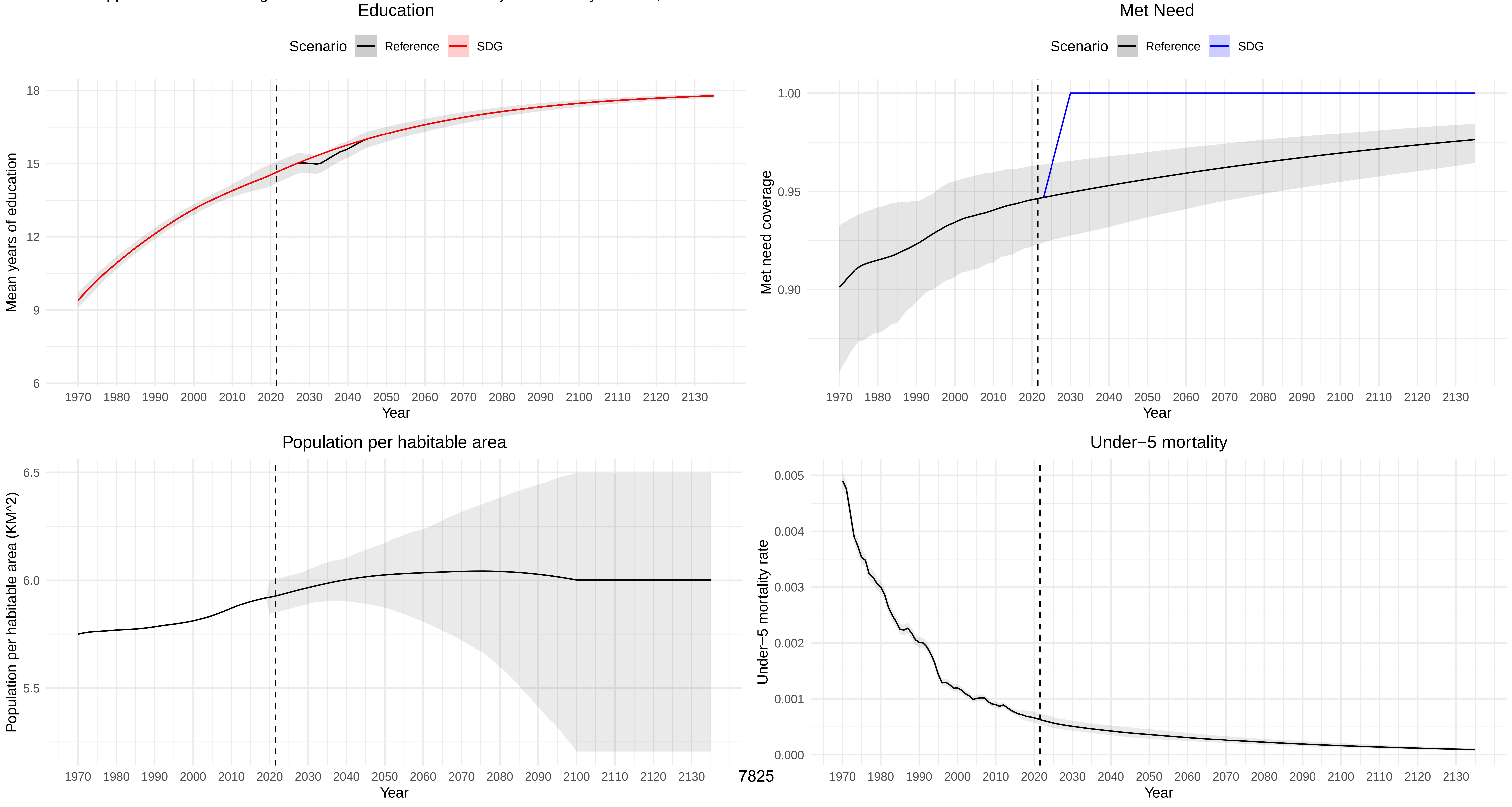

Cyprus

Supplemental Results Figure S2: Covariates used in fertility forecasts by location, 1970-2135

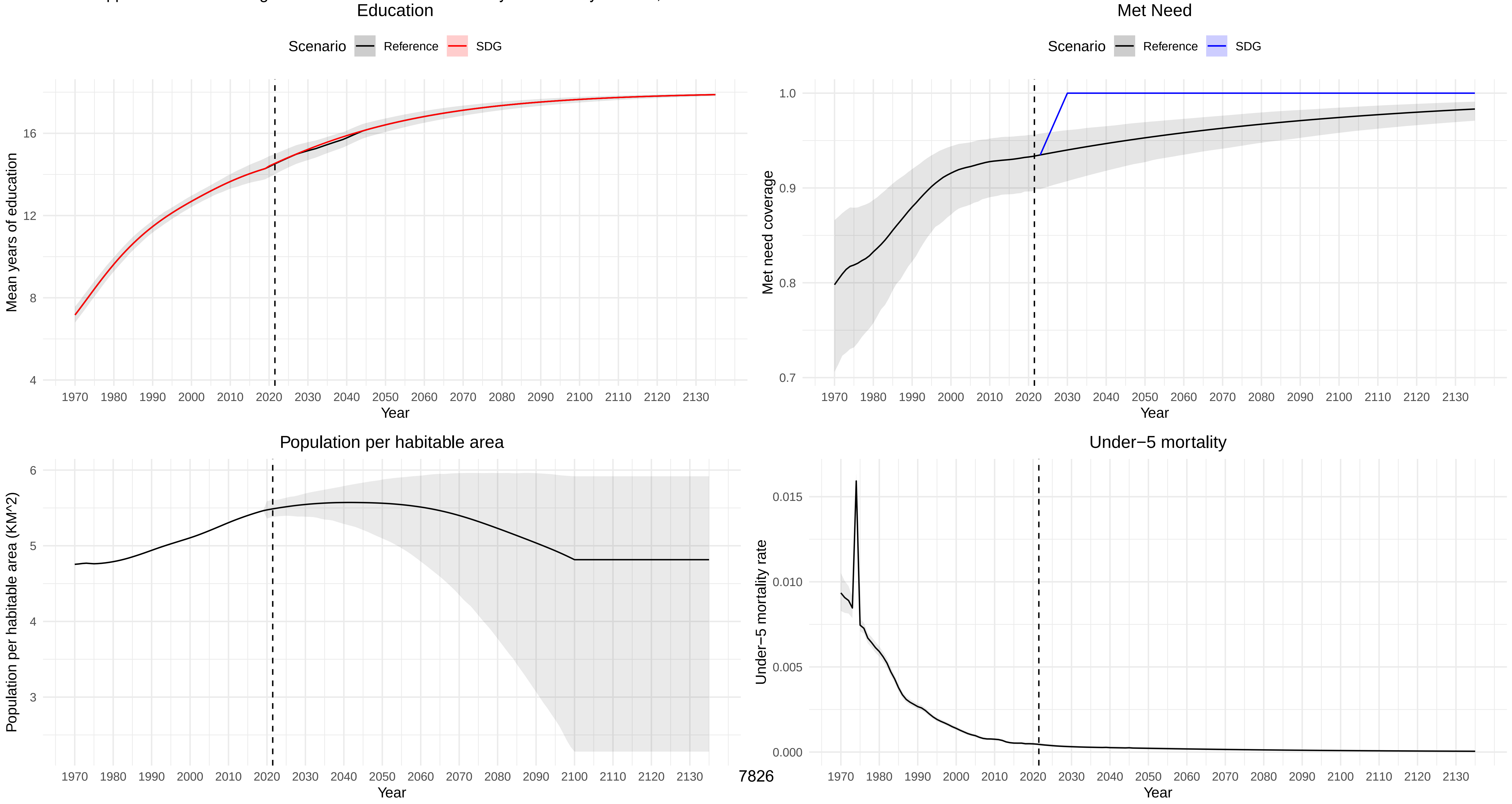

Denmark

Supplemental Results Figure S2: Covariates used in fertility forecasts by location, 1970-2135

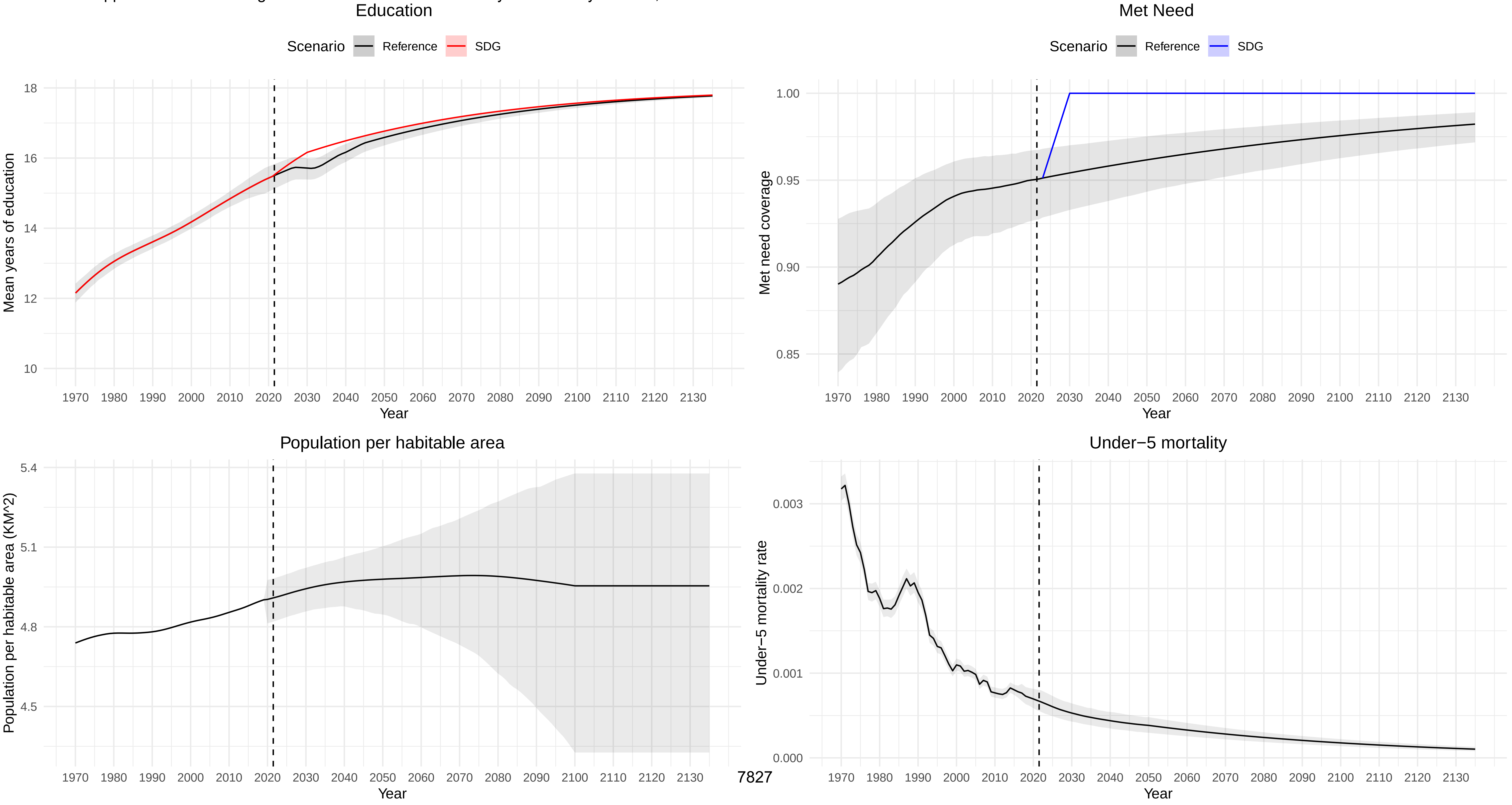

Finland

Supplemental Results Figure S2: Covariates used in fertility forecasts by location, 1970-2135

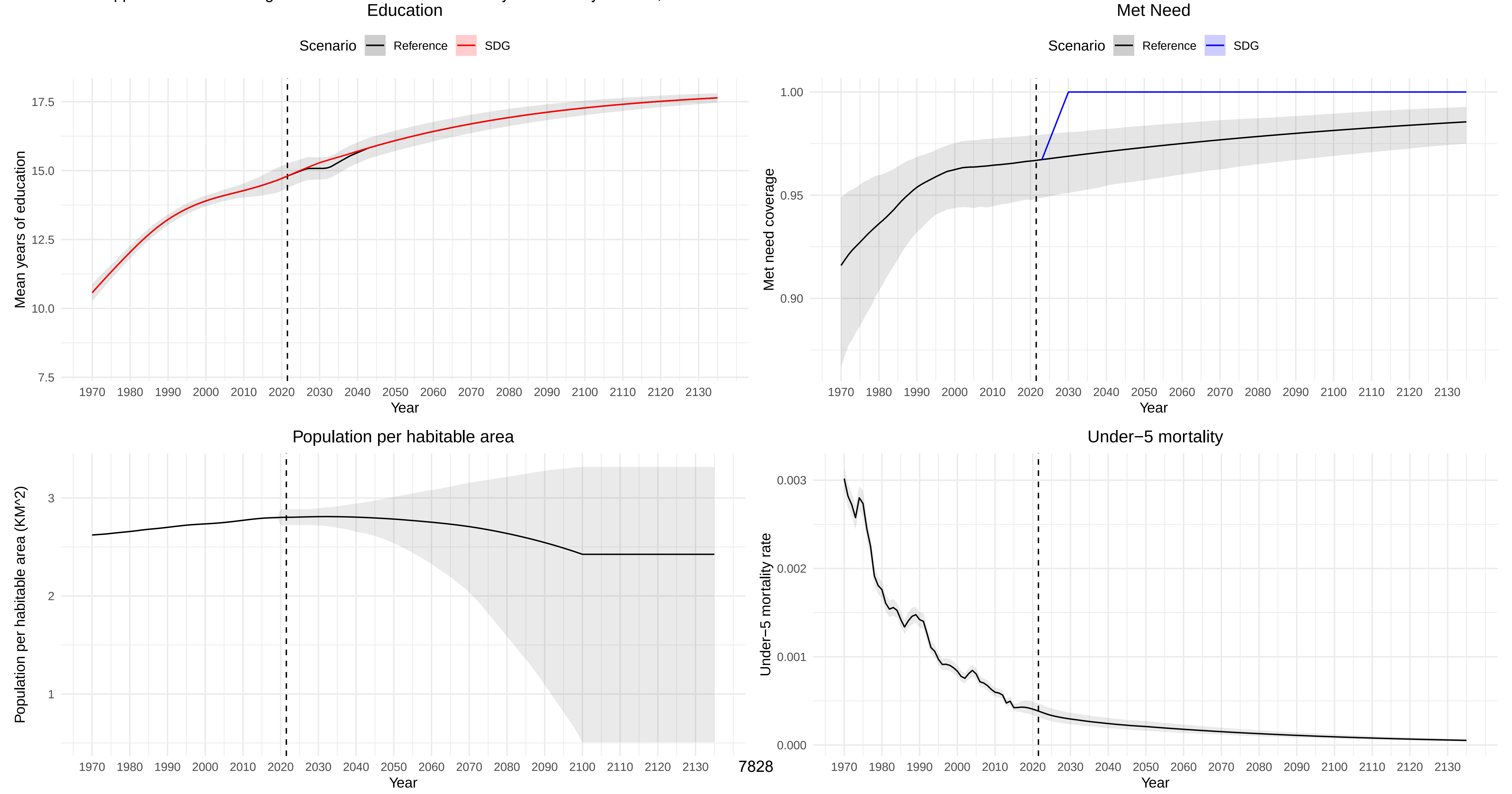

France

Supplemental Results Figure S2: Covariates used in fertility forecasts by location, 1970-2135

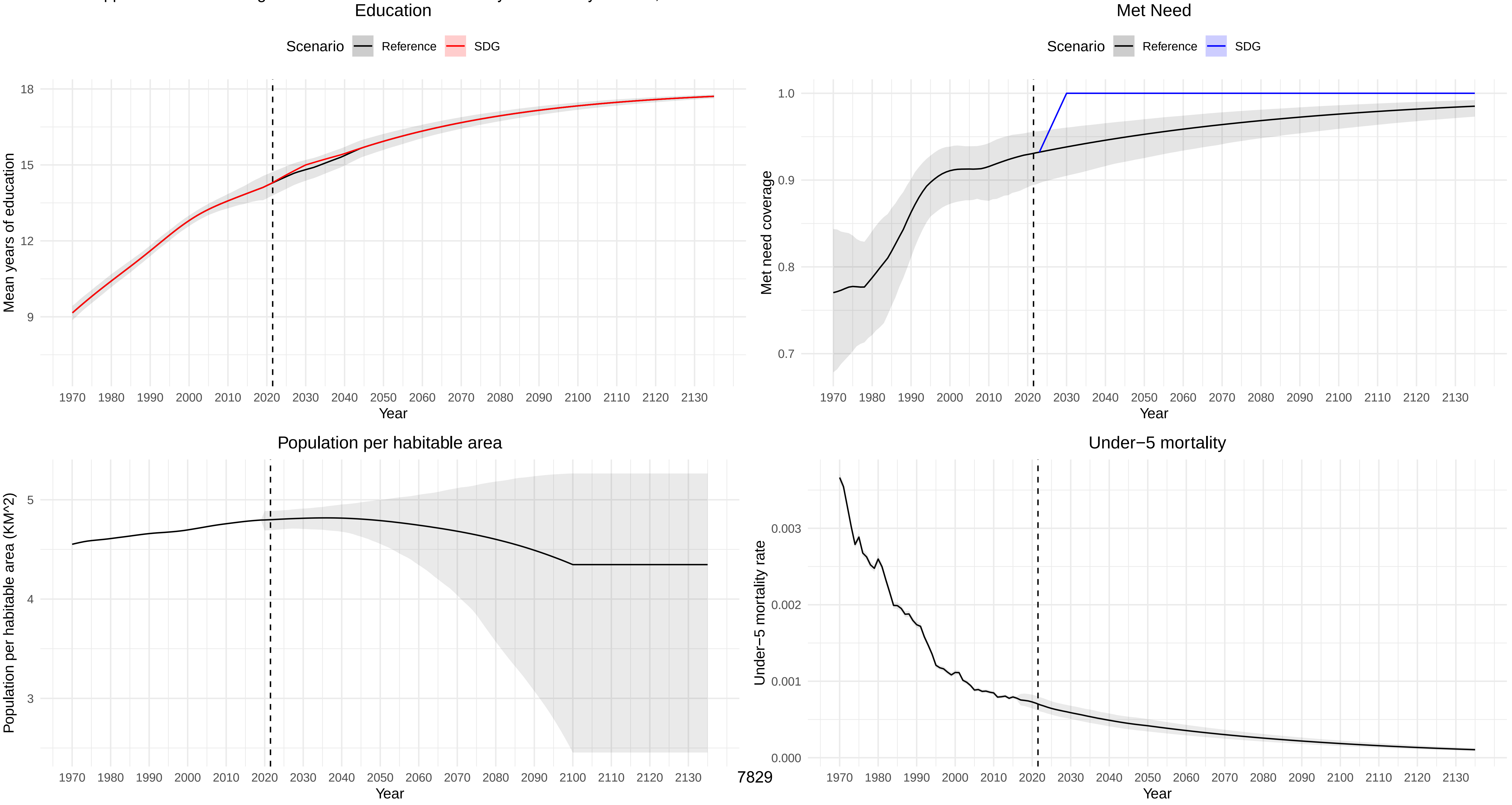

Germany

Supplemental Results Figure S2: Covariates used in fertility forecasts by location, 1970-2135

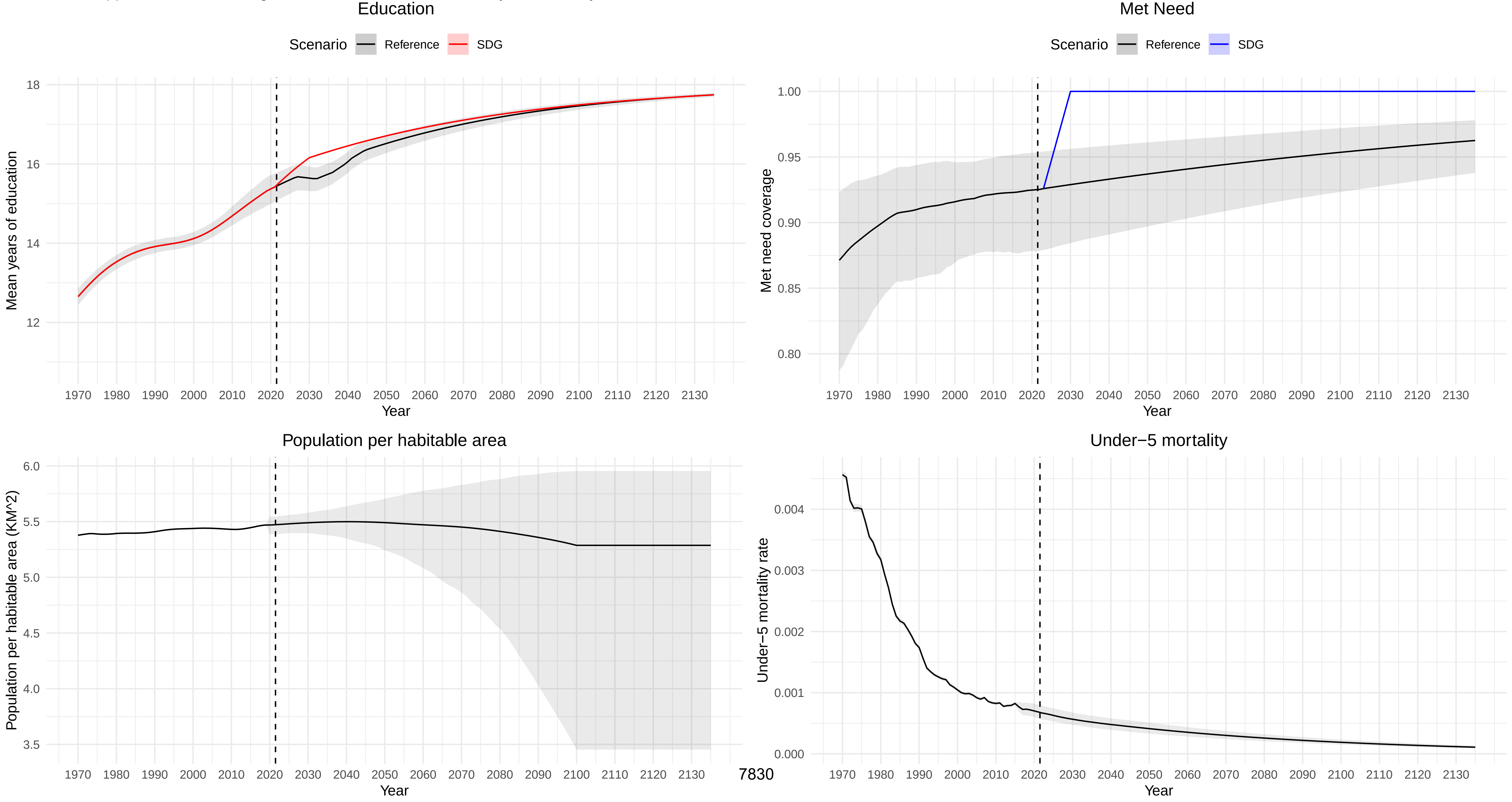

Greece

Supplemental Results Figure S2: Covariates used in fertility forecasts by location, 1970-2135

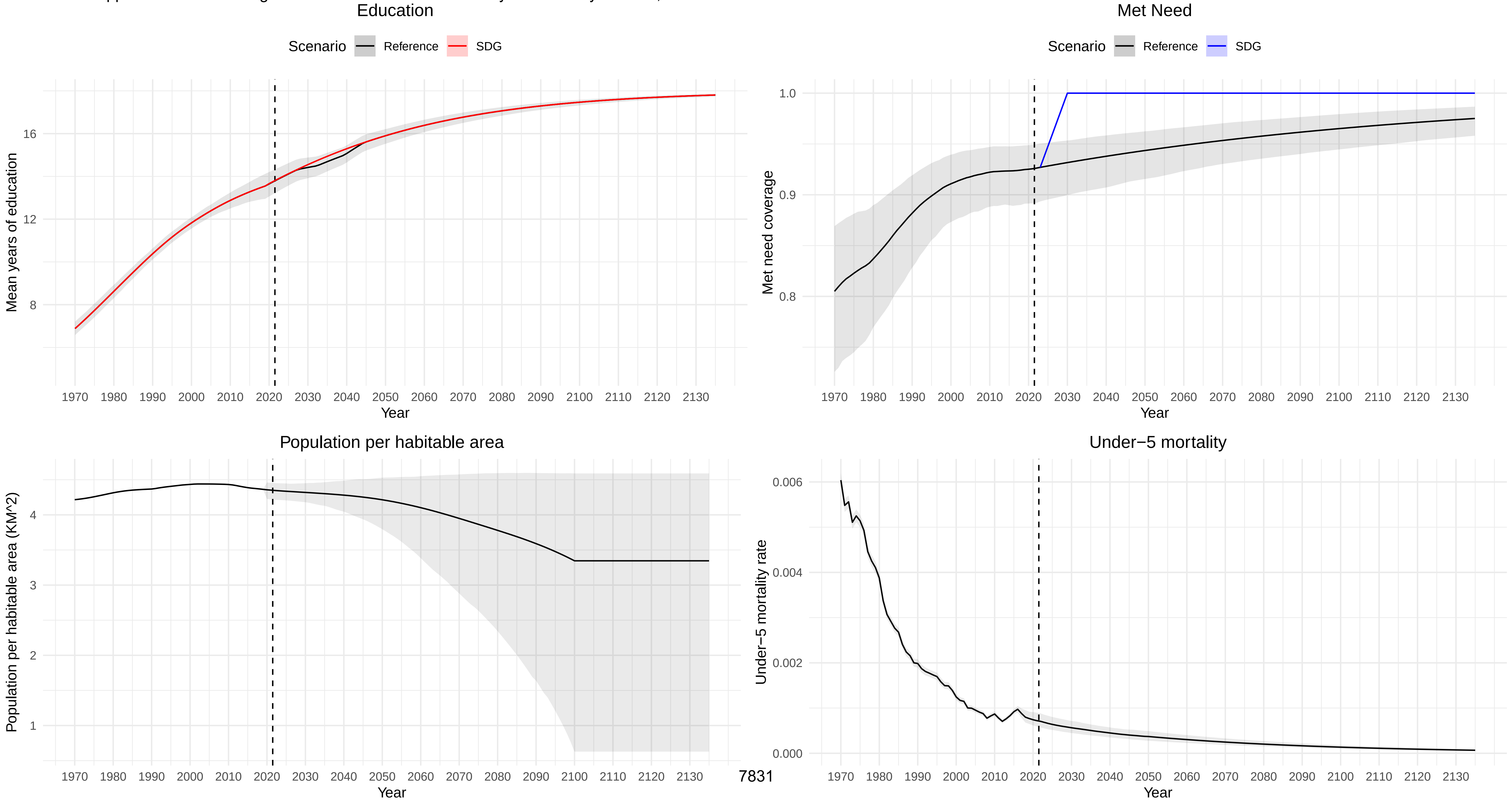

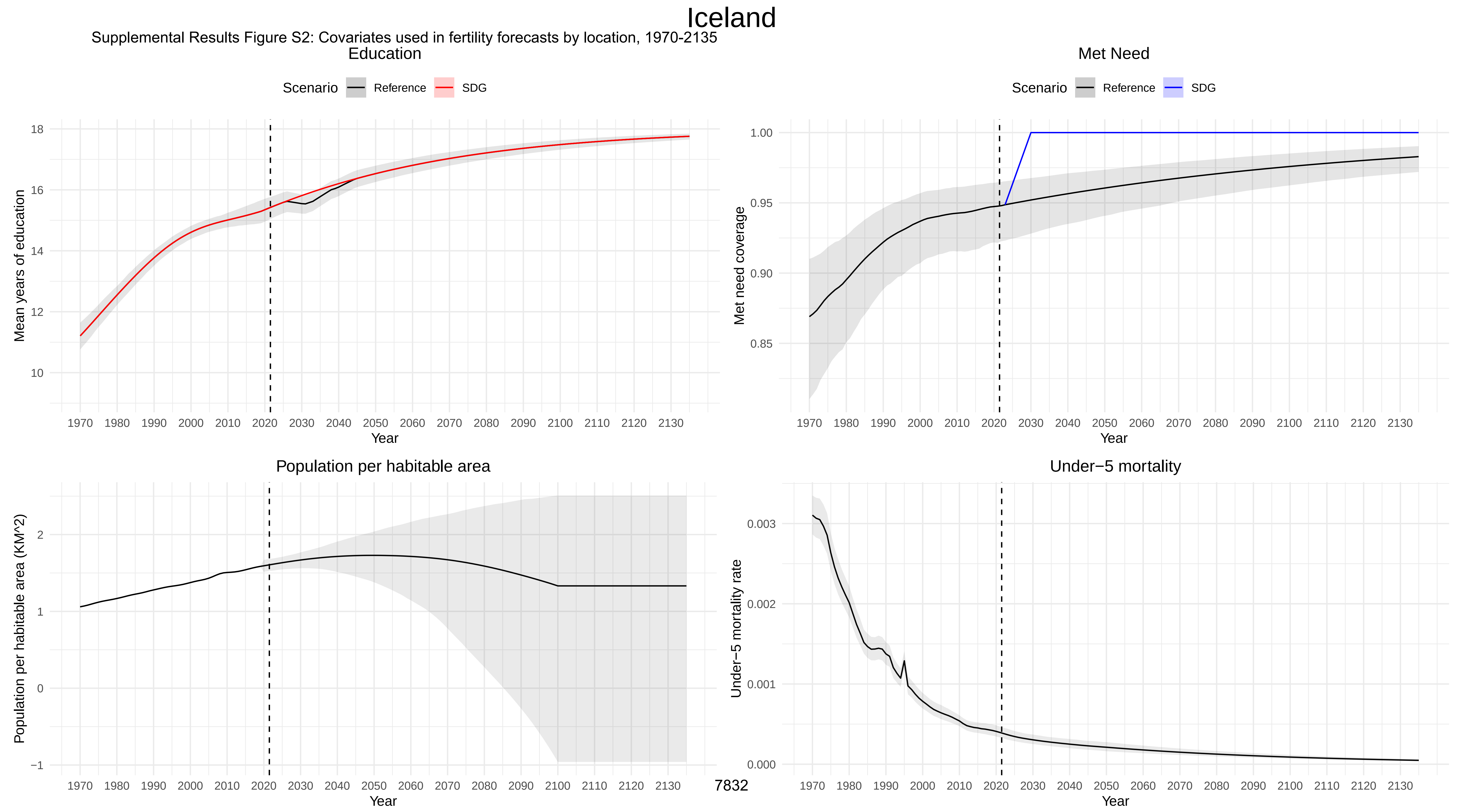
Iceland
Supplemental Results Figure S2: Covariates used in fertility forecasts by location, 1970-2135
Education
Scenario
Reference
SDG
Mean years of education
10
12
14
16
18
Year
1970
1980
1990
2000
2010
2020
2030
2040
2050
2060
2070
2080
2090
2100
2110
2120
2130
Met Need
Scenario
Reference
SDG
Met need coverage
0.85
0.90
0.95
1.00
Year
Population per habitable area
Population per habitable area (KM^2)
−1
0
1
2
Year
Under−5 mortality
Under−5 mortality rate
0.000
0.001
0.002
0.003
Year

Ireland

Supplemental Results Figure S2: Covariates used in fertility forecasts by location, 1970-2135

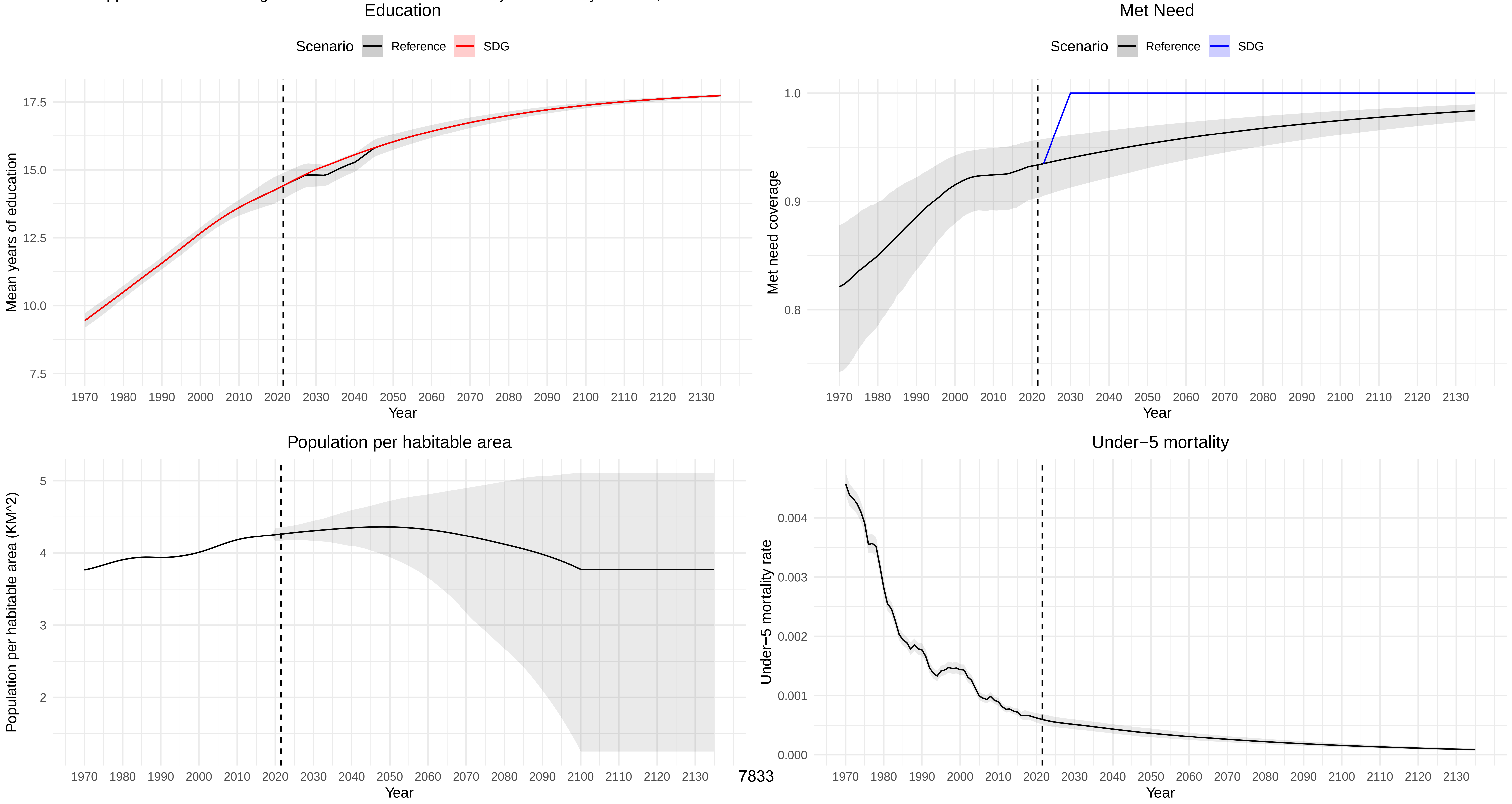

Israel

Supplemental Results Figure S2: Covariates used in fertility forecasts by location, 1970-2135

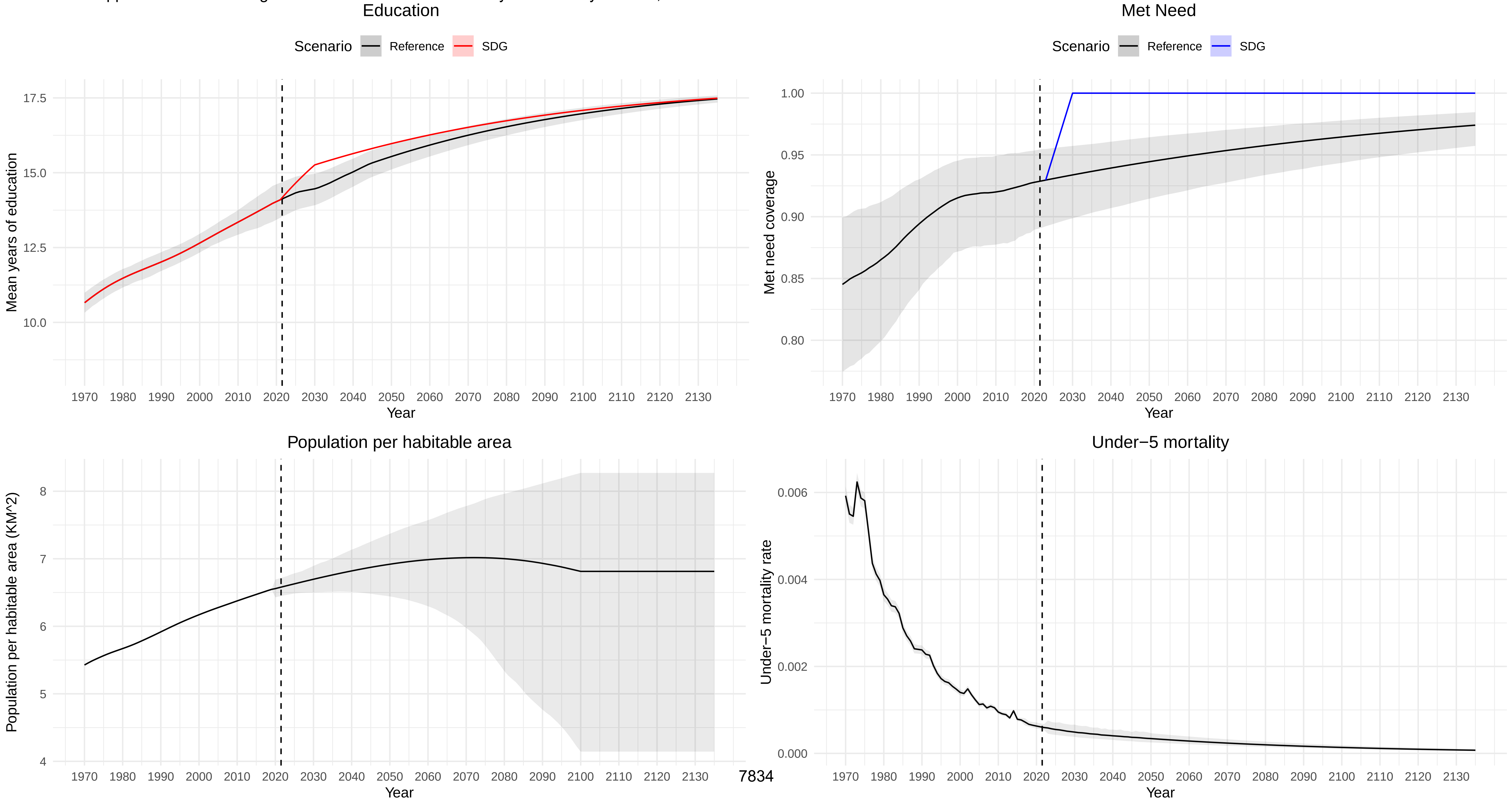

Italy

Supplemental Results Figure S2: Covariates used in fertility forecasts by location, 1970-2135

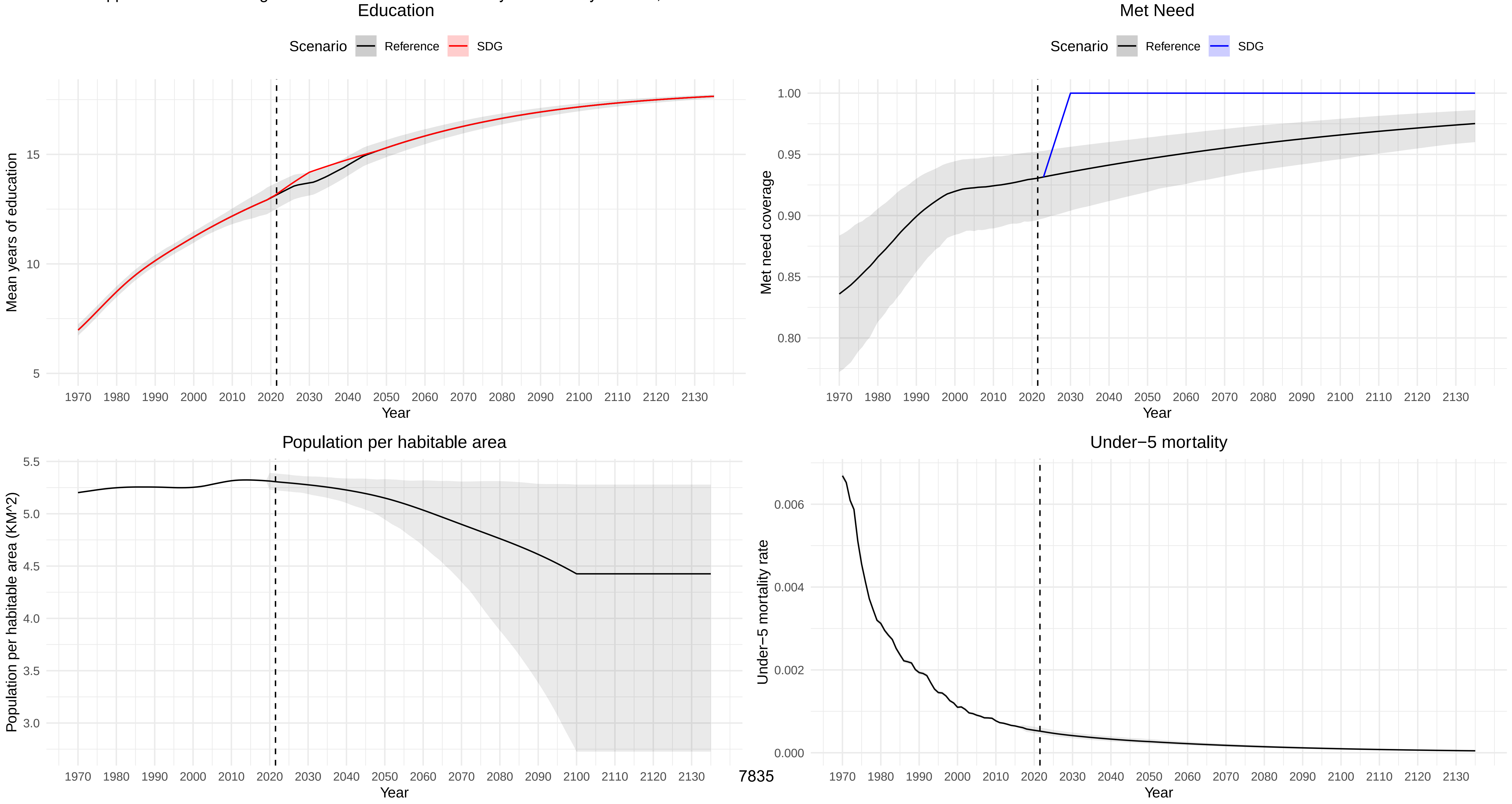

Luxembourg

Supplemental Results Figure S2: Covariates used in fertility forecasts by location, 1970-2135

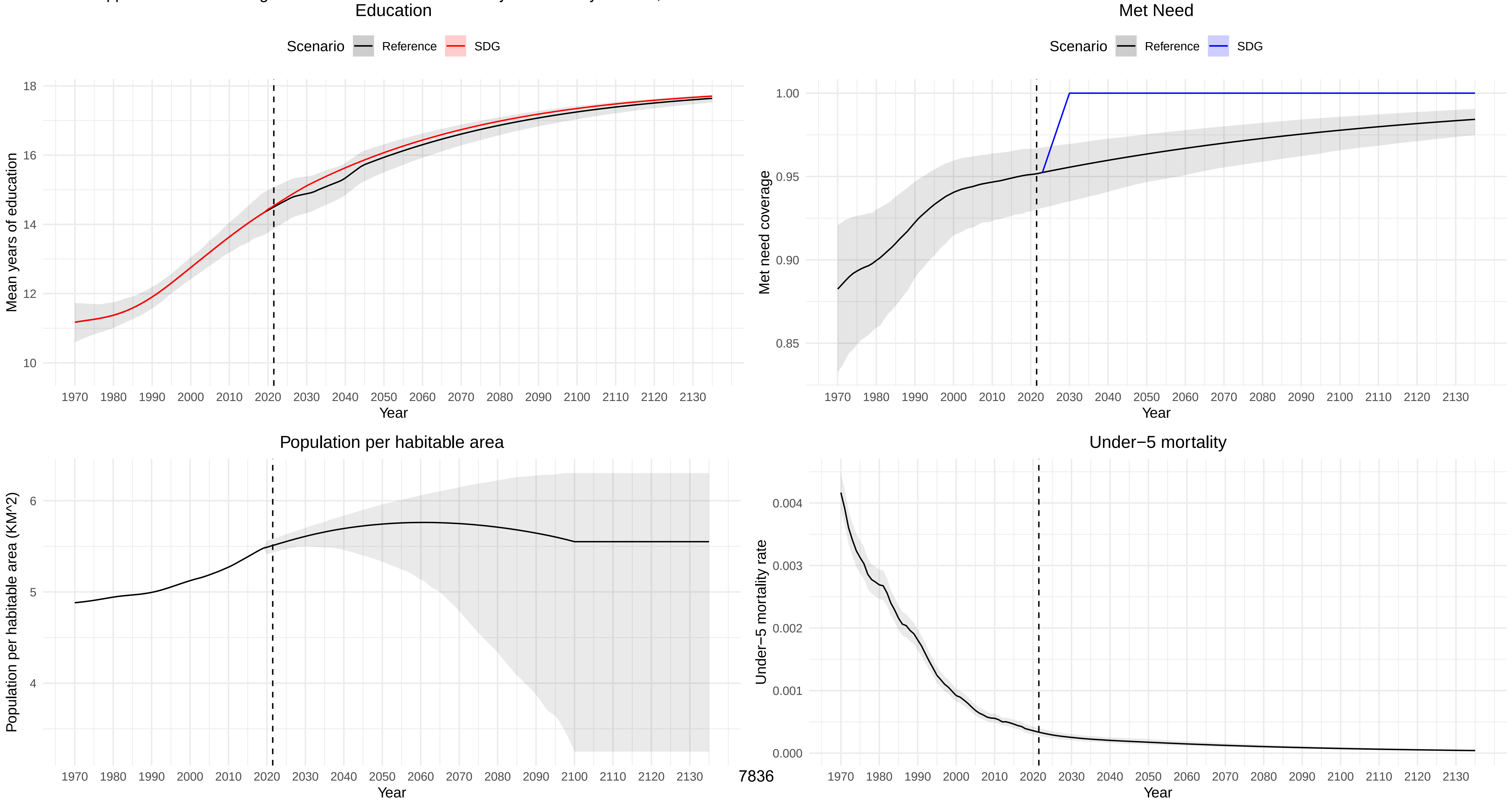

Malta

Supplemental Results Figure S2: Covariates used in fertility forecasts by location, 1970-2135

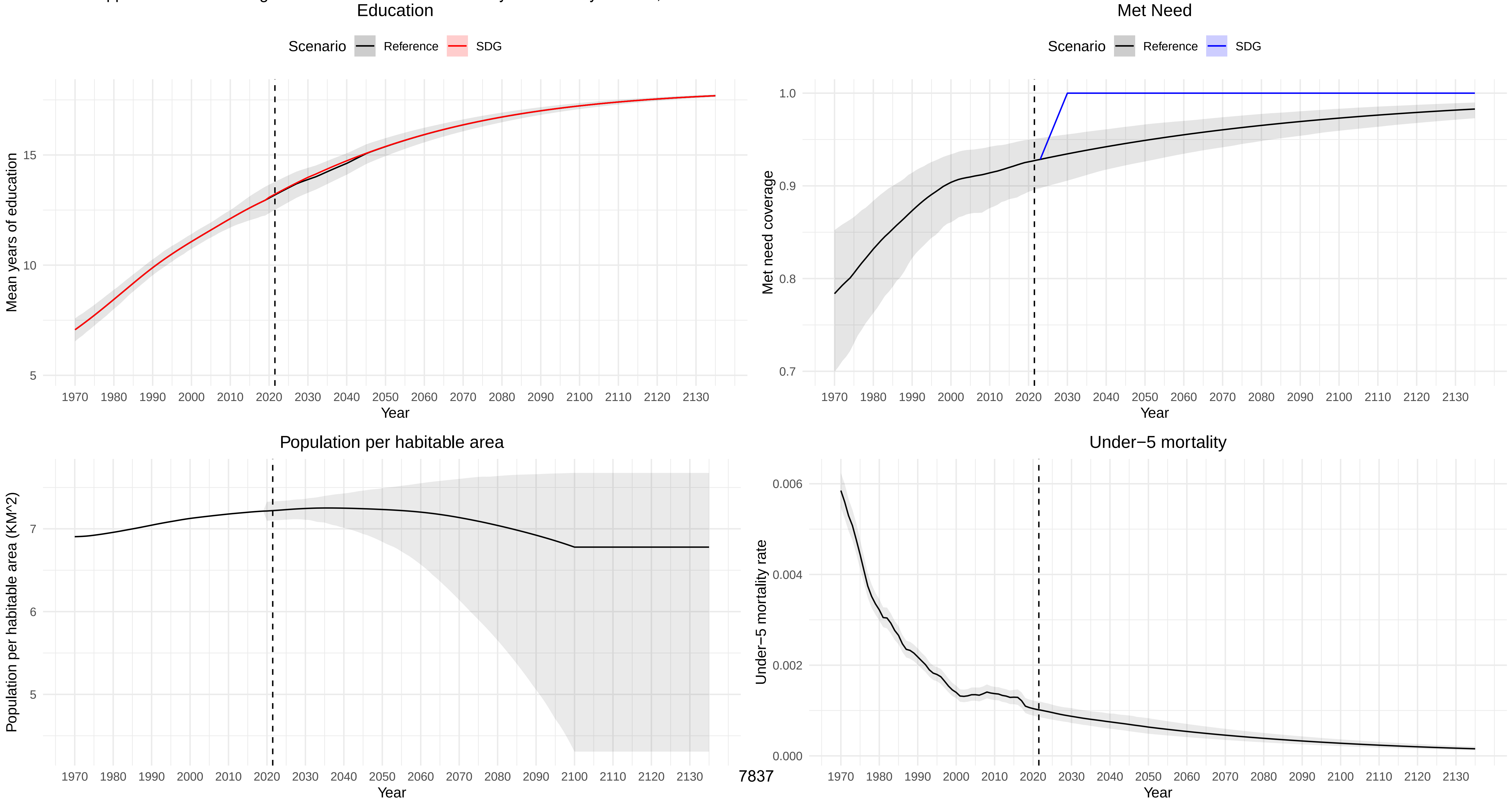

Monaco

Supplemental Results Figure S2: Covariates used in fertility forecasts by location, 1970-2135

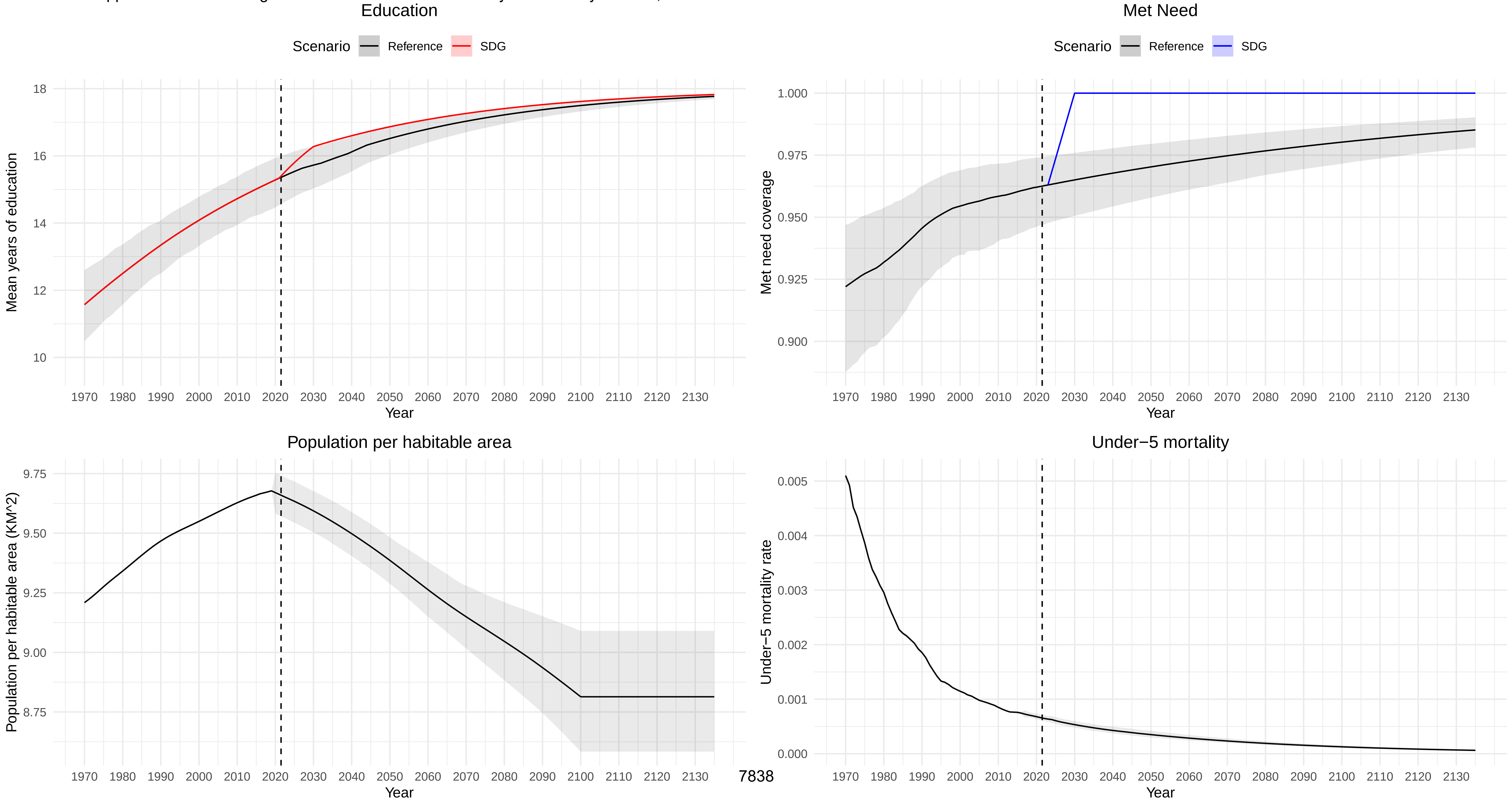

Netherlands

Supplemental Results Figure S2: Covariates used in fertility forecasts by location, 1970-2135

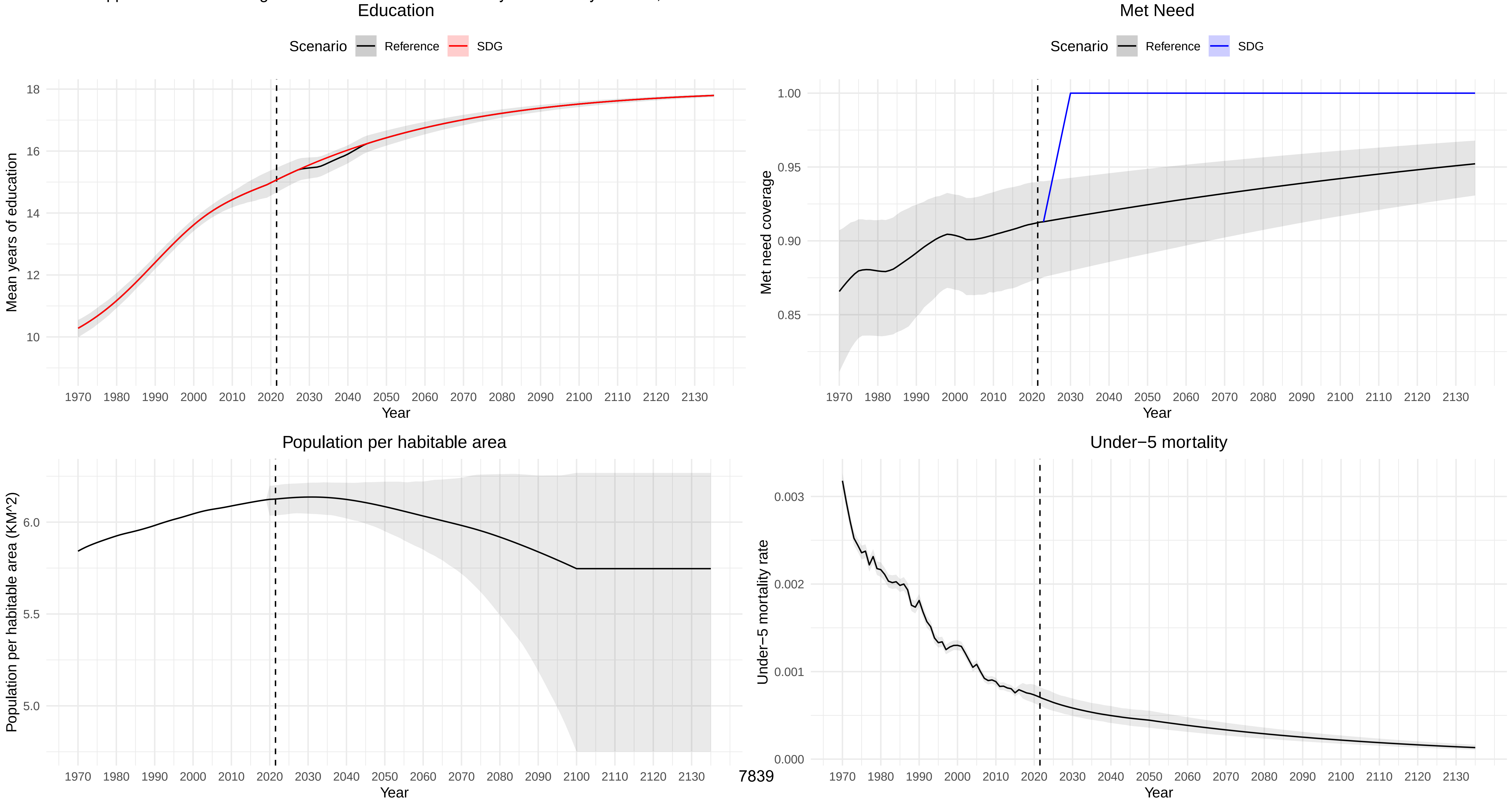

Norway

Supplemental Results Figure S2: Covariates used in fertility forecasts by location, 1970-2135

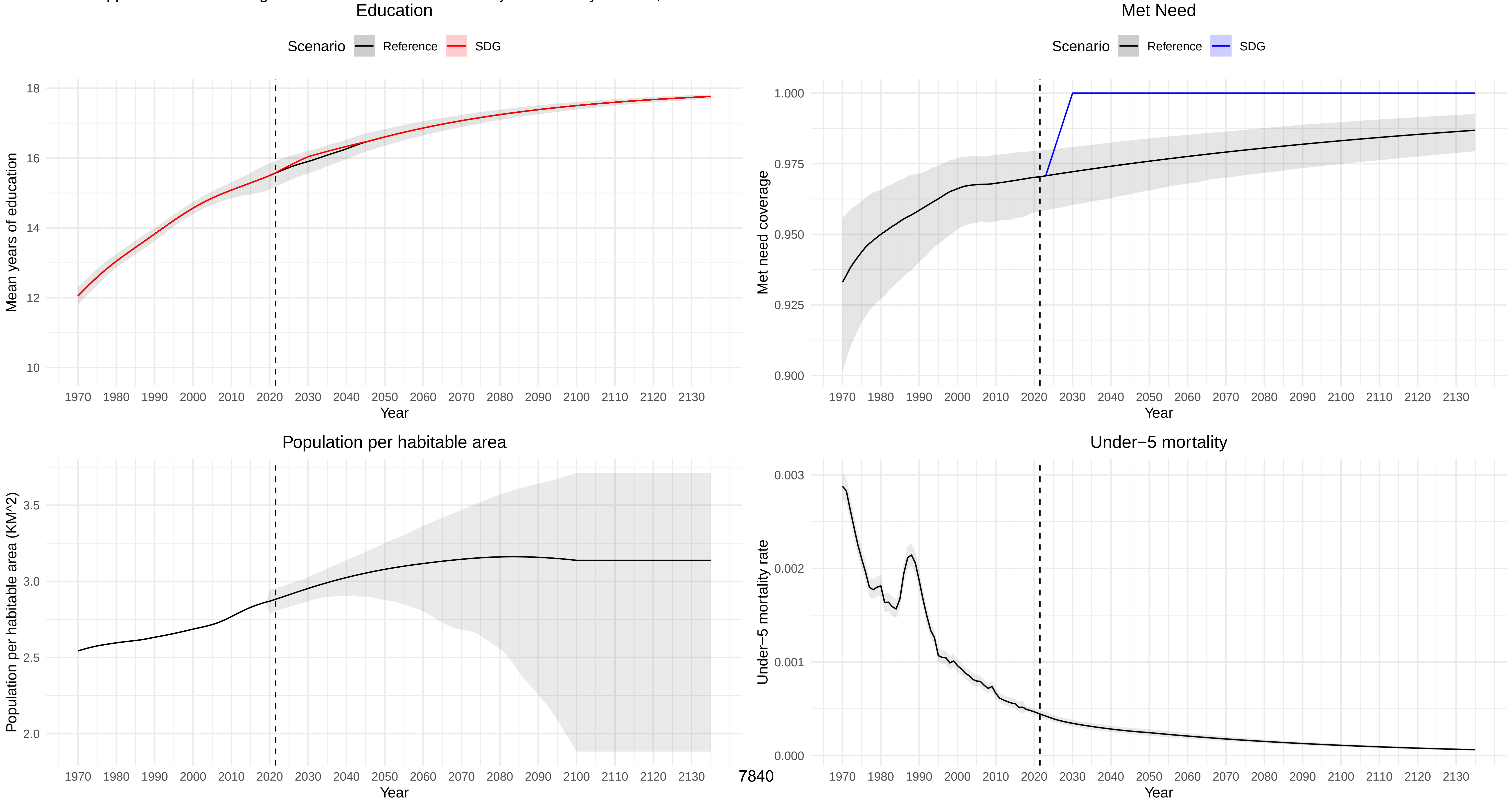

Portugal

Supplemental Results Figure S2: Covariates used in fertility forecasts by location, 1970-2135

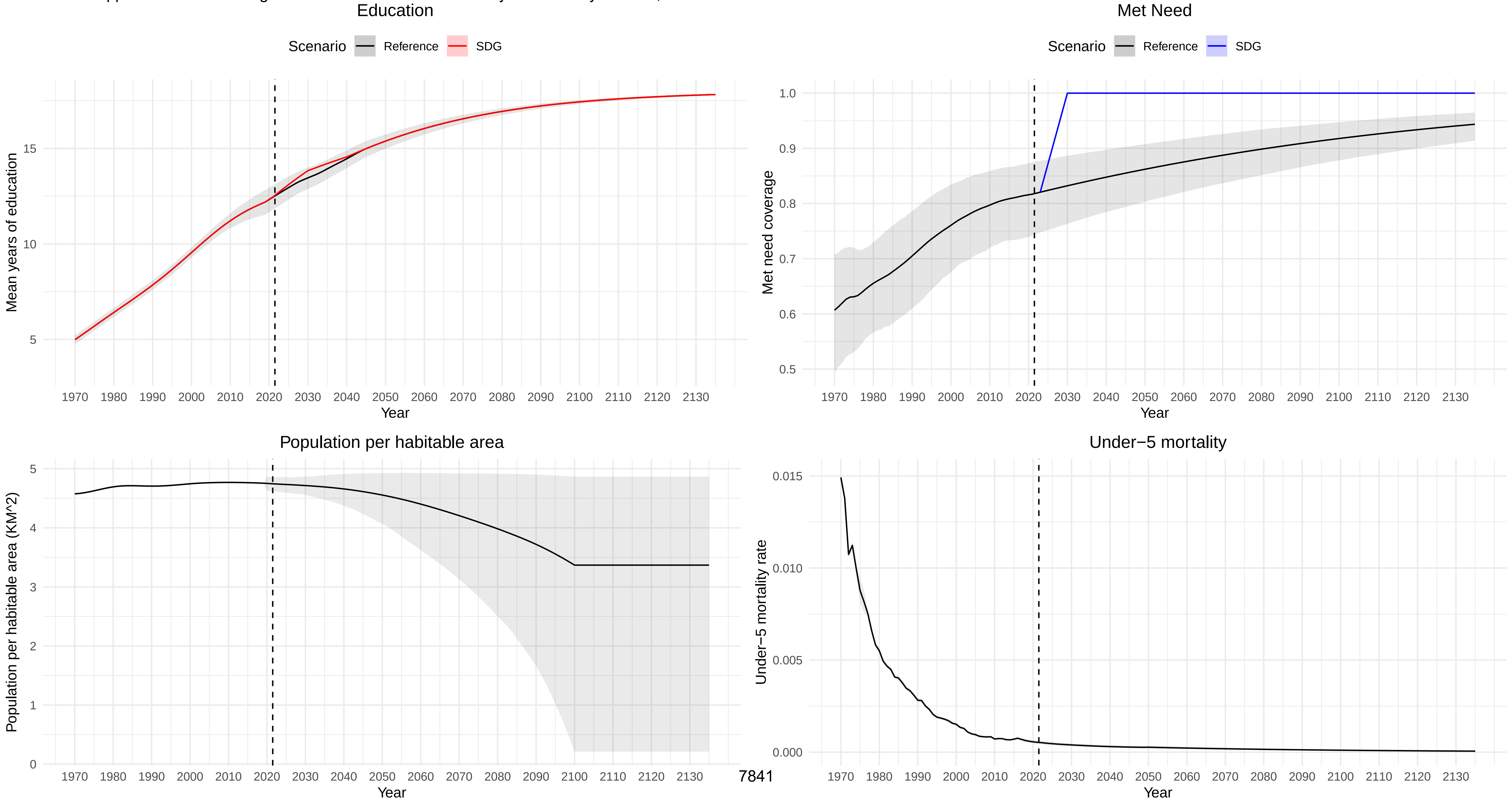

San Marino

Supplemental Results Figure S2: Covariates used in fertility forecasts by location, 1970-2135

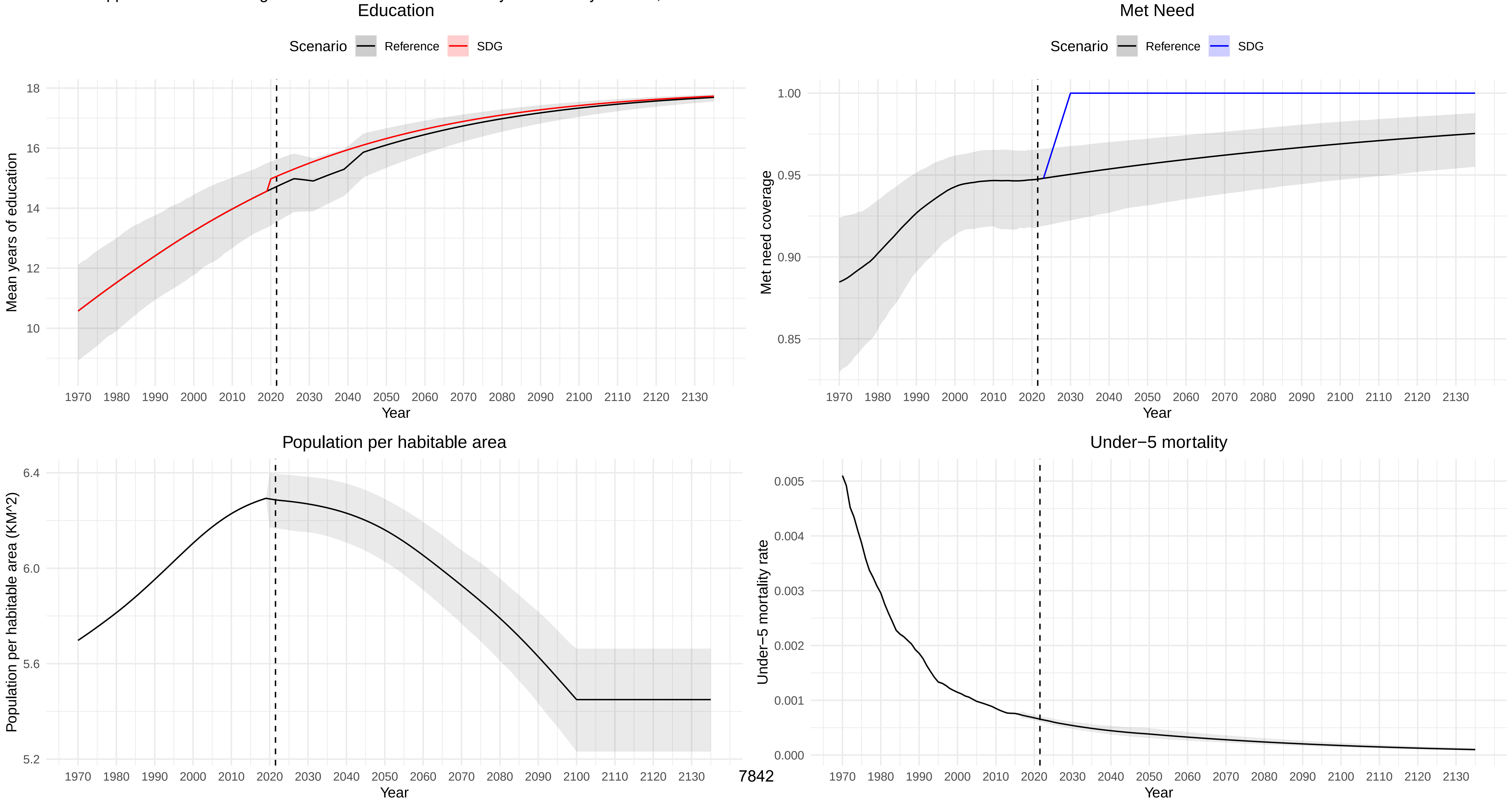

Spain

Supplemental Results Figure S2: Covariates used in fertility forecasts by location, 1970-2135

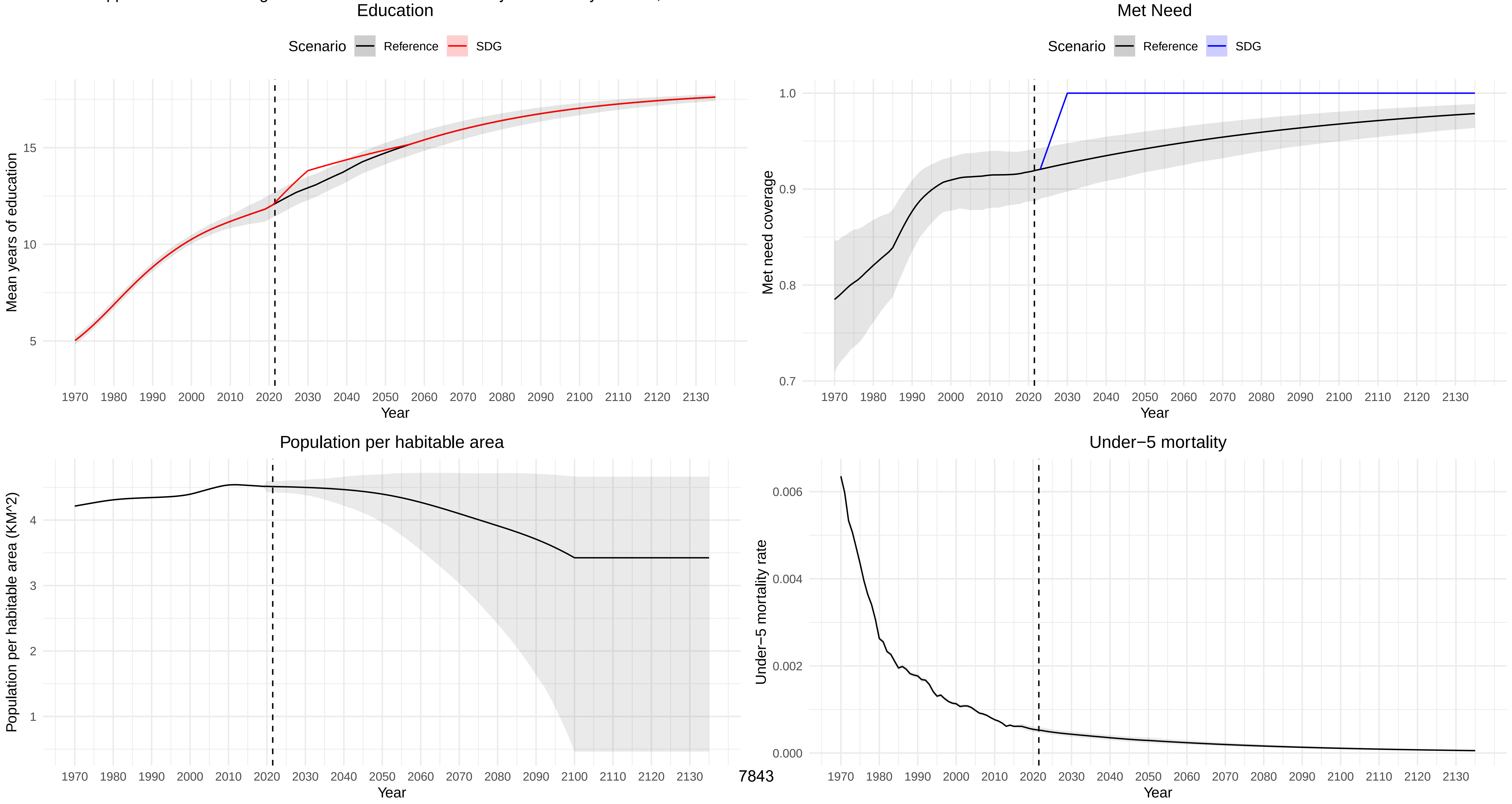

Sweden

Supplemental Results Figure S2: Covariates used in fertility forecasts by location, 1970-2135

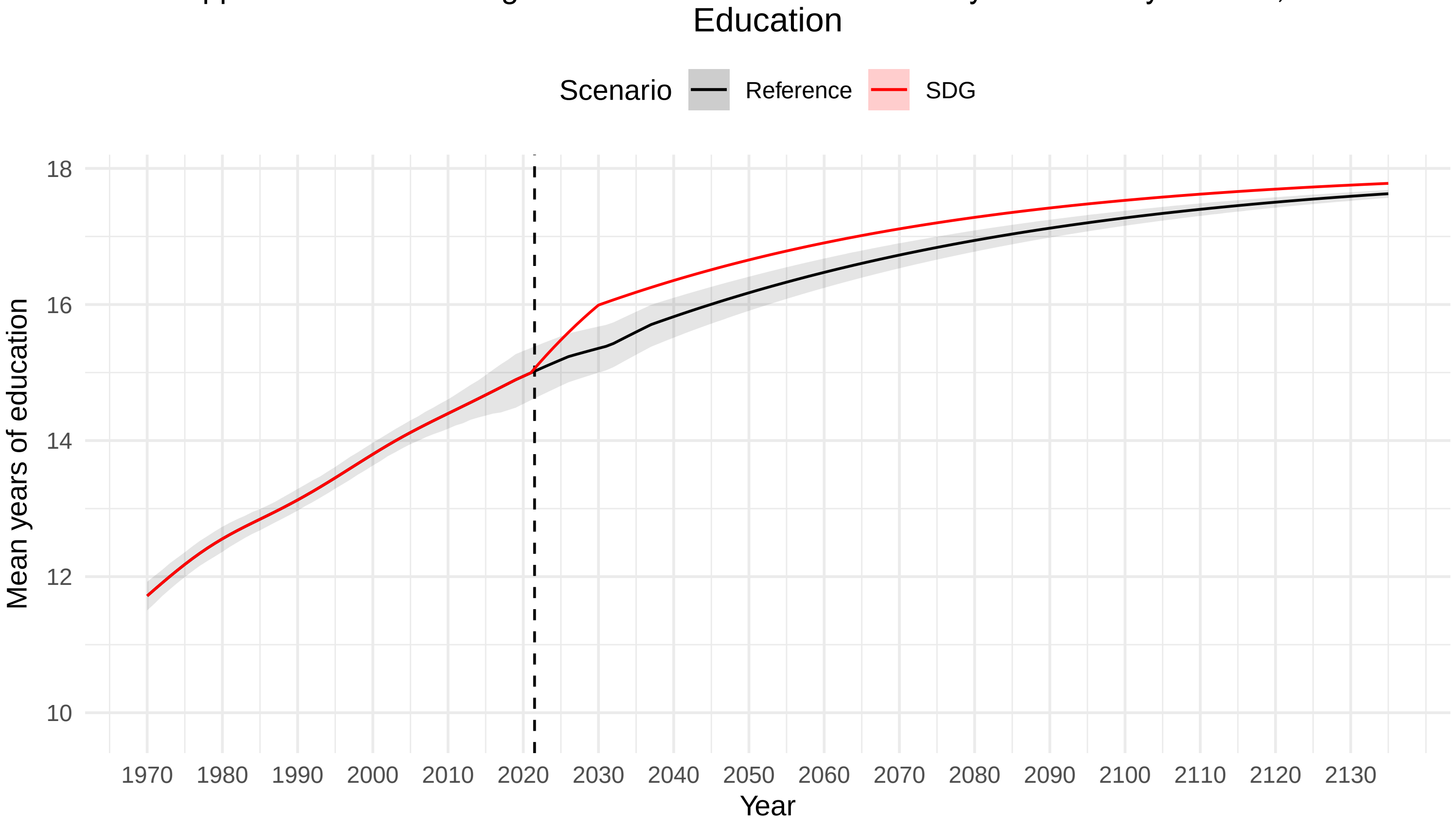

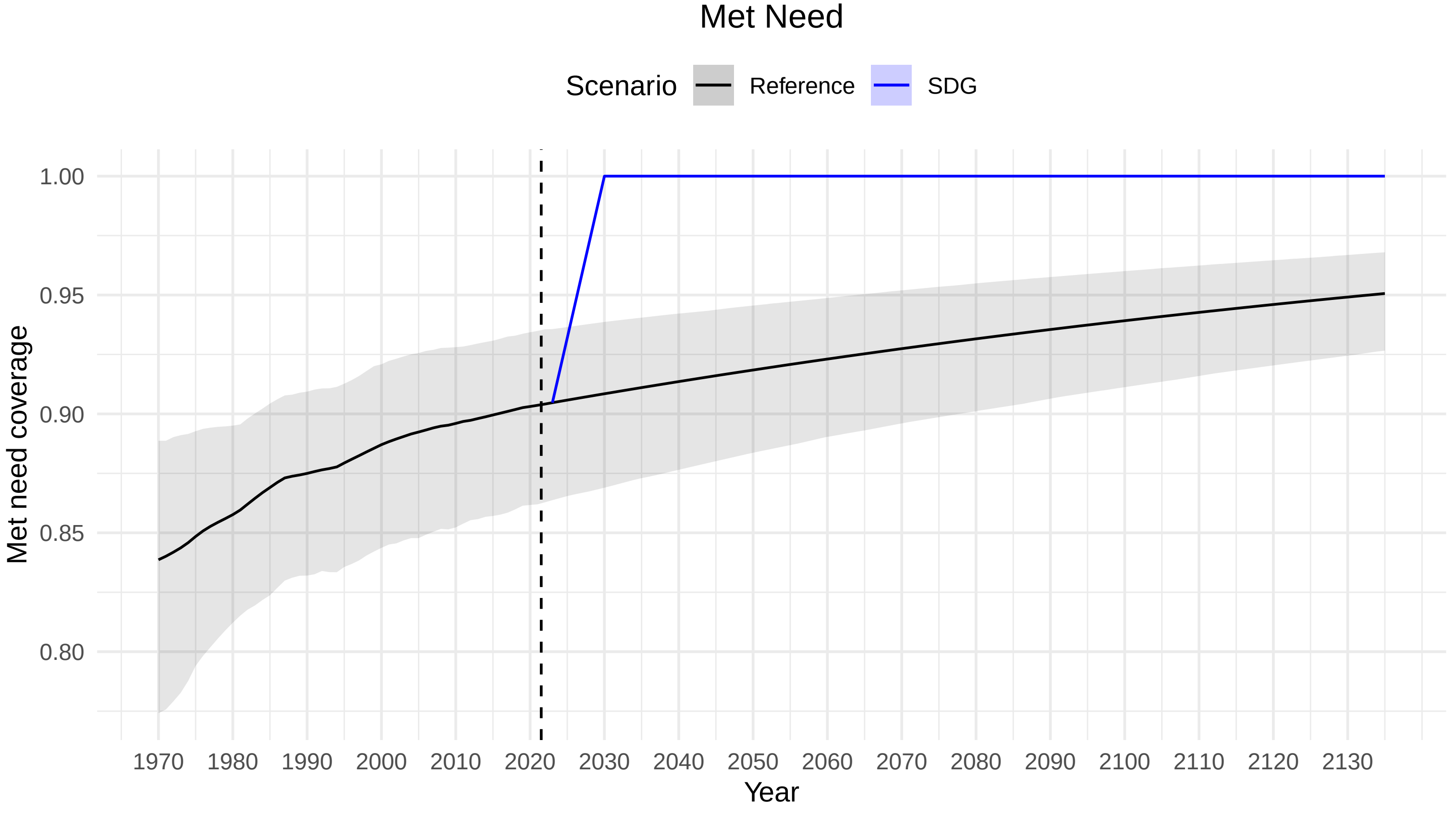

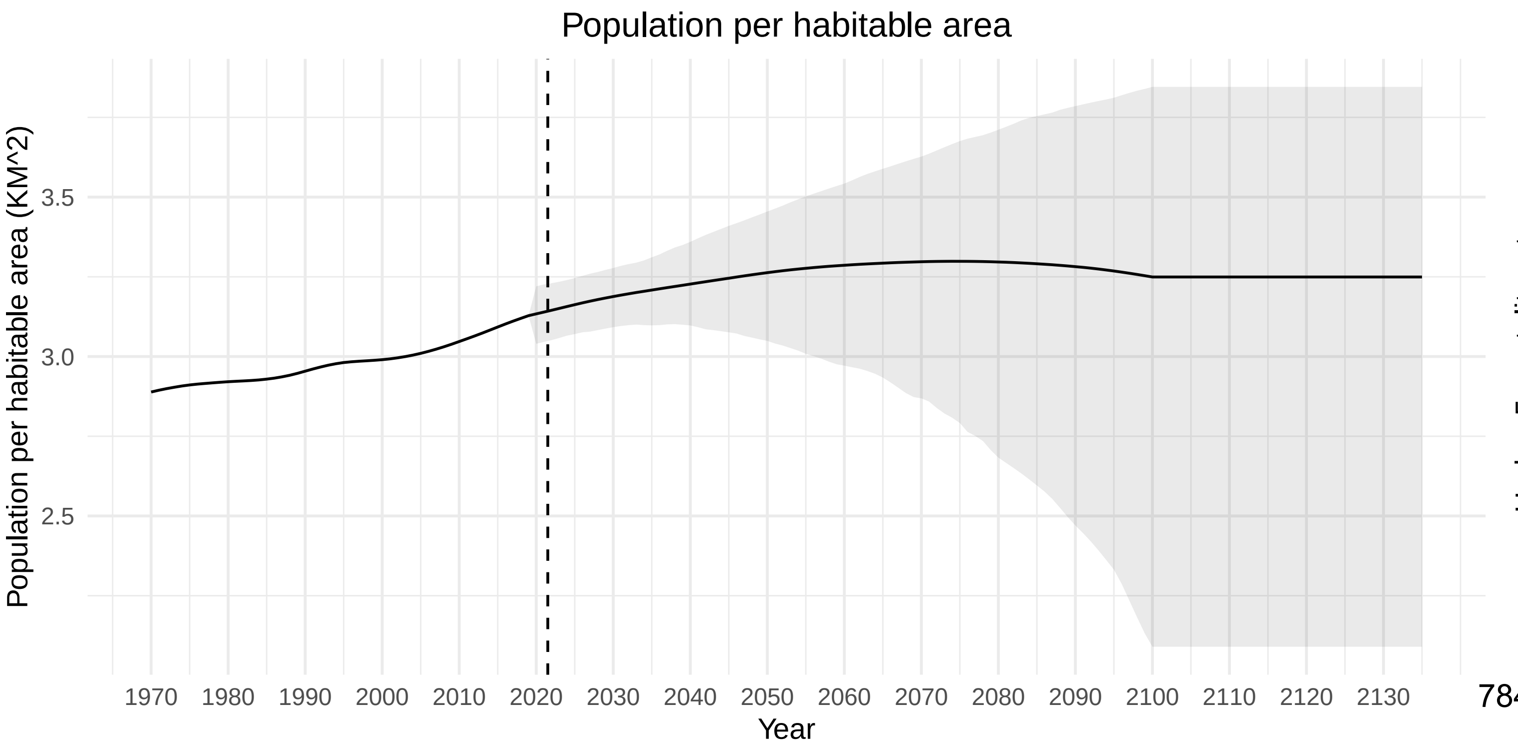

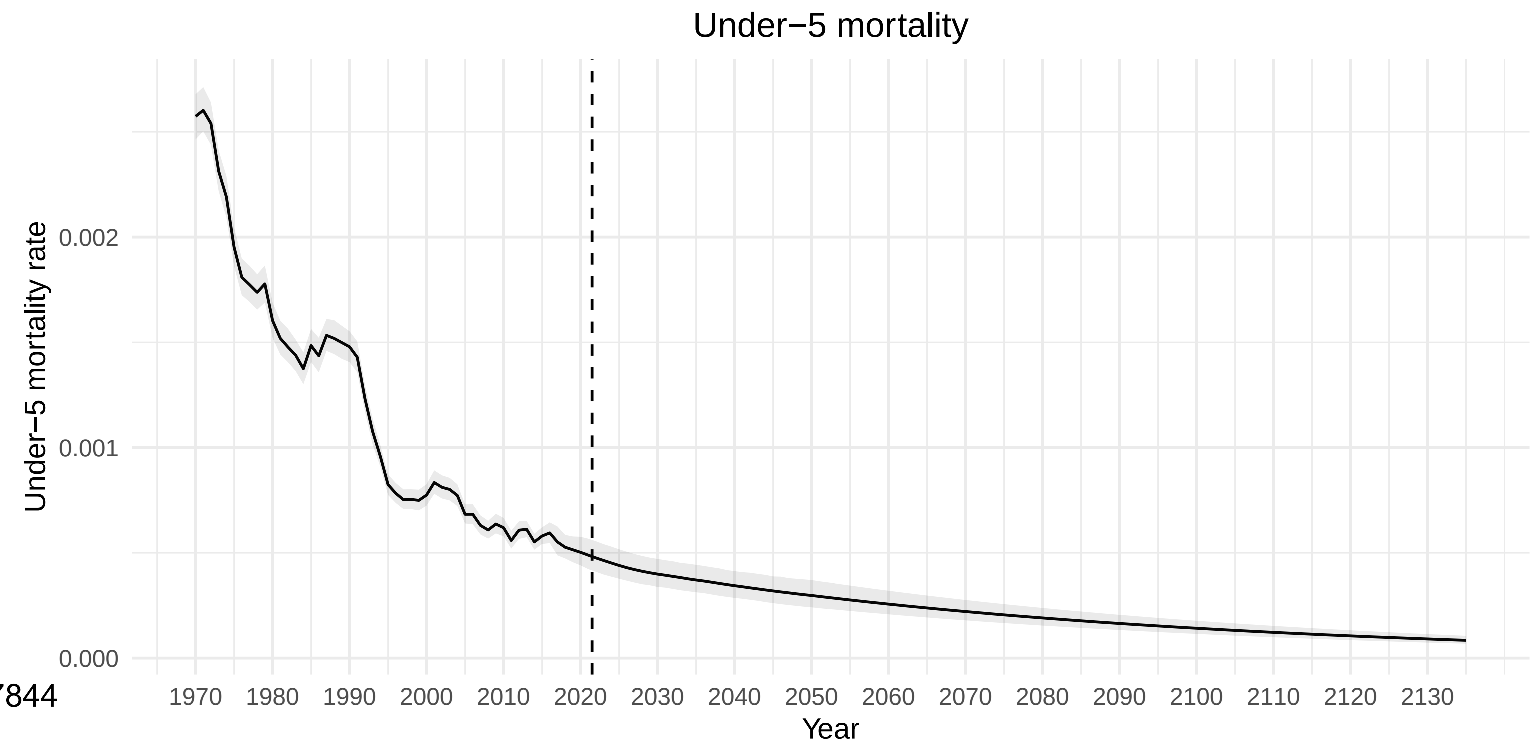

Switzerland

Supplemental Results Figure S2: Covariates used in fertility forecasts by location, 1970-2135

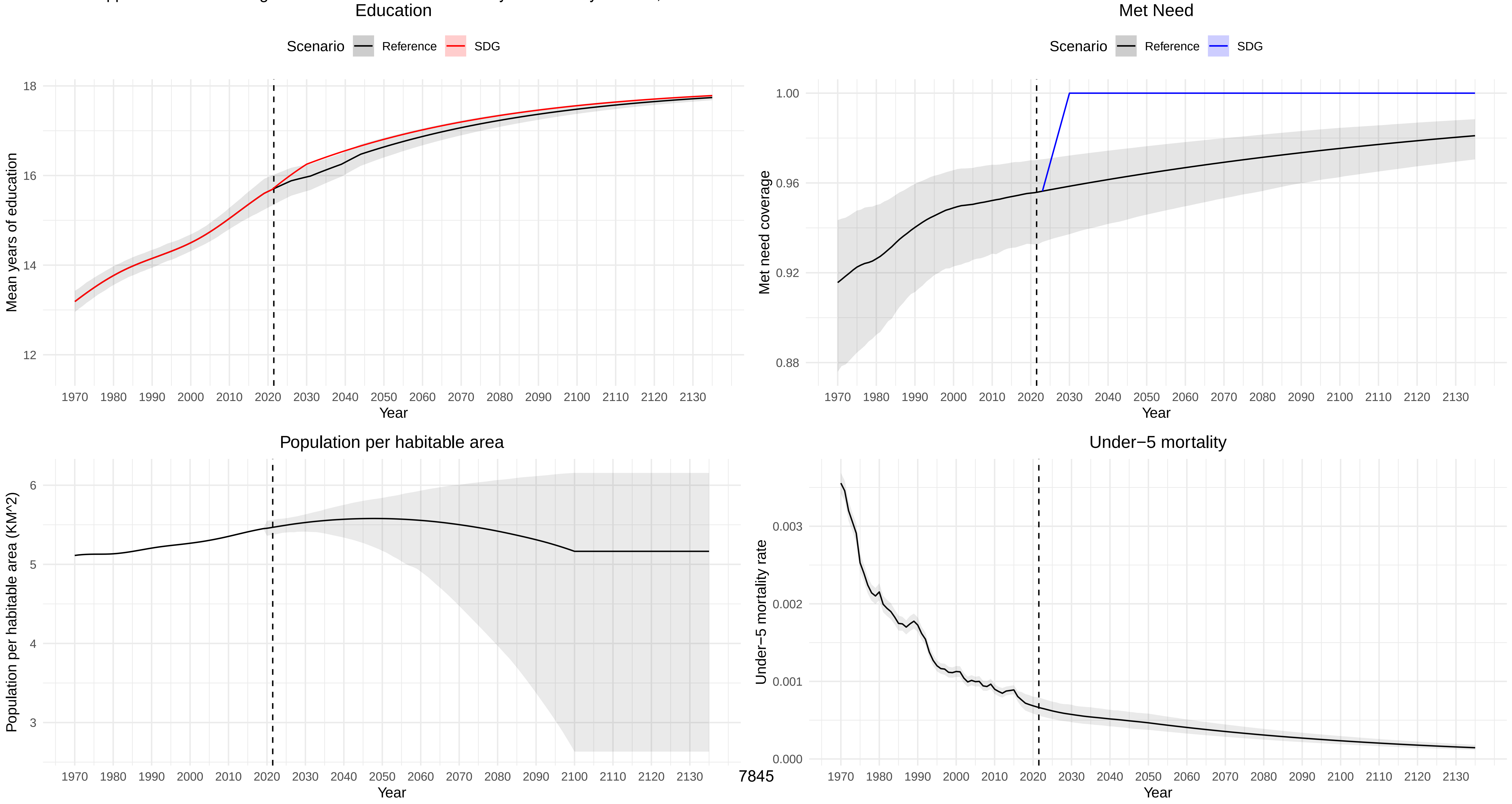

United Kingdom

Supplemental Results Figure S2: Covariates used in fertility forecasts by location, 1970-2135

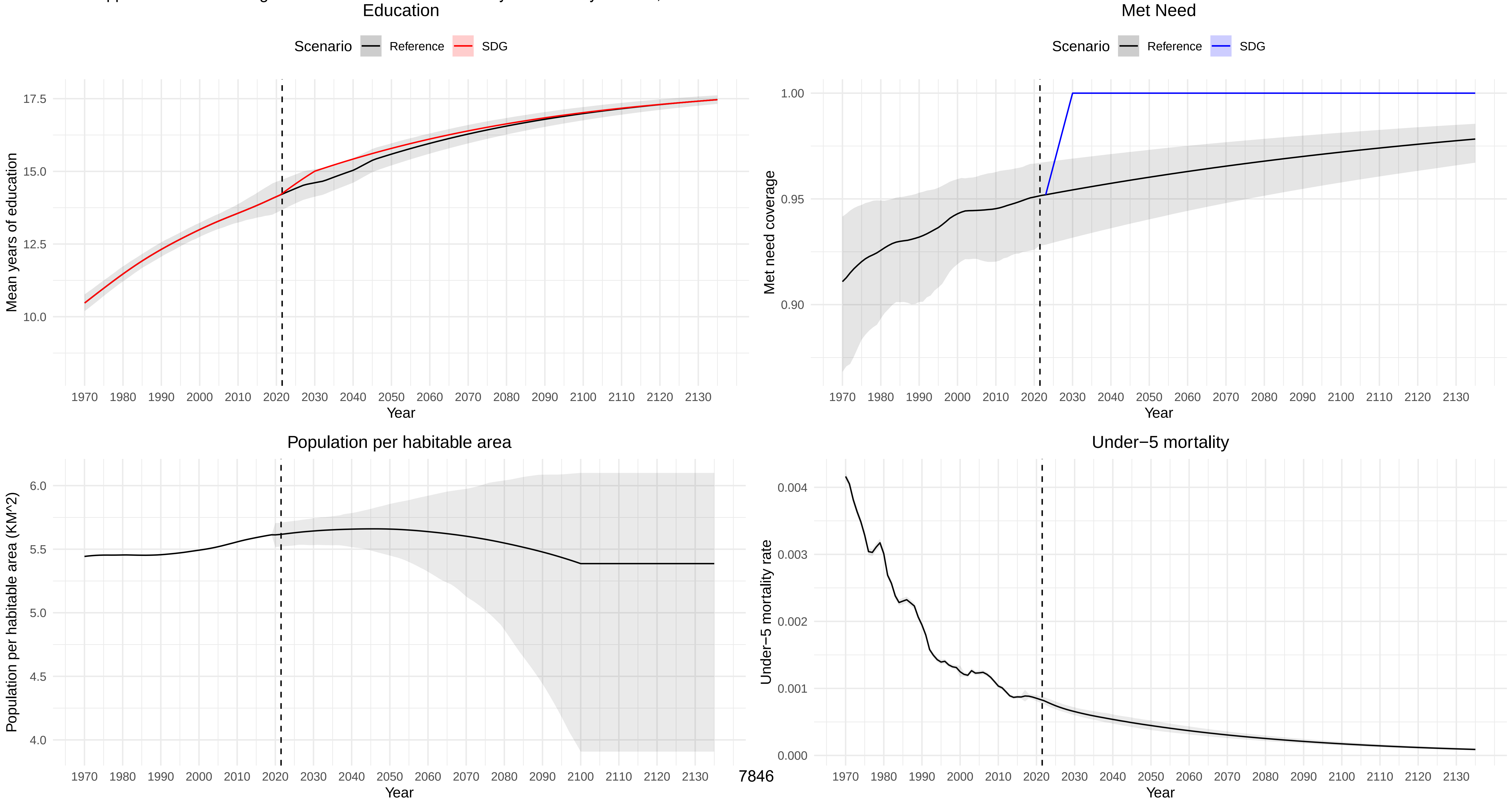

Bolivia (Plurinational State of)

Supplemental Results Figure S2: Covariates used in fertility forecasts by location, 1970-2135

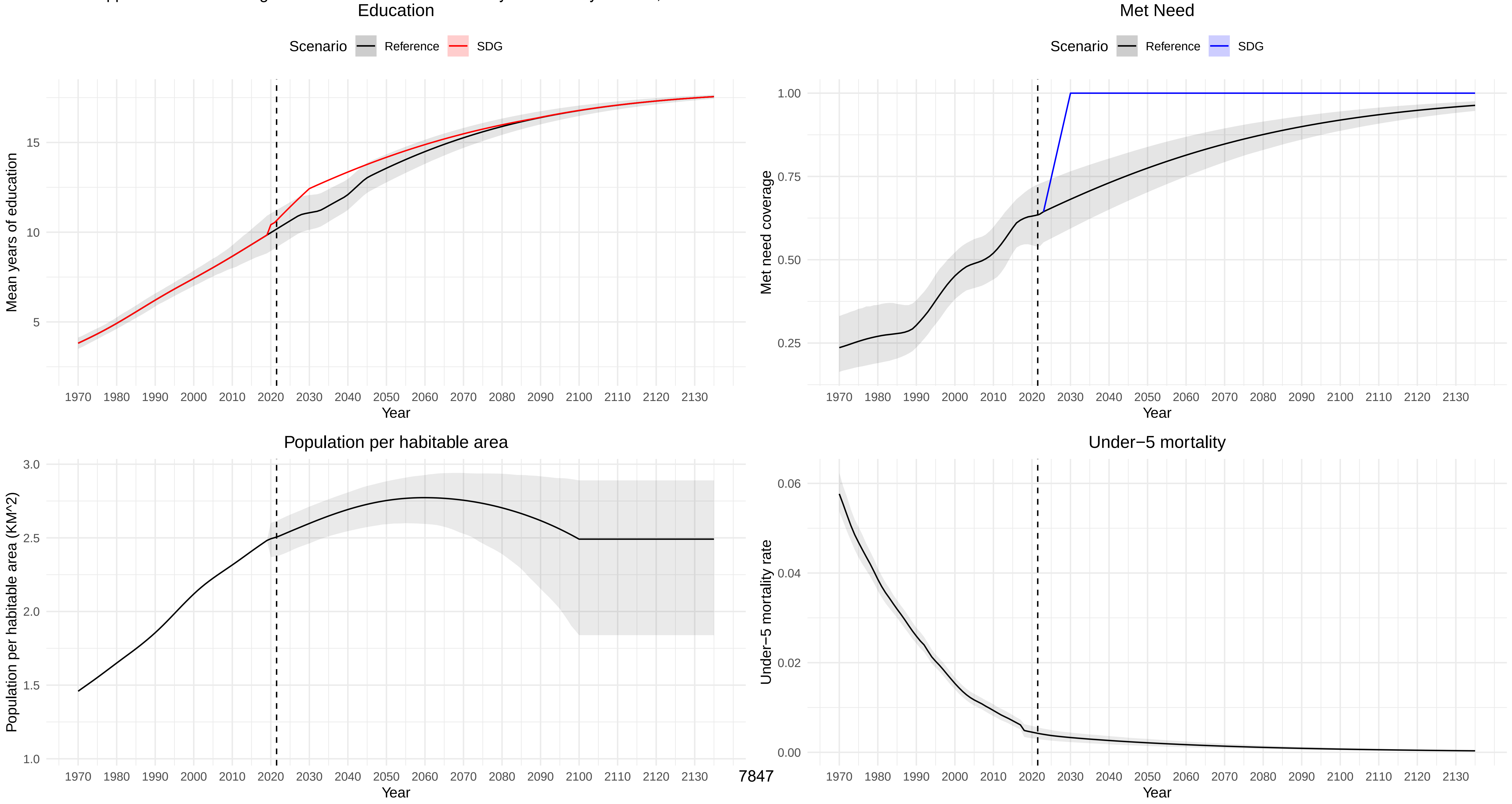

Ecuador

Supplemental Results Figure S2: Covariates used in fertility forecasts by location, 1970-2135

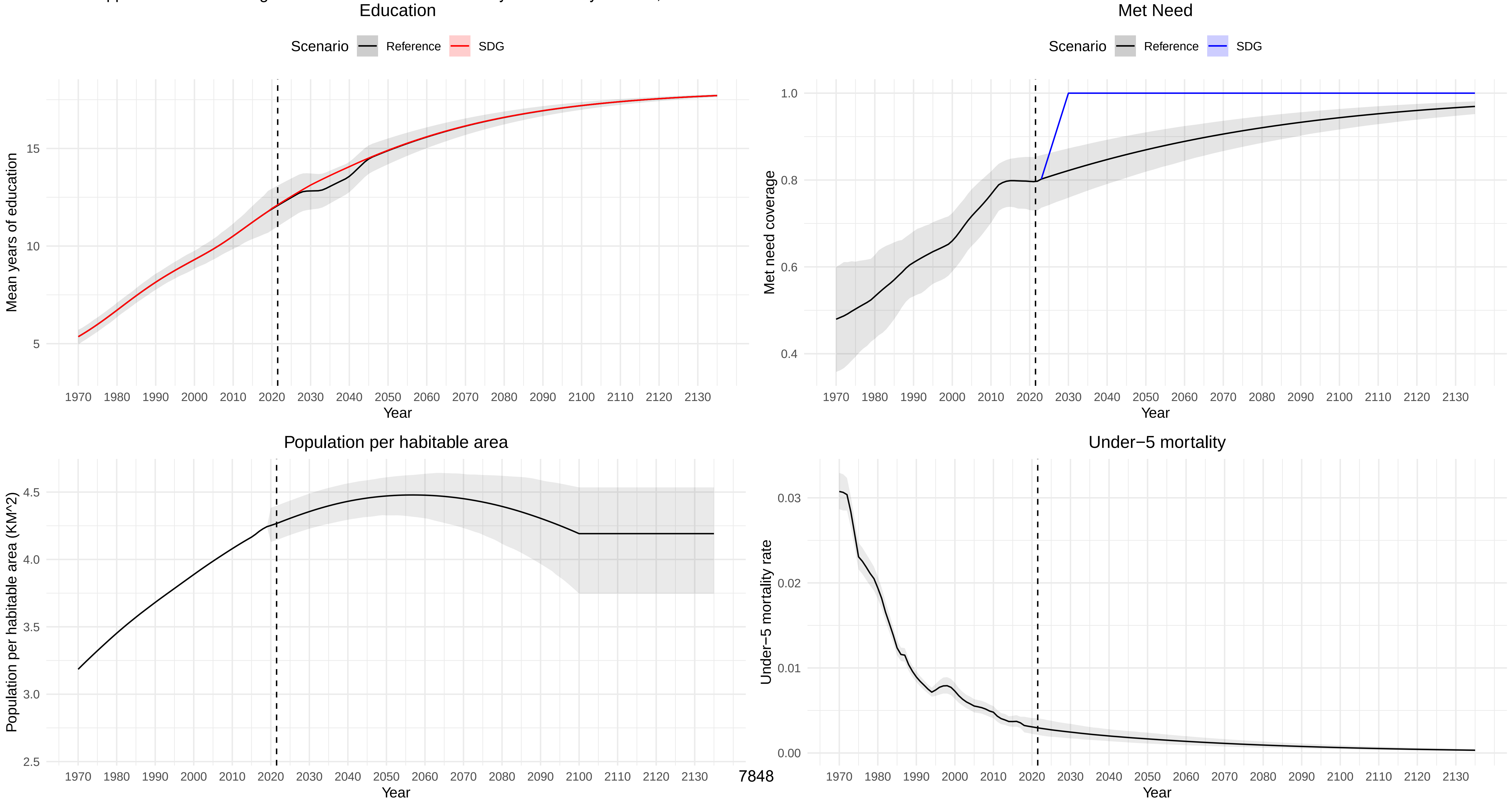

Peru

Supplemental Results Figure S2: Covariates used in fertility forecasts by location, 1970-2135

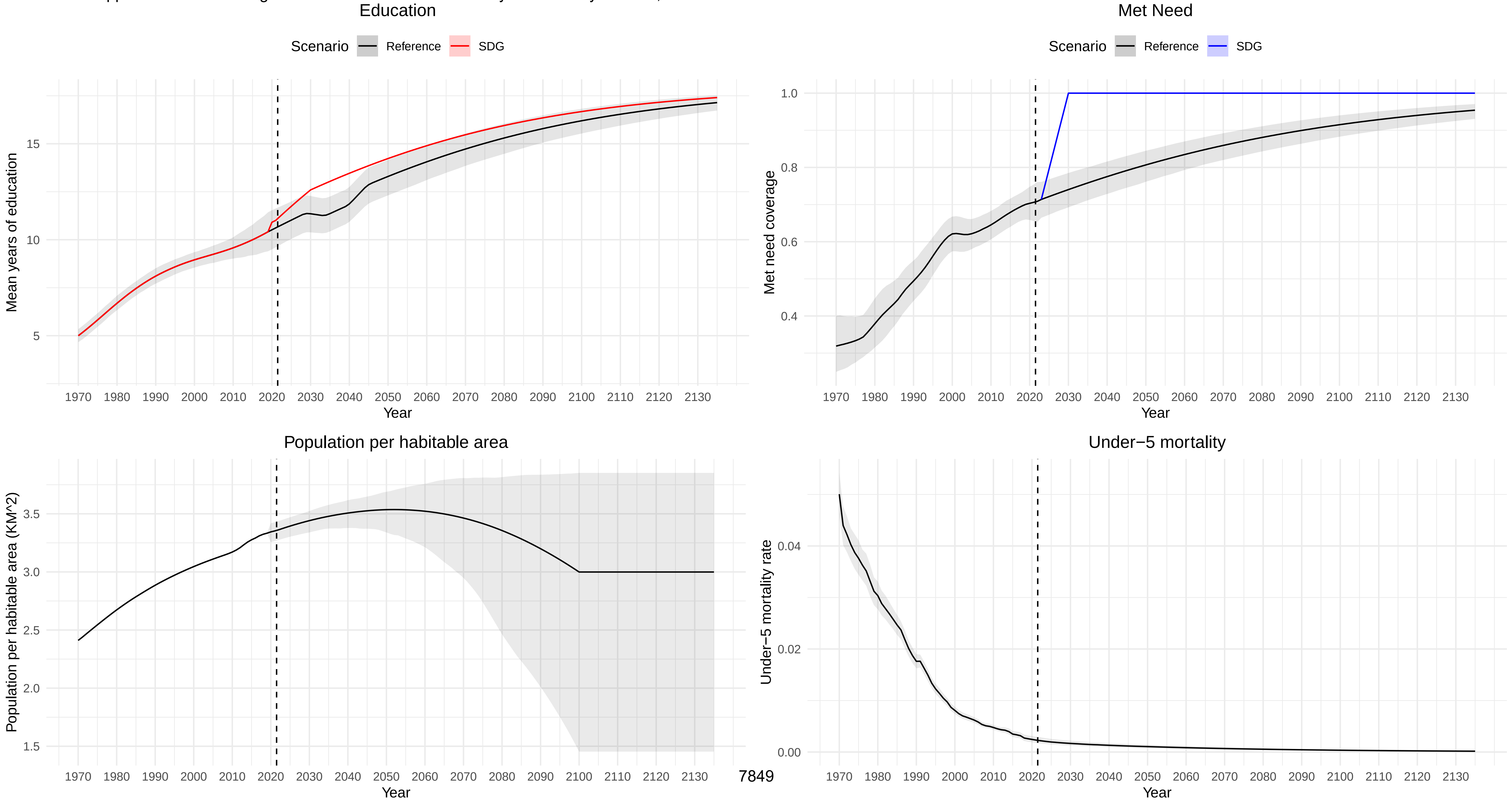

Antigua and Barbuda

Supplemental Results Figure S2: Covariates used in fertility forecasts by location, 1970-2135

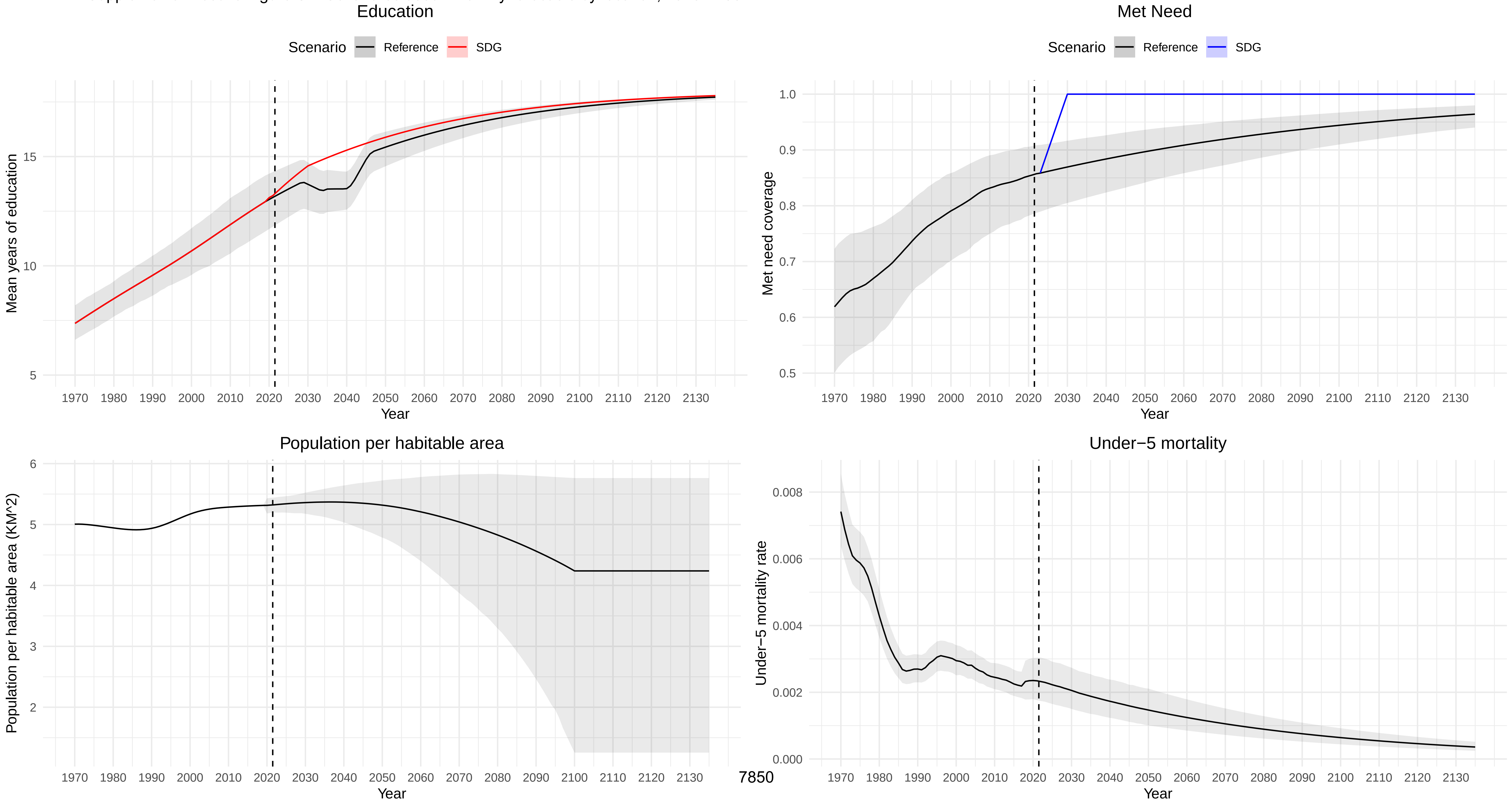

Bahamas

Supplemental Results Figure S2: Covariates used in fertility forecasts by location, 1970-2135

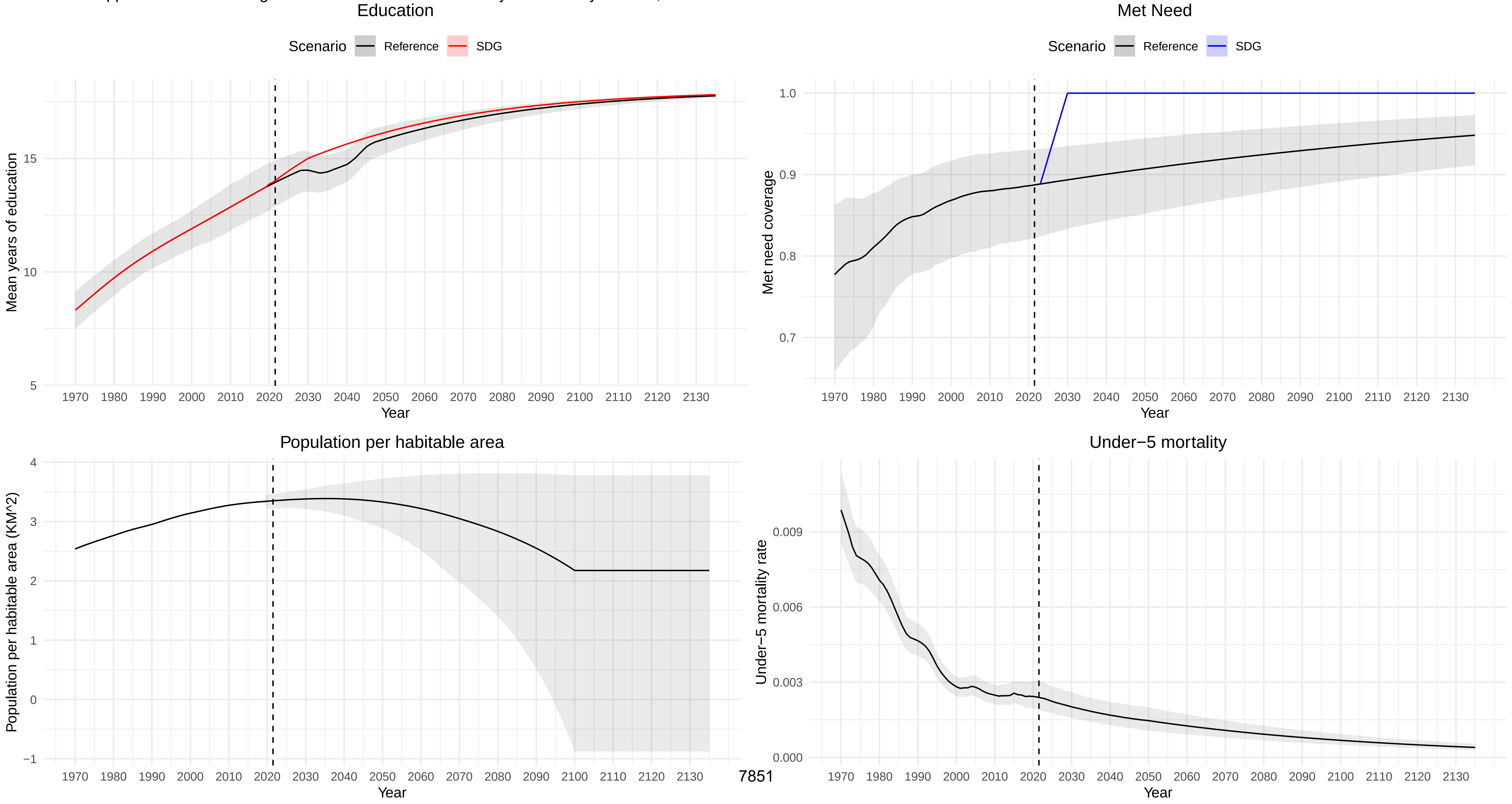

Barbados

Supplemental Results Figure S2: Covariates used in fertility forecasts by location, 1970-2135

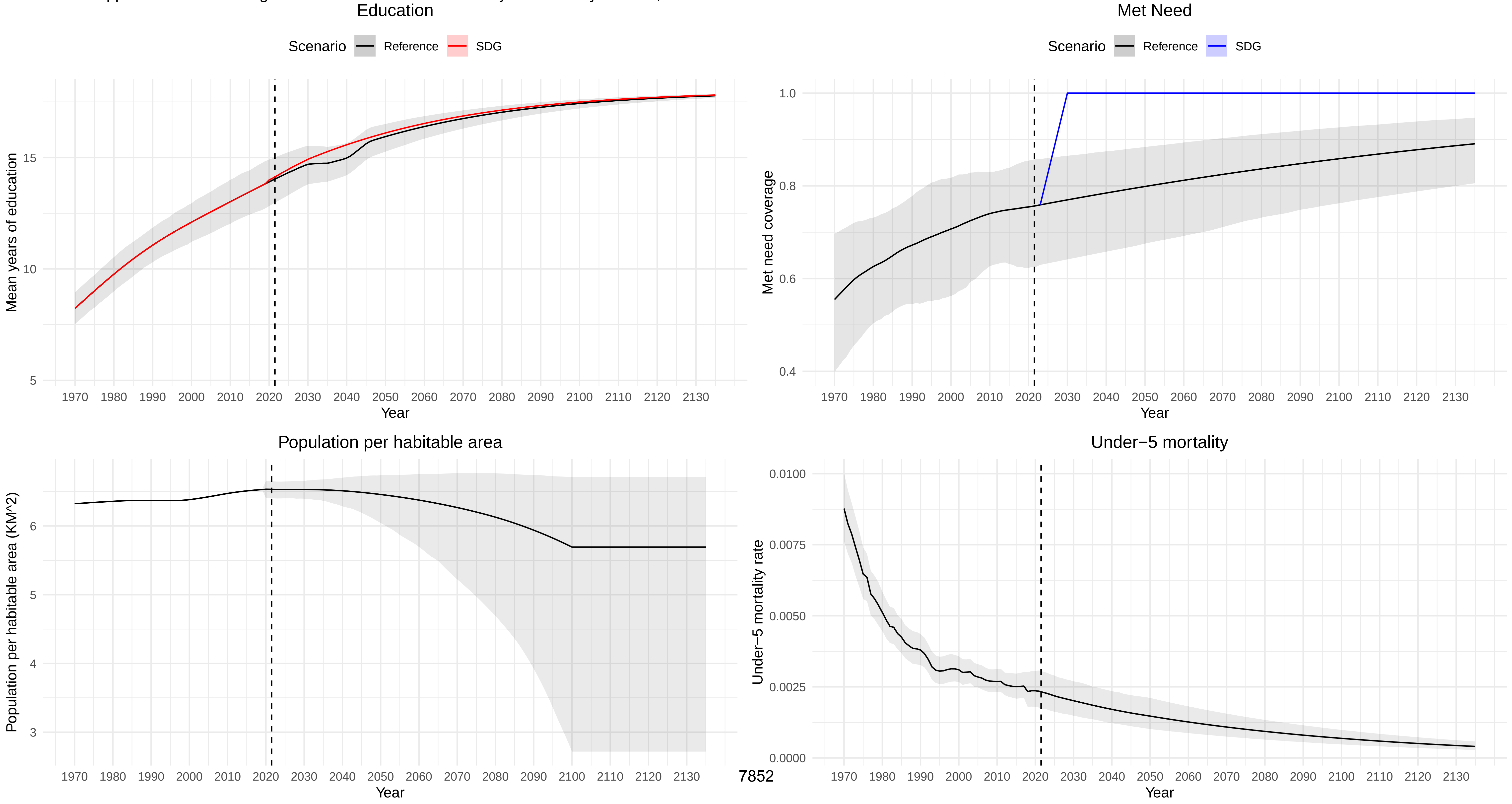

Belize

Supplemental Results Figure S2: Covariates used in fertility forecasts by location, 1970-2135

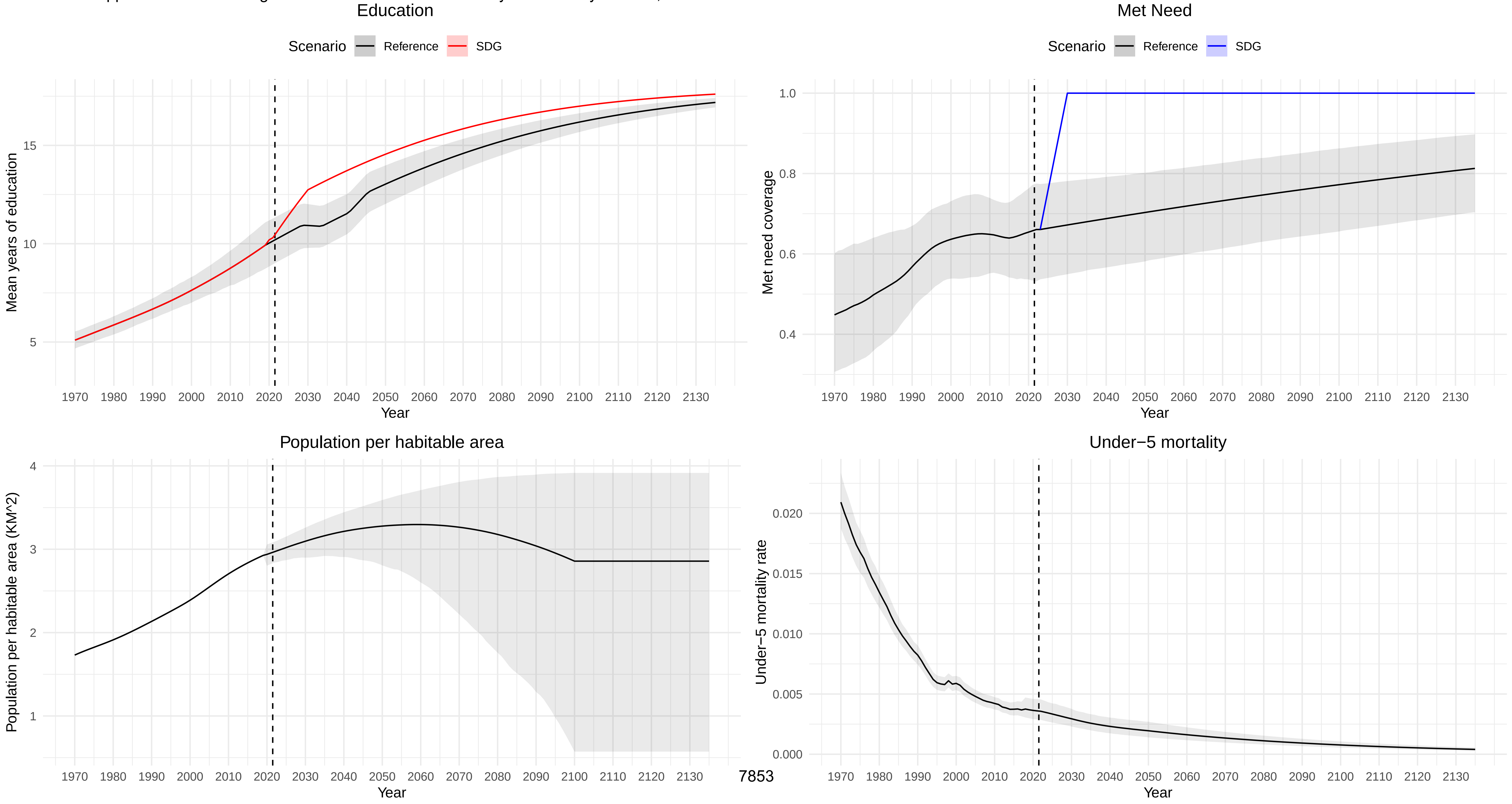

Bermuda

Supplemental Results Figure S2: Covariates used in fertility forecasts by location, 1970-2135

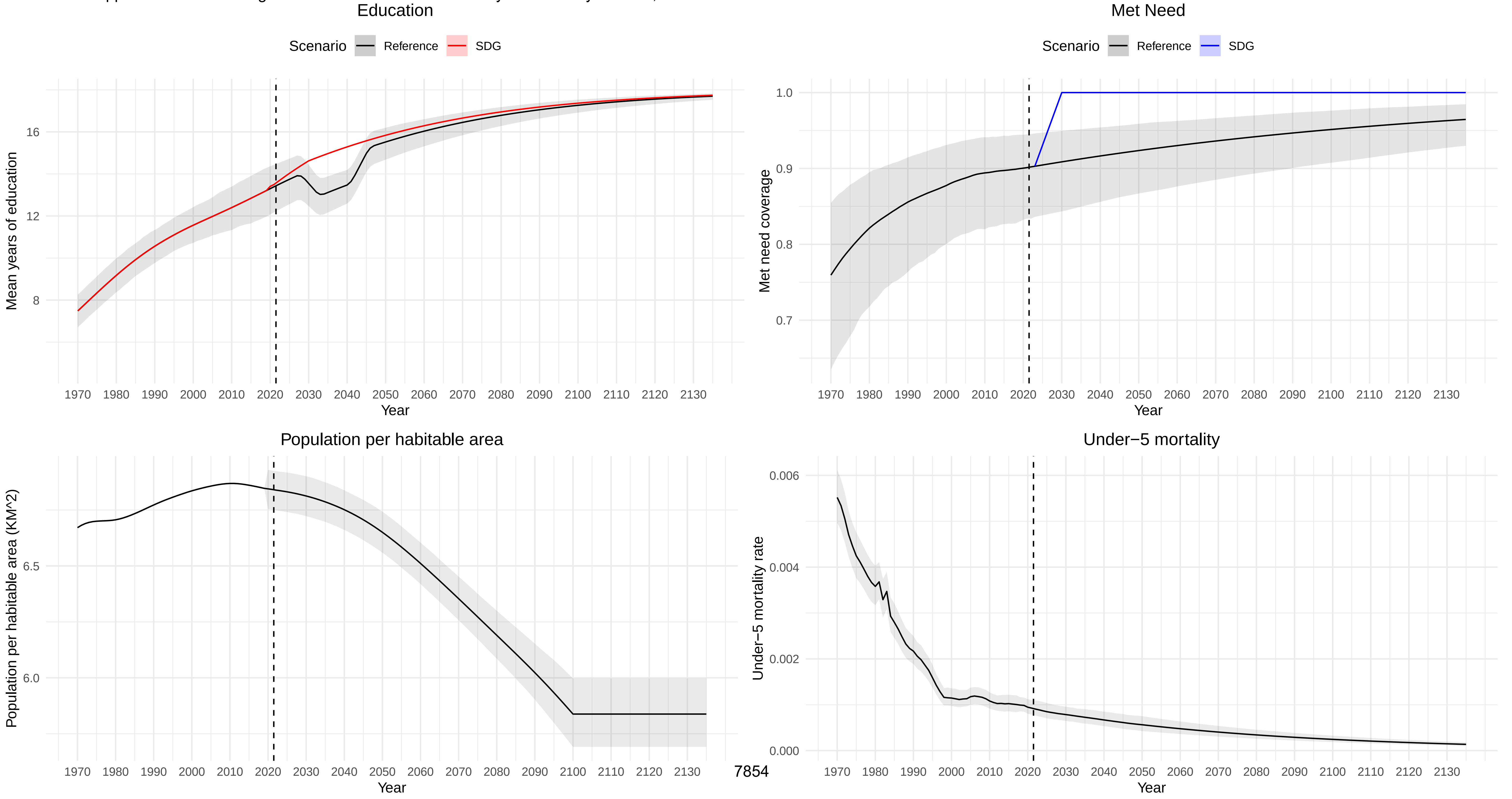

Cuba

Supplemental Results Figure S2: Covariates used in fertility forecasts by location, 1970-2135

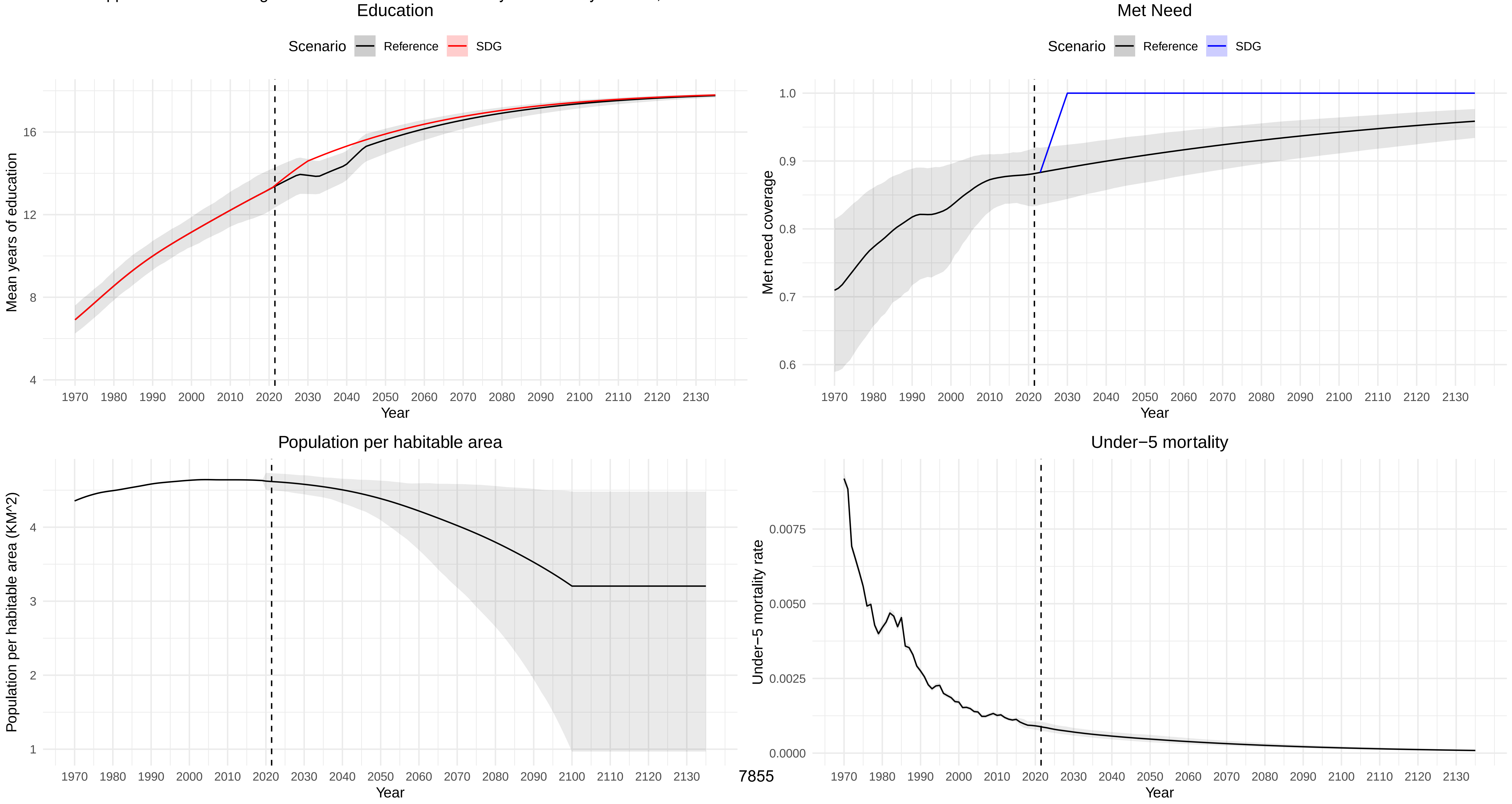

Dominica

Supplemental Results Figure S2: Covariates used in fertility forecasts by location, 1970-2135

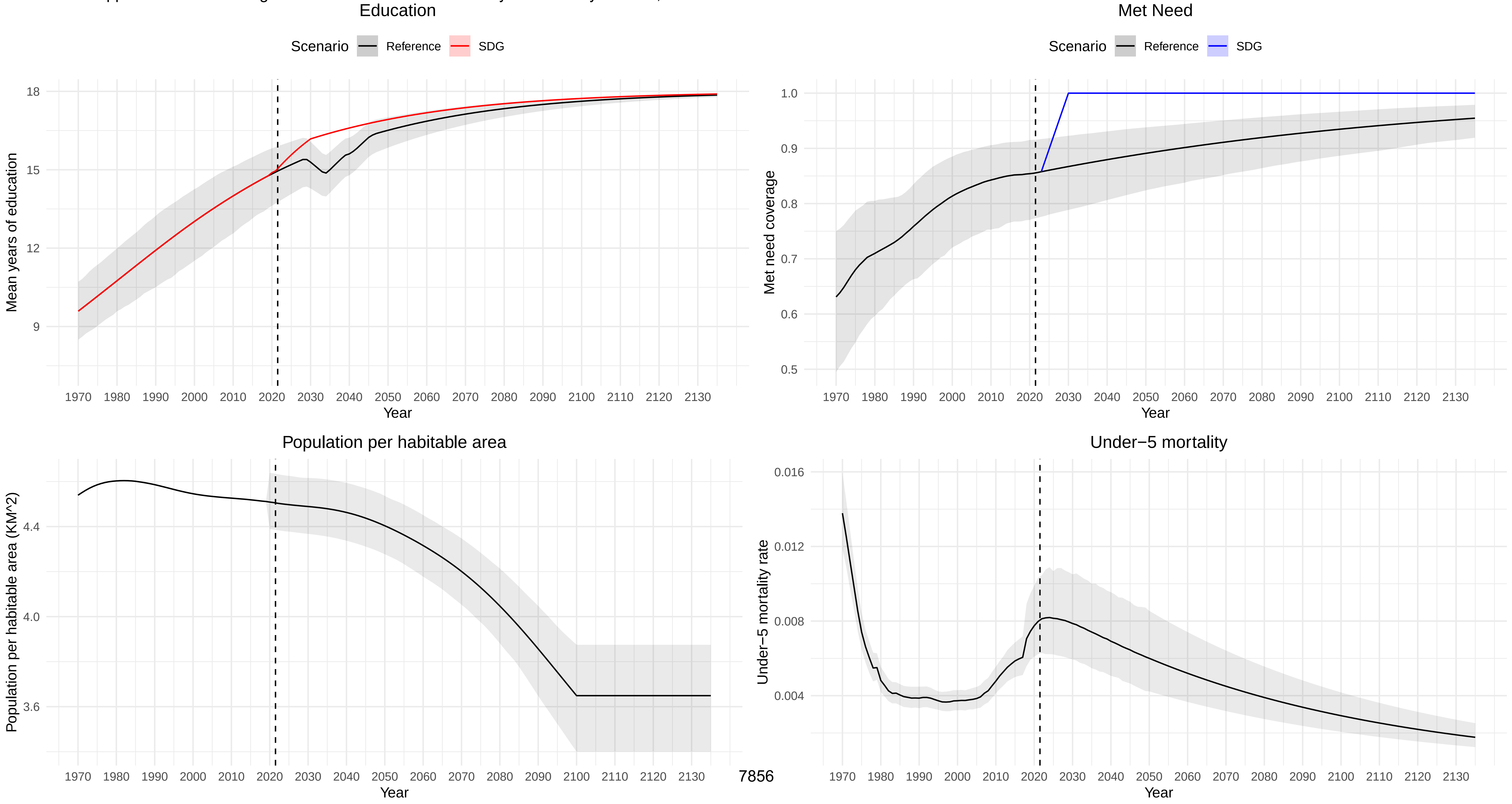

Dominican Republic

Supplemental Results Figure S2: Covariates used in fertility forecasts by location, 1970-2135

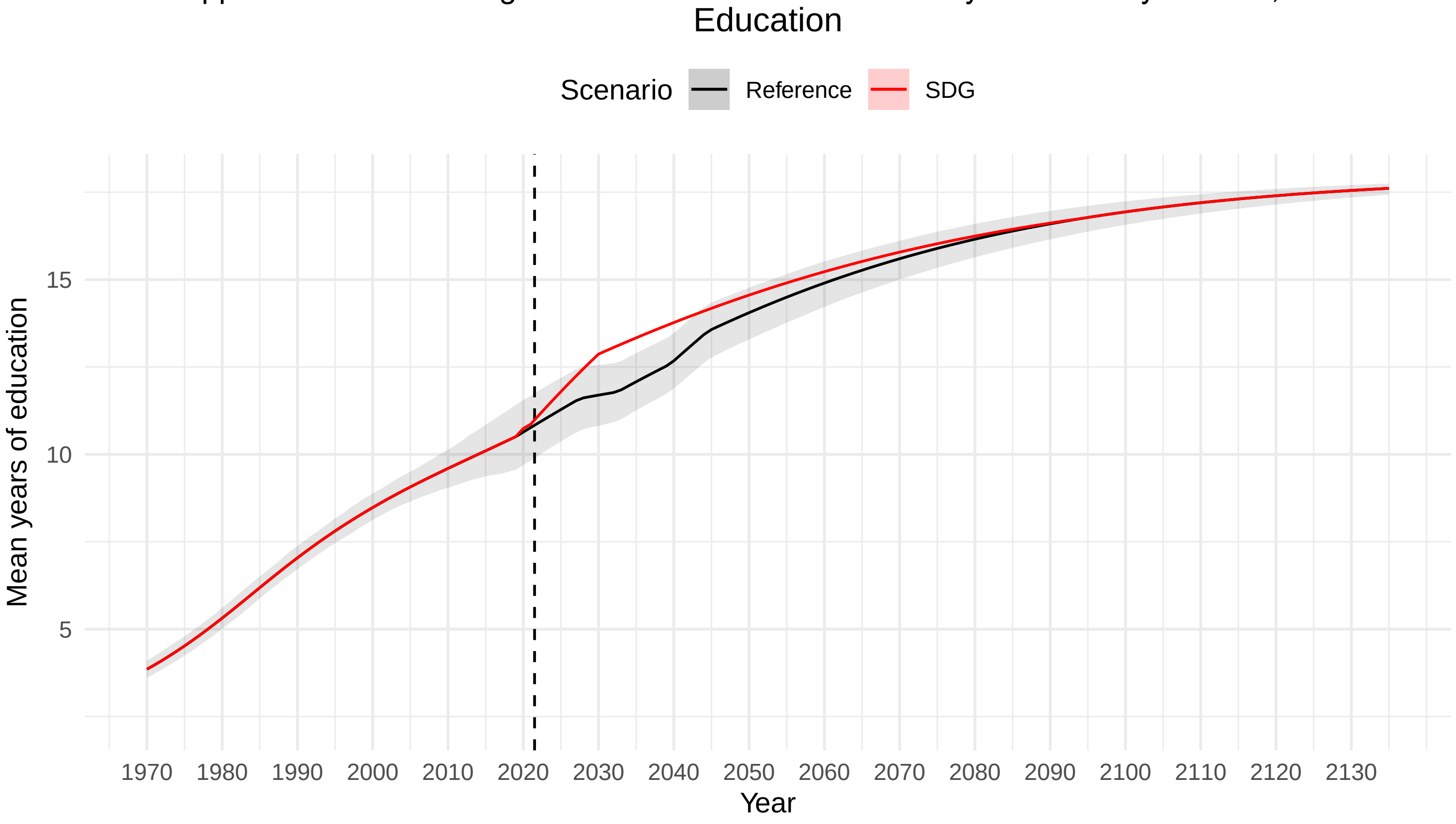

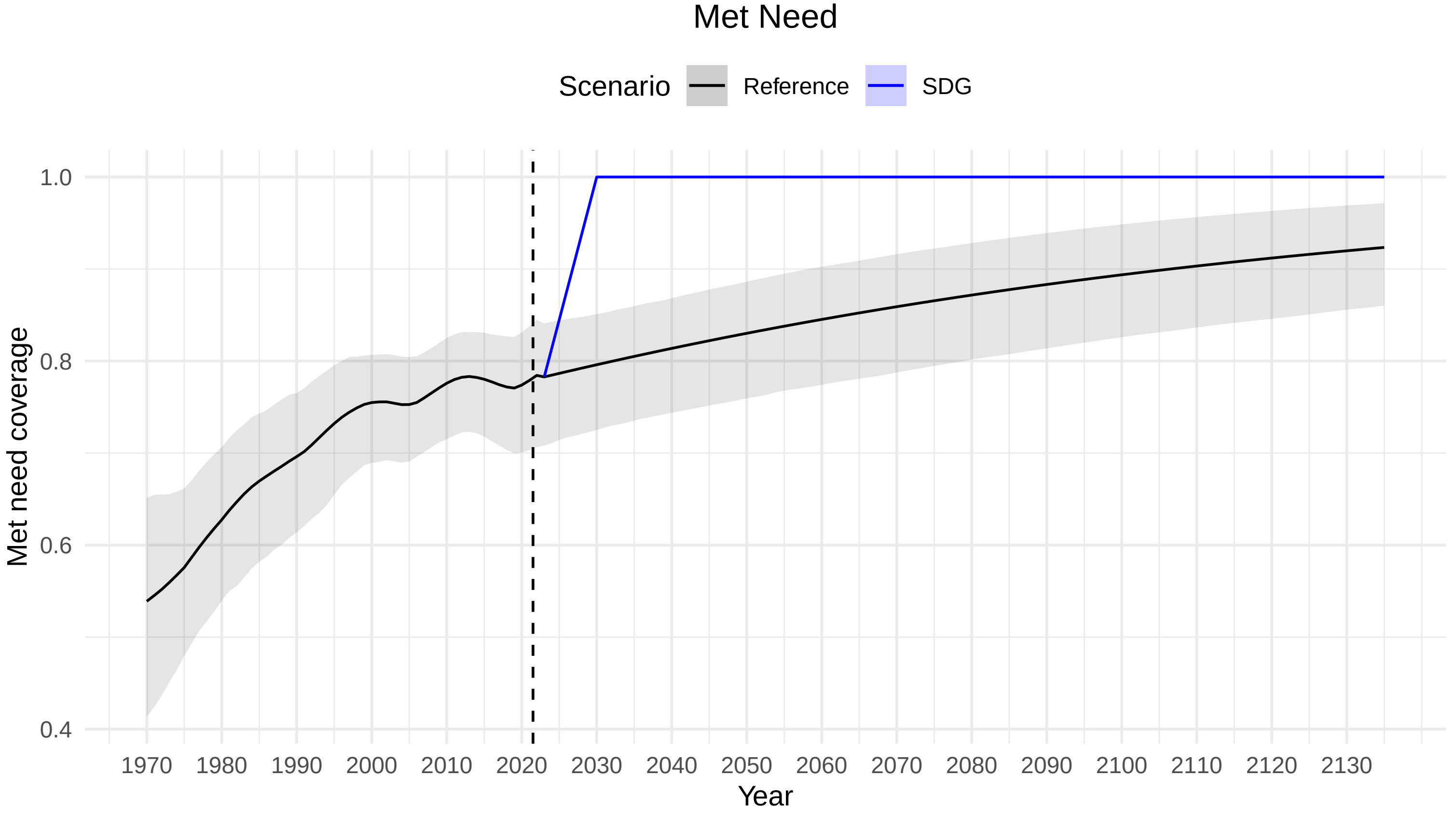

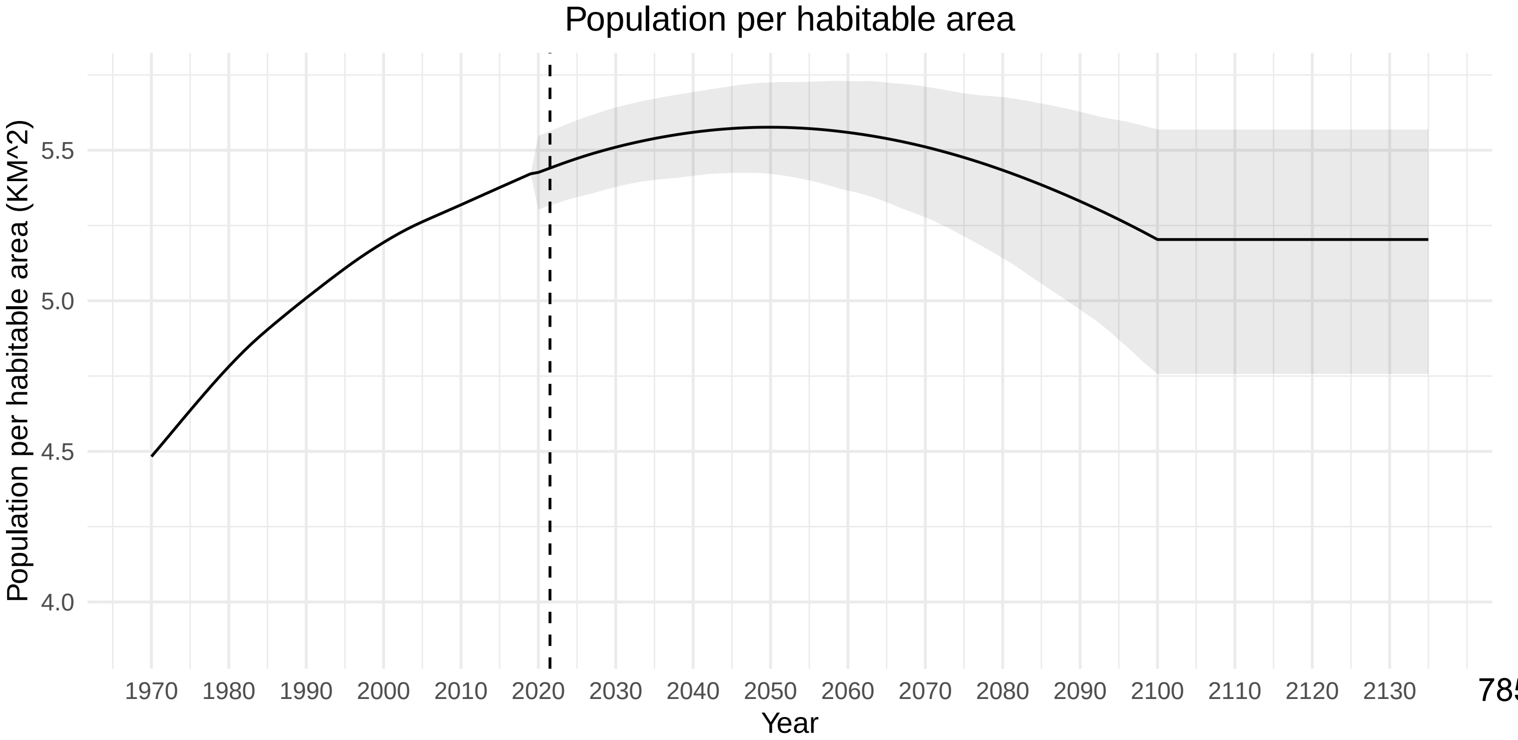

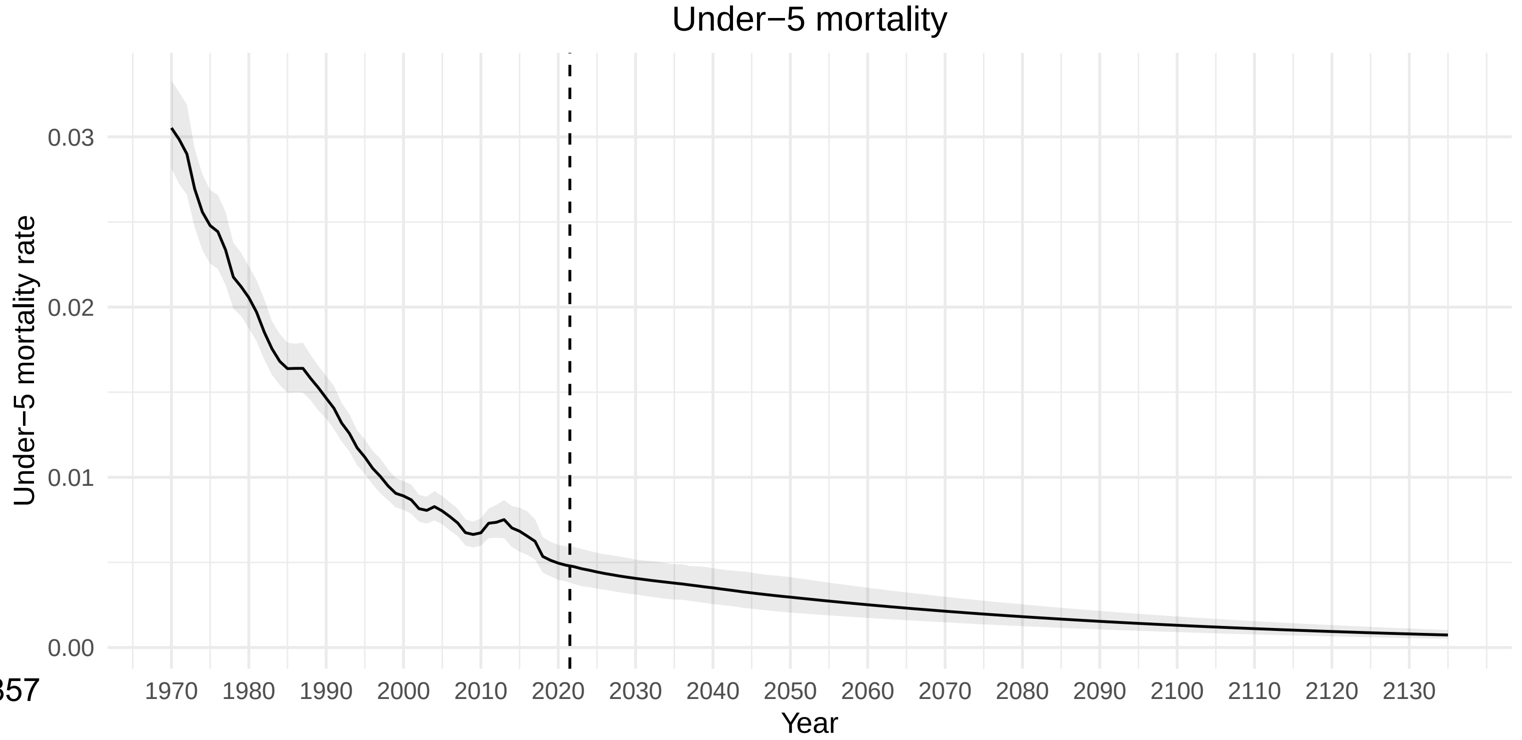

Grenada

Supplemental Results Figure S2: Covariates used in fertility forecasts by location, 1970-2135

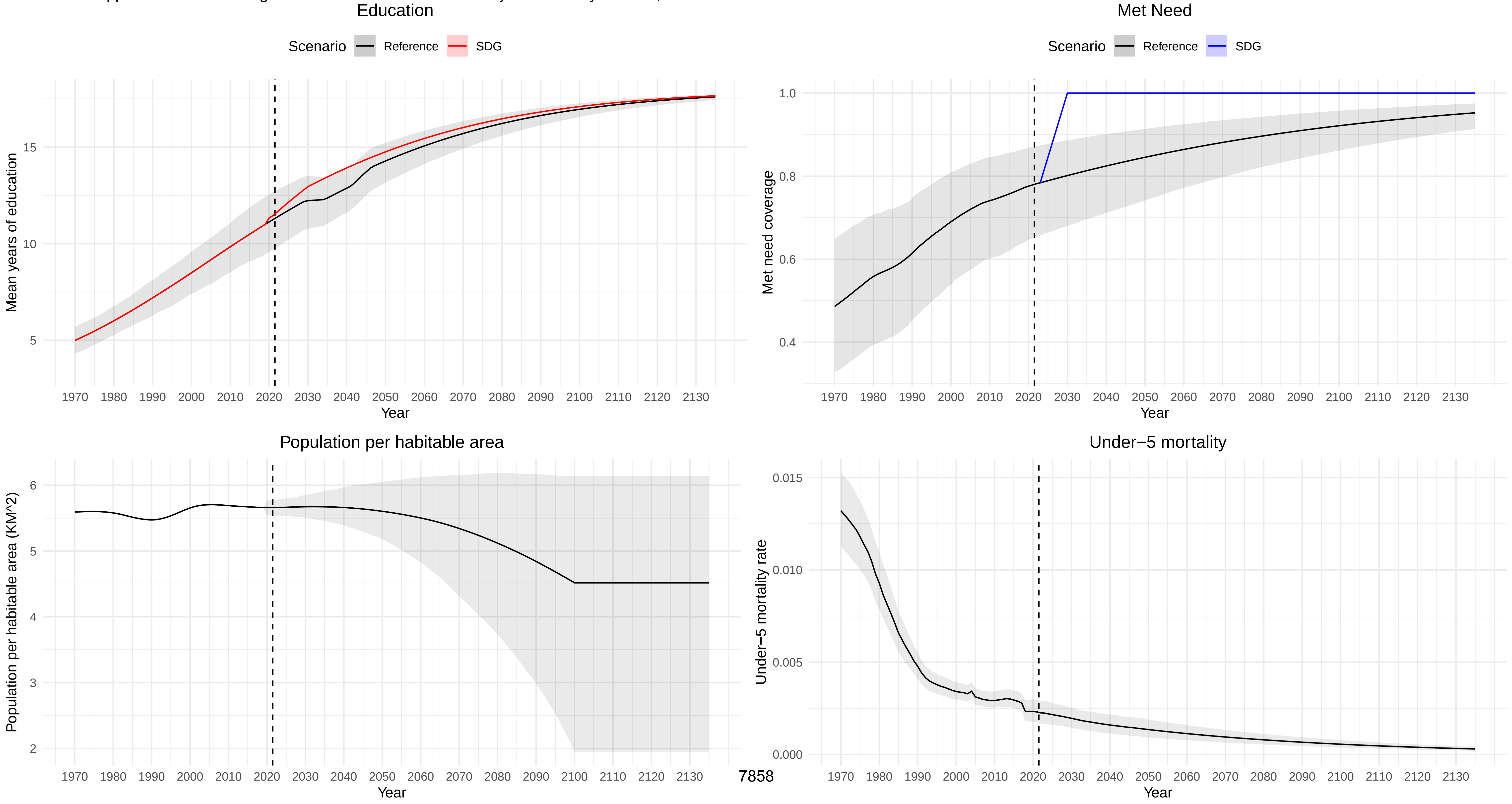

Guyana

Supplemental Results Figure S2: Covariates used in fertility forecasts by location, 1970-2135

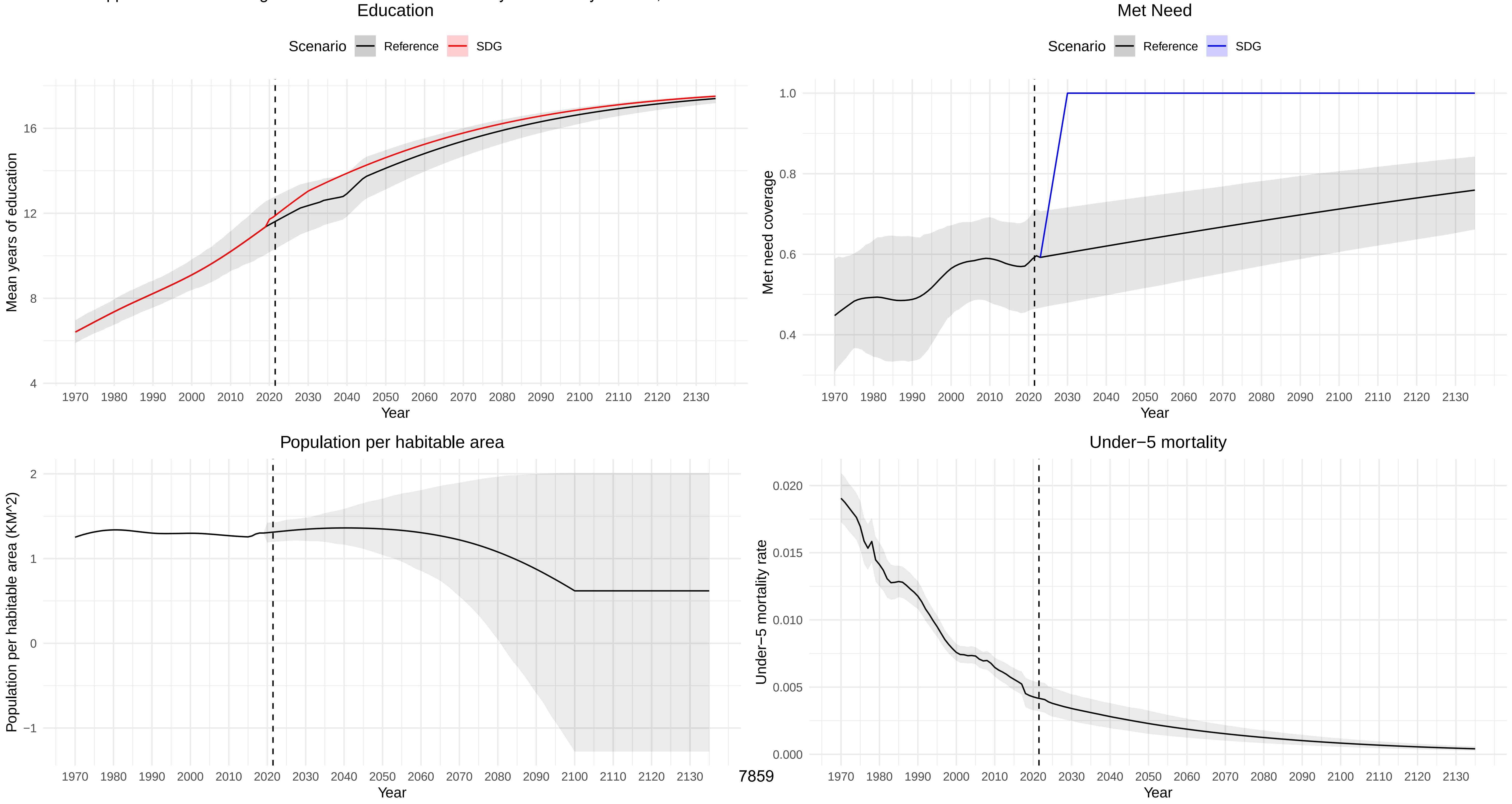

Haiti

Supplemental Results Figure S2: Covariates used in fertility forecasts by location, 1970-2135

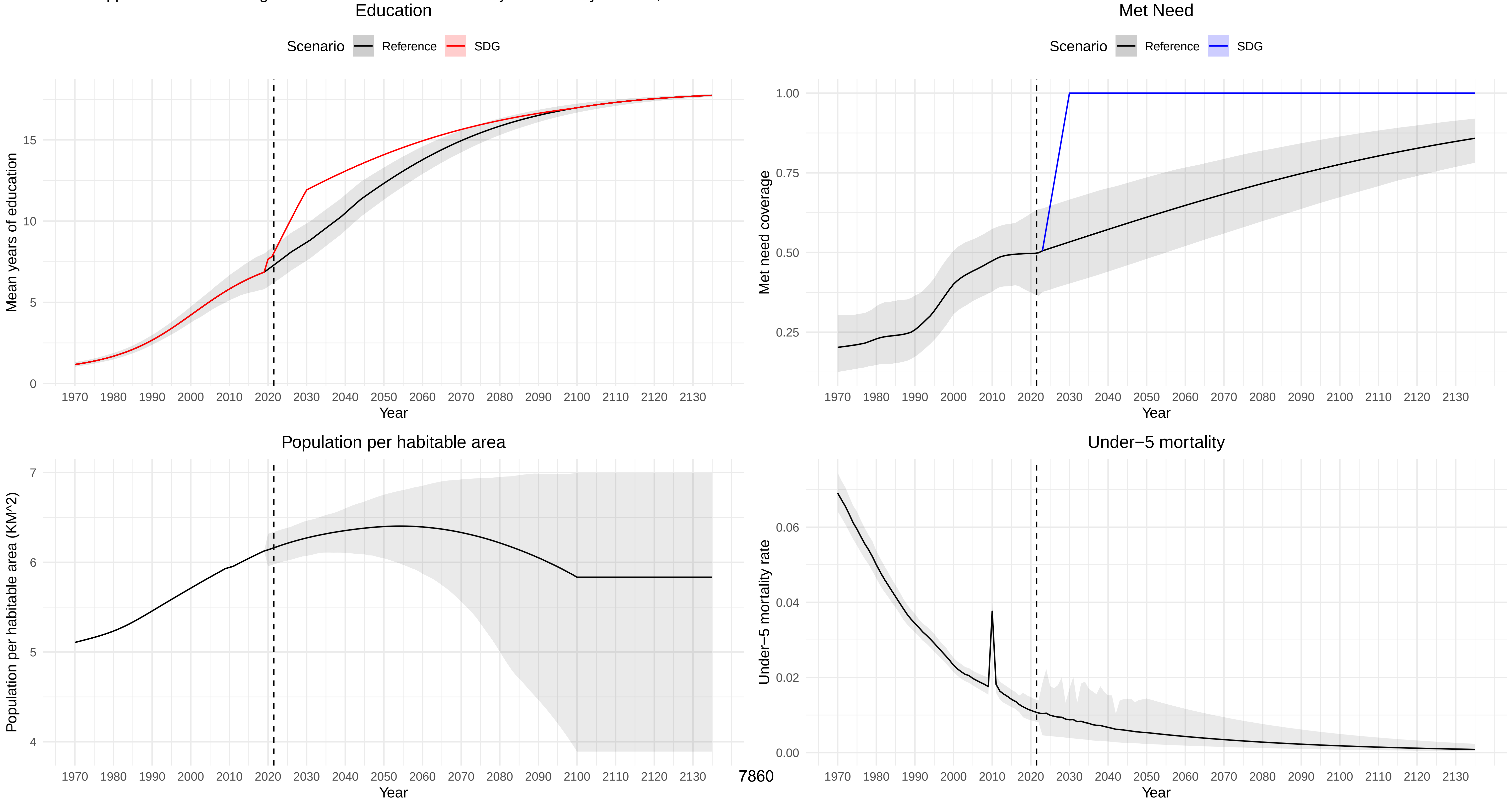

Jamaica

Supplemental Results Figure S2: Covariates used in fertility forecasts by location, 1970-2135

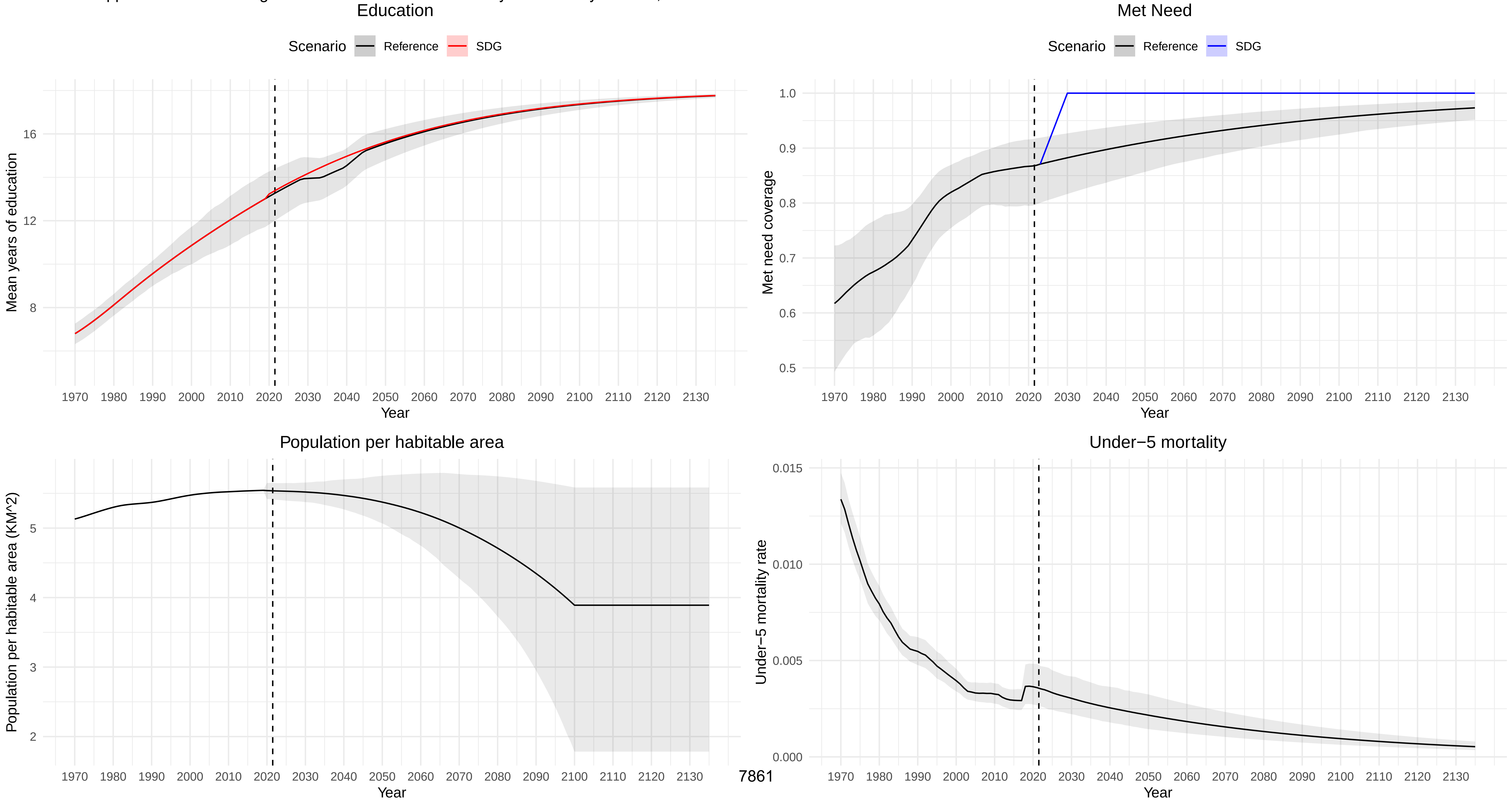

Puerto Rico

Supplemental Results Figure S2: Covariates used in fertility forecasts by location, 1970-2135

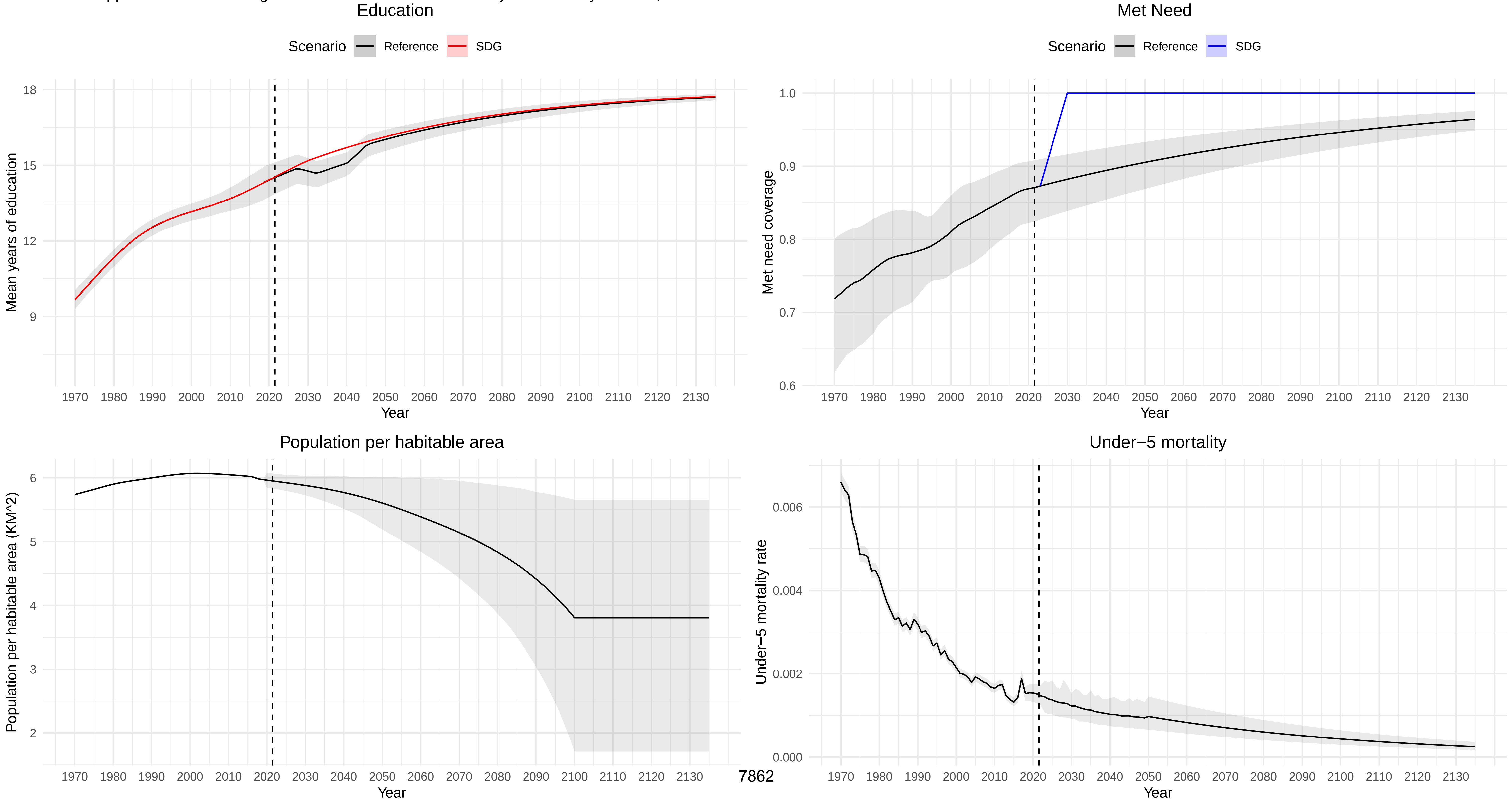

Saint Kitts and Nevis

Supplemental Results Figure S2: Covariates used in fertility forecasts by location, 1970-2135

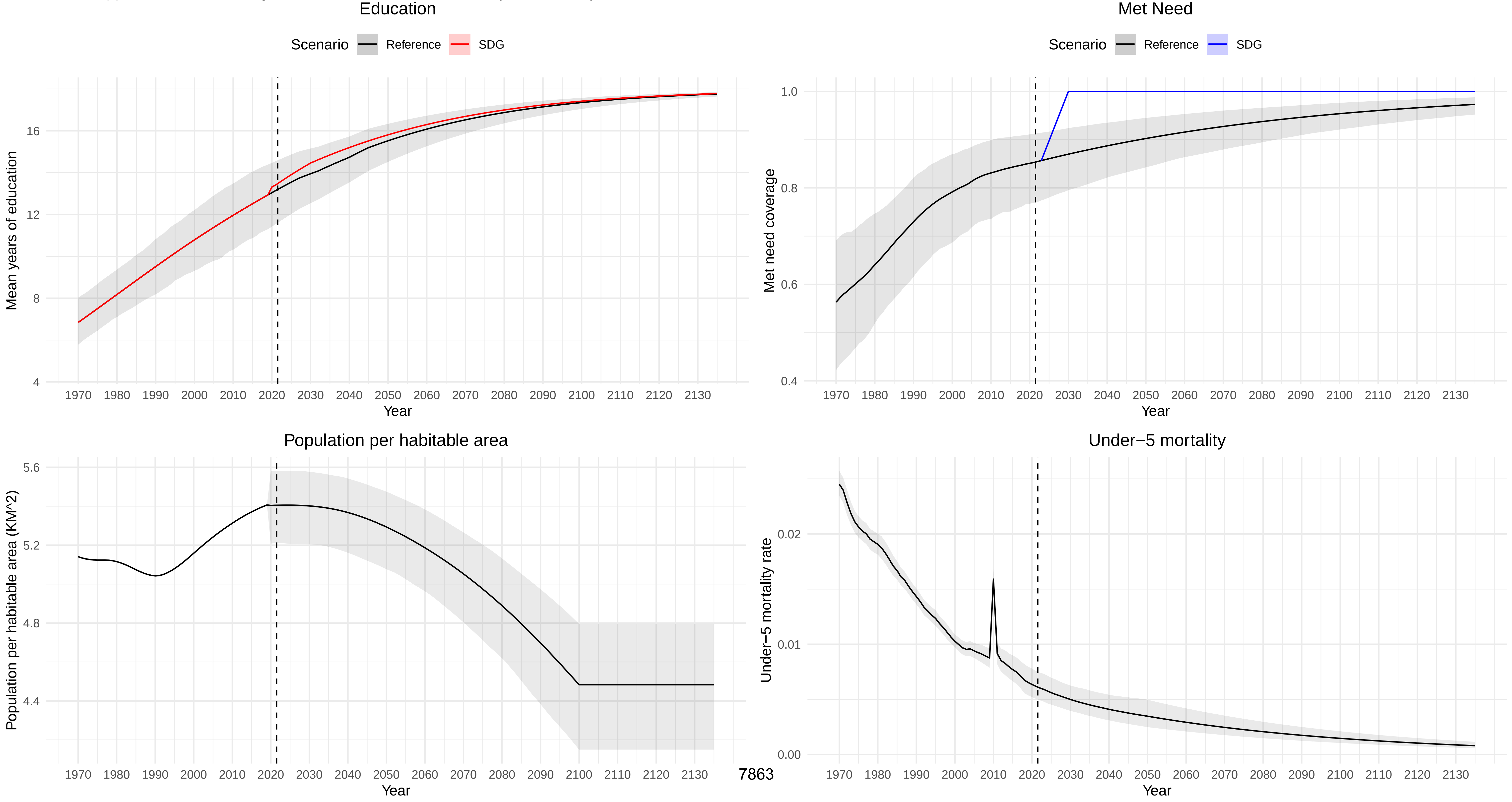

Saint Lucia

Supplemental Results Figure S2: Covariates used in fertility forecasts by location, 1970-2135

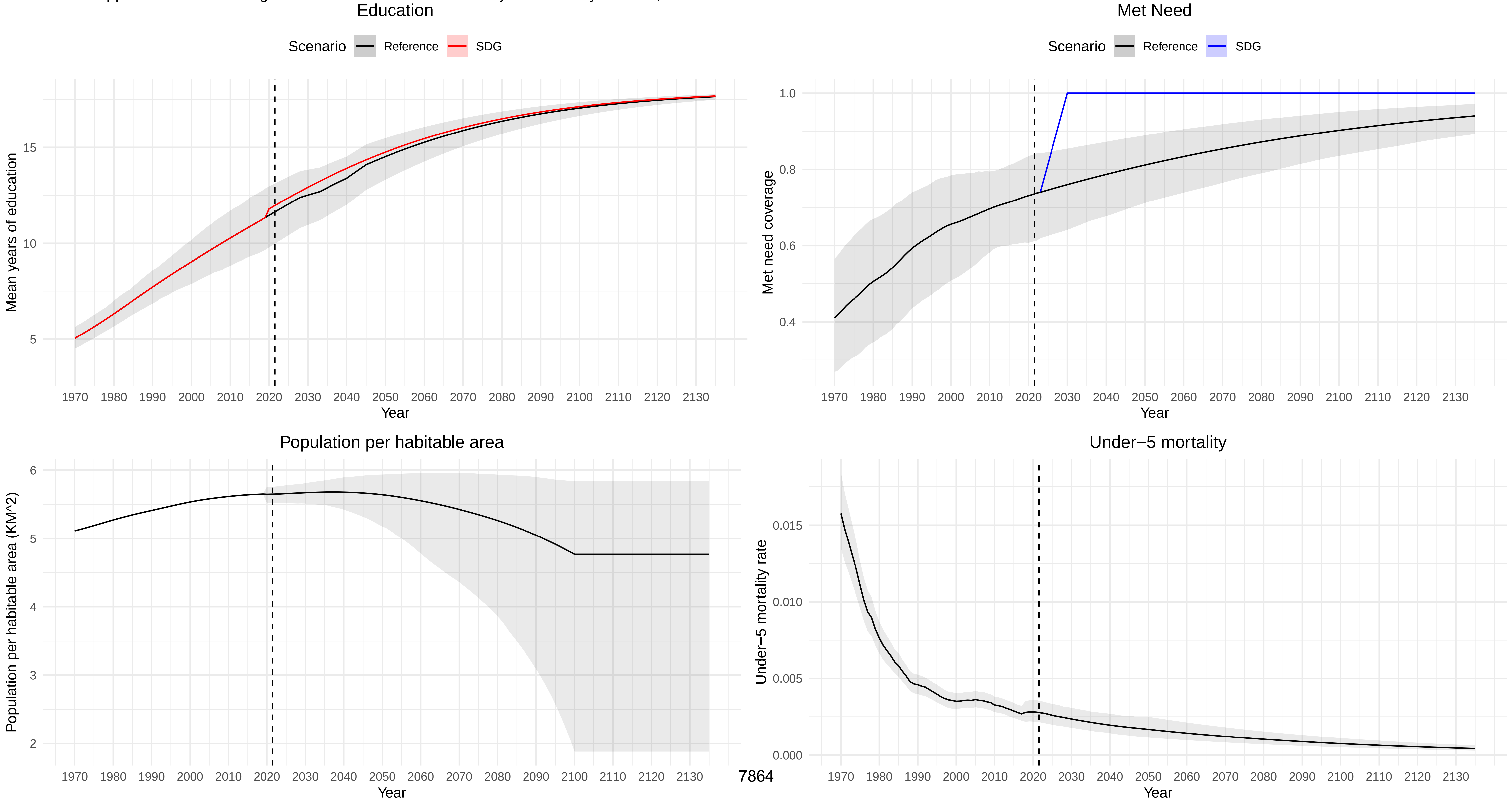

Saint Vincent and the Grenadines

Supplemental Results Figure S2: Covariates used in fertility forecasts by location, 1970-2135

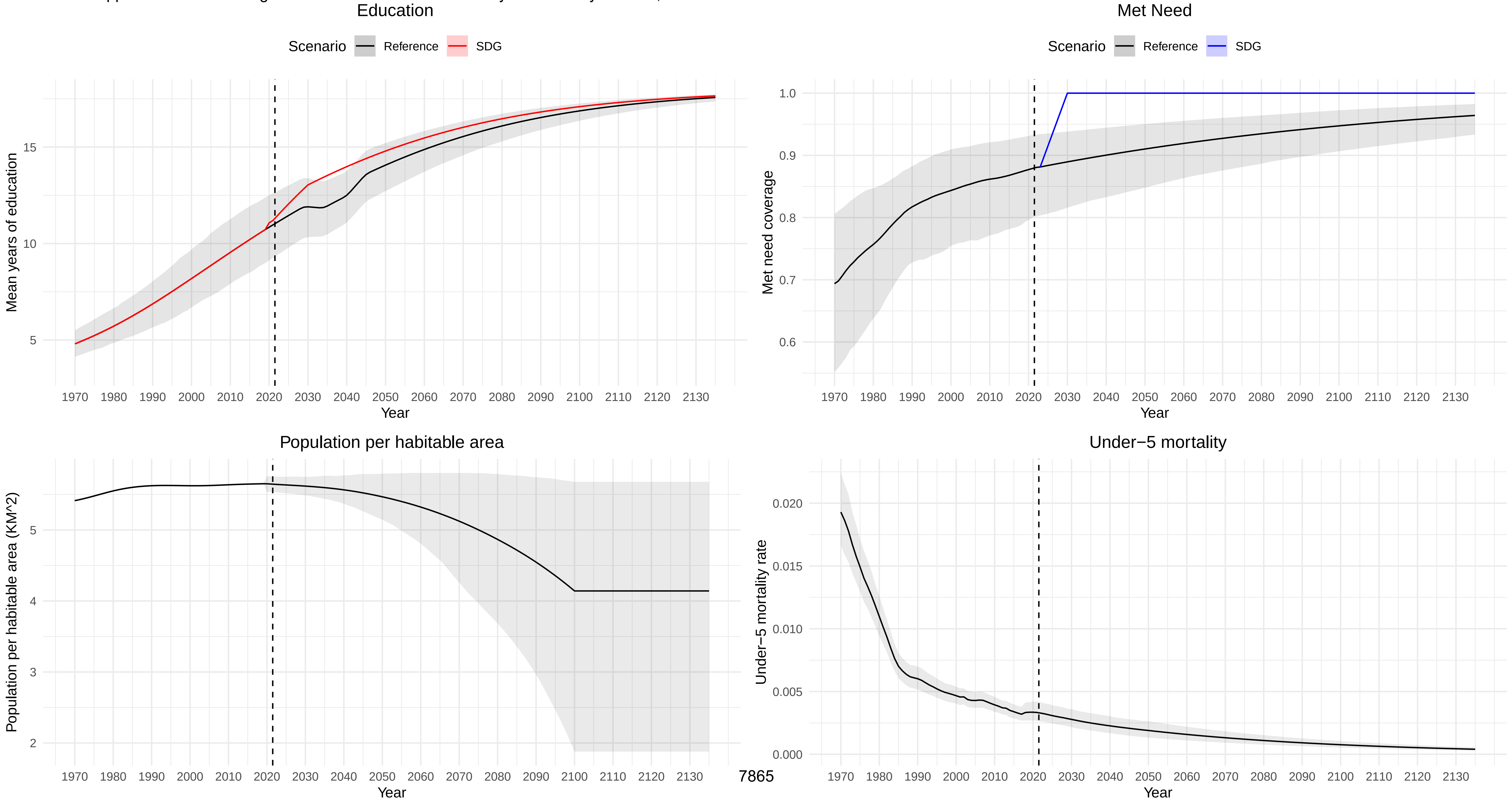

Suriname

Supplemental Results Figure S2: Covariates used in fertility forecasts by location, 1970-2135

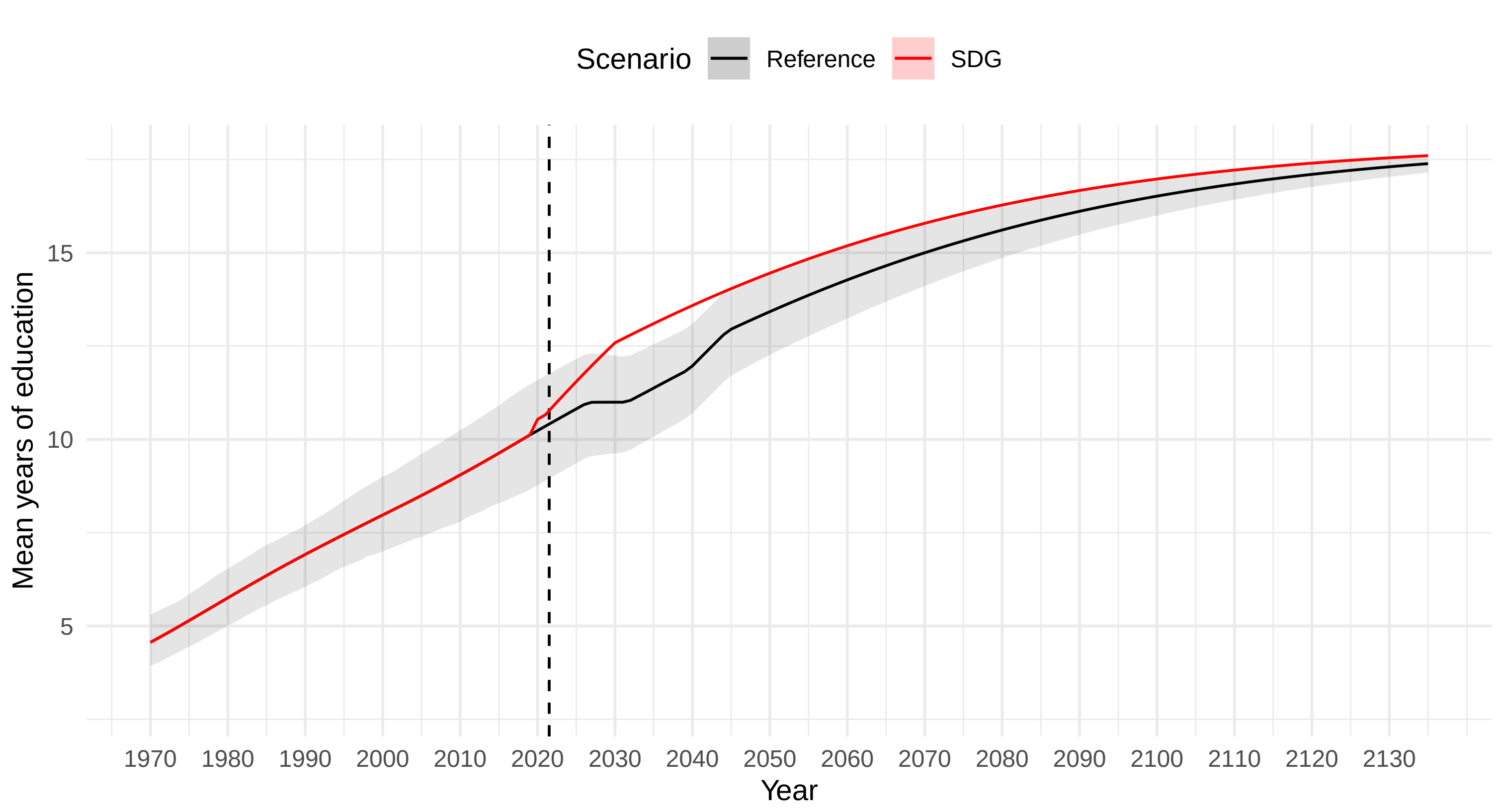

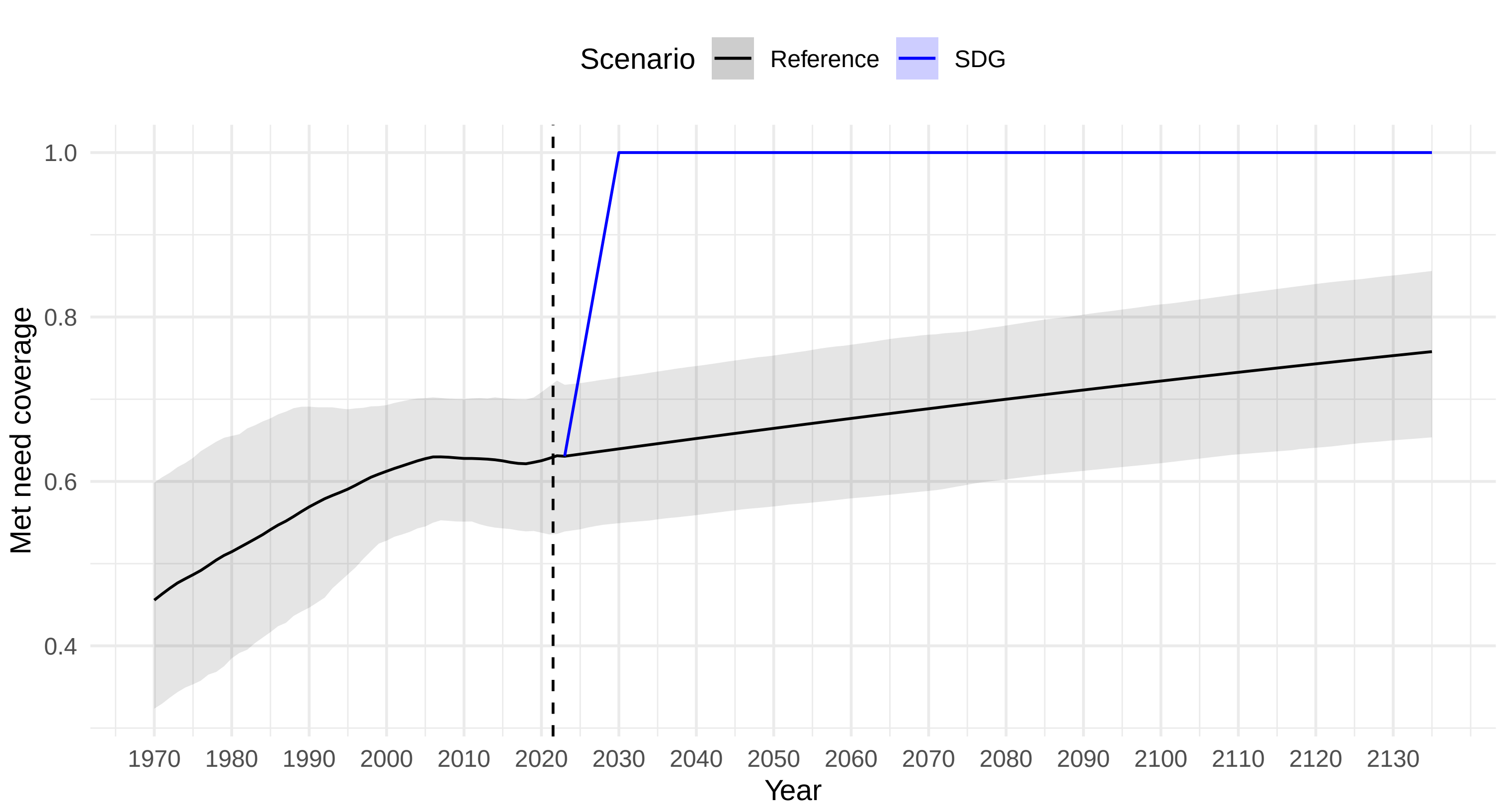

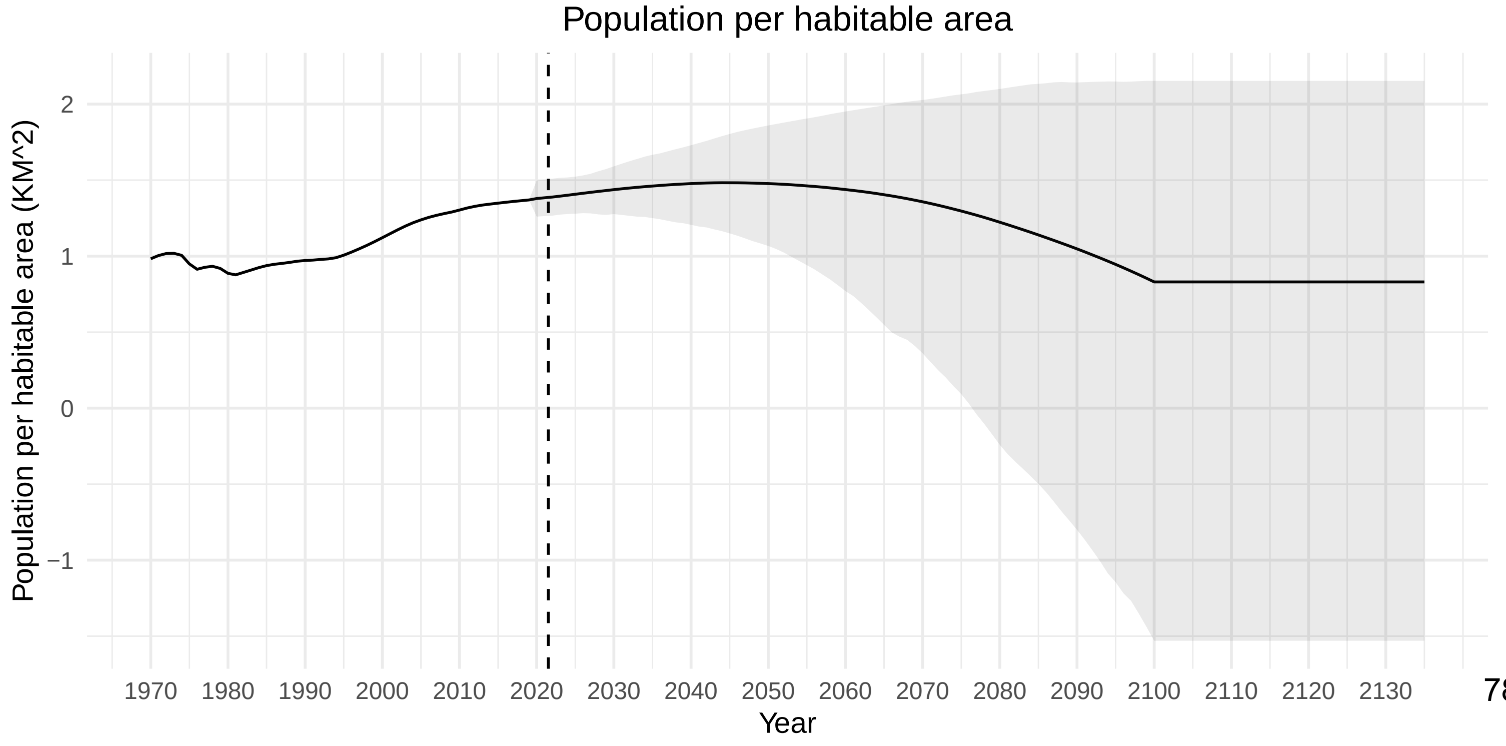

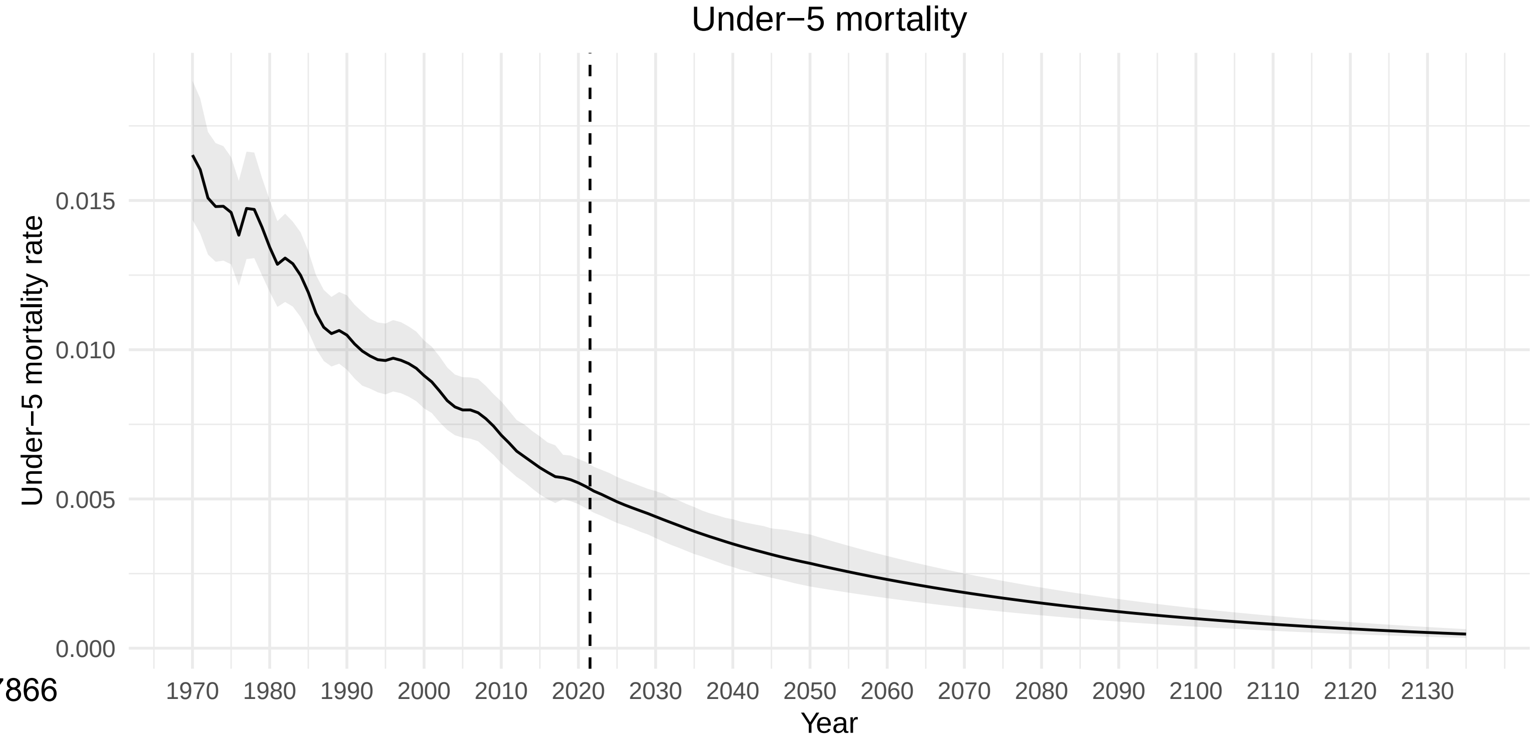

Trinidad and Tobago

Supplemental Results Figure S2: Covariates used in fertility forecasts by location, 1970-2135

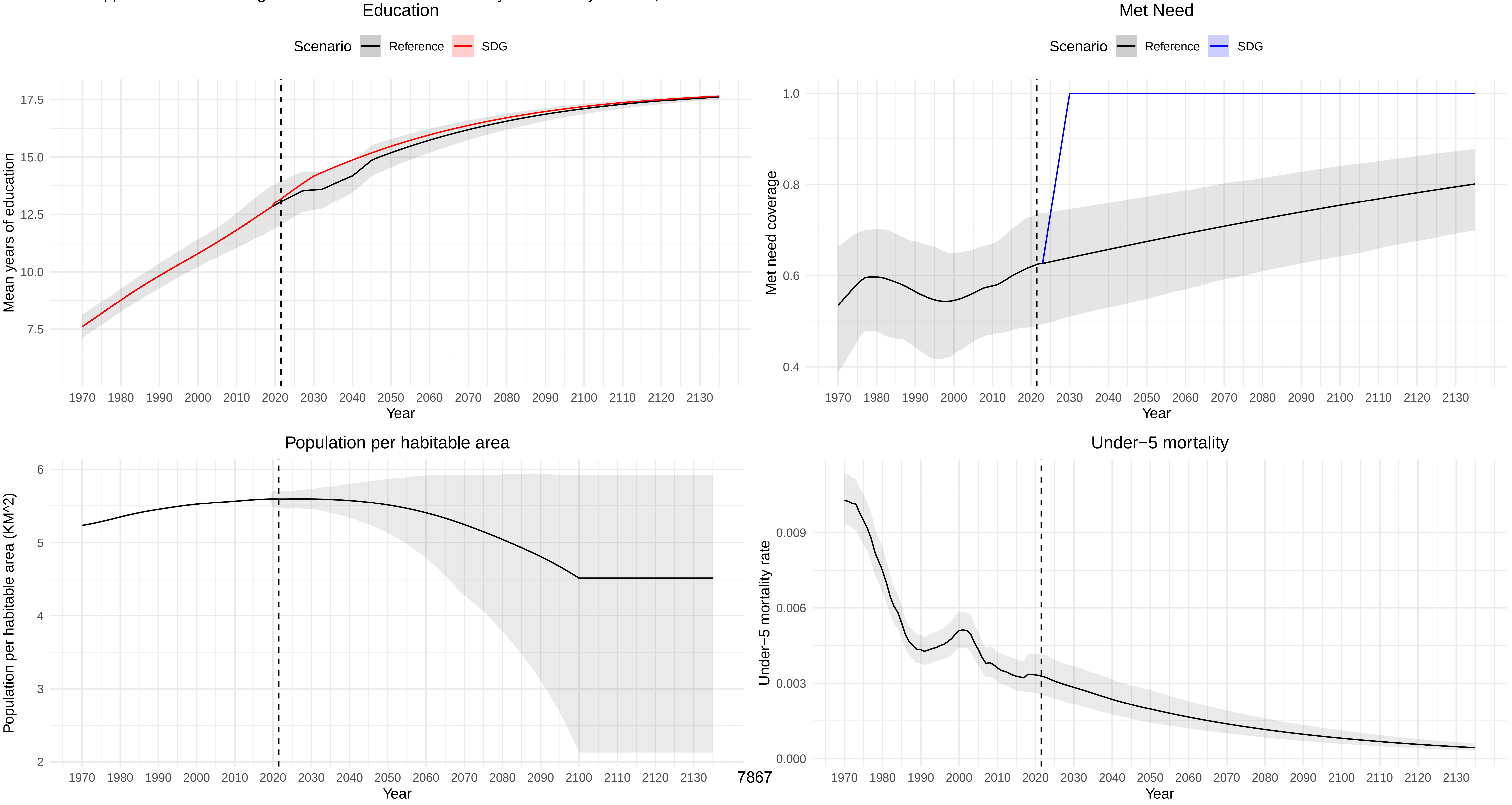

United States Virgin Islands

Supplemental Results Figure S2: Covariates used in fertility forecasts by location, 1970-2135

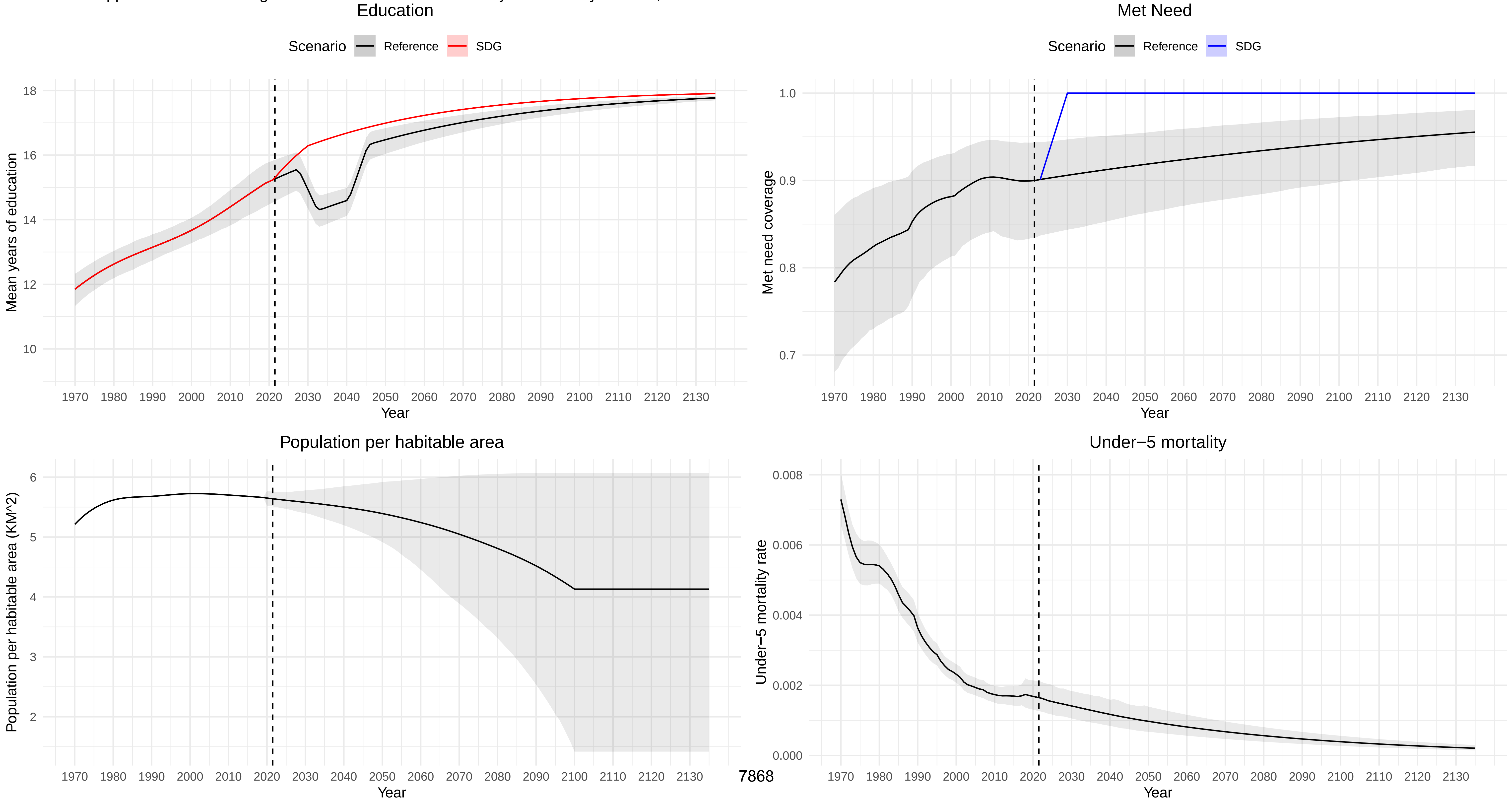

Colombia

Supplemental Results Figure S2: Covariates used in fertility forecasts by location, 1970-2135

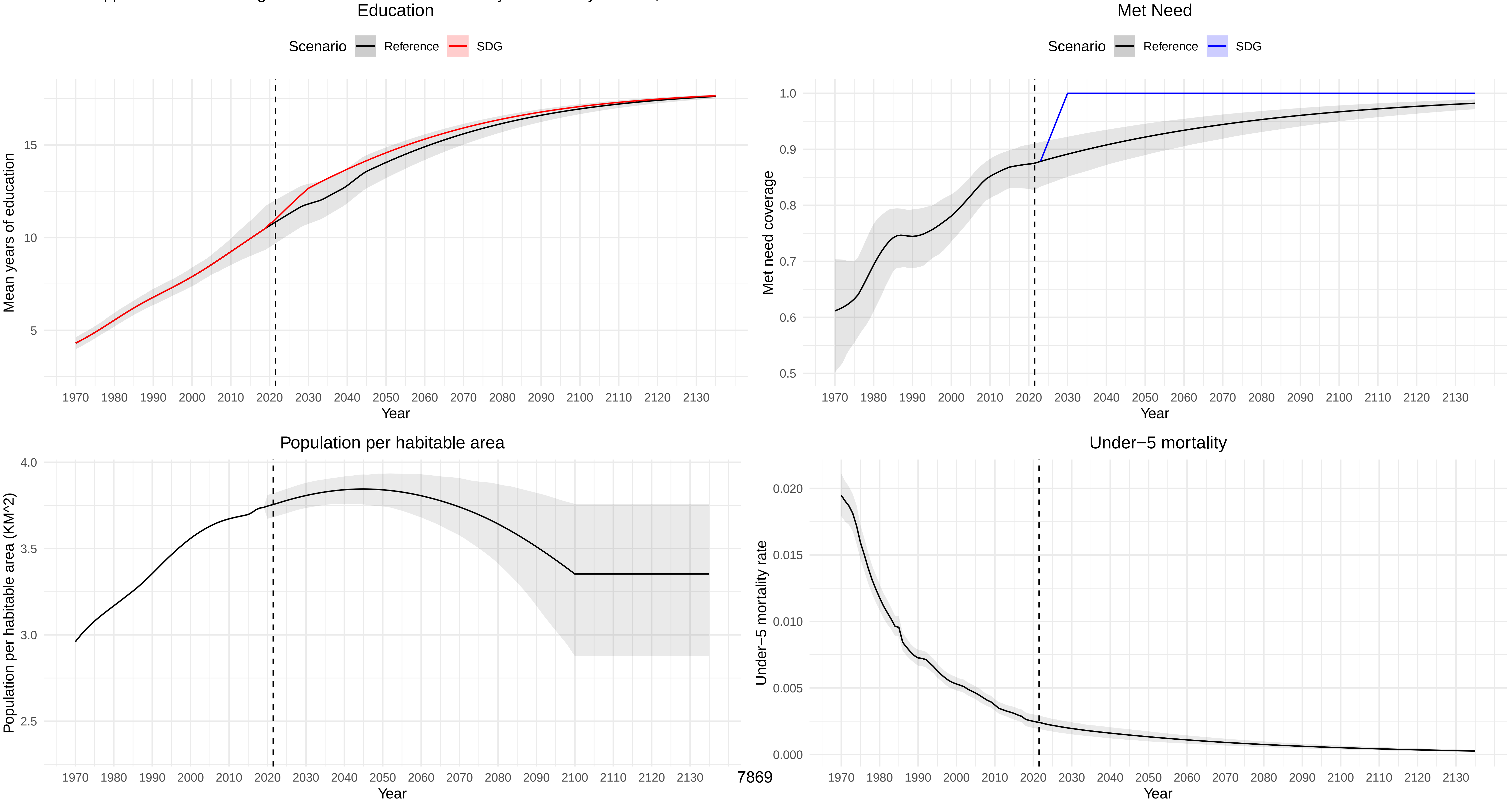

Costa Rica

Supplemental Results Figure S2: Covariates used in fertility forecasts by location, 1970-2135

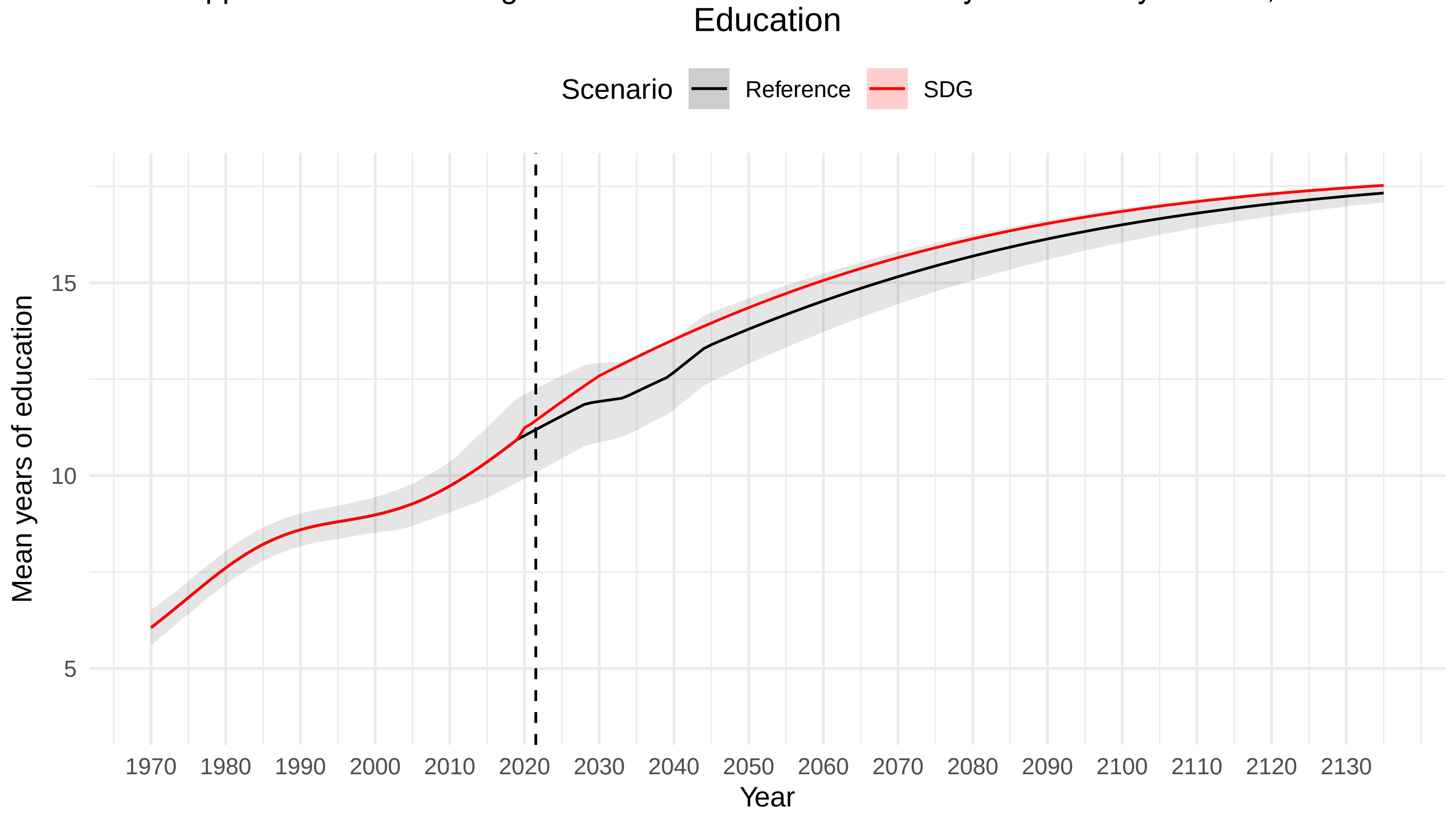

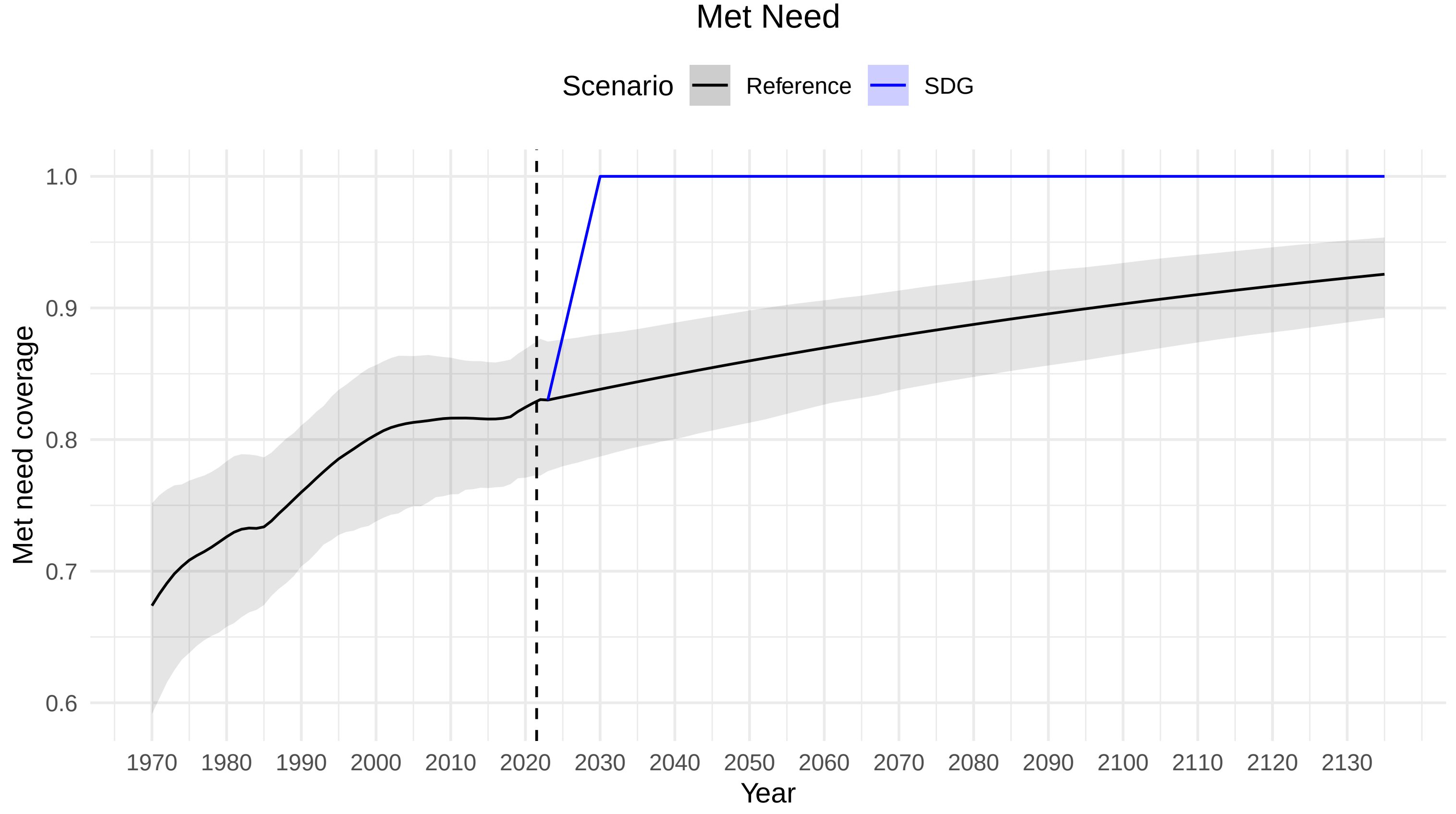

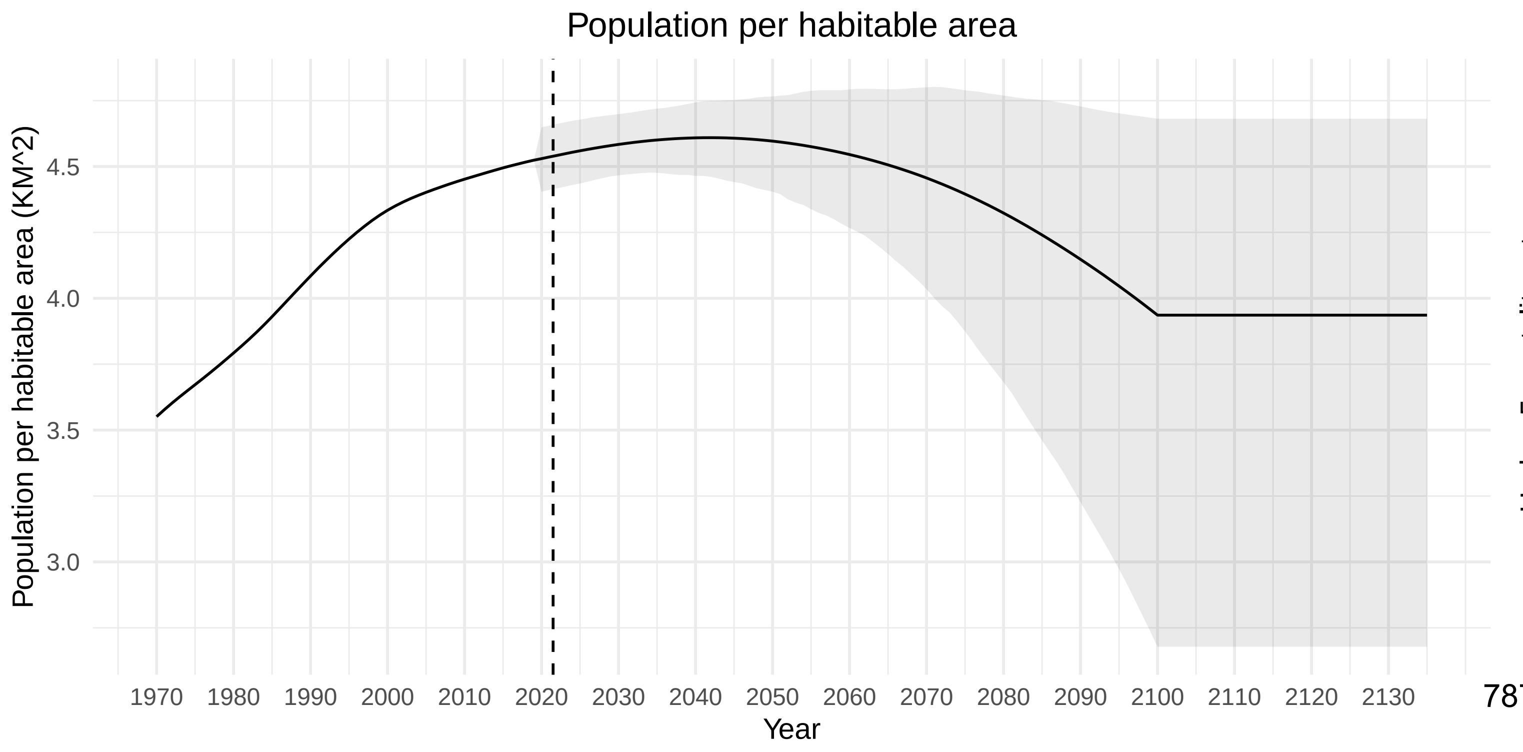

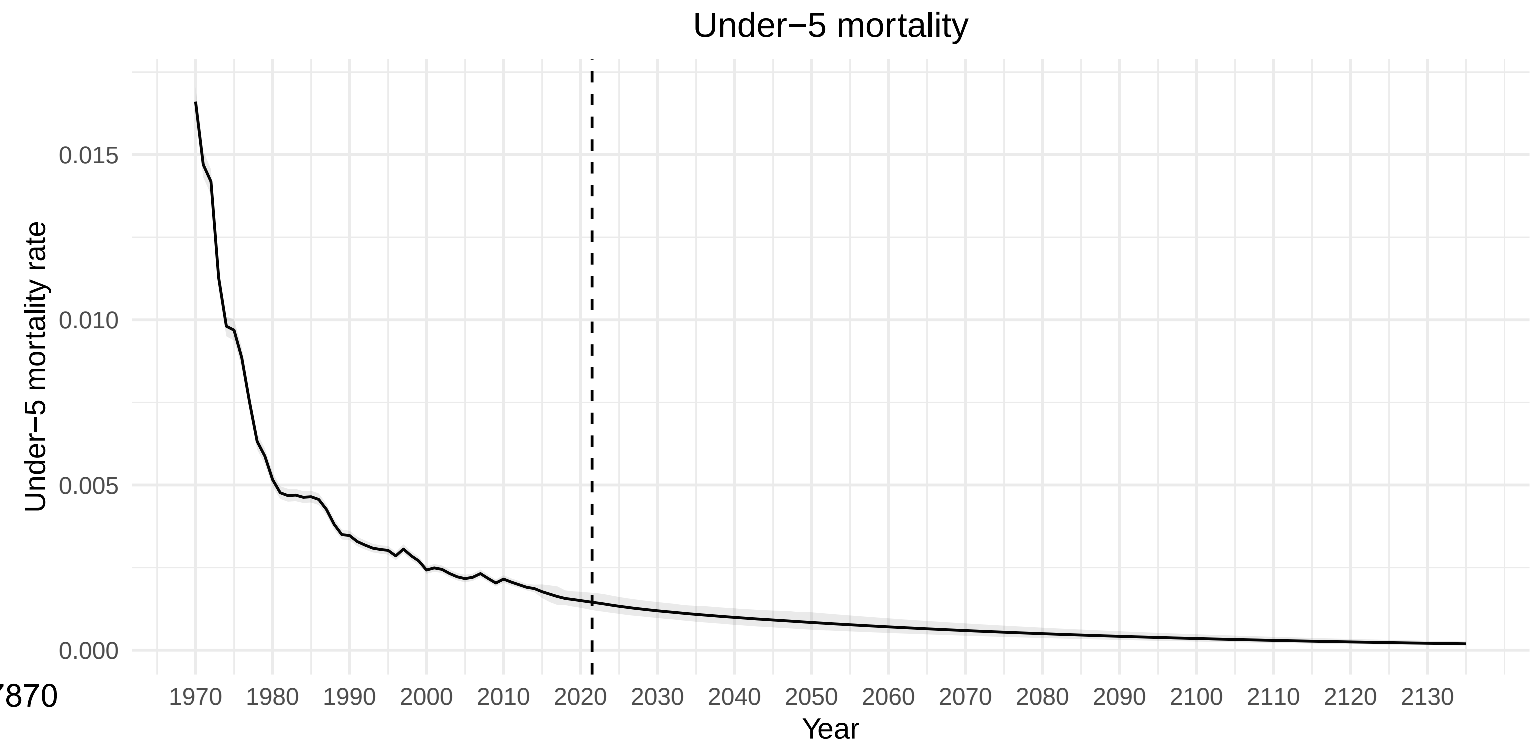

El Salvador

Supplemental Results Figure S2: Covariates used in fertility forecasts by location, 1970-2135

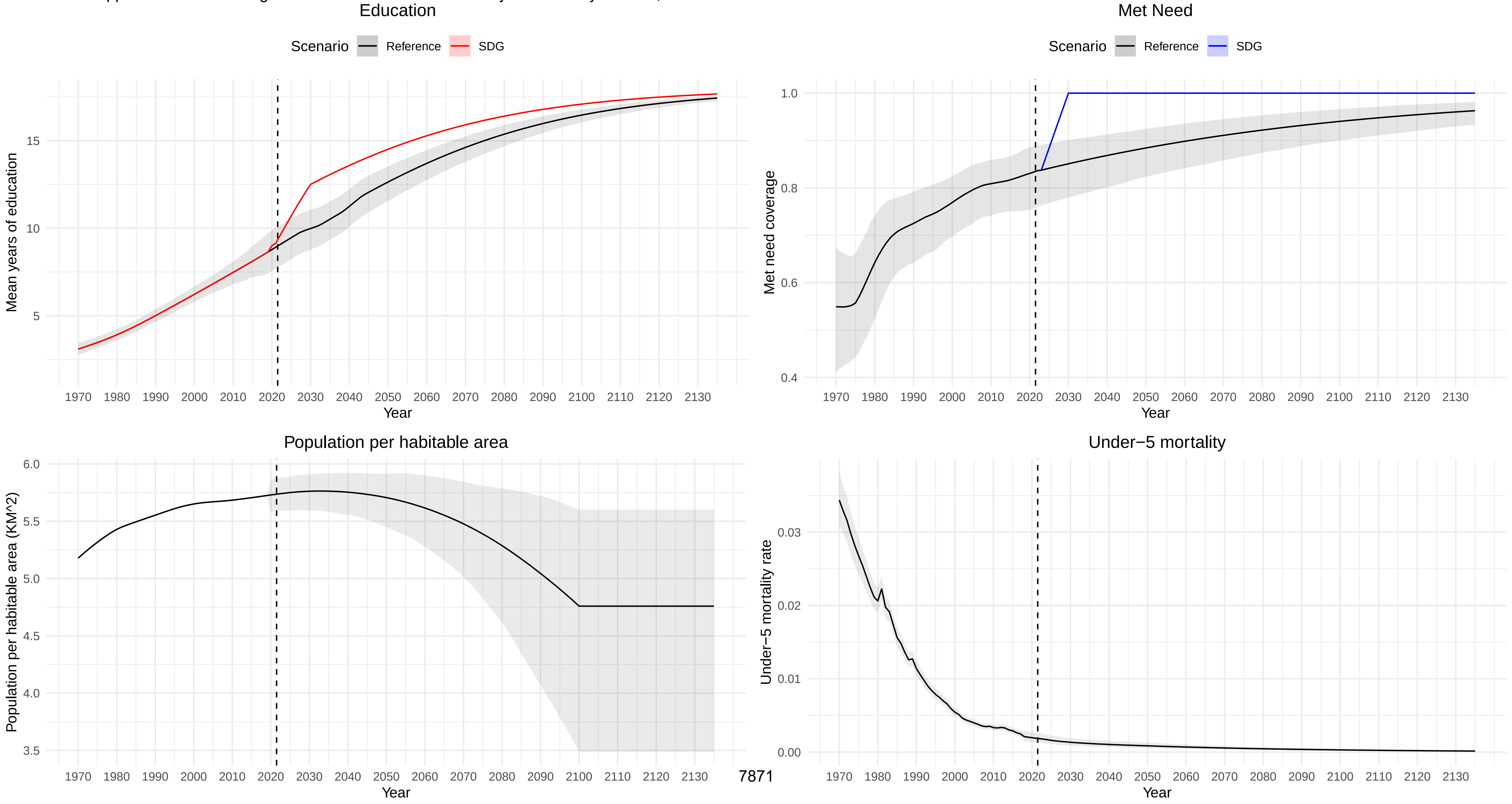

Guatemala

Supplemental Results Figure S2: Covariates used in fertility forecasts by location, 1970-2135

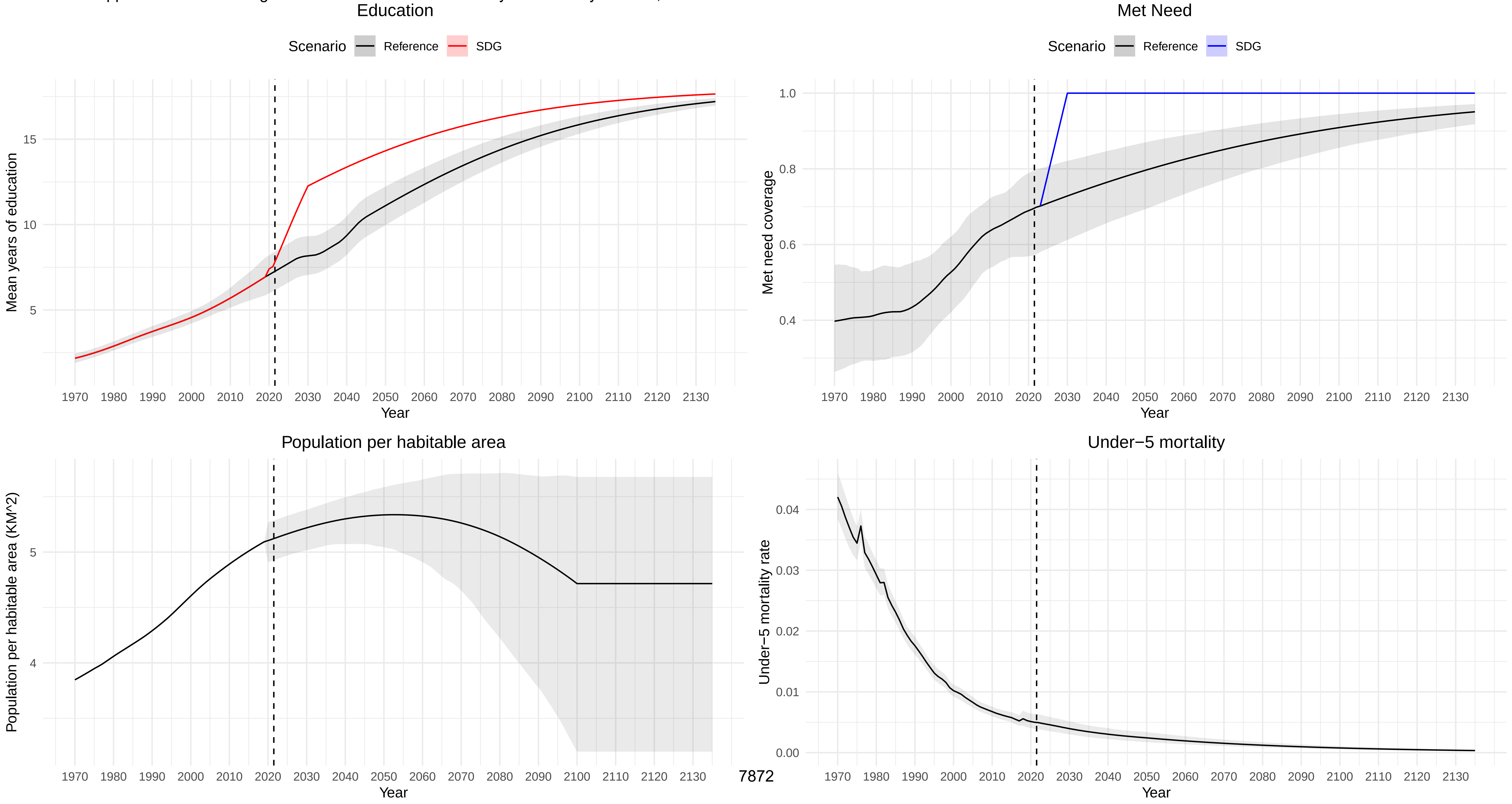

Honduras

Supplemental Results Figure S2: Covariates used in fertility forecasts by location, 1970-2135

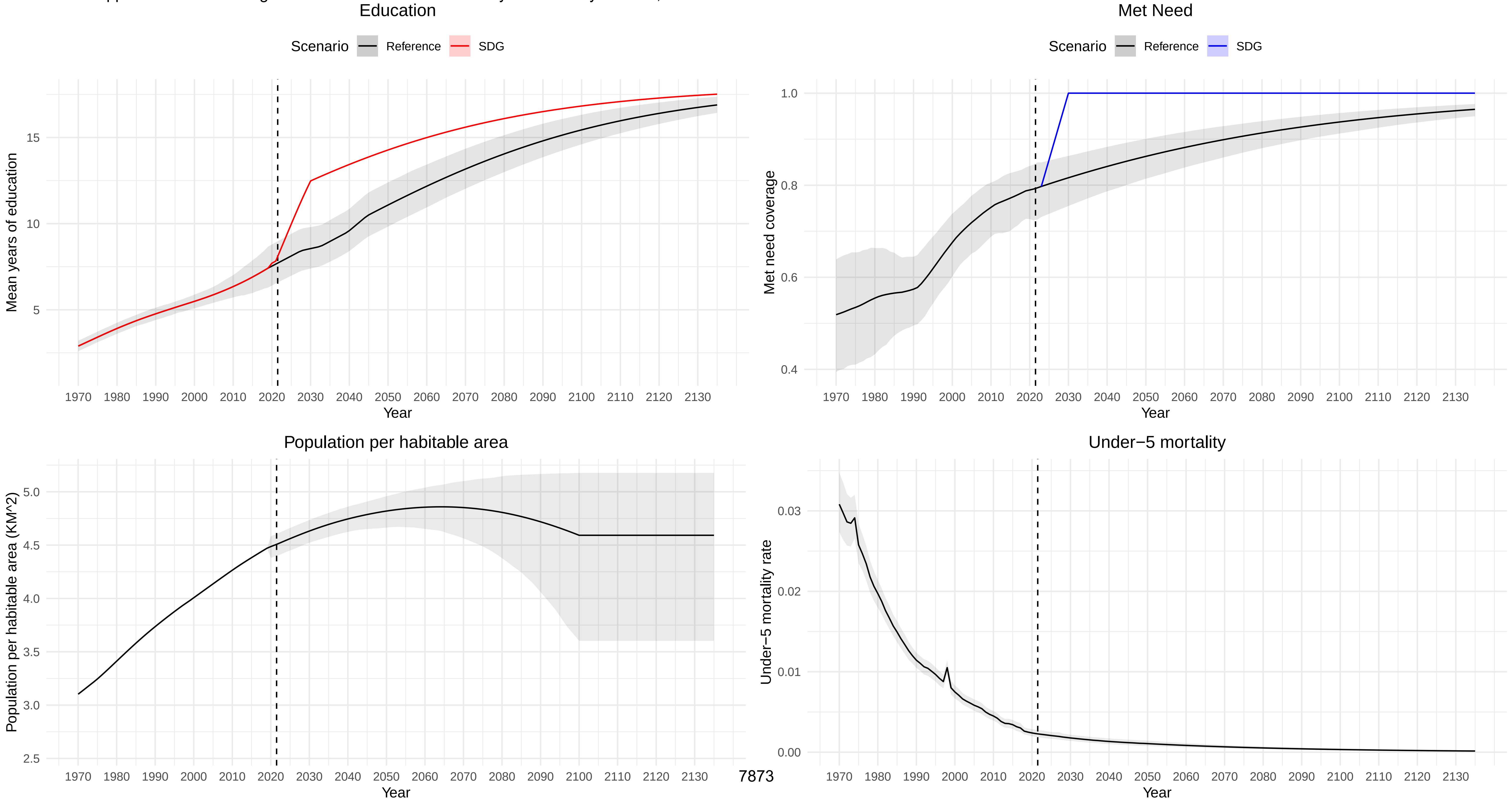

Mexico

Supplemental Results Figure S2: Covariates used in fertility forecasts by location, 1970-2135

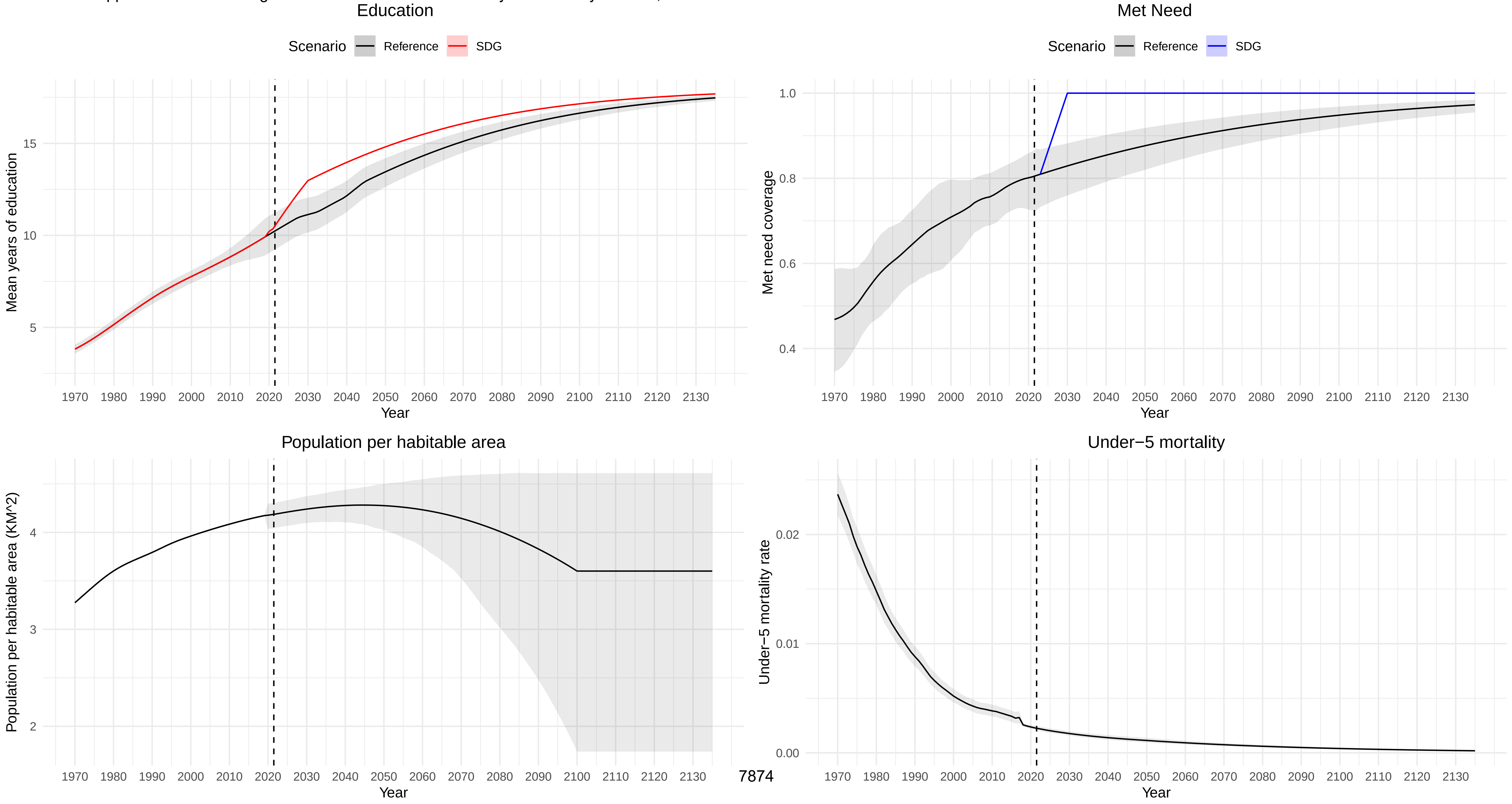

Nicaragua

Supplemental Results Figure S2: Covariates used in fertility forecasts by location, 1970-2135

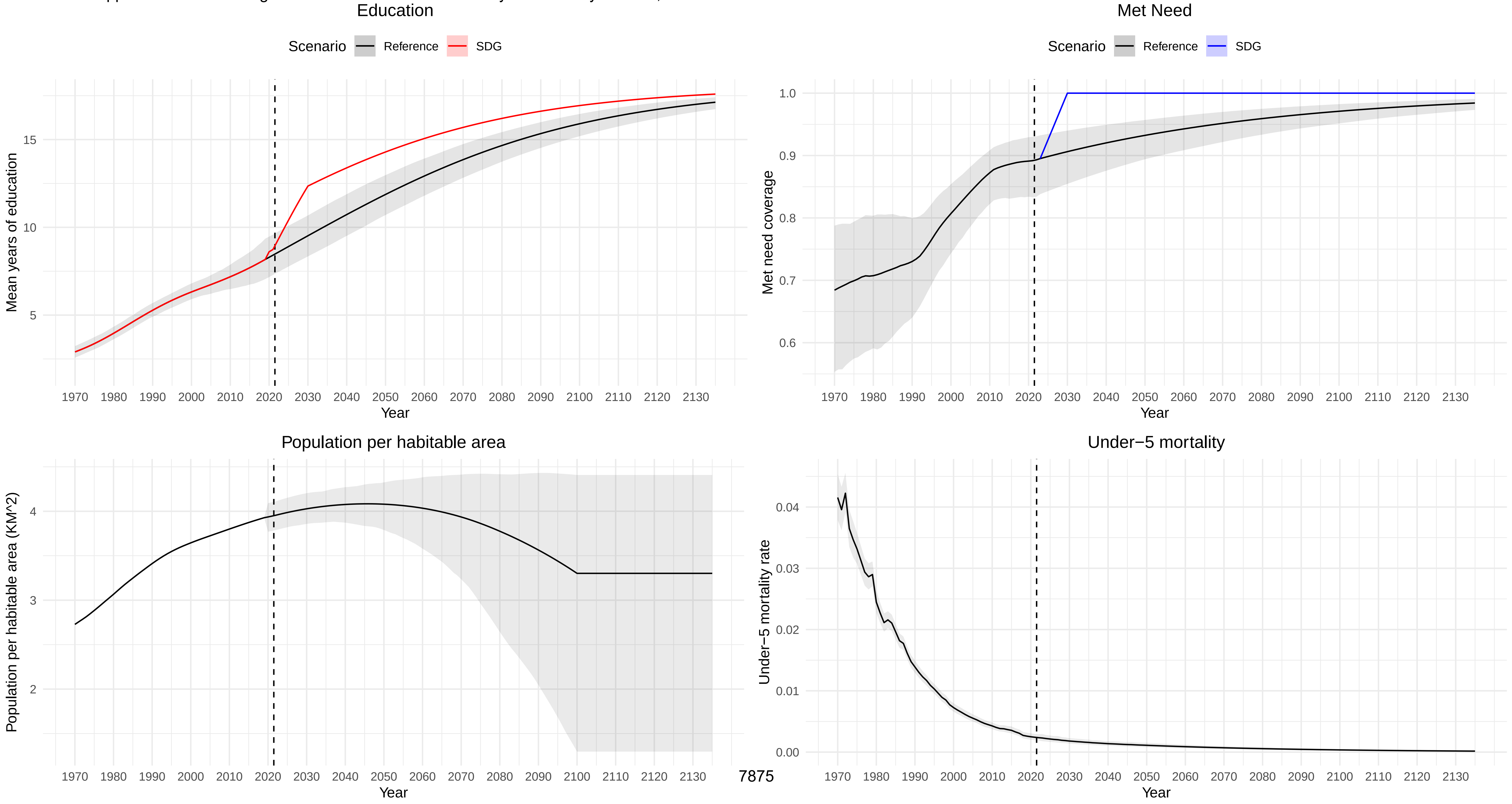

Panama

Supplemental Results Figure S2: Covariates used in fertility forecasts by location, 1970-2135

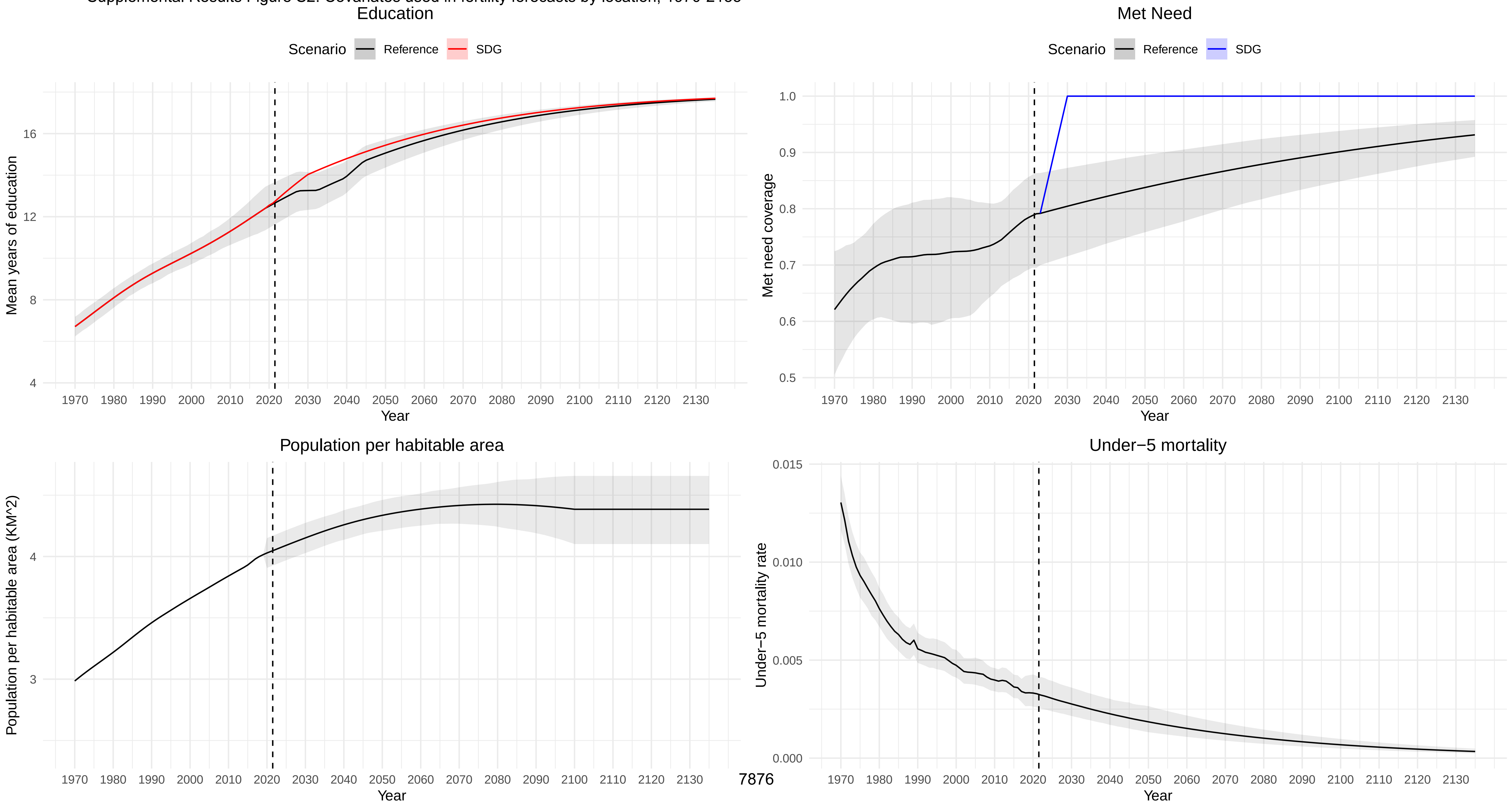

Venezuela (Bolivarian Republic of)

Supplemental Results Figure S2: Covariates used in fertility forecasts by location, 1970-2135

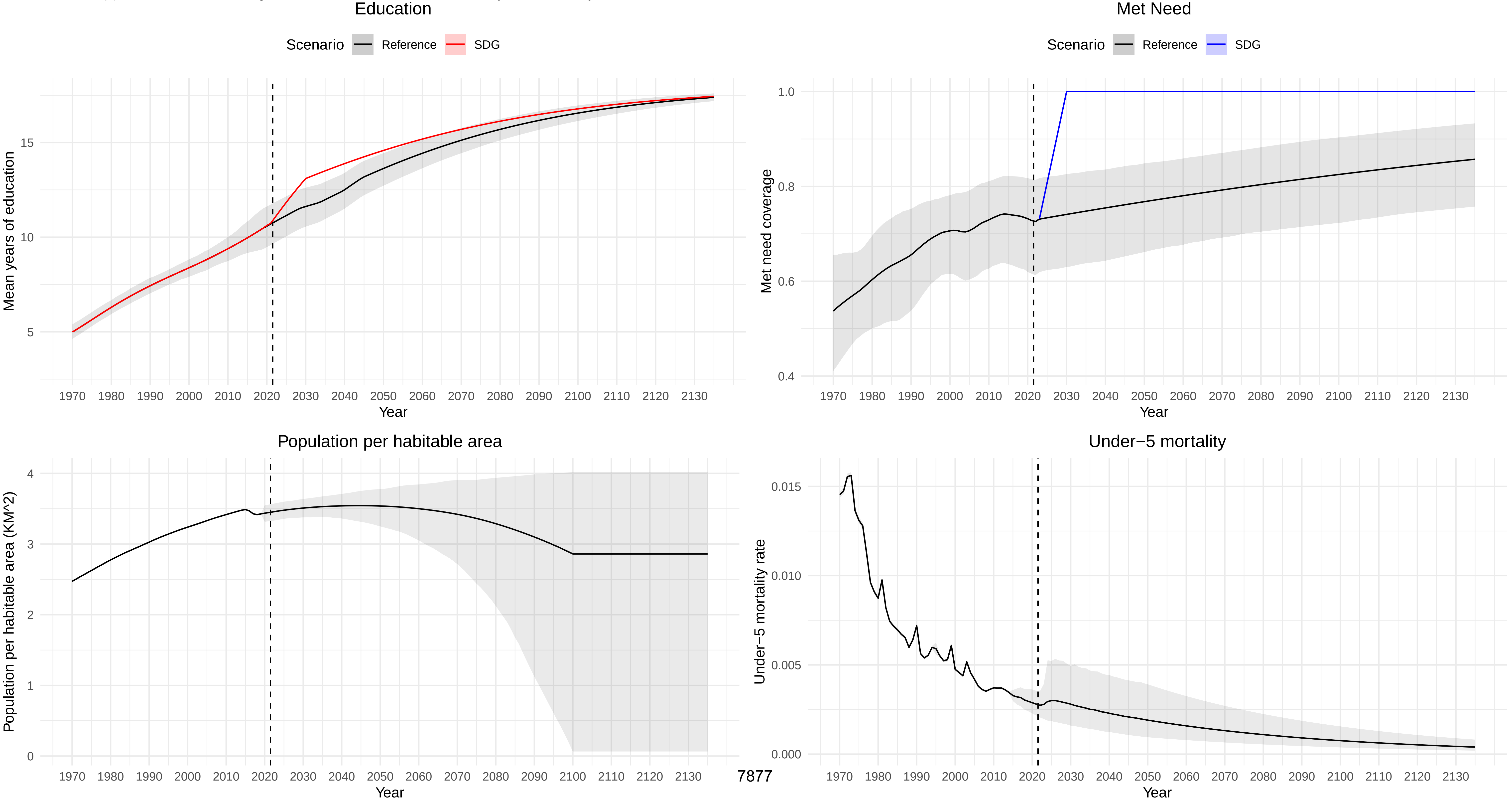

Brazil

Supplemental Results Figure S2: Covariates used in fertility forecasts by location, 1970-2135

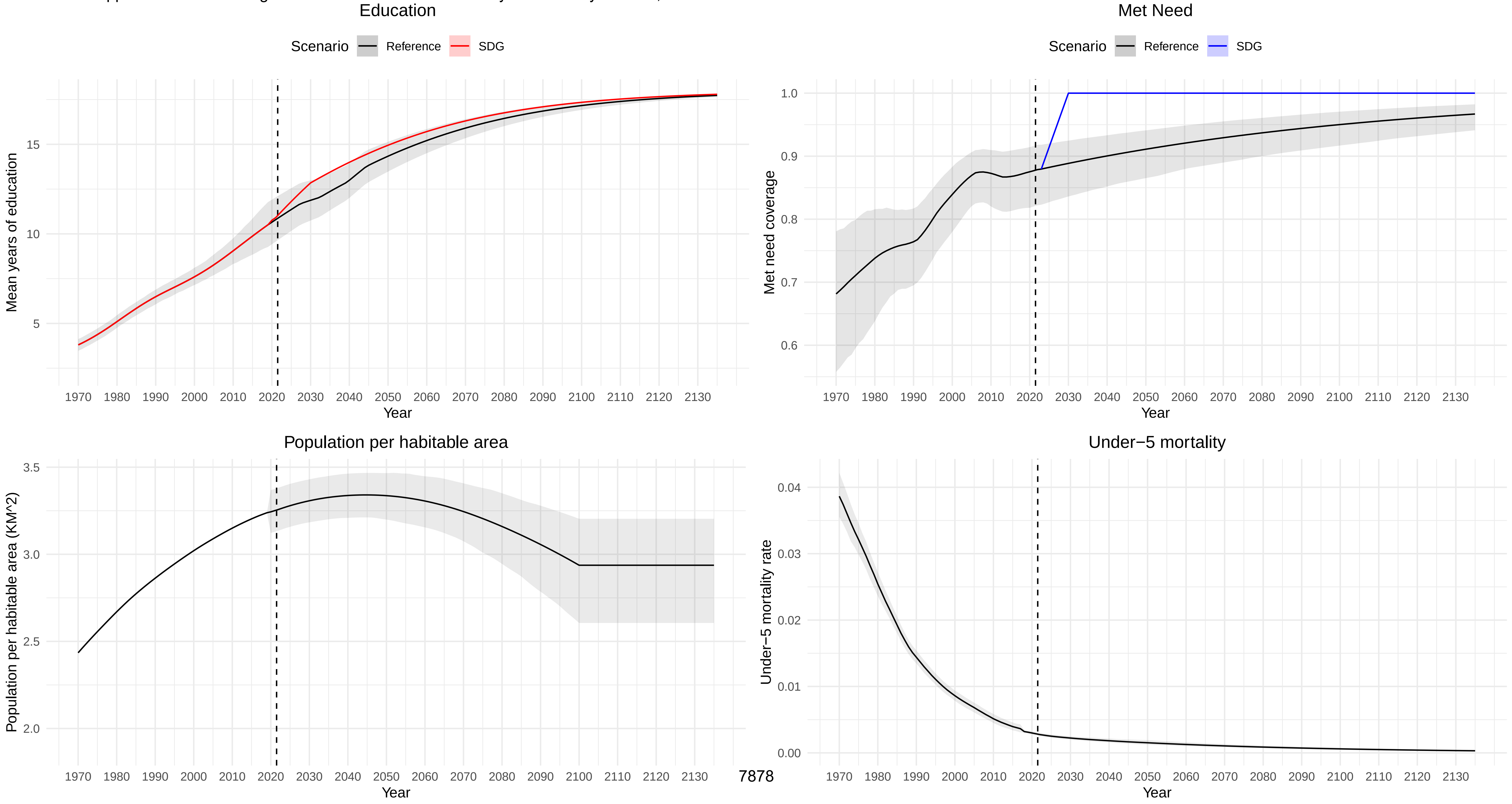

Paraguay

Supplemental Results Figure S2: Covariates used in fertility forecasts by location, 1970-2135

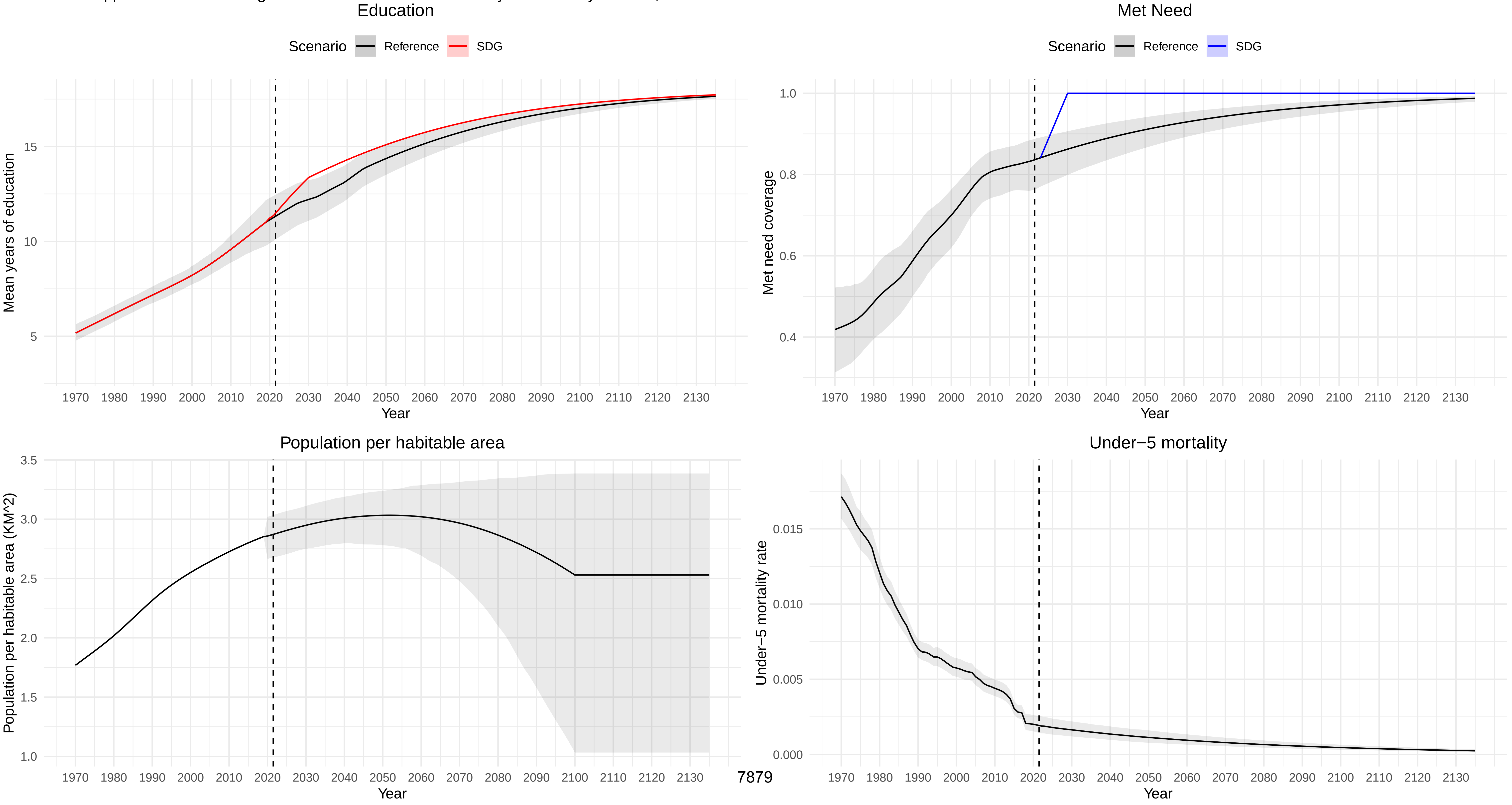

Afghanistan

Supplemental Results Figure S2: Covariates used in fertility forecasts by location, 1970-2135

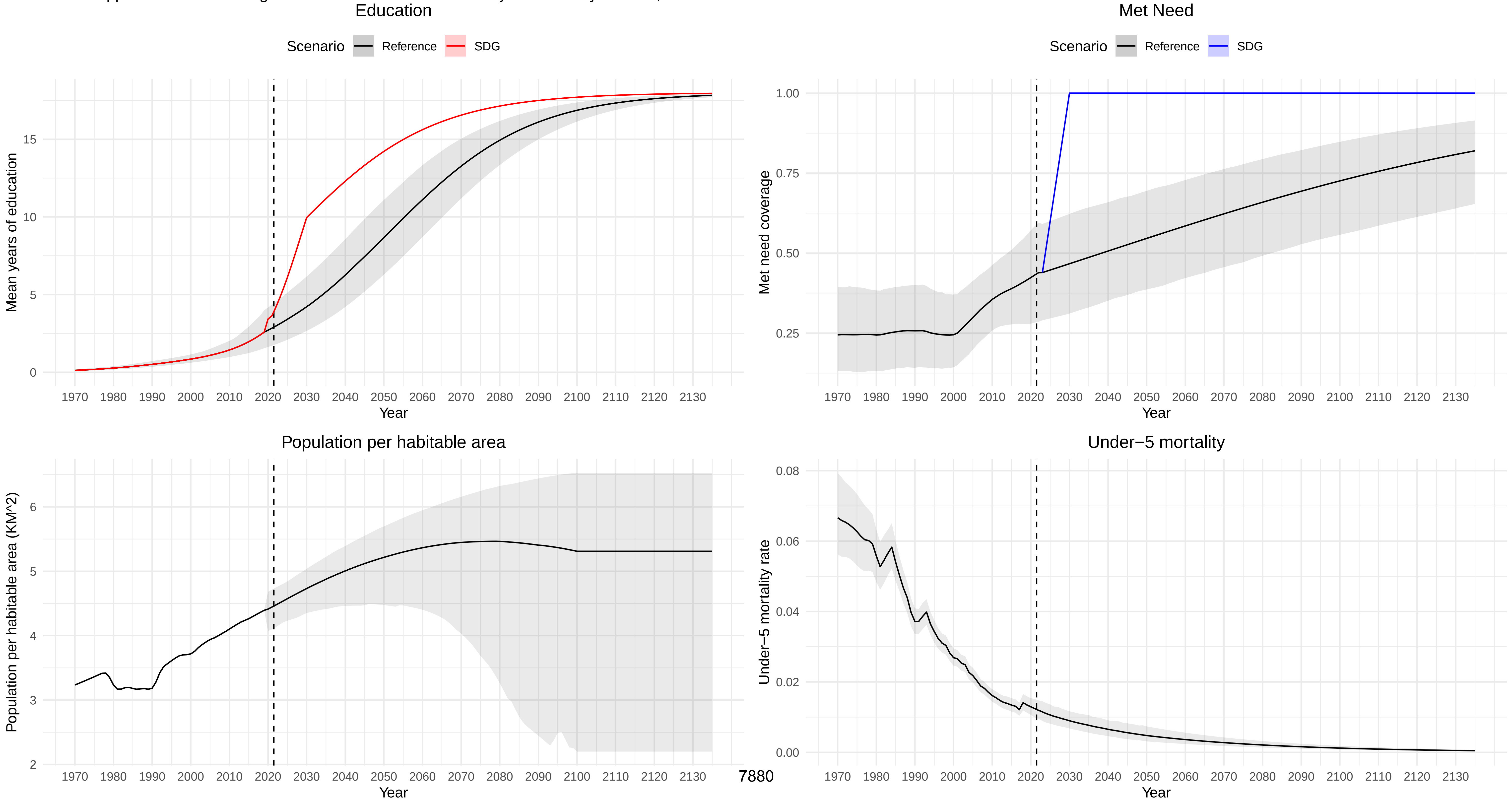

Algeria

Supplemental Results Figure S2: Covariates used in fertility forecasts by location, 1970-2135

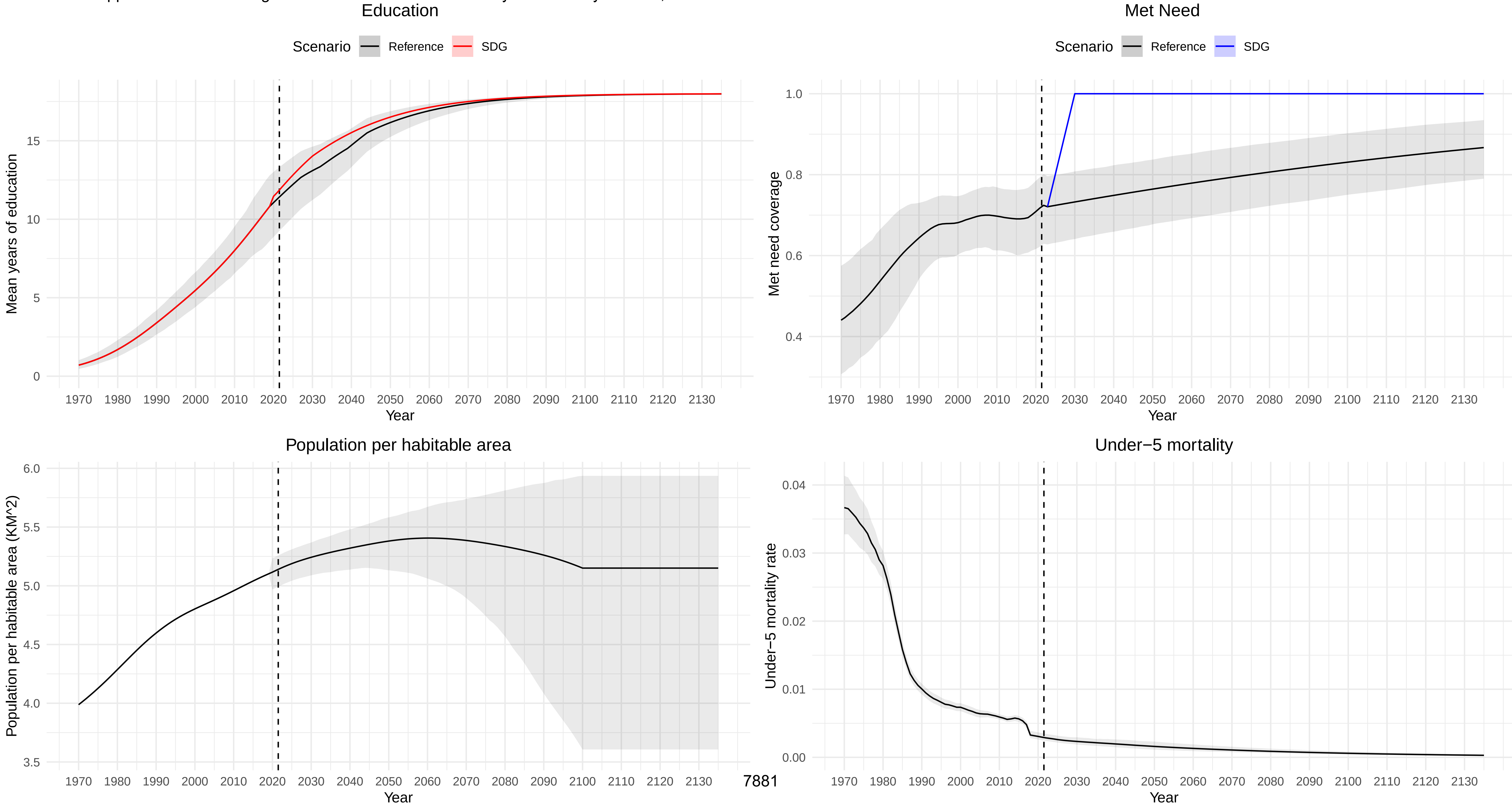

Bahrain

Supplemental Results Figure S2: Covariates used in fertility forecasts by location, 1970-2135

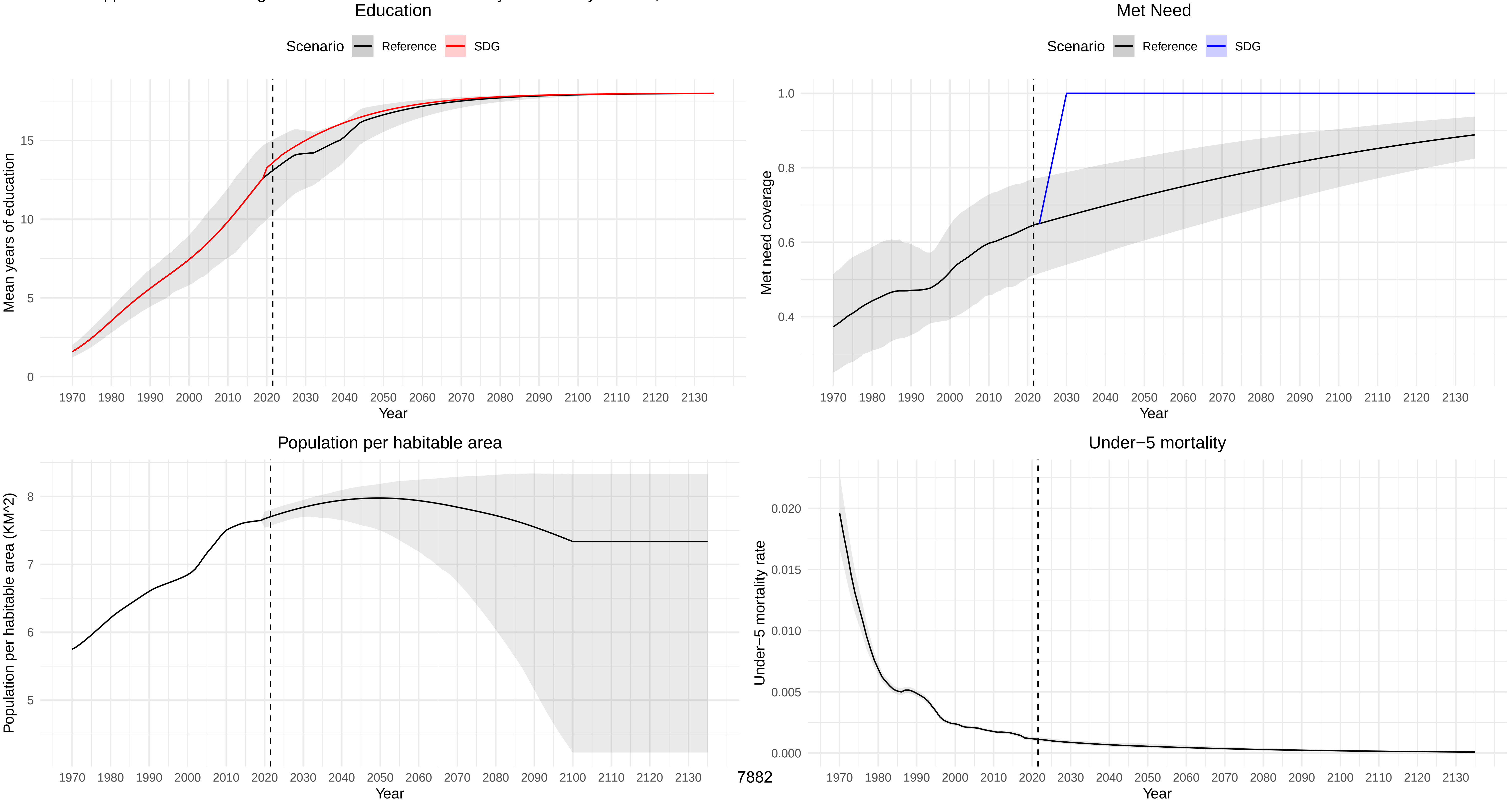

Egypt

Supplemental Results Figure S2: Covariates used in fertility forecasts by location, 1970-2135

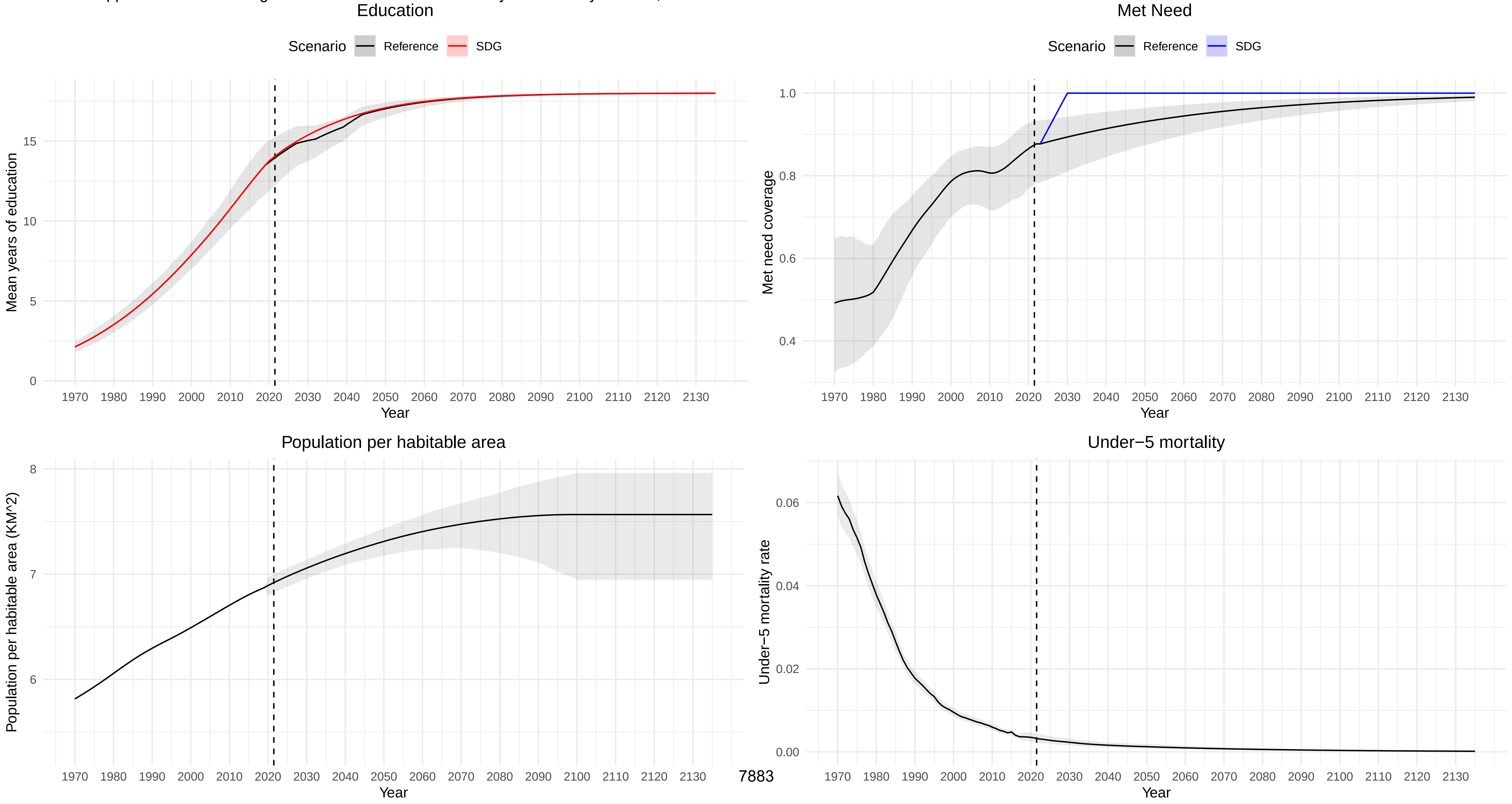

Iran (Islamic Republic of)

Supplemental Results Figure S2: Covariates used in fertility forecasts by location, 1970-2135

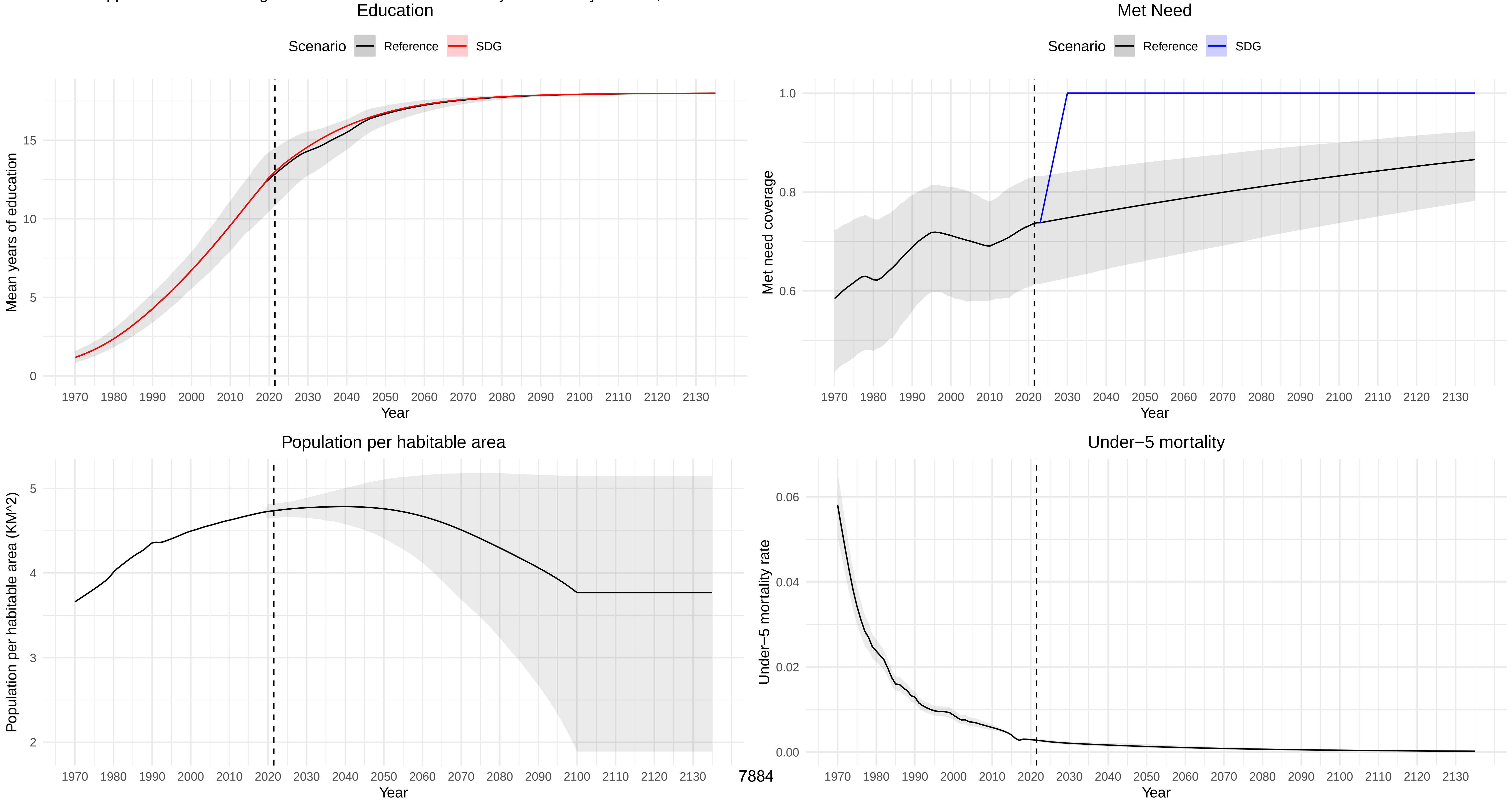

Iraq

Supplemental Results Figure S2: Covariates used in fertility forecasts by location, 1970-2135

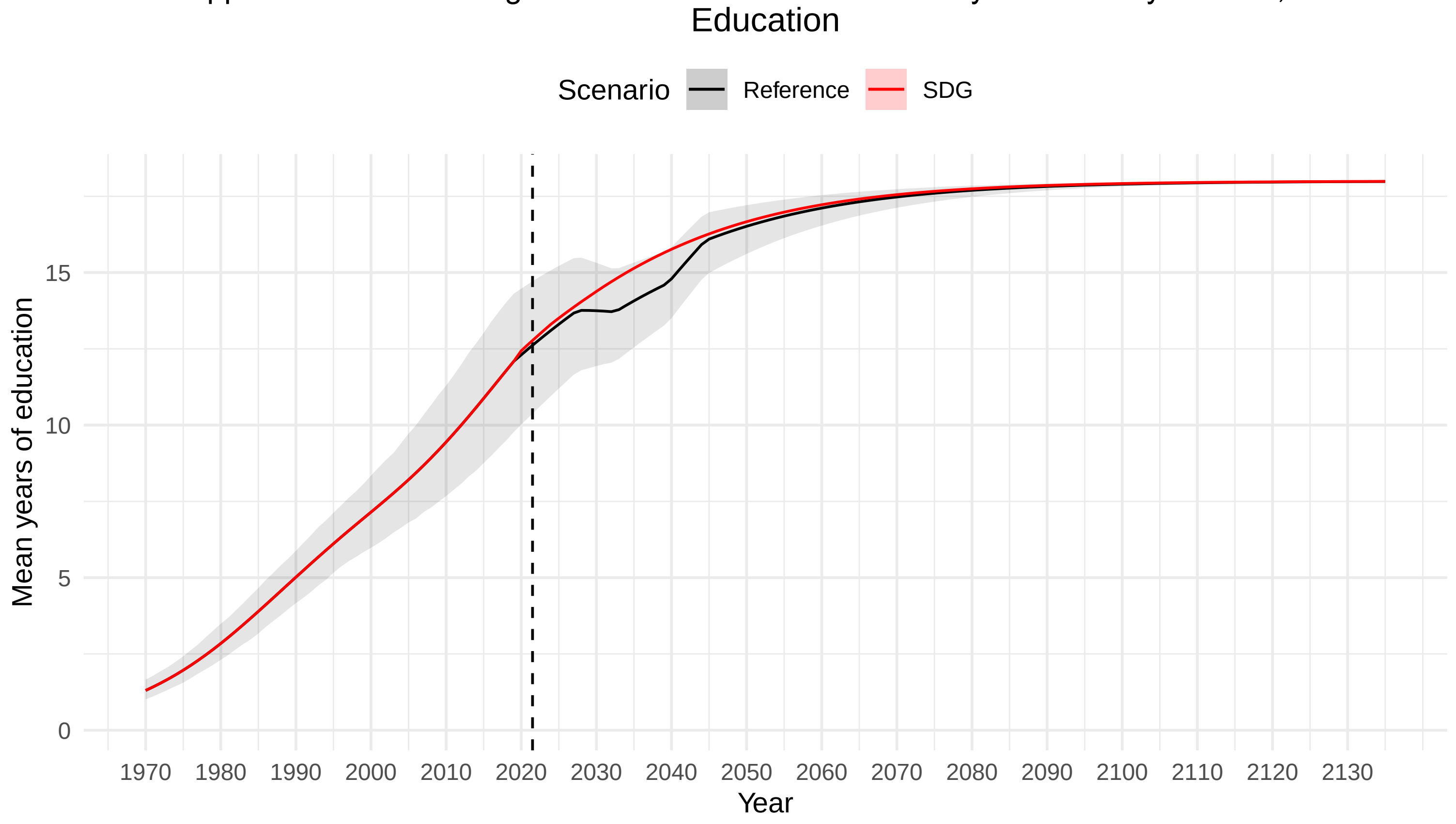

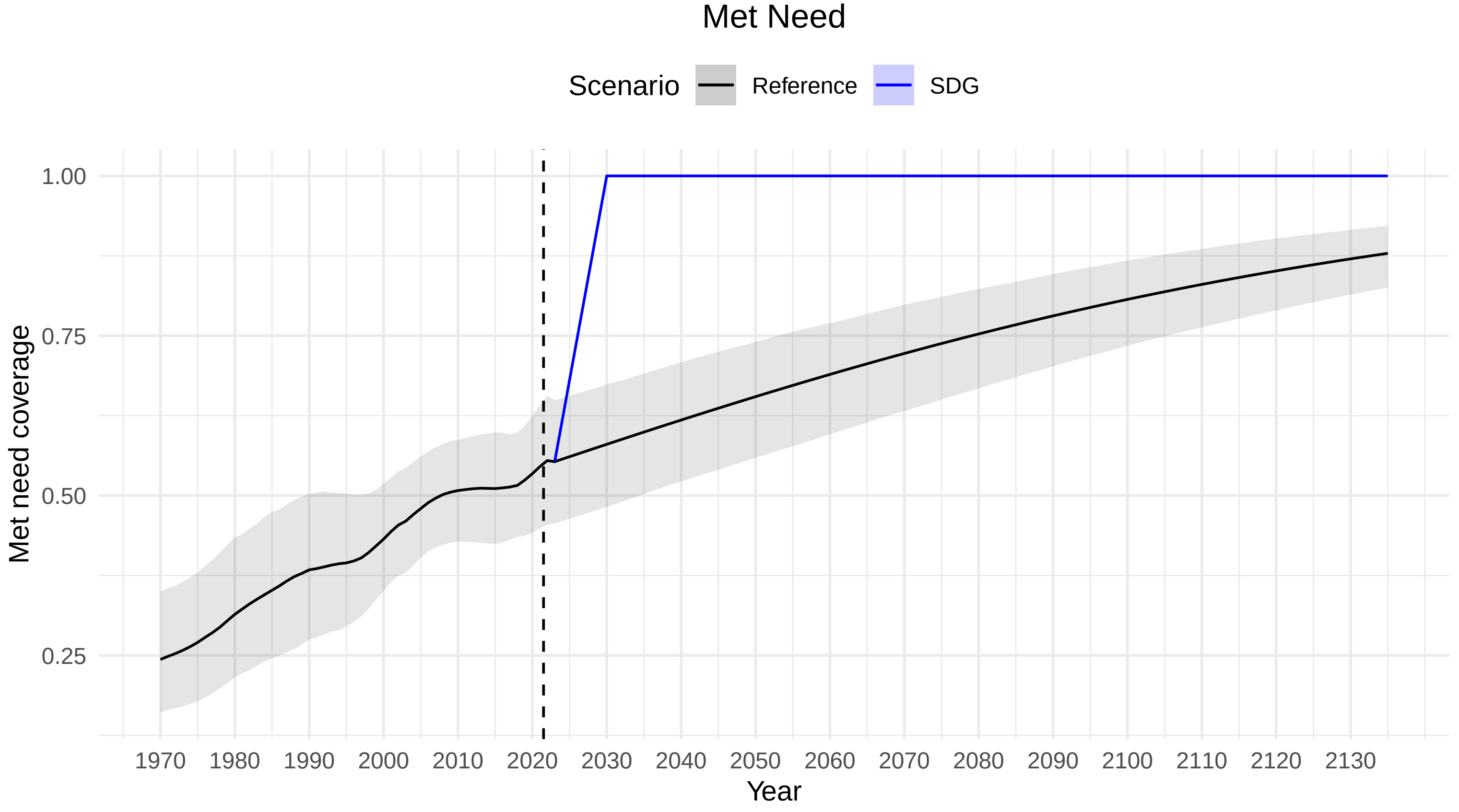

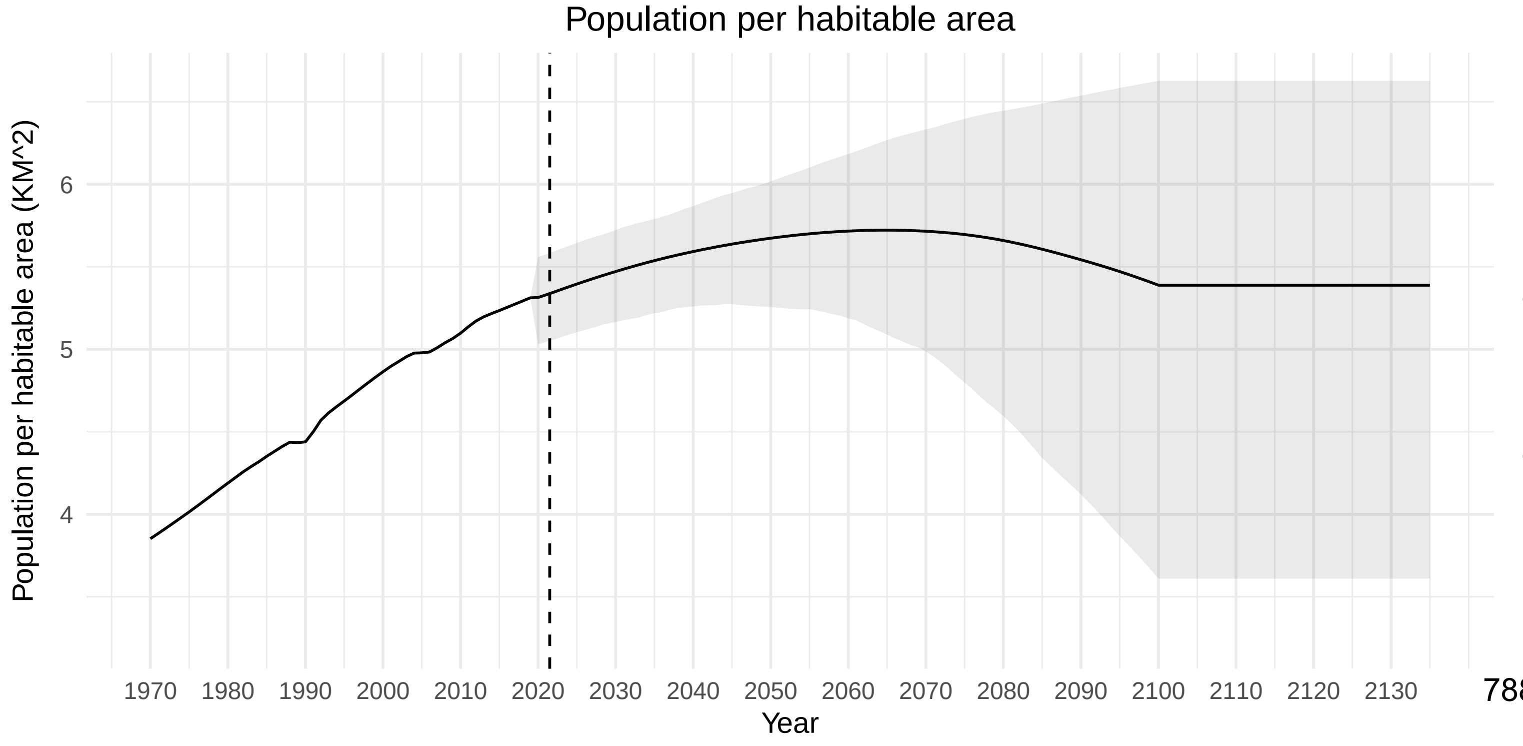

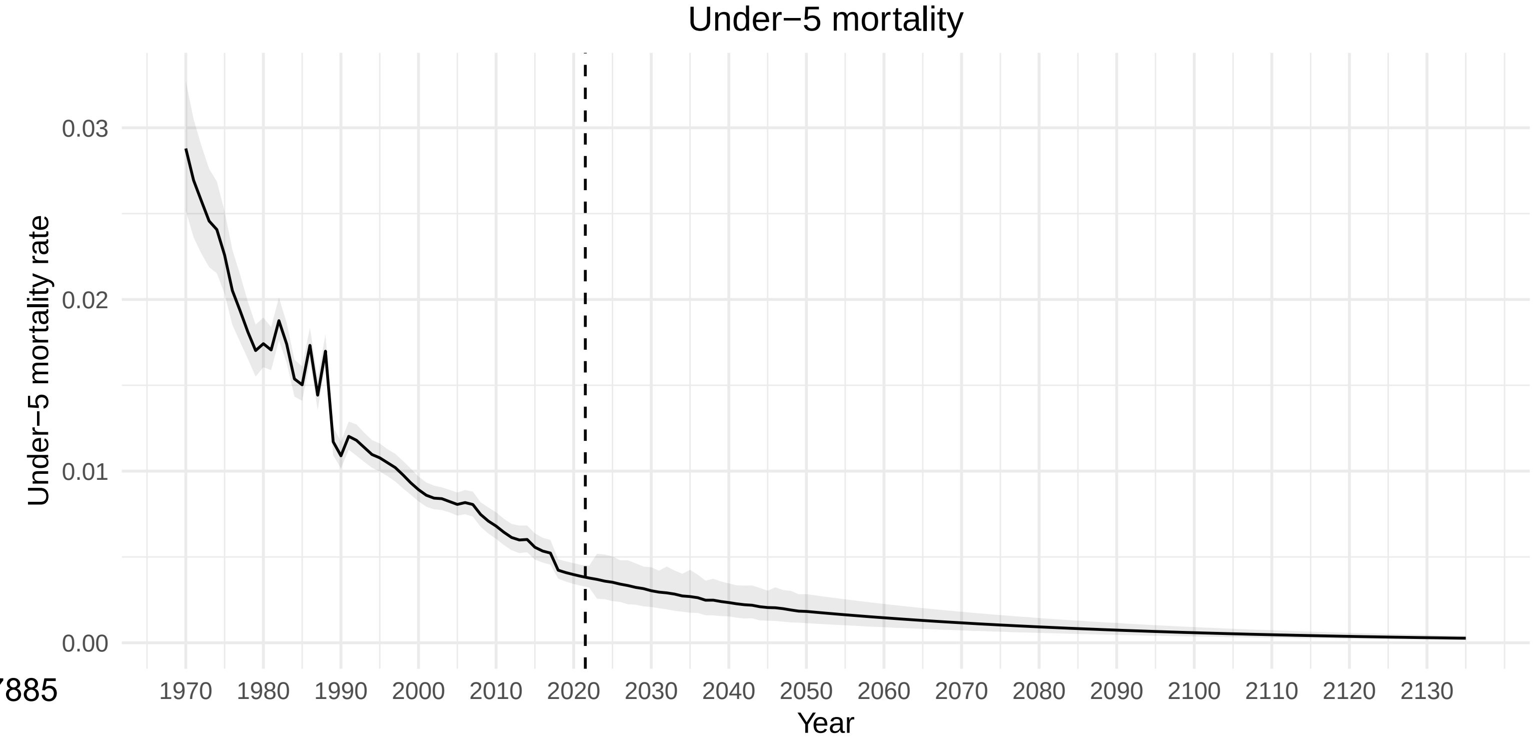

Jordan

Supplemental Results Figure S2: Covariates used in fertility forecasts by location, 1970-2135

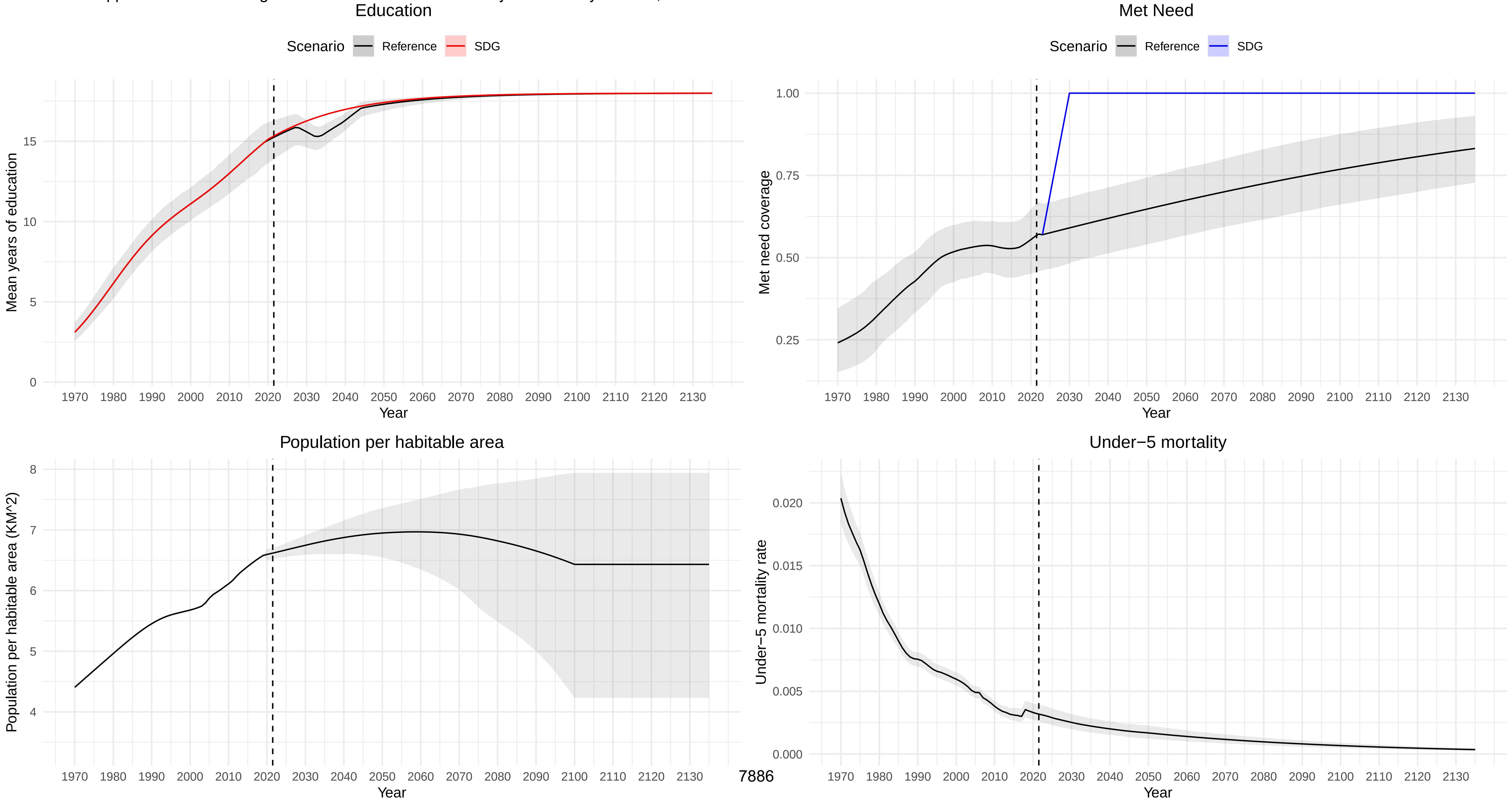

Kuwait

Supplemental Results Figure S2: Covariates used in fertility forecasts by location, 1970-2135

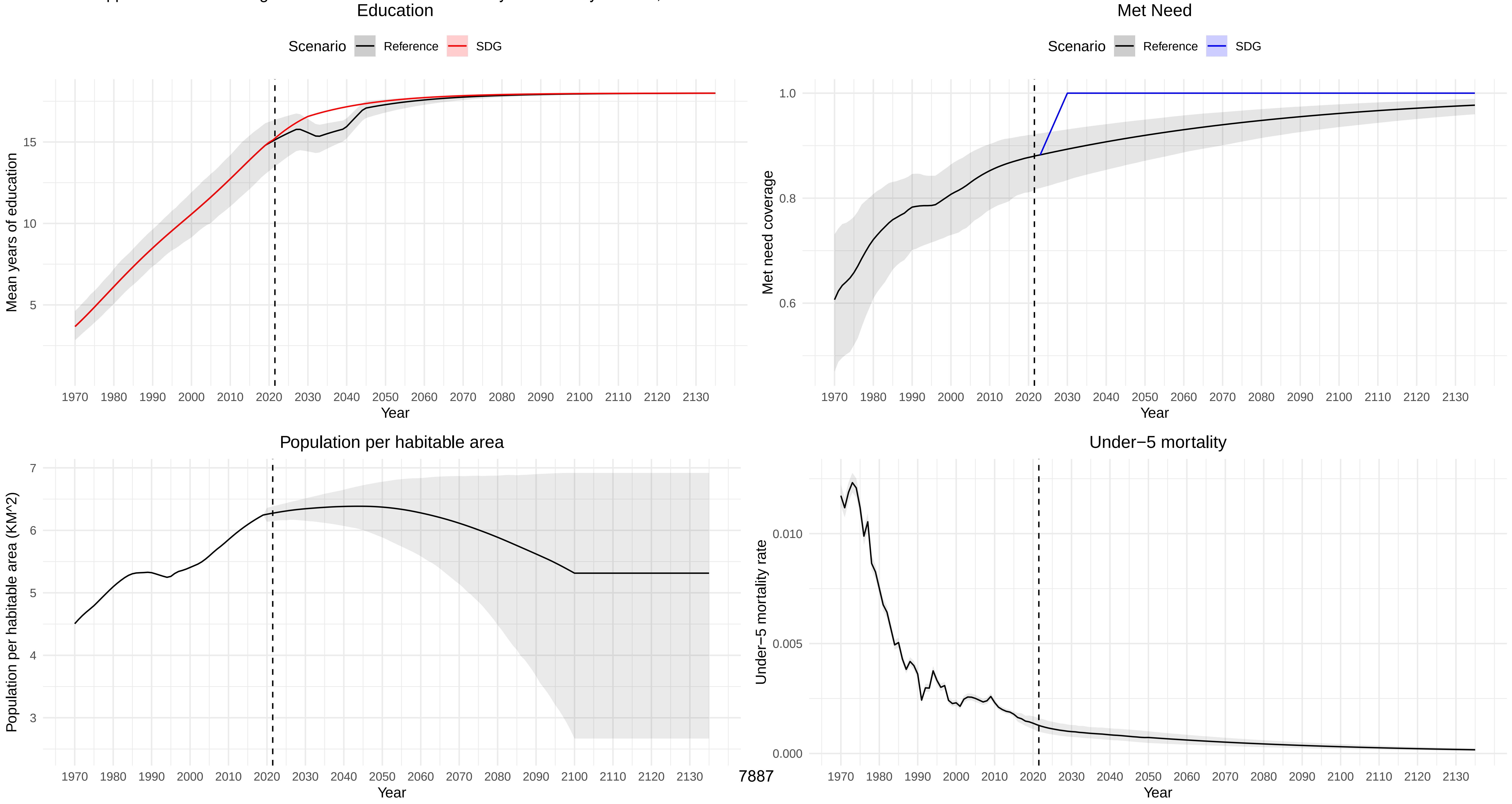

Lebanon

Supplemental Results Figure S2: Covariates used in fertility forecasts by location, 1970-2135

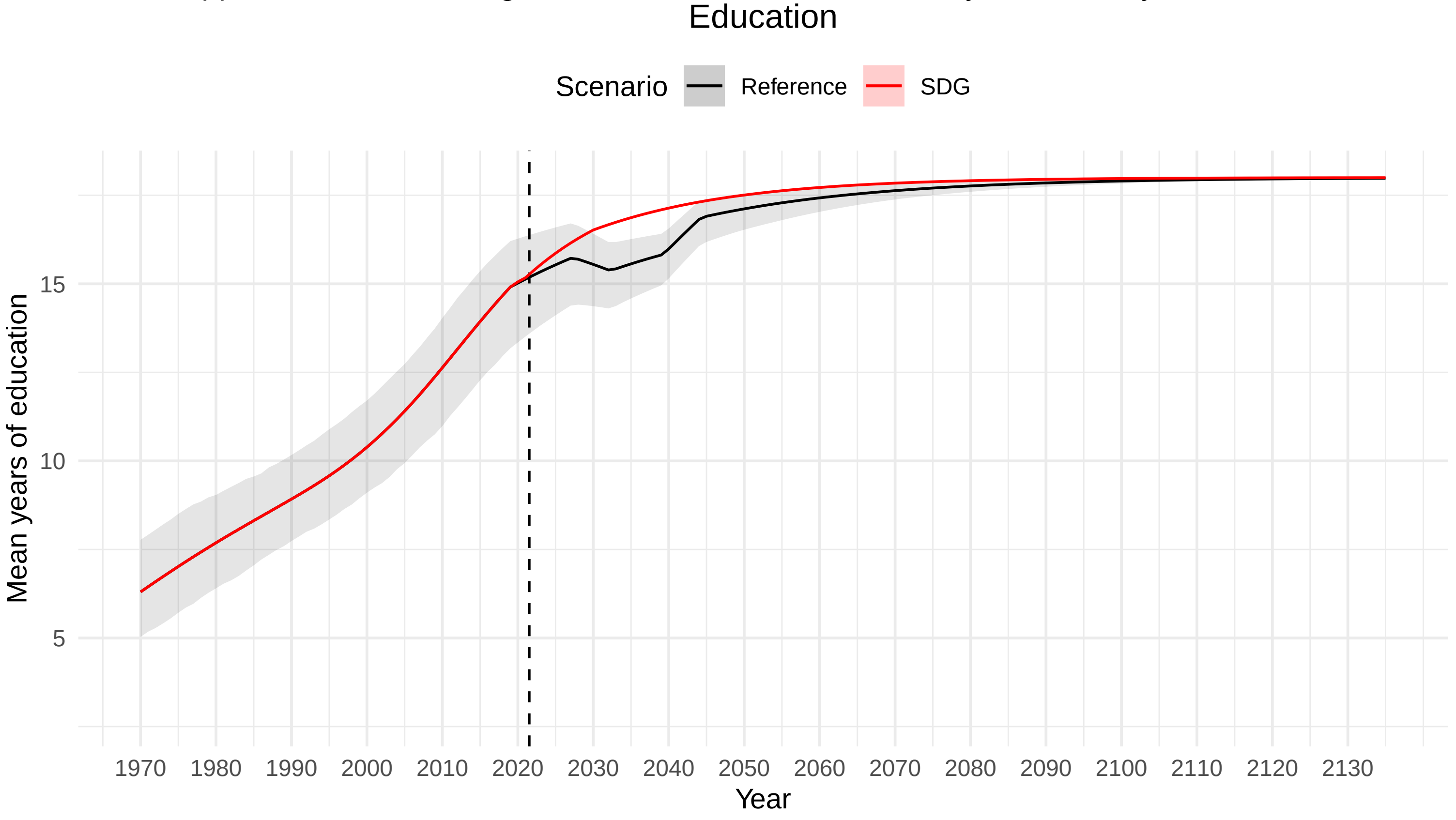

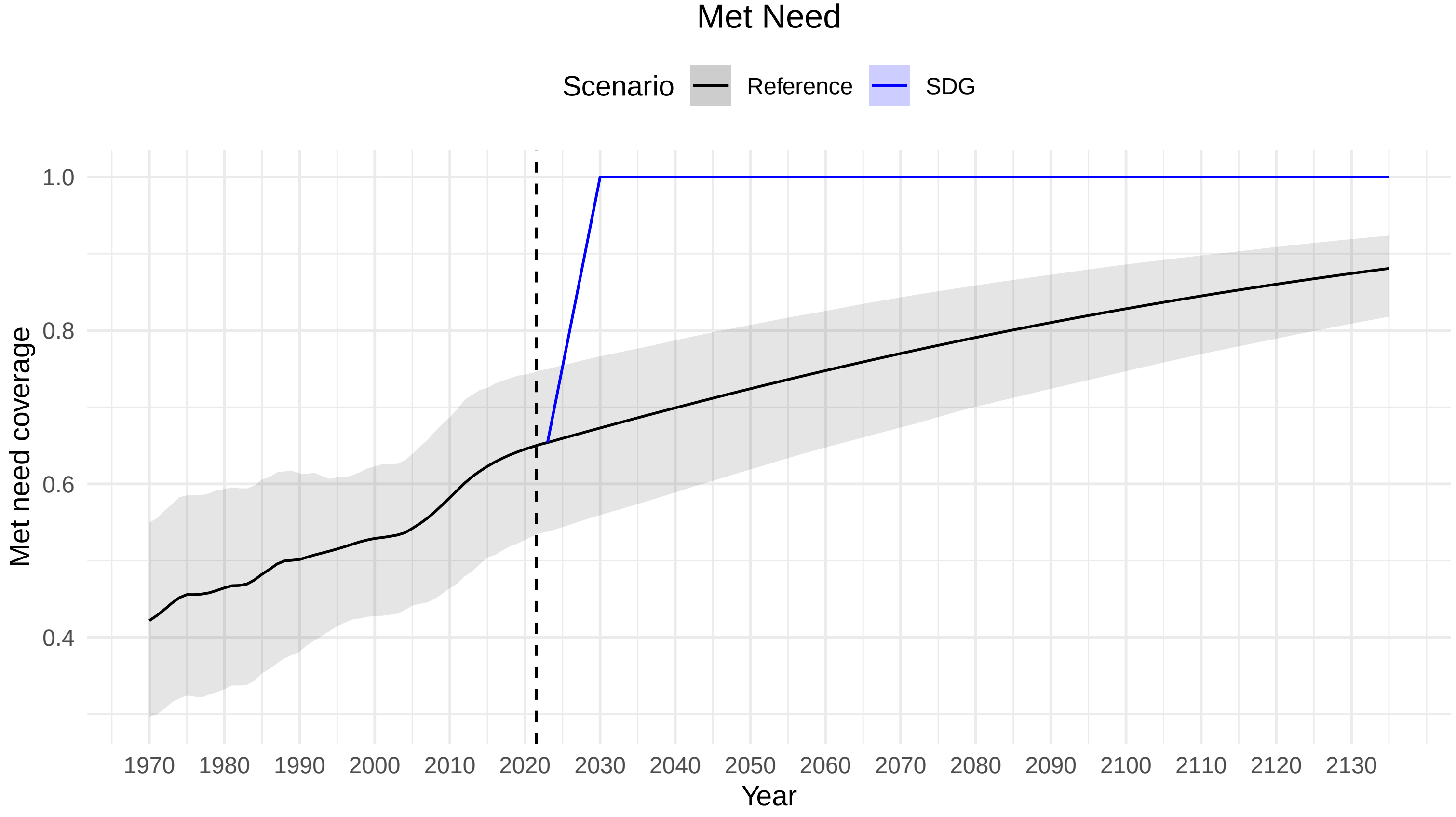

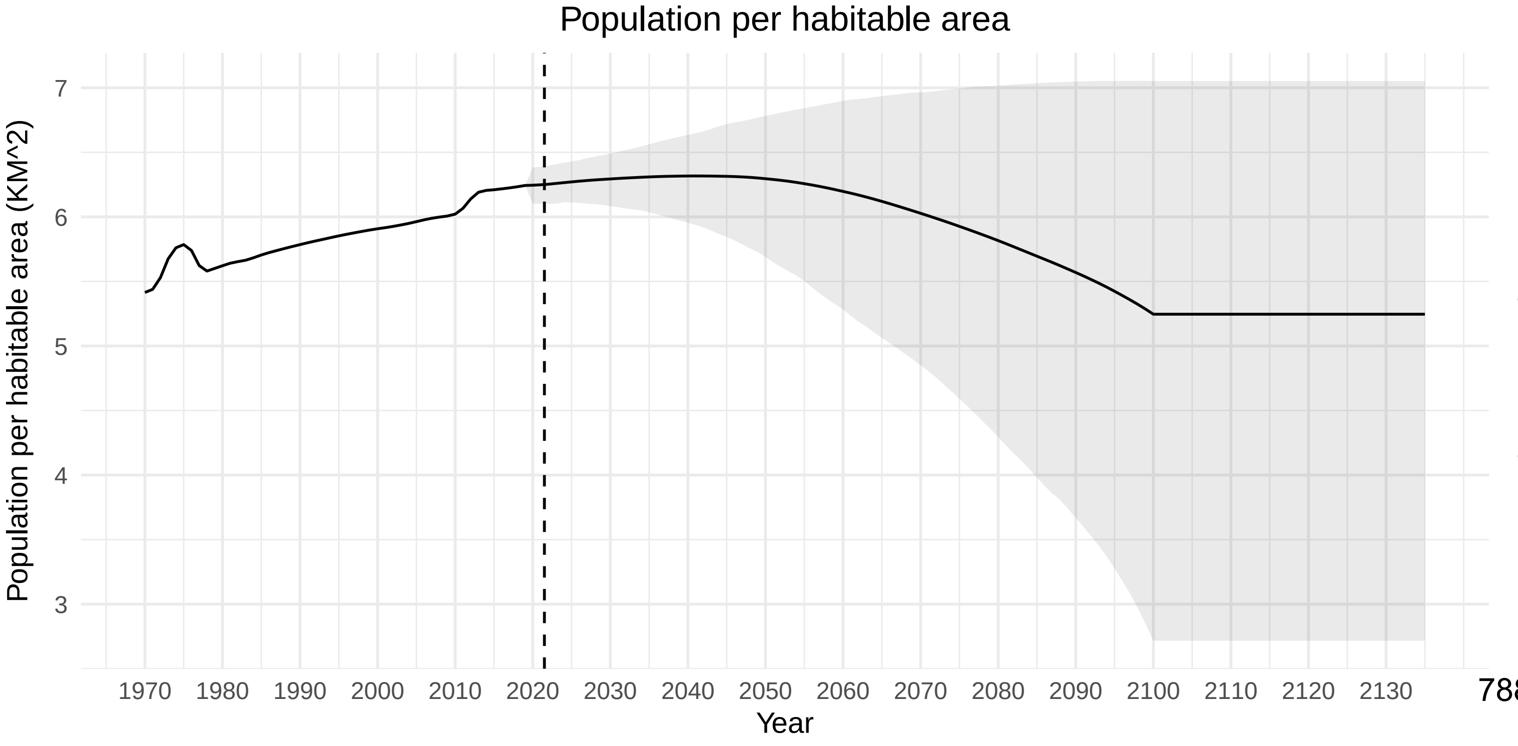

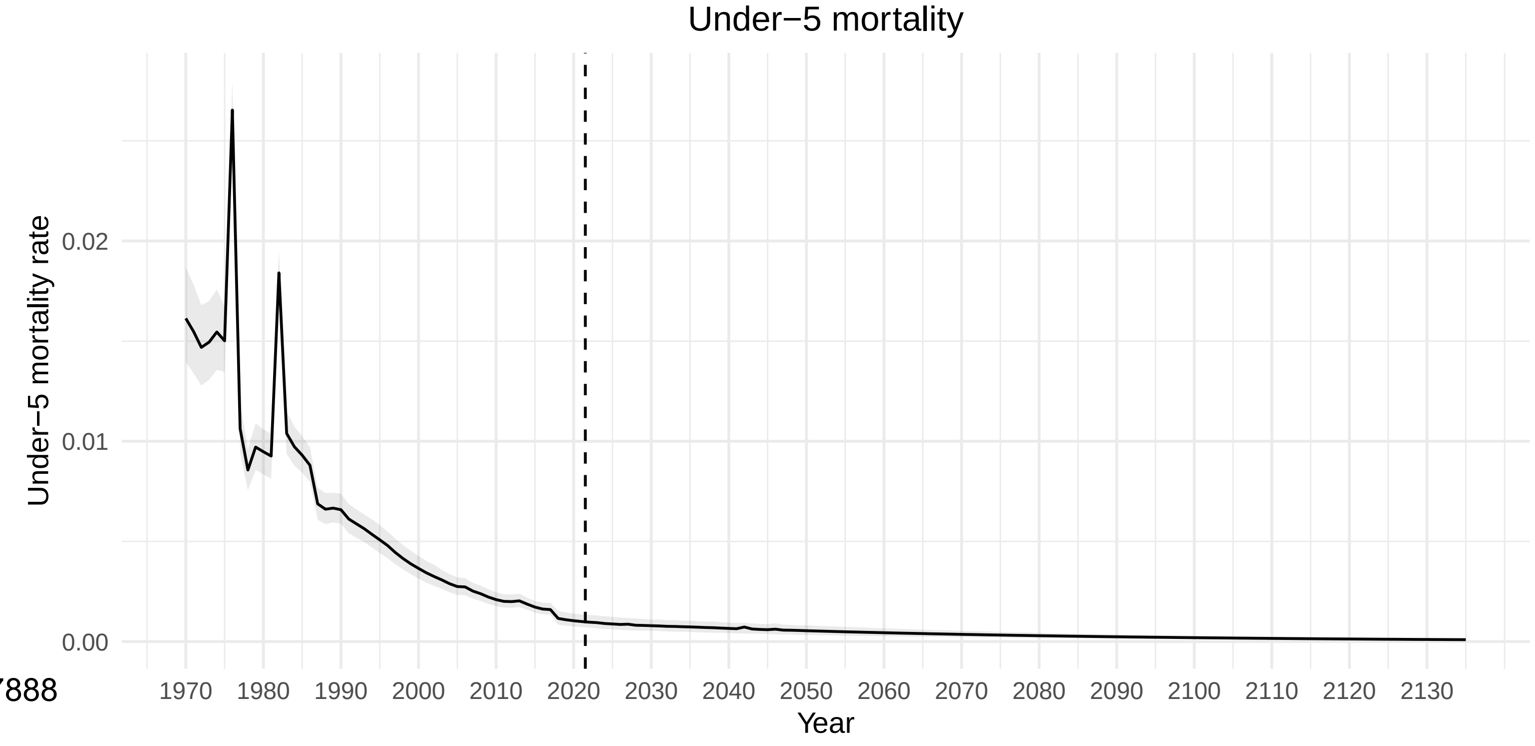

Libya

Supplemental Results Figure S2: Covariates used in fertility forecasts by location, 1970-2135

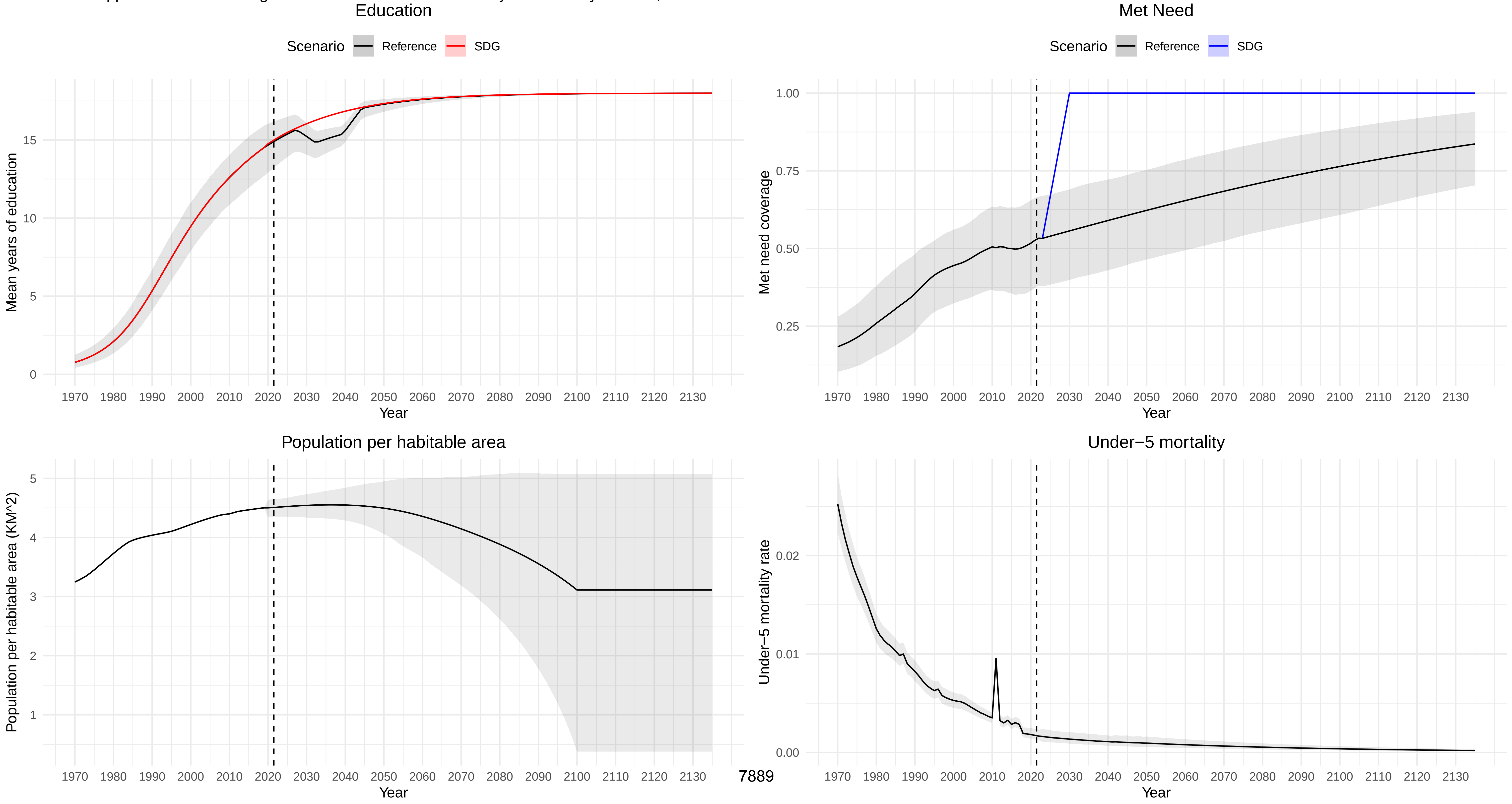

Morocco

Supplemental Results Figure S2: Covariates used in fertility forecasts by location, 1970-2135

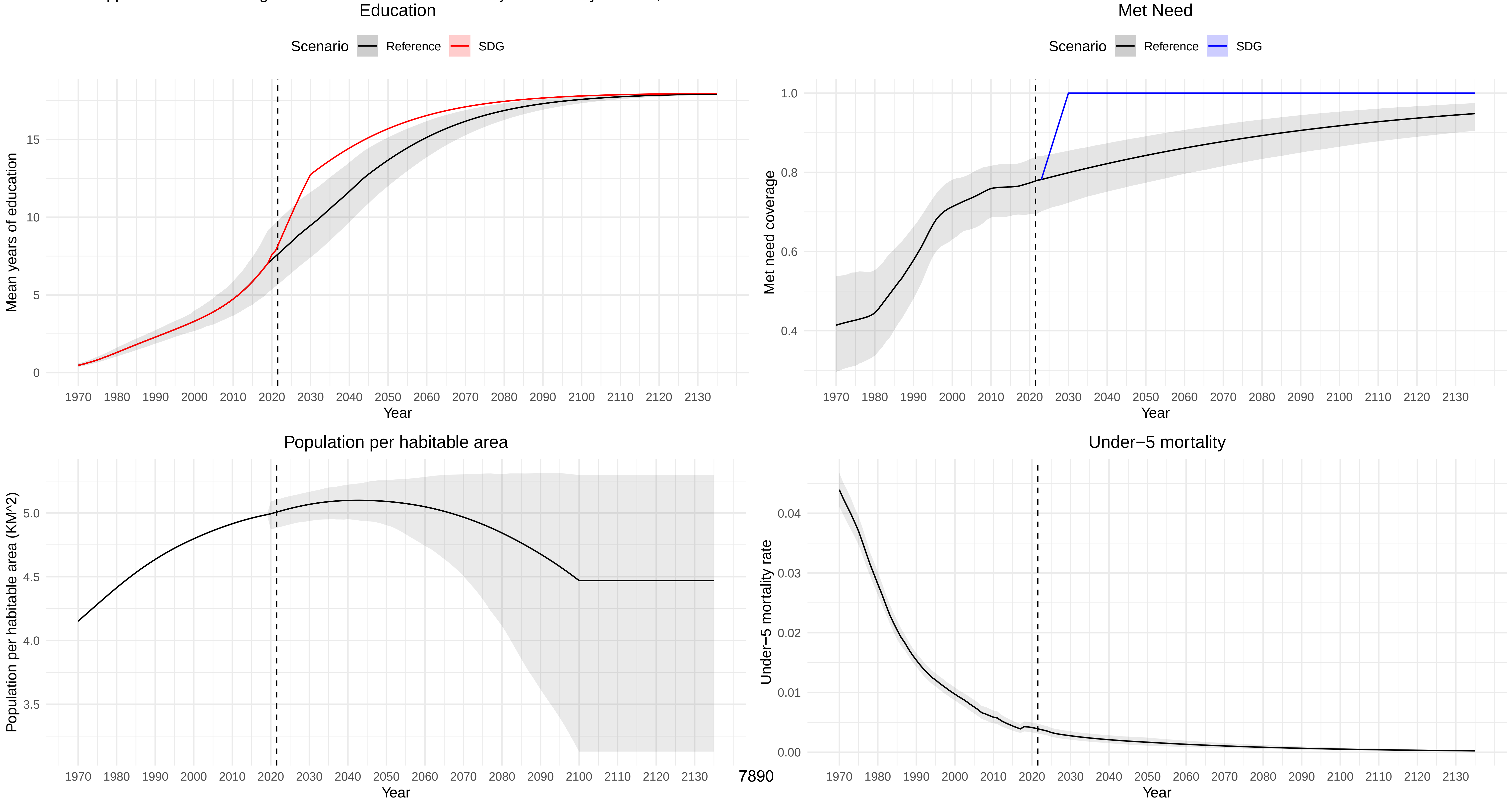

Oman

Supplemental Results Figure S2: Covariates used in fertility forecasts by location, 1970-2135

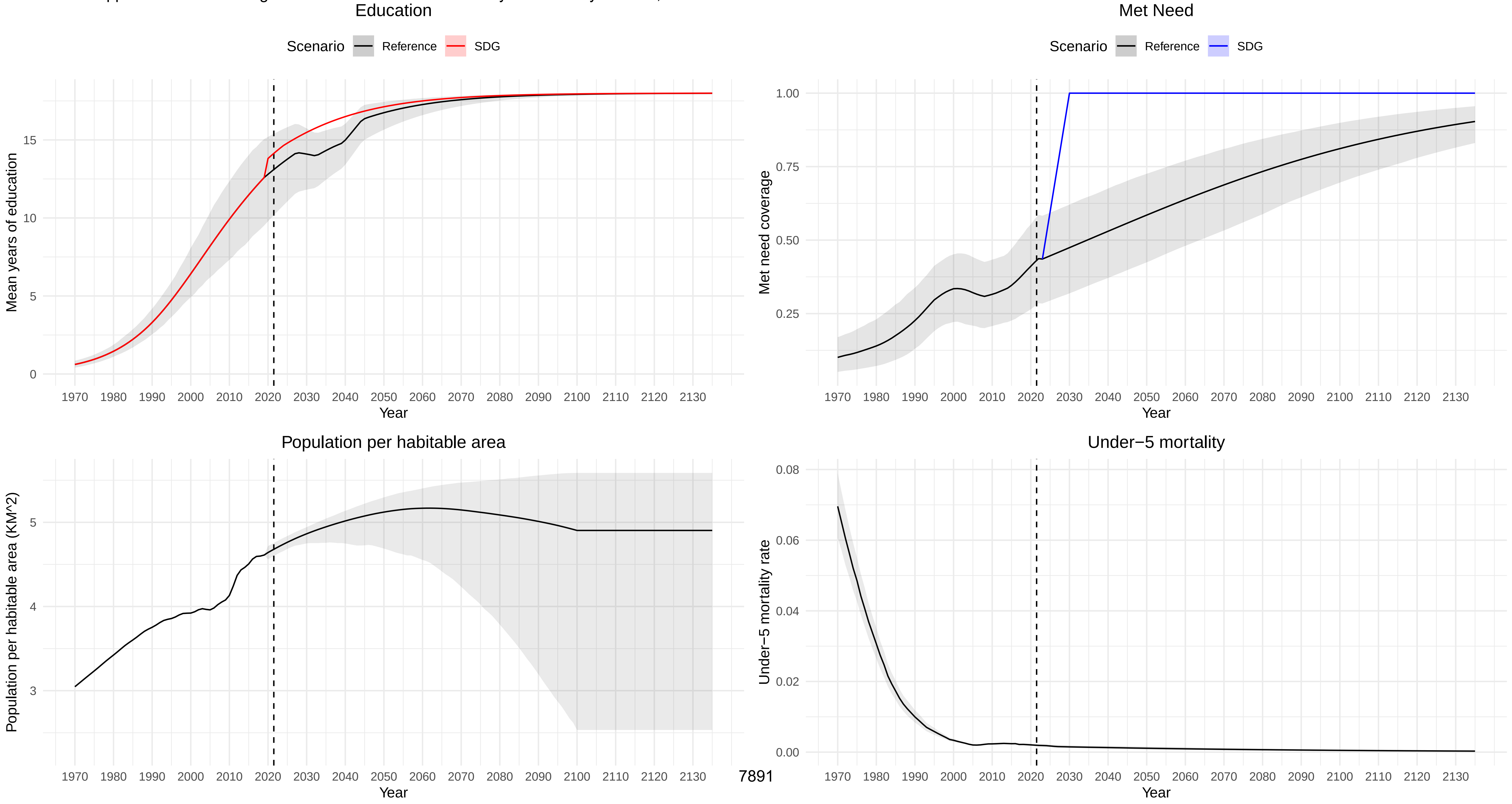

Palestine

Supplemental Results Figure S2: Covariates used in fertility forecasts by location, 1970-2135

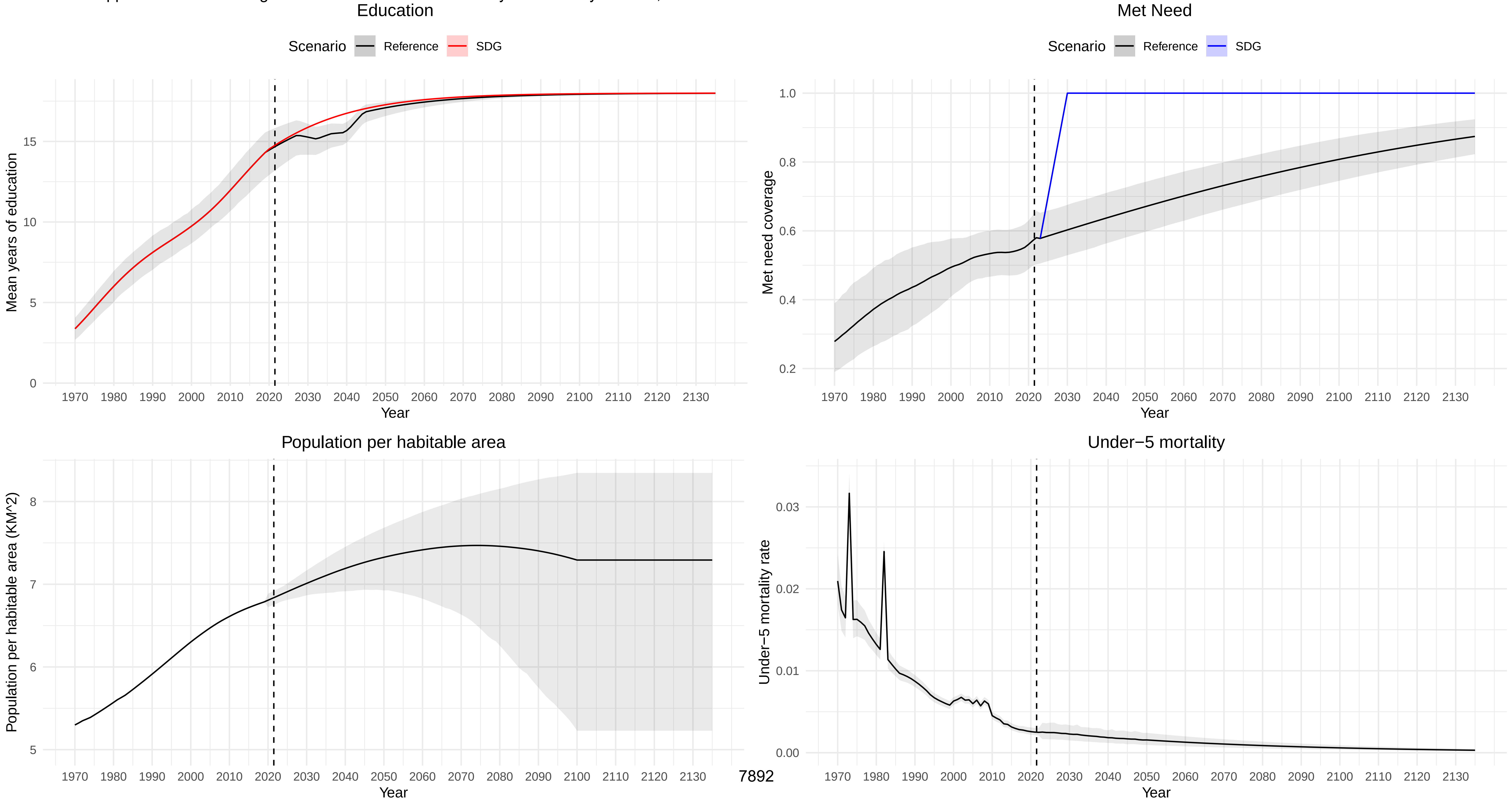

Qatar

Supplemental Results Figure S2: Covariates used in fertility forecasts by location, 1970-2135

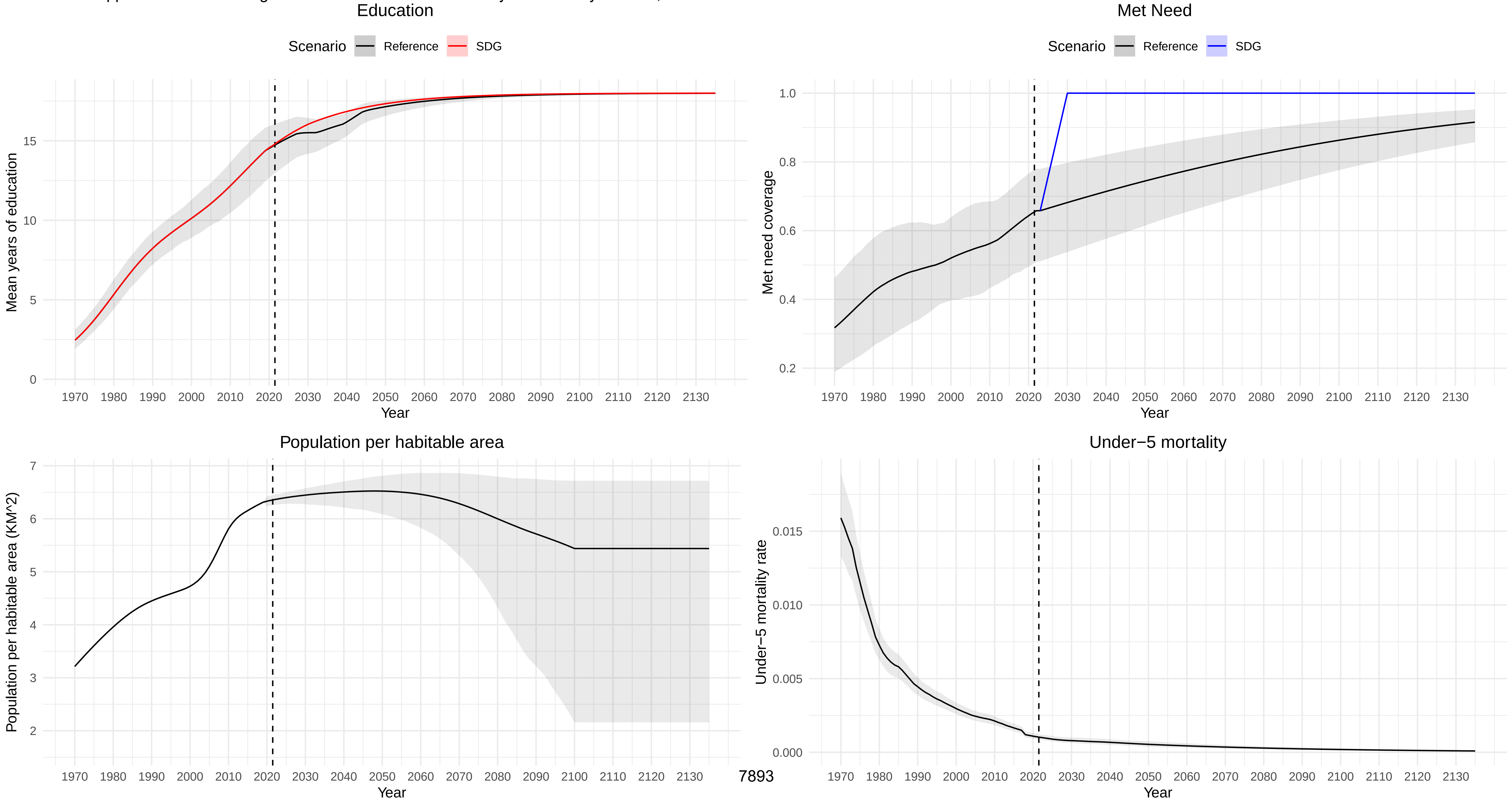

Saudi Arabia

Supplemental Results Figure S2: Covariates used in fertility forecasts by location, 1970-2135

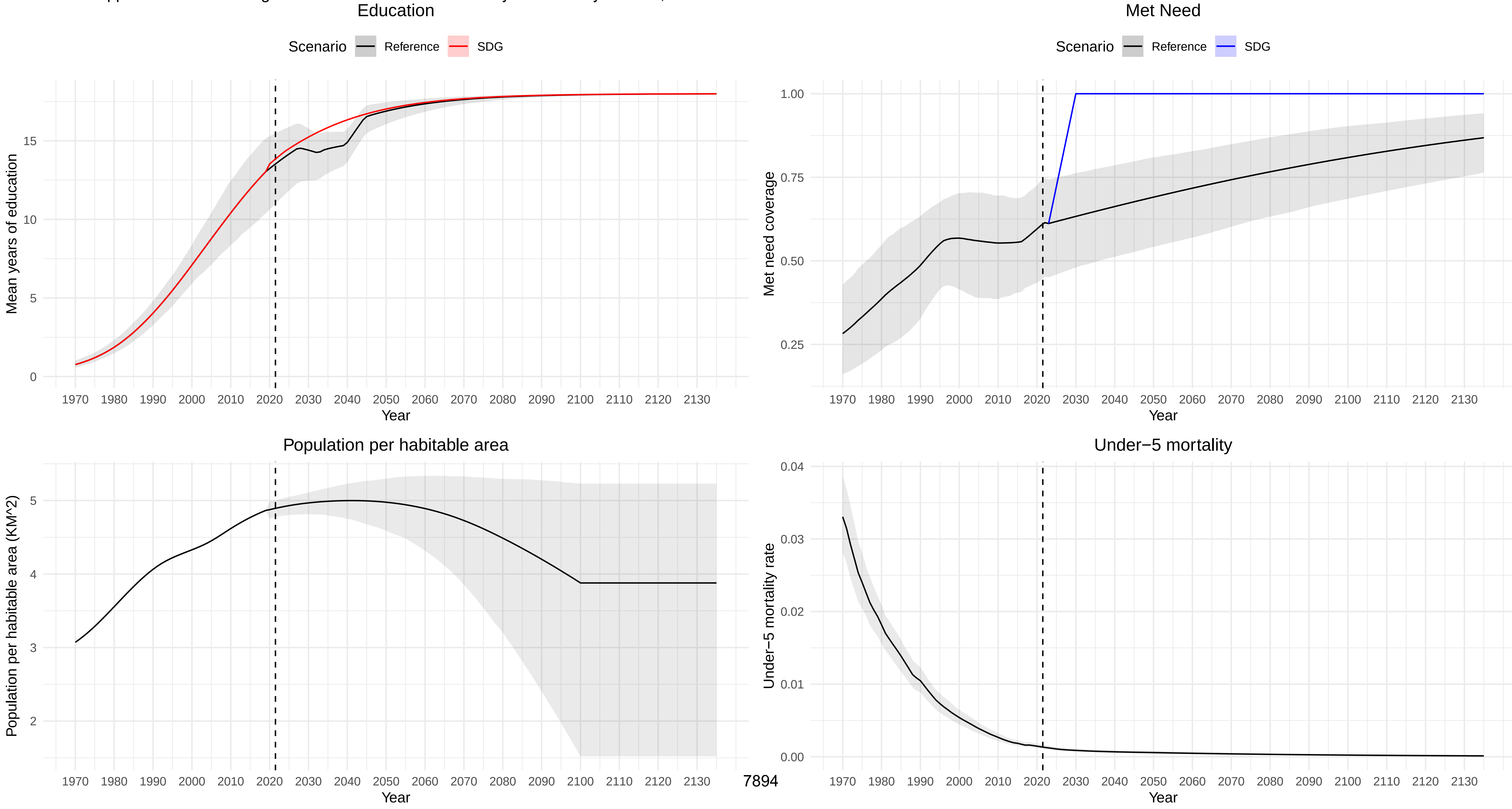

Sudan

Supplemental Results Figure S2: Covariates used in fertility forecasts by location, 1970-2135

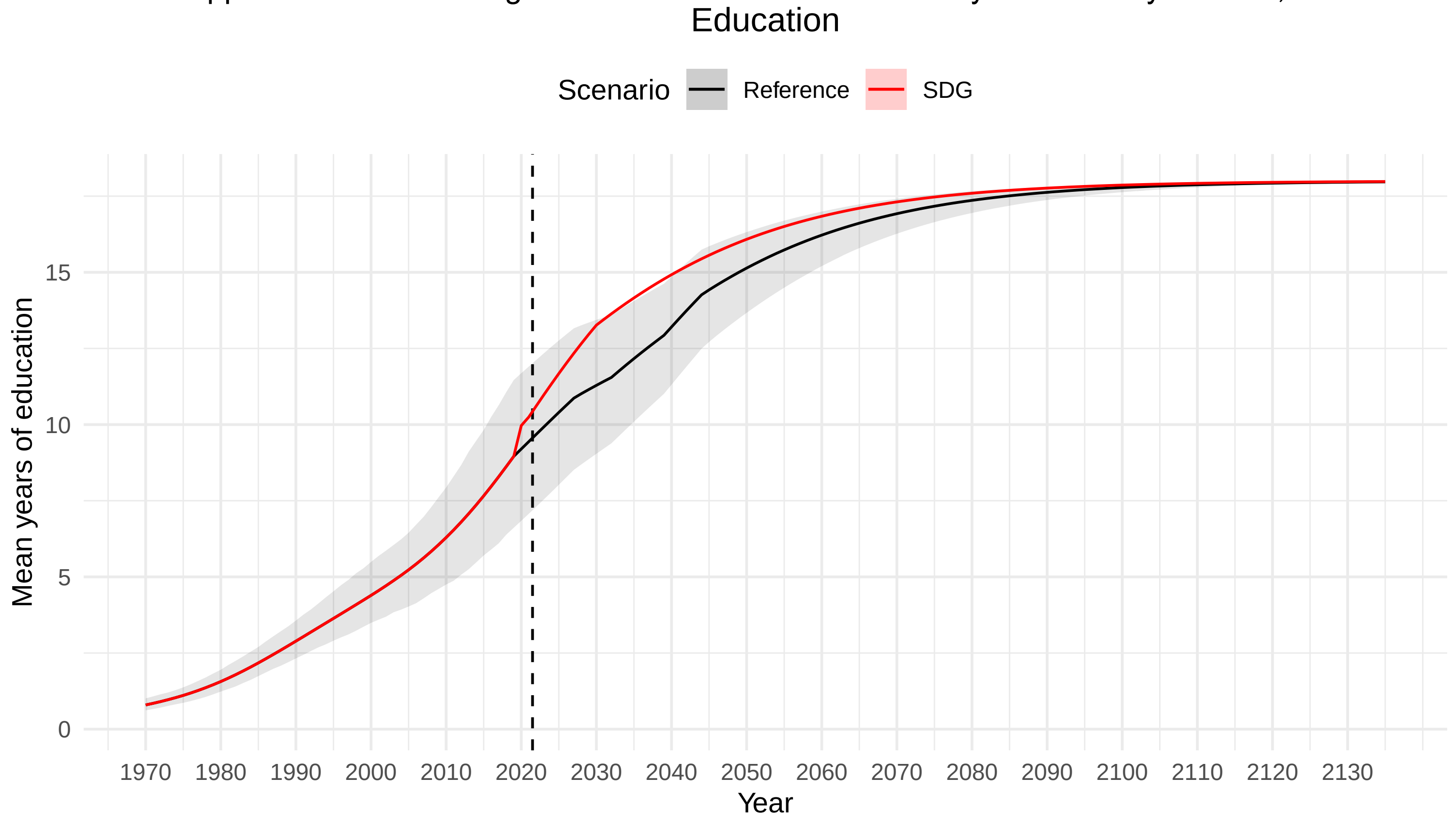

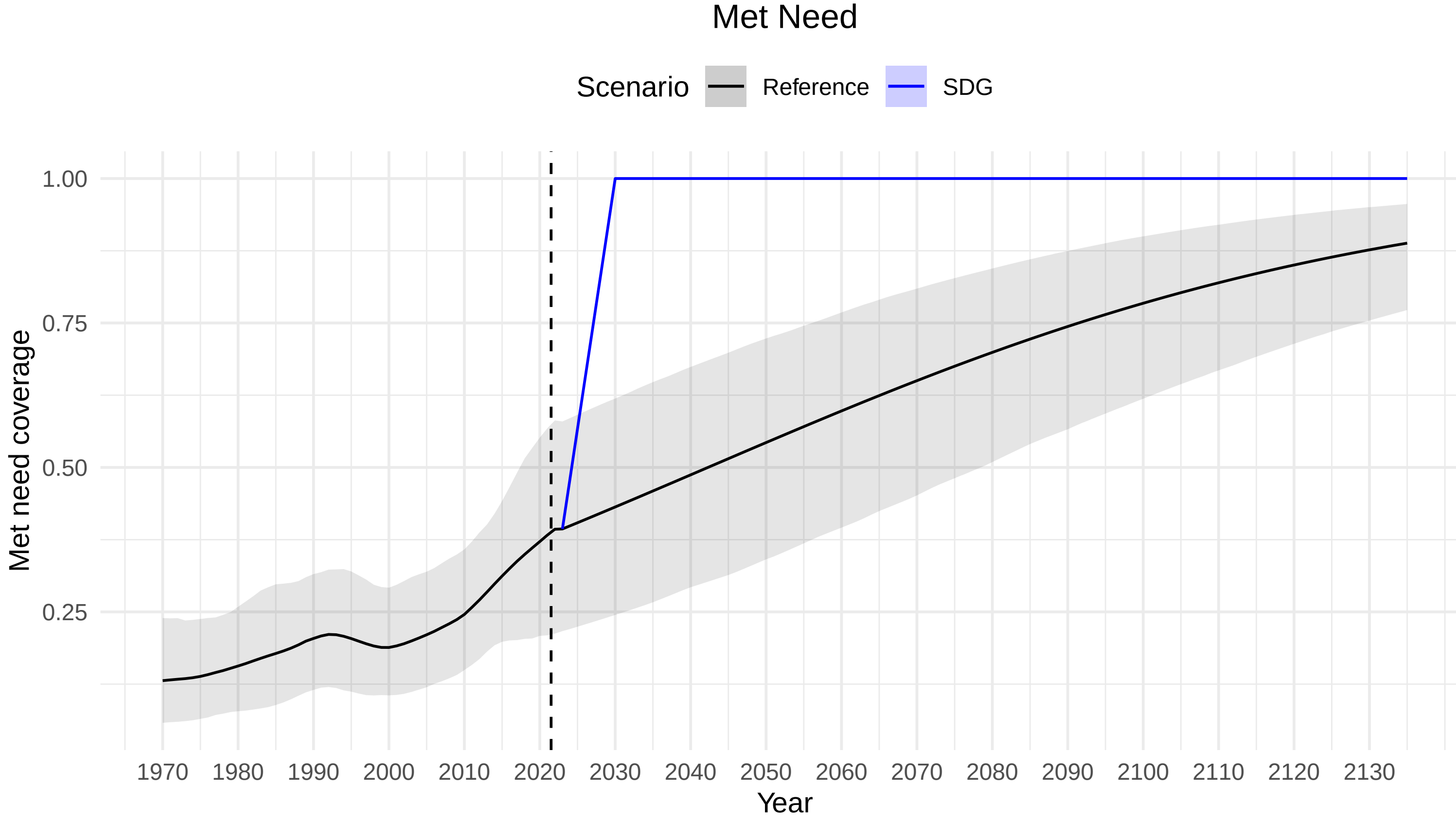

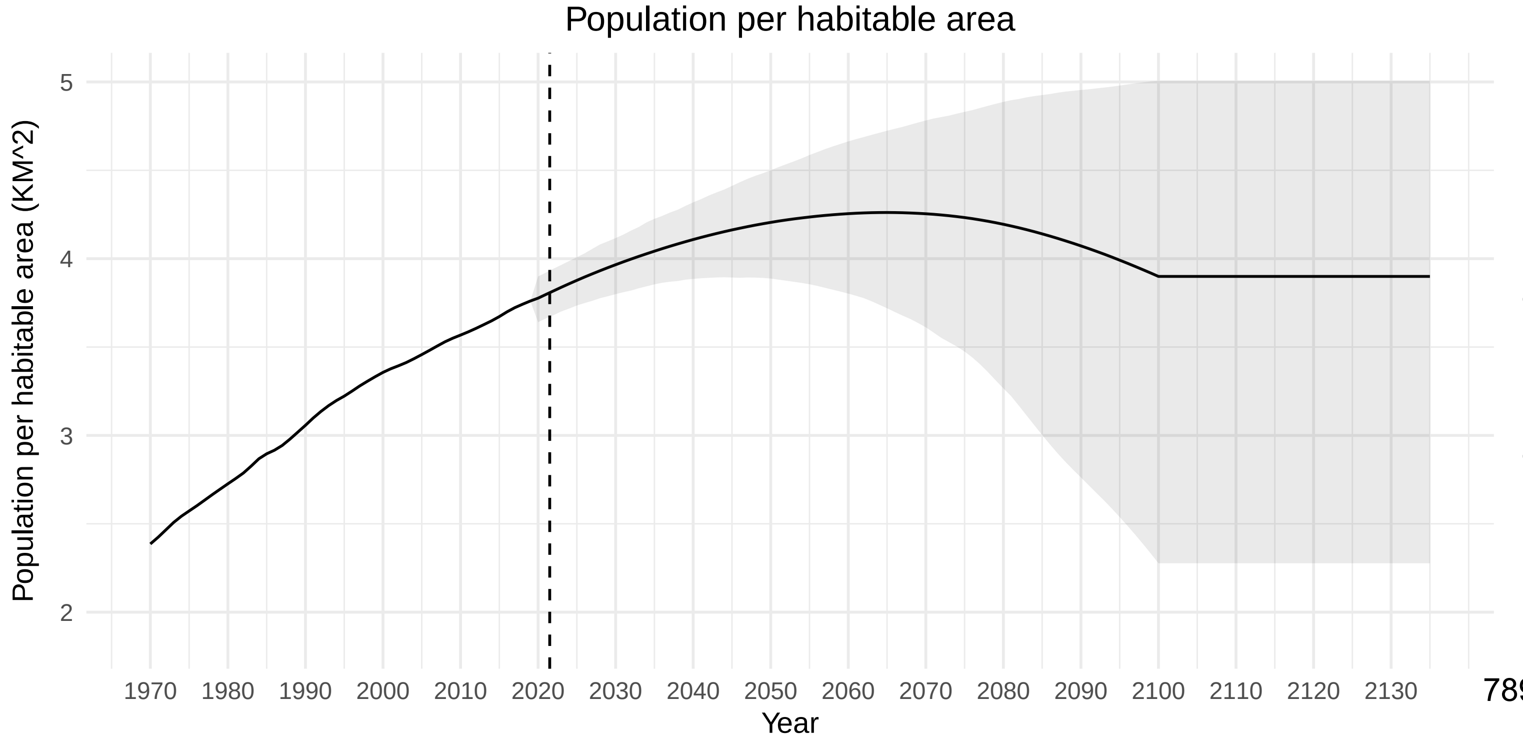

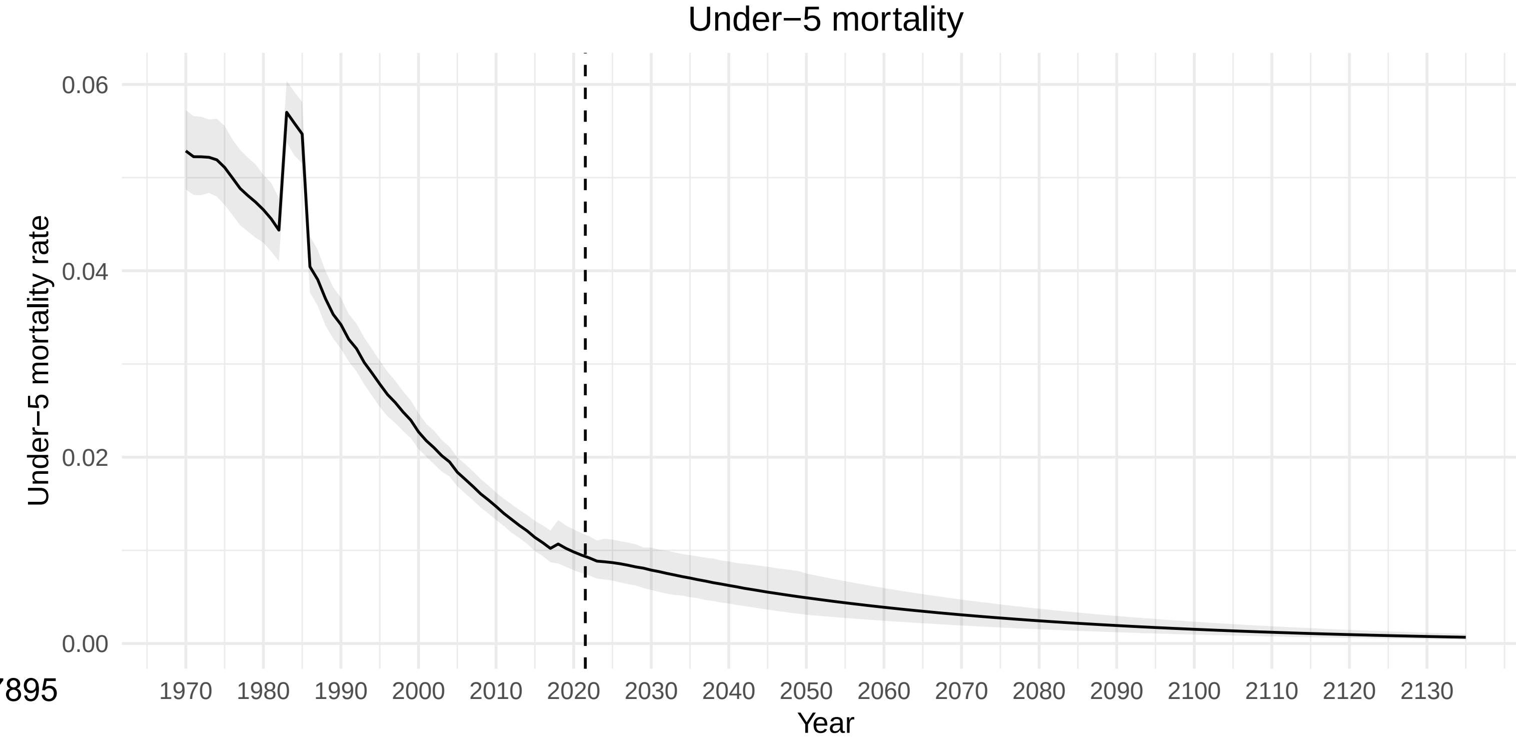

Syrian Arab Republic

Supplemental Results Figure S2: Covariates used in fertility forecasts by location, 1970-2135

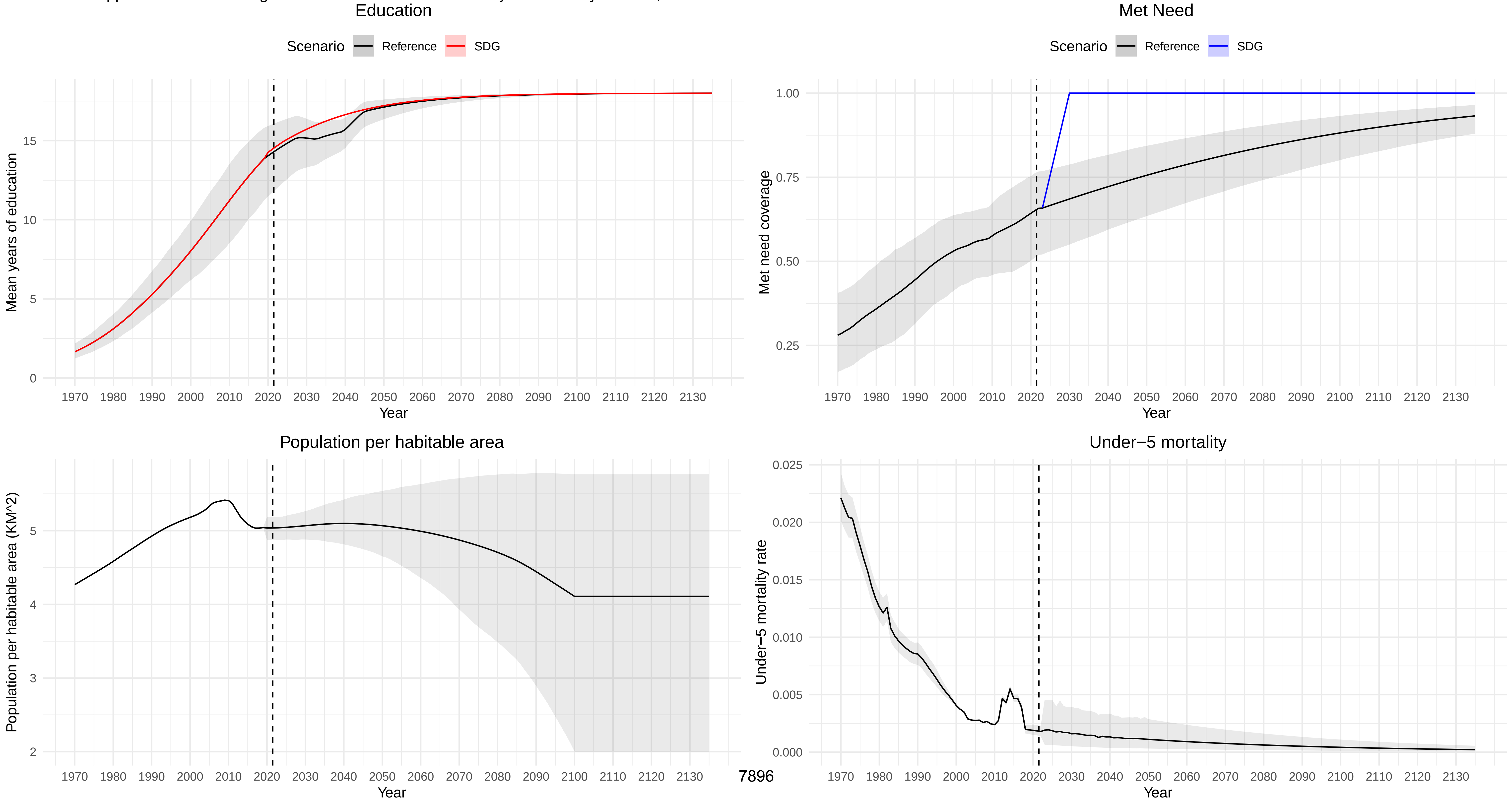

Tunisia

Supplemental Results Figure S2: Covariates used in fertility forecasts by location, 1970-2135

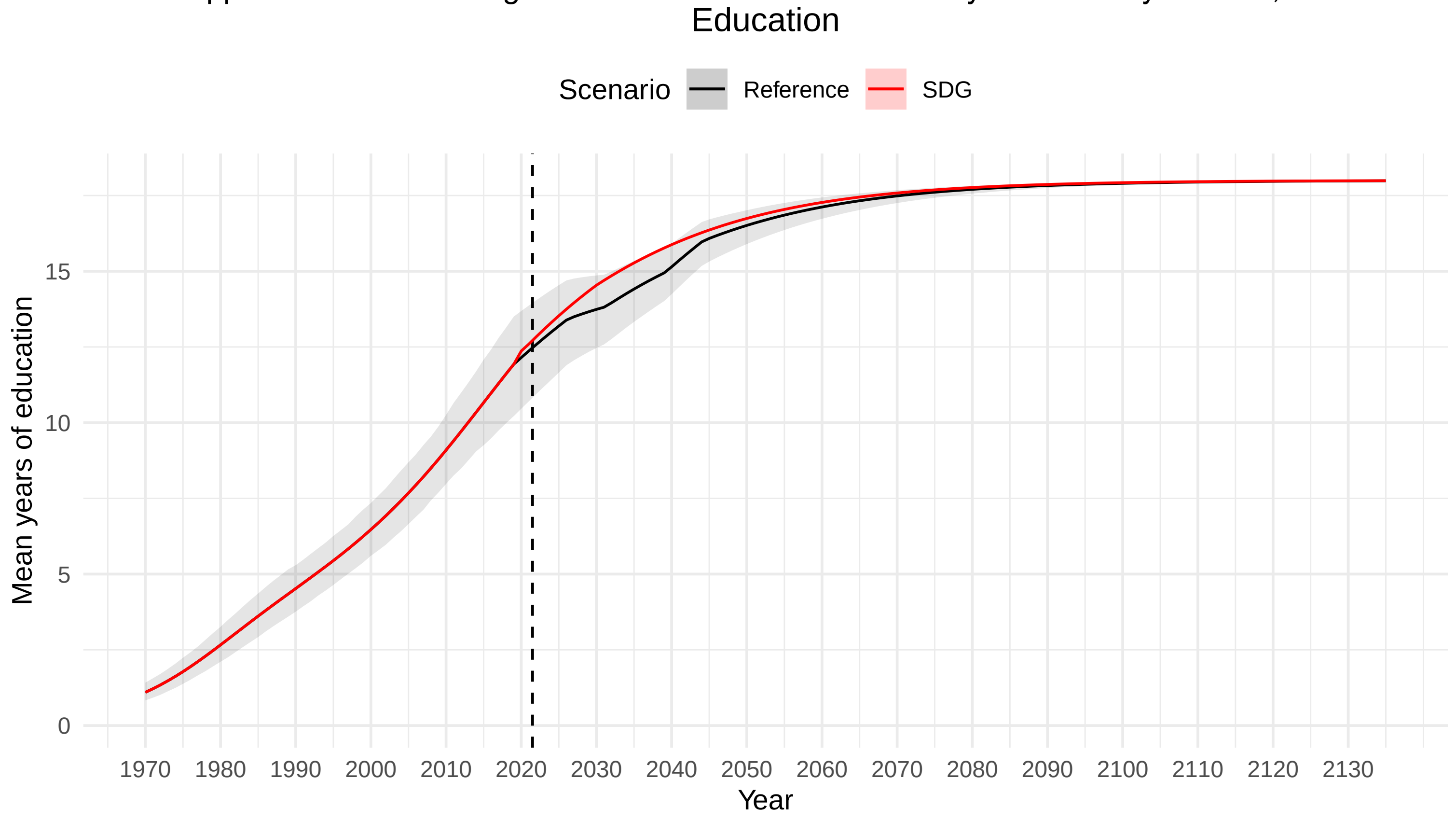

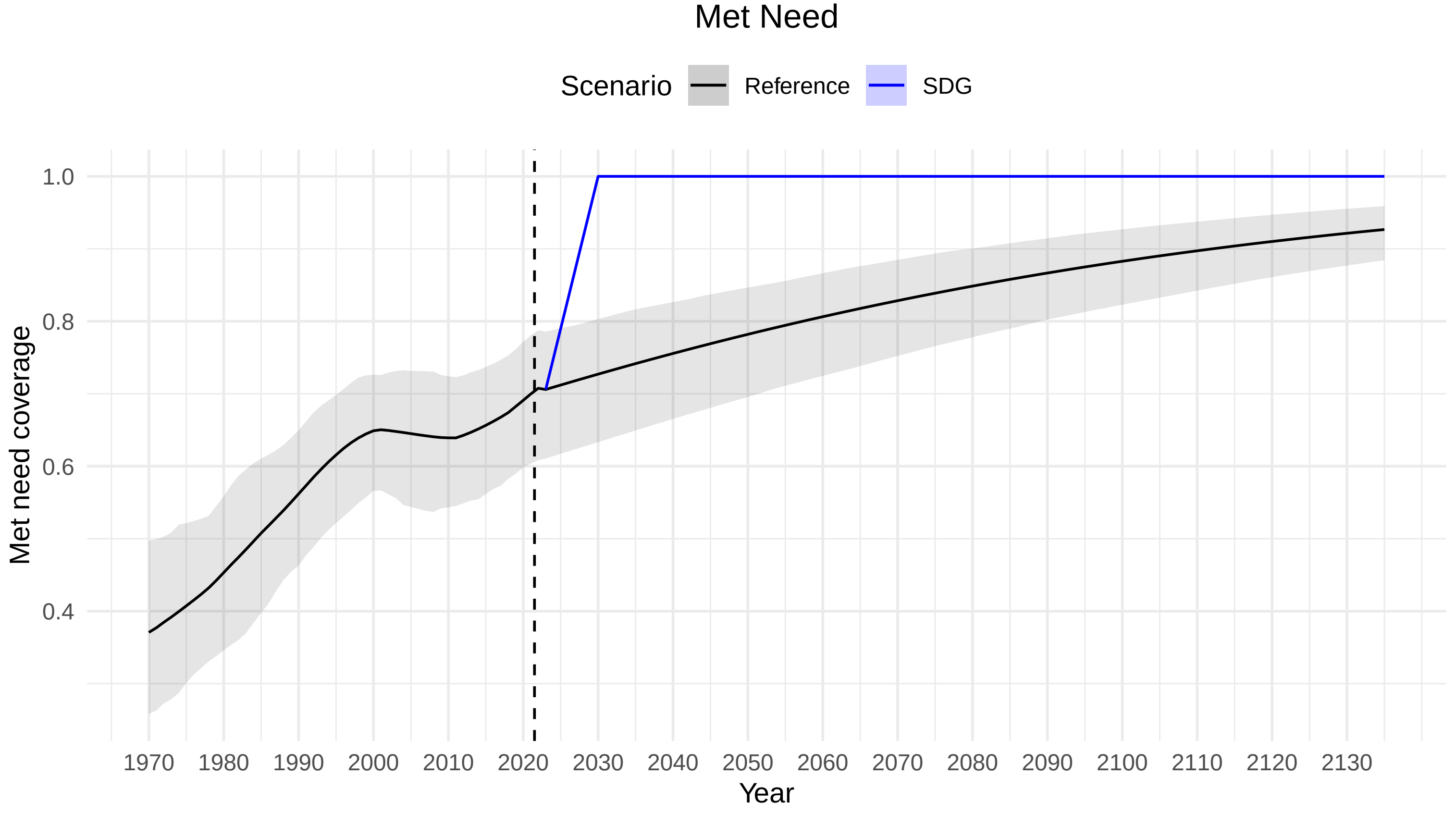

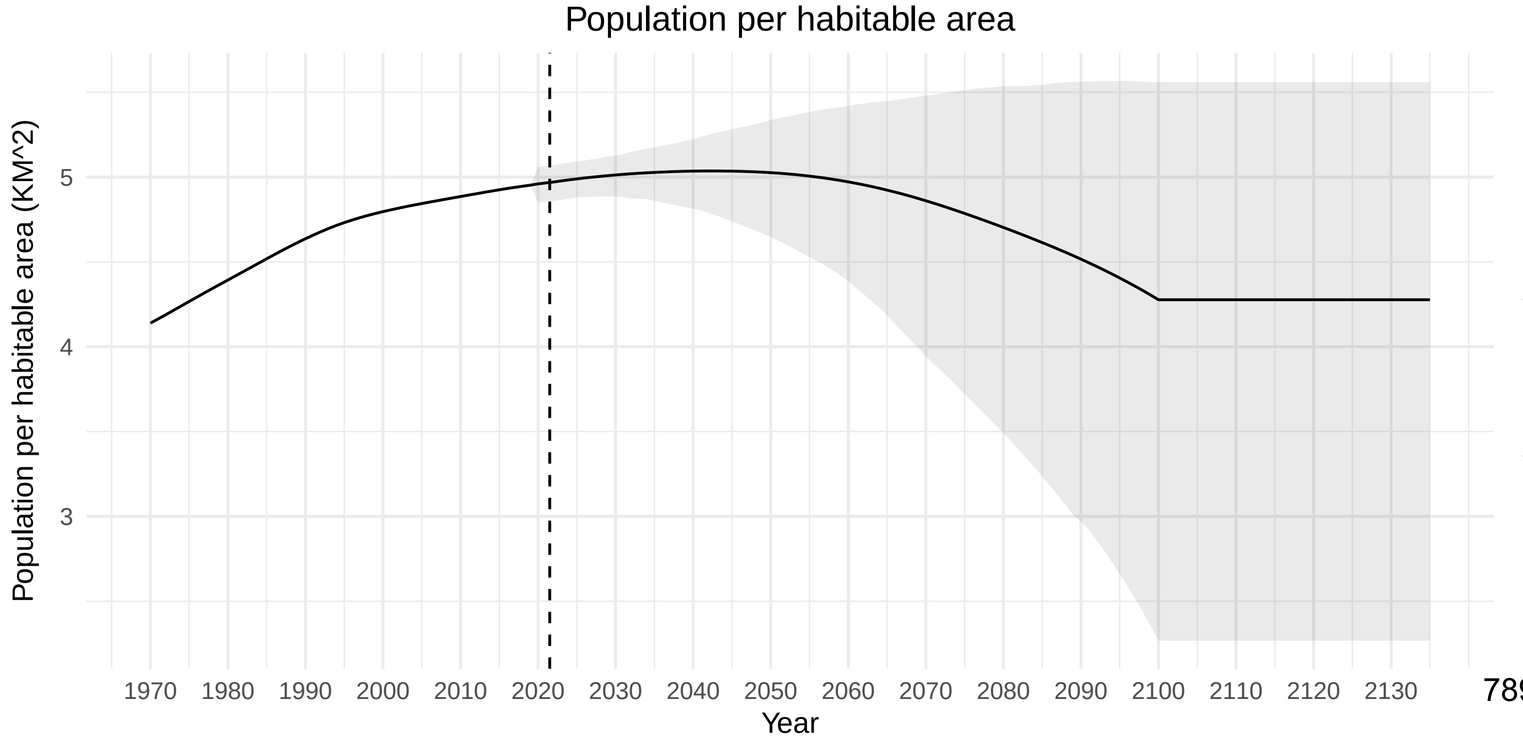

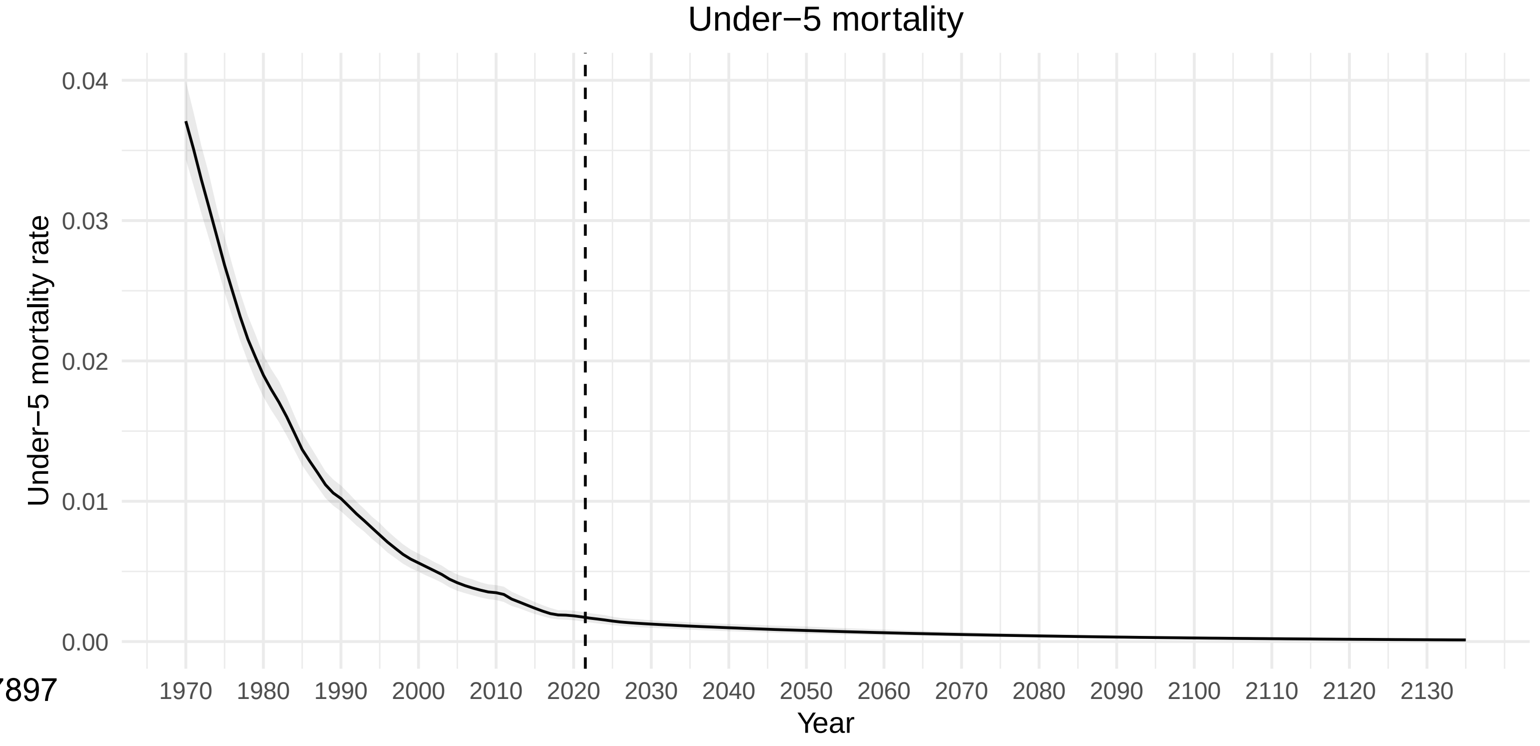

Türkiye

Supplemental Results Figure S2: Covariates used in fertility forecasts by location, 1970-2135

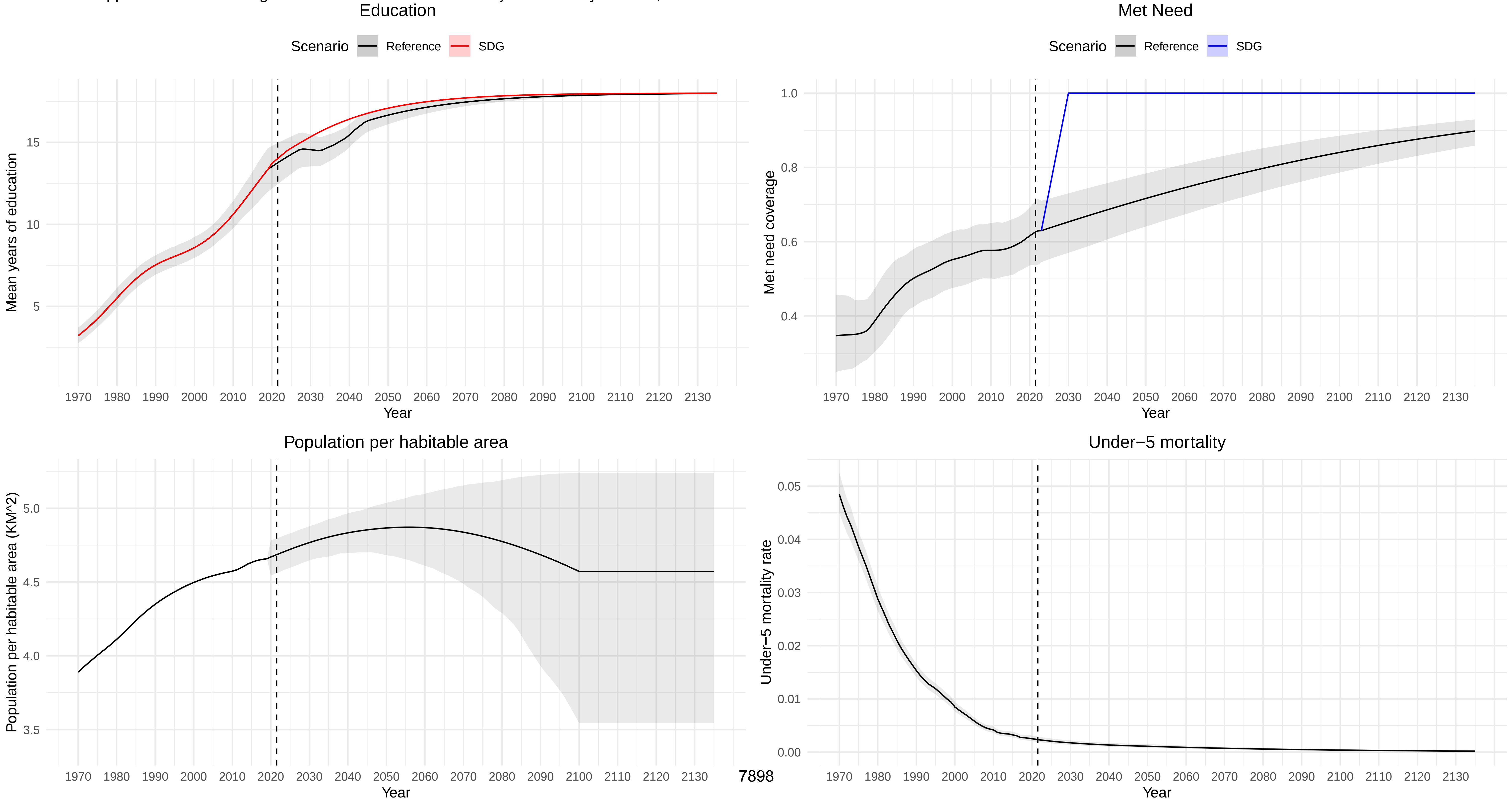

United Arab Emirates

Supplemental Results Figure S2: Covariates used in fertility forecasts by location, 1970-2135

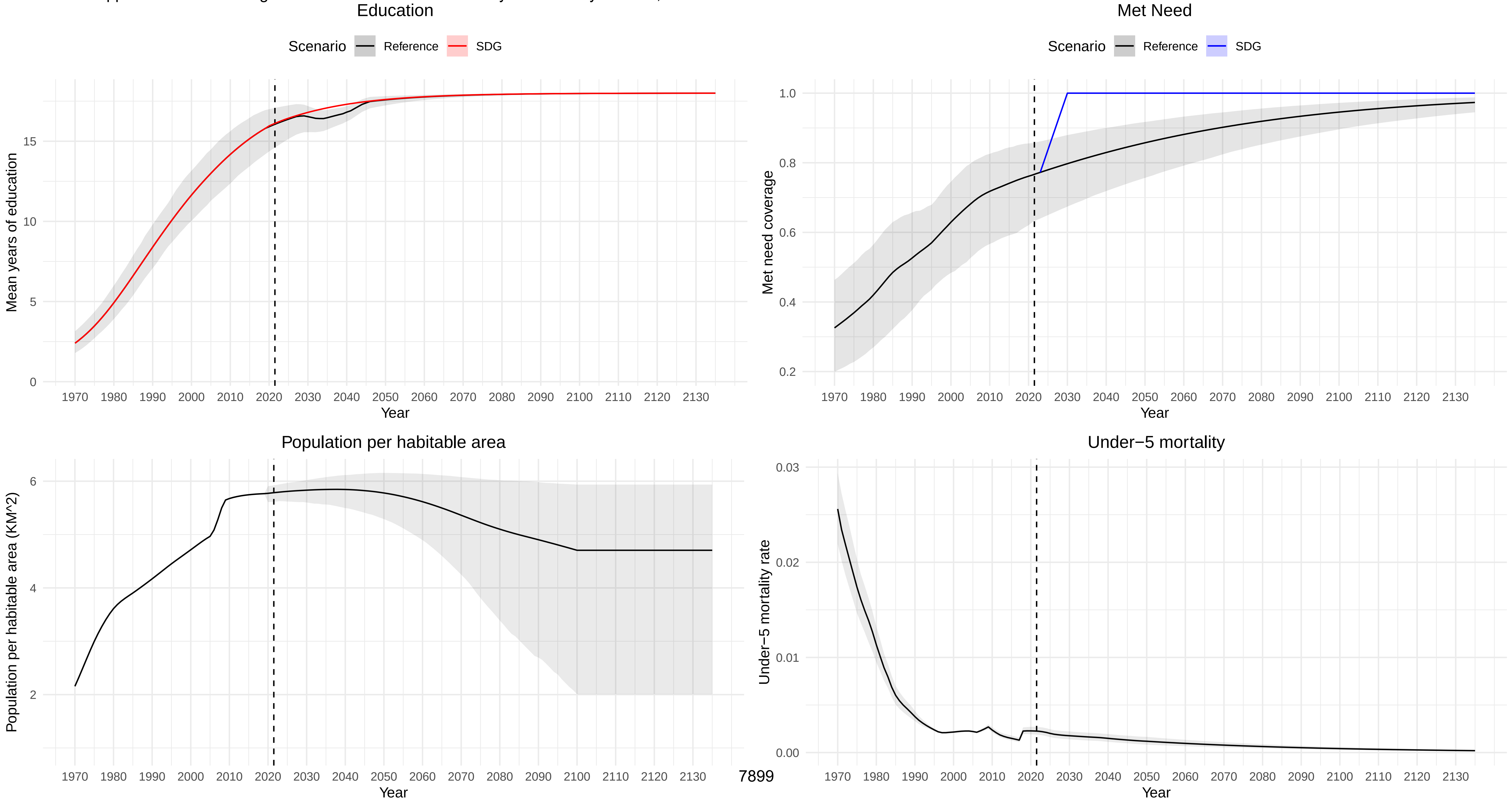

Yemen

Supplemental Results Figure S2: Covariates used in fertility forecasts by location, 1970-2135

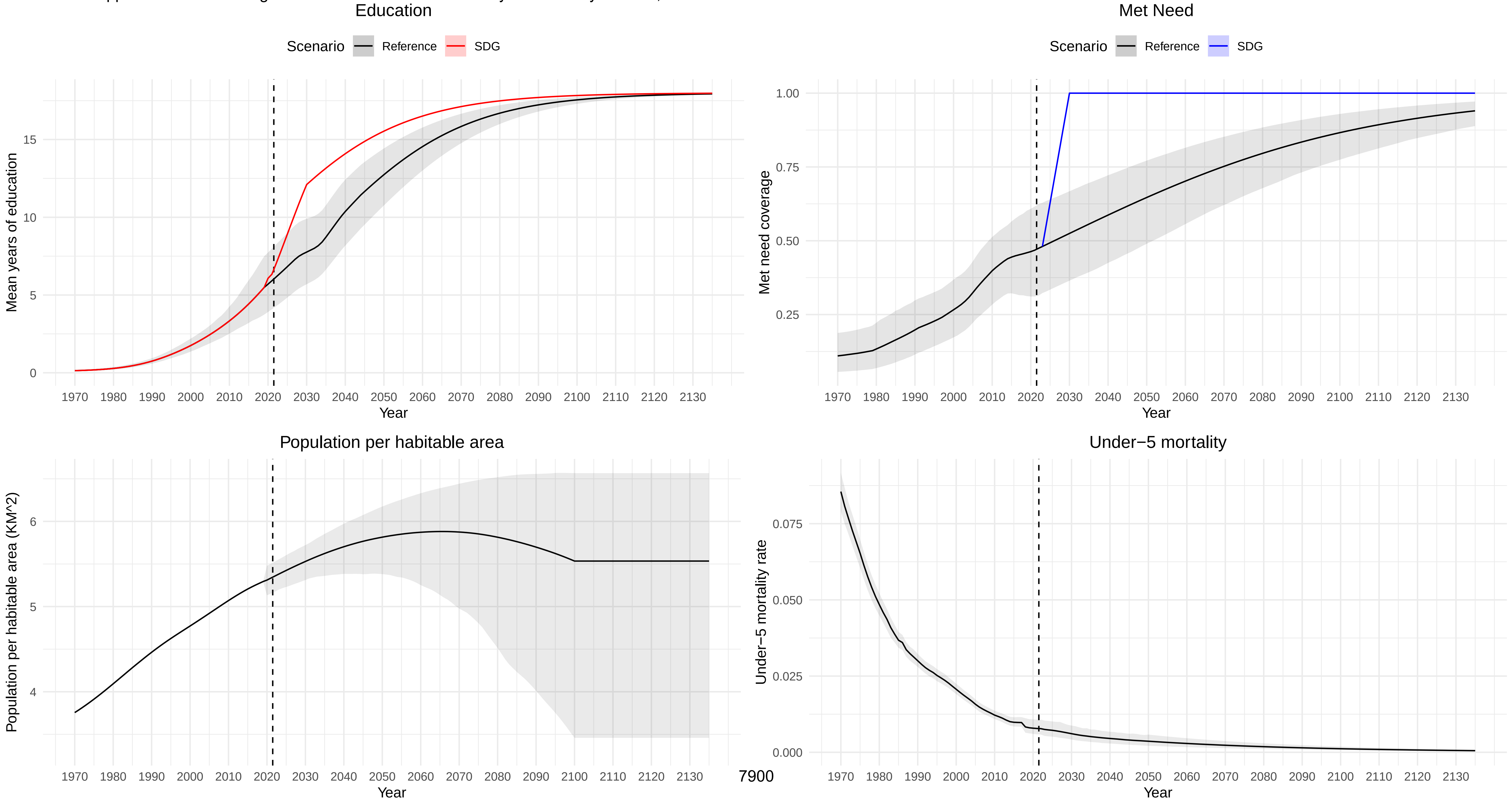

Bangladesh

Supplemental Results Figure S2: Covariates used in fertility forecasts by location, 1970-2135

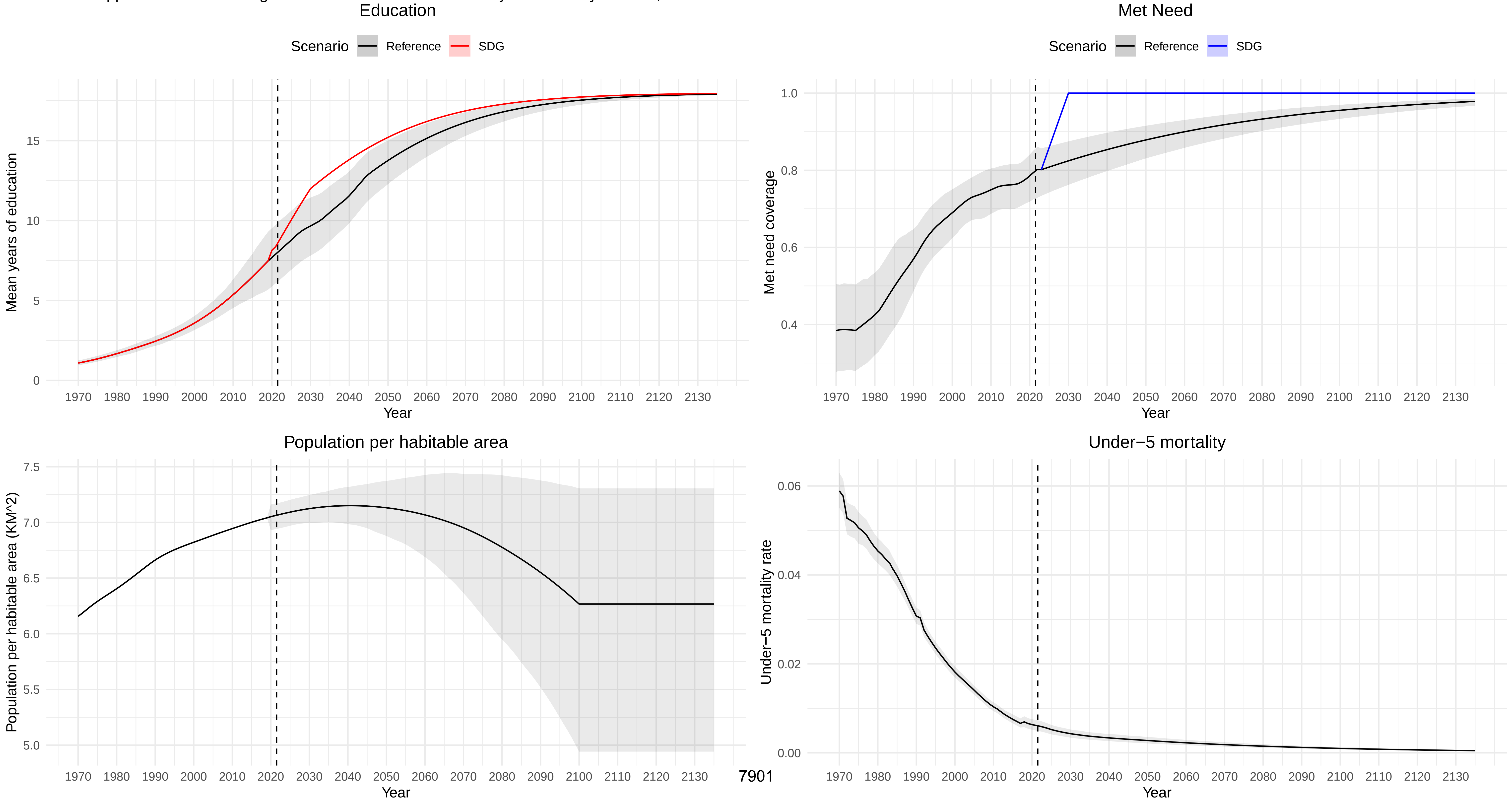

Bhutan

Supplemental Results Figure S2: Covariates used in fertility forecasts by location, 1970-2135

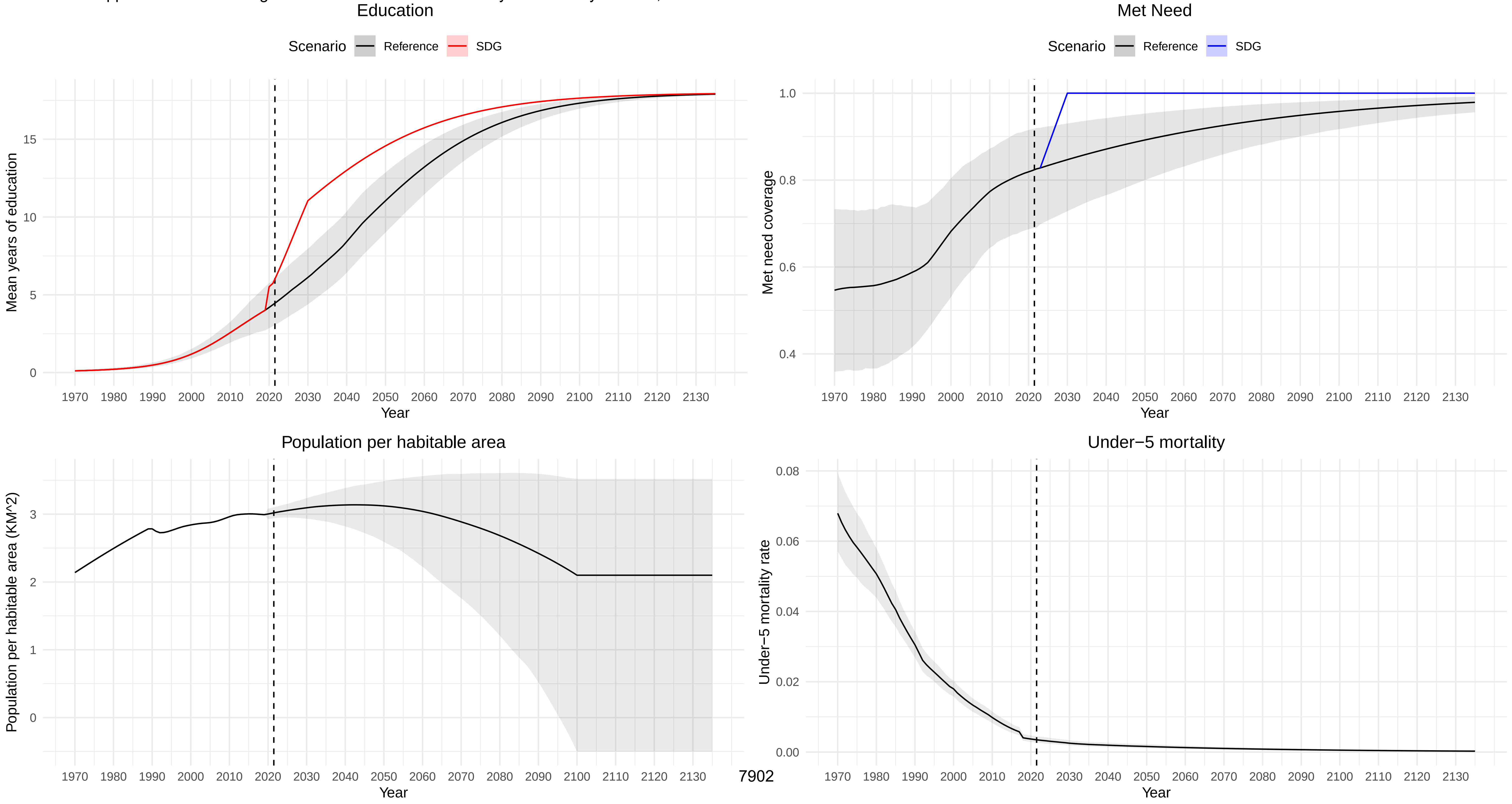

India

Supplemental Results Figure S2: Covariates used in fertility forecasts by location, 1970-2135

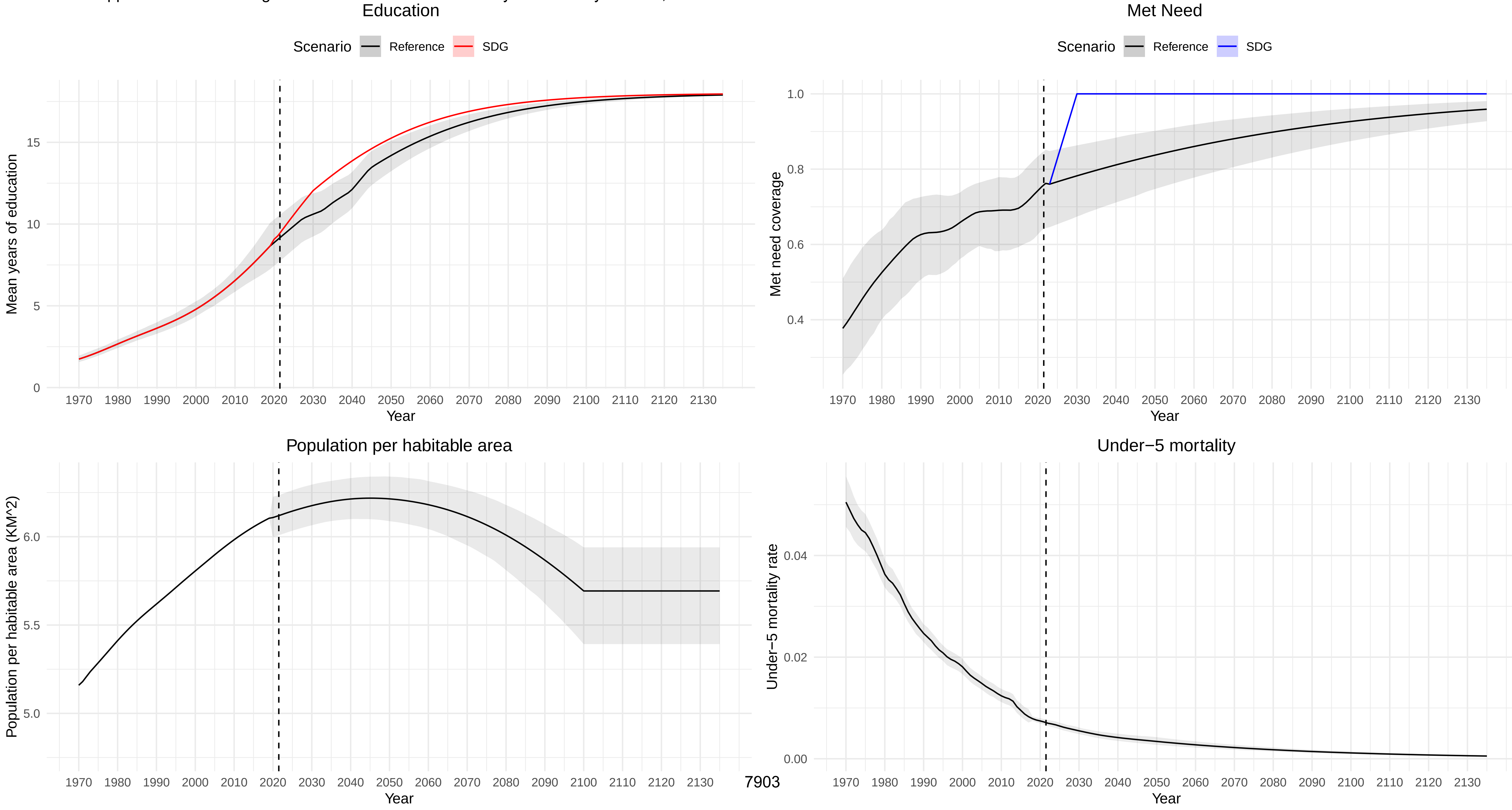

Nepal

Supplemental Results Figure S2: Covariates used in fertility forecasts by location, 1970-2135

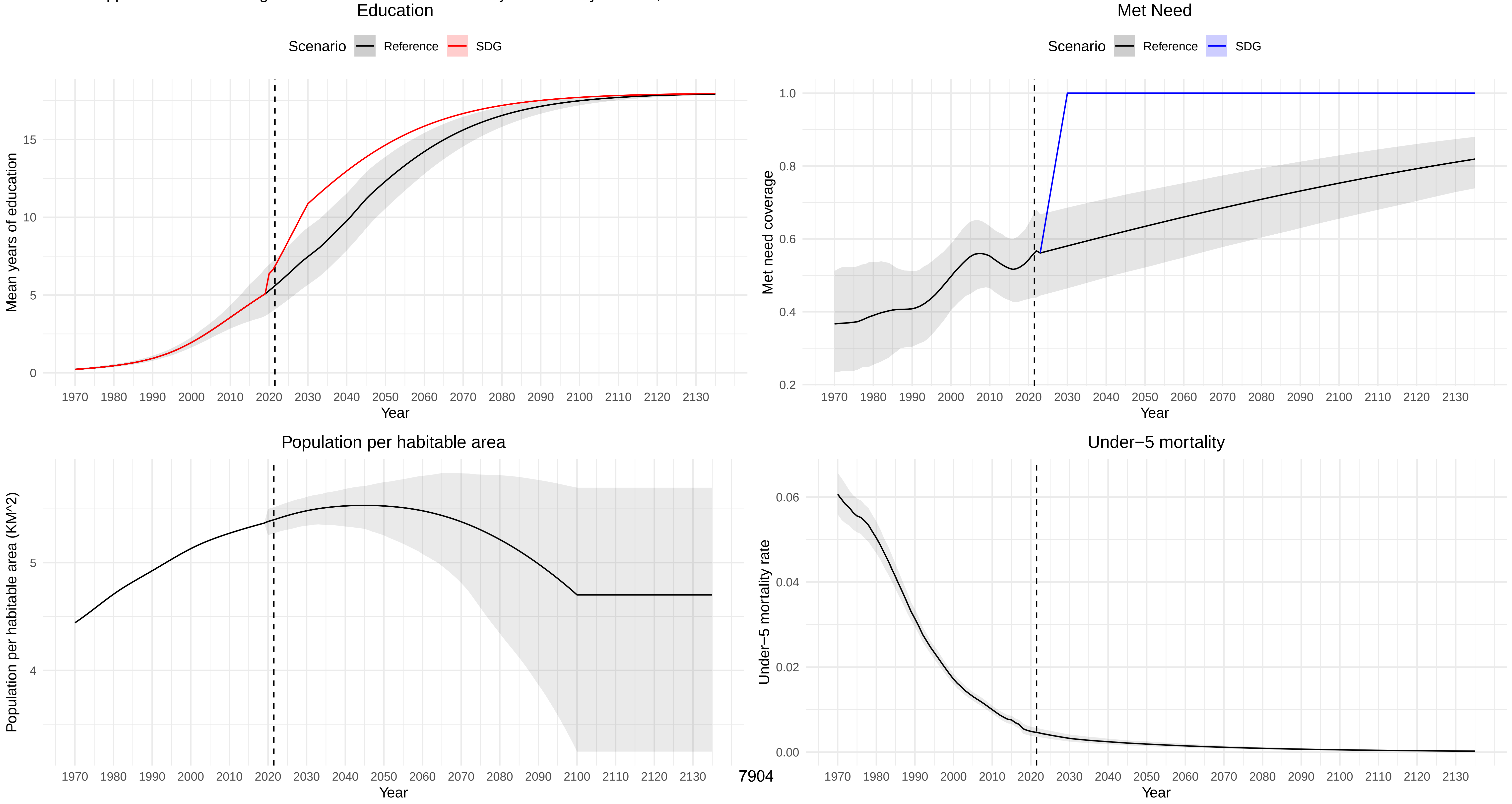

Pakistan

Supplemental Results Figure S2: Covariates used in fertility forecasts by location, 1970-2135

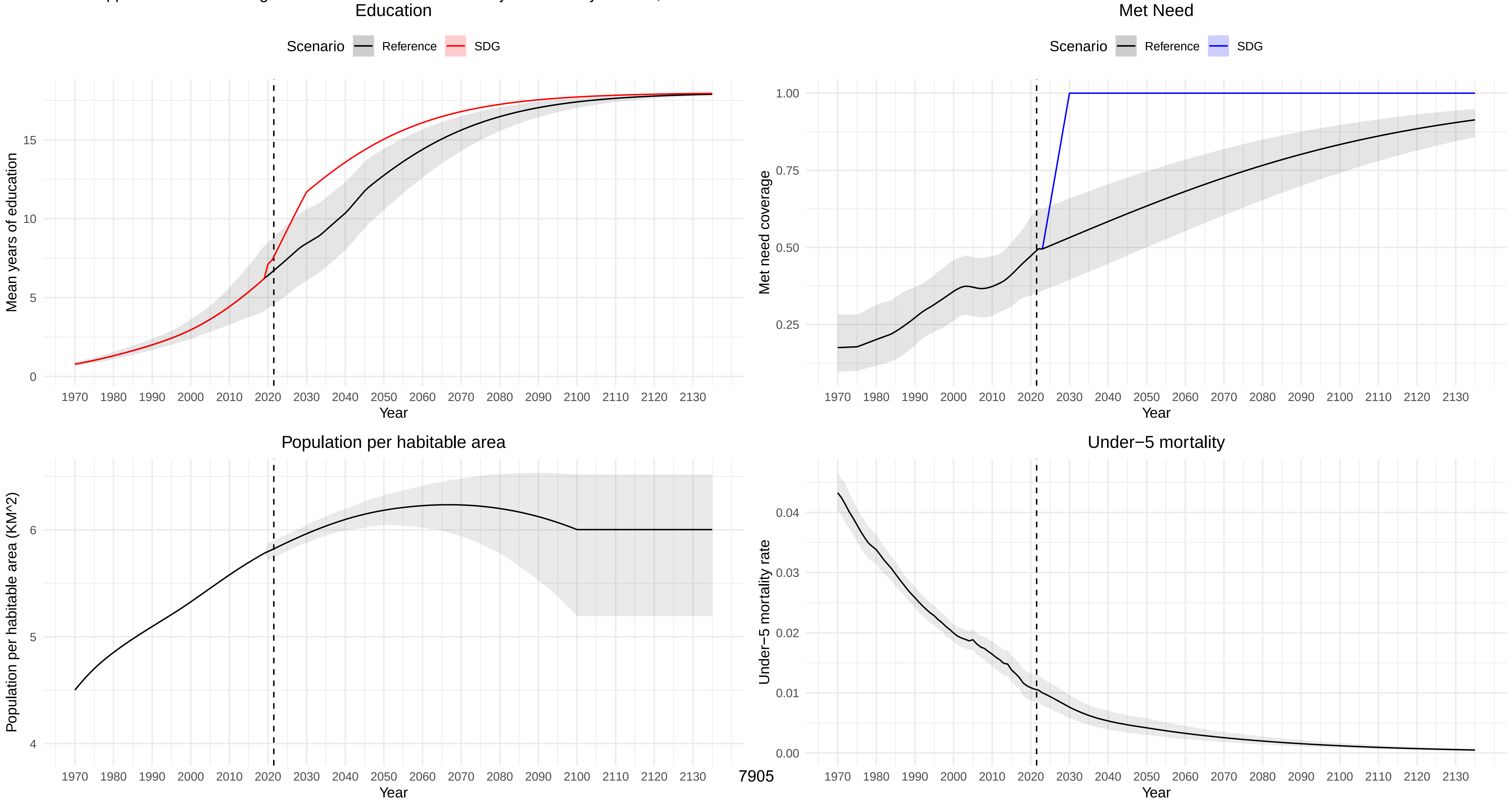

China

Supplemental Results Figure S2: Covariates used in fertility forecasts by location, 1970-2135

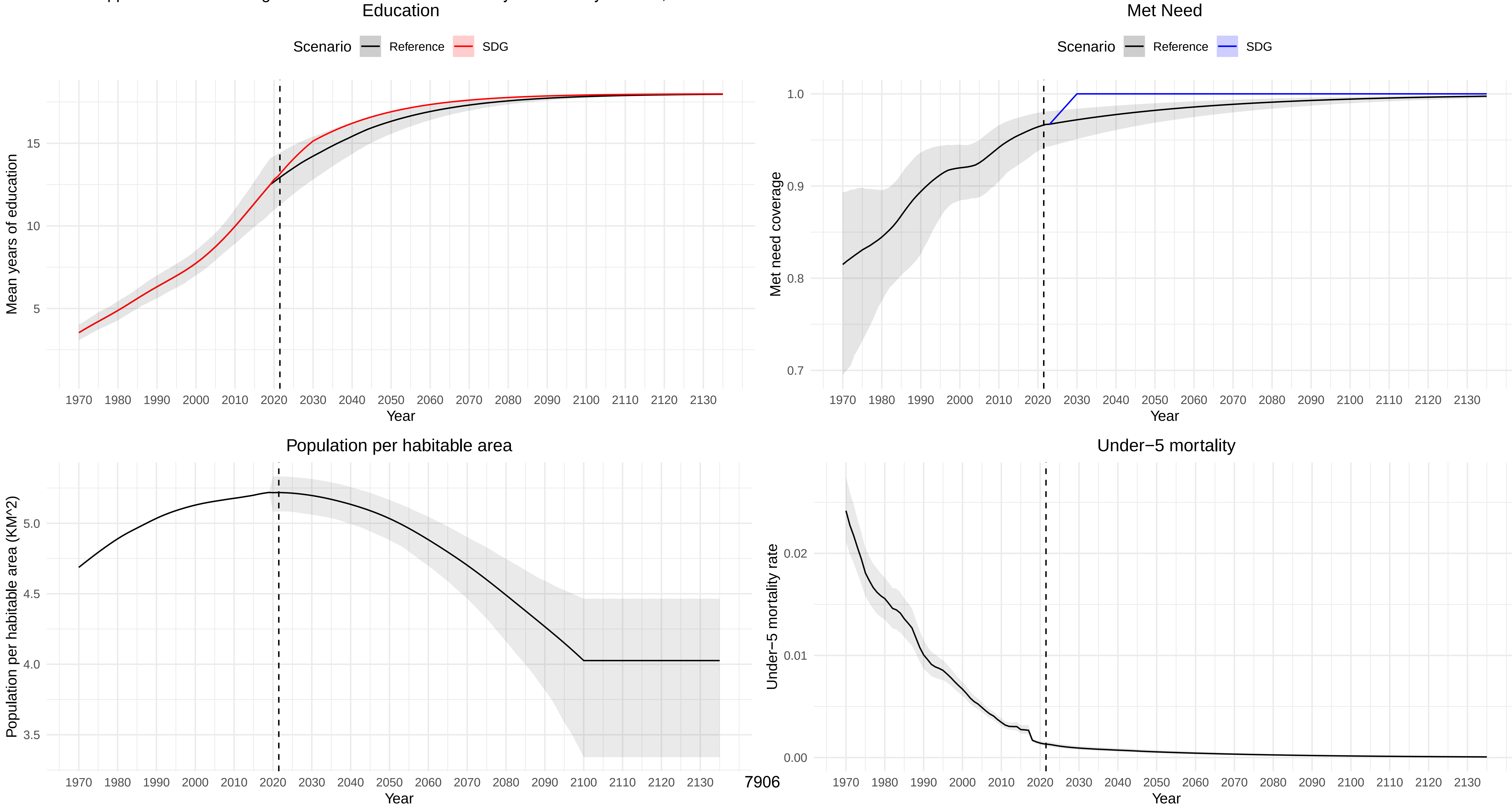

Democratic People's Republic of Korea

Supplemental Results Figure S2: Covariates used in fertility forecasts by location, 1970-2135

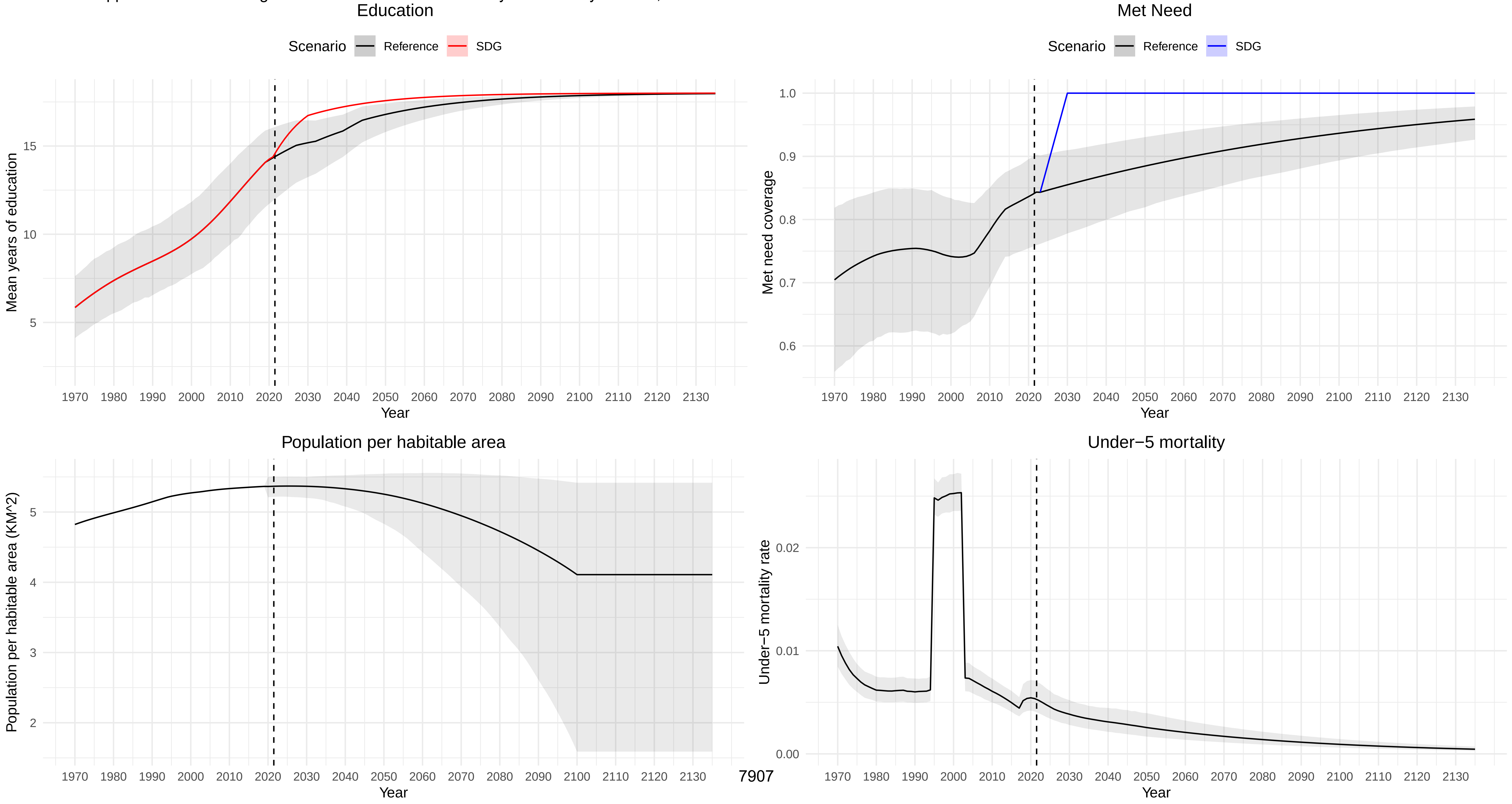

Taiwan (Province of China)

Supplemental Results Figure S2: Covariates used in fertility forecasts by location, 1970-2135

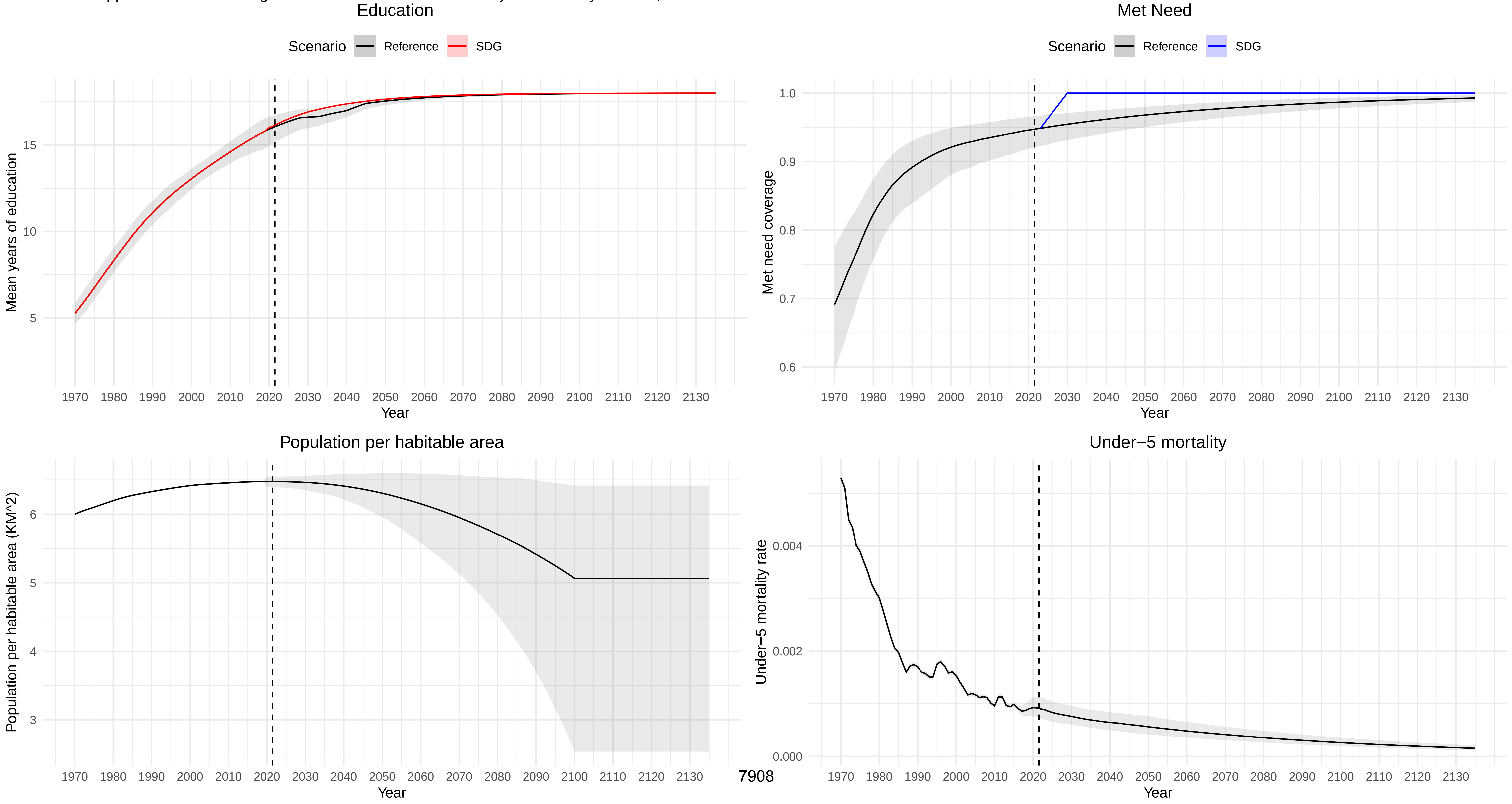

American Samoa

Supplemental Results Figure S2: Covariates used in fertility forecasts by location, 1970-2135

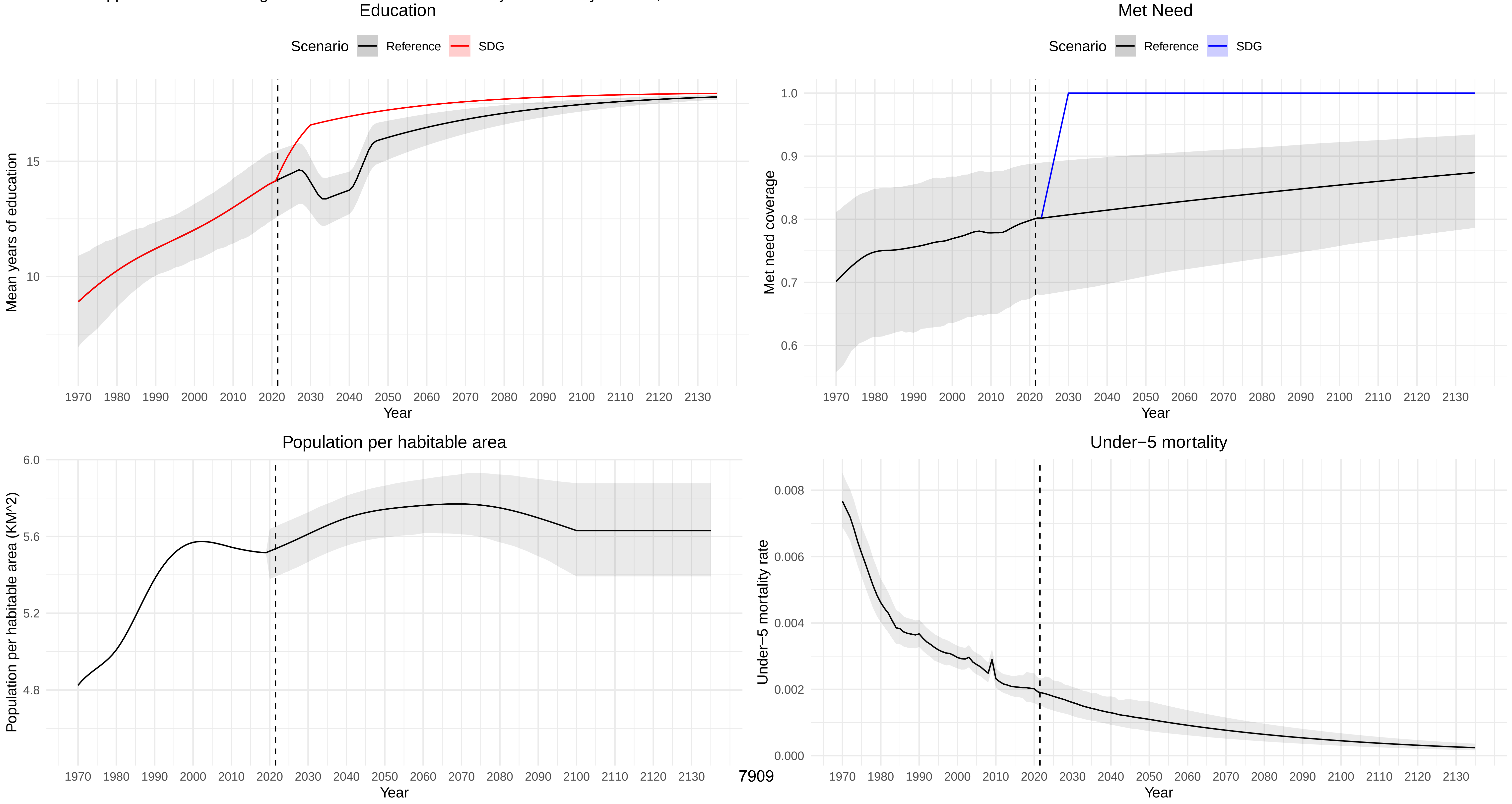

Cook Islands

Supplemental Results Figure S2: Covariates used in fertility forecasts by location, 1970-2135

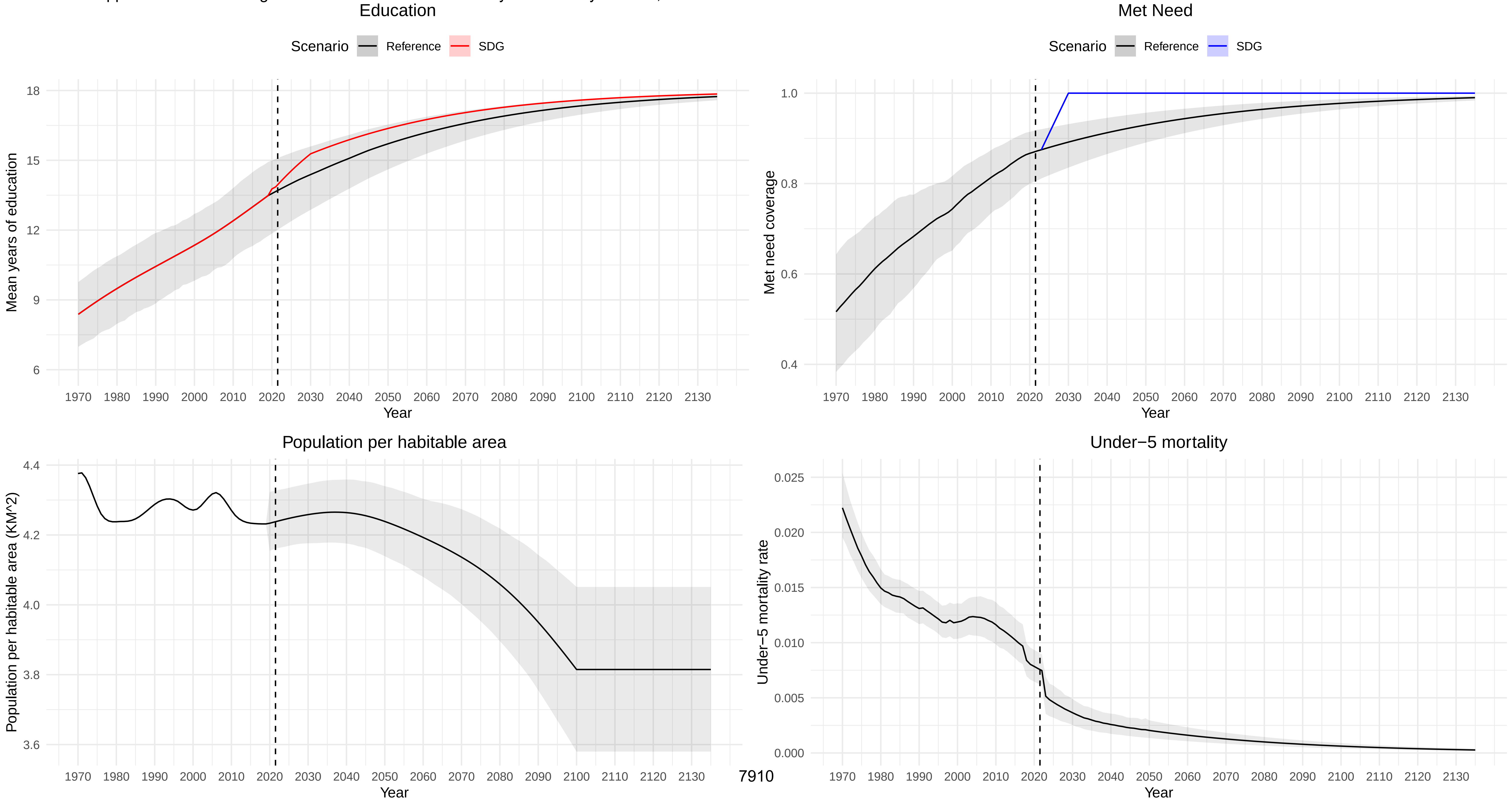

Fiji

Supplemental Results Figure S2: Covariates used in fertility forecasts by location, 1970-2135

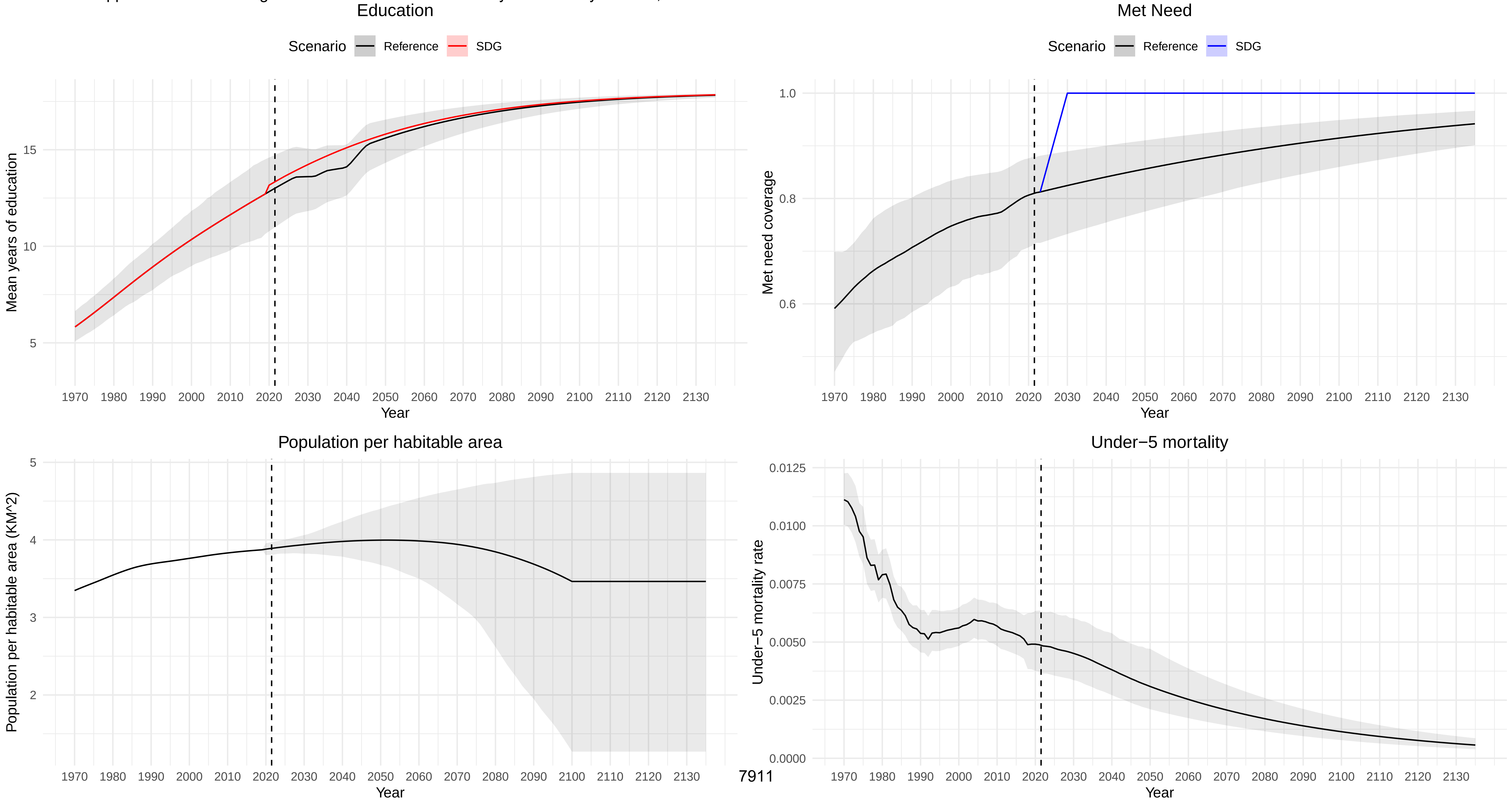

Guam

Supplemental Results Figure S2: Covariates used in fertility forecasts by location, 1970-2135

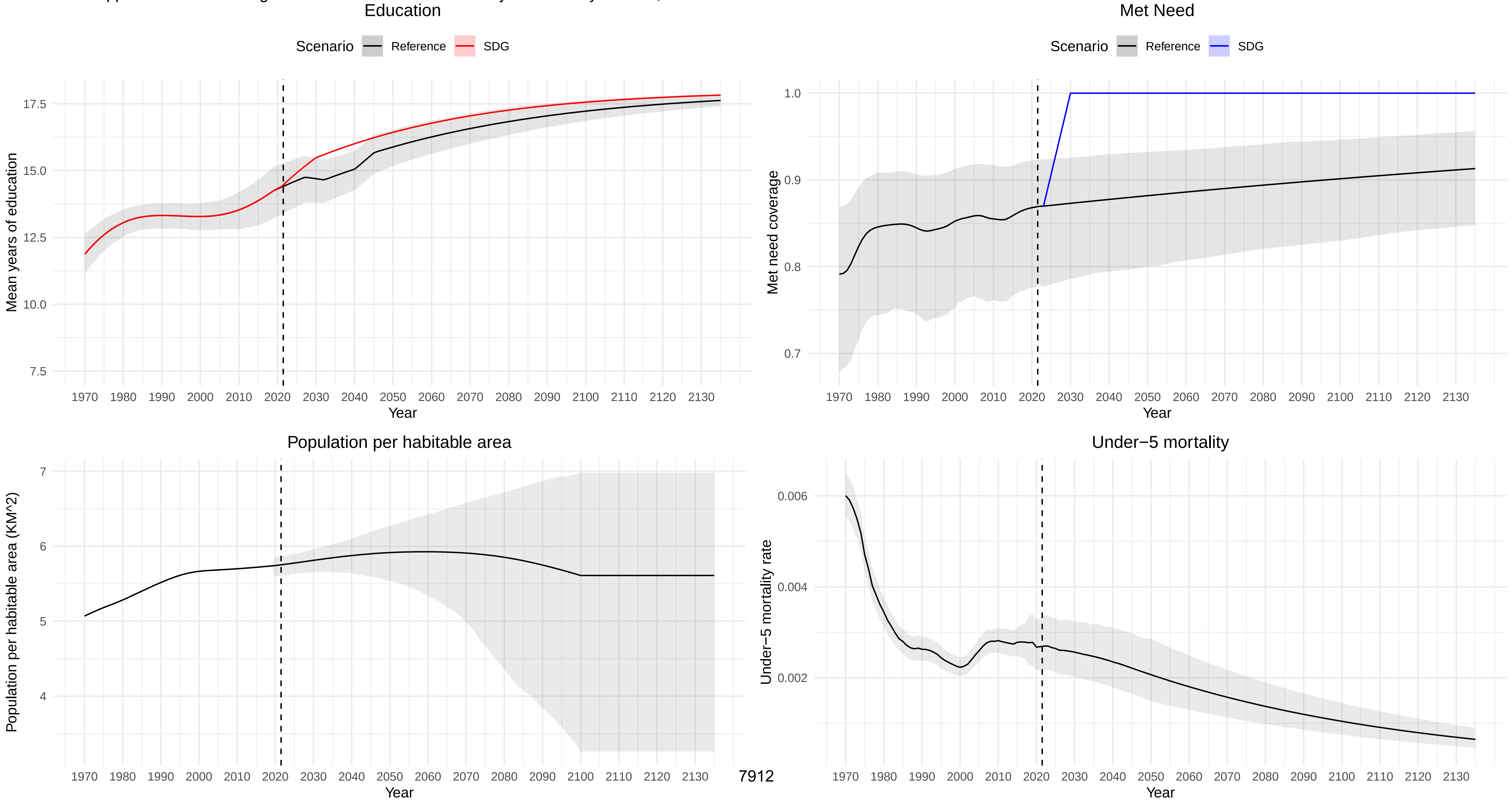

Kiribati

Supplemental Results Figure S2: Covariates used in fertility forecasts by location, 1970-2135

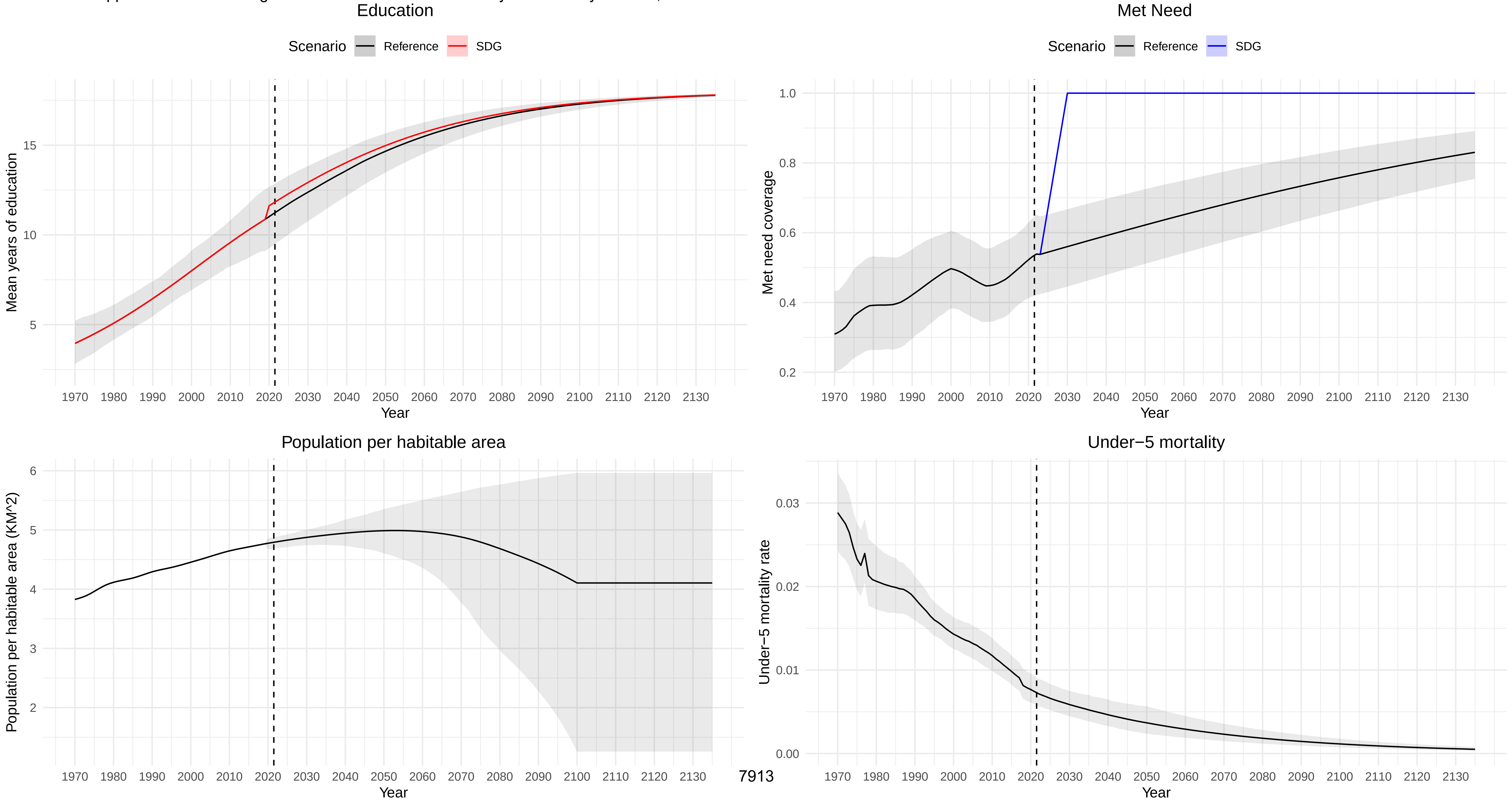

Marshall Islands

Supplemental Results Figure S2: Covariates used in fertility forecasts by location, 1970-2135

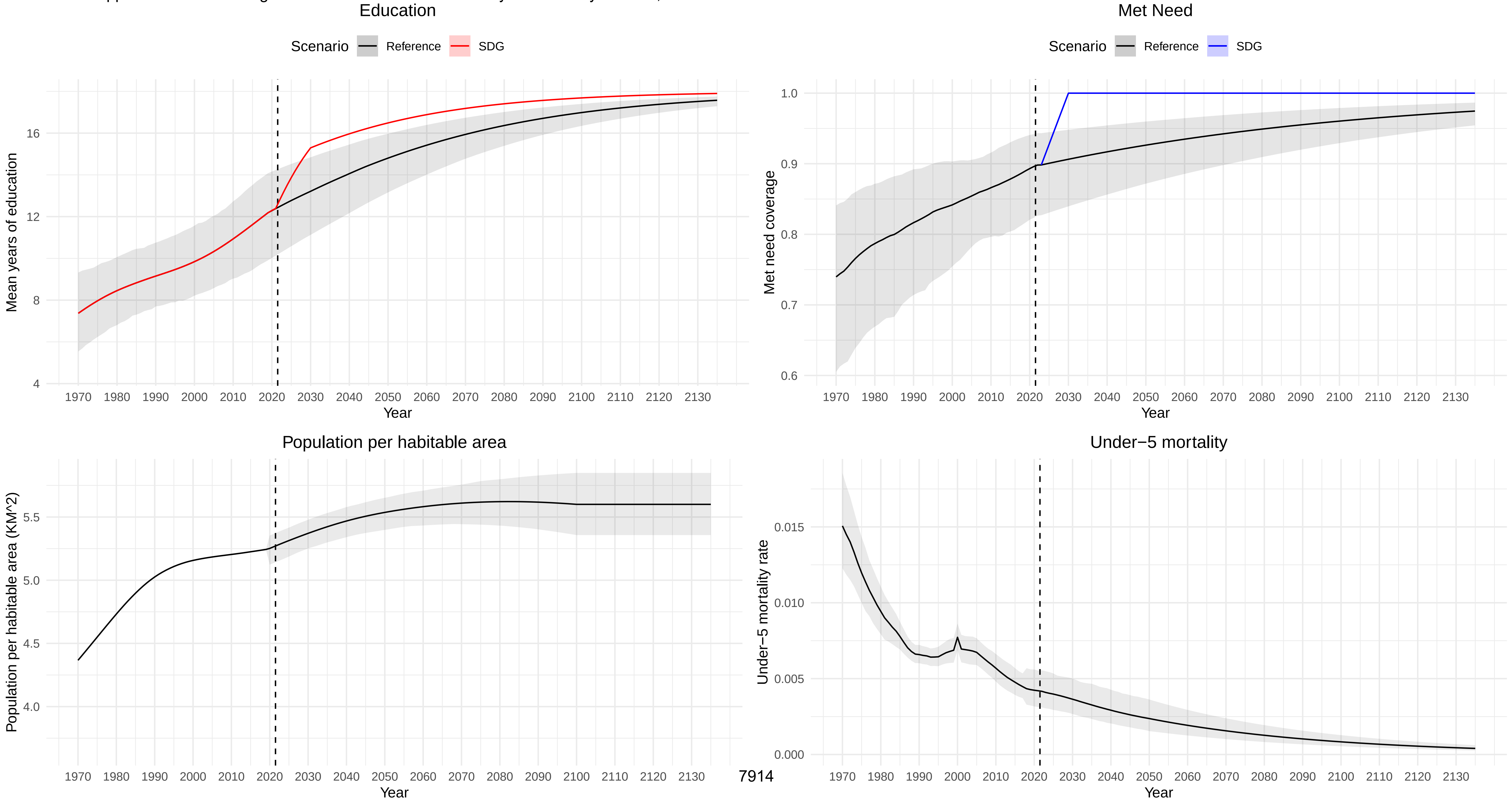

Micronesia (Federated States of)

Supplemental Results Figure S2: Covariates used in fertility forecasts by location, 1970-2135

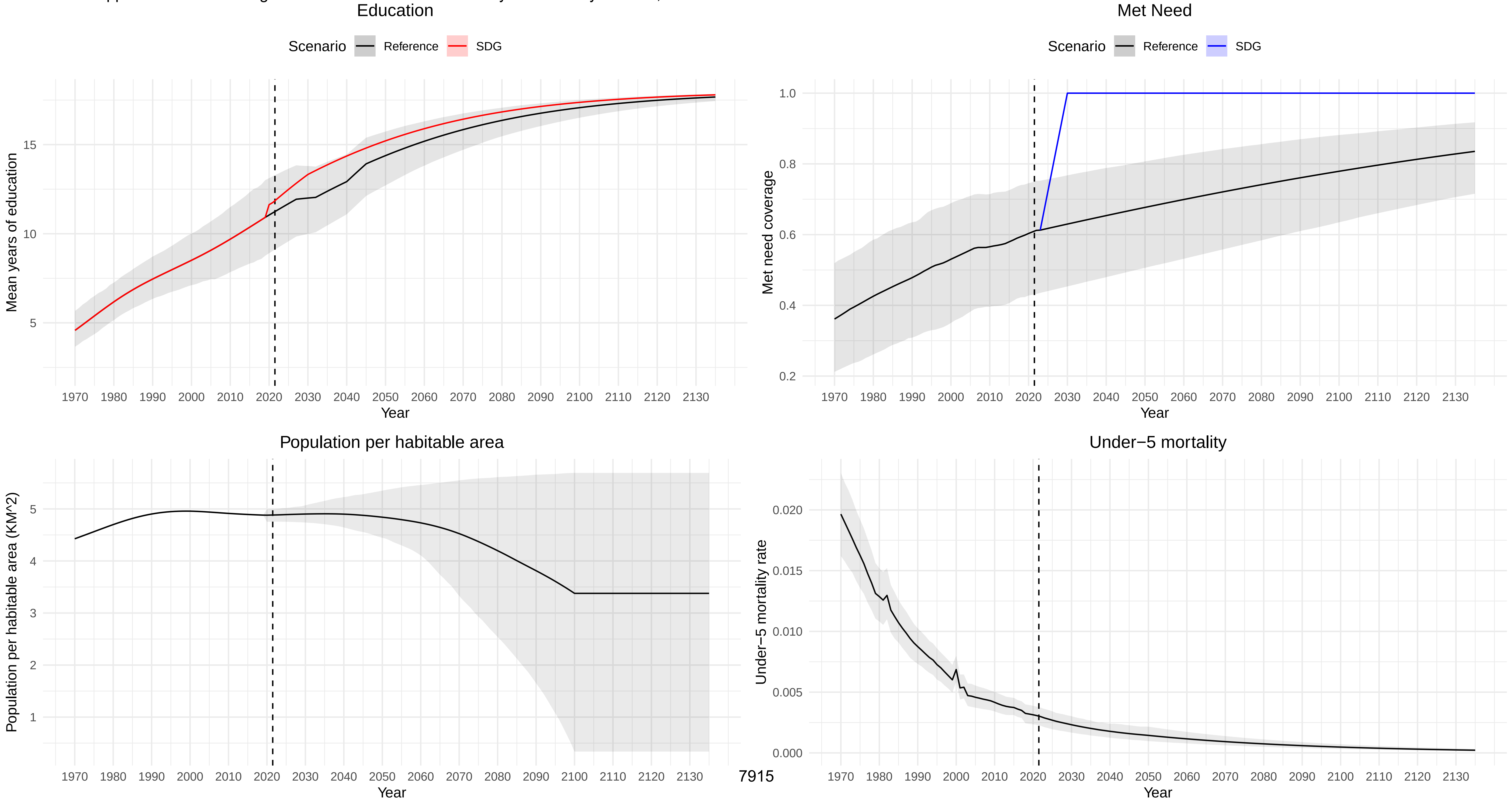

Nauru

Supplemental Results Figure S2: Covariates used in fertility forecasts by location, 1970-2135

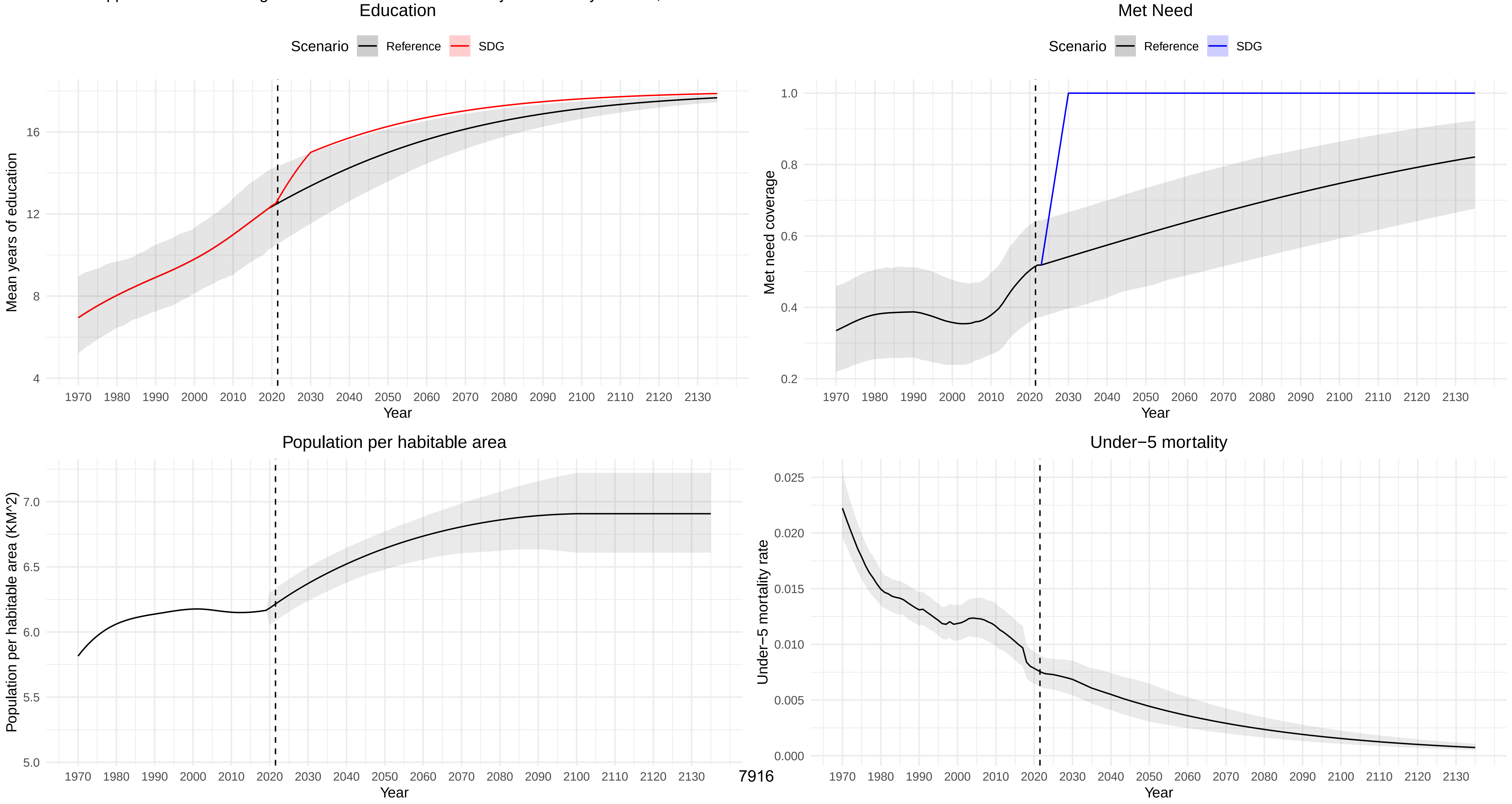

Niue

Supplemental Results Figure S2: Covariates used in fertility forecasts by location, 1970-2135

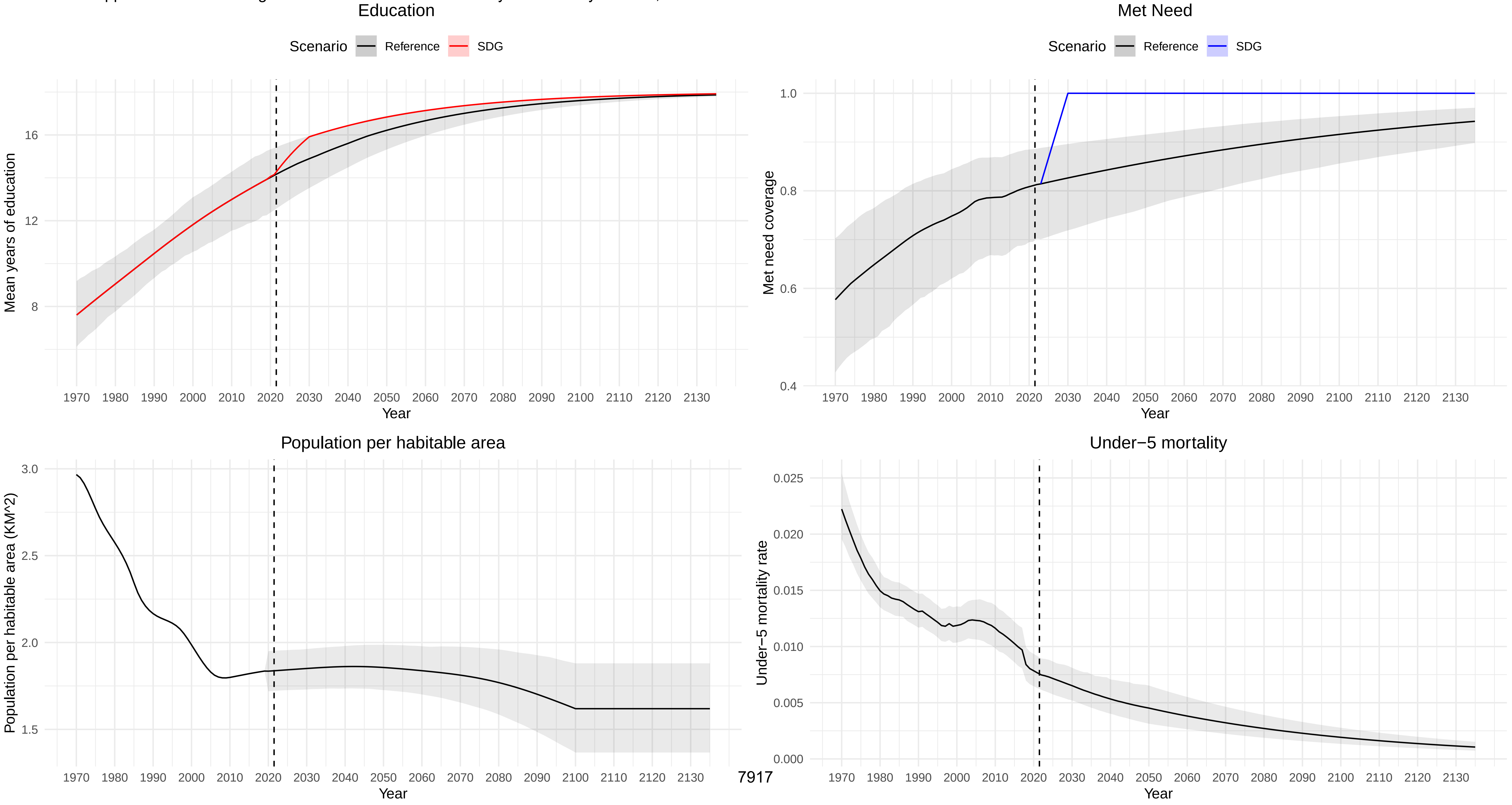

Northern Mariana Islands

Supplemental Results Figure S2: Covariates used in fertility forecasts by location, 1970-2135

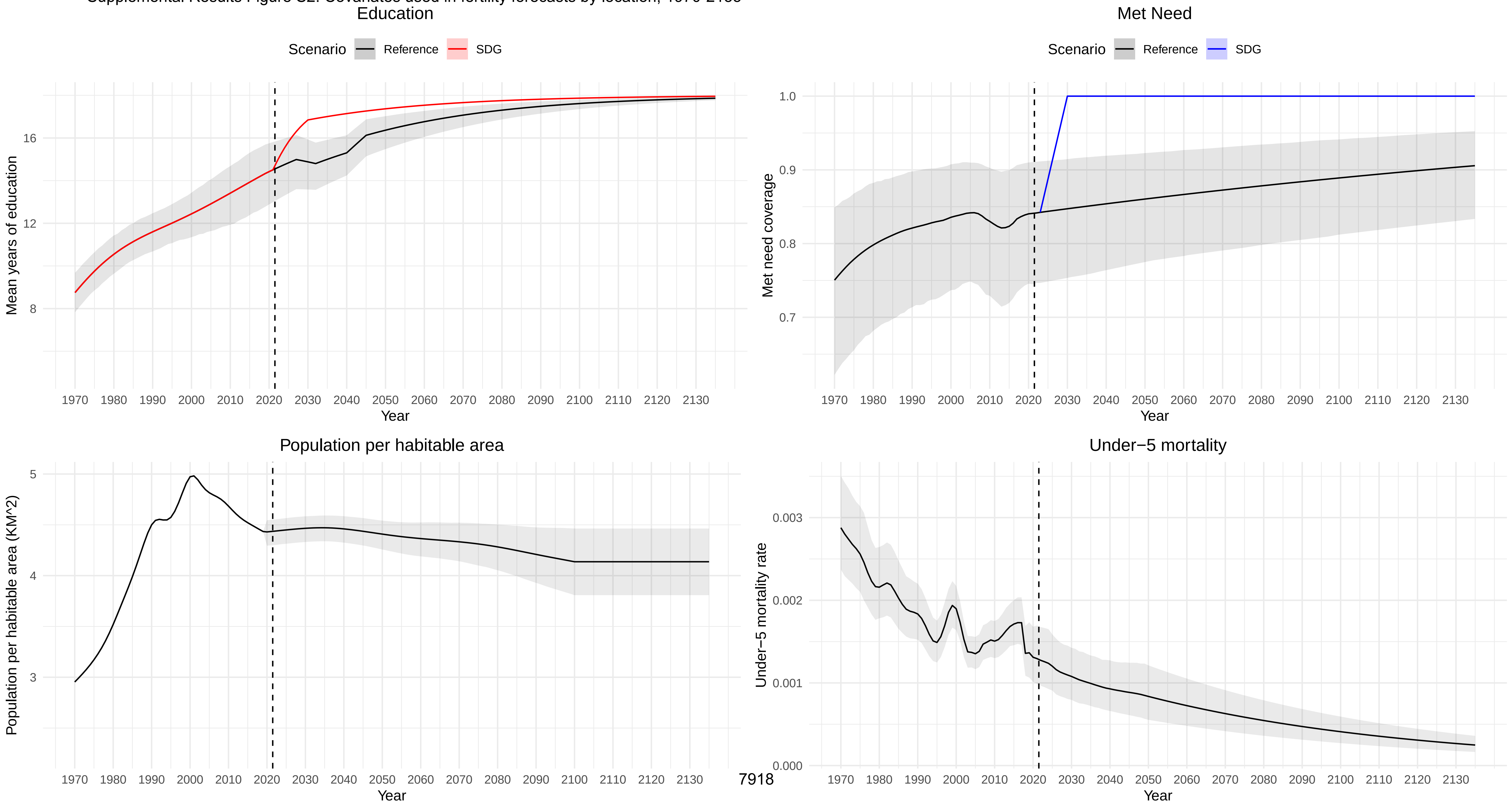

Palau

Supplemental Results Figure S2: Covariates used in fertility forecasts by location, 1970-2135

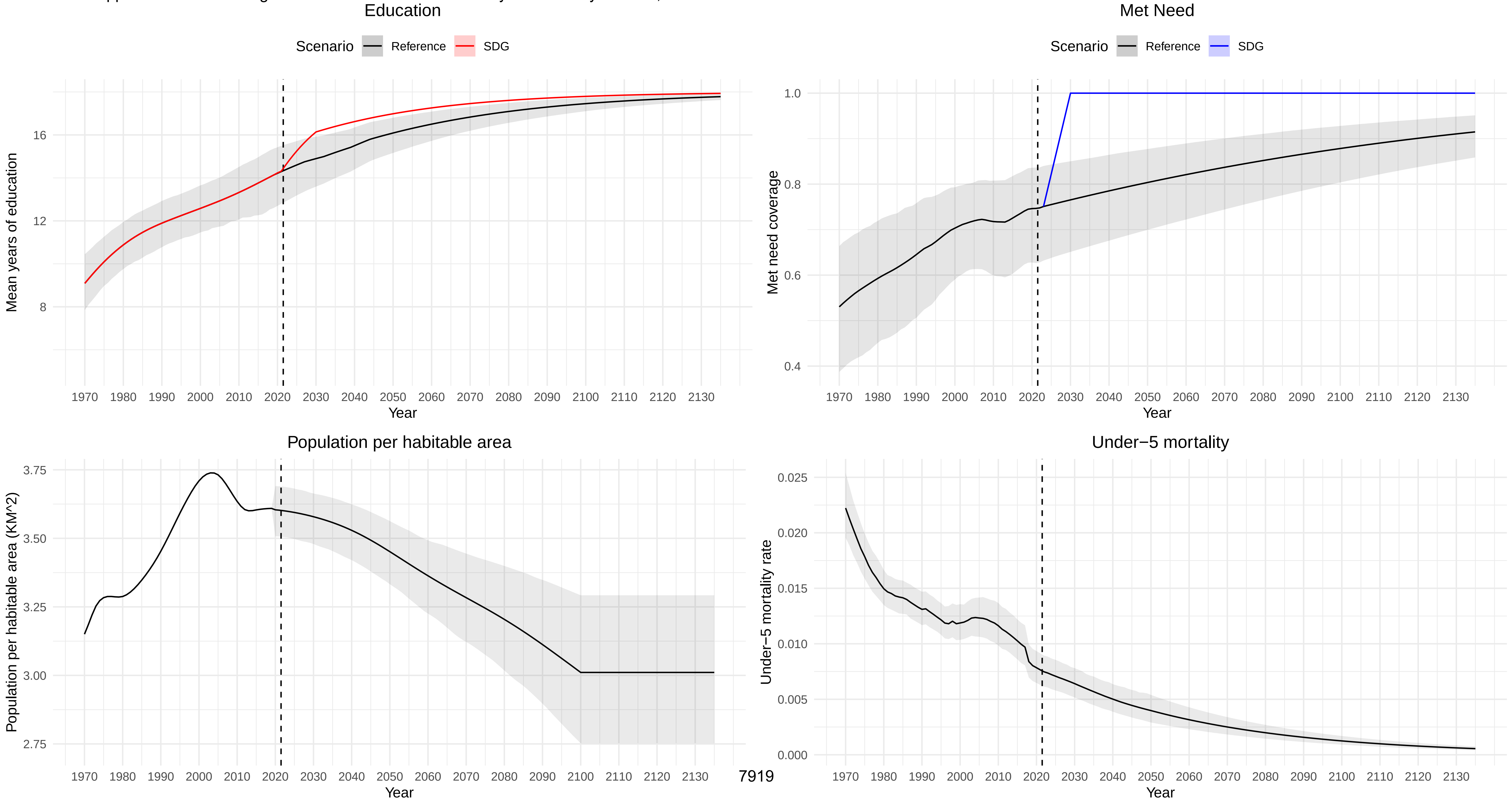

Papua New Guinea

Supplemental Results Figure S2: Covariates used in fertility forecasts by location, 1970-2135

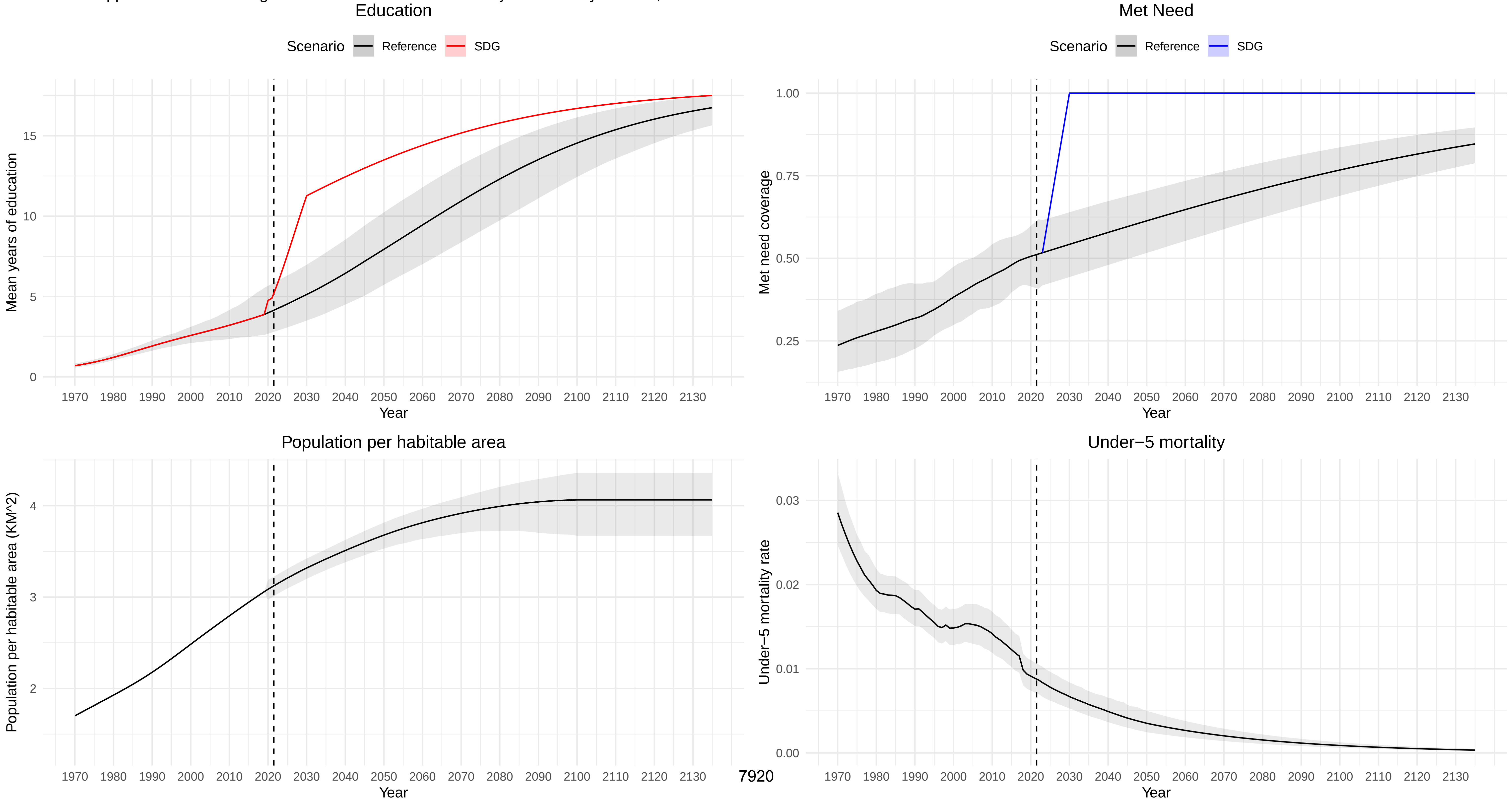

Samoa

Supplemental Results Figure S2: Covariates used in fertility forecasts by location, 1970-2135

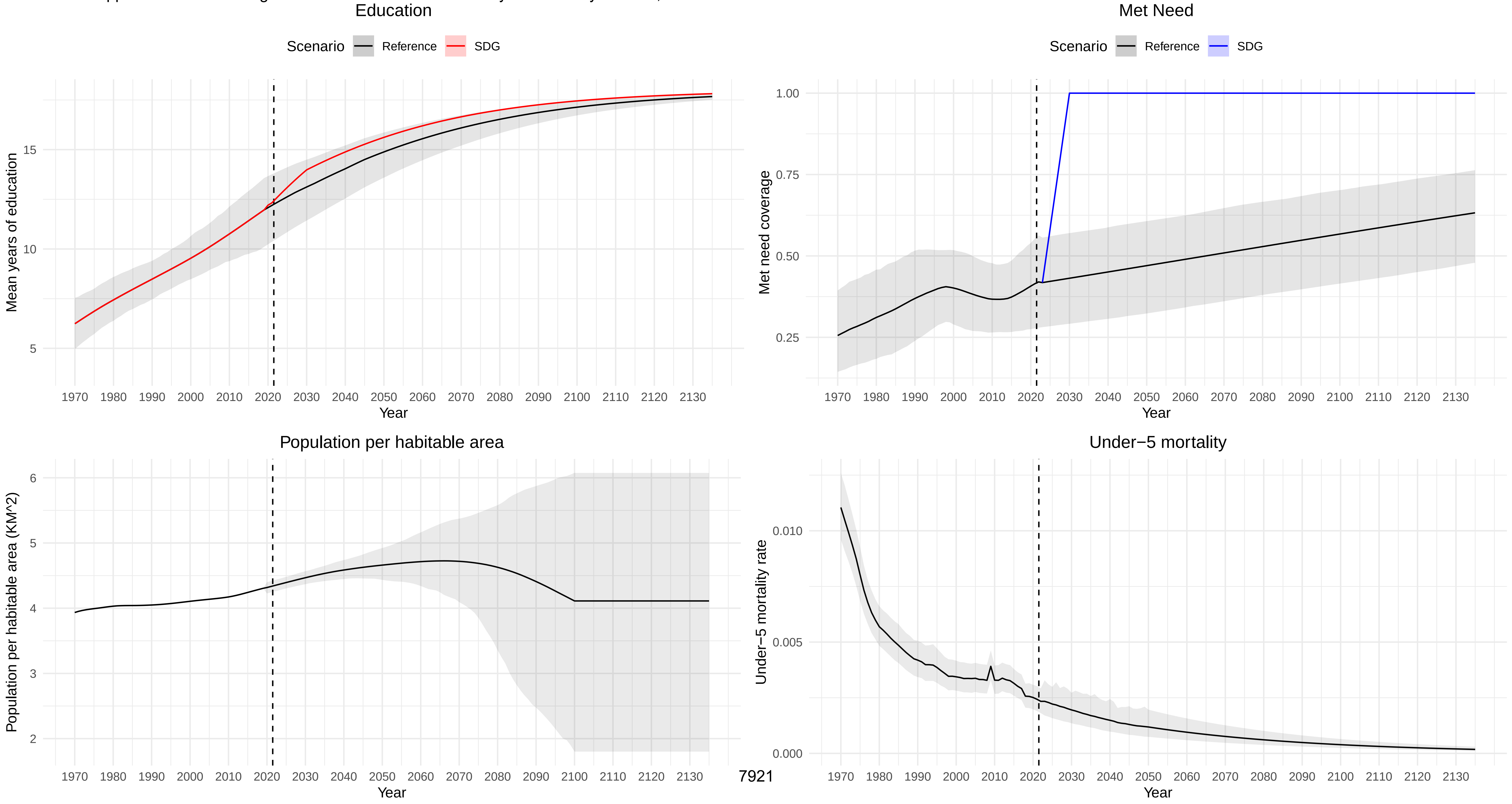

Solomon Islands

Supplemental Results Figure S2: Covariates used in fertility forecasts by location, 1970-2135

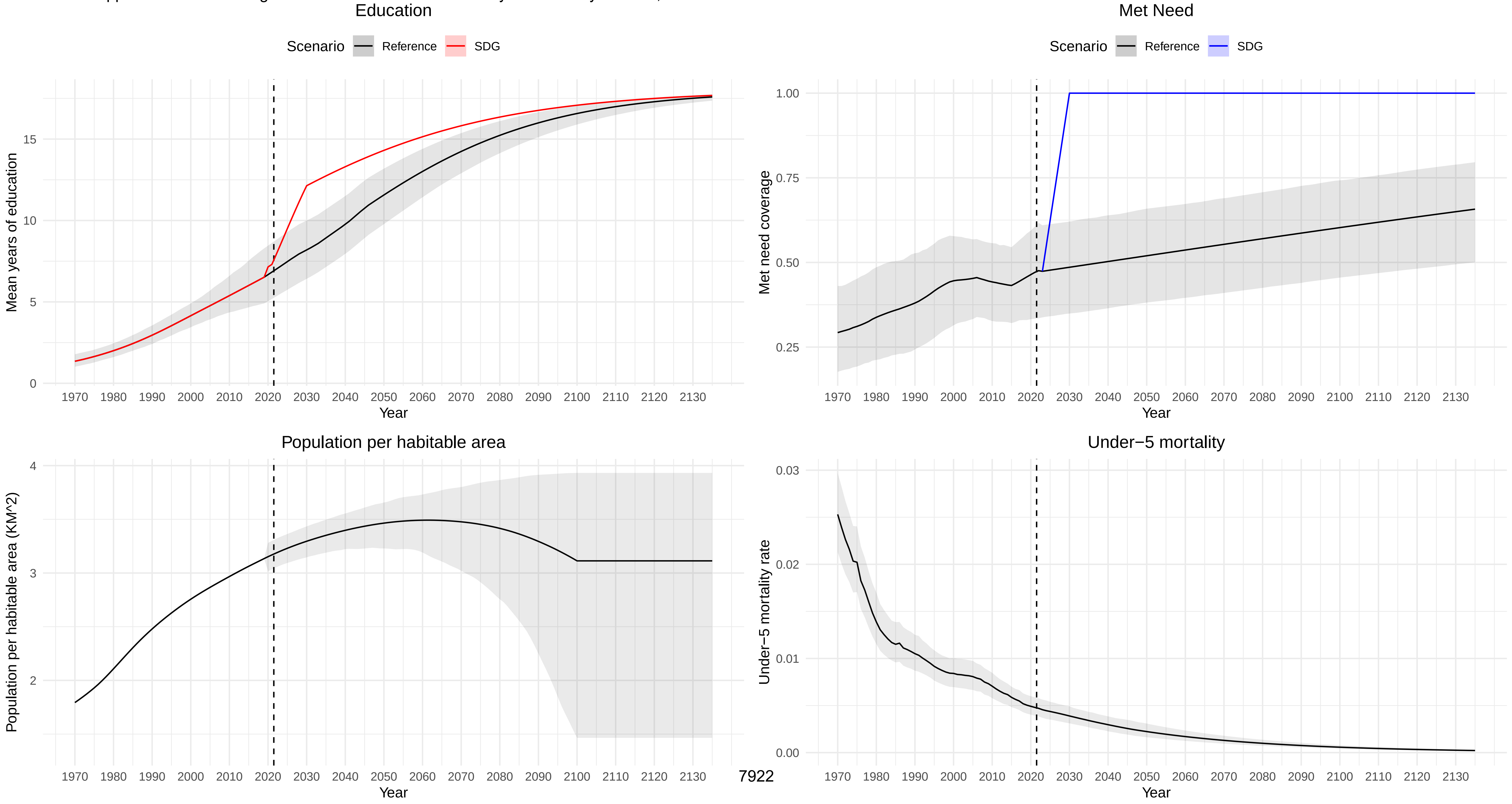

Tokelau

Supplemental Results Figure S2: Covariates used in fertility forecasts by location, 1970-2135

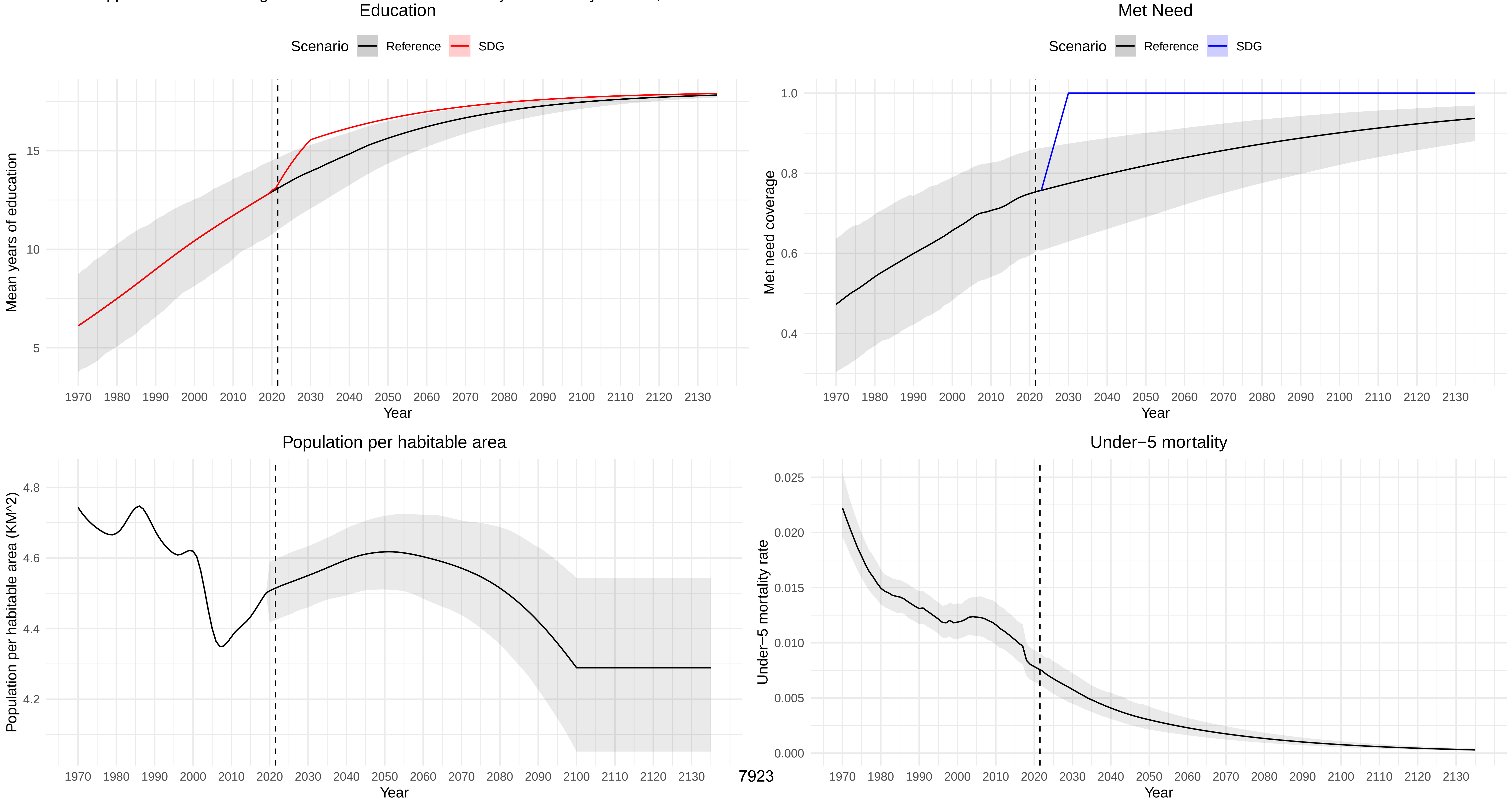

Tonga

Supplemental Results Figure S2: Covariates used in fertility forecasts by location, 1970-2135

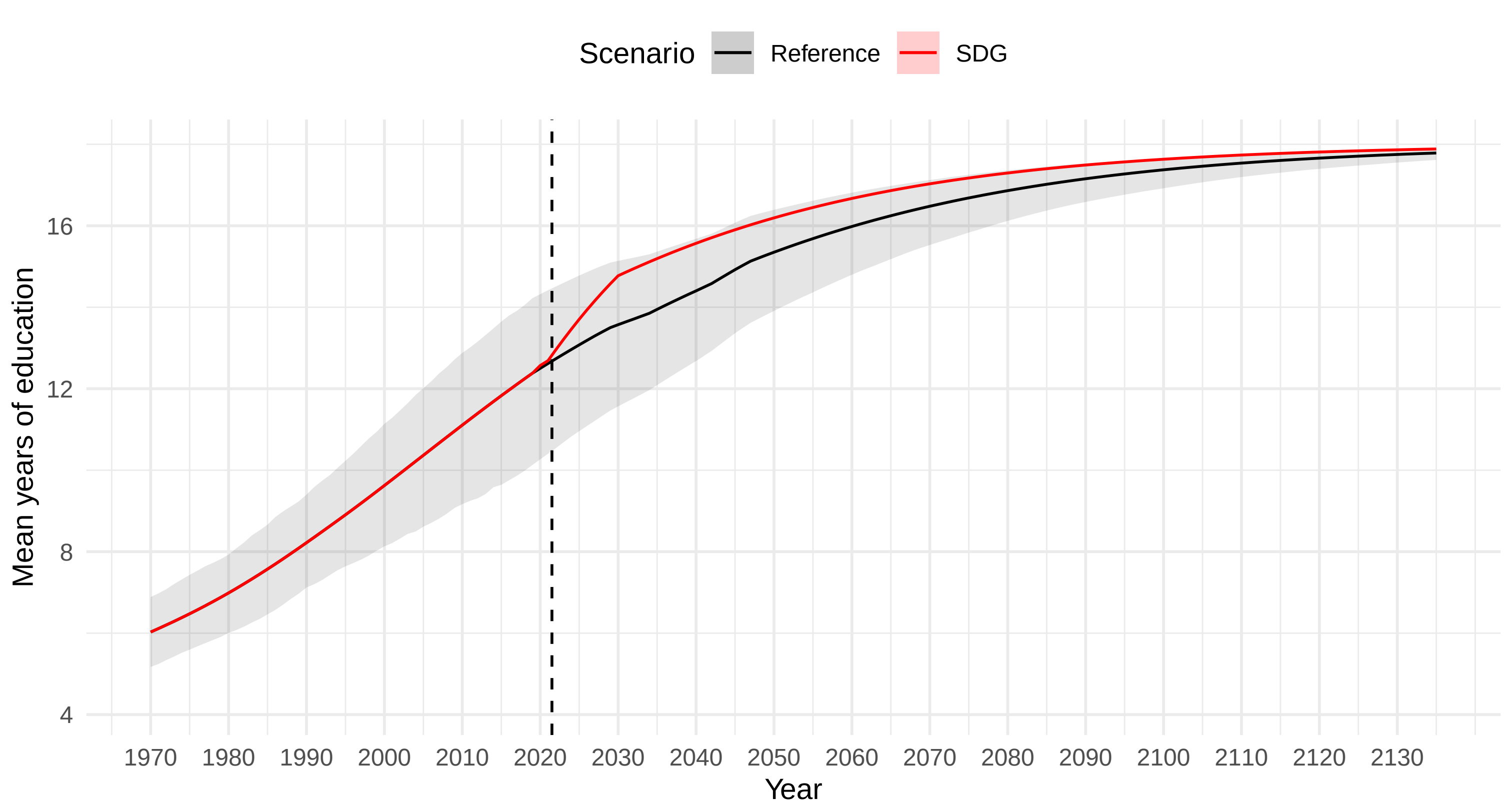

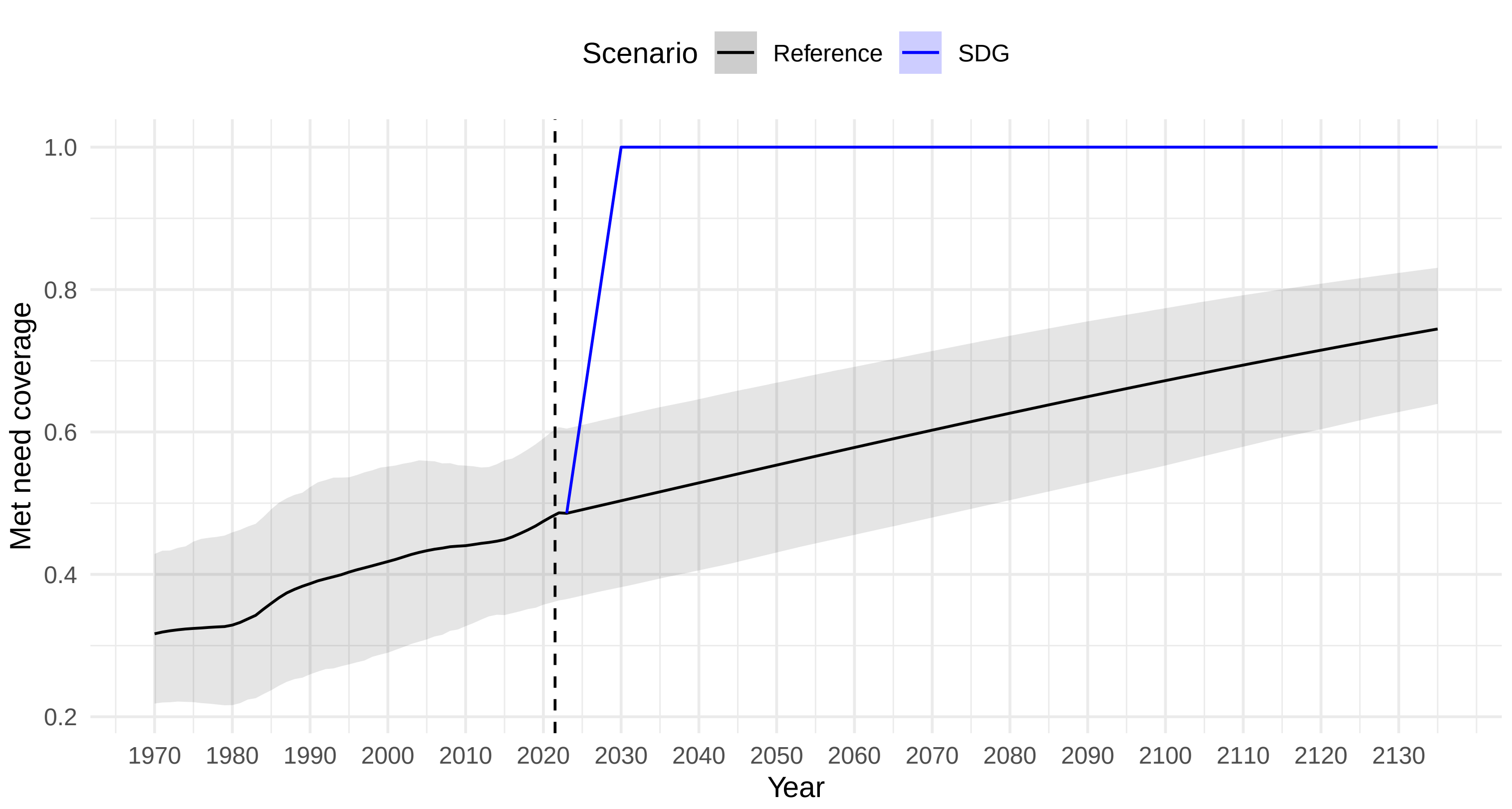

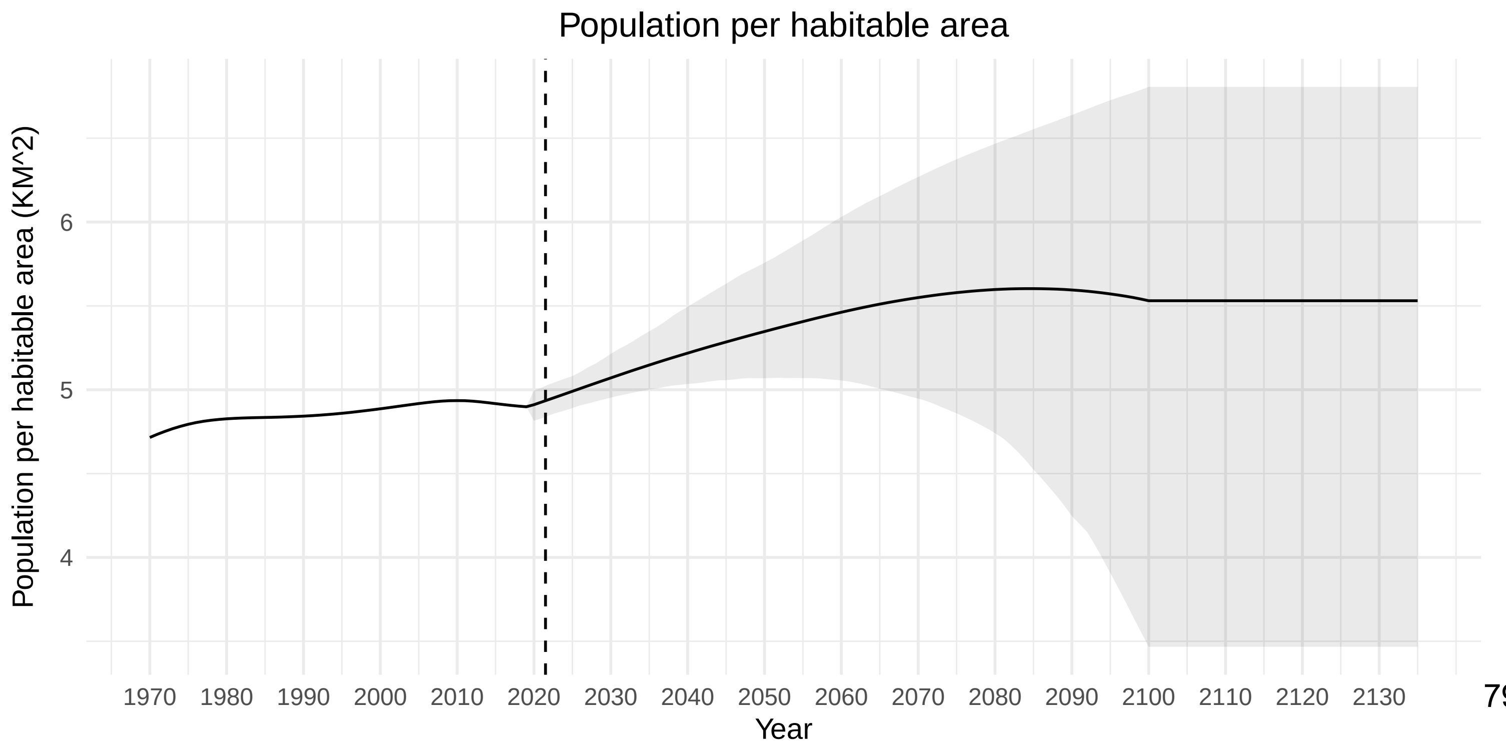

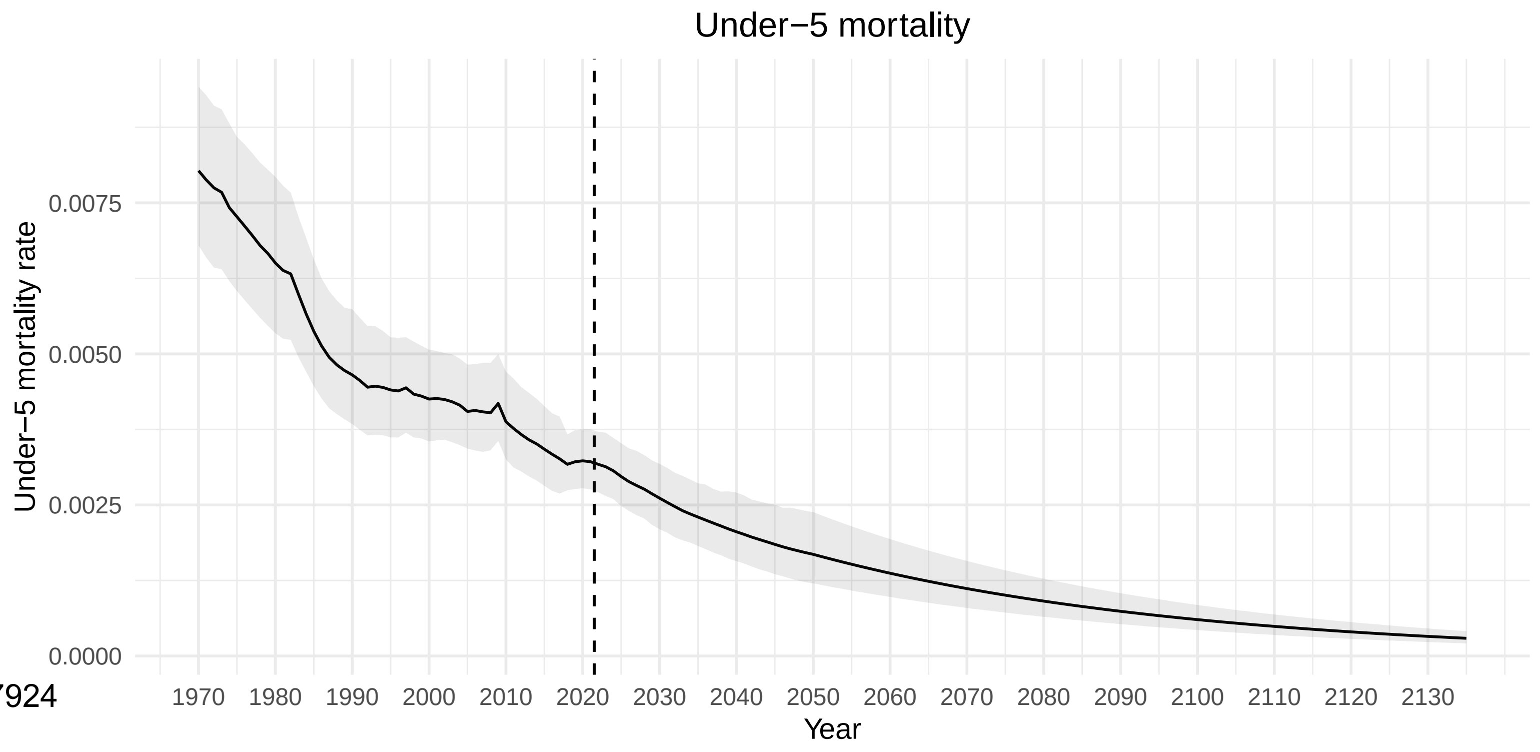

Tuvalu

Supplemental Results Figure S2: Covariates used in fertility forecasts by location, 1970-2135

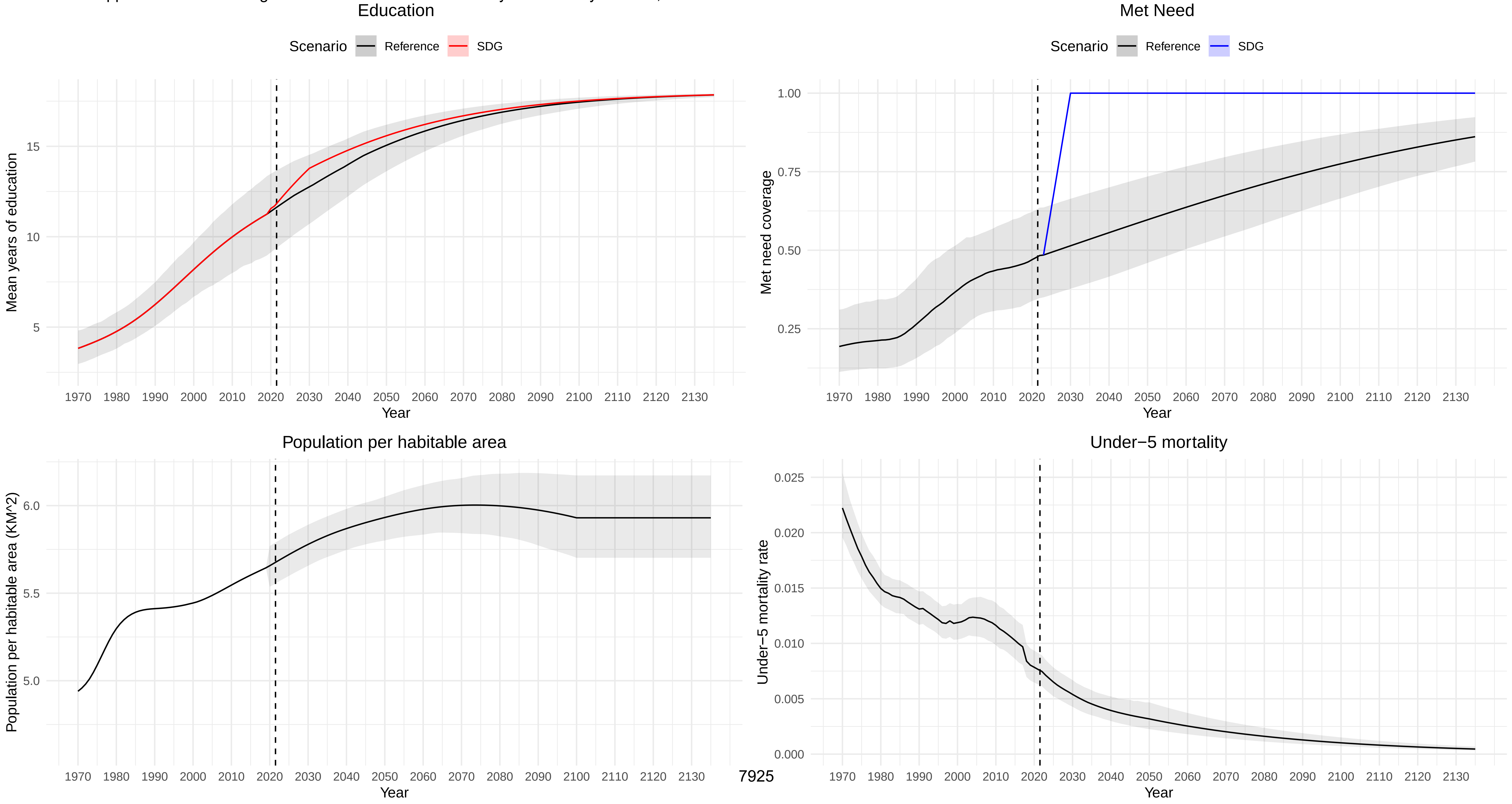

Vanuatu

Supplemental Results Figure S2: Covariates used in fertility forecasts by location, 1970-2135

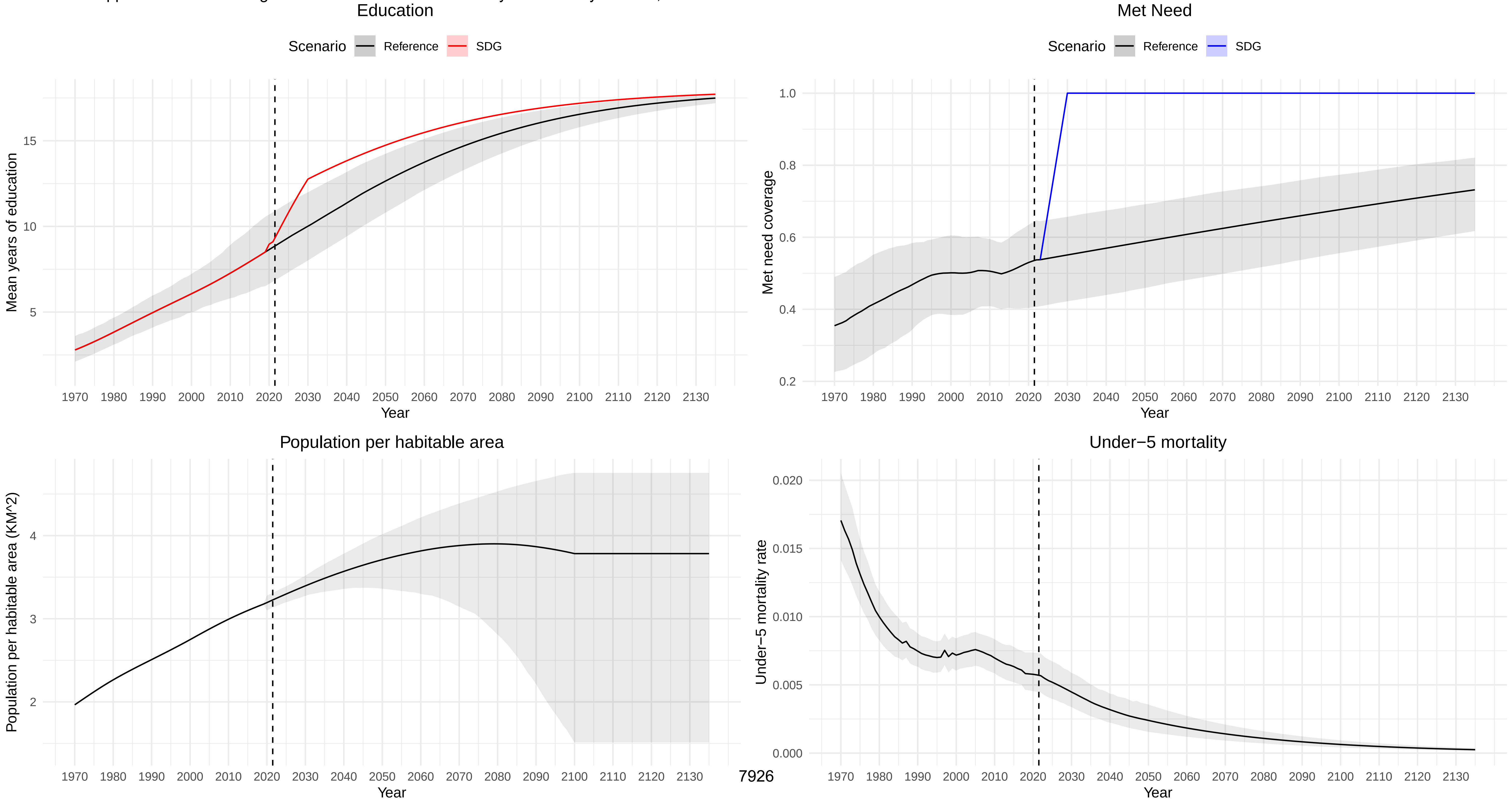

Cambodia

Supplemental Results Figure S2: Covariates used in fertility forecasts by location, 1970-2135

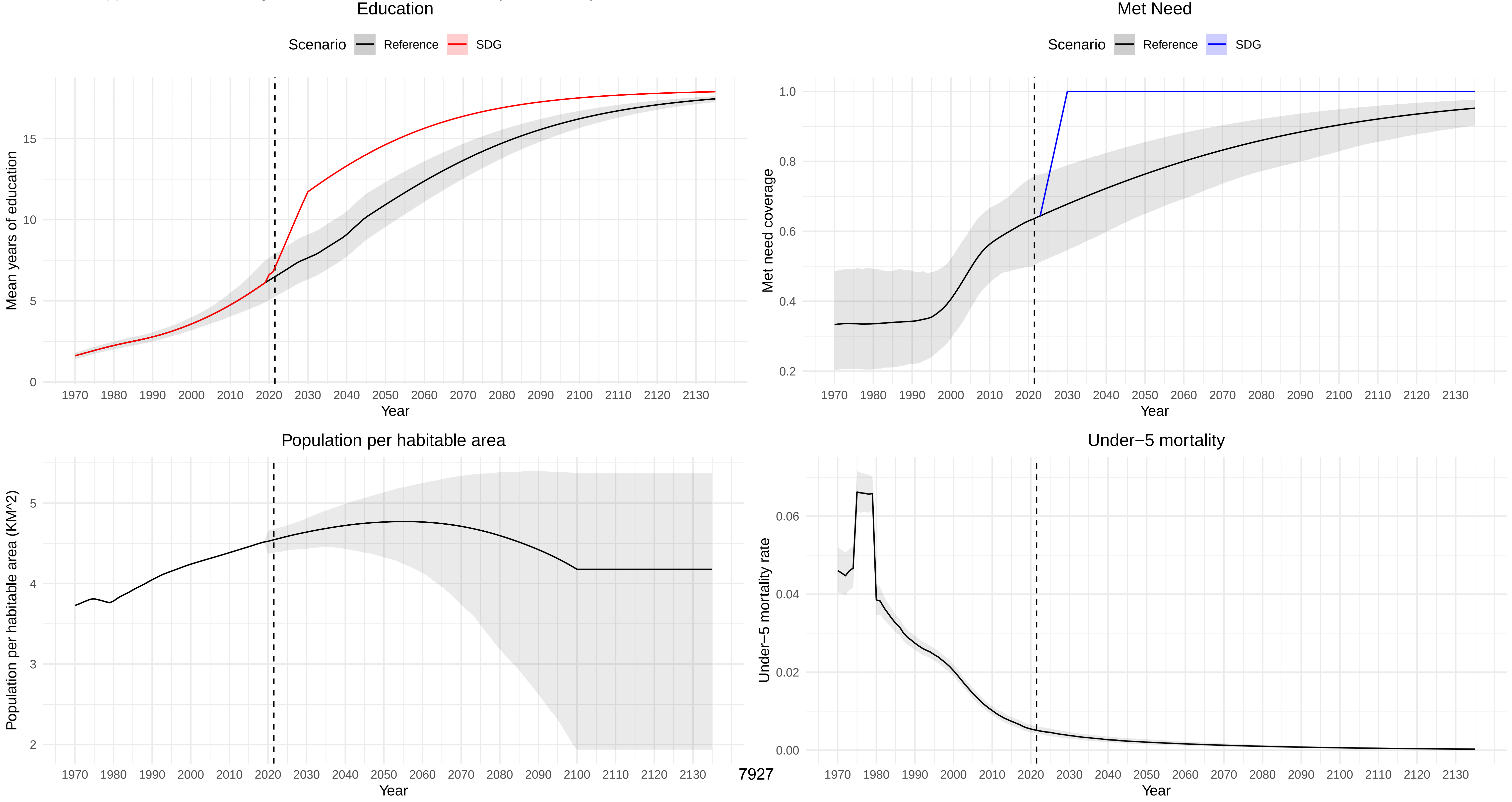

Indonesia

Supplemental Results Figure S2: Covariates used in fertility forecasts by location, 1970-2135

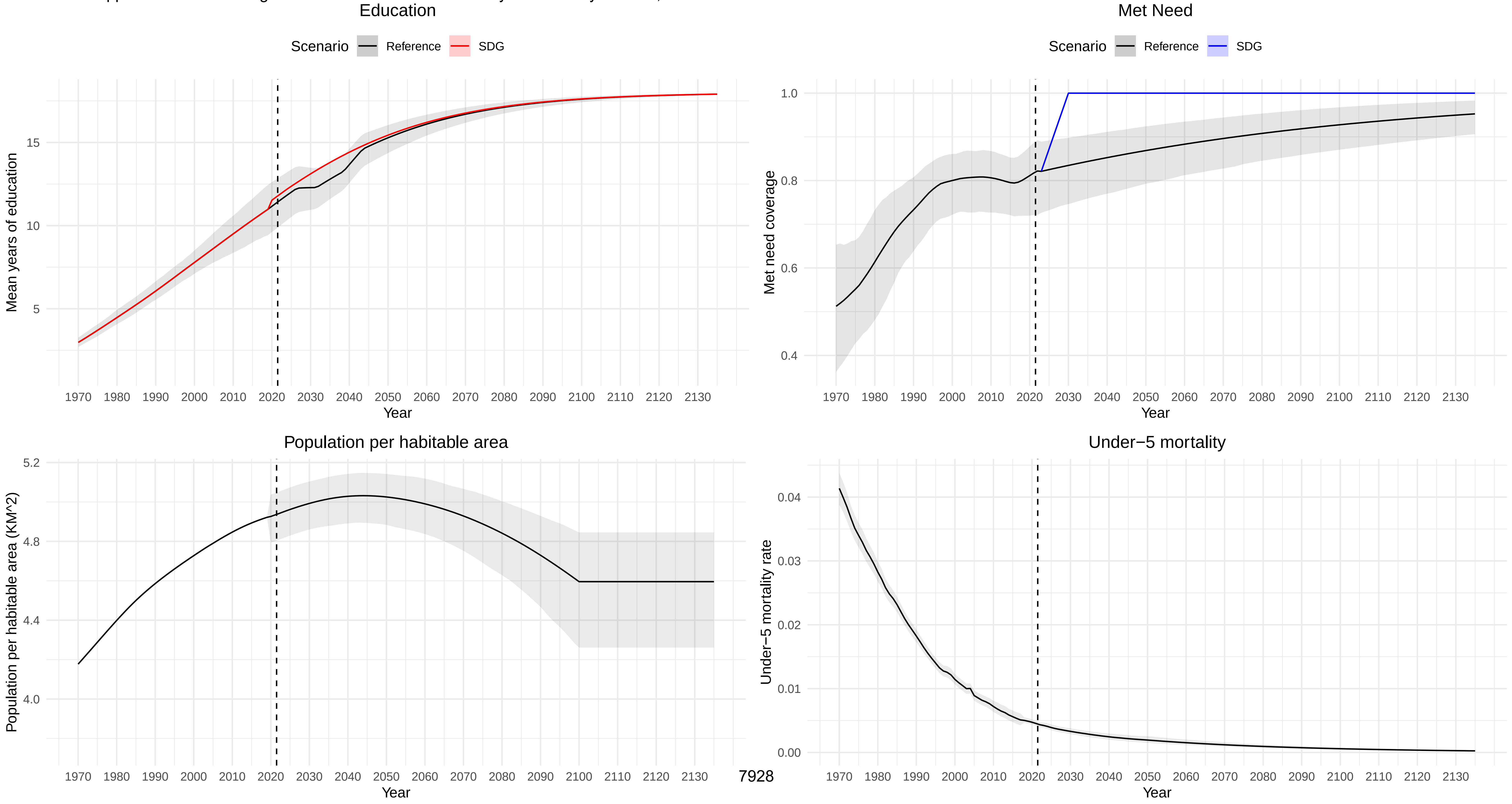

Lao People's Democratic Republic

Supplemental Results Figure S2: Covariates used in fertility forecasts by location, 1970-2135

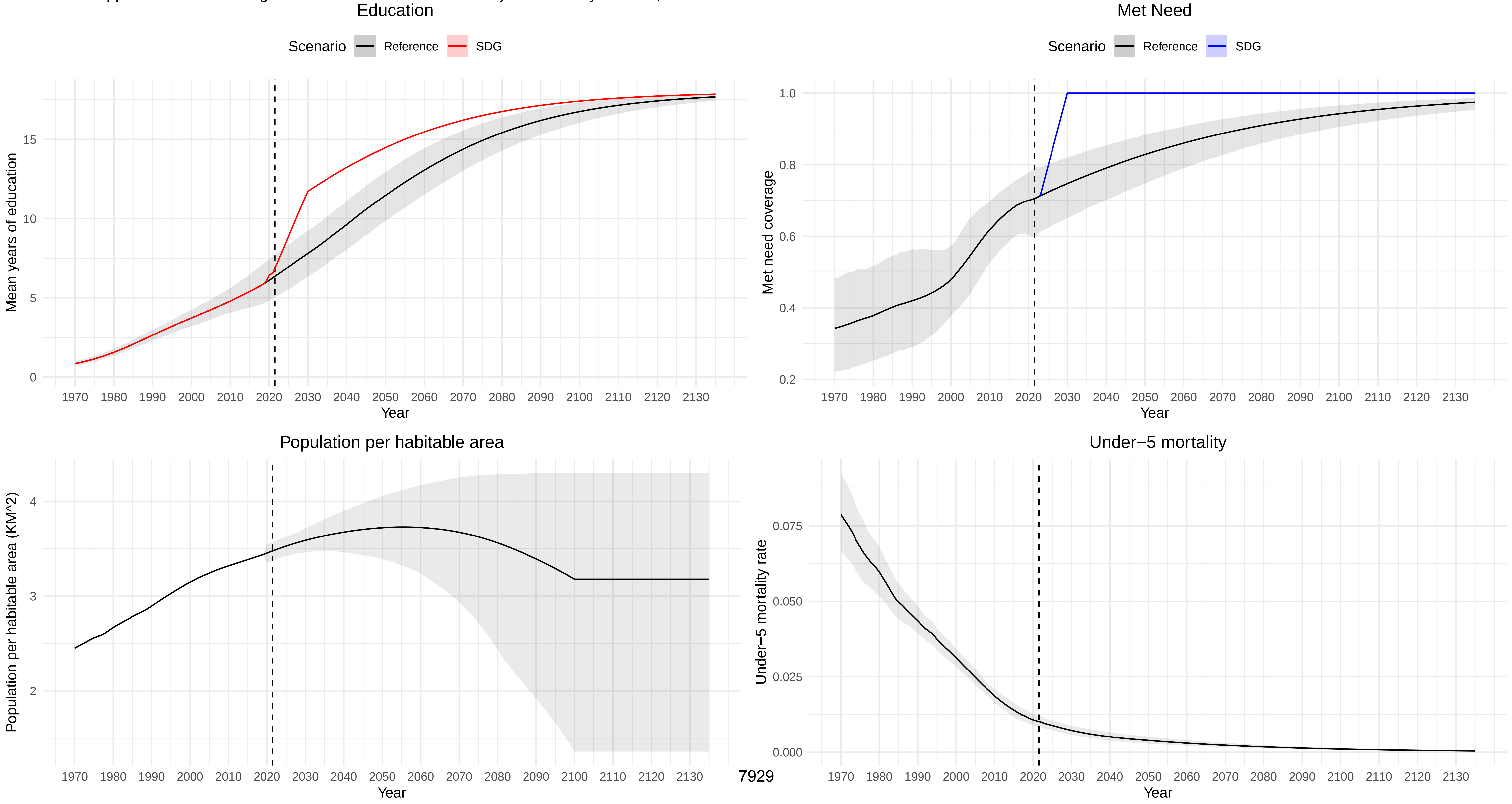

Malaysia

Supplemental Results Figure S2: Covariates used in fertility forecasts by location, 1970-2135

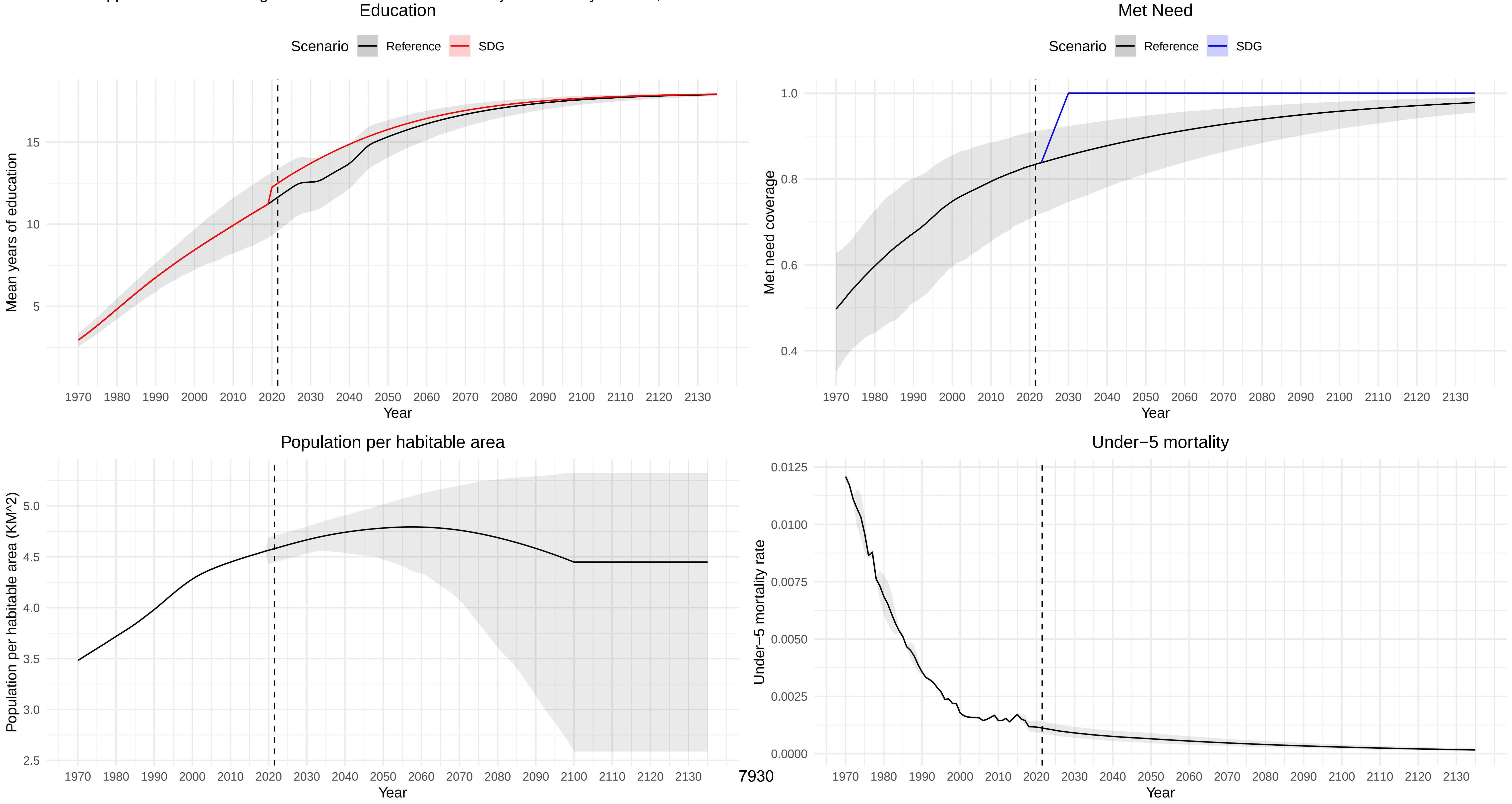

Maldives

Supplemental Results Figure S2: Covariates used in fertility forecasts by location, 1970-2135

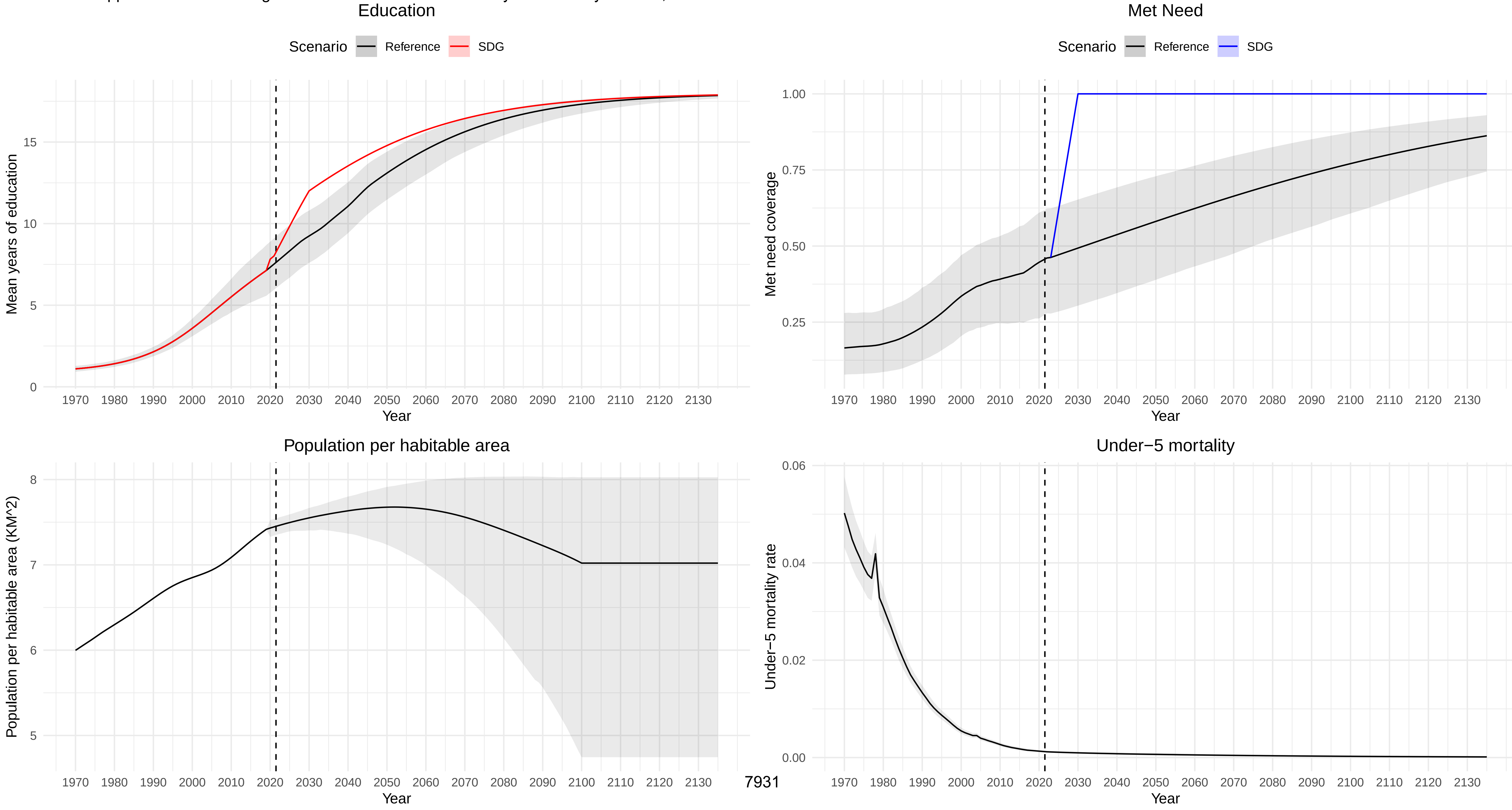

Mauritius

Supplemental Results Figure S2: Covariates used in fertility forecasts by location, 1970-2135

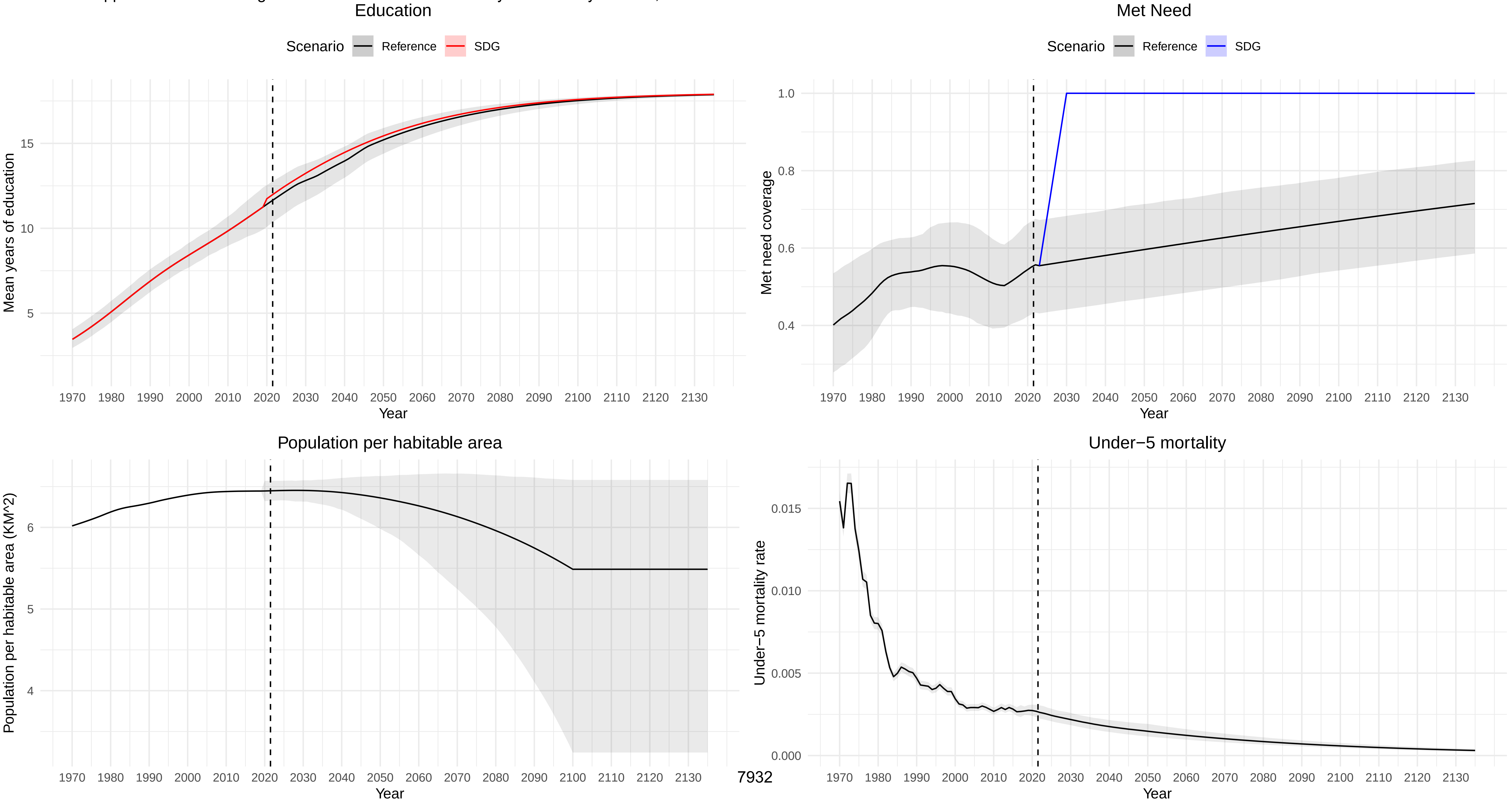

Myanmar

Supplemental Results Figure S2: Covariates used in fertility forecasts by location, 1970-2135

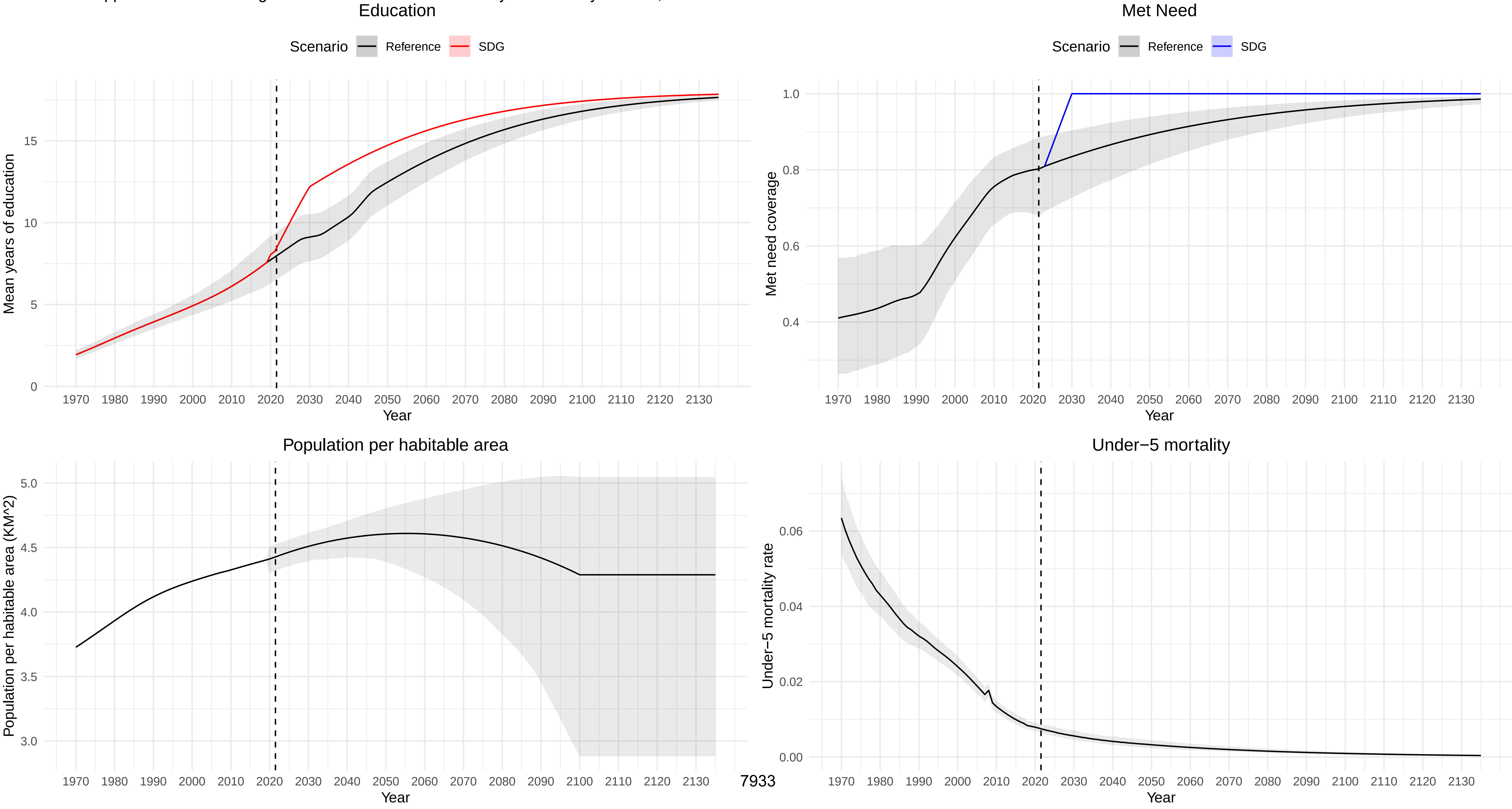

Philippines

Supplemental Results Figure S2: Covariates used in fertility forecasts by location, 1970-2135

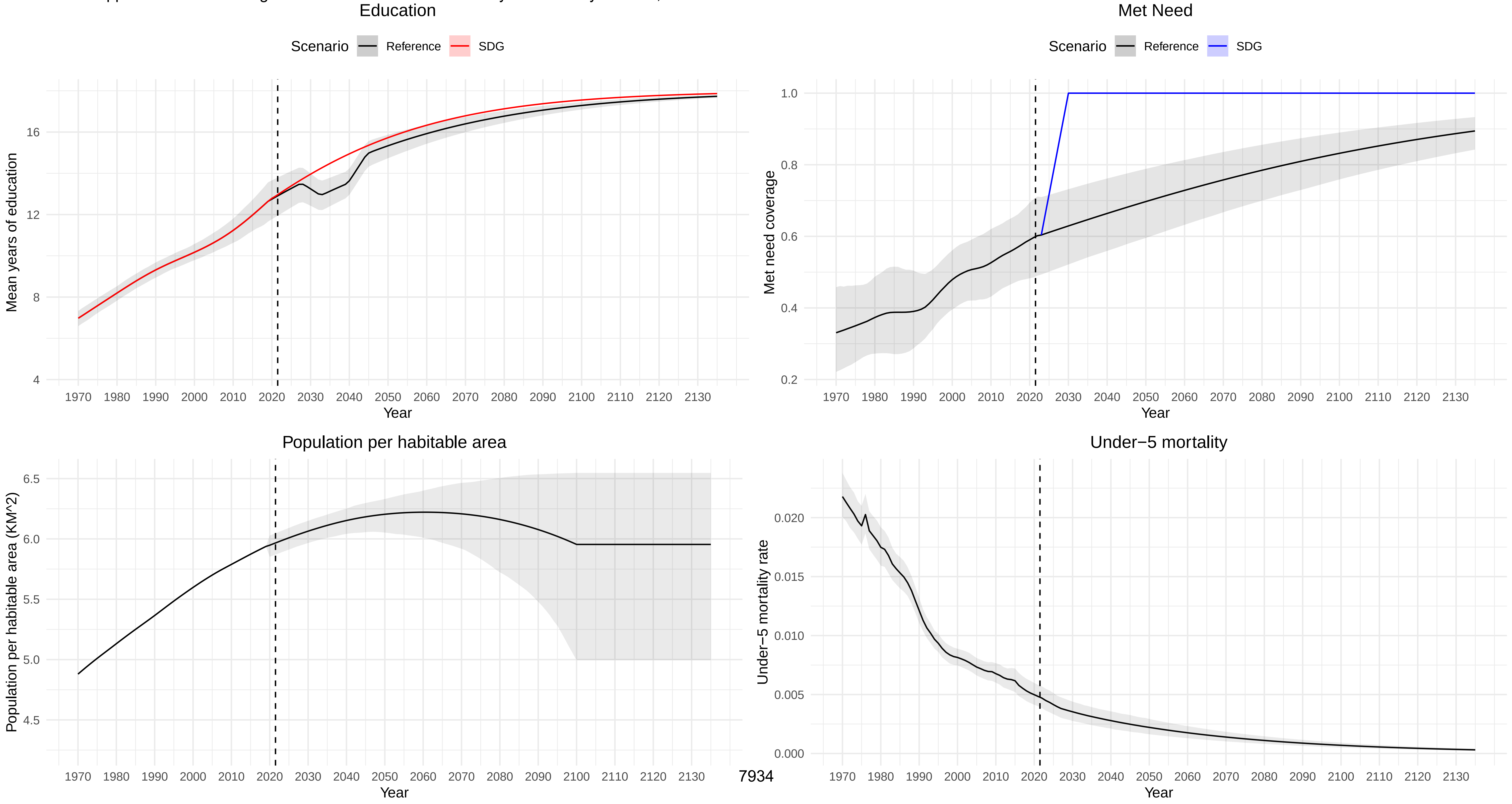

Seychelles

Supplemental Results Figure S2: Covariates used in fertility forecasts by location, 1970-2135

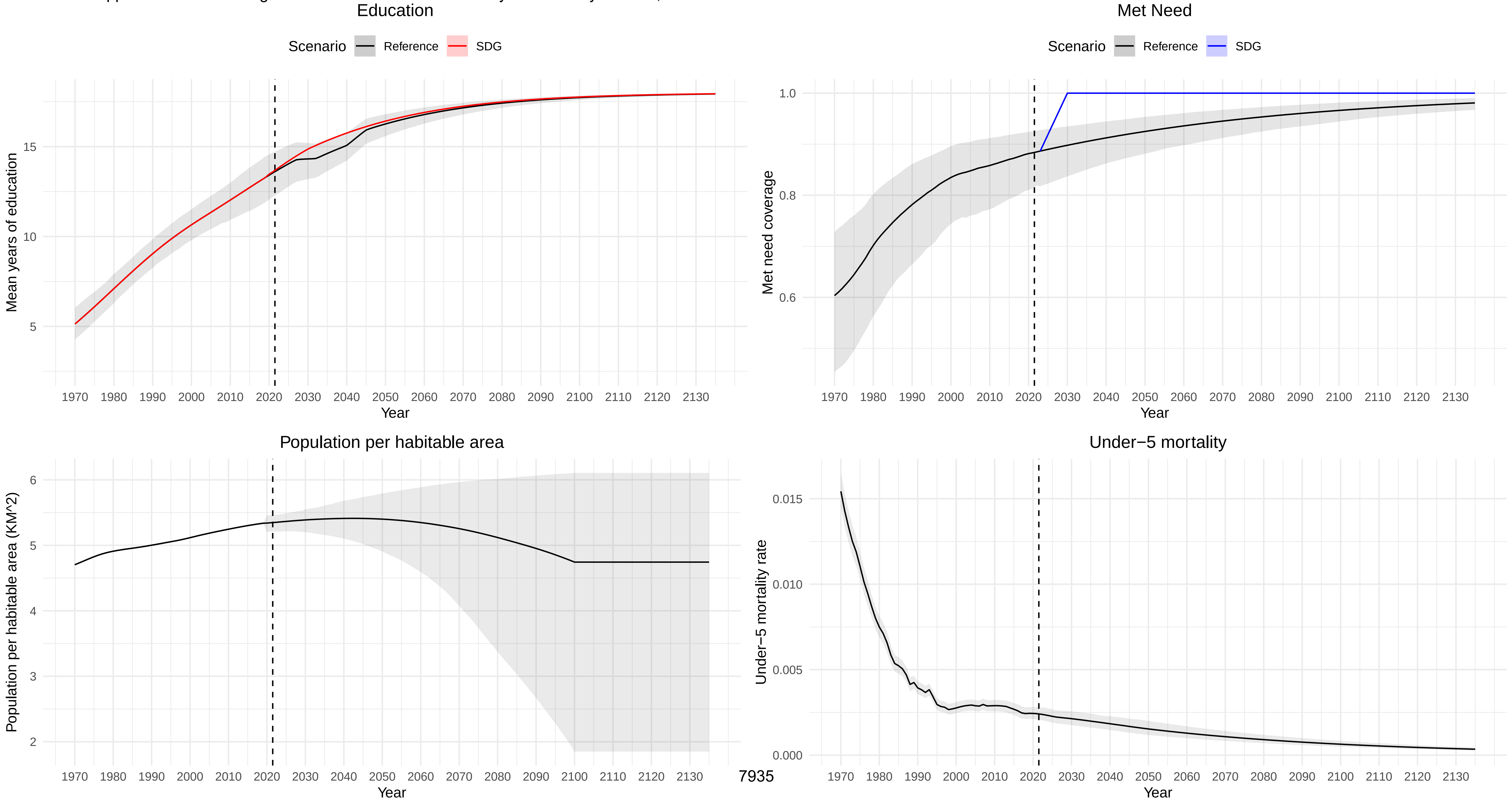

Sri Lanka

Supplemental Results Figure S2: Covariates used in fertility forecasts by location, 1970-2135

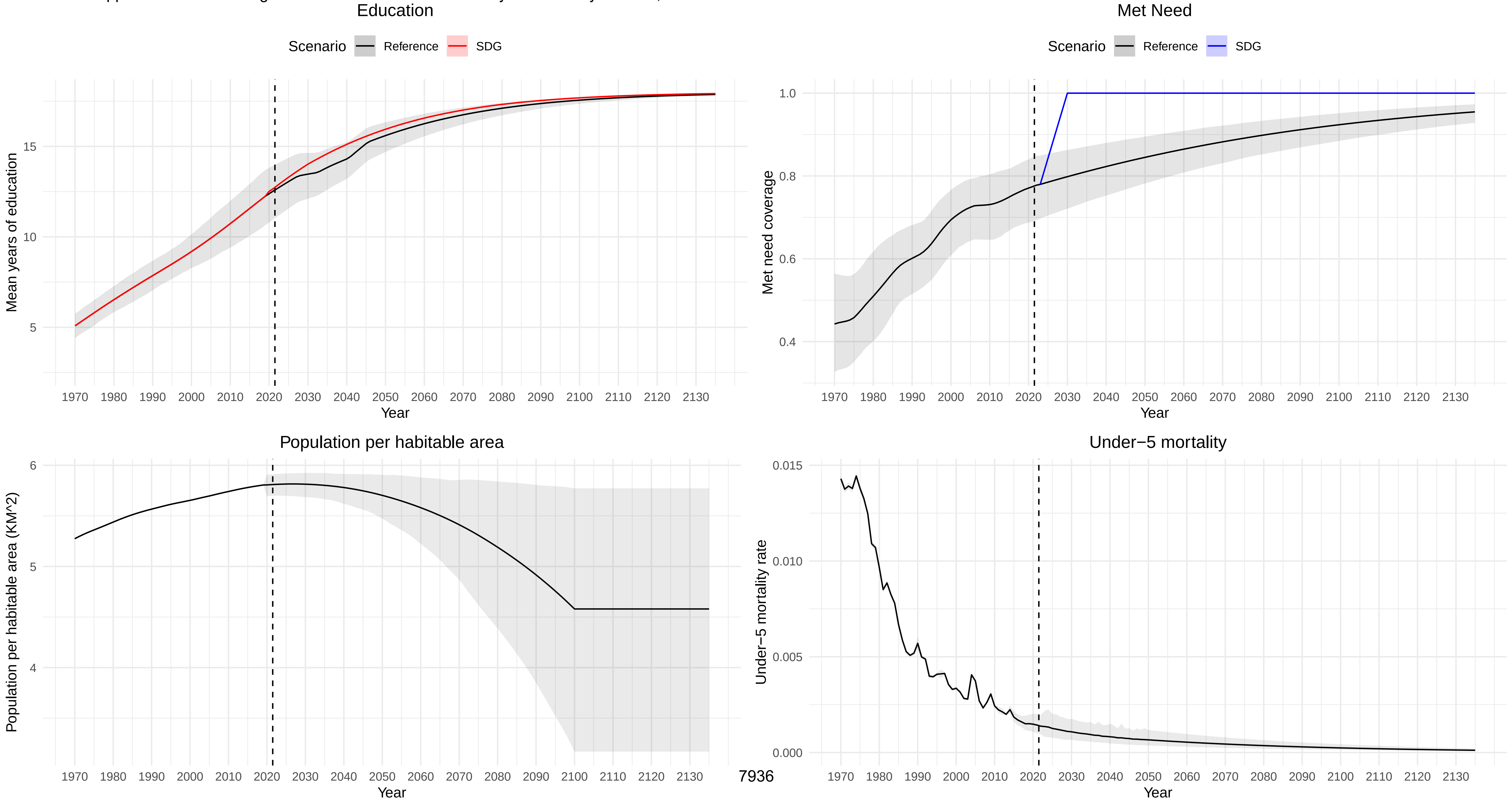

Thailand

Supplemental Results Figure S2: Covariates used in fertility forecasts by location, 1970-2135

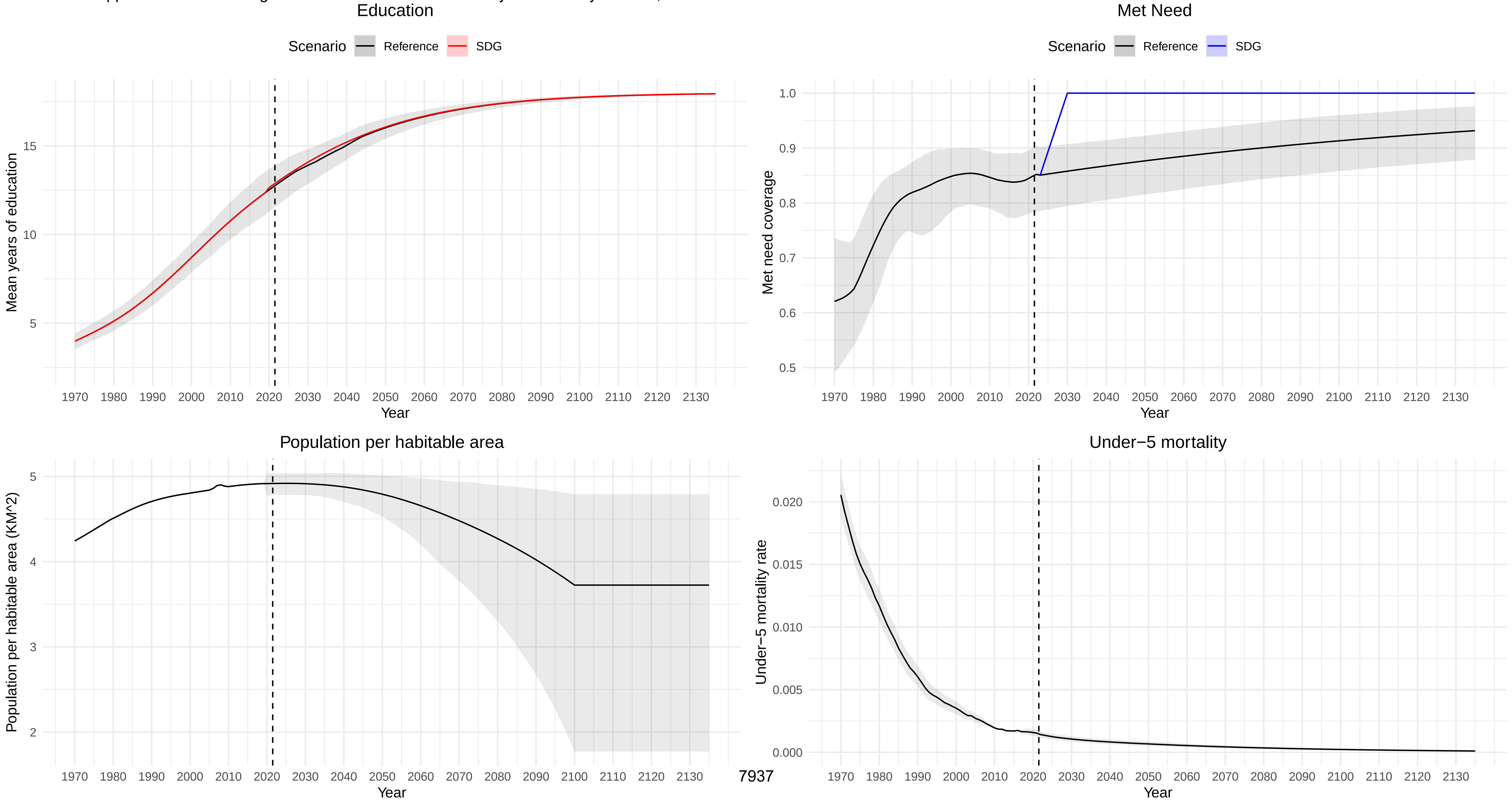

Timor−Leste

Supplemental Results Figure S2: Covariates used in fertility forecasts by location, 1970-2135

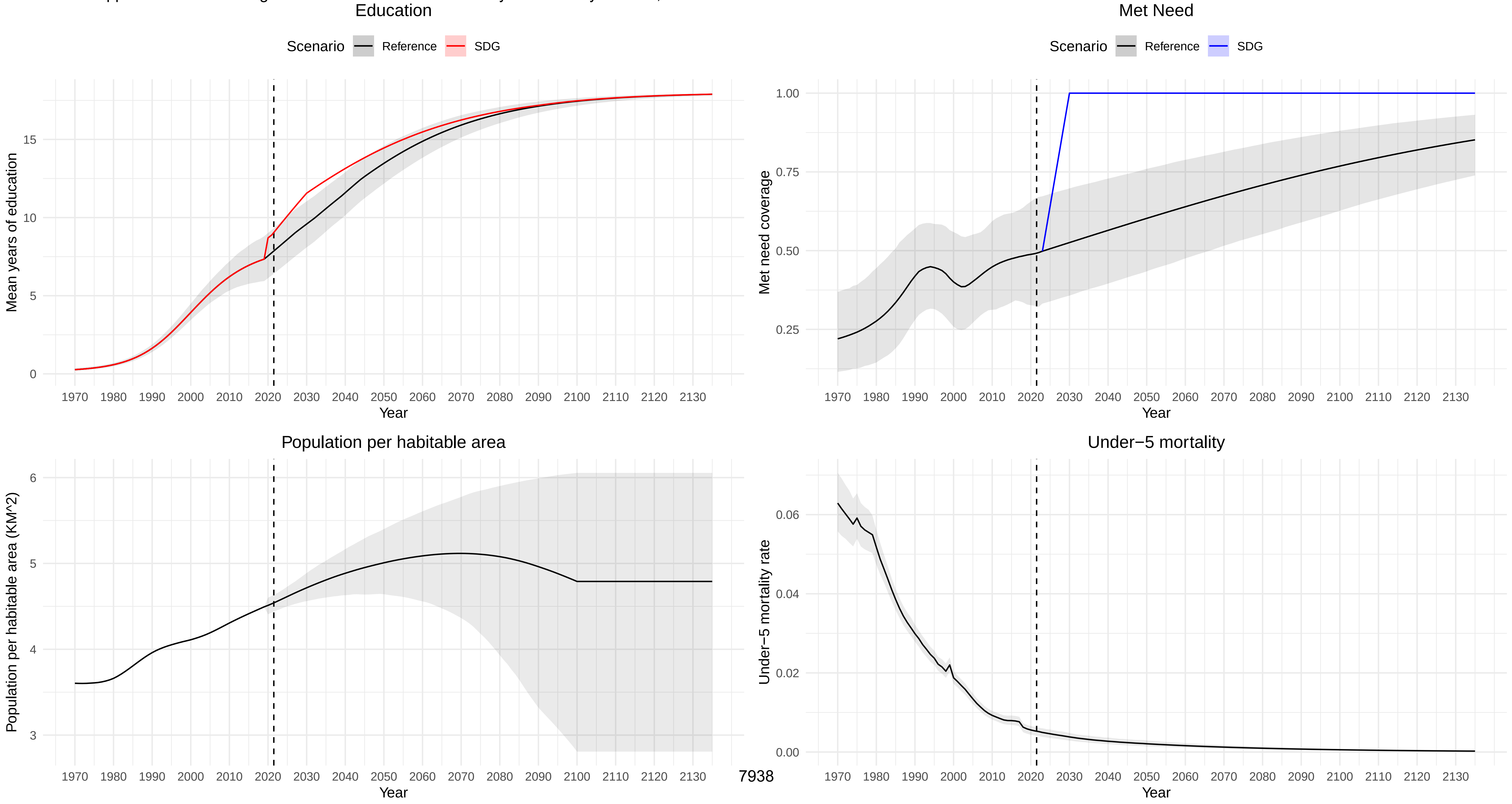

Viet Nam

Supplemental Results Figure S2: Covariates used in fertility forecasts by location, 1970-2135

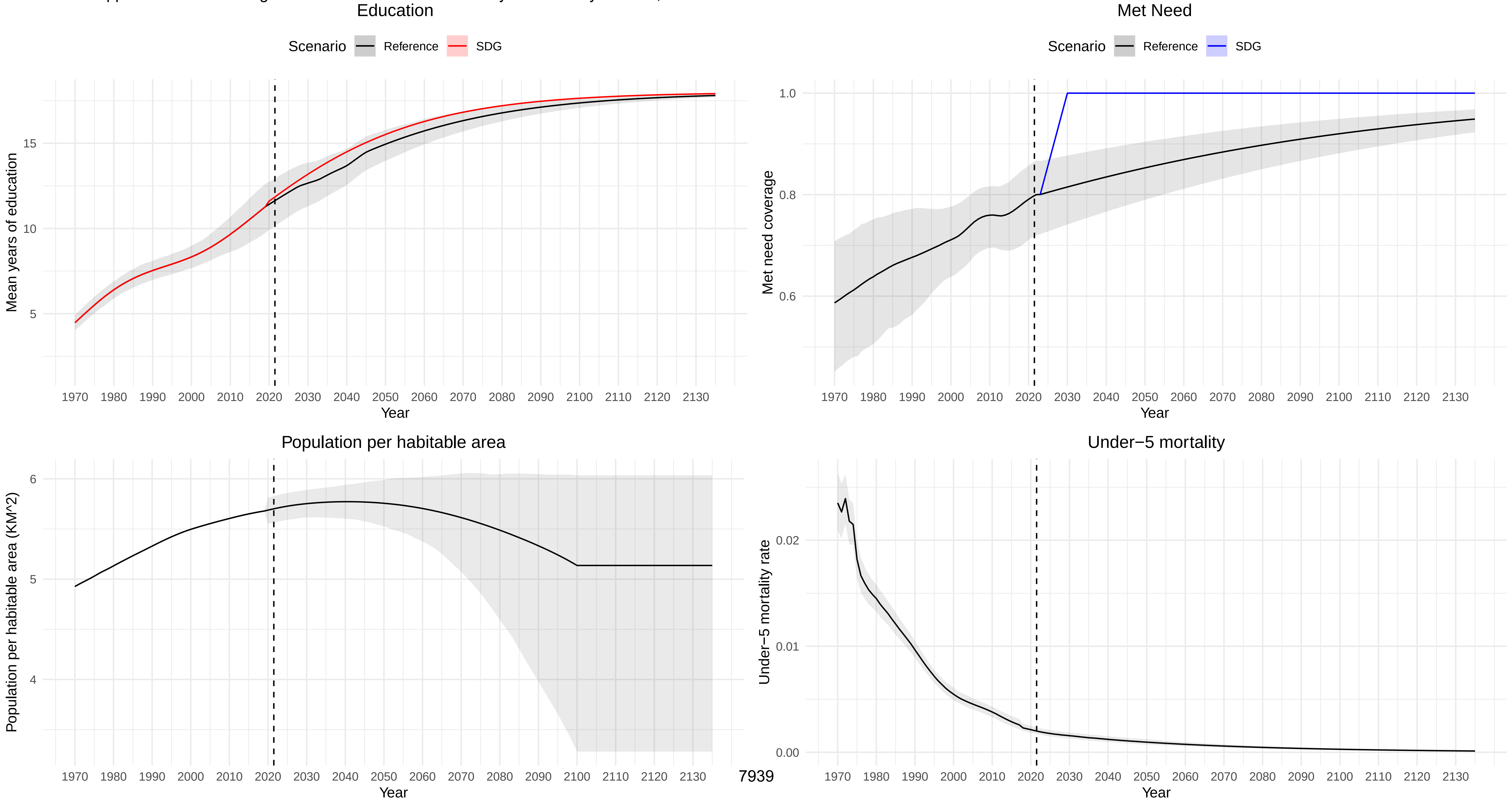

Angola

Supplemental Results Figure S2: Covariates used in fertility forecasts by location, 1970-2135

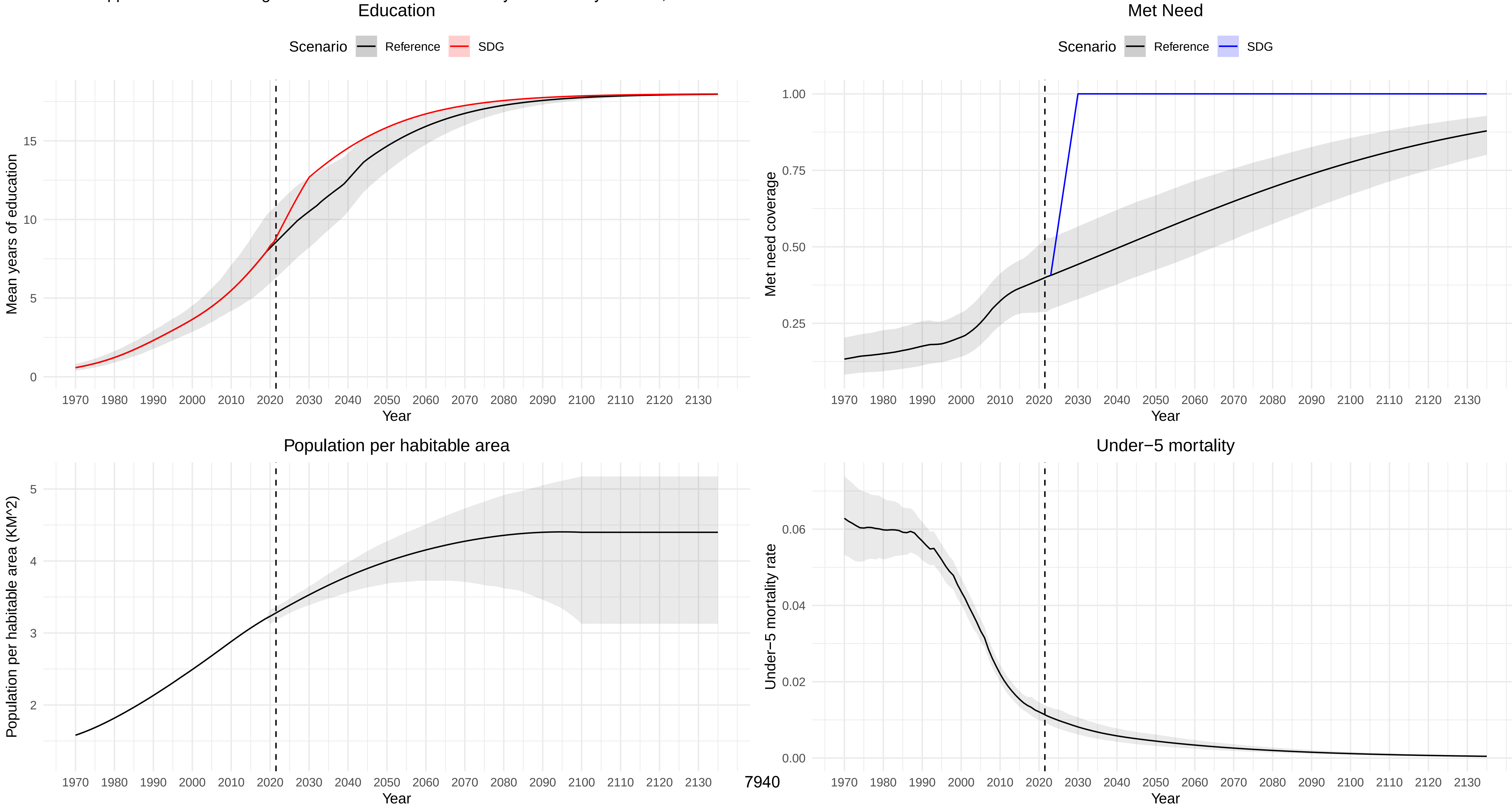

Central African Republic

Supplemental Results Figure S2: Covariates used in fertility forecasts by location, 1970-2135

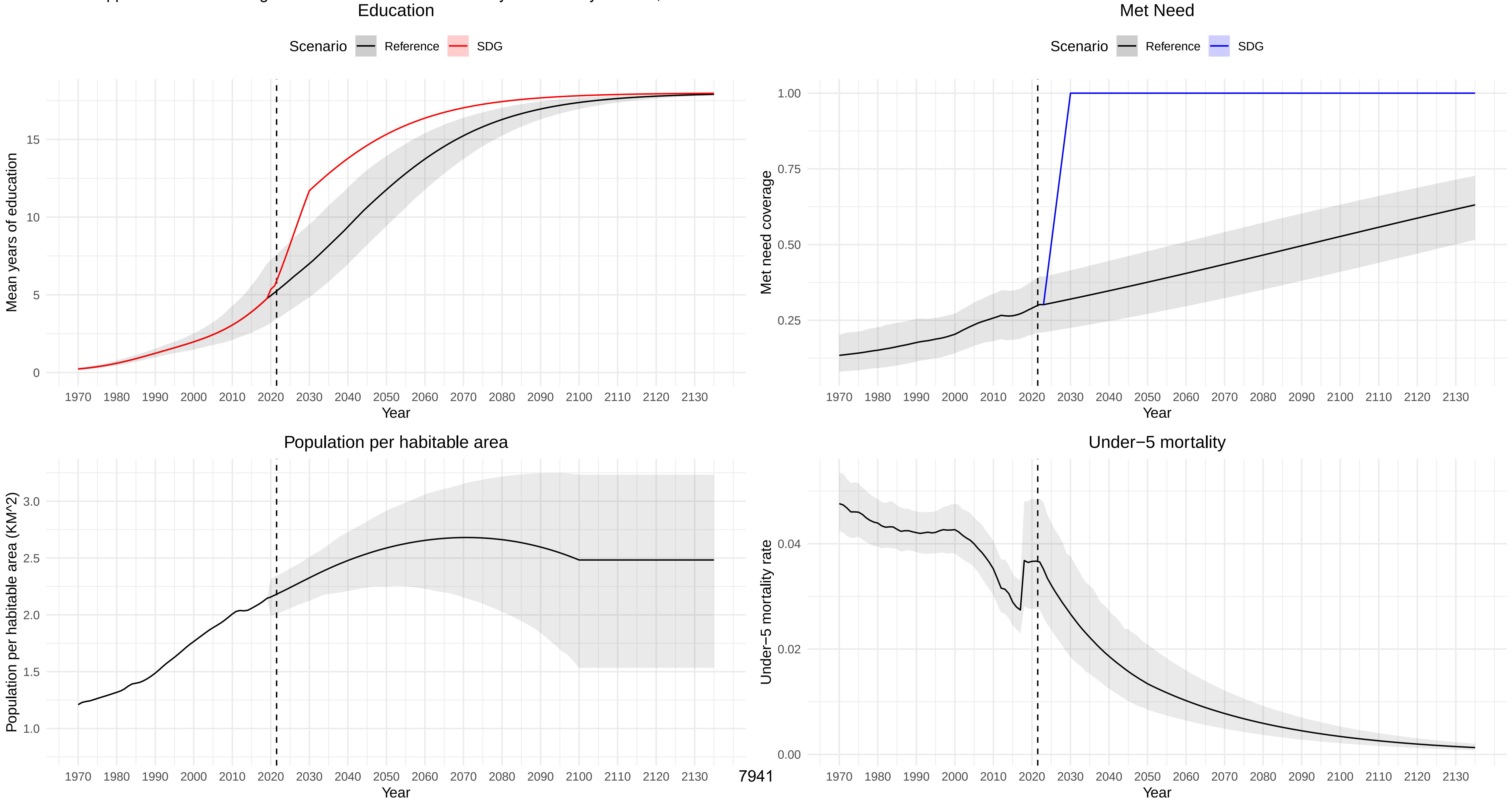

Congo (Brazzaville)

Supplemental Results Figure S2: Covariates used in fertility forecasts by location, 1970-2135

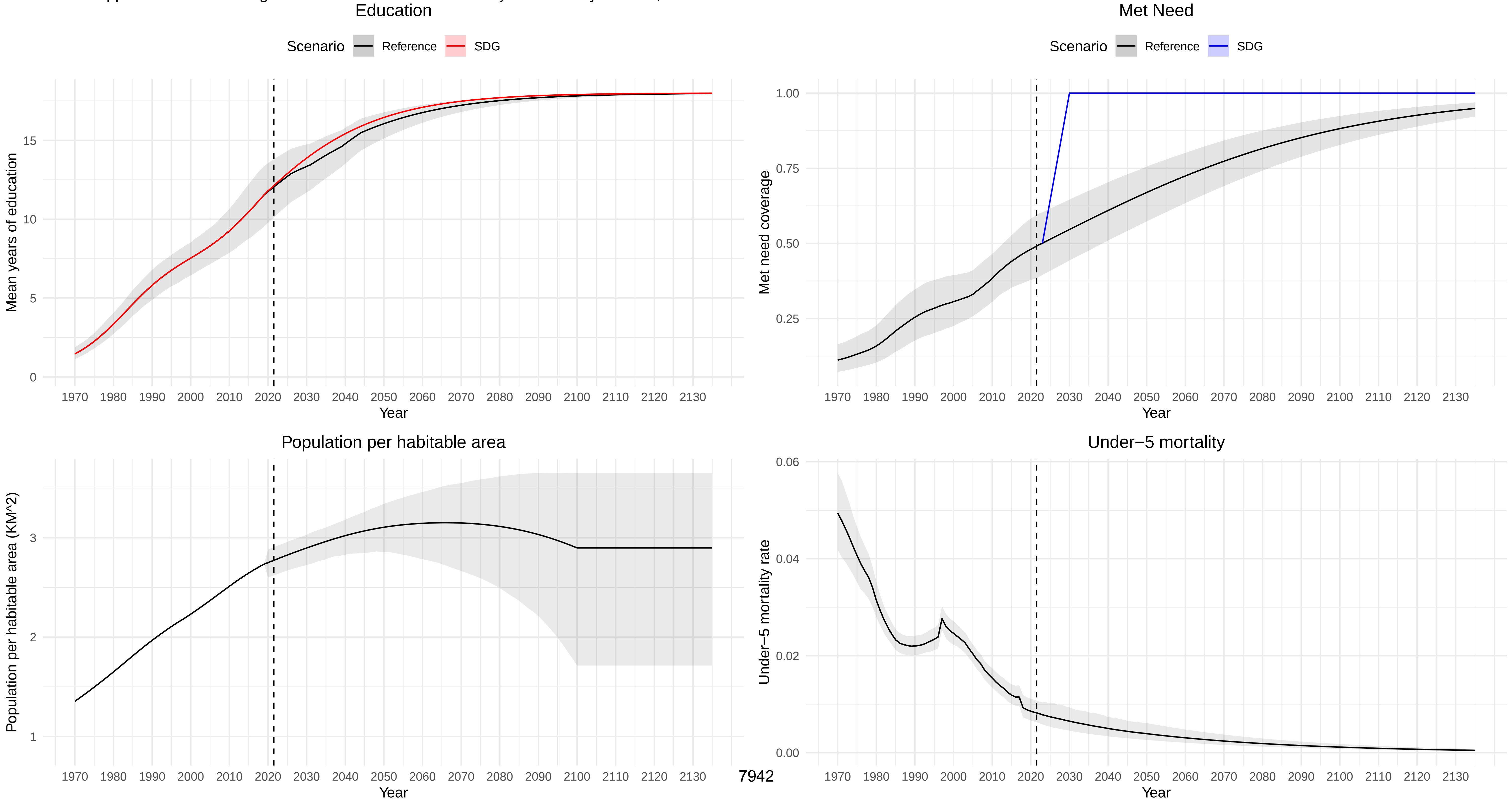

Democratic Republic of the Congo

Supplemental Results Figure S2: Covariates used in fertility forecasts by location, 1970-2135

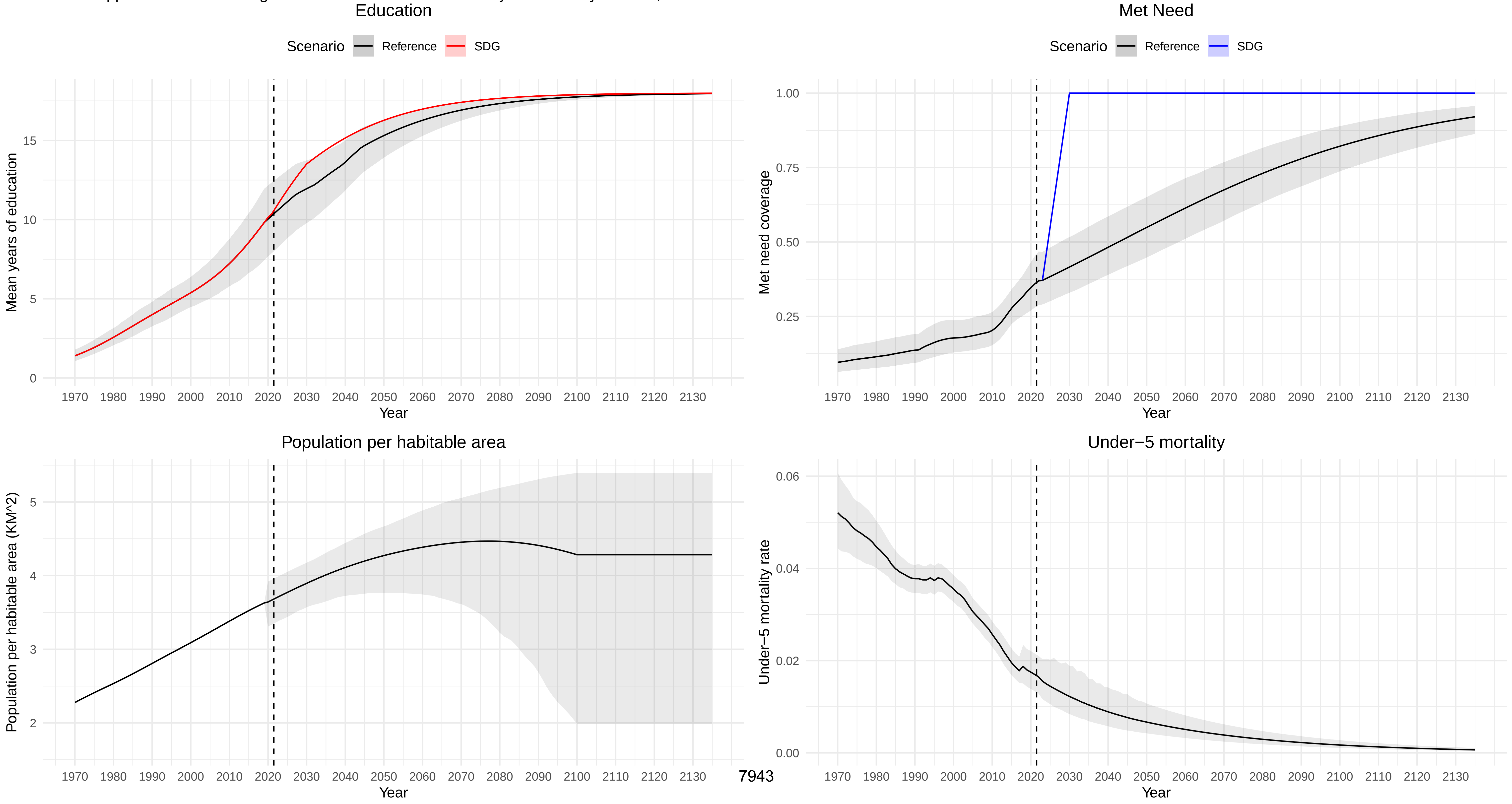

Equatorial Guinea

Supplemental Results Figure S2: Covariates used in fertility forecasts by location, 1970-2135

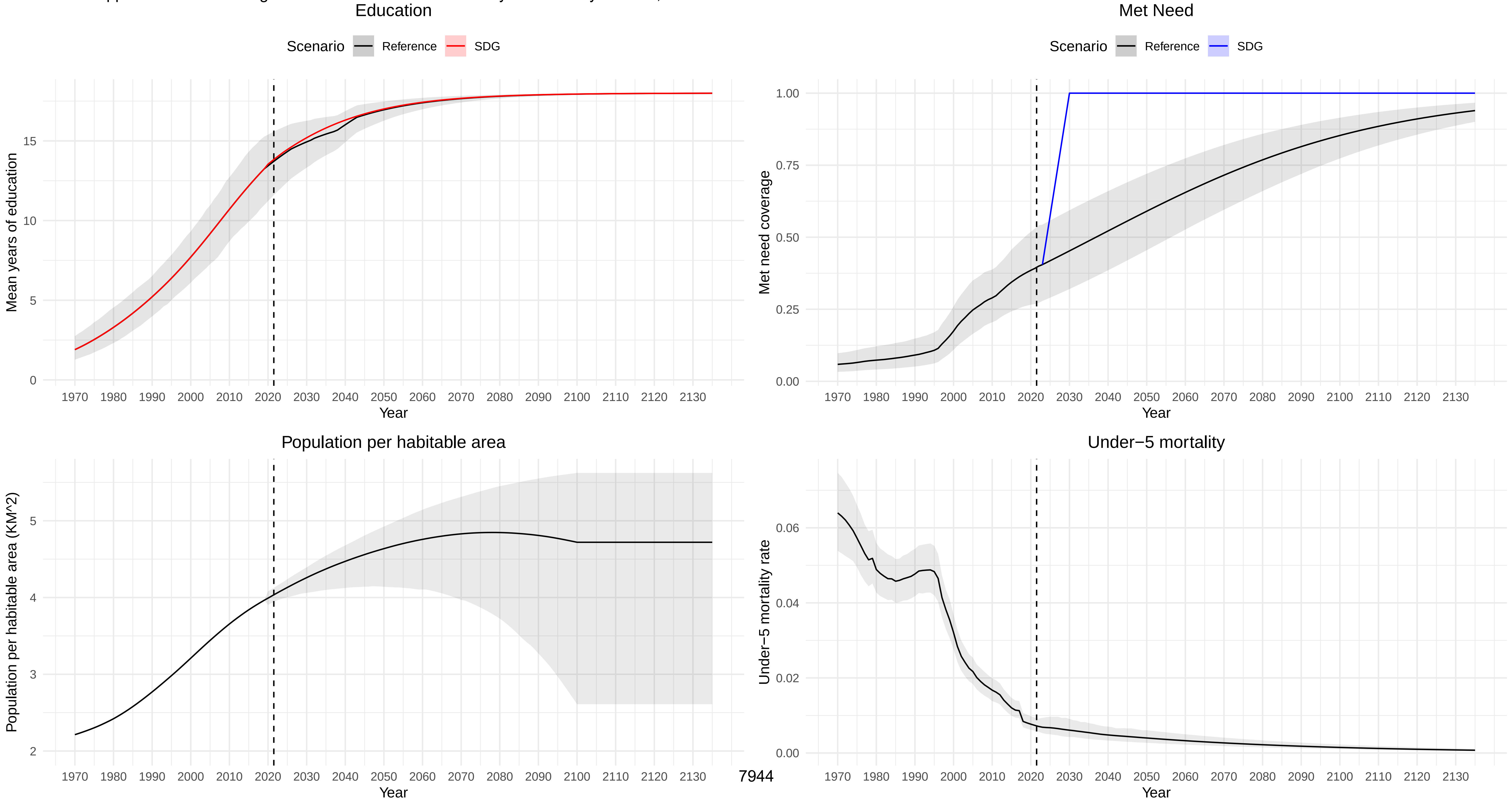

Gabon

Supplemental Results Figure S2: Covariates used in fertility forecasts by location, 1970-2135

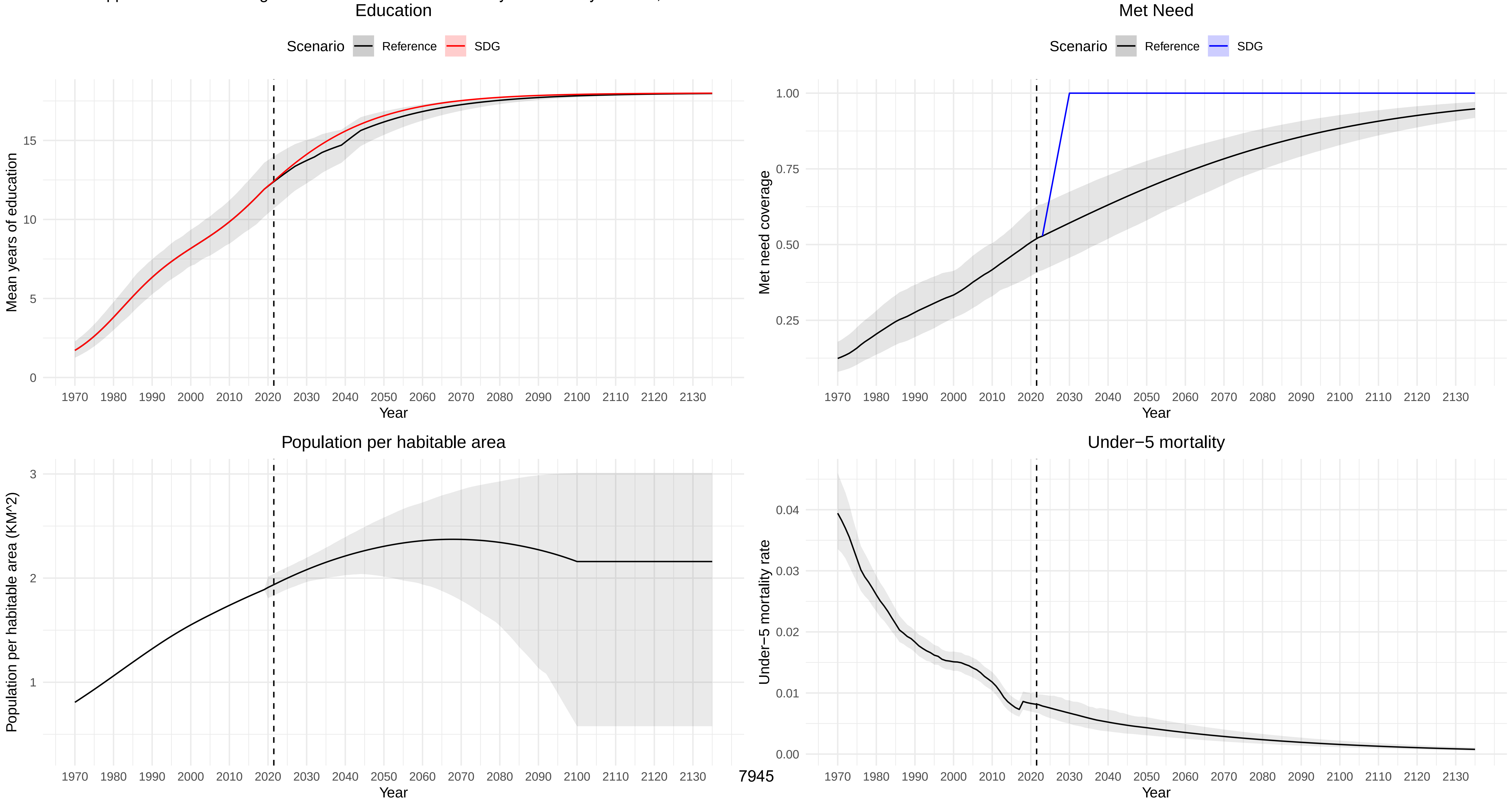

Burundi

Supplemental Results Figure S2: Covariates used in fertility forecasts by location, 1970-2135

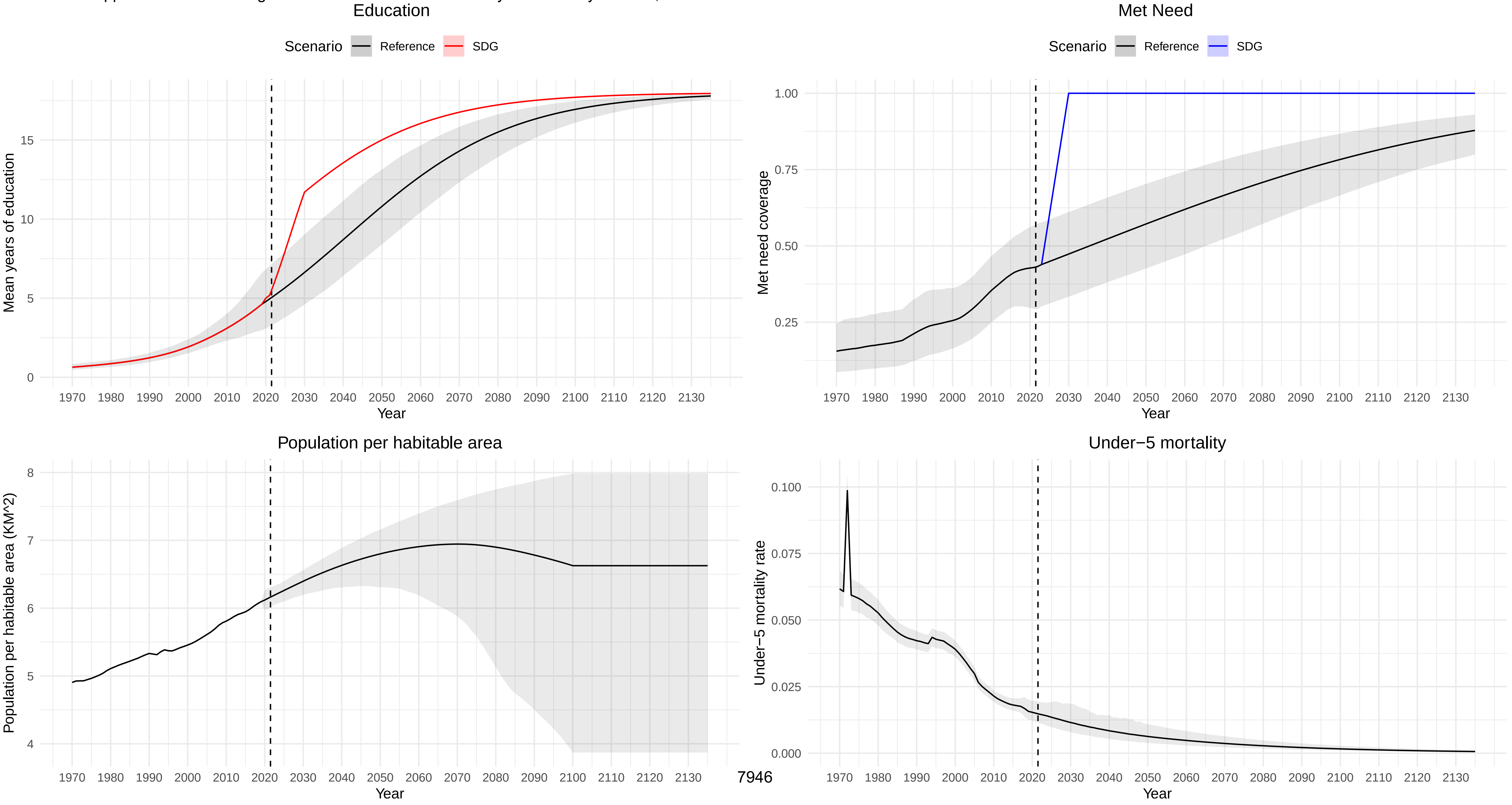

Comoros

Supplemental Results Figure S2: Covariates used in fertility forecasts by location, 1970-2135

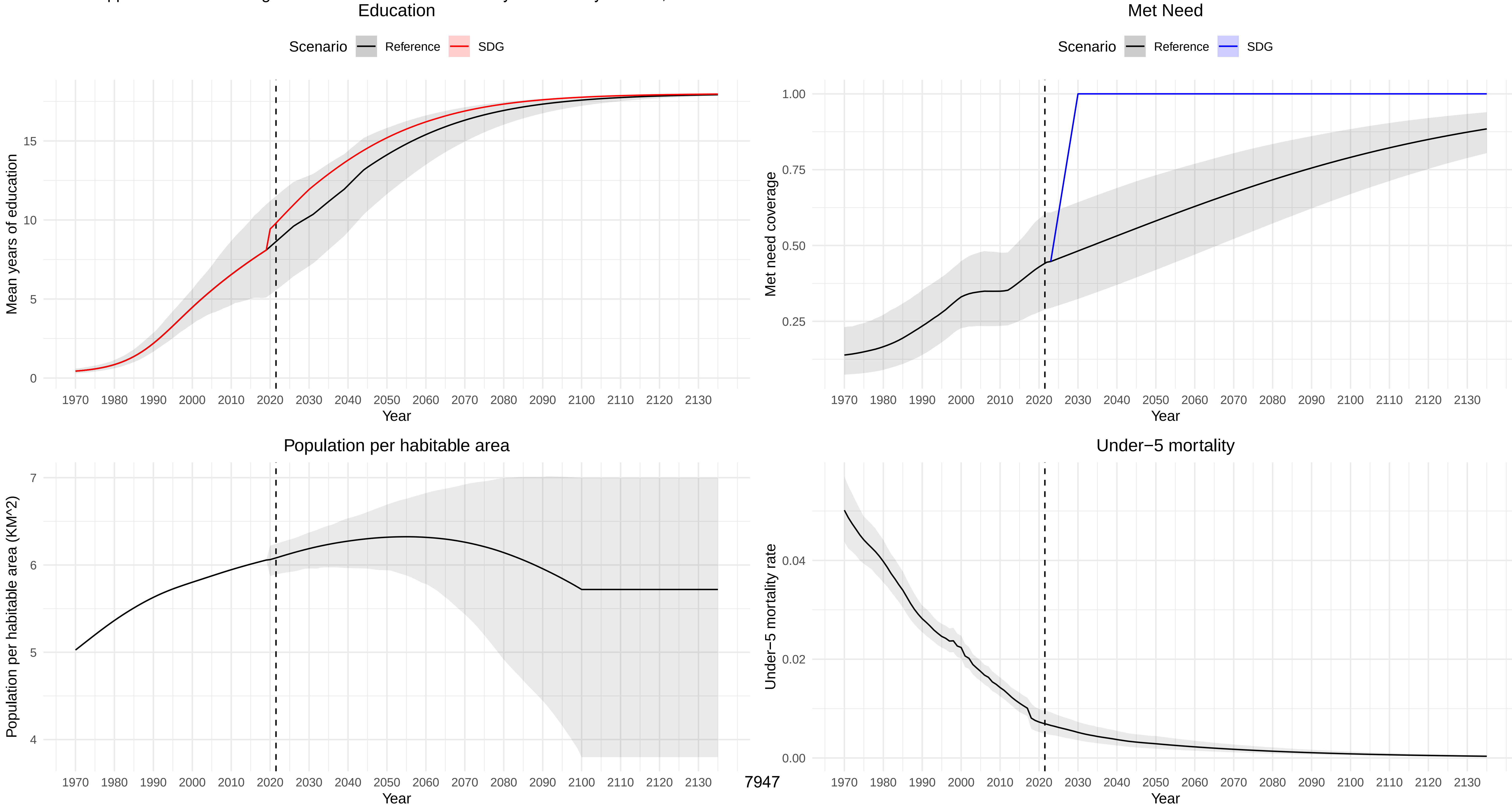

Djibouti

Supplemental Results Figure S2: Covariates used in fertility forecasts by location, 1970-2135

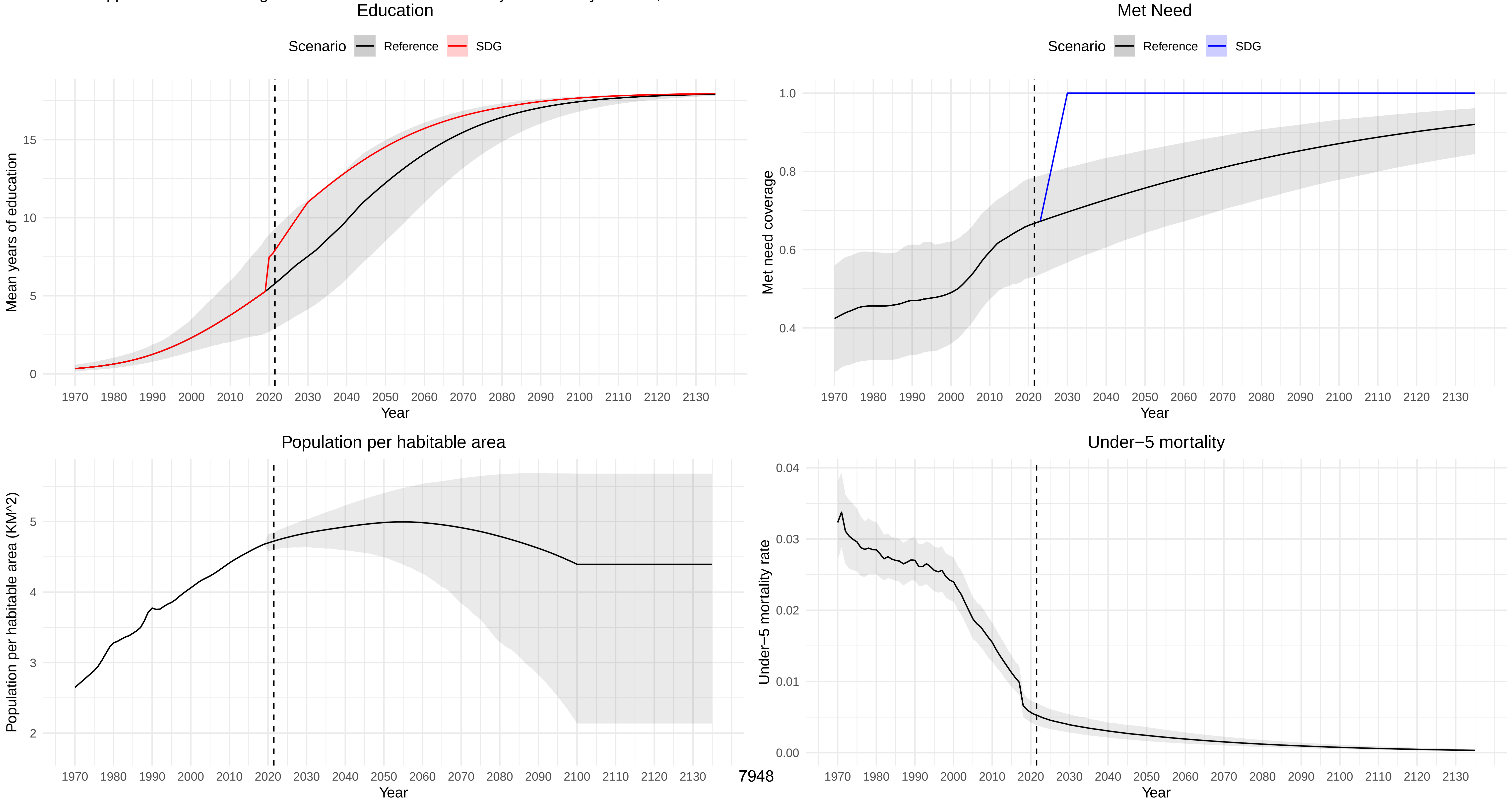

Eritrea

Supplemental Results Figure S2: Covariates used in fertility forecasts by location, 1970-2135

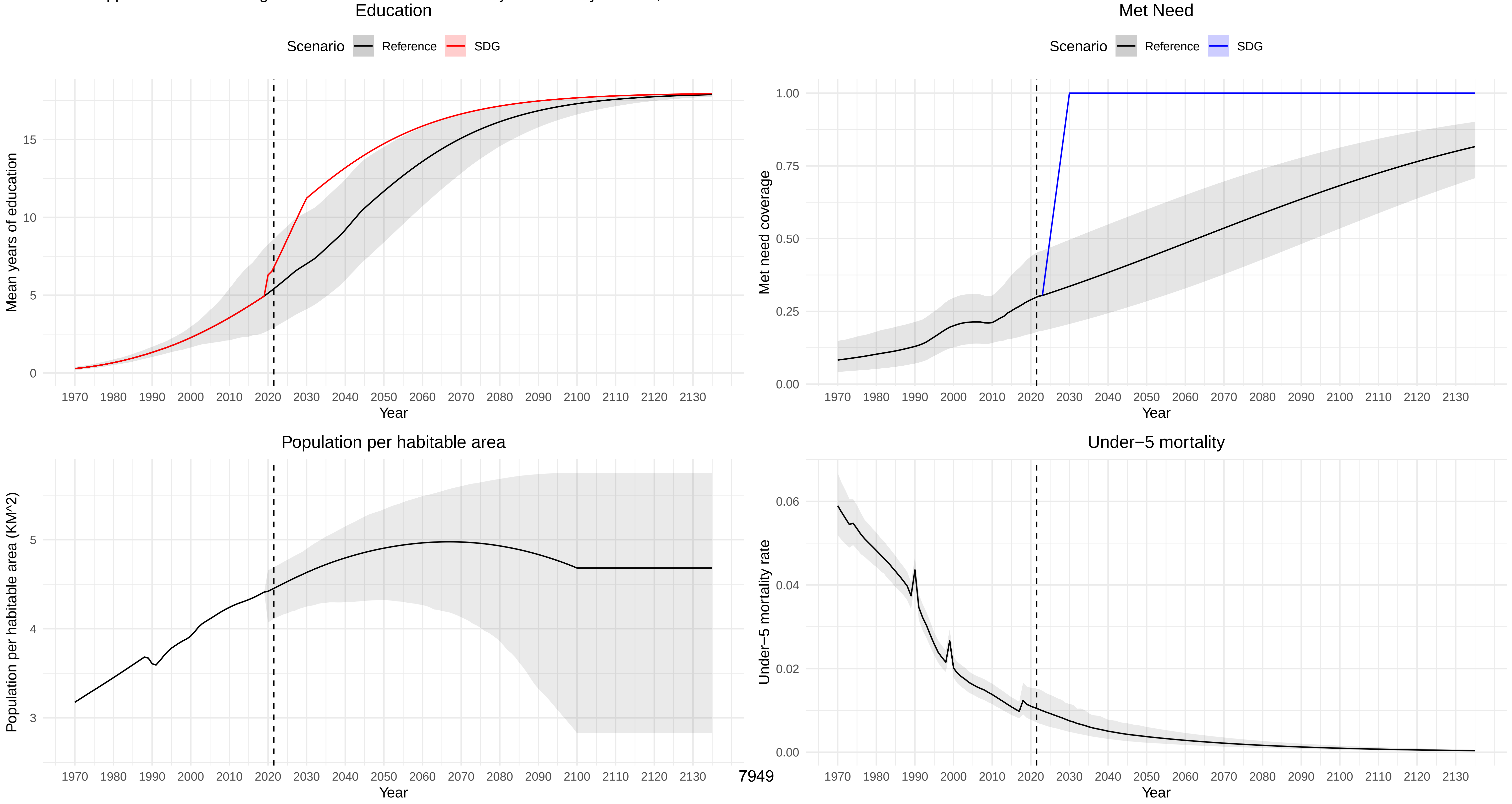

Ethiopia

Supplemental Results Figure S2: Covariates used in fertility forecasts by location, 1970-2135

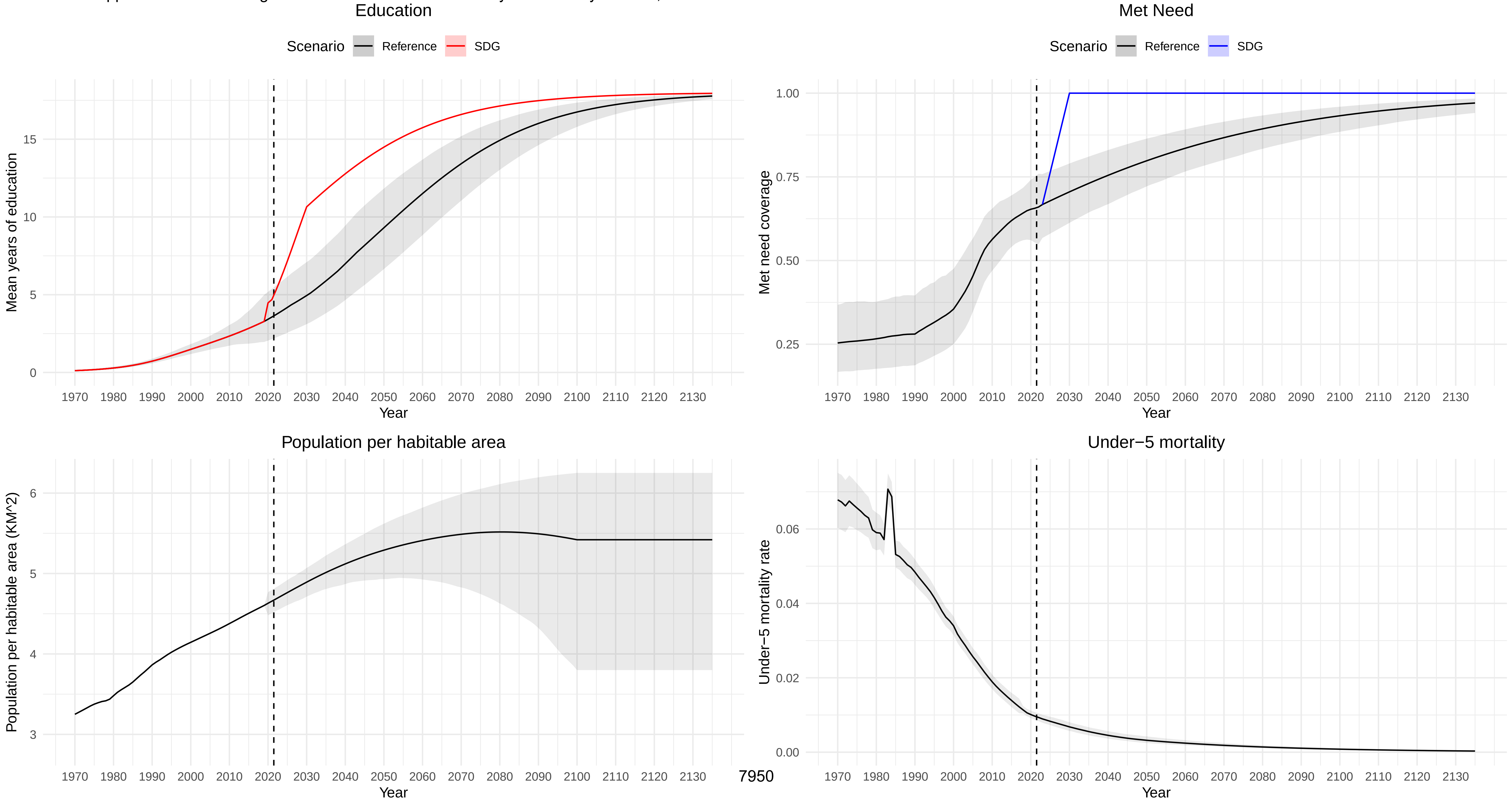

Kenya

Supplemental Results Figure S2: Covariates used in fertility forecasts by location, 1970-2135

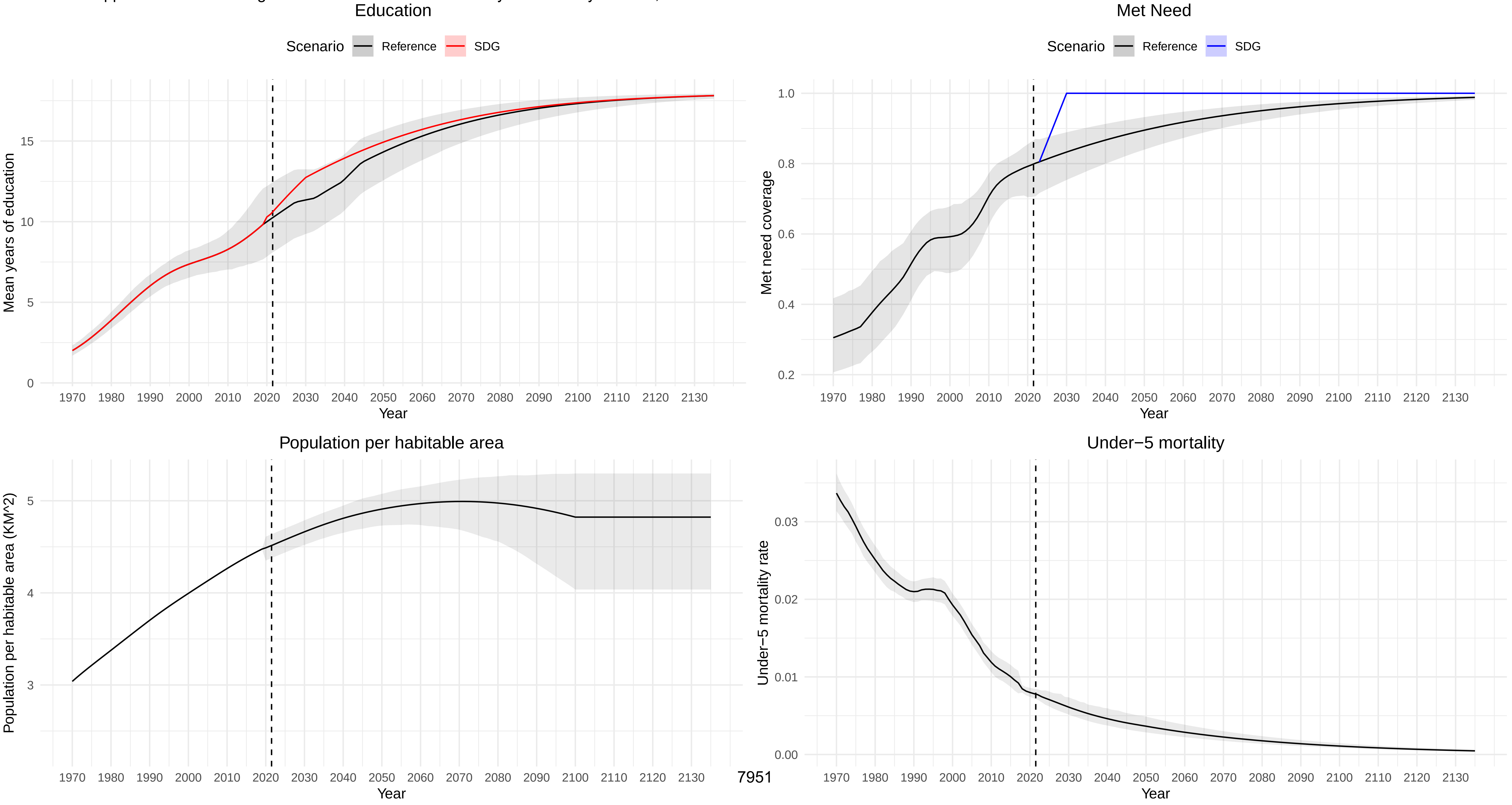

Madagascar

Supplemental Results Figure S2: Covariates used in fertility forecasts by location, 1970-2135

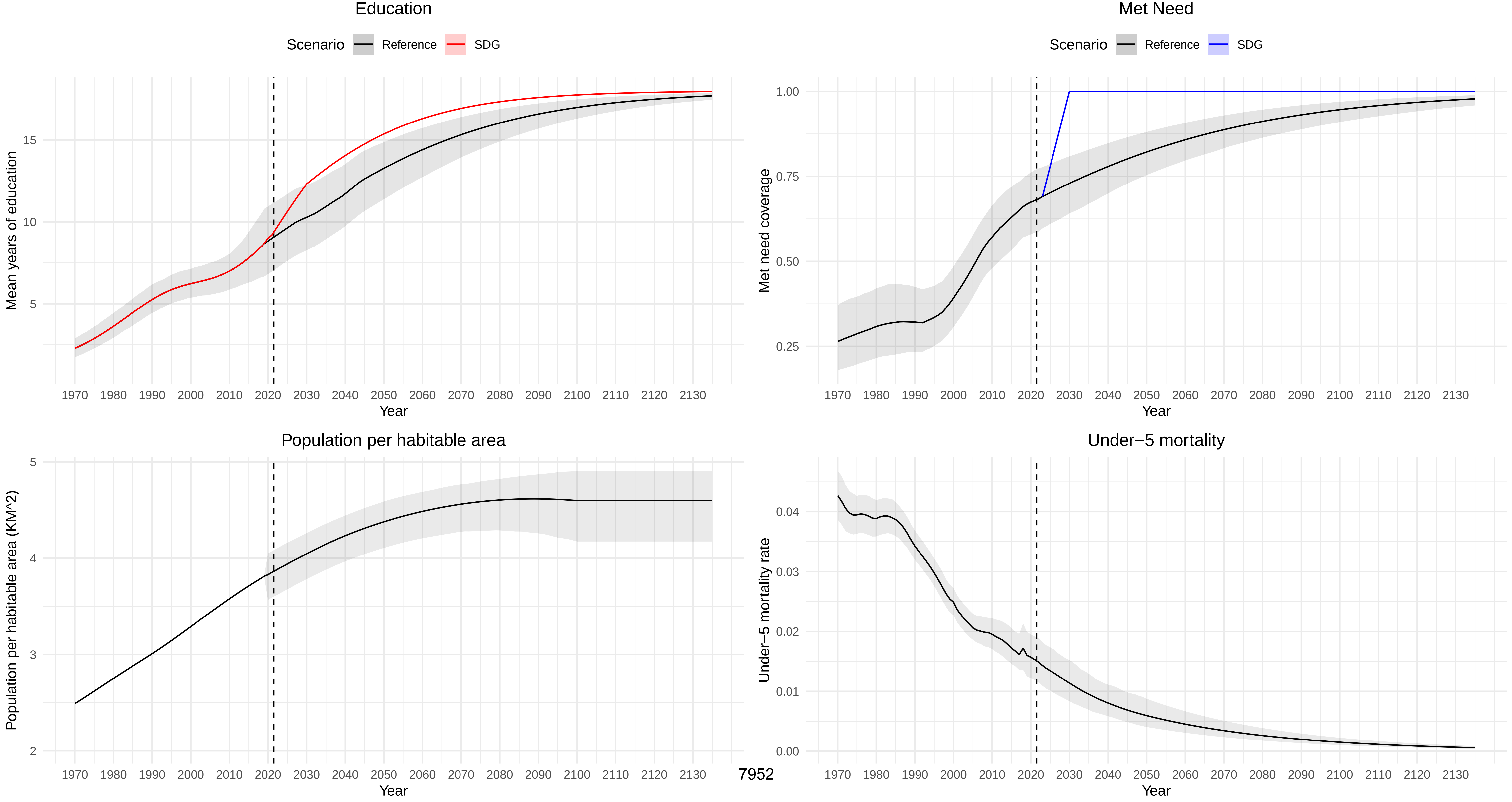

Malawi

Supplemental Results Figure S2: Covariates used in fertility forecasts by location, 1970-2135

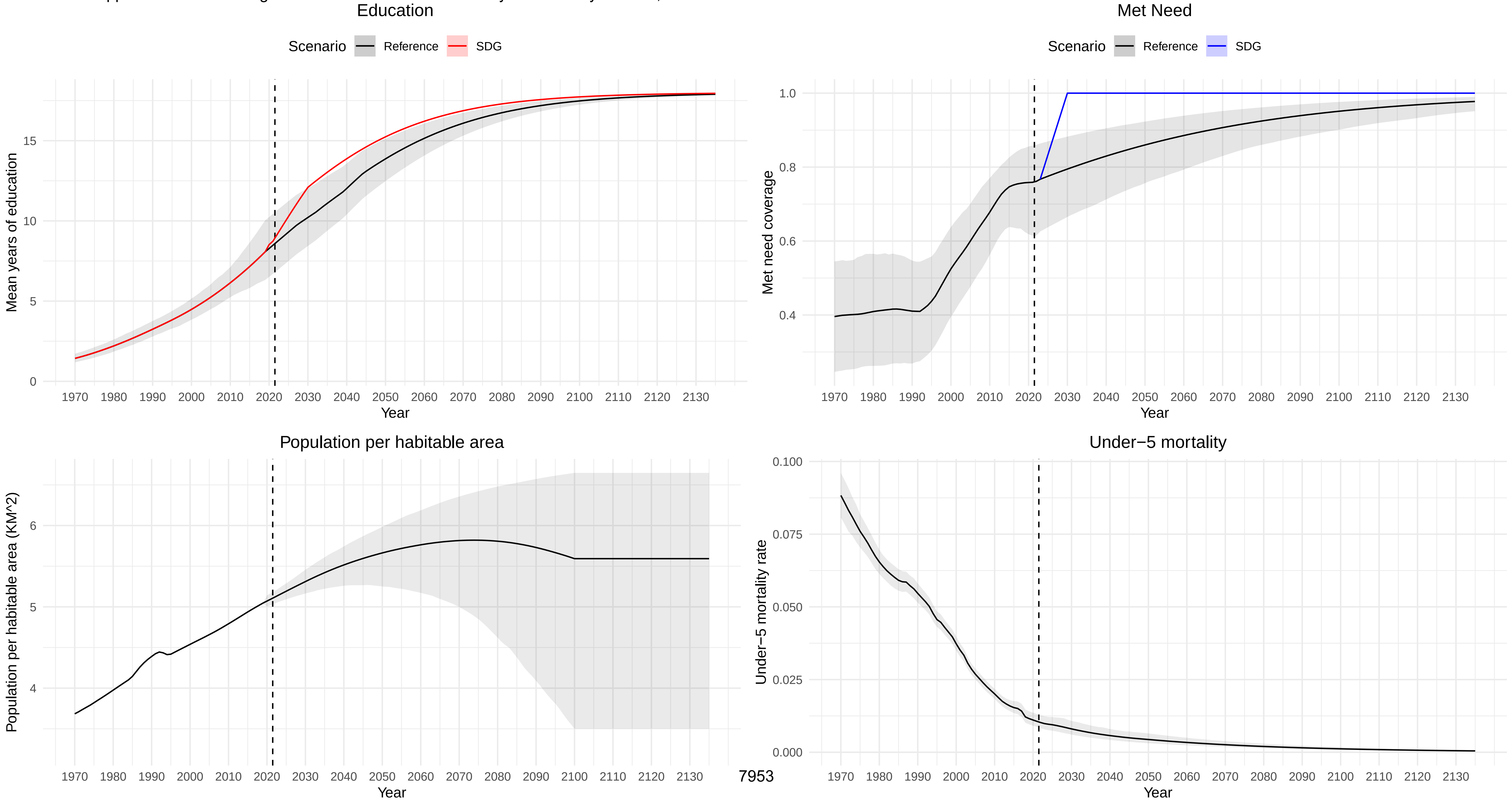

Mozambique

Supplemental Results Figure S2: Covariates used in fertility forecasts by location, 1970-2135

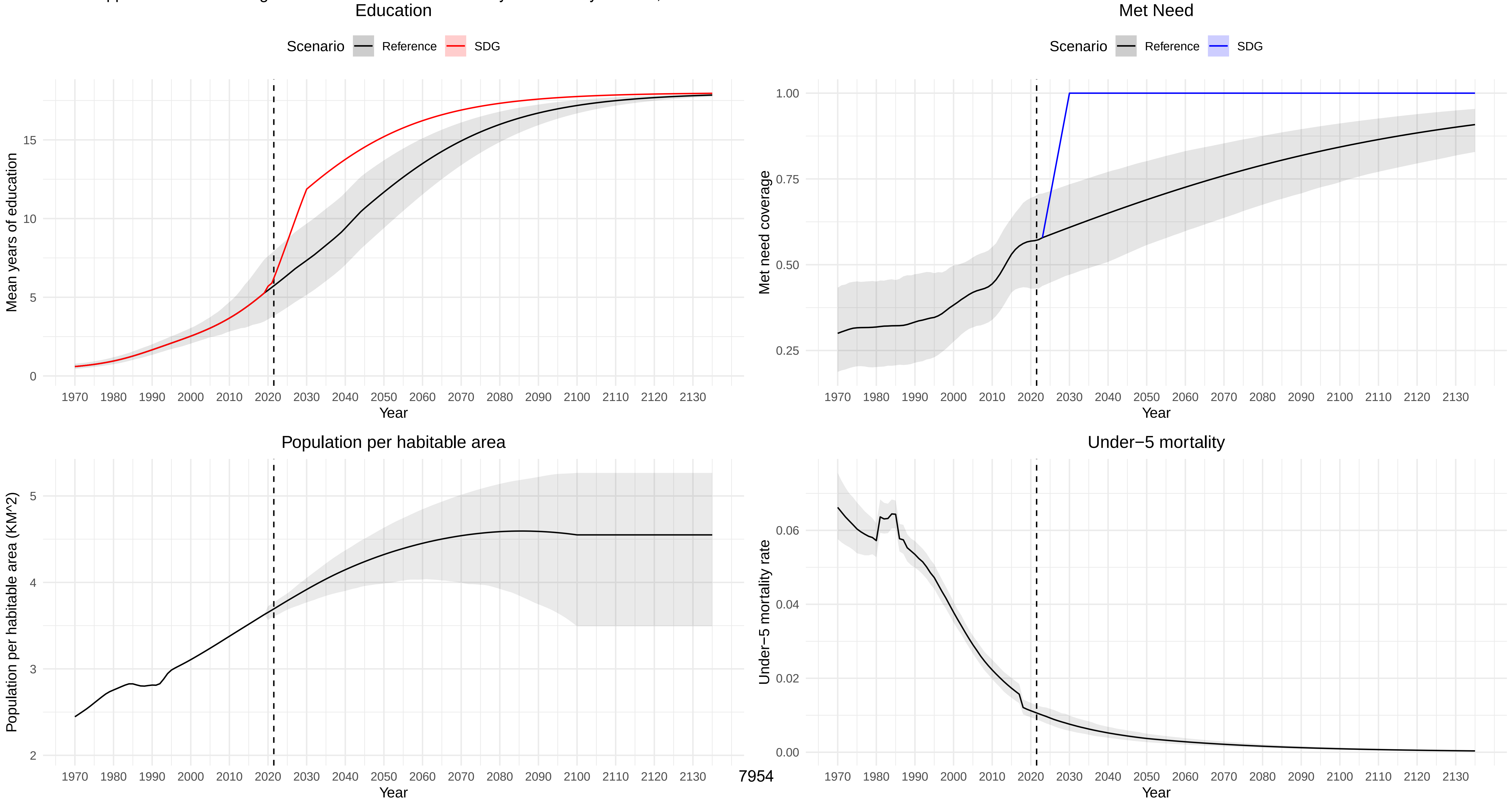

Rwanda

Supplemental Results Figure S2: Covariates used in fertility forecasts by location, 1970-2135

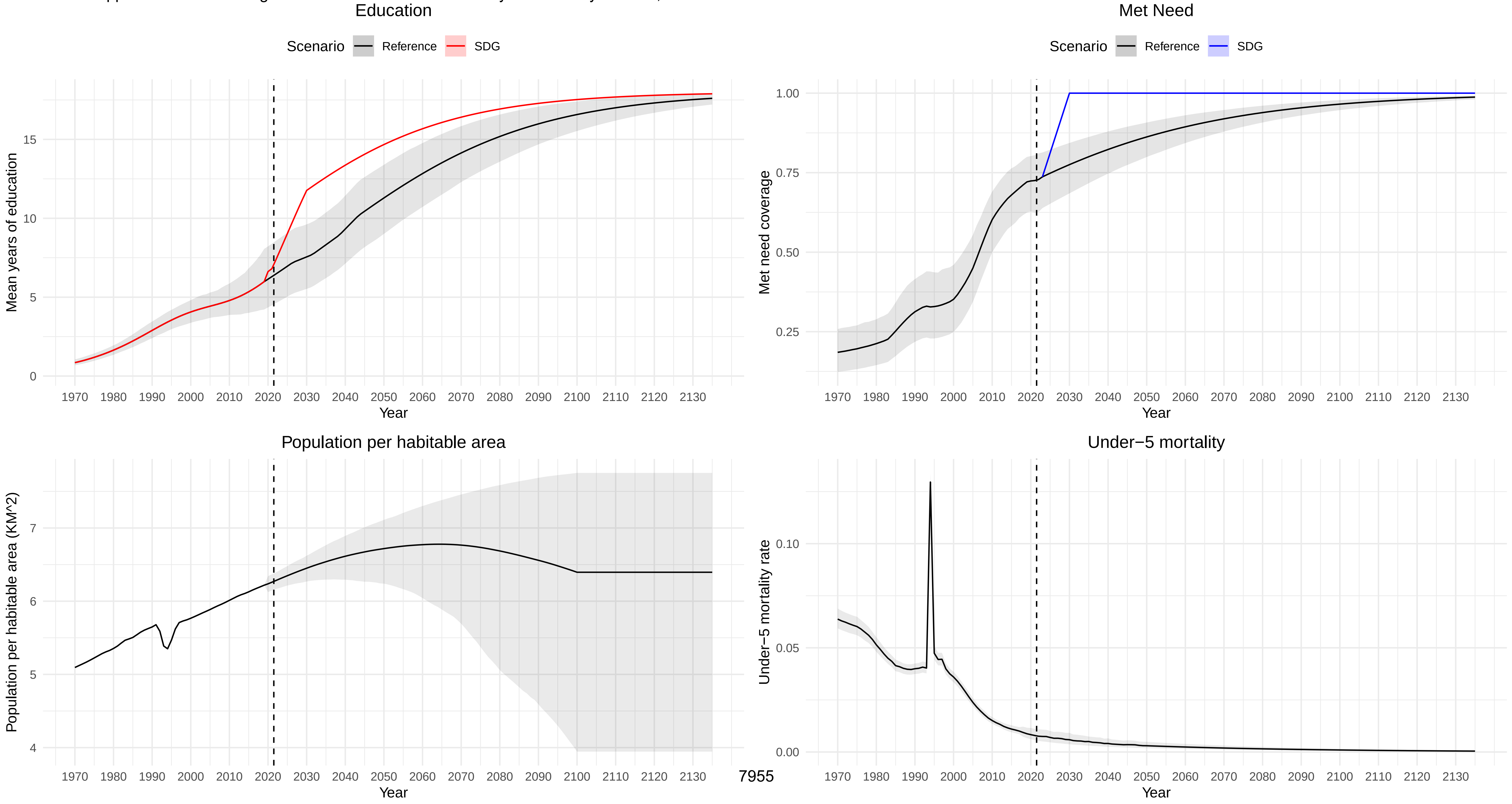

Somalia

Supplemental Results Figure S2: Covariates used in fertility forecasts by location, 1970-2135

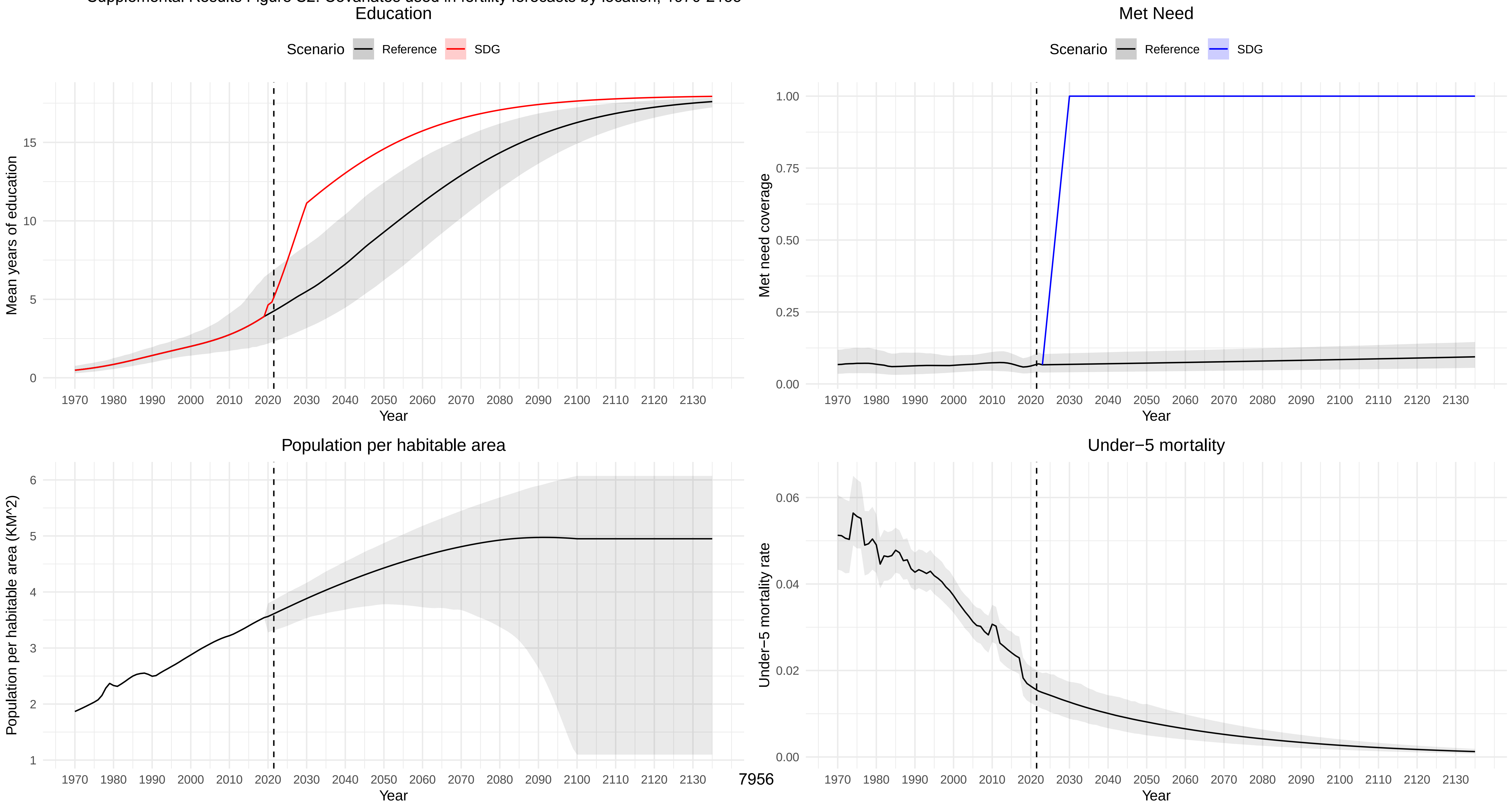

South Sudan

Supplemental Results Figure S2: Covariates used in fertility forecasts by location, 1970-2135

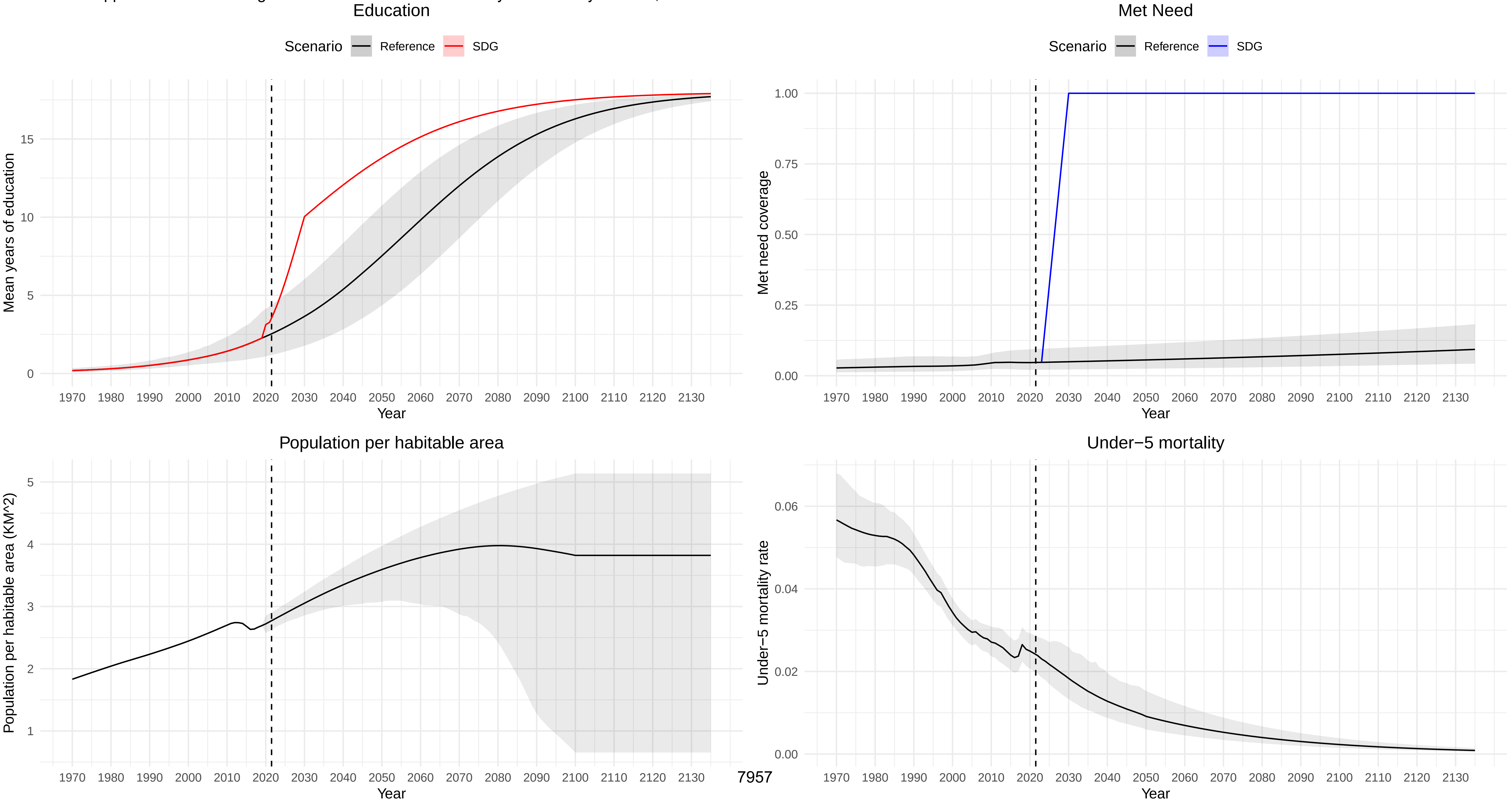

Uganda

Supplemental Results Figure S2: Covariates used in fertility forecasts by location, 1970-2135

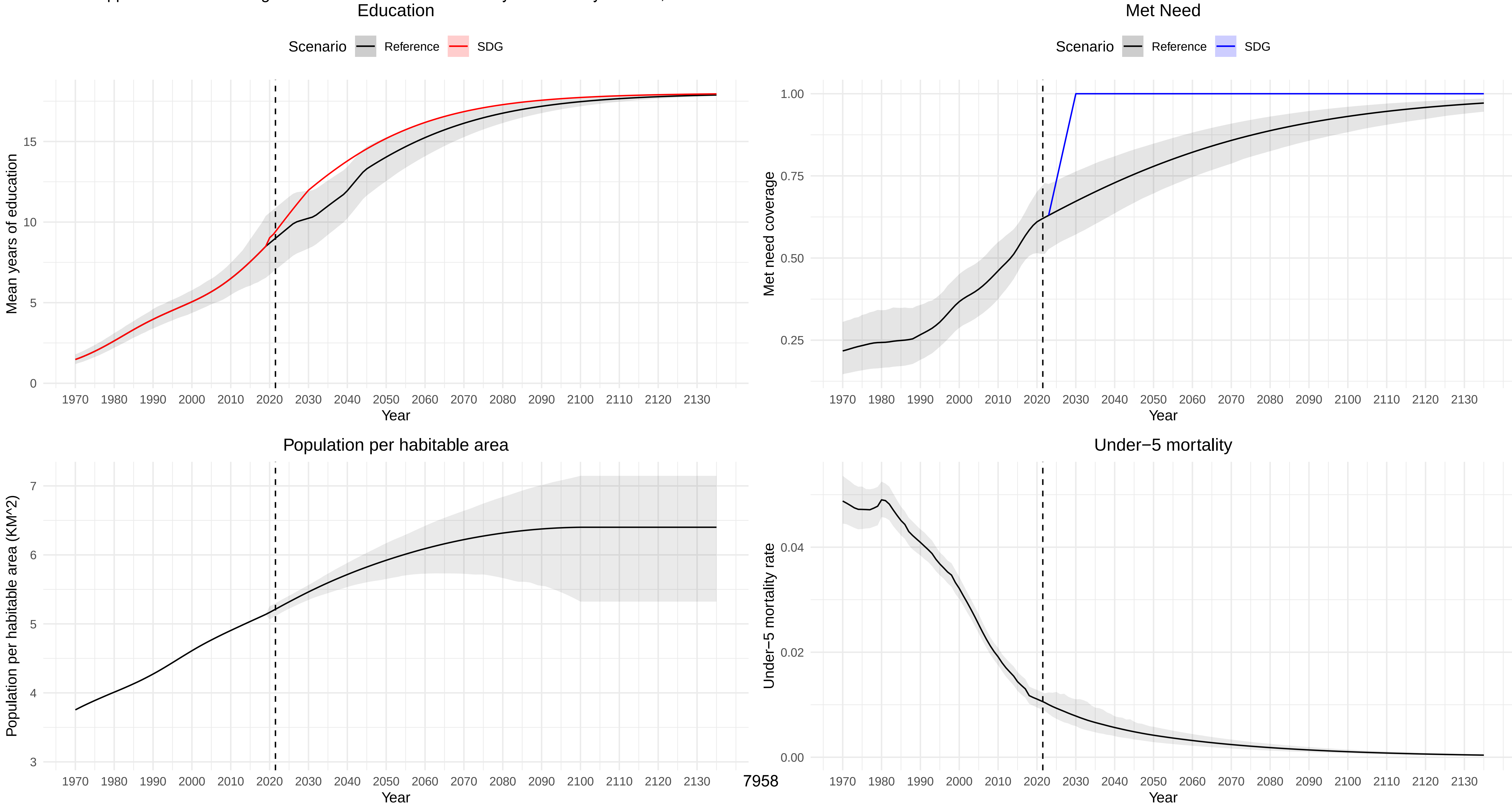

United Republic of Tanzania

Supplemental Results Figure S2: Covariates used in fertility forecasts by location, 1970-2135

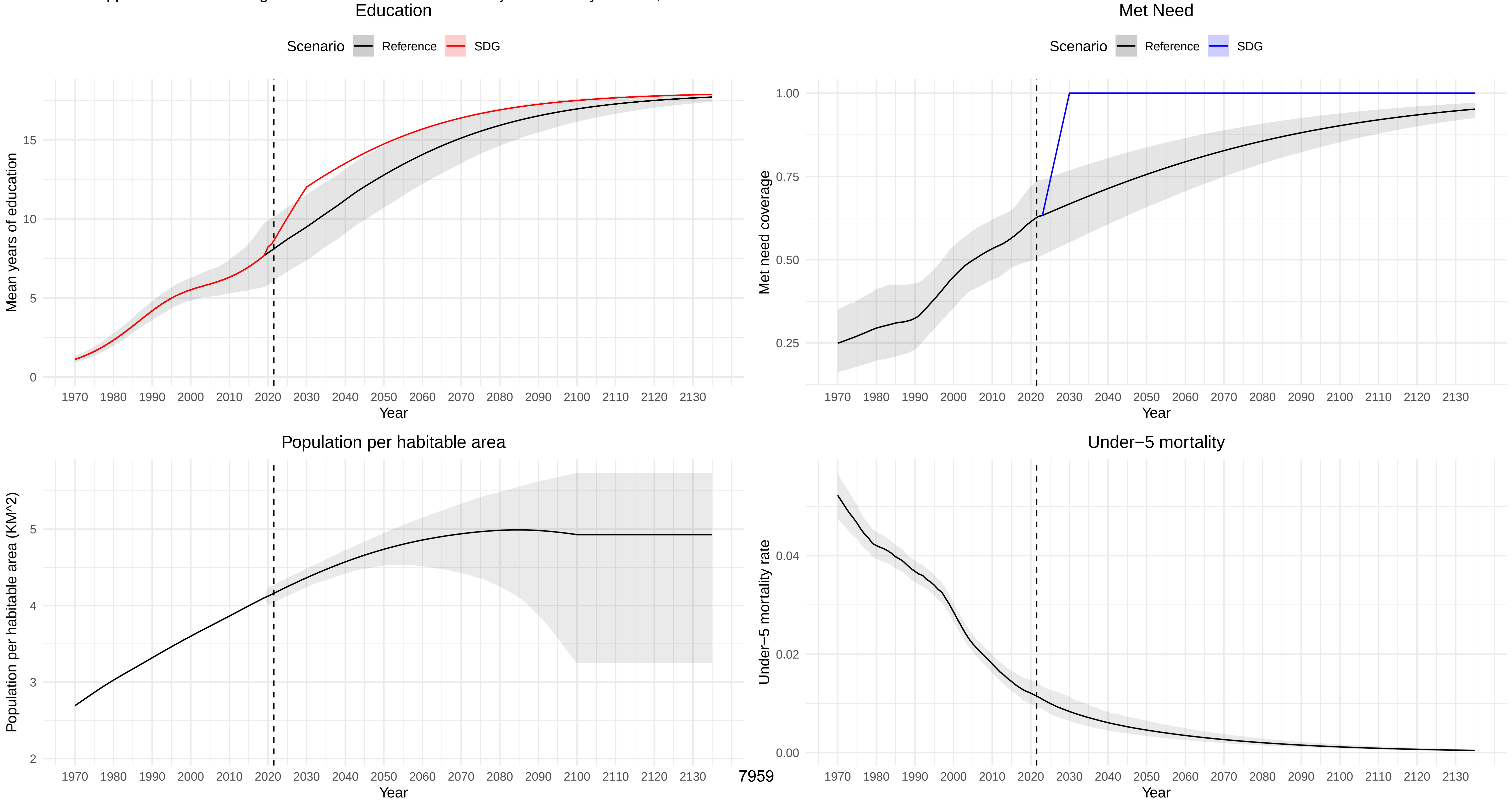

Zambia

Supplemental Results Figure S2: Covariates used in fertility forecasts by location, 1970-2135

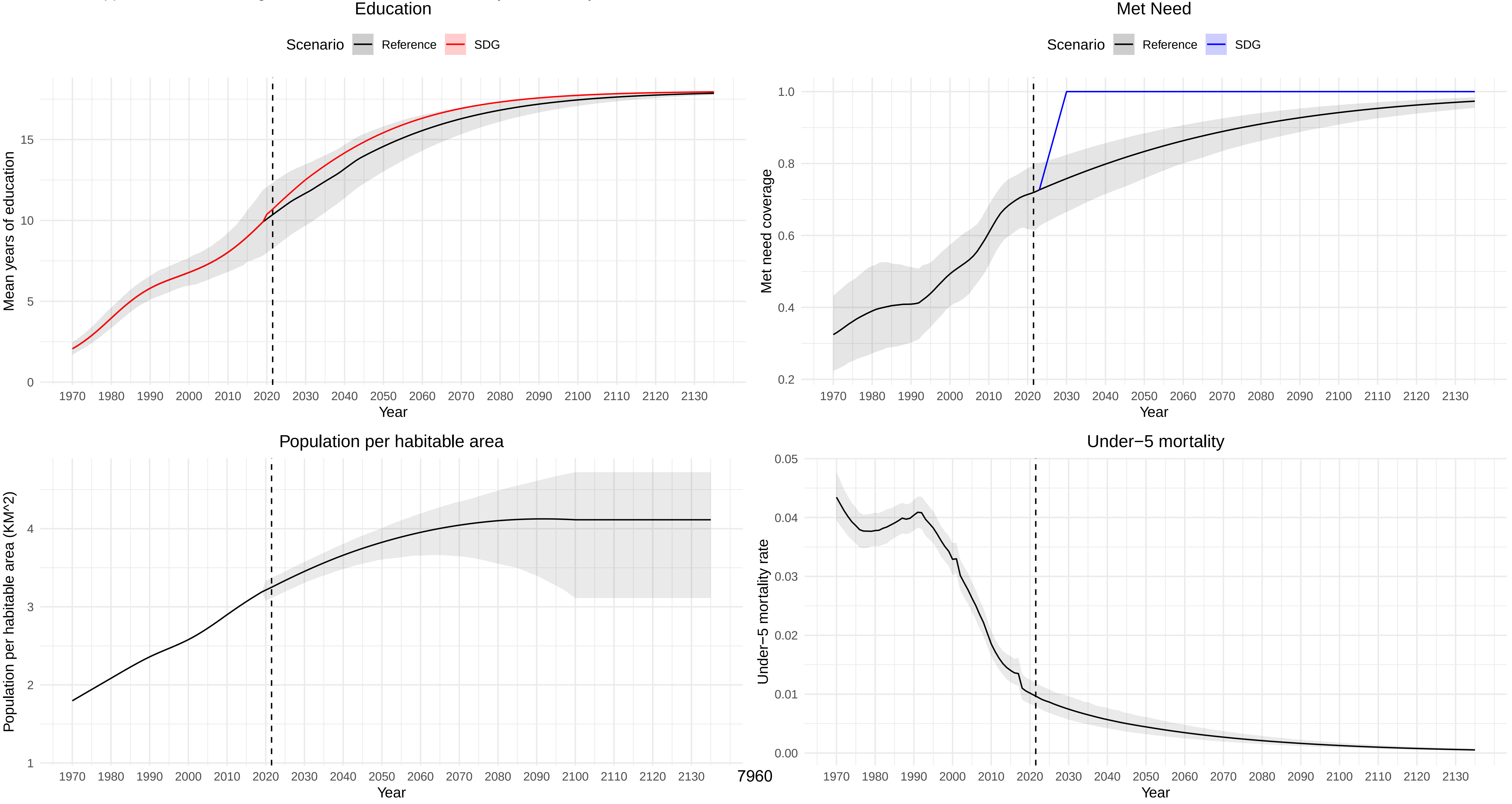

Botswana

Supplemental Results Figure S2: Covariates used in fertility forecasts by location, 1970-2135

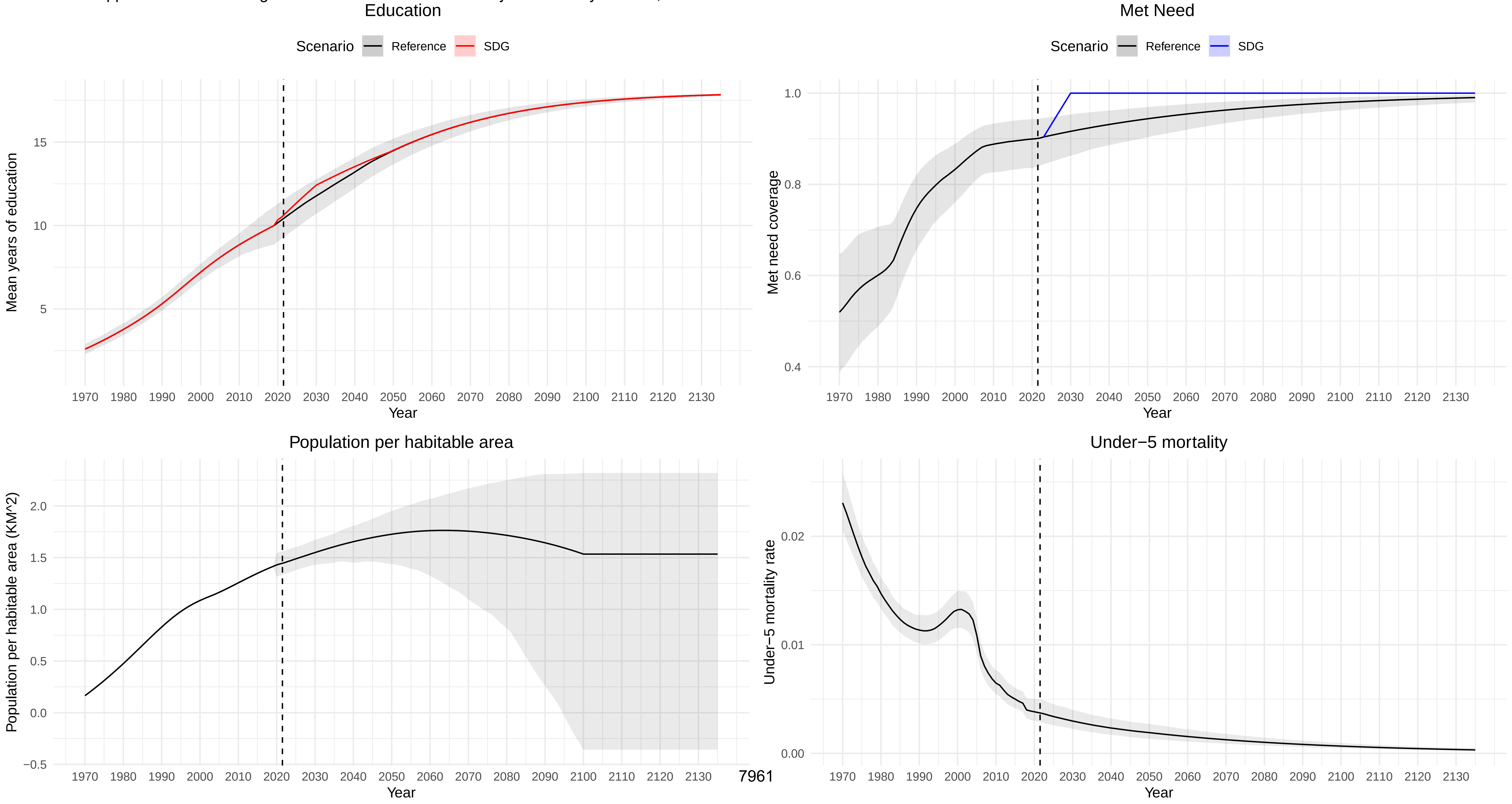

Eswatini

Supplemental Results Figure S2: Covariates used in fertility forecasts by location, 1970-2135

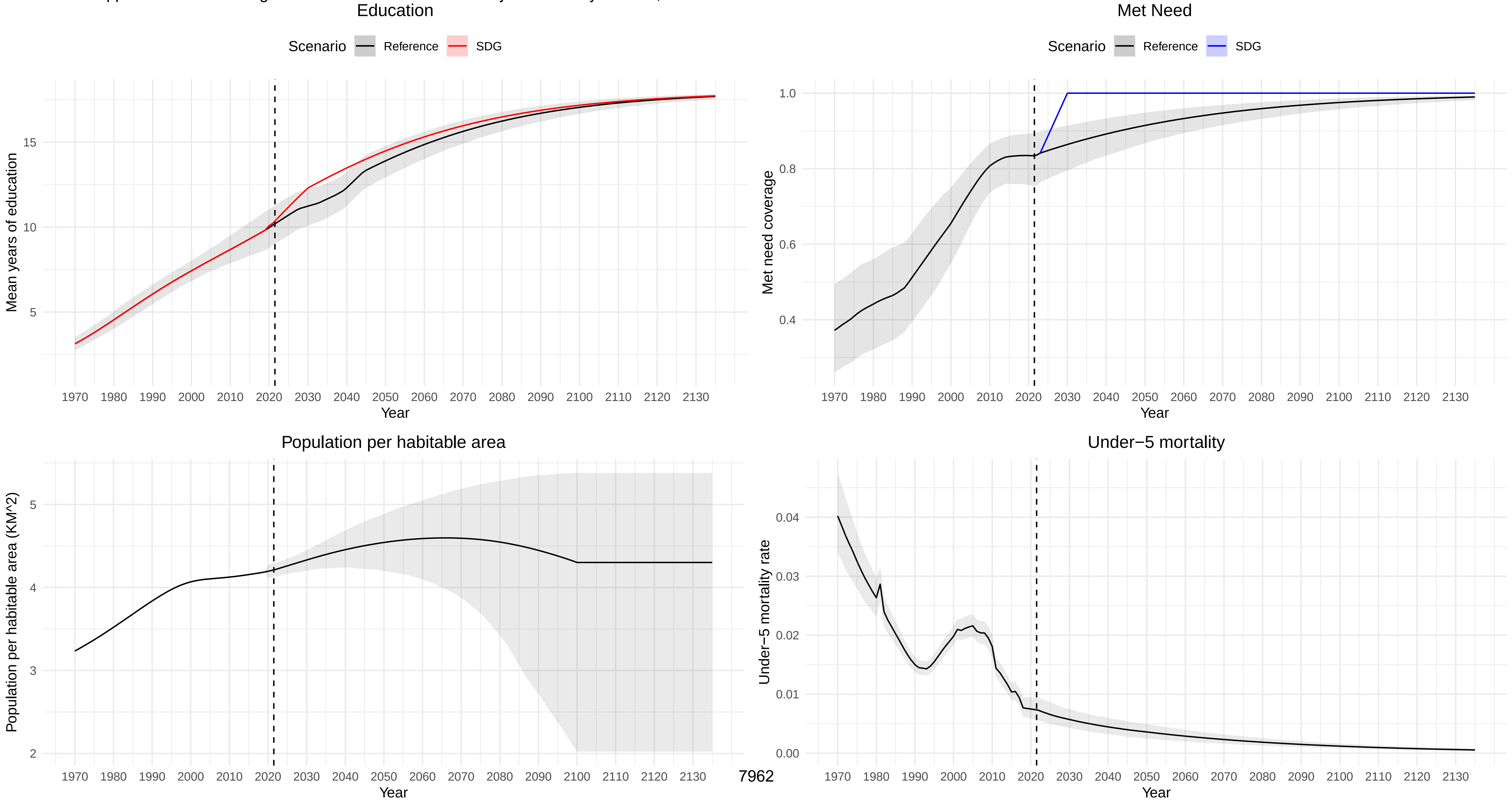

Lesotho

Supplemental Results Figure S2: Covariates used in fertility forecasts by location, 1970-2135

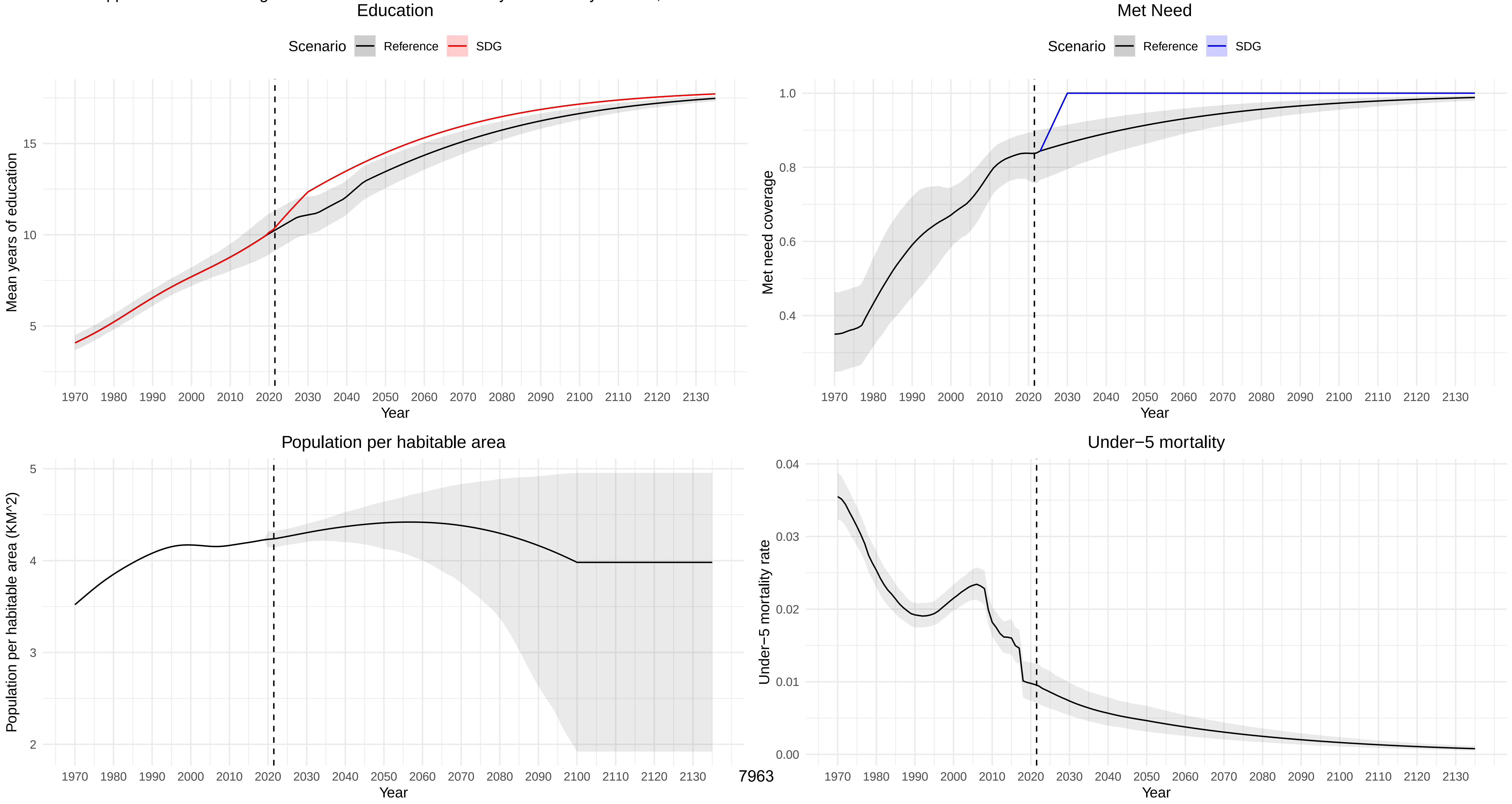

Namibia

Supplemental Results Figure S2: Covariates used in fertility forecasts by location, 1970-2135

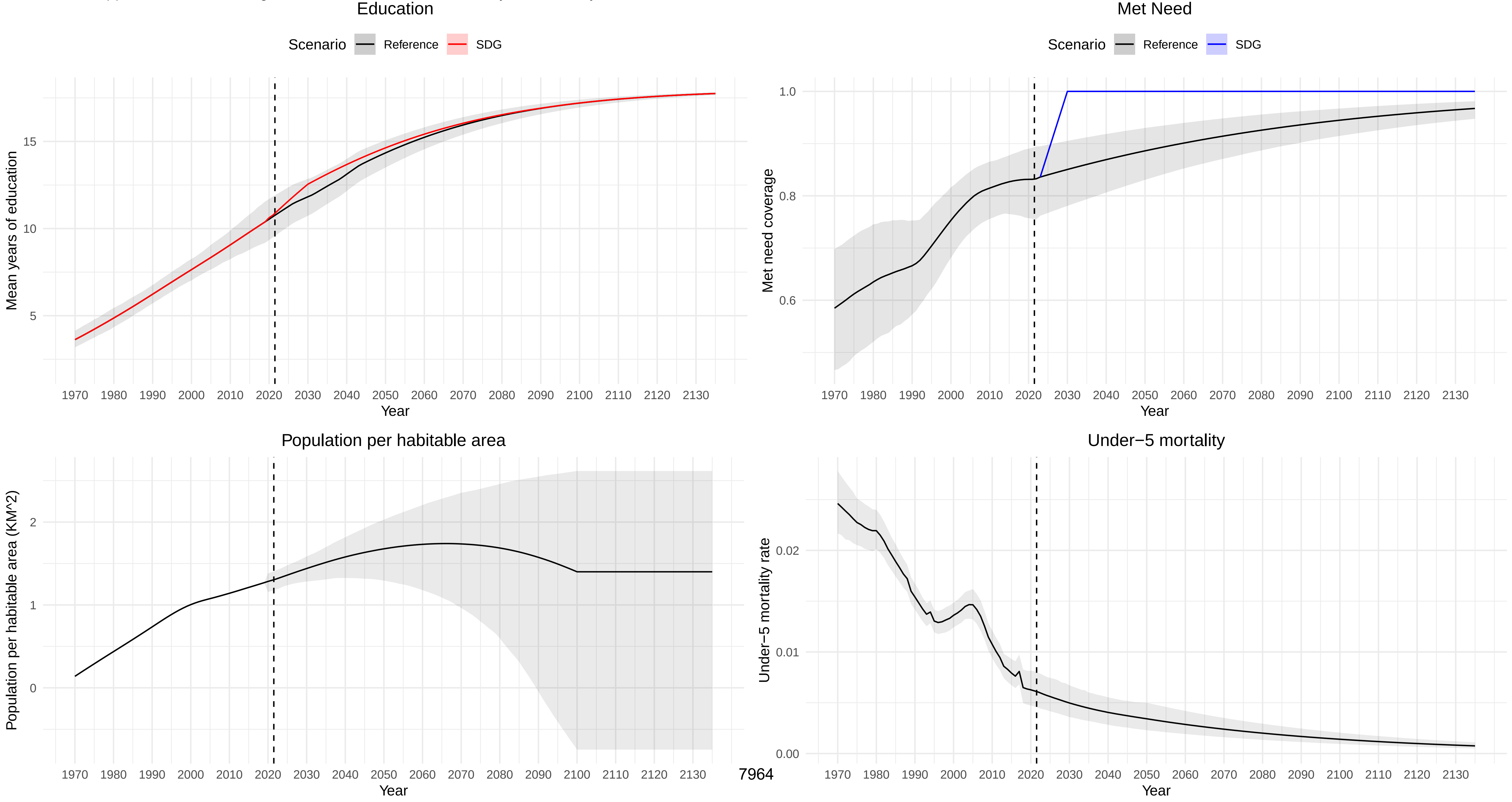

South Africa

Supplemental Results Figure S2: Covariates used in fertility forecasts by location, 1970-2135

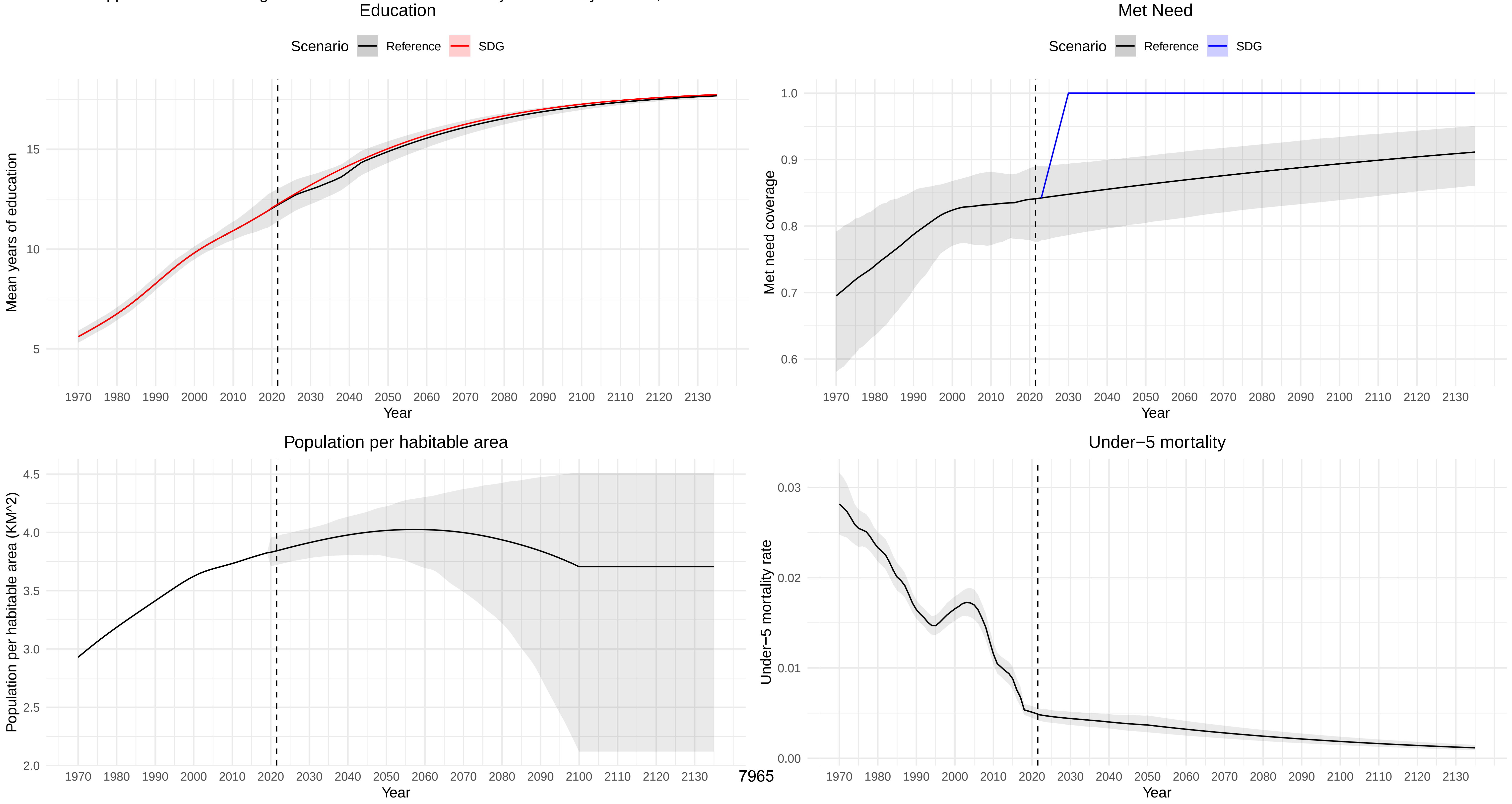

Zimbabwe

Supplemental Results Figure S2: Covariates used in fertility forecasts by location, 1970-2135

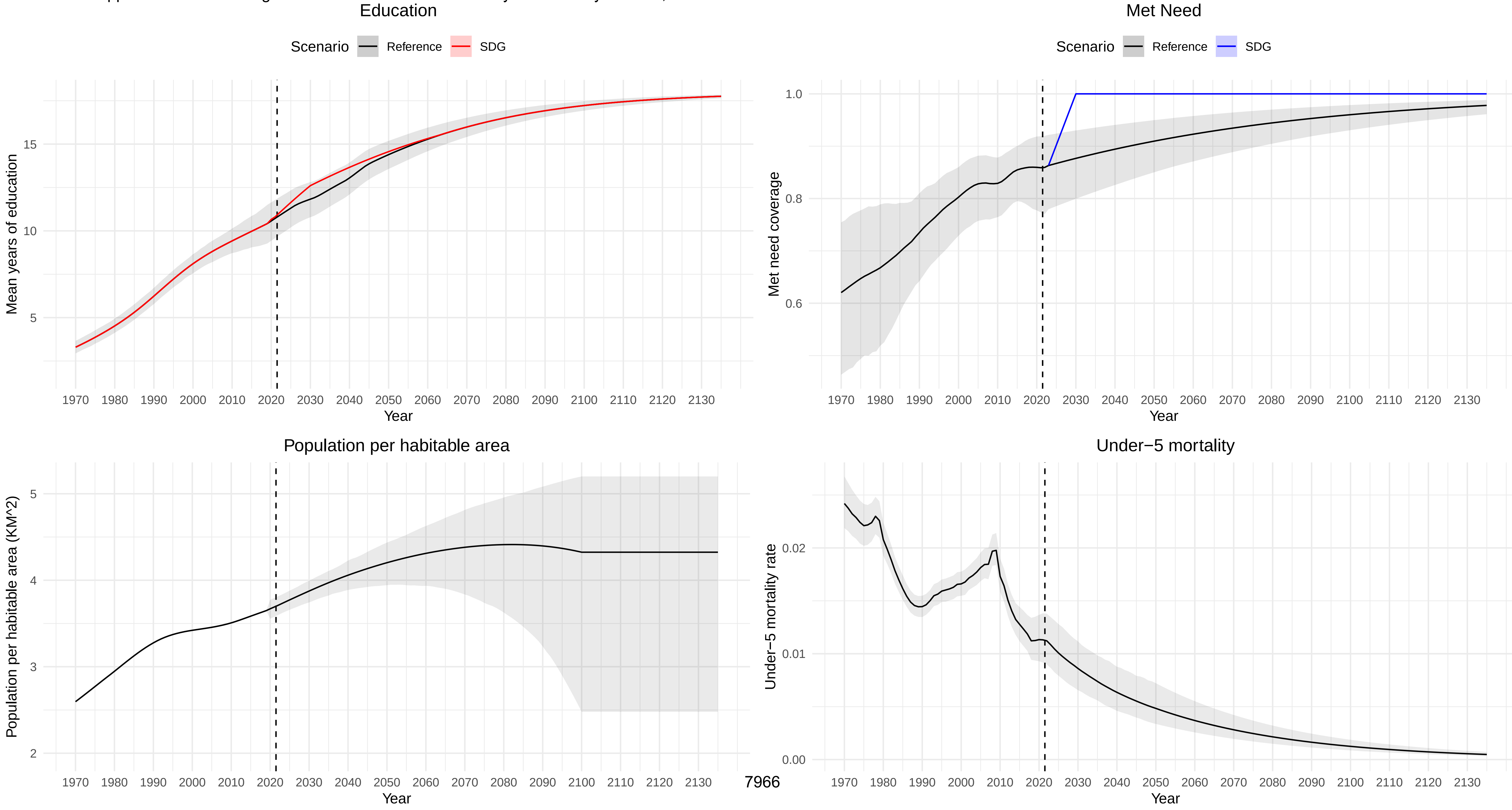

Benin

Supplemental Results Figure S2: Covariates used in fertility forecasts by location, 1970-2135

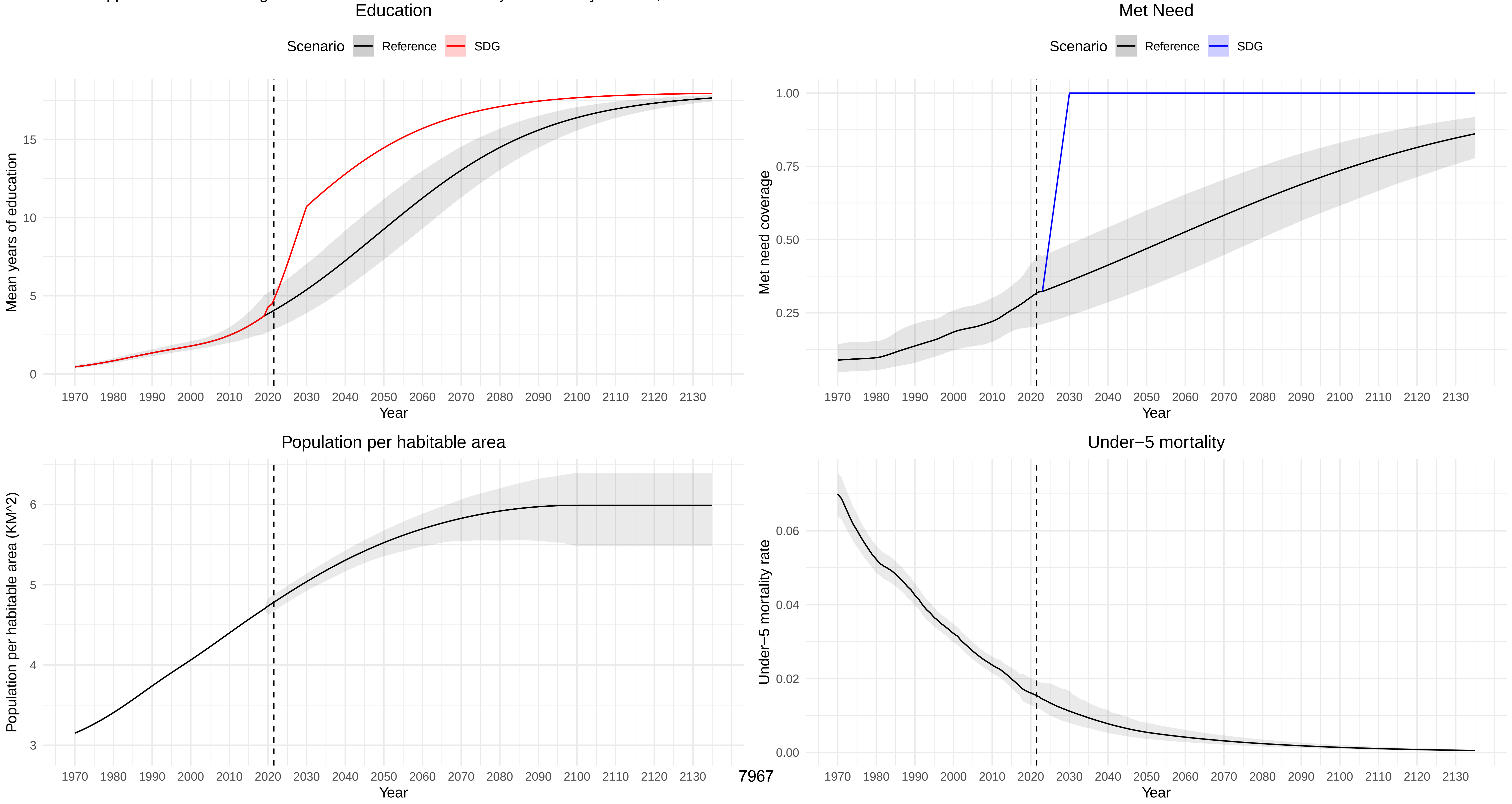

Burkina Faso

Supplemental Results Figure S2: Covariates used in fertility forecasts by location, 1970-2135

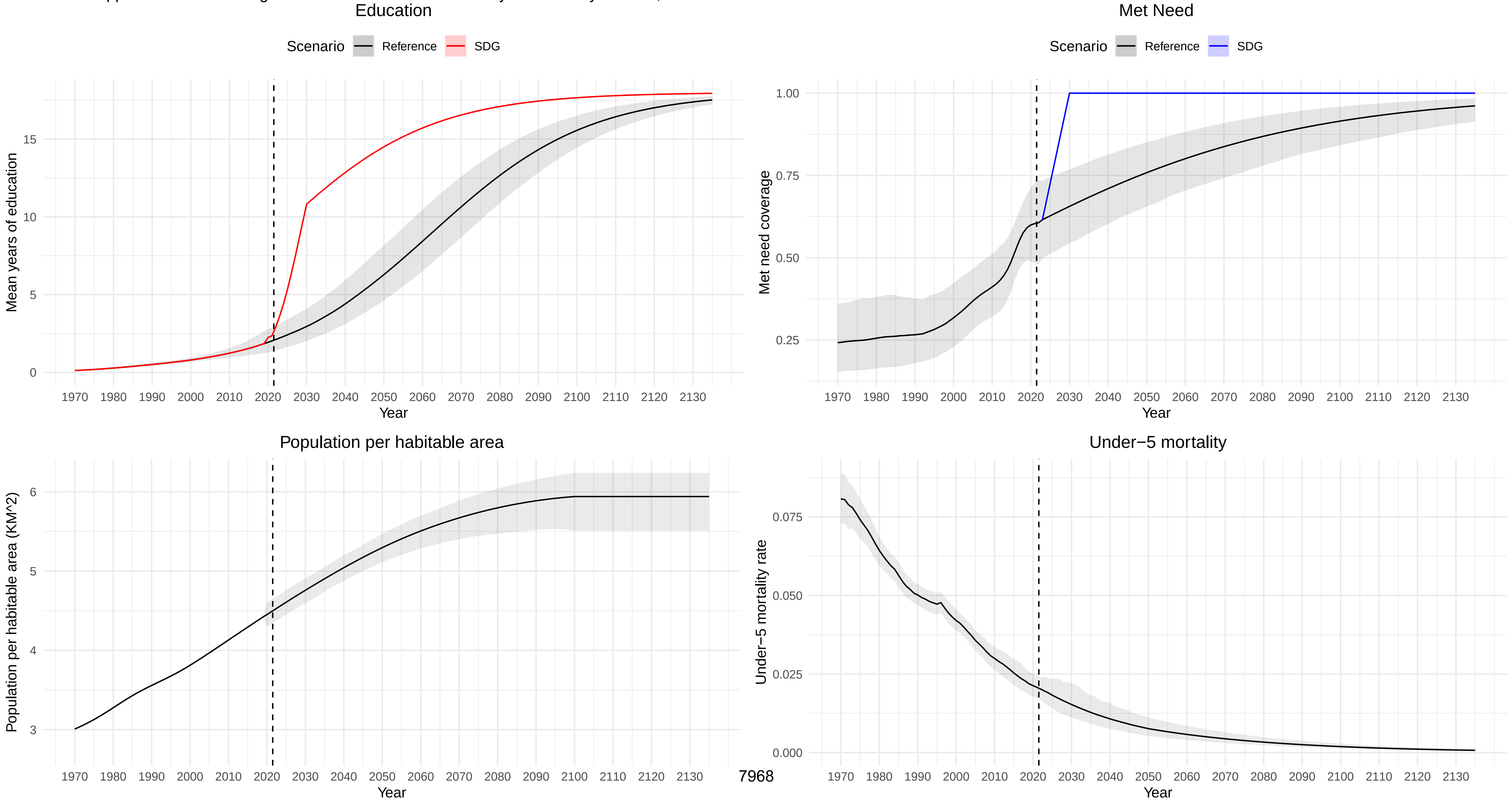

Cabo Verde

Supplemental Results Figure S2: Covariates used in fertility forecasts by location, 1970-2135

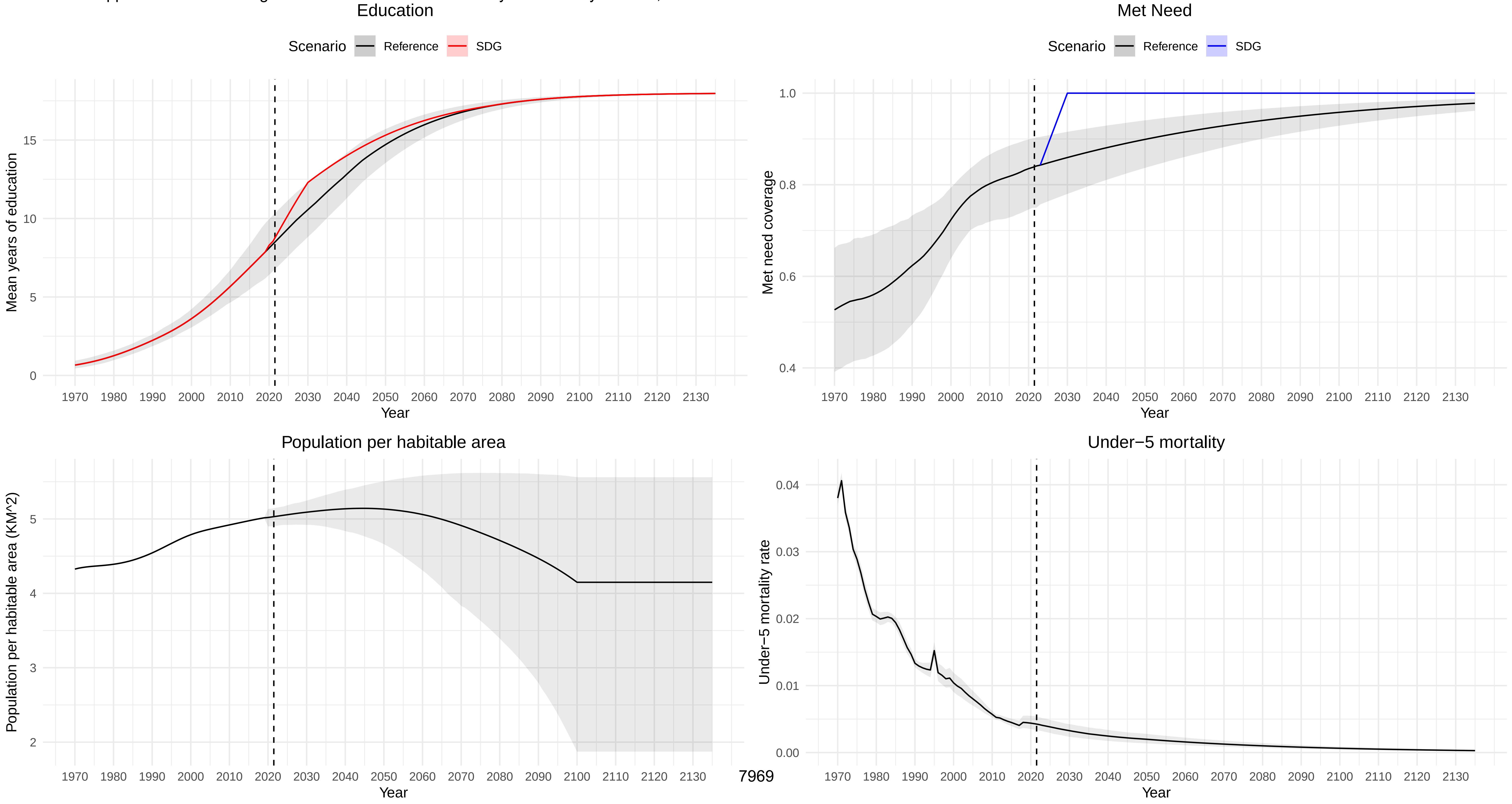

Cameroon

Supplemental Results Figure S2: Covariates used in fertility forecasts by location, 1970-2135

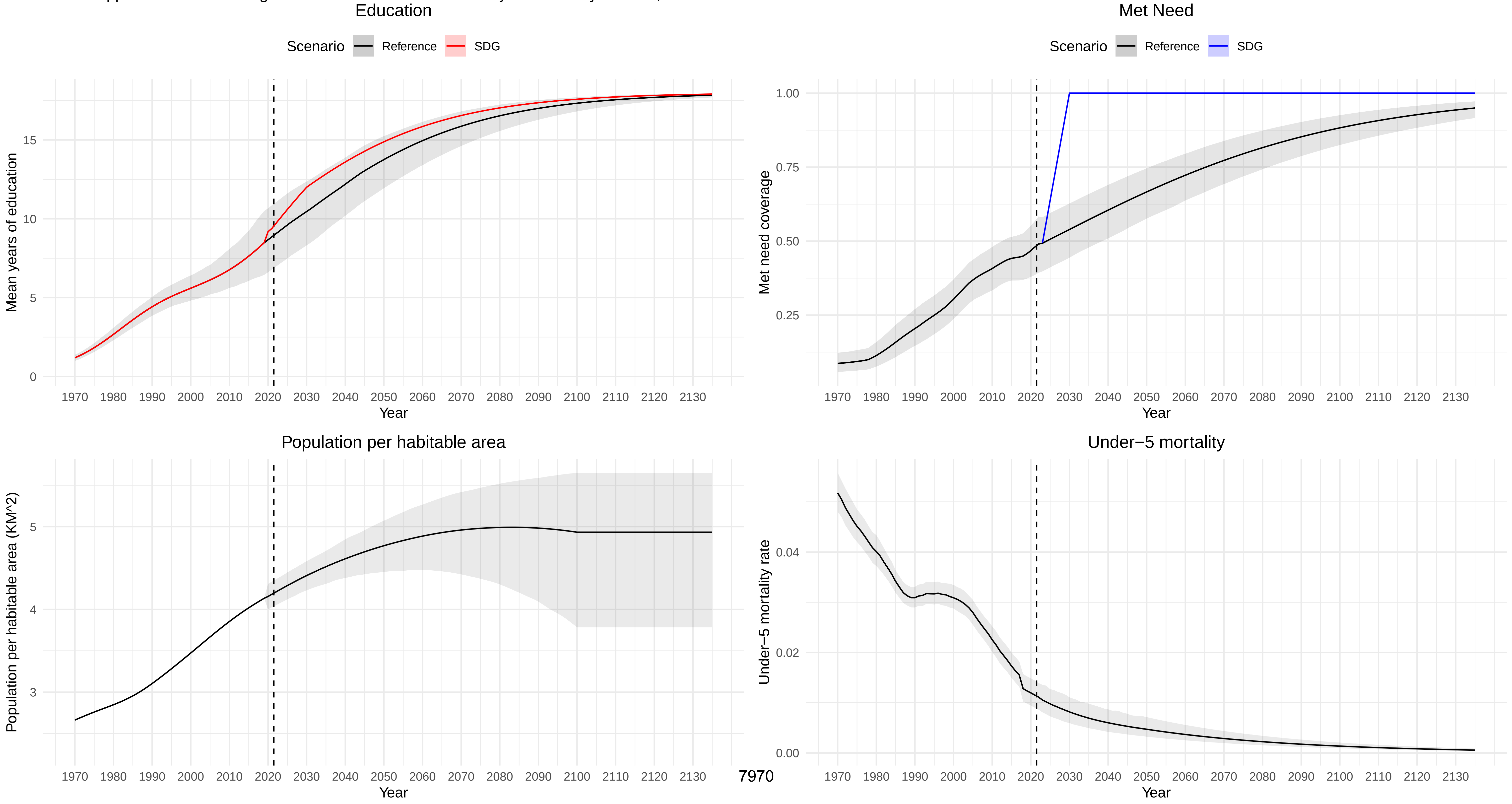

Chad

Supplemental Results Figure S2: Covariates used in fertility forecasts by location, 1970-2135

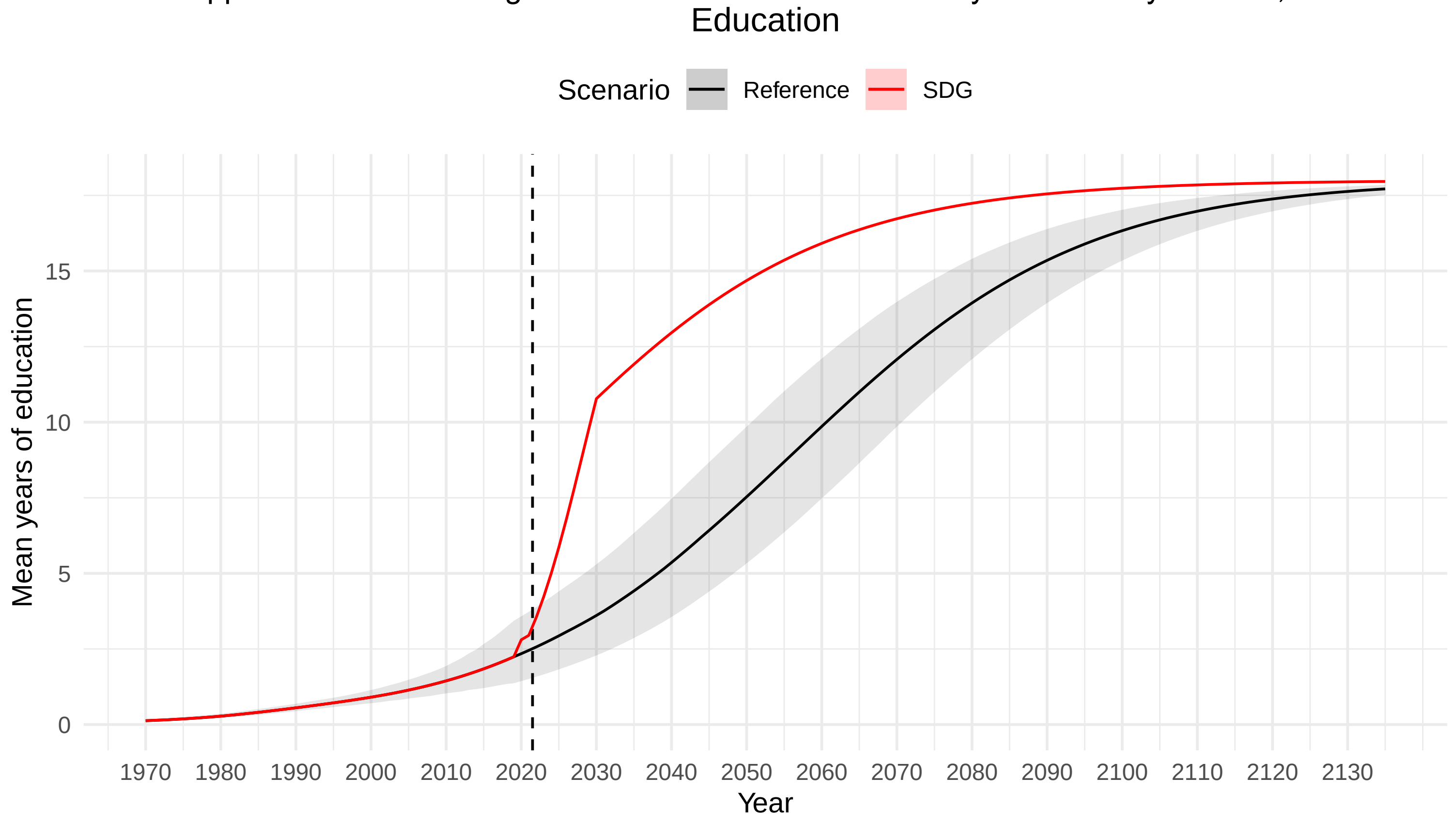

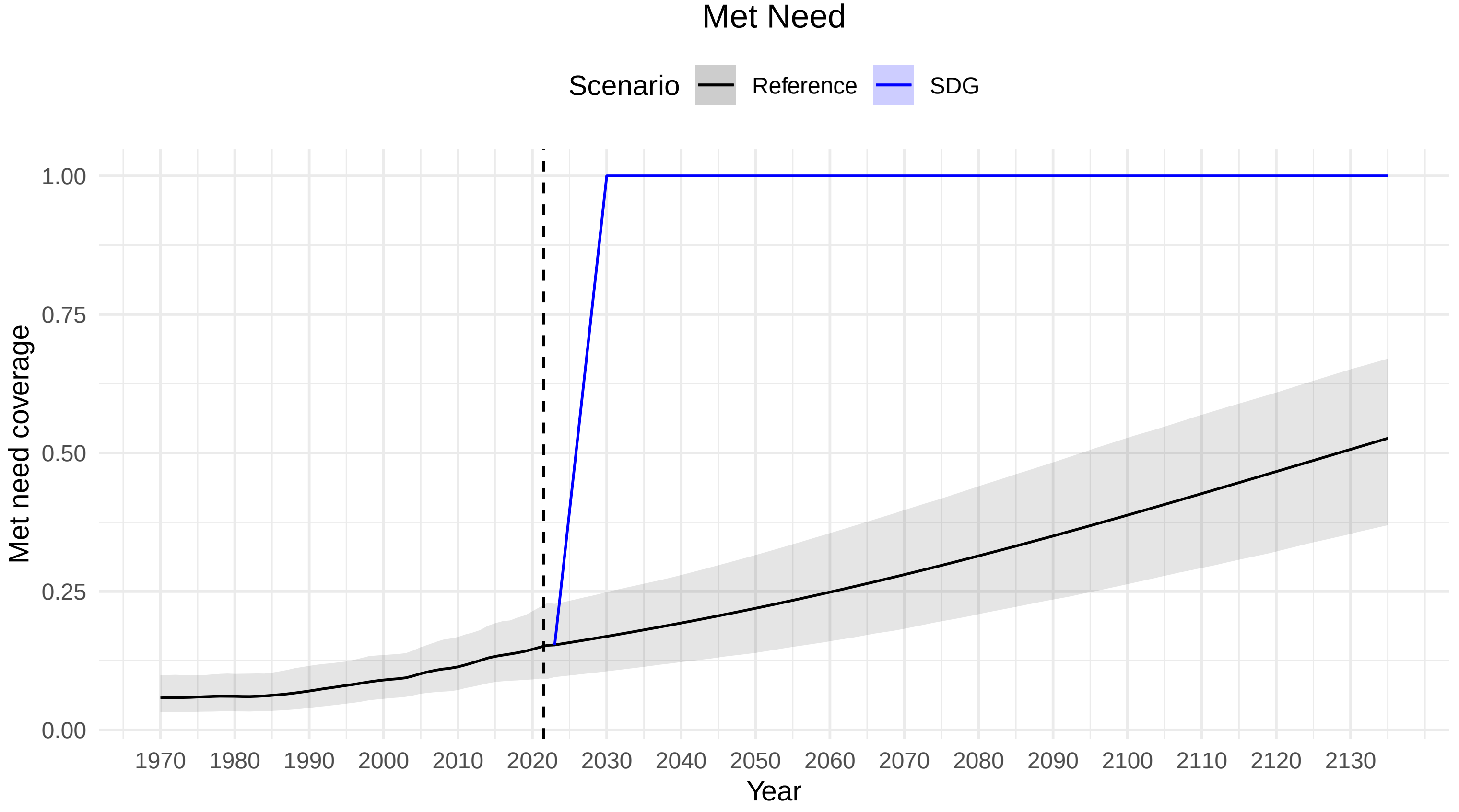

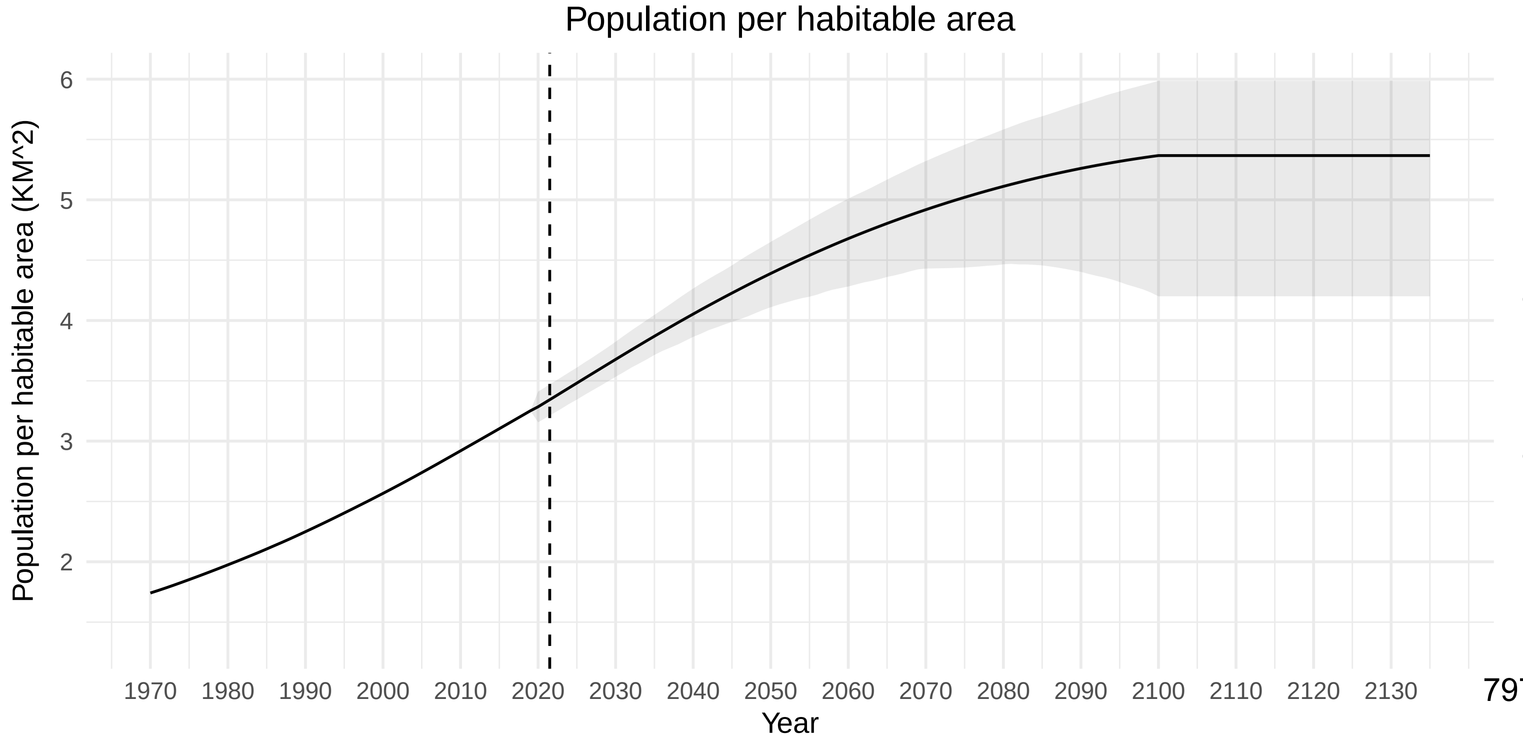

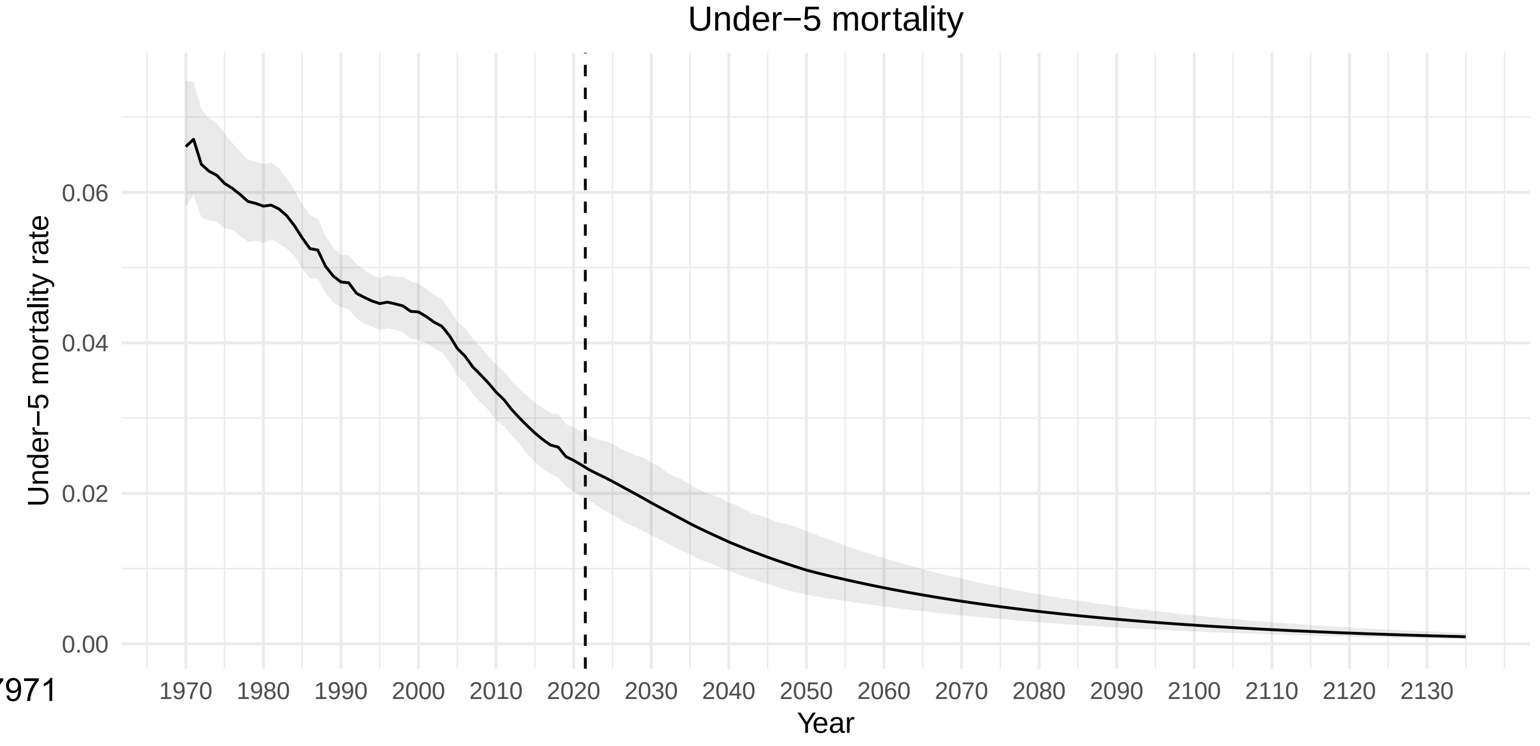

Côte d'Ivoire

Supplemental Results Figure S2: Covariates used in fertility forecasts by location, 1970-2135

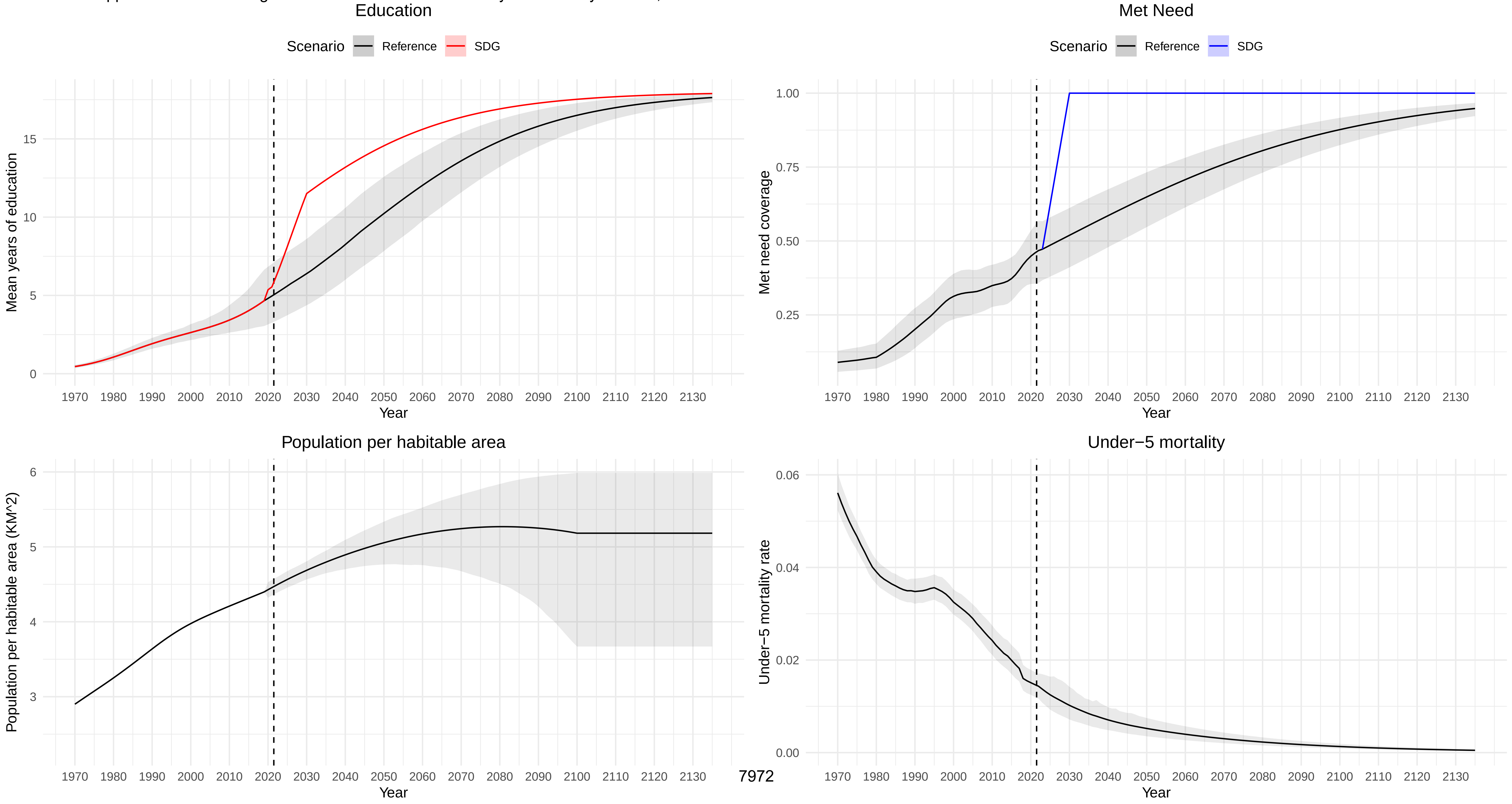

The Gambia

Supplemental Results Figure S2: Covariates used in fertility forecasts by location, 1970-2135

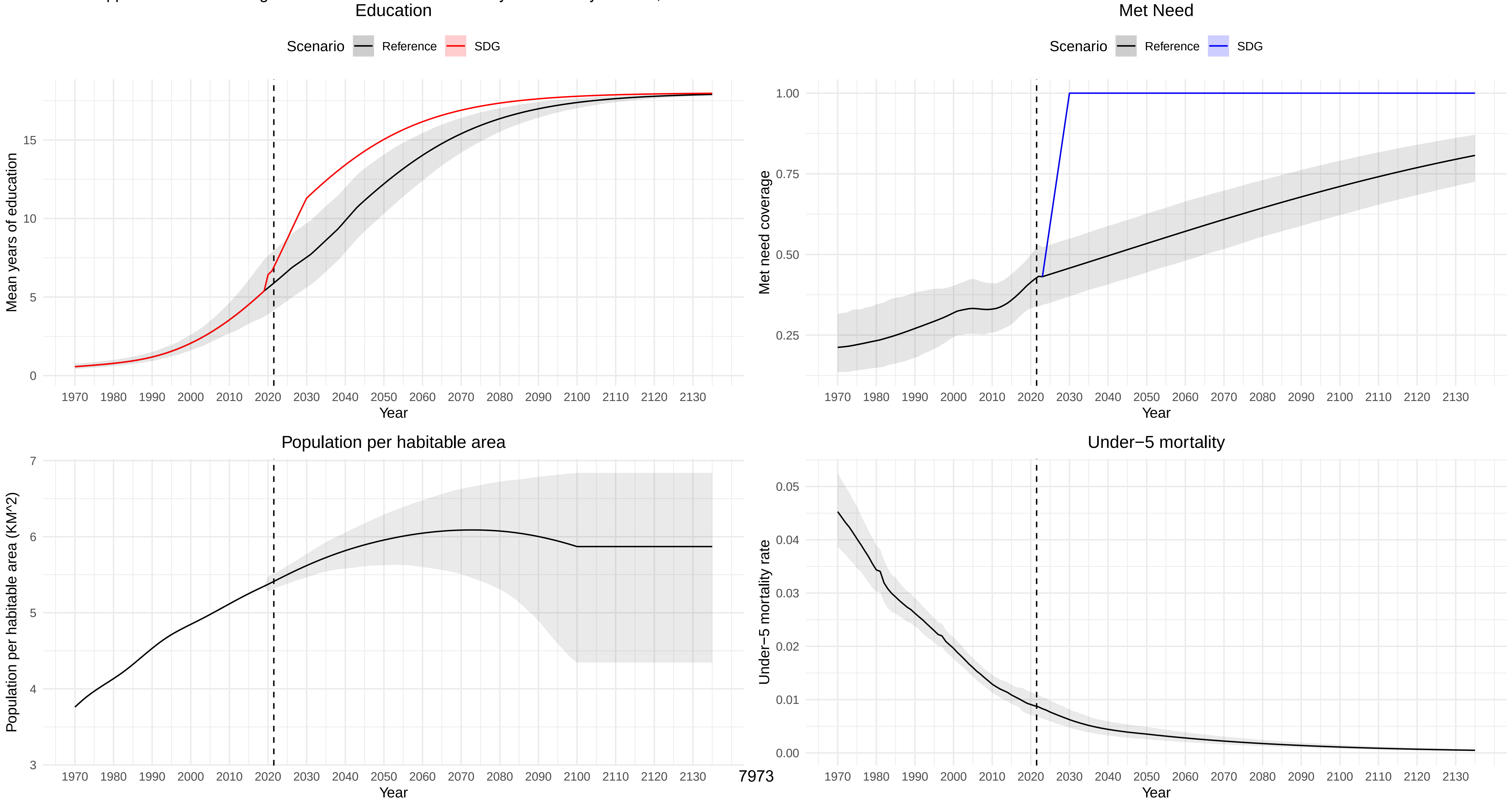

Ghana

Supplemental Results Figure S2: Covariates used in fertility forecasts by location, 1970-2135

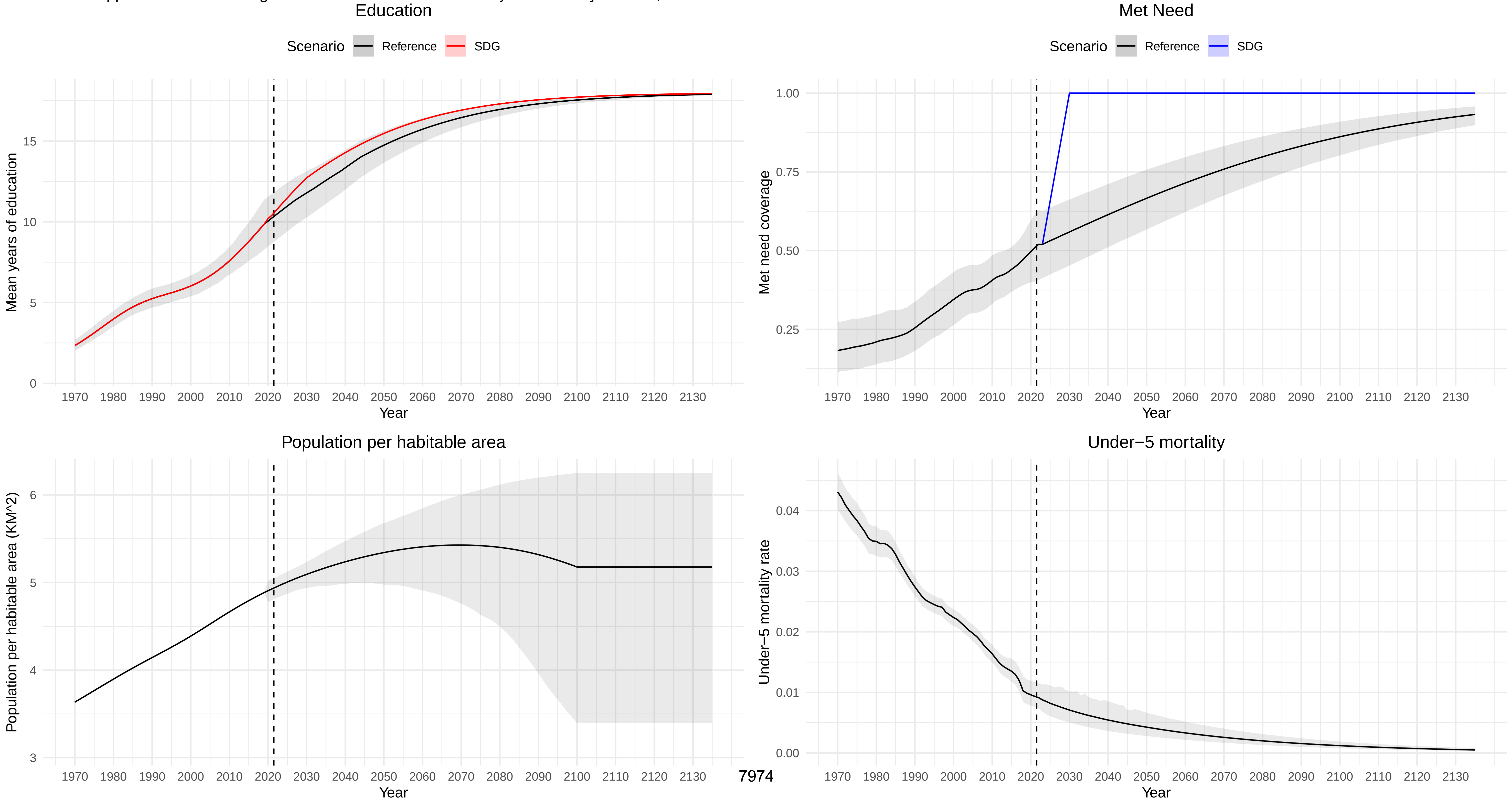

Guinea

Supplemental Results Figure S2: Covariates used in fertility forecasts by location, 1970-2135

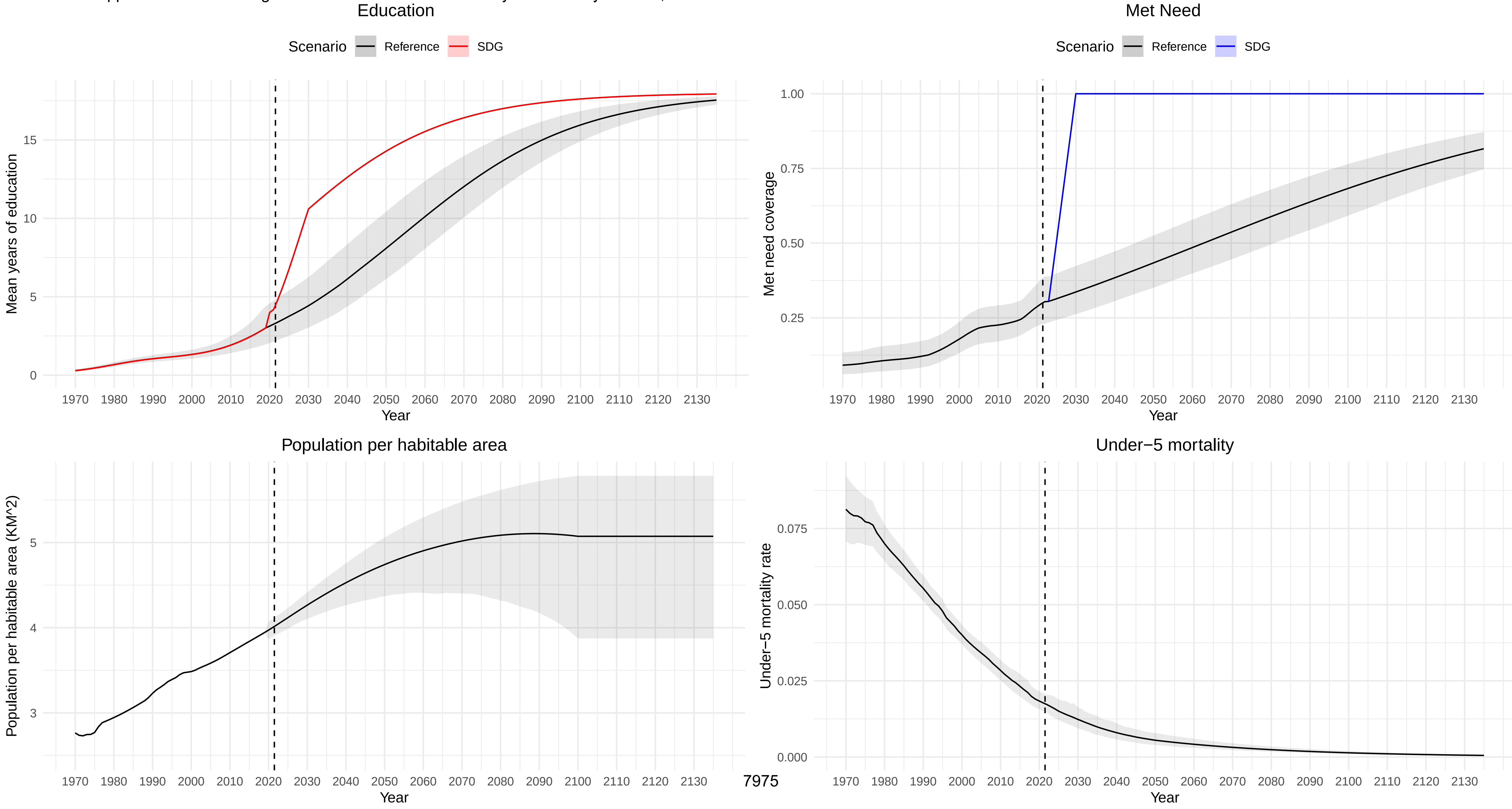

Guinea−Bissau

Supplemental Results Figure S2: Covariates used in fertility forecasts by location, 1970-2135

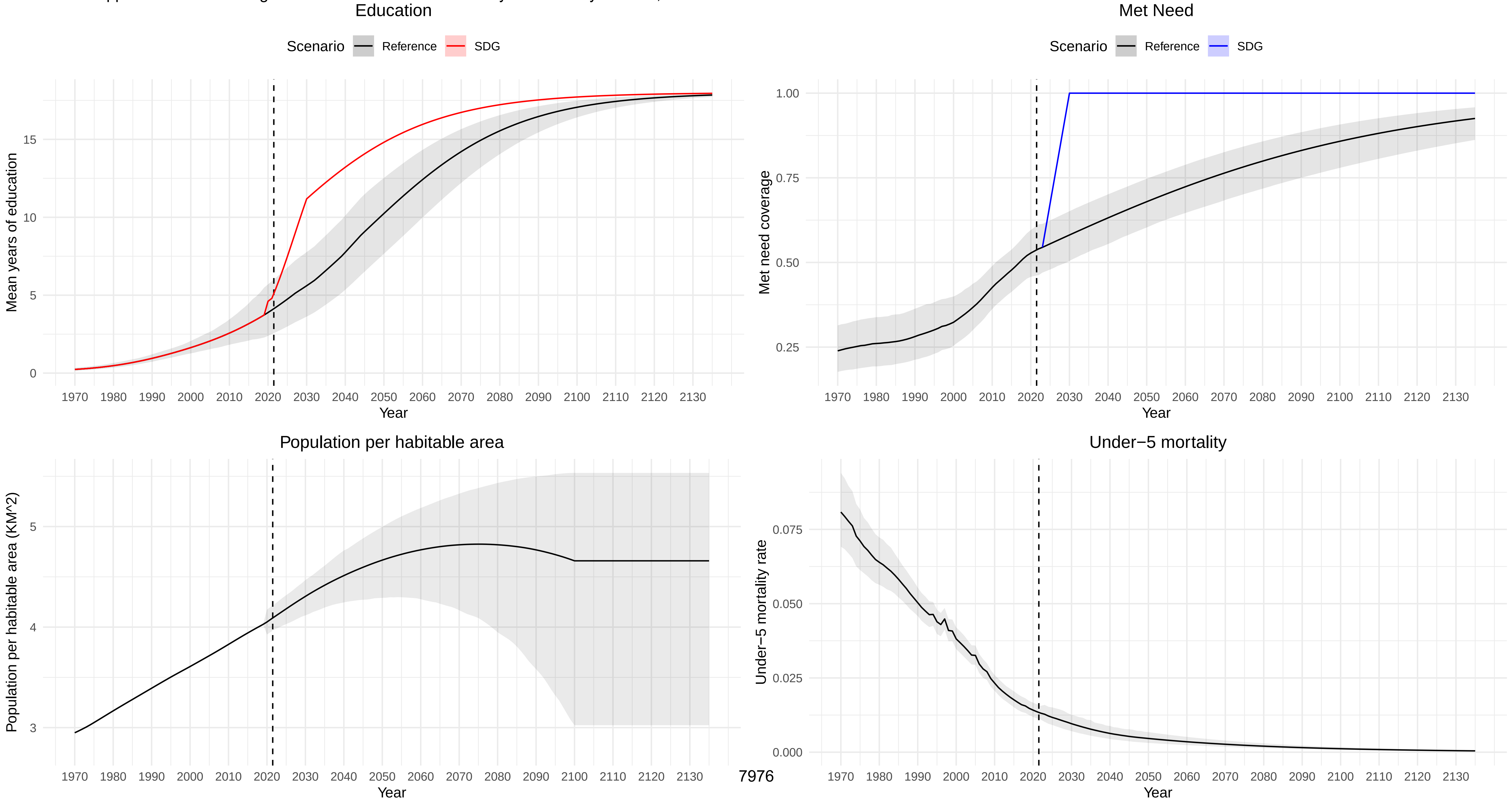

Liberia

Supplemental Results Figure S2: Covariates used in fertility forecasts by location, 1970-2135

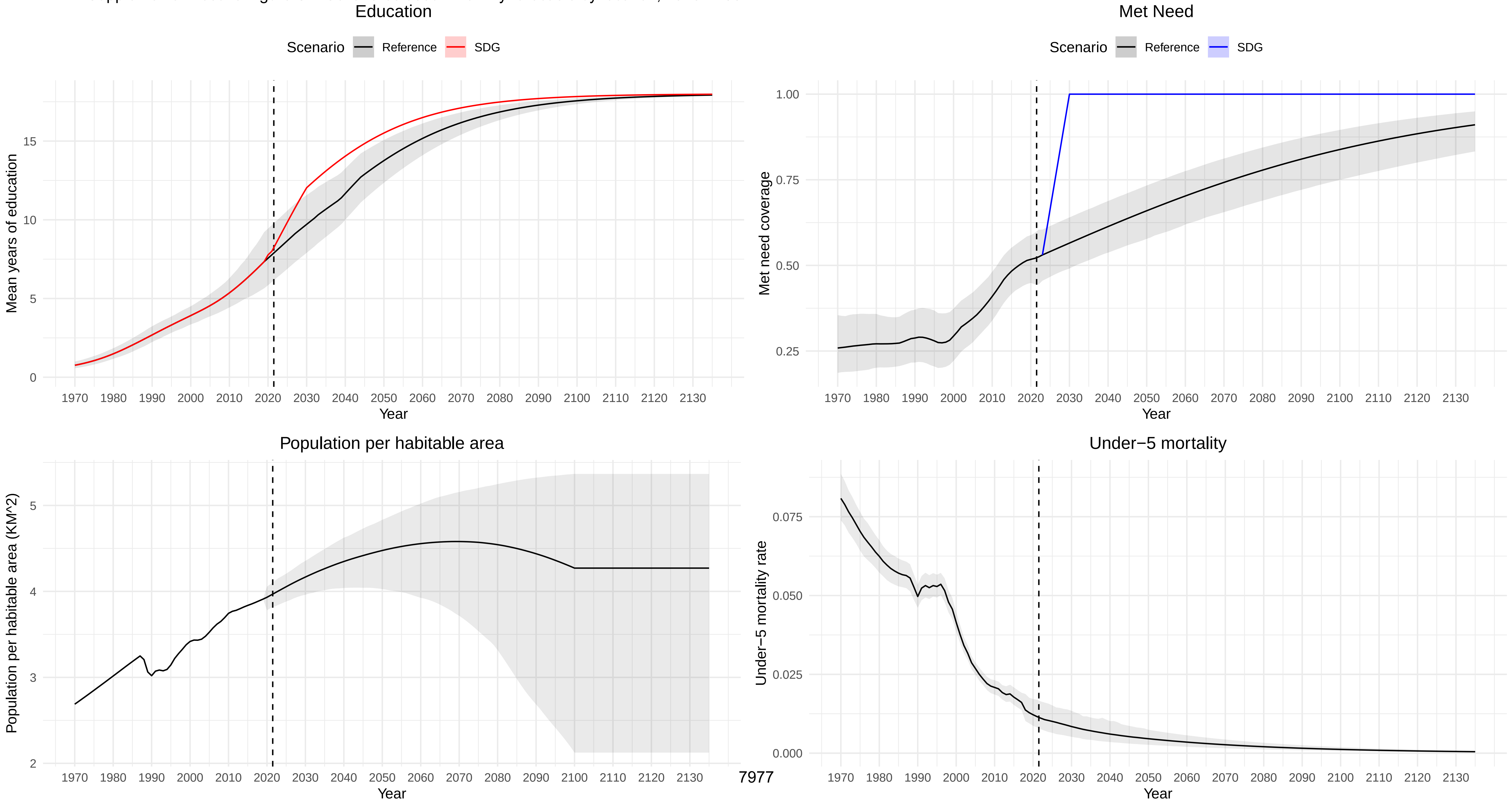

Mali

Supplemental Results Figure S2: Covariates used in fertility forecasts by location, 1970-2135

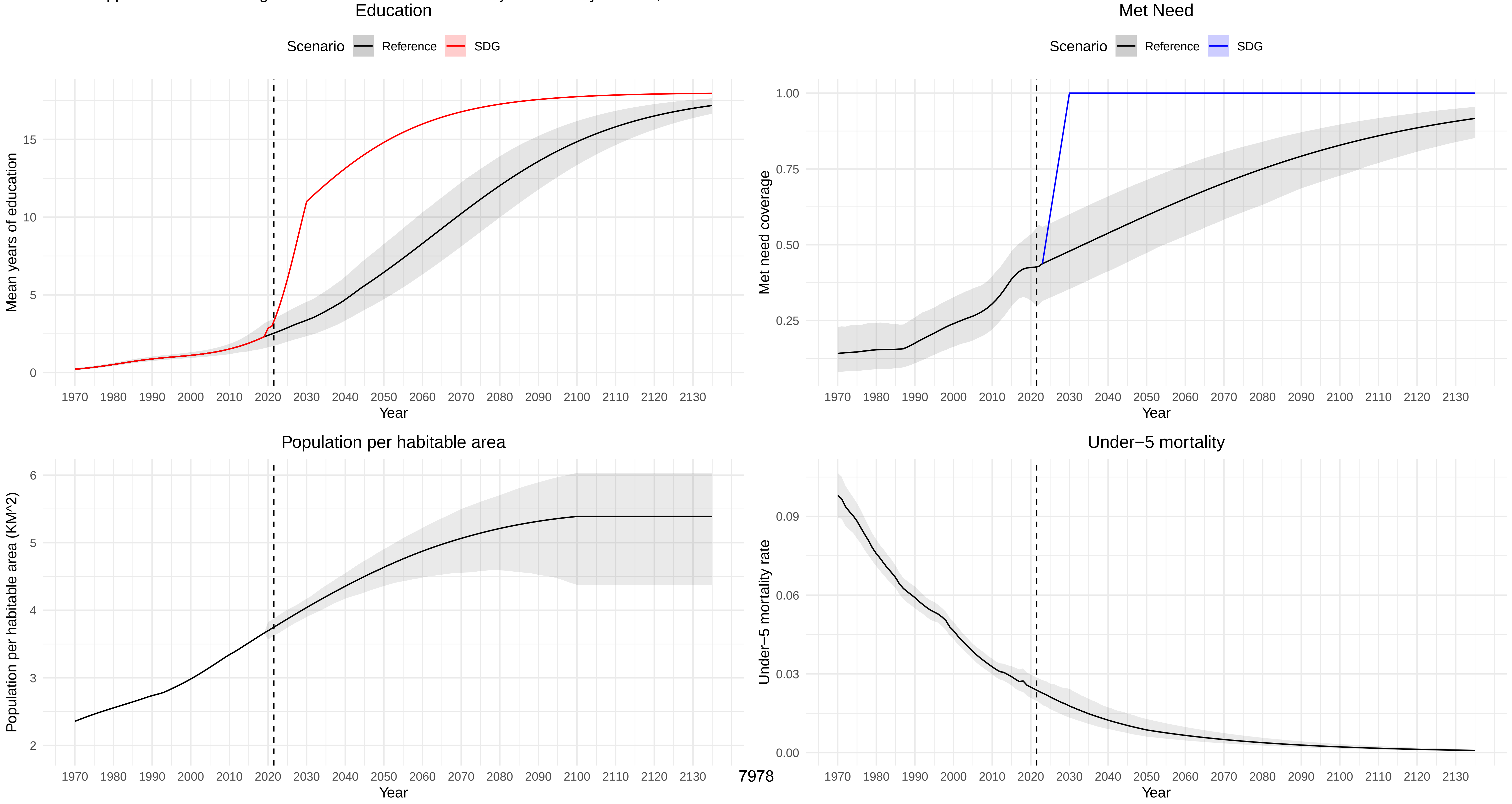

Mauritania

Supplemental Results Figure S2: Covariates used in fertility forecasts by location, 1970-2135

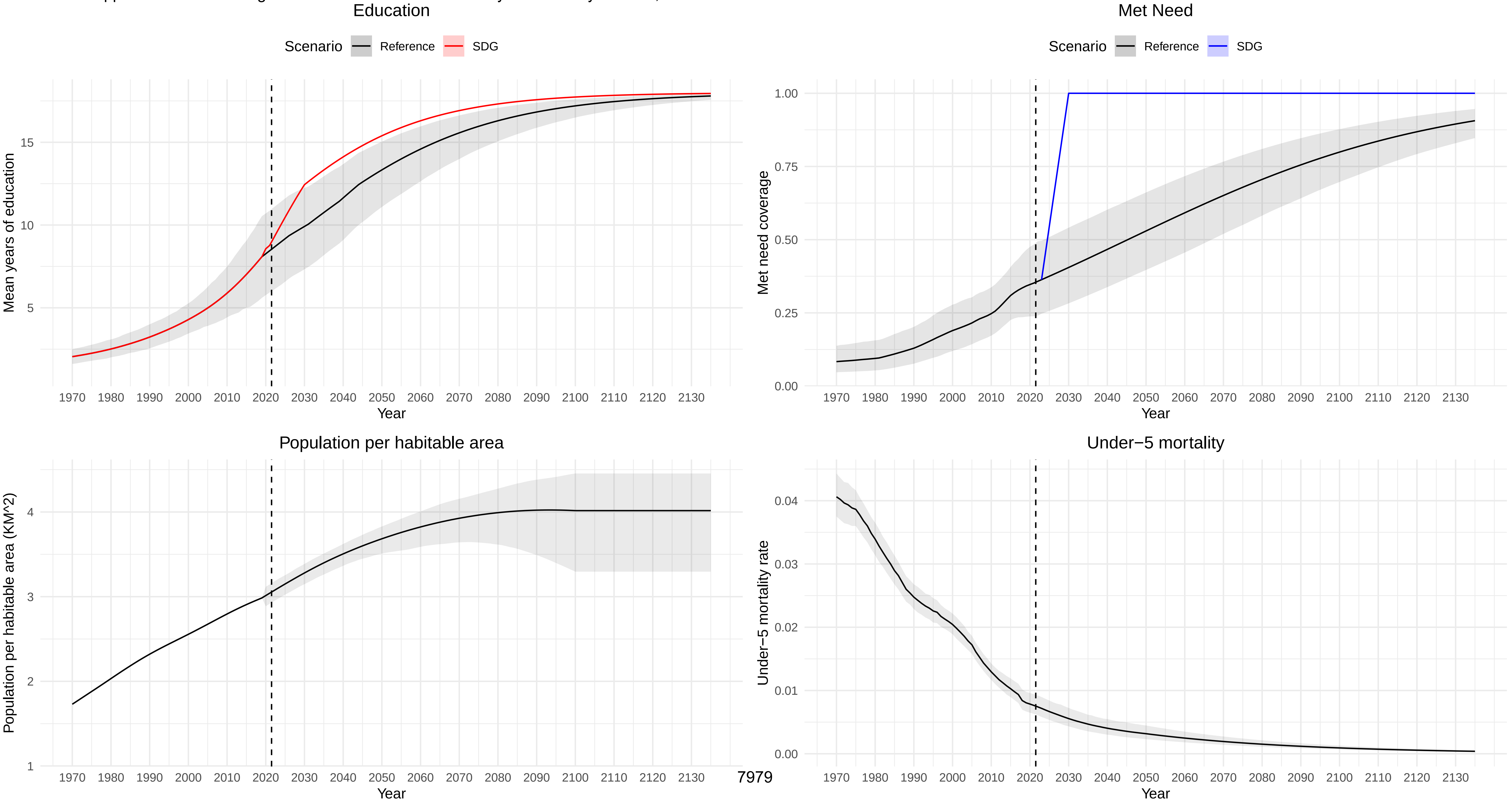

Niger

Supplemental Results Figure S2: Covariates used in fertility forecasts by location, 1970-2135

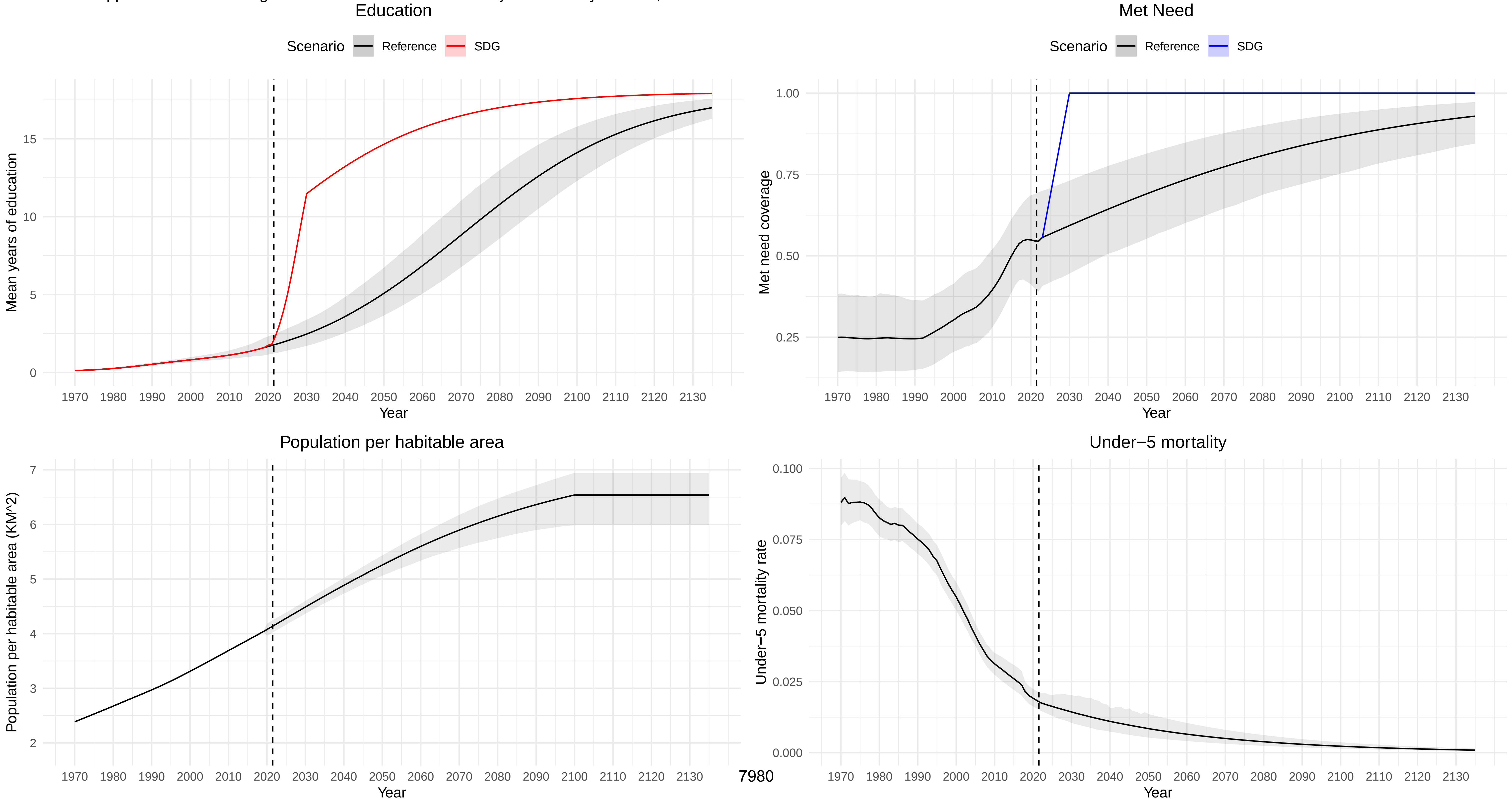

Nigeria

Supplemental Results Figure S2: Covariates used in fertility forecasts by location, 1970-2135

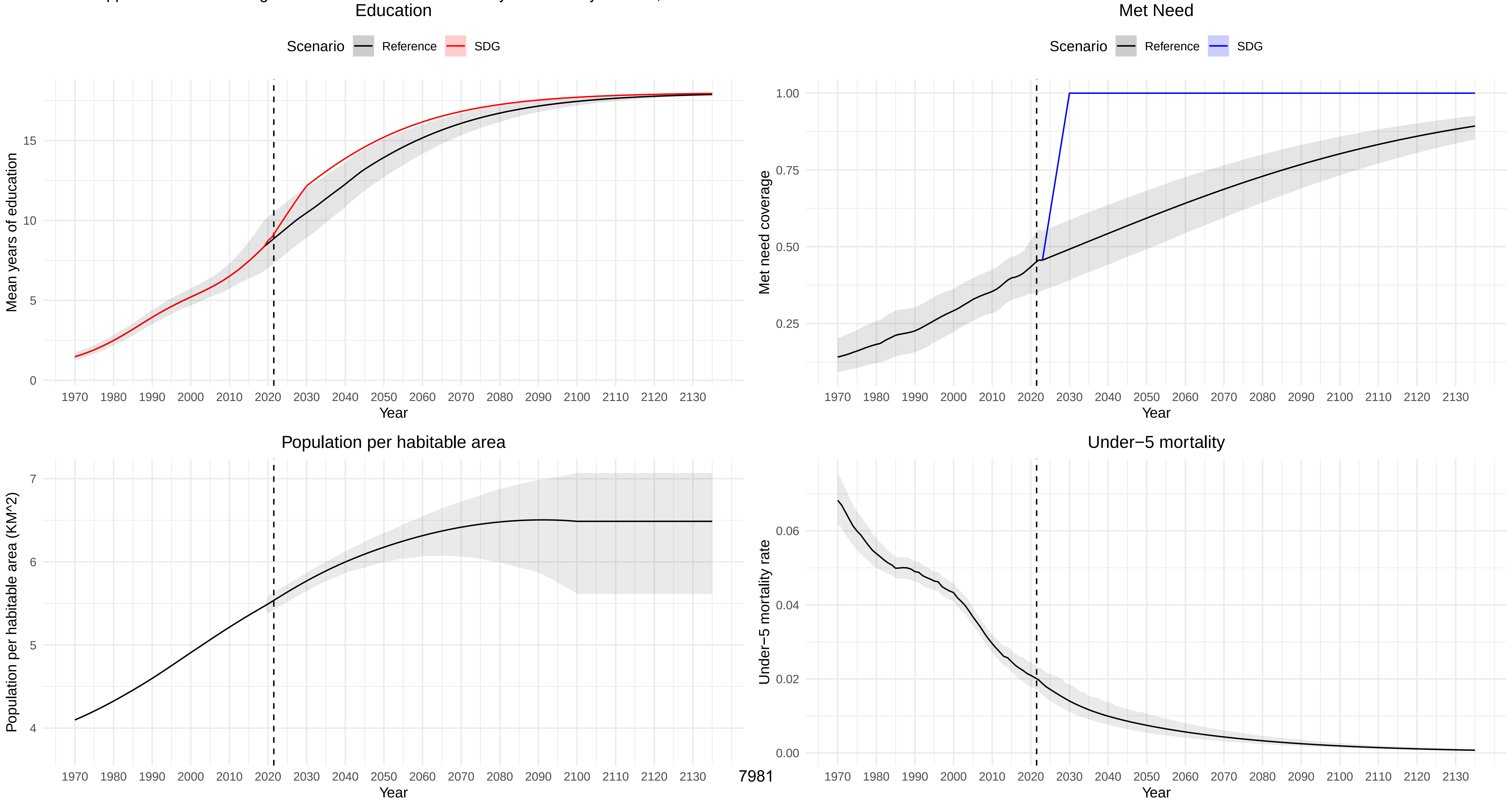

São Tomé and Príncipe

Supplemental Results Figure S2: Covariates used in fertility forecasts by location, 1970-2135

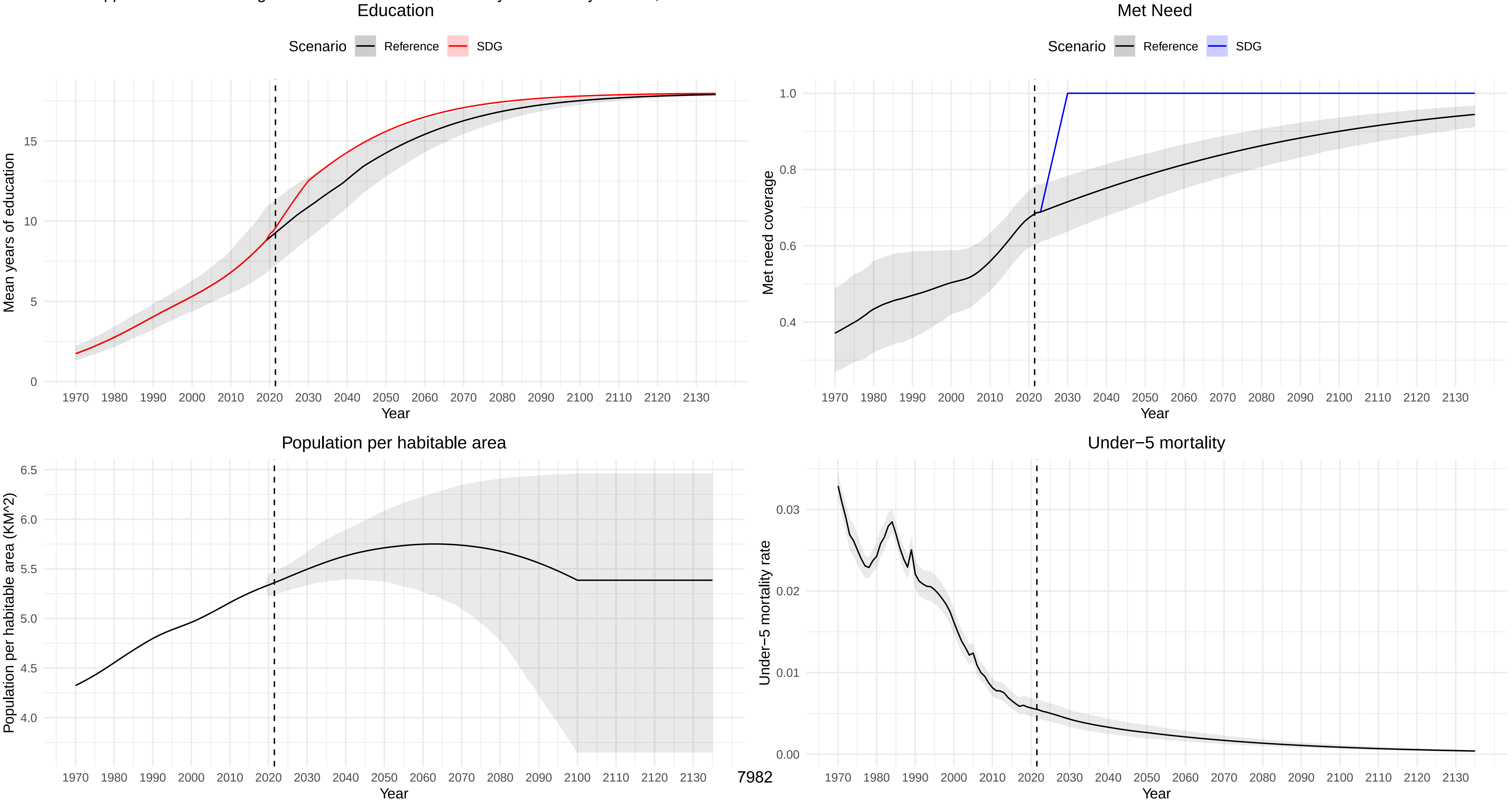

Senegal

Supplemental Results Figure S2: Covariates used in fertility forecasts by location, 1970-2135

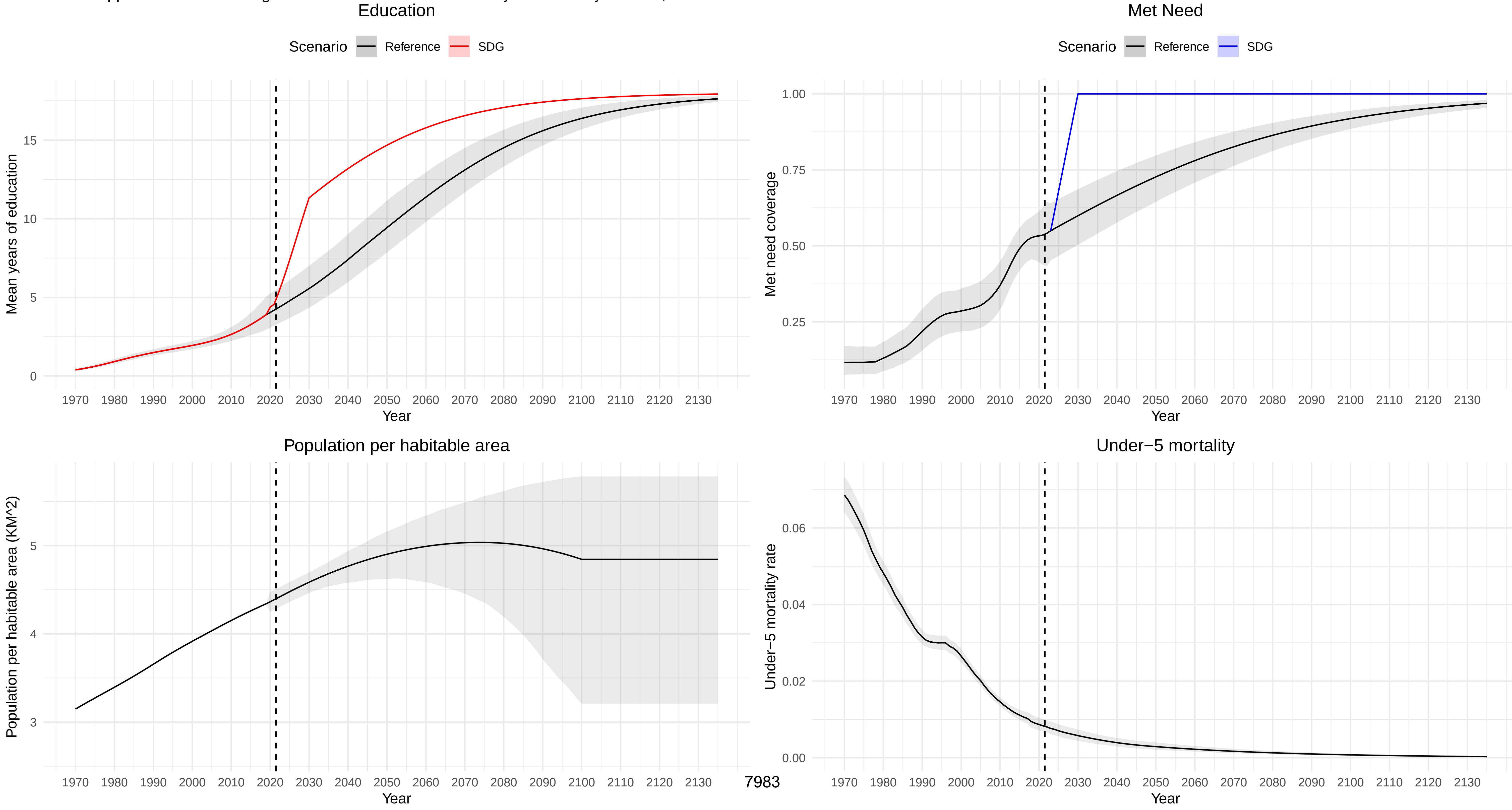

Sierra Leone

Supplemental Results Figure S2: Covariates used in fertility forecasts by location, 1970-2135

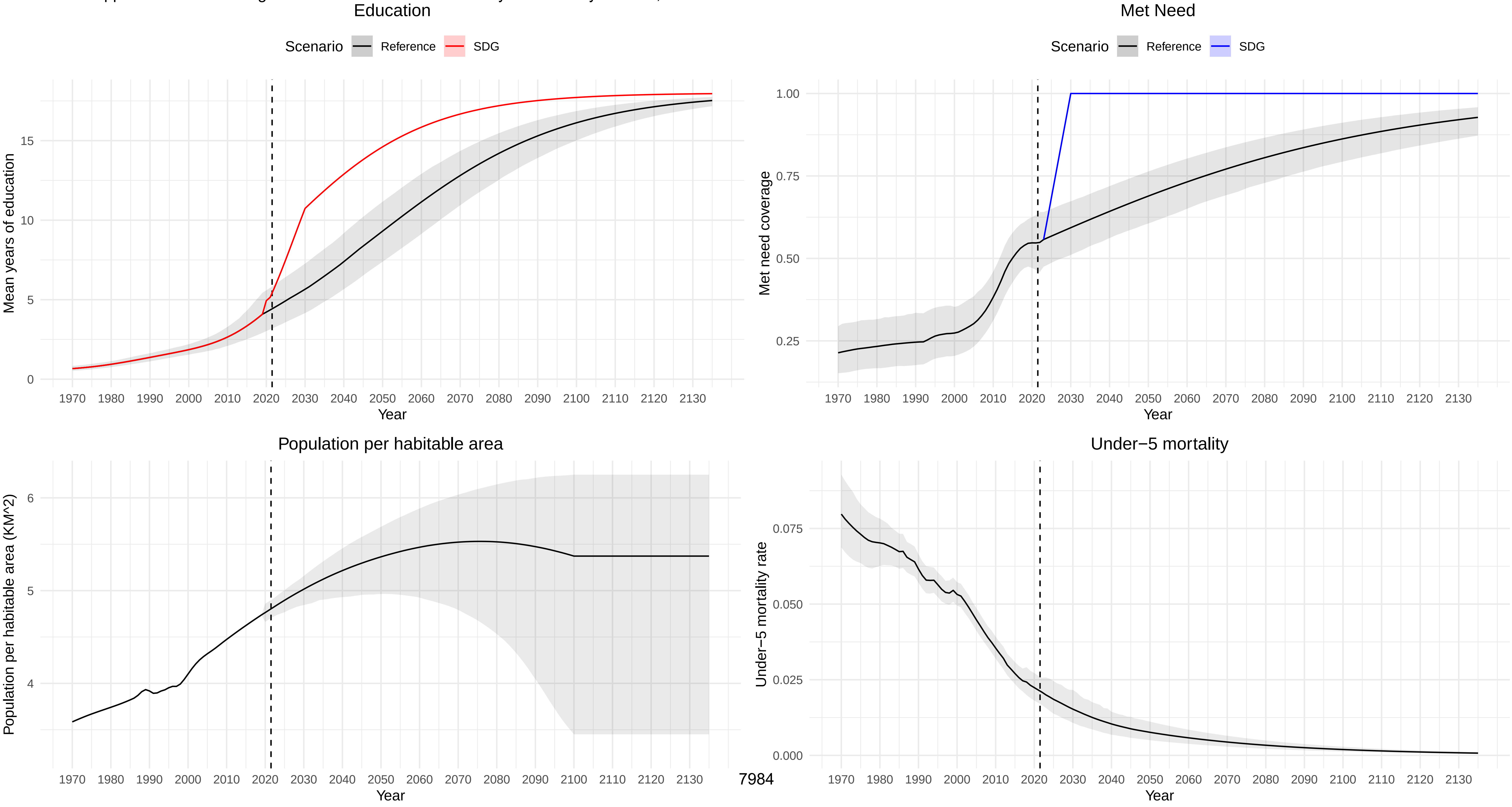

Togo

Supplemental Results Figure S2: Covariates used in fertility forecasts by location, 1970-2135

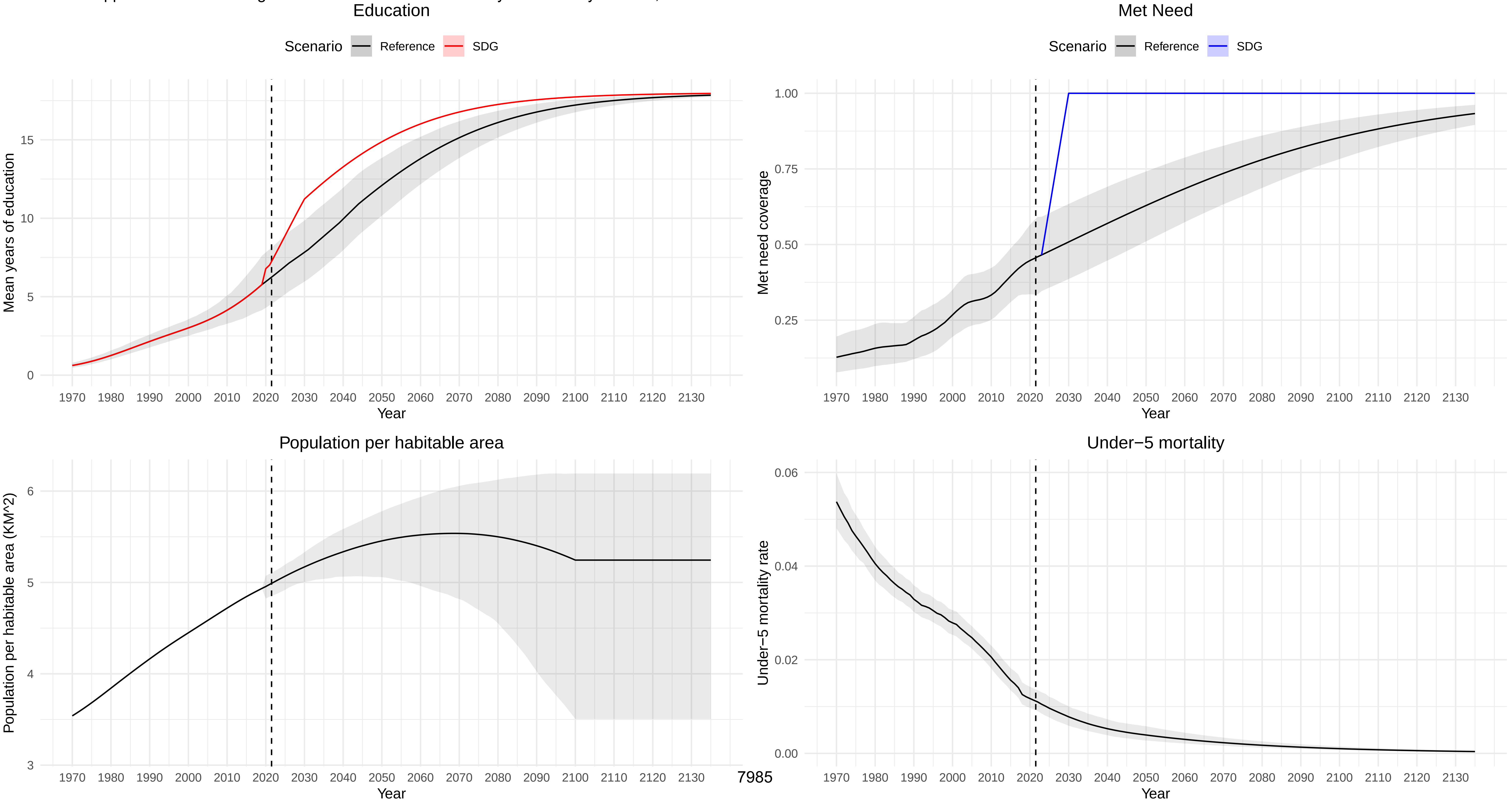

Supplemental Results Figure S3: Lexis diagram of CCF50, single-year age interval ASFR, and TFR by mother's age and location, 1950-2100, Armenia

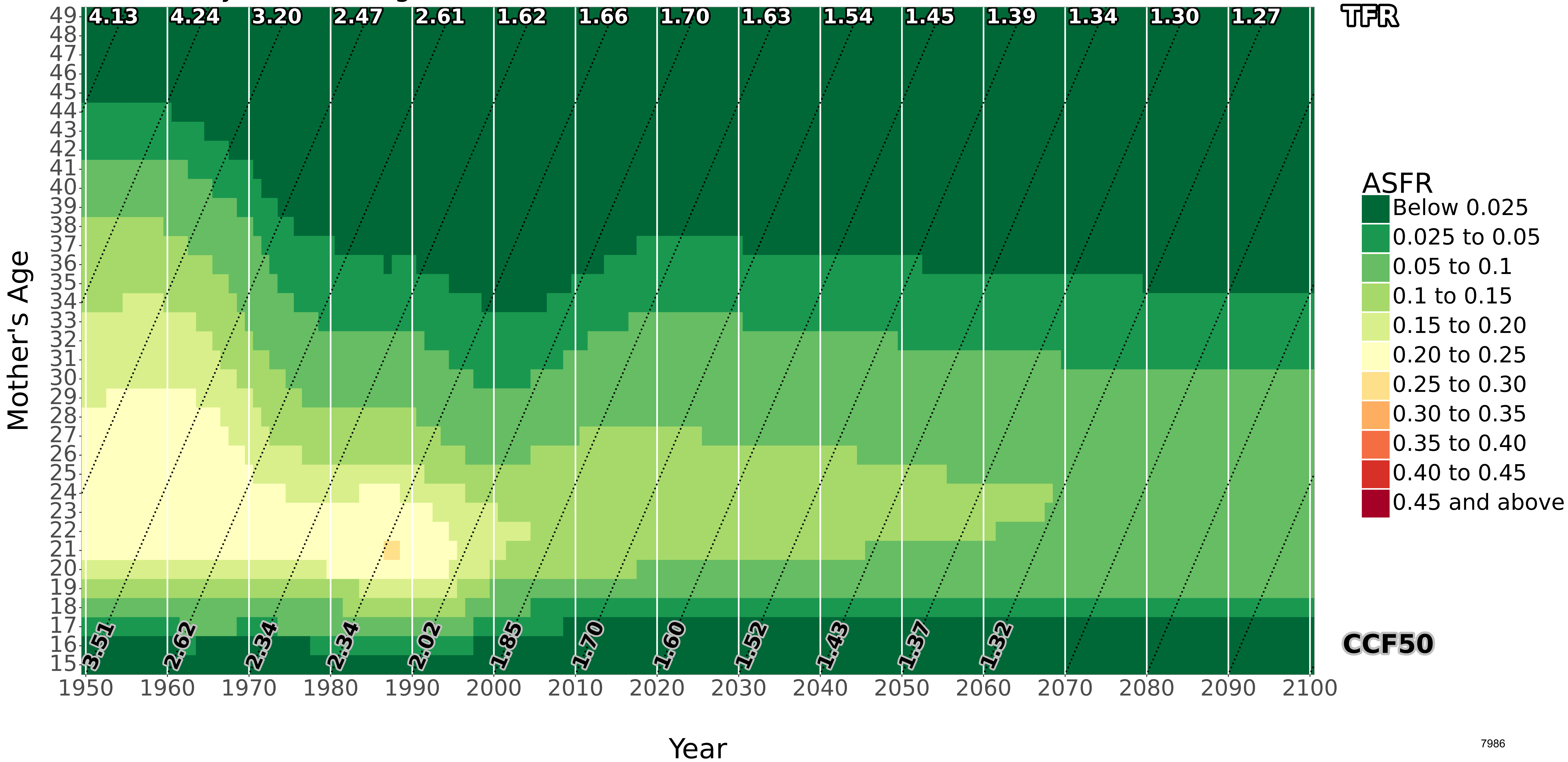

Supplemental Results Figure S3: Lexis diagram of CCF50, single-year age interval ASFR, and TFR by mother's age and location, 1950-2100, Azerbaijan

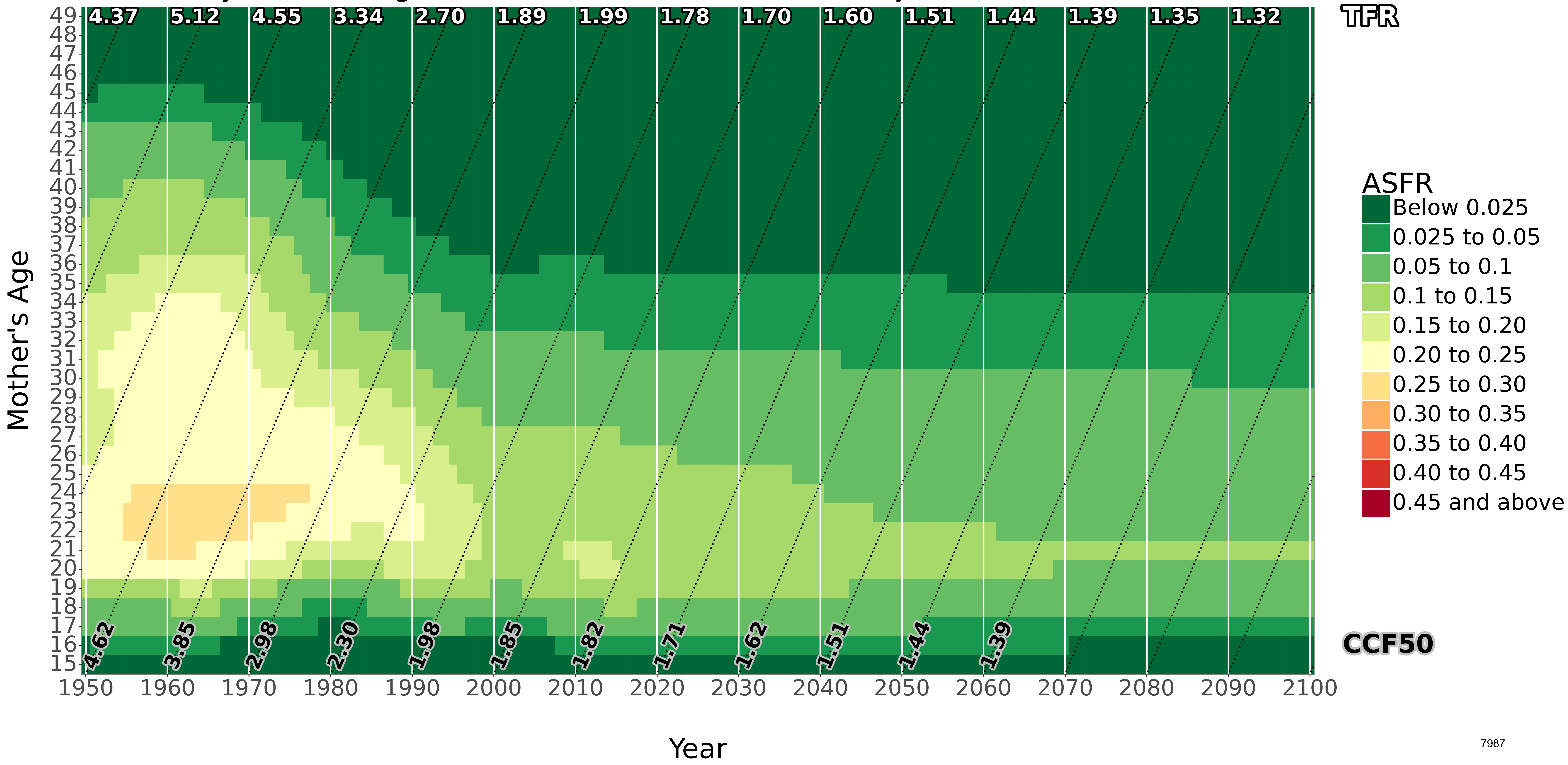

Supplemental Results Figure S3: Lexis diagram of CCF50, single-year age interval ASFR, and TFR by mother's age and location, 1950-2100, Georgia

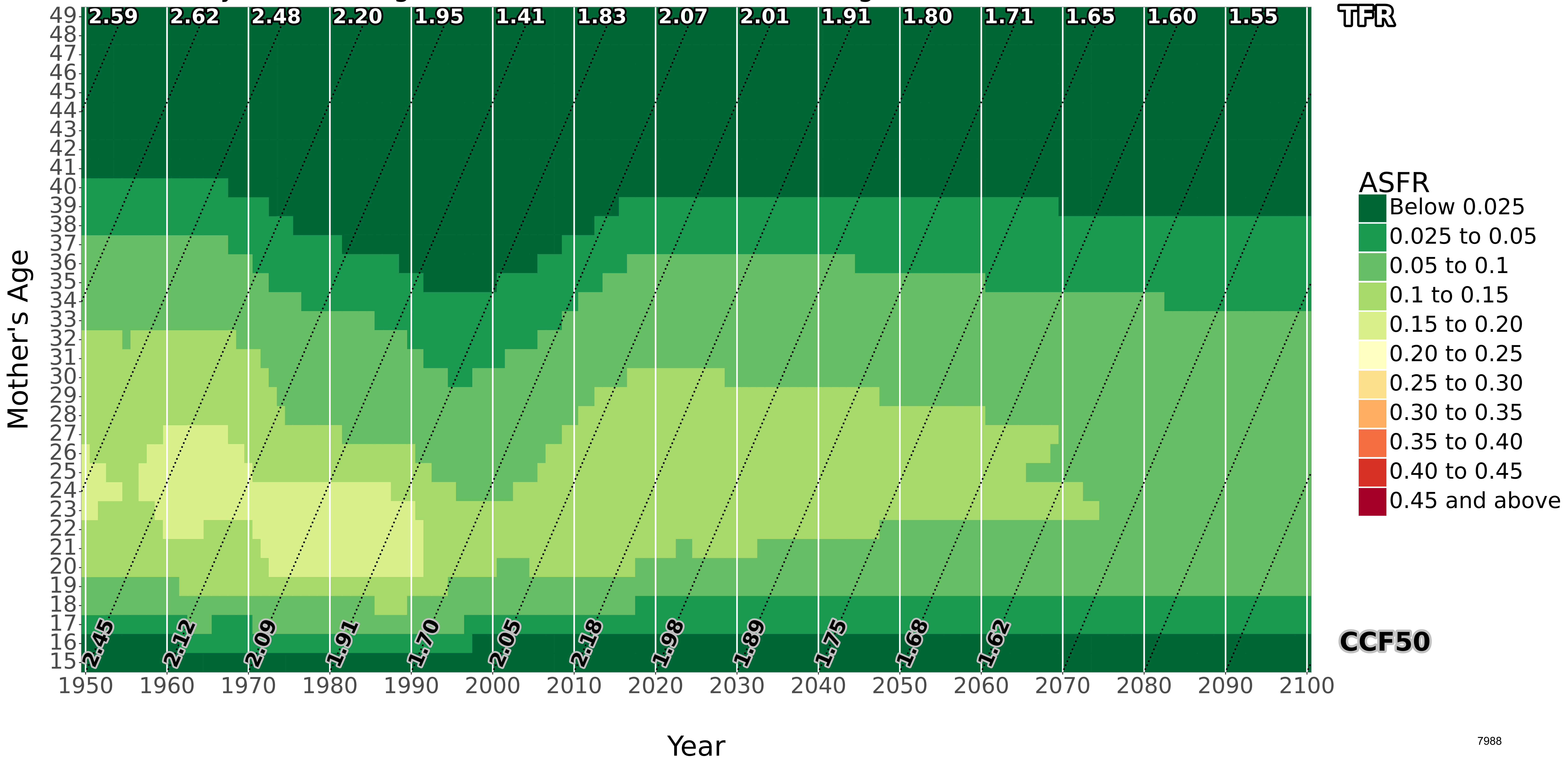

Supplemental Results Figure S3: Lexis diagram of CCF50, single-year age interval ASFR, and TFR by mother's age and location, 1950-2100, Kazakhstan

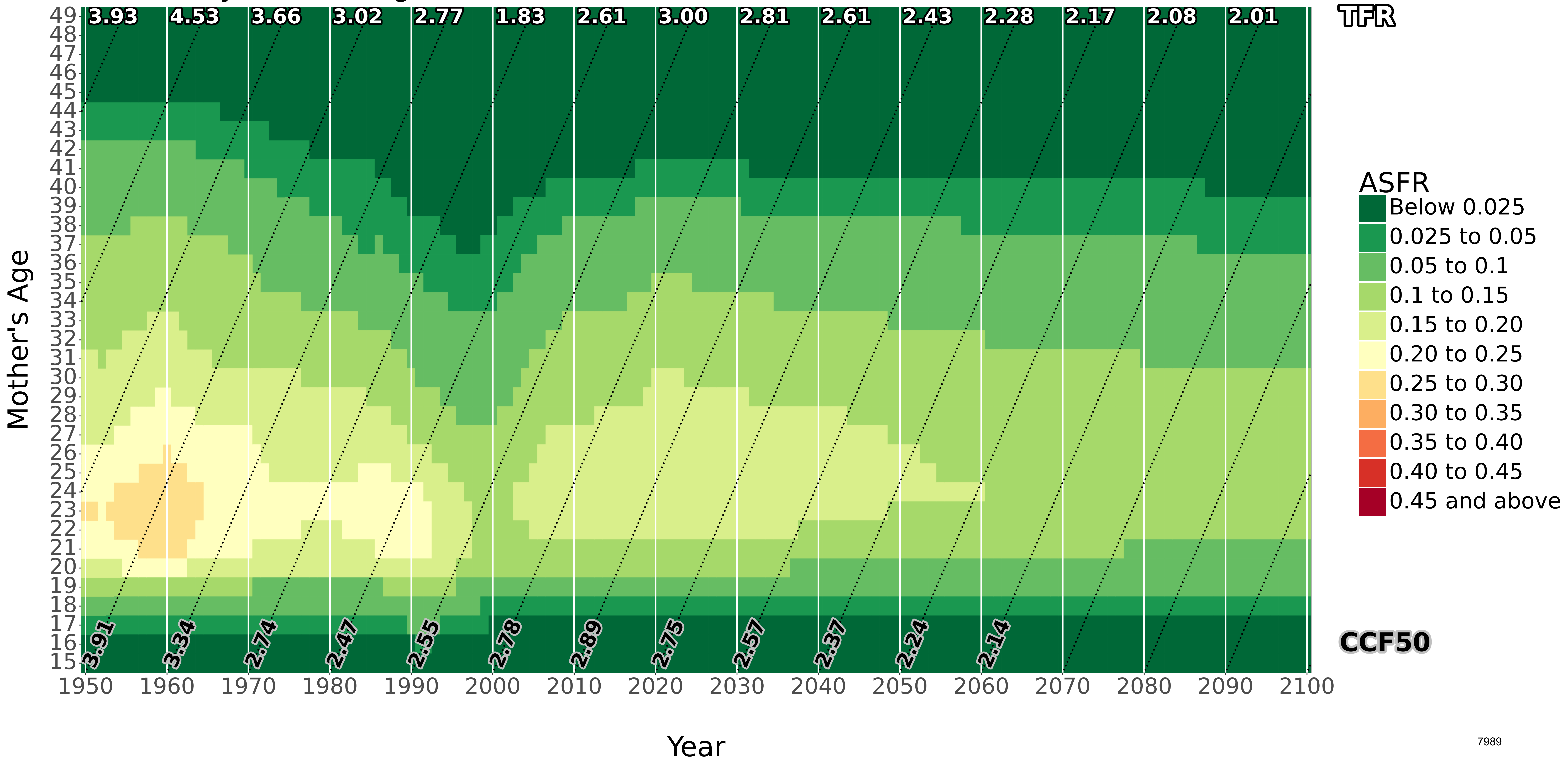

Supplemental Results Figure S3: Lexis diagram of CCF50, single-year age interval ASFR, and TFR by mother's age and location, 1950-2100, Kyrgyzstan

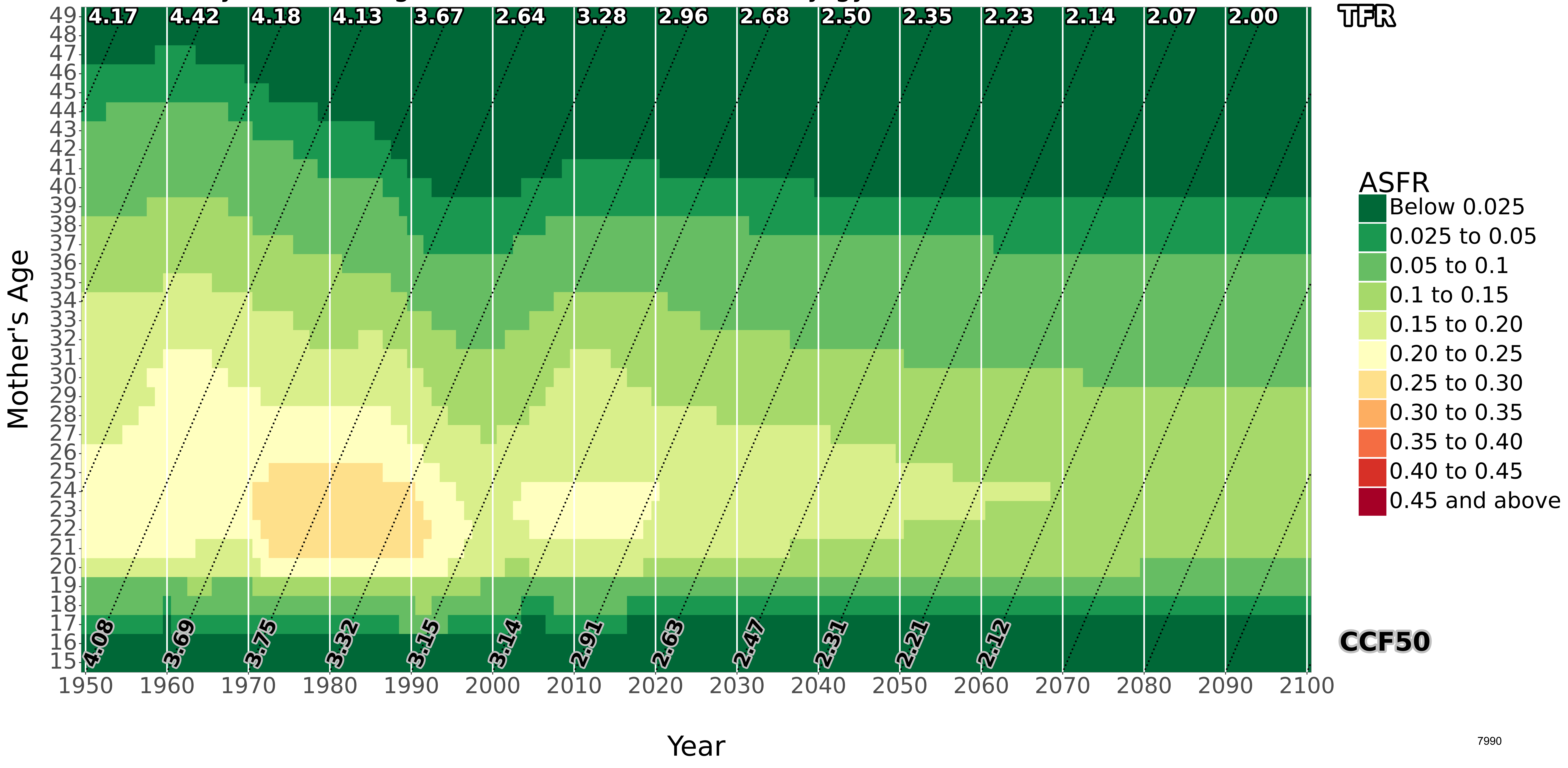

Supplemental Results Figure S3: Lexis diagram of CCF50, single-year age interval ASFR, and TFR by mother's age and location, 1950-2100, Mongolia

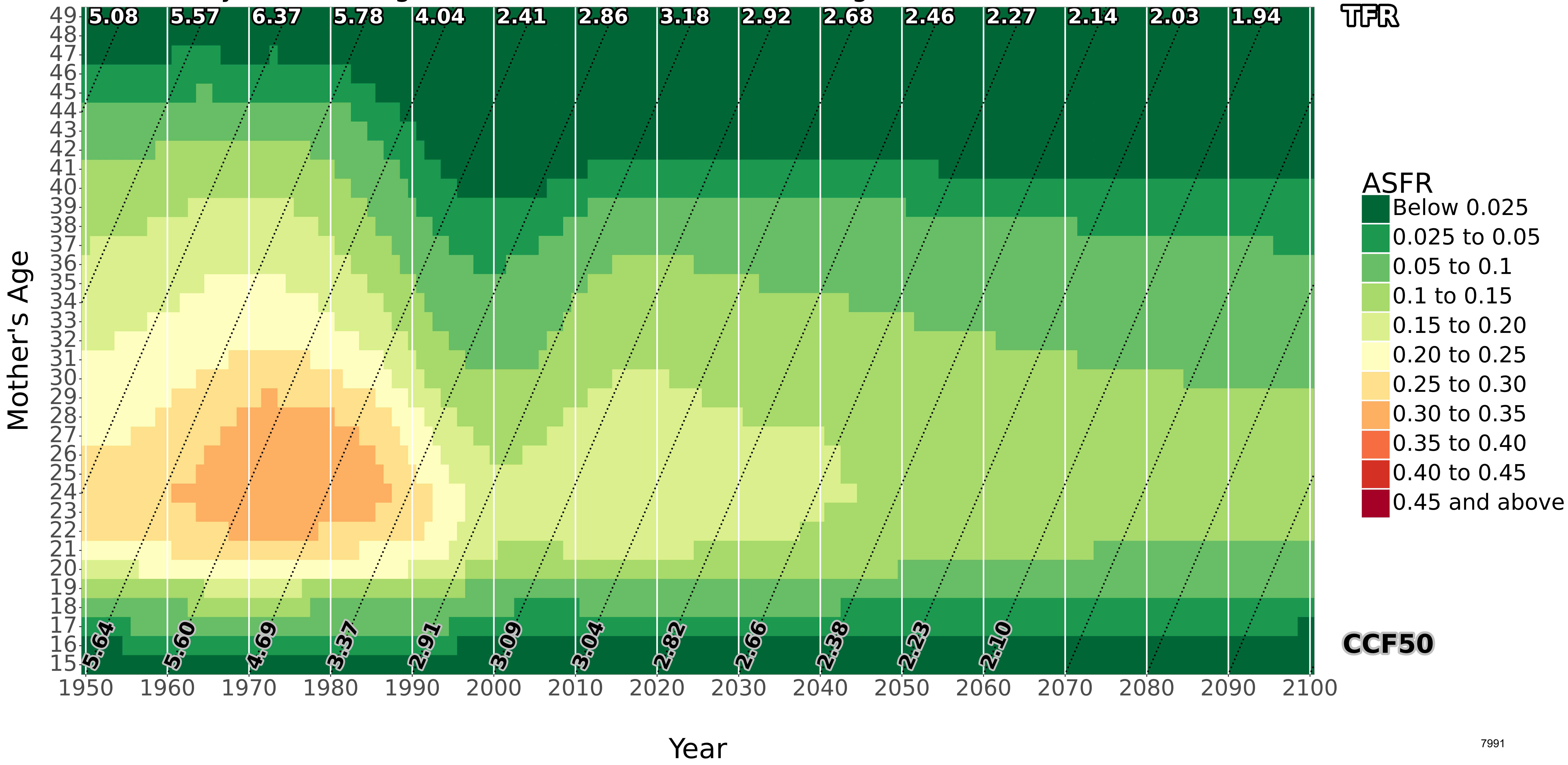

Supplemental Results Figure S3: Lexis diagram of CCF50, single-year age interval ASFR, and TFR by mother's age and location, 1950-2100, Tajikistan

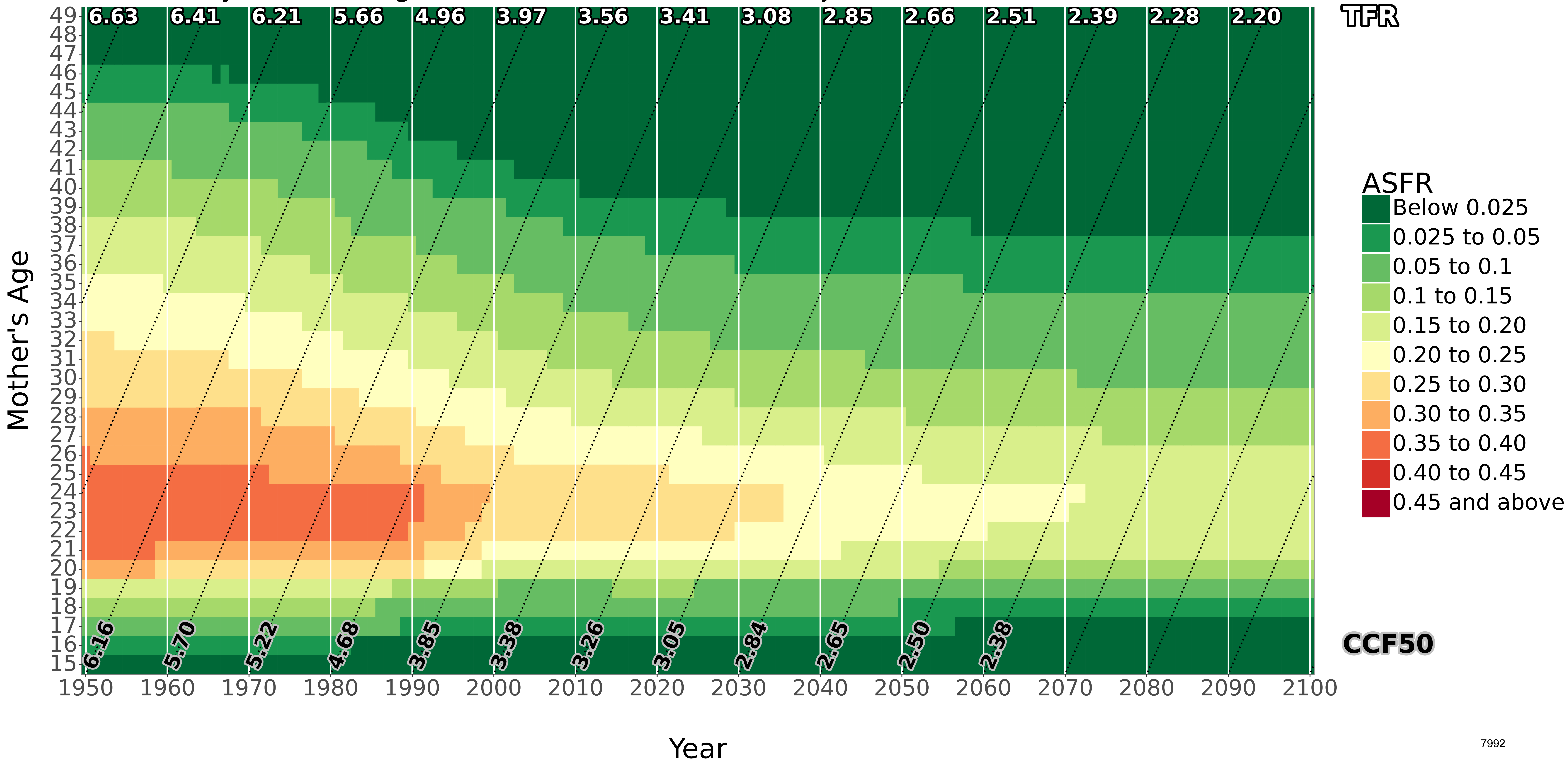

Supplemental Results Figure S3: Lexis diagram of CCF50, single-year age interval ASFR, and TFR by mother's age and location, 1950-2100, Turkmenistan

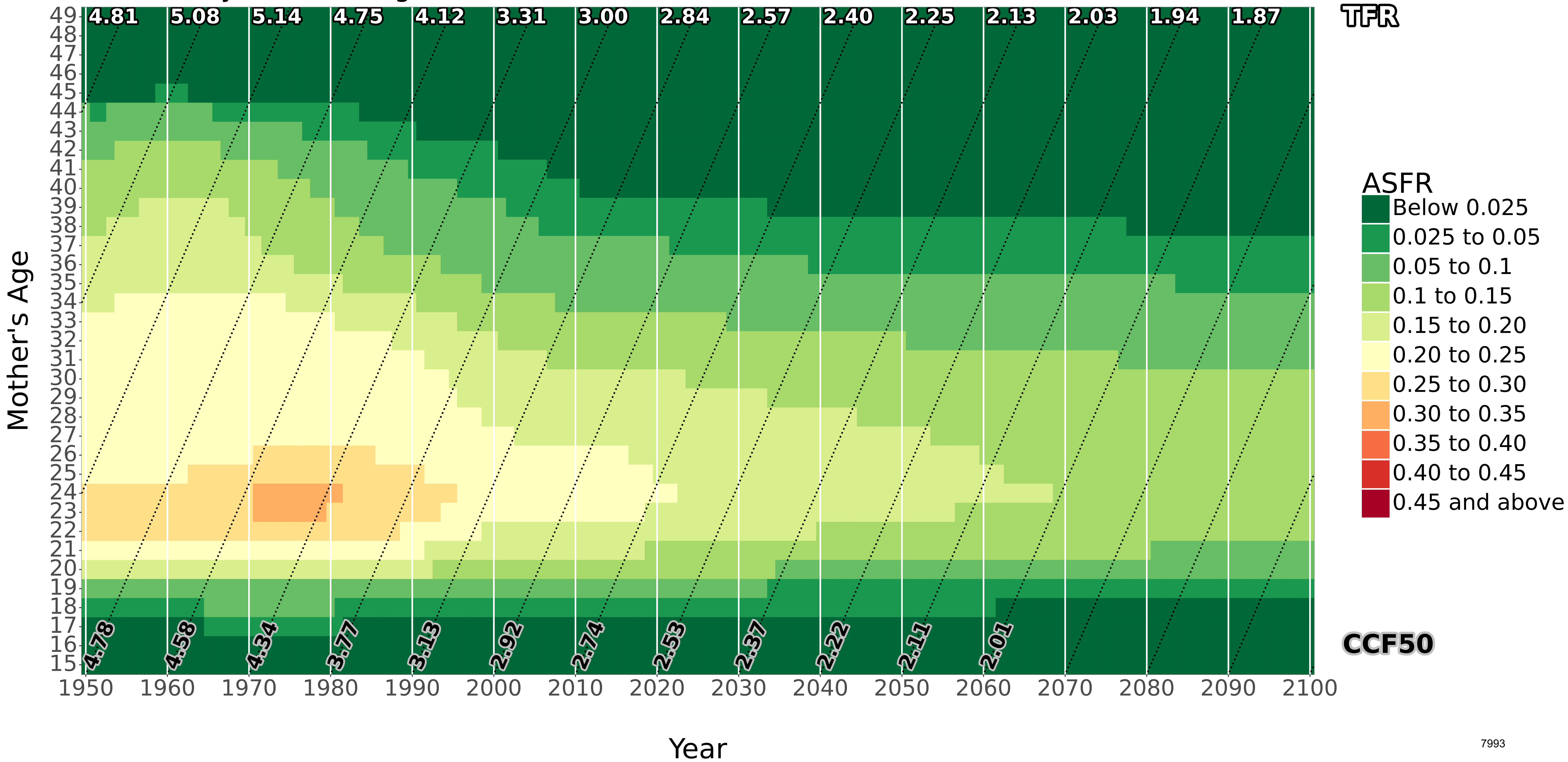

Supplemental Results Figure S3: Lexis diagram of CCF50, single-year age interval ASFR, and TFR by mother's age and location, 1950-2100, Uzbekistan

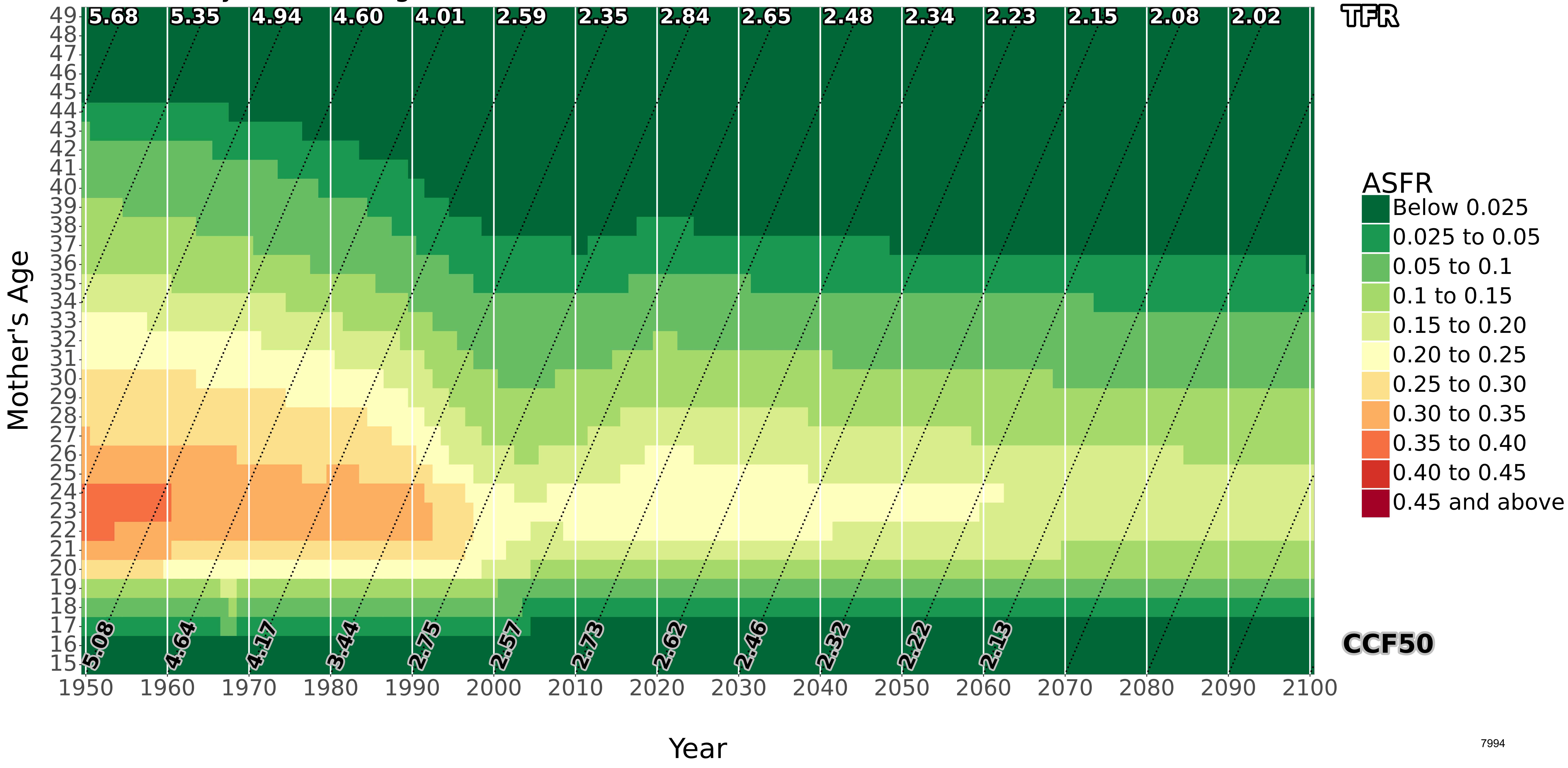

Supplemental Results Figure S3: Lexis diagram of CCF50, single-year age interval ASFR, and TFR by mother's age and location, 1950-2100, Albania

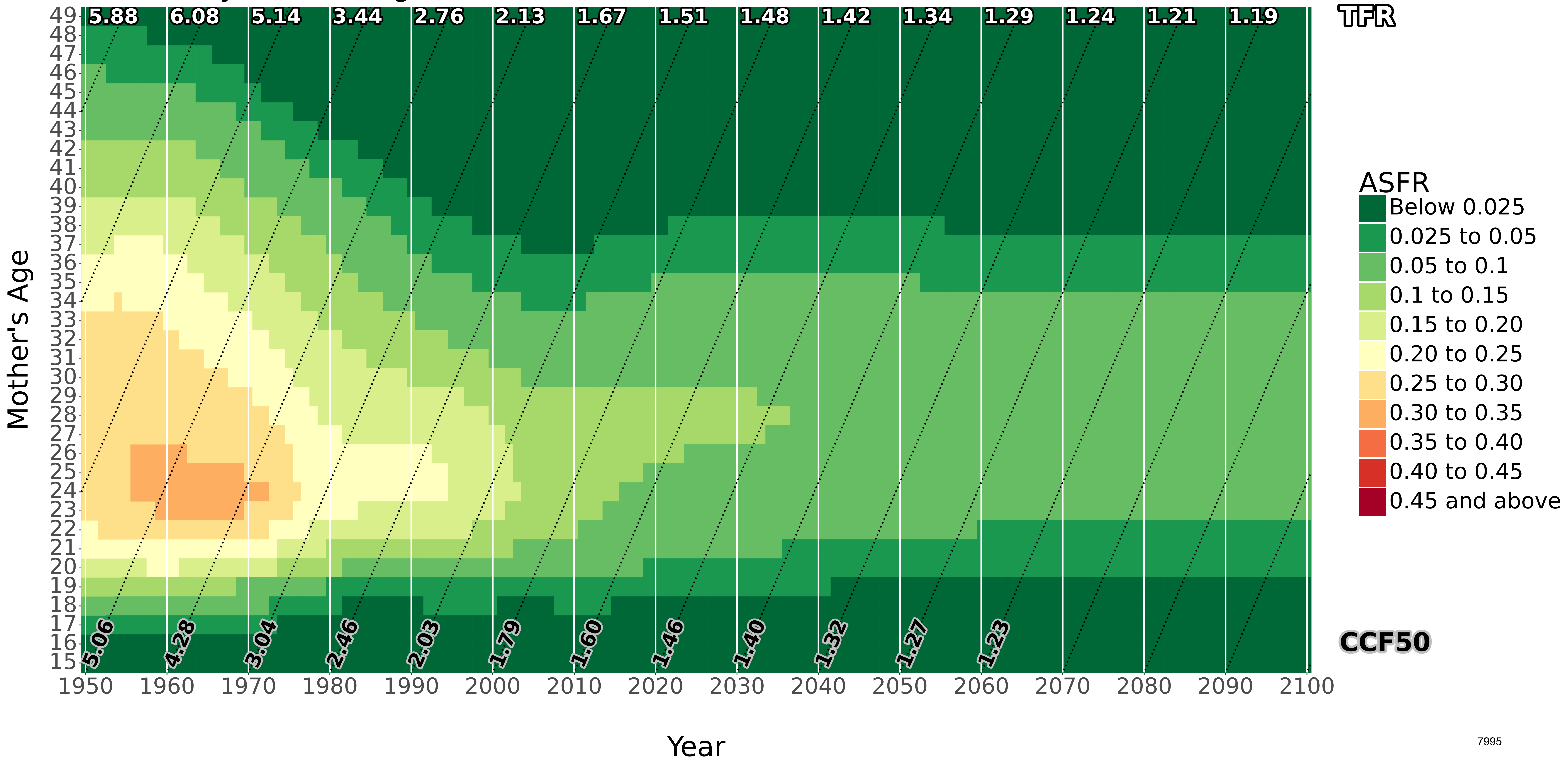

Supplemental Results Figure S3: Lexis diagram of CCF50, single-year age interval ASFR, and TFR by mother's age and location, 1950-2100, Bosnia and Herzegovina

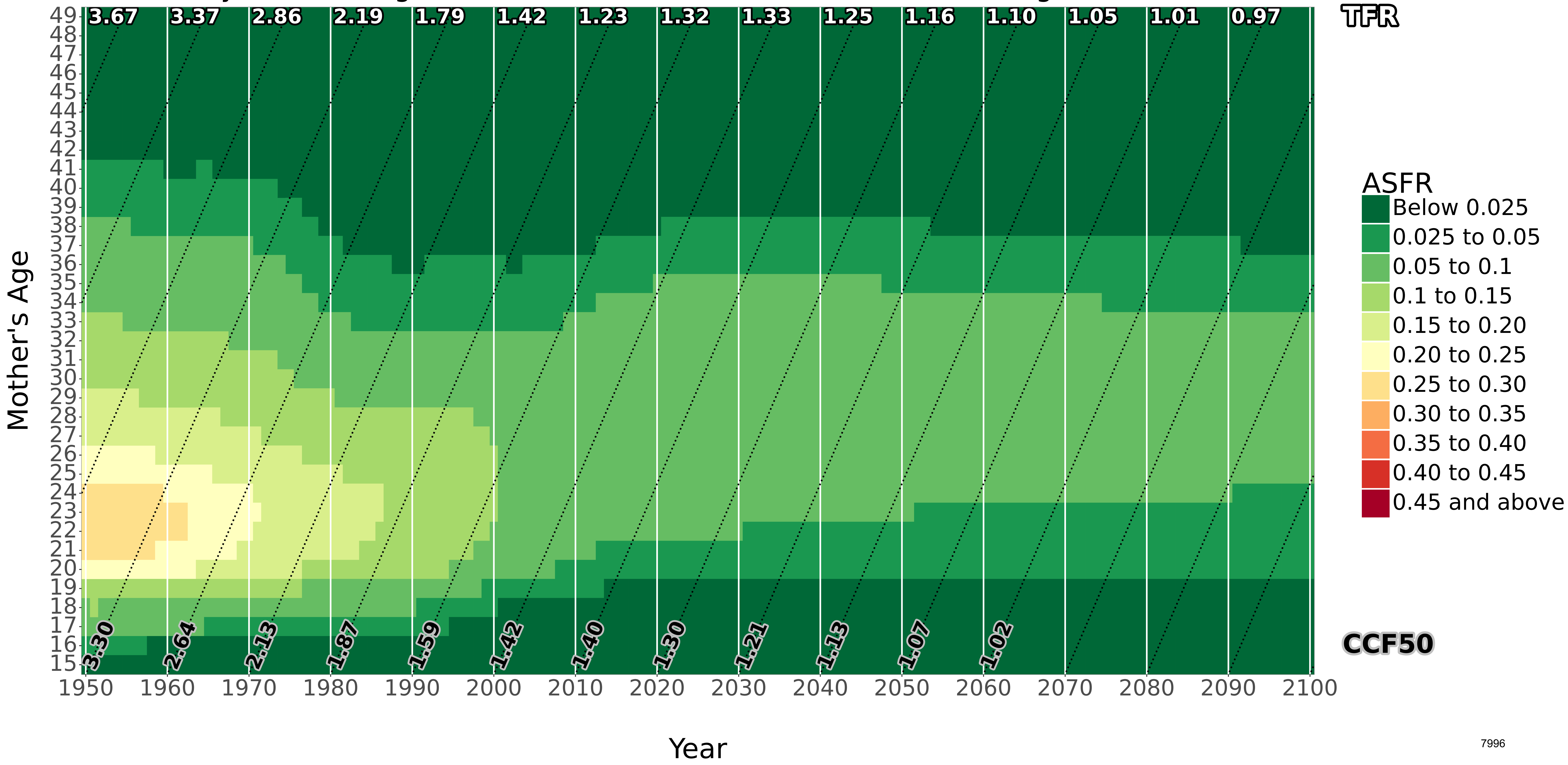

Supplemental Results Figure S3: Lexis diagram of CCF50, single-year age interval ASFR, and TFR by mother's age and location, 1950-2100, Bulgaria

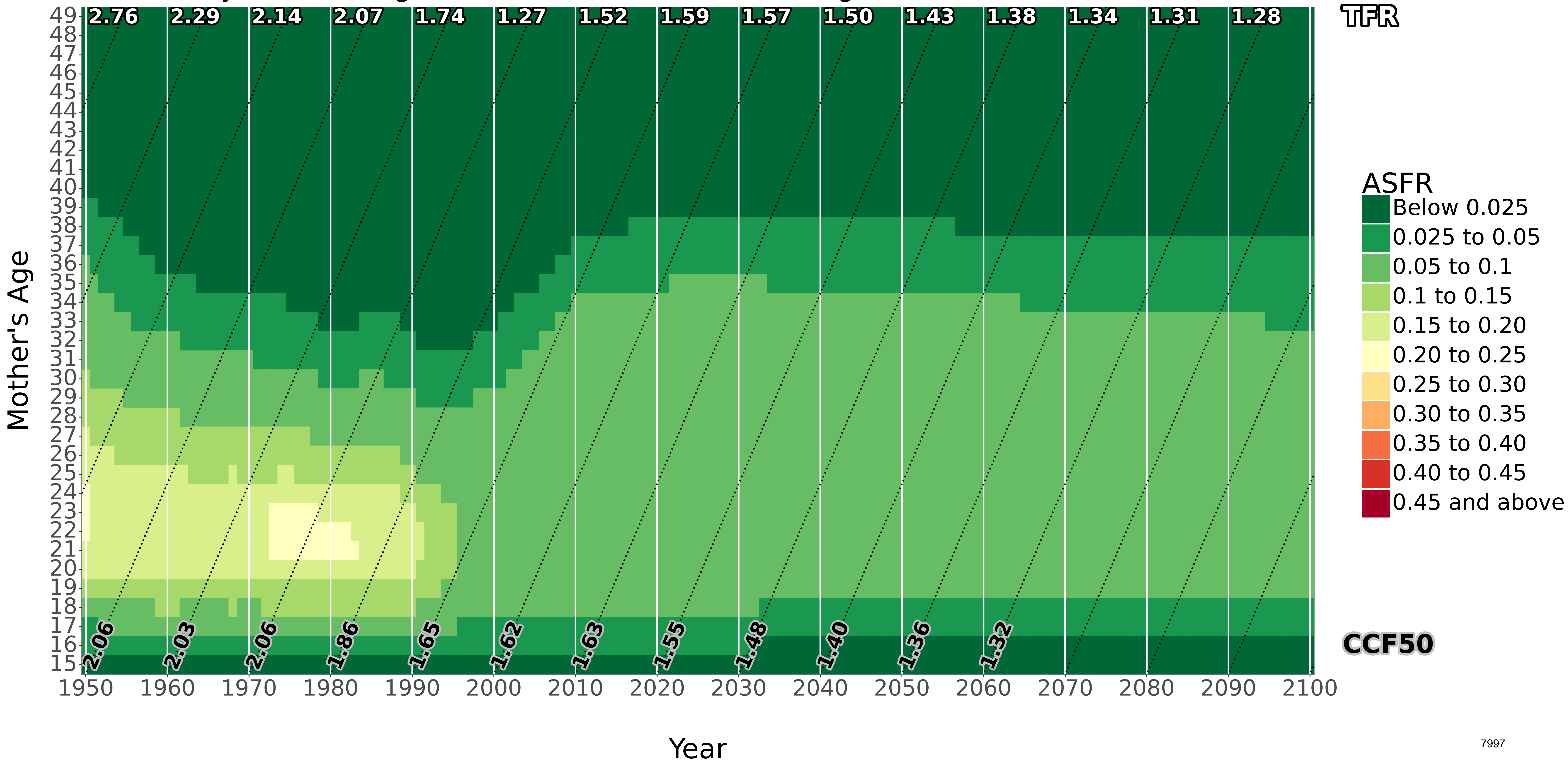

7997

Supplemental Results Figure S3: Lexis diagram of CCF50, single-year age interval ASFR, and TFR by mother's age and location, 1950-2100, Croatia

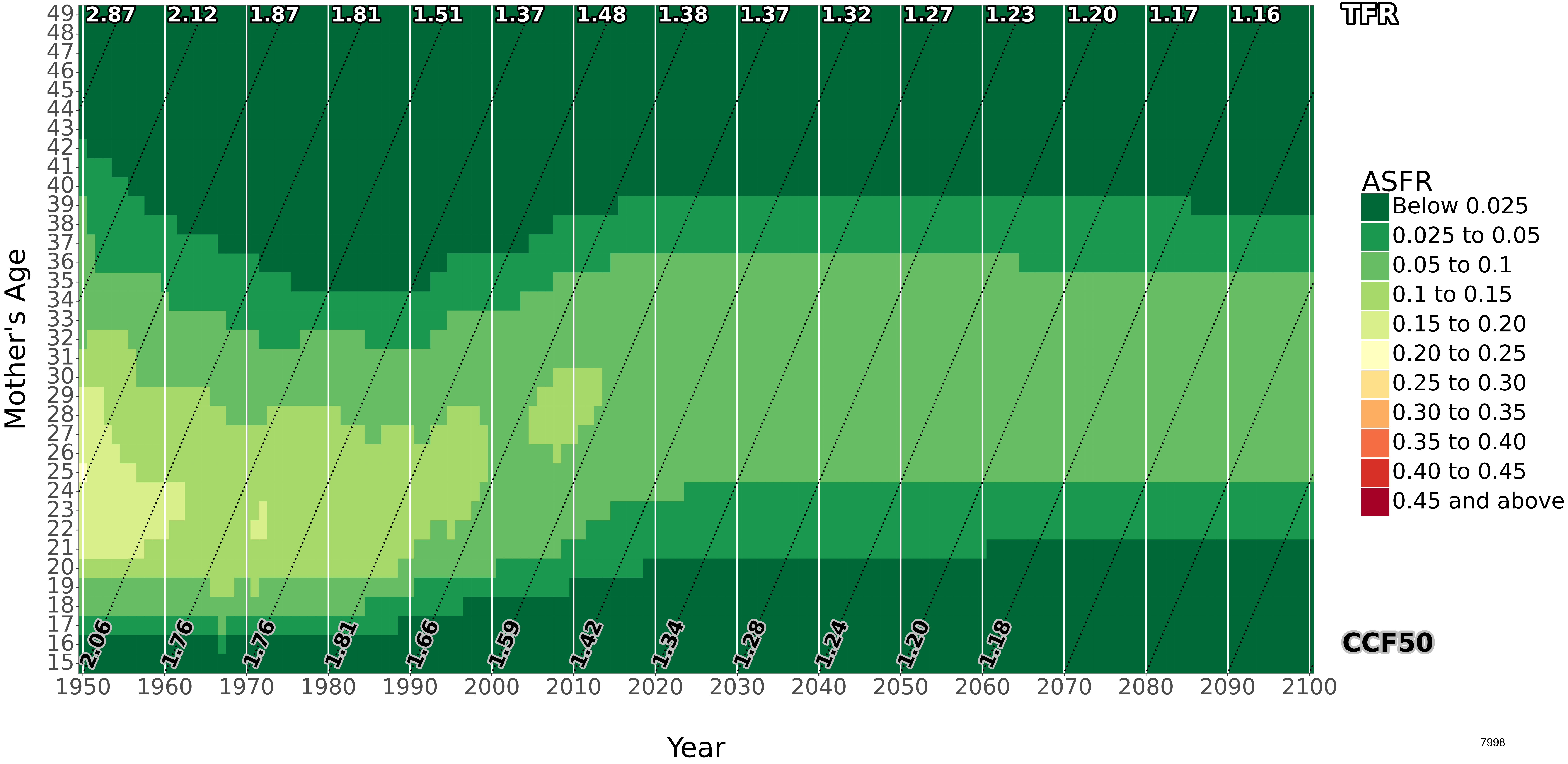

Supplemental Results Figure S3: Lexis diagram of CCF50, single-year age interval ASFR, and TFR by mother's age and location, 1950-2100, Czechia

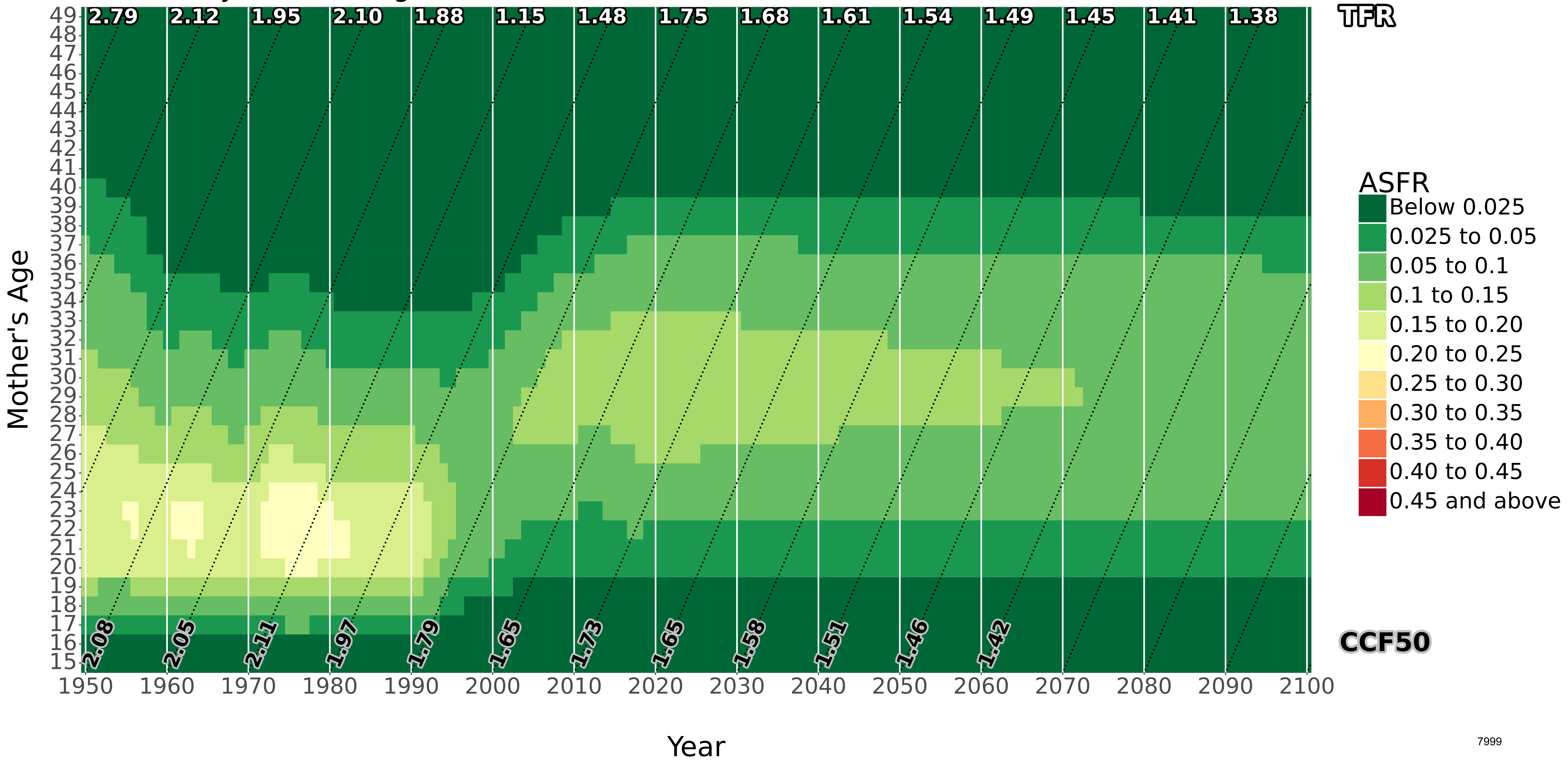

Supplemental Results Figure S3: Lexis diagram of CCF50, single-year age interval ASFR, and TFR by mother's age and location, 1950-2100, Hungary

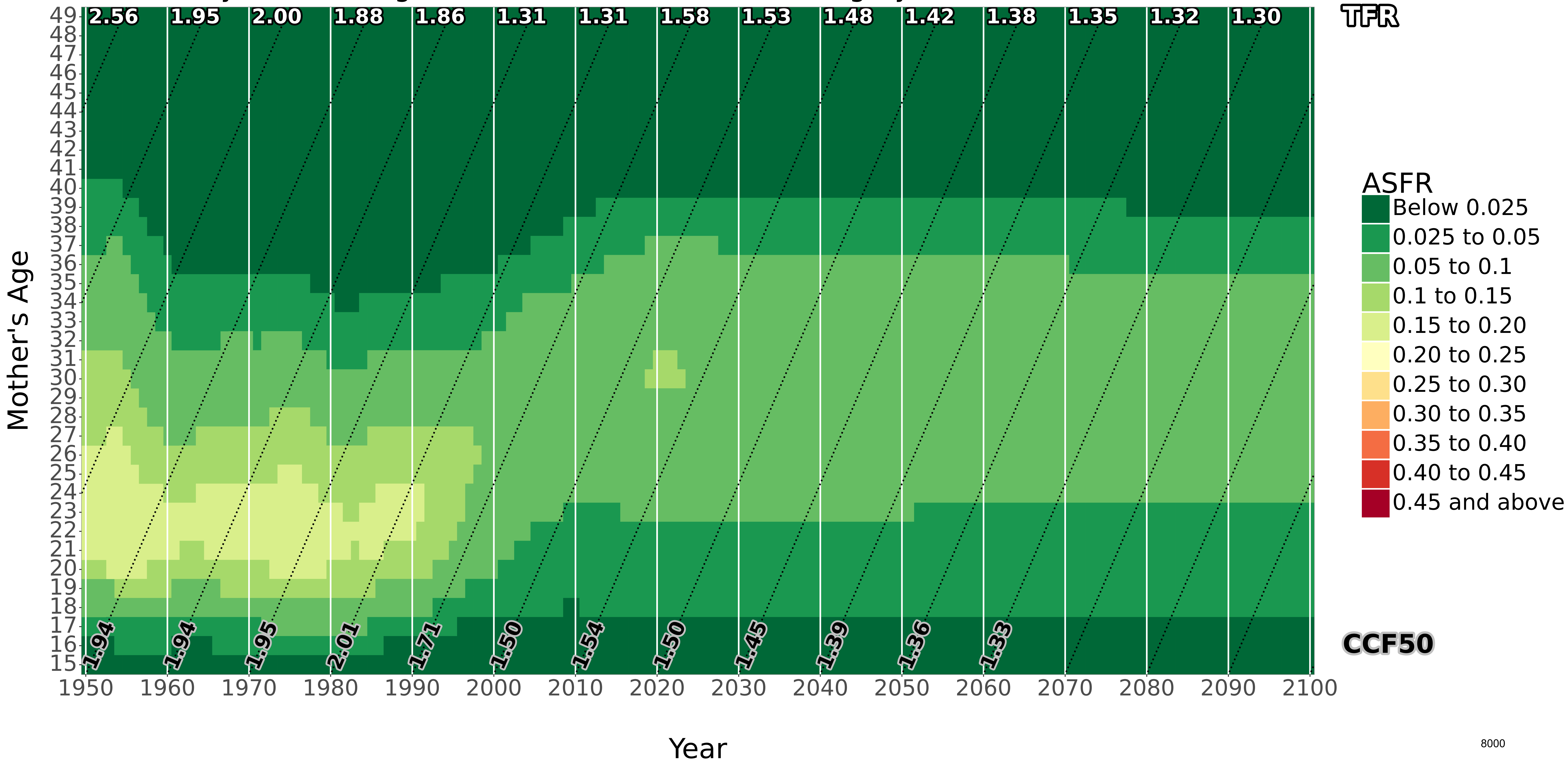

Supplemental Results Figure S3: Lexis diagram of CCF50, single-year age interval ASFR, and TFR by mother's age and location, 1950-2100, Montenegro

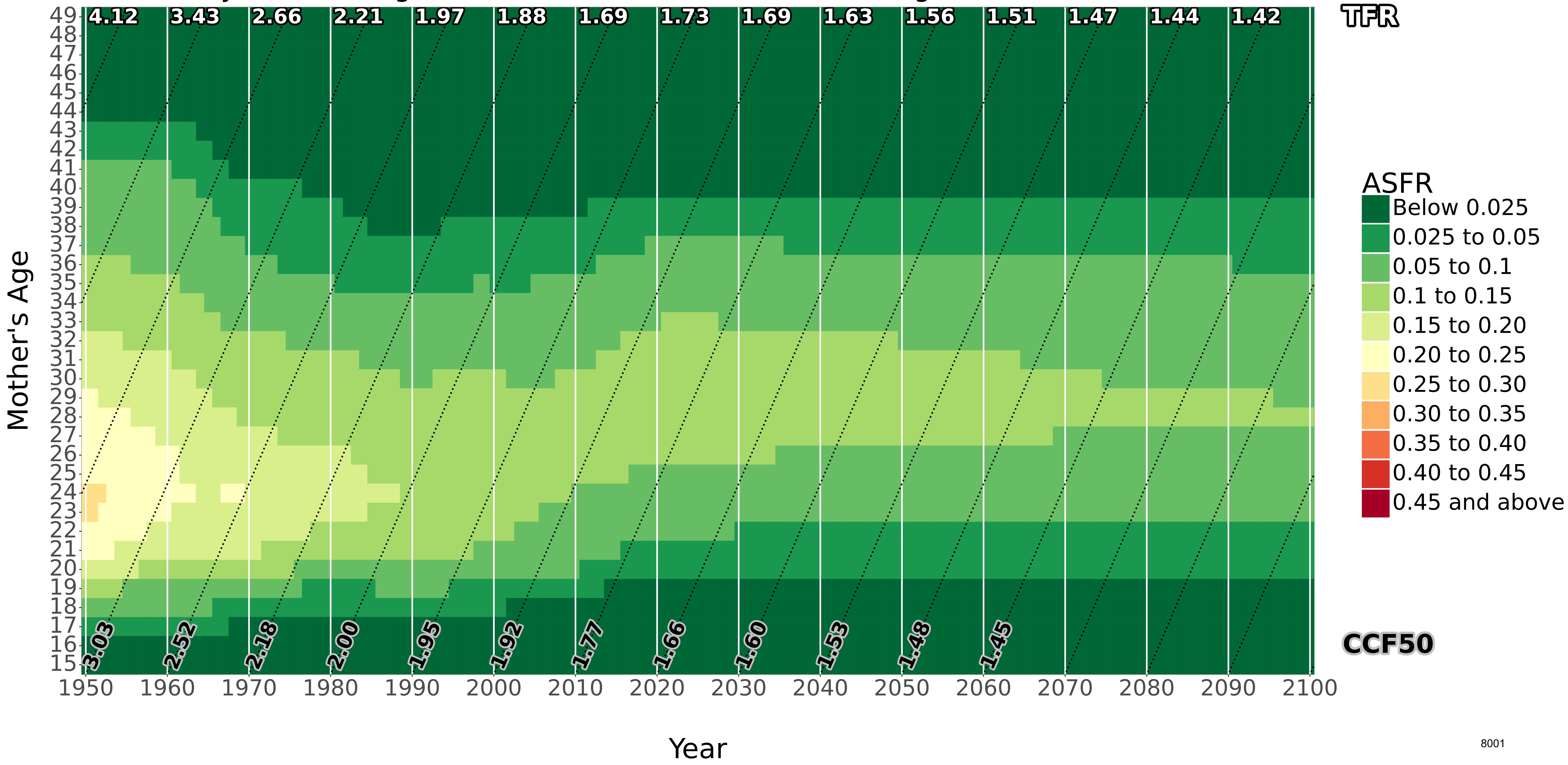

8001

Supplemental Results Figure S3: Lexis diagram of CCF50, single-year age interval ASFR, and TFR by mother's age and location, 1950-2100, North Macedonia

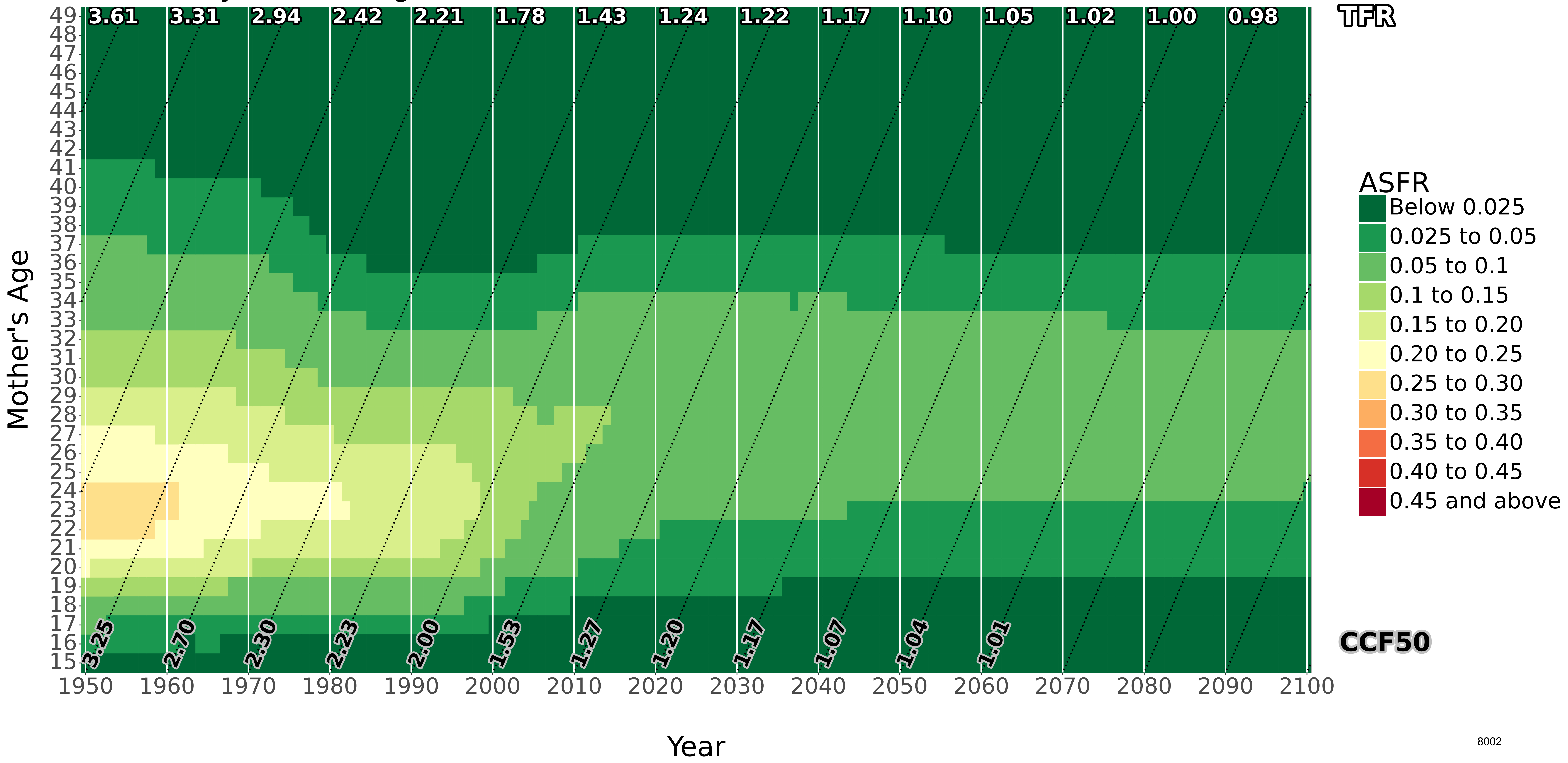

Supplemental Results Figure S3: Lexis diagram of CCF50, single-year age interval ASFR, and TFR by mother's age and location, 1950-2100, Poland

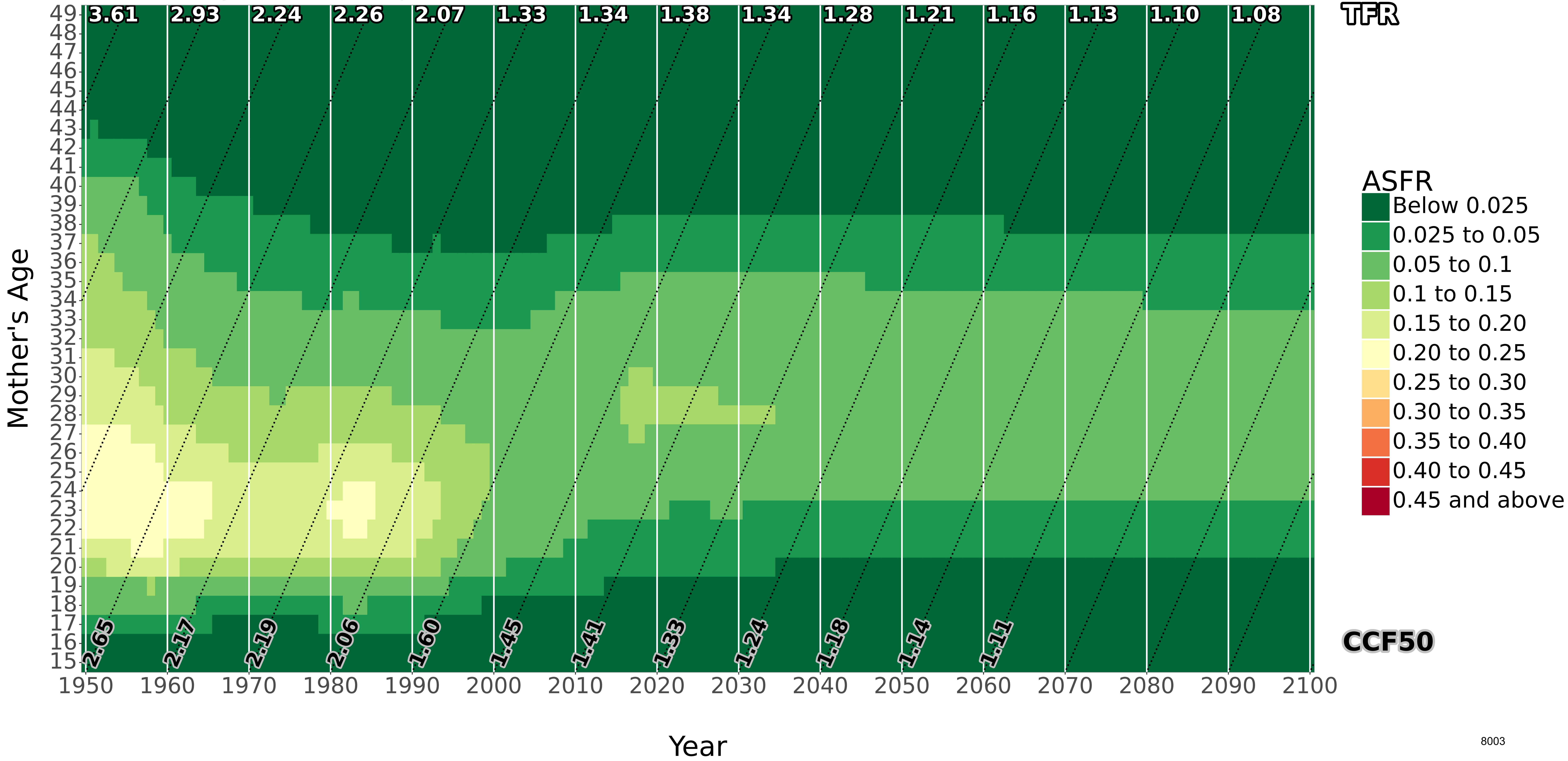

Supplemental Results Figure S3: Lexis diagram of CCF50, single-year age interval ASFR, and TFR by mother's age and location, 1950-2100, Romania

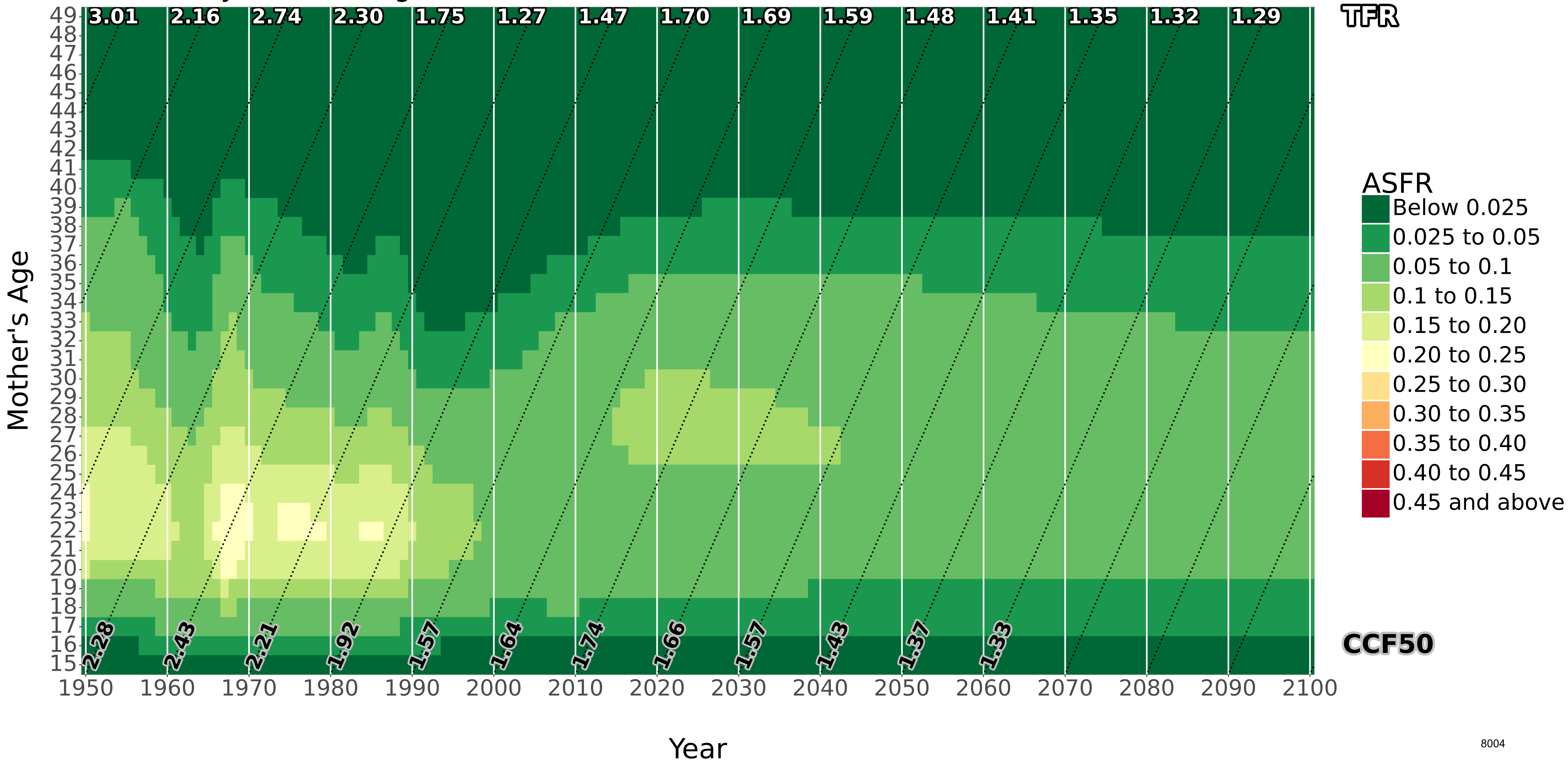

Supplemental Results Figure S3: Lexis diagram of CCF50, single-year age interval ASFR, and TFR by mother's age and location, 1950-2100, Serbia

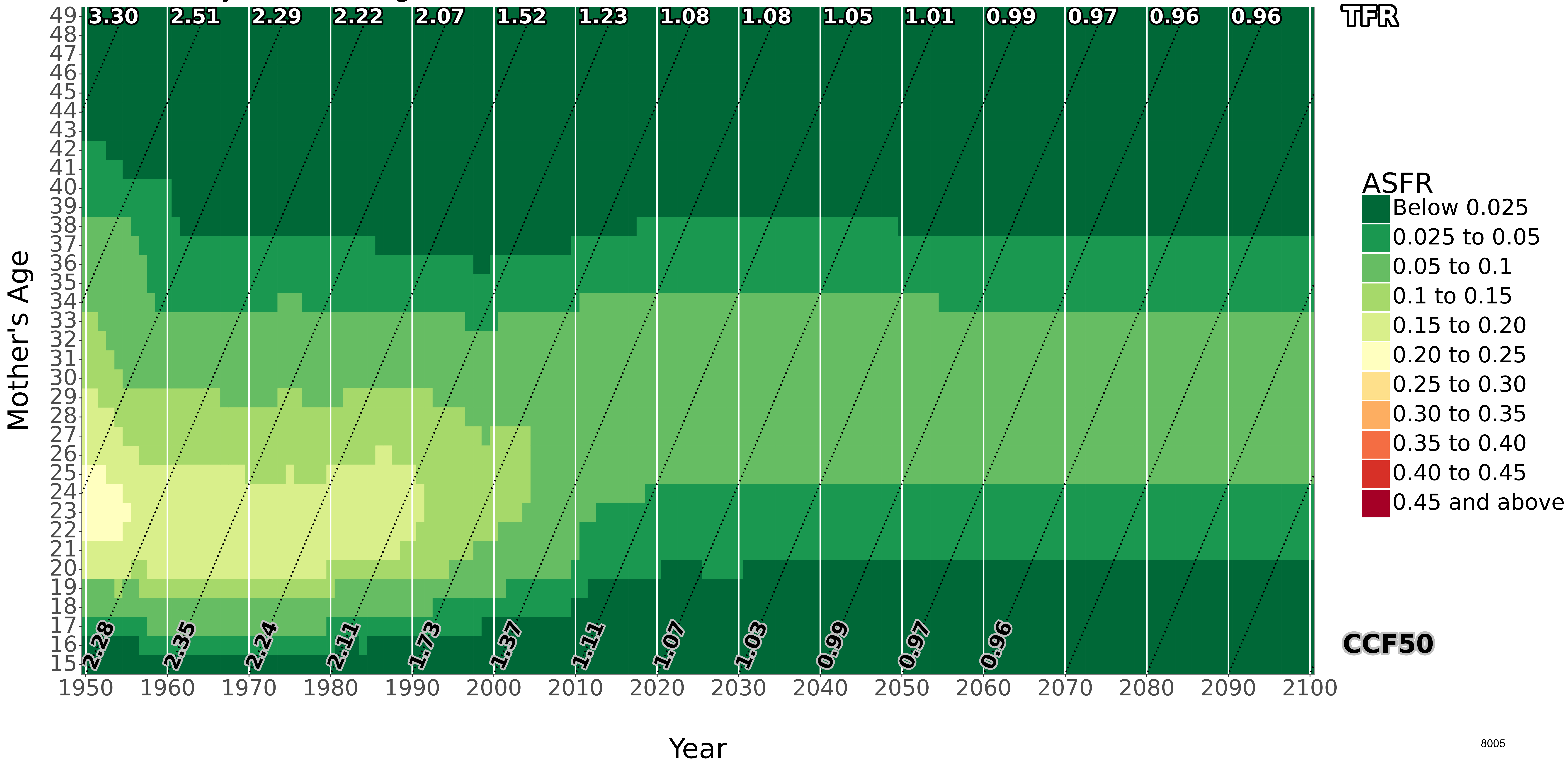

Supplemental Results Figure S3: Lexis diagram of CCF50, single-year age interval ASFR, and TFR by mother's age and location, 1950-2100, Slovakia

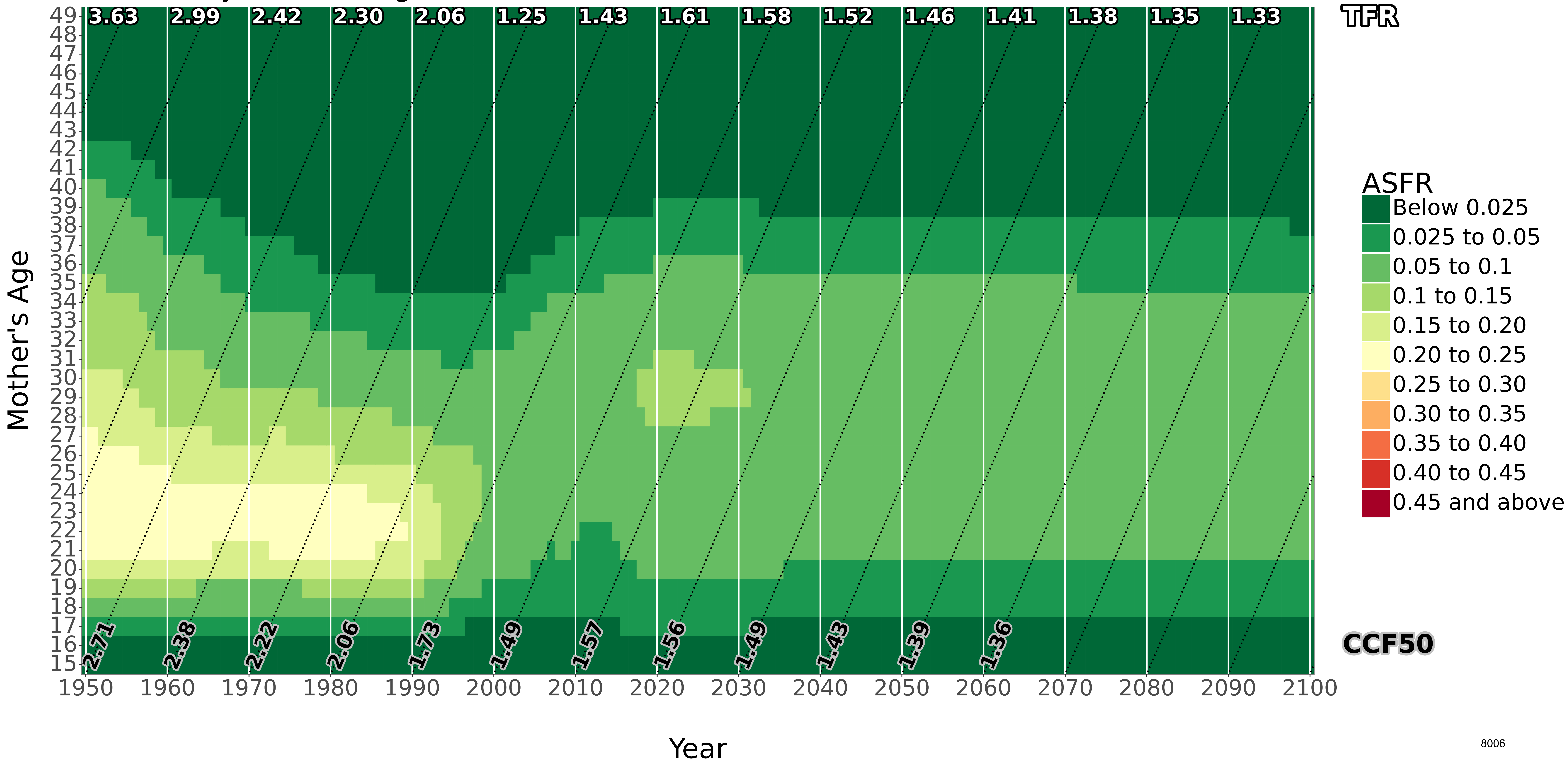

Supplemental Results Figure S3: Lexis diagram of CCF50, single-year age interval ASFR, and TFR by mother's age and location, 1950-2100, Slovenia

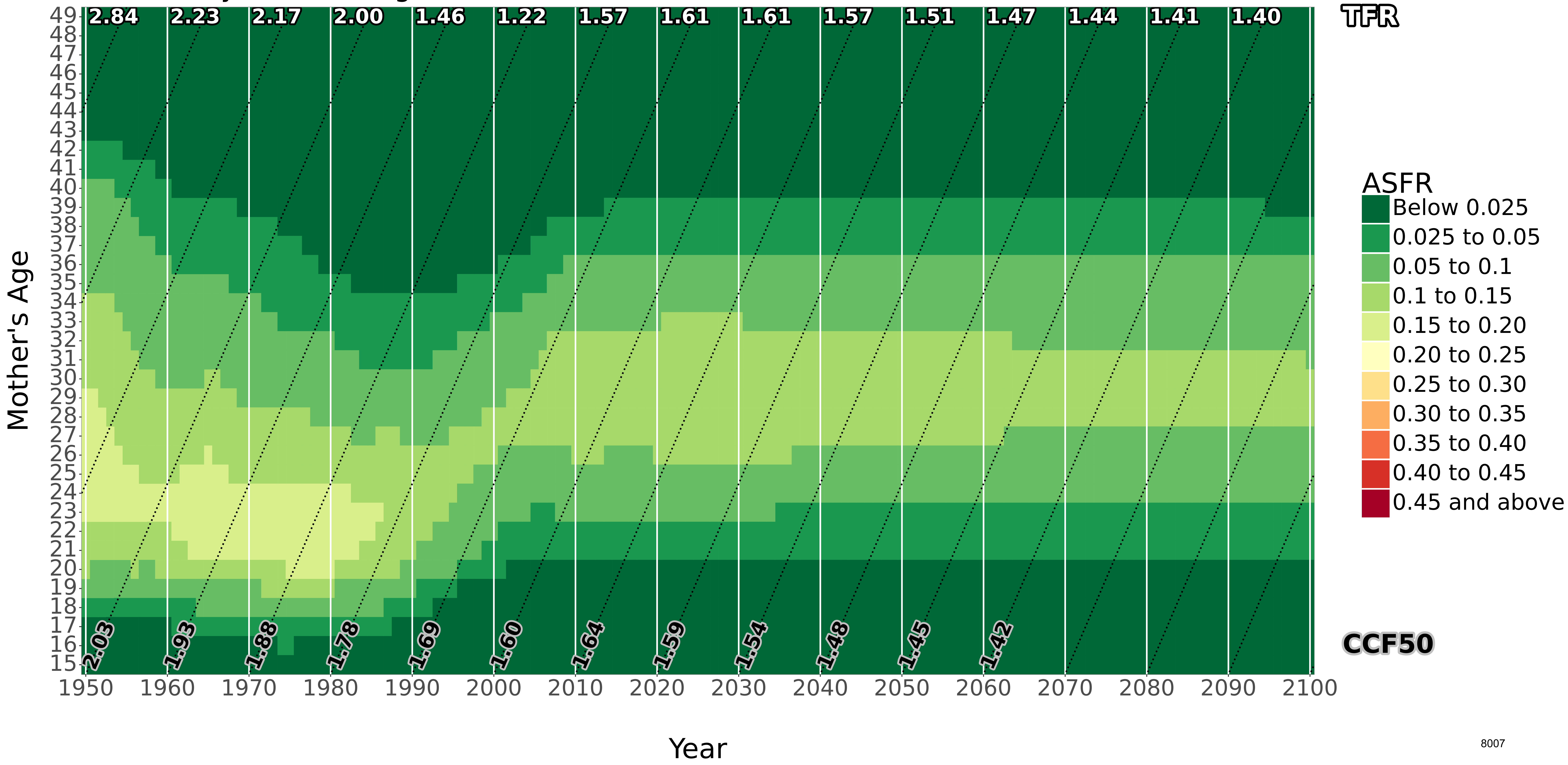

Supplemental Results Figure S3: Lexis diagram of CCF50, single-year age interval ASFR, and TFR by mother's age and location, 1950-2100, Belarus

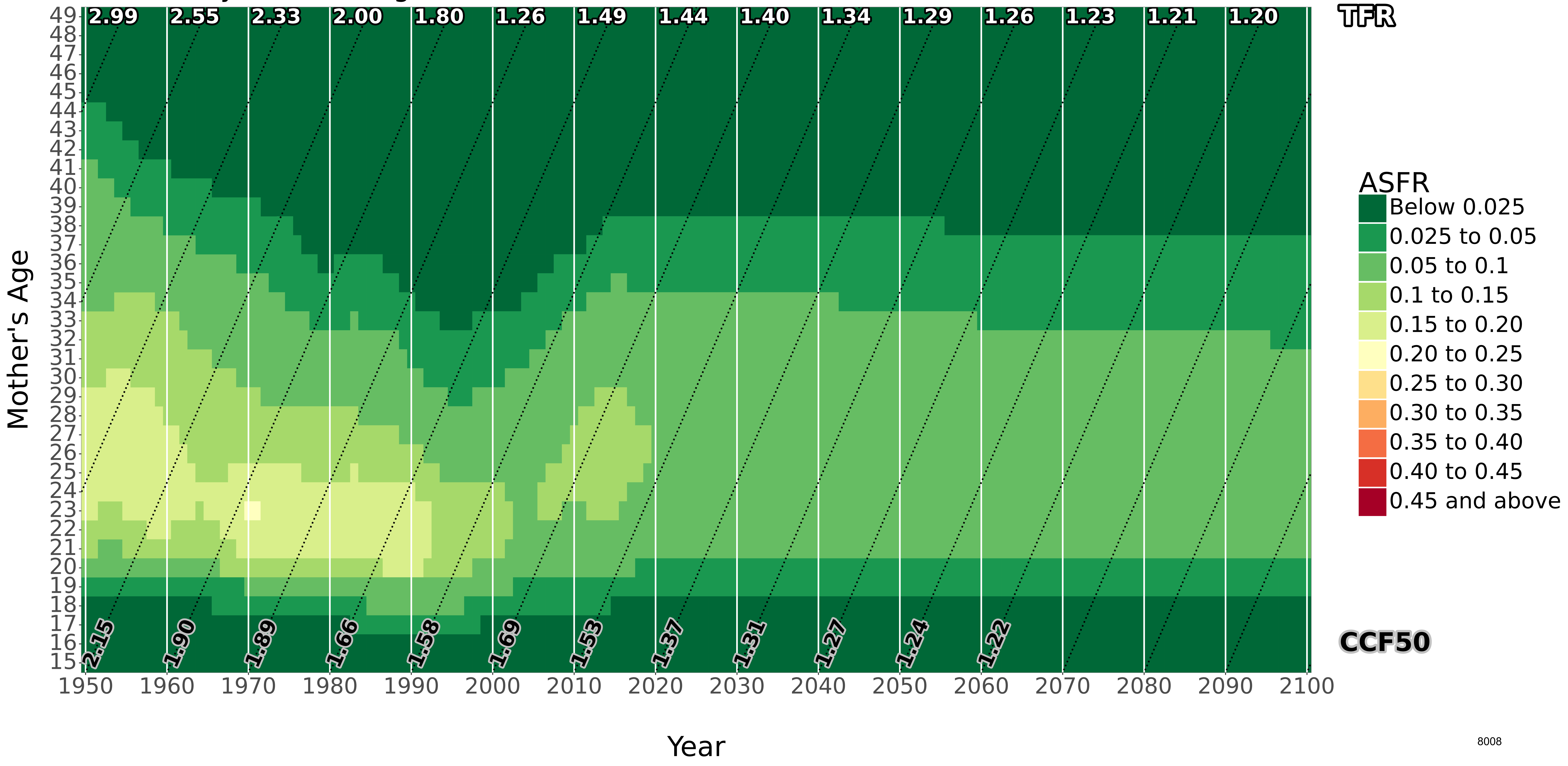

Supplemental Results Figure S3: Lexis diagram of CCF50, single-year age interval ASFR, and TFR by mother's age and location, 1950-2100, Estonia

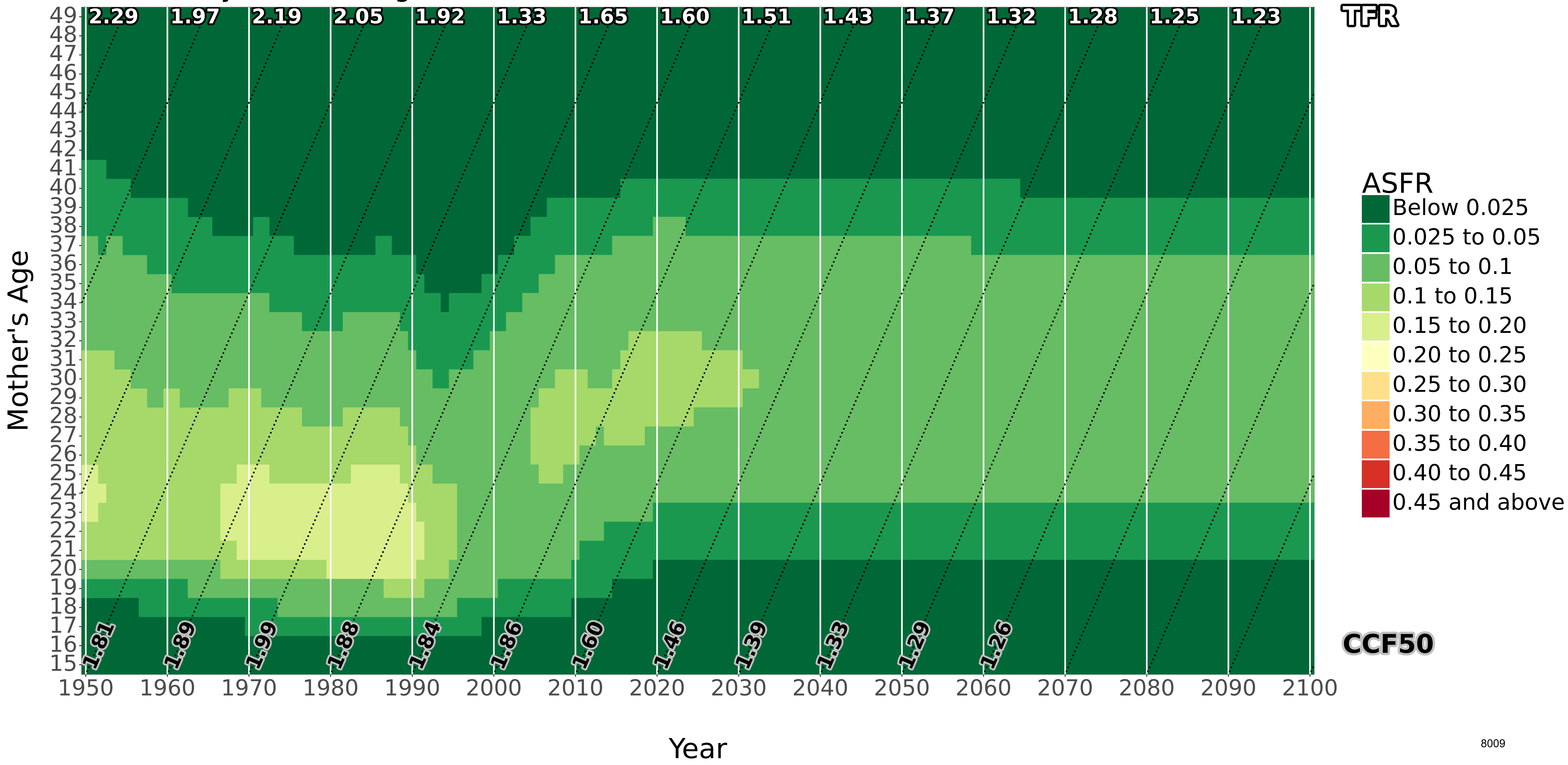

Supplemental Results Figure S3: Lexis diagram of CCF50, single-year age interval ASFR, and TFR by mother's age and location, 1950-2100, Latvia

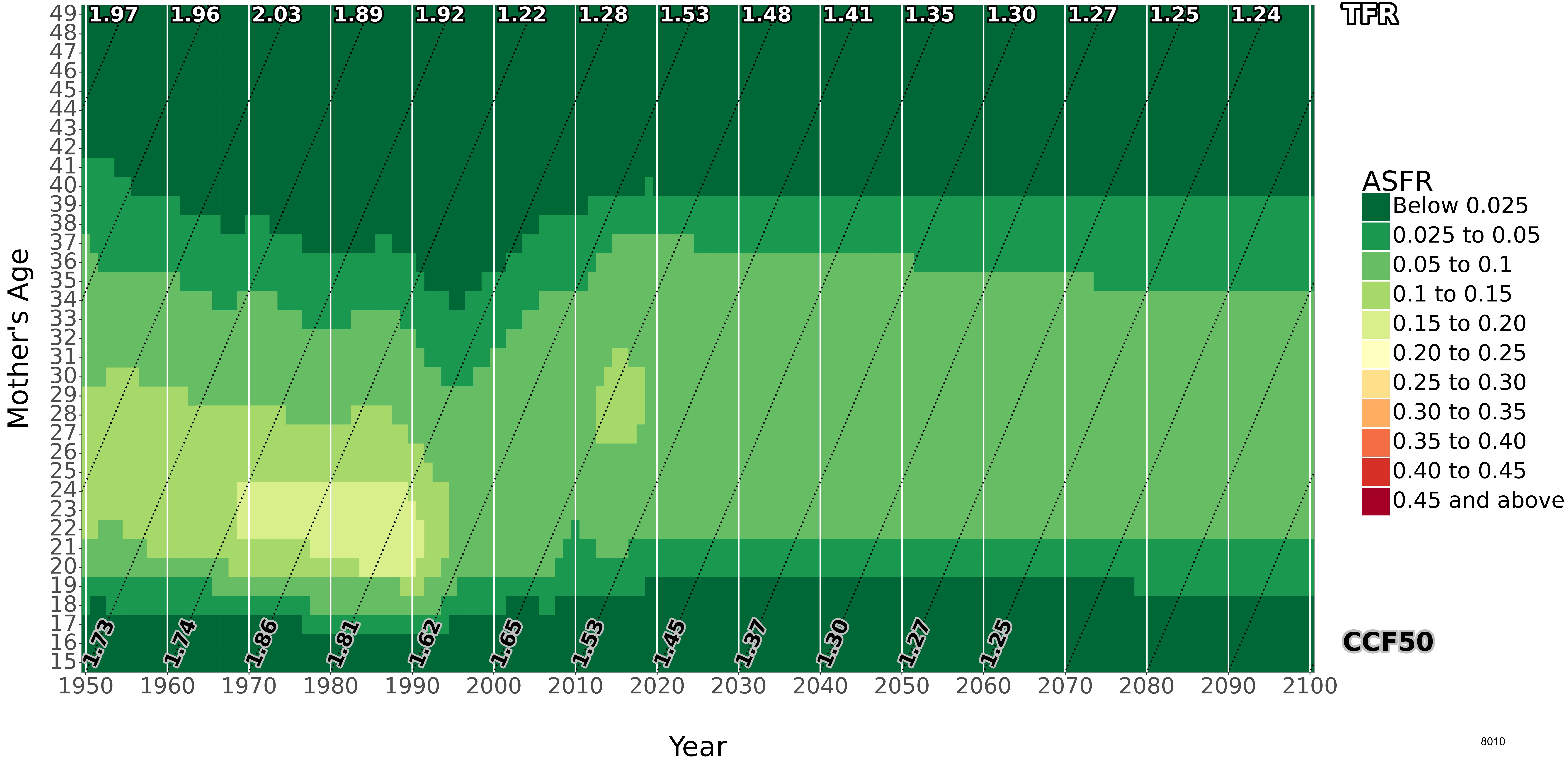

Supplemental Results Figure S3: Lexis diagram of CCF50, single-year age interval ASFR, and TFR by mother's age and location, 1950-2100, Lithuania

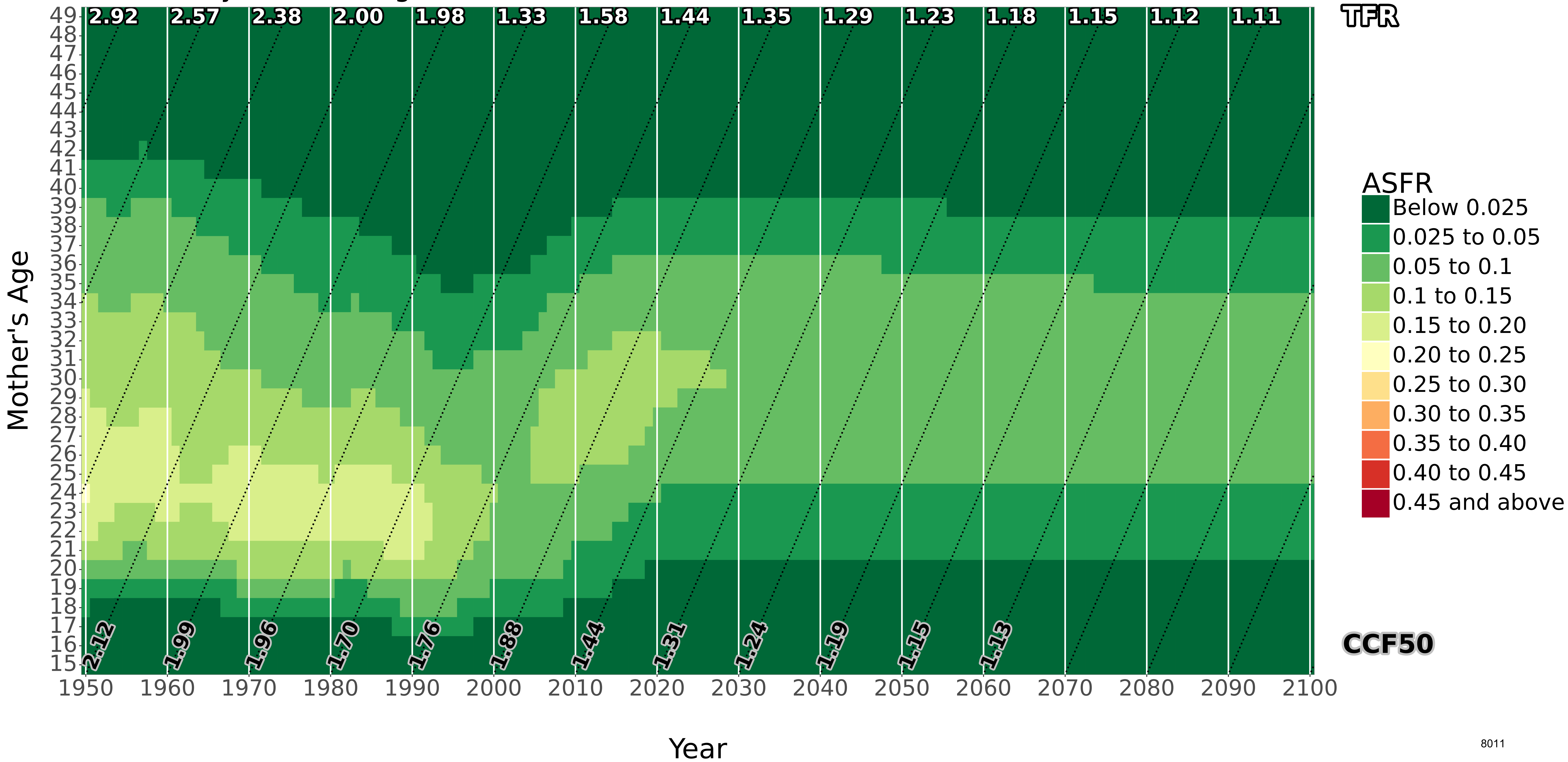

8011

Supplemental Results Figure S3: Lexis diagram of CCF50, single-year age interval ASFR, and TFR by mother's age and location, 1950-2100, Moldova

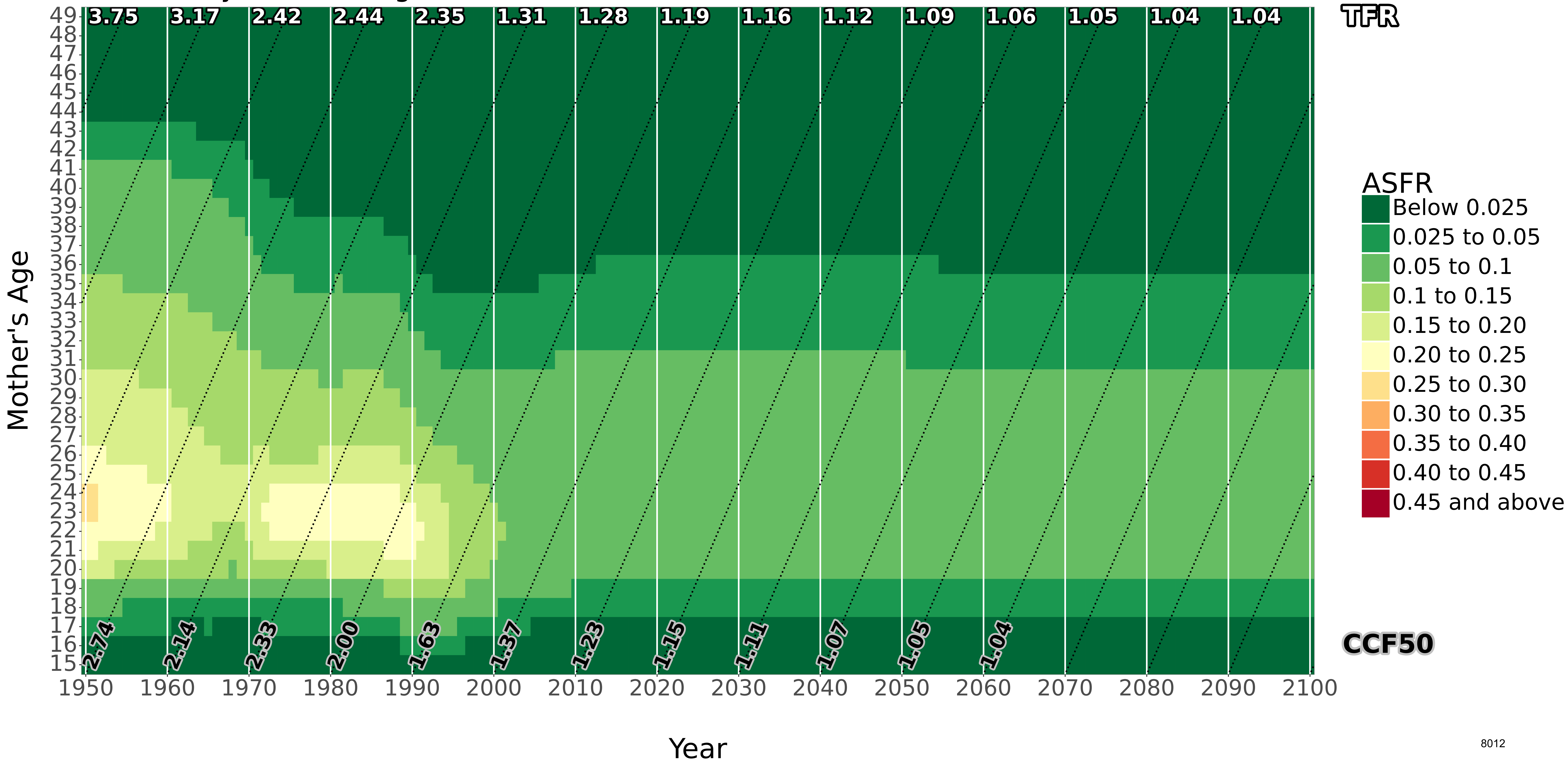

Supplemental Results Figure S3: Lexis diagram of CCF50, single-year age interval ASFR, and TFR by mother's age and location, 1950-2100, Russia

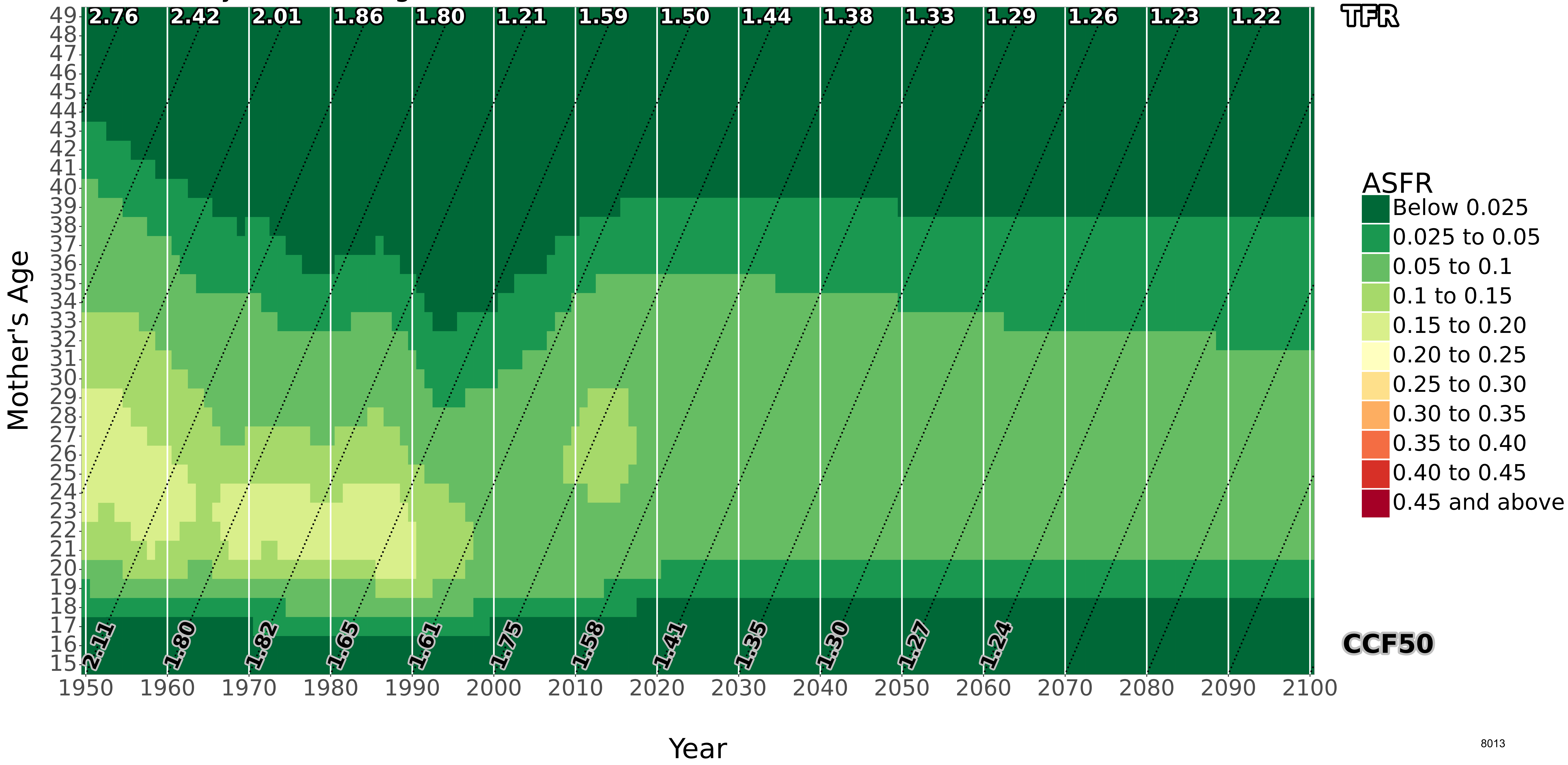

Supplemental Results Figure S3: Lexis diagram of CCF50, single-year age interval ASFR, and TFR by mother's age and location, 1950-2100, Ukraine

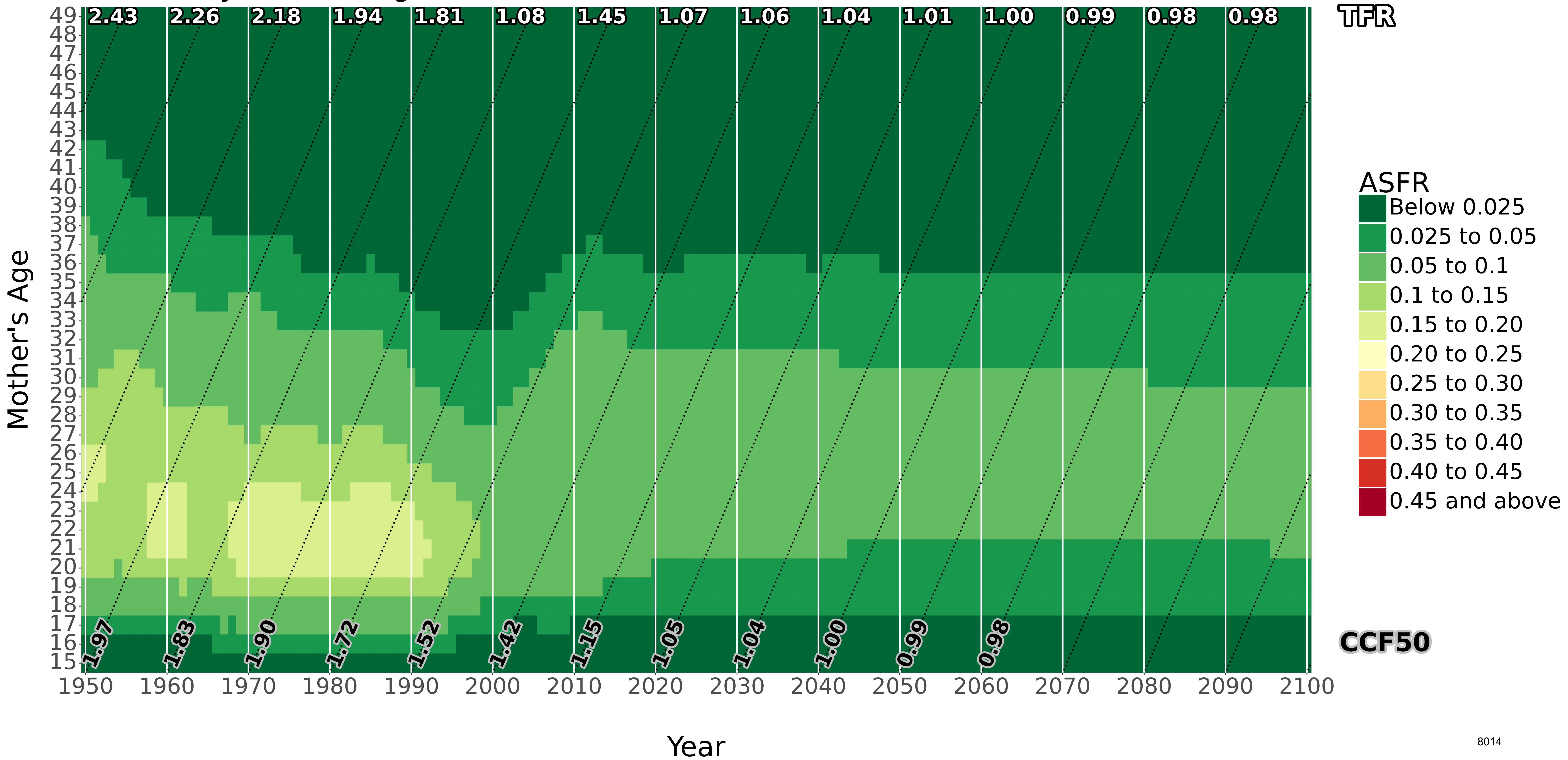

Supplemental Results Figure S3: Lexis diagram of CCF50, single-year age interval ASFR, and TFR by mother's age and location, 1950-2100, Australia

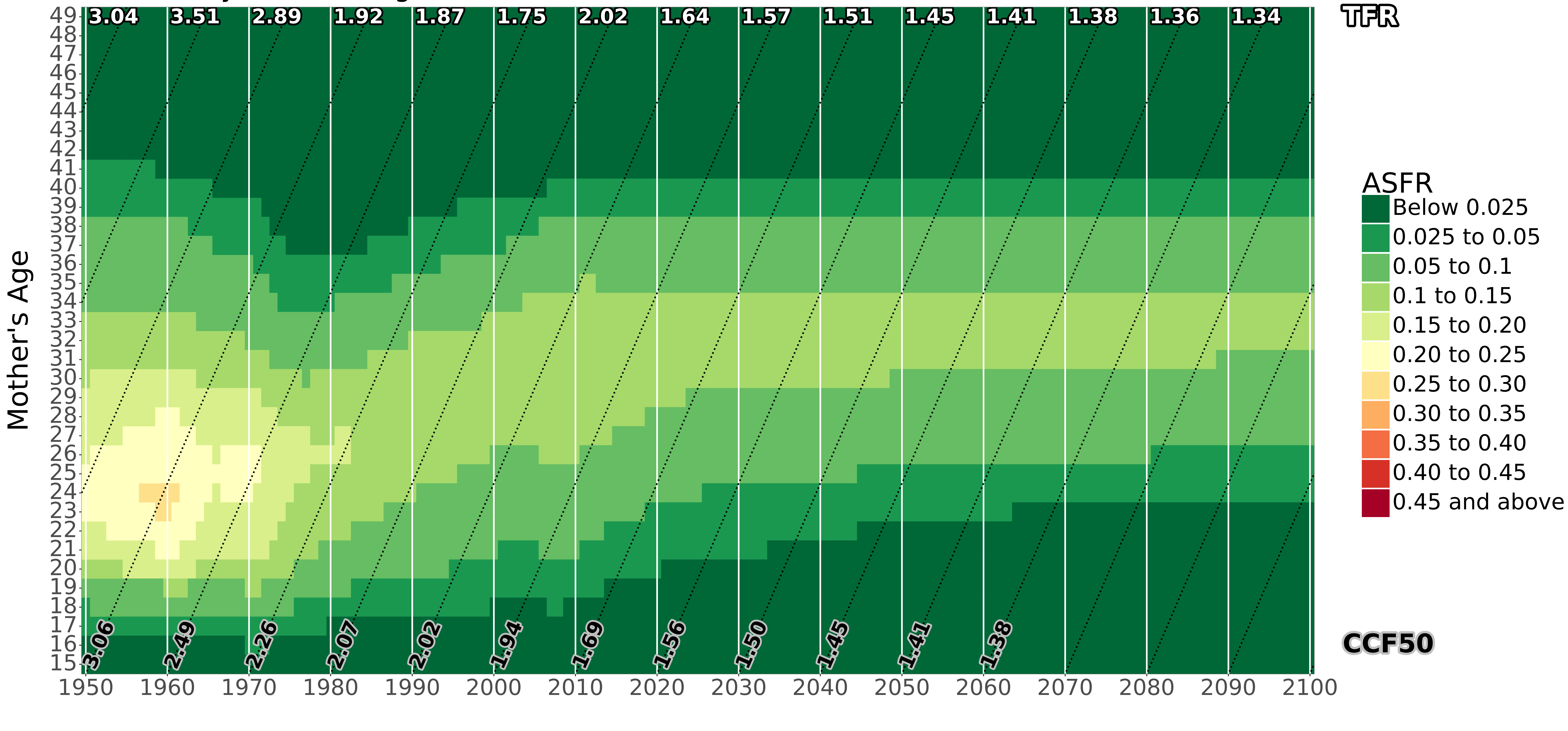

Supplemental Results Figure S3: Lexis diagram of CCF50, single-year age interval ASFR, and TFR by mother's age and location, 1950-2100, New Zealand

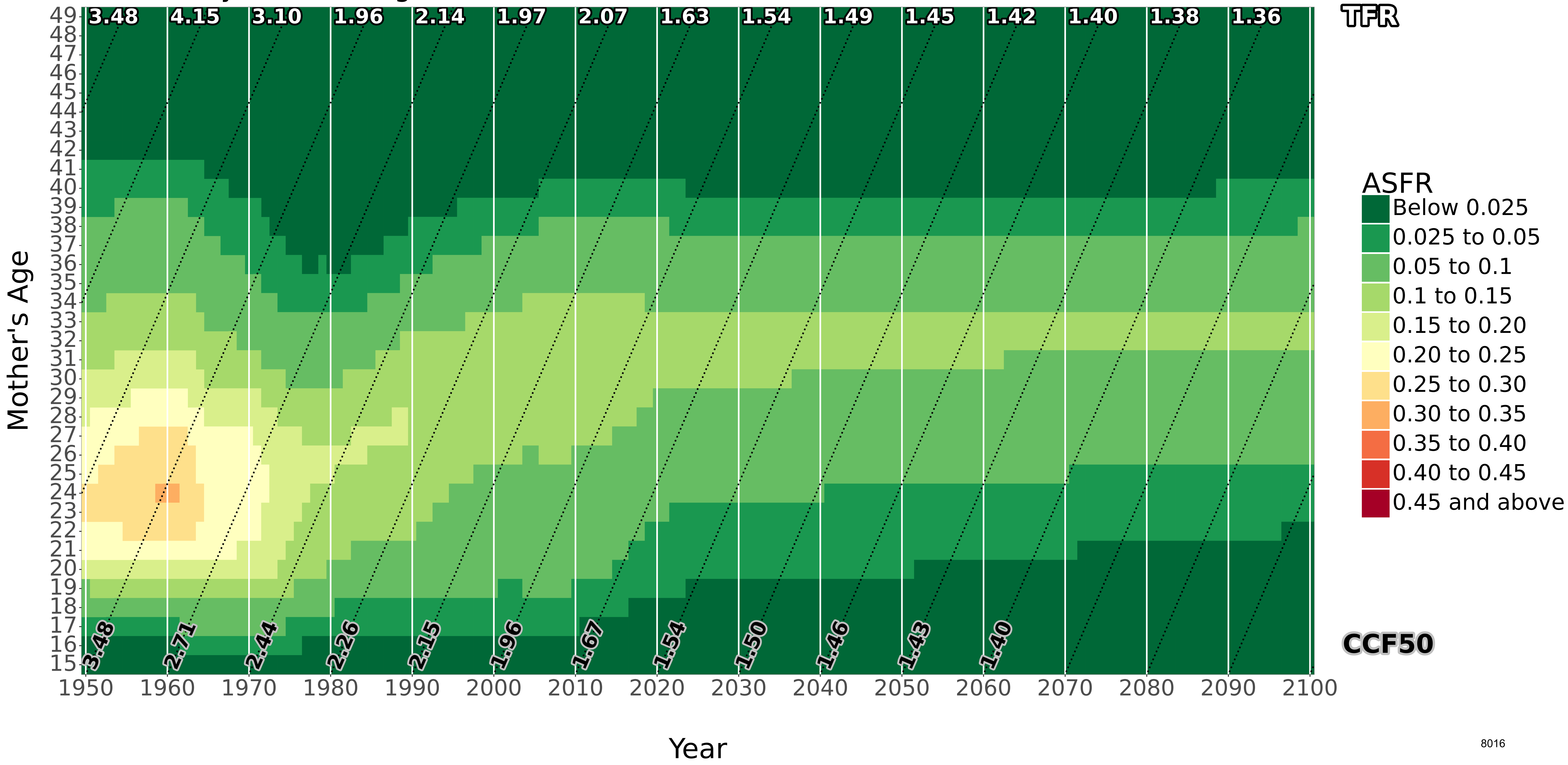

Supplemental Results Figure S3: Lexis diagram of CCF50, single-year age interval ASFR, and TFR by mother's age and location, 1950-2100, Brunei

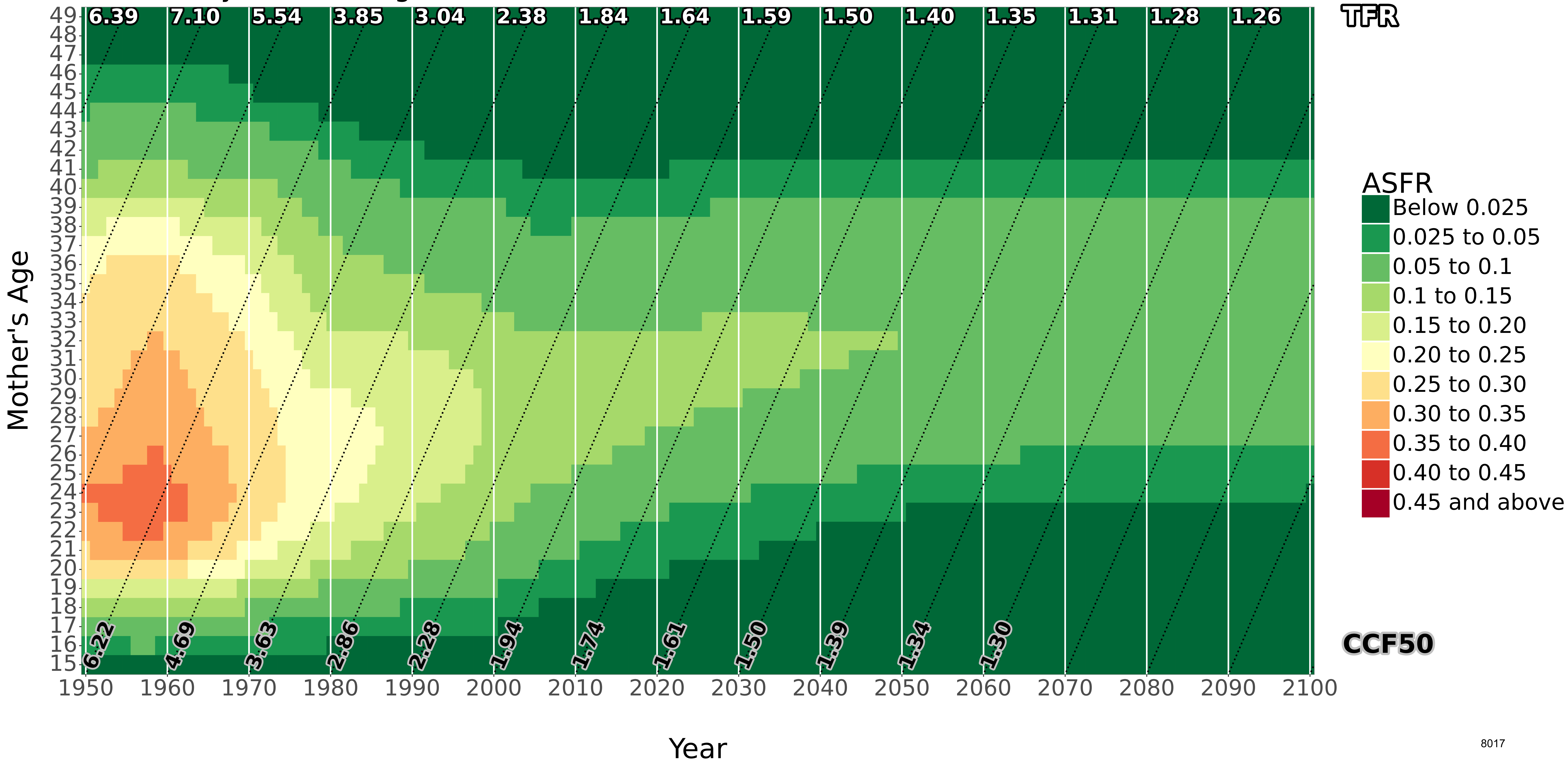

Supplemental Results Figure S3: Lexis diagram of CCF50, single-year age interval ASFR, and TFR by mother's age and location, 1950-2100, Japan

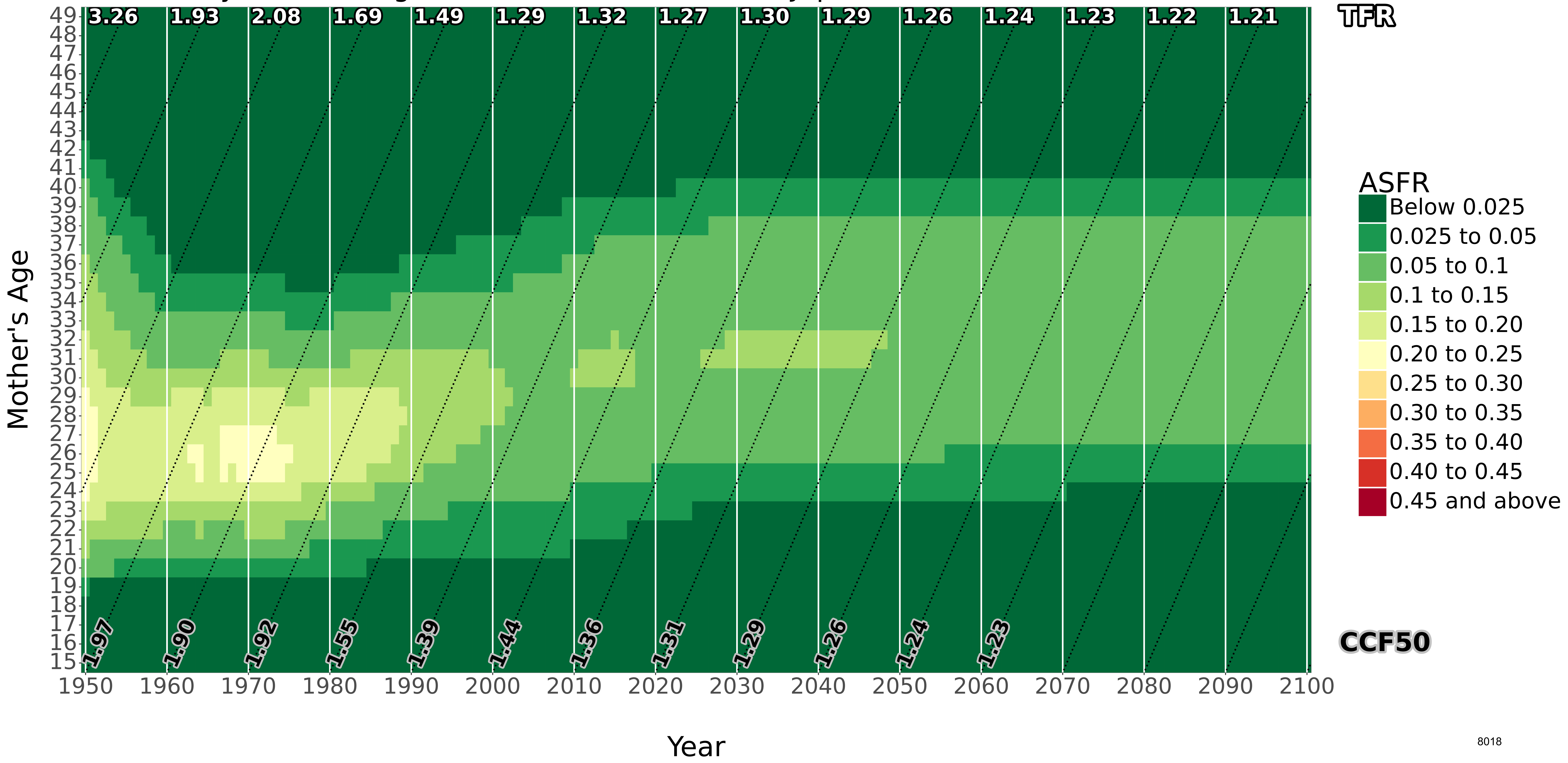

Supplemental Results Figure S3: Lexis diagram of CCF50, single-year age interval ASFR, and TFR by mother's age and location, 1950-2100, South Korea

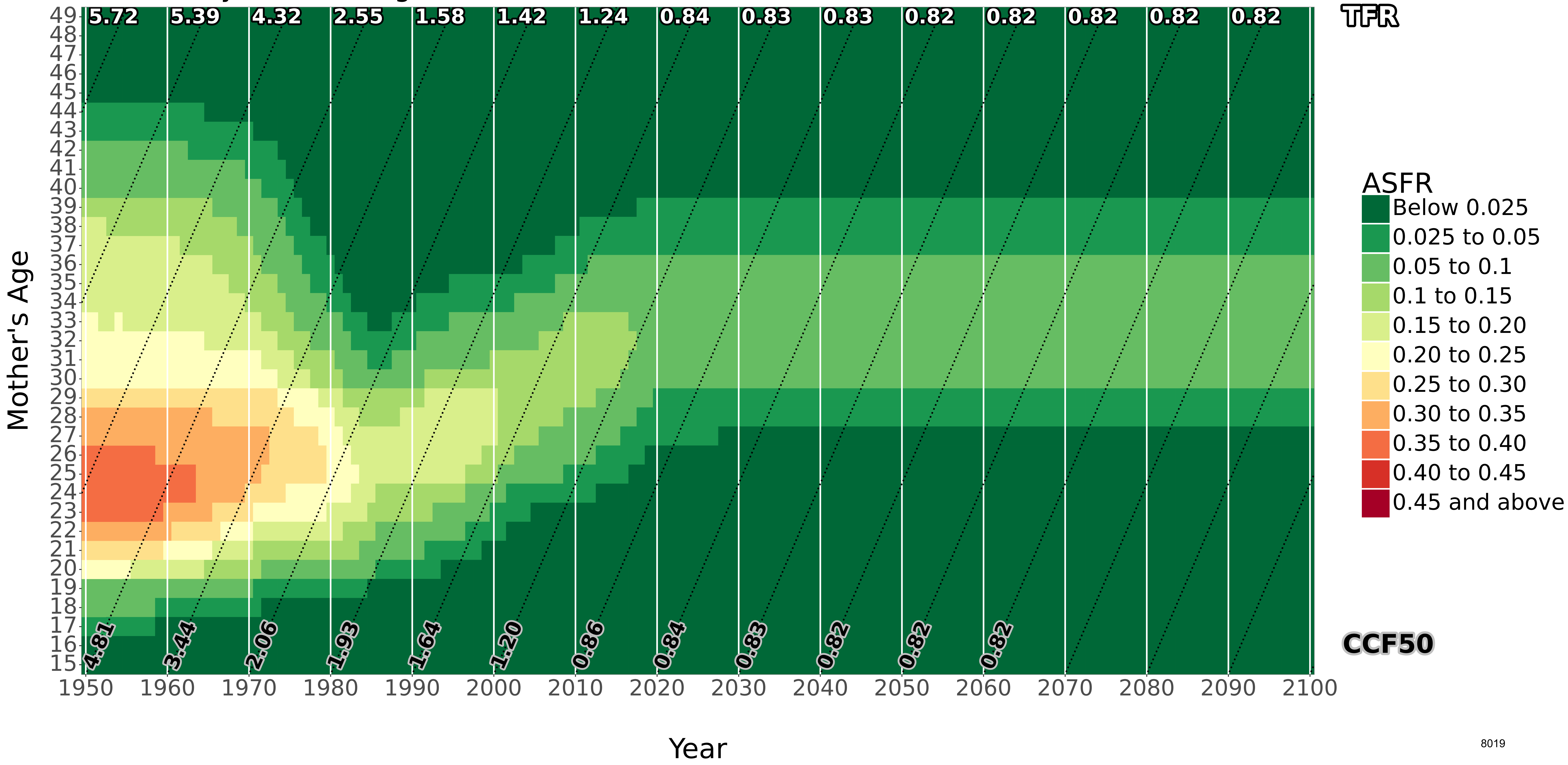

Supplemental Results Figure S3: Lexis diagram of CCF50, single-year age interval ASFR, and TFR by mother's age and location, 1950-2100, Singapore

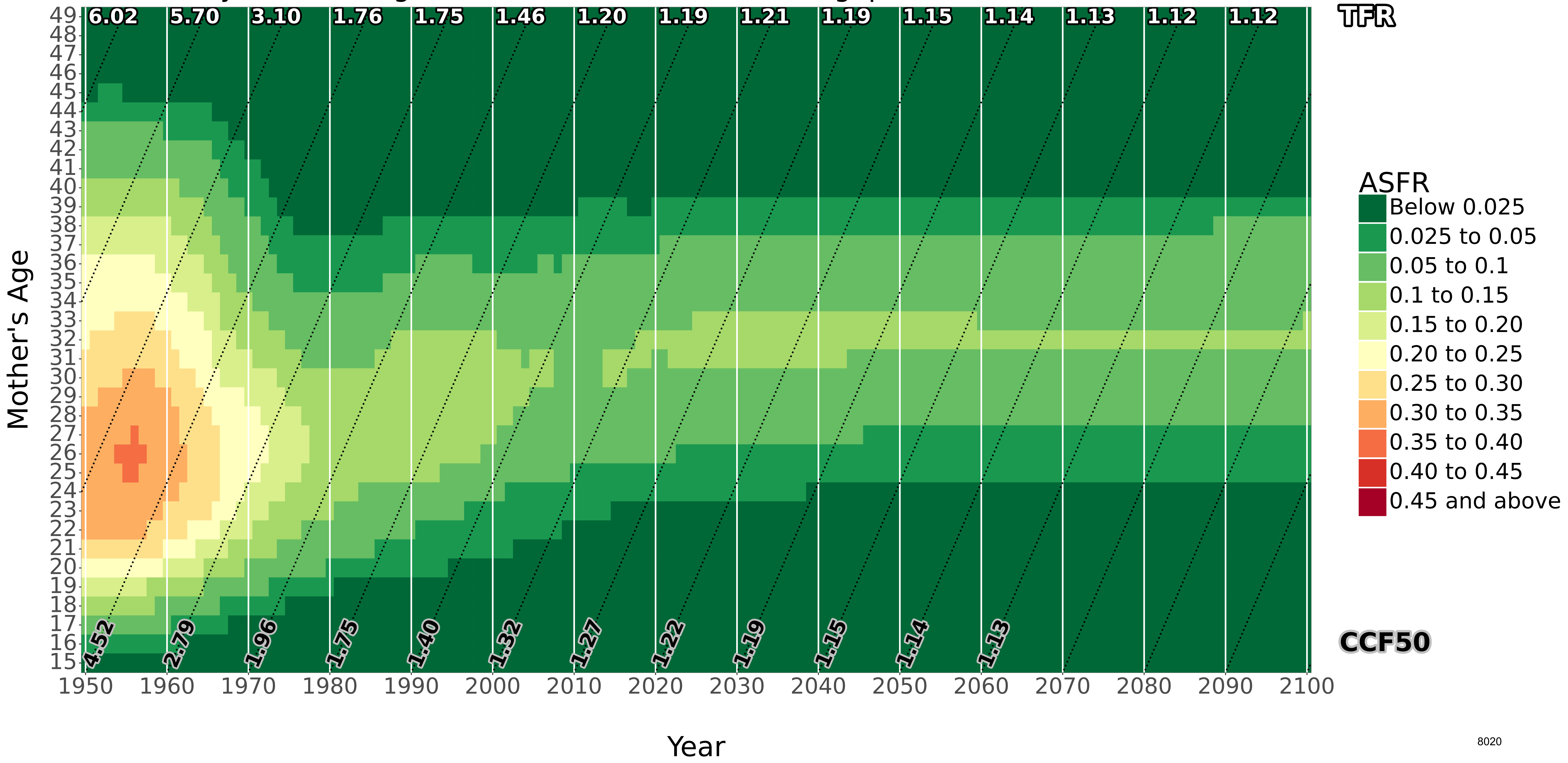

Supplemental Results Figure S3: Lexis diagram of CCF50, single-year age interval ASFR, and TFR by mother's age and location, 1950-2100, Canada

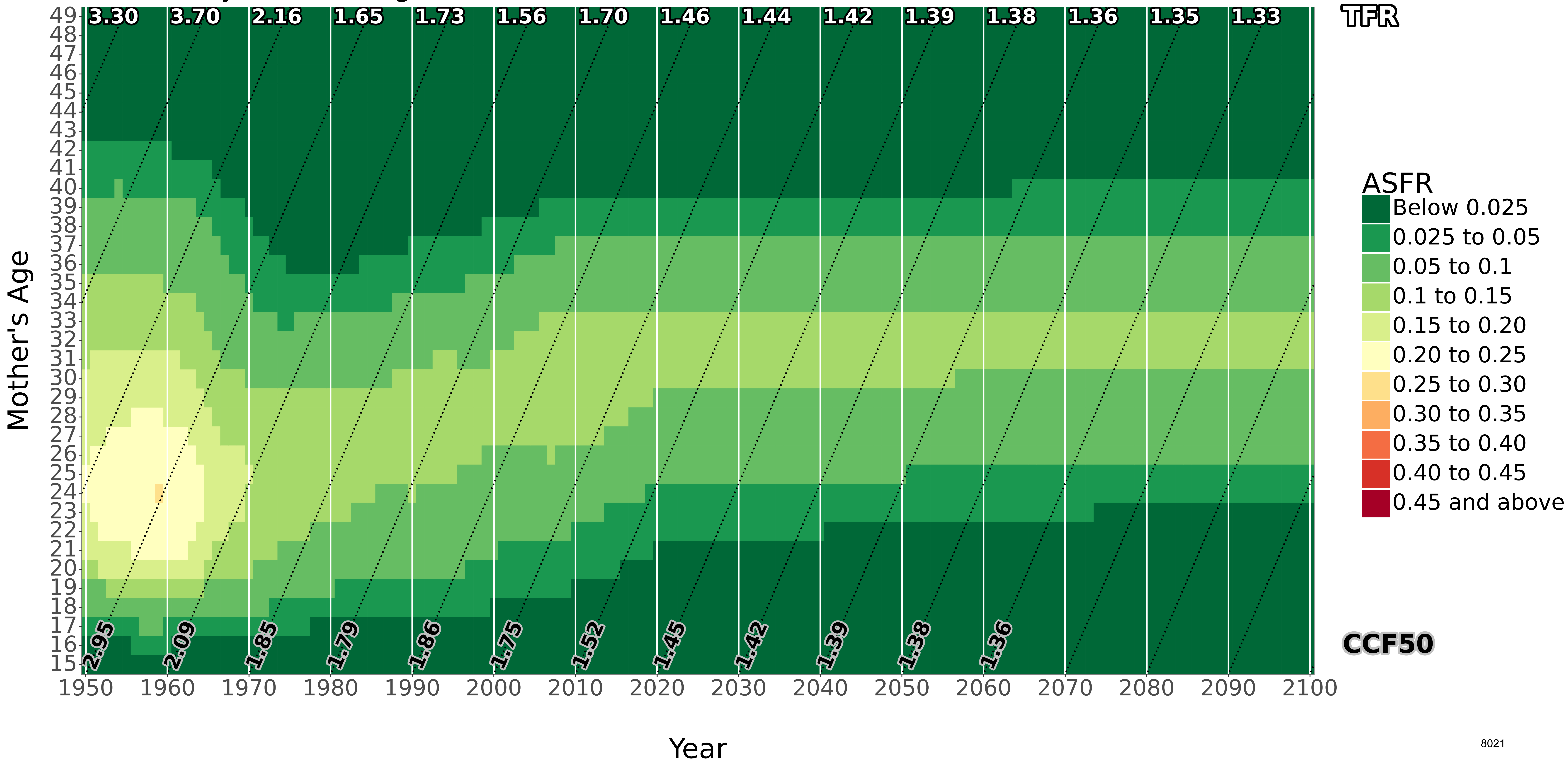

Supplemental Results Figure S3: Lexis diagram of CCF50, single-year age interval ASFR, and TFR by mother's age and location, 1950-2100, Greenland

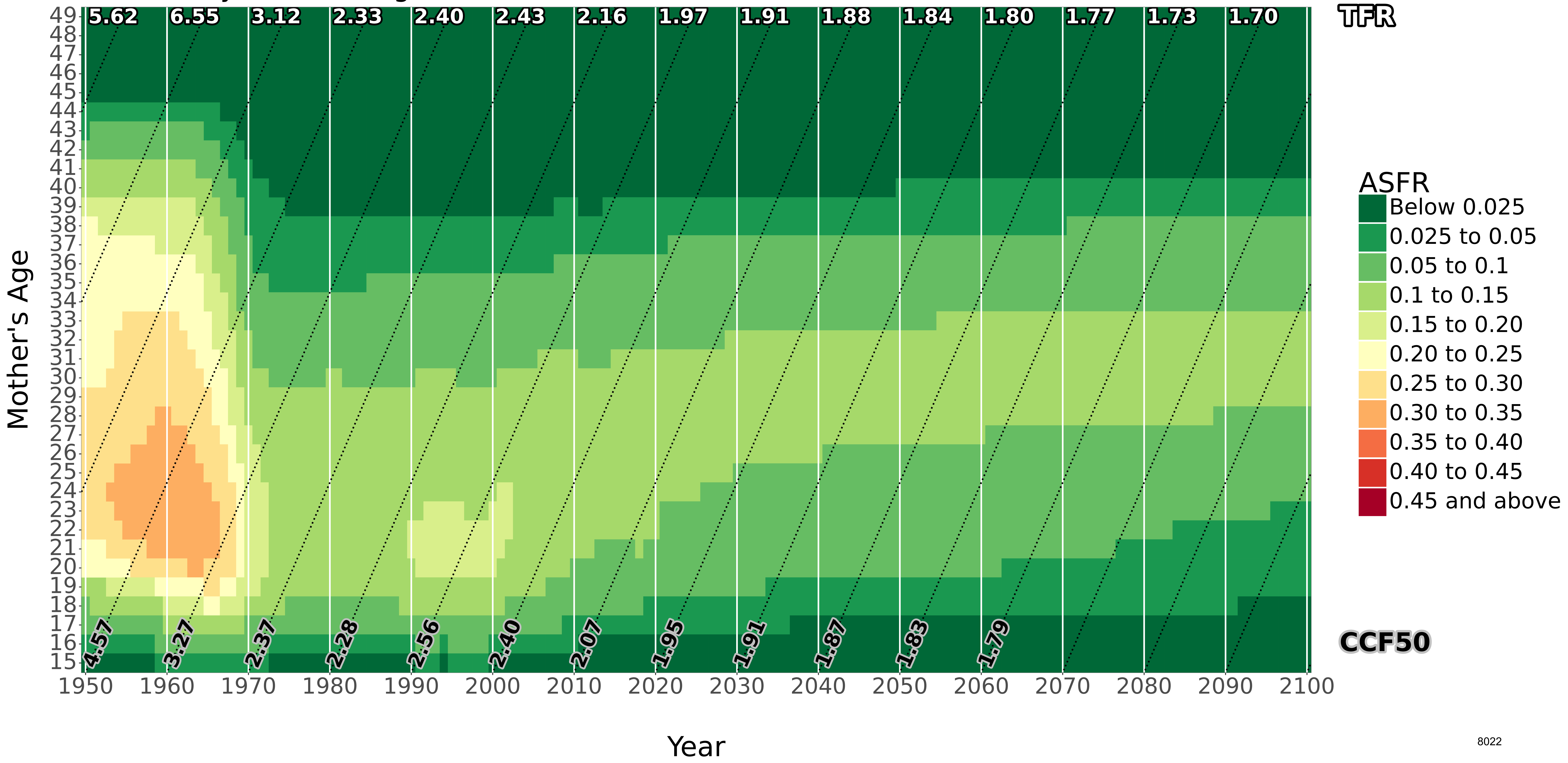

Supplemental Results Figure S3: Lexis diagram of CCF50, single-year age interval ASFR, and TFR by mother's age and location, 1950-2100, USA

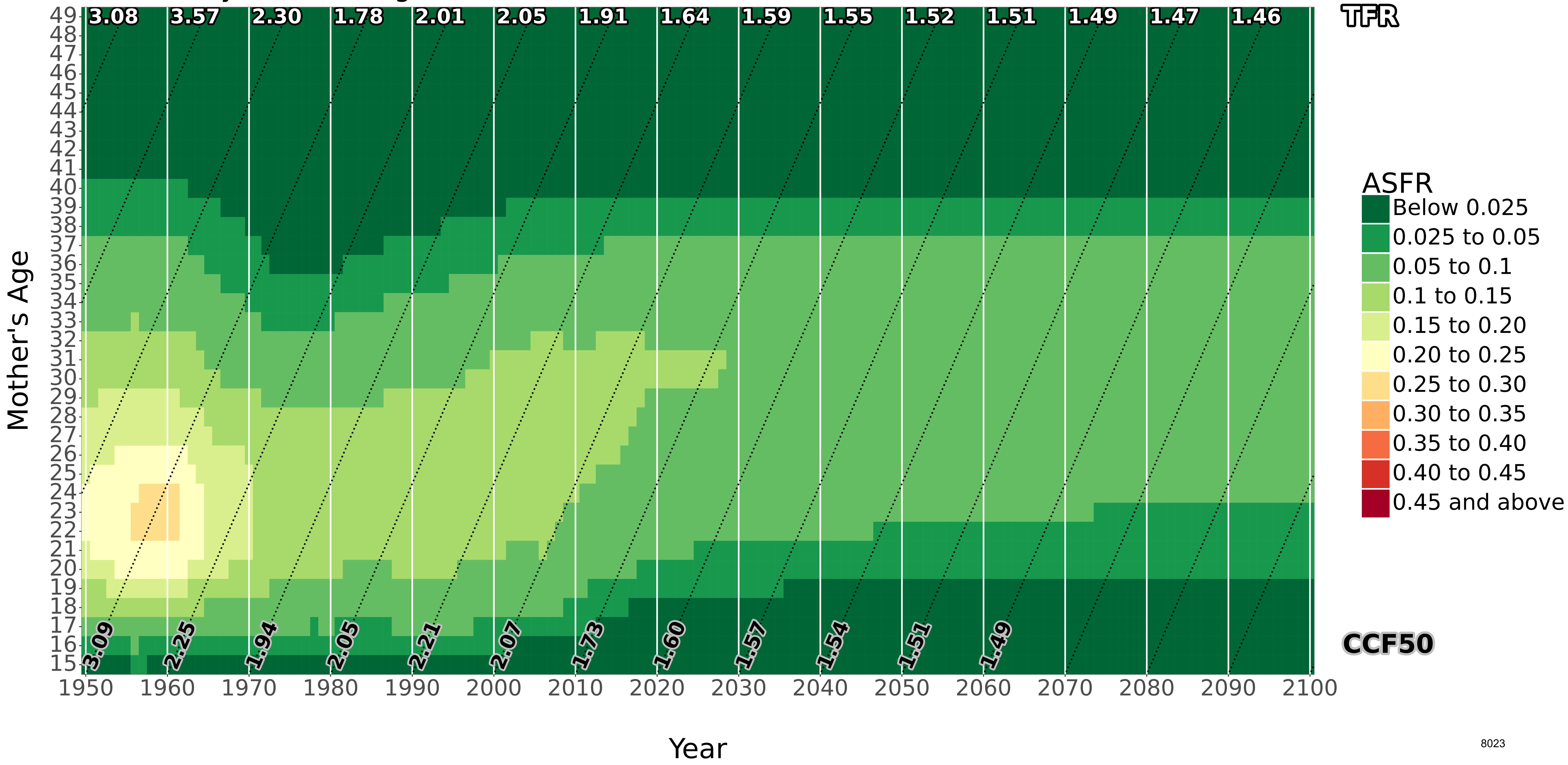

Supplemental Results Figure S3: Lexis diagram of CCF50, single-year age interval ASFR, and TFR by mother's age and location, 1950-2100, Argentina

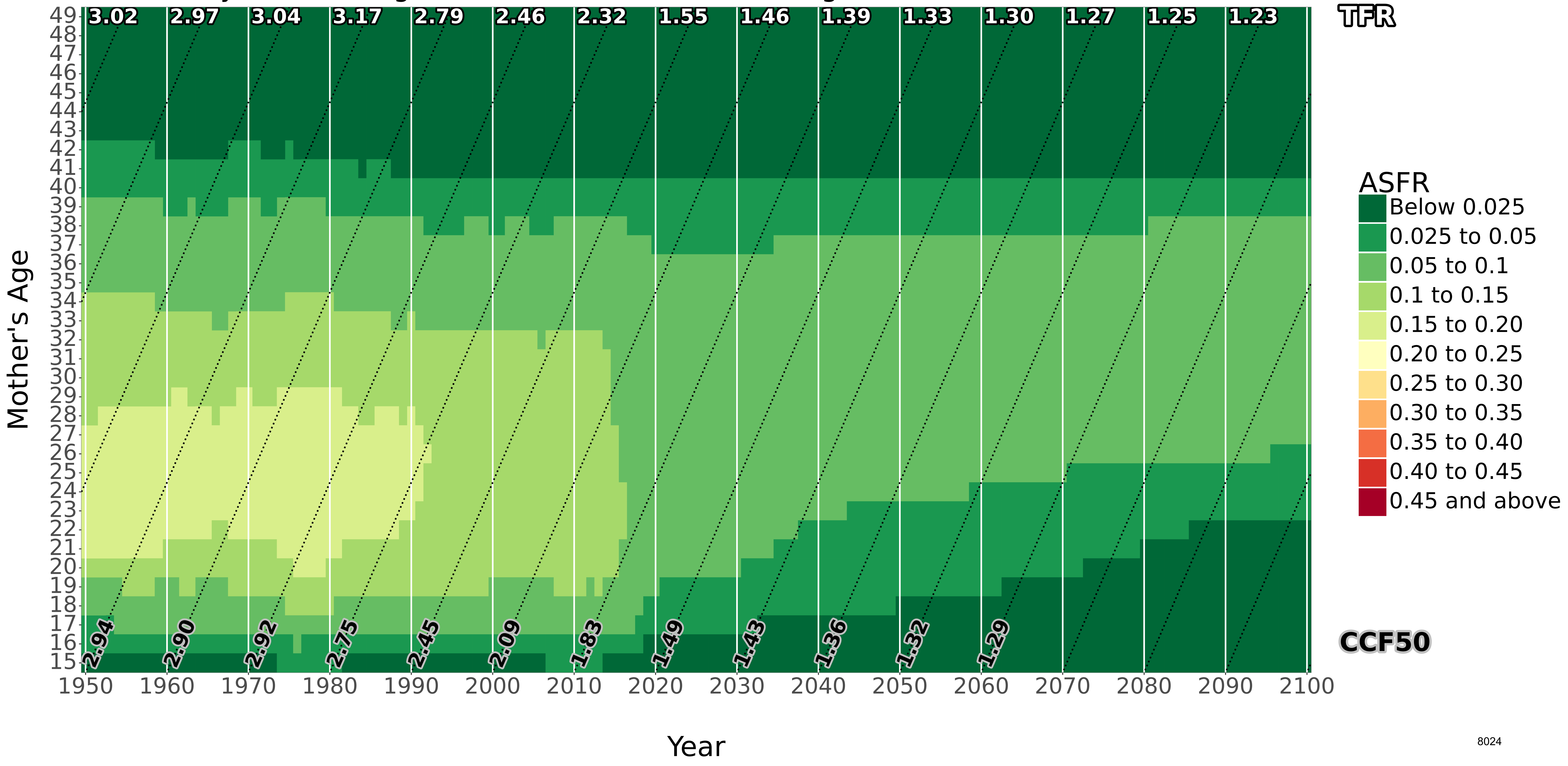

Supplemental Results Figure S3: Lexis diagram of CCF50, single-year age interval ASFR, and TFR by mother's age and location, 1950-2100, Chile

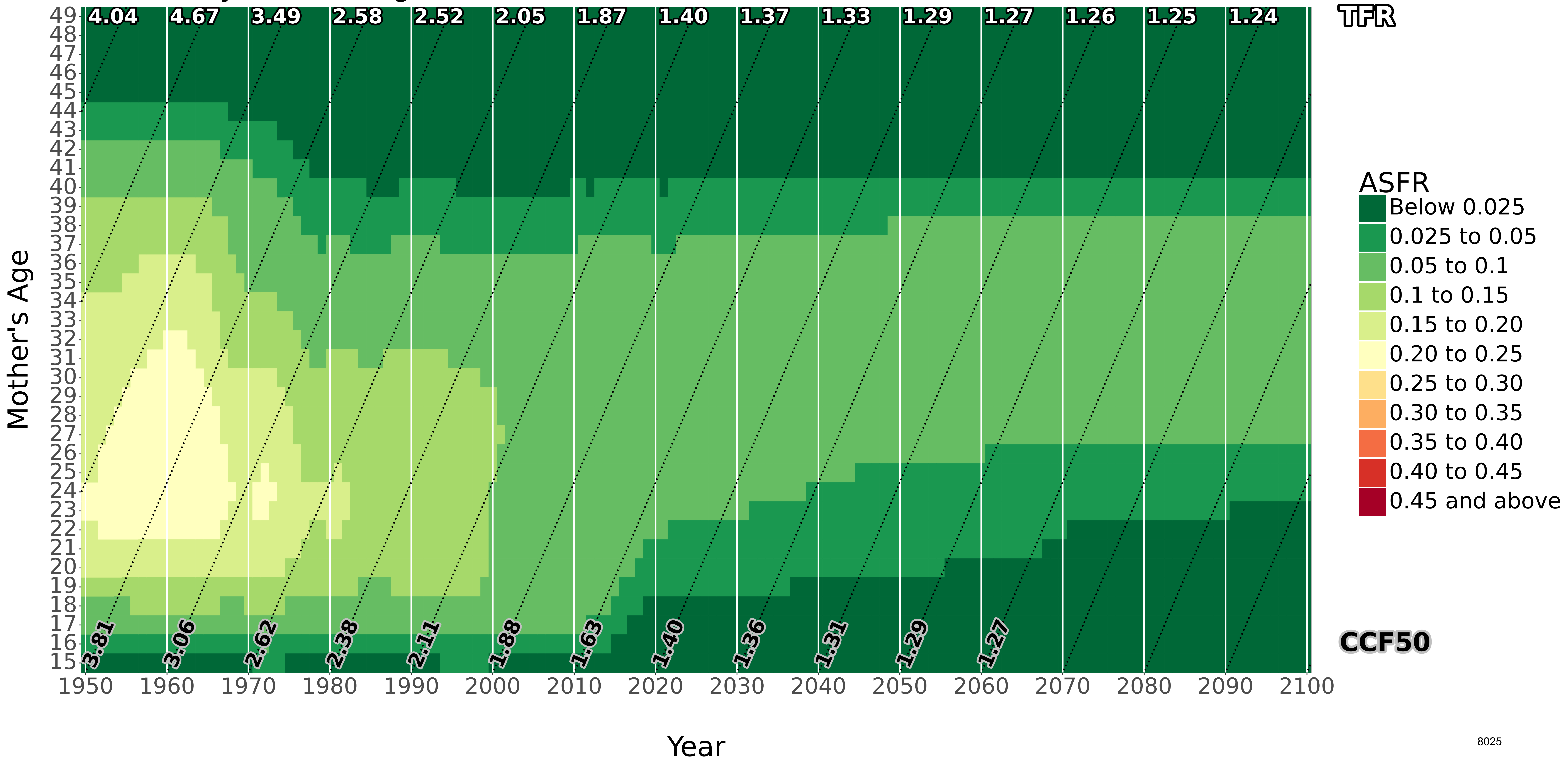

Supplemental Results Figure S3: Lexis diagram of CCF50, single-year age interval ASFR, and TFR by mother's age and location, 1950-2100, Uruguay

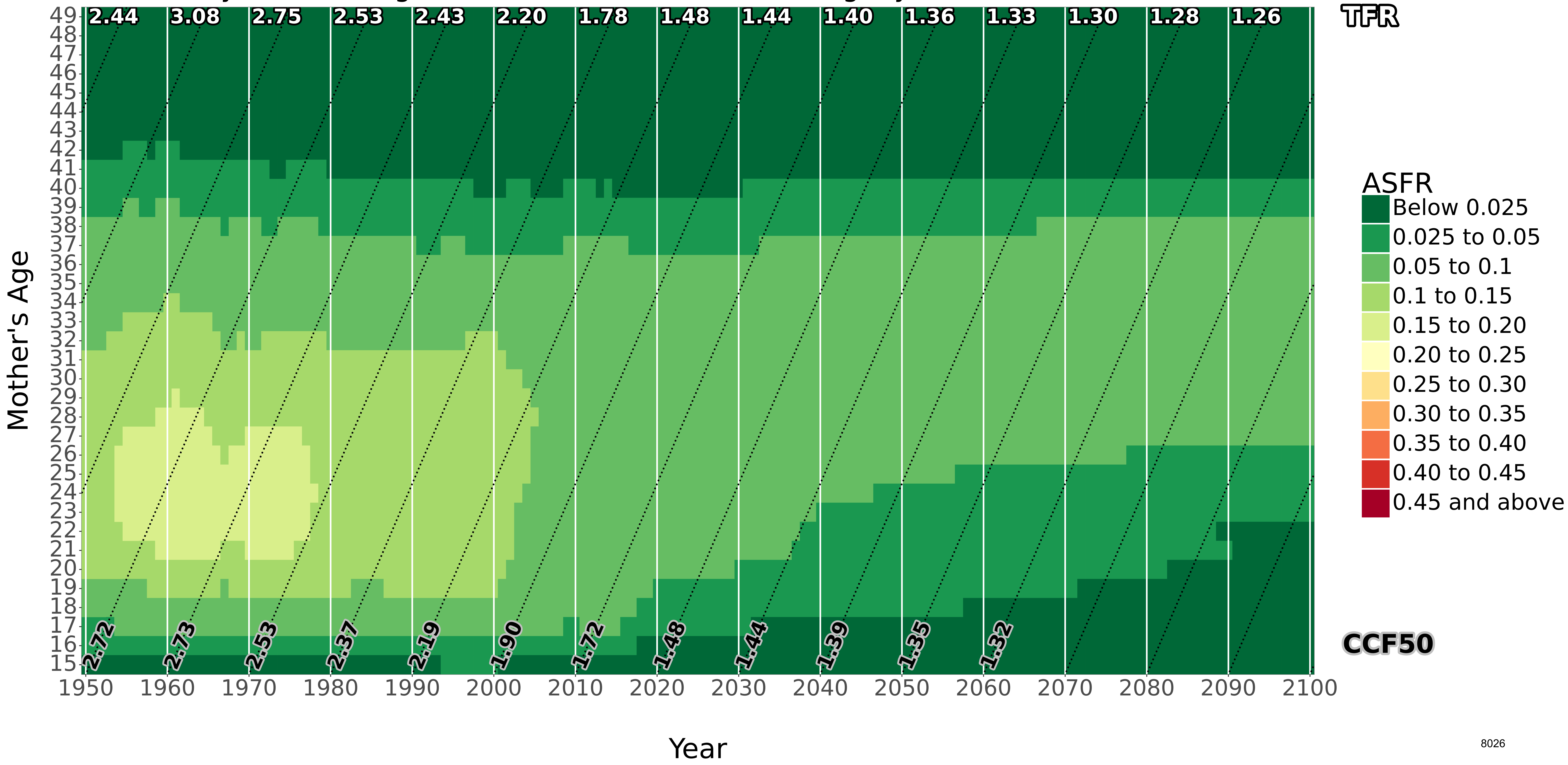

Supplemental Results Figure S3: Lexis diagram of CCF50, single-year age interval ASFR, and TFR by mother's age and location, 1950-2100, Andorra

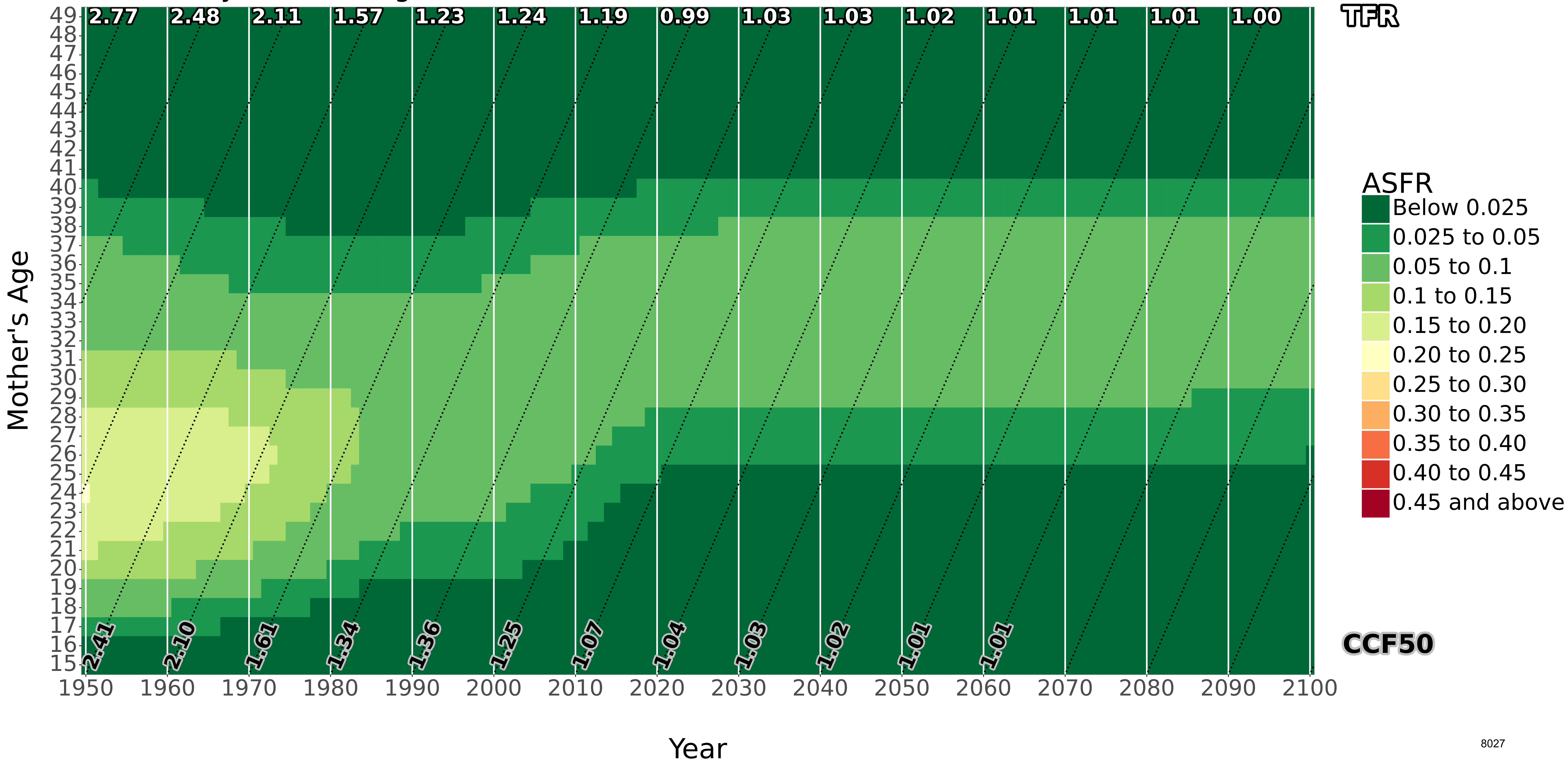

Supplemental Results Figure S3: Lexis diagram of CCF50, single-year age interval ASFR, and TFR by mother's age and location, 1950-2100, Austria

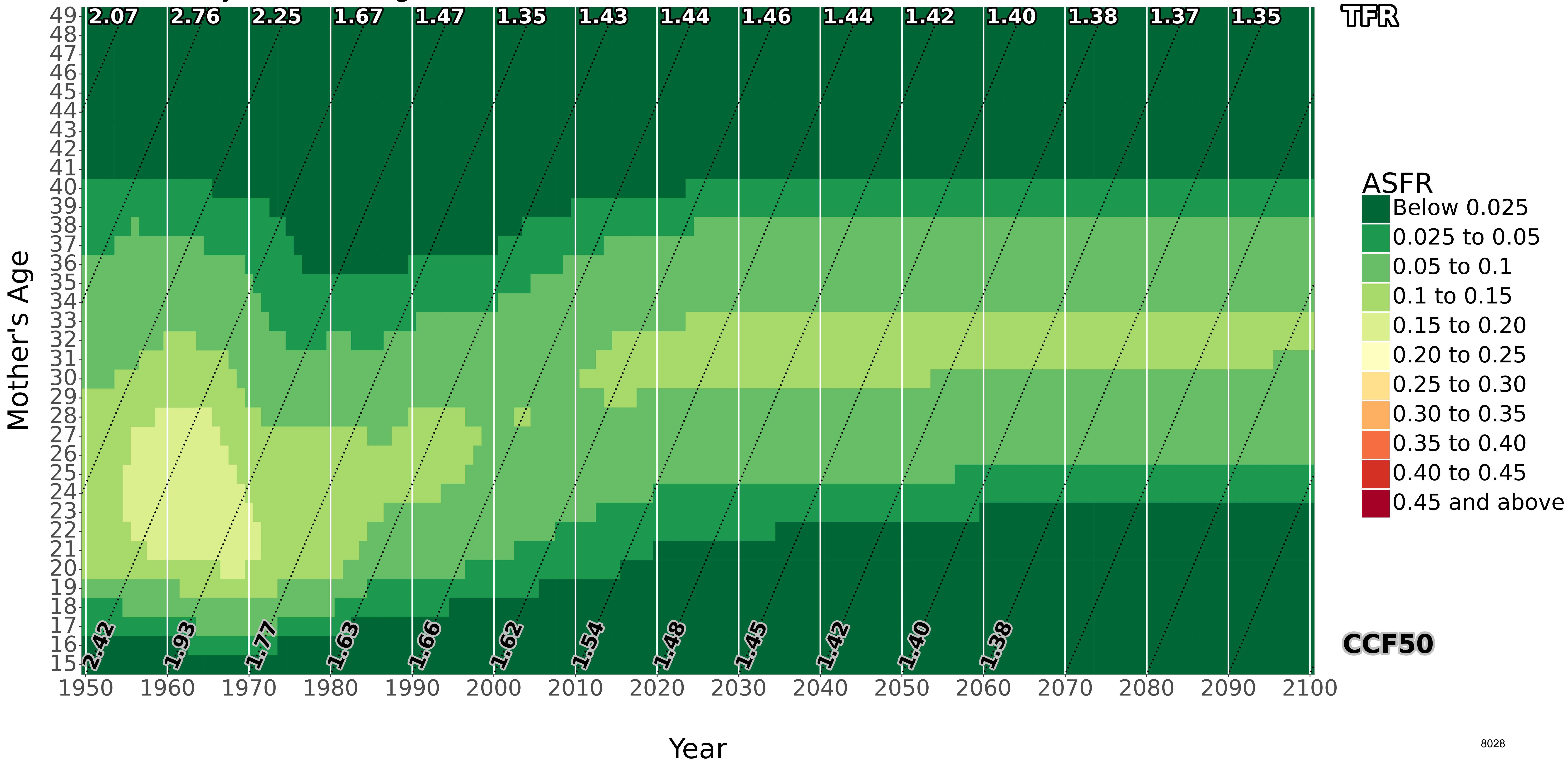

Supplemental Results Figure S3: Lexis diagram of CCF50, single-year age interval ASFR, and TFR by mother's age and location, 1950-2100, Belgium

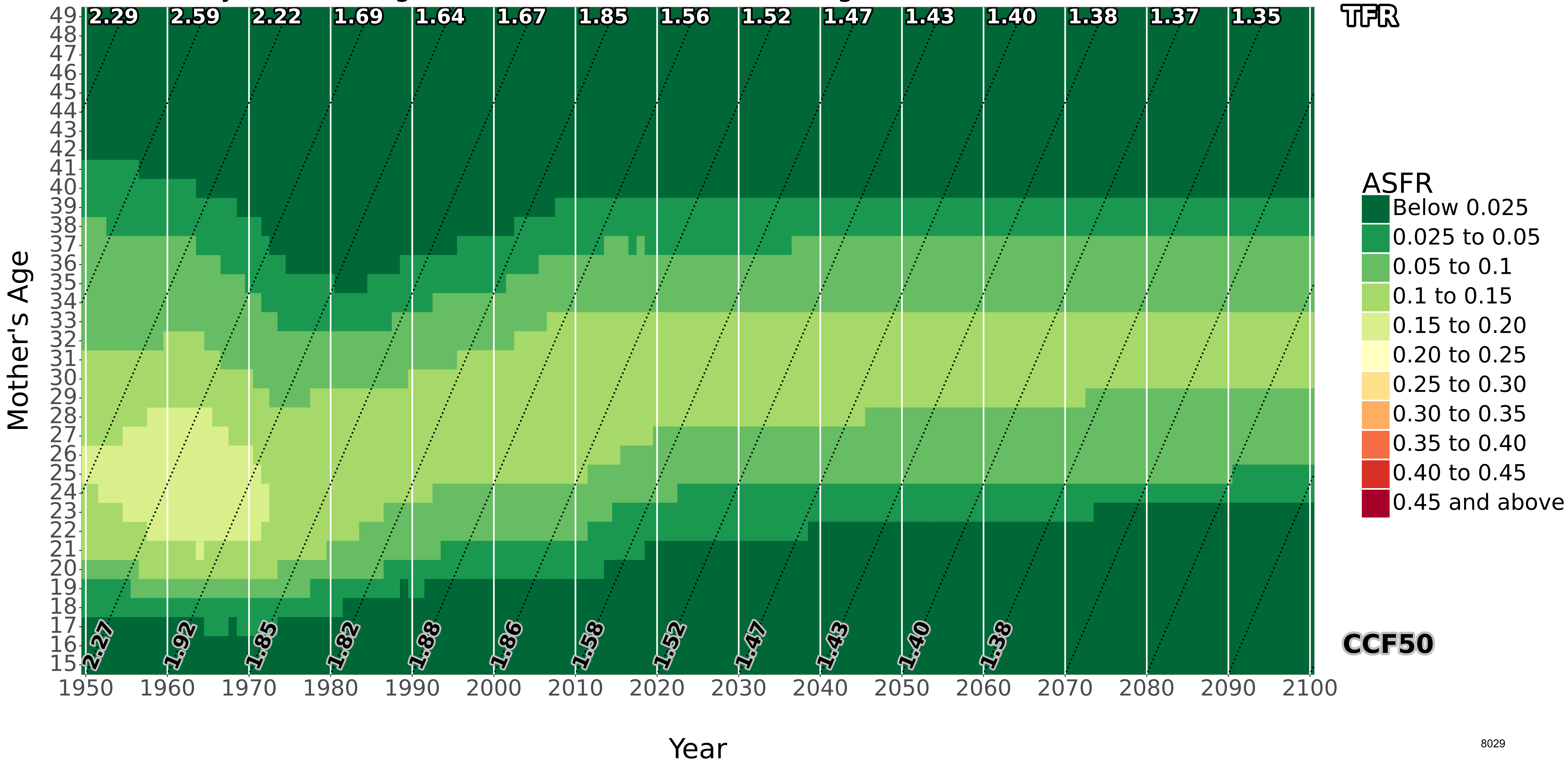

Supplemental Results Figure S3: Lexis diagram of CCF50, single-year age interval ASFR, and TFR by mother's age and location, 1950-2100, Cyprus

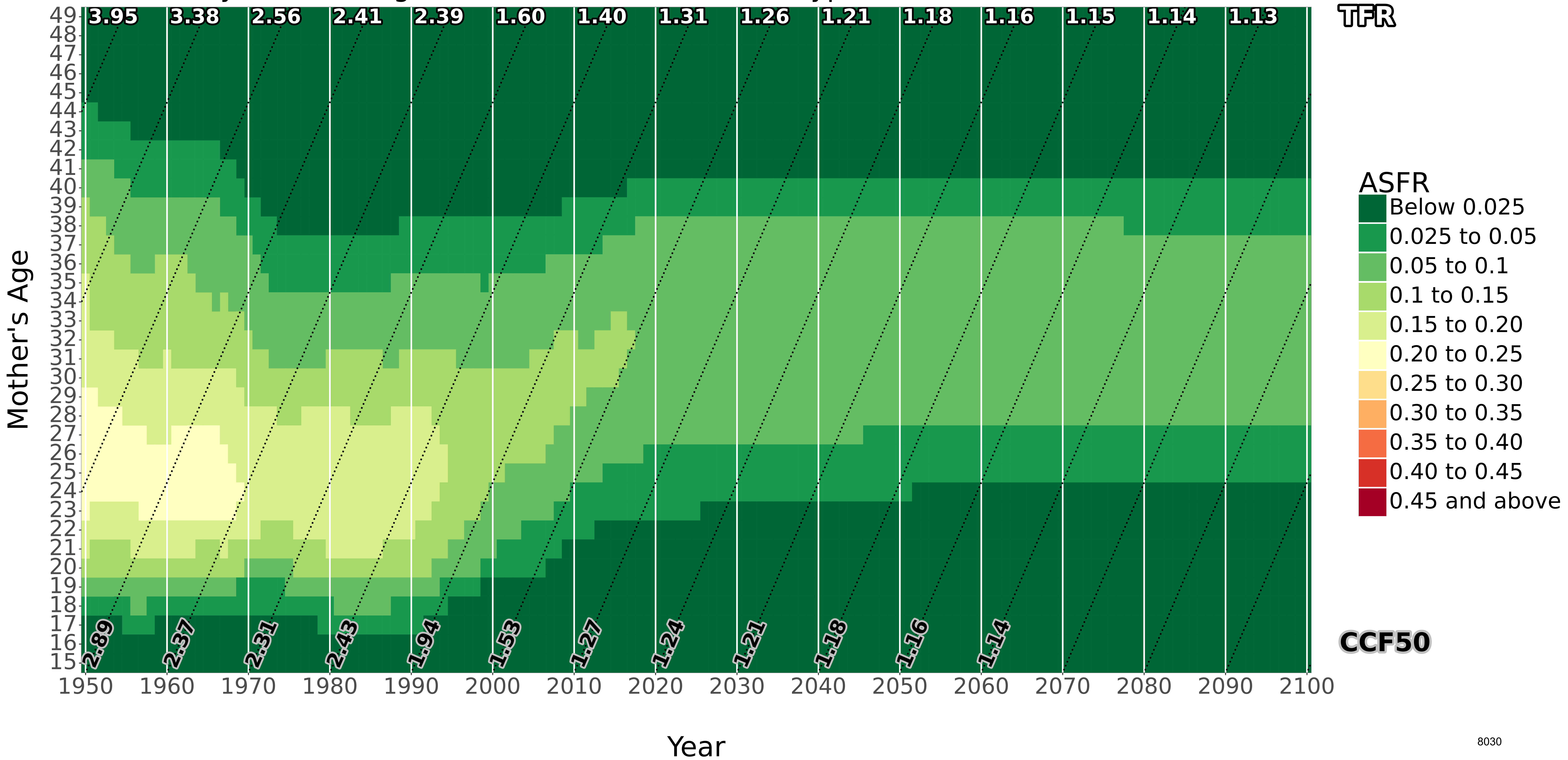

Supplemental Results Figure S3: Lexis diagram of CCF50, single-year age interval ASFR, and TFR by mother's age and location, 1950-2100, Denmark

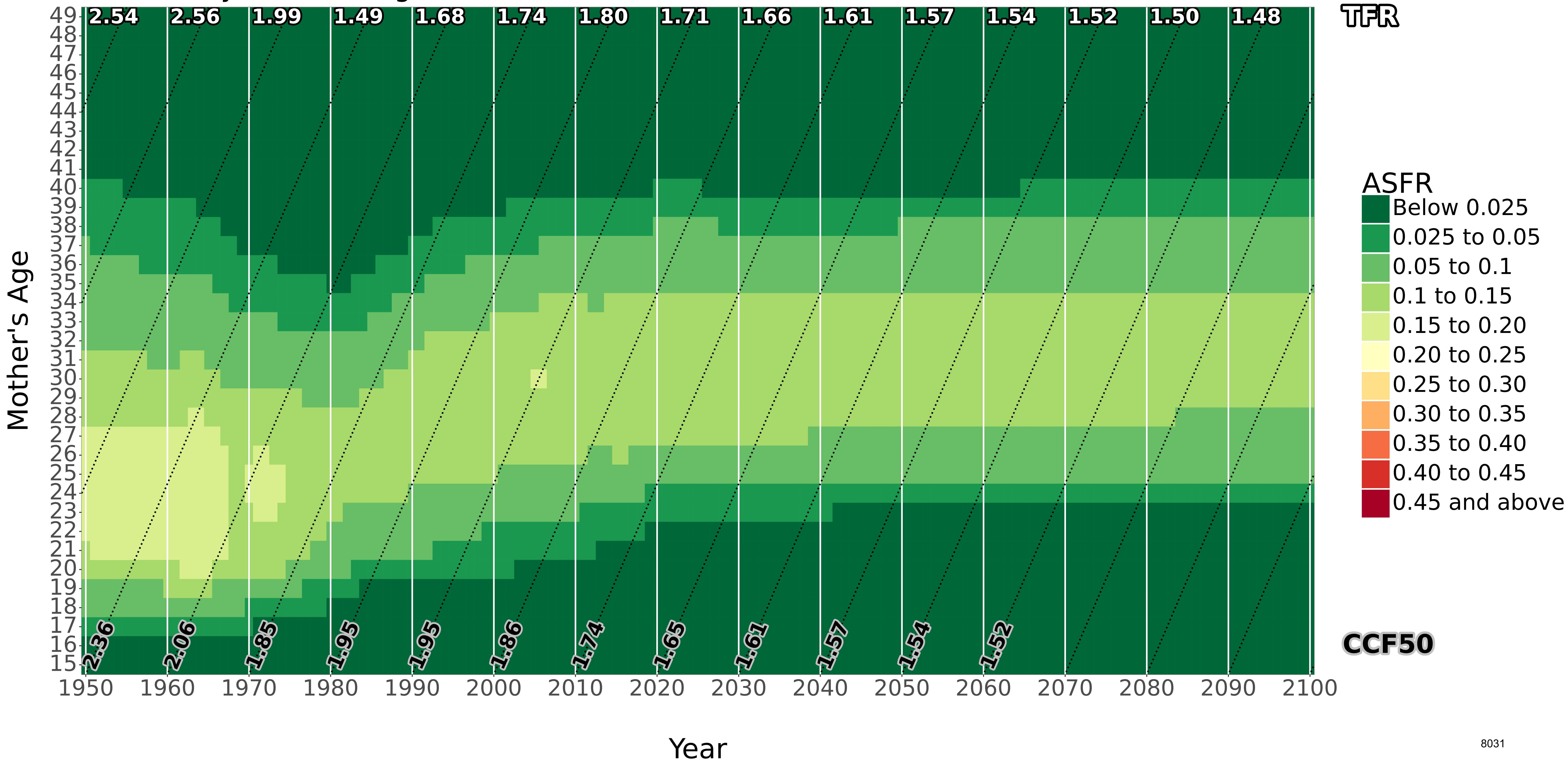

Supplemental Results Figure S3: Lexis diagram of CCF50, single-year age interval ASFR, and TFR by mother's age and location, 1950-2100, Finland

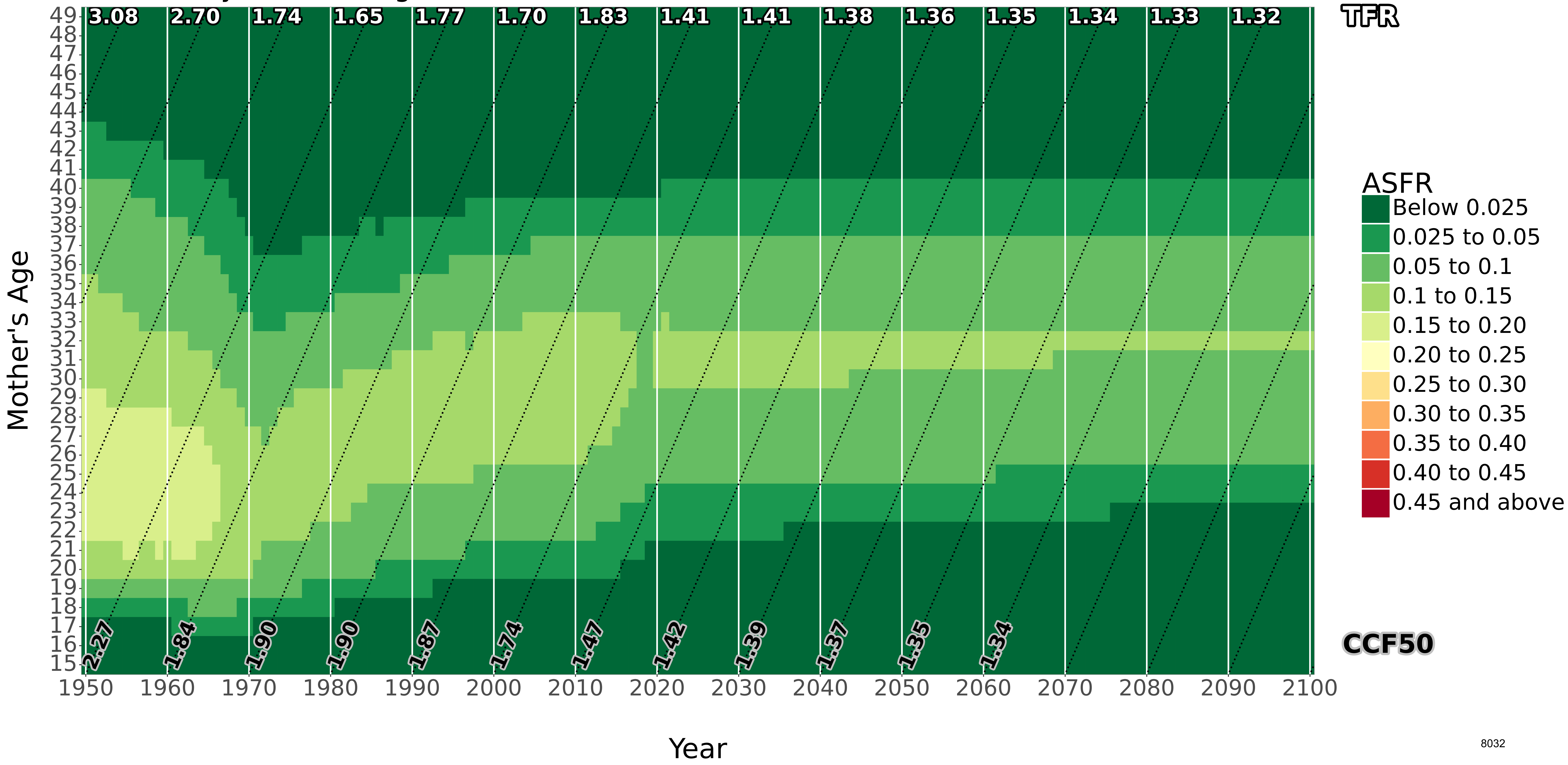

Supplemental Results Figure S3: Lexis diagram of CCF50, single-year age interval ASFR, and TFR by mother's age and location, 1950-2100, France

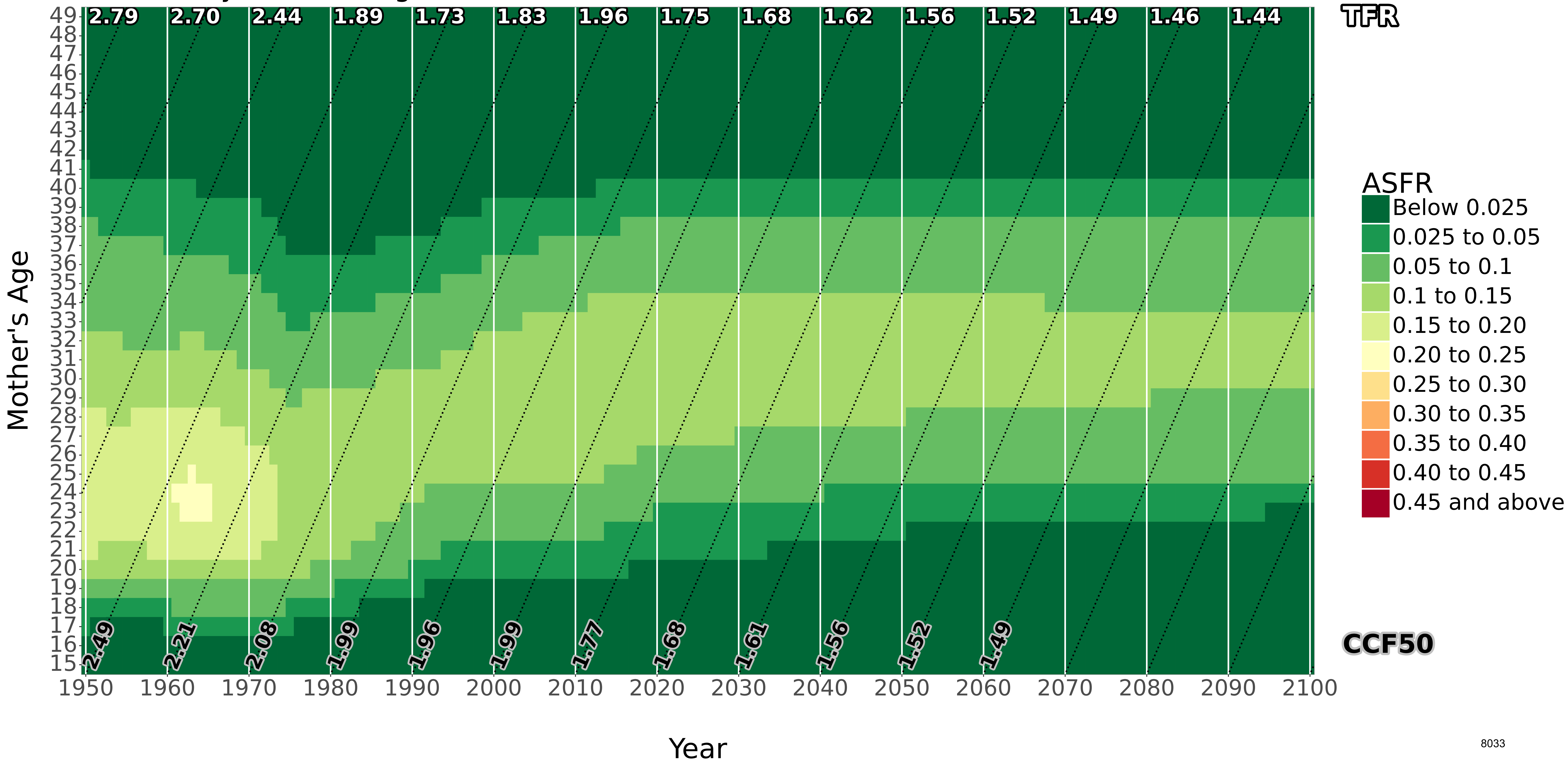

Supplemental Results Figure S3: Lexis diagram of CCF50, single-year age interval ASFR, and TFR by mother's age and location, 1950-2100, Germany

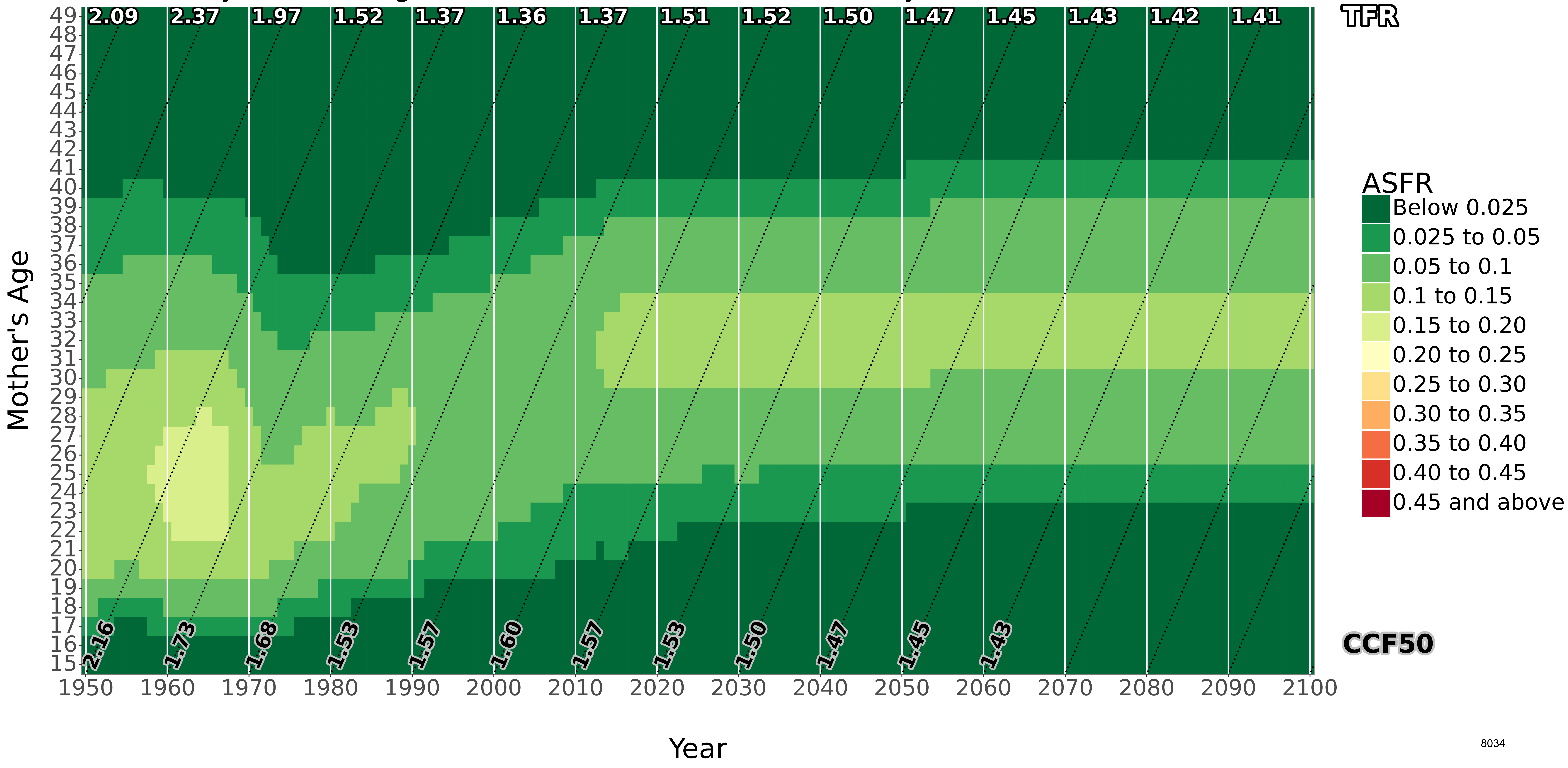

Supplemental Results Figure S3: Lexis diagram of CCF50, single-year age interval ASFR, and TFR by mother's age and location, 1950-2100, Greece

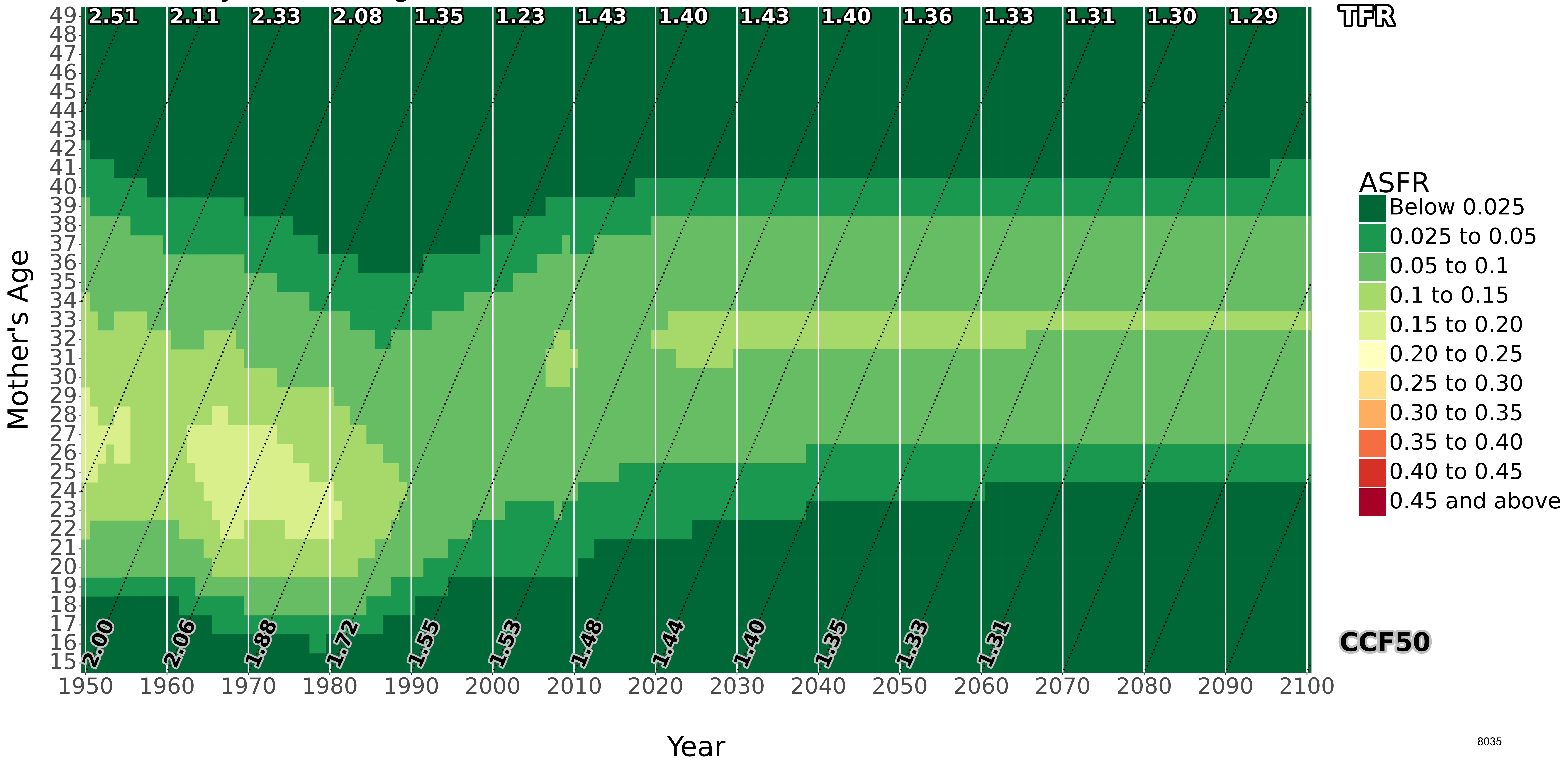

Supplemental Results Figure S3: Lexis diagram of CCF50, single-year age interval ASFR, and TFR by mother's age and location, 1950-2100, Iceland

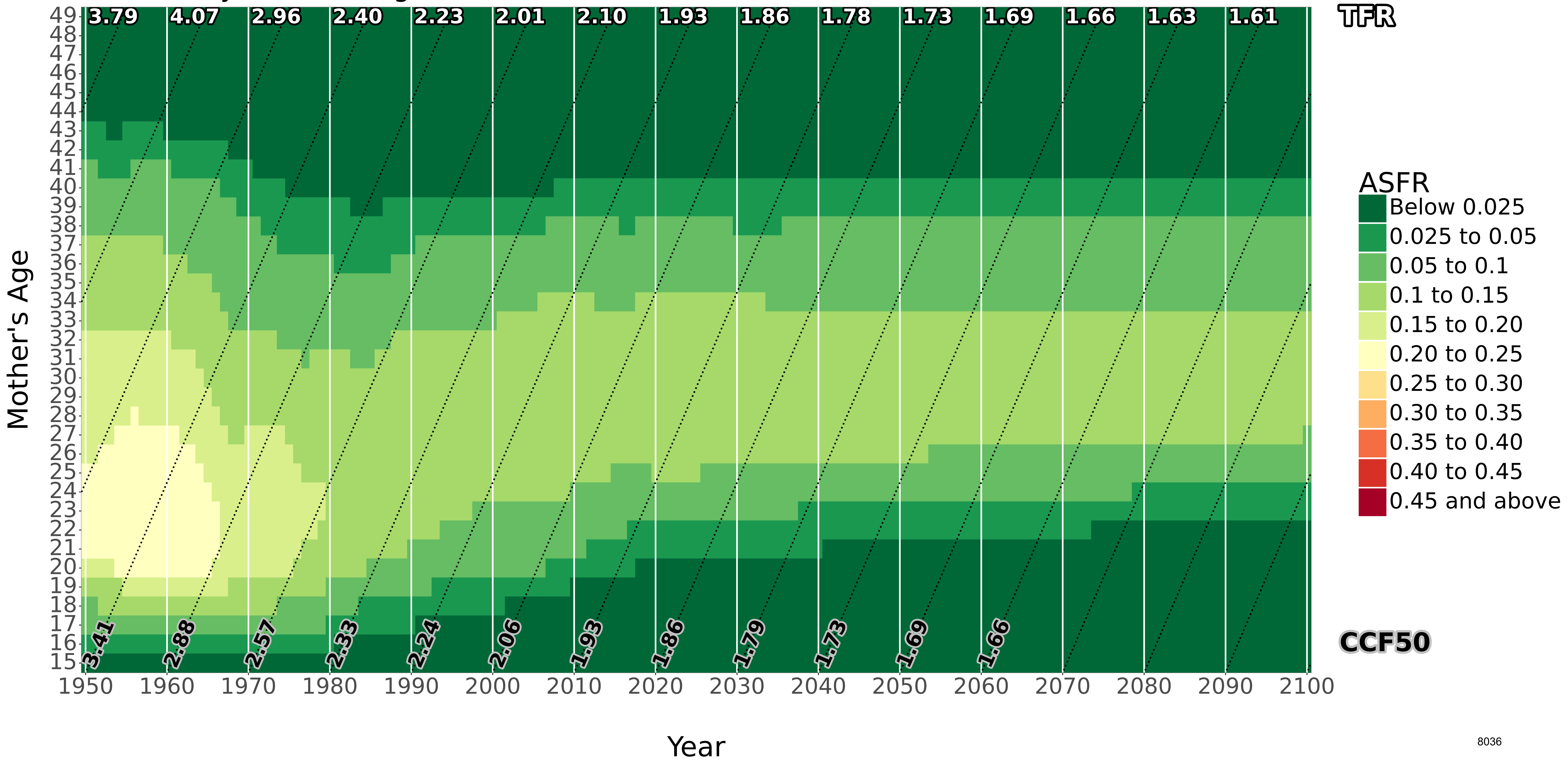

Supplemental Results Figure S3: Lexis diagram of CCF50, single-year age interval ASFR, and TFR by mother's age and location, 1950-2100, Ireland

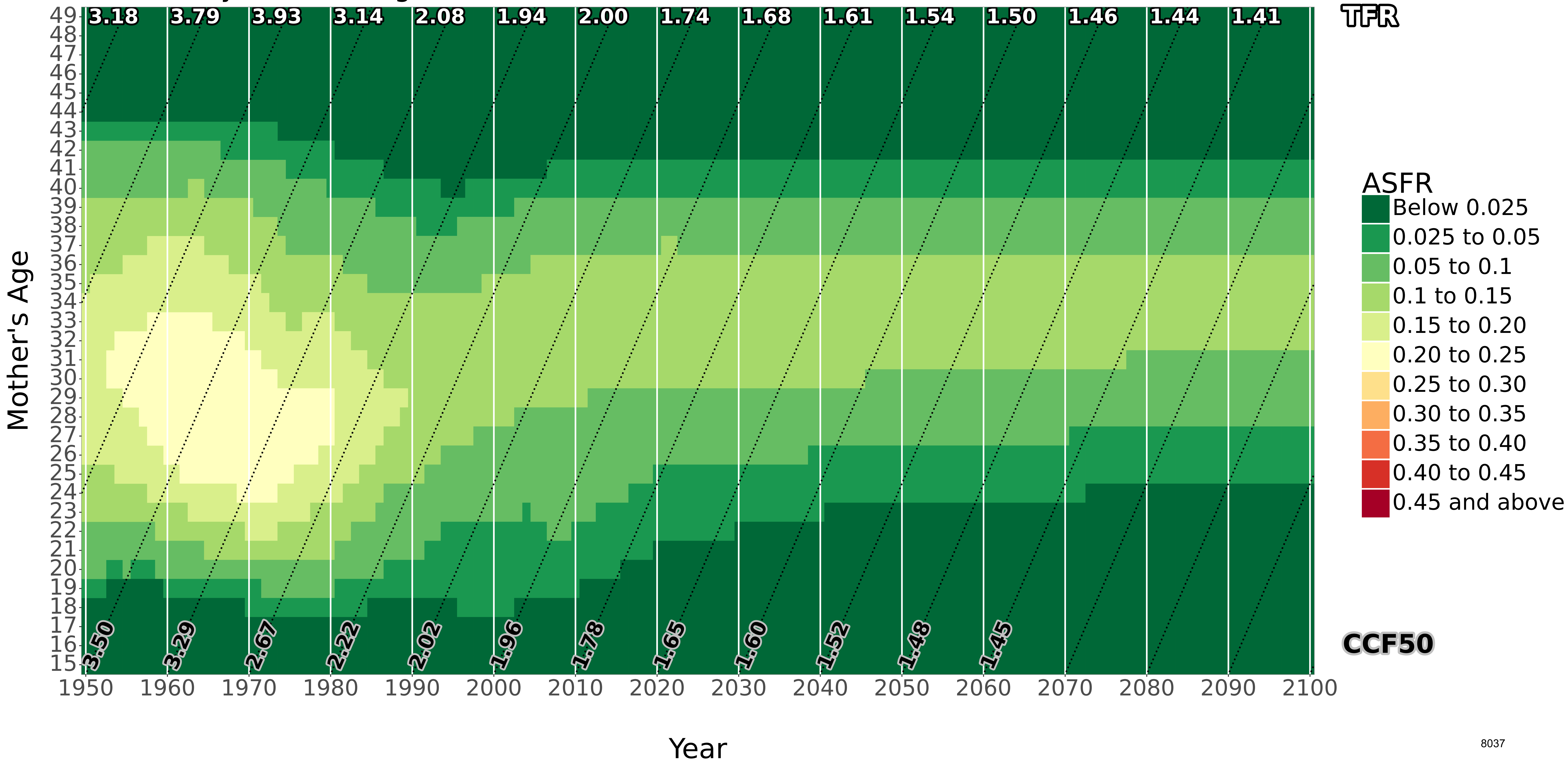

Supplemental Results Figure S3: Lexis diagram of CCF50, single-year age interval ASFR, and TFR by mother's age and location, 1950-2100, Israel

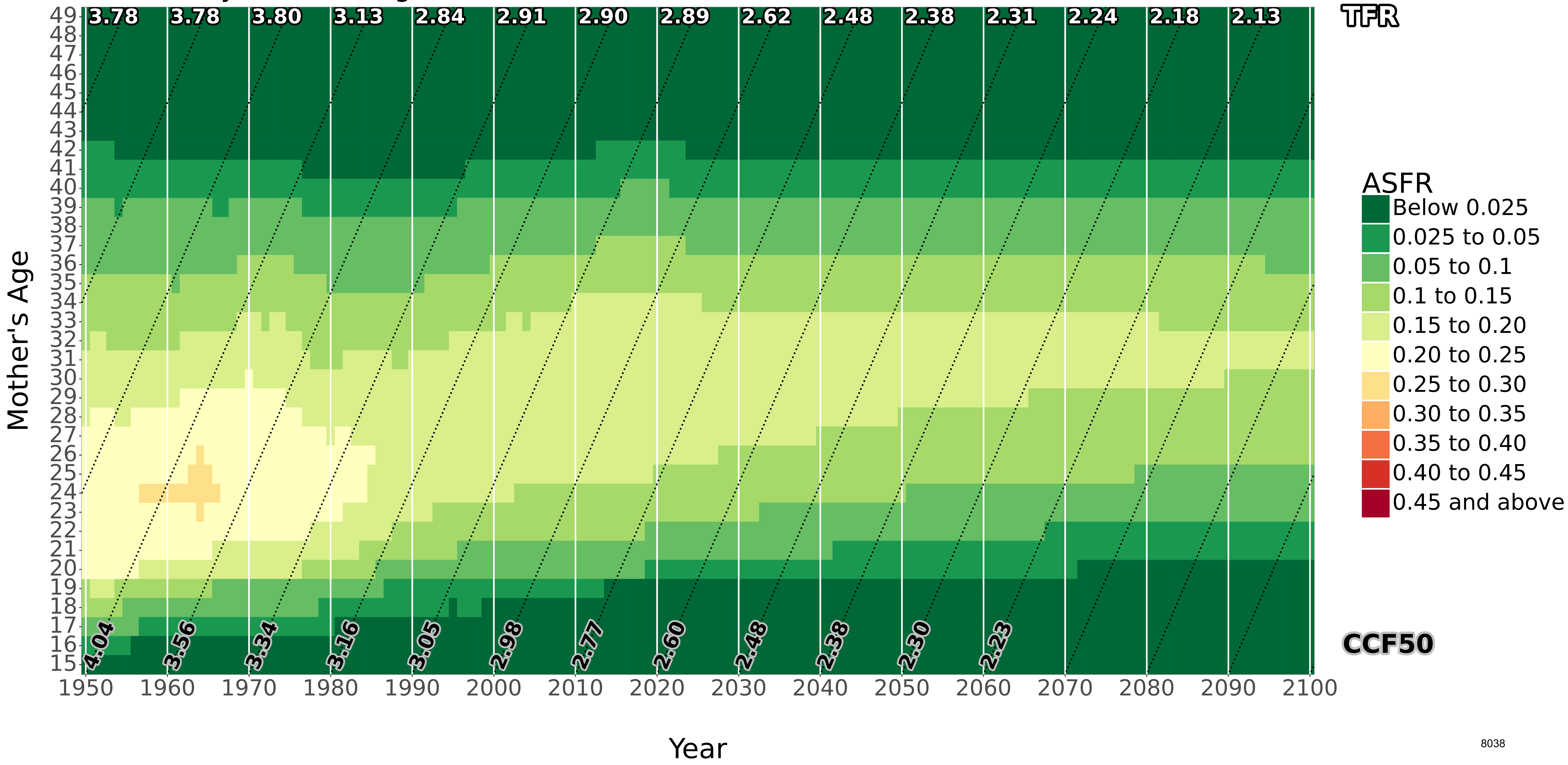

Supplemental Results Figure S3: Lexis diagram of CCF50, single-year age interval ASFR, and TFR by mother's age and location, 1950-2100, Italy

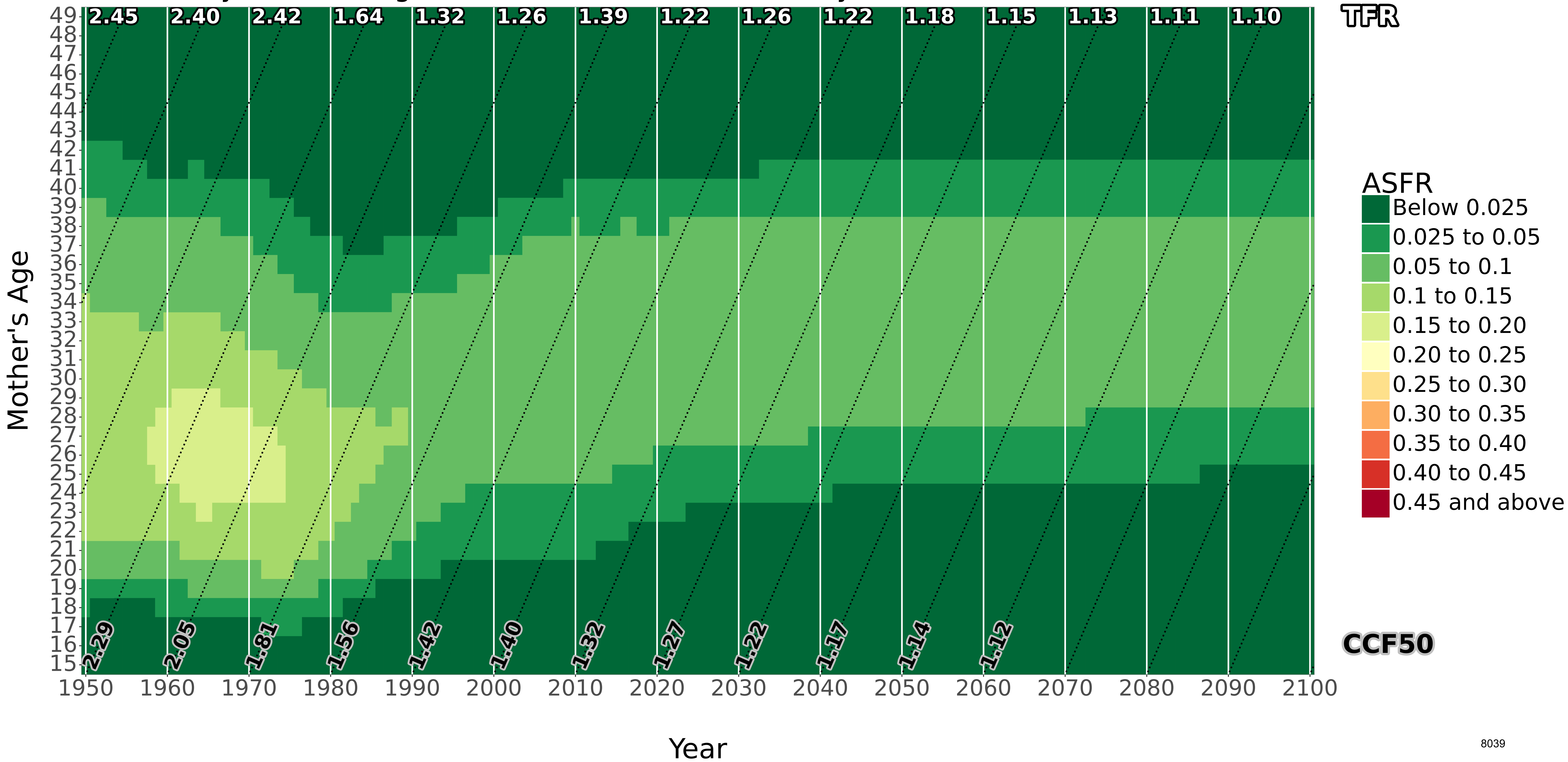

Supplemental Results Figure S3: Lexis diagram of CCF50, single-year age interval ASFR, and TFR by mother's age and location, 1950-2100, Luxembourg

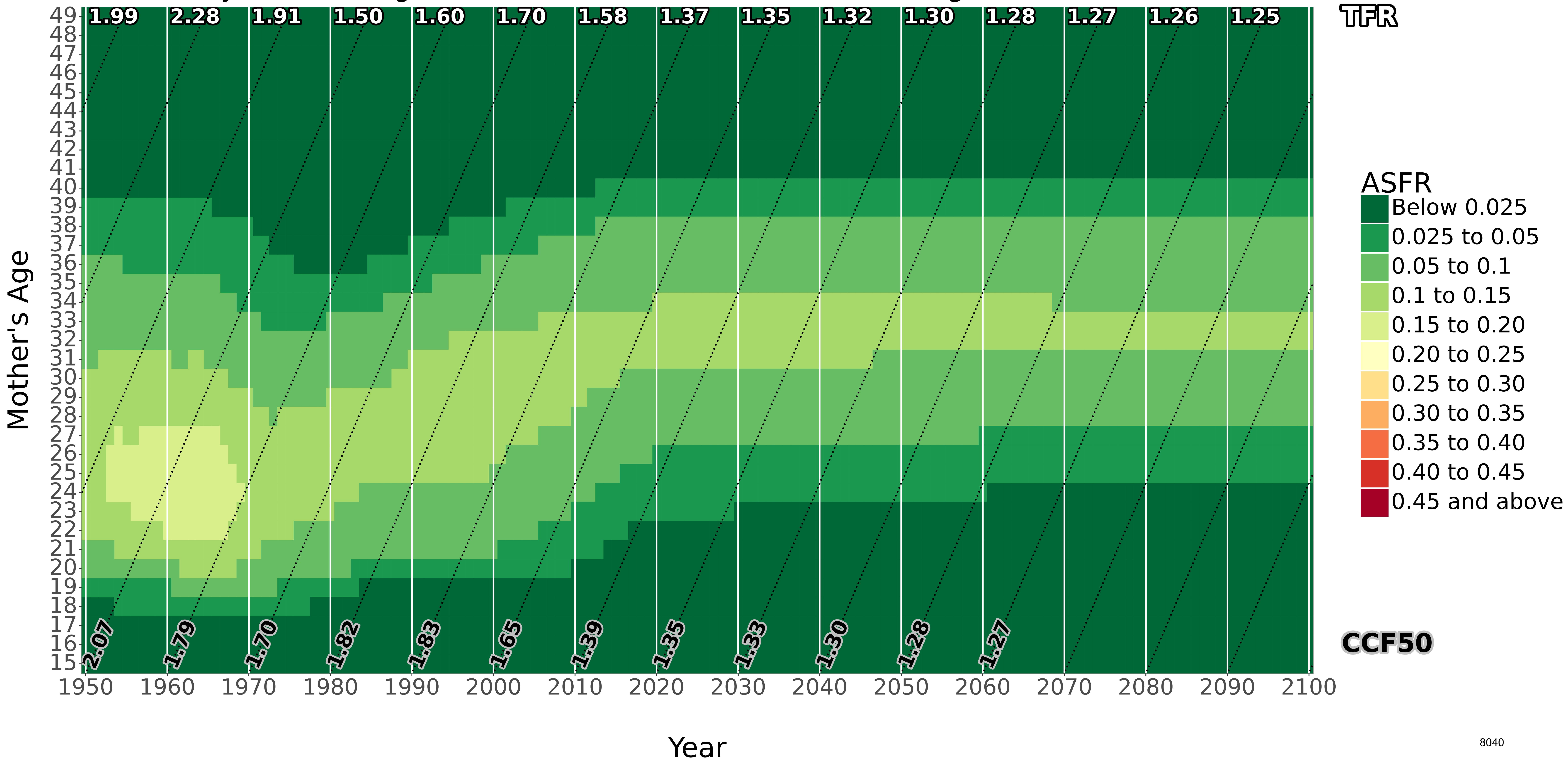

Supplemental Results Figure S3: Lexis diagram of CCF50, single-year age interval ASFR, and TFR by mother's age and location, 1950-2100, Malta

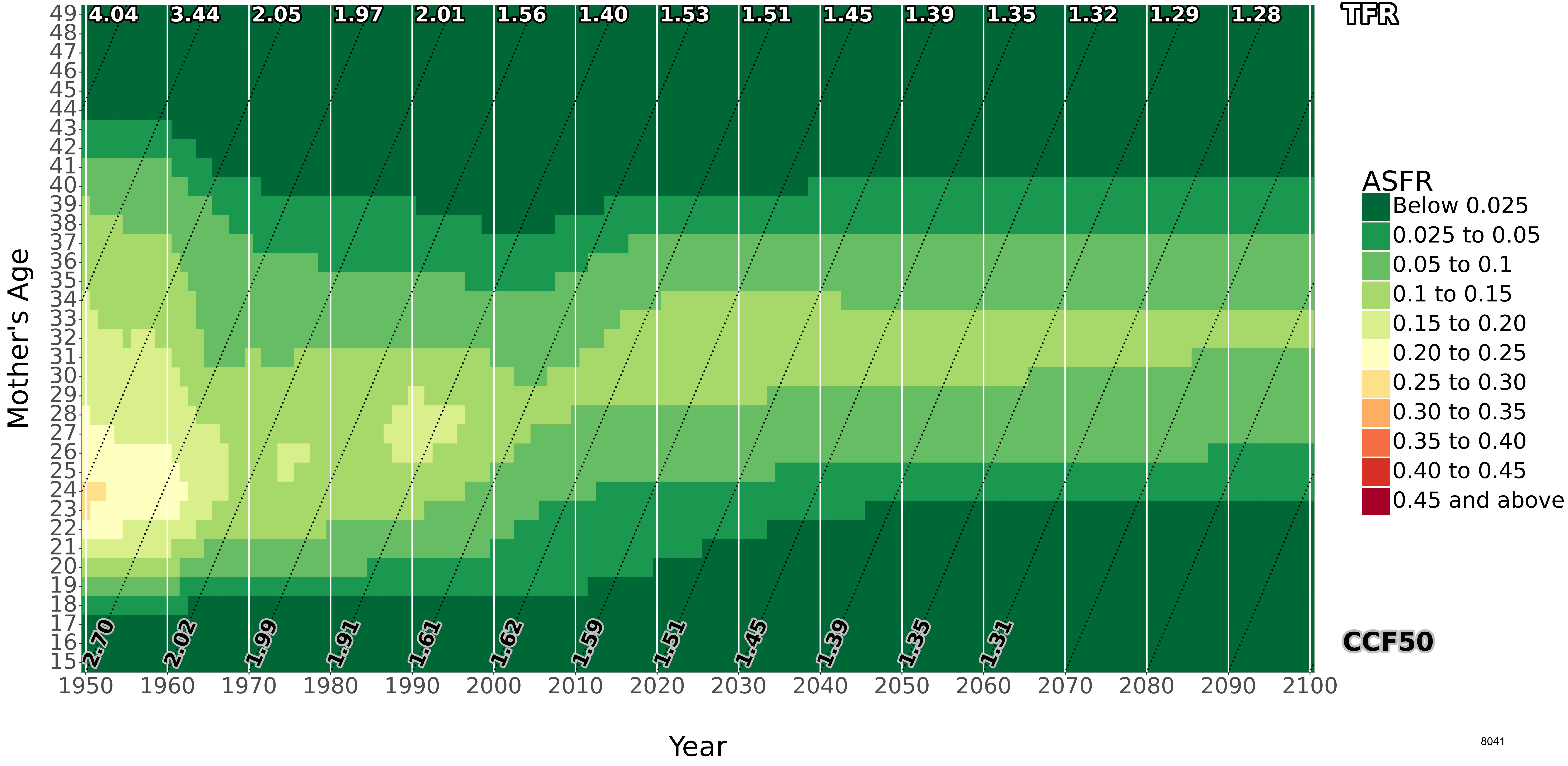

Supplemental Results Figure S3: Lexis diagram of CCF50, single-year age interval ASFR, and TFR by mother's age and location, 1950-2100, Monaco

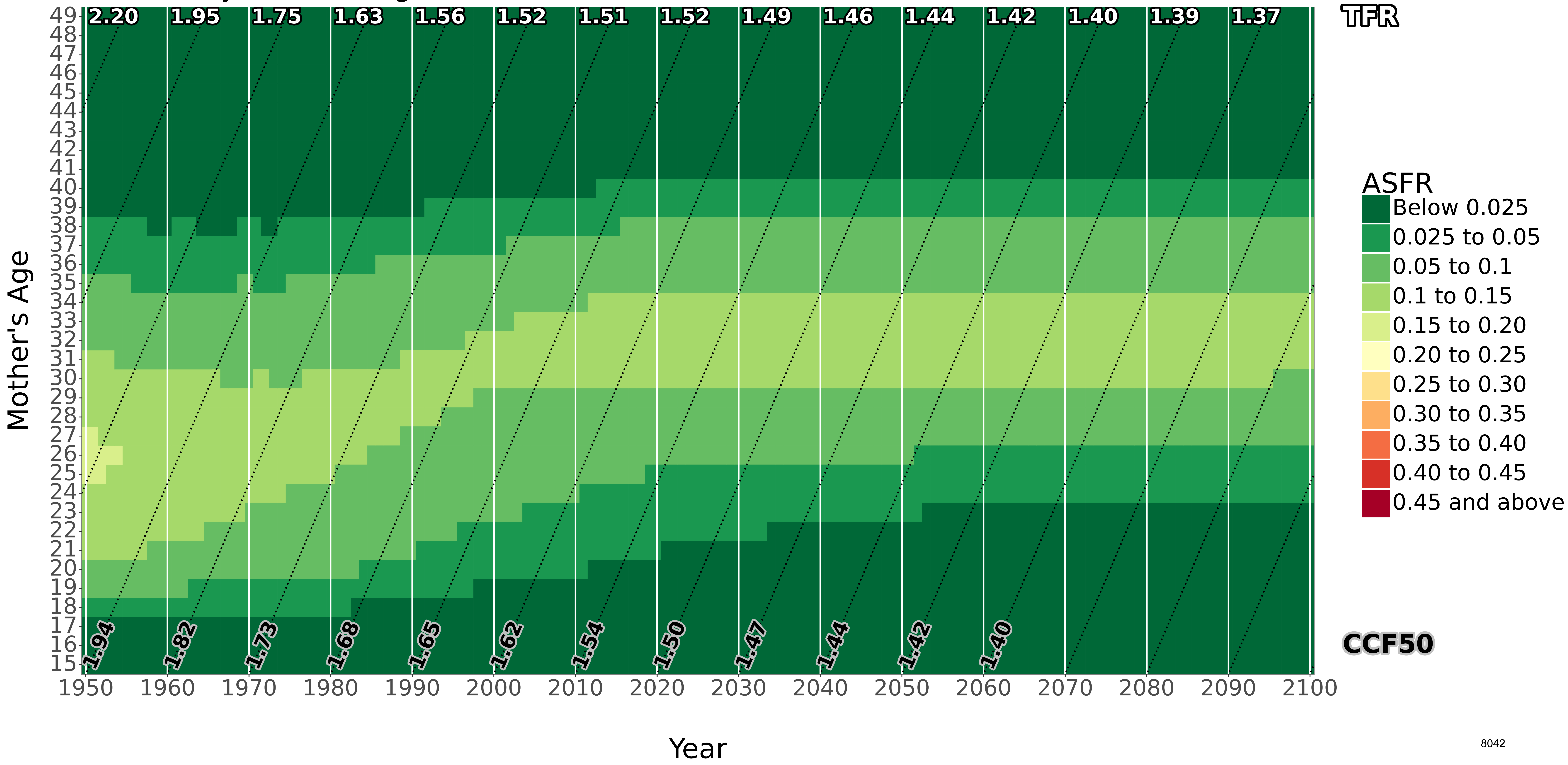

Supplemental Results Figure S3: Lexis diagram of CCF50, single-year age interval ASFR, and TFR by mother's age and location, 1950-2100, Netherlands

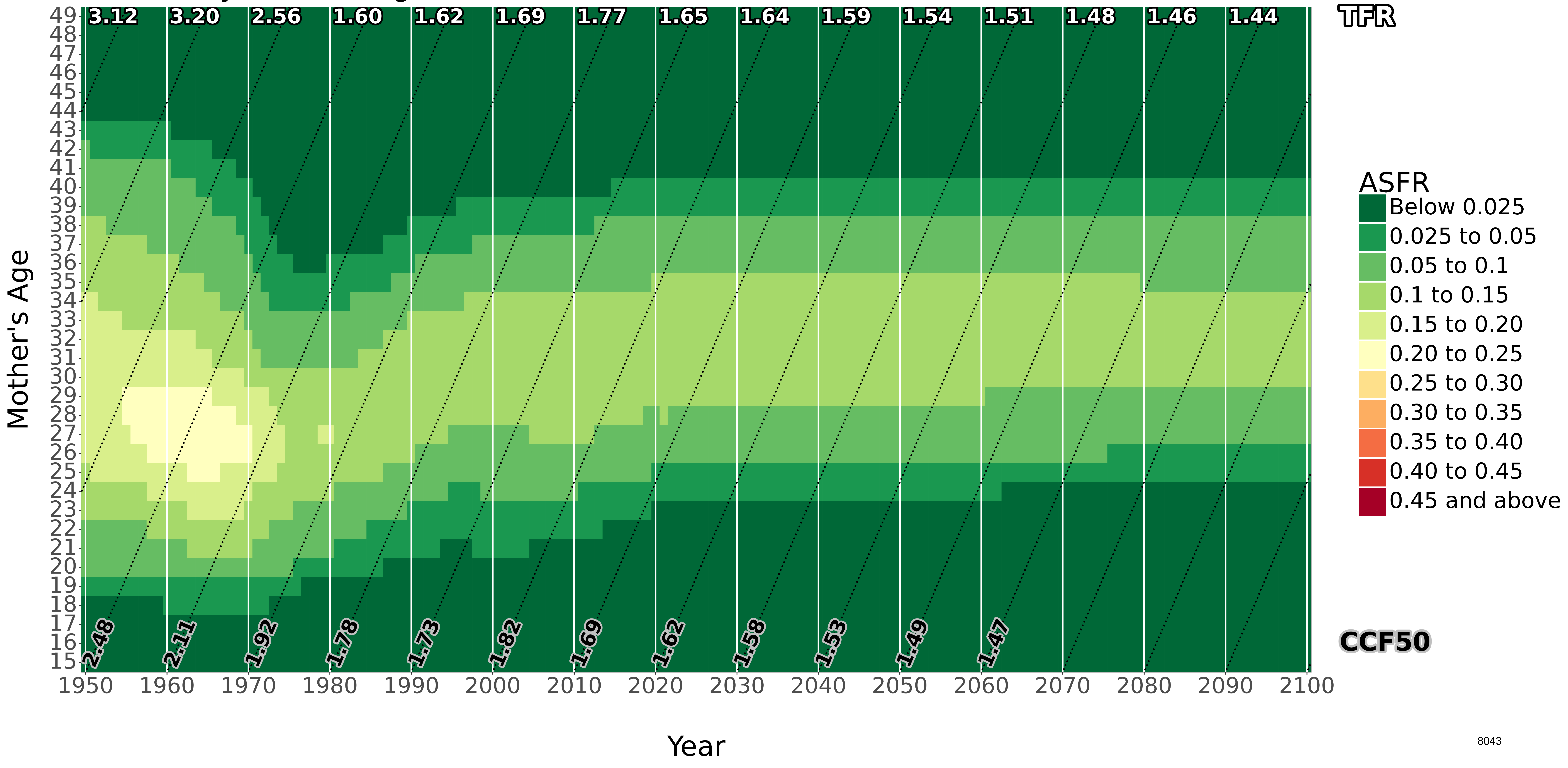

Supplemental Results Figure S3: Lexis diagram of CCF50, single-year age interval ASFR, and TFR by mother's age and location, 1950-2100, Norway

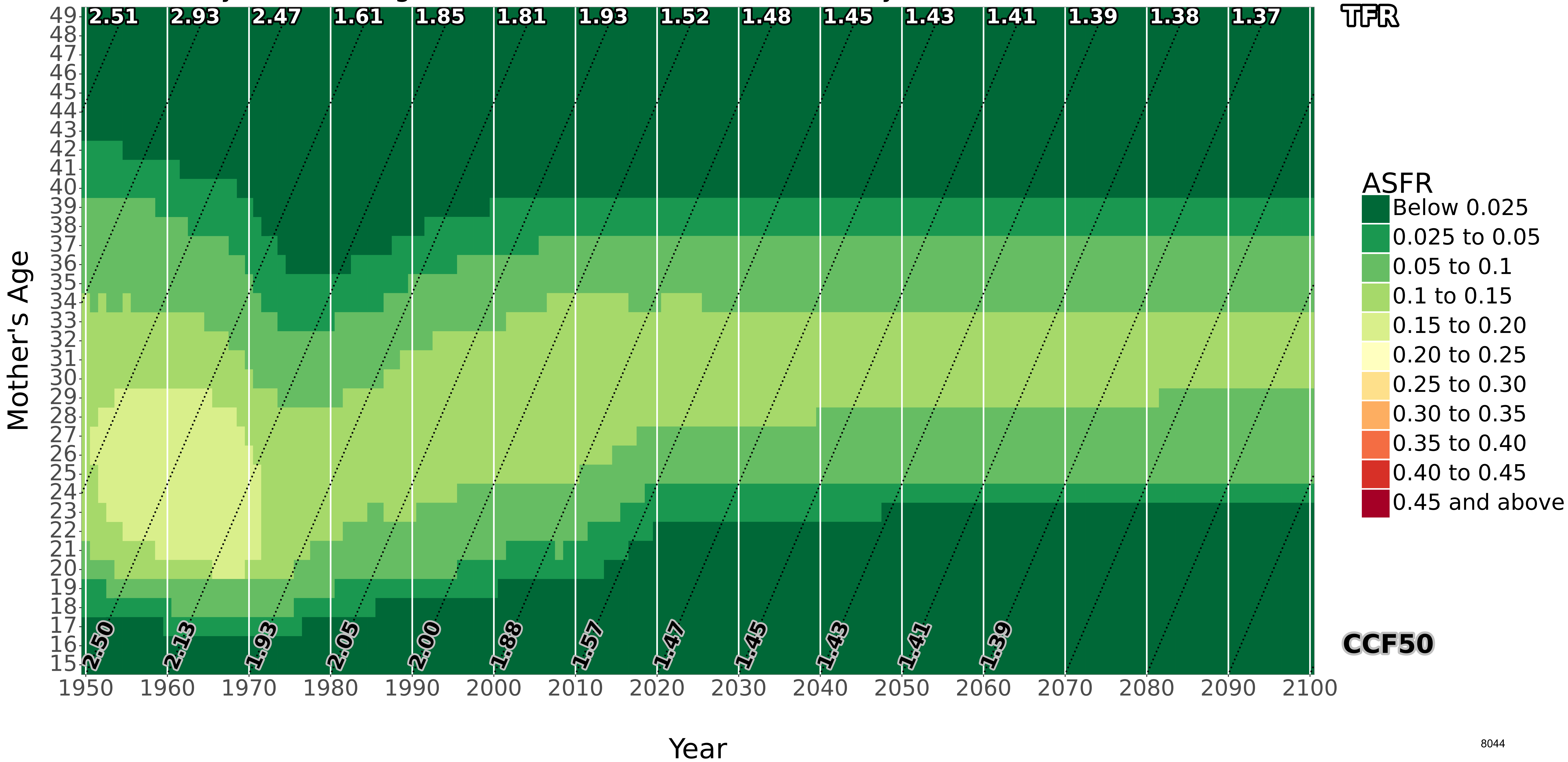

Supplemental Results Figure S3: Lexis diagram of CCF50, single-year age interval ASFR, and TFR by mother's age and location, 1950-2100, Portugal

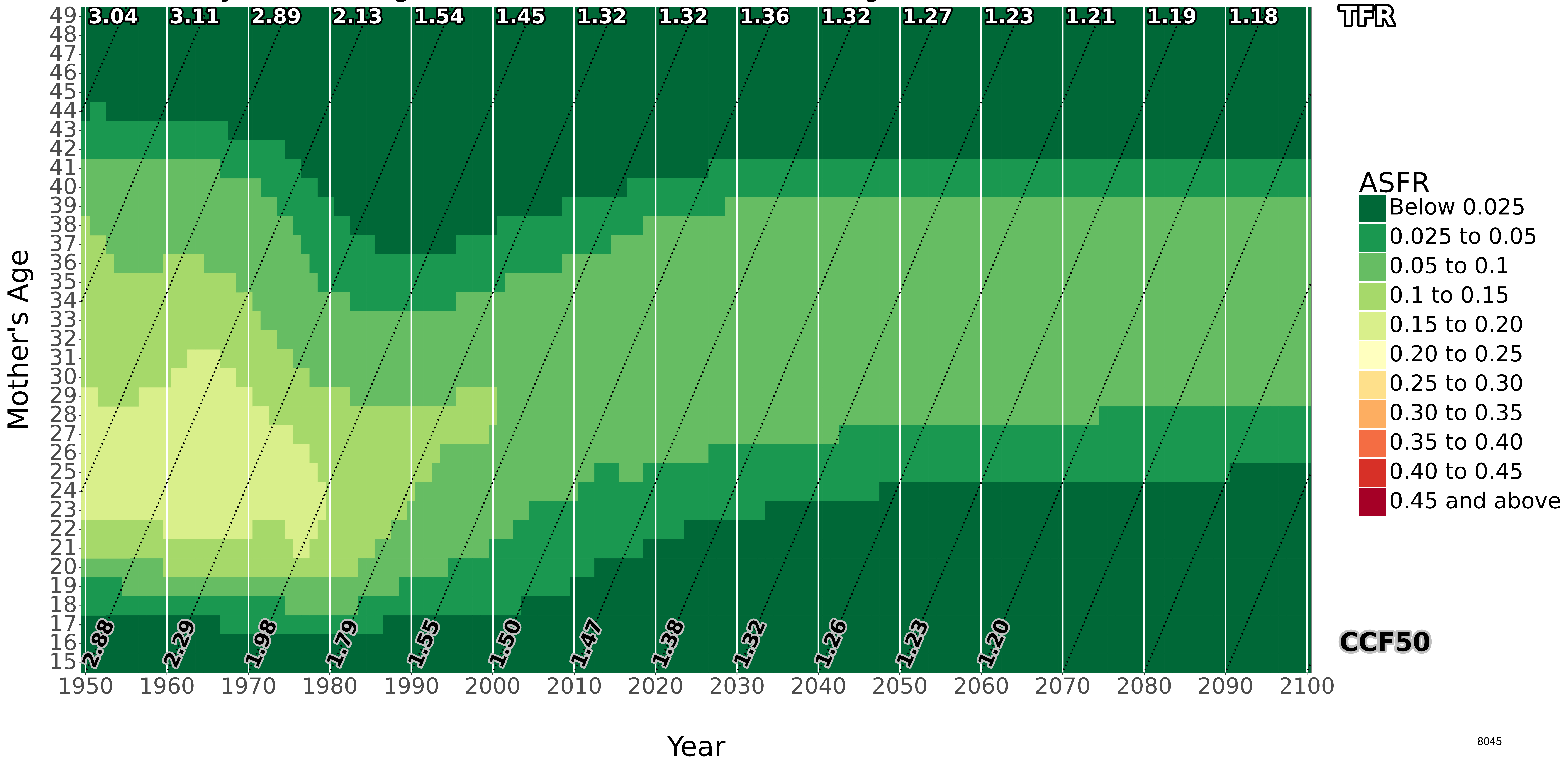

8045

Supplemental Results Figure S3: Lexis diagram of CCF50, single-year age interval ASFR, and TFR by mother's age and location, 1950-2100, San Marino

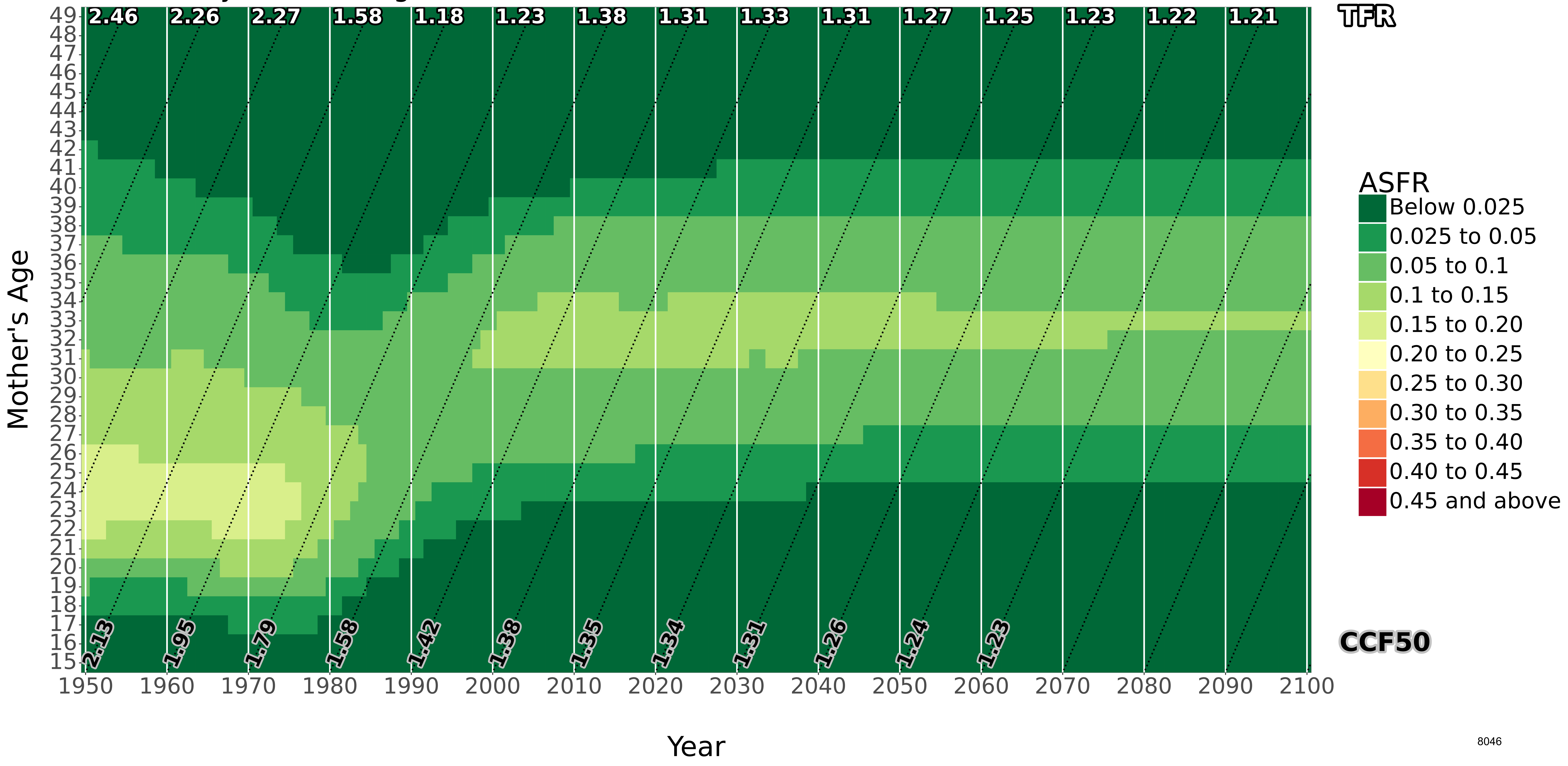

Supplemental Results Figure S3: Lexis diagram of CCF50, single-year age interval ASFR, and TFR by mother's age and location, 1950-2100, Spain

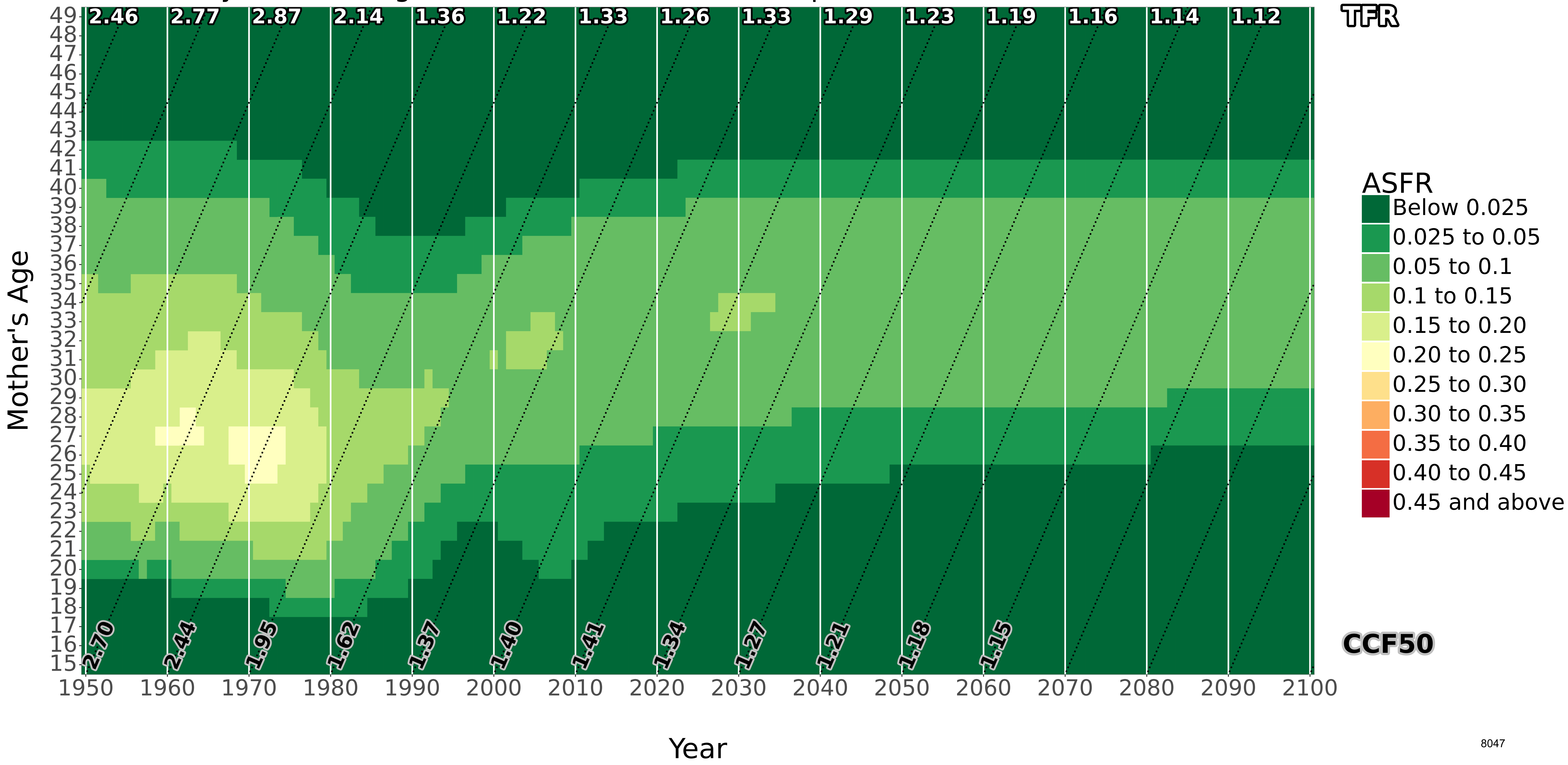

8047

Supplemental Results Figure S3: Lexis diagram of CCF50, single-year age interval ASFR, and TFR by mother's age and location, 1950-2100, Sweden

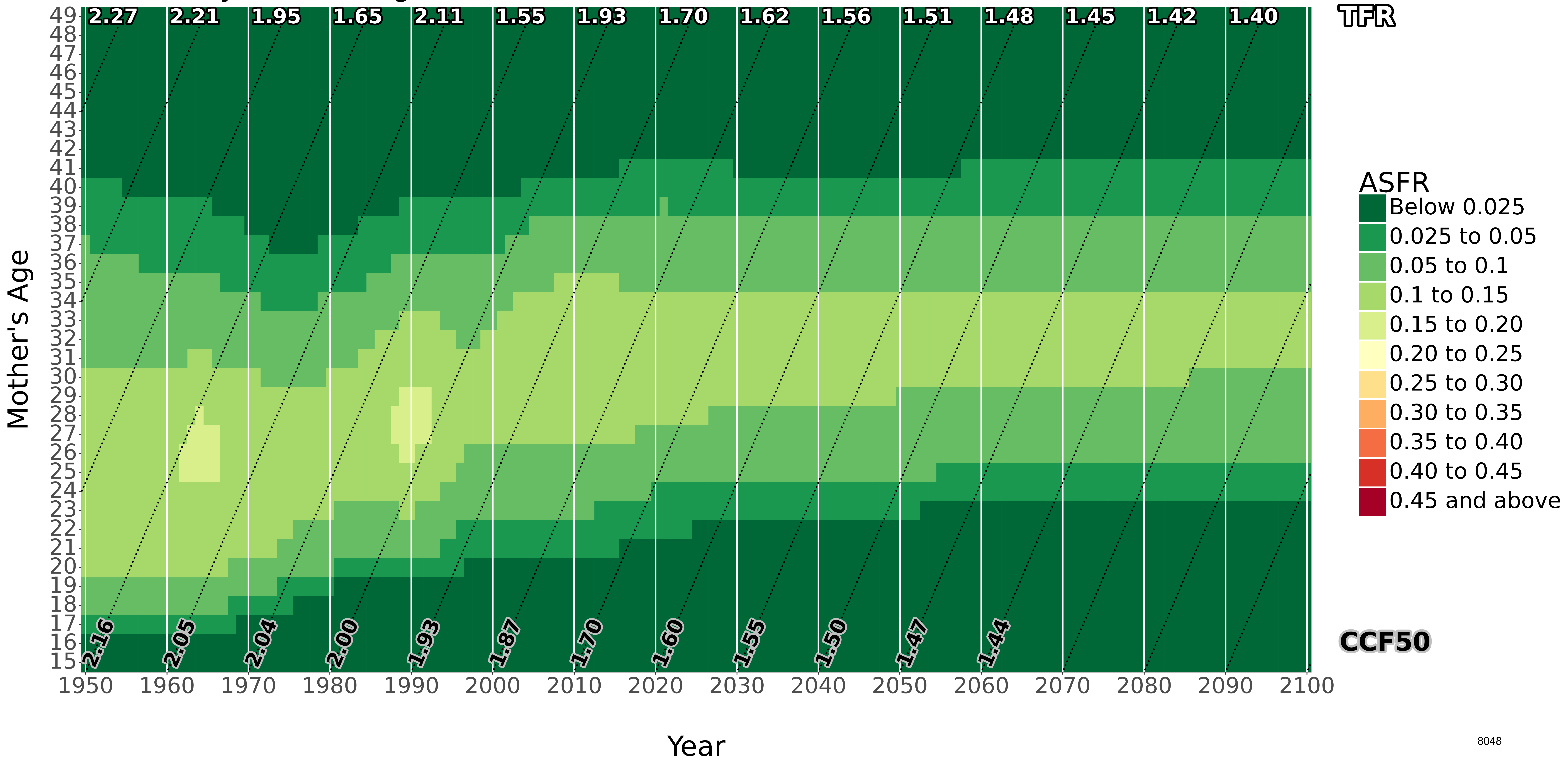

Supplemental Results Figure S3: Lexis diagram of CCF50, single-year age interval ASFR, and TFR by mother's age and location, 1950-2100, Switzerland

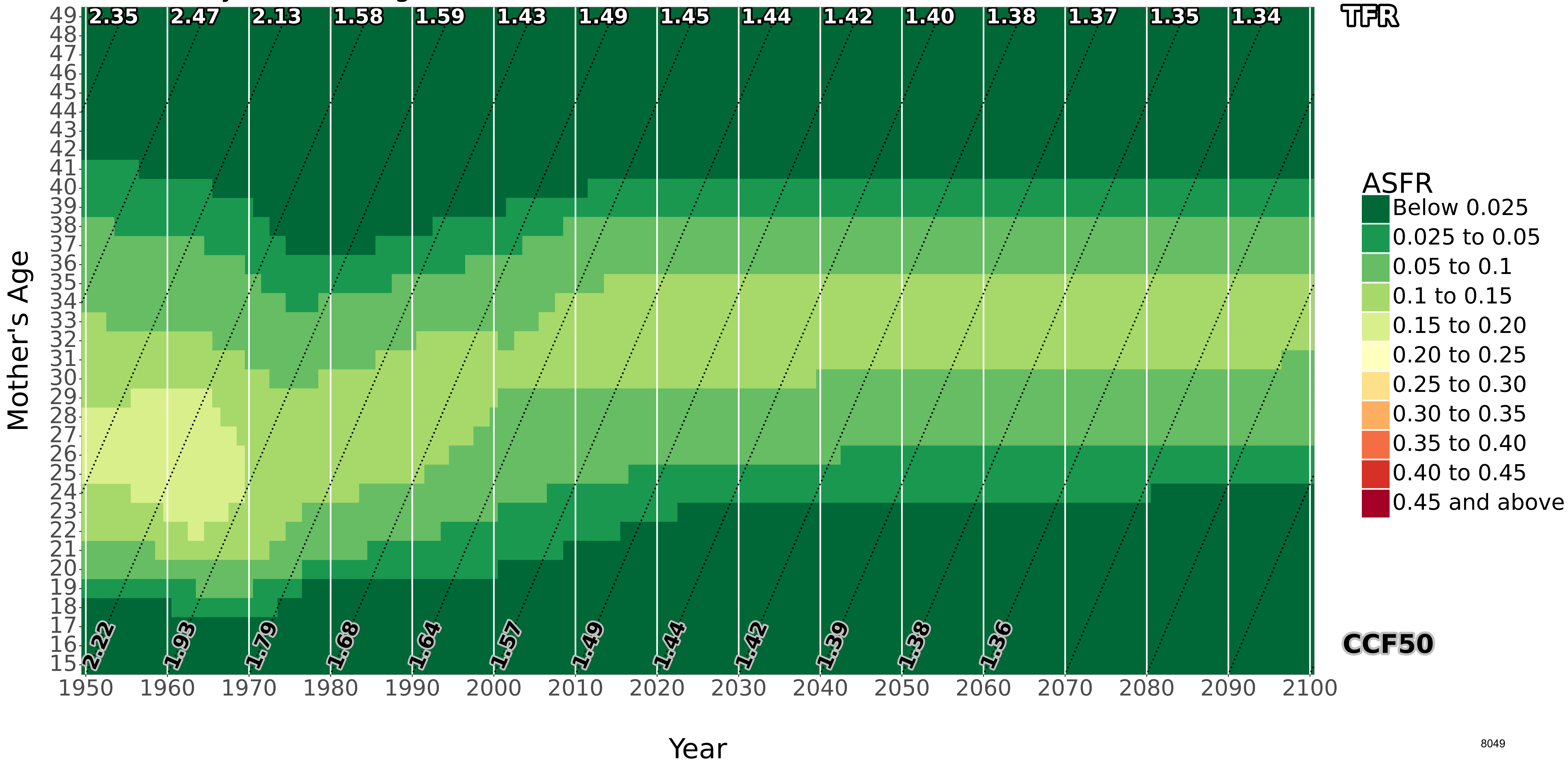

8049

Supplemental Results Figure S3: Lexis diagram of CCF50, single-year age interval ASFR, and TFR by mother's age and location, 1950-2100, UK

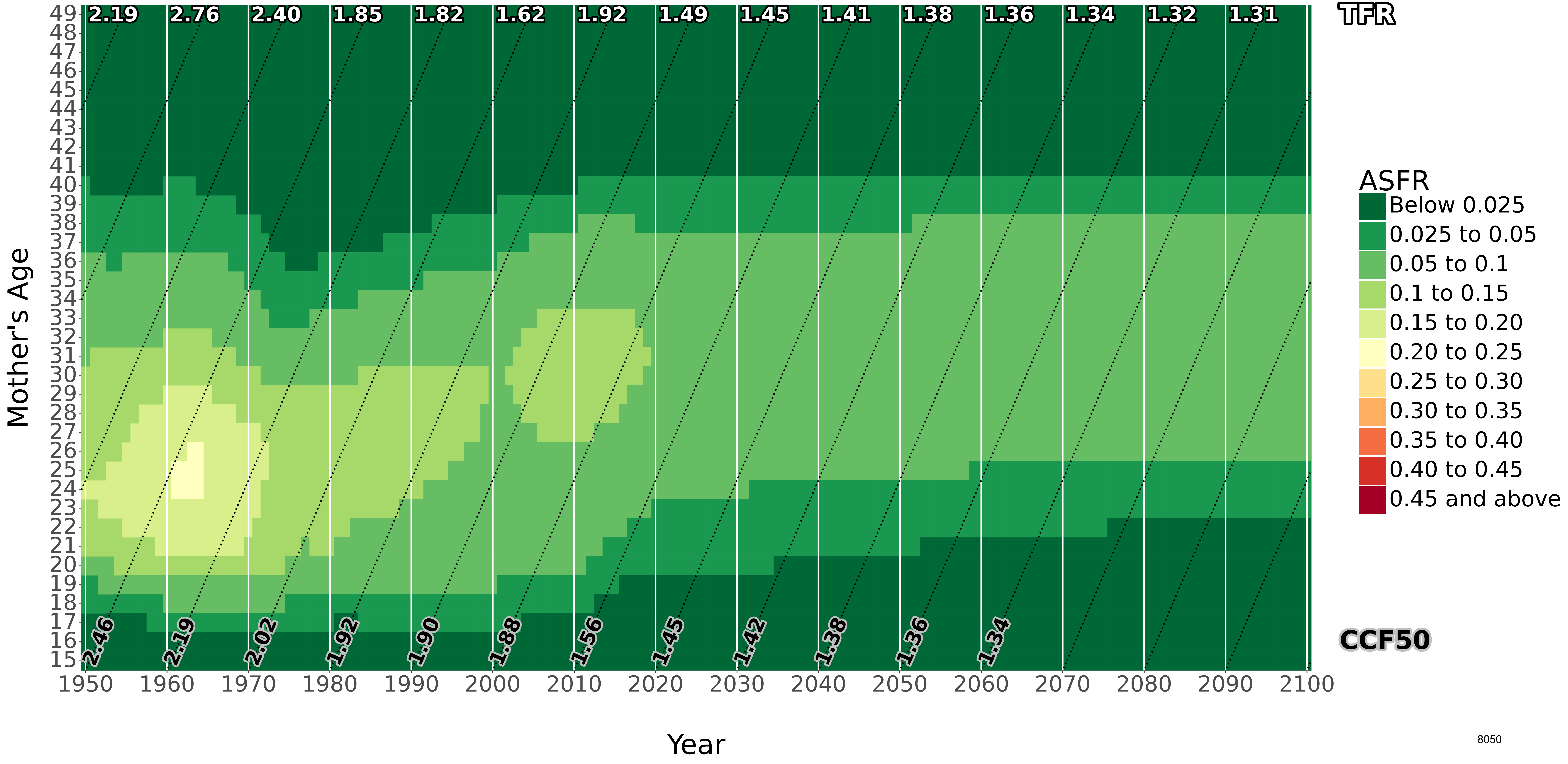

Supplemental Results Figure S3: Lexis diagram of CCF50, single-year age interval ASFR, and TFR by mother's age and location, 1950-2100, Bolivia

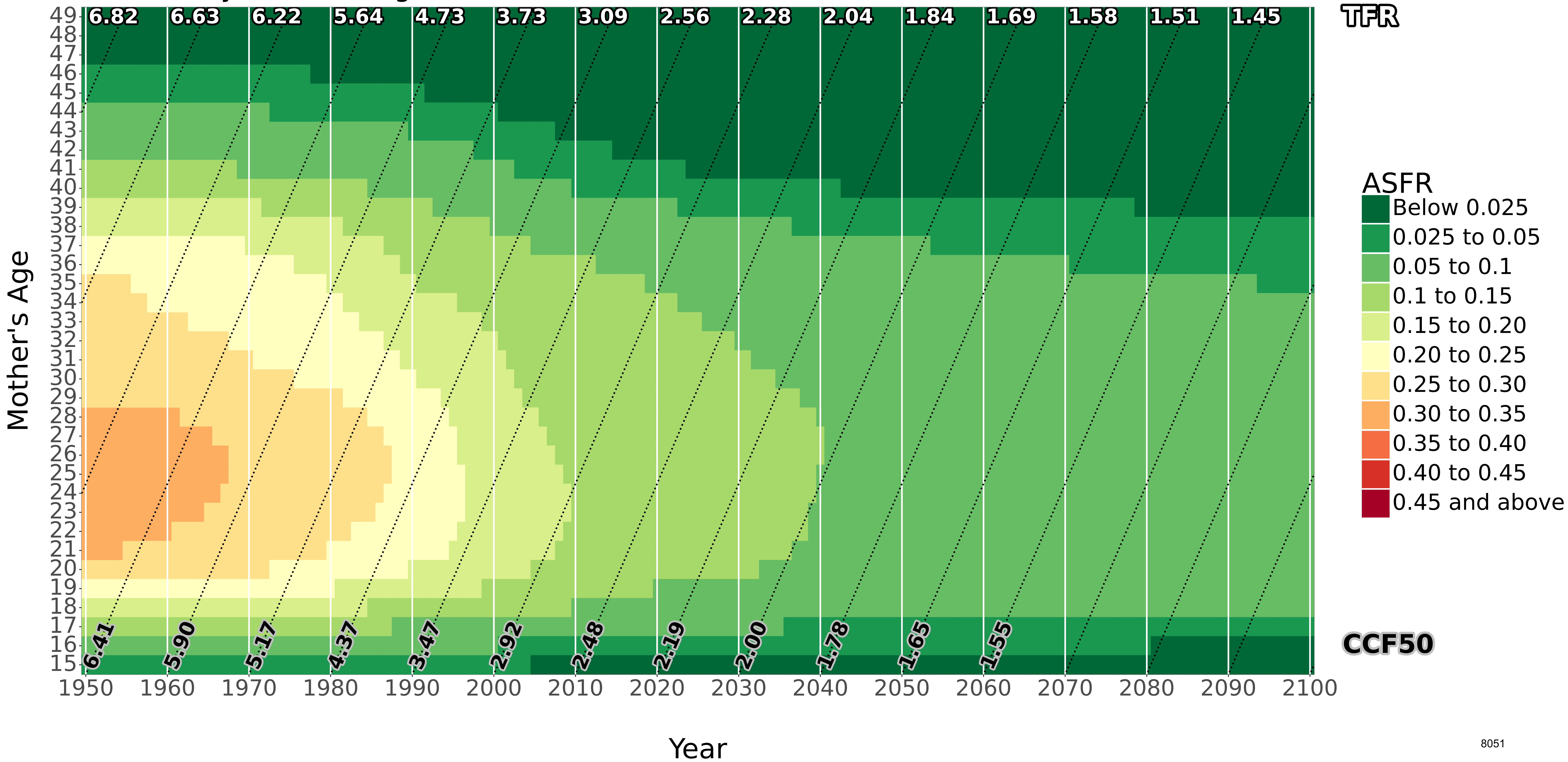

Supplemental Results Figure S3: Lexis diagram of CCF50, single-year age interval ASFR, and TFR by mother's age and location, 1950-2100, Ecuador

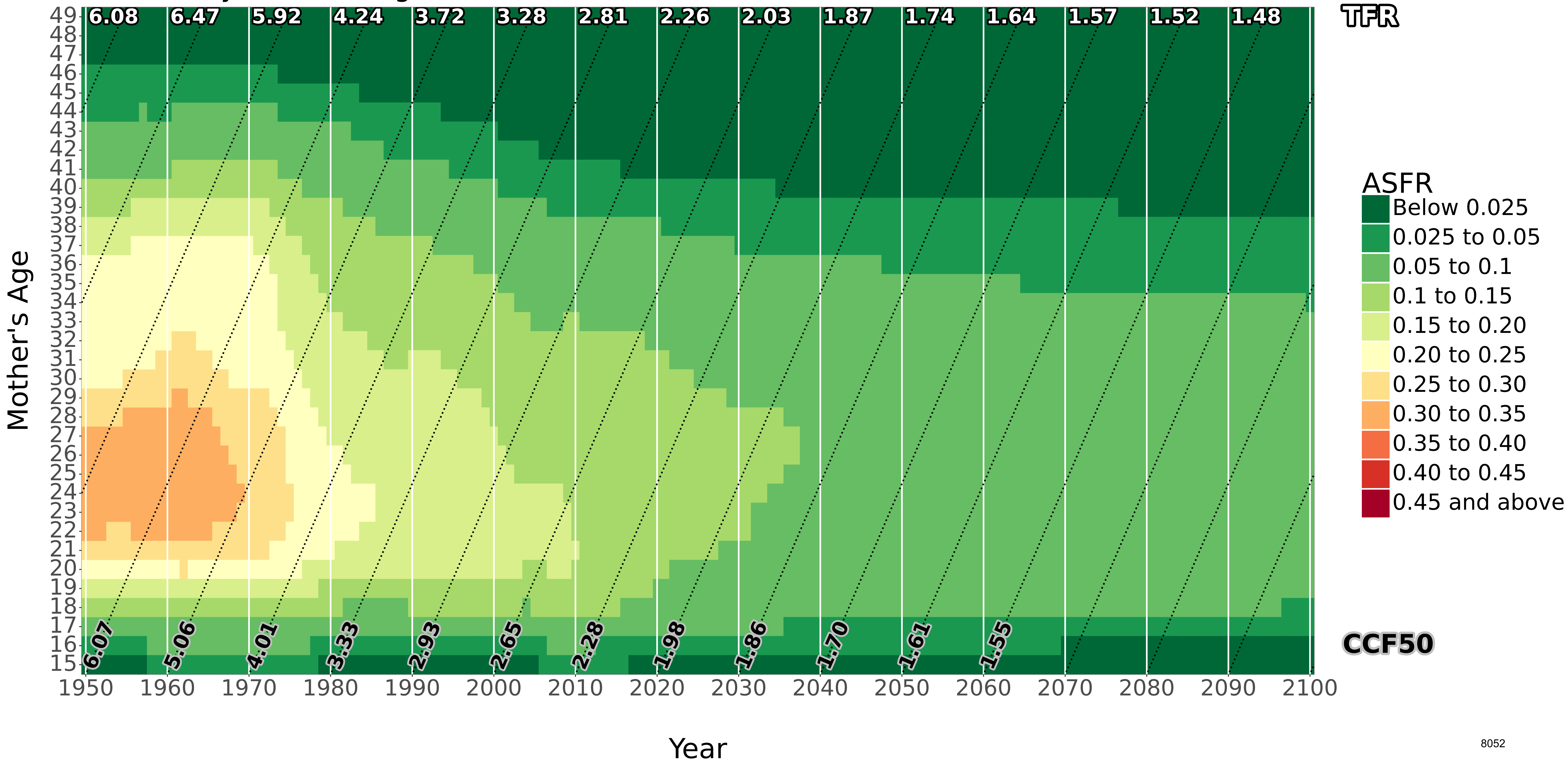

Supplemental Results Figure S3: Lexis diagram of CCF50, single-year age interval ASFR, and TFR by mother's age and location, 1950-2100, Peru

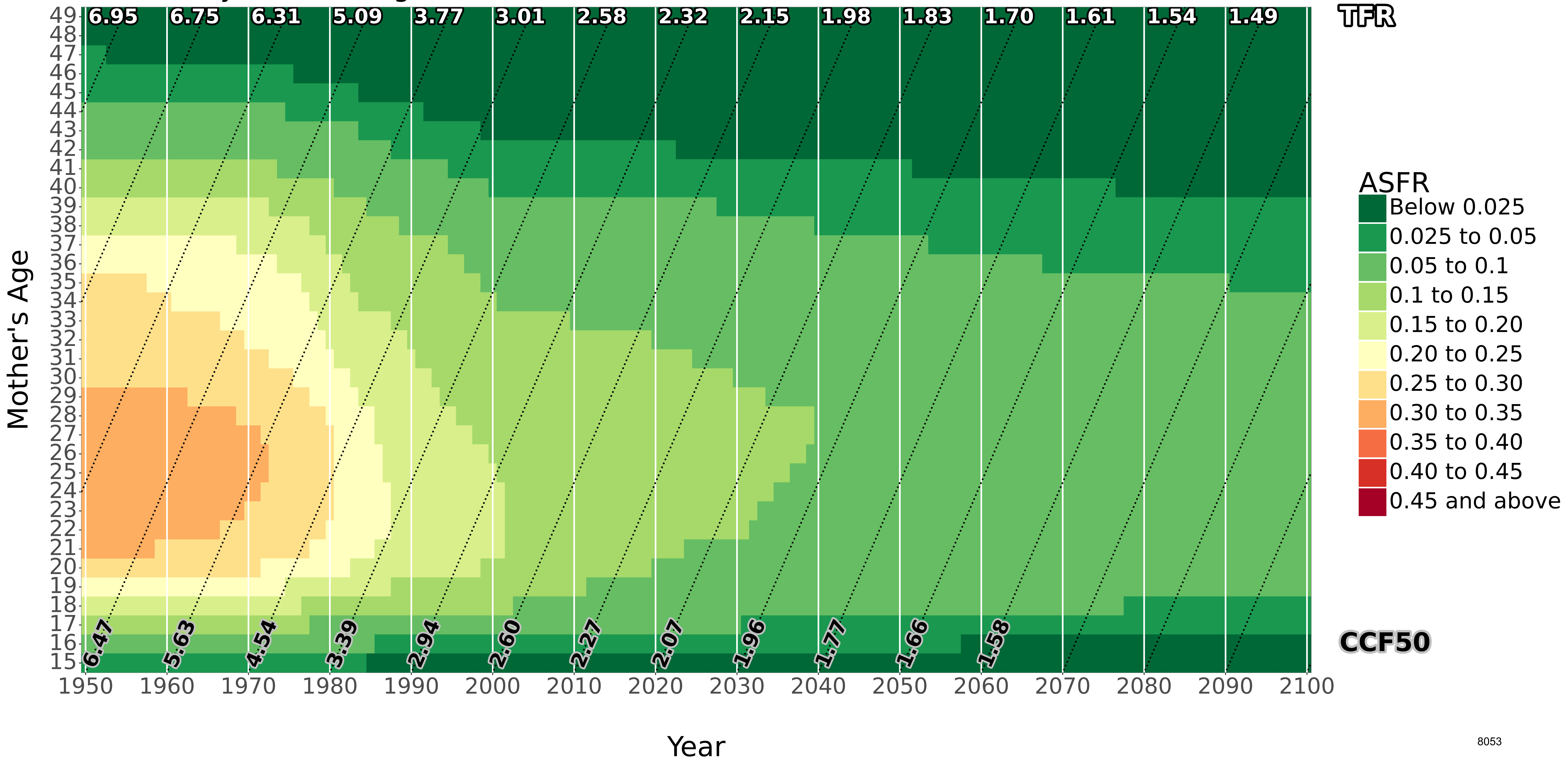

Supplemental Results Figure S3: Lexis diagram of CCF50, single-year age interval ASFR, and TFR by mother's age and location, 1950-2100, Antigua and Barbuda

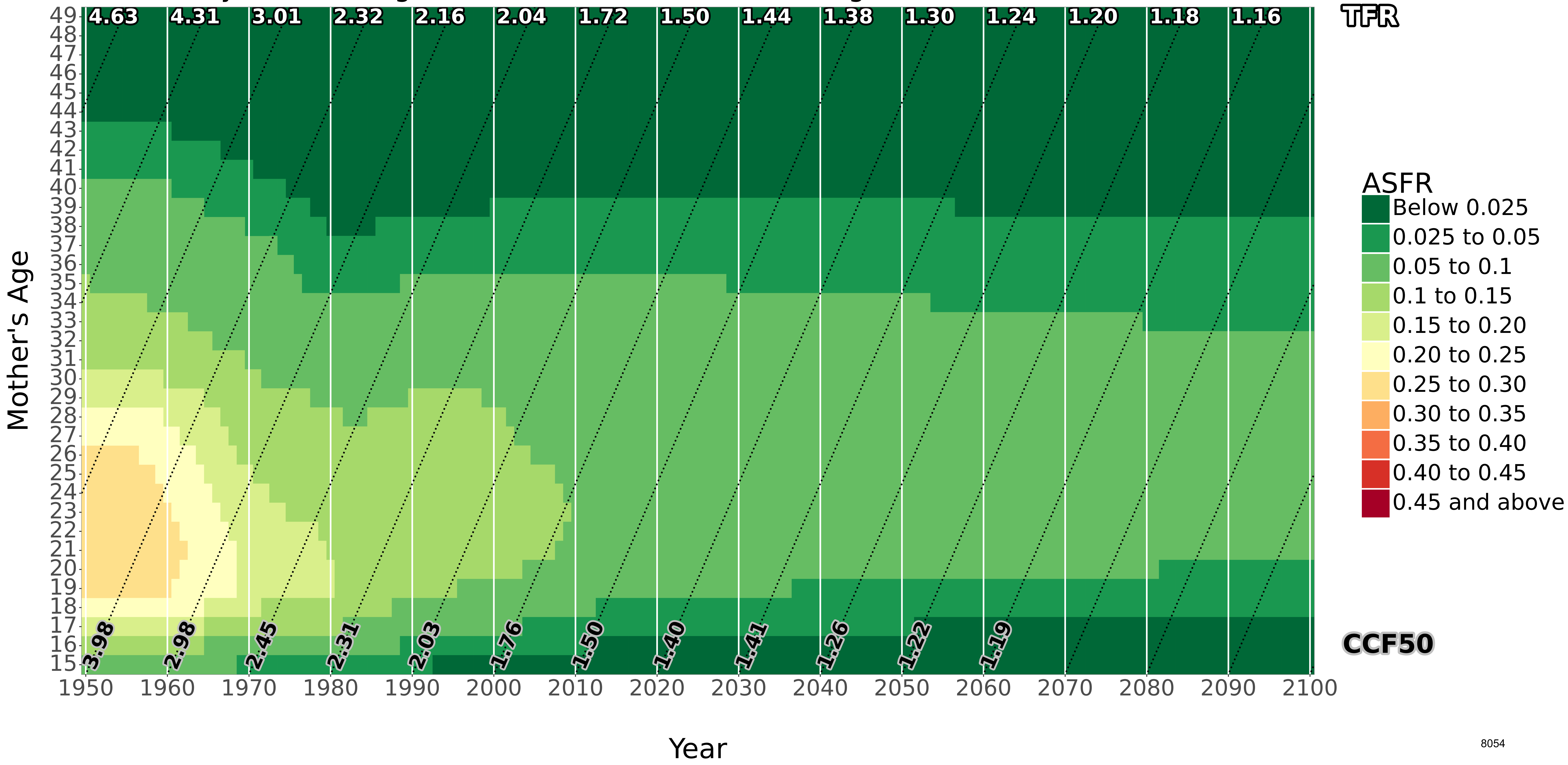

Supplemental Results Figure S3: Lexis diagram of CCF50, single-year age interval ASFR, and TFR by mother's age and location, 1950-2100, The Bahamas

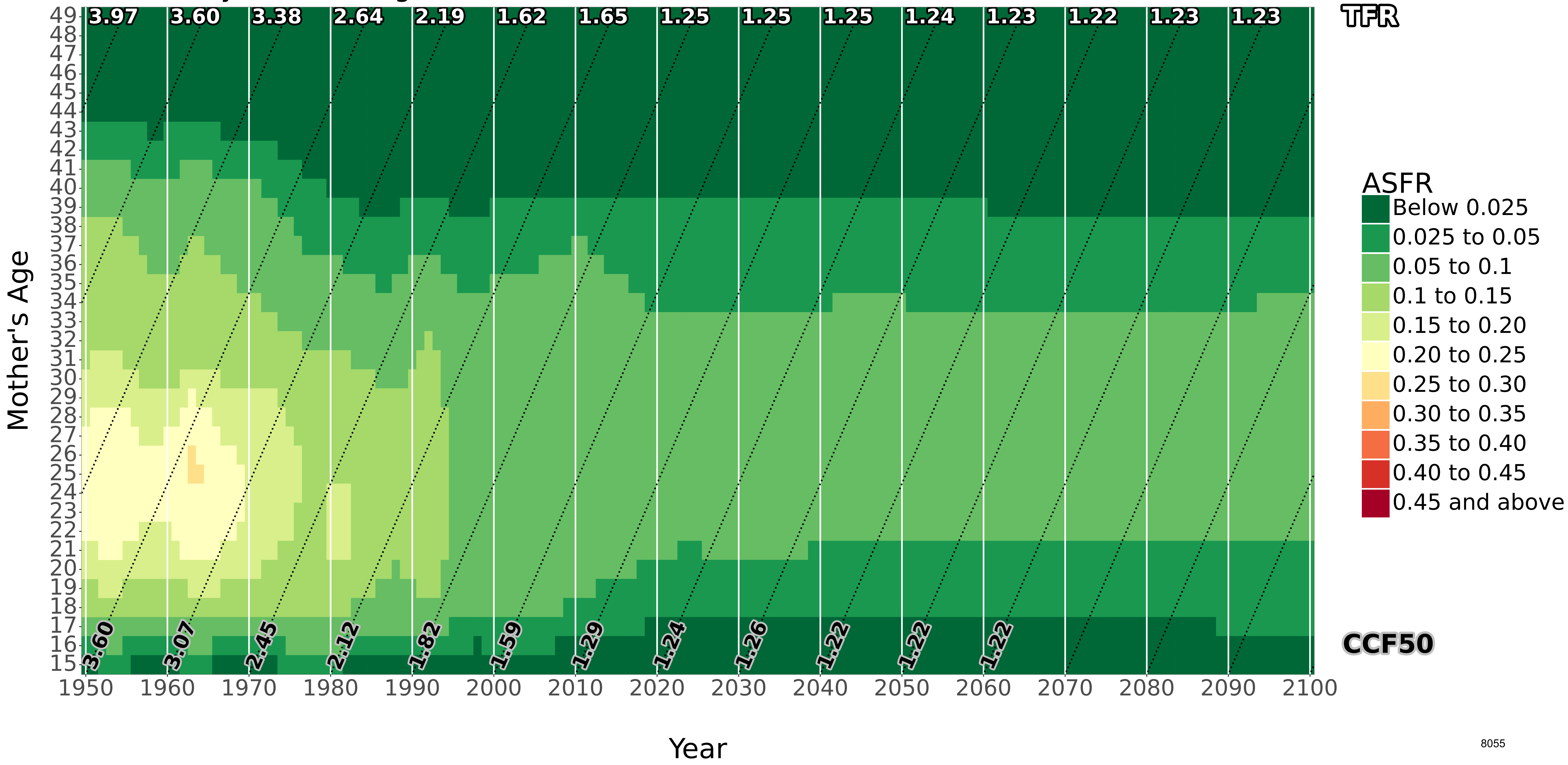

Supplemental Results Figure S3: Lexis diagram of CCF50, single-year age interval ASFR, and TFR by mother's age and location, 1950-2100, Barbados

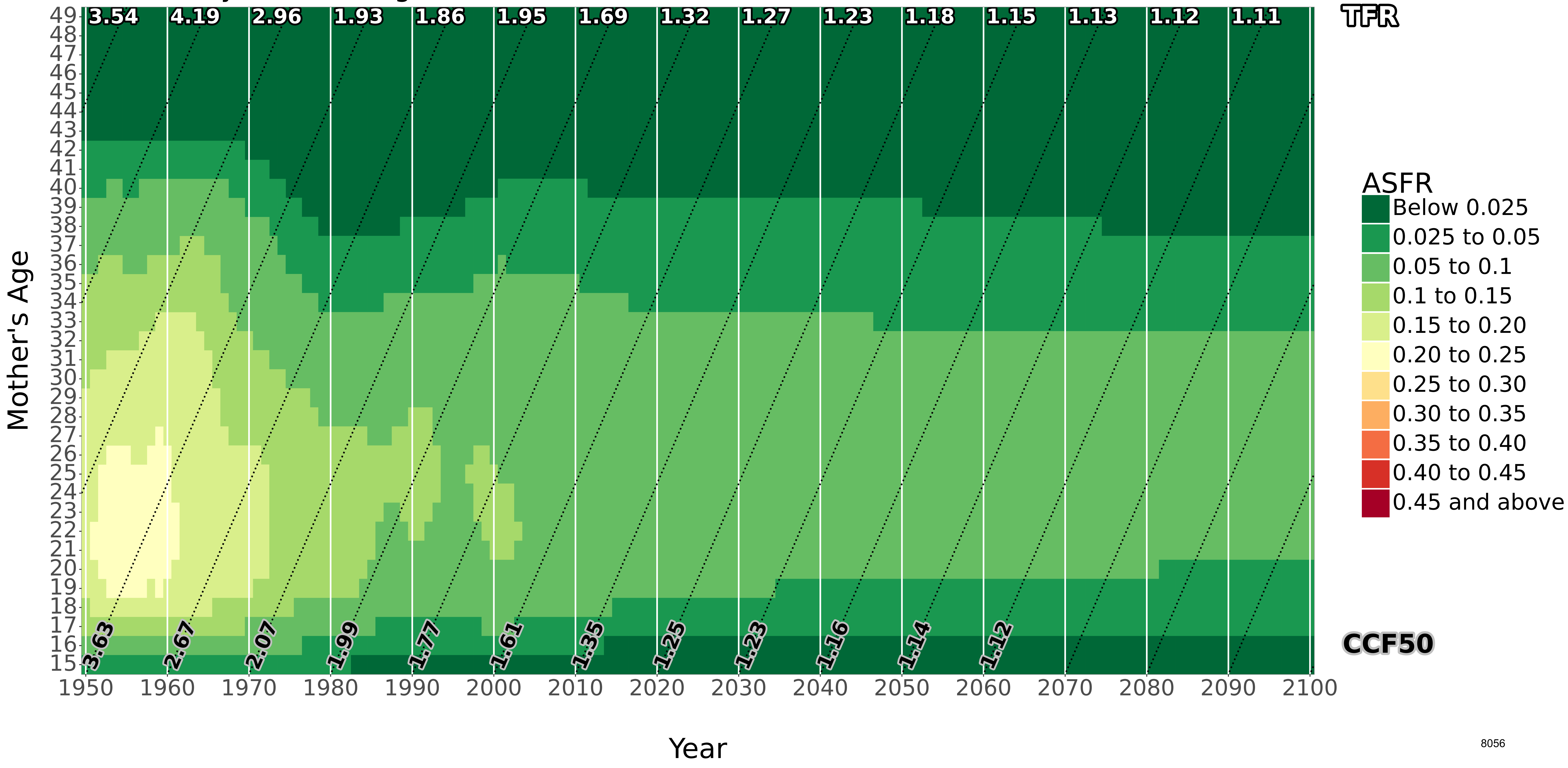

Supplemental Results Figure S3: Lexis diagram of CCF50, single-year age interval ASFR, and TFR by mother's age and location, 1950-2100, Belize

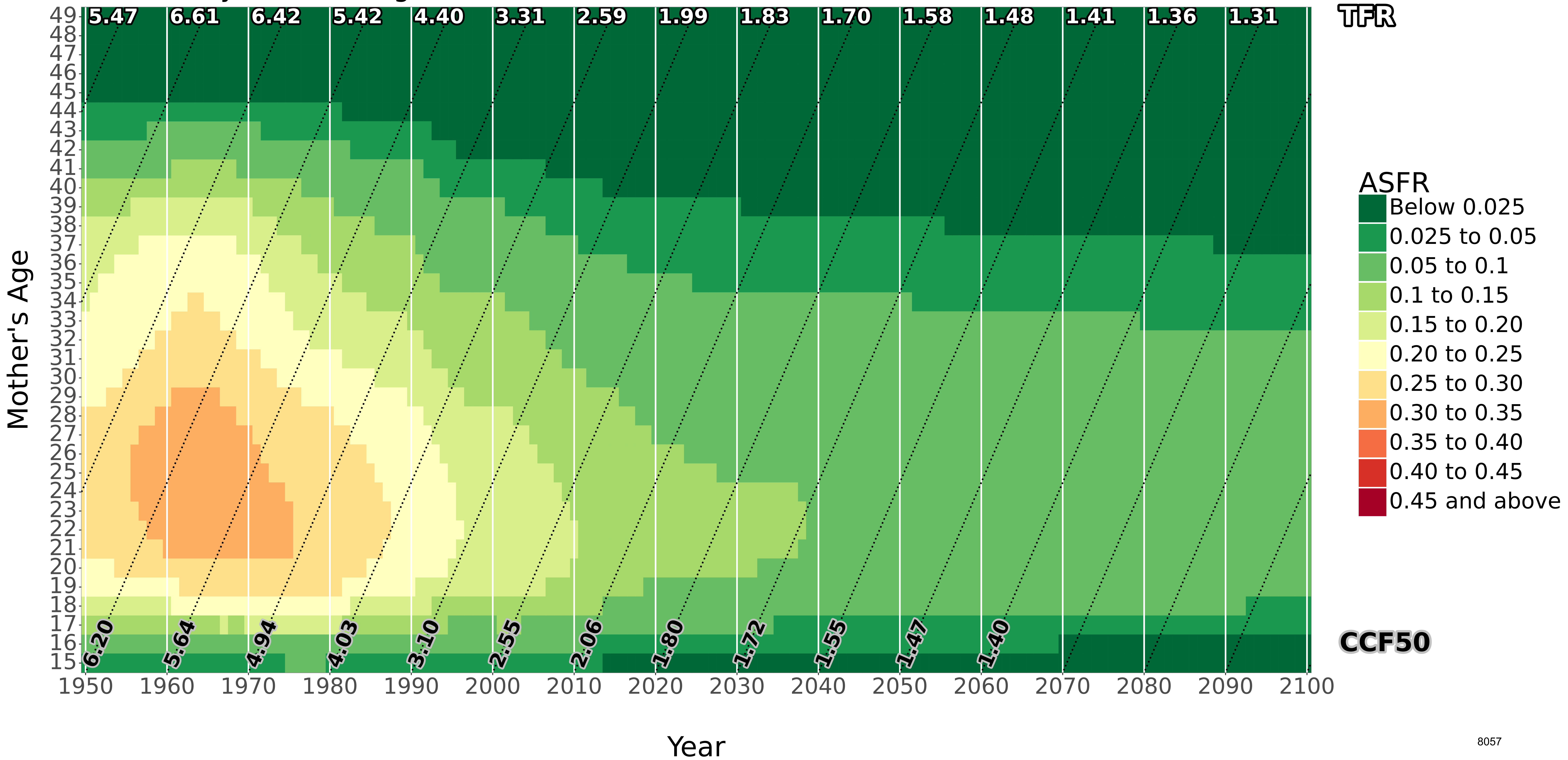

Supplemental Results Figure S3: Lexis diagram of CCF50, single-year age interval ASFR, and TFR by mother's age and location, 1950-2100, Bermuda

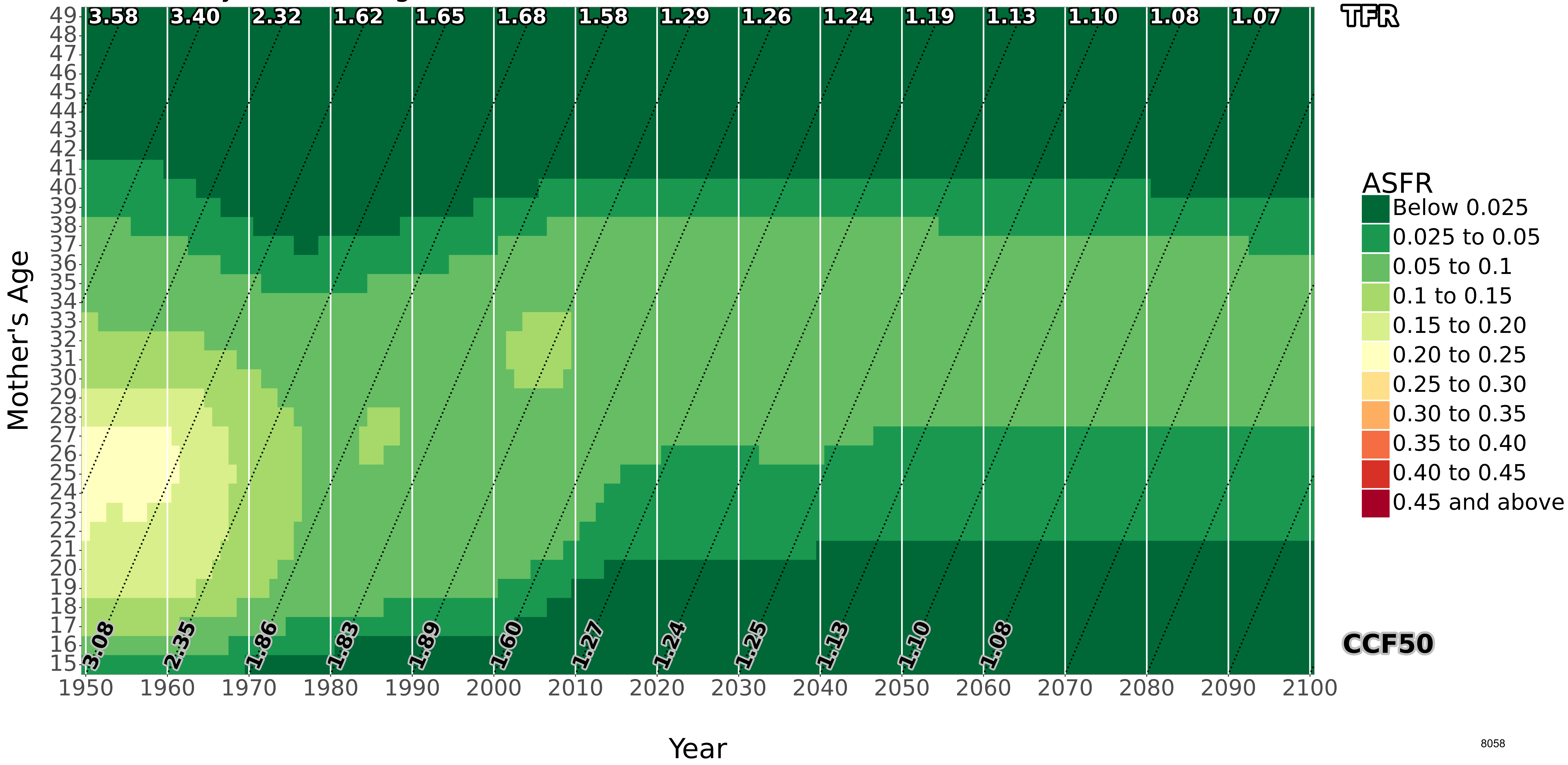

Supplemental Results Figure S3: Lexis diagram of CCF50, single-year age interval ASFR, and TFR by mother's age and location, 1950-2100, Cuba

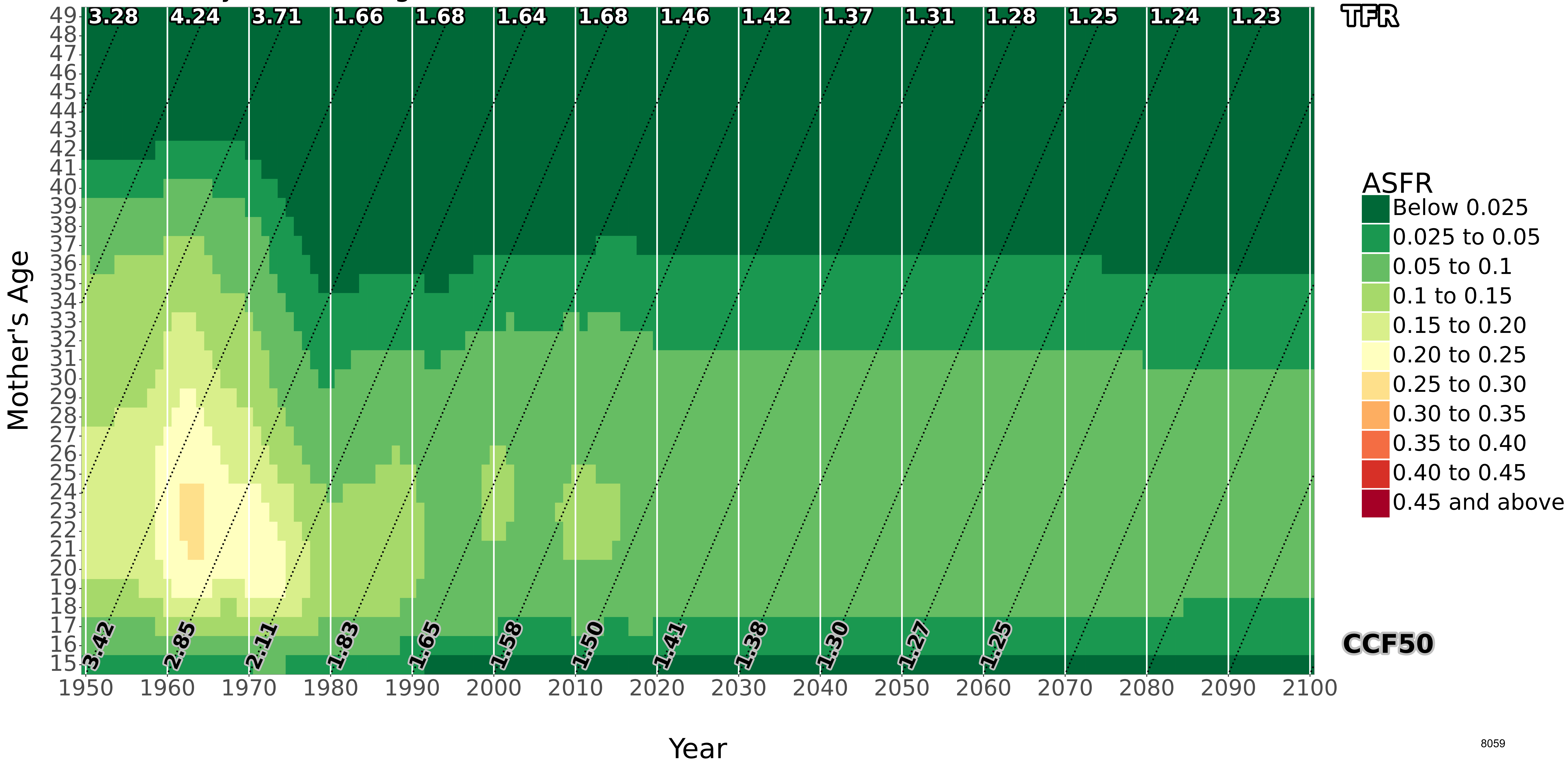

Supplemental Results Figure S3: Lexis diagram of CCF50, single-year age interval ASFR, and TFR by mother's age and location, 1950-2100, Dominica

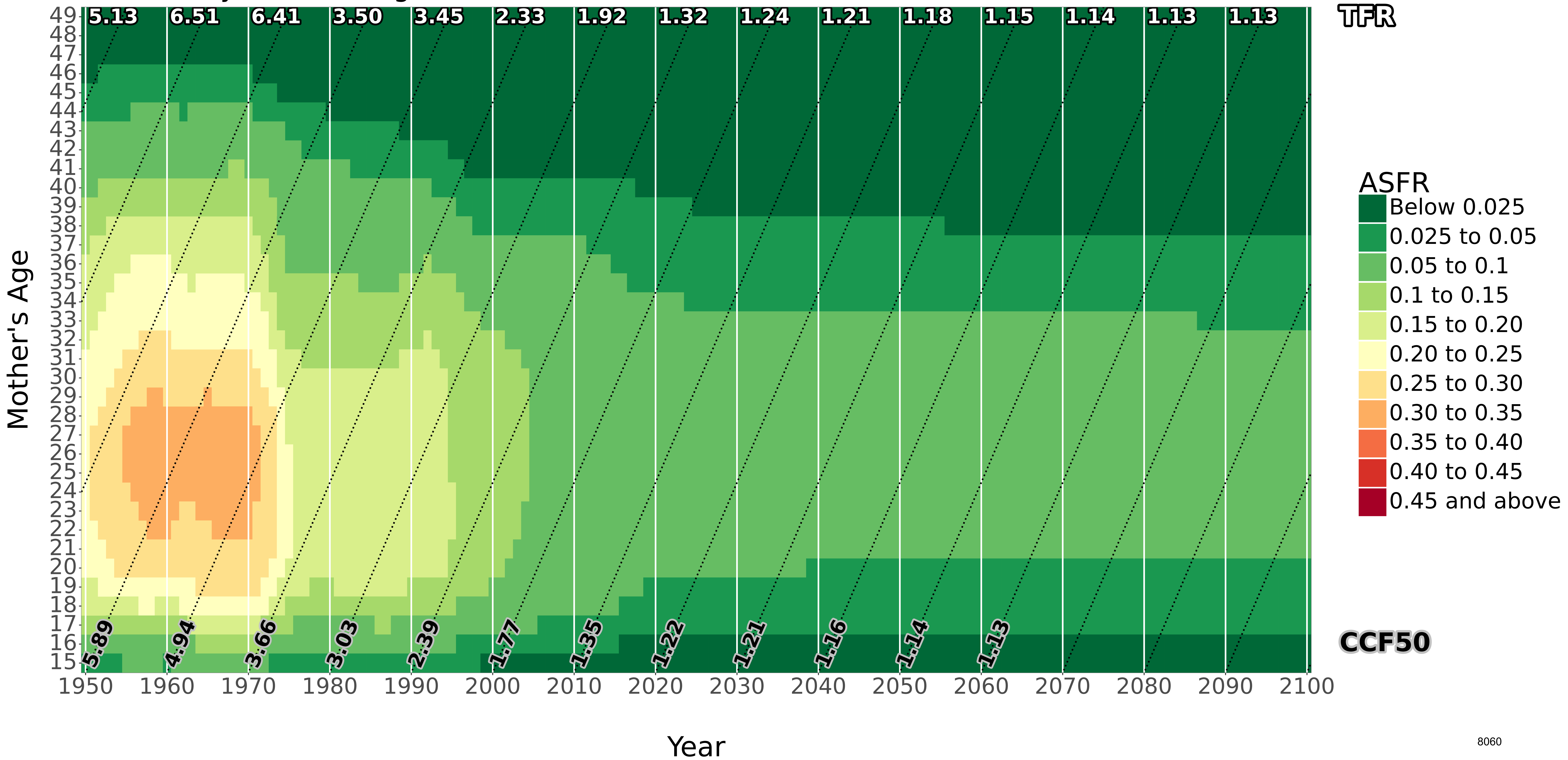

Supplemental Results Figure S3: Lexis diagram of CCF50, single-year age interval ASFR, and TFR by mother's age and location, 1950-2100, Dominican Republic

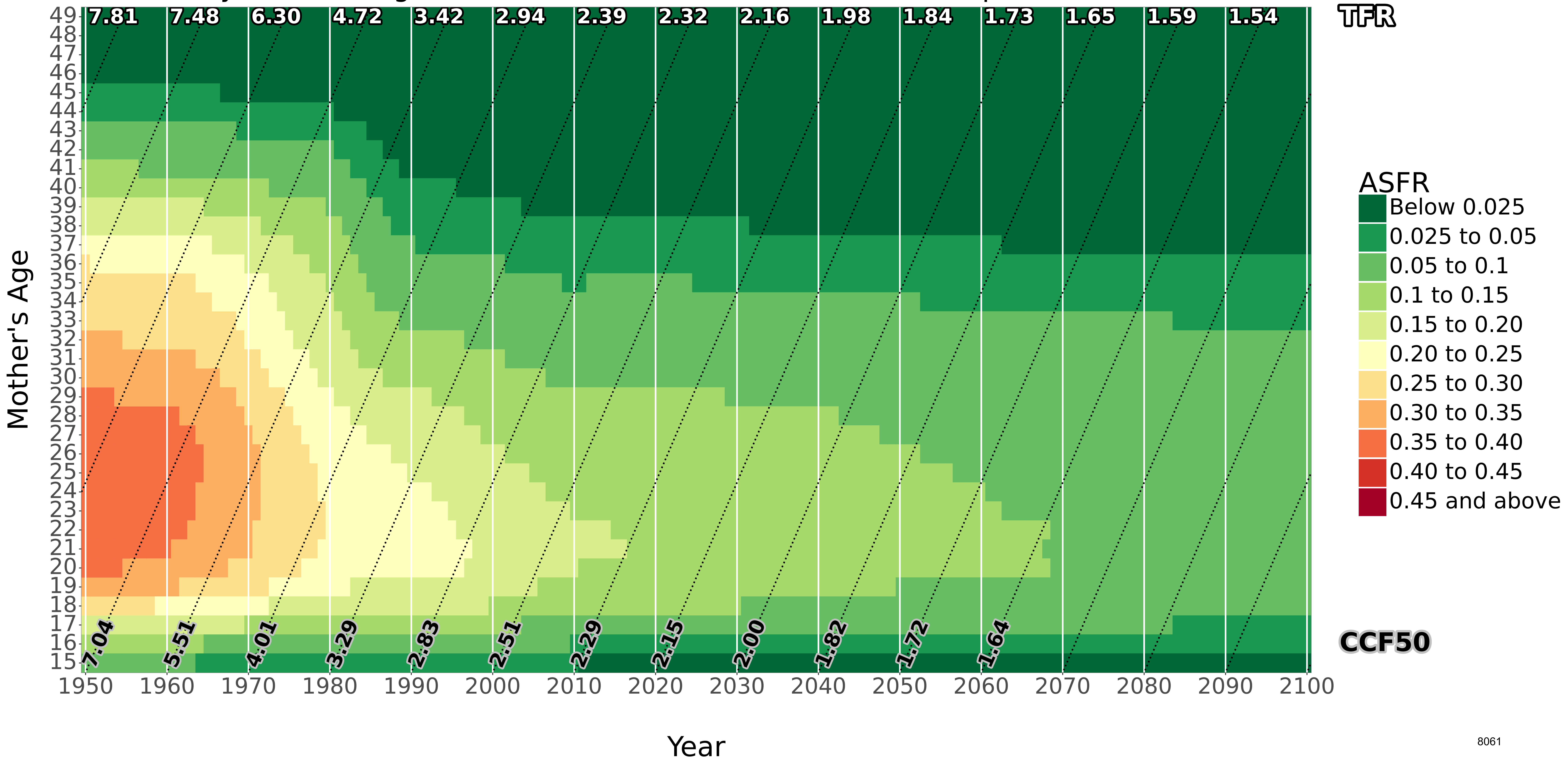

Supplemental Results Figure S3: Lexis diagram of CCF50, single-year age interval ASFR, and TFR by mother's age and location, 1950-2100, Grenada

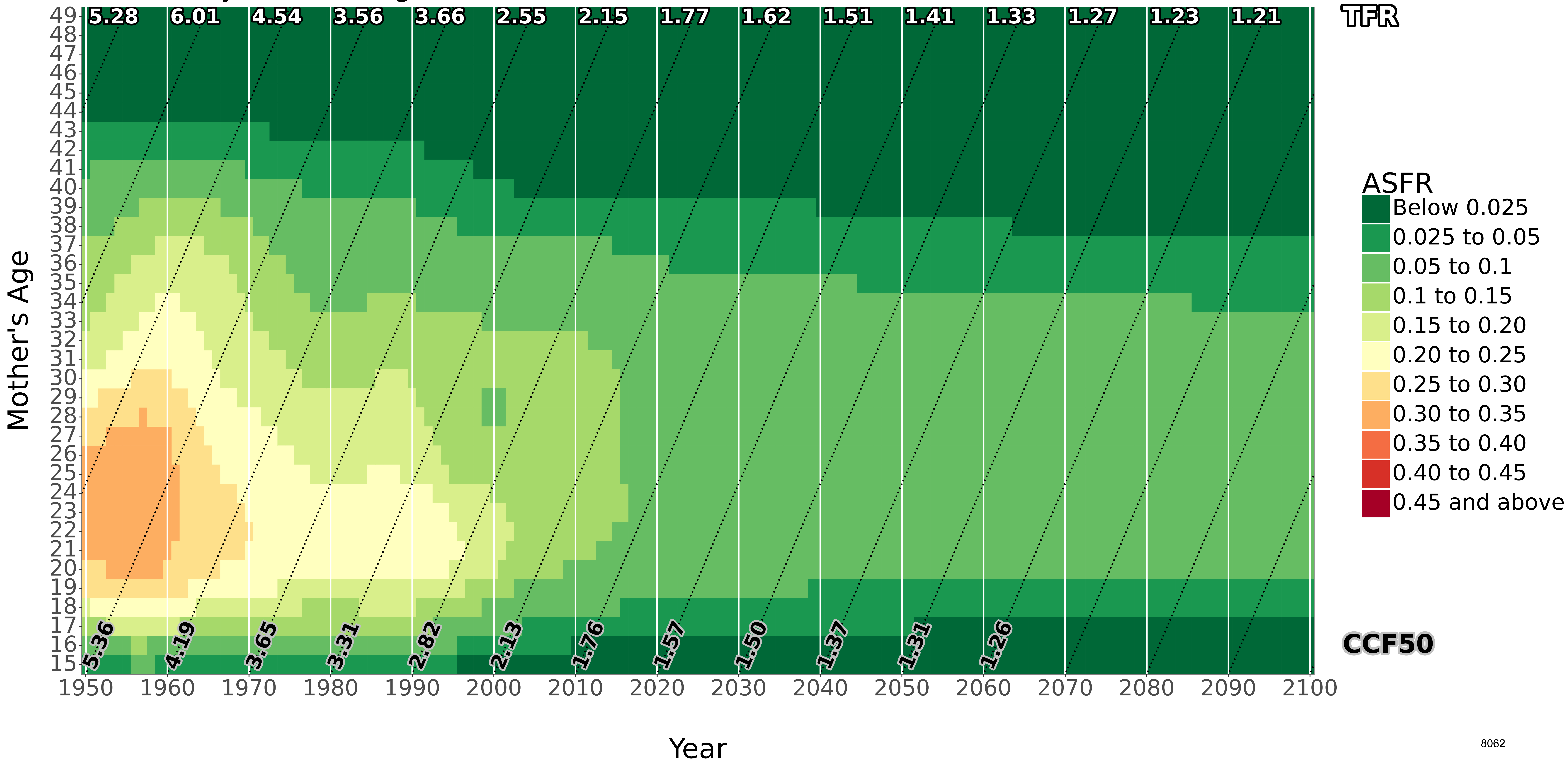

Supplemental Results Figure S3: Lexis diagram of CCF50, single-year age interval ASFR, and TFR by mother's age and location, 1950-2100, Guyana

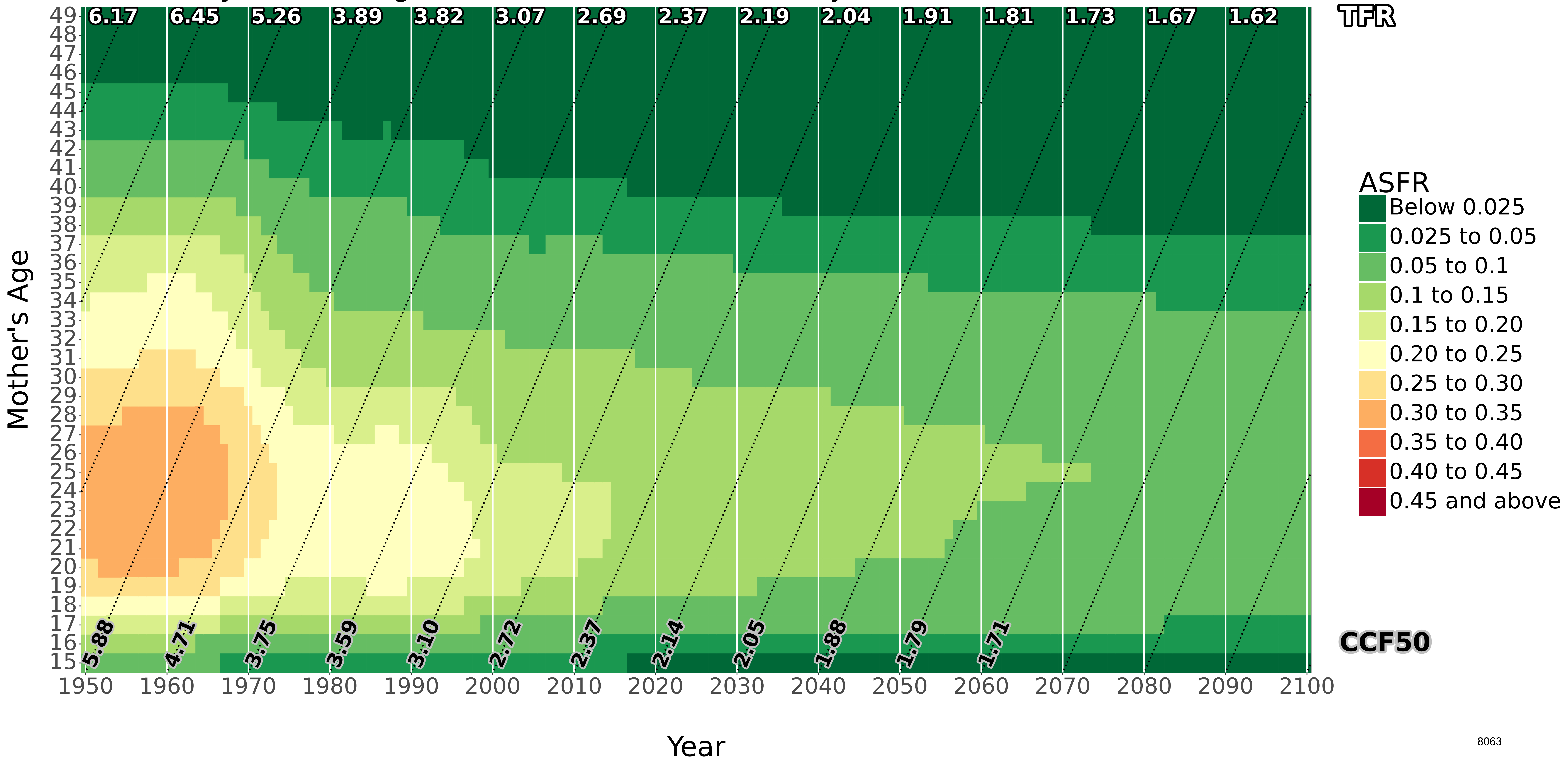

Supplemental Results Figure S3: Lexis diagram of CCF50, single-year age interval ASFR, and TFR by mother's age and location, 1950-2100, Haiti

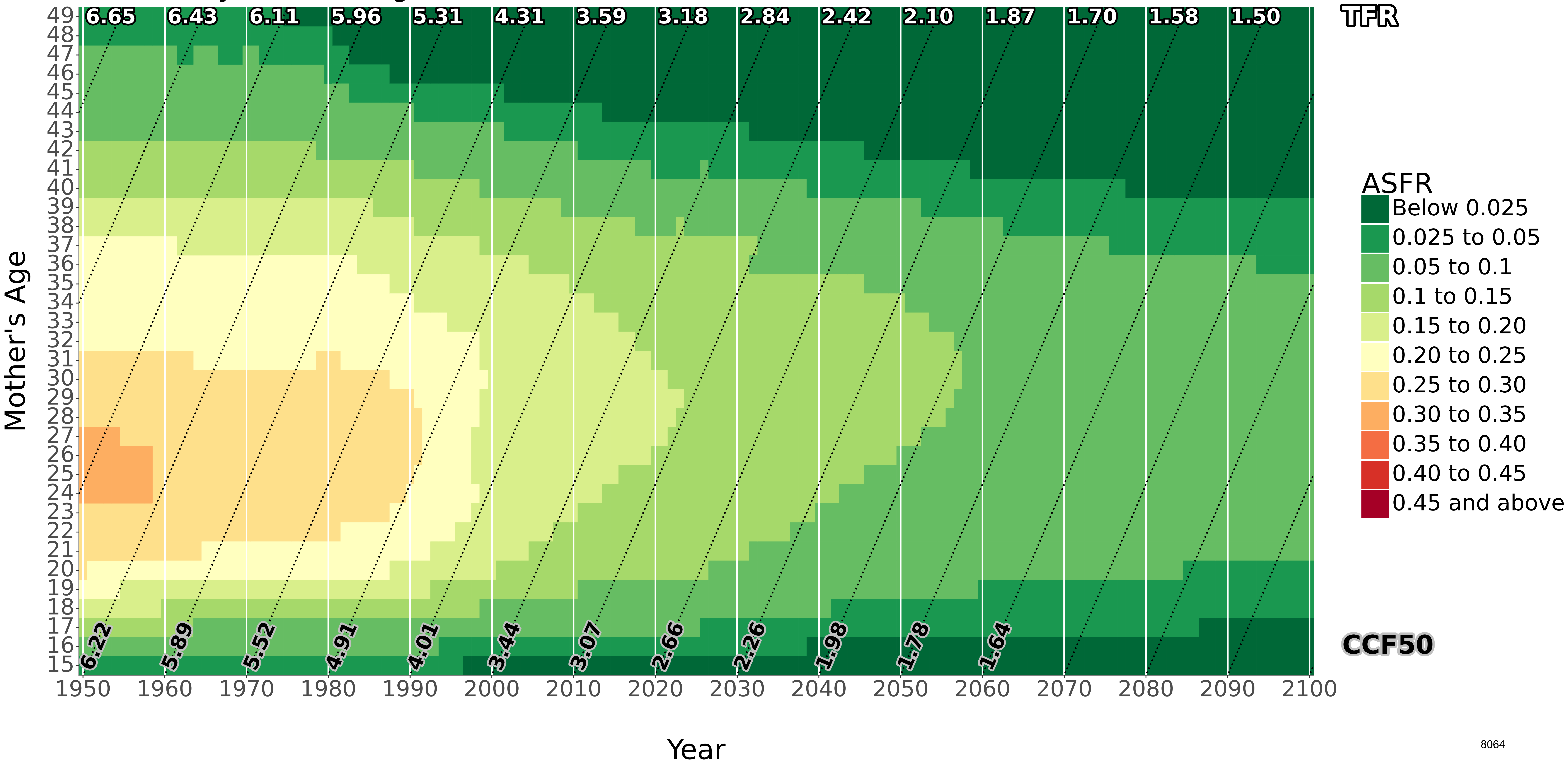

Supplemental Results Figure S3: Lexis diagram of CCF50, single-year age interval ASFR, and TFR by mother's age and location, 1950-2100, Jamaica

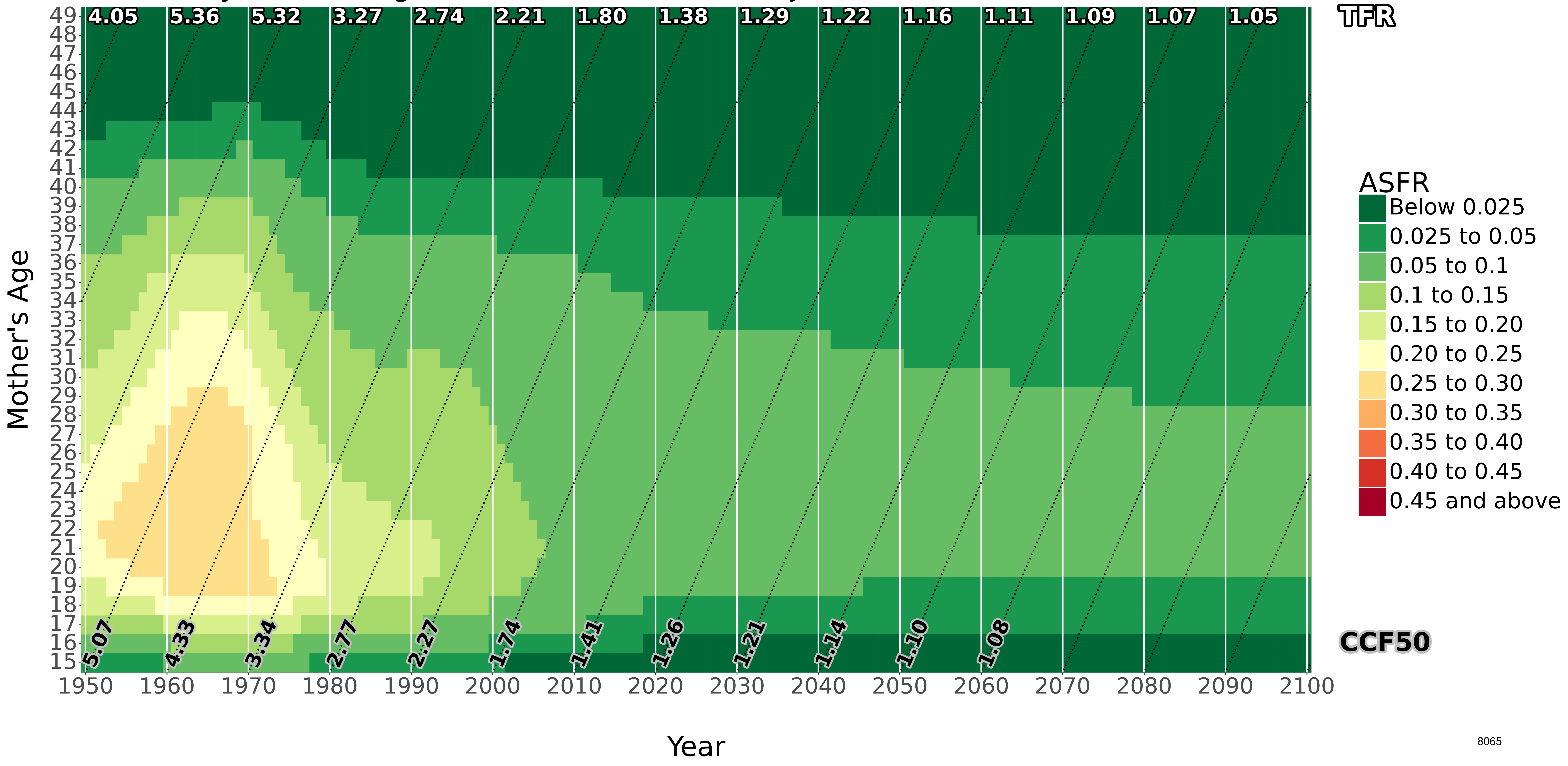

Supplemental Results Figure S3: Lexis diagram of CCF50, single-year age interval ASFR, and TFR by mother's age and location, 1950-2100, Puerto Rico

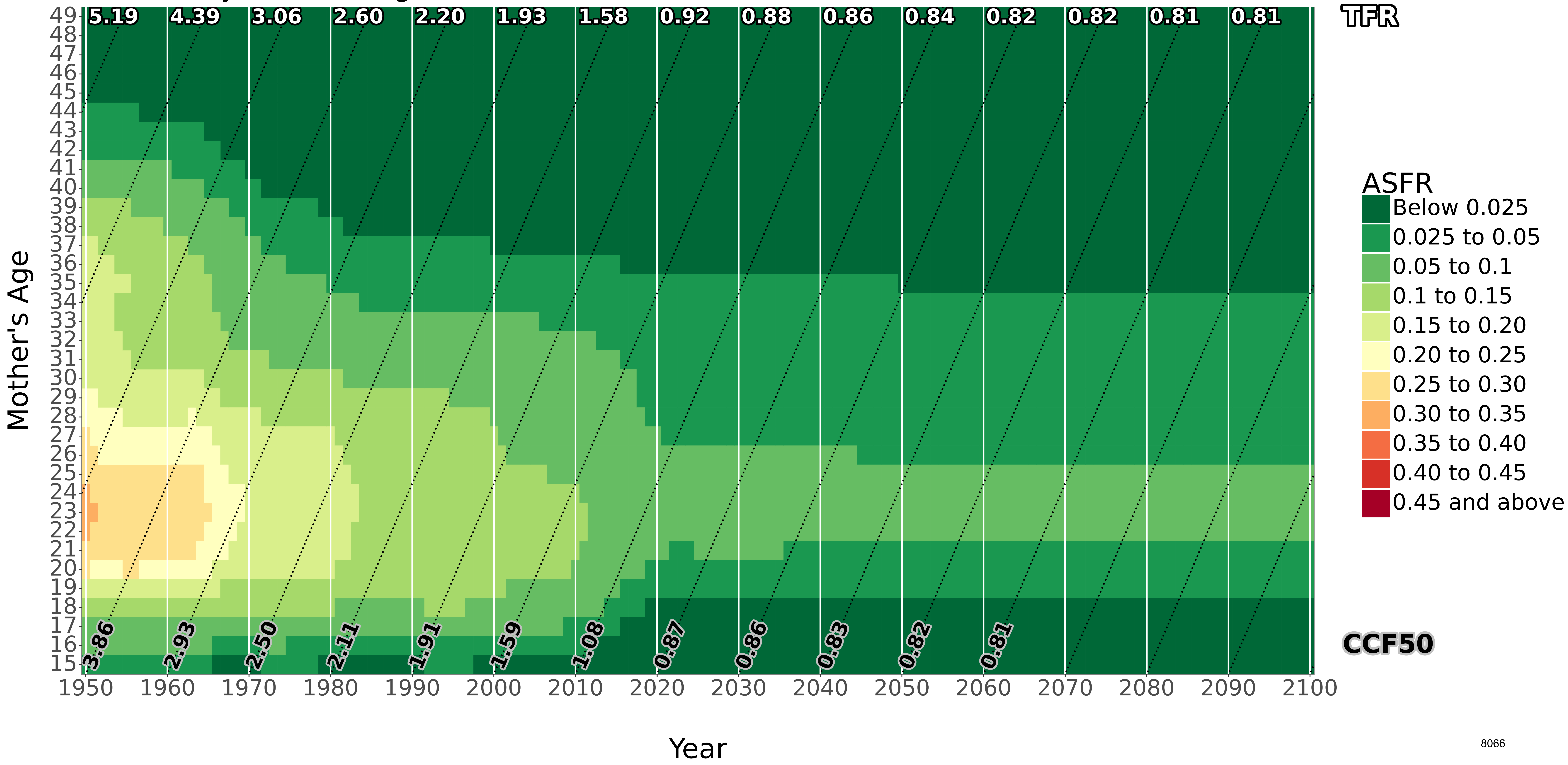

Supplemental Results Figure S3: Lexis diagram of CCF50, single-year age interval ASFR, and TFR by mother's age and location, 1950-2100, Saint Kitts and Nevis

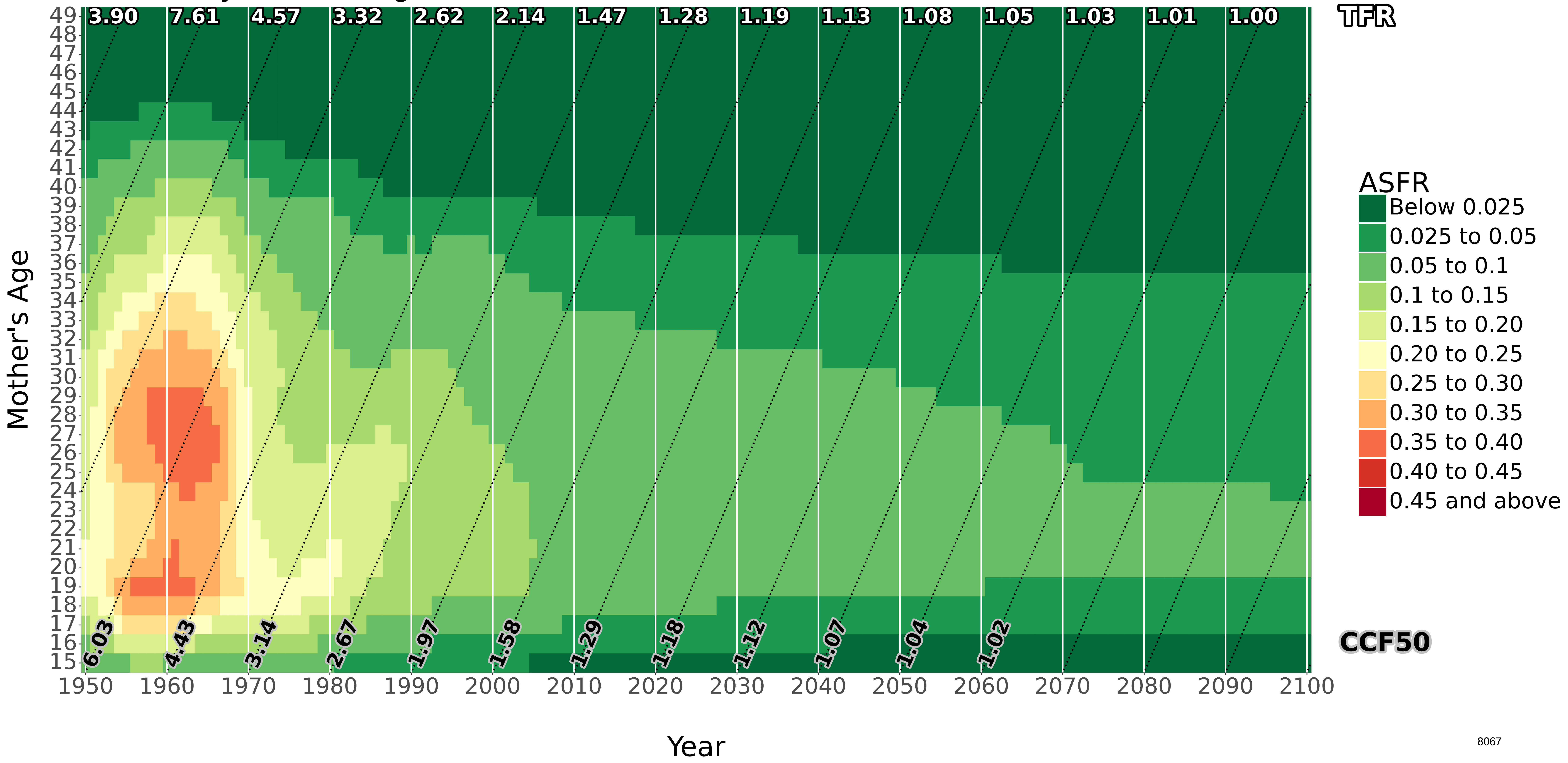

Supplemental Results Figure S3: Lexis diagram of CCF50, single-year age interval ASFR, and TFR by mother's age and location, 1950-2100, Saint Lucia

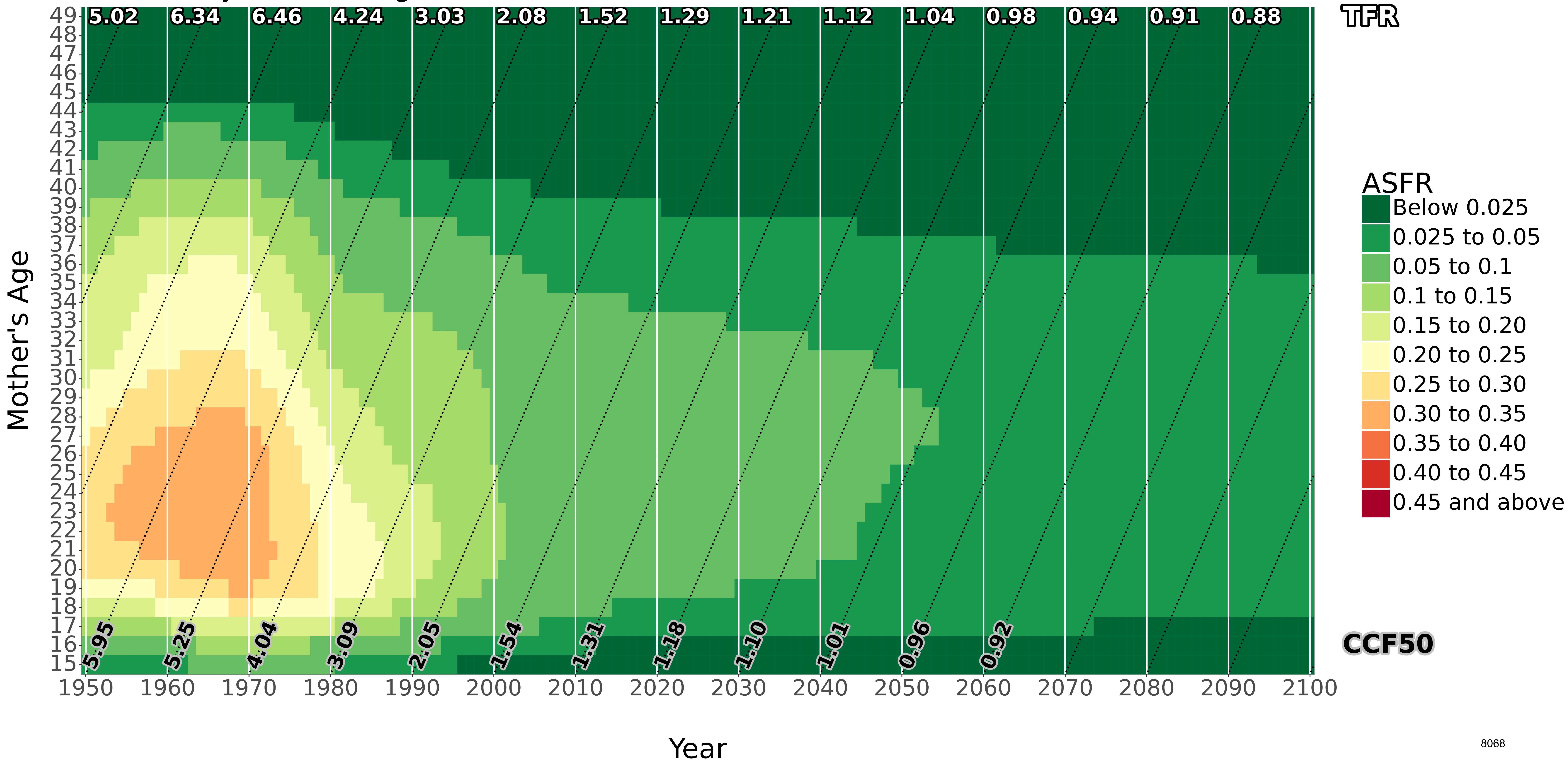

Supplemental Results Figure S3: Lexis diagram of CCF50, single-year age interval ASFR, and TFR by mother's age and location, 1950-2100, Saint Vincent and the Grenadines

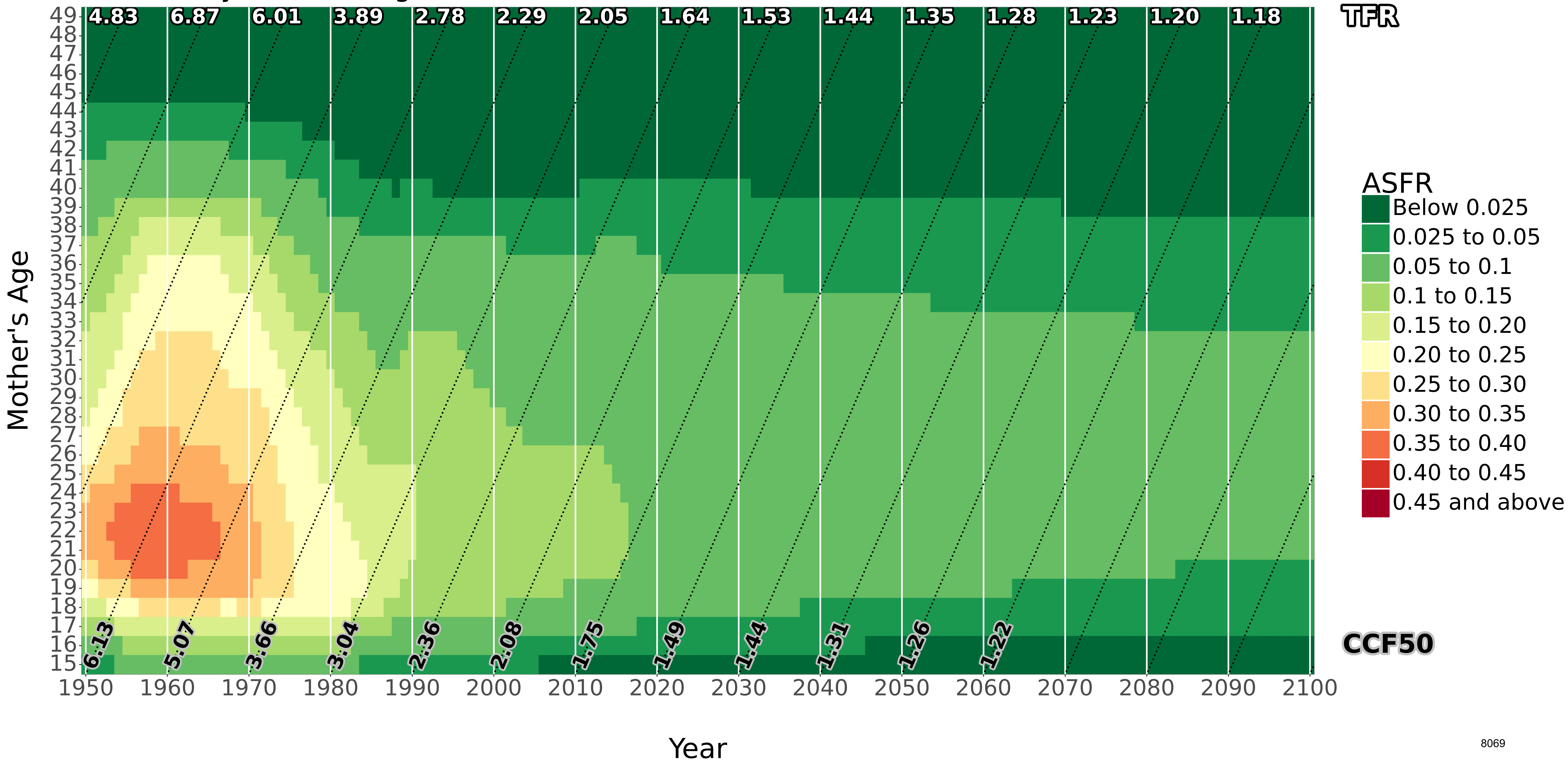

Supplemental Results Figure S3: Lexis diagram of CCF50, single-year age interval ASFR, and TFR by mother's age and location, 1950-2100, Suriname

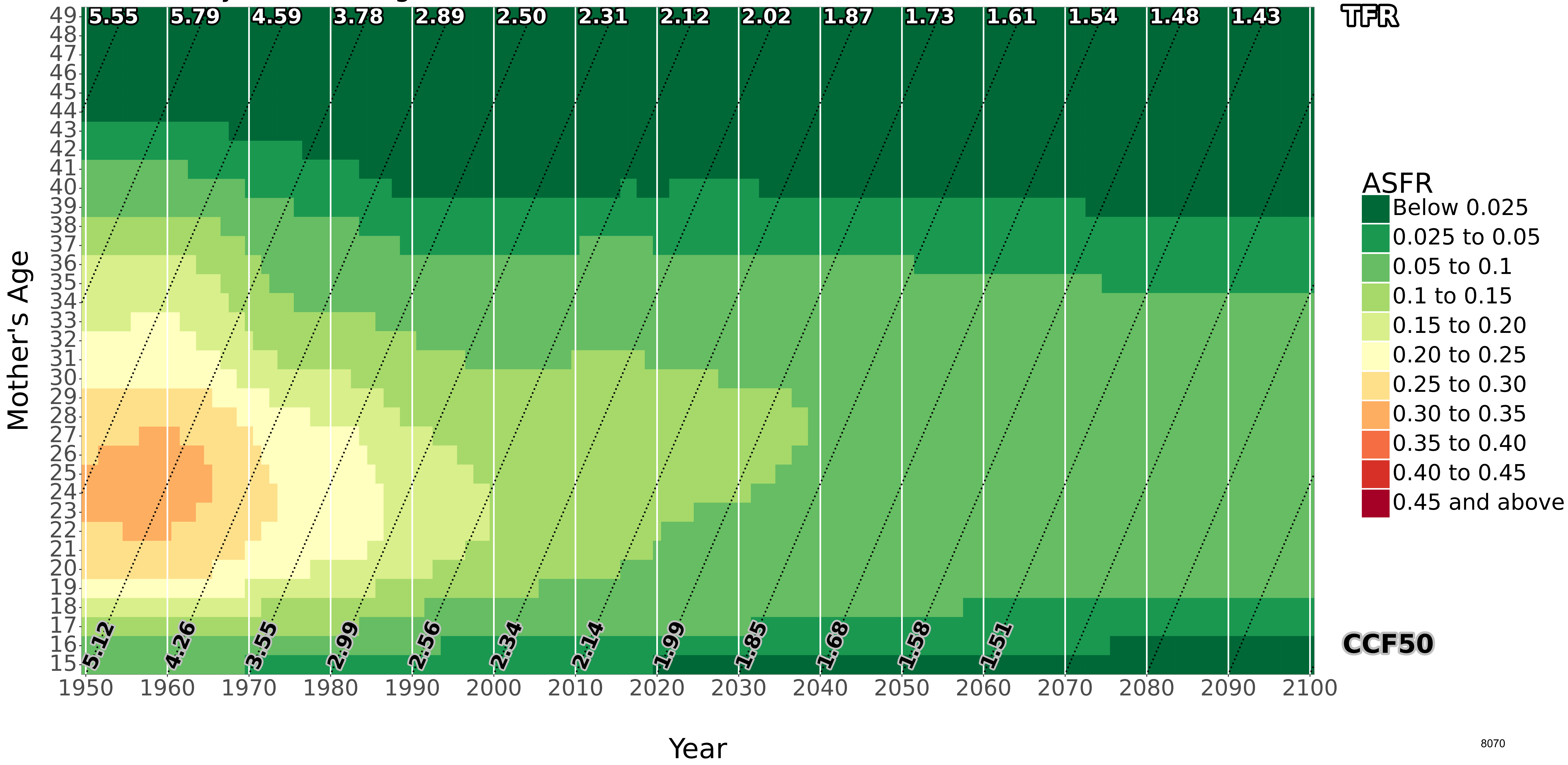

8070

Supplemental Results Figure S3: Lexis diagram of CCF50, single-year age interval ASFR, and TFR by mother's age and location, 1950-2100, Trinidad and Tobago

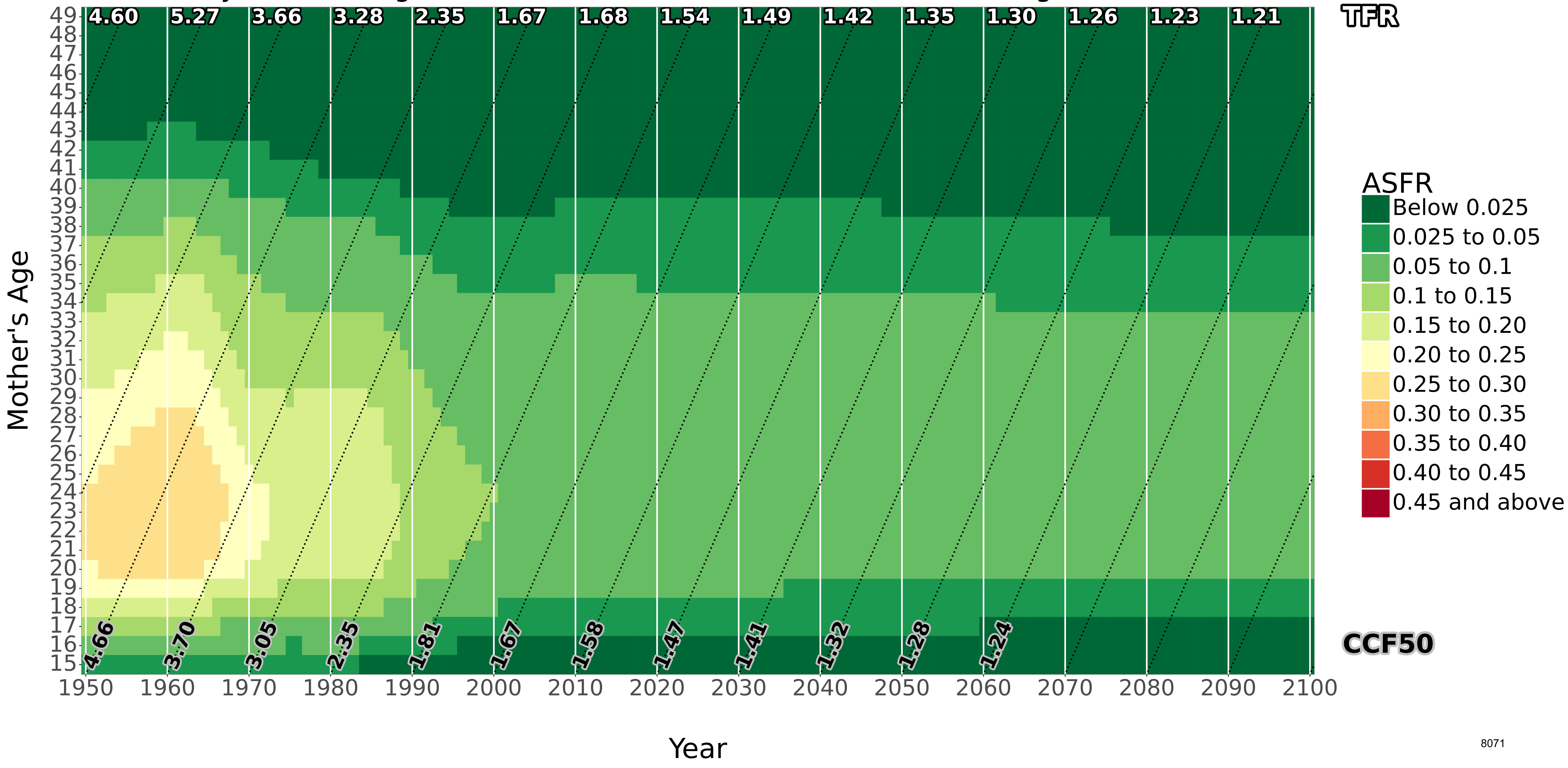

8071

Supplemental Results Figure S3: Lexis diagram of CCF50, single-year age interval ASFR, and TFR by mother's age and location, 1950-2100, Virgin Islands

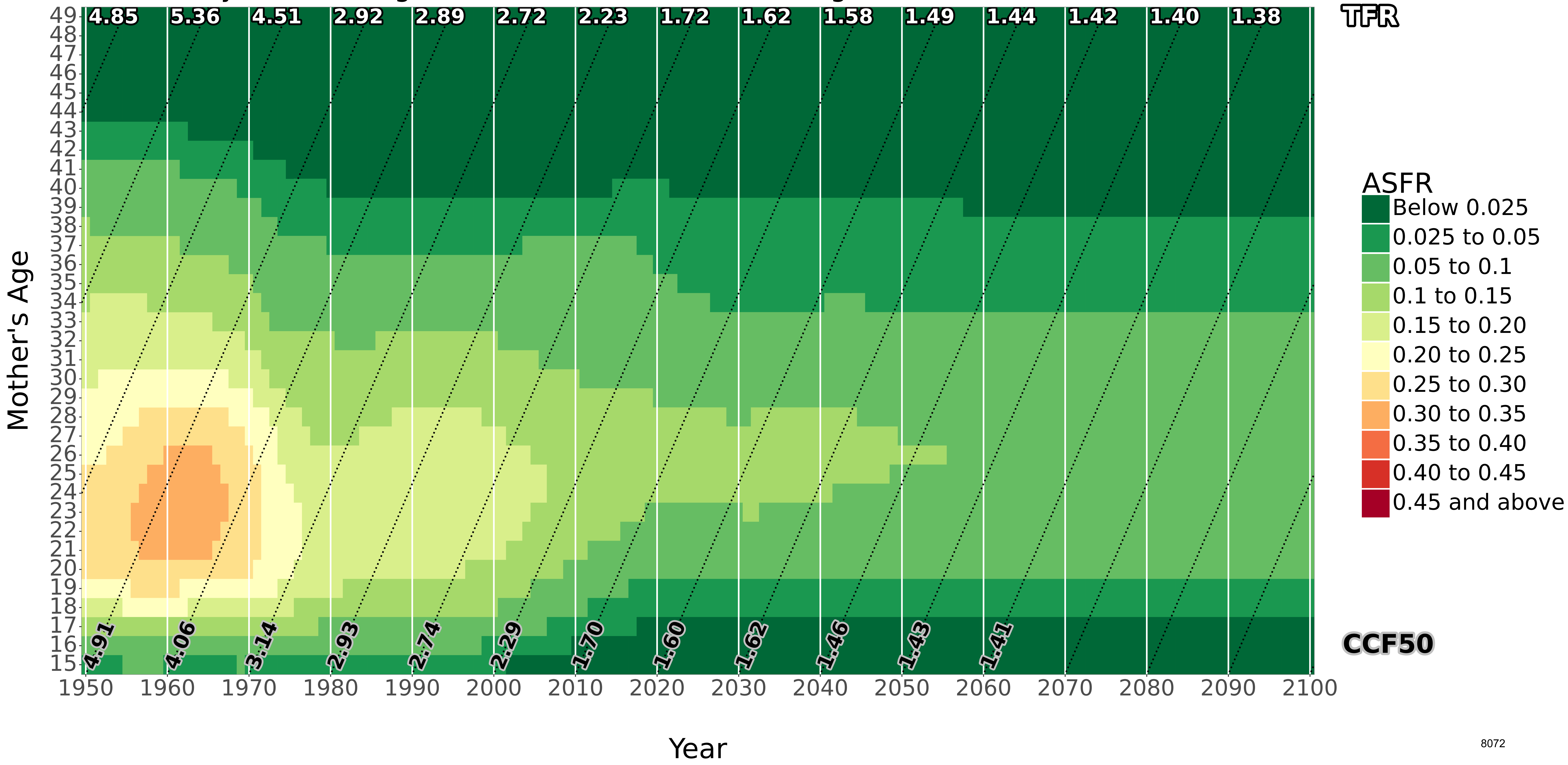

Supplemental Results Figure S3: Lexis diagram of CCF50, single-year age interval ASFR, and TFR by mother's age and location, 1950-2100, Colombia

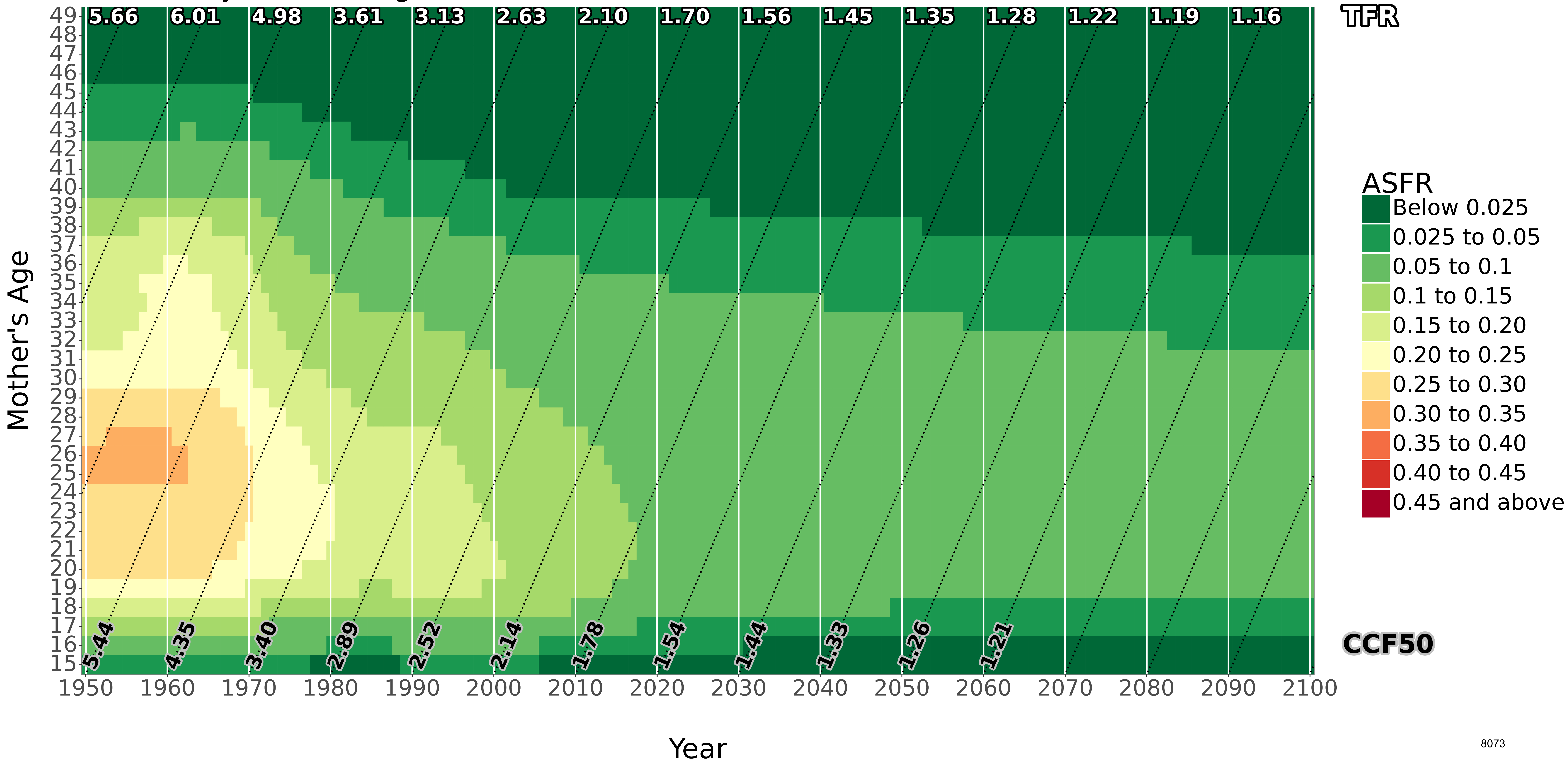

8073

Supplemental Results Figure S3: Lexis diagram of CCF50, single-year age interval ASFR, and TFR by mother's age and location, 1950-2100, Costa Rica

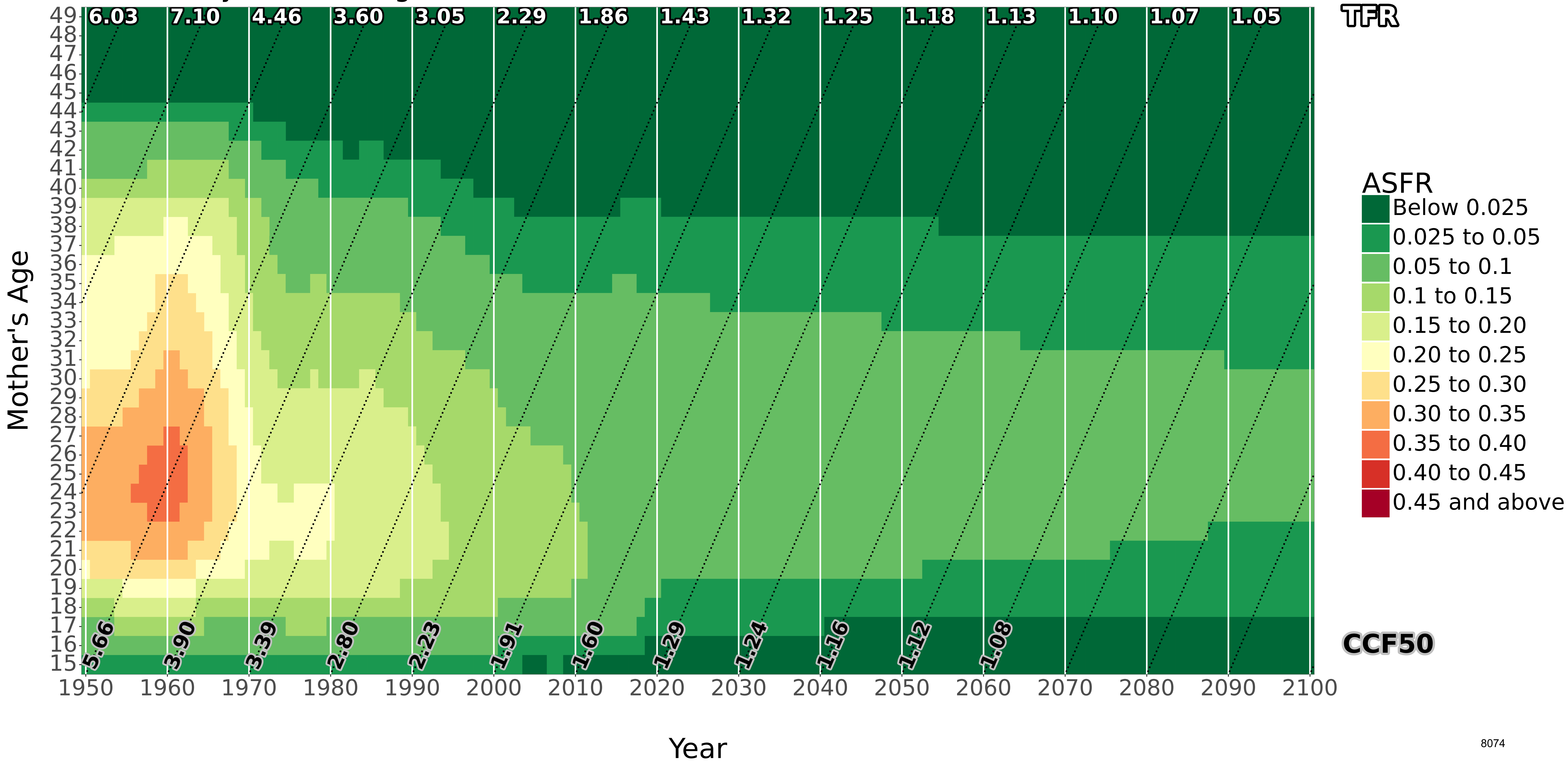

8074

Supplemental Results Figure S3: Lexis diagram of CCF50, single-year age interval ASFR, and TFR by mother's age and location, 1950-2100, El Salvador

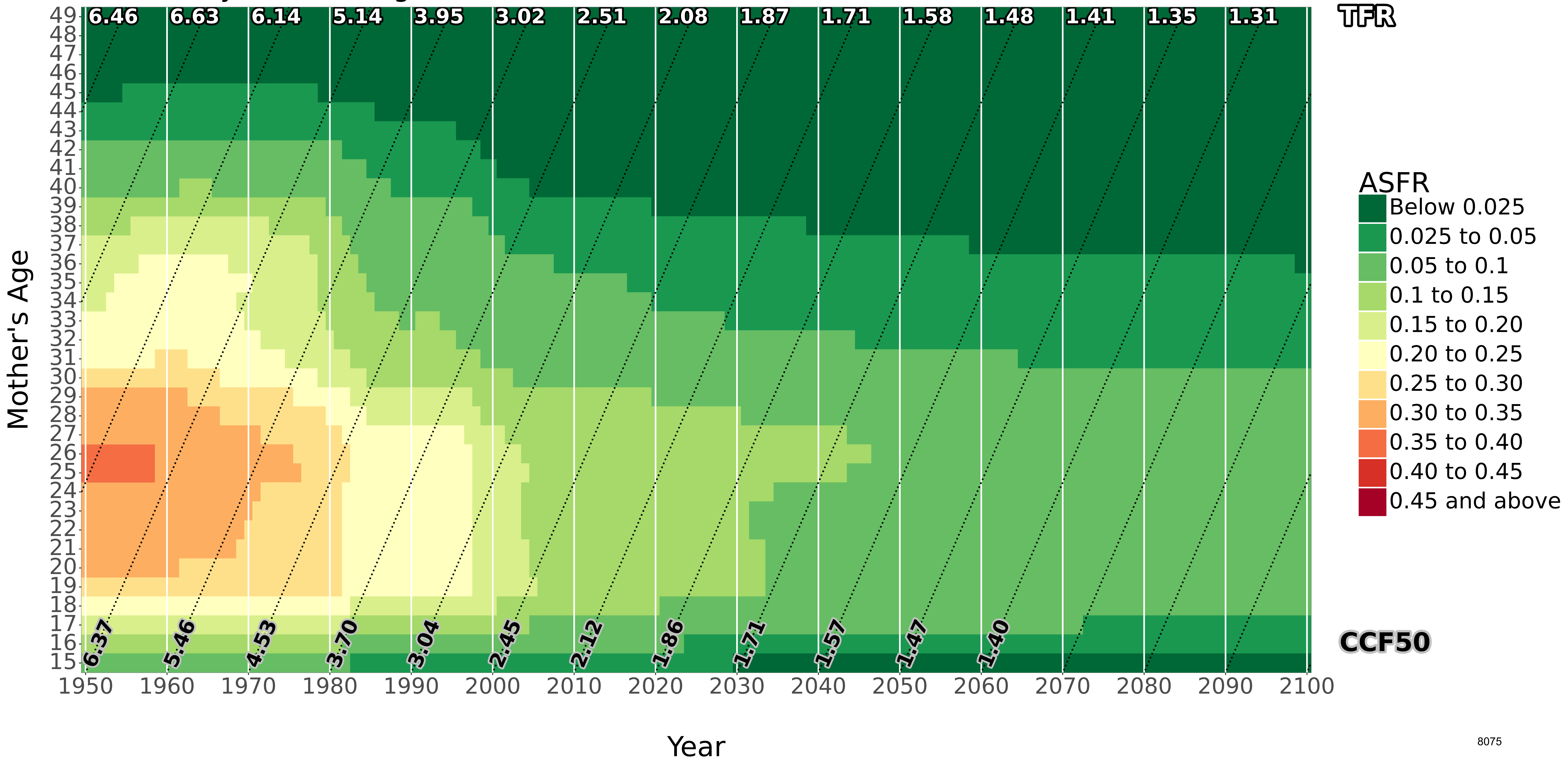

Supplemental Results Figure S3: Lexis diagram of CCF50, single-year age interval ASFR, and TFR by mother's age and location, 1950-2100, Guatemala

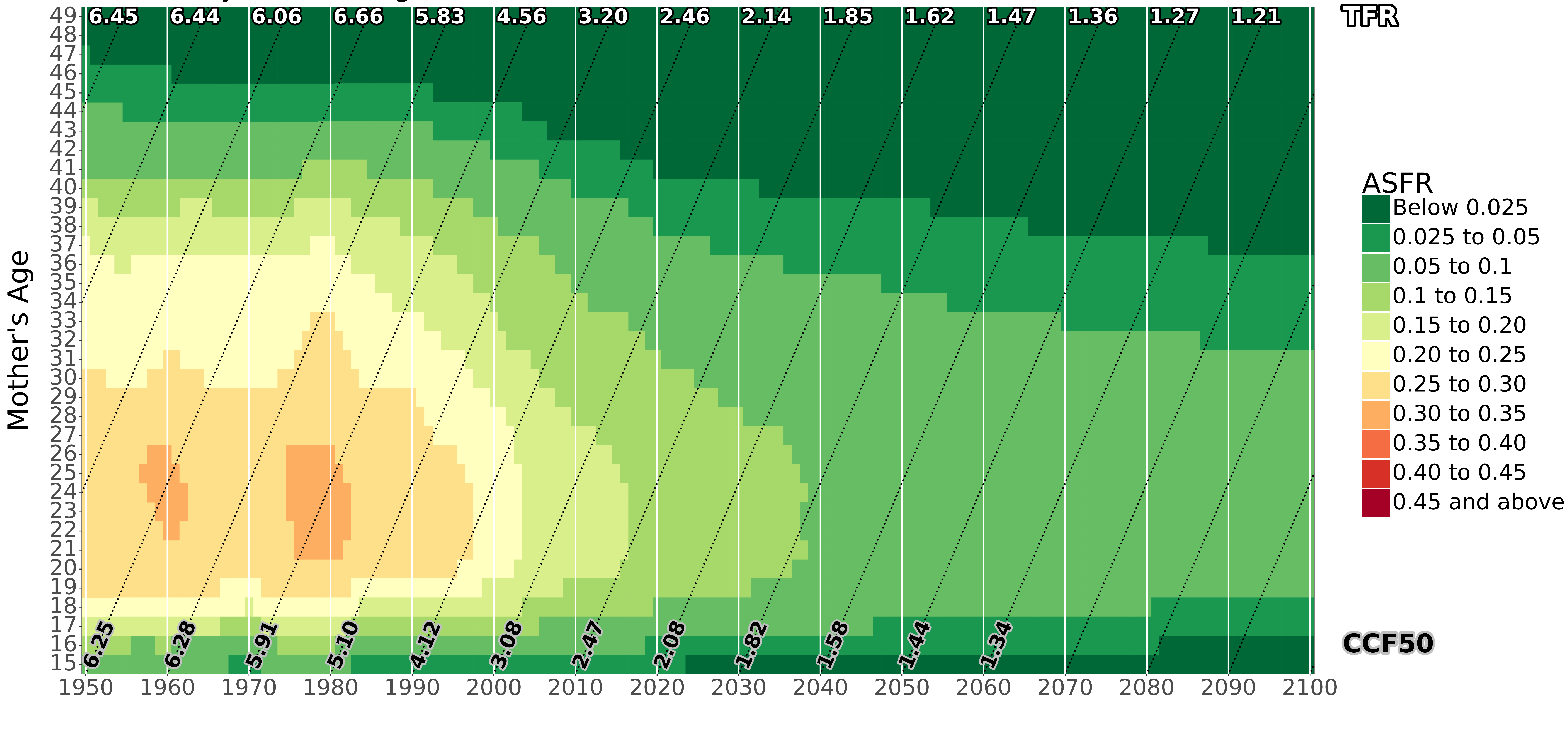

Supplemental Results Figure S3: Lexis diagram of CCF50, single-year age interval ASFR, and TFR by mother's age and location, 1950-2100, Honduras

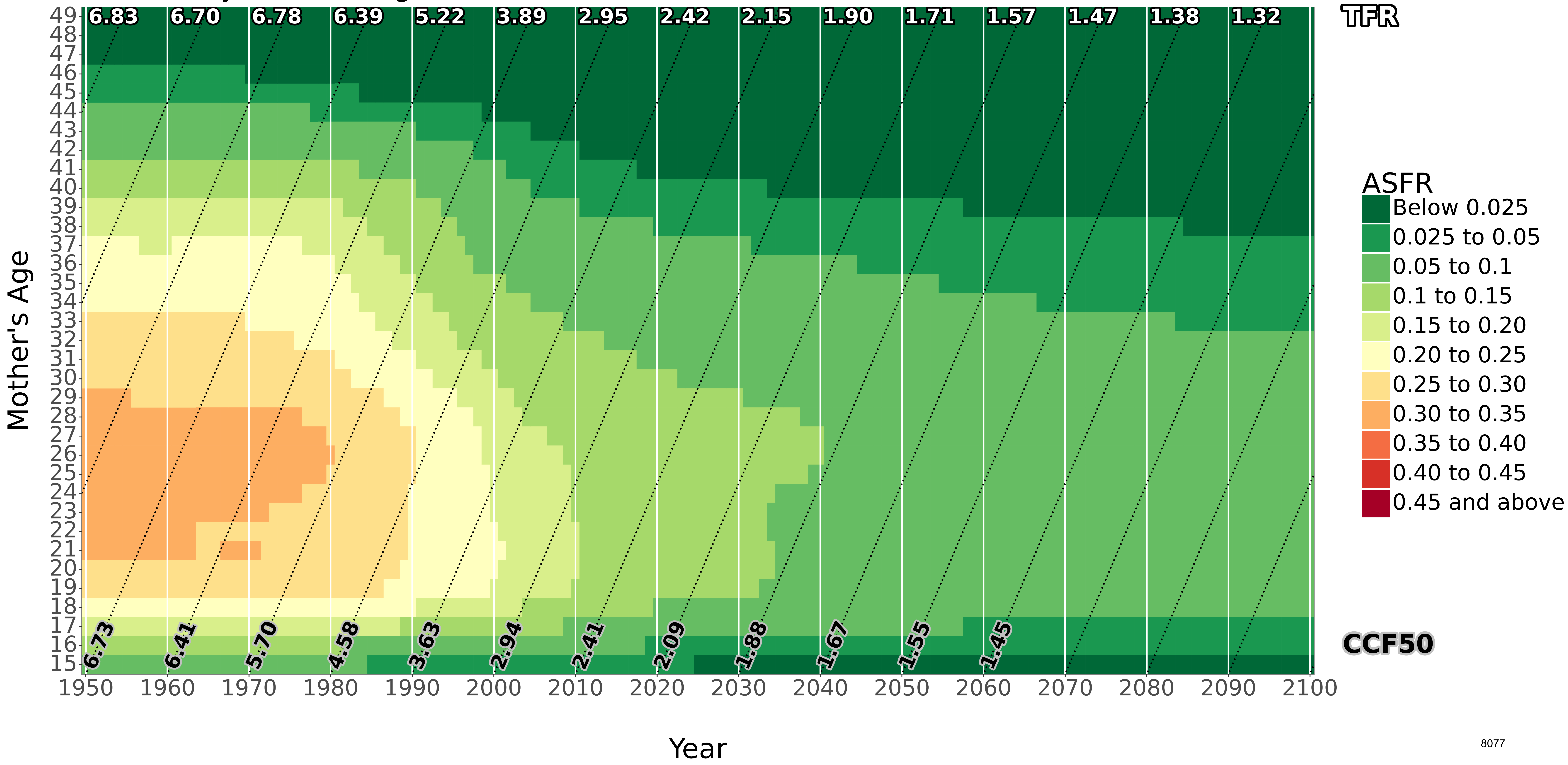

Supplemental Results Figure S3: Lexis diagram of CCF50, single-year age interval ASFR, and TFR by mother's age and location, 1950-2100, Mexico

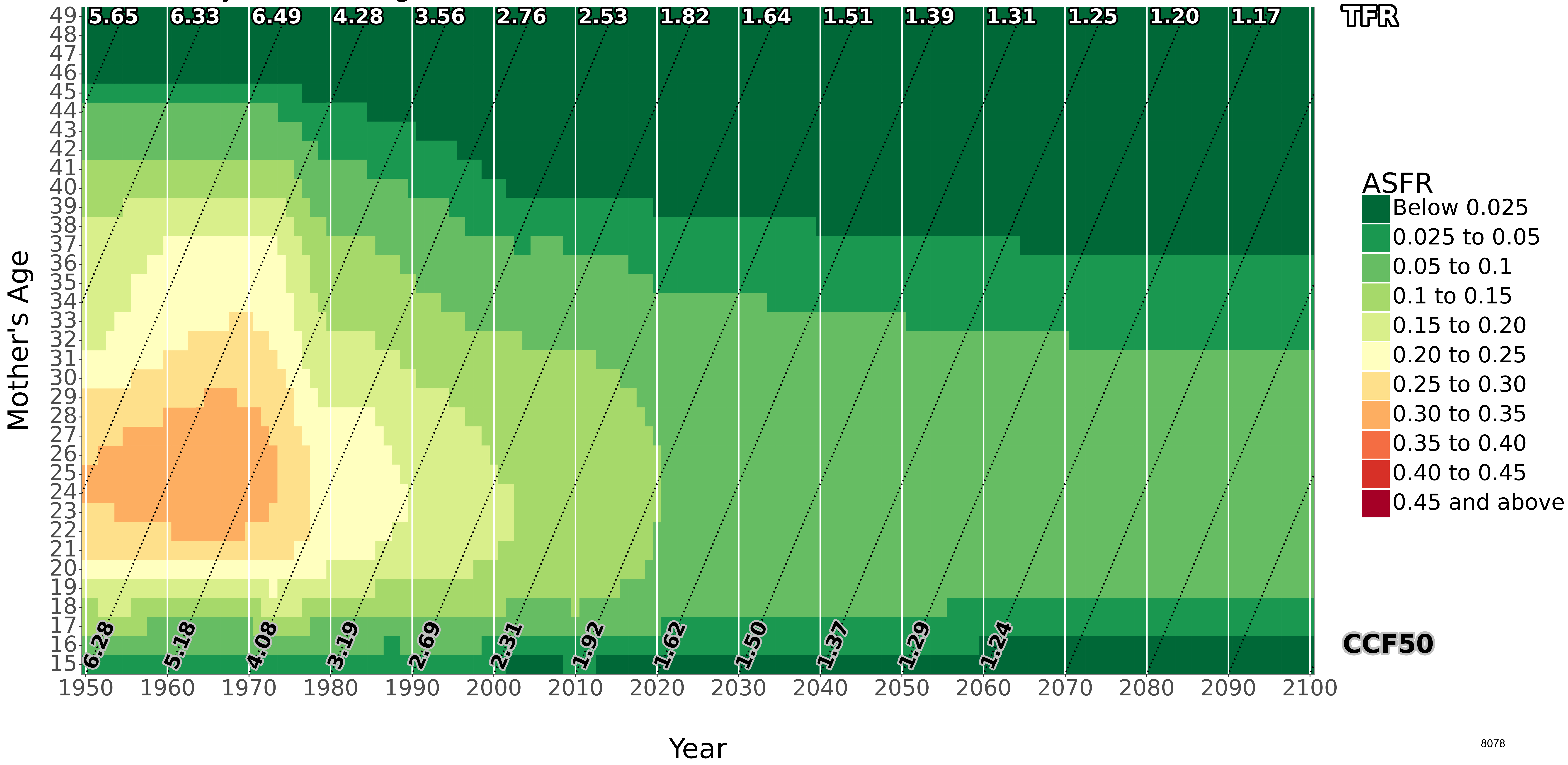

Supplemental Results Figure S3: Lexis diagram of CCF50, single-year age interval ASFR, and TFR by mother's age and location, 1950-2100, Nicaragua

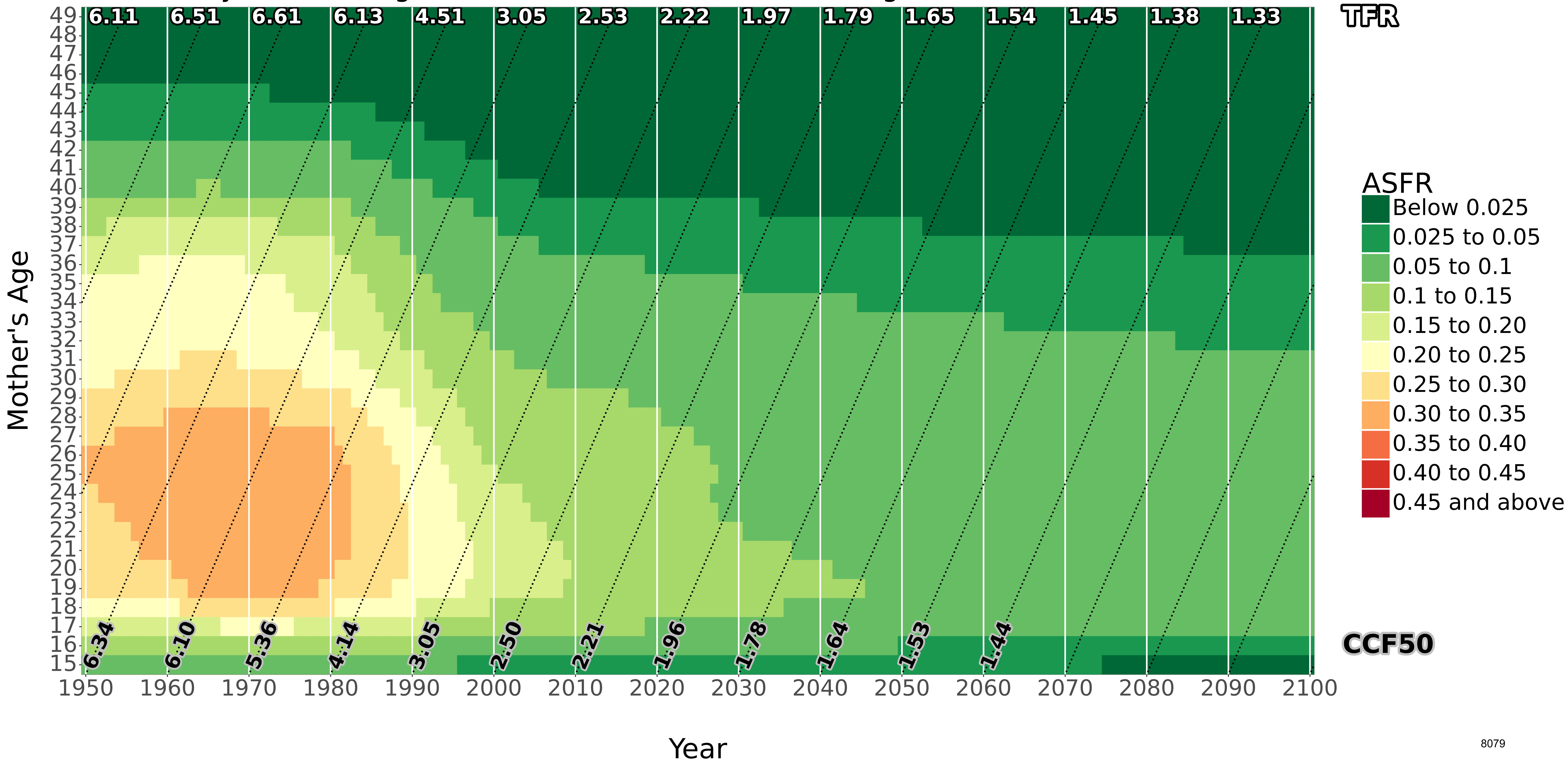

Supplemental Results Figure S3: Lexis diagram of CCF50, single-year age interval ASFR, and TFR by mother's age and location, 1950-2100, Panama

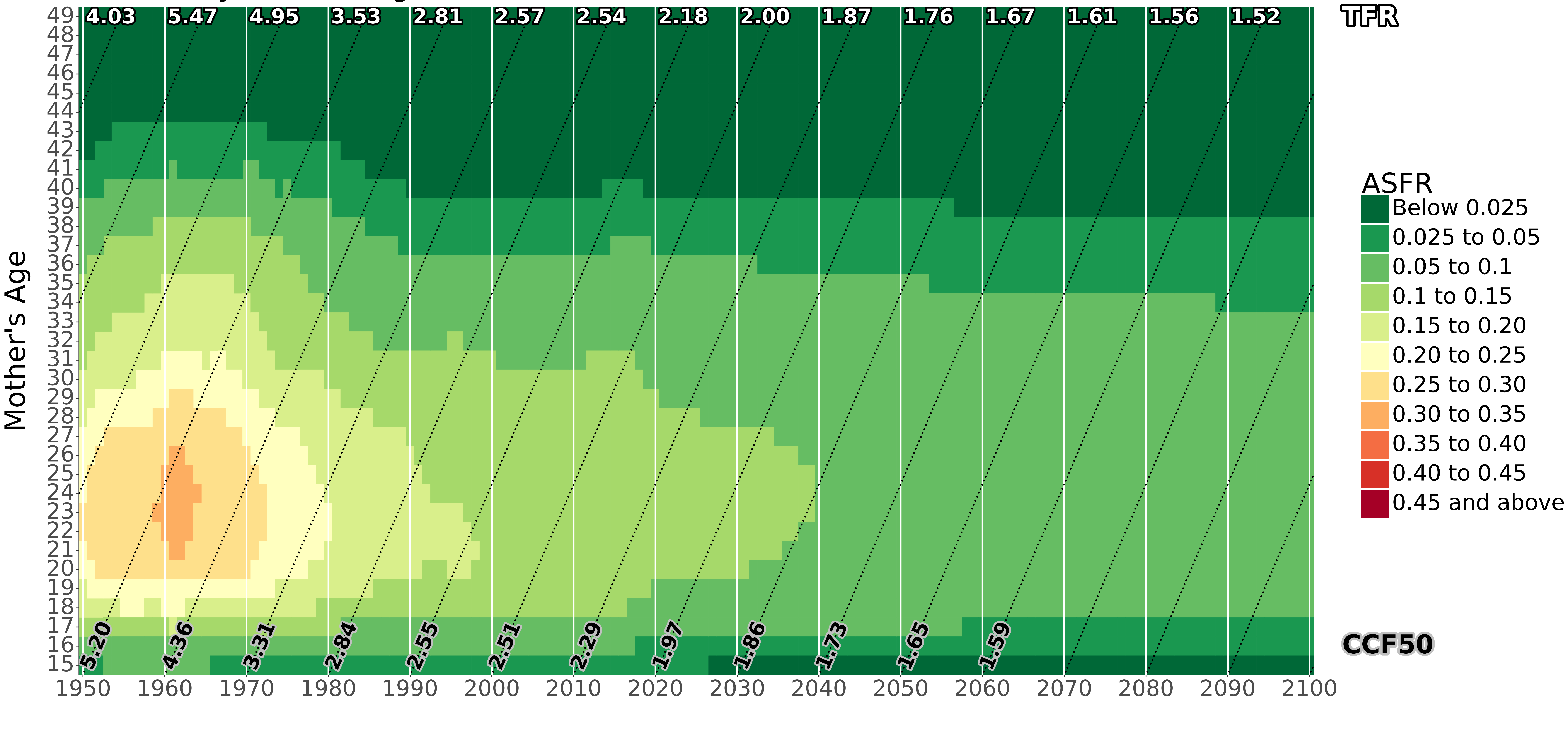

Supplemental Results Figure S3: Lexis diagram of CCF50, single-year age interval ASFR, and TFR by mother's age and location, 1950-2100, Venezuela

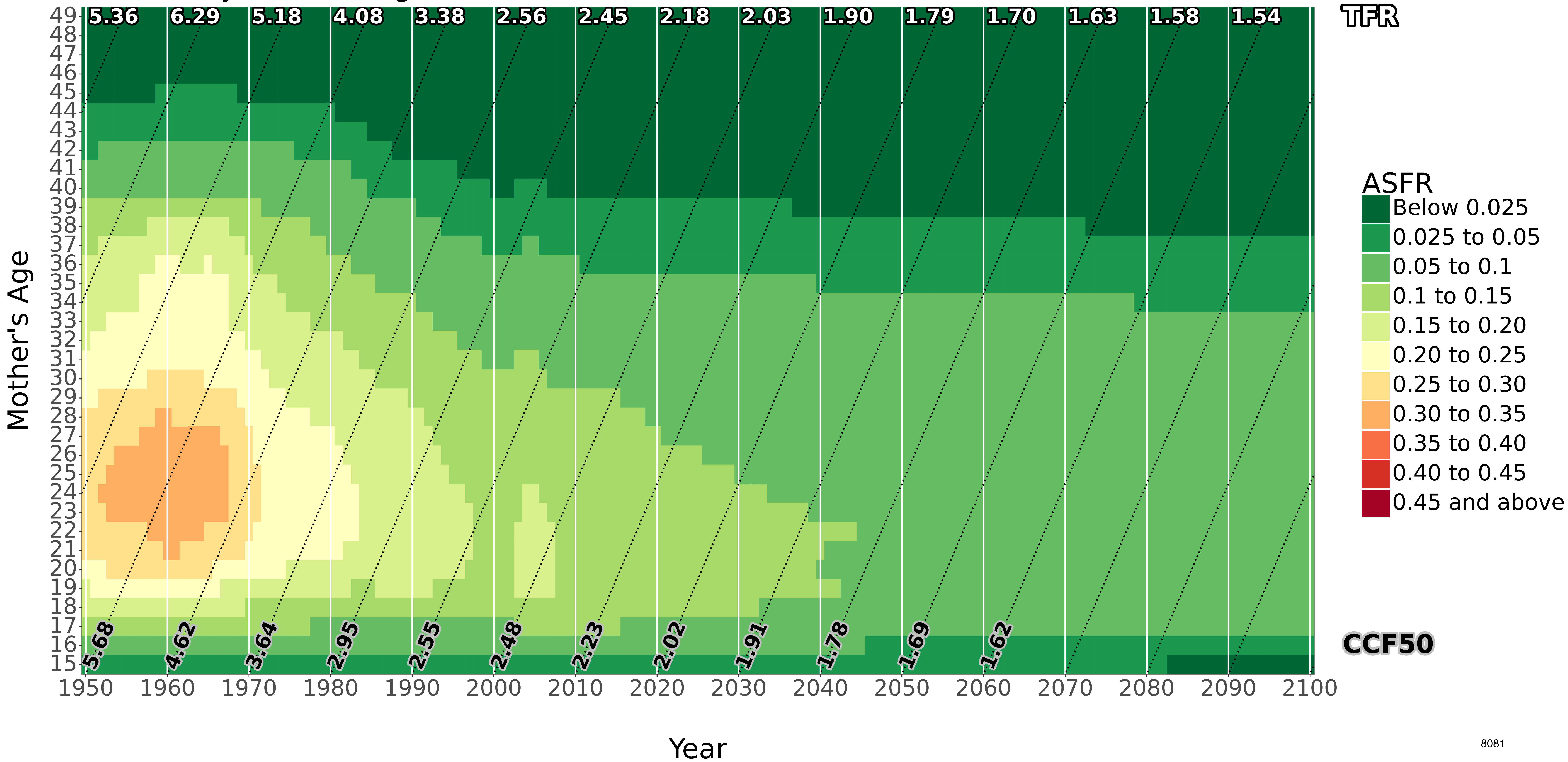

Supplemental Results Figure S3: Lexis diagram of CCF50, single-year age interval ASFR, and TFR by mother's age and location, 1950-2100, Brazil

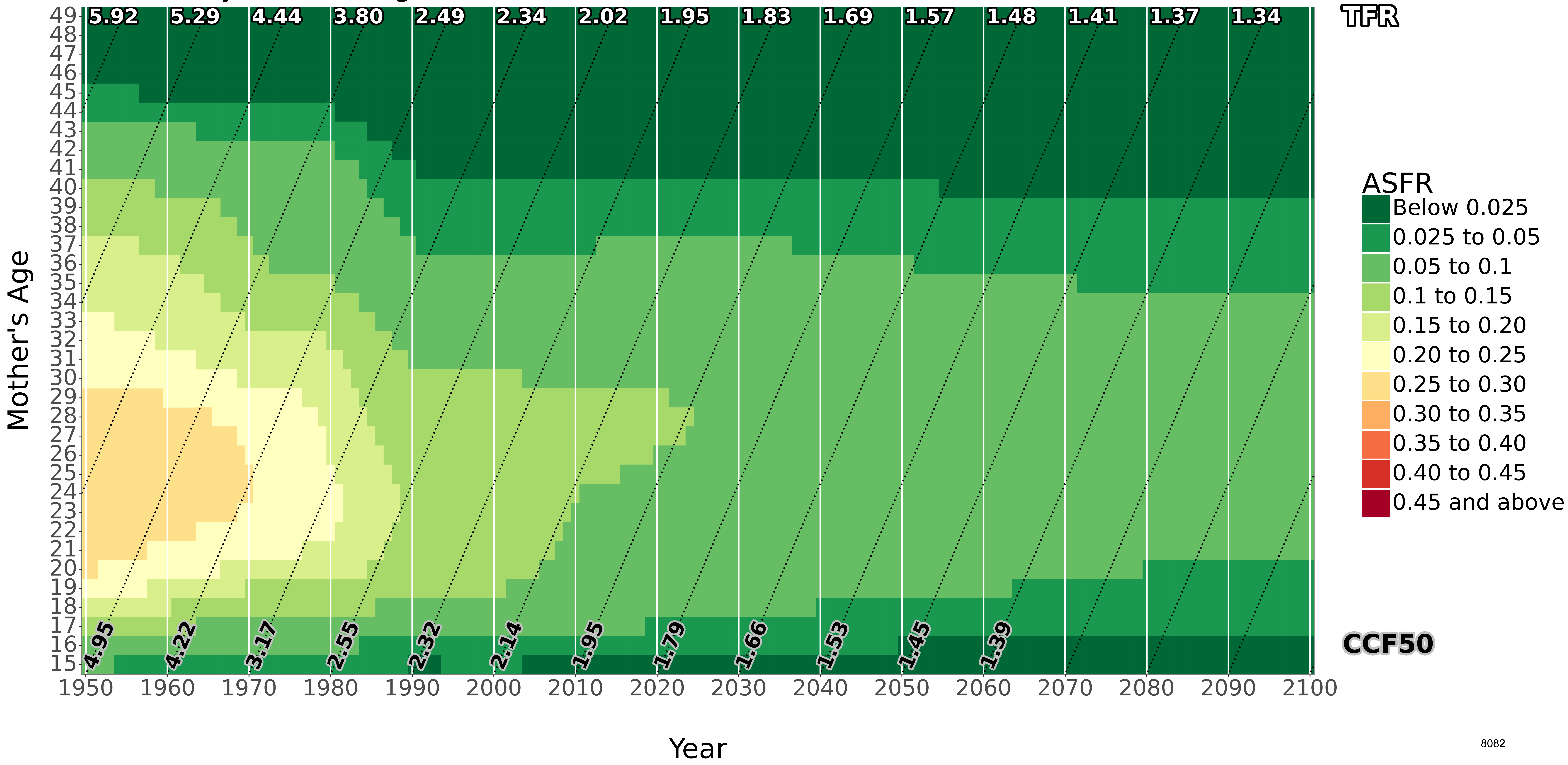

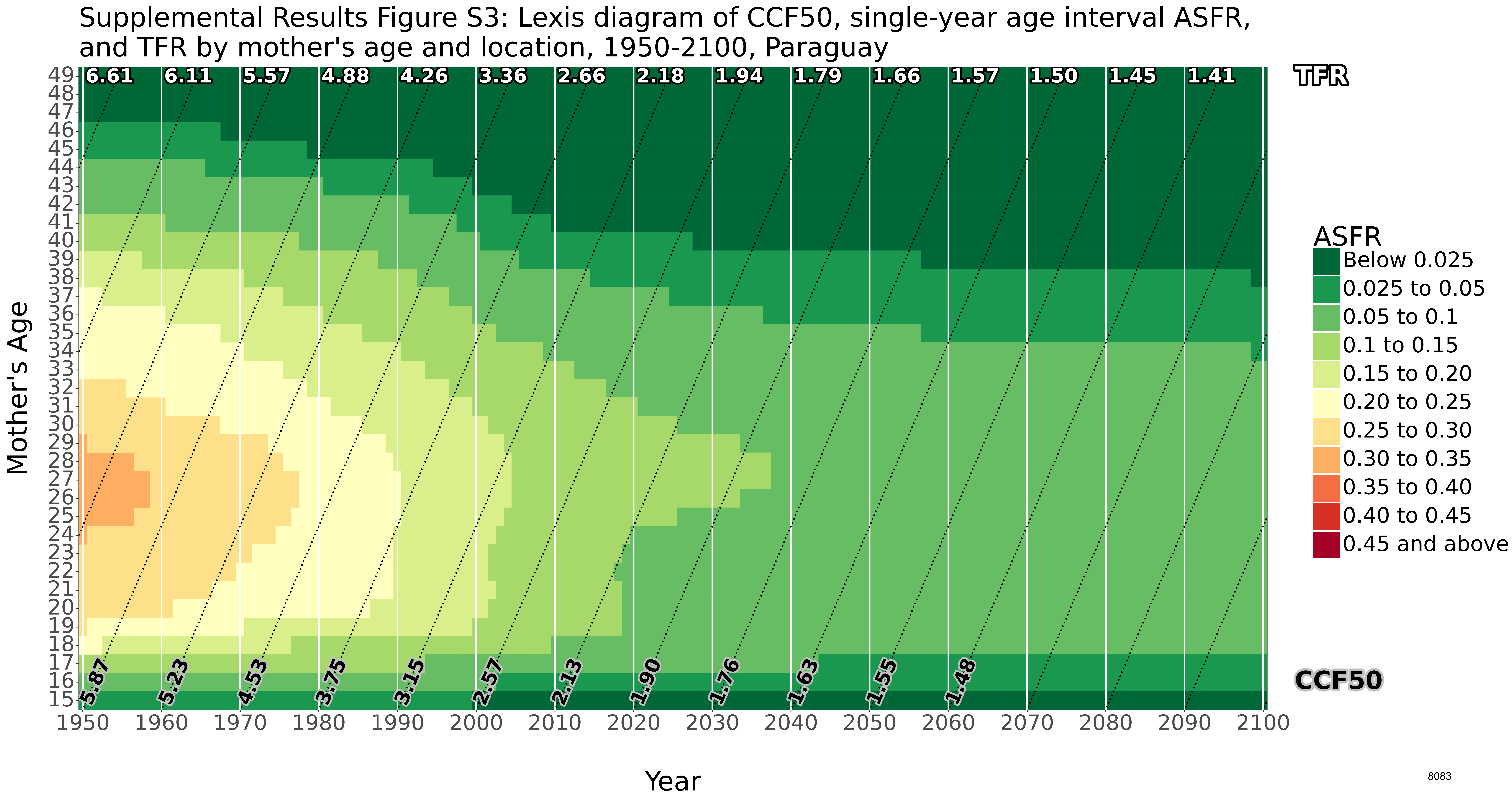

Supplemental Results Figure S3: Lexis diagram of CCF50, single-year age interval ASFR, and TFR by mother's age and location, 1950-2100, Paraguay
TFR
6.61
6.11
5.57
4.88
4.26
3.36
2.66
2.18
1.94
1.79
1.66
1.57
1.50
1.45
1.41
CCF50
5.87
5.23
4.53
3.75
3.15
2.57
2.13
1.90
1.76
1.63
1.55
1.48
Mother's Age
Year
ASFR
Below 0.025
0.025 to 0.05
0.05 to 0.1
0.1 to 0.15
0.15 to 0.20
0.20 to 0.25
0.25 to 0.30
0.30 to 0.35
0.35 to 0.40
0.40 to 0.45
0.45 and above

Supplemental Results Figure S3: Lexis diagram of CCF50, single-year age interval ASFR, and TFR by mother's age and location, 1950-2100, Afghanistan

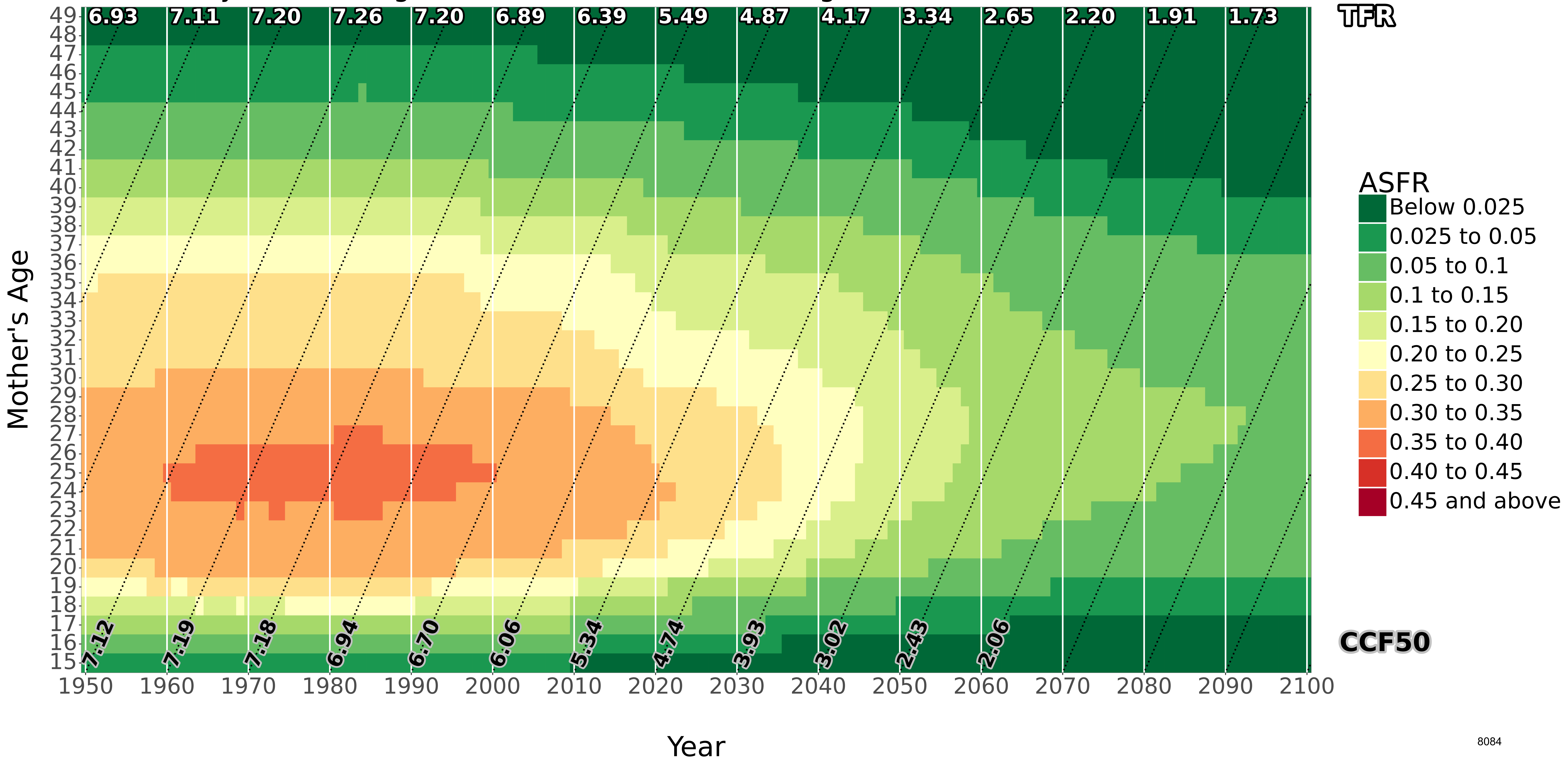

Supplemental Results Figure S3: Lexis diagram of CCF50, single-year age interval ASFR, and TFR by mother's age and location, 1950-2100, Algeria

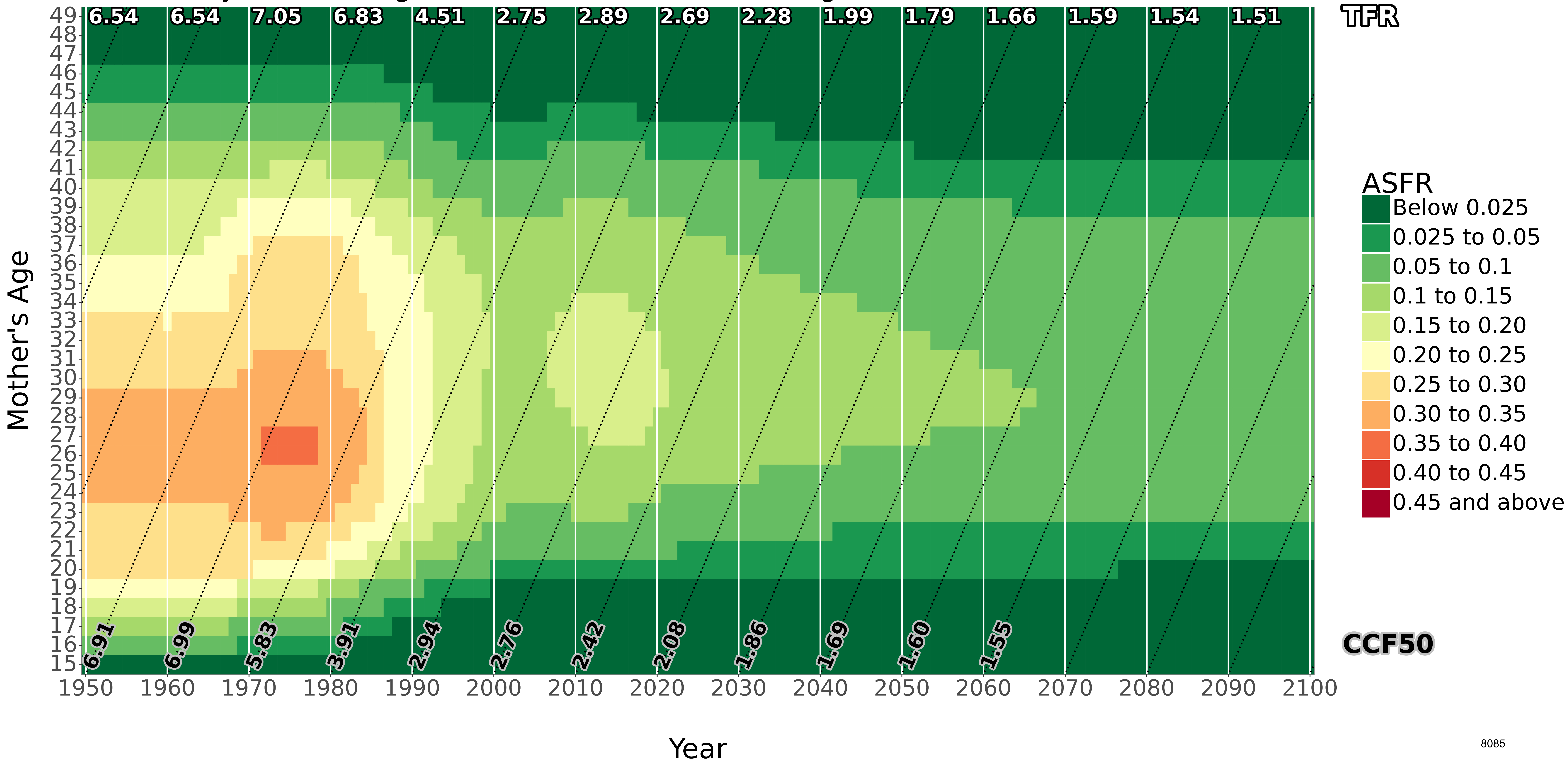

Supplemental Results Figure S3: Lexis diagram of CCF50, single-year age interval ASFR, and TFR by mother's age and location, 1950-2100, Bahrain

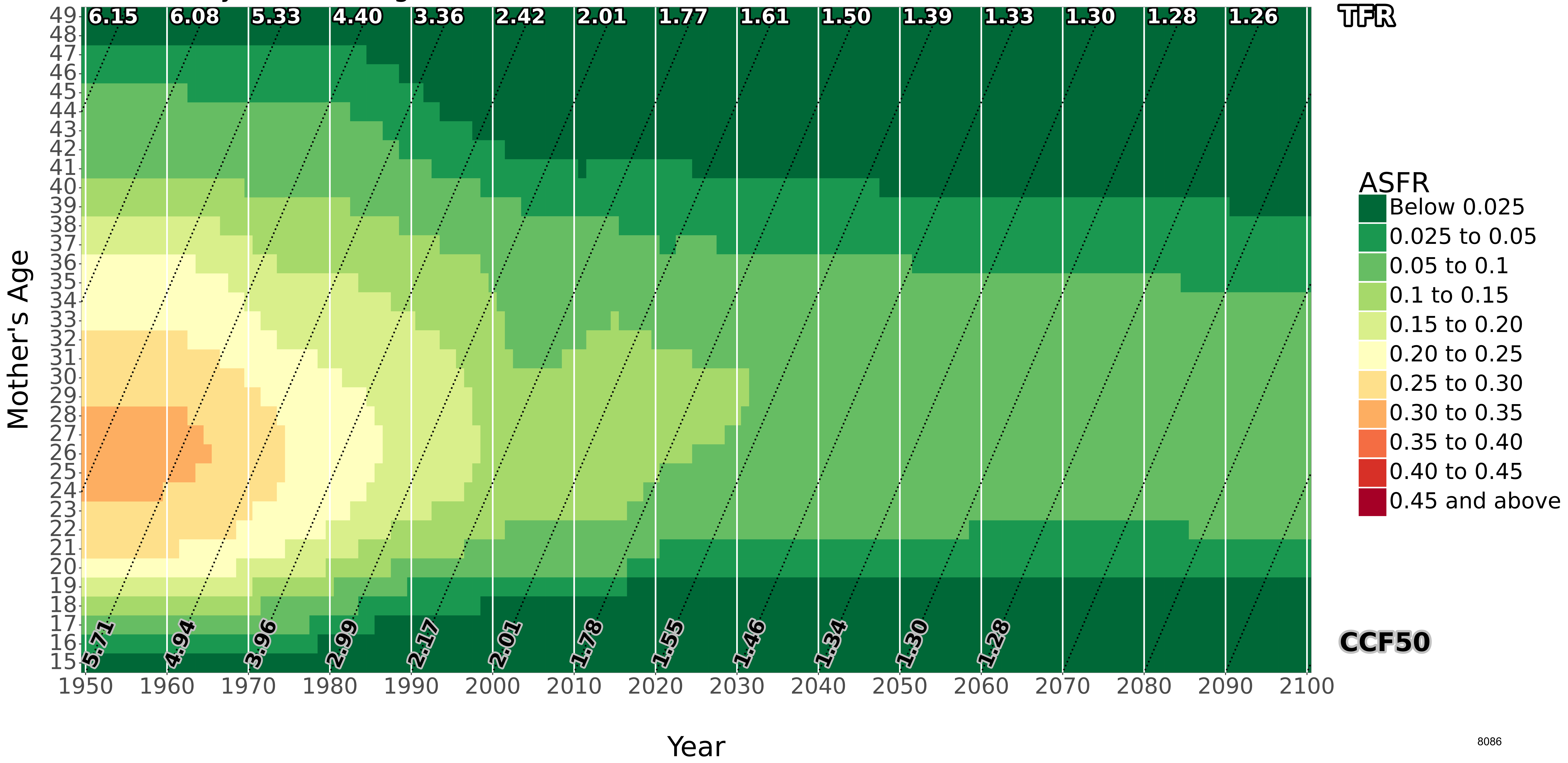

Supplemental Results Figure S3: Lexis diagram of CCF50, single-year age interval ASFR, and TFR by mother's age and location, 1950-2100, Egypt

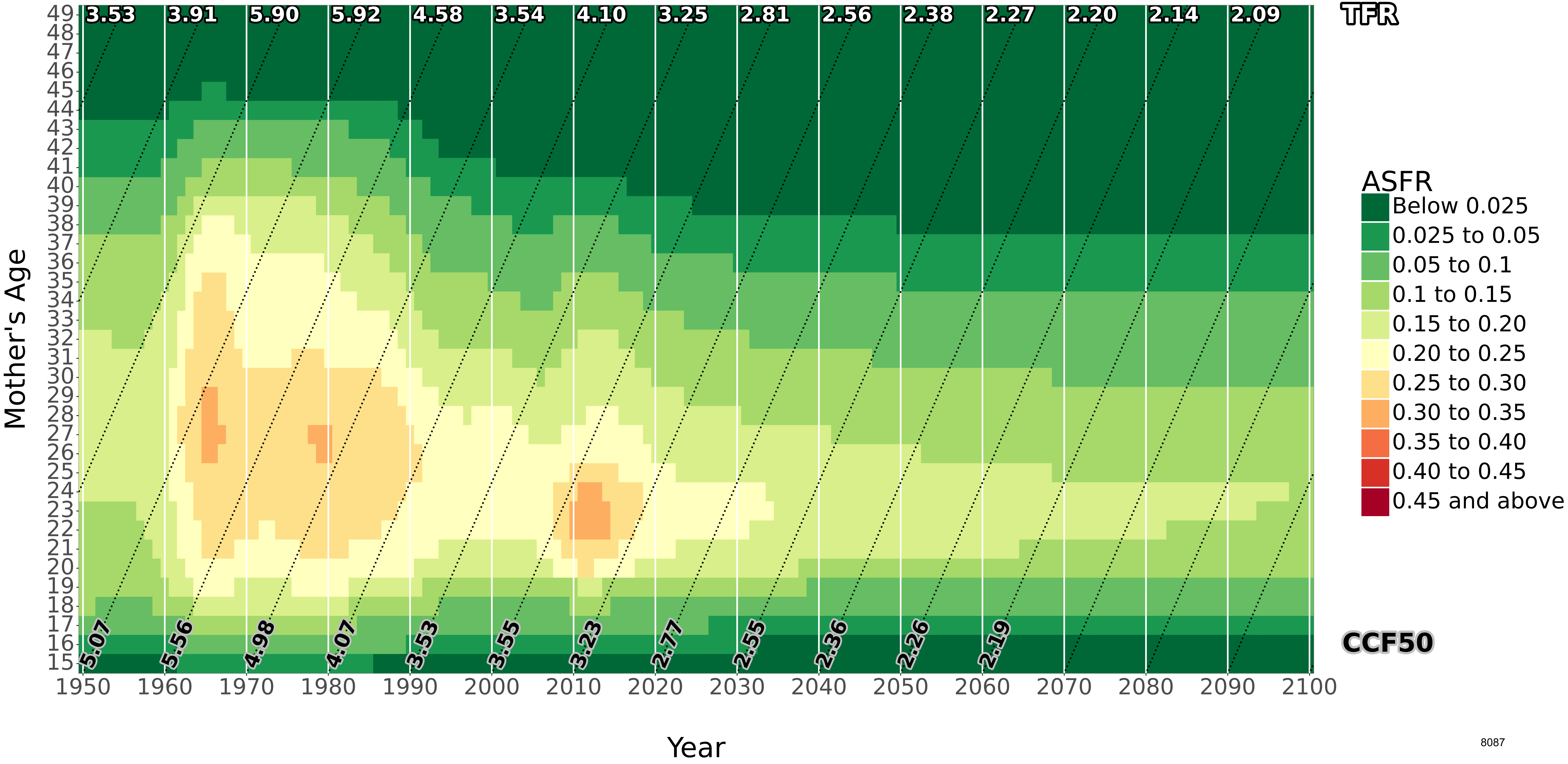

Supplemental Results Figure S3: Lexis diagram of CCF50, single-year age interval ASFR, and TFR by mother's age and location, 1950-2100, Iran

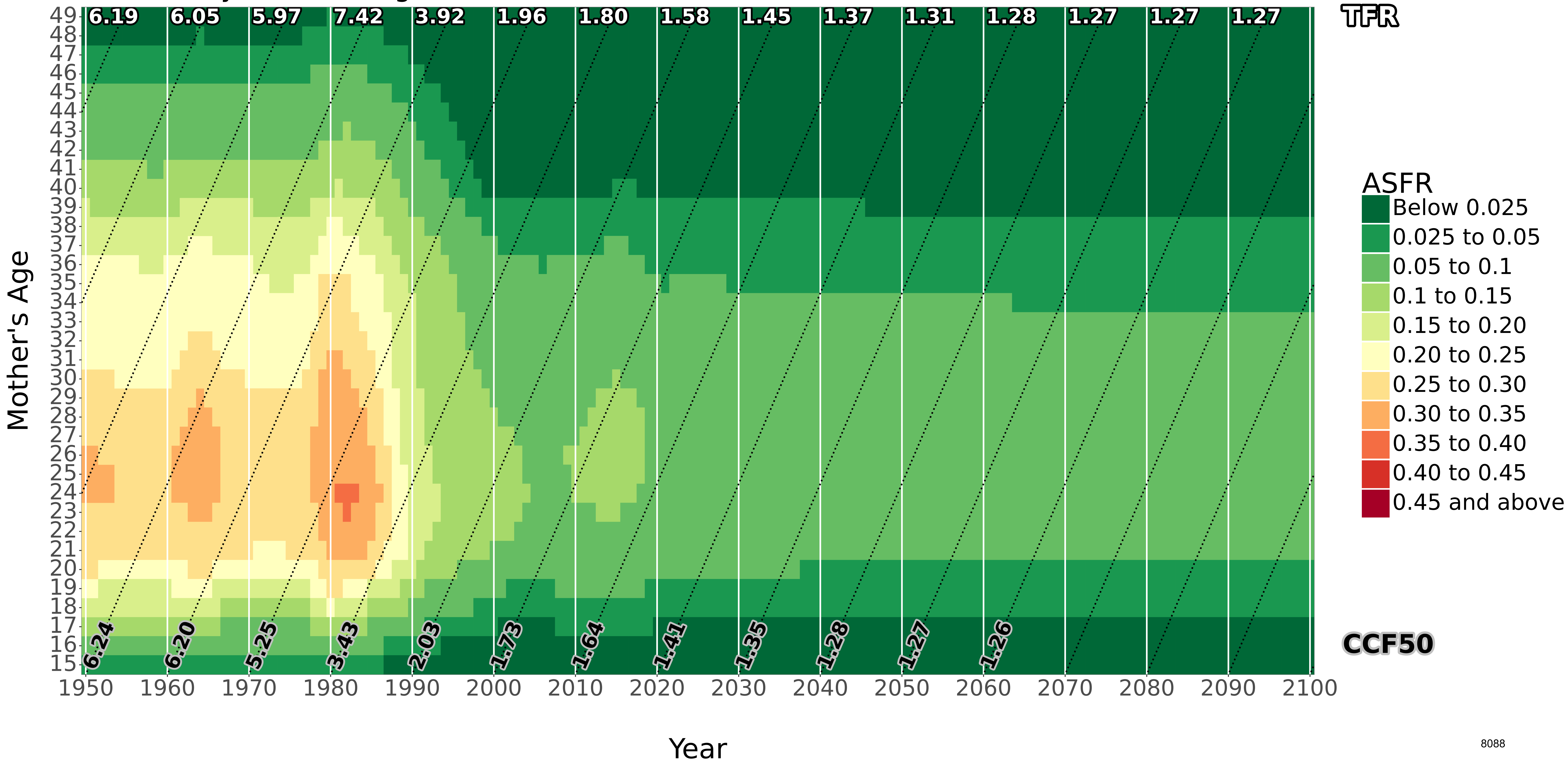

Supplemental Results Figure S3: Lexis diagram of CCF50, single-year age interval ASFR, and TFR by mother's age and location, 1950-2100, Iraq

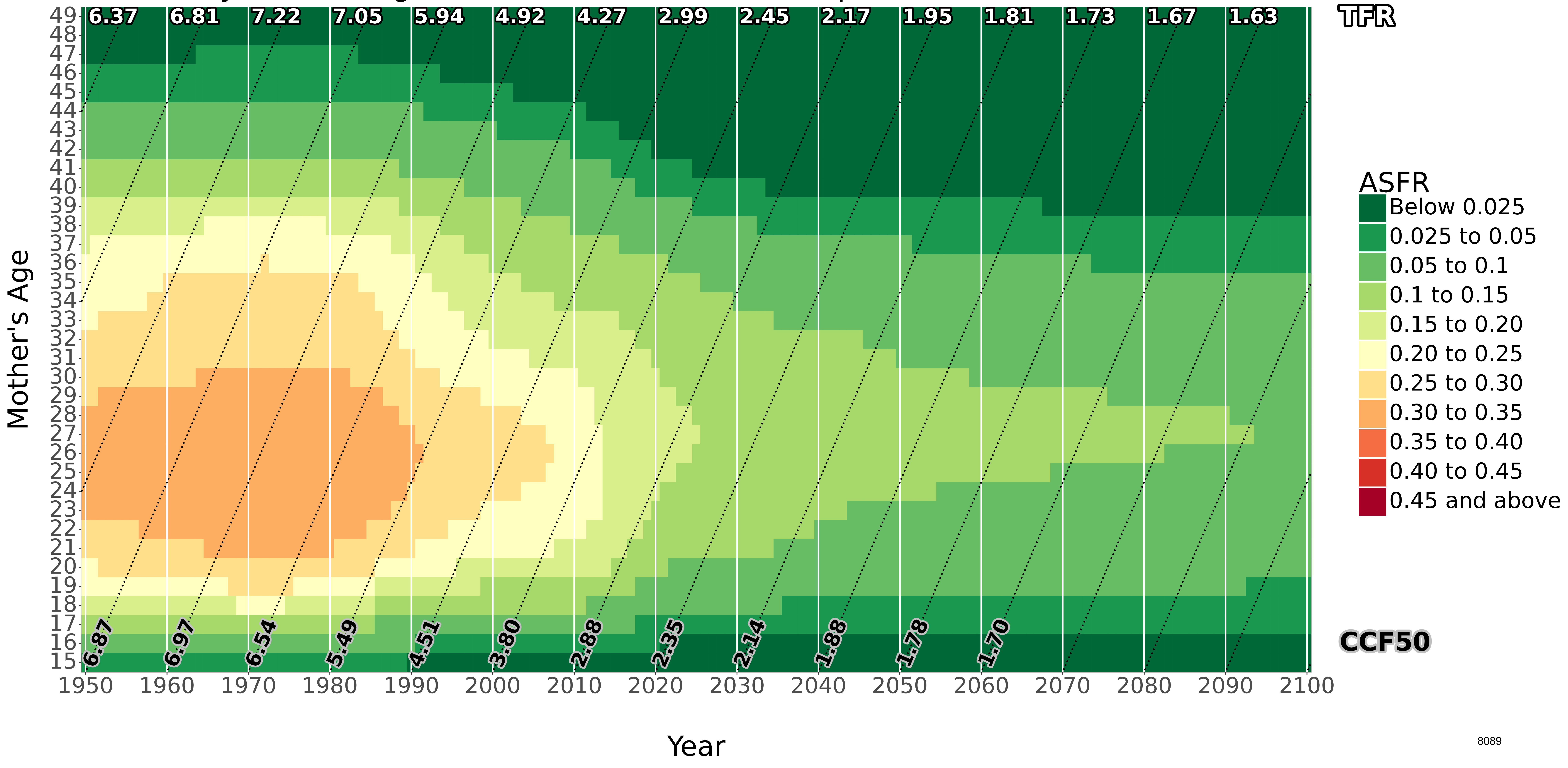

Supplemental Results Figure S3: Lexis diagram of CCF50, single-year age interval ASFR, and TFR by mother's age and location, 1950-2100, Jordan

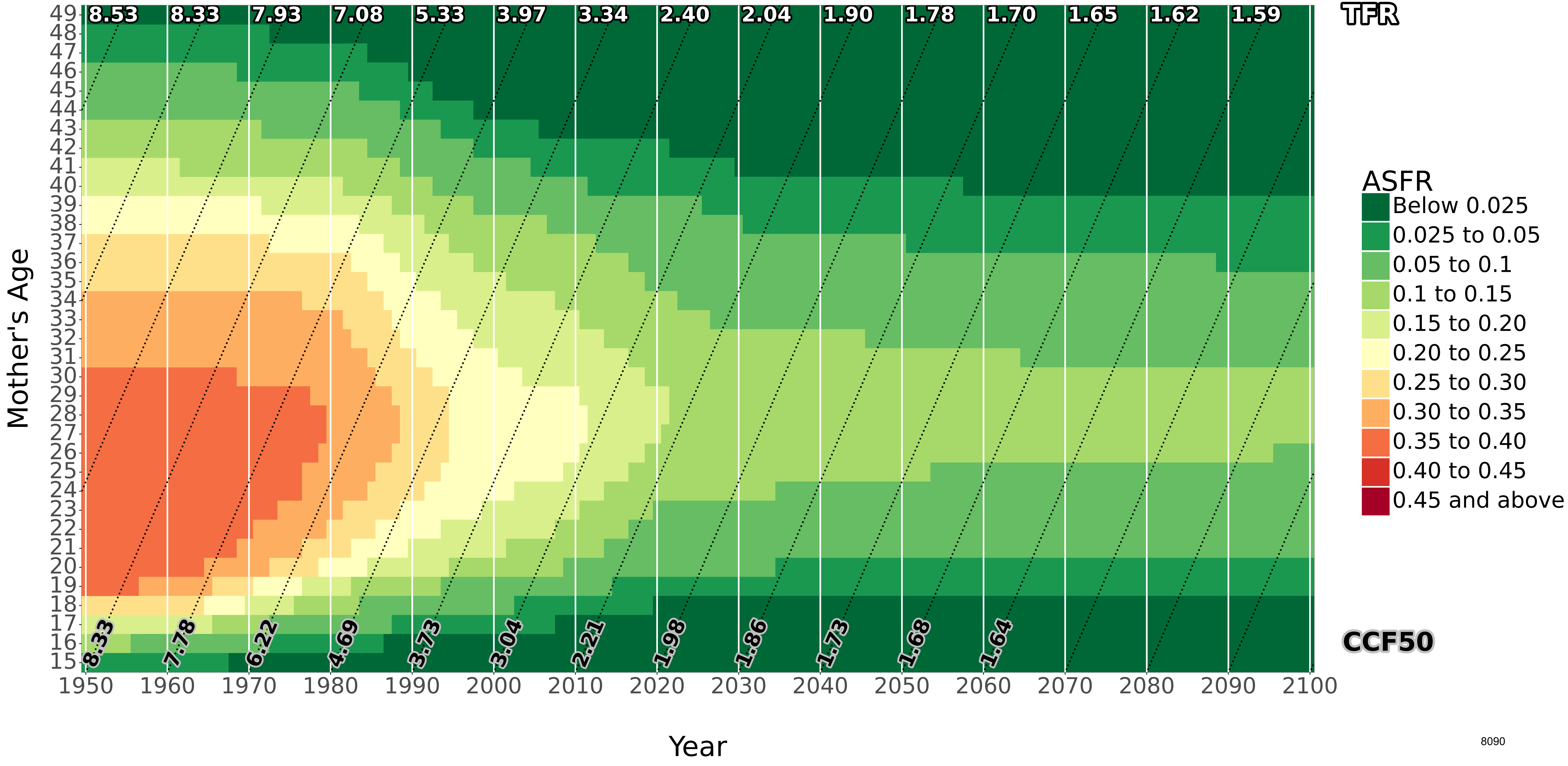

Supplemental Results Figure S3: Lexis diagram of CCF50, single-year age interval ASFR, and TFR by mother's age and location, 1950-2100, Kuwait

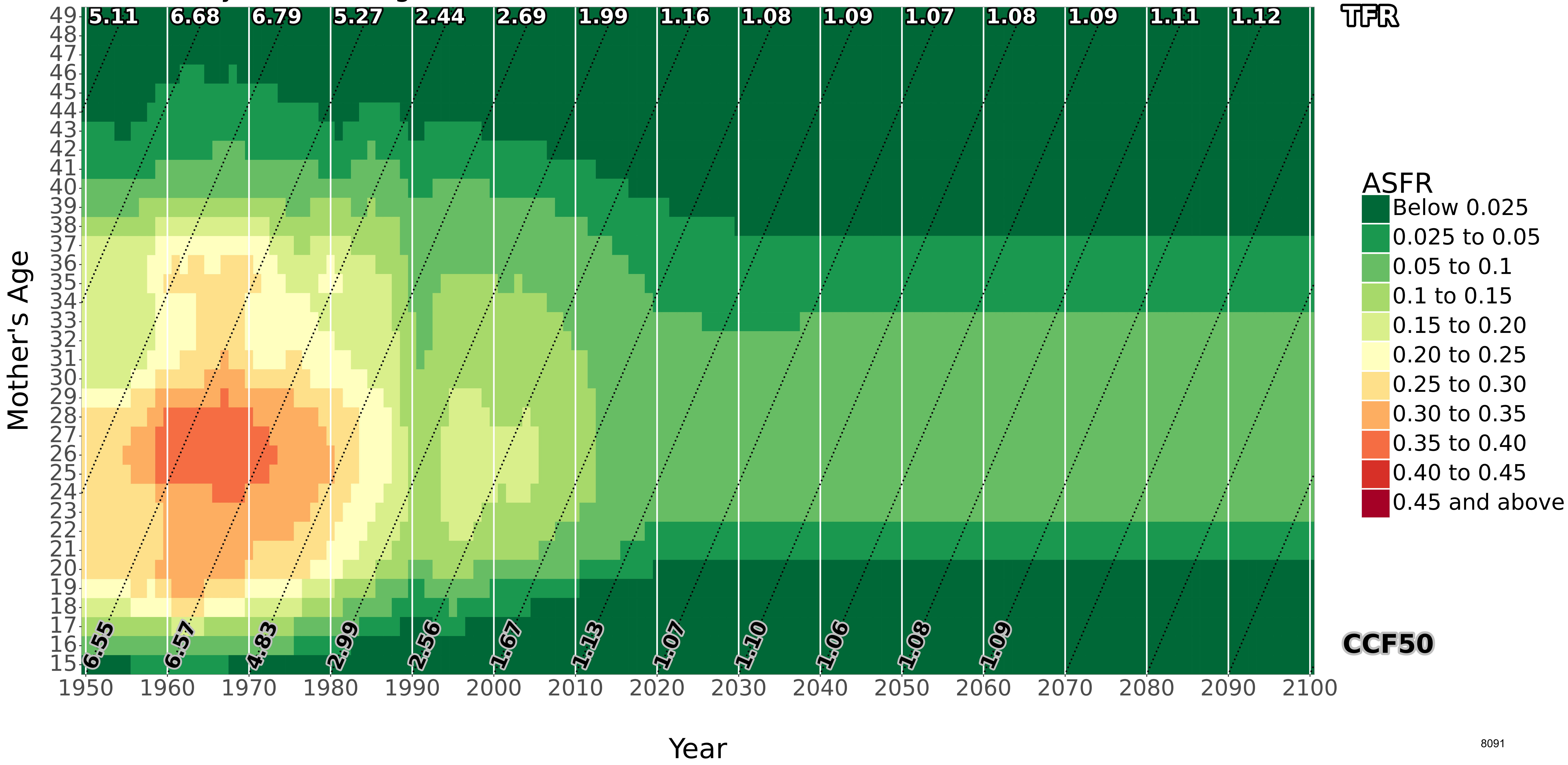

Supplemental Results Figure S3: Lexis diagram of CCF50, single-year age interval ASFR, and TFR by mother's age and location, 1950-2100, Lebanon

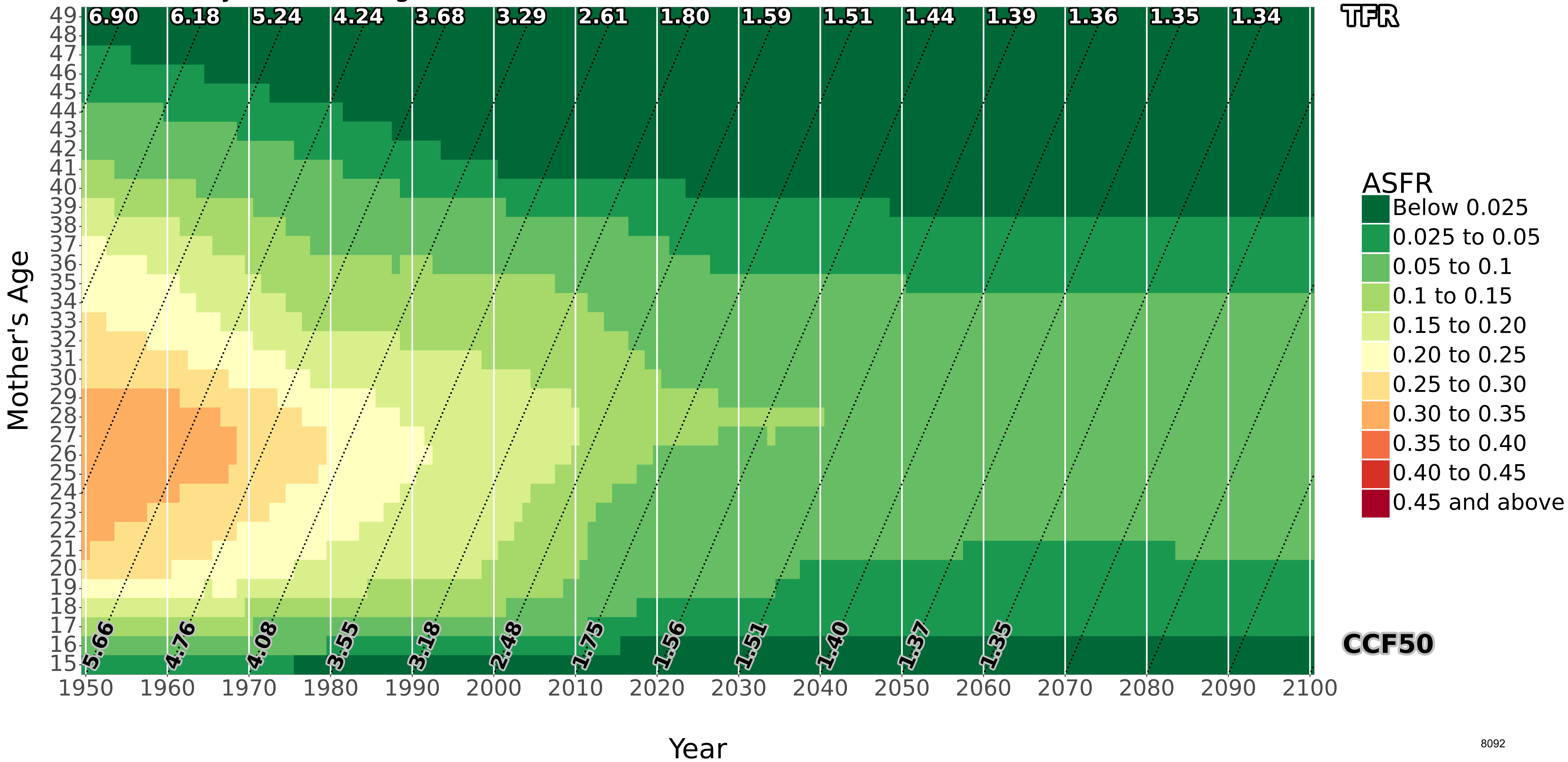

Supplemental Results Figure S3: Lexis diagram of CCF50, single-year age interval ASFR, and TFR by mother's age and location, 1950-2100, Libya

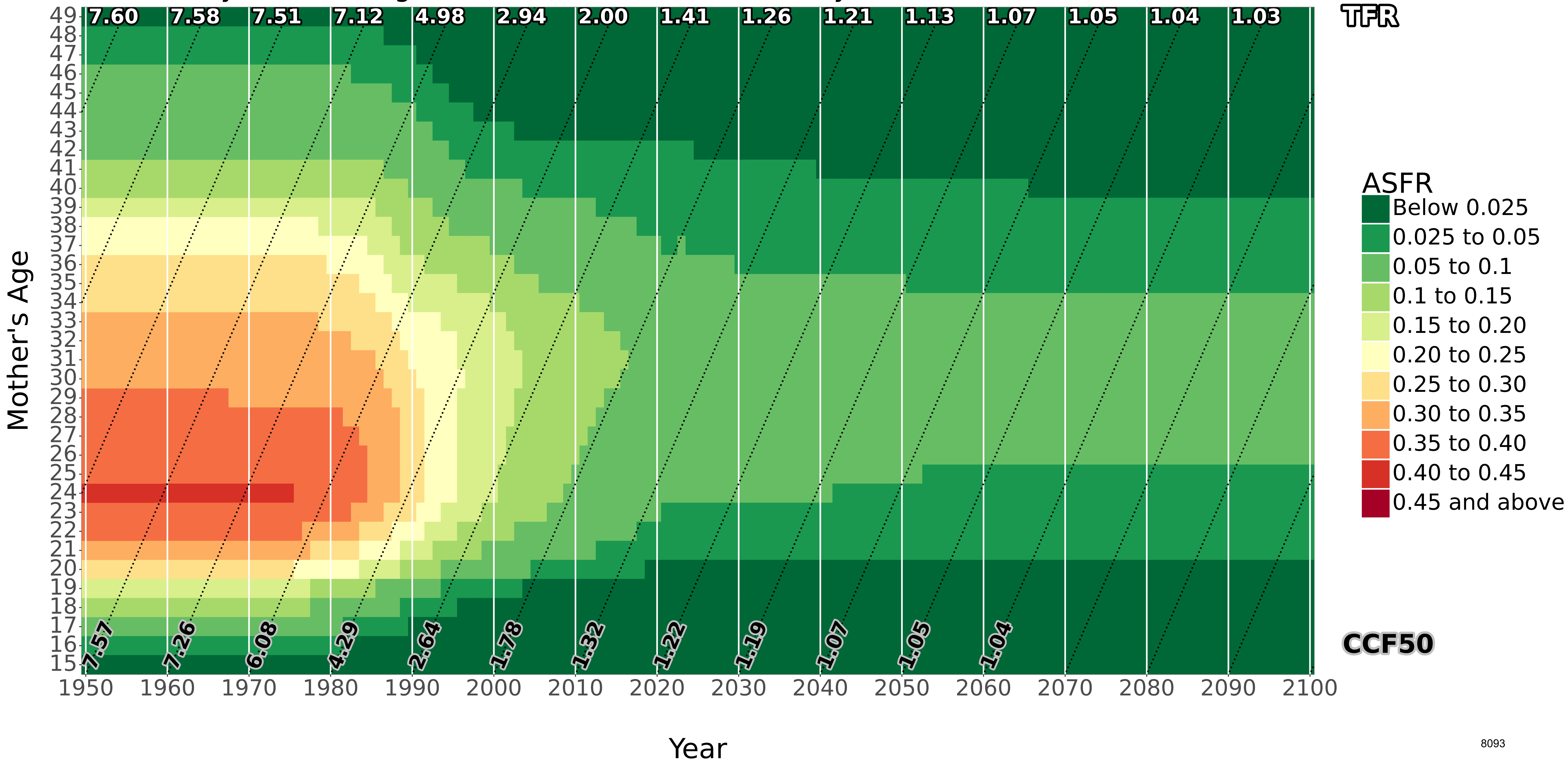

Supplemental Results Figure S3: Lexis diagram of CCF50, single-year age interval ASFR, and TFR by mother's age and location, 1950-2100, Morocco

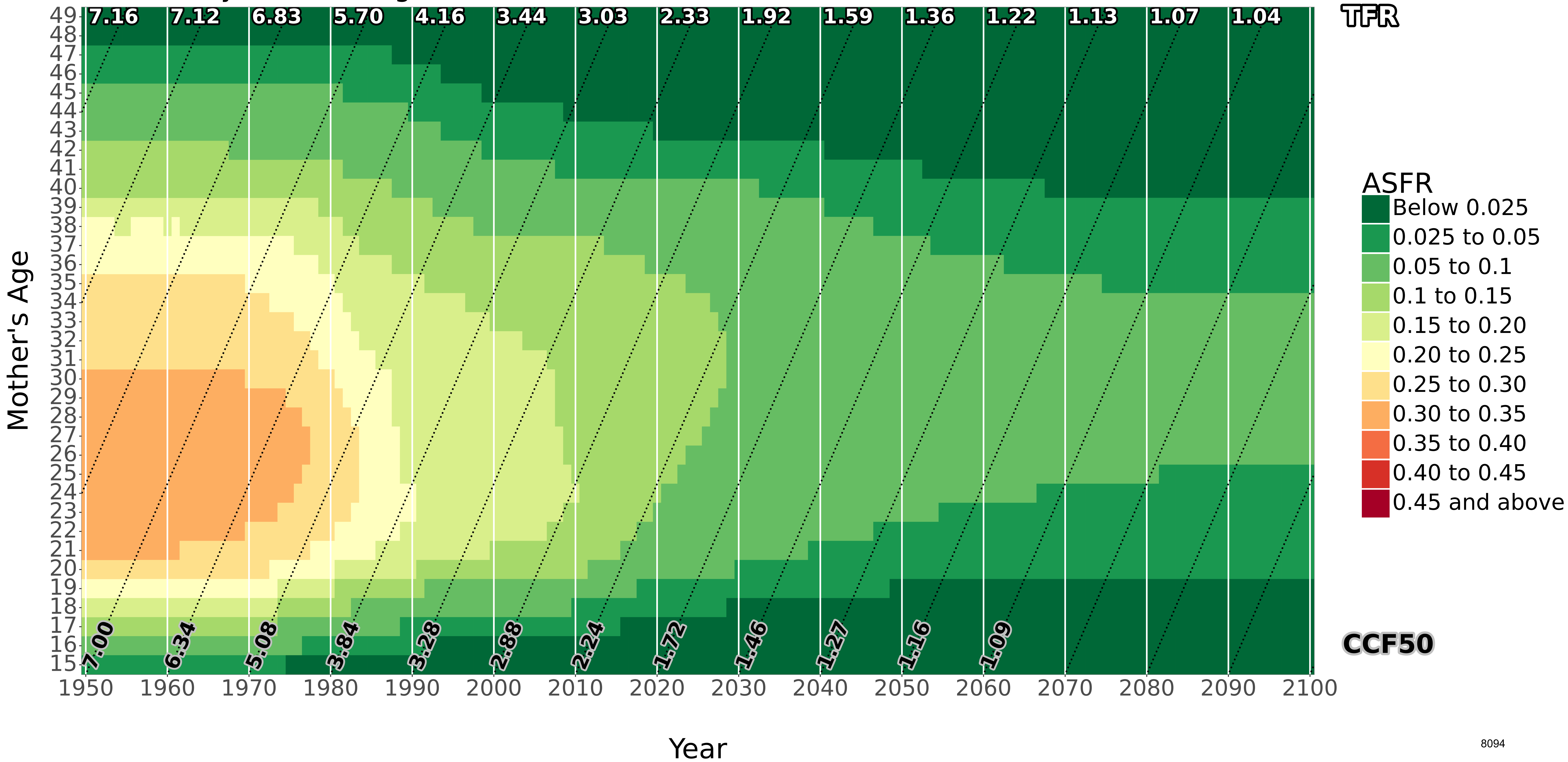

Supplemental Results Figure S3: Lexis diagram of CCF50, single-year age interval ASFR, and TFR by mother's age and location, 1950-2100, Oman

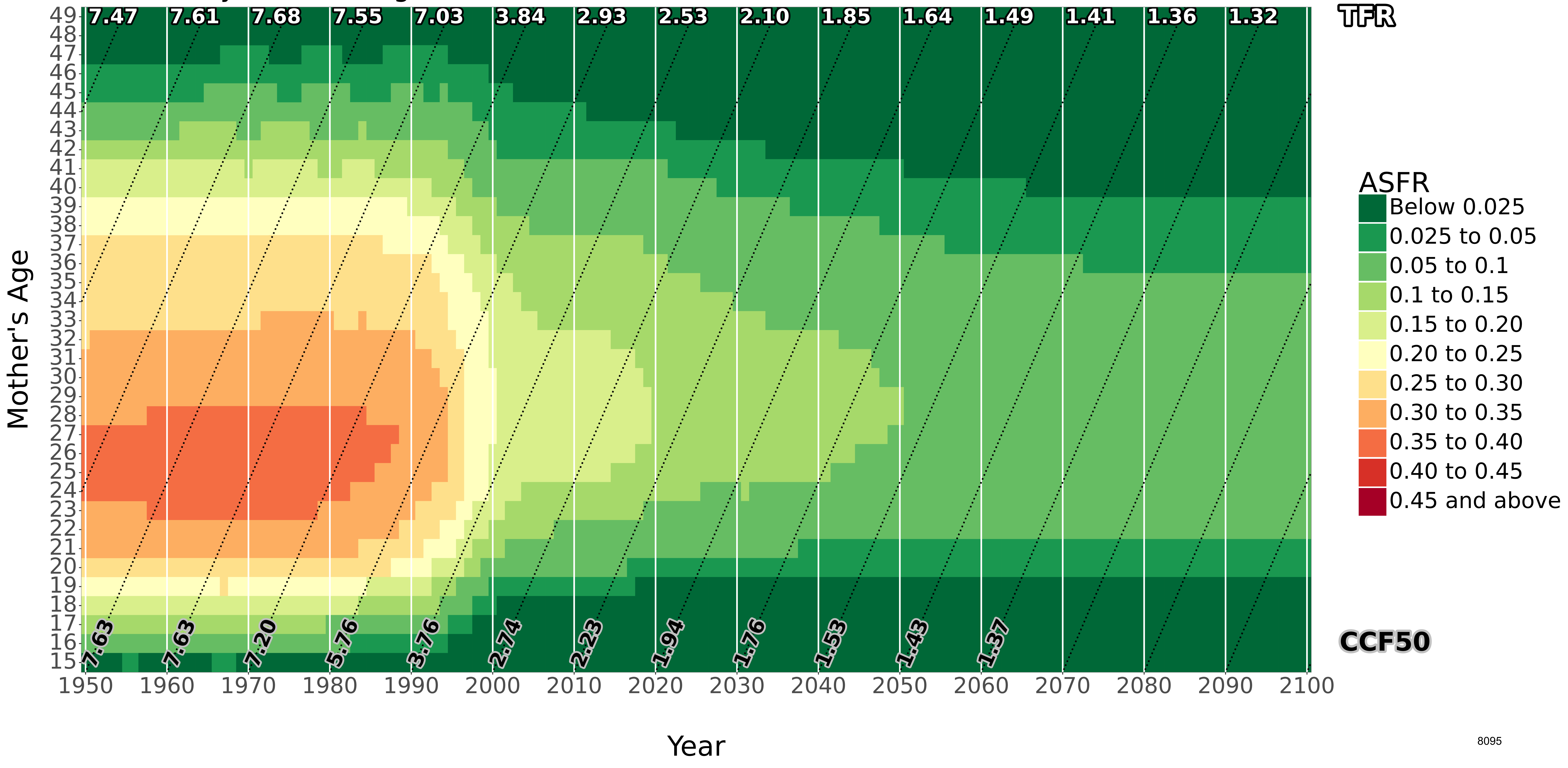

Supplemental Results Figure S3: Lexis diagram of CCF50, single-year age interval ASFR, and TFR by mother's age and location, 1950-2100, Palestine

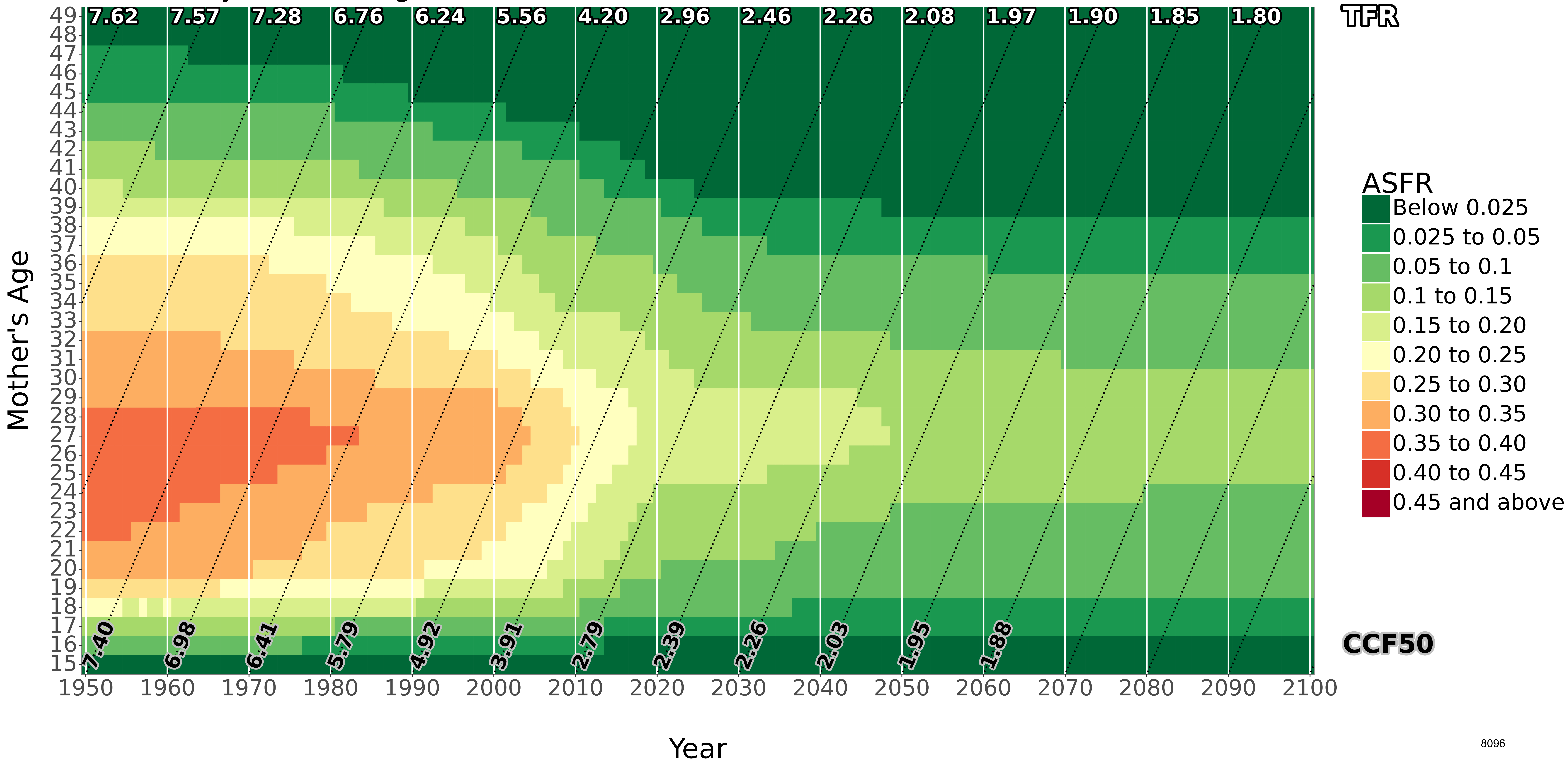

Supplemental Results Figure S3: Lexis diagram of CCF50, single-year age interval ASFR, and TFR by mother's age and location, 1950-2100, Qatar

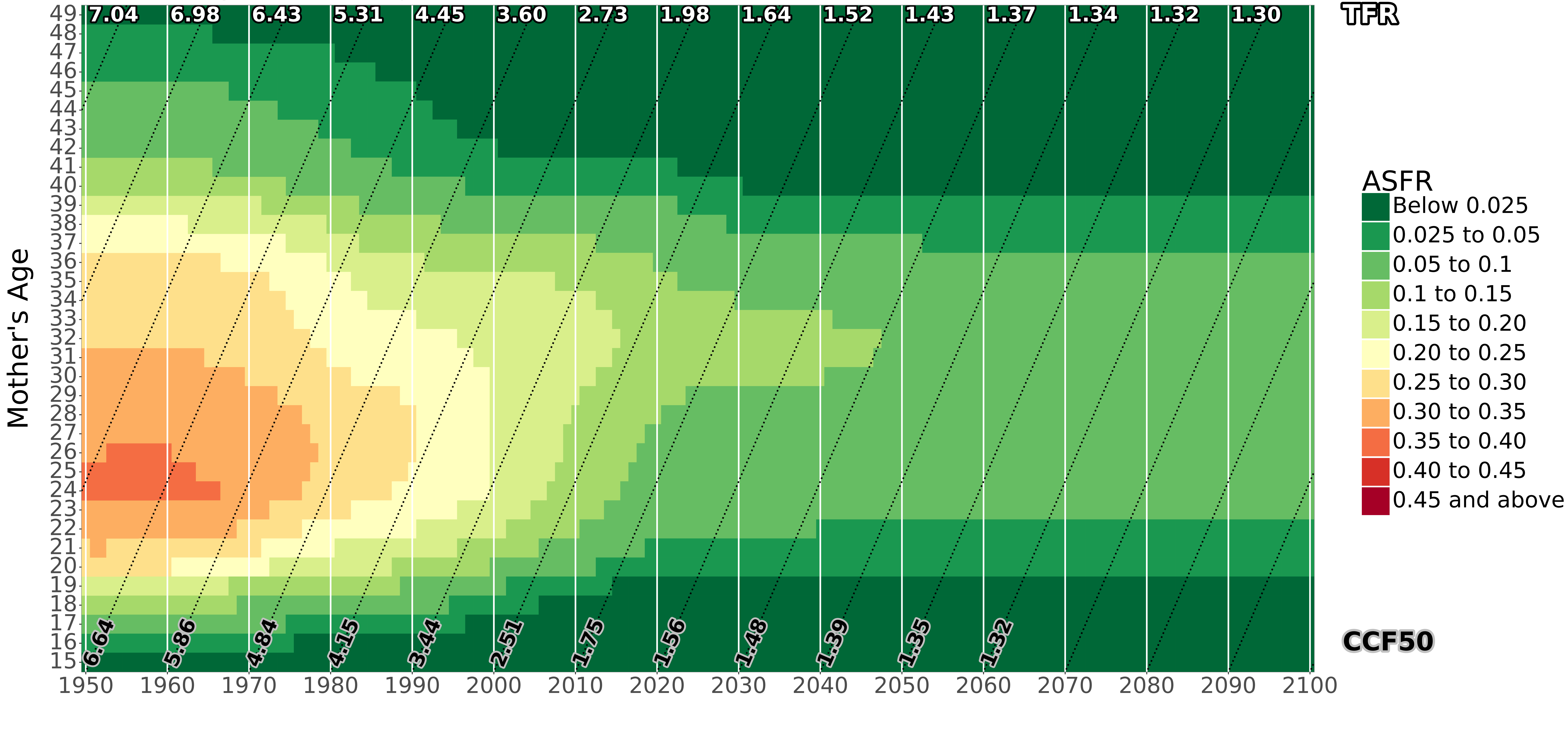

Supplemental Results Figure S3: Lexis diagram of CCF50, single-year age interval ASFR, and TFR by mother's age and location, 1950-2100, Saudi Arabia

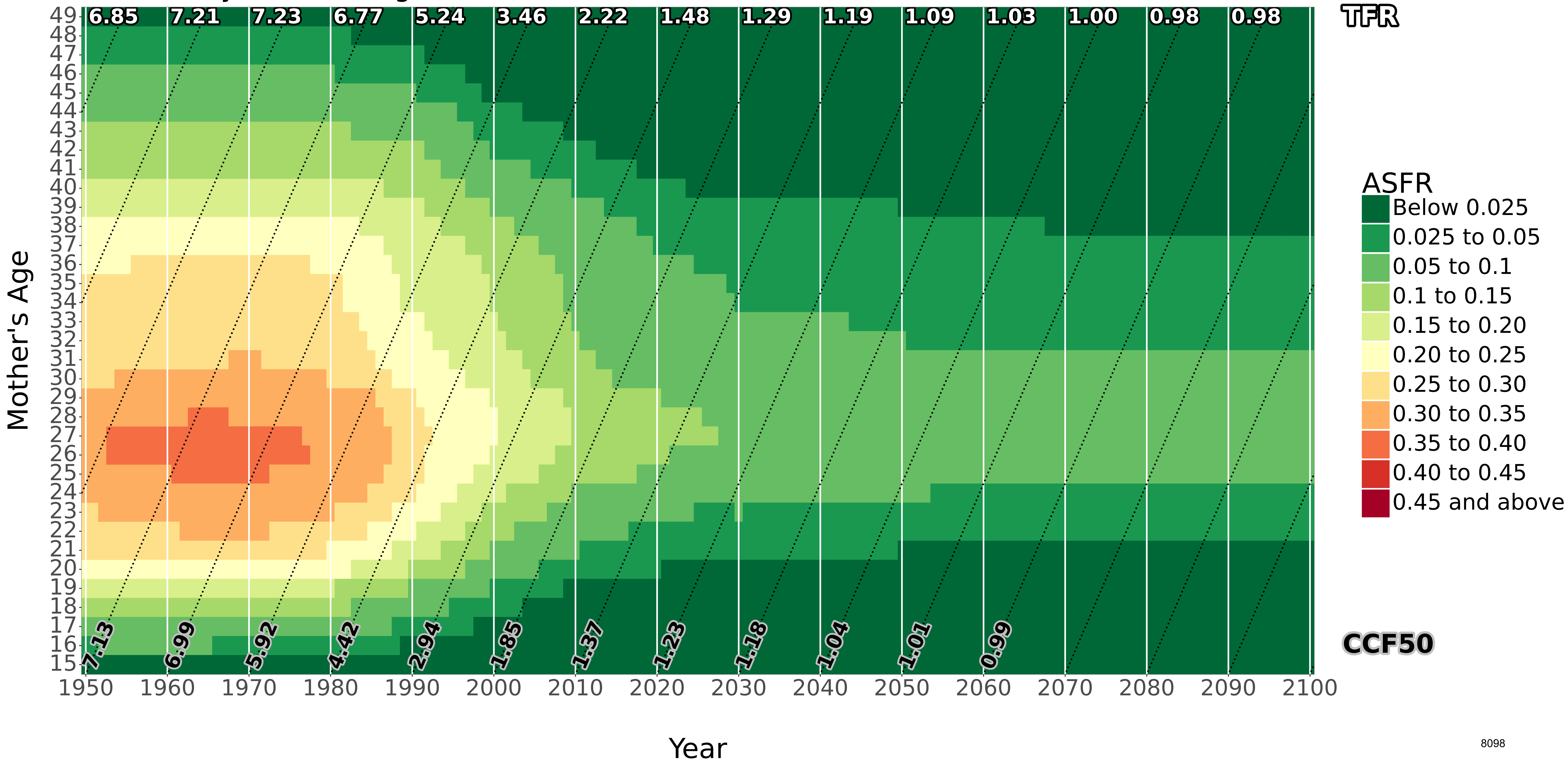

8098

Supplemental Results Figure S3: Lexis diagram of CCF50, single-year age interval ASFR, and TFR by mother's age and location, 1950-2100, Sudan

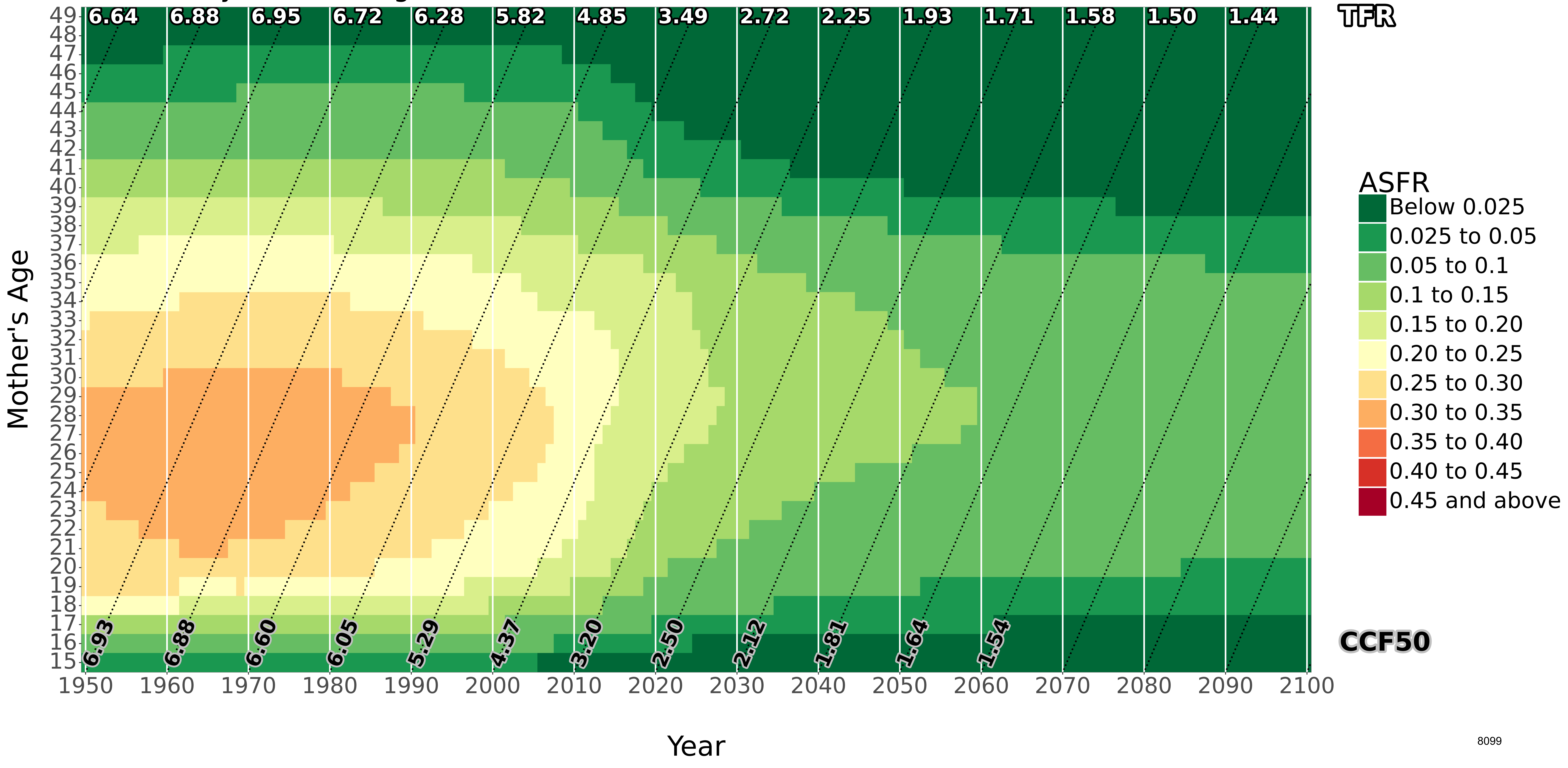

Supplemental Results Figure S3: Lexis diagram of CCF50, single-year age interval ASFR, and TFR by mother's age and location, 1950-2100, Syria

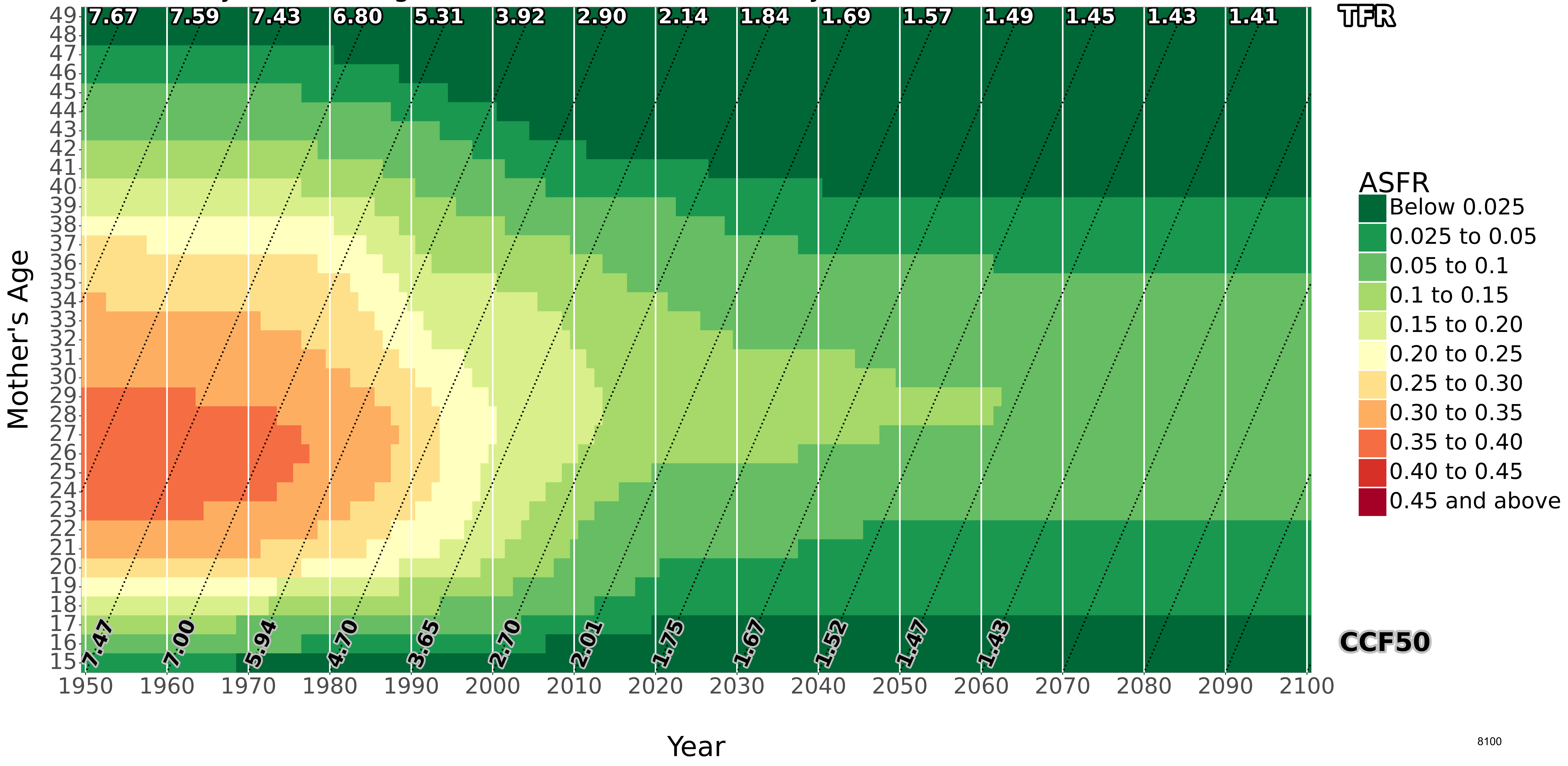

Supplemental Results Figure S3: Lexis diagram of CCF50, single-year age interval ASFR, and TFR by mother's age and location, 1950-2100, Tunisia

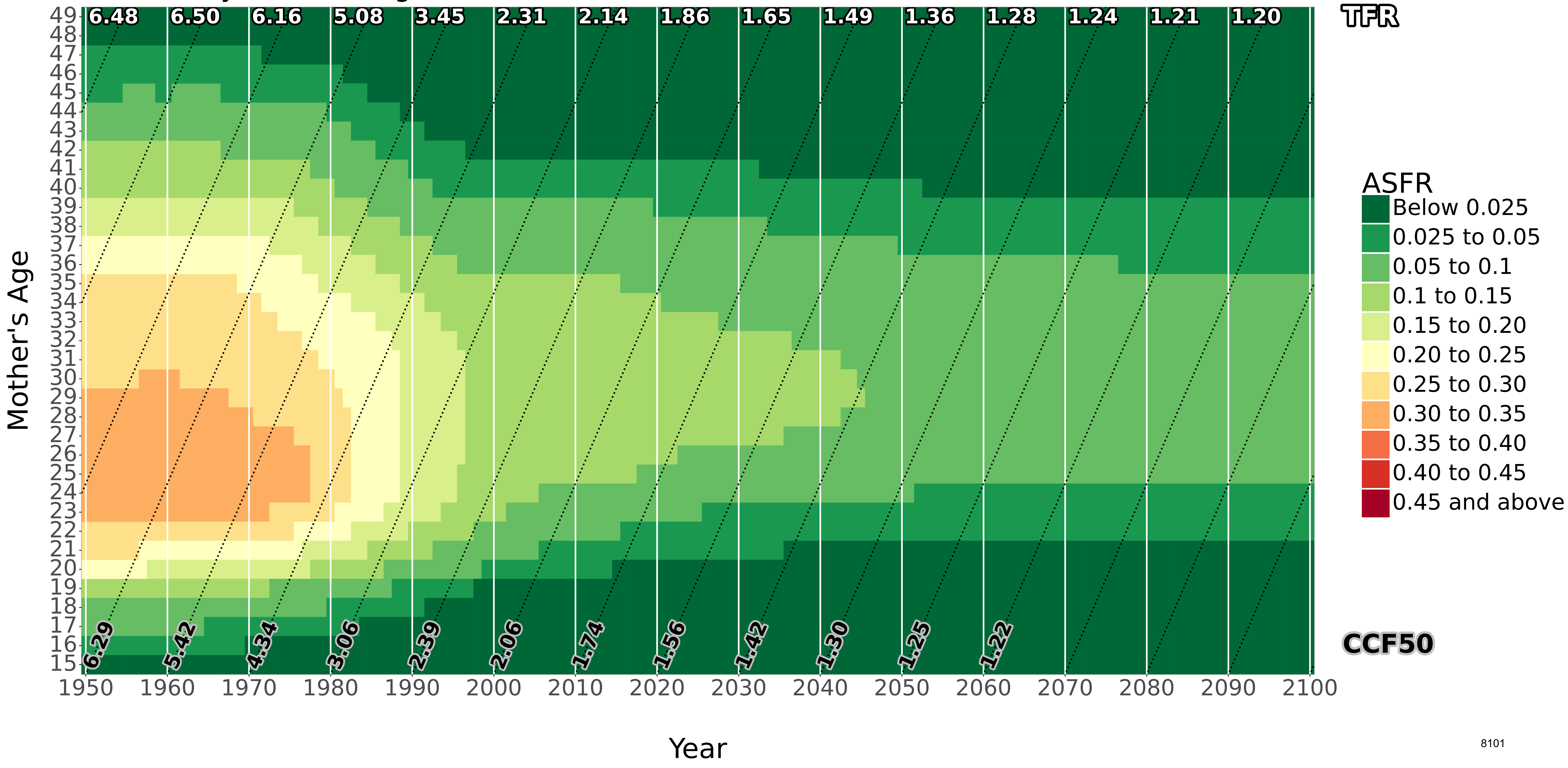

Supplemental Results Figure S3: Lexis diagram of CCF50, single-year age interval ASFR, and TFR by mother's age and location, 1950-2100, Türkiye

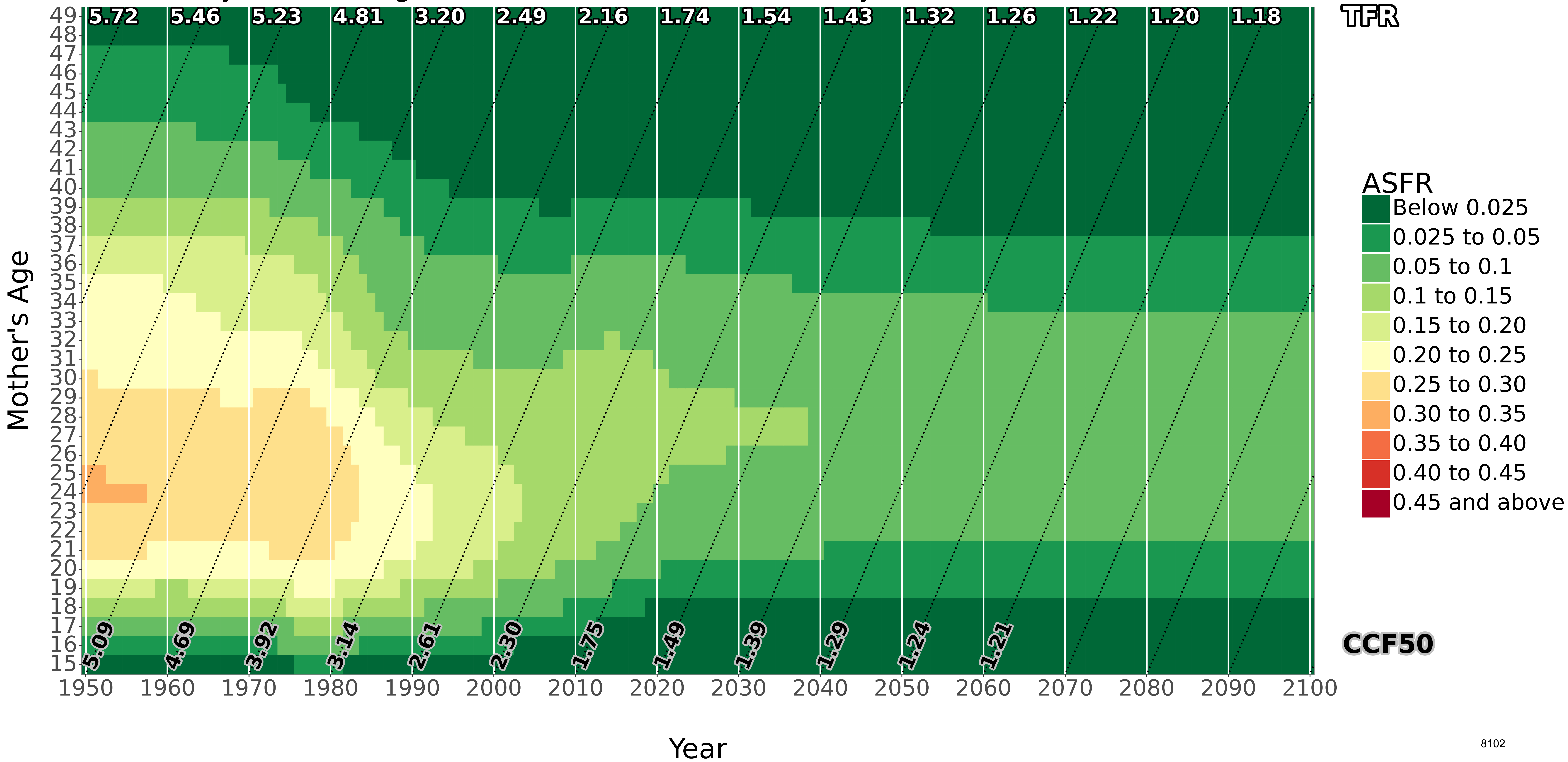

Supplemental Results Figure S3: Lexis diagram of CCF50, single-year age interval ASFR, and TFR by mother's age and location, 1950-2100, United Arab Emirates

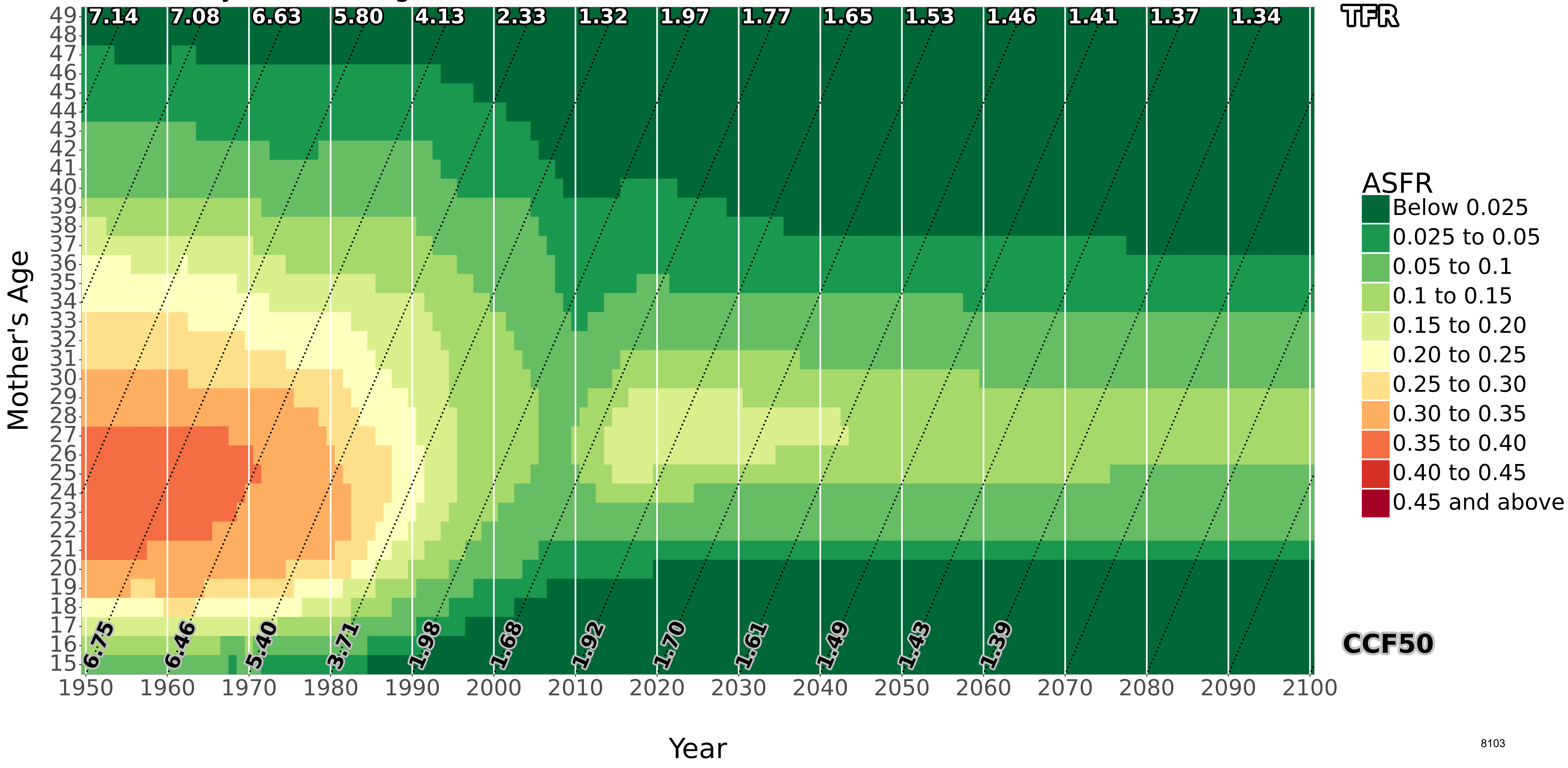

Supplemental Results Figure S3: Lexis diagram of CCF50, single-year age interval ASFR, and TFR by mother's age and location, 1950-2100, Yemen

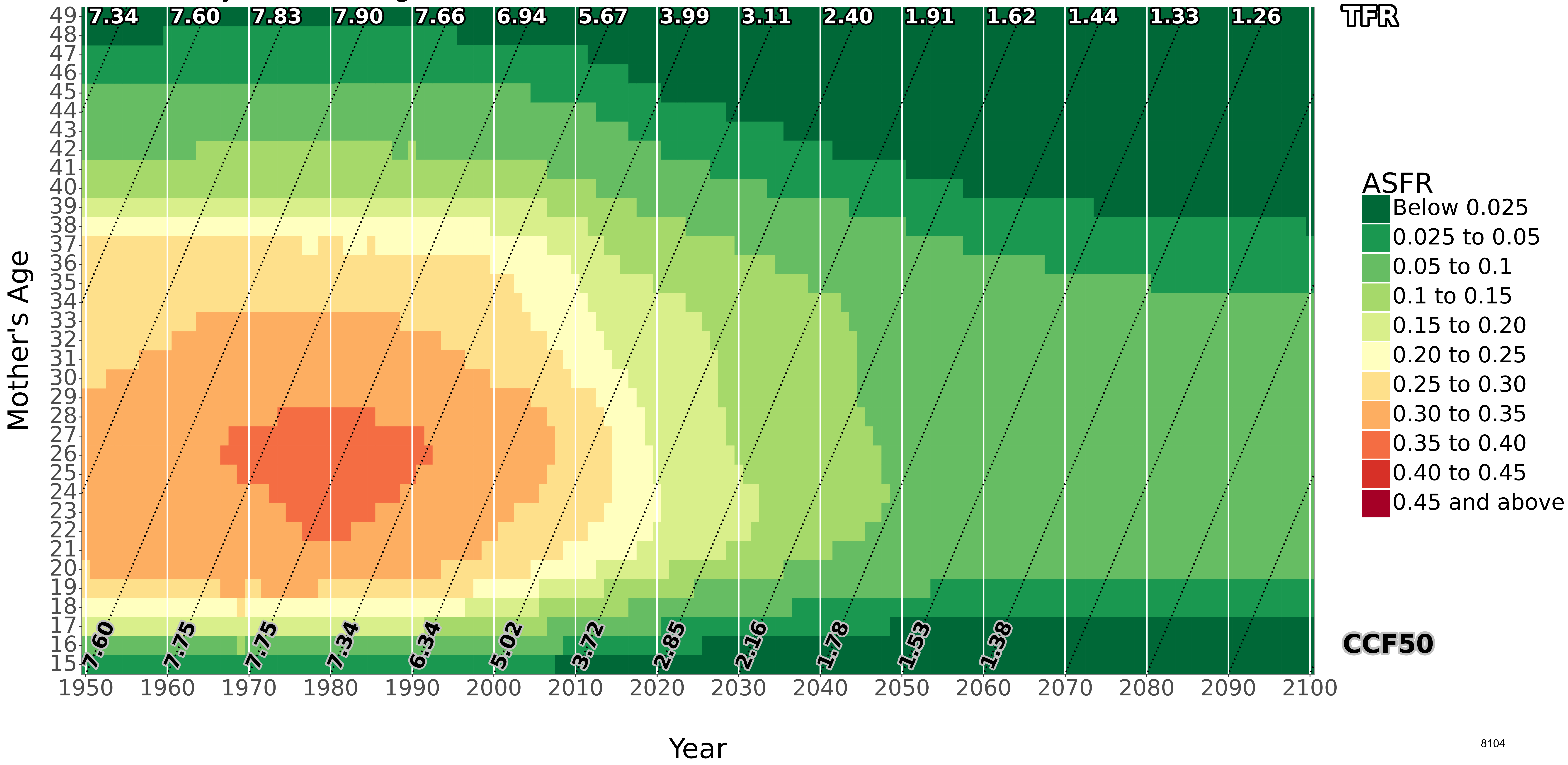

Supplemental Results Figure S3: Lexis diagram of CCF50, single-year age interval ASFR, and TFR by mother's age and location, 1950-2100, Bangladesh

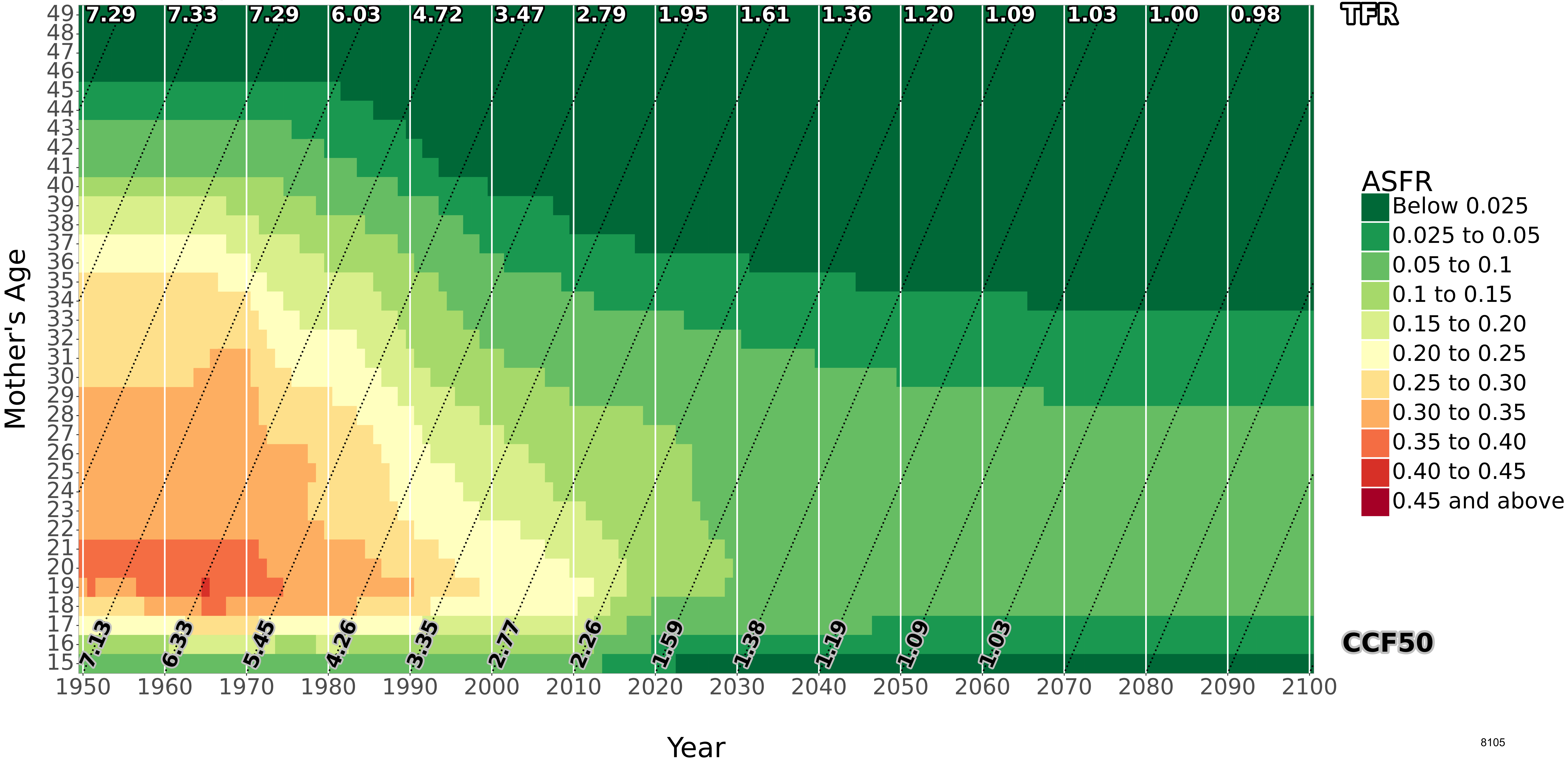

Supplemental Results Figure S3: Lexis diagram of CCF50, single-year age interval ASFR, and TFR by mother's age and location, 1950-2100, Bhutan

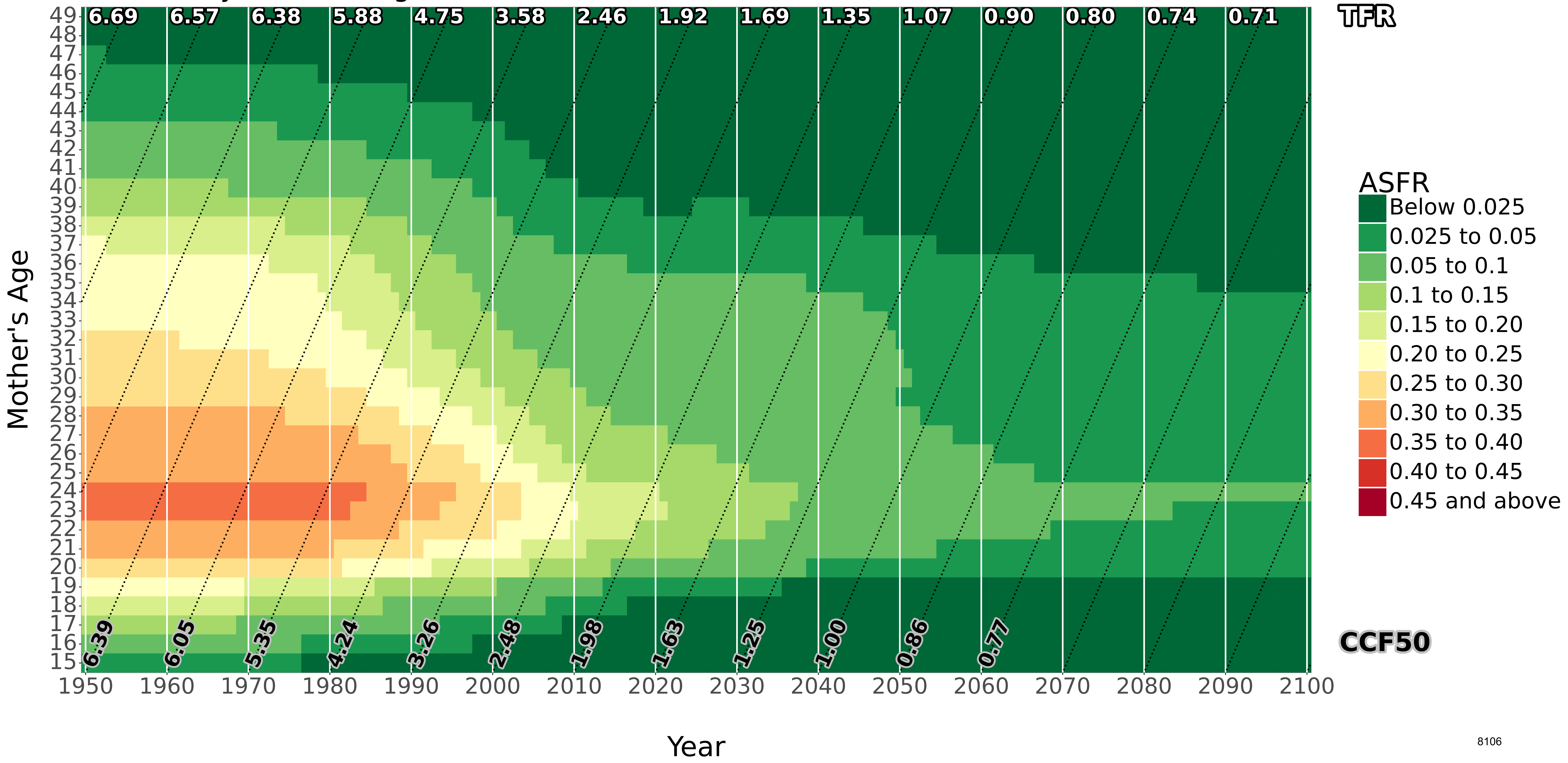

8106

Supplemental Results Figure S3: Lexis diagram of CCF50, single-year age interval ASFR, and TFR by mother's age and location, 1950-2100, India

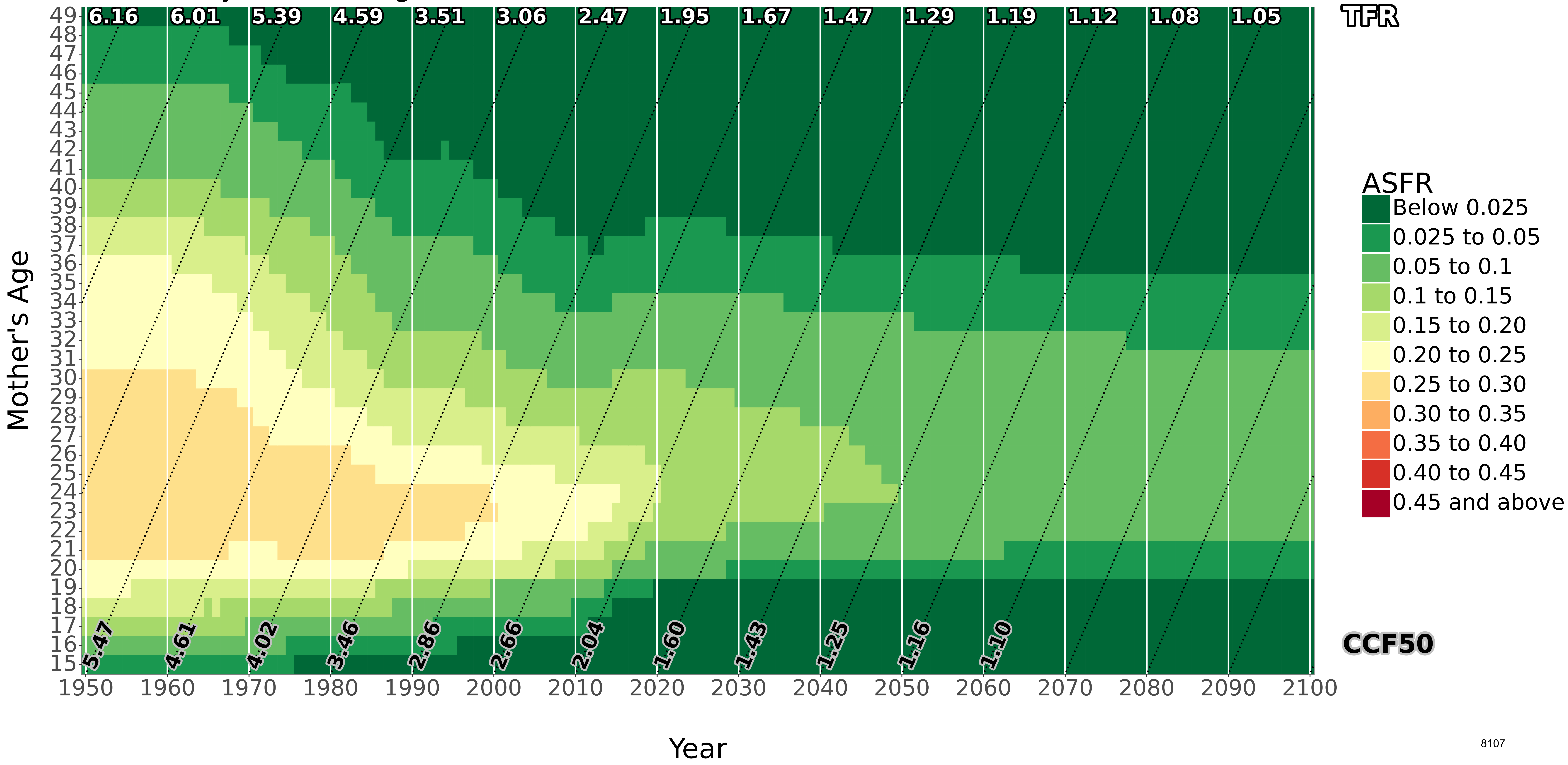

Supplemental Results Figure S3: Lexis diagram of CCF50, single-year age interval ASFR, and TFR by mother's age and location, 1950-2100, Nepal

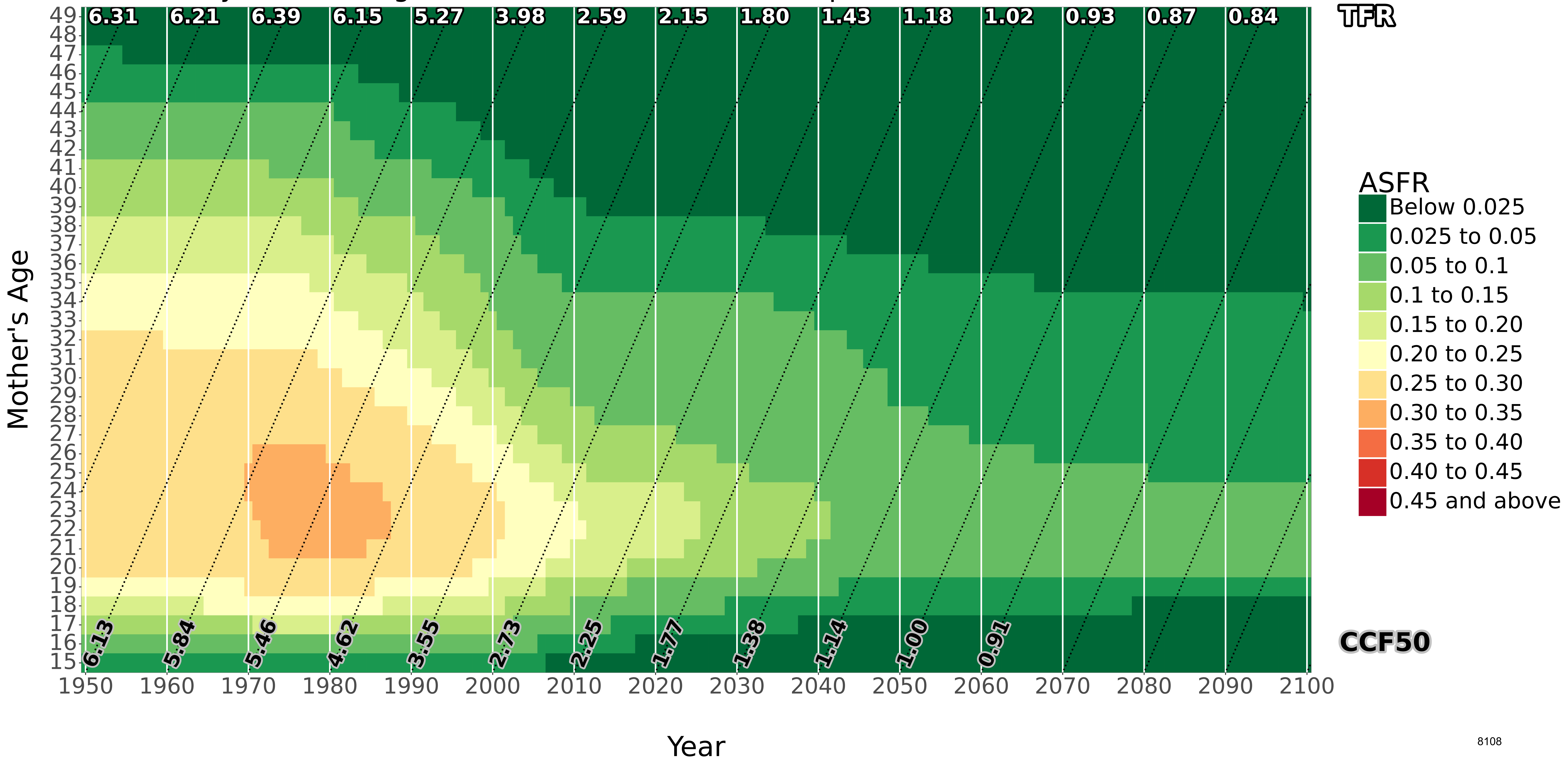

Supplemental Results Figure S3: Lexis diagram of CCF50, single-year age interval ASFR, and TFR by mother's age and location, 1950-2100, Pakistan

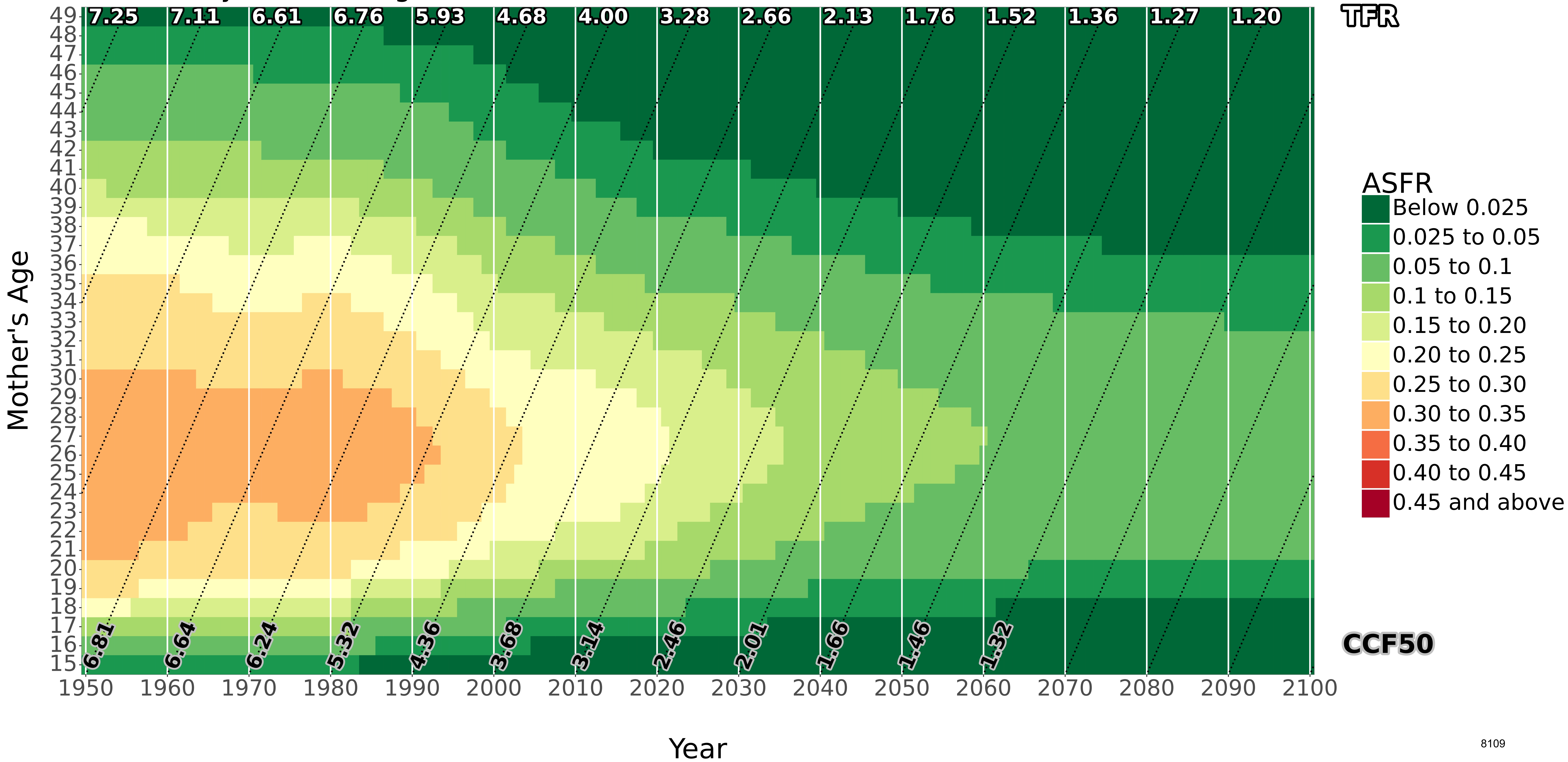

Supplemental Results Figure S3: Lexis diagram of CCF50, single-year age interval ASFR, and TFR by mother's age and location, 1950-2100, China

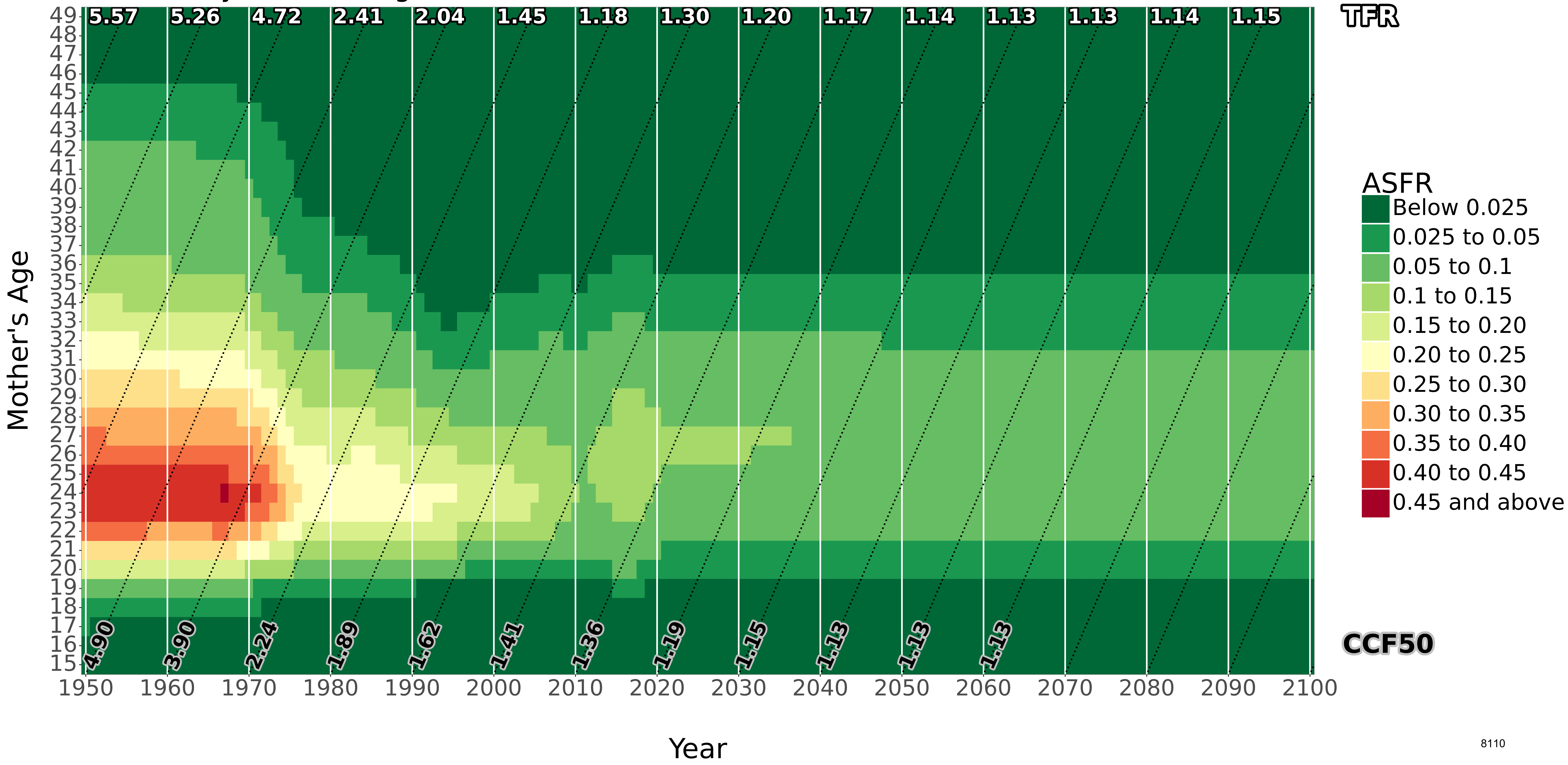

Supplemental Results Figure S3: Lexis diagram of CCF50, single-year age interval ASFR, and TFR by mother's age and location, 1950-2100, North Korea

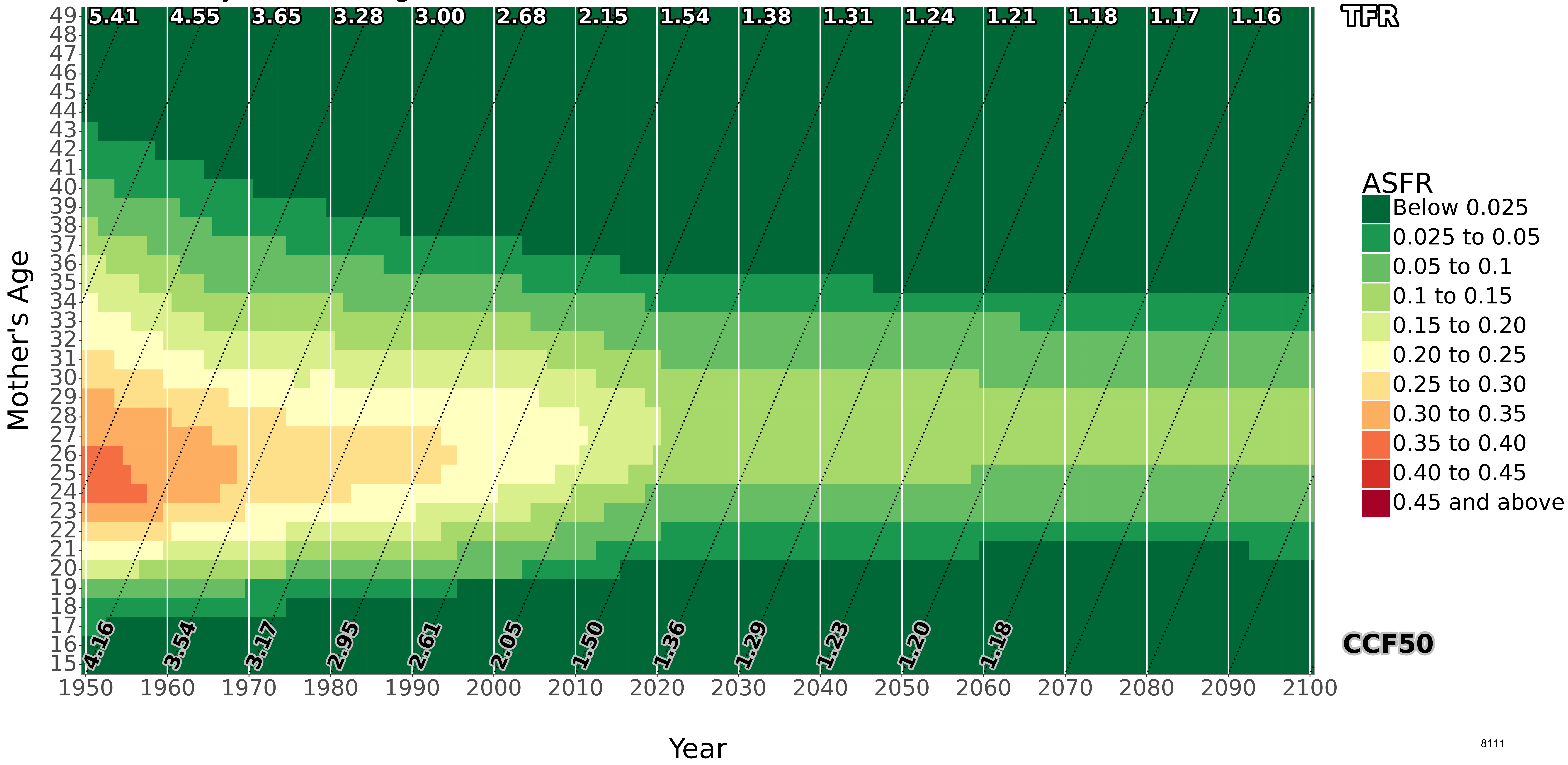

8111

Supplemental Results Figure S3: Lexis diagram of CCF50, single-year age interval ASFR, and TFR by mother's age and location, 1950-2100, Taiwan (province of China)

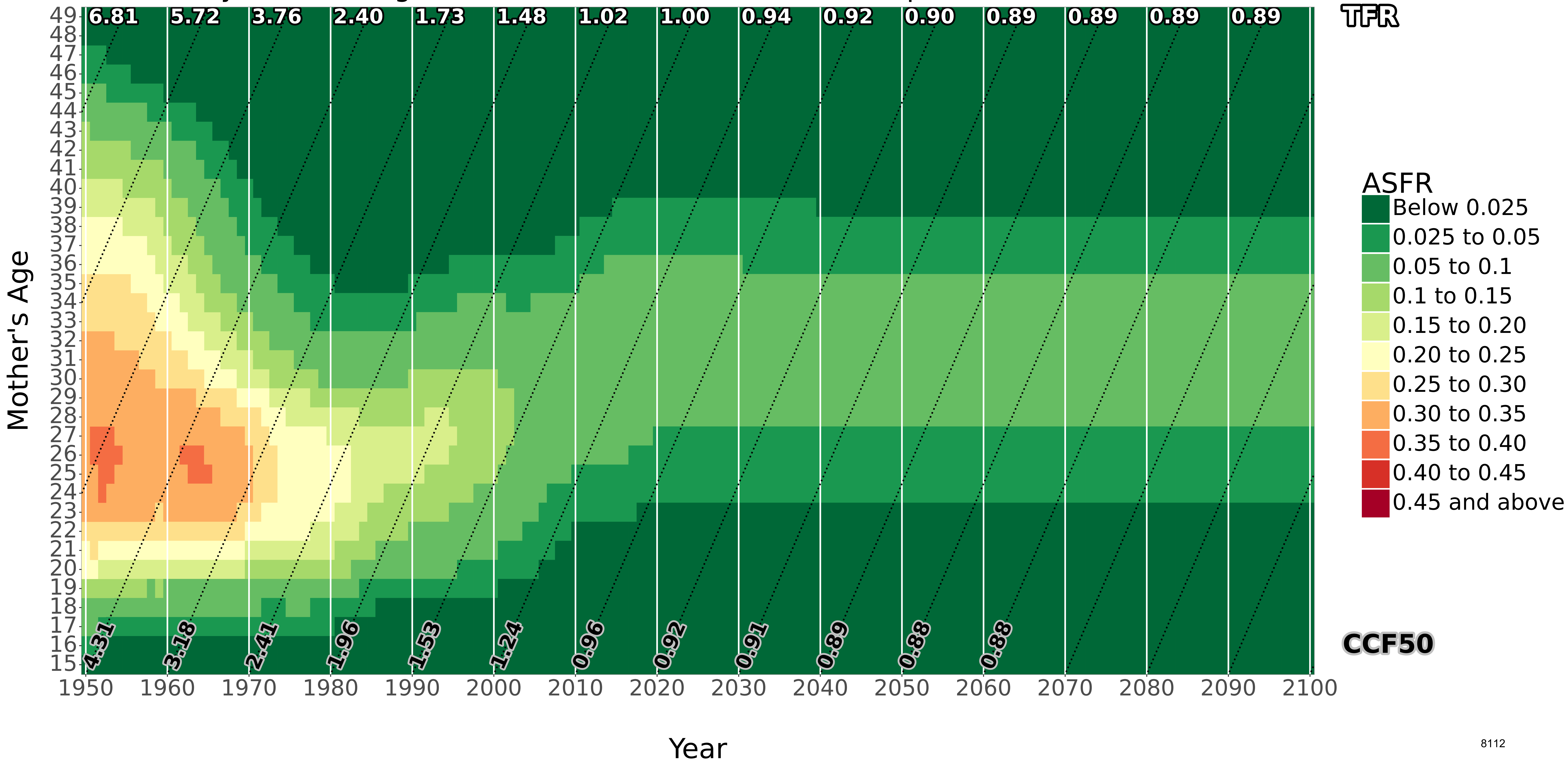

Supplemental Results Figure S3: Lexis diagram of CCF50, single-year age interval ASFR, and TFR by mother's age and location, 1950-2100, American Samoa

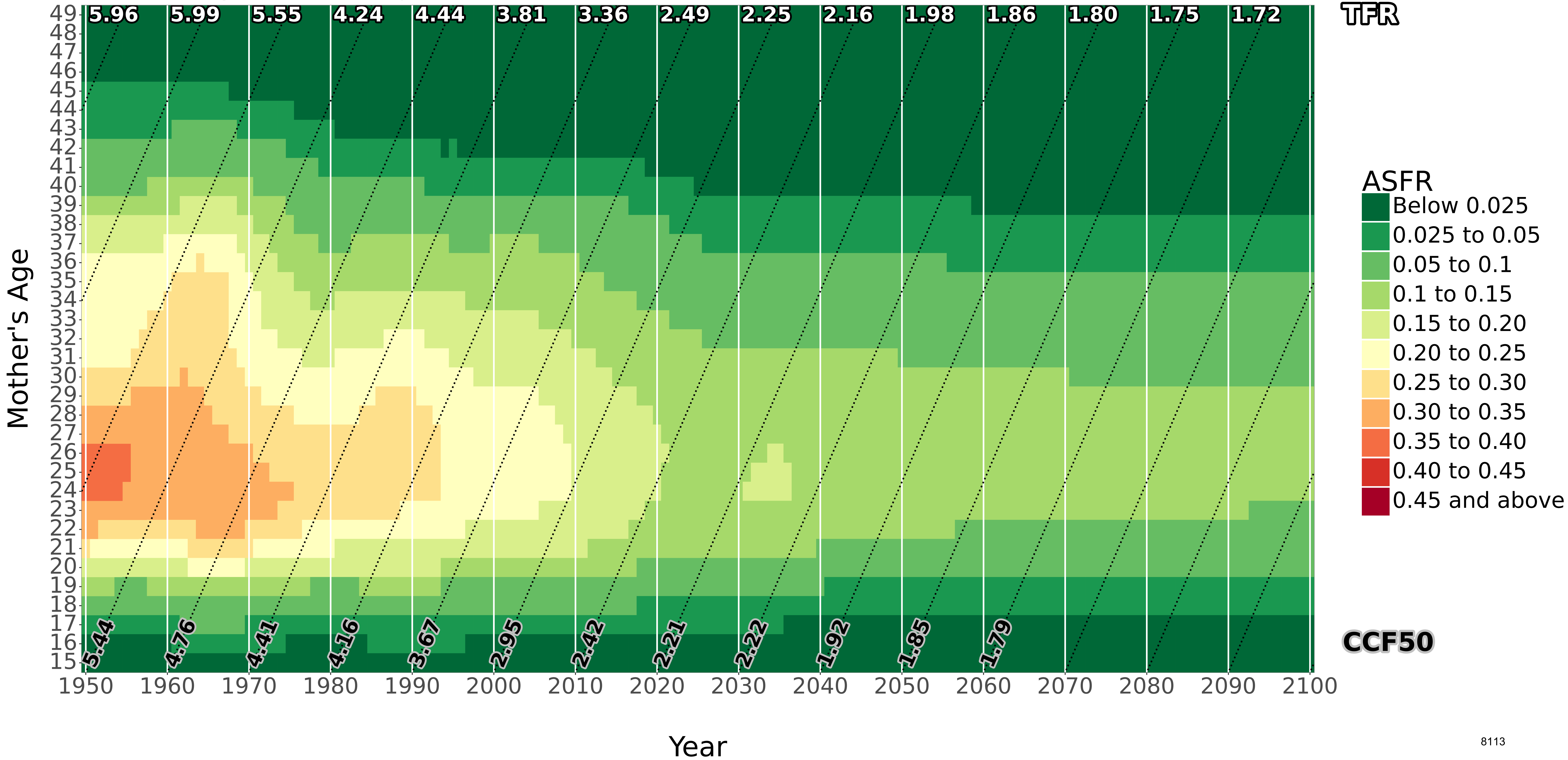

Supplemental Results Figure S3: Lexis diagram of CCF50, single-year age interval ASFR, and TFR by mother's age and location, 1950-2100, Cook Islands

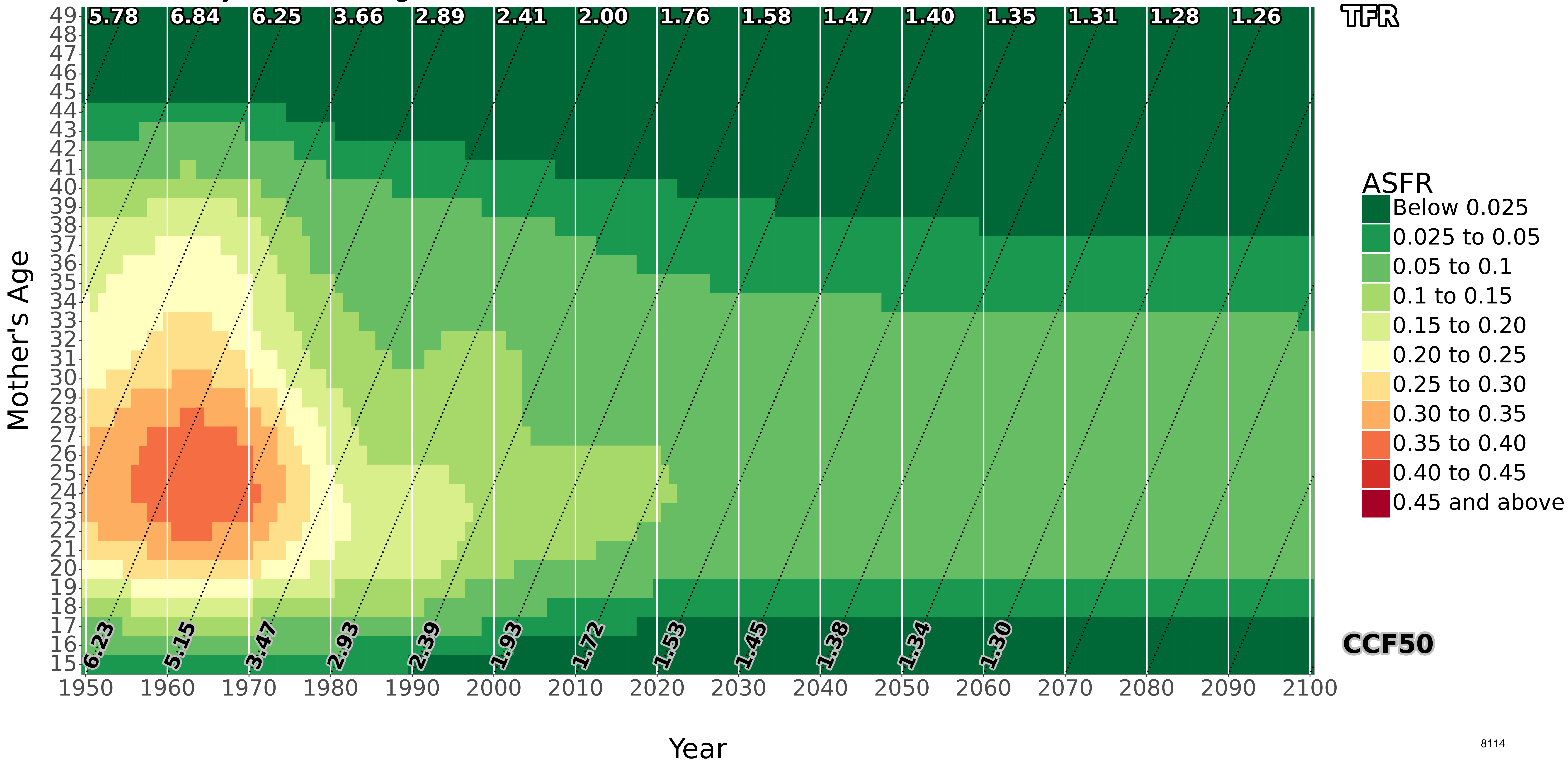

8114

Supplemental Results Figure S3: Lexis diagram of CCF50, single-year age interval ASFR, and TFR by mother's age and location, 1950-2100, Fiji

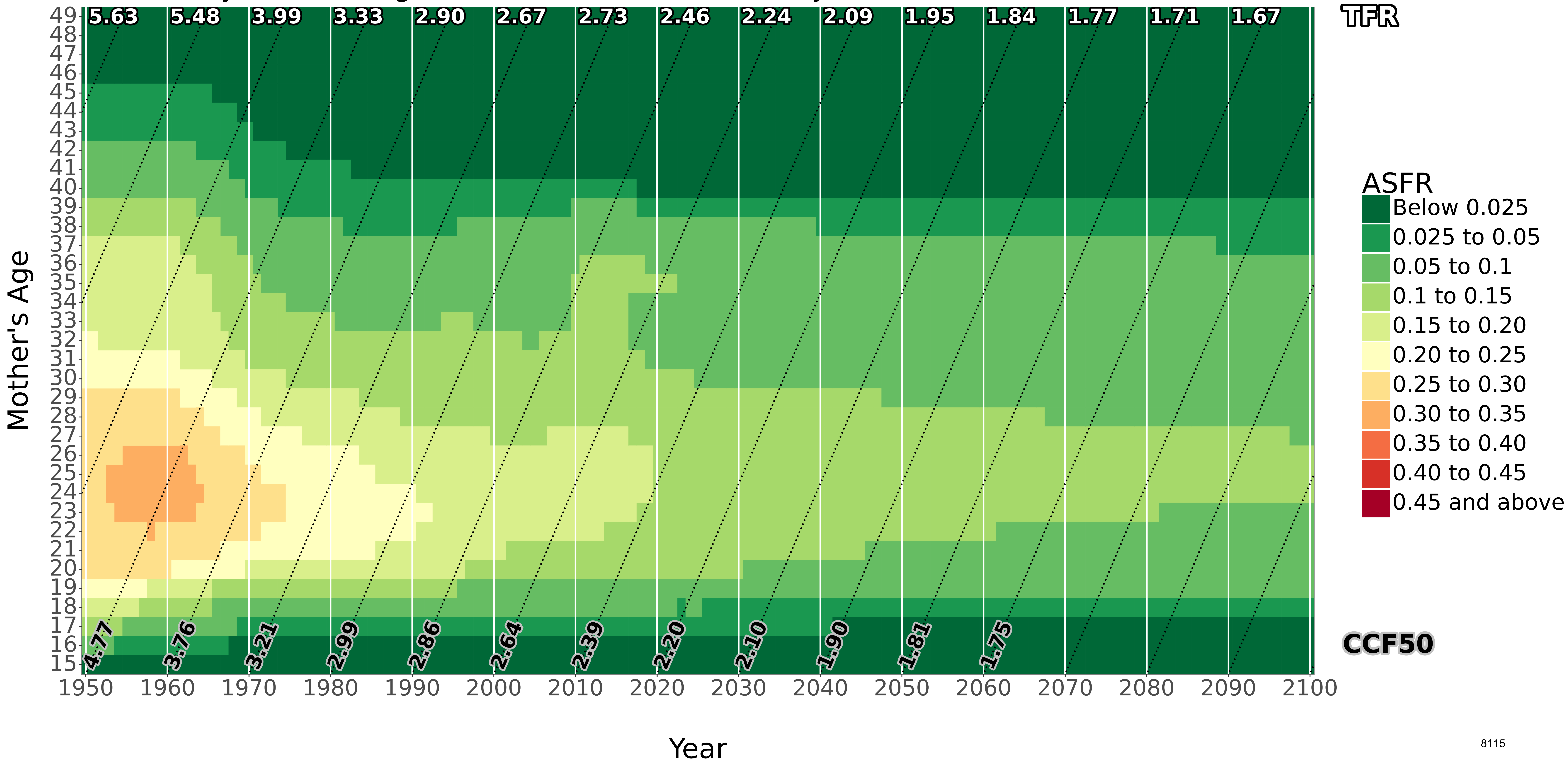

Supplemental Results Figure S3: Lexis diagram of CCF50, single-year age interval ASFR, and TFR by mother's age and location, 1950-2100, Guam

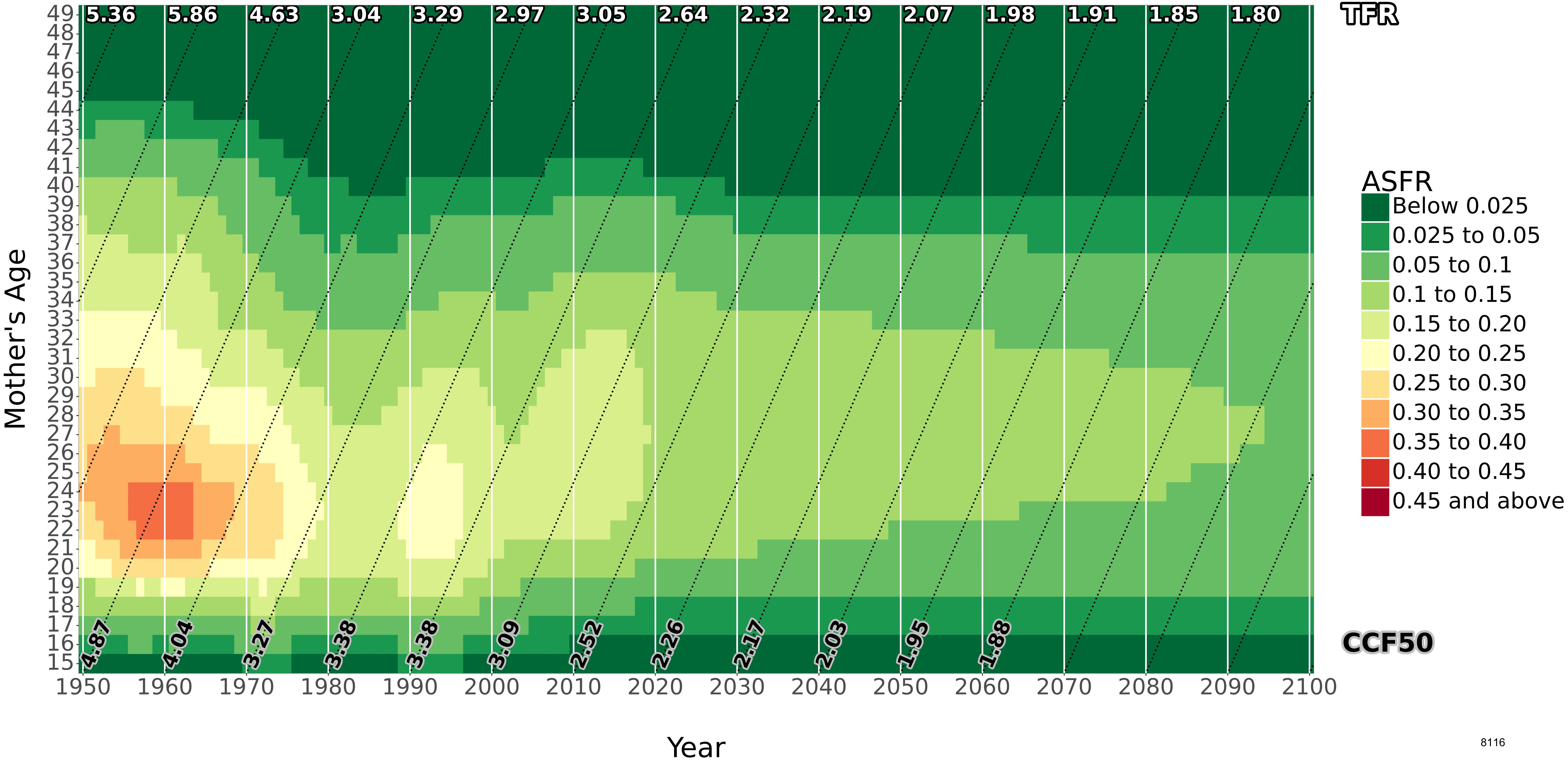

Supplemental Results Figure S3: Lexis diagram of CCF50, single-year age interval ASFR, and TFR by mother's age and location, 1950-2100, Kiribati

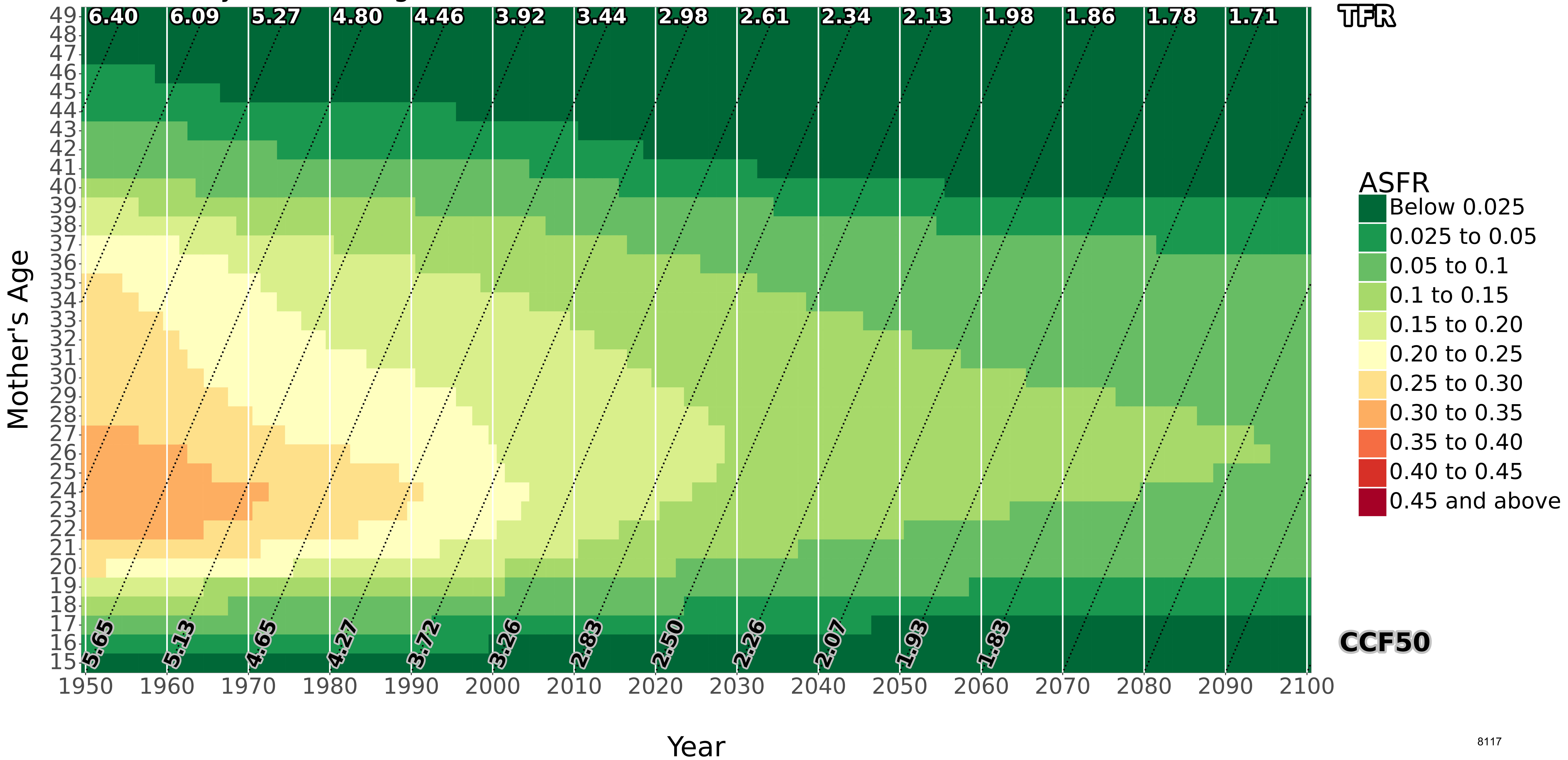

Supplemental Results Figure S3: Lexis diagram of CCF50, single-year age interval ASFR, and TFR by mother's age and location, 1950-2100, Marshall Islands

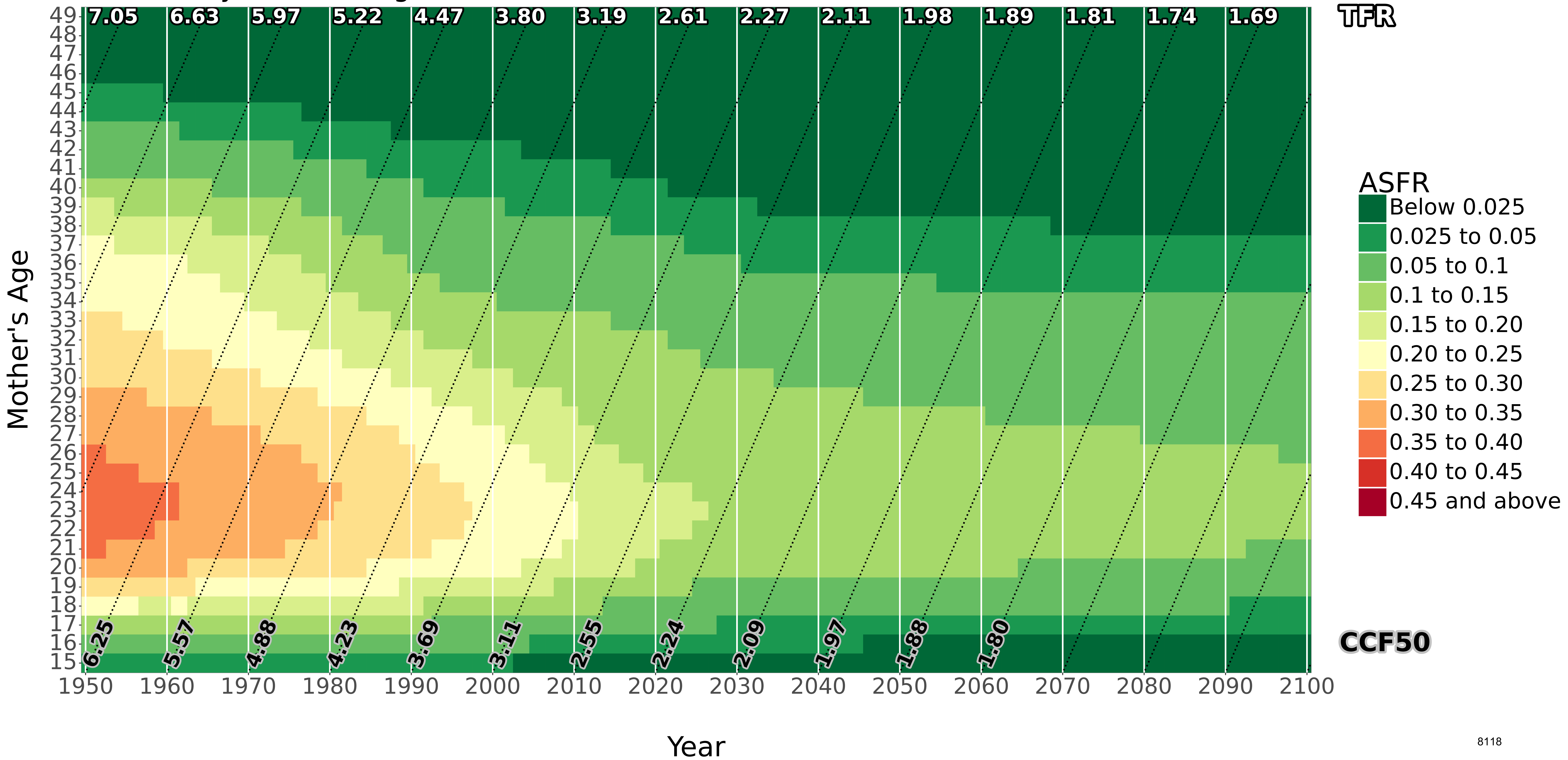

Supplemental Results Figure S3: Lexis diagram of CCF50, single-year age interval ASFR, and TFR by mother's age and location, 1950-2100, Federated States of Micronesia

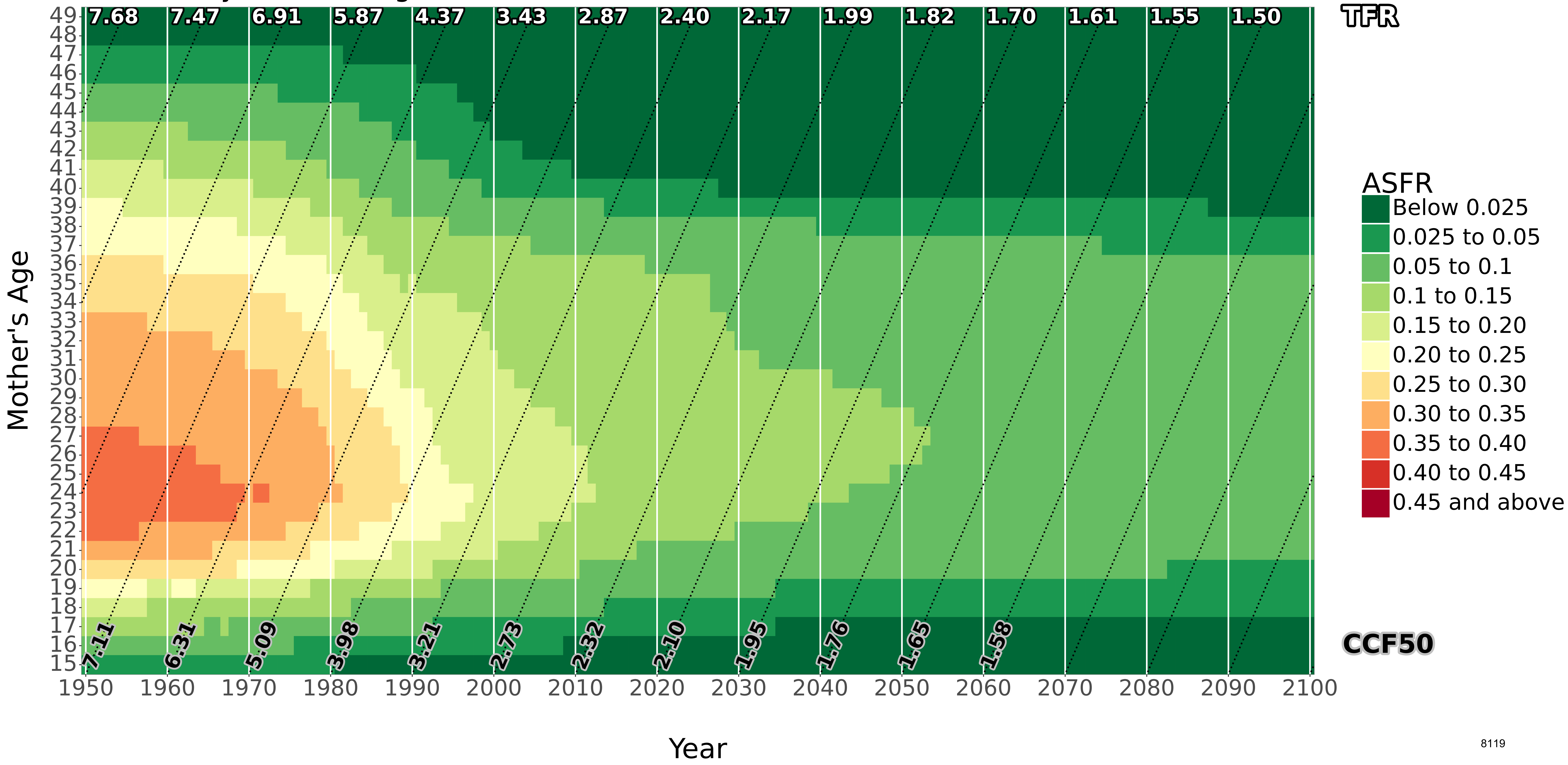

Supplemental Results Figure S3: Lexis diagram of CCF50, single-year age interval ASFR, and TFR by mother's age and location, 1950-2100, Nauru

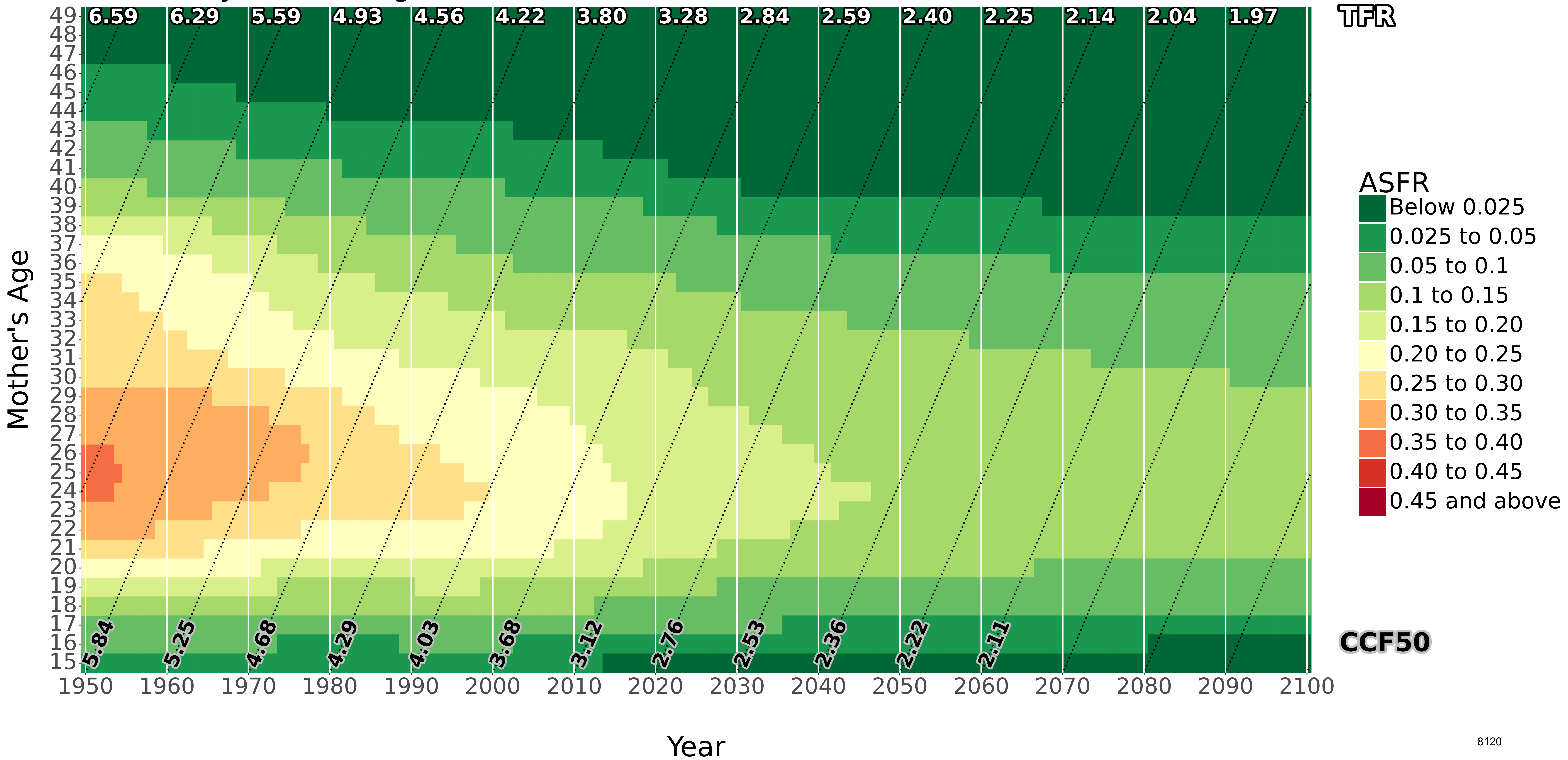

Supplemental Results Figure S3: Lexis diagram of CCF50, single-year age interval ASFR, and TFR by mother's age and location, 1950-2100, Niue

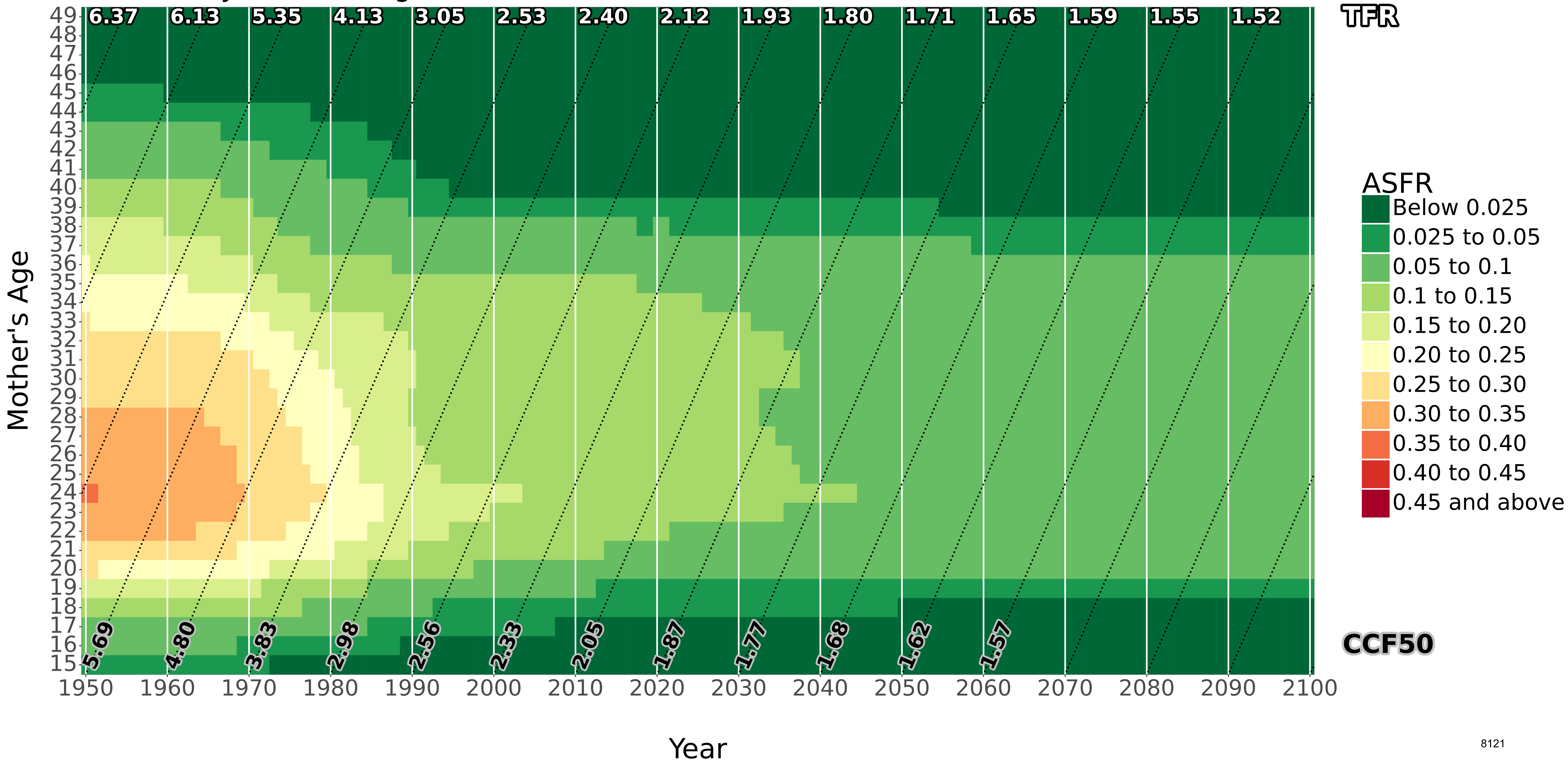

Supplemental Results Figure S3: Lexis diagram of CCF50, single-year age interval ASFR, and TFR by mother's age and location, 1950-2100, Northern Mariana Islands

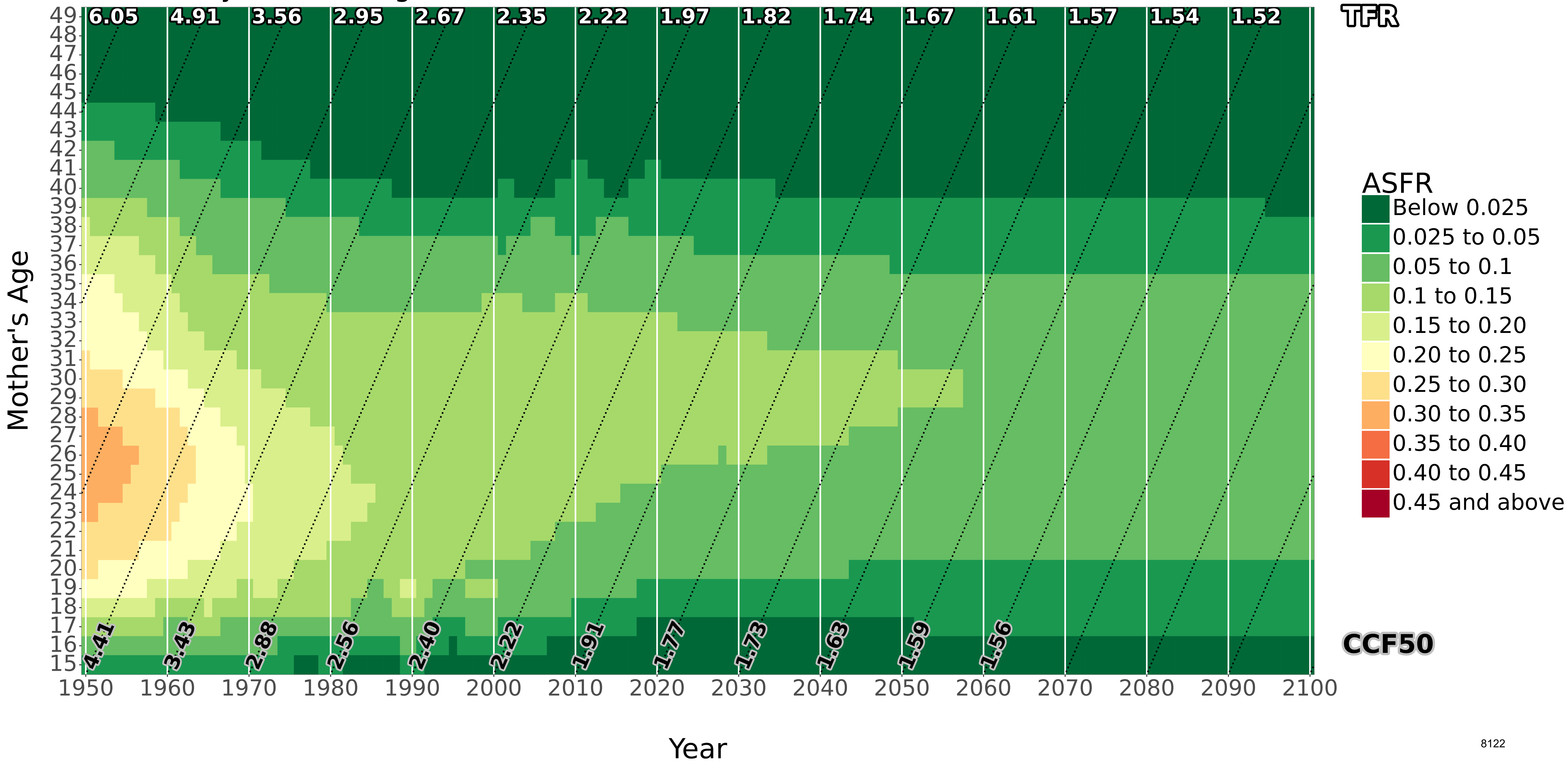

Supplemental Results Figure S3: Lexis diagram of CCF50, single-year age interval ASFR, and TFR by mother's age and location, 1950-2100, Palau

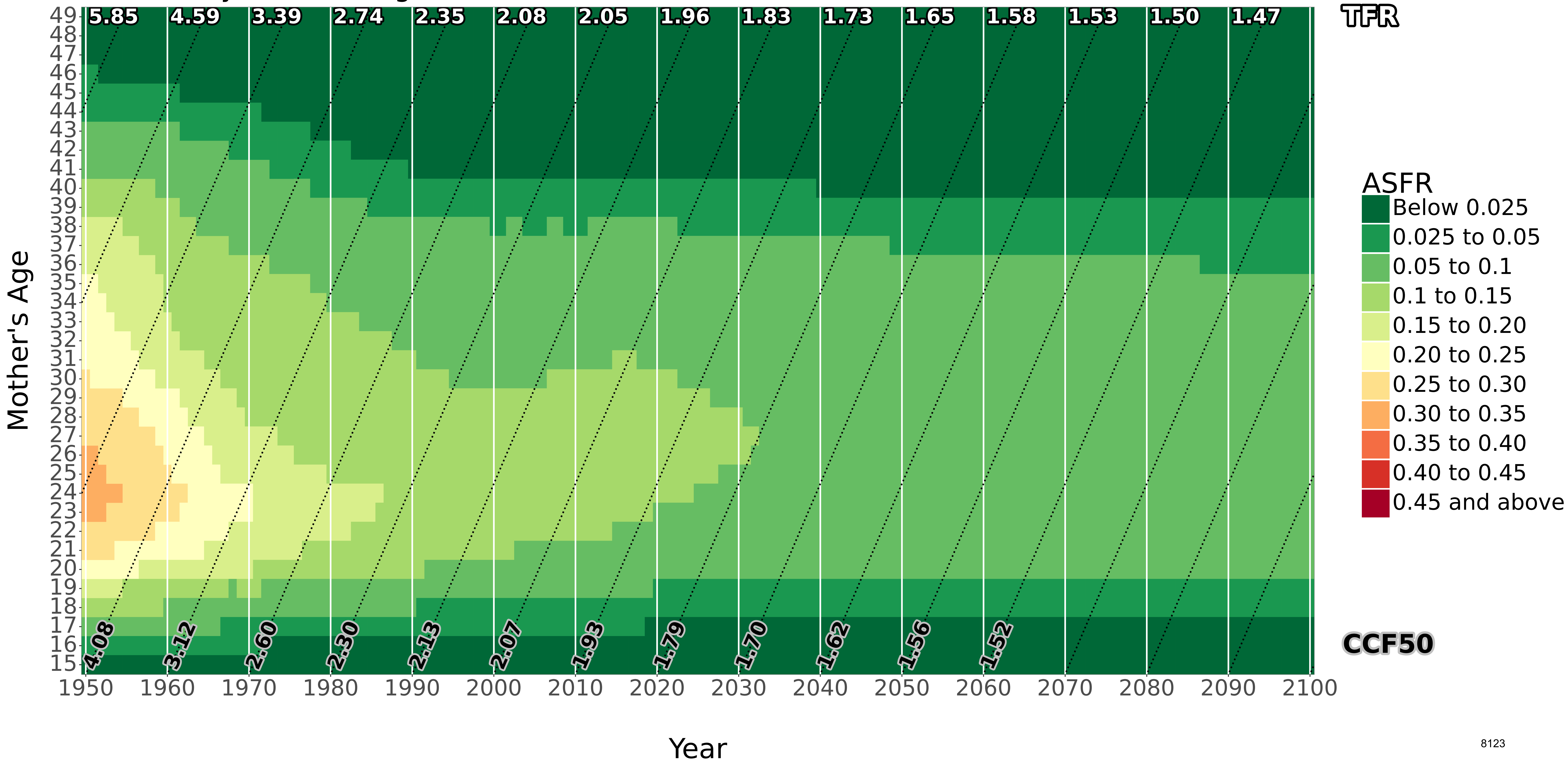

Supplemental Results Figure S3: Lexis diagram of CCF50, single-year age interval ASFR, and TFR by mother's age and location, 1950-2100, Papua New Guinea

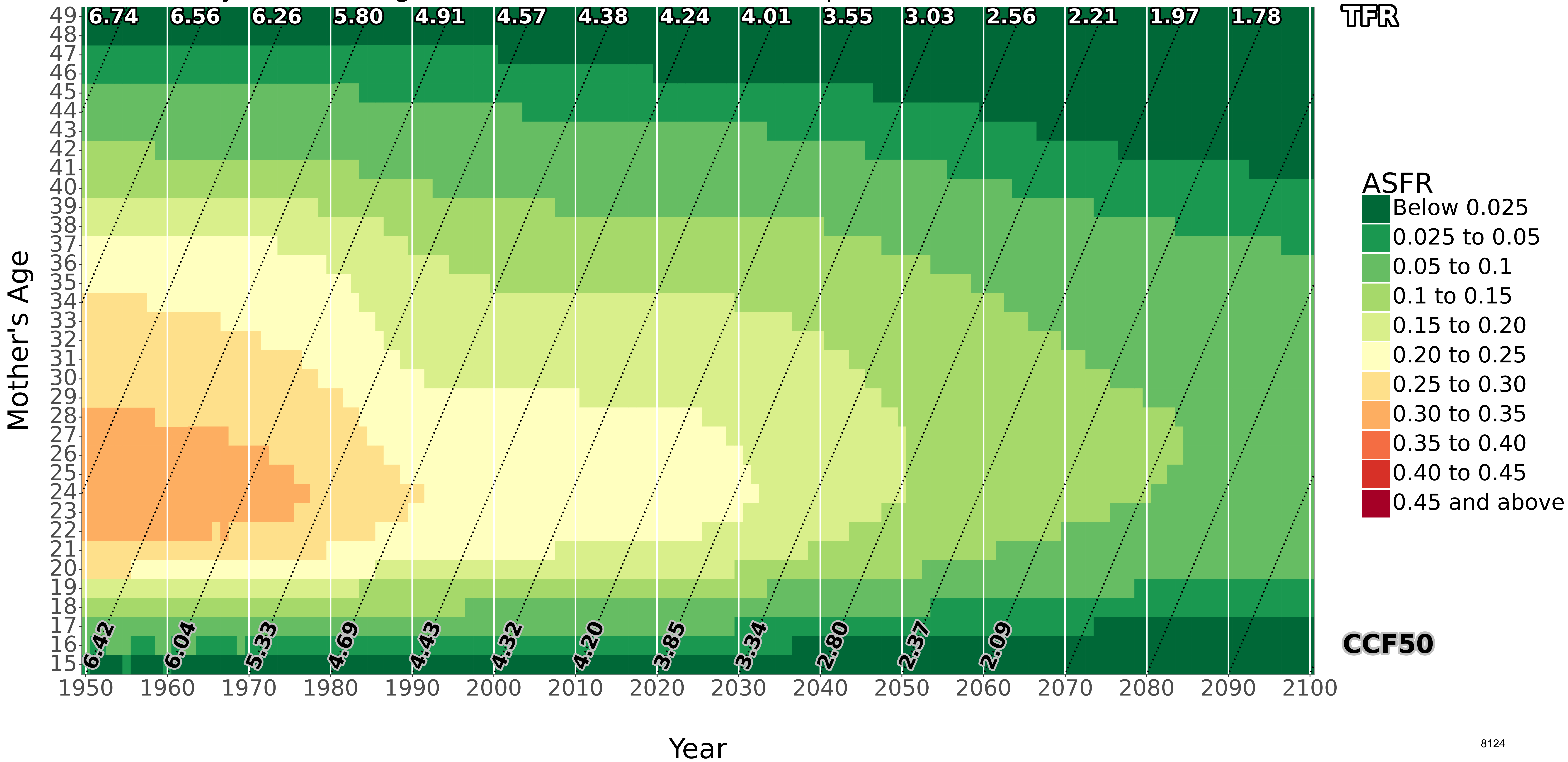

Supplemental Results Figure S3: Lexis diagram of CCF50, single-year age interval ASFR, and TFR by mother's age and location, 1950-2100, Samoa

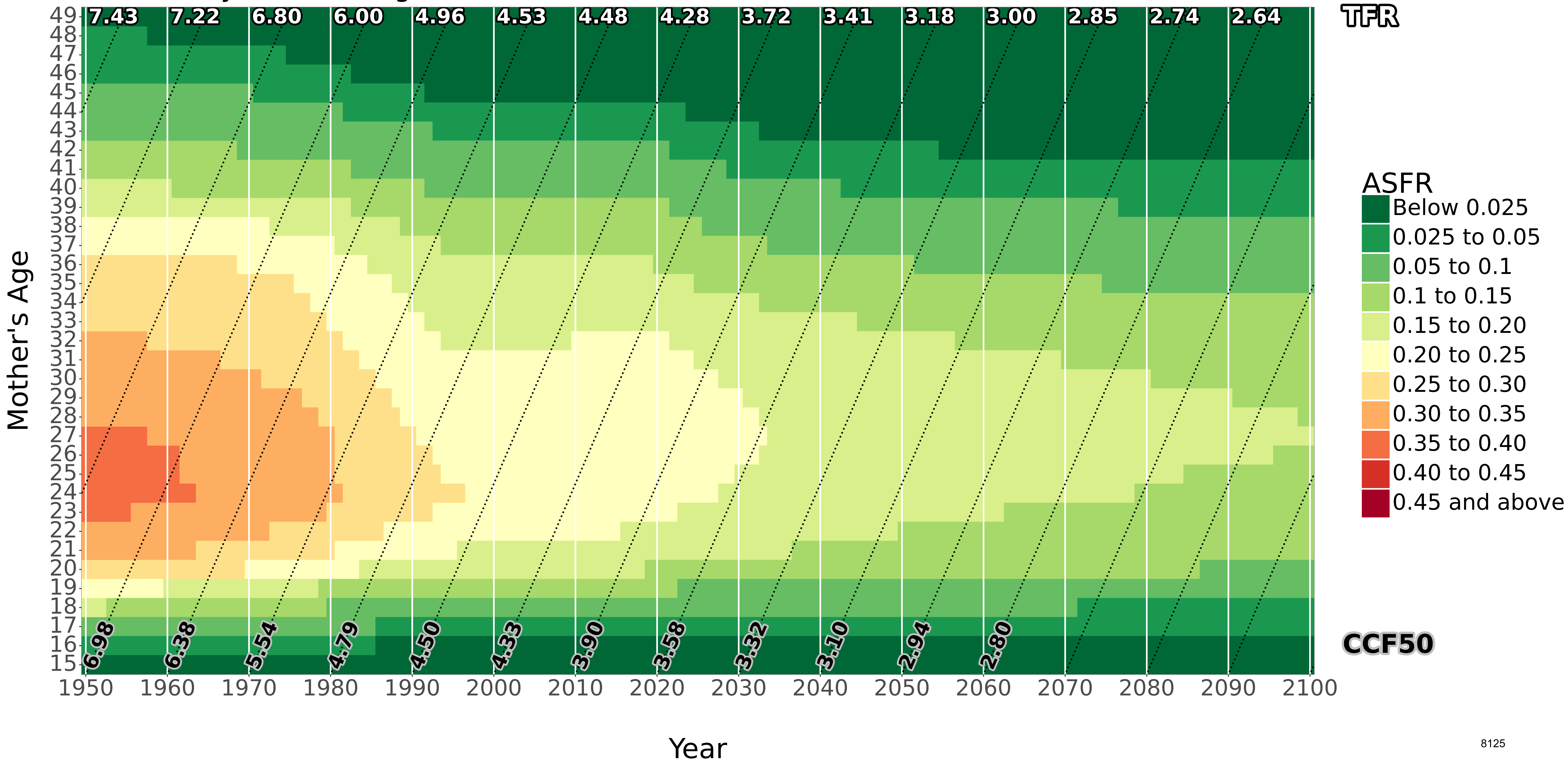

Supplemental Results Figure S3: Lexis diagram of CCF50, single-year age interval ASFR, and TFR by mother's age and location, 1950-2100, Solomon Islands

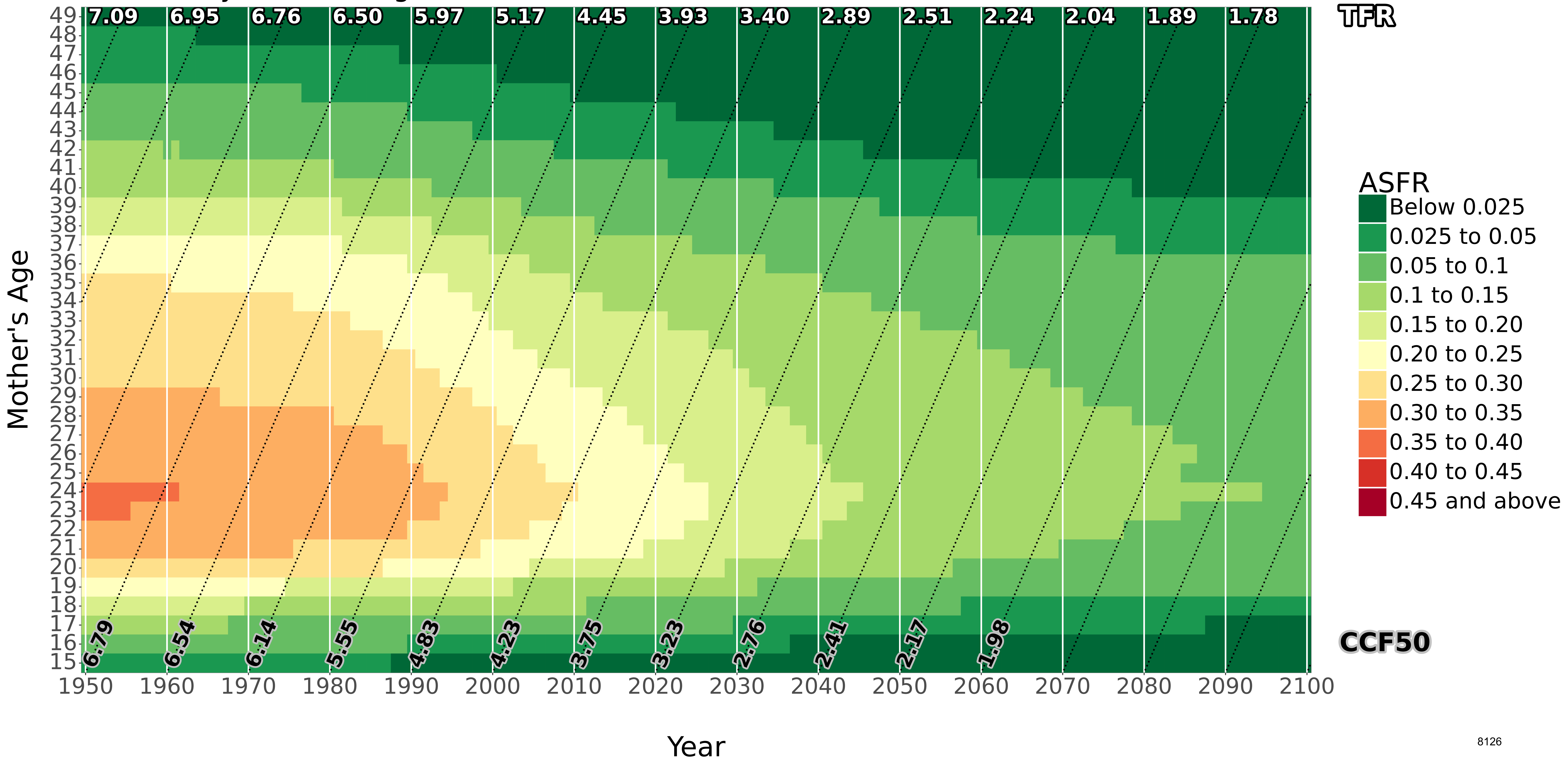

Supplemental Results Figure S3: Lexis diagram of CCF50, single-year age interval ASFR, and TFR by mother's age and location, 1950-2100, Tokelau

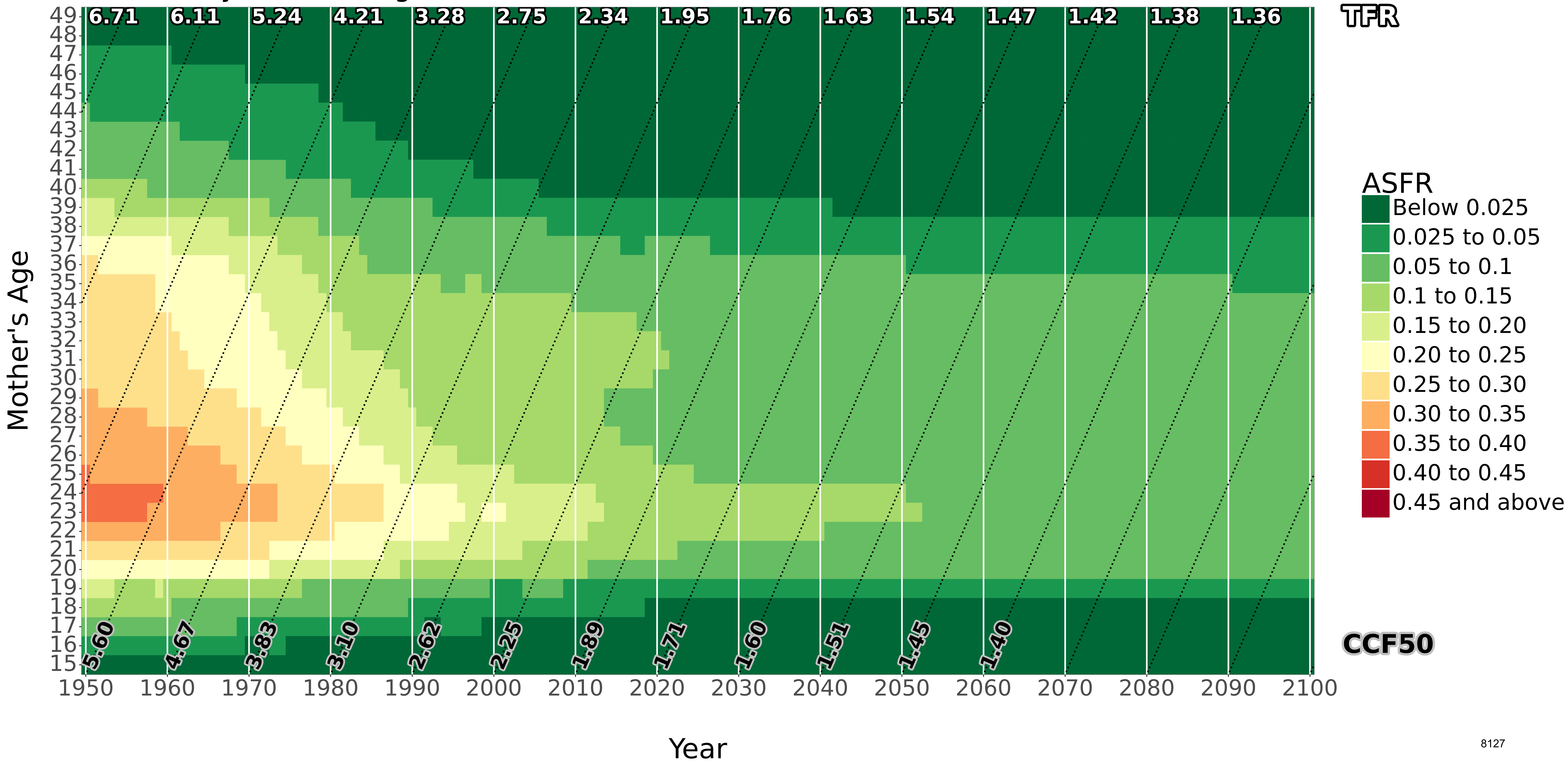

Supplemental Results Figure S3: Lexis diagram of CCF50, single-year age interval ASFR, and TFR by mother's age and location, 1950-2100, Tonga

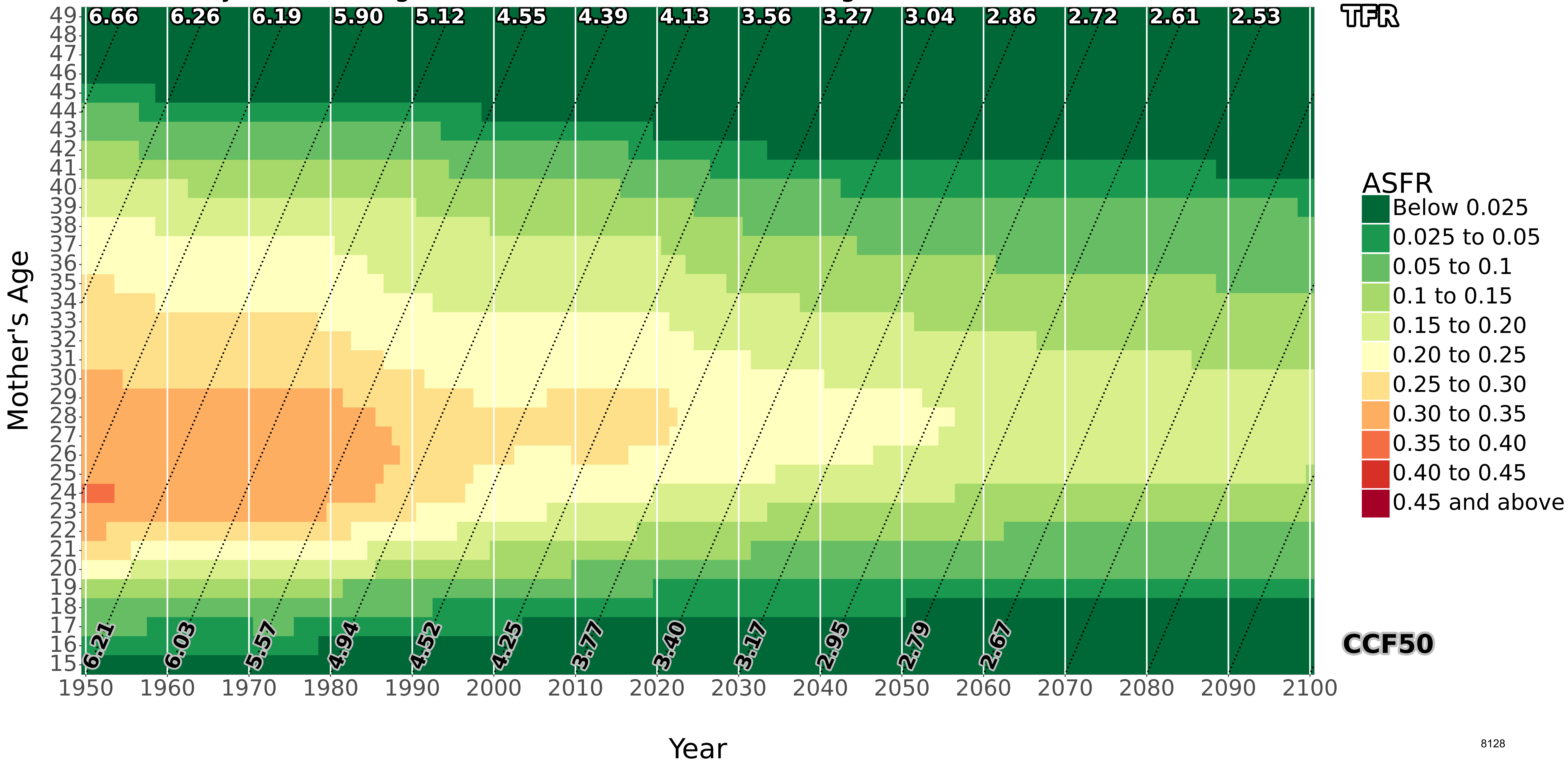

Supplemental Results Figure S3: Lexis diagram of CCF50, single-year age interval ASFR, and TFR by mother's age and location, 1950-2100, Tuvalu

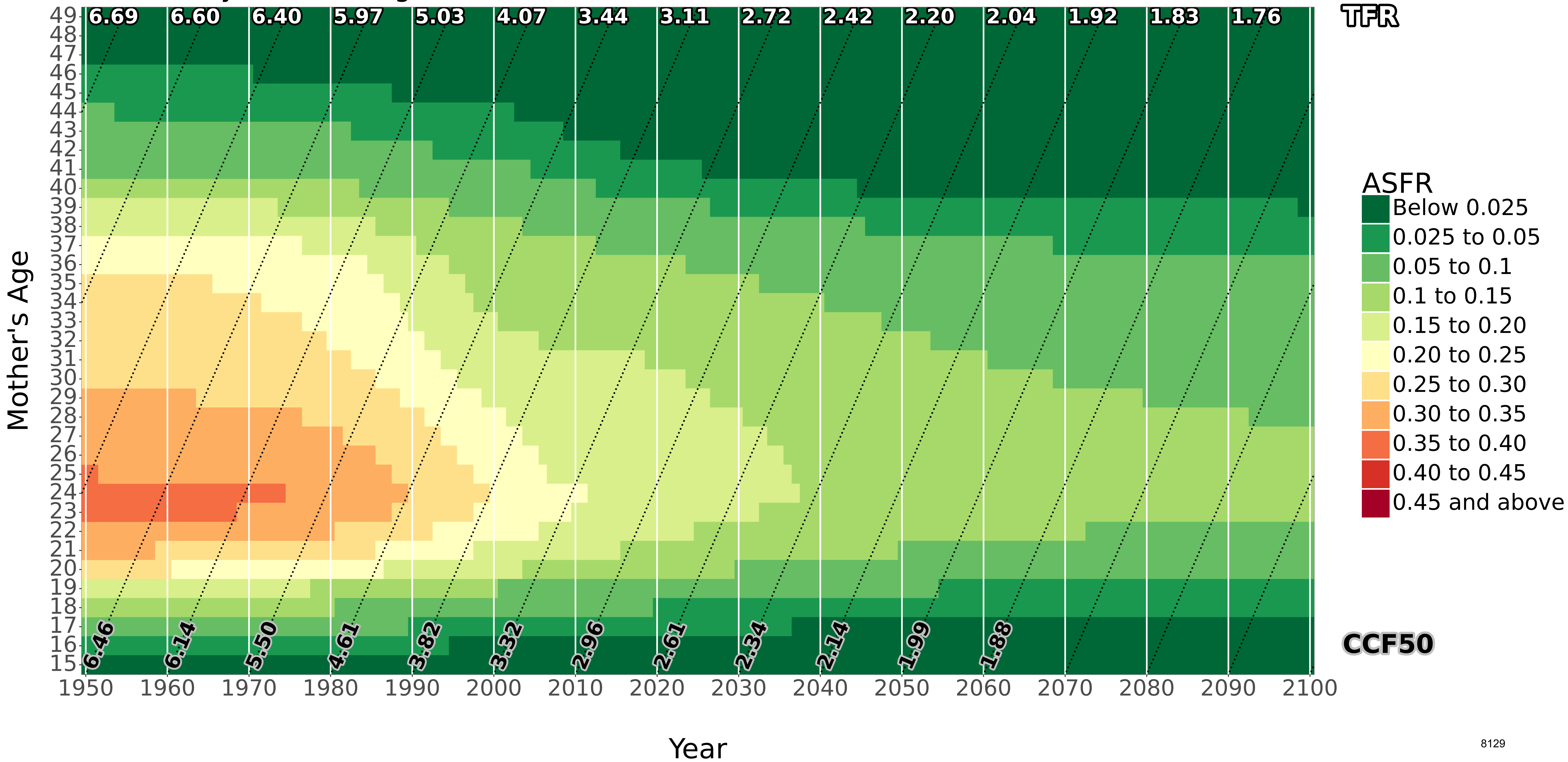

Supplemental Results Figure S3: Lexis diagram of CCF50, single-year age interval ASFR, and TFR by mother's age and location, 1950-2100, Vanuatu

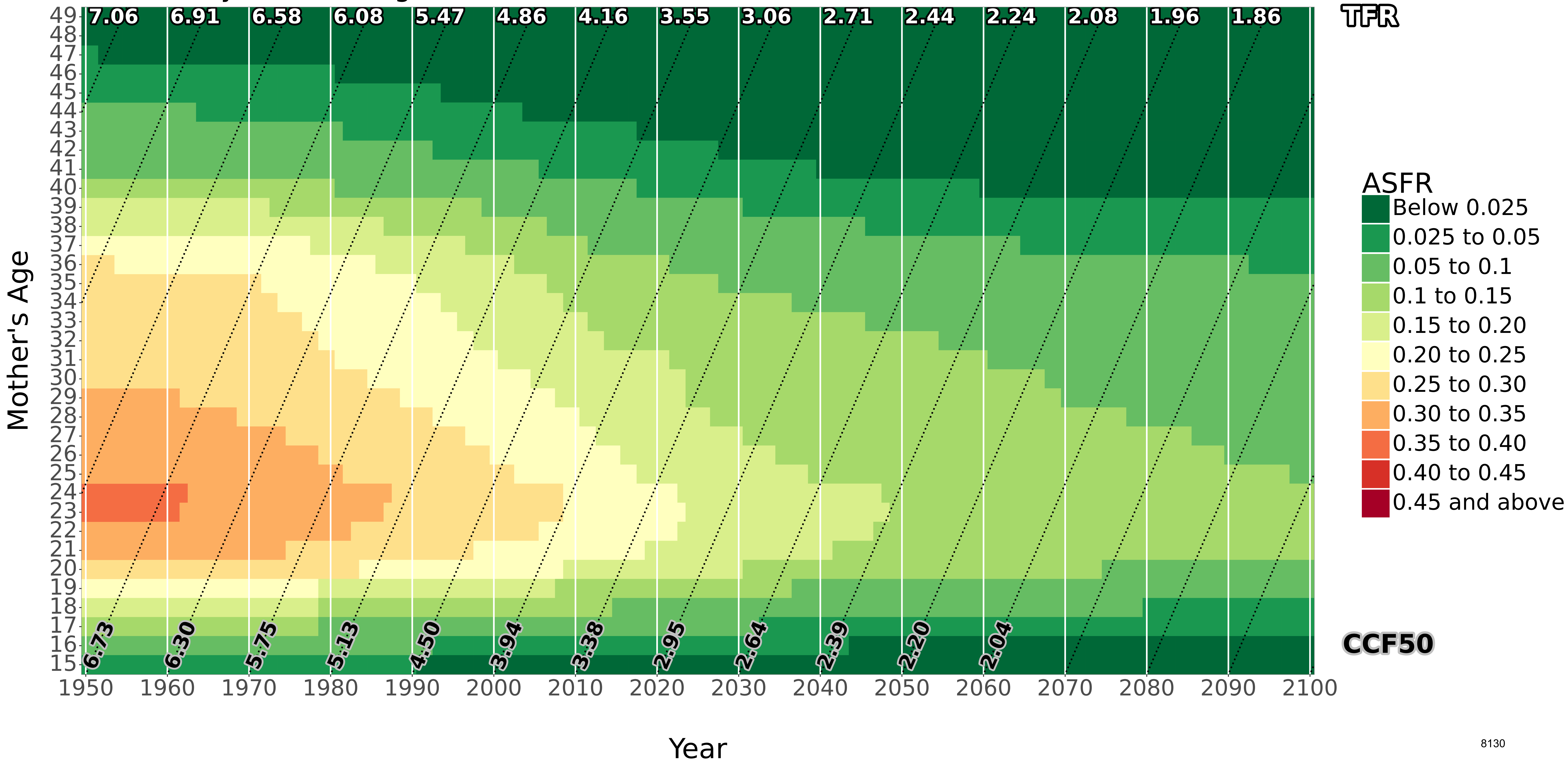

Supplemental Results Figure S3: Lexis diagram of CCF50, single-year age interval ASFR, and TFR by mother's age and location, 1950-2100, Cambodia

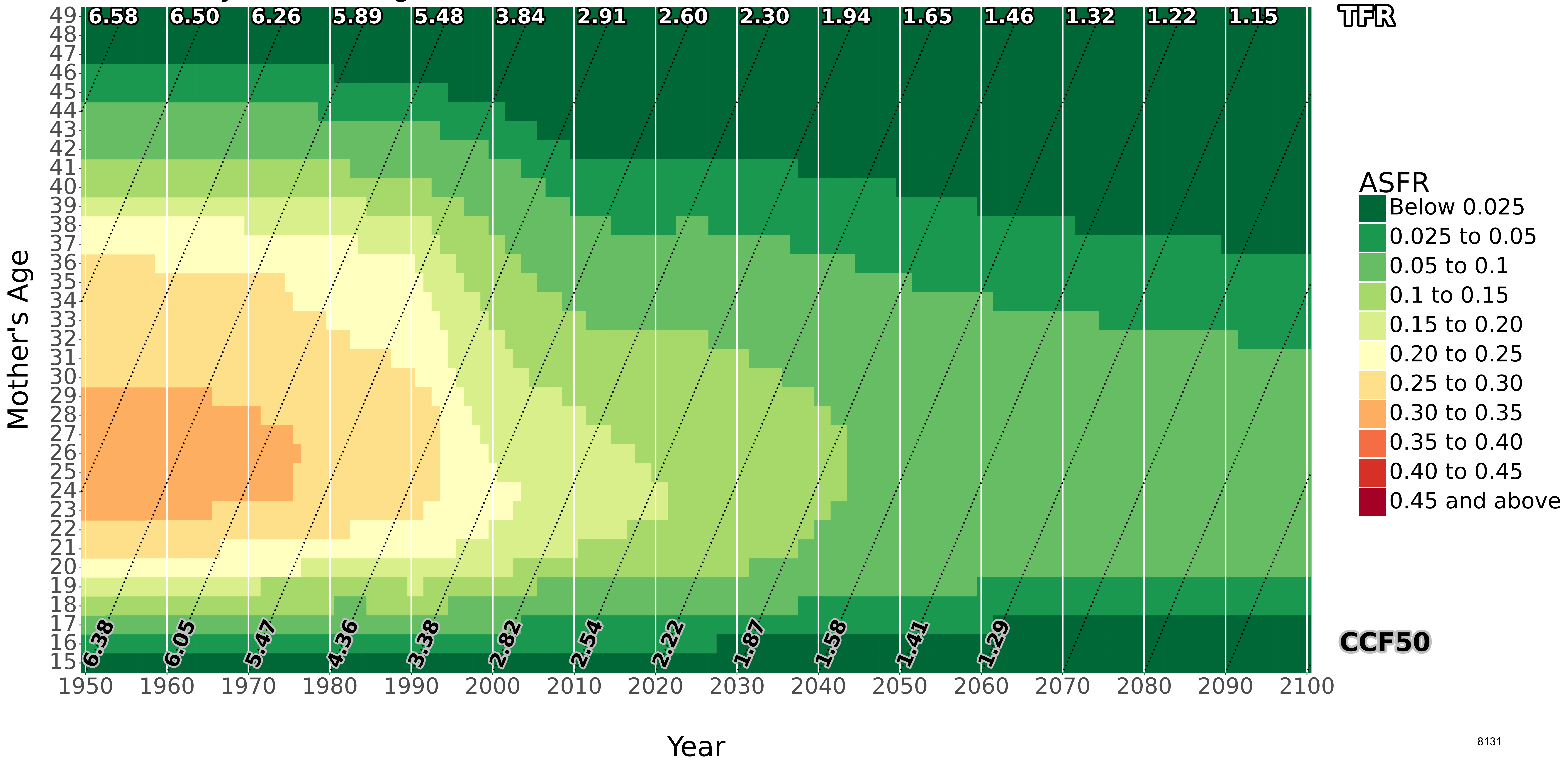

Supplemental Results Figure S3: Lexis diagram of CCF50, single-year age interval ASFR, and TFR by mother's age and location, 1950-2100, Indonesia

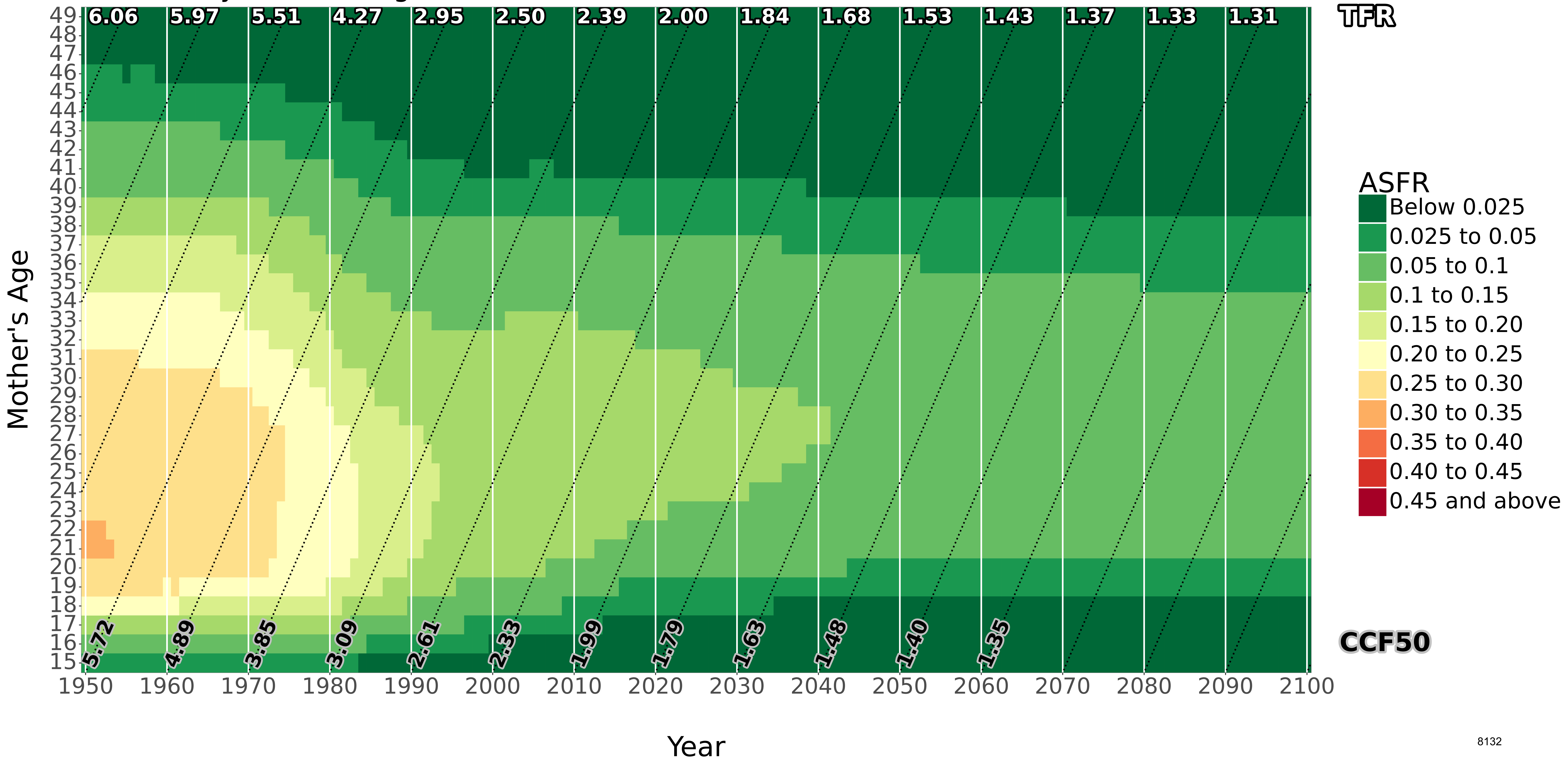

Supplemental Results Figure S3: Lexis diagram of CCF50, single-year age interval ASFR, and TFR by mother's age and location, 1950-2100, Laos

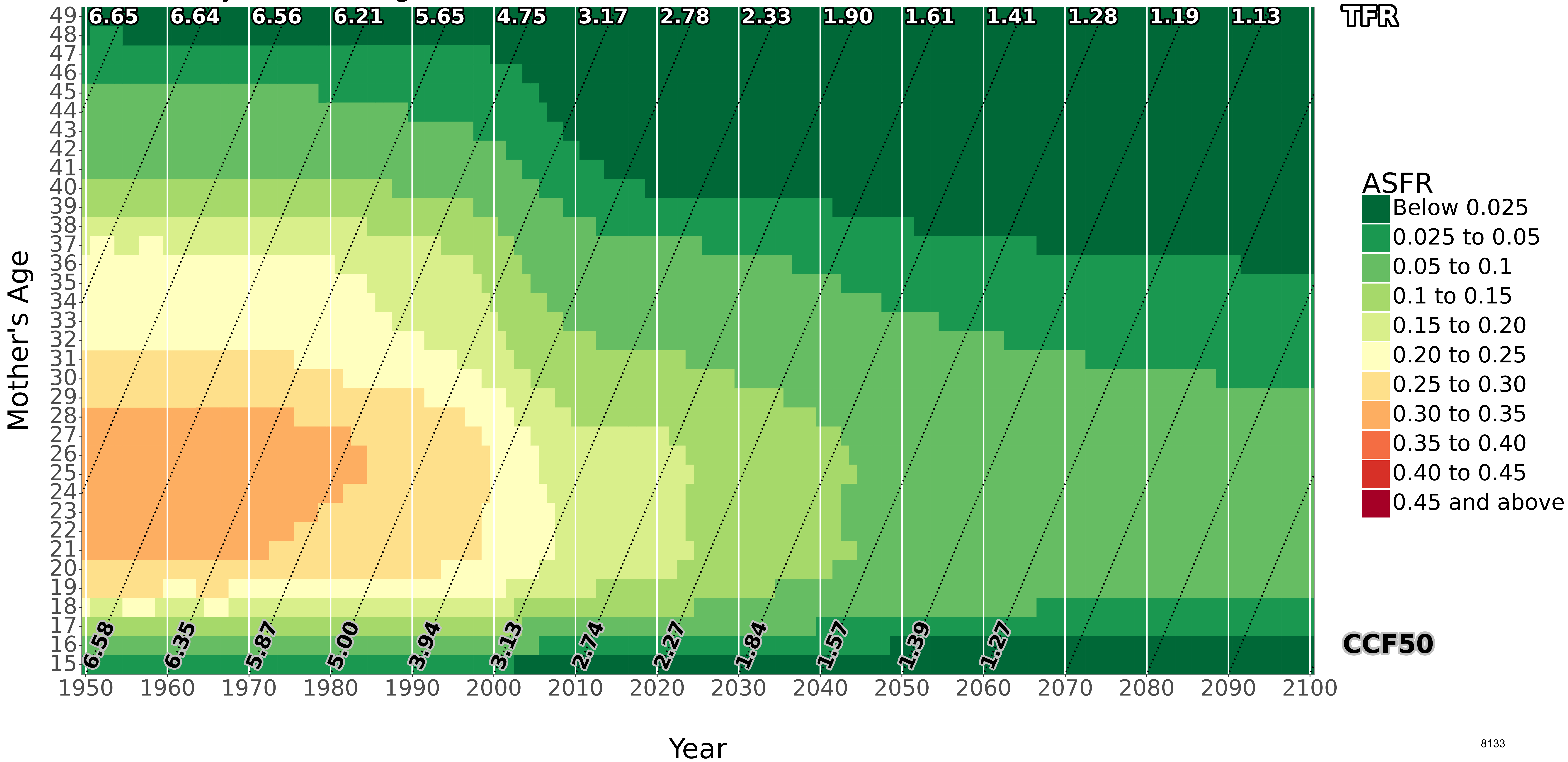

Supplemental Results Figure S3: Lexis diagram of CCF50, single-year age interval ASFR, and TFR by mother's age and location, 1950-2100, Malaysia

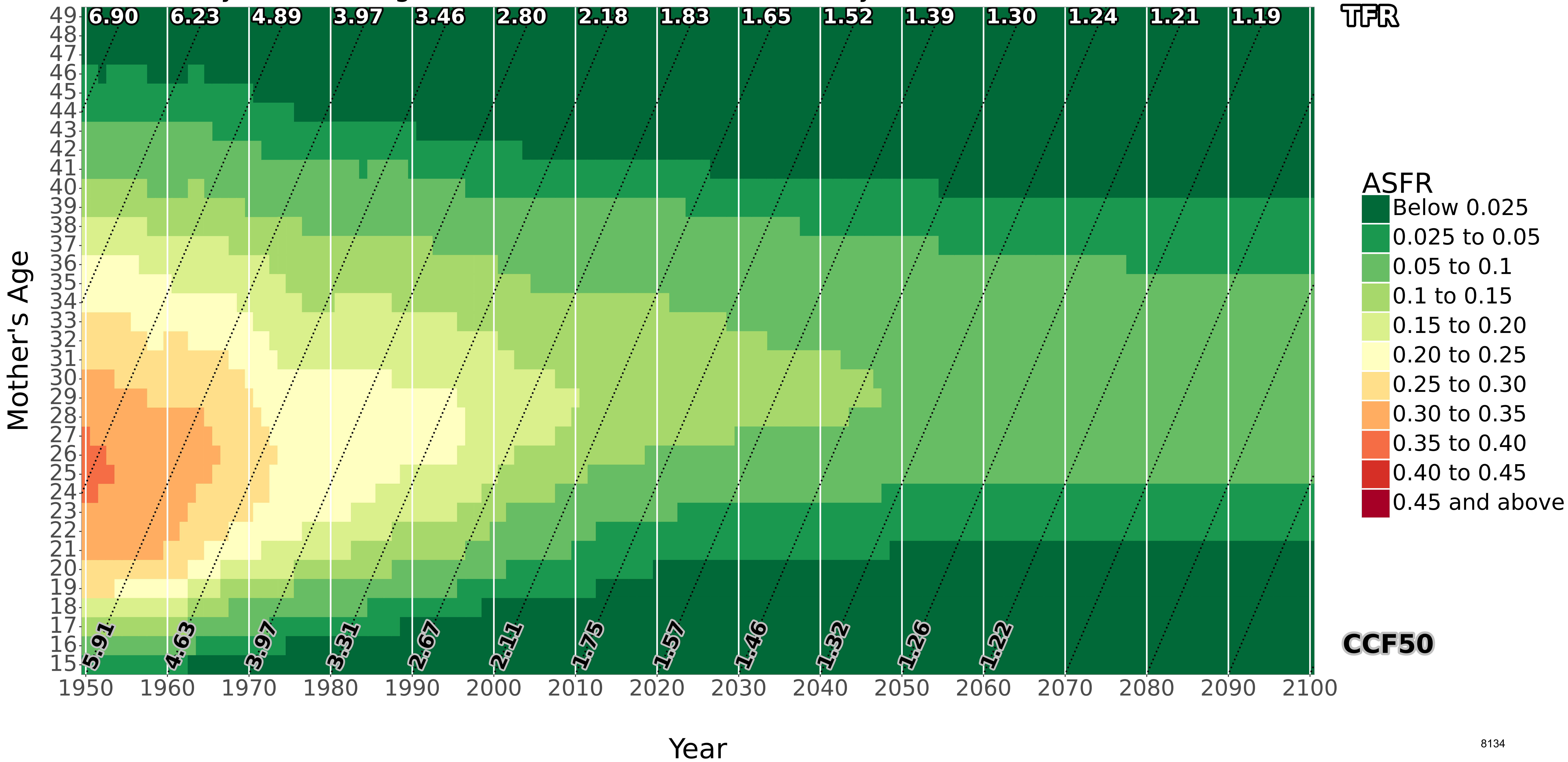

Supplemental Results Figure S3: Lexis diagram of CCF50, single-year age interval ASFR, and TFR by mother's age and location, 1950-2100, Maldives

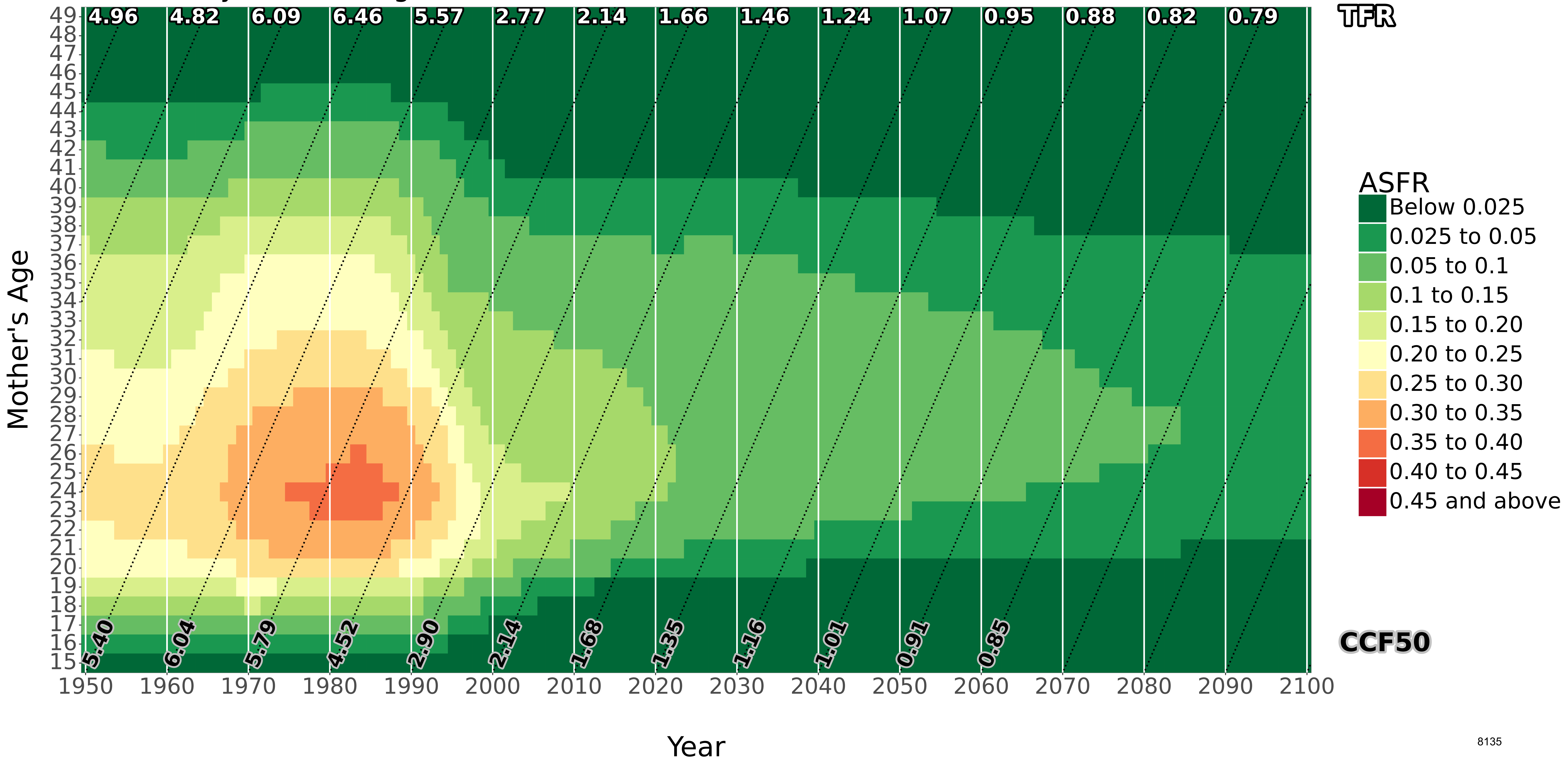

Supplemental Results Figure S3: Lexis diagram of CCF50, single-year age interval ASFR, and TFR by mother's age and location, 1950-2100, Mauritius

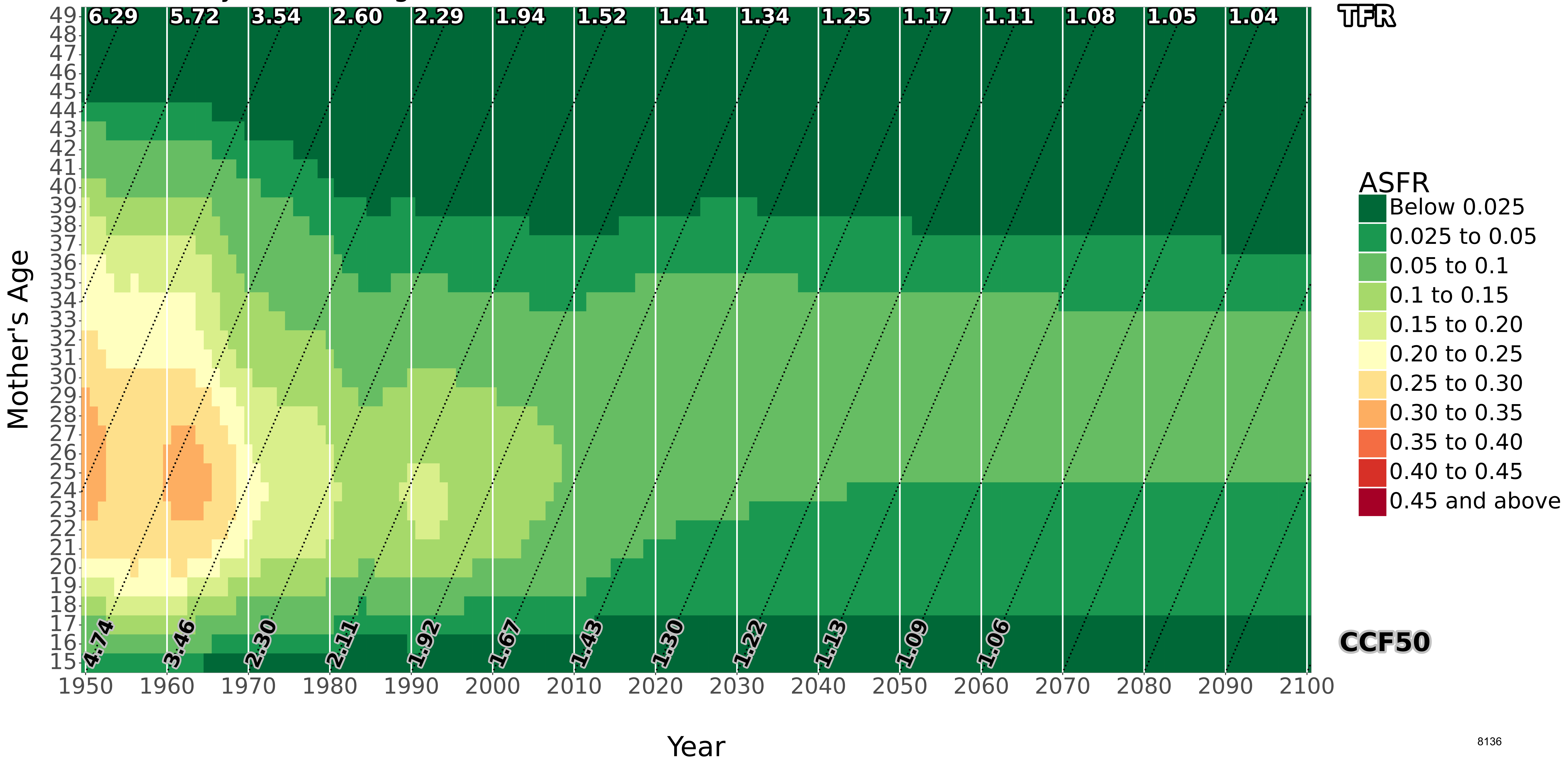

Supplemental Results Figure S3: Lexis diagram of CCF50, single-year age interval ASFR, and TFR by mother's age and location, 1950-2100, Myanmar

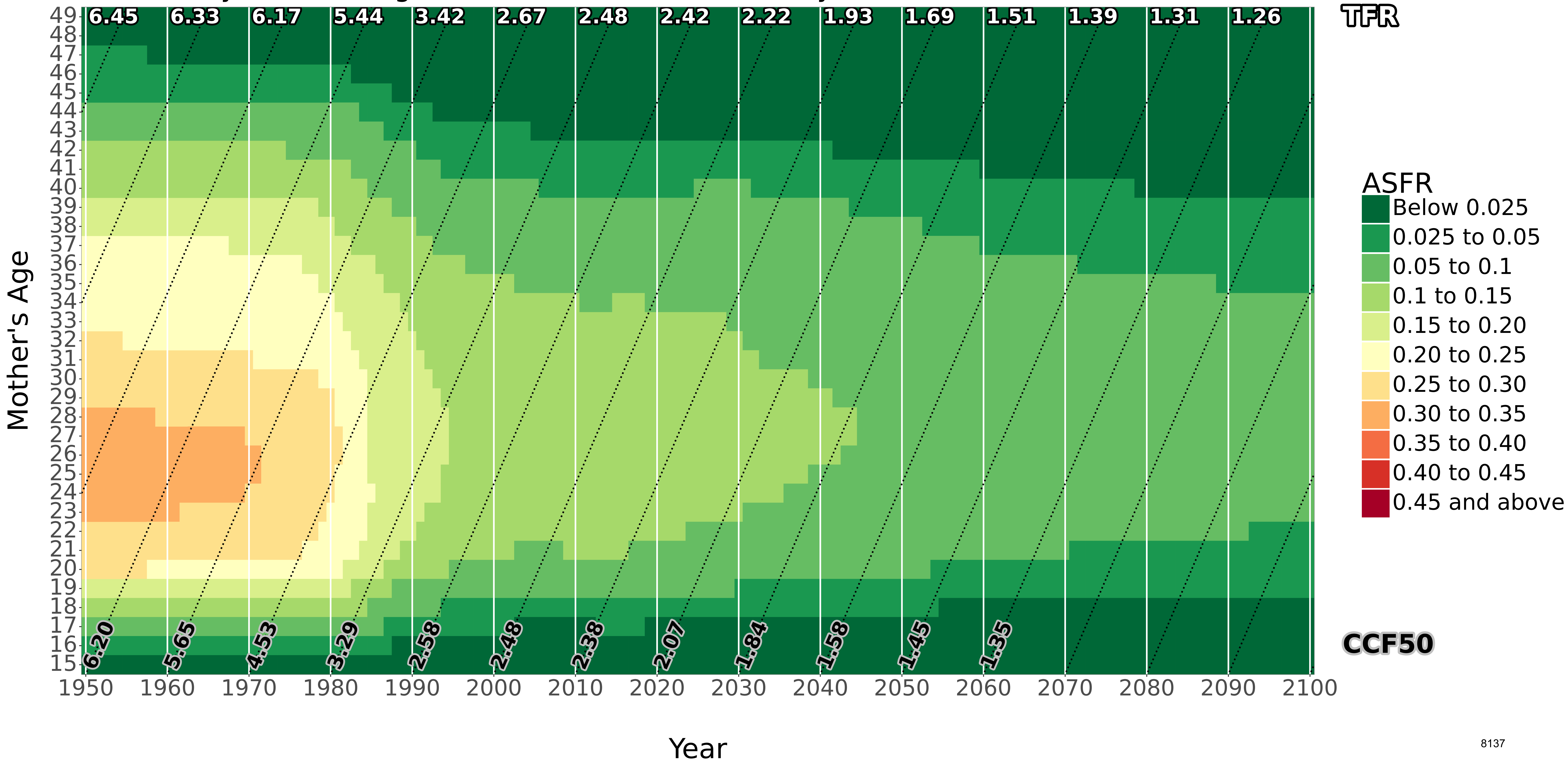

Supplemental Results Figure S3: Lexis diagram of CCF50, single-year age interval ASFR, and TFR by mother's age and location, 1950-2100, Philippines

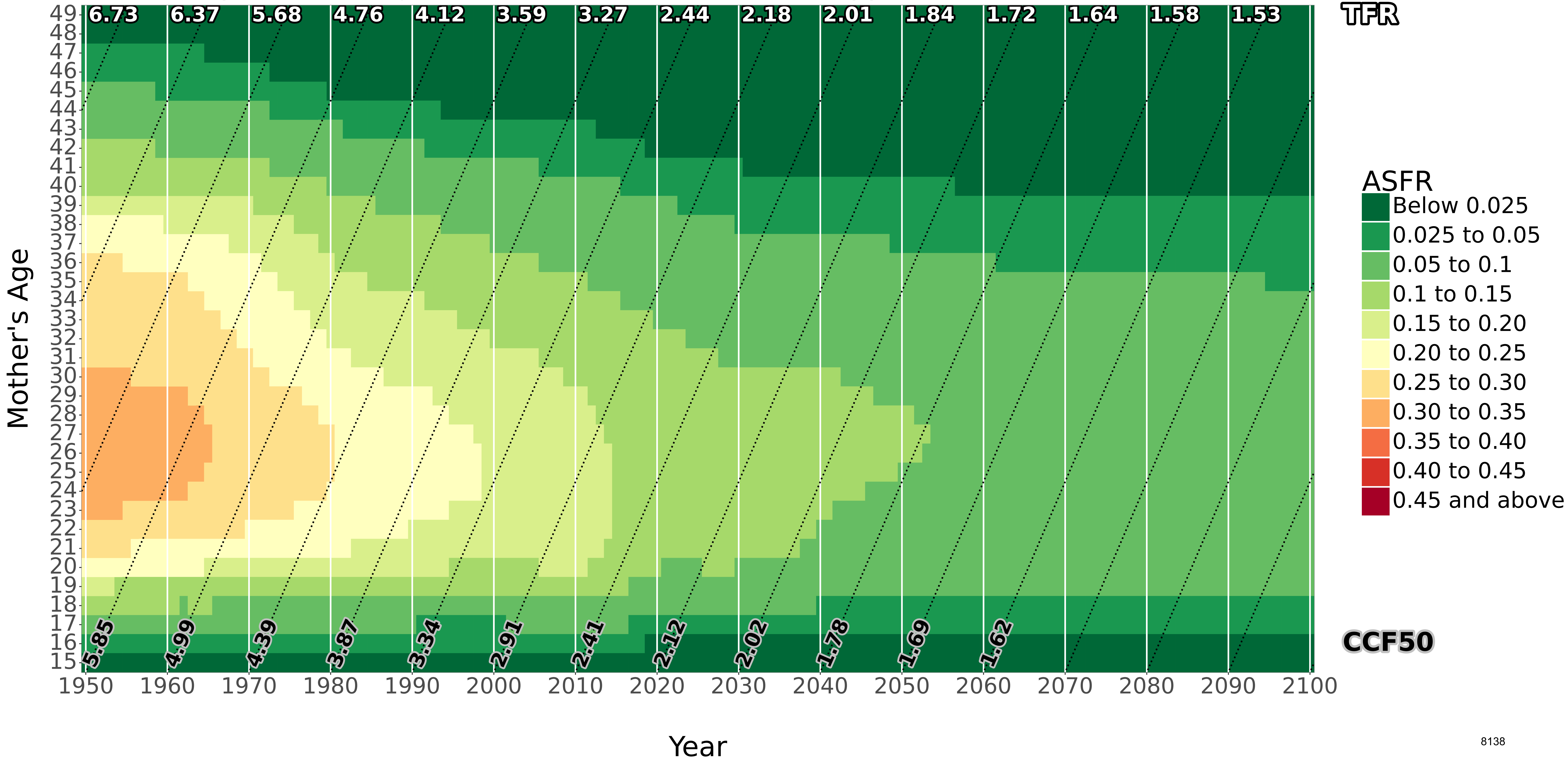

Supplemental Results Figure S3: Lexis diagram of CCF50, single-year age interval ASFR, and TFR by mother's age and location, 1950-2100, Seychelles

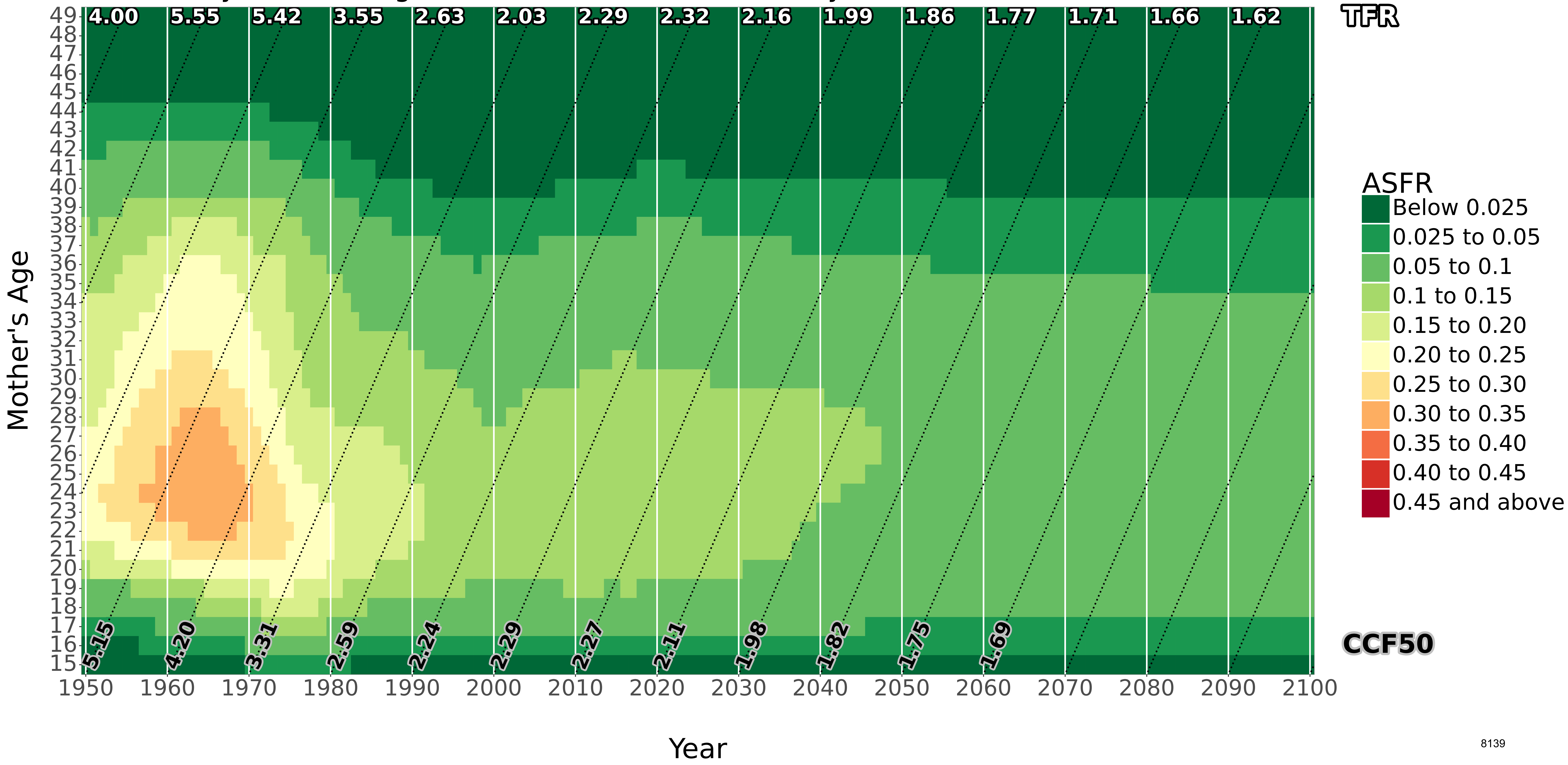

Supplemental Results Figure S3: Lexis diagram of CCF50, single-year age interval ASFR, and TFR by mother's age and location, 1950-2100, Sri Lanka

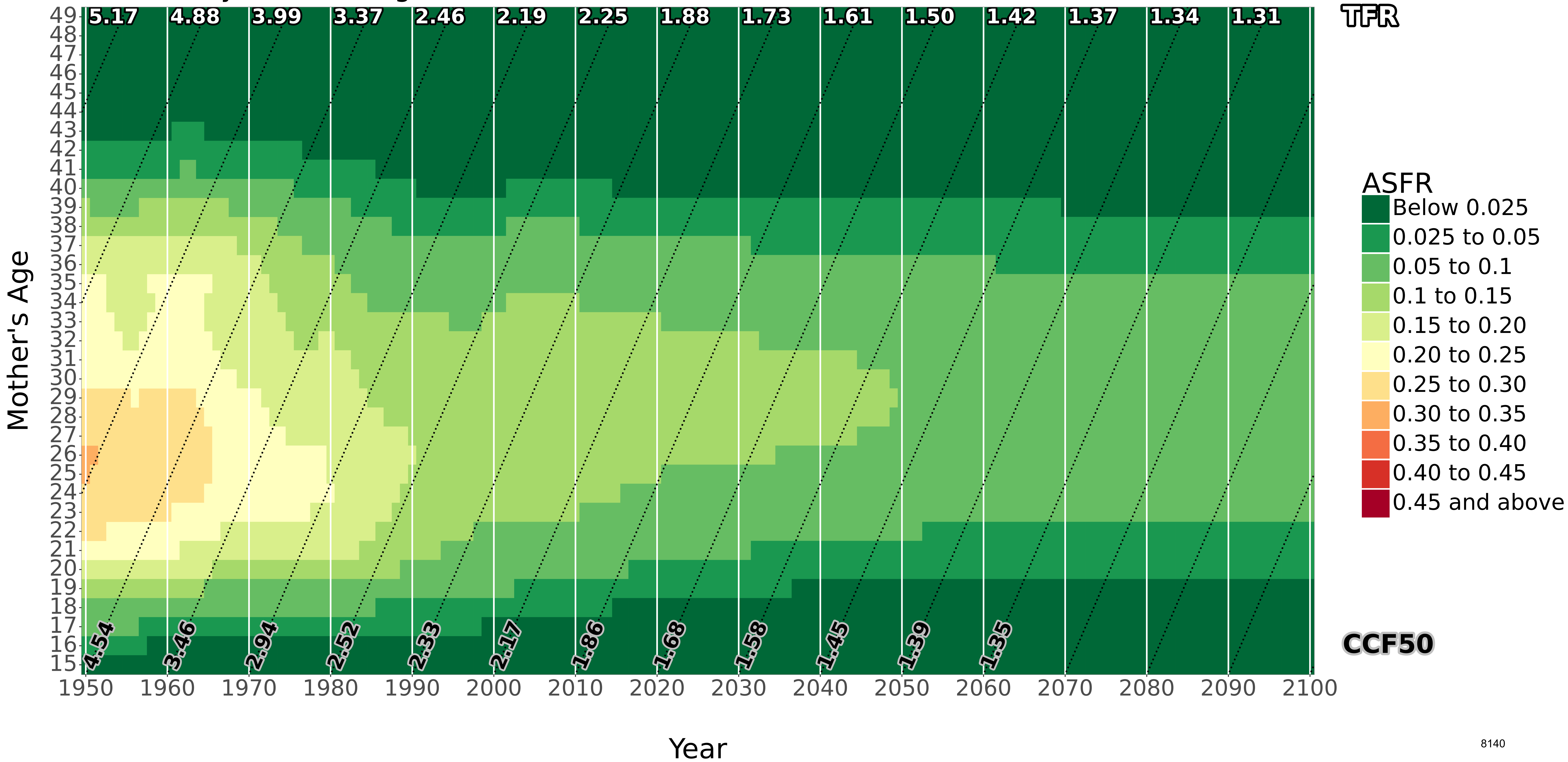

Supplemental Results Figure S3: Lexis diagram of CCF50, single-year age interval ASFR, and TFR by mother's age and location, 1950-2100, Thailand

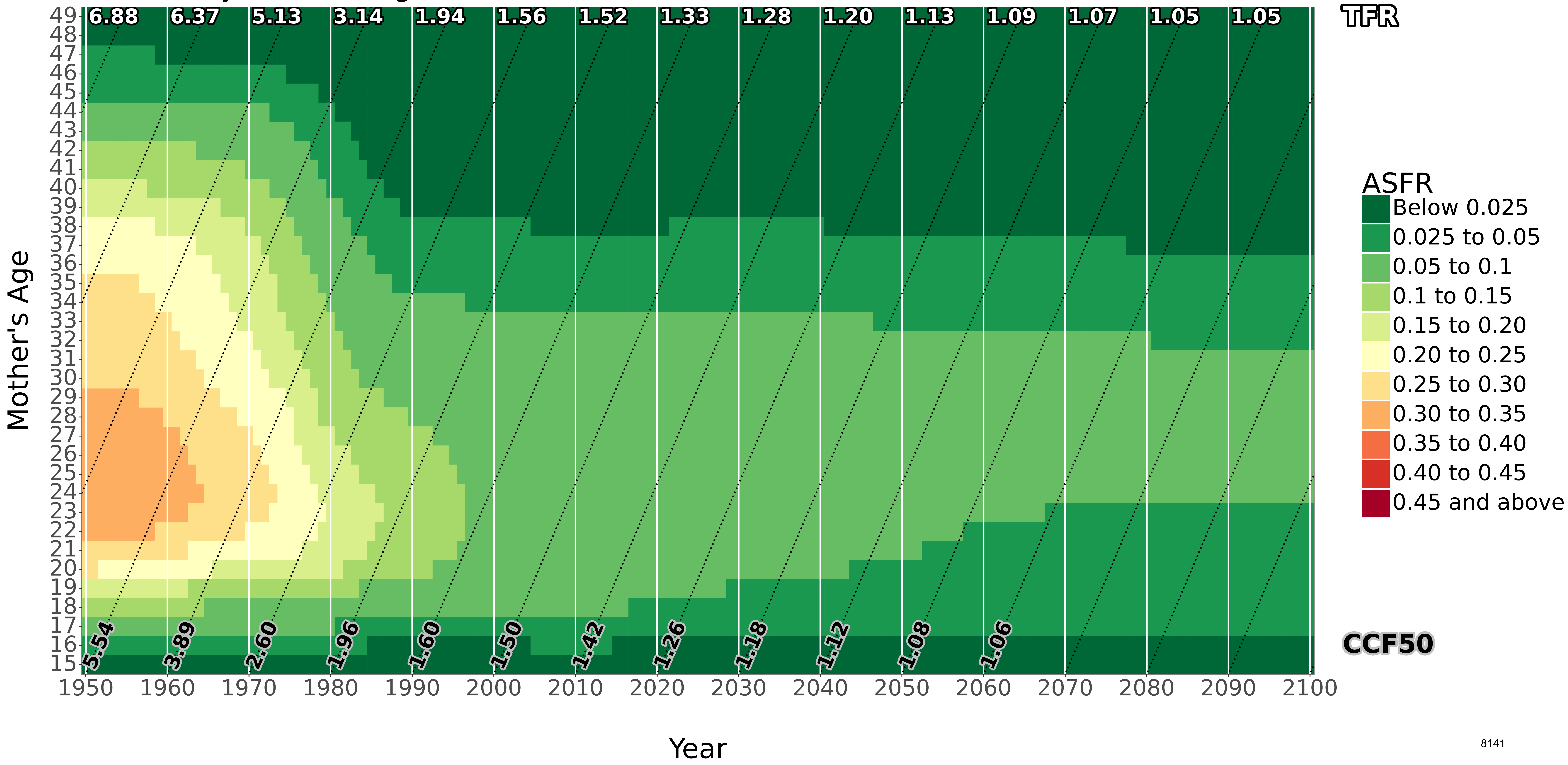

Supplemental Results Figure S3: Lexis diagram of CCF50, single-year age interval ASFR, and TFR by mother's age and location, 1950-2100, Timor-Leste

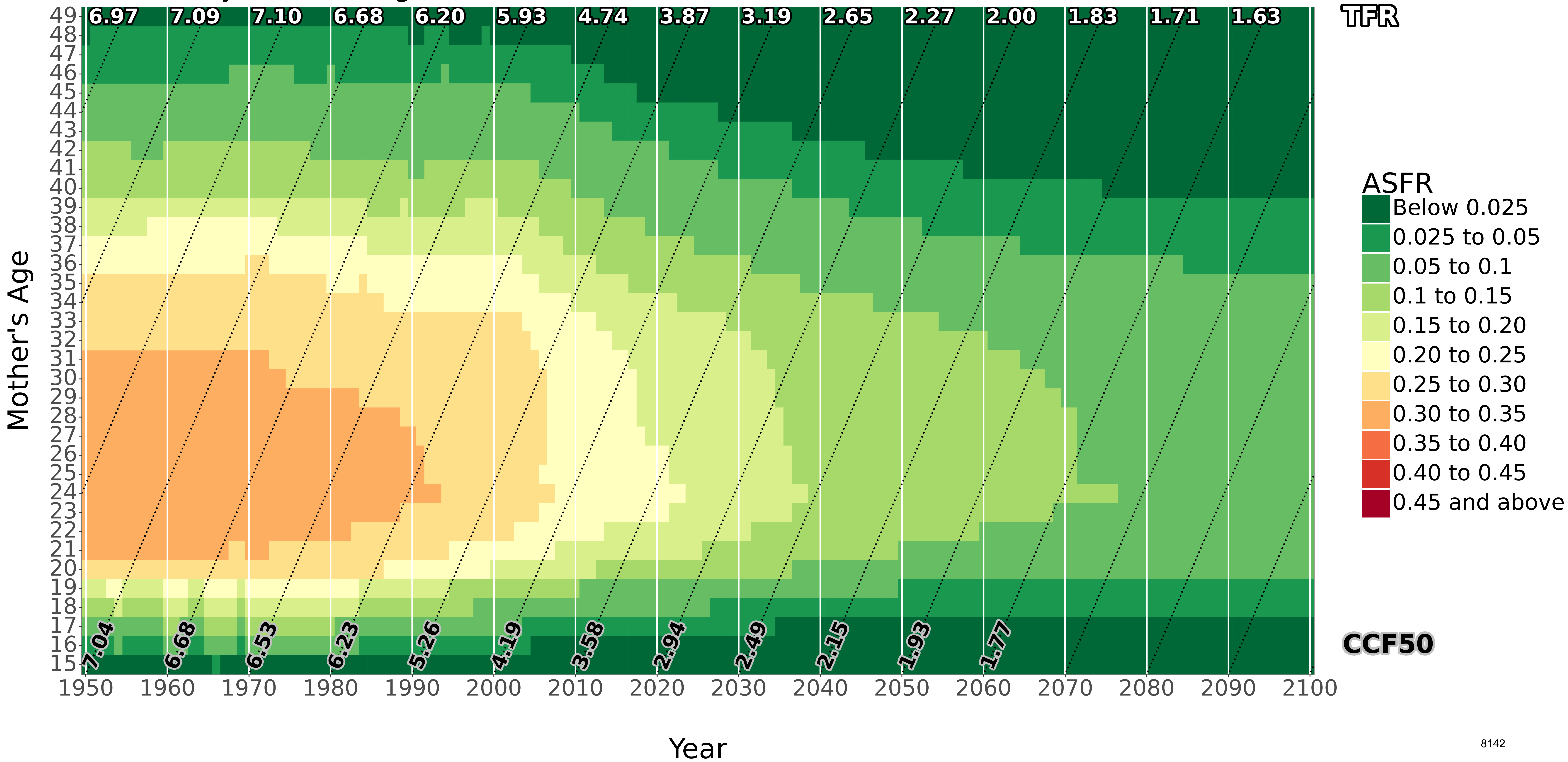

Supplemental Results Figure S3: Lexis diagram of CCF50, single-year age interval ASFR, and TFR by mother's age and location, 1950-2100, Viet Nam

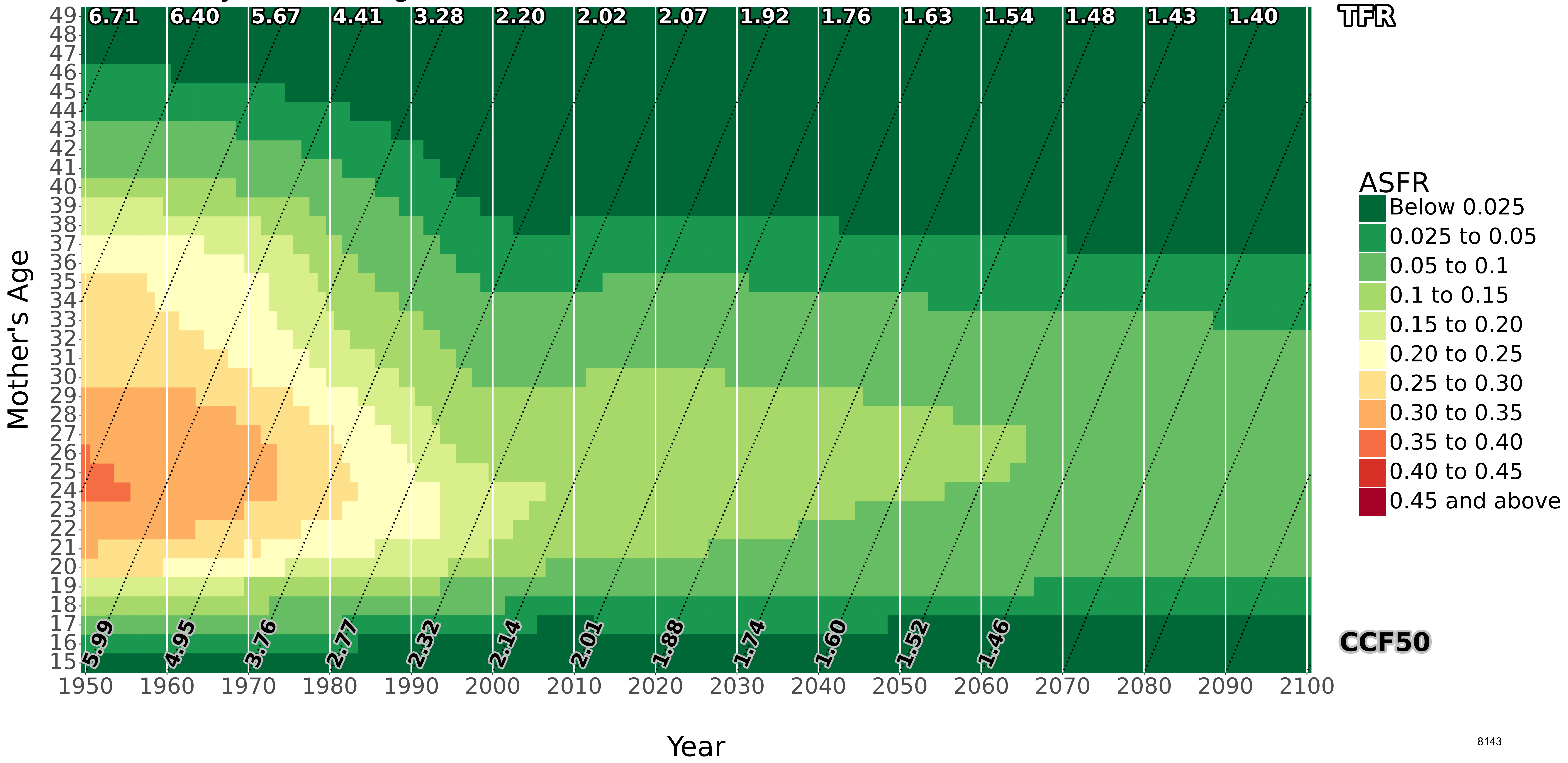

Supplemental Results Figure S3: Lexis diagram of CCF50, single-year age interval ASFR, and TFR by mother's age and location, 1950-2100, Angola

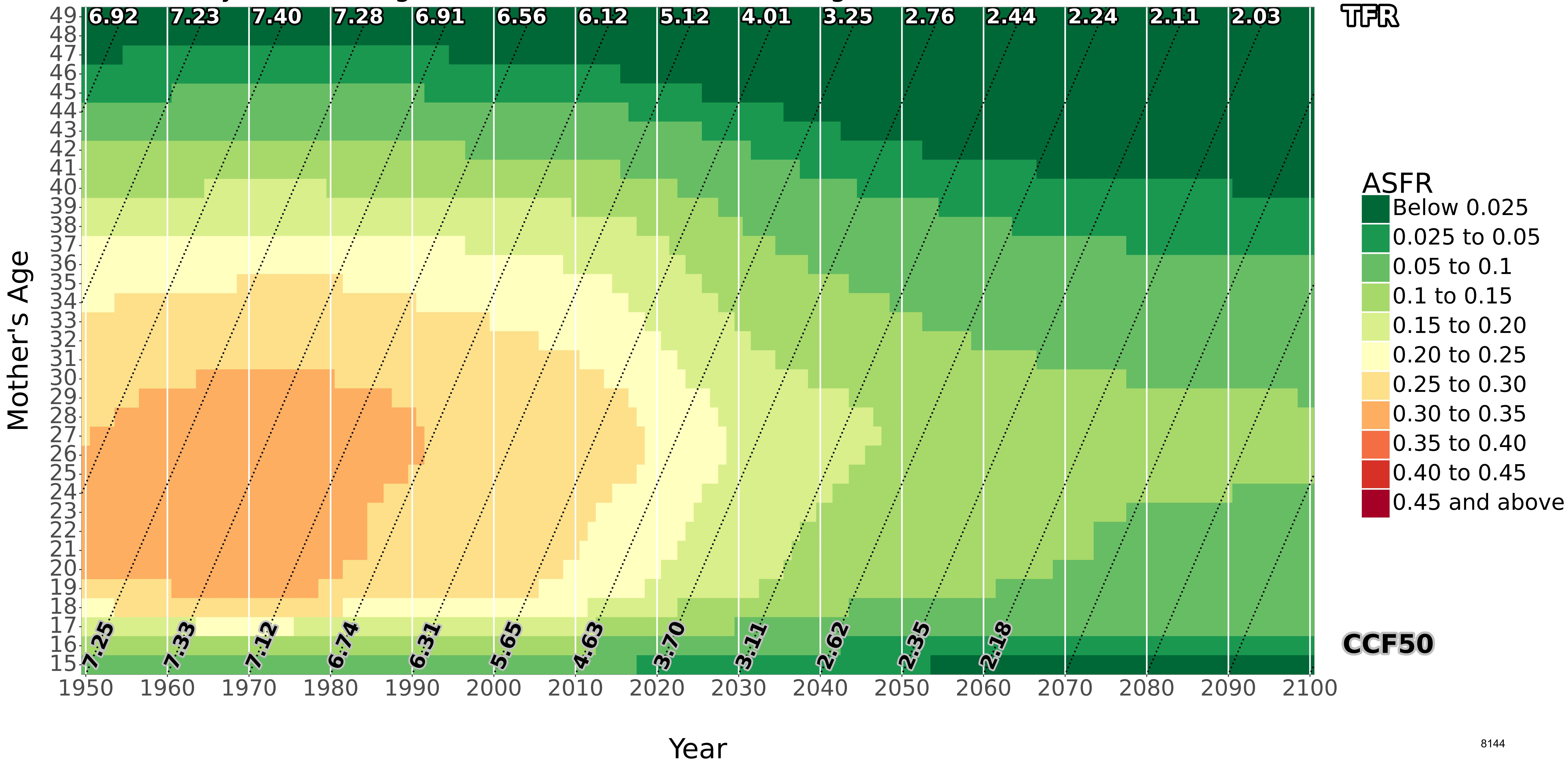

Supplemental Results Figure S3: Lexis diagram of CCF50, single-year age interval ASFR, and TFR by mother's age and location, 1950-2100, Central African Republic

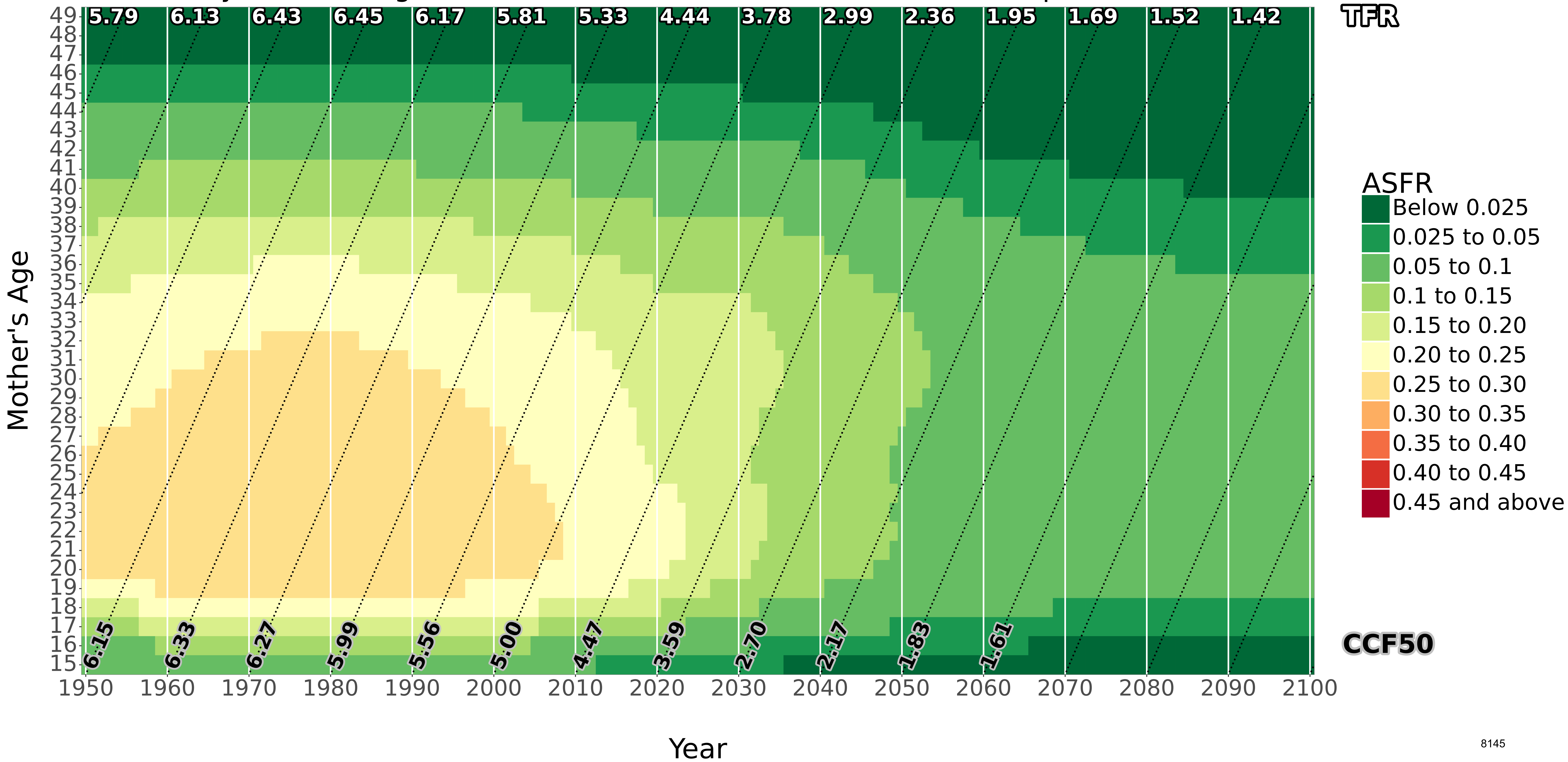

Supplemental Results Figure S3: Lexis diagram of CCF50, single-year age interval ASFR, and TFR by mother's age and location, 1950-2100, Congo (Brazzaville)

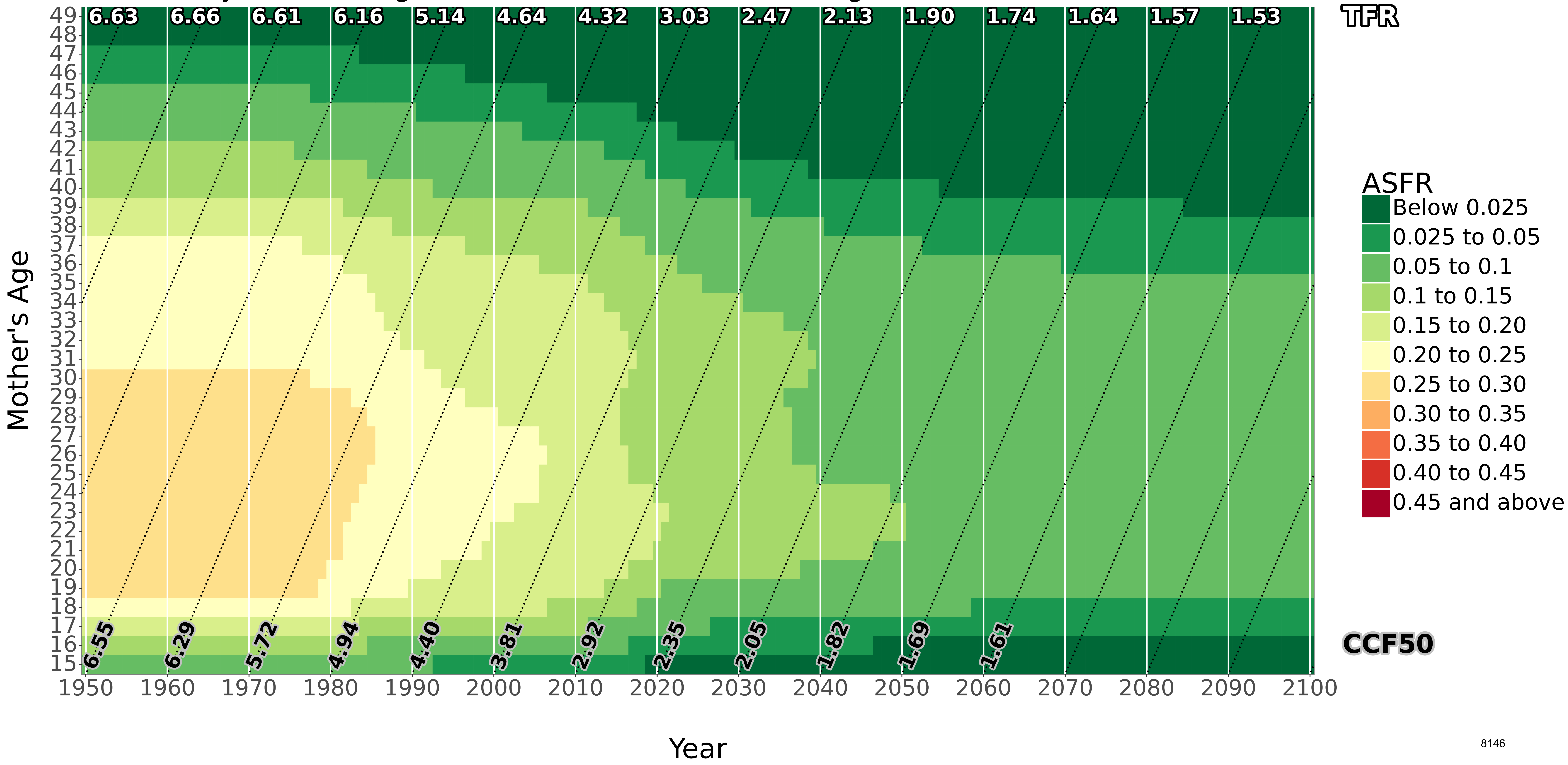

Supplemental Results Figure S3: Lexis diagram of CCF50, single-year age interval ASFR, and TFR by mother's age and location, 1950-2100, DR Congo

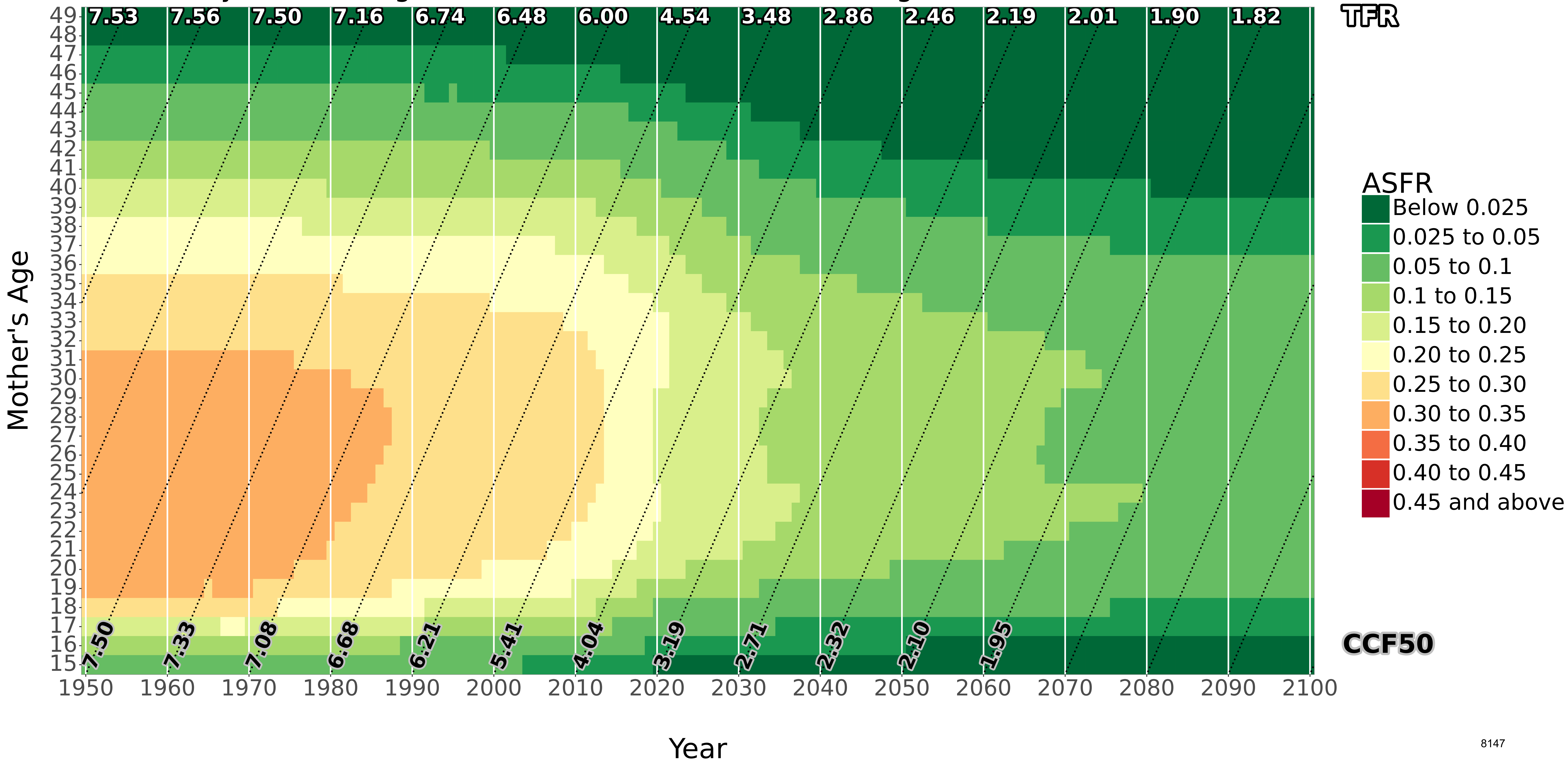

Supplemental Results Figure S3: Lexis diagram of CCF50, single-year age interval ASFR, and TFR by mother's age and location, 1950-2100, Equatorial Guinea

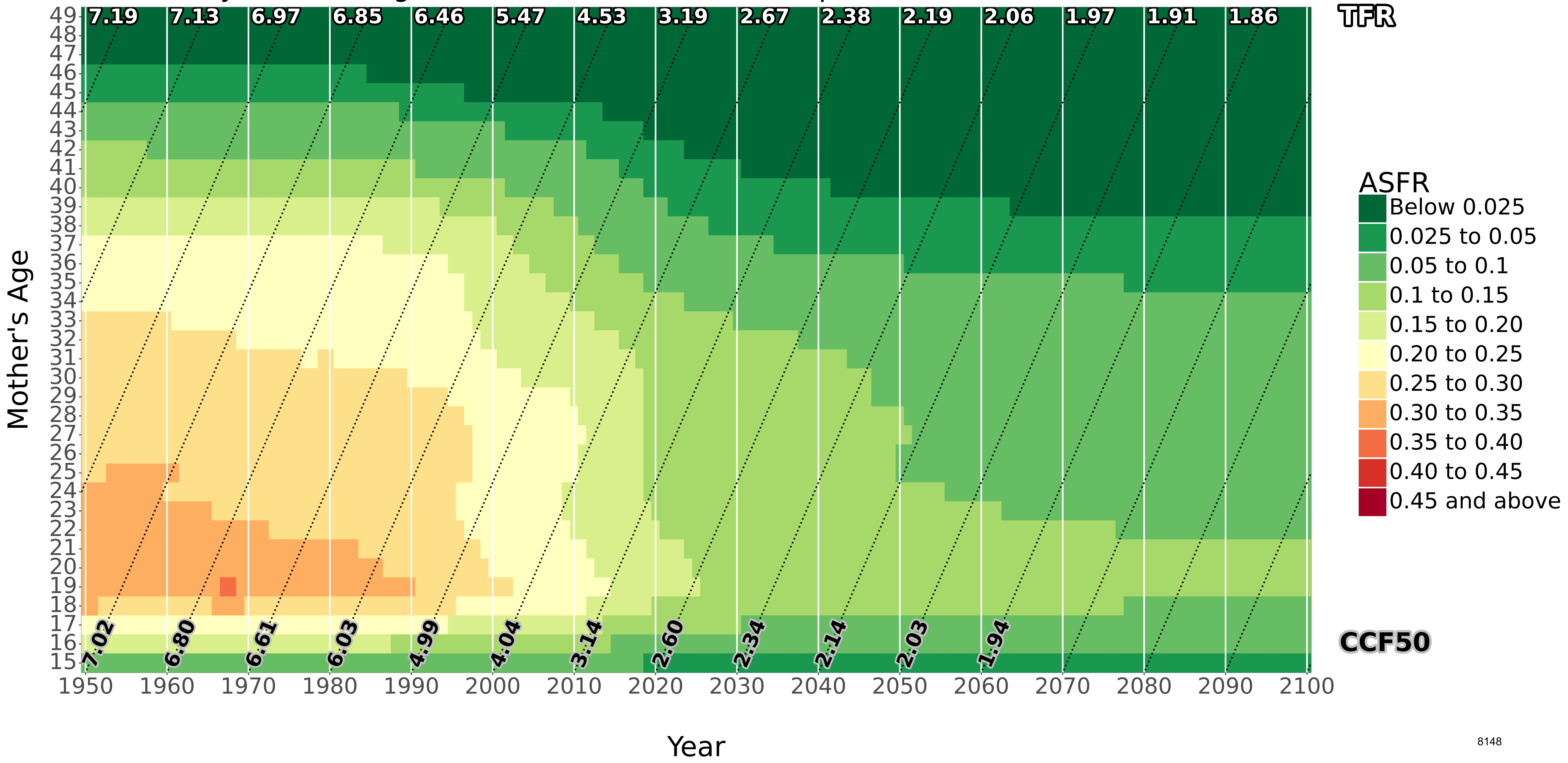

Supplemental Results Figure S3: Lexis diagram of CCF50, single-year age interval ASFR, and TFR by mother's age and location, 1950-2100, Gabon

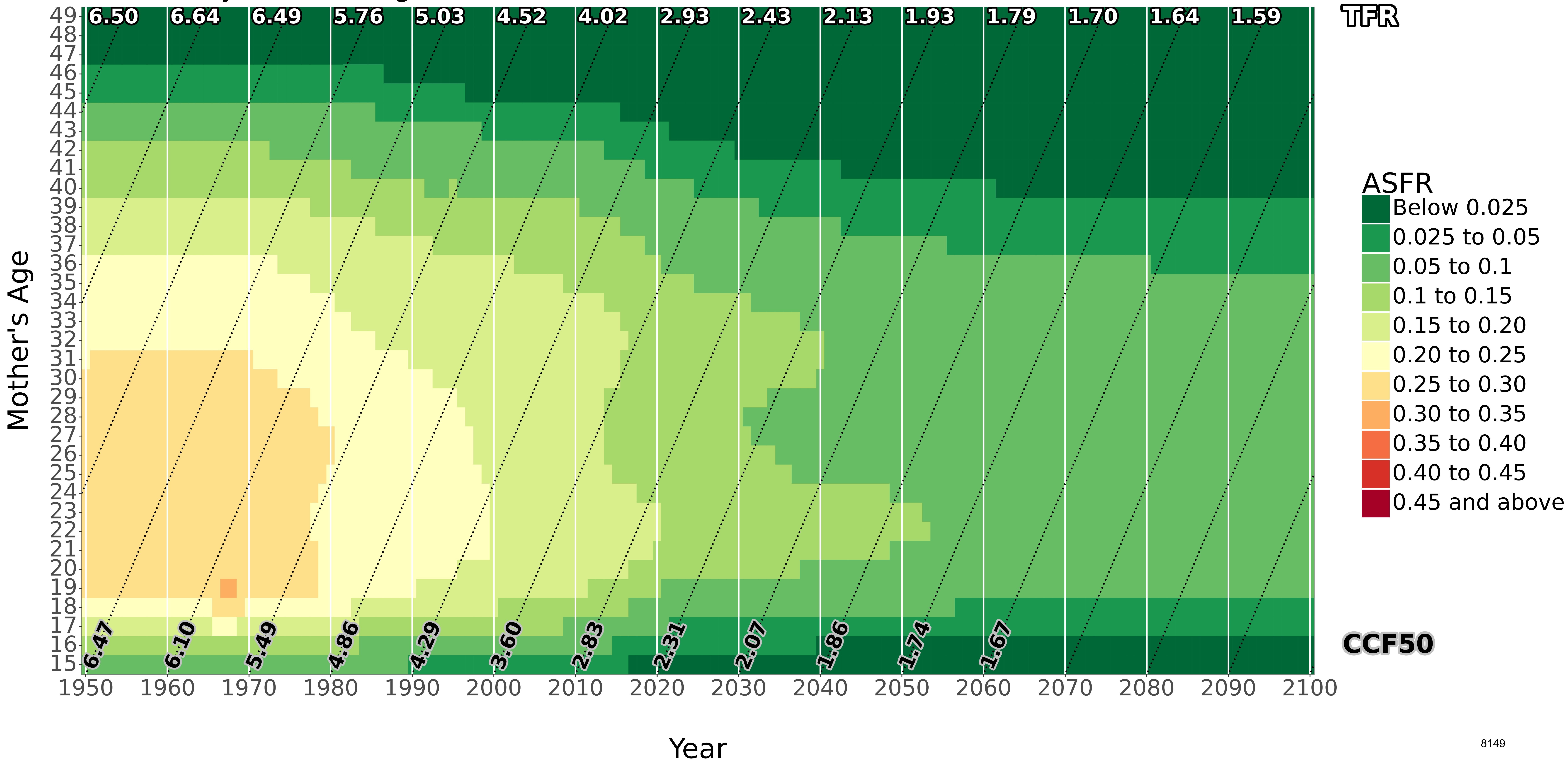

Supplemental Results Figure S3: Lexis diagram of CCF50, single-year age interval ASFR, and TFR by mother's age and location, 1950-2100, Burundi

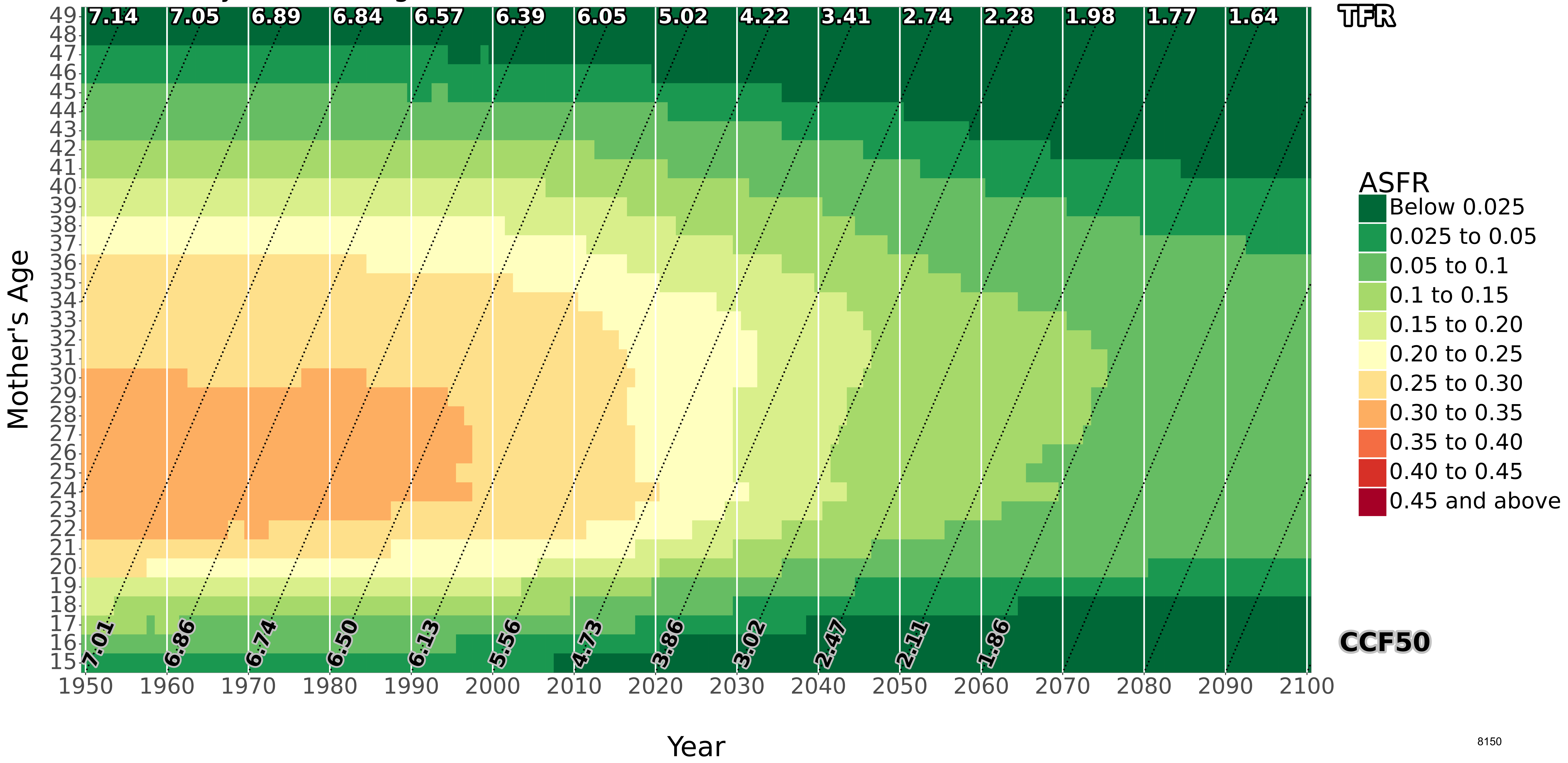

Supplemental Results Figure S3: Lexis diagram of CCF50, single-year age interval ASFR, and TFR by mother's age and location, 1950-2100, Comoros

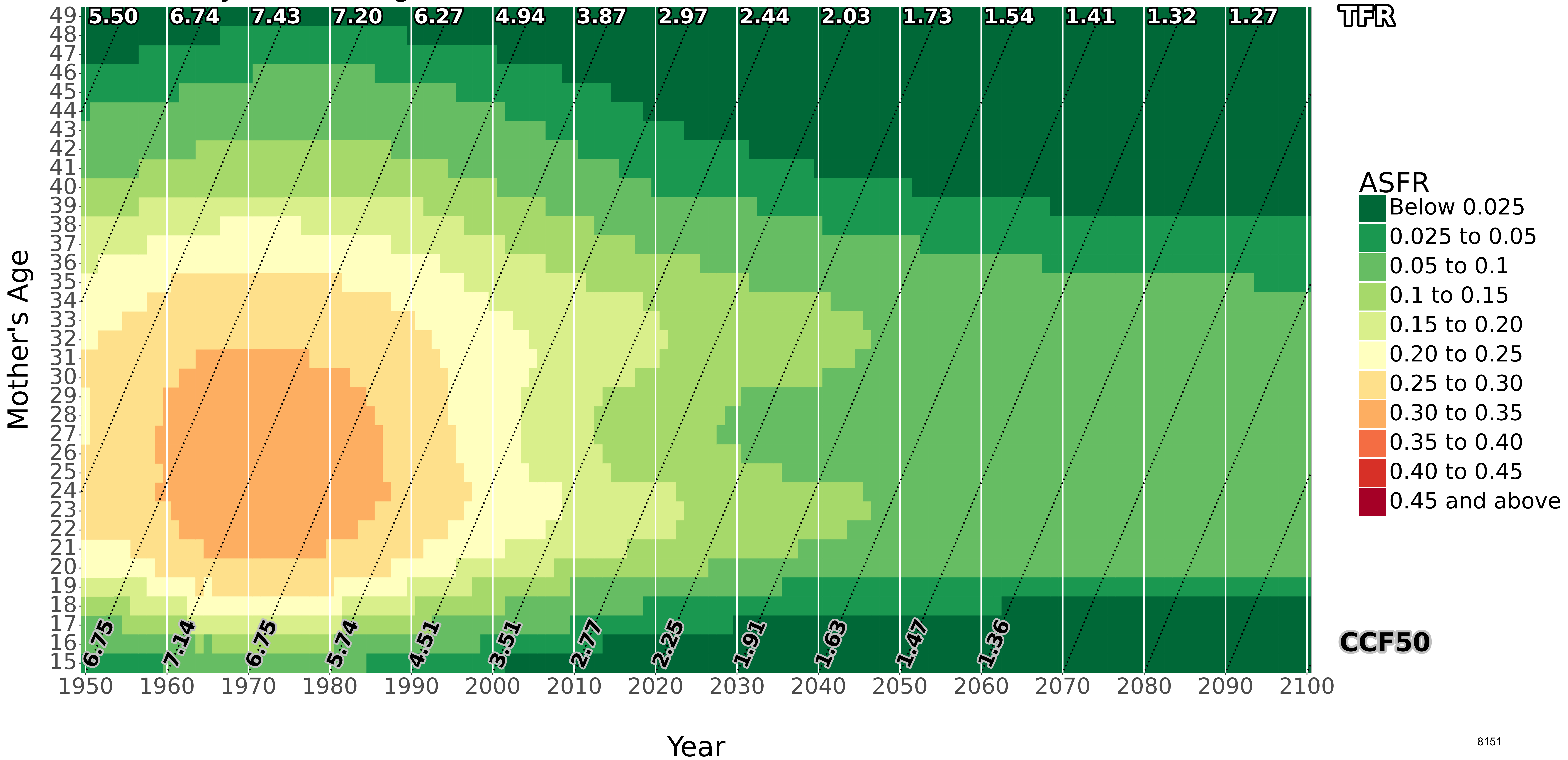

Supplemental Results Figure S3: Lexis diagram of CCF50, single-year age interval ASFR, and TFR by mother's age and location, 1950-2100, Djibouti

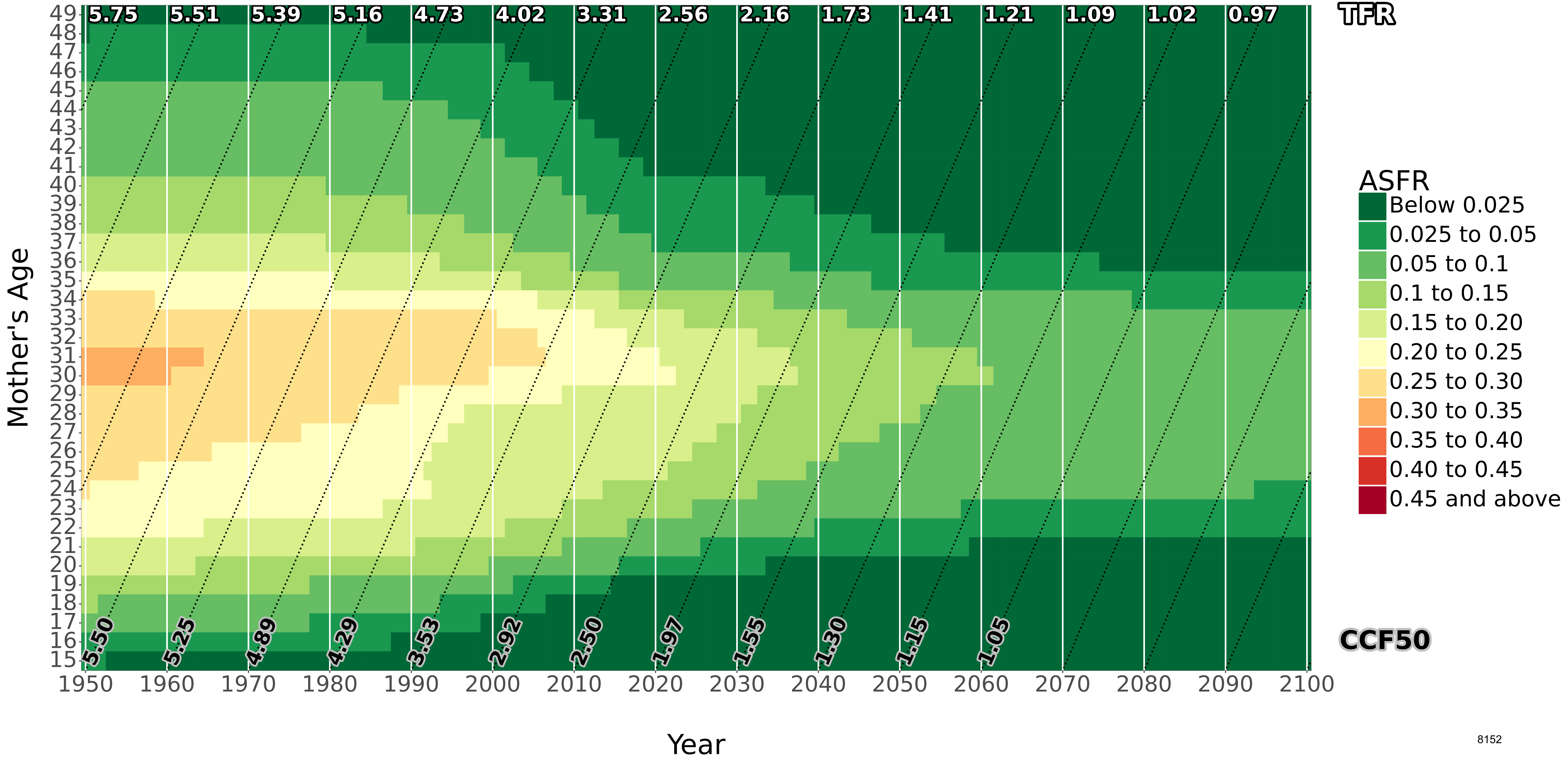

Supplemental Results Figure S3: Lexis diagram of CCF50, single-year age interval ASFR, and TFR by mother's age and location, 1950-2100, Eritrea

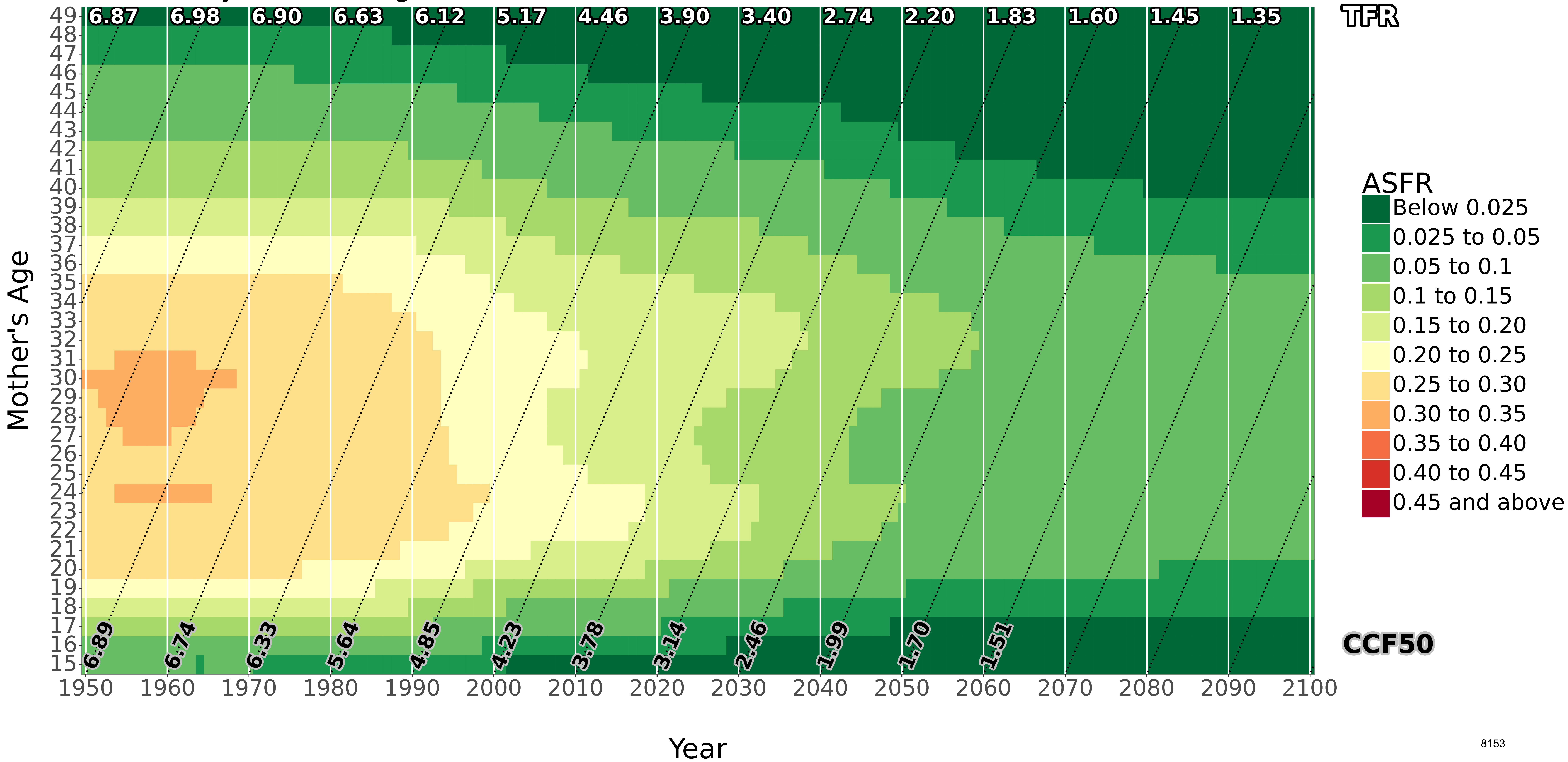

Supplemental Results Figure S3: Lexis diagram of CCF50, single-year age interval ASFR, and TFR by mother's age and location, 1950-2100, Ethiopia

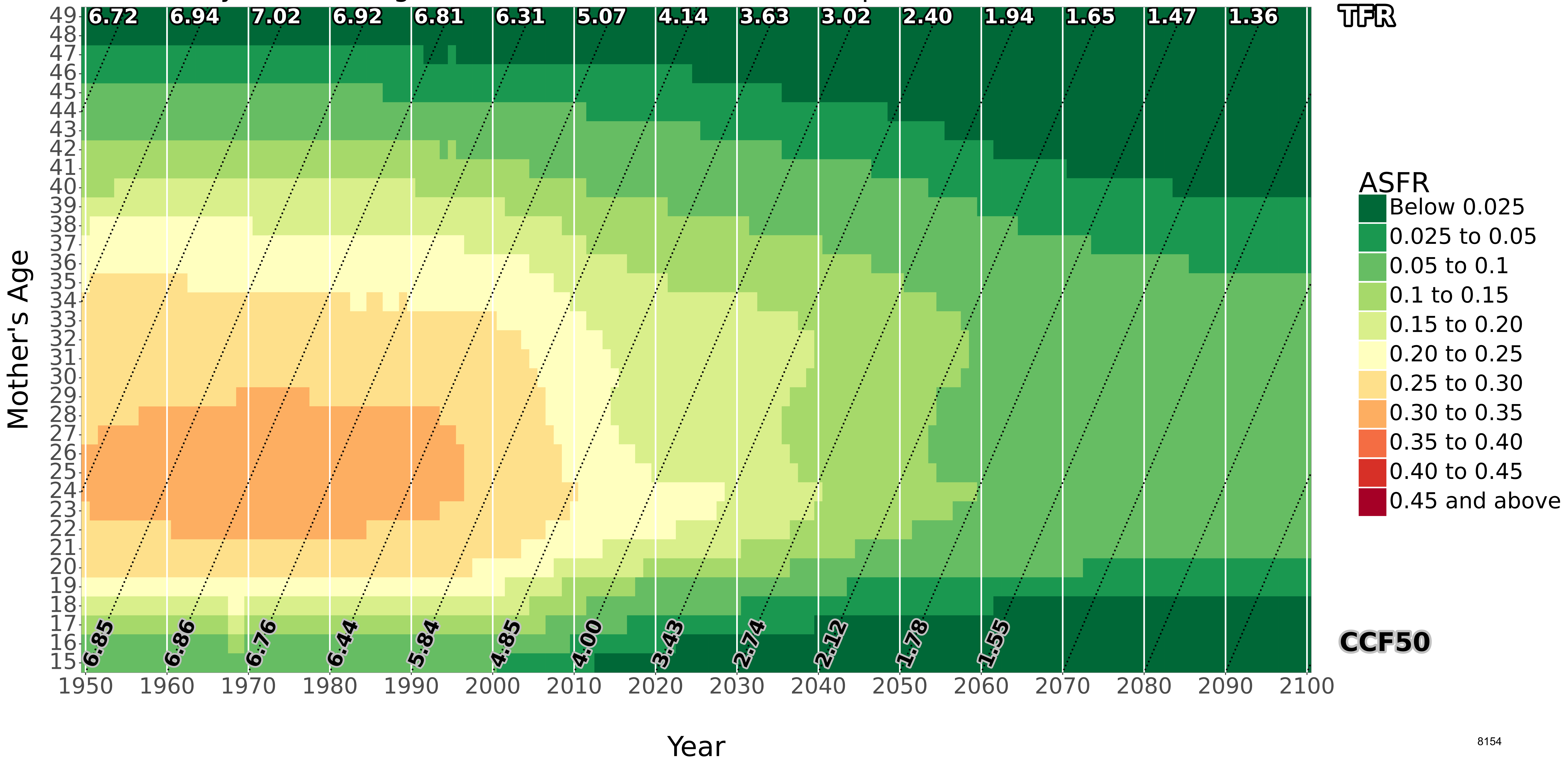

Supplemental Results Figure S3: Lexis diagram of CCF50, single-year age interval ASFR, and TFR by mother's age and location, 1950-2100, Kenya

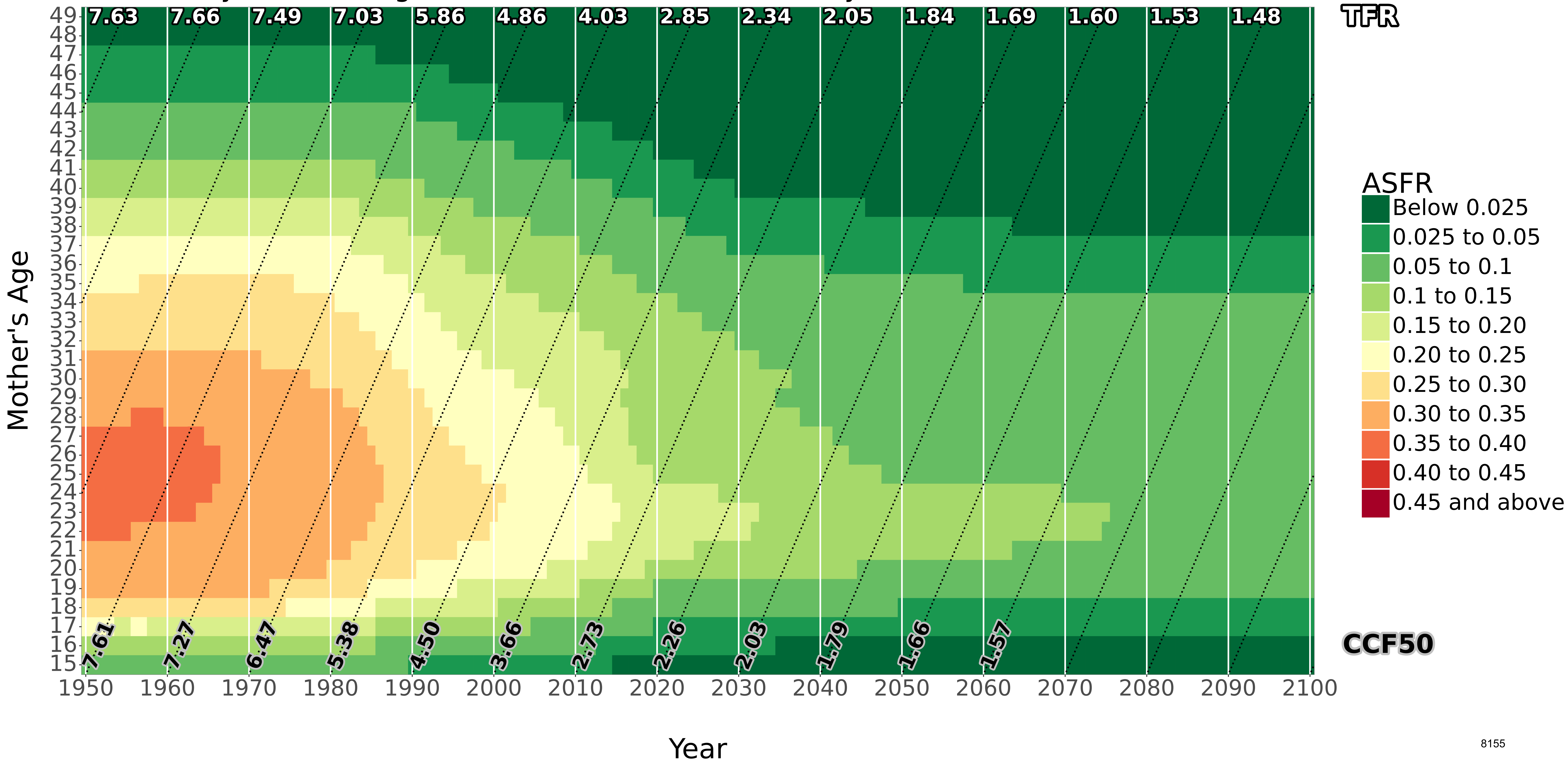

Supplemental Results Figure S3: Lexis diagram of CCF50, single-year age interval ASFR, and TFR by mother's age and location, 1950-2100, Madagascar

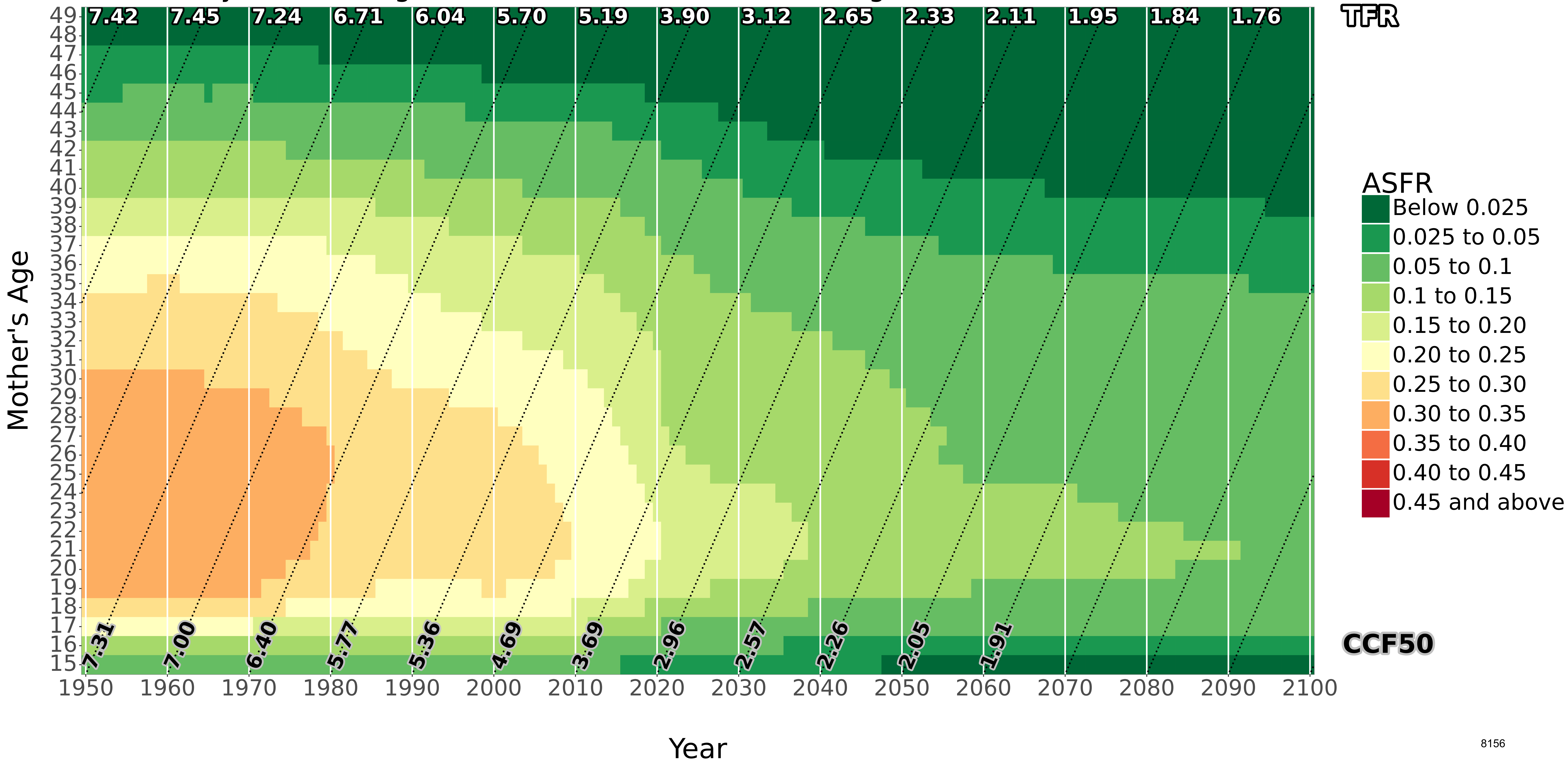

Supplemental Results Figure S3: Lexis diagram of CCF50, single-year age interval ASFR, and TFR by mother's age and location, 1950-2100, Malawi

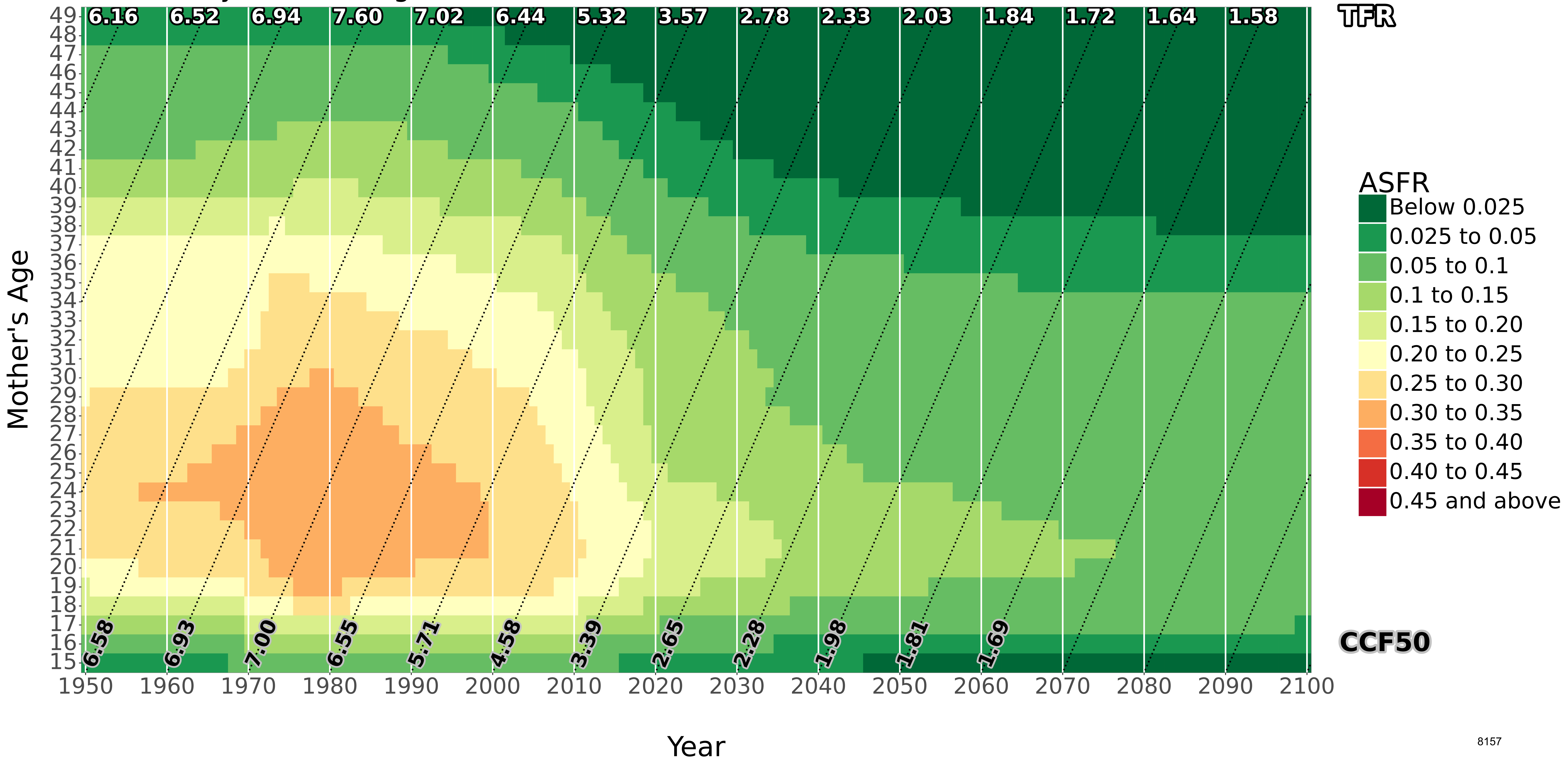

Supplemental Results Figure S3: Lexis diagram of CCF50, single-year age interval ASFR, and TFR by mother's age and location, 1950-2100, Mozambique

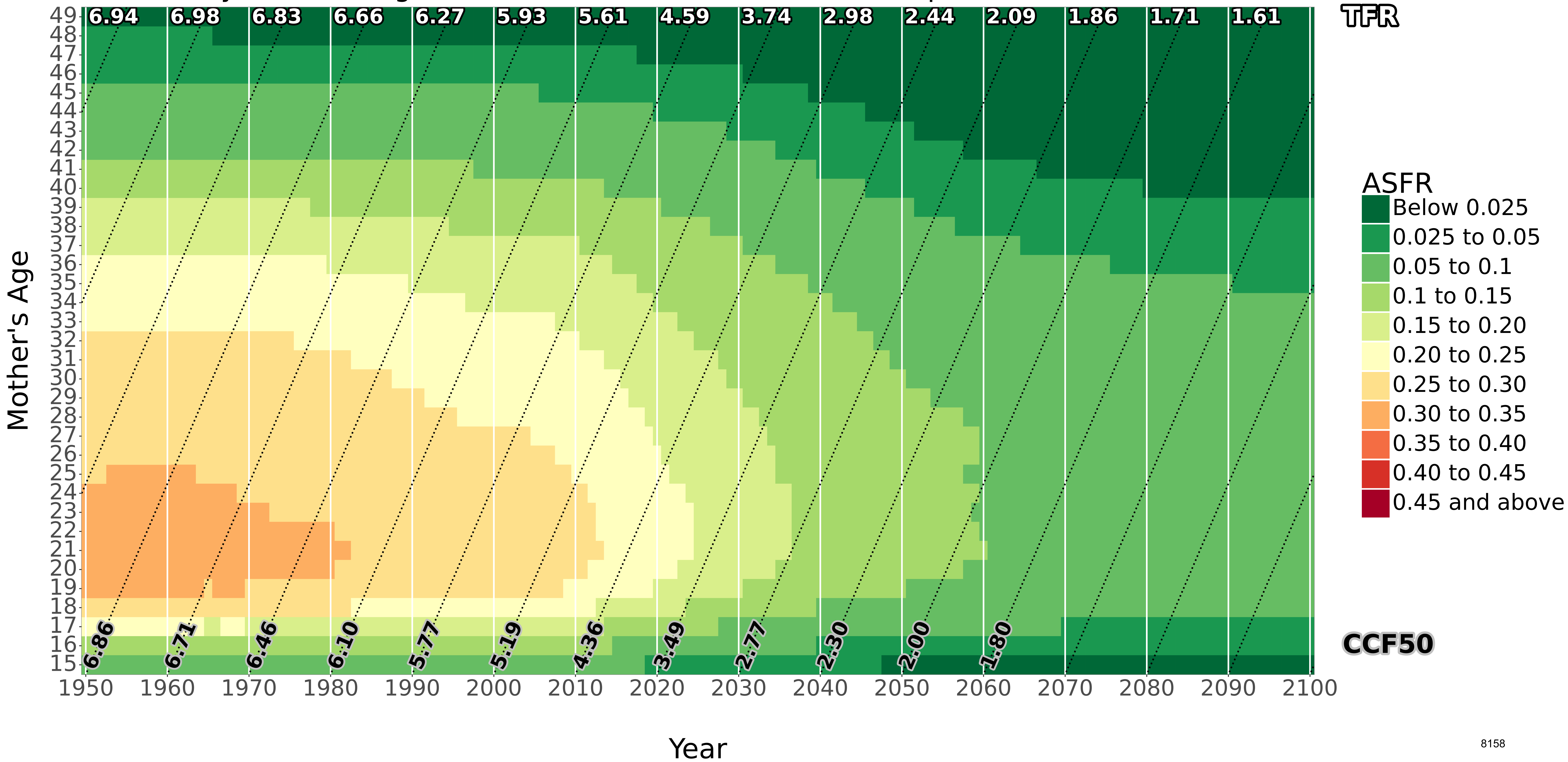

Supplemental Results Figure S3: Lexis diagram of CCF50, single-year age interval ASFR, and TFR by mother's age and location, 1950-2100, Rwanda

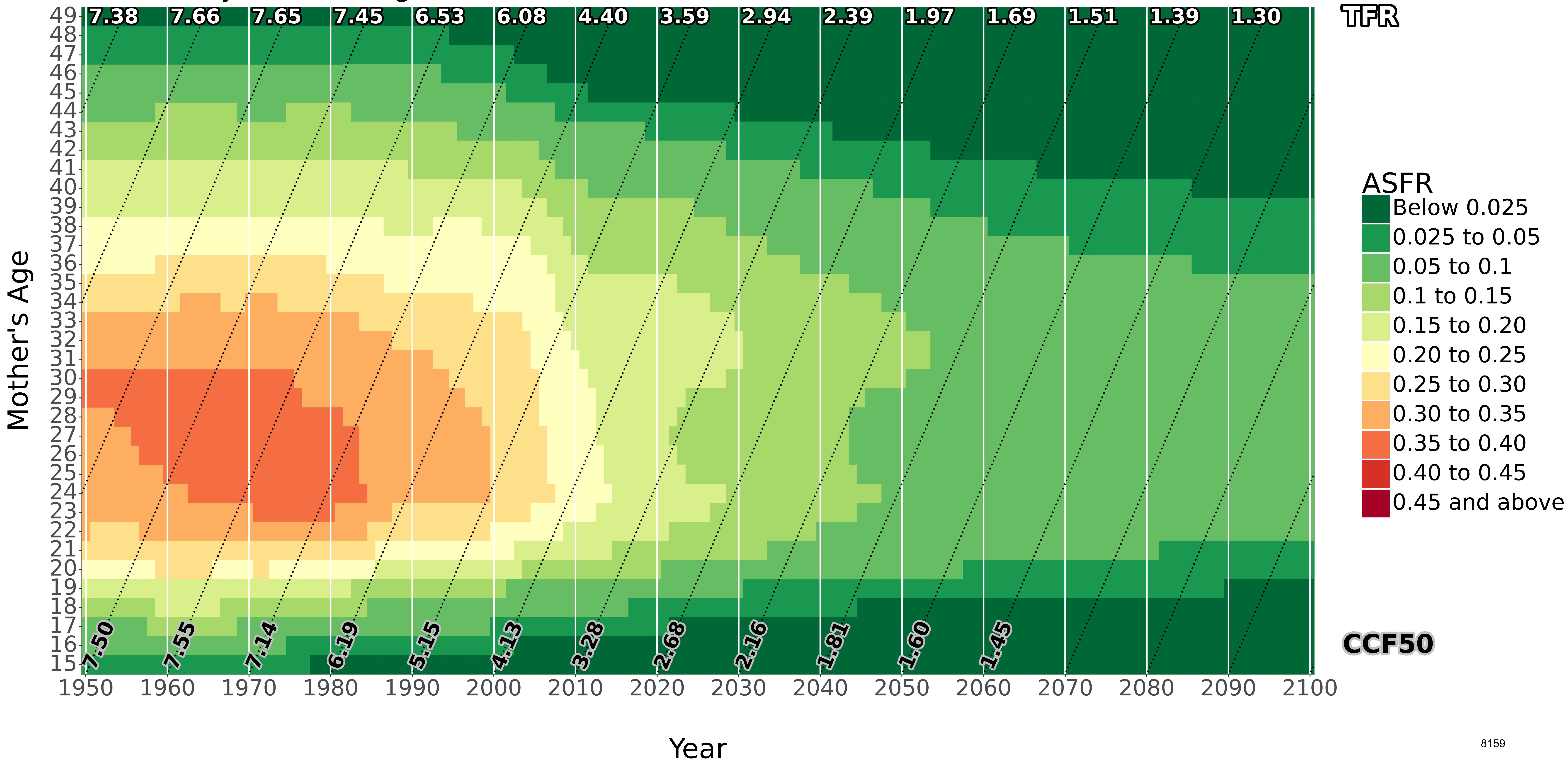

Supplemental Results Figure S3: Lexis diagram of CCF50, single-year age interval ASFR, and TFR by mother's age and location, 1950-2100, Somalia

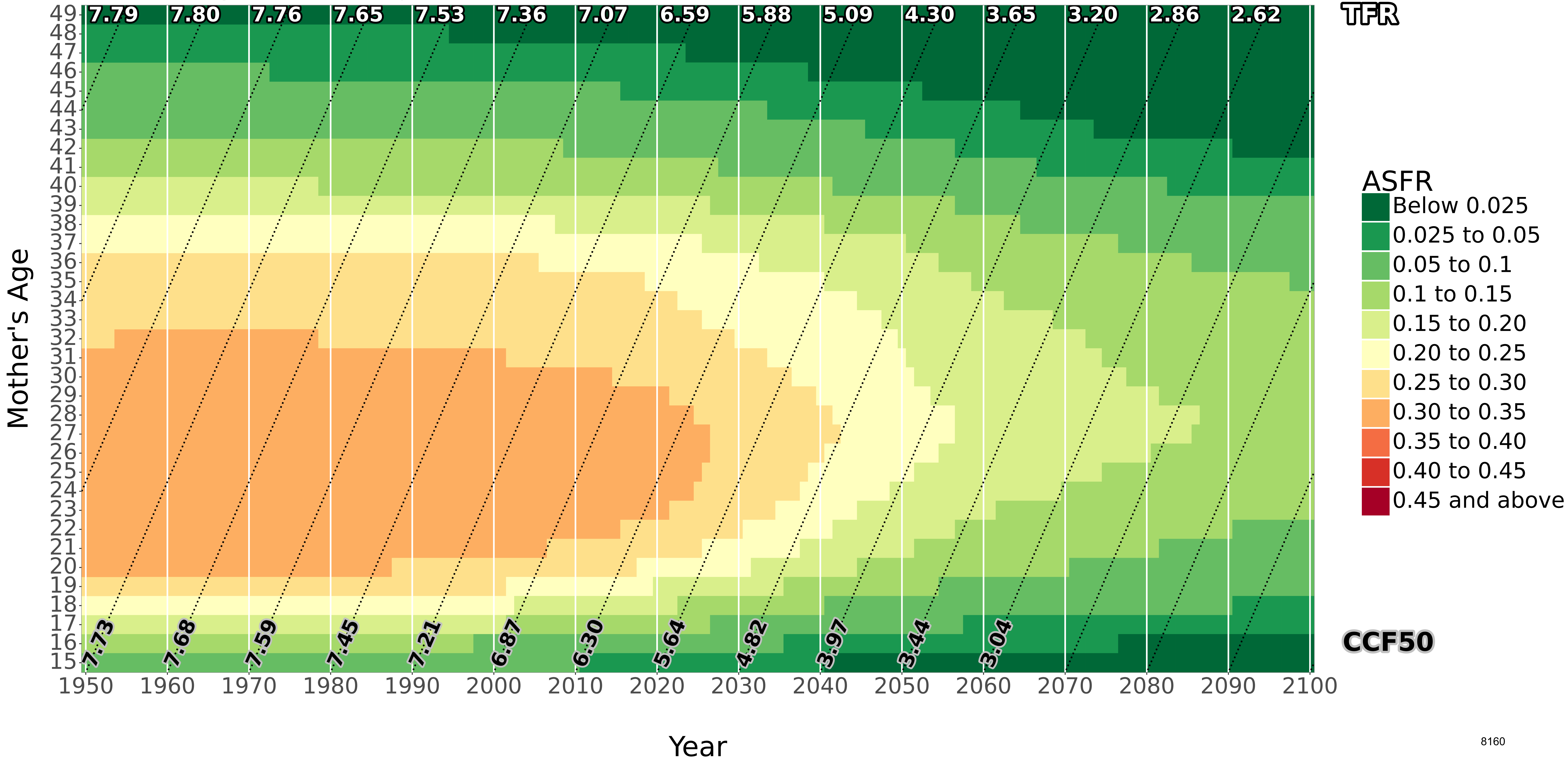

Supplemental Results Figure S3: Lexis diagram of CCF50, single-year age interval ASFR, and TFR by mother's age and location, 1950-2100, South Sudan

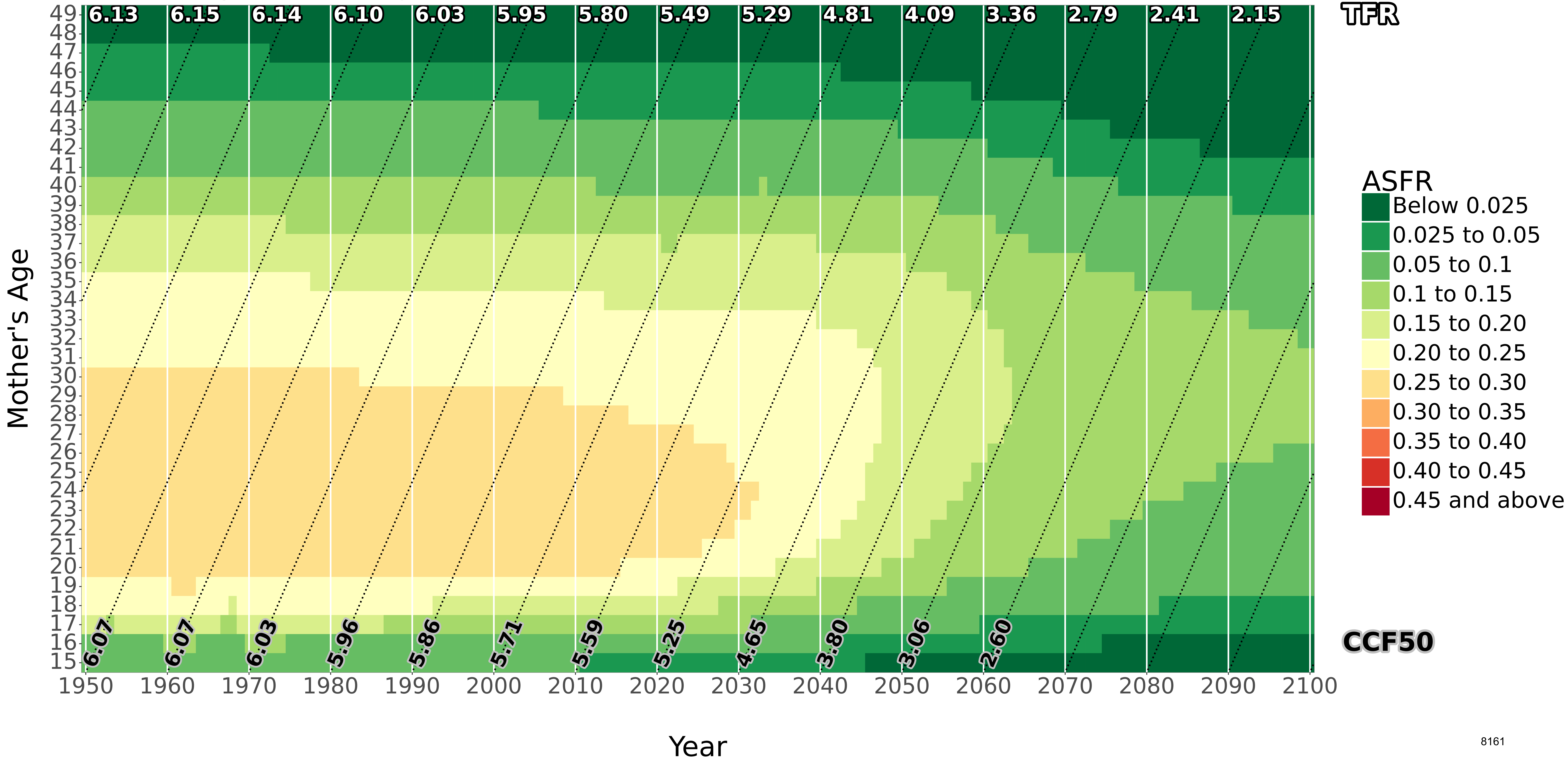

Supplemental Results Figure S3: Lexis diagram of CCF50, single-year age interval ASFR, and TFR by mother's age and location, 1950-2100, Uganda

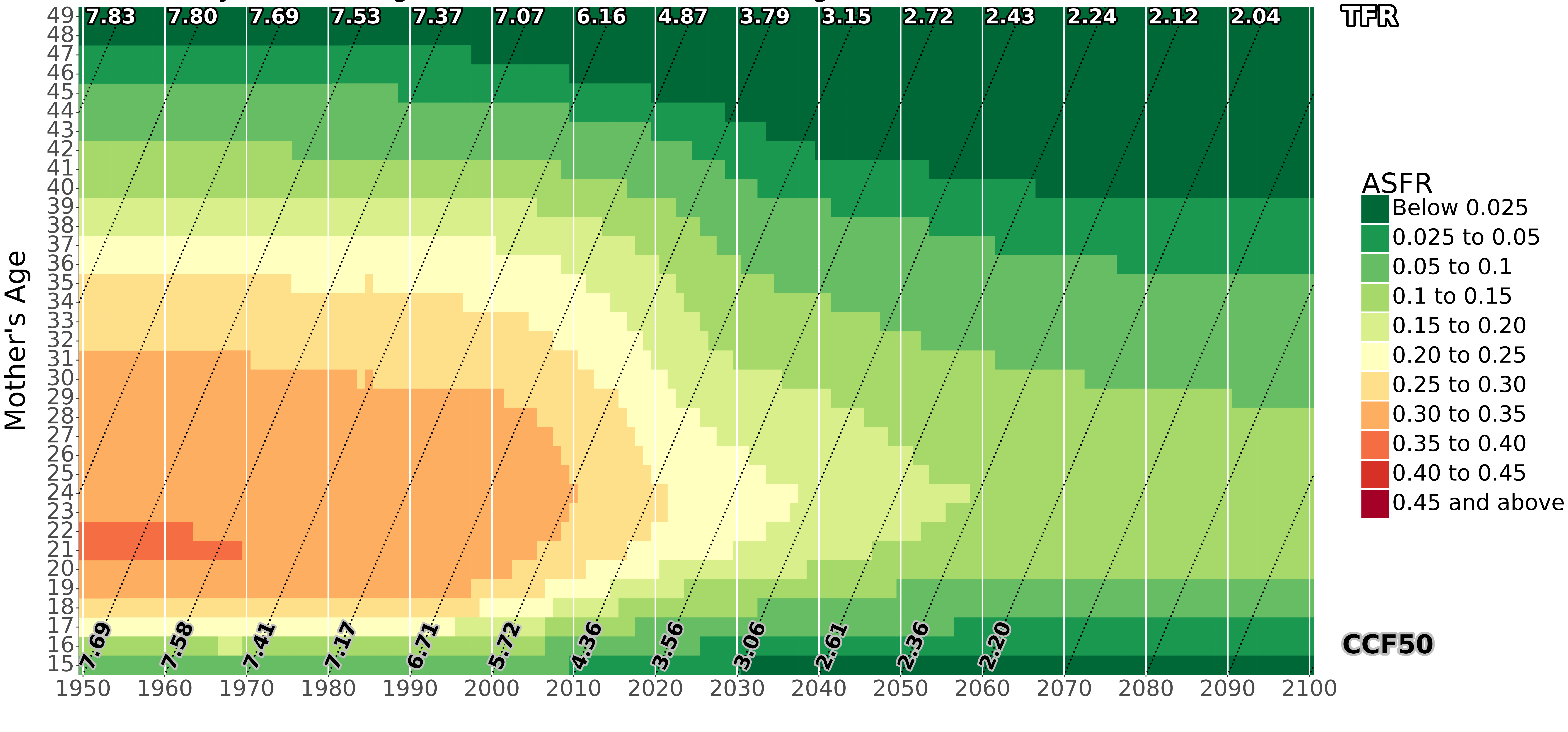

Supplemental Results Figure S3: Lexis diagram of CCF50, single-year age interval ASFR, and TFR by mother's age and location, 1950-2100, Tanzania

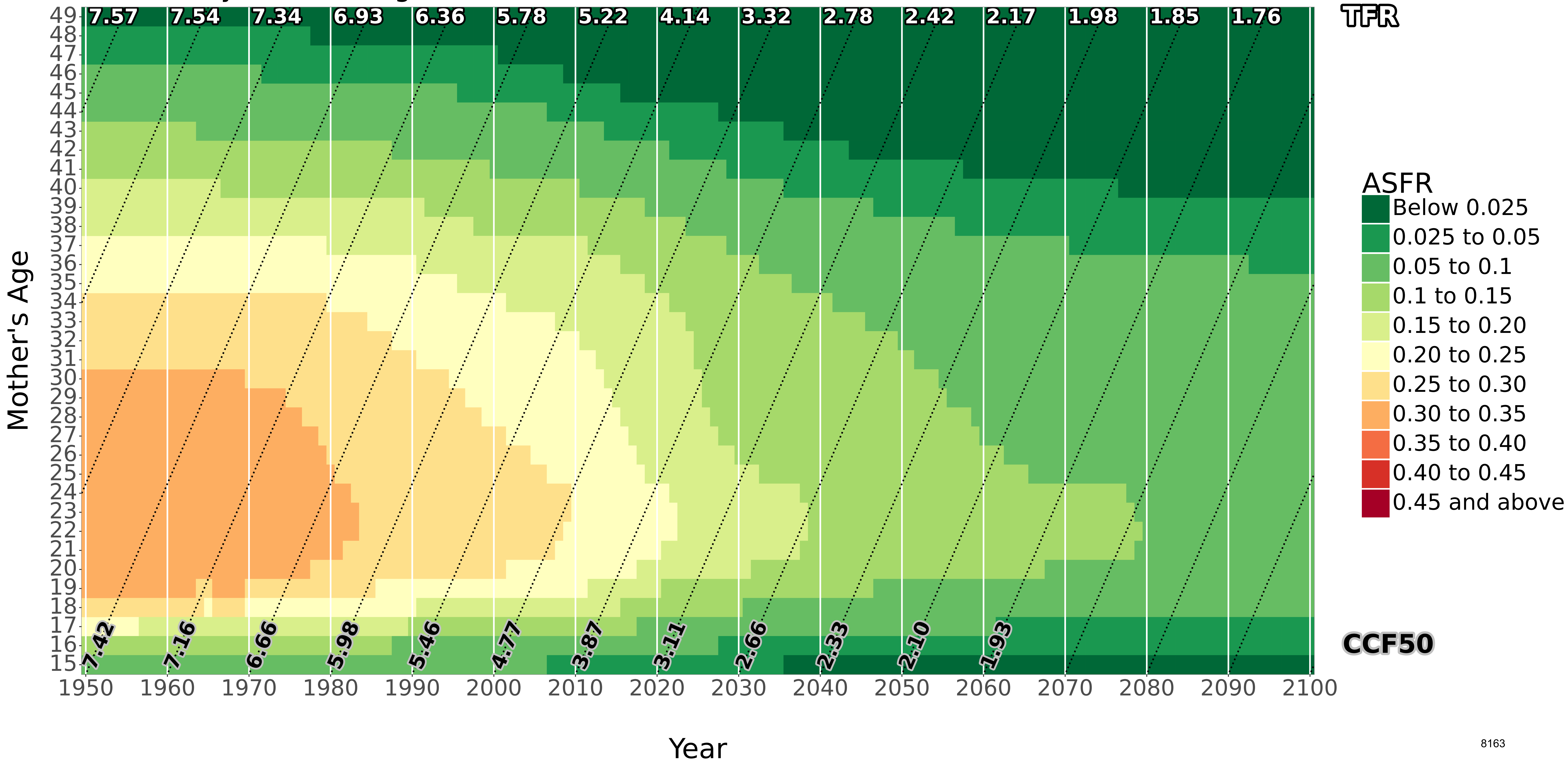

Supplemental Results Figure S3: Lexis diagram of CCF50, single-year age interval ASFR, and TFR by mother's age and location, 1950-2100, Zambia

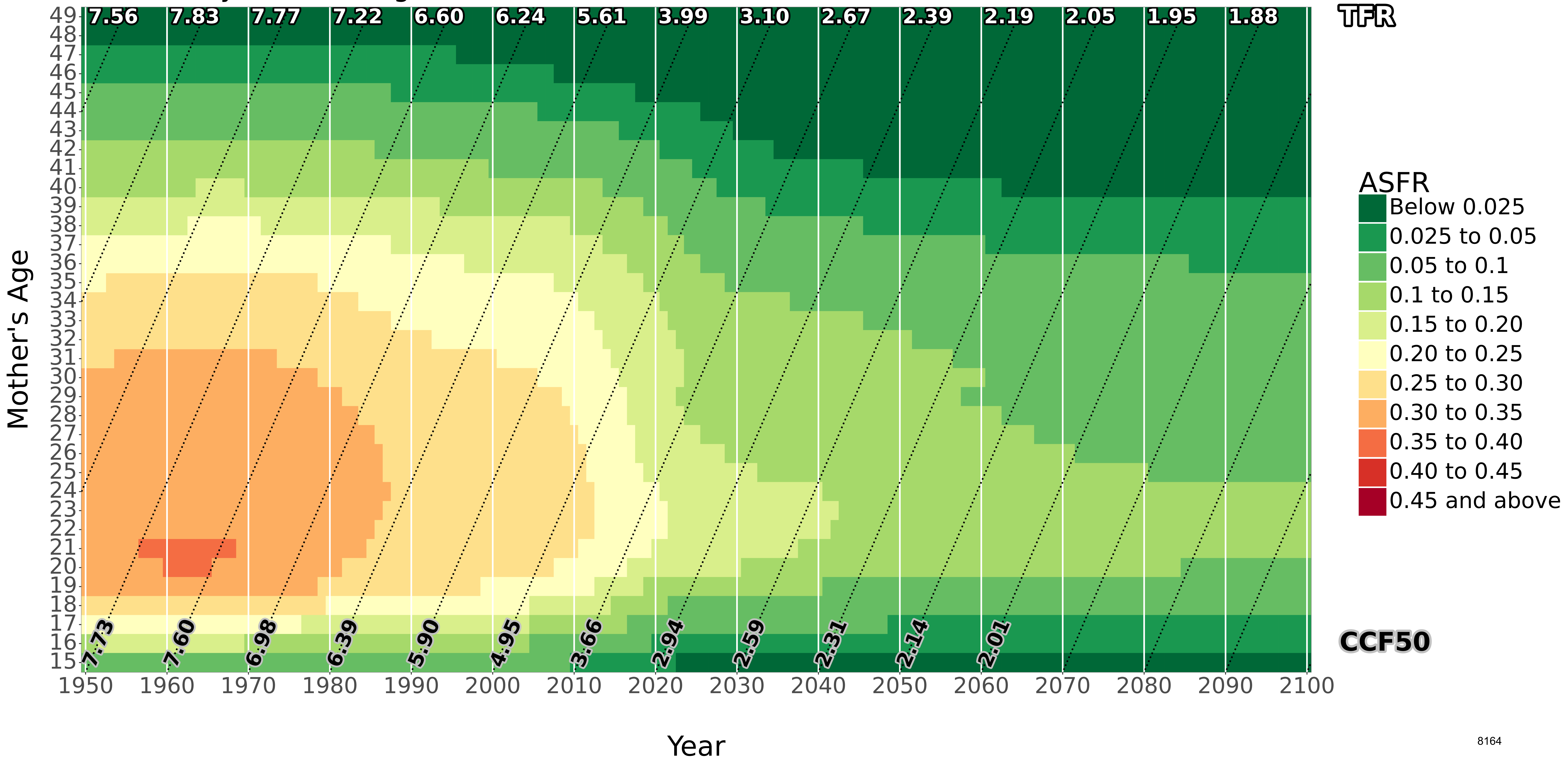

Supplemental Results Figure S3: Lexis diagram of CCF50, single-year age interval ASFR, and TFR by mother's age and location, 1950-2100, Botswana

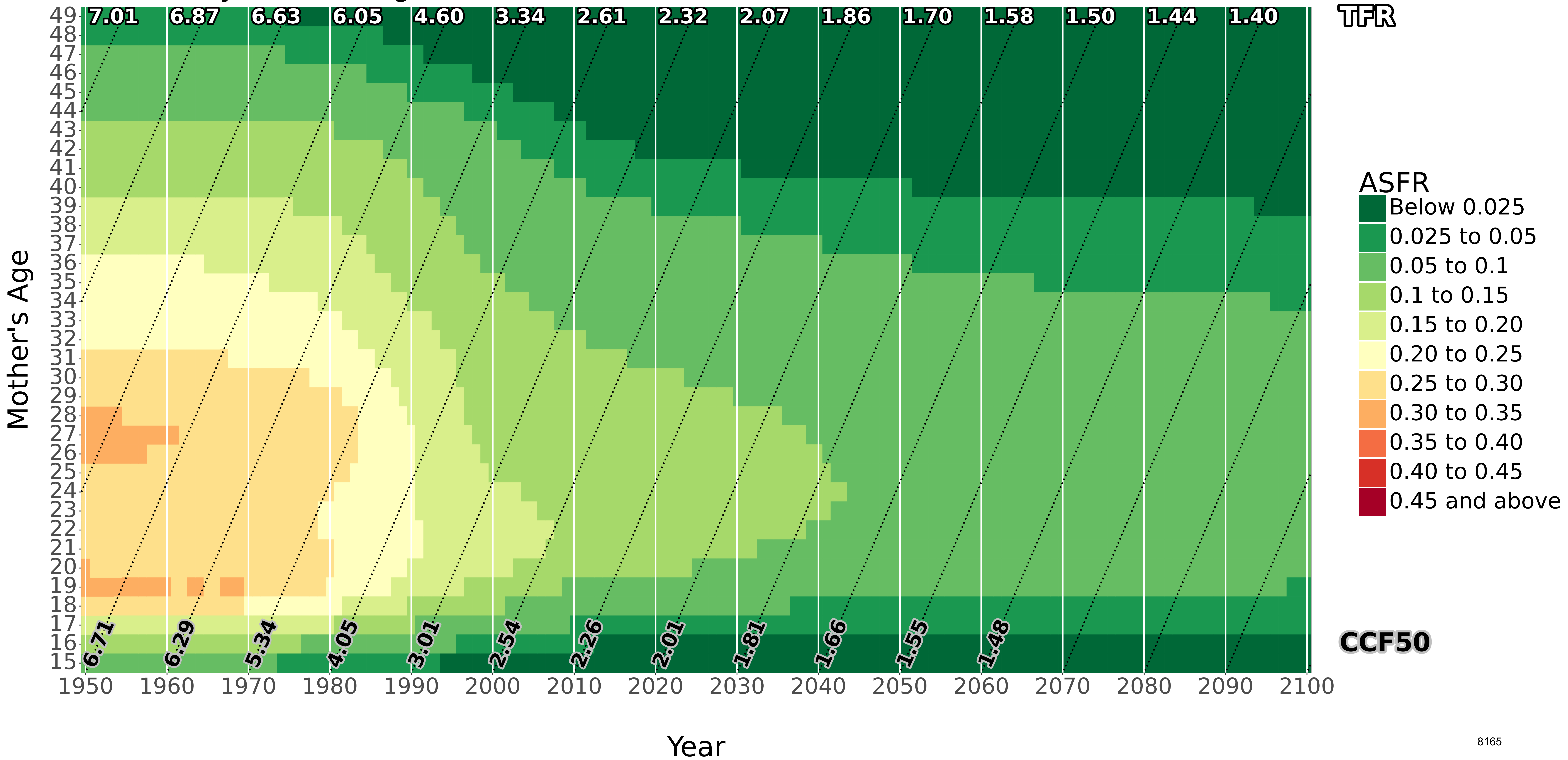

Supplemental Results Figure S3: Lexis diagram of CCF50, single-year age interval ASFR, and TFR by mother's age and location, 1950-2100, Eswatini

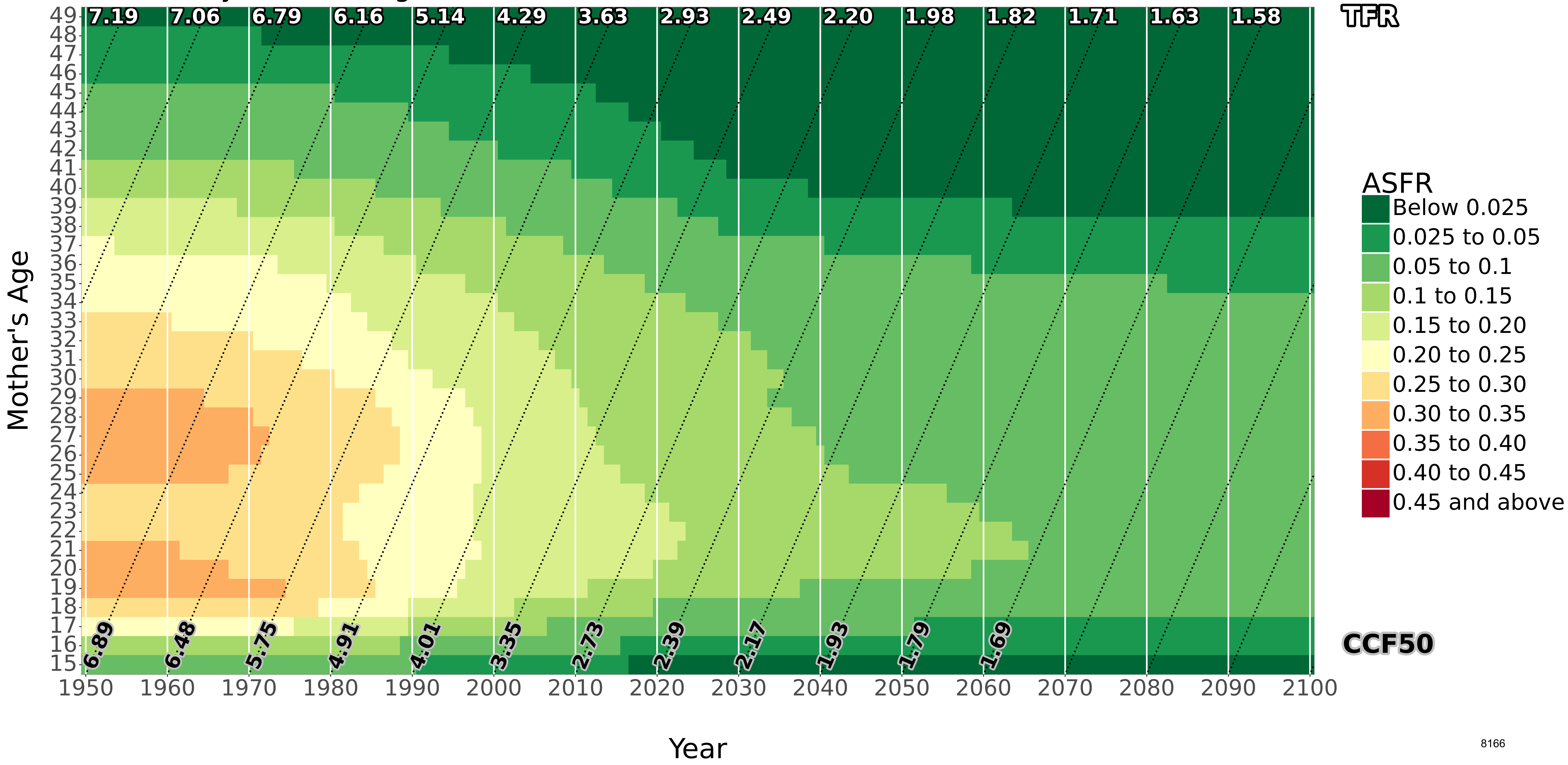

Supplemental Results Figure S3: Lexis diagram of CCF50, single-year age interval ASFR, and TFR by mother's age and location, 1950-2100, Lesotho

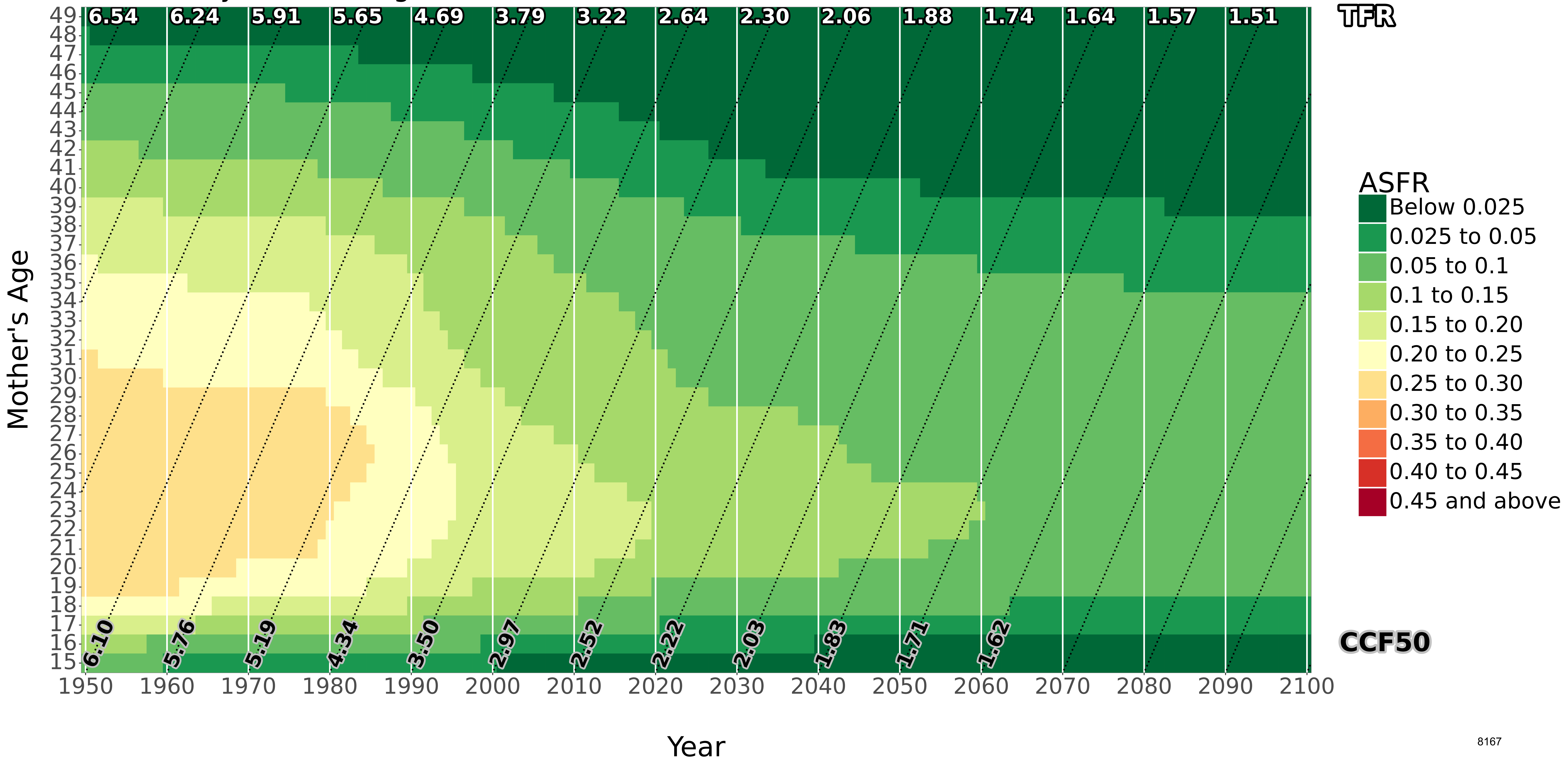

Supplemental Results Figure S3: Lexis diagram of CCF50, single-year age interval ASFR, and TFR by mother's age and location, 1950-2100, Namibia

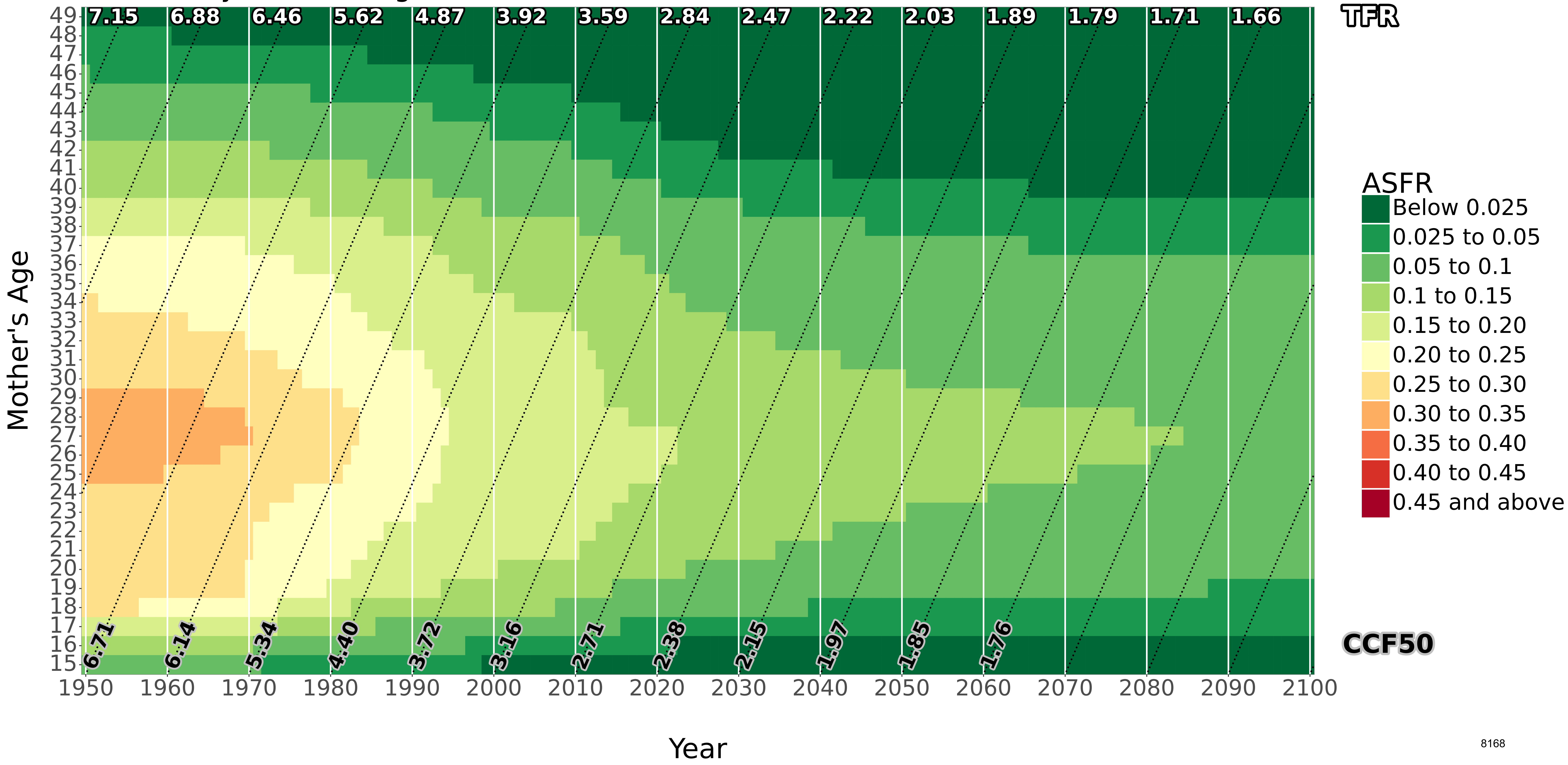

8168

Supplemental Results Figure S3: Lexis diagram of CCF50, single-year age interval ASFR, and TFR by mother's age and location, 1950-2100, South Africa

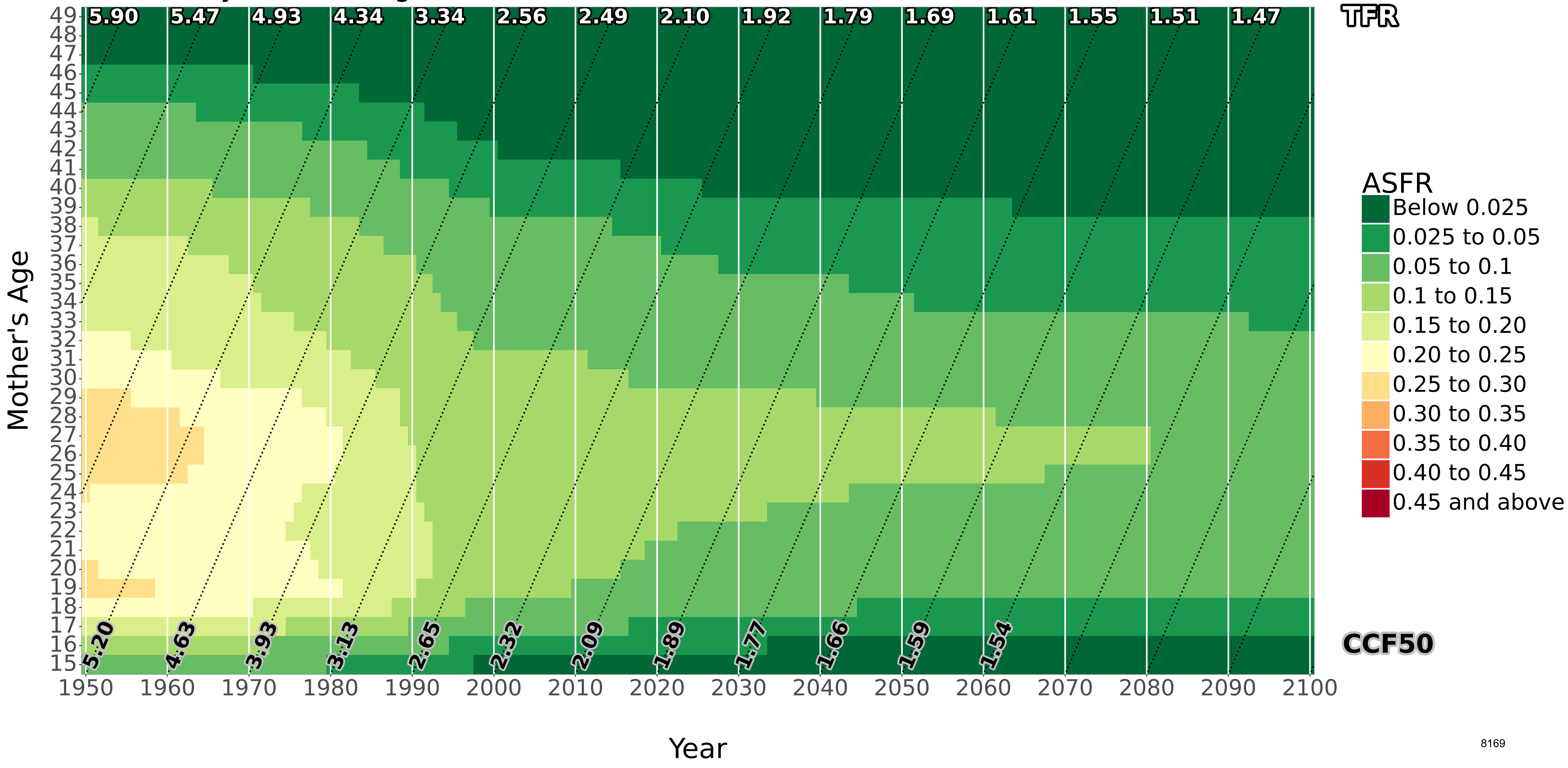

Supplemental Results Figure S3: Lexis diagram of CCF50, single-year age interval ASFR, and TFR by mother's age and location, 1950-2100, Zimbabwe

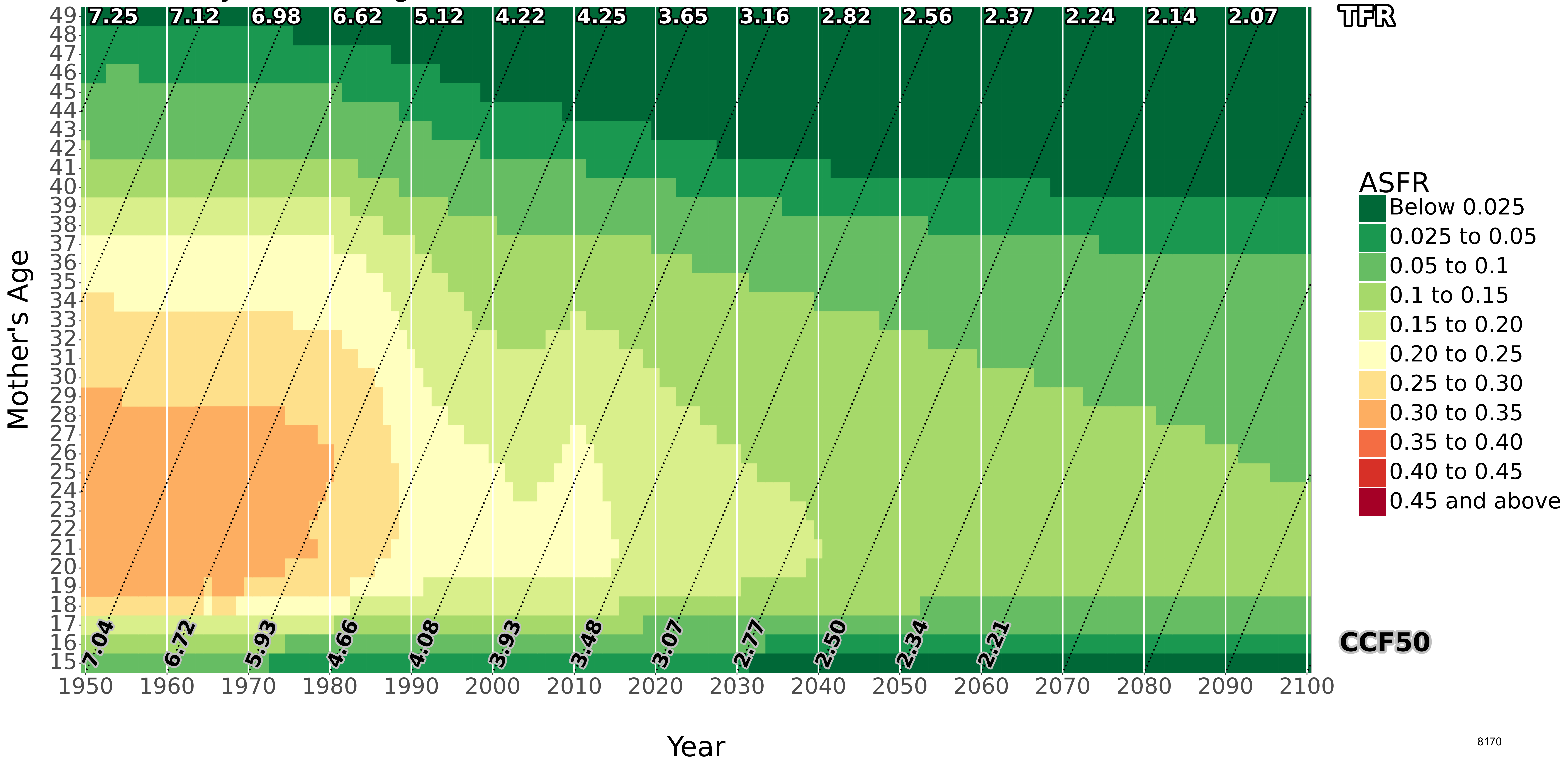

Supplemental Results Figure S3: Lexis diagram of CCF50, single-year age interval ASFR, and TFR by mother's age and location, 1950-2100, Benin

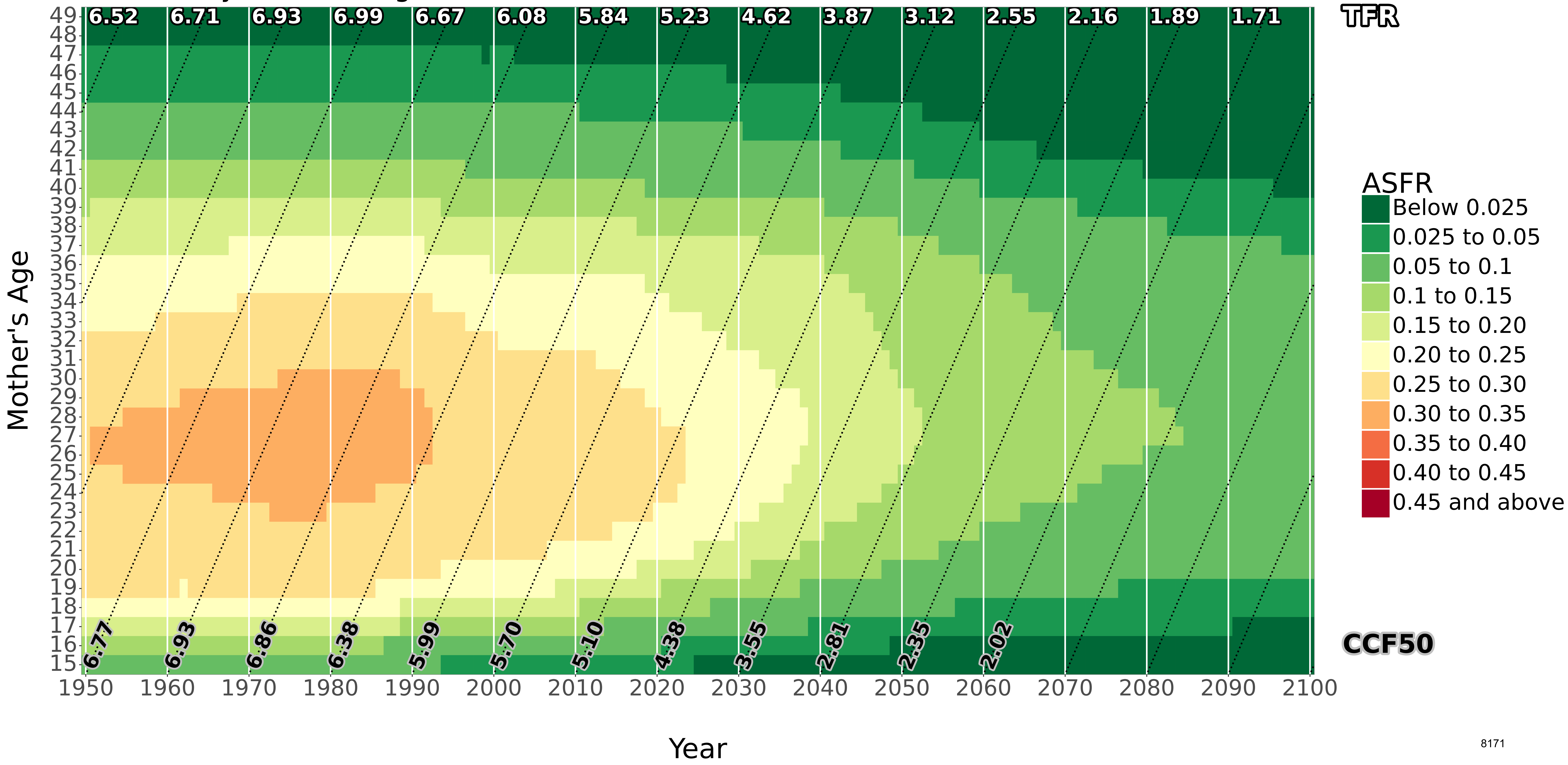

8171

Supplemental Results Figure S3: Lexis diagram of CCF50, single-year age interval ASFR, and TFR by mother's age and location, 1950-2100, Burkina Faso

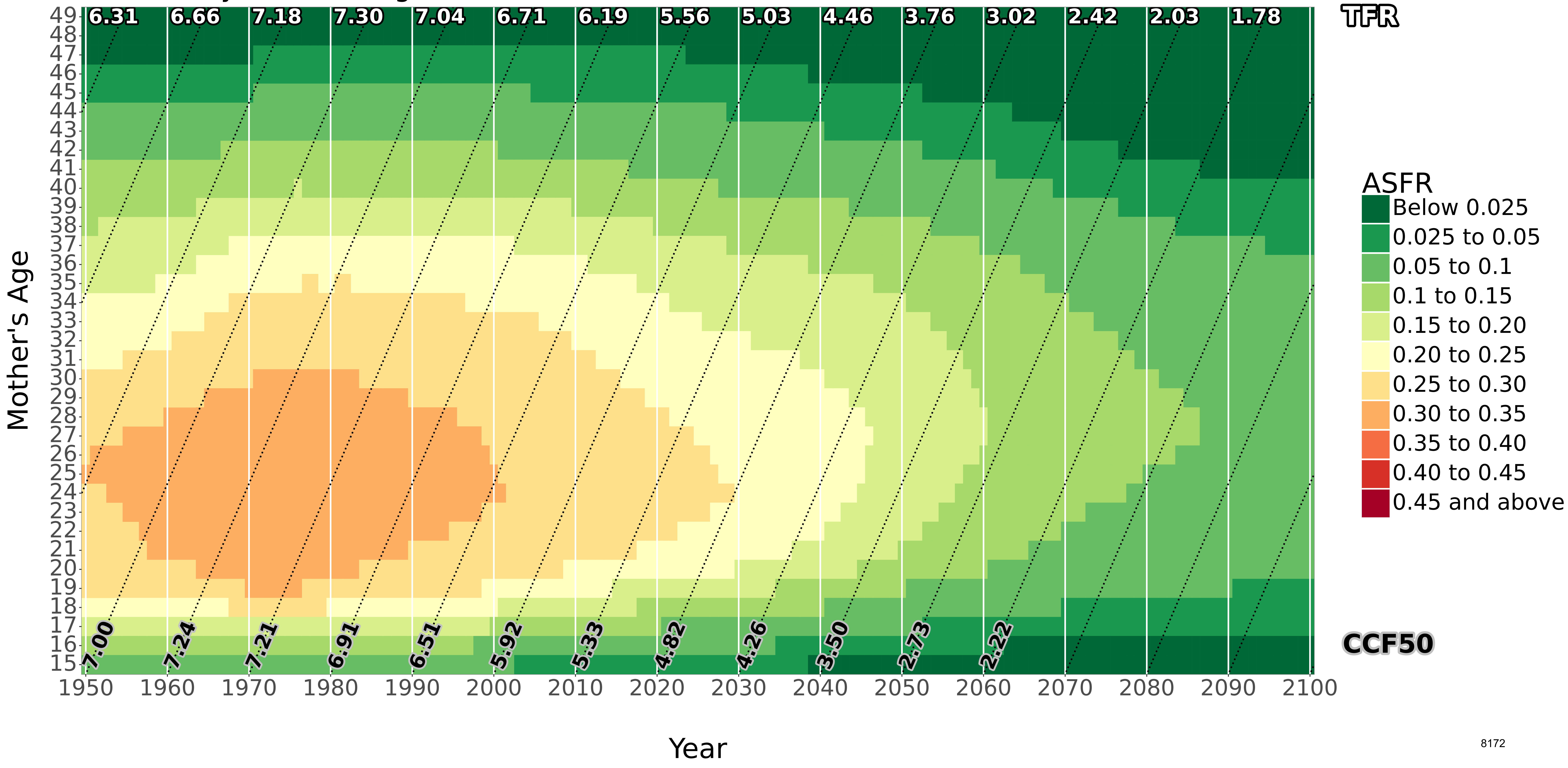

Supplemental Results Figure S3: Lexis diagram of CCF50, single-year age interval ASFR, and TFR by mother's age and location, 1950-2100, Cape Verde

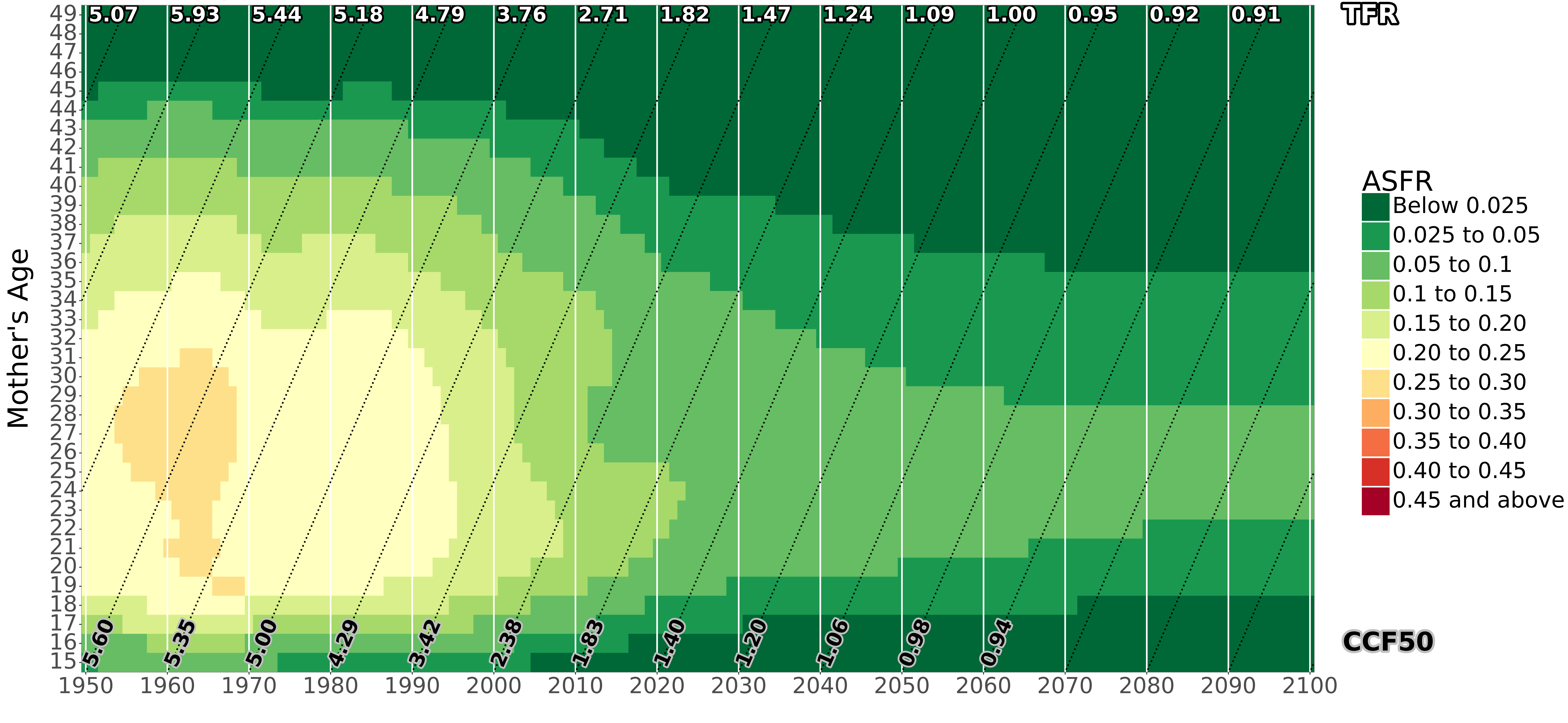

Supplemental Results Figure S3: Lexis diagram of CCF50, single-year age interval ASFR, and TFR by mother's age and location, 1950-2100, Cameroon

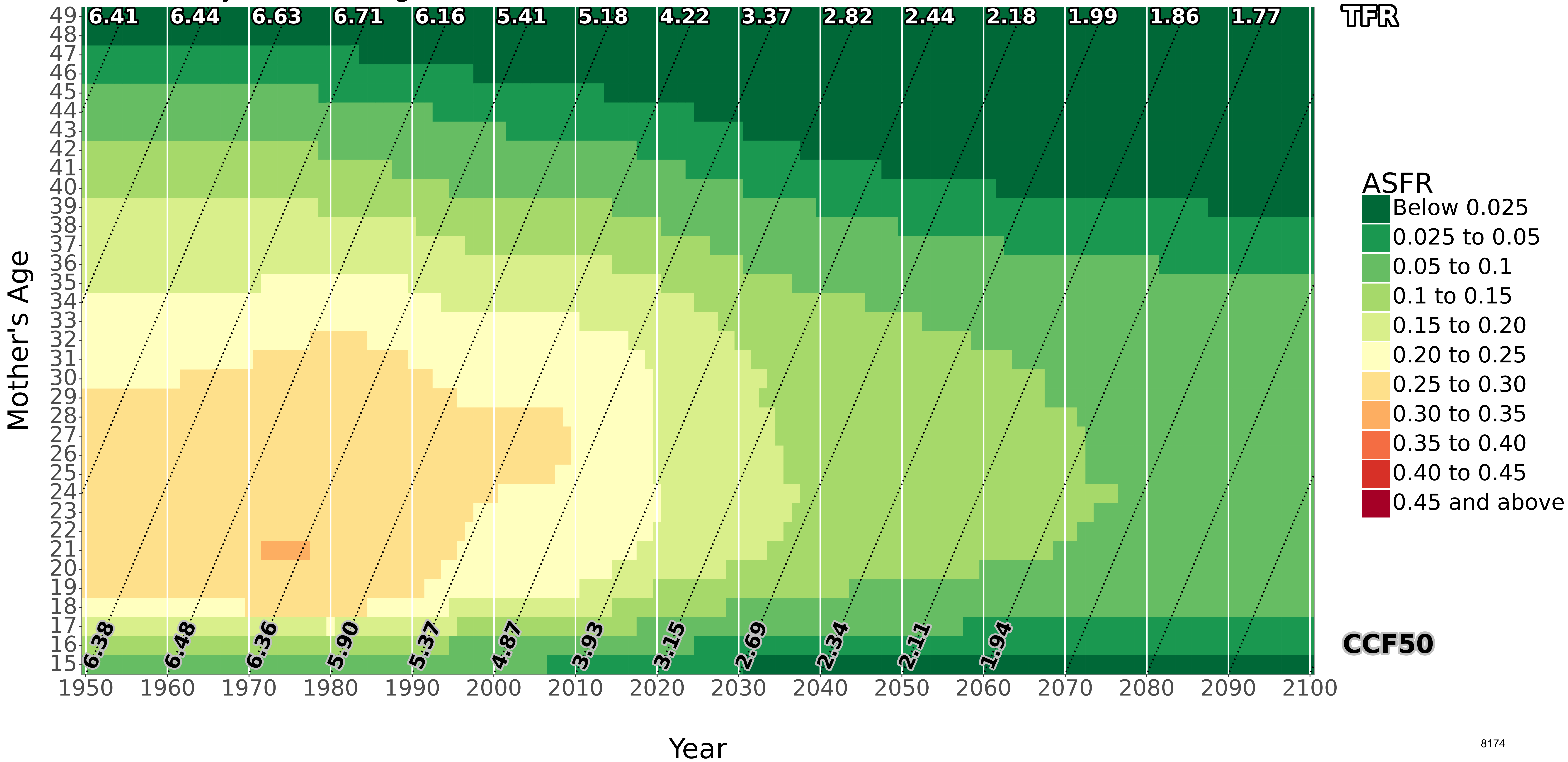

Supplemental Results Figure S3: Lexis diagram of CCF50, single-year age interval ASFR, and TFR by mother's age and location, 1950-2100, Chad

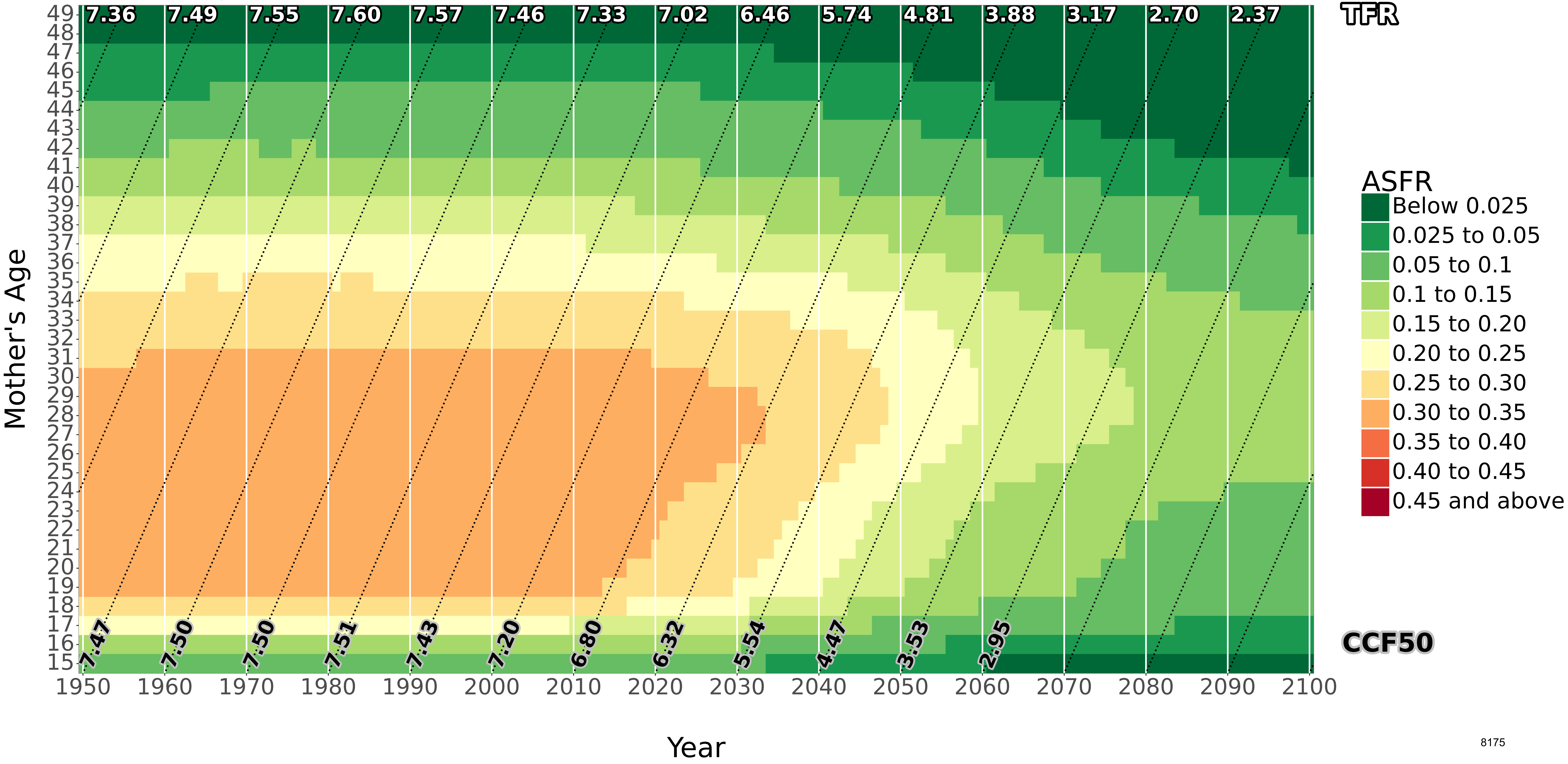

Supplemental Results Figure S3: Lexis diagram of CCF50, single-year age interval ASFR, and TFR by mother's age and location, 1950-2100, Côte d'Ivoire

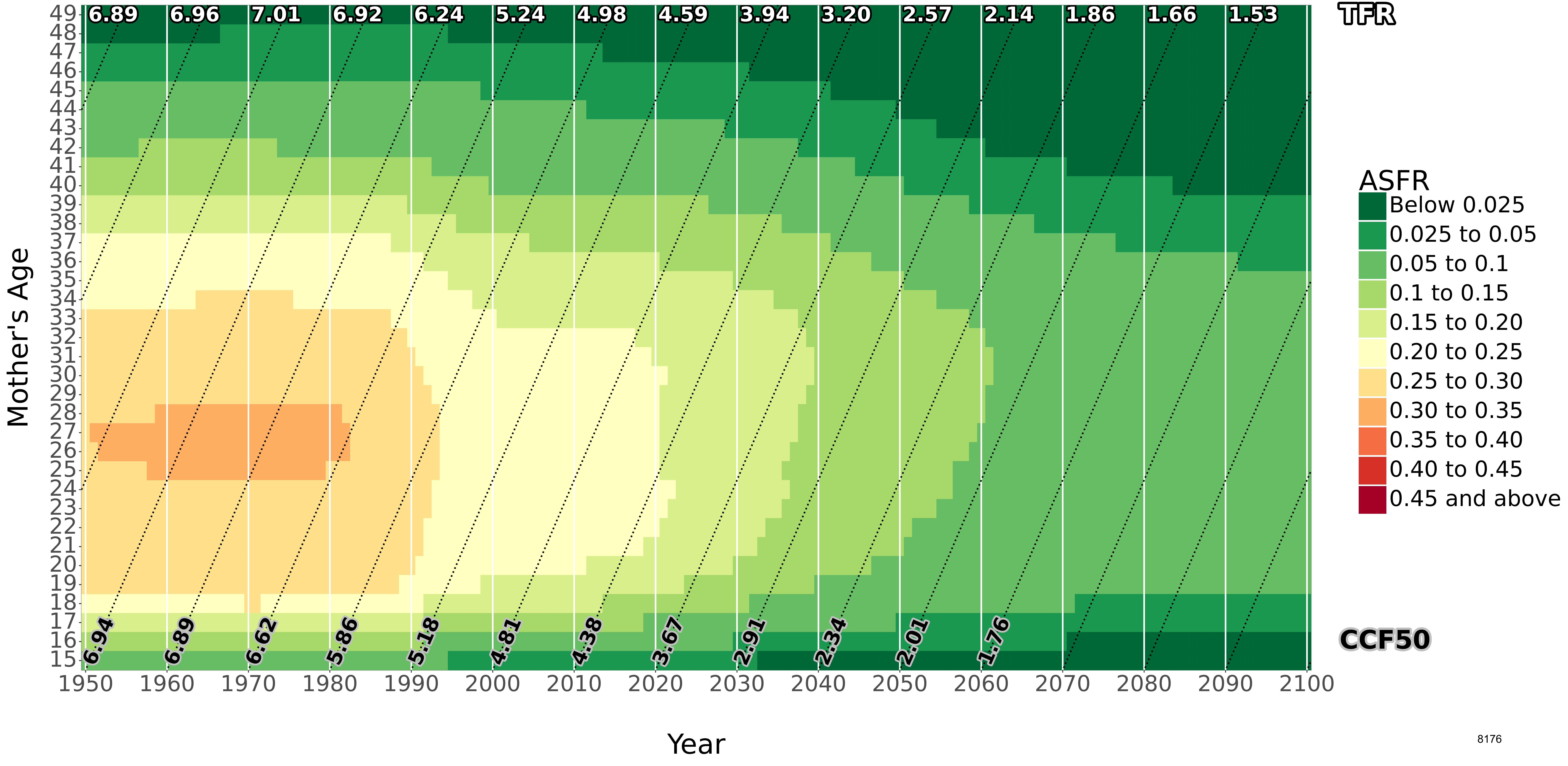

Supplemental Results Figure S3: Lexis diagram of CCF50, single-year age interval ASFR, and TFR by mother's age and location, 1950-2100, The Gambia

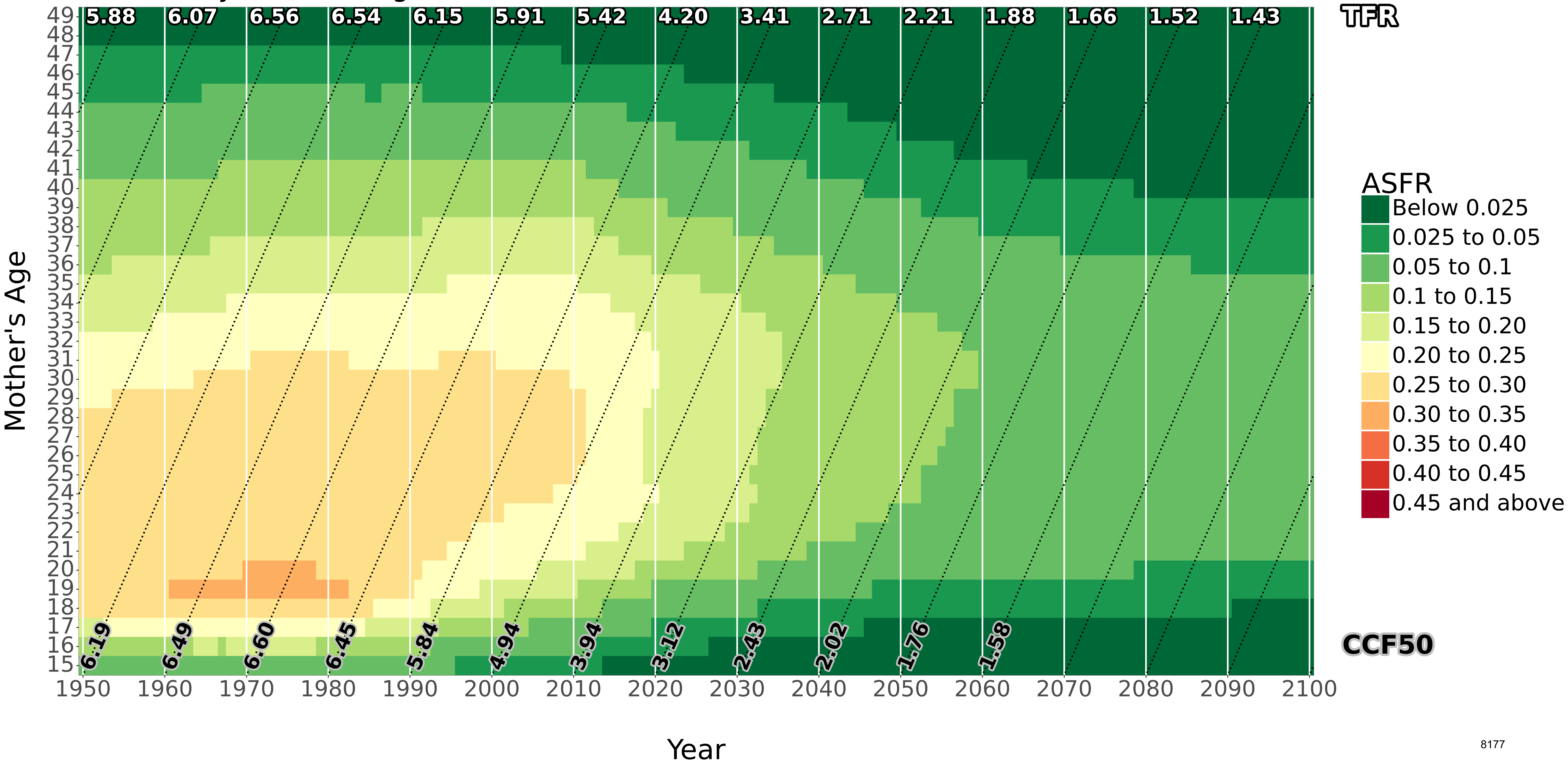

Supplemental Results Figure S3: Lexis diagram of CCF50, single-year age interval ASFR, and TFR by mother's age and location, 1950-2100, Ghana

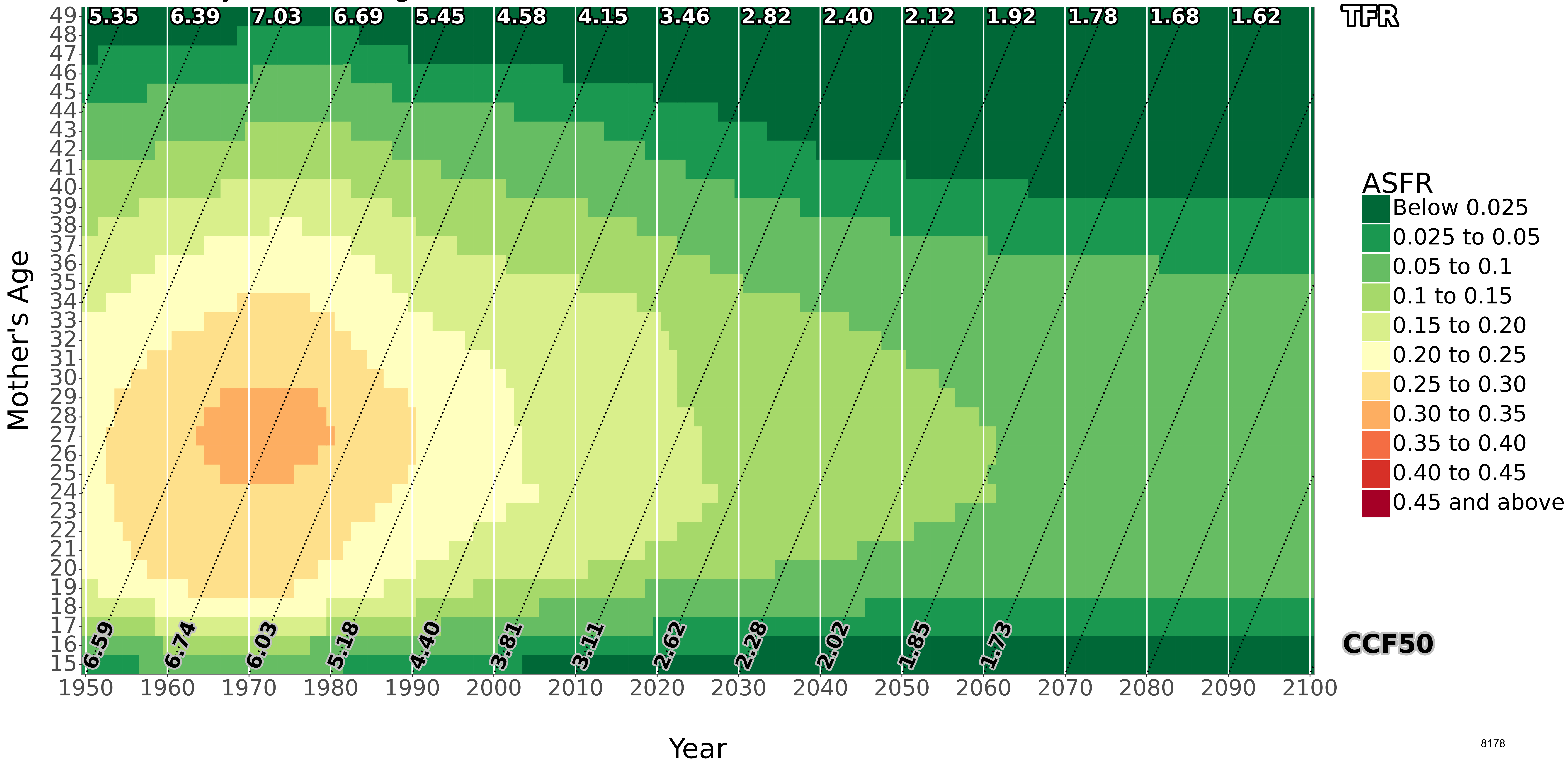

Supplemental Results Figure S3: Lexis diagram of CCF50, single-year age interval ASFR, and TFR by mother's age and location, 1950-2100, Guinea

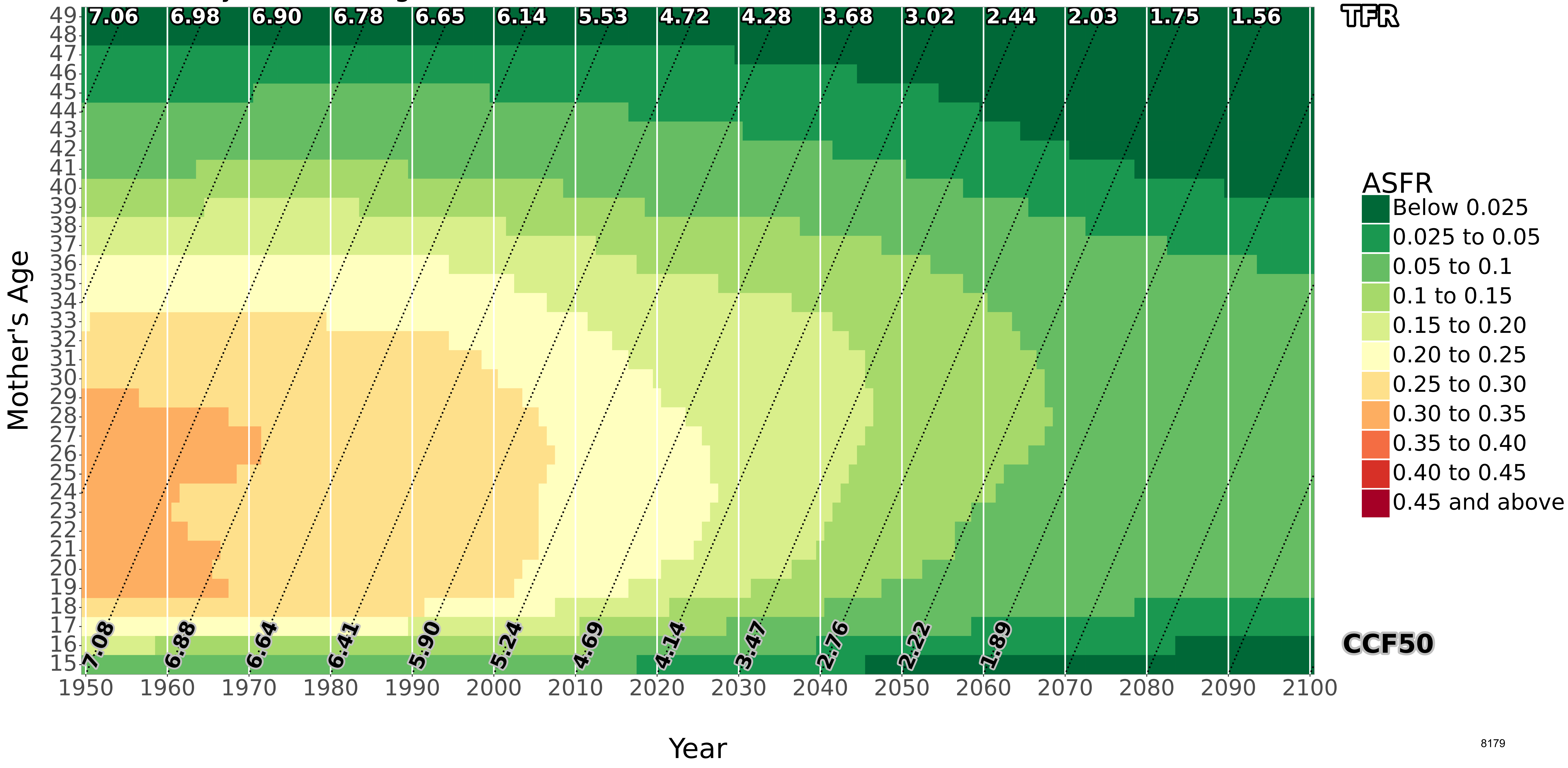

Supplemental Results Figure S3: Lexis diagram of CCF50, single-year age interval ASFR, and TFR by mother's age and location, 1950-2100, Guinea-Bissau

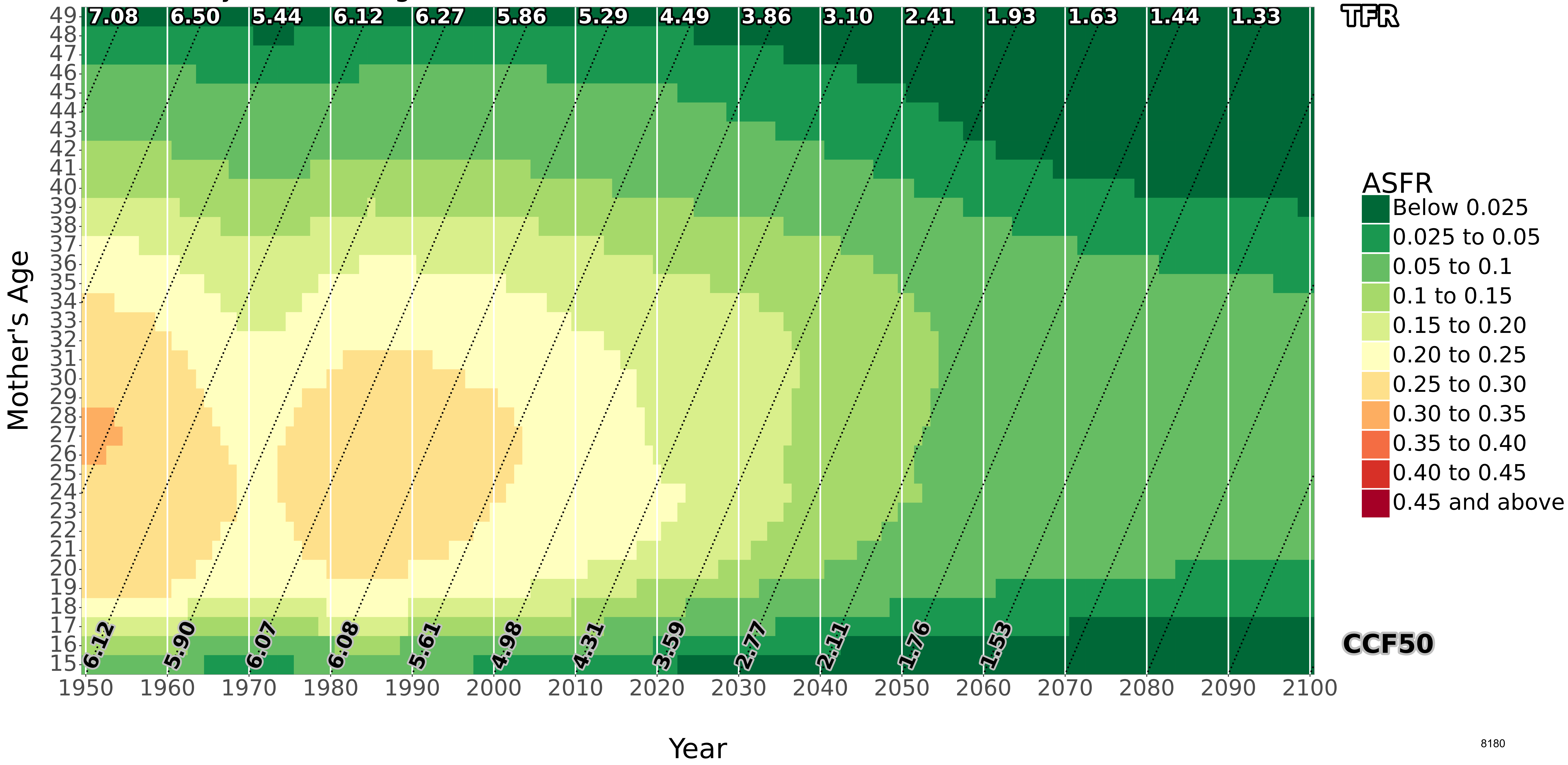

Supplemental Results Figure S3: Lexis diagram of CCF50, single-year age interval ASFR, and TFR by mother's age and location, 1950-2100, Liberia

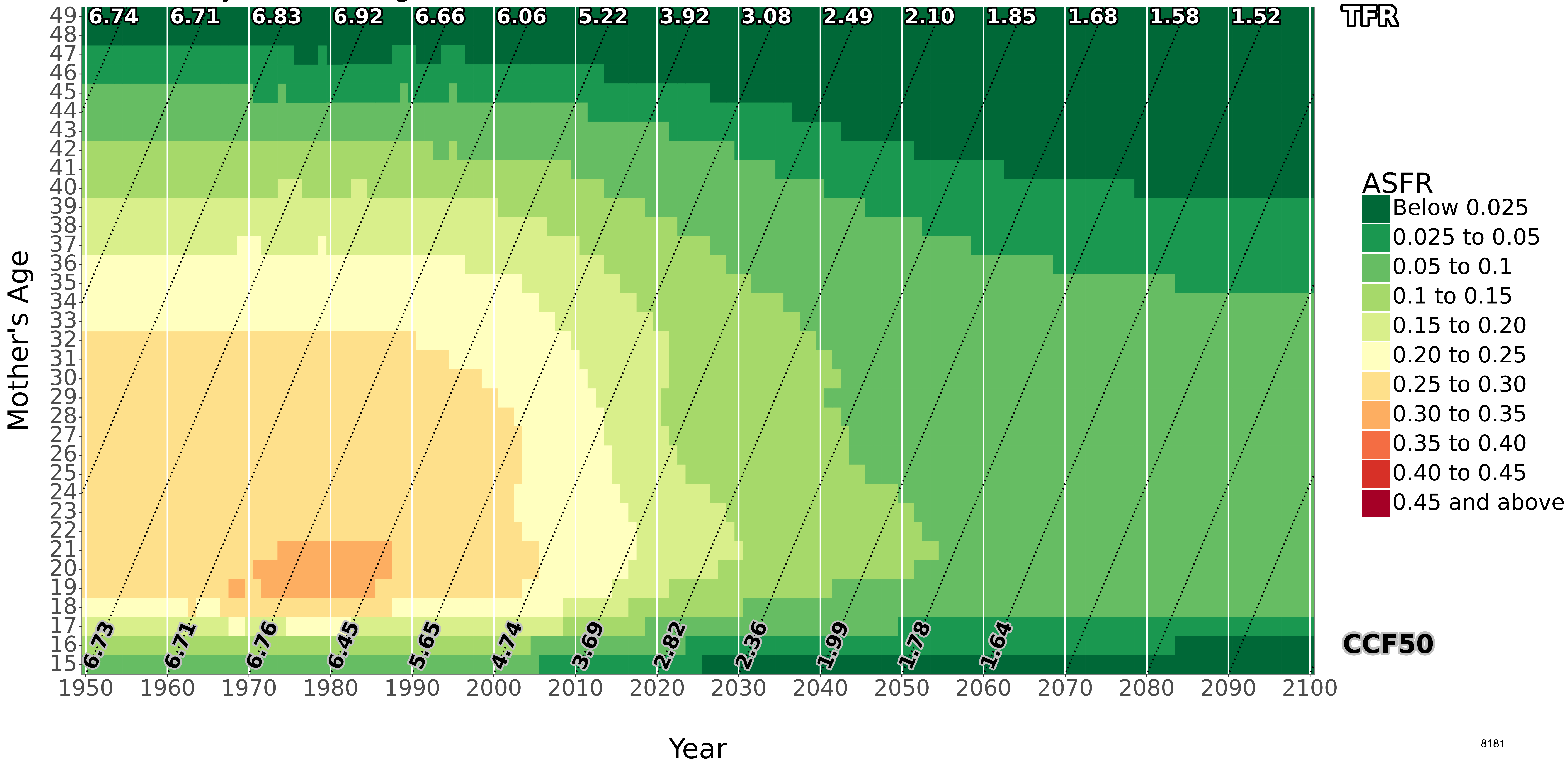

Supplemental Results Figure S3: Lexis diagram of CCF50, single-year age interval ASFR, and TFR by mother's age and location, 1950-2100, Mali

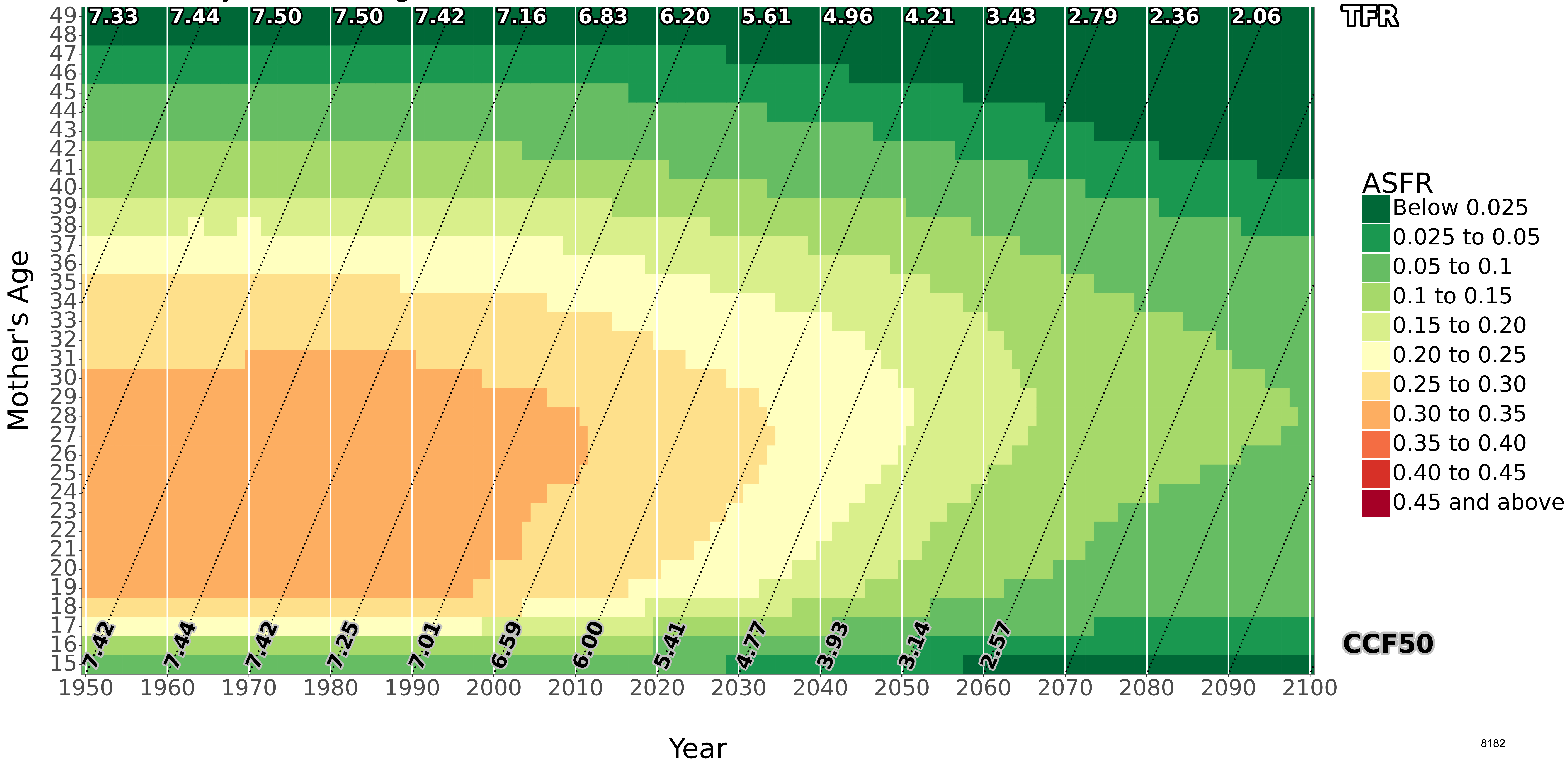

Supplemental Results Figure S3: Lexis diagram of CCF50, single-year age interval ASFR, and TFR by mother's age and location, 1950-2100, Mauritania

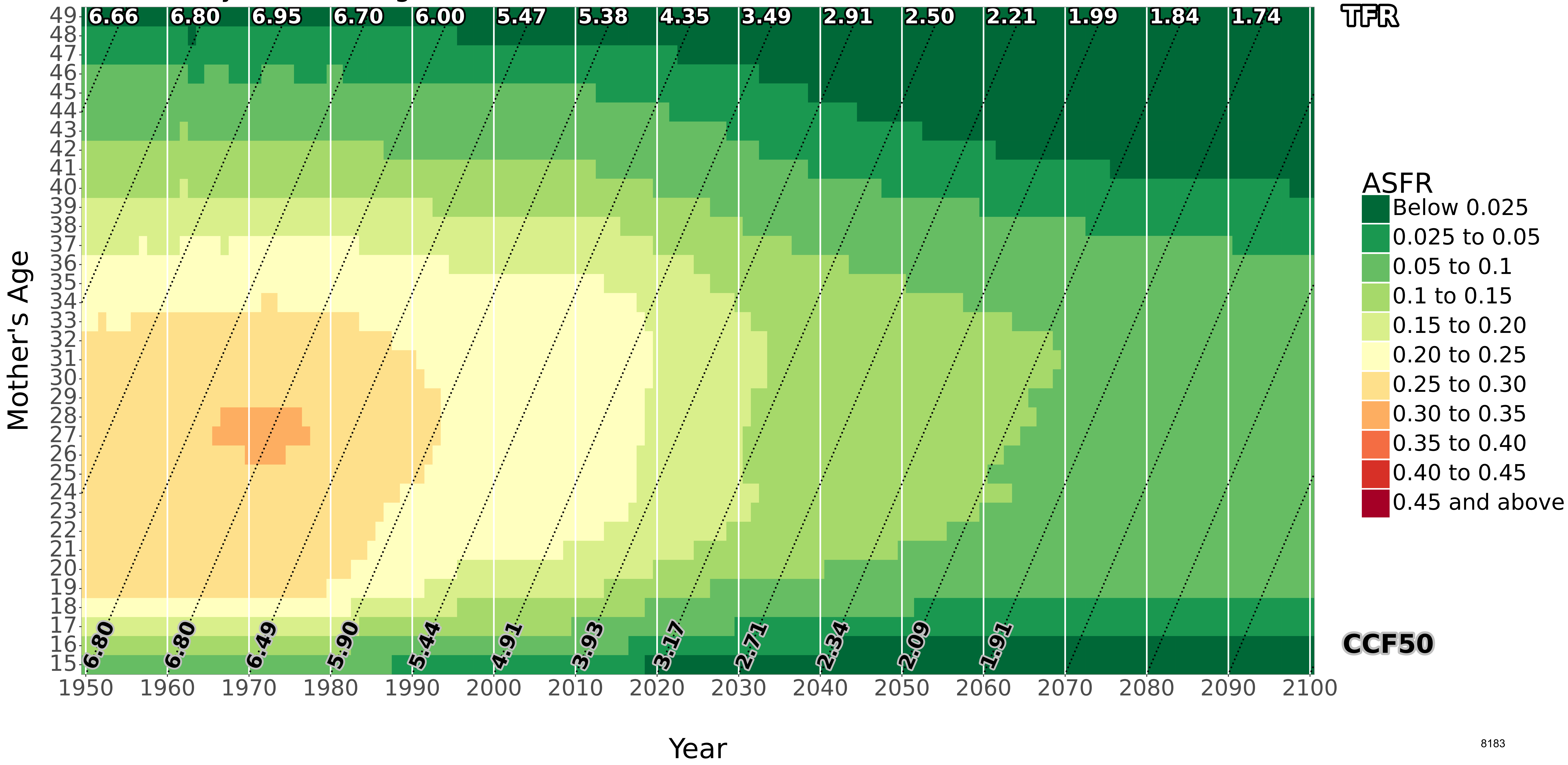

Supplemental Results Figure S3: Lexis diagram of CCF50, single-year age interval ASFR, and TFR by mother's age and location, 1950-2100, Niger

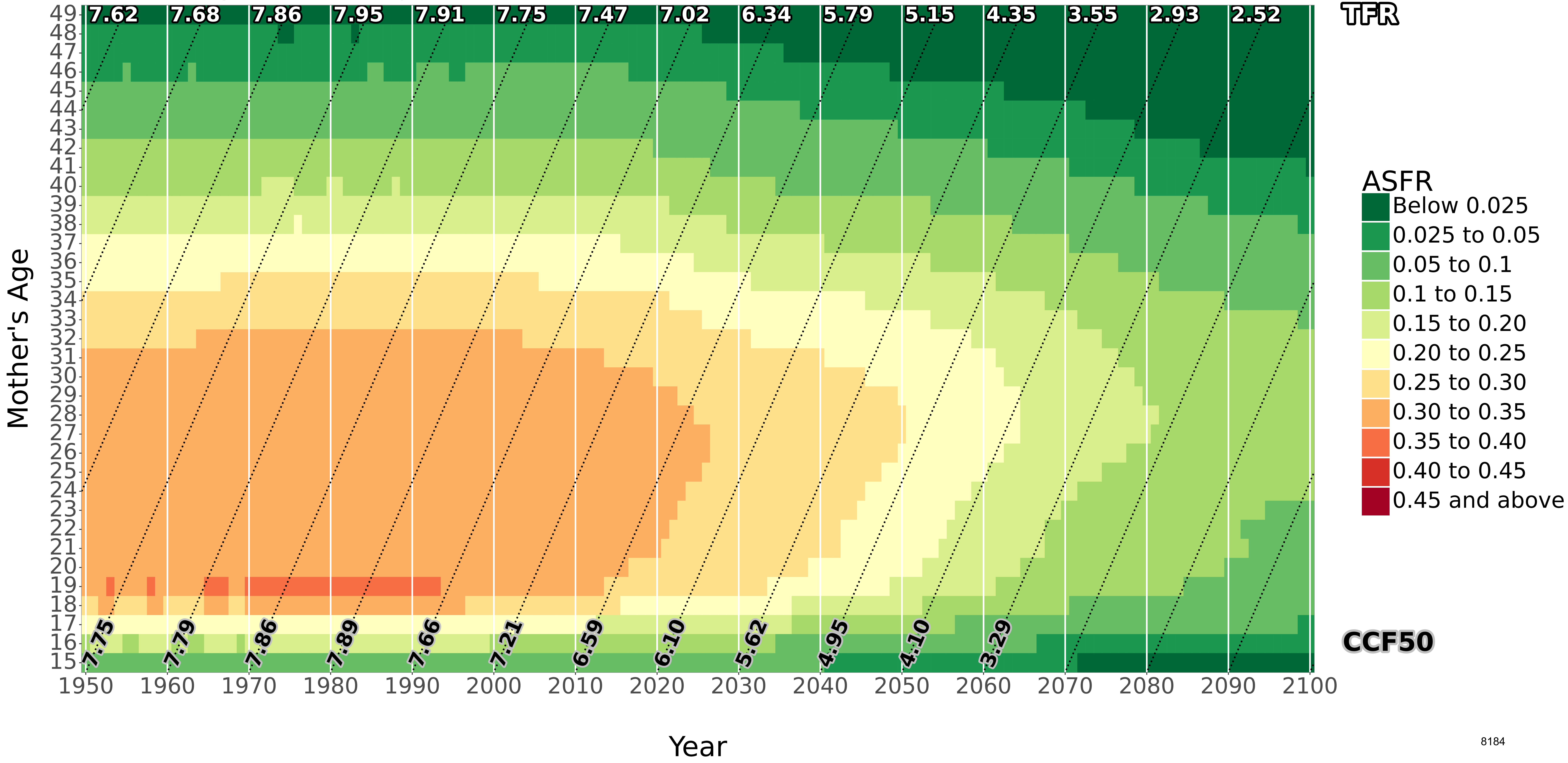

Supplemental Results Figure S3: Lexis diagram of CCF50, single-year age interval ASFR, and TFR by mother's age and location, 1950-2100, Nigeria

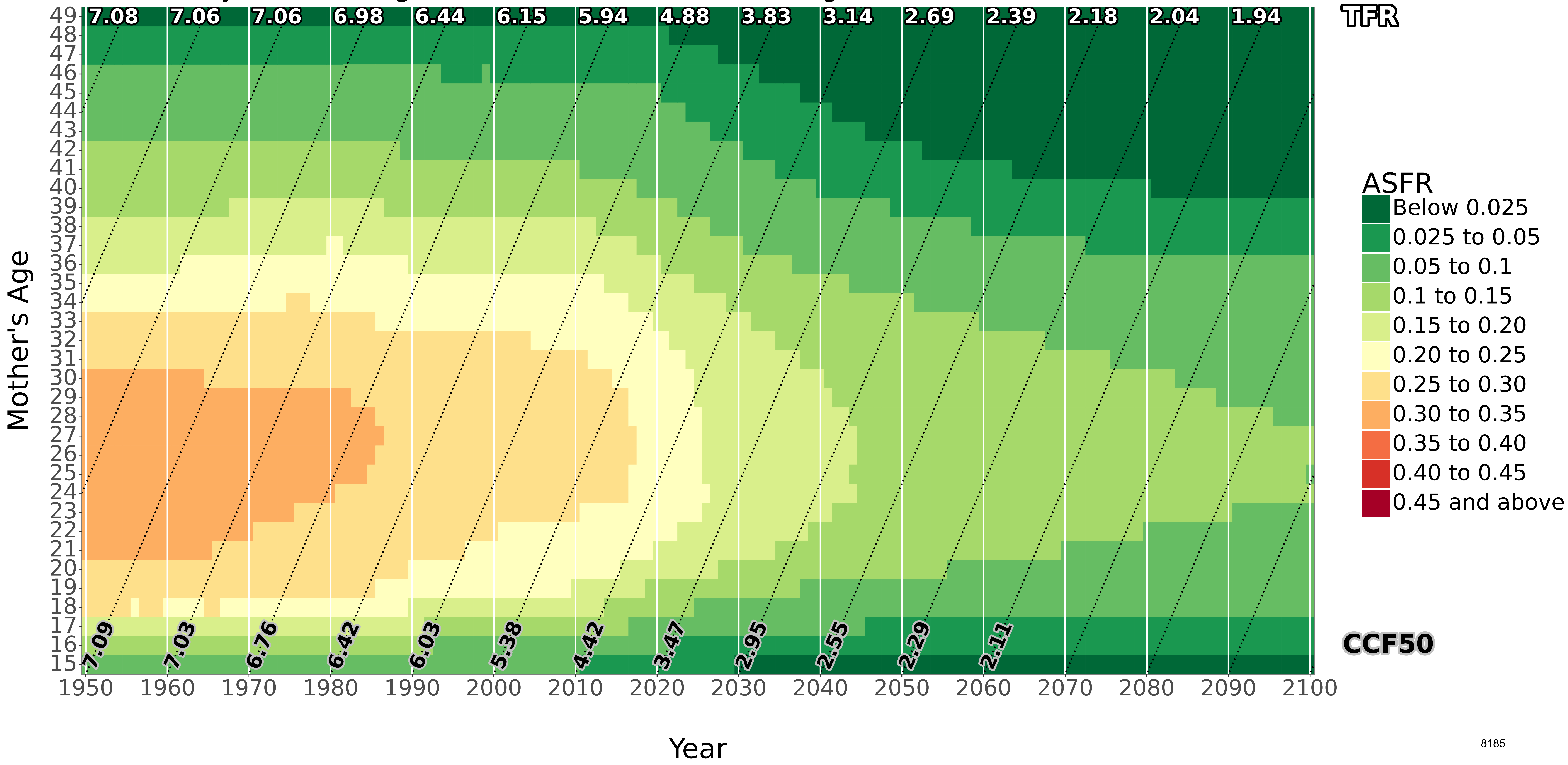

Supplemental Results Figure S3: Lexis diagram of CCF50, single-year age interval ASFR, and TFR by mother's age and location, 1950-2100, São Tomé and Príncipe

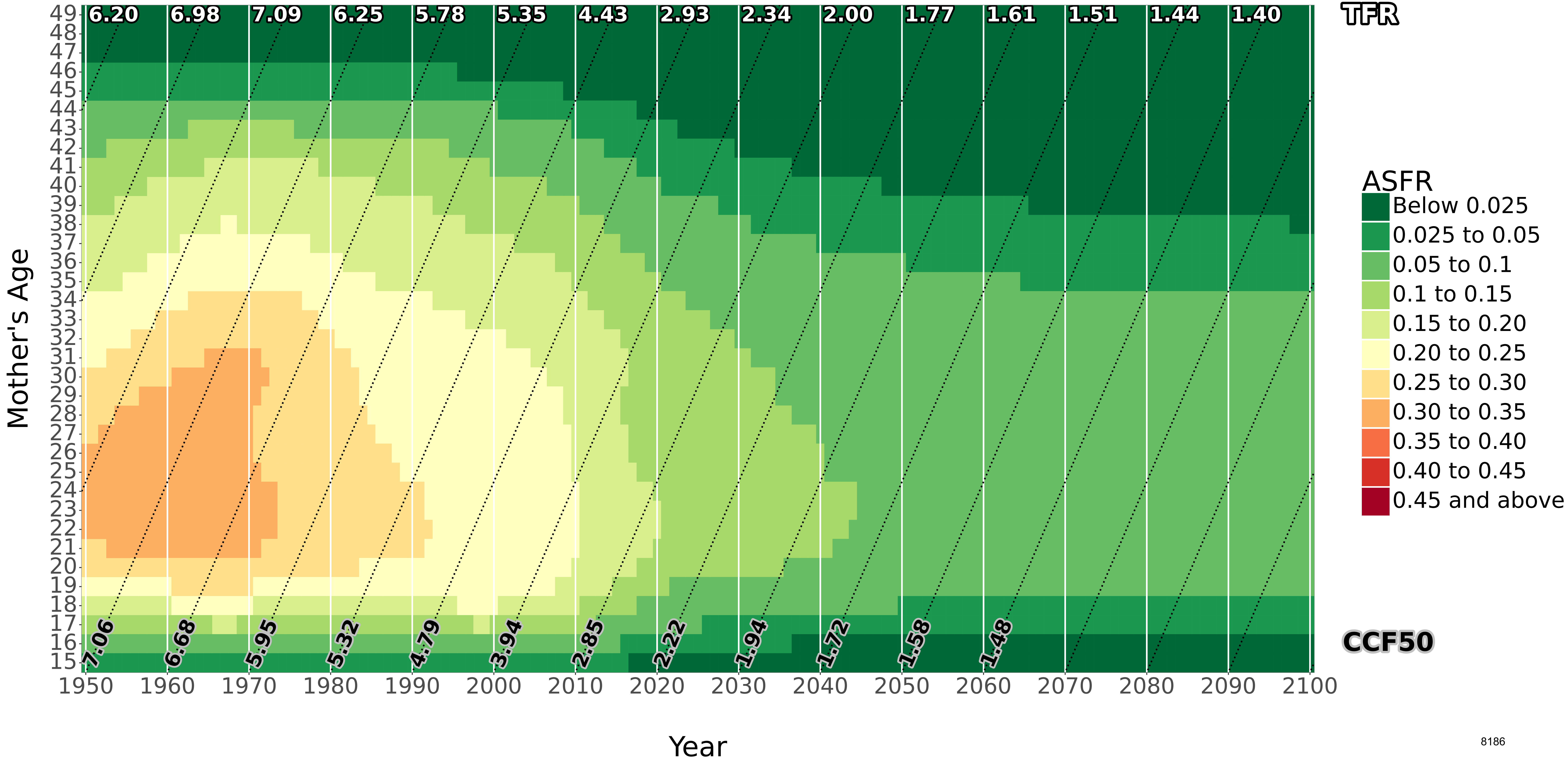

Supplemental Results Figure S3: Lexis diagram of CCF50, single-year age interval ASFR, and TFR by mother's age and location, 1950-2100, Senegal

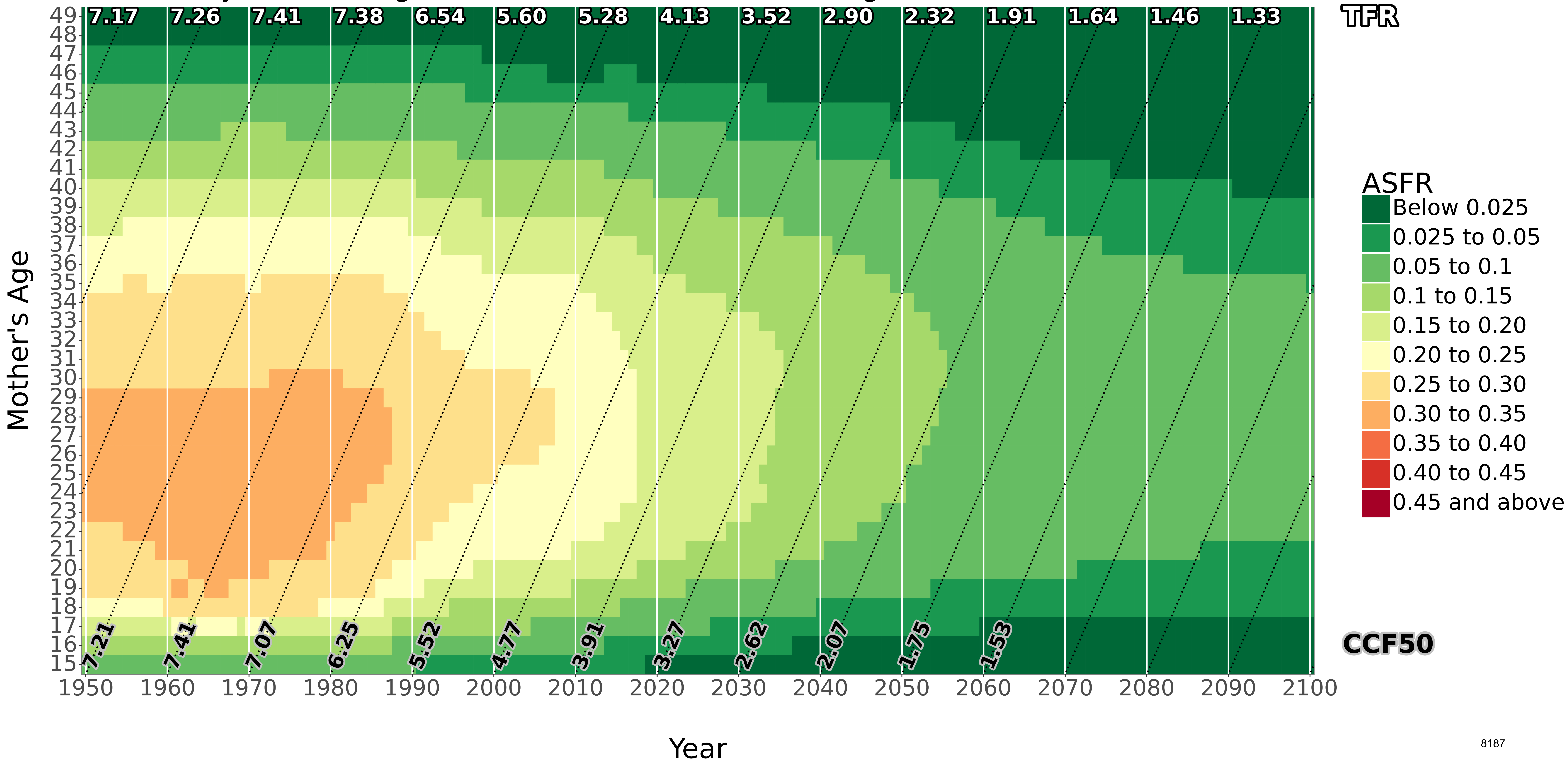

Supplemental Results Figure S3: Lexis diagram of CCF50, single-year age interval ASFR, and TFR by mother's age and location, 1950-2100, Sierra Leone

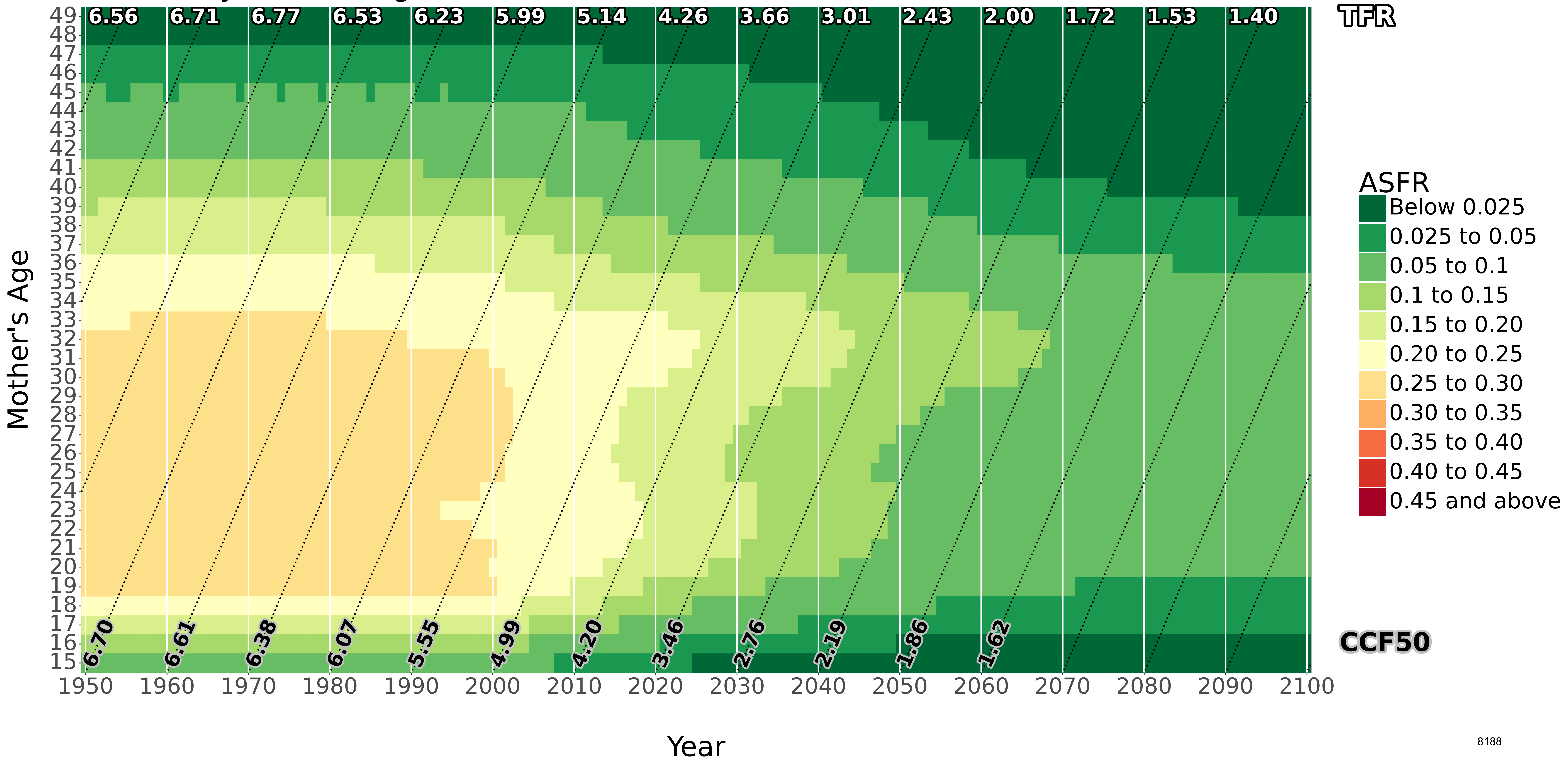

Supplemental Results Figure S3: Lexis diagram of CCF50, single-year age interval ASFR, and TFR by mother's age and location, 1950-2100, Togo

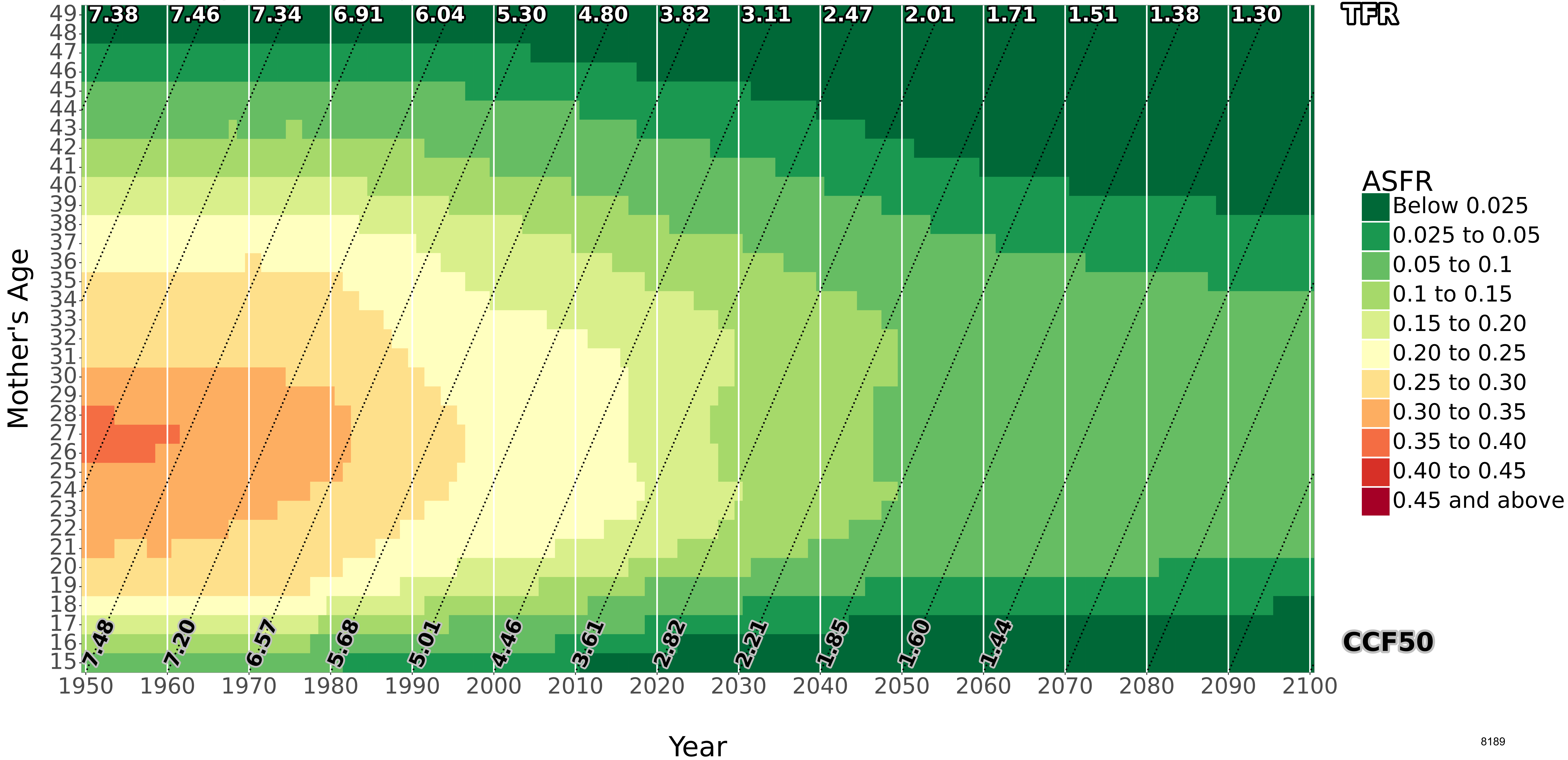

Supplemental Results Figure S3: Lexis diagram of CCF50, single-year age interval ASFR, and TFR by mother's age and location, 1950-2100, Global

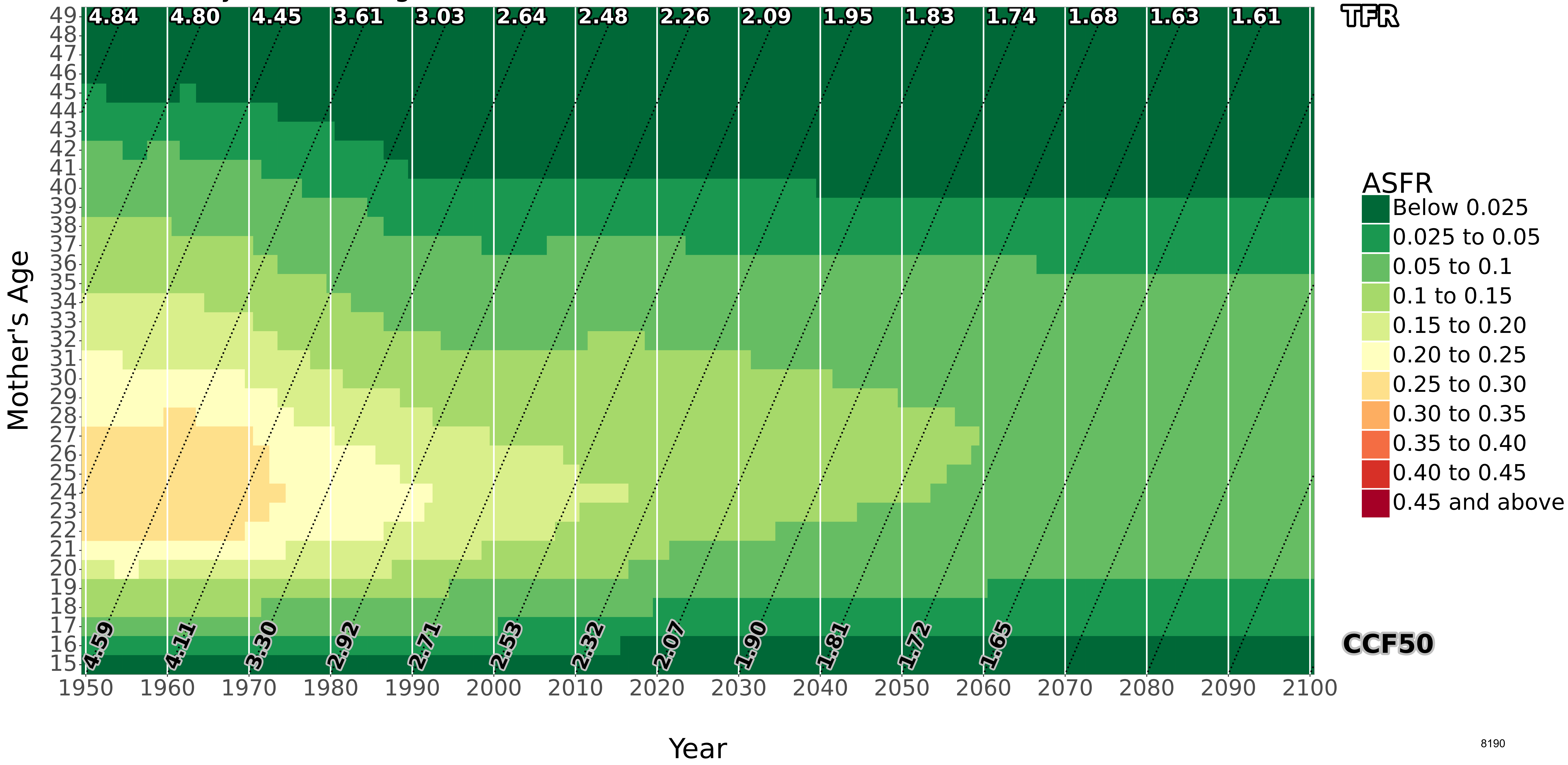

Supplemental Results Figure S3: Lexis diagram of CCF50, single-year age interval ASFR, and TFR by mother's age and location, 1950-2100, Central Europe, Eastern Europe, and Central Asia

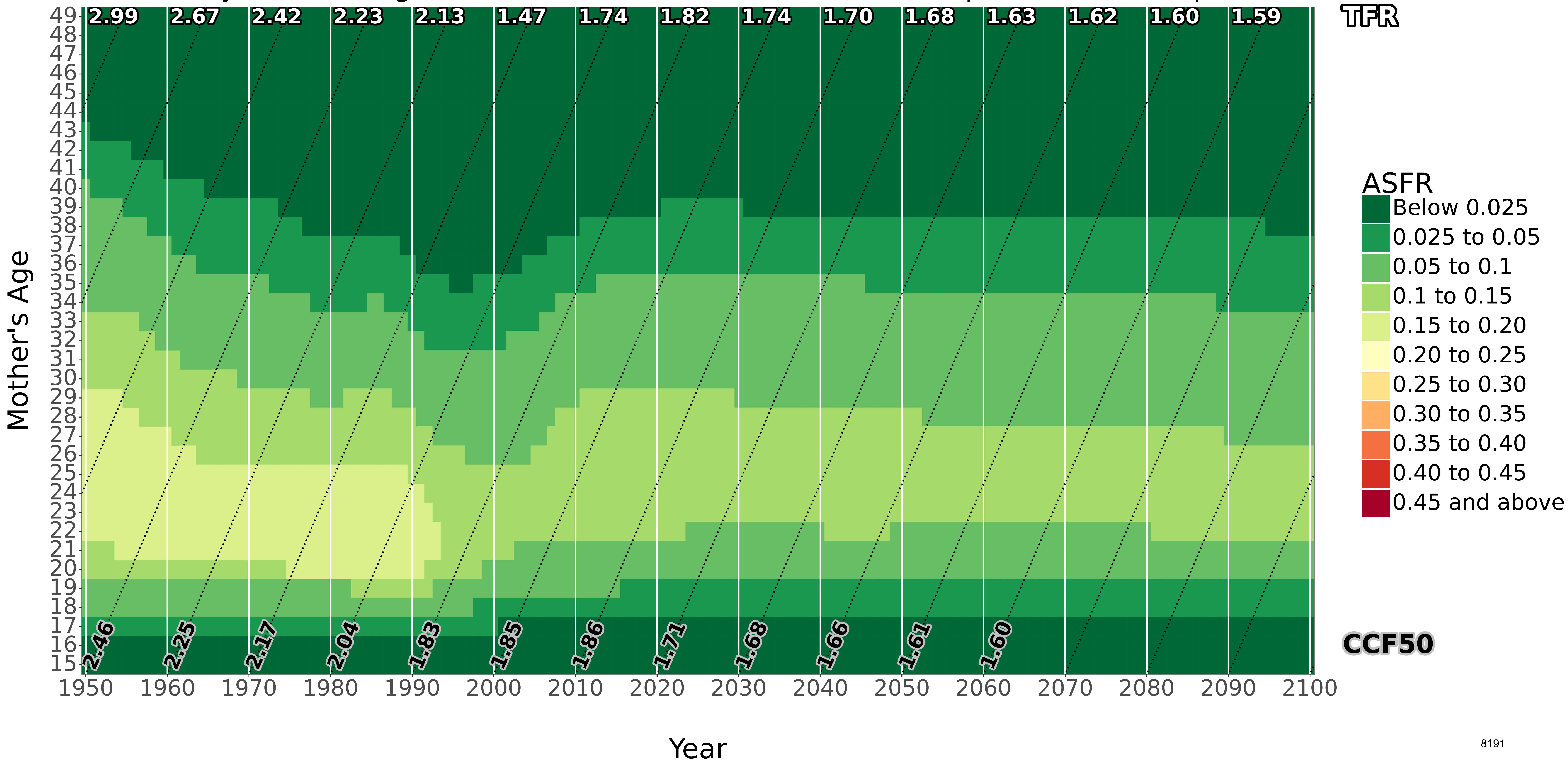

Supplemental Results Figure S3: Lexis diagram of CCF50, single-year age interval ASFR, and TFR by mother's age and location, 1950-2100, High income

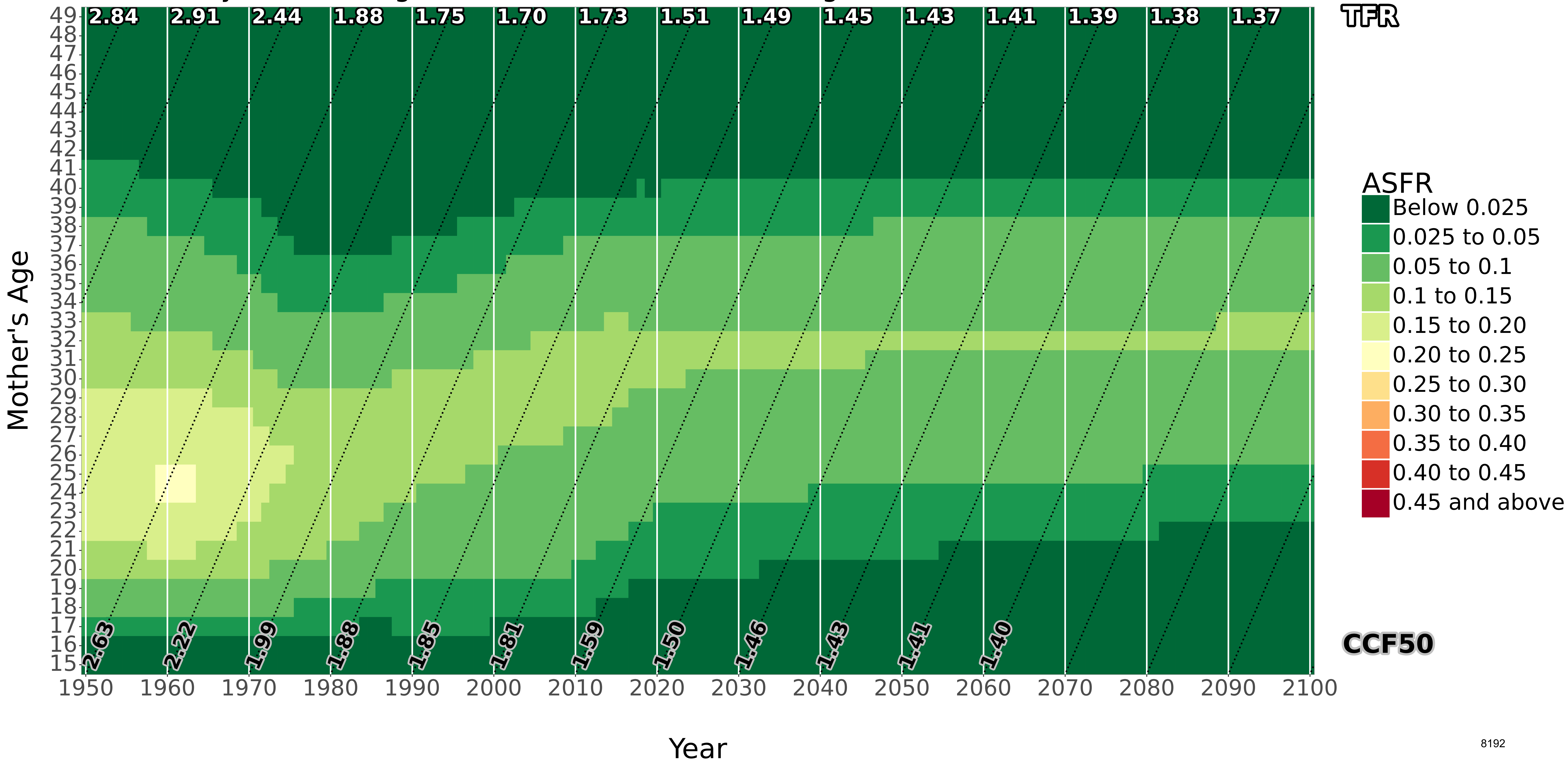

Supplemental Results Figure S3: Lexis diagram of CCF50, single-year age interval ASFR, and TFR by mother's age and location, 1950-2100, Latin America and Caribbean

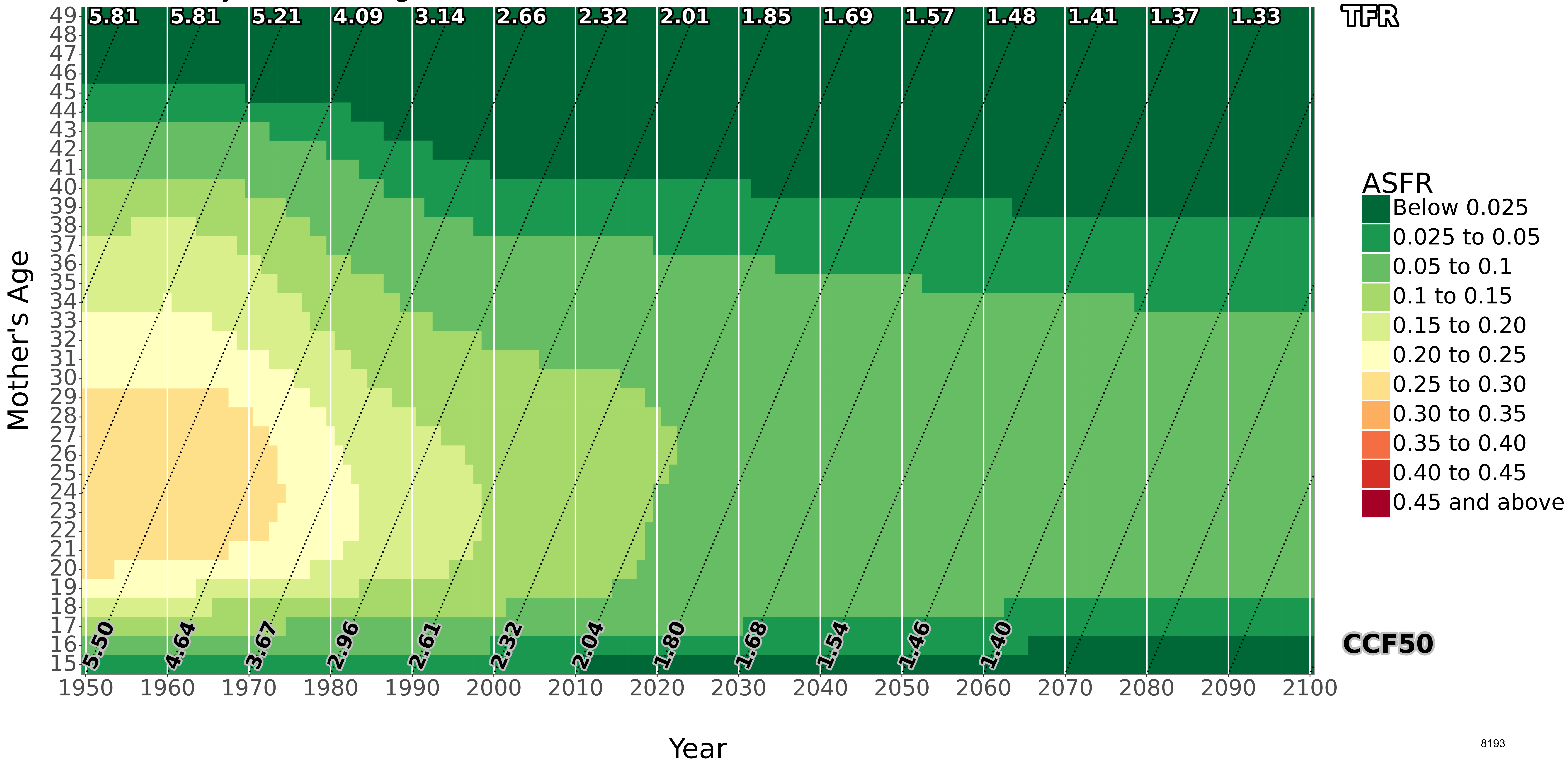

Supplemental Results Figure S3: Lexis diagram of CCF50, single-year age interval ASFR, and TFR by mother's age and location, 1950-2100, North Africa and Middle East

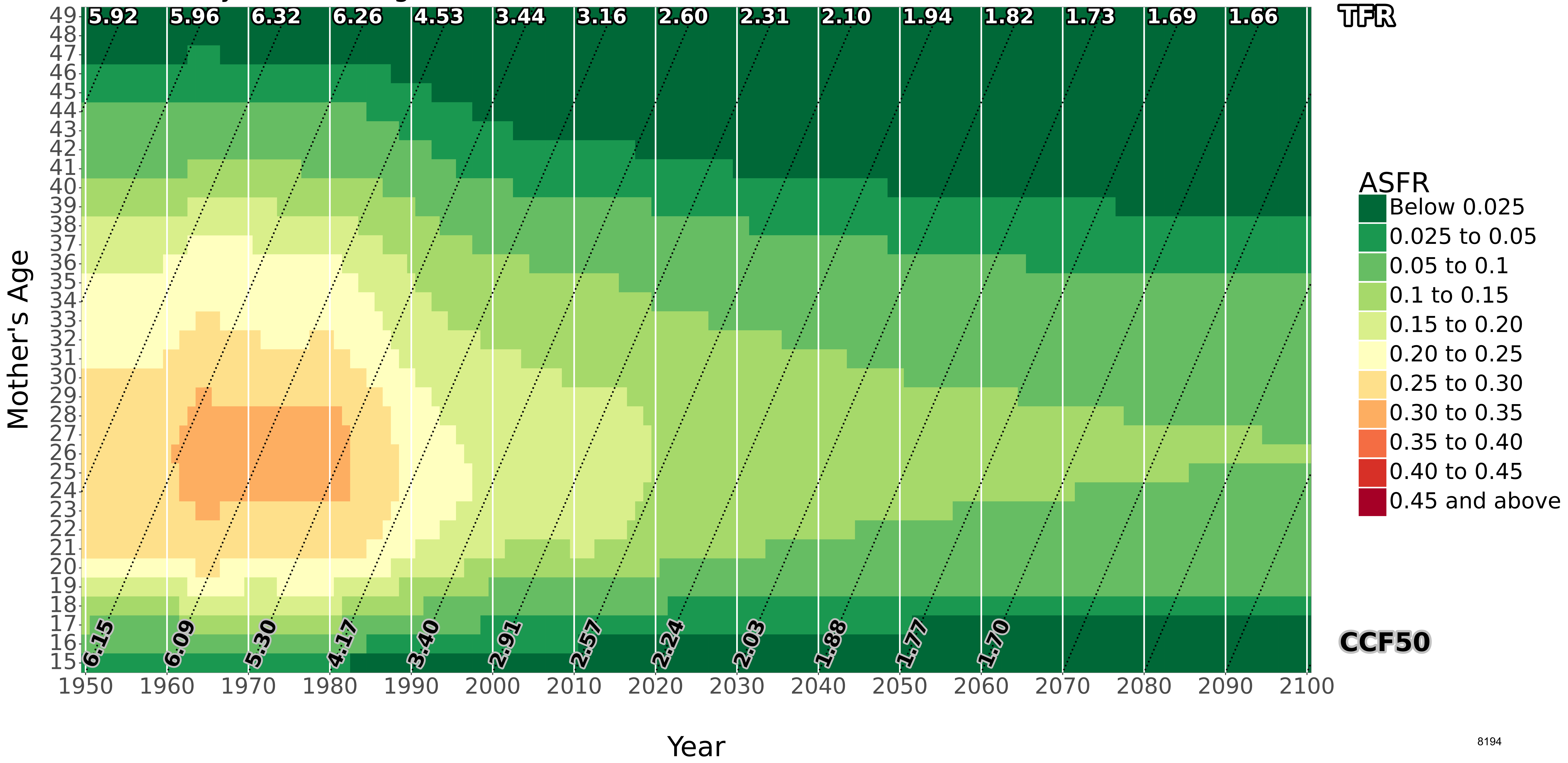

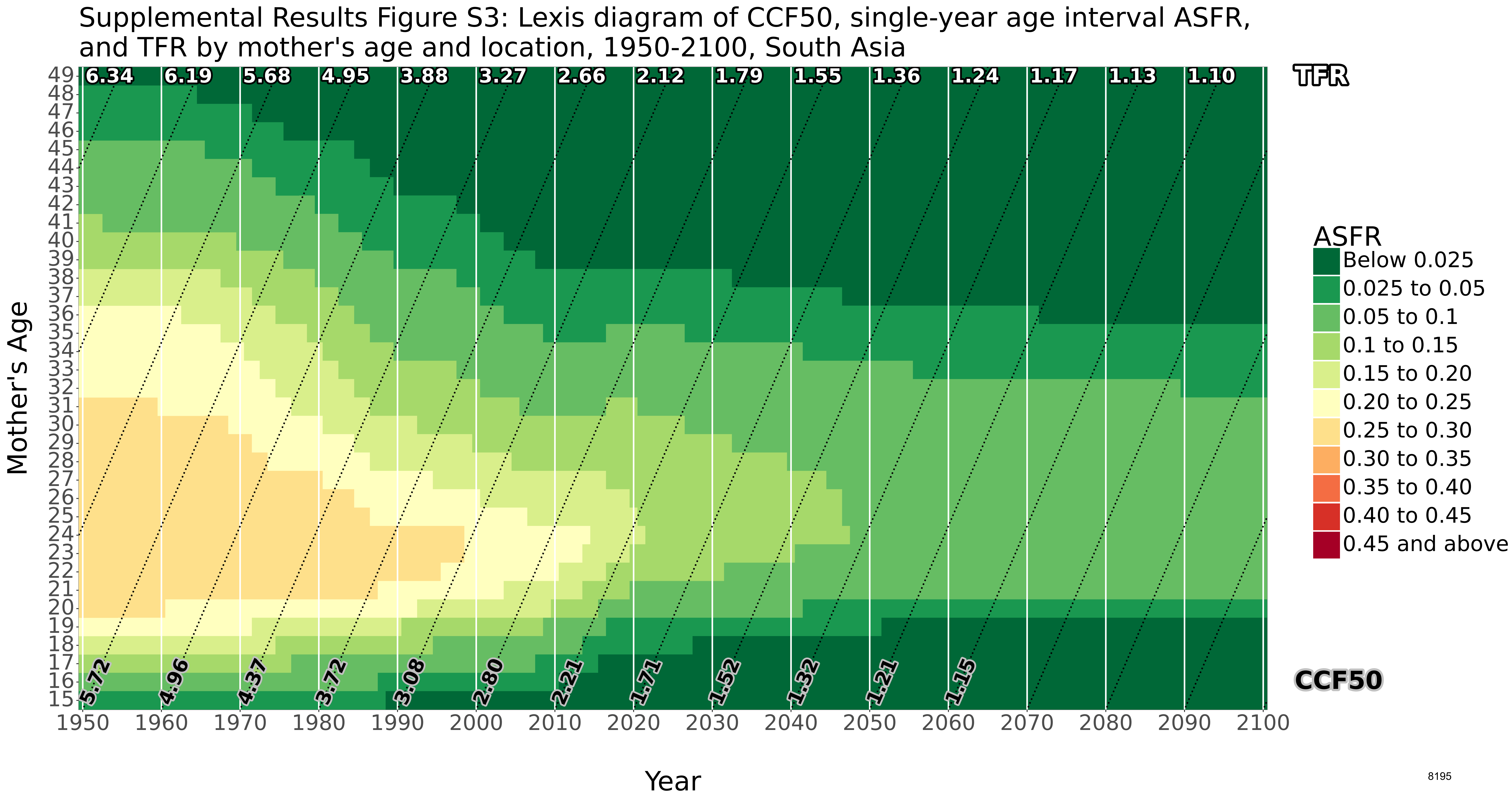

Supplemental Results Figure S3: Lexis diagram of CCF50, single-year age interval ASFR, and TFR by mother's age and location, 1950-2100, South Asia
TFR
6.34
6.19
5.68
4.95
3.88
3.27
2.66
2.12
1.79
1.55
1.36
1.24
1.17
1.13
1.10
CCF50
5.72
4.96
4.37
3.72
3.08
2.80
2.21
1.71
1.52
1.32
1.21
1.15
Mother's Age
Year
ASFR
Below 0.025
0.025 to 0.05
0.05 to 0.1
0.1 to 0.15
0.15 to 0.20
0.20 to 0.25
0.25 to 0.30
0.30 to 0.35
0.35 to 0.40
0.40 to 0.45
0.45 and above

Supplemental Results Figure S3: Lexis diagram of CCF50, single-year age interval ASFR, and TFR by mother's age and location, 1950-2100, Southeast Asia, East Asia, and Oceania

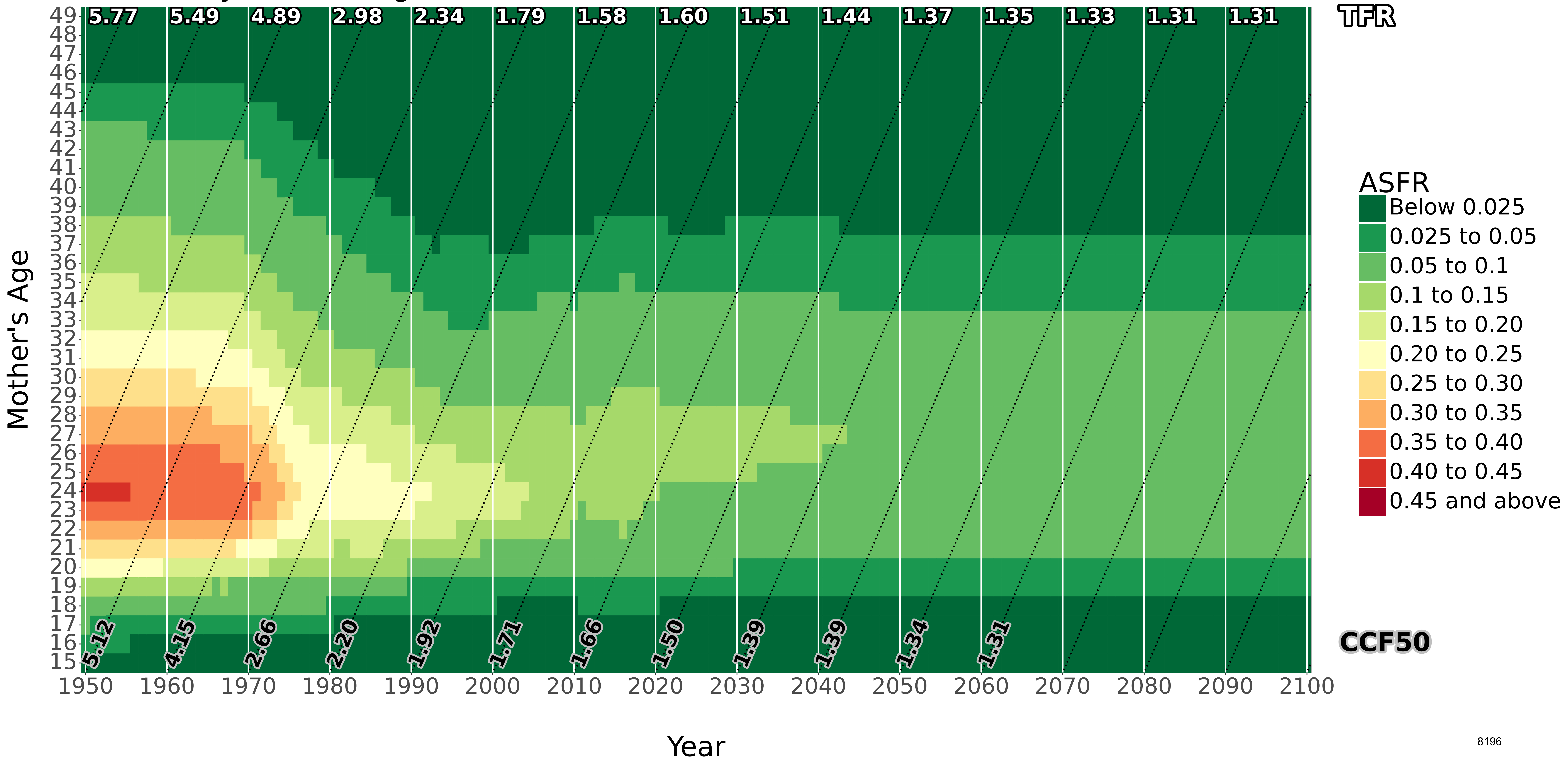

8196

Supplemental Results Figure S3: Lexis diagram of CCF50, single-year age interval ASFR, and TFR by mother's age and location, 1950-2100, Sub-Saharan Africa

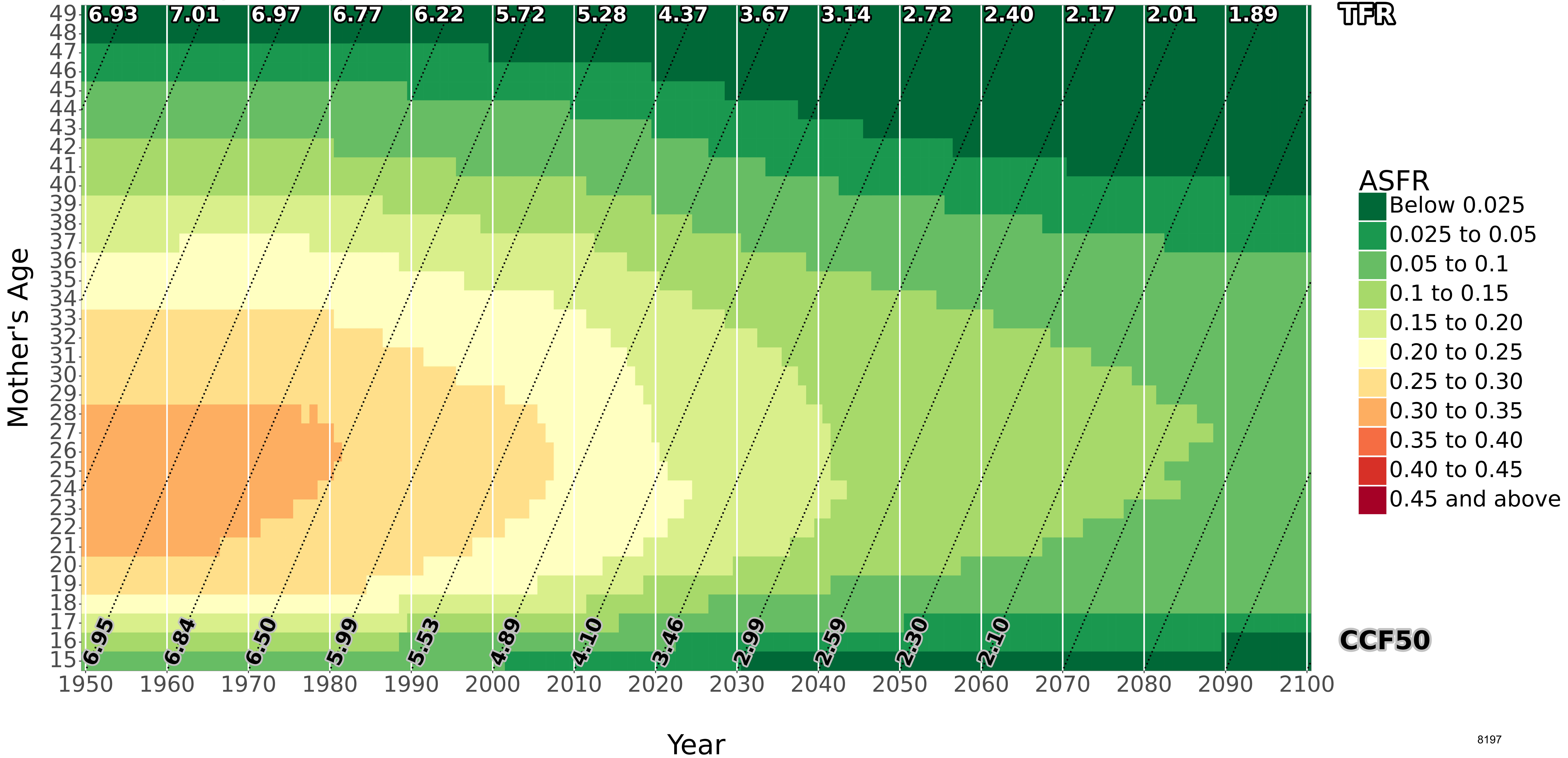

Supplemental Results Figure S3: Lexis diagram of CCF50, single-year age interval ASFR, and TFR by mother's age and location, 1950-2100, Central Asia

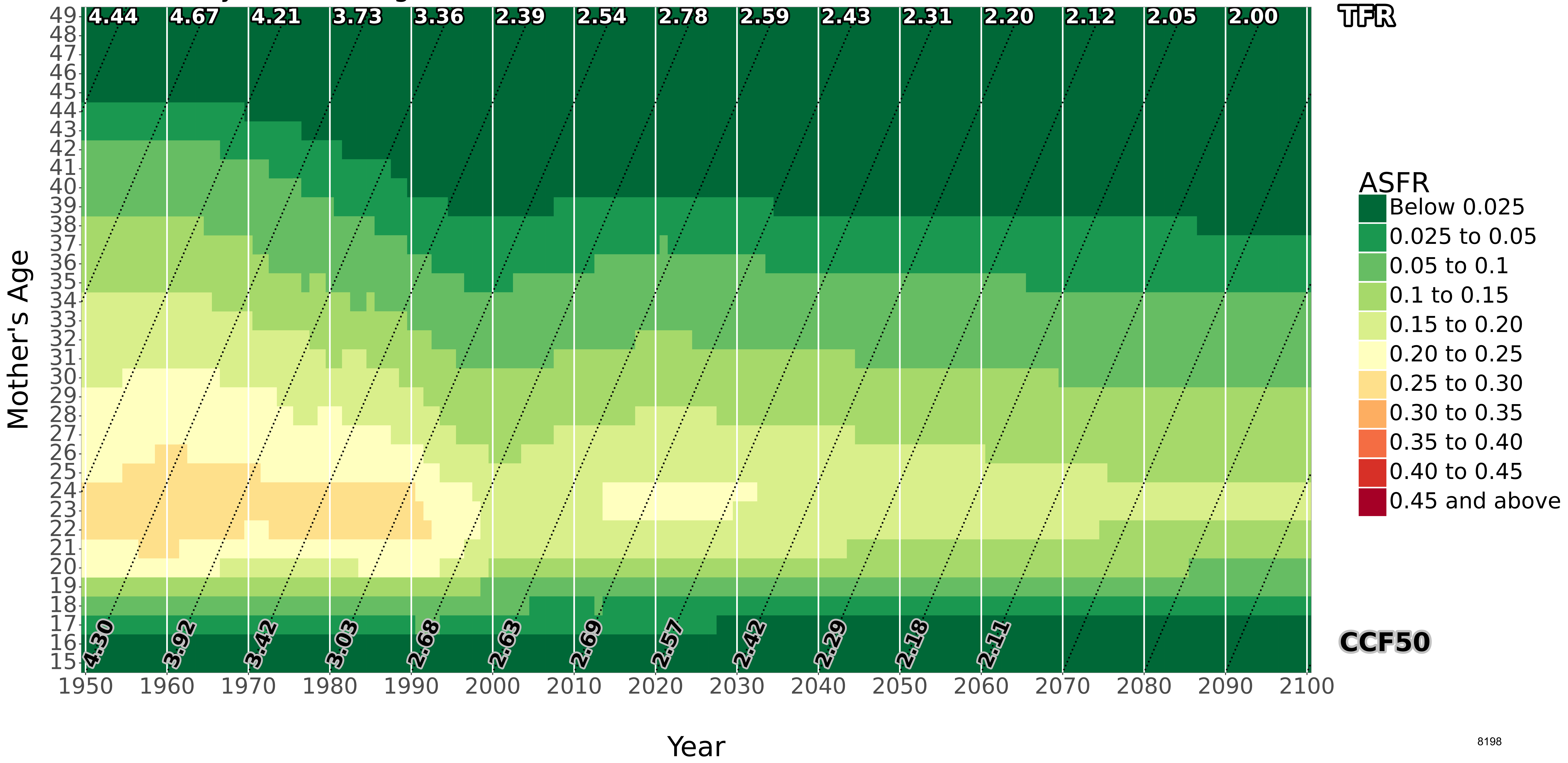

Supplemental Results Figure S3: Lexis diagram of CCF50, single-year age interval ASFR, and TFR by mother's age and location, 1950-2100, Central Europe

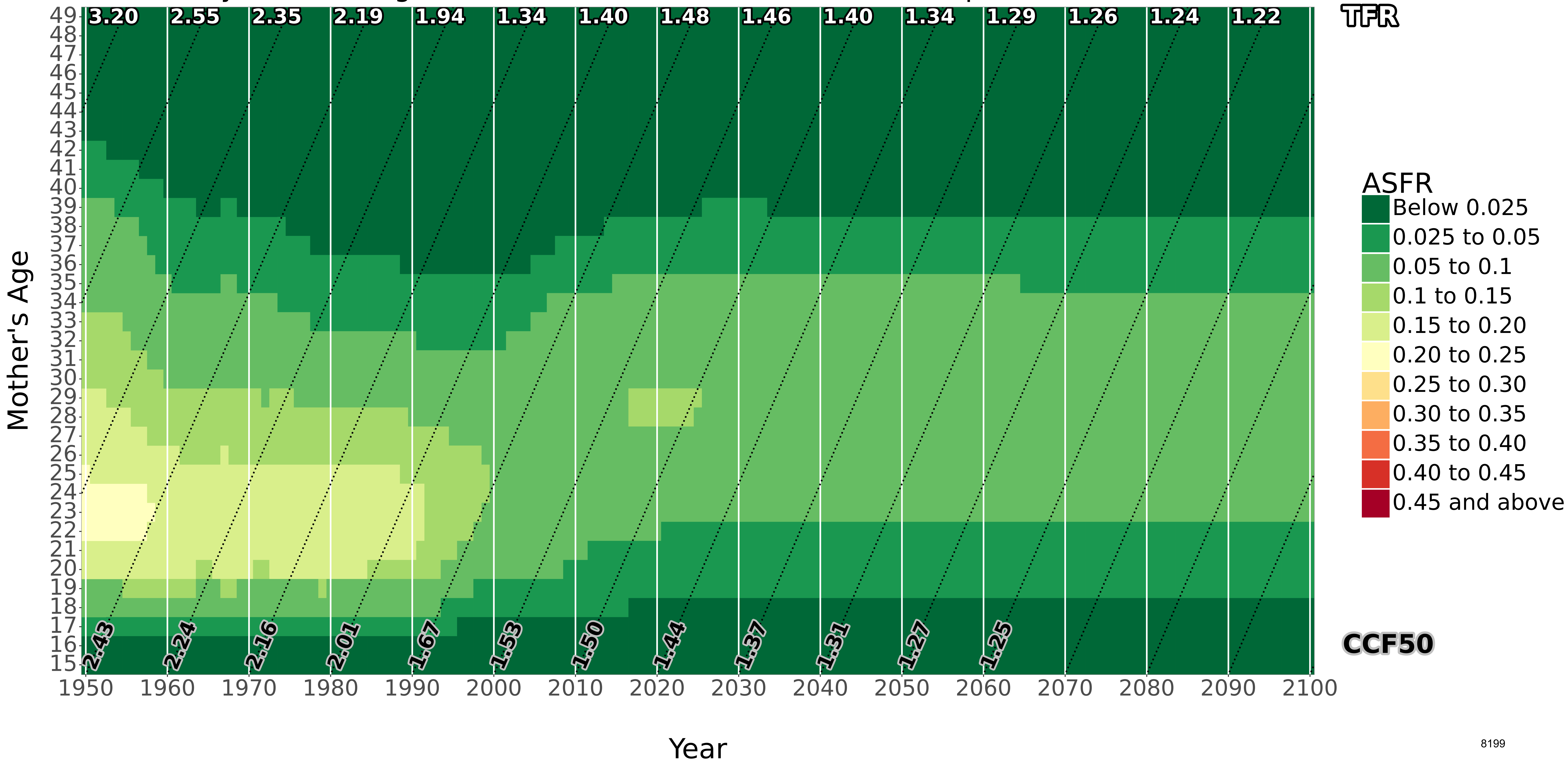

Supplemental Results Figure S3: Lexis diagram of CCF50, single-year age interval ASFR, and TFR by mother's age and location, 1950-2100, Eastern Europe

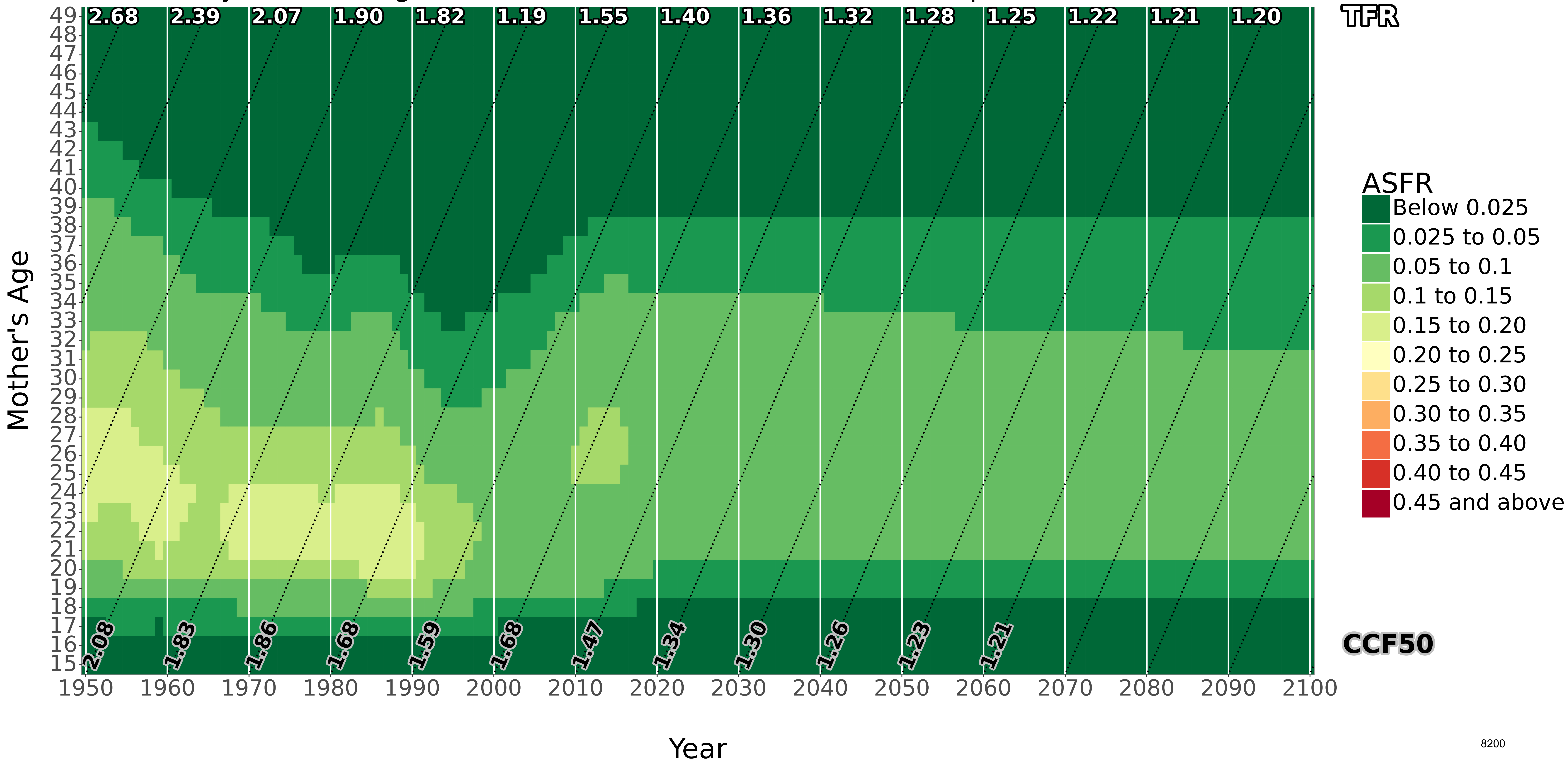

Supplemental Results Figure S3: Lexis diagram of CCF50, single-year age interval ASFR, and TFR by mother's age and location, 1950-2100, Australasia

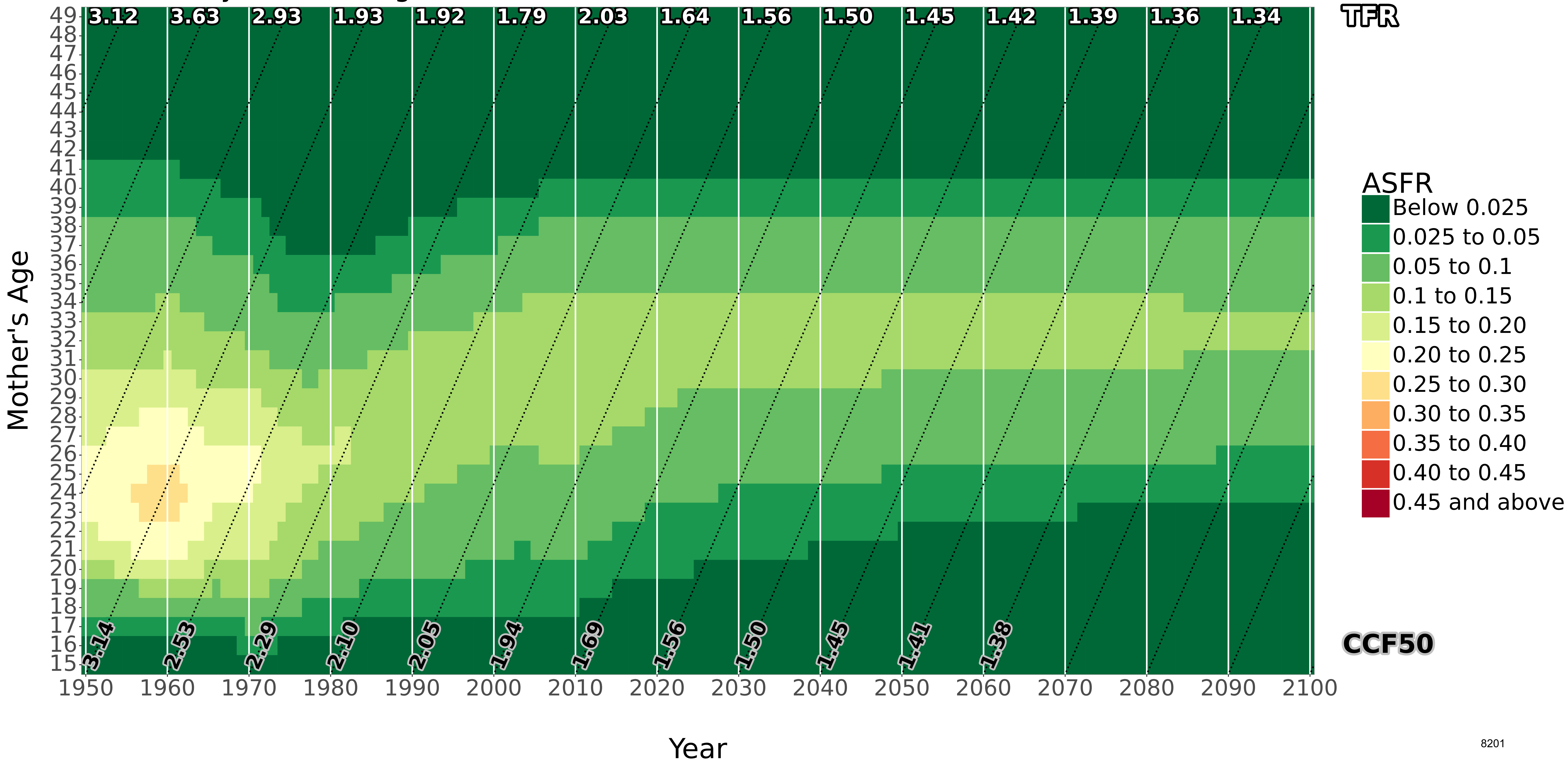

Supplemental Results Figure S3: Lexis diagram of CCF50, single-year age interval ASFR, and TFR by mother's age and location, 1950-2100, High-income Asia Pacific

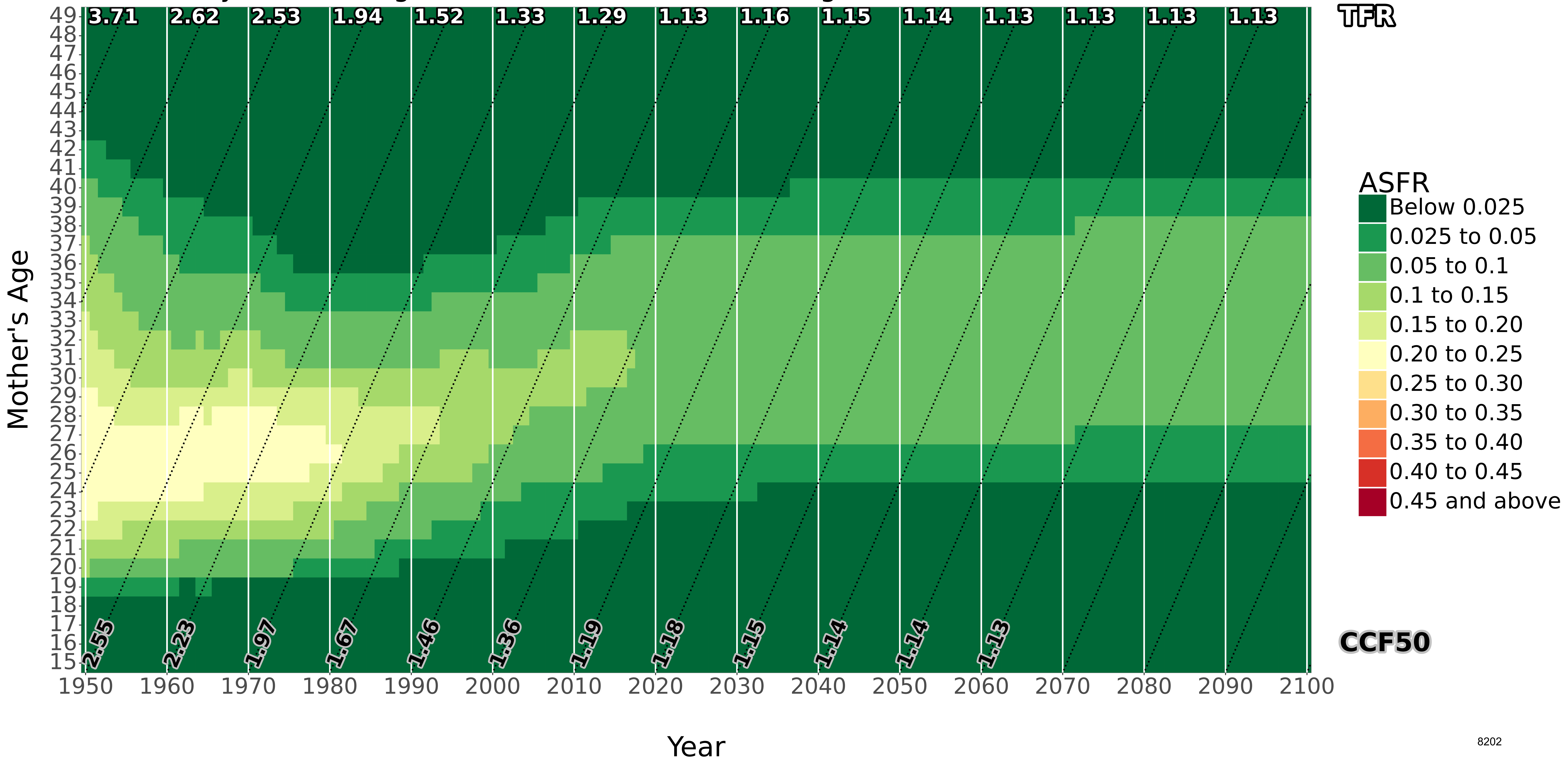

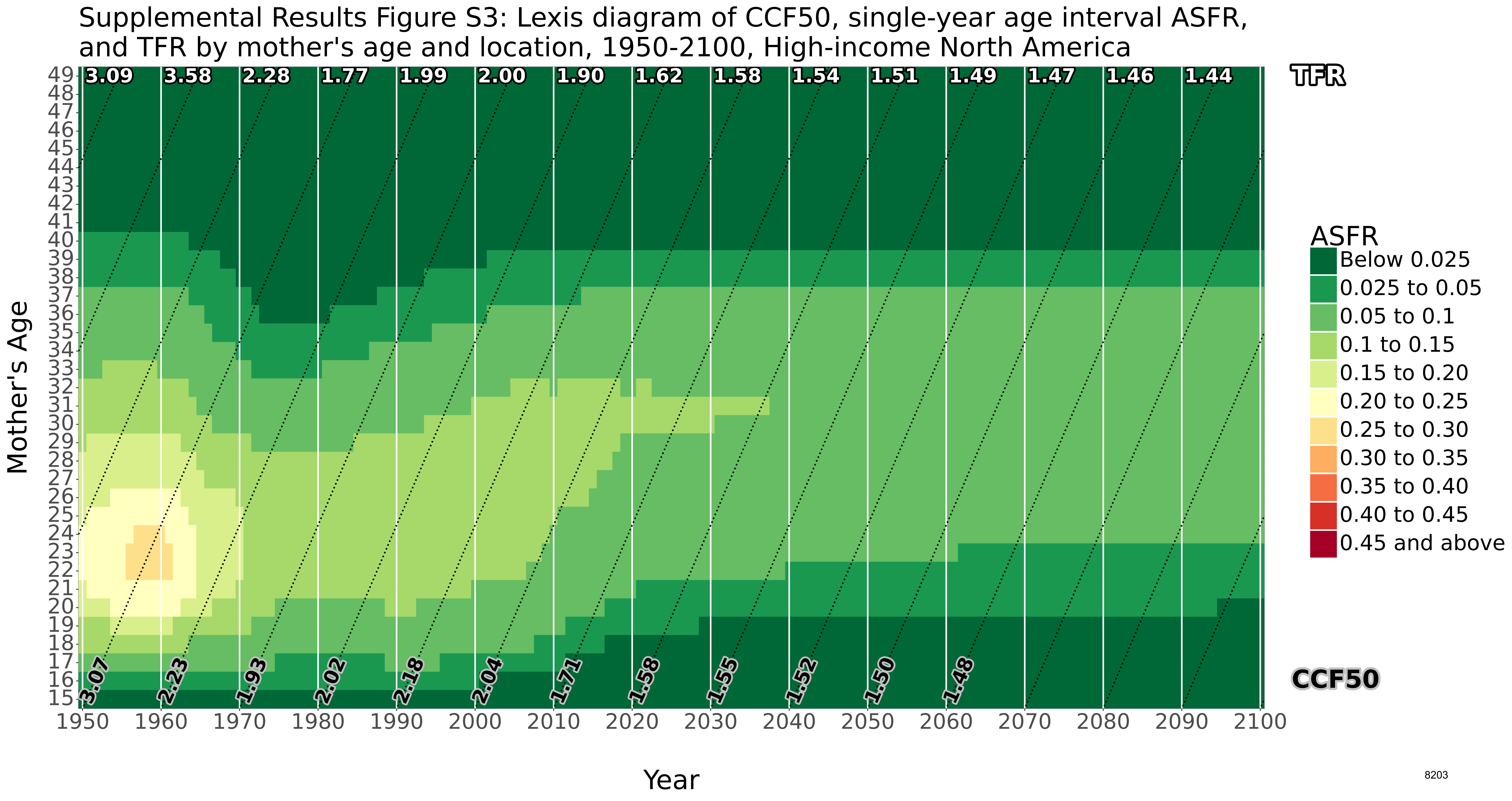

Supplemental Results Figure S3: Lexis diagram of CCF50, single-year age interval ASFR, and TFR by mother's age and location, 1950-2100, High-income North America
TFR
3.09
3.58
2.28
1.77
1.99
2.00
1.90
1.62
1.58
1.54
1.51
1.49
1.47
1.46
1.44
CCF50
3.07
2.23
1.93
2.02
2.18
2.04
1.71
1.58
1.55
1.52
1.50
1.48
Mother's Age
Year
ASFR
Below 0.025
0.025 to 0.05
0.05 to 0.1
0.1 to 0.15
0.15 to 0.20
0.20 to 0.25
0.25 to 0.30
0.30 to 0.35
0.35 to 0.40
0.40 to 0.45
0.45 and above

Supplemental Results Figure S3: Lexis diagram of CCF50, single-year age interval ASFR, and TFR by mother's age and location, 1950-2100, Southern Latin America

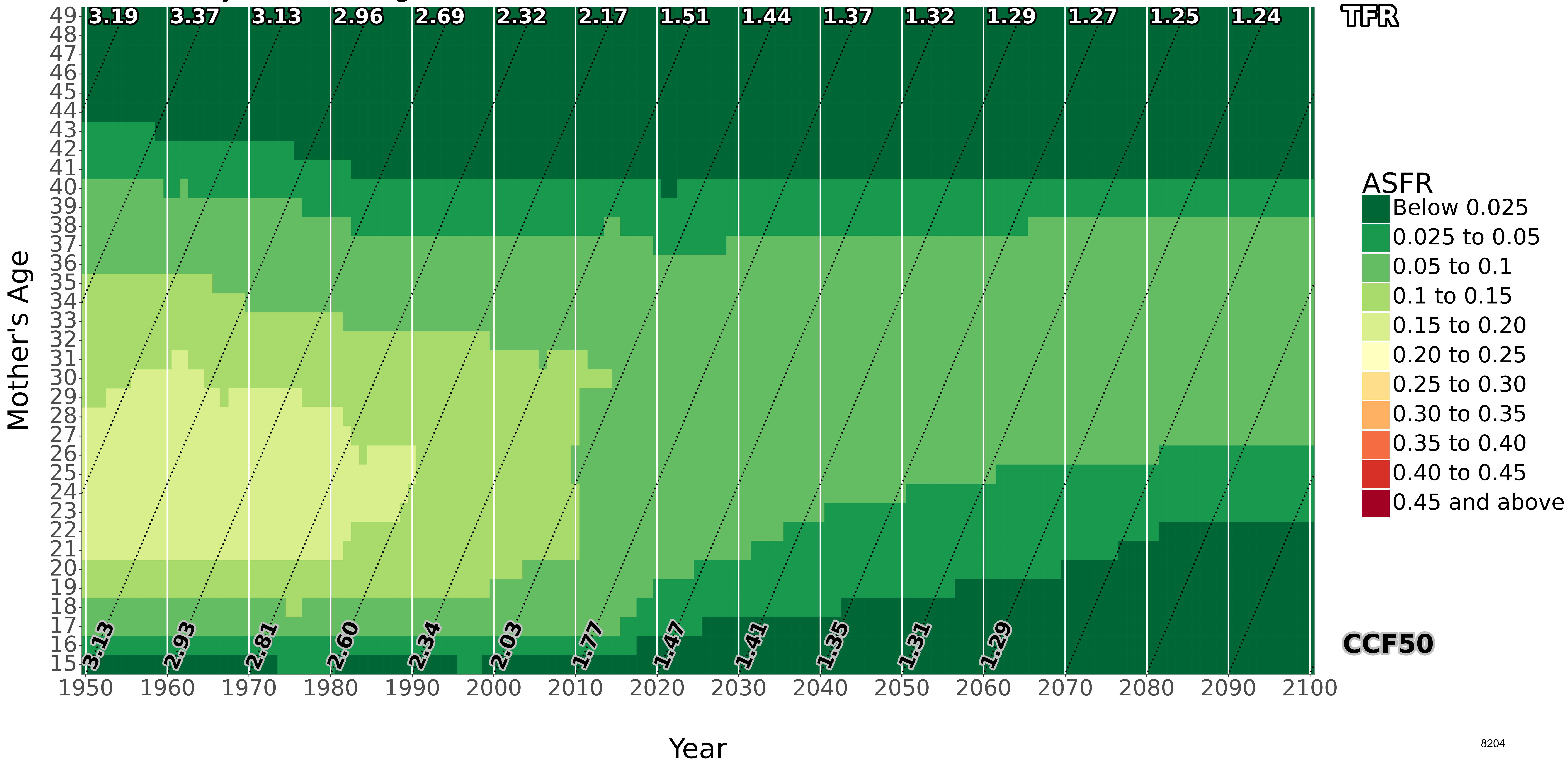

Supplemental Results Figure S3: Lexis diagram of CCF50, single-year age interval ASFR, and TFR by mother's age and location, 1950-2100, Western Europe

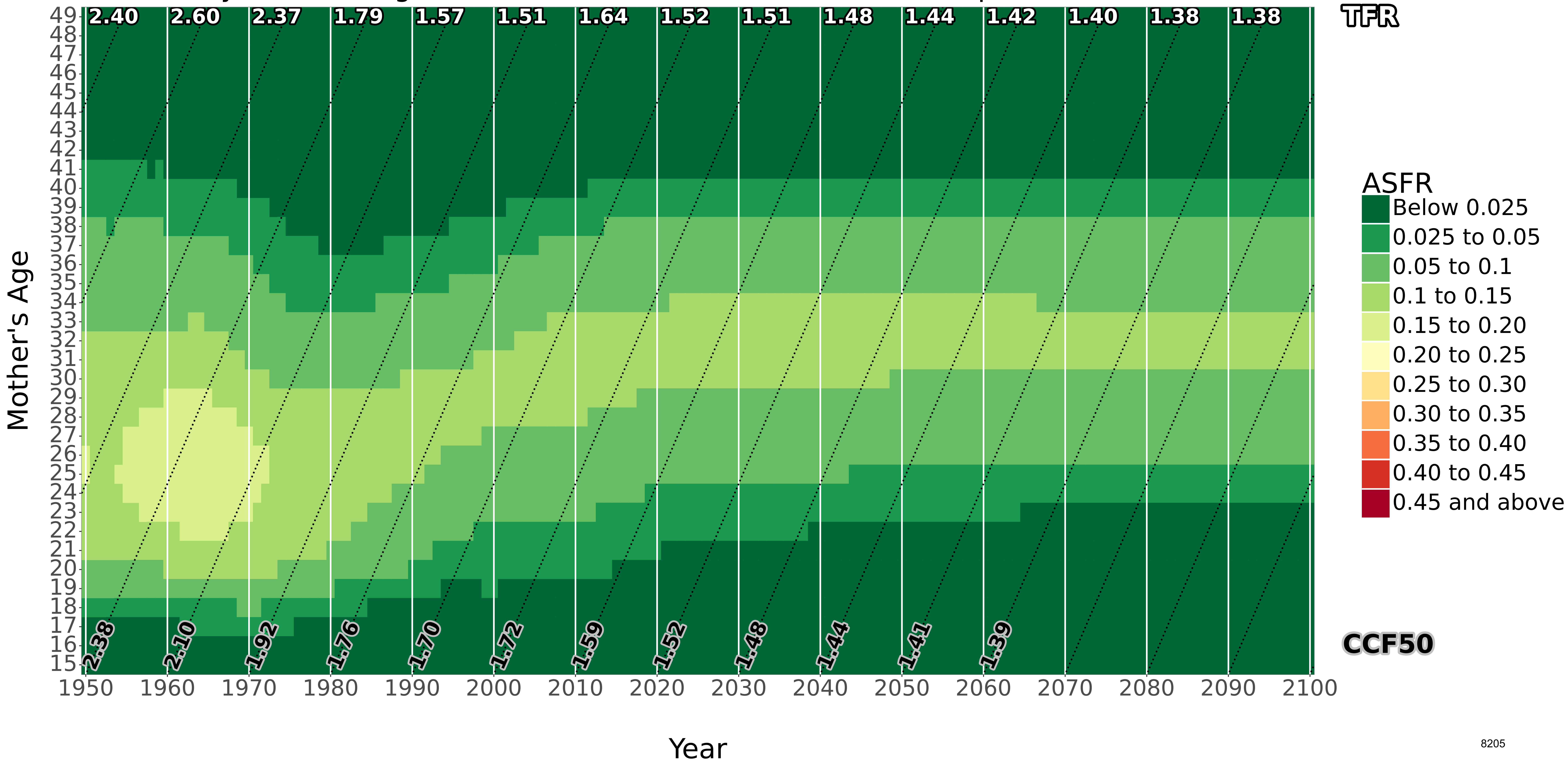

Supplemental Results Figure S3: Lexis diagram of CCF50, single-year age interval ASFR, and TFR by mother's age and location, 1950-2100, Andean Latin America

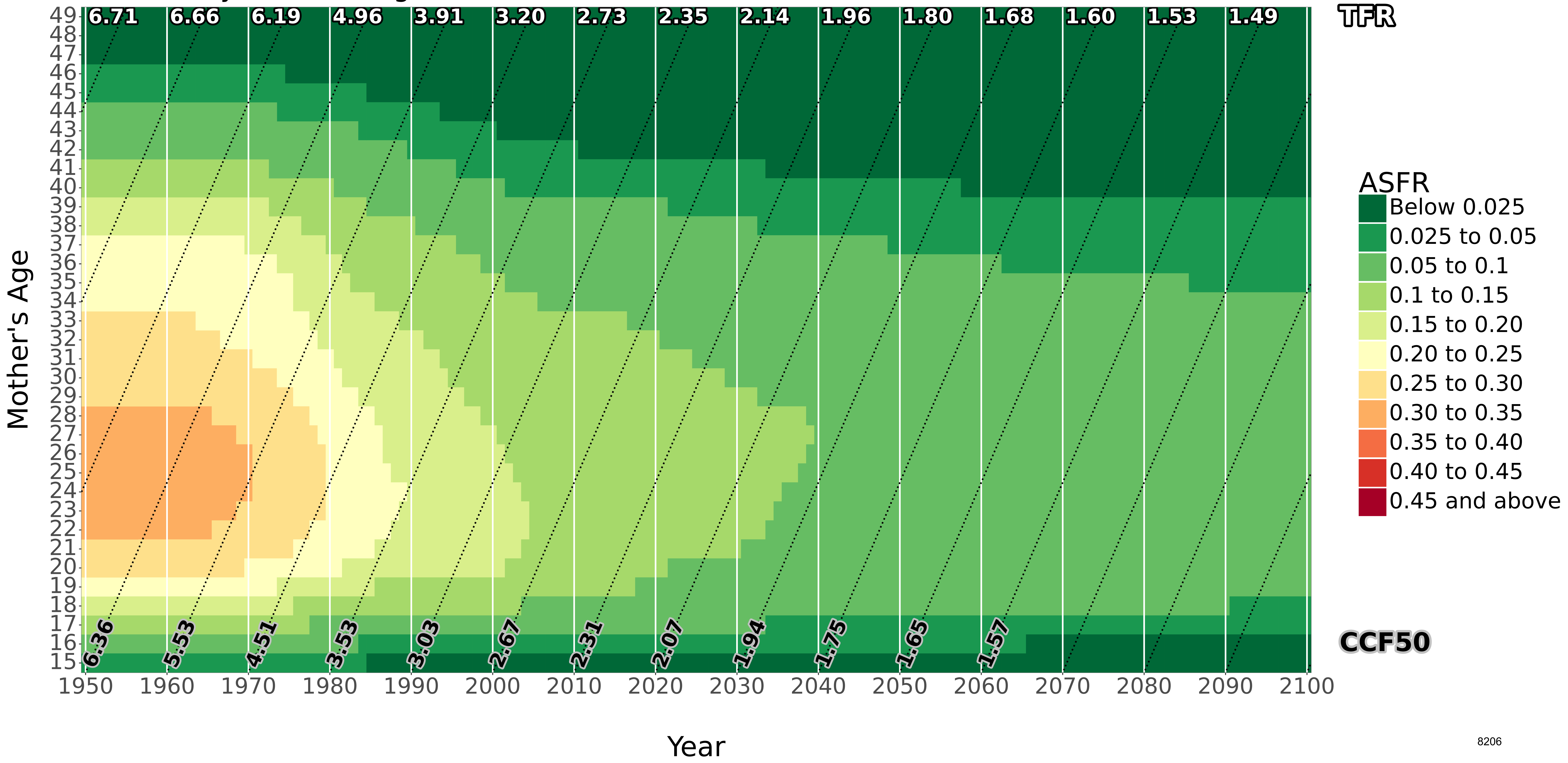

Supplemental Results Figure S3: Lexis diagram of CCF50, single-year age interval ASFR, and TFR by mother's age and location, 1950-2100, Caribbean

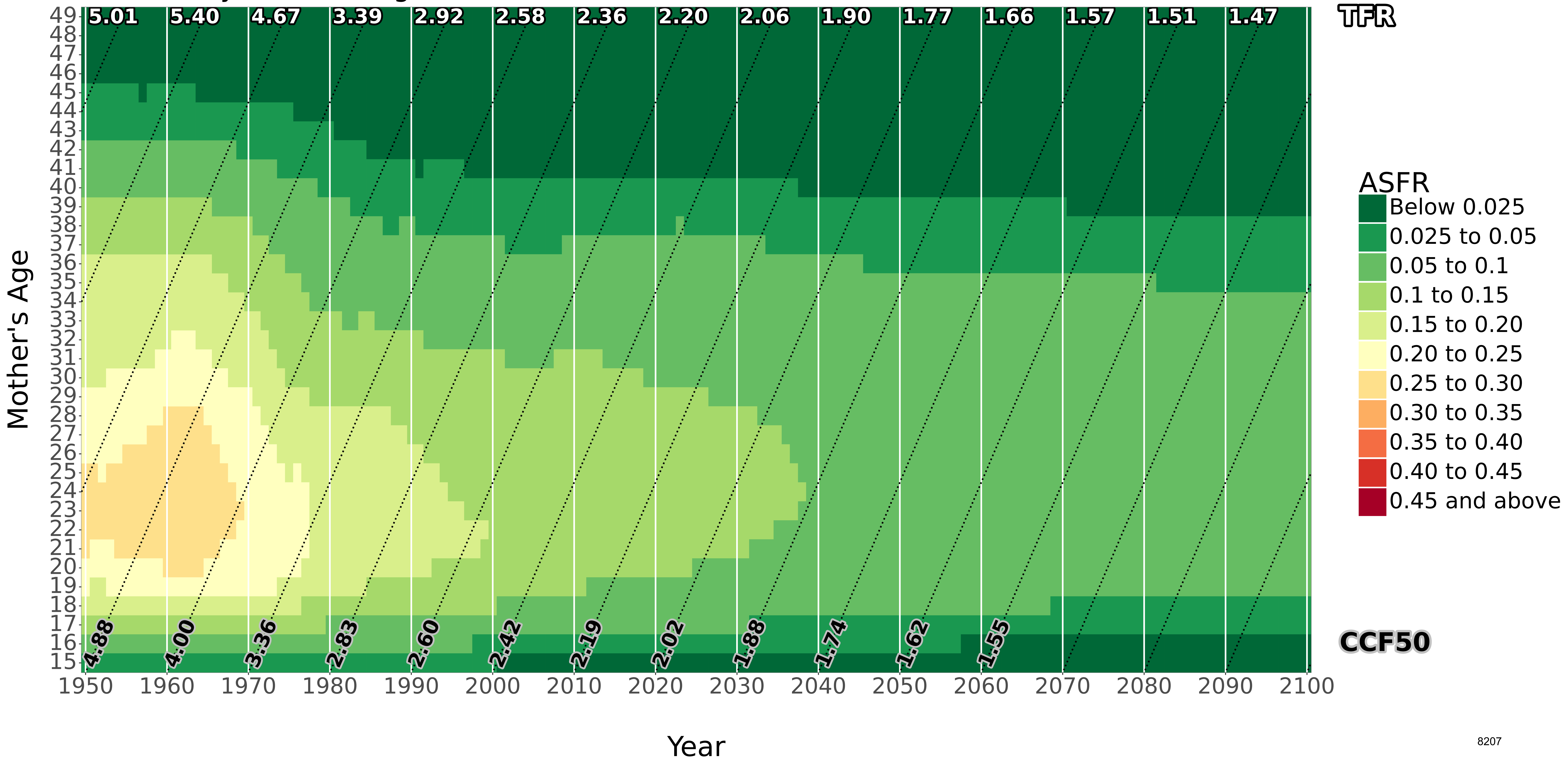

Supplemental Results Figure S3: Lexis diagram of CCF50, single-year age interval ASFR, and TFR by mother's age and location, 1950-2100, Central Latin America

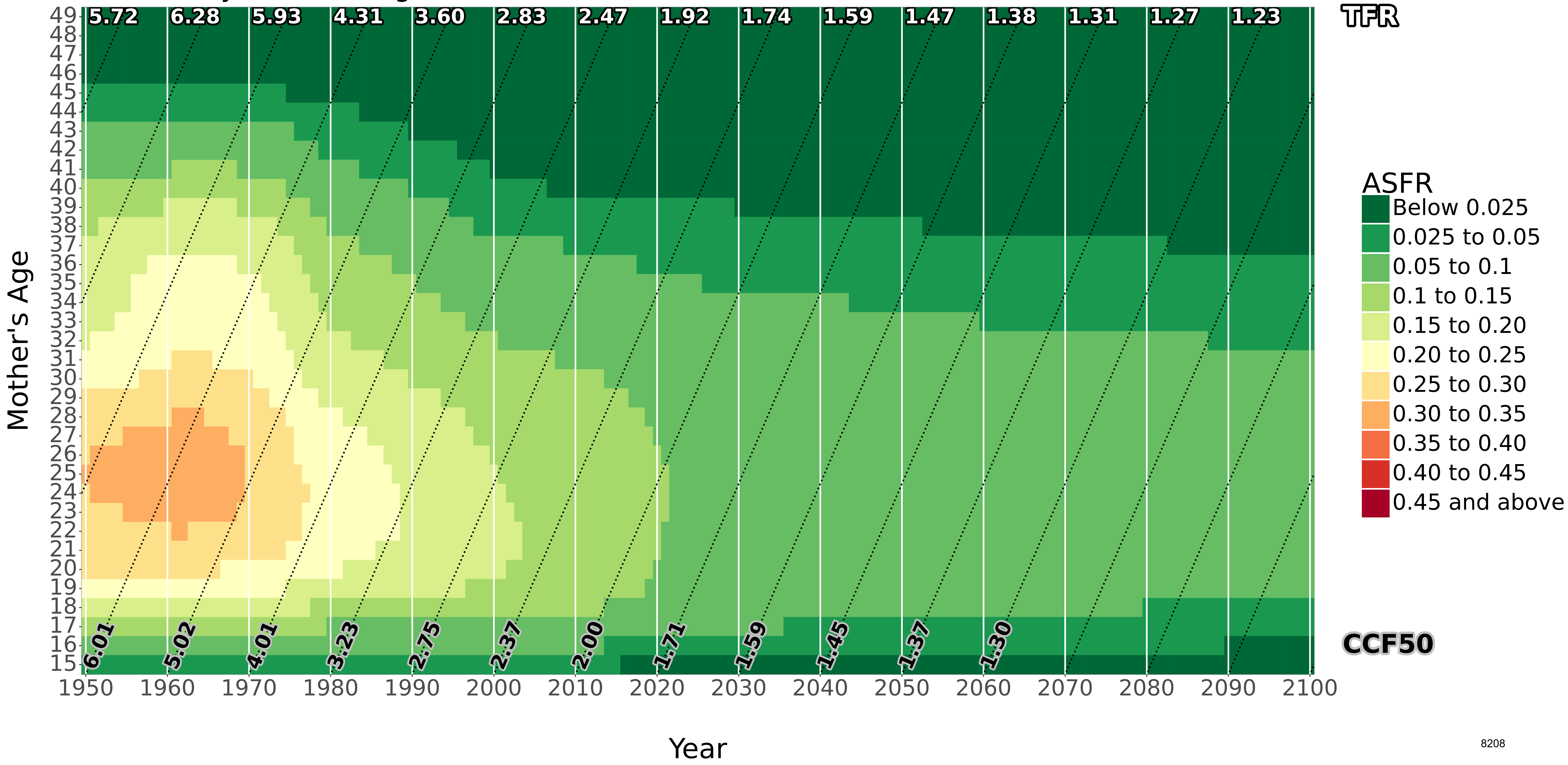

Supplemental Results Figure S3: Lexis diagram of CCF50, single-year age interval ASFR, and TFR by mother's age and location, 1950-2100, Tropical Latin America

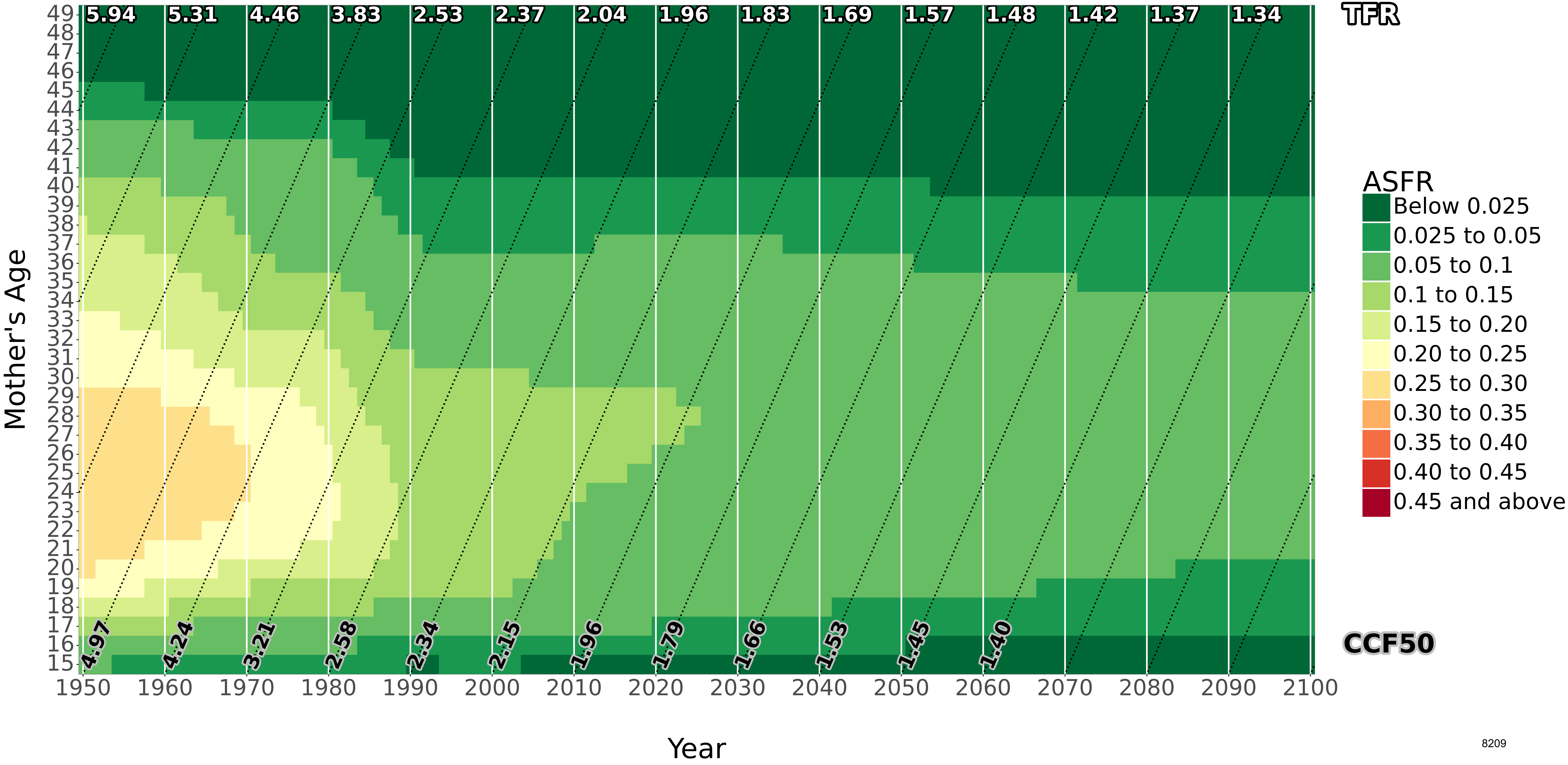

Supplemental Results Figure S3: Lexis diagram of CCF50, single-year age interval ASFR, and TFR by mother's age and location, 1950-2100, North Africa and Middle East

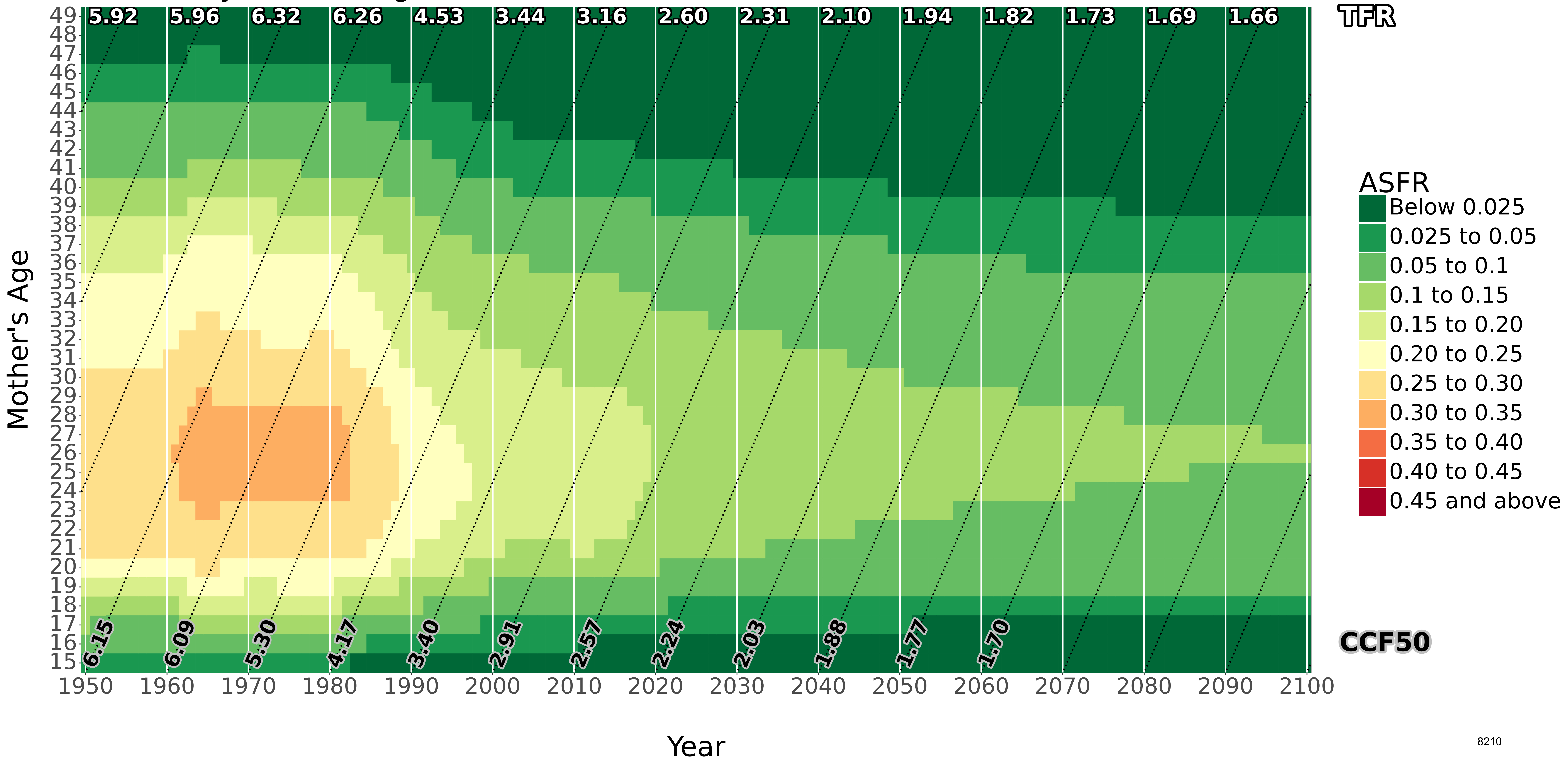

Supplemental Results Figure S3: Lexis diagram of CCF50, single-year age interval ASFR, and TFR by mother's age and location, 1950-2100, South Asia

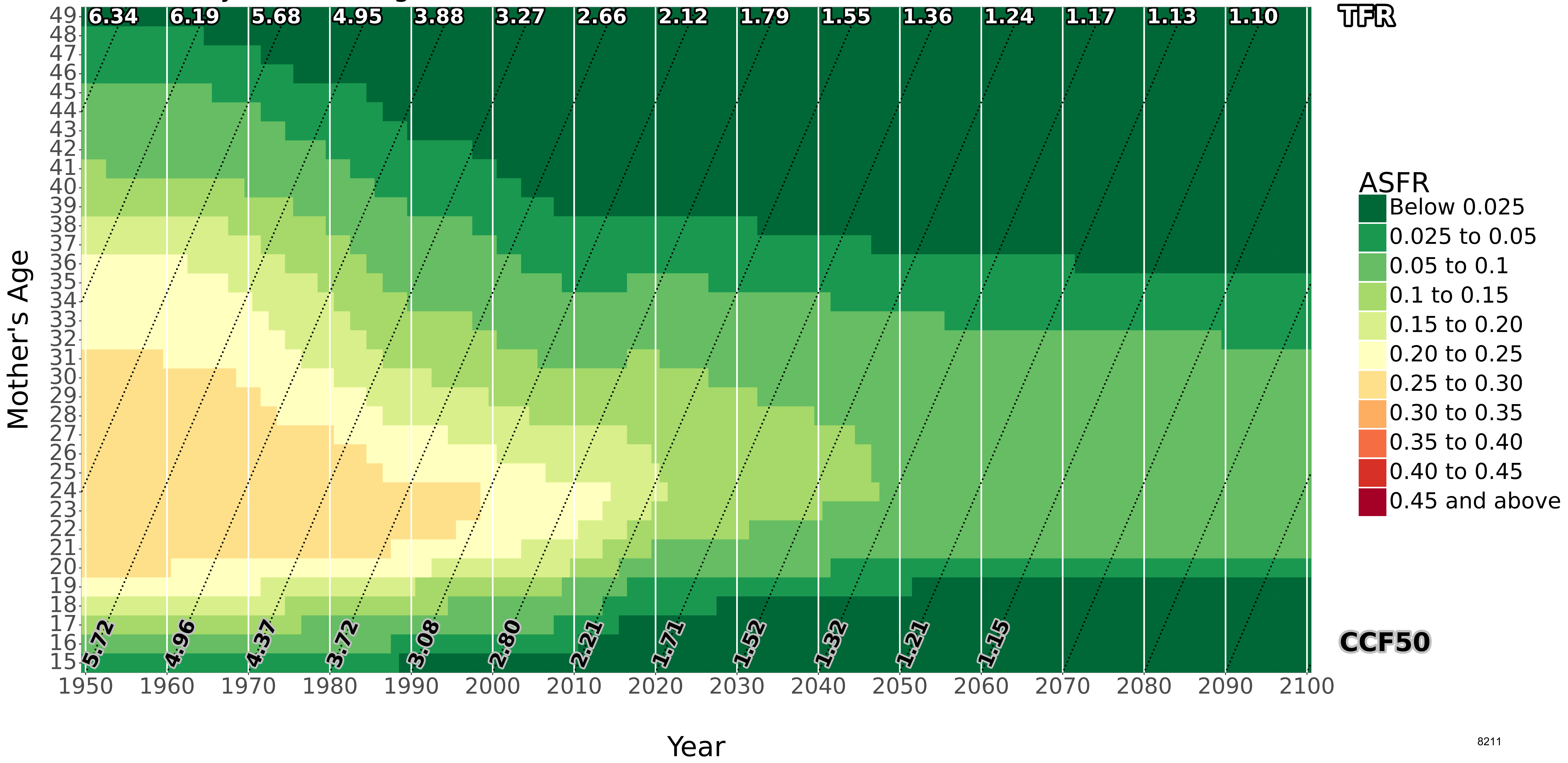

Supplemental Results Figure S3: Lexis diagram of CCF50, single-year age interval ASFR, and TFR by mother's age and location, 1950-2100, East Asia

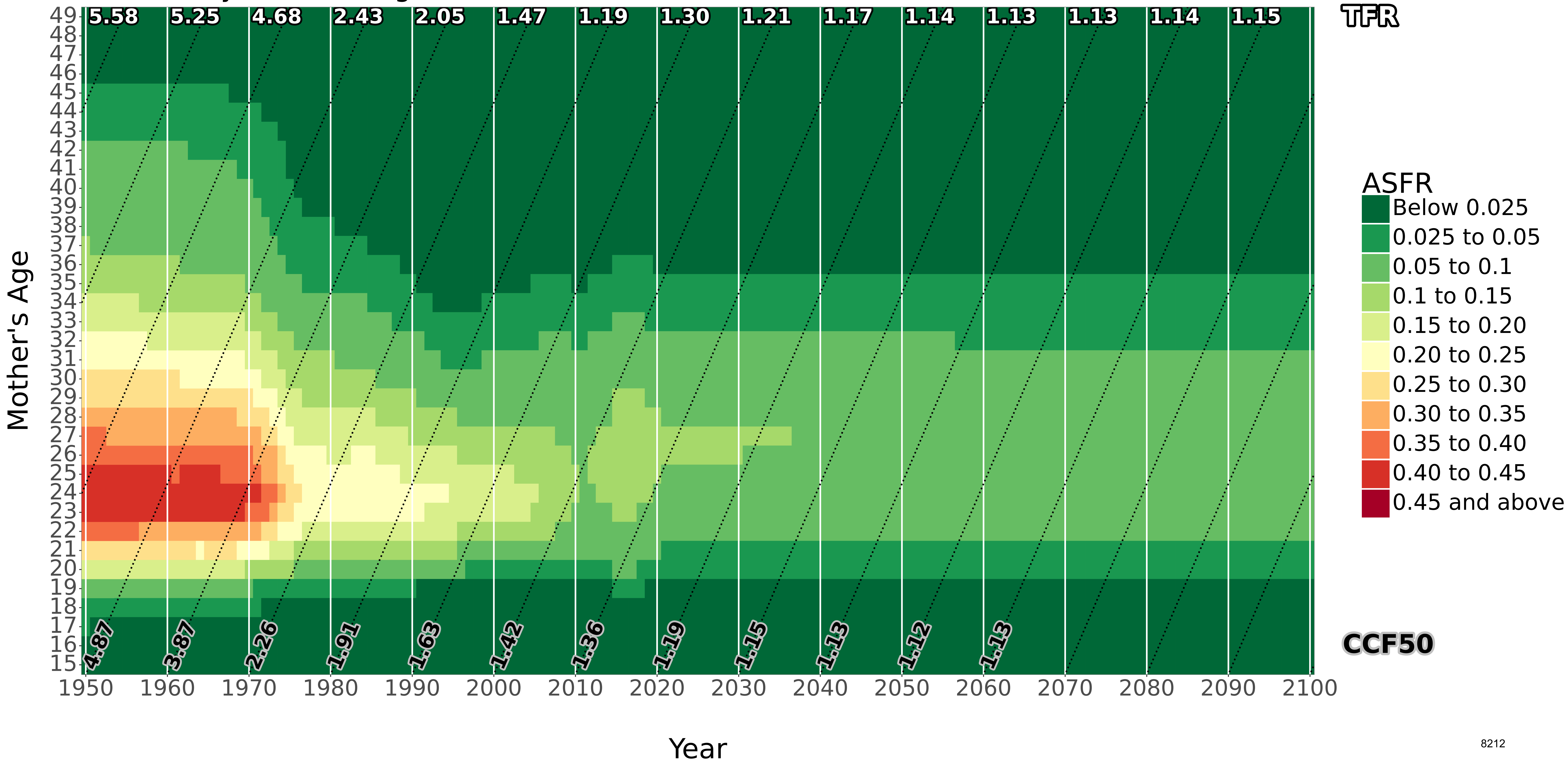

Supplemental Results Figure S3: Lexis diagram of CCF50, single-year age interval ASFR, and TFR by mother's age and location, 1950-2100, Oceania

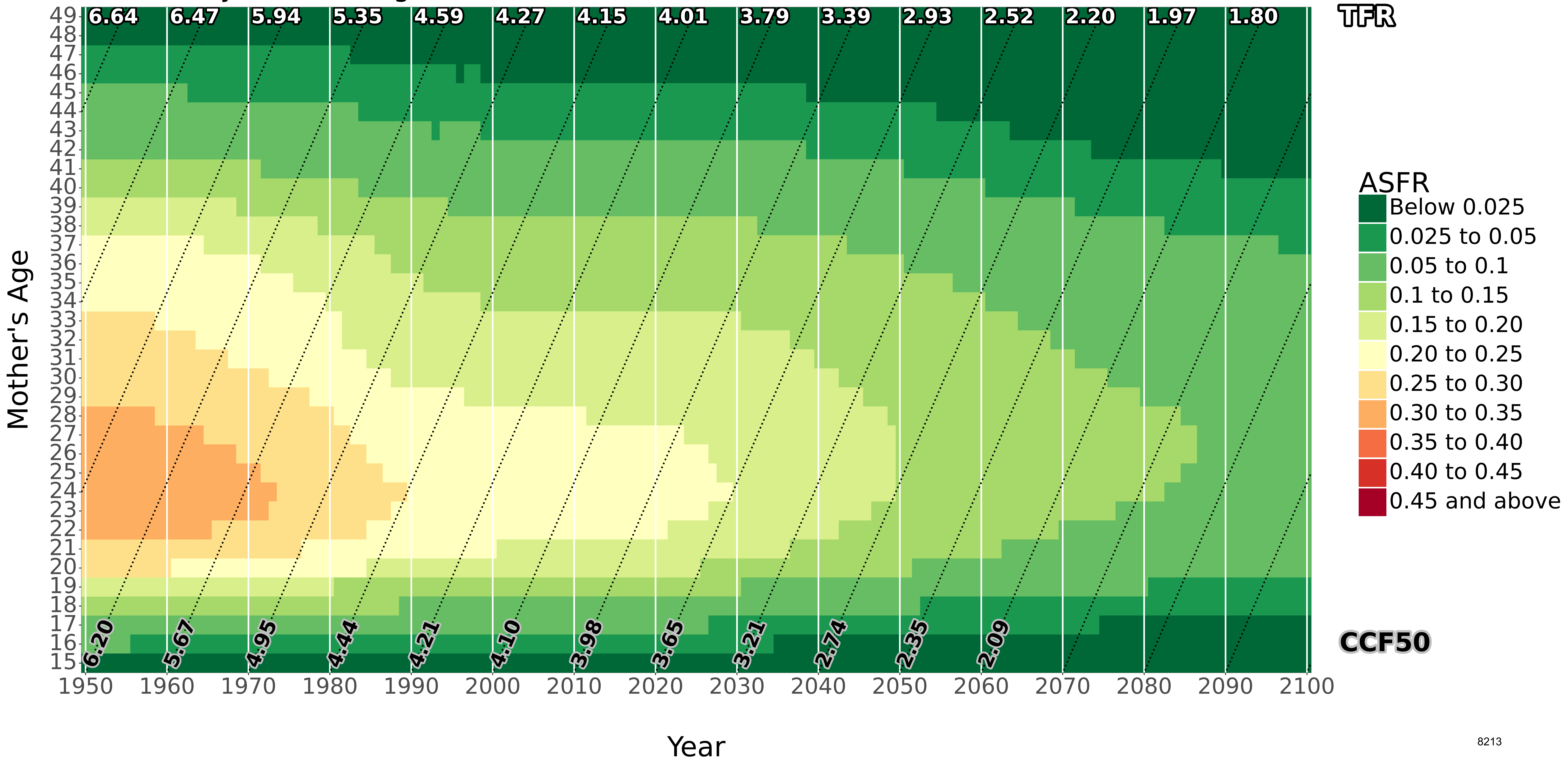

Supplemental Results Figure S3: Lexis diagram of CCF50, single-year age interval ASFR, and TFR by mother's age and location, 1950-2100, Southeast Asia

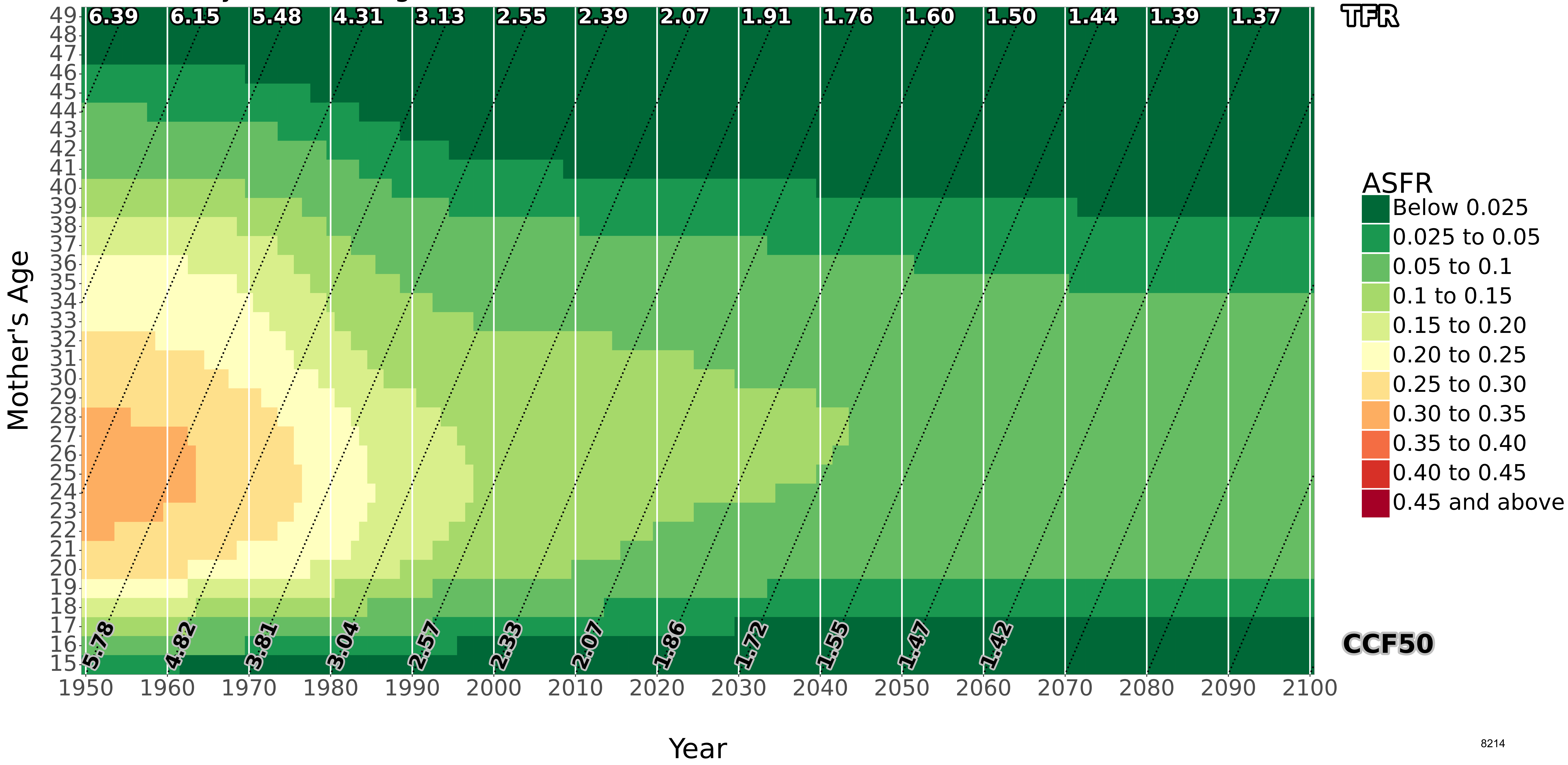

Supplemental Results Figure S3: Lexis diagram of CCF50, single-year age interval ASFR, and TFR by mother's age and location, 1950-2100, Central sub-Saharan Africa

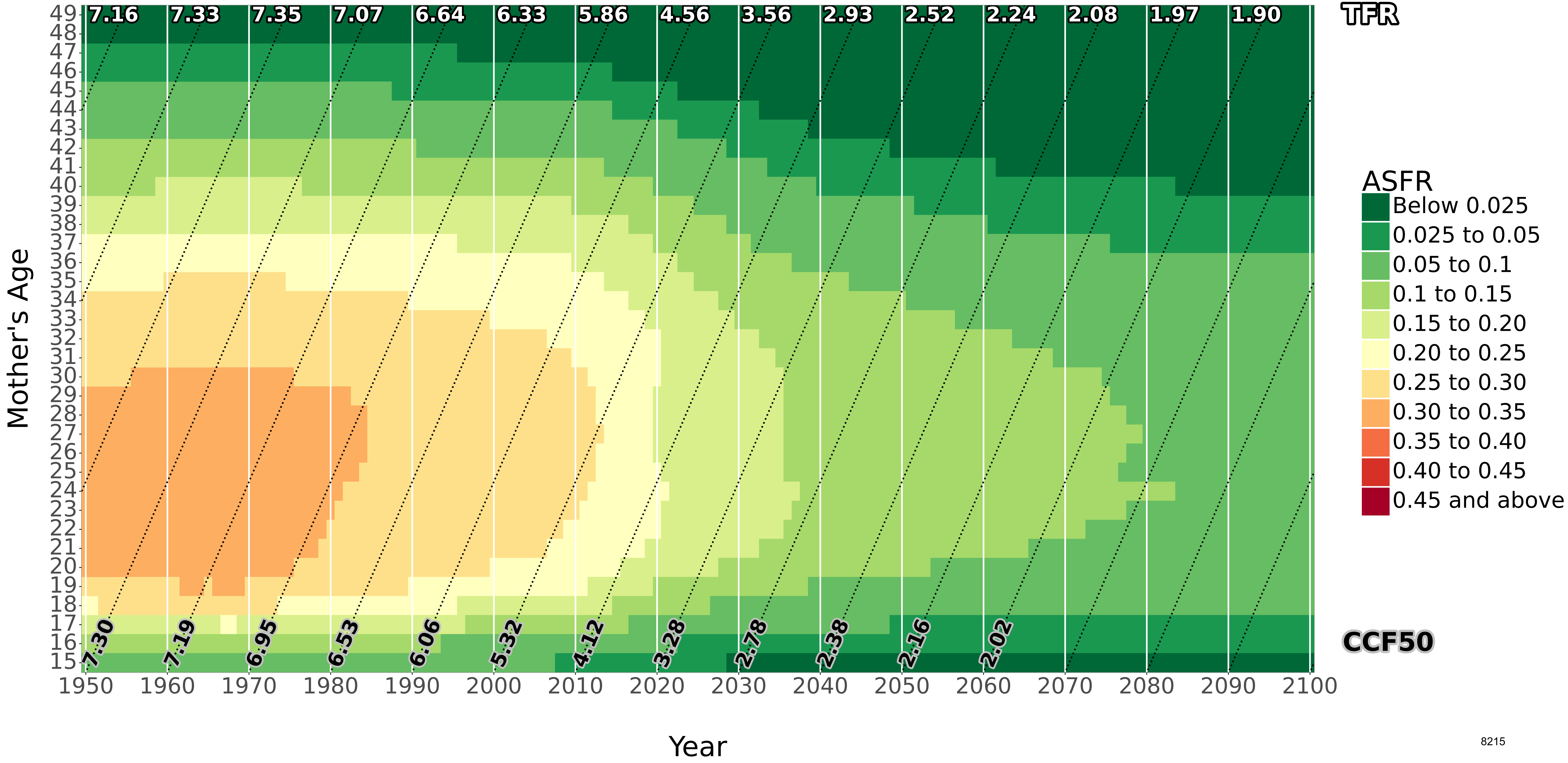

Supplemental Results Figure S3: Lexis diagram of CCF50, single-year age interval ASFR, and TFR by mother's age and location, 1950-2100, Eastern sub-Saharan Africa

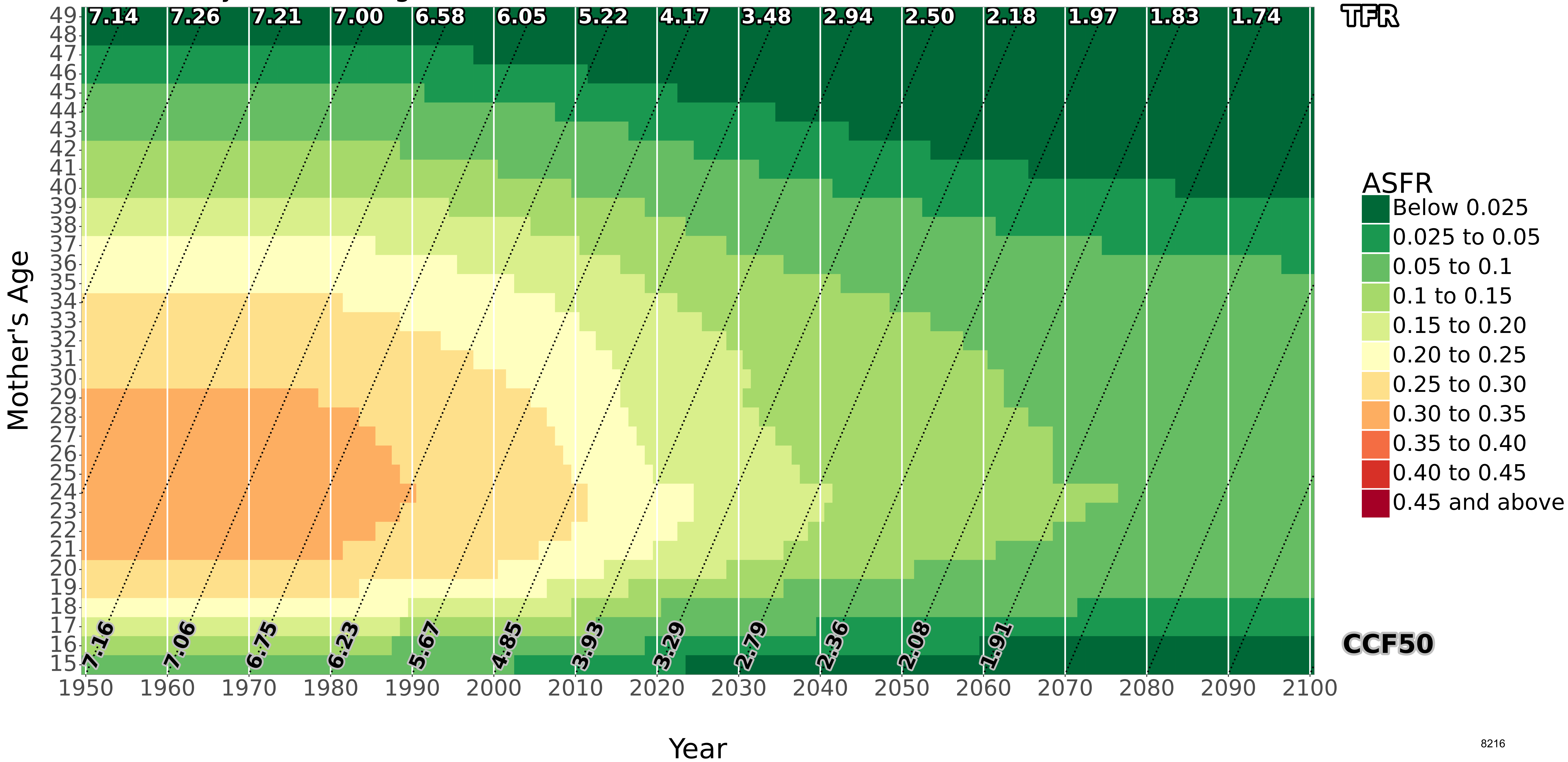

Supplemental Results Figure S3: Lexis diagram of CCF50, single-year age interval ASFR, and TFR by mother's age and location, 1950-2100, Southern sub-Saharan Africa

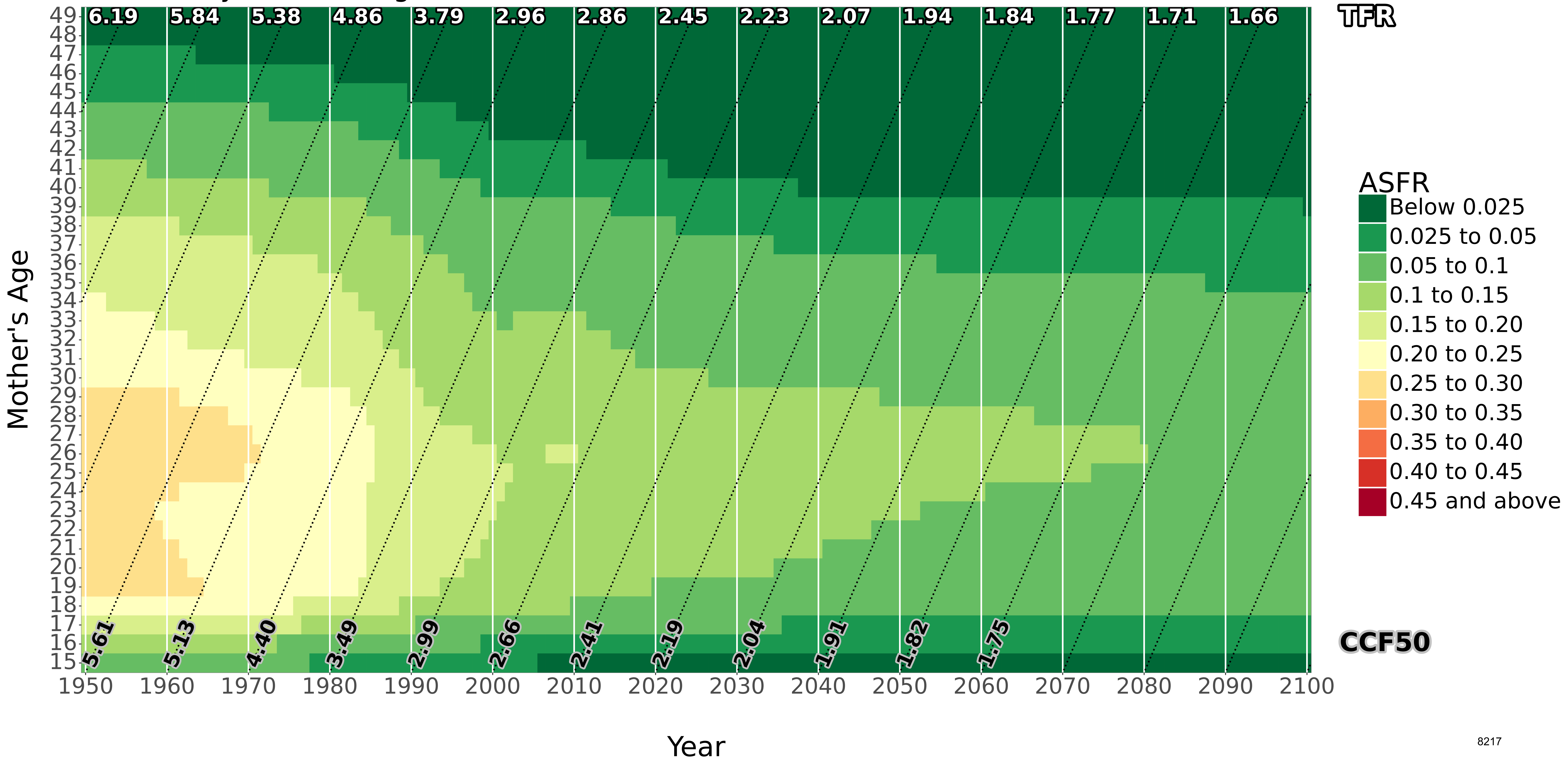

8217

Supplemental Results Figure S3: Lexis diagram of CCF50, single-year age interval ASFR, and TFR by mother's age and location, 1950-2100, Western sub-Saharan Africa

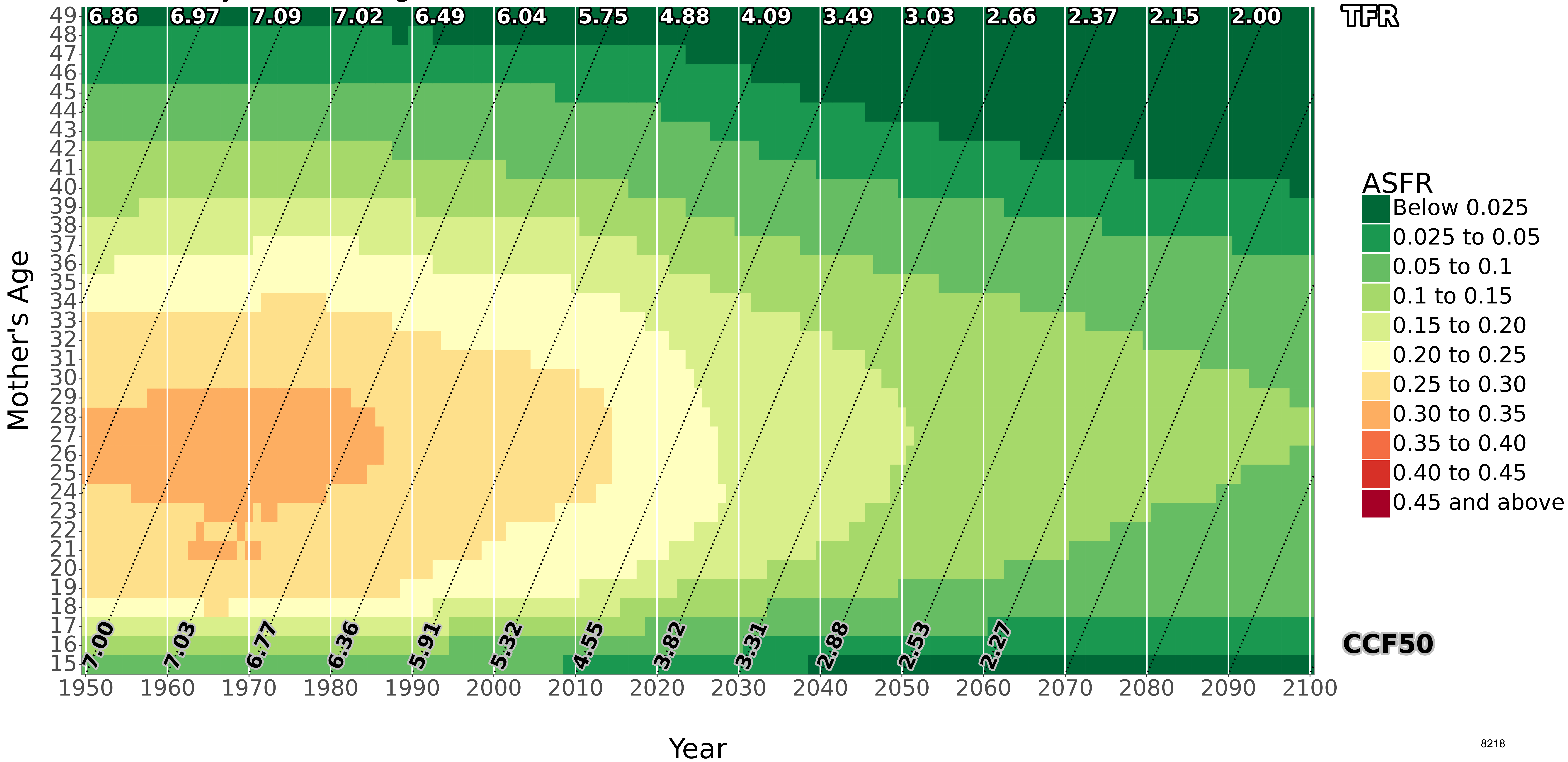

Supplemental Results Figure S4: Number of livebirths (millions) by 2021 World Bank income group

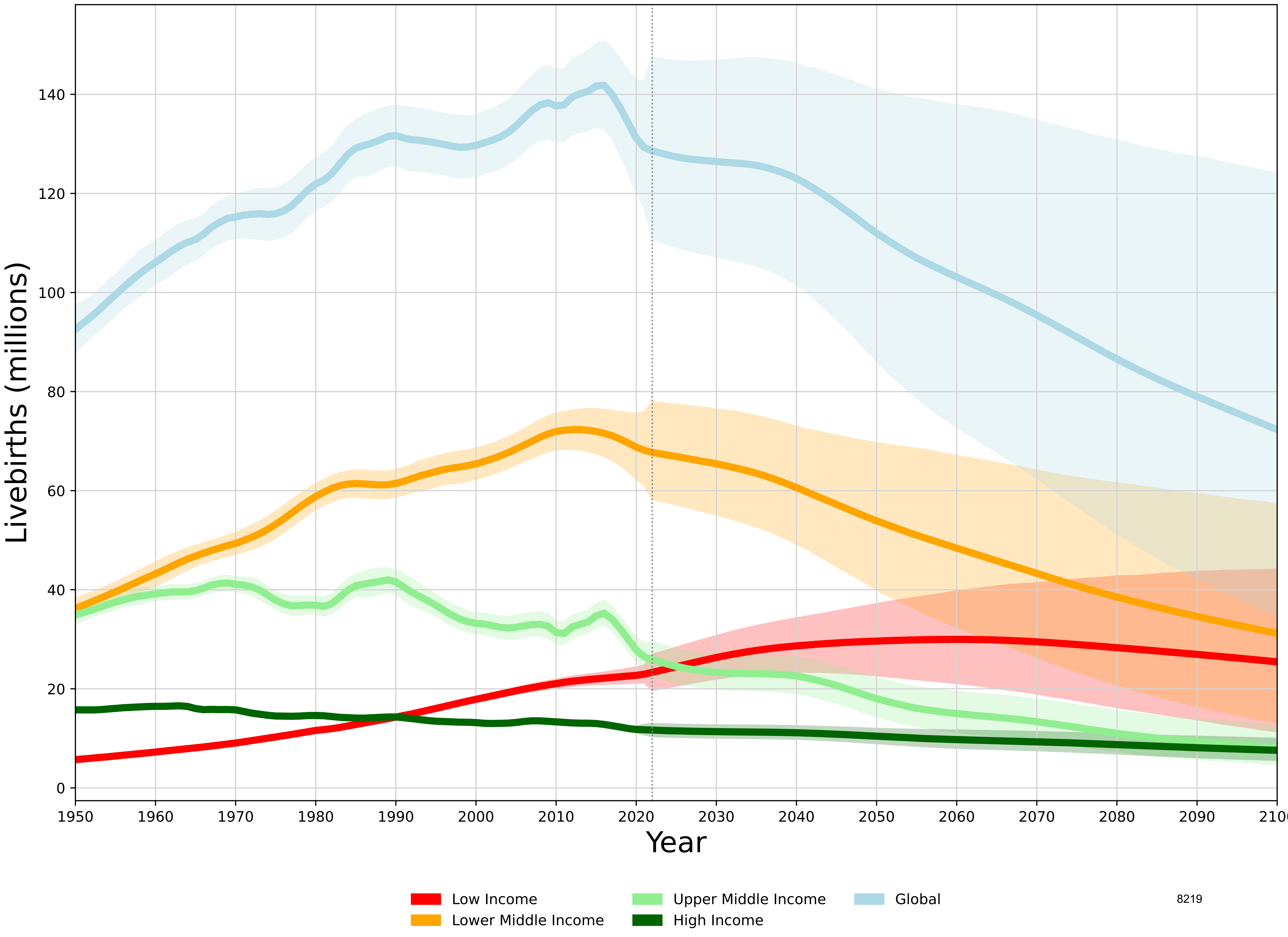

Supplemental Results Figure S5: TFR for reference and alternative scenario forecasts by location, 1950– 2100

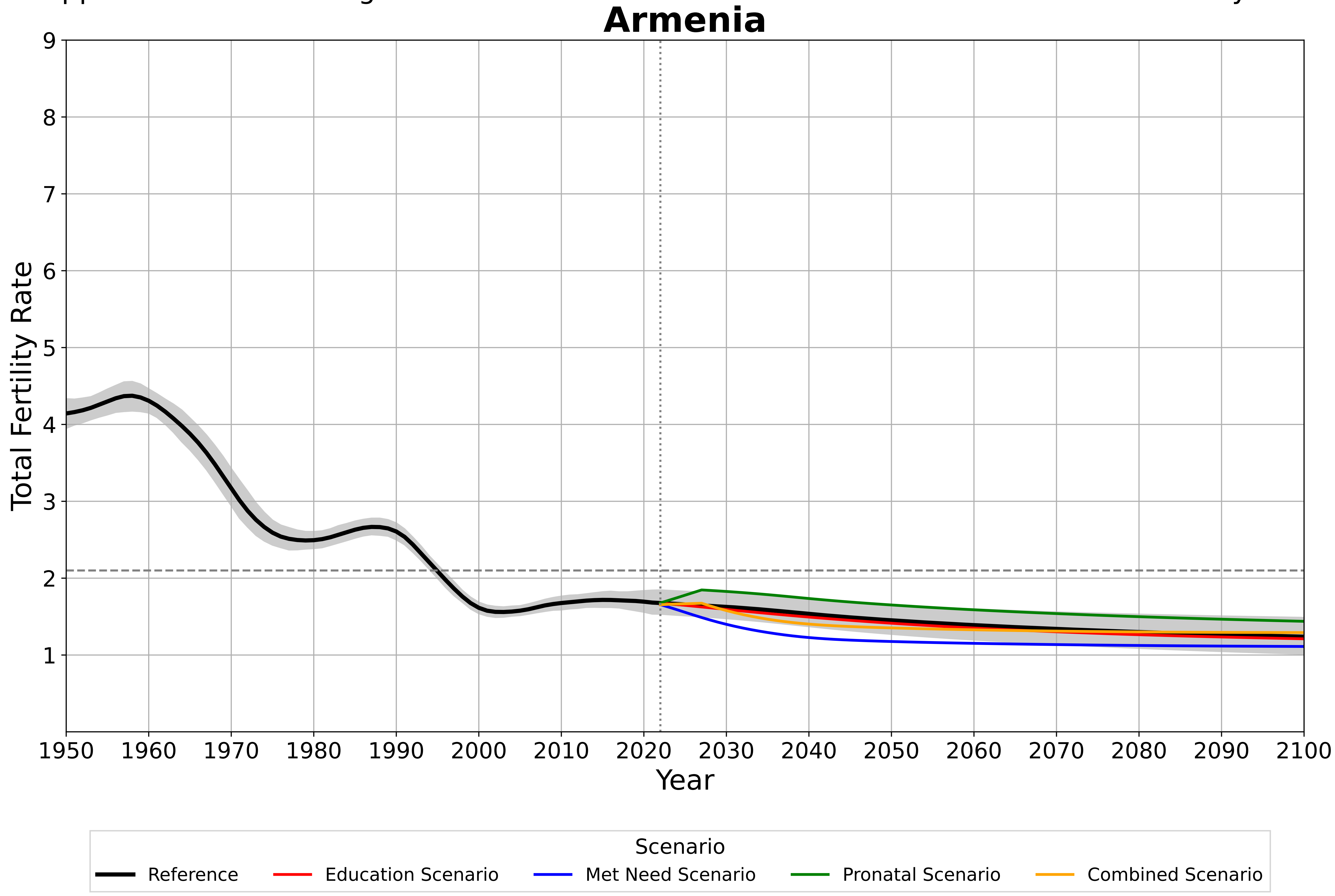

Supplemental Results Figure S5: TFR for reference and alternative scenario forecasts by location, 1950– 2100

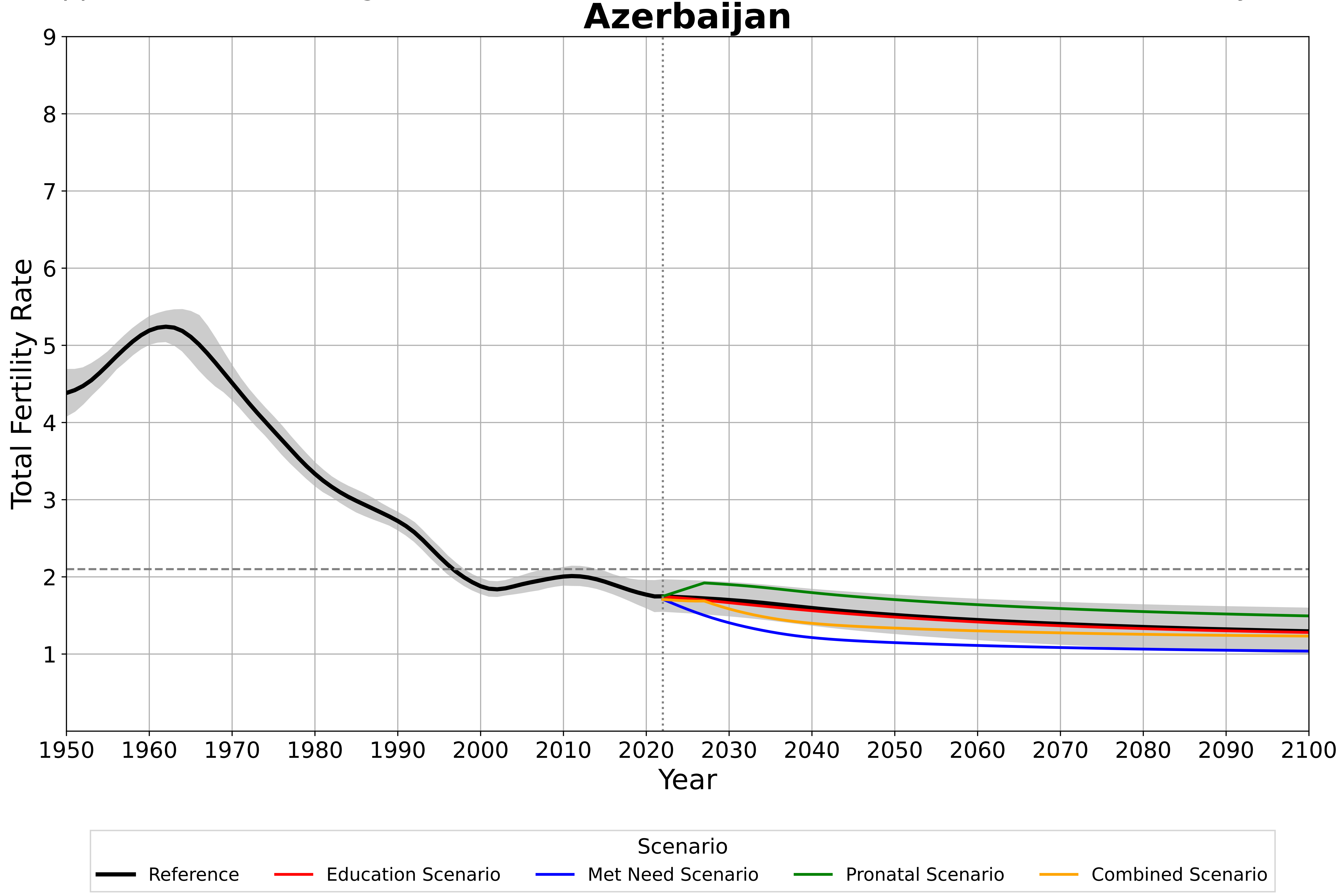

Supplemental Results Figure S5: TFR for reference and alternative scenario forecasts by location, 1950– 2100

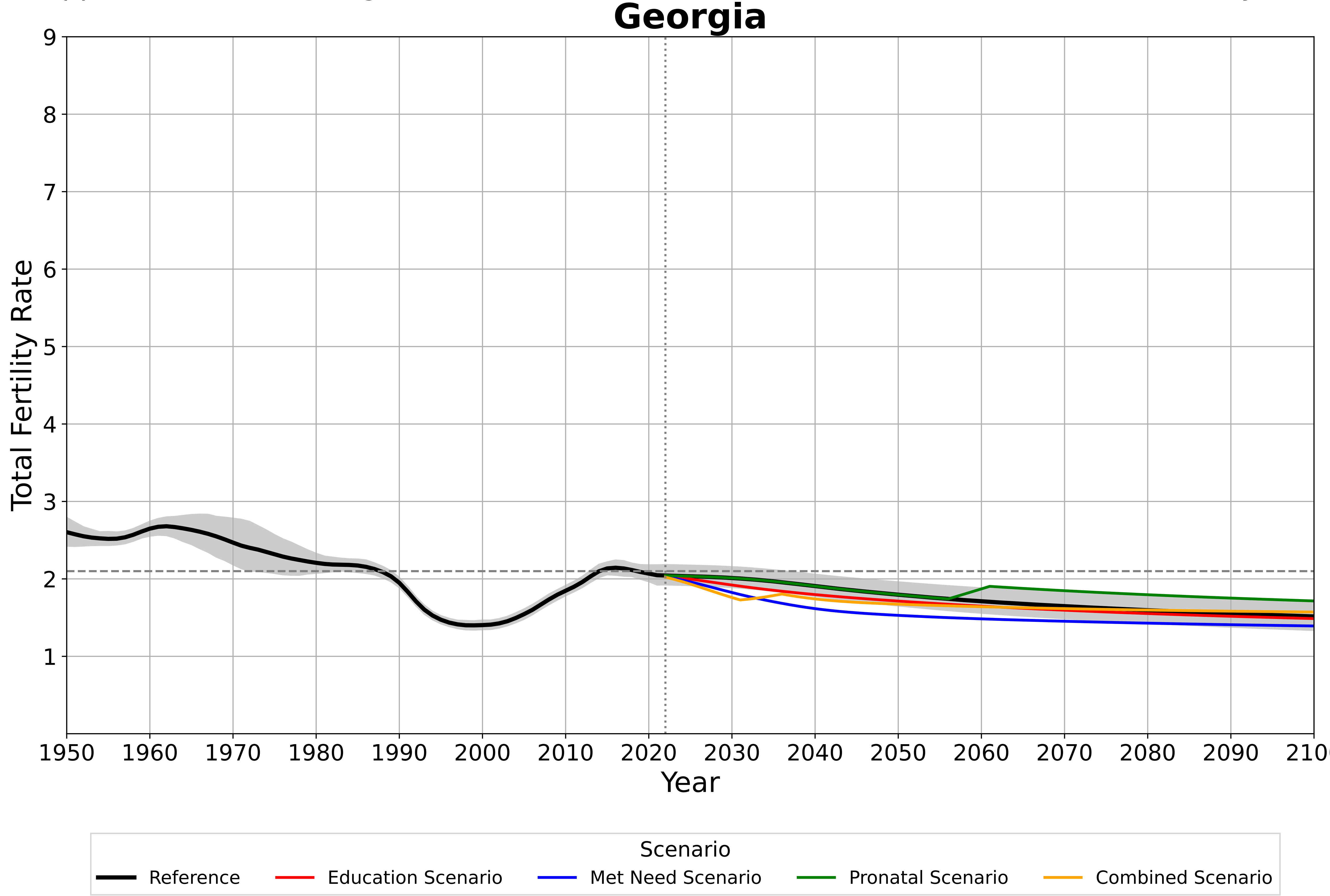

Supplemental Results Figure S5: TFR for reference and alternative scenario forecasts by location, 1950– 2100

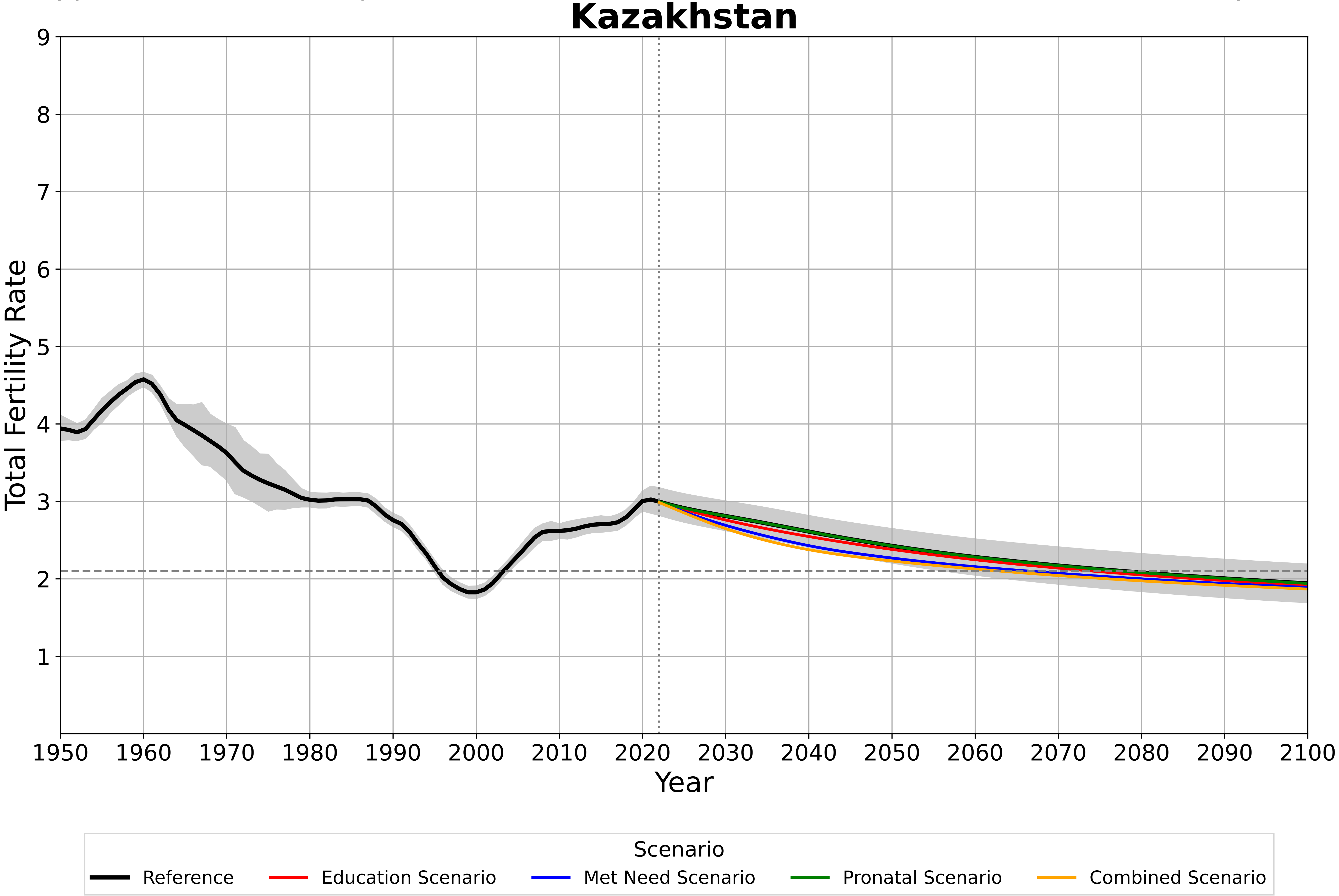

Supplemental Results Figure S5: TFR for reference and alternative scenario forecasts by location, 1950– 2100

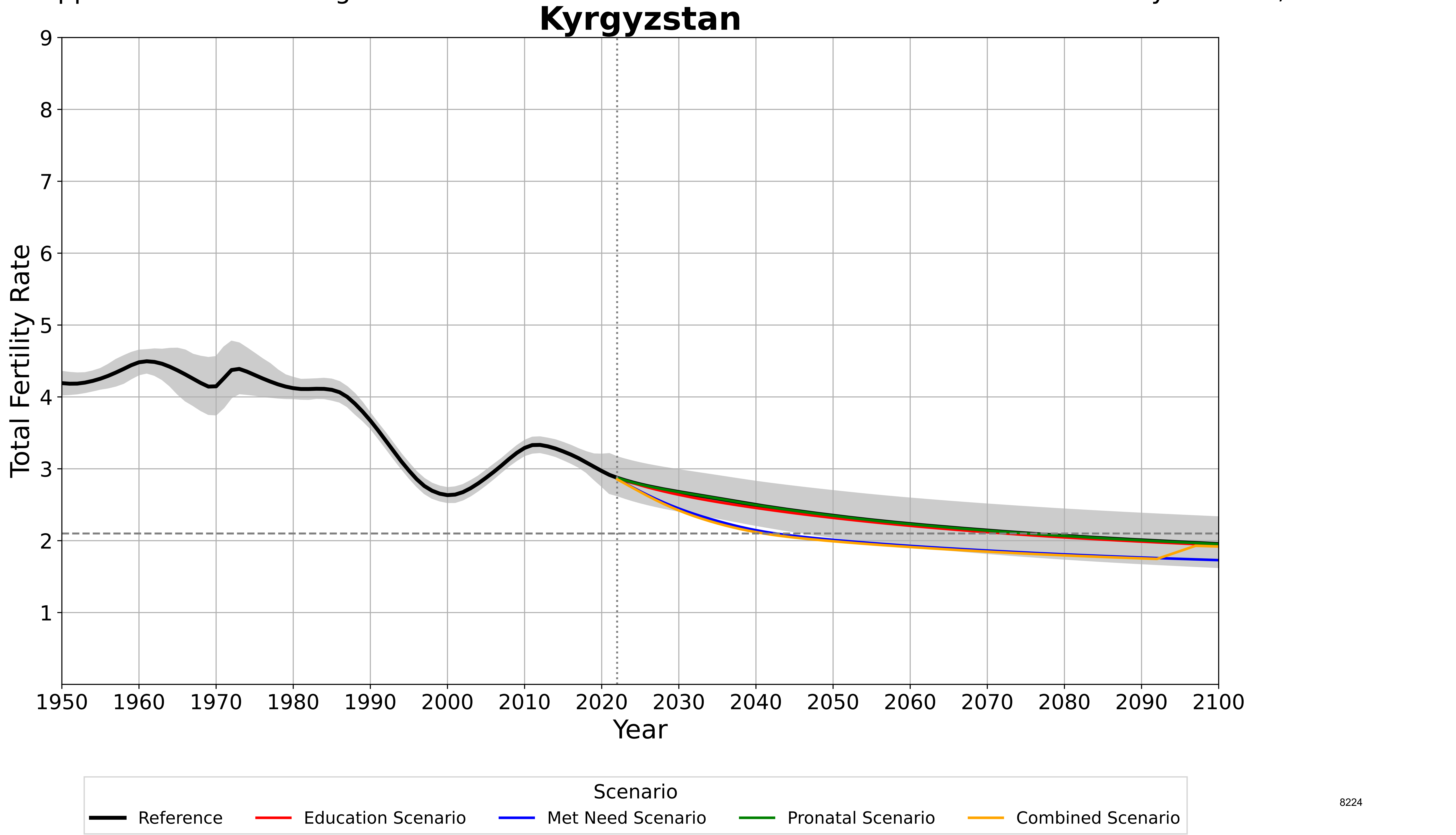

Supplemental Results Figure S5: TFR for reference and alternative scenario forecasts by location, 1950– 2100

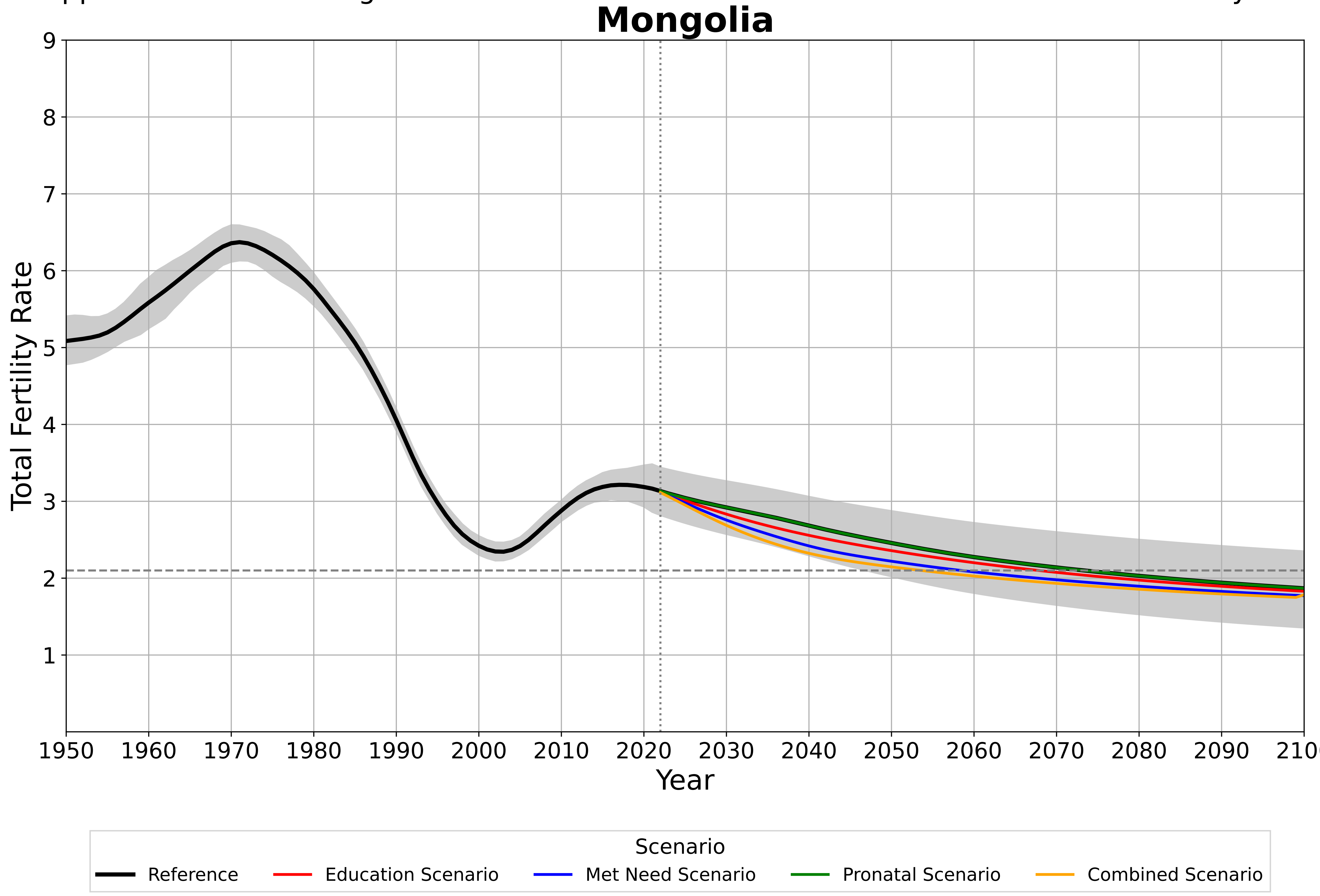

Supplemental Results Figure S5: TFR for reference and alternative scenario forecasts by location, 1950– 2100

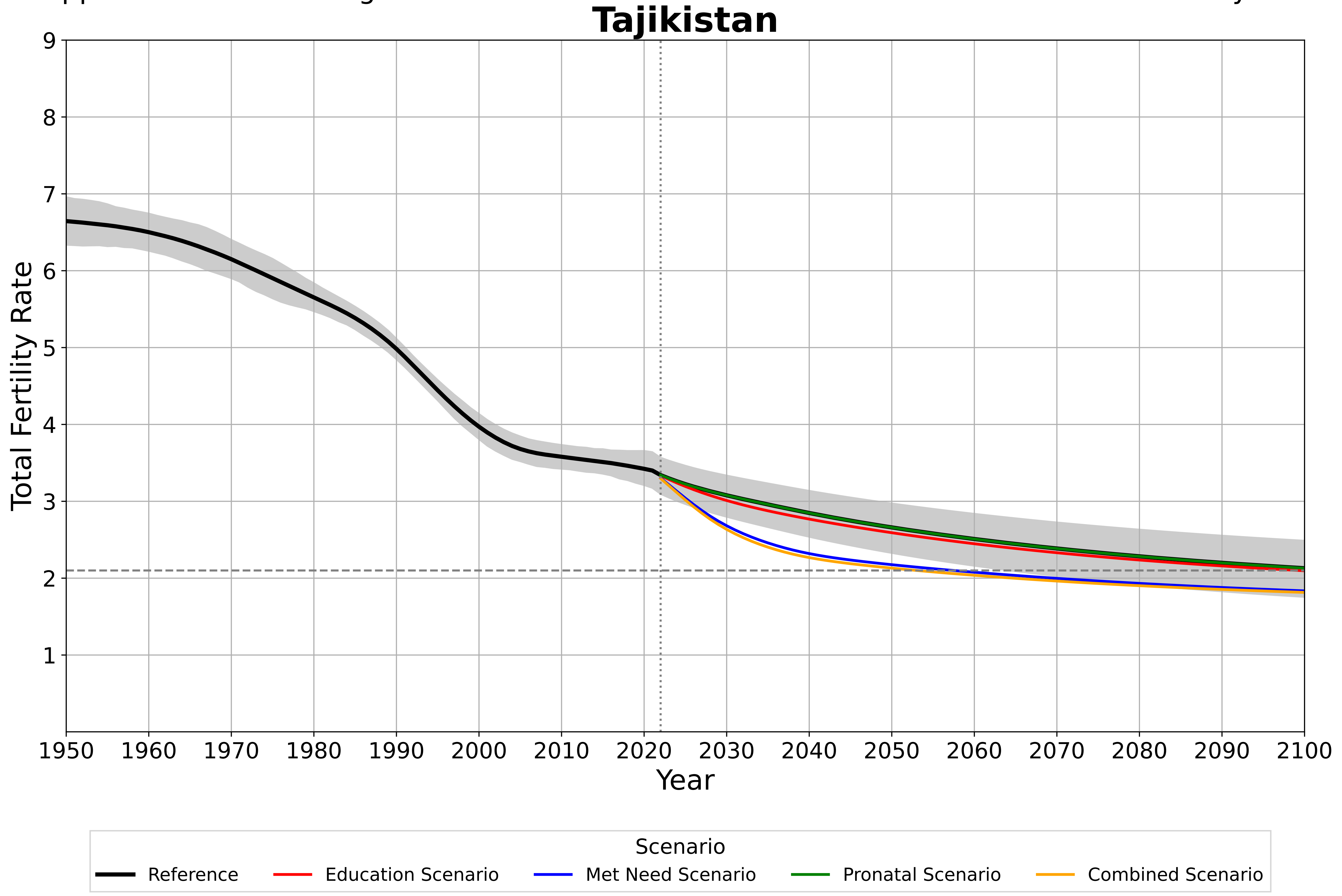

8226

Supplemental Results Figure S5: TFR for reference and alternative scenario forecasts by location, 1950– 2100

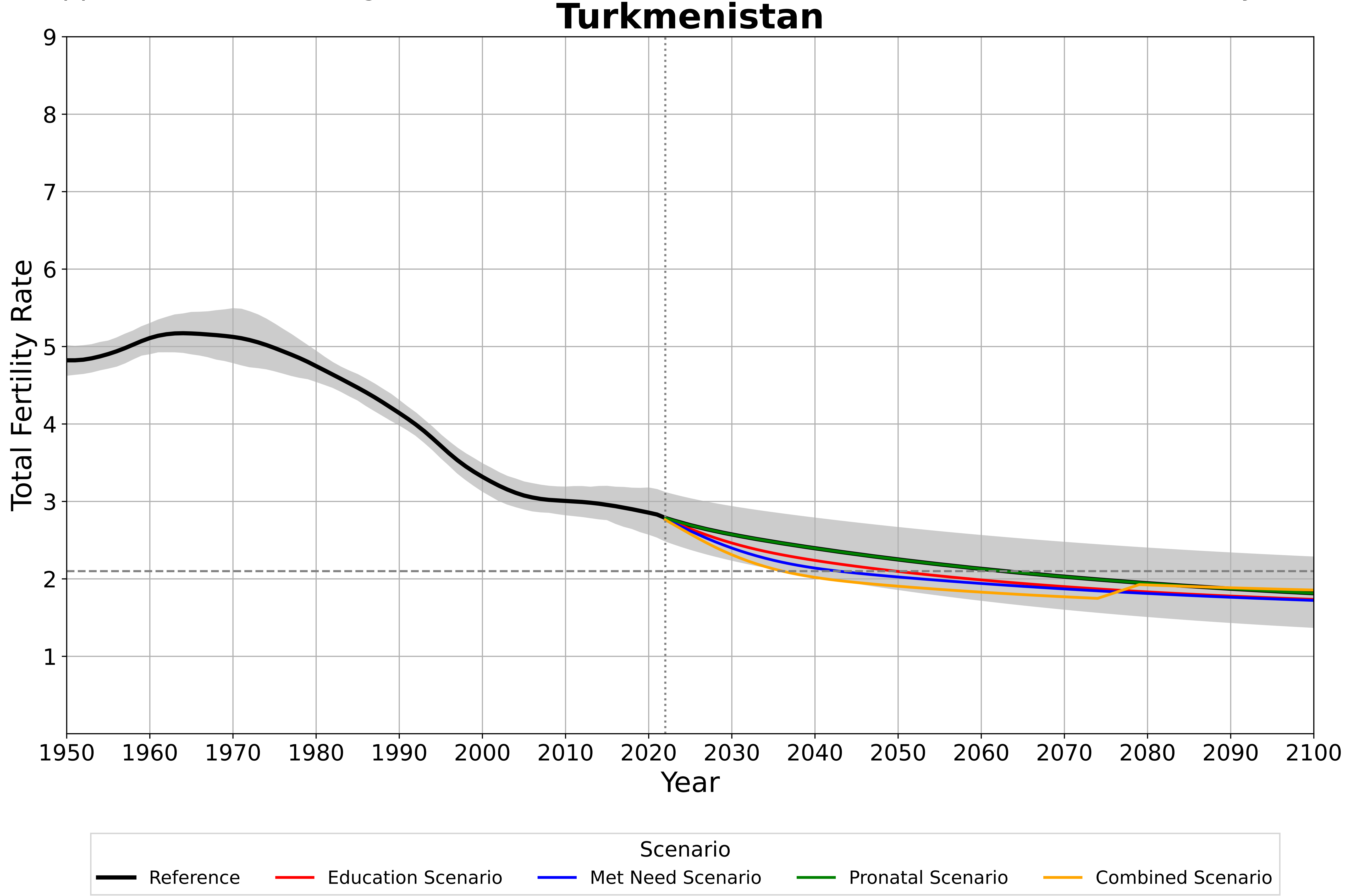

Supplemental Results Figure S5: TFR for reference and alternative scenario forecasts by location, 1950– 2100

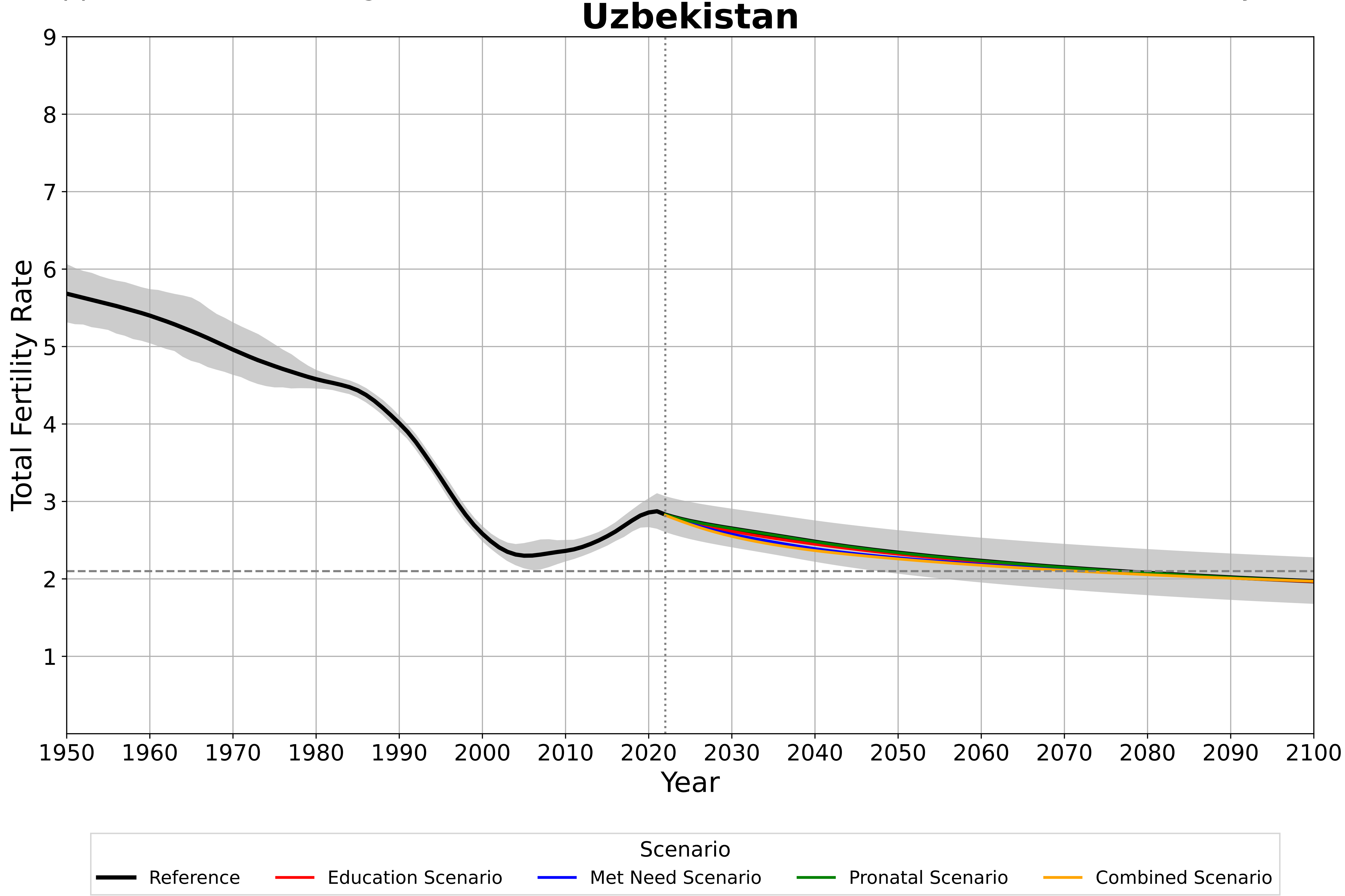

Supplemental Results Figure S5: TFR for reference and alternative scenario forecasts by location, 1950– 2100

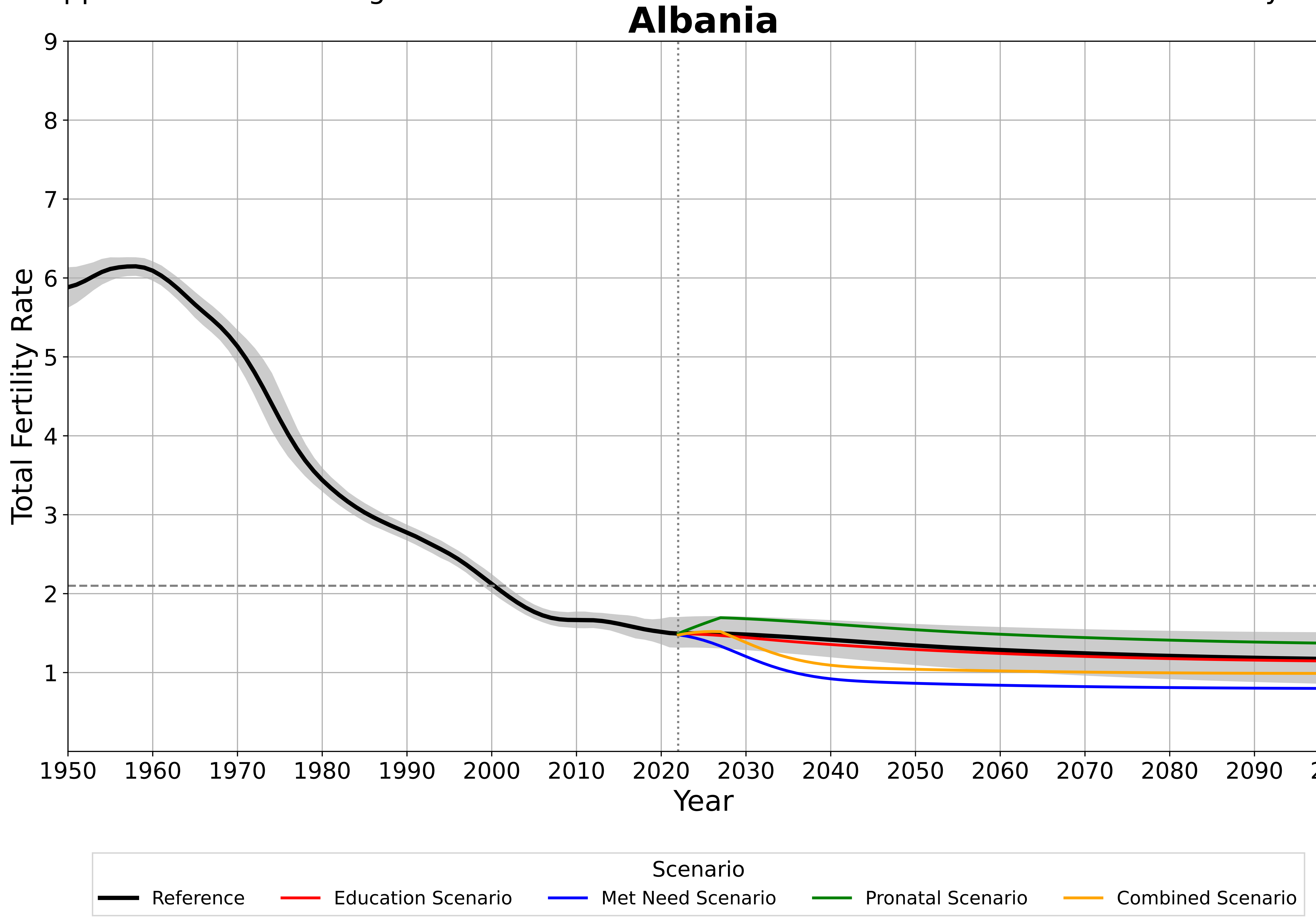

Supplemental Results Figure S5: TFR for reference and alternative scenario forecasts by location, 1950– 2100

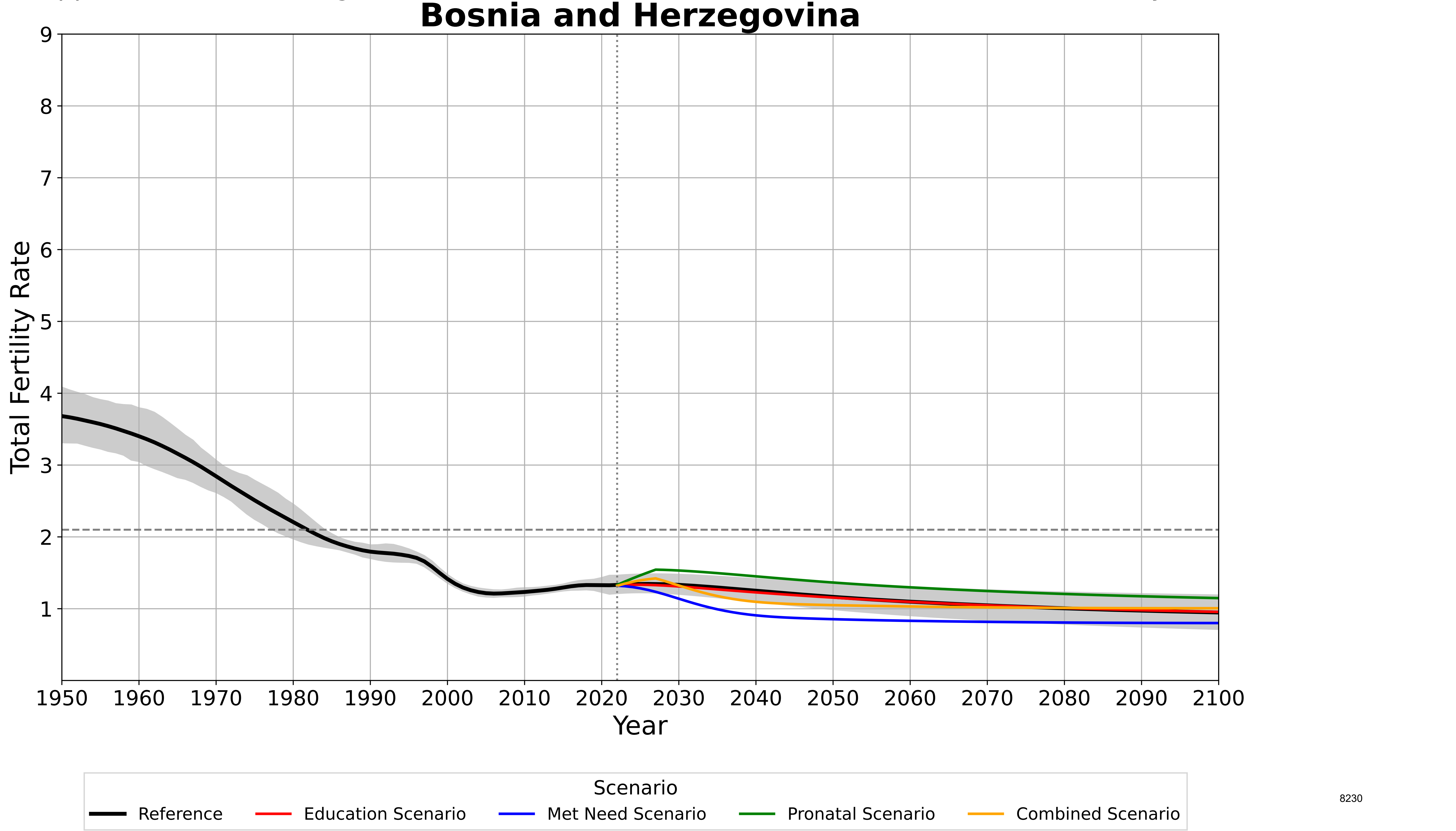

Supplemental Results Figure S5: TFR for reference and alternative scenario forecasts by location, 1950– 2100

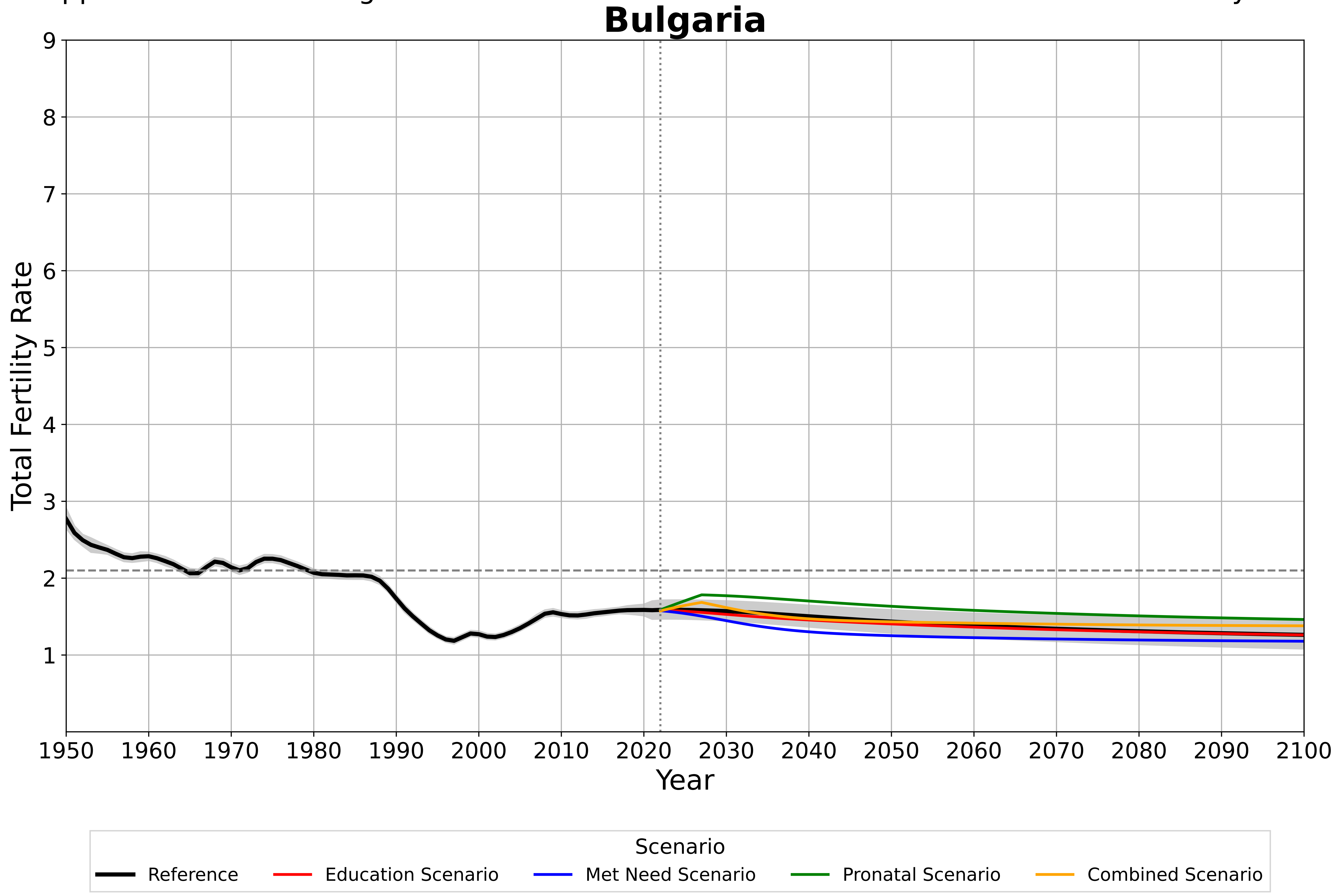

Supplemental Results Figure S5: TFR for reference and alternative scenario forecasts by location, 1950– 2100

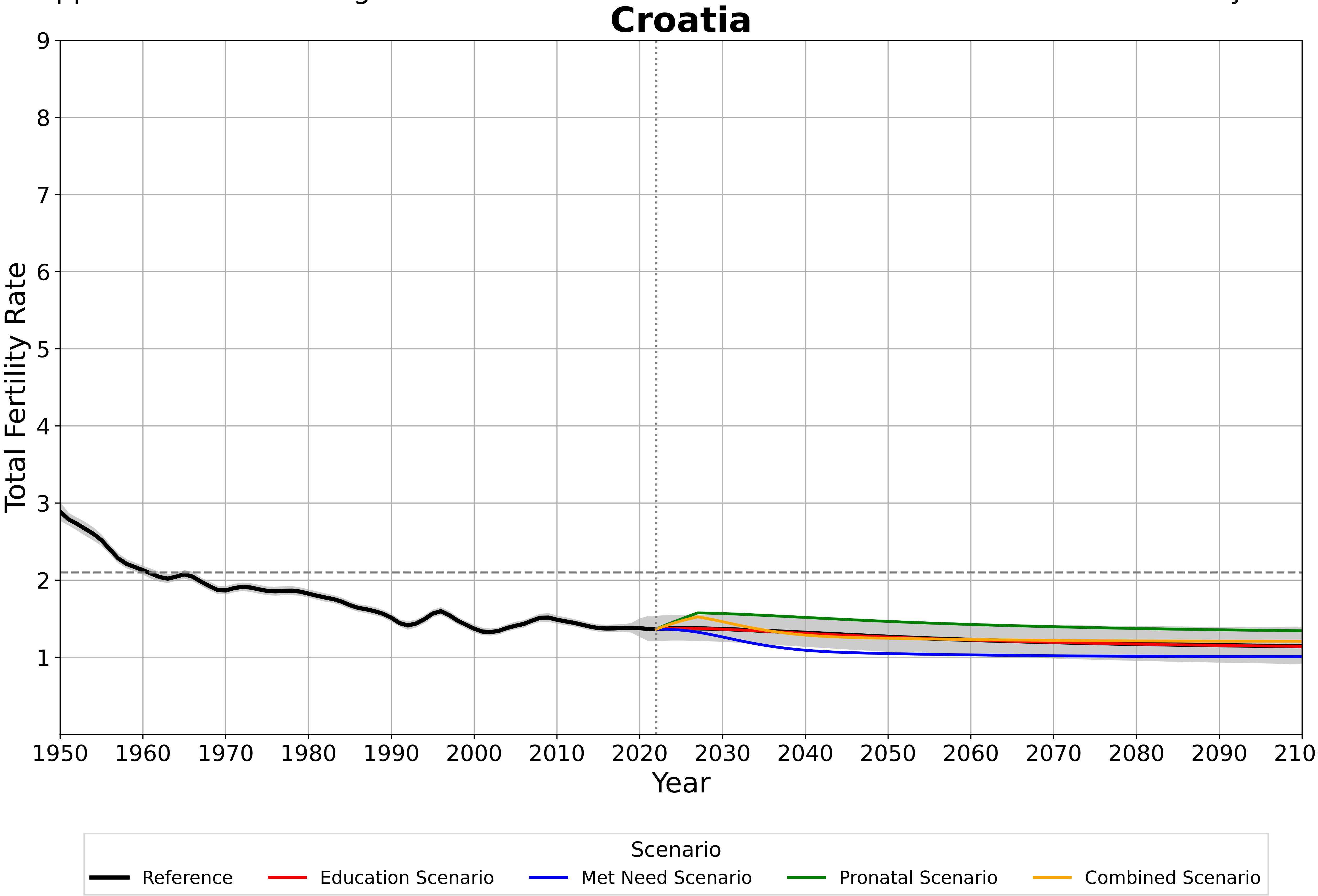

Supplemental Results Figure S5: TFR for reference and alternative scenario forecasts by location, 1950– 2100

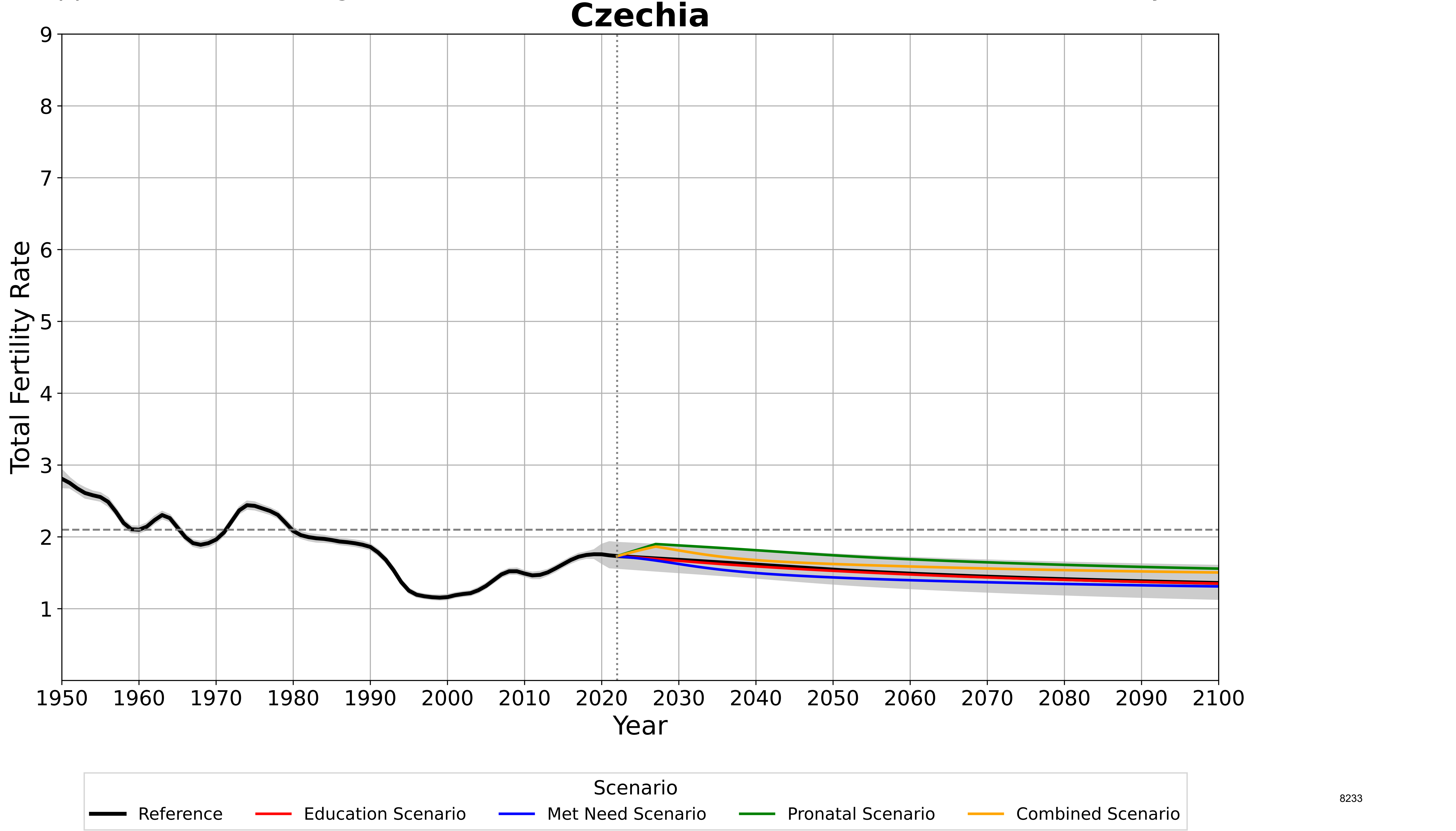

Supplemental Results Figure S5: TFR for reference and alternative scenario forecasts by location, 1950– 2100

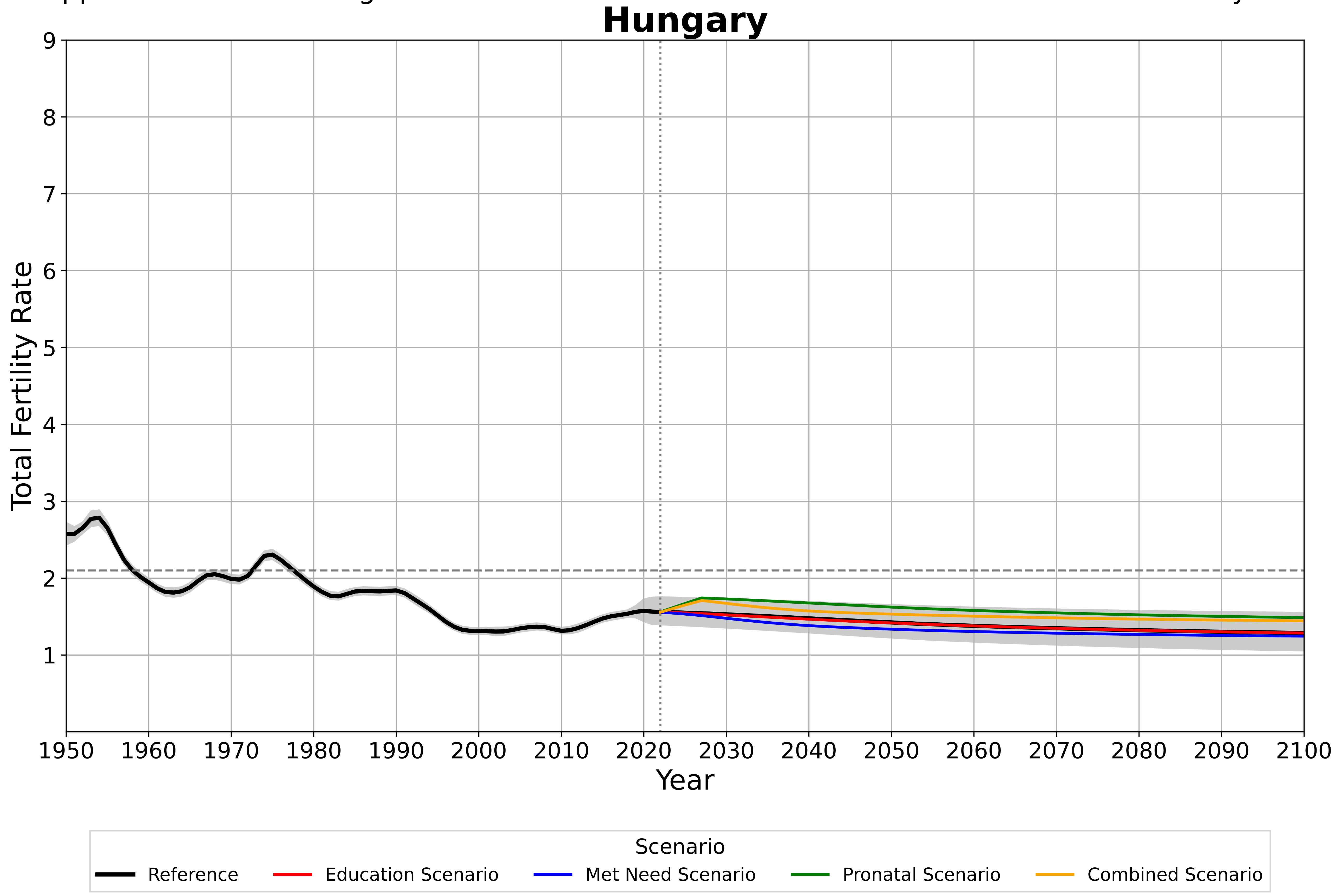

Supplemental Results Figure S5: TFR for reference and alternative scenario forecasts by location, 1950– 2100

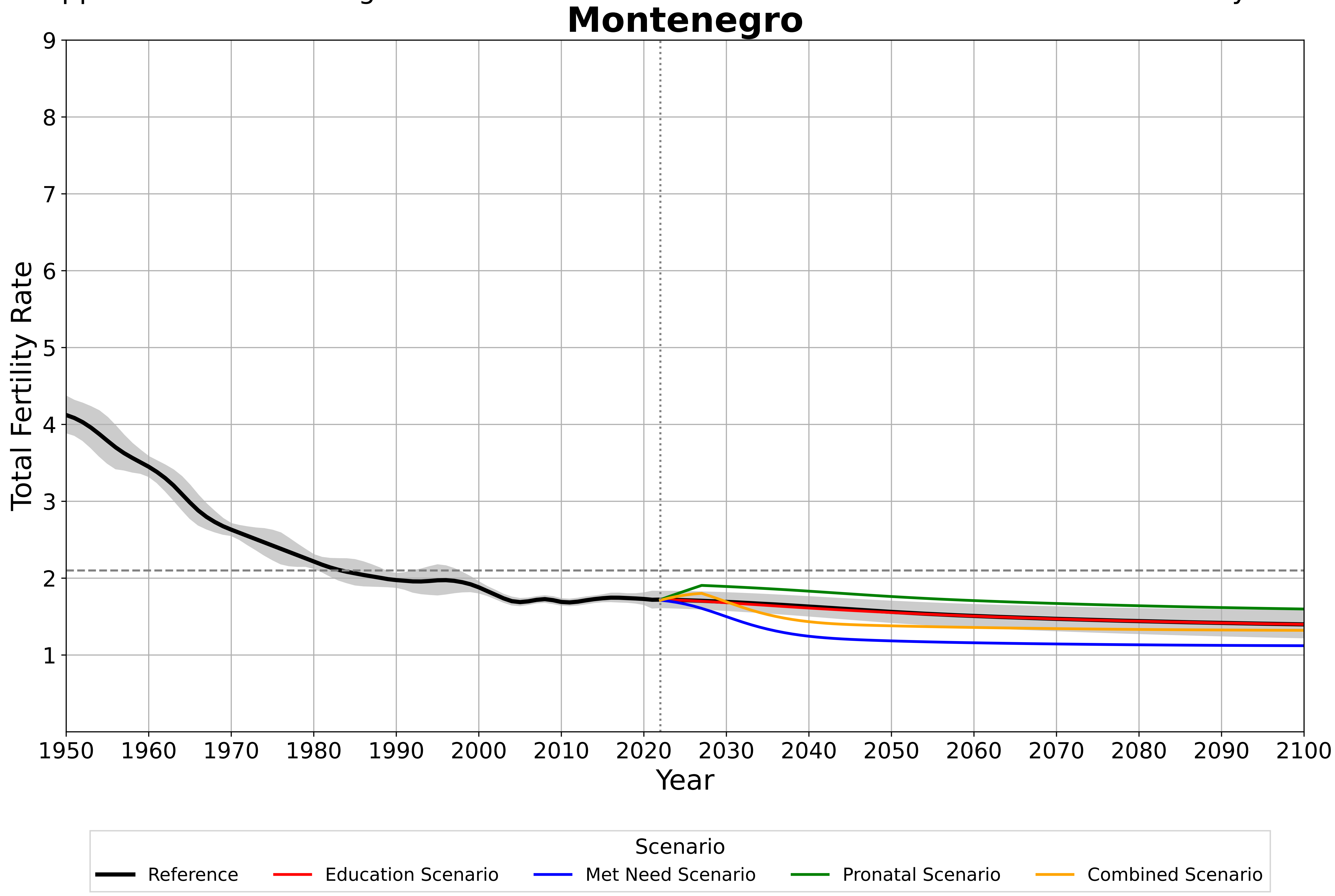

Supplemental Results Figure S5: TFR for reference and alternative scenario forecasts by location, 1950– 2100

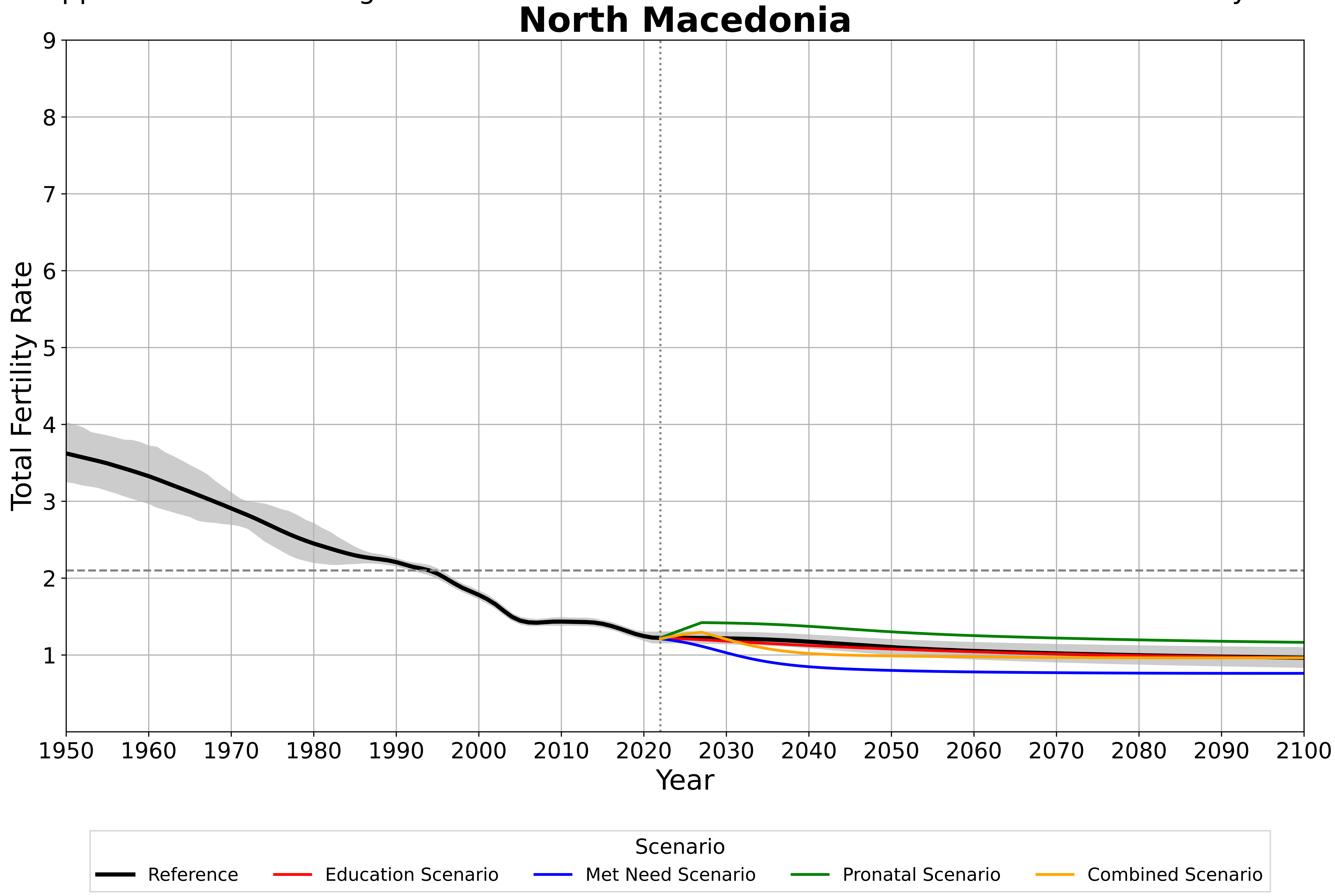

Supplemental Results Figure S5: TFR for reference and alternative scenario forecasts by location, 1950– 2100

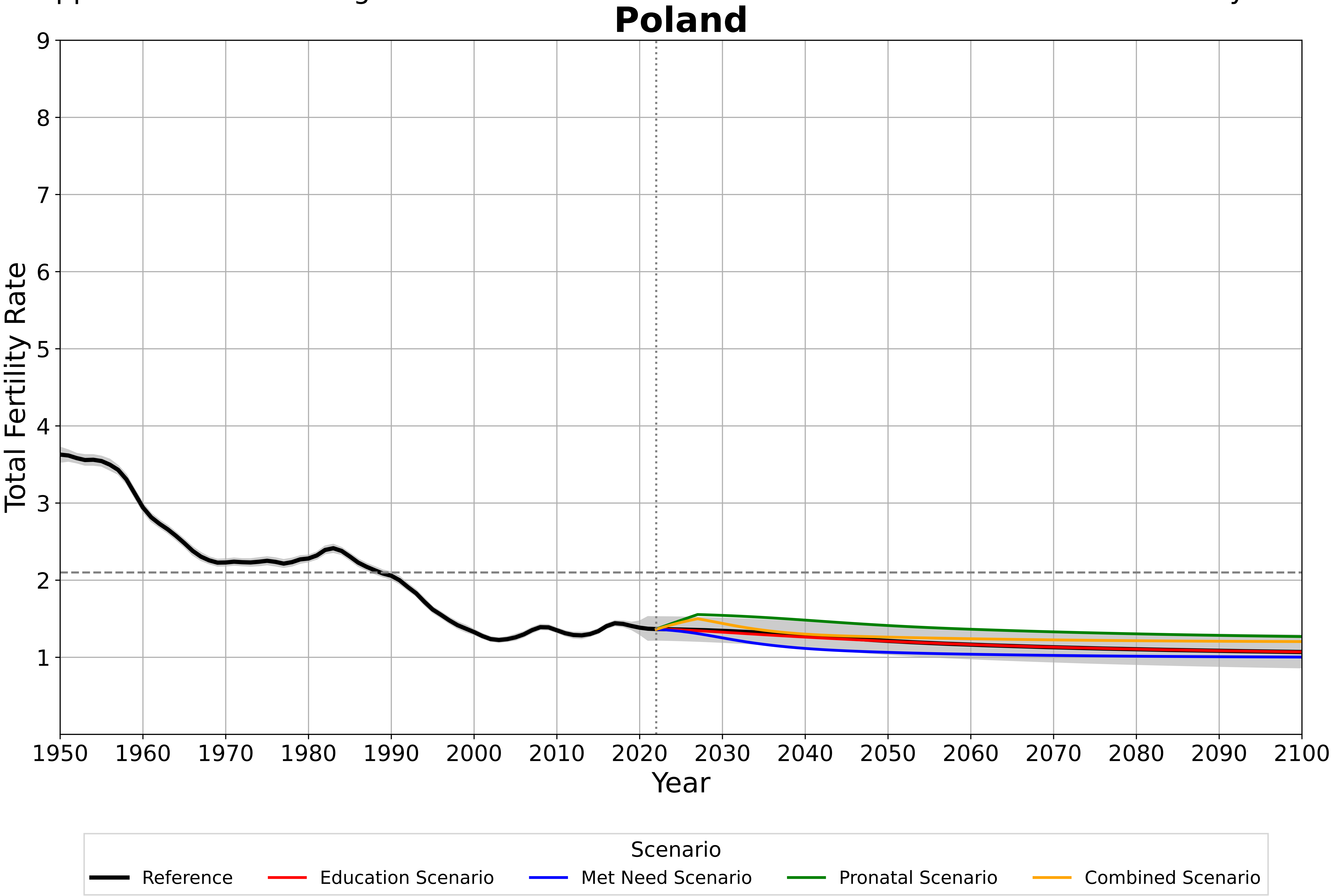

Supplemental Results Figure S5: TFR for reference and alternative scenario forecasts by location, 1950– 2100

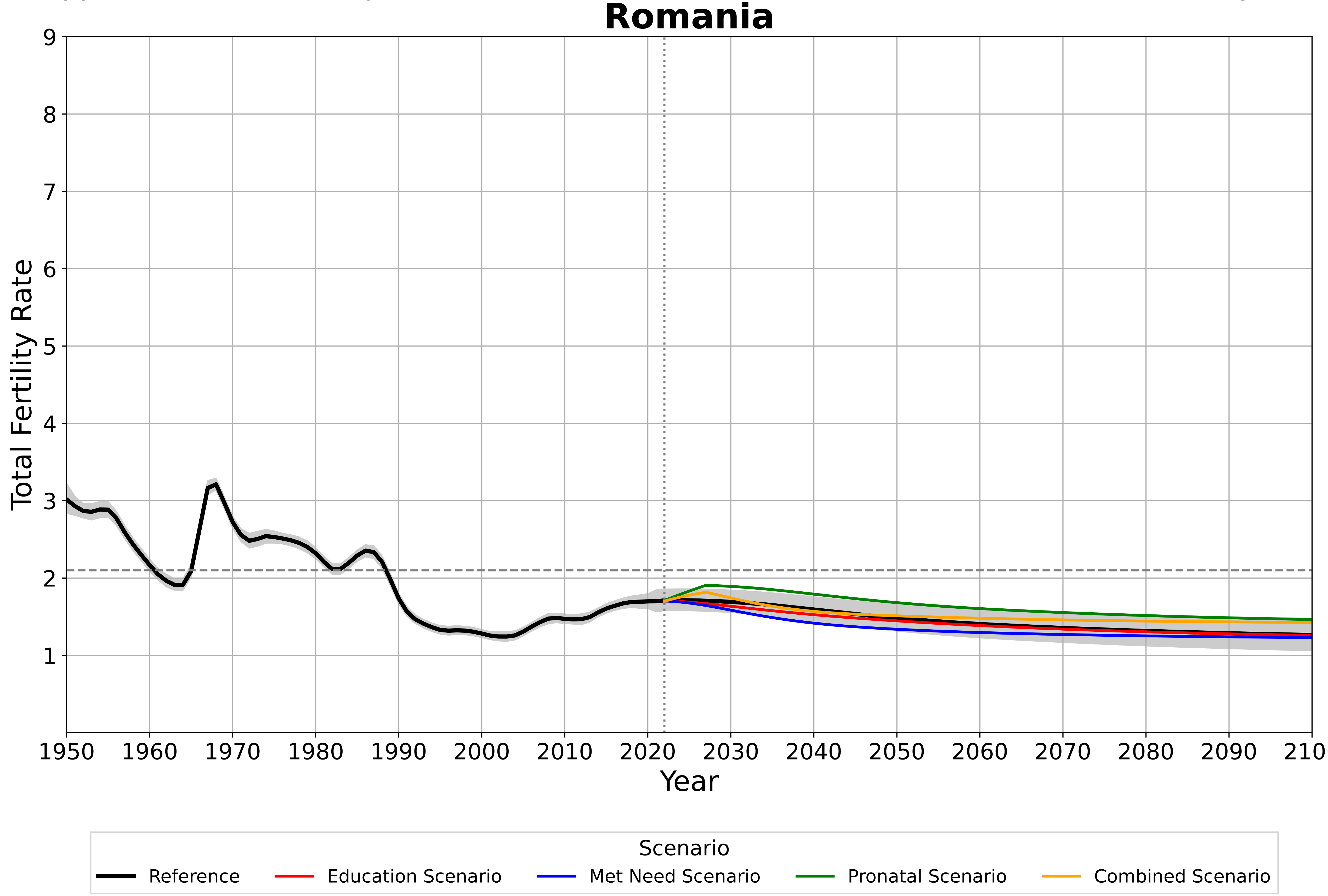

Supplemental Results Figure S5: TFR for reference and alternative scenario forecasts by location, 1950– 2100

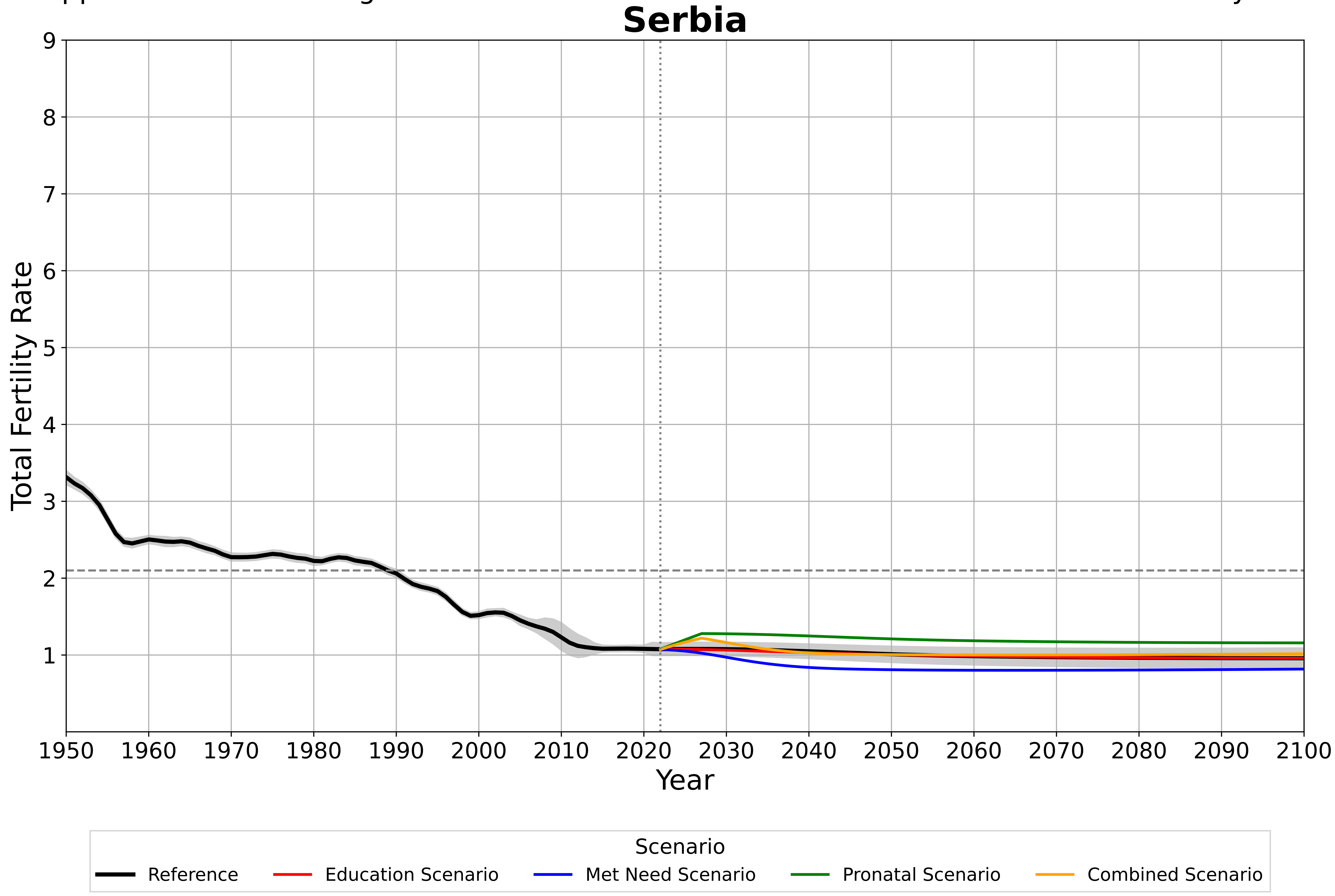

Supplemental Results Figure S5: TFR for reference and alternative scenario forecasts by location, 1950– 2100

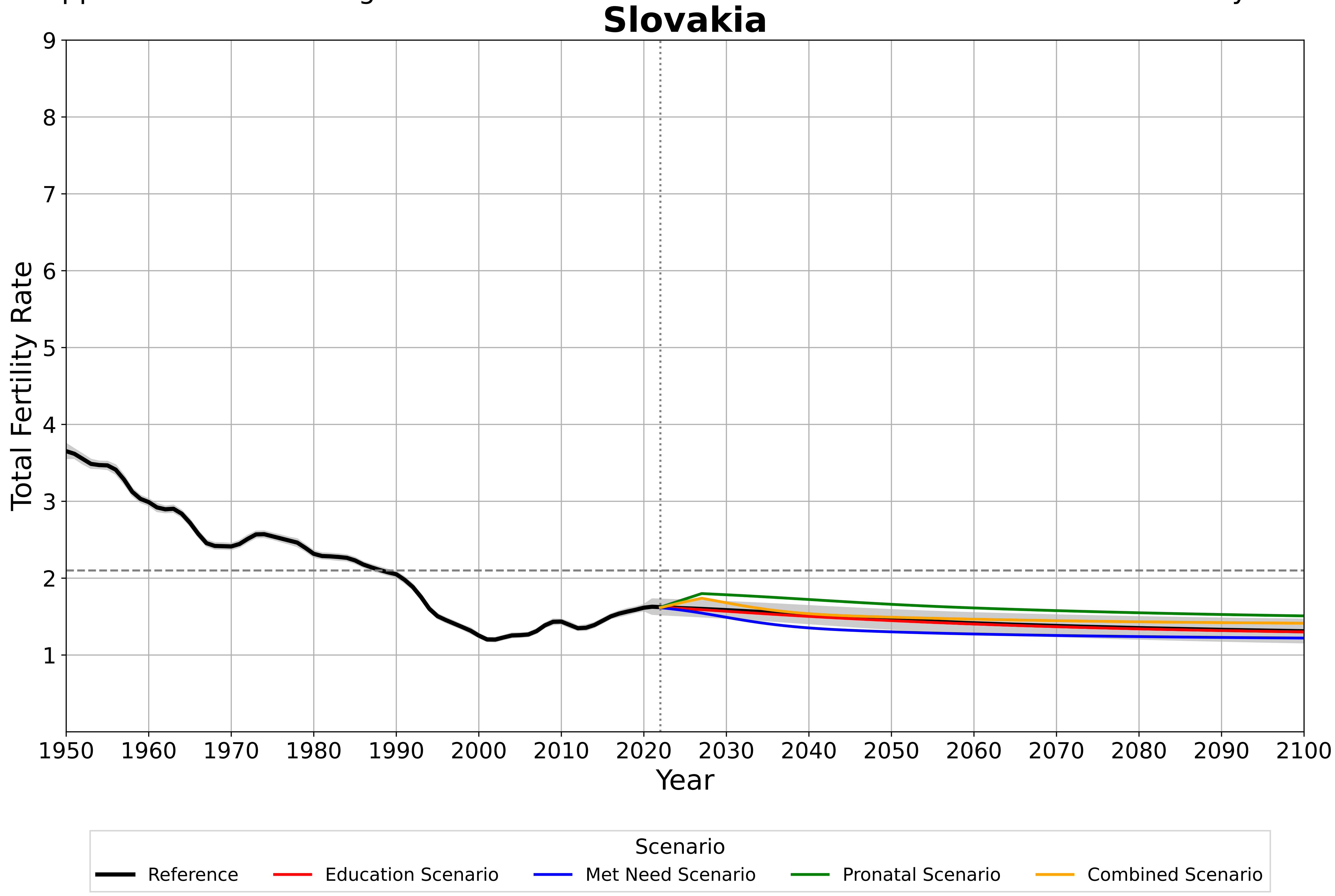

8240

Supplemental Results Figure S5: TFR for reference and alternative scenario forecasts by location, 1950– 2100

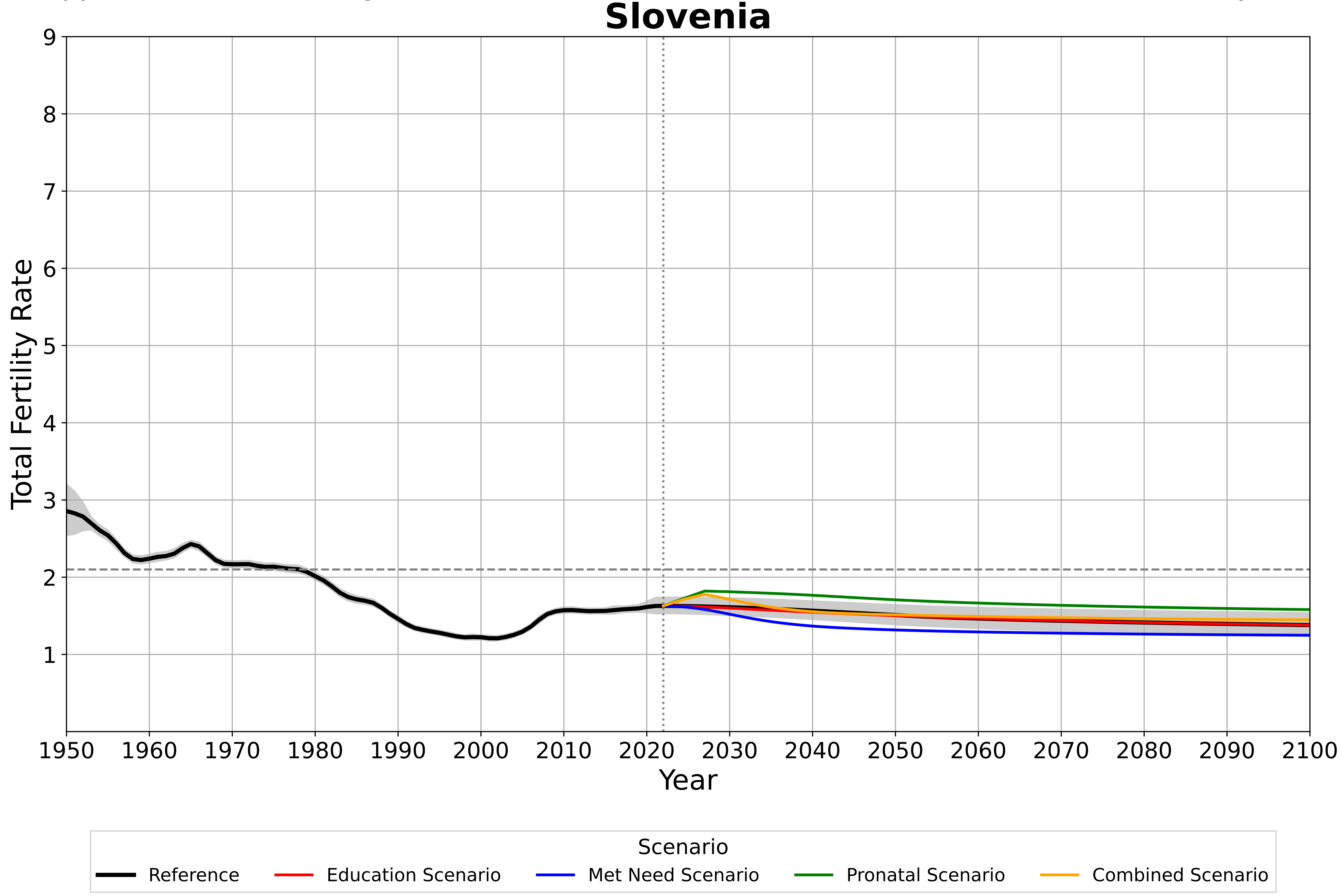

Supplemental Results Figure S5: TFR for reference and alternative scenario forecasts by location, 1950– 2100

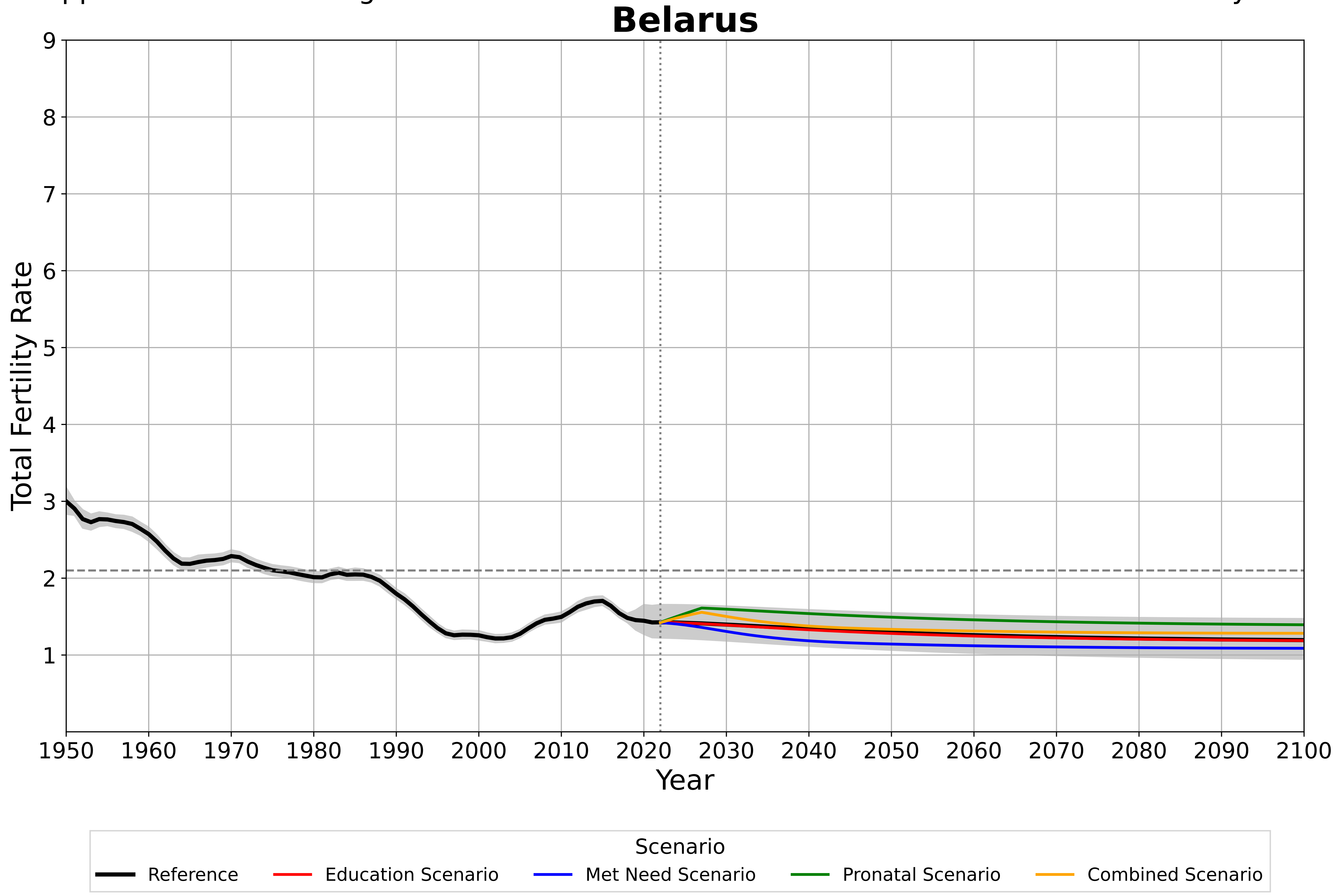

Supplemental Results Figure S5: TFR for reference and alternative scenario forecasts by location, 1950– 2100

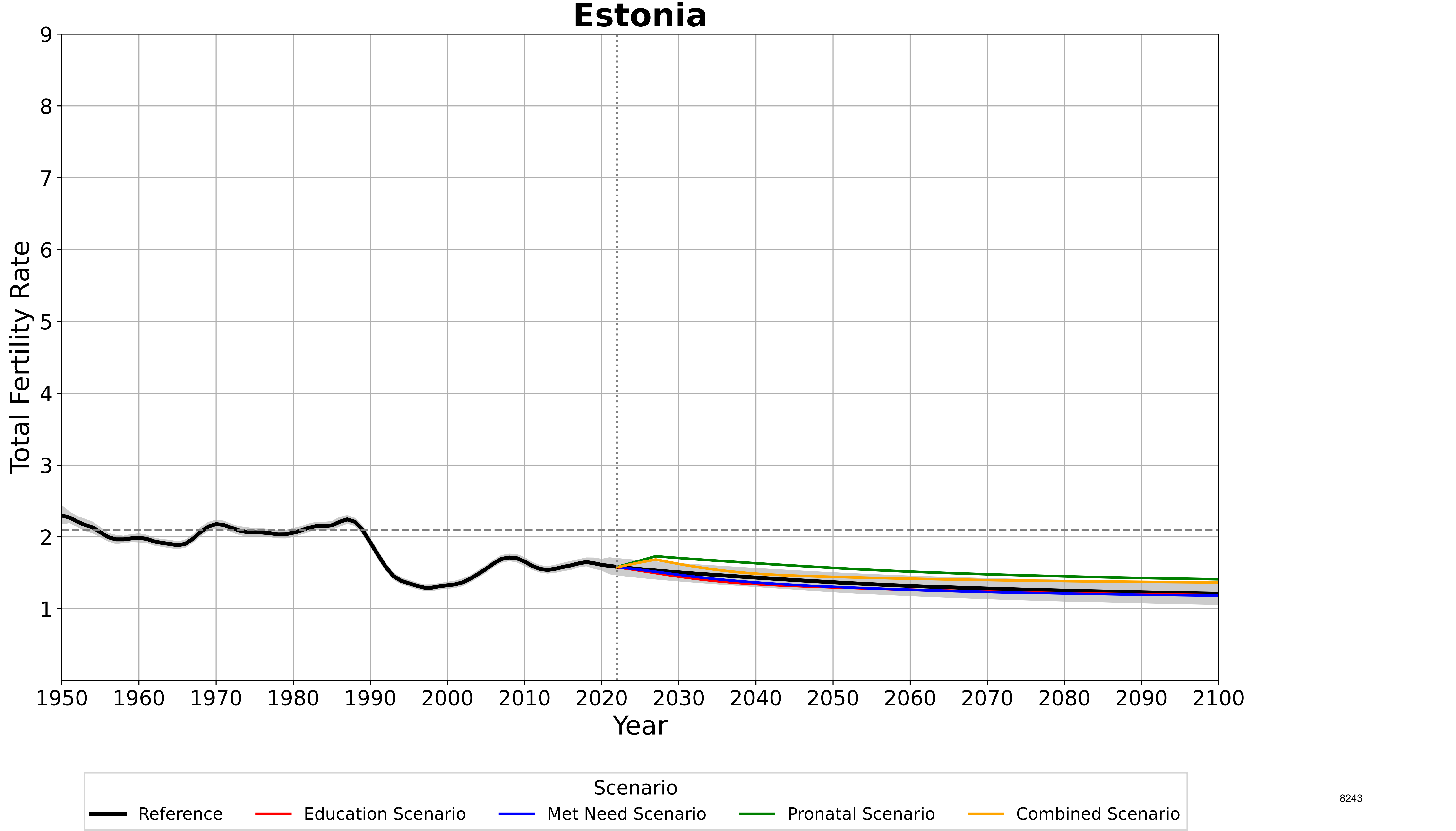

Supplemental Results Figure S5: TFR for reference and alternative scenario forecasts by location, 1950– 2100

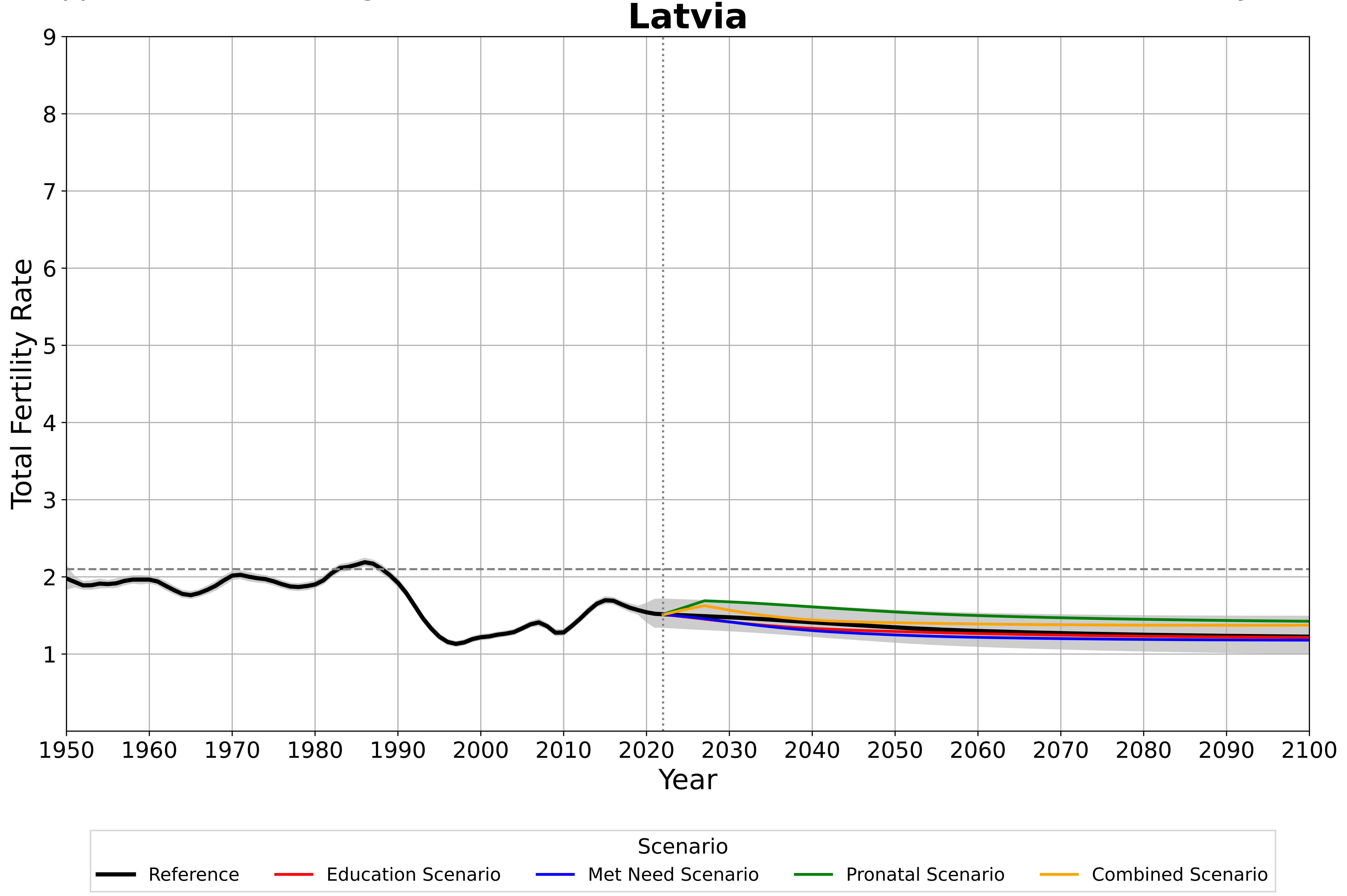

Supplemental Results Figure S5: TFR for reference and alternative scenario forecasts by location, 1950– 2100

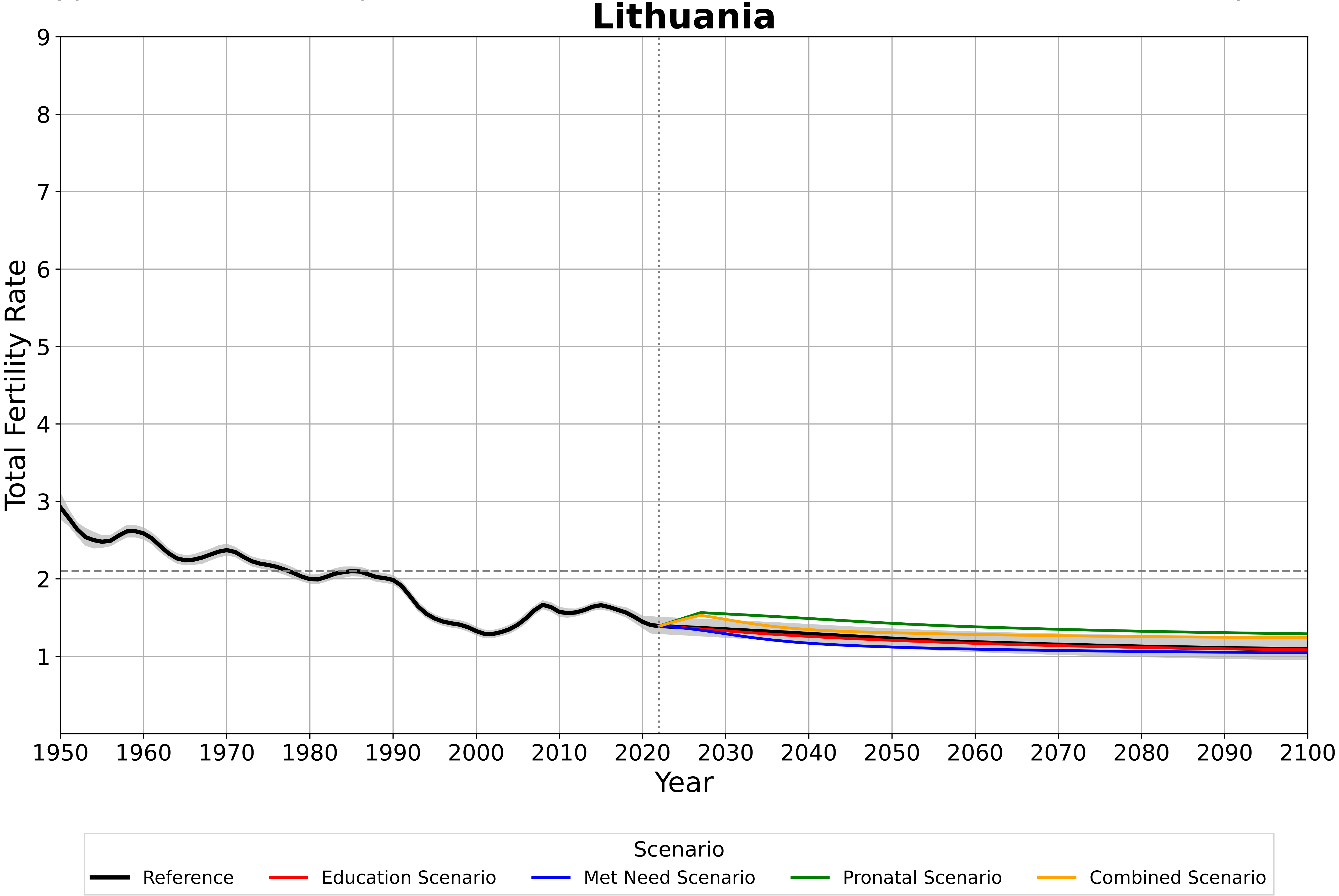

8245

Supplemental Results Figure S5: TFR for reference and alternative scenario forecasts by location, 1950– 2100

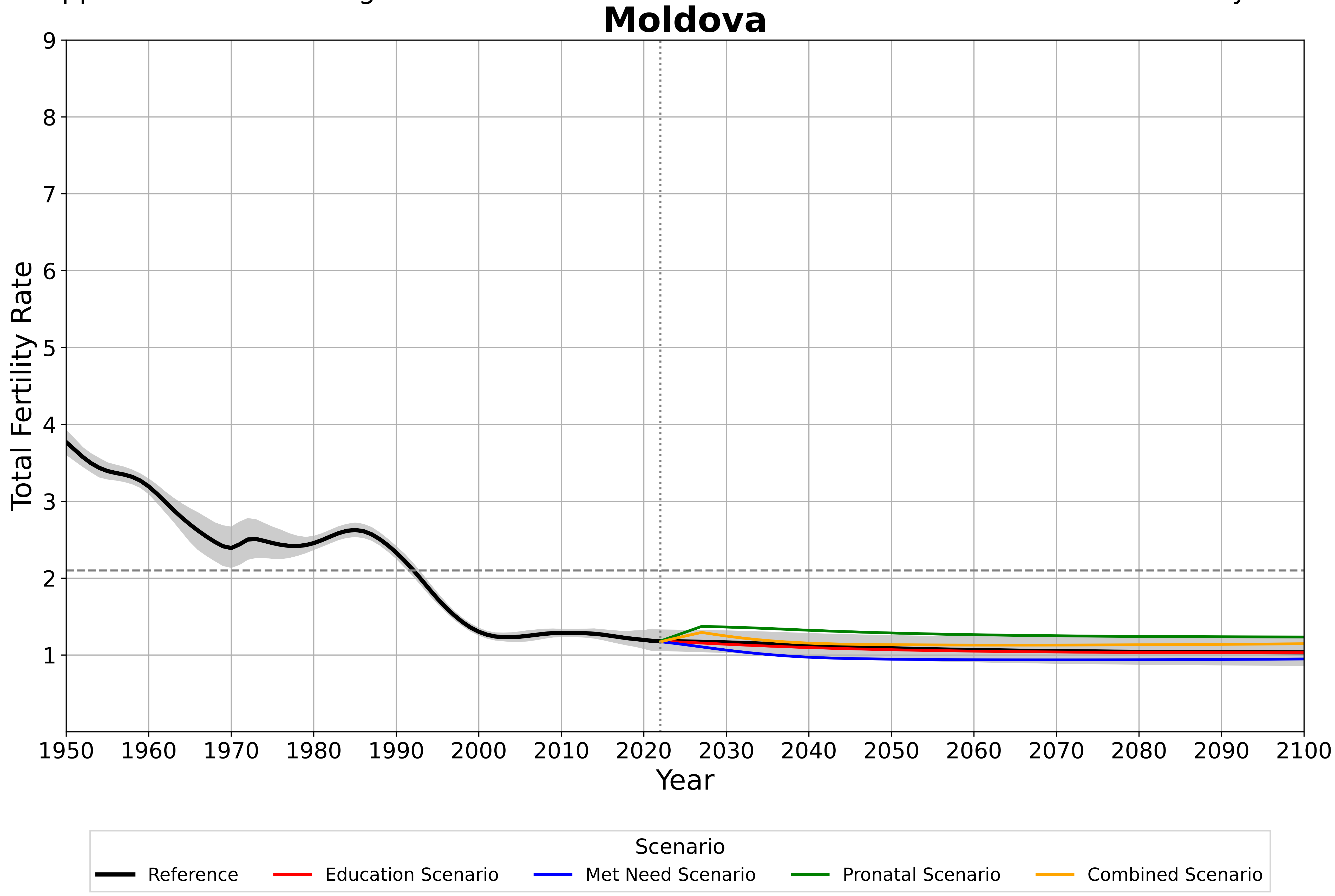

Supplemental Results Figure S5: TFR for reference and alternative scenario forecasts by location, 1950– 2100

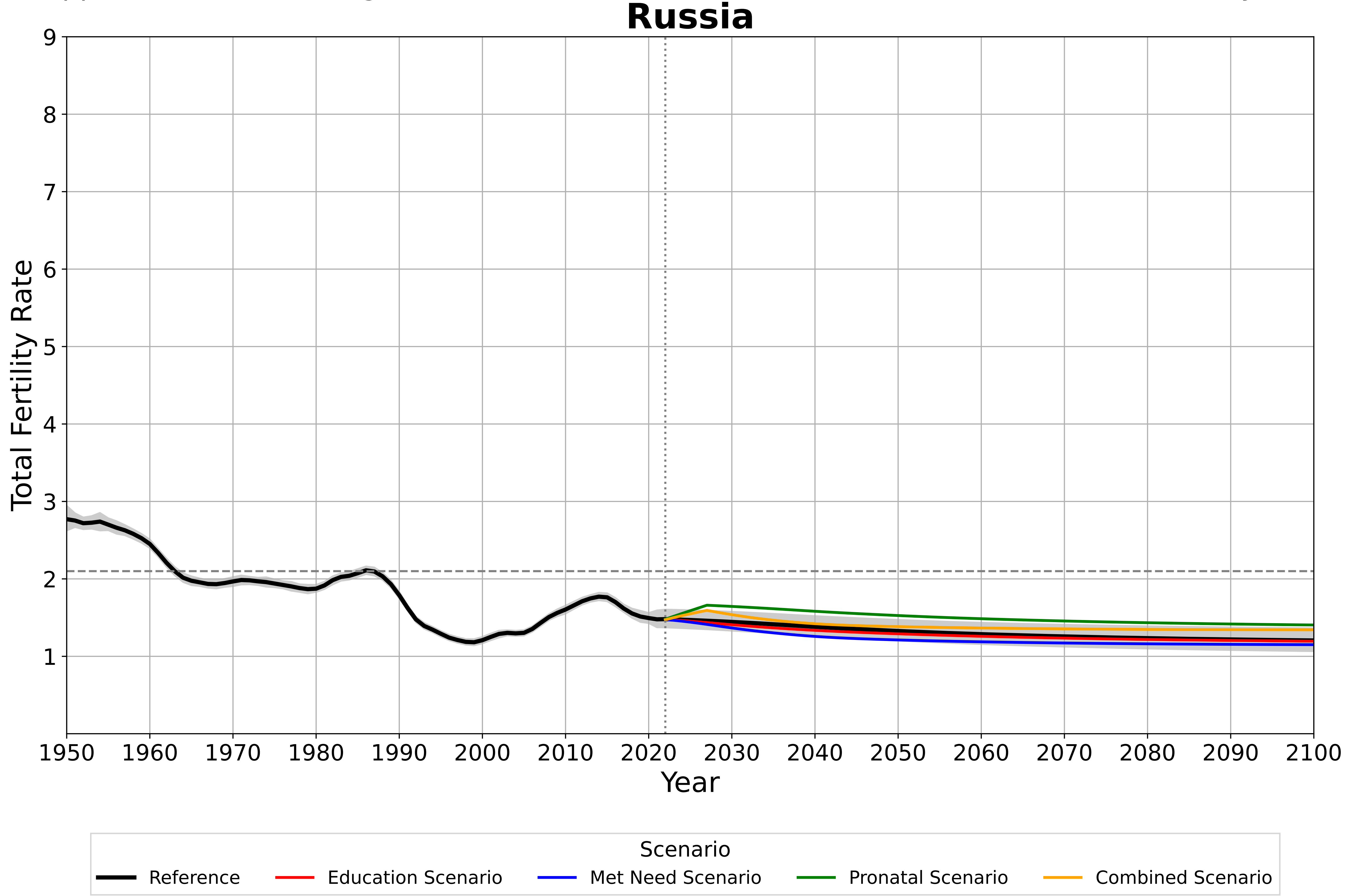

Supplemental Results Figure S5: TFR for reference and alternative scenario forecasts by location, 1950– 2100

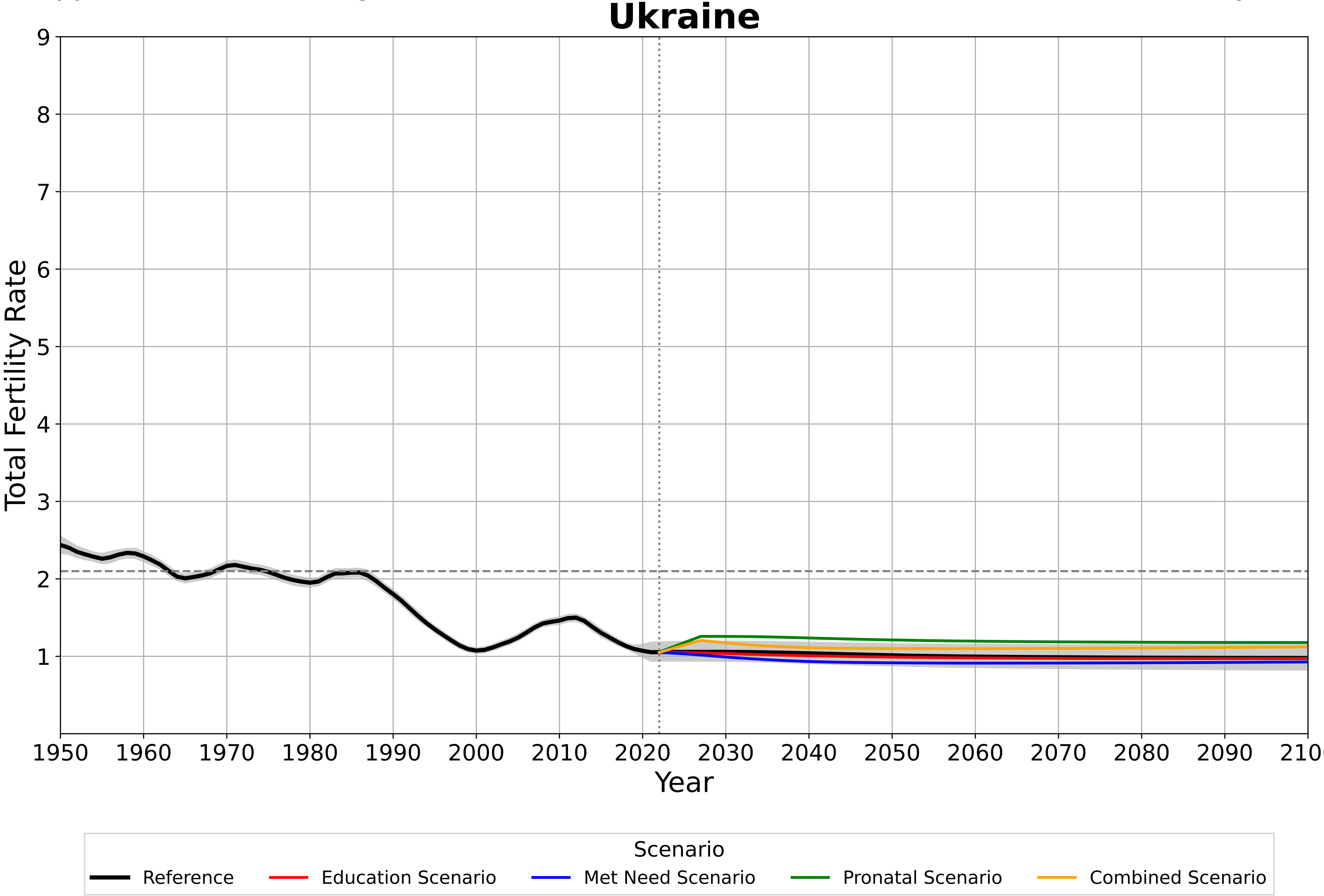

Supplemental Results Figure S5: TFR for reference and alternative scenario forecasts by location, 1950– 2100

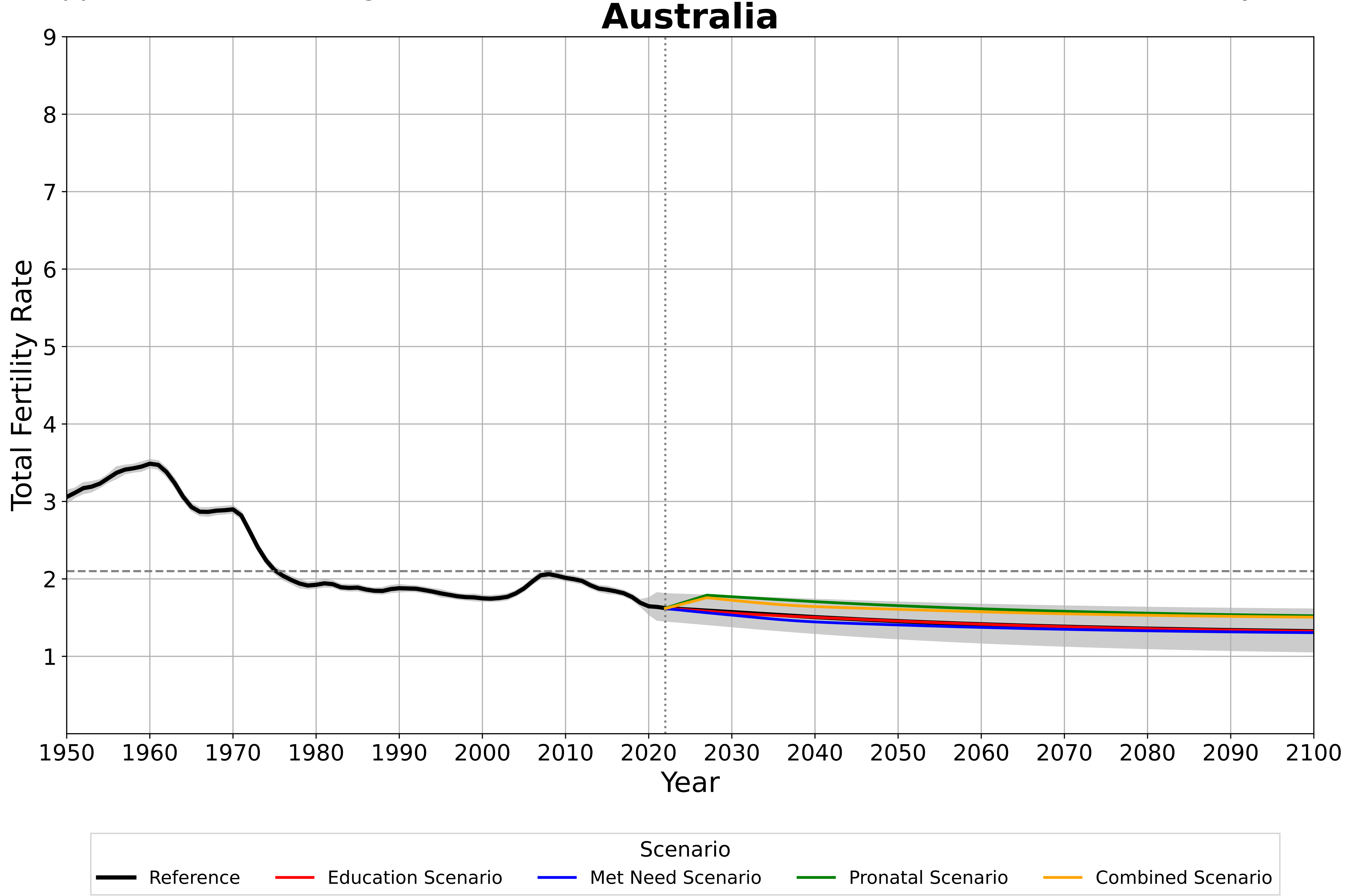

Supplemental Results Figure S5: TFR for reference and alternative scenario forecasts by location, 1950– 2100

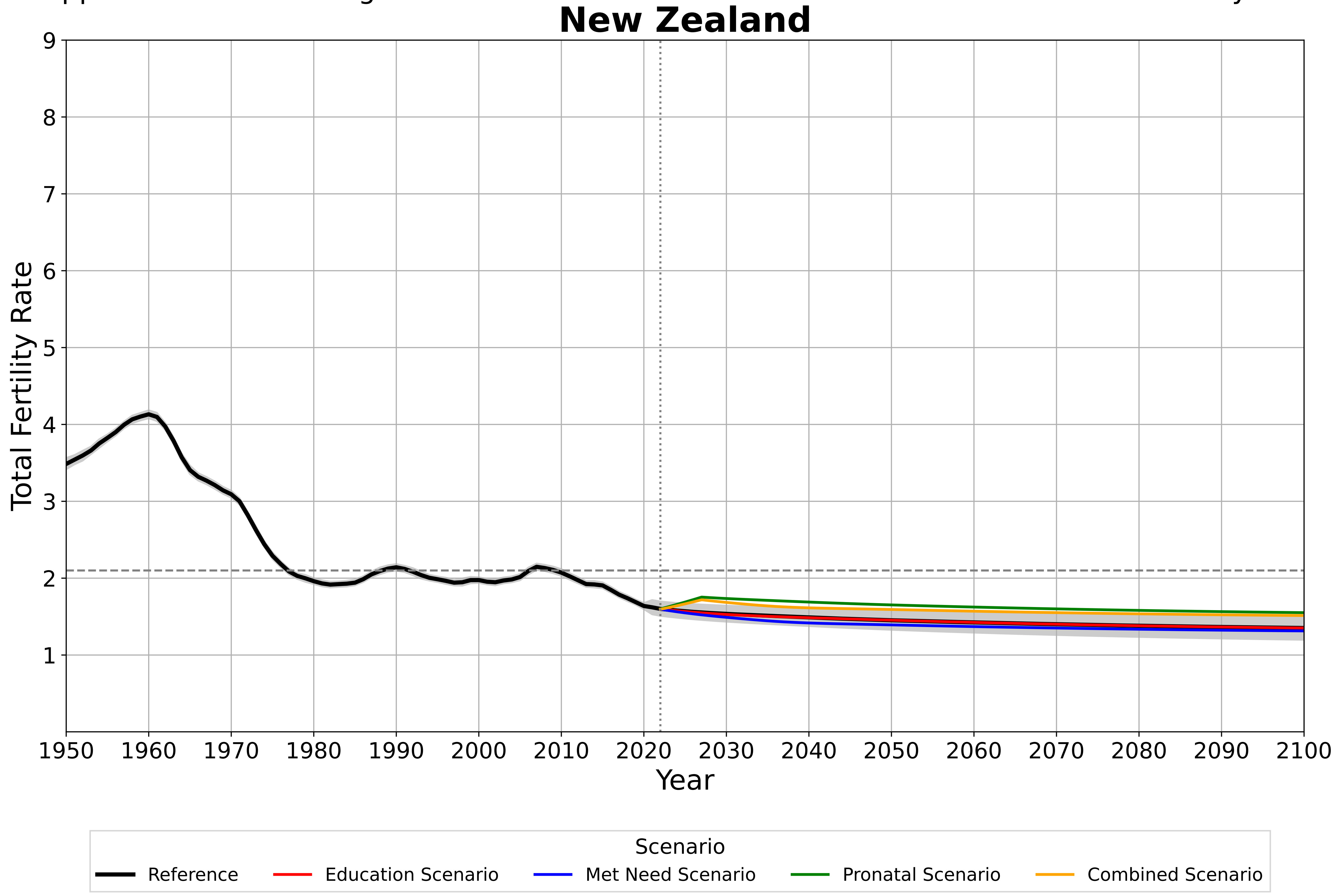

Supplemental Results Figure S5: TFR for reference and alternative scenario forecasts by location, 1950– 2100

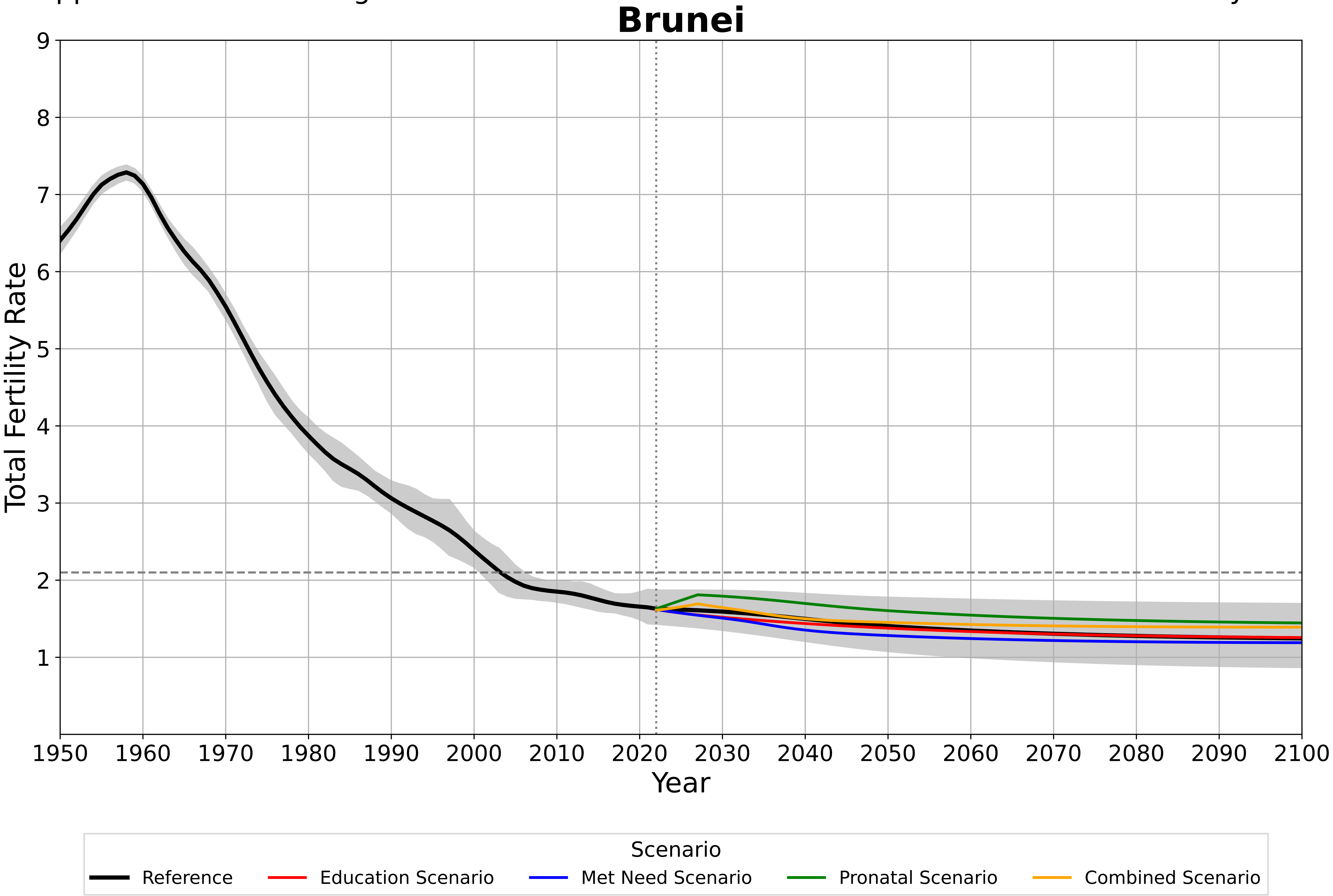

Supplemental Results Figure S5: TFR for reference and alternative scenario forecasts by location, 1950– 2100

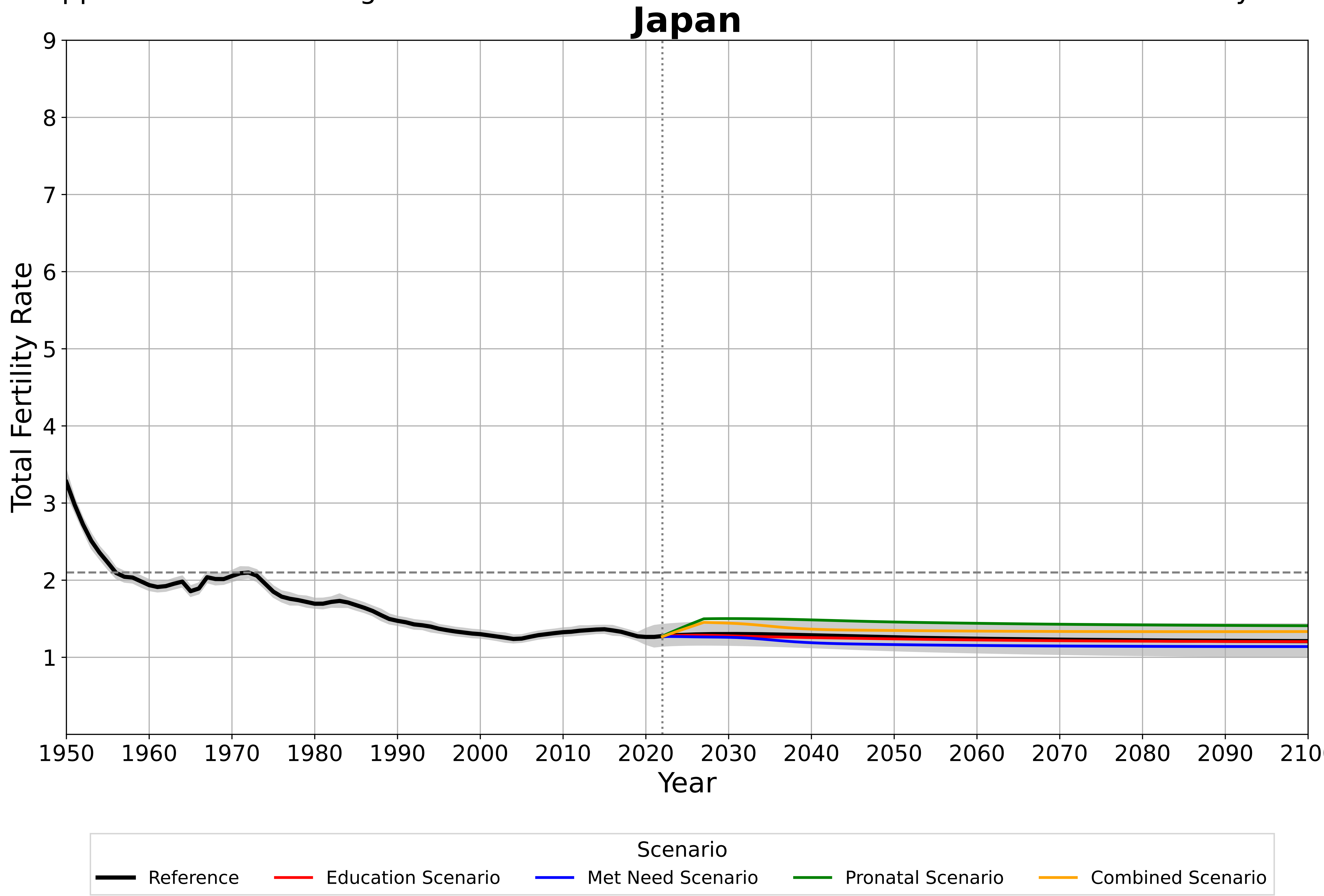

Supplemental Results Figure S5: TFR for reference and alternative scenario forecasts by location, 1950– 2100

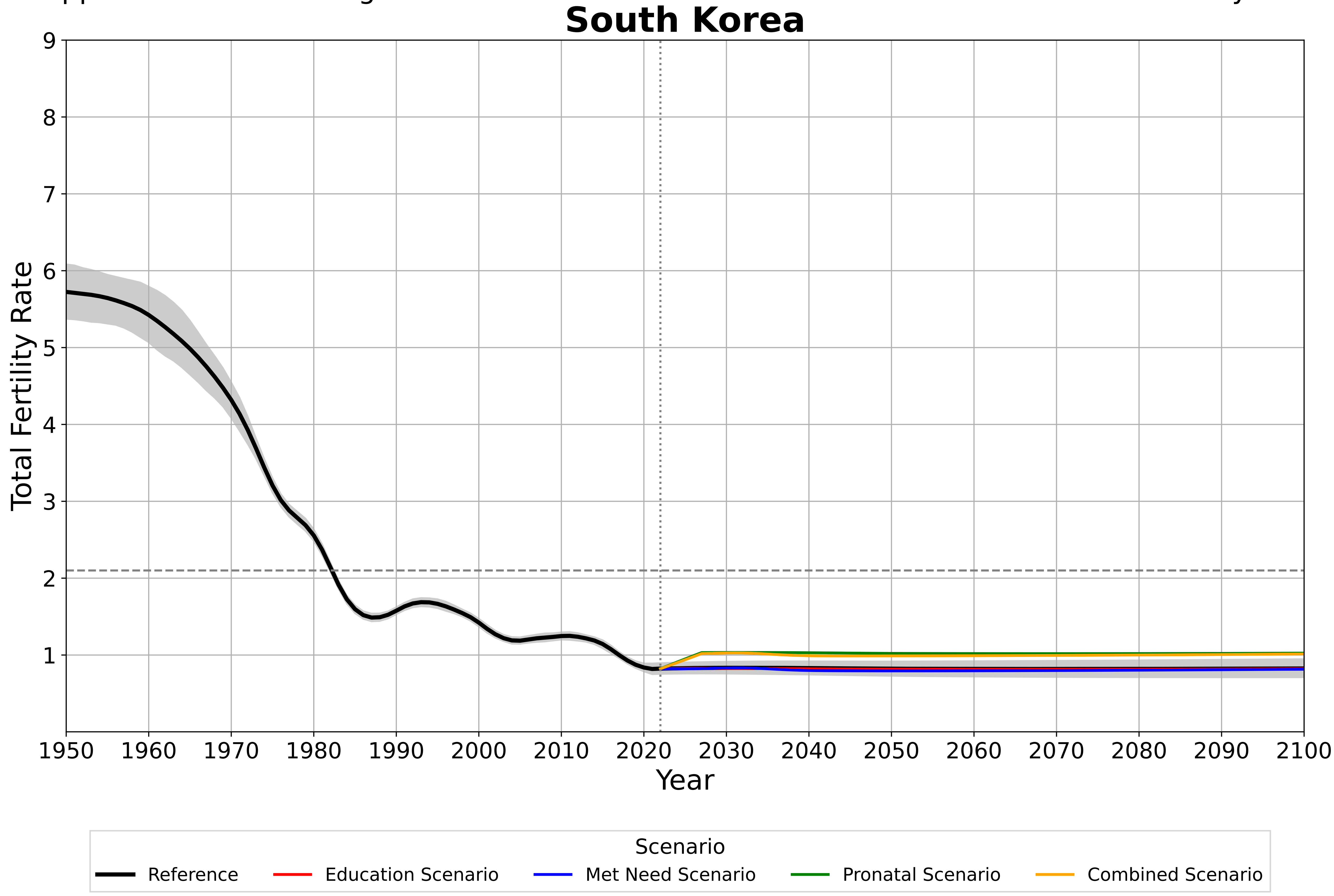

Supplemental Results Figure S5: TFR for reference and alternative scenario forecasts by location, 1950– 2100

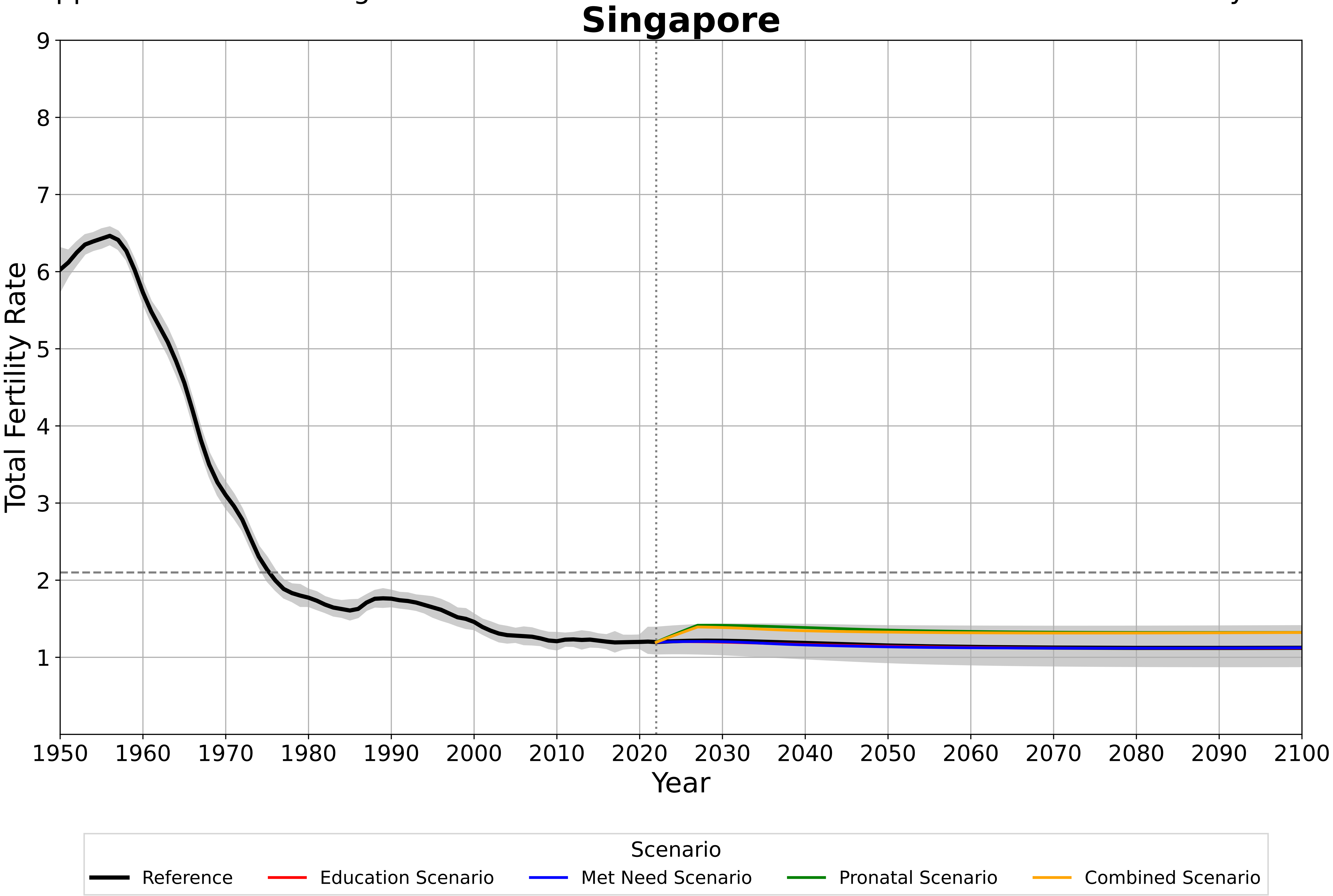

Supplemental Results Figure S5: TFR for reference and alternative scenario forecasts by location, 1950– 2100

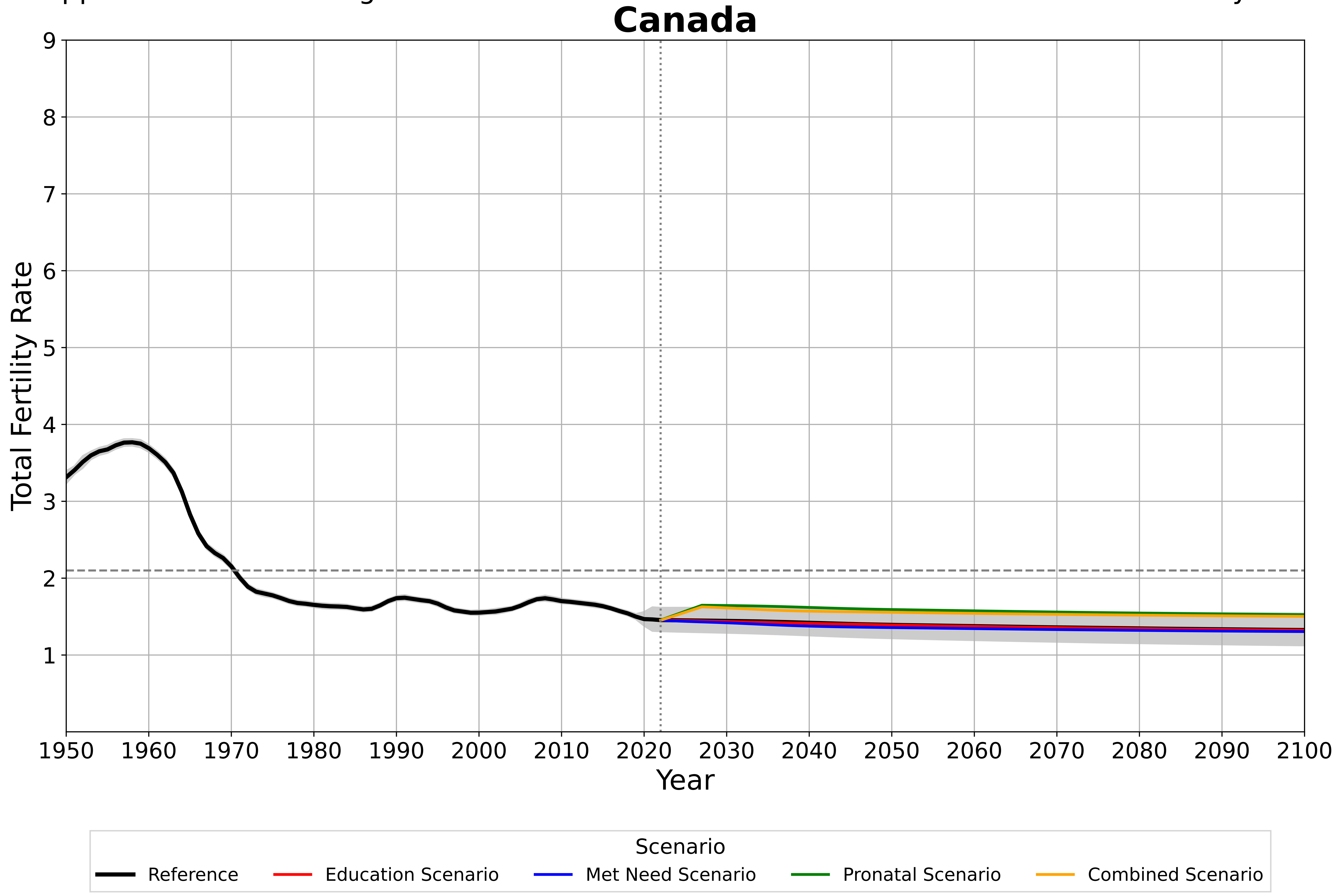

8255

Supplemental Results Figure S5: TFR for reference and alternative scenario forecasts by location, 1950– 2100

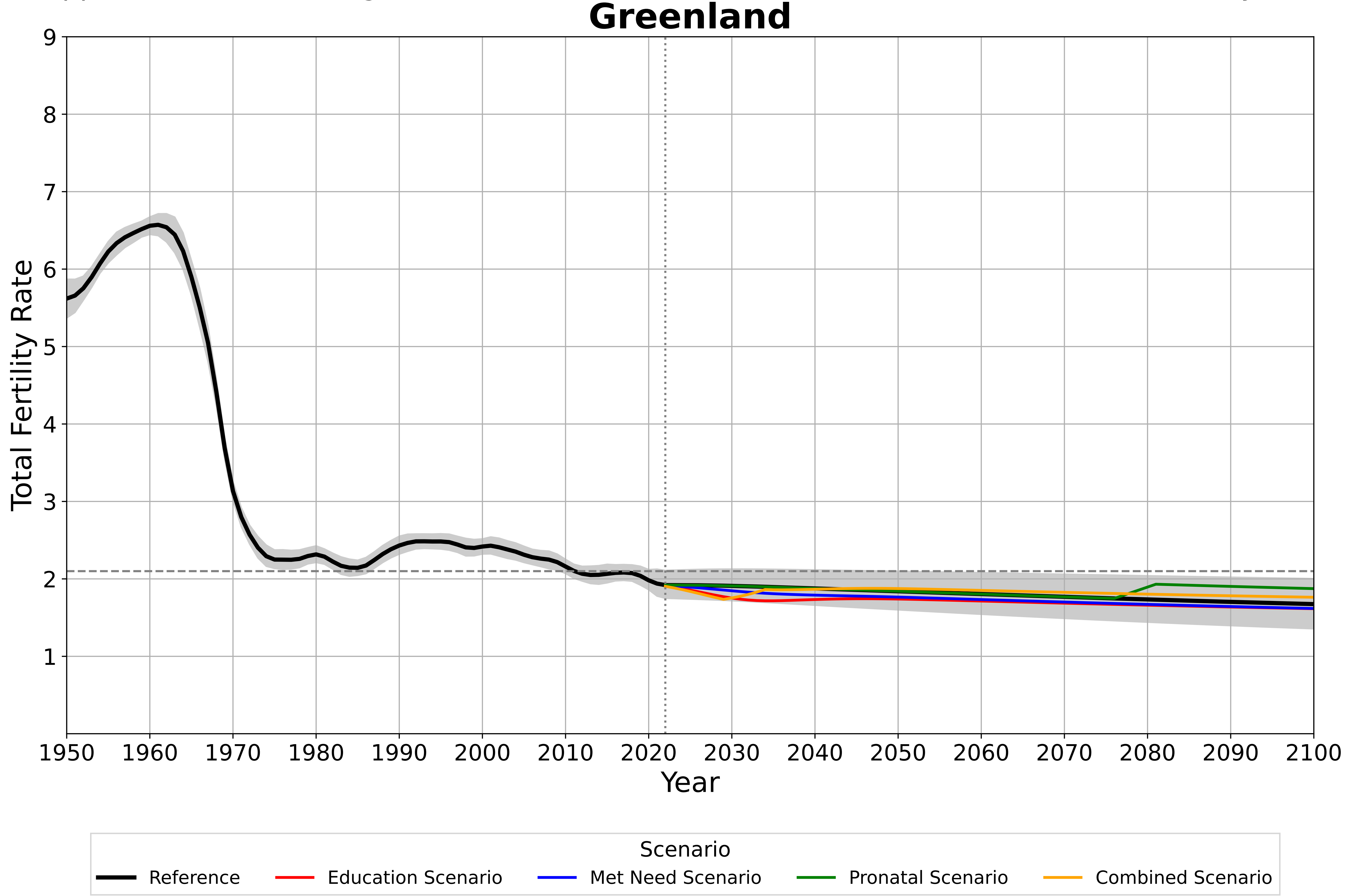

Supplemental Results Figure S5: TFR for reference and alternative scenario forecasts by location, 1950– 2100

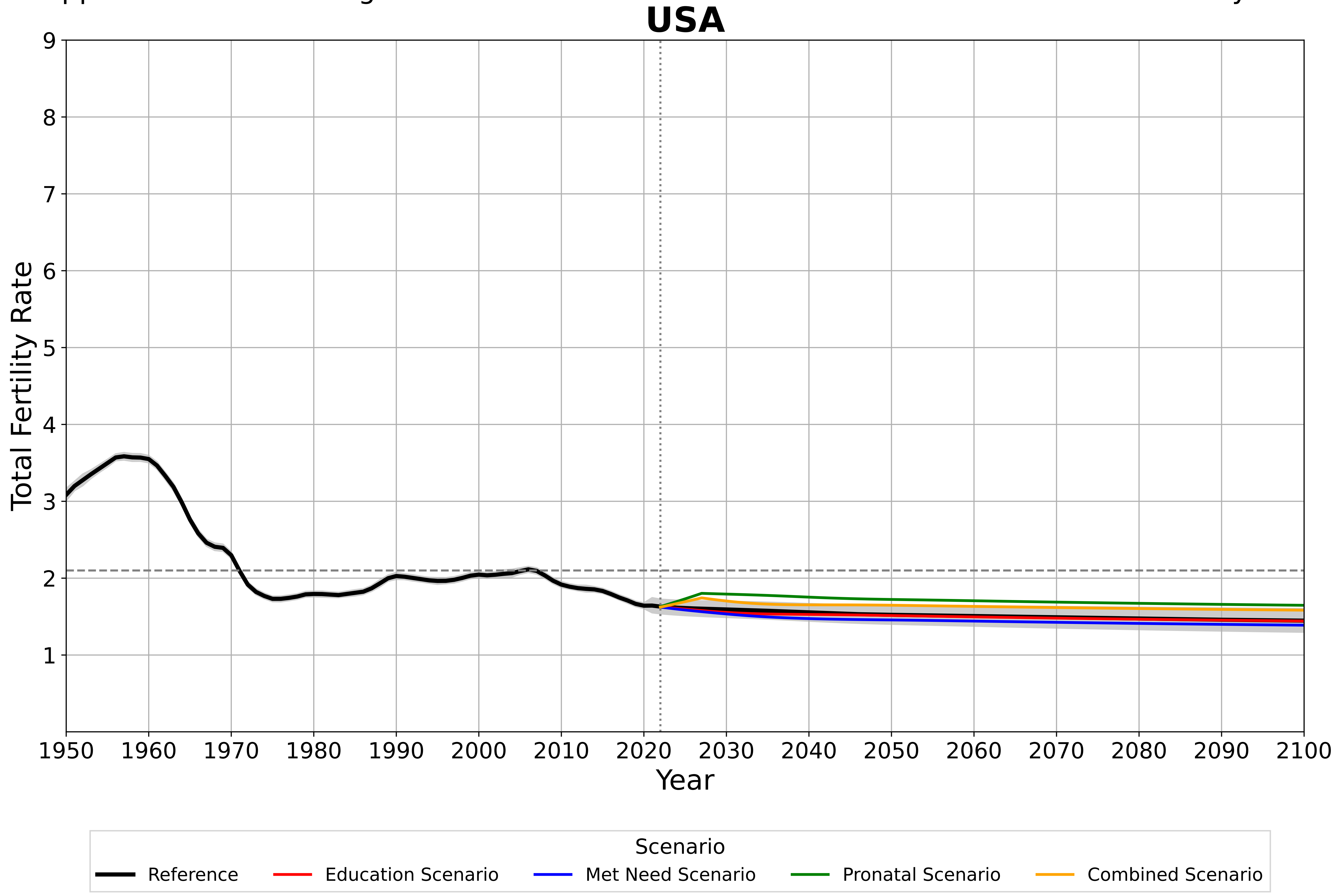

Supplemental Results Figure S5: TFR for reference and alternative scenario forecasts by location, 1950– 2100

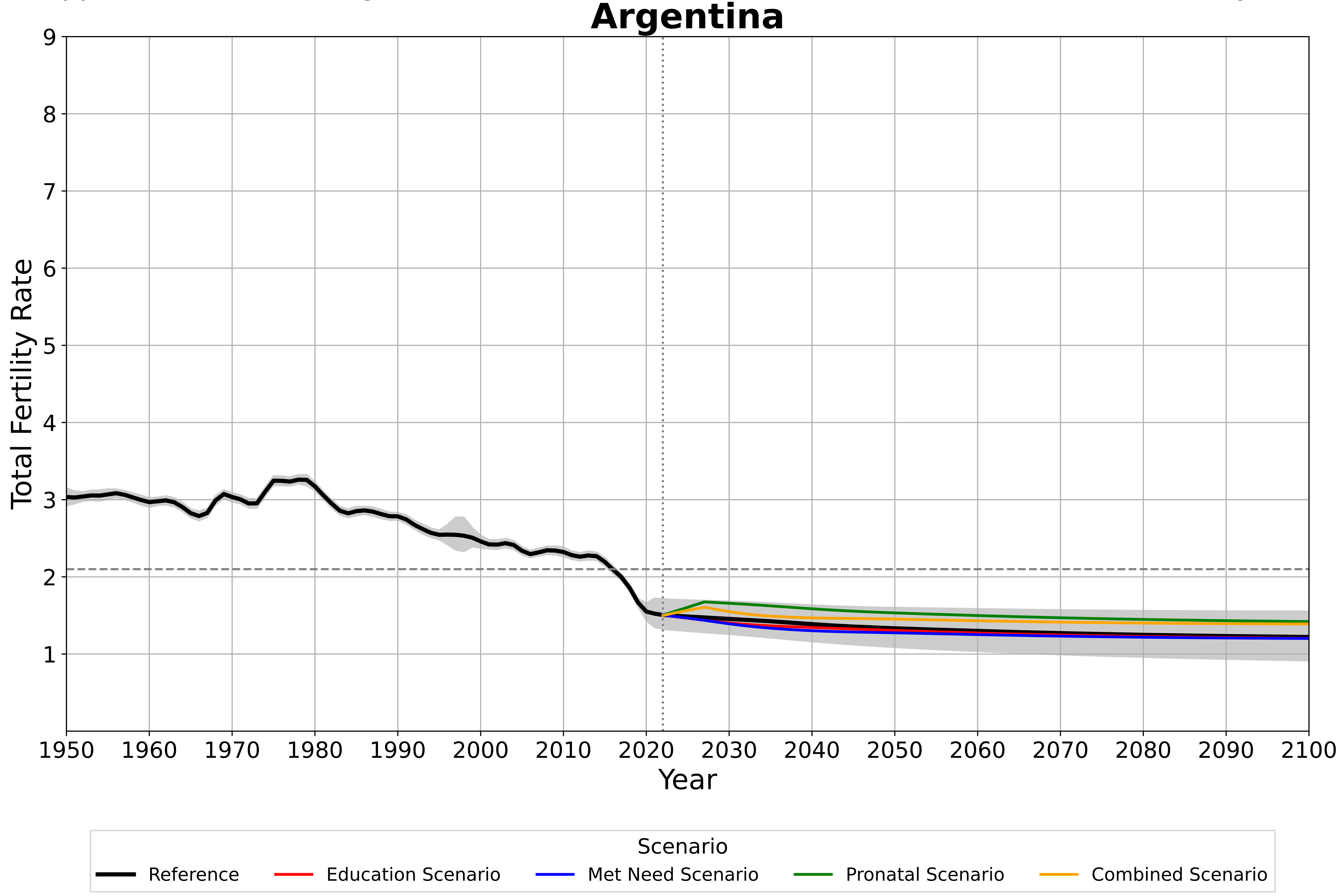

Supplemental Results Figure S5: TFR for reference and alternative scenario forecasts by location, 1950– 2100

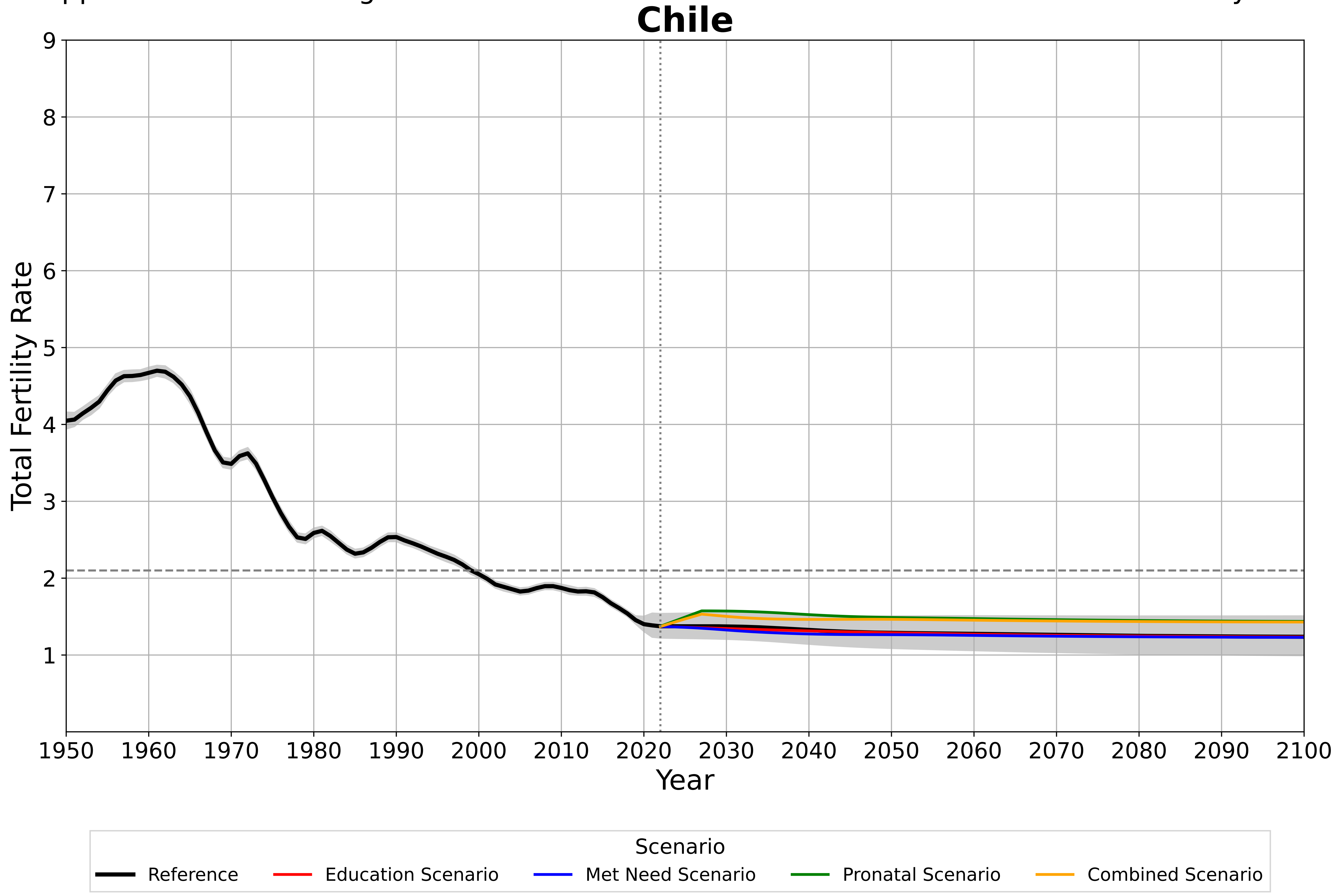

Supplemental Results Figure S5: TFR for reference and alternative scenario forecasts by location, 1950– 2100

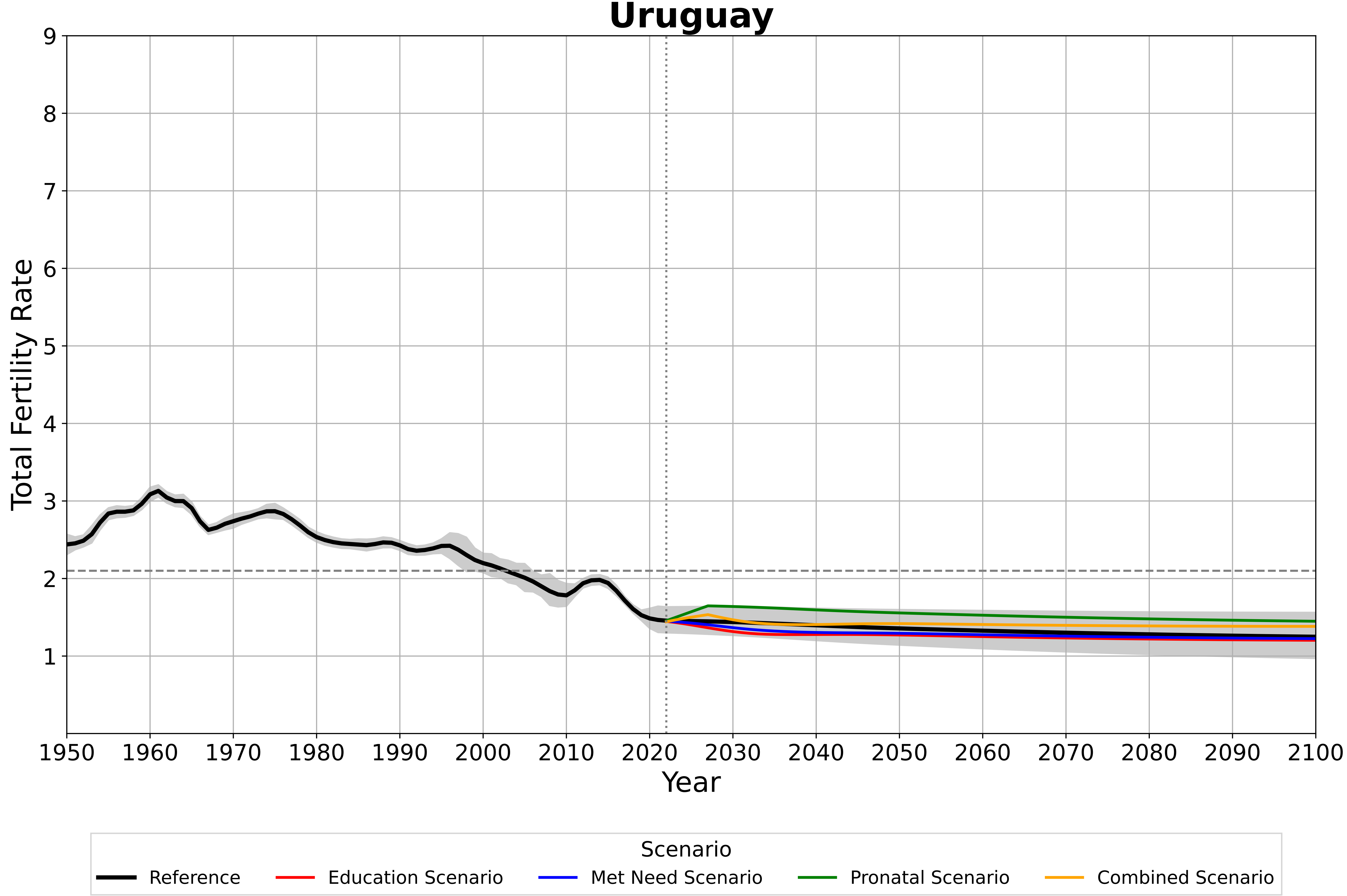

Supplemental Results Figure S5: TFR for reference and alternative scenario forecasts by location, 1950– 2100

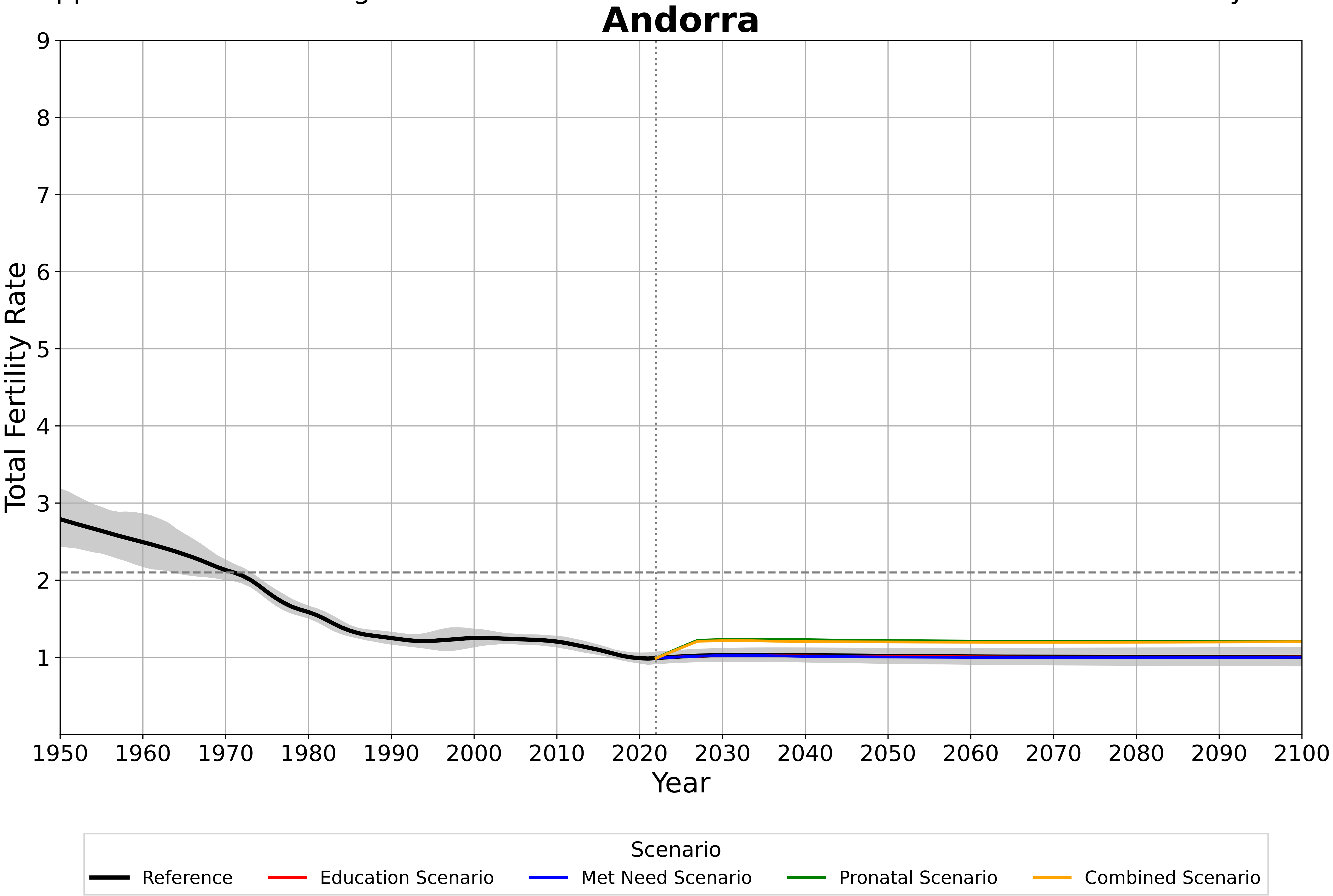

Supplemental Results Figure S5: TFR for reference and alternative scenario forecasts by location, 1950– 2100

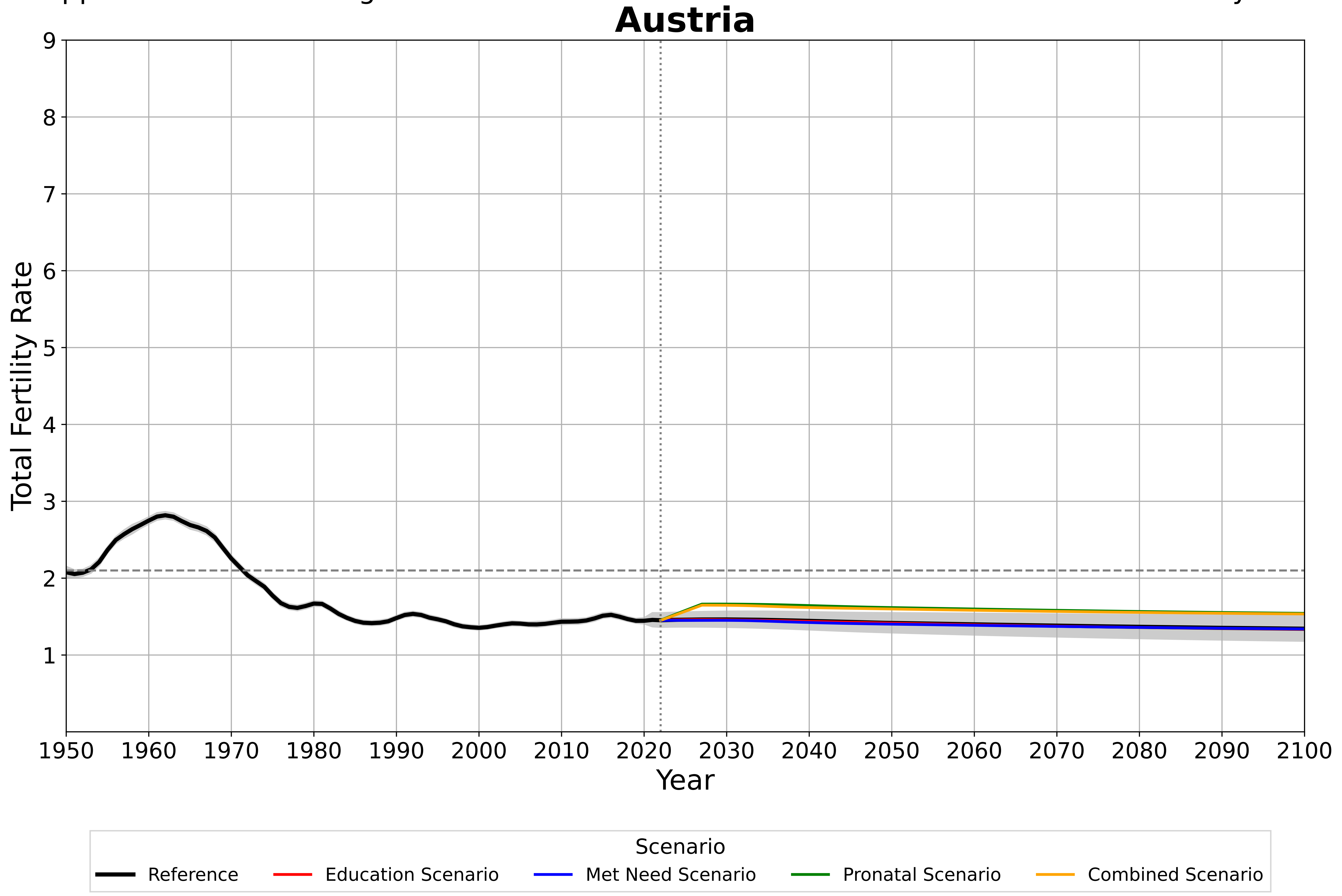

Supplemental Results Figure S5: TFR for reference and alternative scenario forecasts by location, 1950– 2100

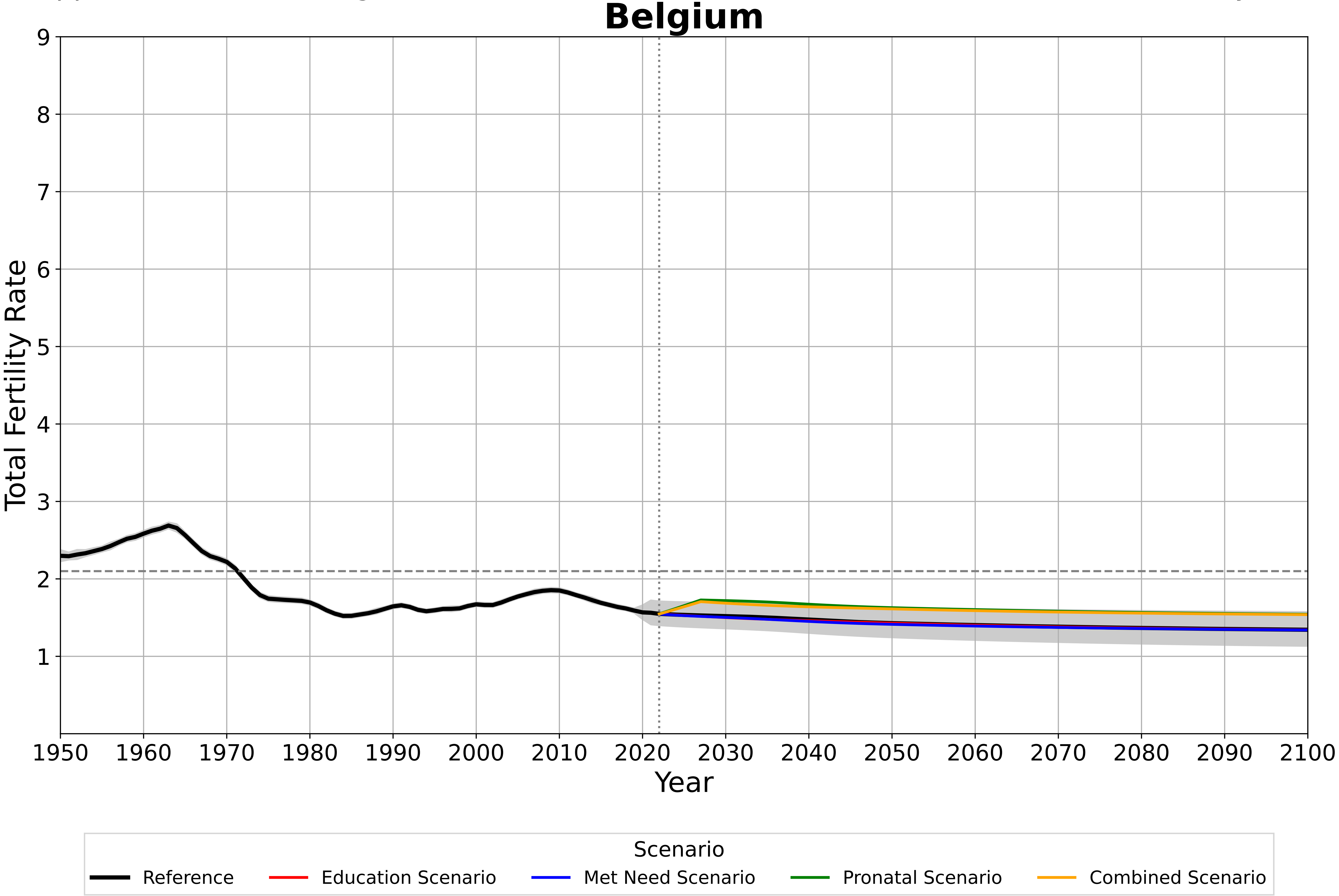

Supplemental Results Figure S5: TFR for reference and alternative scenario forecasts by location, 1950– 2100

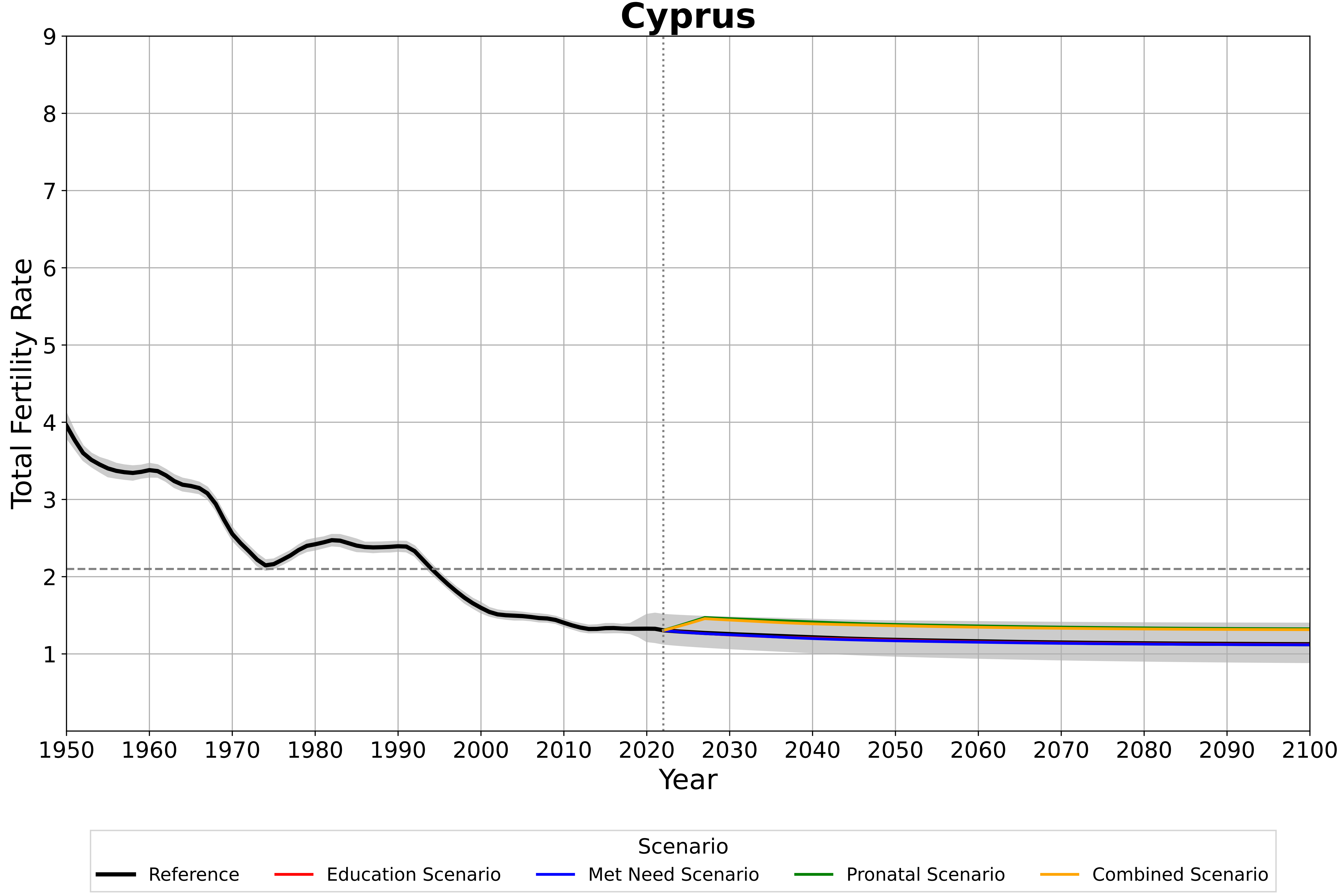

Supplemental Results Figure S5: TFR for reference and alternative scenario forecasts by location, 1950– 2100

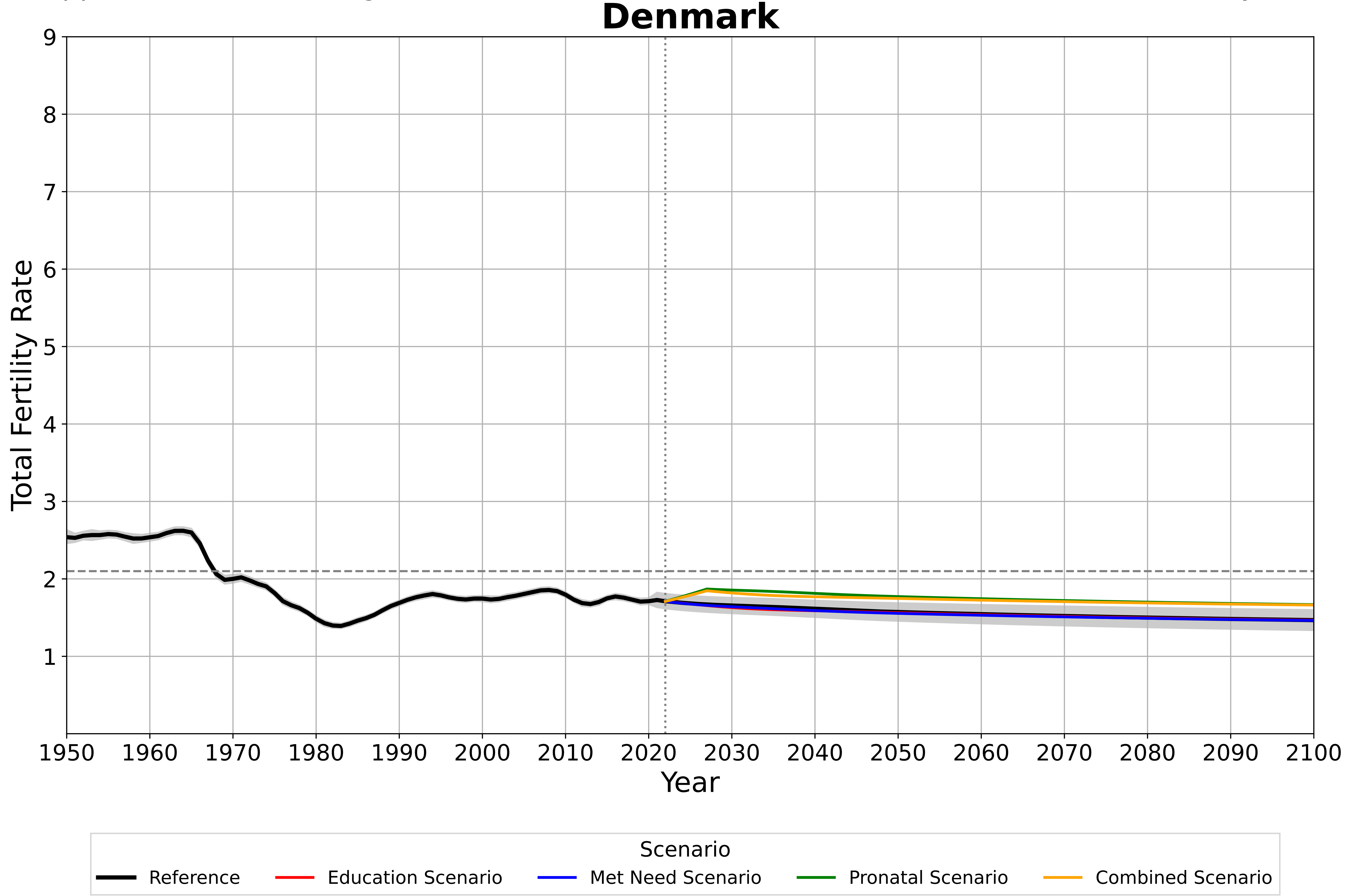

Supplemental Results Figure S5: TFR for reference and alternative scenario forecasts by location, 1950– 2100

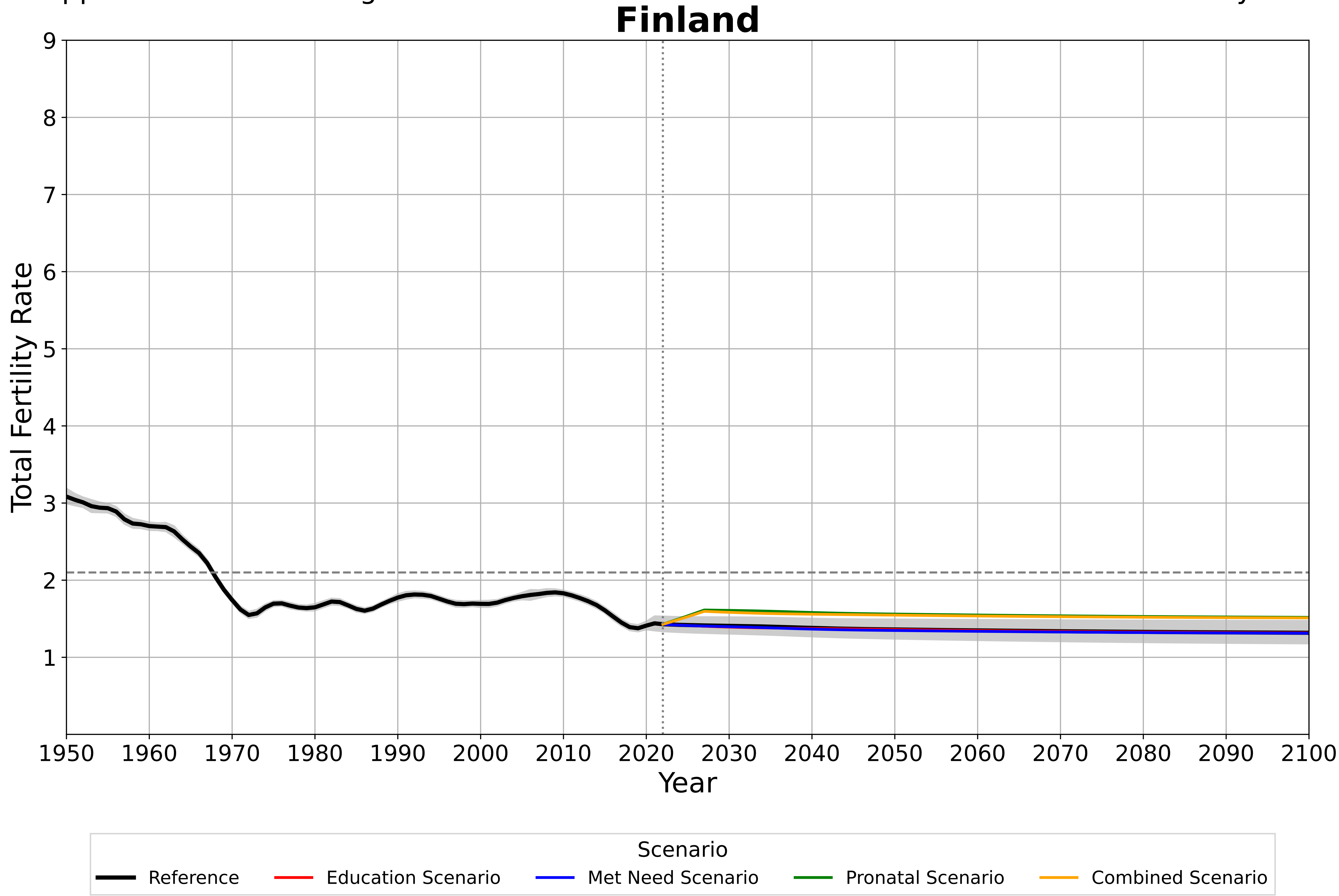

Supplemental Results Figure S5: TFR for reference and alternative scenario forecasts by location, 1950– 2100

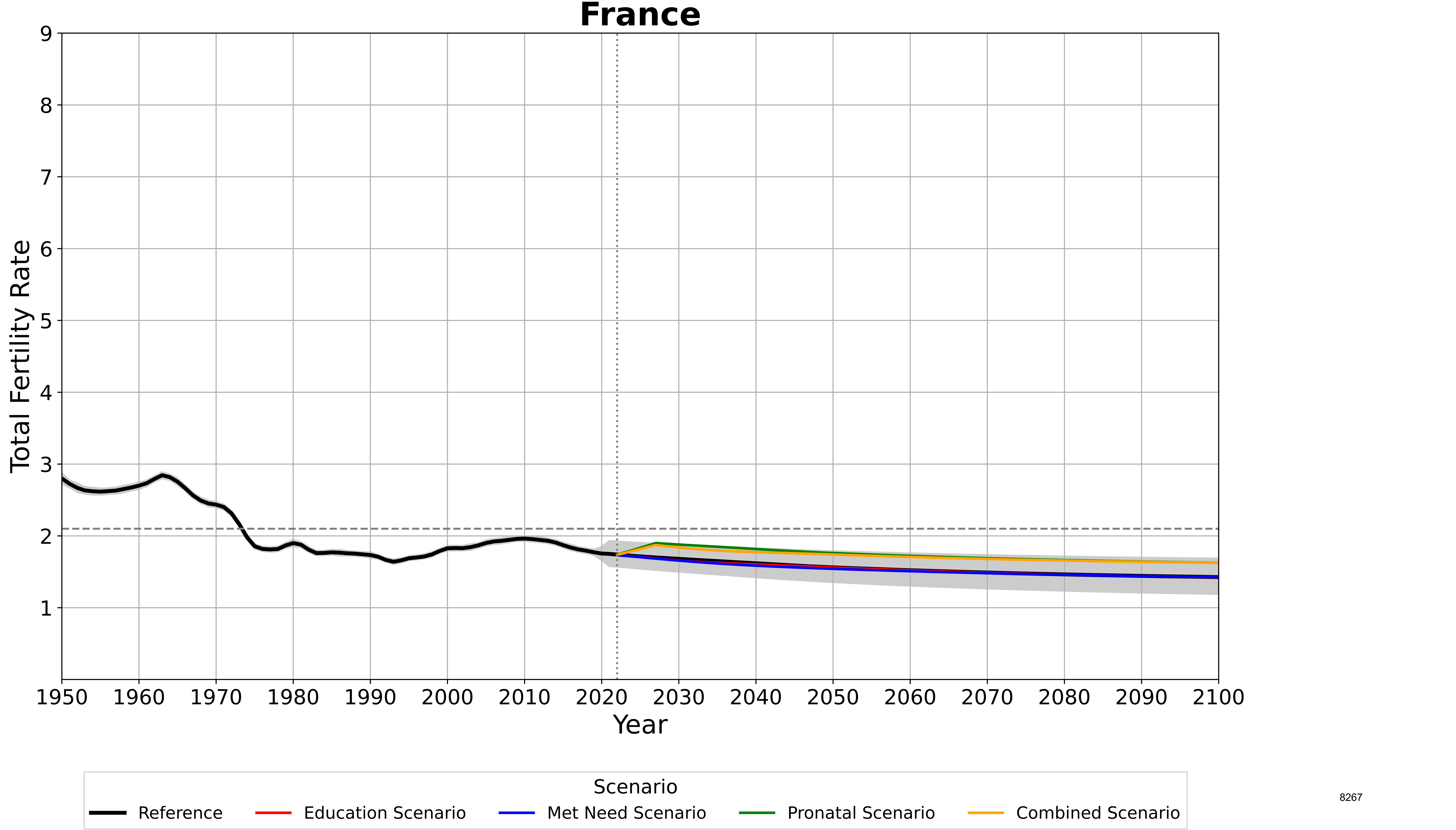

Supplemental Results Figure S5: TFR for reference and alternative scenario forecasts by location, 1950– 2100

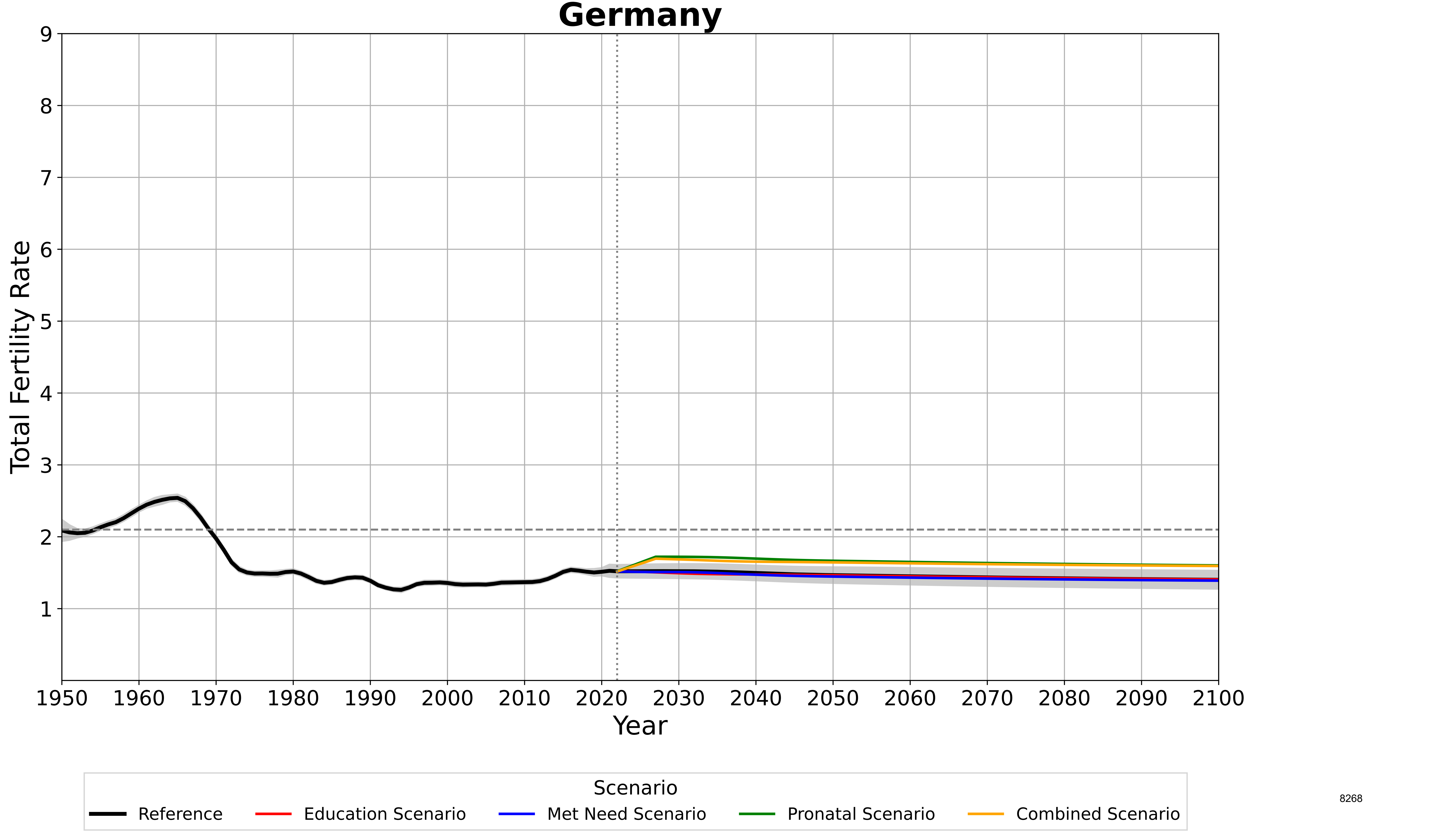

Supplemental Results Figure S5: TFR for reference and alternative scenario forecasts by location, 1950– 2100

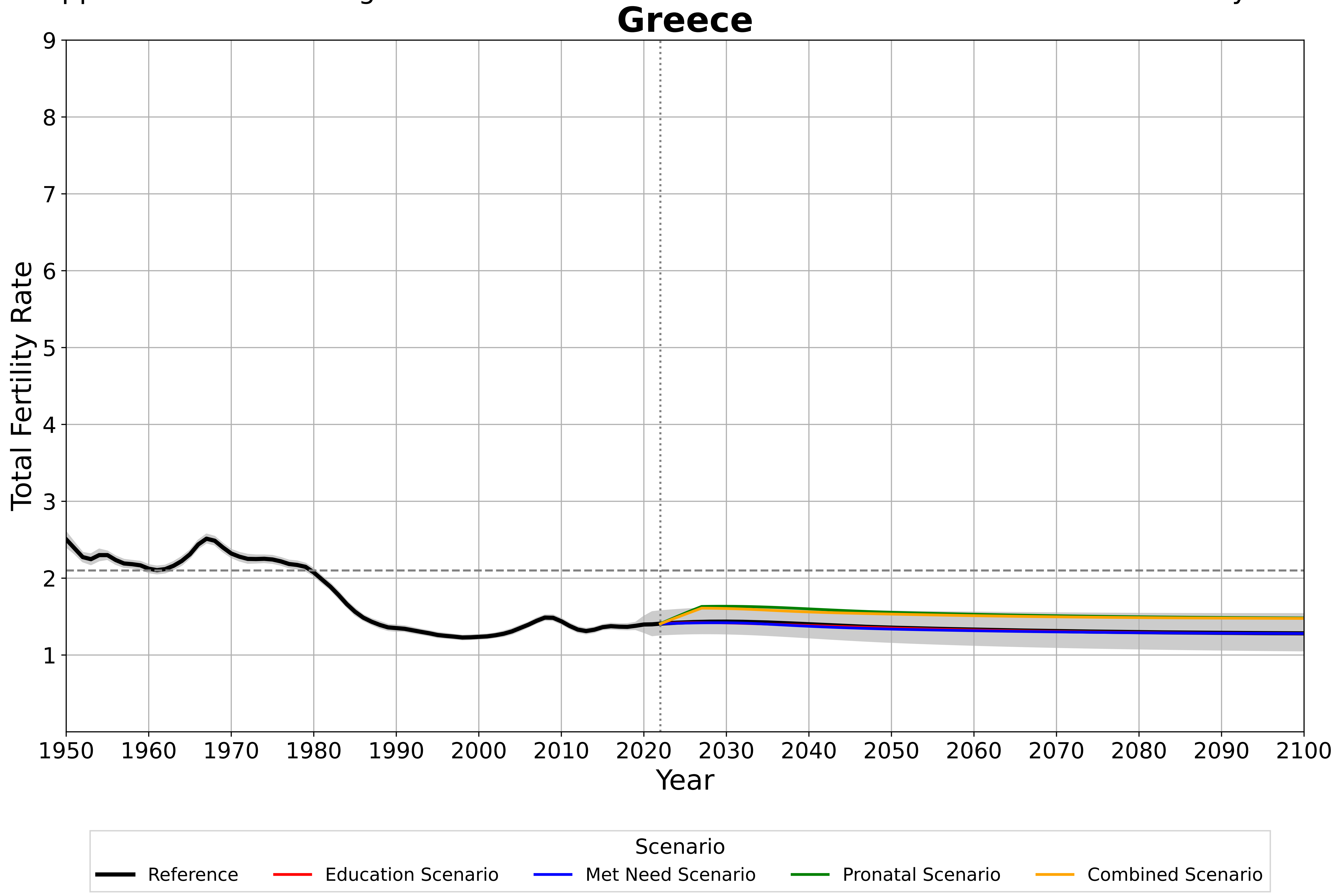

Supplemental Results Figure S5: TFR for reference and alternative scenario forecasts by location, 1950– 2100

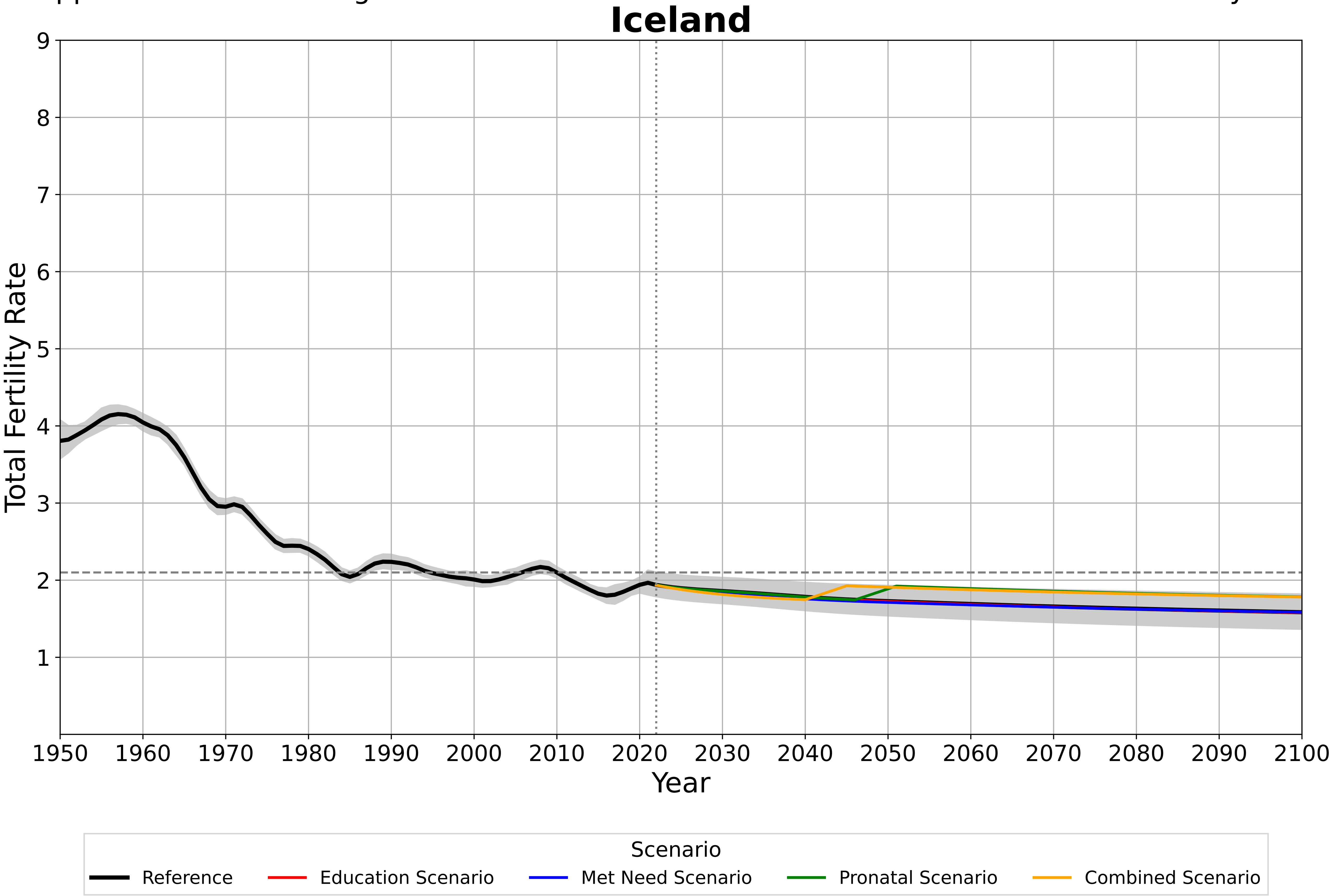

8270

Supplemental Results Figure S5: TFR for reference and alternative scenario forecasts by location, 1950– 2100

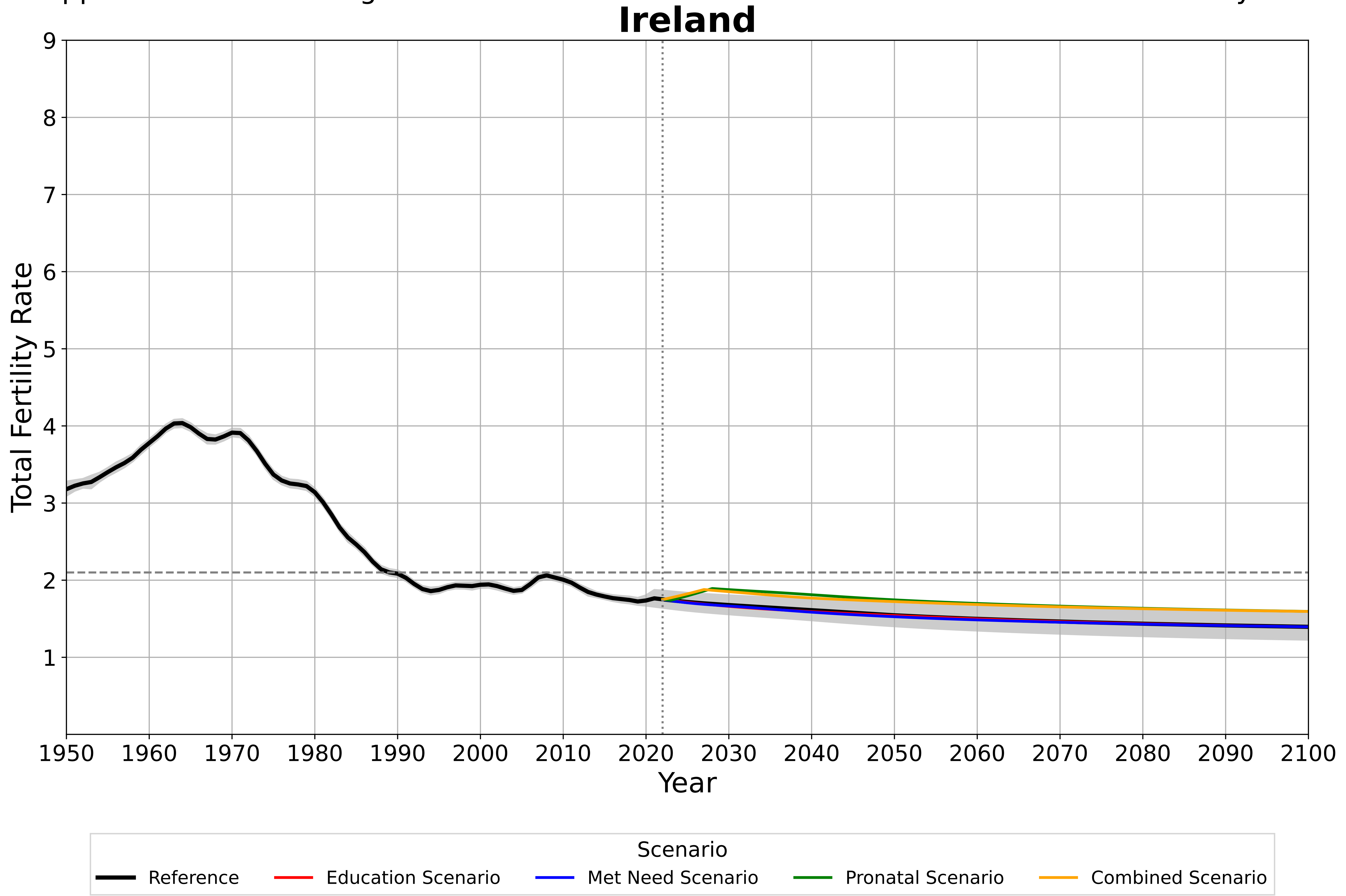

Supplemental Results Figure S5: TFR for reference and alternative scenario forecasts by location, 1950– 2100

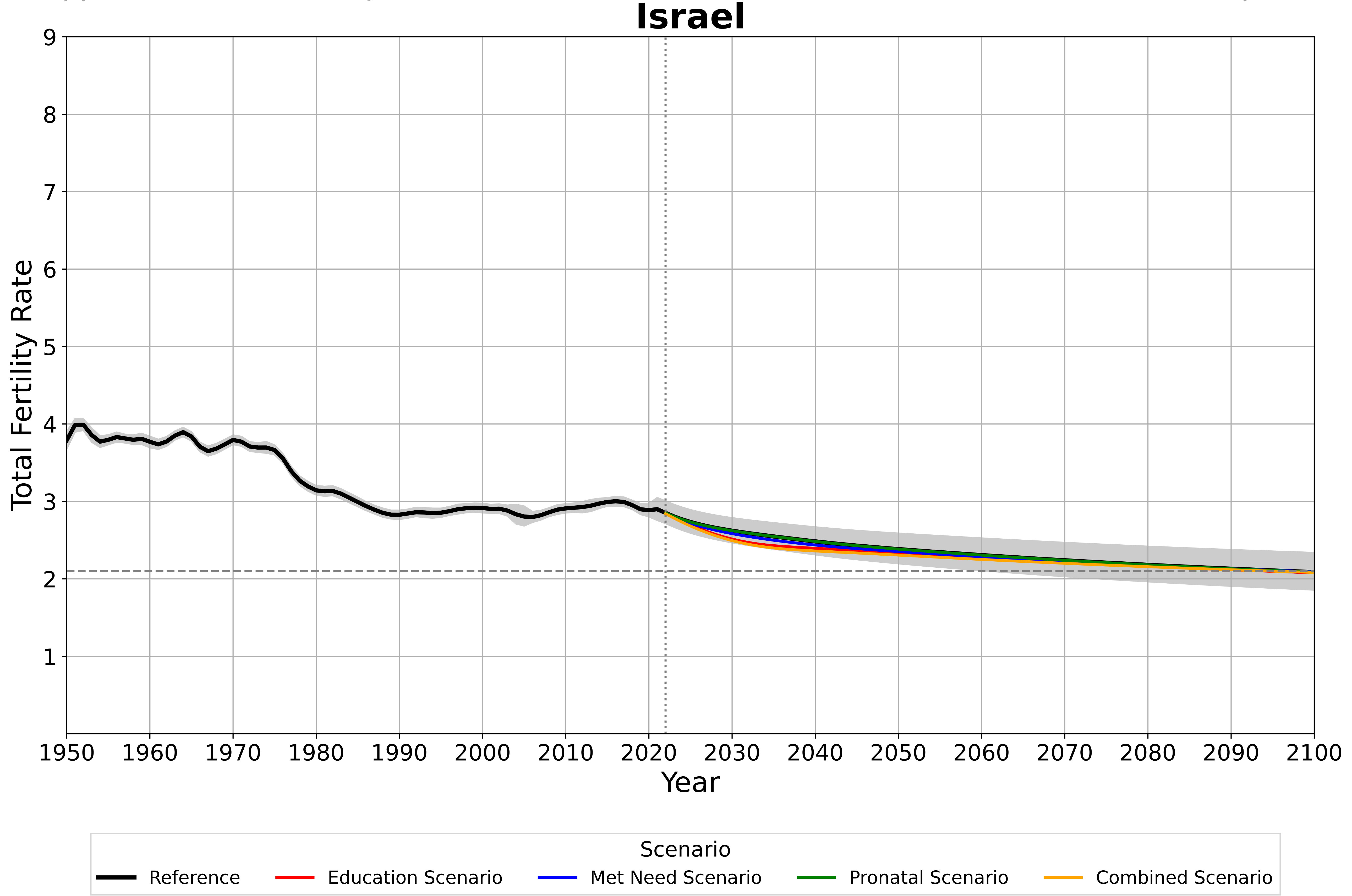

Supplemental Results Figure S5: TFR for reference and alternative scenario forecasts by location, 1950– 2100

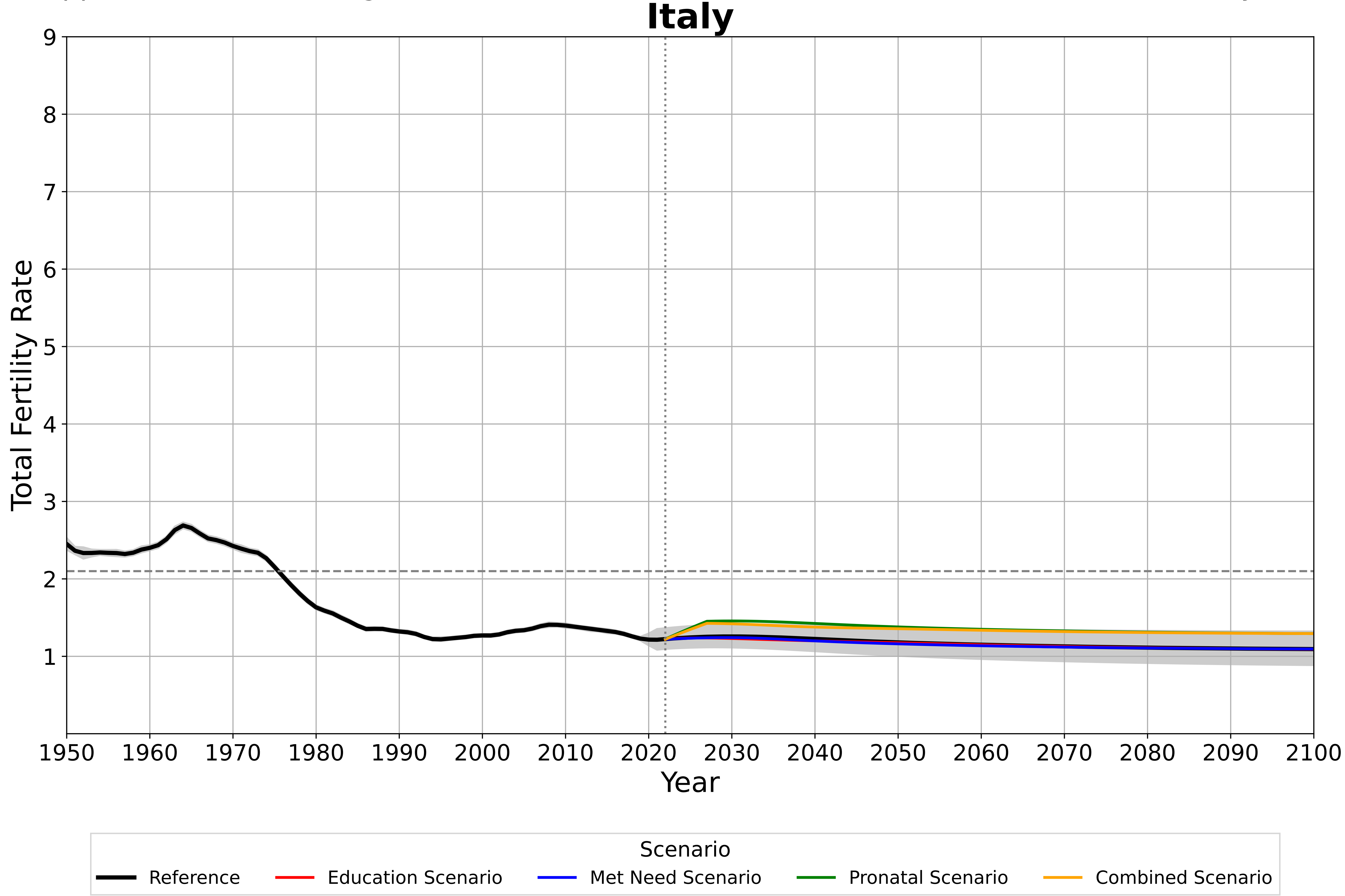

Supplemental Results Figure S5: TFR for reference and alternative scenario forecasts by location, 1950– 2100

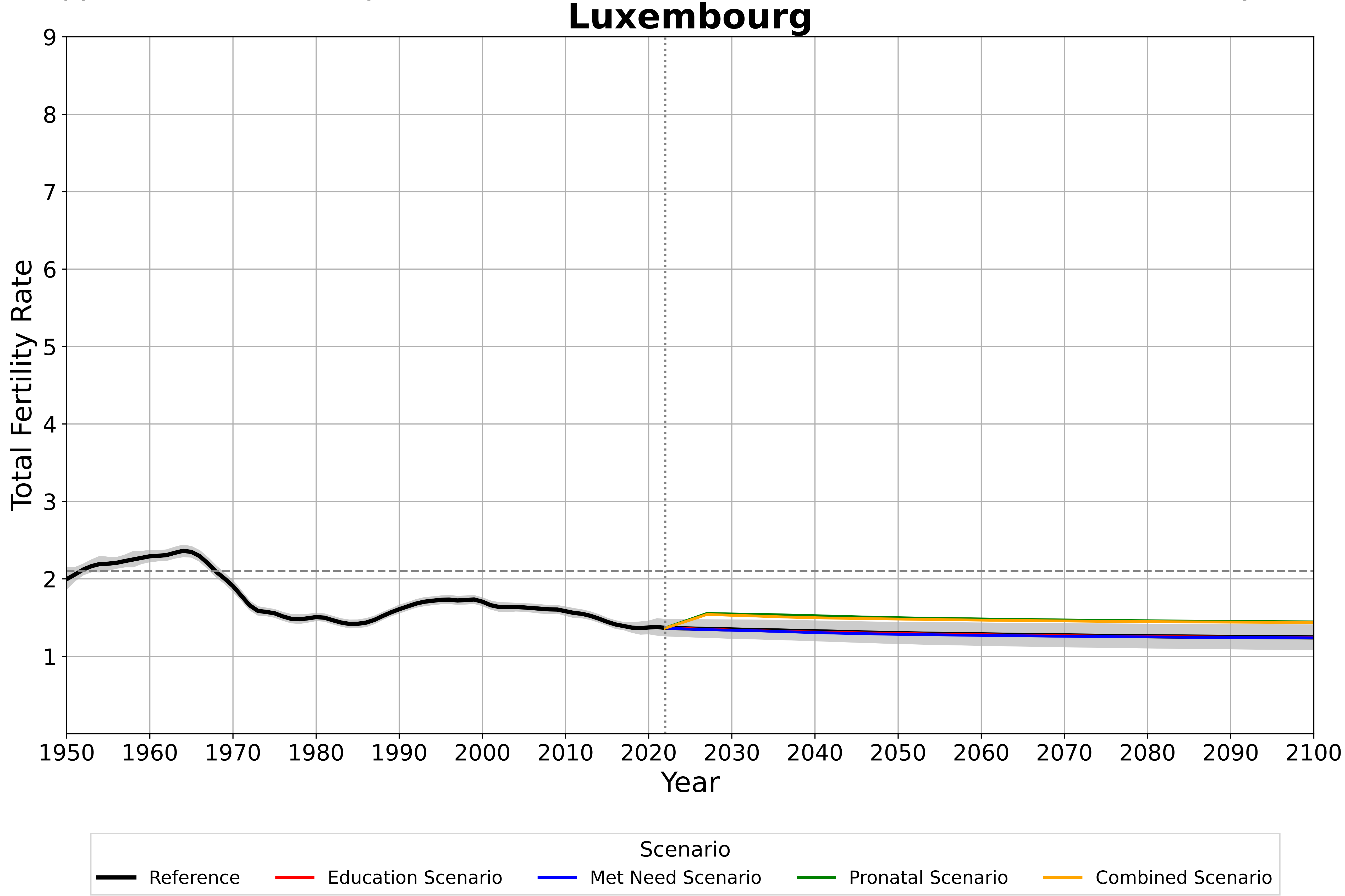

Supplemental Results Figure S5: TFR for reference and alternative scenario forecasts by location, 1950– 2100

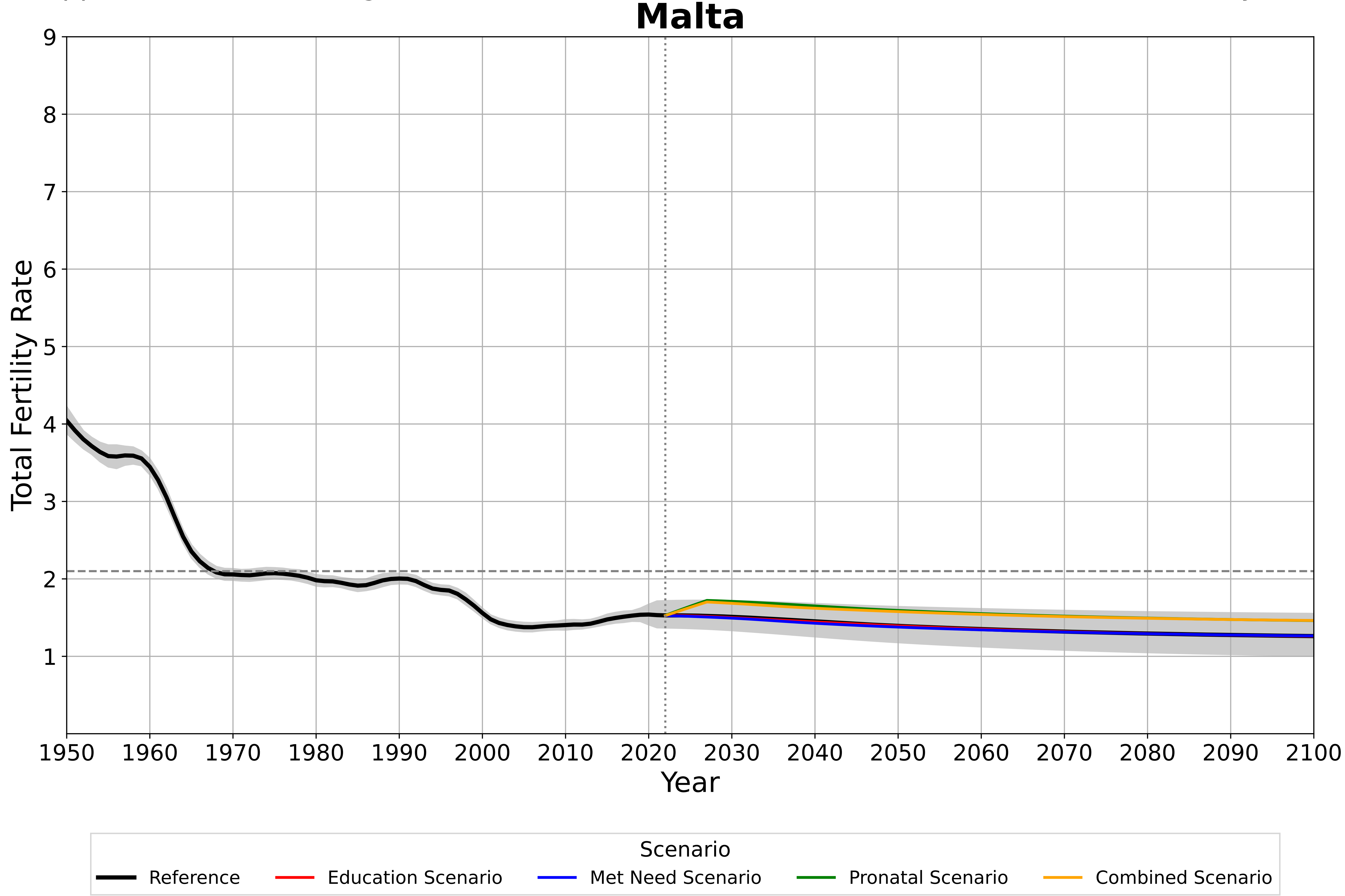

Supplemental Results Figure S5: TFR for reference and alternative scenario forecasts by location, 1950– 2100

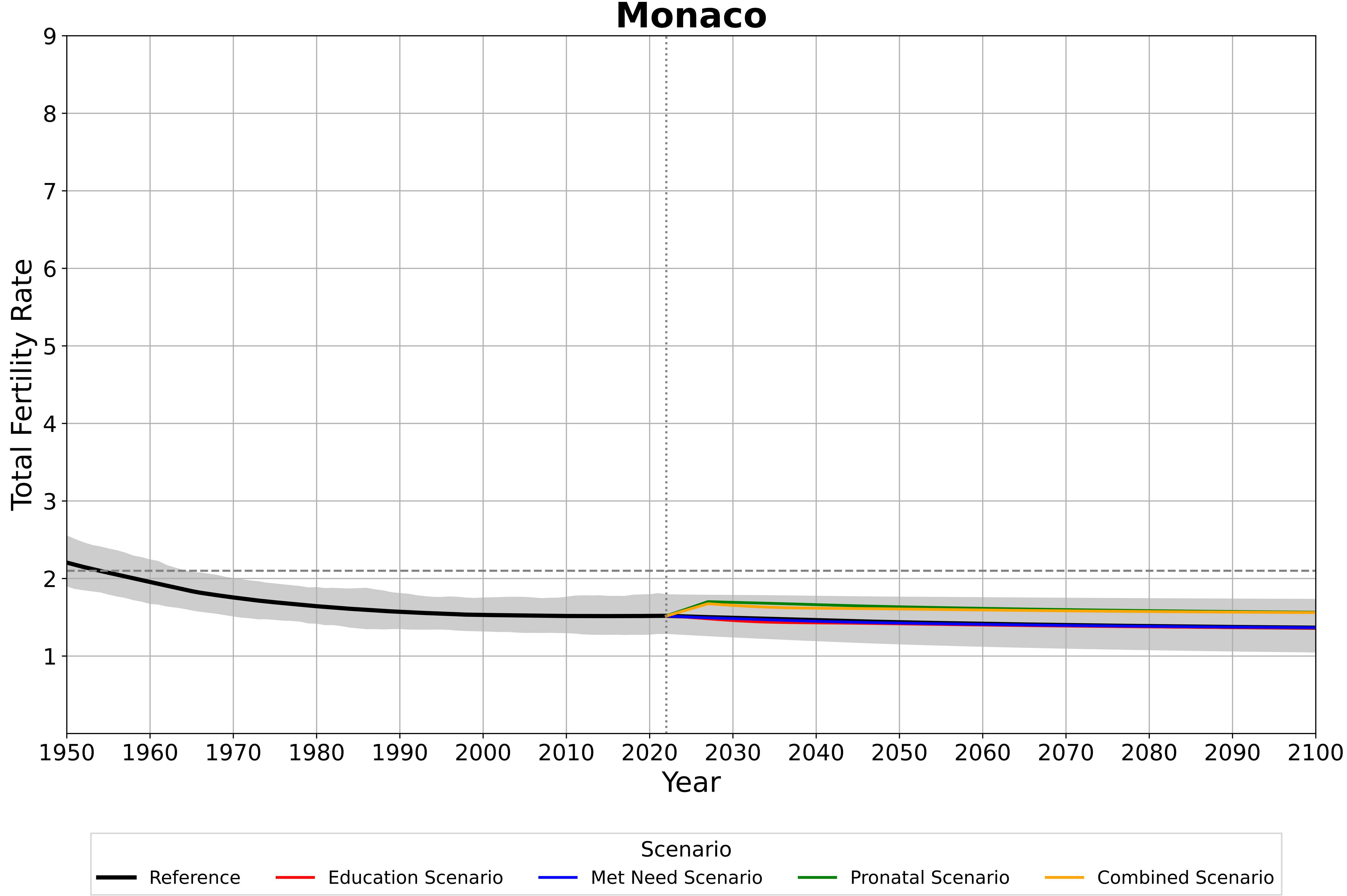

8276

Supplemental Results Figure S5: TFR for reference and alternative scenario forecasts by location, 1950– 2100

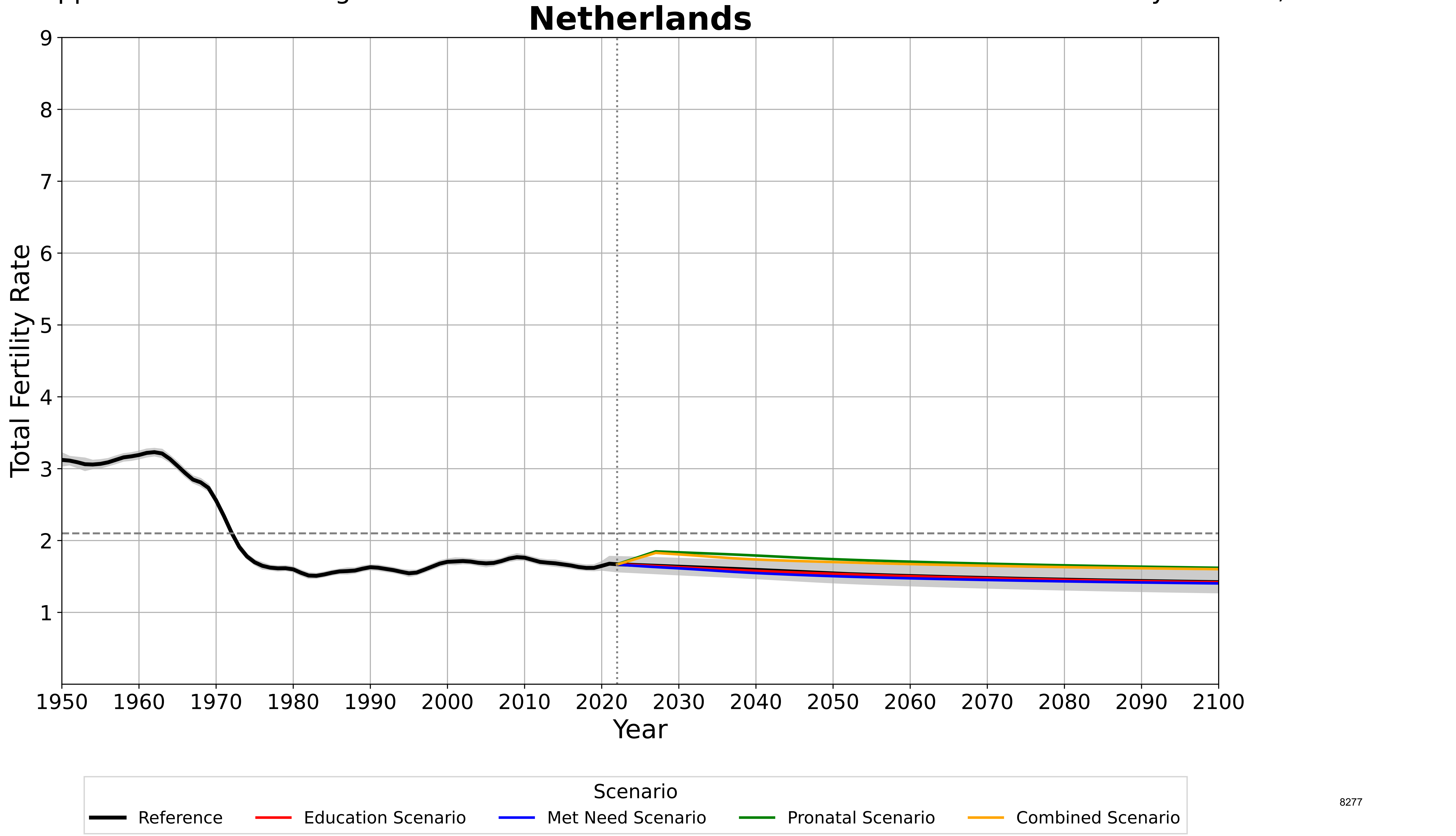

8277

Supplemental Results Figure S5: TFR for reference and alternative scenario forecasts by location, 1950– 2100

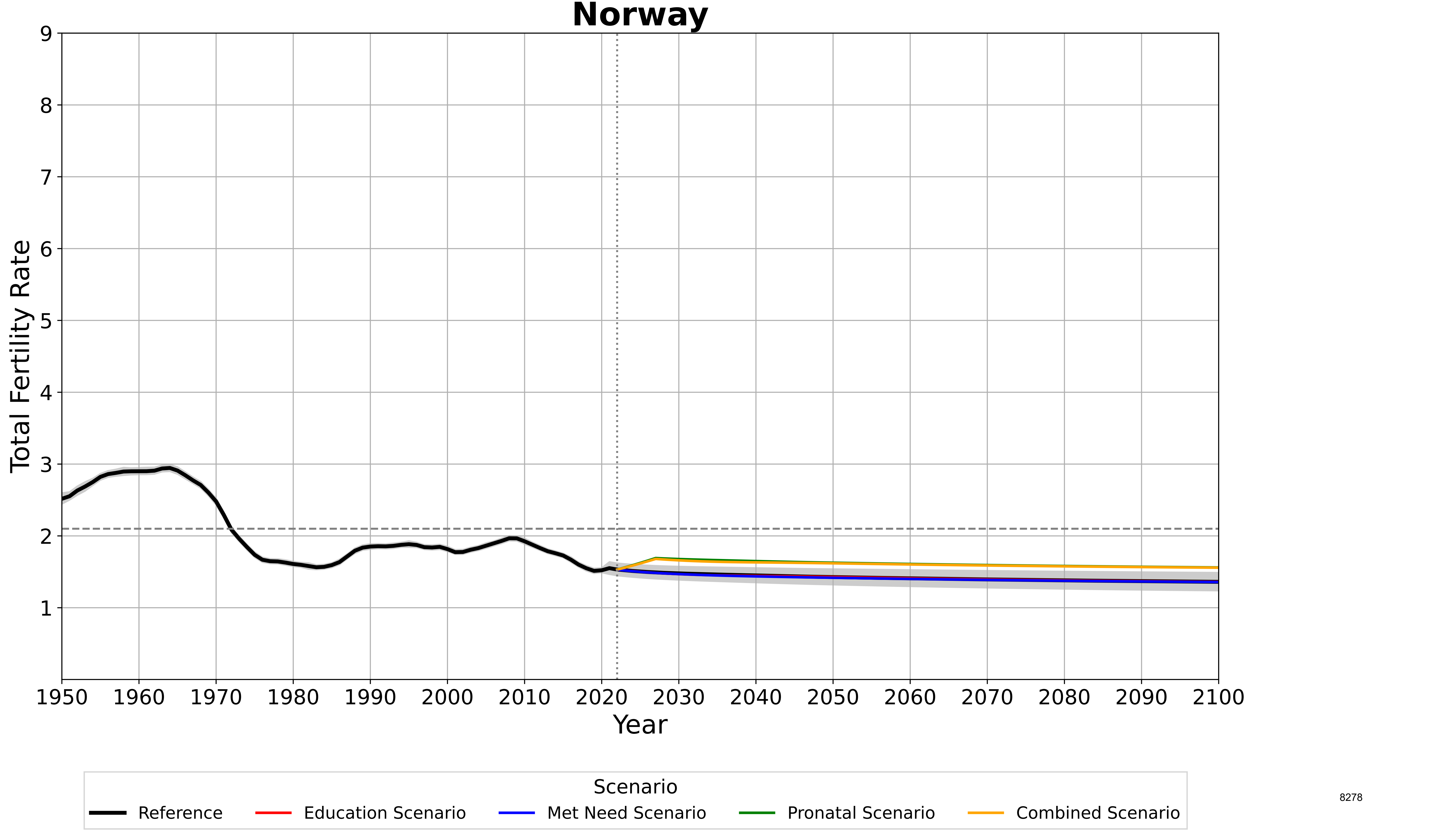

Supplemental Results Figure S5: TFR for reference and alternative scenario forecasts by location, 1950– 2100

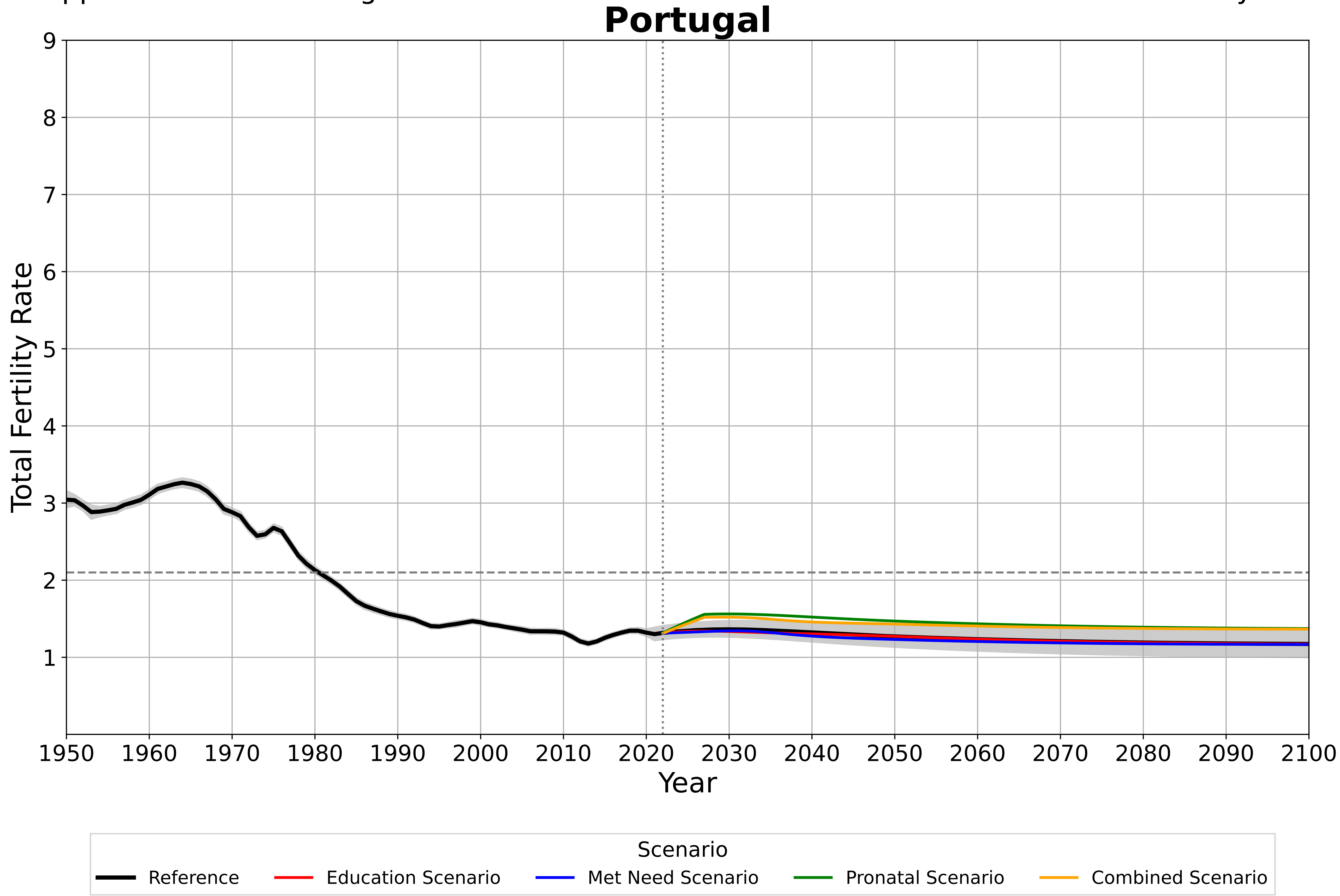

Supplemental Results Figure S5: TFR for reference and alternative scenario forecasts by location, 1950– 2100

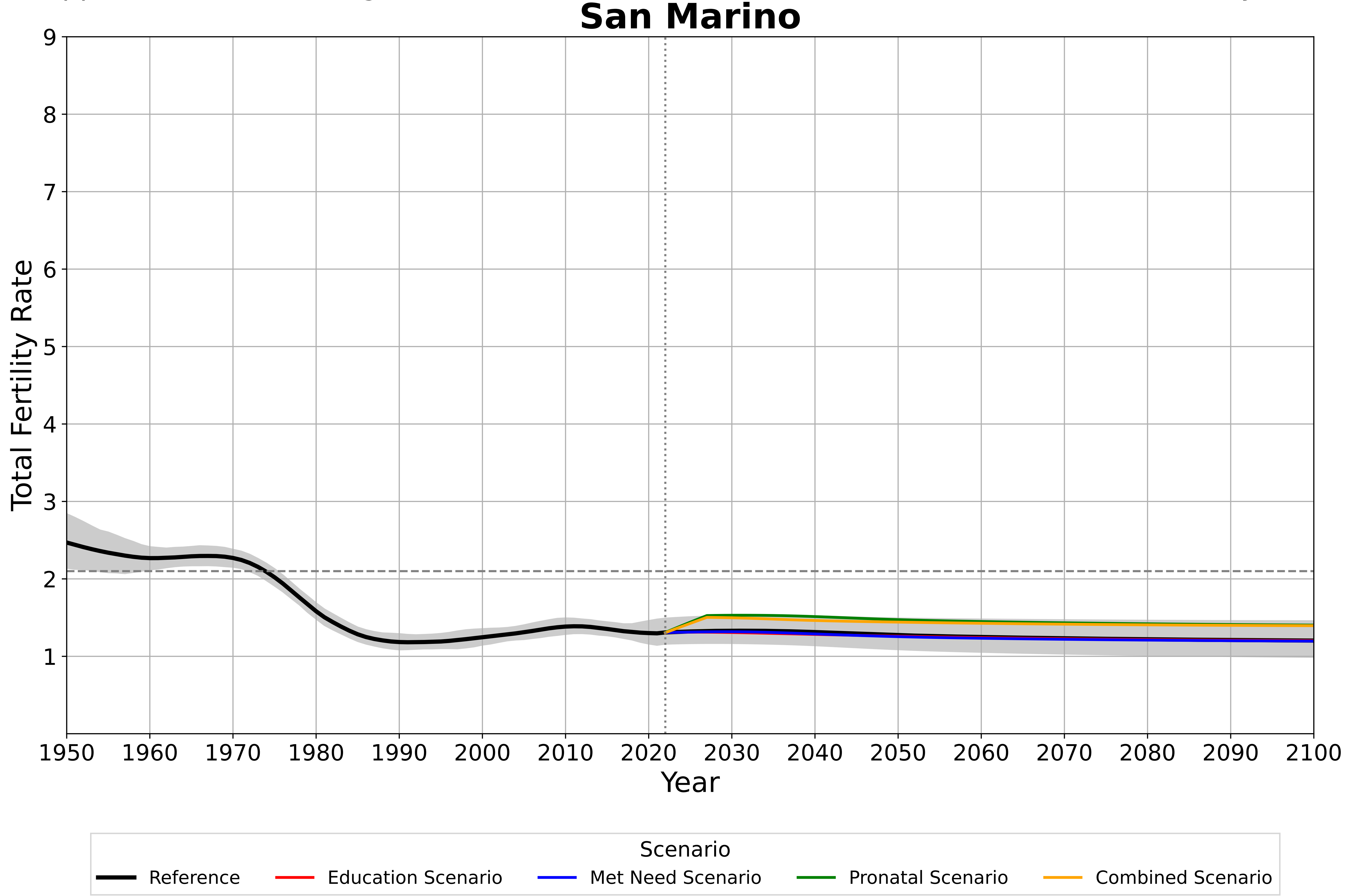

8280

Supplemental Results Figure S5: TFR for reference and alternative scenario forecasts by location, 1950– 2100

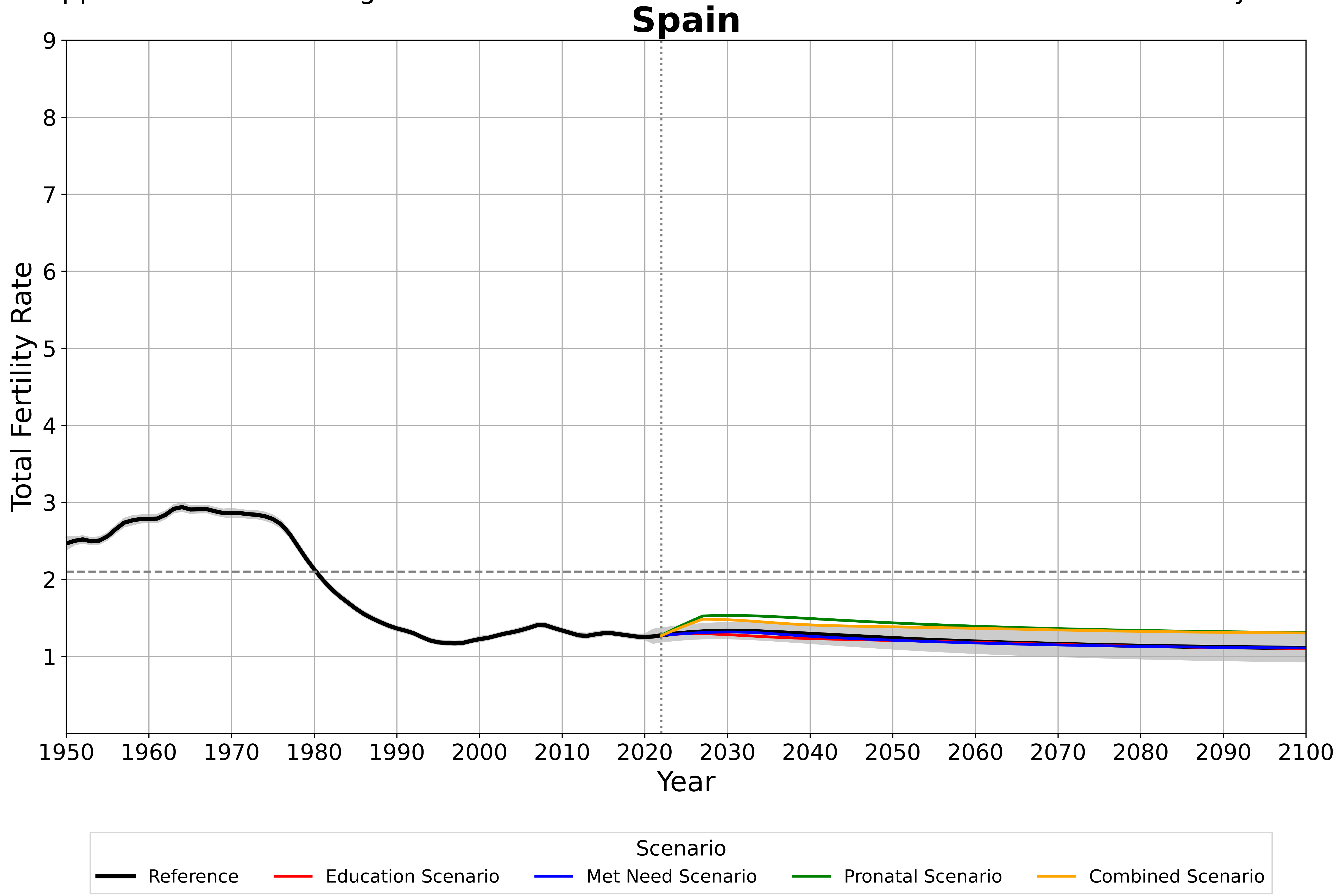

Supplemental Results Figure S5: TFR for reference and alternative scenario forecasts by location, 1950– 2100

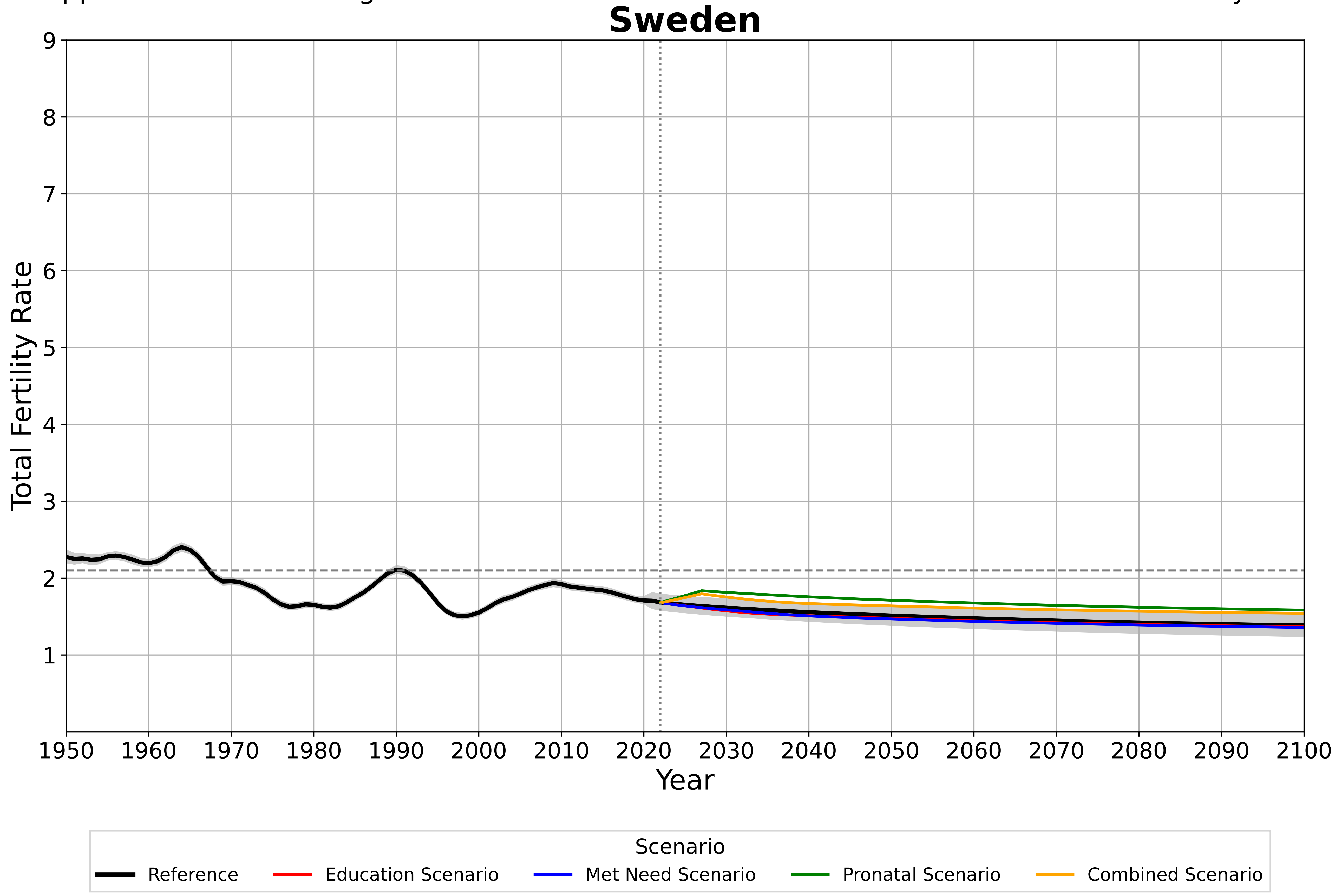

Supplemental Results Figure S5: TFR for reference and alternative scenario forecasts by location, 1950– 2100

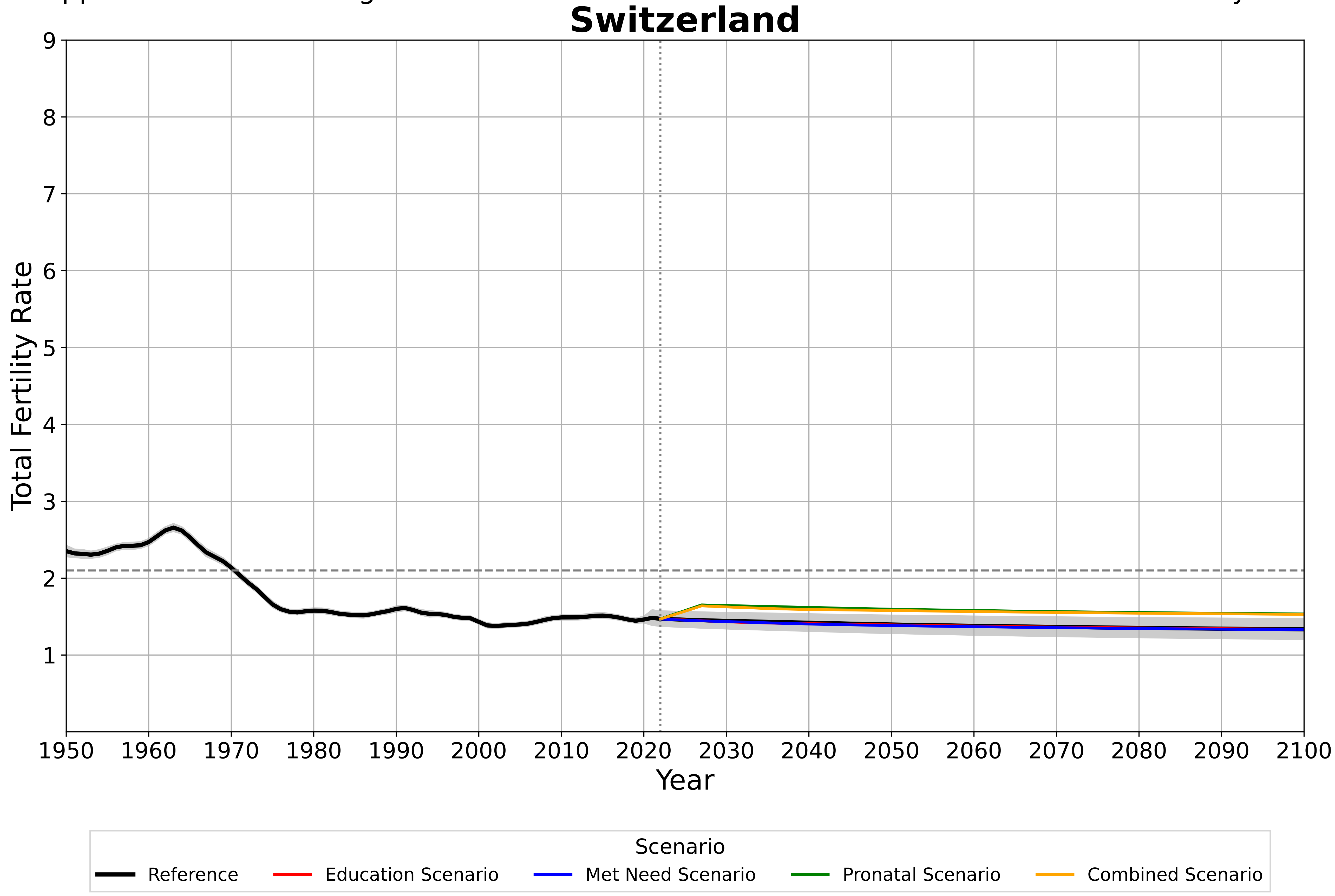

Supplemental Results Figure S5: TFR for reference and alternative scenario forecasts by location, 1950– 2100

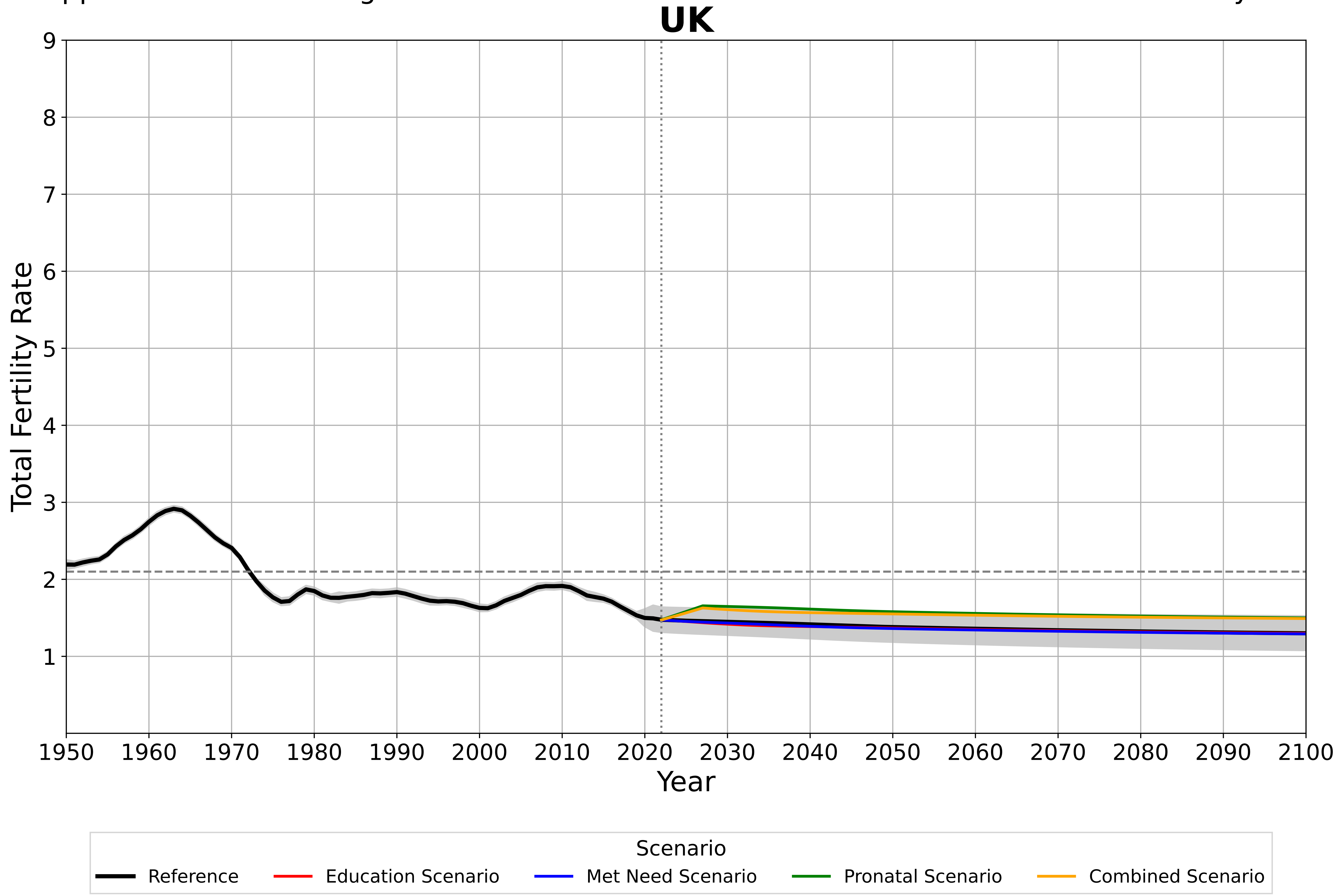

Supplemental Results Figure S5: TFR for reference and alternative scenario forecasts by location, 1950– 2100

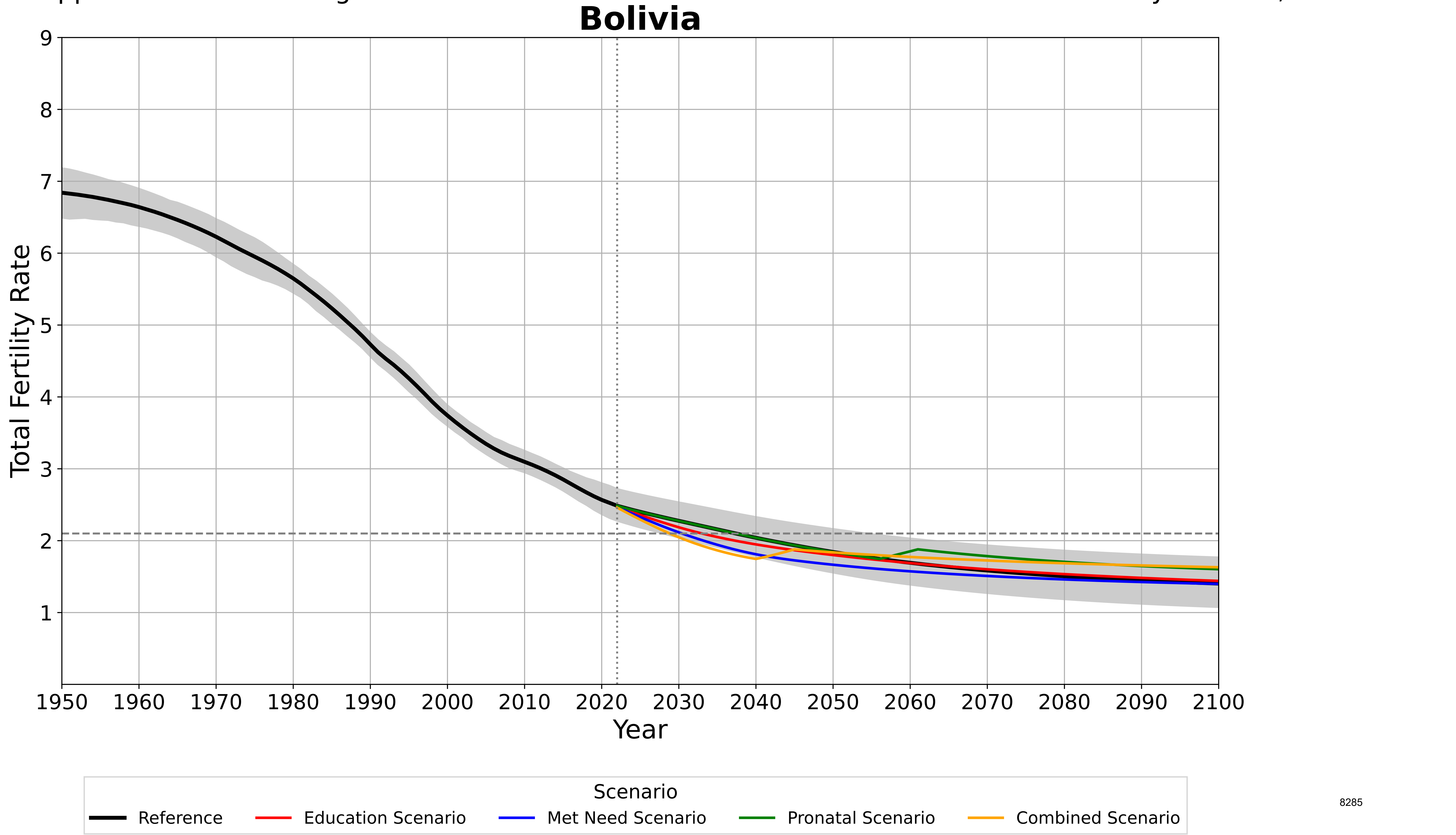

8285

Supplemental Results Figure S5: TFR for reference and alternative scenario forecasts by location, 1950– 2100

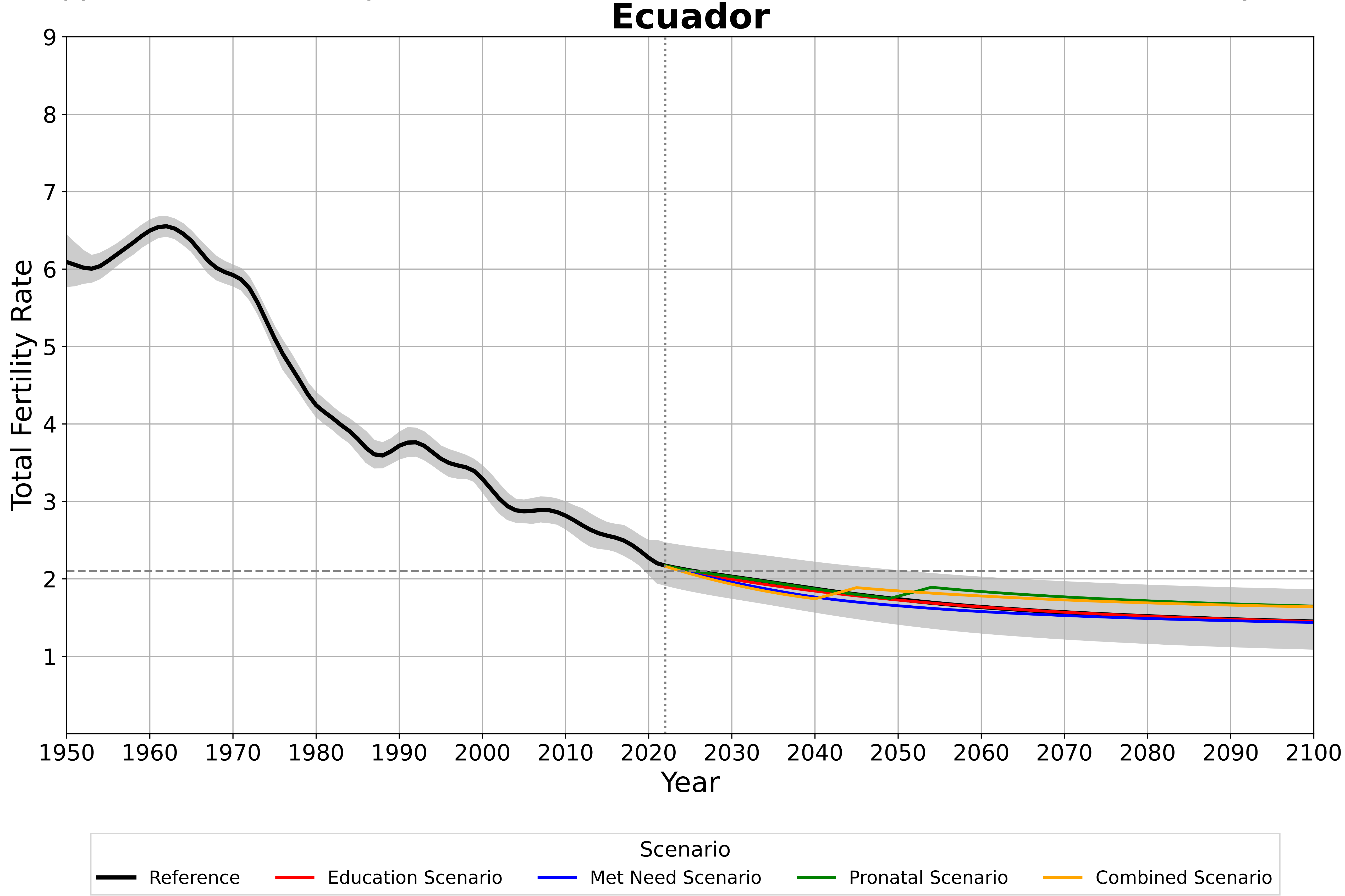

Supplemental Results Figure S5: TFR for reference and alternative scenario forecasts by location, 1950– 2100

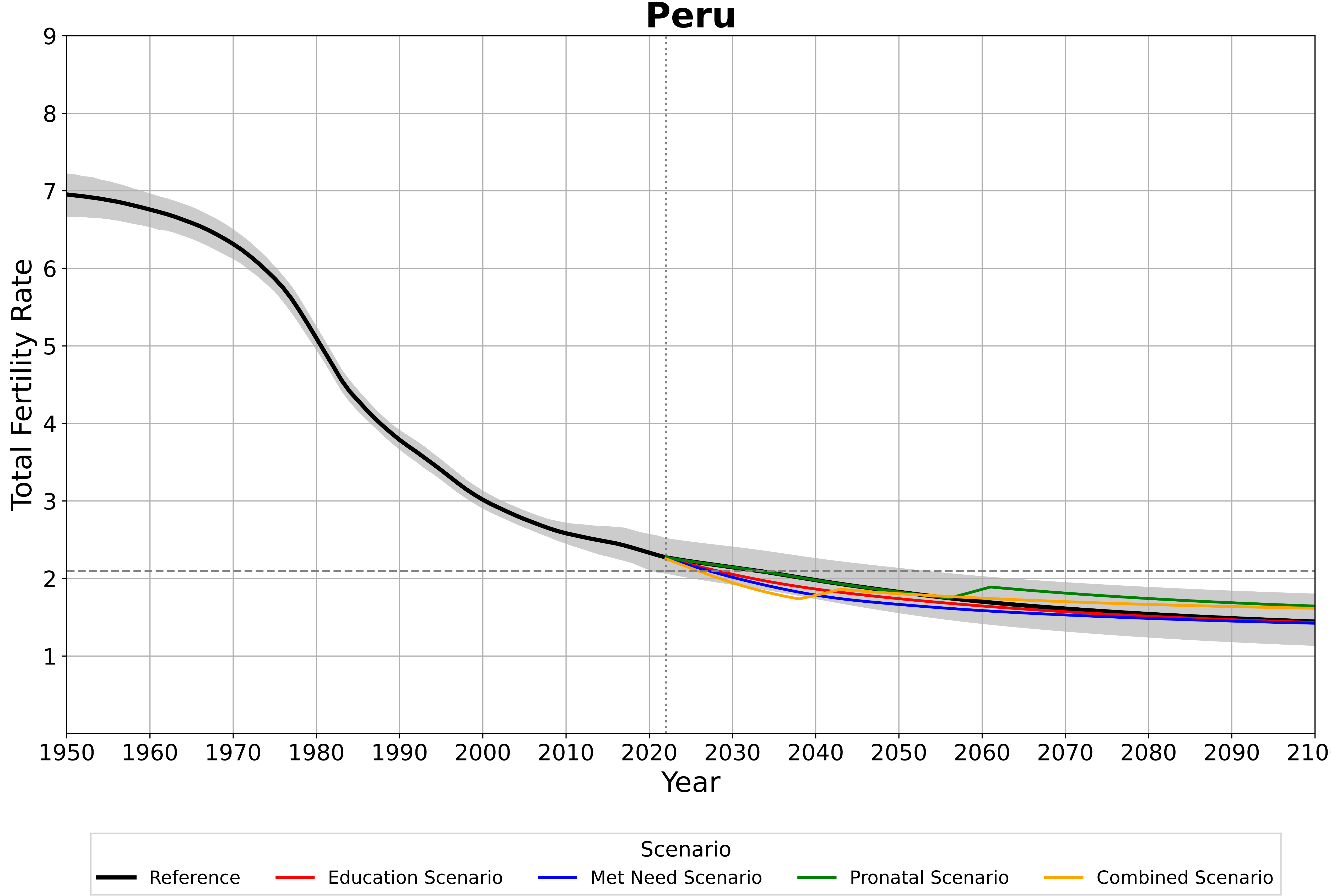

Supplemental Results Figure S5: TFR for reference and alternative scenario forecasts by location, 1950– 2100

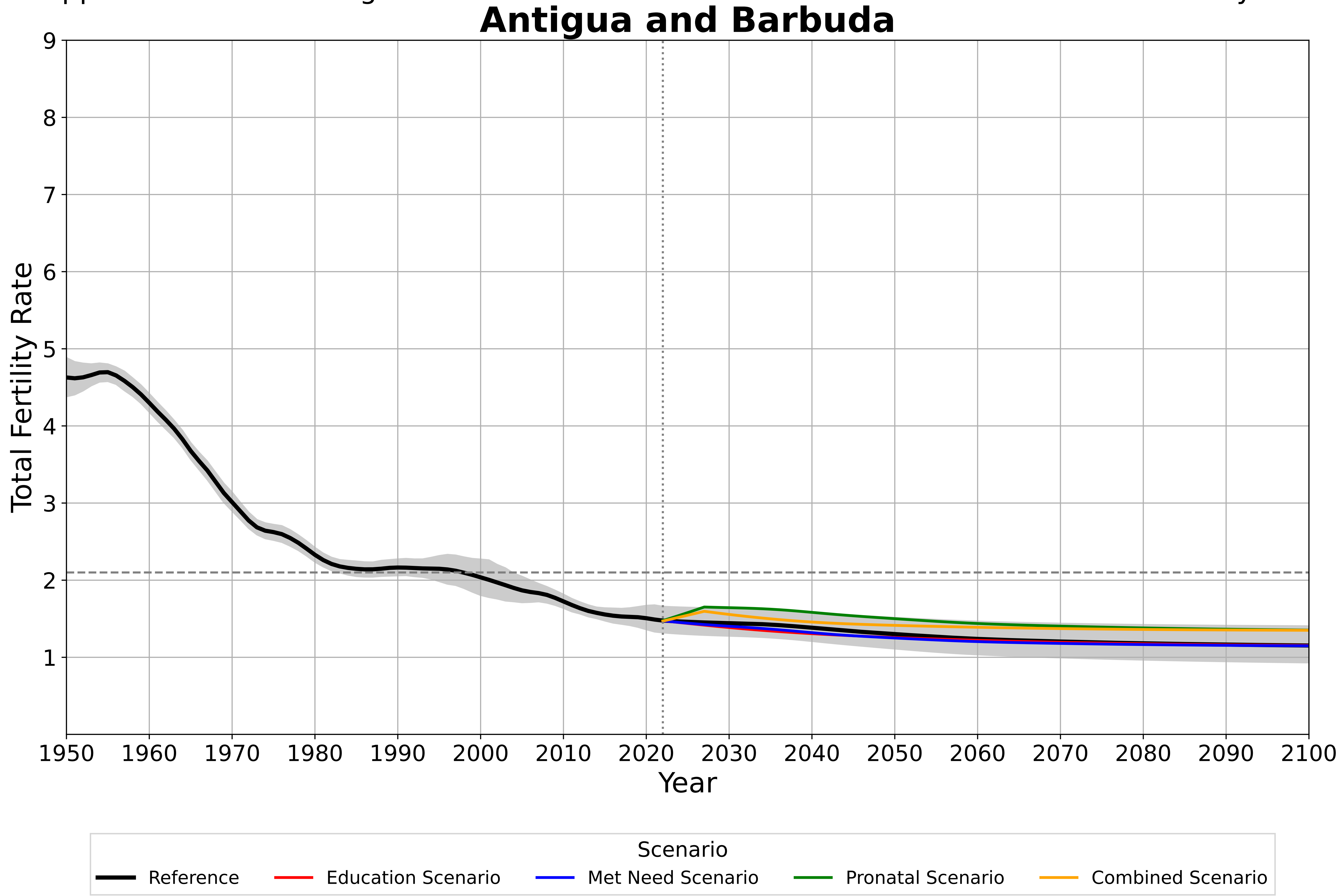

Supplemental Results Figure S5: TFR for reference and alternative scenario forecasts by location, 1950– 2100

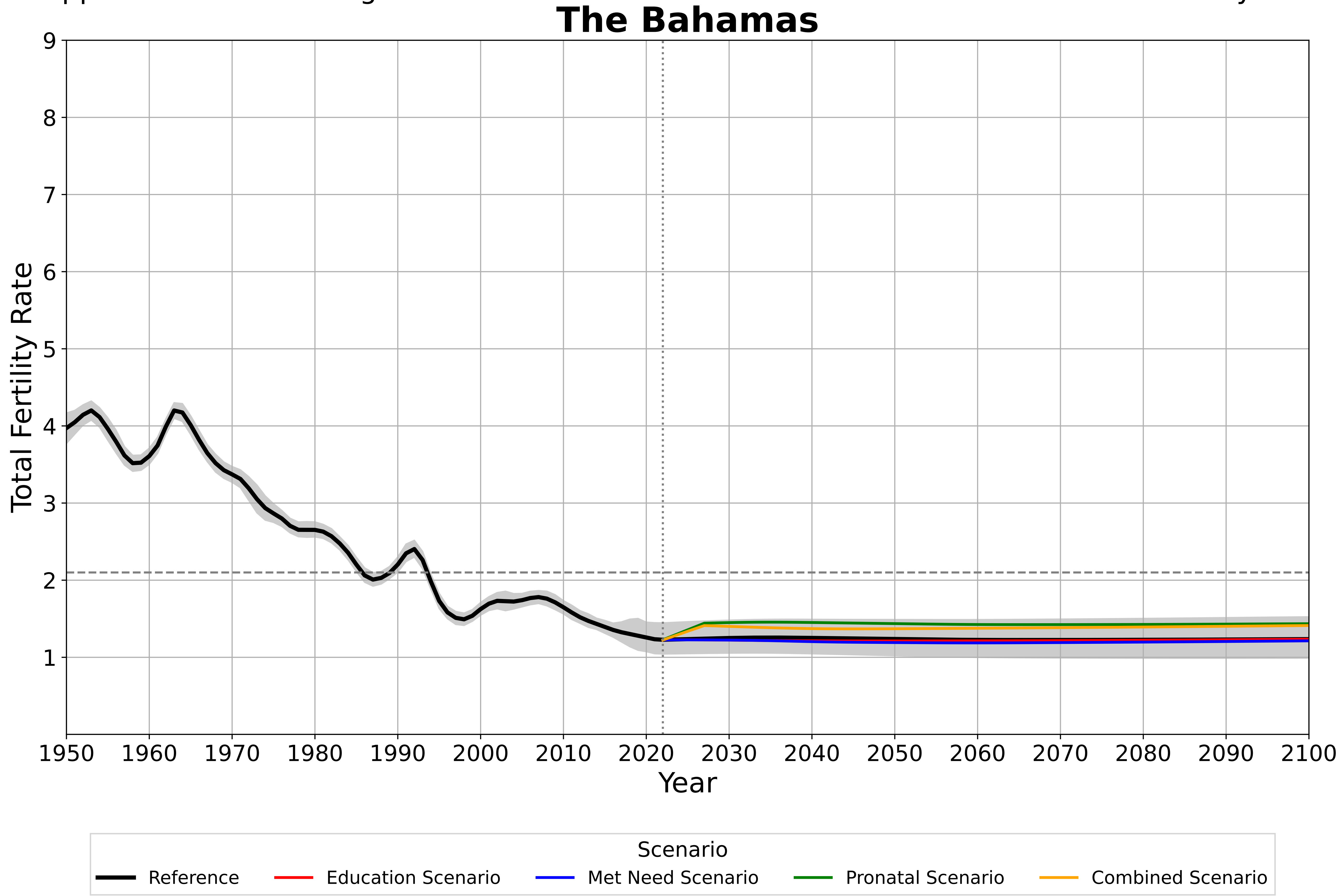

8289

Supplemental Results Figure S5: TFR for reference and alternative scenario forecasts by location, 1950– 2100

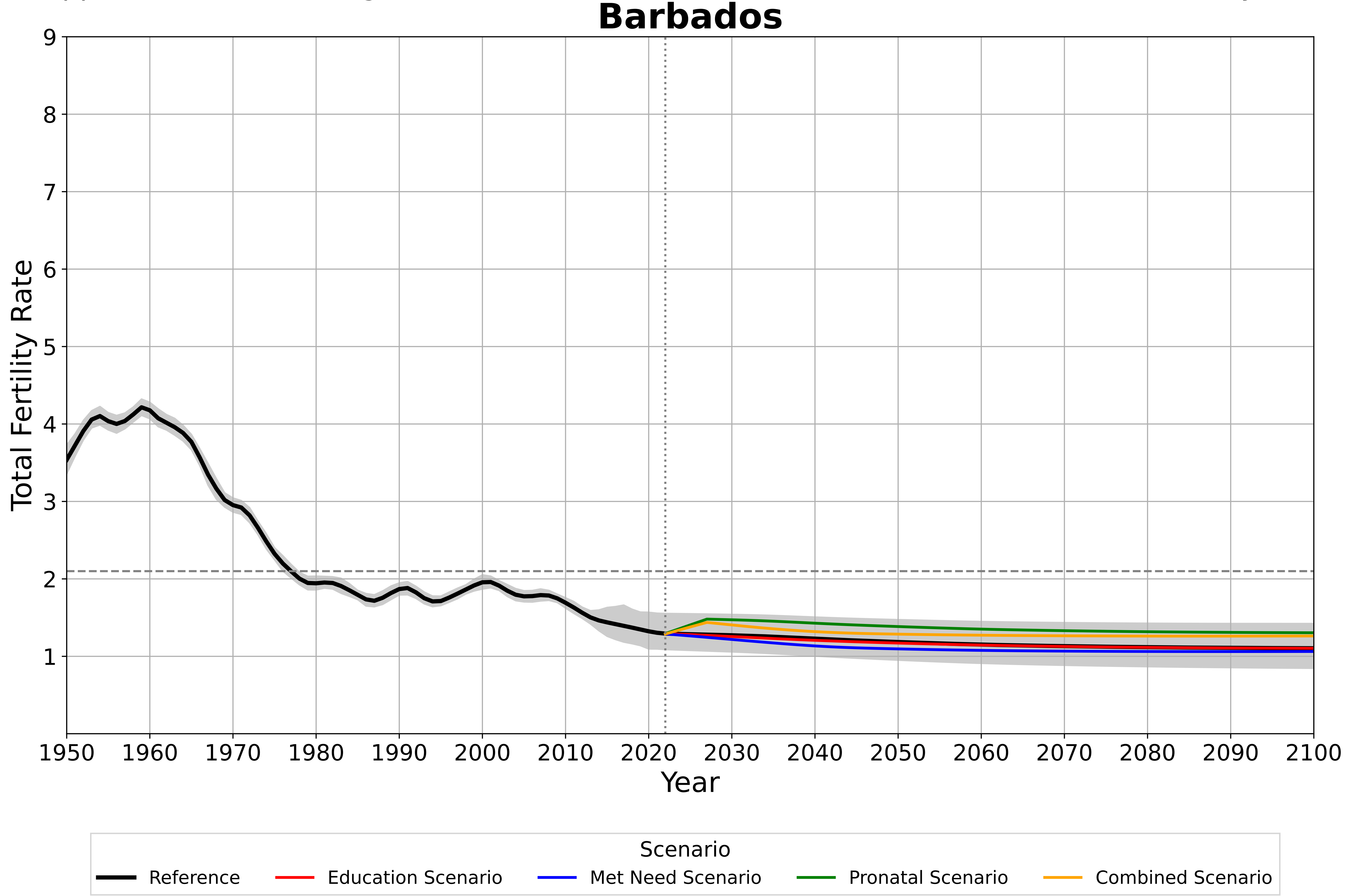

8290

Supplemental Results Figure S5: TFR for reference and alternative scenario forecasts by location, 1950– 2100

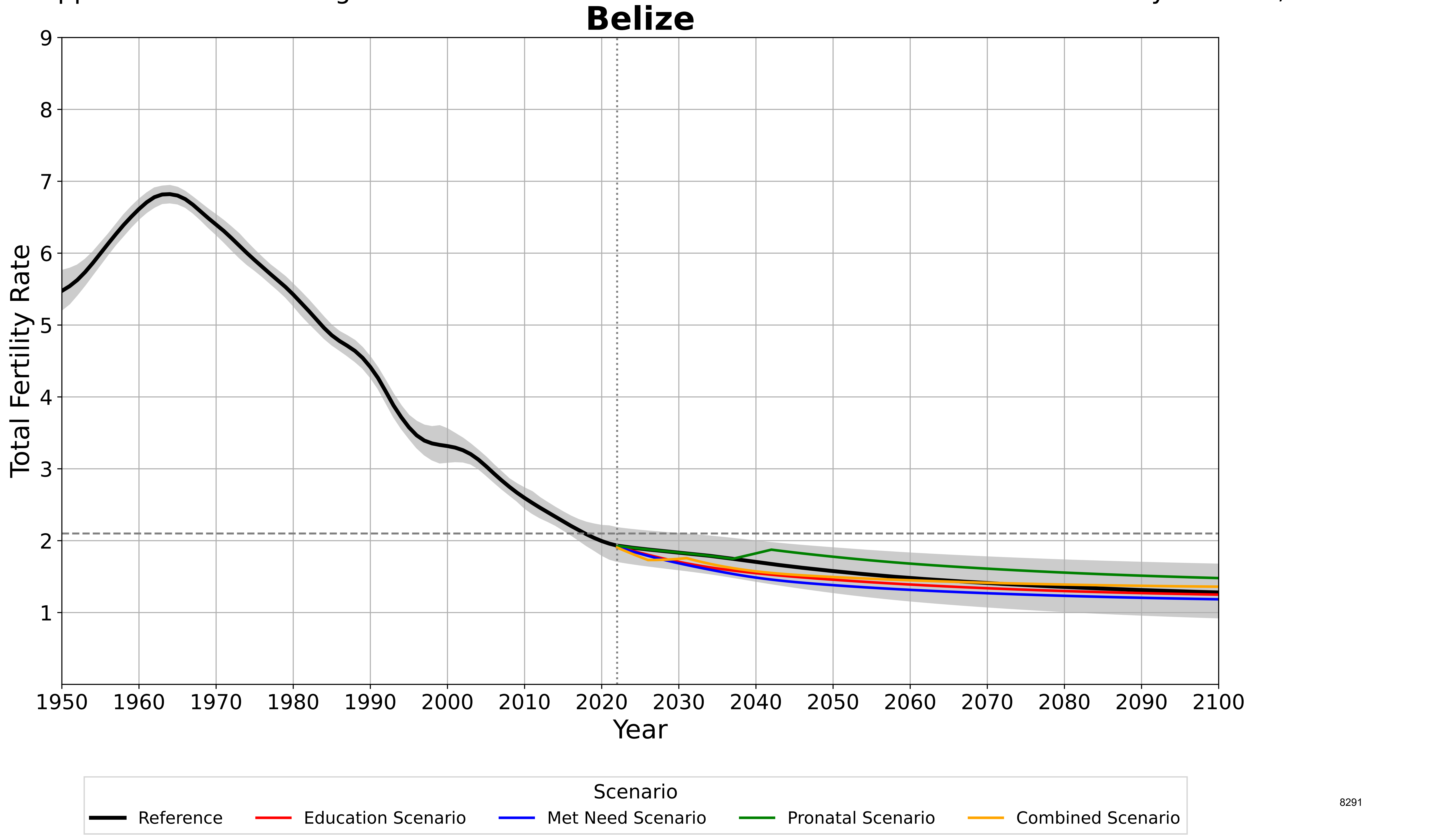

Supplemental Results Figure S5: TFR for reference and alternative scenario forecasts by location, 1950– 2100

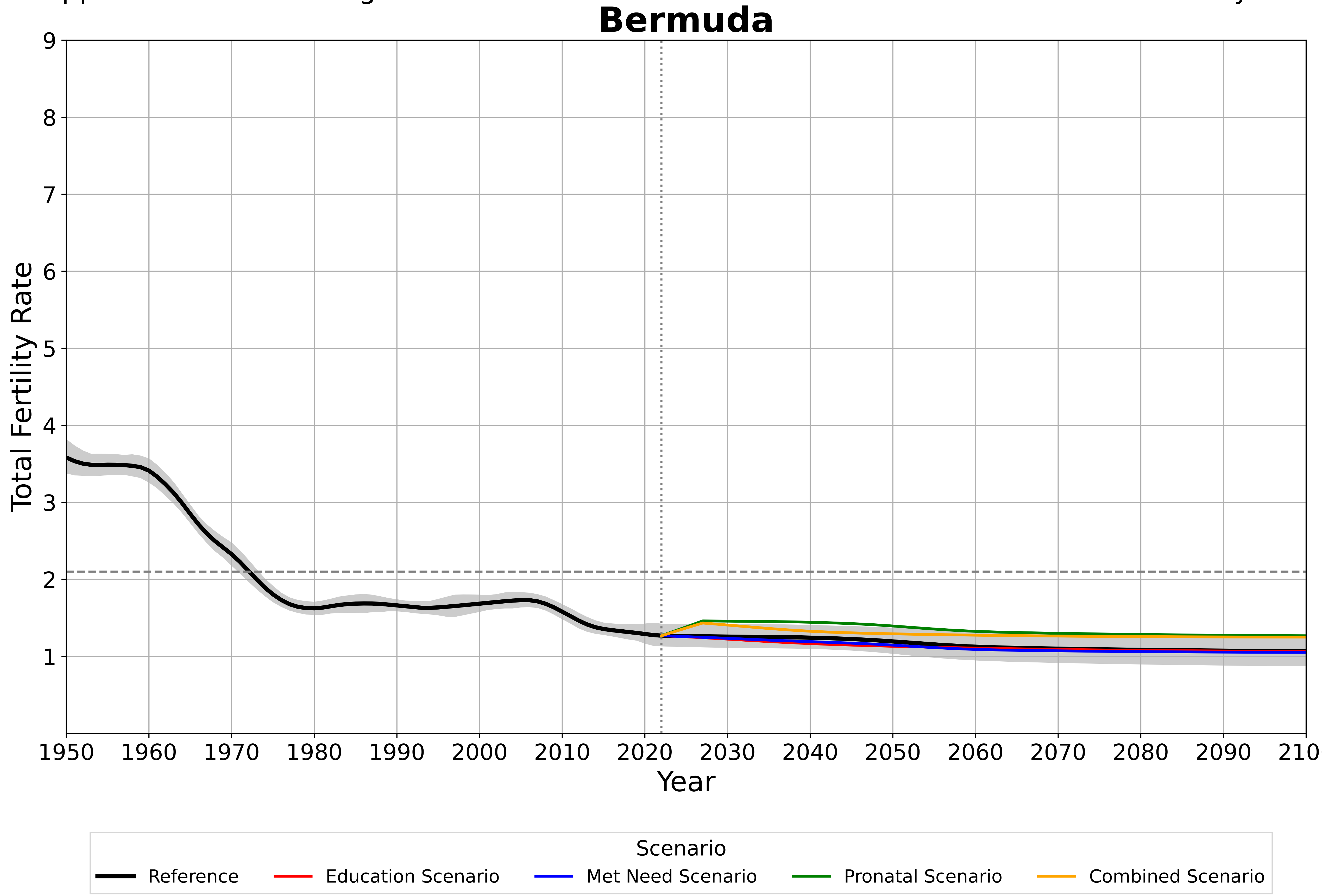

8292

Supplemental Results Figure S5: TFR for reference and alternative scenario forecasts by location, 1950– 2100

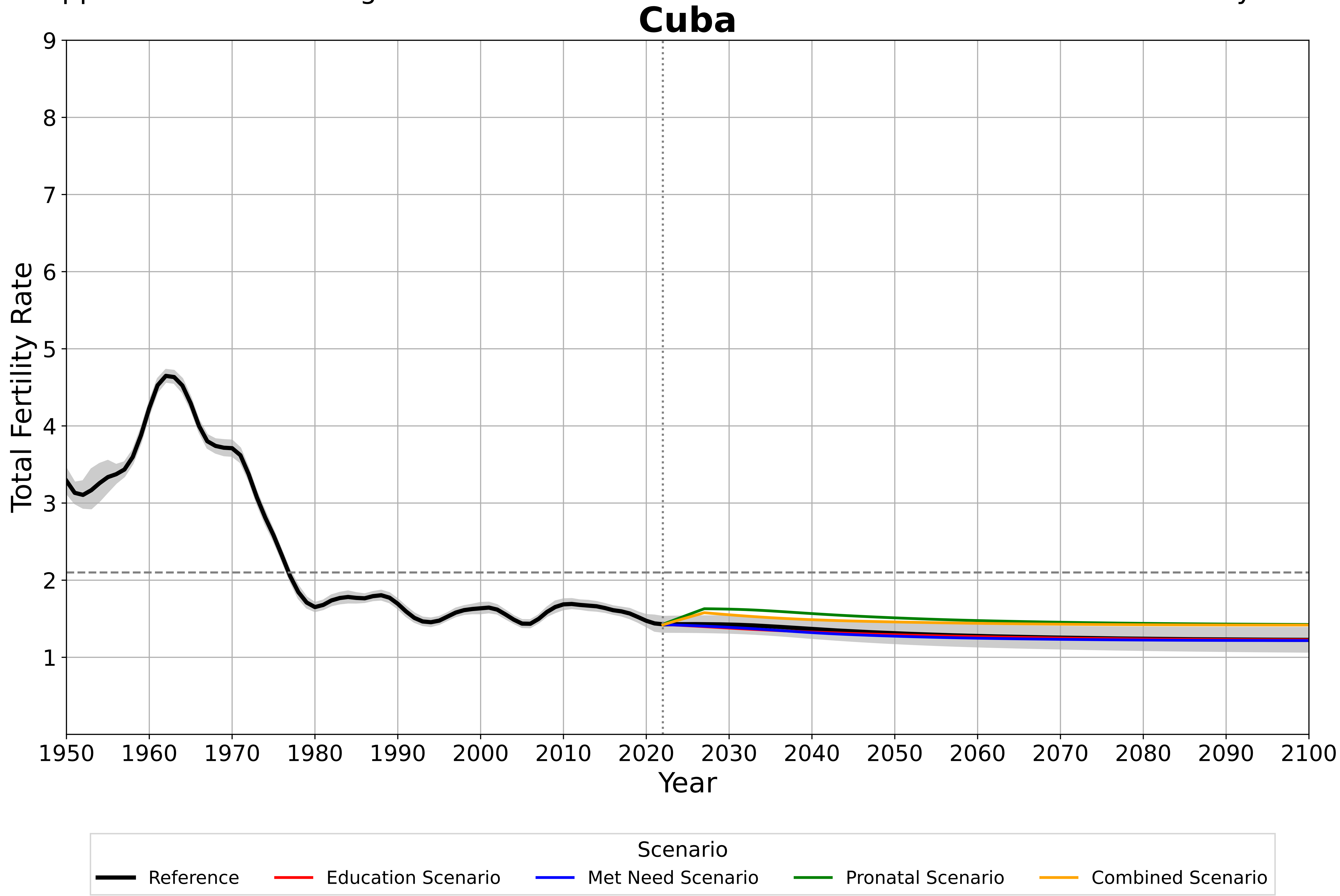

Supplemental Results Figure S5: TFR for reference and alternative scenario forecasts by location, 1950– 2100

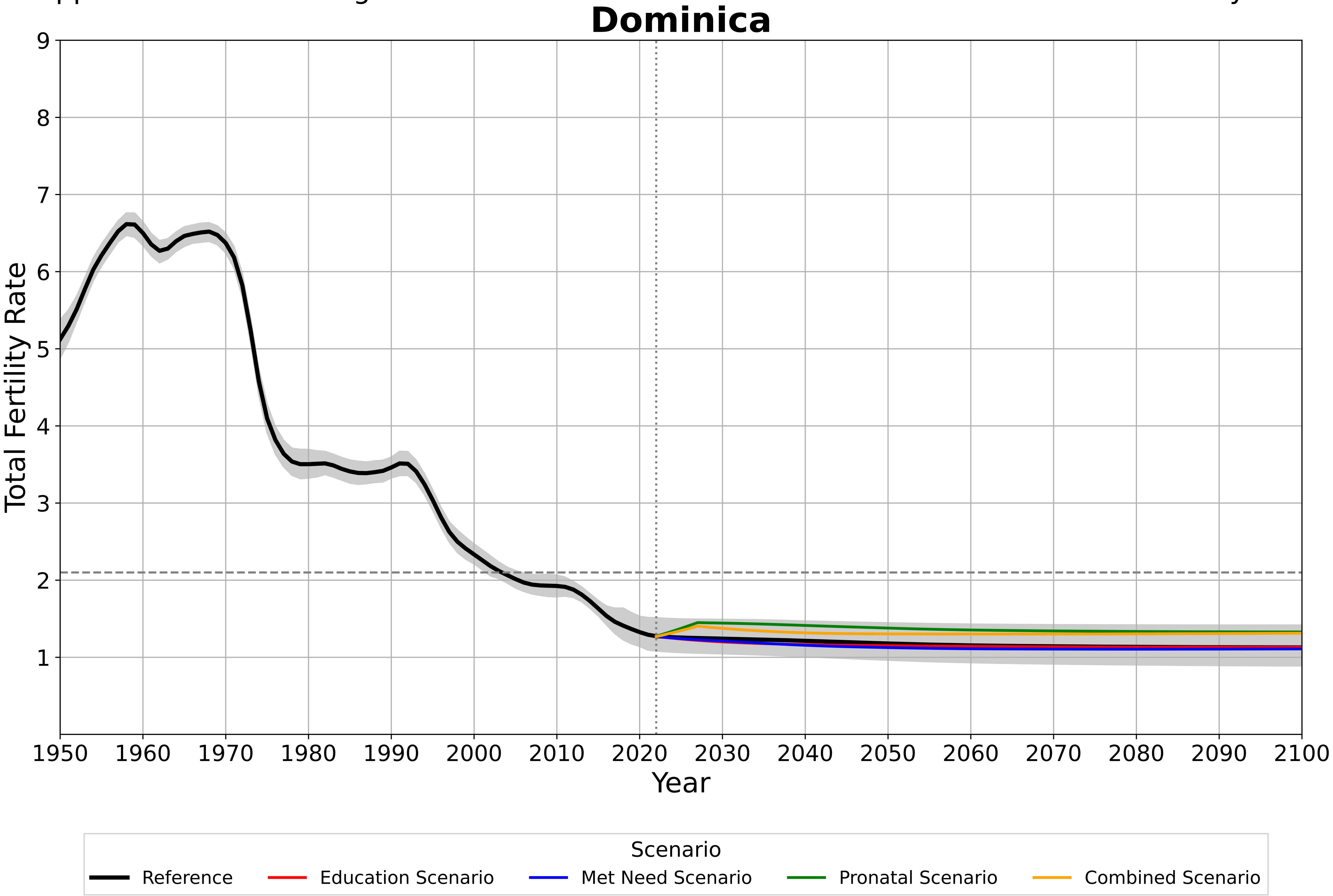

Supplemental Results Figure S5: TFR for reference and alternative scenario forecasts by location, 1950– 2100

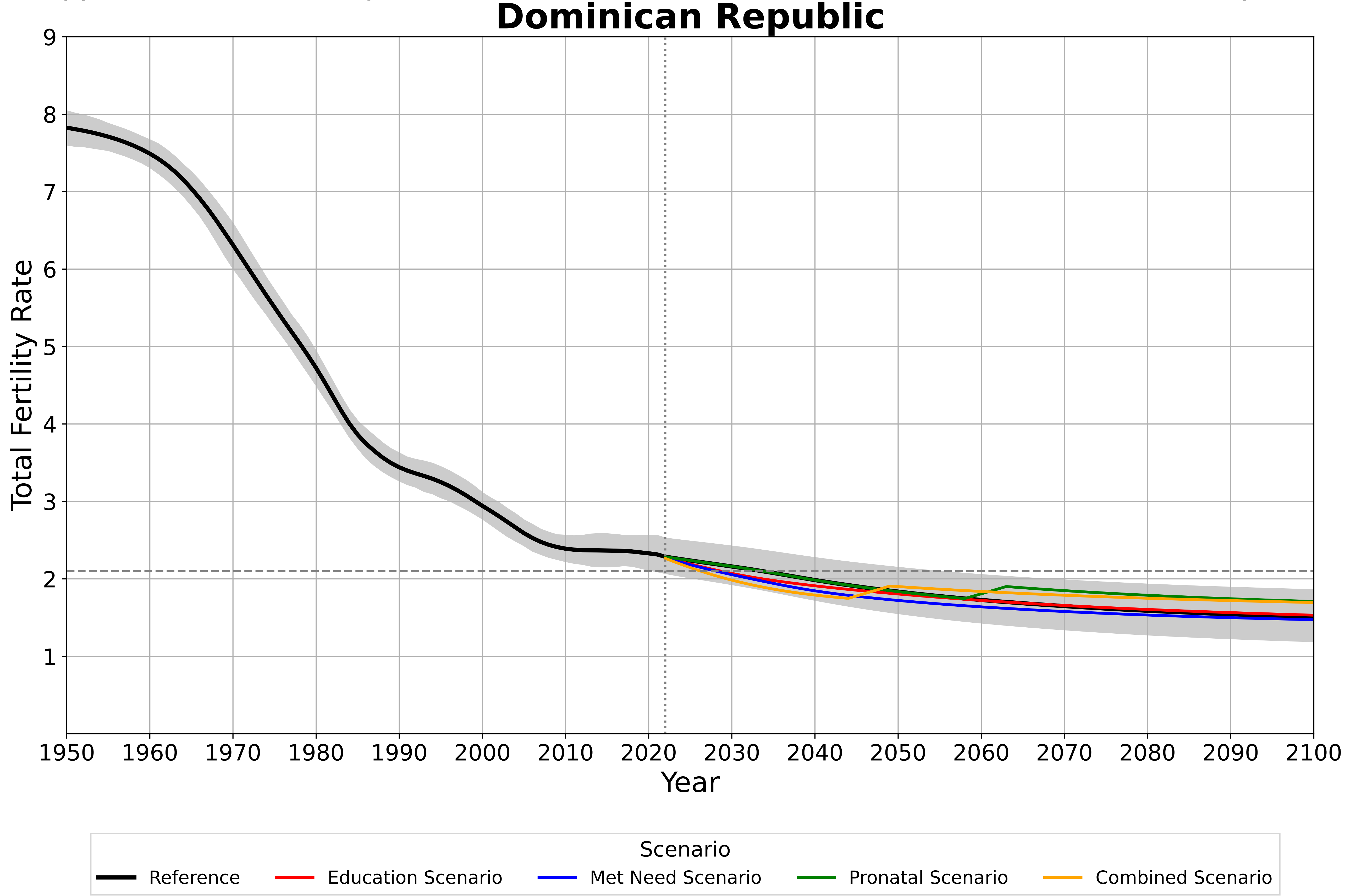

Supplemental Results Figure S5: TFR for reference and alternative scenario forecasts by location, 1950– 2100

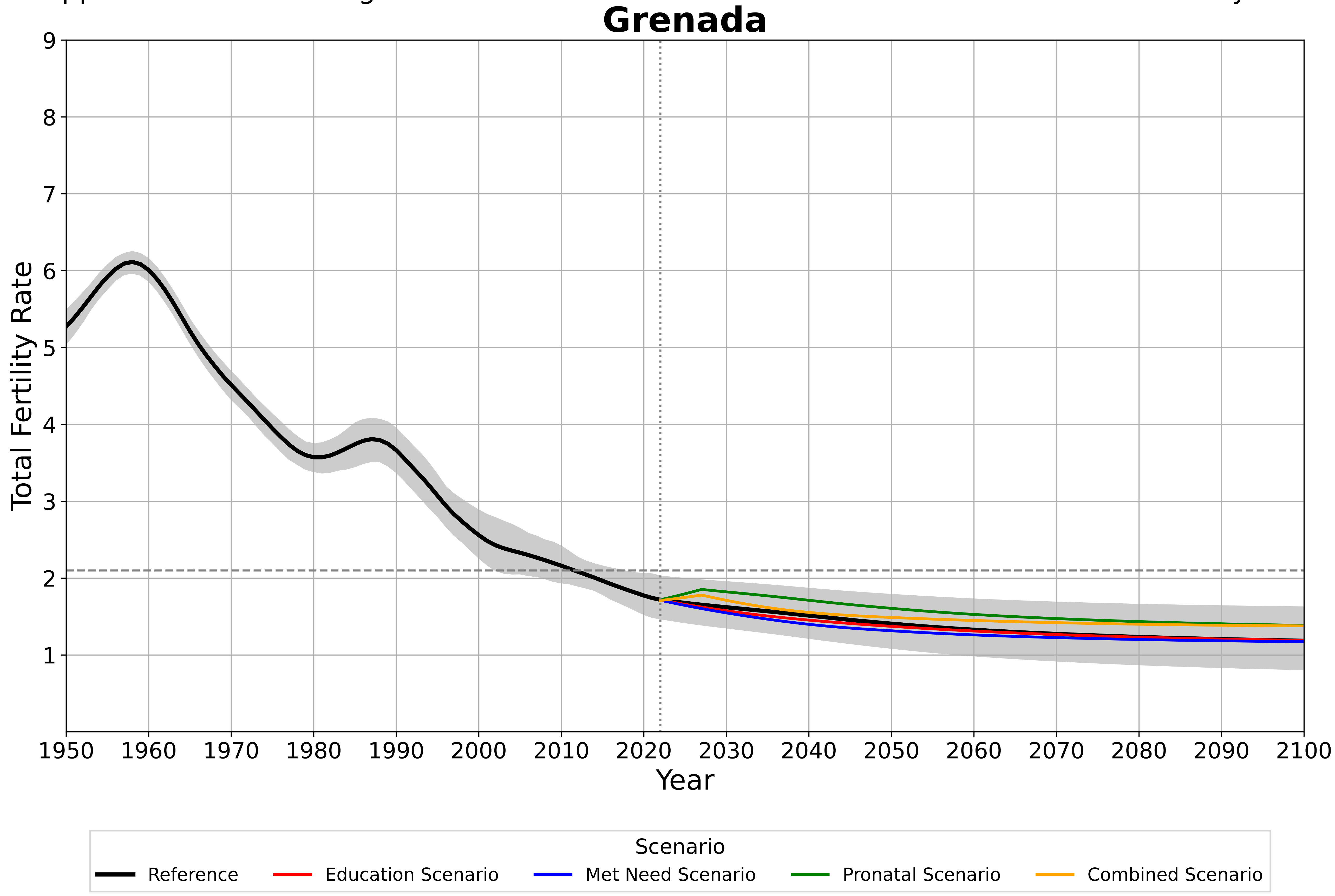

8296

Supplemental Results Figure S5: TFR for reference and alternative scenario forecasts by location, 1950– 2100

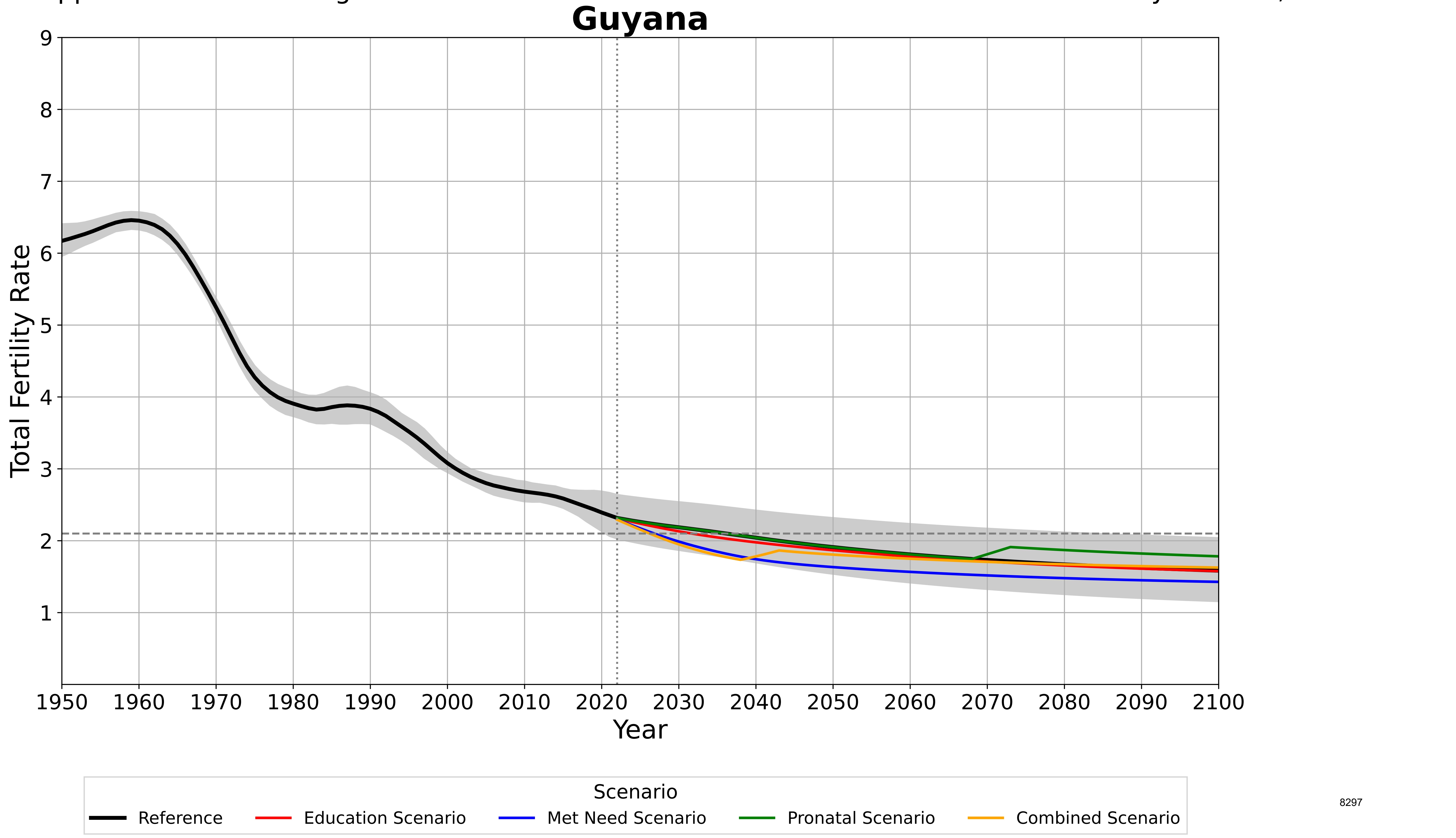

Supplemental Results Figure S5: TFR for reference and alternative scenario forecasts by location, 1950– 2100

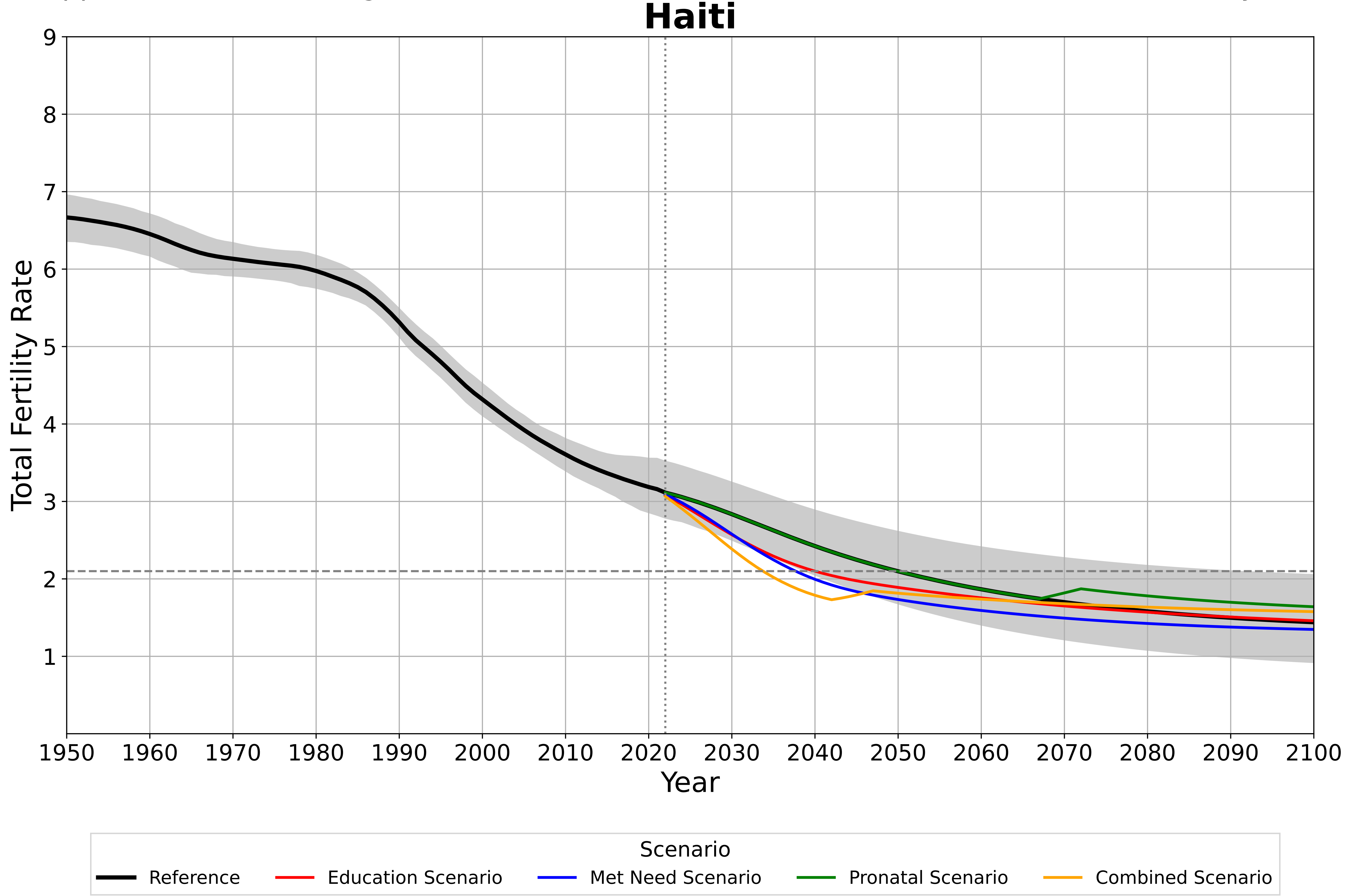

8298

Supplemental Results Figure S5: TFR for reference and alternative scenario forecasts by location, 1950– 2100

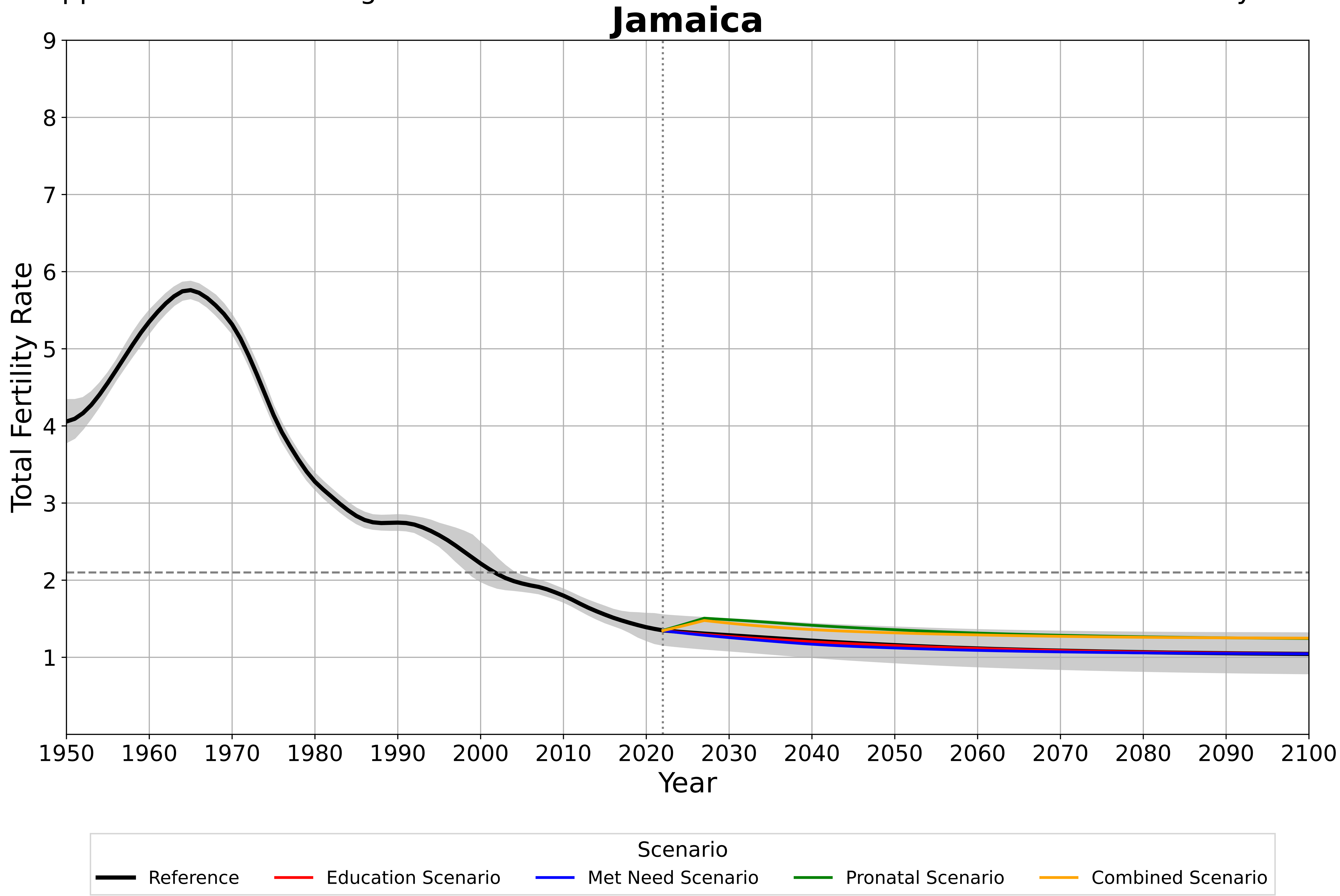

Supplemental Results Figure S5: TFR for reference and alternative scenario forecasts by location, 1950– 2100

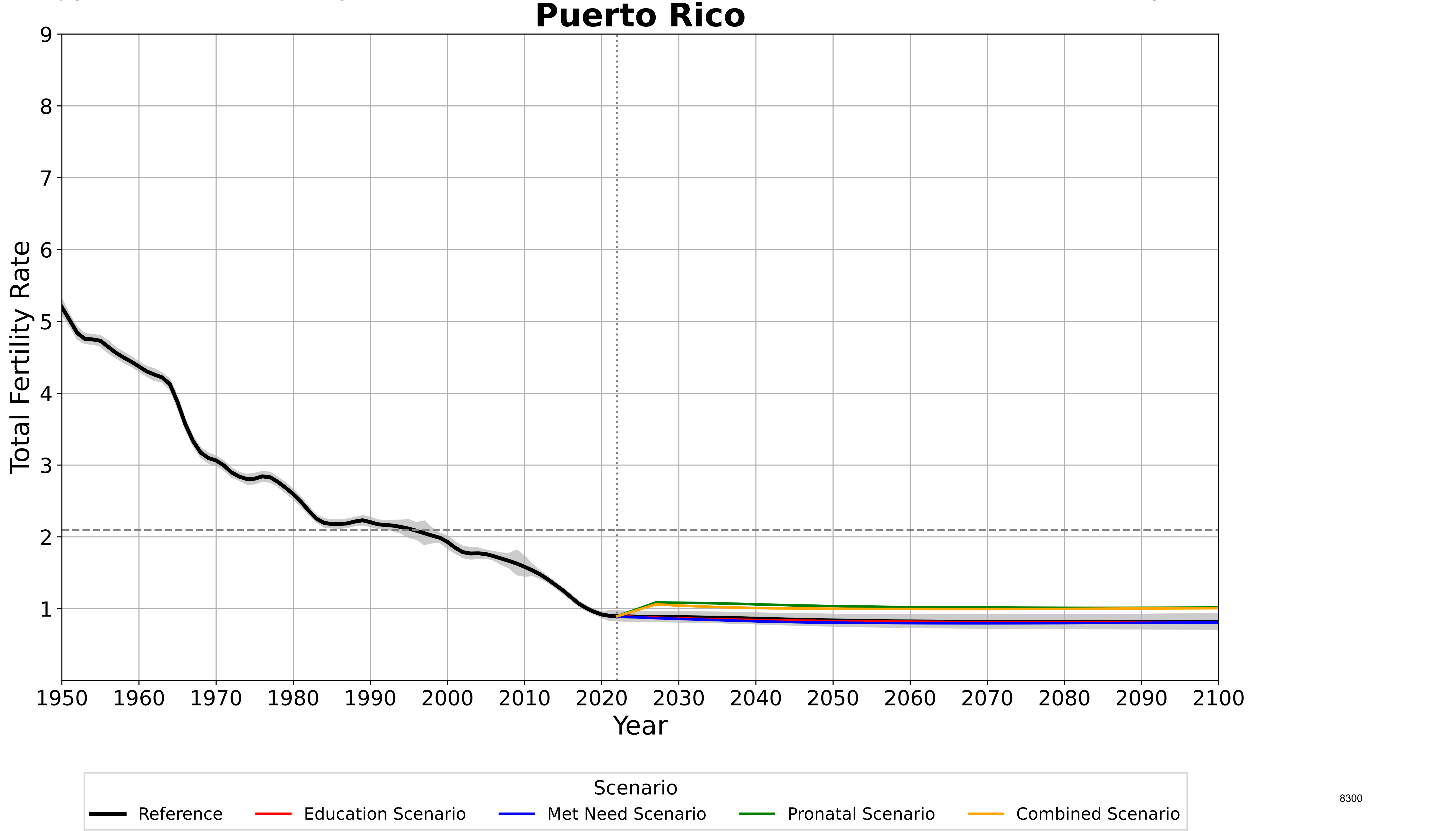

Supplemental Results Figure S5: TFR for reference and alternative scenario forecasts by location, 1950– 2100

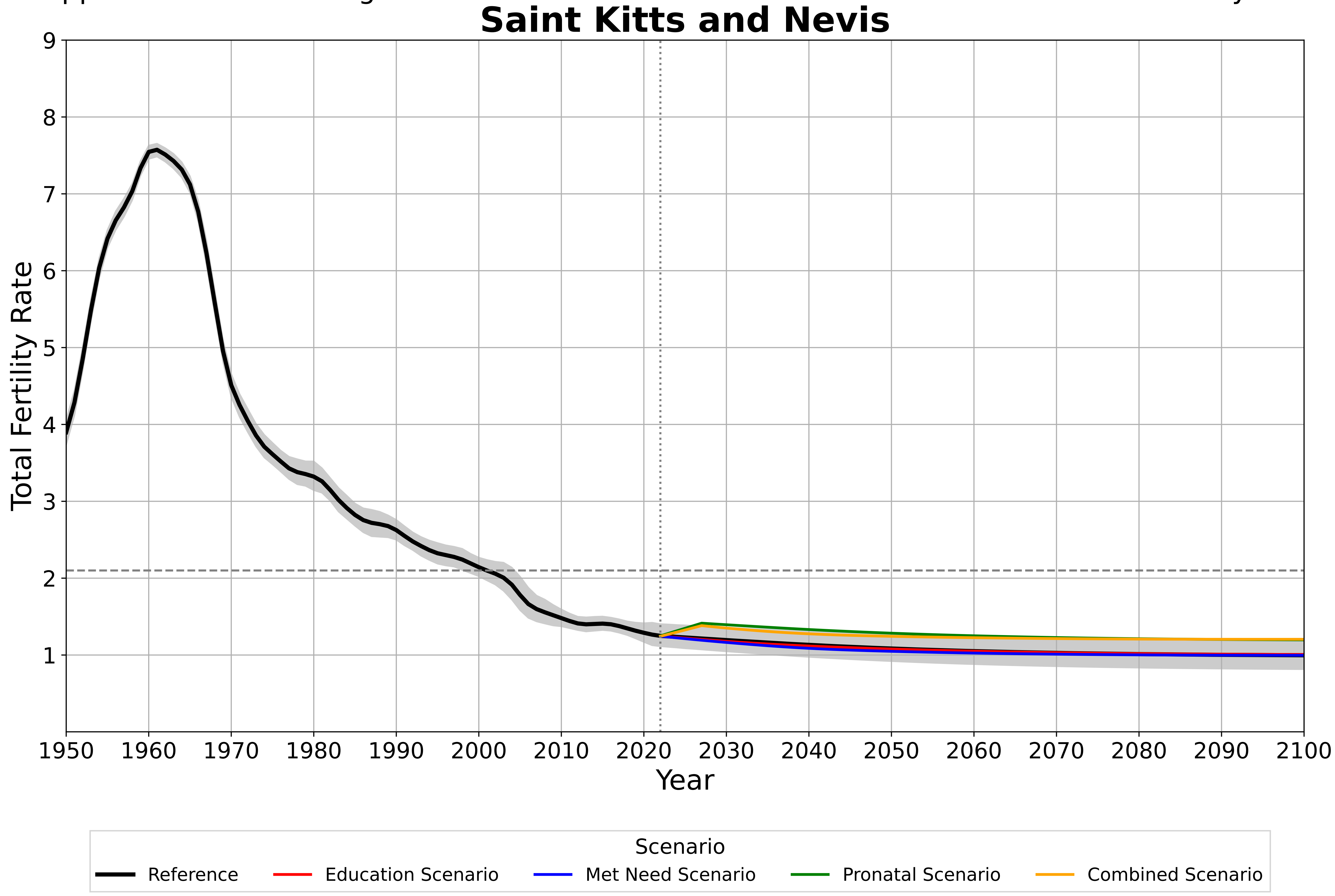

8301

Supplemental Results Figure S5: TFR for reference and alternative scenario forecasts by location, 1950– 2100

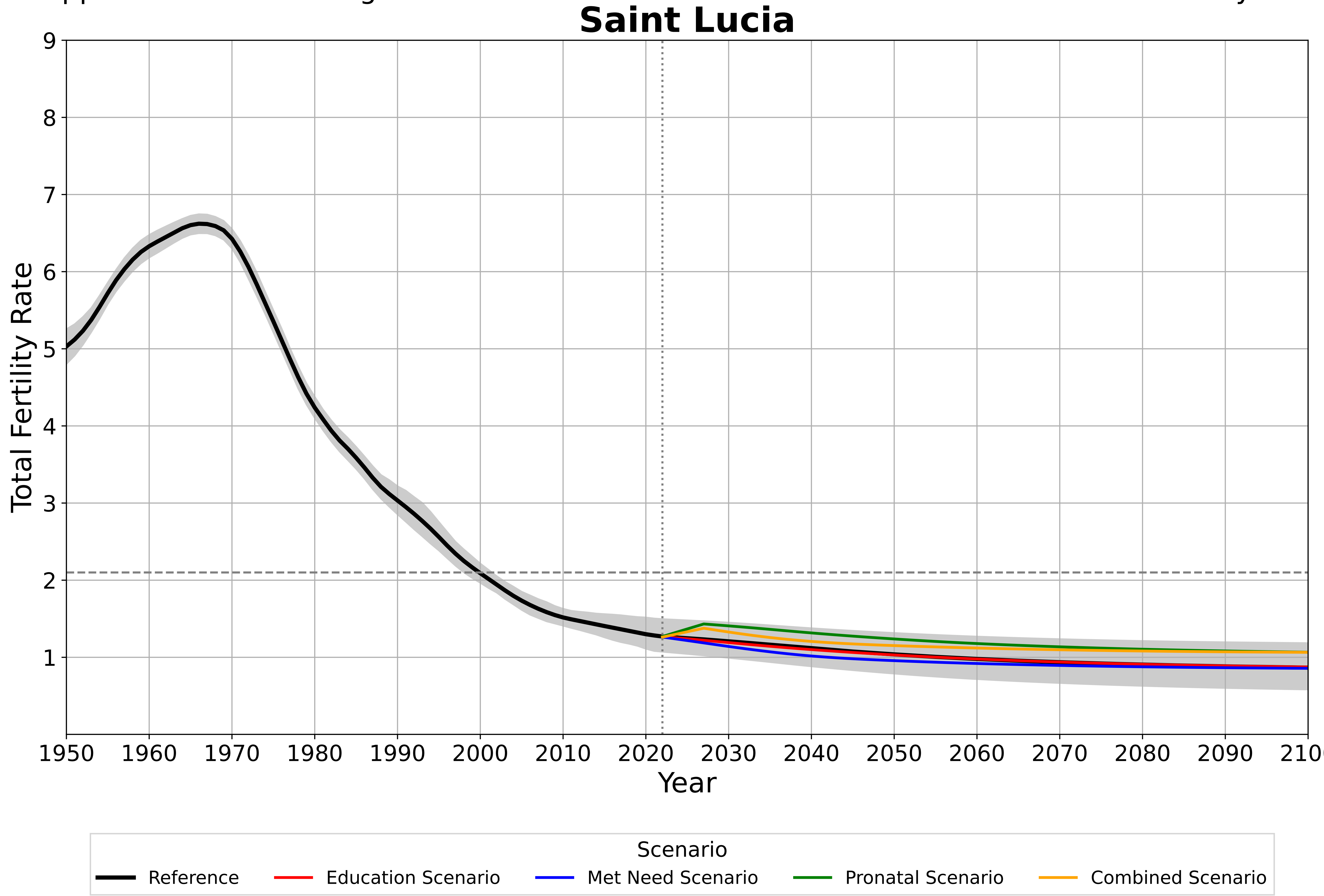

Supplemental Results Figure S5: TFR for reference and alternative scenario forecasts by location, 1950– 2100

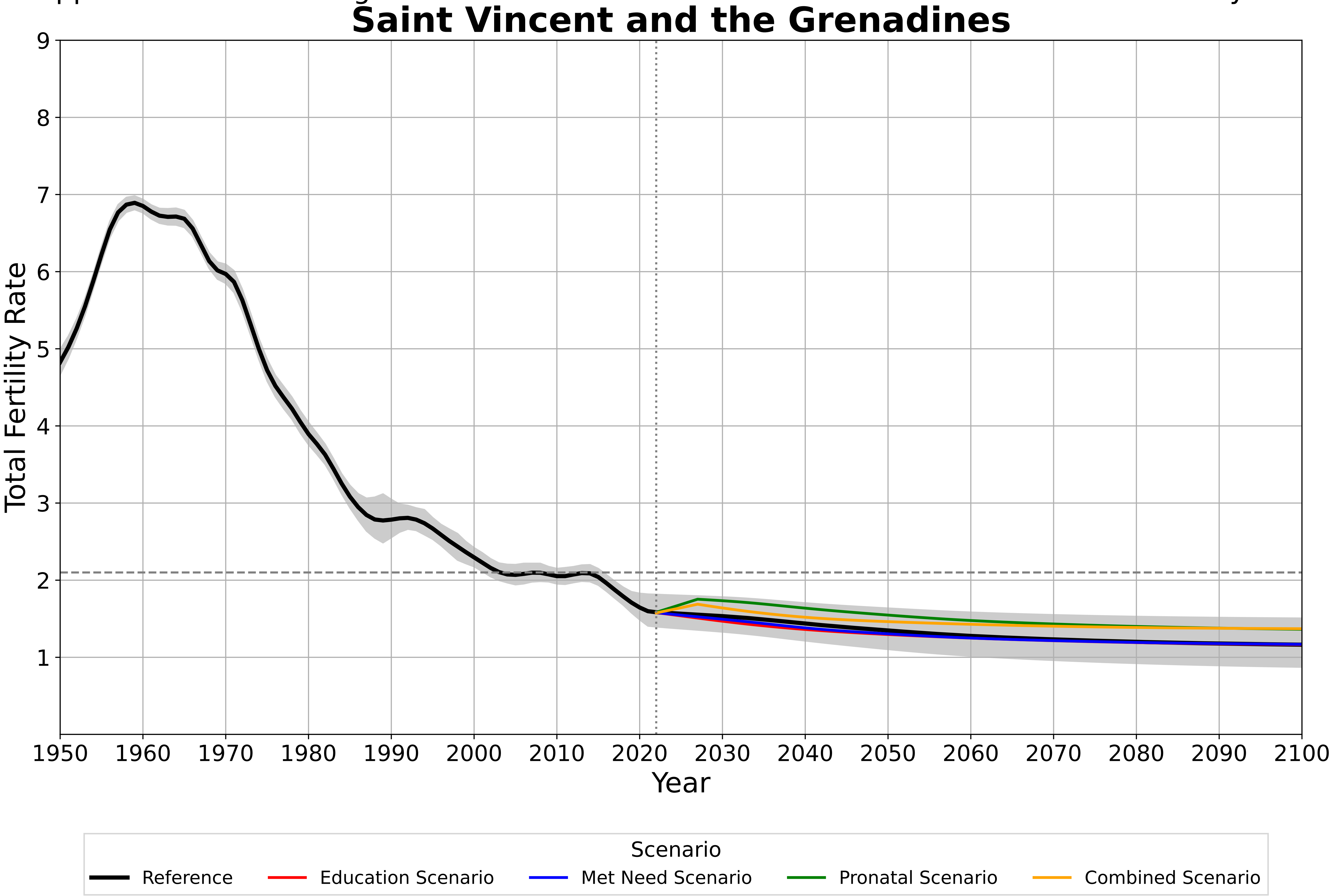

Supplemental Results Figure S5: TFR for reference and alternative scenario forecasts by location, 1950– 2100

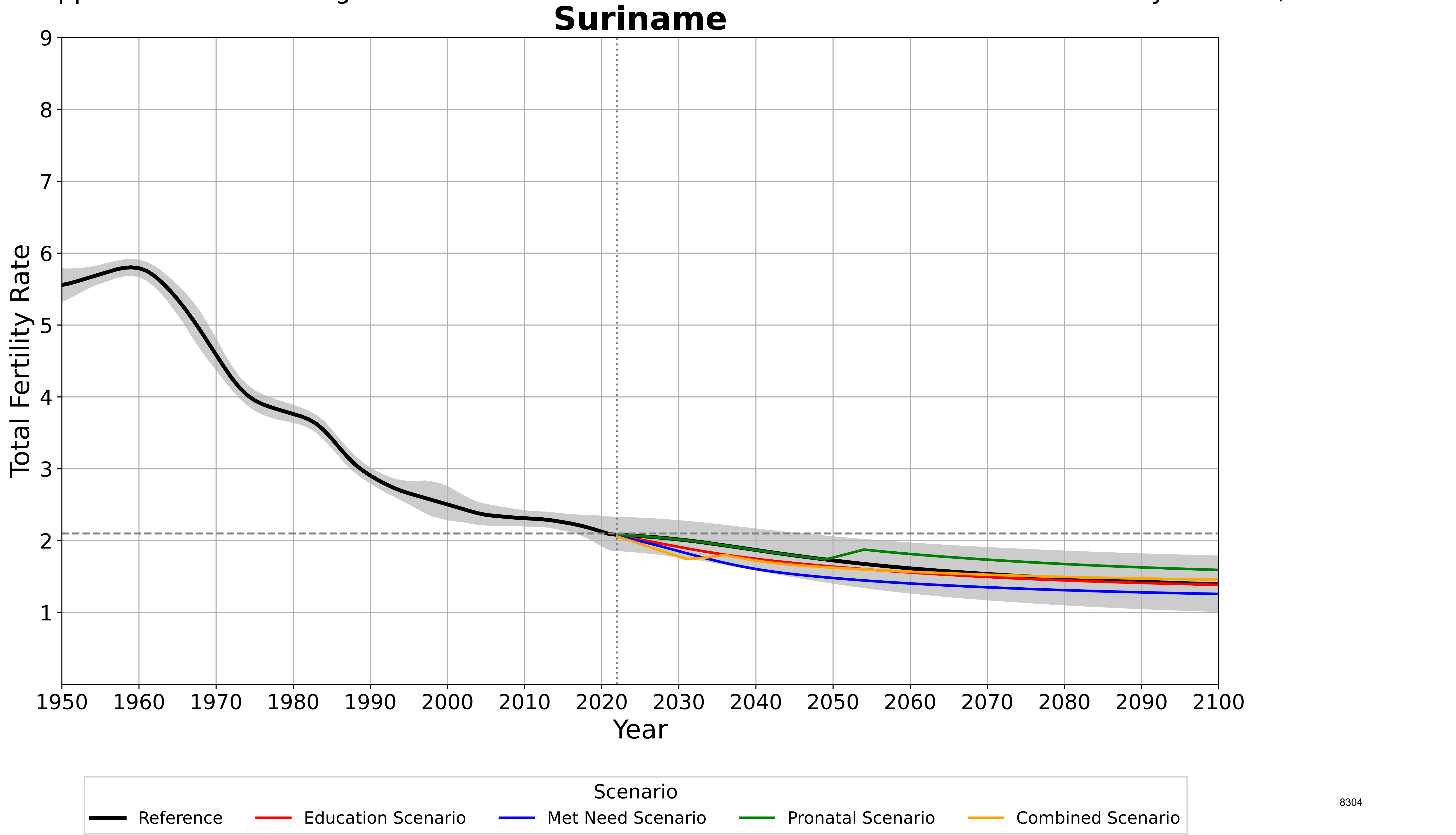

8304

Supplemental Results Figure S5: TFR for reference and alternative scenario forecasts by location, 1950– 2100

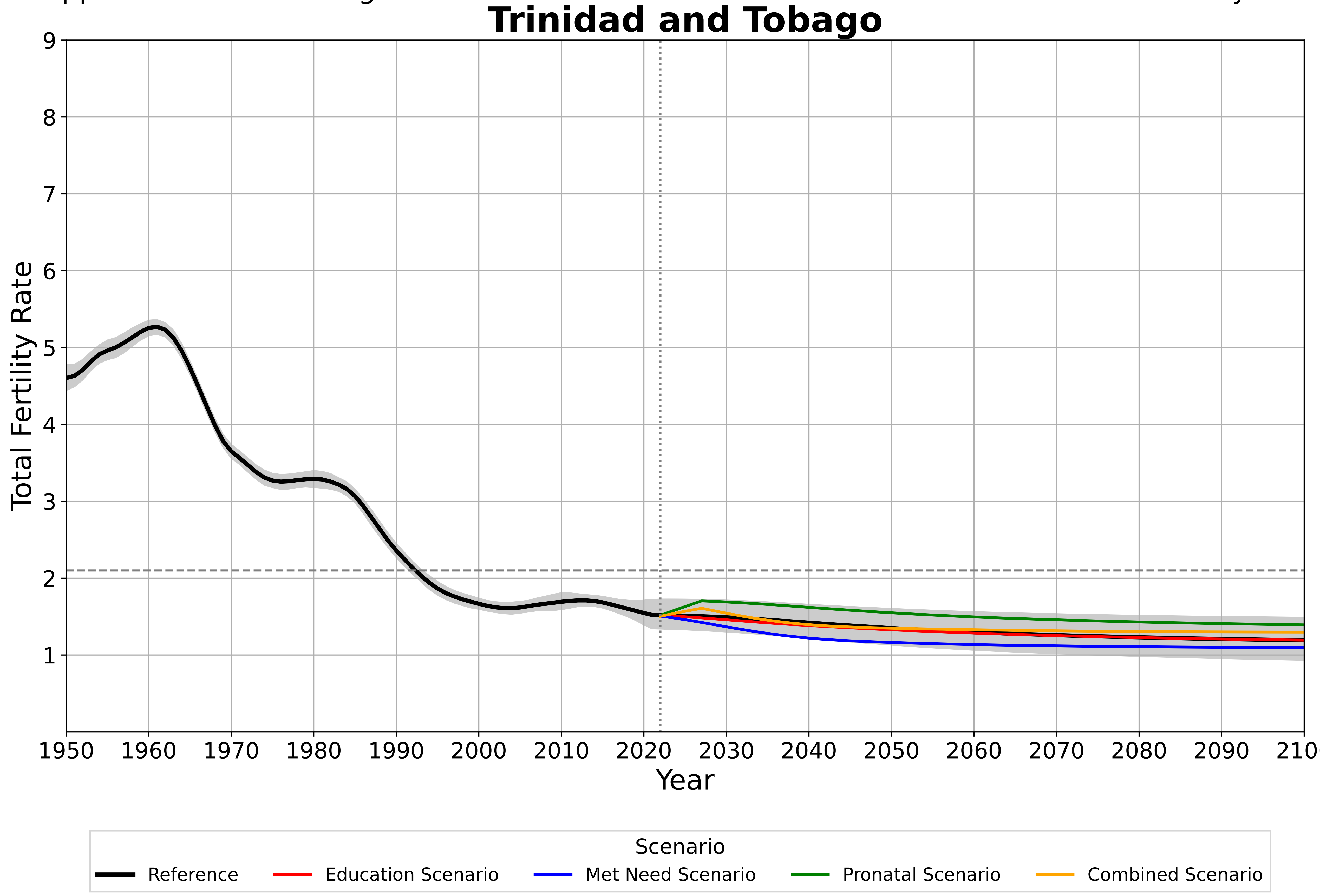

8305

Supplemental Results Figure S5: TFR for reference and alternative scenario forecasts by location, 1950– 2100

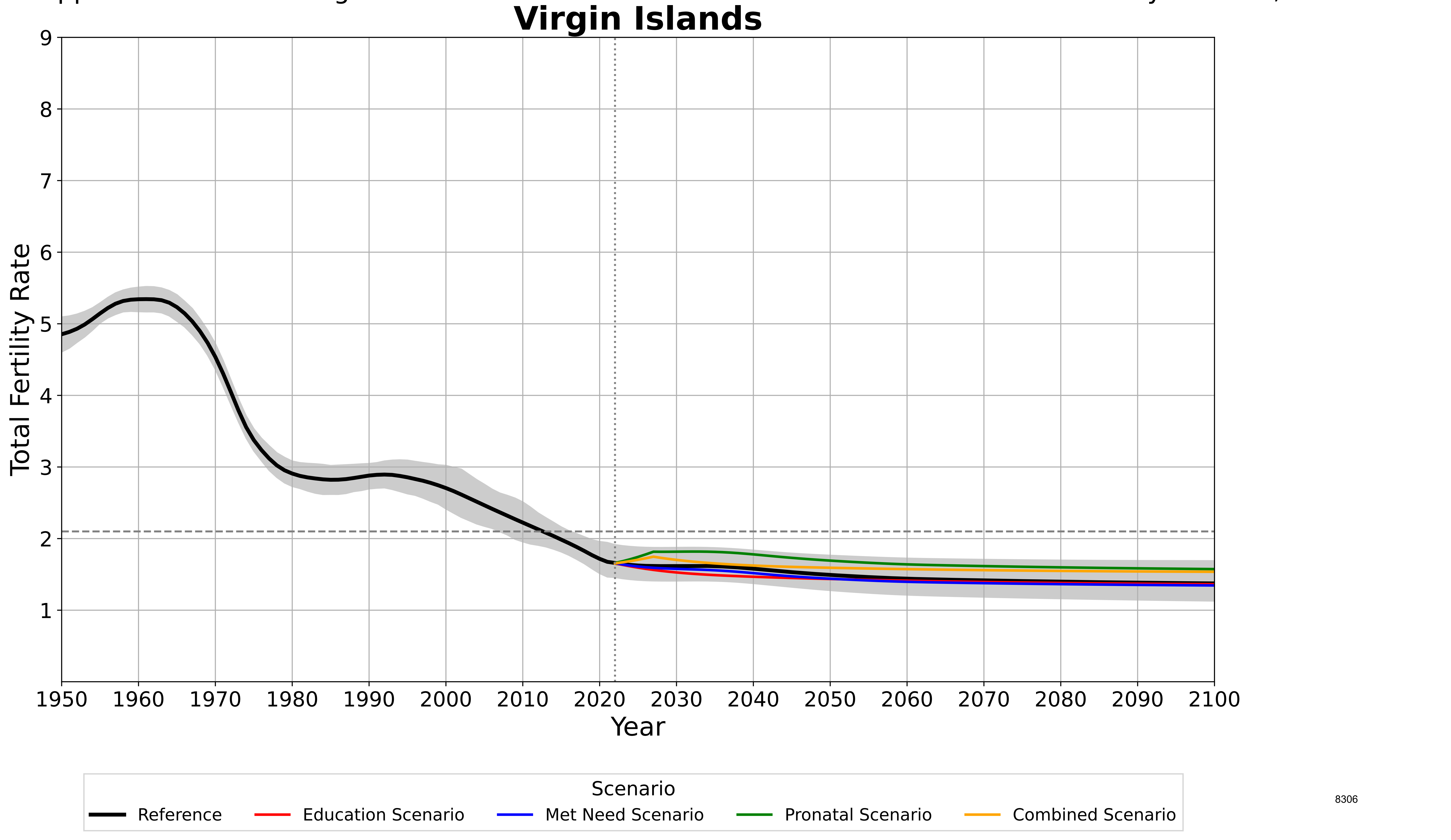

Supplemental Results Figure S5: TFR for reference and alternative scenario forecasts by location, 1950– 2100

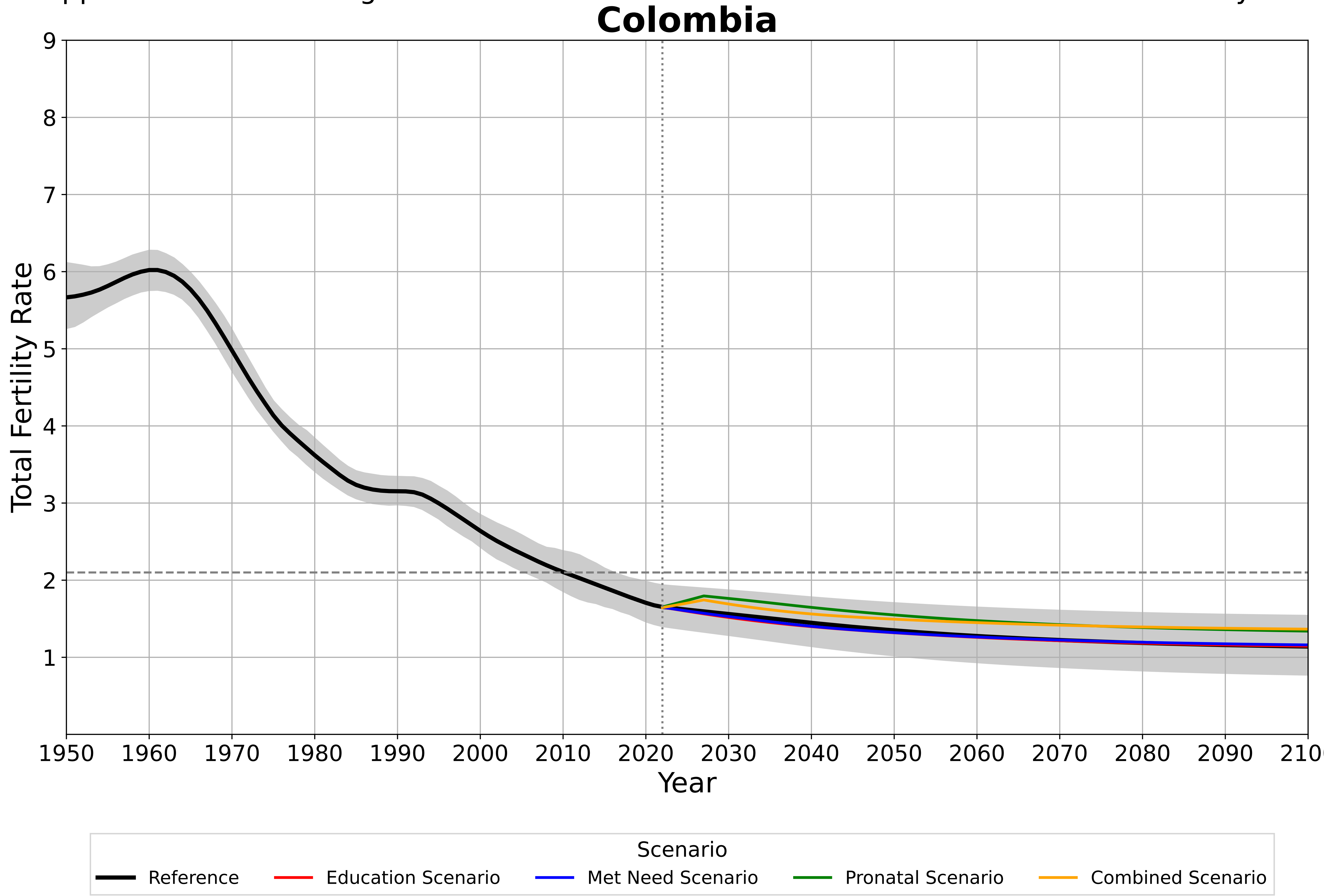

Supplemental Results Figure S5: TFR for reference and alternative scenario forecasts by location, 1950– 2100

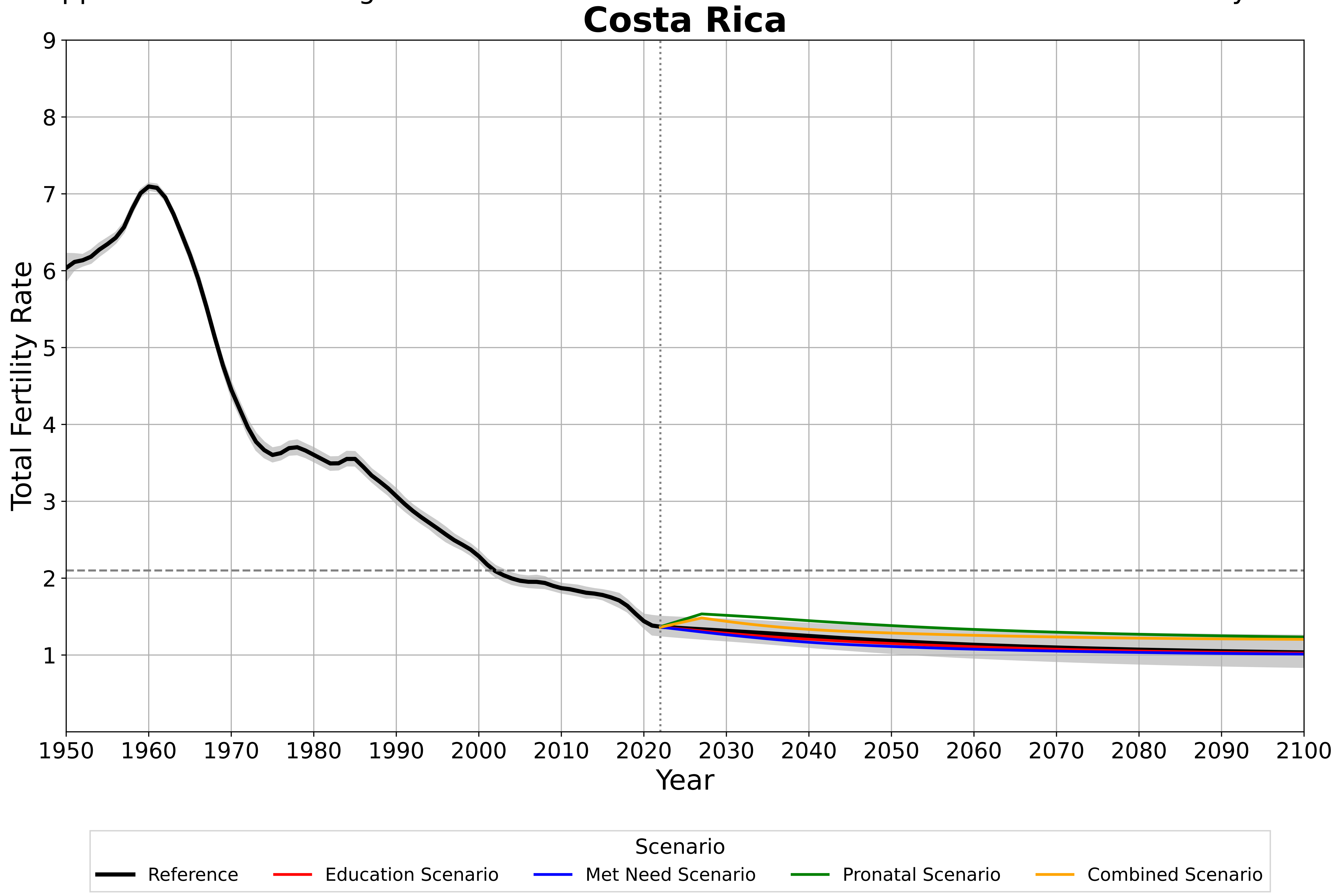

Supplemental Results Figure S5: TFR for reference and alternative scenario forecasts by location, 1950– 2100

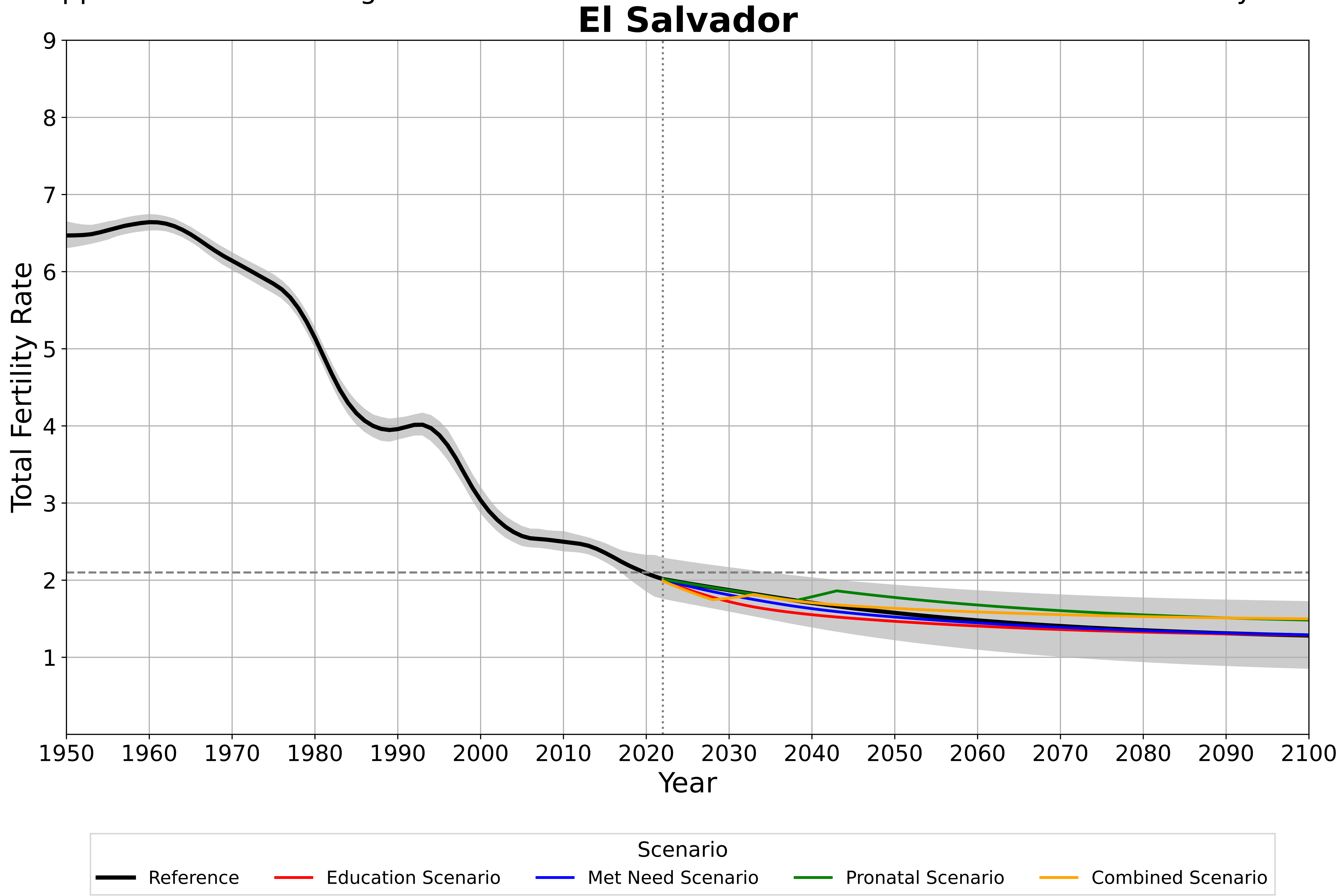

Supplemental Results Figure S5: TFR for reference and alternative scenario forecasts by location, 1950– 2100

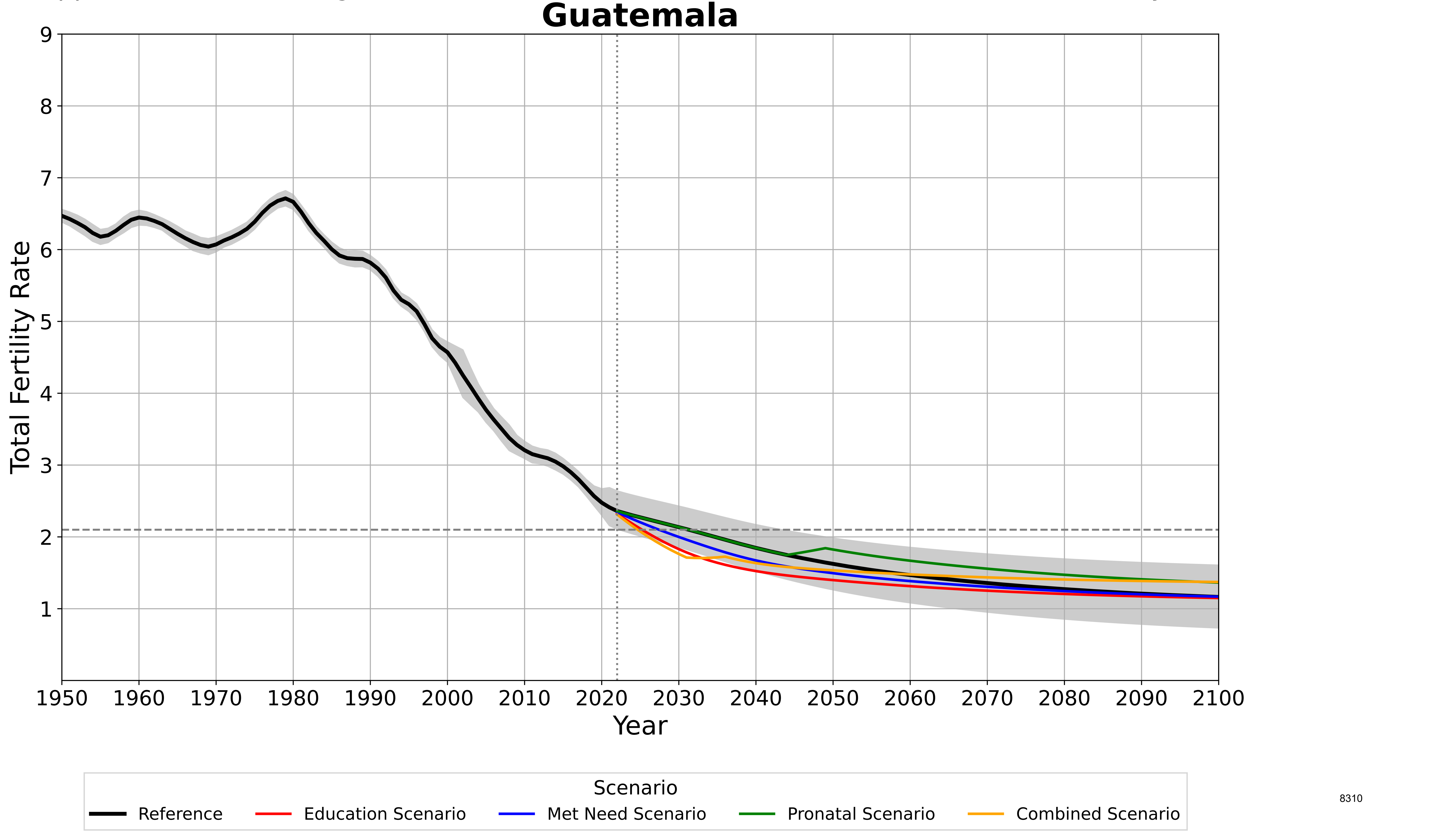

Supplemental Results Figure S5: TFR for reference and alternative scenario forecasts by location, 1950– 2100

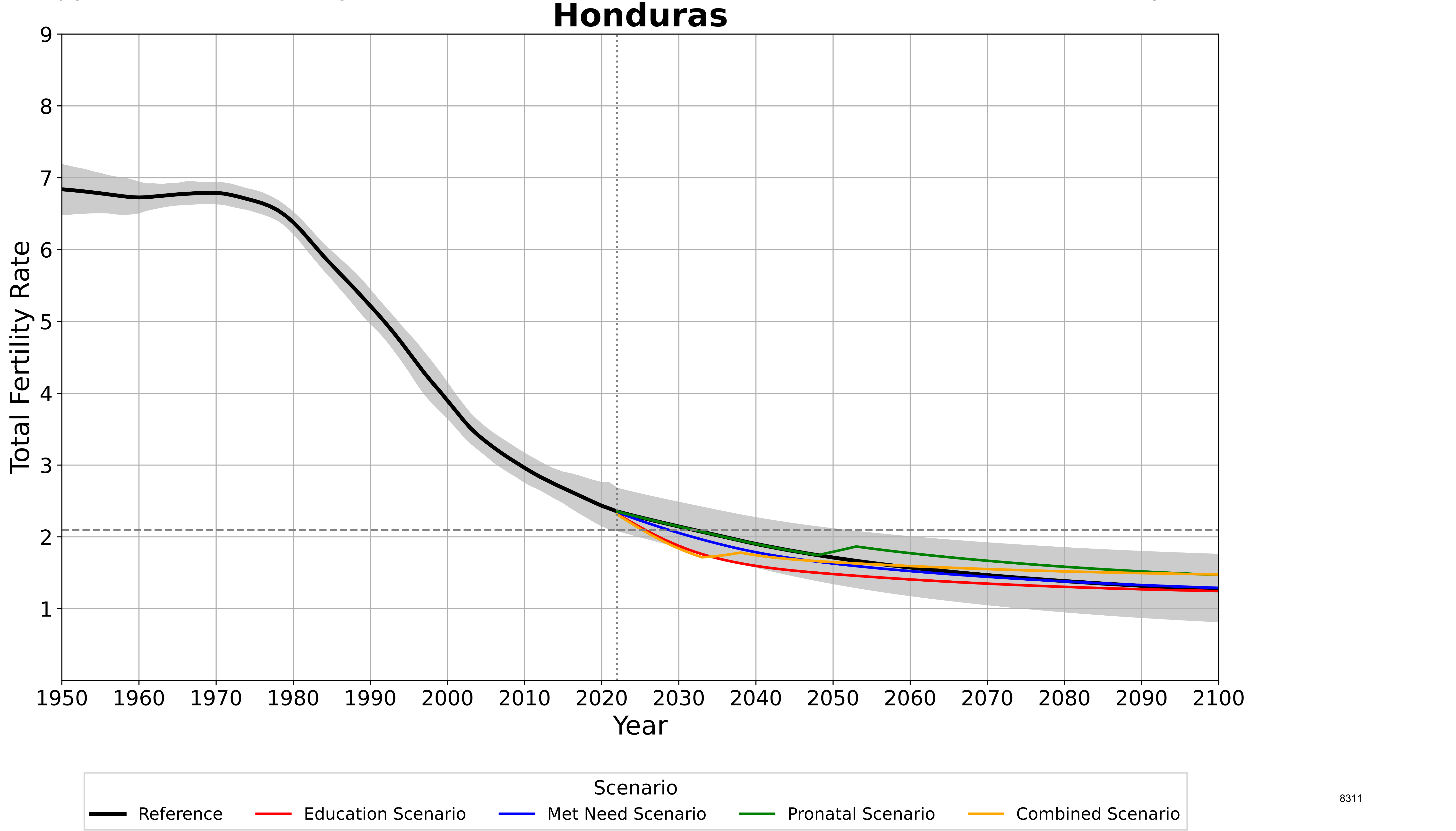

8311

Supplemental Results Figure S5: TFR for reference and alternative scenario forecasts by location, 1950– 2100

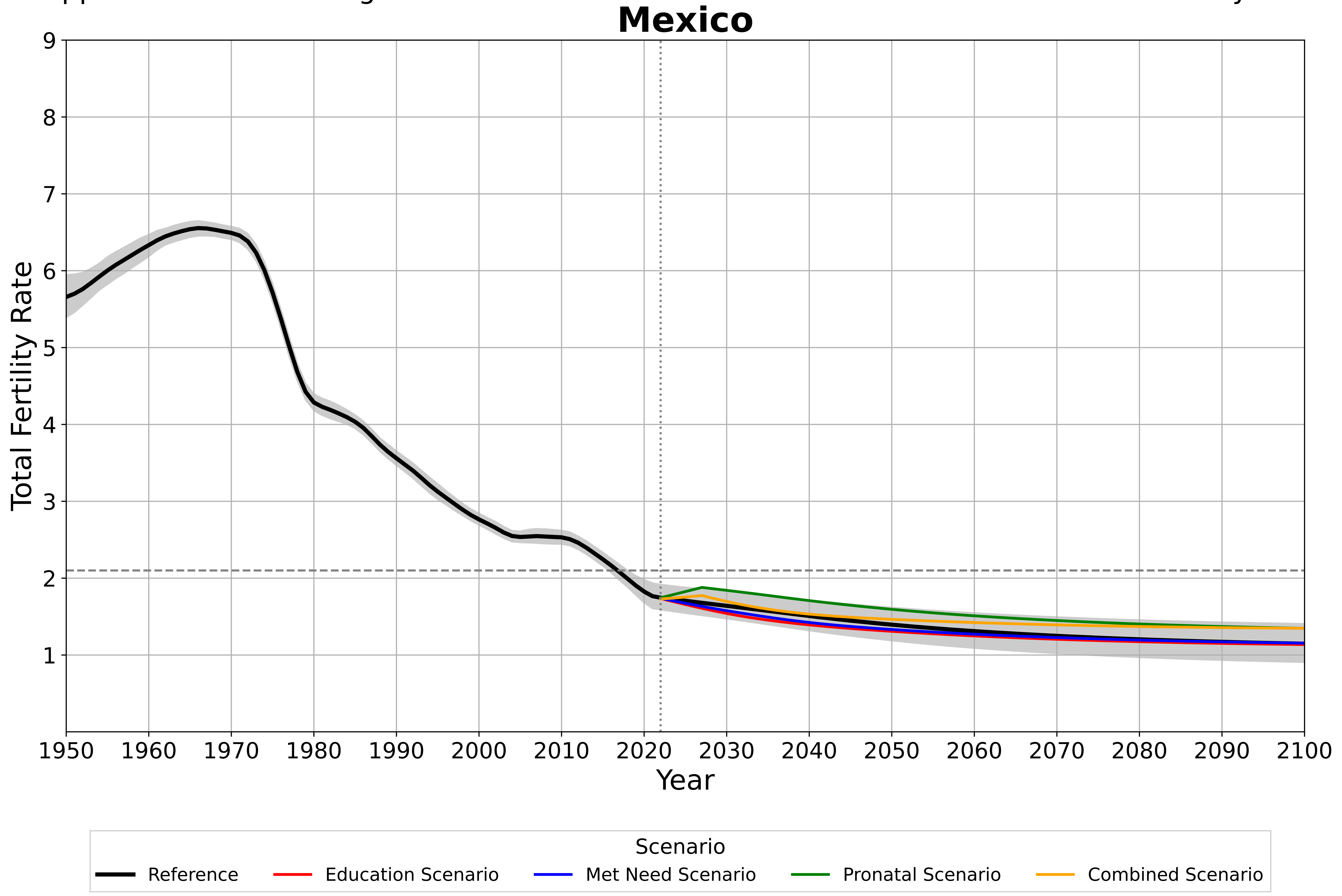

Supplemental Results Figure S5: TFR for reference and alternative scenario forecasts by location, 1950– 2100

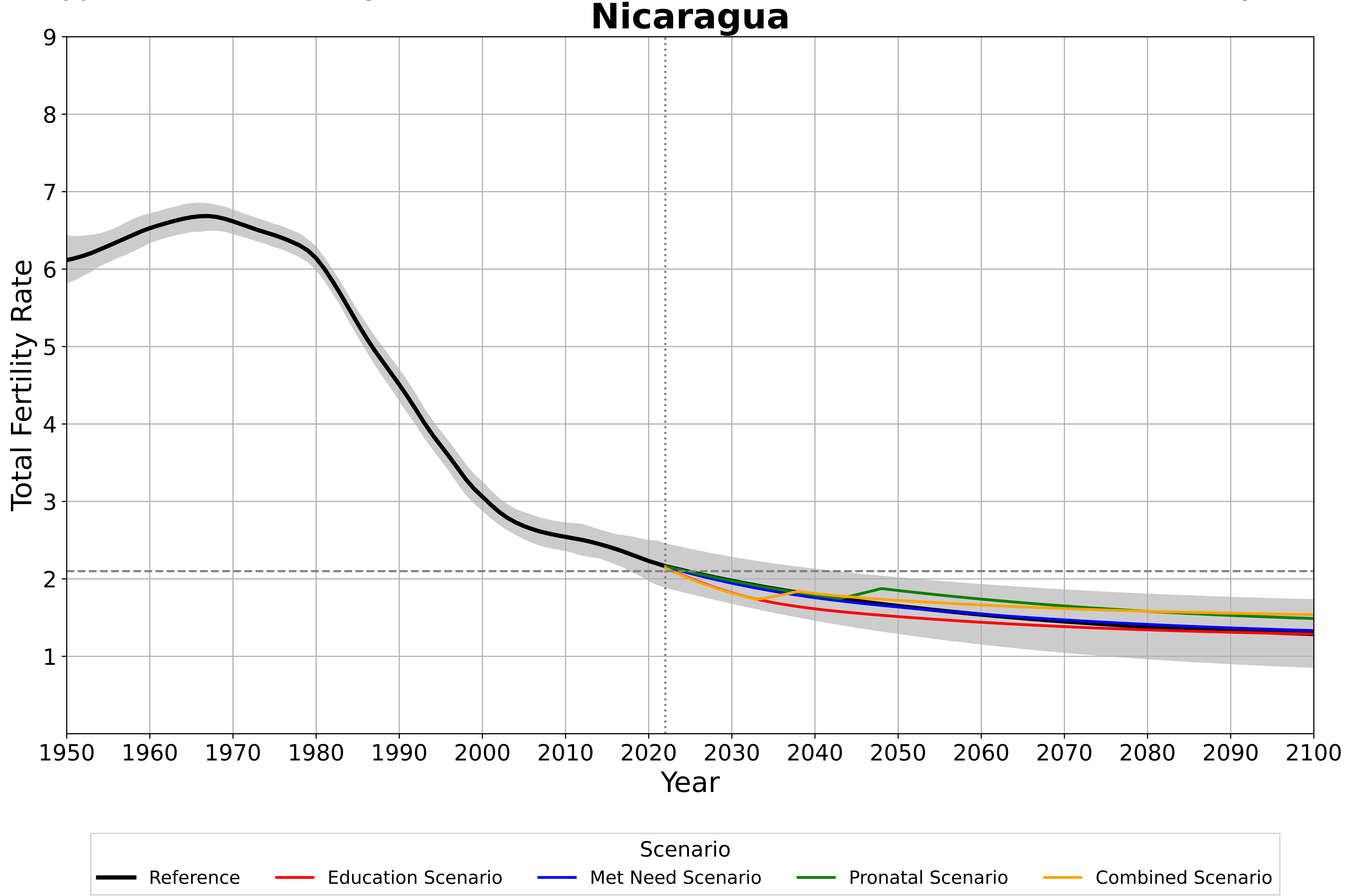

Supplemental Results Figure S5: TFR for reference and alternative scenario forecasts by location, 1950– 2100

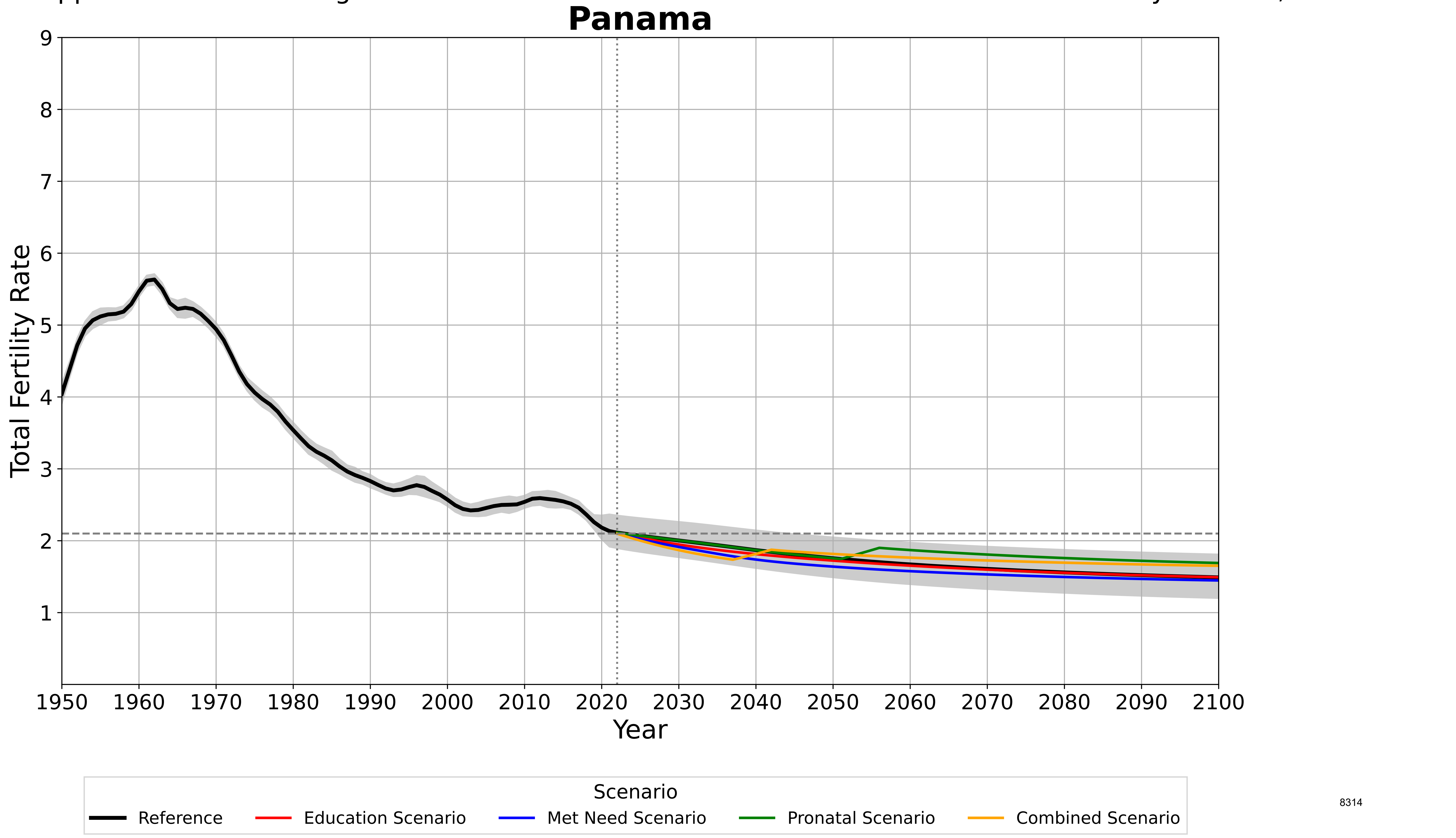

Supplemental Results Figure S5: TFR for reference and alternative scenario forecasts by location, 1950– 2100

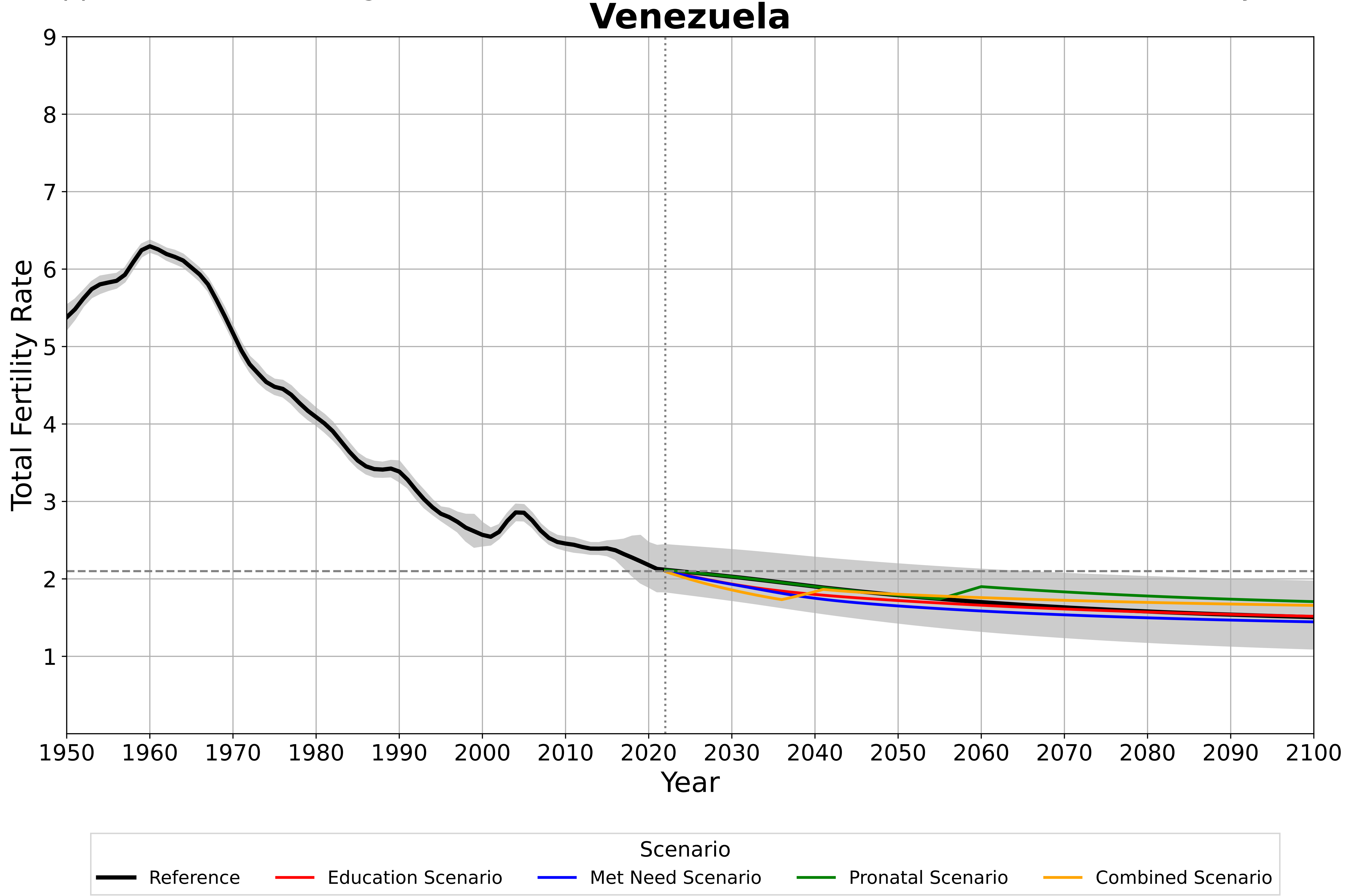

Supplemental Results Figure S5: TFR for reference and alternative scenario forecasts by location, 1950– 2100

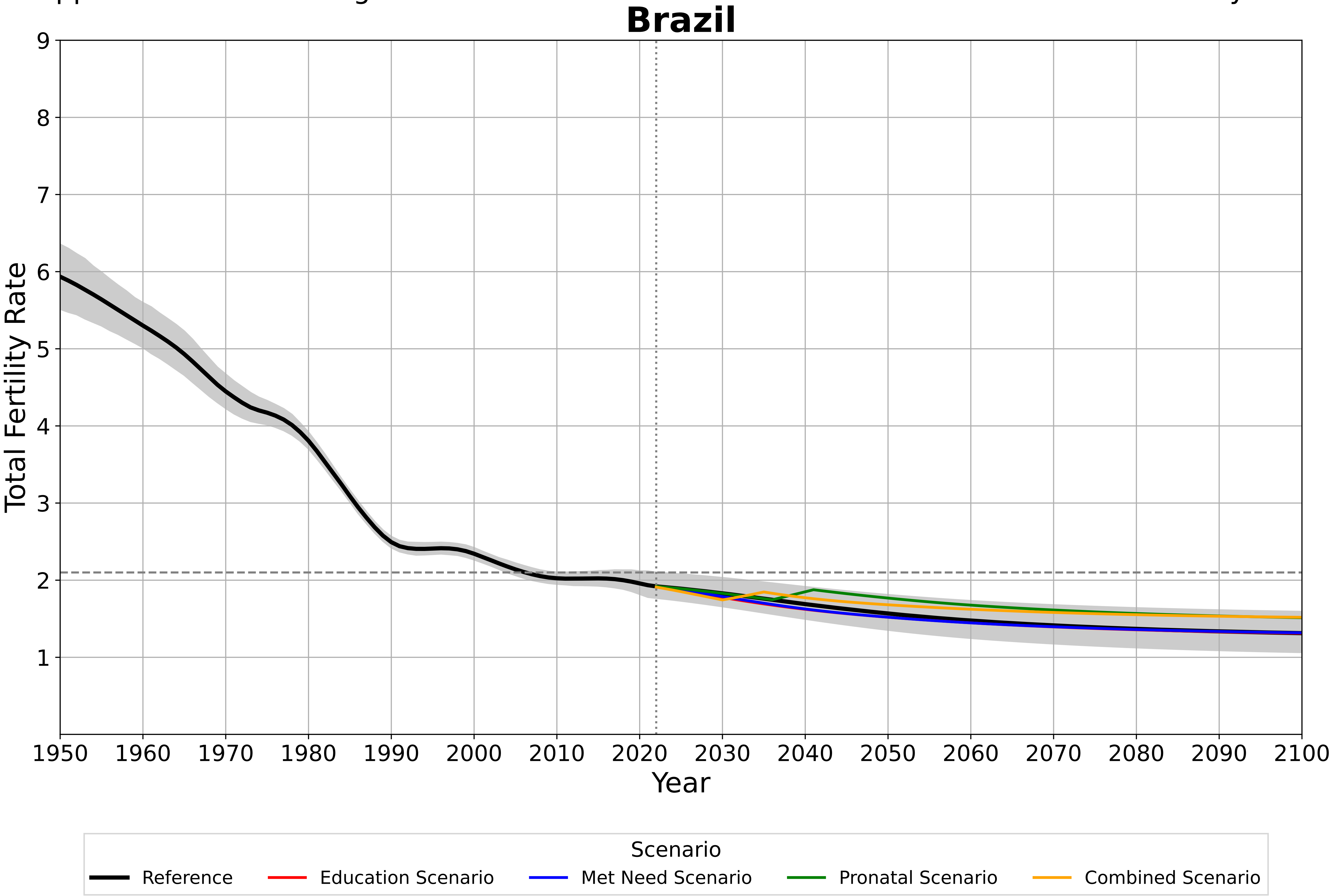

Supplemental Results Figure S5: TFR for reference and alternative scenario forecasts by location, 1950– 2100

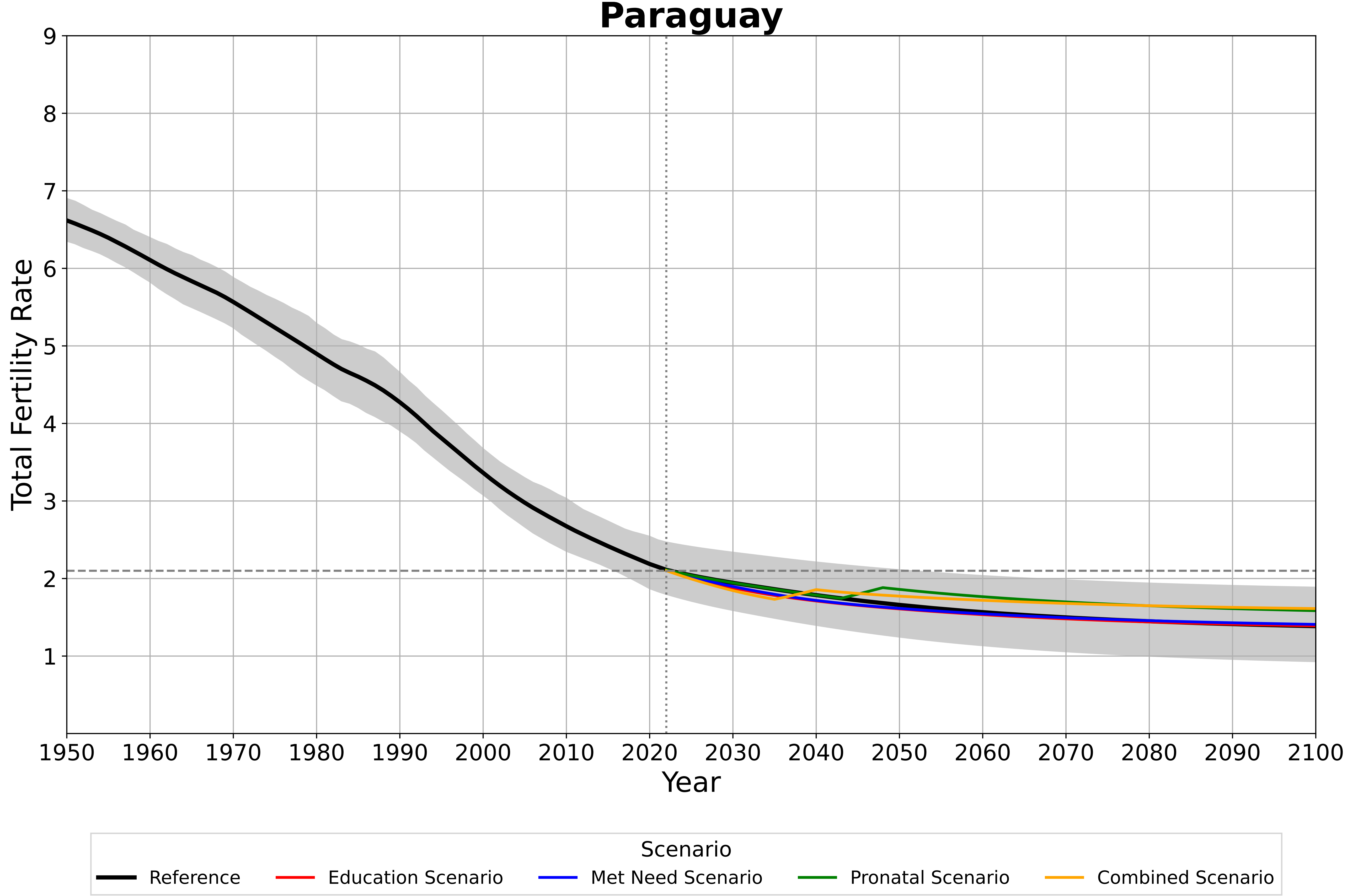

Supplemental Results Figure S5: TFR for reference and alternative scenario forecasts by location, 1950– 2100

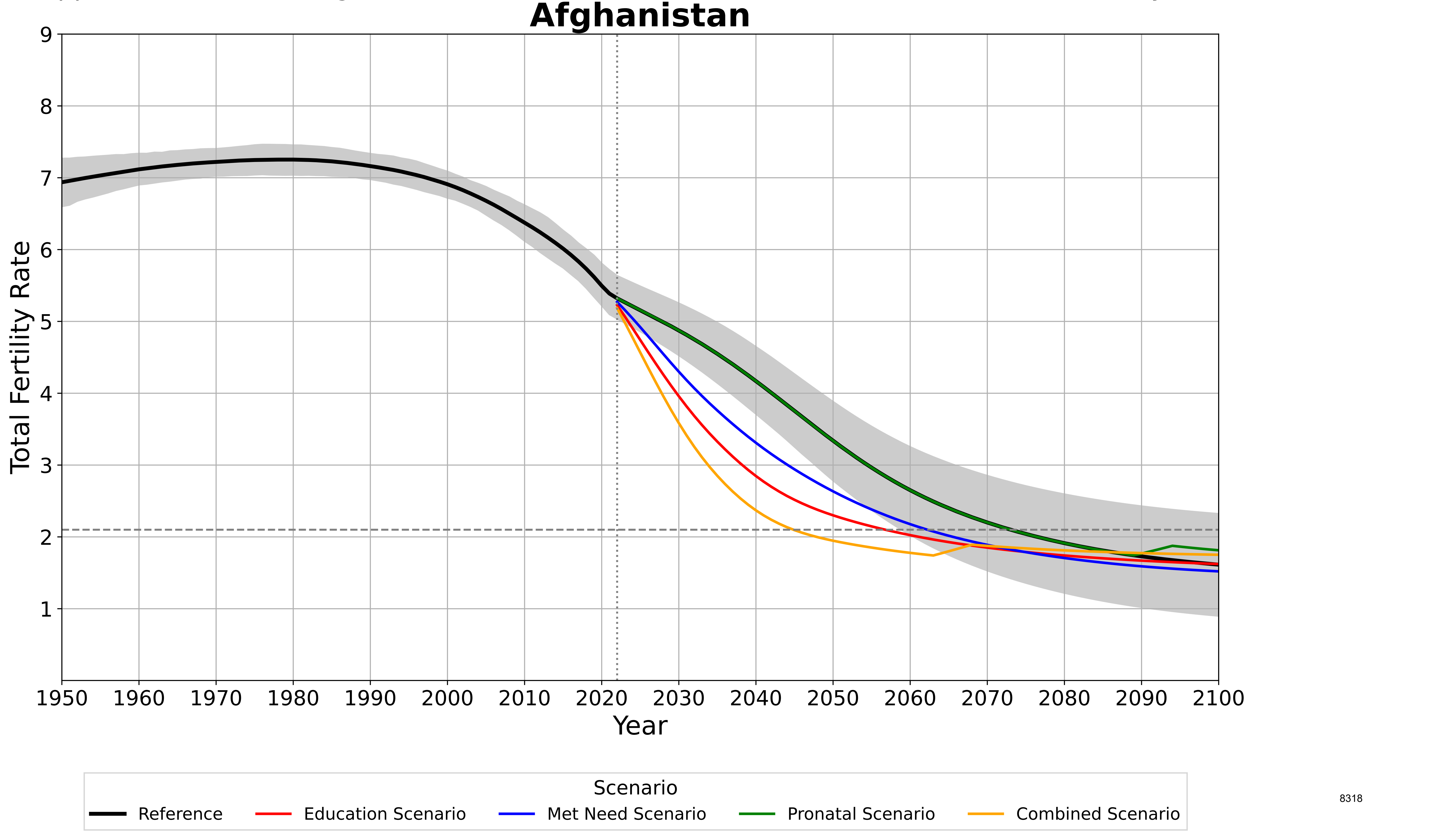

Supplemental Results Figure S5: TFR for reference and alternative scenario forecasts by location, 1950– 2100

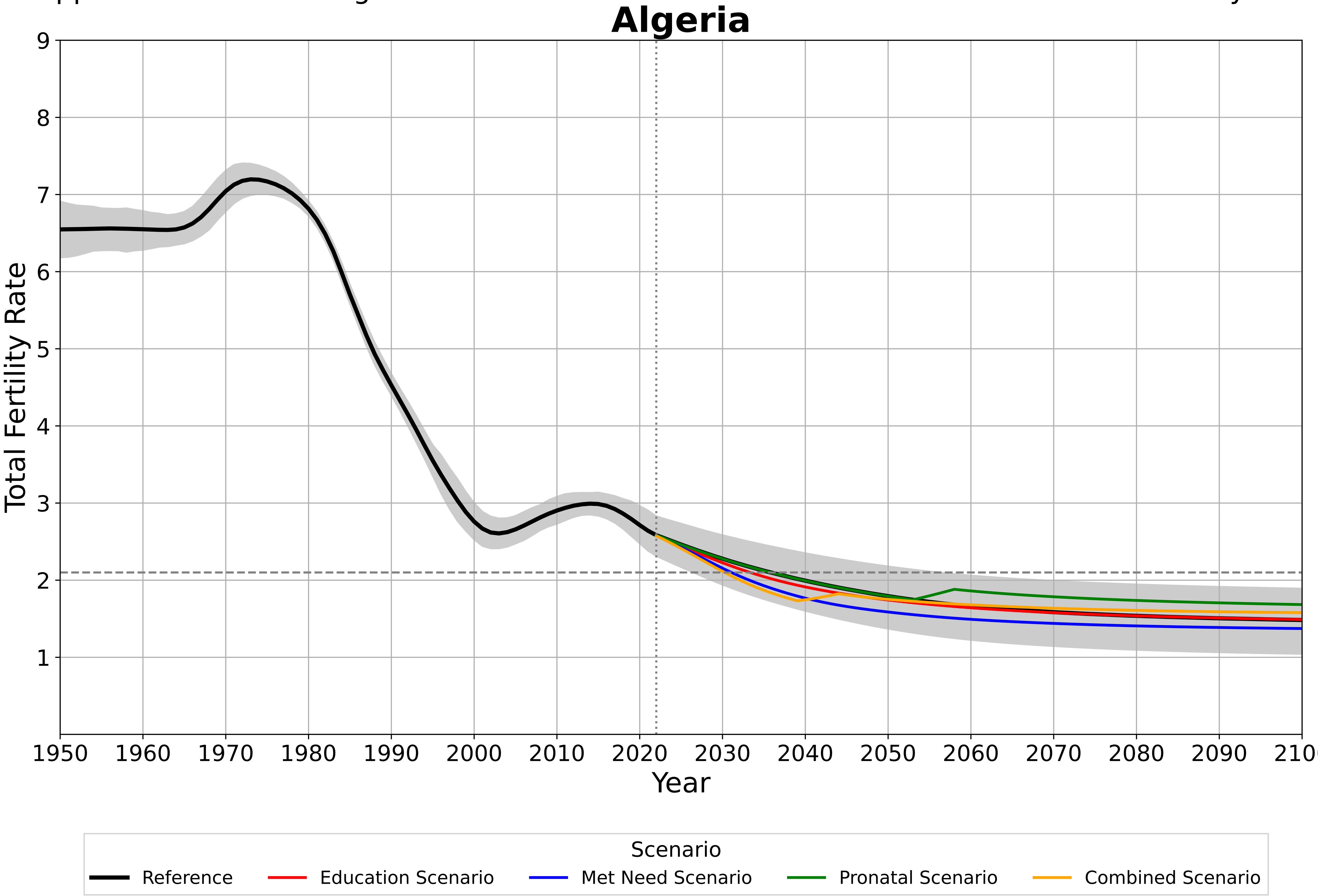

Supplemental Results Figure S5: TFR for reference and alternative scenario forecasts by location, 1950– 2100

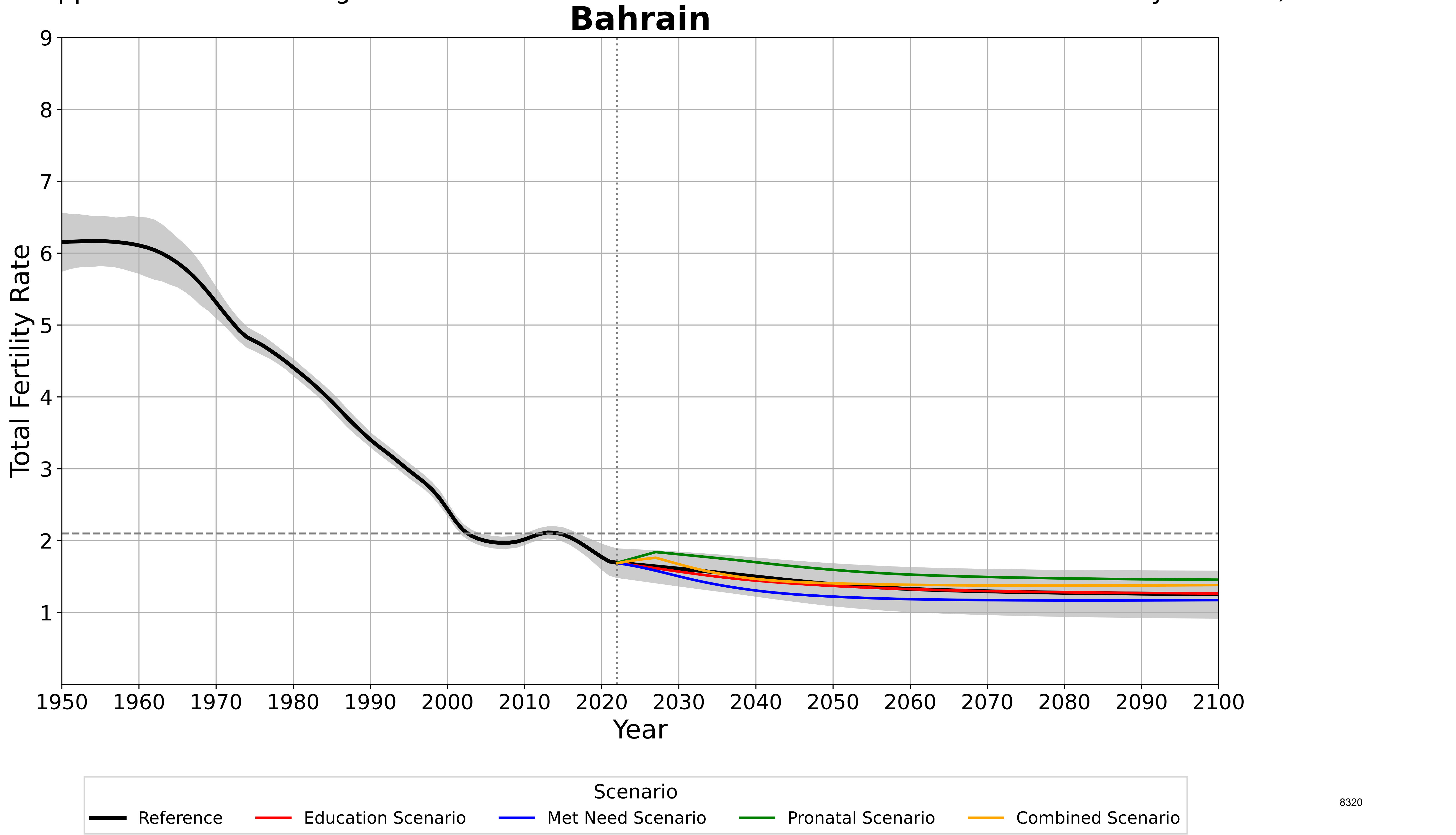

Supplemental Results Figure S5: TFR for reference and alternative scenario forecasts by location, 1950– 2100

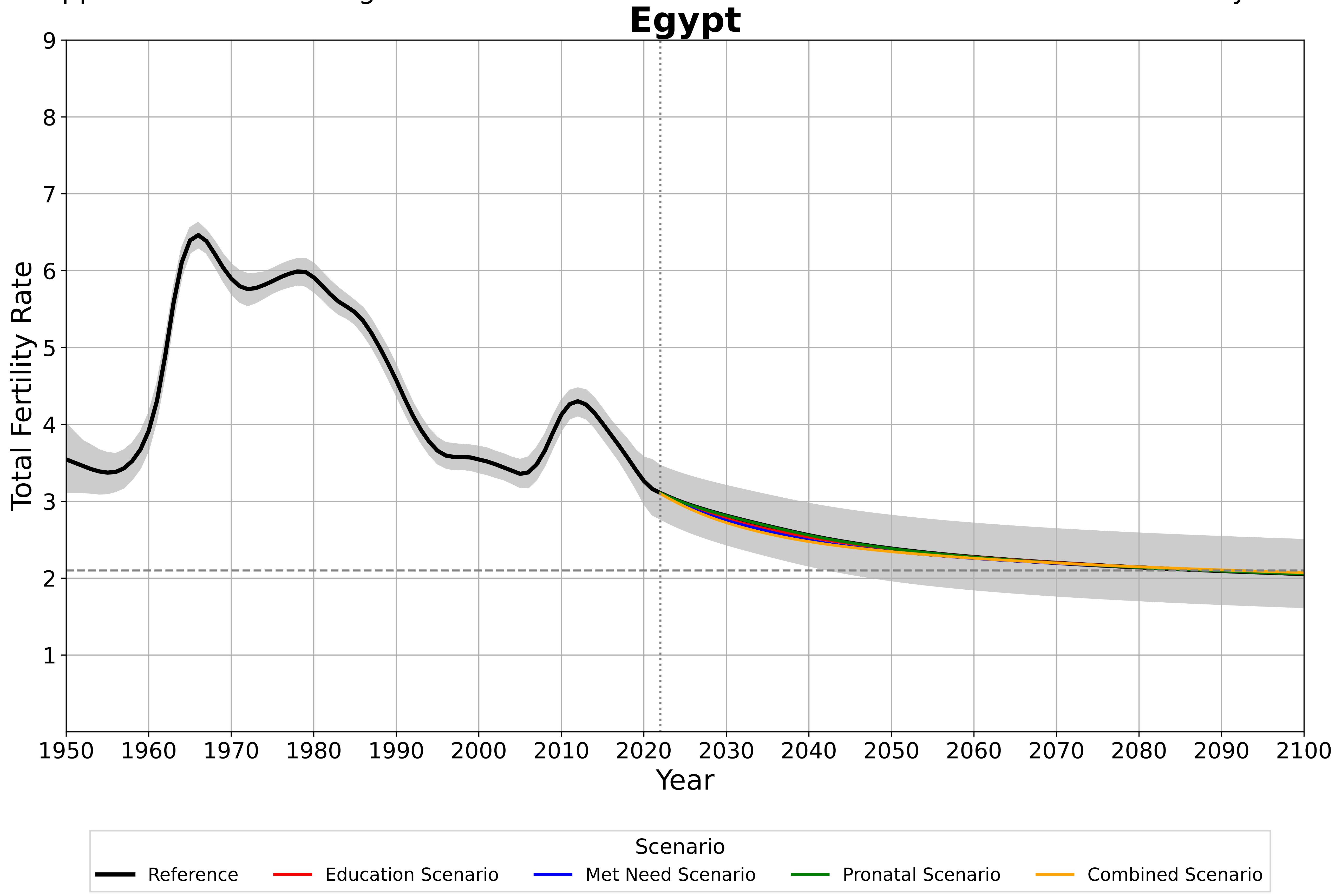

8321

Supplemental Results Figure S5: TFR for reference and alternative scenario forecasts by location, 1950– 2100

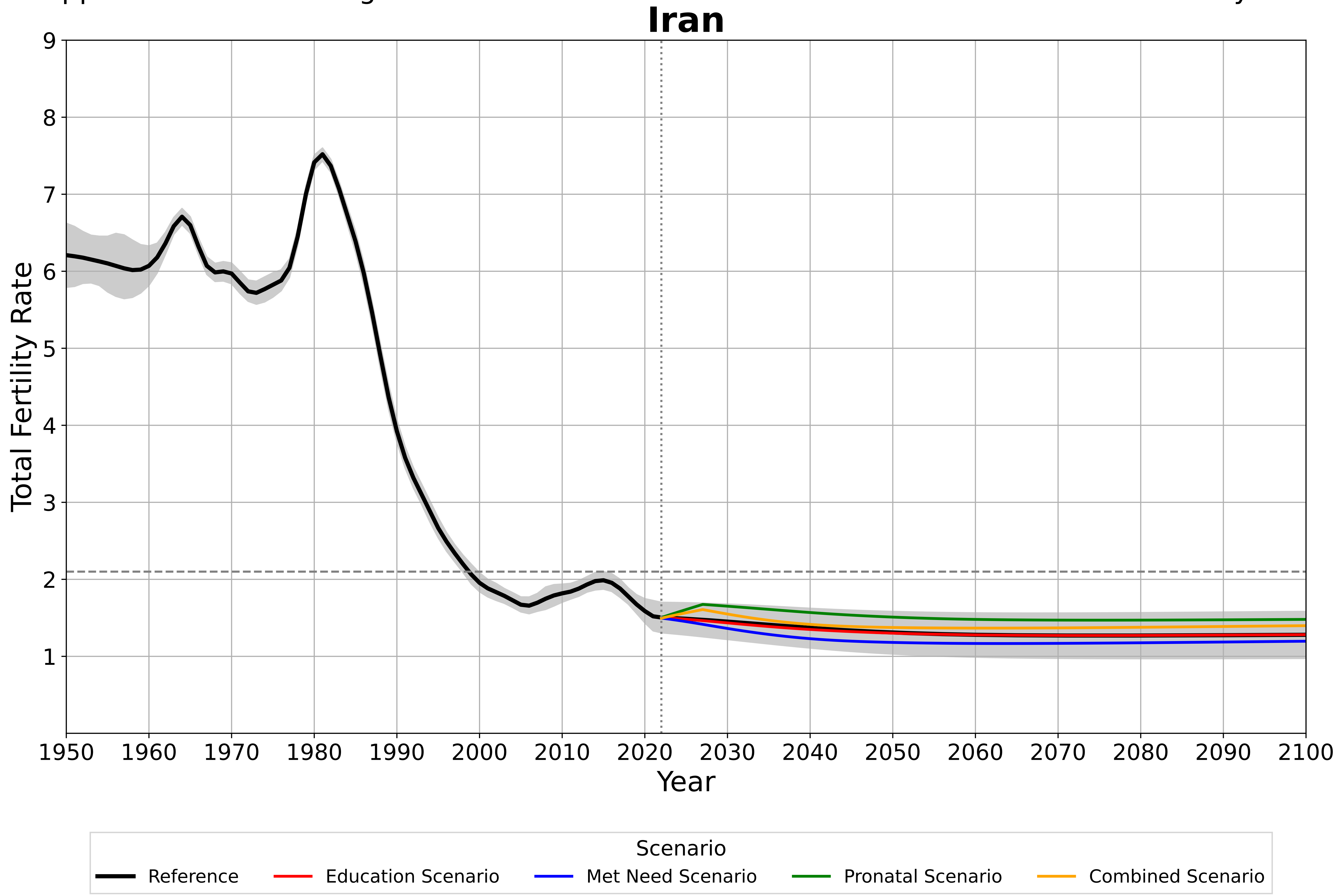

Supplemental Results Figure S5: TFR for reference and alternative scenario forecasts by location, 1950– 2100

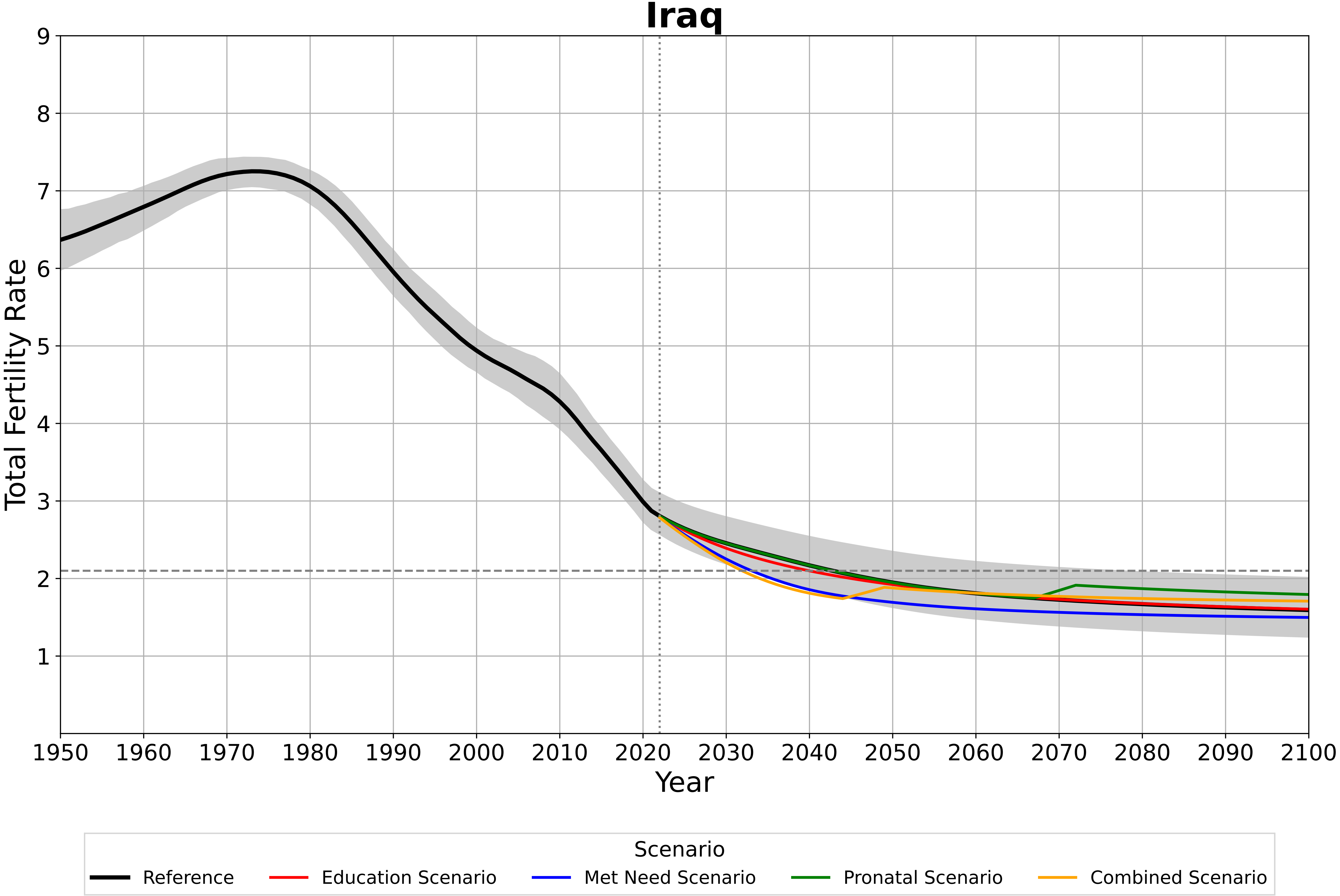

Supplemental Results Figure S5: TFR for reference and alternative scenario forecasts by location, 1950– 2100

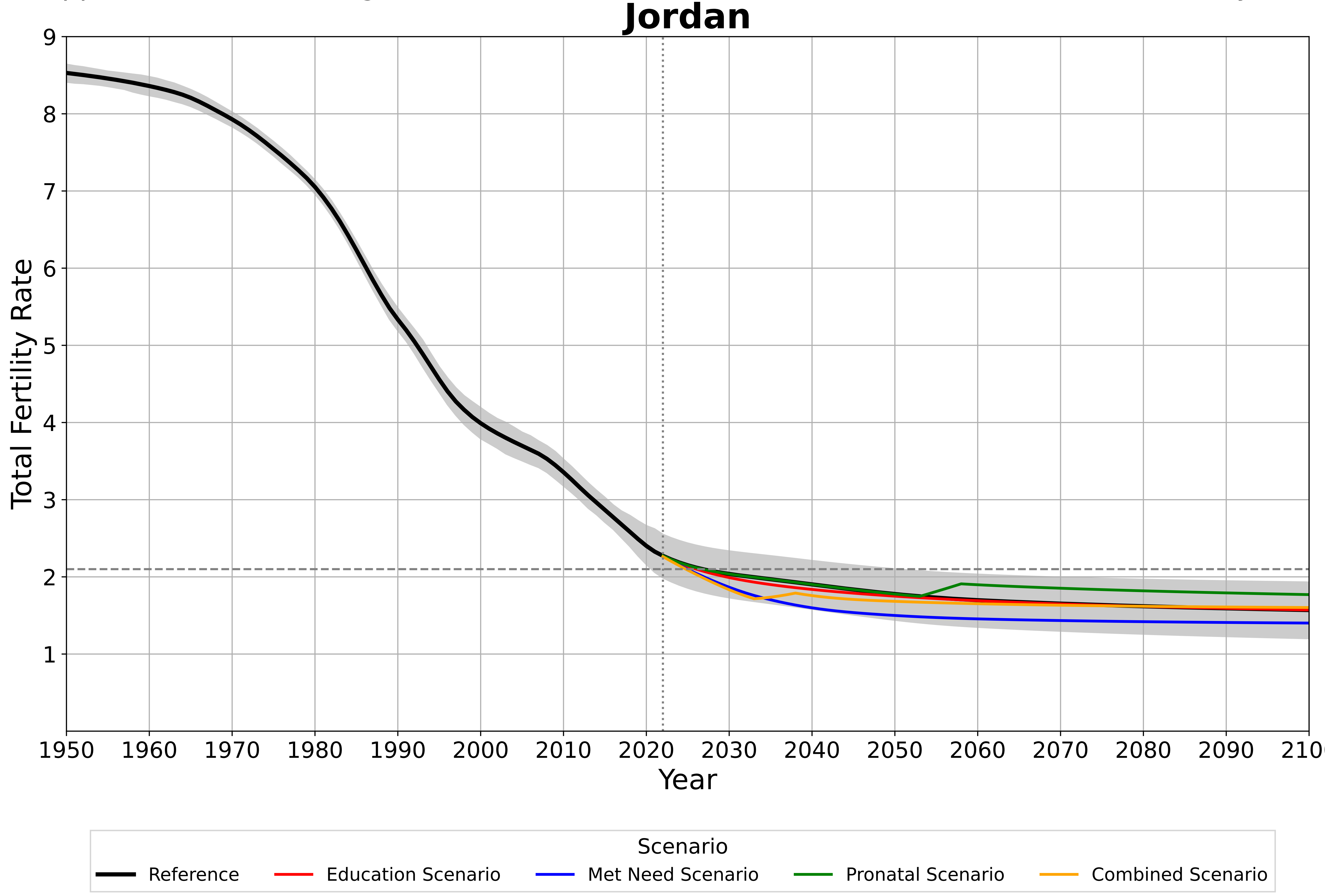

Supplemental Results Figure S5: TFR for reference and alternative scenario forecasts by location, 1950– 2100

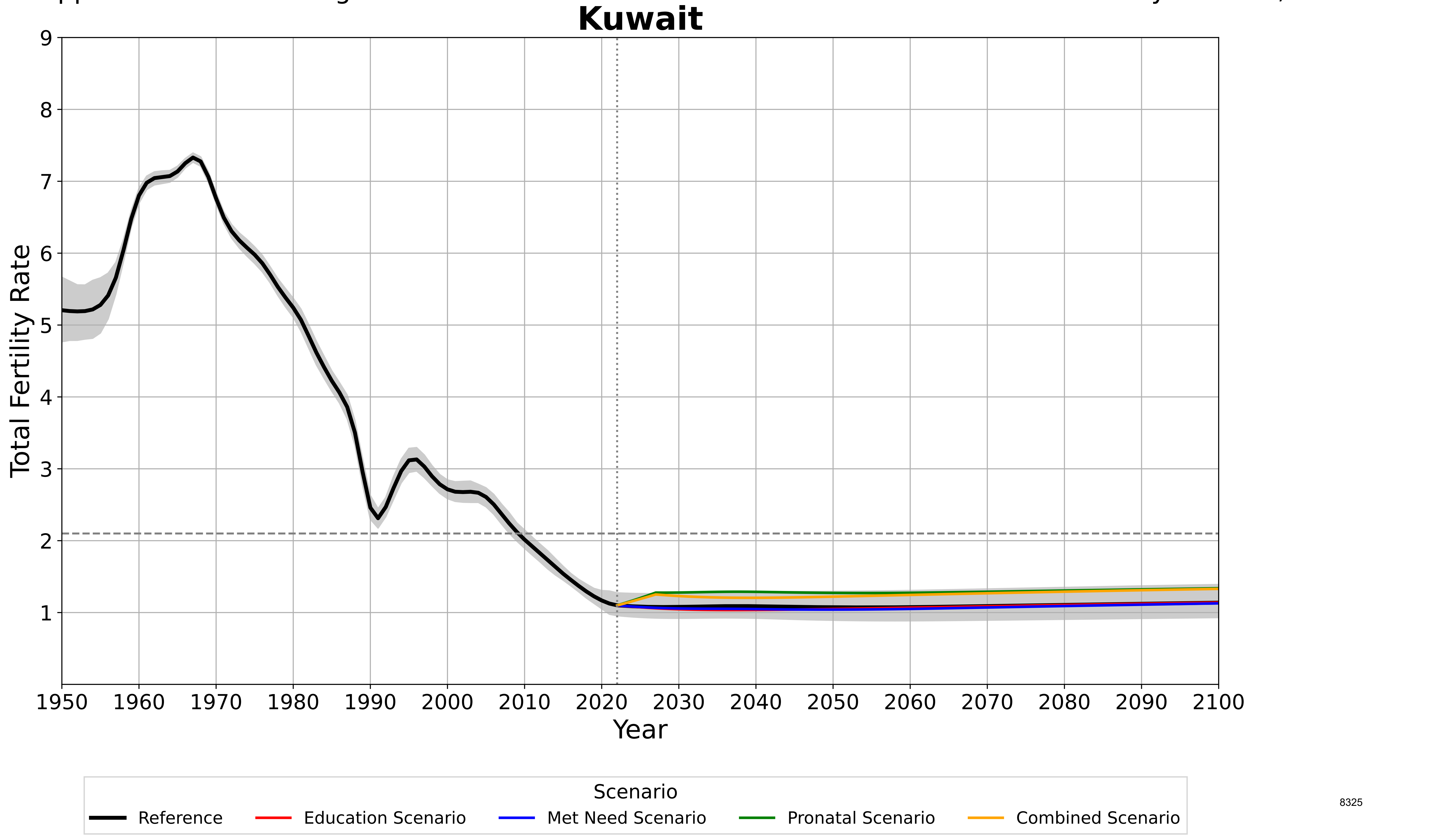

Supplemental Results Figure S5: TFR for reference and alternative scenario forecasts by location, 1950– 2100

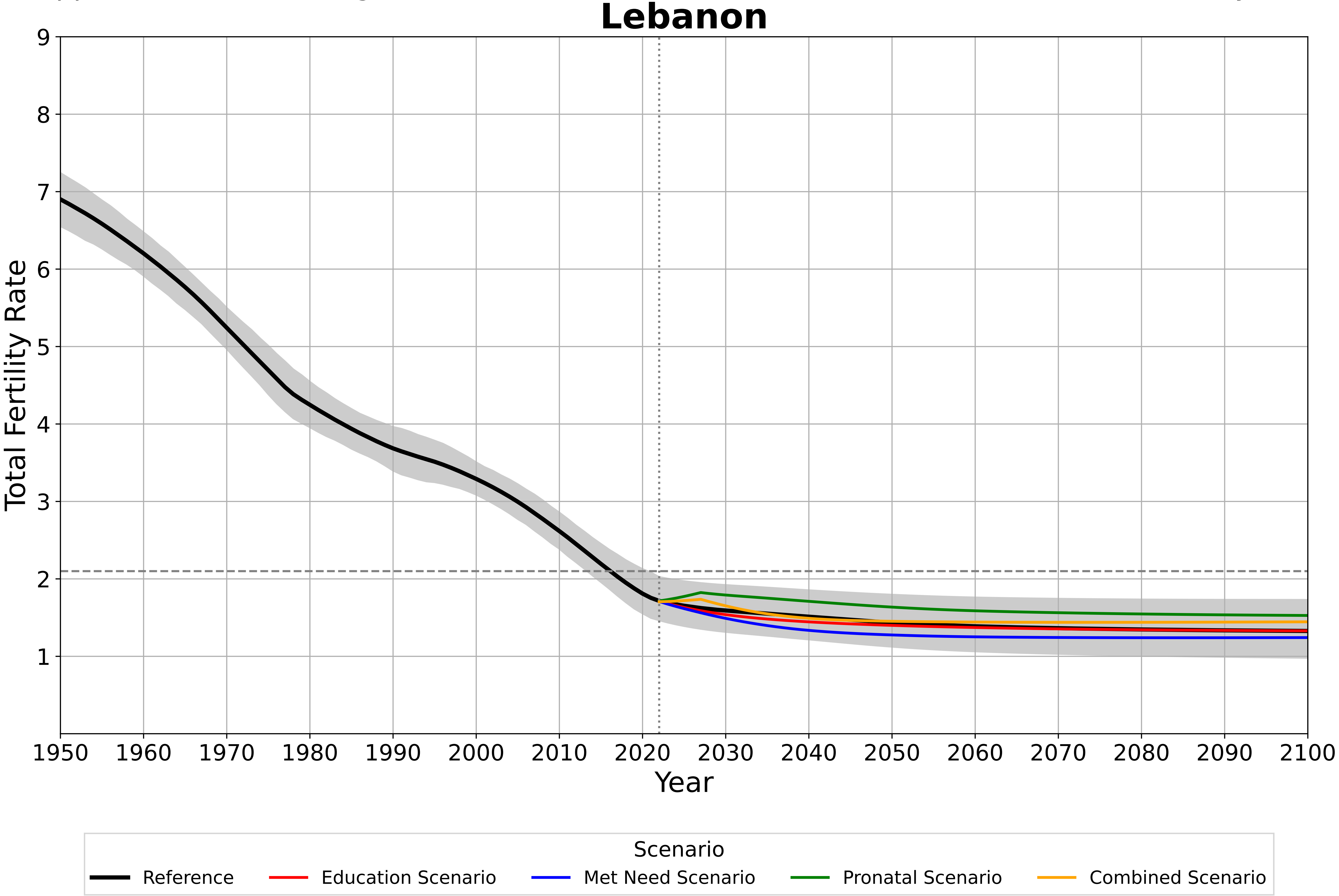

Supplemental Results Figure S5: TFR for reference and alternative scenario forecasts by location, 1950– 2100

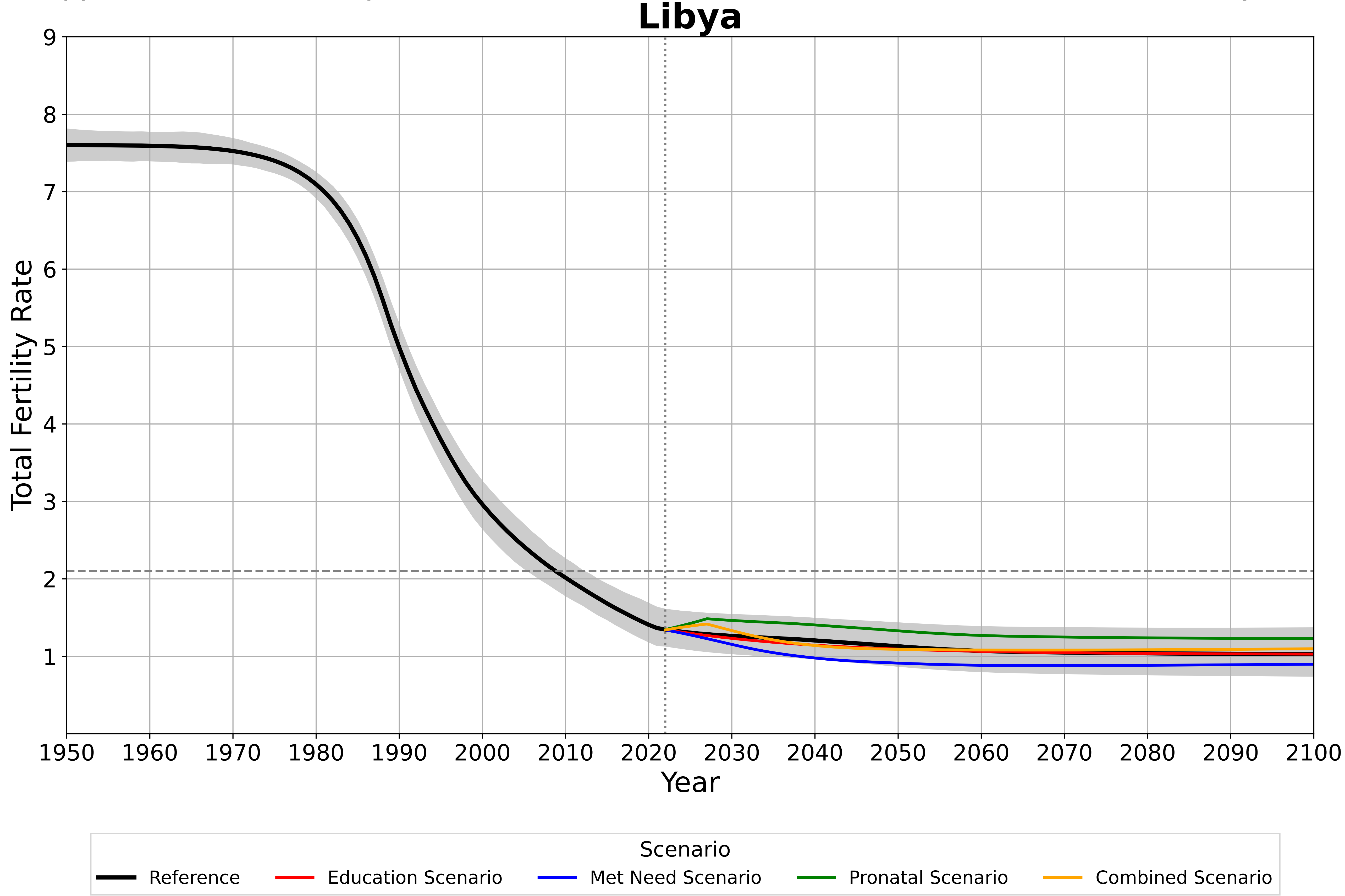

Supplemental Results Figure S5: TFR for reference and alternative scenario forecasts by location, 1950– 2100

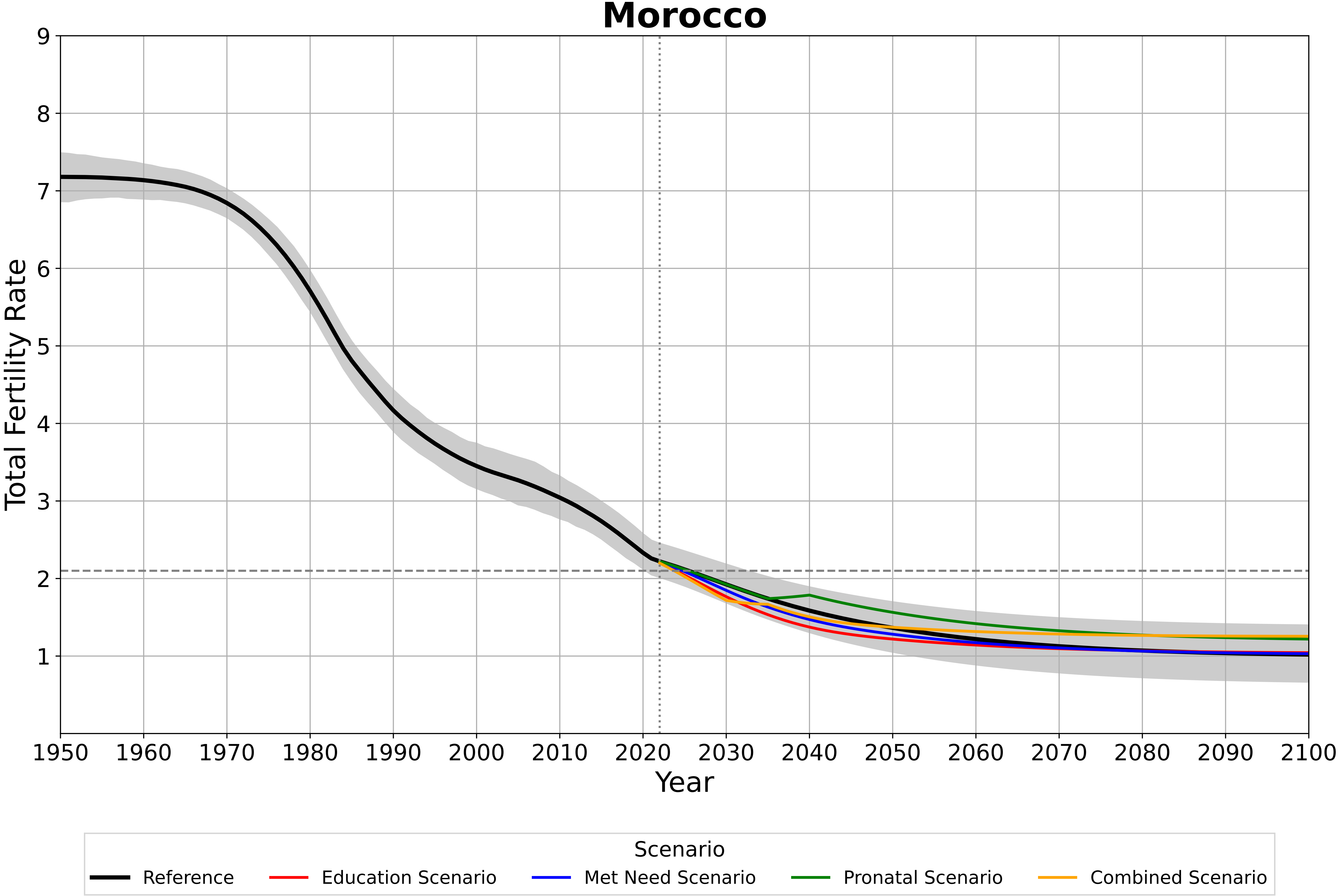

Supplemental Results Figure S5: TFR for reference and alternative scenario forecasts by location, 1950– 2100

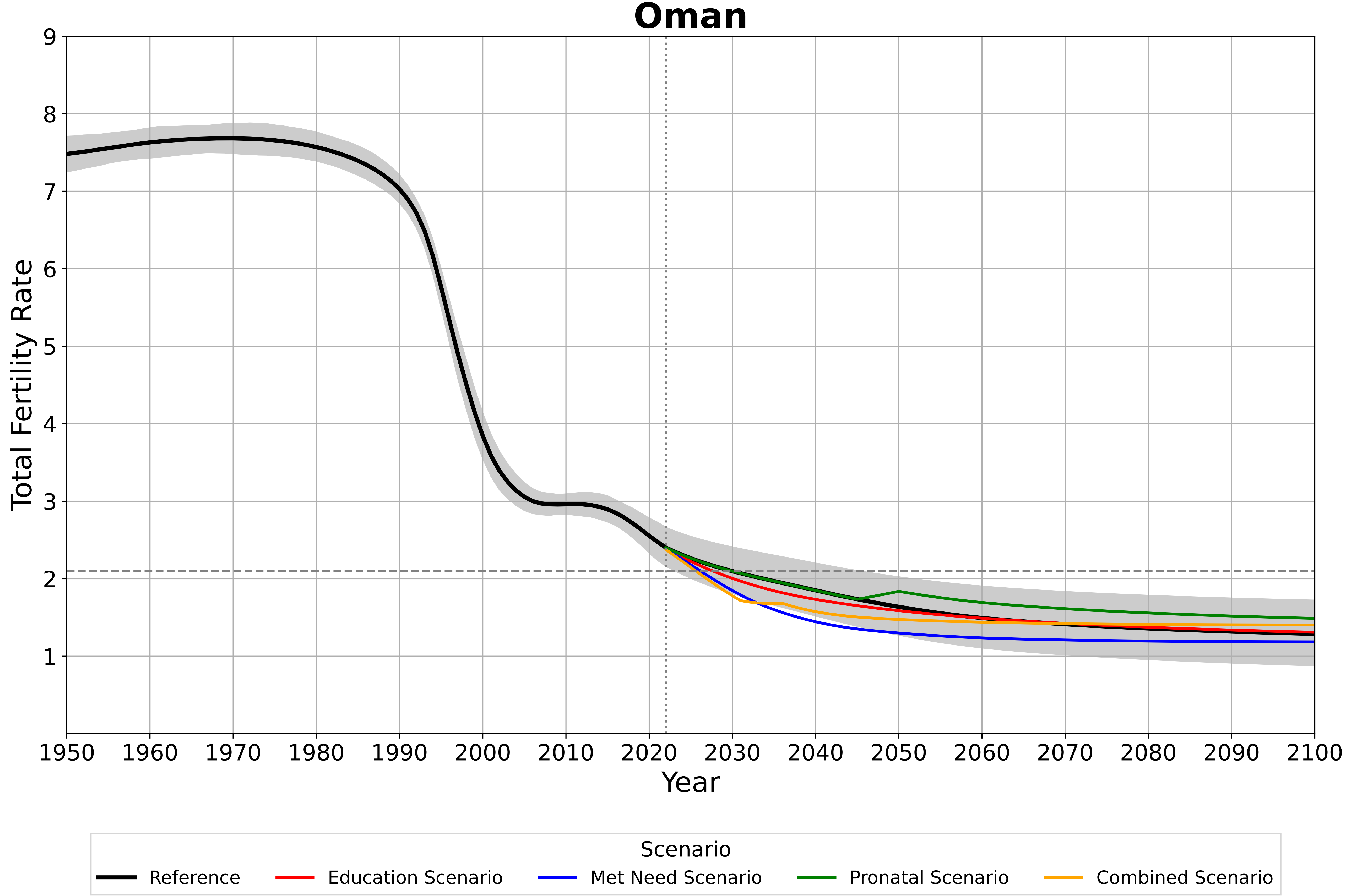

Supplemental Results Figure S5: TFR for reference and alternative scenario forecasts by location, 1950– 2100

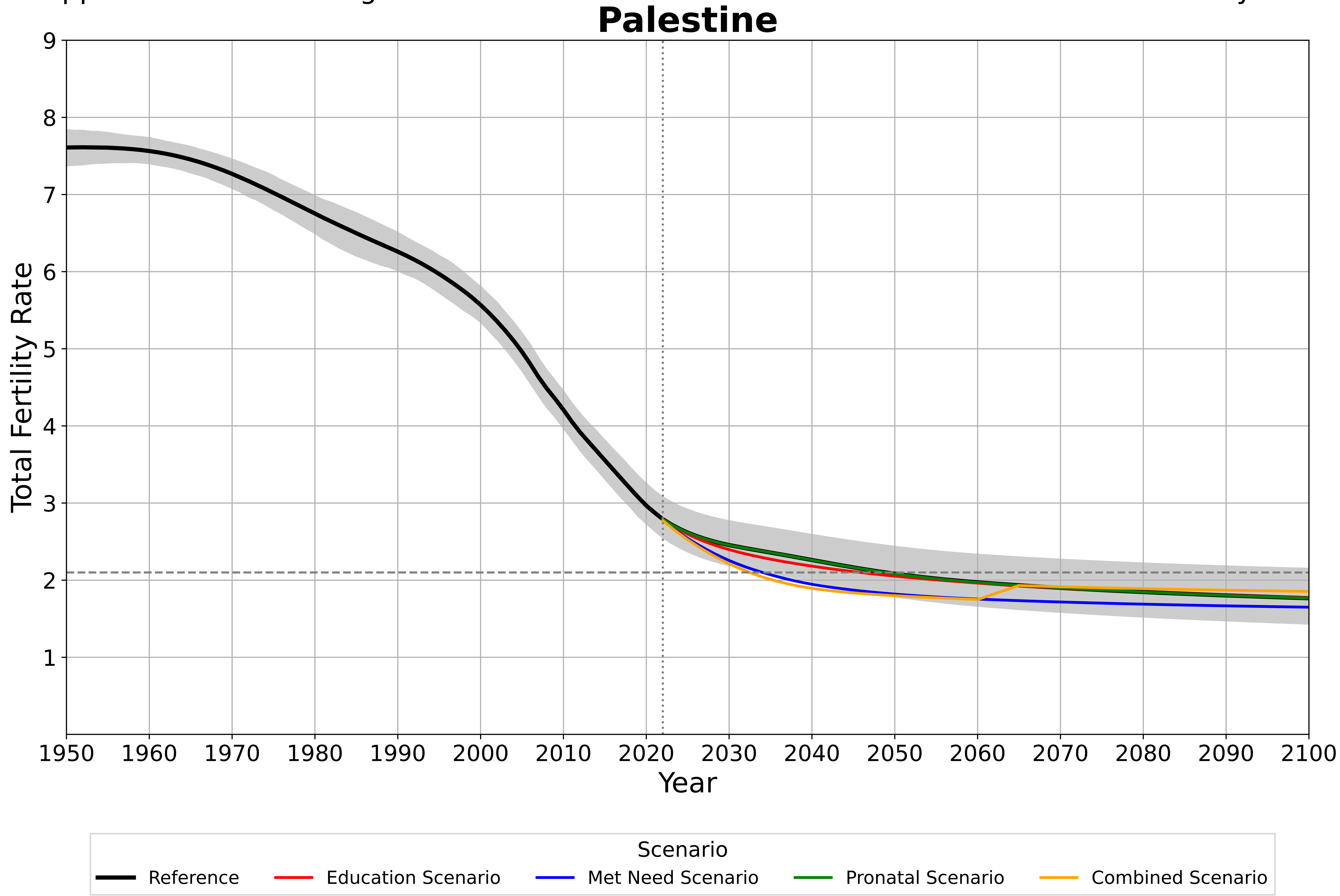

Supplemental Results Figure S5: TFR for reference and alternative scenario forecasts by location, 1950– 2100

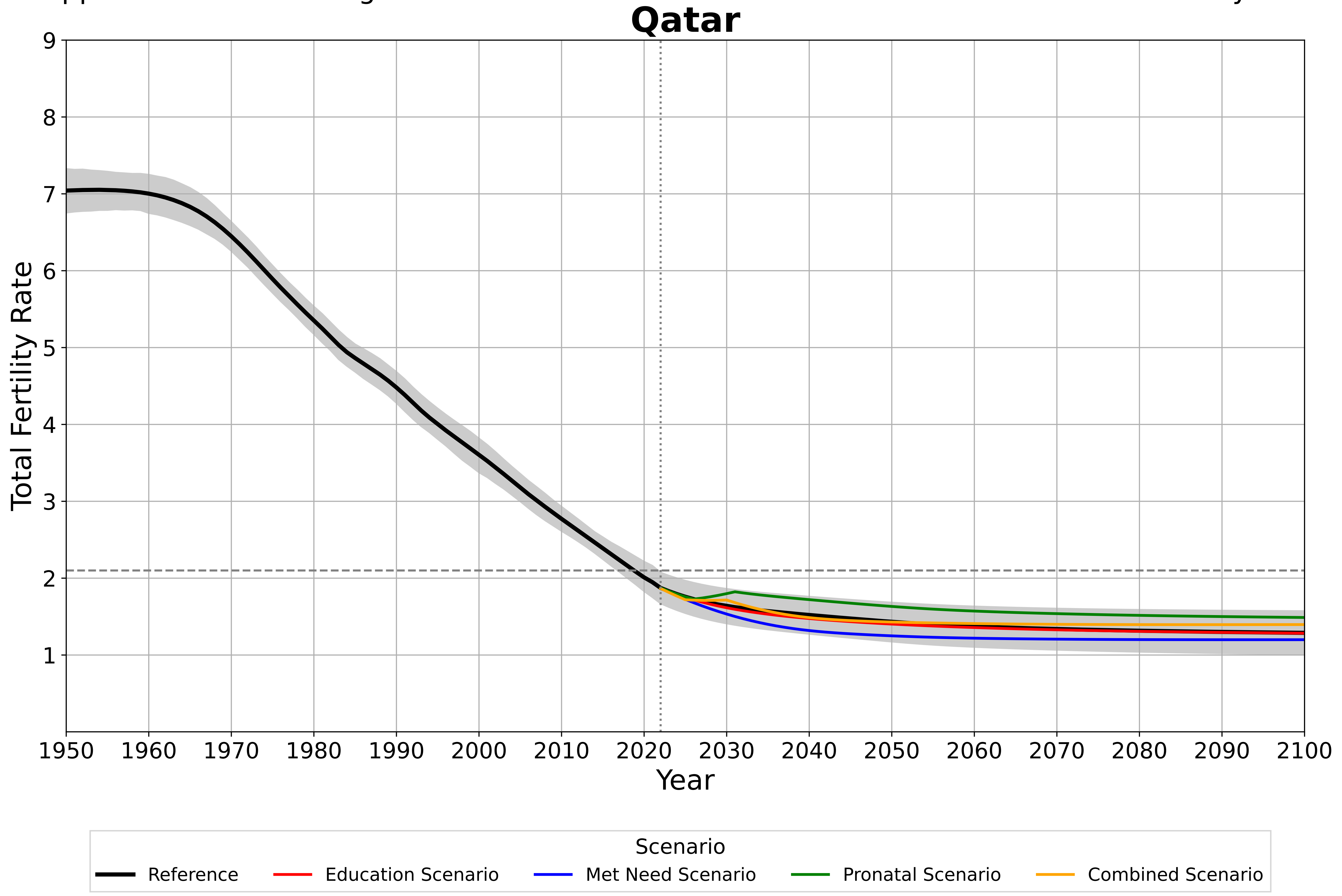

Supplemental Results Figure S5: TFR for reference and alternative scenario forecasts by location, 1950– 2100

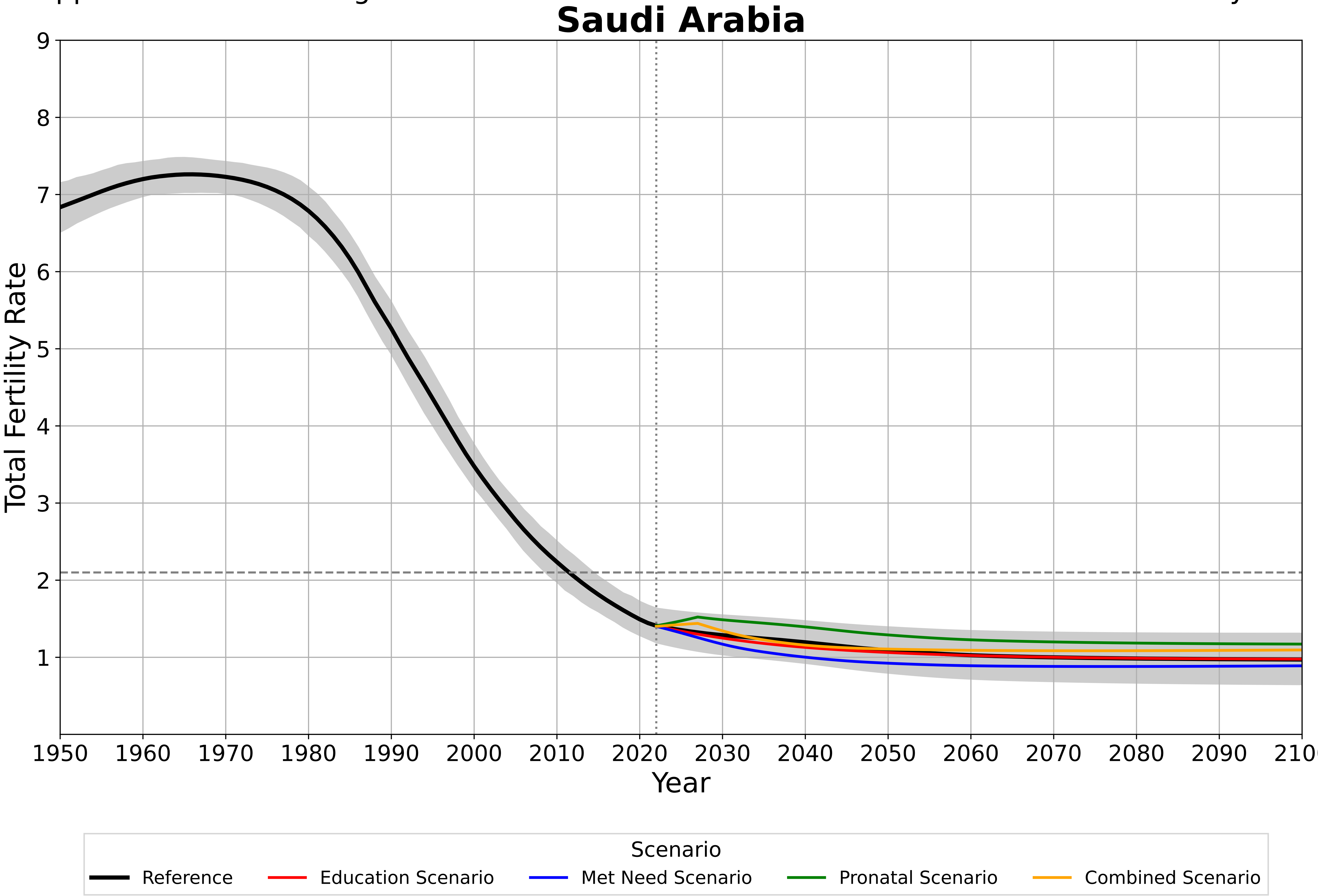

Supplemental Results Figure S5: TFR for reference and alternative scenario forecasts by location, 1950– 2100

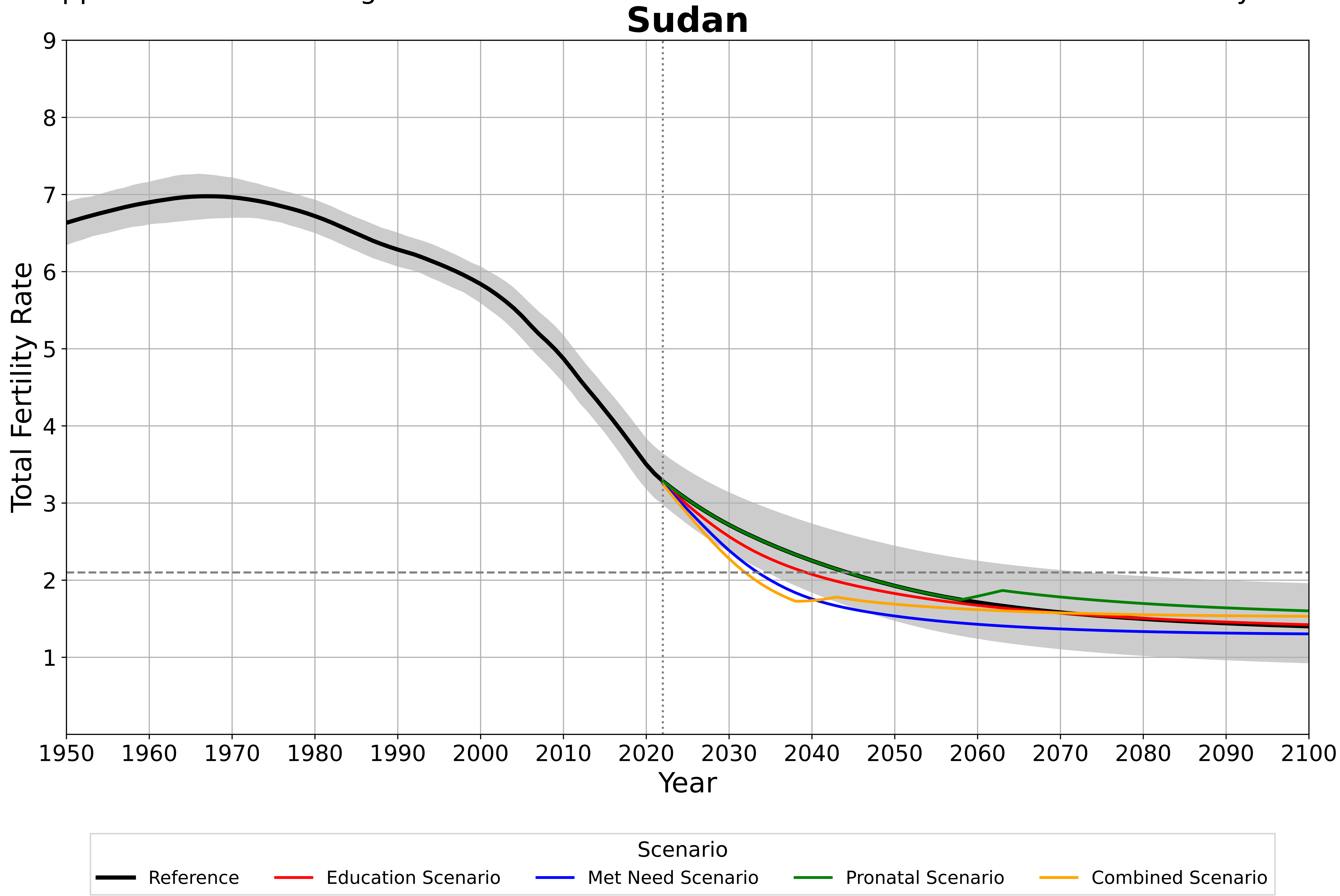

Supplemental Results Figure S5: TFR for reference and alternative scenario forecasts by location, 1950– 2100

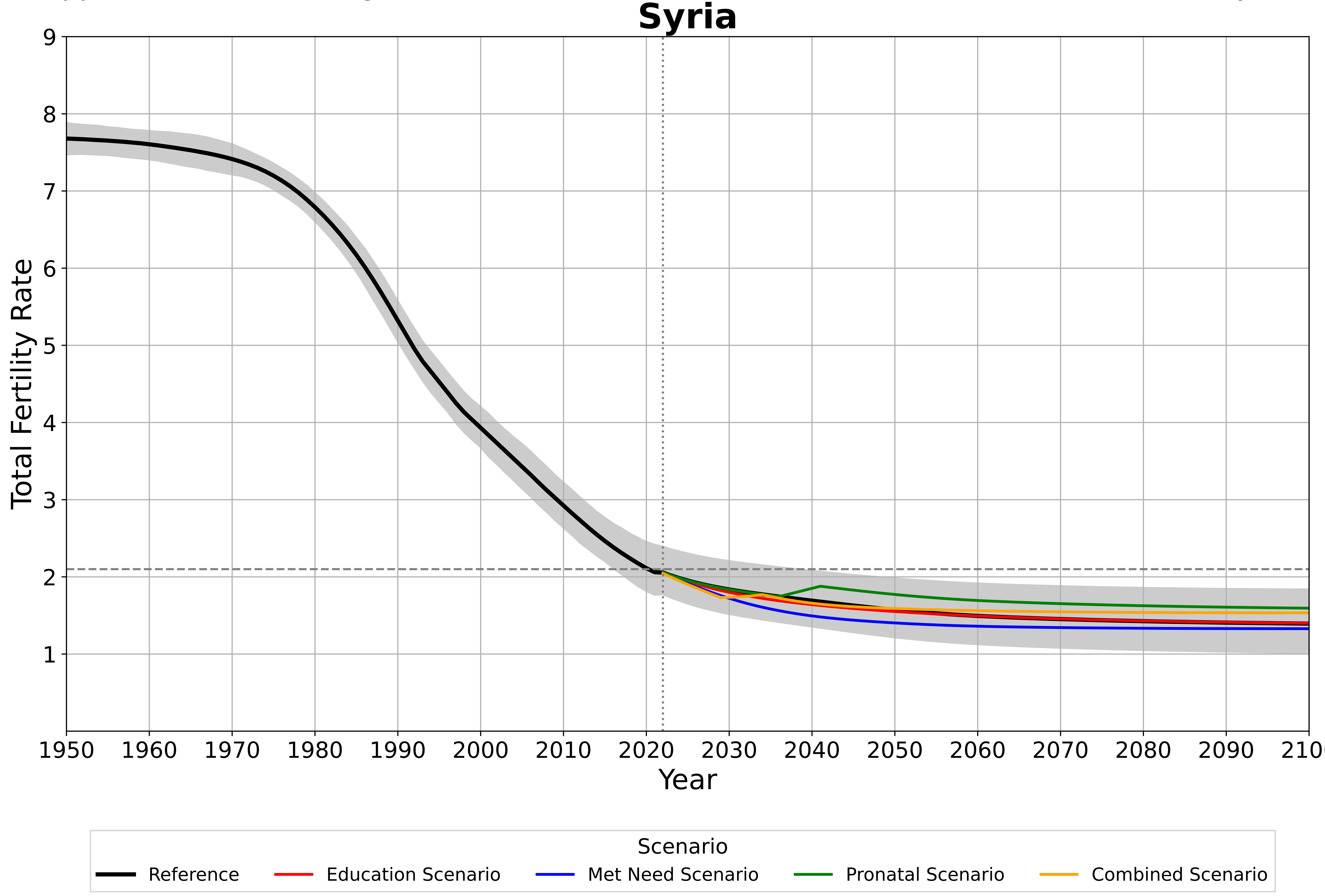

Supplemental Results Figure S5: TFR for reference and alternative scenario forecasts by location, 1950– 2100

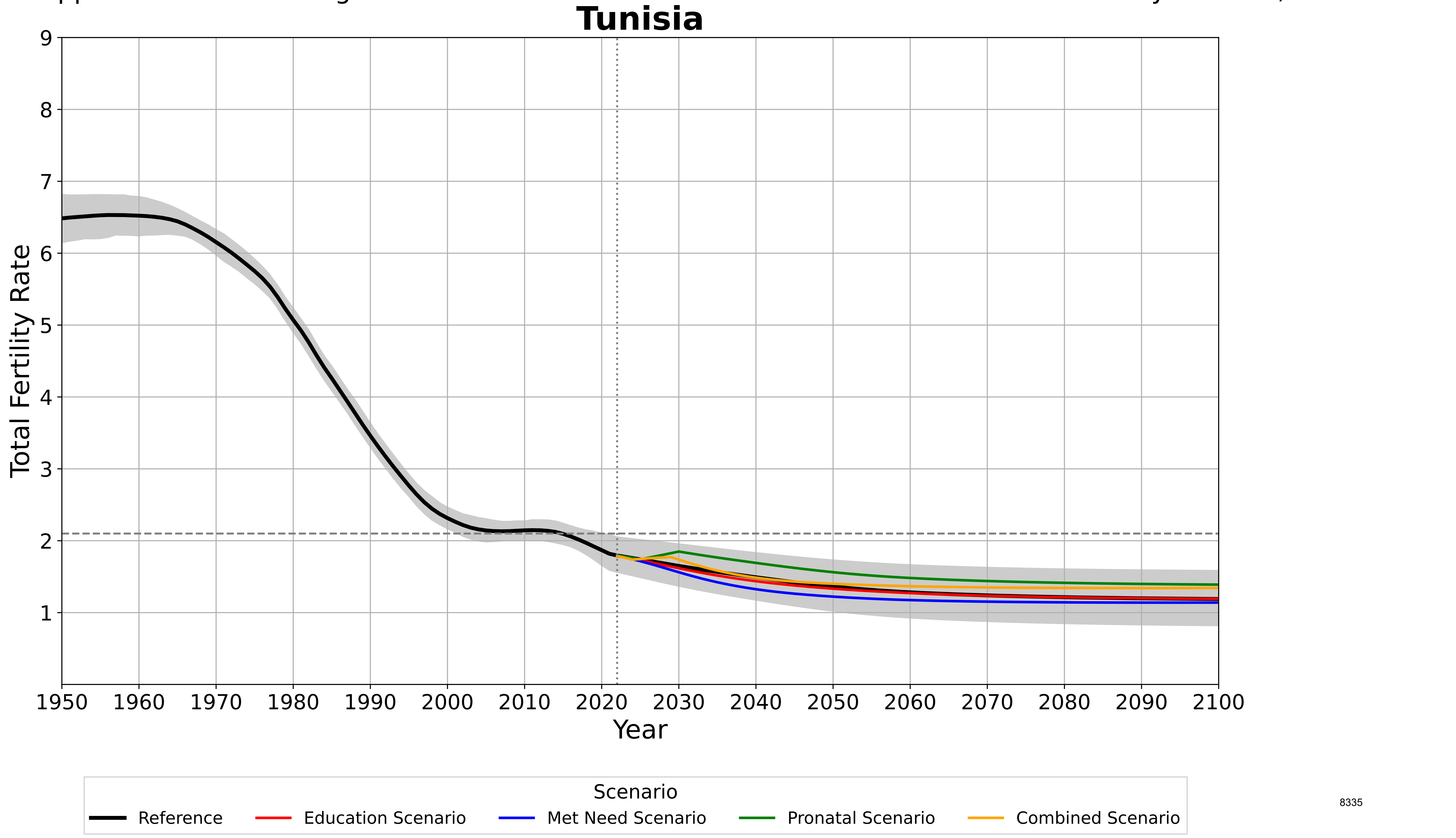

Supplemental Results Figure S5: TFR for reference and alternative scenario forecasts by location, 1950– 2100

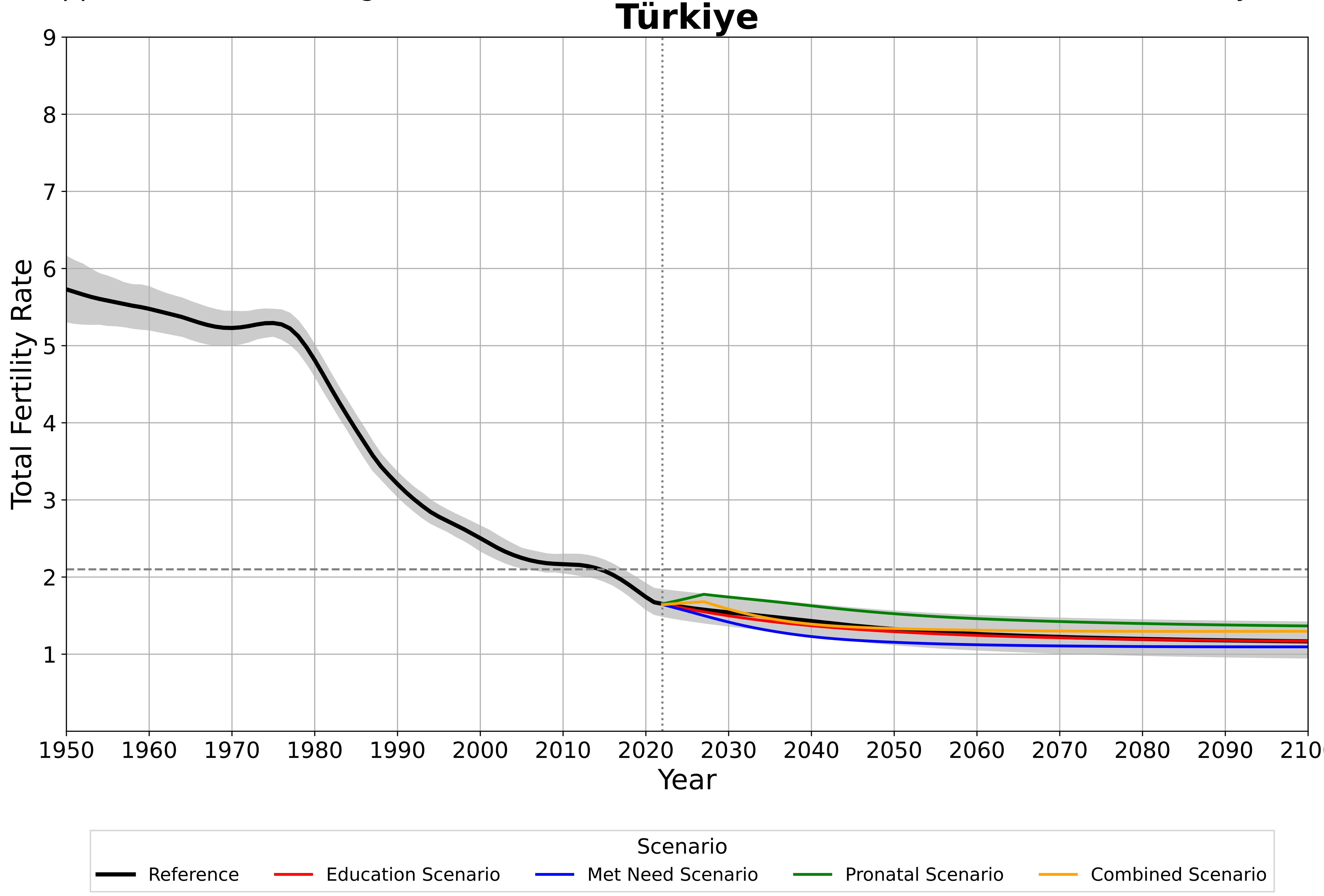

Supplemental Results Figure S5: TFR for reference and alternative scenario forecasts by location, 1950– 2100

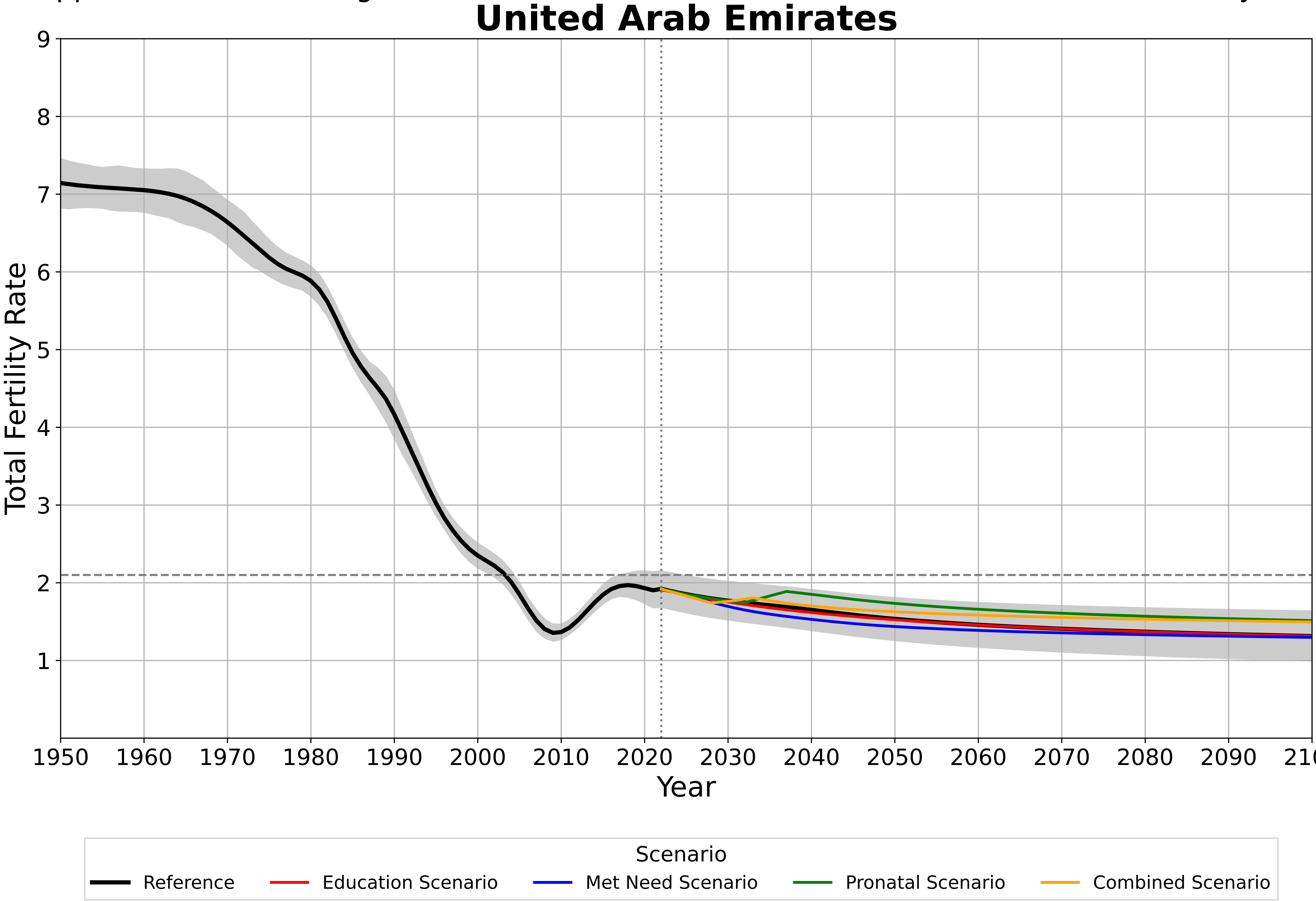

8337

Supplemental Results Figure S5: TFR for reference and alternative scenario forecasts by location, 1950– 2100

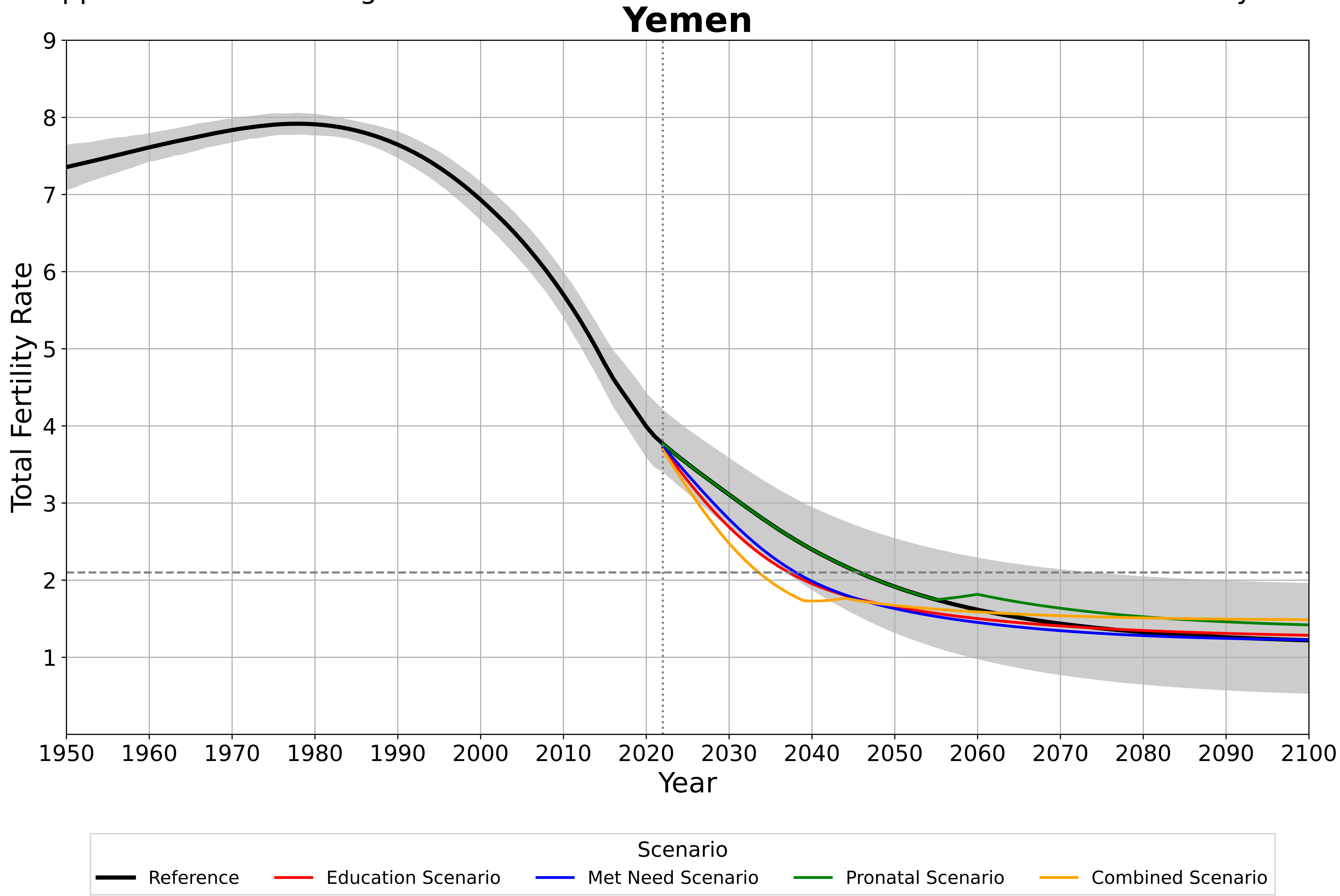

8338

Supplemental Results Figure S5: TFR for reference and alternative scenario forecasts by location, 1950– 2100

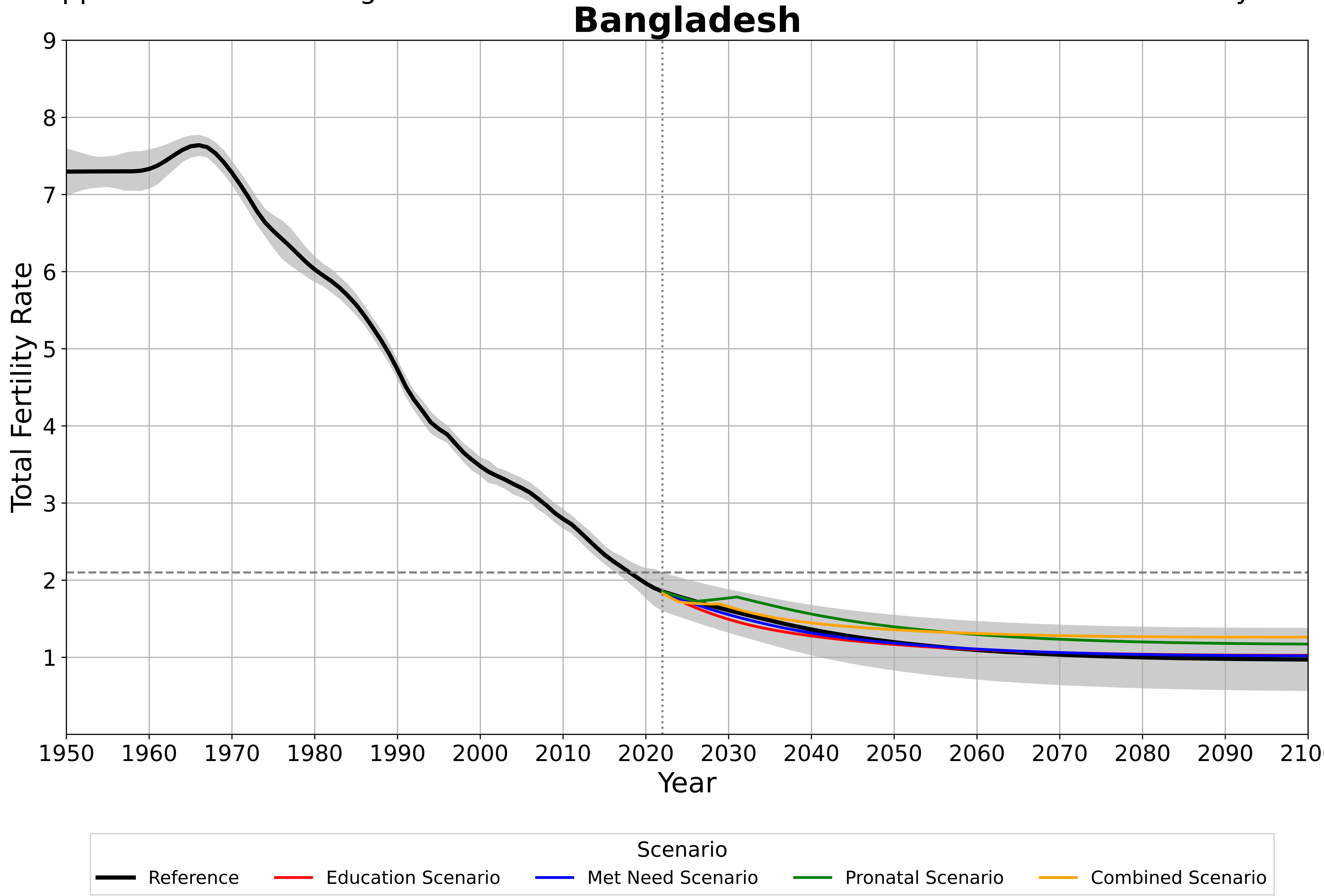

Supplemental Results Figure S5: TFR for reference and alternative scenario forecasts by location, 1950– 2100

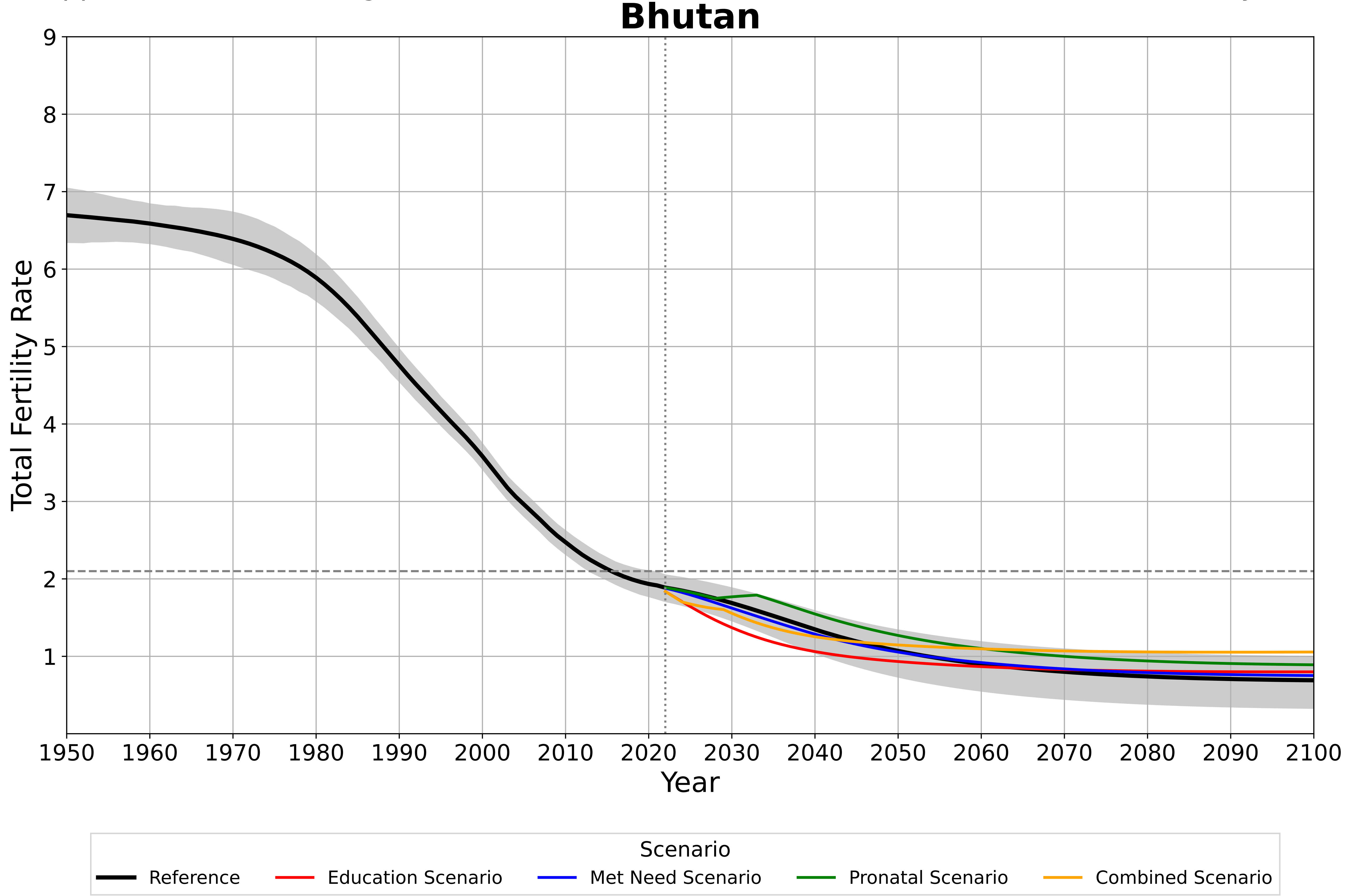

Supplemental Results Figure S5: TFR for reference and alternative scenario forecasts by location, 1950– 2100

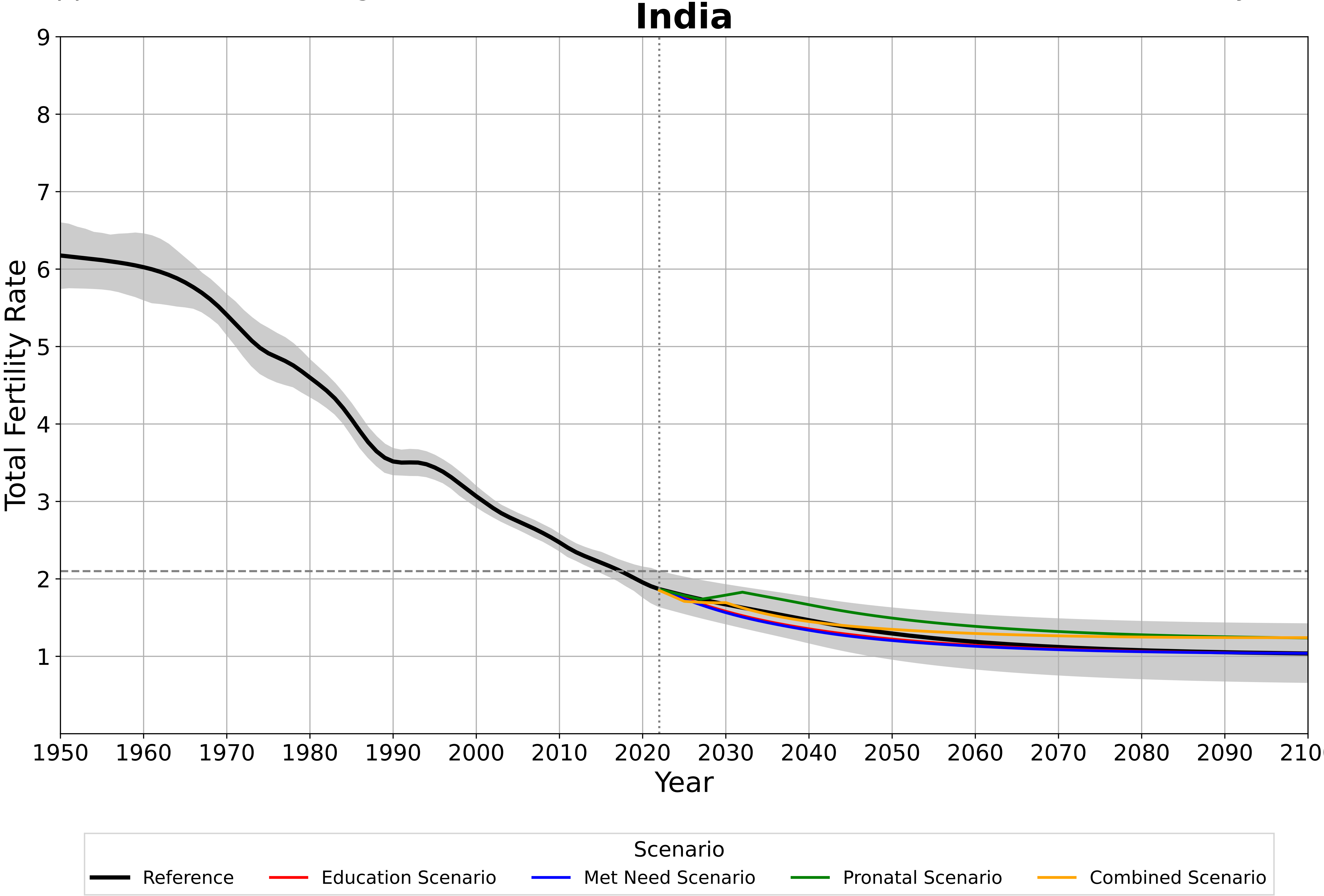

Supplemental Results Figure S5: TFR for reference and alternative scenario forecasts by location, 1950– 2100

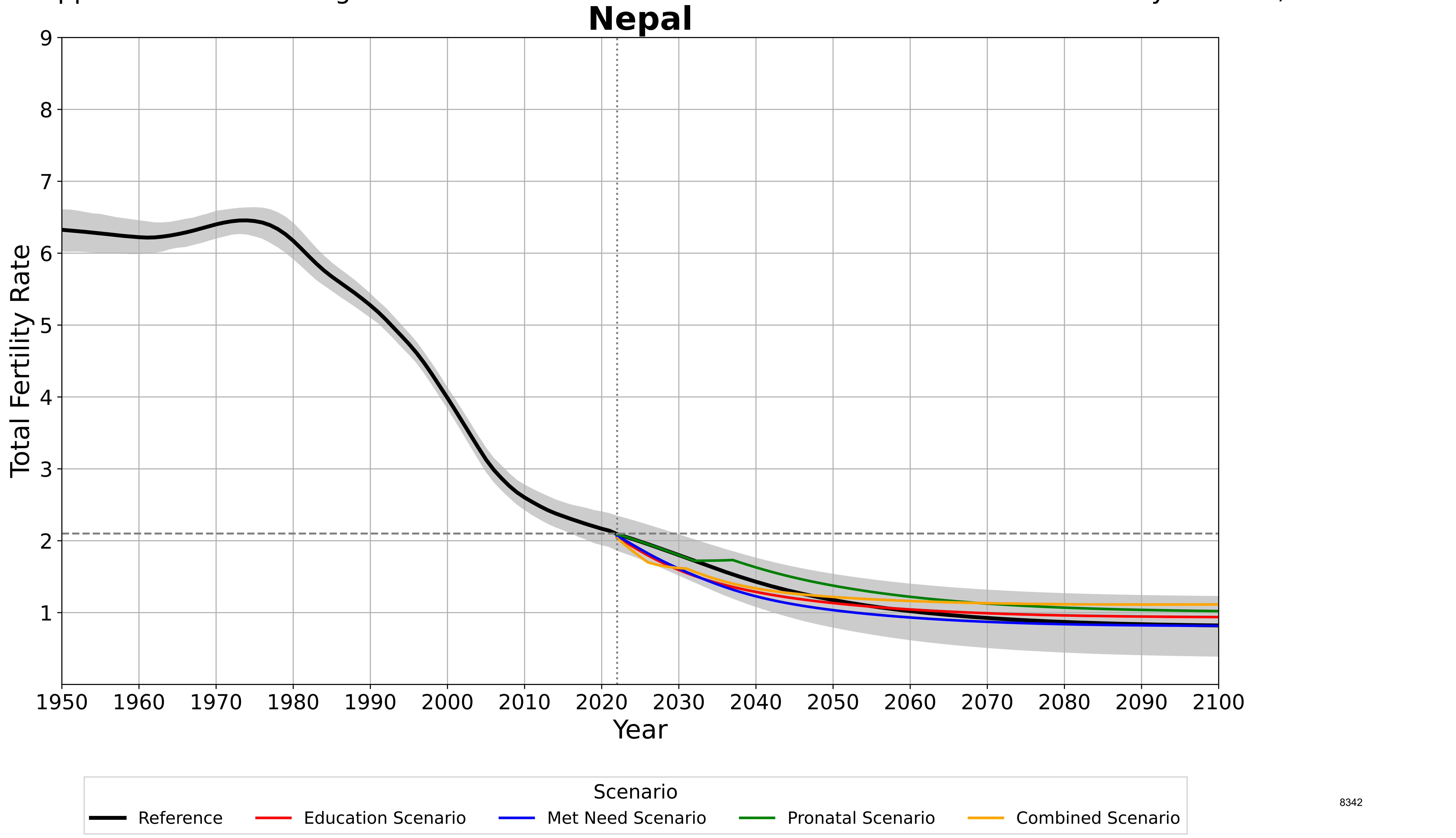

Supplemental Results Figure S5: TFR for reference and alternative scenario forecasts by location, 1950– 2100

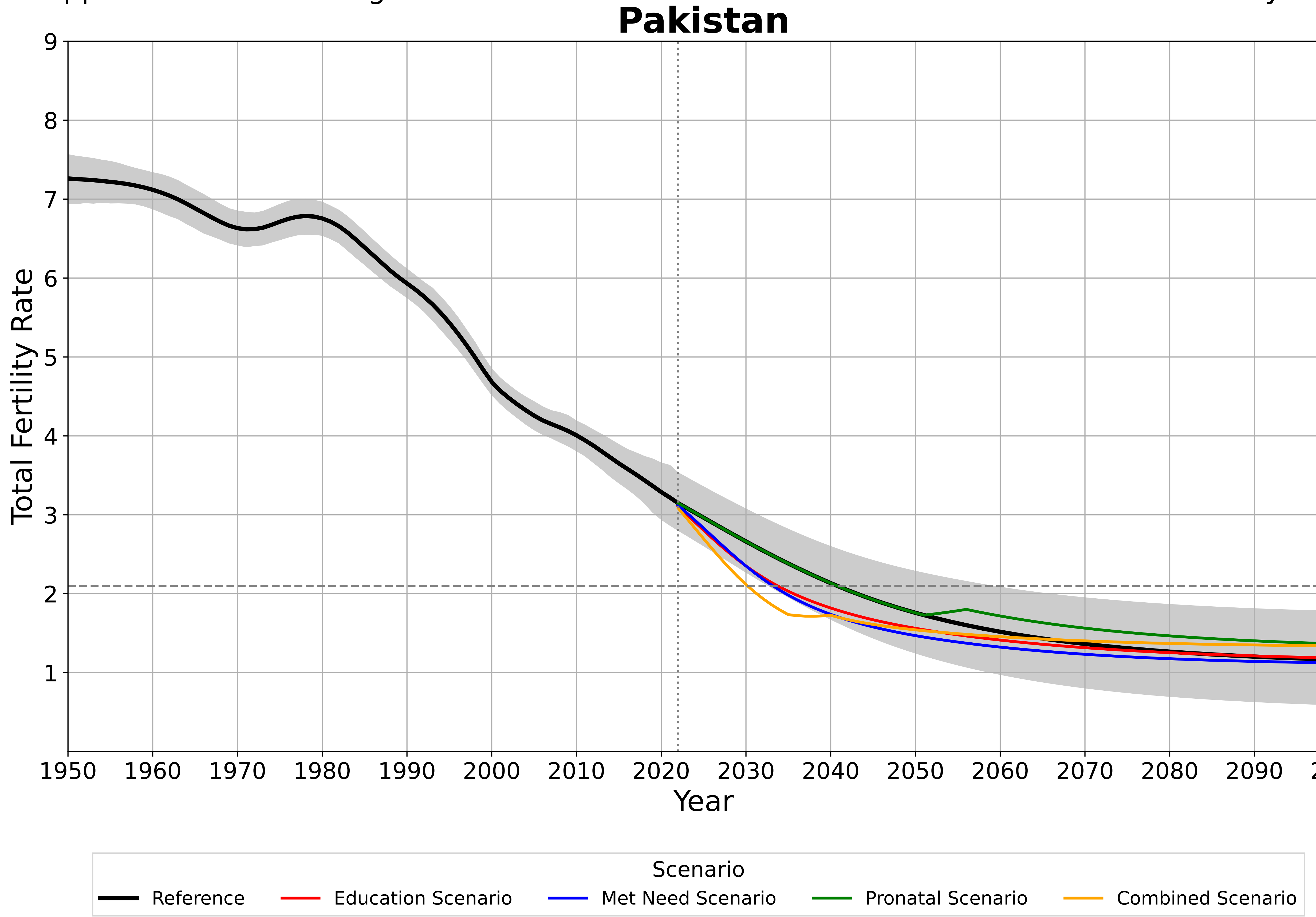

Supplemental Results Figure S5: TFR for reference and alternative scenario forecasts by location, 1950– 2100

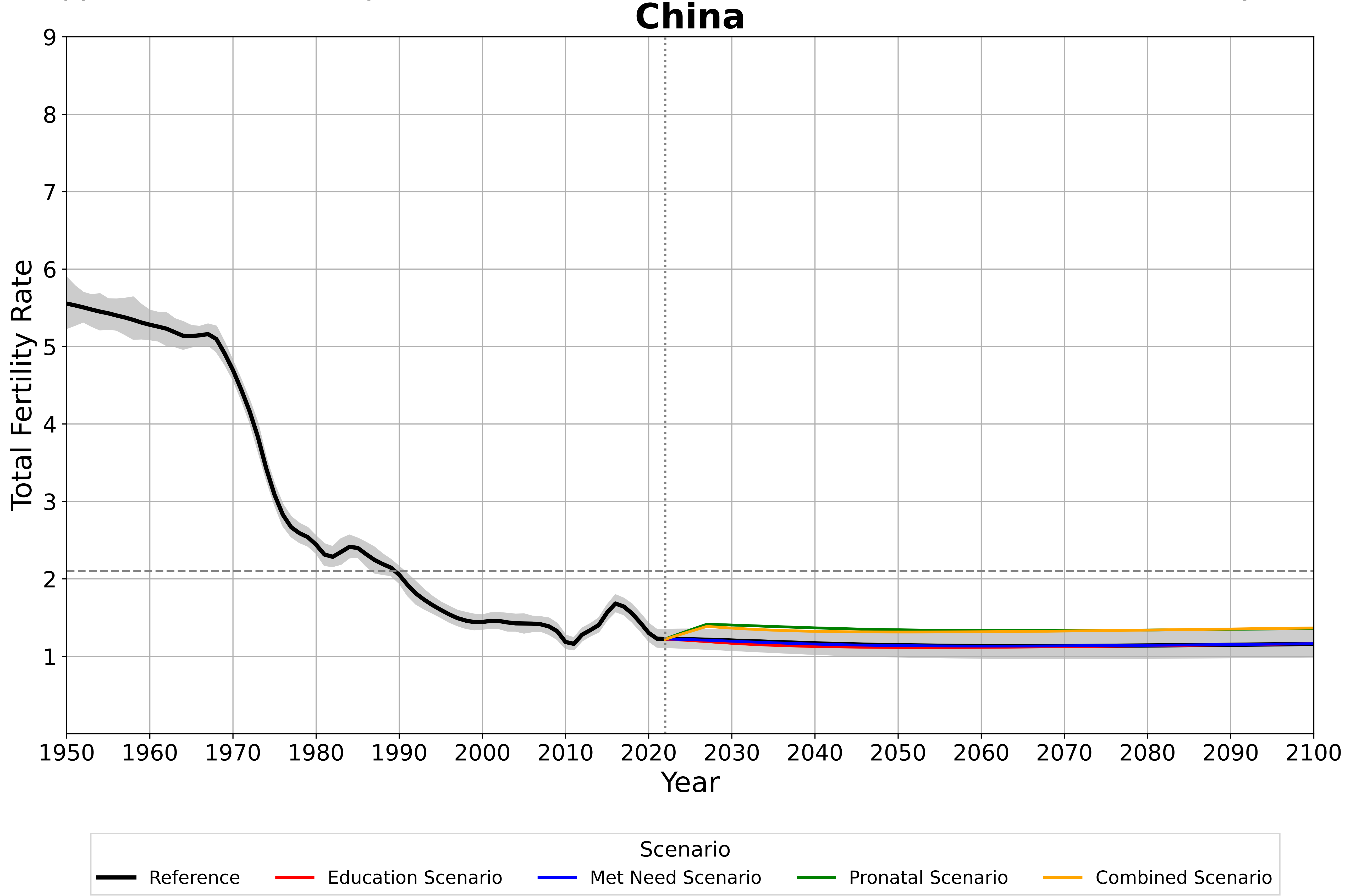

8344

Supplemental Results Figure S5: TFR for reference and alternative scenario forecasts by location, 1950– 2100

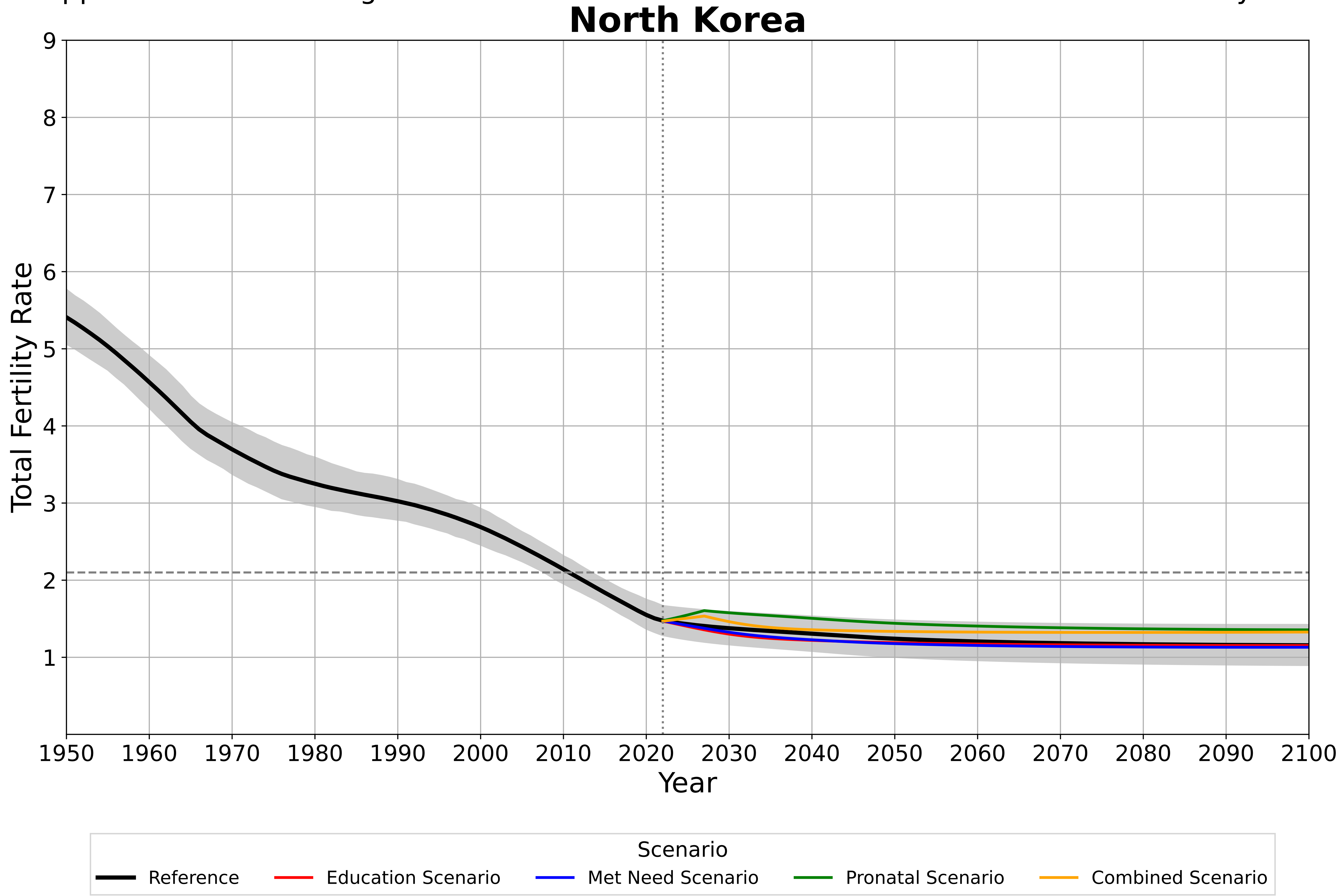

Supplemental Results Figure S5: TFR for reference and alternative scenario forecasts by location, 1950– 2100

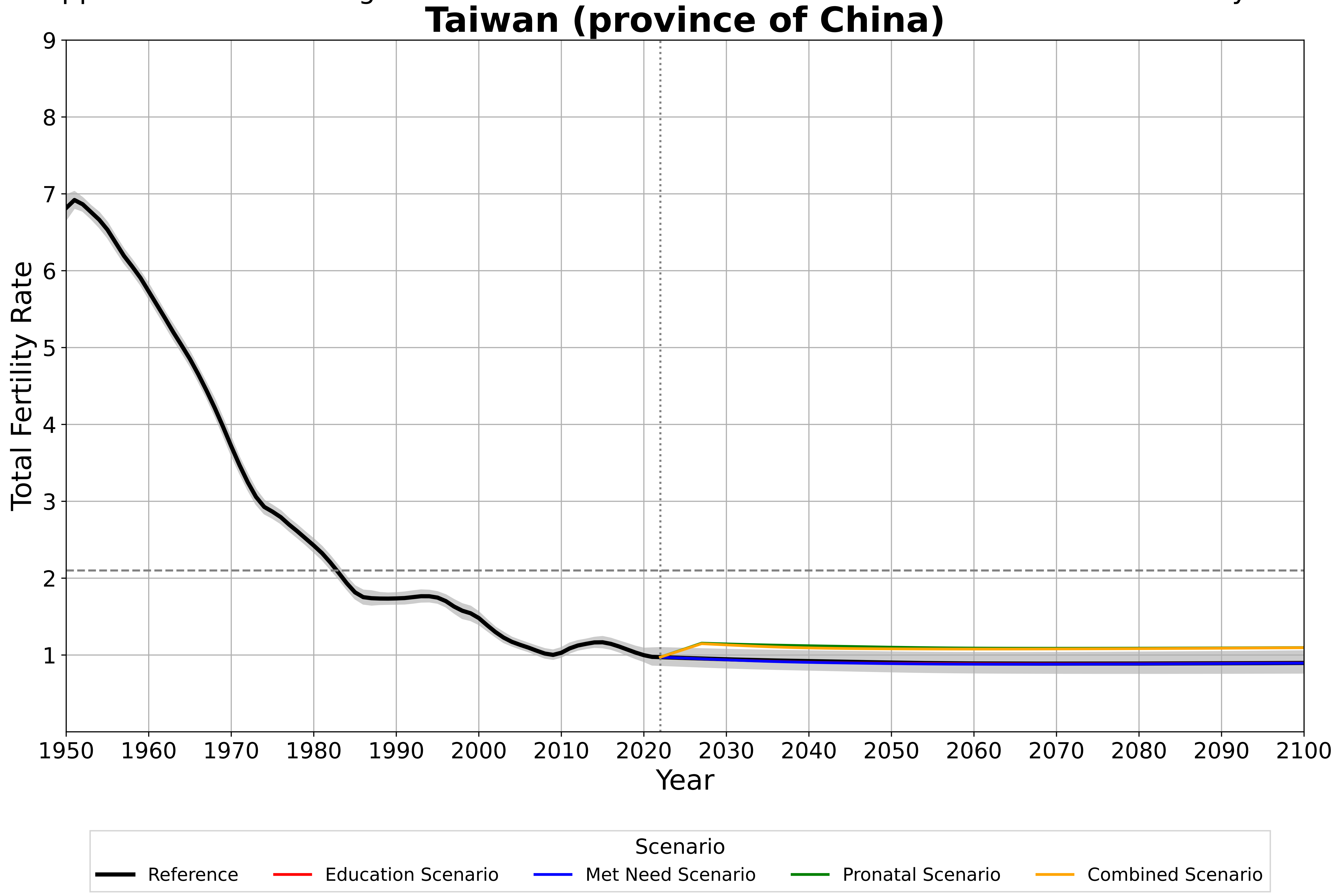

Supplemental Results Figure S5: TFR for reference and alternative scenario forecasts by location, 1950– 2100

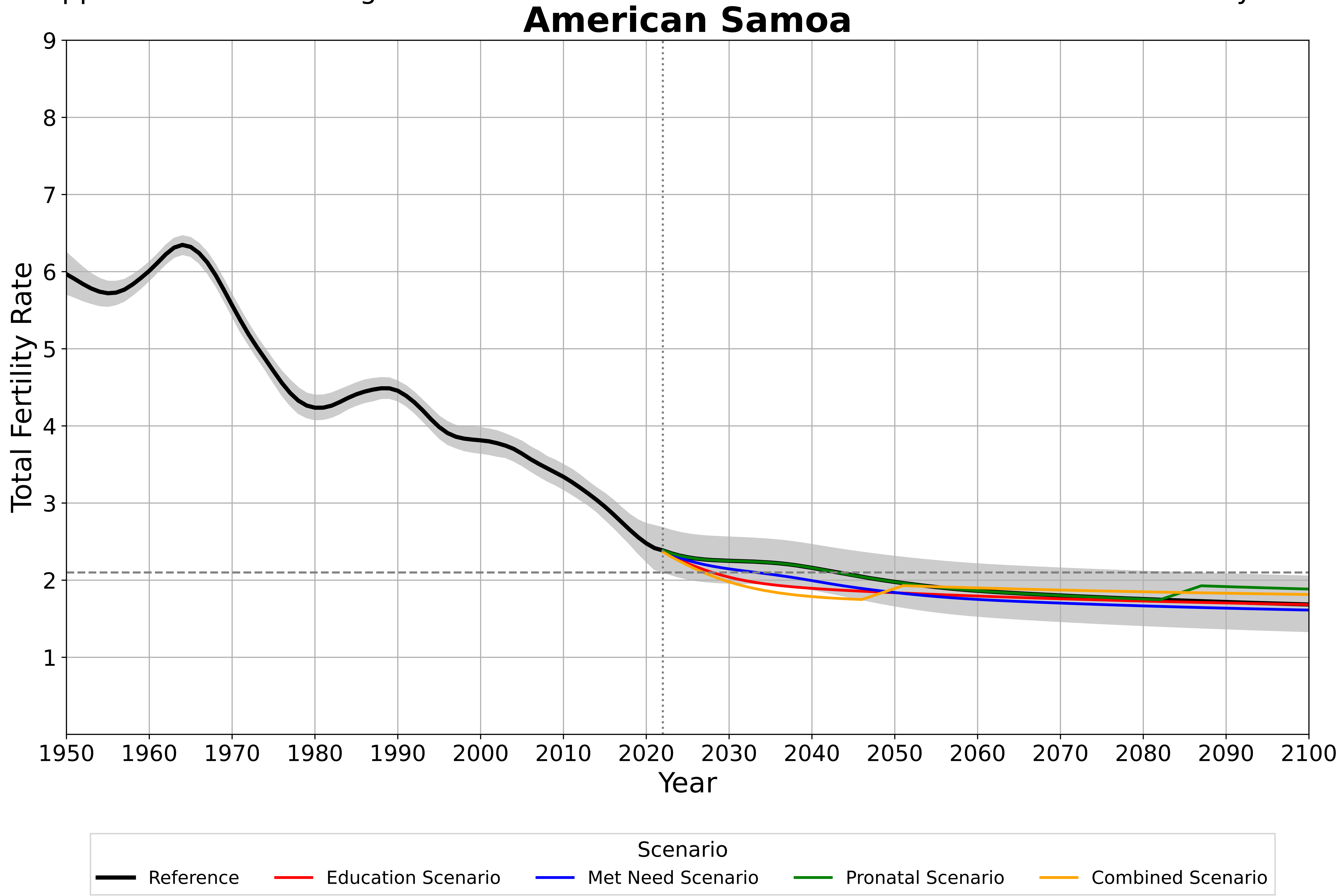

Supplemental Results Figure S5: TFR for reference and alternative scenario forecasts by location, 1950– 2100

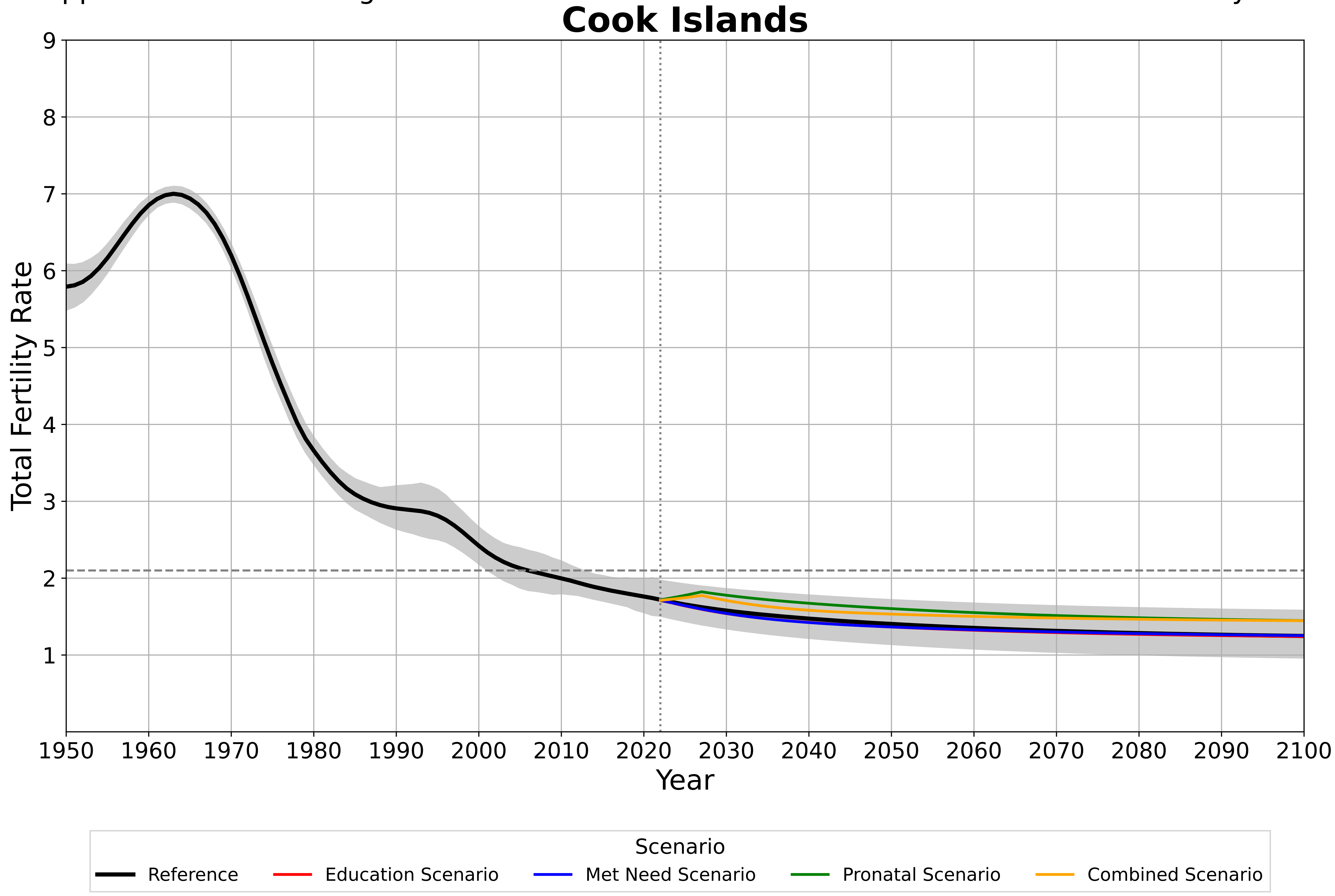

Supplemental Results Figure S5: TFR for reference and alternative scenario forecasts by location, 1950– 2100

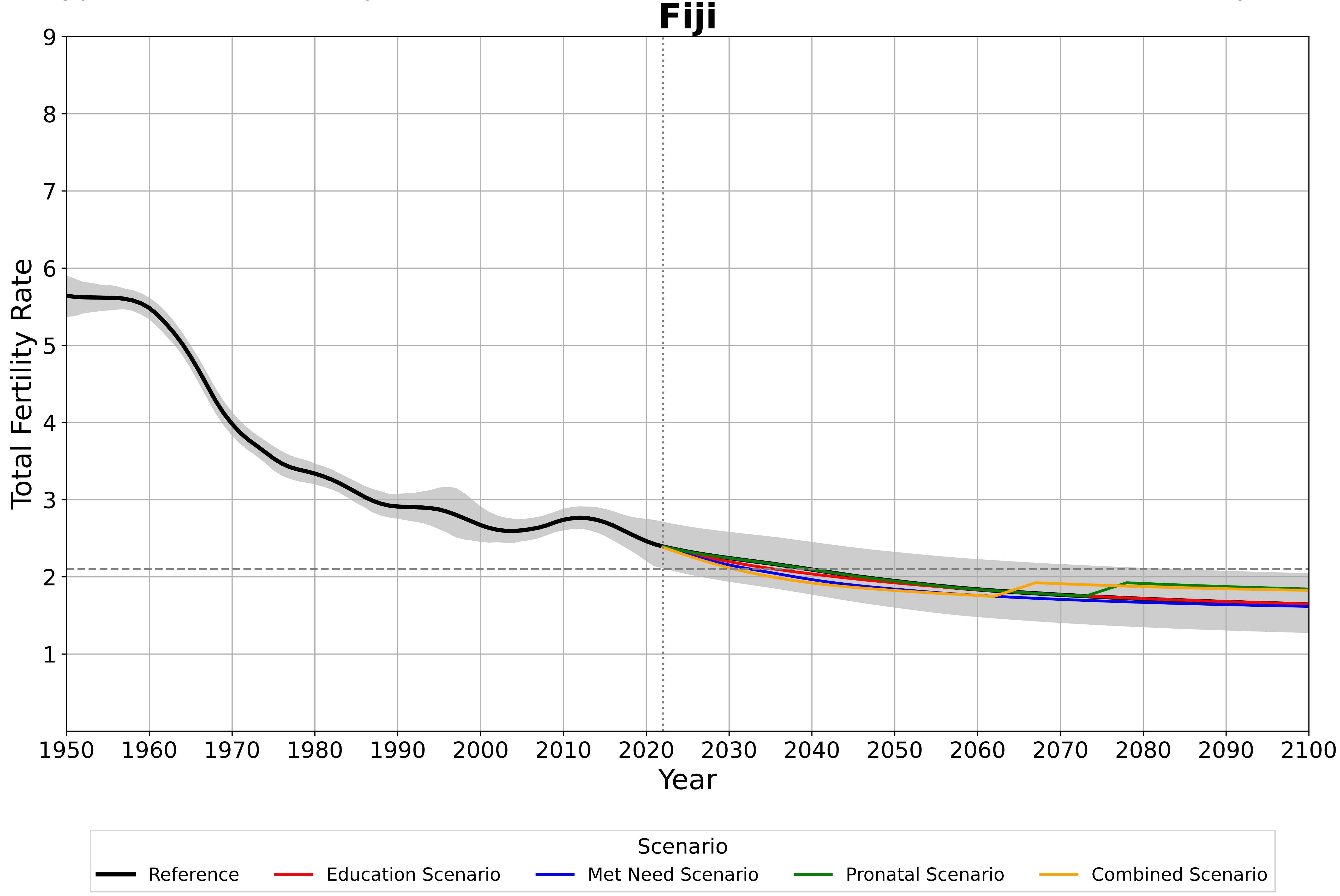

8349

Supplemental Results Figure S5: TFR for reference and alternative scenario forecasts by location, 1950– 2100

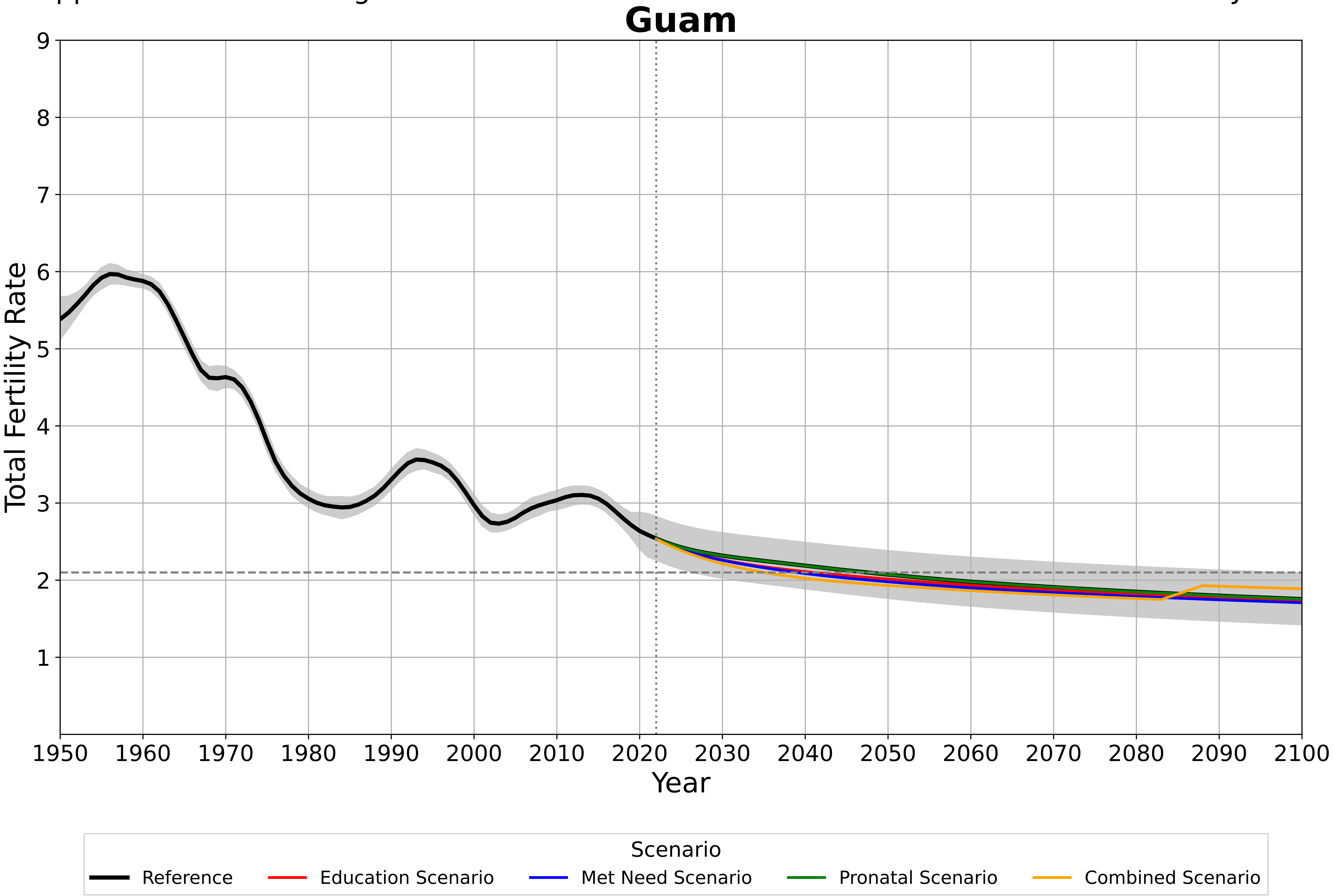

Supplemental Results Figure S5: TFR for reference and alternative scenario forecasts by location, 1950– 2100

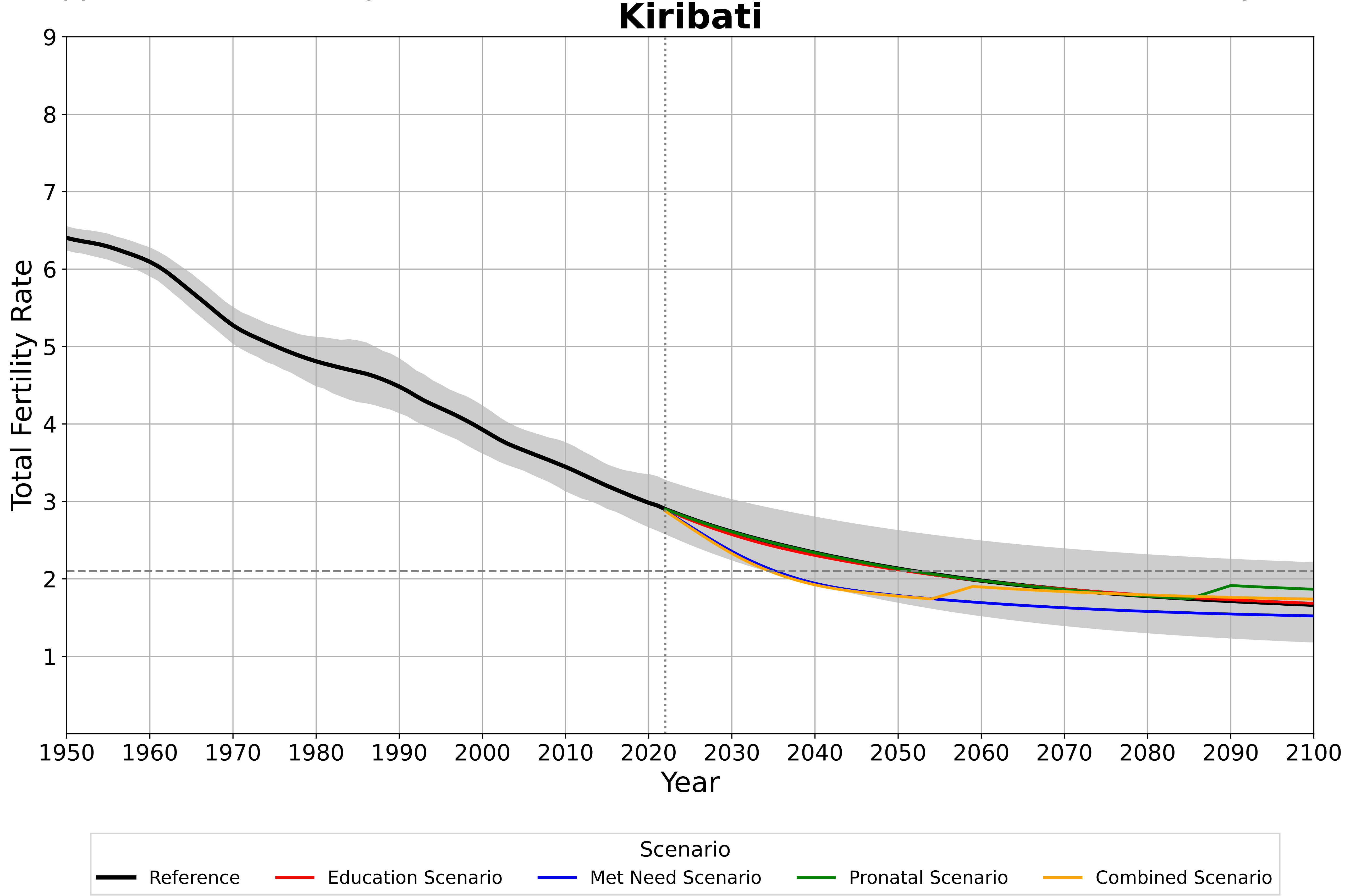

Supplemental Results Figure S5: TFR for reference and alternative scenario forecasts by location, 1950– 2100

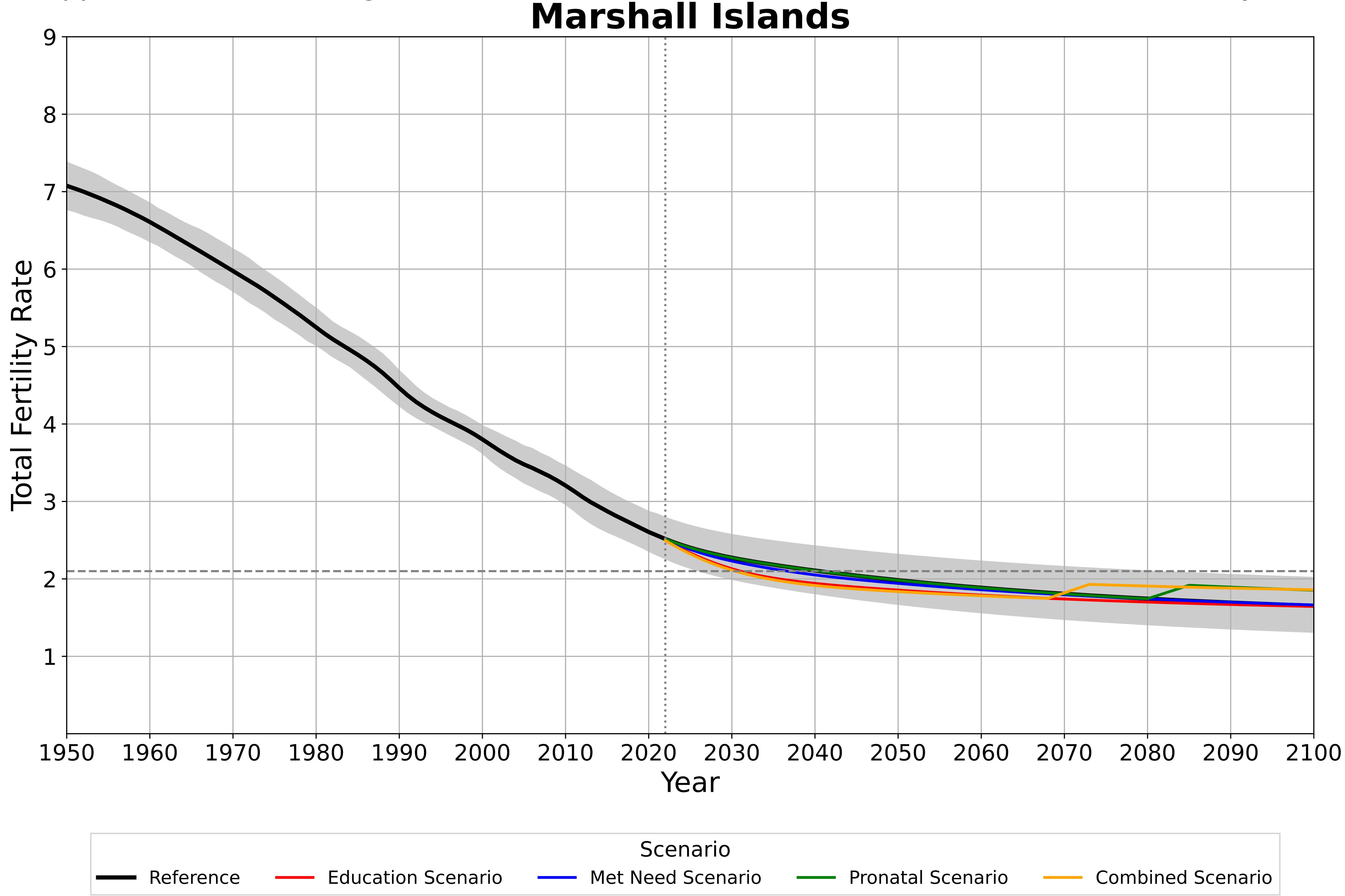

Supplemental Results Figure S5: TFR for reference and alternative scenario forecasts by location, 1950– 2100

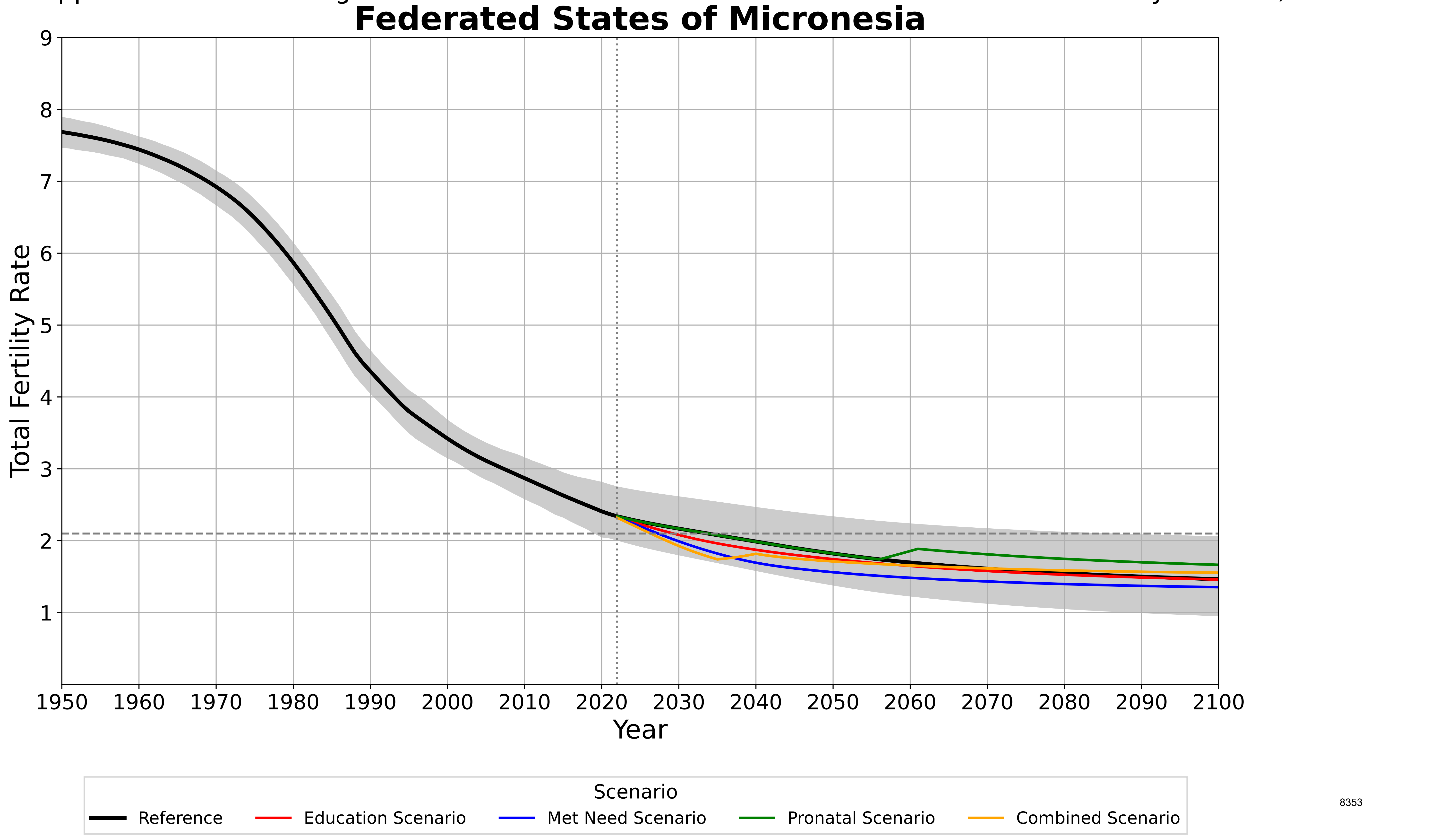

Supplemental Results Figure S5: TFR for reference and alternative scenario forecasts by location, 1950– 2100

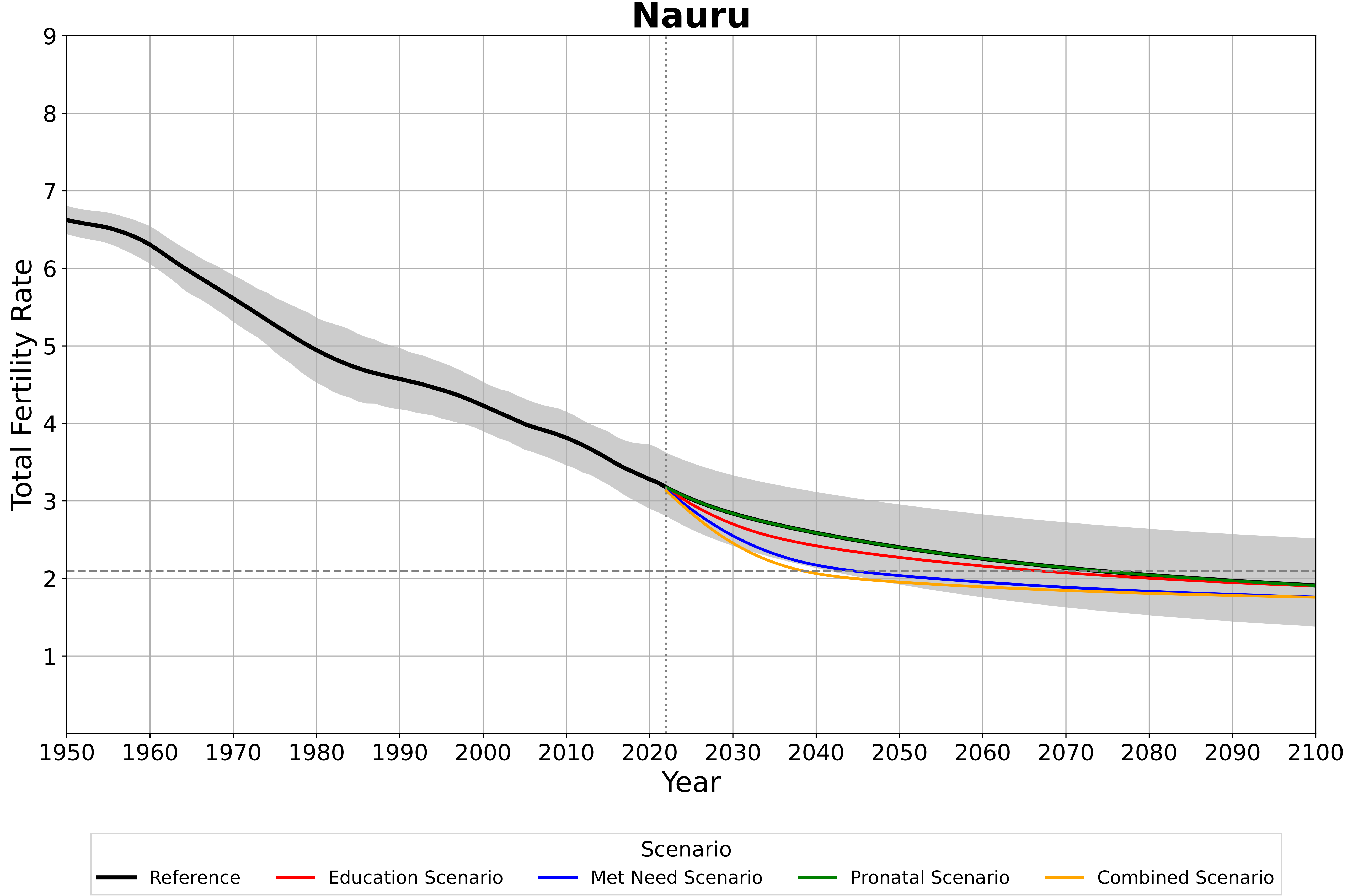

Supplemental Results Figure S5: TFR for reference and alternative scenario forecasts by location, 1950– 2100

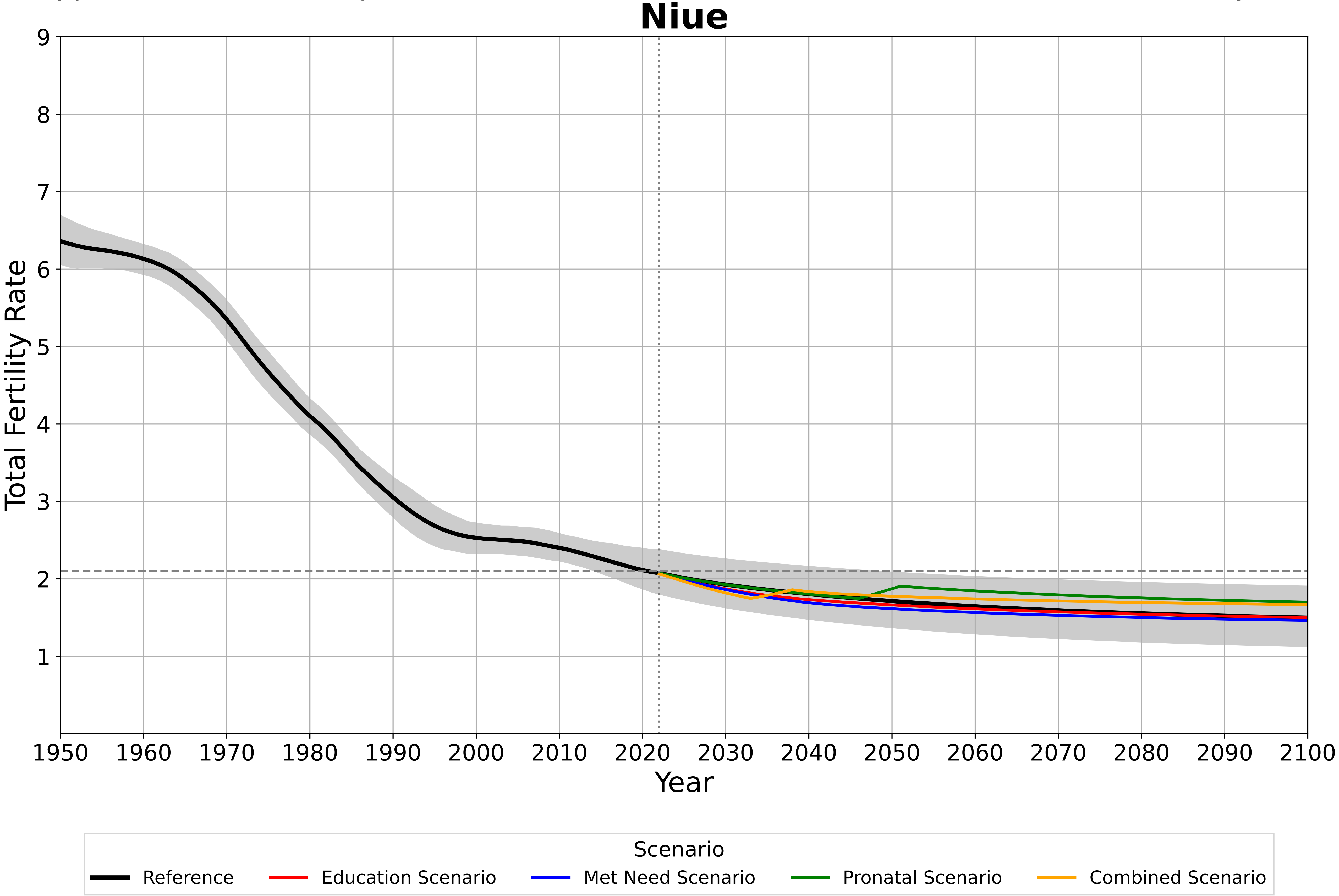

Supplemental Results Figure S5: TFR for reference and alternative scenario forecasts by location, 1950– 2100

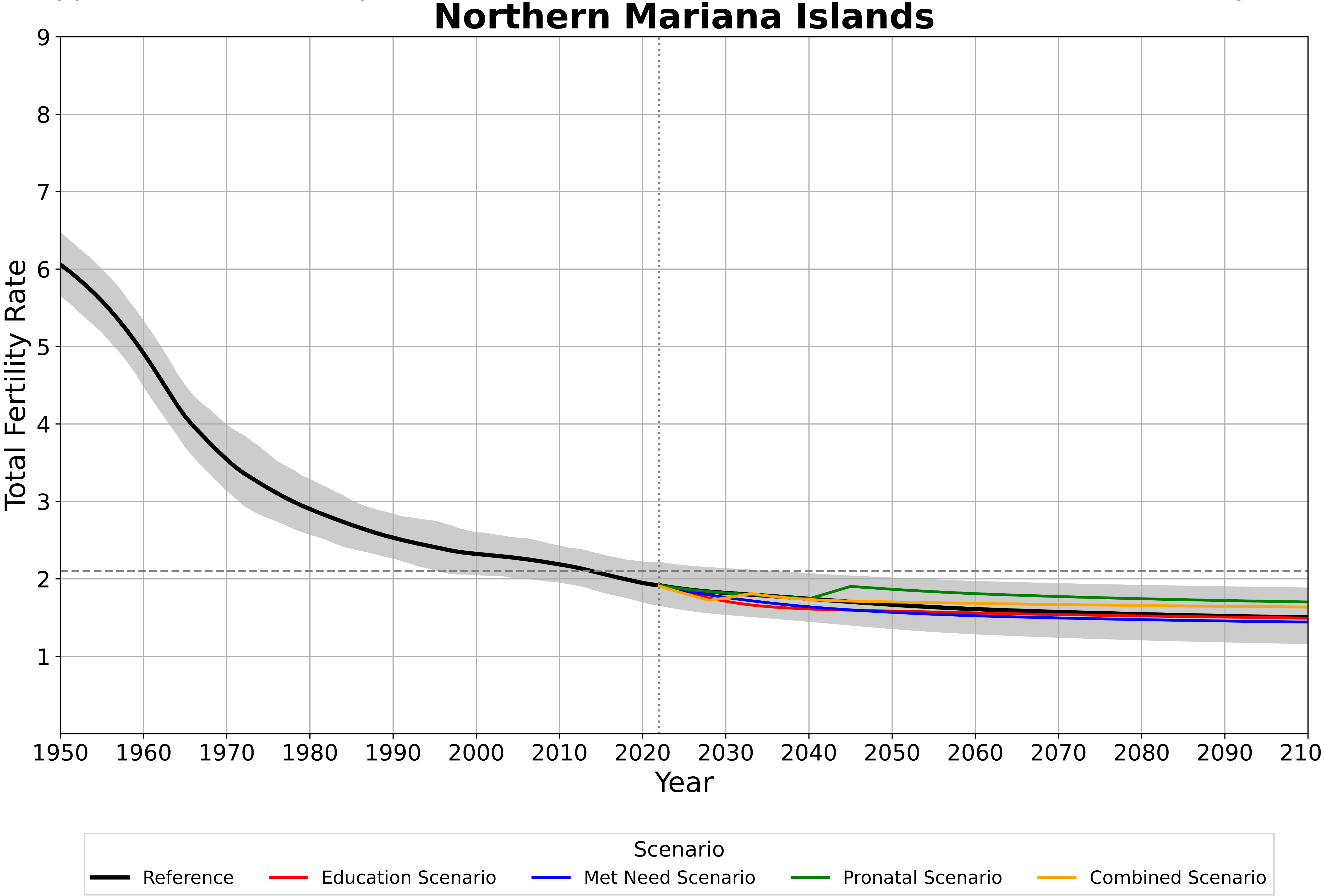

Supplemental Results Figure S5: TFR for reference and alternative scenario forecasts by location, 1950– 2100

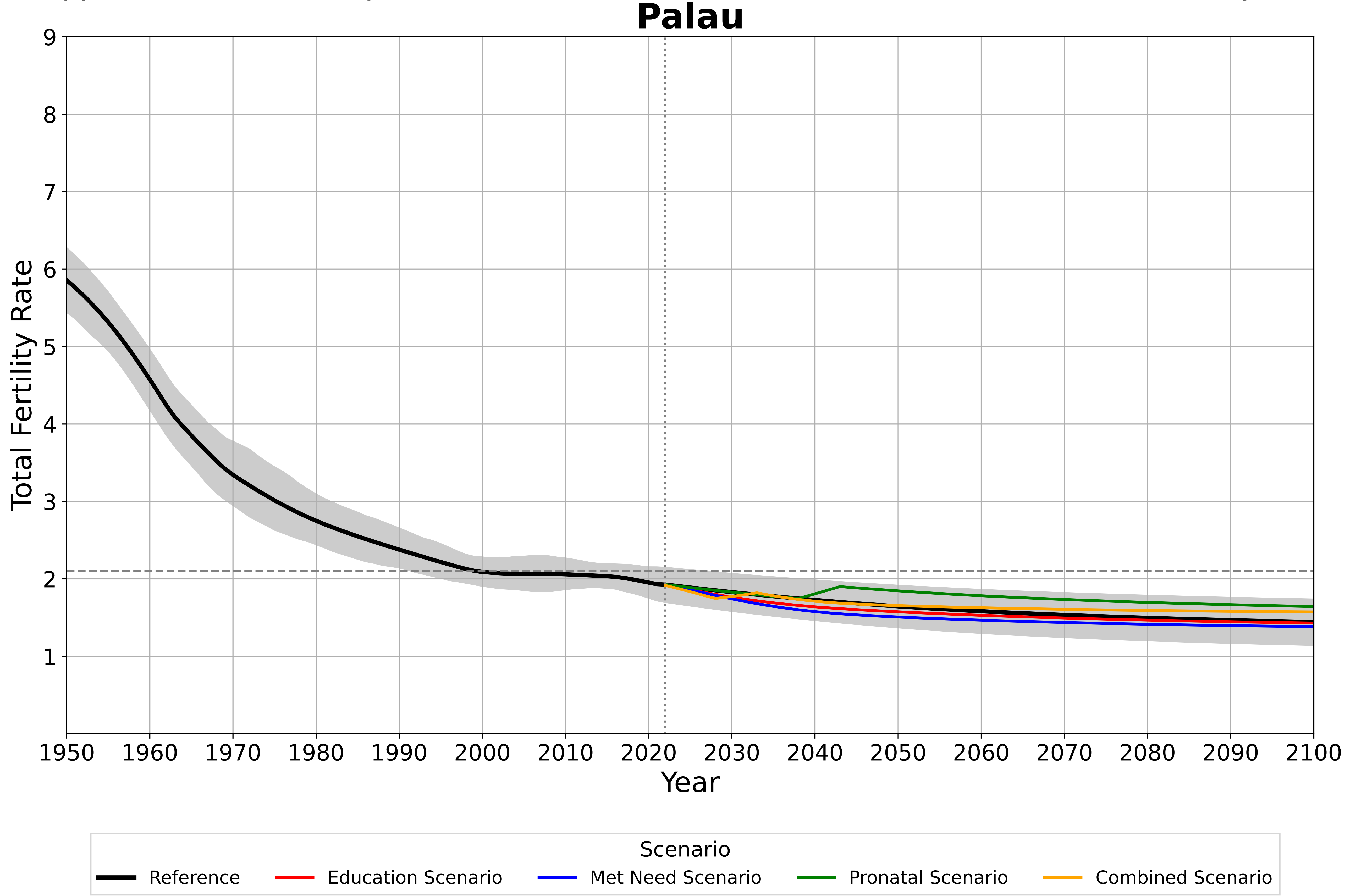

Supplemental Results Figure S5: TFR for reference and alternative scenario forecasts by location, 1950– 2100

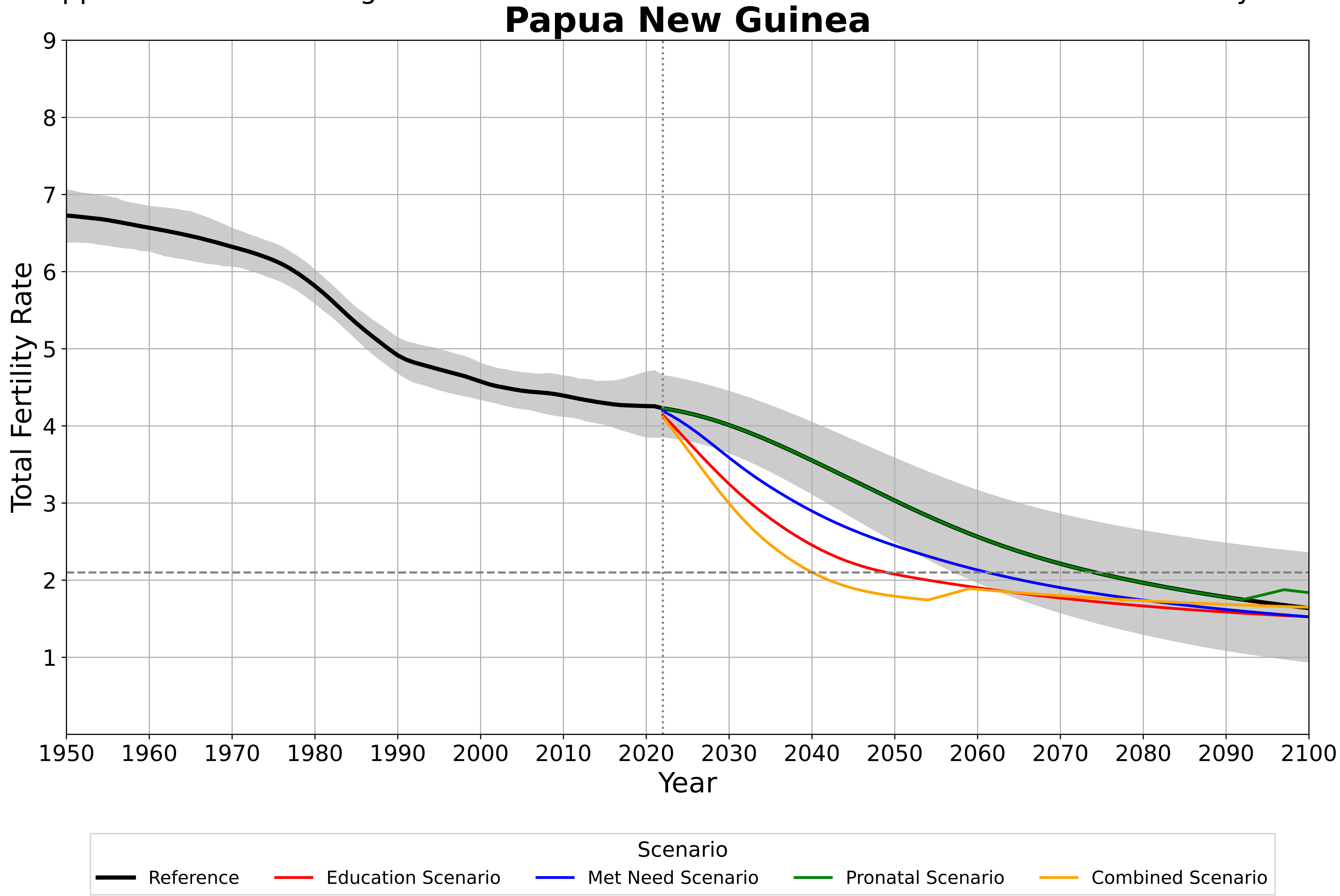

Supplemental Results Figure S5: TFR for reference and alternative scenario forecasts by location, 1950– 2100

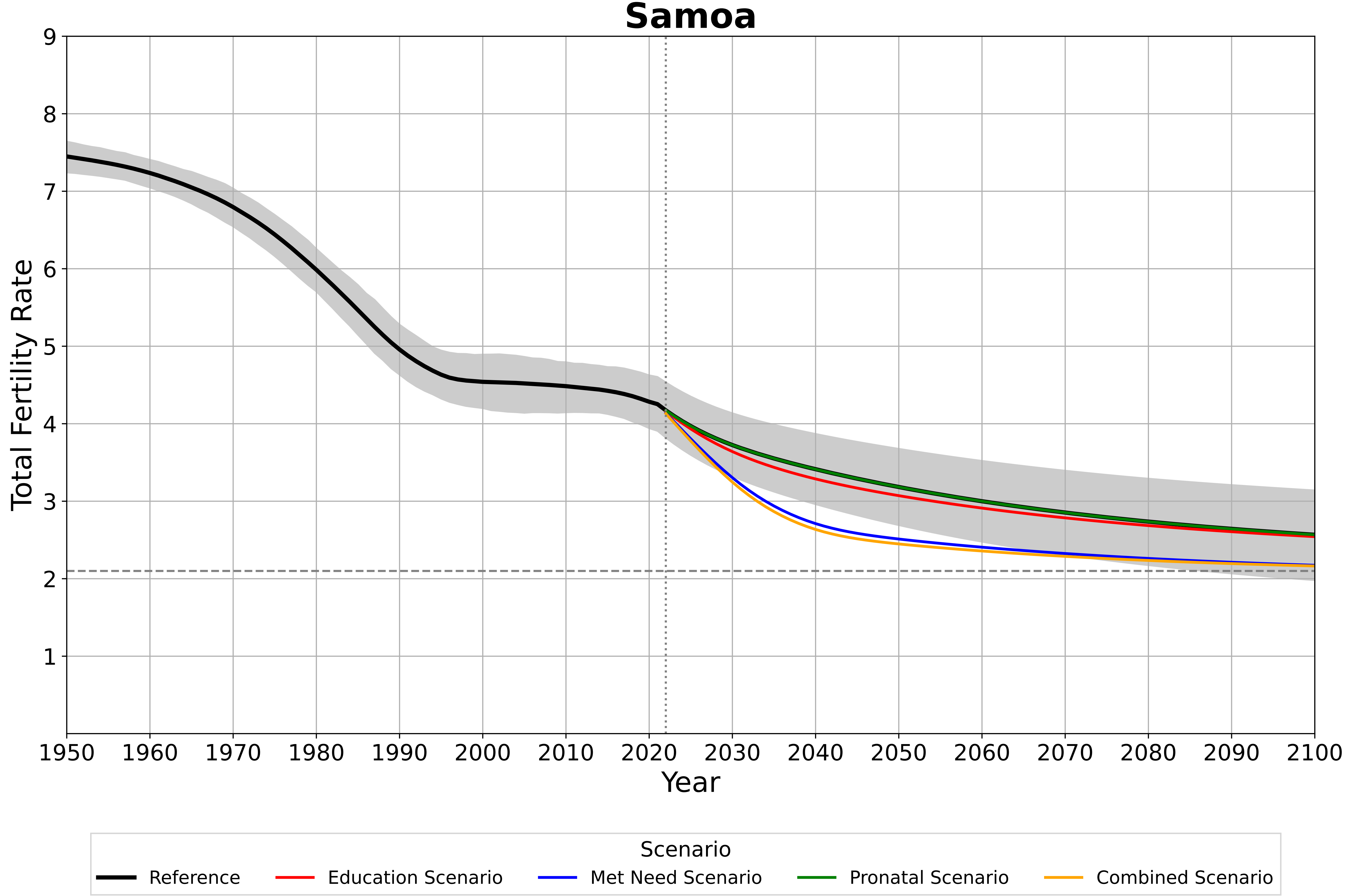

Supplemental Results Figure S5: TFR for reference and alternative scenario forecasts by location, 1950– 2100

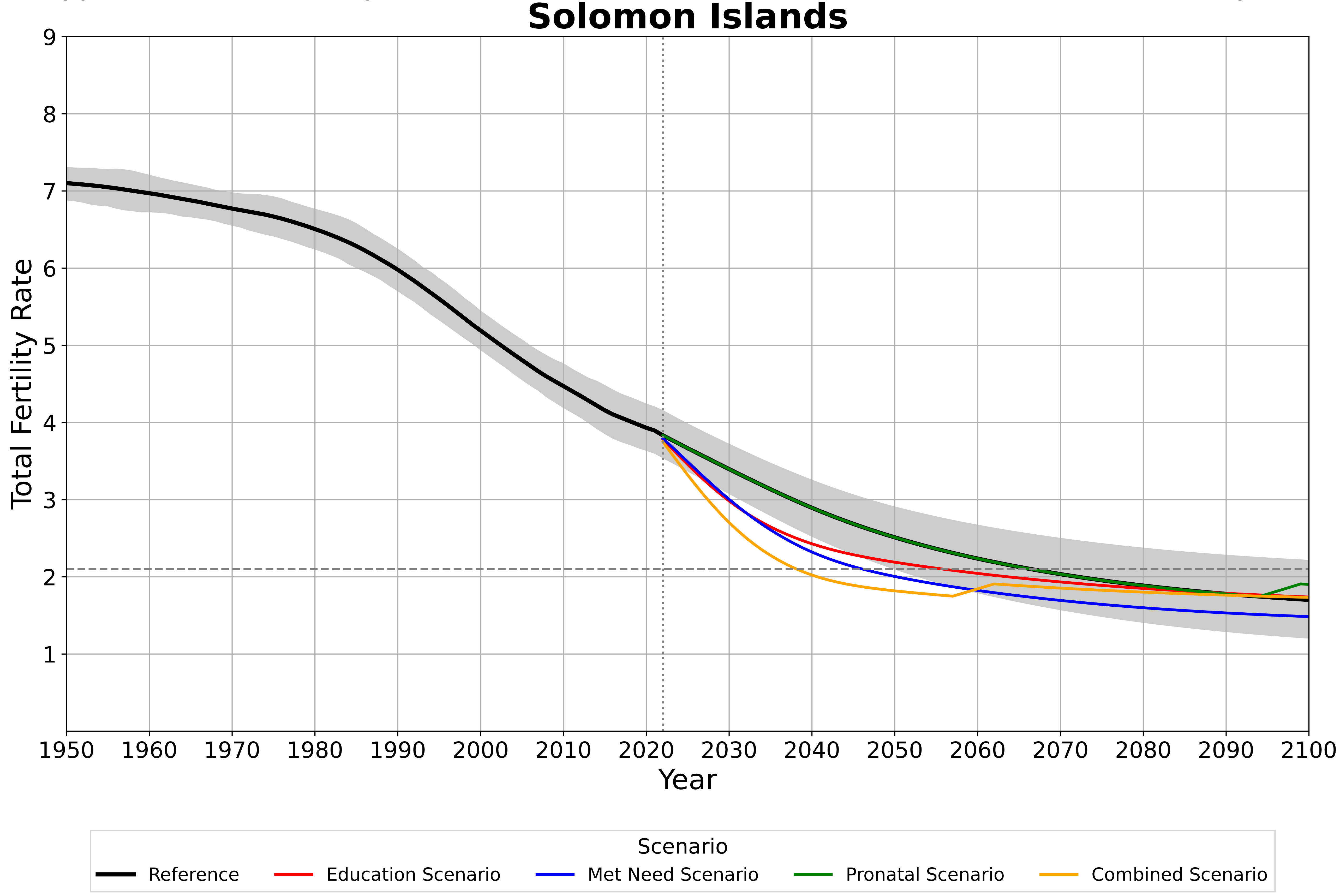

Supplemental Results Figure S5: TFR for reference and alternative scenario forecasts by location, 1950– 2100

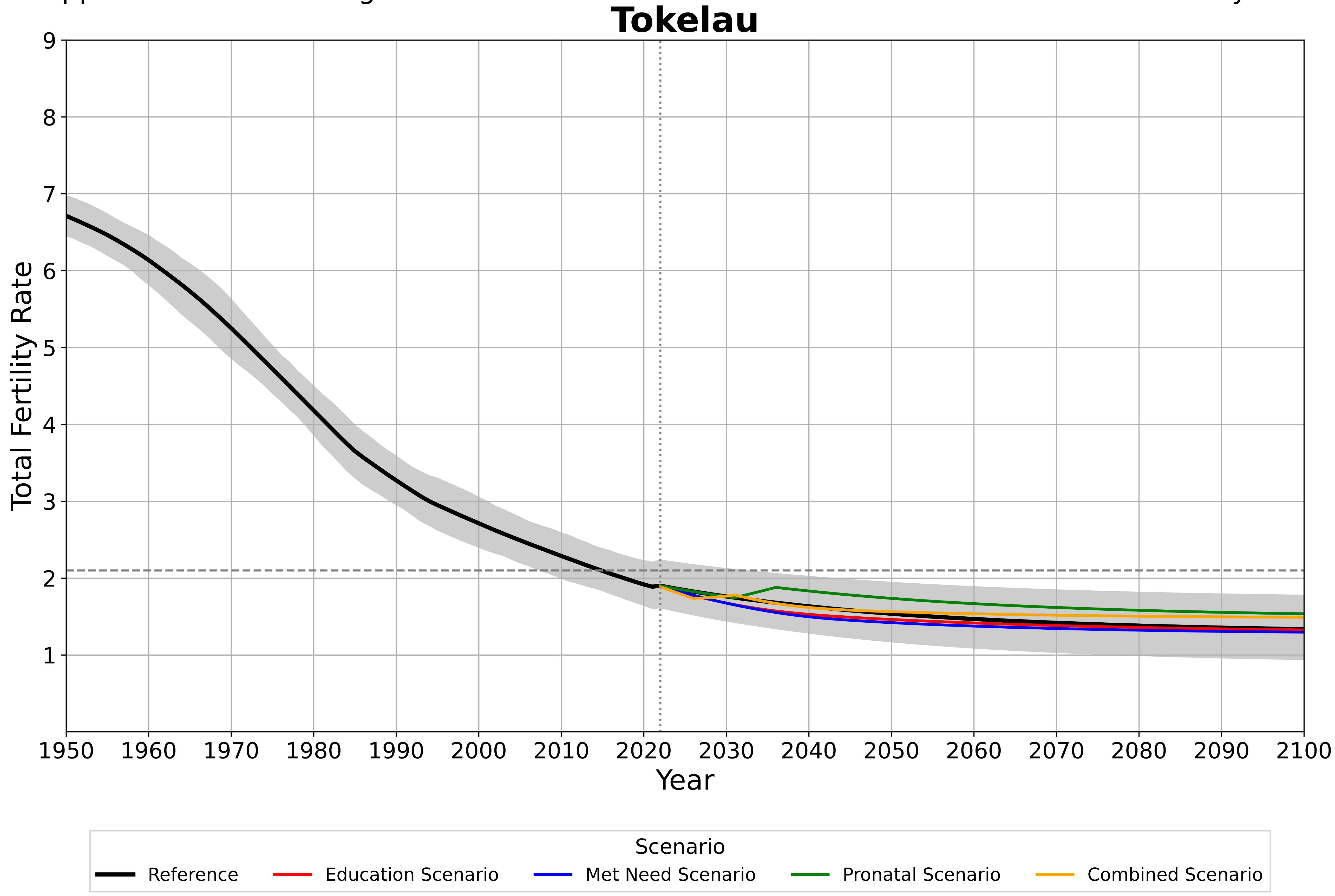

Supplemental Results Figure S5: TFR for reference and alternative scenario forecasts by location, 1950– 2100

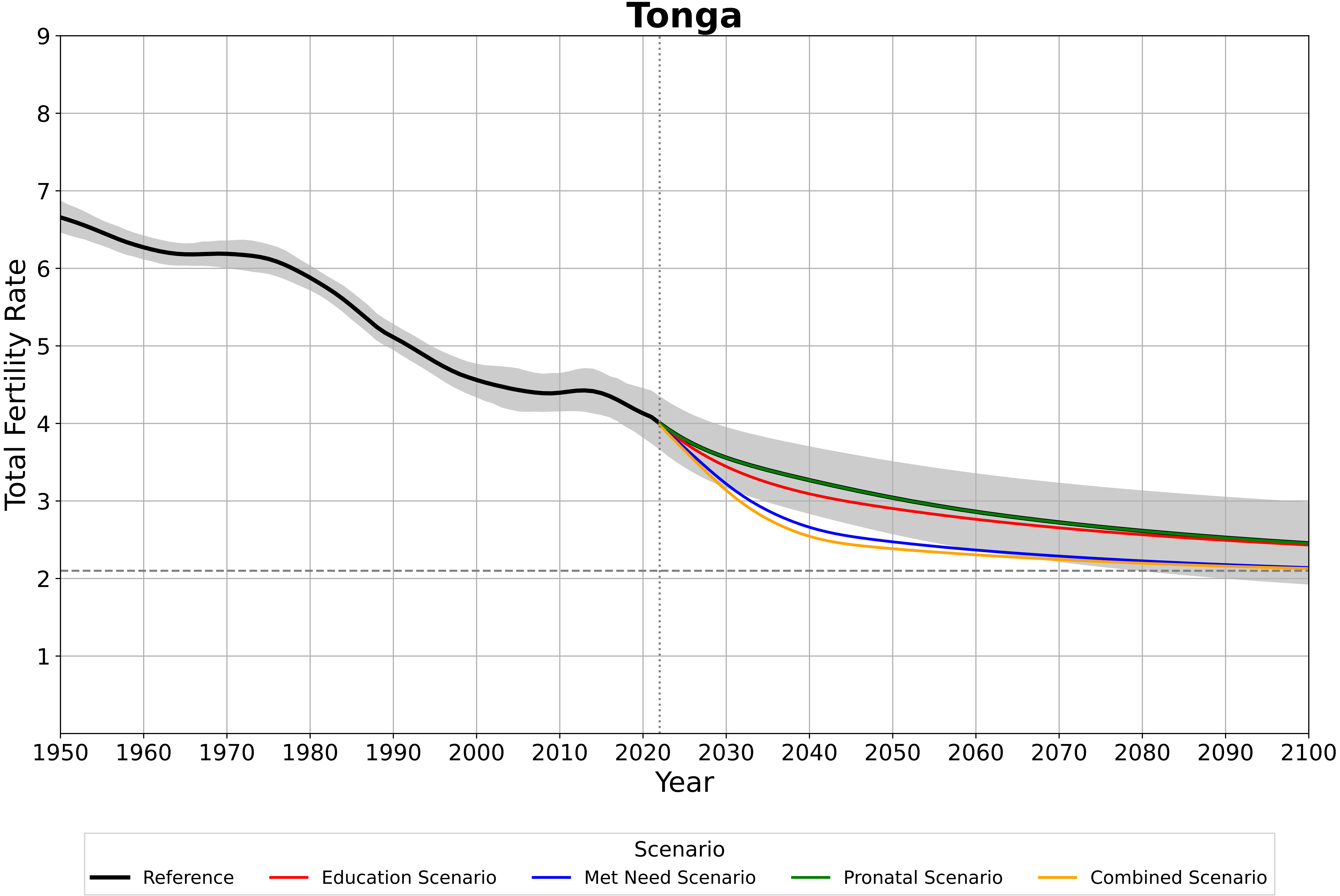

Supplemental Results Figure S5: TFR for reference and alternative scenario forecasts by location, 1950– 2100

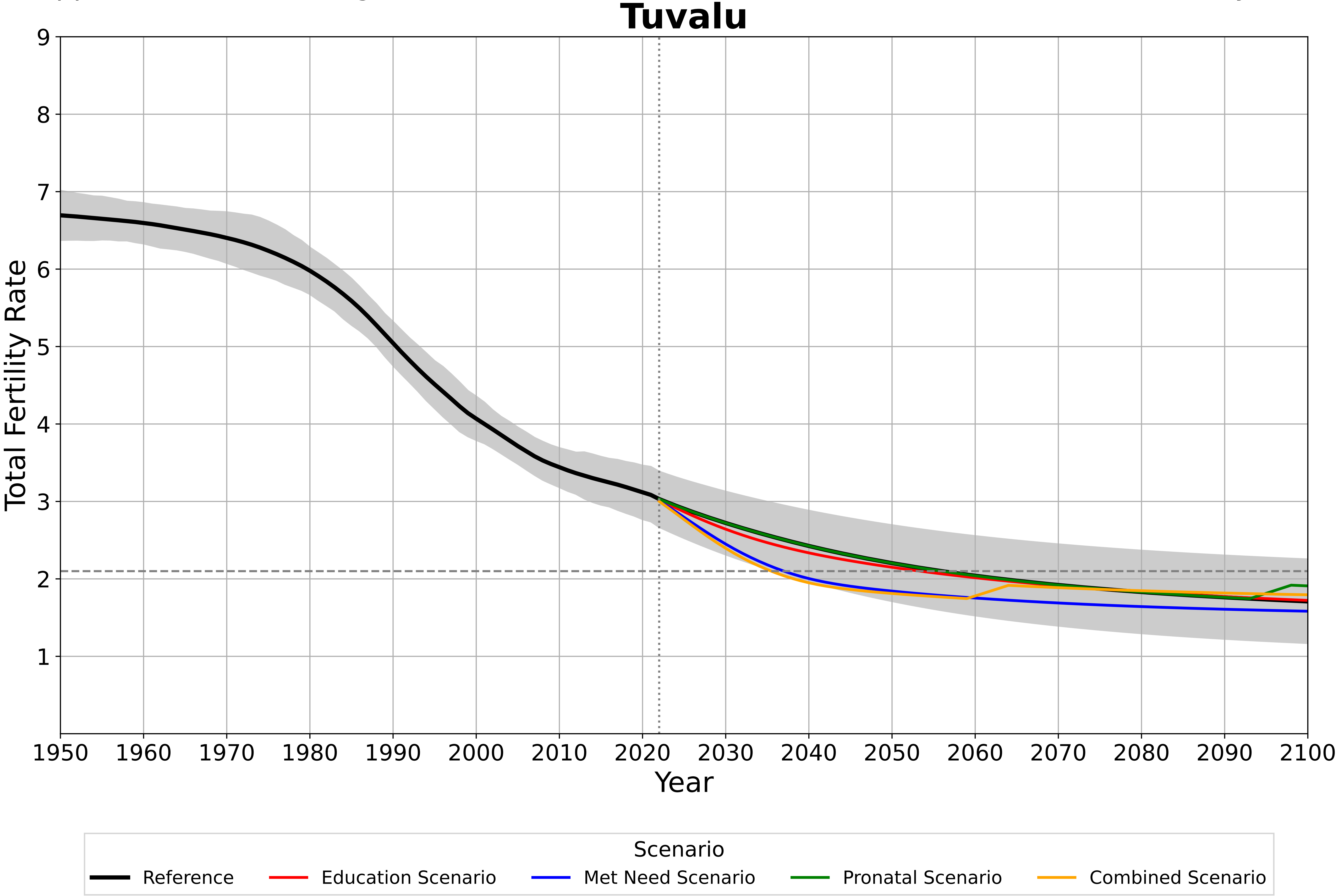

8363

Supplemental Results Figure S5: TFR for reference and alternative scenario forecasts by location, 1950– 2100

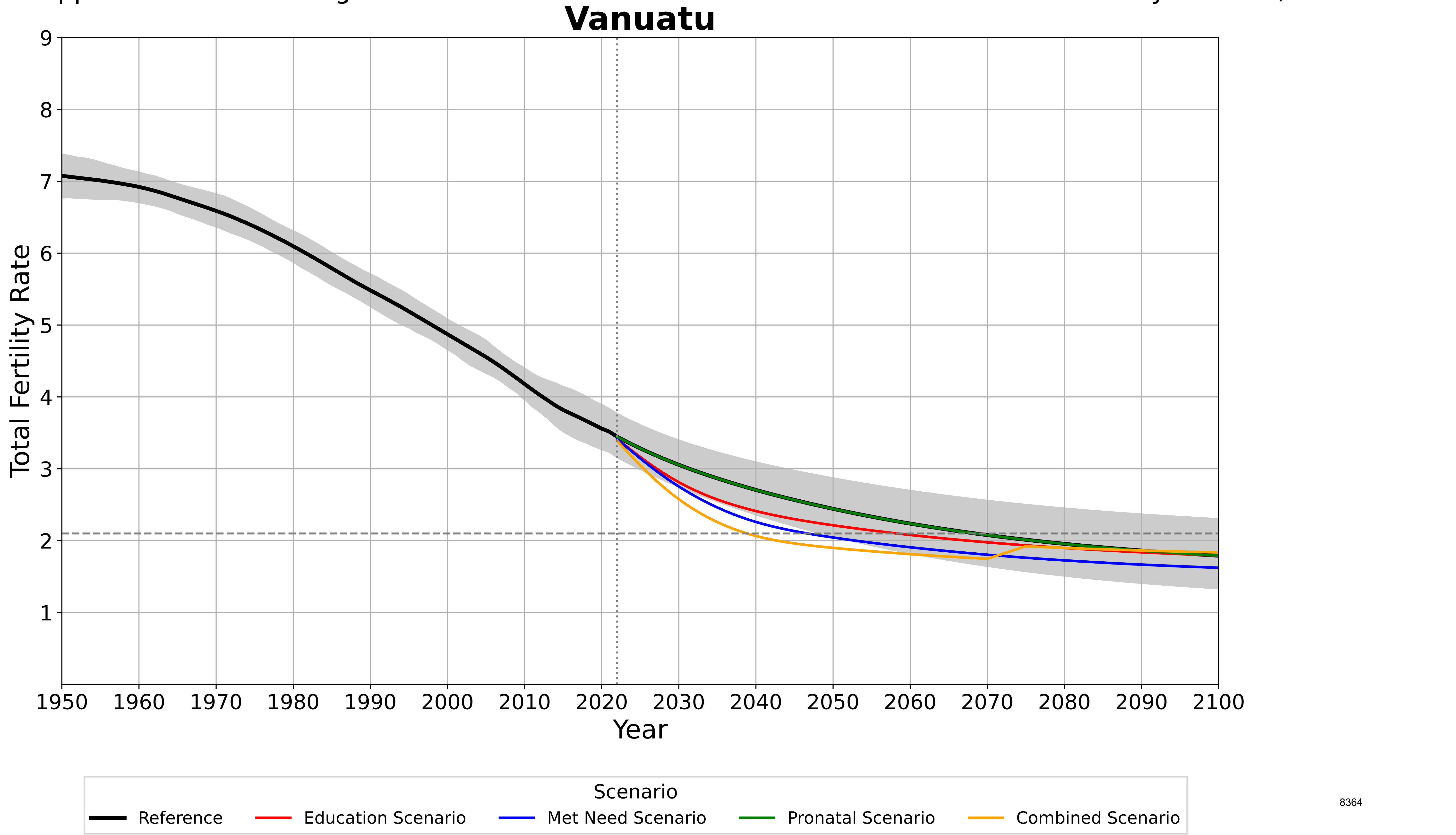

8364

Supplemental Results Figure S5: TFR for reference and alternative scenario forecasts by location, 1950– 2100

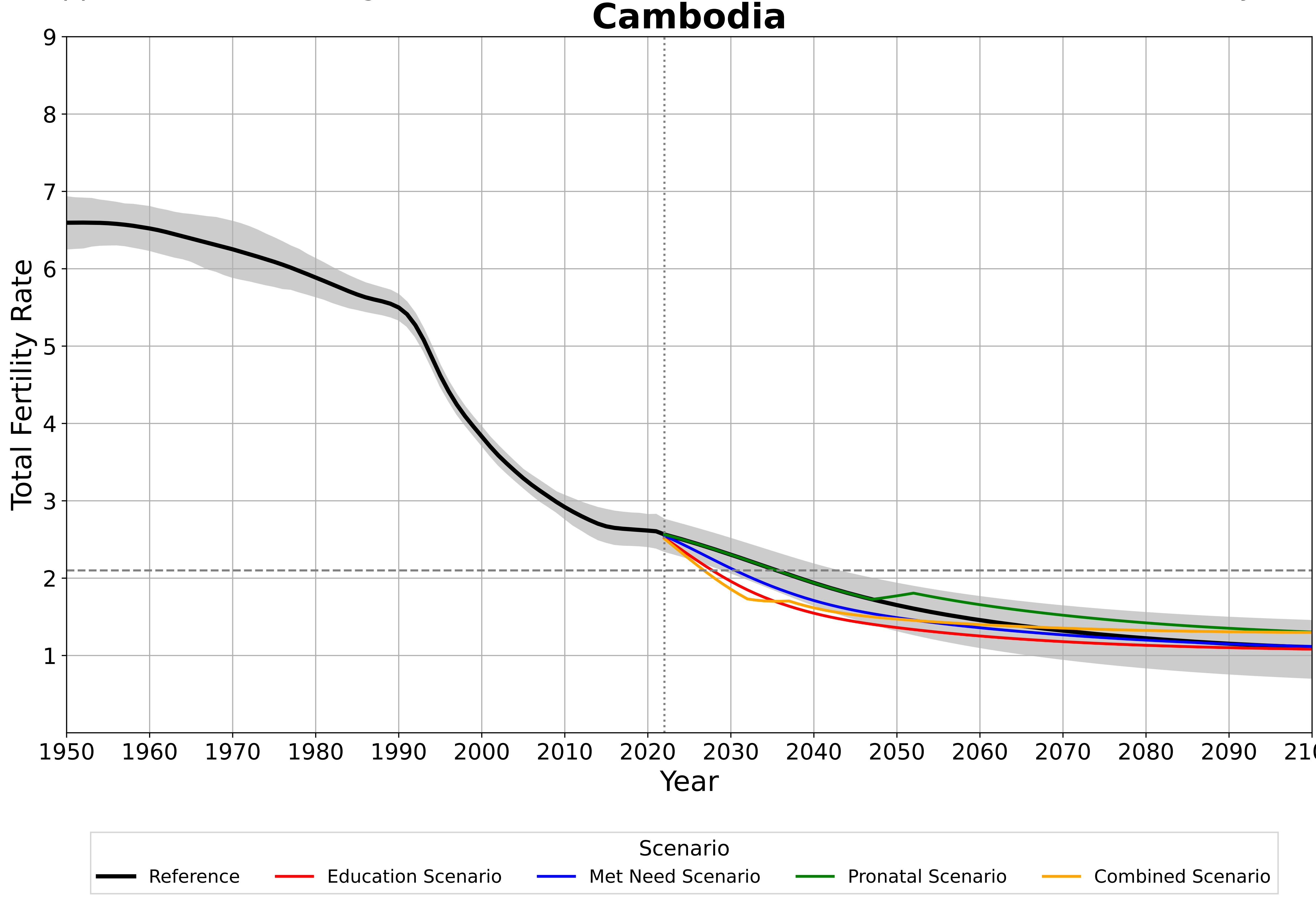

Supplemental Results Figure S5: TFR for reference and alternative scenario forecasts by location, 1950– 2100

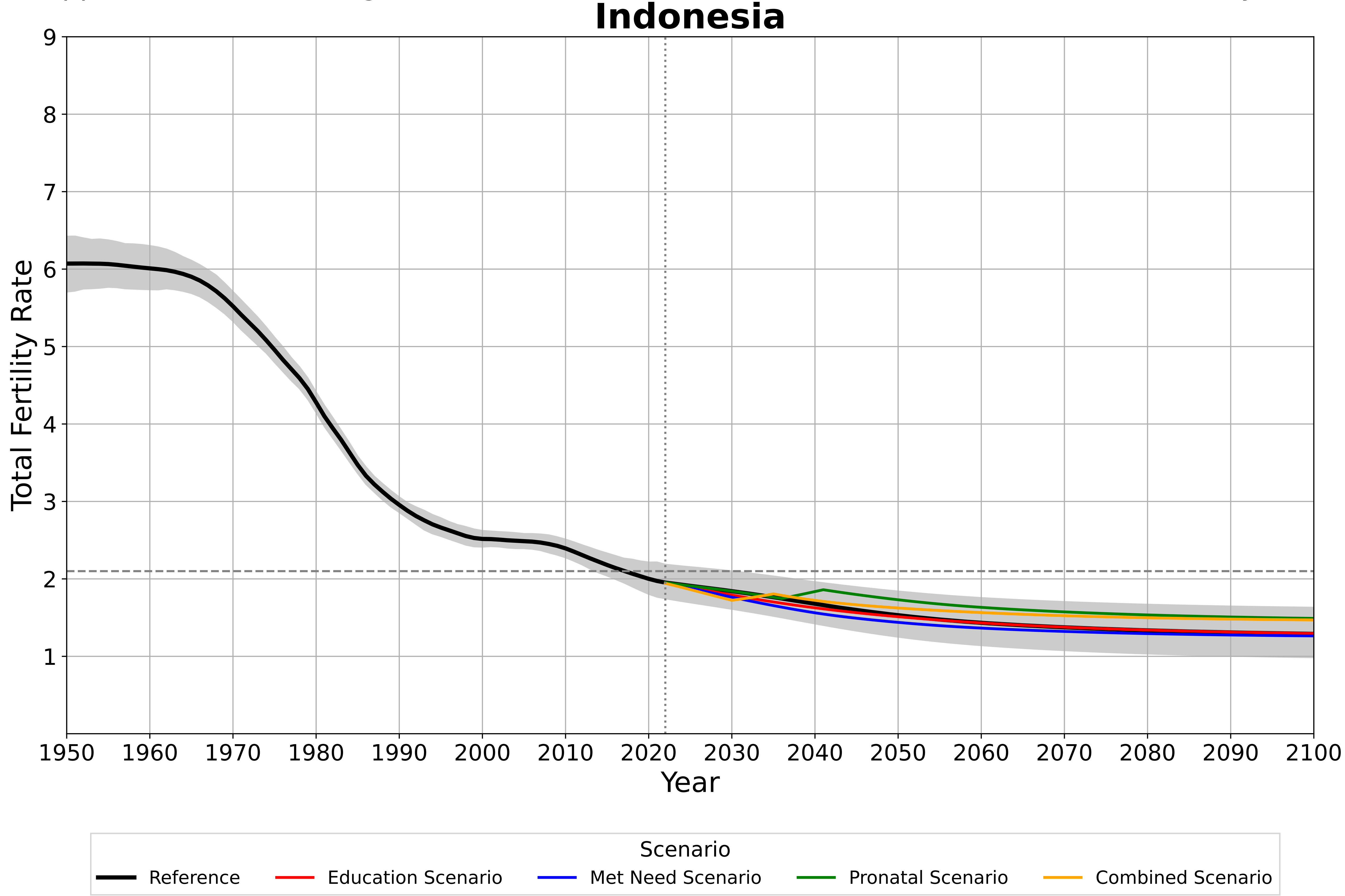

Supplemental Results Figure S5: TFR for reference and alternative scenario forecasts by location, 1950– 2100

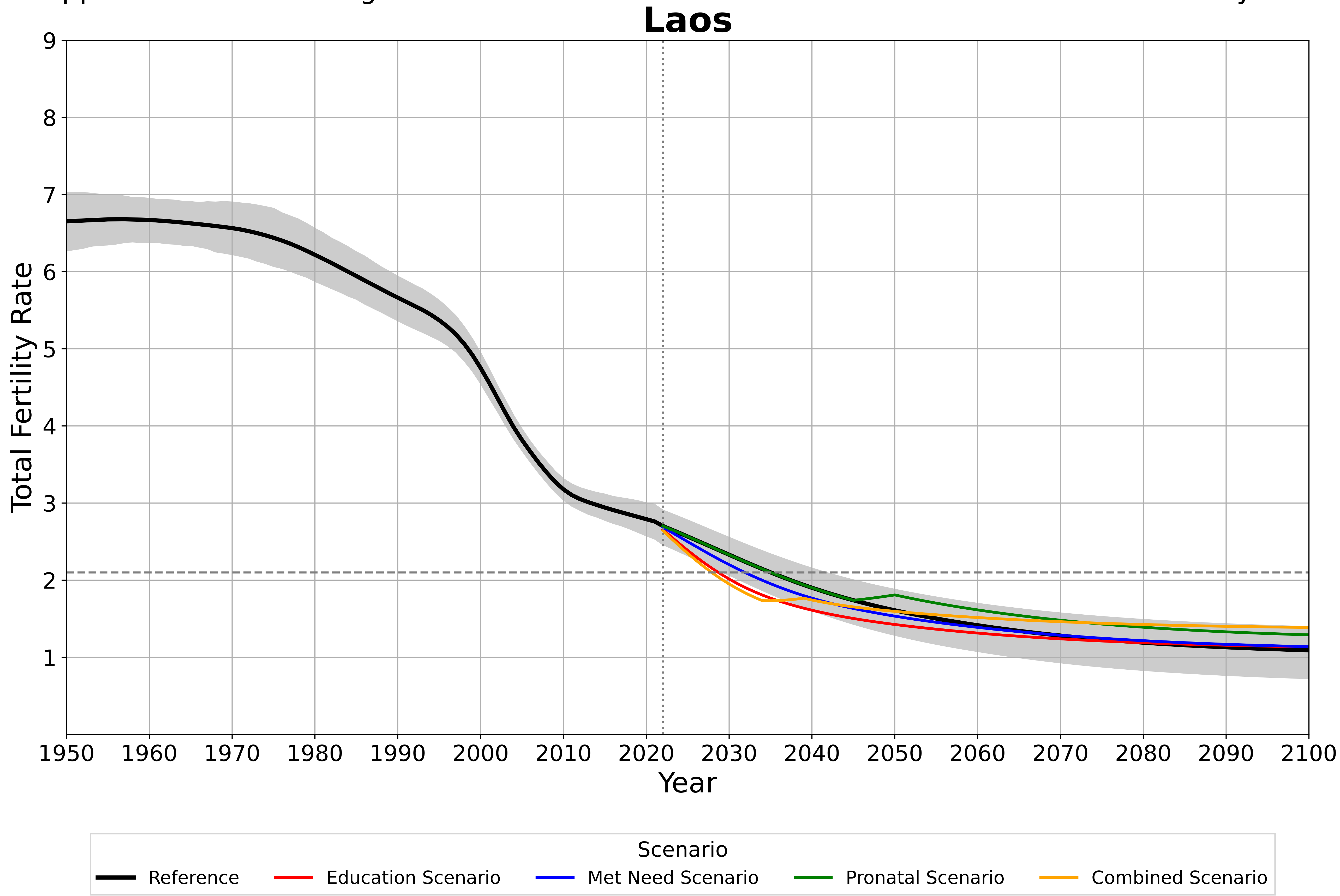

Supplemental Results Figure S5: TFR for reference and alternative scenario forecasts by location, 1950– 2100

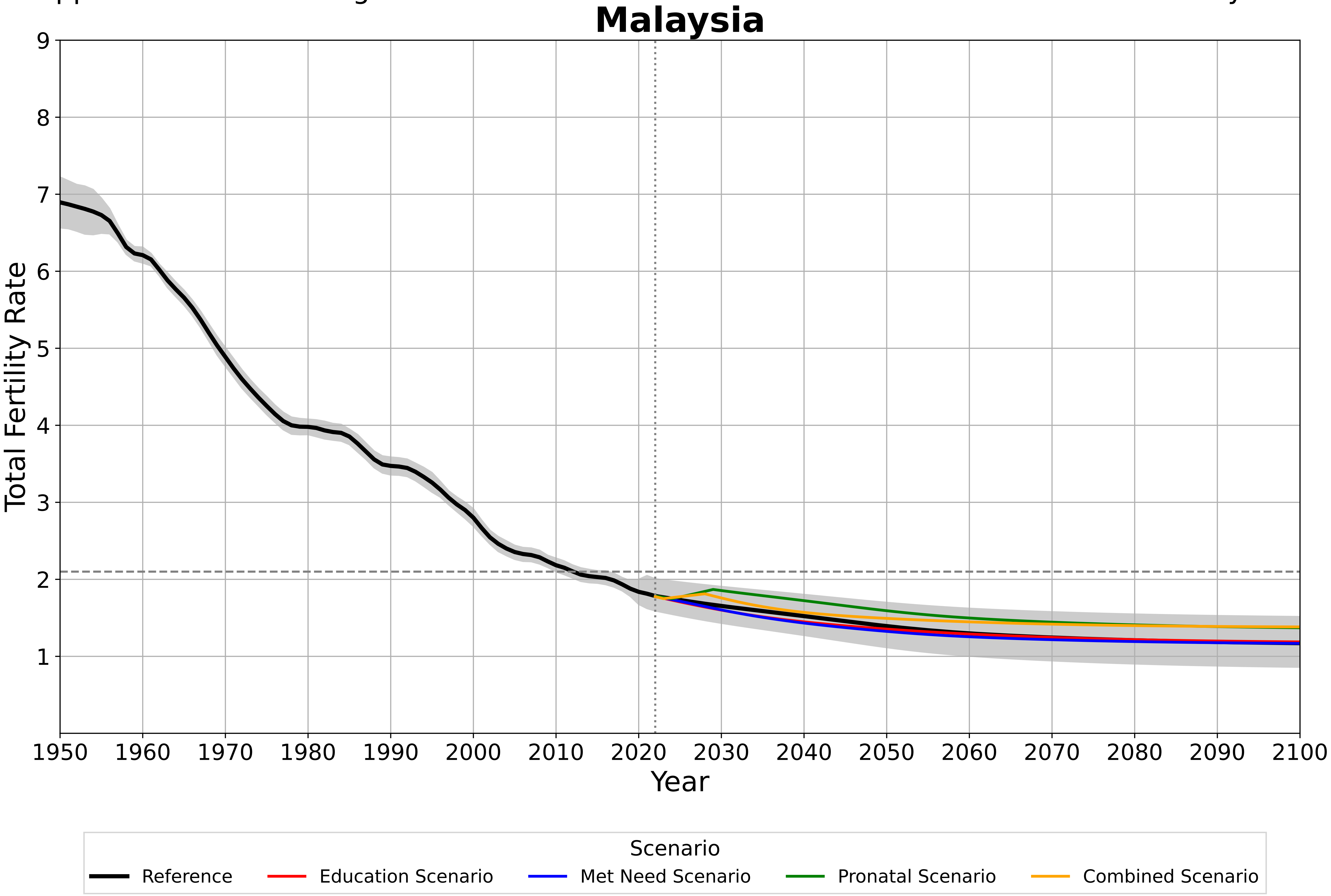

Supplemental Results Figure S5: TFR for reference and alternative scenario forecasts by location, 1950– 2100

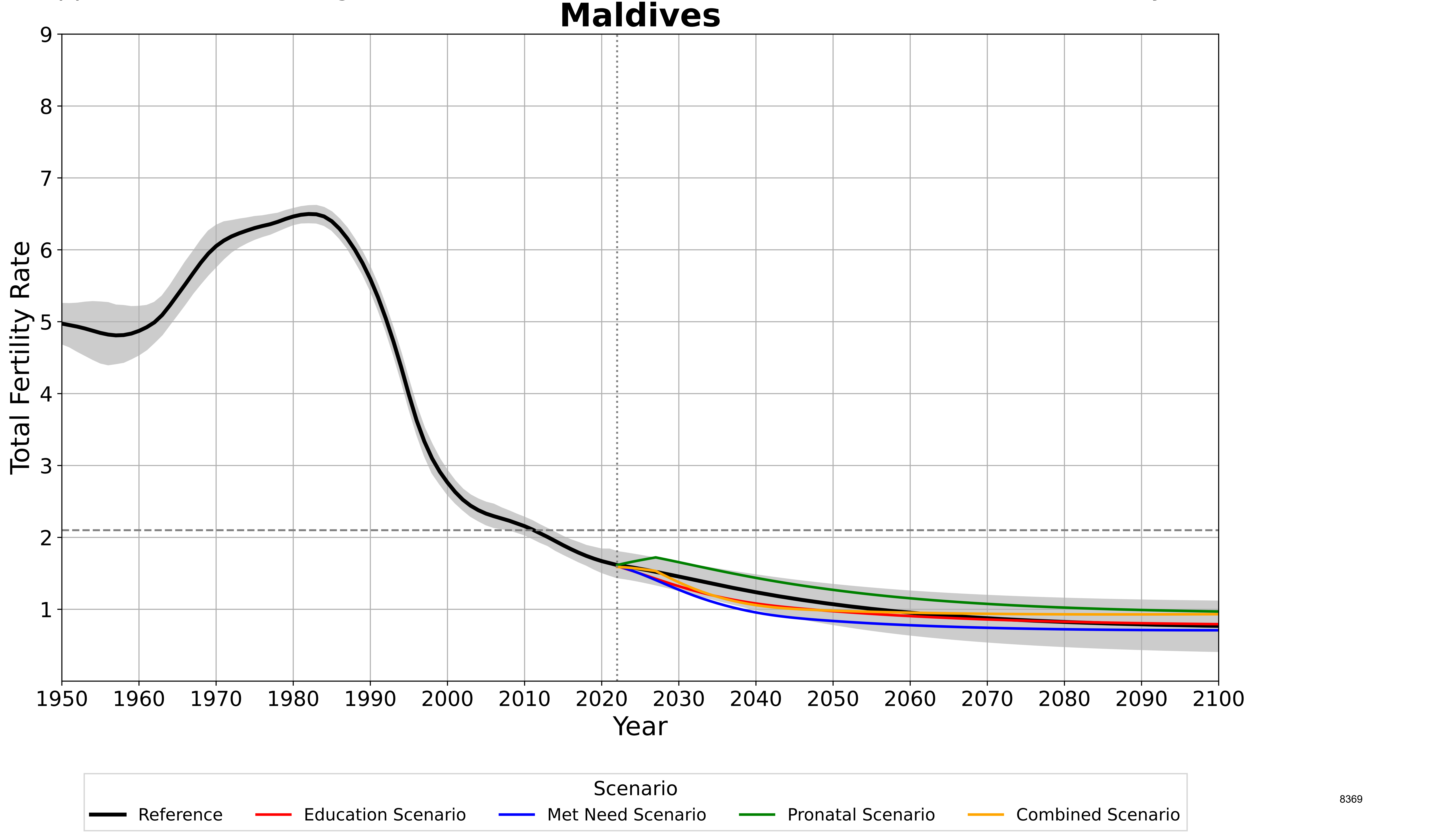

Supplemental Results Figure S5: TFR for reference and alternative scenario forecasts by location, 1950– 2100

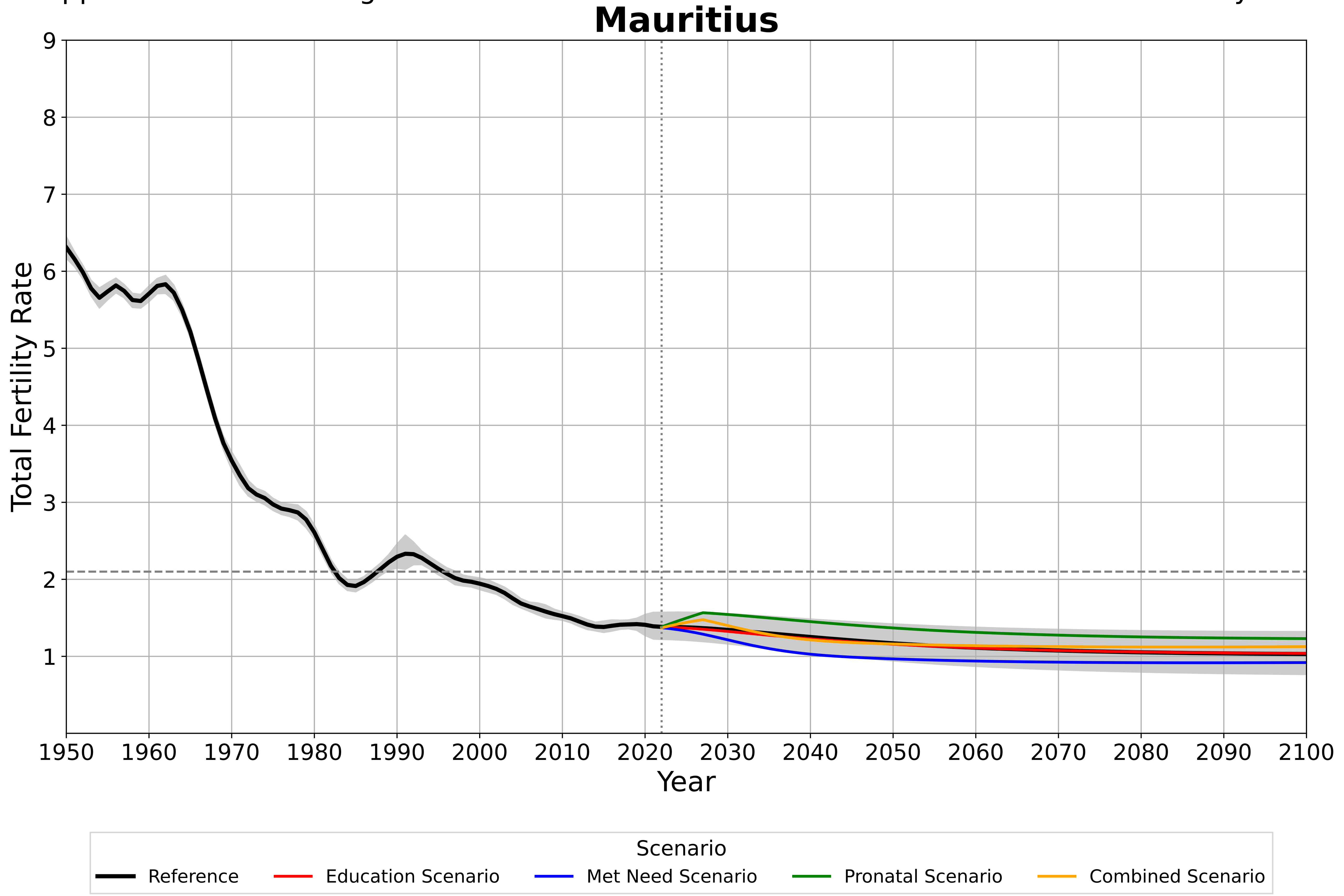

8370

Supplemental Results Figure S5: TFR for reference and alternative scenario forecasts by location, 1950– 2100

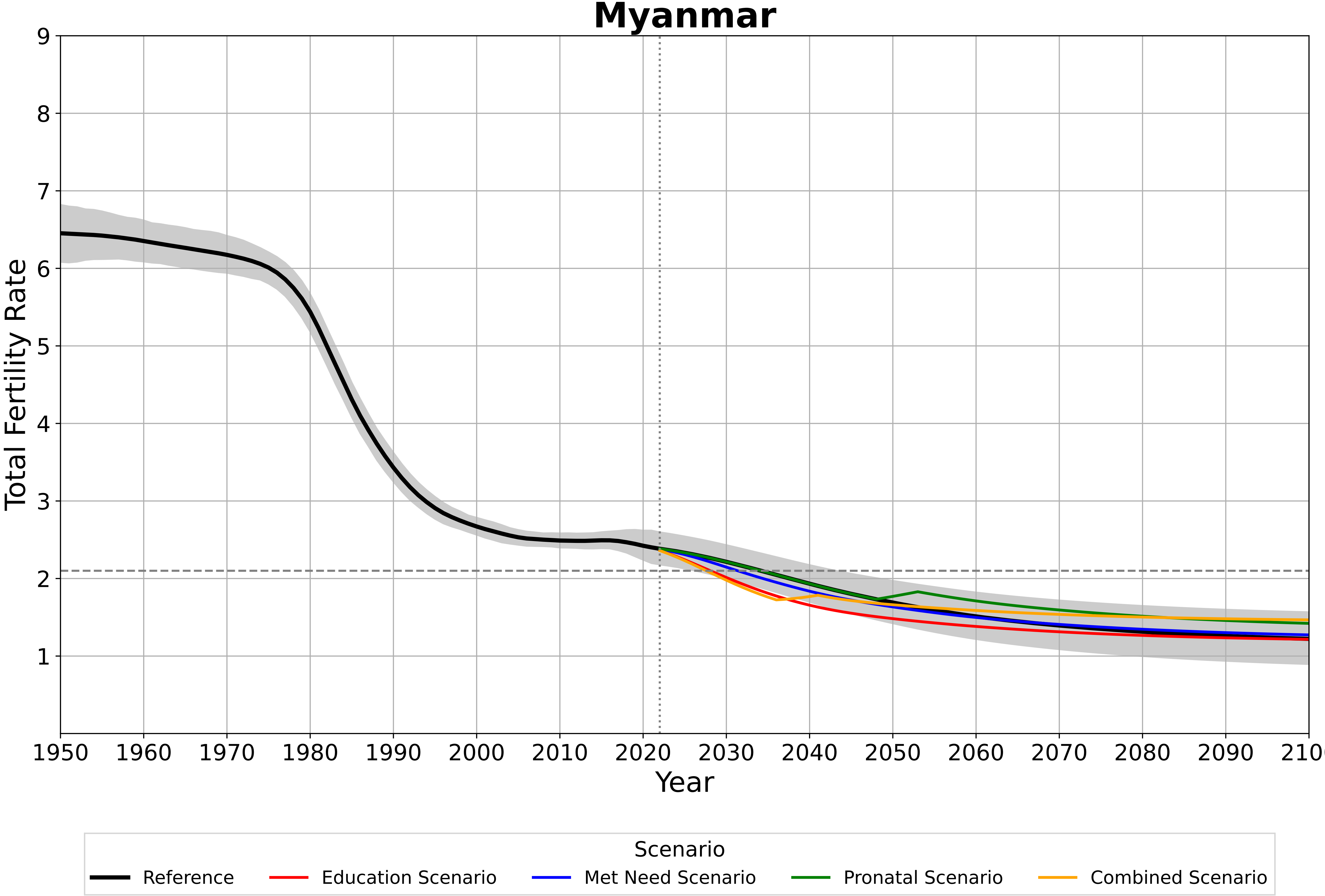

Supplemental Results Figure S5: TFR for reference and alternative scenario forecasts by location, 1950– 2100

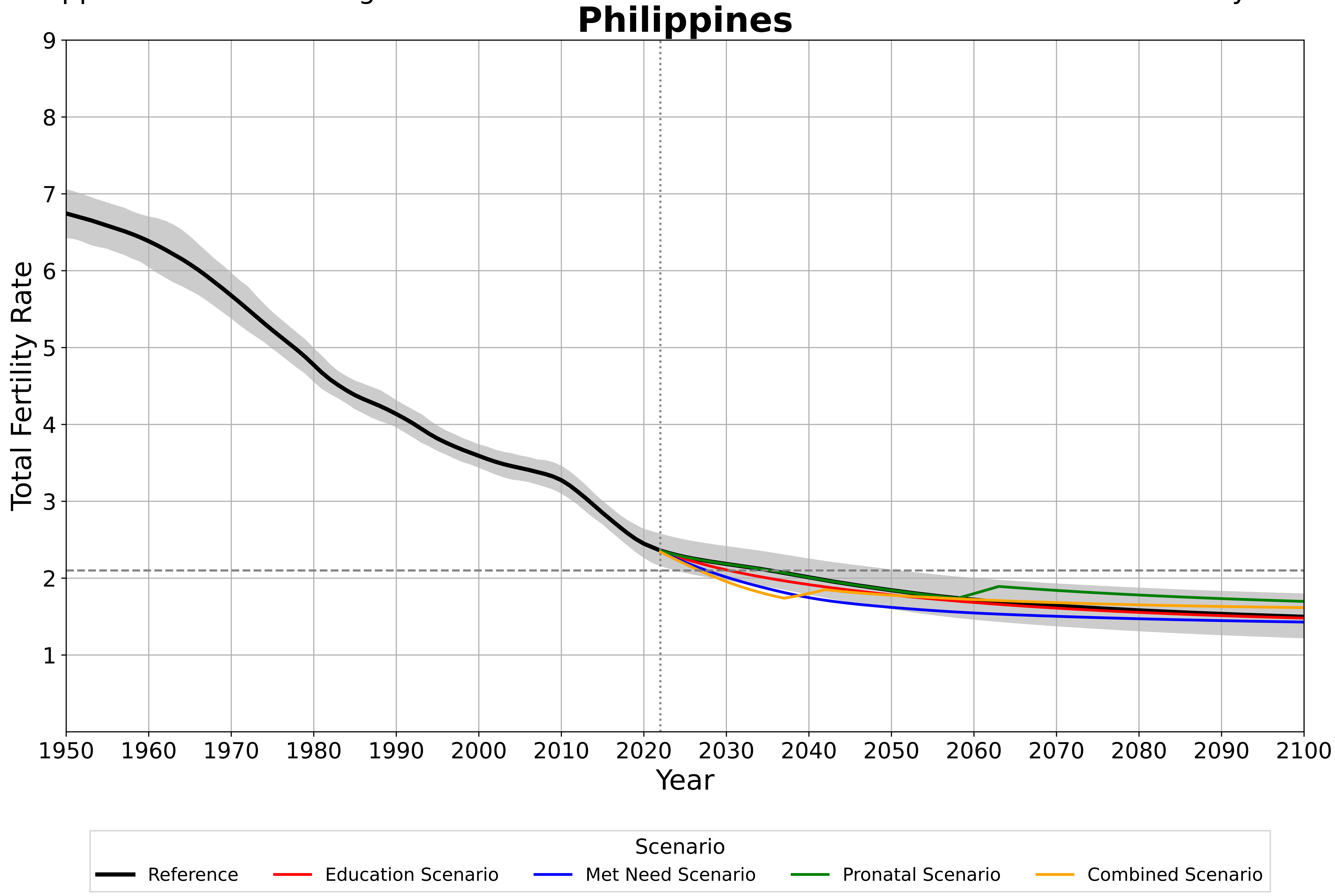

Supplemental Results Figure S5: TFR for reference and alternative scenario forecasts by location, 1950– 2100

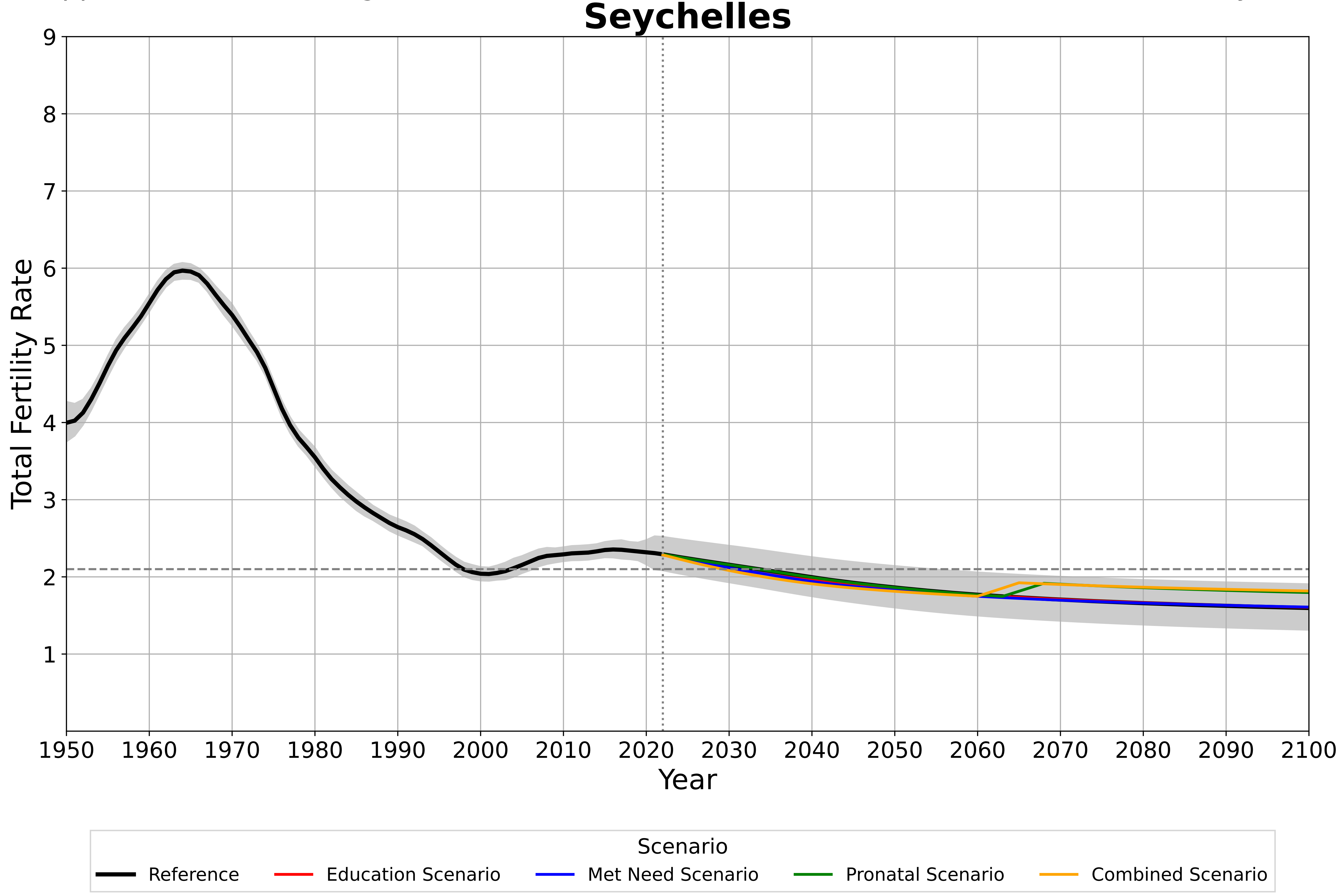

Supplemental Results Figure S5: TFR for reference and alternative scenario forecasts by location, 1950– 2100

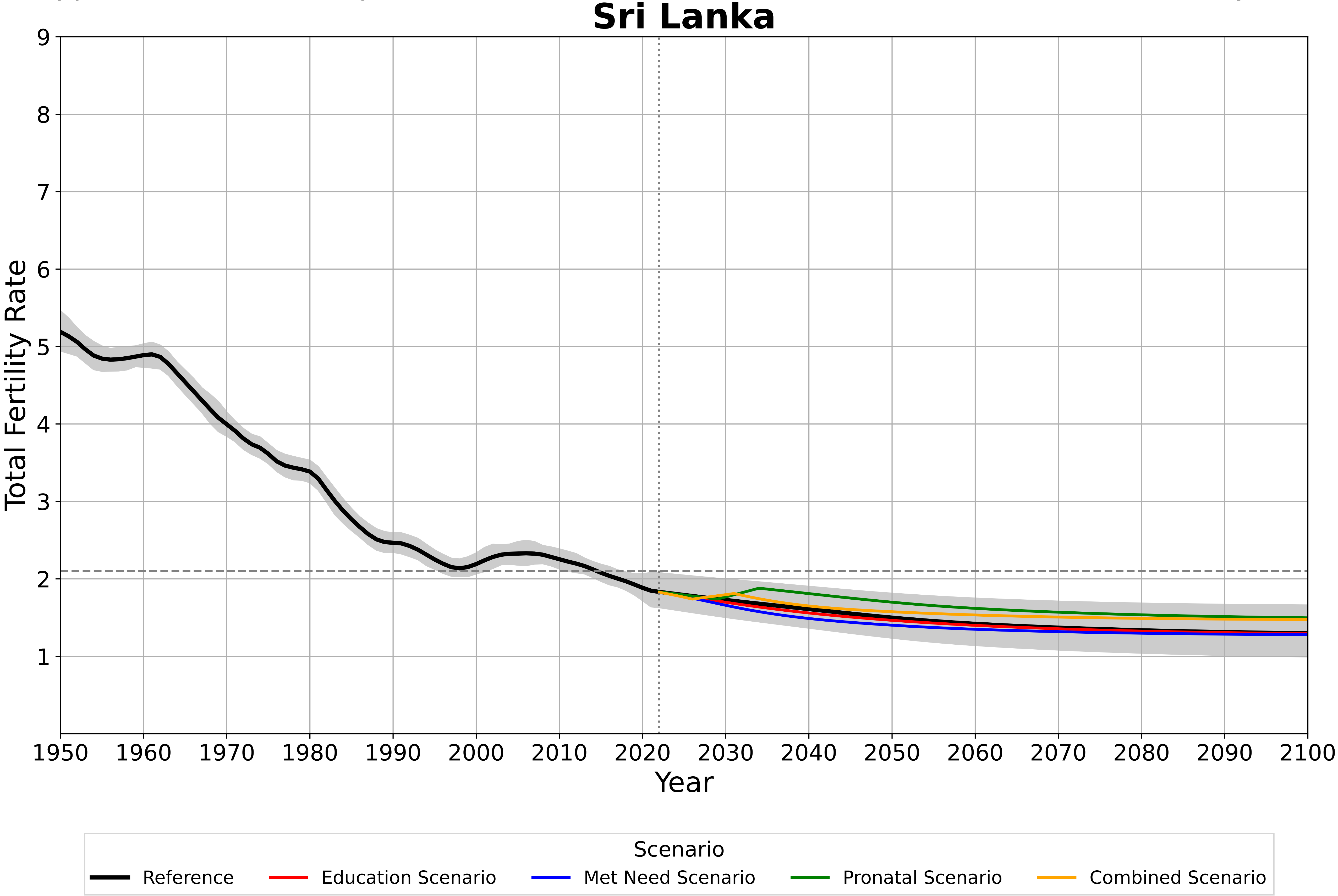

Supplemental Results Figure S5: TFR for reference and alternative scenario forecasts by location, 1950– 2100

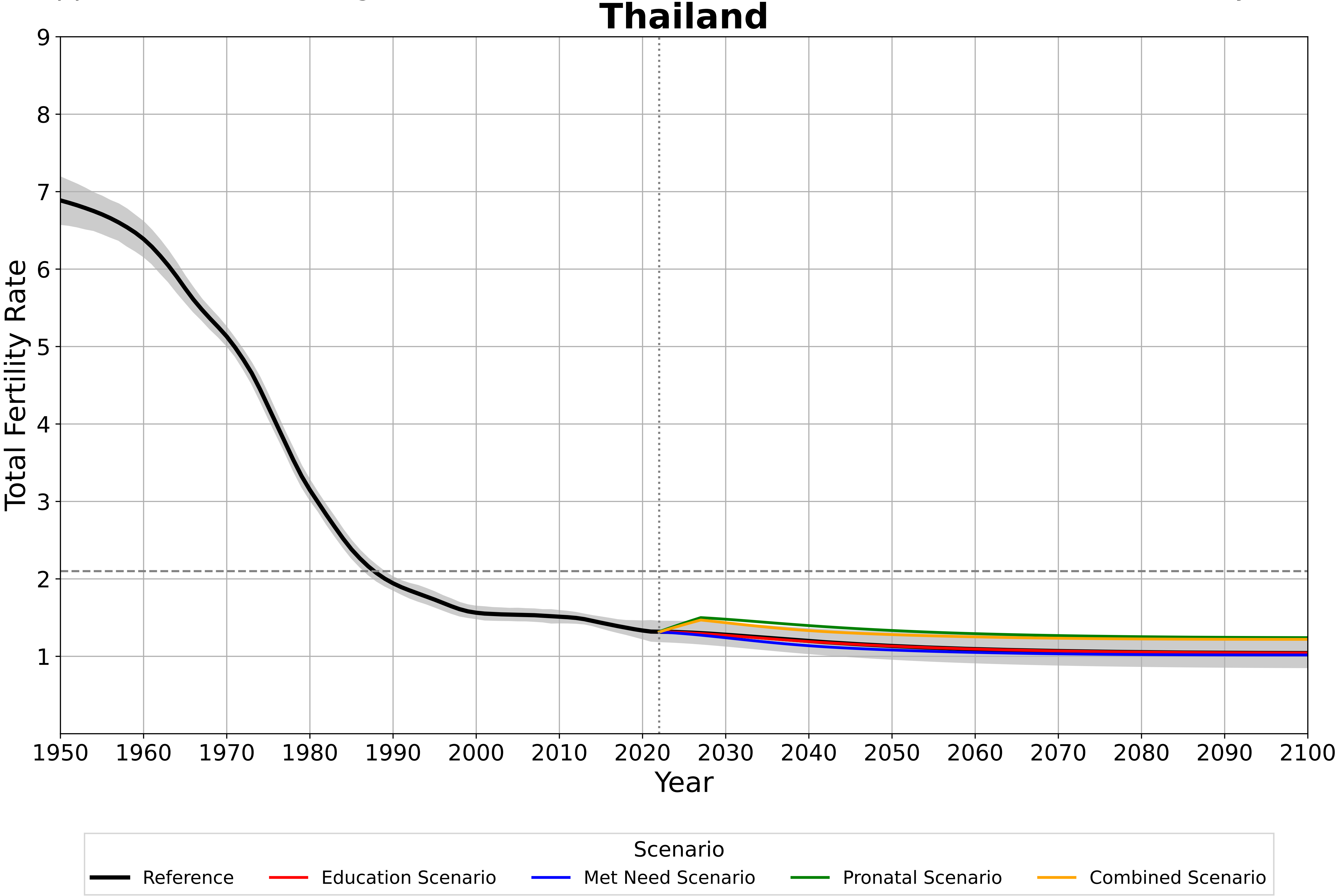

8375

Supplemental Results Figure S5: TFR for reference and alternative scenario forecasts by location, 1950– 2100

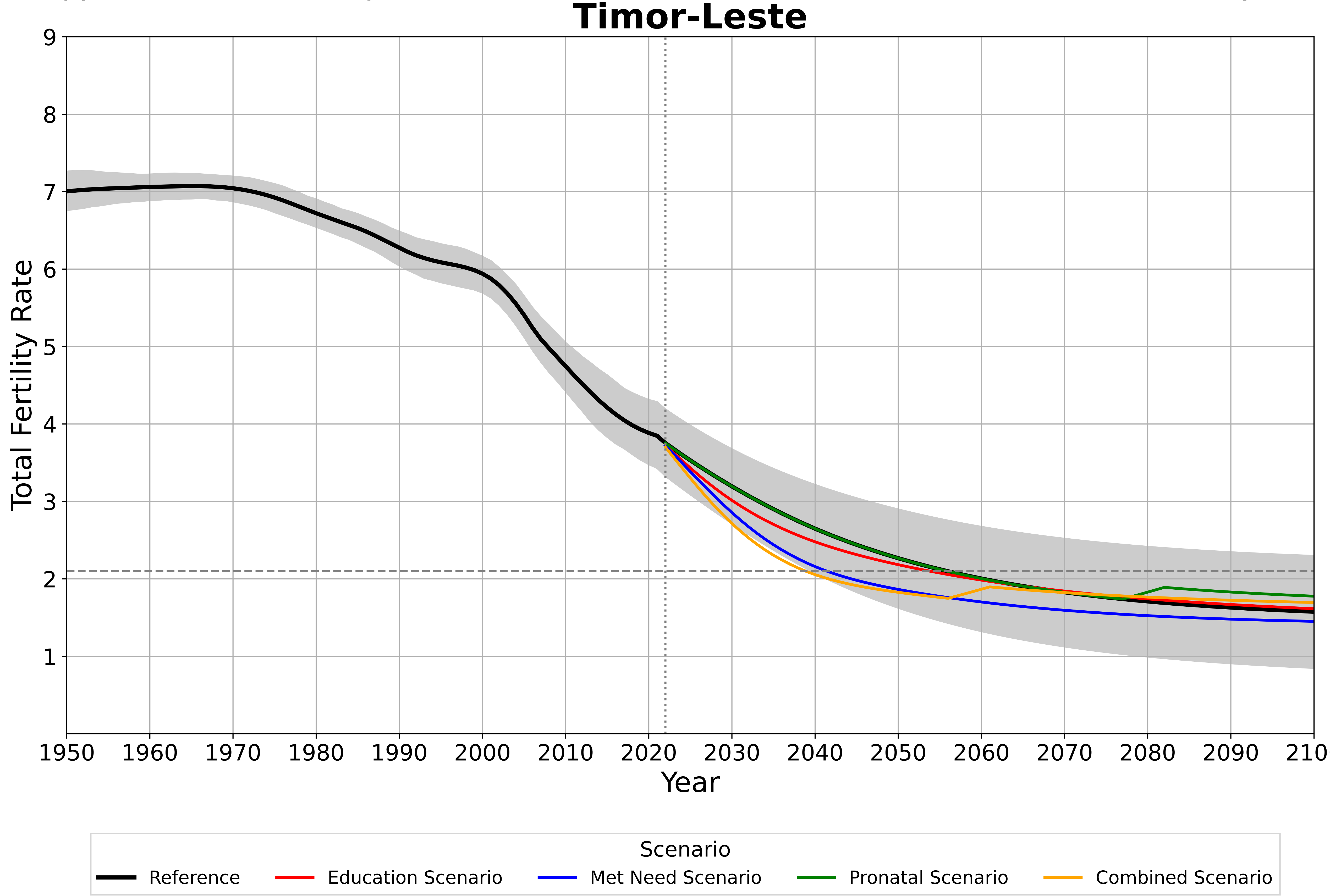

Supplemental Results Figure S5: TFR for reference and alternative scenario forecasts by location, 1950– 2100

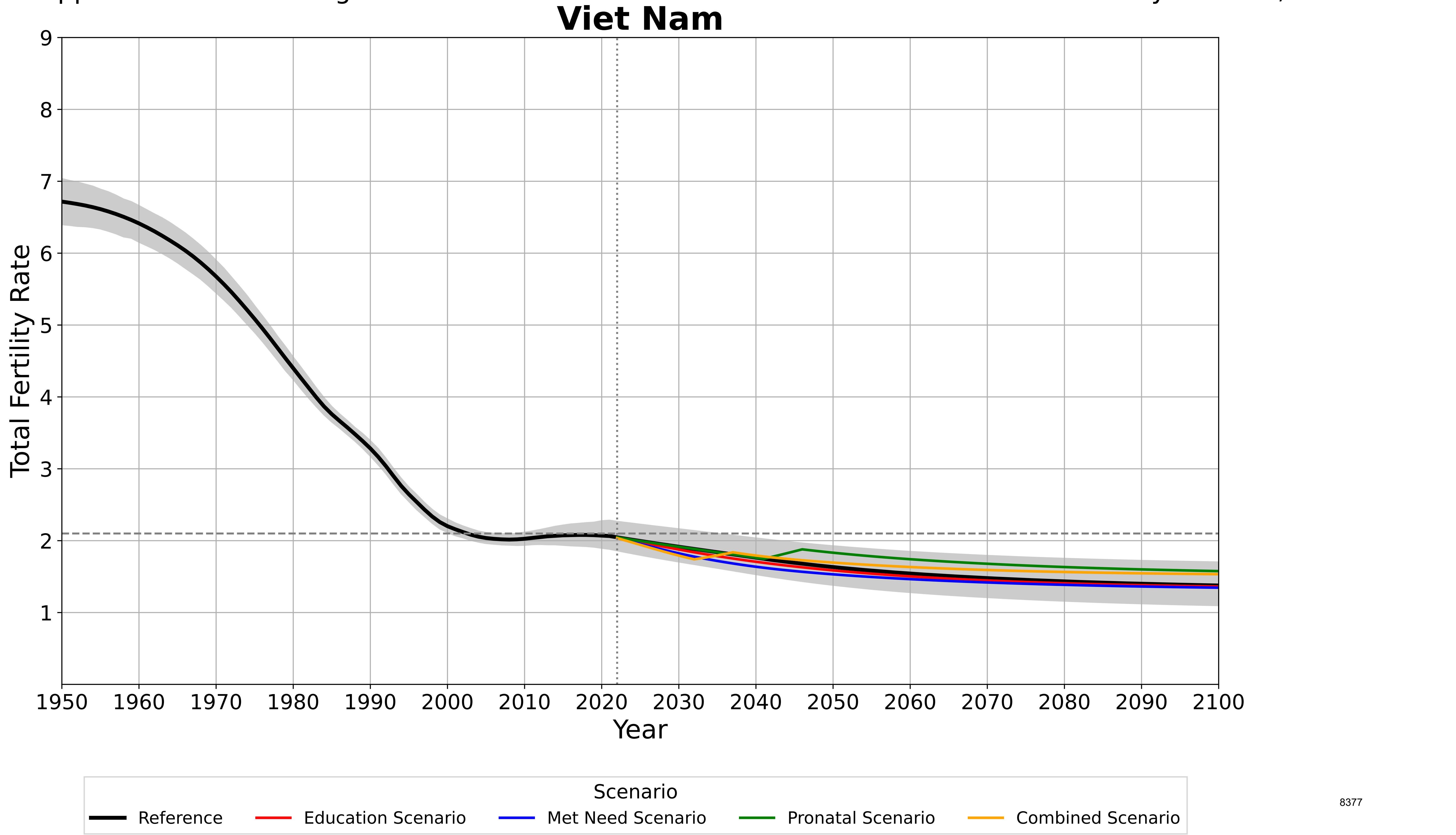

8377

Supplemental Results Figure S5: TFR for reference and alternative scenario forecasts by location, 1950– 2100

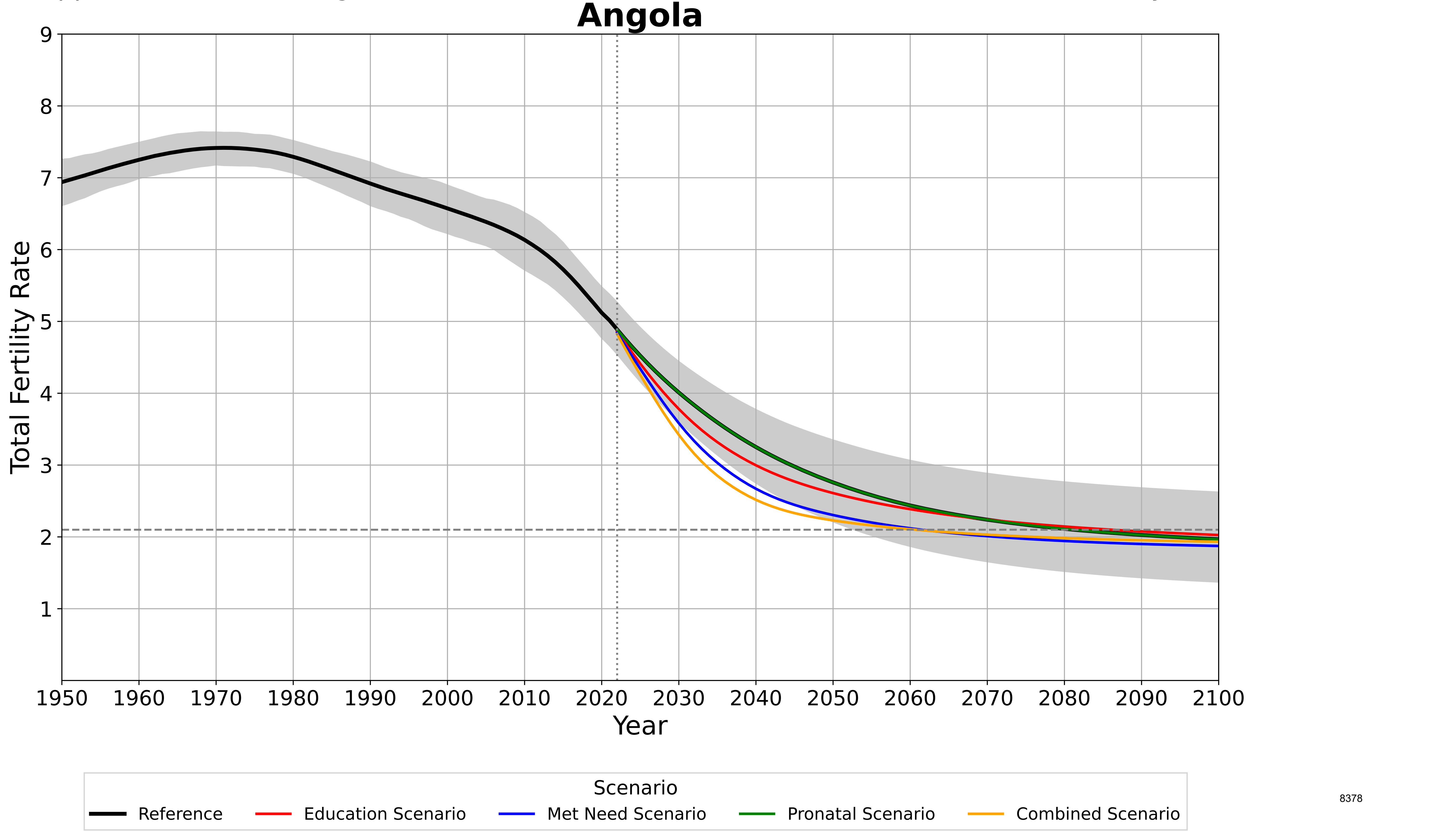

Supplemental Results Figure S5: TFR for reference and alternative scenario forecasts by location, 1950– 2100

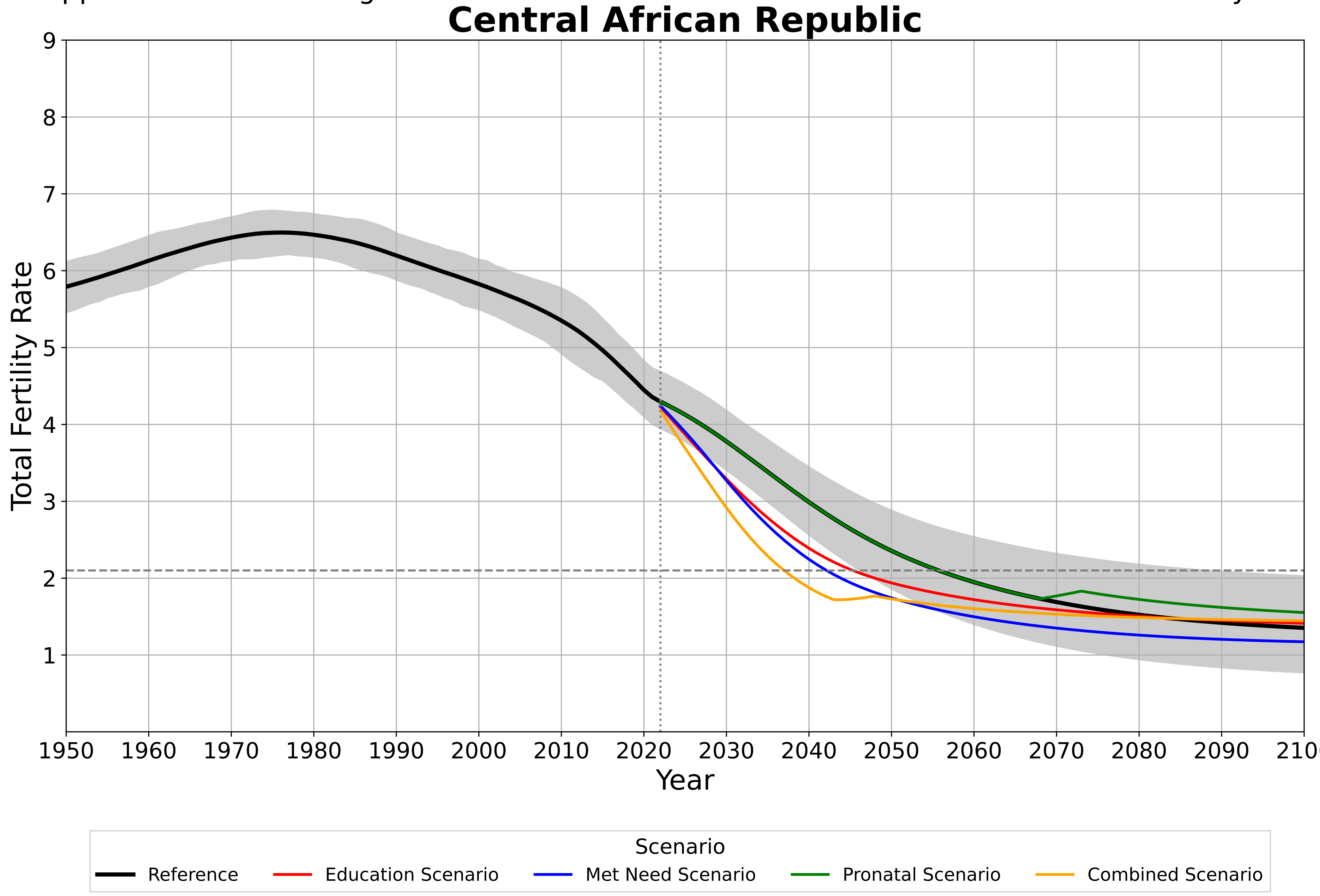

8379

Supplemental Results Figure S5: TFR for reference and alternative scenario forecasts by location, 1950– 2100

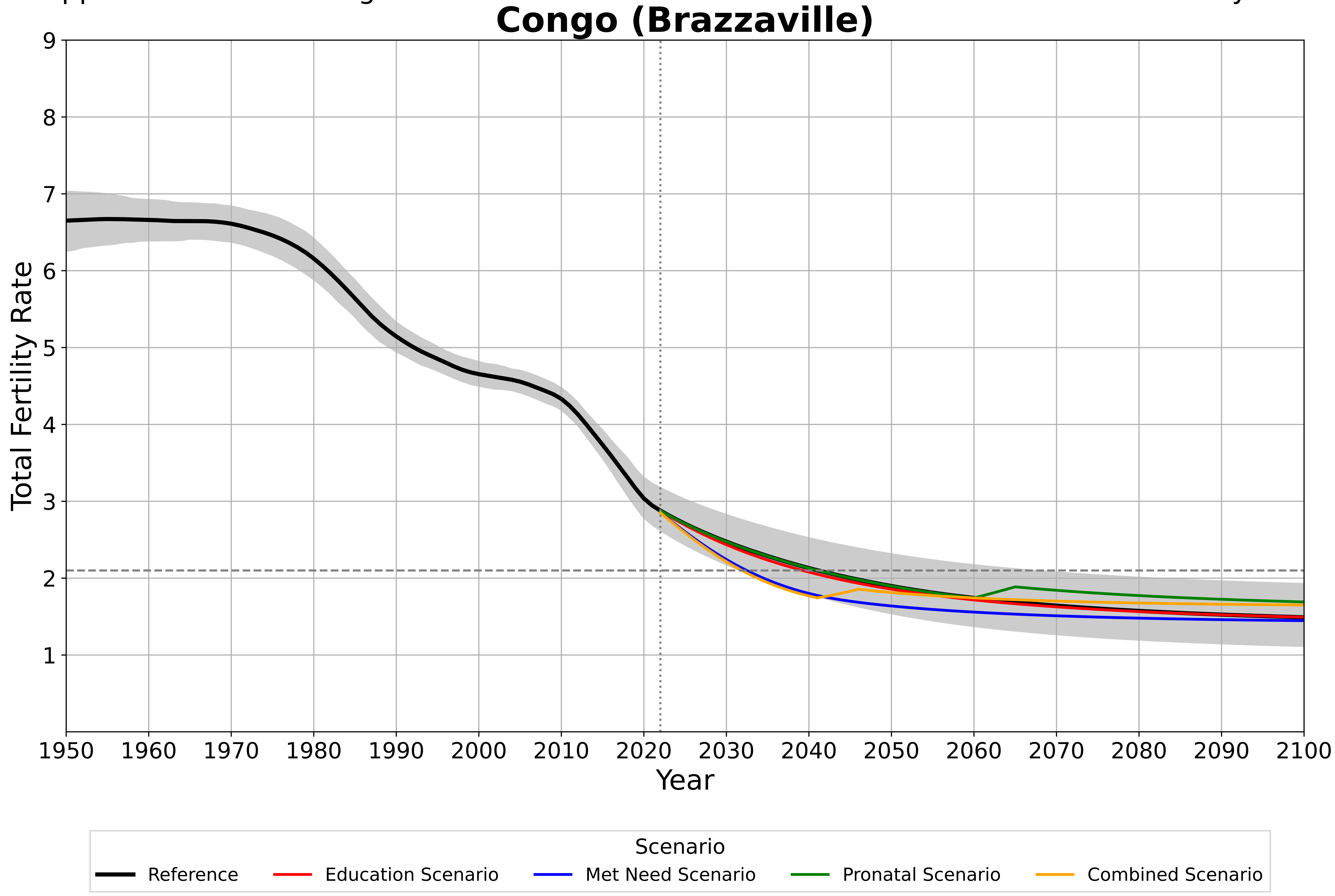

Supplemental Results Figure S5: TFR for reference and alternative scenario forecasts by location, 1950– 2100

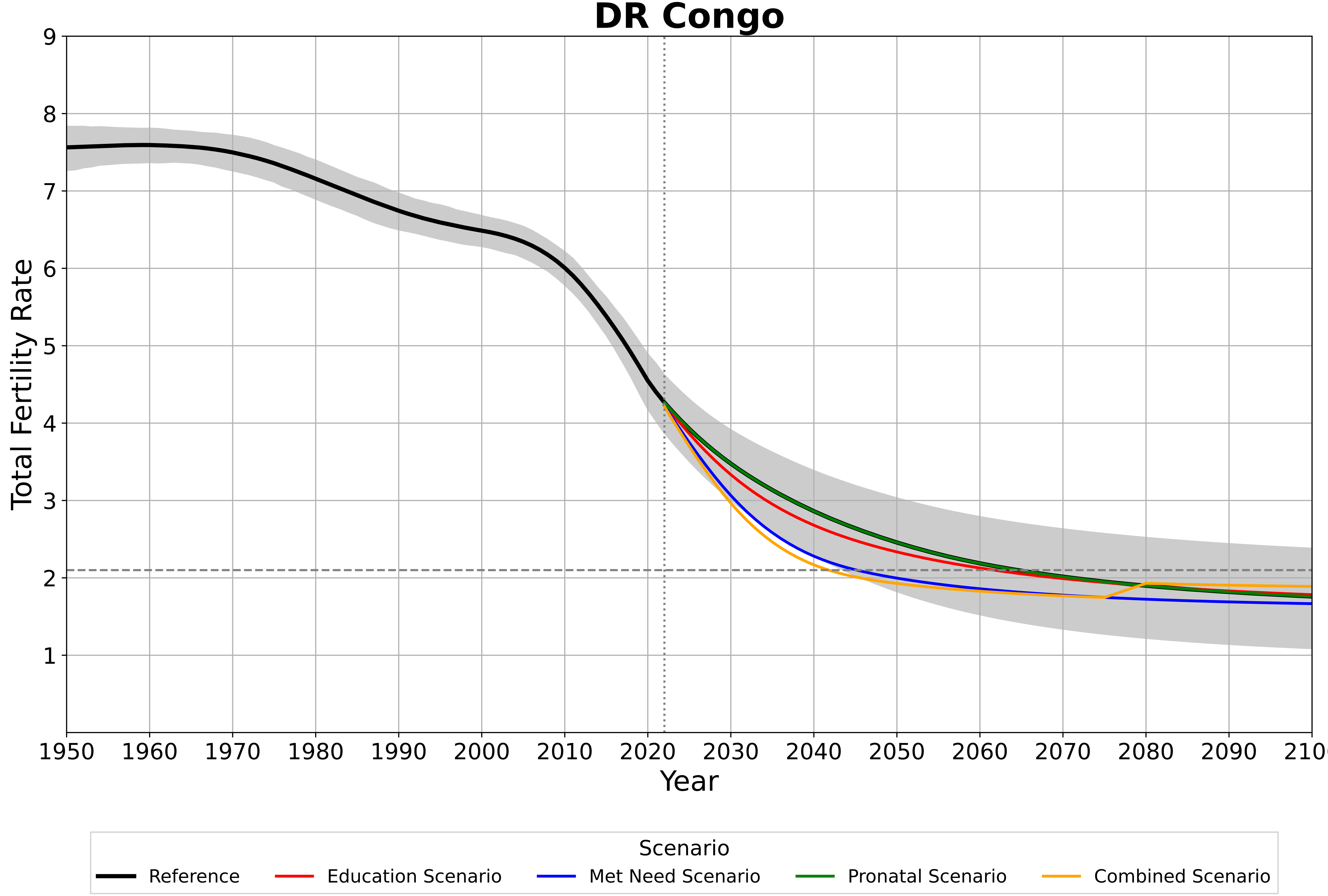

Supplemental Results Figure S5: TFR for reference and alternative scenario forecasts by location, 1950– 2100

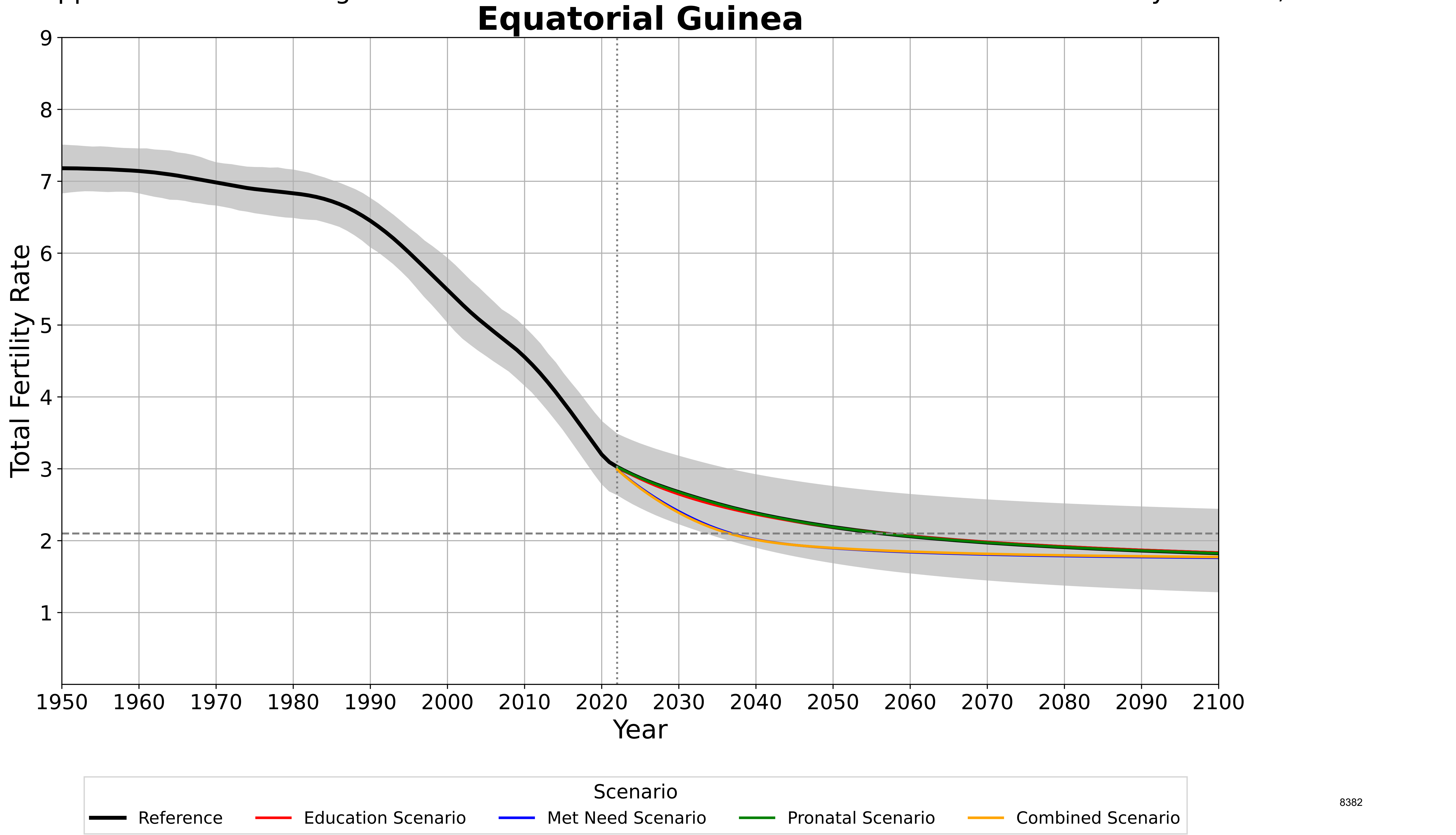

Supplemental Results Figure S5: TFR for reference and alternative scenario forecasts by location, 1950– 2100

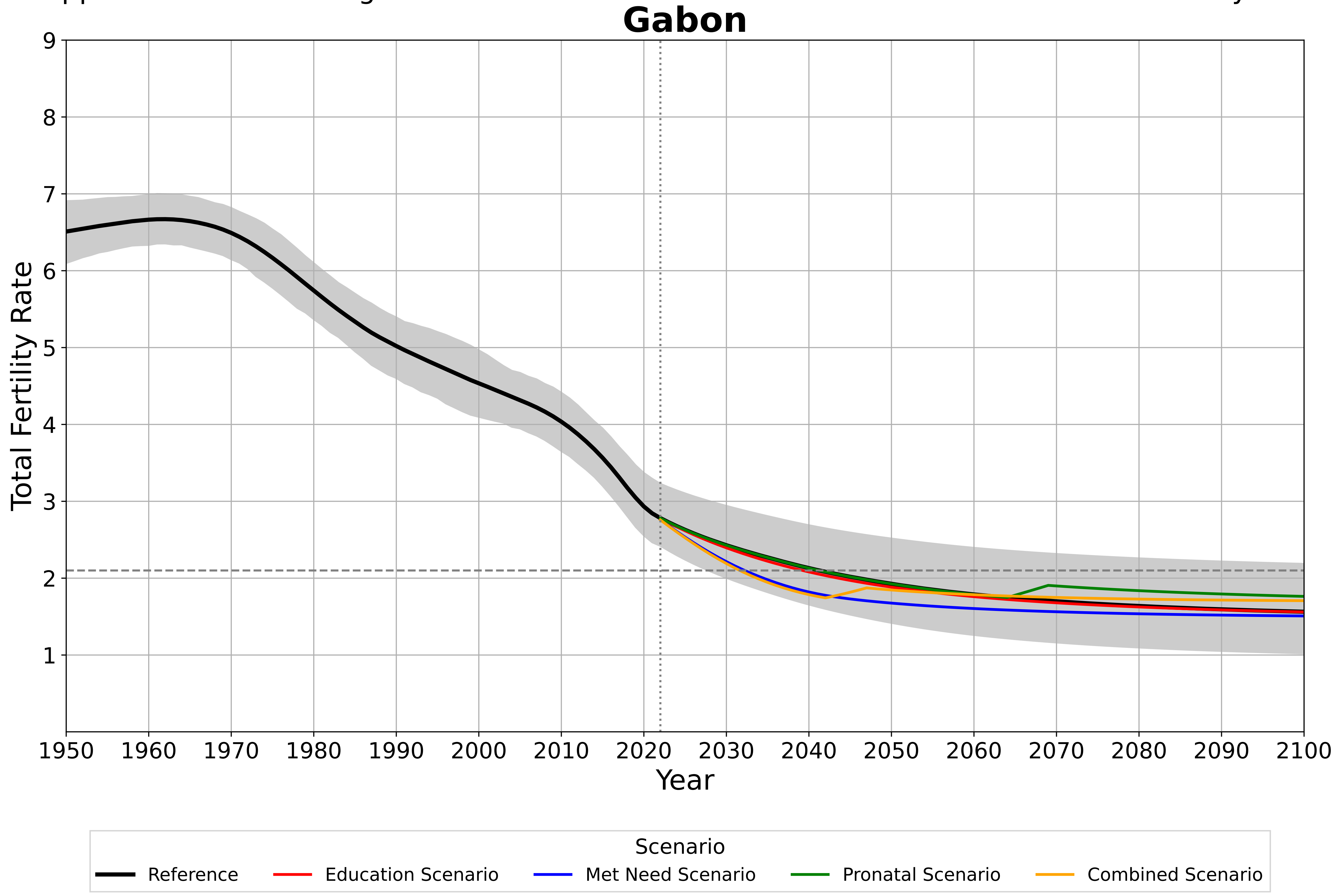

Supplemental Results Figure S5: TFR for reference and alternative scenario forecasts by location, 1950– 2100

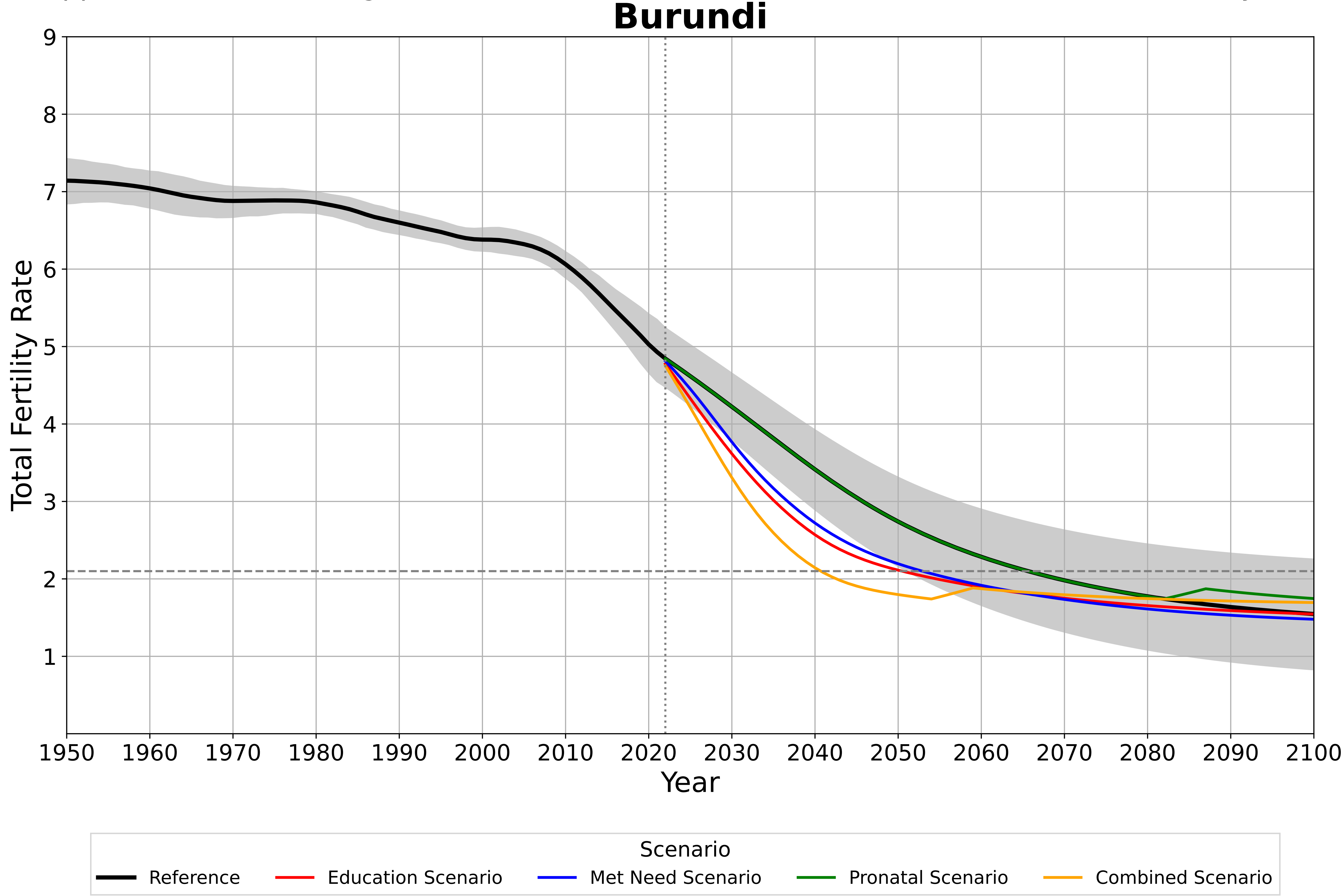

8384

Supplemental Results Figure S5: TFR for reference and alternative scenario forecasts by location, 1950– 2100

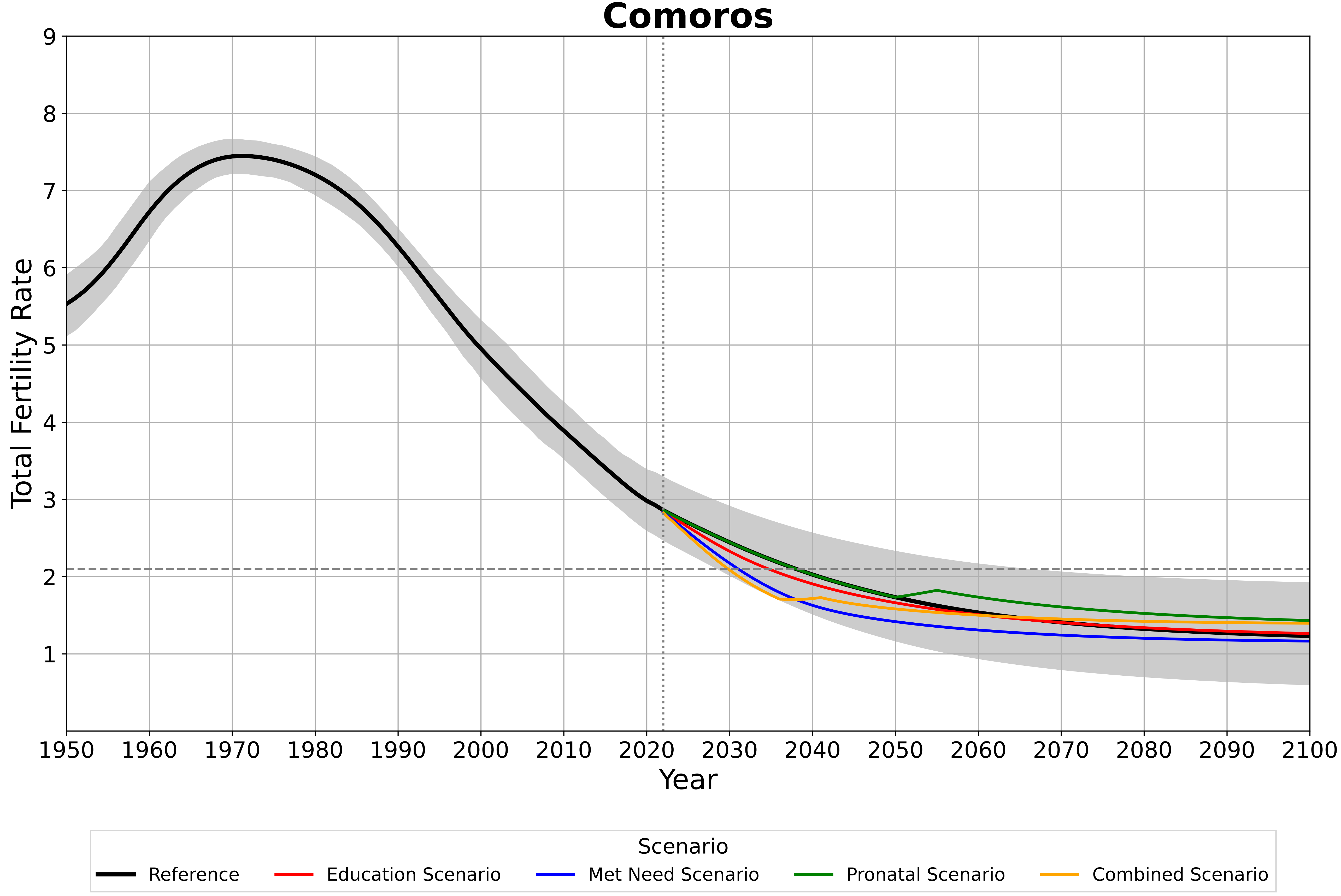

Supplemental Results Figure S5: TFR for reference and alternative scenario forecasts by location, 1950– 2100

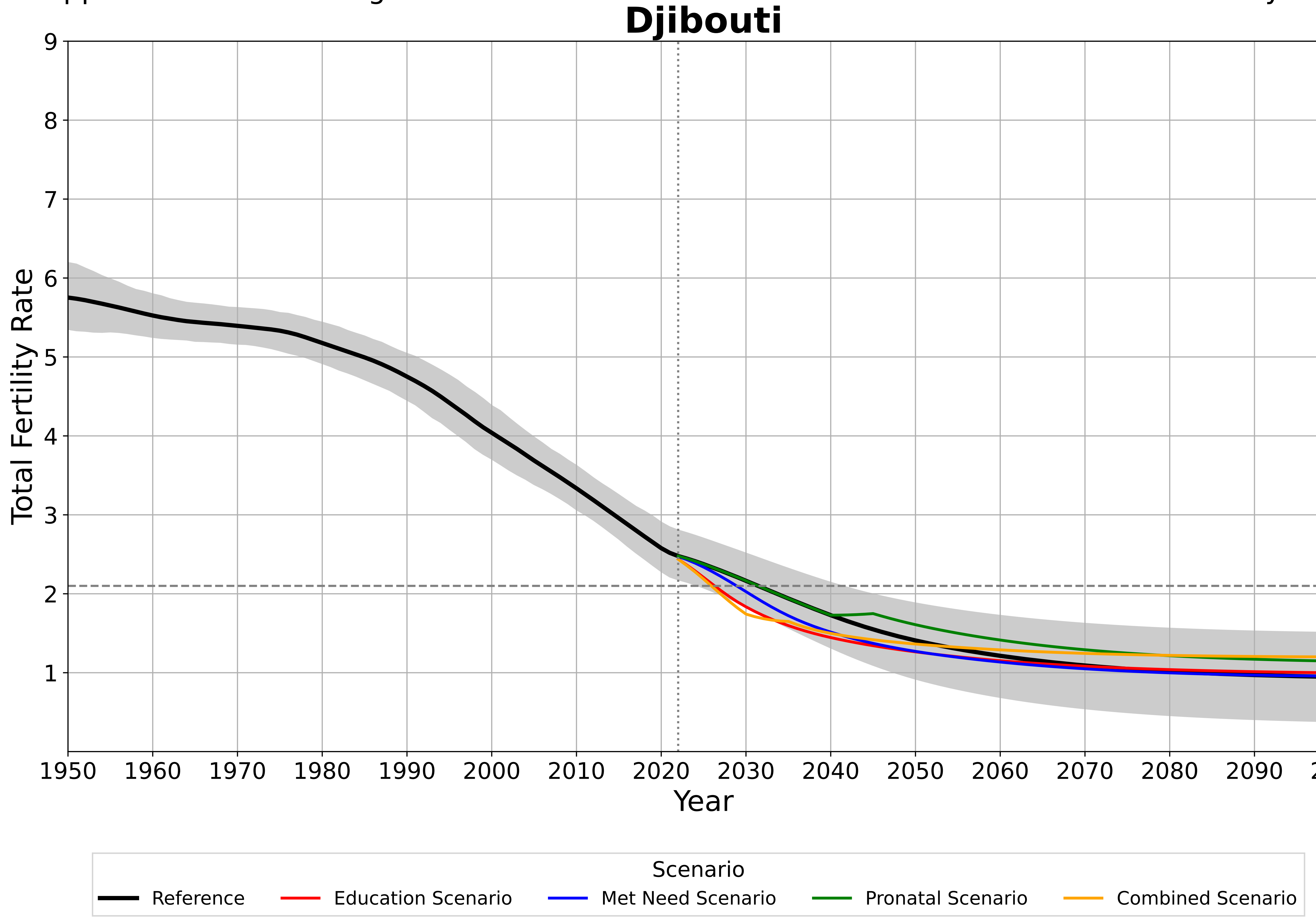

Supplemental Results Figure S5: TFR for reference and alternative scenario forecasts by location, 1950– 2100

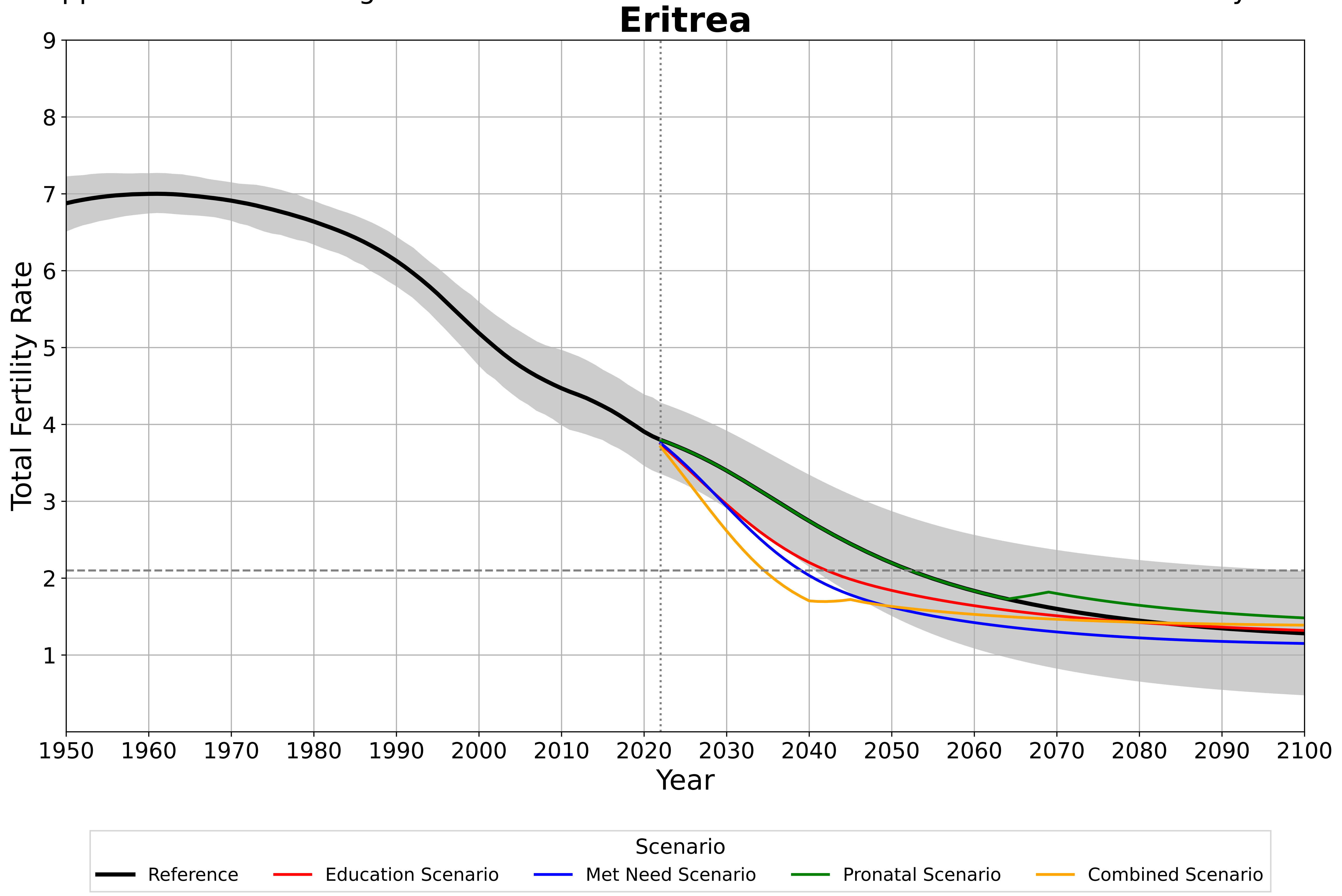

Supplemental Results Figure S5: TFR for reference and alternative scenario forecasts by location, 1950– 2100

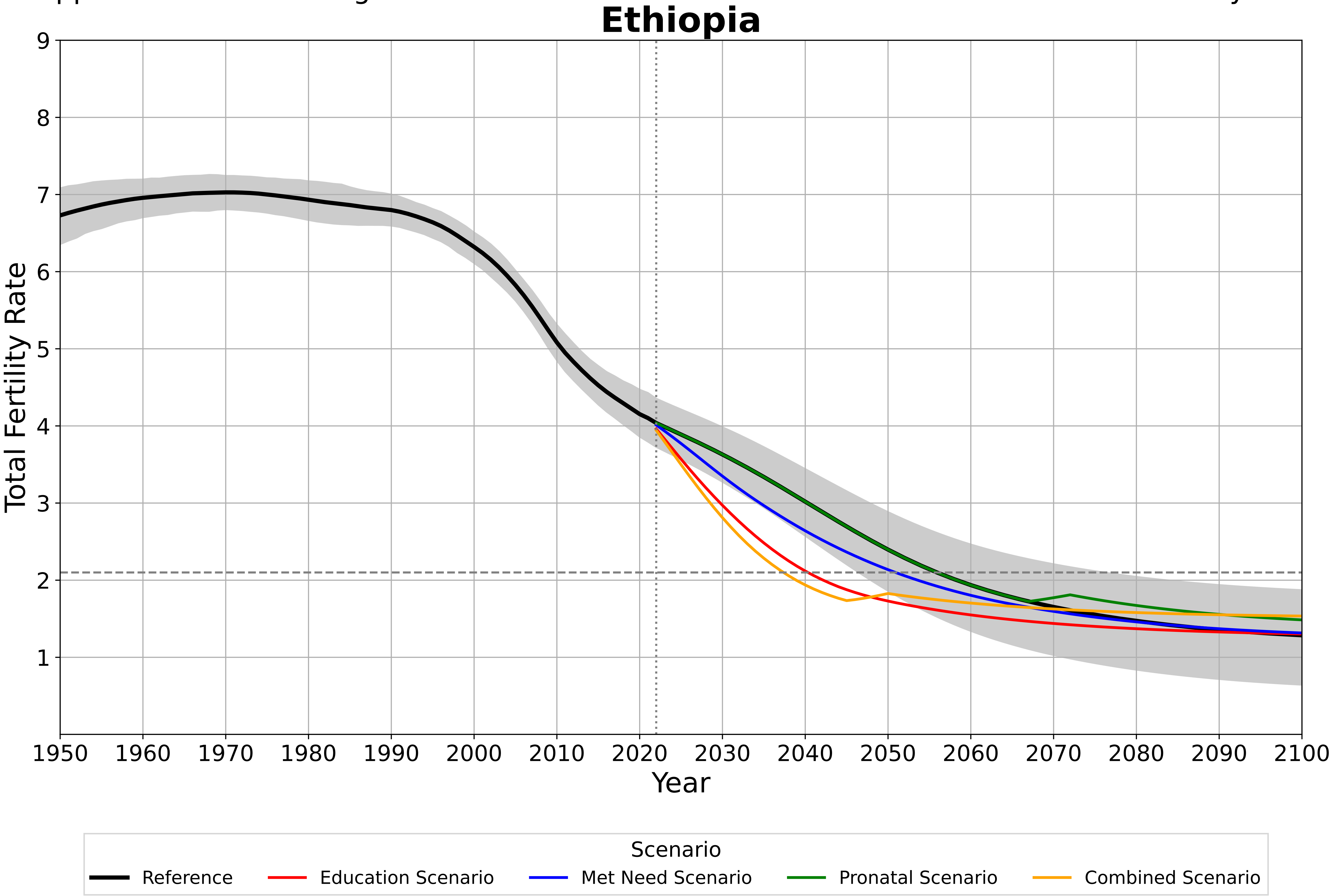

Supplemental Results Figure S5: TFR for reference and alternative scenario forecasts by location, 1950– 2100

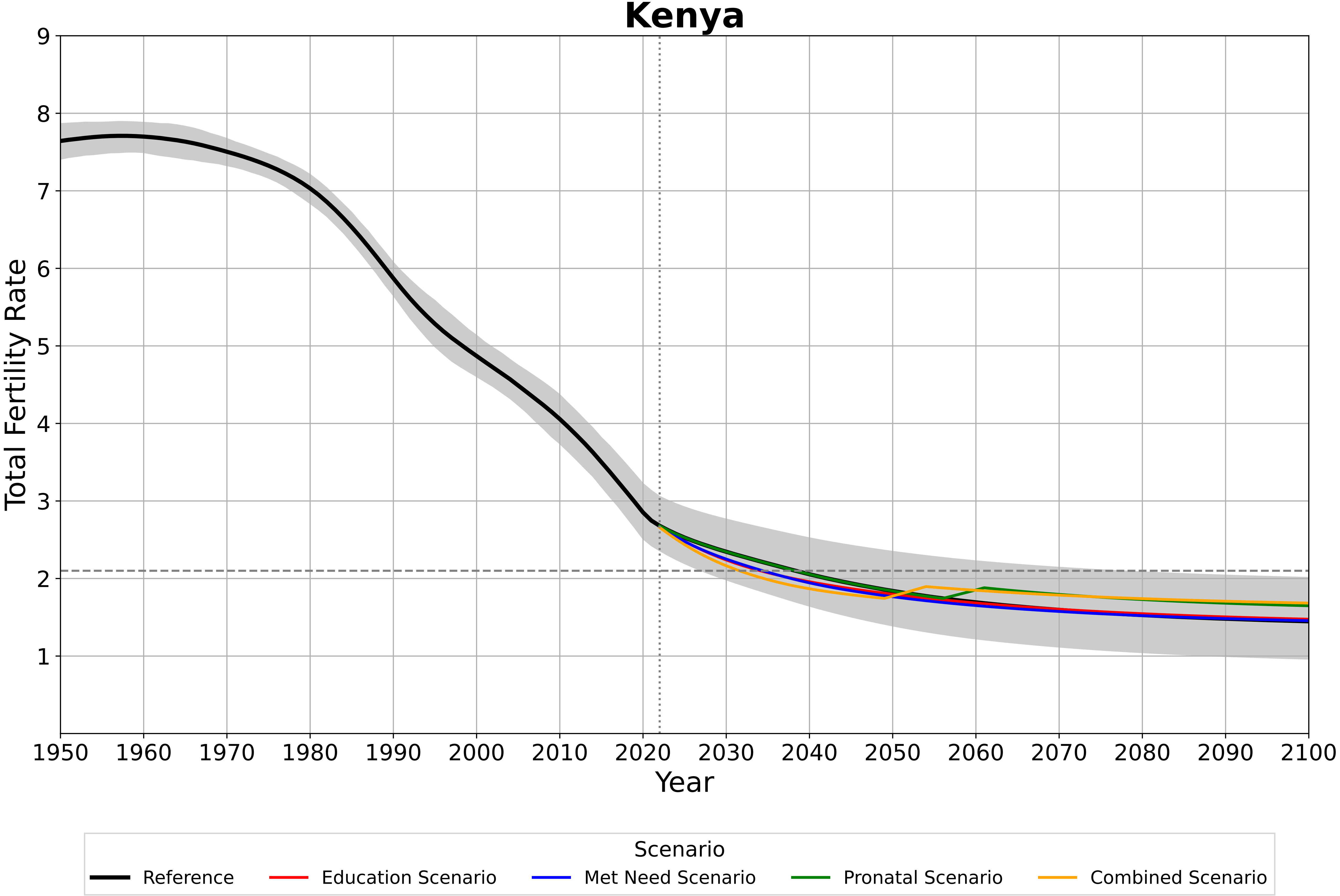

Supplemental Results Figure S5: TFR for reference and alternative scenario forecasts by location, 1950– 2100

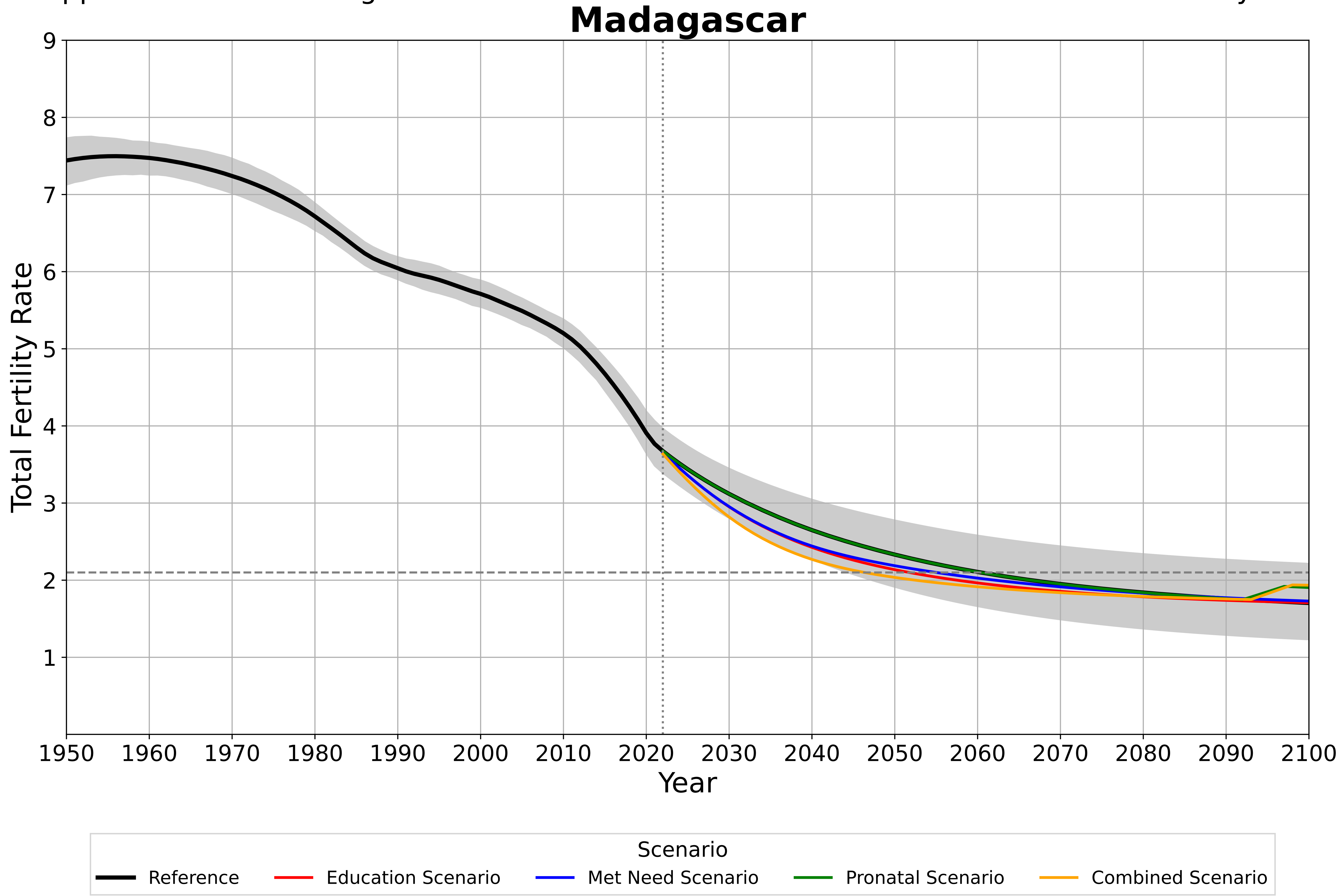

8390

Supplemental Results Figure S5: TFR for reference and alternative scenario forecasts by location, 1950– 2100

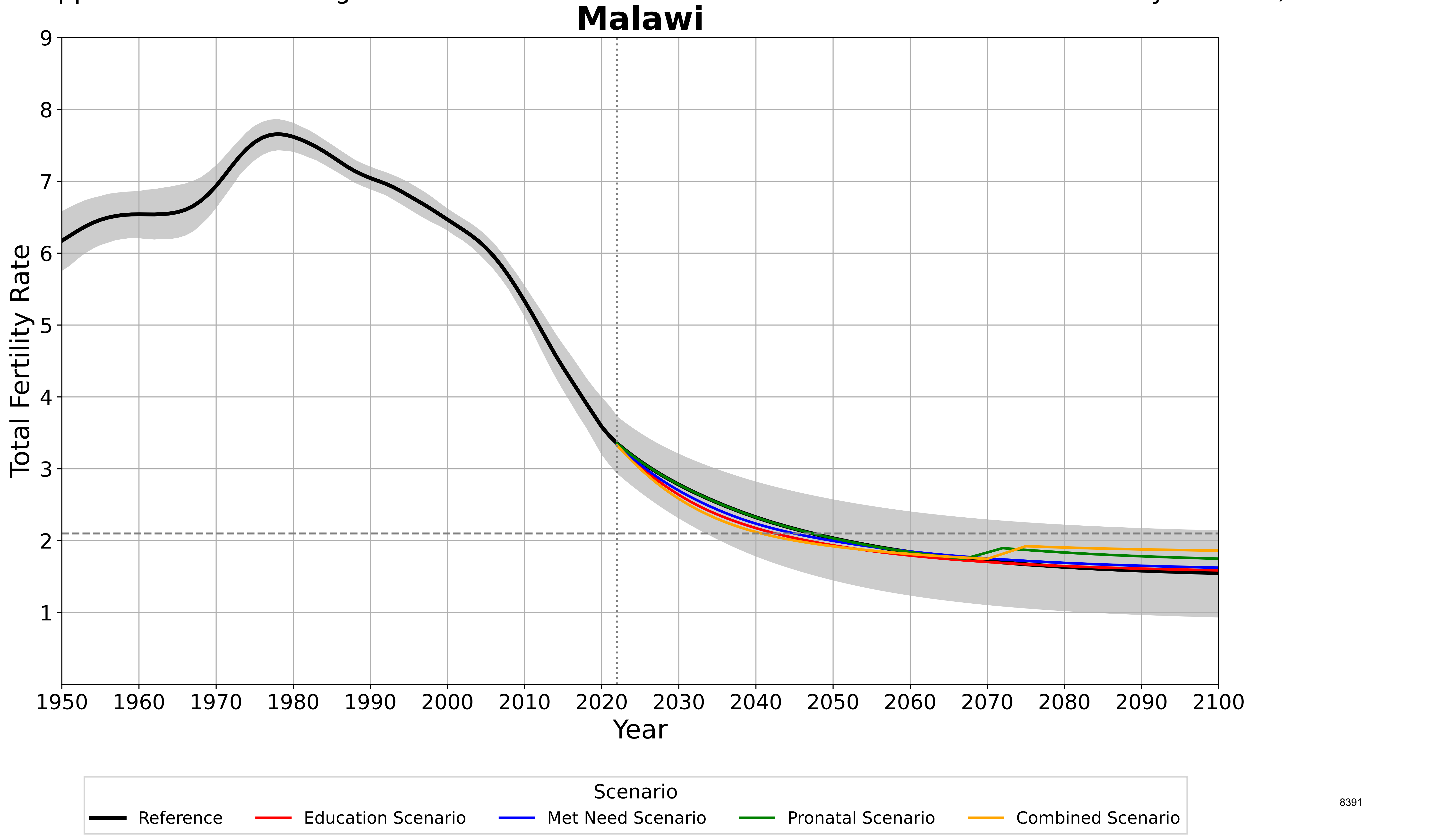

Supplemental Results Figure S5: TFR for reference and alternative scenario forecasts by location, 1950– 2100

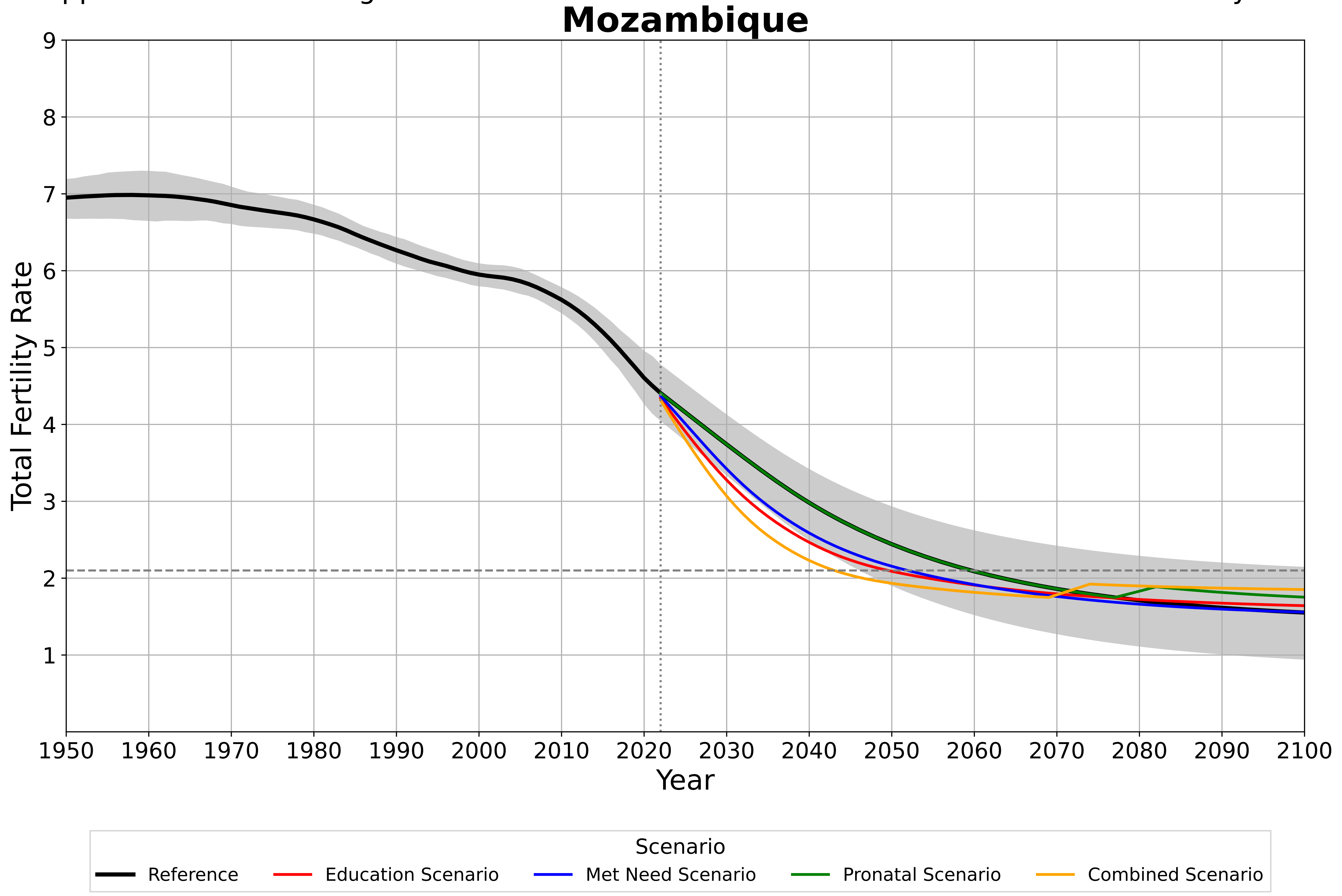

Supplemental Results Figure S5: TFR for reference and alternative scenario forecasts by location, 1950– 2100

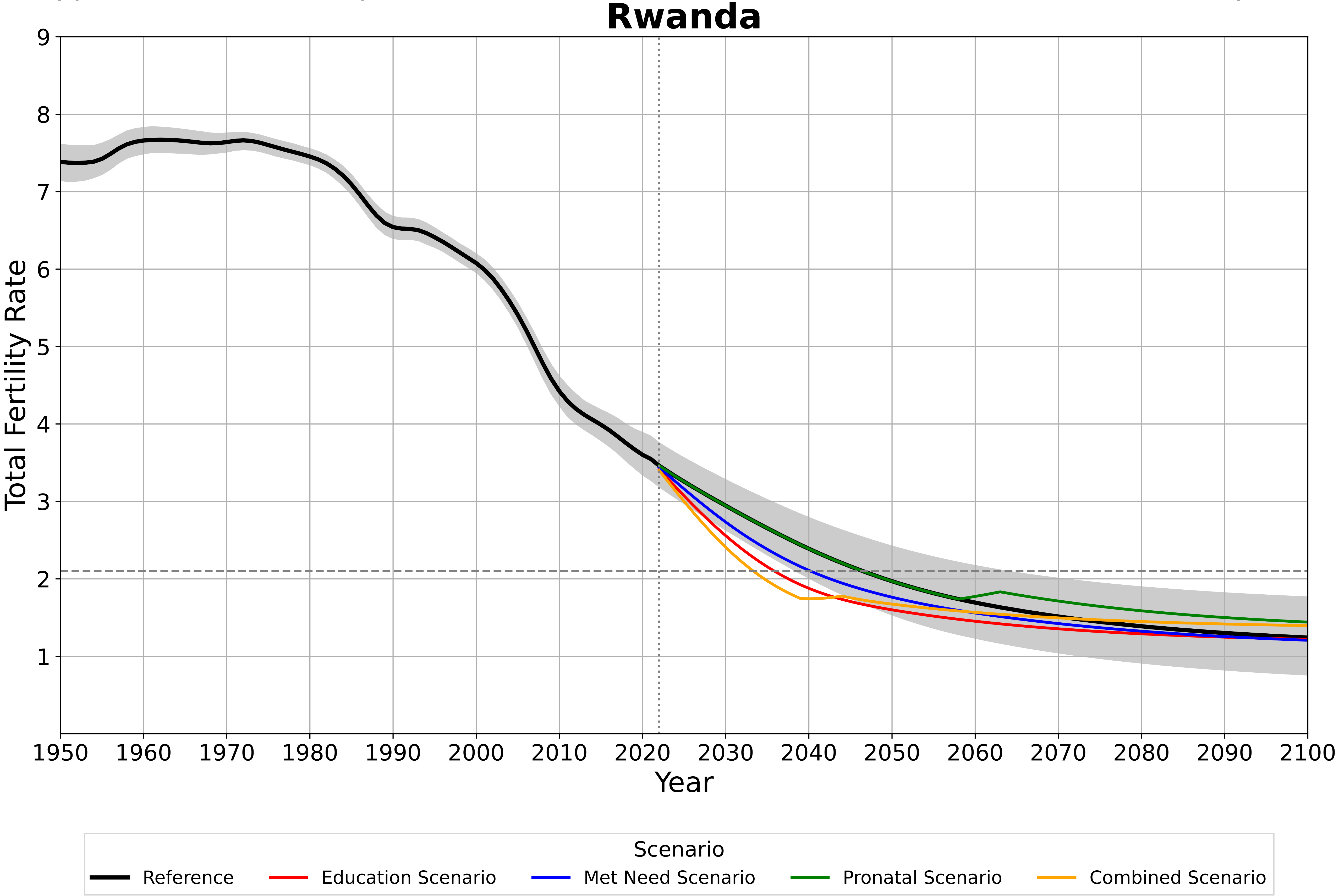

Supplemental Results Figure S5: TFR for reference and alternative scenario forecasts by location, 1950– 2100

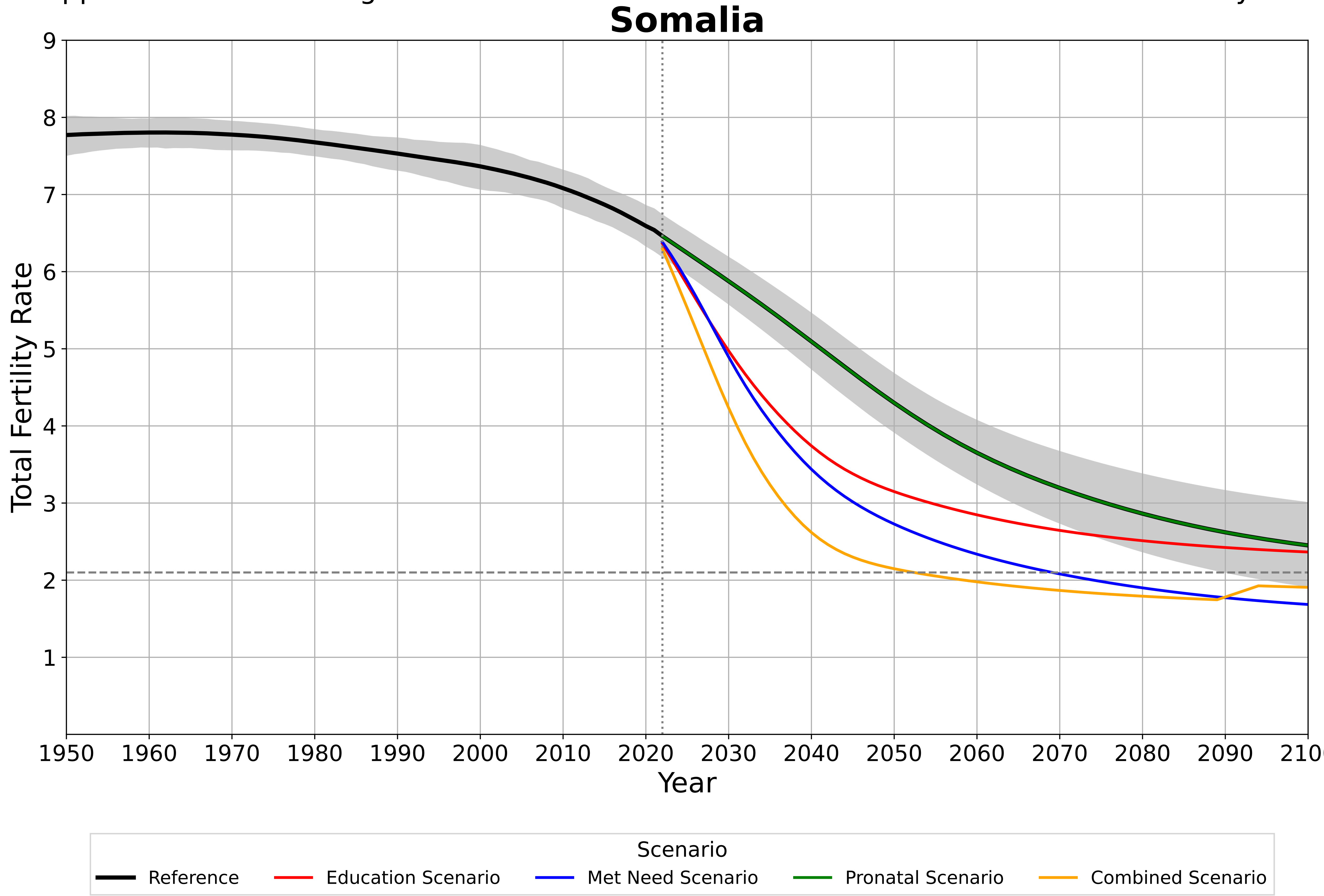

8394

Supplemental Results Figure S5: TFR for reference and alternative scenario forecasts by location, 1950– 2100

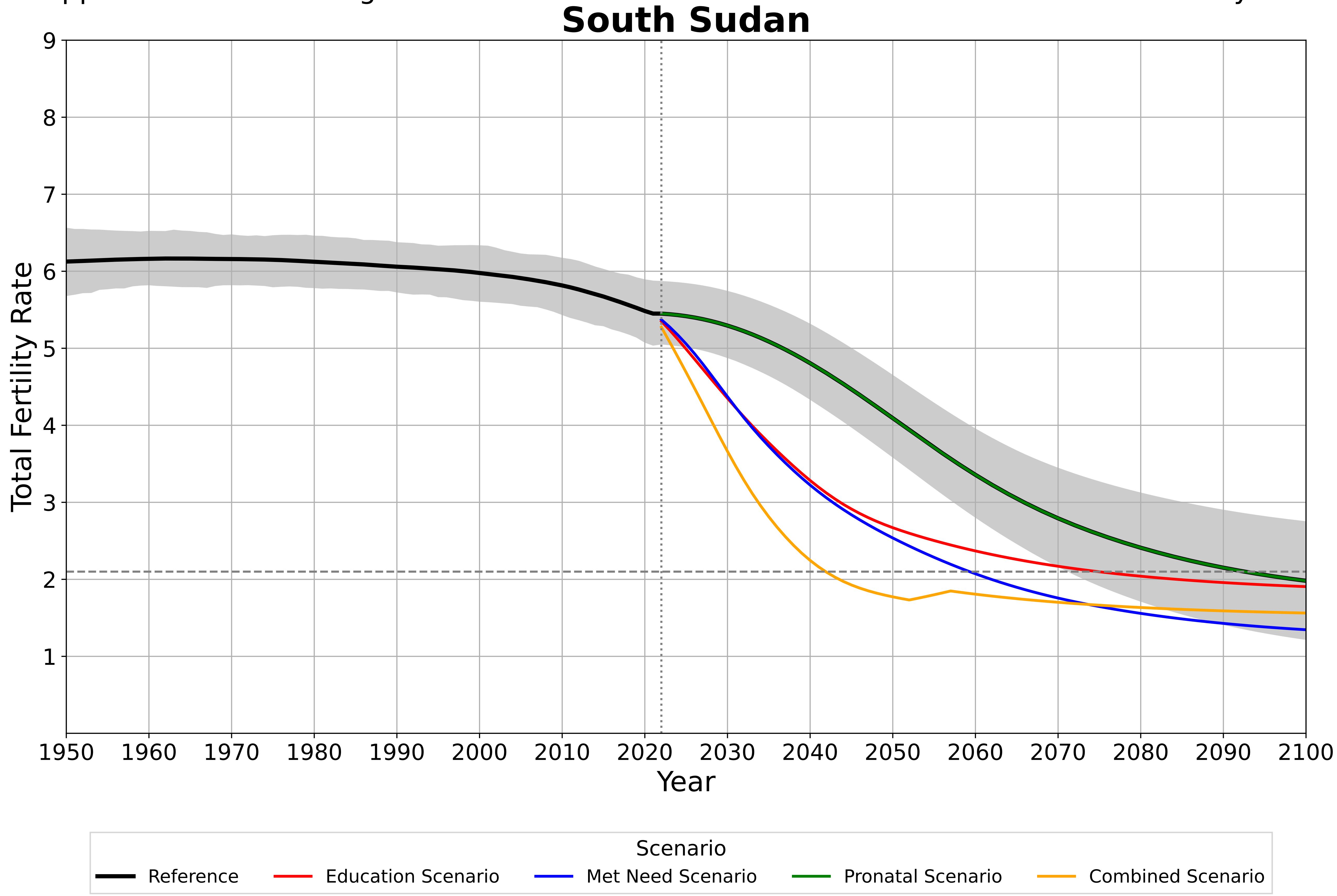

Supplemental Results Figure S5: TFR for reference and alternative scenario forecasts by location, 1950– 2100

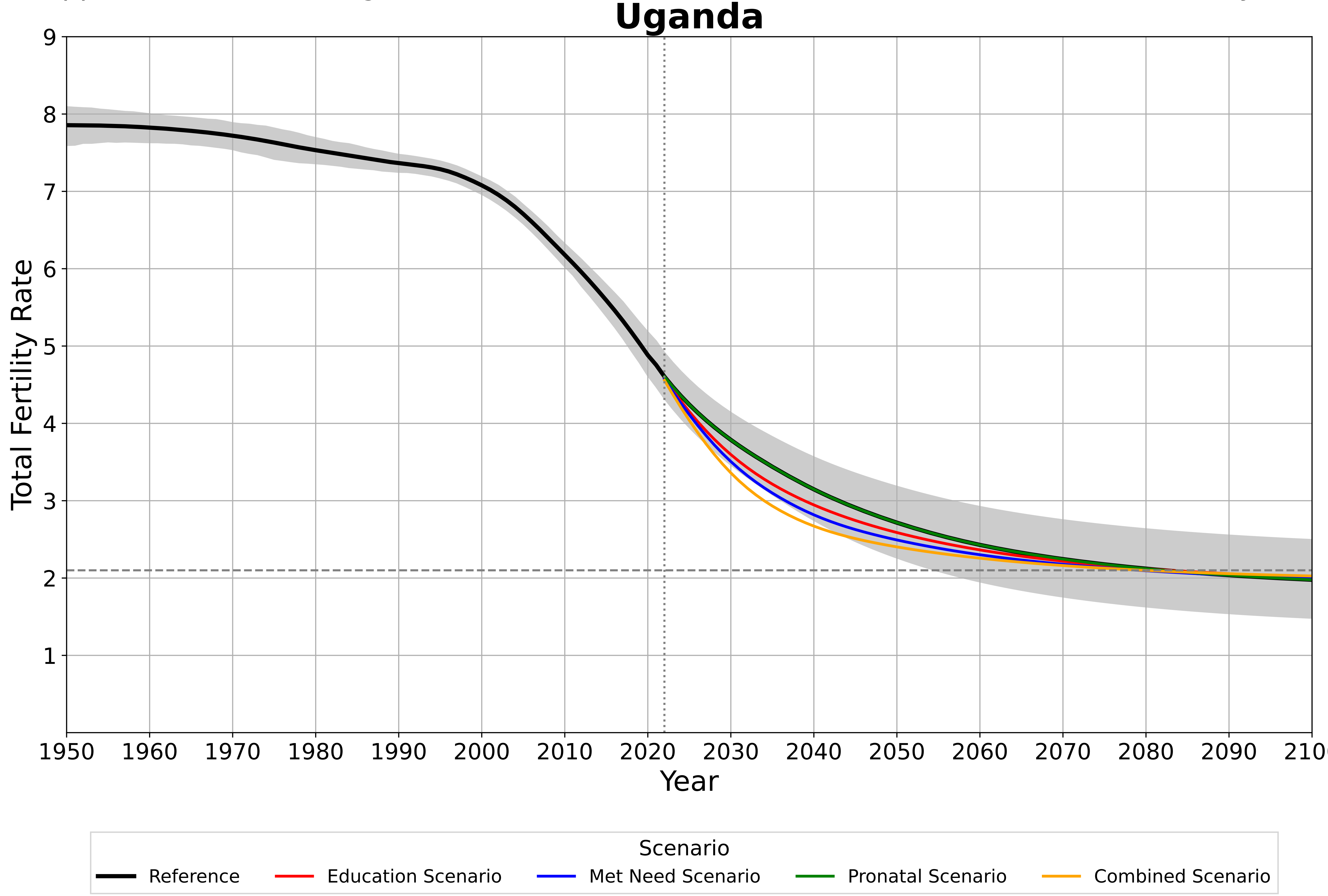

8396

Supplemental Results Figure S5: TFR for reference and alternative scenario forecasts by location, 1950– 2100

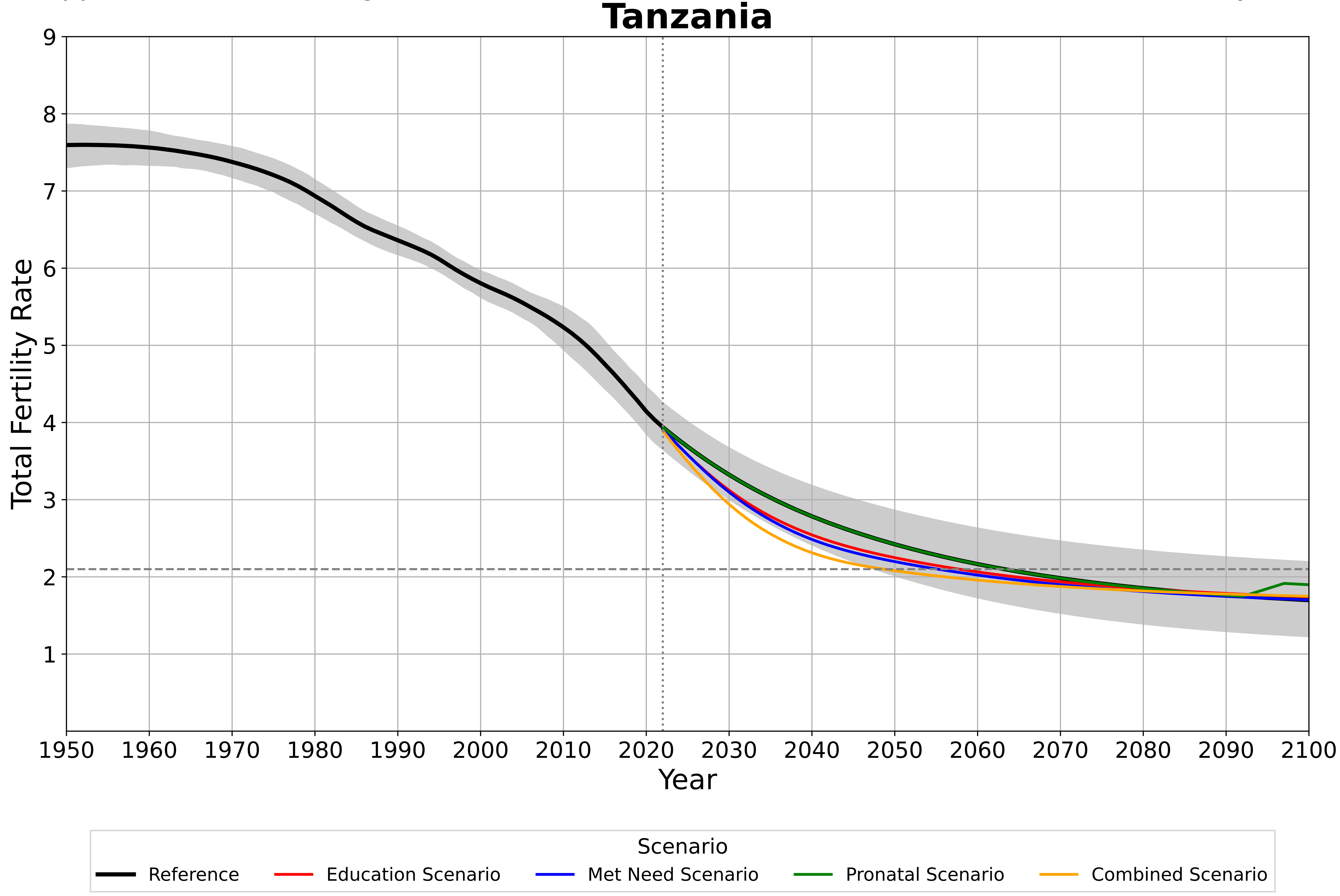

Supplemental Results Figure S5: TFR for reference and alternative scenario forecasts by location, 1950– 2100

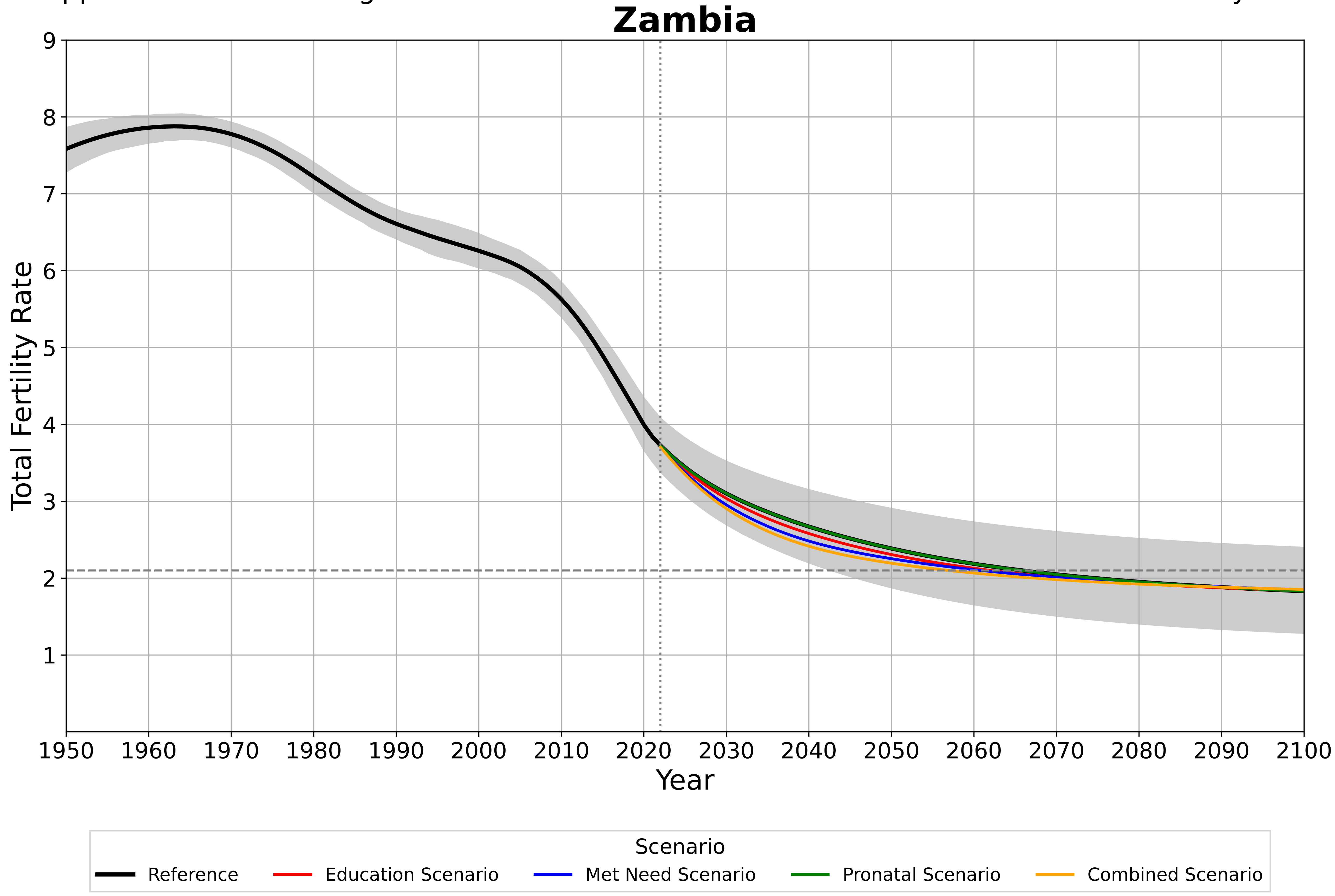

Supplemental Results Figure S5: TFR for reference and alternative scenario forecasts by location, 1950– 2100

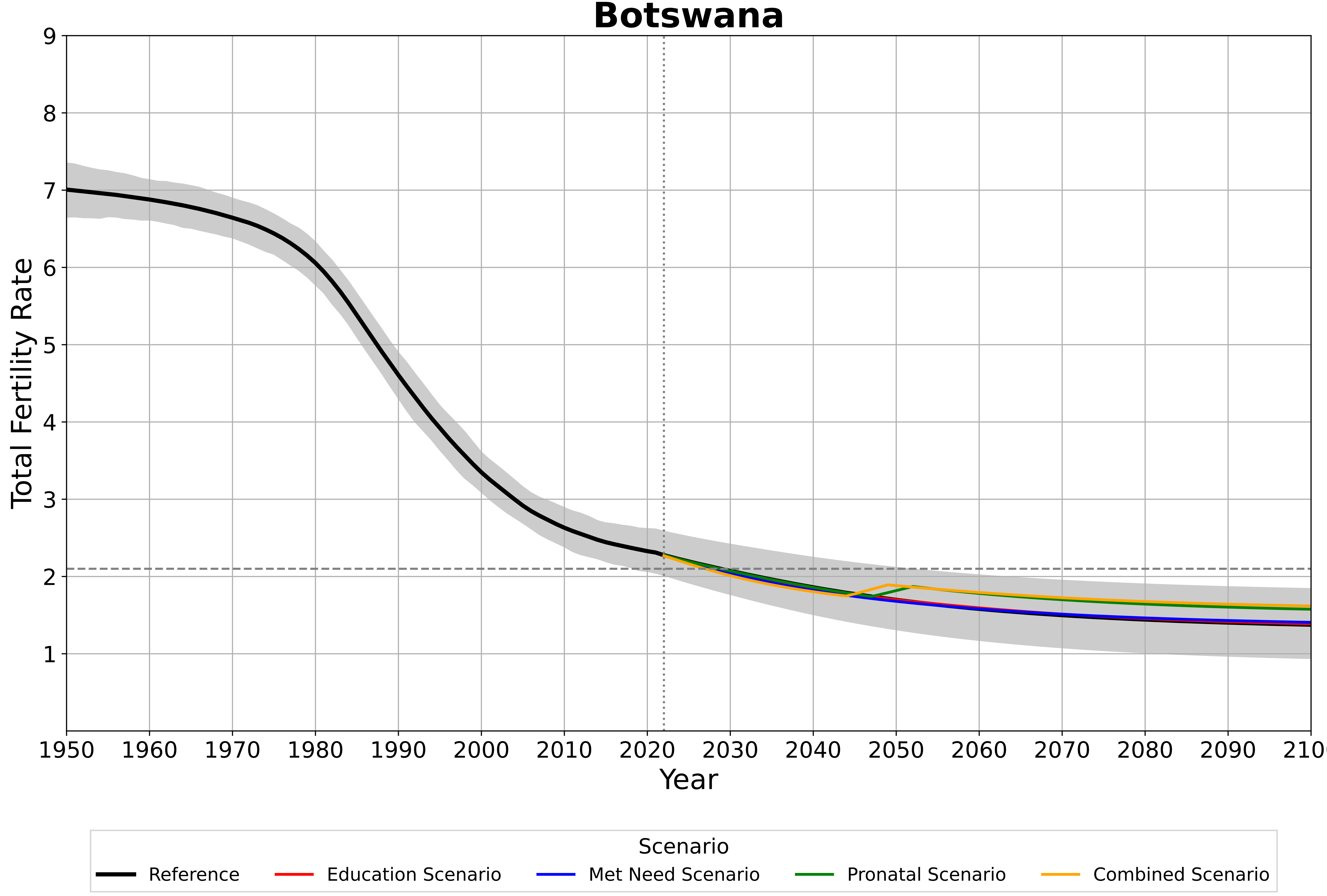

Supplemental Results Figure S5: TFR for reference and alternative scenario forecasts by location, 1950– 2100

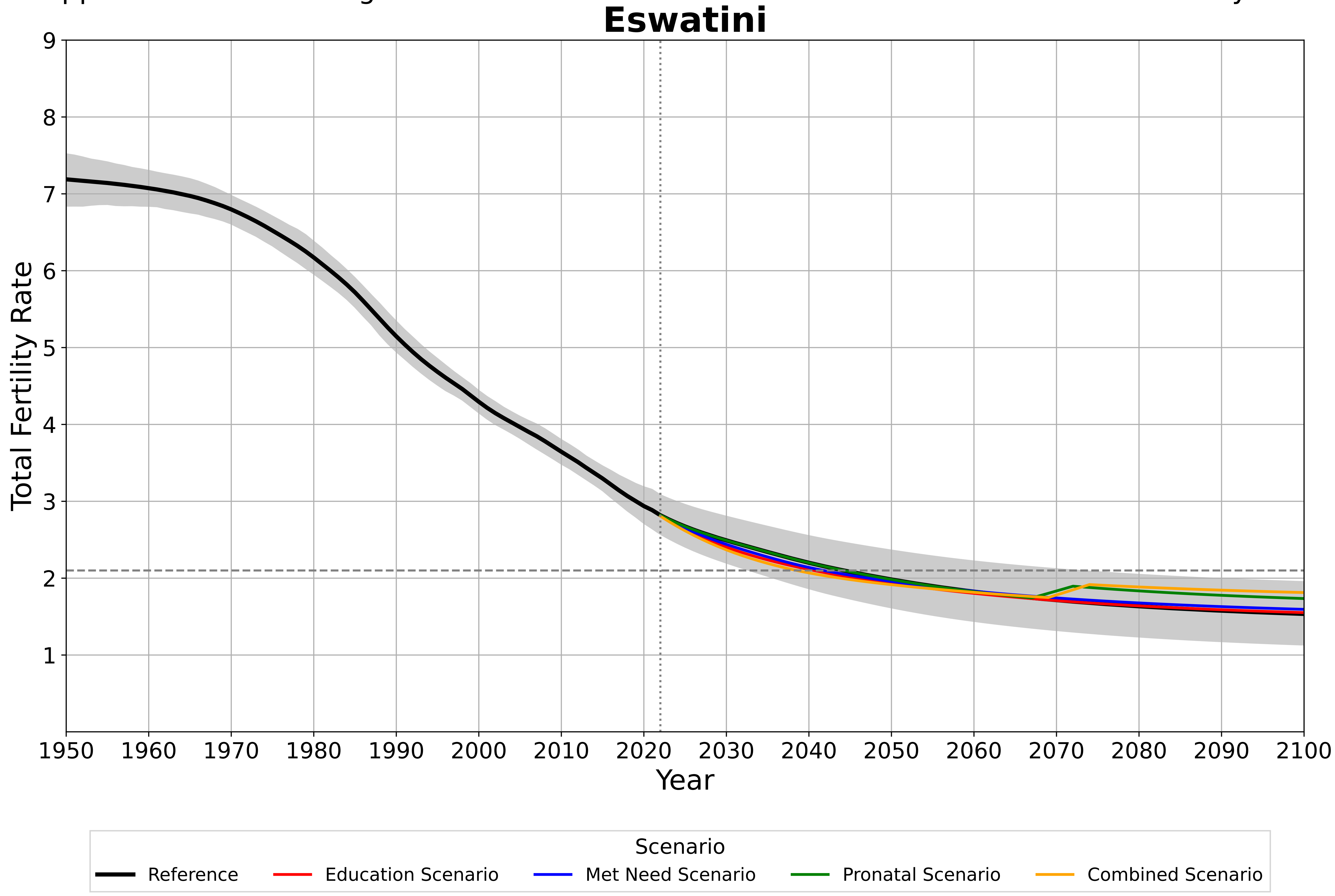

Supplemental Results Figure S5: TFR for reference and alternative scenario forecasts by location, 1950– 2100

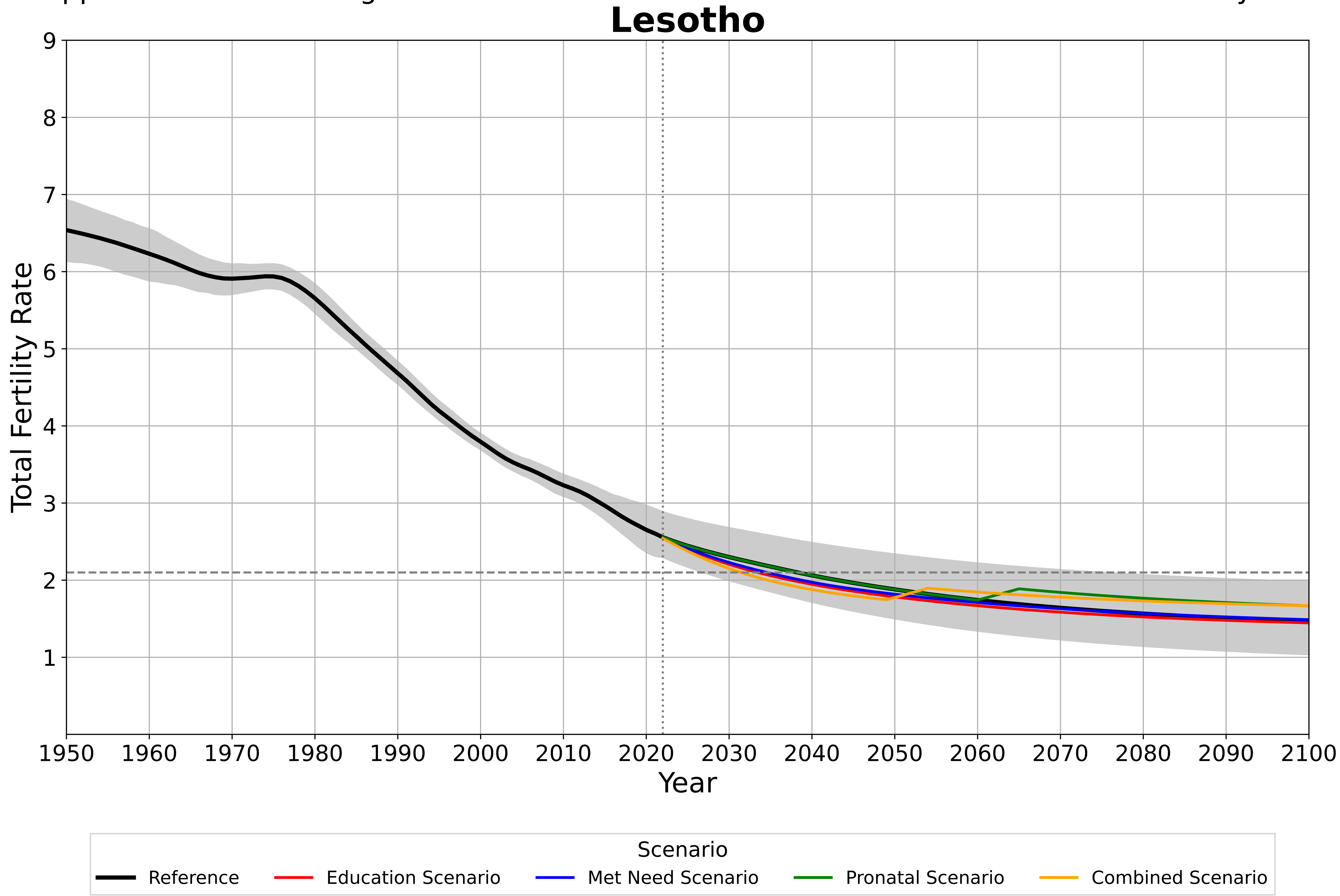

8401

Supplemental Results Figure S5: TFR for reference and alternative scenario forecasts by location, 1950– 2100

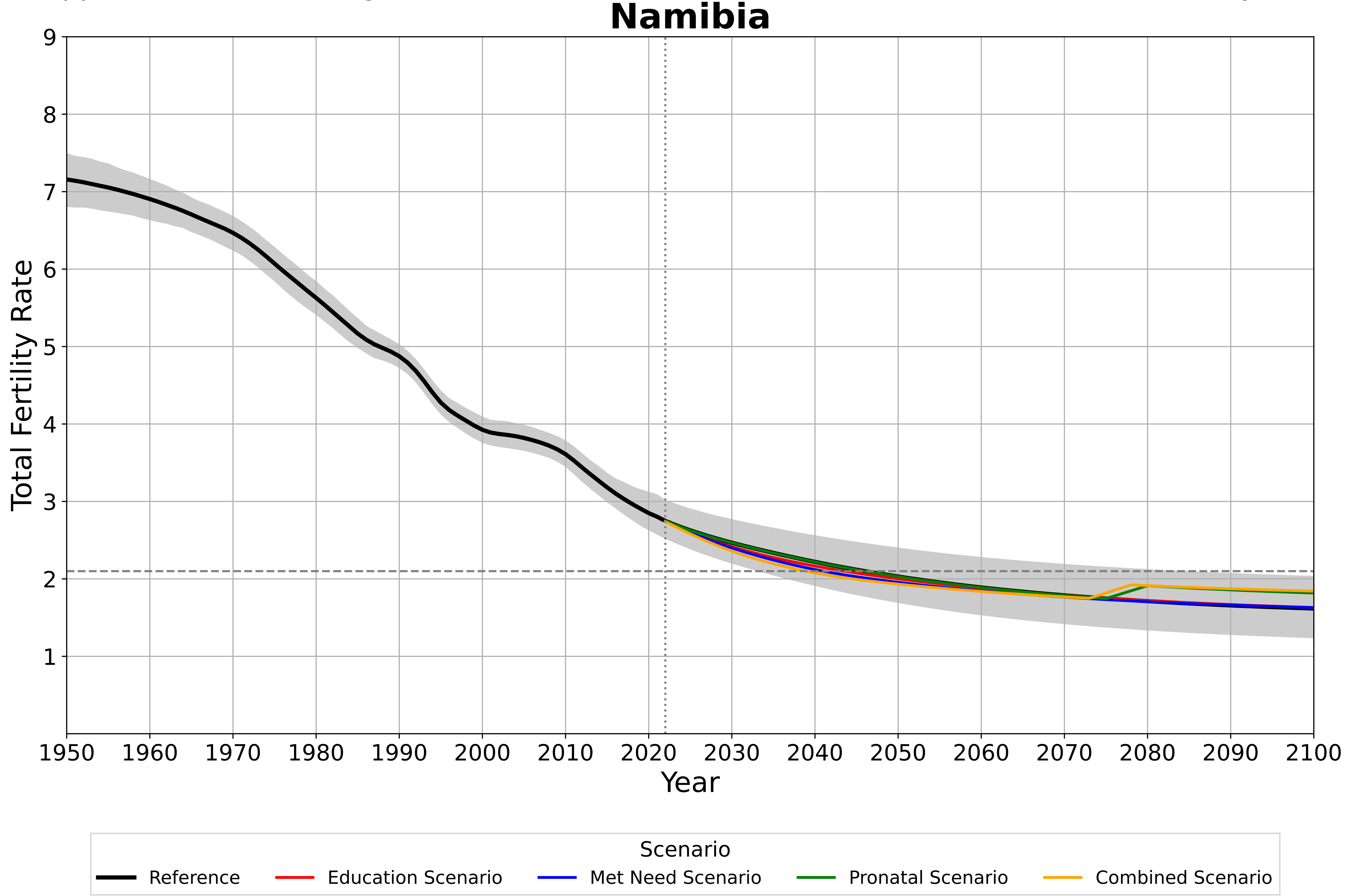

8402

Supplemental Results Figure S5: TFR for reference and alternative scenario forecasts by location, 1950– 2100

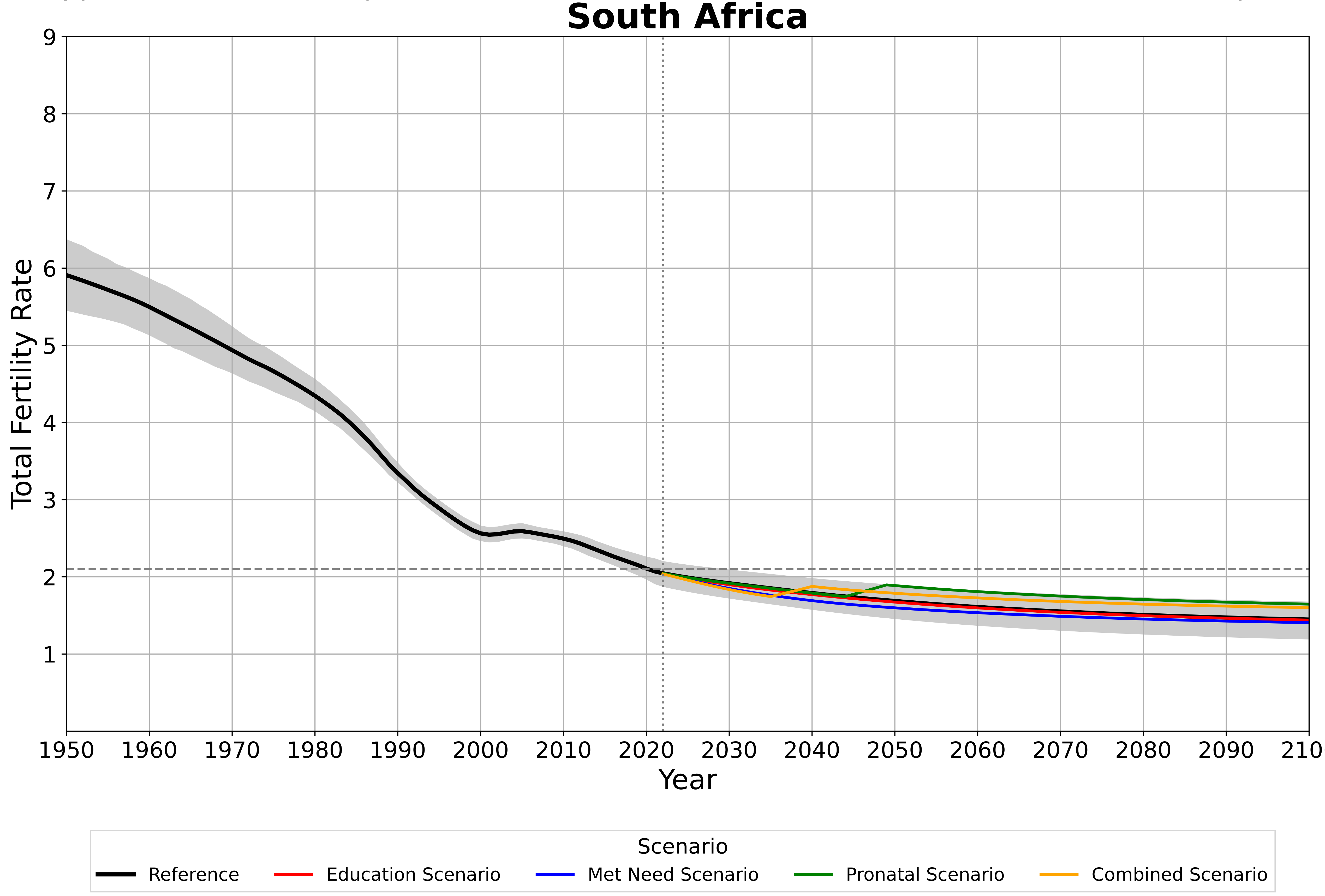

Supplemental Results Figure S5: TFR for reference and alternative scenario forecasts by location, 1950– 2100

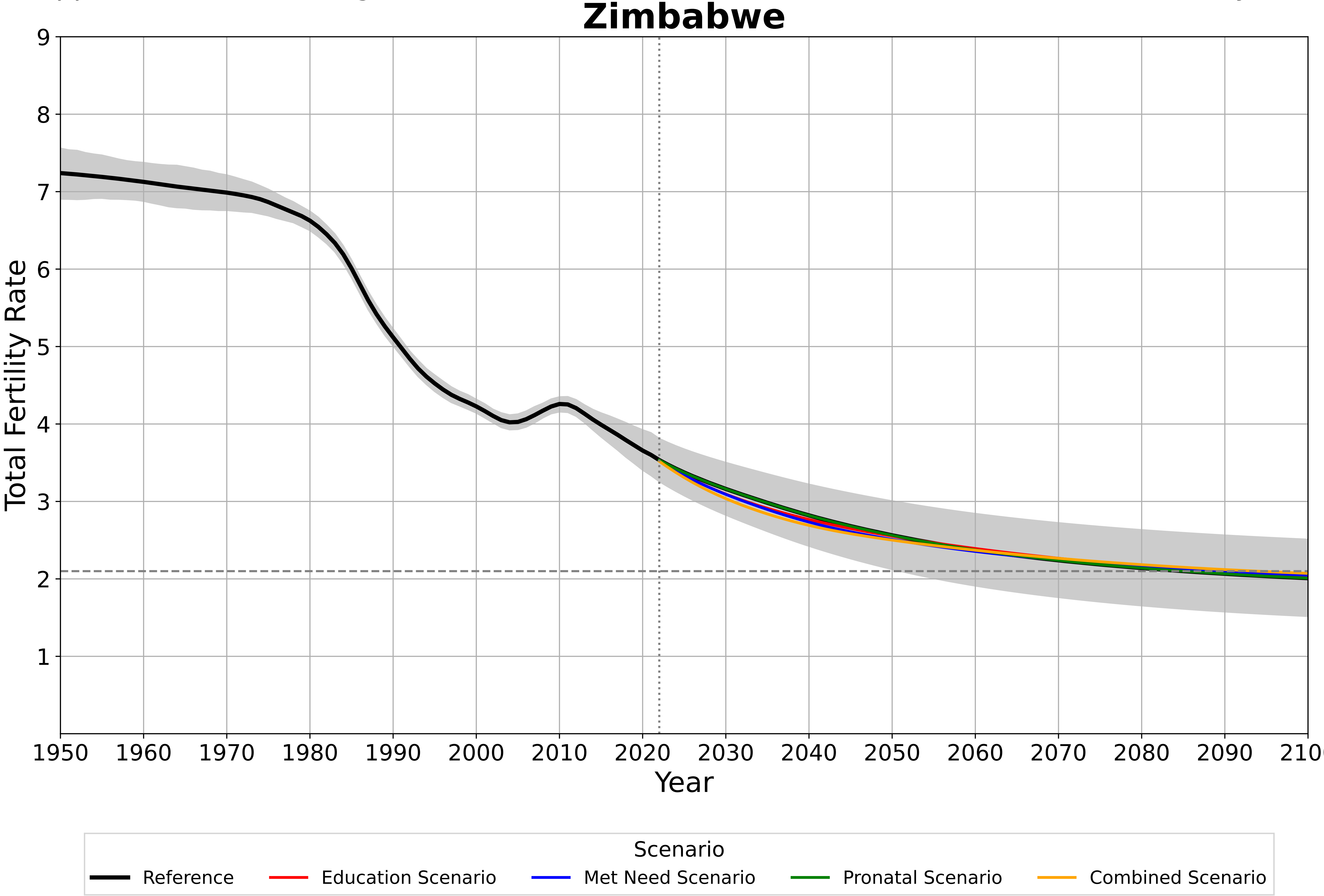

Supplemental Results Figure S5: TFR for reference and alternative scenario forecasts by location, 1950– 2100

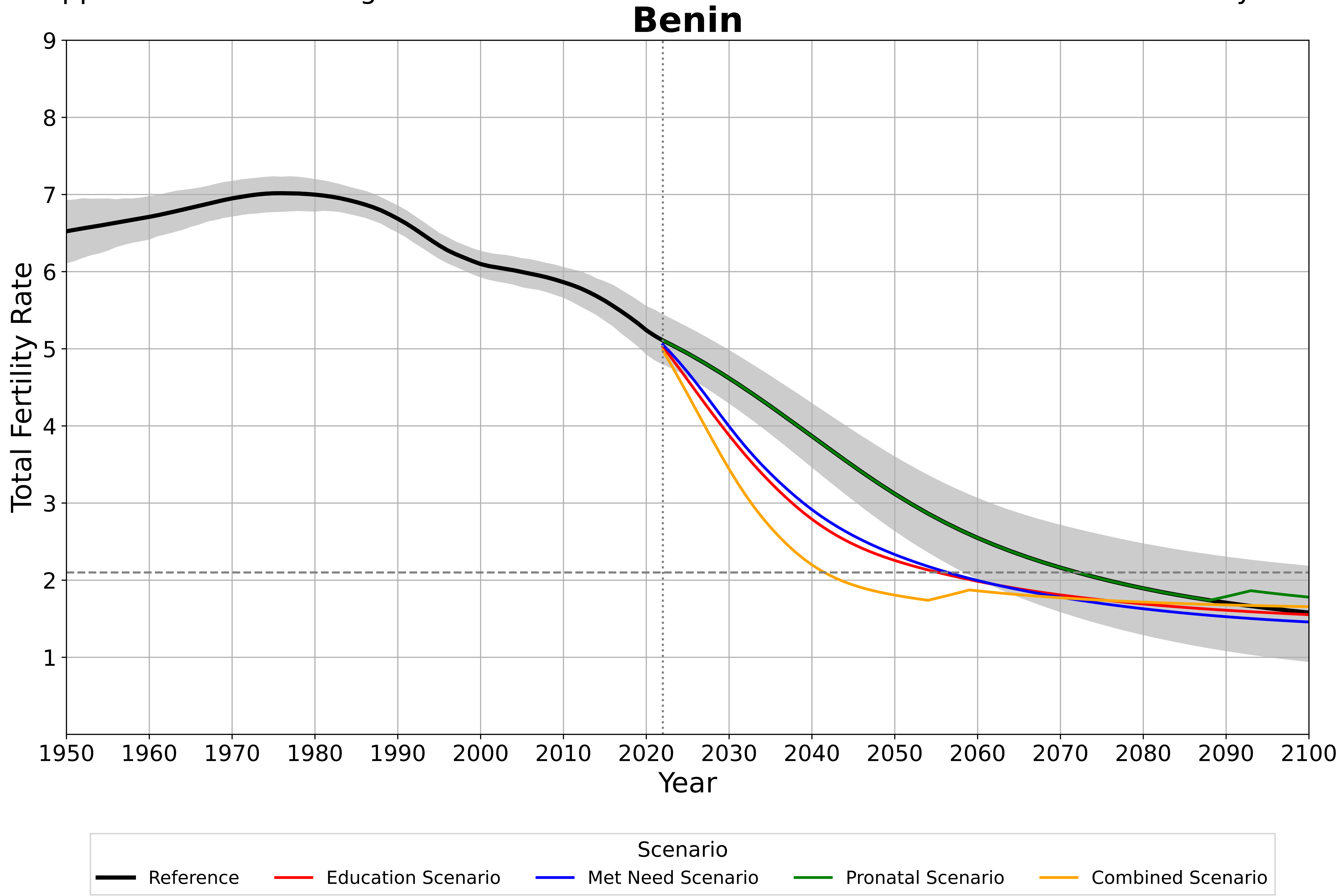

Supplemental Results Figure S5: TFR for reference and alternative scenario forecasts by location, 1950– 2100

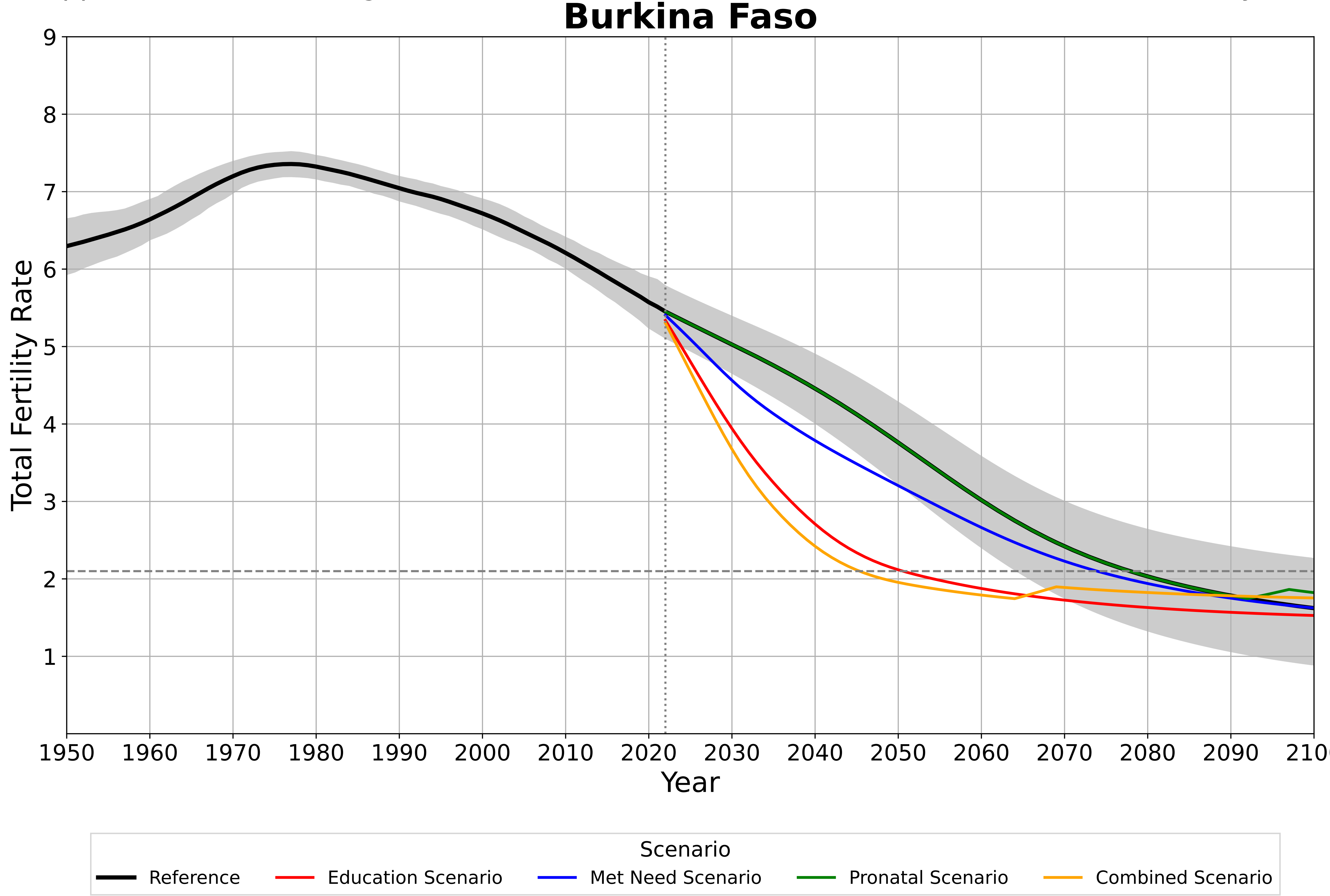

8406

Supplemental Results Figure S5: TFR for reference and alternative scenario forecasts by location, 1950– 2100

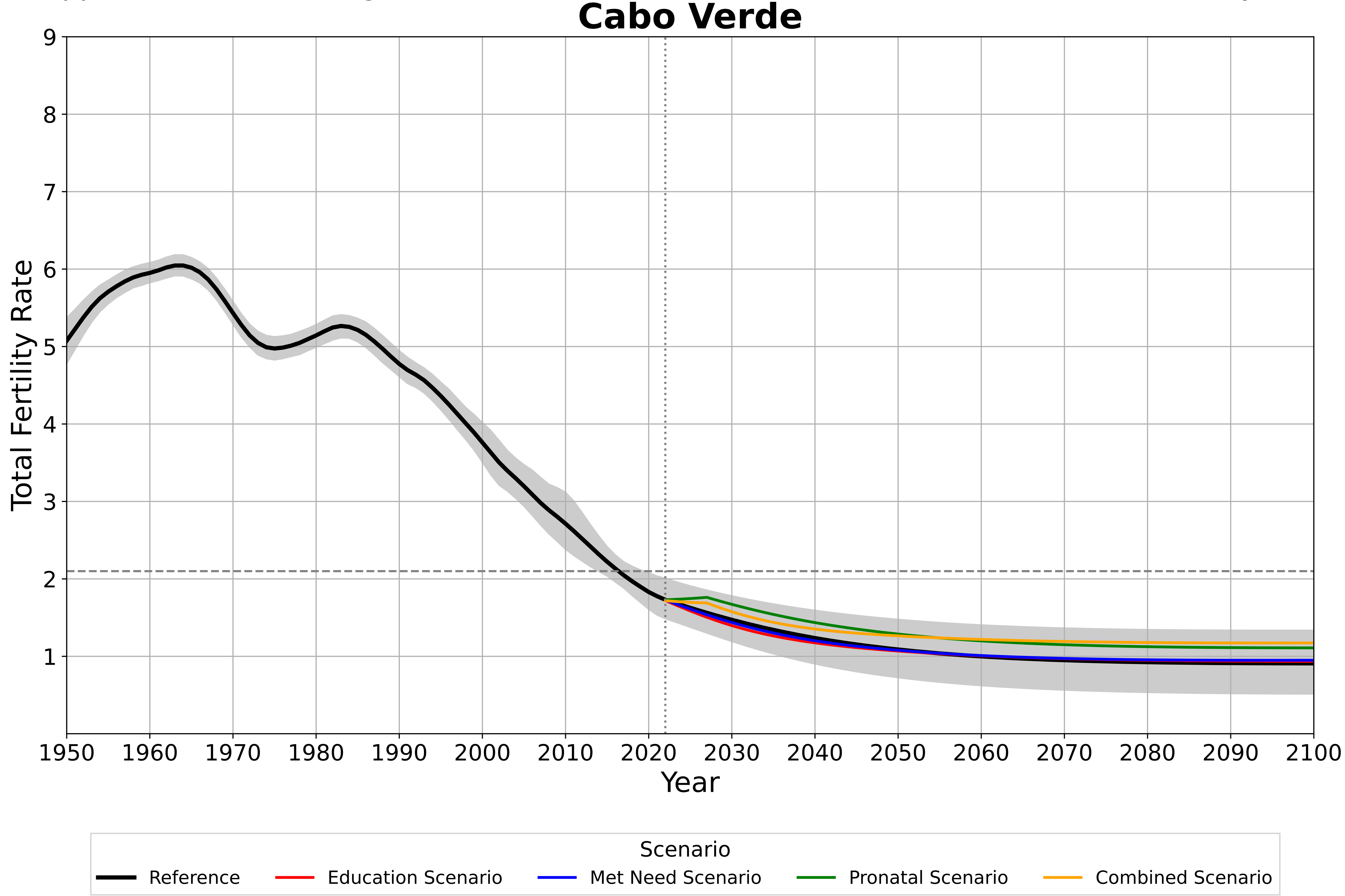

8407

Supplemental Results Figure S5: TFR for reference and alternative scenario forecasts by location, 1950– 2100

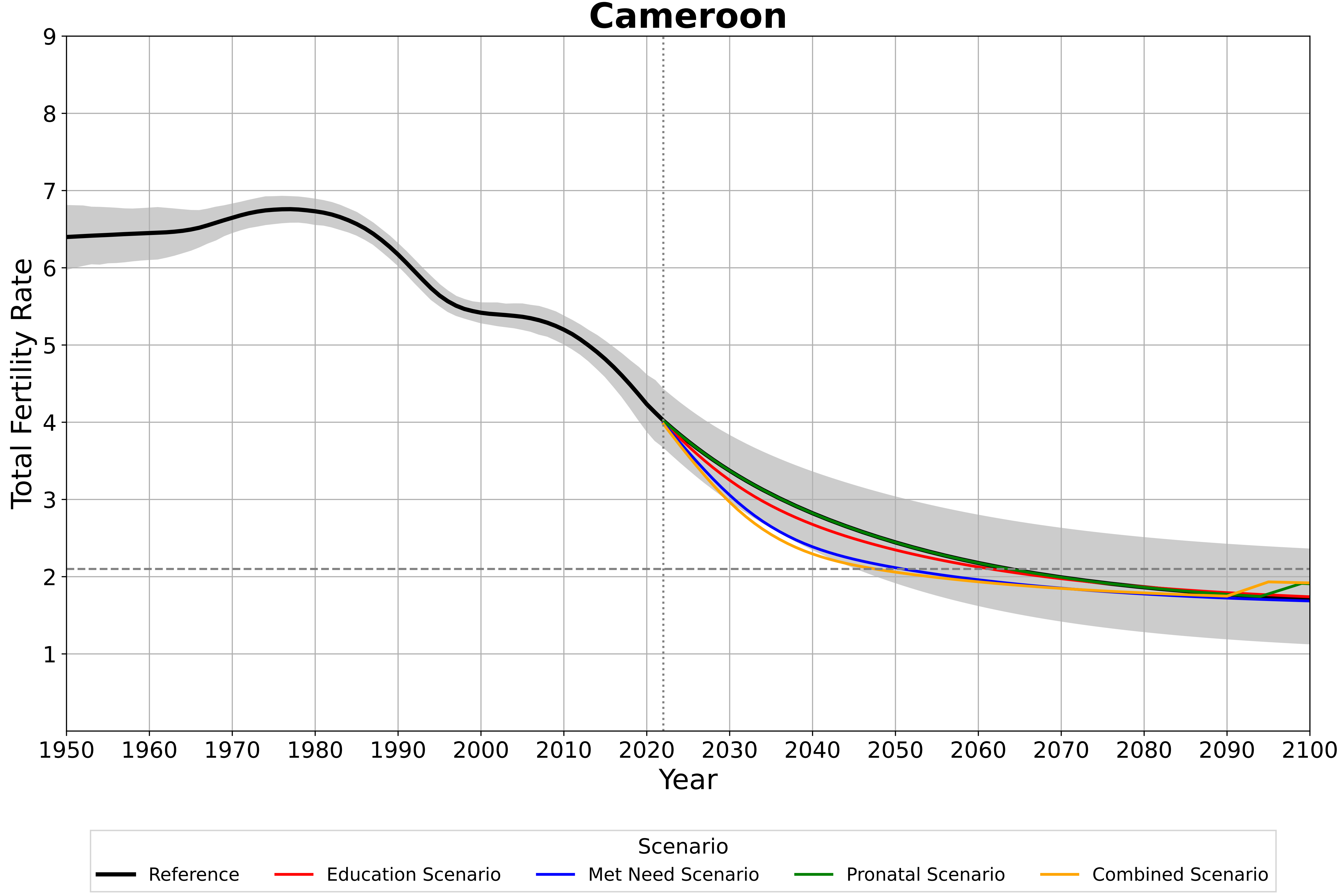

Supplemental Results Figure S5: TFR for reference and alternative scenario forecasts by location, 1950– 2100

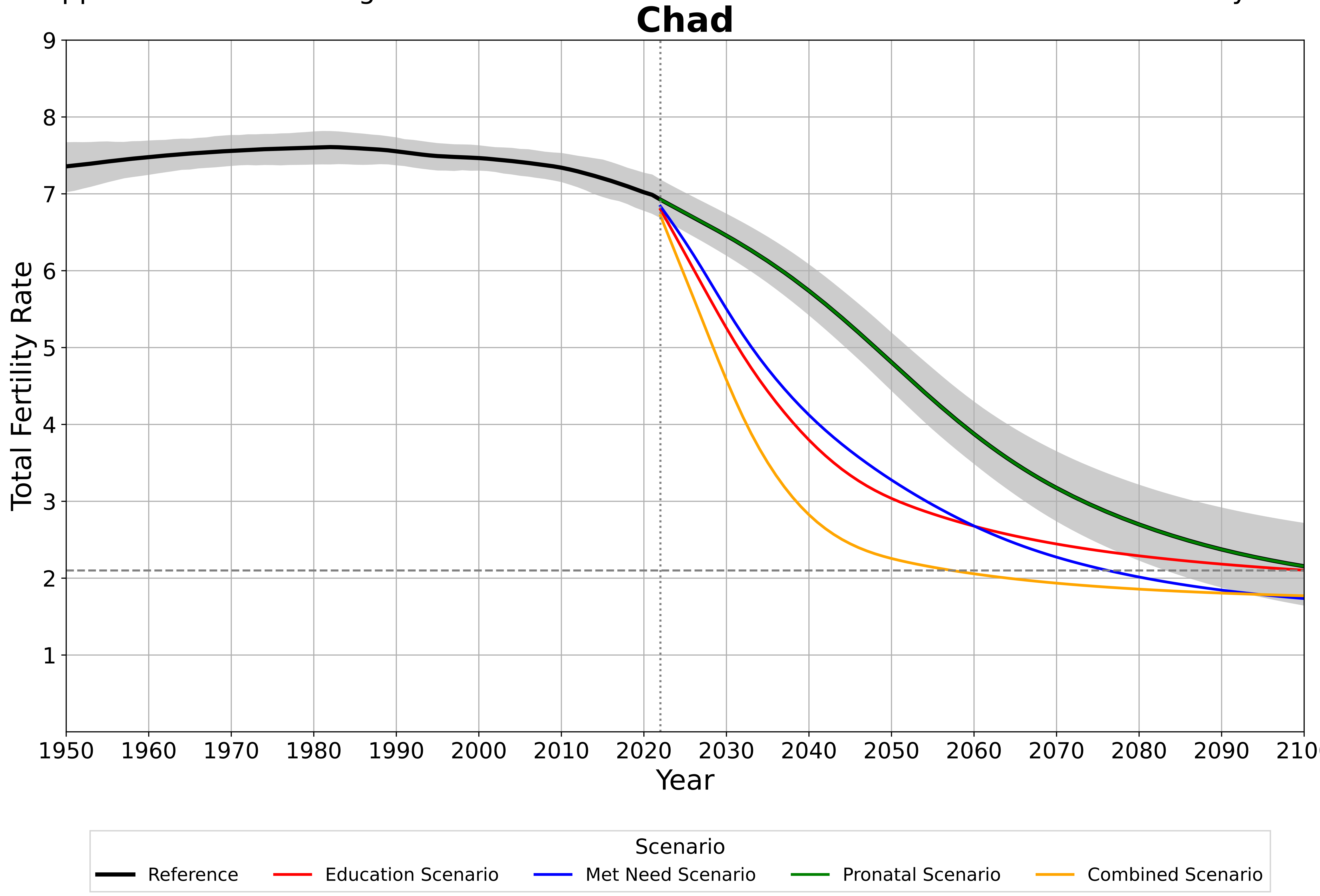

Supplemental Results Figure S5: TFR for reference and alternative scenario forecasts by location, 1950– 2100

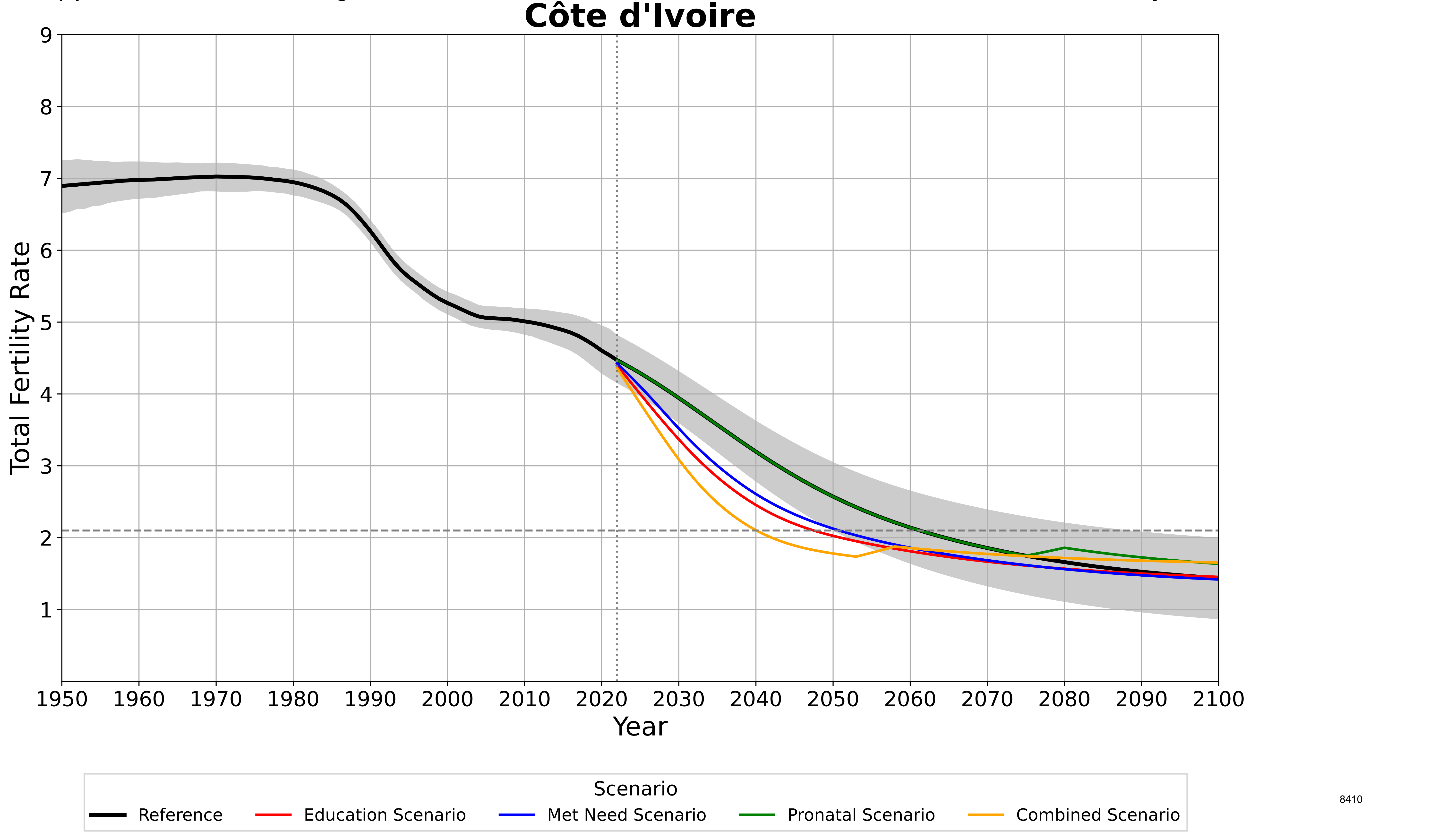

Supplemental Results Figure S5: TFR for reference and alternative scenario forecasts by location, 1950– 2100

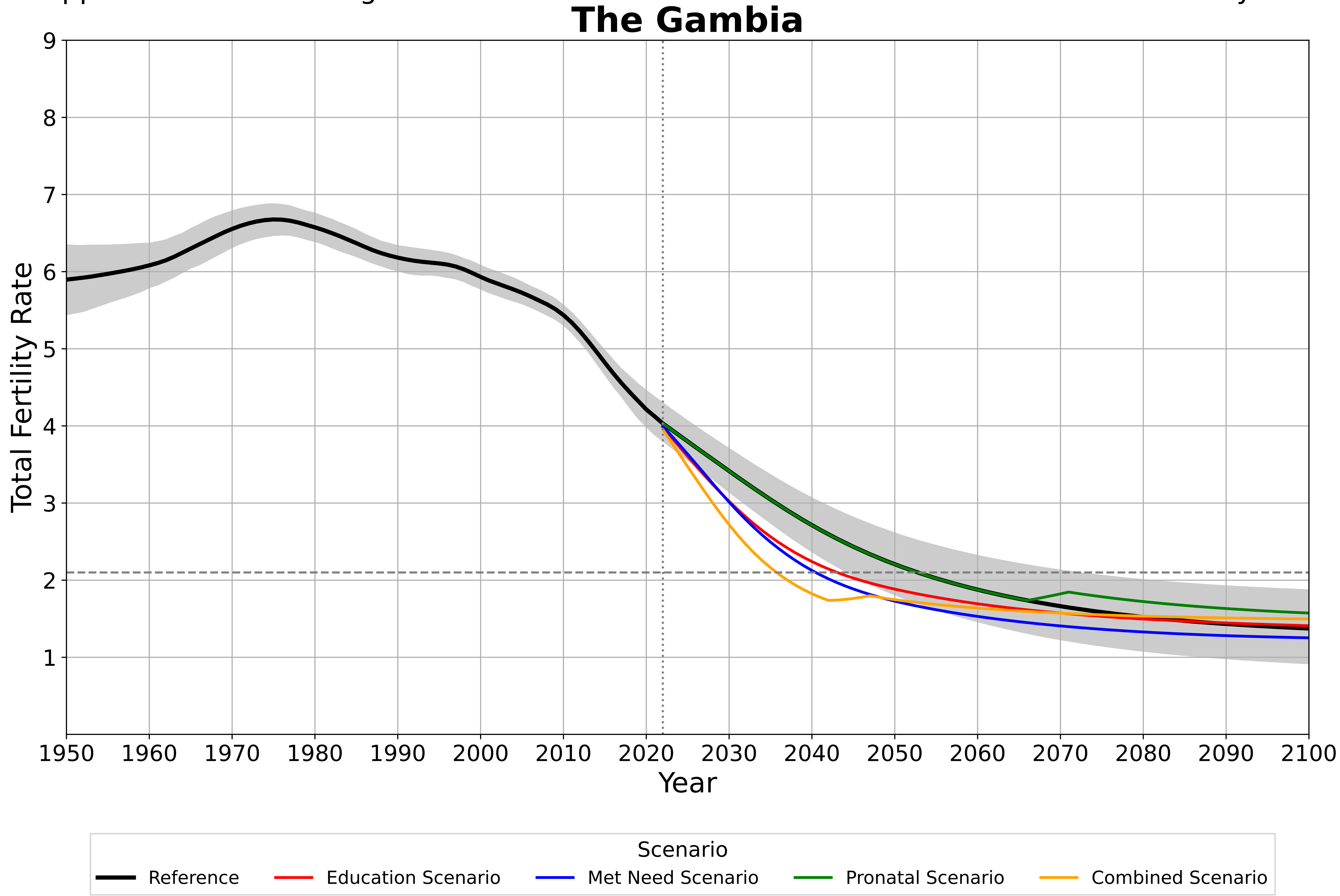

Supplemental Results Figure S5: TFR for reference and alternative scenario forecasts by location, 1950– 2100

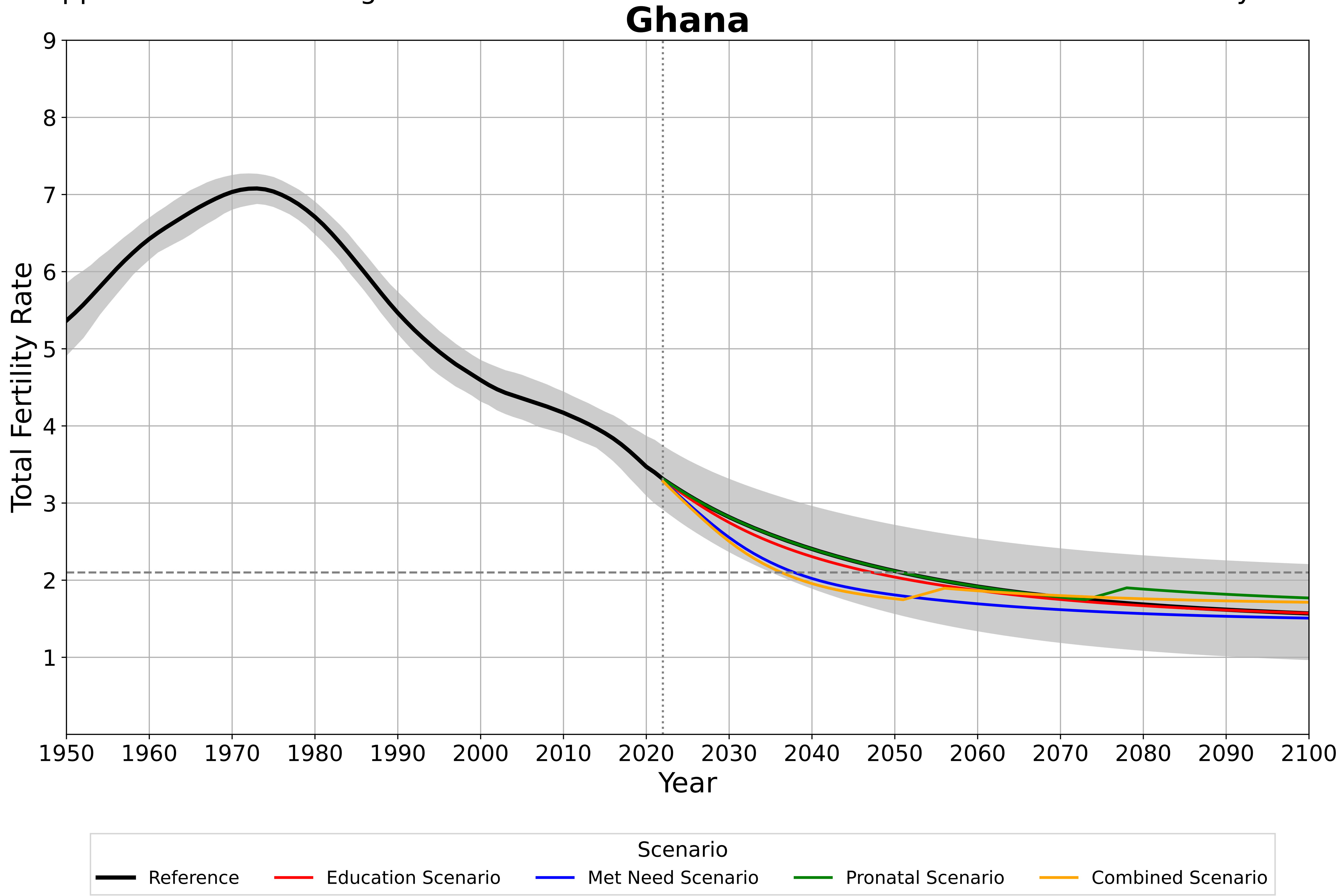

Supplemental Results Figure S5: TFR for reference and alternative scenario forecasts by location, 1950– 2100

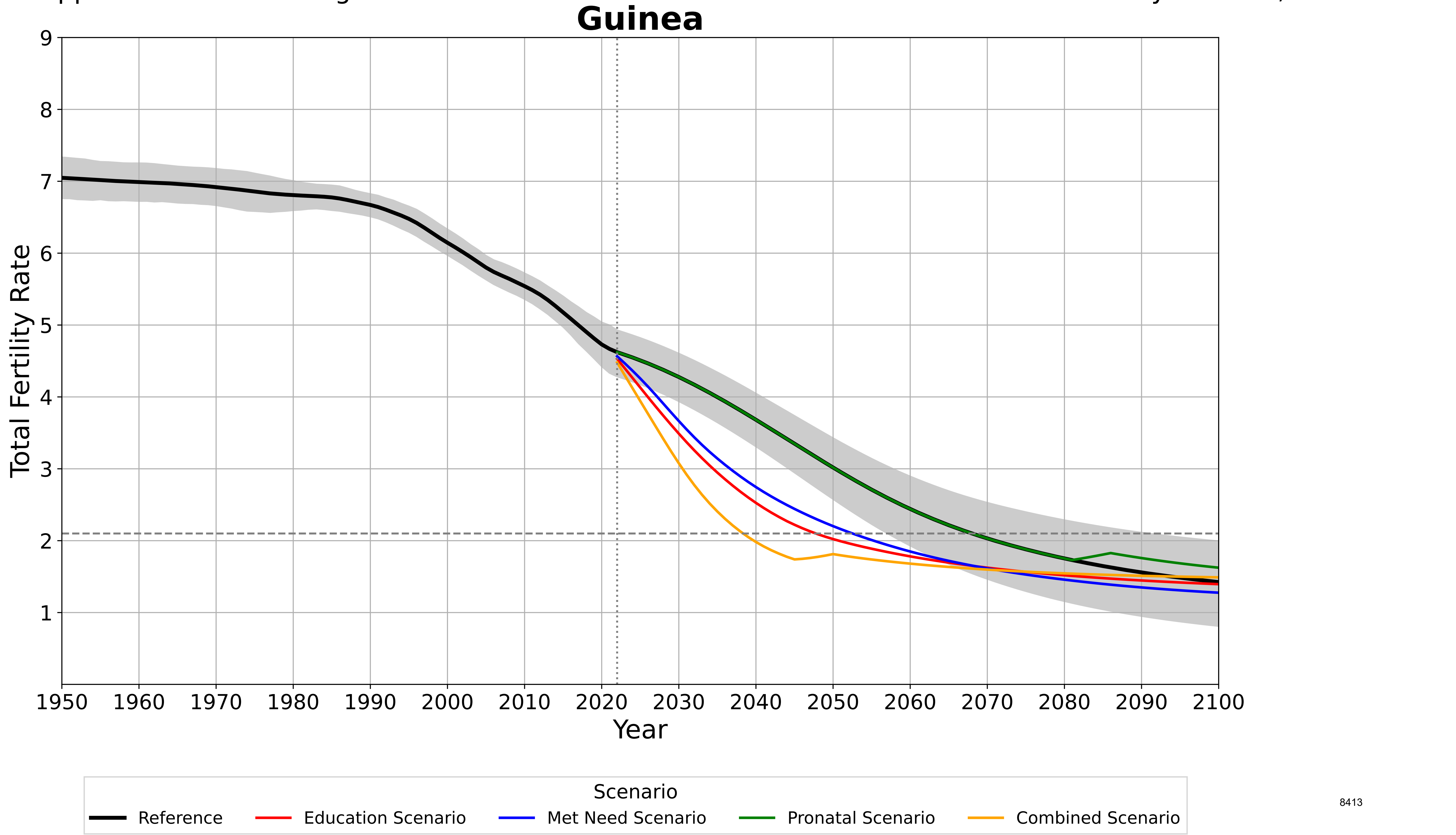

Supplemental Results Figure S5: TFR for reference and alternative scenario forecasts by location, 1950– 2100

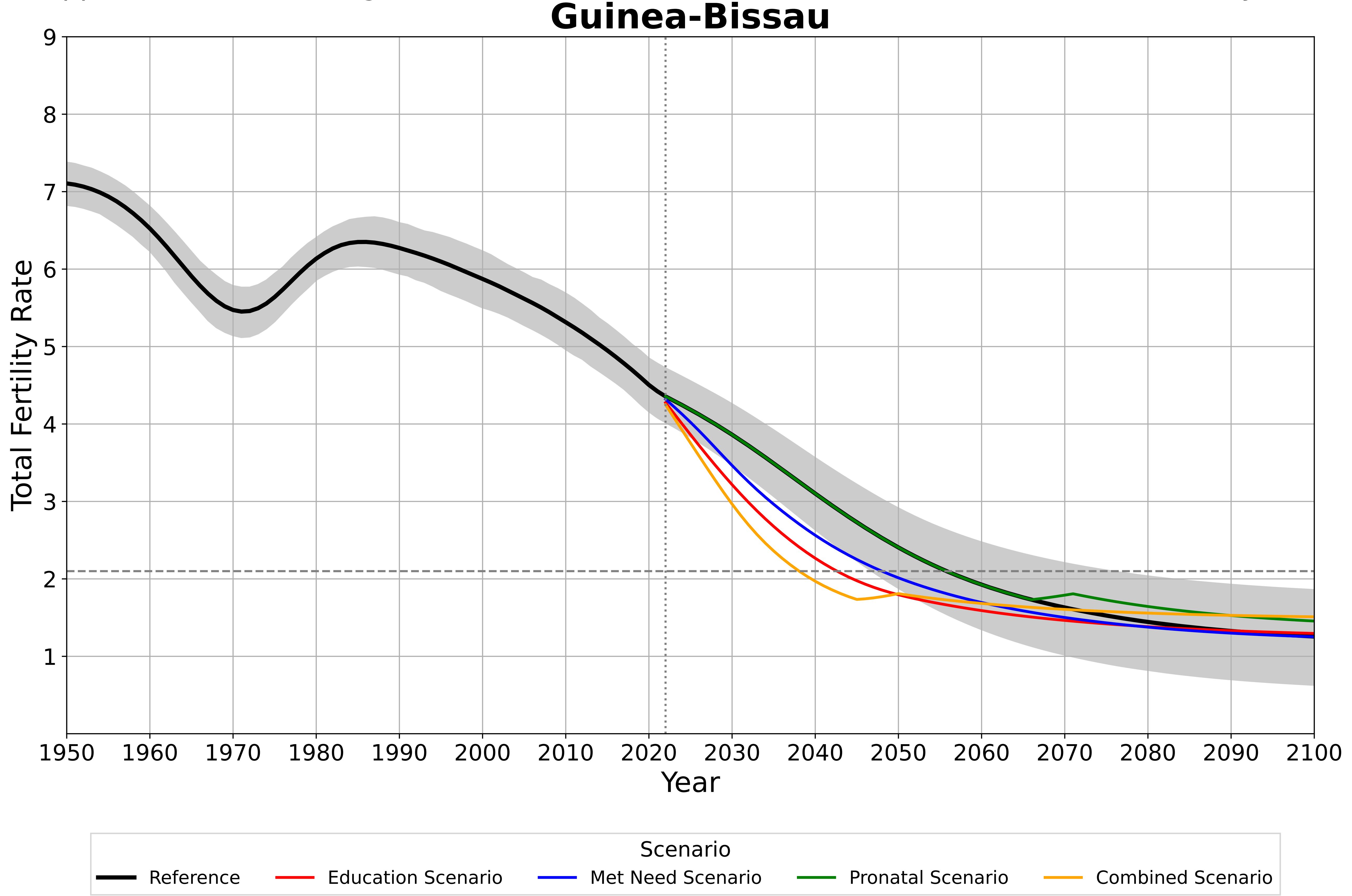

Supplemental Results Figure S5: TFR for reference and alternative scenario forecasts by location, 1950– 2100

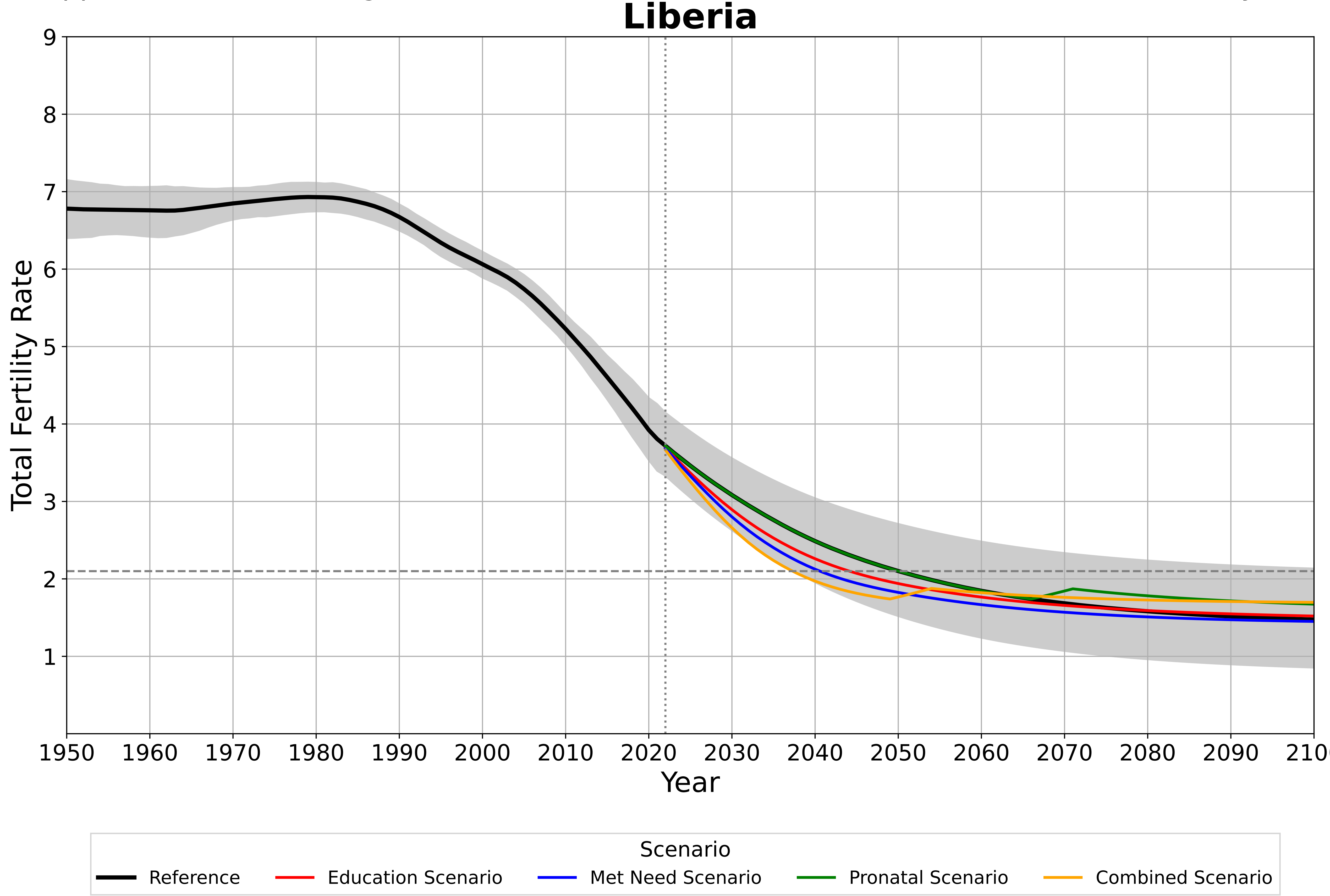

Supplemental Results Figure S5: TFR for reference and alternative scenario forecasts by location, 1950– 2100

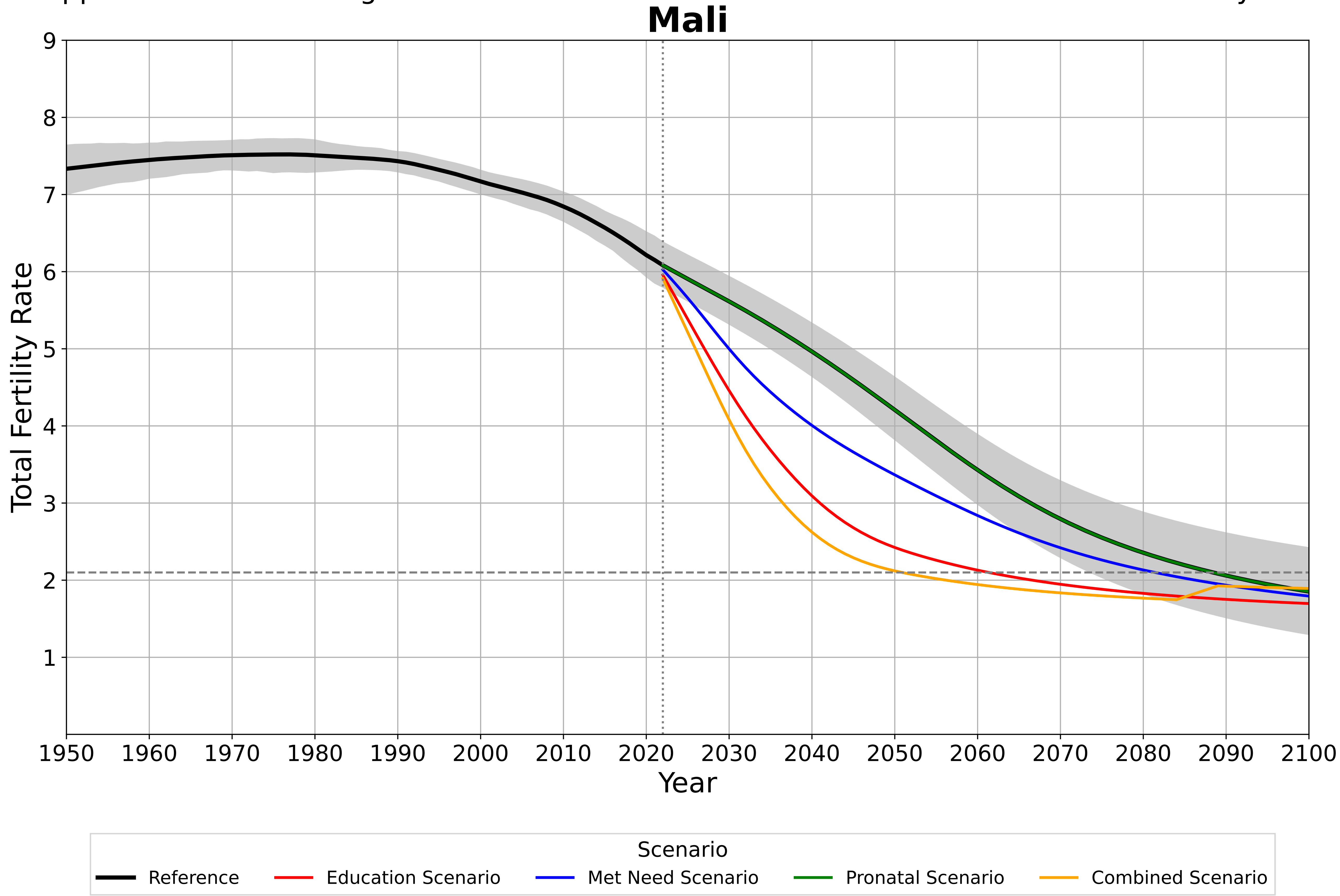

Supplemental Results Figure S5: TFR for reference and alternative scenario forecasts by location, 1950– 2100

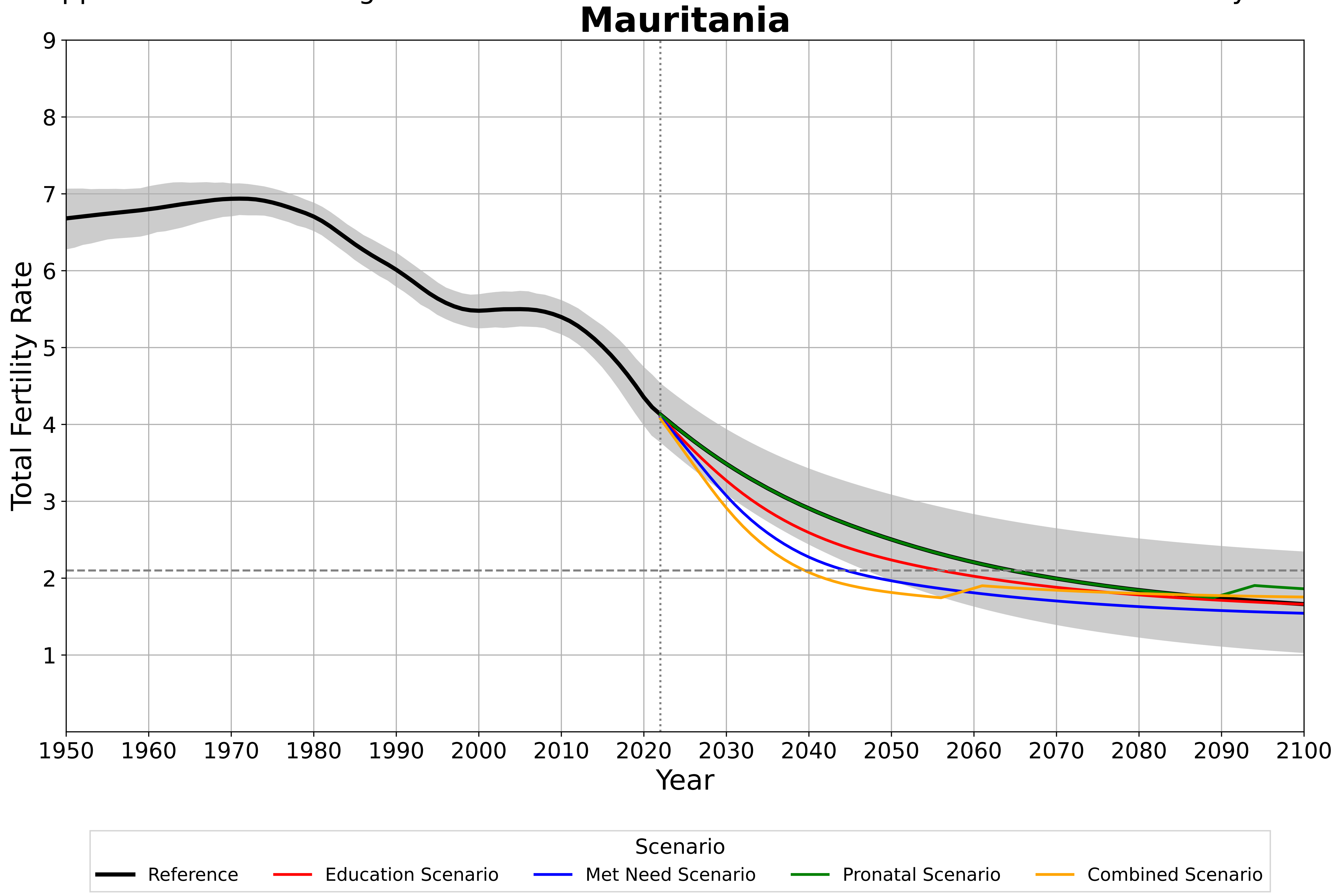

Supplemental Results Figure S5: TFR for reference and alternative scenario forecasts by location, 1950– 2100

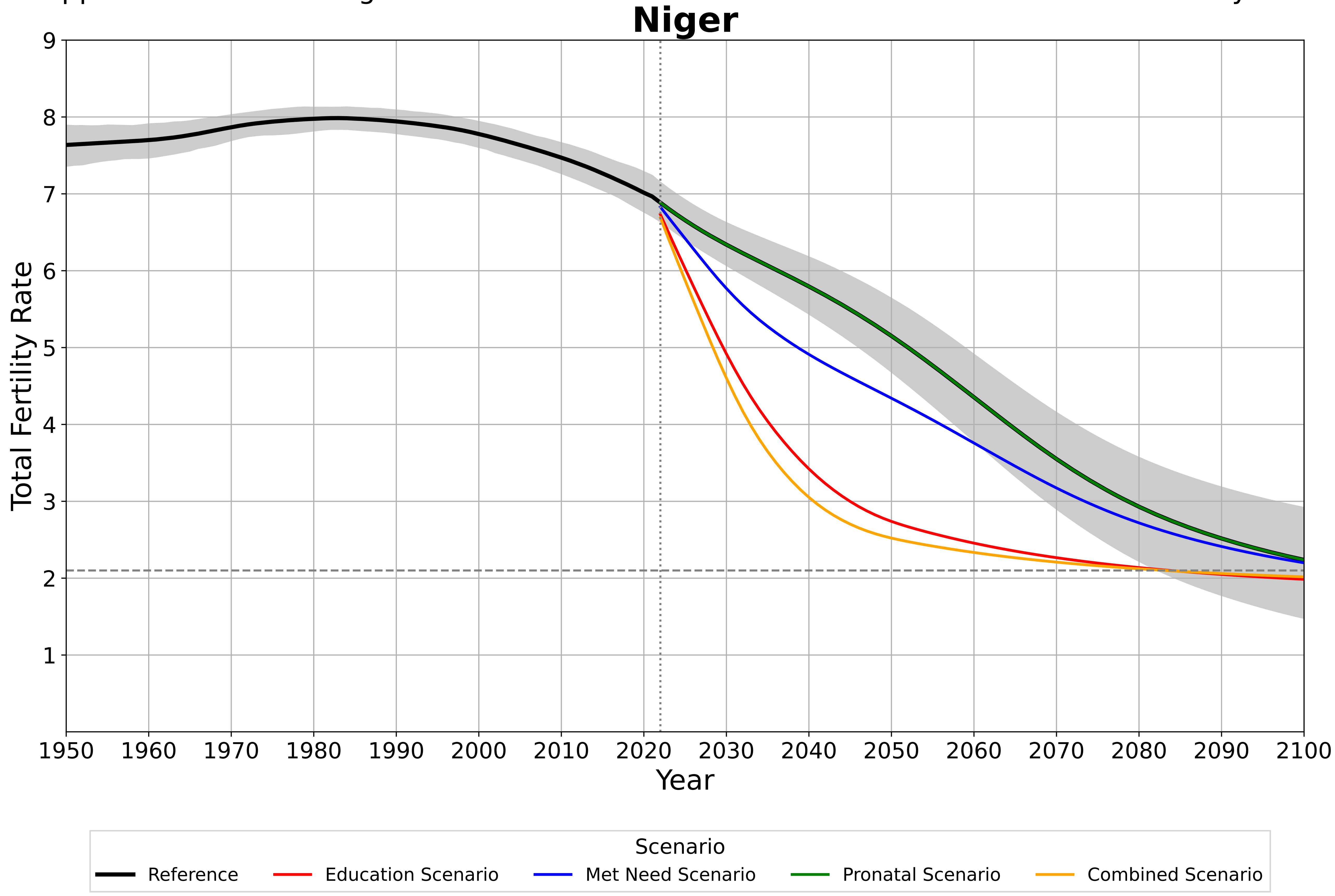

Supplemental Results Figure S5: TFR for reference and alternative scenario forecasts by location, 1950– 2100

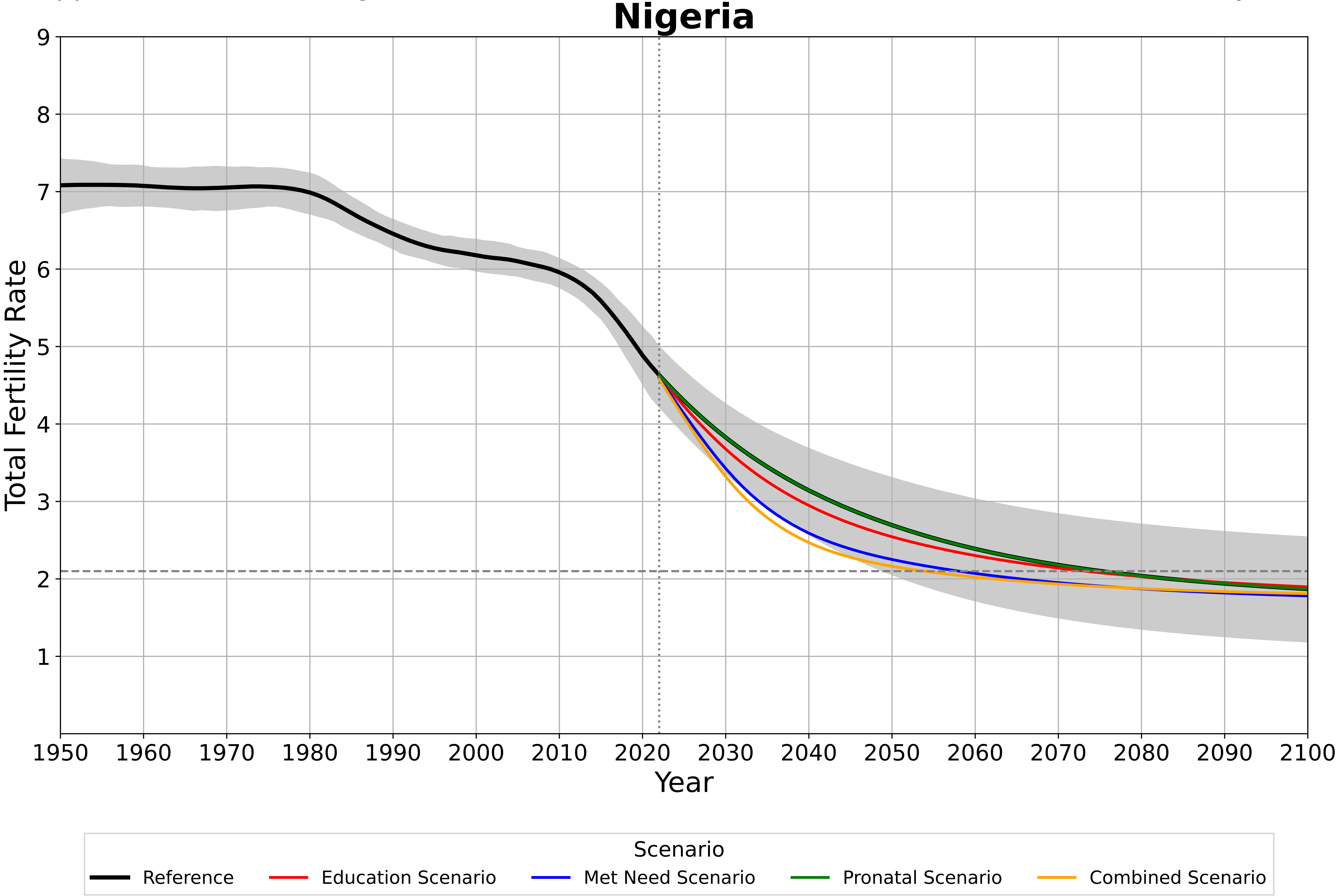

8419

Supplemental Results Figure S5: TFR for reference and alternative scenario forecasts by location, 1950– 2100

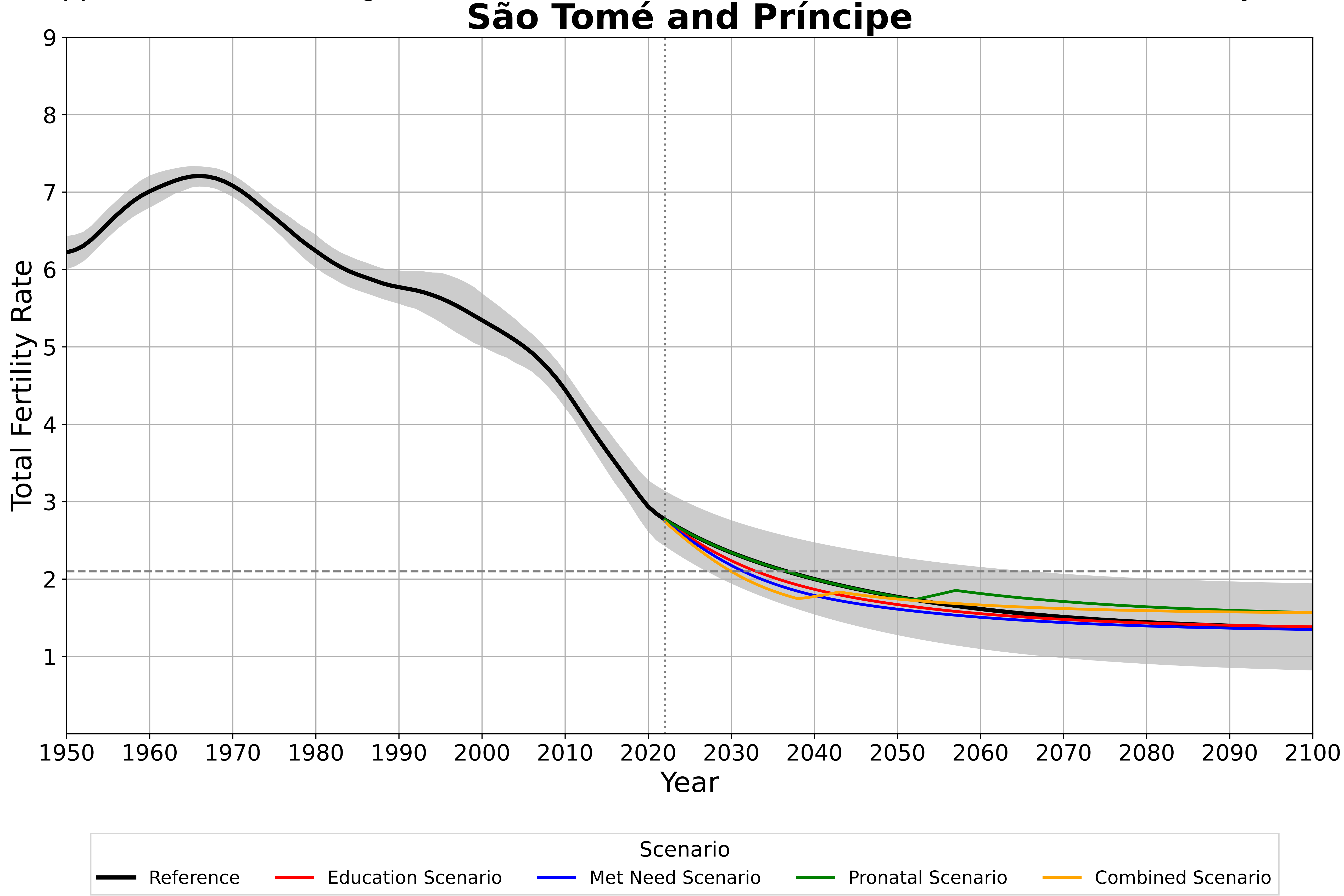

Supplemental Results Figure S5: TFR for reference and alternative scenario forecasts by location, 1950– 2100

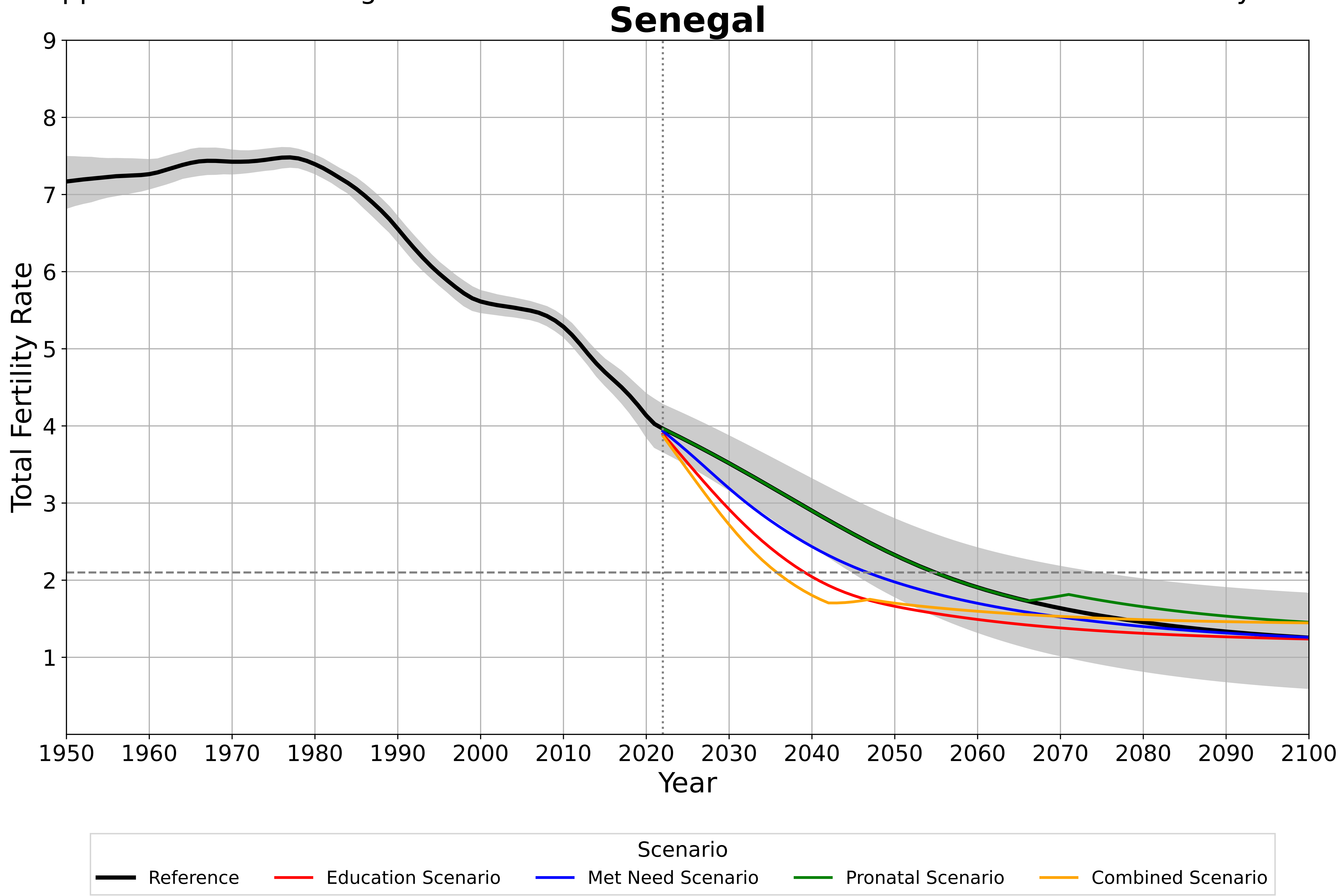

Supplemental Results Figure S5: TFR for reference and alternative scenario forecasts by location, 1950– 2100

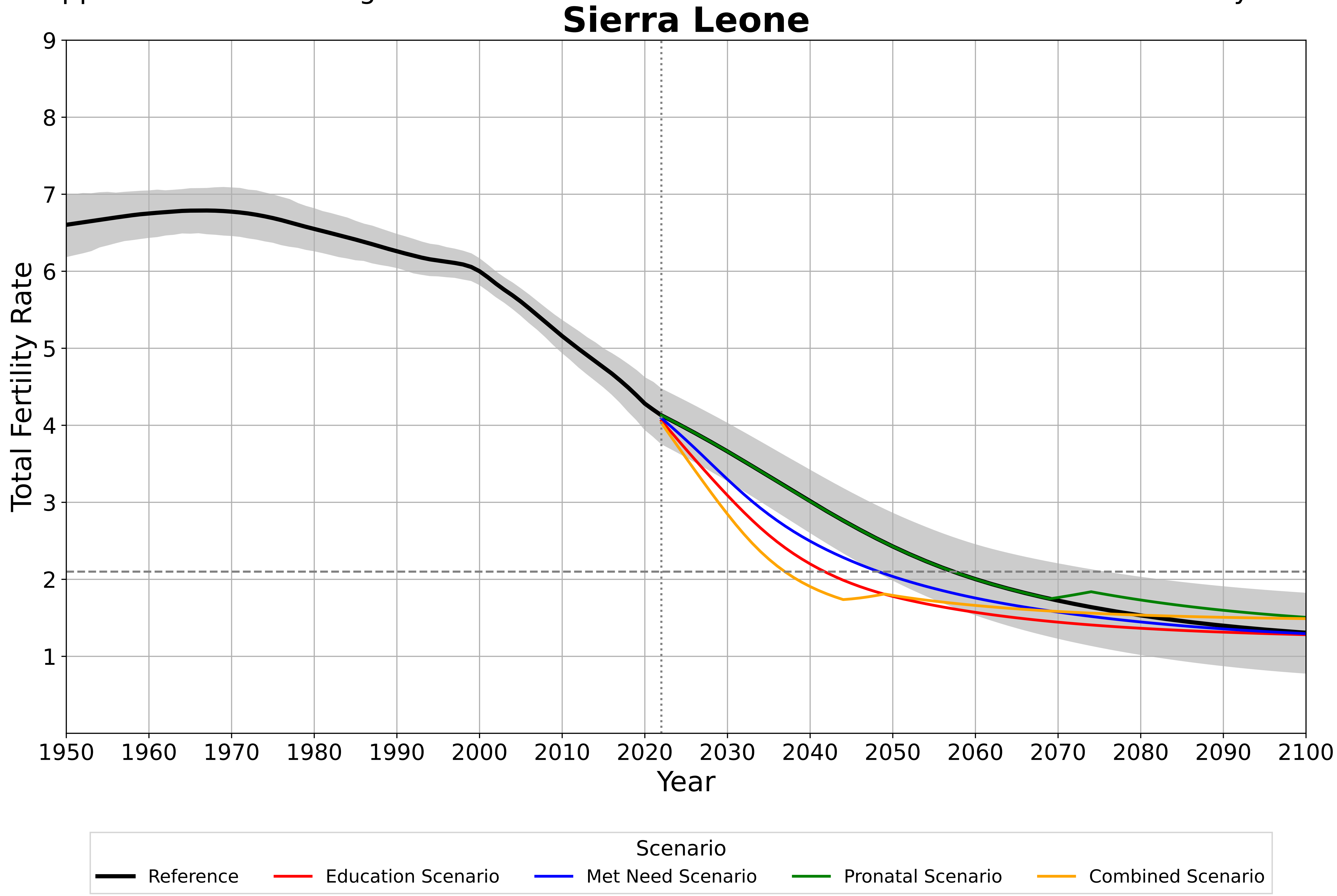

8422

Supplemental Results Figure S5: TFR for reference and alternative scenario forecasts by location, 1950– 2100

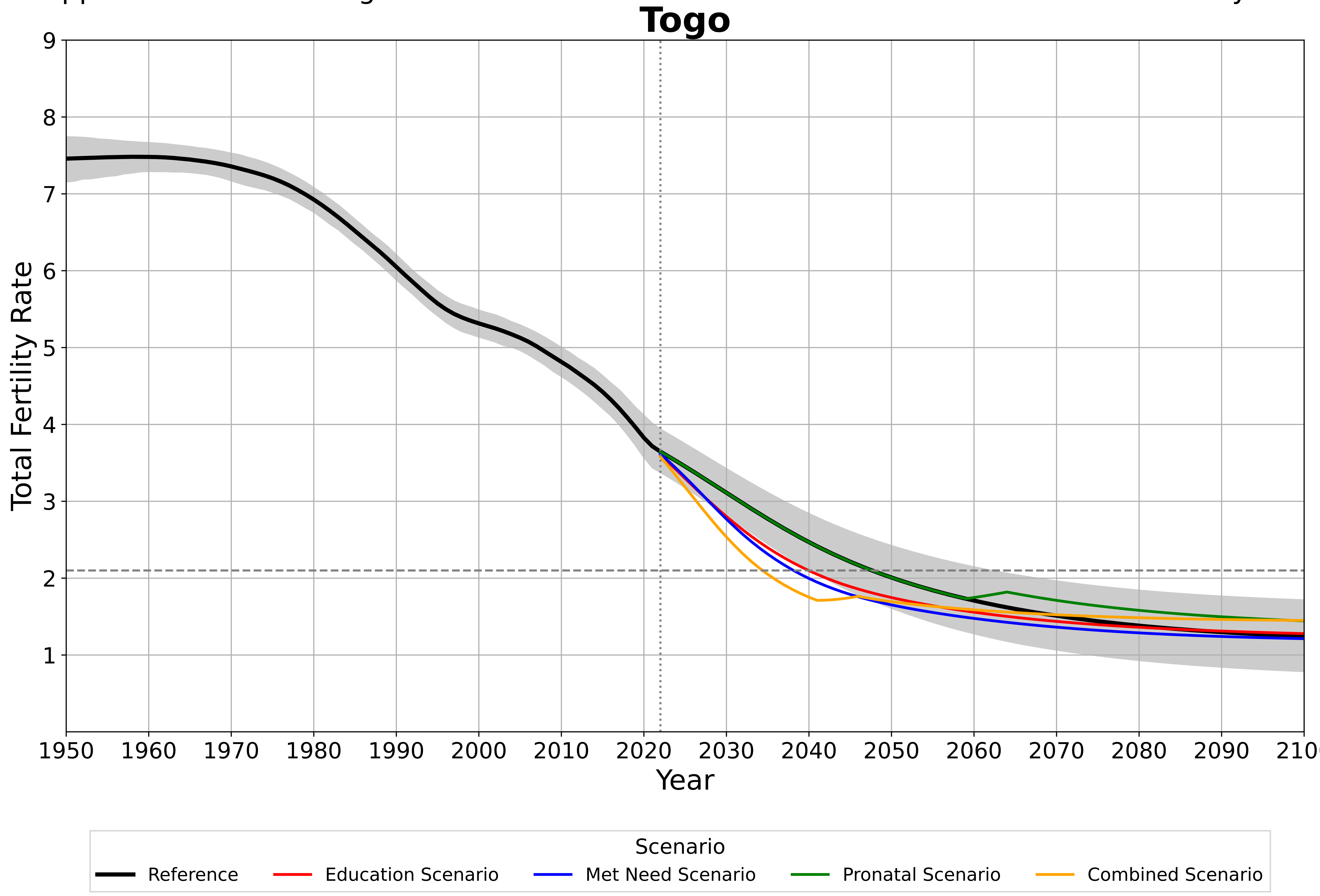

8423

Supplemental Results Figure S5: TFR for reference and alternative scenario forecasts by location, 1950– 2100

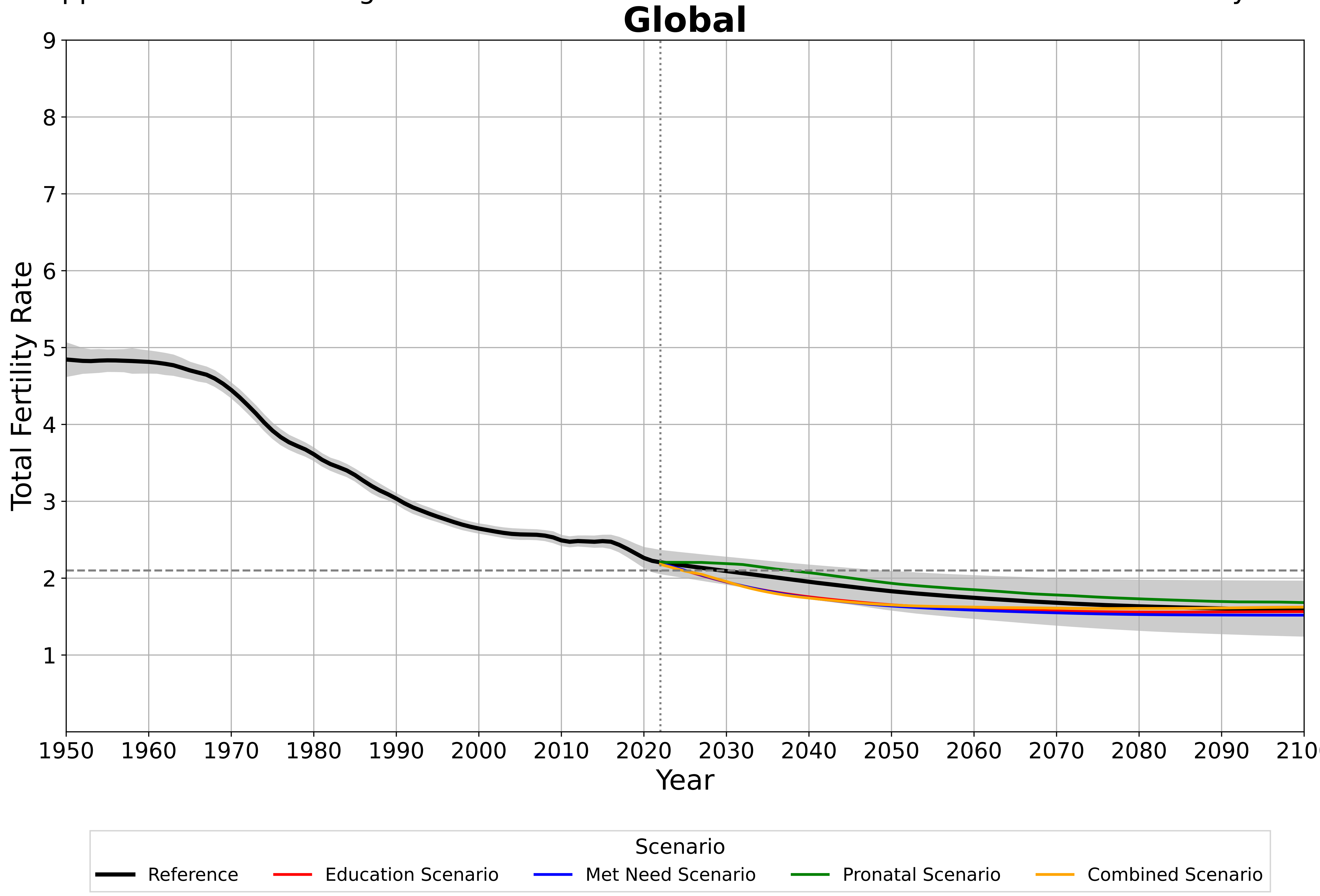

Supplemental Results Figure S5: TFR for reference and alternative scenario forecasts by location, 1950– 2100

Central Europe, eastern Europe, and central Asia

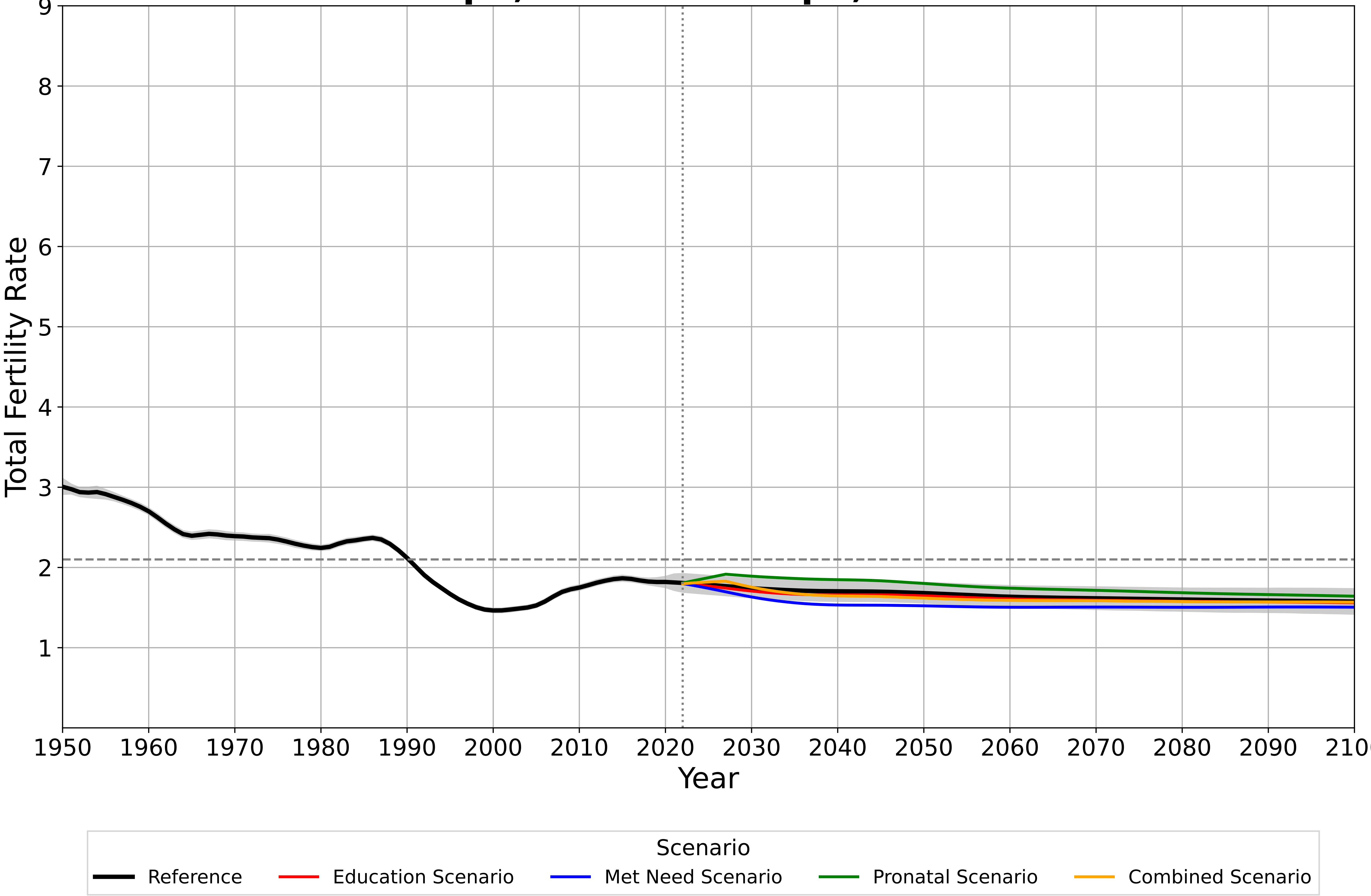

Supplemental Results Figure S5: TFR for reference and alternative scenario forecasts by location, 1950– 2100

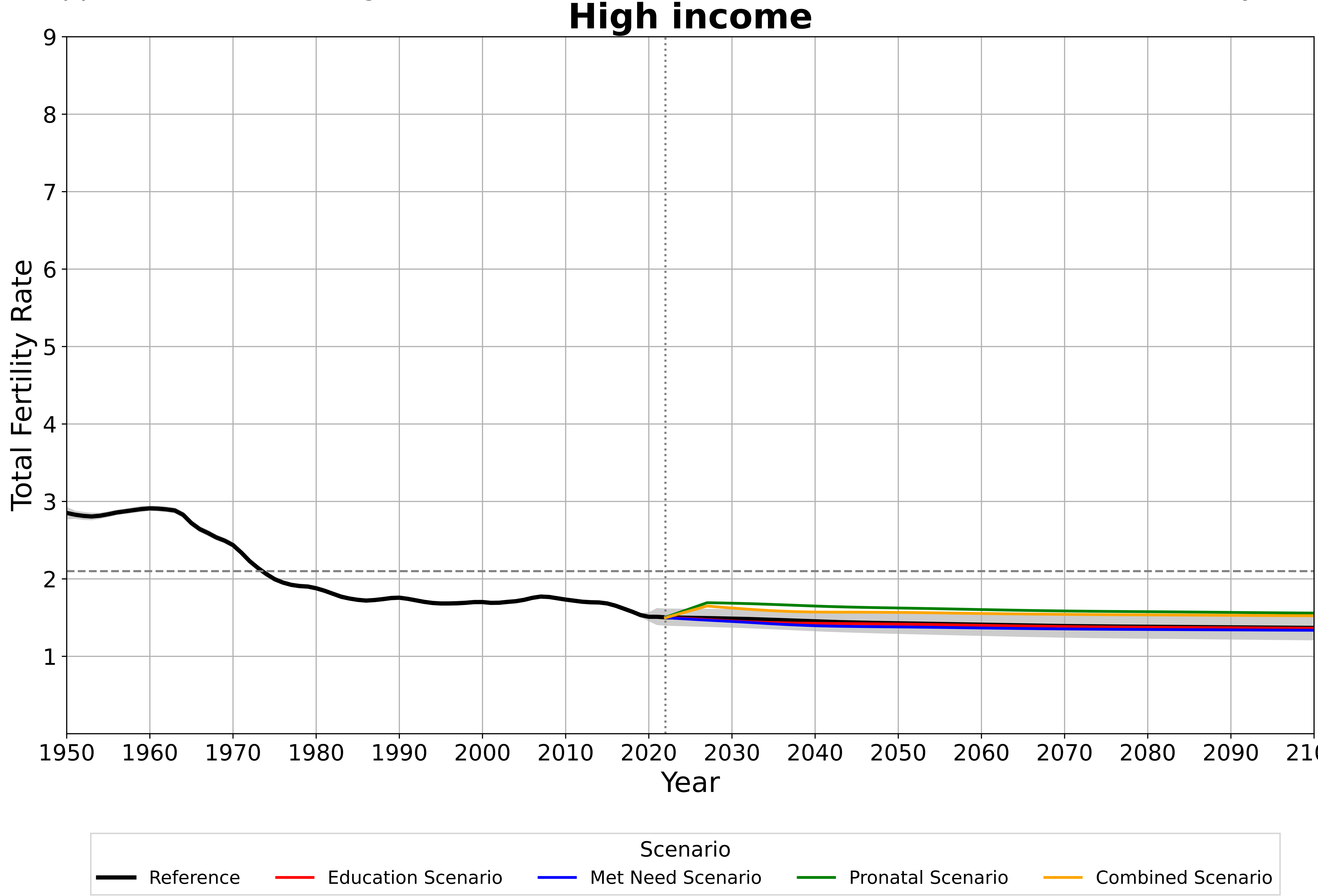

Supplemental Results Figure S5: TFR for reference and alternative scenario forecasts by location, 1950– 2100

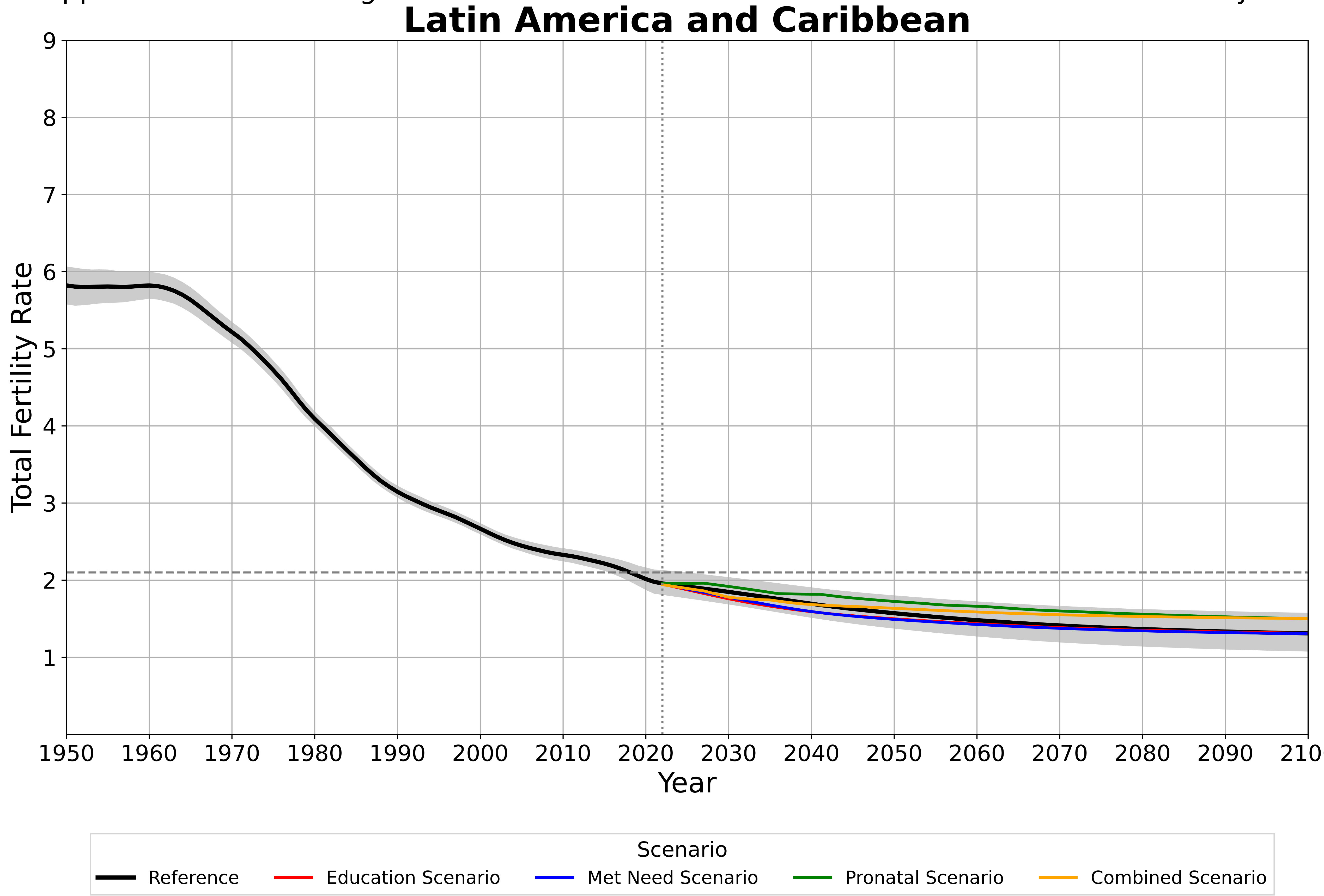

Supplemental Results Figure S5: TFR for reference and alternative scenario forecasts by location, 1950– 2100

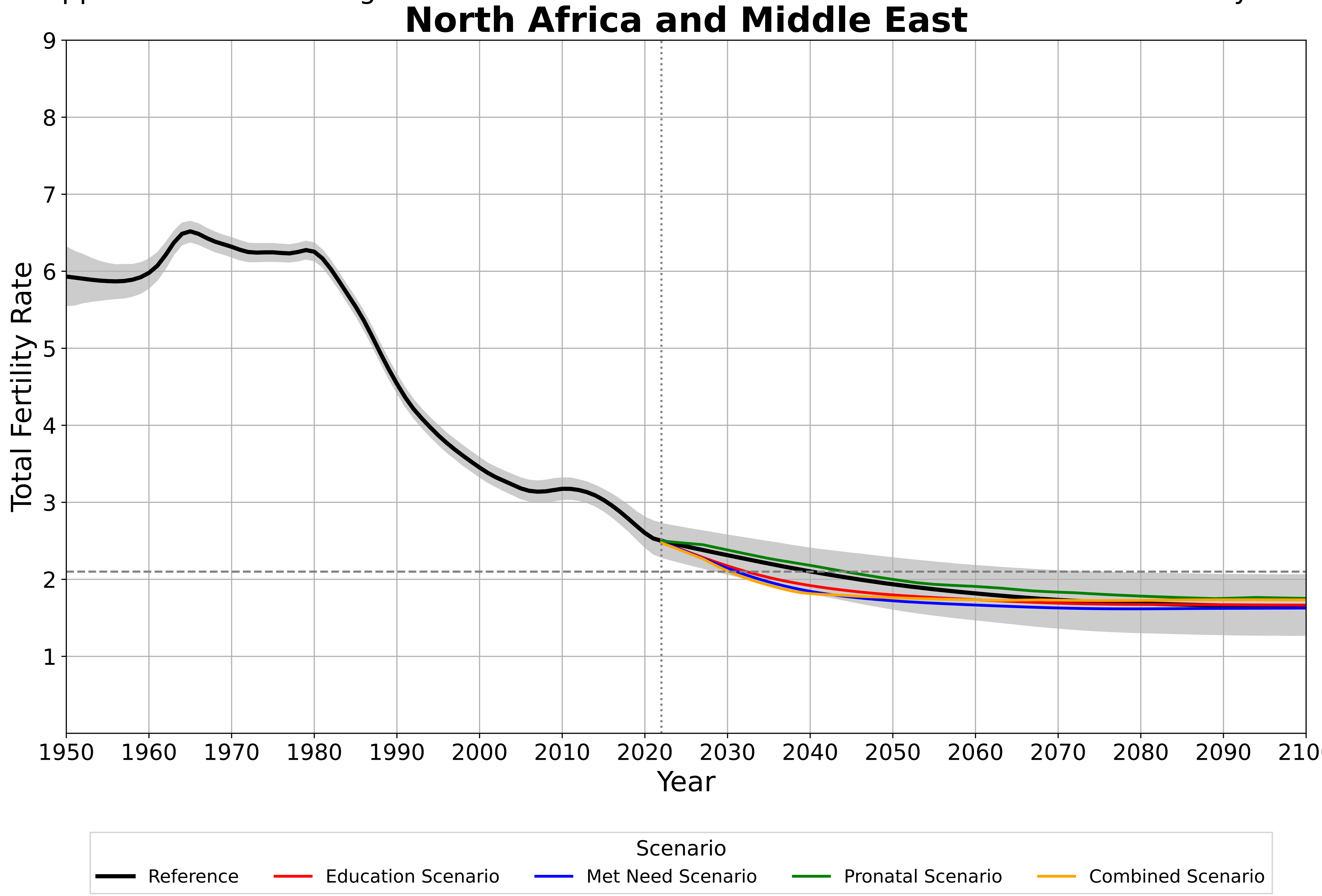

Supplemental Results Figure S5: TFR for reference and alternative scenario forecasts by location, 1950– 2100

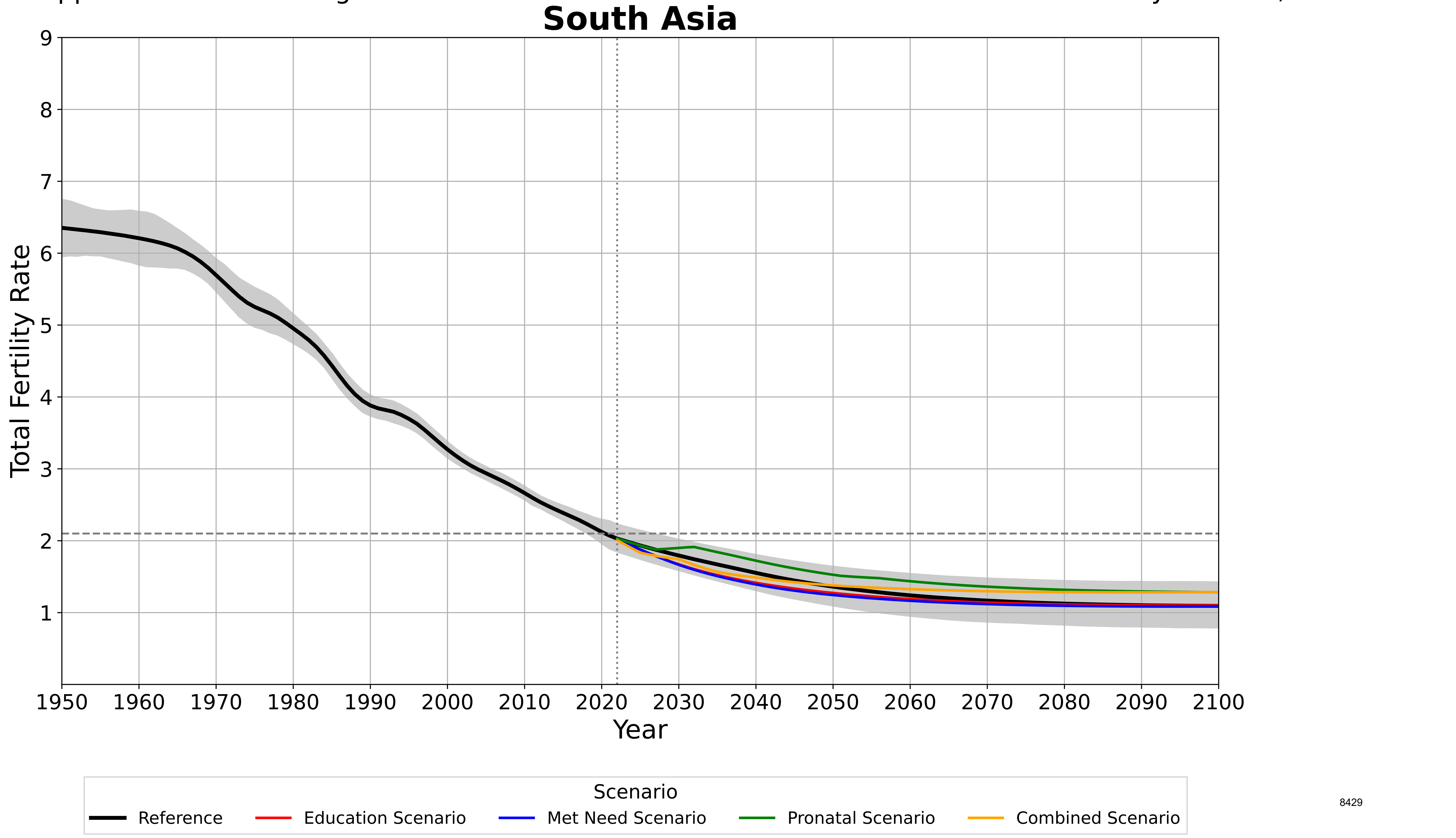

Supplemental Results Figure S5: TFR for reference and alternative scenario forecasts by location, 1950– 2100

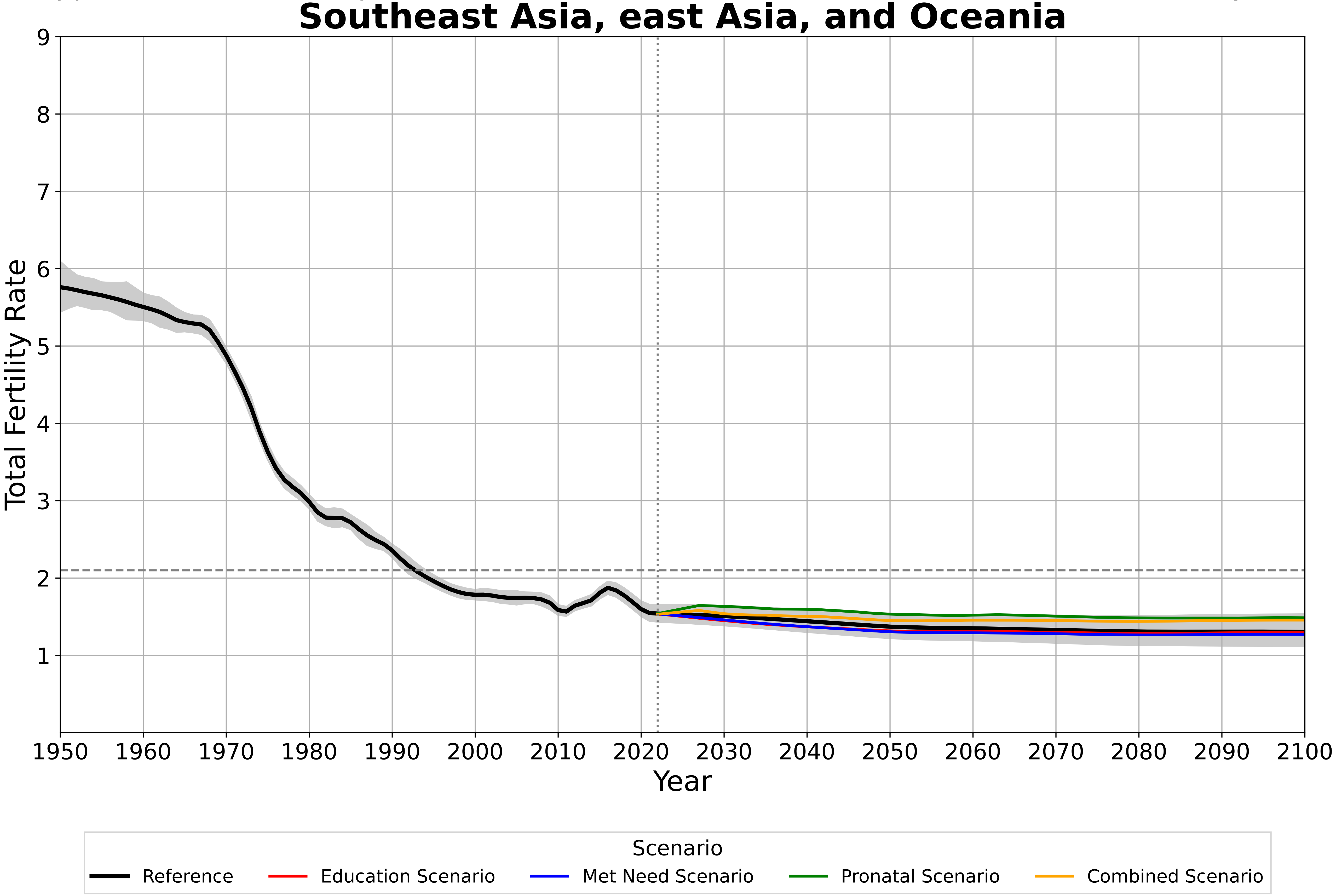

Supplemental Results Figure S5: TFR for reference and alternative scenario forecasts by location, 1950– 2100

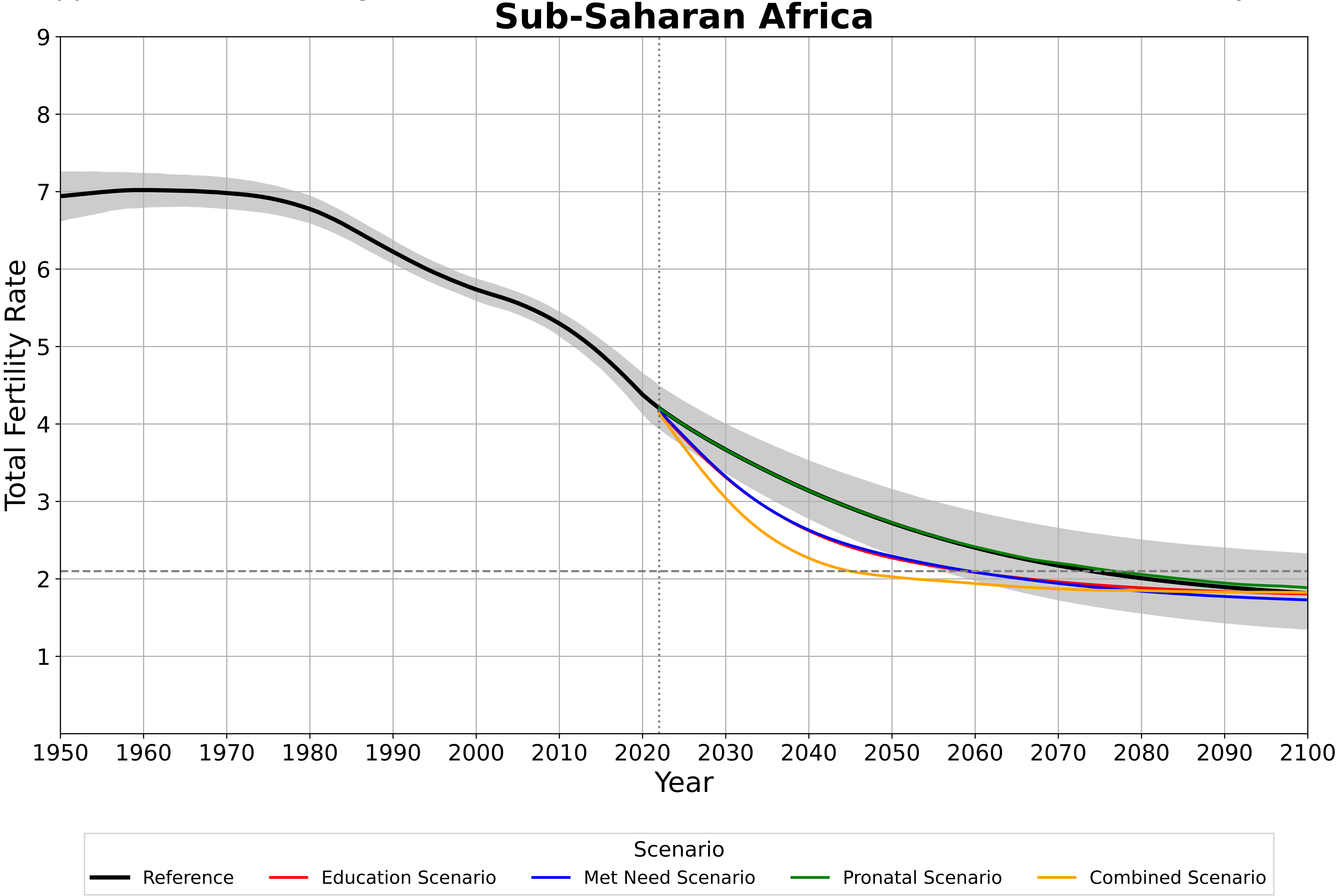

8431

Supplemental Results Figure S5: TFR for reference and alternative scenario forecasts by location, 1950– 2100

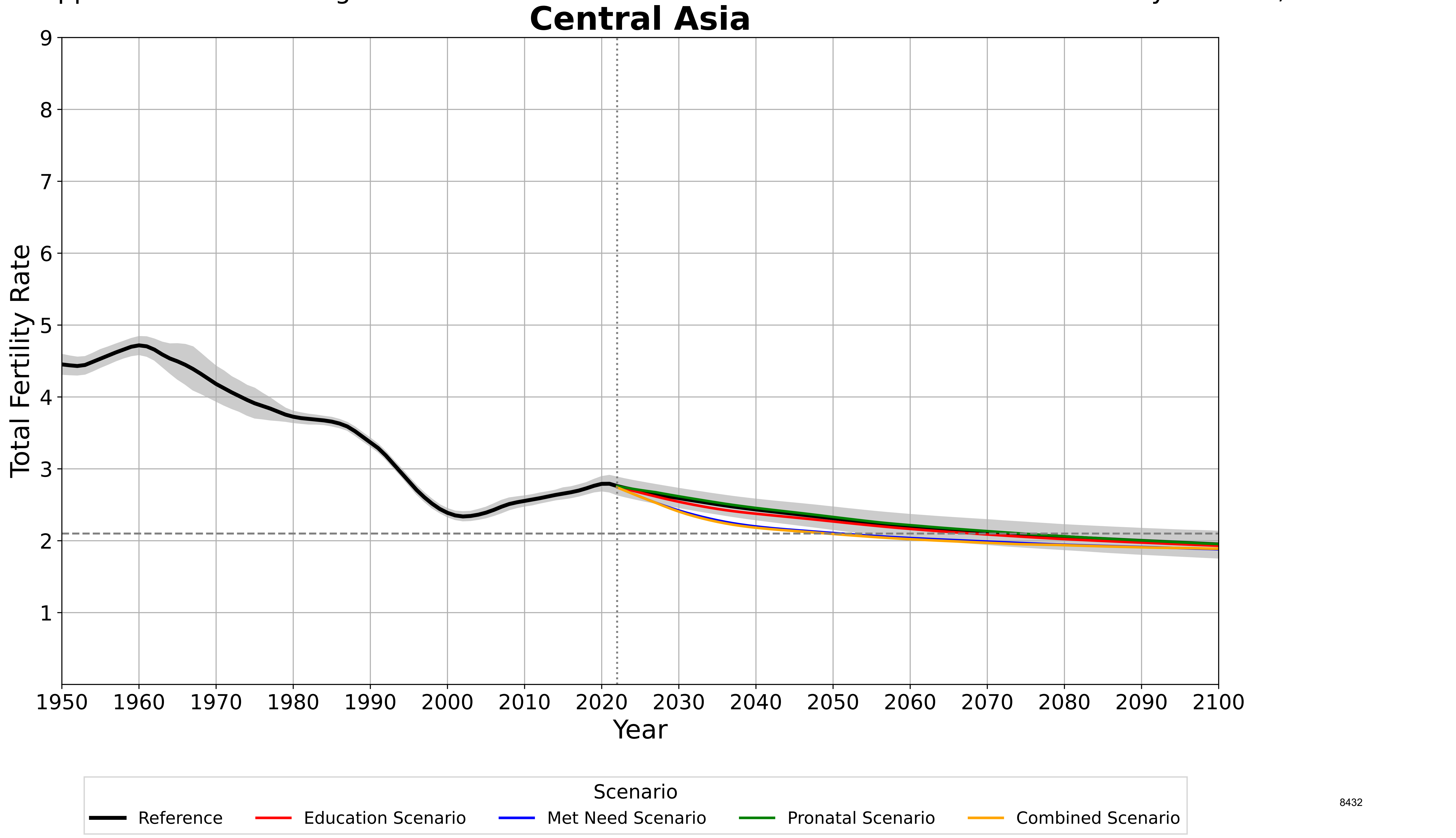

Supplemental Results Figure S5: TFR for reference and alternative scenario forecasts by location, 1950– 2100

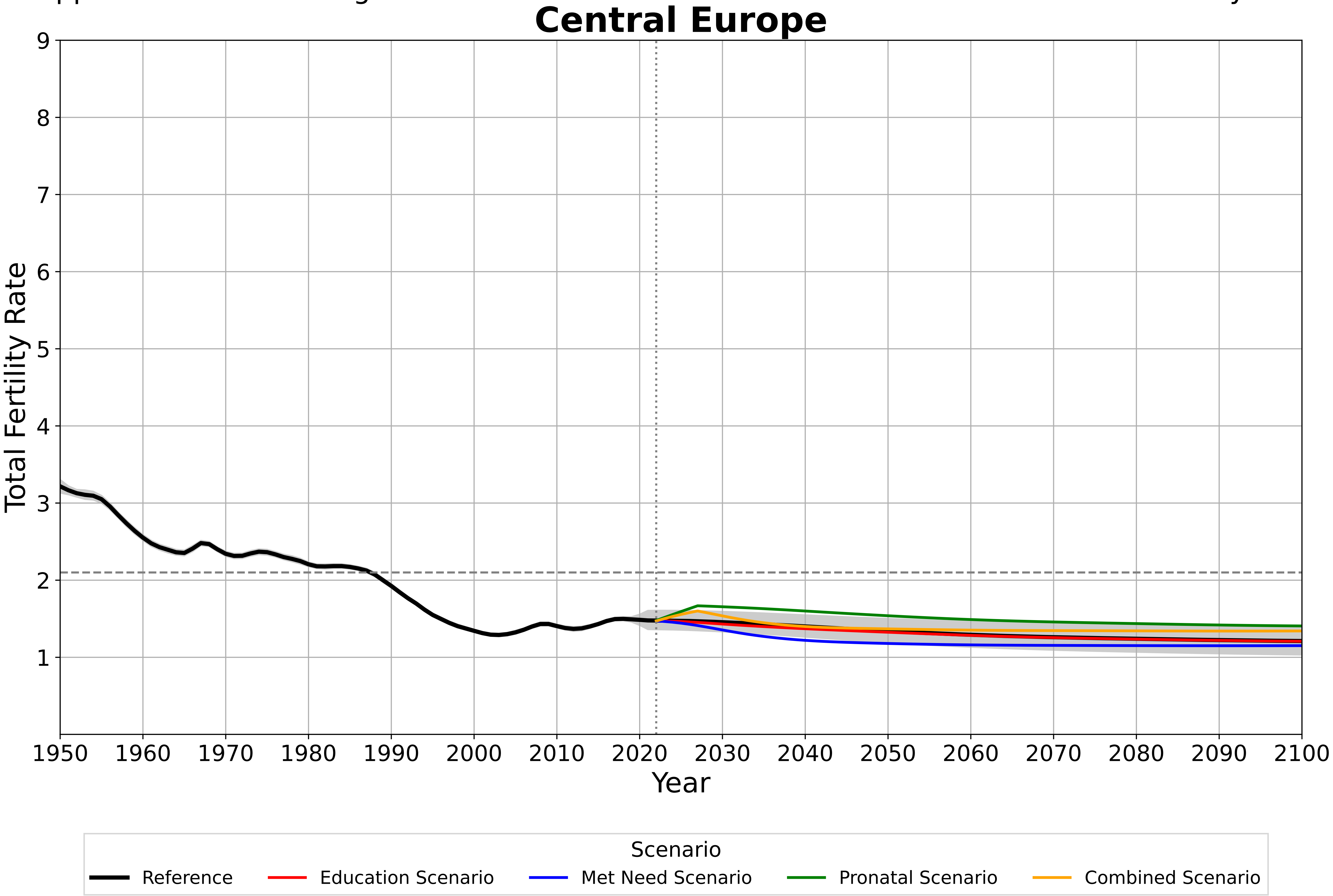

8433

Supplemental Results Figure S5: TFR for reference and alternative scenario forecasts by location, 1950– 2100

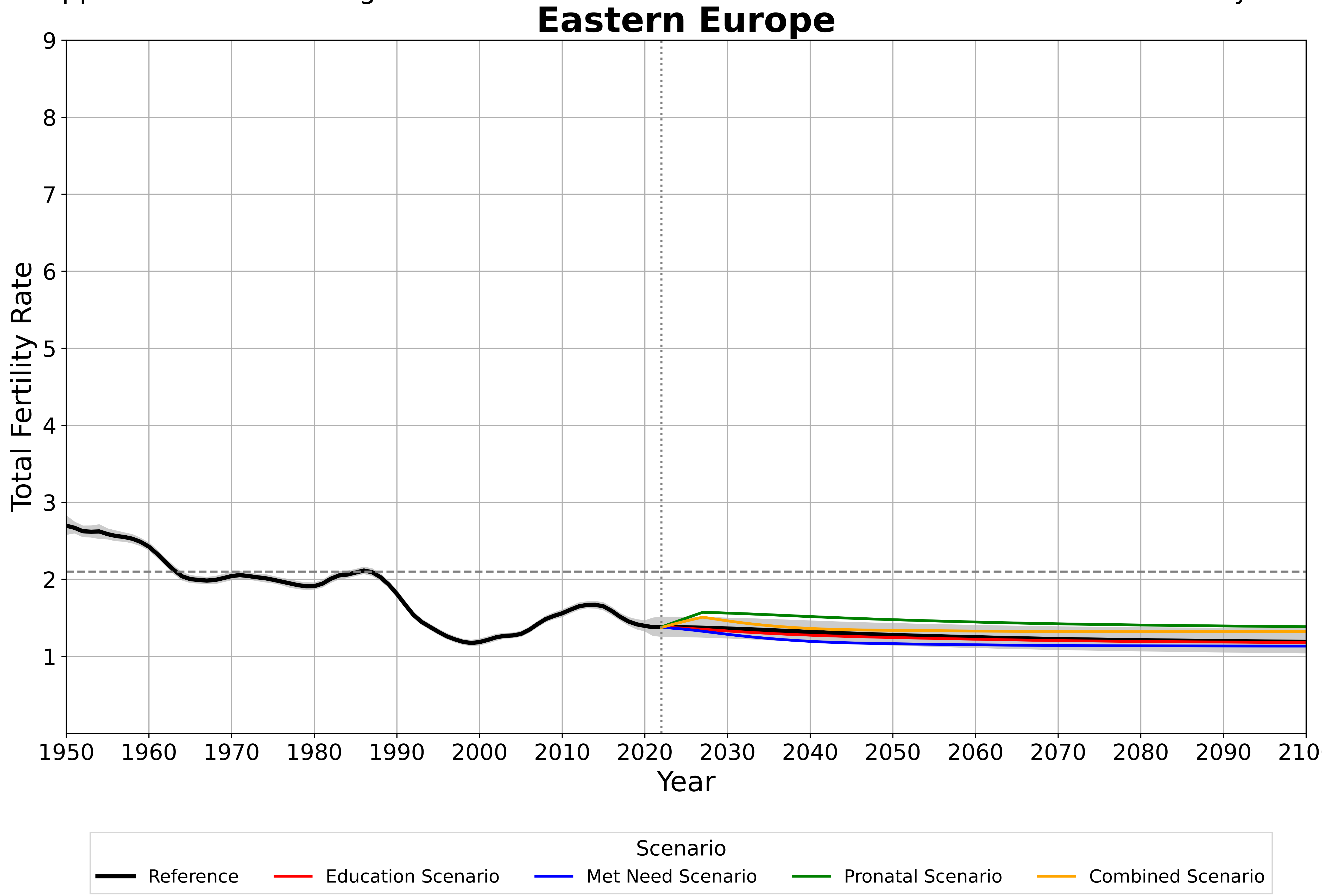

Supplemental Results Figure S5: TFR for reference and alternative scenario forecasts by location, 1950– 2100

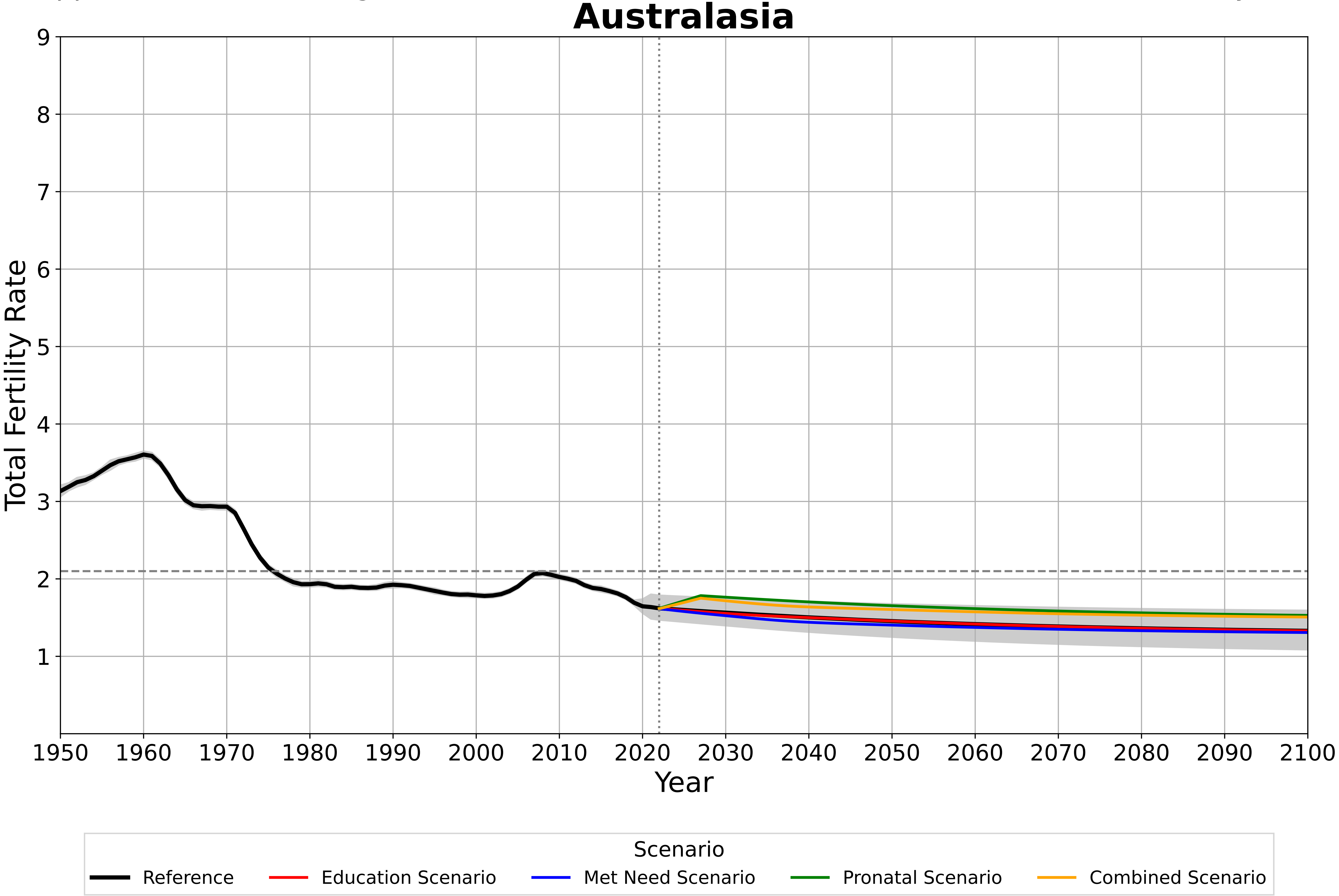

Supplemental Results Figure S5: TFR for reference and alternative scenario forecasts by location, 1950– 2100

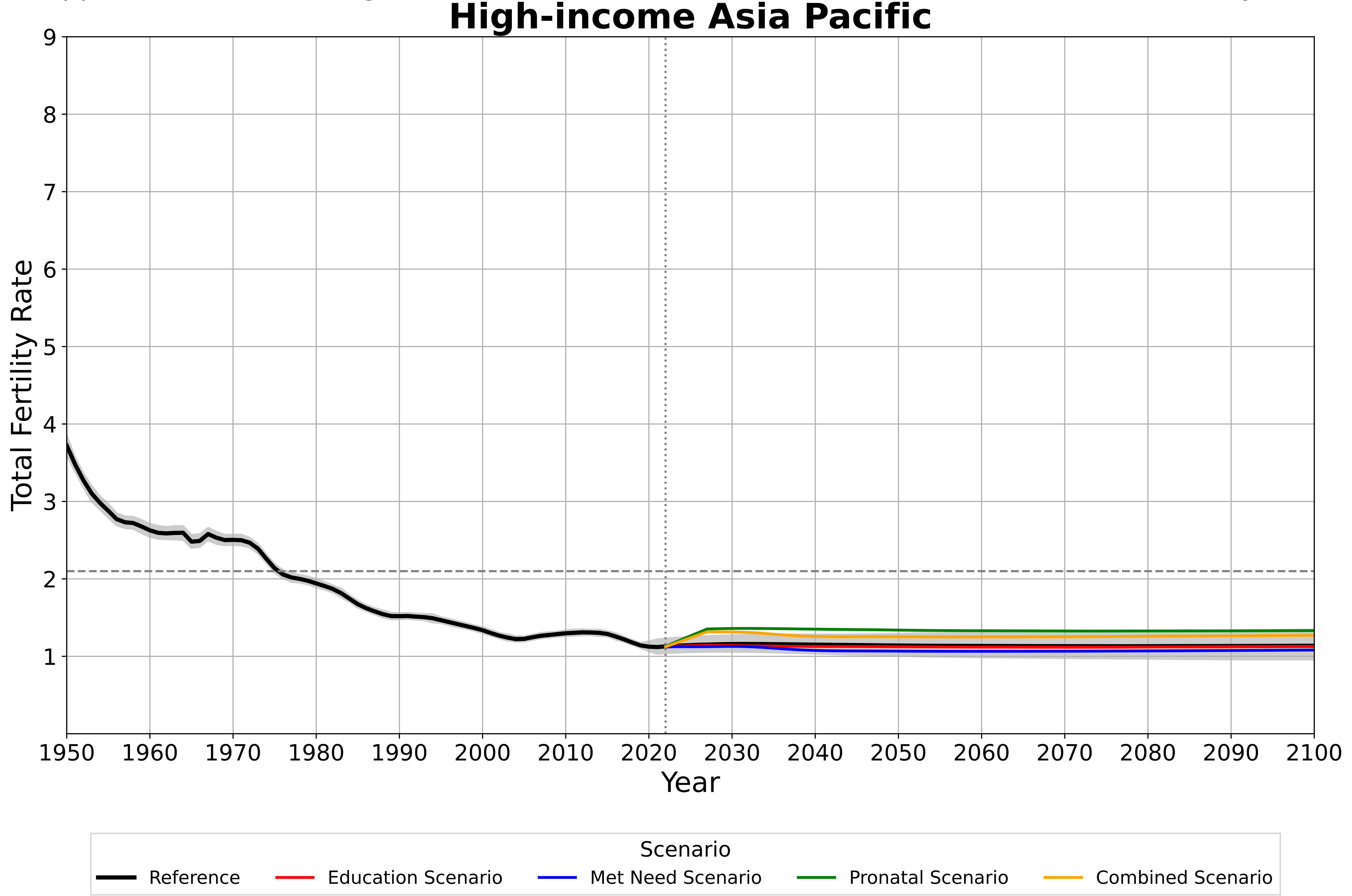

Supplemental Results Figure S5: TFR for reference and alternative scenario forecasts by location, 1950– 2100

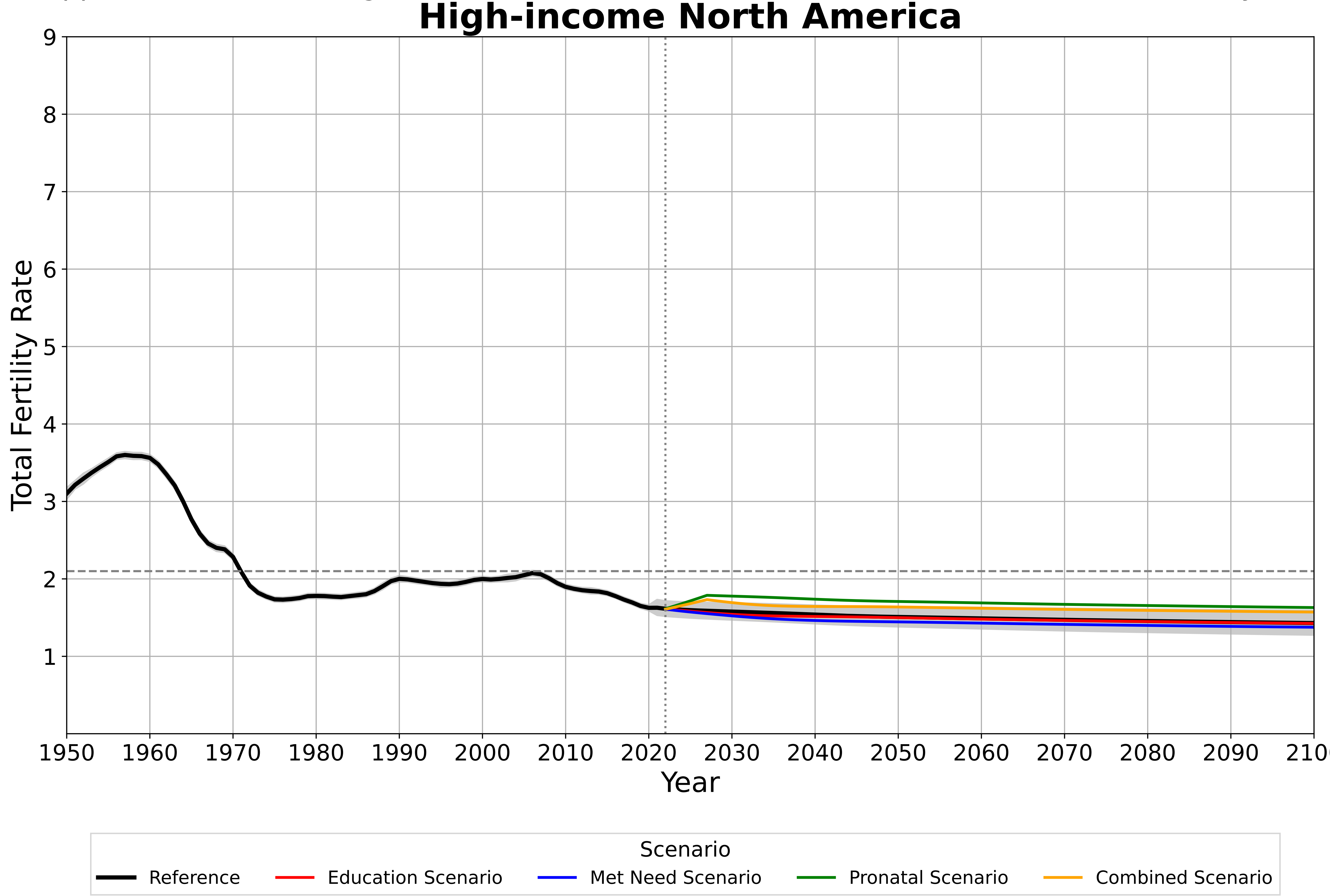

Supplemental Results Figure S5: TFR for reference and alternative scenario forecasts by location, 1950– 2100

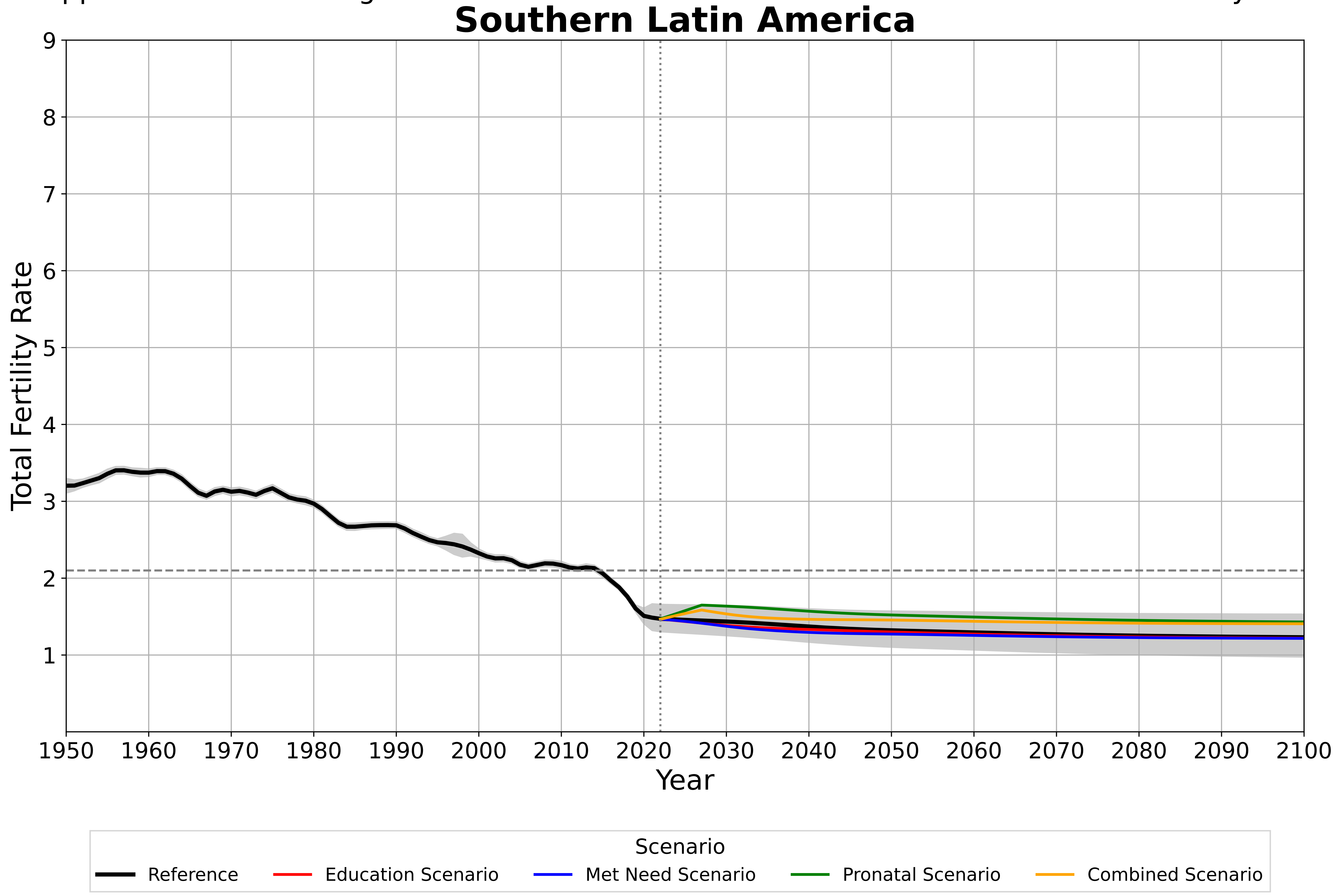

Supplemental Results Figure S5: TFR for reference and alternative scenario forecasts by location, 1950– 2100

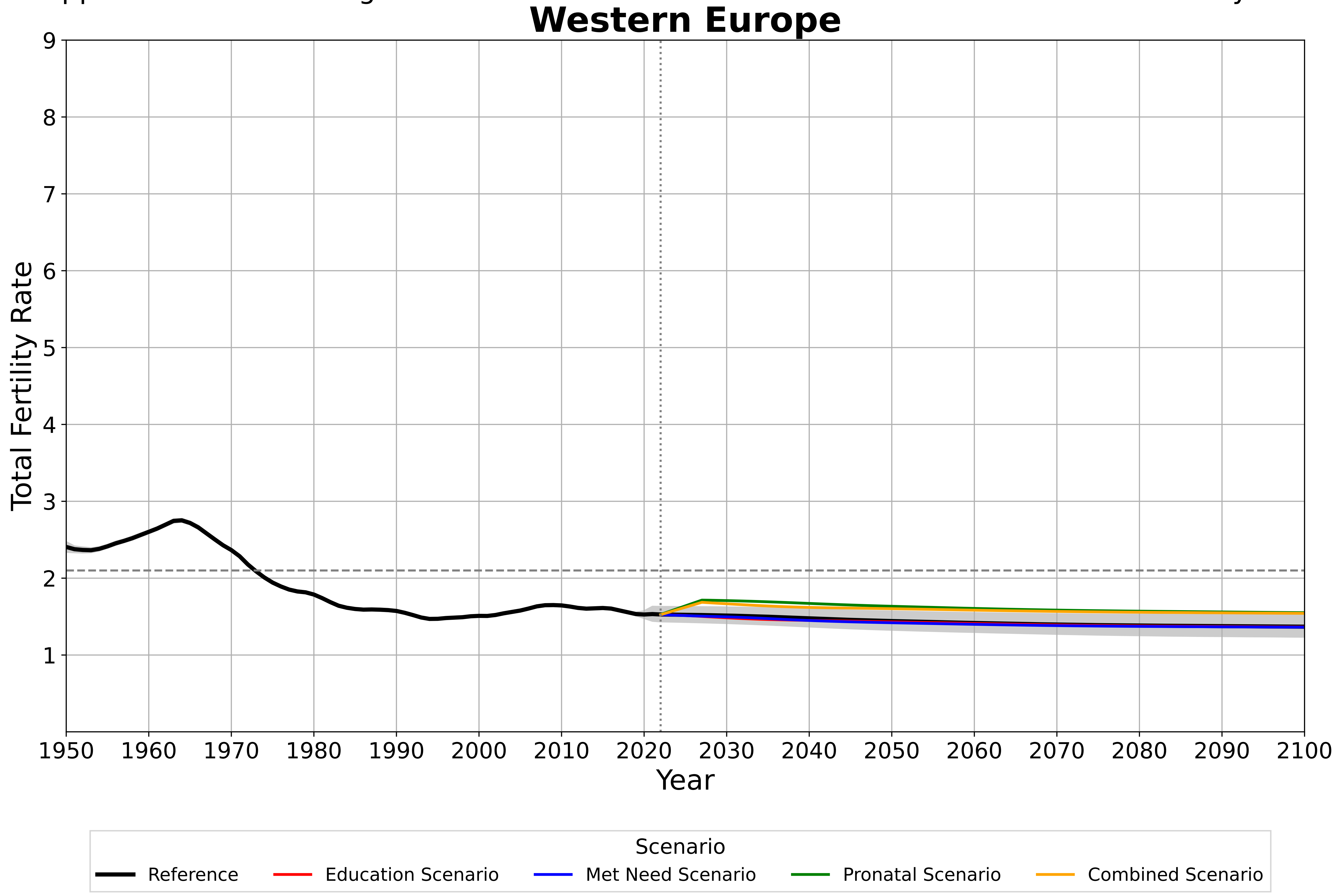

Supplemental Results Figure S5: TFR for reference and alternative scenario forecasts by location, 1950– 2100

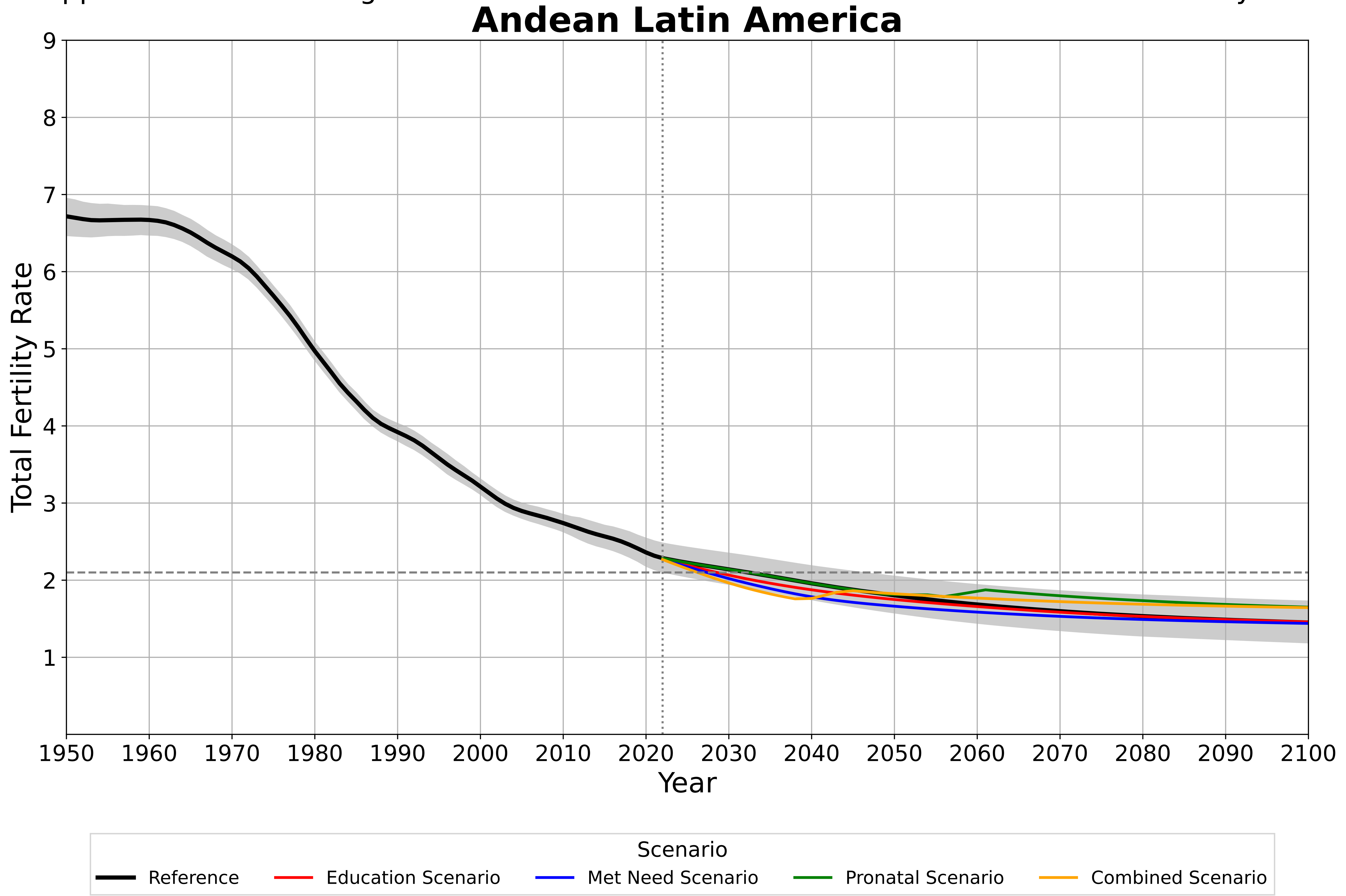

Supplemental Results Figure S5: TFR for reference and alternative scenario forecasts by location, 1950– 2100

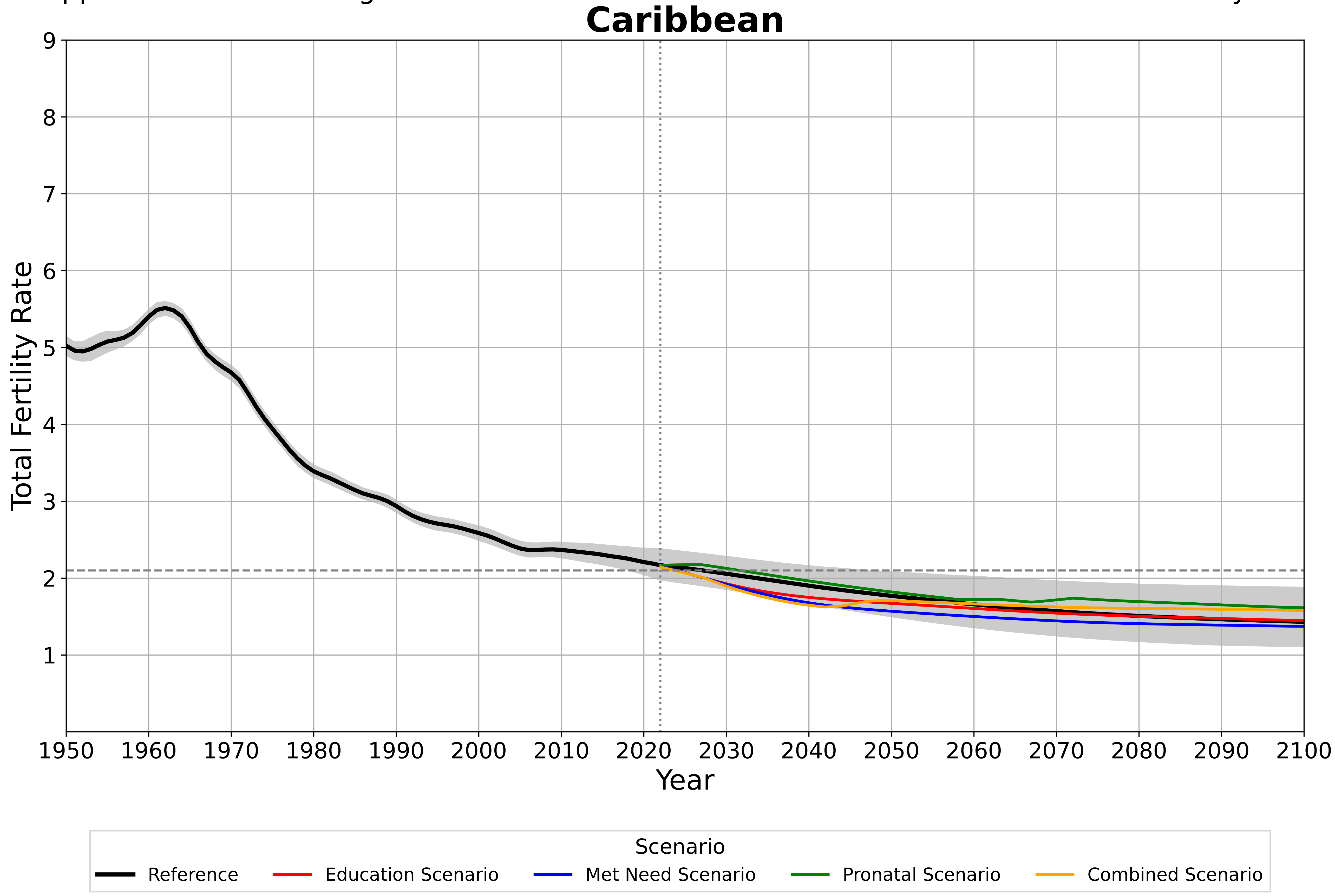

8441

Supplemental Results Figure S5: TFR for reference and alternative scenario forecasts by location, 1950– 2100

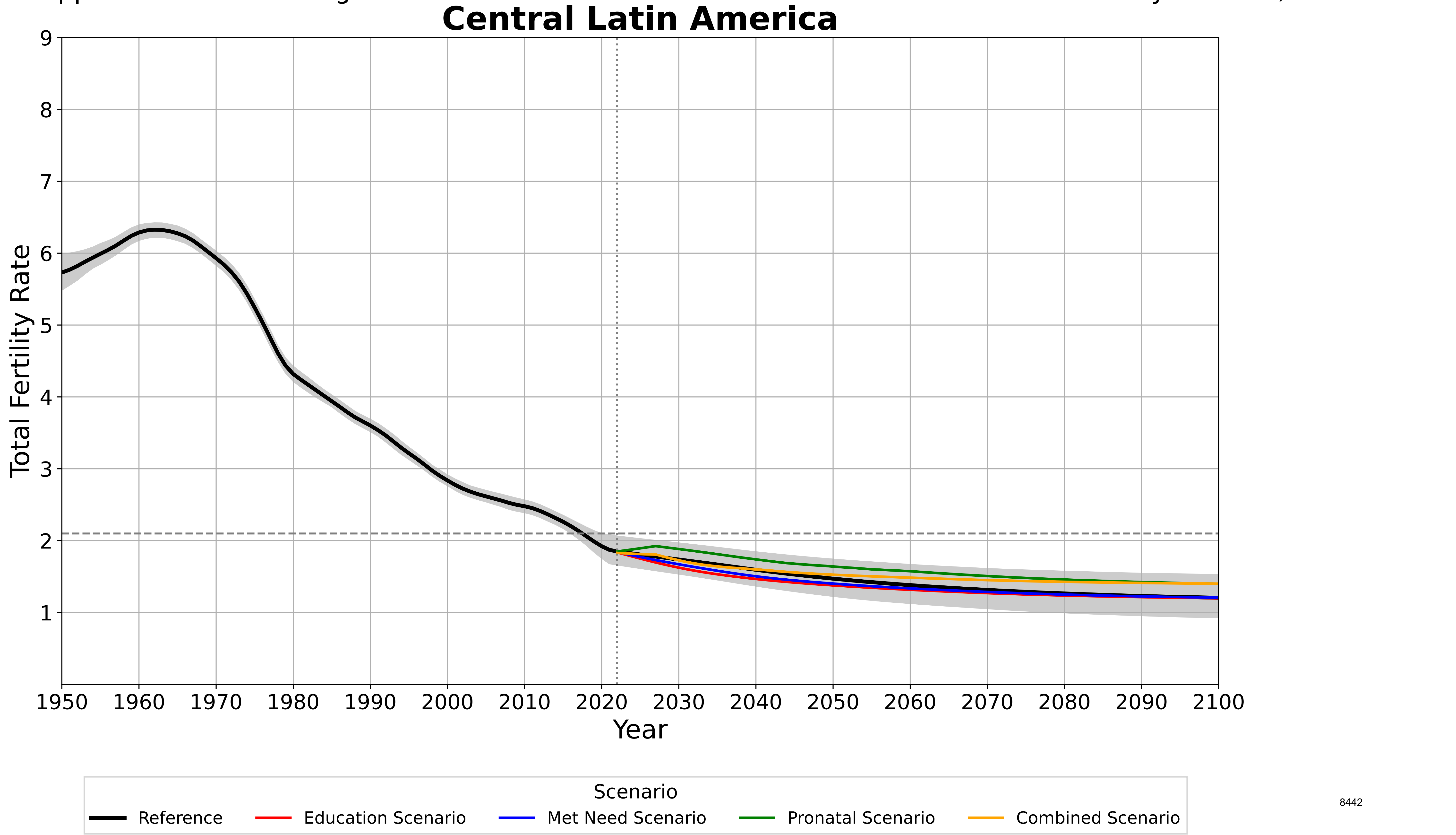

Supplemental Results Figure S5: TFR for reference and alternative scenario forecasts by location, 1950– 2100

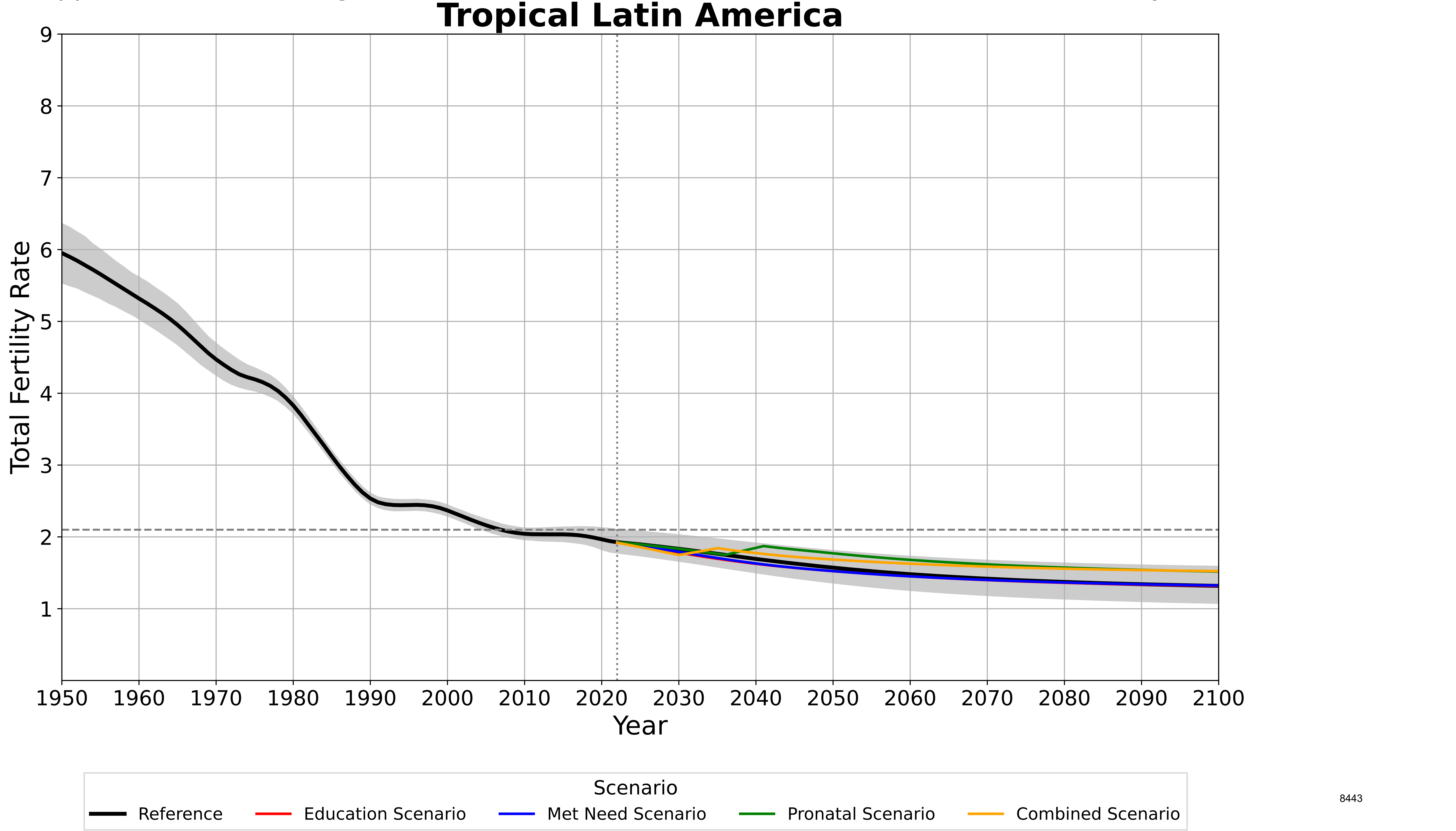

8443

Supplemental Results Figure S5: TFR for reference and alternative scenario forecasts by location, 1950– 2100

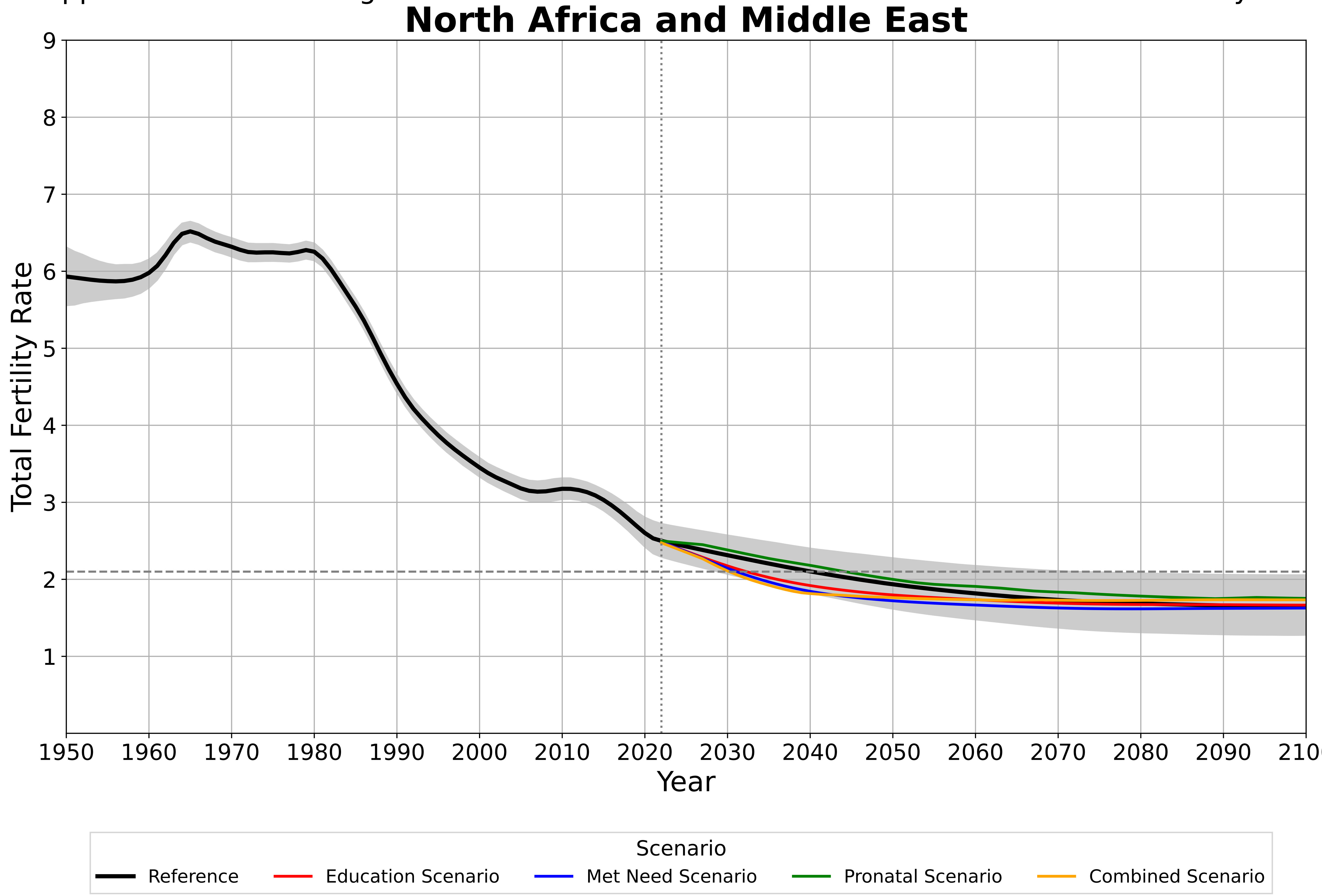

Supplemental Results Figure S5: TFR for reference and alternative scenario forecasts by location, 1950– 2100

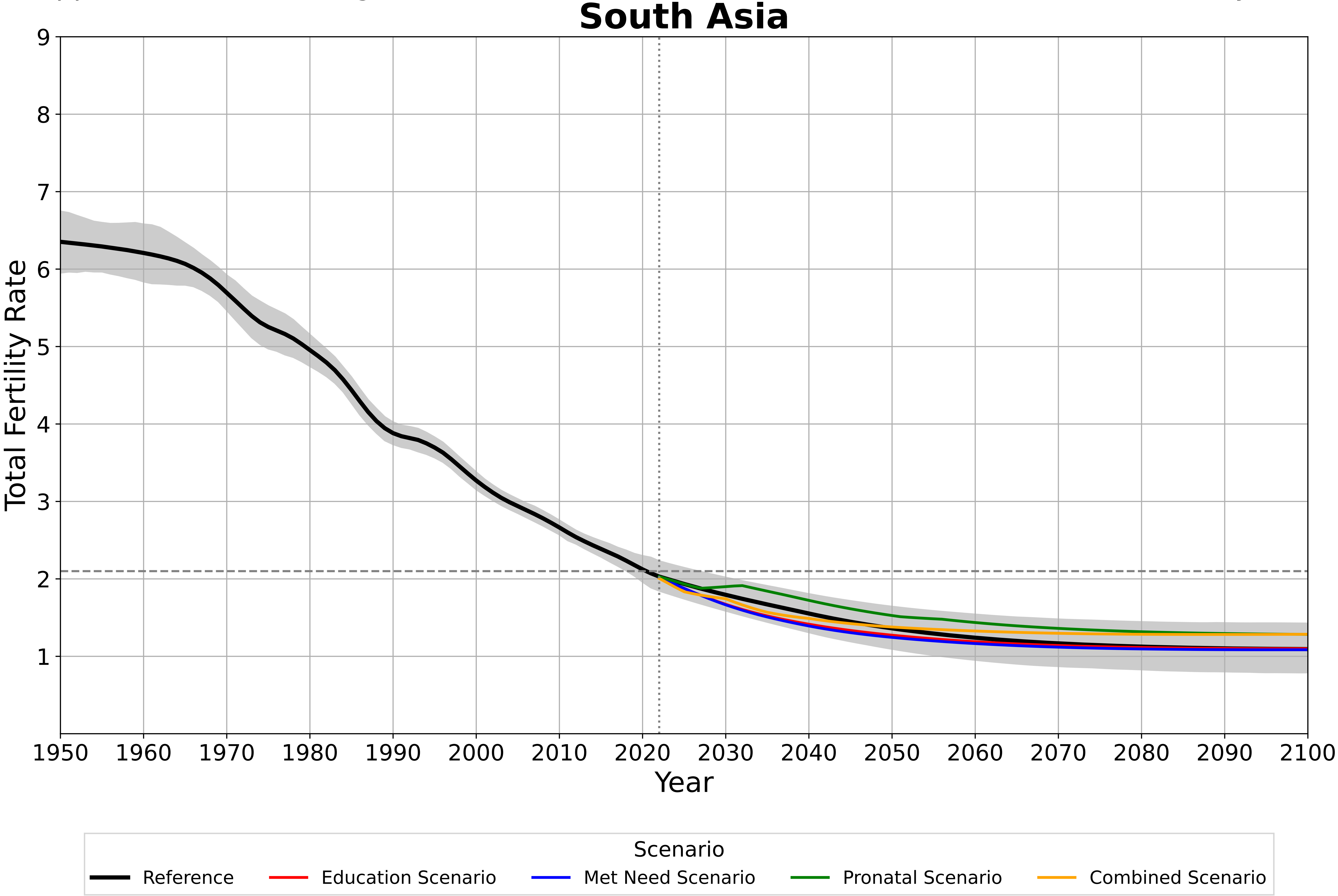

8445

Supplemental Results Figure S5: TFR for reference and alternative scenario forecasts by location, 1950– 2100

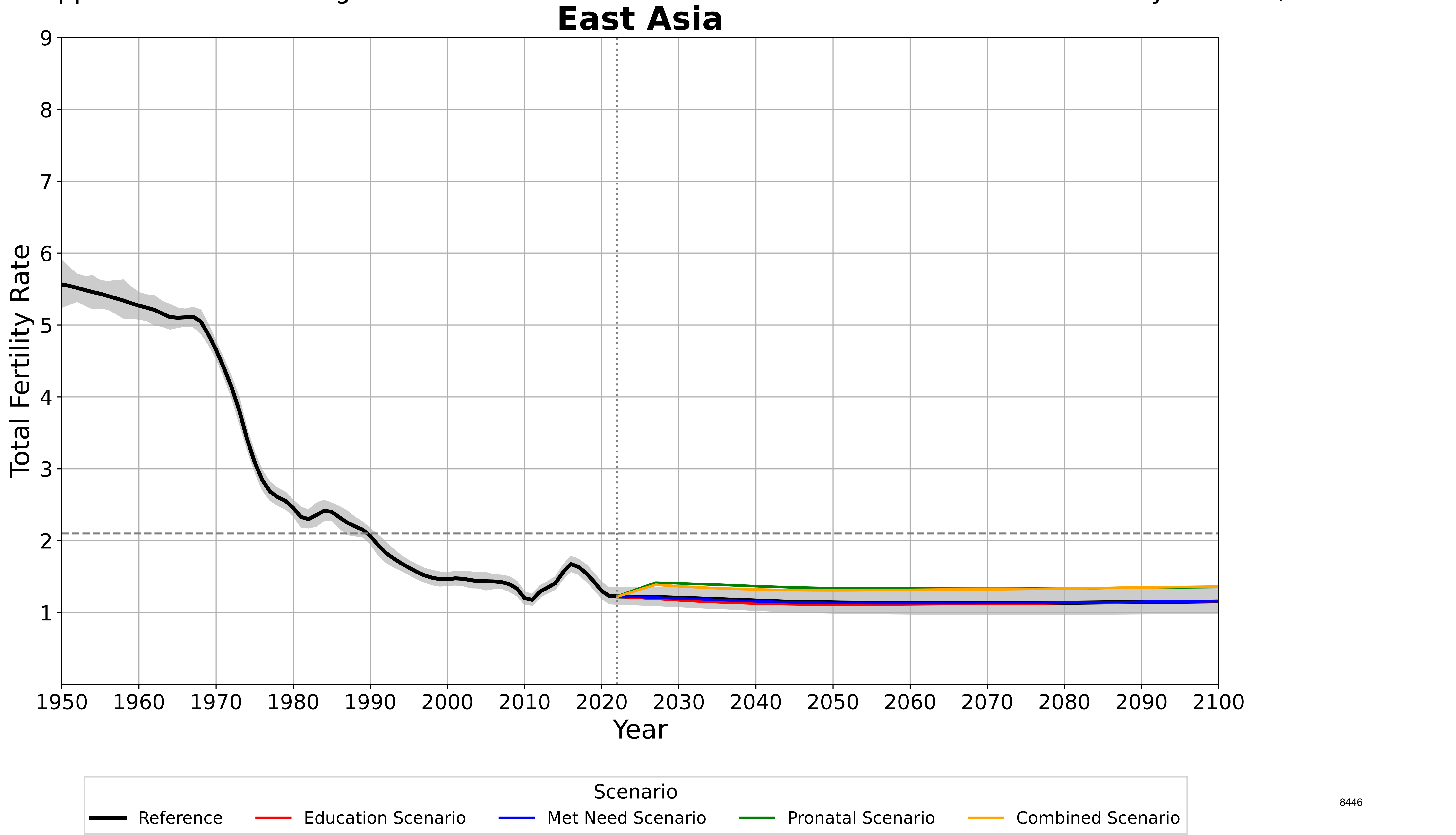

Supplemental Results Figure S5: TFR for reference and alternative scenario forecasts by location, 1950– 2100

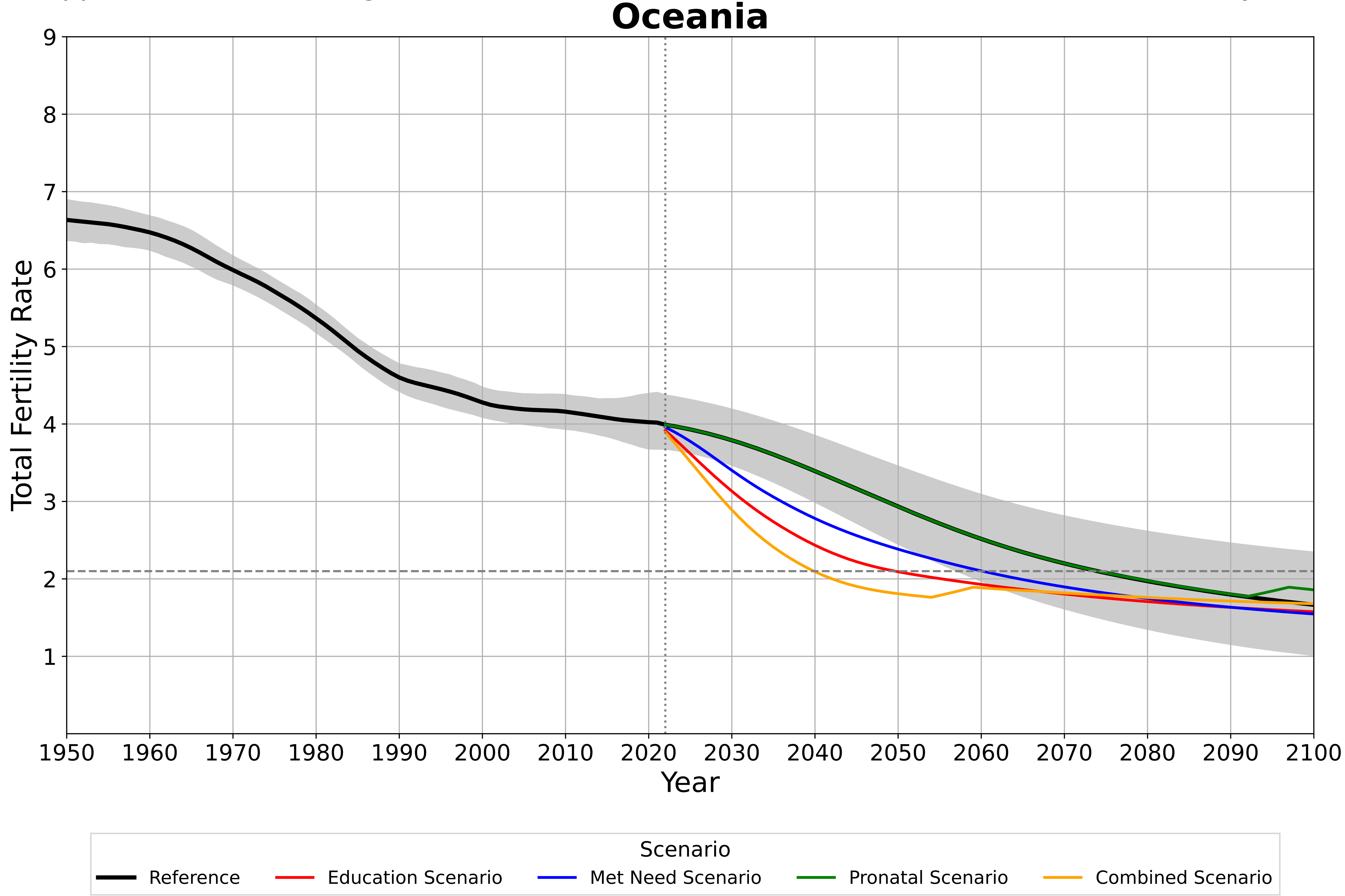

Supplemental Results Figure S5: TFR for reference and alternative scenario forecasts by location, 1950– 2100

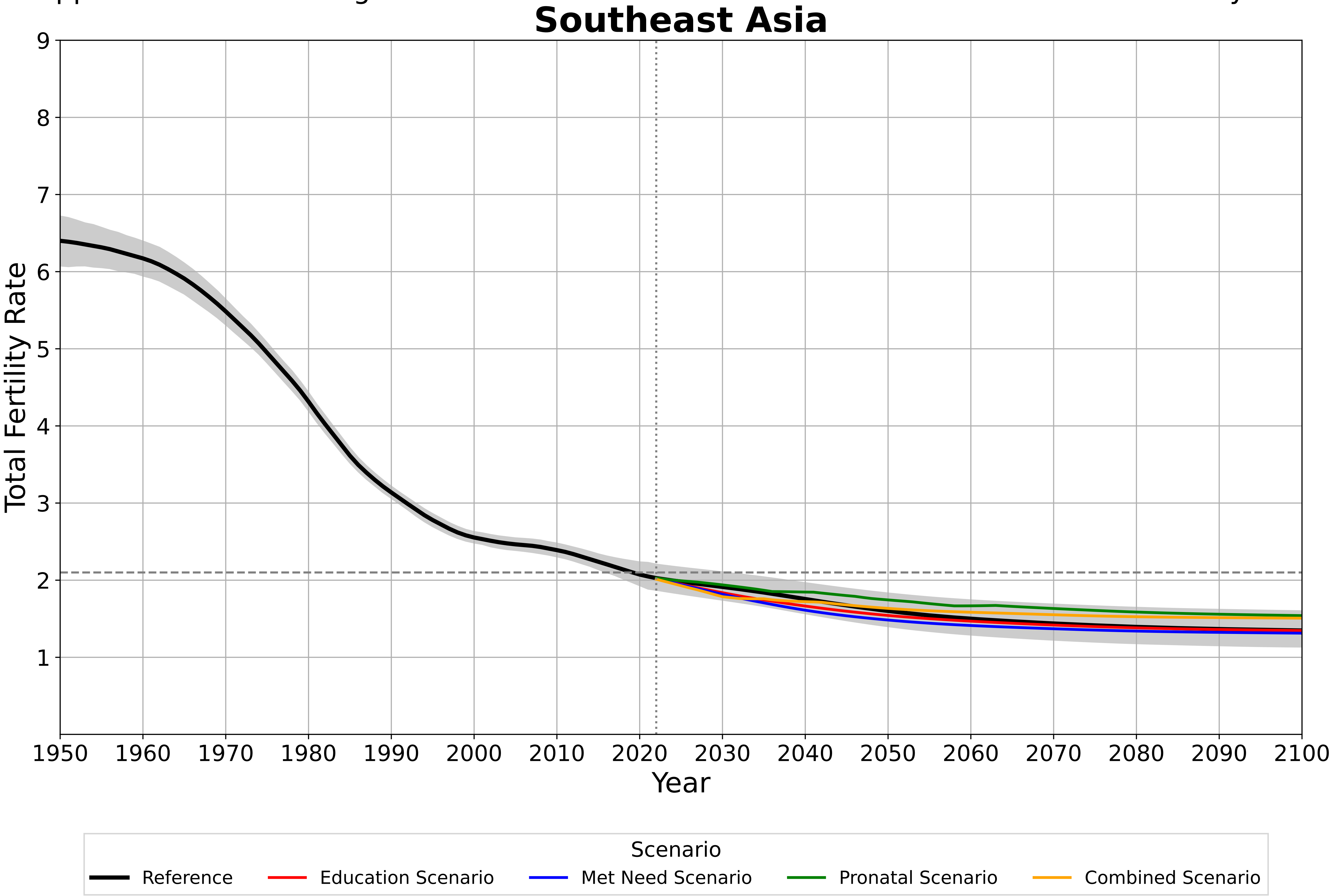

Supplemental Results Figure S5: TFR for reference and alternative scenario forecasts by location, 1950– 2100

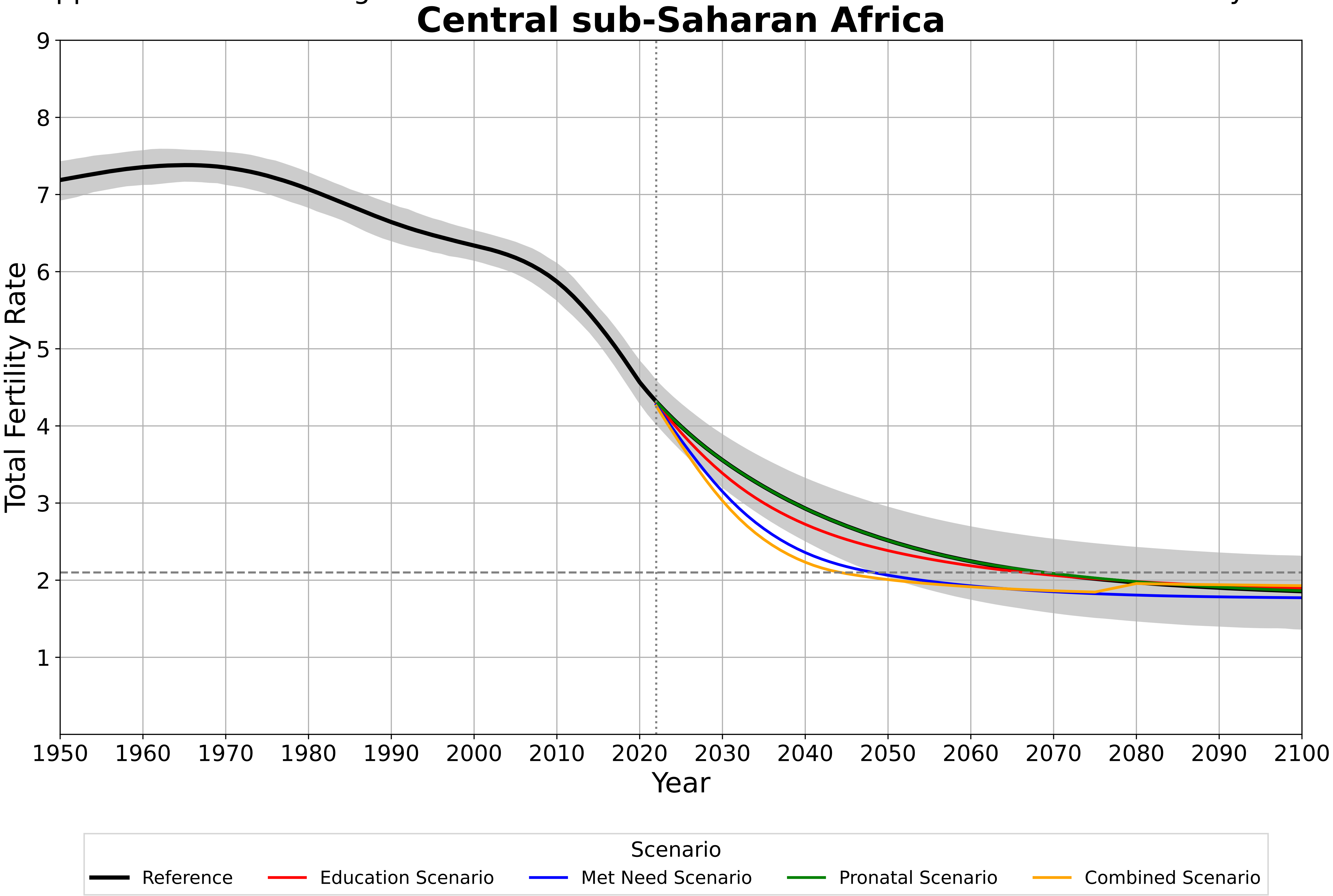

Supplemental Results Figure S5: TFR for reference and alternative scenario forecasts by location, 1950– 2100

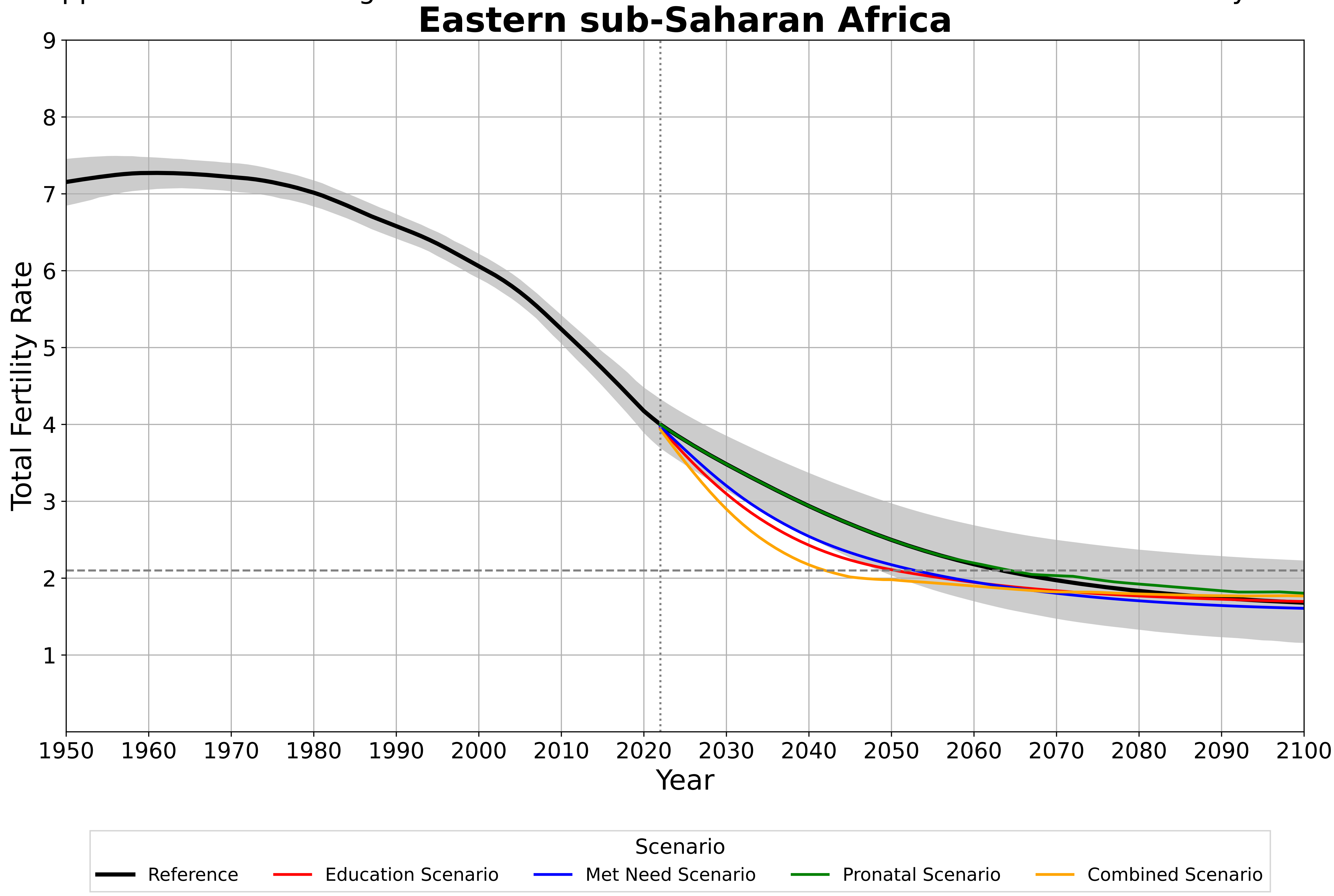

Supplemental Results Figure S5: TFR for reference and alternative scenario forecasts by location, 1950– 2100

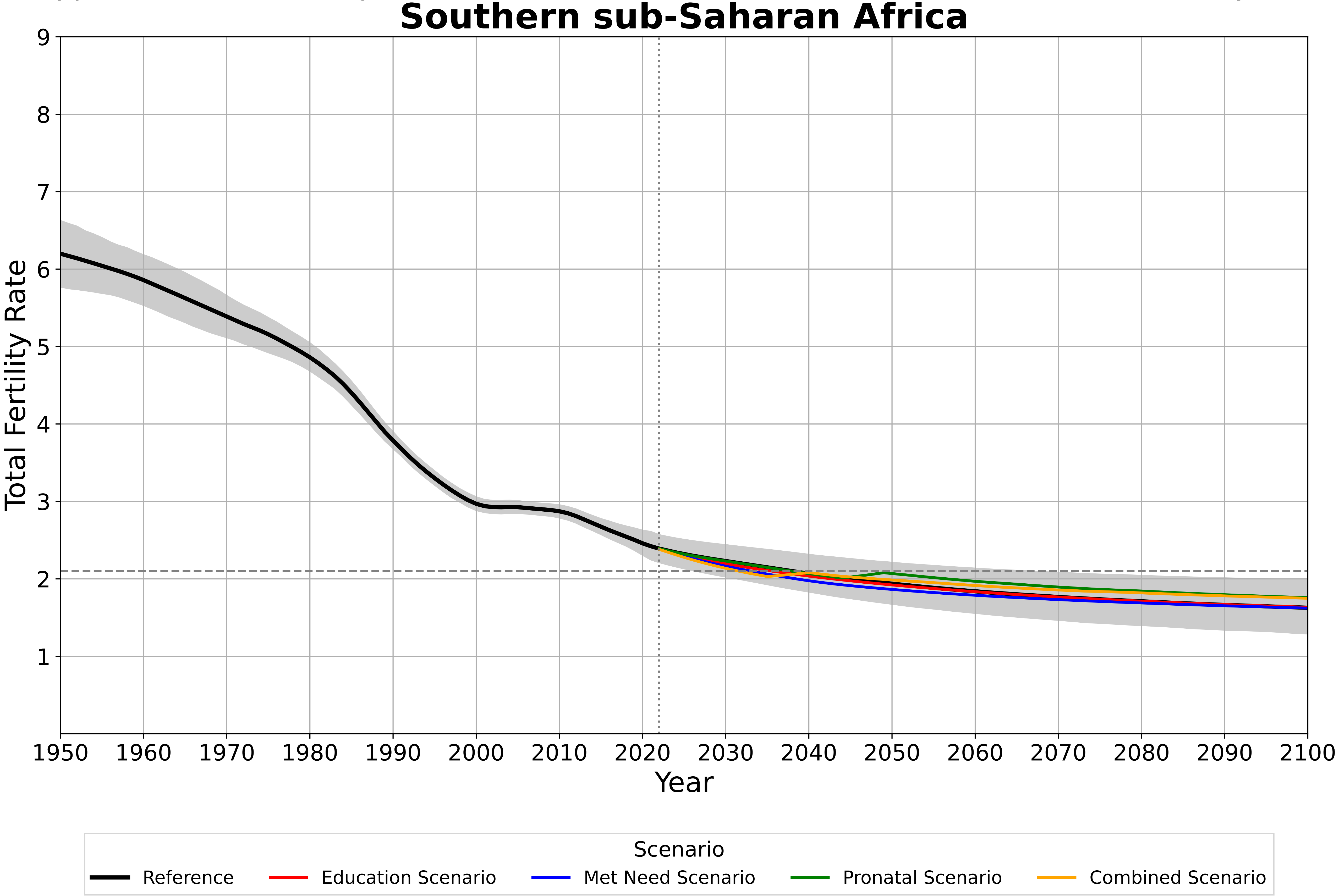

8451

Supplemental Results Figure S5: TFR for reference and alternative scenario forecasts by location, 1950– 2100

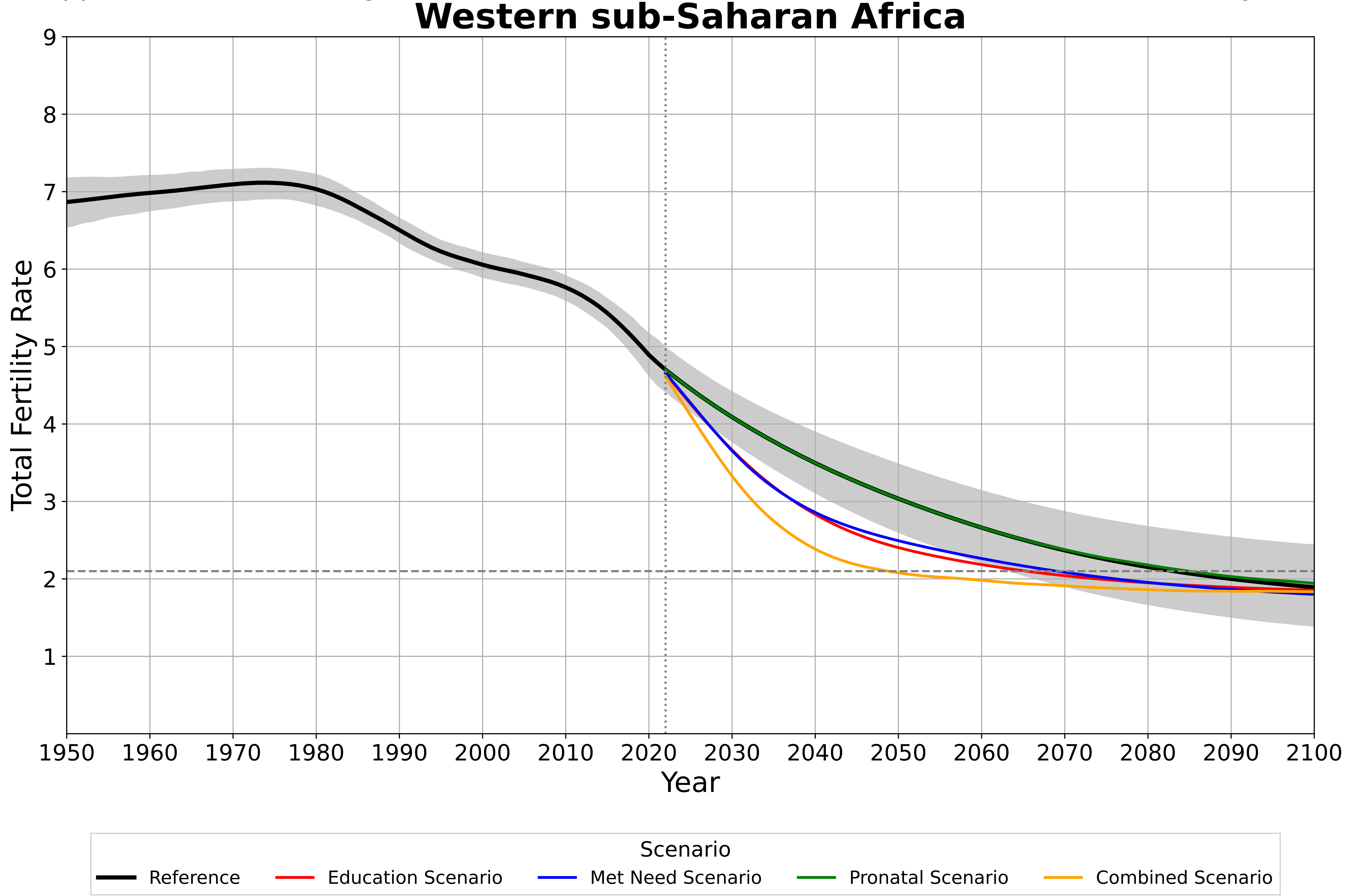

Supplemental Results Figure S6: Forecasts of TFR, globally and by country and territory, for the IHME reference scenario and the UNPD medium variant scenario, 1950–2100

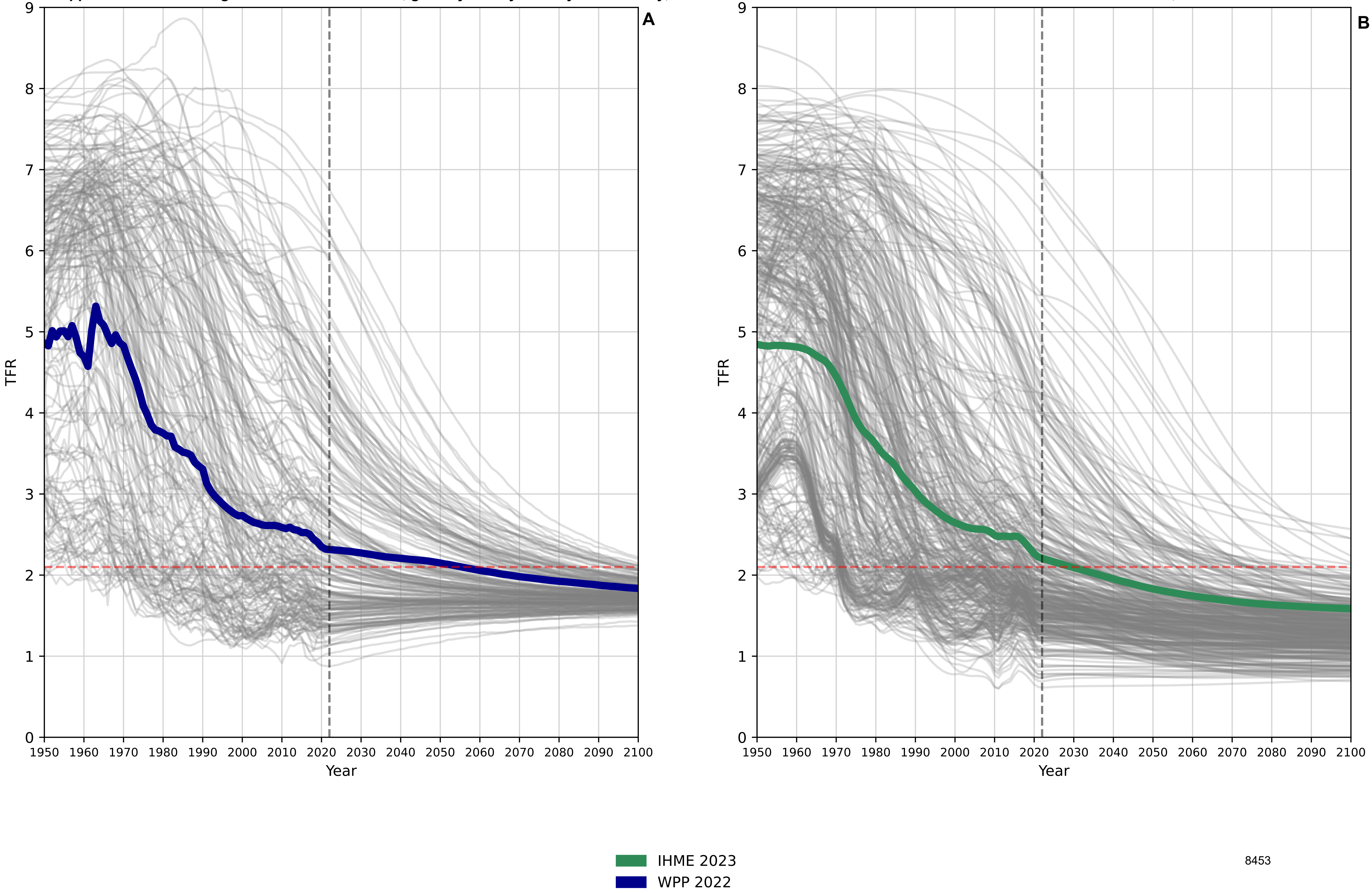

Supplemental Results Figure S7: TFR for IHME reference scenario and the UNPD medium variant scenario by location, 1950– 2100

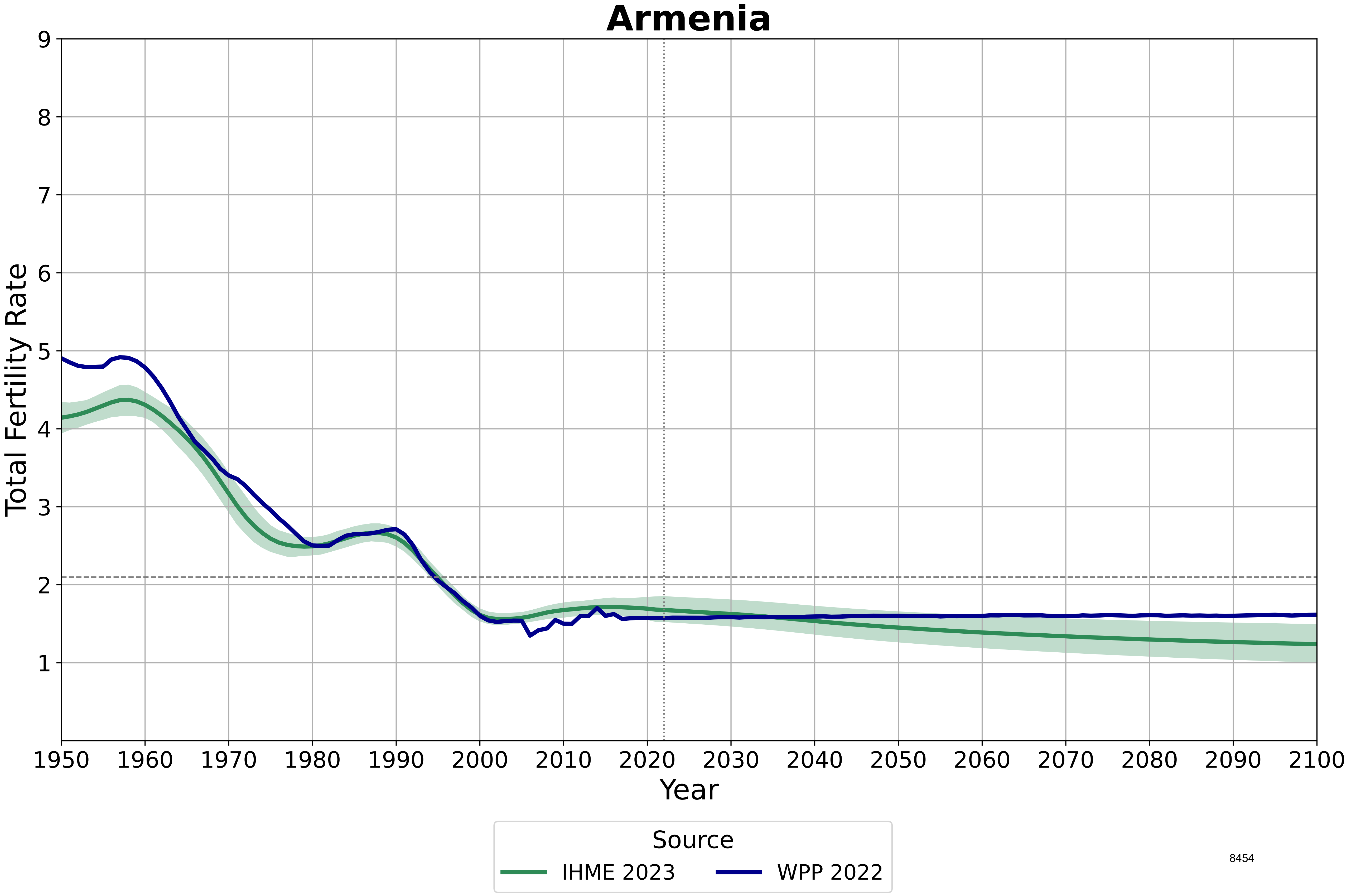

Supplemental Results Figure S7: TFR for IHME reference scenario and the UNPD medium variant scenario by location, 1950– 2100

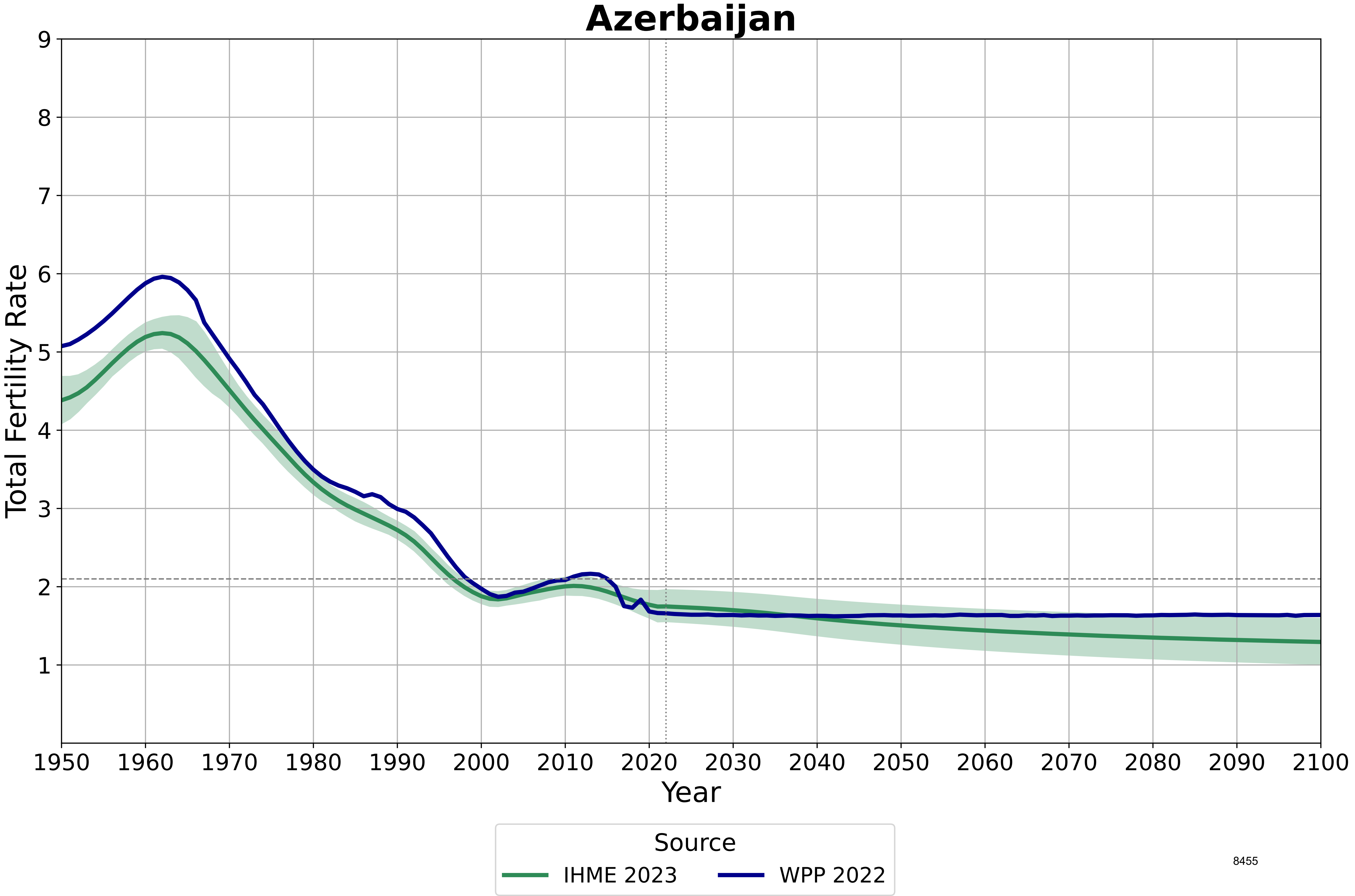

Supplemental Results Figure S7: TFR for IHME reference scenario and the UNPD medium variant scenario by location, 1950– 2100

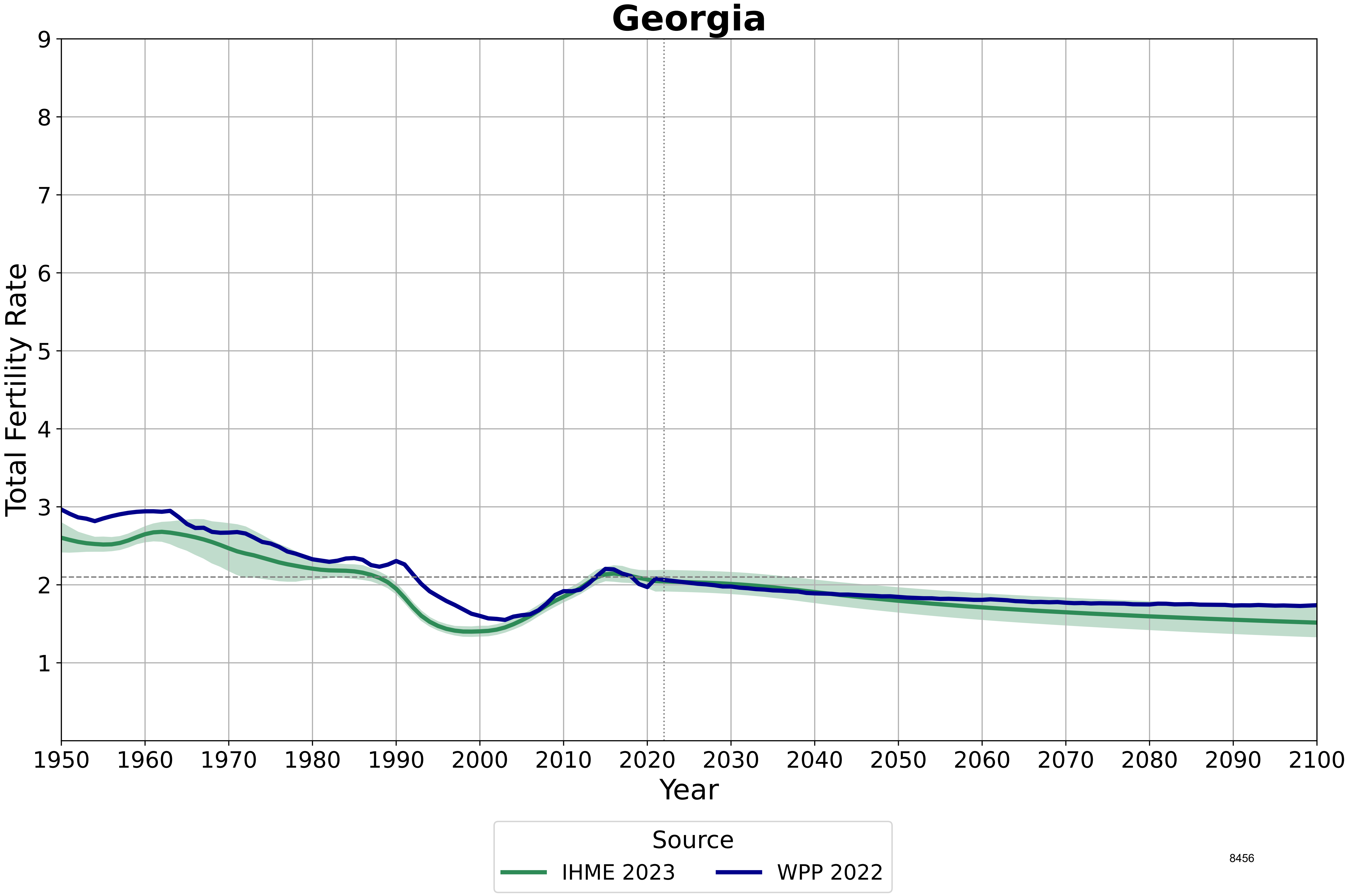

Supplemental Results Figure S7: TFR for IHME reference scenario and the UNPD medium variant scenario by location, 1950– 2100

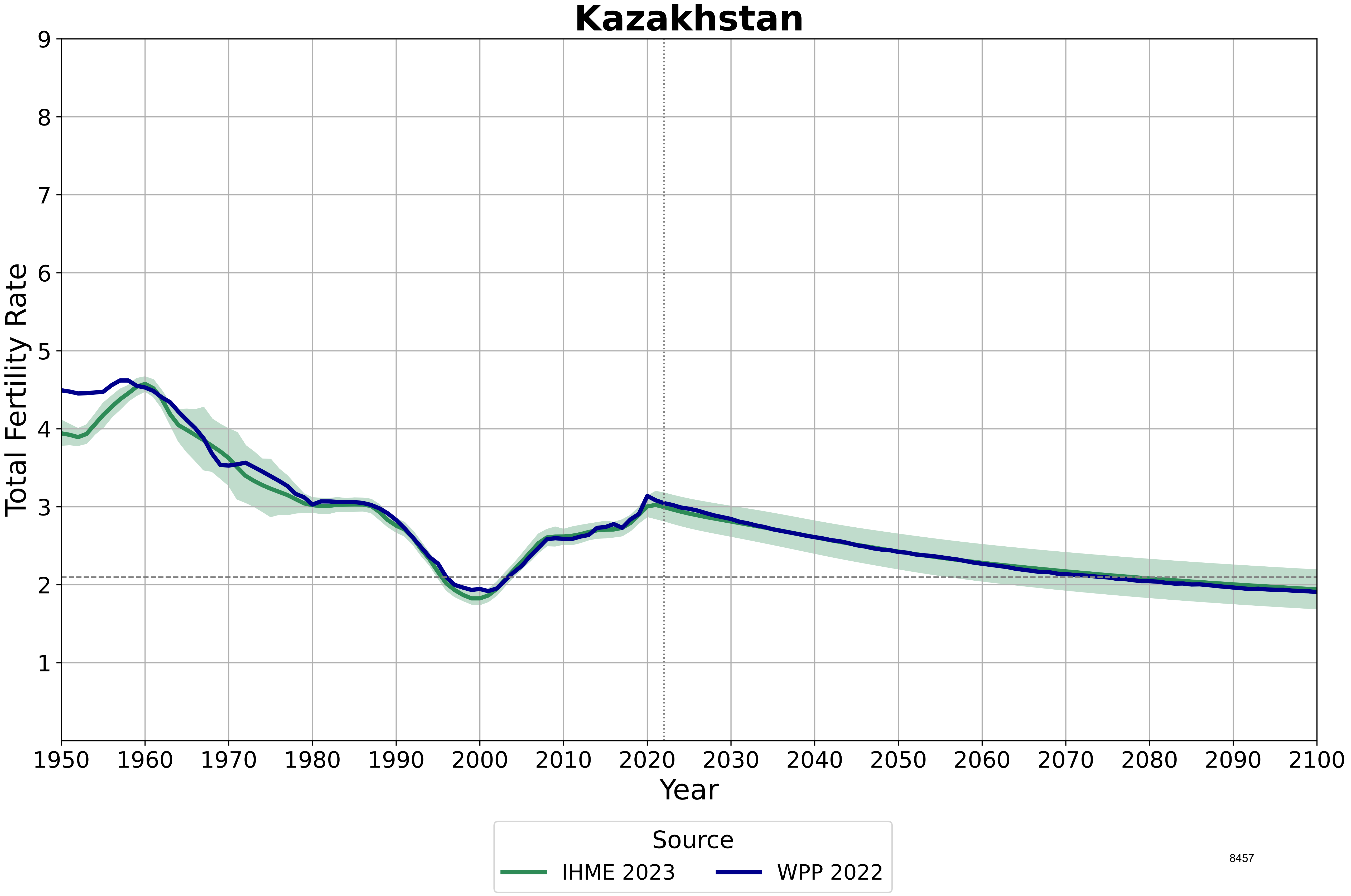

Supplemental Results Figure S7: TFR for IHME reference scenario and the UNPD medium variant scenario by location, 1950– 2100

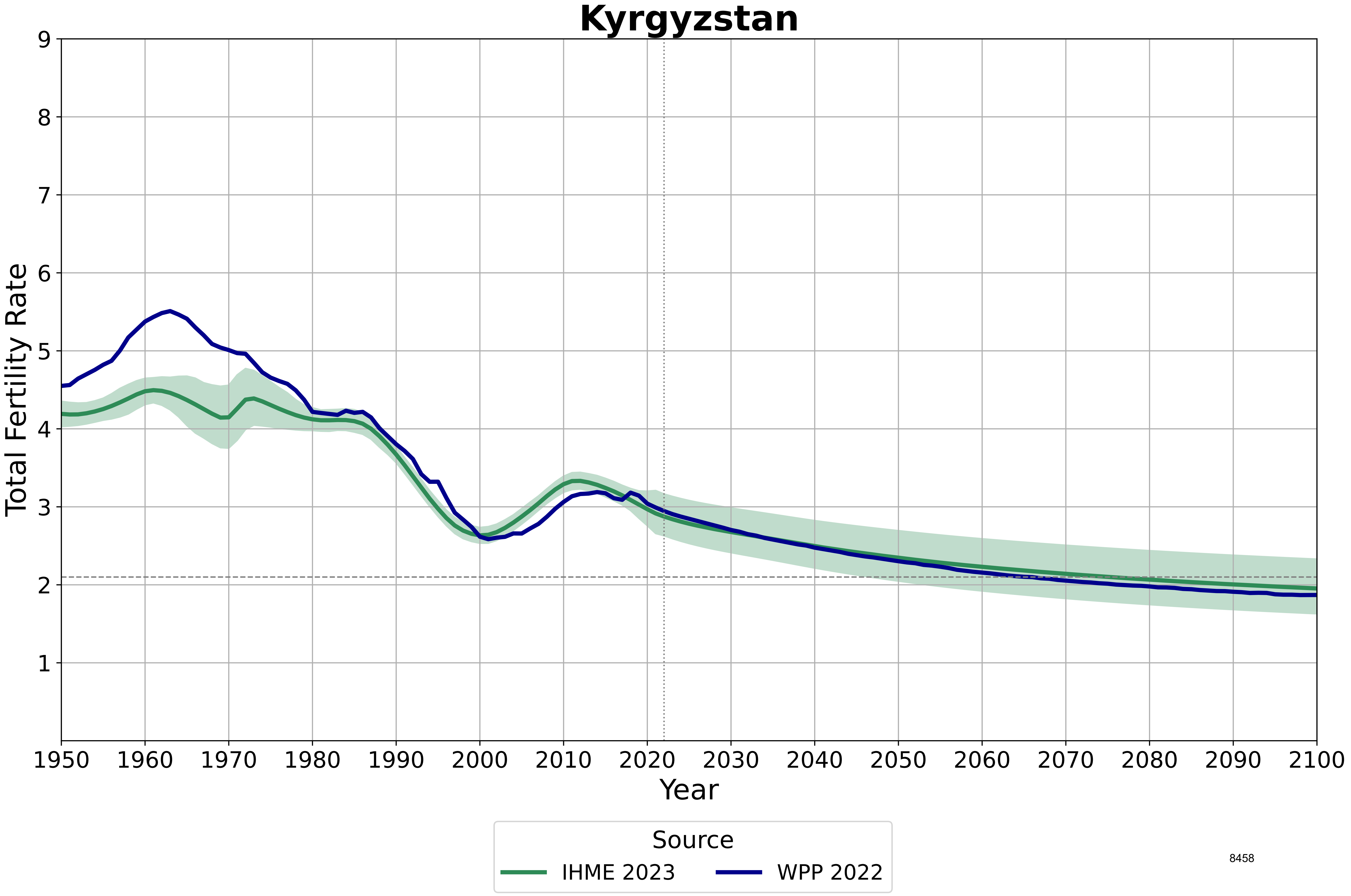

Supplemental Results Figure S7: TFR for IHME reference scenario and the UNPD medium variant scenario by location, 1950– 2100

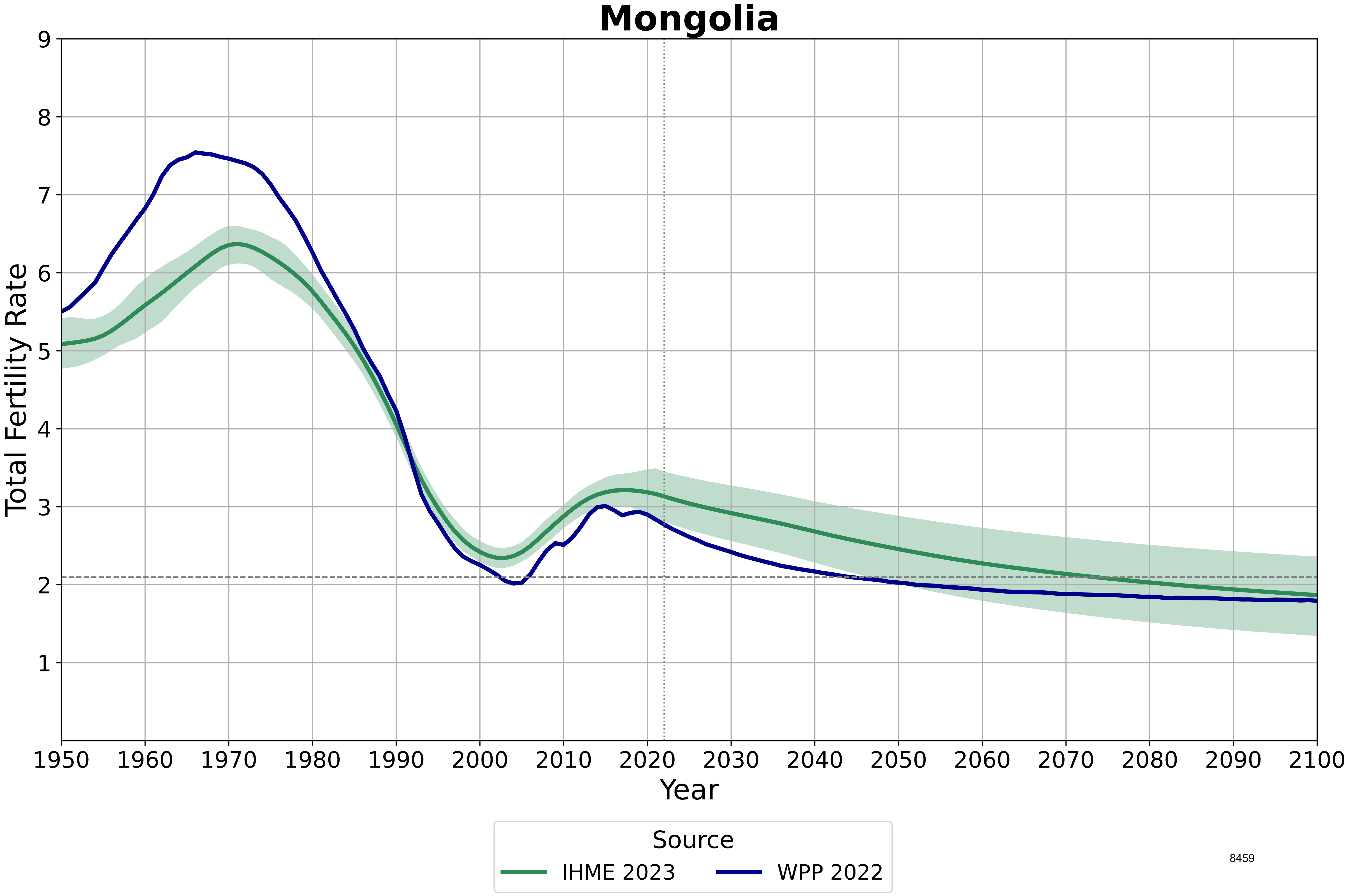

Supplemental Results Figure S7: TFR for IHME reference scenario and the UNPD medium variant scenario by location, 1950– 2100

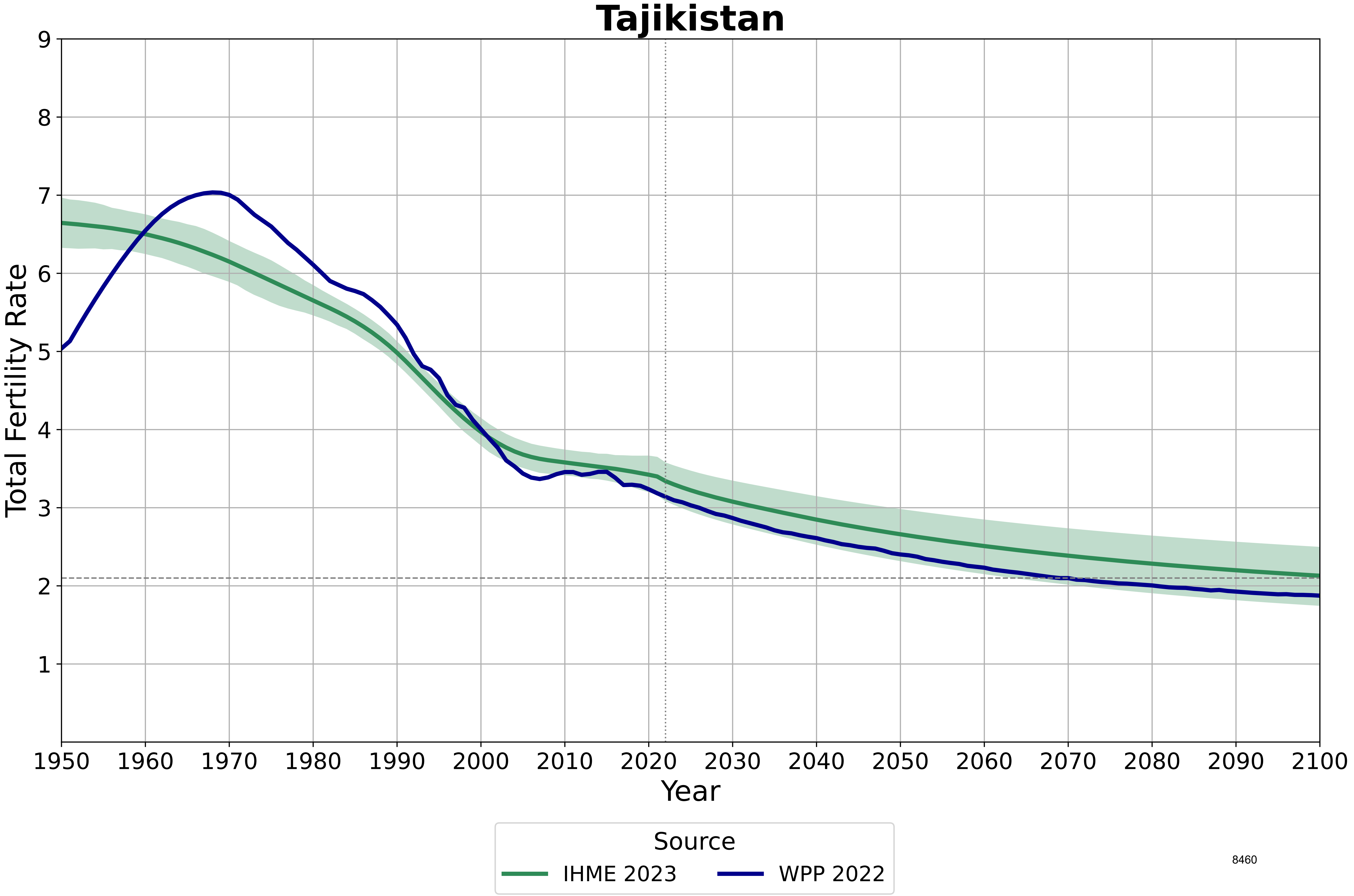

Supplemental Results Figure S7: TFR for IHME reference scenario and the UNPD medium variant scenario by location, 1950– 2100

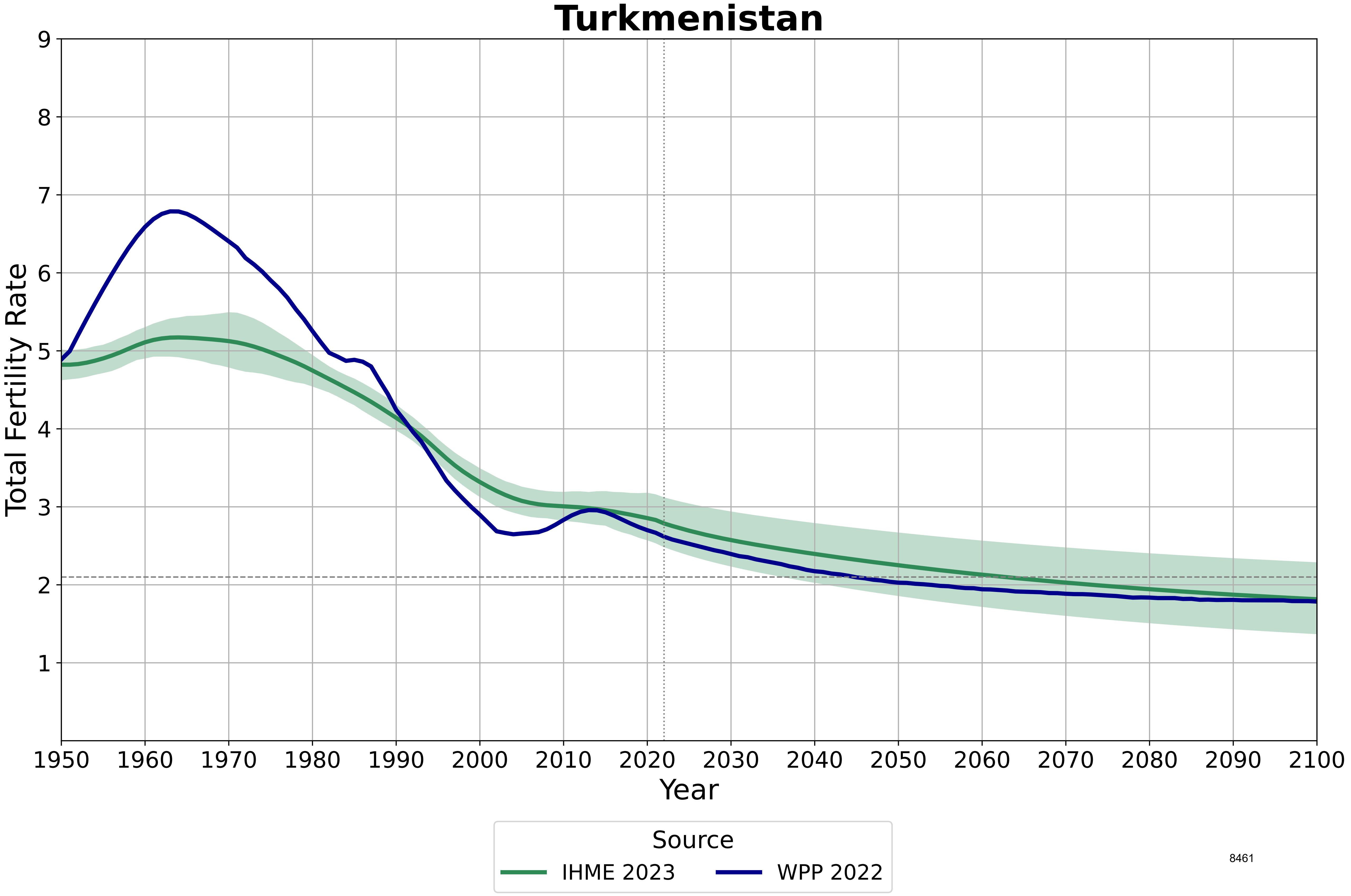

Supplemental Results Figure S7: TFR for IHME reference scenario and the UNPD medium variant scenario by location, 1950– 2100

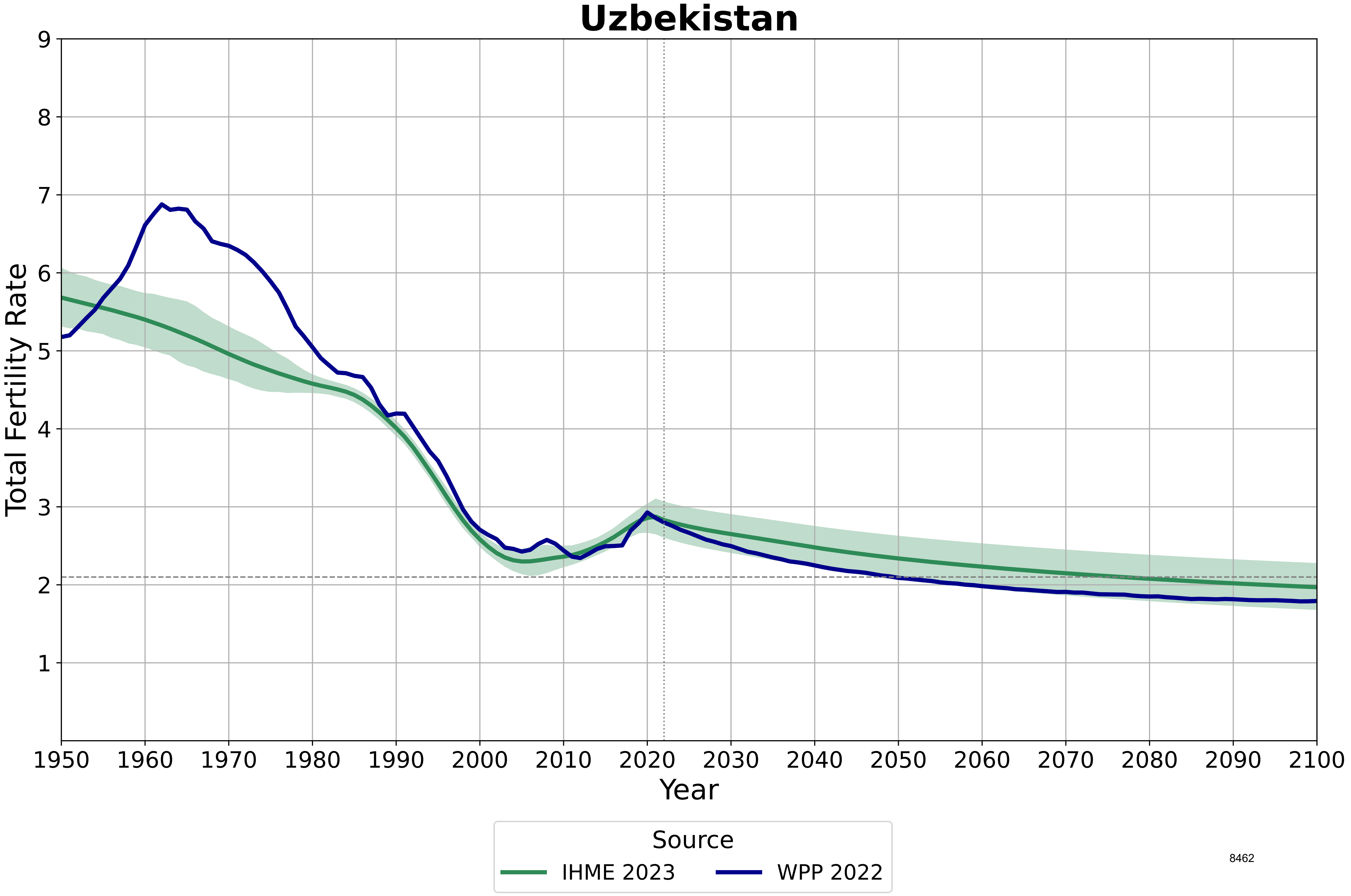

Supplemental Results Figure S7: TFR for IHME reference scenario and the UNPD medium variant scenario by location, 1950– 2100

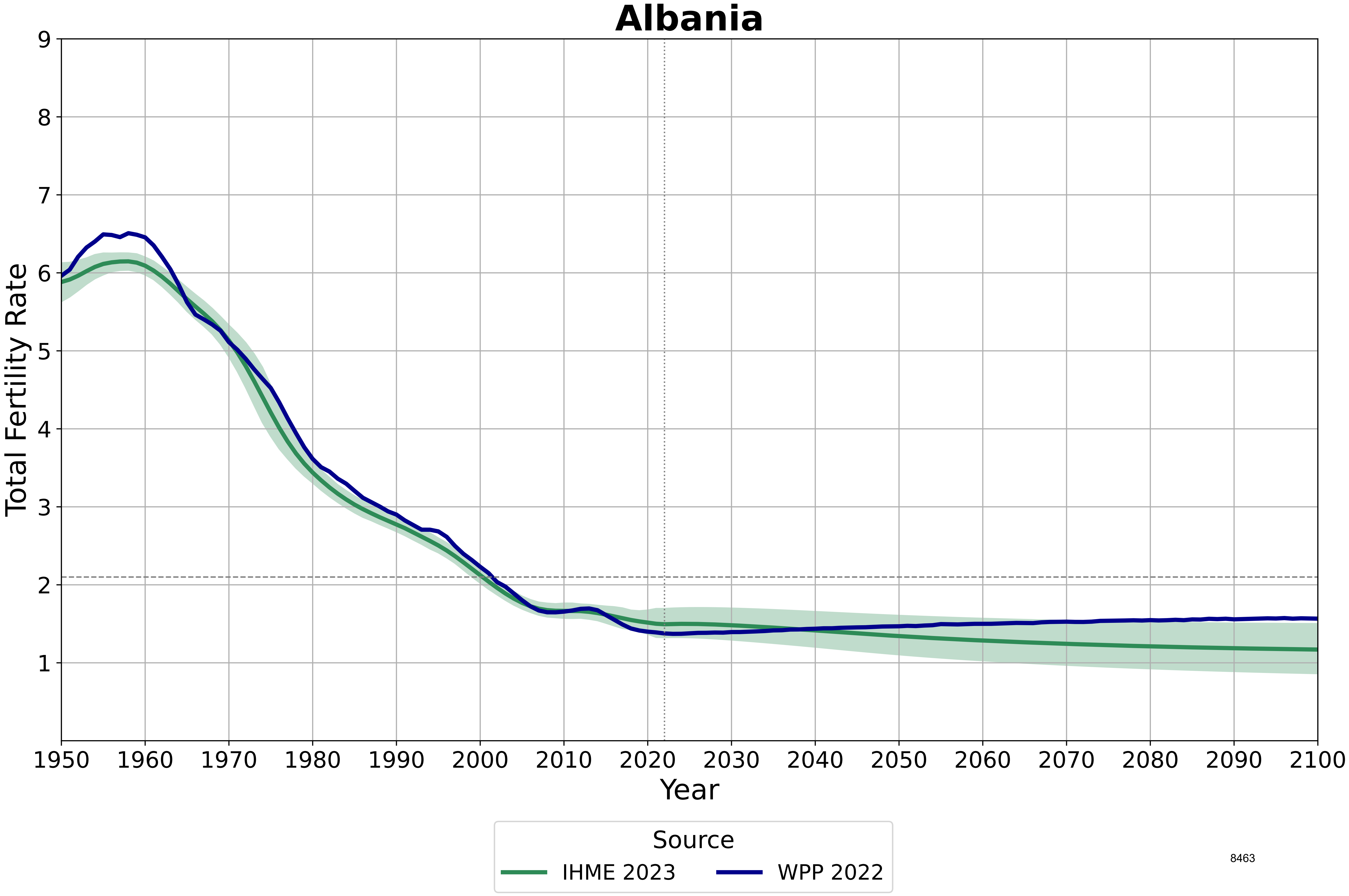

Supplemental Results Figure S7: TFR for IHME reference scenario and the UNPD medium variant scenario by location, 1950– 2100

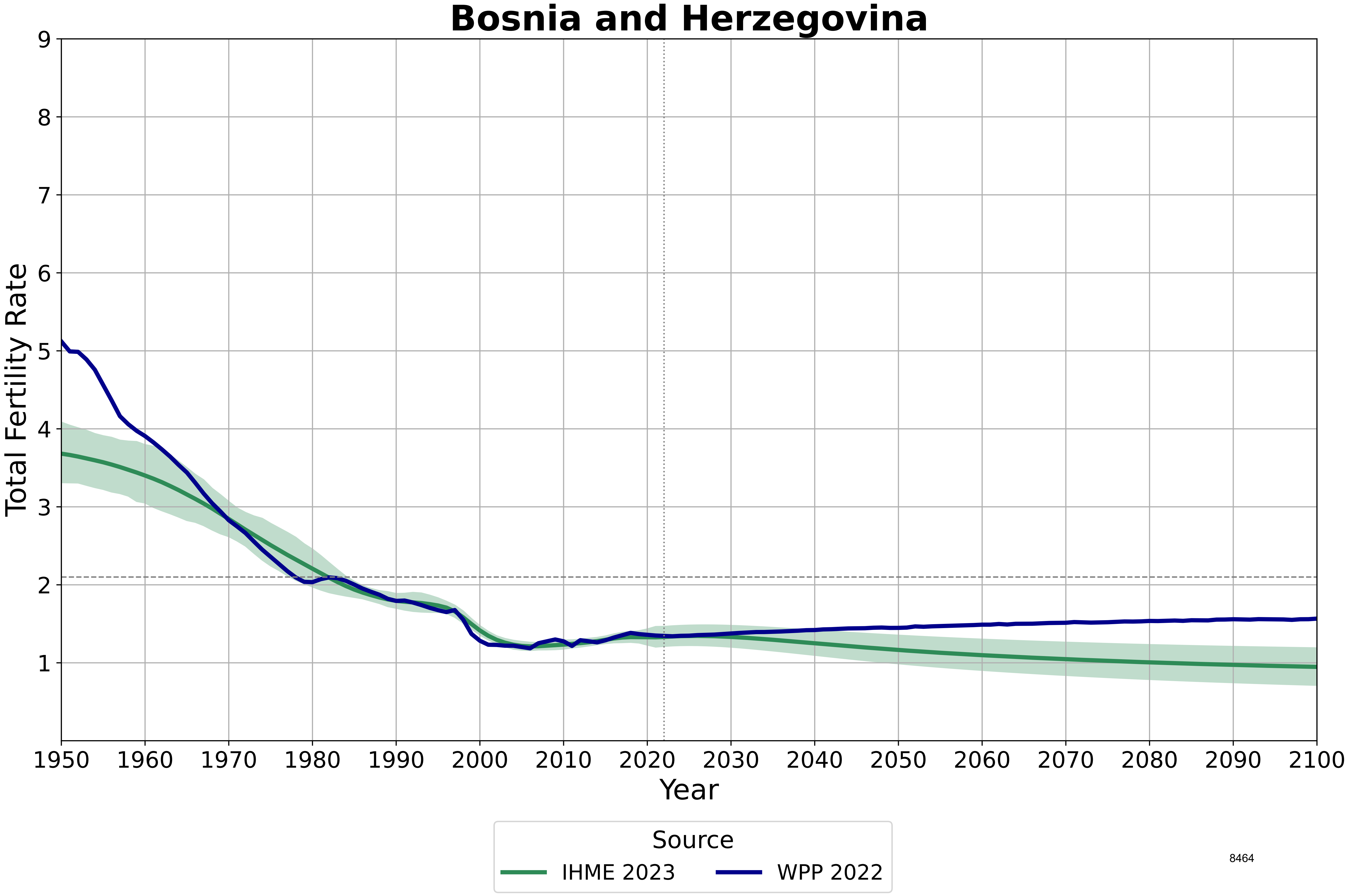

Supplemental Results Figure S7: TFR for IHME reference scenario and the UNPD medium variant scenario by location, 1950– 2100

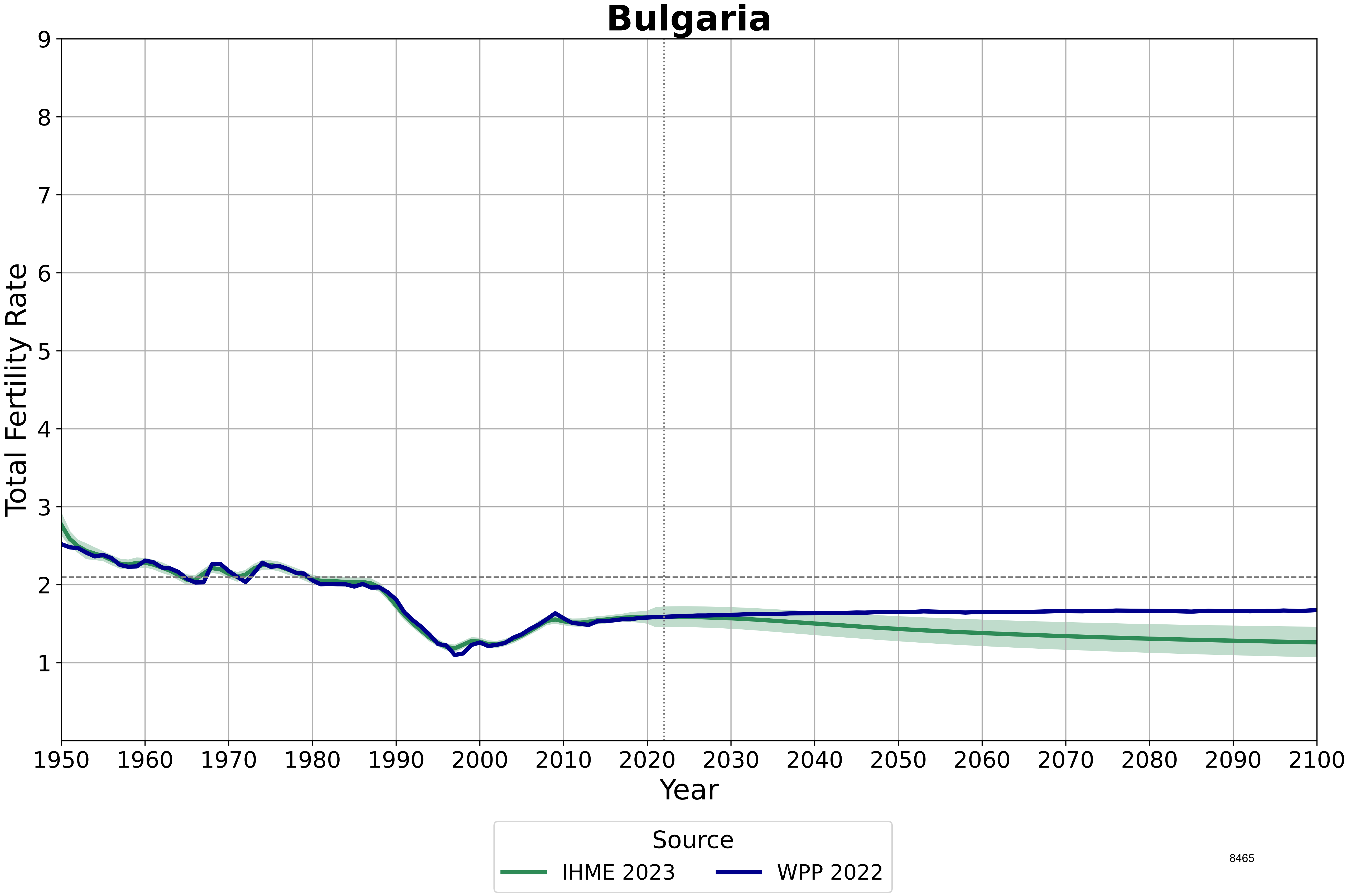

Supplemental Results Figure S7: TFR for IHME reference scenario and the UNPD medium variant scenario by location, 1950– 2100

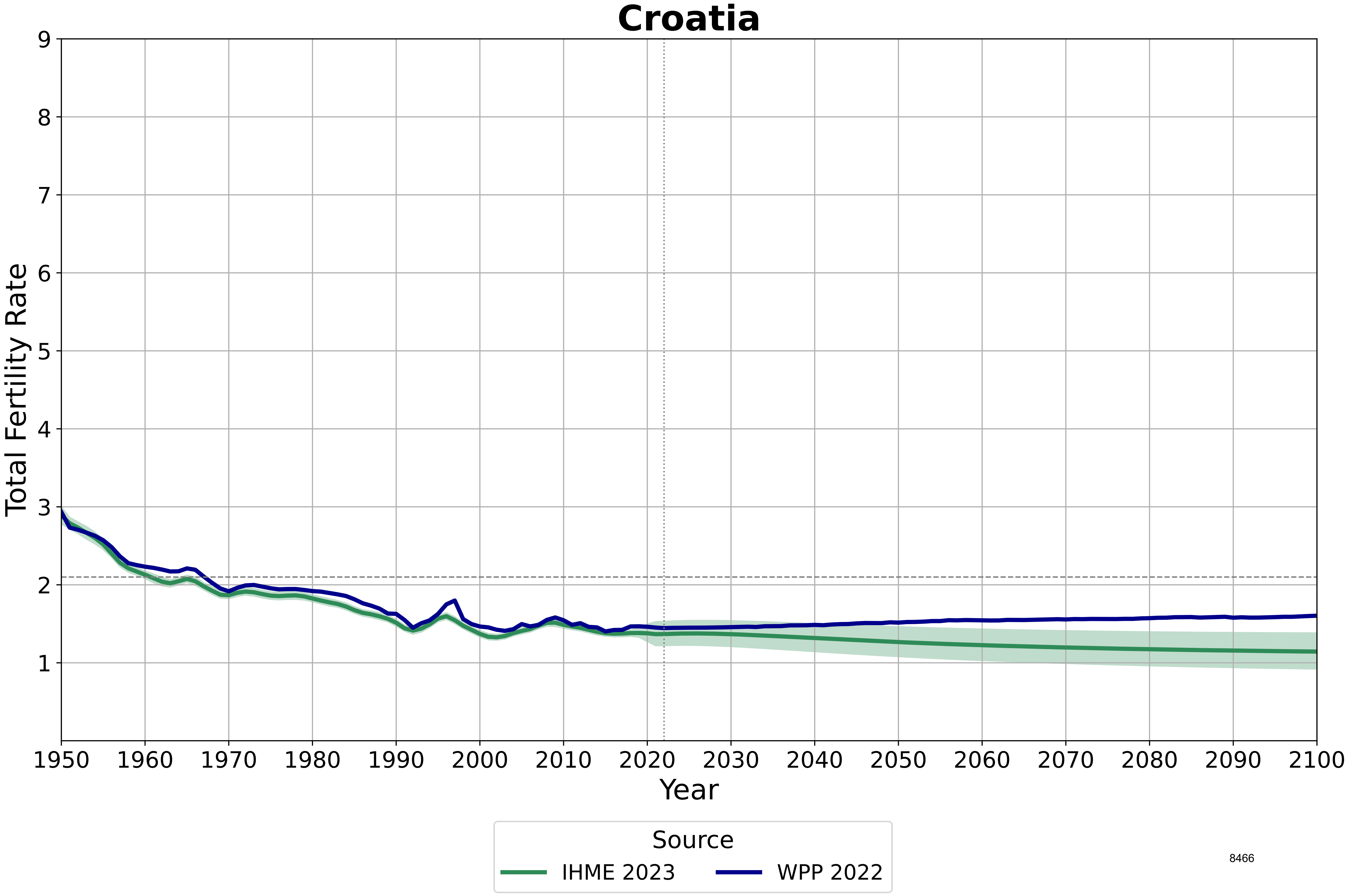

Supplemental Results Figure S7: TFR for IHME reference scenario and the UNPD medium variant scenario by location, 1950– 2100

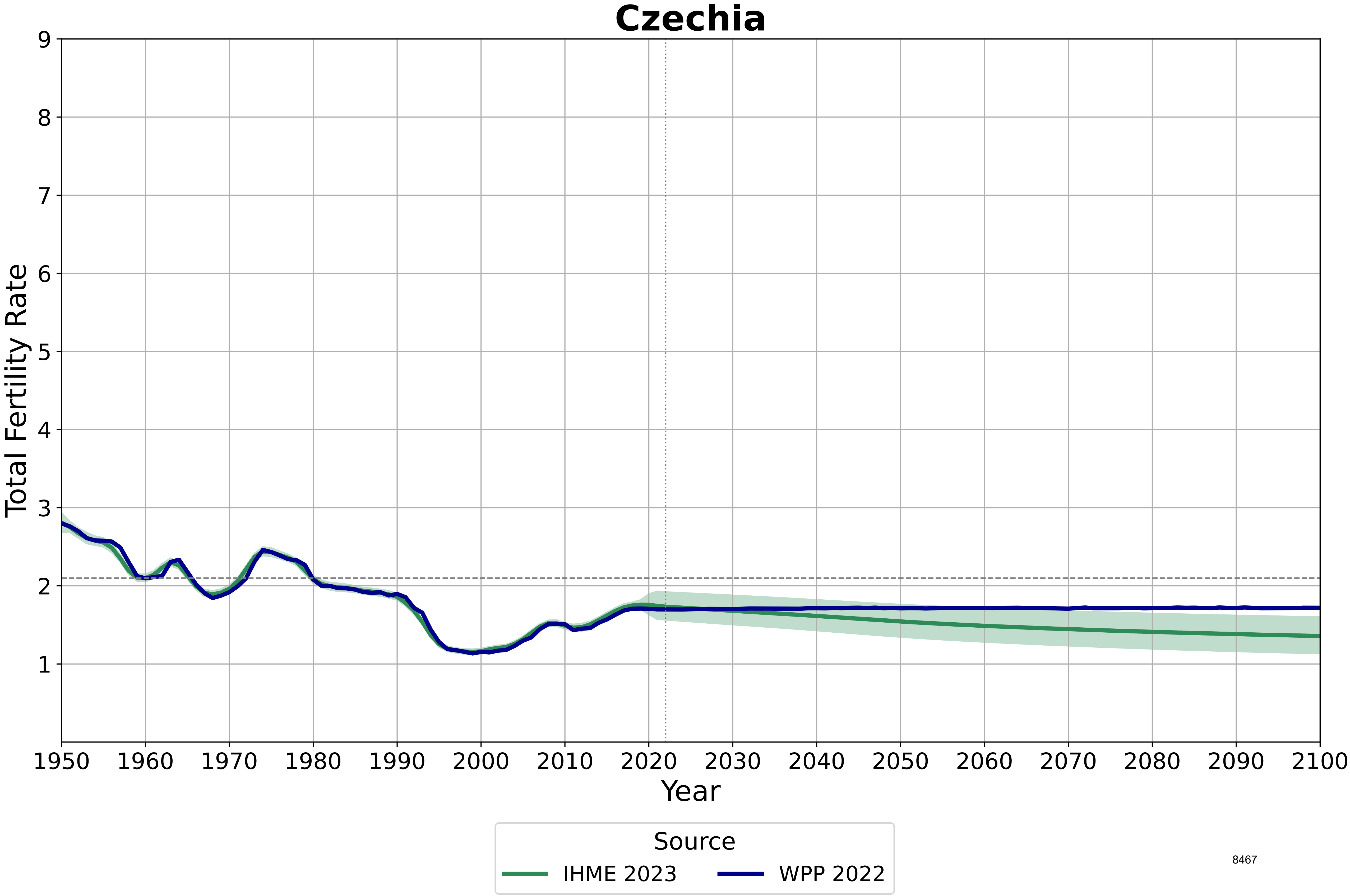

Supplemental Results Figure S7: TFR for IHME reference scenario and the UNPD medium variant scenario by location, 1950– 2100

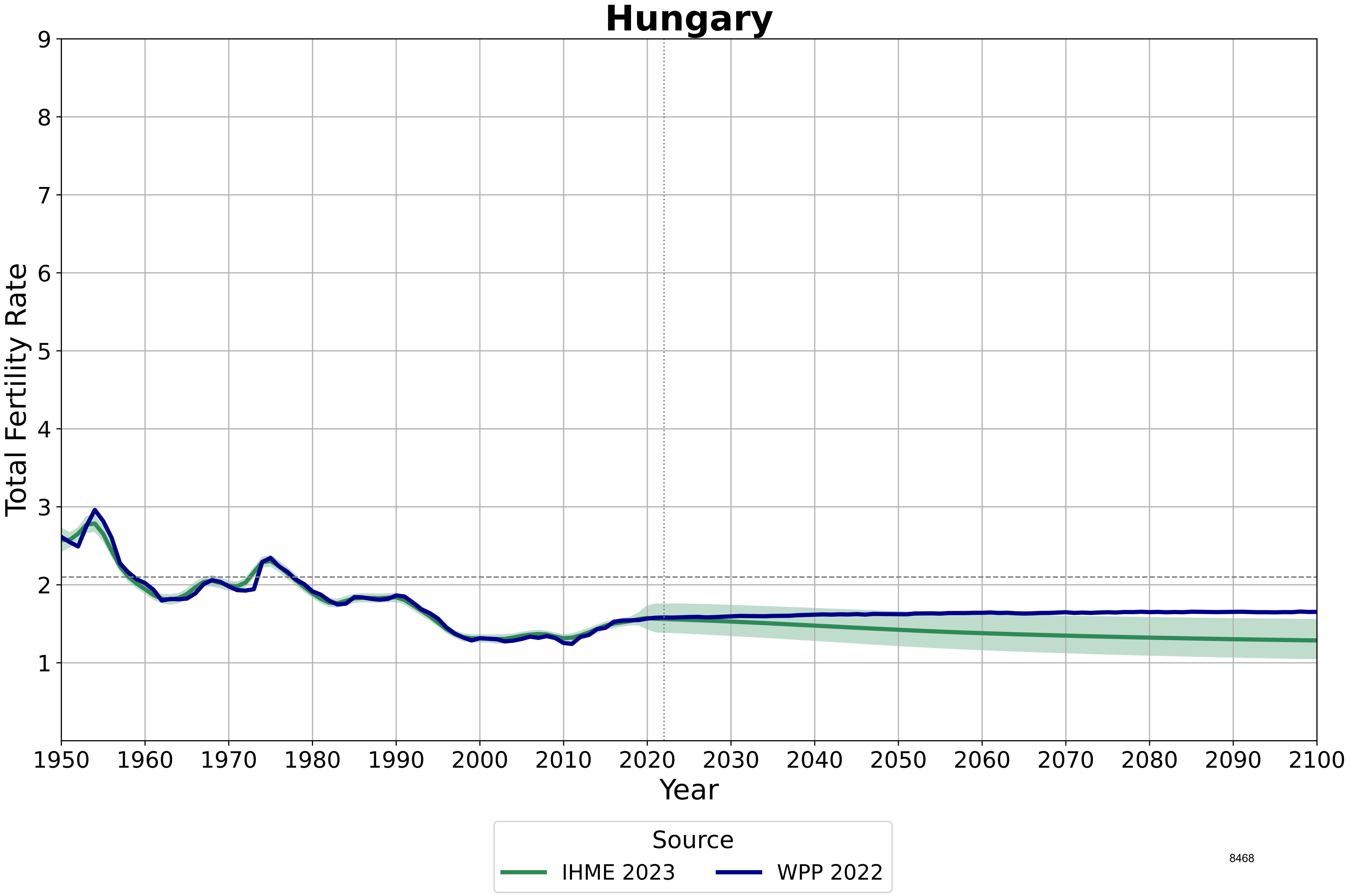

Supplemental Results Figure S7: TFR for IHME reference scenario and the UNPD medium variant scenario by location, 1950– 2100

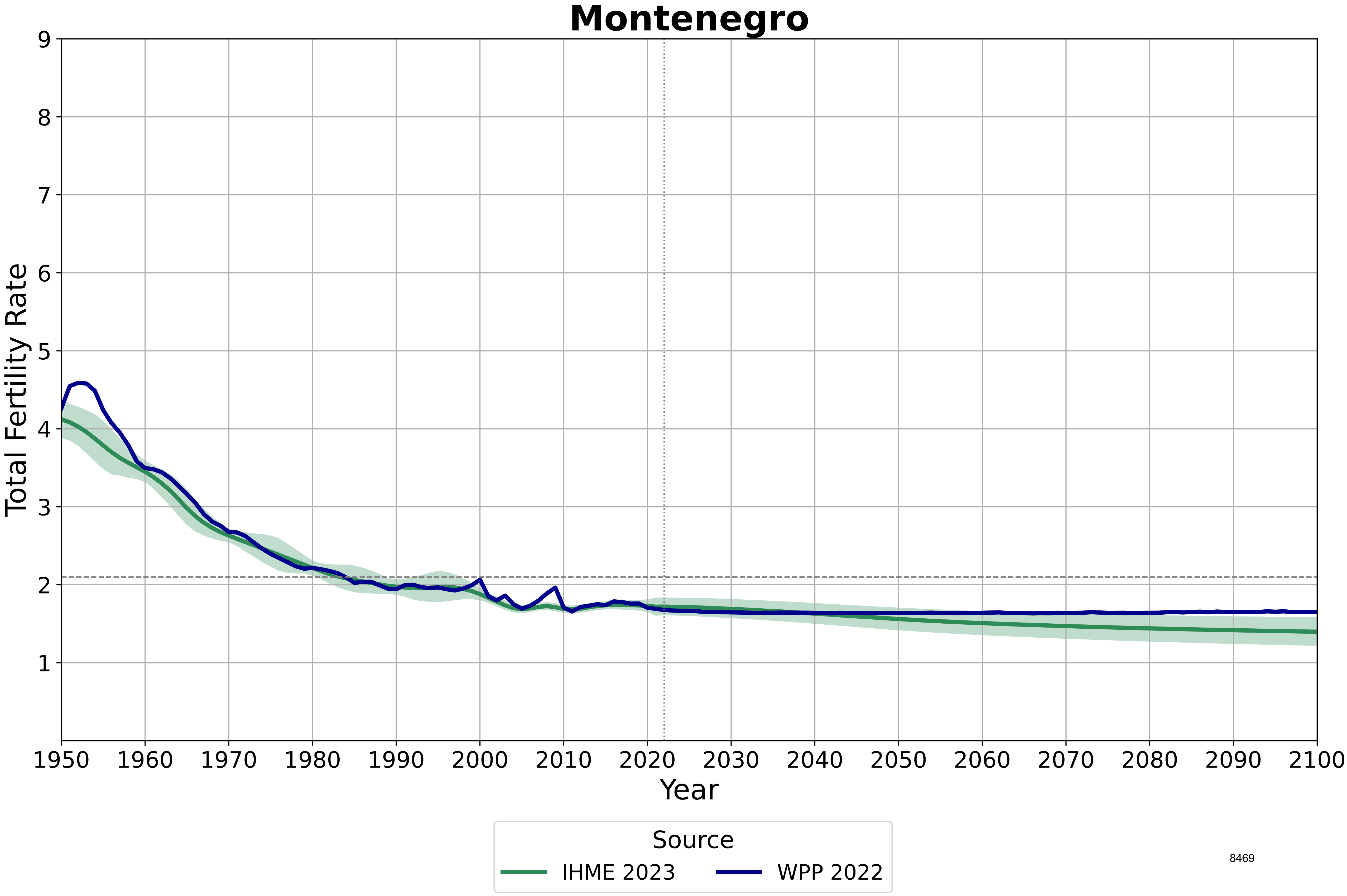

Supplemental Results Figure S7: TFR for IHME reference scenario and the UNPD medium variant scenario by location, 1950– 2100

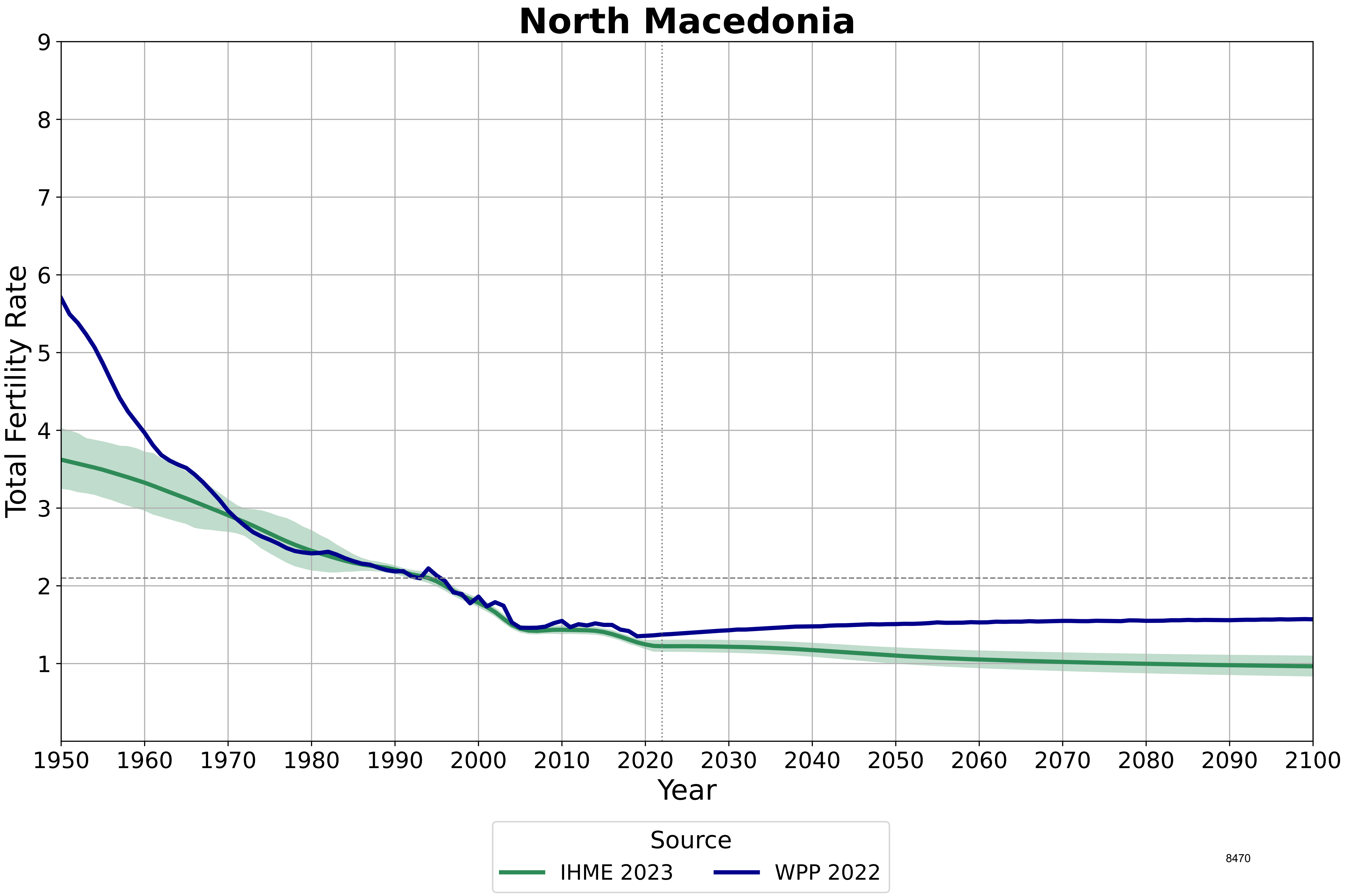

Supplemental Results Figure S7: TFR for IHME reference scenario and the UNPD medium variant scenario by location, 1950– 2100

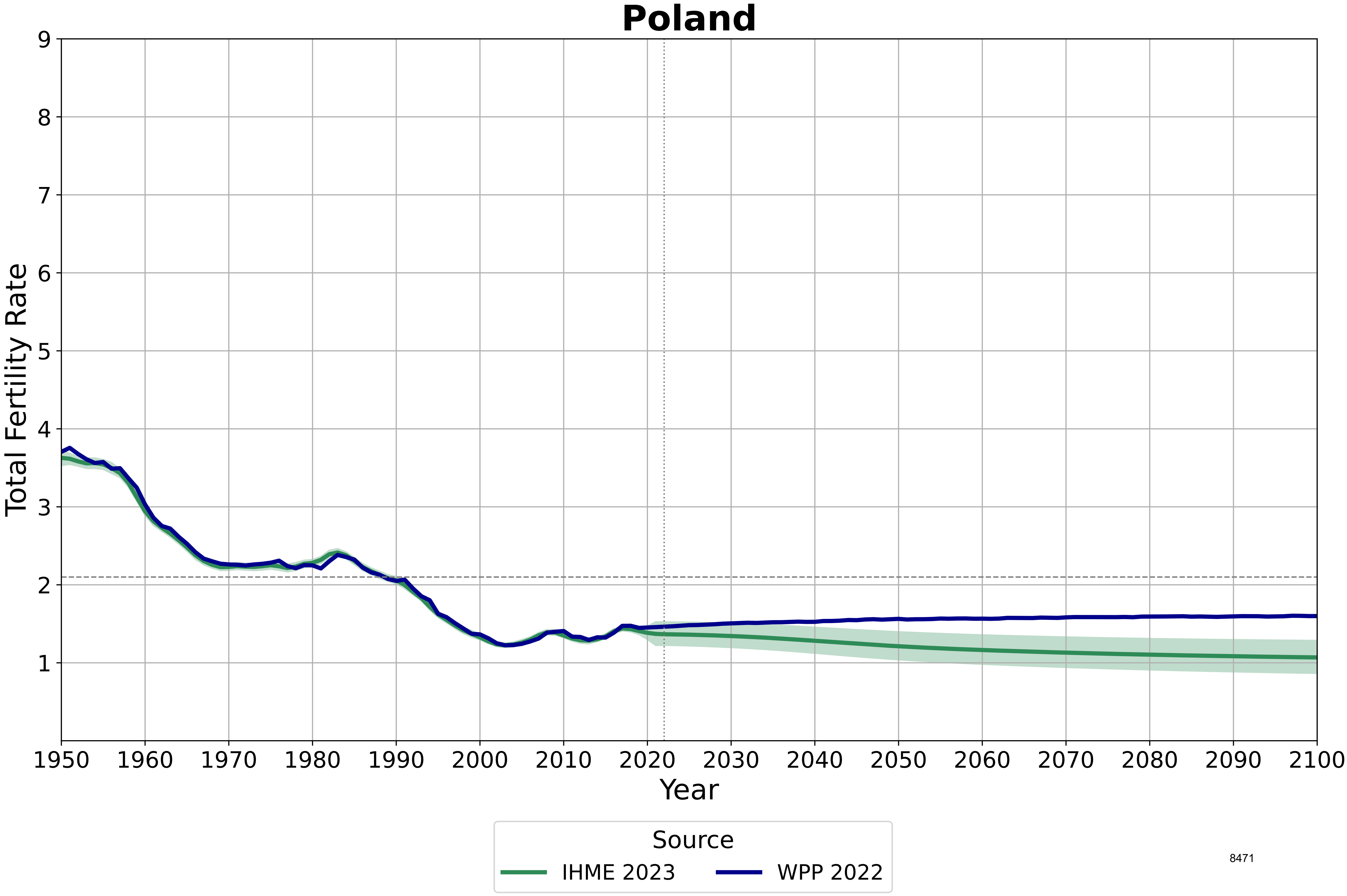

Supplemental Results Figure S7: TFR for IHME reference scenario and the UNPD medium variant scenario by location, 1950– 2100

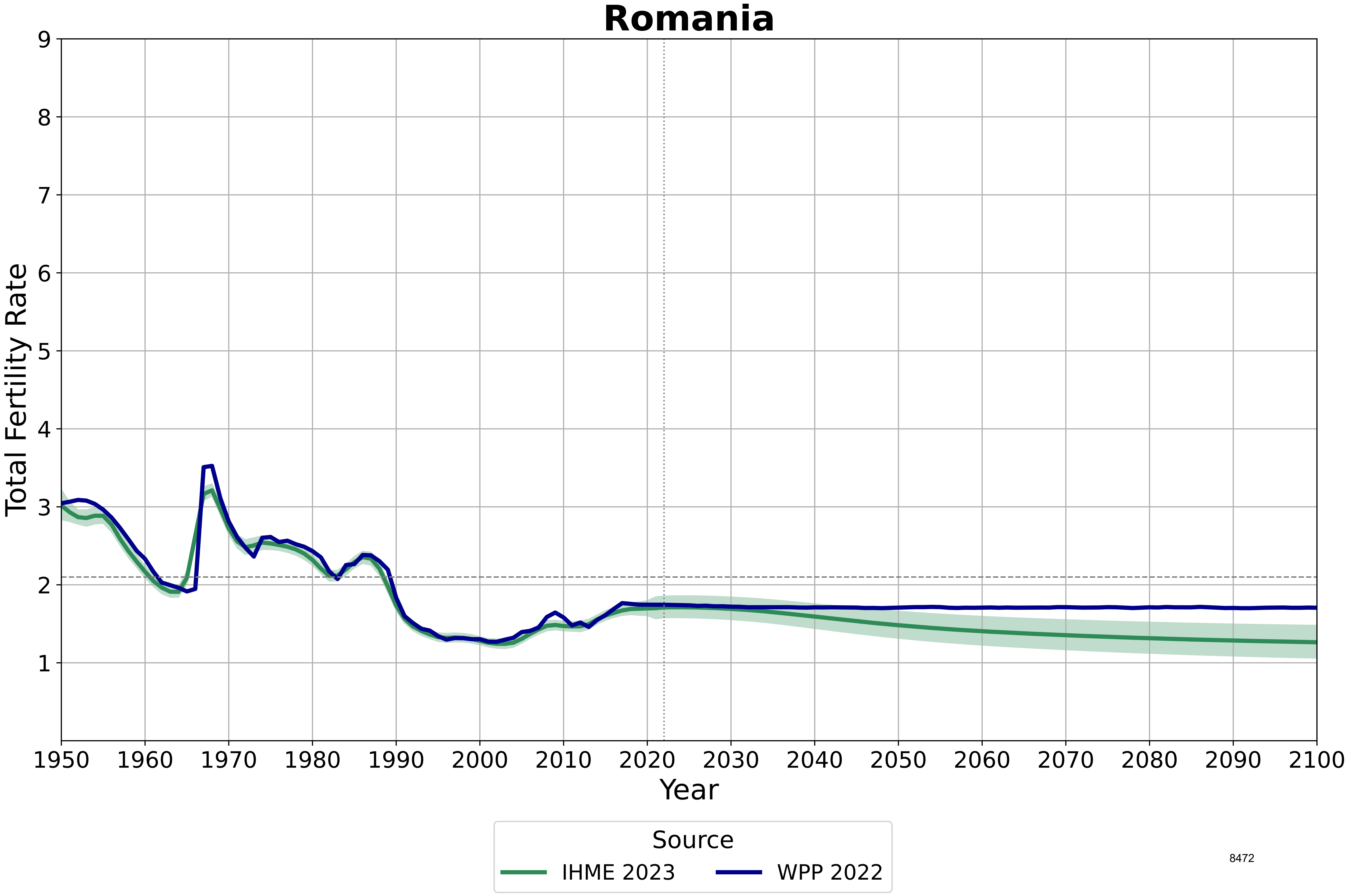

8472

Supplemental Results Figure S7: TFR for IHME reference scenario and the UNPD medium variant scenario by location, 1950– 2100

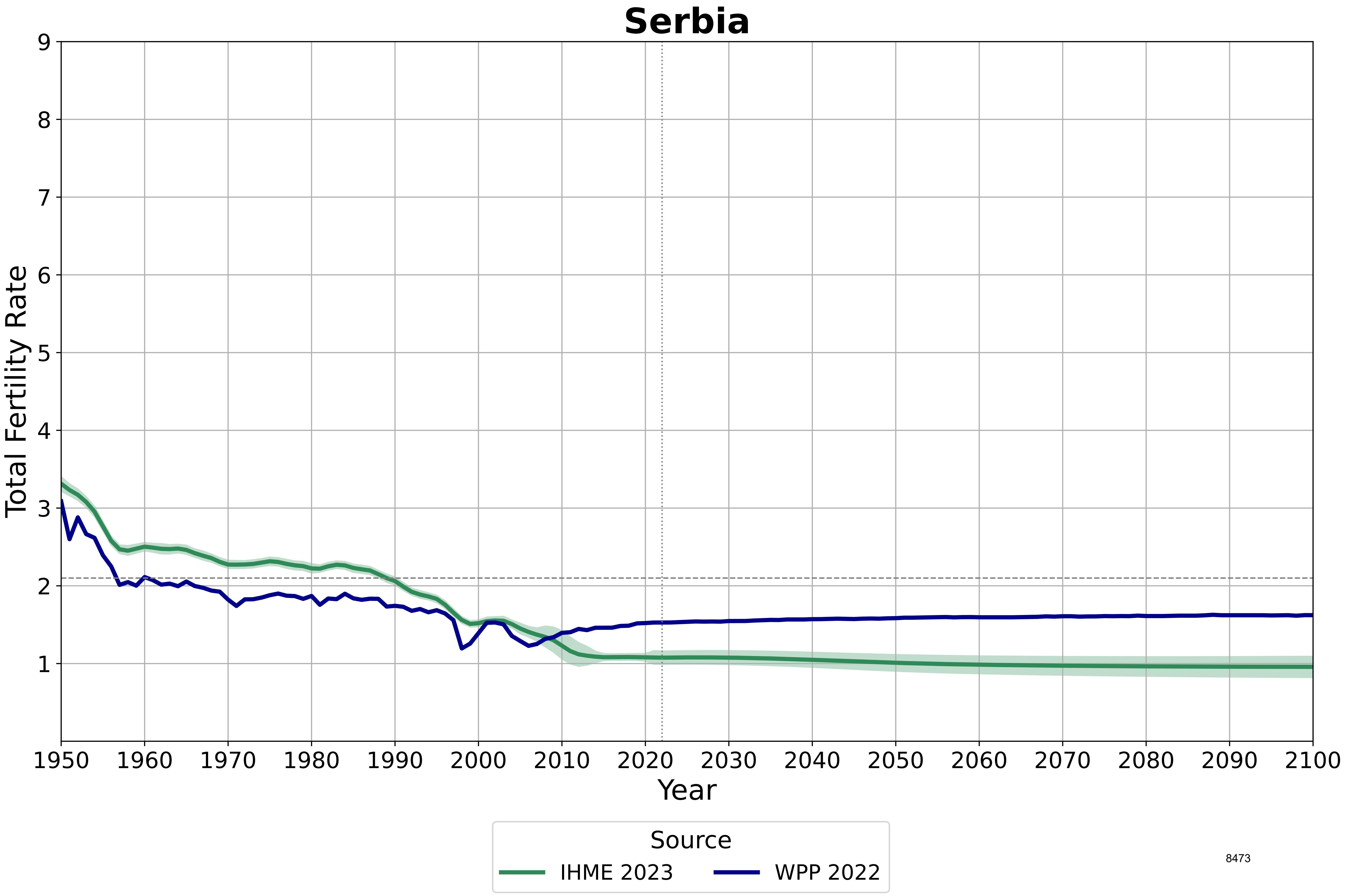

Supplemental Results Figure S7: TFR for IHME reference scenario and the UNPD medium variant scenario by location, 1950– 2100

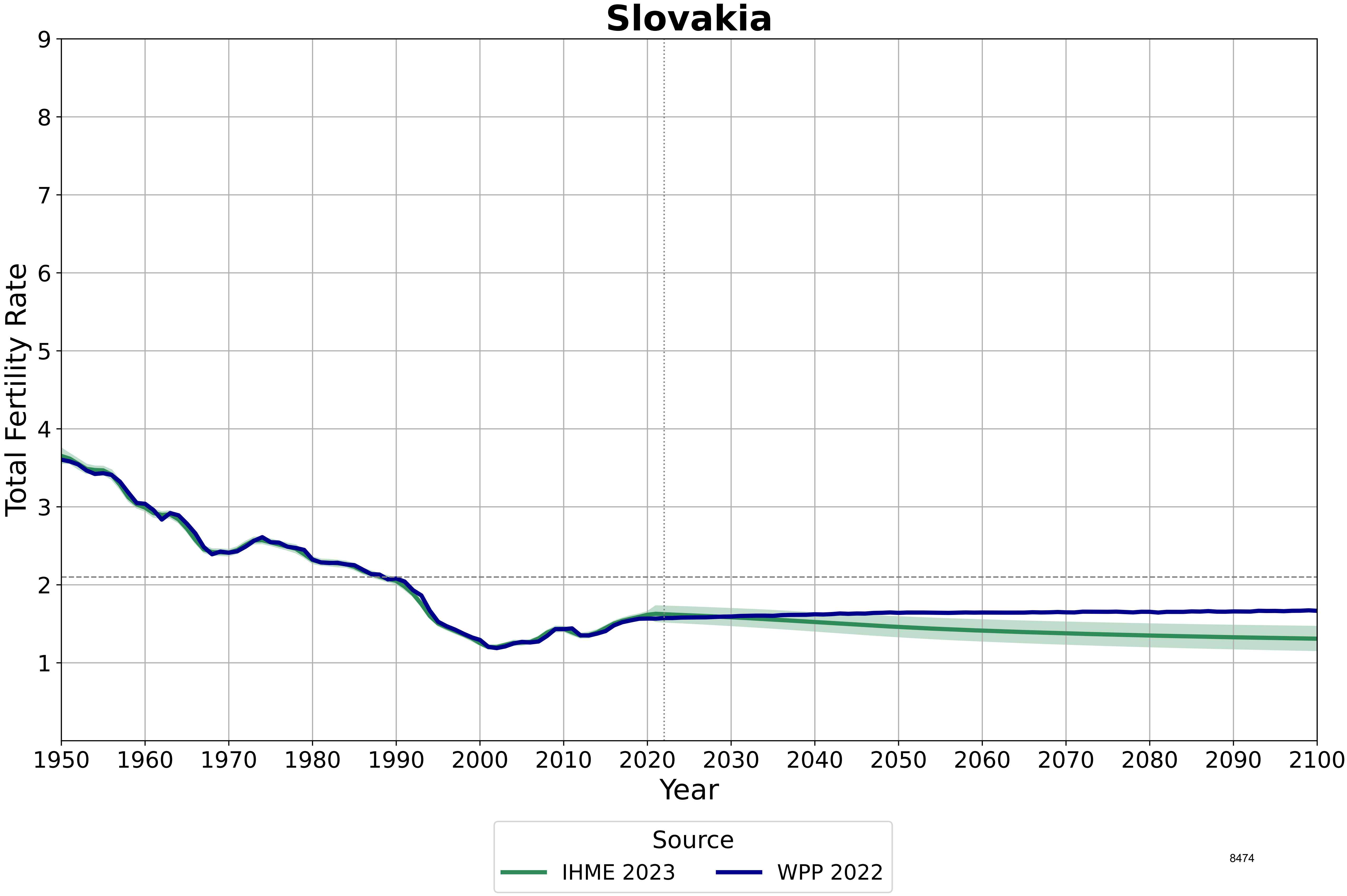

Supplemental Results Figure S7: TFR for IHME reference scenario and the UNPD medium variant scenario by location, 1950– 2100

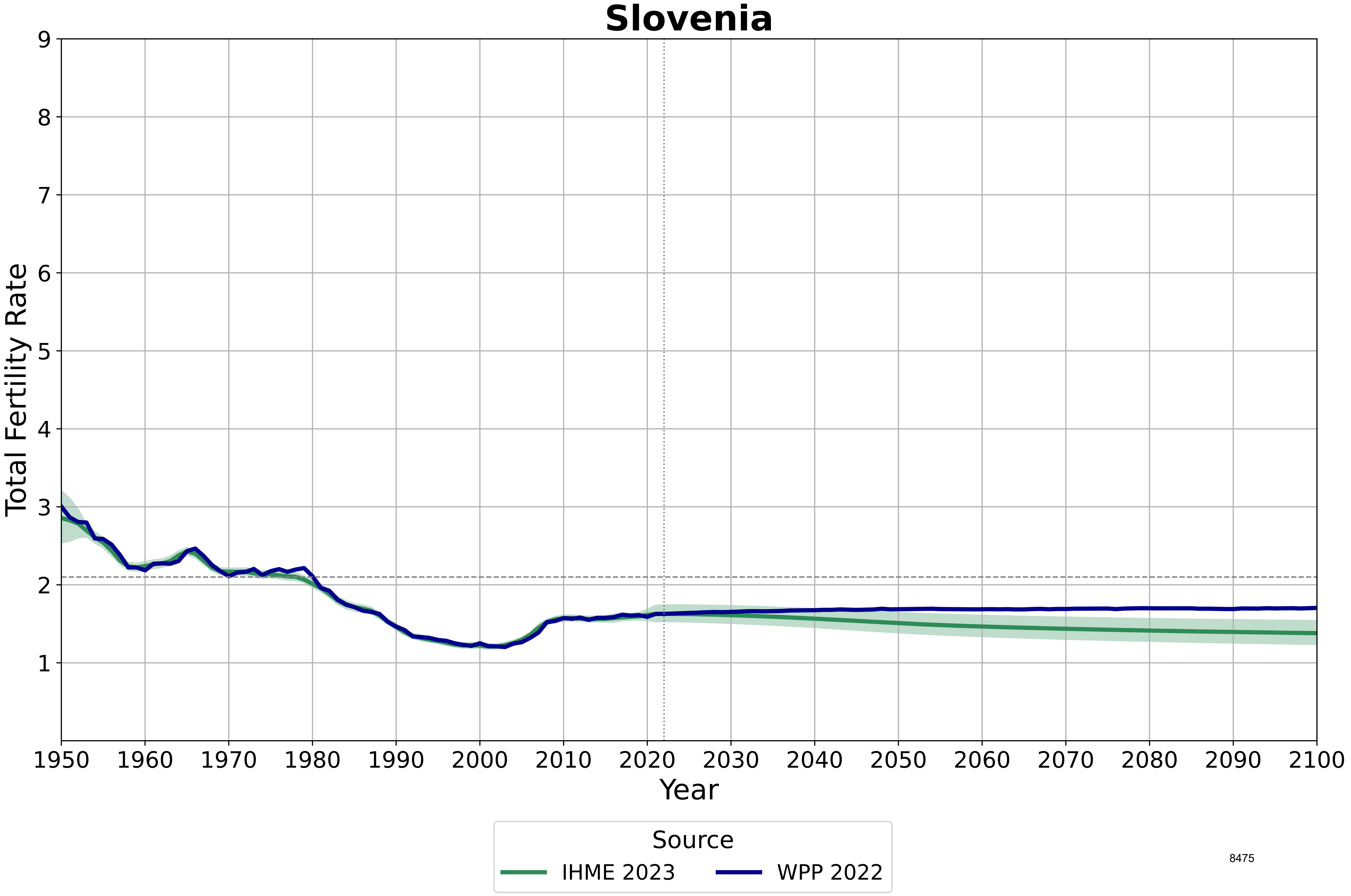

Supplemental Results Figure S7: TFR for IHME reference scenario and the UNPD medium variant scenario by location, 1950– 2100

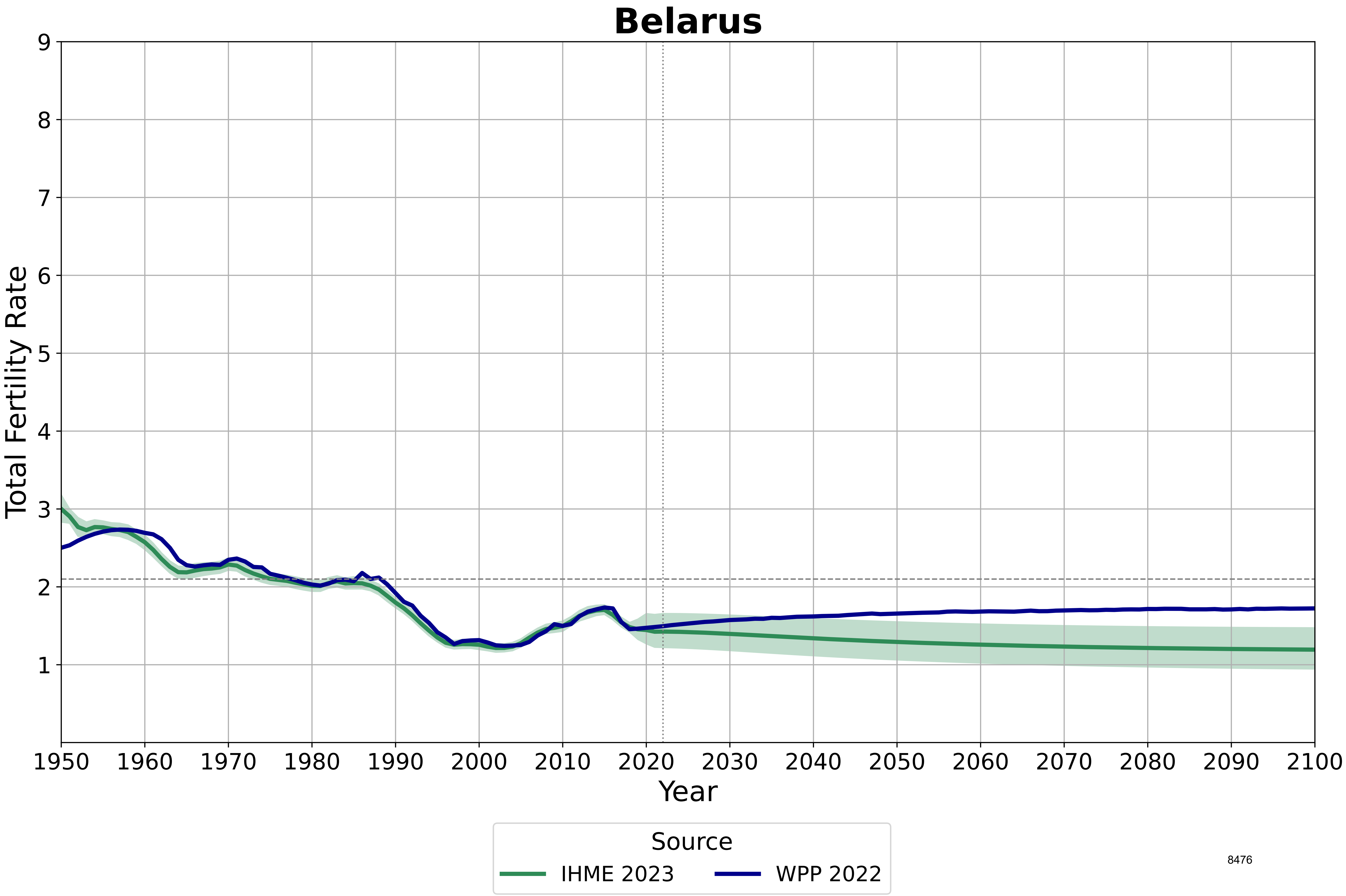

Supplemental Results Figure S7: TFR for IHME reference scenario and the UNPD medium variant scenario by location, 1950– 2100

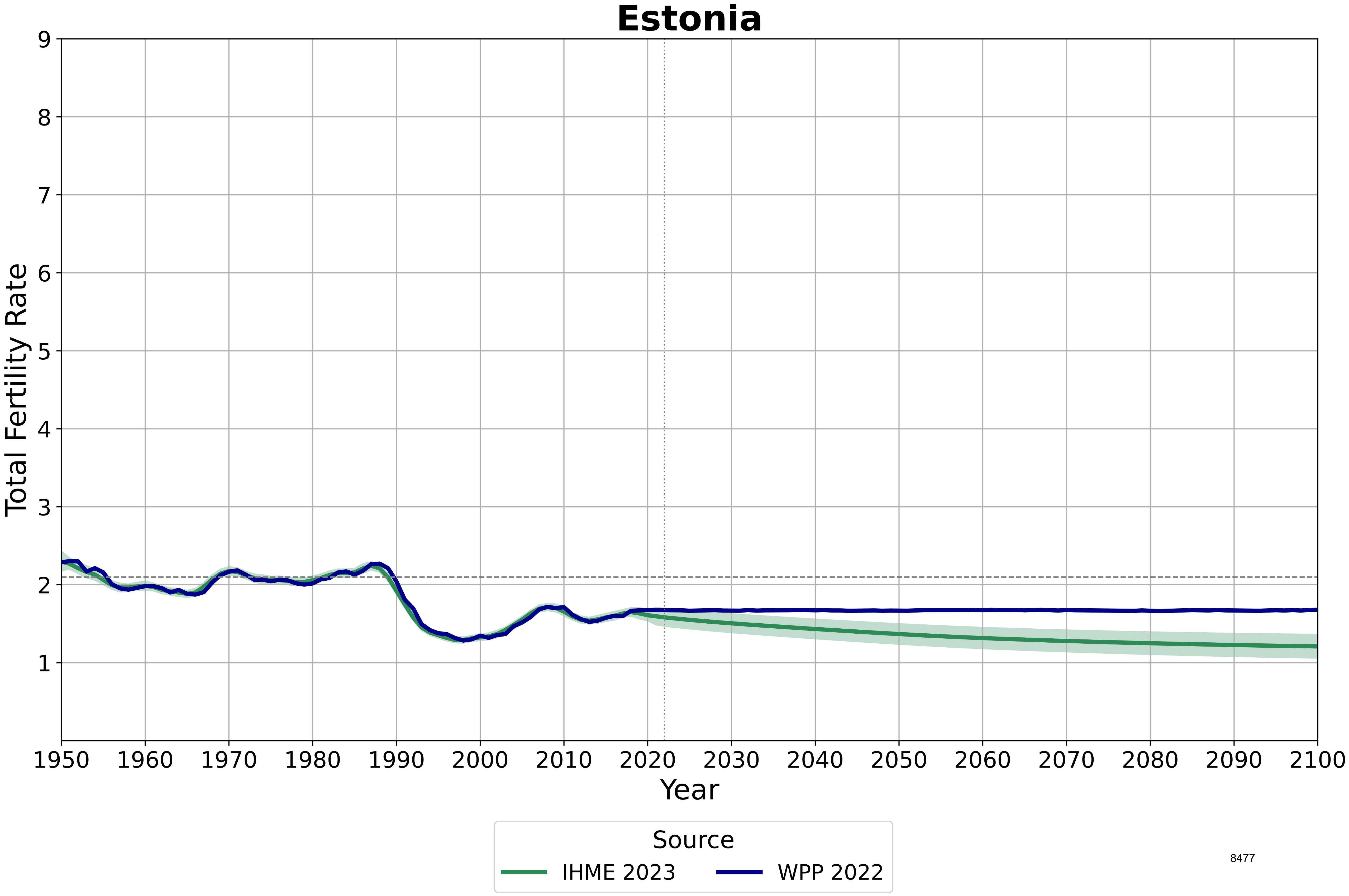

Supplemental Results Figure S7: TFR for IHME reference scenario and the UNPD medium variant scenario by location, 1950– 2100

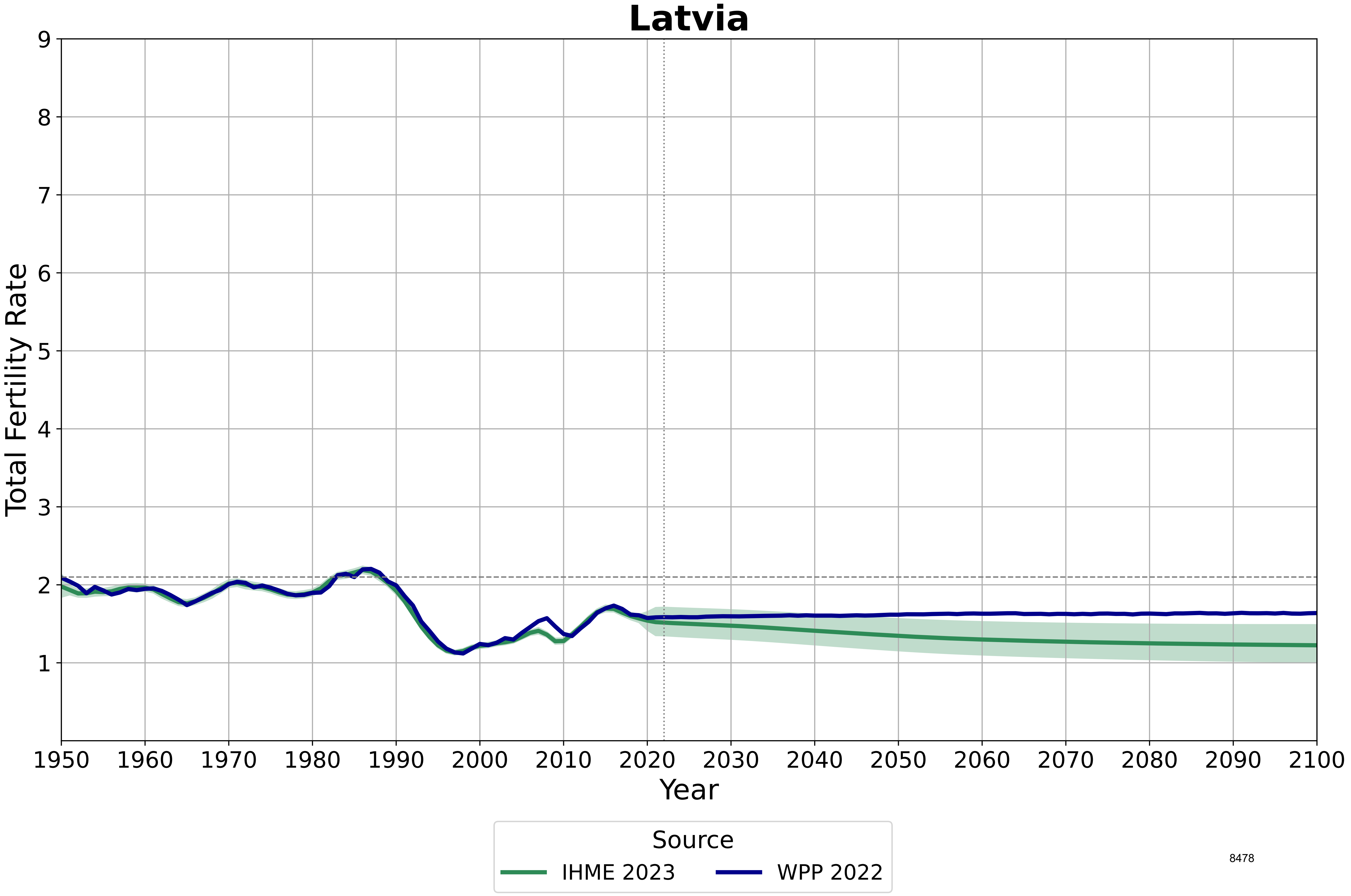

Supplemental Results Figure S7: TFR for IHME reference scenario and the UNPD medium variant scenario by location, 1950– 2100

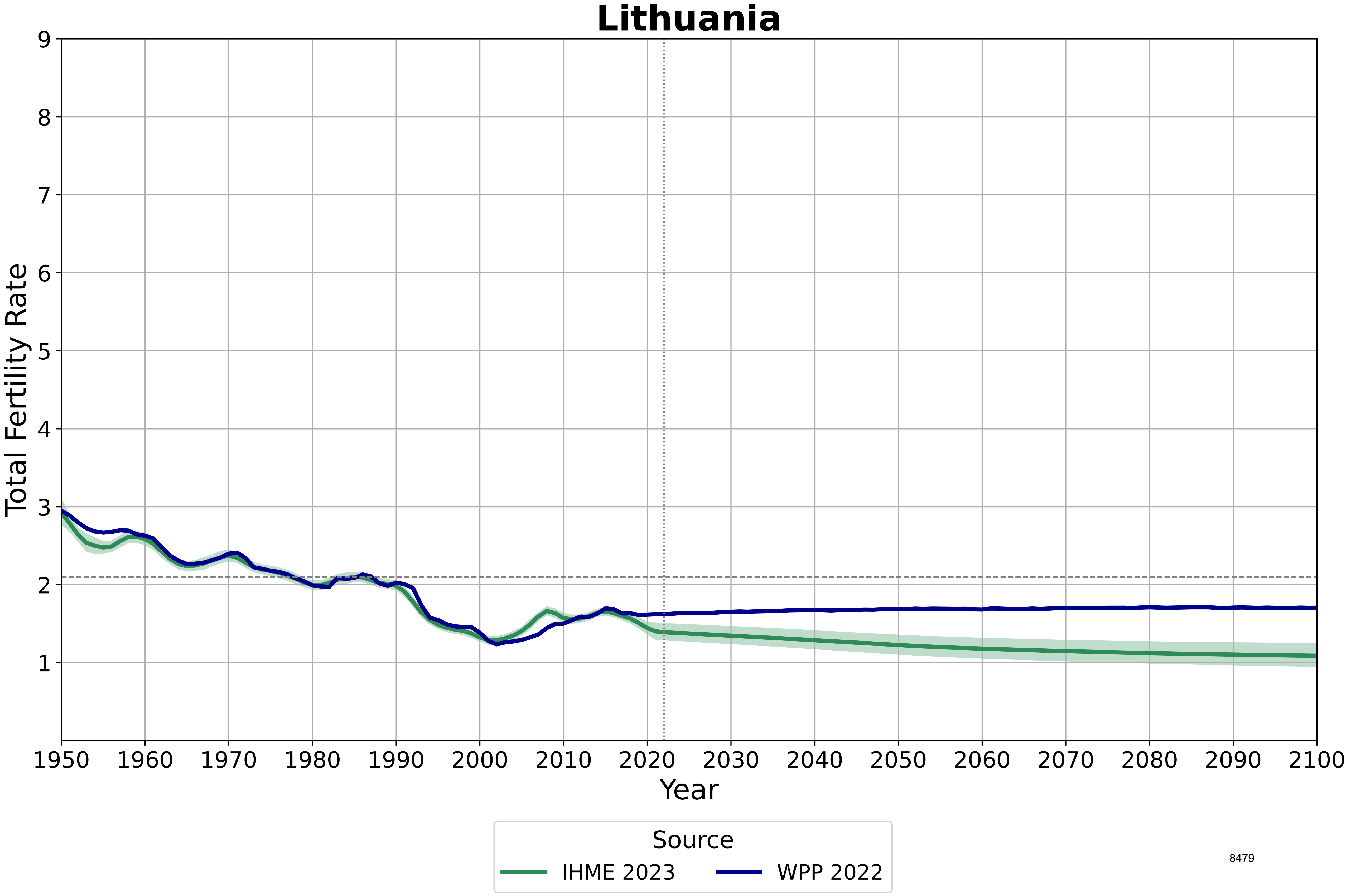

Supplemental Results Figure S7: TFR for IHME reference scenario and the UNPD medium variant scenario by location, 1950– 2100

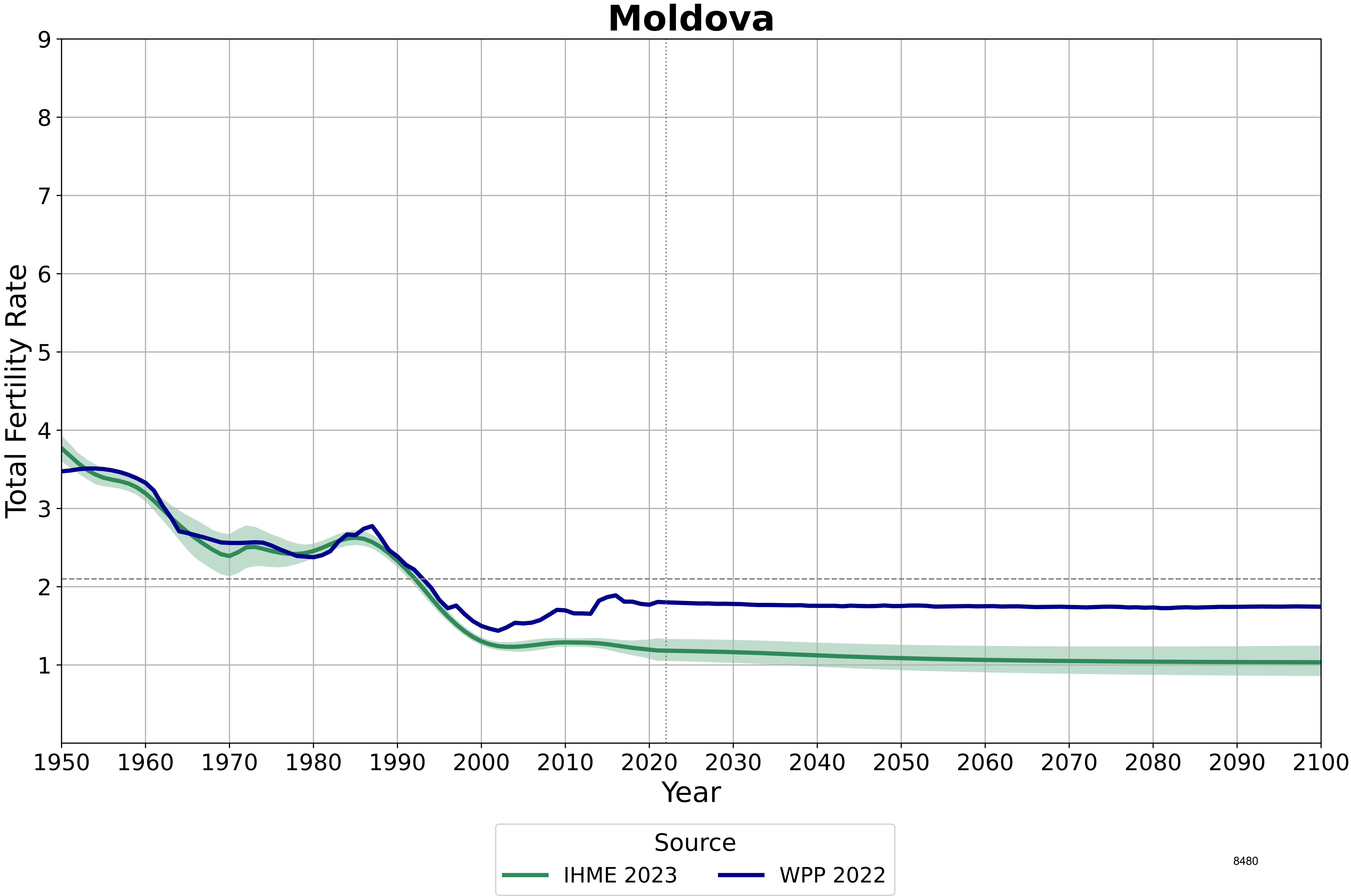

Supplemental Results Figure S7: TFR for IHME reference scenario and the UNPD medium variant scenario by location, 1950– 2100

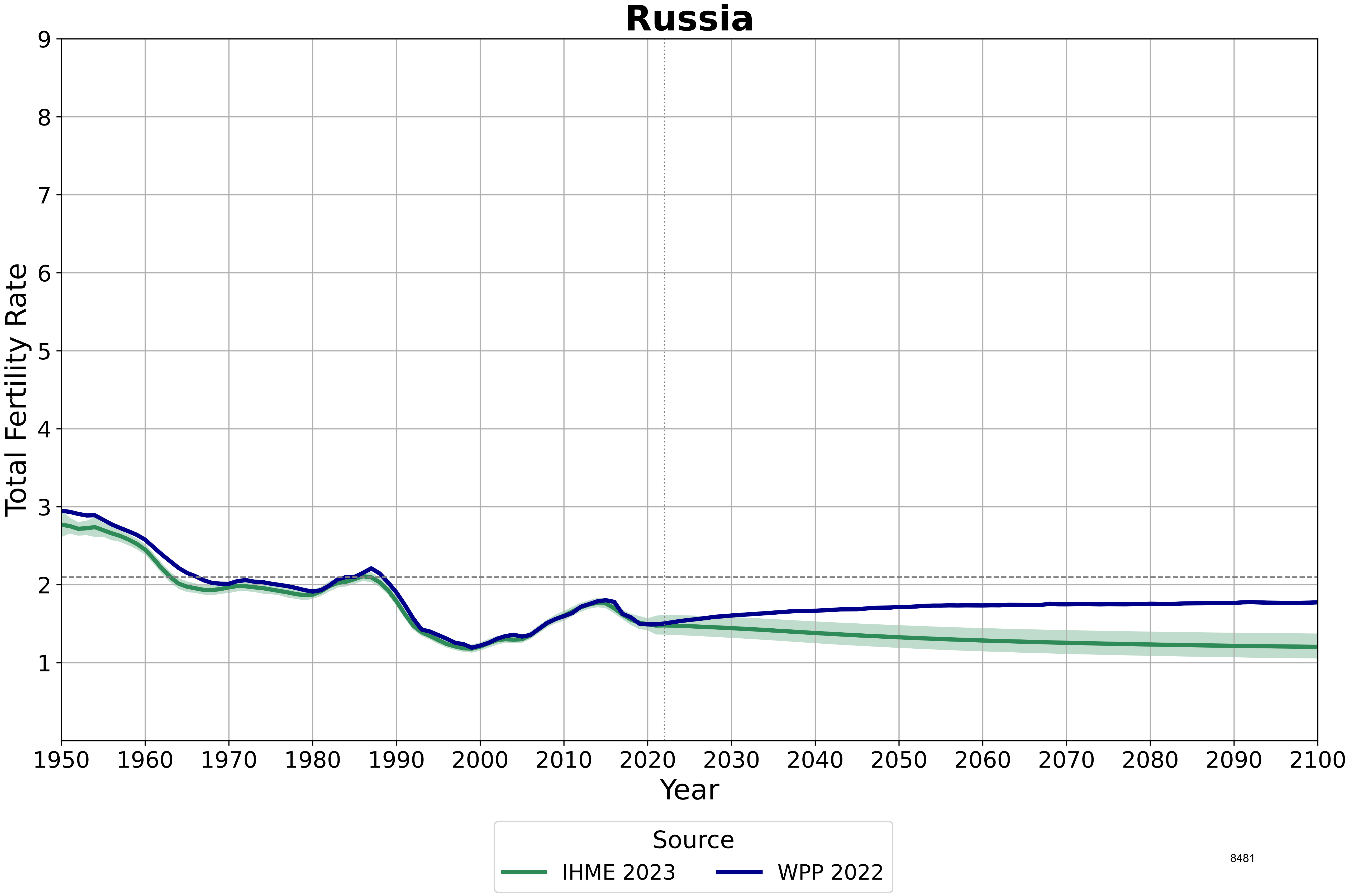

Supplemental Results Figure S7: TFR for IHME reference scenario and the UNPD medium variant scenario by location, 1950– 2100

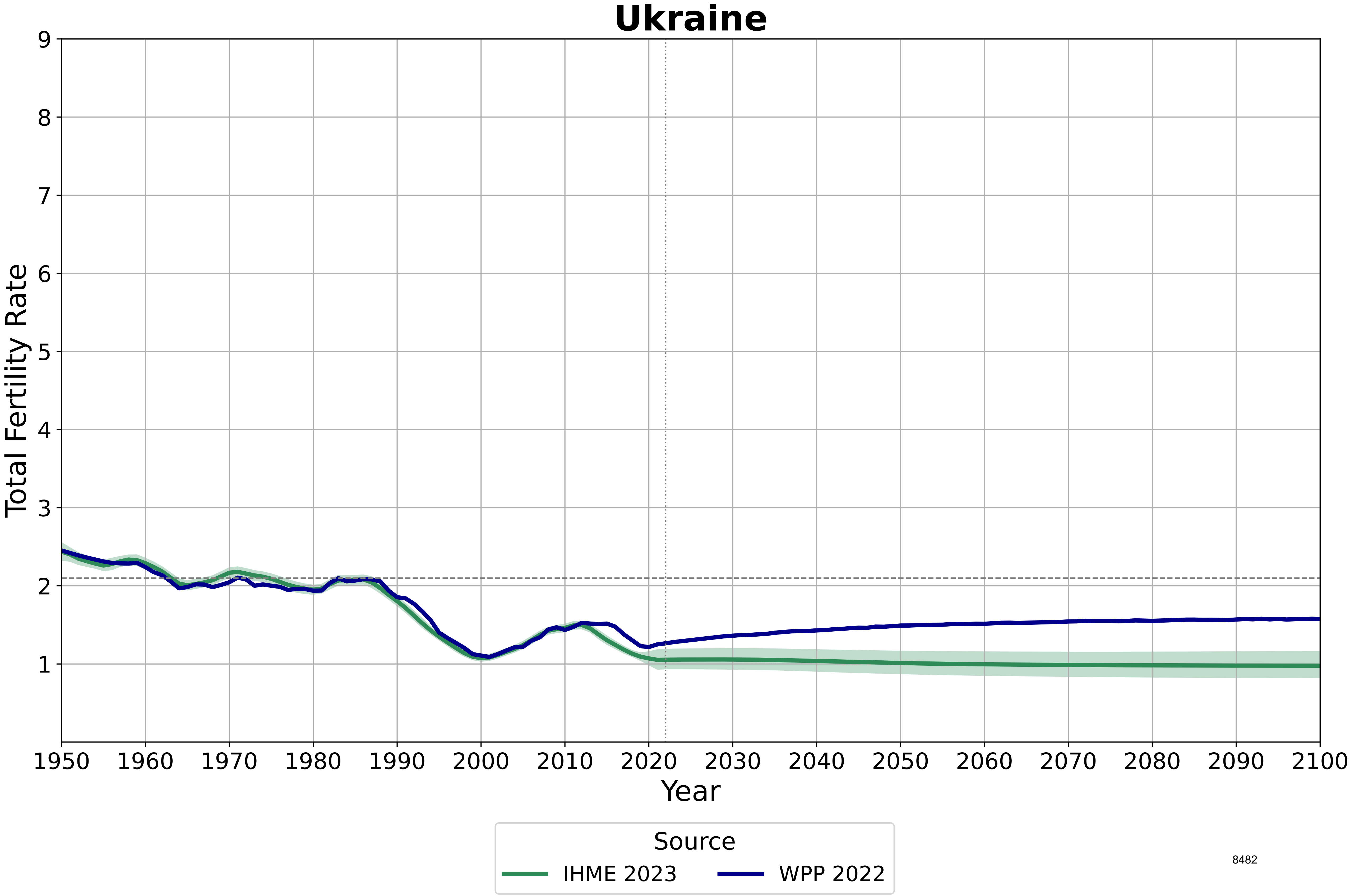

Supplemental Results Figure S7: TFR for IHME reference scenario and the UNPD medium variant scenario by location, 1950– 2100

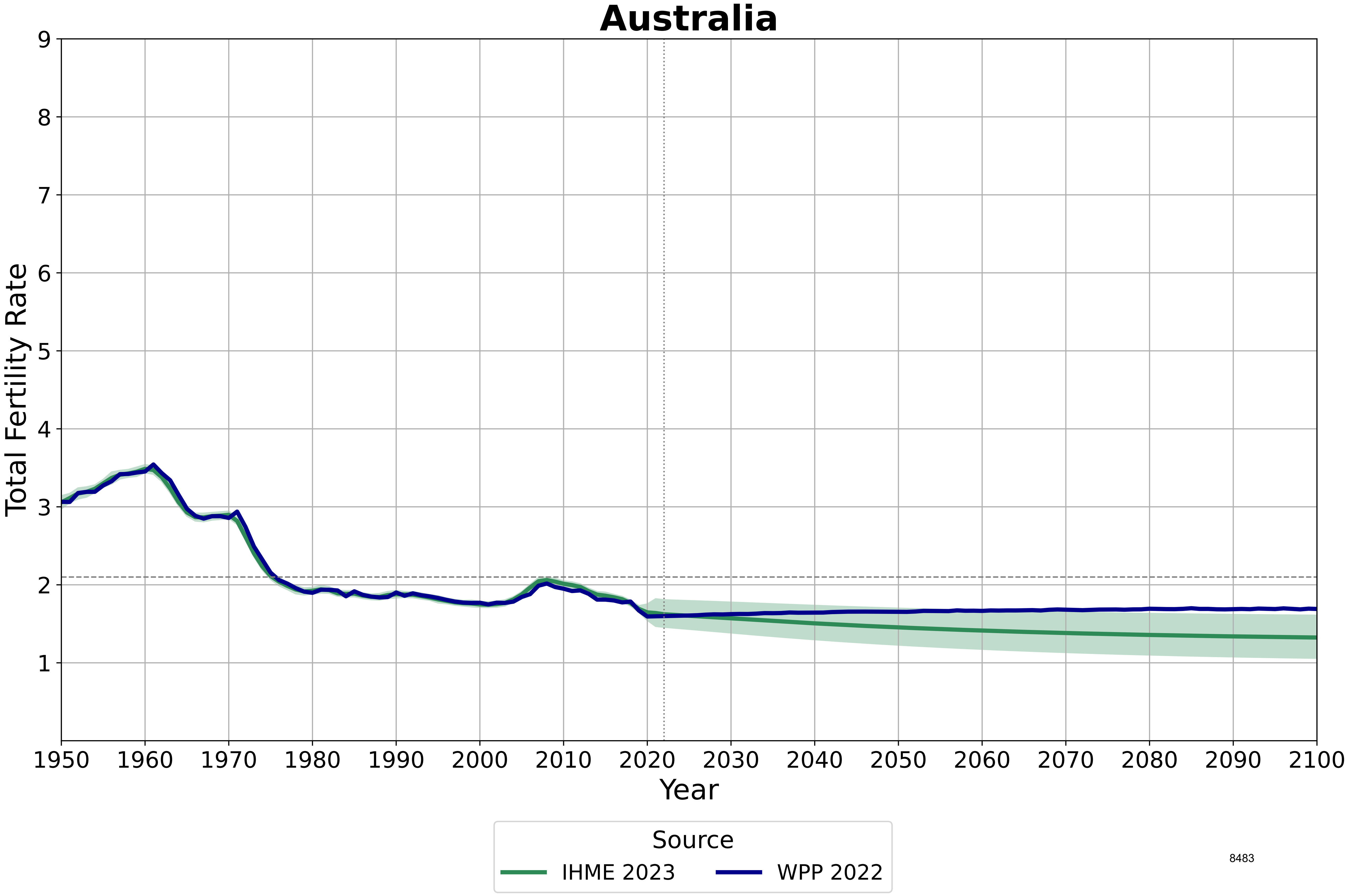

Supplemental Results Figure S7: TFR for IHME reference scenario and the UNPD medium variant scenario by location, 1950– 2100

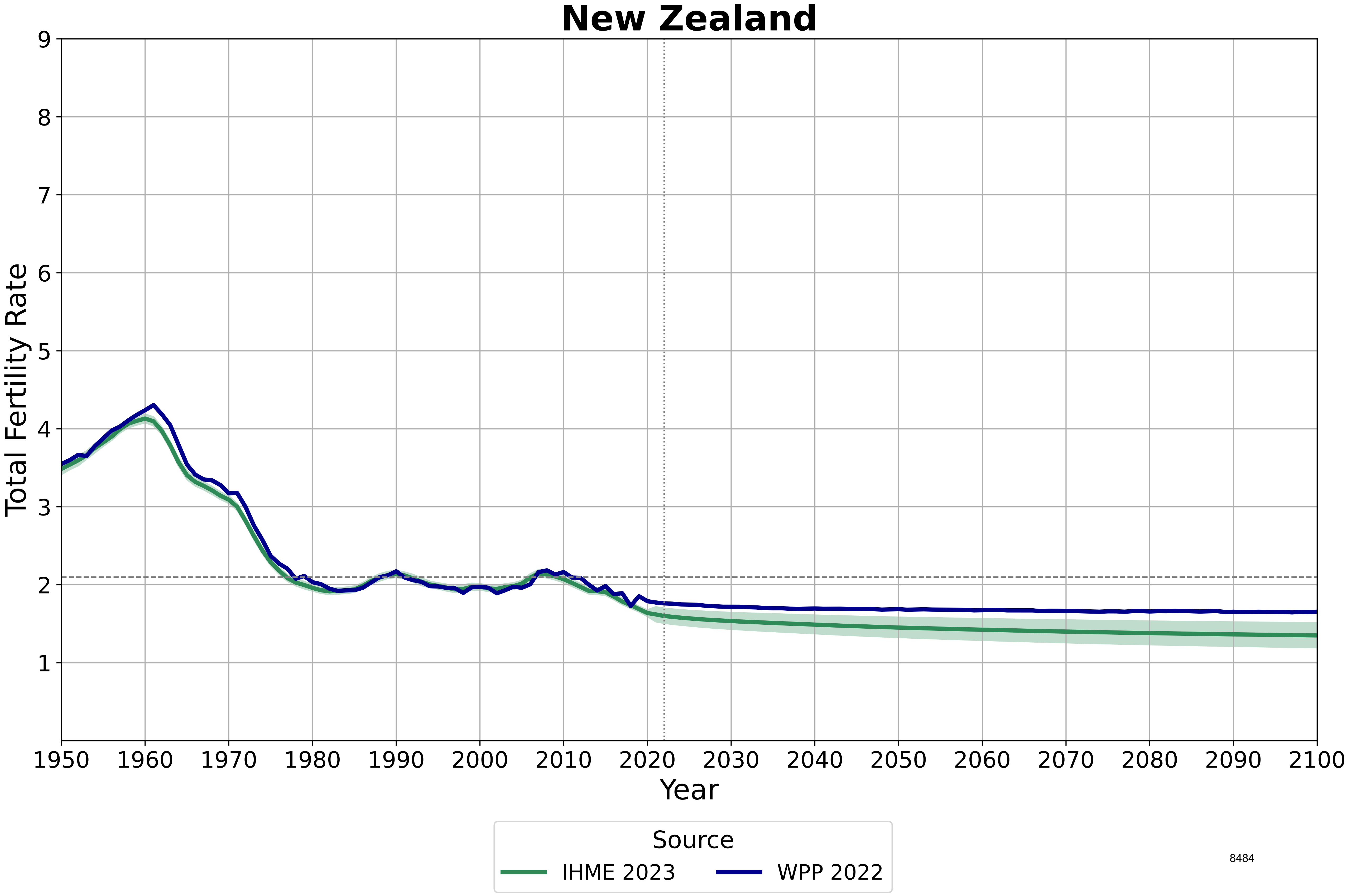

Supplemental Results Figure S7: TFR for IHME reference scenario and the UNPD medium variant scenario by location, 1950– 2100

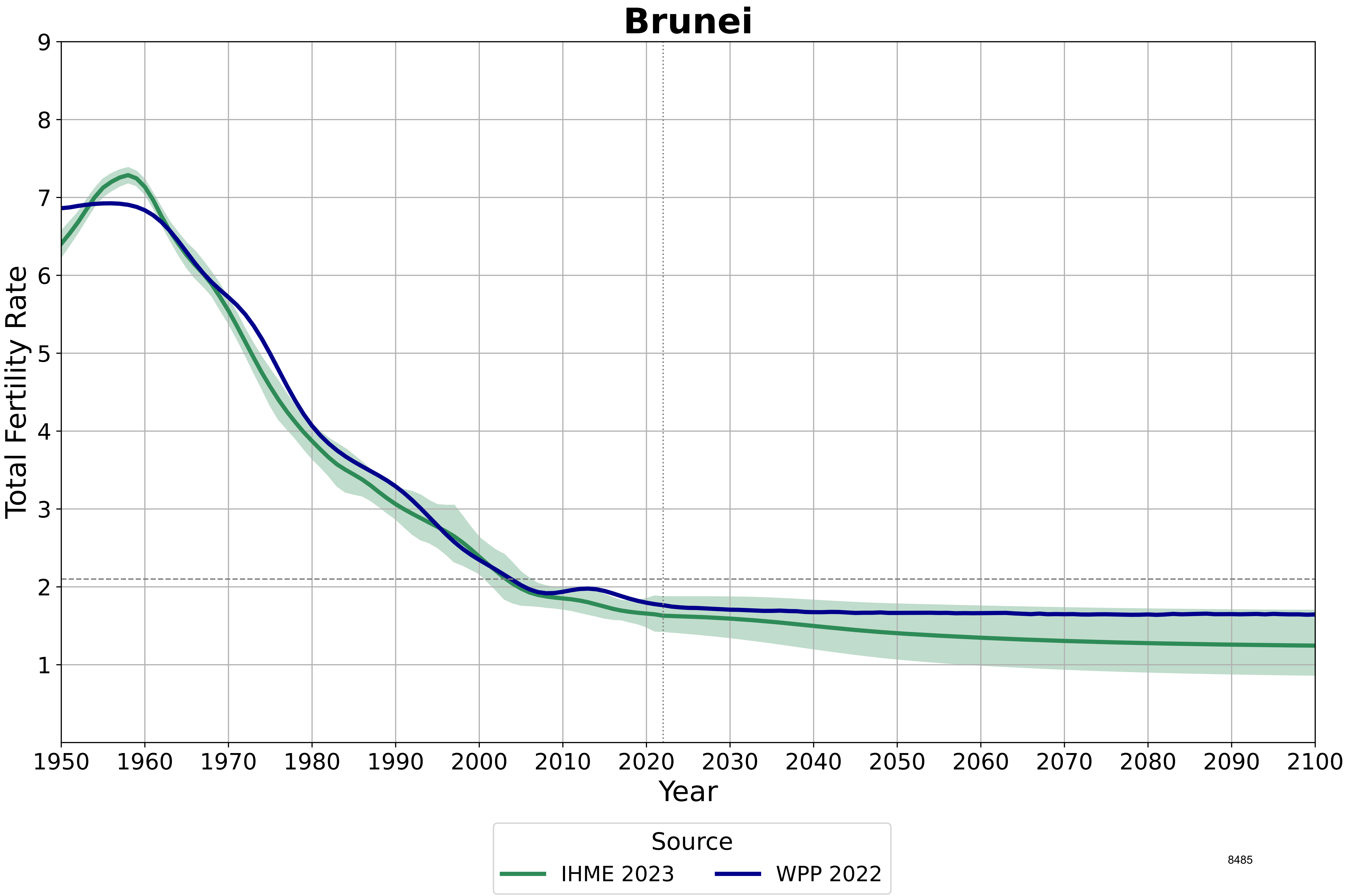

Supplemental Results Figure S7: TFR for IHME reference scenario and the UNPD medium variant scenario by location, 1950– 2100

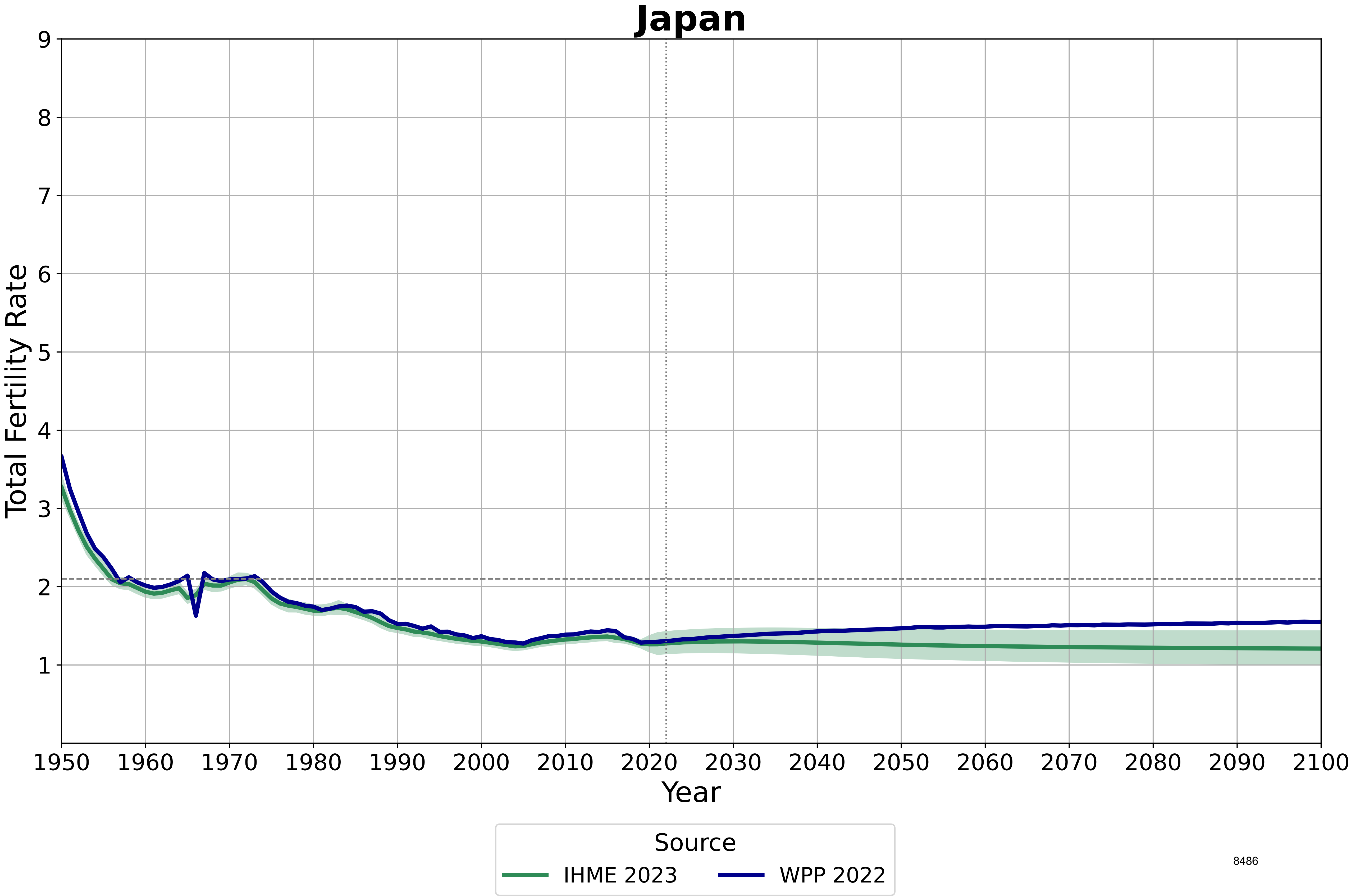

Supplemental Results Figure S7: TFR for IHME reference scenario and the UNPD medium variant scenario by location, 1950– 2100

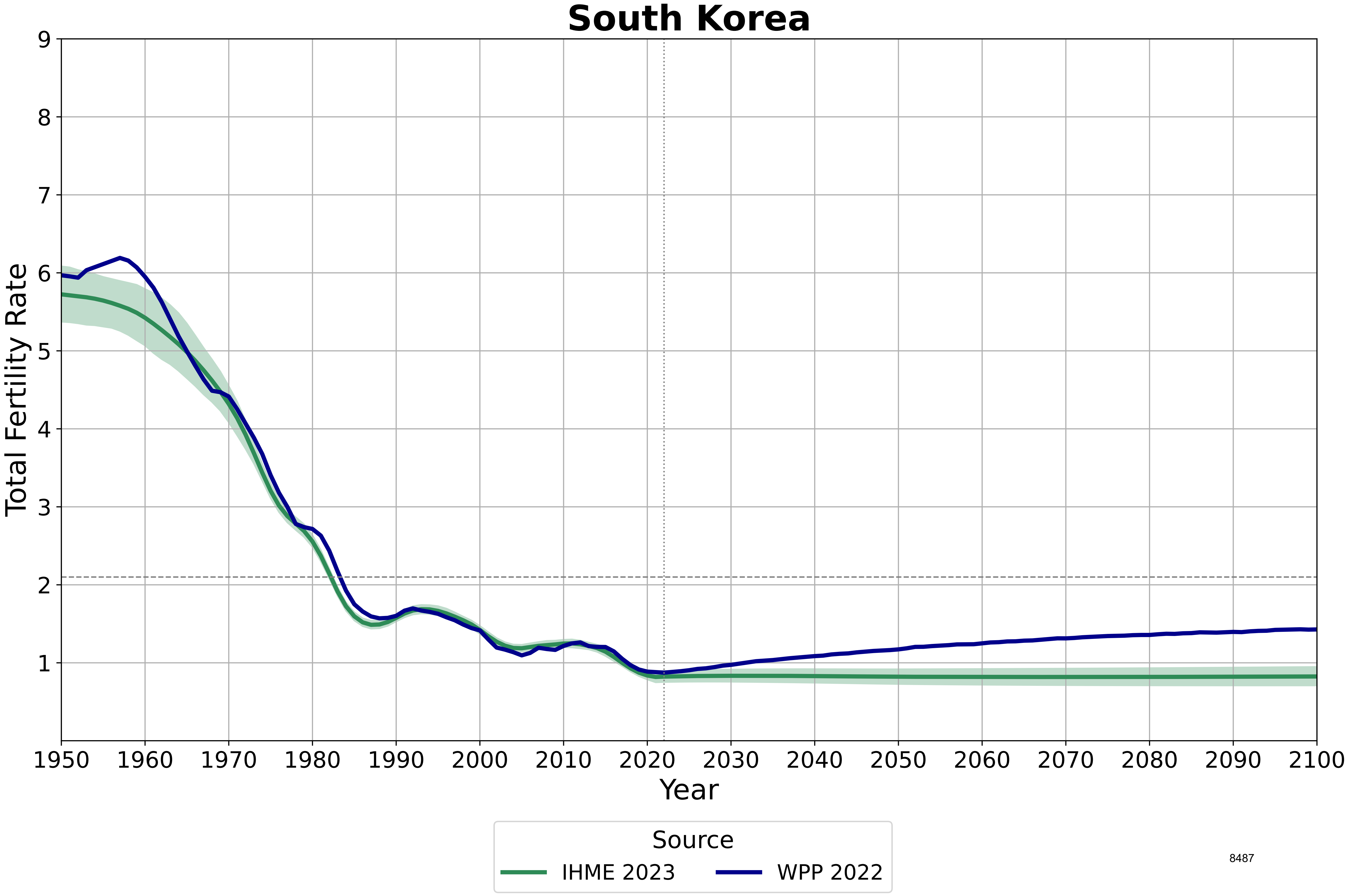

Supplemental Results Figure S7: TFR for IHME reference scenario and the UNPD medium variant scenario by location, 1950– 2100

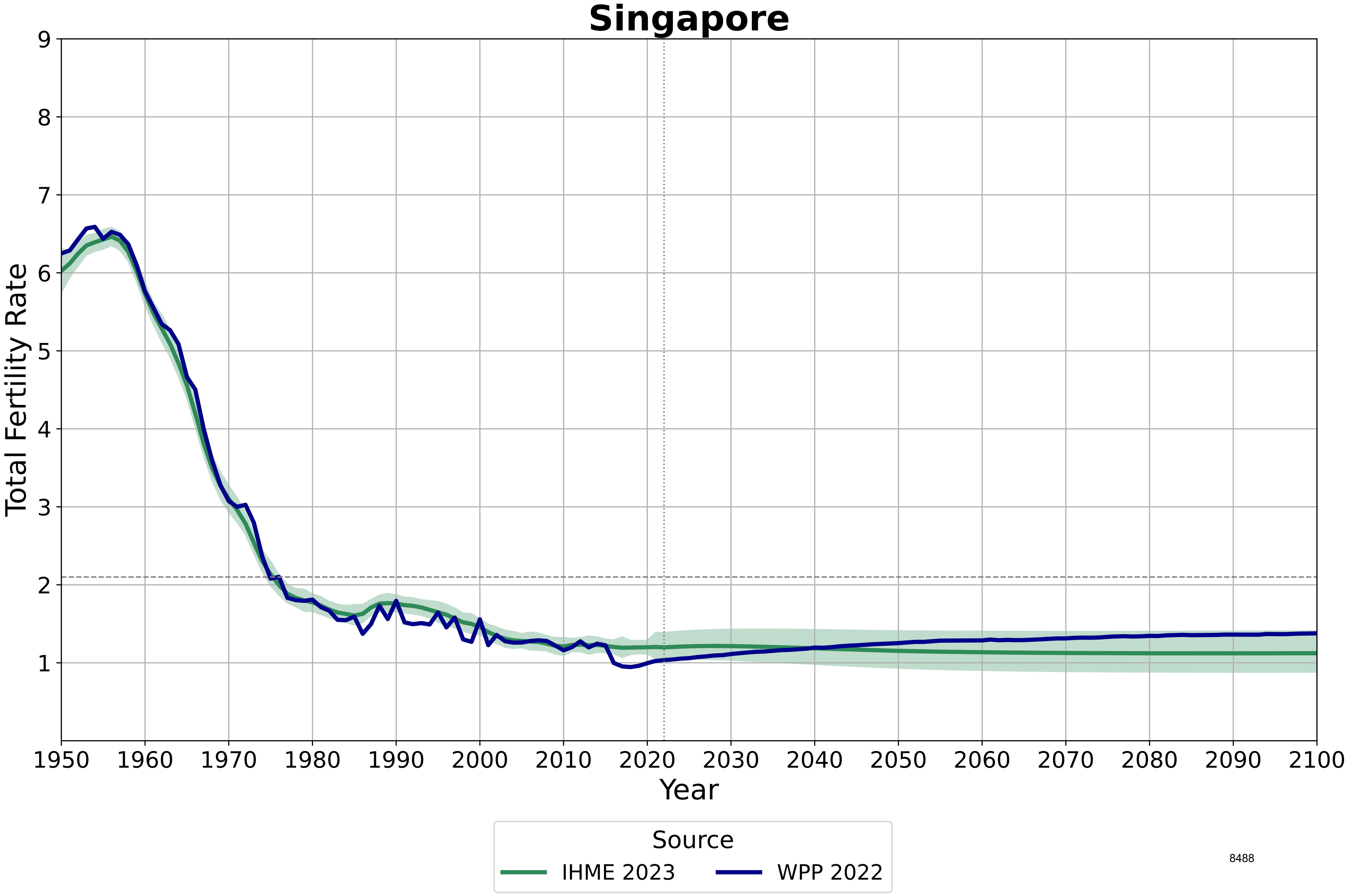

Supplemental Results Figure S7: TFR for IHME reference scenario and the UNPD medium variant scenario by location, 1950– 2100

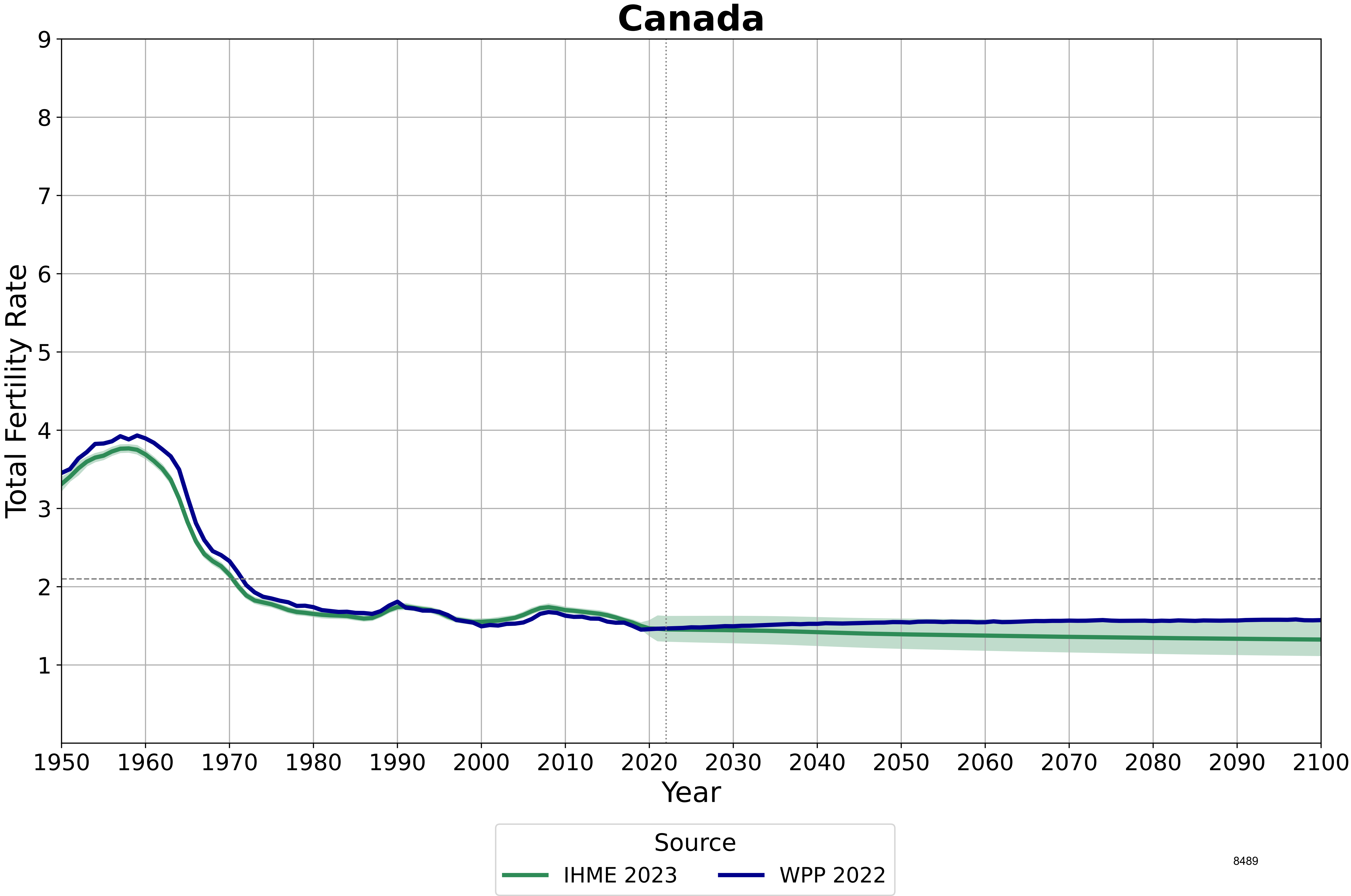

Supplemental Results Figure S7: TFR for IHME reference scenario and the UNPD medium variant scenario by location, 1950– 2100

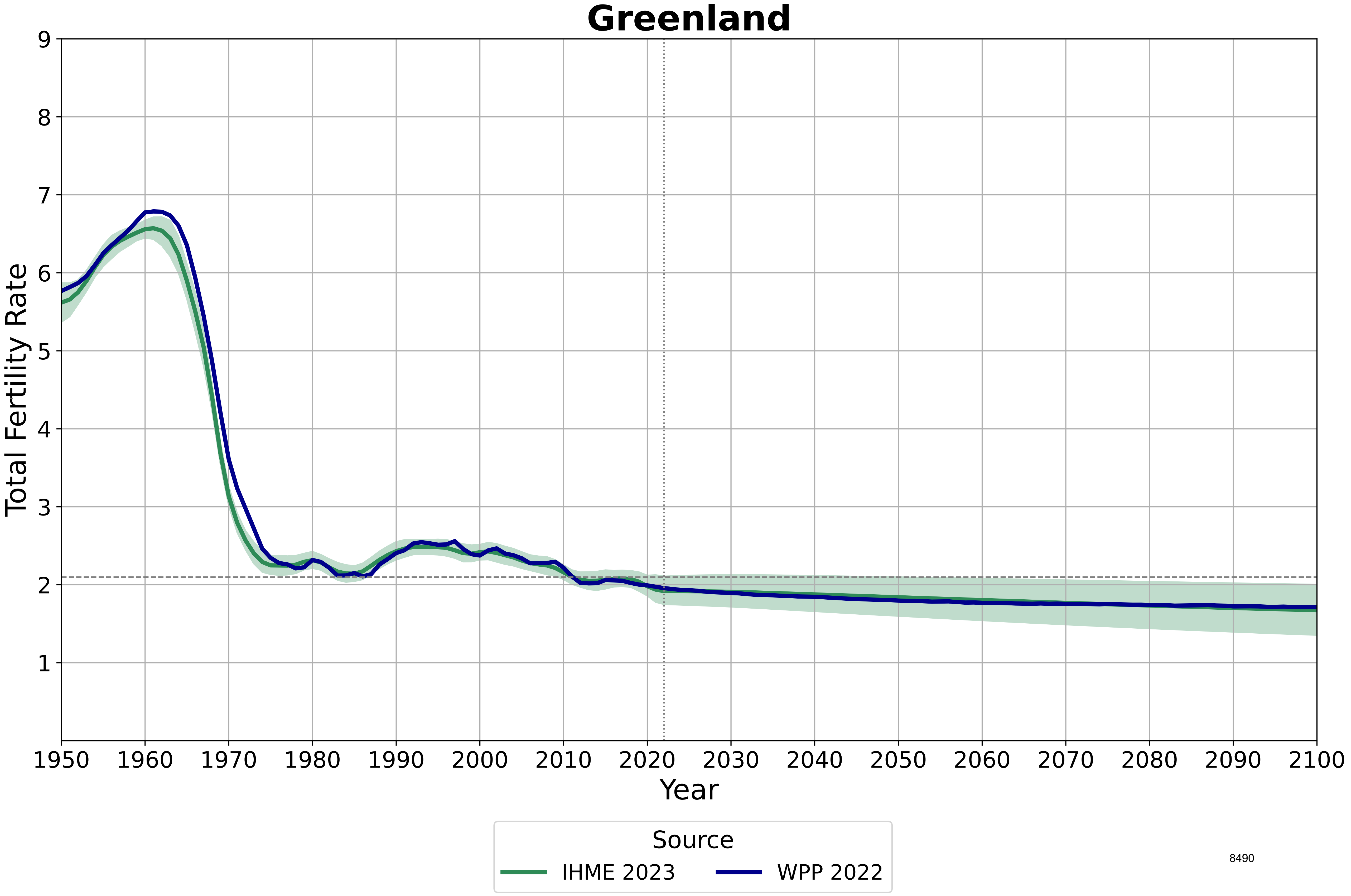

Supplemental Results Figure S7: TFR for IHME reference scenario and the UNPD medium variant scenario by location, 1950– 2100

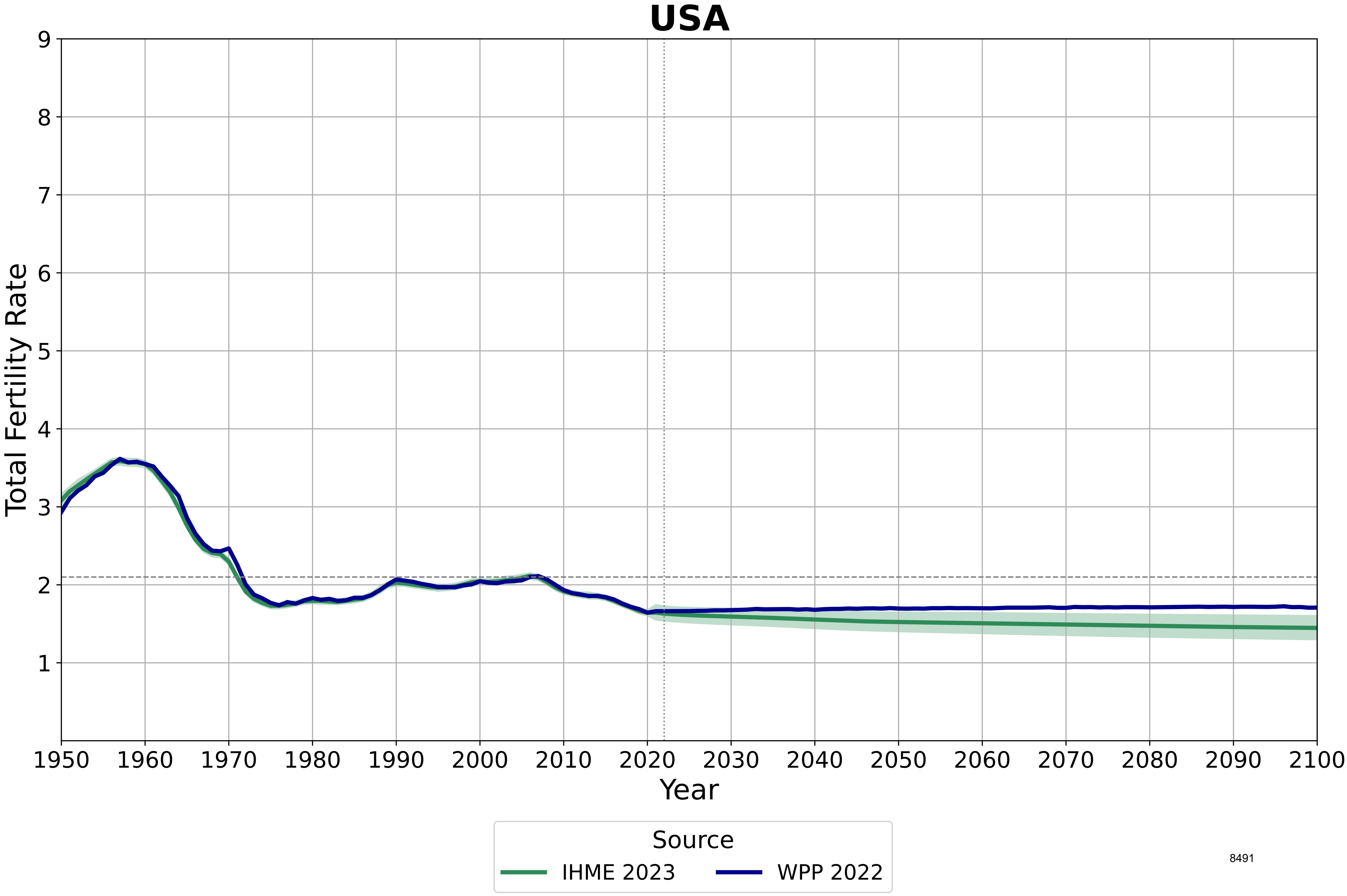

Supplemental Results Figure S7: TFR for IHME reference scenario and the UNPD medium variant scenario by location, 1950– 2100

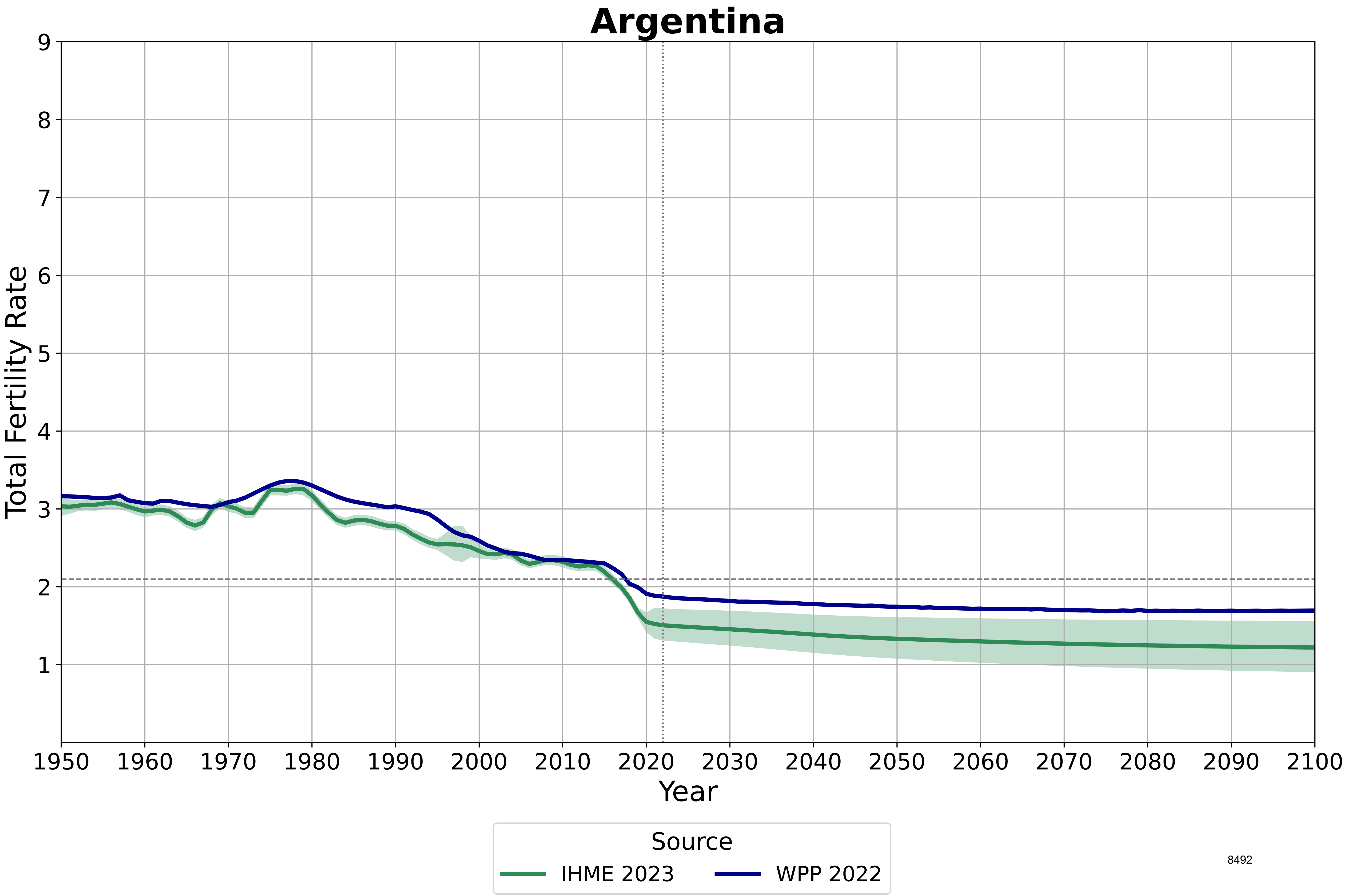

Supplemental Results Figure S7: TFR for IHME reference scenario and the UNPD medium variant scenario by location, 1950– 2100

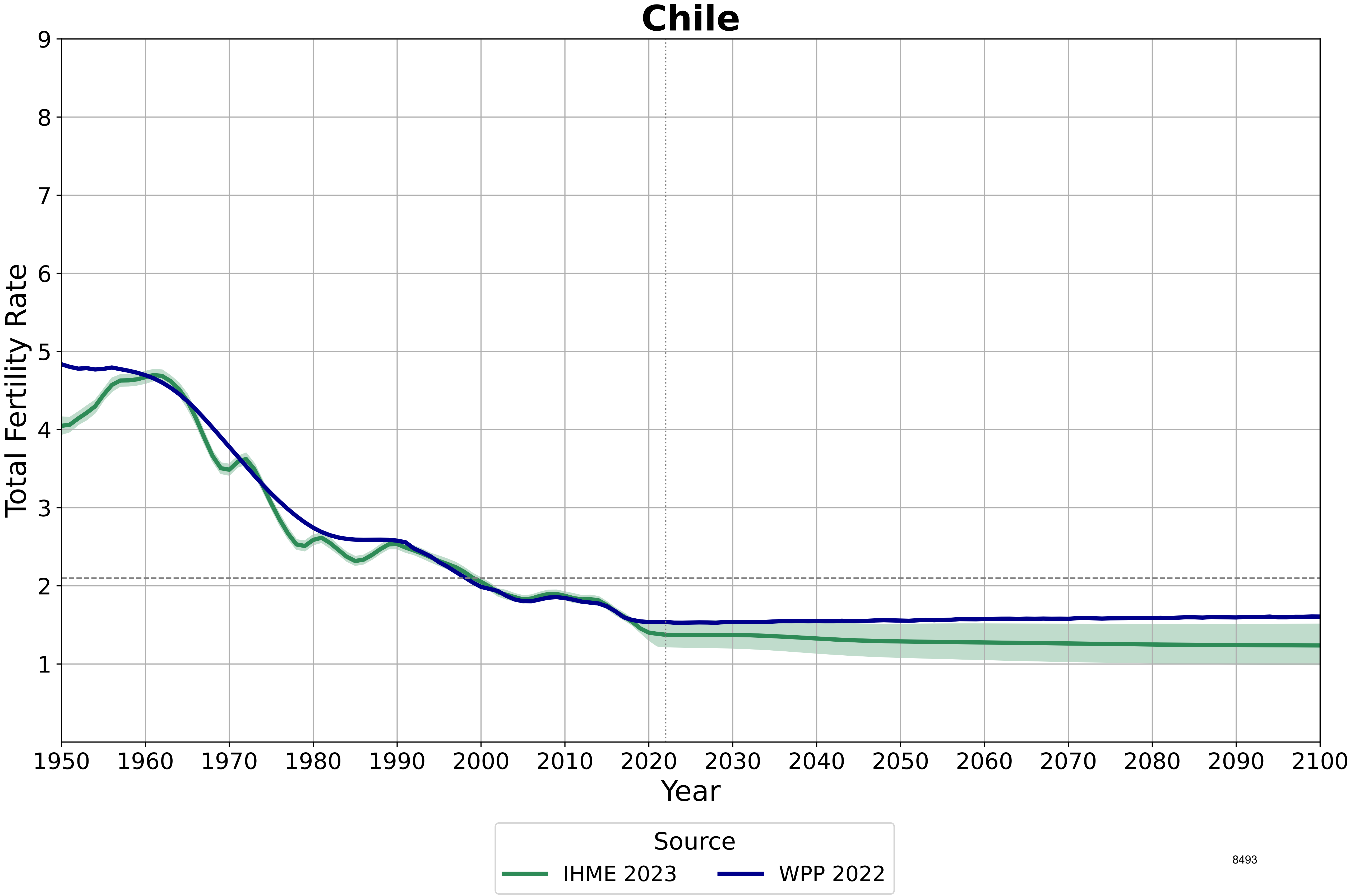

Supplemental Results Figure S7: TFR for IHME reference scenario and the UNPD medium variant scenario by location, 1950– 2100

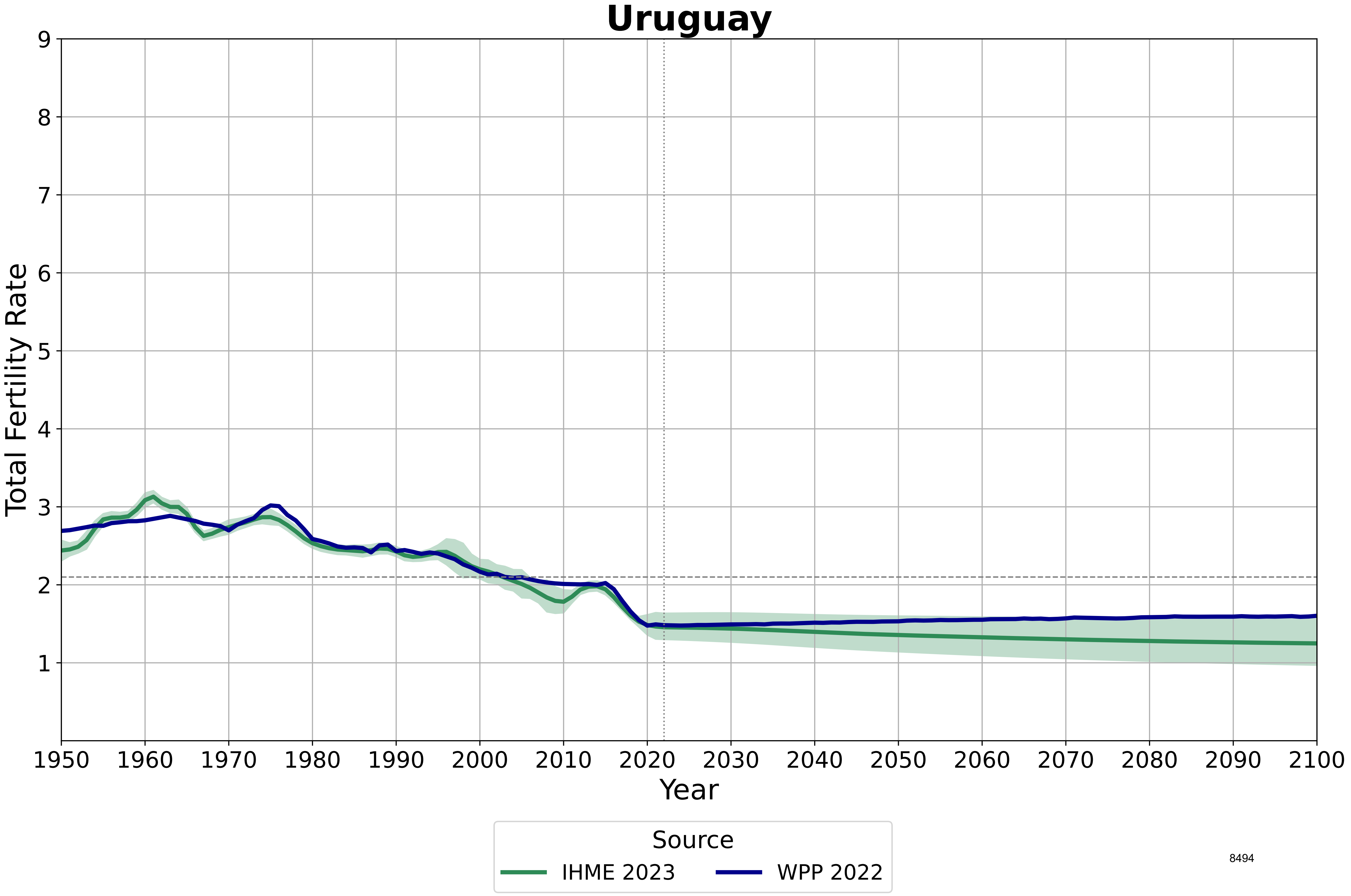

Supplemental Results Figure S7: TFR for IHME reference scenario and the UNPD medium variant scenario by location, 1950– 2100

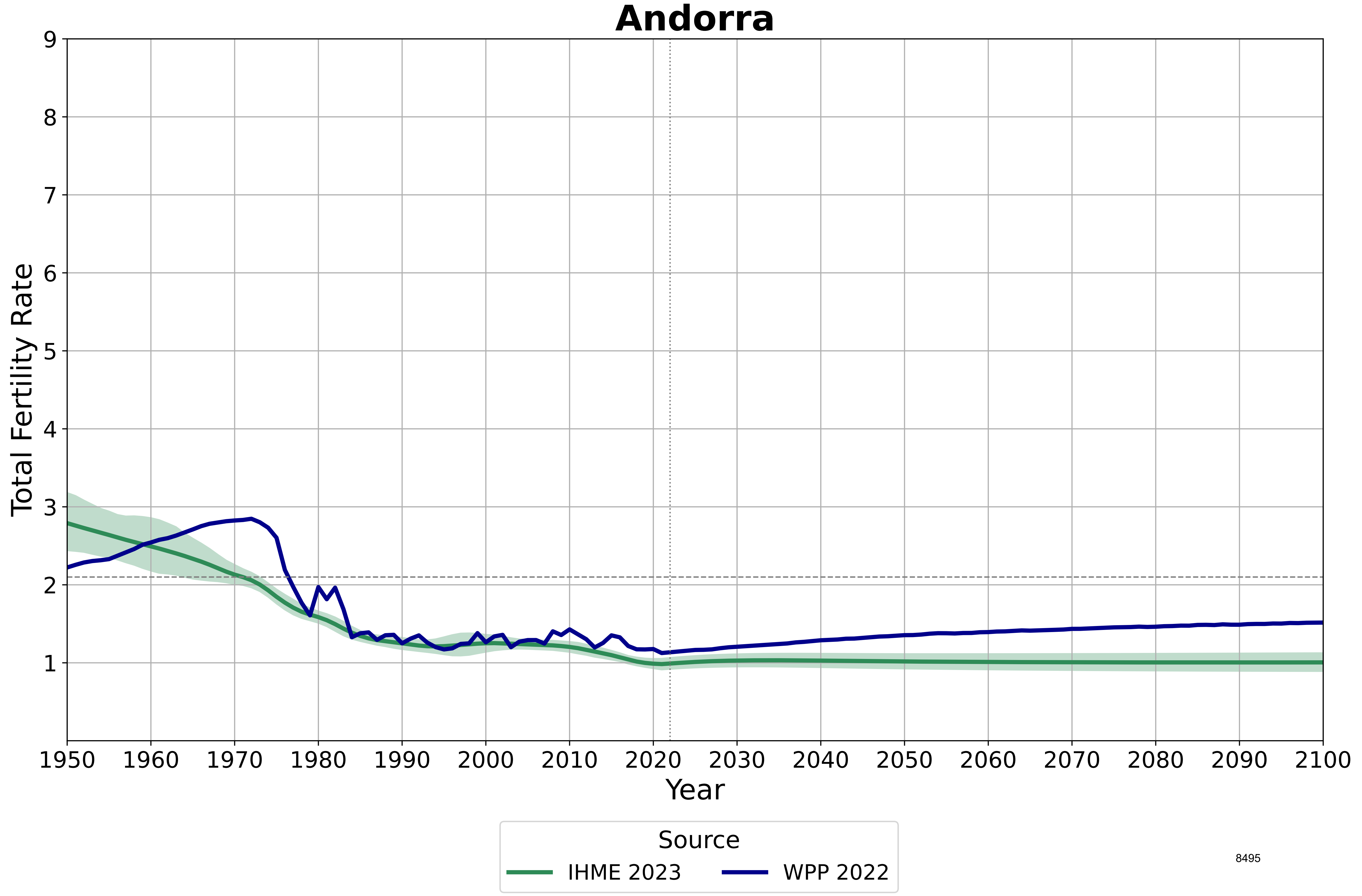

Supplemental Results Figure S7: TFR for IHME reference scenario and the UNPD medium variant scenario by location, 1950– 2100

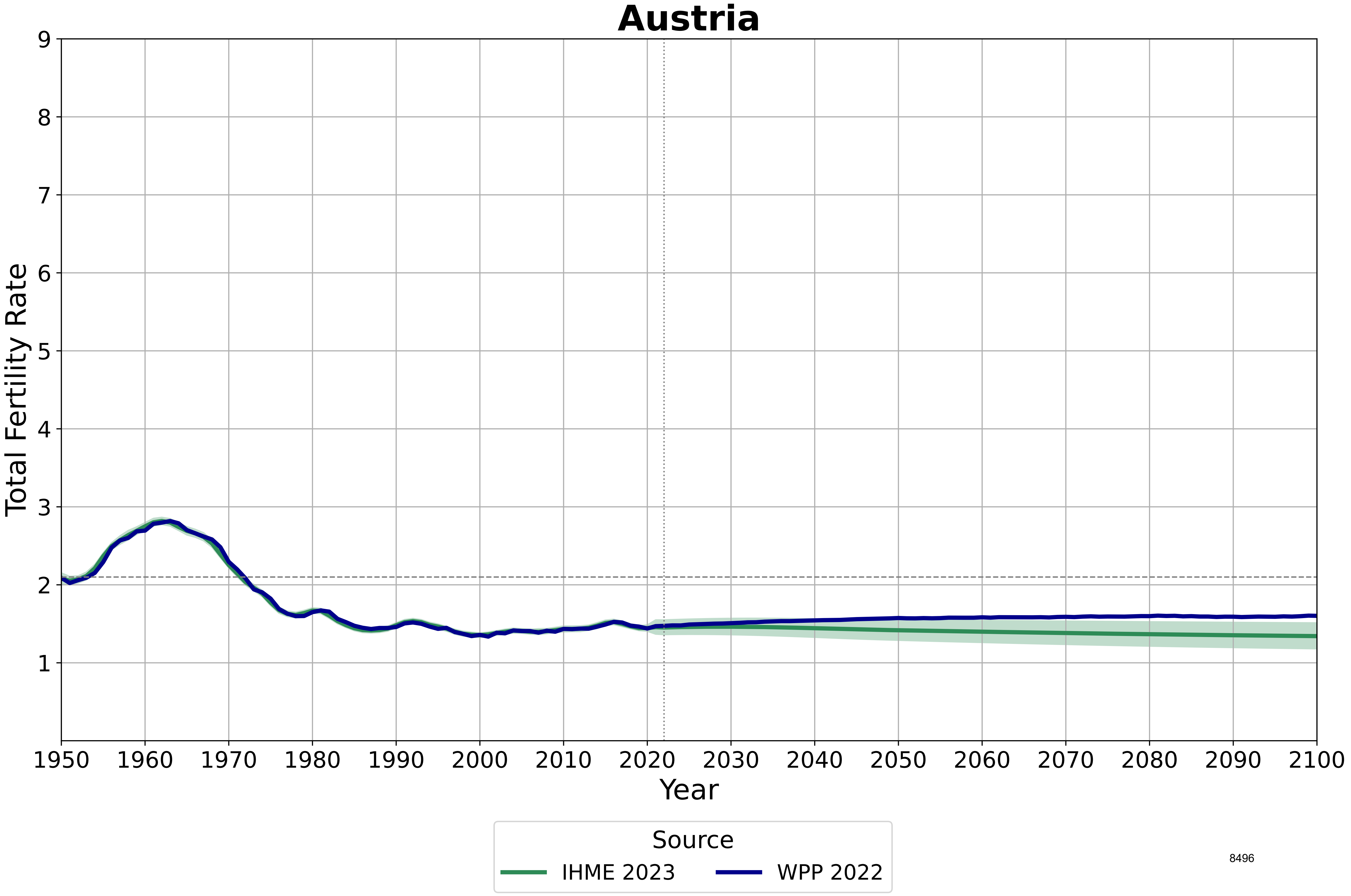

Supplemental Results Figure S7: TFR for IHME reference scenario and the UNPD medium variant scenario by location, 1950– 2100

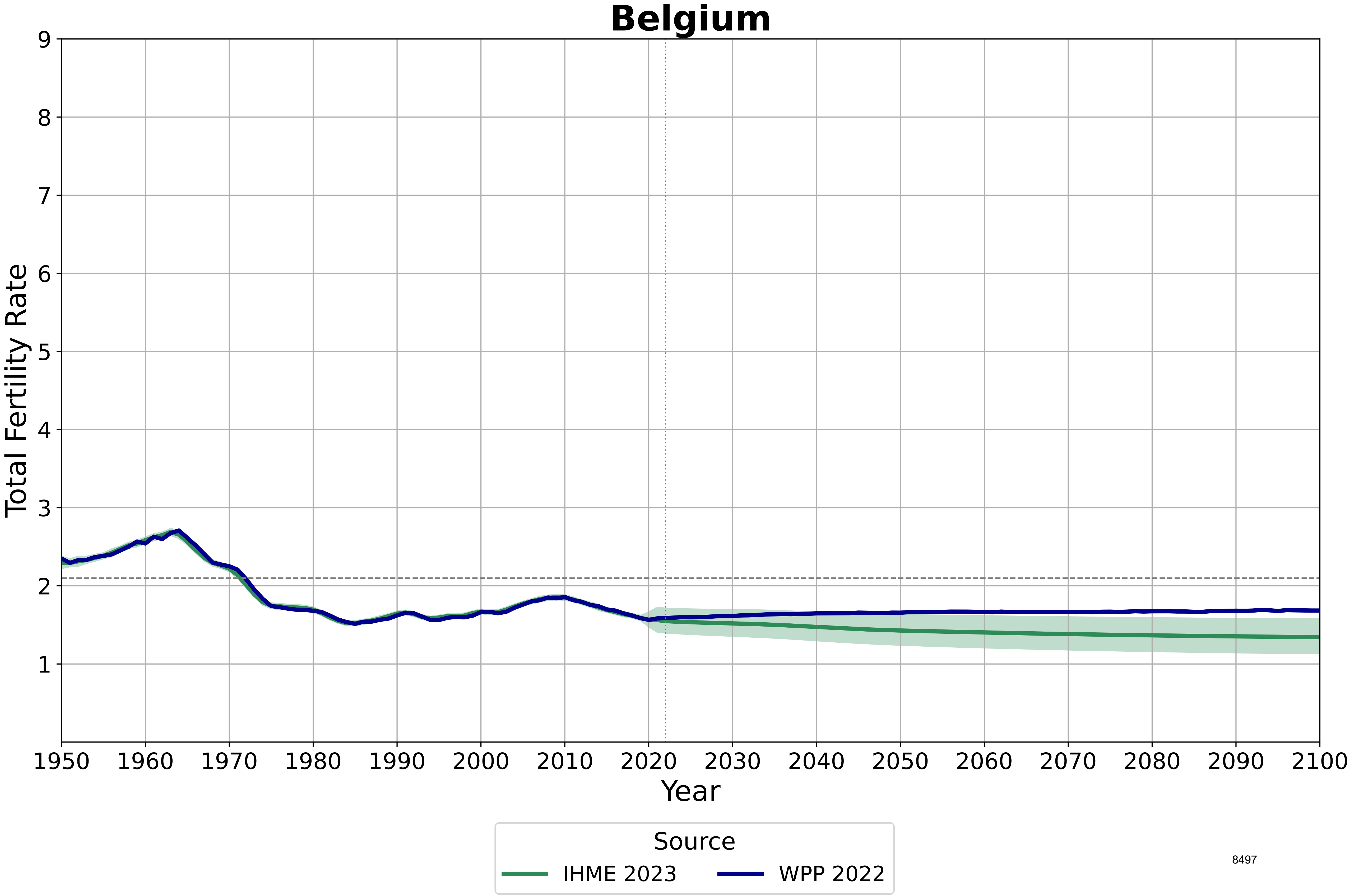

Supplemental Results Figure S7: TFR for IHME reference scenario and the UNPD medium variant scenario by location, 1950– 2100

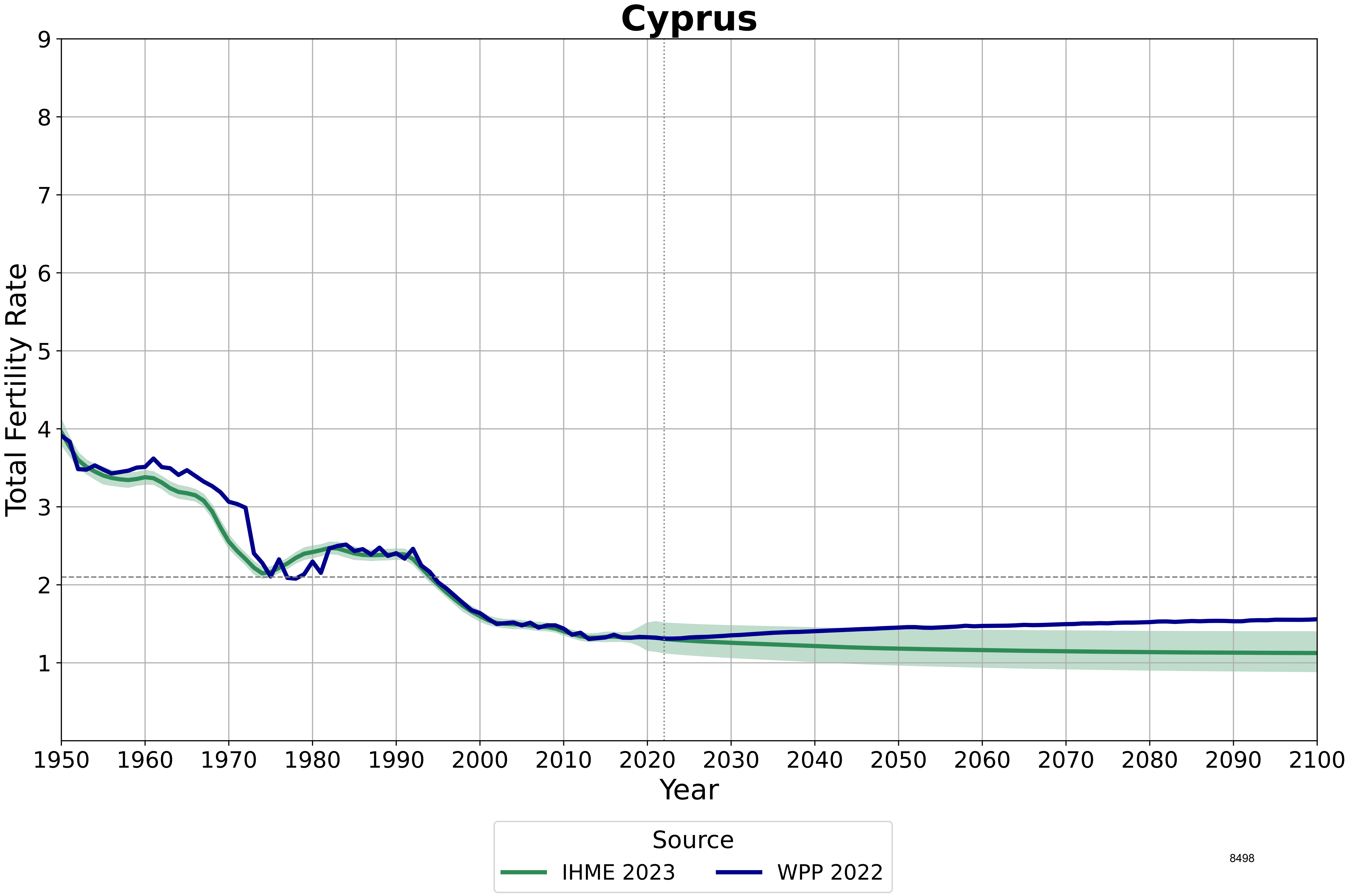

Supplemental Results Figure S7: TFR for IHME reference scenario and the UNPD medium variant scenario by location, 1950– 2100

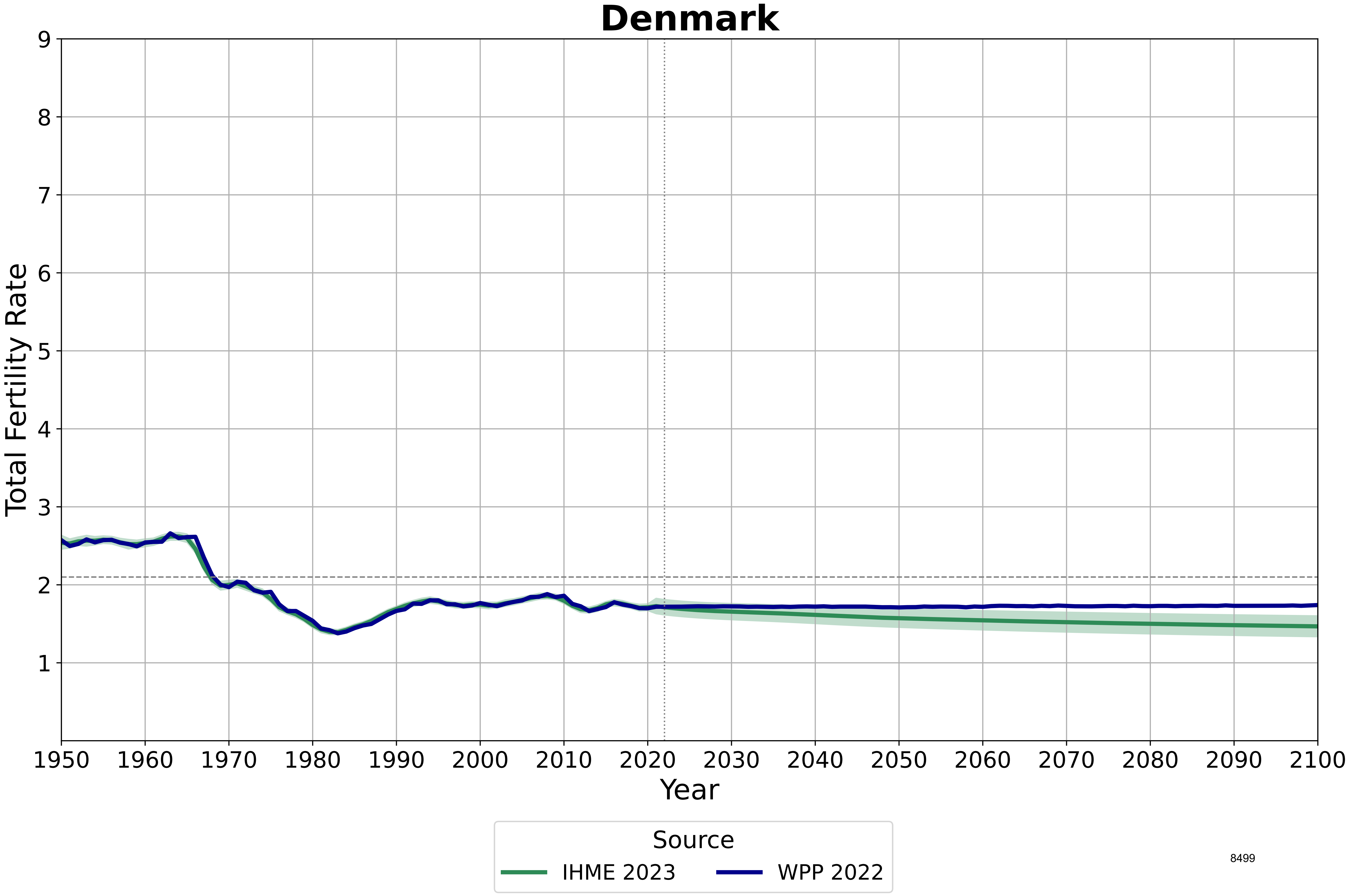

Supplemental Results Figure S7: TFR for IHME reference scenario and the UNPD medium variant scenario by location, 1950– 2100

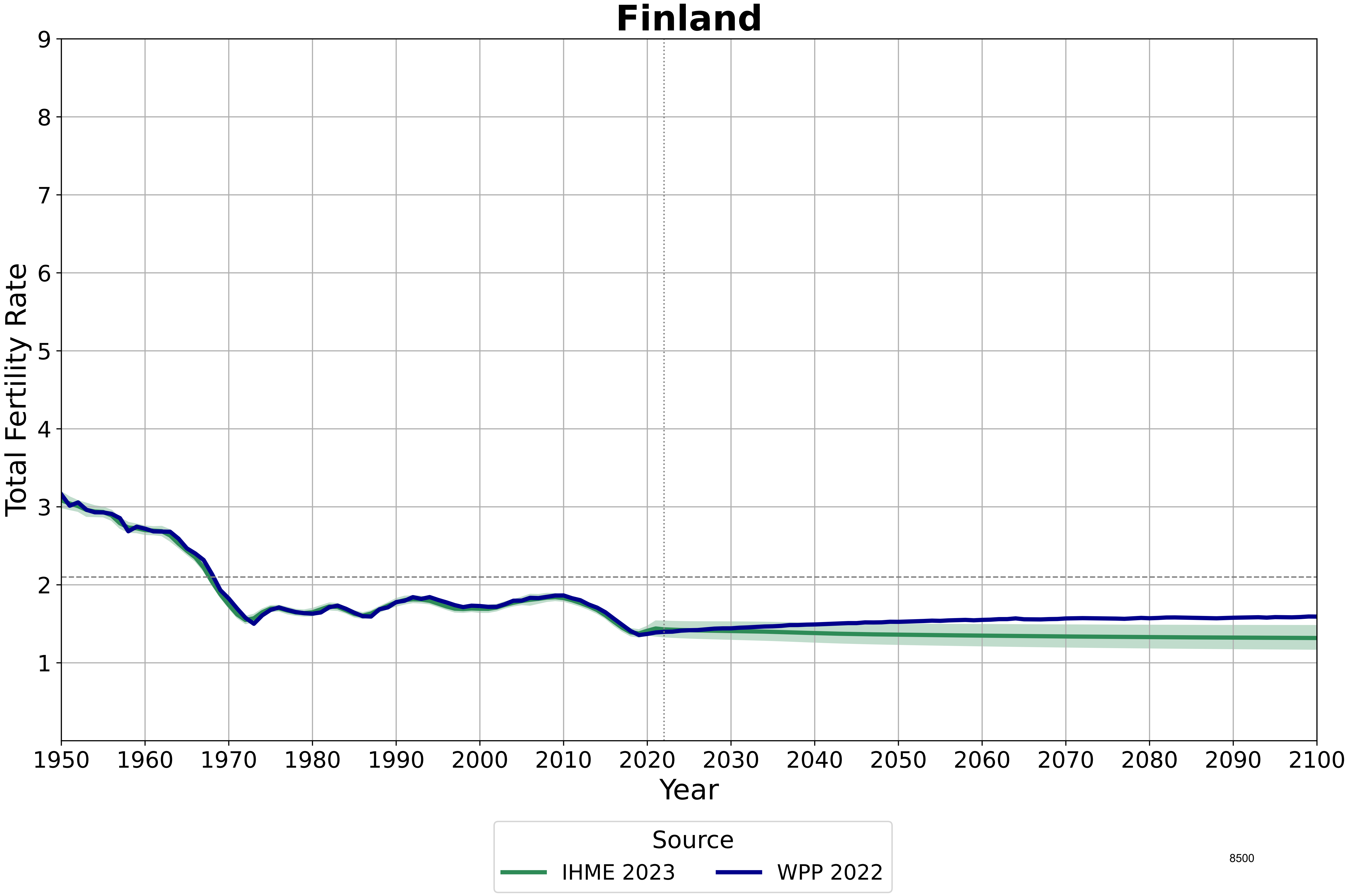

Supplemental Results Figure S7: TFR for IHME reference scenario and the UNPD medium variant scenario by location, 1950– 2100

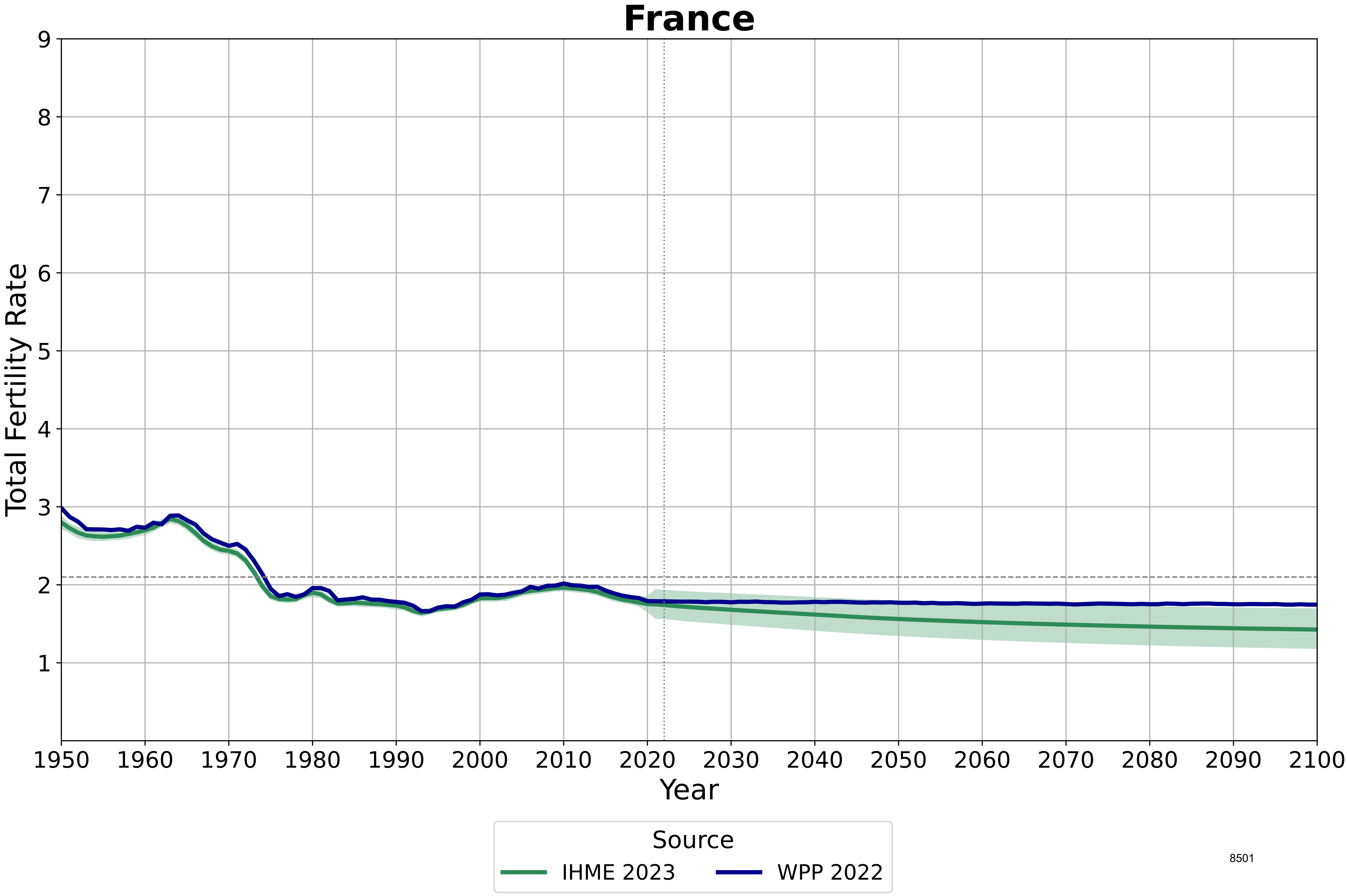

Supplemental Results Figure S7: TFR for IHME reference scenario and the UNPD medium variant scenario by location, 1950– 2100

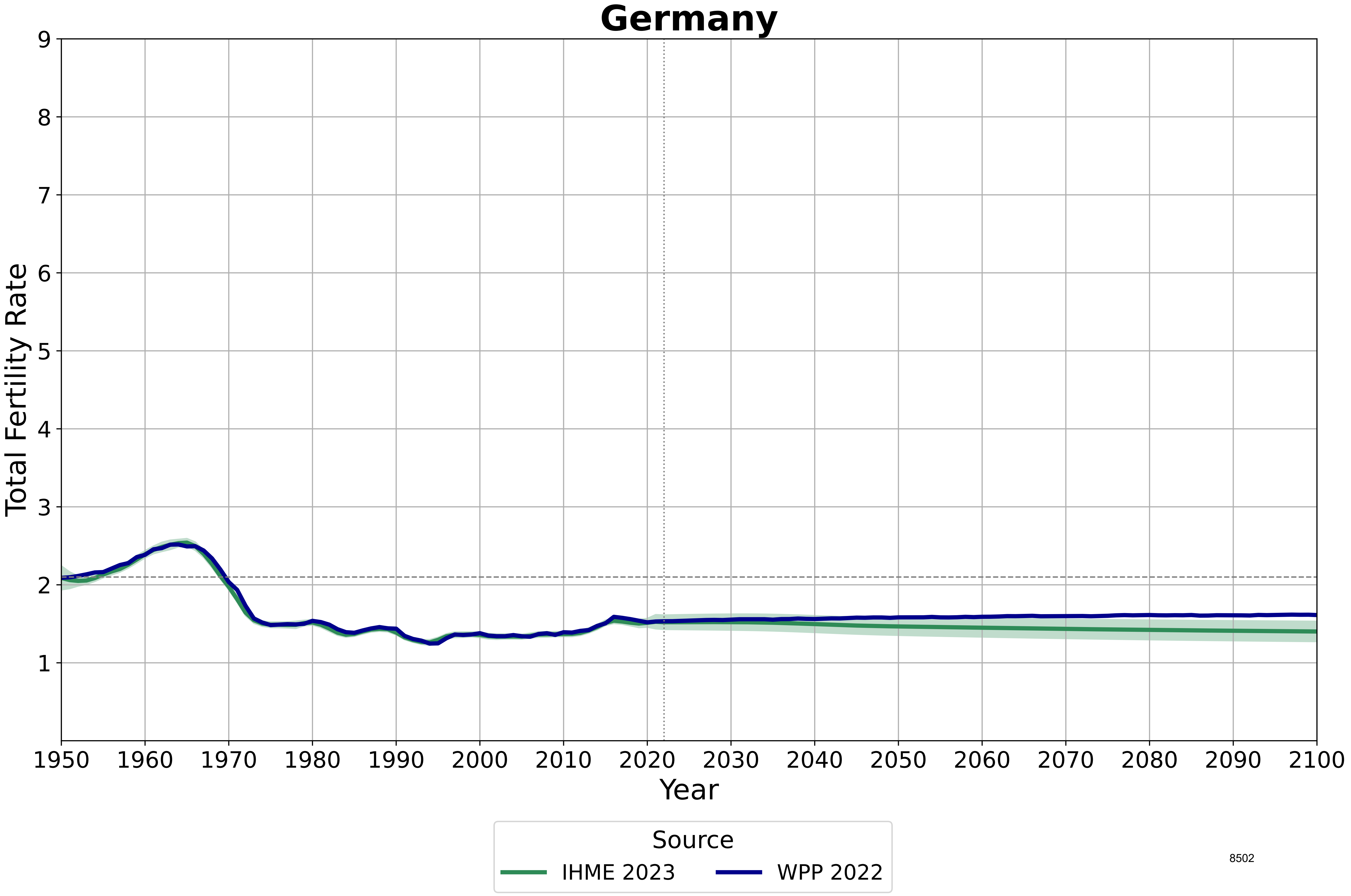

Supplemental Results Figure S7: TFR for IHME reference scenario and the UNPD medium variant scenario by location, 1950– 2100

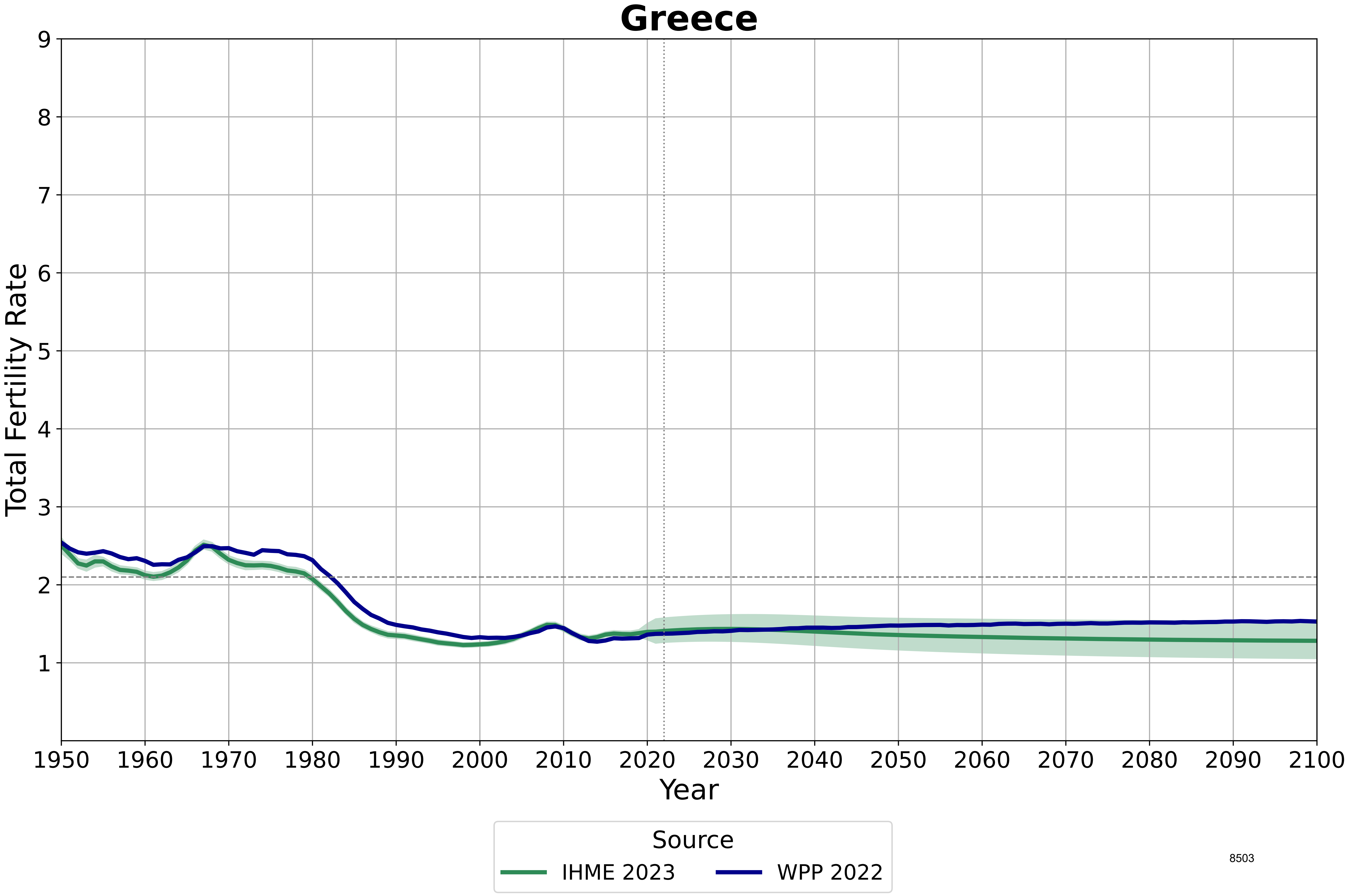

Supplemental Results Figure S7: TFR for IHME reference scenario and the UNPD medium variant scenario by location, 1950– 2100

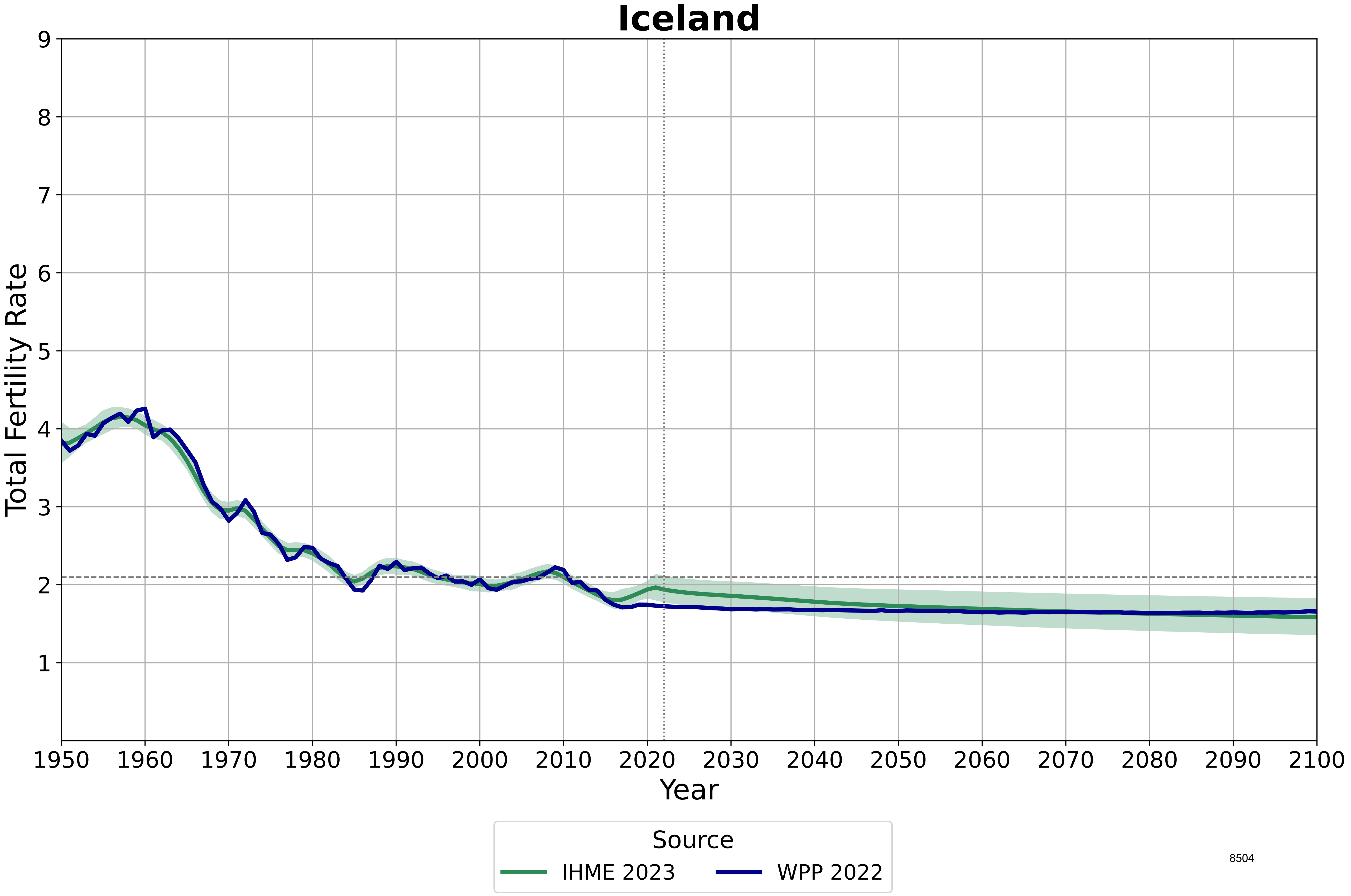

8504

Supplemental Results Figure S7: TFR for IHME reference scenario and the UNPD medium variant scenario by location, 1950– 2100

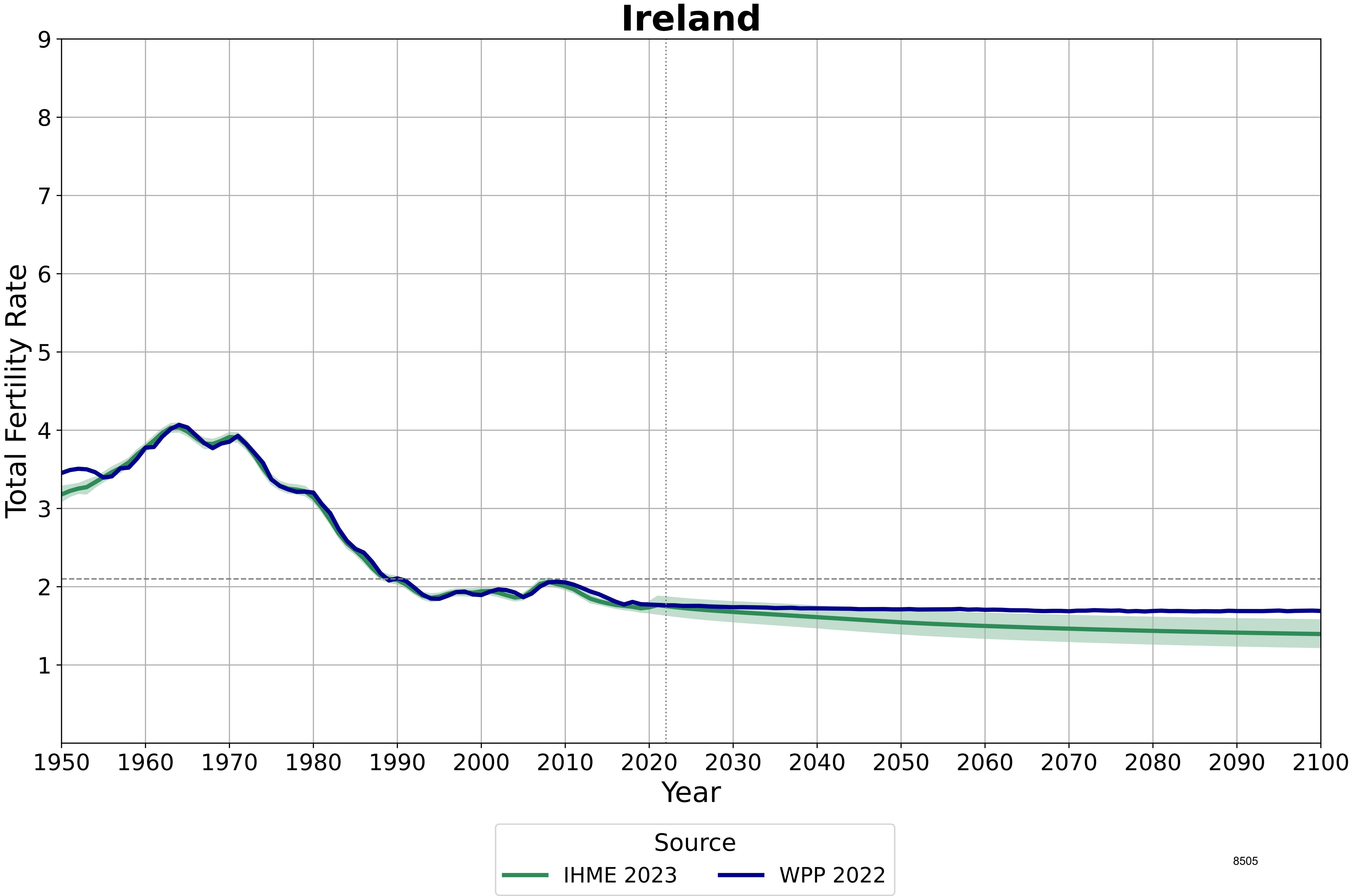

Supplemental Results Figure S7: TFR for IHME reference scenario and the UNPD medium variant scenario by location, 1950– 2100

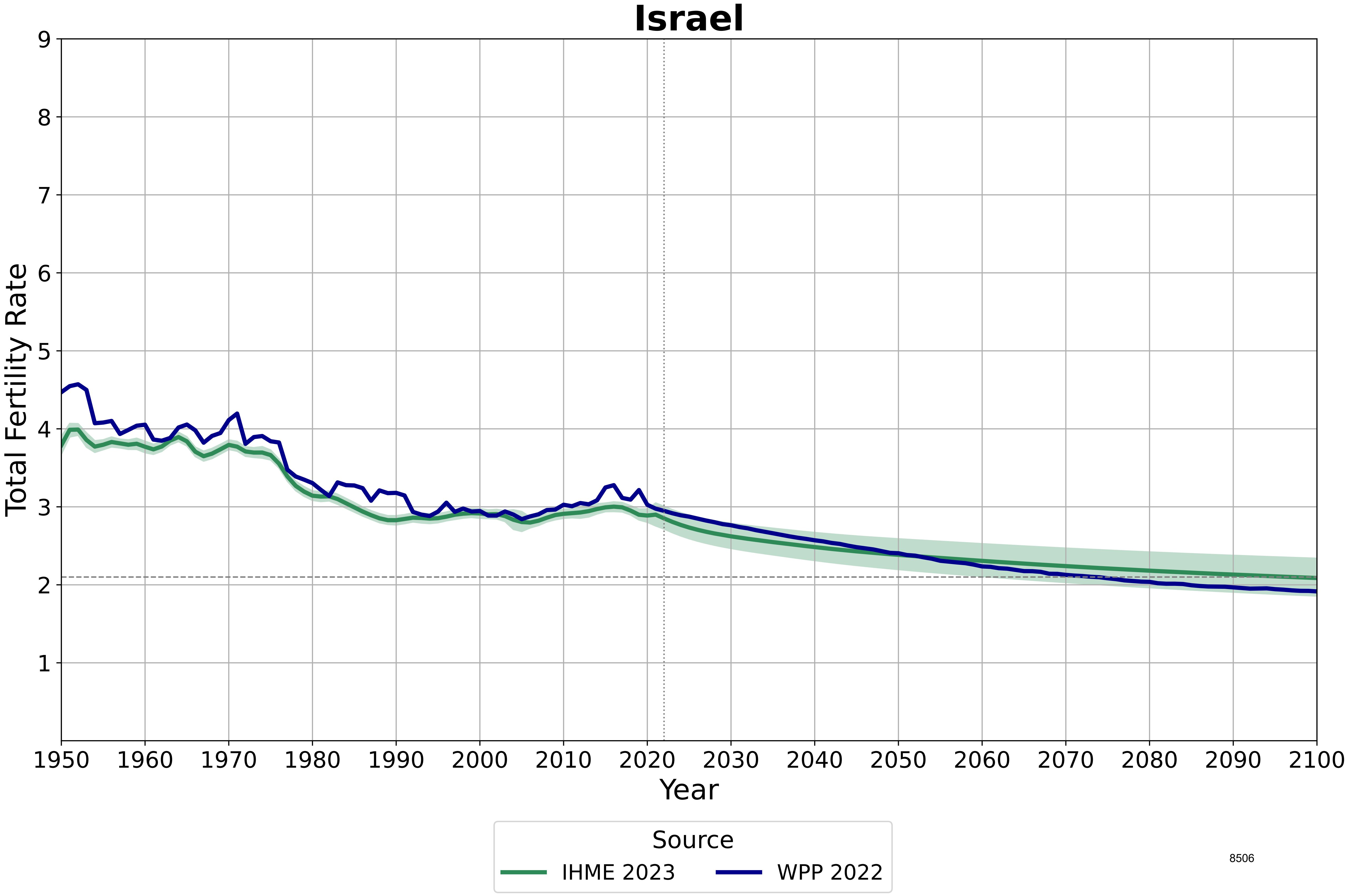

Supplemental Results Figure S7: TFR for IHME reference scenario and the UNPD medium variant scenario by location, 1950– 2100

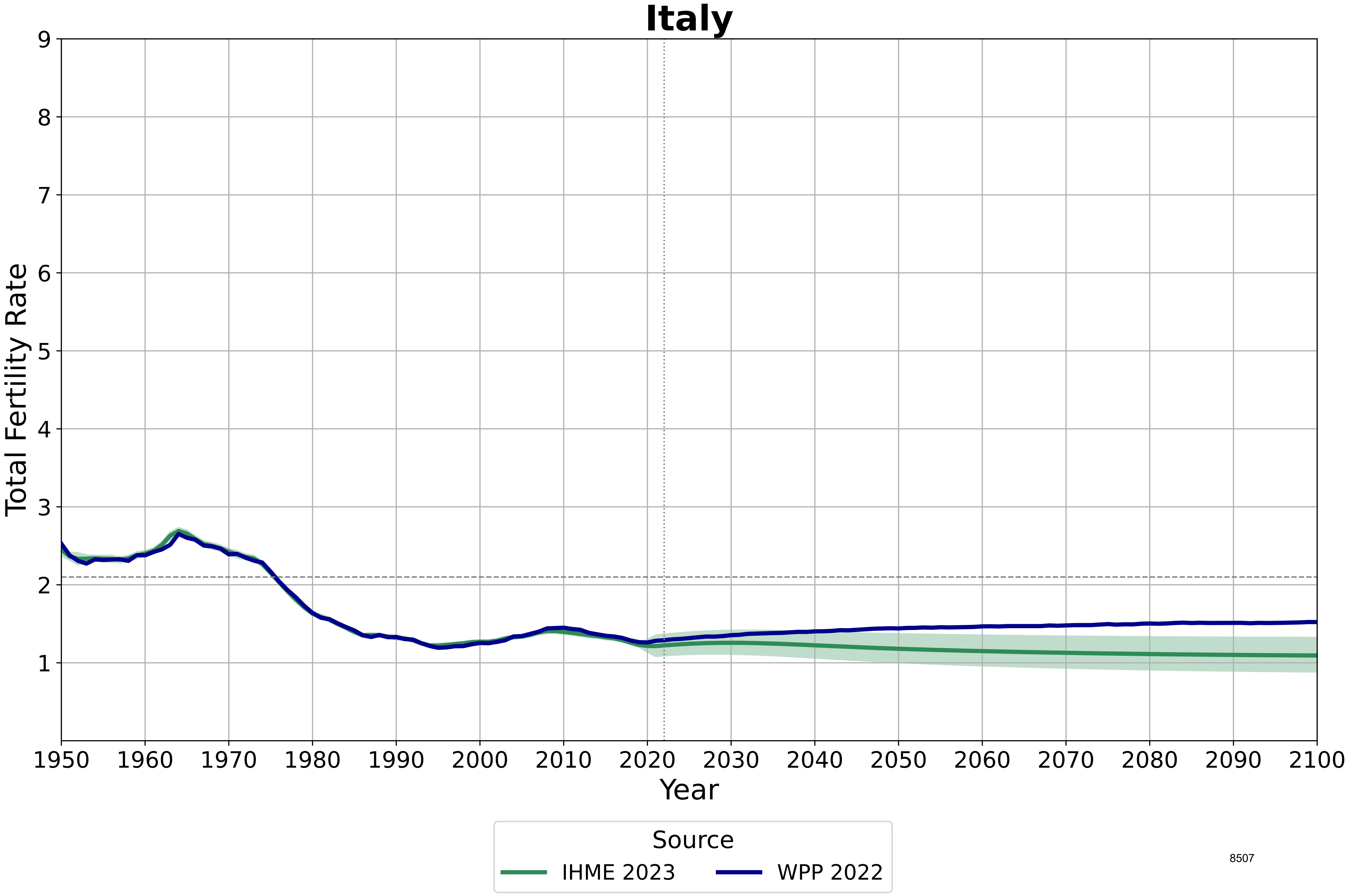

Supplemental Results Figure S7: TFR for IHME reference scenario and the UNPD medium variant scenario by location, 1950– 2100

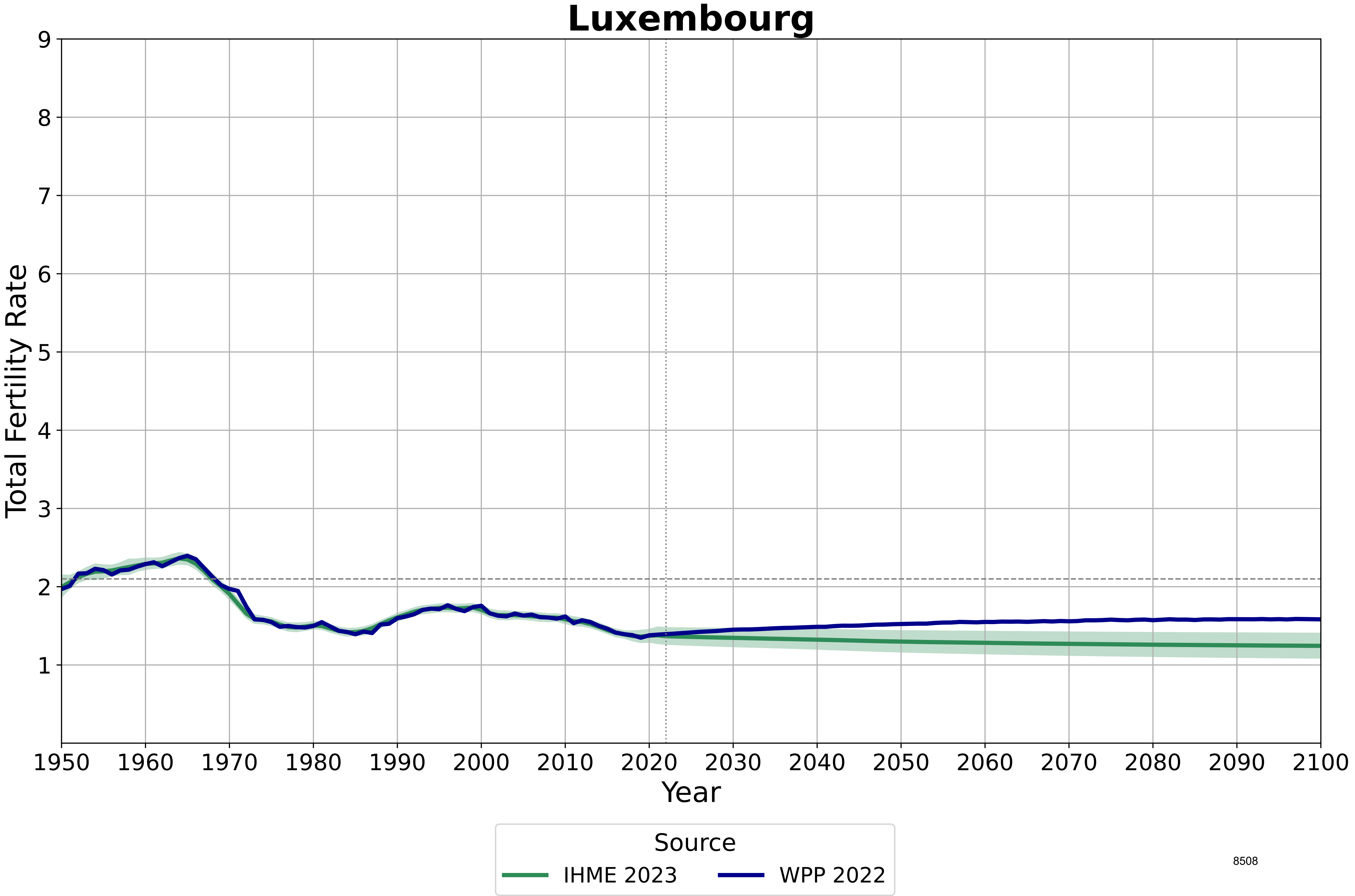

Supplemental Results Figure S7: TFR for IHME reference scenario and the UNPD medium variant scenario by location, 1950– 2100

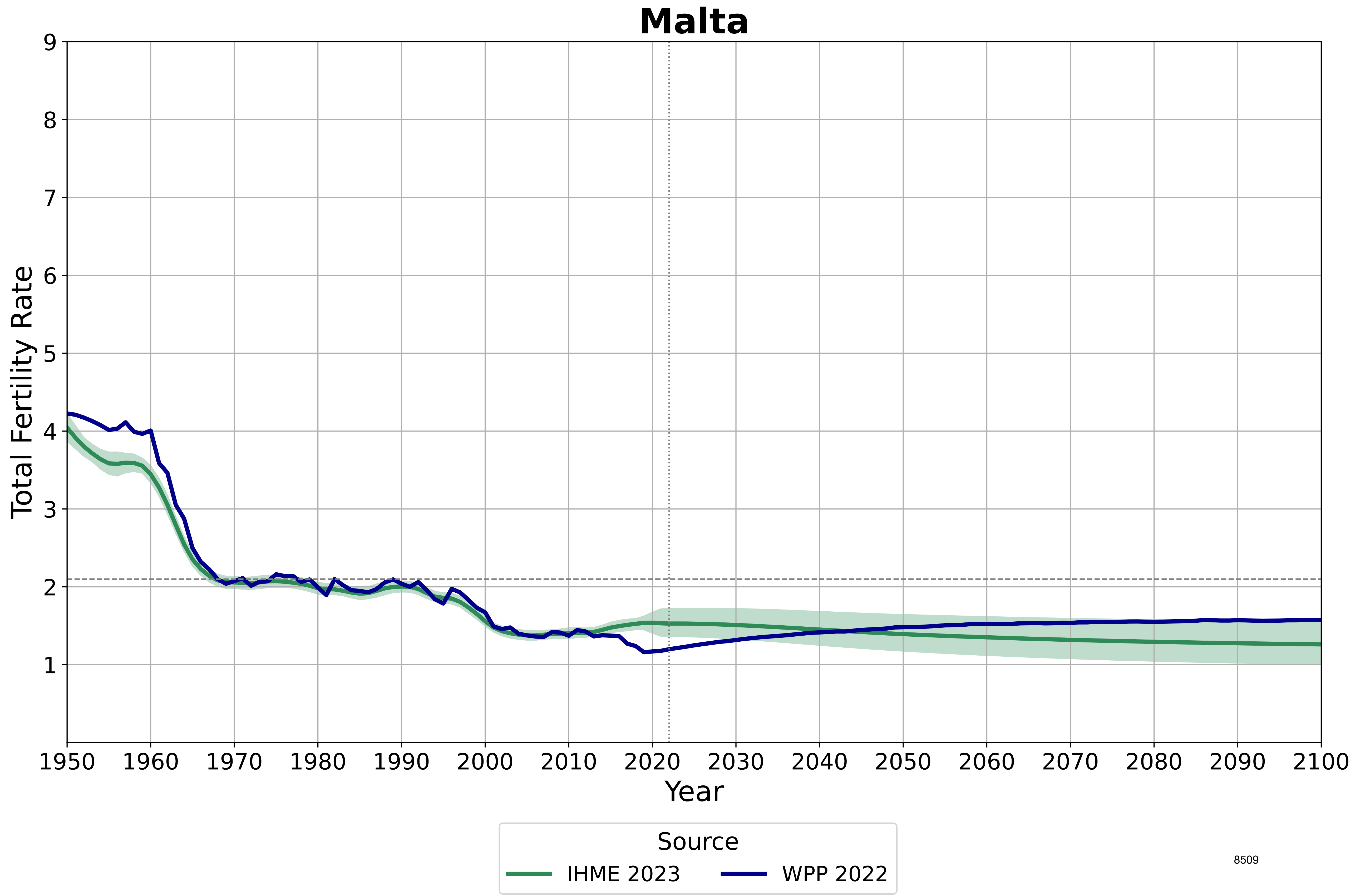

Supplemental Results Figure S7: TFR for IHME reference scenario and the UNPD medium variant scenario by location, 1950– 2100

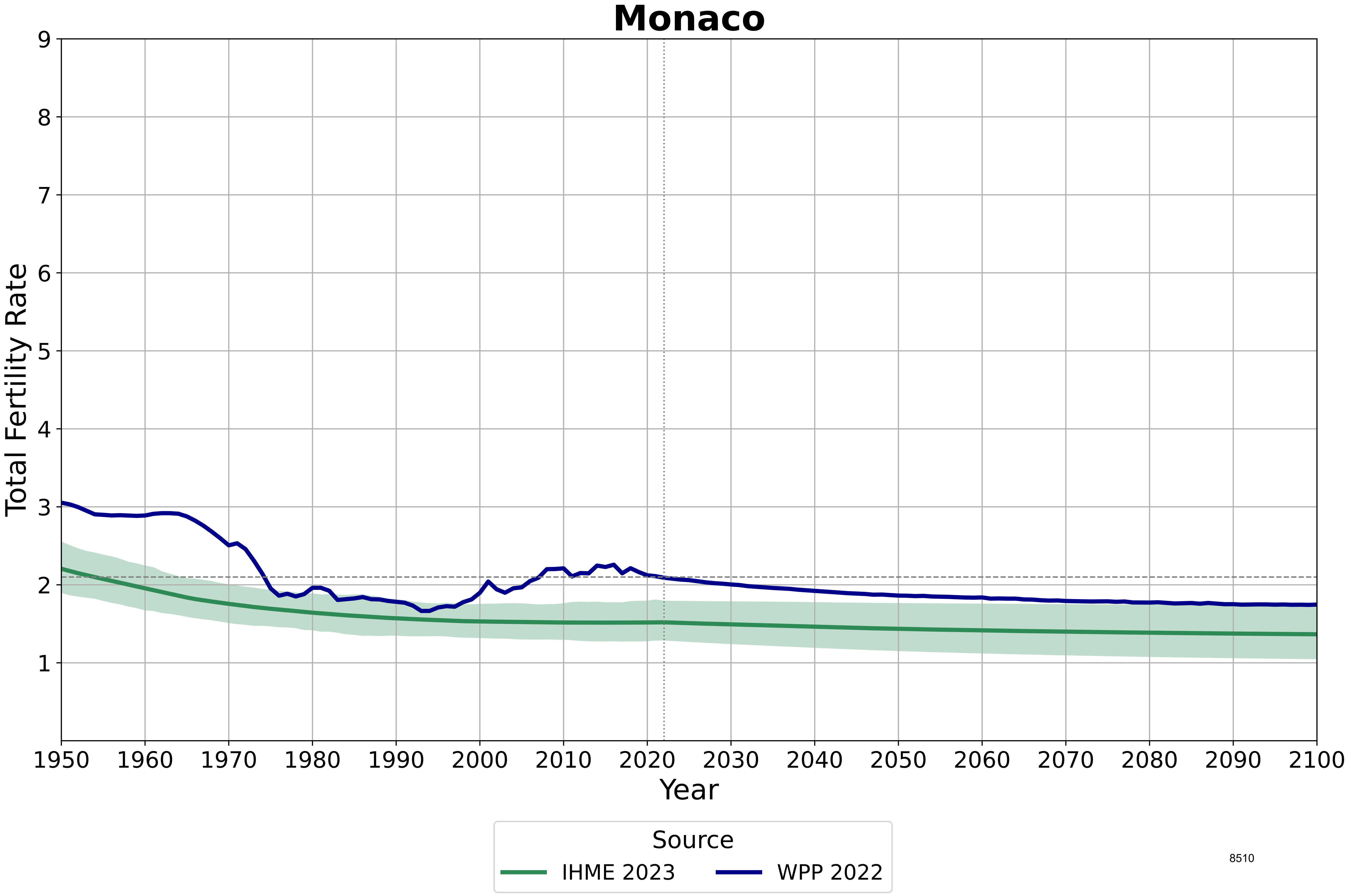

Supplemental Results Figure S7: TFR for IHME reference scenario and the UNPD medium variant scenario by location, 1950– 2100

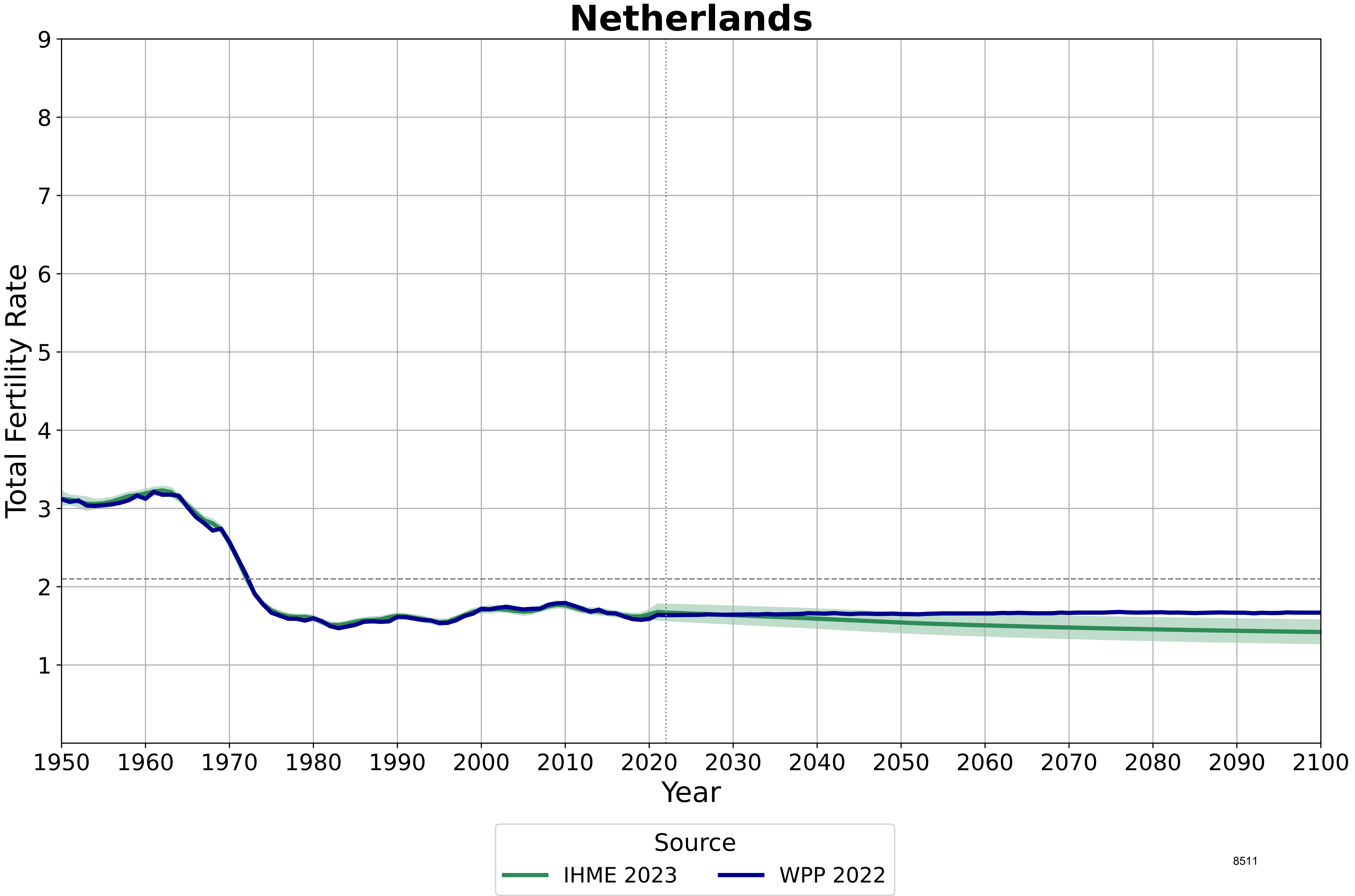

8511

Supplemental Results Figure S7: TFR for IHME reference scenario and the UNPD medium variant scenario by location, 1950– 2100

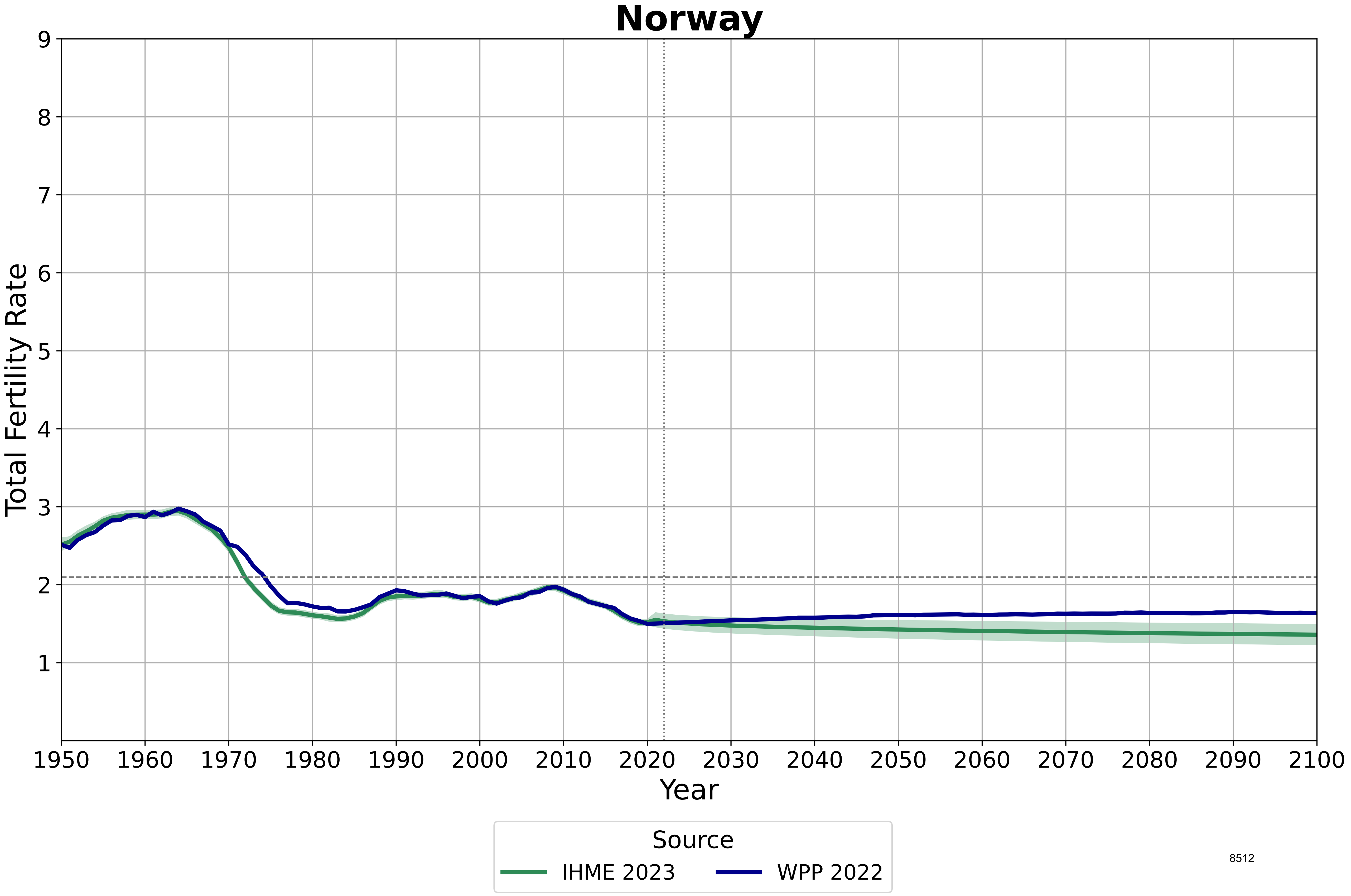

Supplemental Results Figure S7: TFR for IHME reference scenario and the UNPD medium variant scenario by location, 1950– 2100

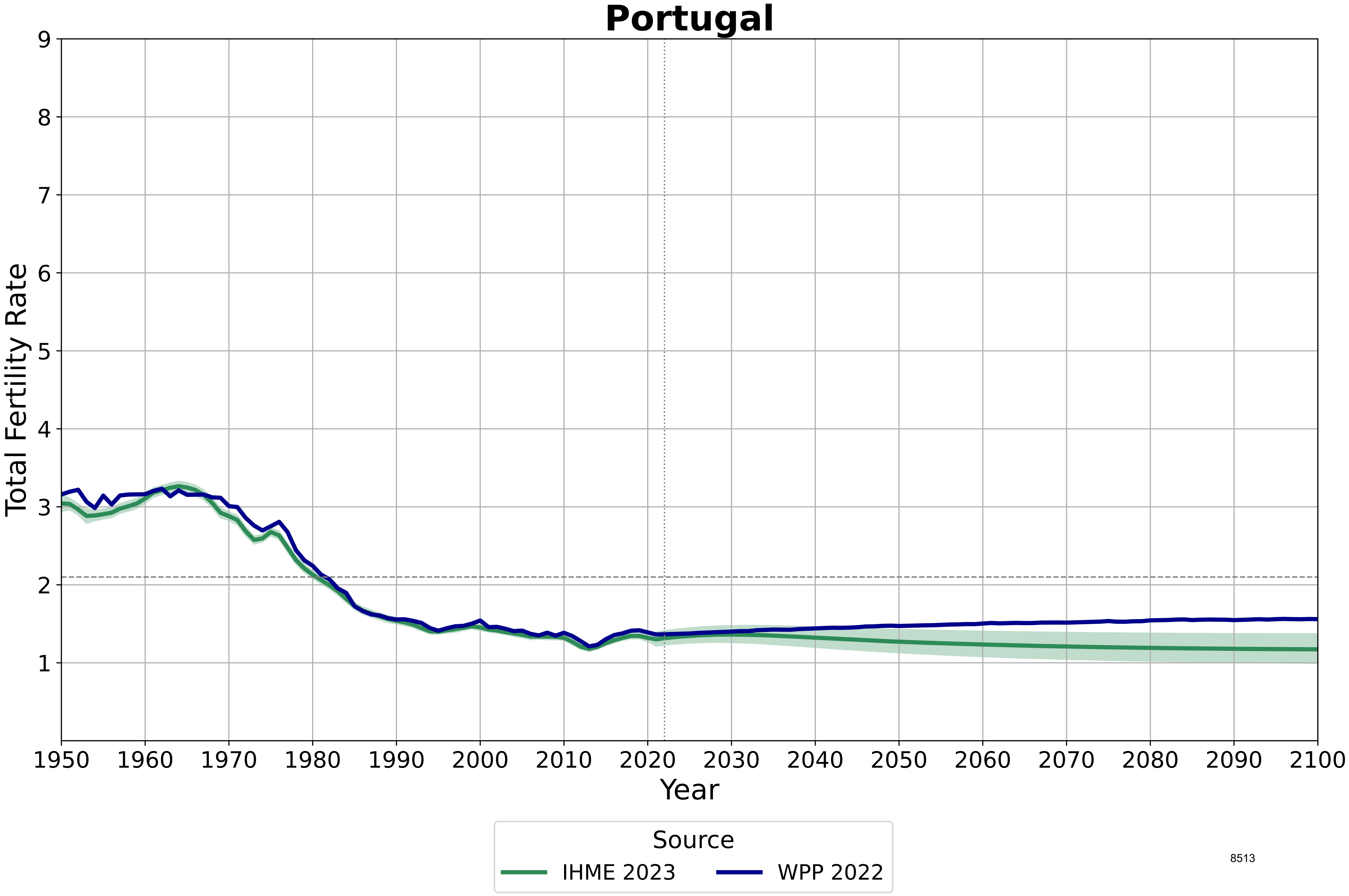

Supplemental Results Figure S7: TFR for IHME reference scenario and the UNPD medium variant scenario by location, 1950– 2100

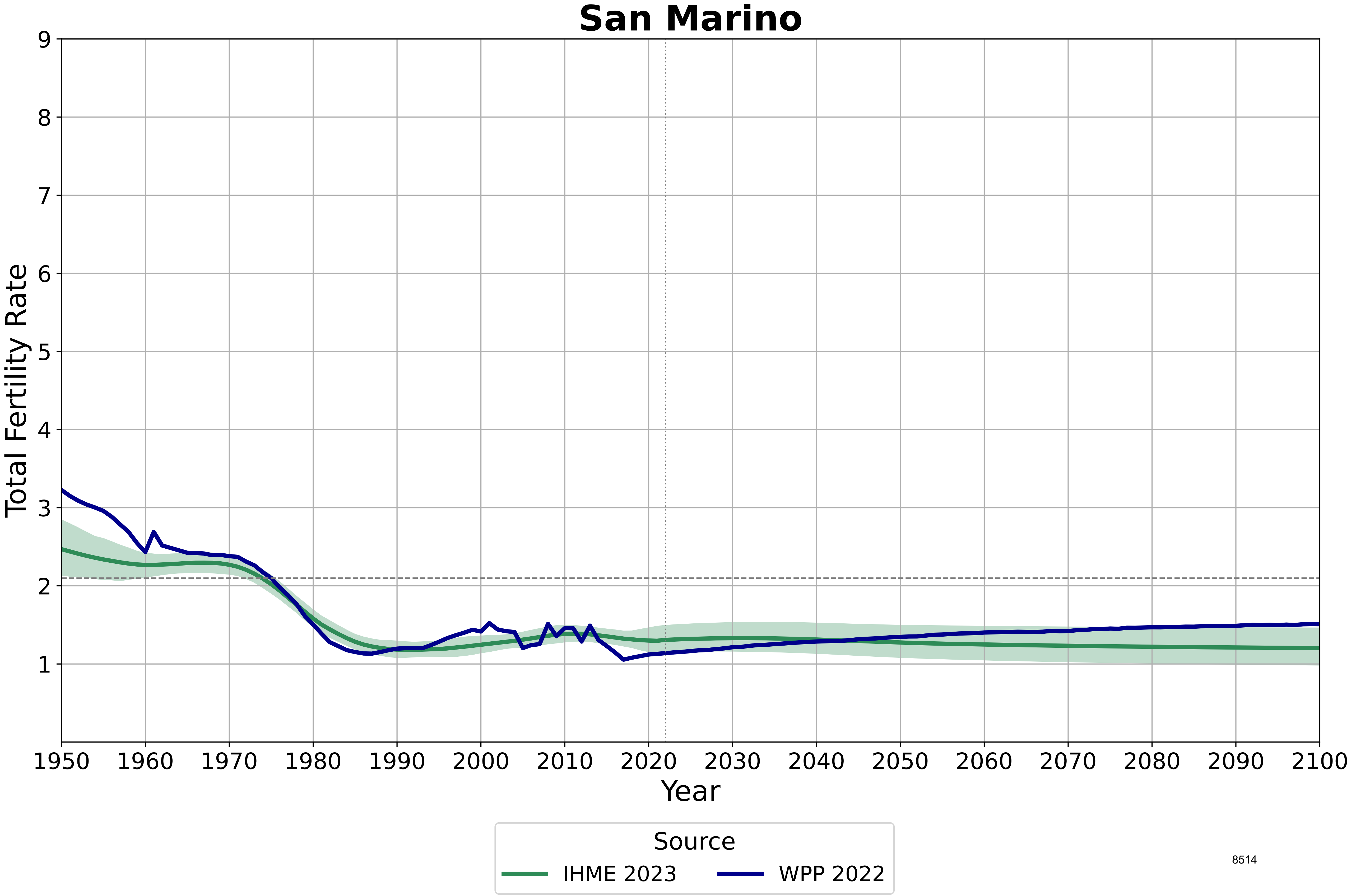

Supplemental Results Figure S7: TFR for IHME reference scenario and the UNPD medium variant scenario by location, 1950– 2100

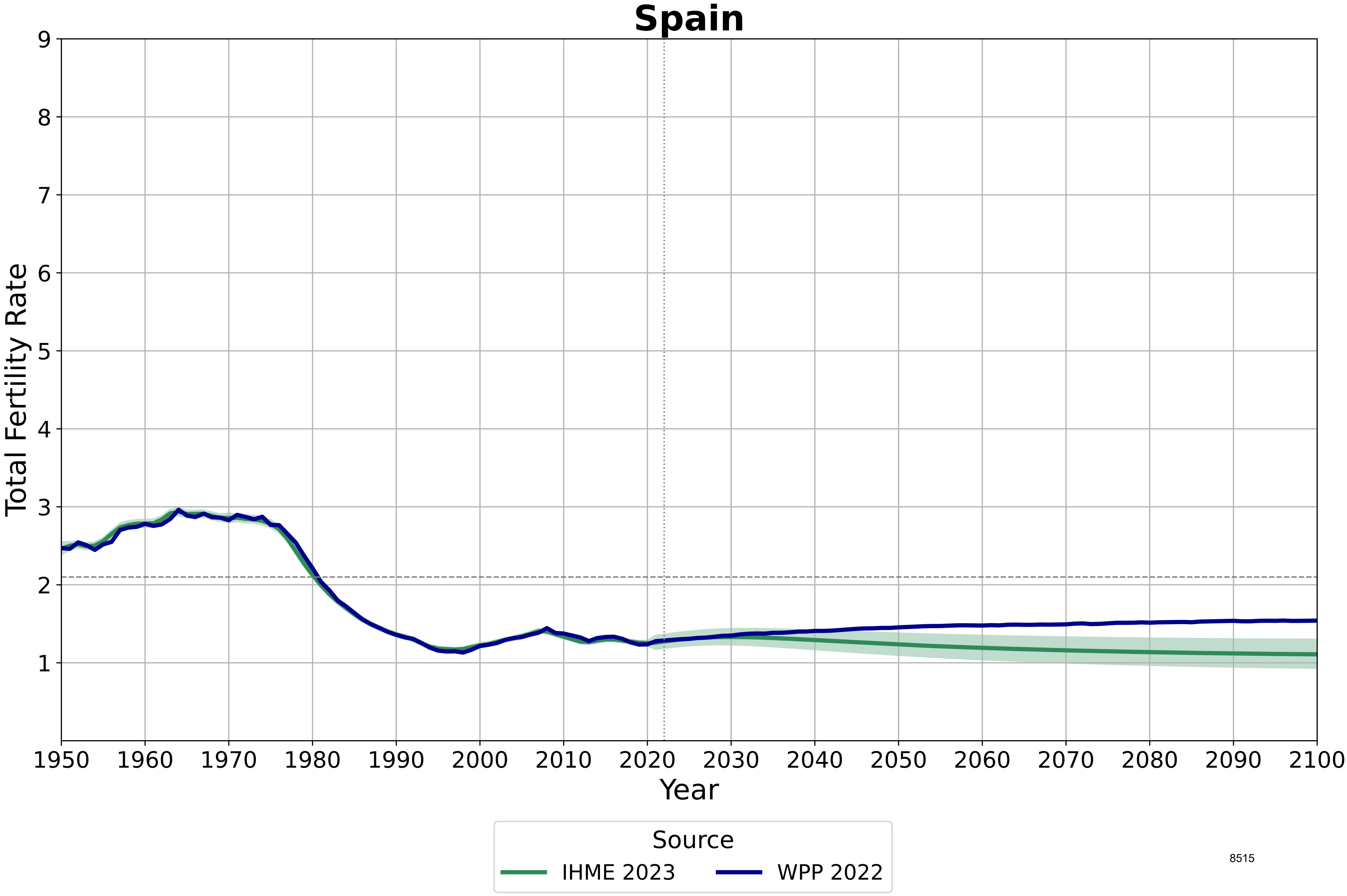

Supplemental Results Figure S7: TFR for IHME reference scenario and the UNPD medium variant scenario by location, 1950– 2100

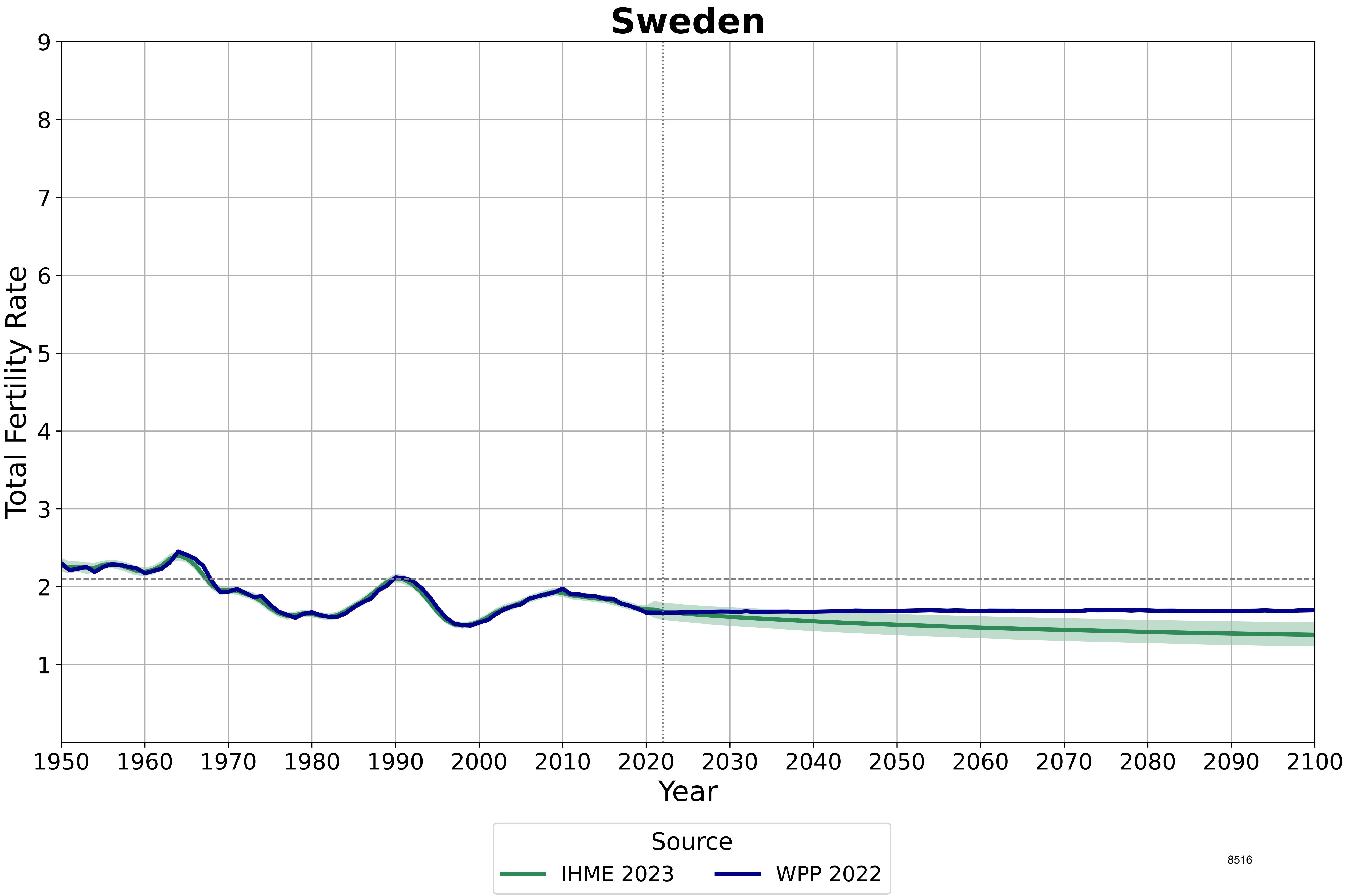

Supplemental Results Figure S7: TFR for IHME reference scenario and the UNPD medium variant scenario by location, 1950– 2100

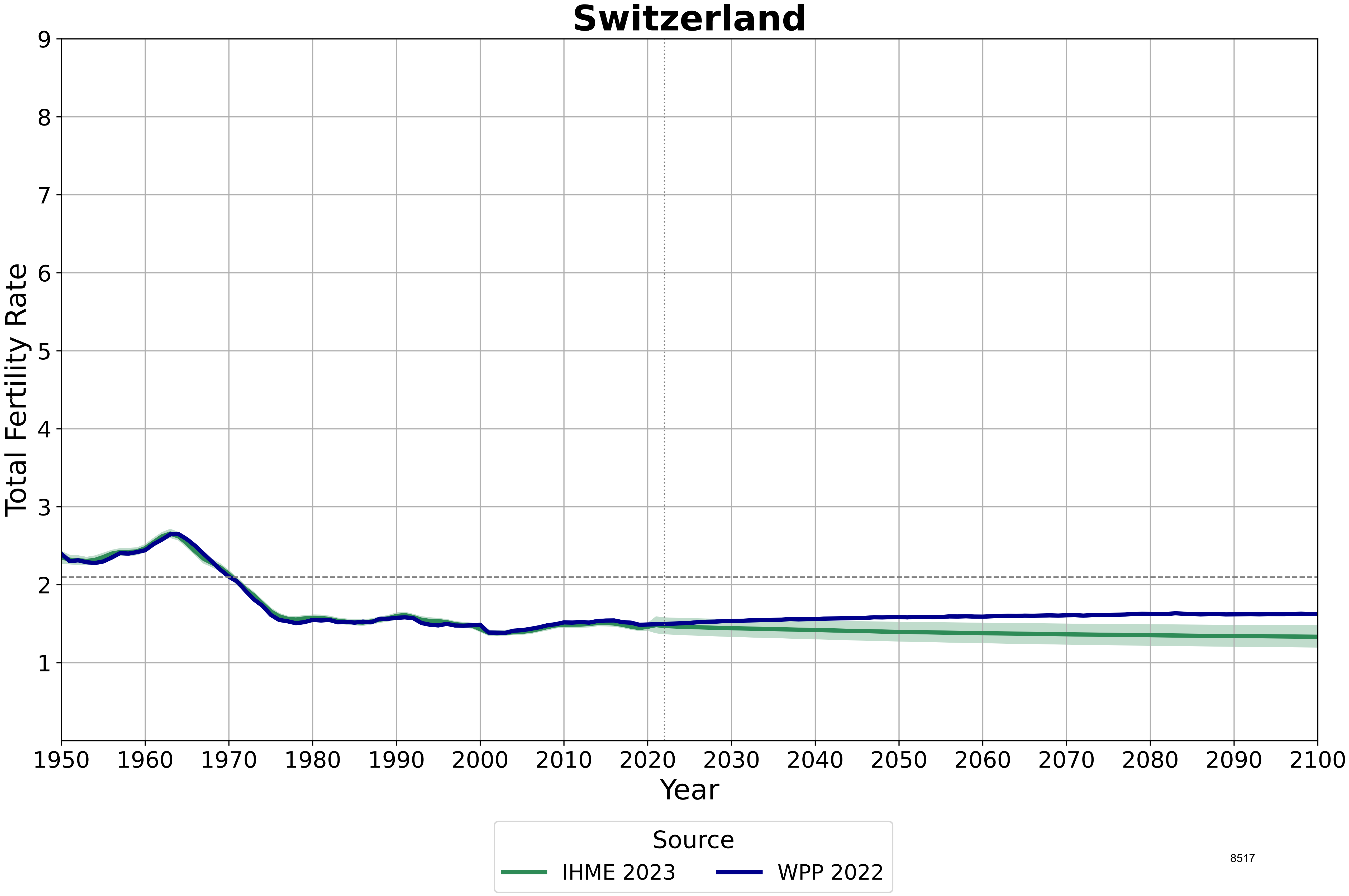

Supplemental Results Figure S7: TFR for IHME reference scenario and the UNPD medium variant scenario by location, 1950– 2100

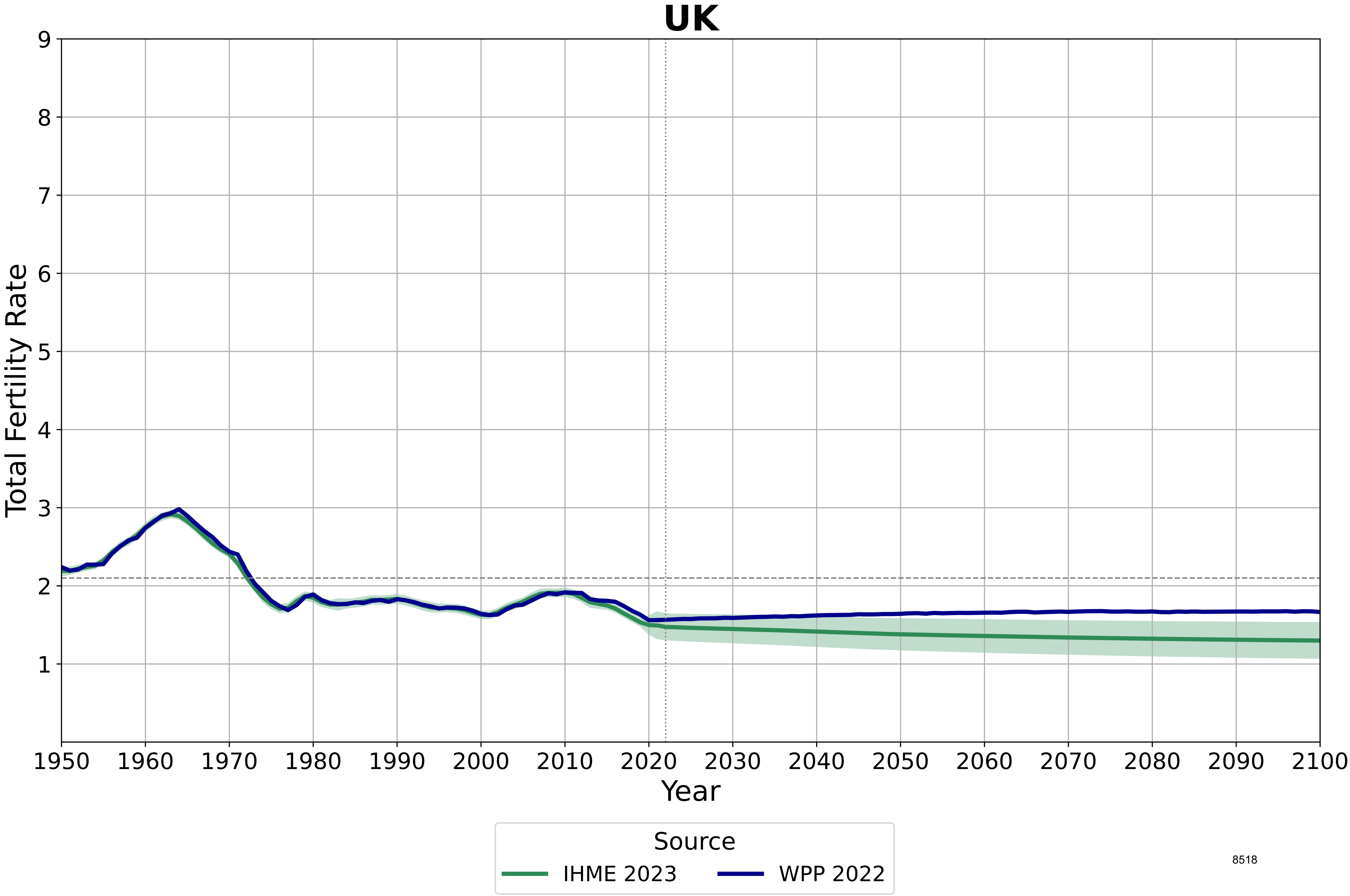

Supplemental Results Figure S7: TFR for IHME reference scenario and the UNPD medium variant scenario by location, 1950– 2100

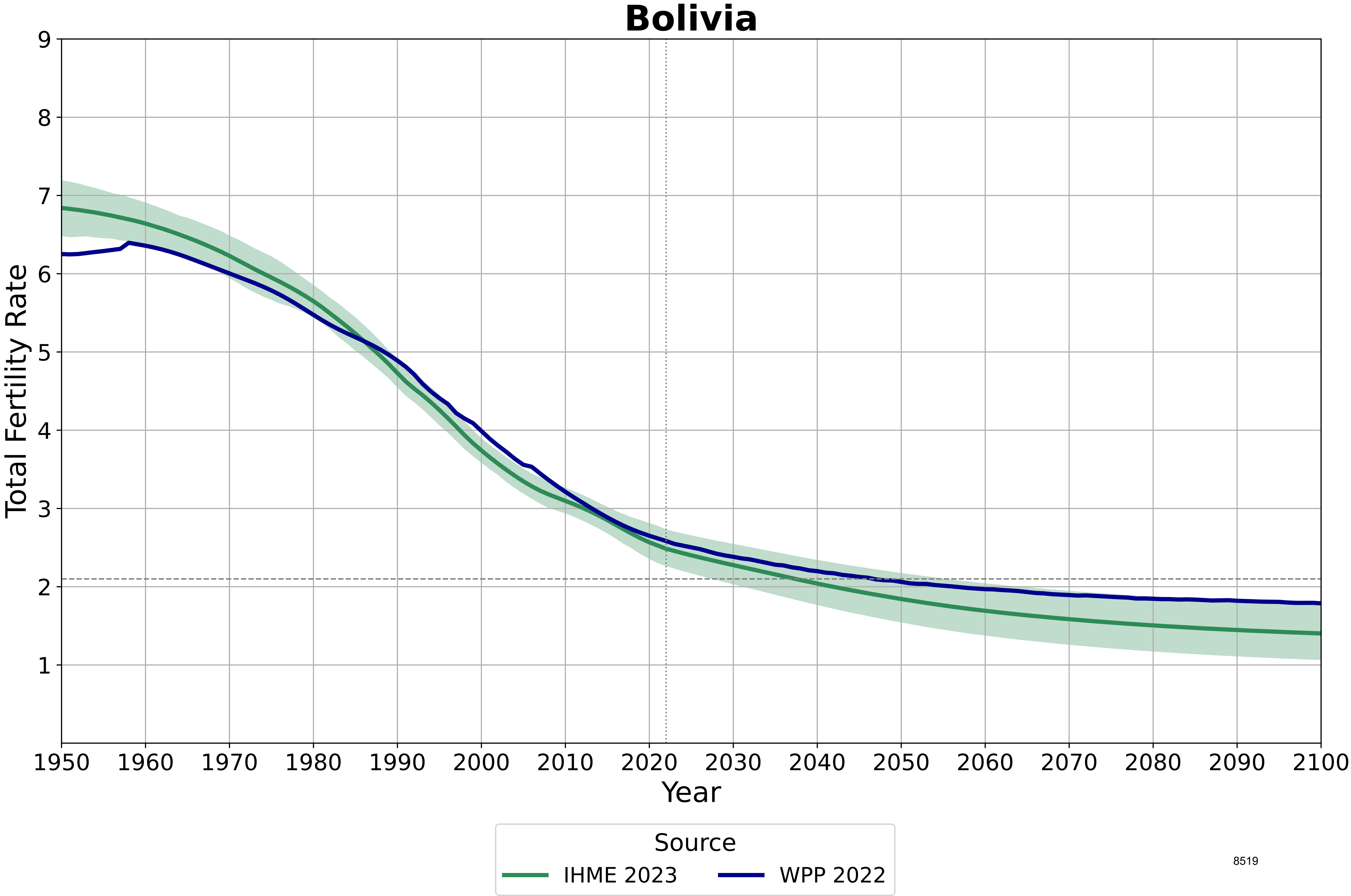

Supplemental Results Figure S7: TFR for IHME reference scenario and the UNPD medium variant scenario by location, 1950– 2100

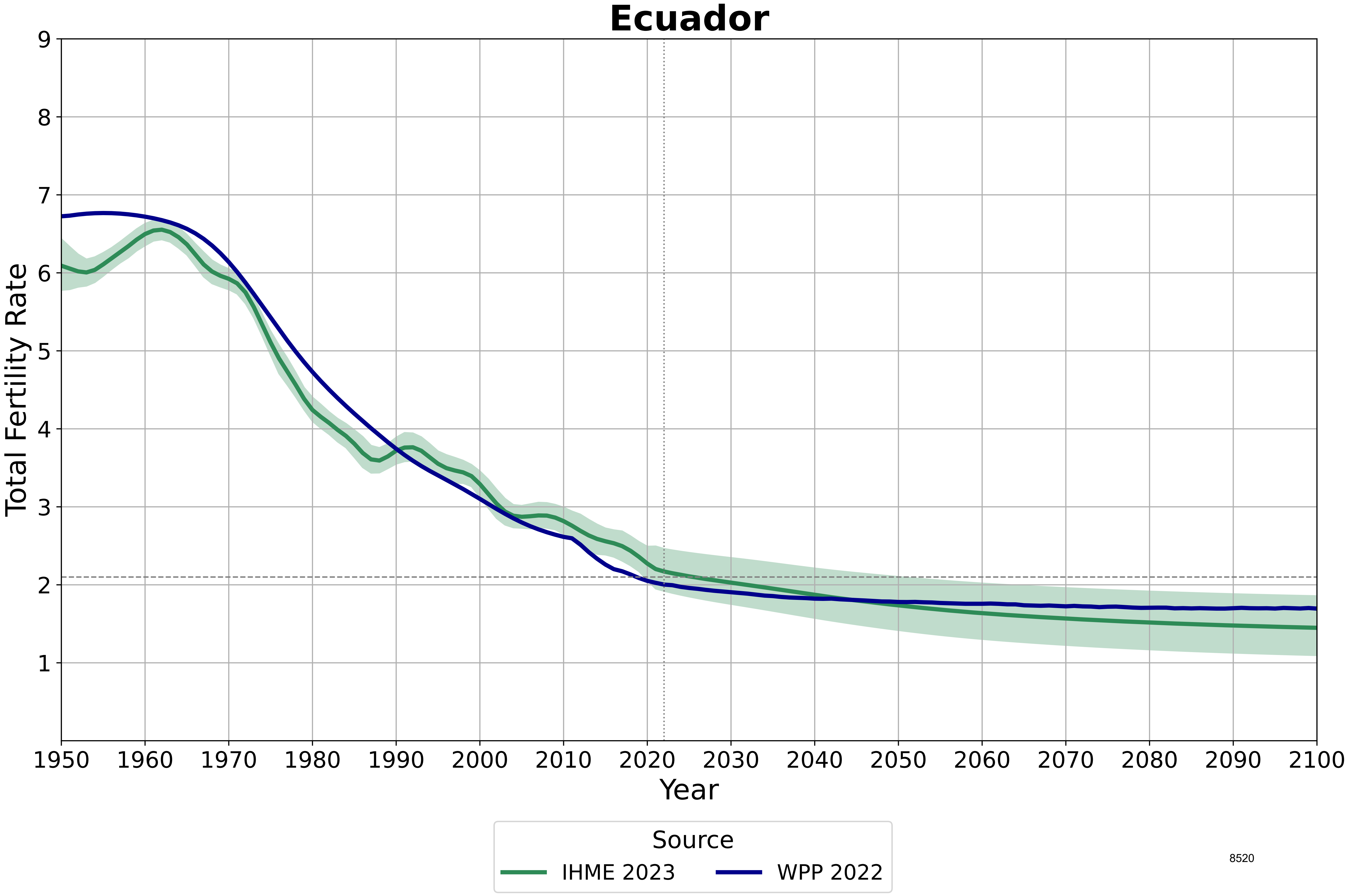

Supplemental Results Figure S7: TFR for IHME reference scenario and the UNPD medium variant scenario by location, 1950– 2100

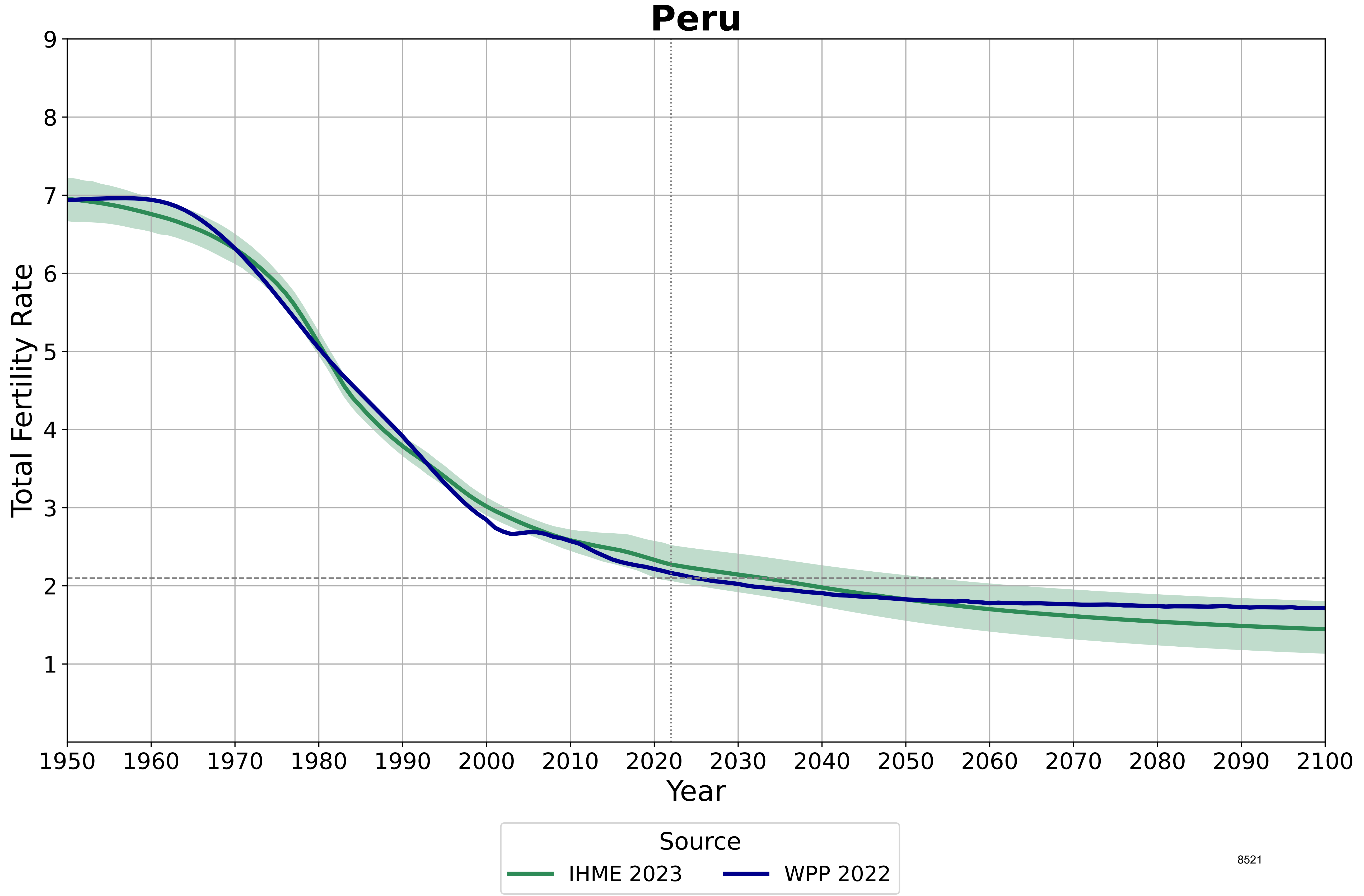

Supplemental Results Figure S7: TFR for IHME reference scenario and the UNPD medium variant scenario by location, 1950– 2100

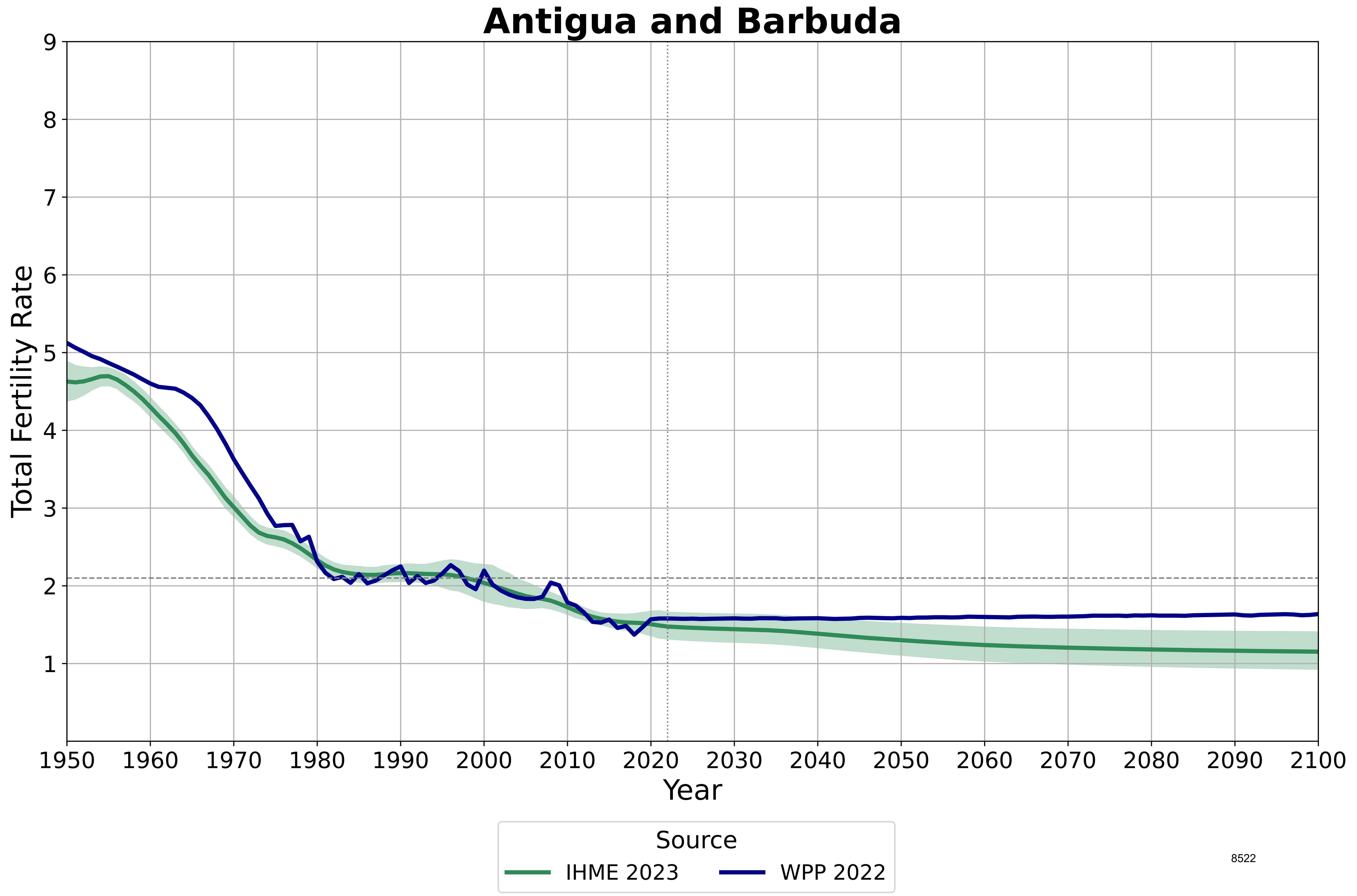

Supplemental Results Figure S7: TFR for IHME reference scenario and the UNPD medium variant scenario by location, 1950– 2100

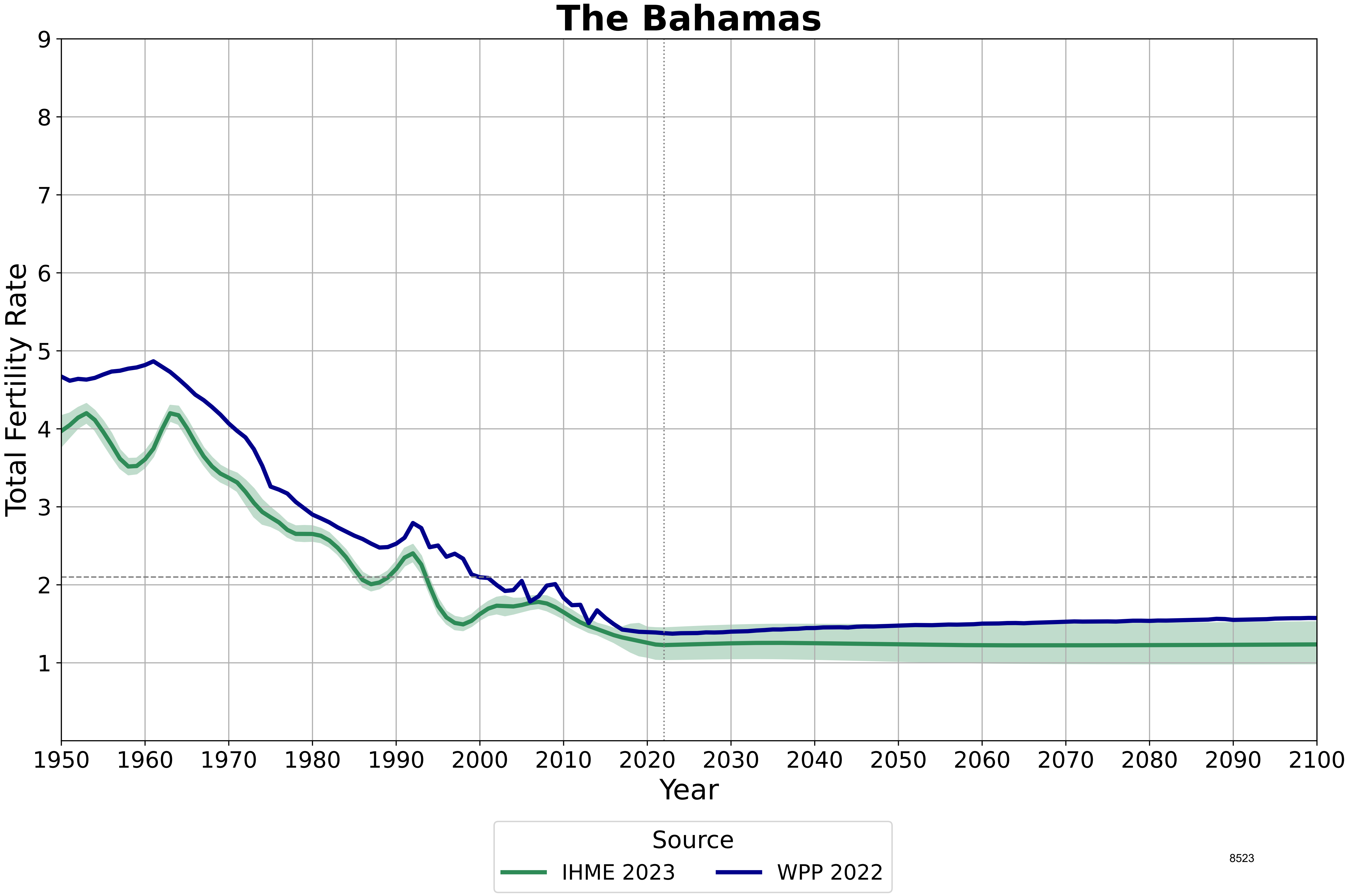

Supplemental Results Figure S7: TFR for IHME reference scenario and the UNPD medium variant scenario by location, 1950– 2100

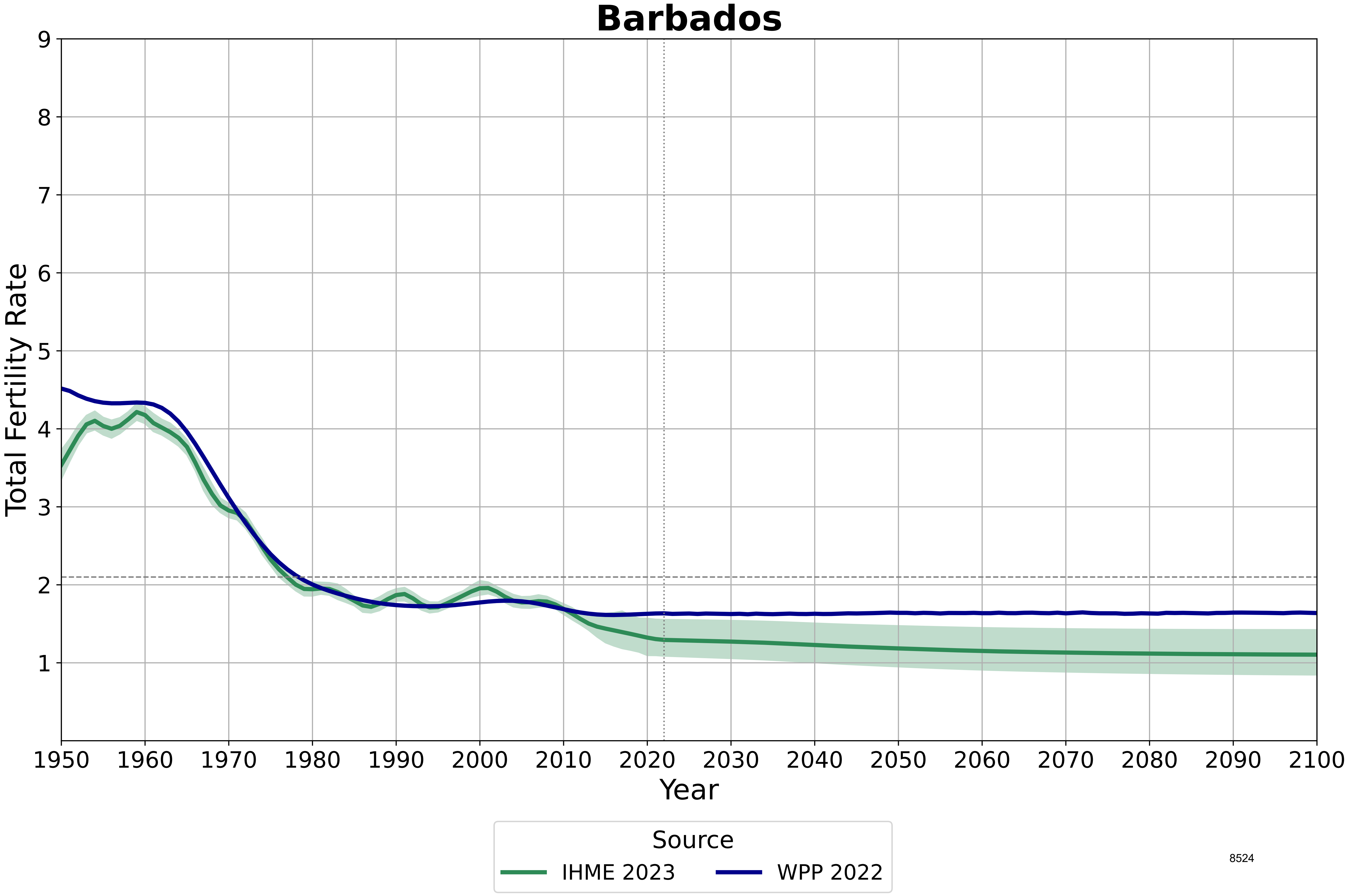

Supplemental Results Figure S7: TFR for IHME reference scenario and the UNPD medium variant scenario by location, 1950– 2100

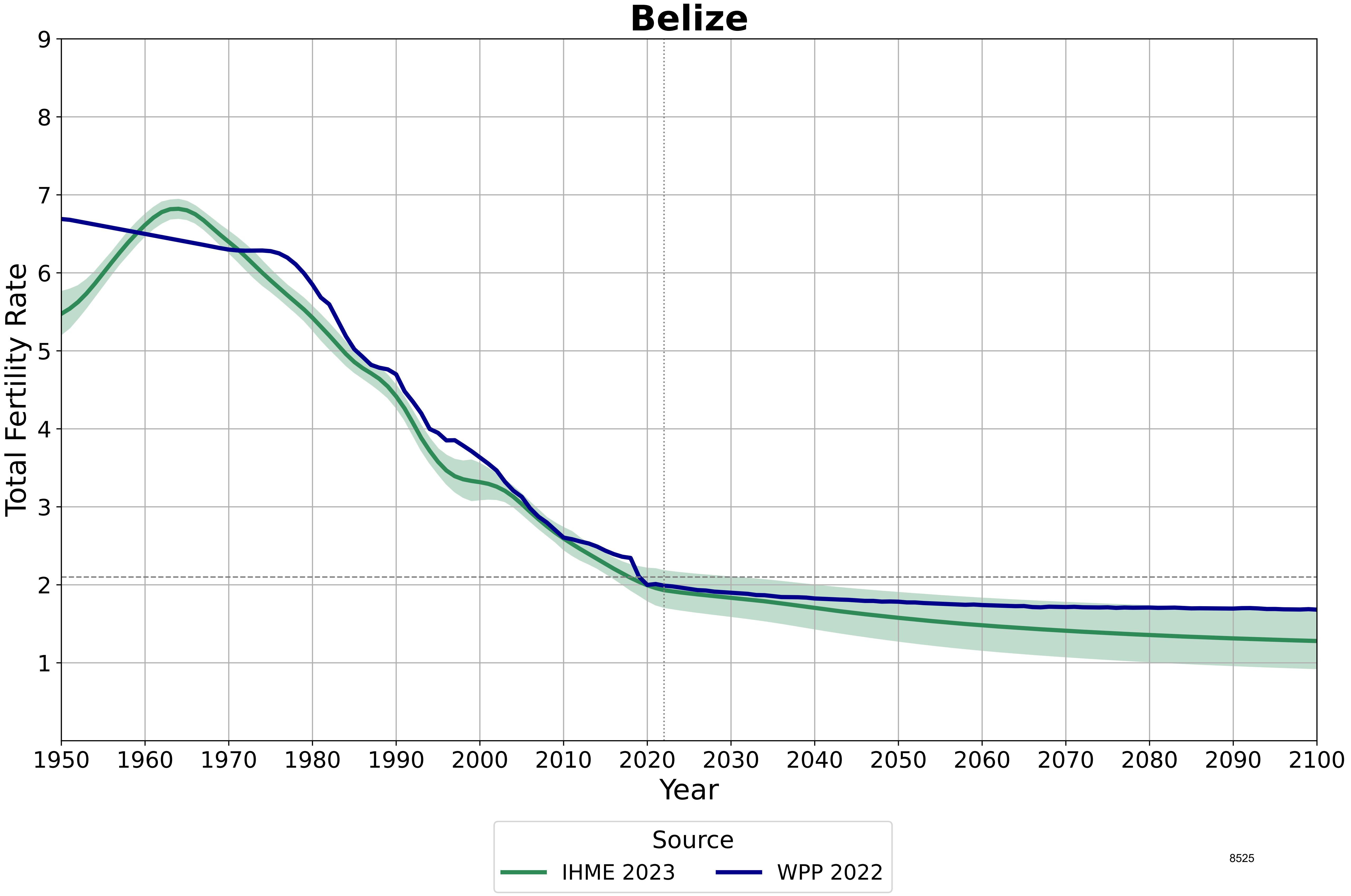

Supplemental Results Figure S7: TFR for IHME reference scenario and the UNPD medium variant scenario by location, 1950– 2100

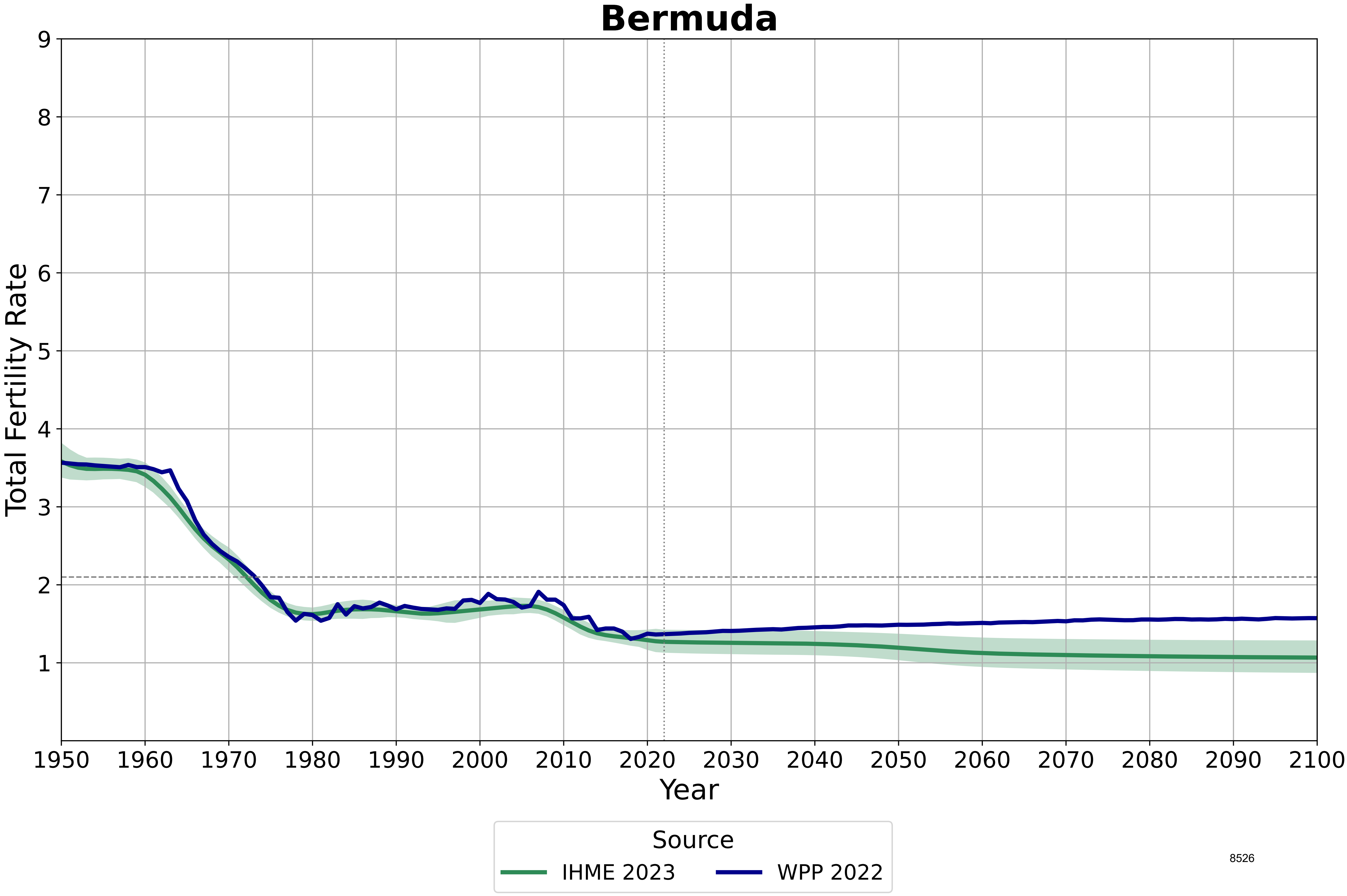

Supplemental Results Figure S7: TFR for IHME reference scenario and the UNPD medium variant scenario by location, 1950– 2100

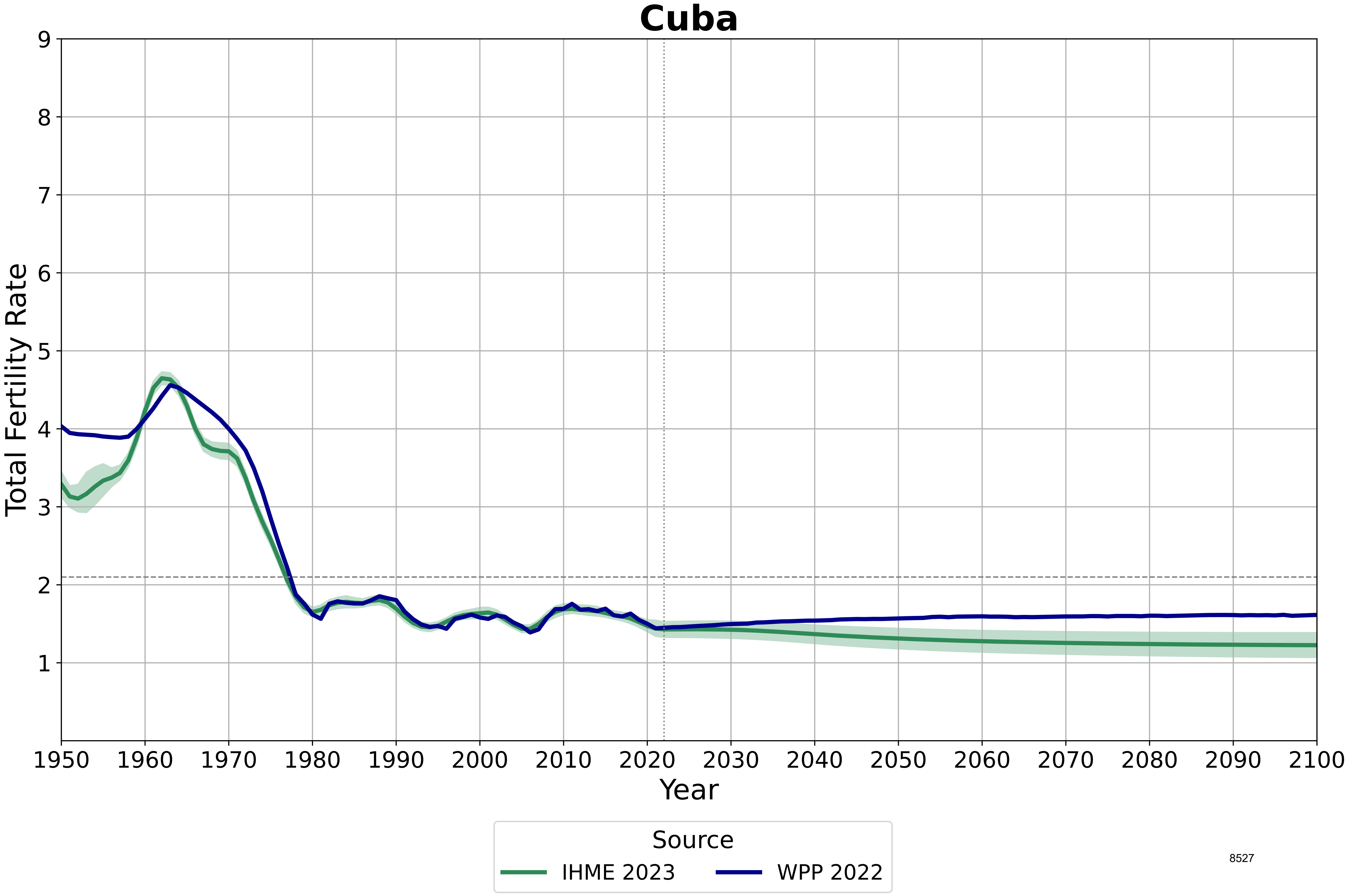

Supplemental Results Figure S7: TFR for IHME reference scenario and the UNPD medium variant scenario by location, 1950– 2100

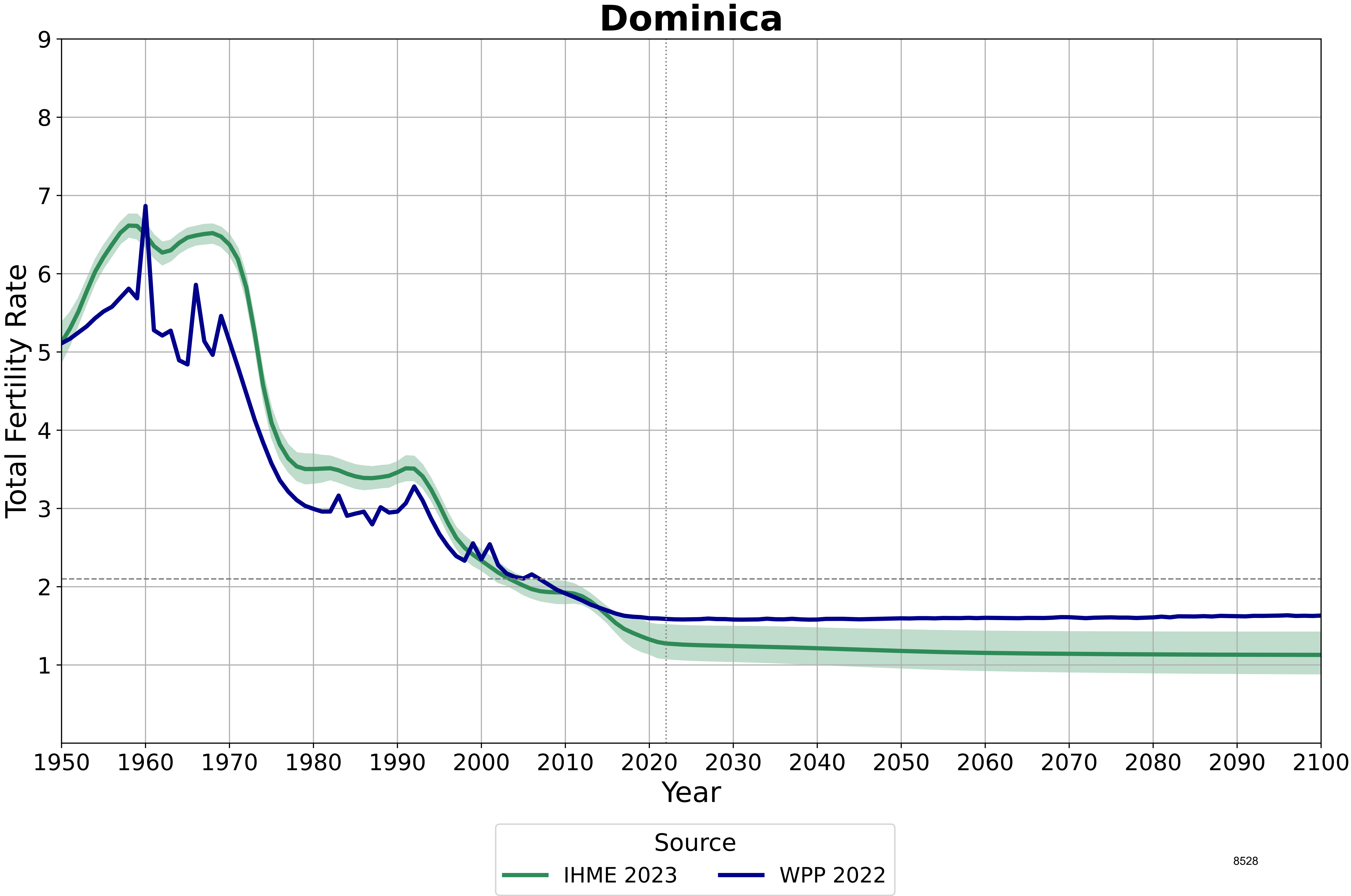

8528

Supplemental Results Figure S7: TFR for IHME reference scenario and the UNPD medium variant scenario by location, 1950– 2100

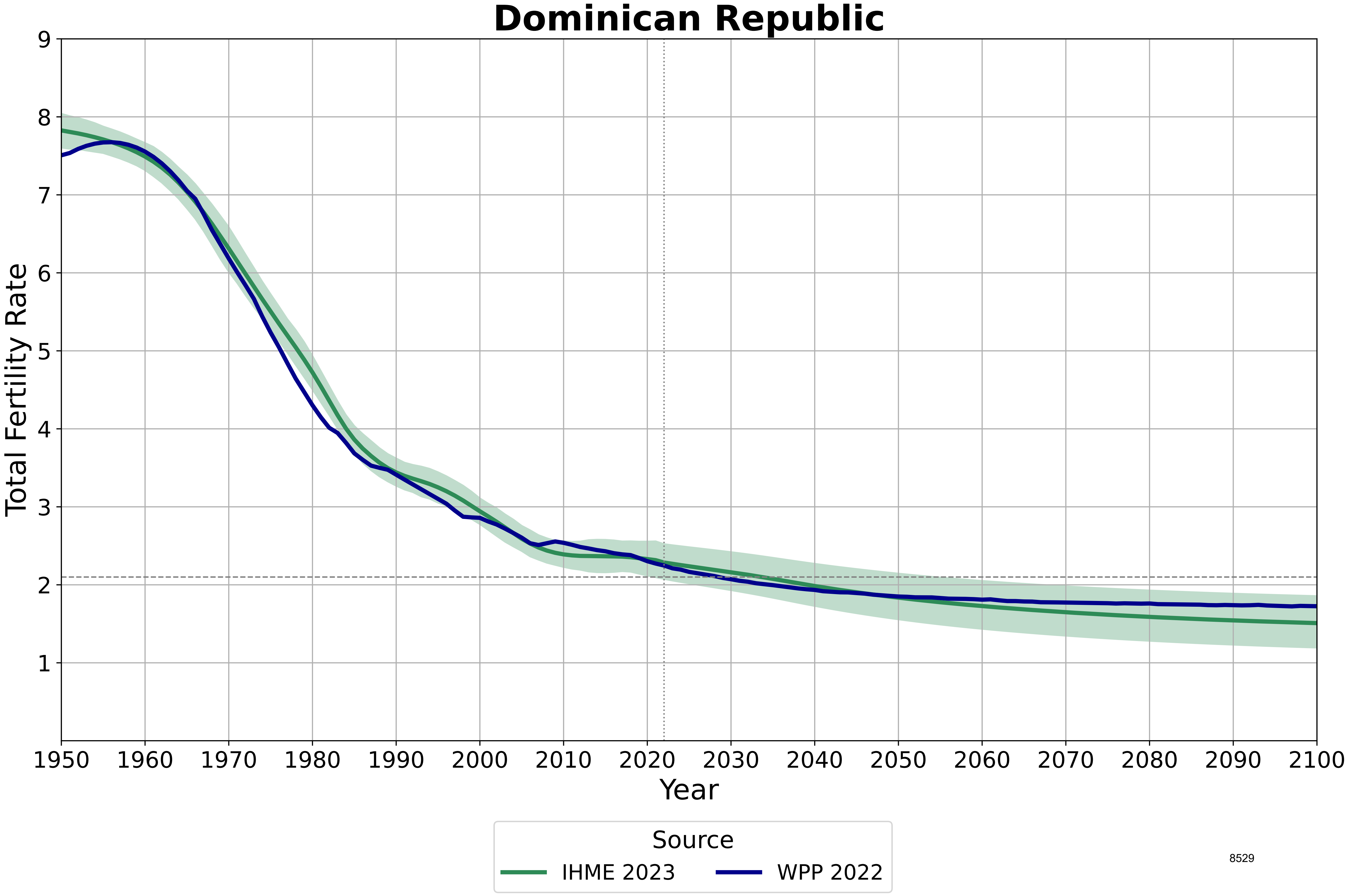

Supplemental Results Figure S7: TFR for IHME reference scenario and the UNPD medium variant scenario by location, 1950– 2100

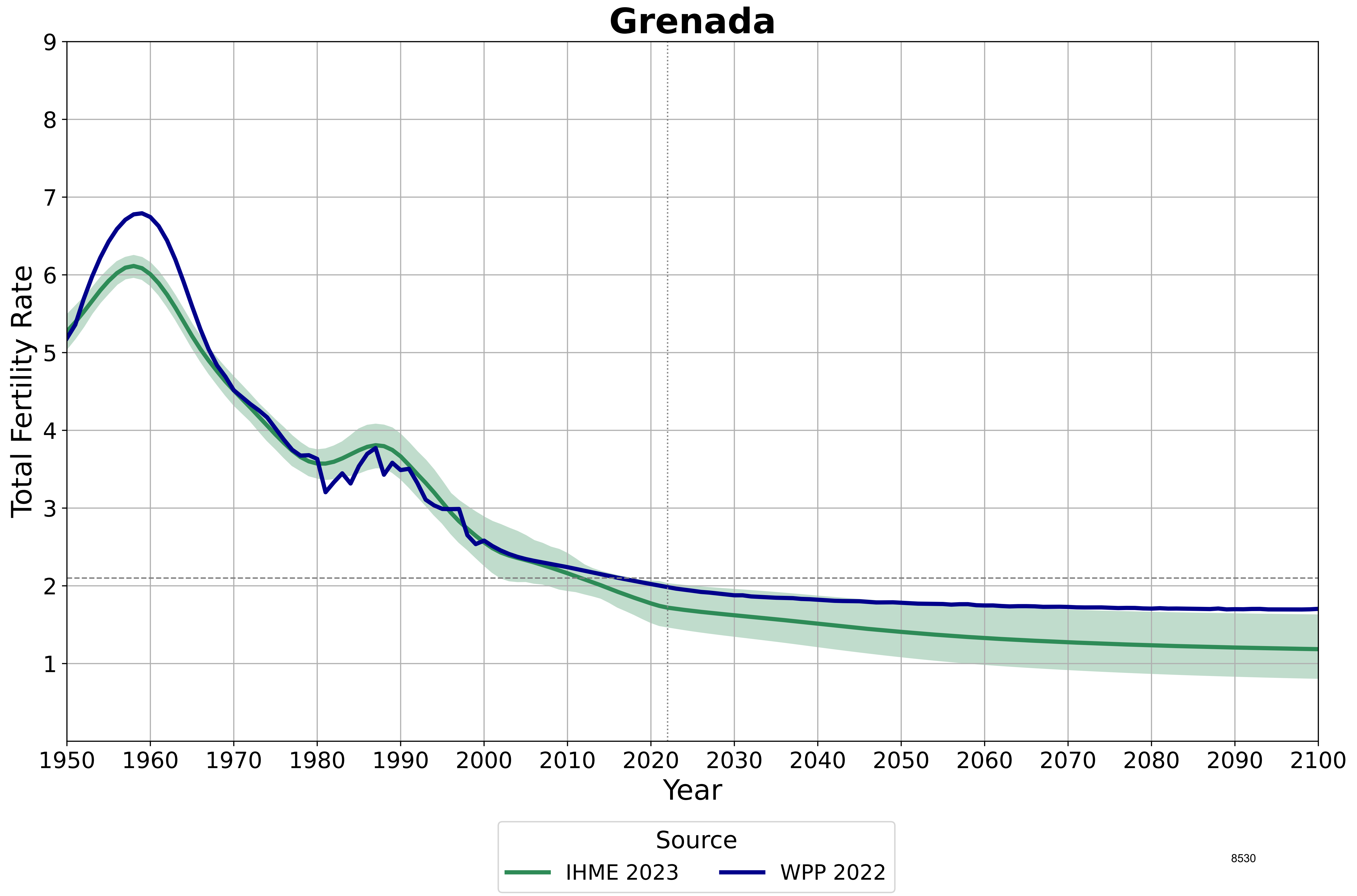

Supplemental Results Figure S7: TFR for IHME reference scenario and the UNPD medium variant scenario by location, 1950– 2100

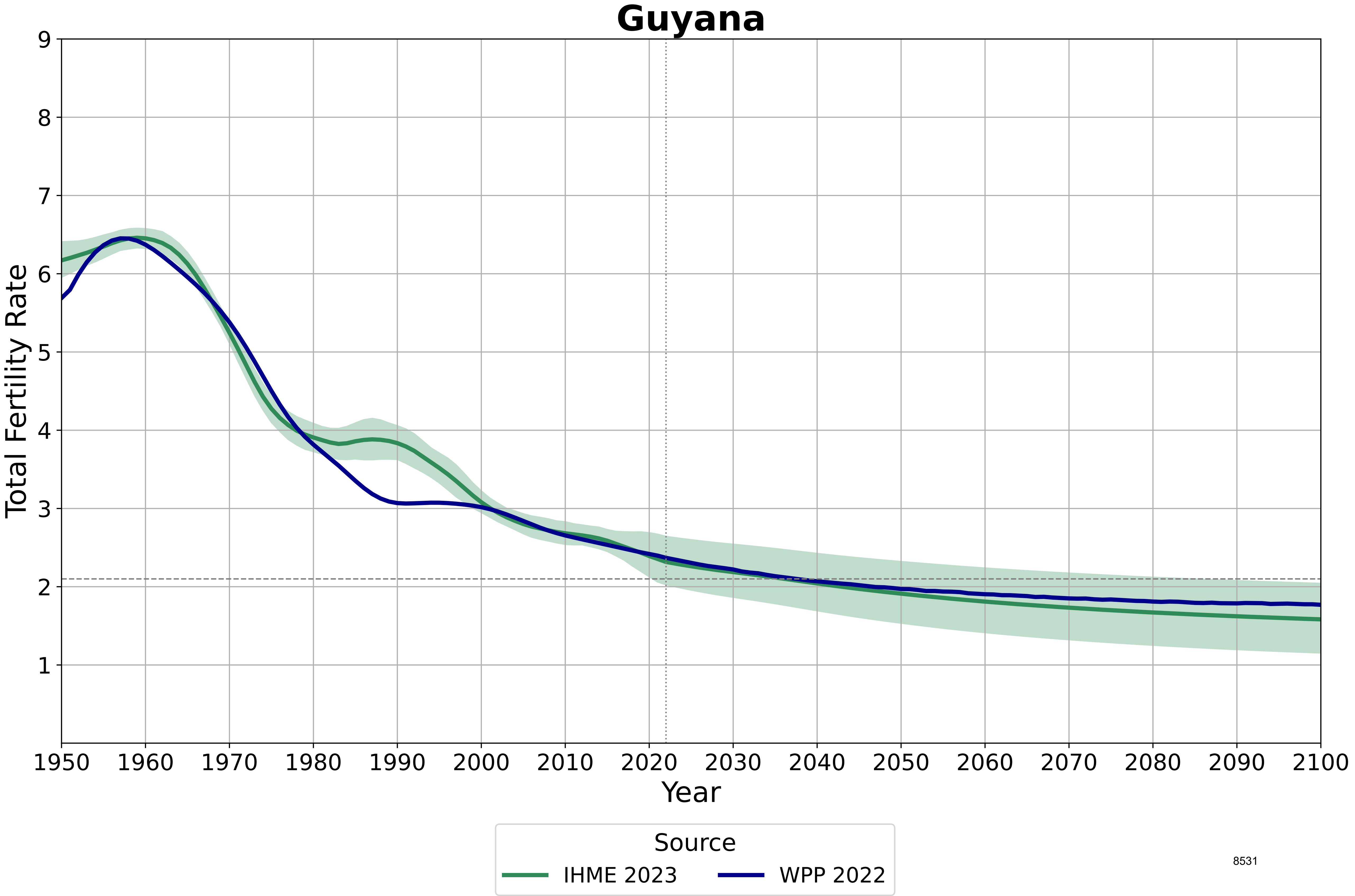

Supplemental Results Figure S7: TFR for IHME reference scenario and the UNPD medium variant scenario by location, 1950– 2100

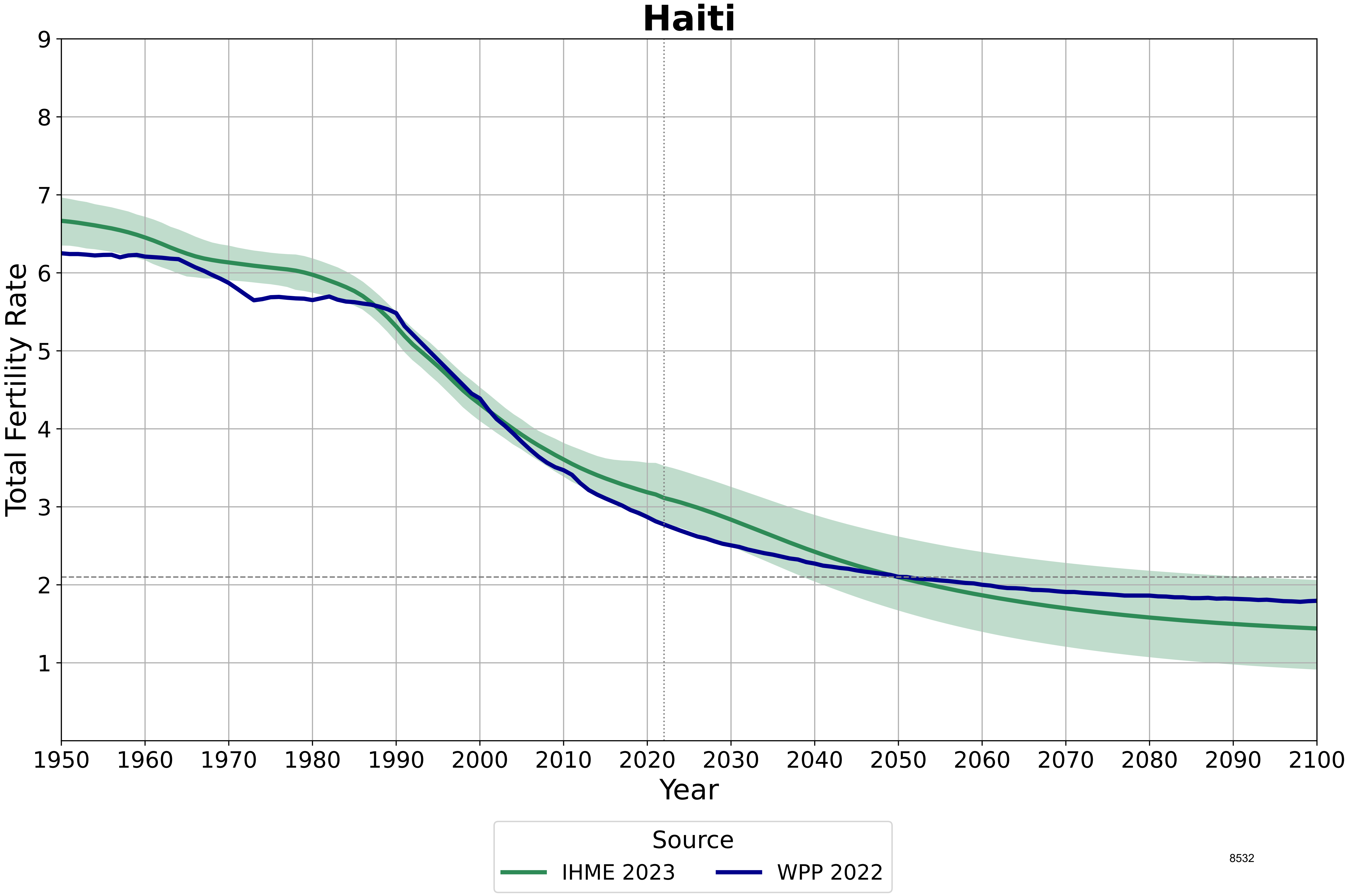

Supplemental Results Figure S7: TFR for IHME reference scenario and the UNPD medium variant scenario by location, 1950– 2100

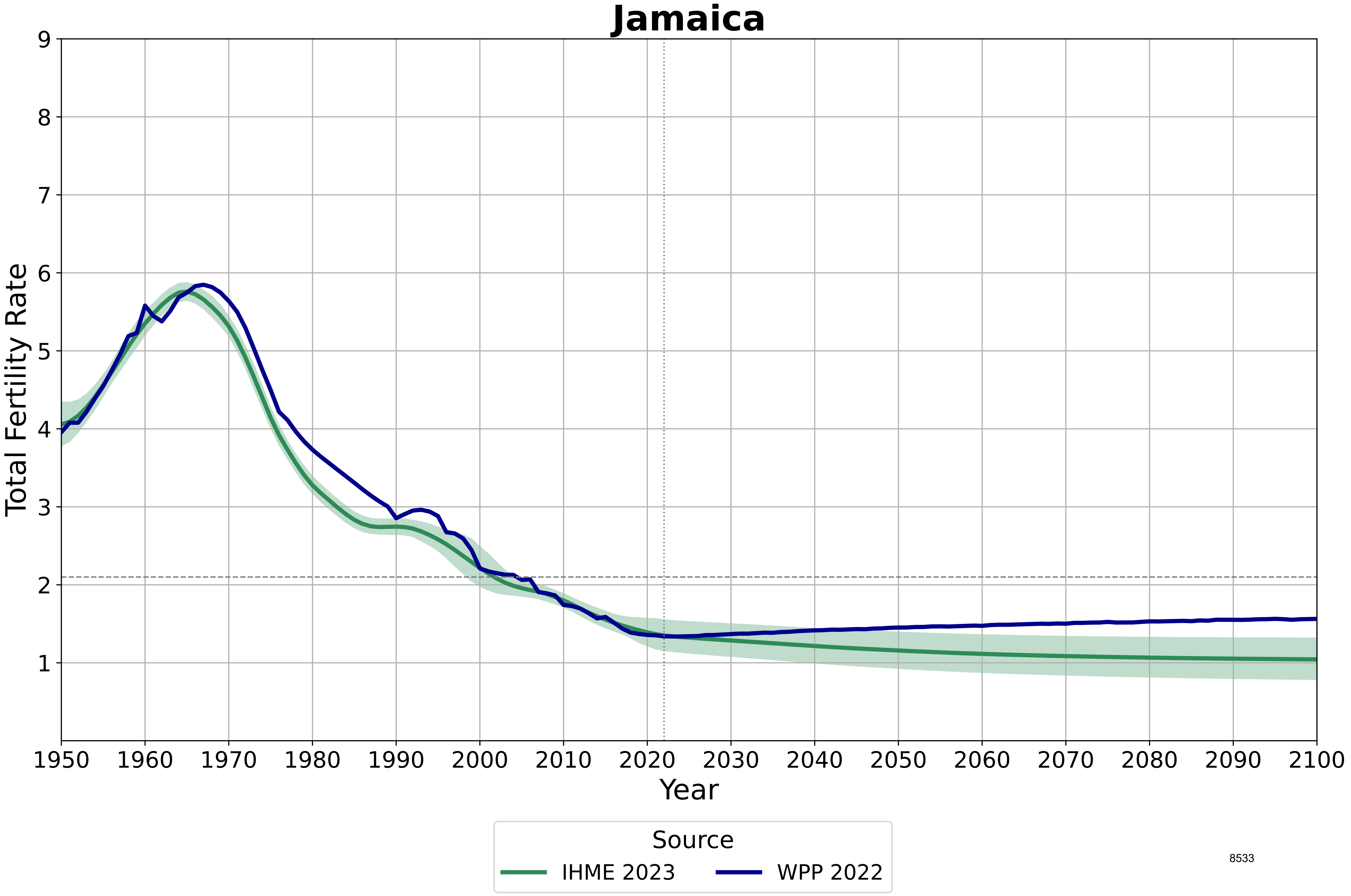

Supplemental Results Figure S7: TFR for IHME reference scenario and the UNPD medium variant scenario by location, 1950– 2100

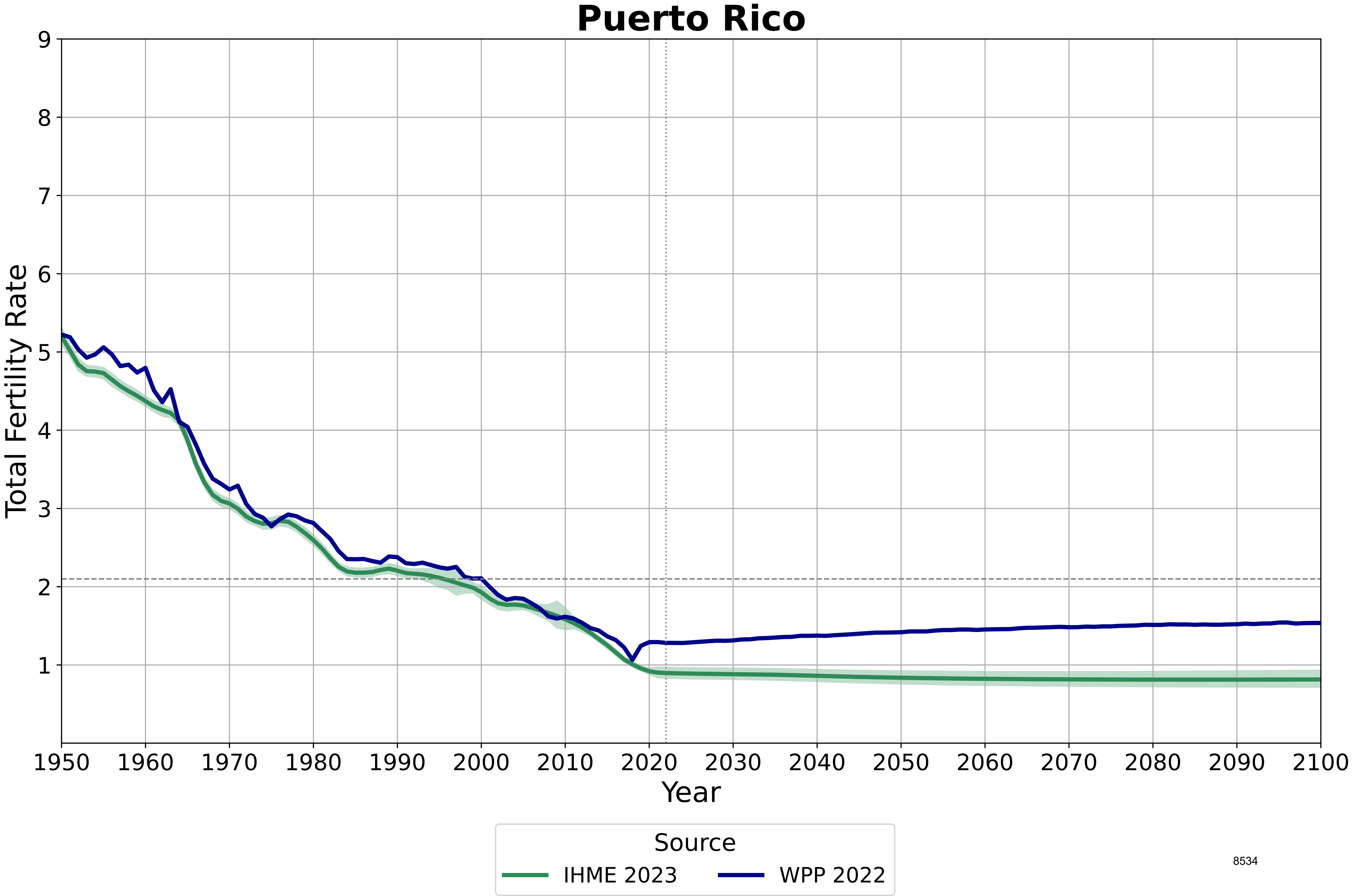

Supplemental Results Figure S7: TFR for IHME reference scenario and the UNPD medium variant scenario by location, 1950– 2100

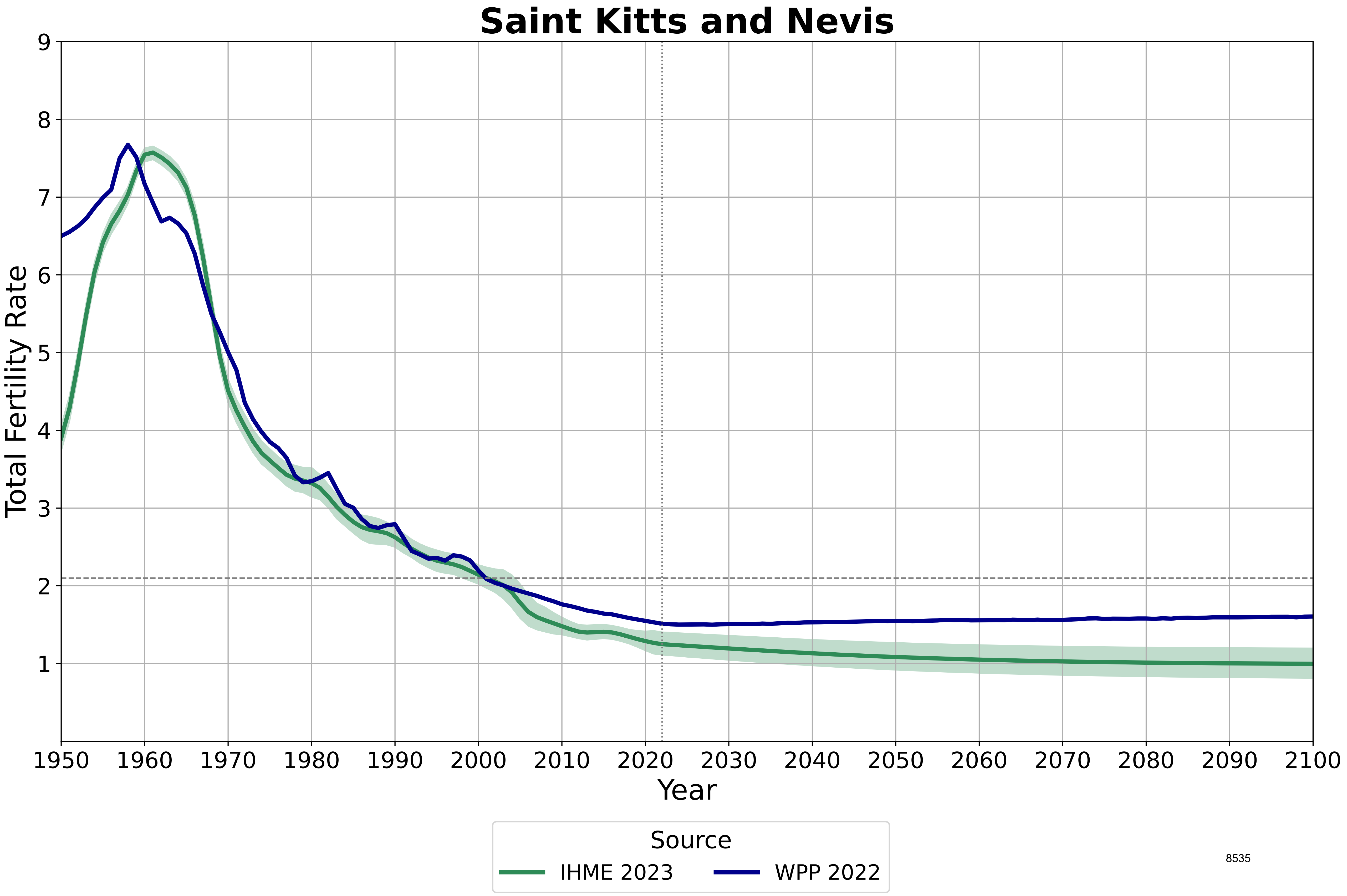

Supplemental Results Figure S7: TFR for IHME reference scenario and the UNPD medium variant scenario by location, 1950– 2100

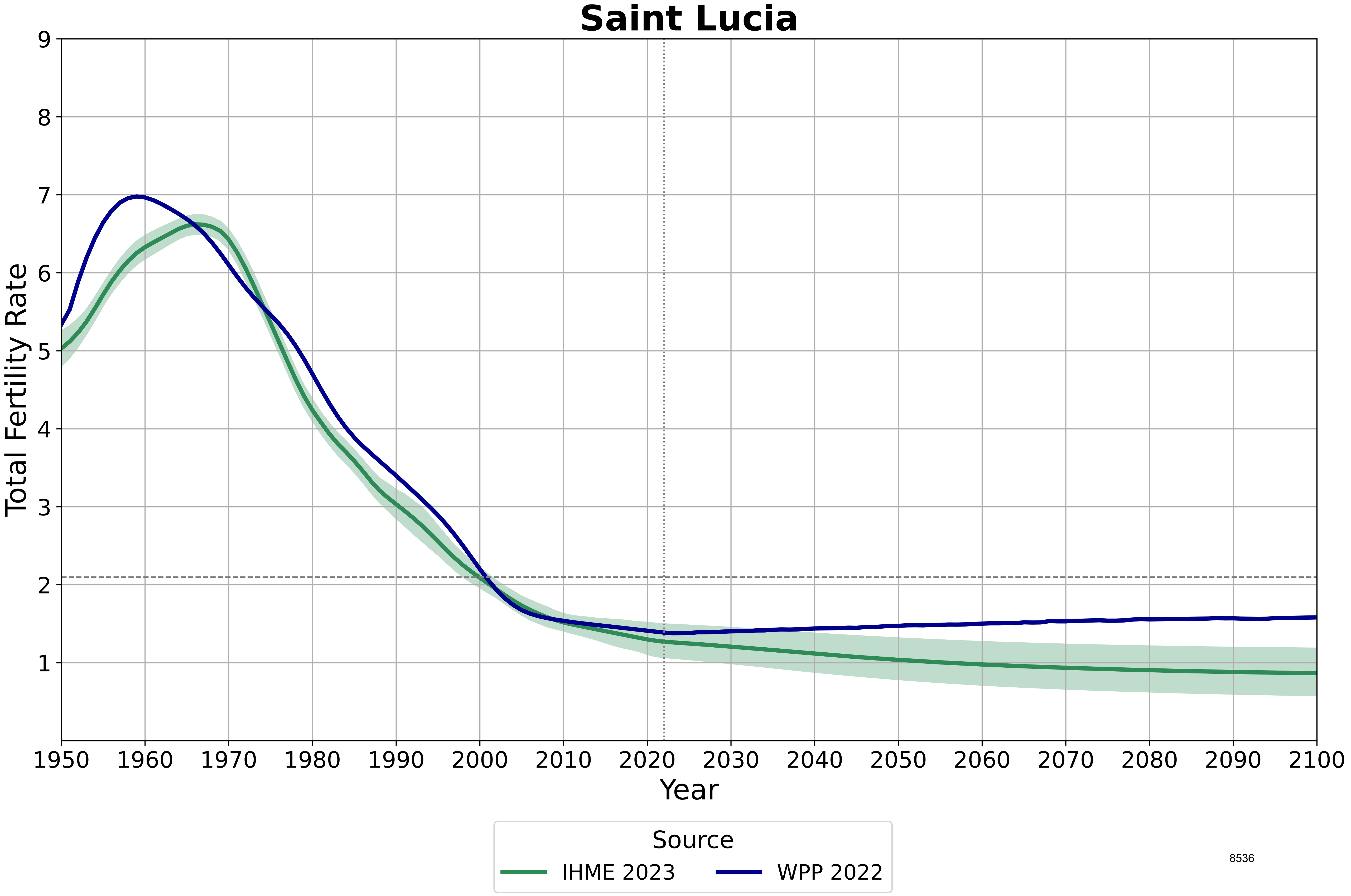

Supplemental Results Figure S7: TFR for IHME reference scenario and the UNPD medium variant scenario by location, 1950– 2100

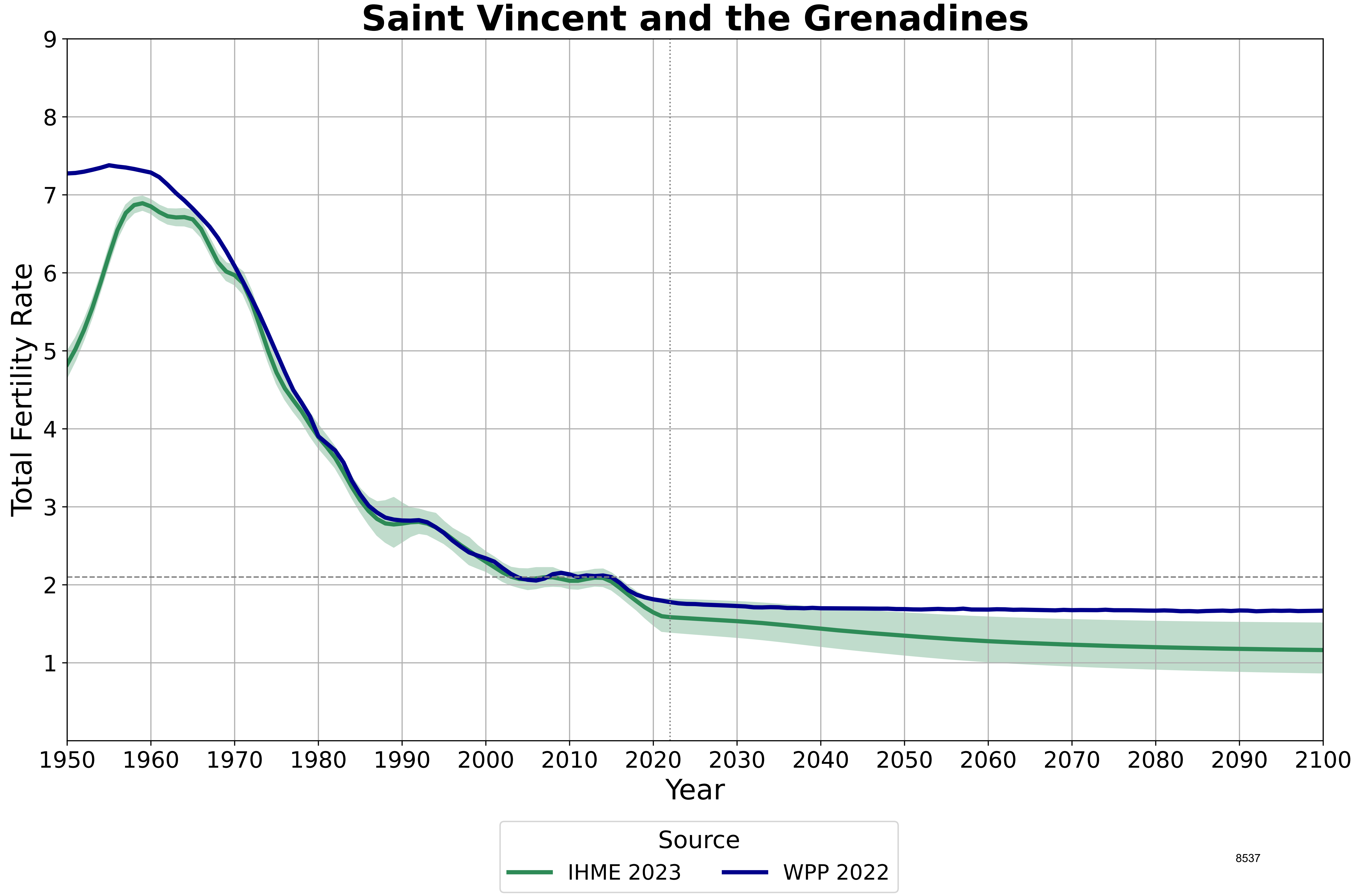

8537

Supplemental Results Figure S7: TFR for IHME reference scenario and the UNPD medium variant scenario by location, 1950– 2100

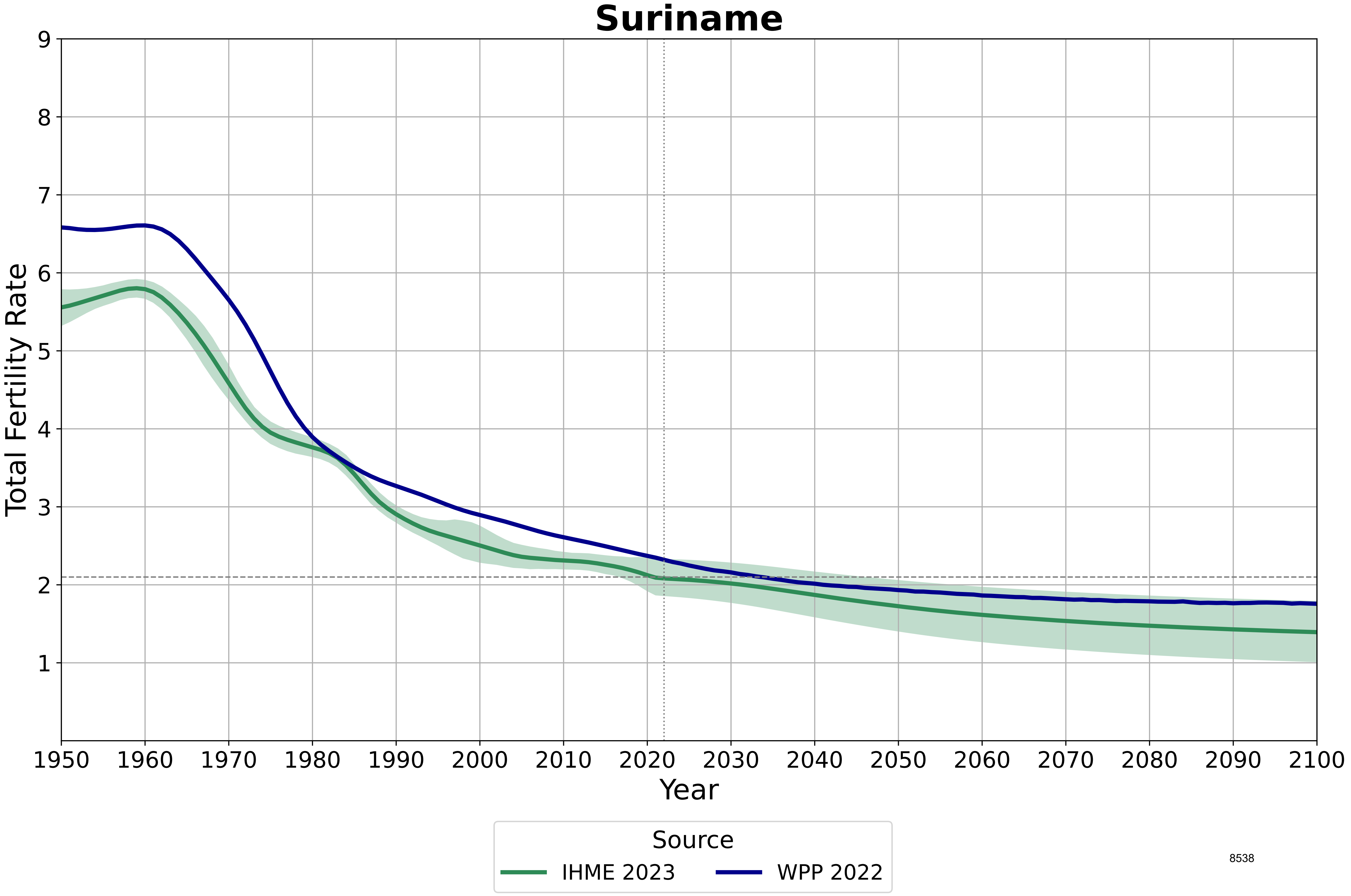

Supplemental Results Figure S7: TFR for IHME reference scenario and the UNPD medium variant scenario by location, 1950– 2100

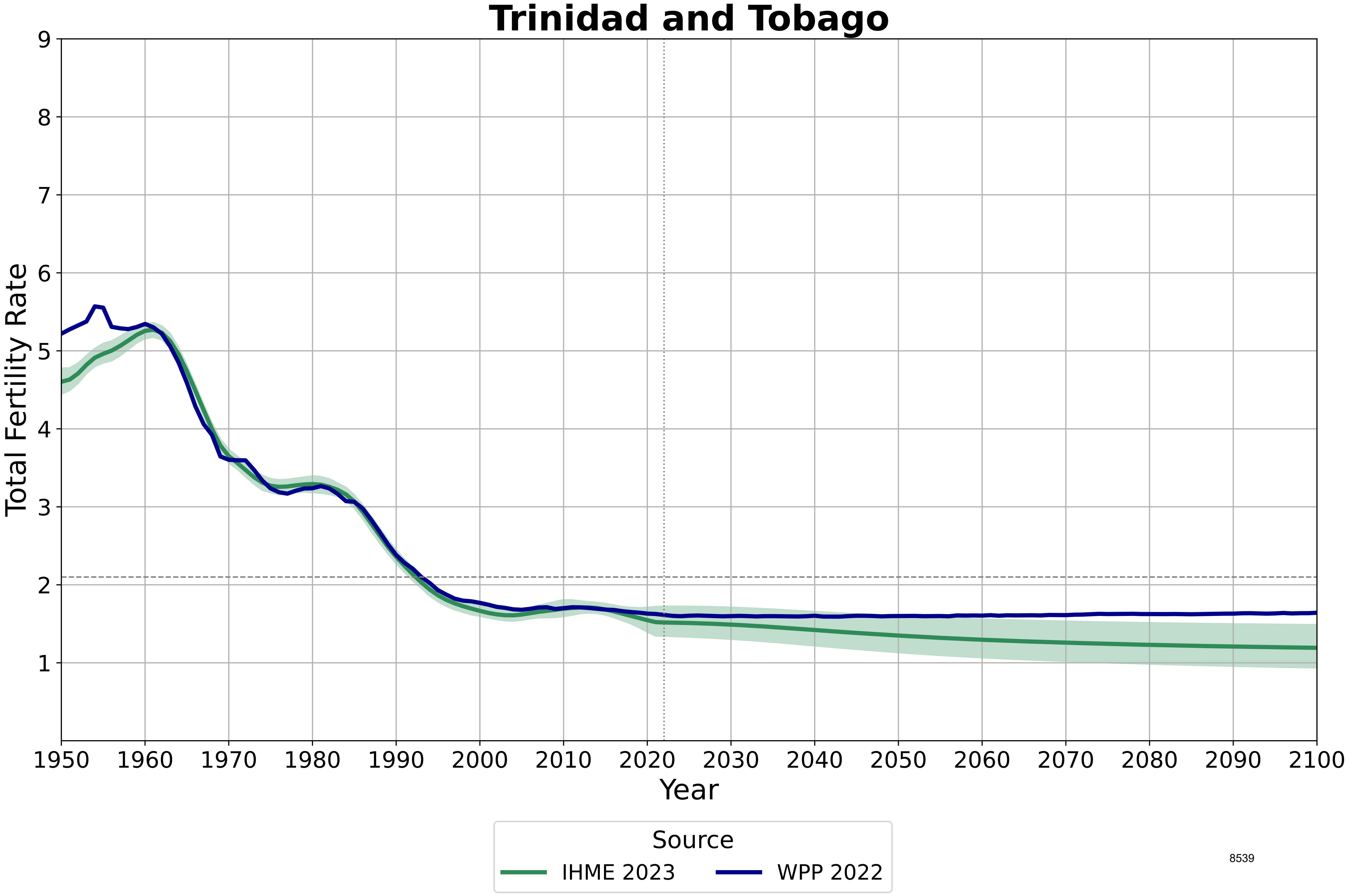

Supplemental Results Figure S7: TFR for IHME reference scenario and the UNPD medium variant scenario by location, 1950– 2100

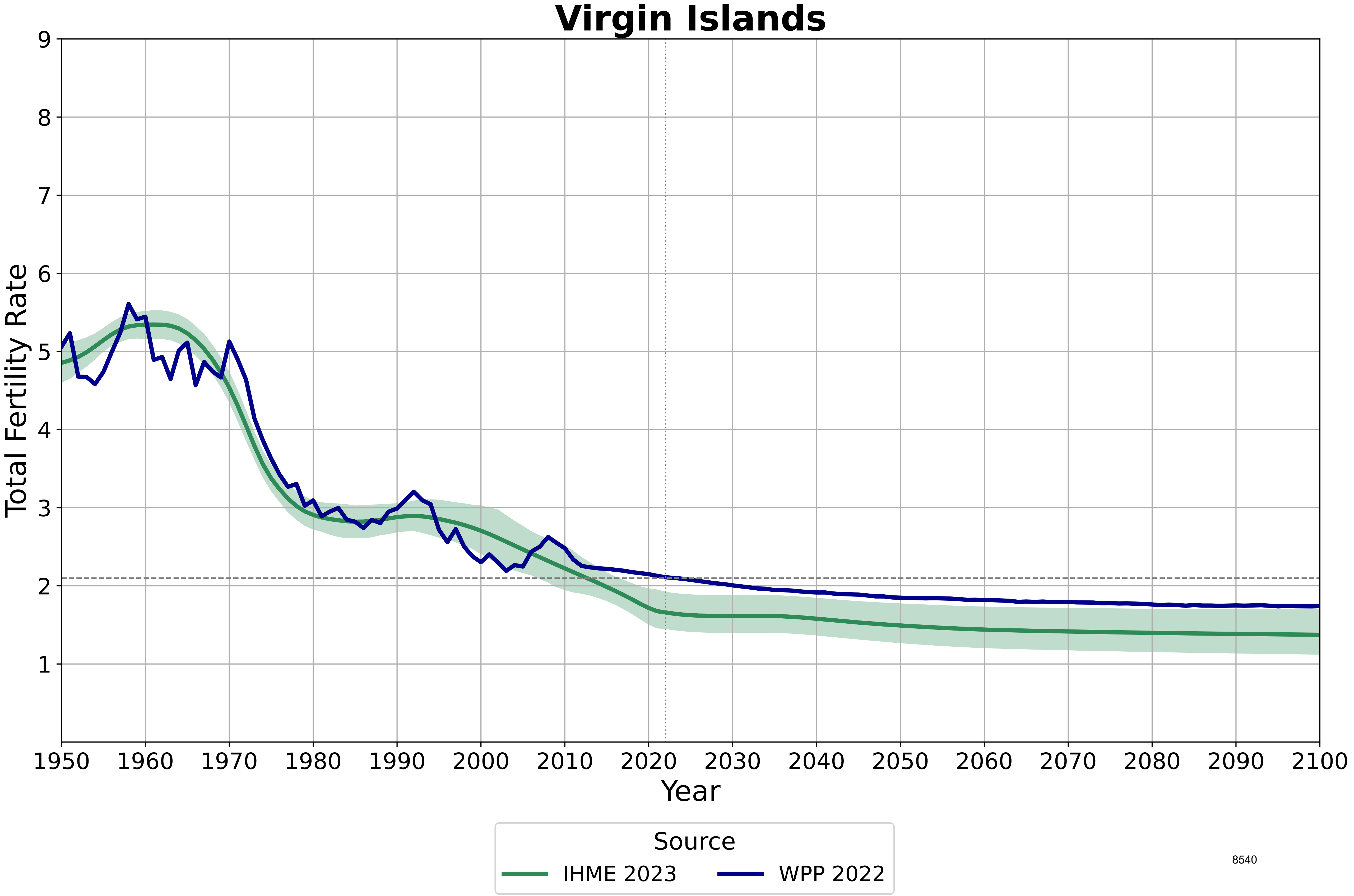

Supplemental Results Figure S7: TFR for IHME reference scenario and the UNPD medium variant scenario by location, 1950– 2100

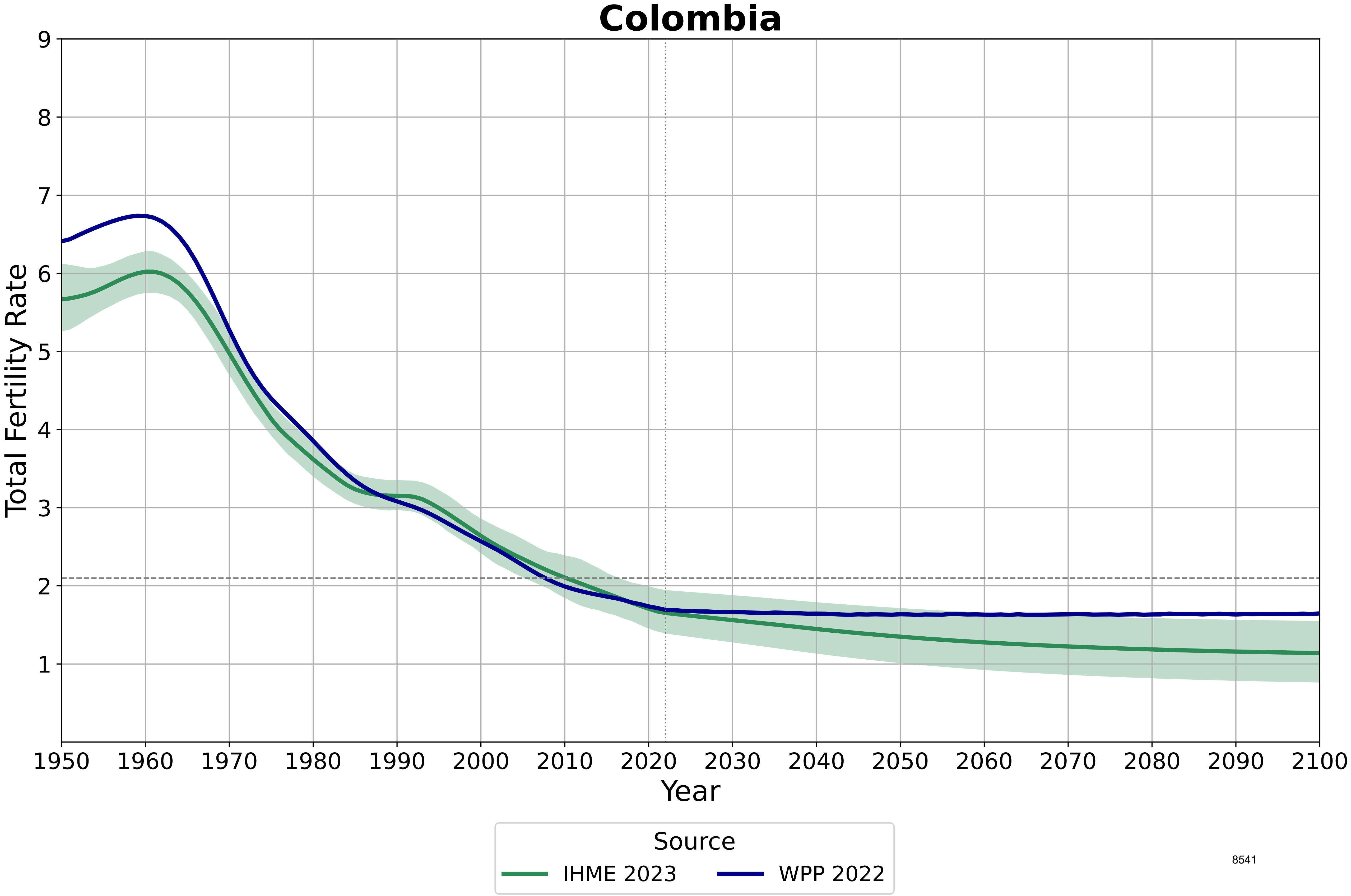

Supplemental Results Figure S7: TFR for IHME reference scenario and the UNPD medium variant scenario by location, 1950– 2100

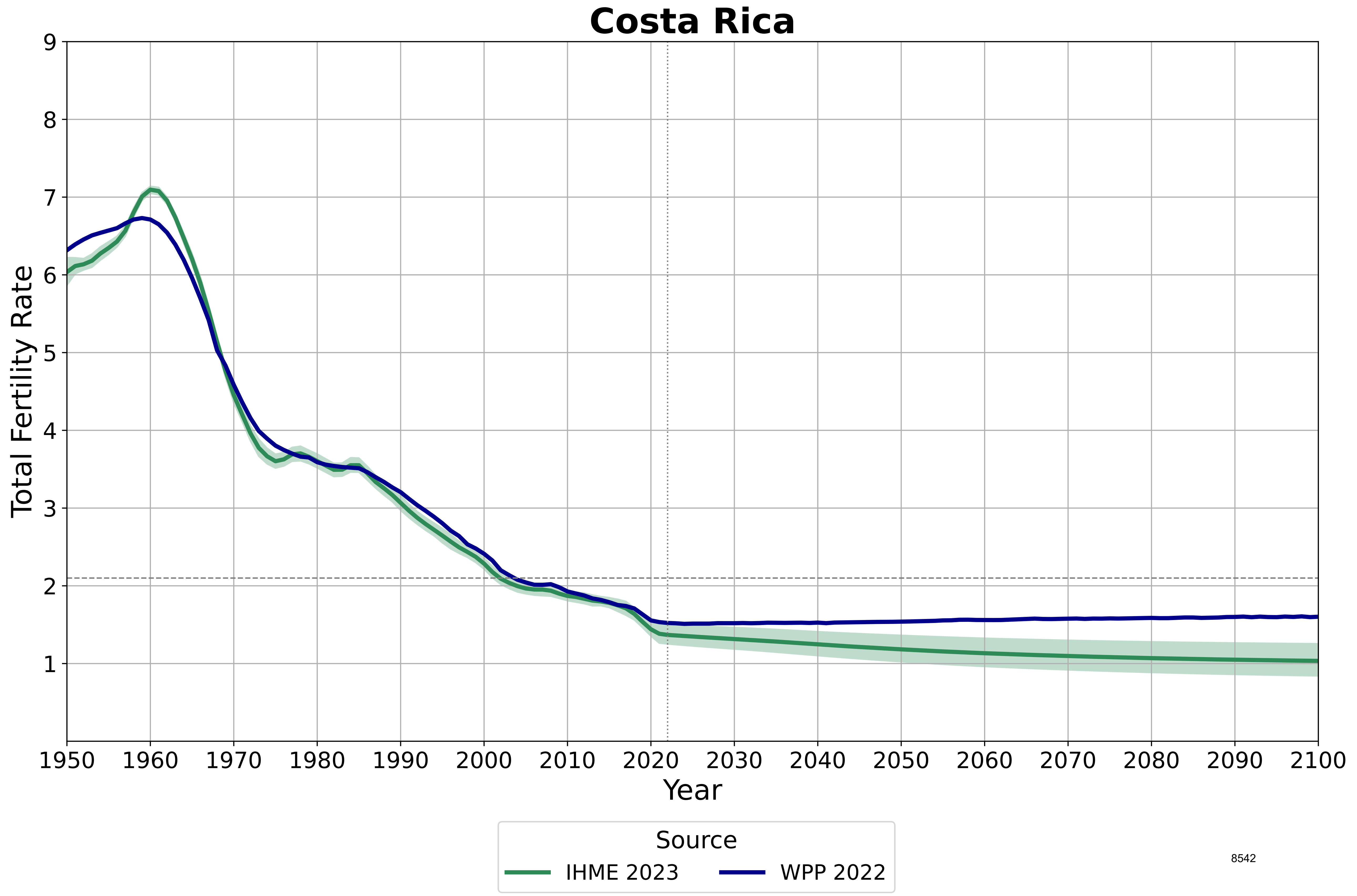

Supplemental Results Figure S7: TFR for IHME reference scenario and the UNPD medium variant scenario by location, 1950– 2100

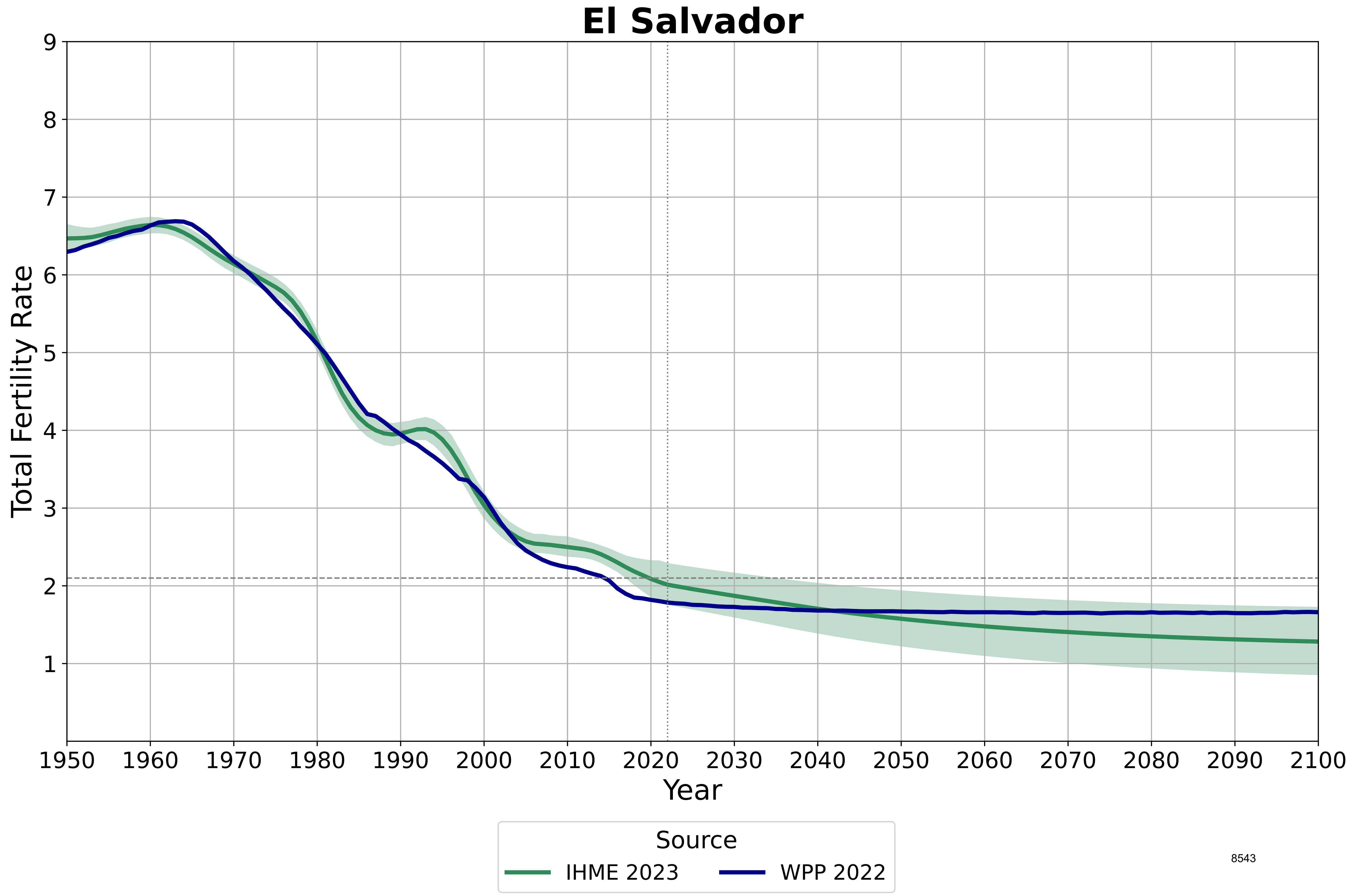

Supplemental Results Figure S7: TFR for IHME reference scenario and the UNPD medium variant scenario by location, 1950– 2100

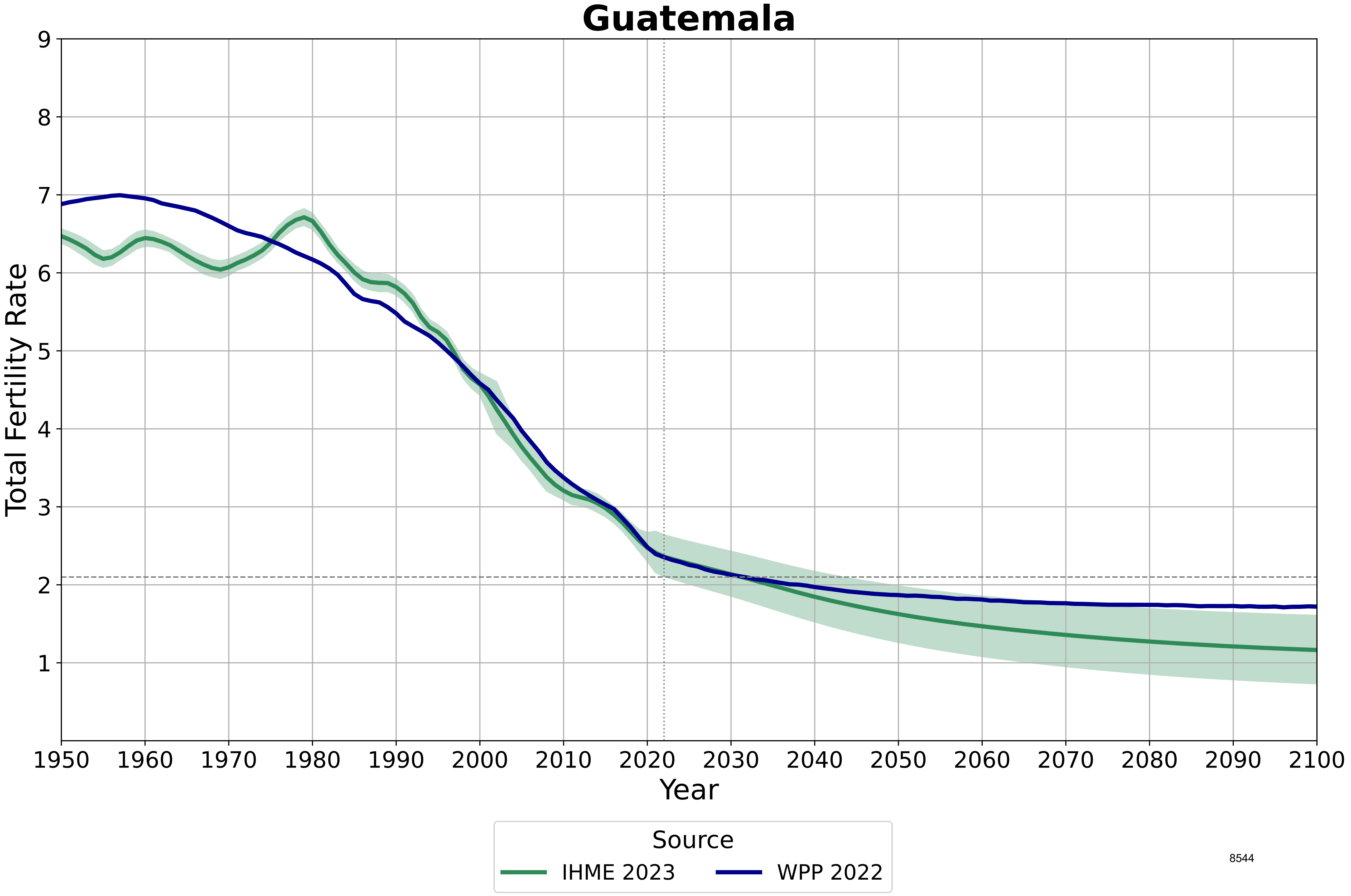

Supplemental Results Figure S7: TFR for IHME reference scenario and the UNPD medium variant scenario by location, 1950– 2100

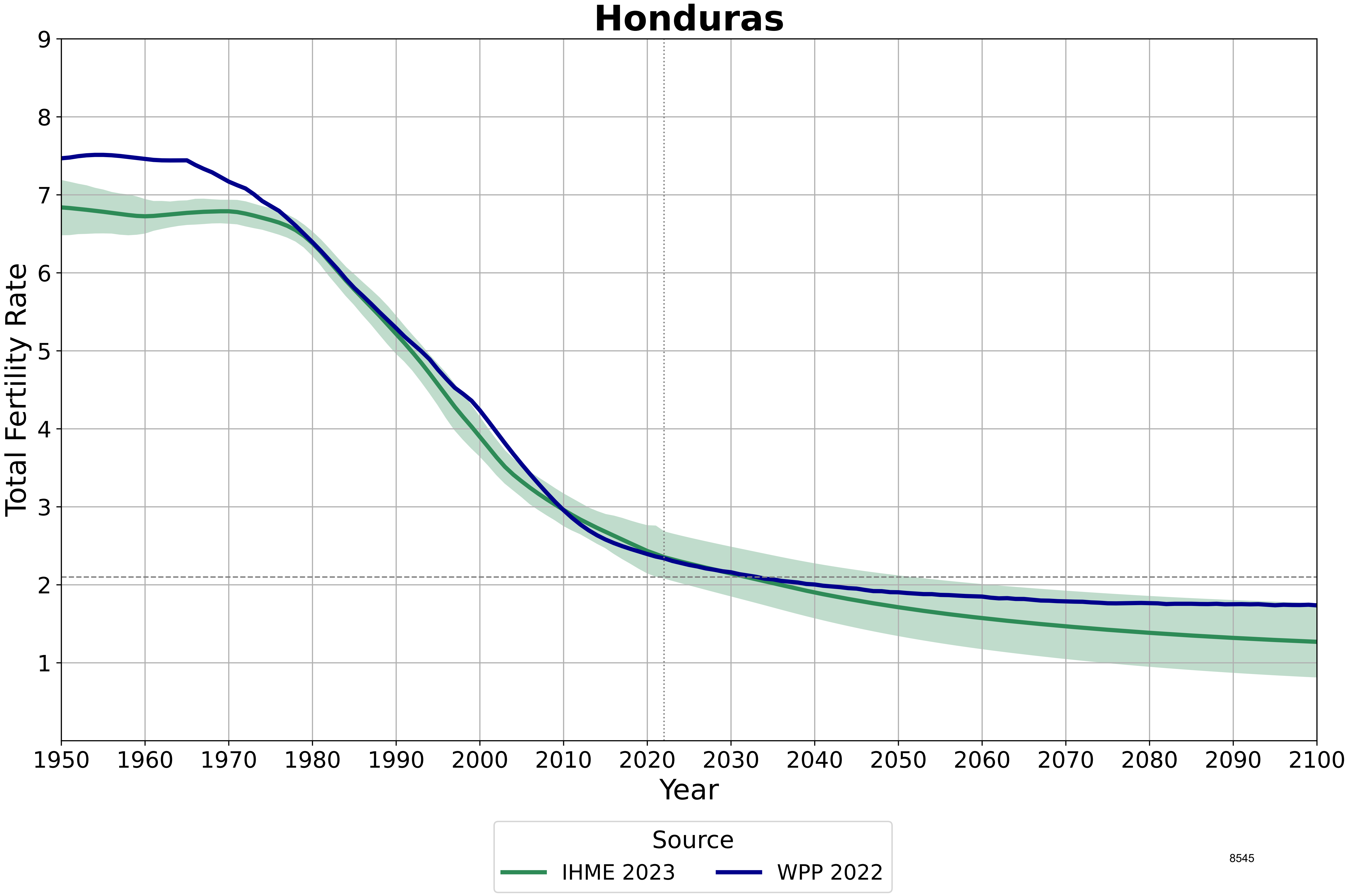

Supplemental Results Figure S7: TFR for IHME reference scenario and the UNPD medium variant scenario by location, 1950– 2100

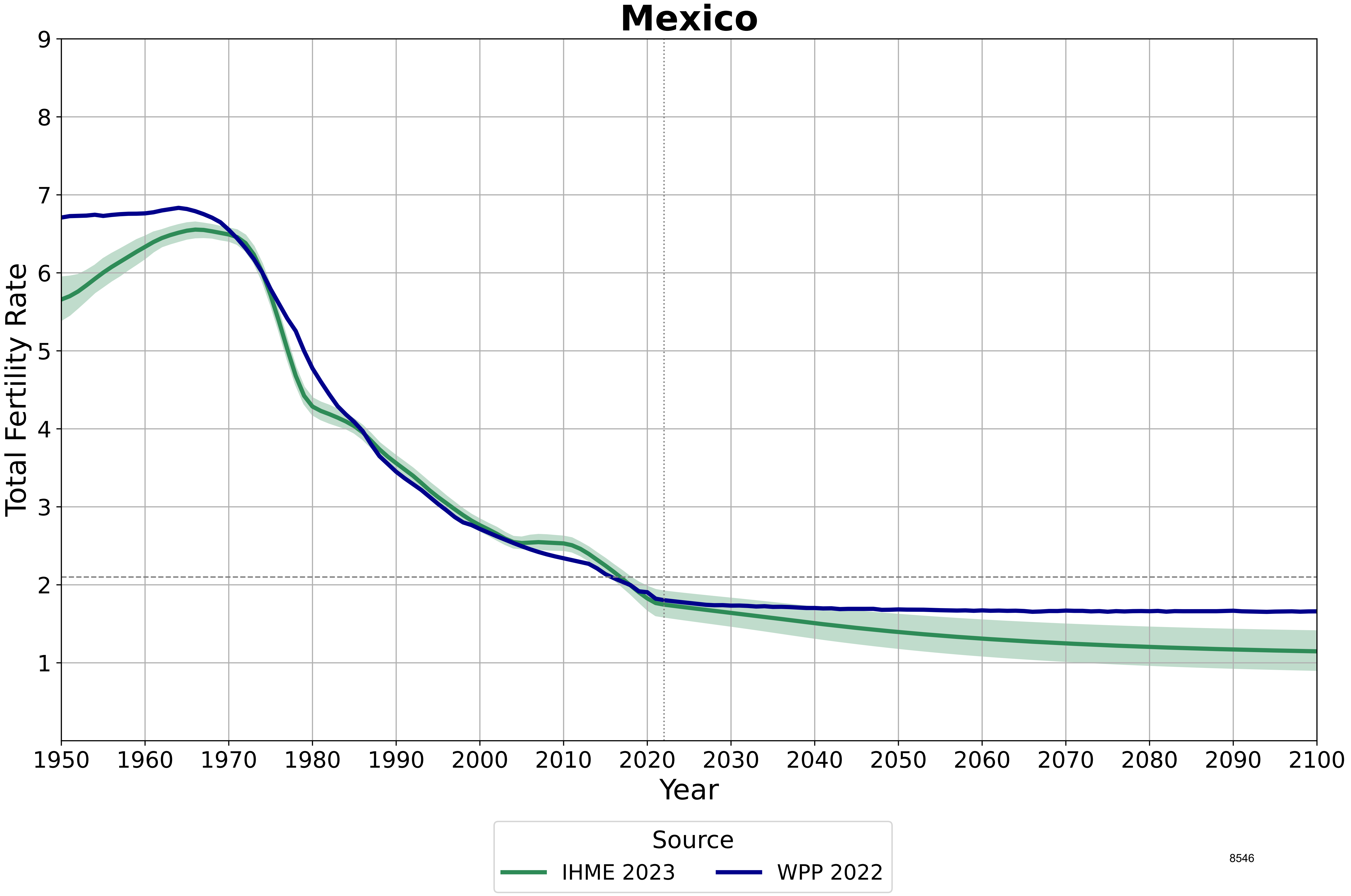

Supplemental Results Figure S7: TFR for IHME reference scenario and the UNPD medium variant scenario by location, 1950– 2100

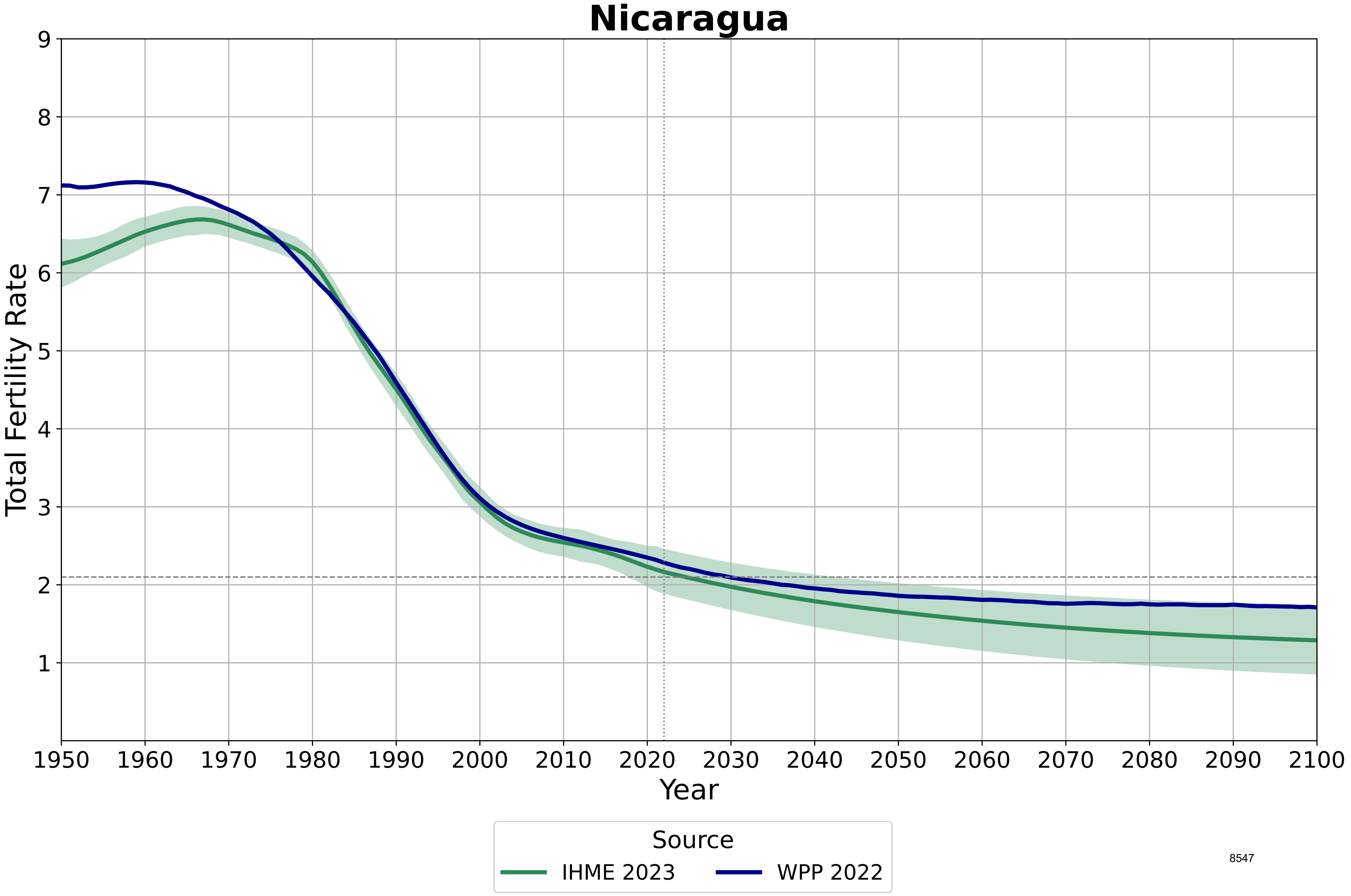

Supplemental Results Figure S7: TFR for IHME reference scenario and the UNPD medium variant scenario by location, 1950– 2100

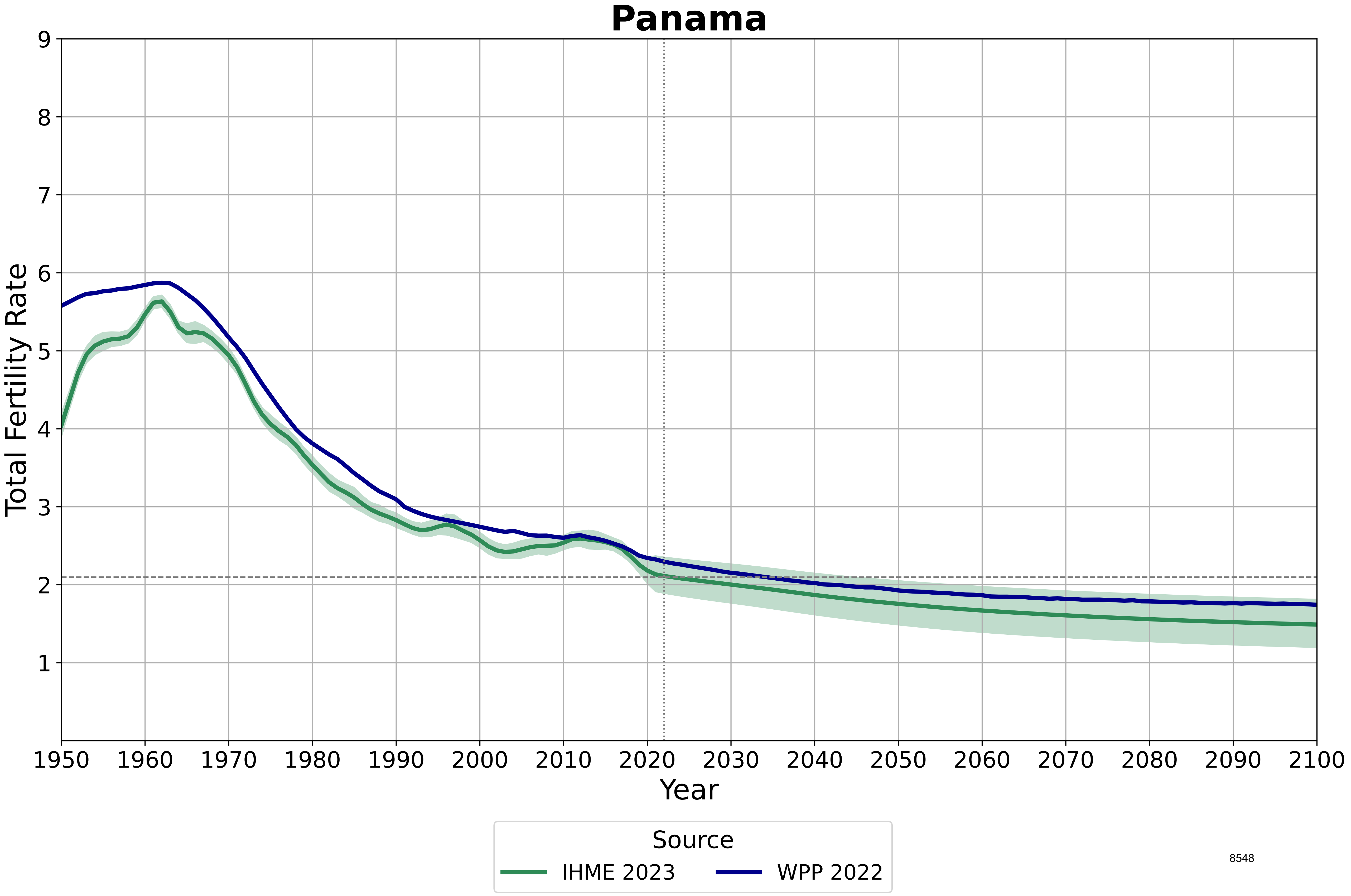

Supplemental Results Figure S7: TFR for IHME reference scenario and the UNPD medium variant scenario by location, 1950– 2100

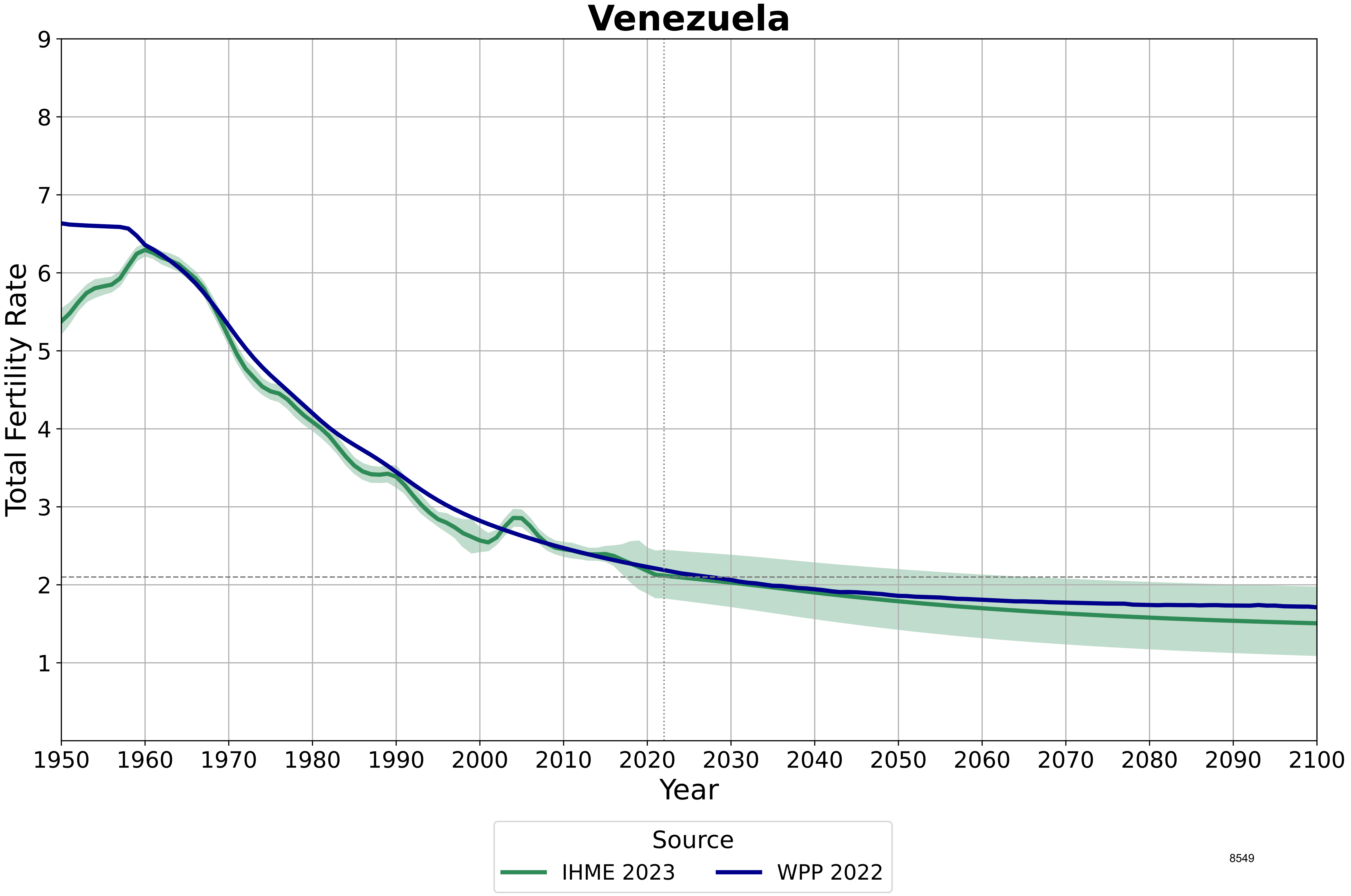

Supplemental Results Figure S7: TFR for IHME reference scenario and the UNPD medium variant scenario by location, 1950– 2100

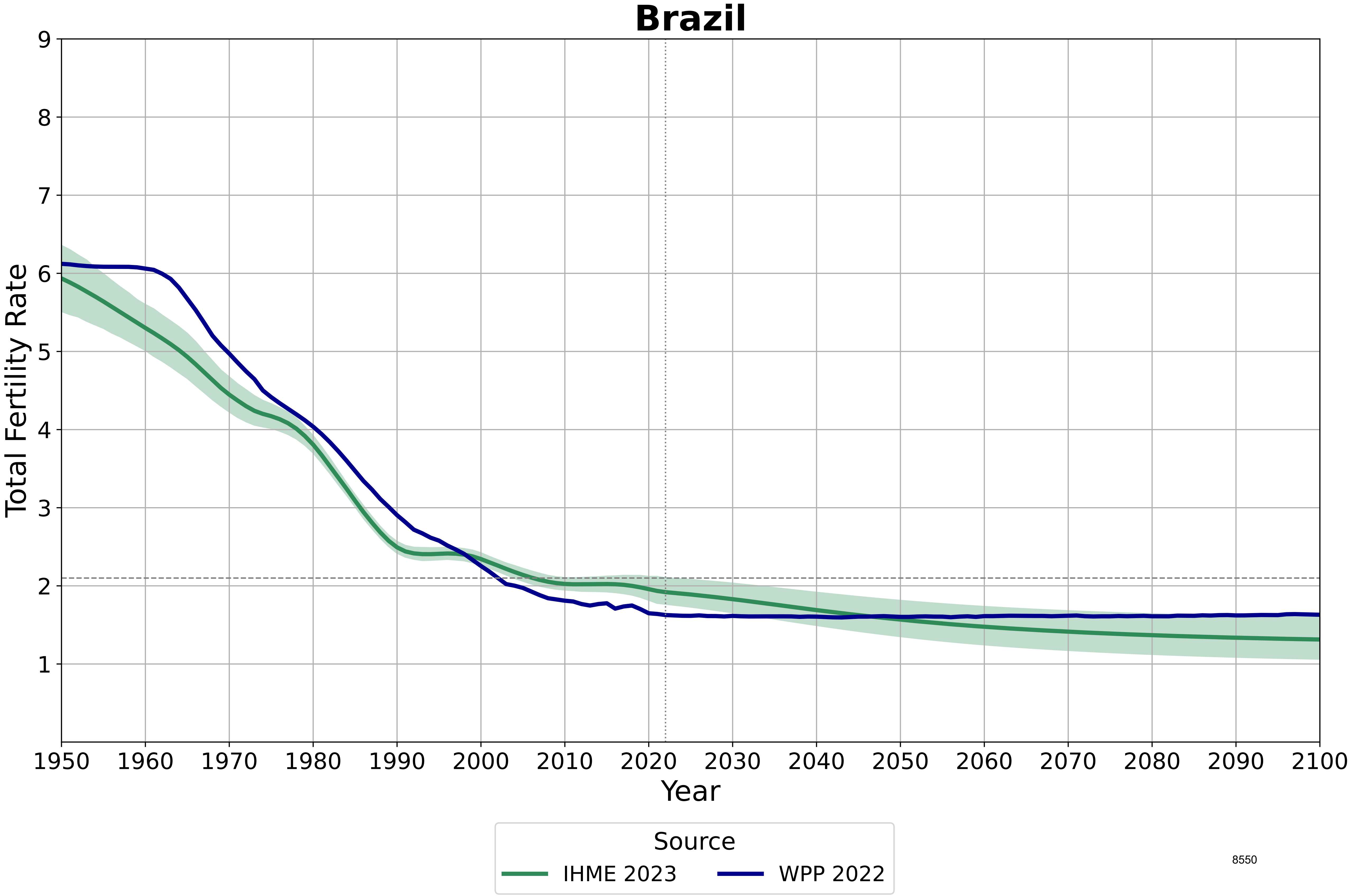

Supplemental Results Figure S7: TFR for IHME reference scenario and the UNPD medium variant scenario by location, 1950– 2100

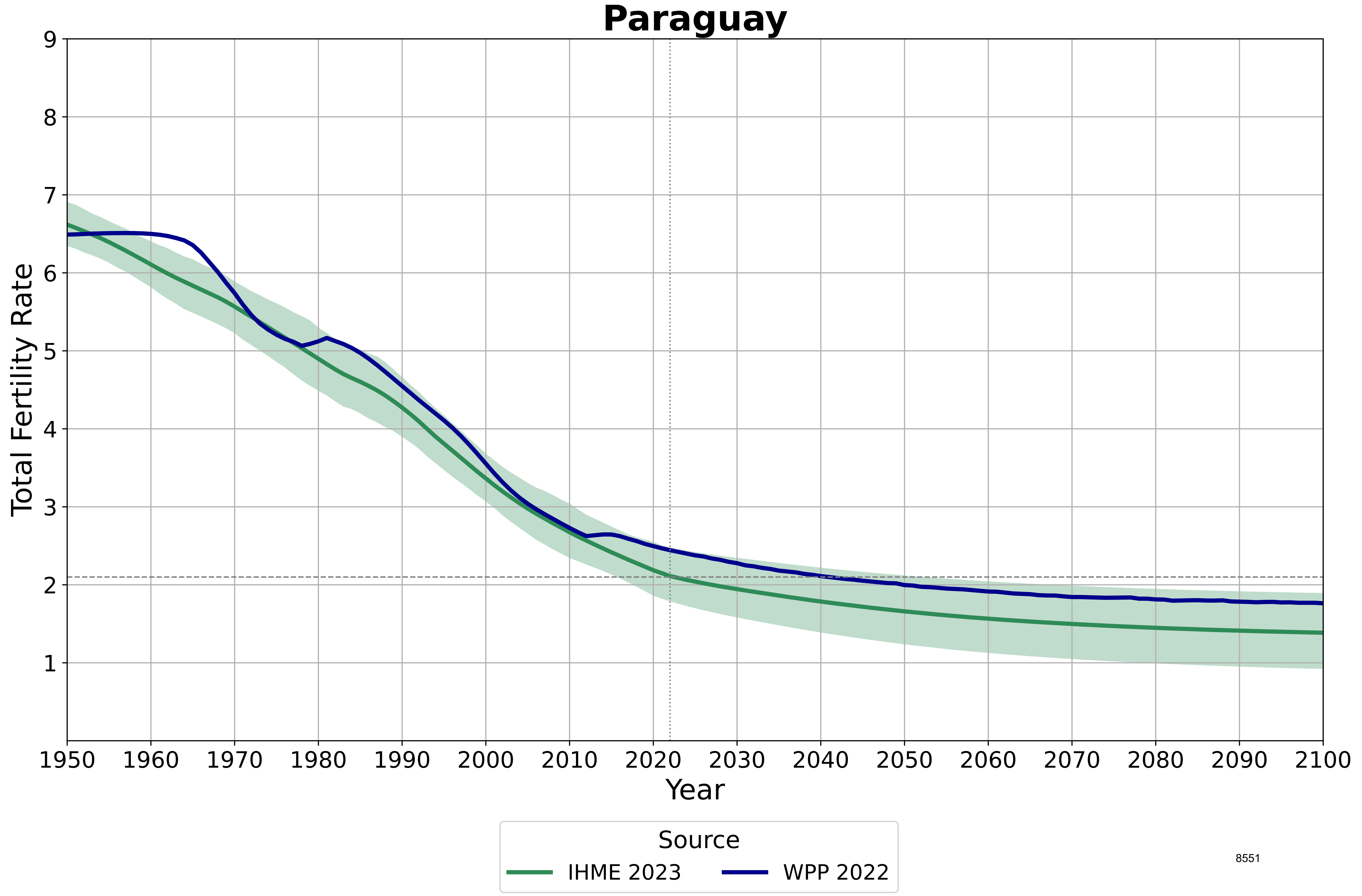

Supplemental Results Figure S7: TFR for IHME reference scenario and the UNPD medium variant scenario by location, 1950– 2100

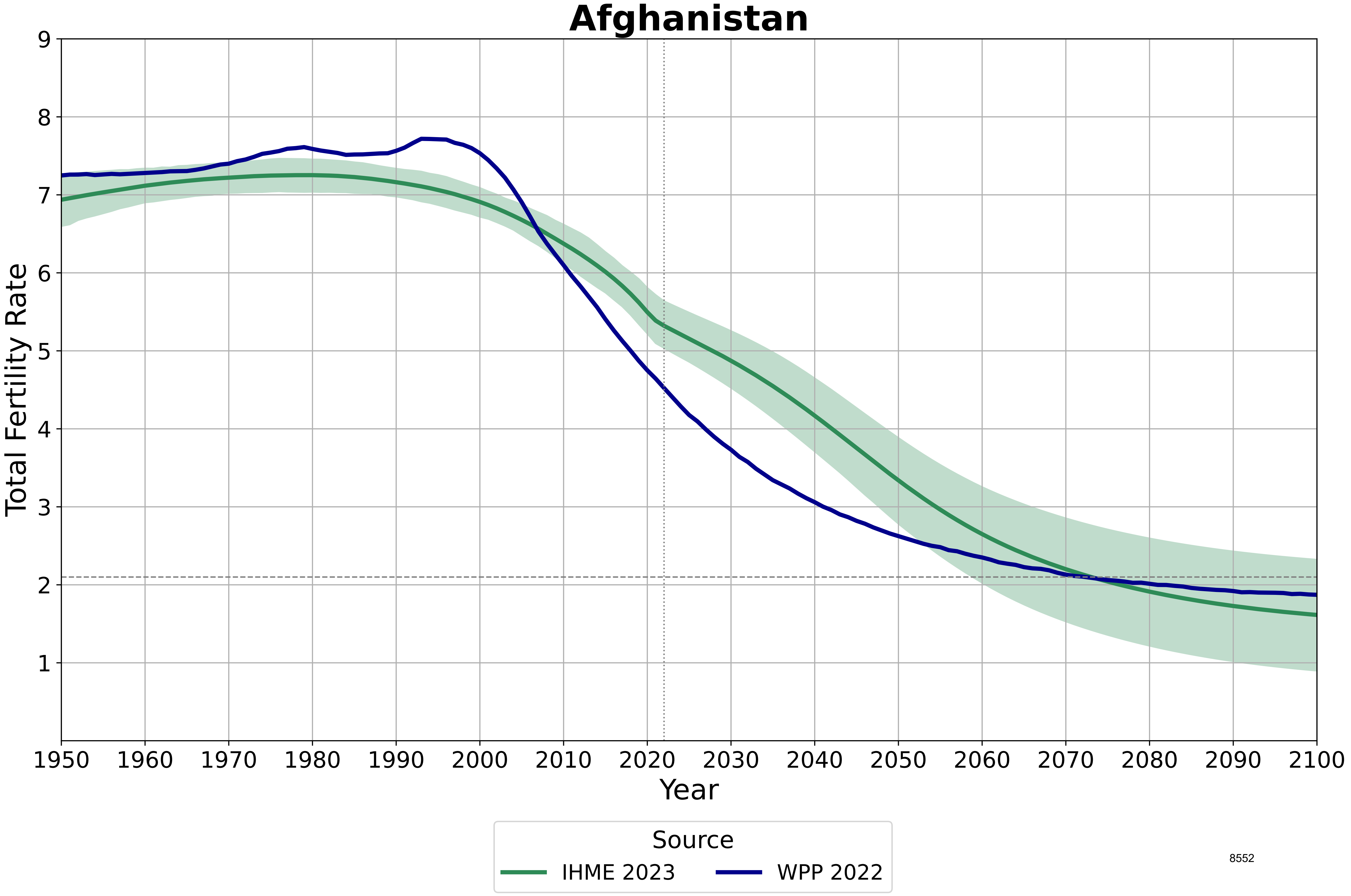

Supplemental Results Figure S7: TFR for IHME reference scenario and the UNPD medium variant scenario by location, 1950– 2100

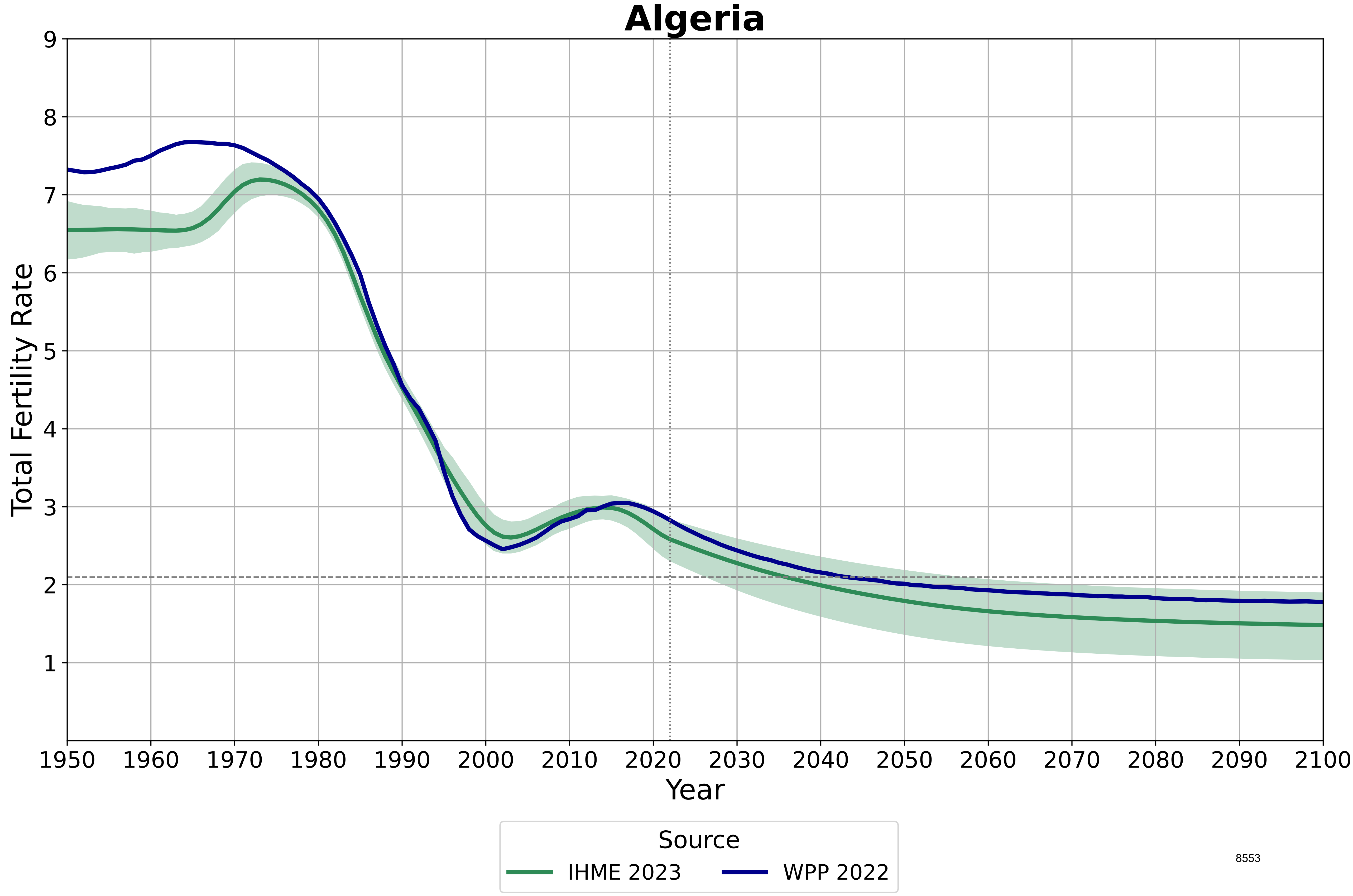

Supplemental Results Figure S7: TFR for IHME reference scenario and the UNPD medium variant scenario by location, 1950– 2100

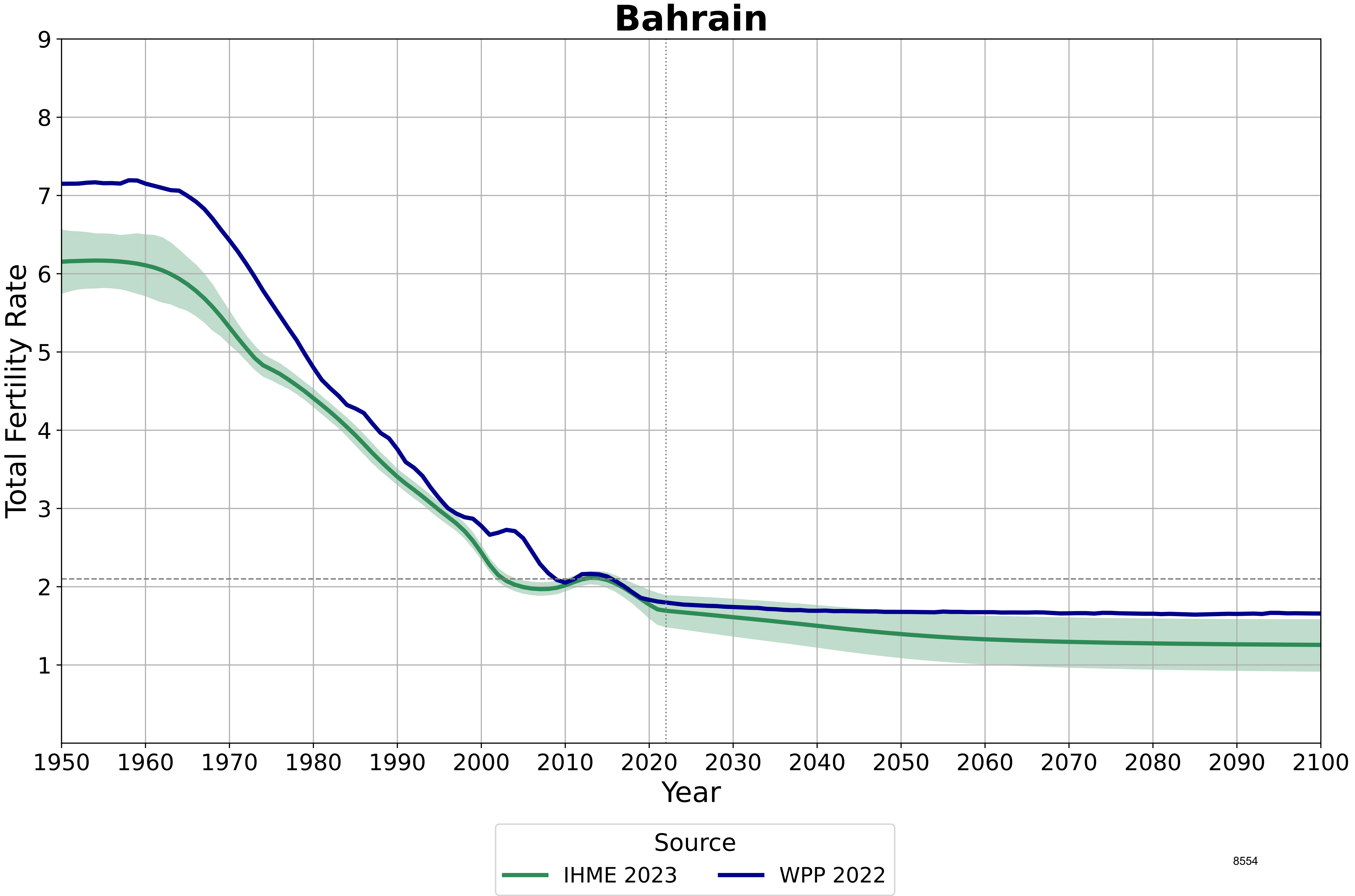

Supplemental Results Figure S7: TFR for IHME reference scenario and the UNPD medium variant scenario by location, 1950– 2100

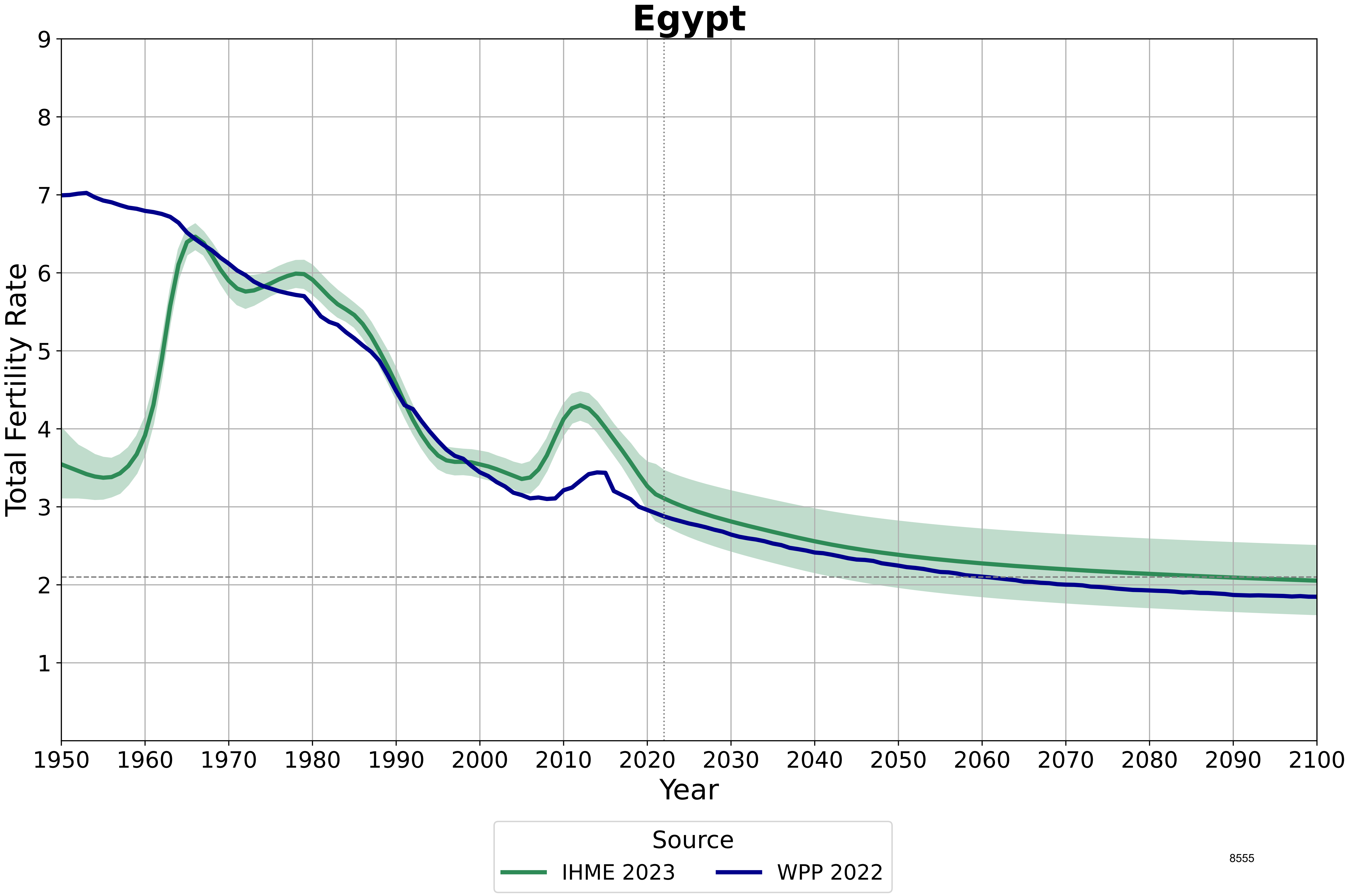

Supplemental Results Figure S7: TFR for IHME reference scenario and the UNPD medium variant scenario by location, 1950– 2100

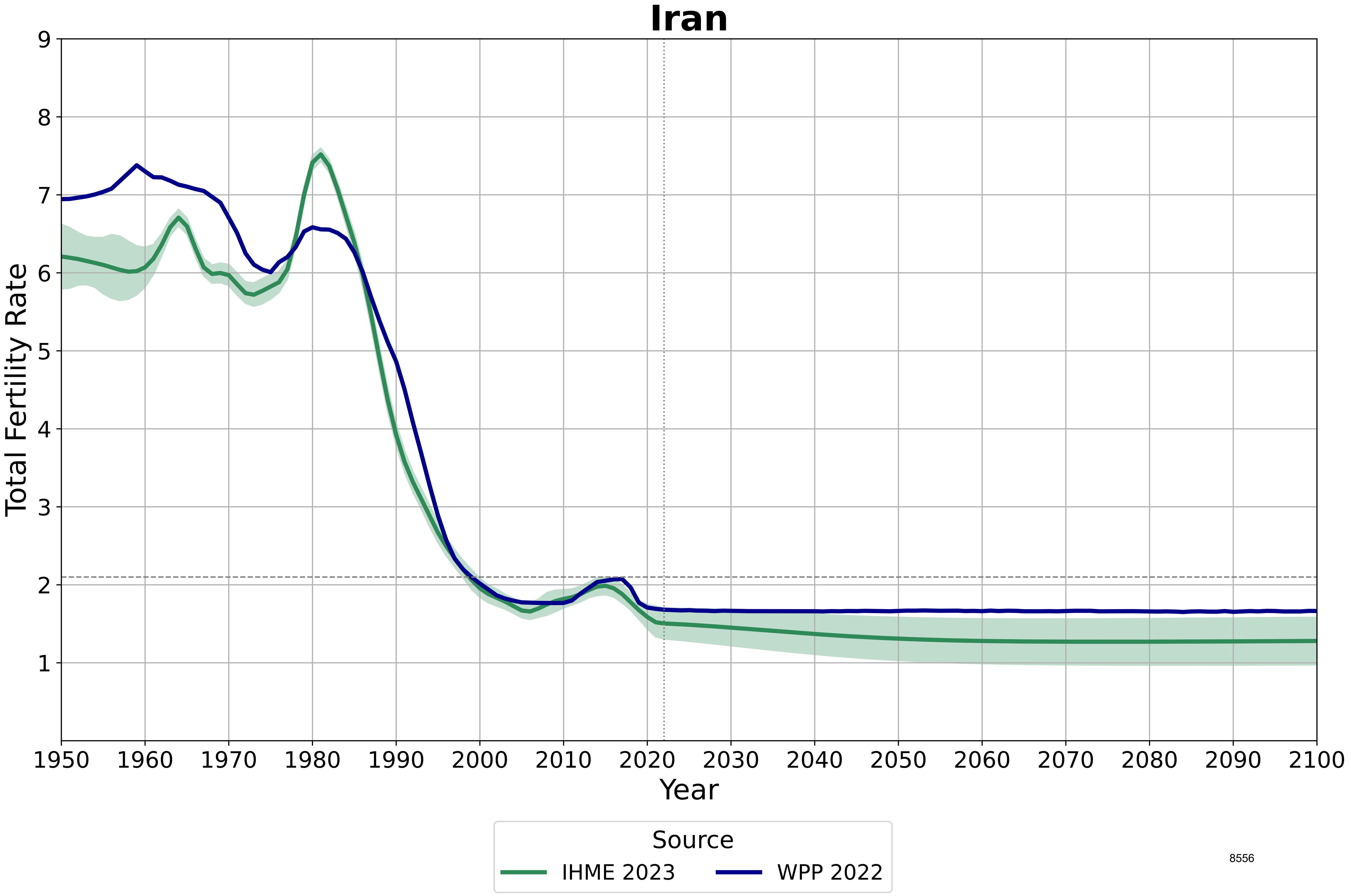

Supplemental Results Figure S7: TFR for IHME reference scenario and the UNPD medium variant scenario by location, 1950– 2100

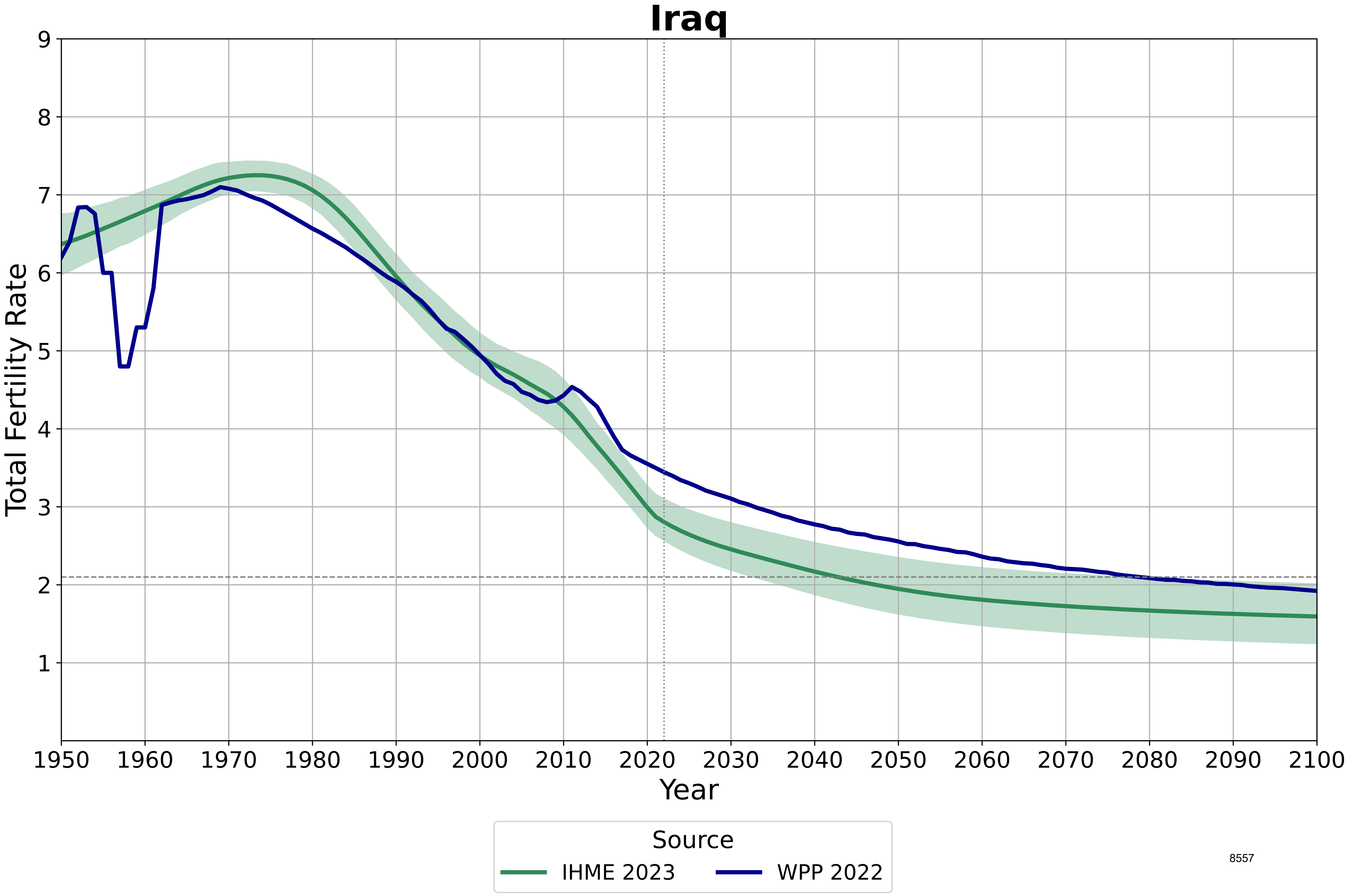

Supplemental Results Figure S7: TFR for IHME reference scenario and the UNPD medium variant scenario by location, 1950– 2100

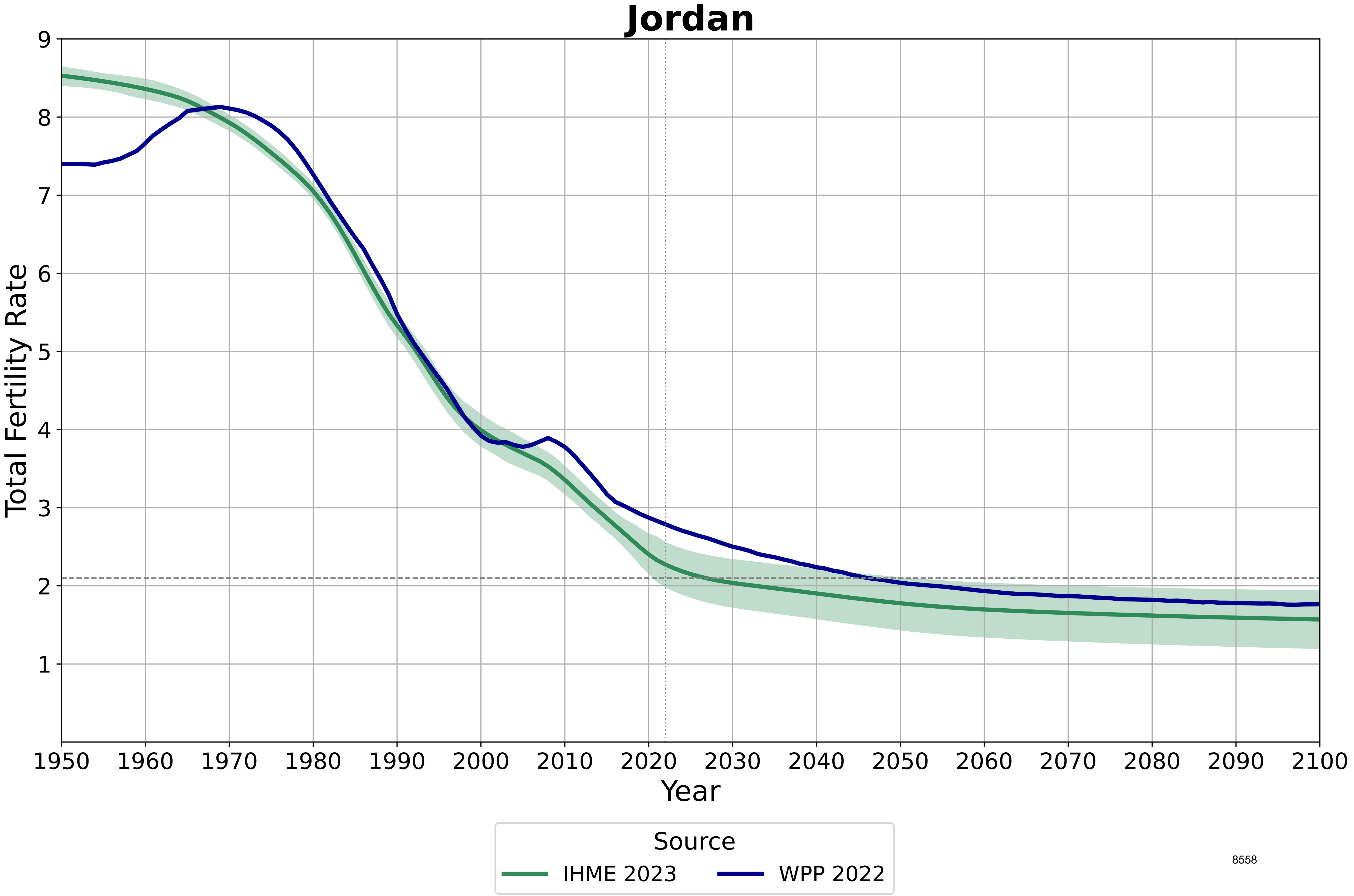

Supplemental Results Figure S7: TFR for IHME reference scenario and the UNPD medium variant scenario by location, 1950– 2100

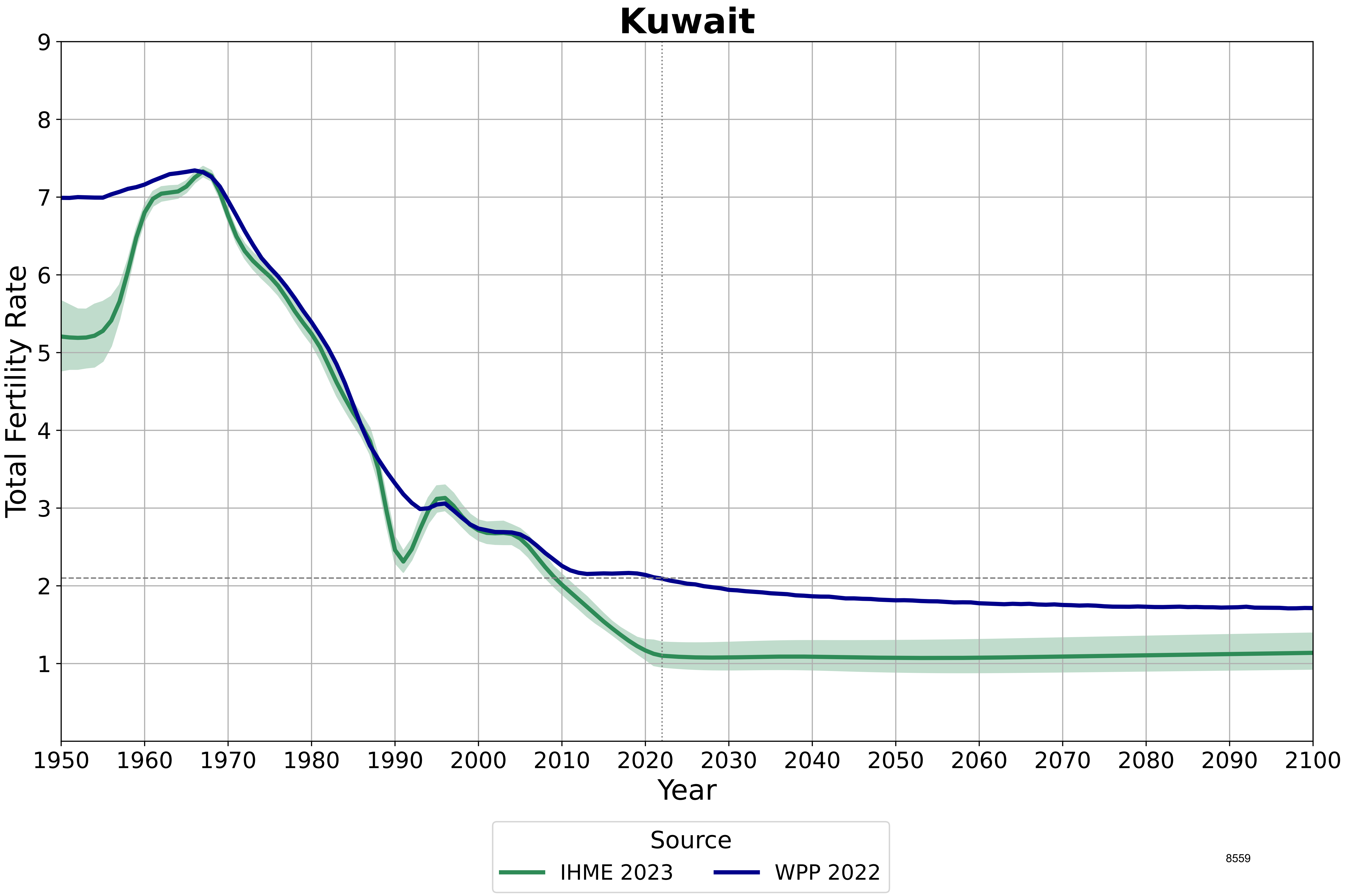

Supplemental Results Figure S7: TFR for IHME reference scenario and the UNPD medium variant scenario by location, 1950– 2100

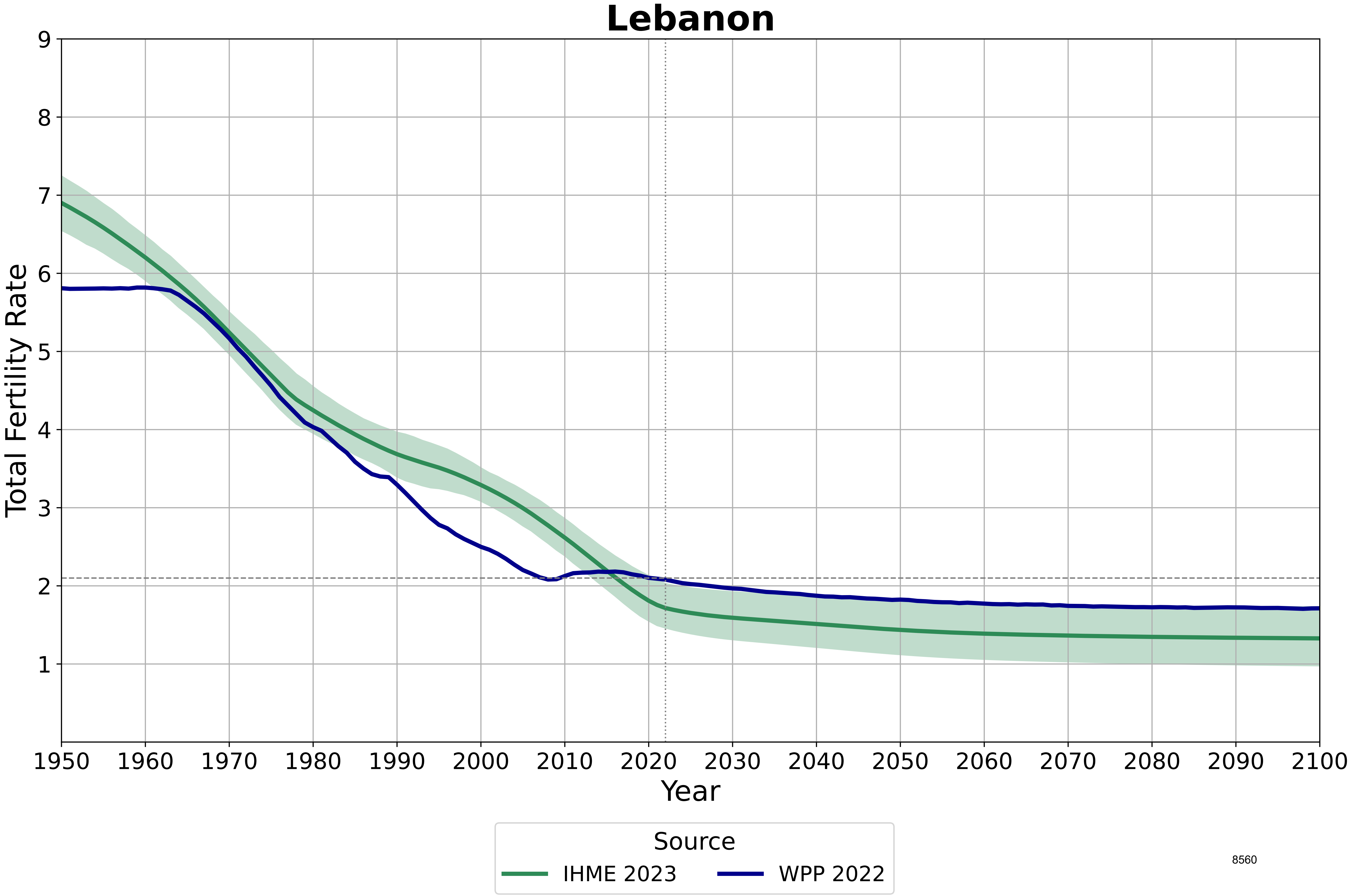

8560

Supplemental Results Figure S7: TFR for IHME reference scenario and the UNPD medium variant scenario by location, 1950– 2100

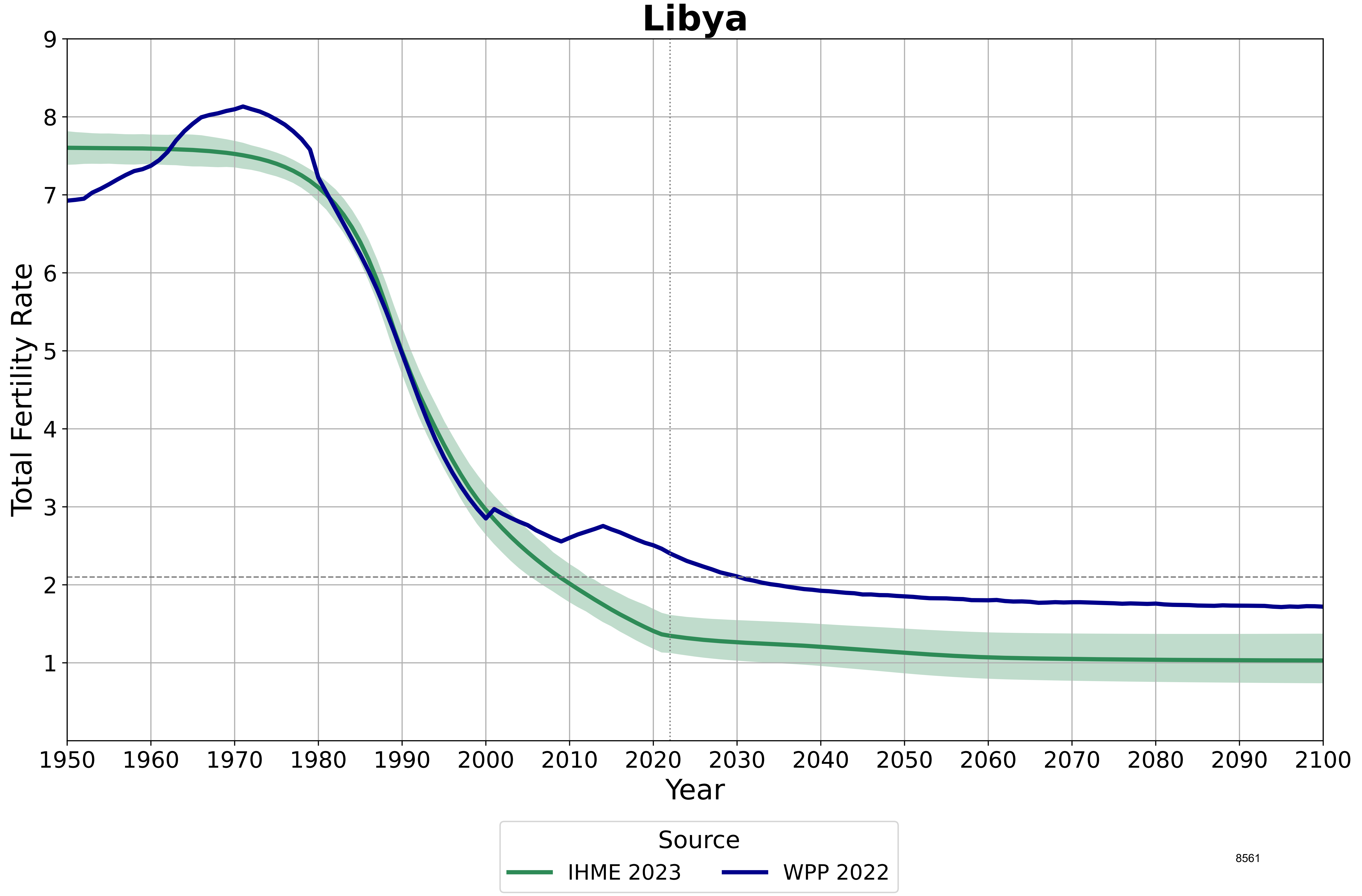

Supplemental Results Figure S7: TFR for IHME reference scenario and the UNPD medium variant scenario by location, 1950– 2100

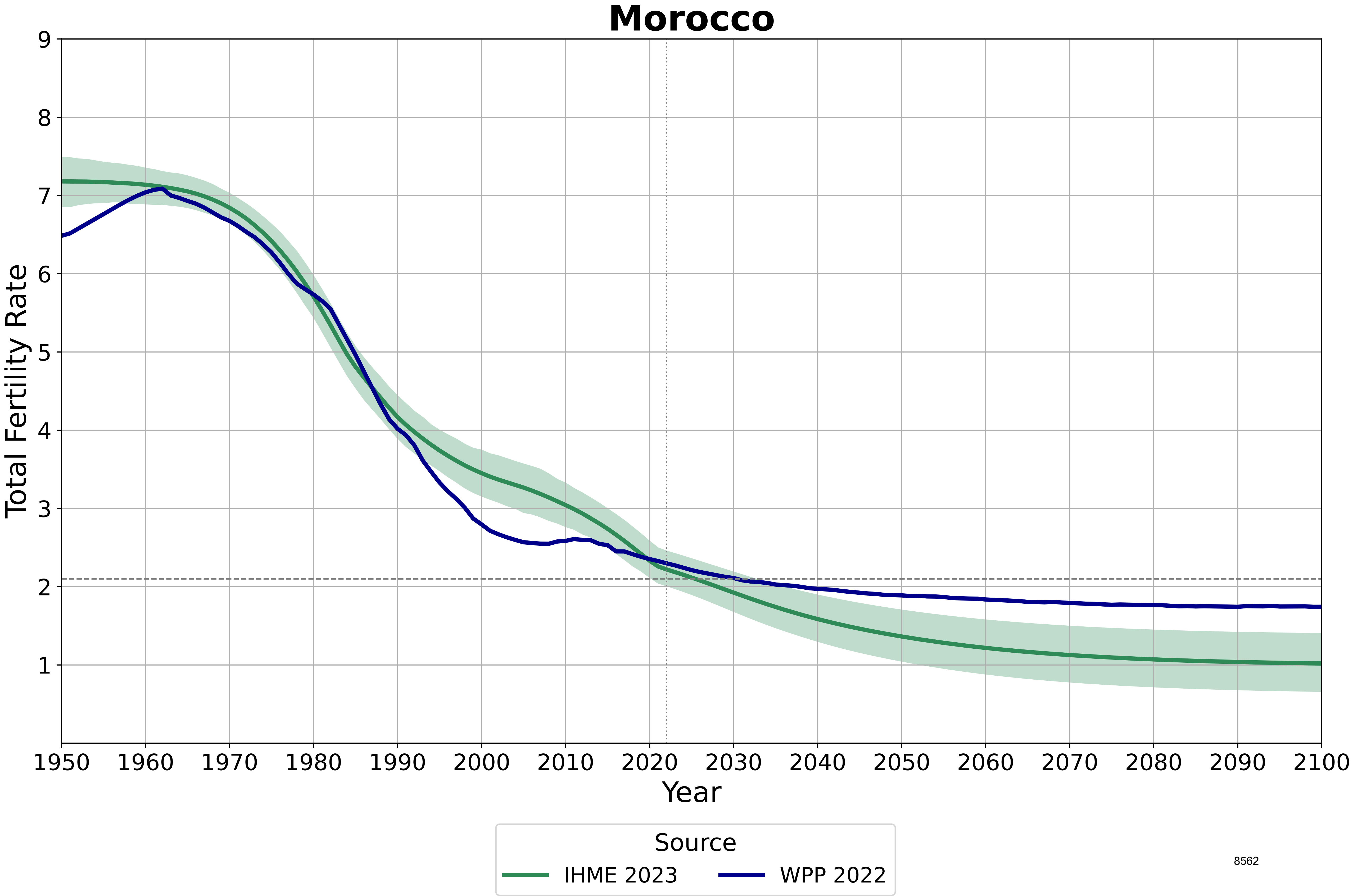

Supplemental Results Figure S7: TFR for IHME reference scenario and the UNPD medium variant scenario by location, 1950– 2100

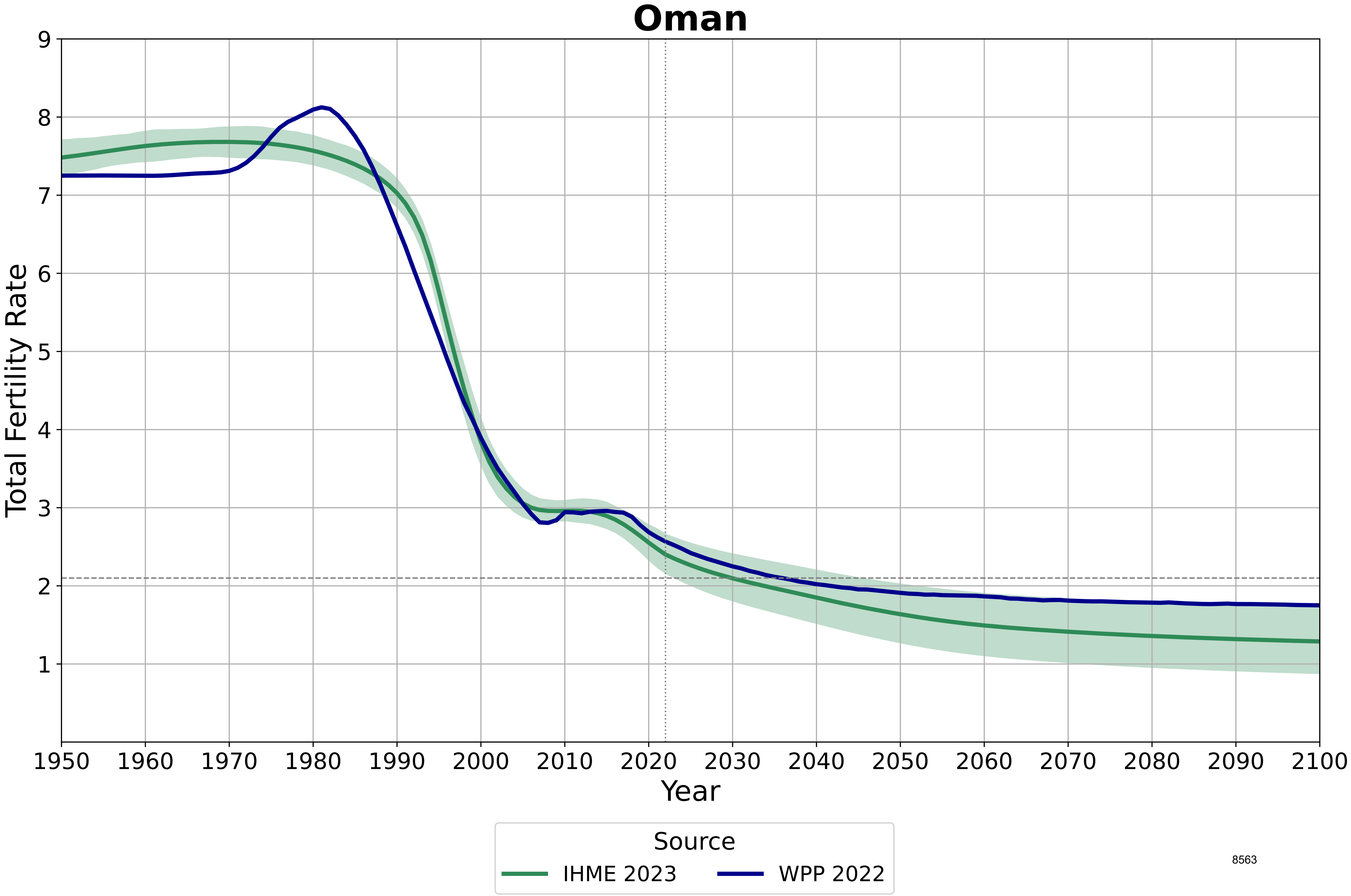

Supplemental Results Figure S7: TFR for IHME reference scenario and the UNPD medium variant scenario by location, 1950– 2100

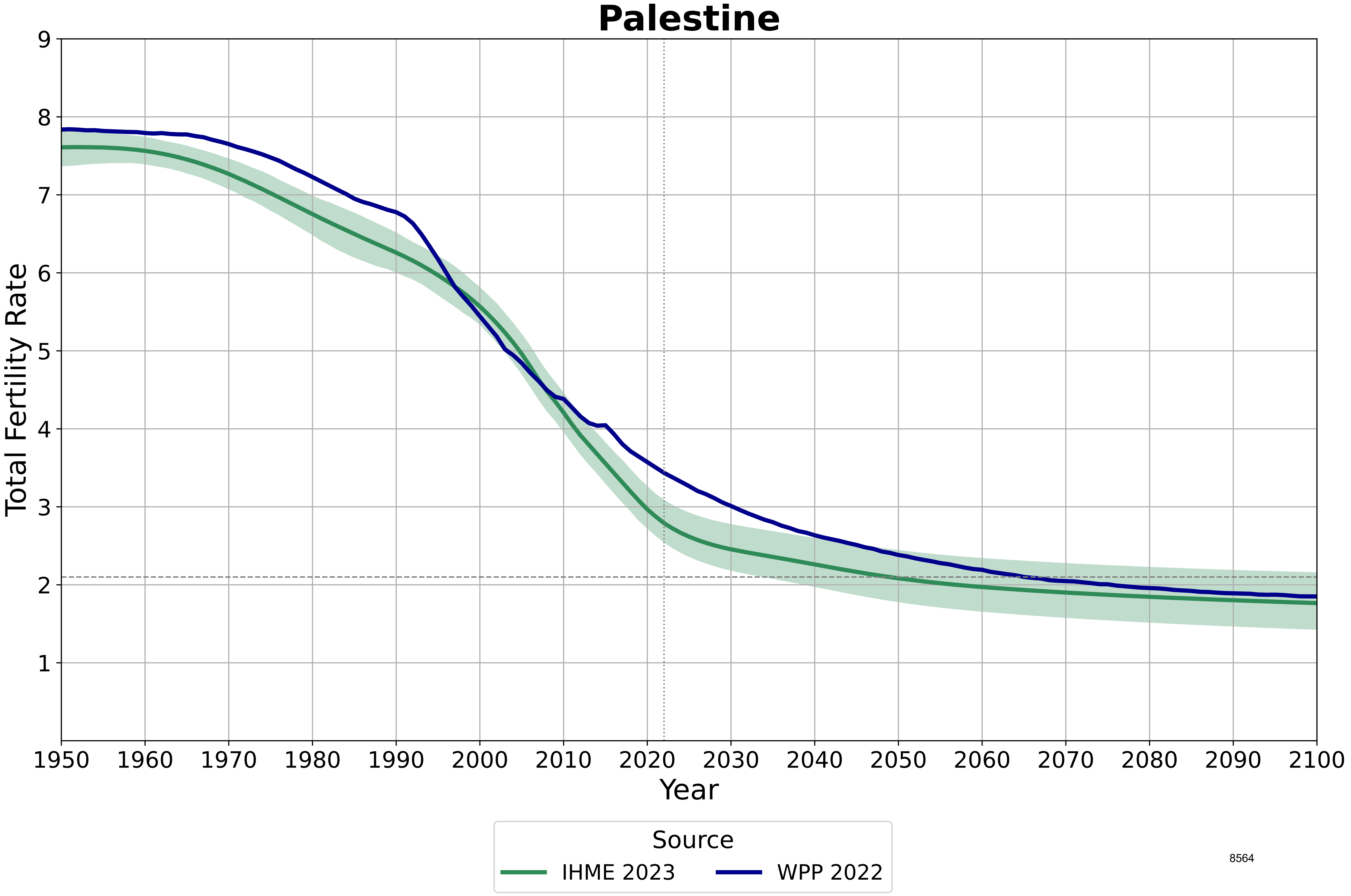

Supplemental Results Figure S7: TFR for IHME reference scenario and the UNPD medium variant scenario by location, 1950– 2100

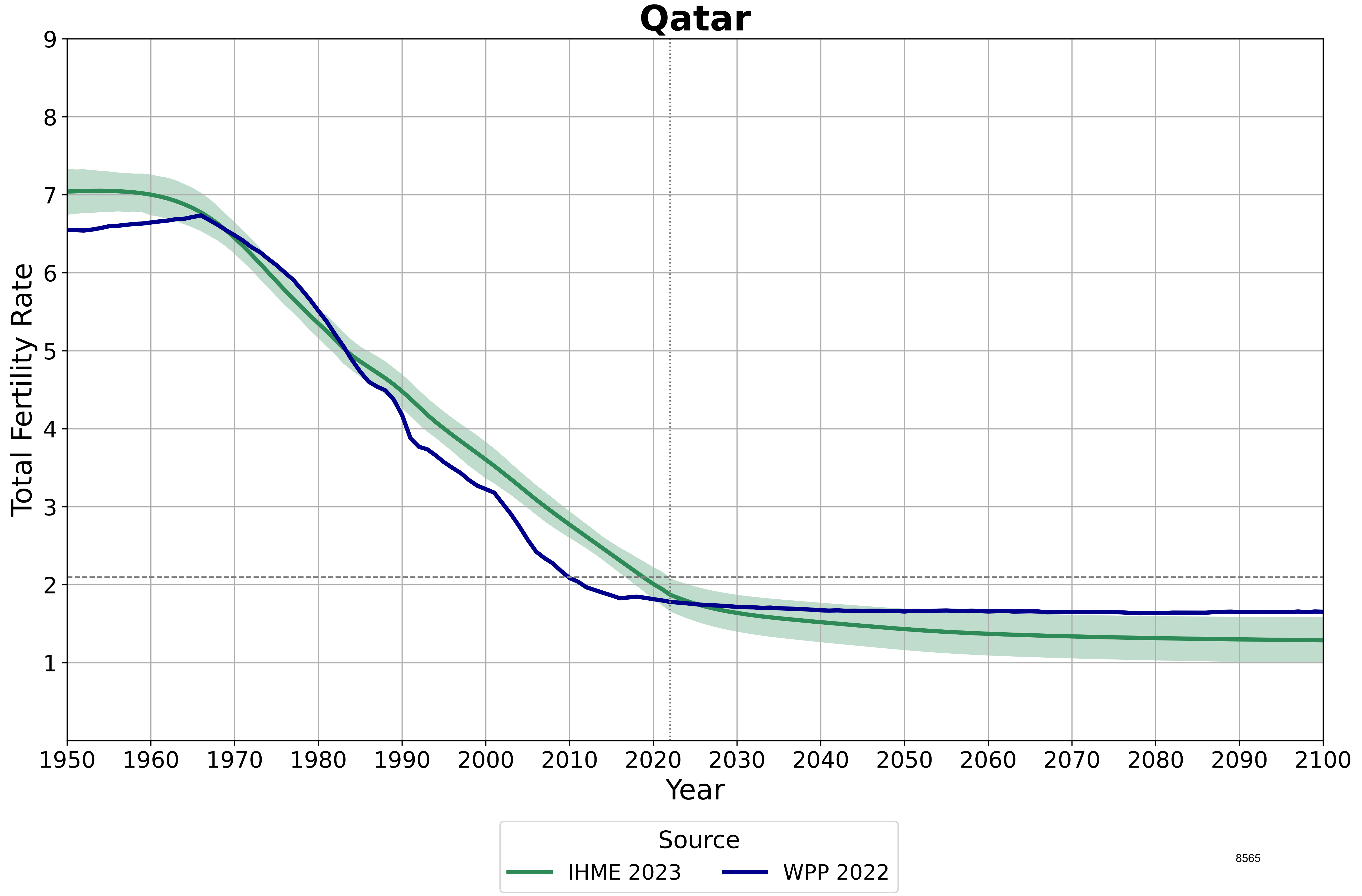

Supplemental Results Figure S7: TFR for IHME reference scenario and the UNPD medium variant scenario by location, 1950– 2100

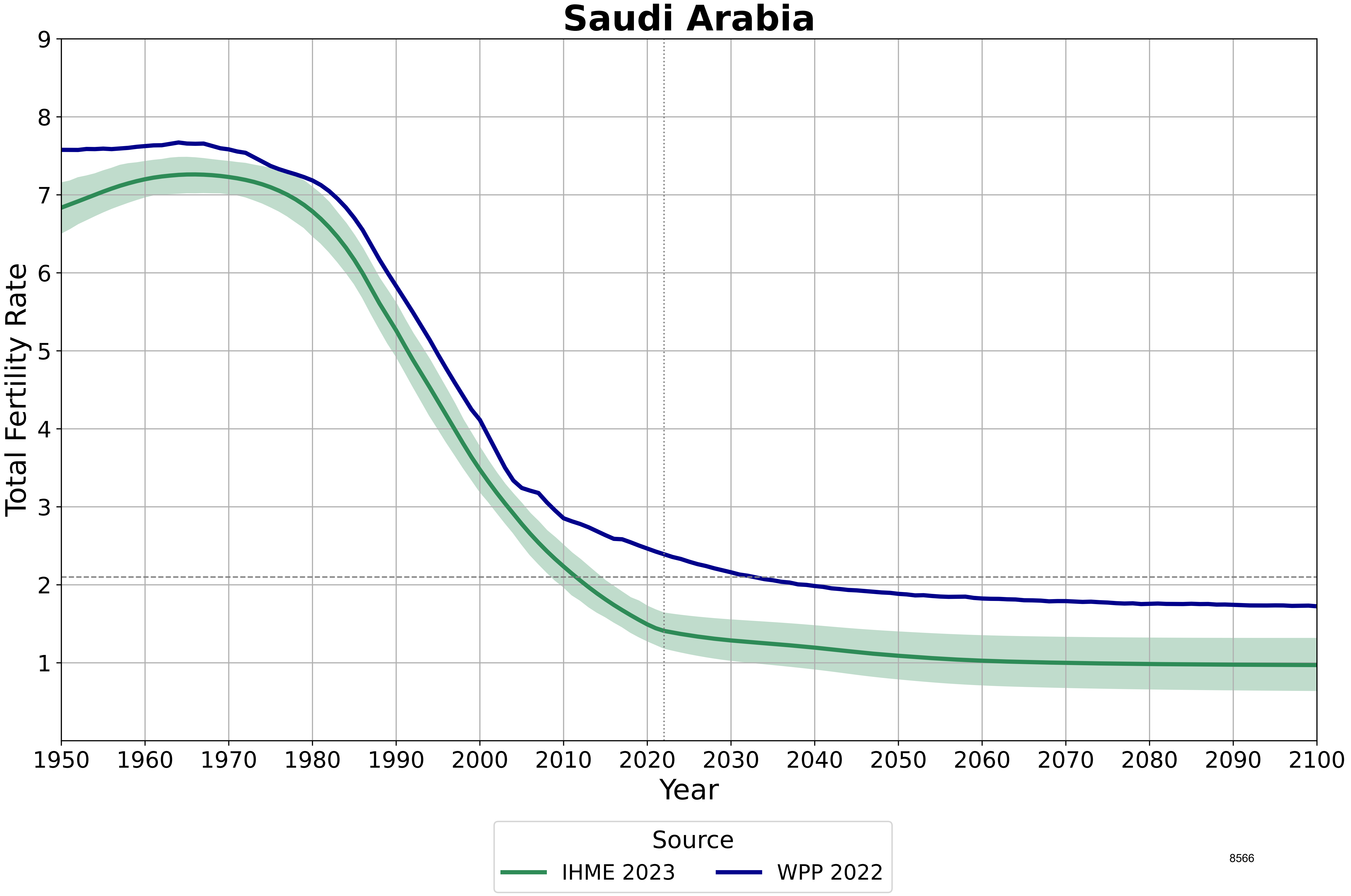

Supplemental Results Figure S7: TFR for IHME reference scenario and the UNPD medium variant scenario by location, 1950– 2100

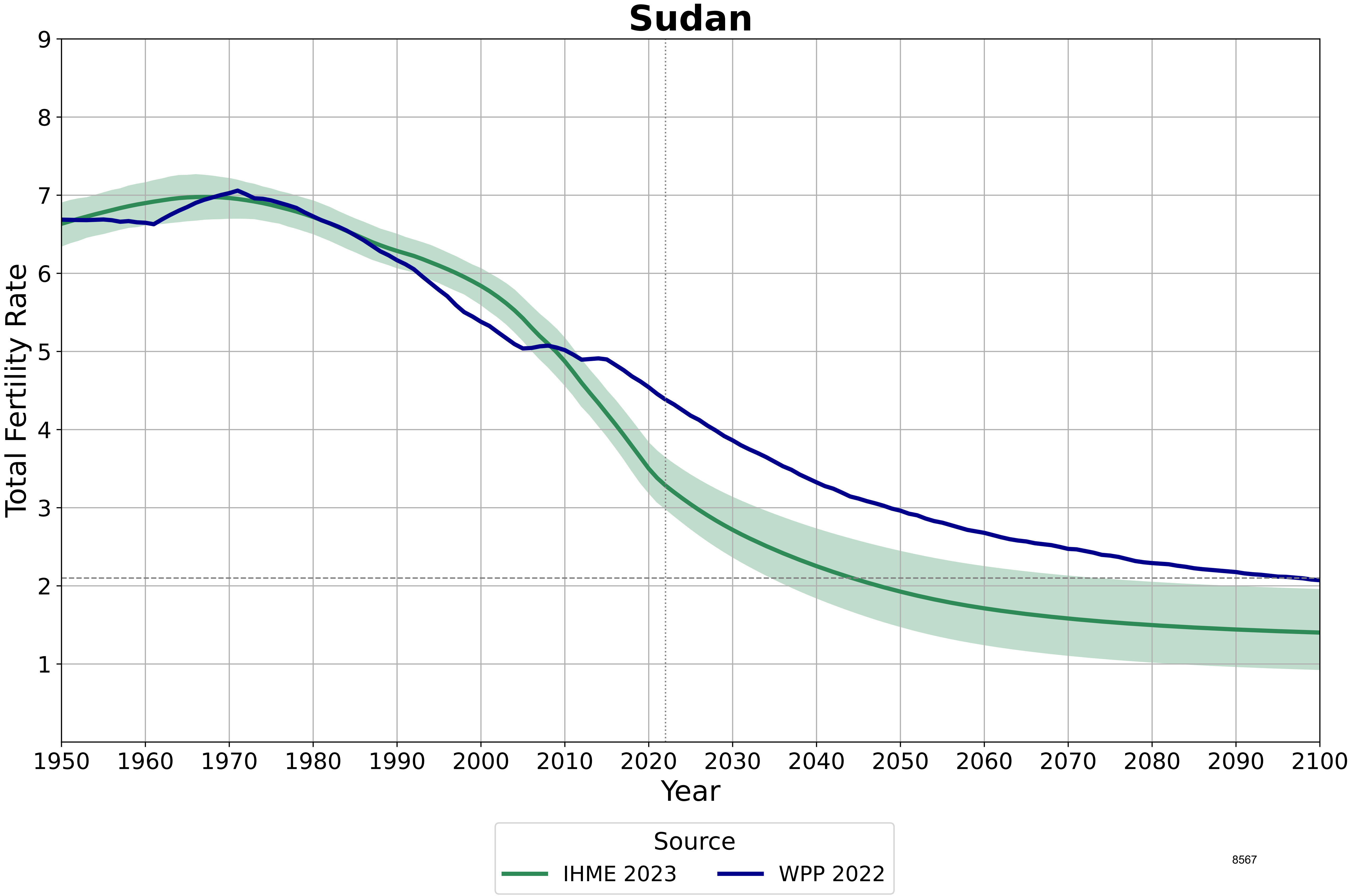

Supplemental Results Figure S7: TFR for IHME reference scenario and the UNPD medium variant scenario by location, 1950– 2100

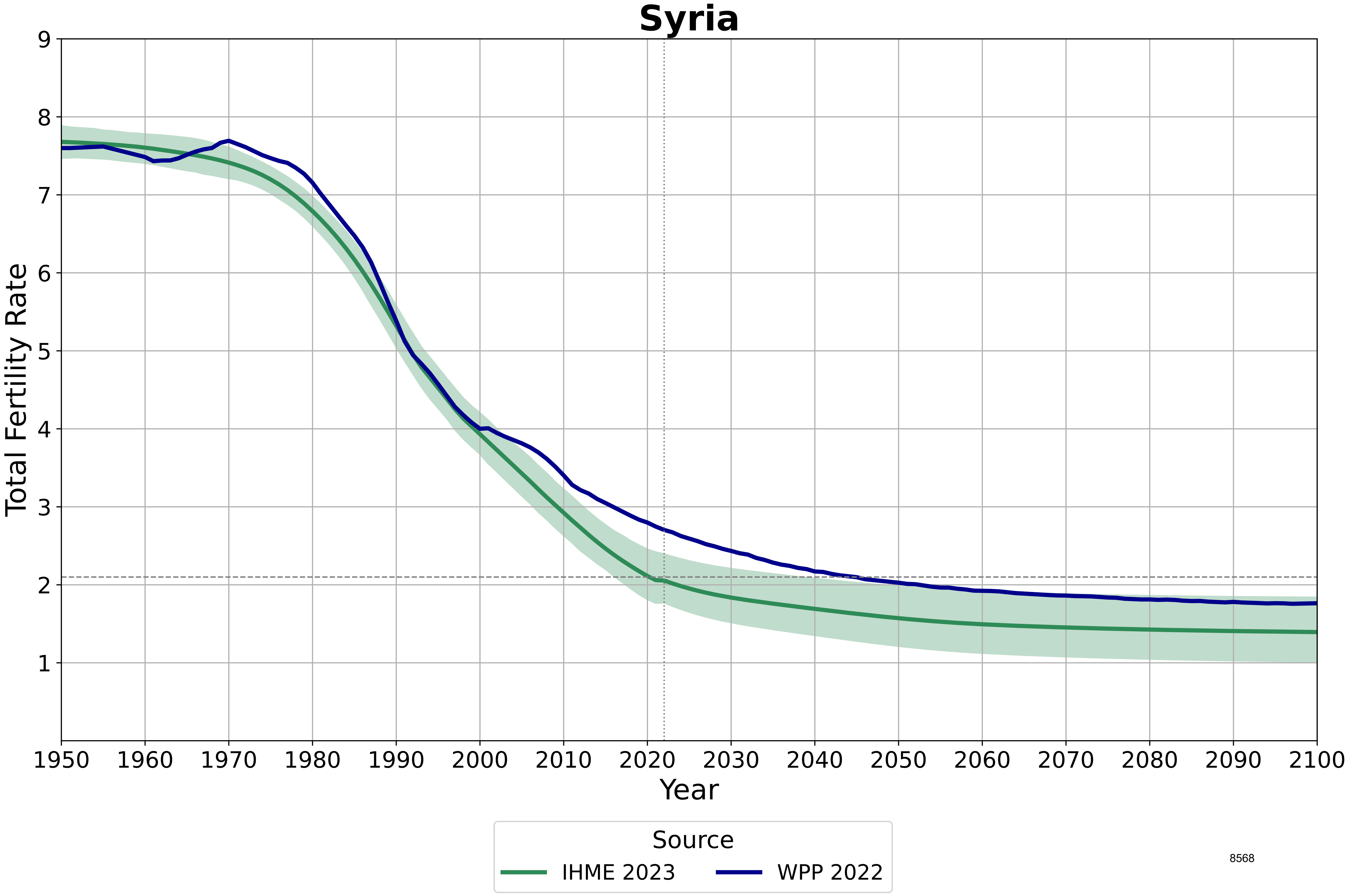

Supplemental Results Figure S7: TFR for IHME reference scenario and the UNPD medium variant scenario by location, 1950– 2100

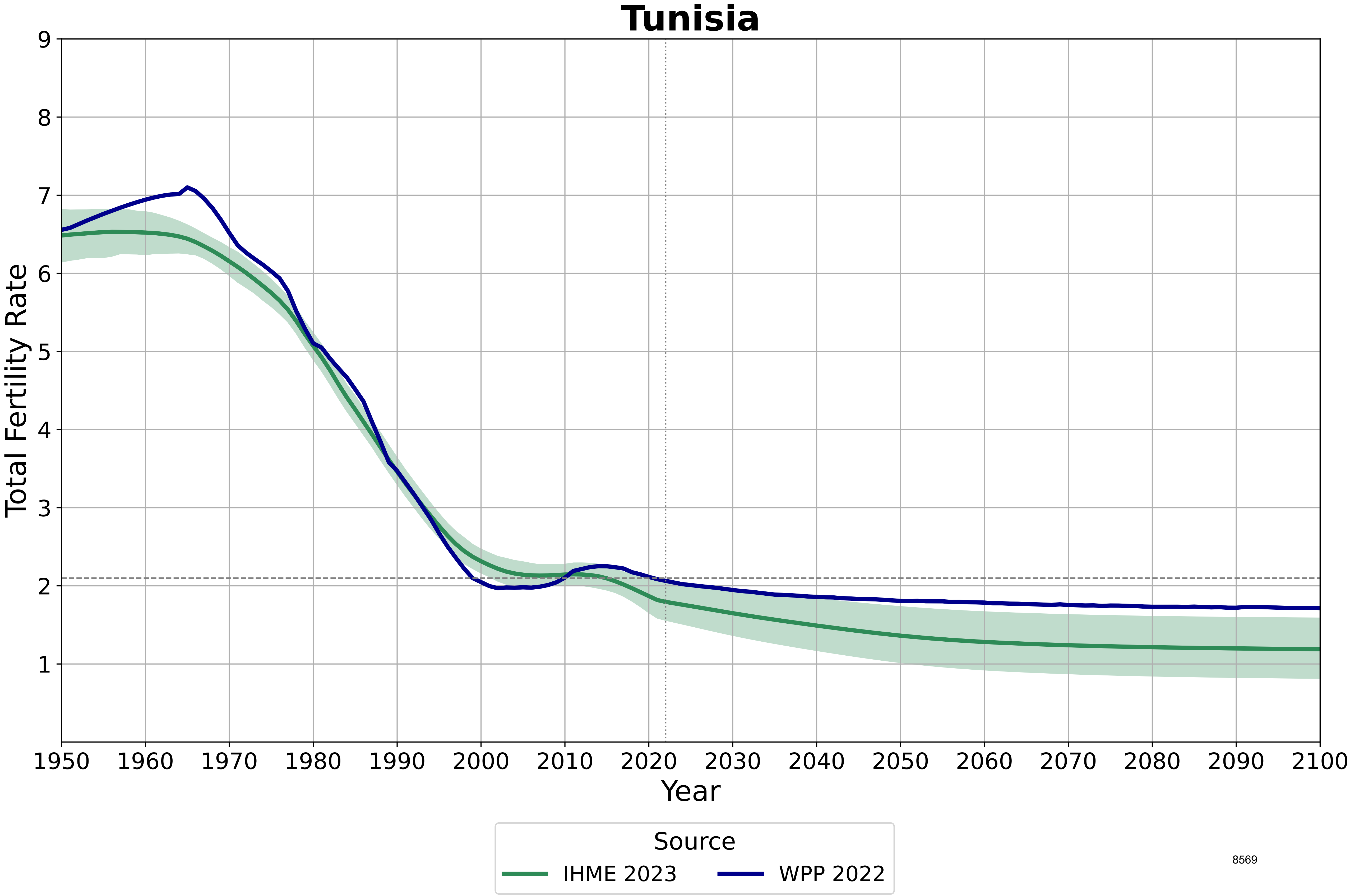

Supplemental Results Figure S7: TFR for IHME reference scenario and the UNPD medium variant scenario by location, 1950– 2100

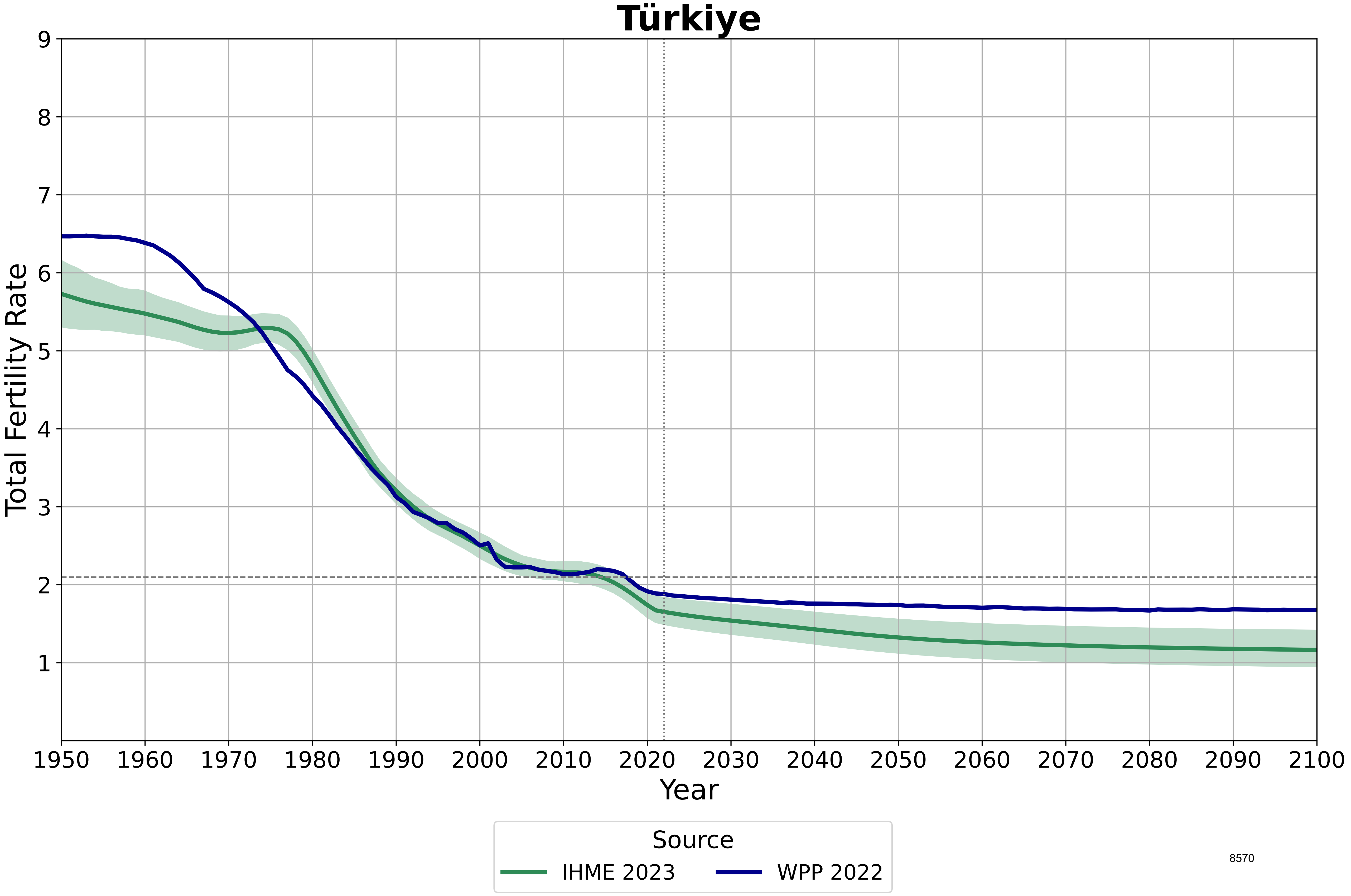

Supplemental Results Figure S7: TFR for IHME reference scenario and the UNPD medium variant scenario by location, 1950– 2100

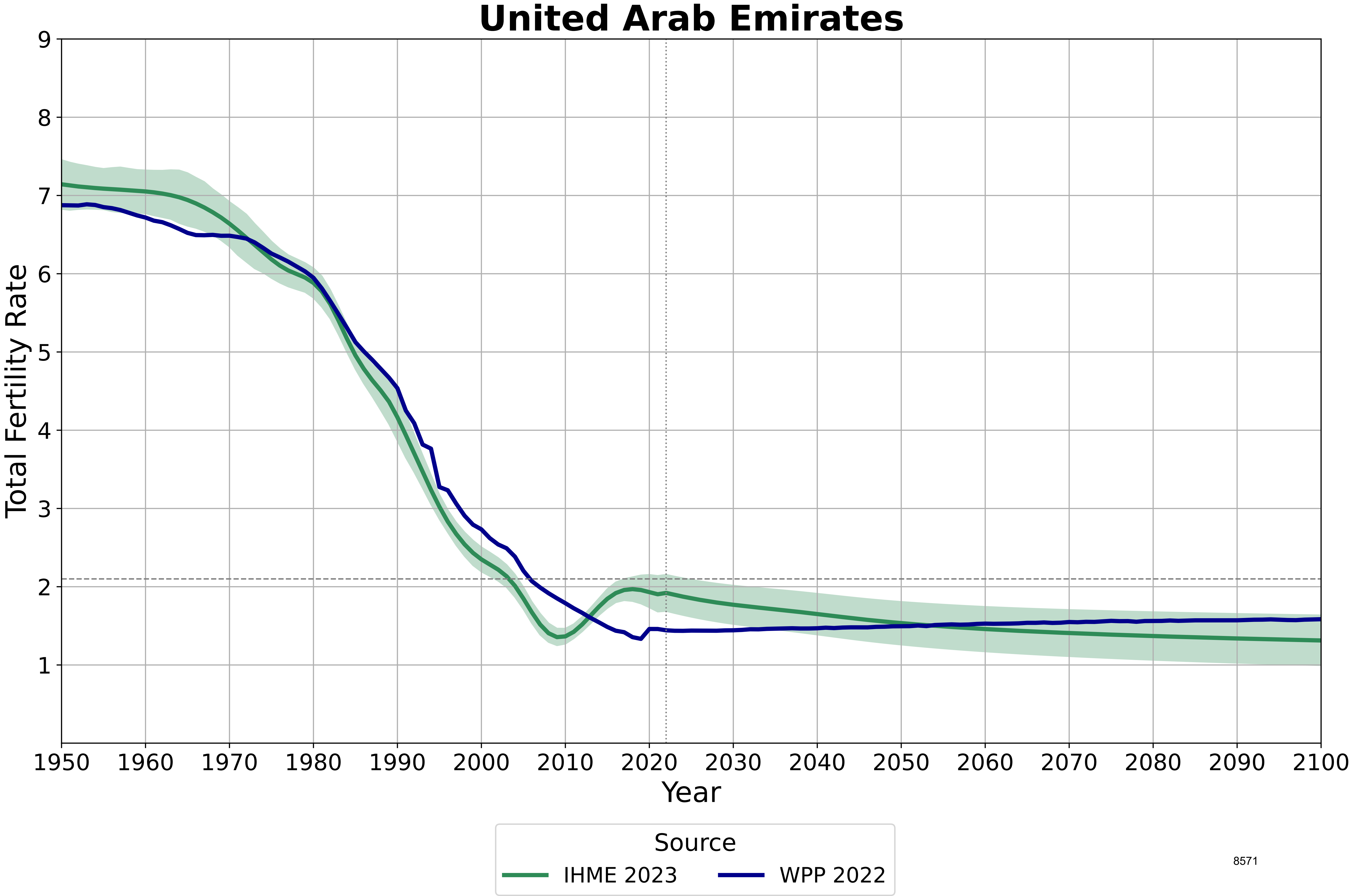

Supplemental Results Figure S7: TFR for IHME reference scenario and the UNPD medium variant scenario by location, 1950– 2100

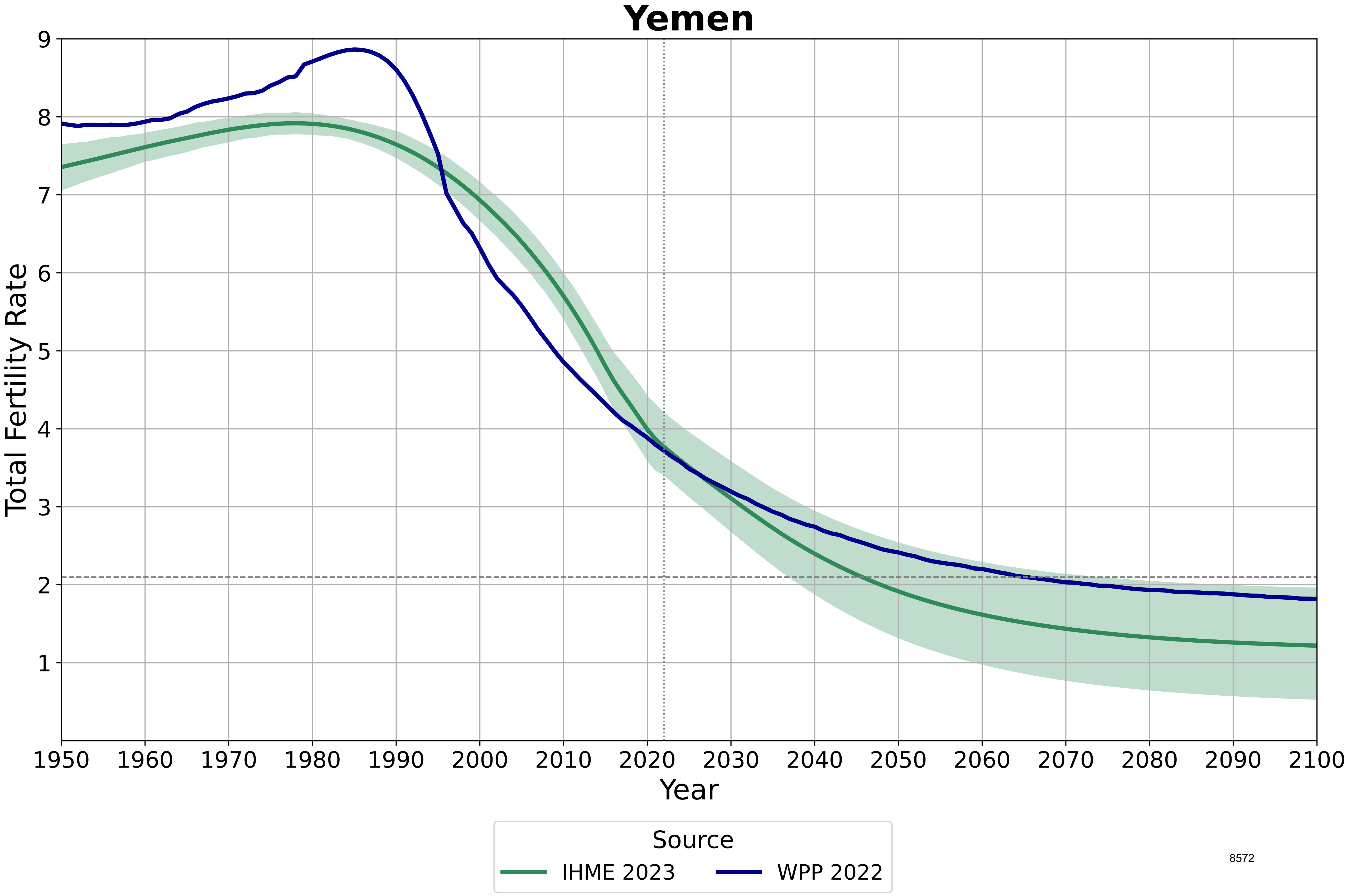

8572

Supplemental Results Figure S7: TFR for IHME reference scenario and the UNPD medium variant scenario by location, 1950– 2100

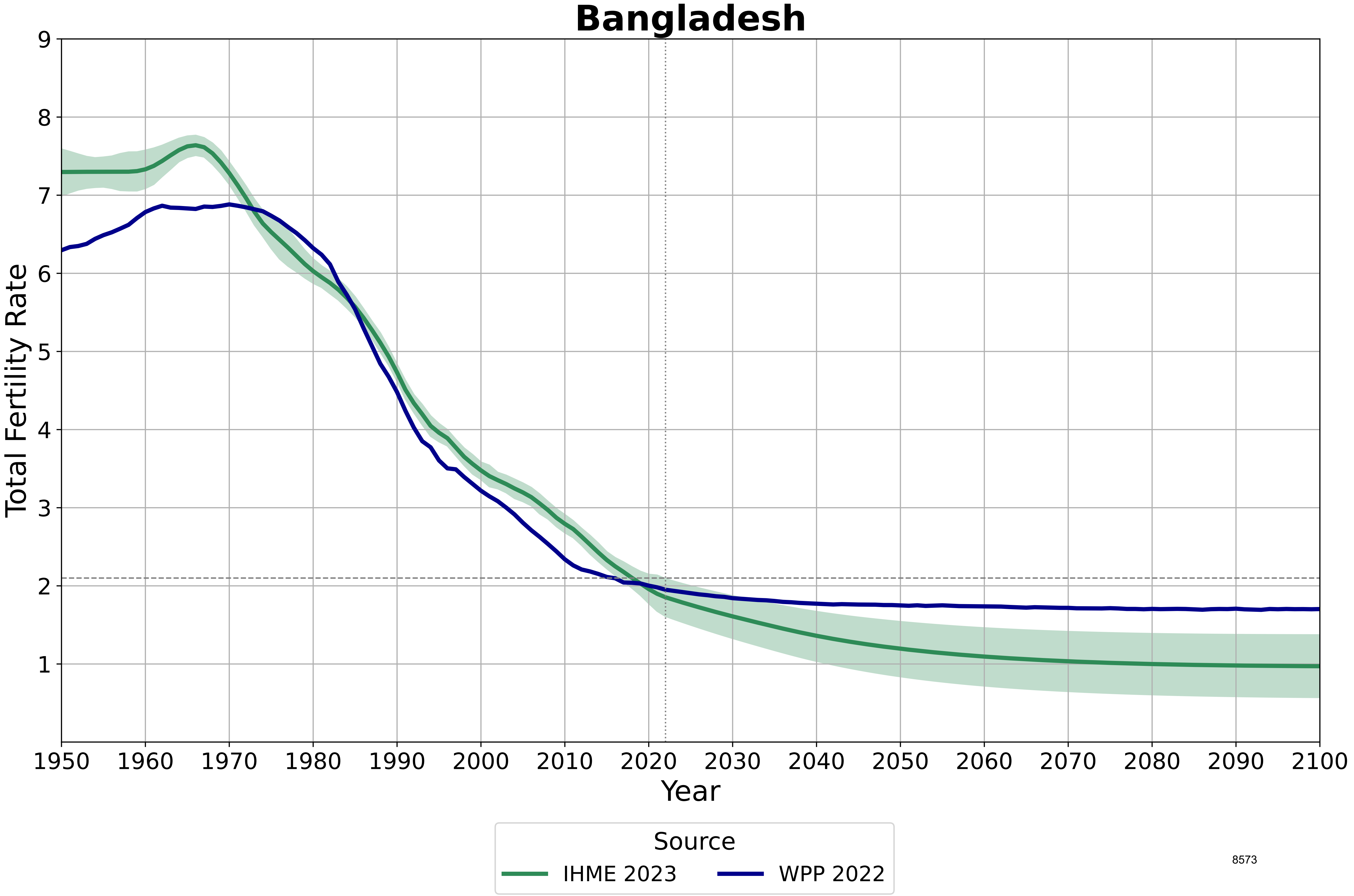

Supplemental Results Figure S7: TFR for IHME reference scenario and the UNPD medium variant scenario by location, 1950– 2100

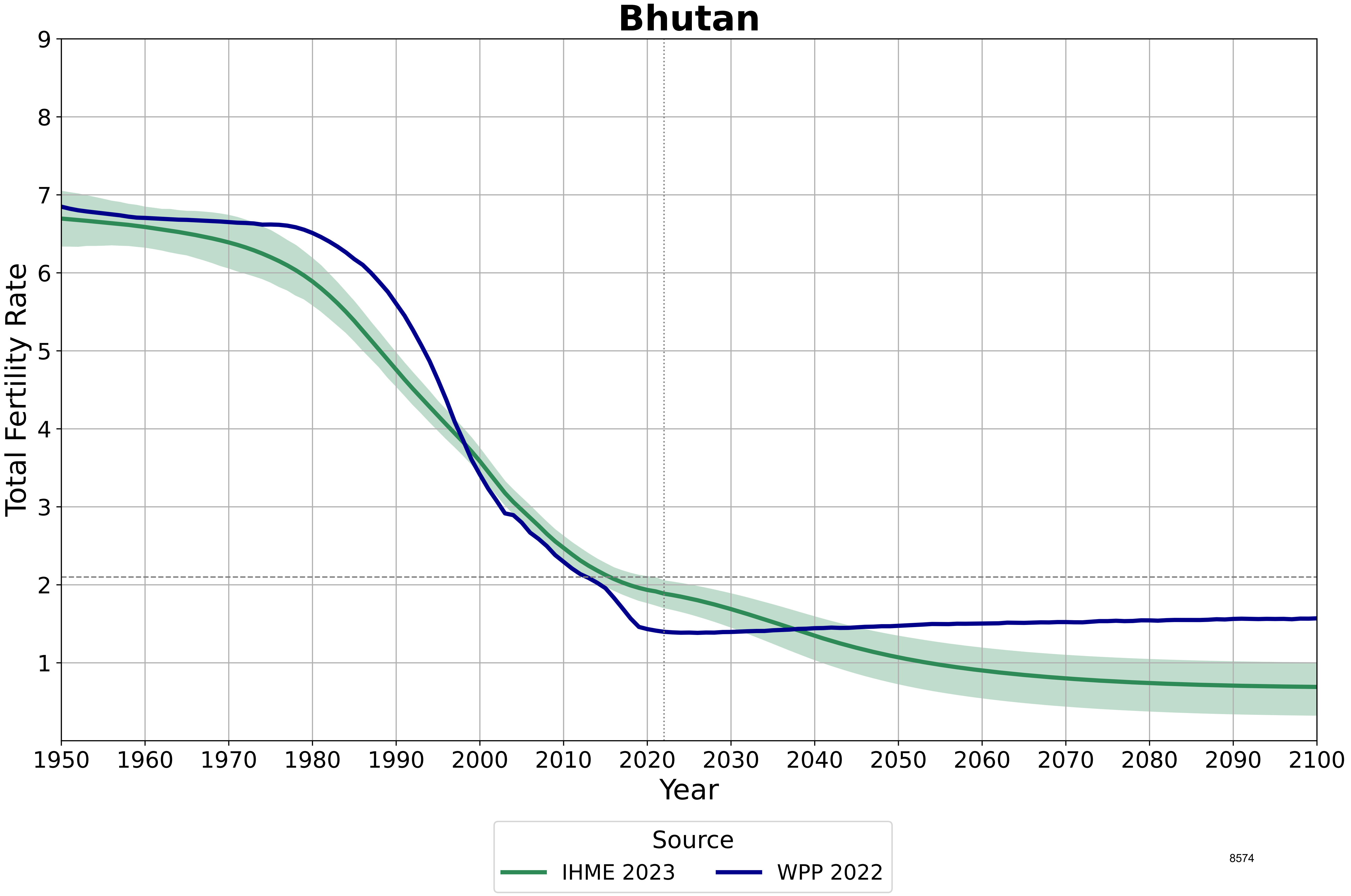

Supplemental Results Figure S7: TFR for IHME reference scenario and the UNPD medium variant scenario by location, 1950– 2100

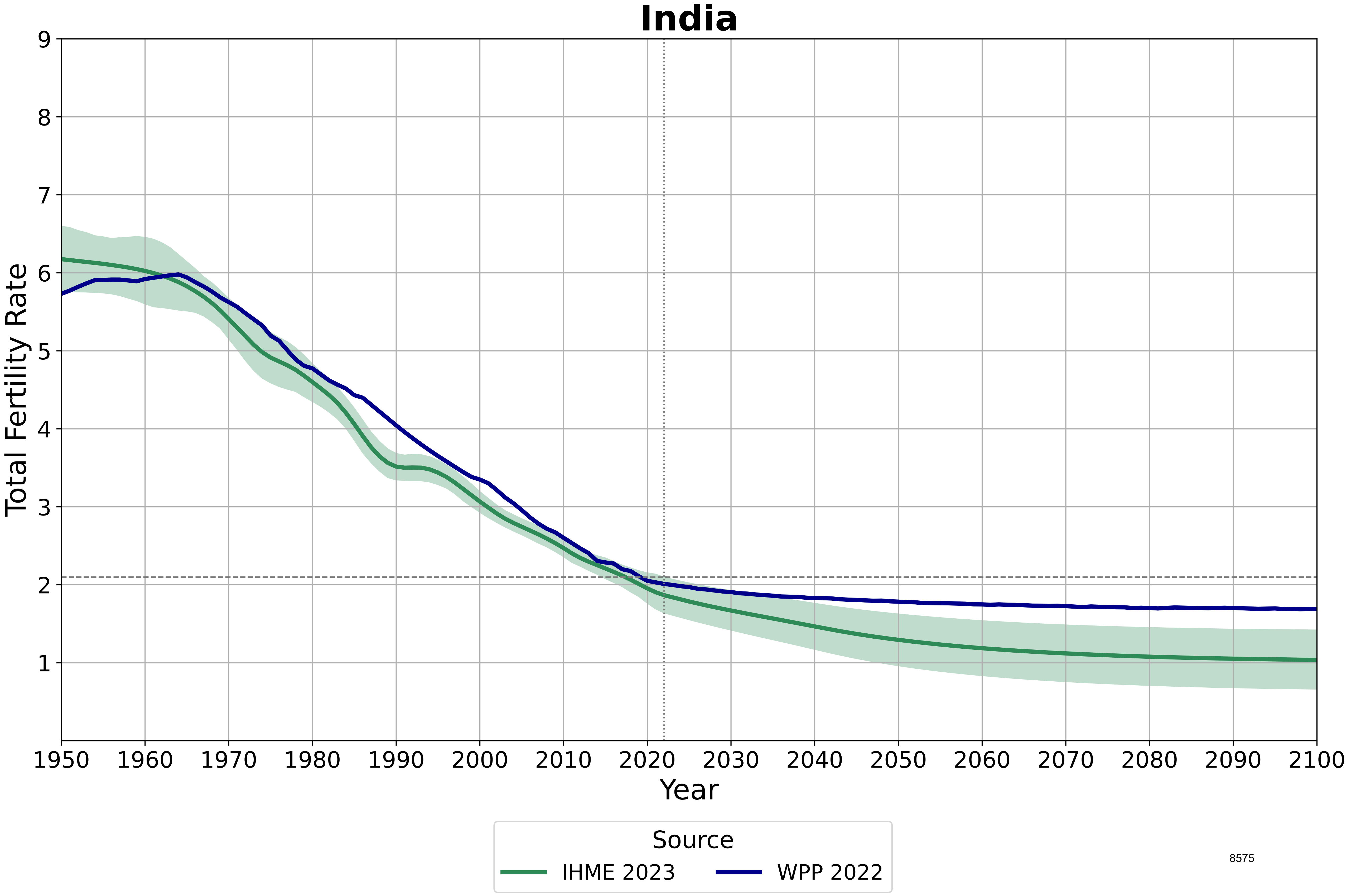

8575

Supplemental Results Figure S7: TFR for IHME reference scenario and the UNPD medium variant scenario by location, 1950– 2100

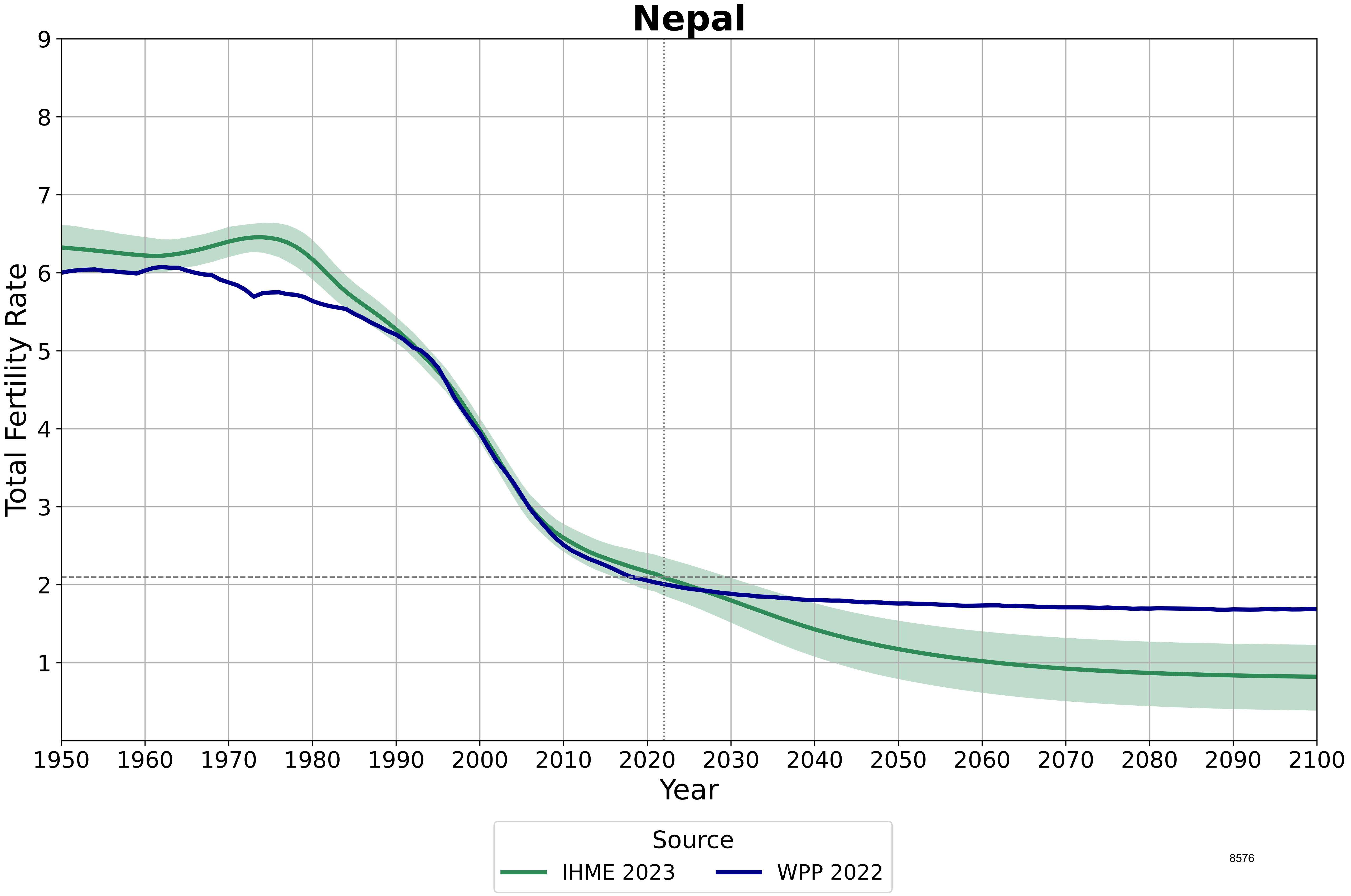

Supplemental Results Figure S7: TFR for IHME reference scenario and the UNPD medium variant scenario by location, 1950– 2100

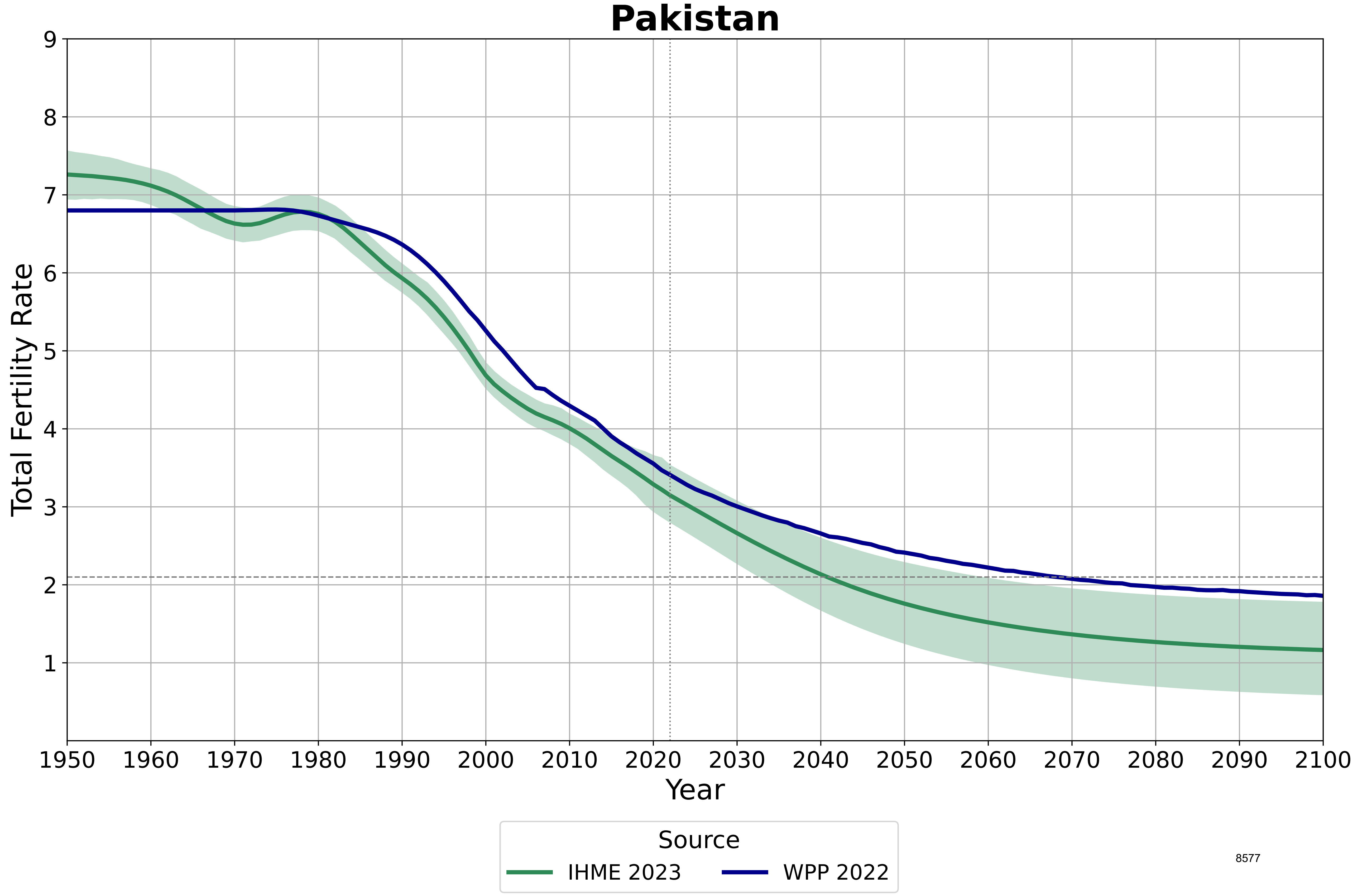

Supplemental Results Figure S7: TFR for IHME reference scenario and the UNPD medium variant scenario by location, 1950– 2100

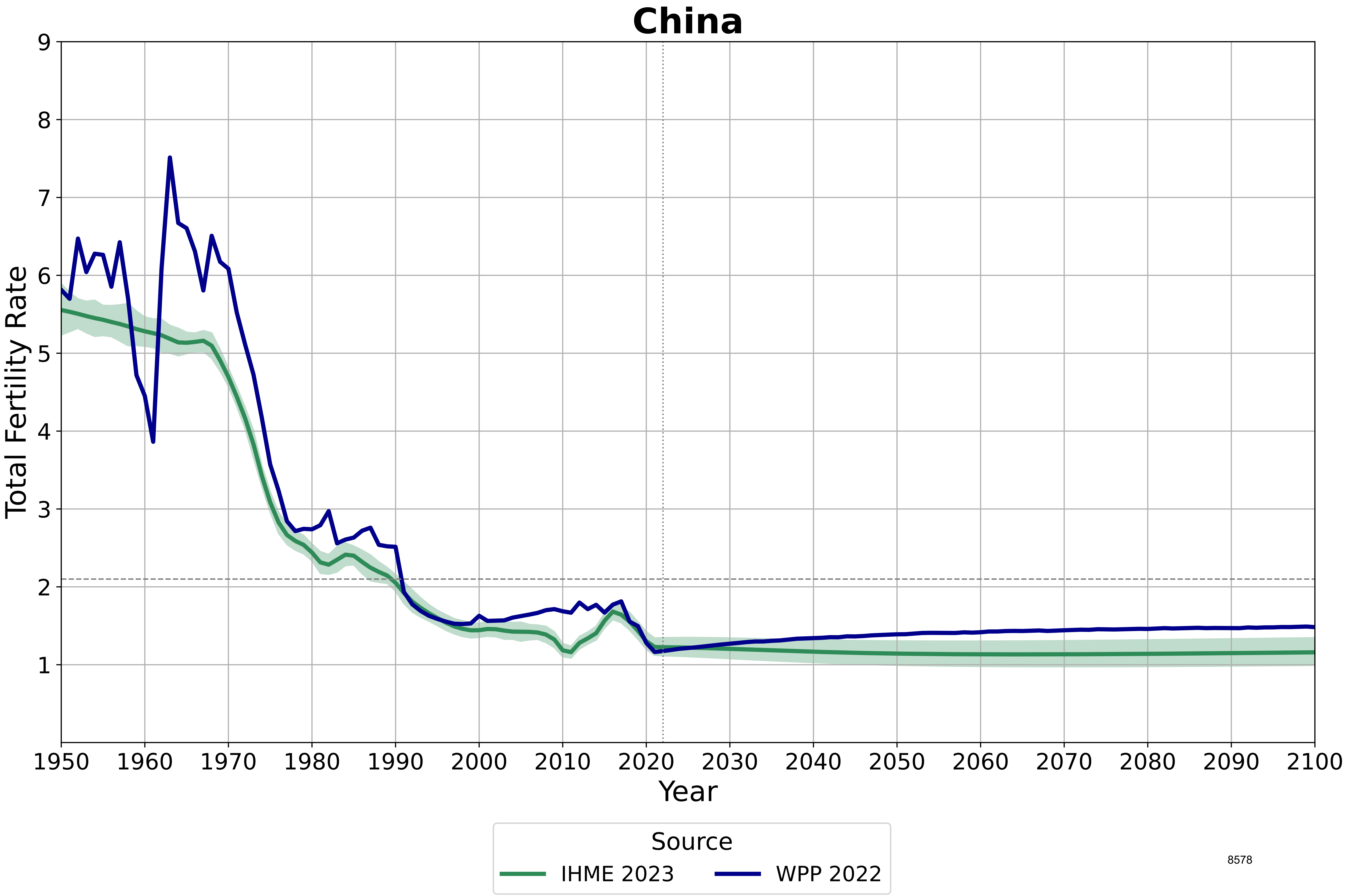

Supplemental Results Figure S7: TFR for IHME reference scenario and the UNPD medium variant scenario by location, 1950– 2100

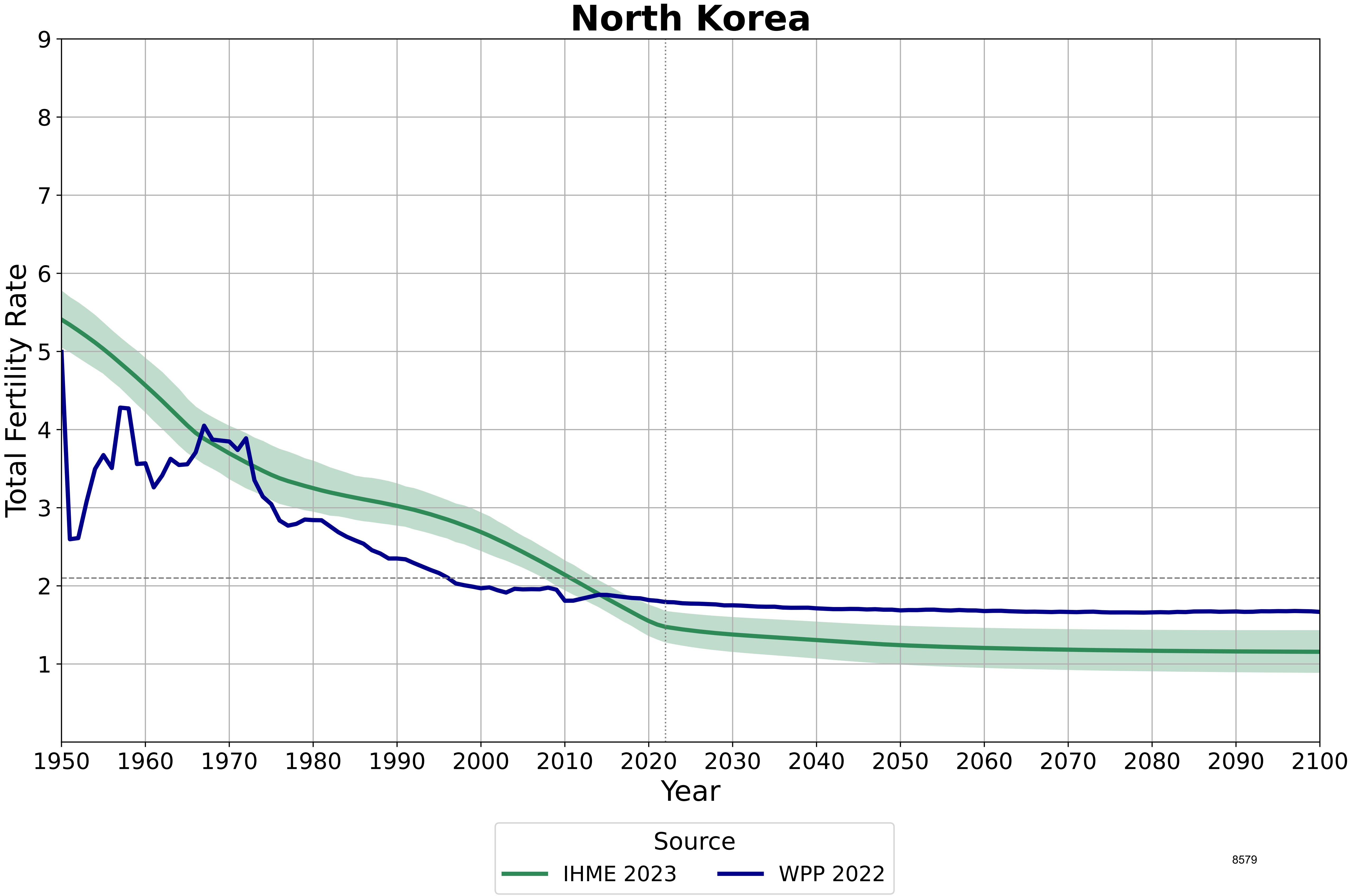

Supplemental Results Figure S7: TFR for IHME reference scenario and the UNPD medium variant scenario by location, 1950– 2100

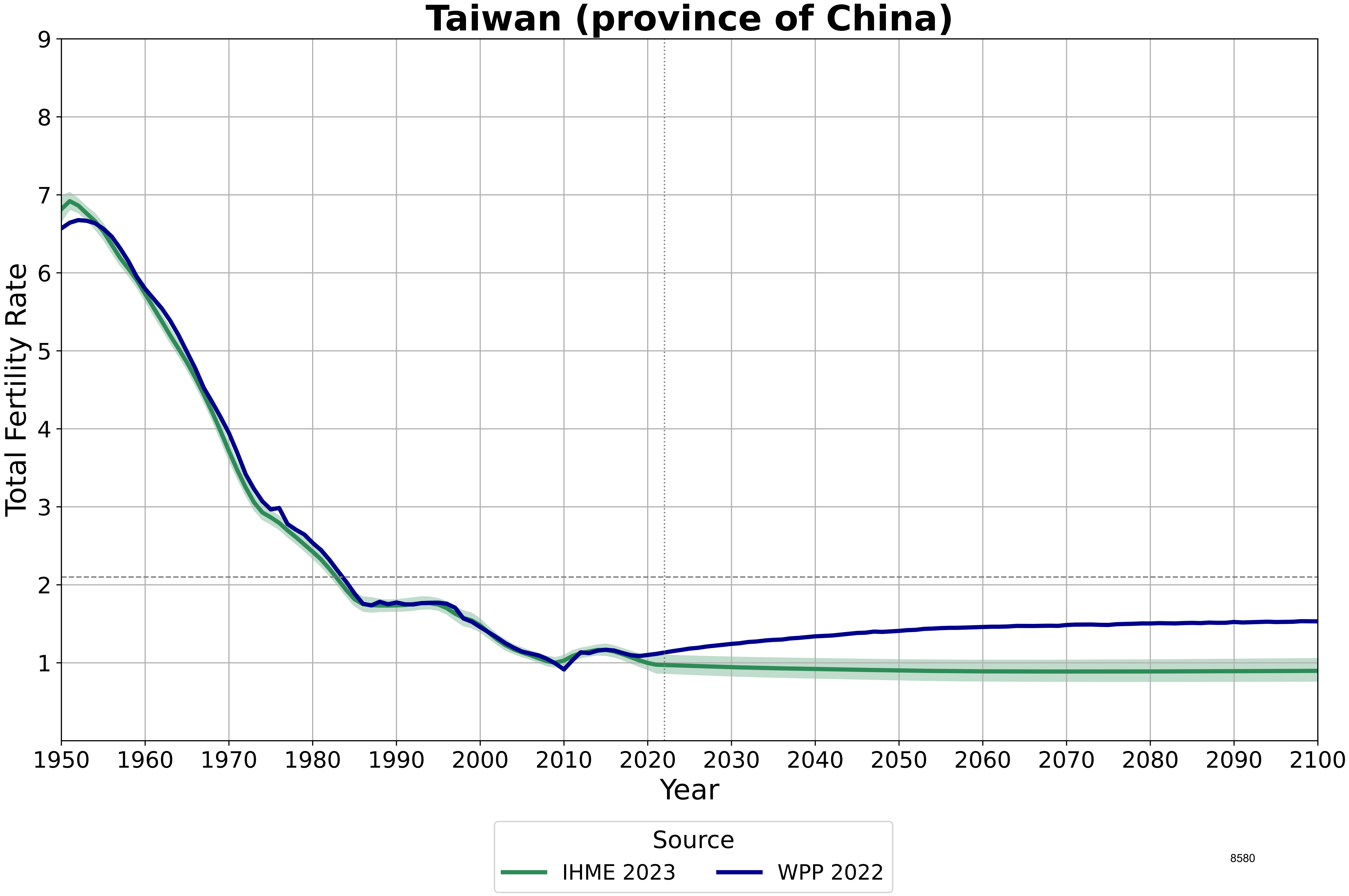

Supplemental Results Figure S7: TFR for IHME reference scenario and the UNPD medium variant scenario by location, 1950– 2100

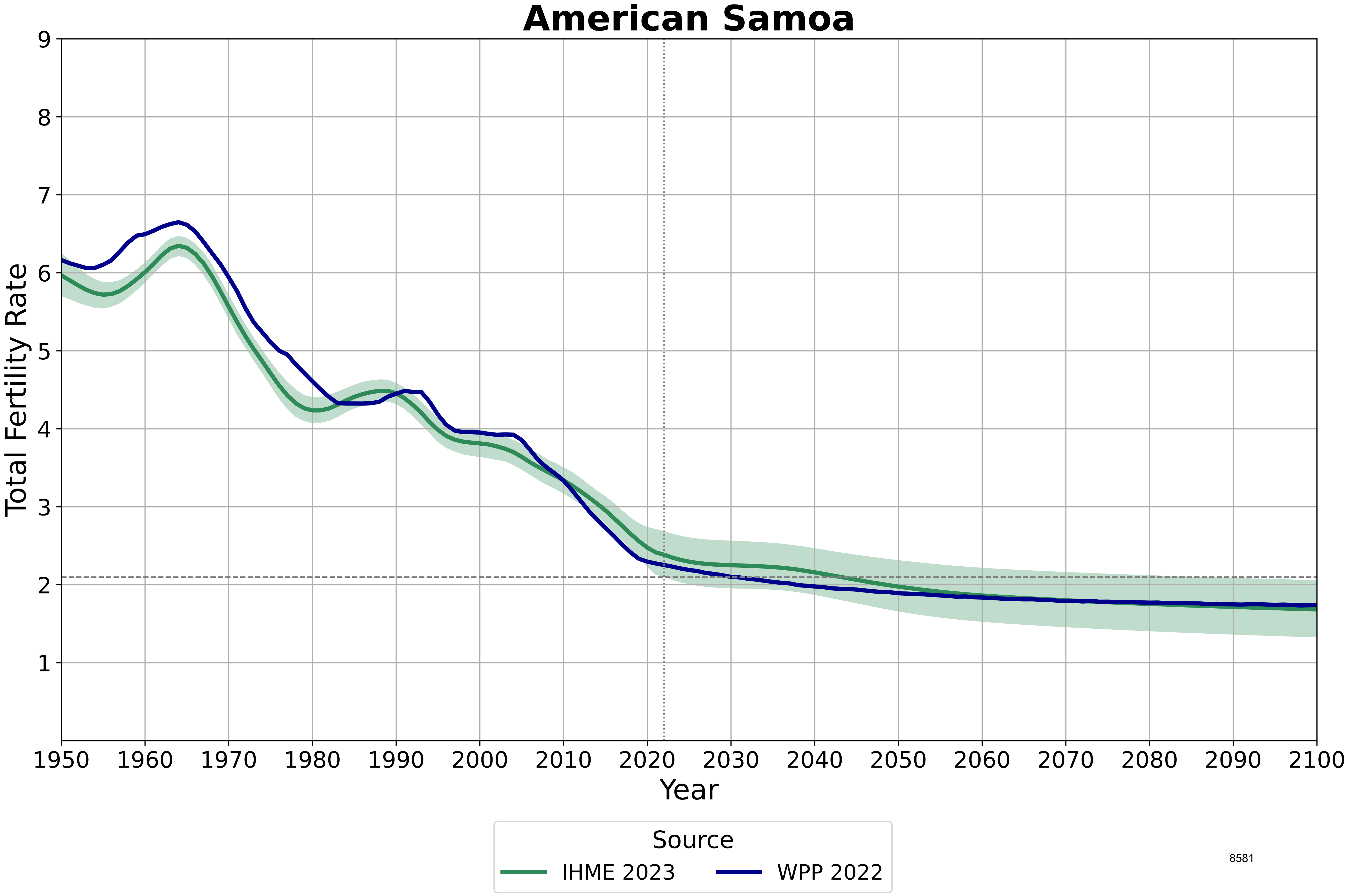

Supplemental Results Figure S7: TFR for IHME reference scenario and the UNPD medium variant scenario by location, 1950– 2100

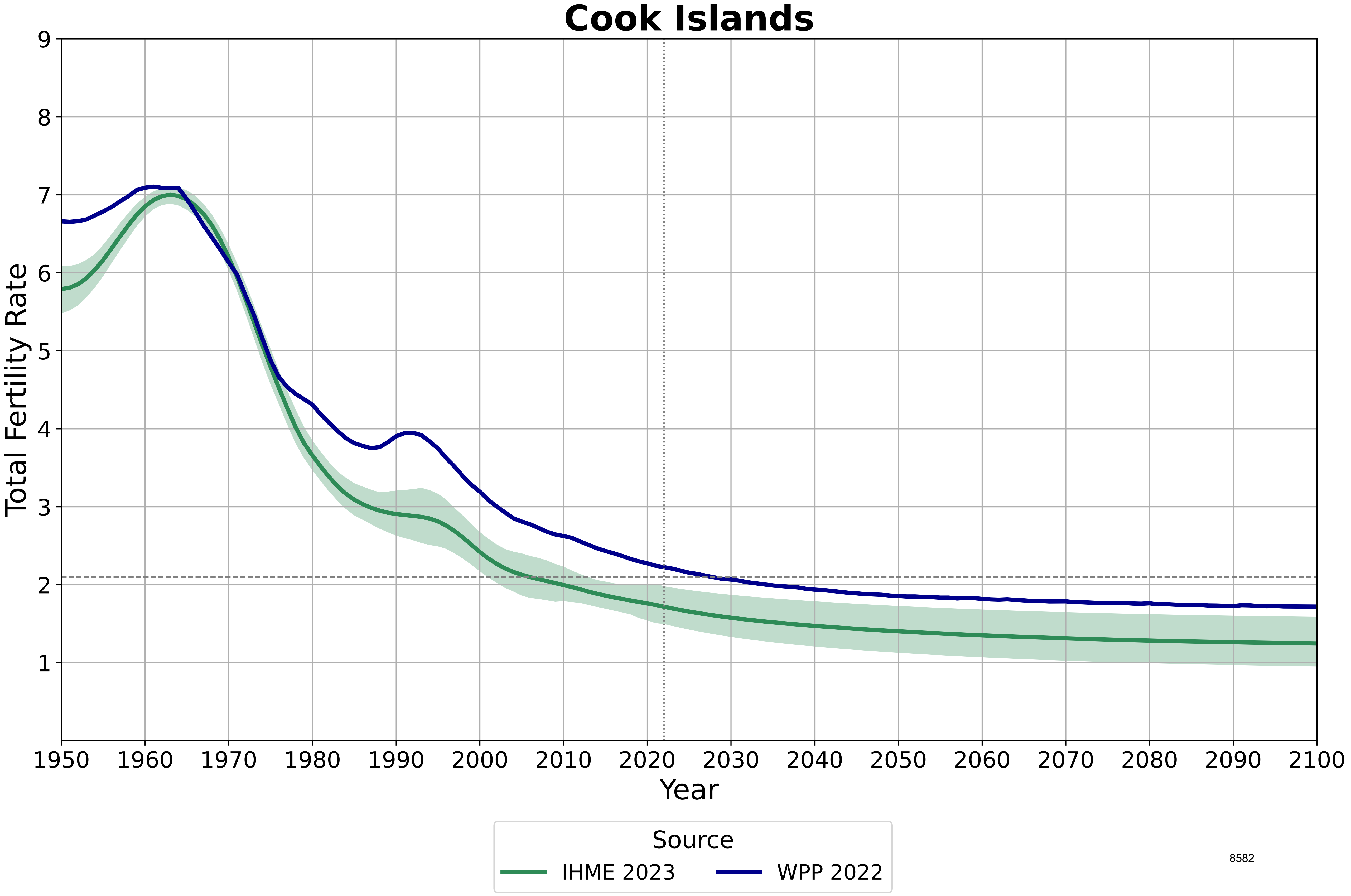

Supplemental Results Figure S7: TFR for IHME reference scenario and the UNPD medium variant scenario by location, 1950– 2100

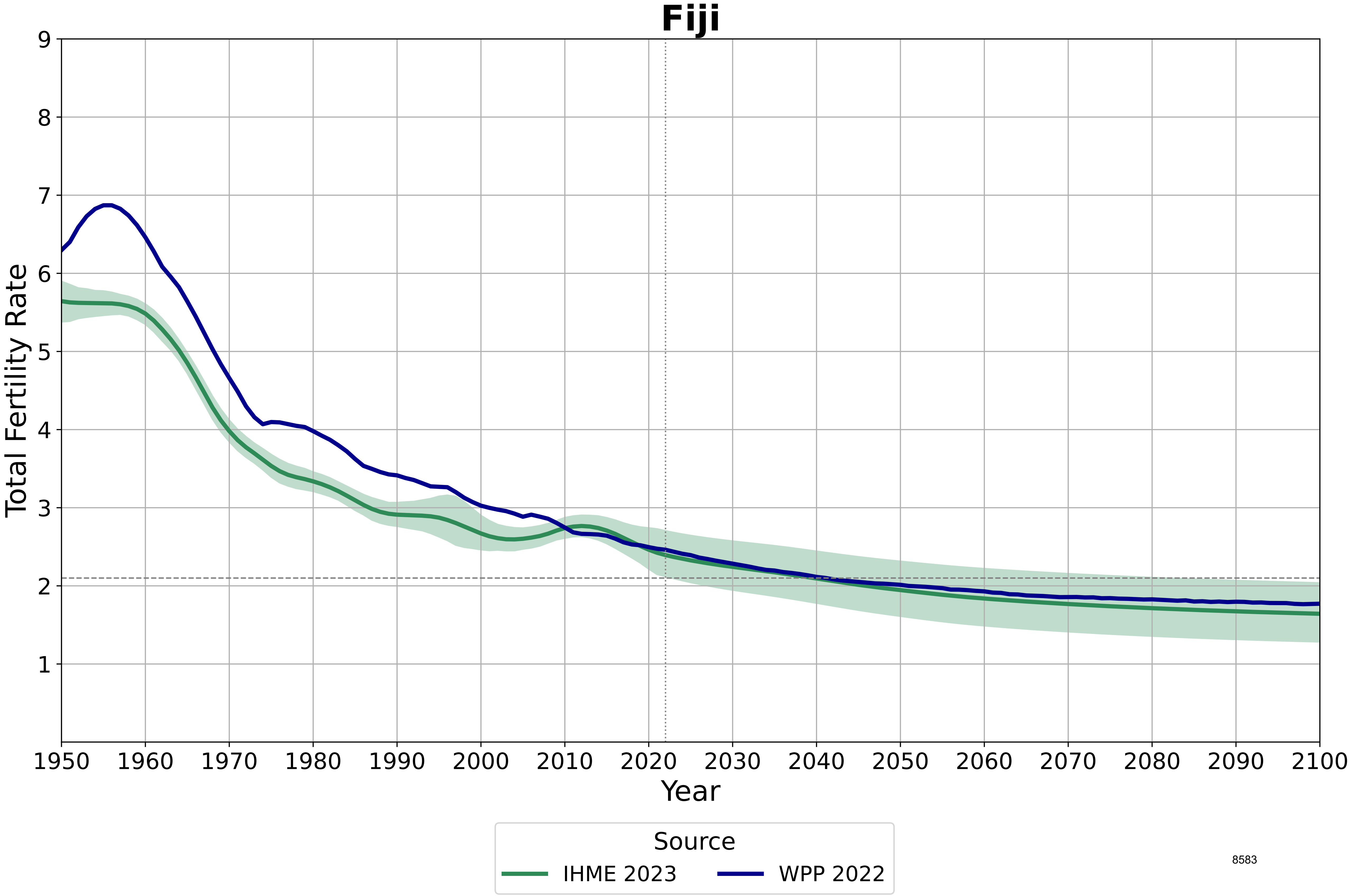

Supplemental Results Figure S7: TFR for IHME reference scenario and the UNPD medium variant scenario by location, 1950– 2100

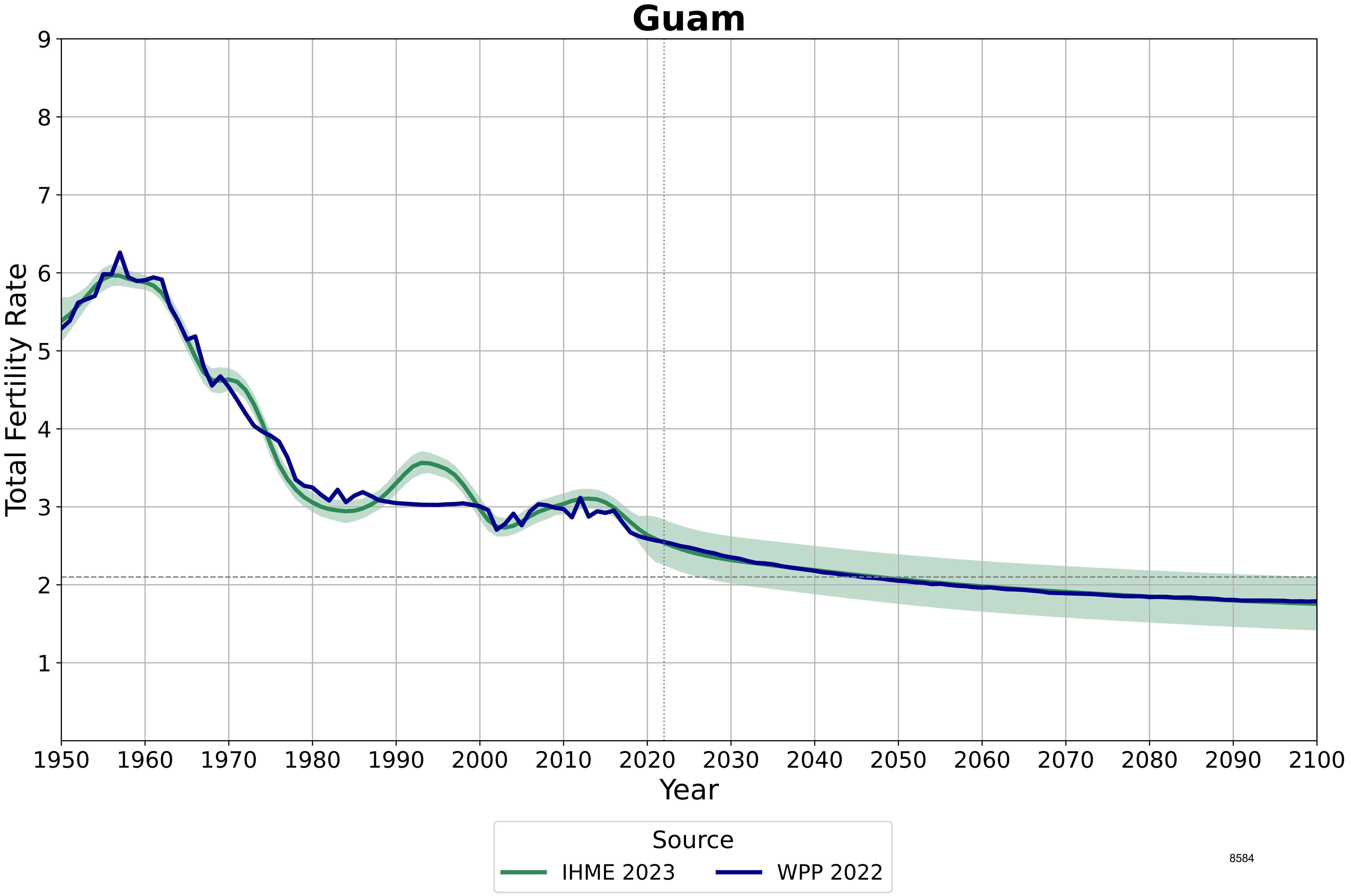

Supplemental Results Figure S7: TFR for IHME reference scenario and the UNPD medium variant scenario by location, 1950– 2100

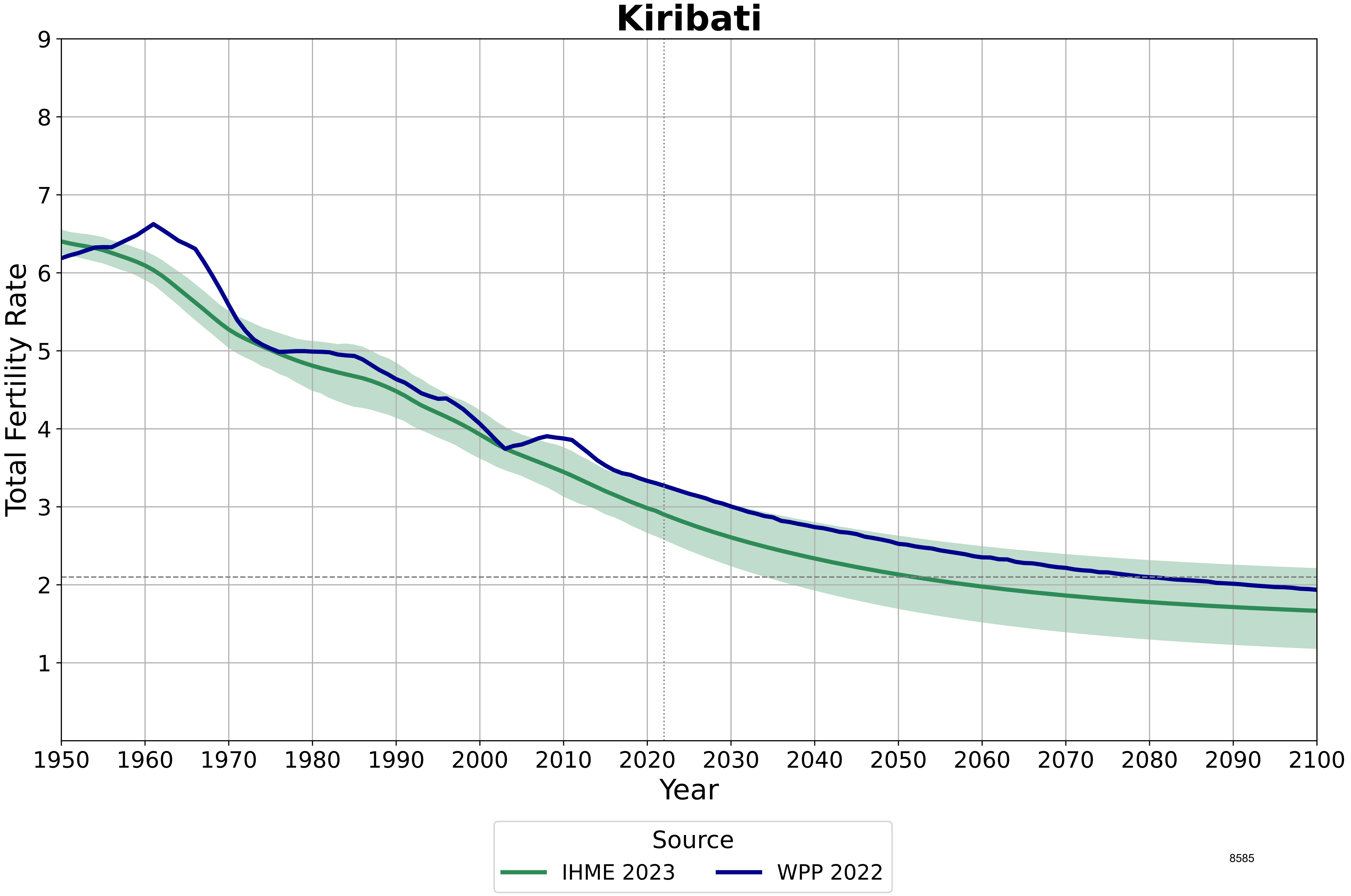

Supplemental Results Figure S7: TFR for IHME reference scenario and the UNPD medium variant scenario by location, 1950– 2100

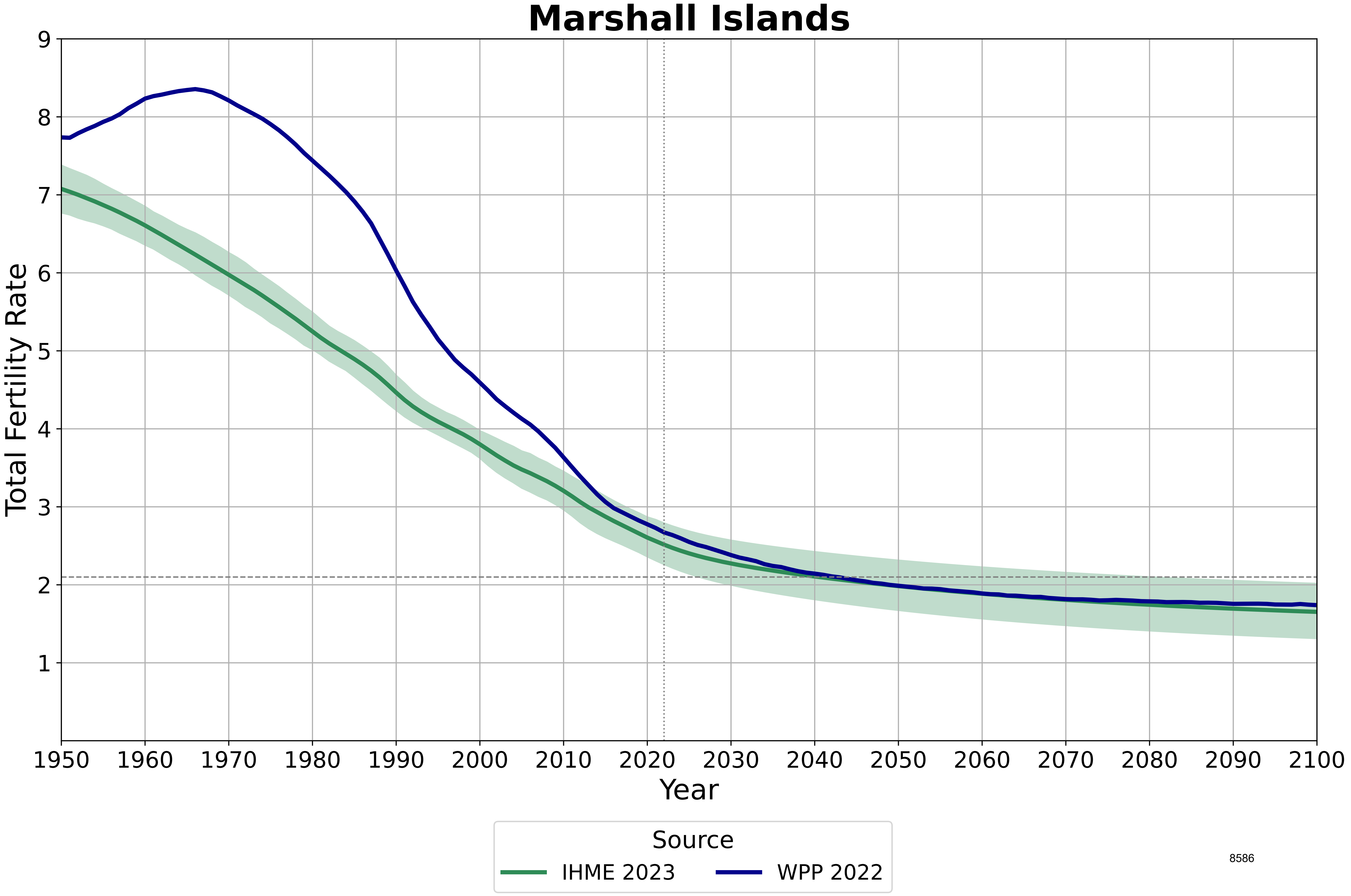

Supplemental Results Figure S7: TFR for IHME reference scenario and the UNPD medium variant scenario by location, 1950– 2100

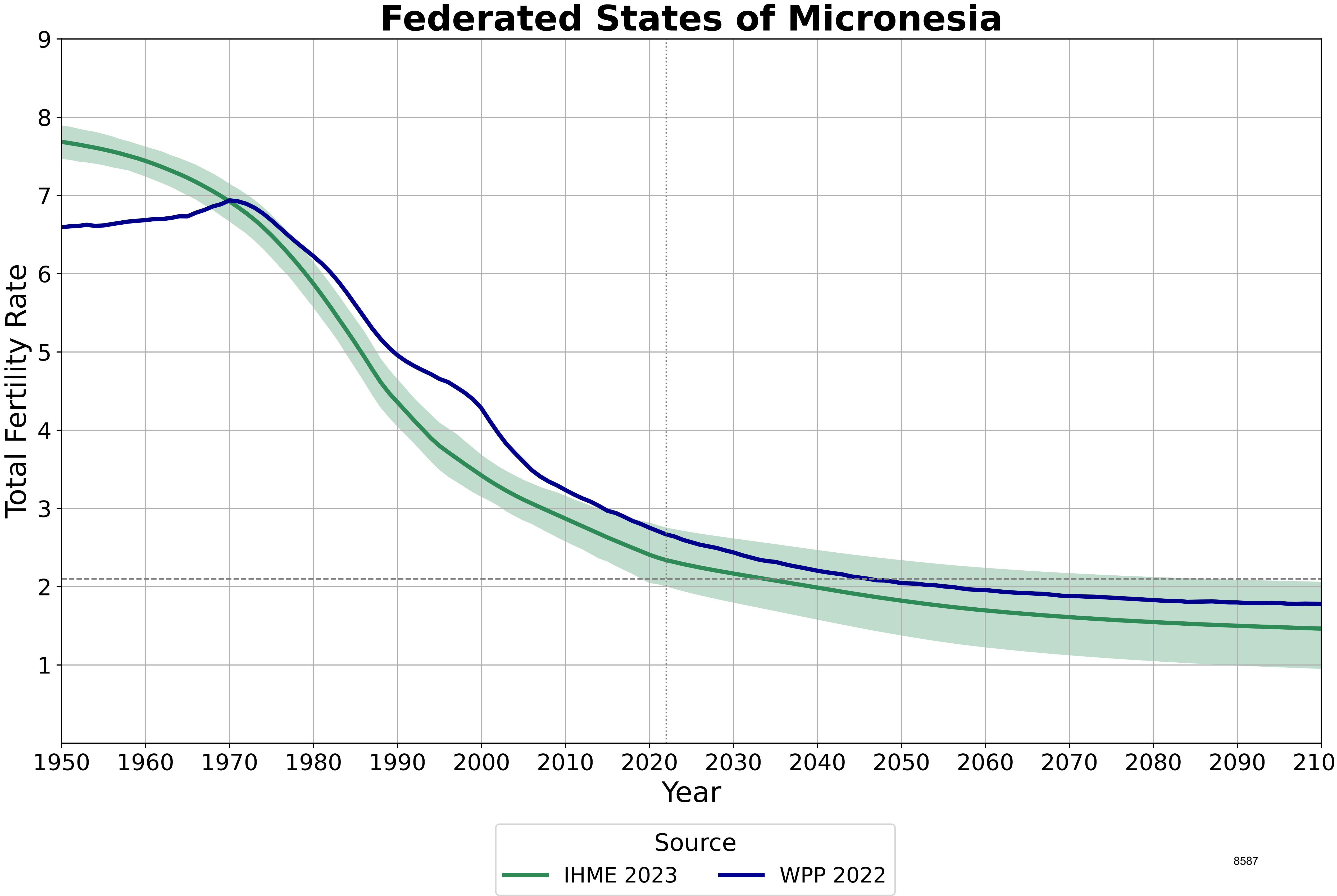

Supplemental Results Figure S7: TFR for IHME reference scenario and the UNPD medium variant scenario by location, 1950– 2100

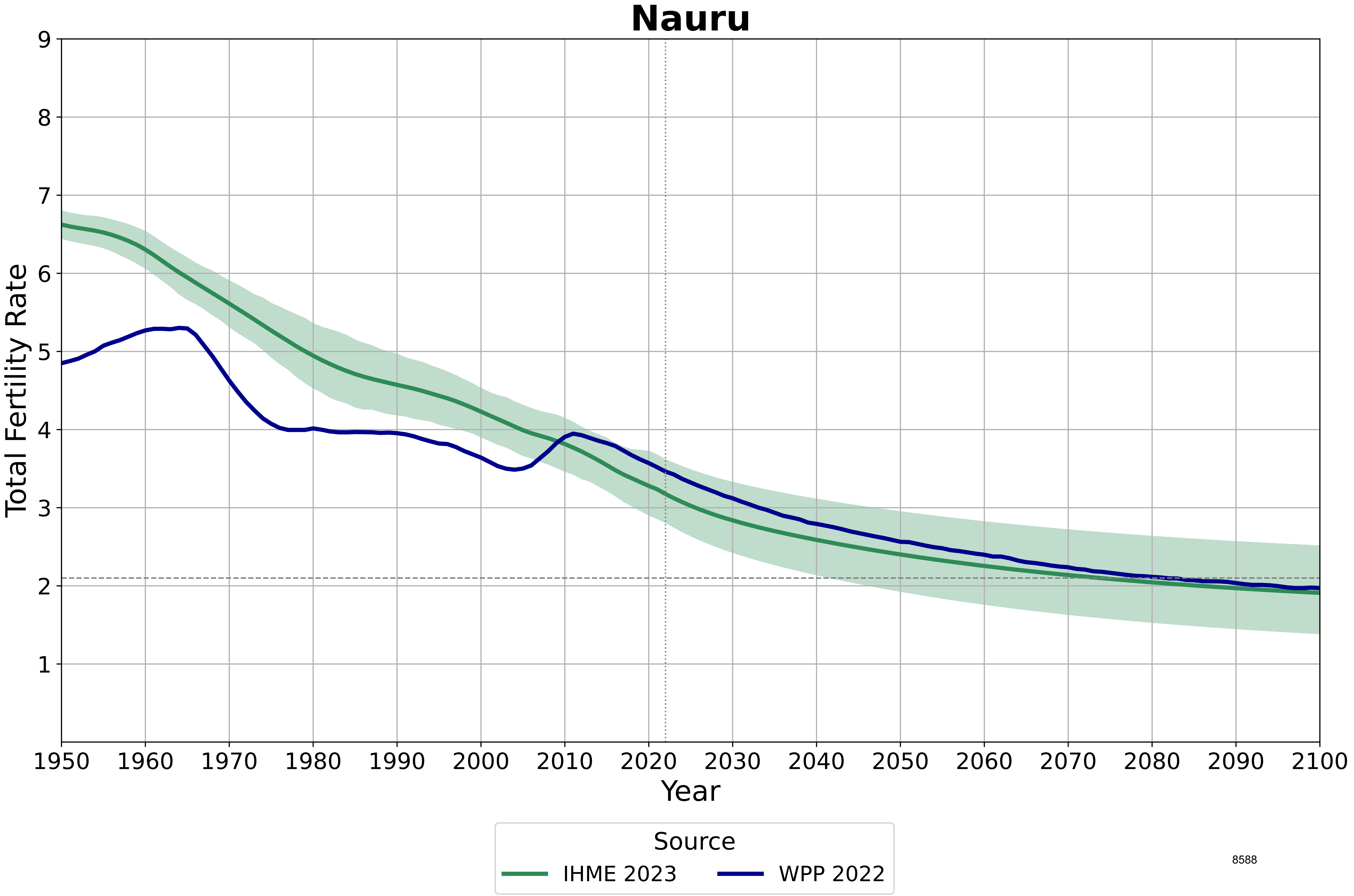

Supplemental Results Figure S7: TFR for IHME reference scenario and the UNPD medium variant scenario by location, 1950– 2100

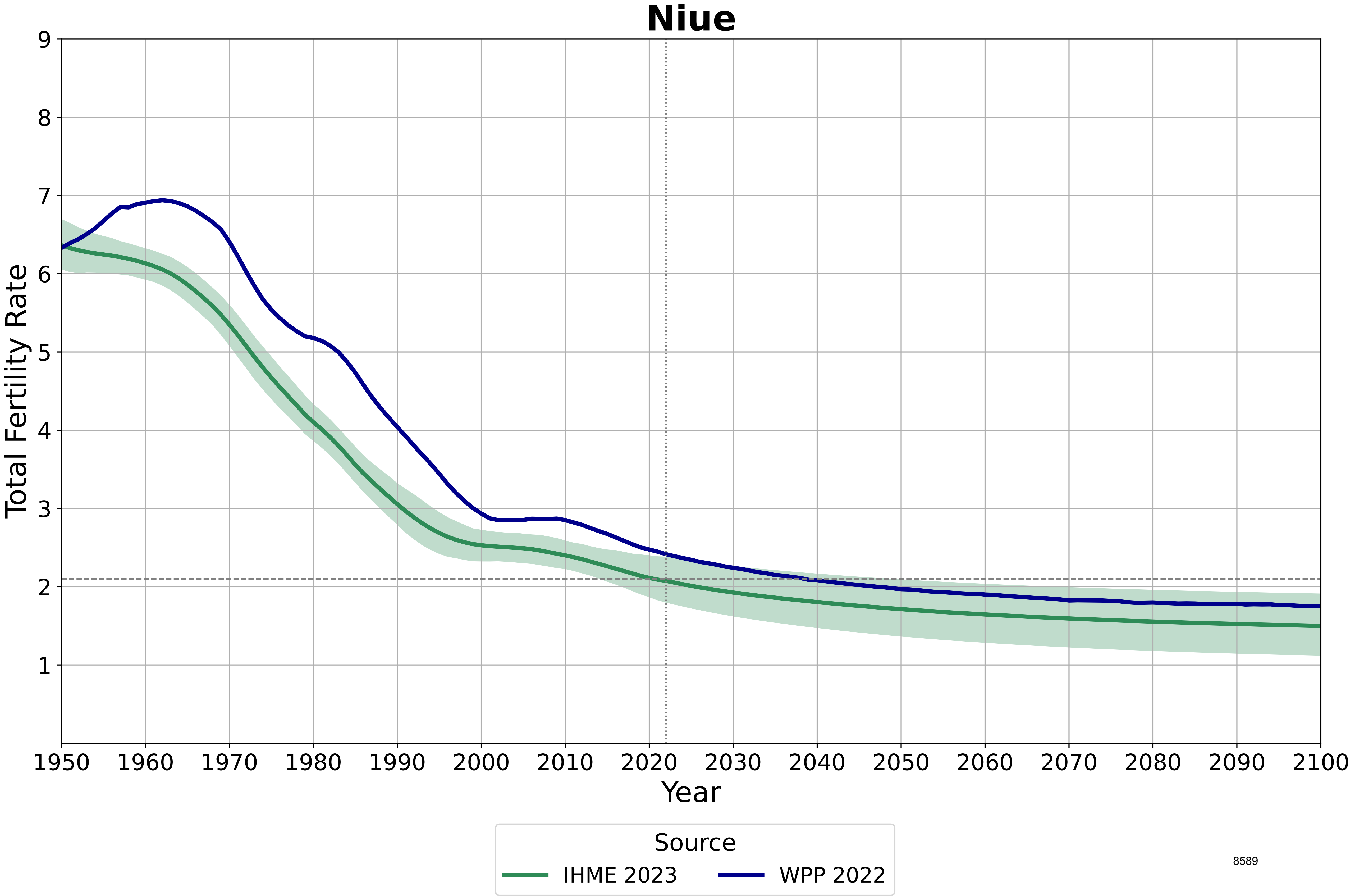

Supplemental Results Figure S7: TFR for IHME reference scenario and the UNPD medium variant scenario by location, 1950– 2100

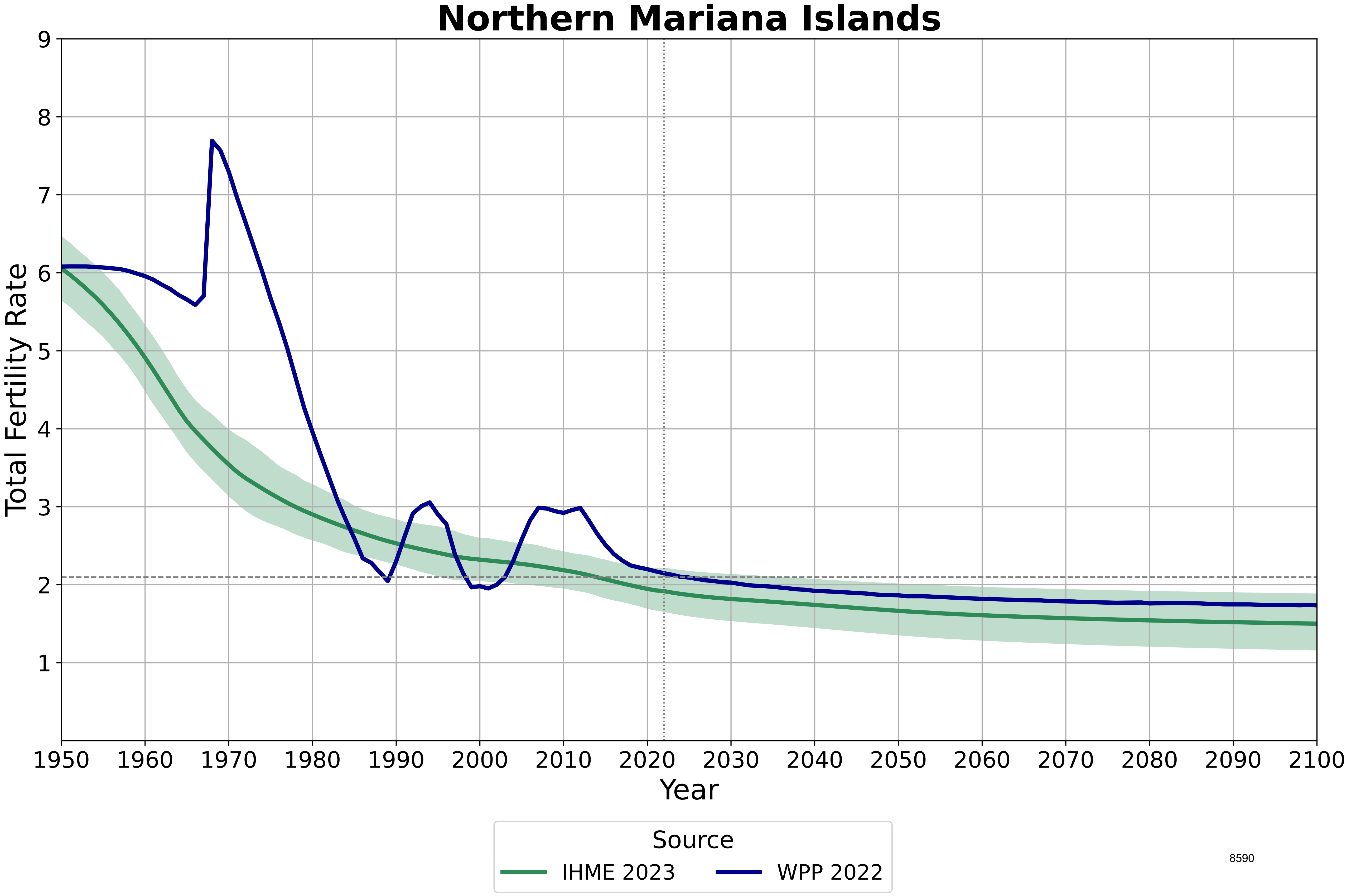

Supplemental Results Figure S7: TFR for IHME reference scenario and the UNPD medium variant scenario by location, 1950– 2100

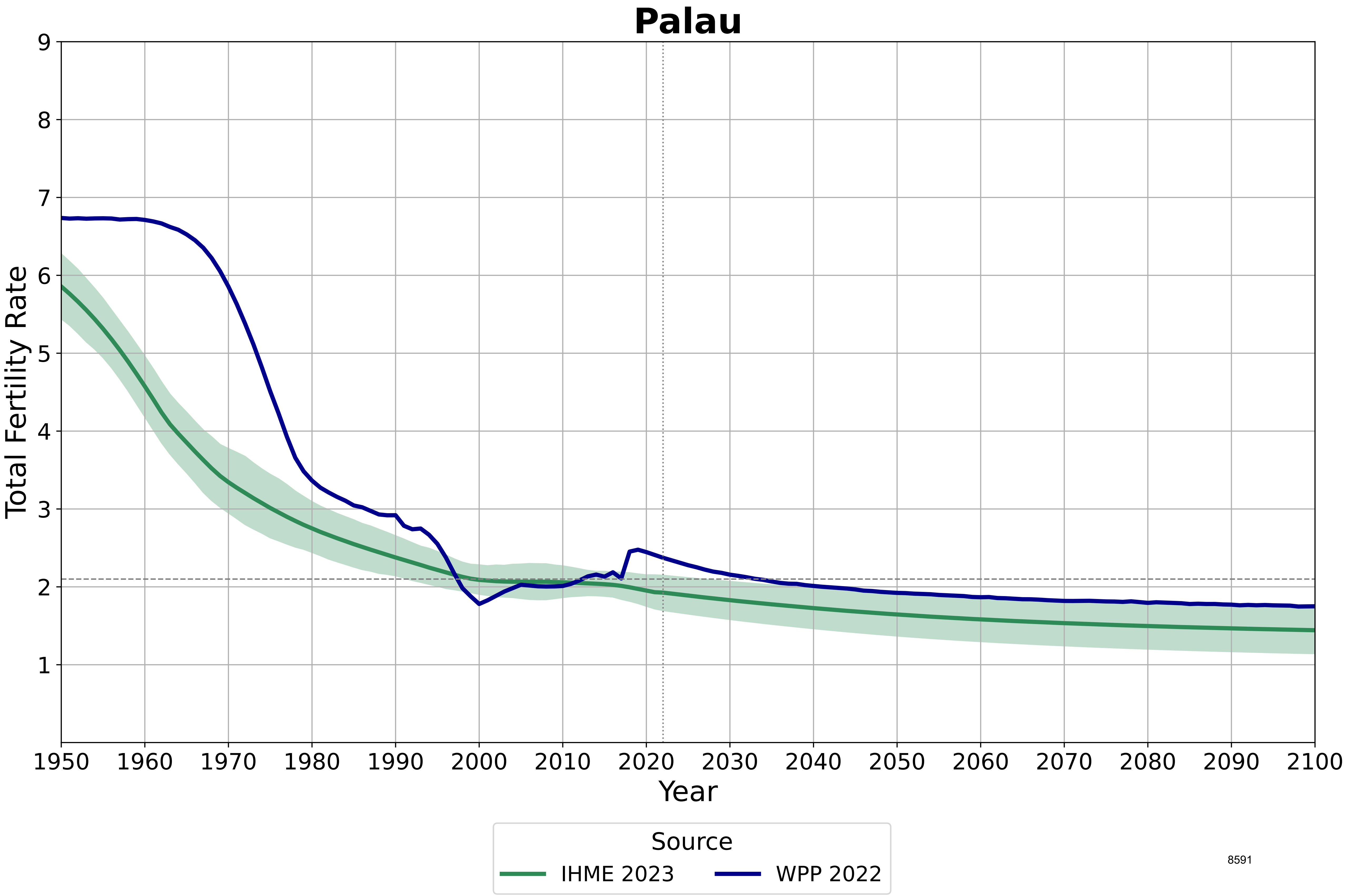

Supplemental Results Figure S7: TFR for IHME reference scenario and the UNPD medium variant scenario by location, 1950– 2100

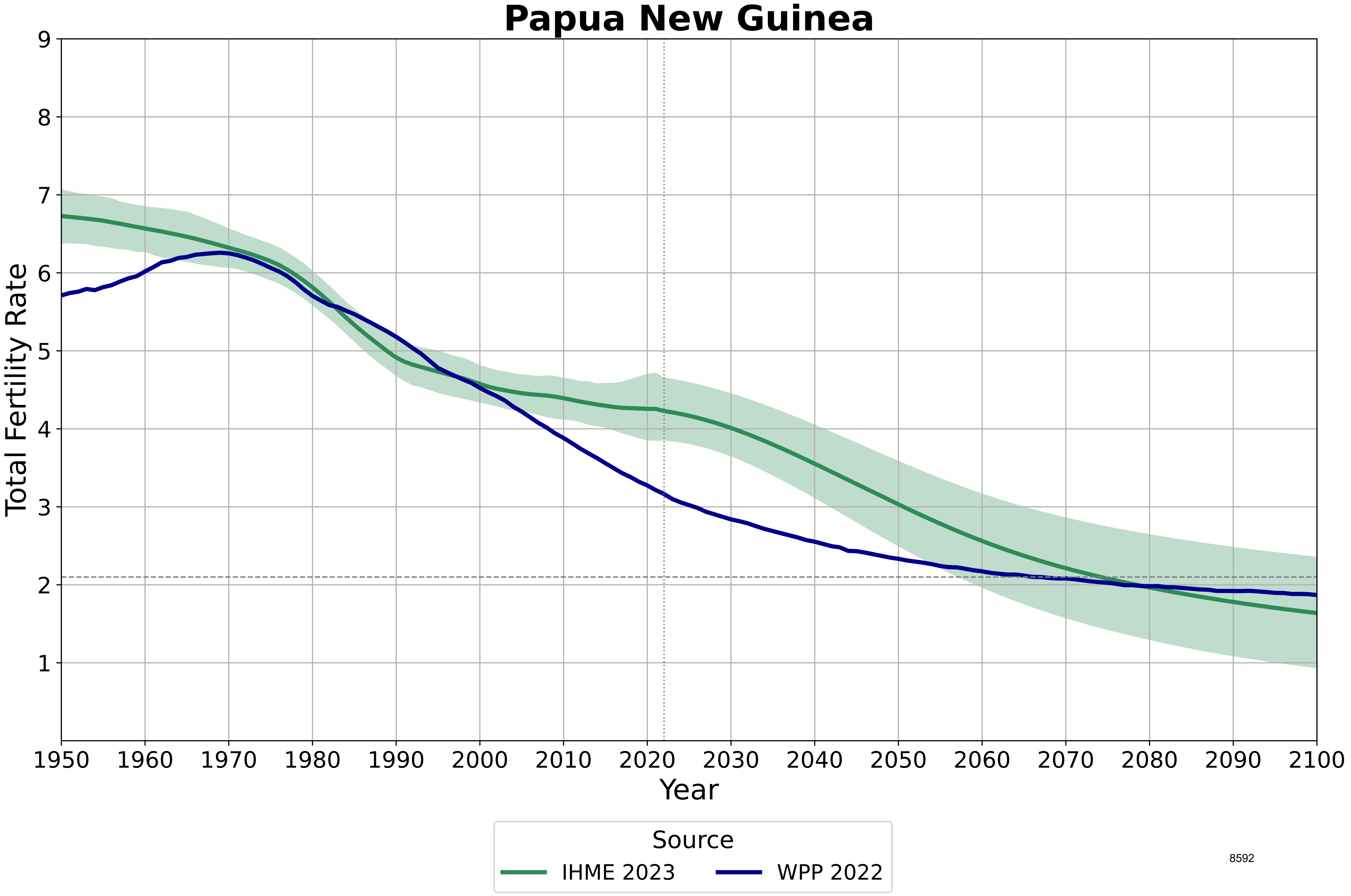

Supplemental Results Figure S7: TFR for IHME reference scenario and the UNPD medium variant scenario by location, 1950– 2100

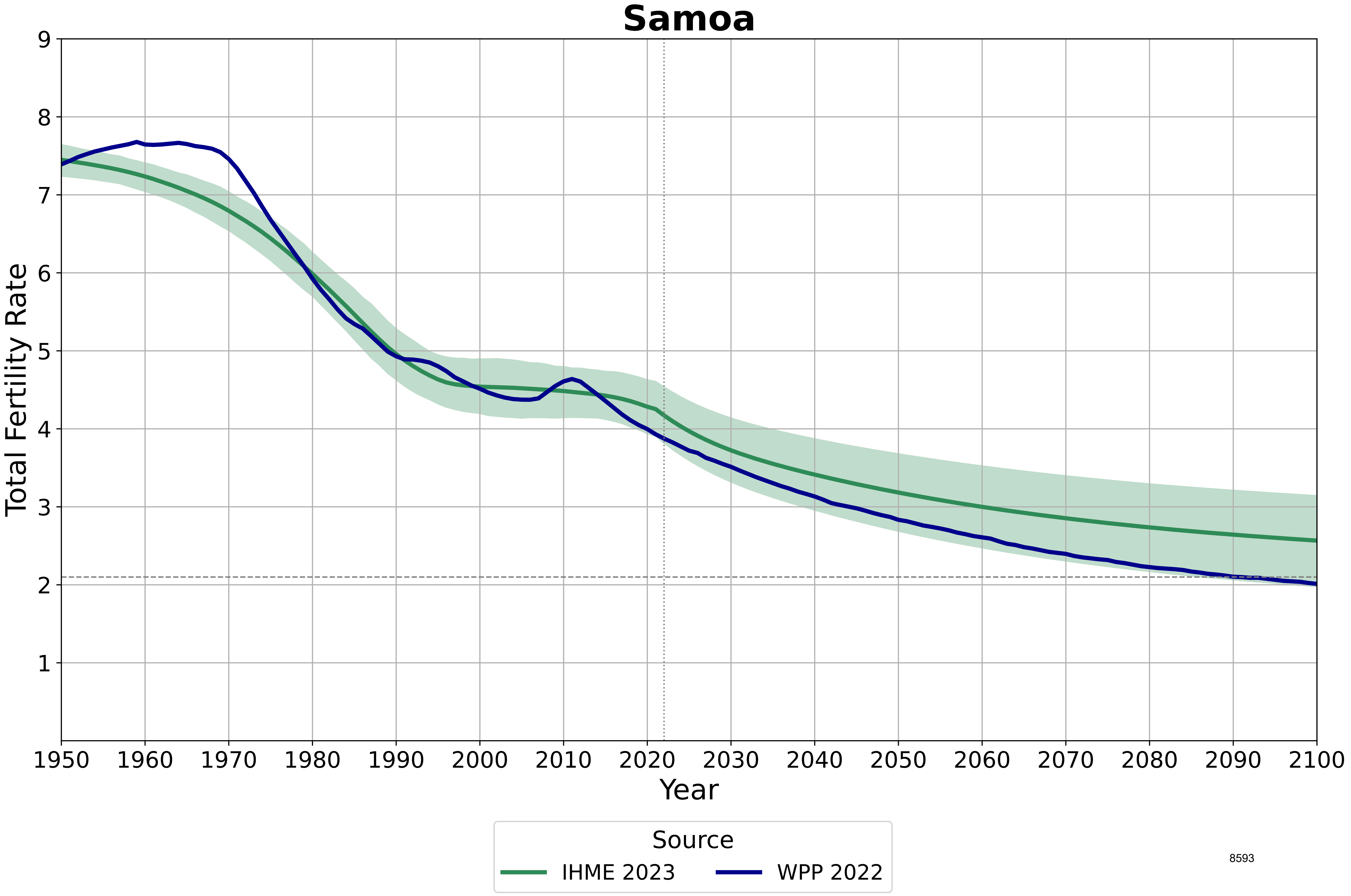

Supplemental Results Figure S7: TFR for IHME reference scenario and the UNPD medium variant scenario by location, 1950– 2100

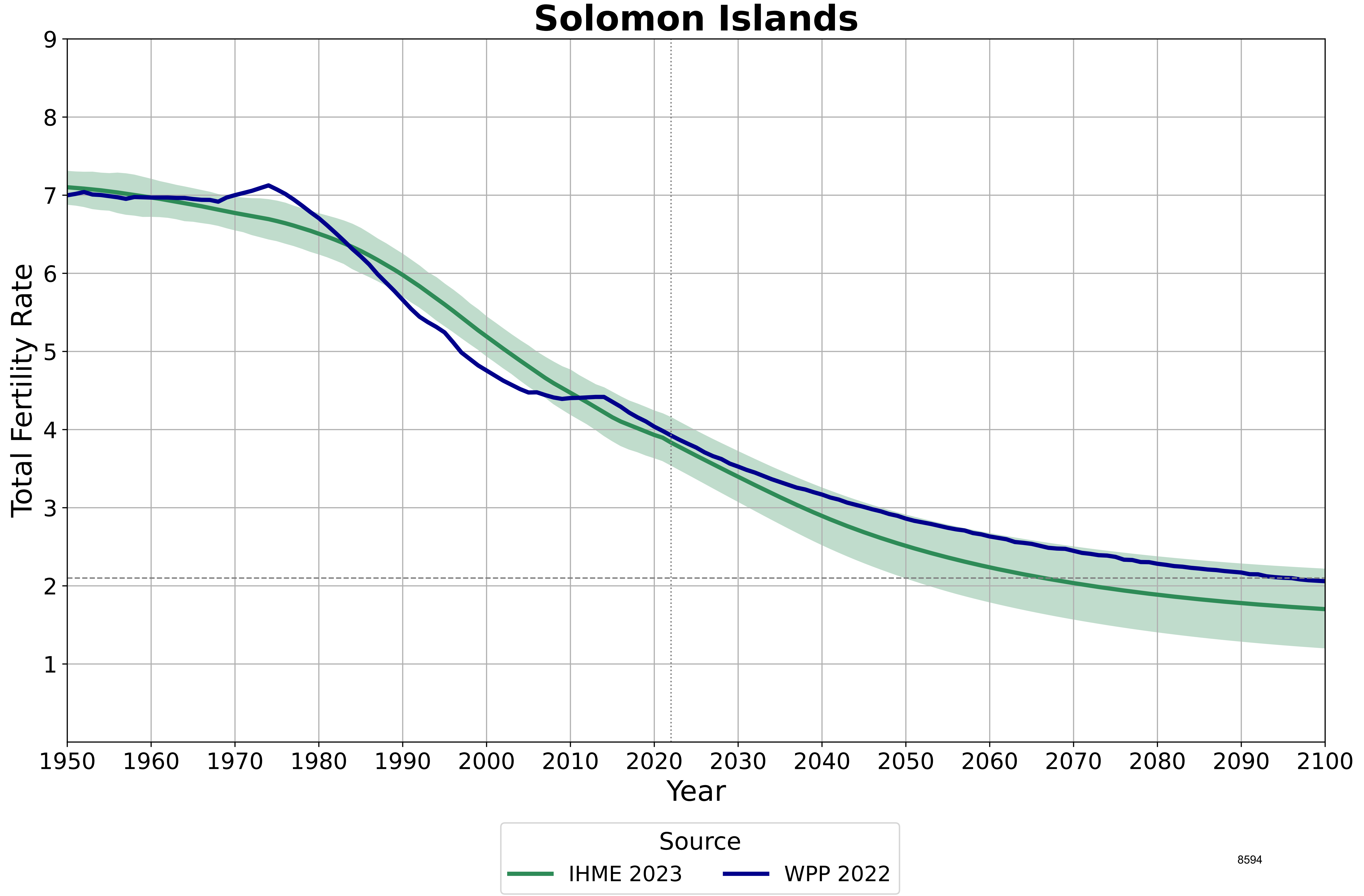

Supplemental Results Figure S7: TFR for IHME reference scenario and the UNPD medium variant scenario by location, 1950– 2100

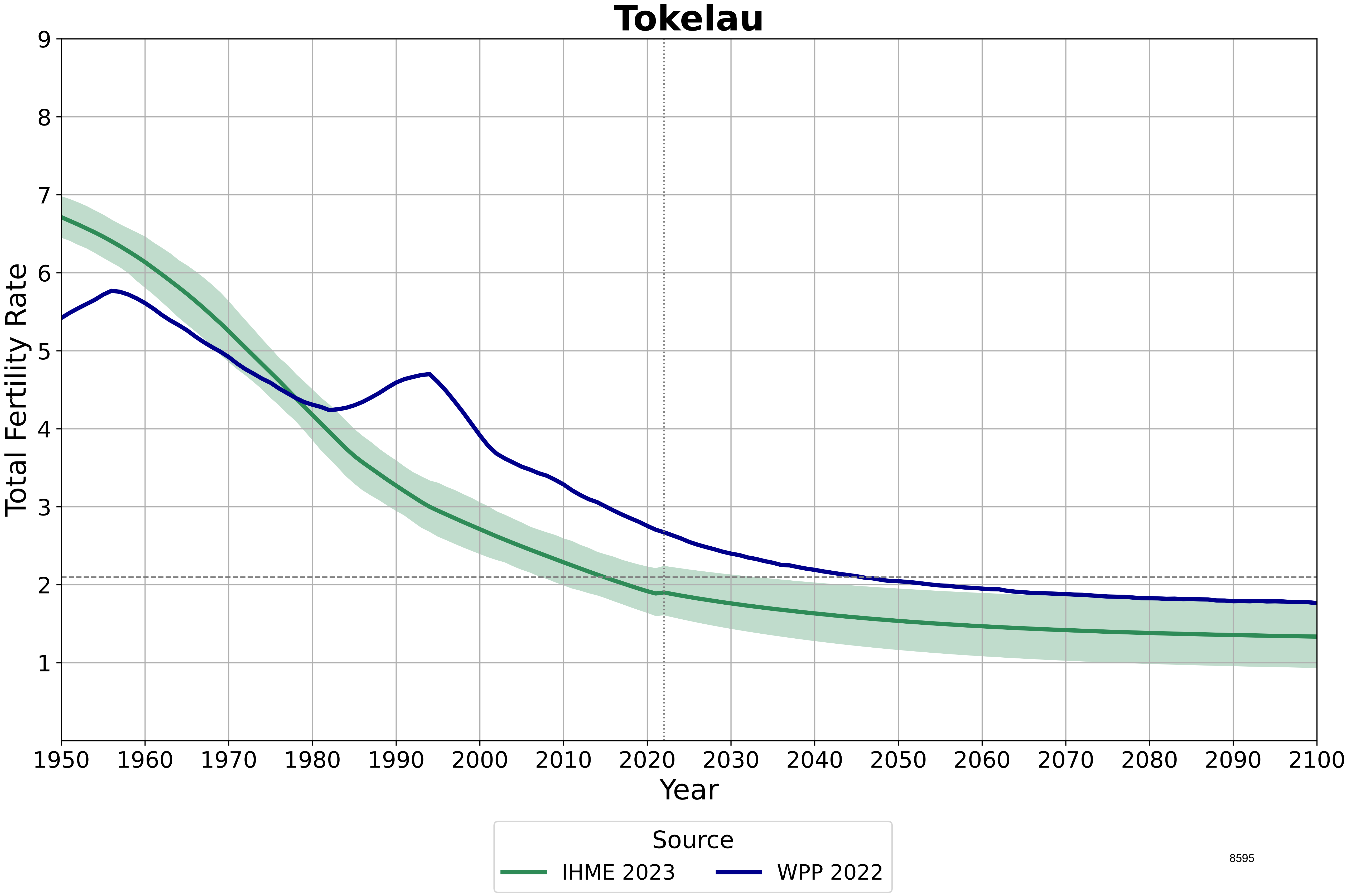

Supplemental Results Figure S7: TFR for IHME reference scenario and the UNPD medium variant scenario by location, 1950– 2100

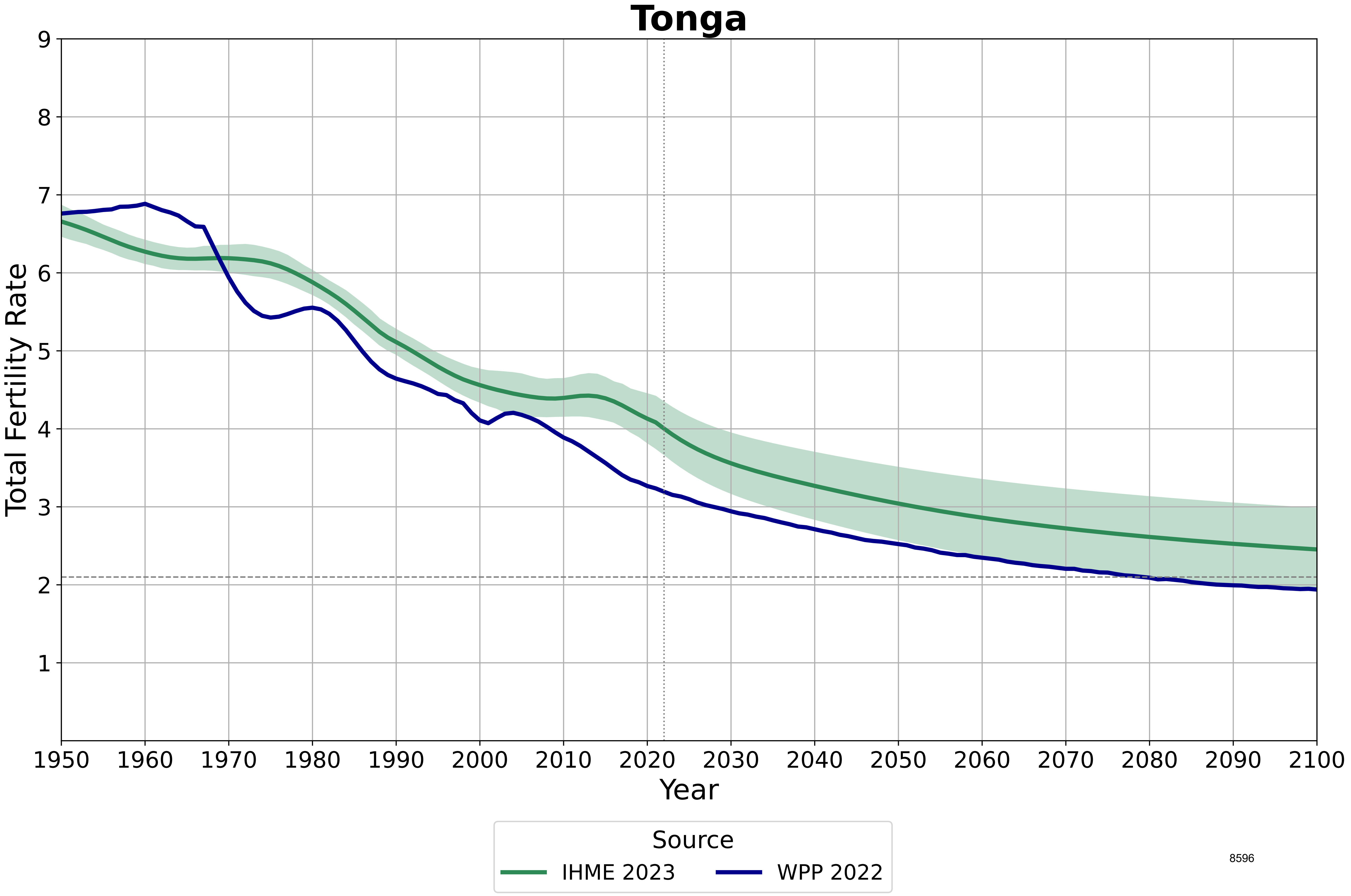

Supplemental Results Figure S7: TFR for IHME reference scenario and the UNPD medium variant scenario by location, 1950– 2100

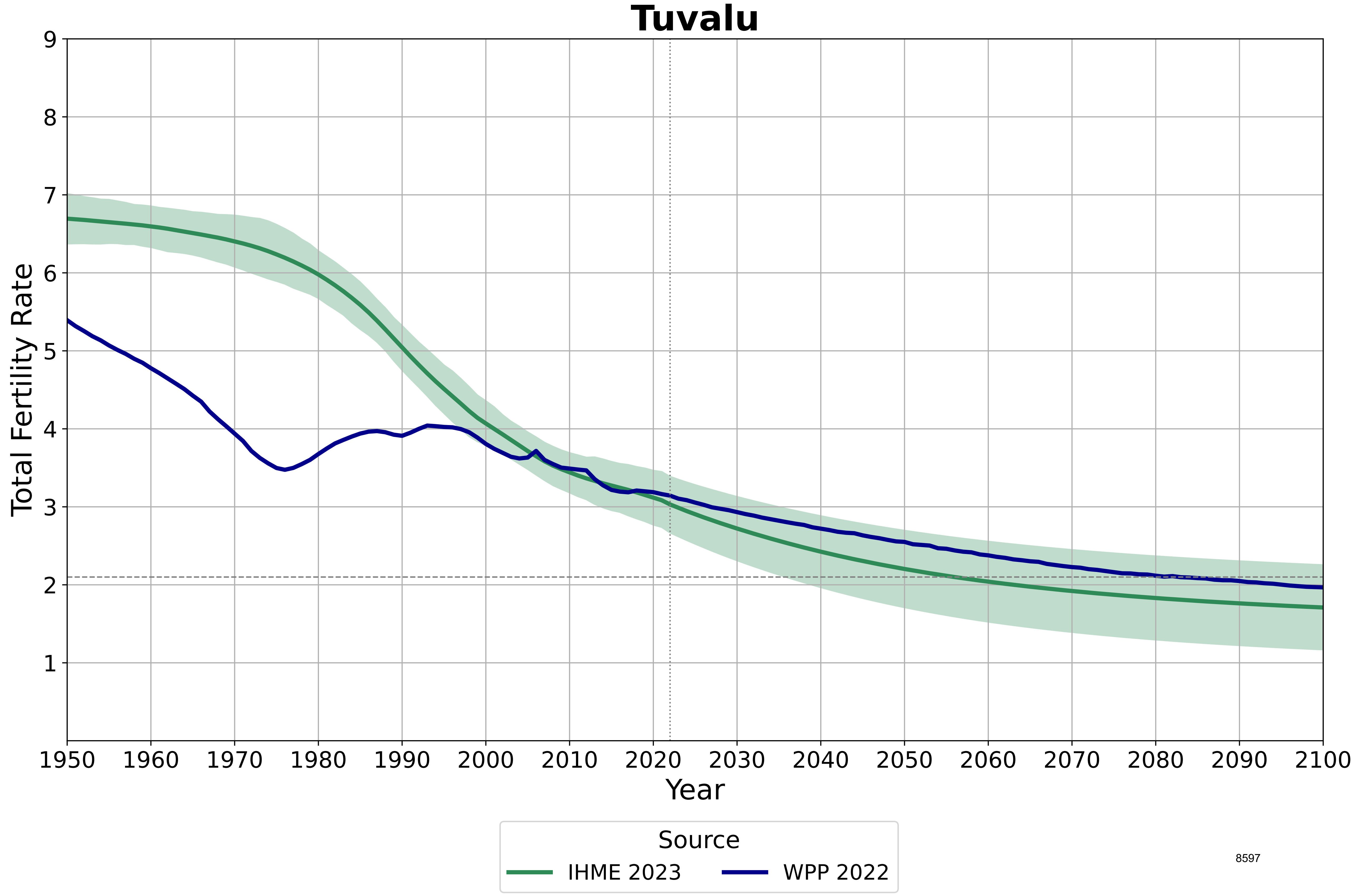

Supplemental Results Figure S7: TFR for IHME reference scenario and the UNPD medium variant scenario by location, 1950– 2100

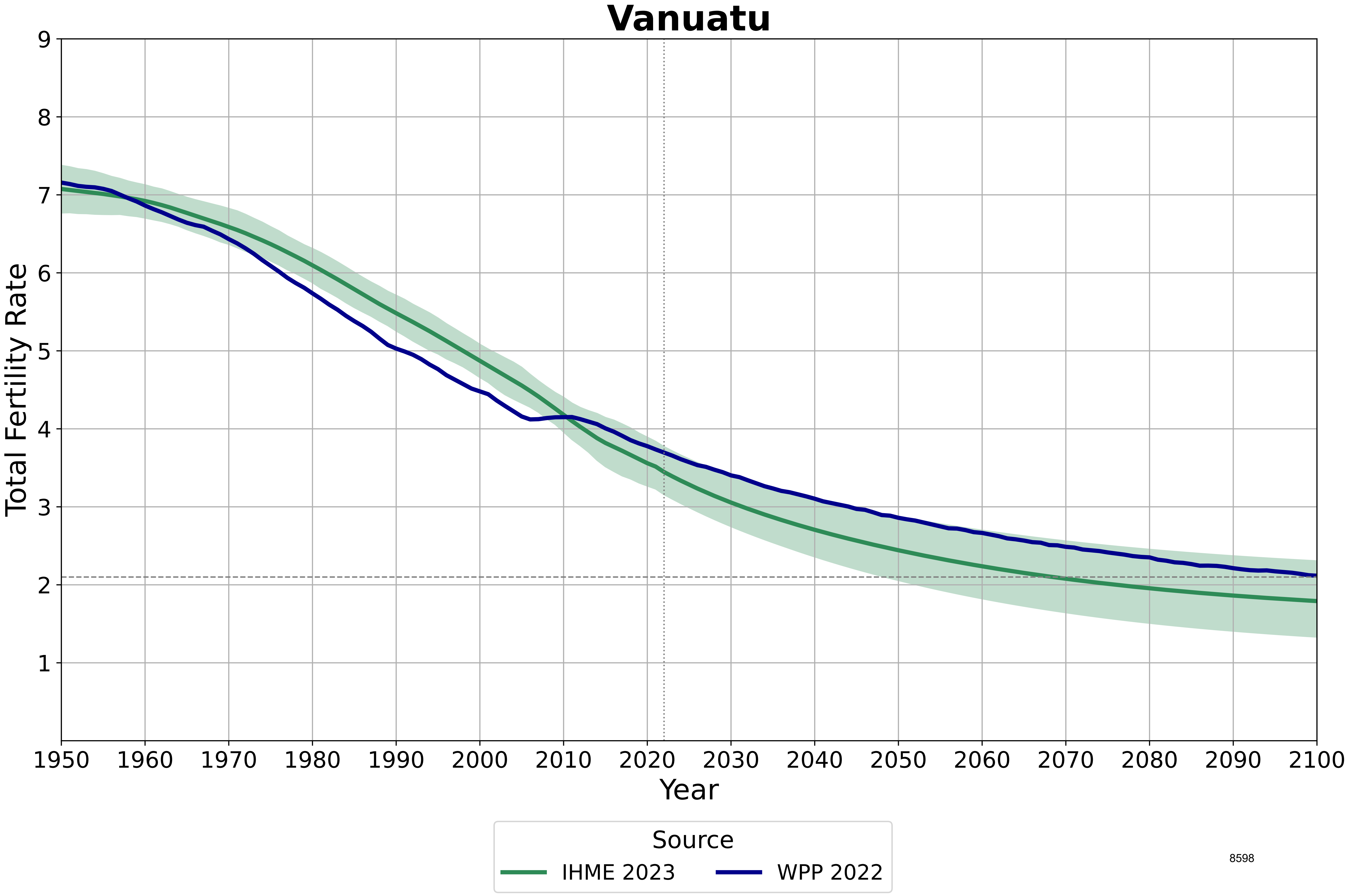

Supplemental Results Figure S7: TFR for IHME reference scenario and the UNPD medium variant scenario by location, 1950– 2100

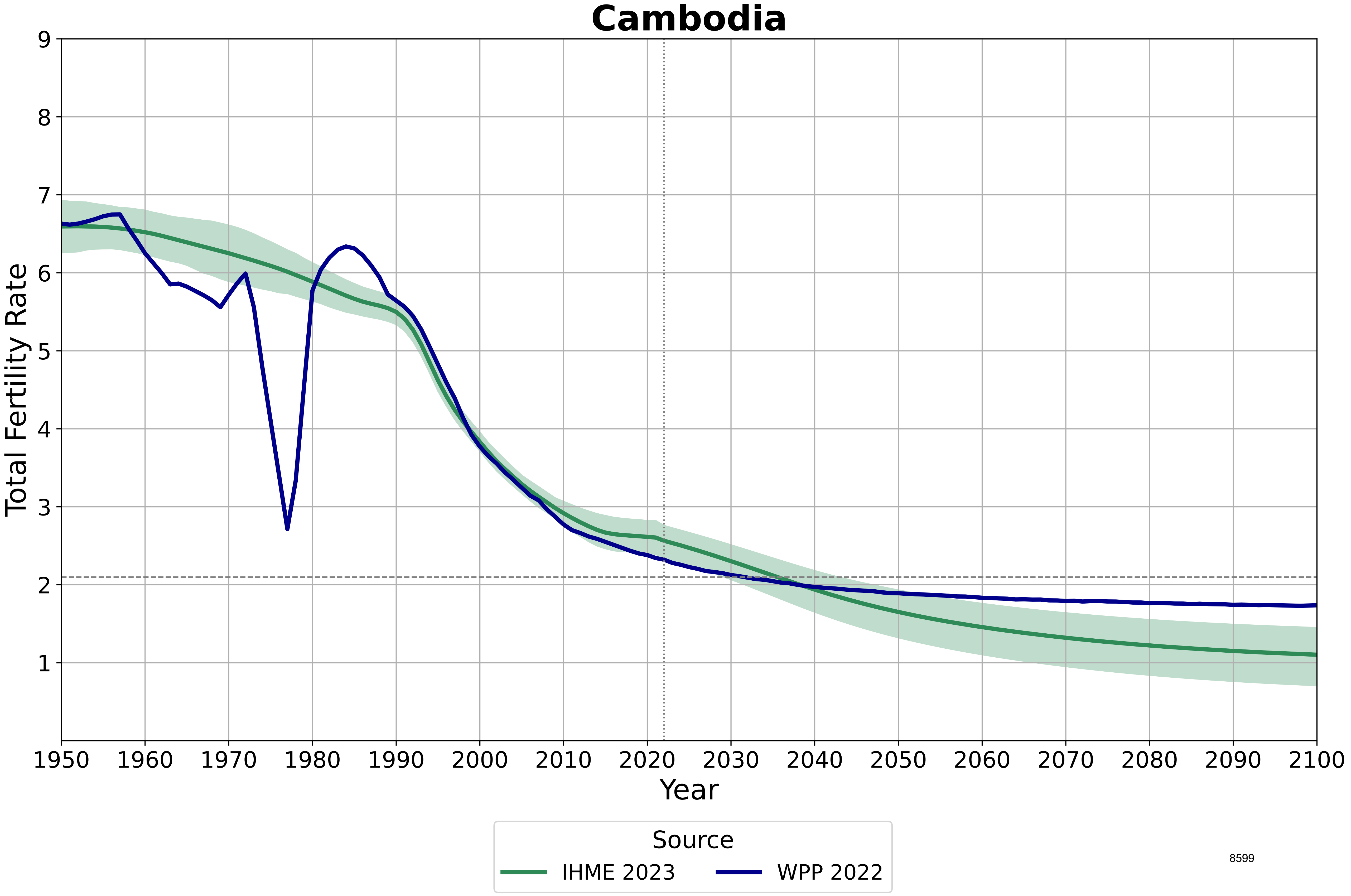

Supplemental Results Figure S7: TFR for IHME reference scenario and the UNPD medium variant scenario by location, 1950– 2100

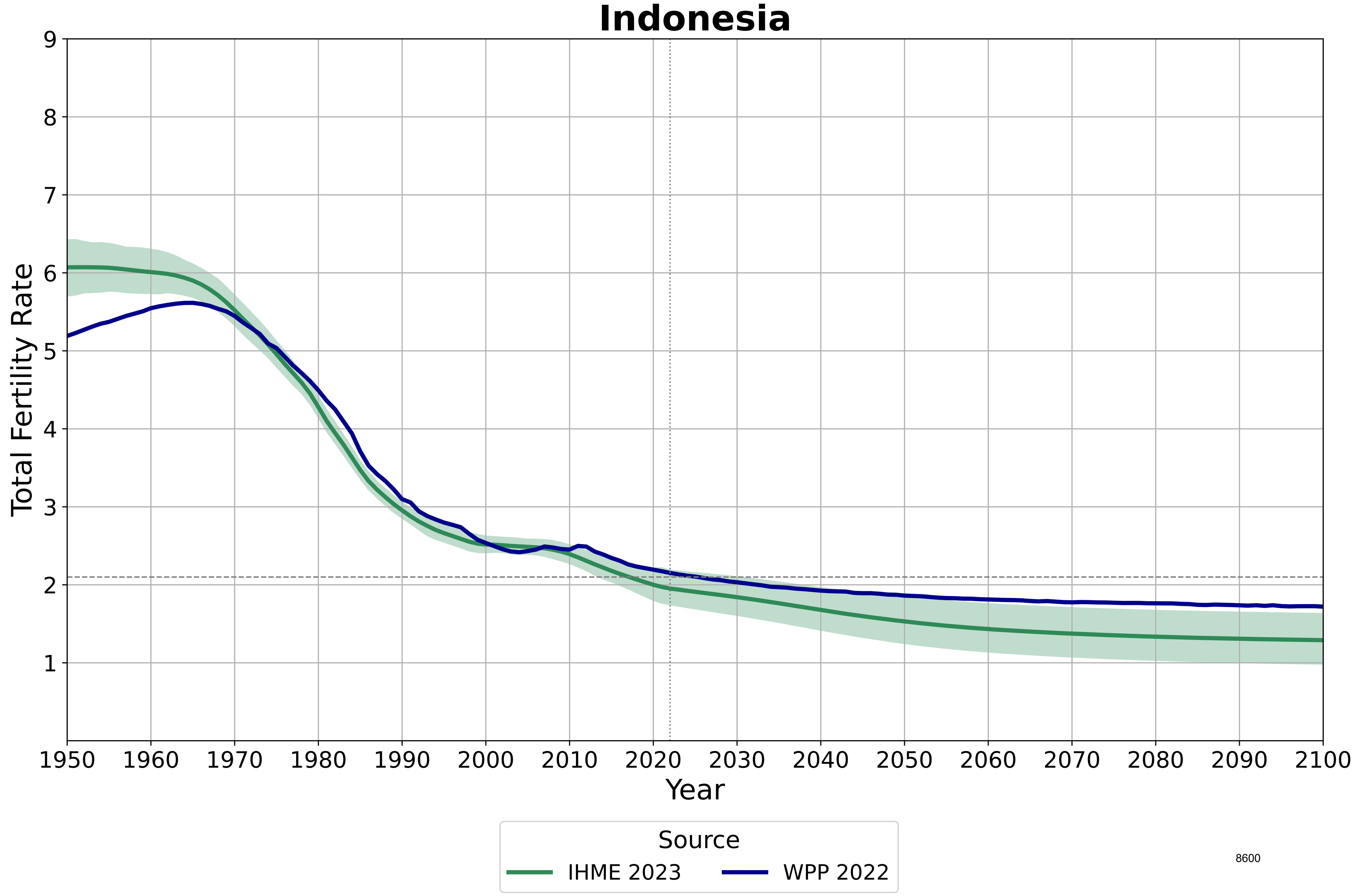

Supplemental Results Figure S7: TFR for IHME reference scenario and the UNPD medium variant scenario by location, 1950– 2100

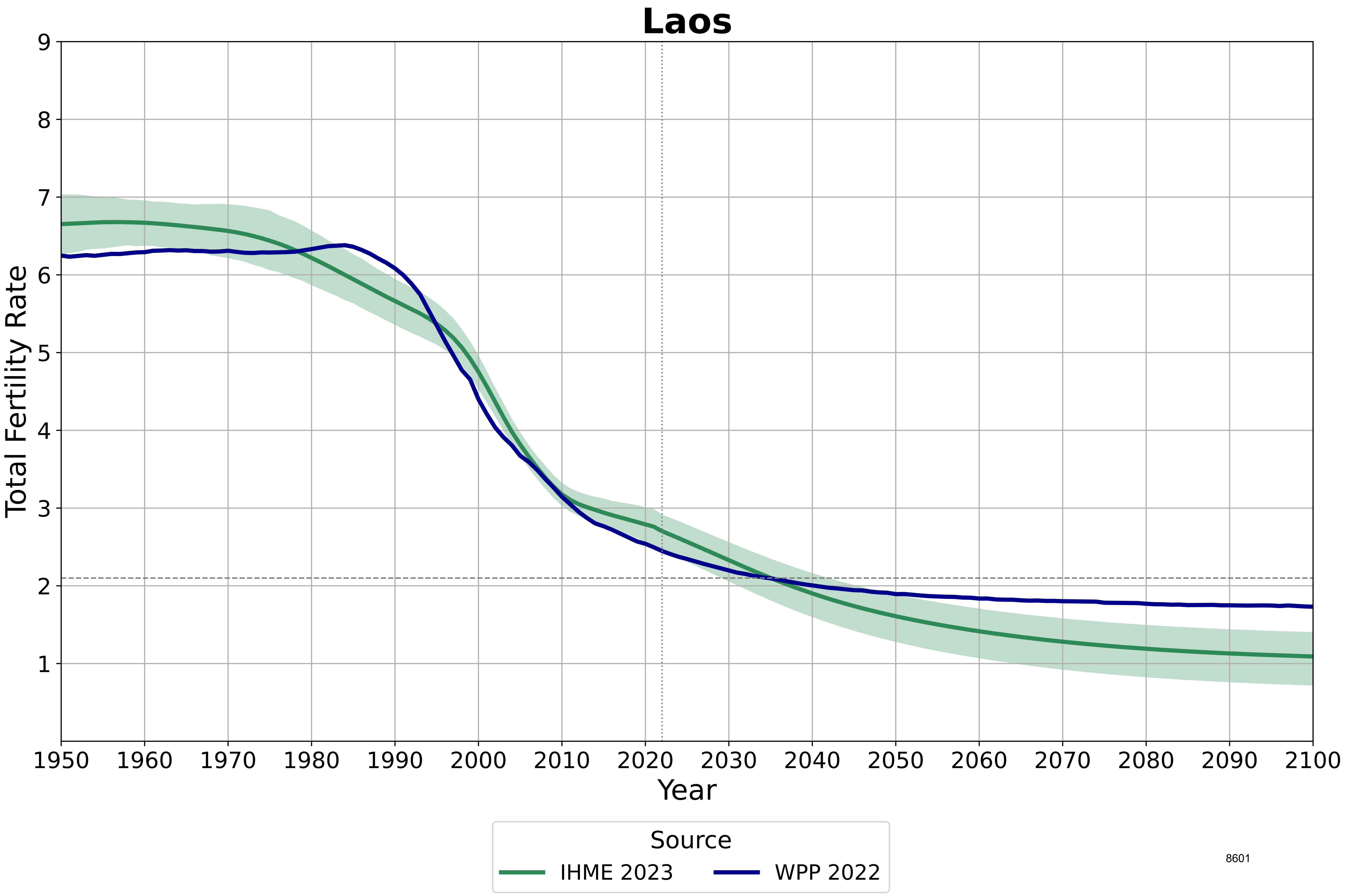

Supplemental Results Figure S7: TFR for IHME reference scenario and the UNPD medium variant scenario by location, 1950– 2100

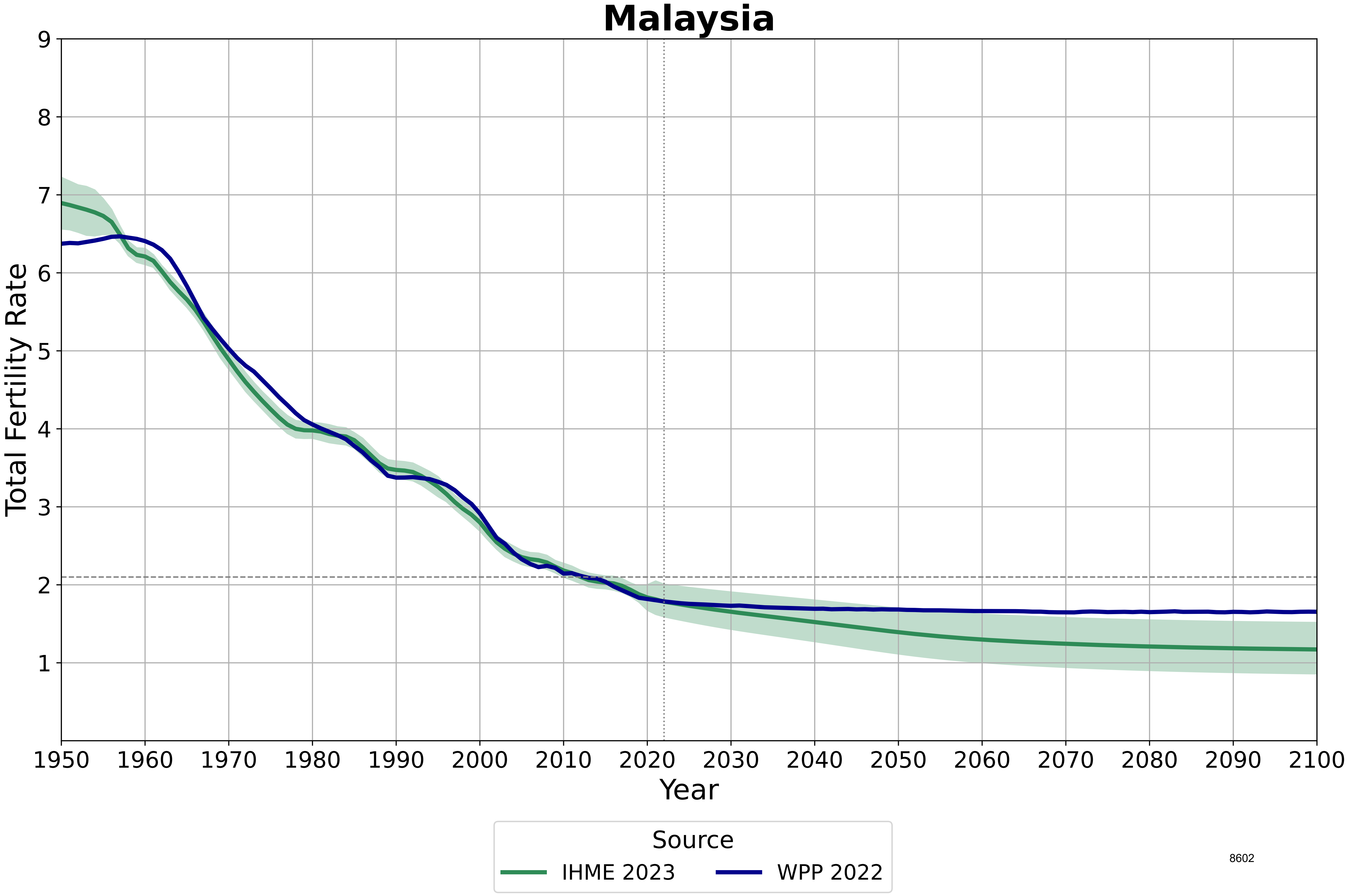

Supplemental Results Figure S7: TFR for IHME reference scenario and the UNPD medium variant scenario by location, 1950– 2100

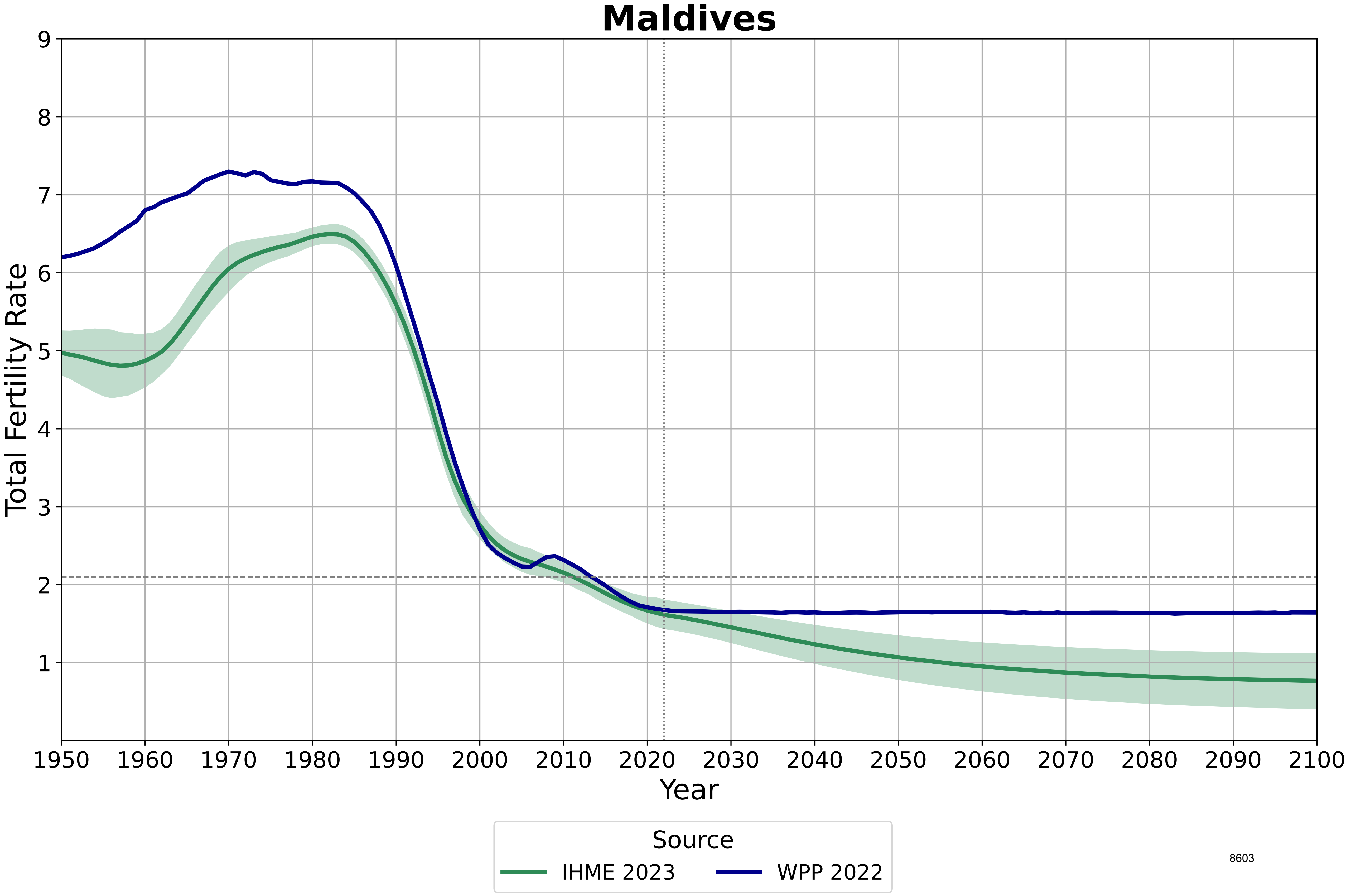

Supplemental Results Figure S7: TFR for IHME reference scenario and the UNPD medium variant scenario by location, 1950– 2100

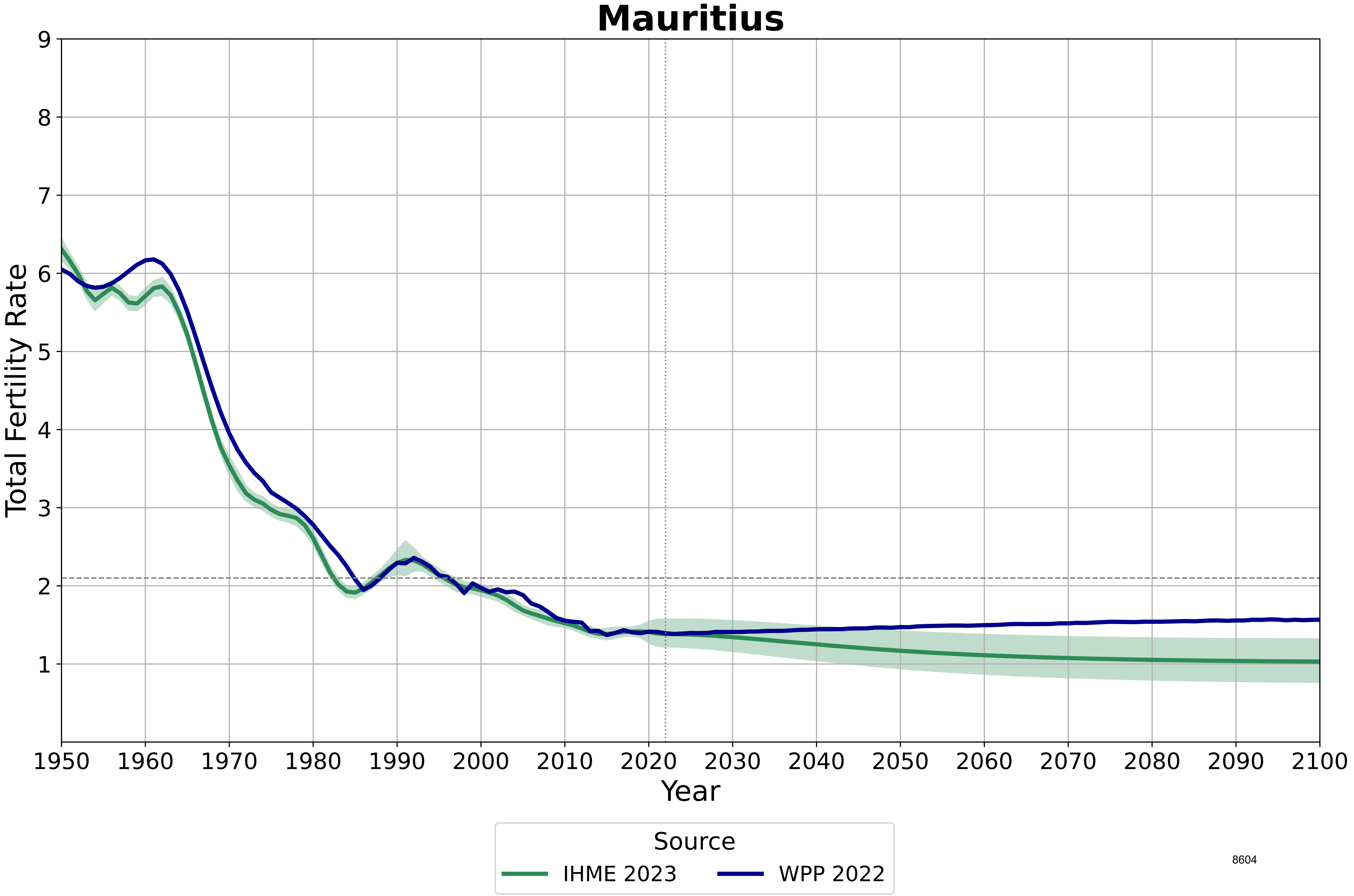

Supplemental Results Figure S7: TFR for IHME reference scenario and the UNPD medium variant scenario by location, 1950– 2100

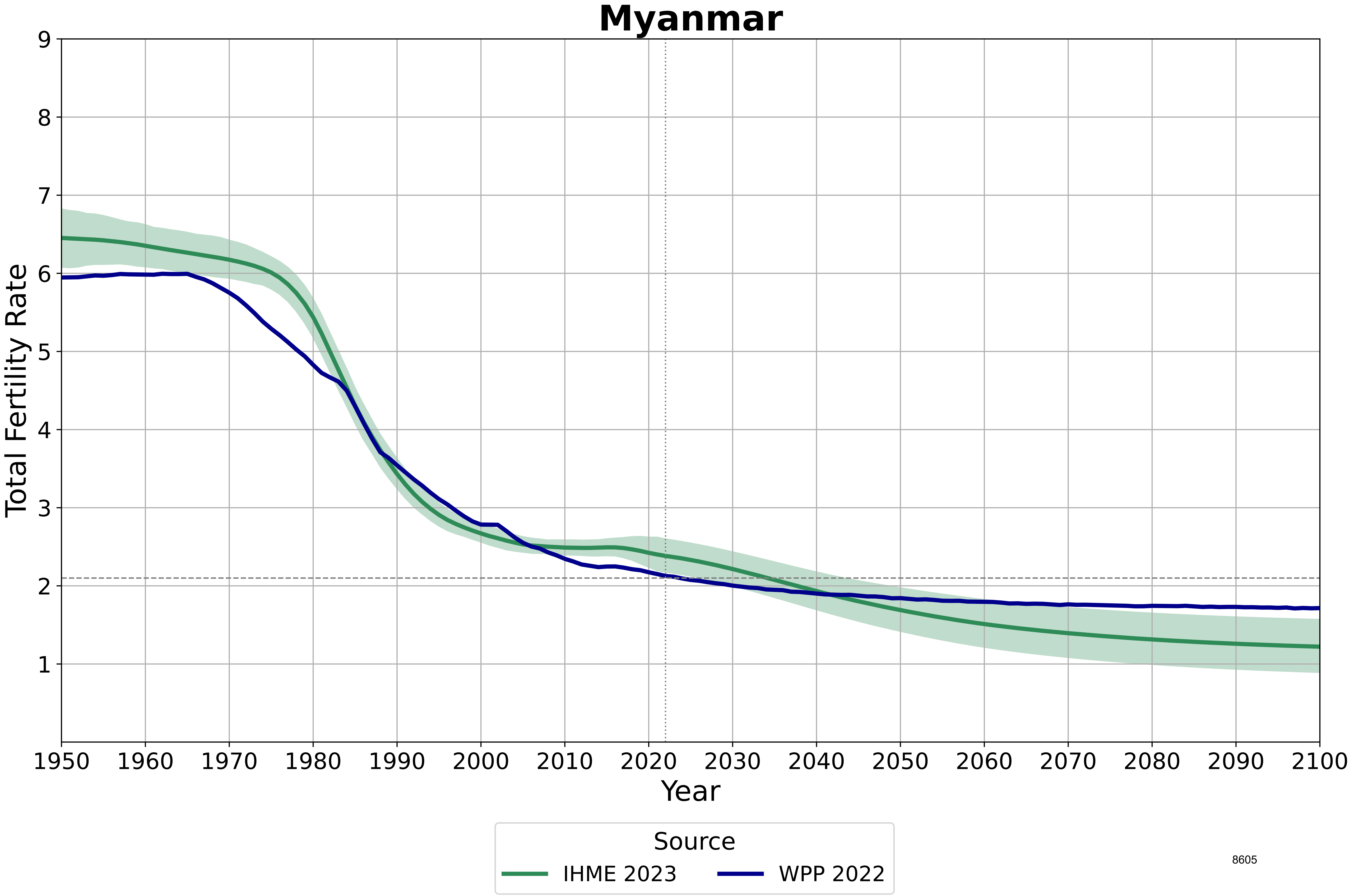

Supplemental Results Figure S7: TFR for IHME reference scenario and the UNPD medium variant scenario by location, 1950– 2100

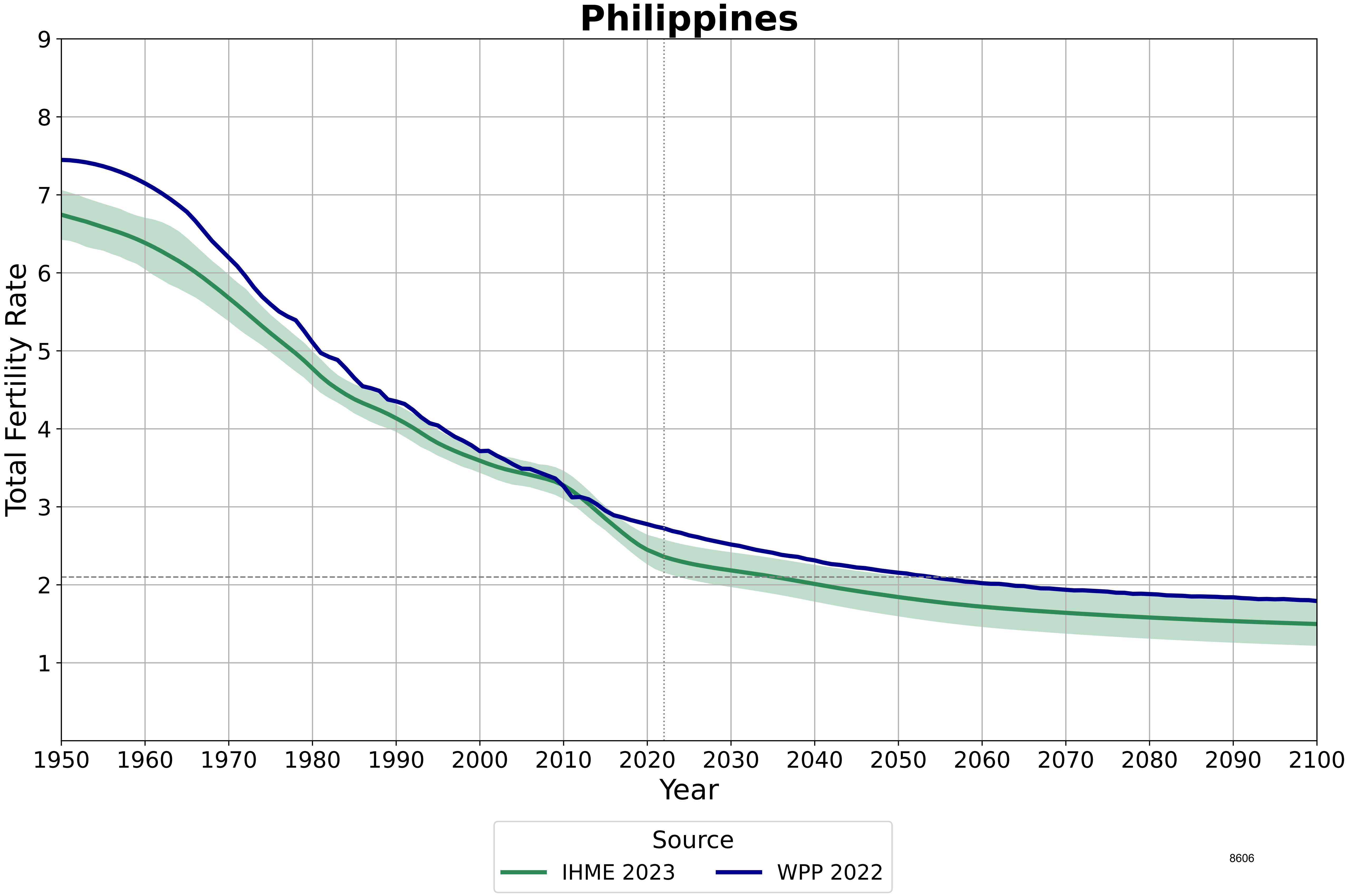

Supplemental Results Figure S7: TFR for IHME reference scenario and the UNPD medium variant scenario by location, 1950– 2100

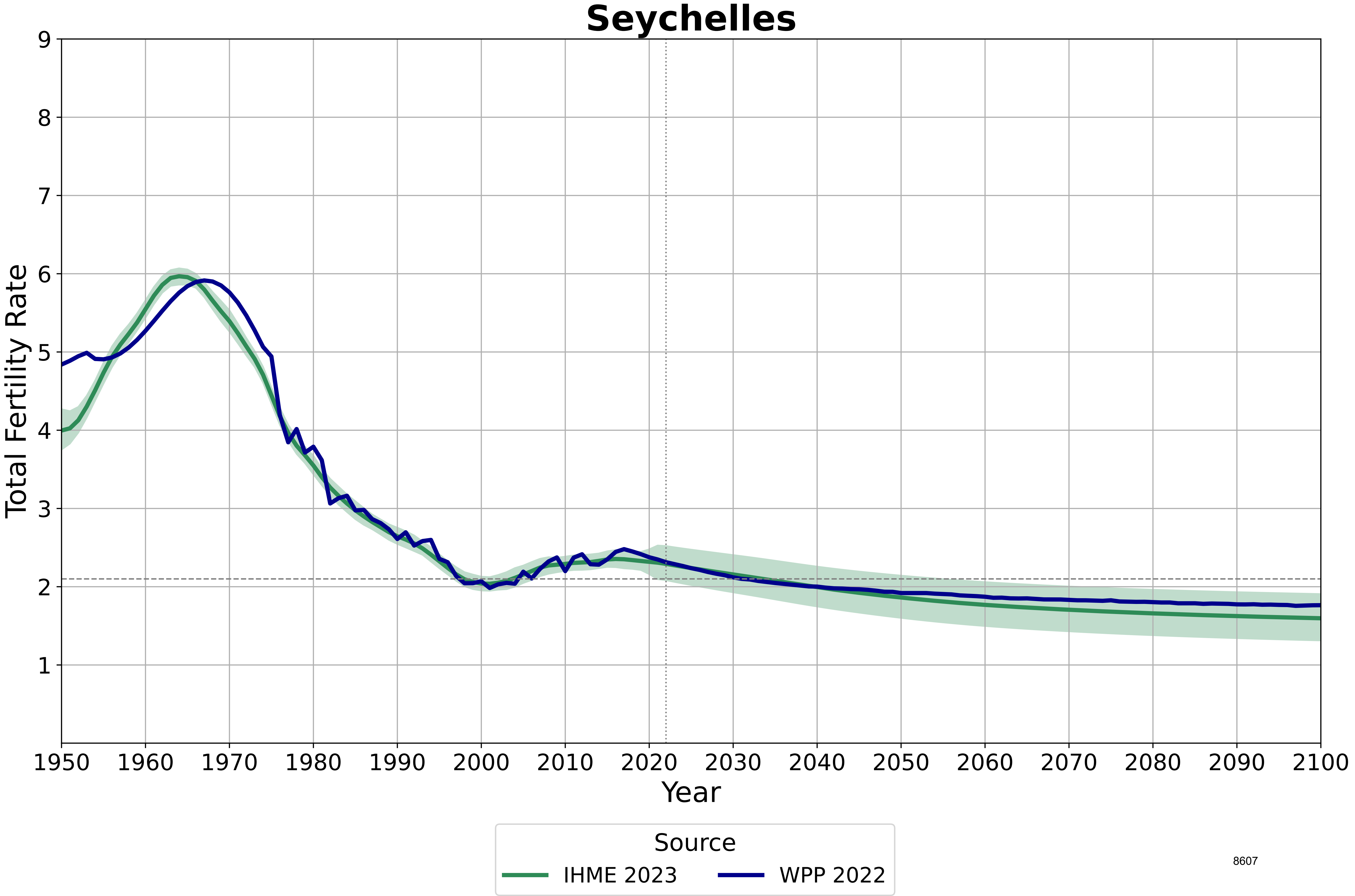

Supplemental Results Figure S7: TFR for IHME reference scenario and the UNPD medium variant scenario by location, 1950– 2100

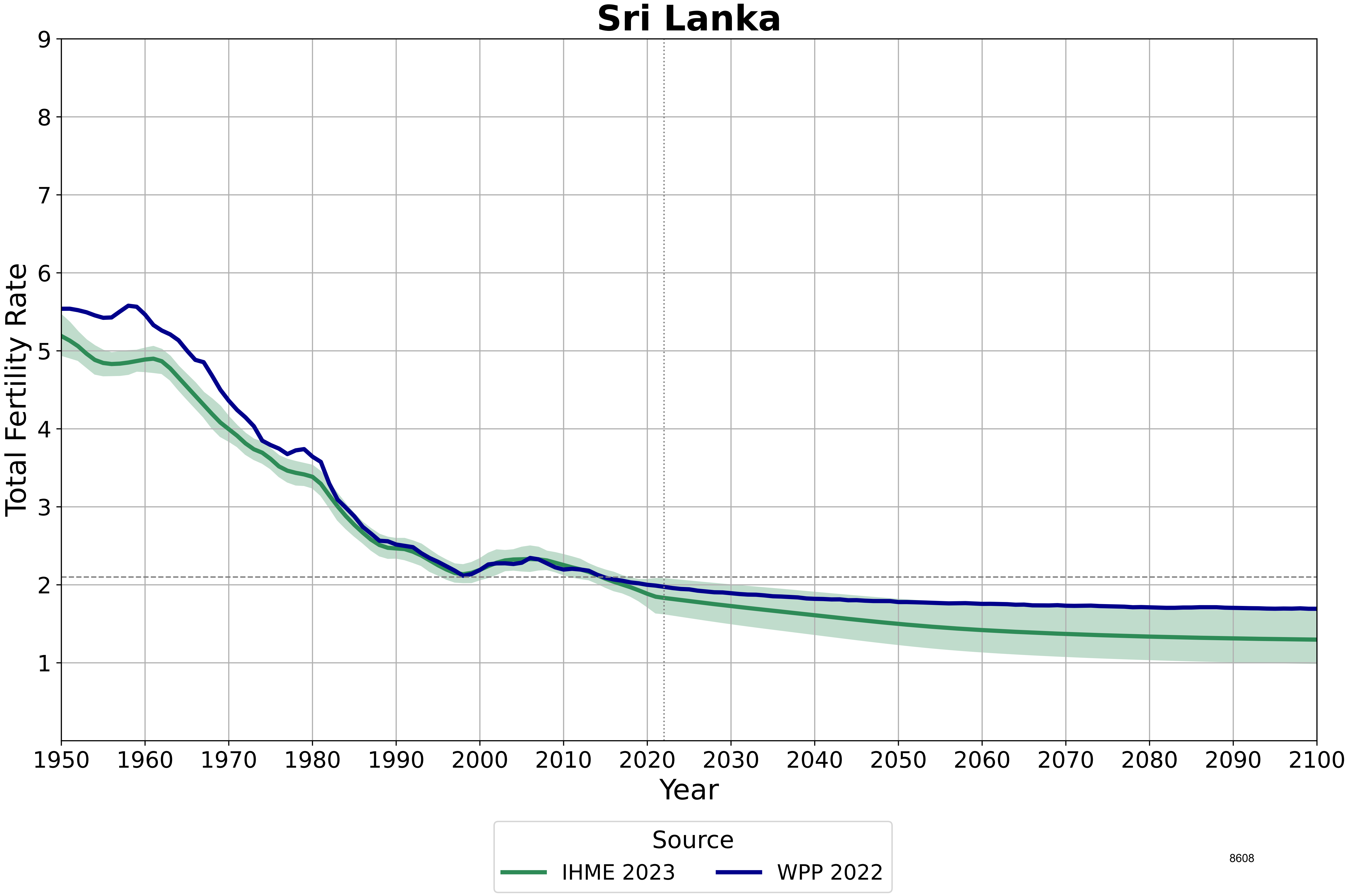

Supplemental Results Figure S7: TFR for IHME reference scenario and the UNPD medium variant scenario by location, 1950– 2100

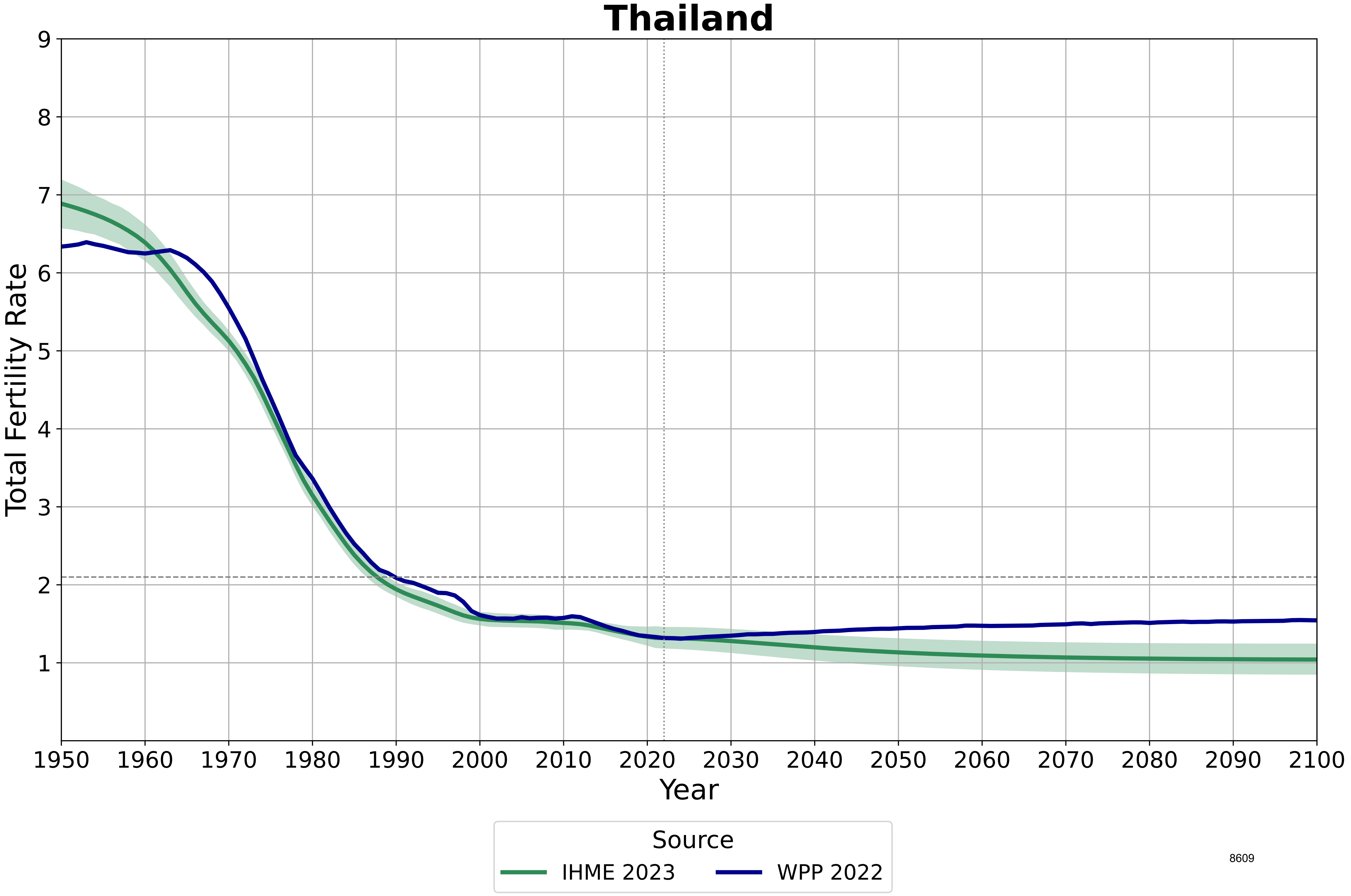

Supplemental Results Figure S7: TFR for IHME reference scenario and the UNPD medium variant scenario by location, 1950– 2100

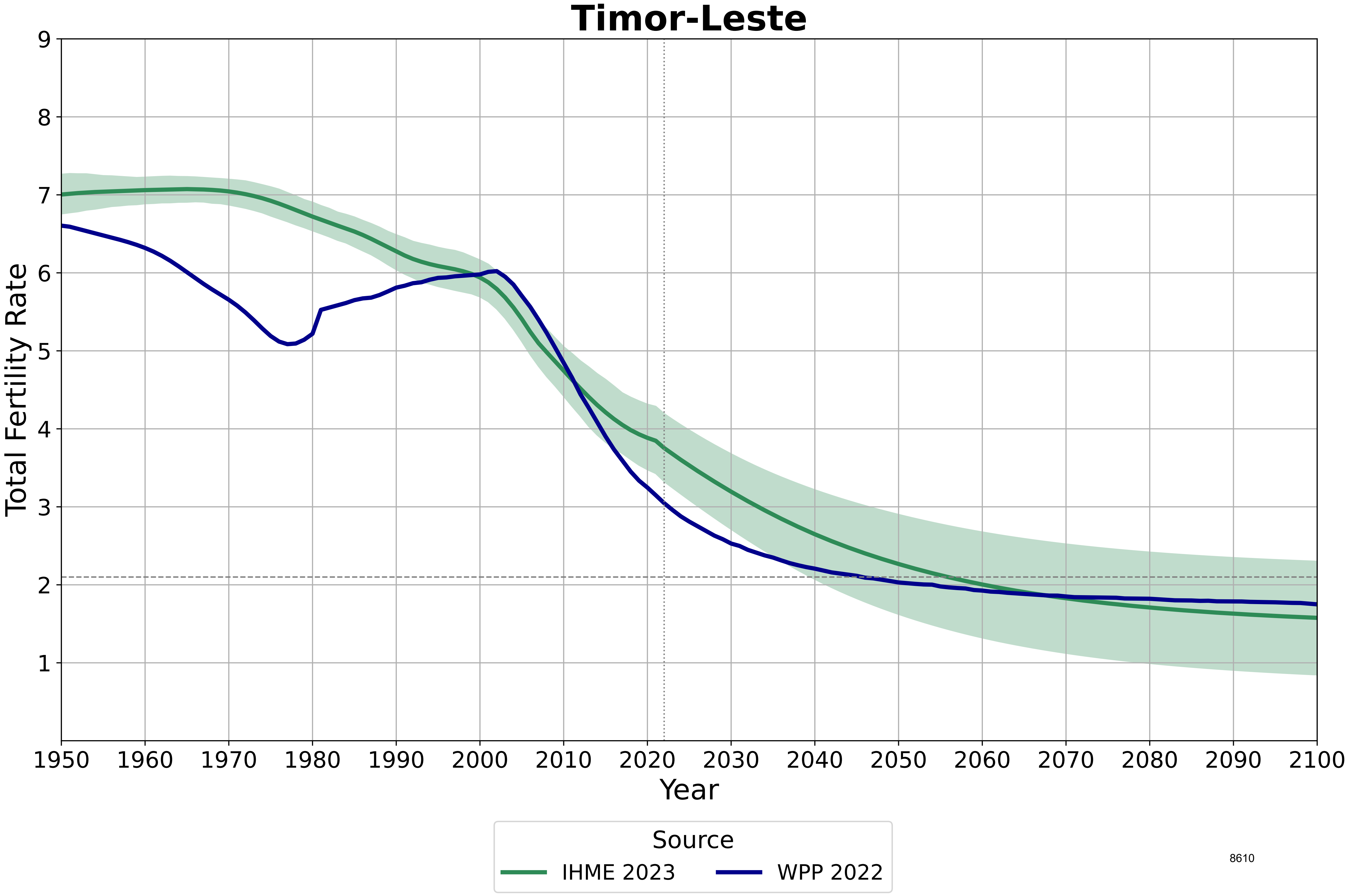

Supplemental Results Figure S7: TFR for IHME reference scenario and the UNPD medium variant scenario by location, 1950– 2100

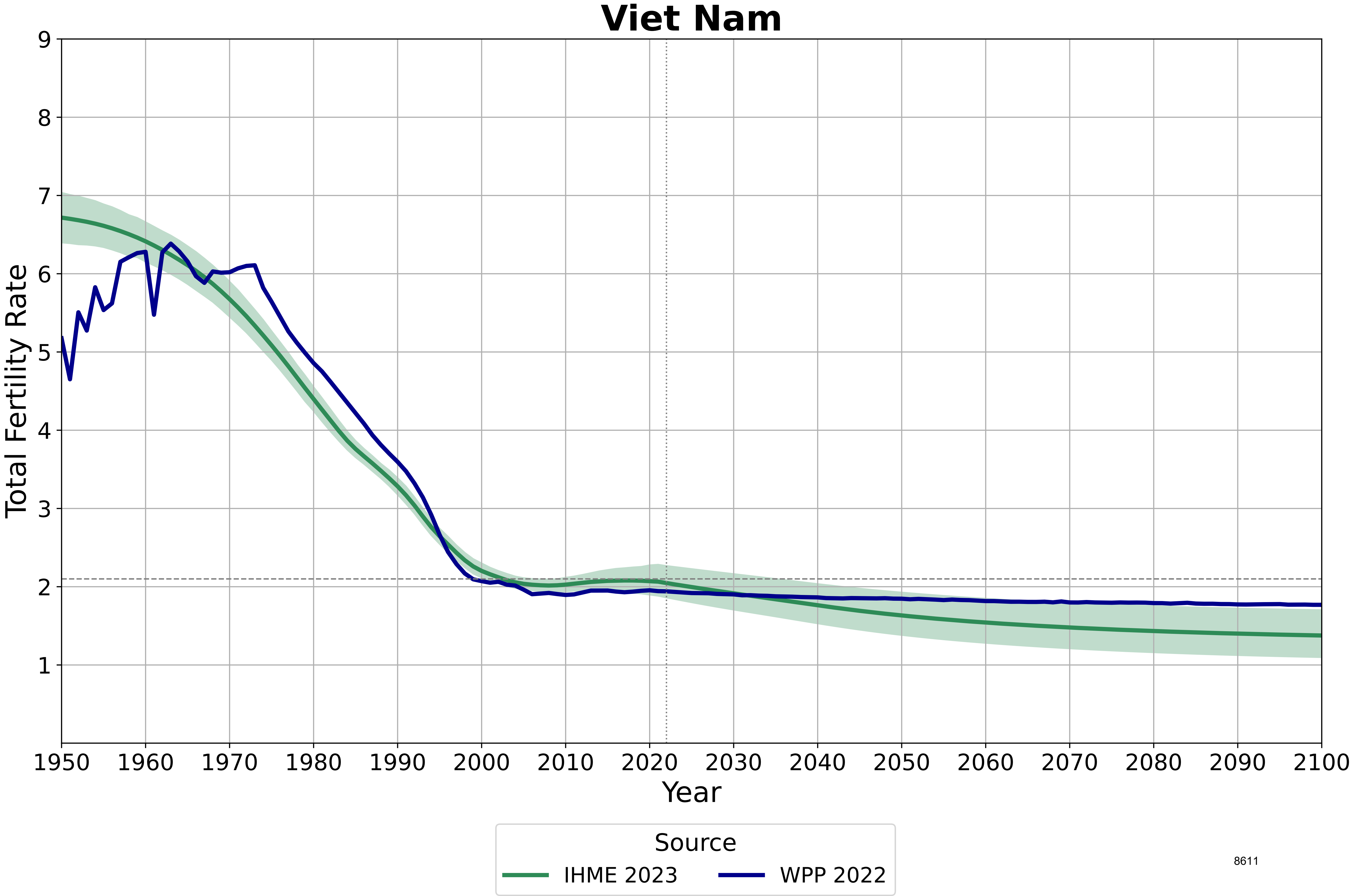

Supplemental Results Figure S7: TFR for IHME reference scenario and the UNPD medium variant scenario by location, 1950– 2100

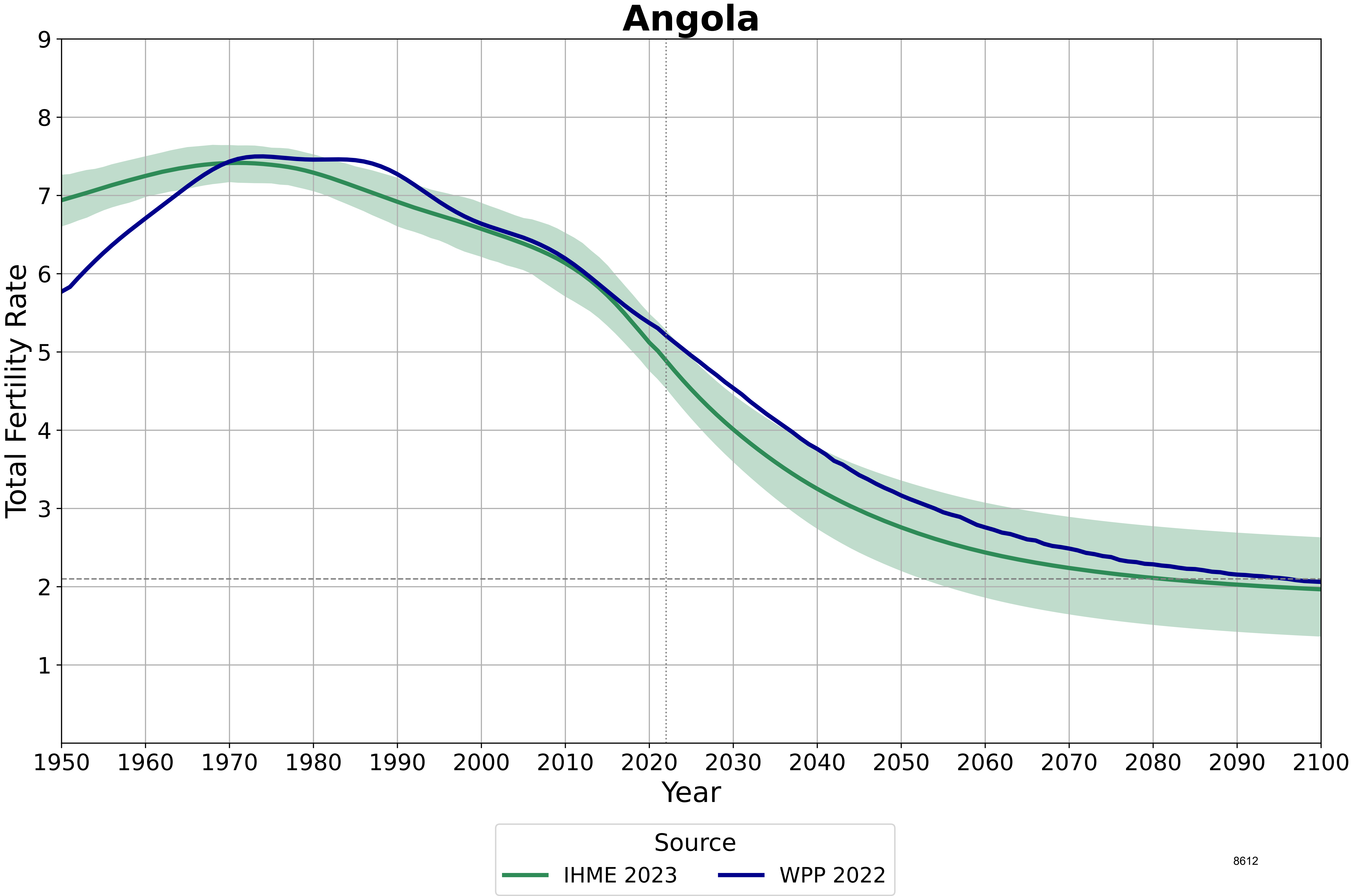

Supplemental Results Figure S7: TFR for IHME reference scenario and the UNPD medium variant scenario by location, 1950– 2100

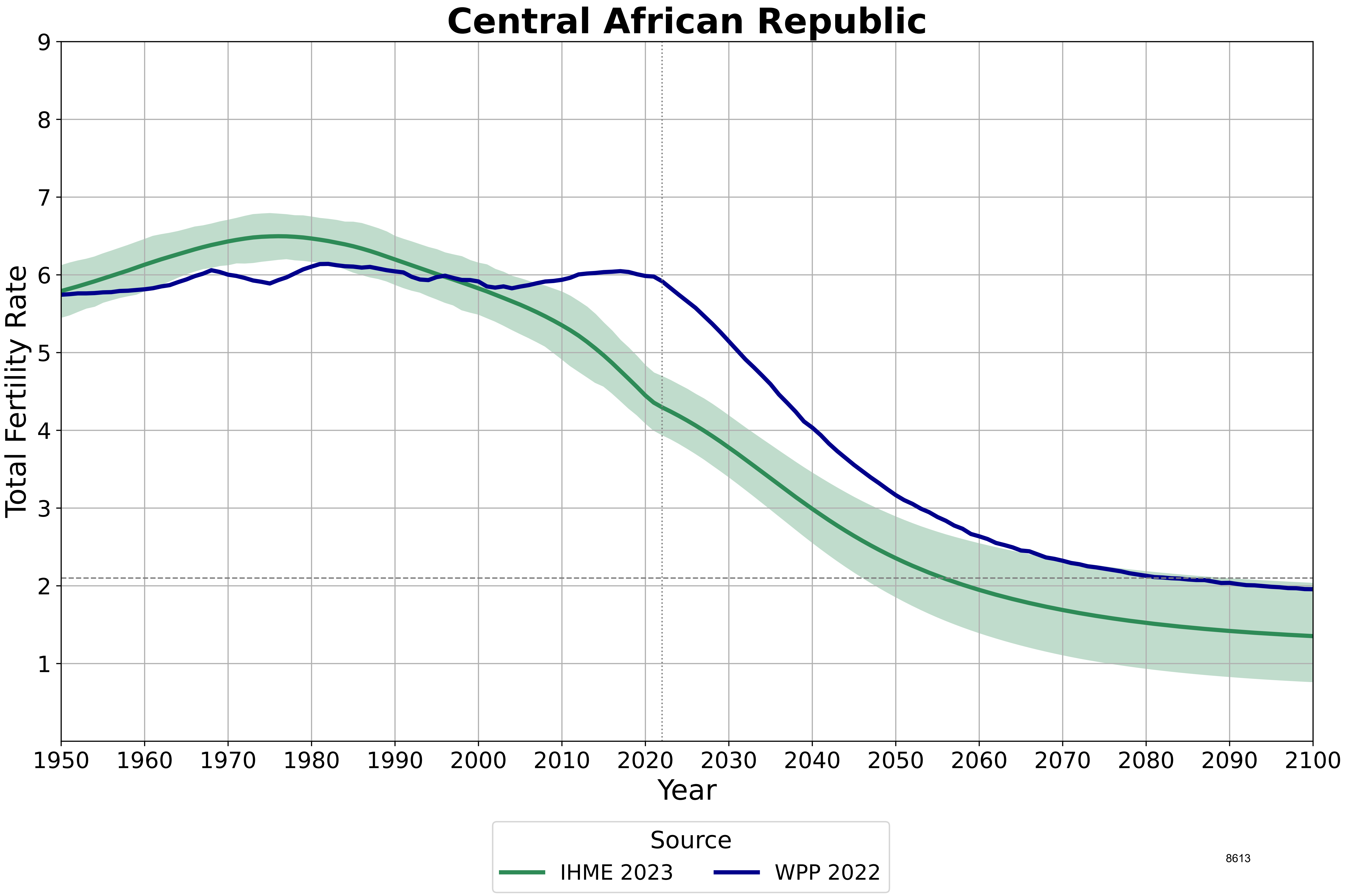

Supplemental Results Figure S7: TFR for IHME reference scenario and the UNPD medium variant scenario by location, 1950– 2100

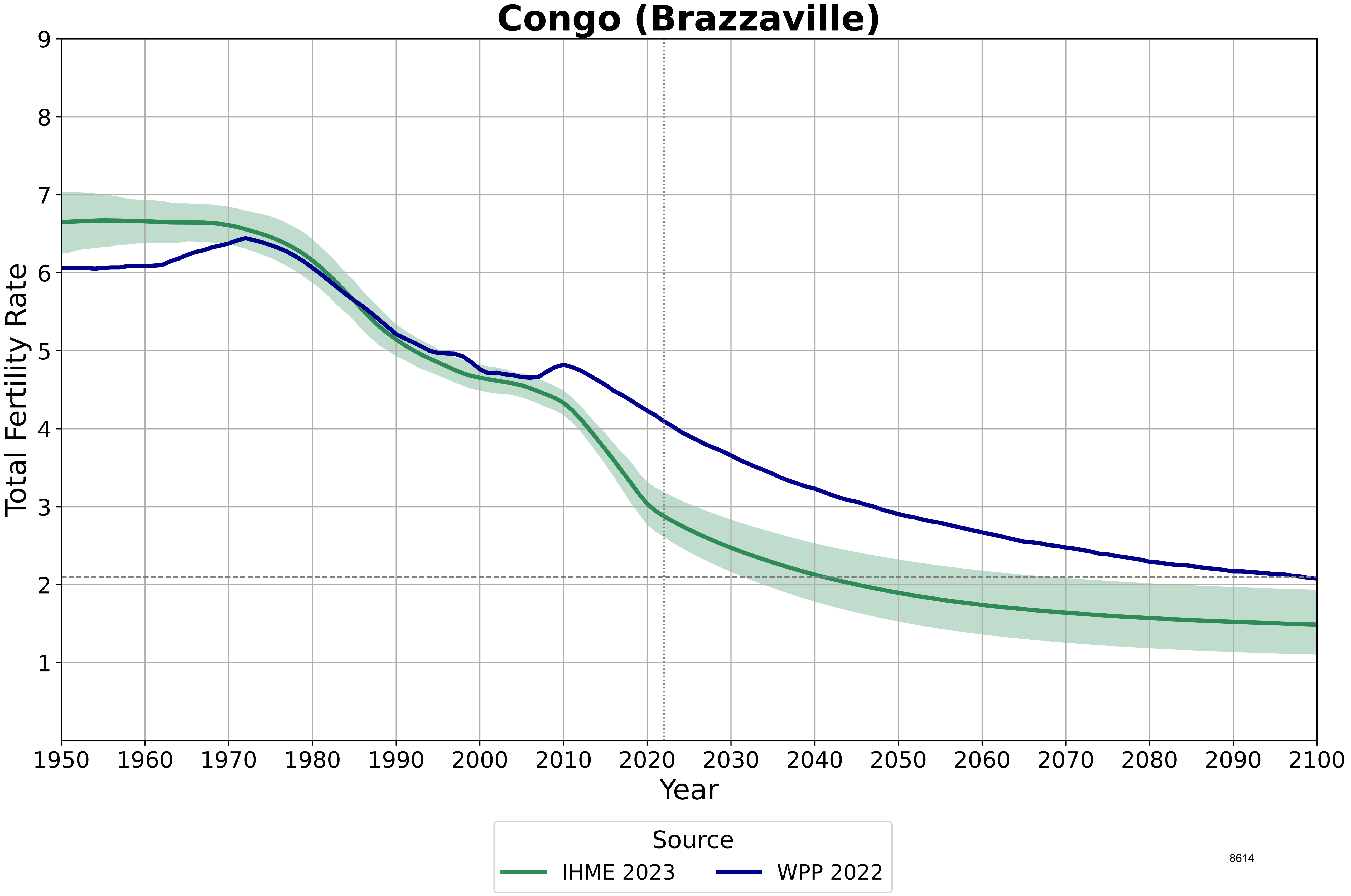

Supplemental Results Figure S7: TFR for IHME reference scenario and the UNPD medium variant scenario by location, 1950– 2100

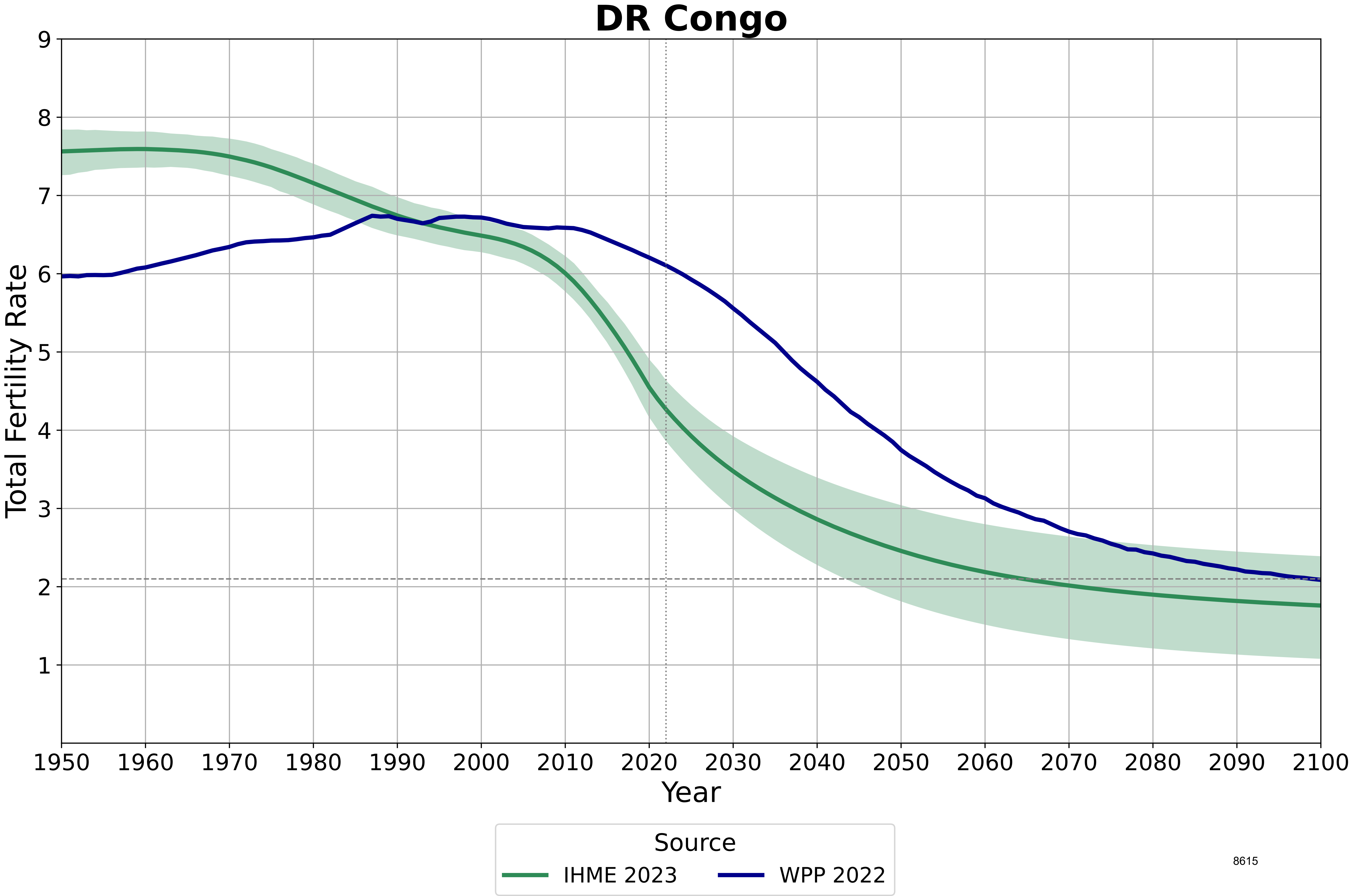

Supplemental Results Figure S7: TFR for IHME reference scenario and the UNPD medium variant scenario by location, 1950– 2100

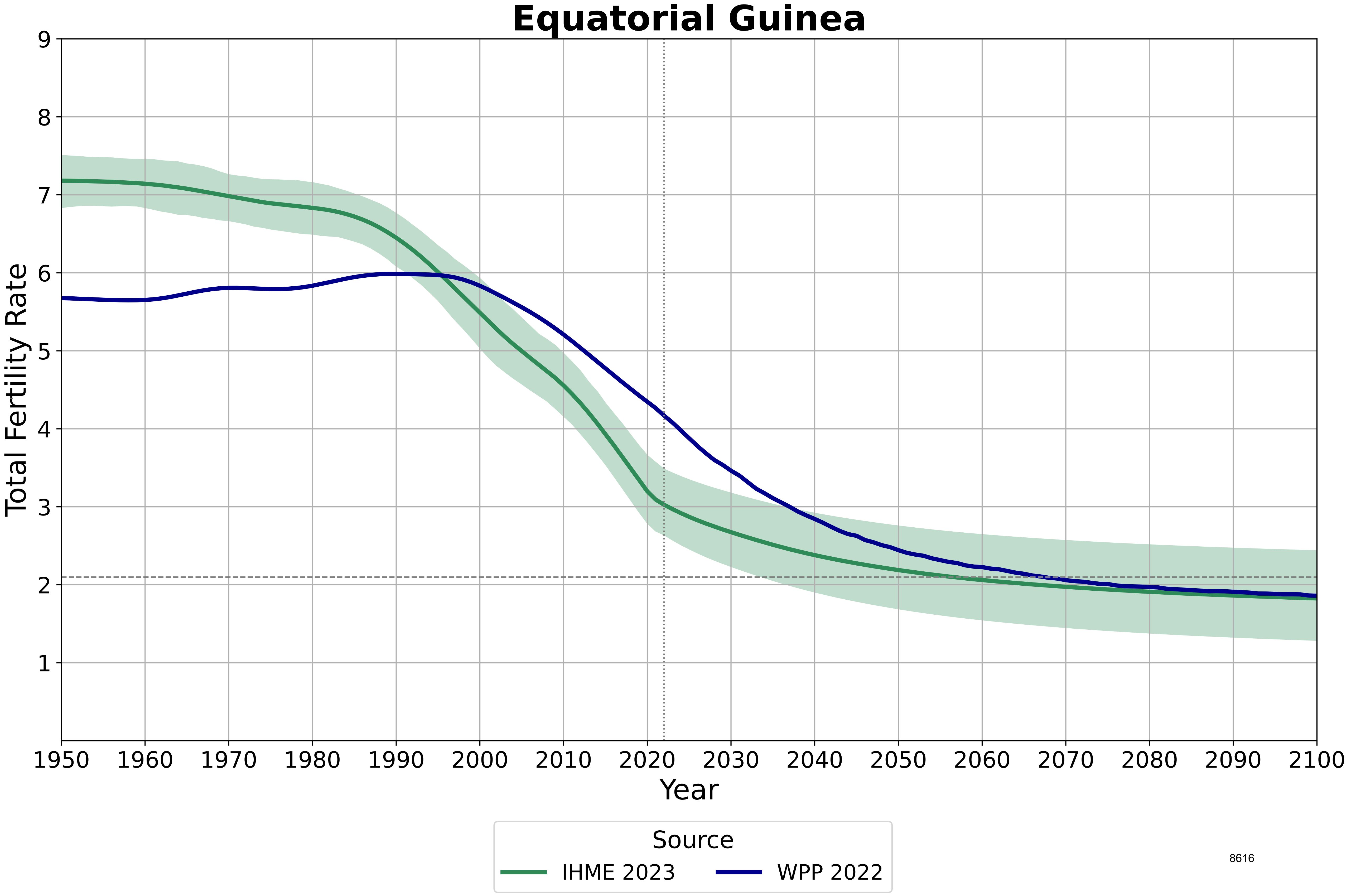

Supplemental Results Figure S7: TFR for IHME reference scenario and the UNPD medium variant scenario by location, 1950– 2100

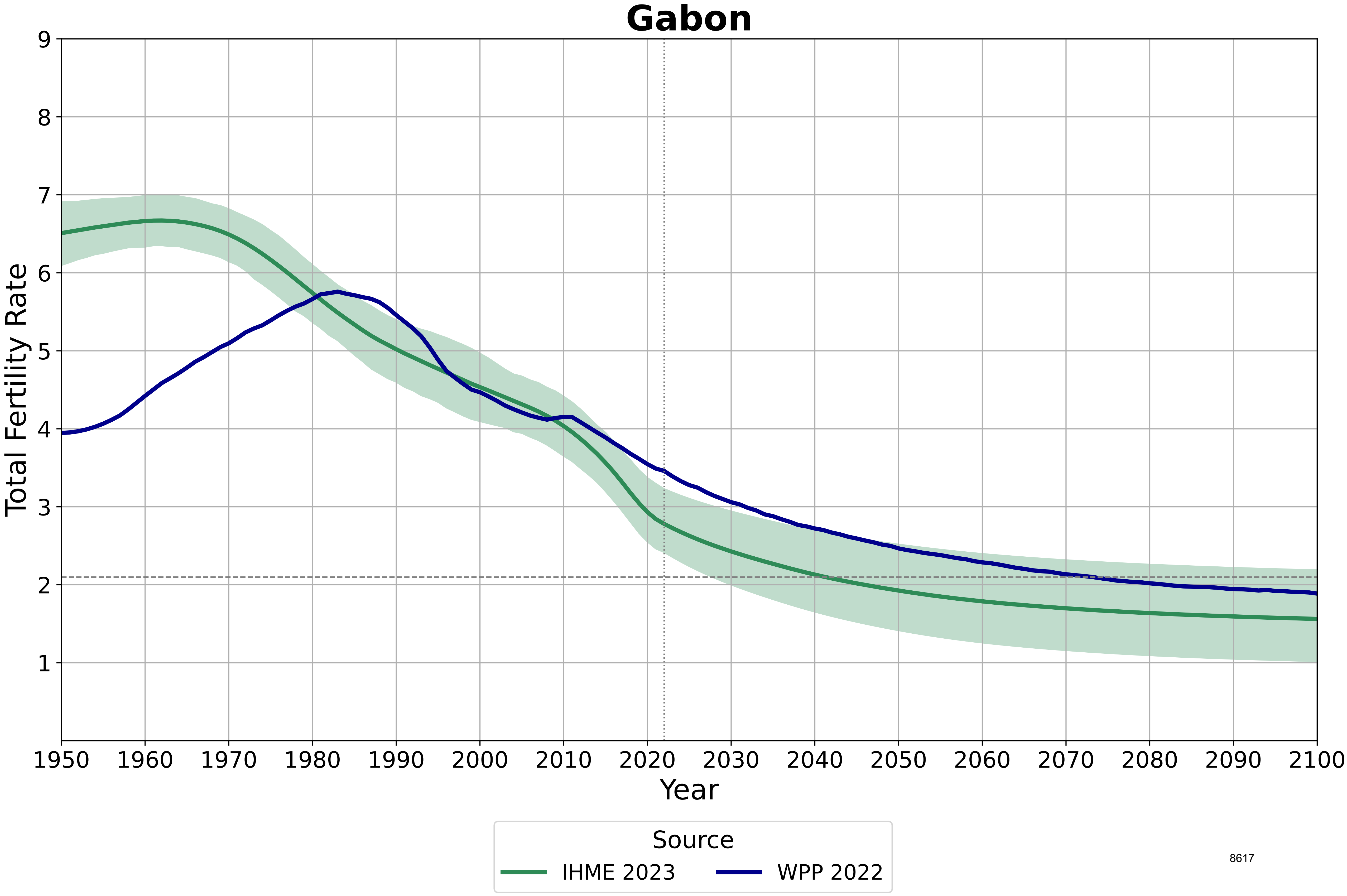

8617

Supplemental Results Figure S7: TFR for IHME reference scenario and the UNPD medium variant scenario by location, 1950– 2100

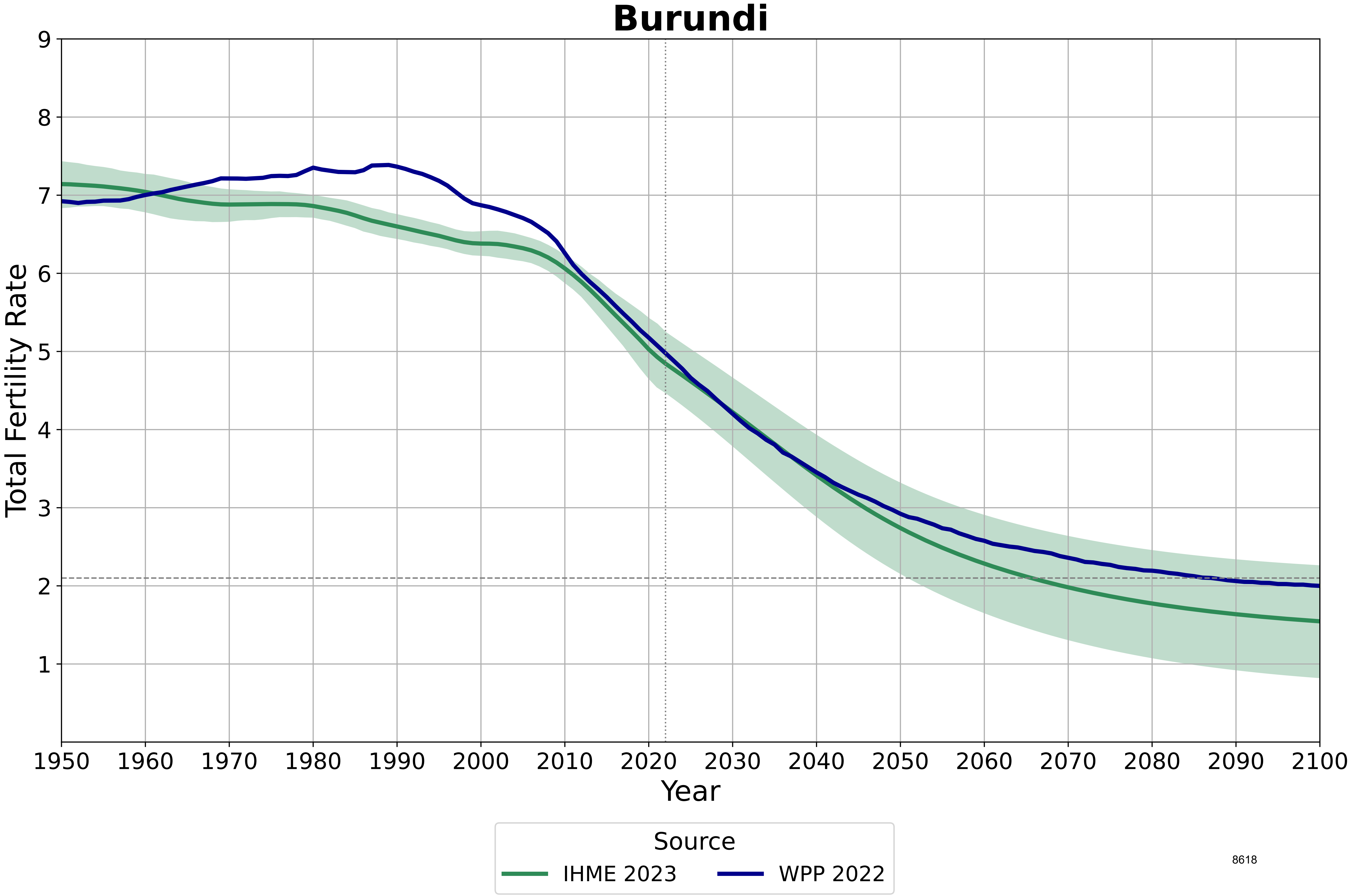

Supplemental Results Figure S7: TFR for IHME reference scenario and the UNPD medium variant scenario by location, 1950– 2100

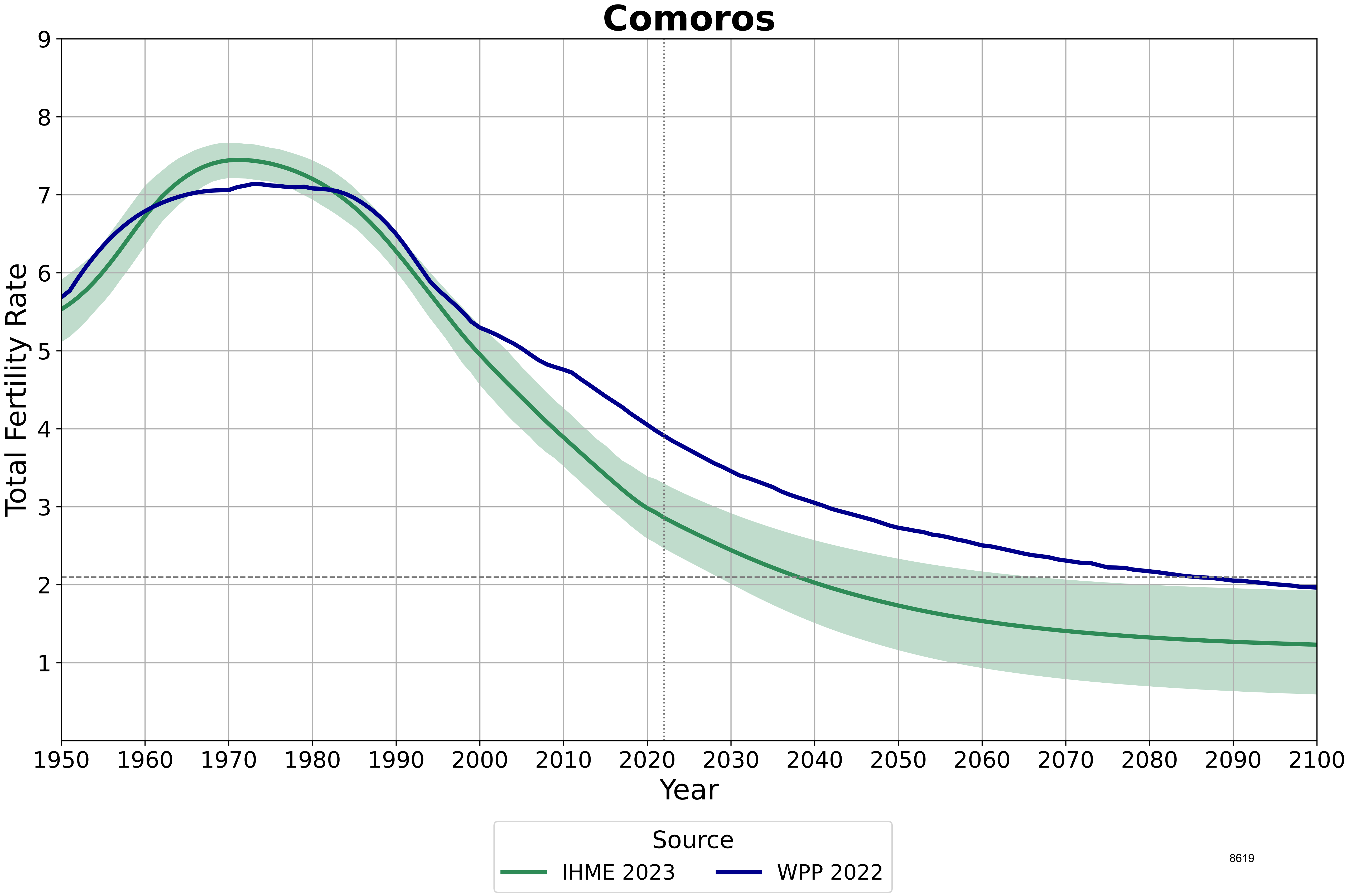

8619

Supplemental Results Figure S7: TFR for IHME reference scenario and the UNPD medium variant scenario by location, 1950– 2100

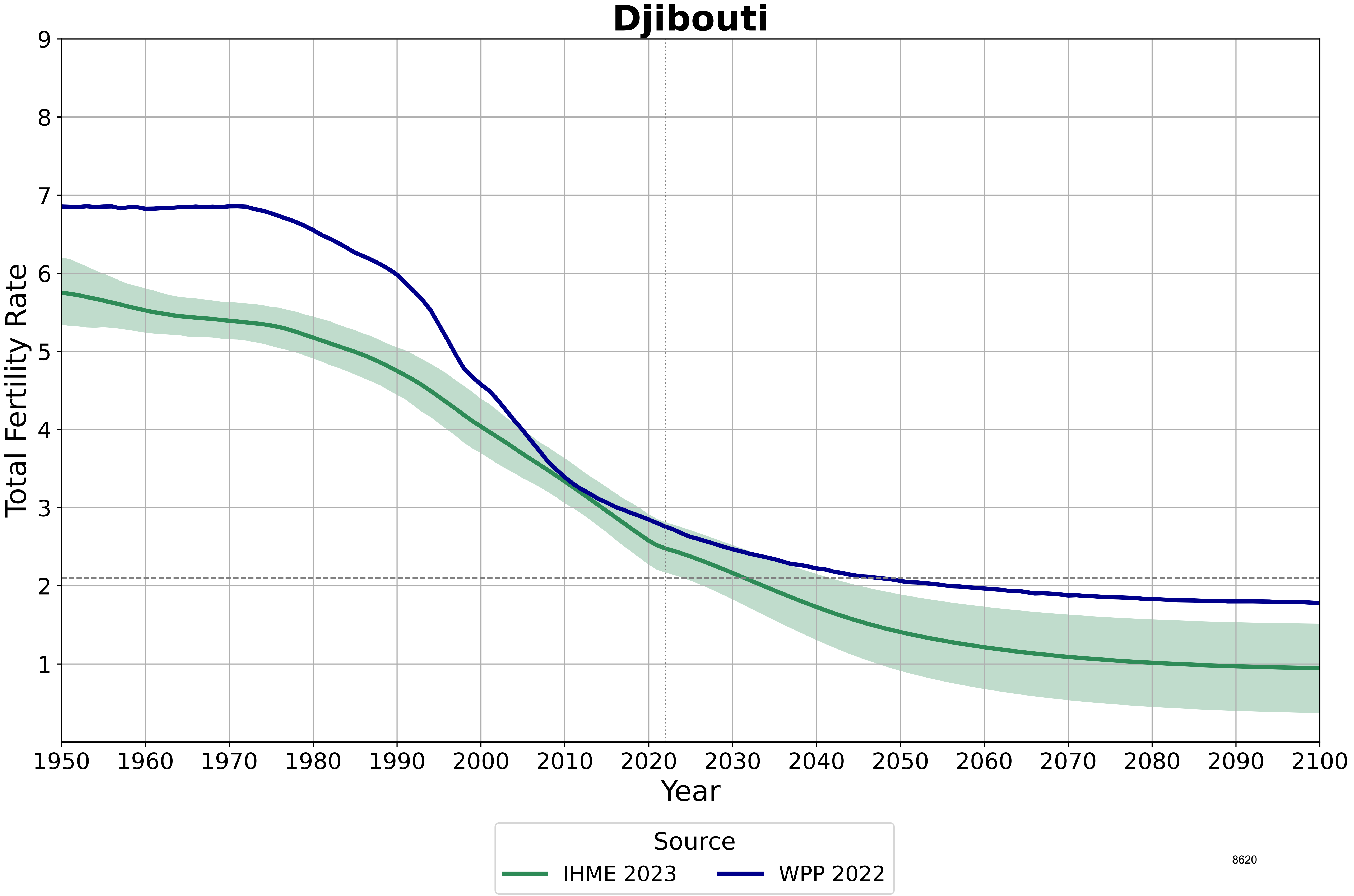

Supplemental Results Figure S7: TFR for IHME reference scenario and the UNPD medium variant scenario by location, 1950– 2100

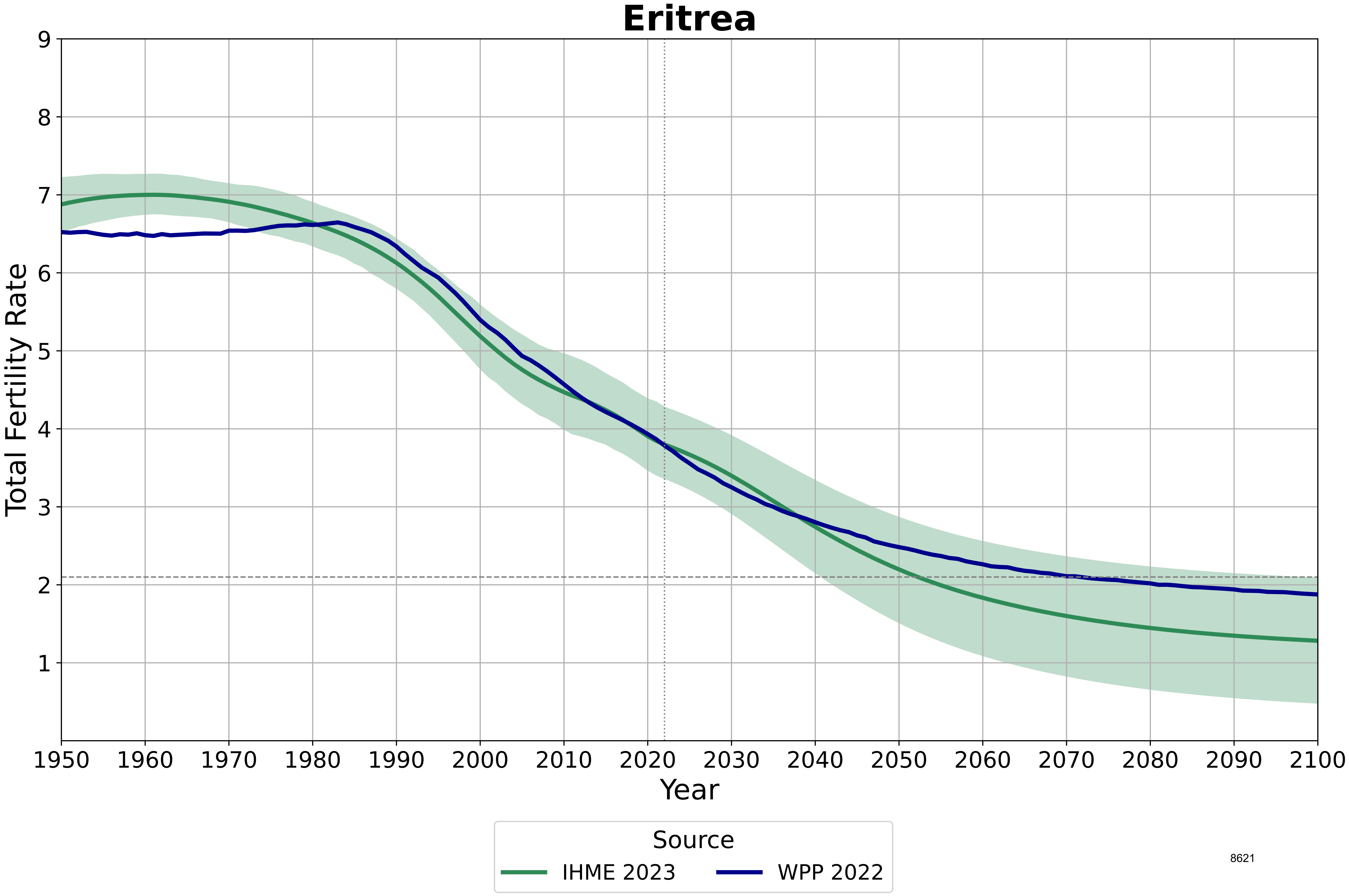

Supplemental Results Figure S7: TFR for IHME reference scenario and the UNPD medium variant scenario by location, 1950– 2100

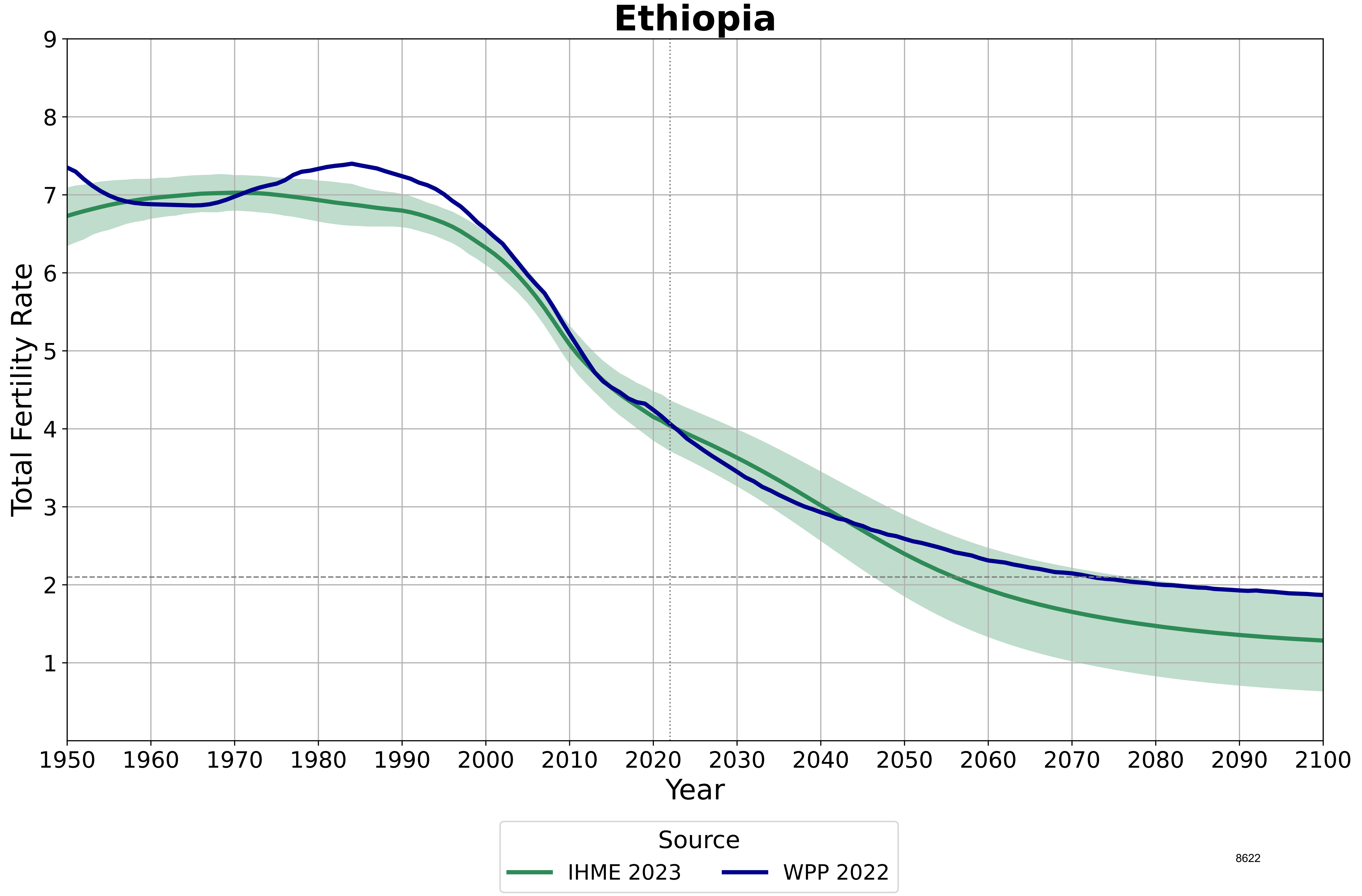

Supplemental Results Figure S7: TFR for IHME reference scenario and the UNPD medium variant scenario by location, 1950– 2100

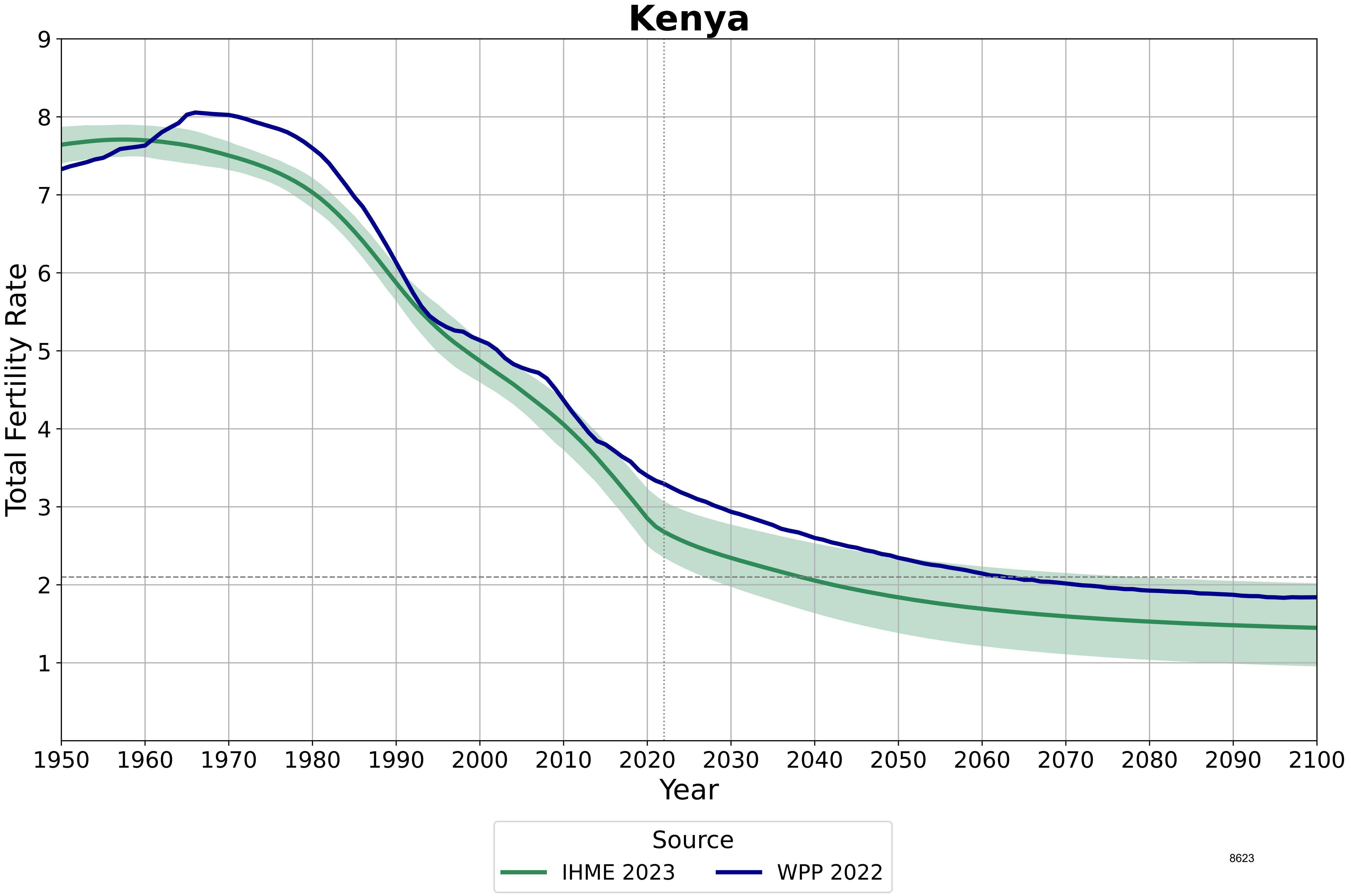

Supplemental Results Figure S7: TFR for IHME reference scenario and the UNPD medium variant scenario by location, 1950– 2100

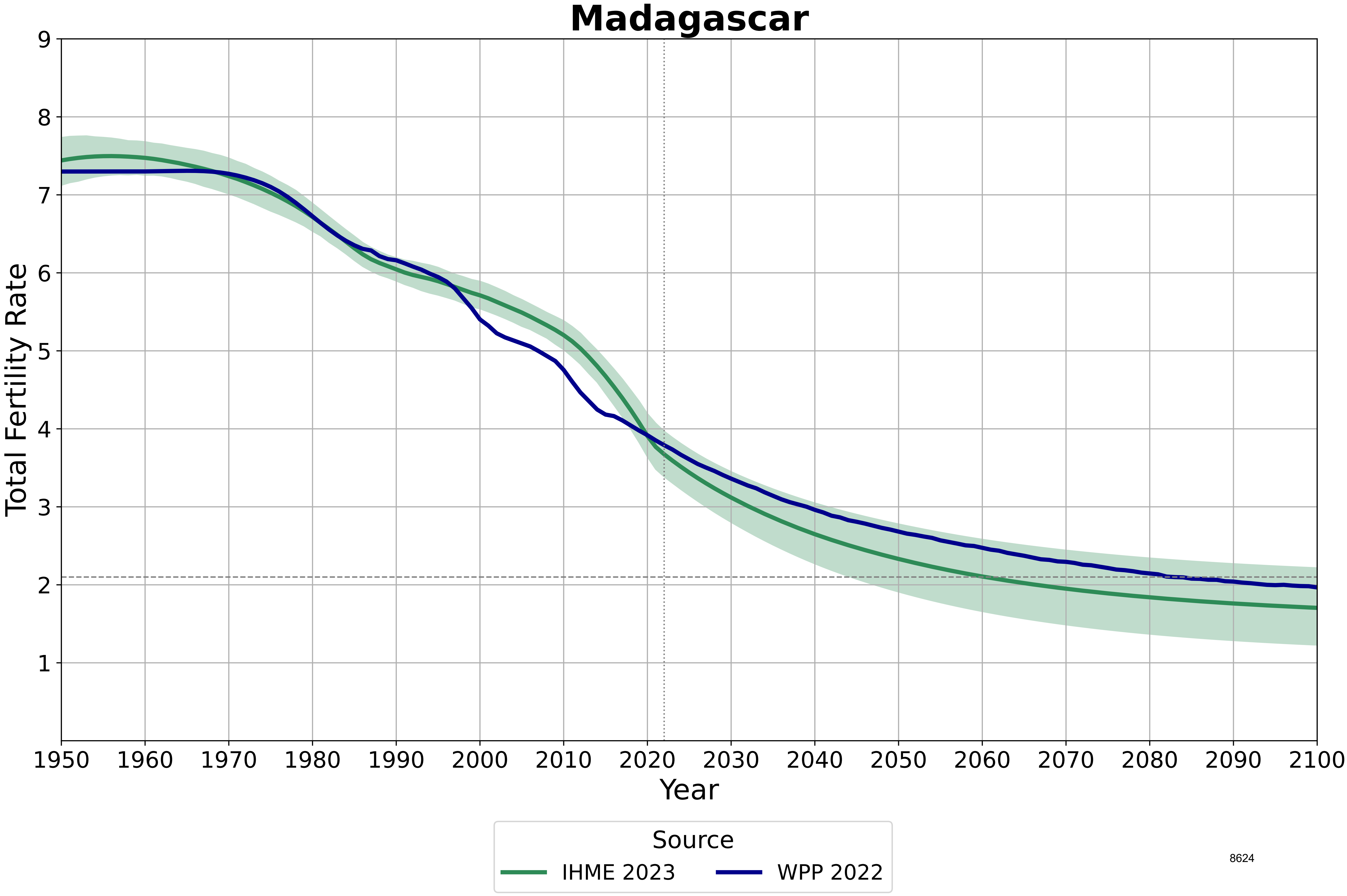

Supplemental Results Figure S7: TFR for IHME reference scenario and the UNPD medium variant scenario by location, 1950– 2100

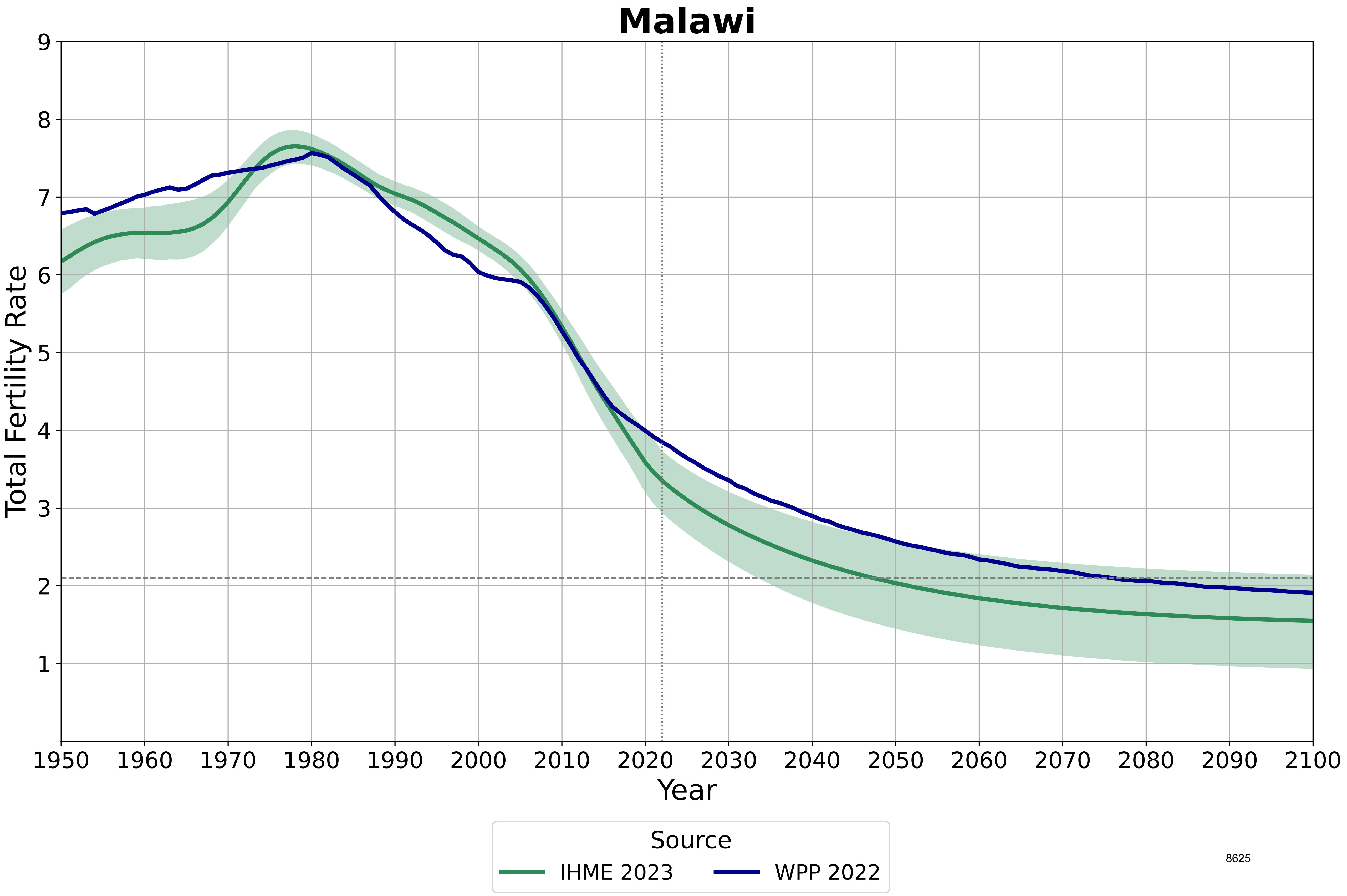

Supplemental Results Figure S7: TFR for IHME reference scenario and the UNPD medium variant scenario by location, 1950– 2100

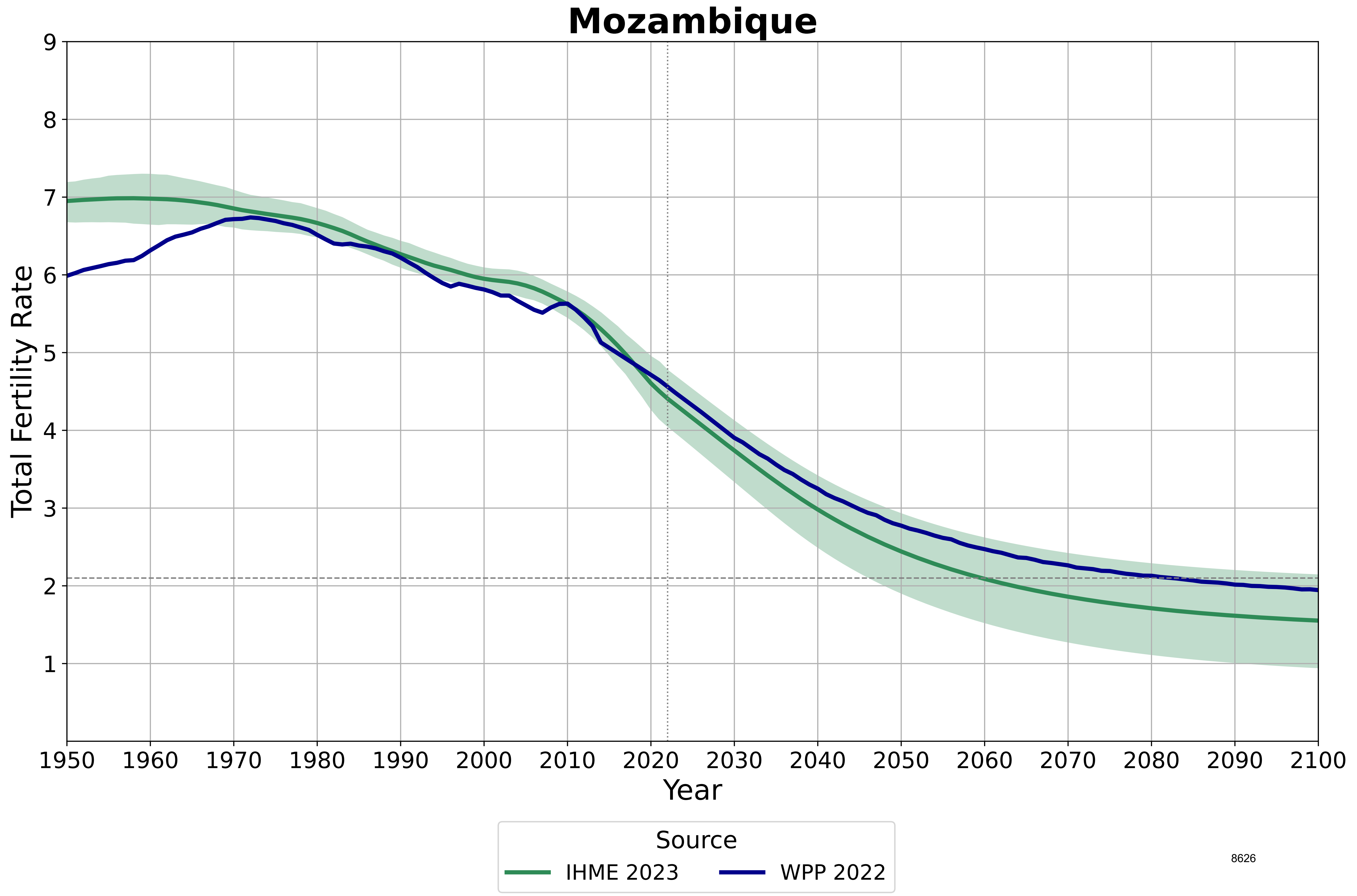

Supplemental Results Figure S7: TFR for IHME reference scenario and the UNPD medium variant scenario by location, 1950– 2100

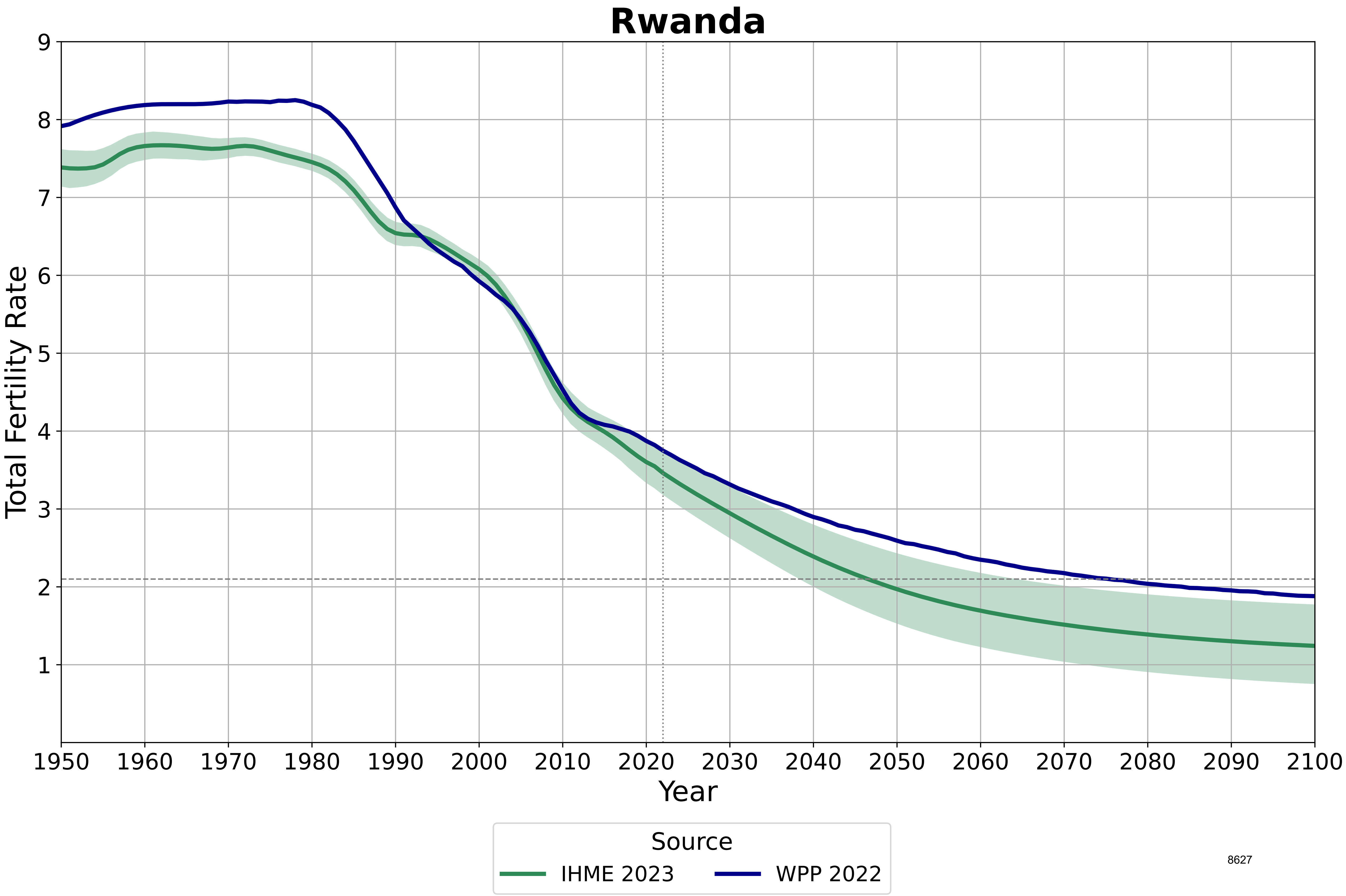

Supplemental Results Figure S7: TFR for IHME reference scenario and the UNPD medium variant scenario by location, 1950– 2100

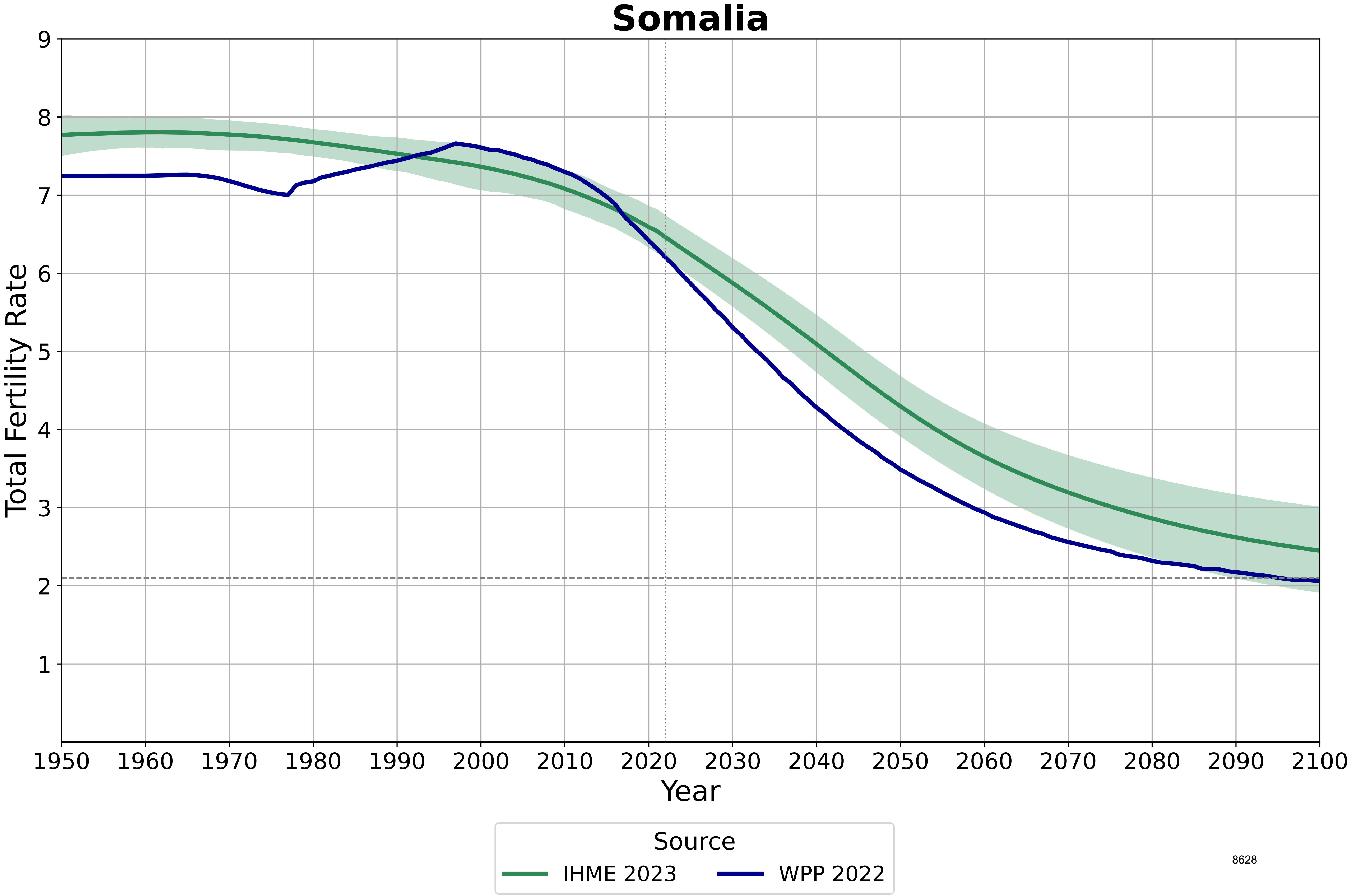

Supplemental Results Figure S7: TFR for IHME reference scenario and the UNPD medium variant scenario by location, 1950– 2100

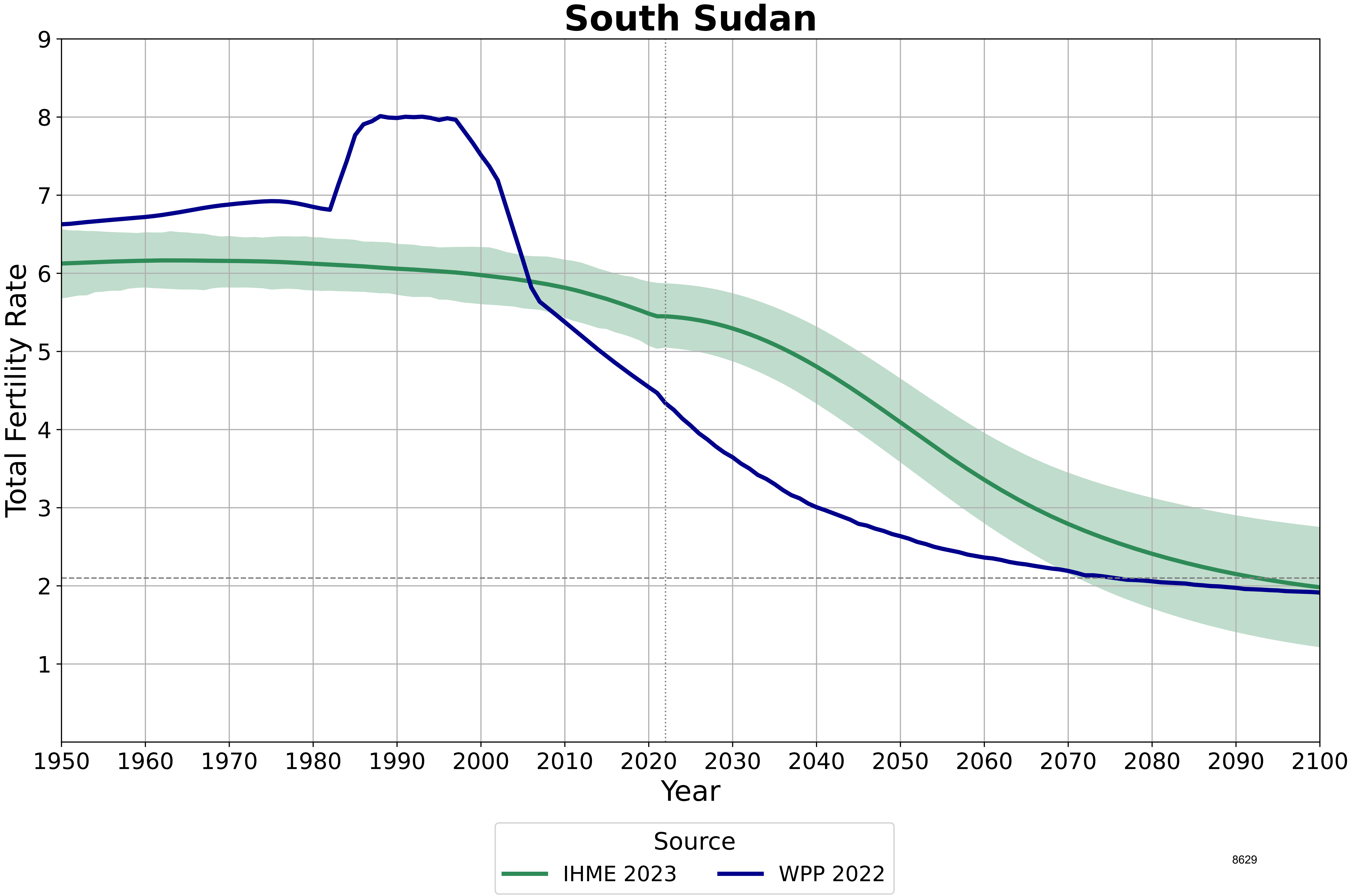

Supplemental Results Figure S7: TFR for IHME reference scenario and the UNPD medium variant scenario by location, 1950– 2100

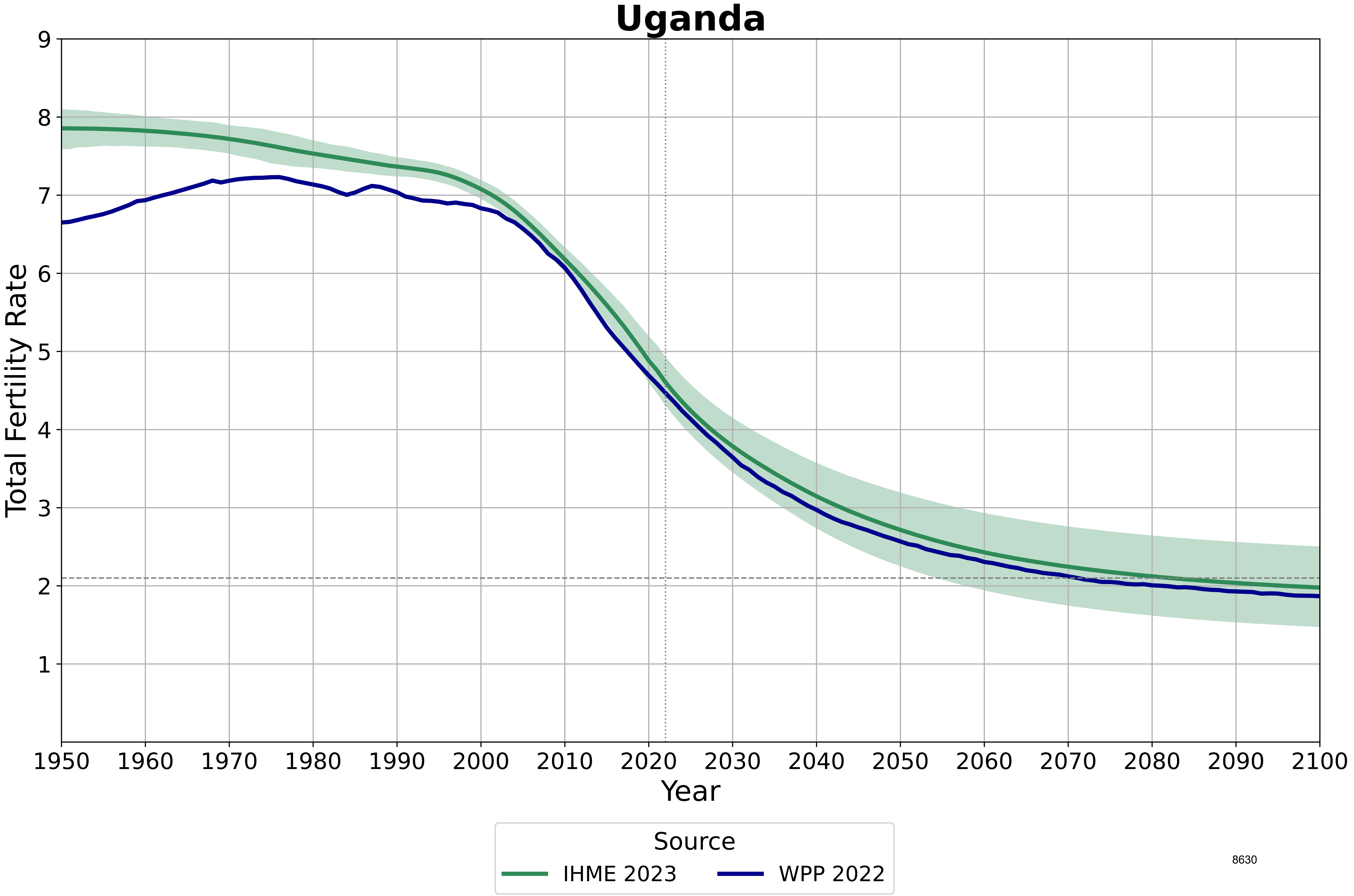

Supplemental Results Figure S7: TFR for IHME reference scenario and the UNPD medium variant scenario by location, 1950– 2100

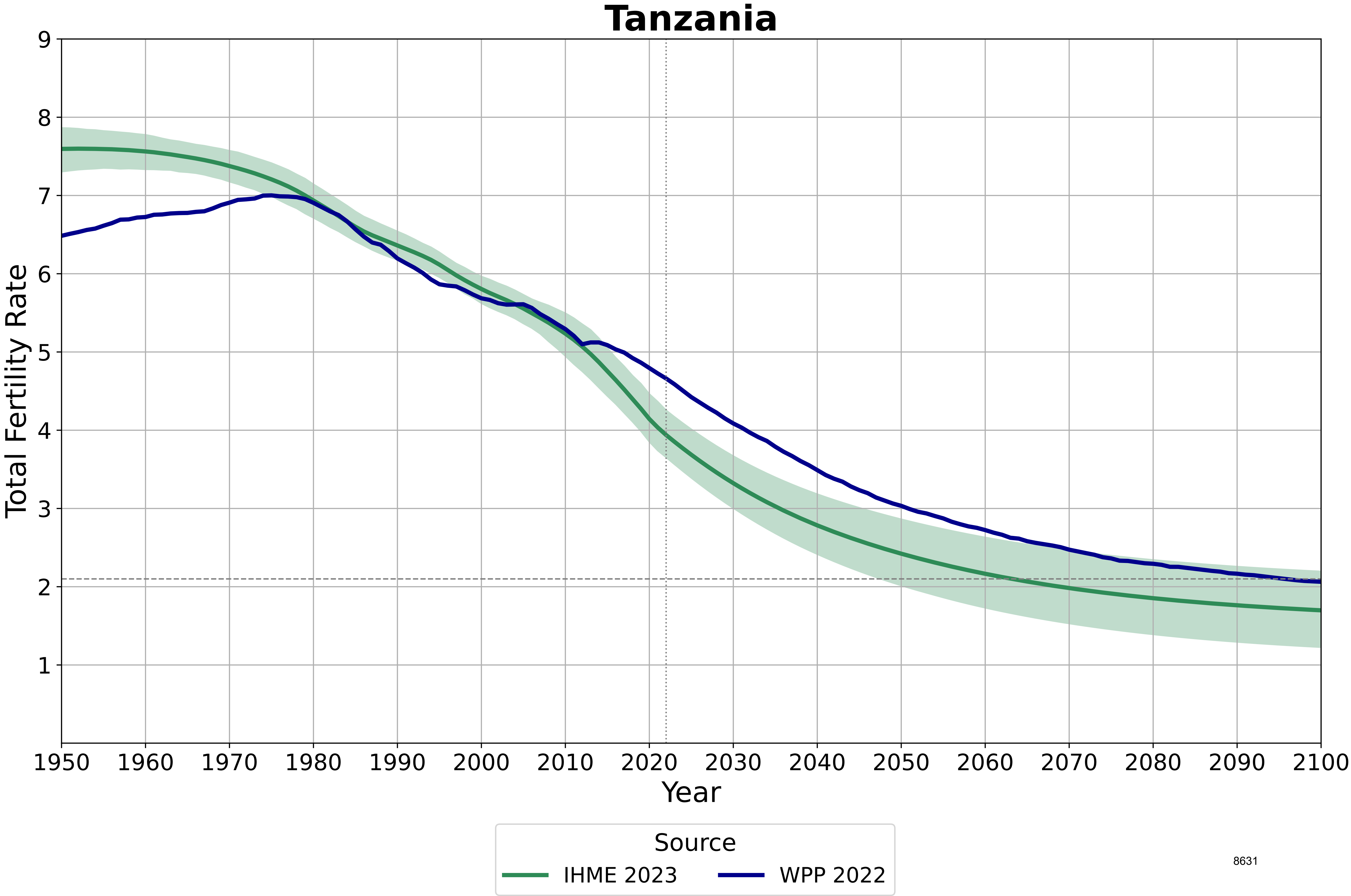

Supplemental Results Figure S7: TFR for IHME reference scenario and the UNPD medium variant scenario by location, 1950– 2100

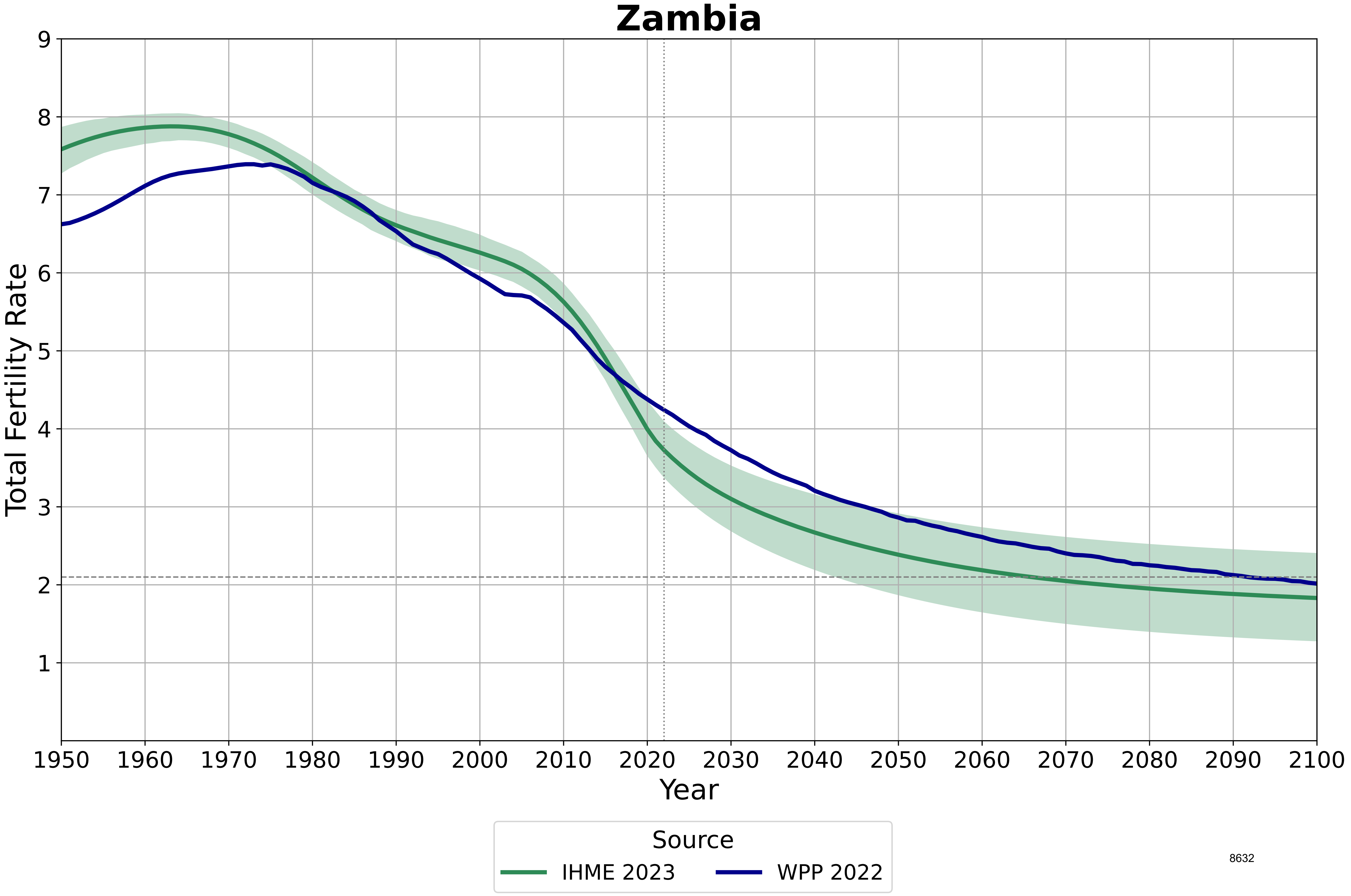

Supplemental Results Figure S7: TFR for IHME reference scenario and the UNPD medium variant scenario by location, 1950– 2100

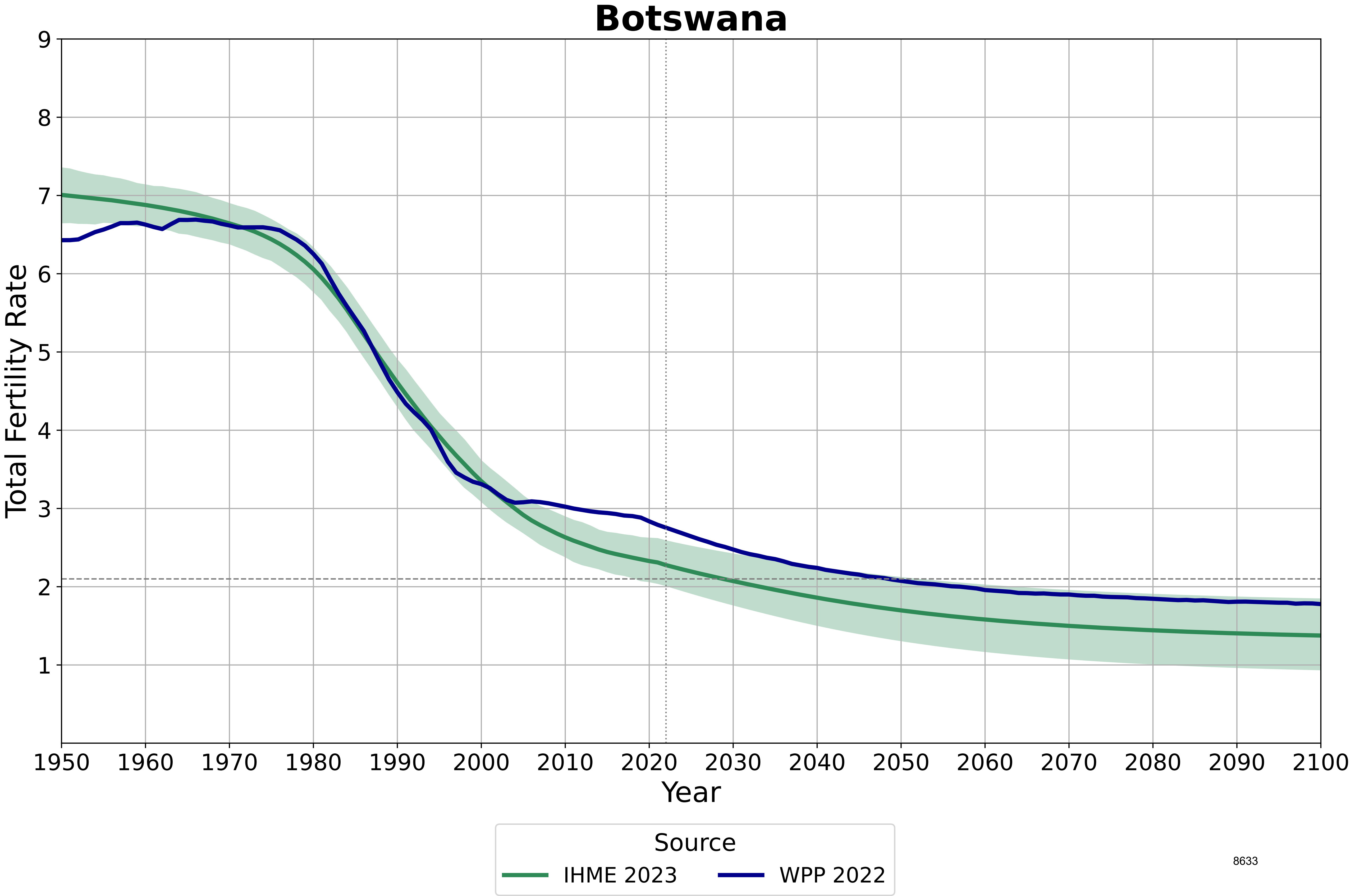

Supplemental Results Figure S7: TFR for IHME reference scenario and the UNPD medium variant scenario by location, 1950– 2100

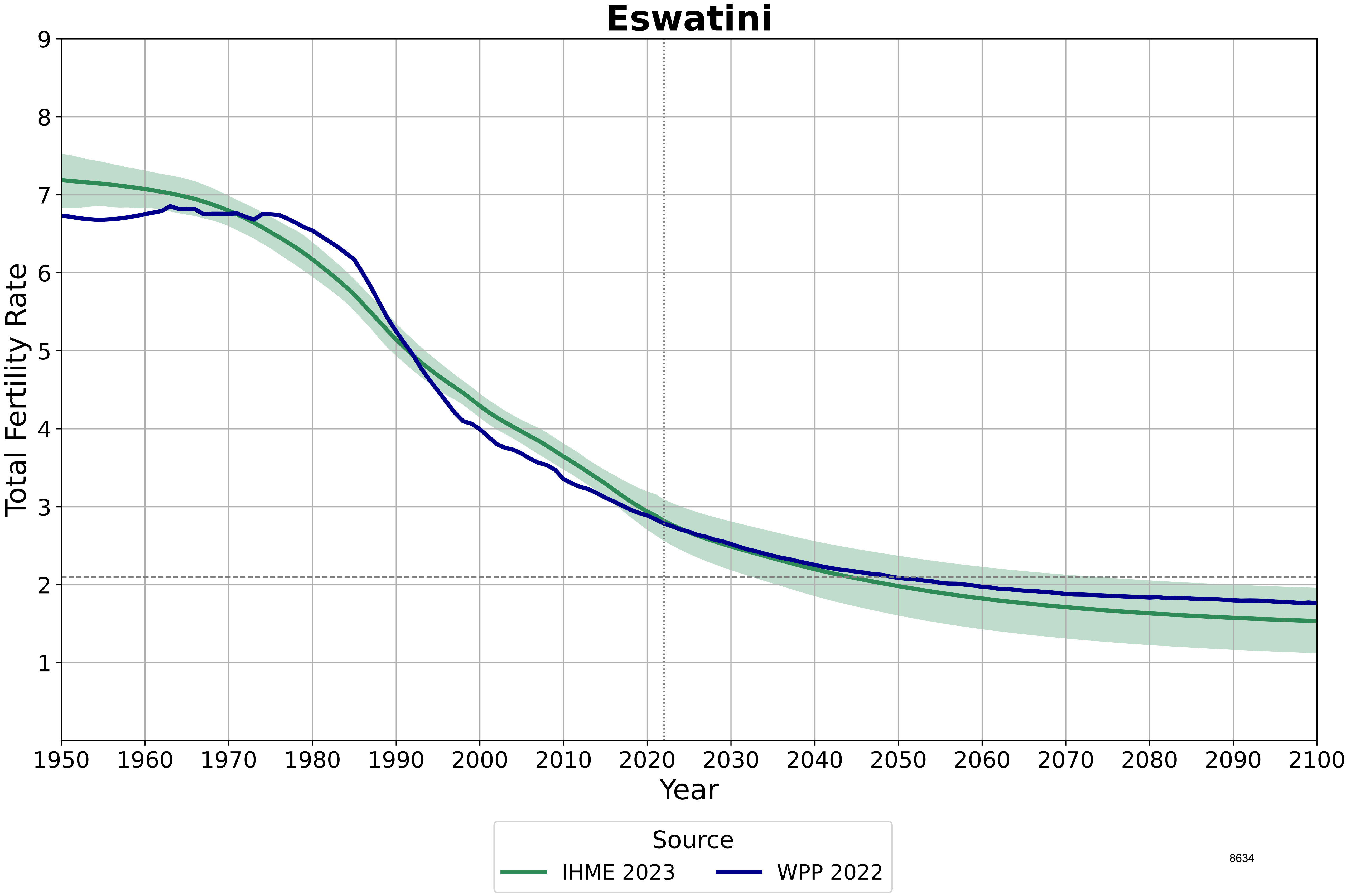

Supplemental Results Figure S7: TFR for IHME reference scenario and the UNPD medium variant scenario by location, 1950– 2100

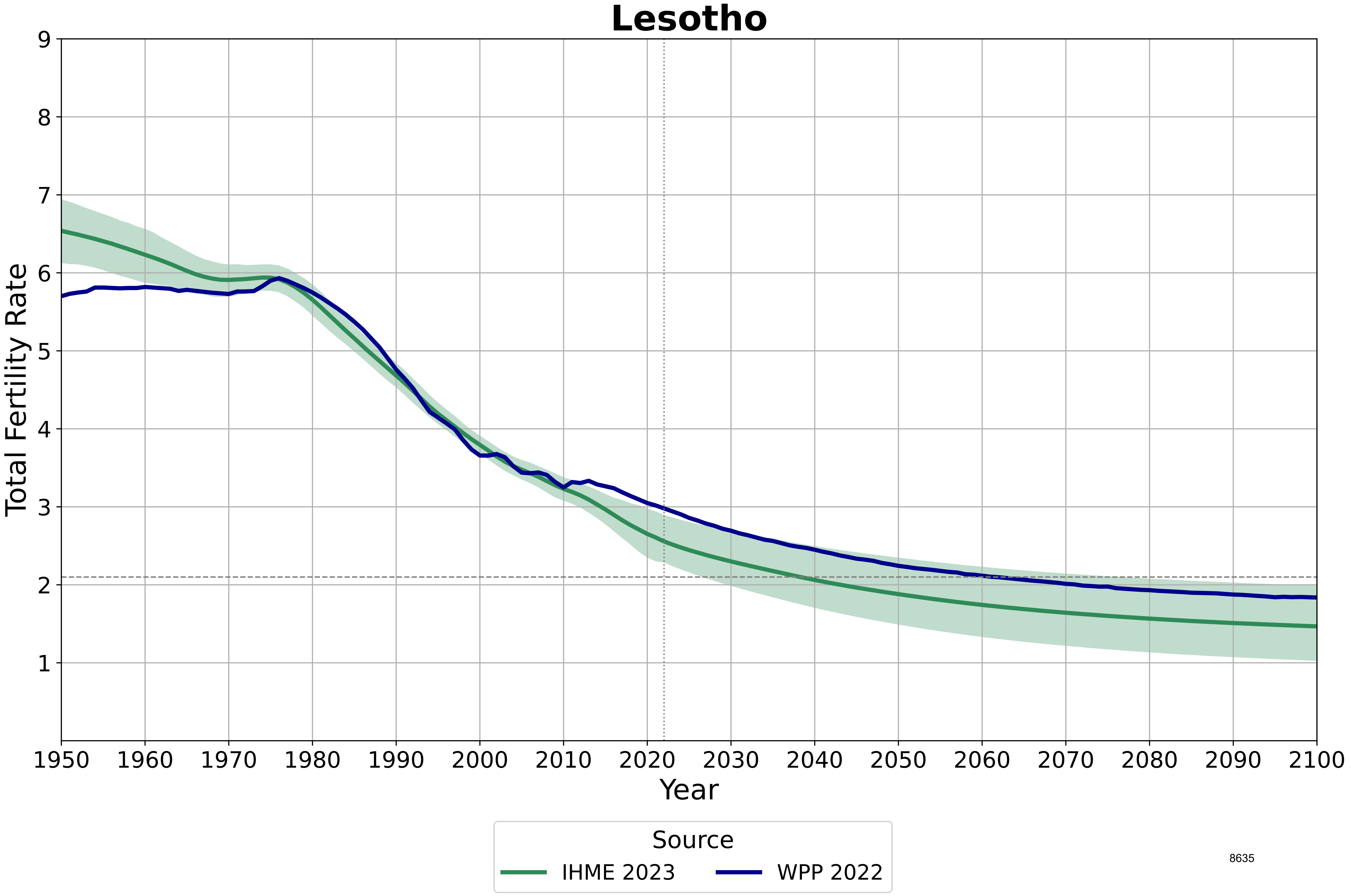

Supplemental Results Figure S7: TFR for IHME reference scenario and the UNPD medium variant scenario by location, 1950– 2100

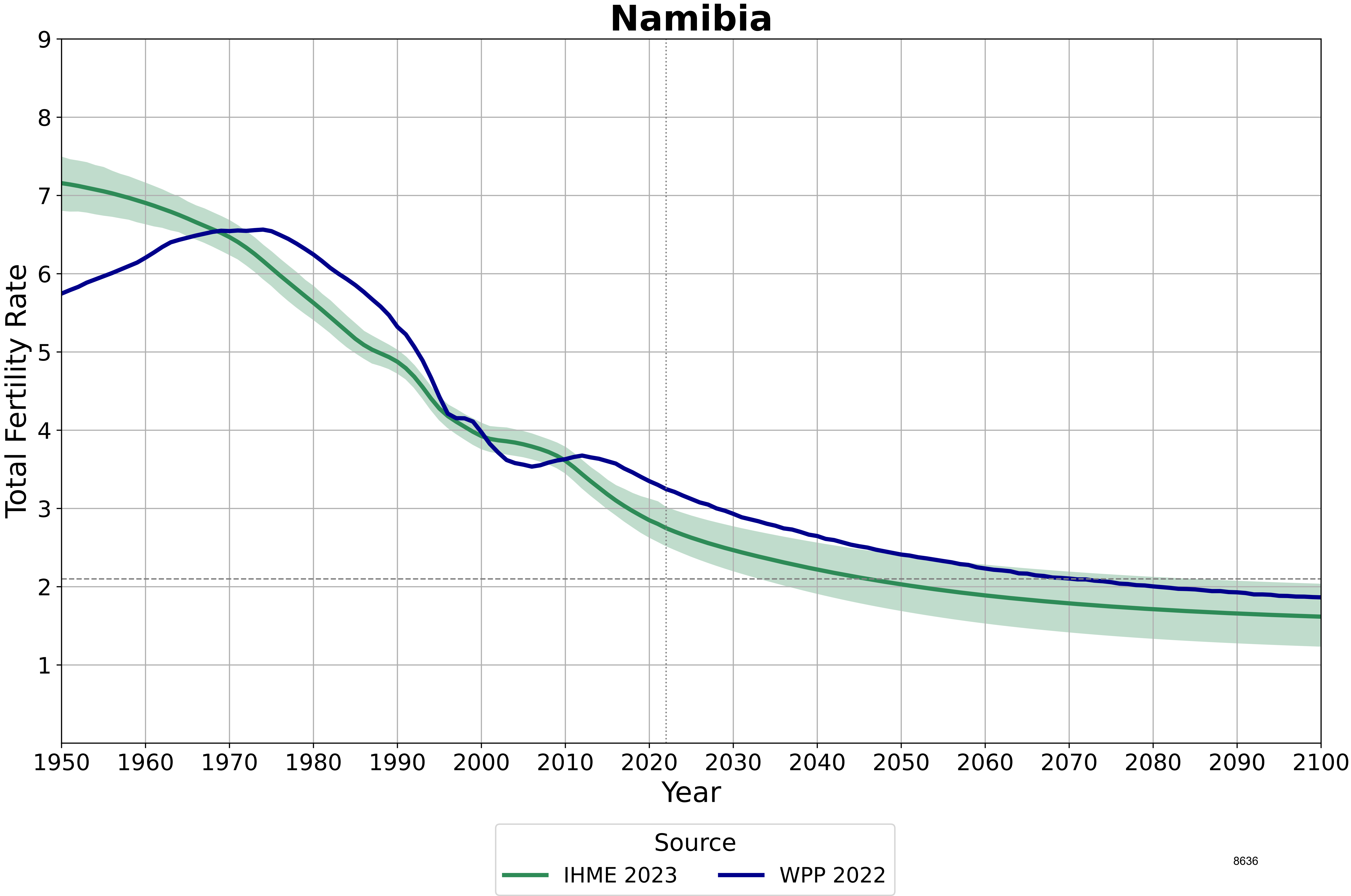

Supplemental Results Figure S7: TFR for IHME reference scenario and the UNPD medium variant scenario by location, 1950– 2100

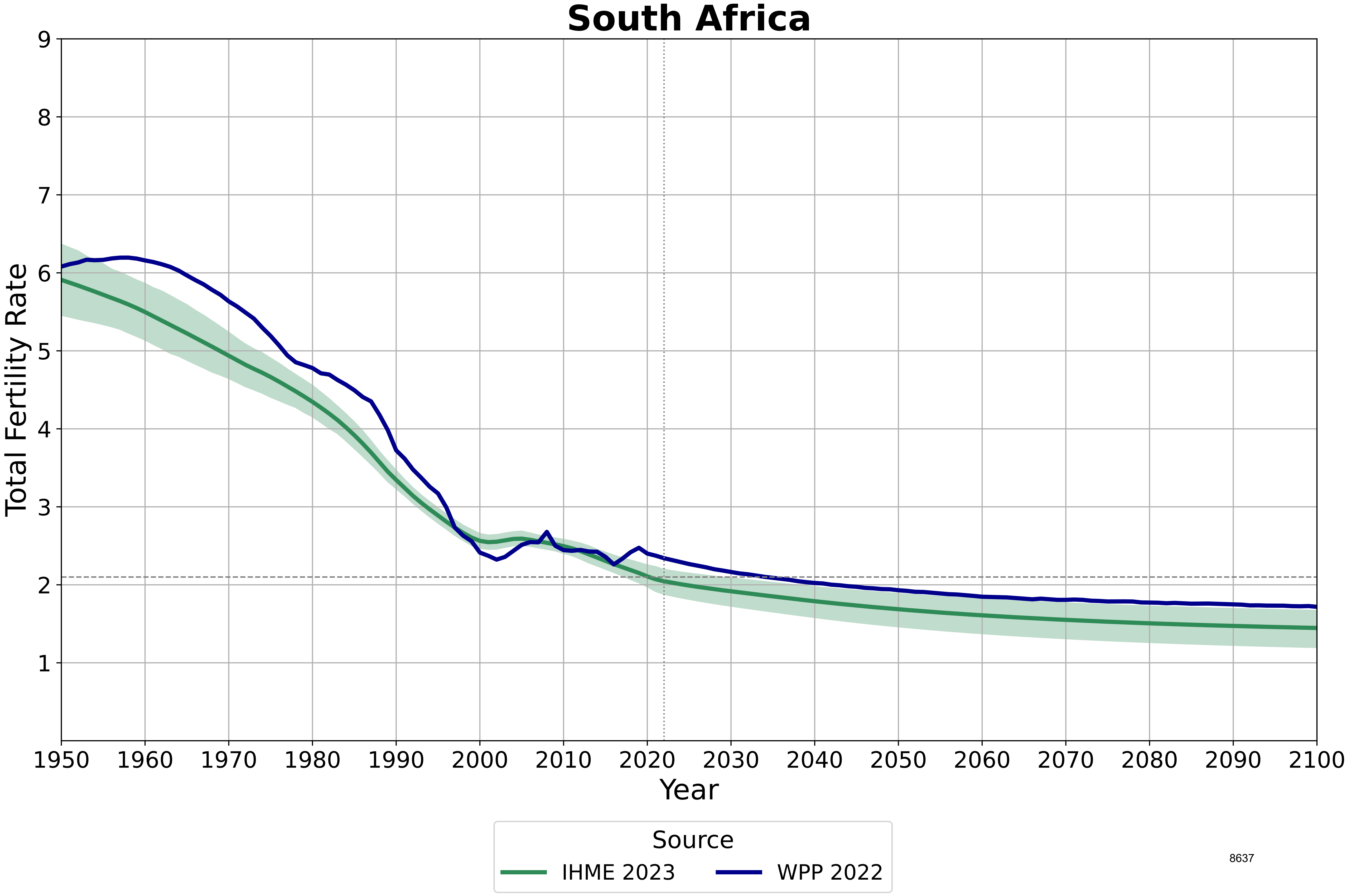

Supplemental Results Figure S7: TFR for IHME reference scenario and the UNPD medium variant scenario by location, 1950– 2100

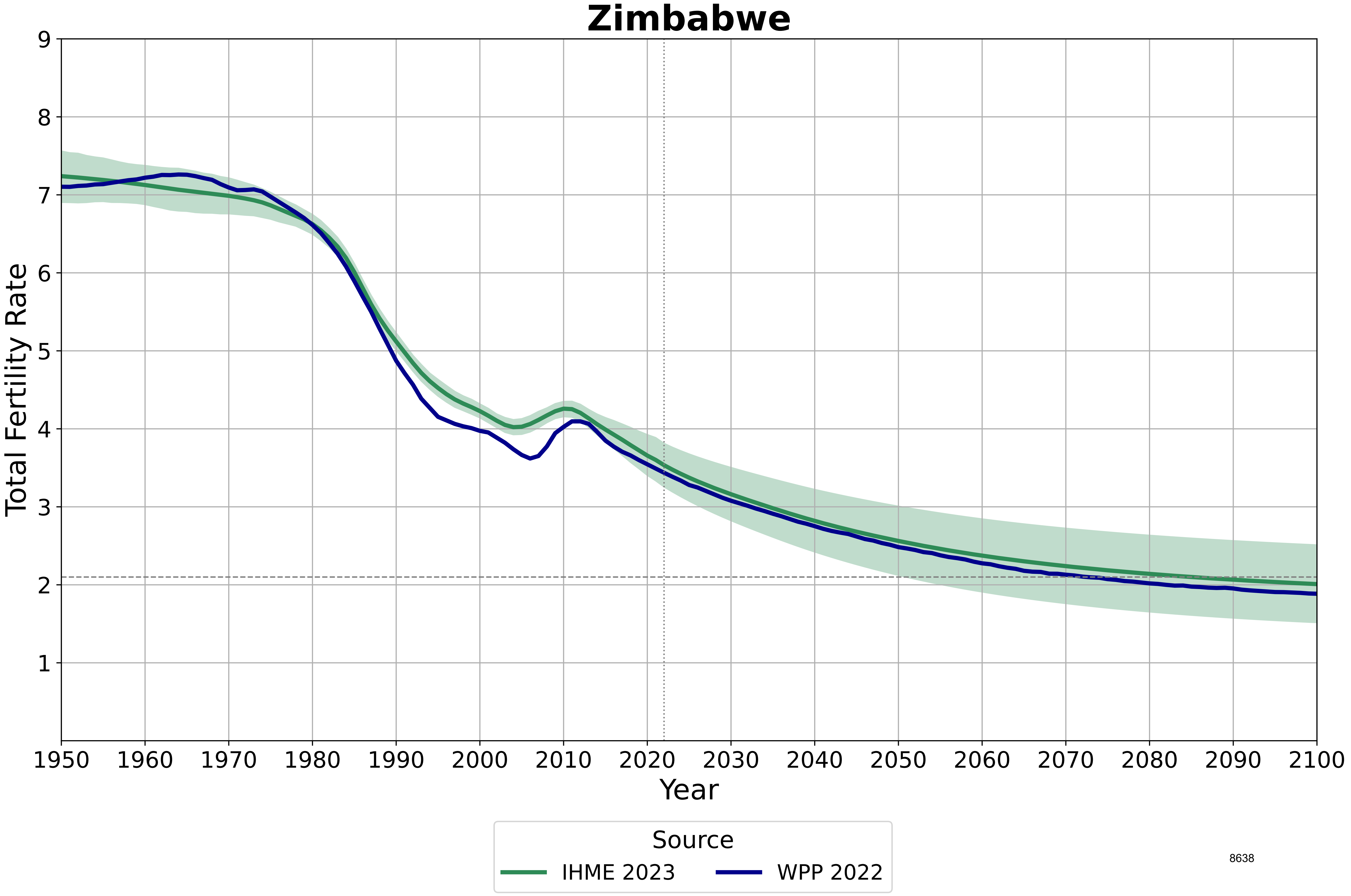

Supplemental Results Figure S7: TFR for IHME reference scenario and the UNPD medium variant scenario by location, 1950– 2100

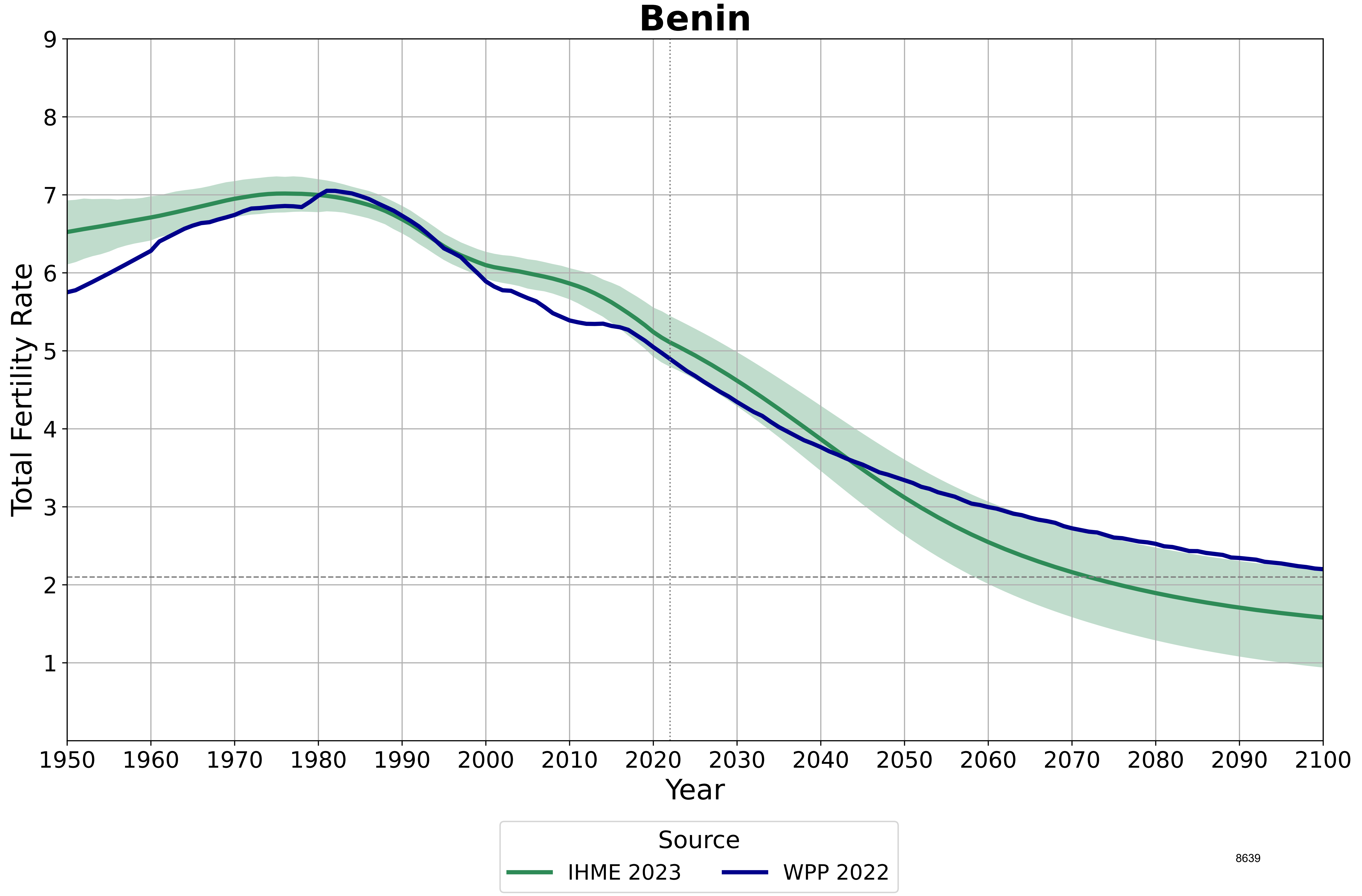

Supplemental Results Figure S7: TFR for IHME reference scenario and the UNPD medium variant scenario by location, 1950– 2100

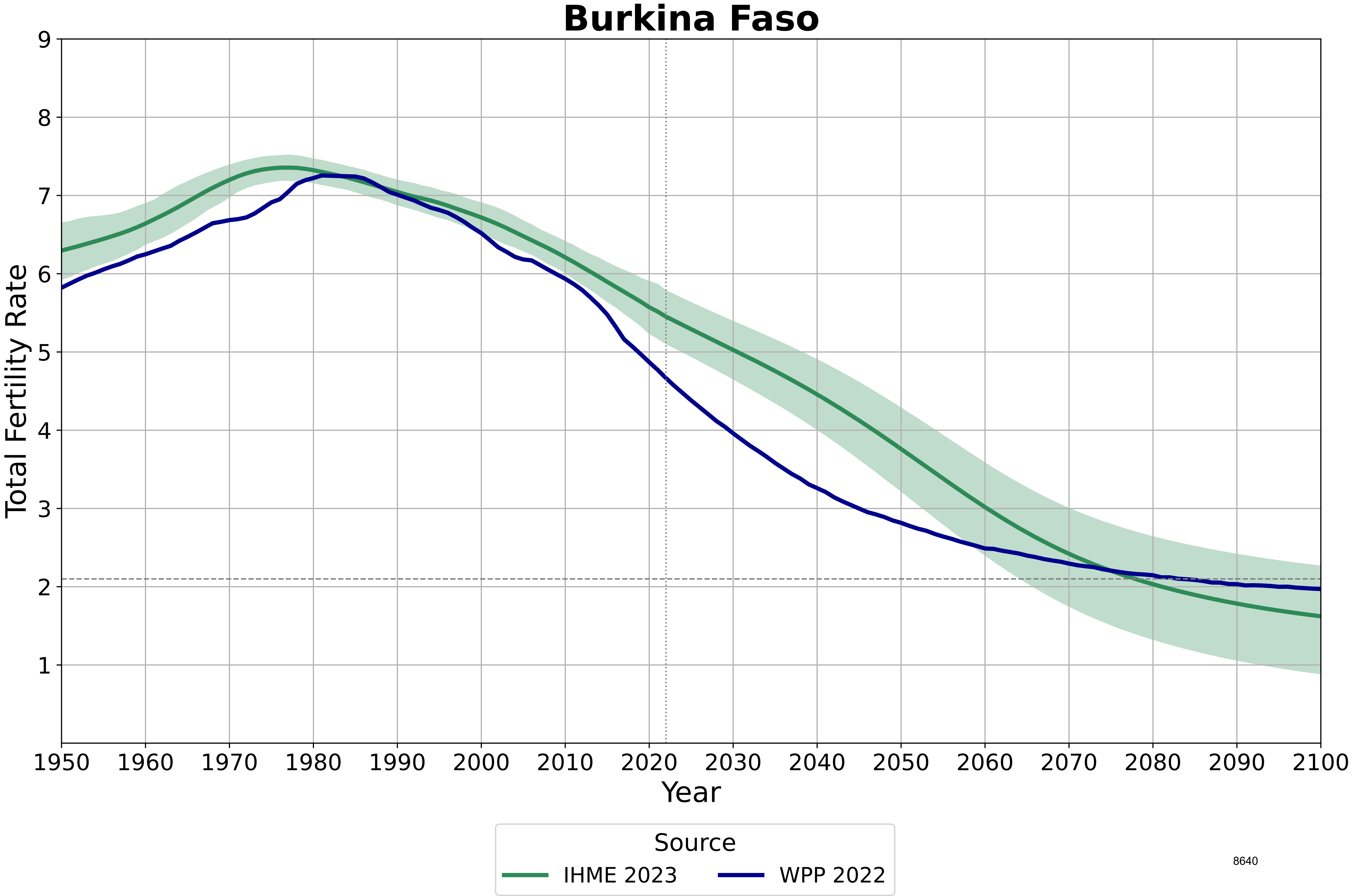

Supplemental Results Figure S7: TFR for IHME reference scenario and the UNPD medium variant scenario by location, 1950– 2100

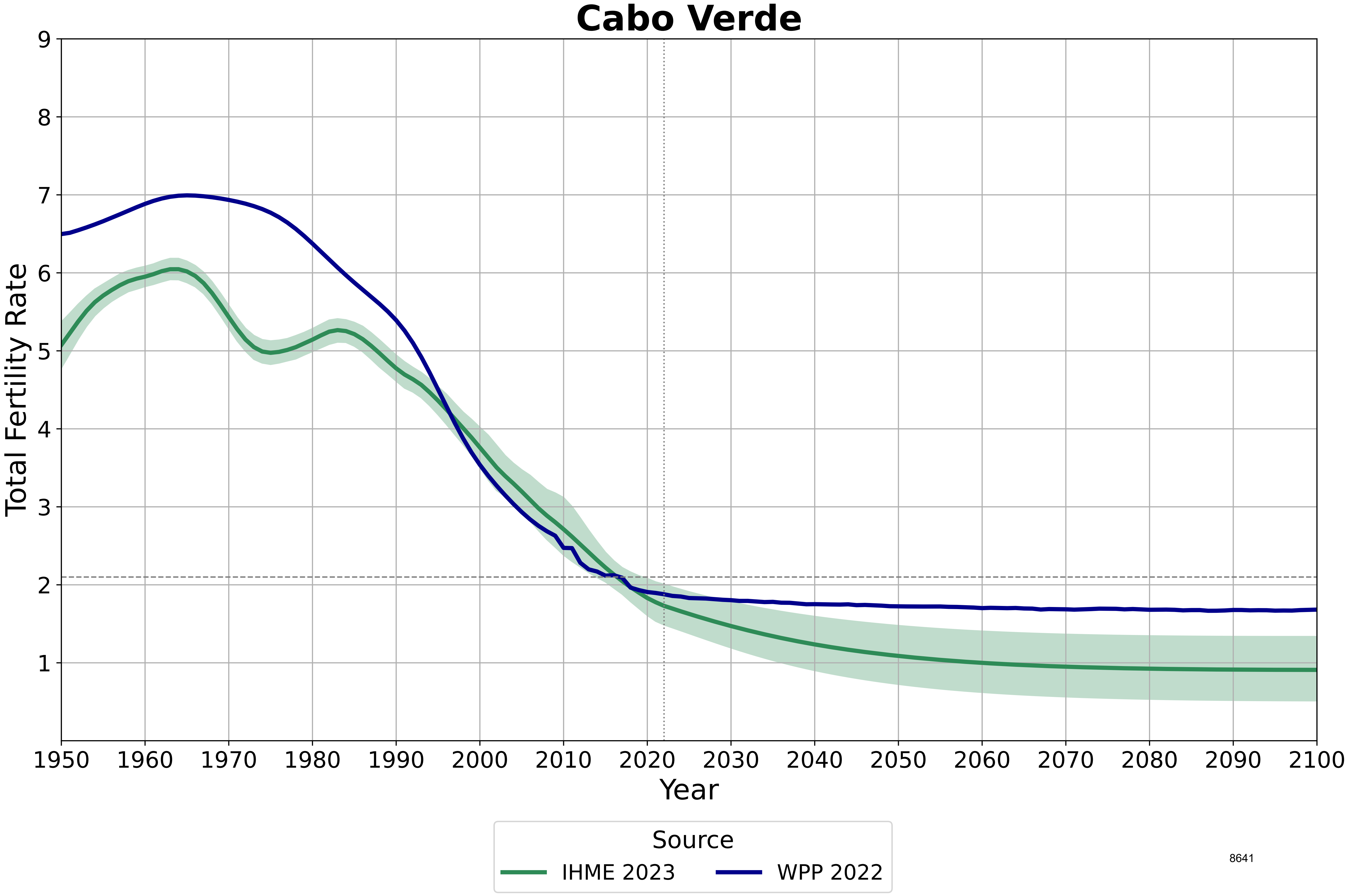

Supplemental Results Figure S7: TFR for IHME reference scenario and the UNPD medium variant scenario by location, 1950– 2100

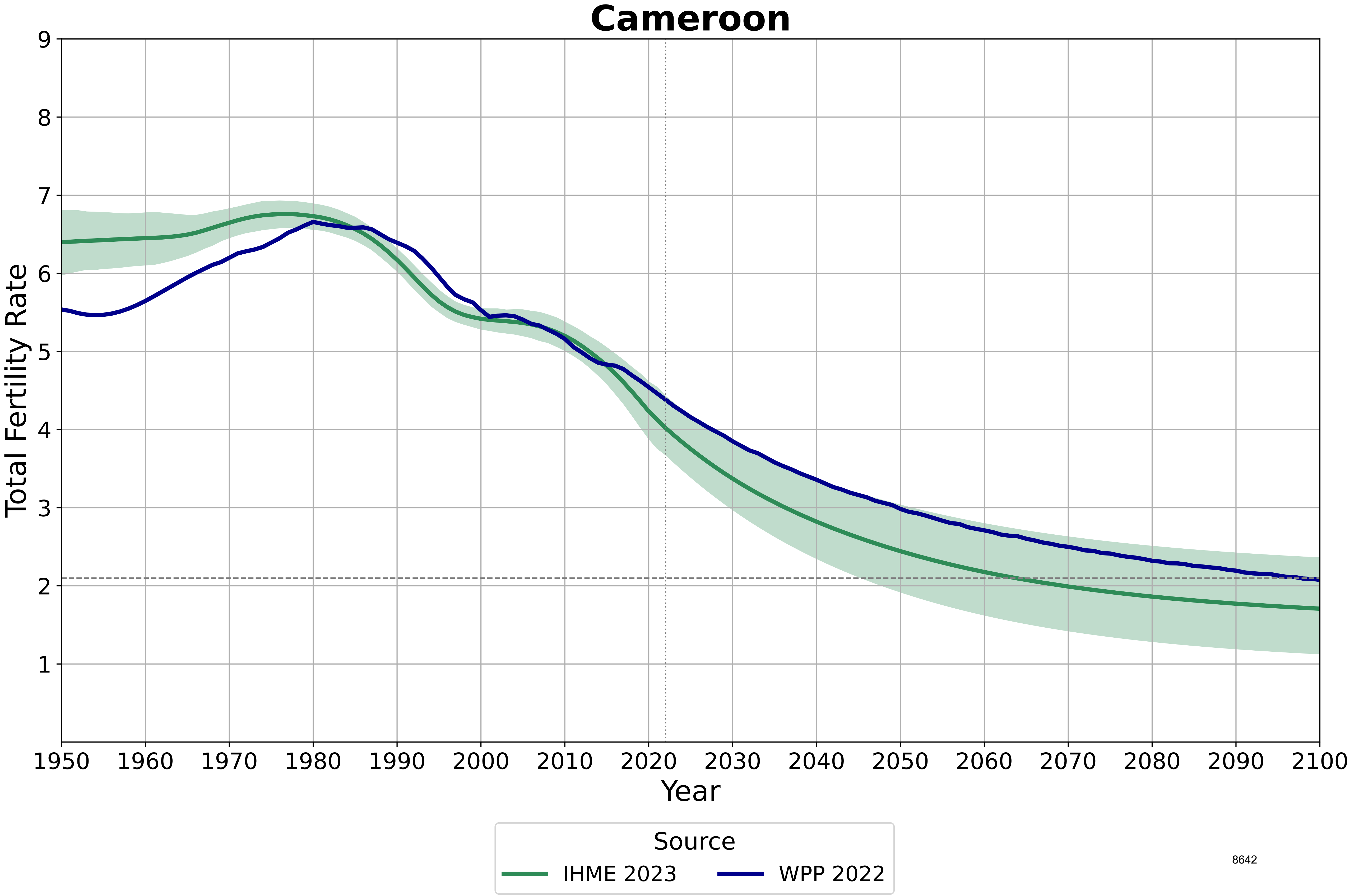

Supplemental Results Figure S7: TFR for IHME reference scenario and the UNPD medium variant scenario by location, 1950– 2100

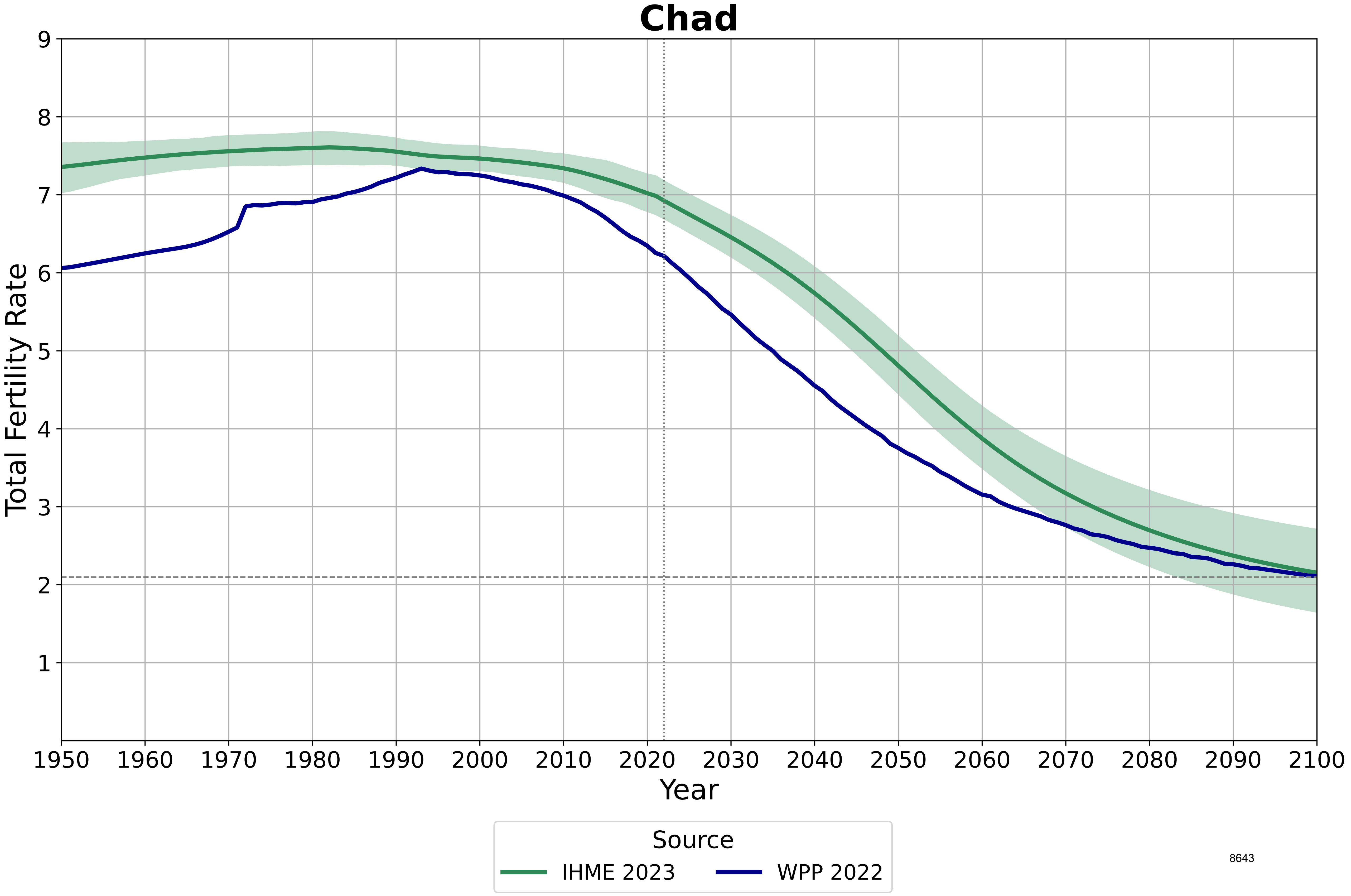

Supplemental Results Figure S7: TFR for IHME reference scenario and the UNPD medium variant scenario by location, 1950– 2100

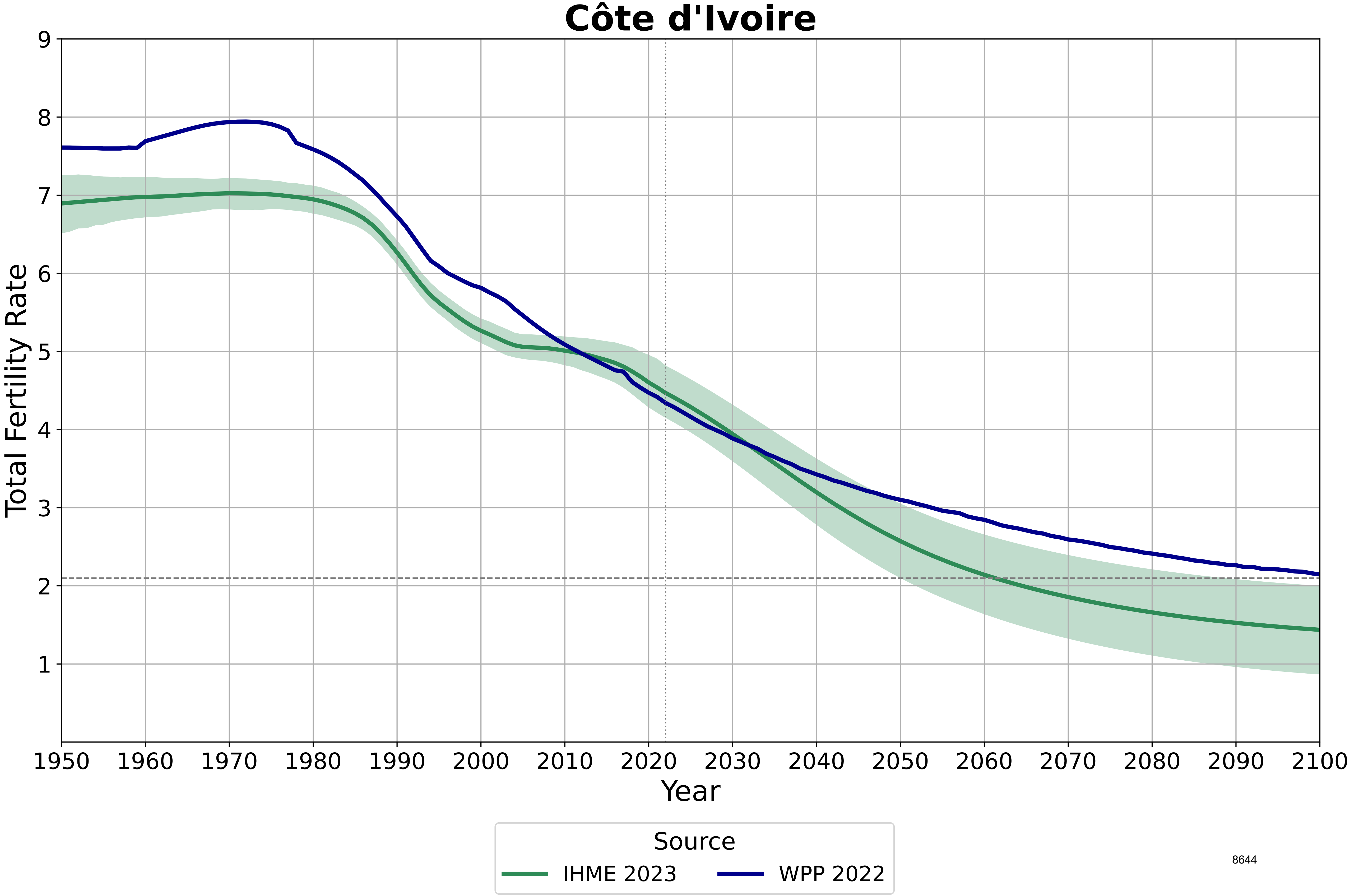

8644

Supplemental Results Figure S7: TFR for IHME reference scenario and the UNPD medium variant scenario by location, 1950– 2100

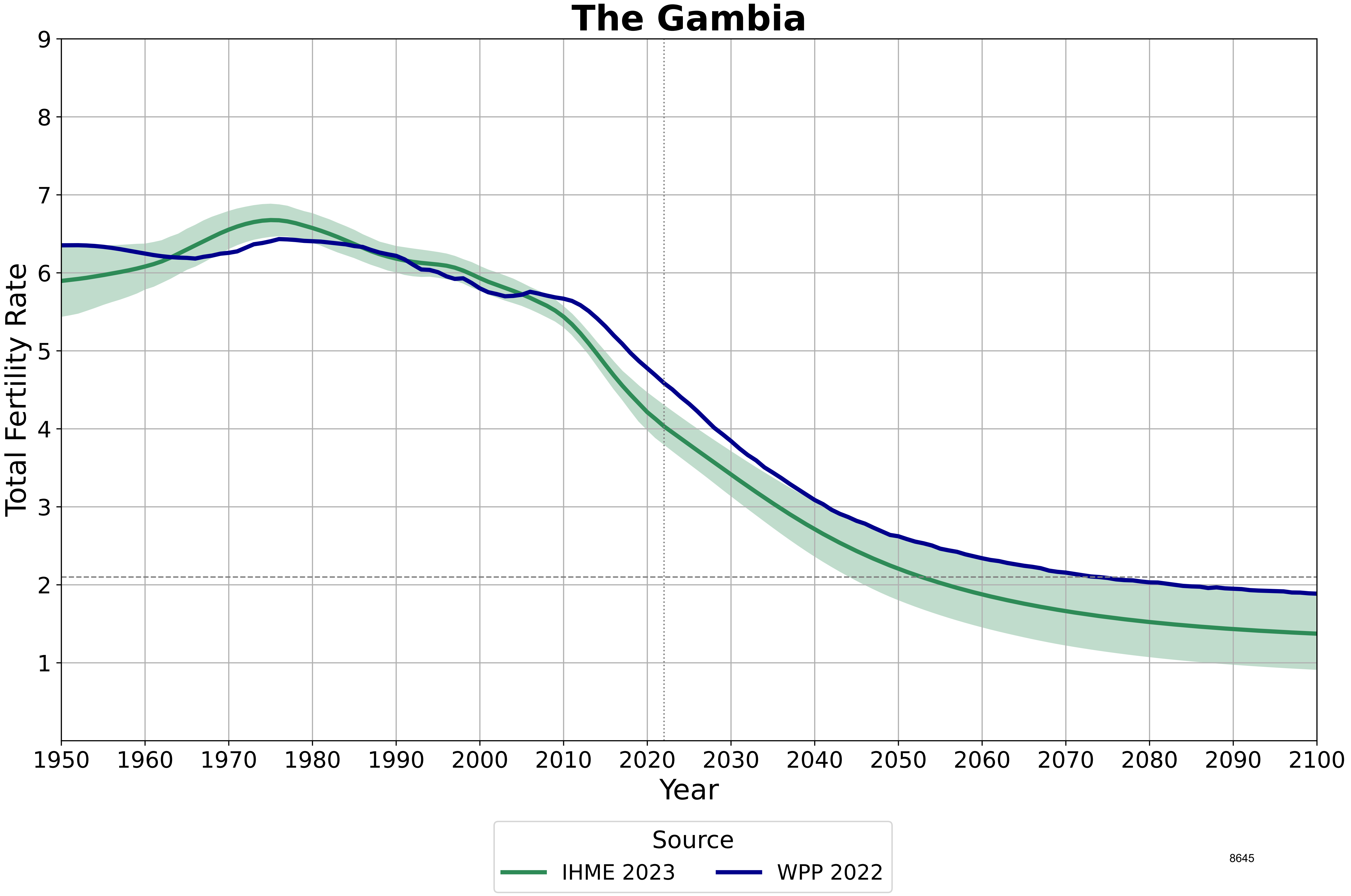

8645

Supplemental Results Figure S7: TFR for IHME reference scenario and the UNPD medium variant scenario by location, 1950– 2100

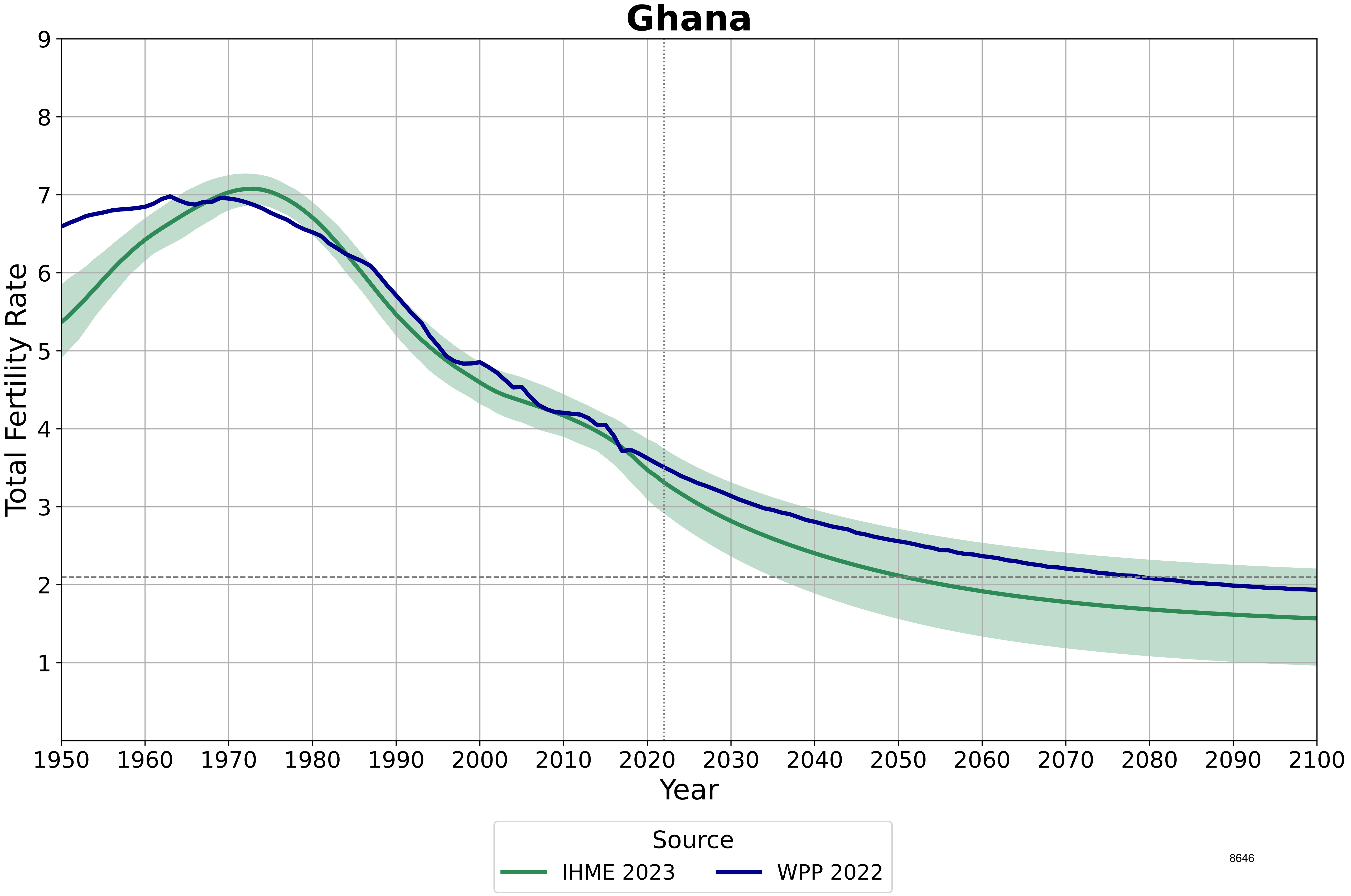

Supplemental Results Figure S7: TFR for IHME reference scenario and the UNPD medium variant scenario by location, 1950– 2100

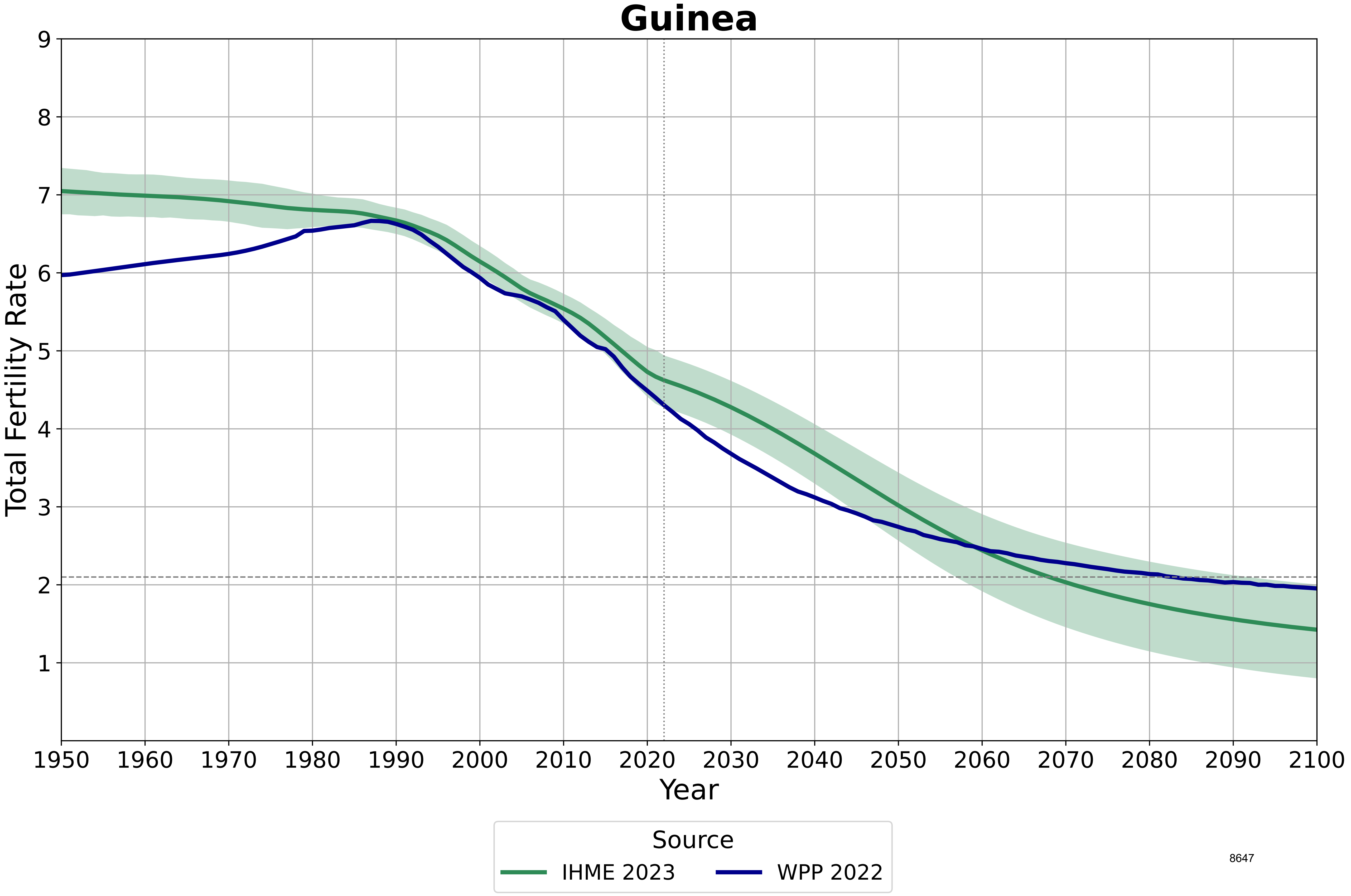

Supplemental Results Figure S7: TFR for IHME reference scenario and the UNPD medium variant scenario by location, 1950– 2100

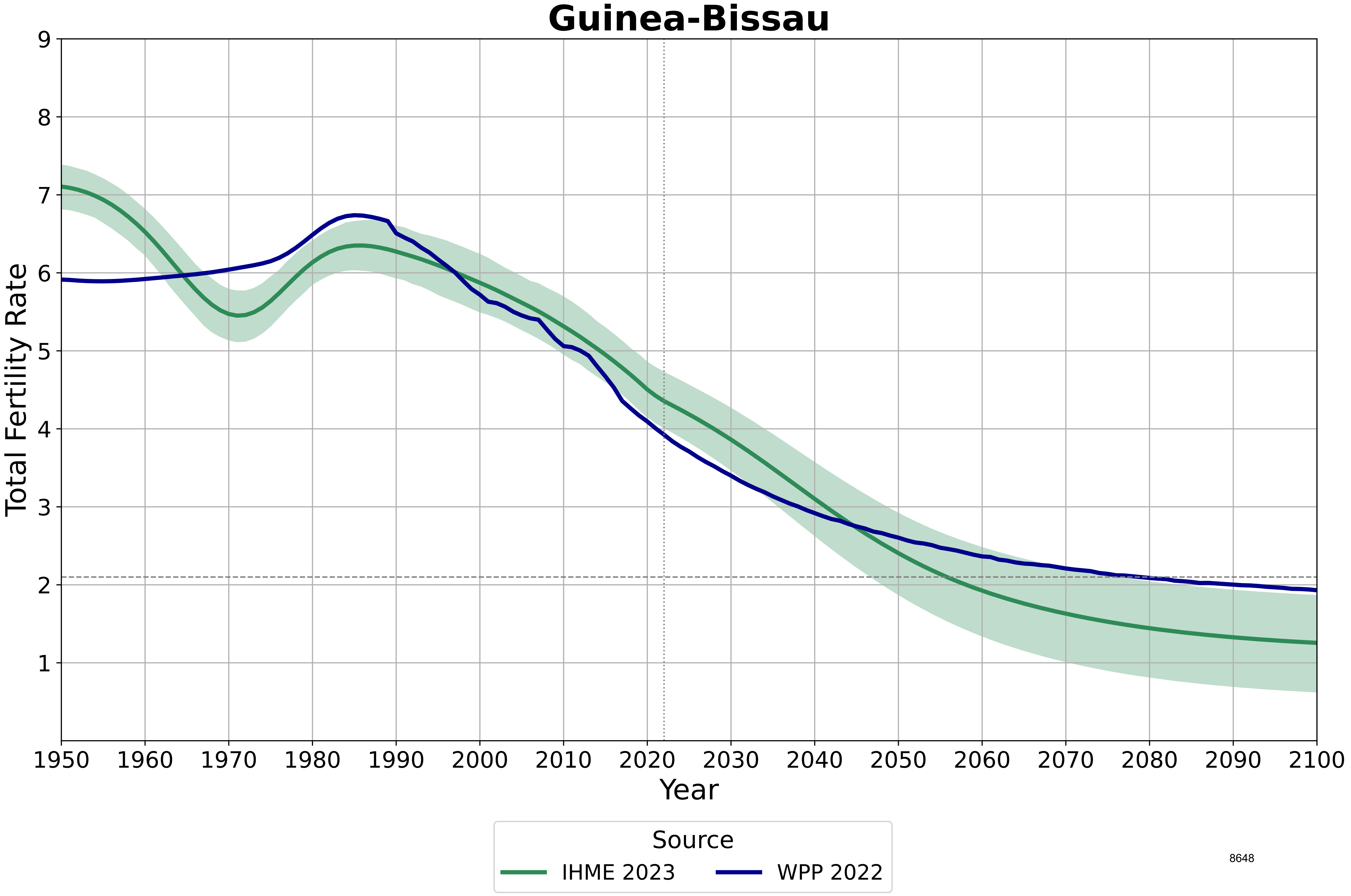

Supplemental Results Figure S7: TFR for IHME reference scenario and the UNPD medium variant scenario by location, 1950– 2100

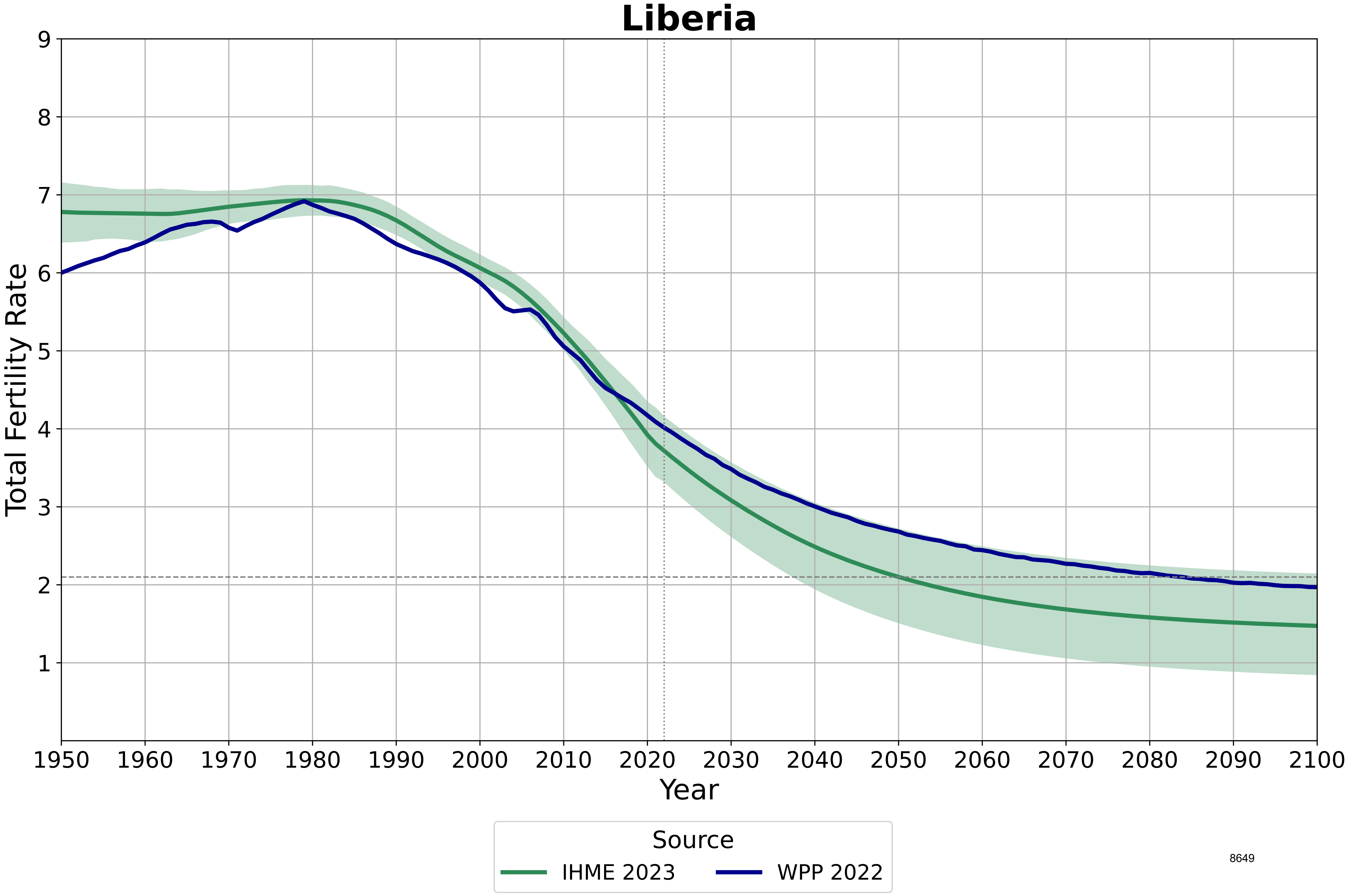

Supplemental Results Figure S7: TFR for IHME reference scenario and the UNPD medium variant scenario by location, 1950– 2100

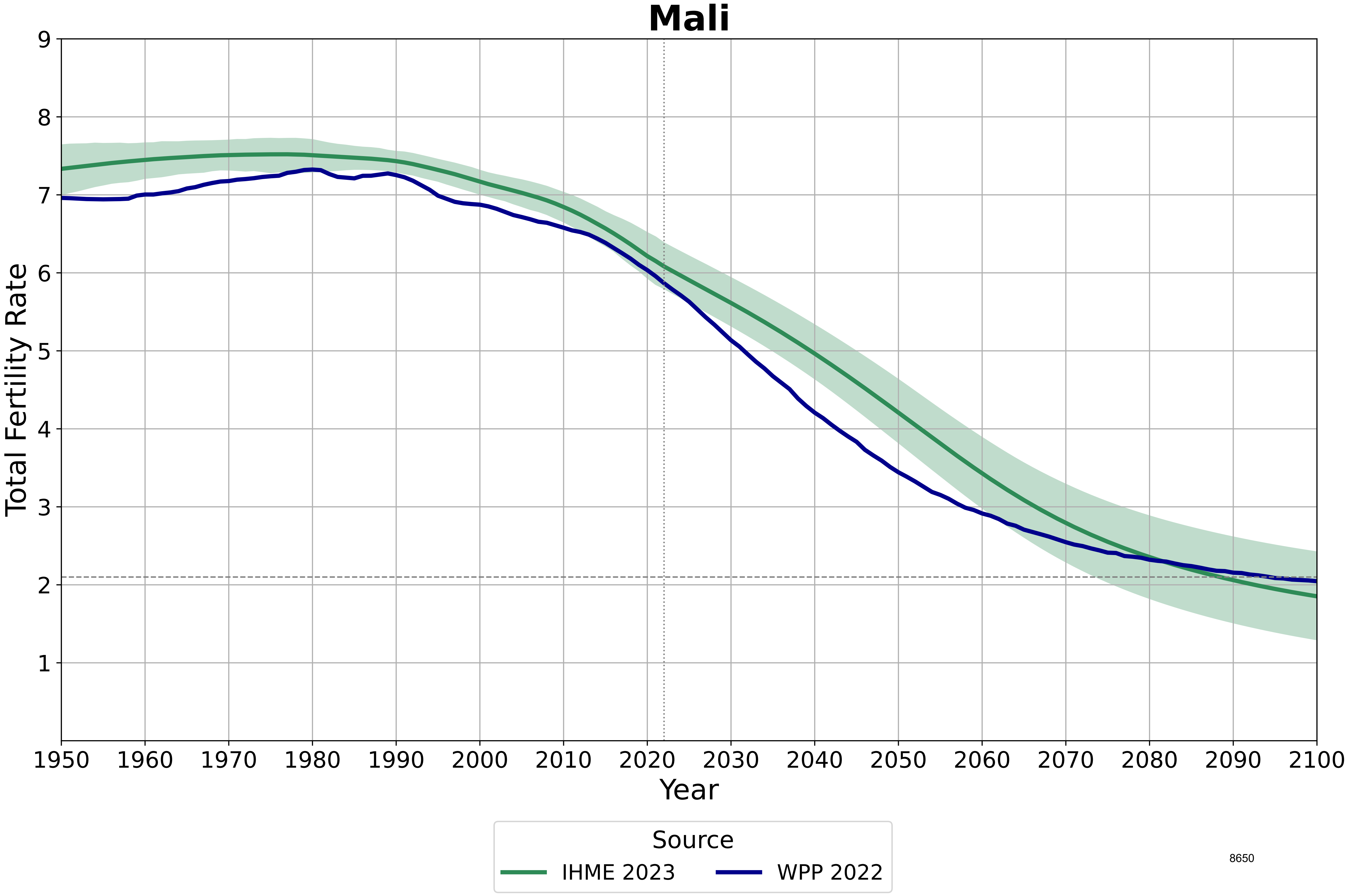

Supplemental Results Figure S7: TFR for IHME reference scenario and the UNPD medium variant scenario by location, 1950– 2100

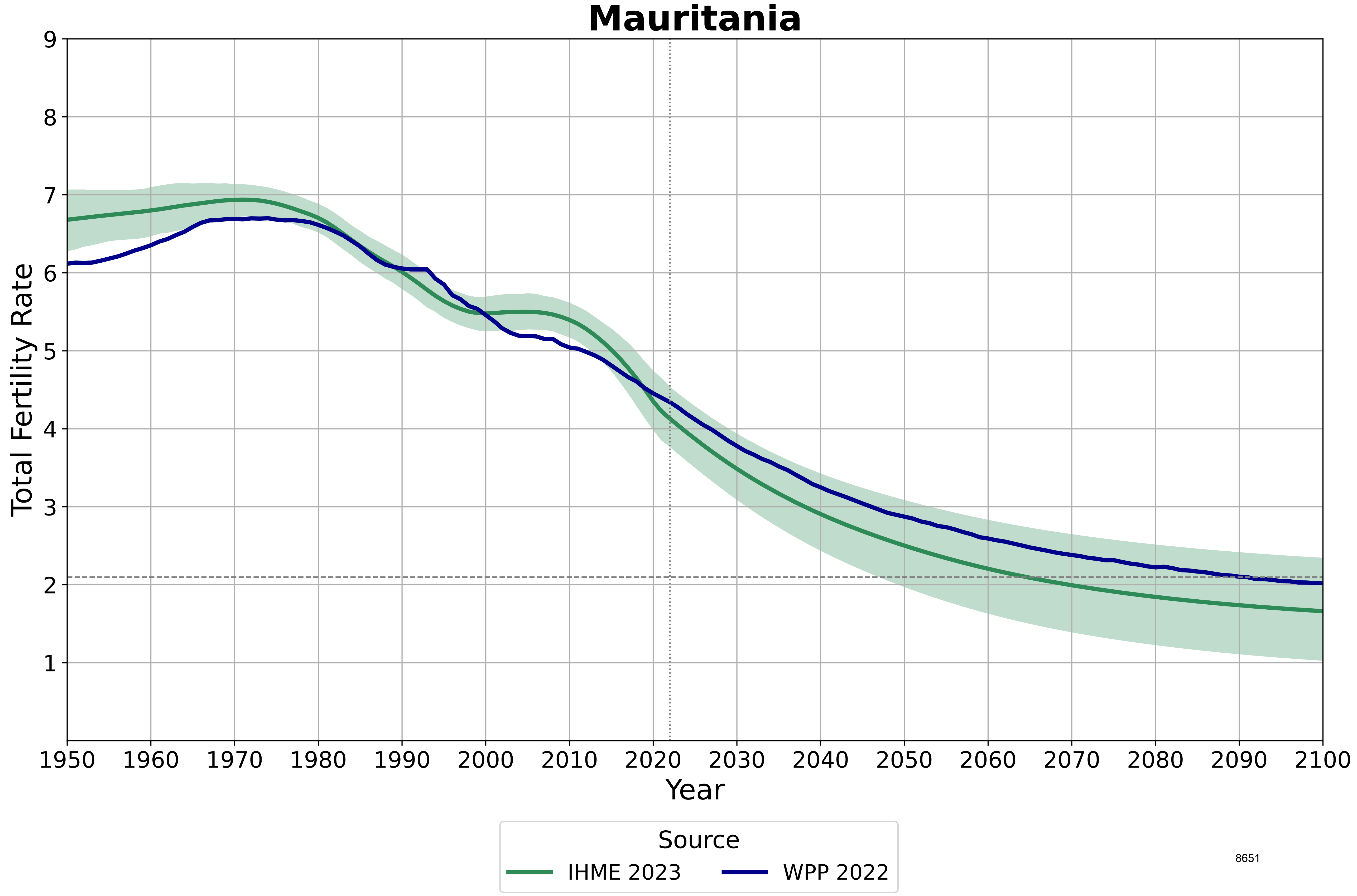

Supplemental Results Figure S7: TFR for IHME reference scenario and the UNPD medium variant scenario by location, 1950– 2100

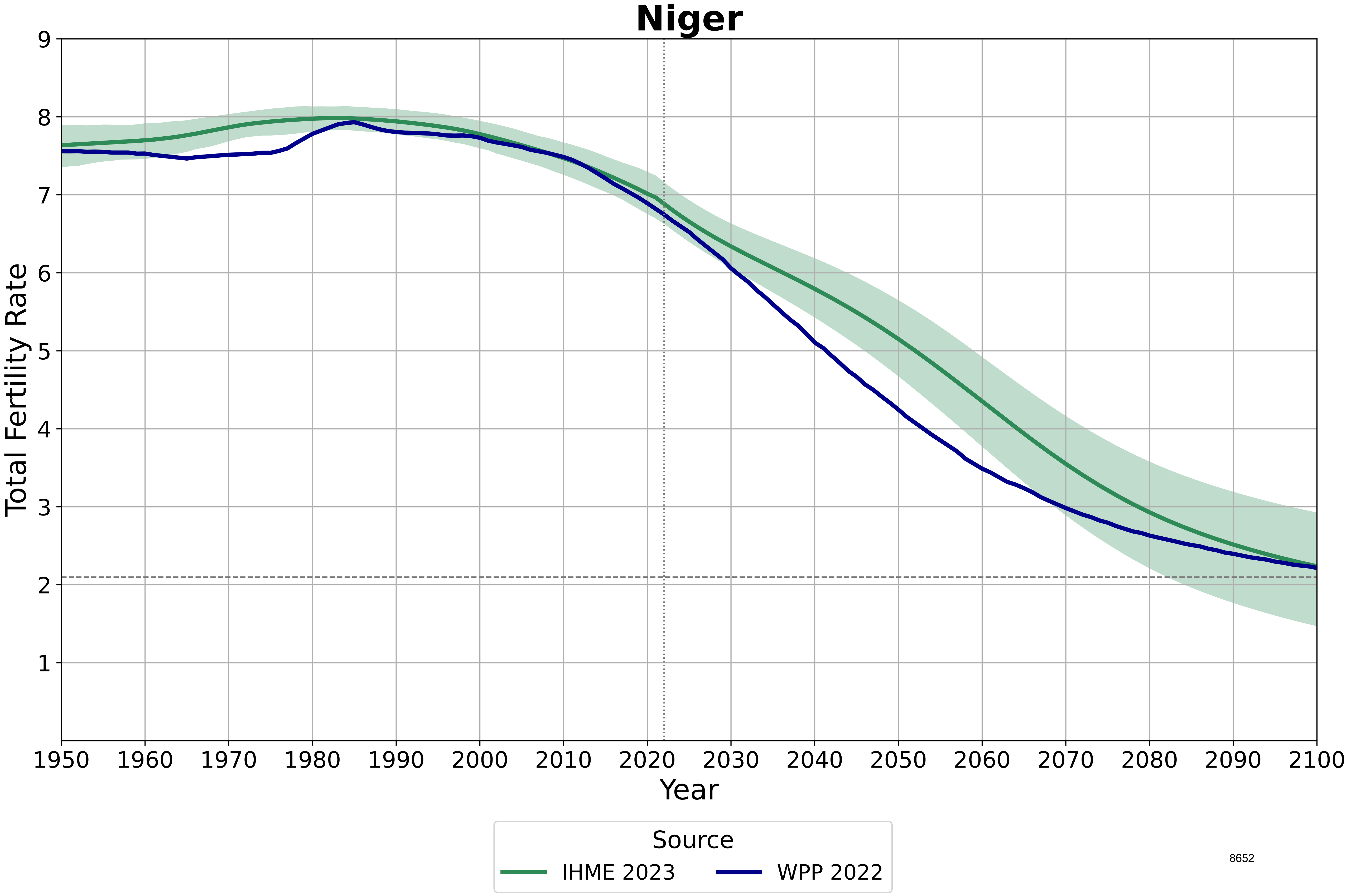

Supplemental Results Figure S7: TFR for IHME reference scenario and the UNPD medium variant scenario by location, 1950– 2100

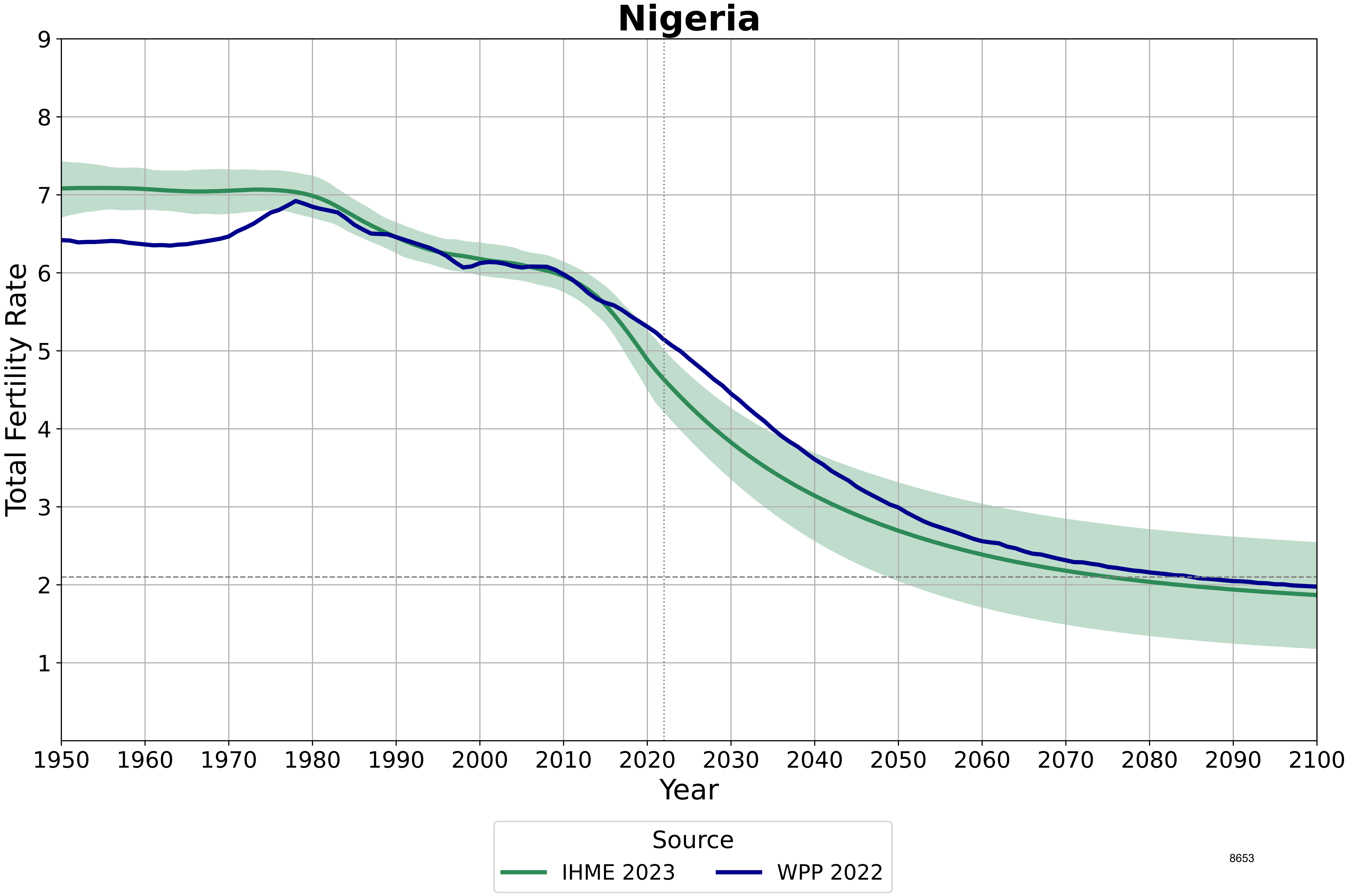

Supplemental Results Figure S7: TFR for IHME reference scenario and the UNPD medium variant scenario by location, 1950– 2100

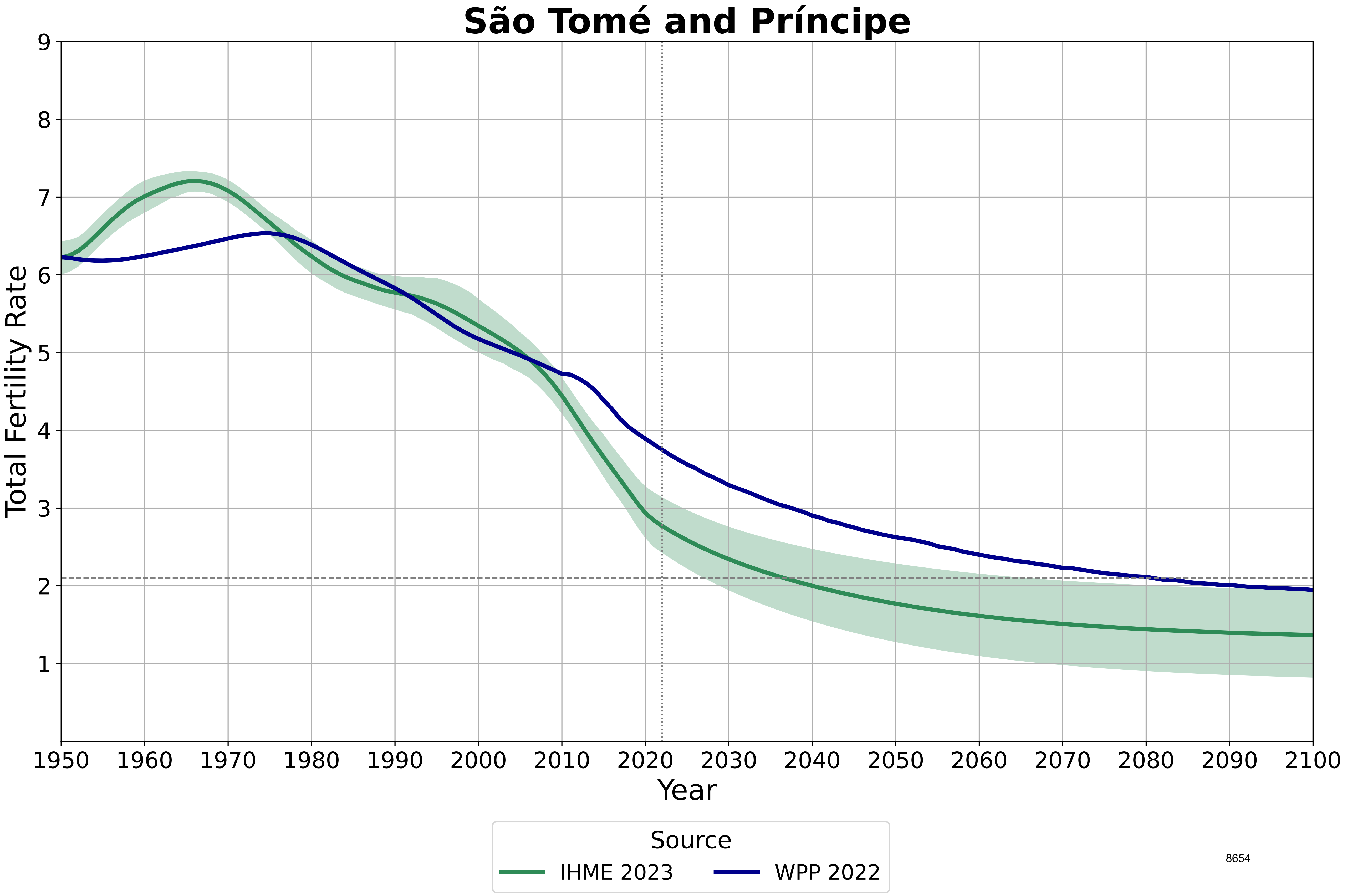

Supplemental Results Figure S7: TFR for IHME reference scenario and the UNPD medium variant scenario by location, 1950– 2100

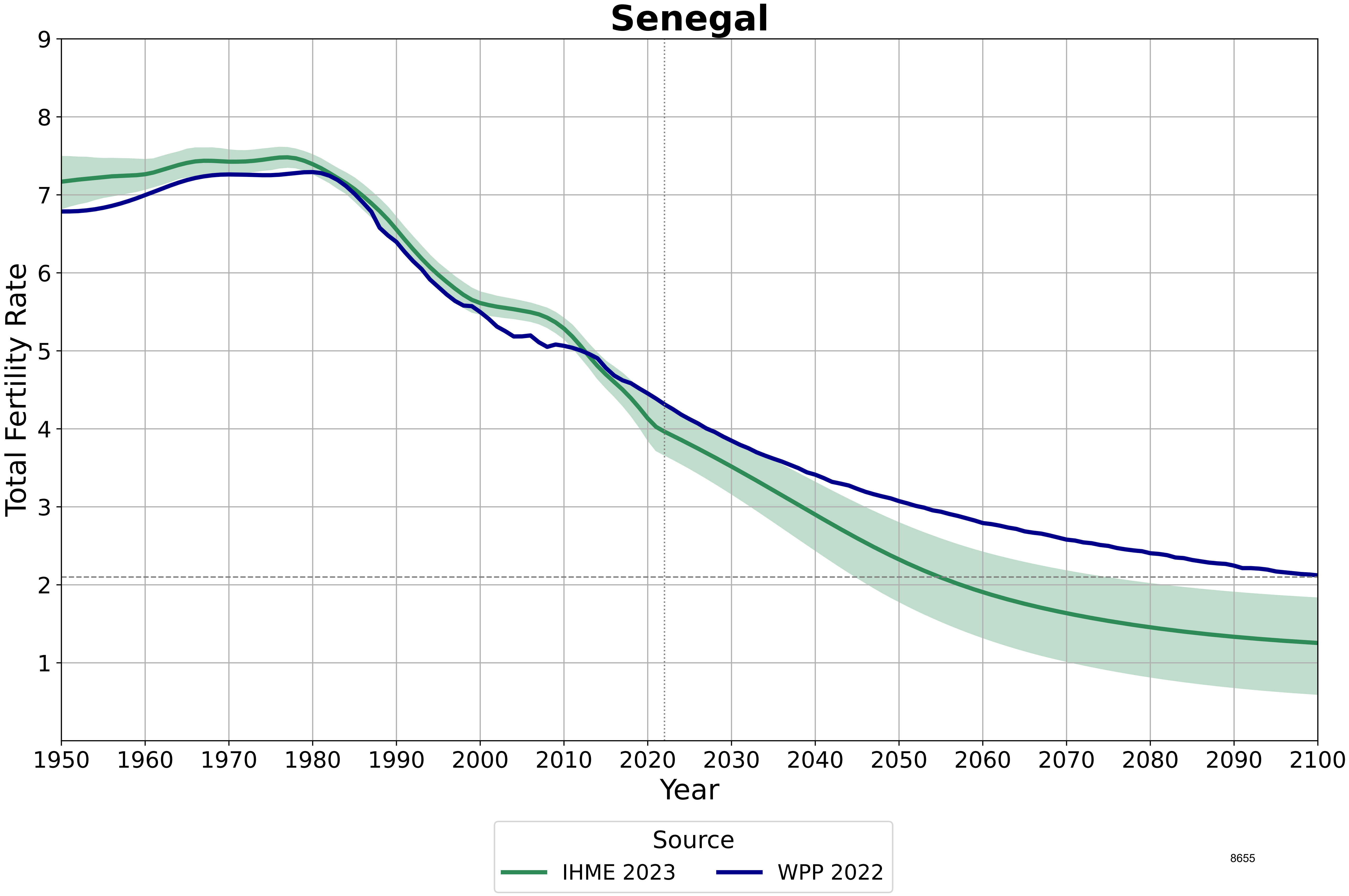

Supplemental Results Figure S7: TFR for IHME reference scenario and the UNPD medium variant scenario by location, 1950– 2100

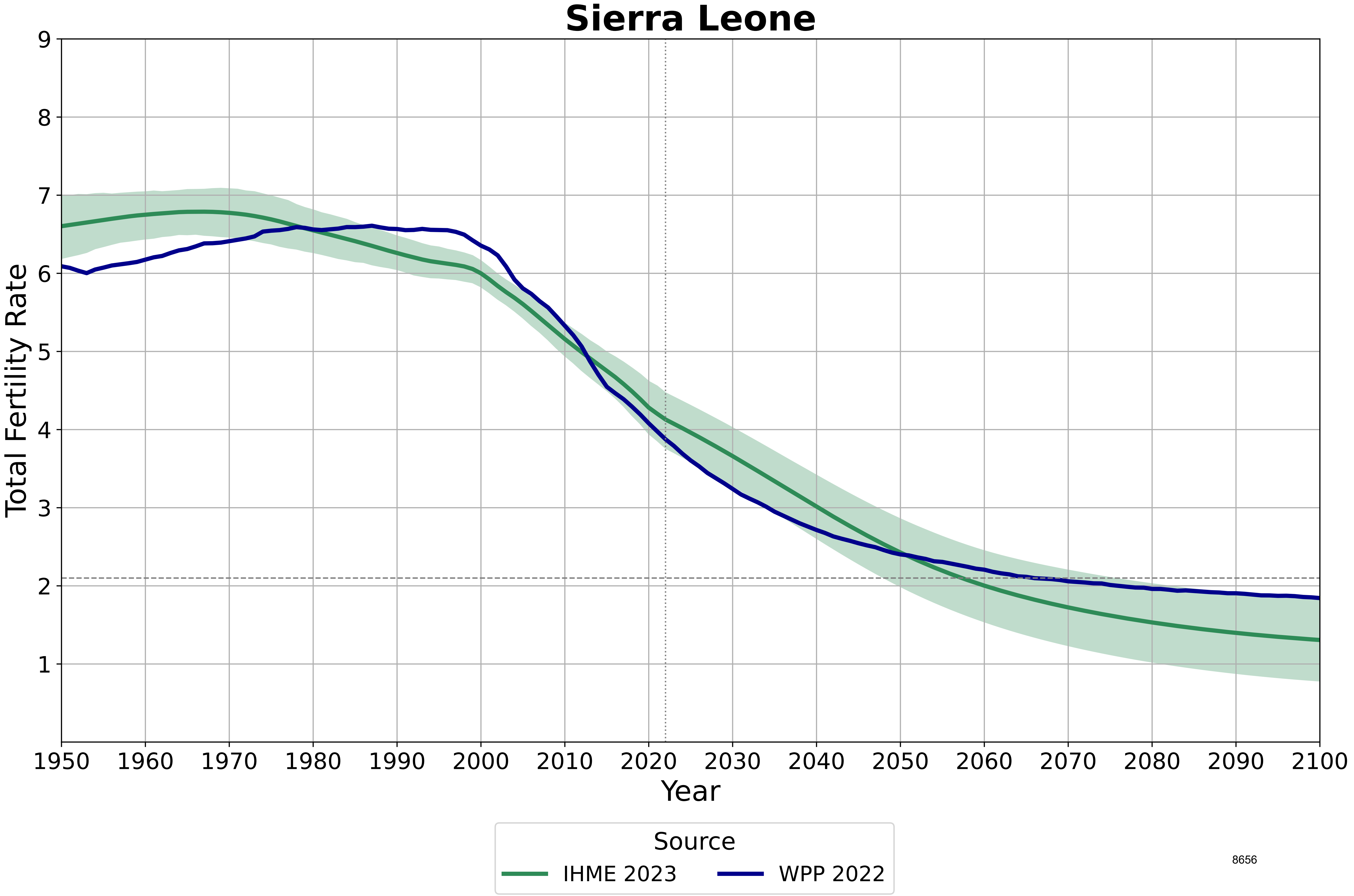

Supplemental Results Figure S7: TFR for IHME reference scenario and the UNPD medium variant scenario by location, 1950– 2100

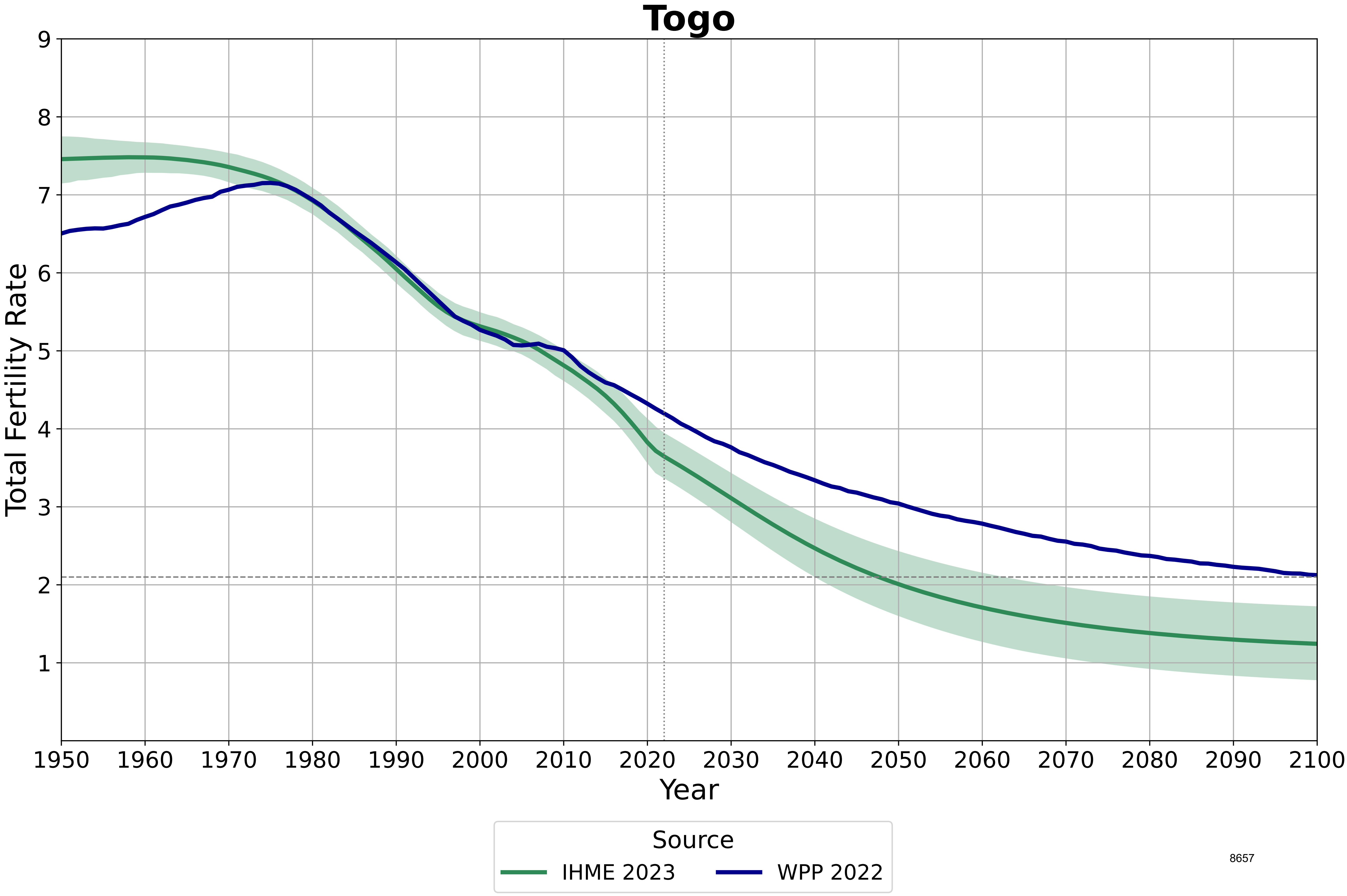

Supplemental Results Figure S7: TFR for IHME reference scenario and the UNPD medium variant scenario by location, 1950– 2100

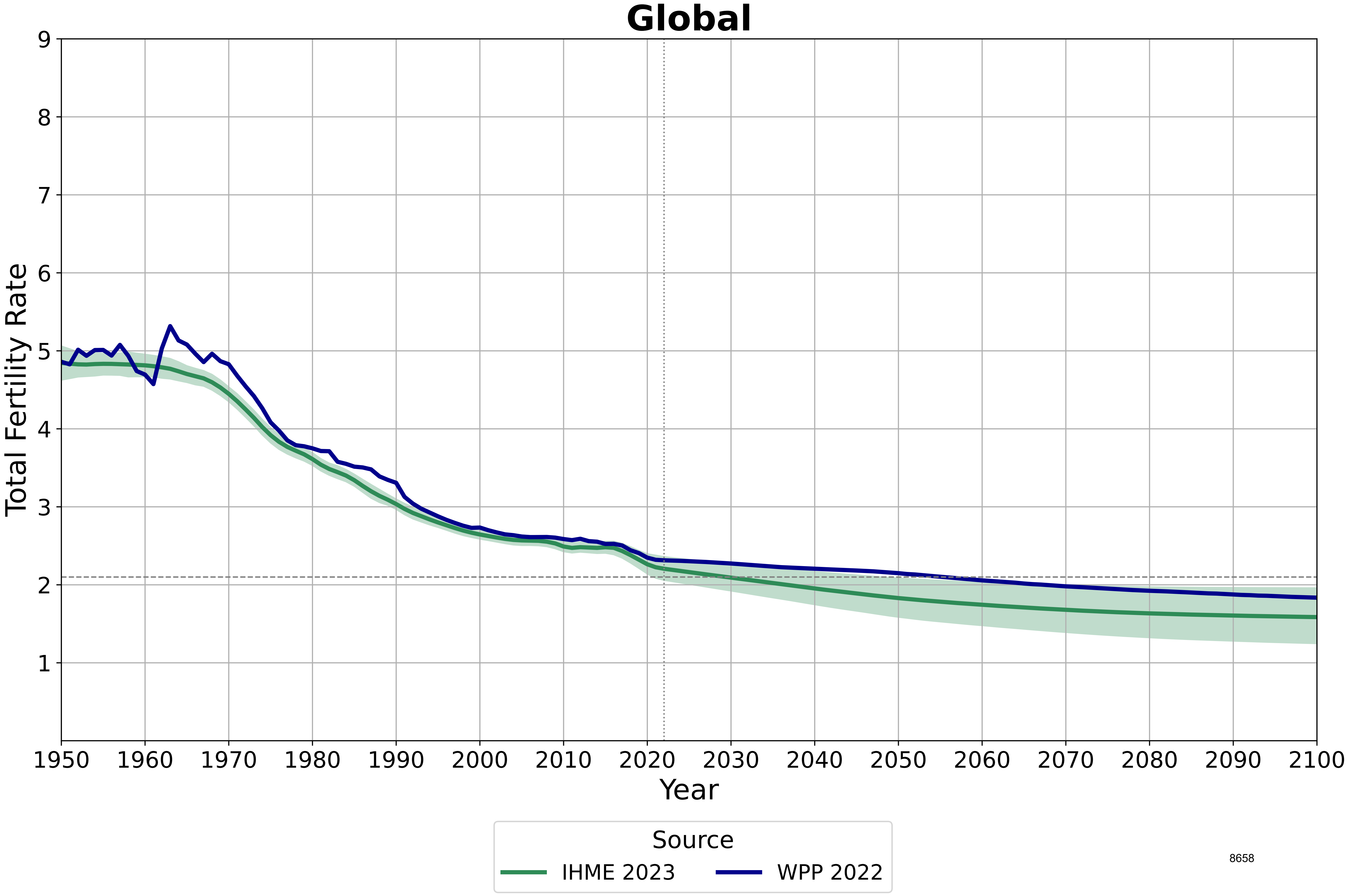

Supplemental Results Figure S8: Five-year interval ASFR from IHME reference scenario and the UNPD medium variant scenario by location, 1950-2100

Armenia

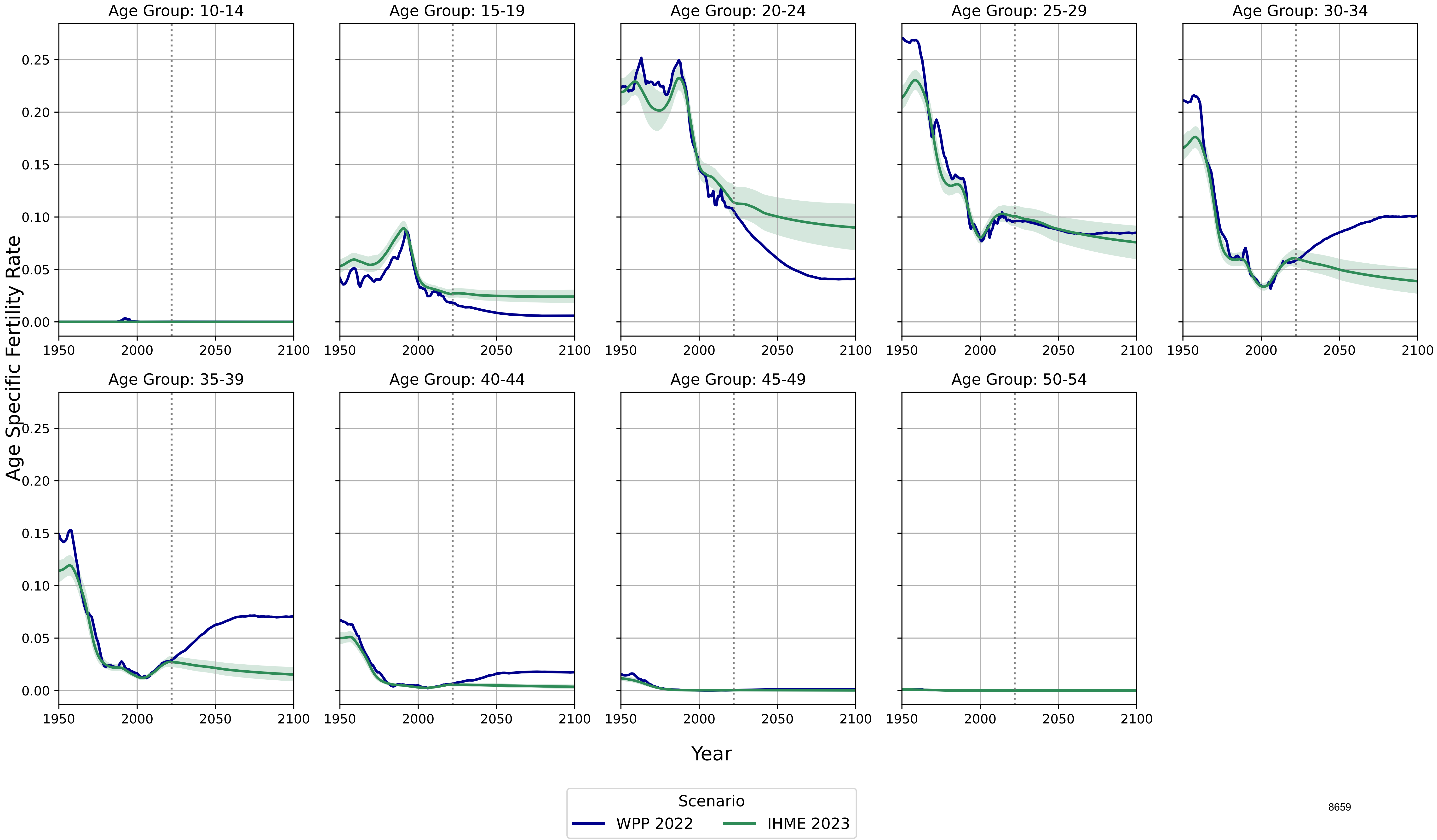

Supplemental Results Figure S8: Five-year interval ASFR from IHME reference scenario and the UNPD medium variant scenario by location, 1950-2100

Azerbaijan

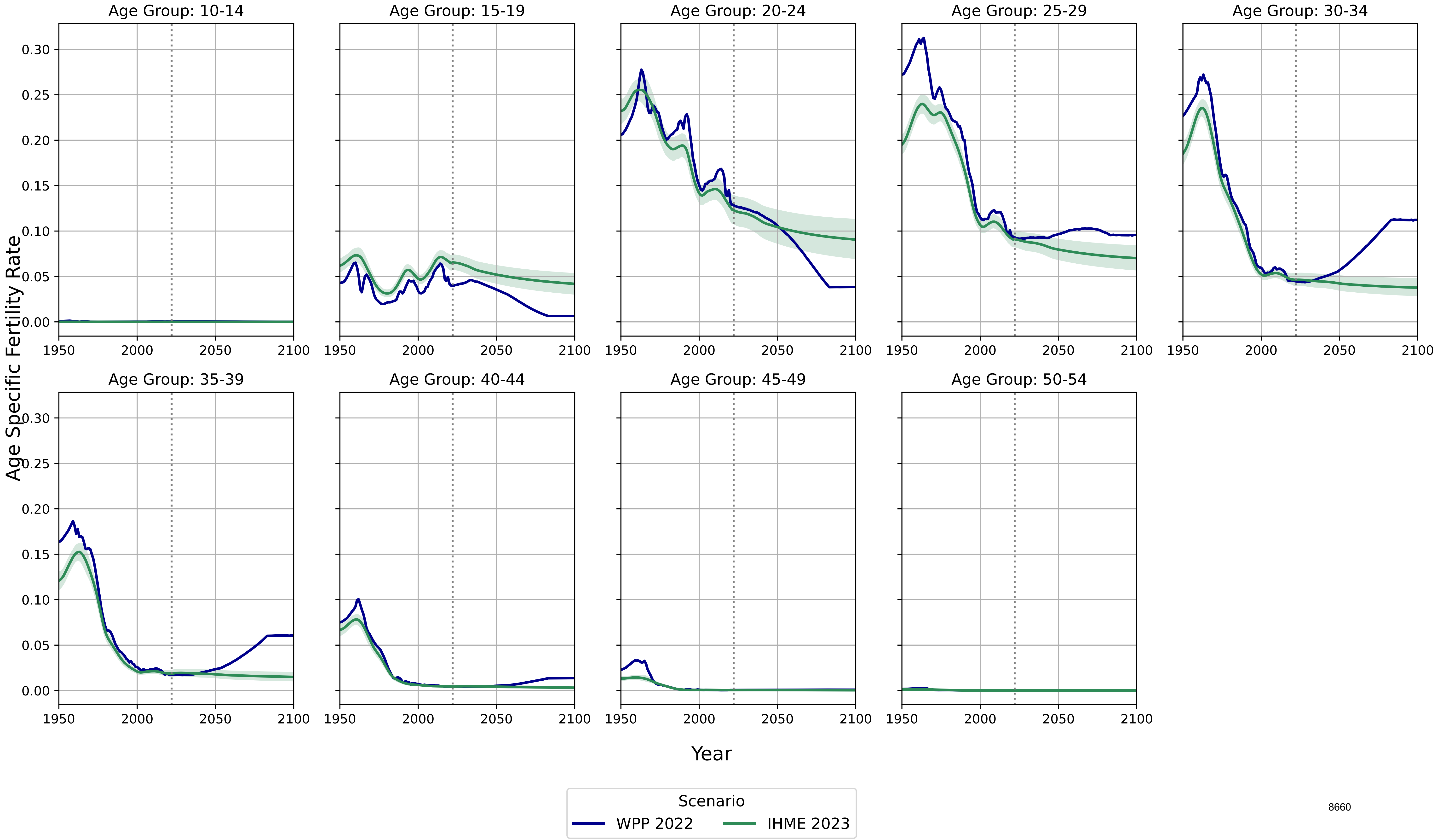

8660

Supplemental Results Figure S8: Five-year interval ASFR from IHME reference scenario and the UNPD medium variant scenario by location, 1950-2100

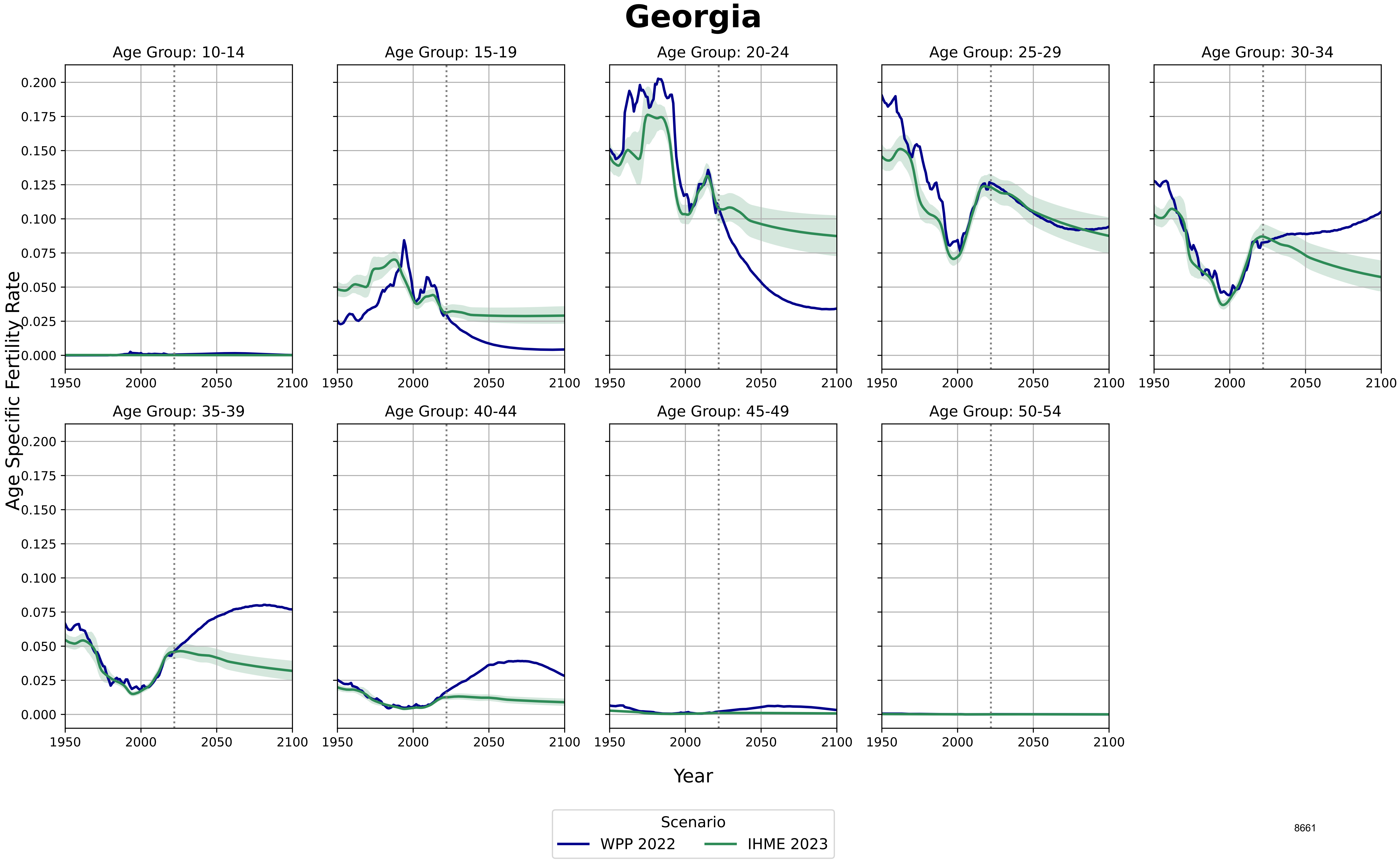

Supplemental Results Figure S8: Five-year interval ASFR from IHME reference scenario and the UNPD medium variant scenario by location, 1950-2100

Kazakhstan

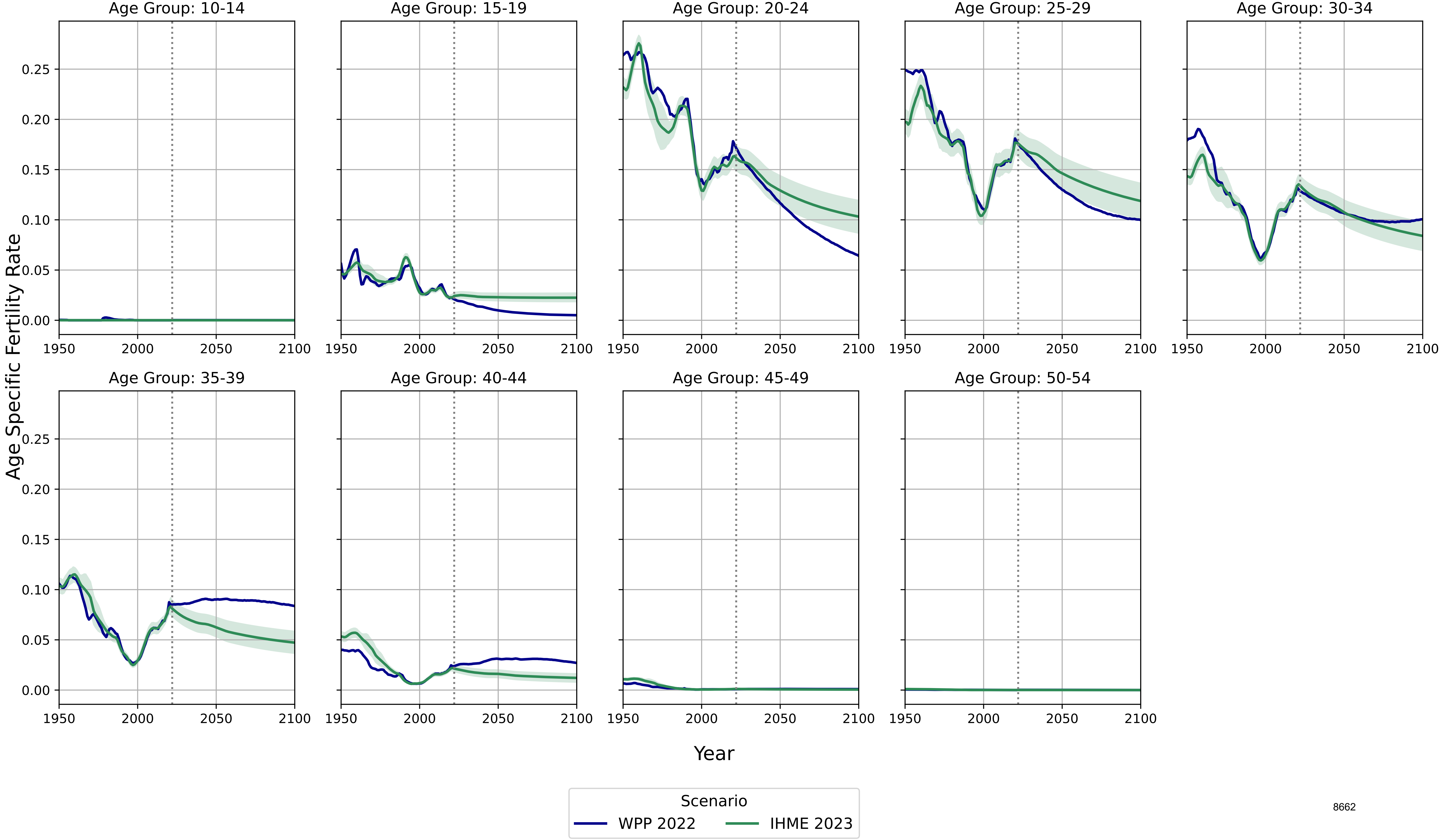

Supplemental Results Figure S8: Five-year interval ASFR from IHME reference scenario and the UNPD medium variant scenario by location, 1950-2100

Kyrgyzstan

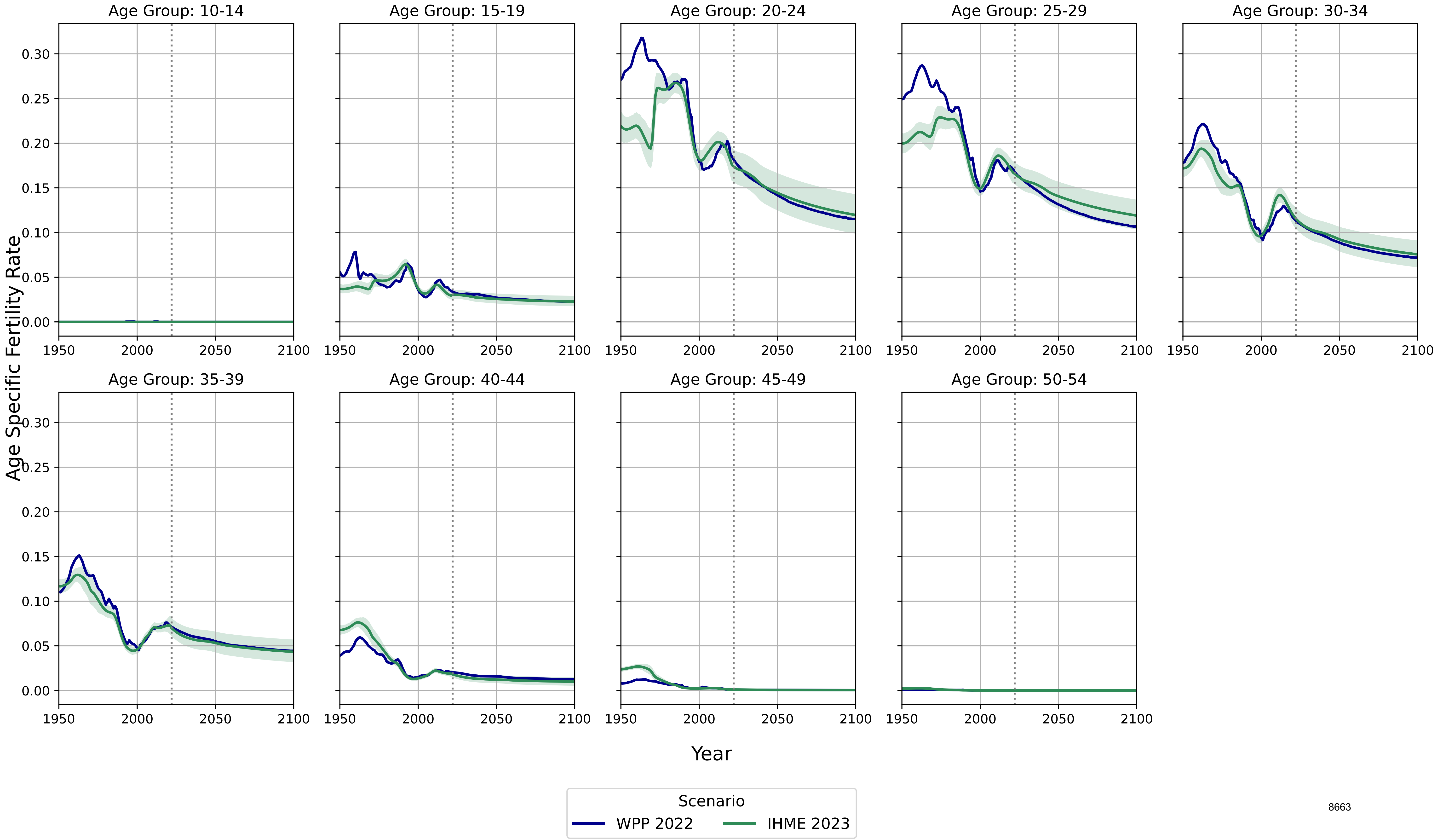

Supplemental Results Figure S8: Five-year interval ASFR from IHME reference scenario and the UNPD medium variant scenario by location, 1950-2100

Mongolia

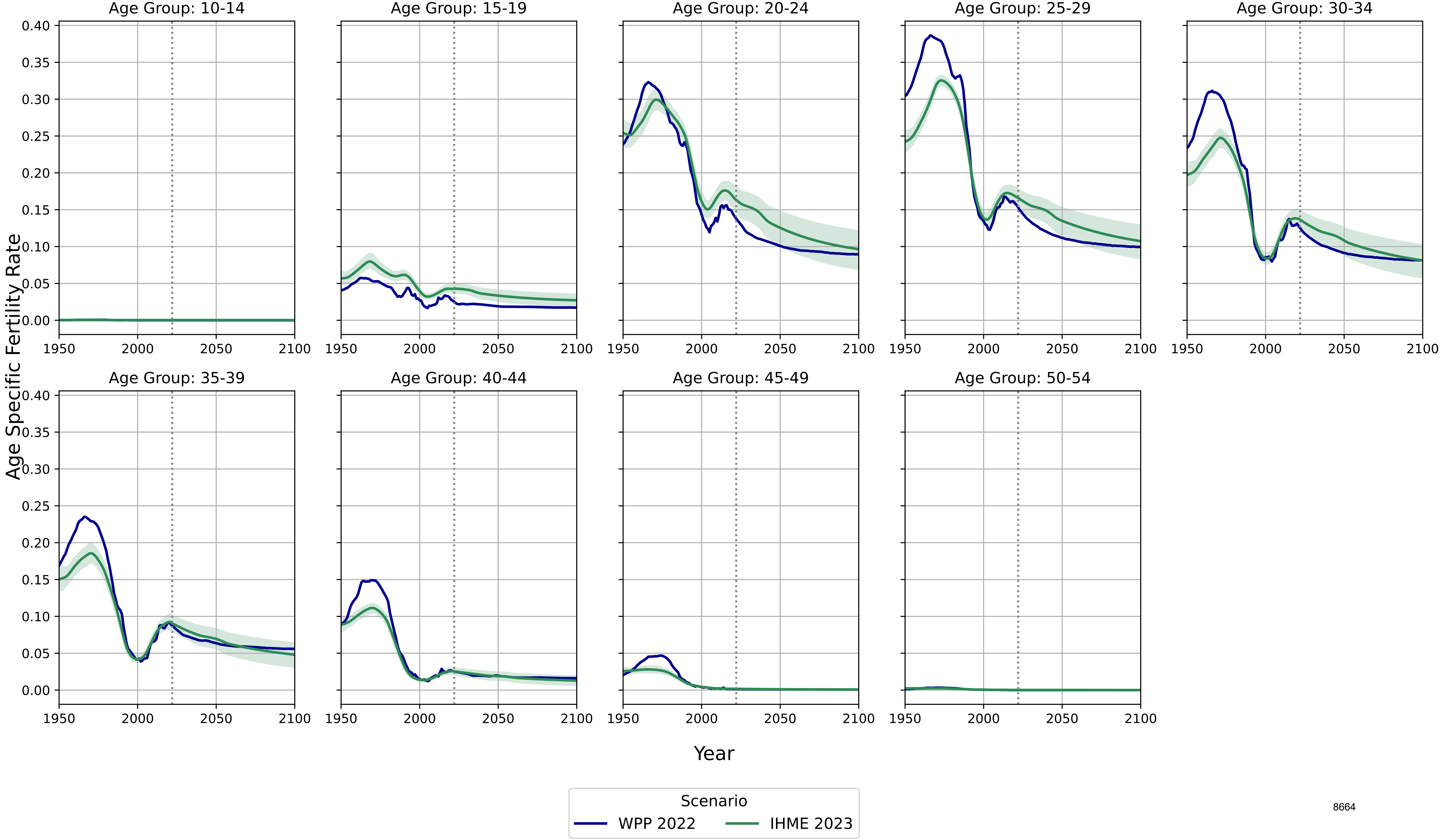

Supplemental Results Figure S8: Five-year interval ASFR from IHME reference scenario and the UNPD medium variant scenario by location, 1950-2100

Tajikistan

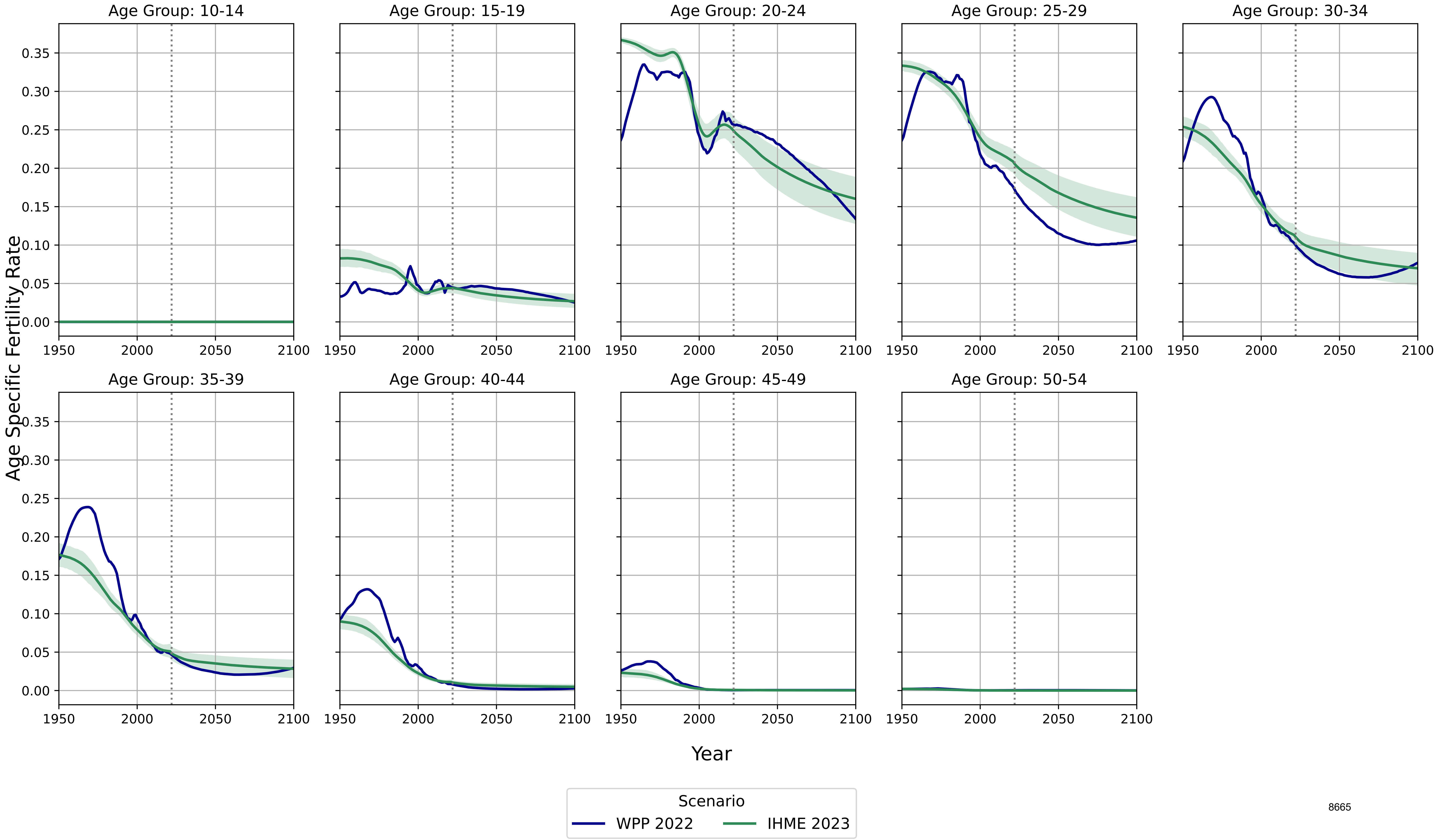

8665

Supplemental Results Figure S8: Five-year interval ASFR from IHME reference scenario and the UNPD medium variant scenario by location, 1950-2100

Turkmenistan

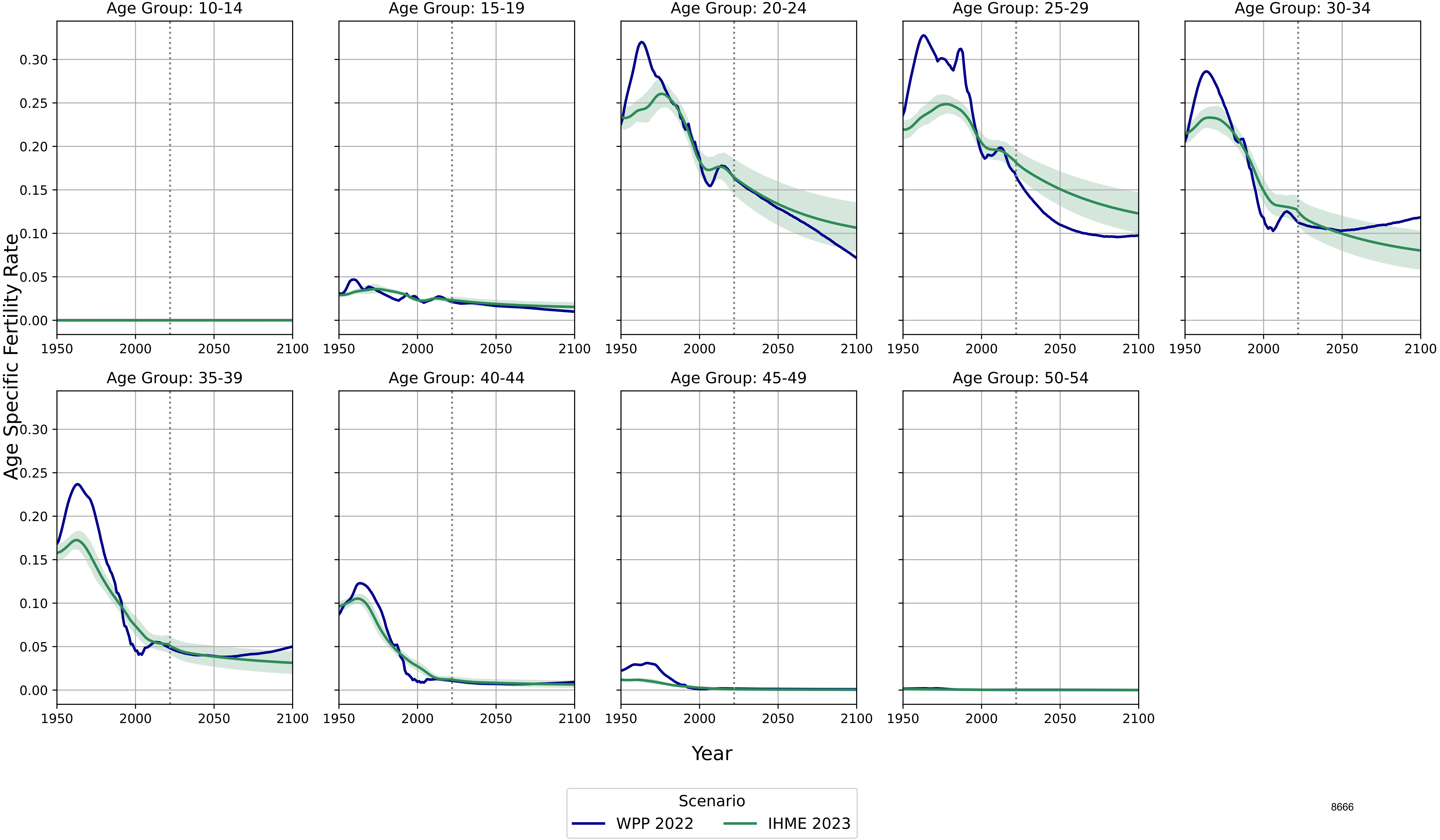

Supplemental Results Figure S8: Five-year interval ASFR from IHME reference scenario and the UNPD medium variant scenario by location, 1950-2100

Uzbekistan

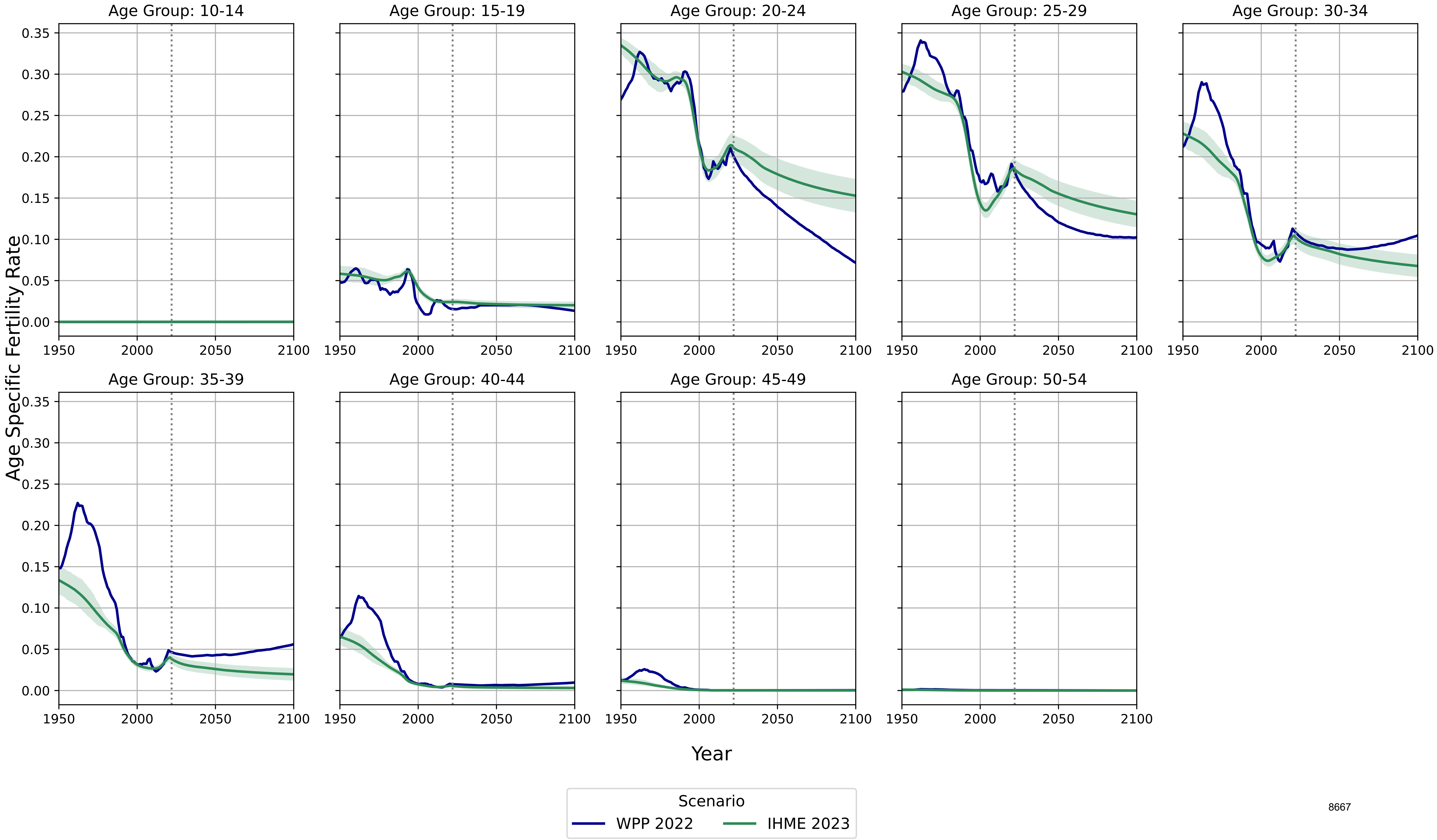

Supplemental Results Figure S8: Five-year interval ASFR from IHME reference scenario and the UNPD medium variant scenario by location, 1950-2100

Albania

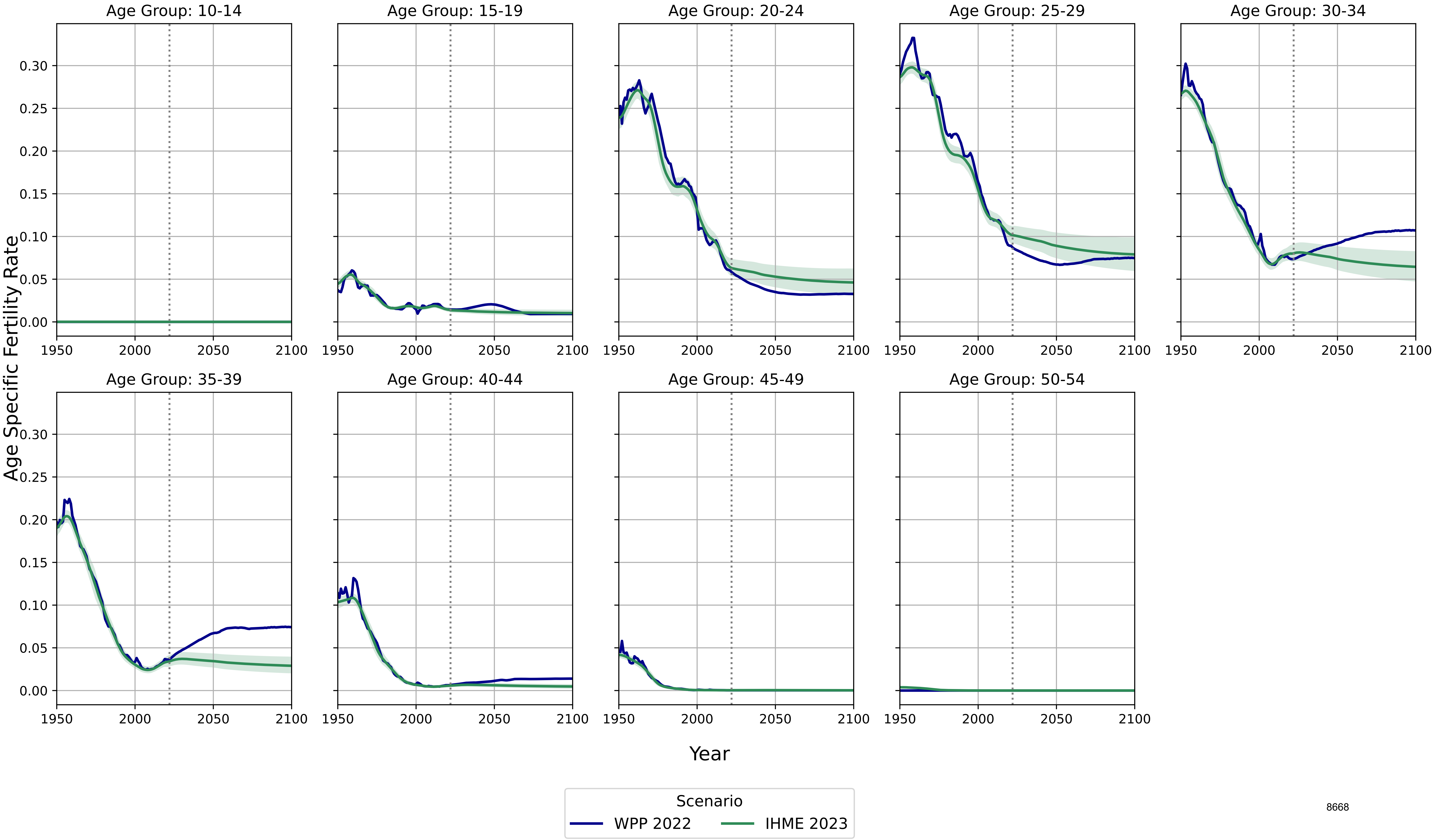

Supplemental Results Figure S8: Five-year interval ASFR from IHME reference scenario and the UNPD medium variant scenario by location, 1950-2100

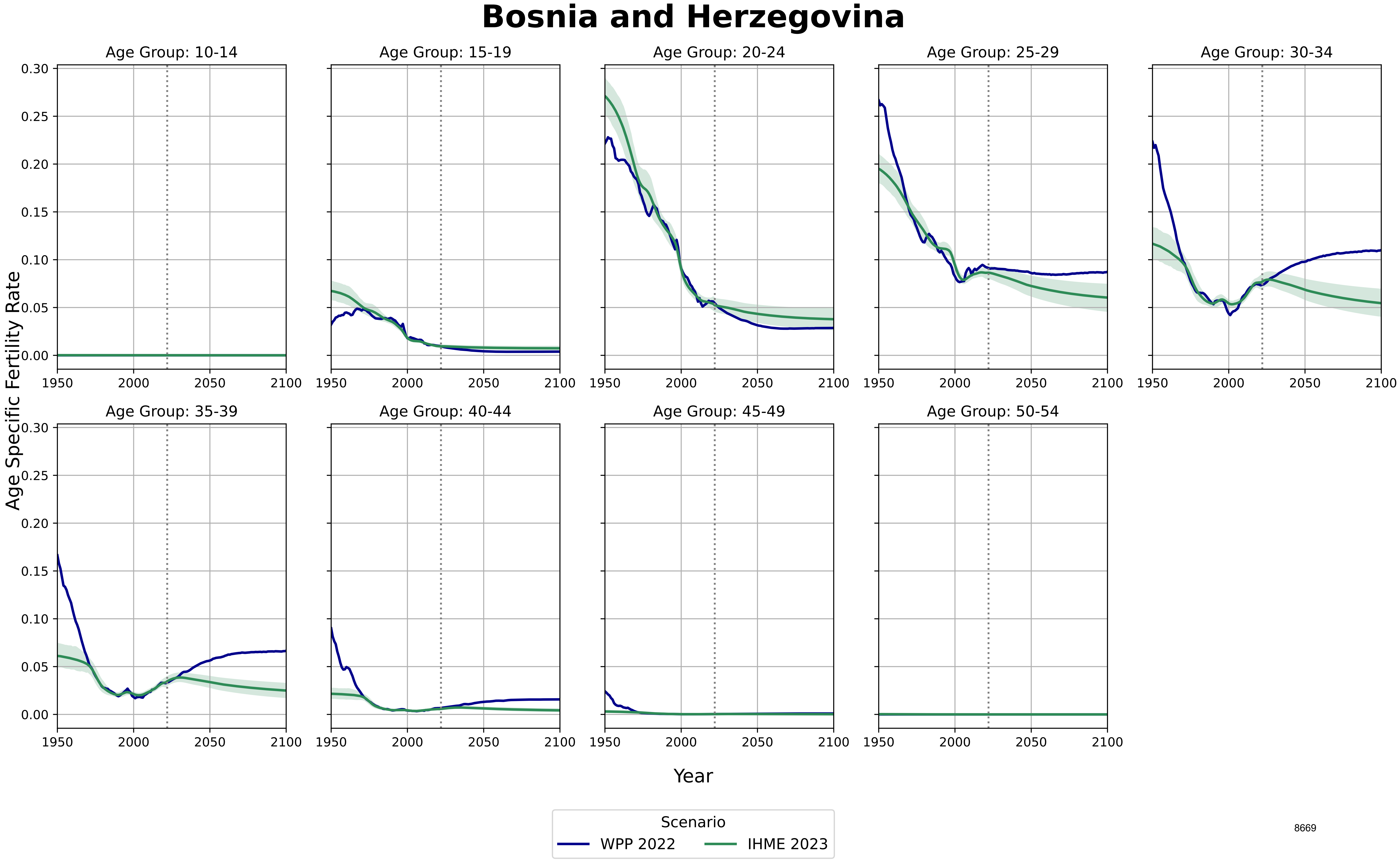

Supplemental Results Figure S8: Five-year interval ASFR from IHME reference scenario and the UNPD medium variant scenario by location, 1950-2100

Bulgaria

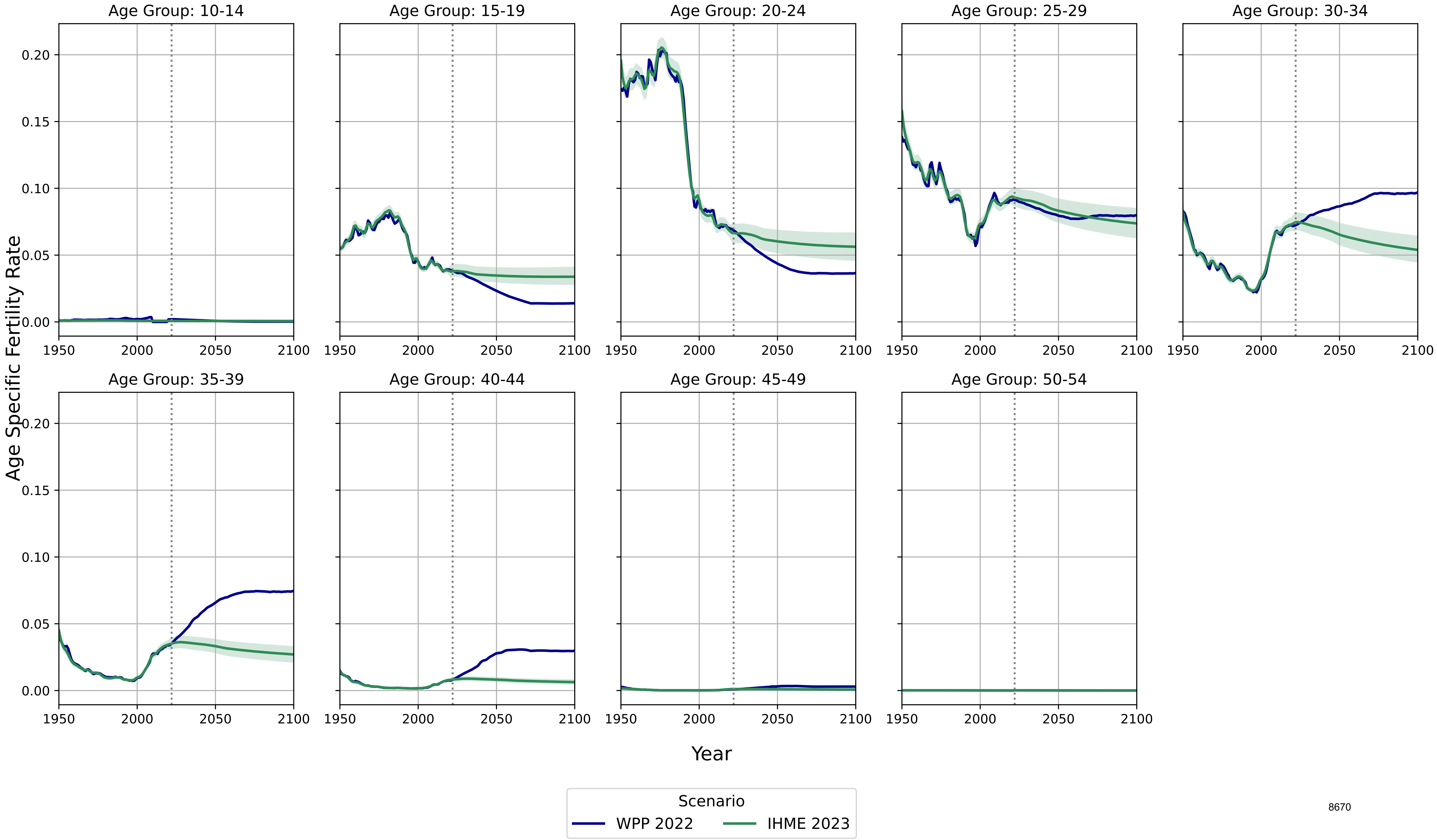

Supplemental Results Figure S8: Five-year interval ASFR from IHME reference scenario and the UNPD medium variant scenario by location, 1950-2100

Croatia

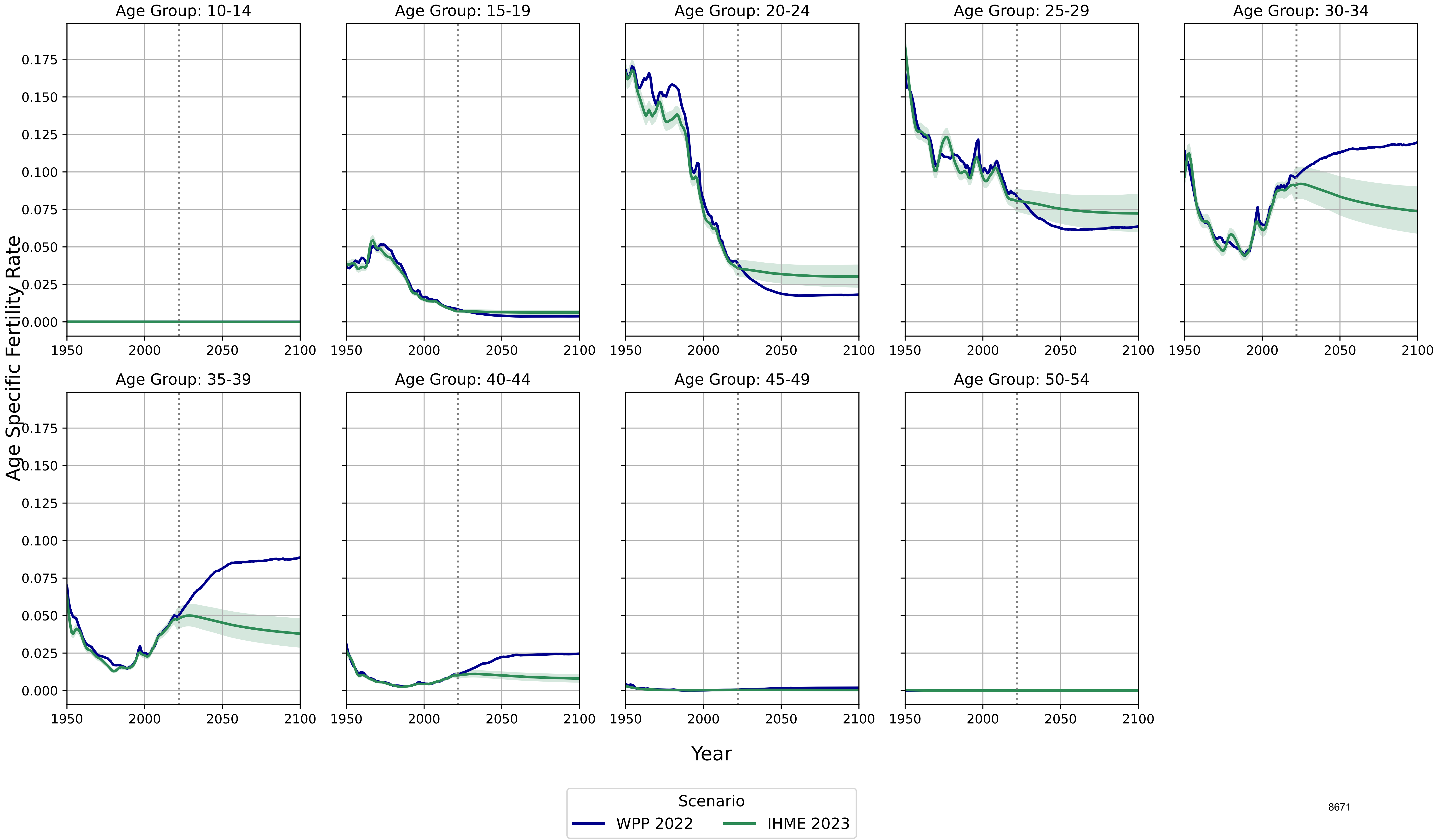

Supplemental Results Figure S8: Five-year interval ASFR from IHME reference scenario and the UNPD medium variant scenario by location, 1950-2100

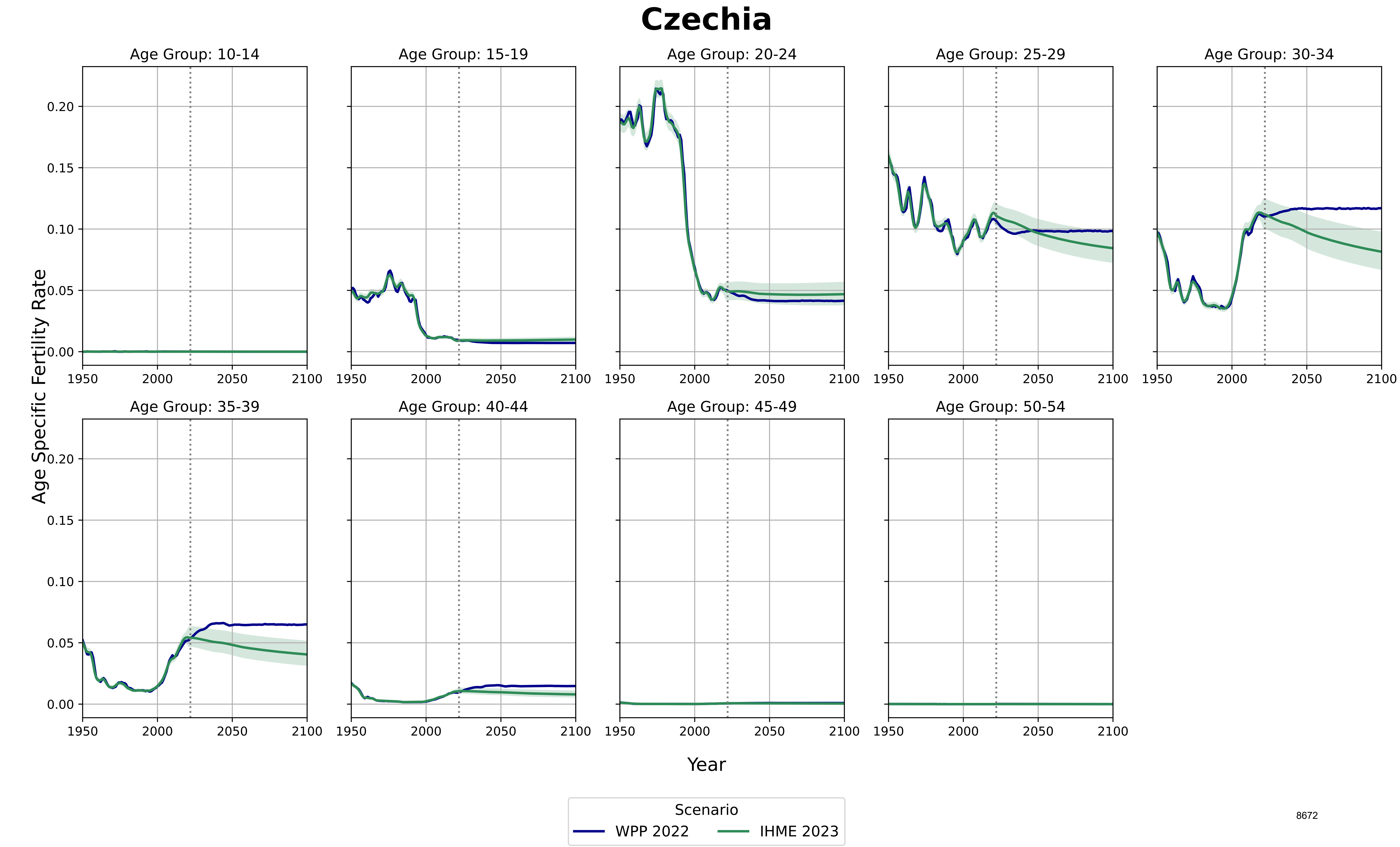

Supplemental Results Figure S8: Five-year interval ASFR from IHME reference scenario and the UNPD medium variant scenario by location, 1950-2100

Hungary

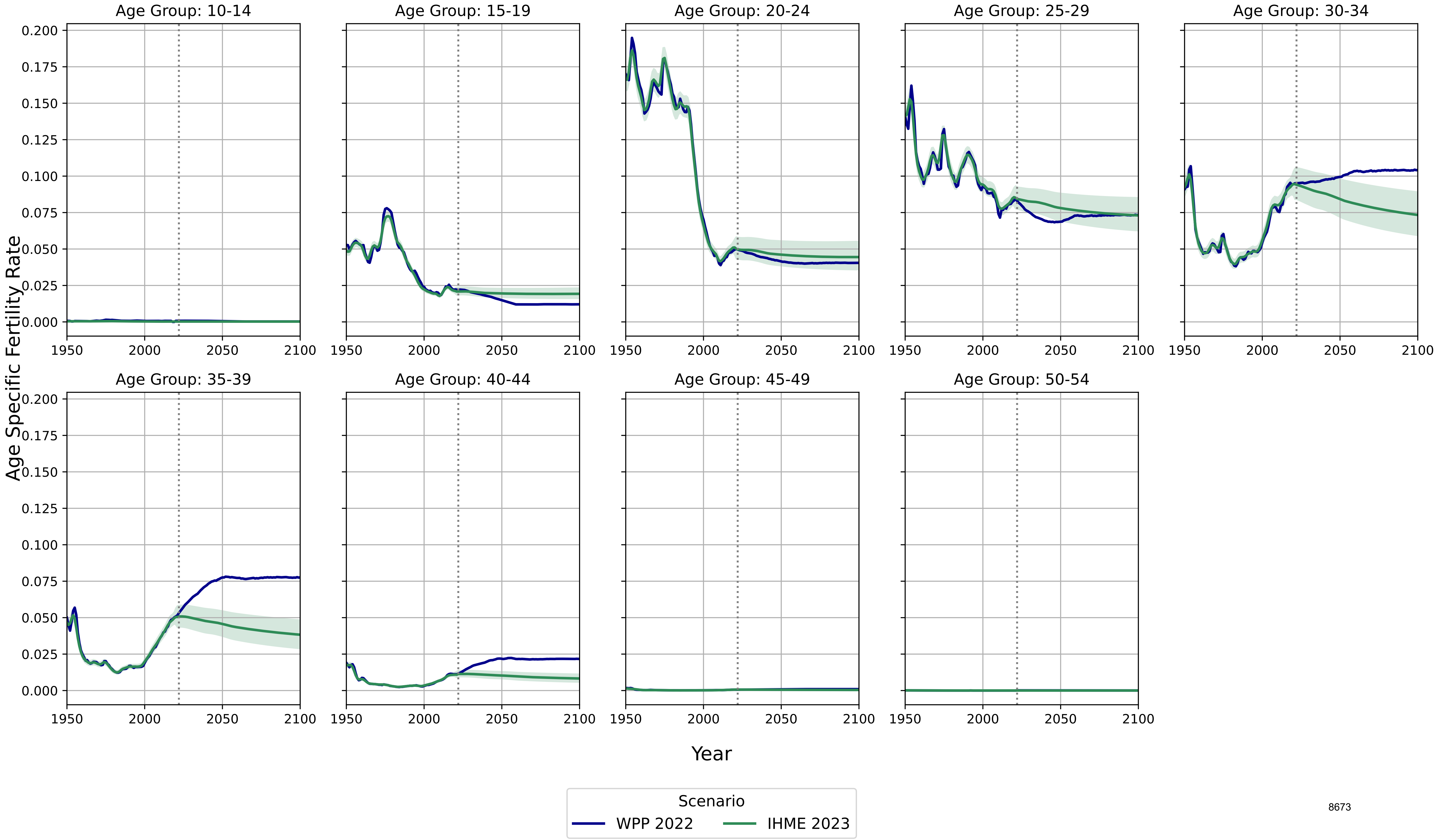

Supplemental Results Figure S8: Five-year interval ASFR from IHME reference scenario and the UNPD medium variant scenario by location, 1950-2100

Montenegro

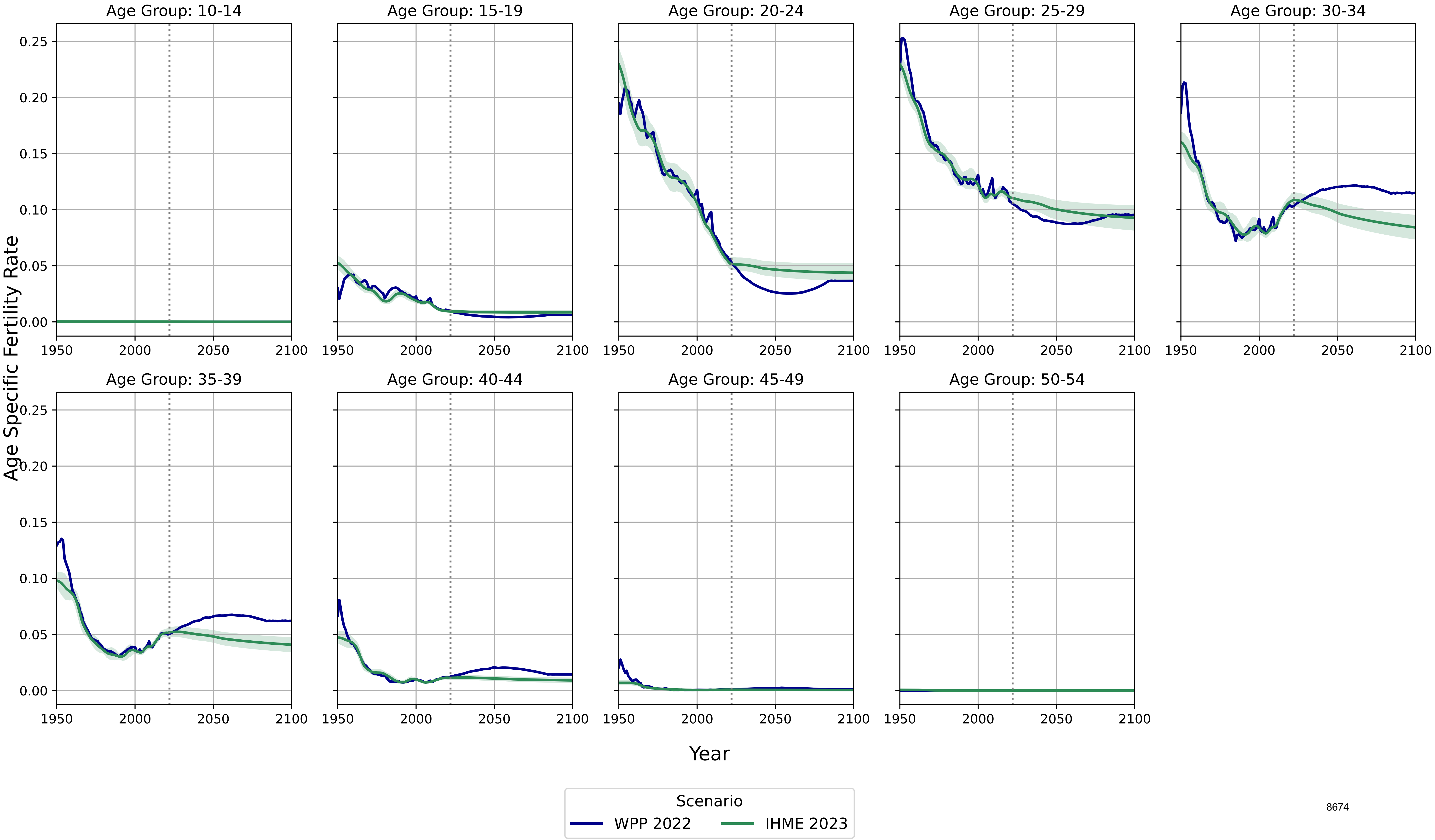

Supplemental Results Figure S8: Five-year interval ASFR from IHME reference scenario and the UNPD medium variant scenario by location, 1950-2100

North Macedonia

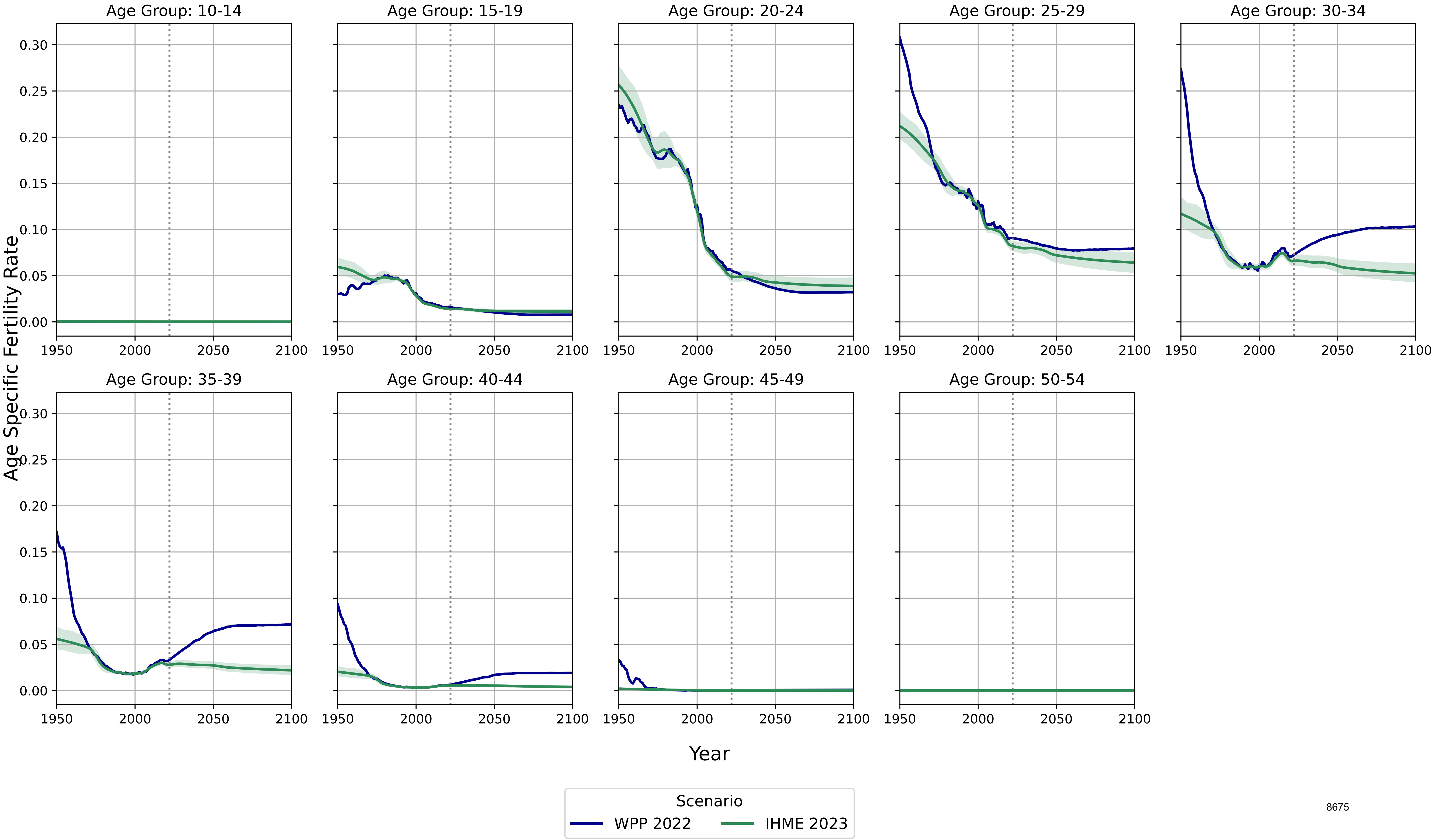

Supplemental Results Figure S8: Five-year interval ASFR from IHME reference scenario and the UNPD medium variant scenario by location, 1950-2100

Poland

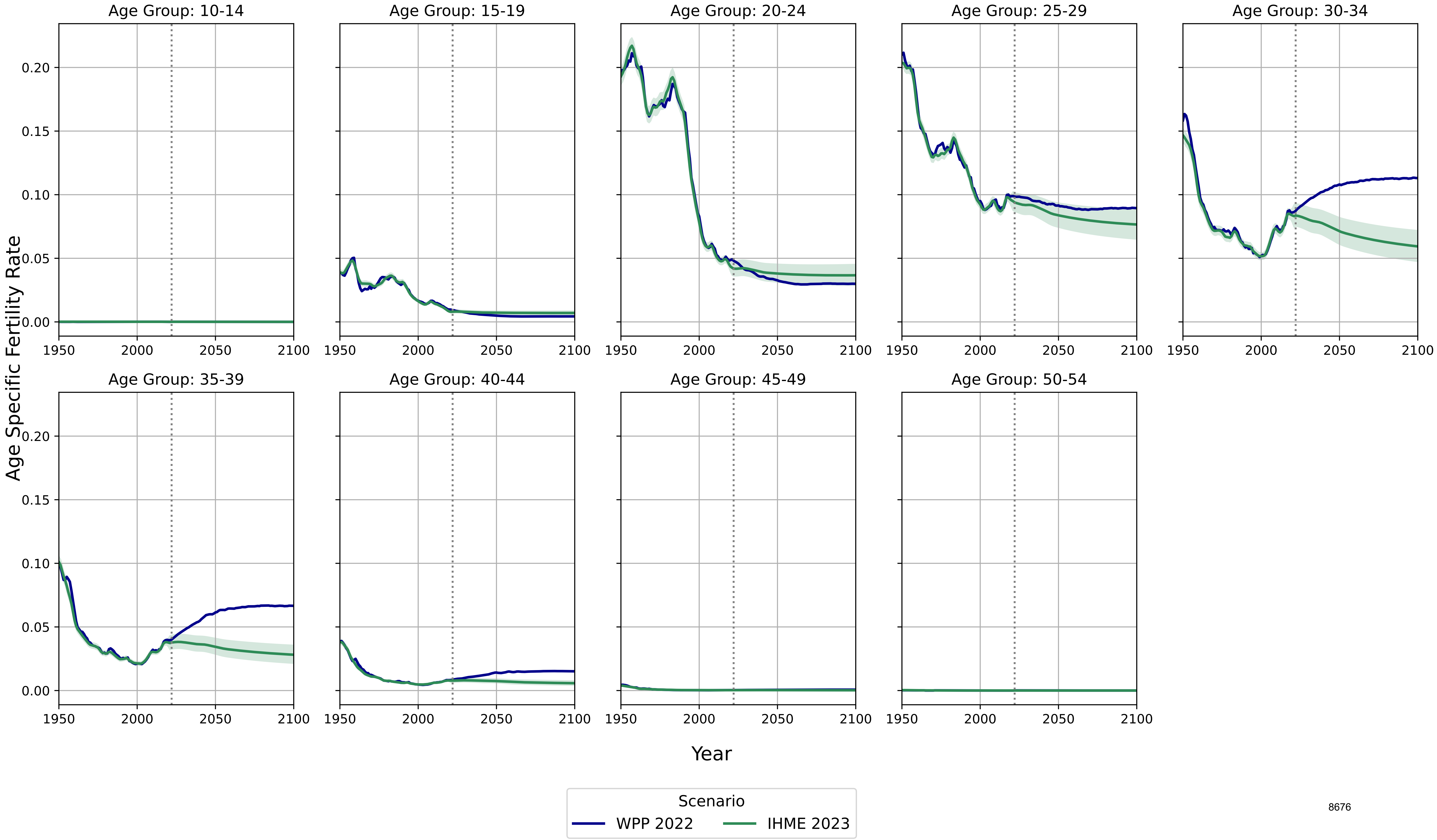

Supplemental Results Figure S8: Five-year interval ASFR from IHME reference scenario and the UNPD medium variant scenario by location, 1950-2100

Romania

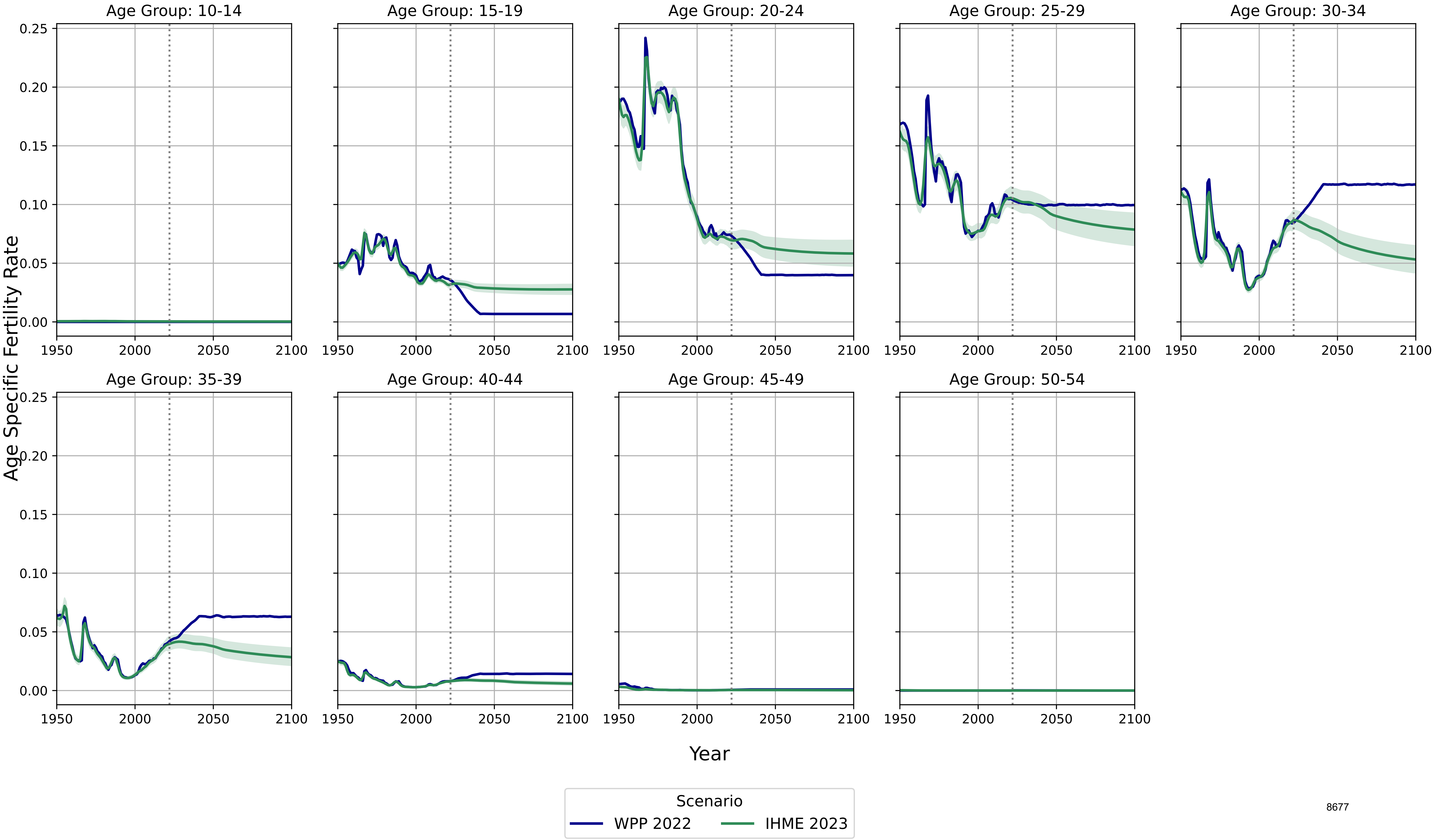

Supplemental Results Figure S8: Five-year interval ASFR from IHME reference scenario and the UNPD medium variant scenario by location, 1950-2100

Serbia

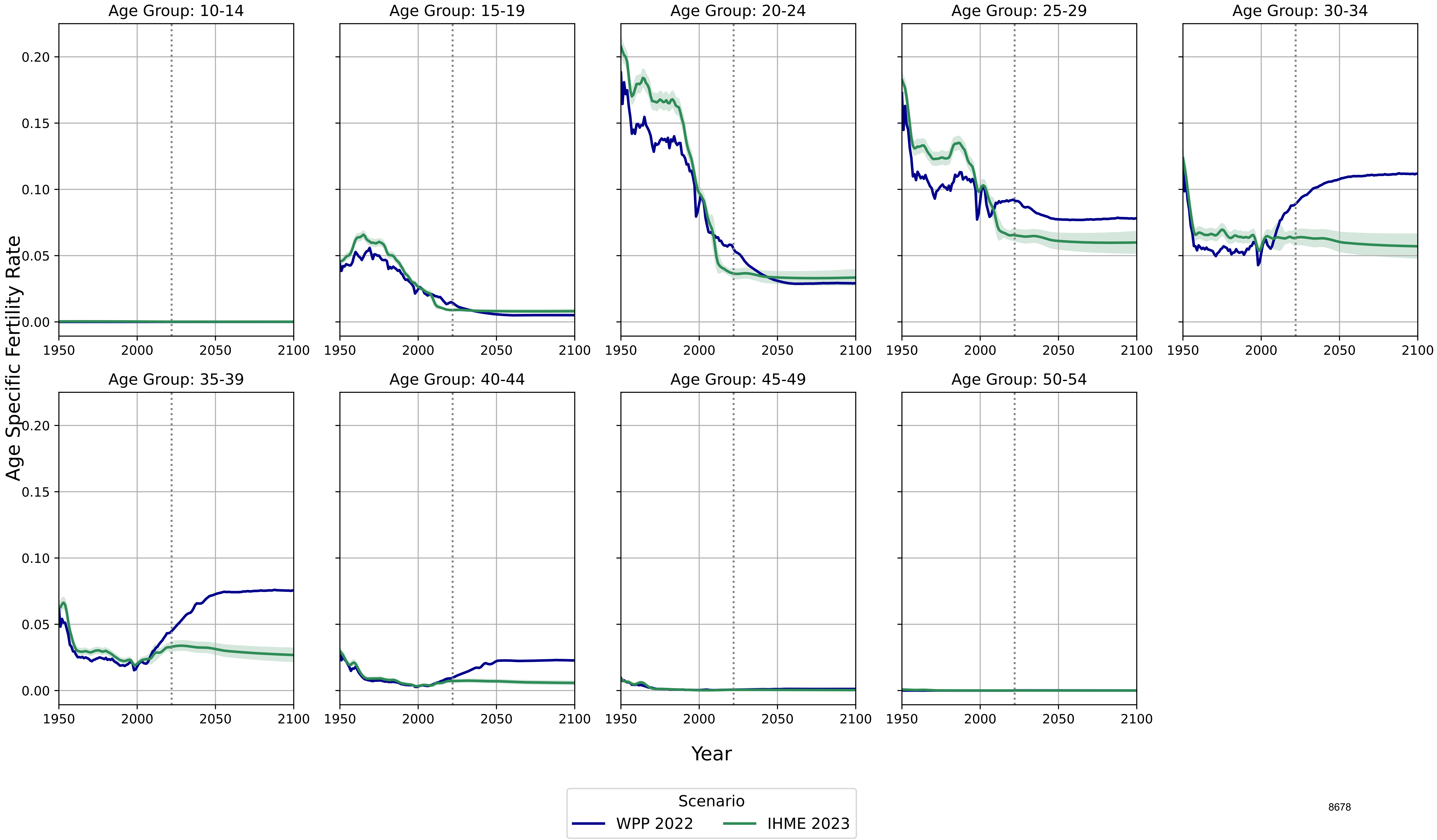

Supplemental Results Figure S8: Five-year interval ASFR from IHME reference scenario and the UNPD medium variant scenario by location, 1950-2100

Slovakia

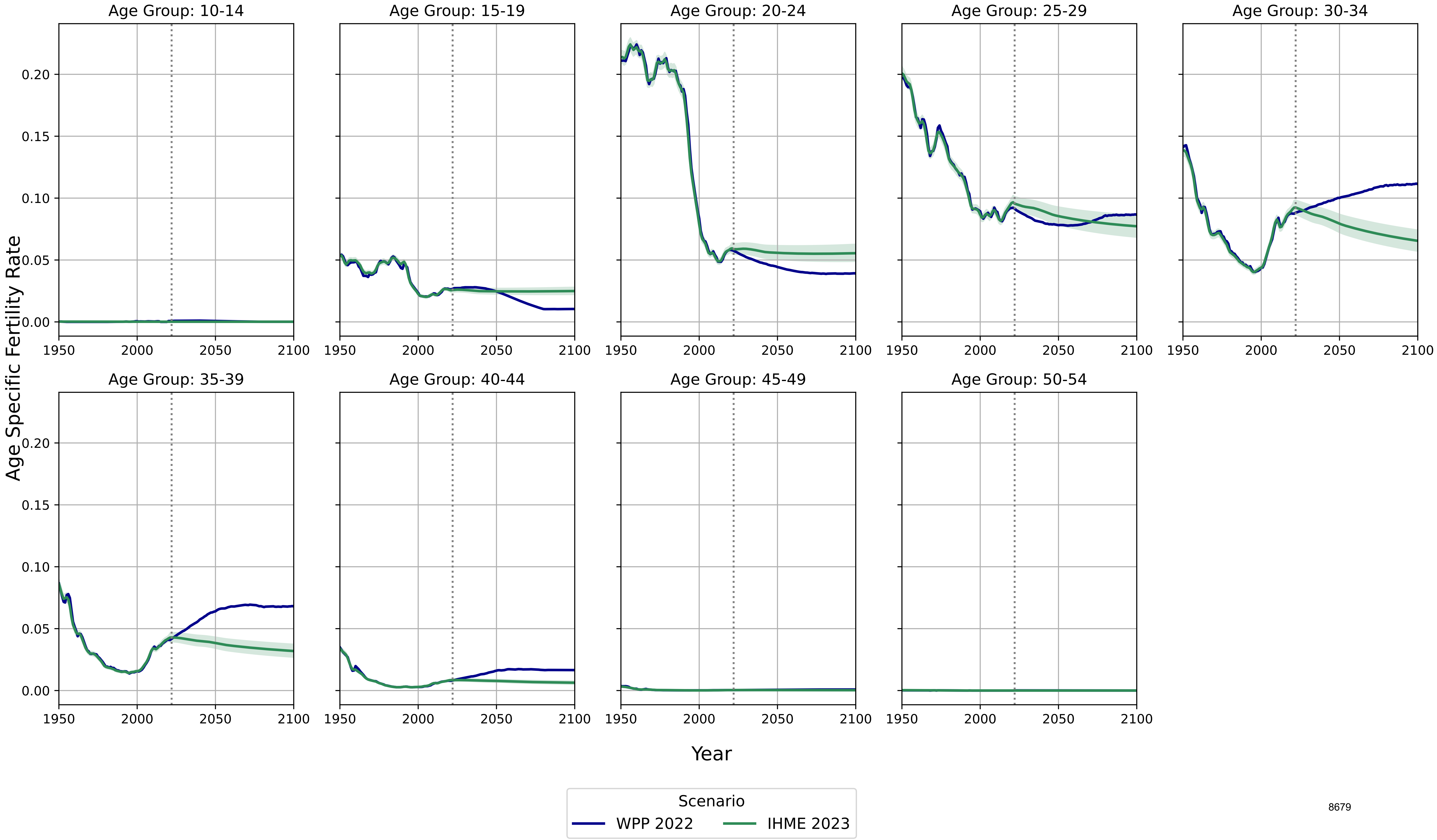

Supplemental Results Figure S8: Five-year interval ASFR from IHME reference scenario and the UNPD medium variant scenario by location, 1950-2100

Slovenia

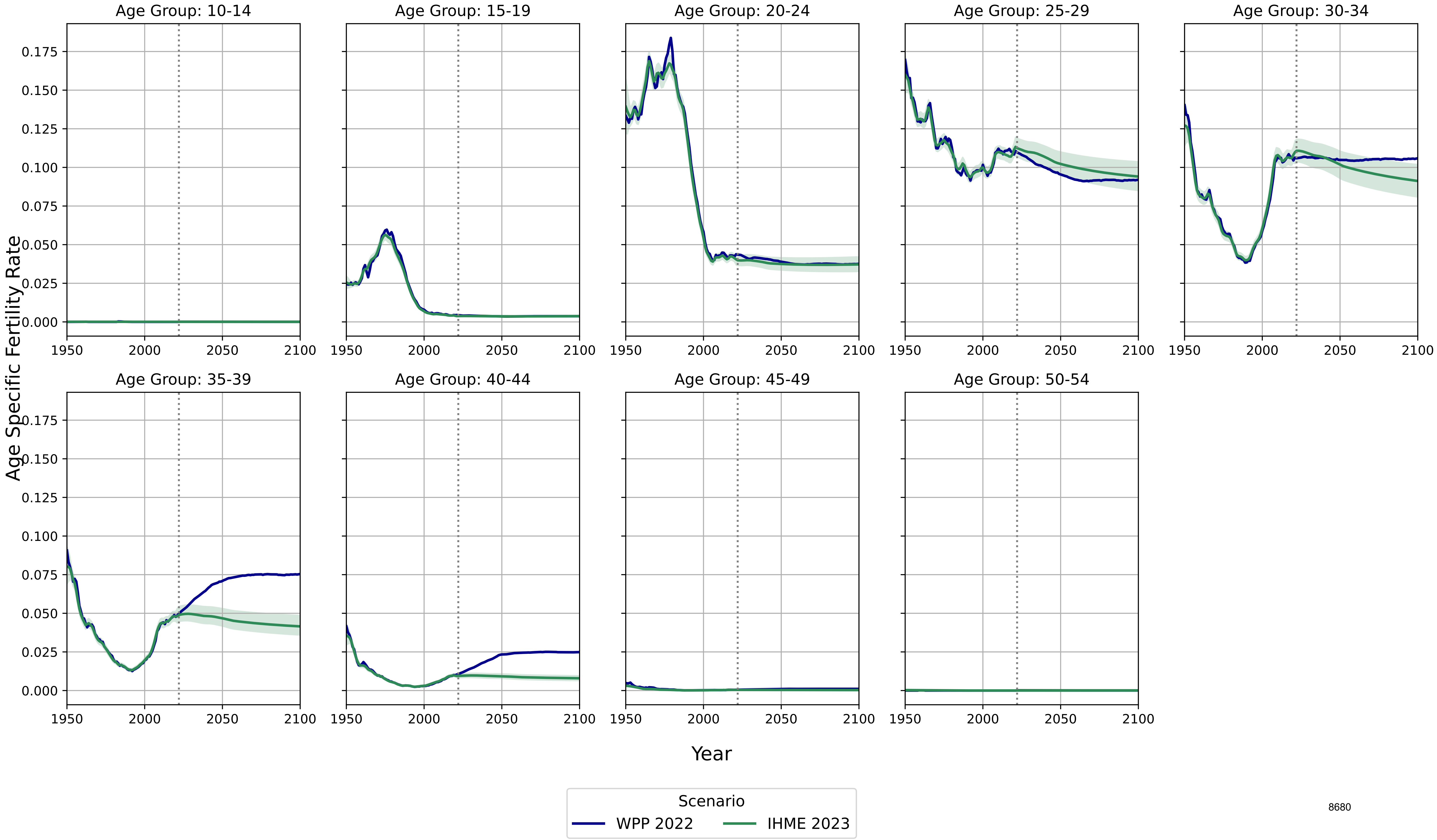

Supplemental Results Figure S8: Five-year interval ASFR from IHME reference scenario and the UNPD medium variant scenario by location, 1950-2100

Belarus

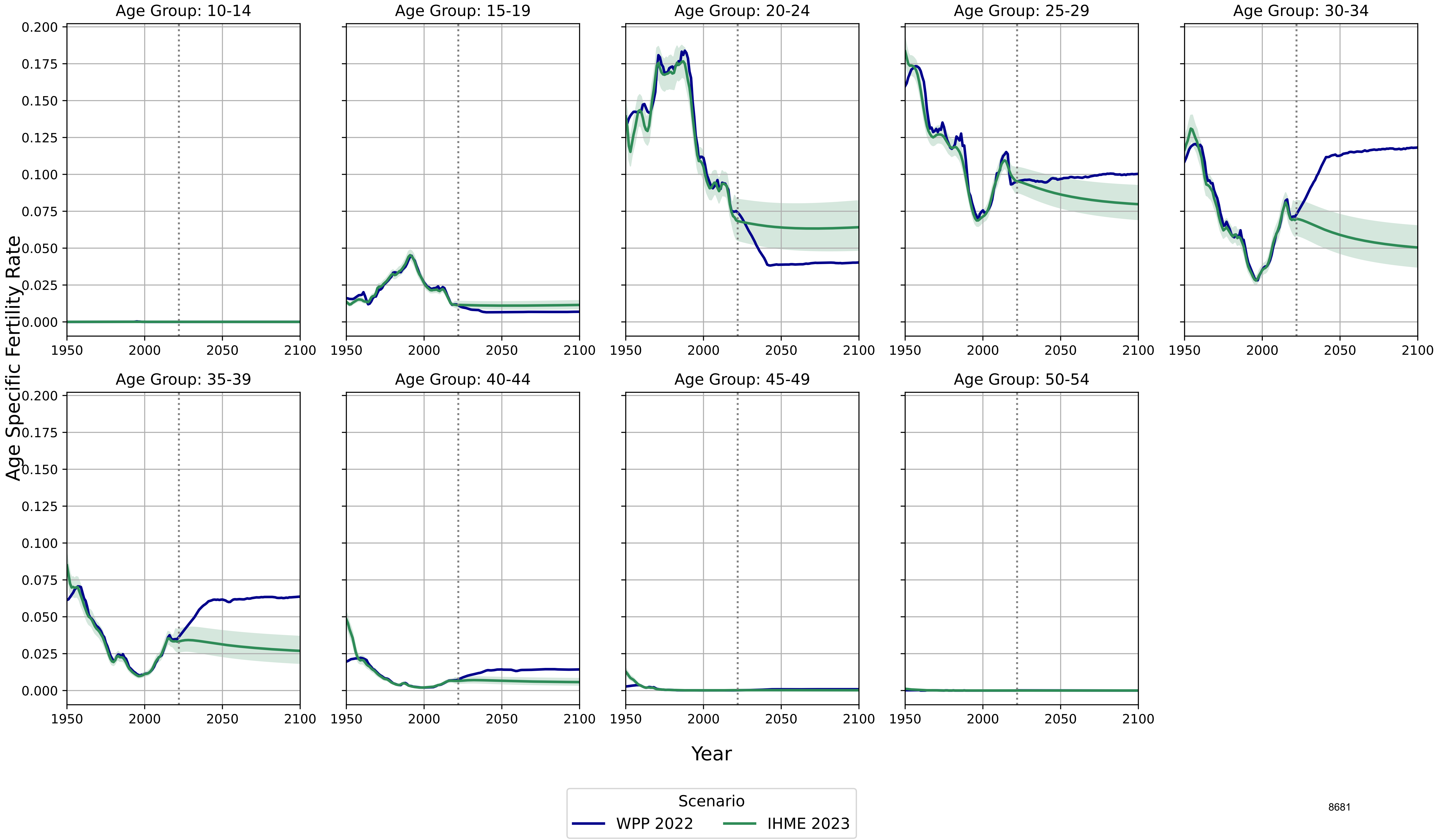

Supplemental Results Figure S8: Five-year interval ASFR from IHME reference scenario and the UNPD medium variant scenario by location, 1950-2100

Estonia

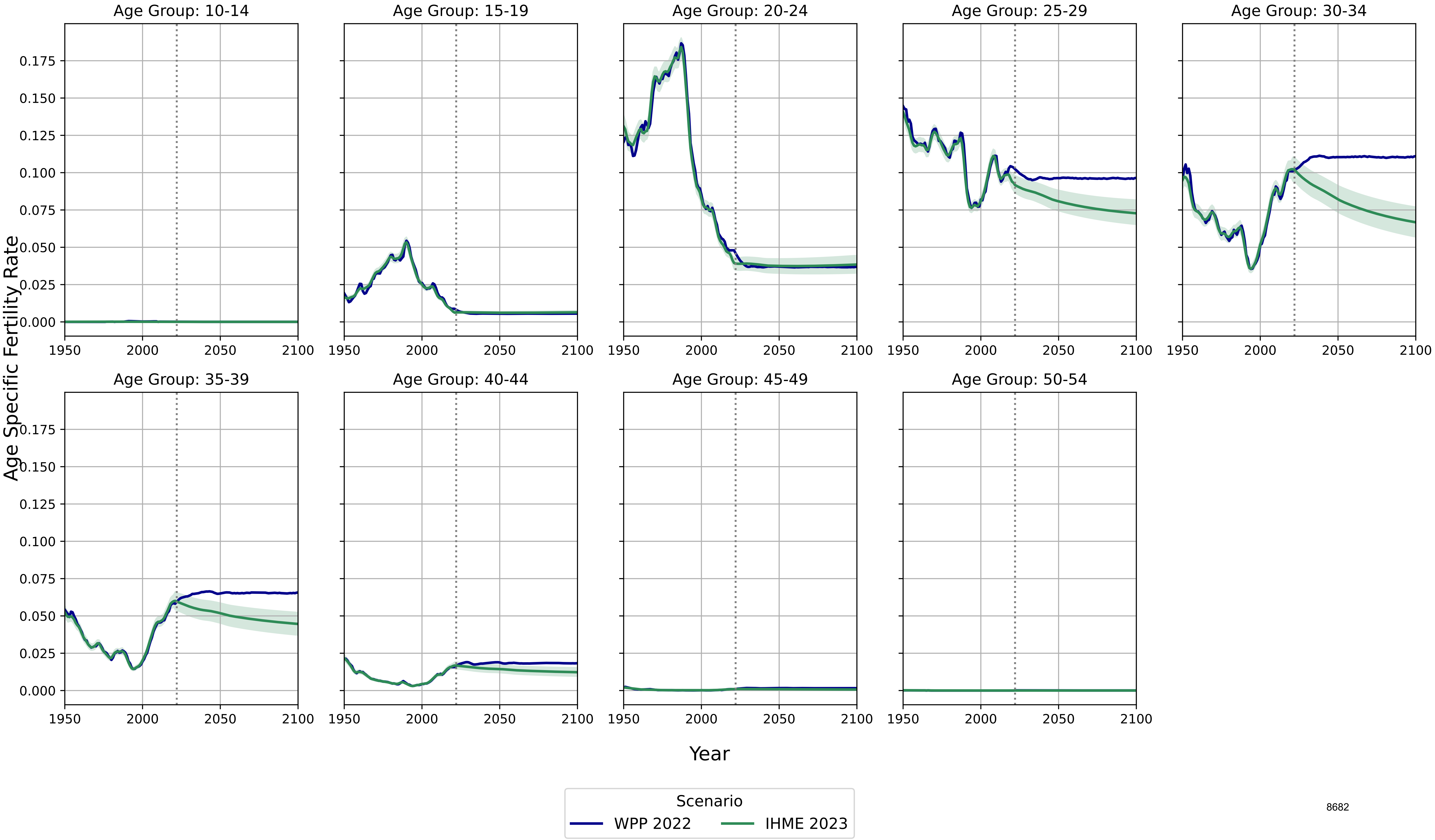

Supplemental Results Figure S8: Five-year interval ASFR from IHME reference scenario and the UNPD medium variant scenario by location, 1950-2100

Latvia

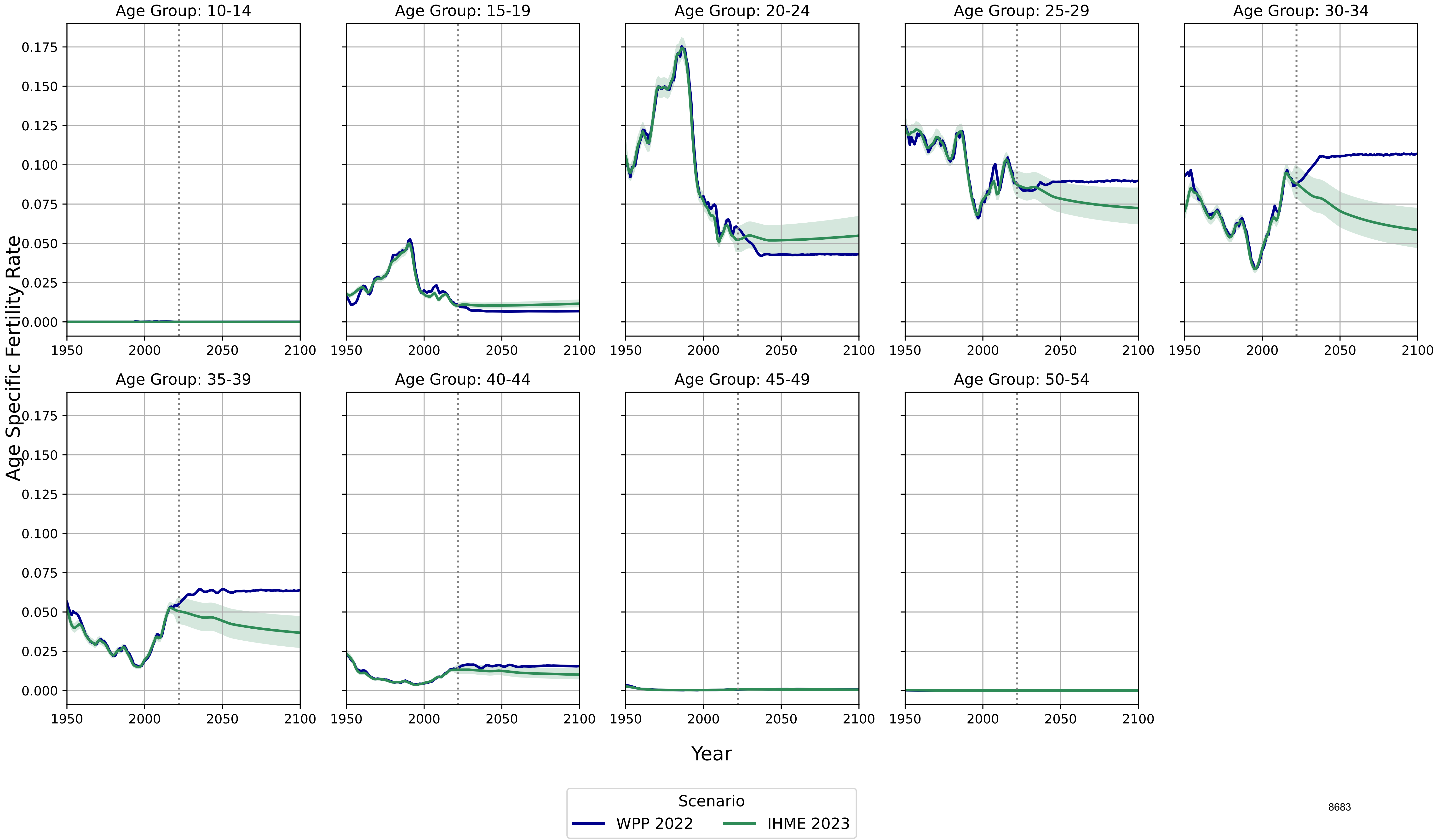

Supplemental Results Figure S8: Five-year interval ASFR from IHME reference scenario and the UNPD medium variant scenario by location, 1950-2100

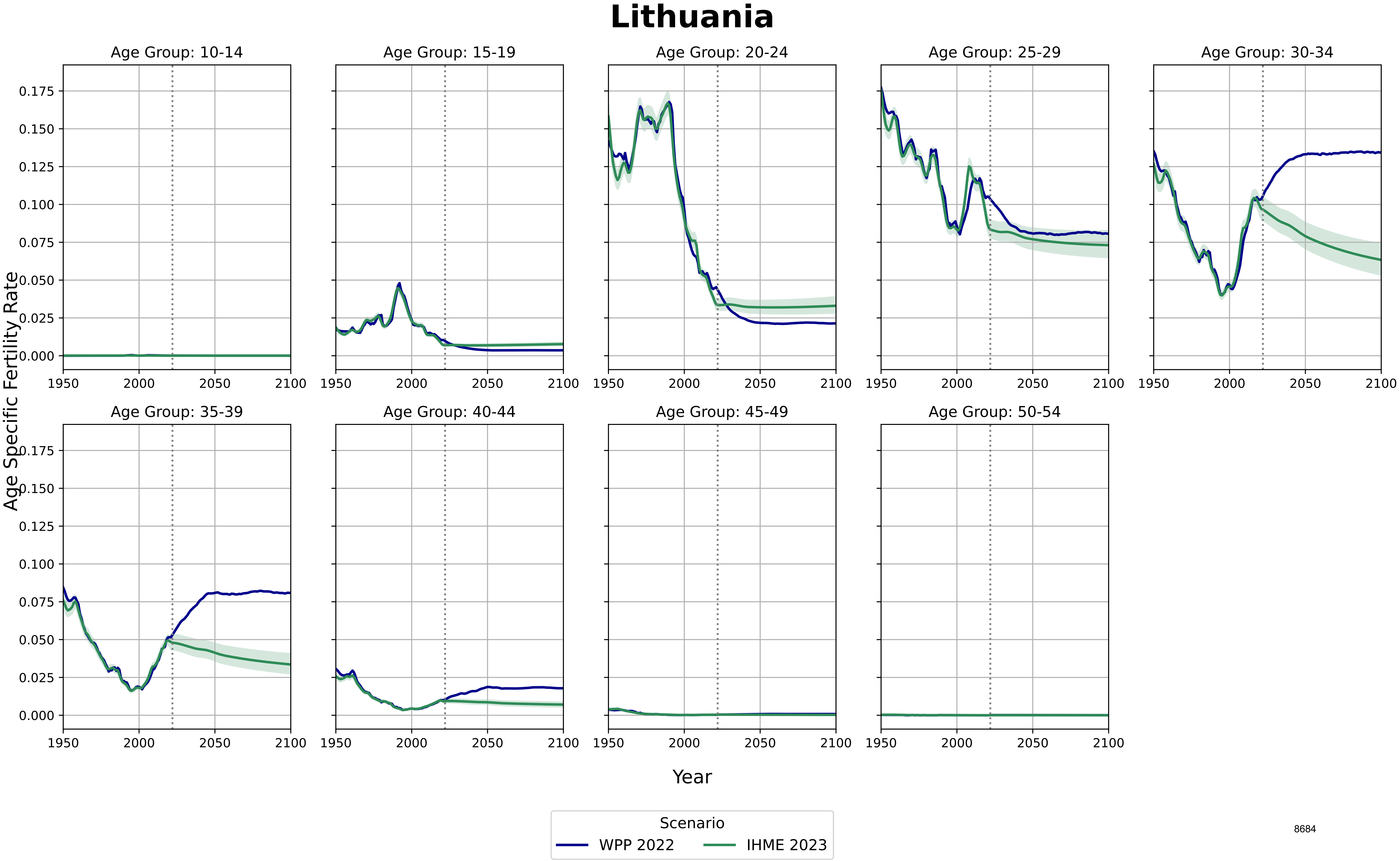

Supplemental Results Figure S8: Five-year interval ASFR from IHME reference scenario and the UNPD medium variant scenario by location, 1950-2100

Moldova

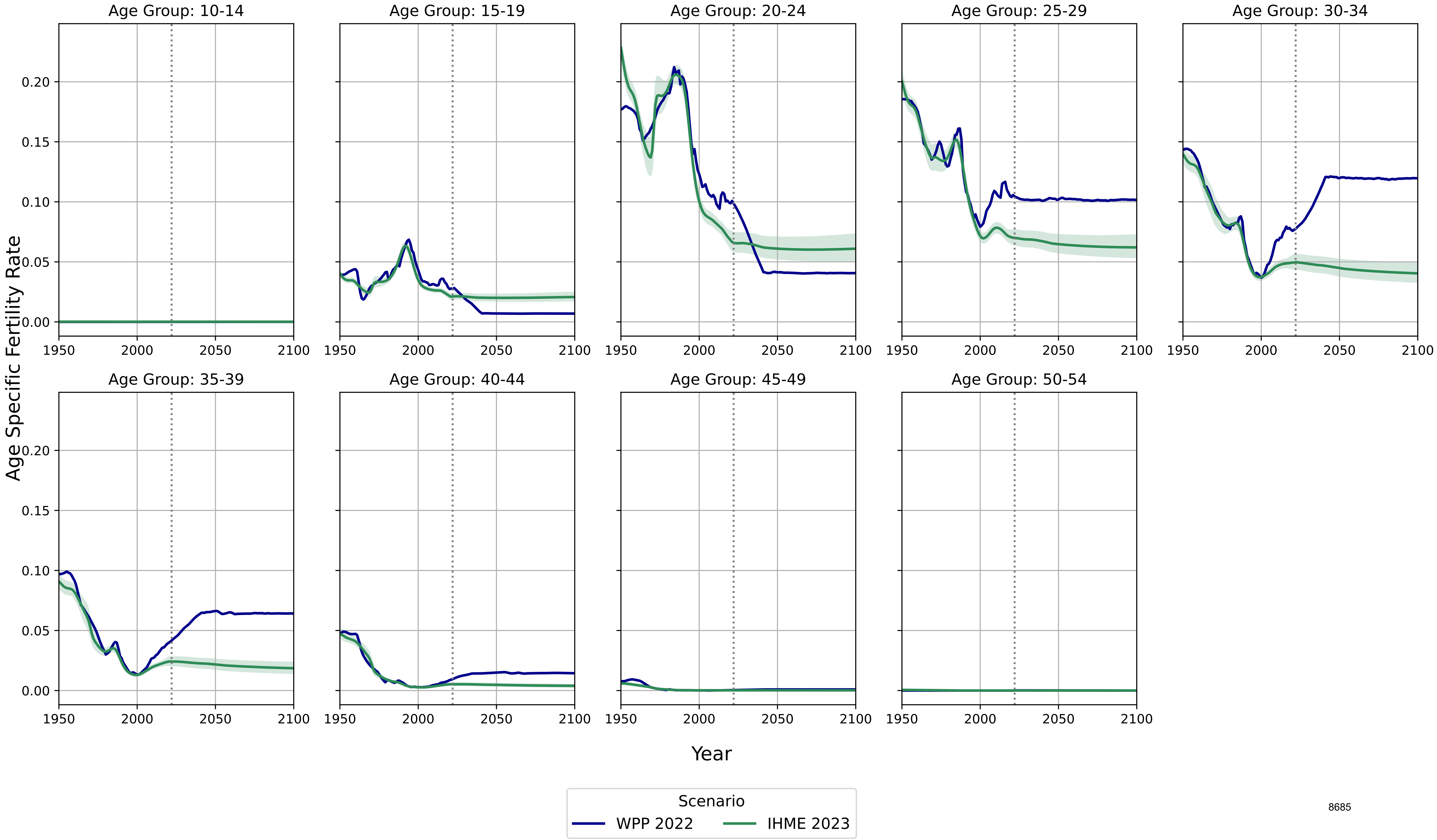

Supplemental Results Figure S8: Five-year interval ASFR from IHME reference scenario and the UNPD medium variant scenario by location, 1950-2100

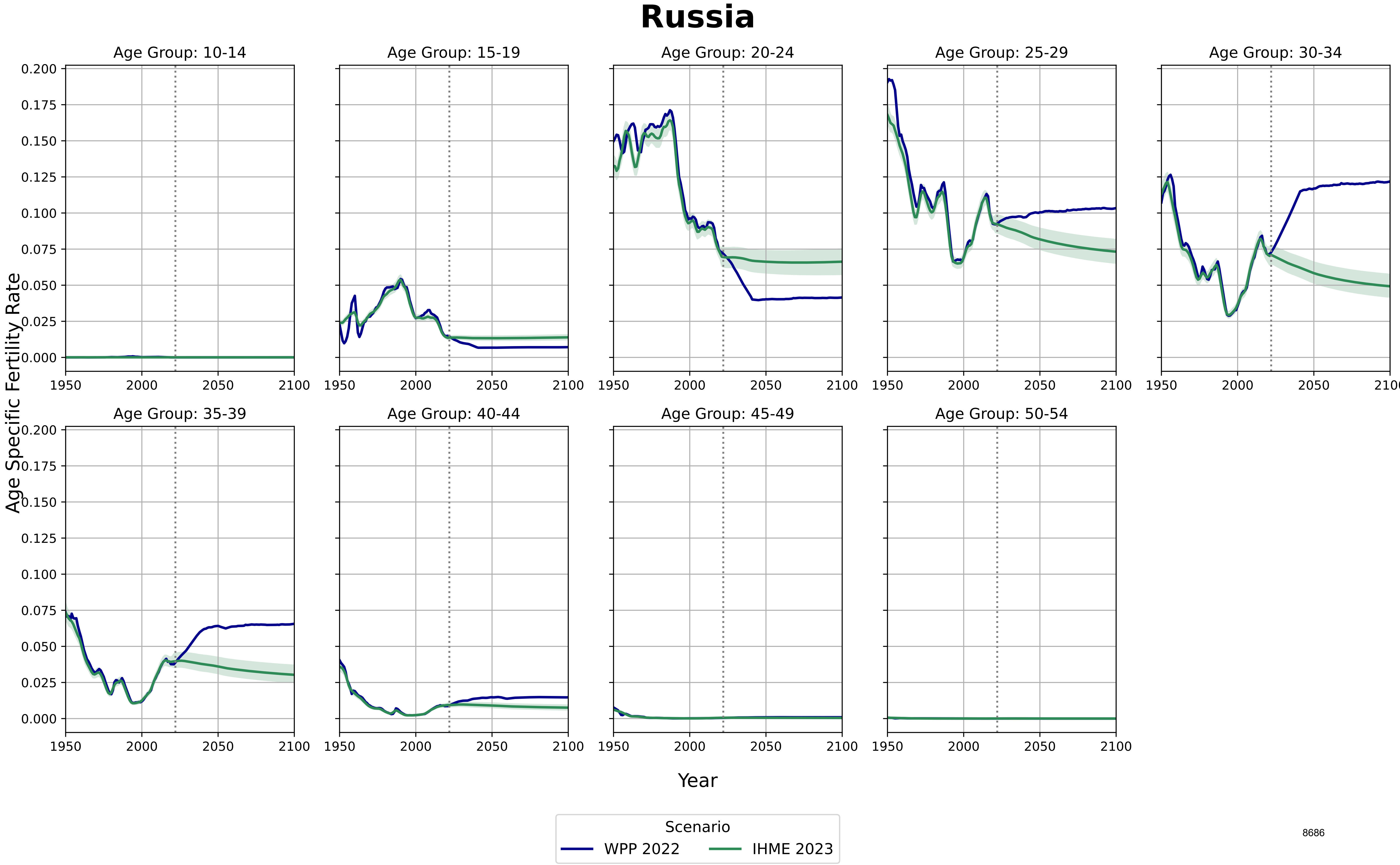

Supplemental Results Figure S8: Five-year interval ASFR from IHME reference scenario and the UNPD medium variant scenario by location, 1950-2100

Ukraine

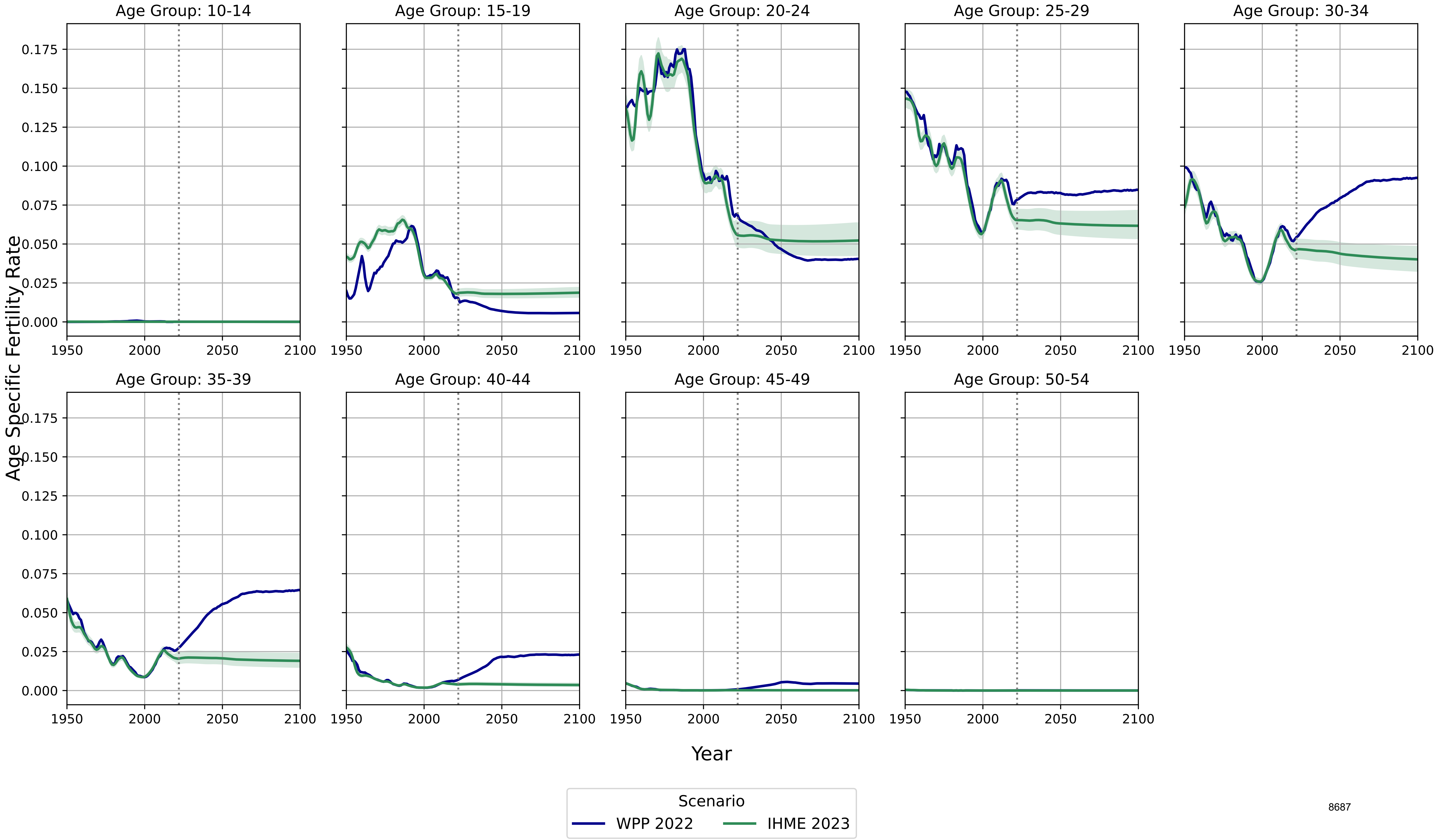

Supplemental Results Figure S8: Five-year interval ASFR from IHME reference scenario and the UNPD medium variant scenario by location, 1950-2100

Australia

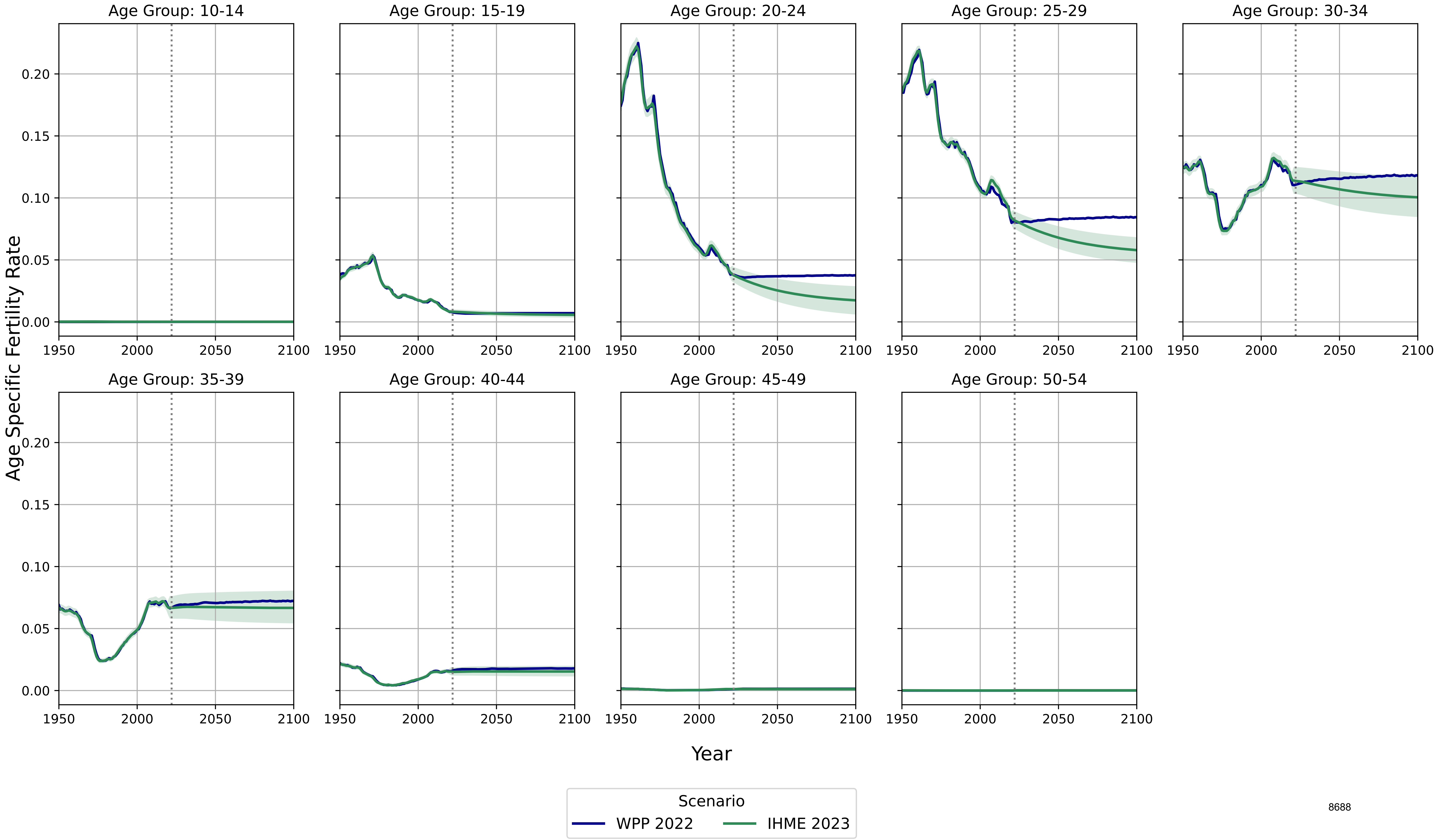

Supplemental Results Figure S8: Five-year interval ASFR from IHME reference scenario and the UNPD medium variant scenario by location, 1950-2100

New Zealand

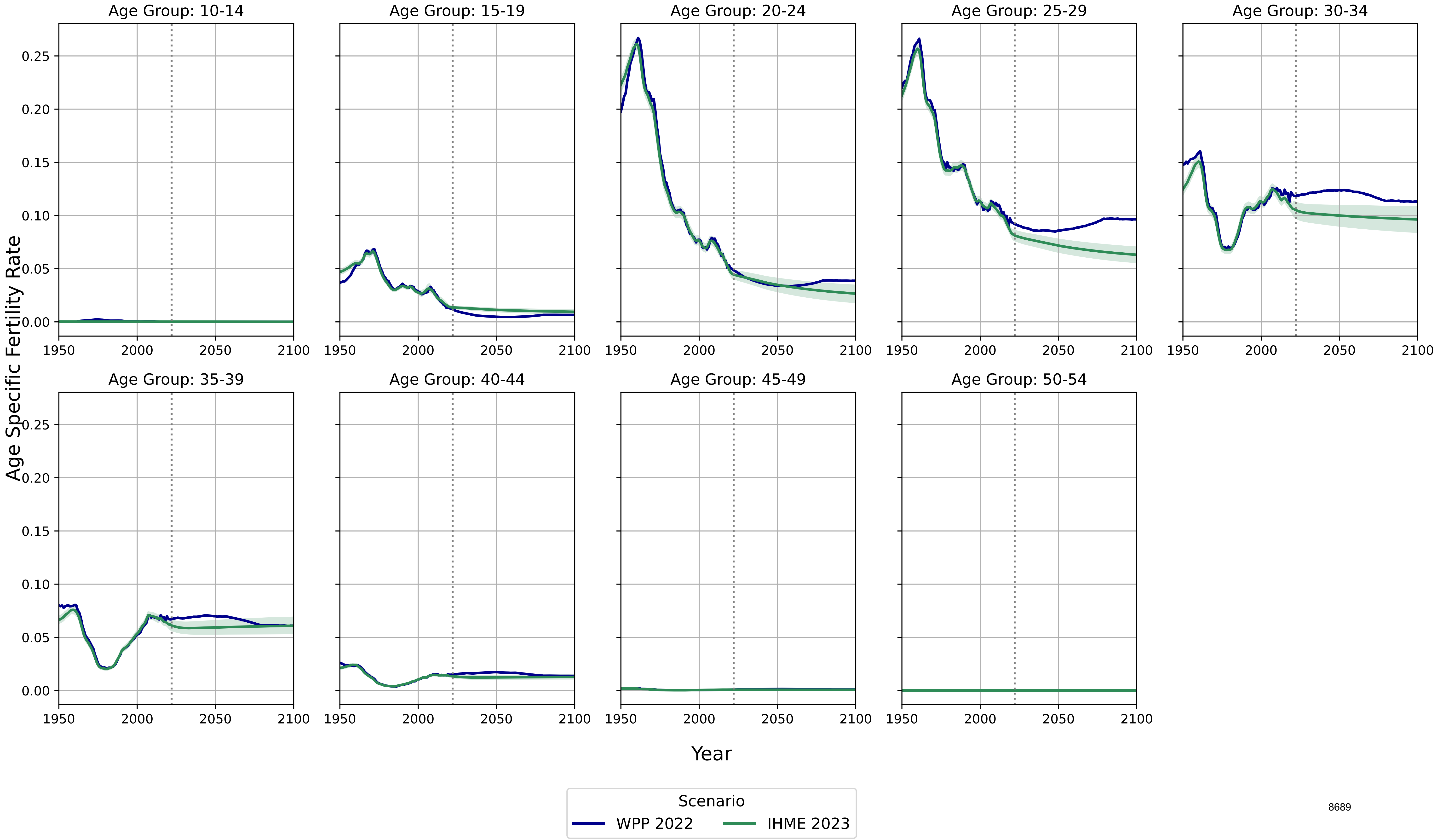

Supplemental Results Figure S8: Five-year interval ASFR from IHME reference scenario and the UNPD medium variant scenario by location, 1950-2100

Brunei

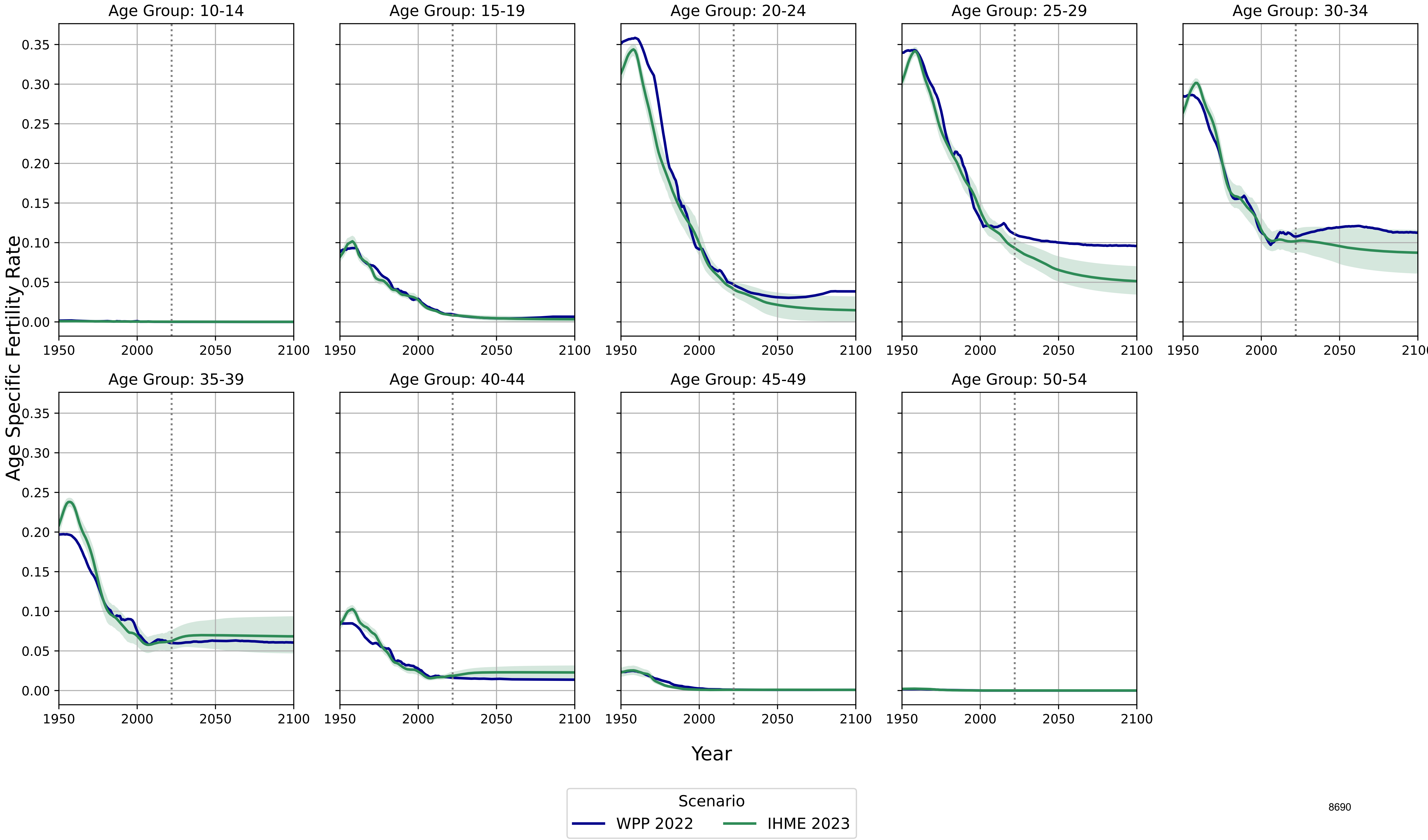

8690

Supplemental Results Figure S8: Five-year interval ASFR from IHME reference scenario and the UNPD medium variant scenario by location, 1950-2100

Japan

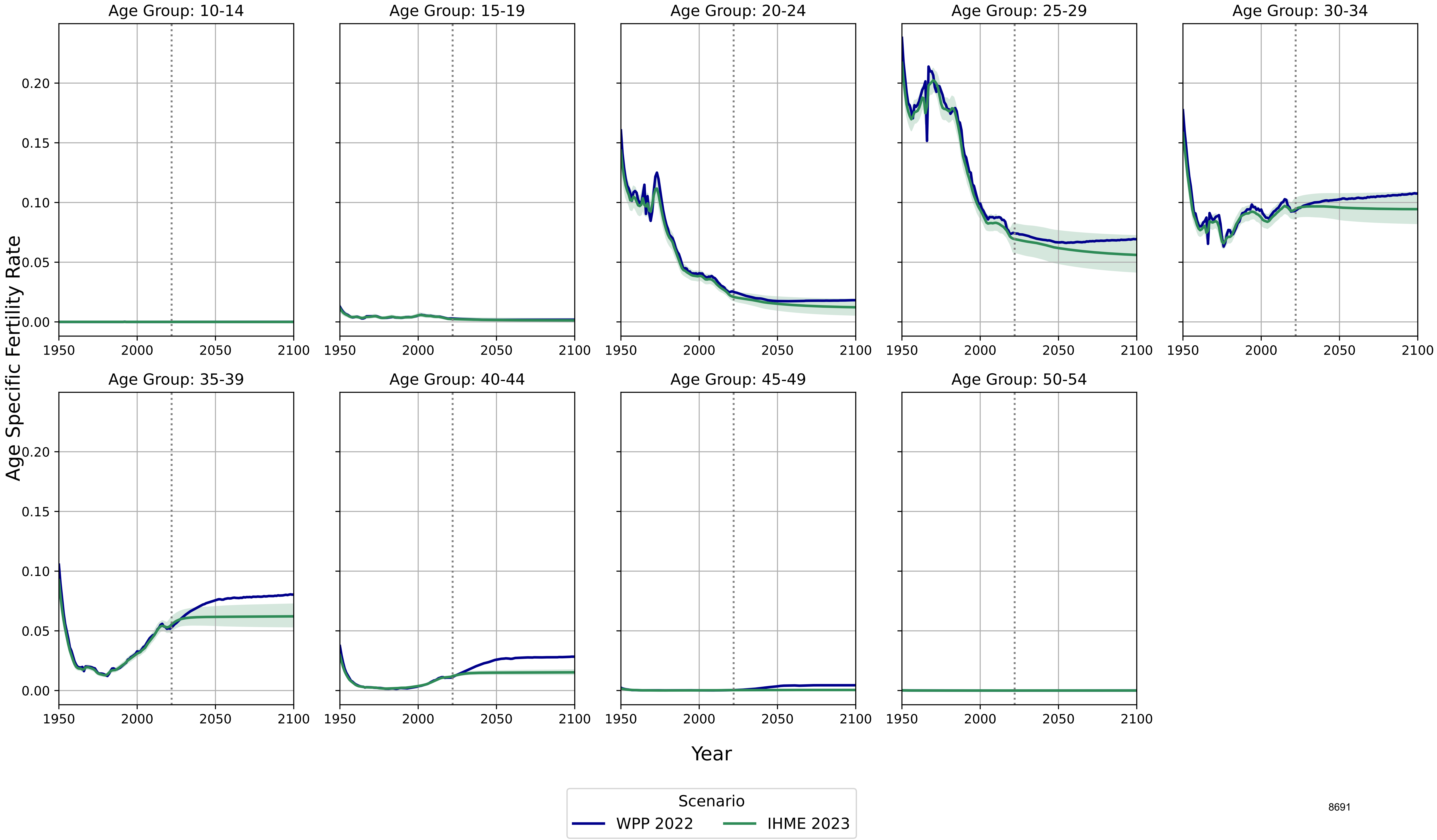

Supplemental Results Figure S8: Five-year interval ASFR from IHME reference scenario and the UNPD medium variant scenario by location, 1950-2100

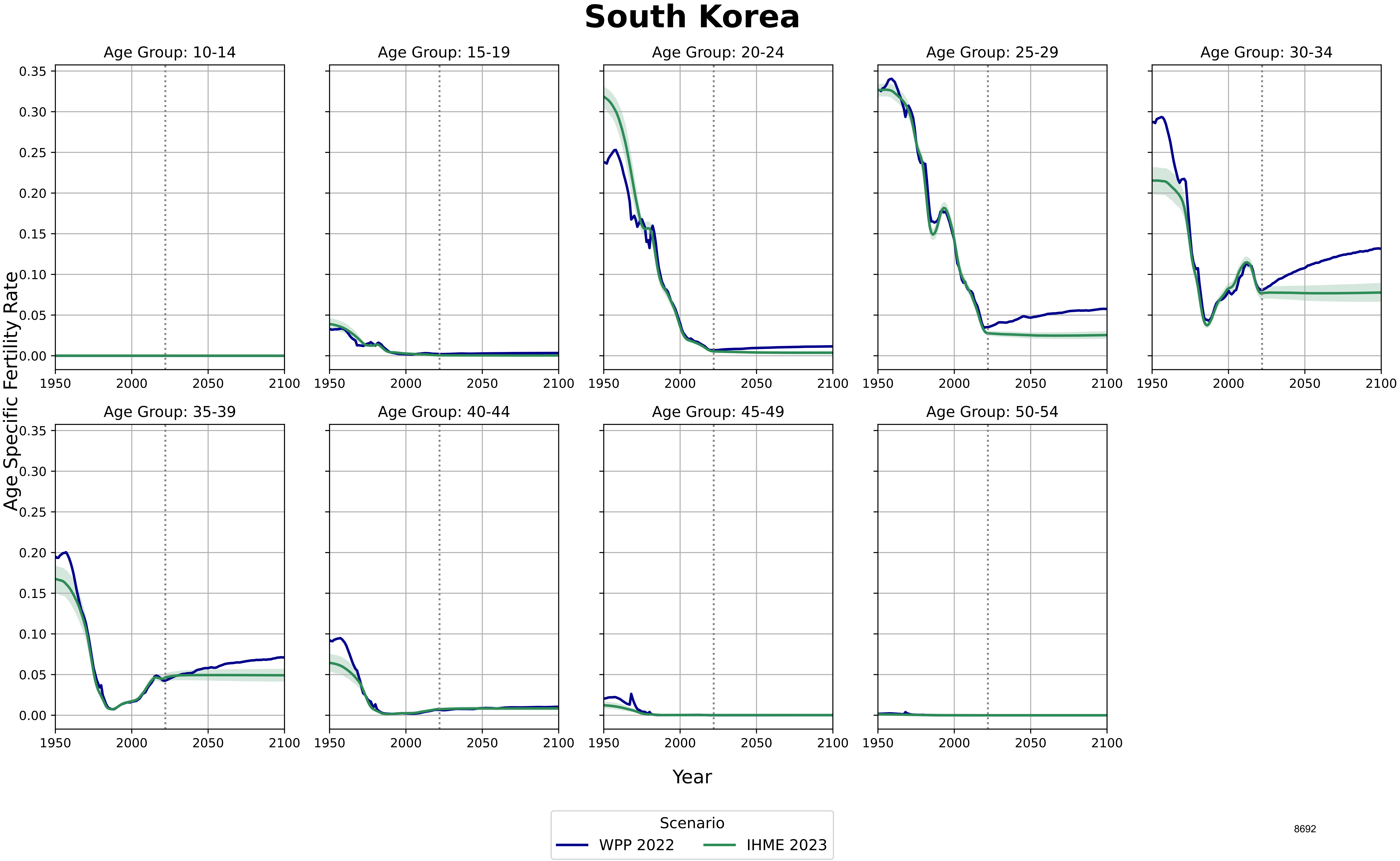

Supplemental Results Figure S8: Five-year interval ASFR from IHME reference scenario and the UNPD medium variant scenario by location, 1950-2100

Singapore

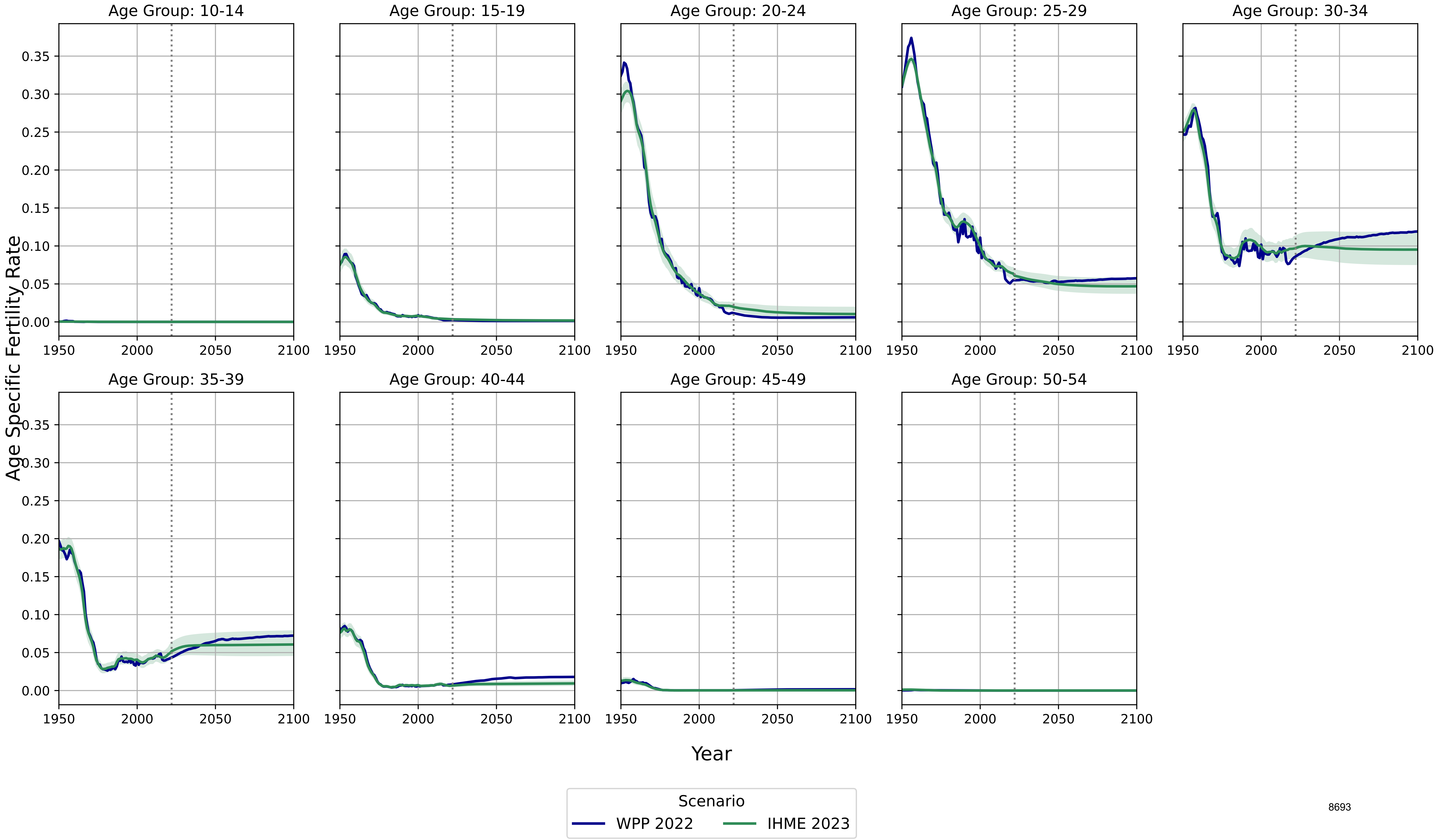

Supplemental Results Figure S8: Five-year interval ASFR from IHME reference scenario and the UNPD medium variant scenario by location, 1950-2100

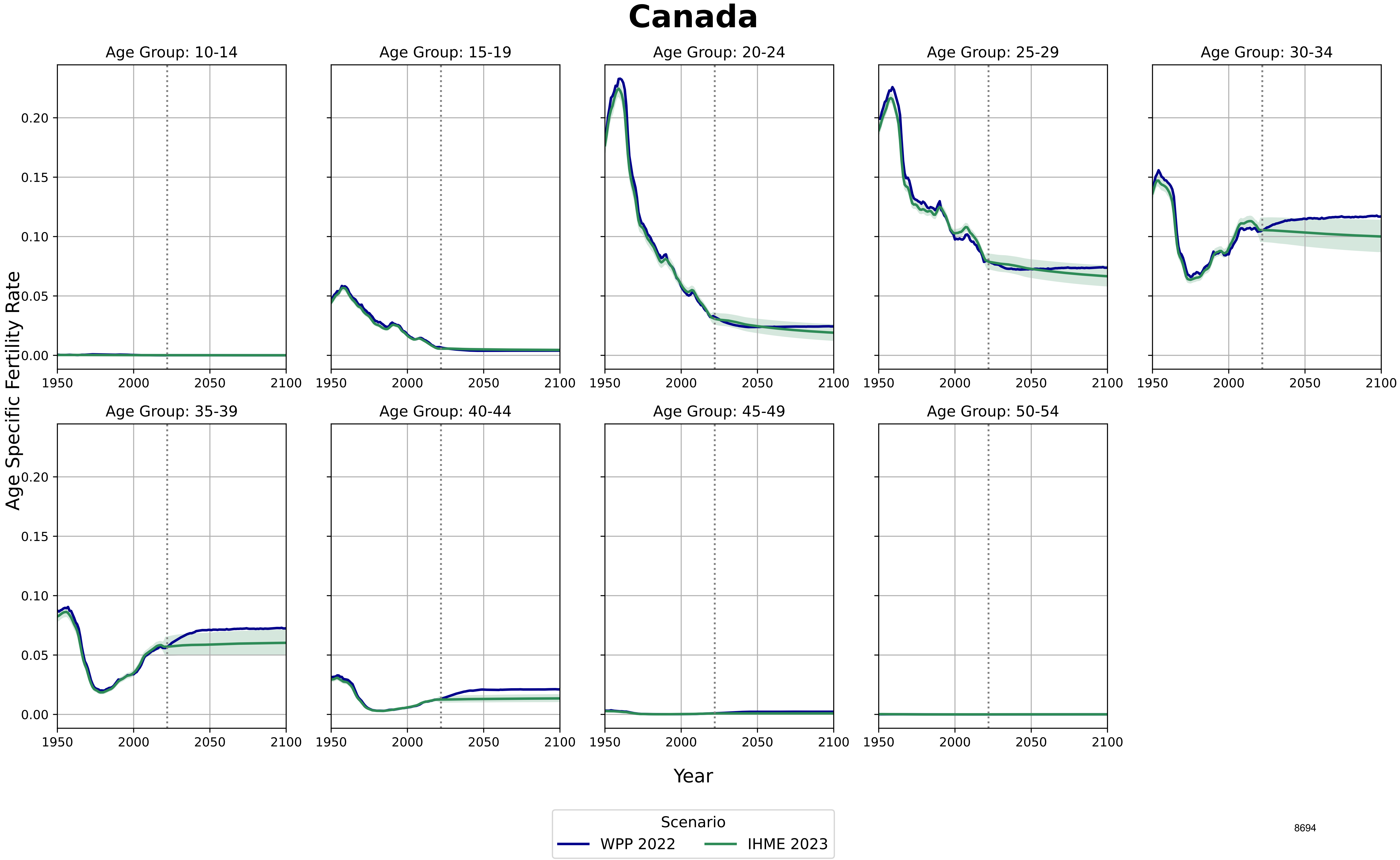

8694

Supplemental Results Figure S8: Five-year interval ASFR from IHME reference scenario and the UNPD medium variant scenario by location, 1950-2100

Greenland

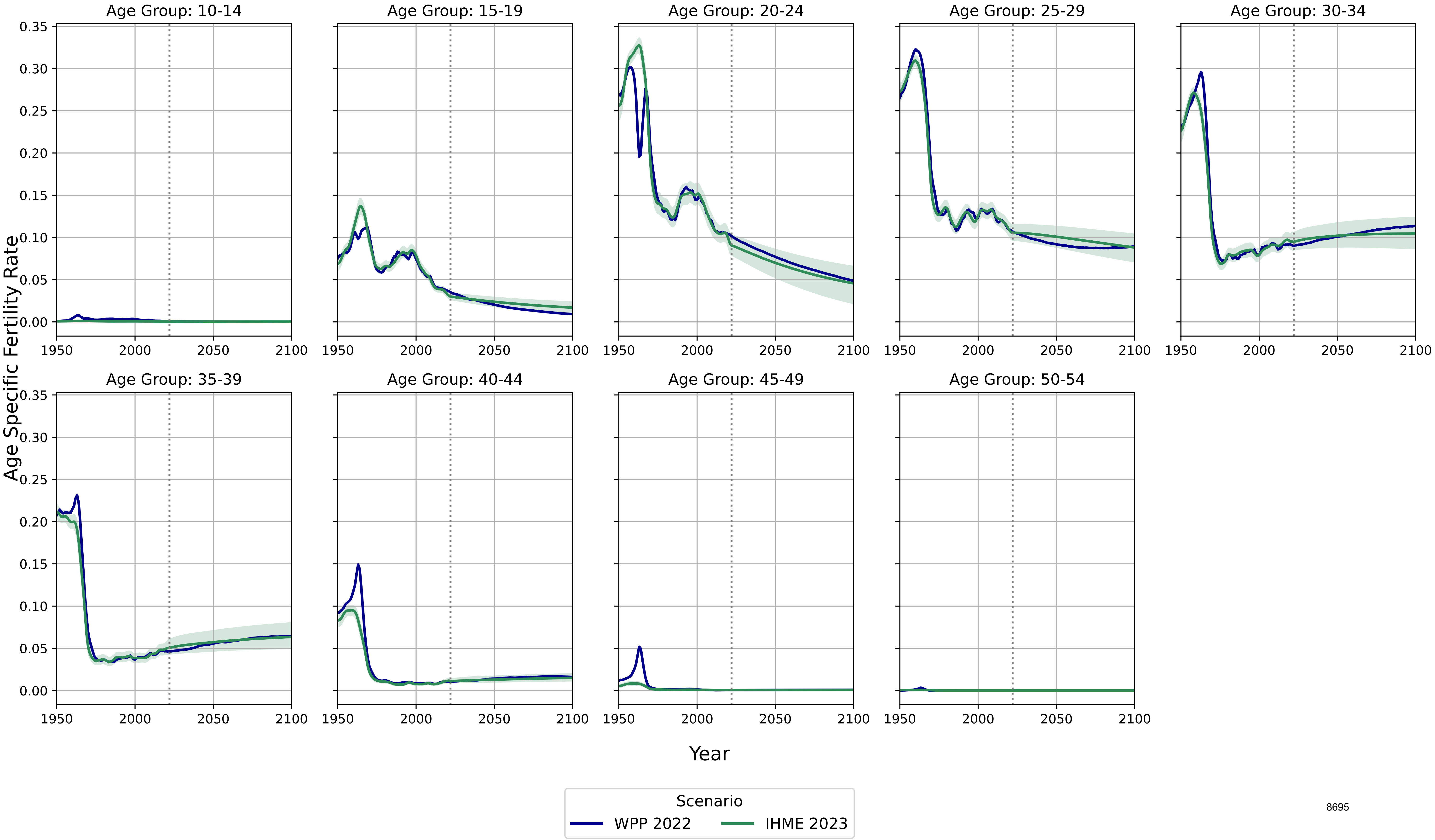

Supplemental Results Figure S8: Five-year interval ASFR from IHME reference scenario and the UNPD medium variant scenario by location, 1950-2100

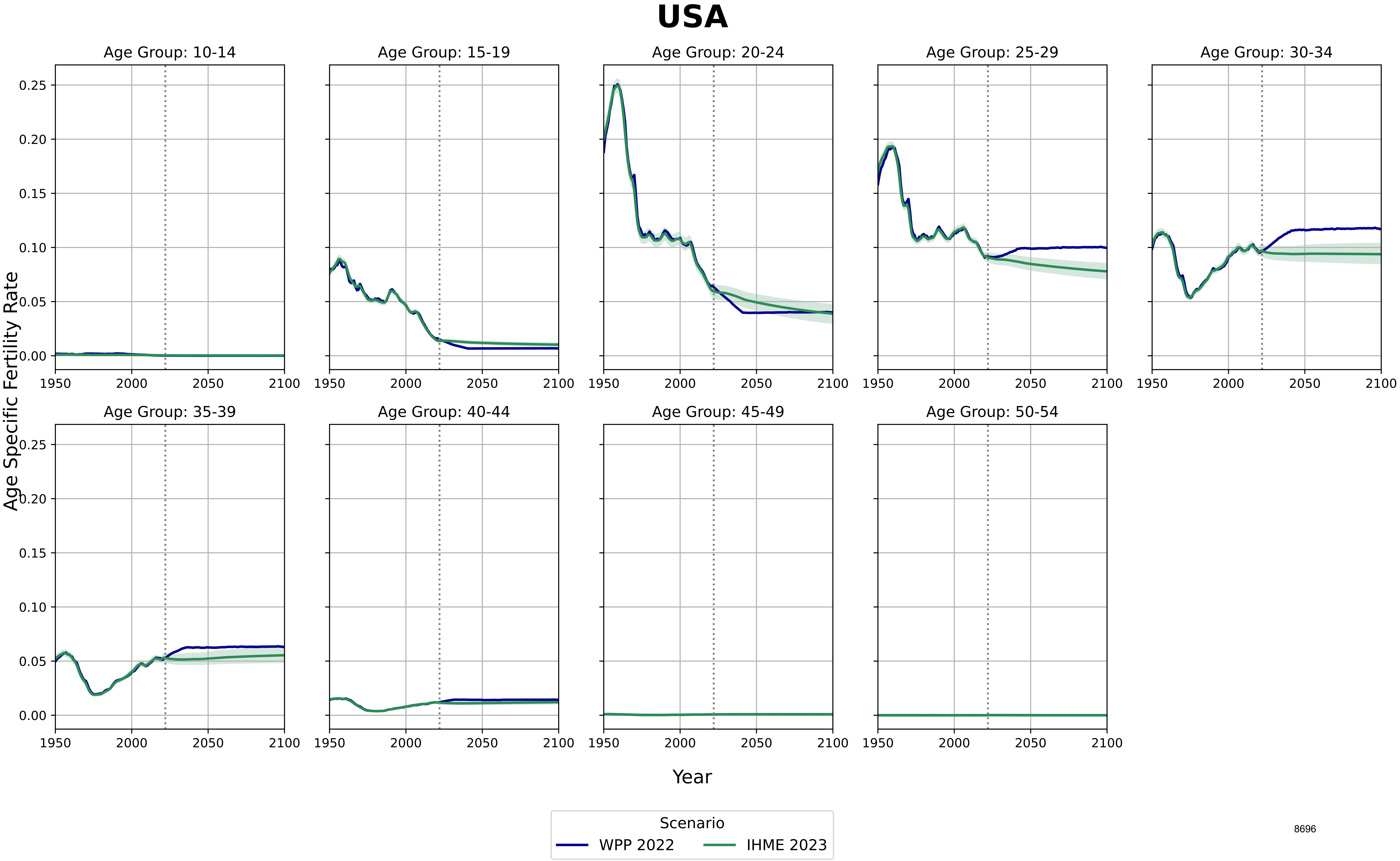

Supplemental Results Figure S8: Five-year interval ASFR from IHME reference scenario and the UNPD medium variant scenario by location, 1950-2100

Argentina

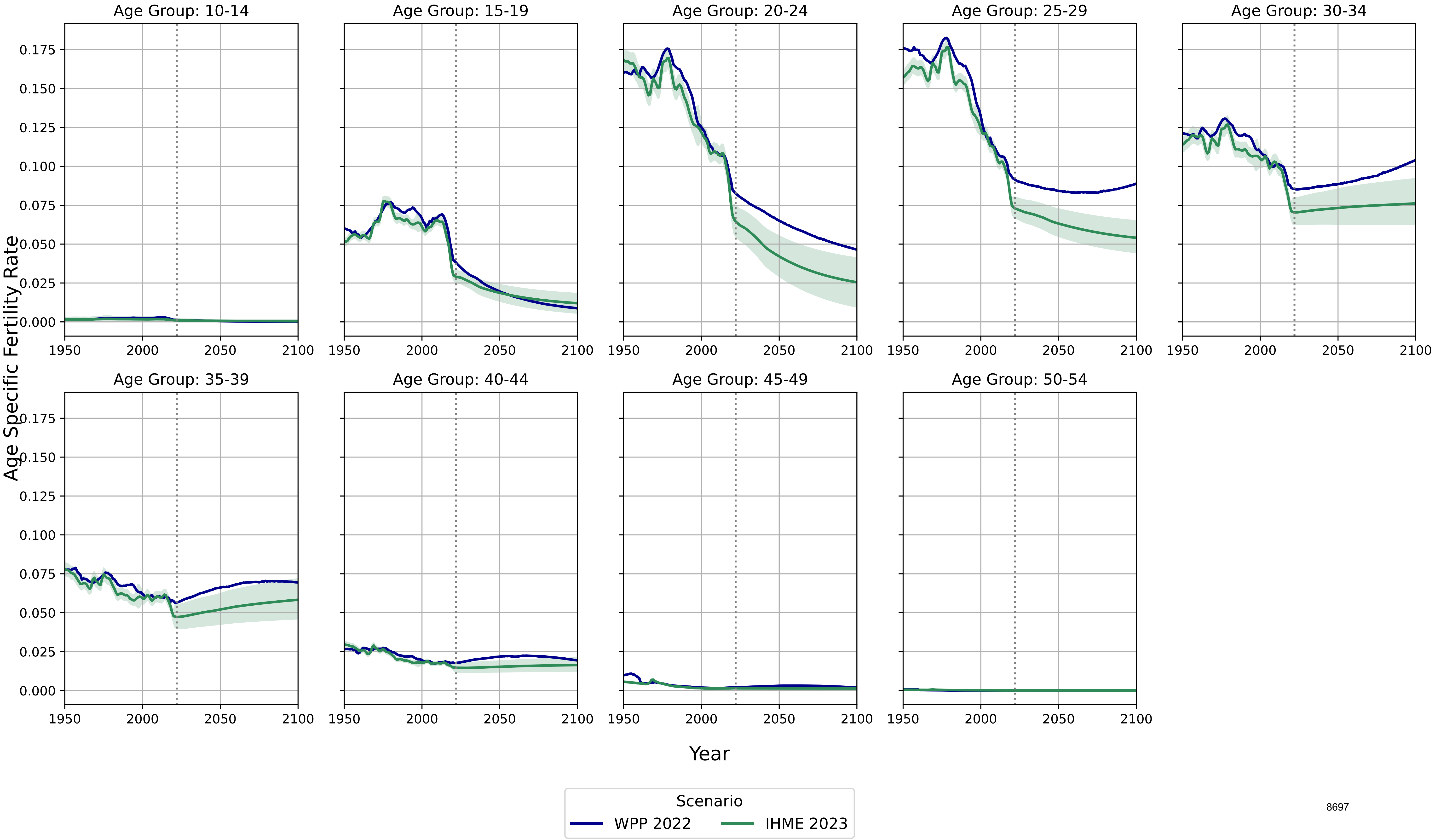

Supplemental Results Figure S8: Five-year interval ASFR from IHME reference scenario and the UNPD medium variant scenario by location, 1950-2100

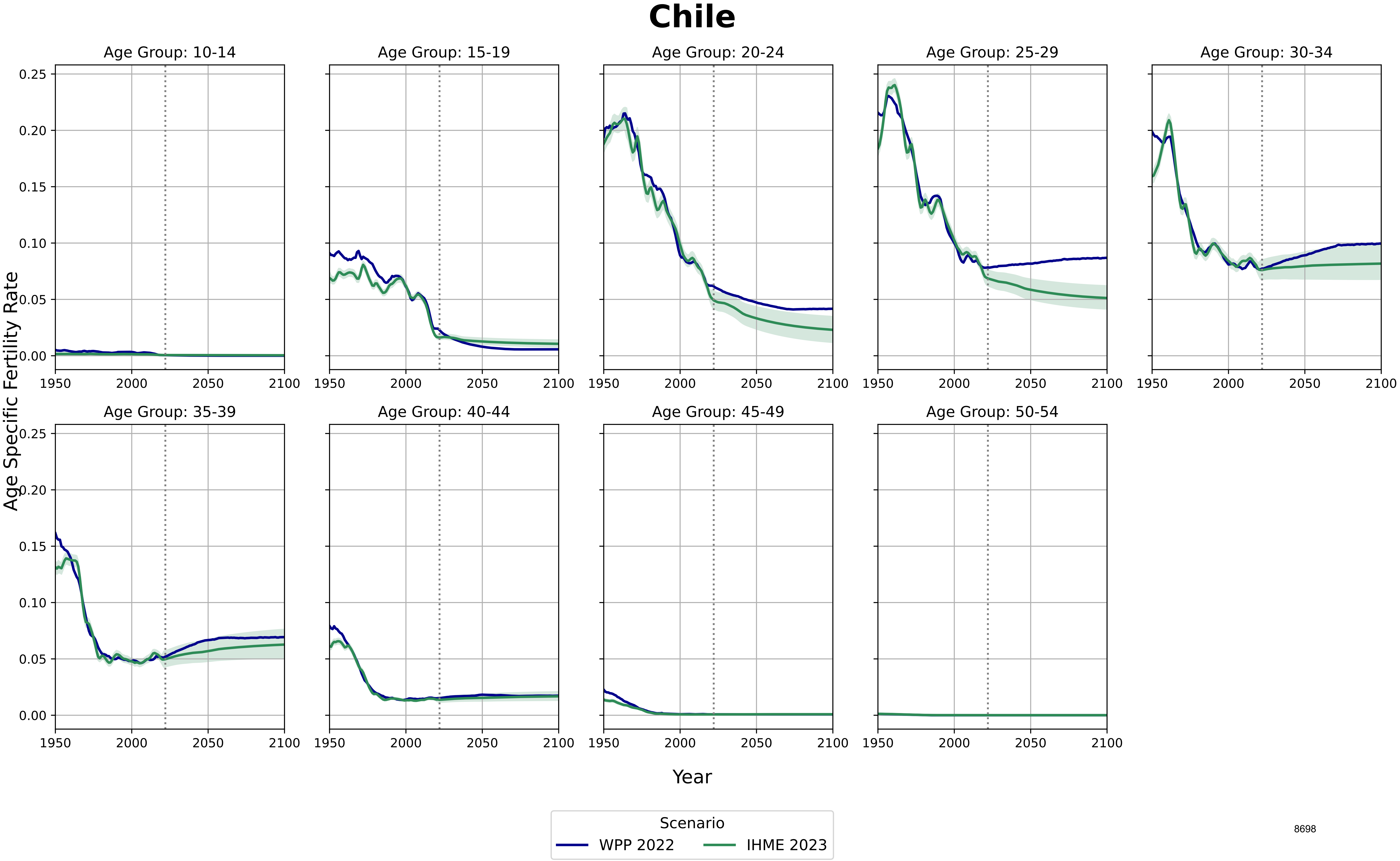

Supplemental Results Figure S8: Five-year interval ASFR from IHME reference scenario and the UNPD medium variant scenario by location, 1950-2100

Uruguay

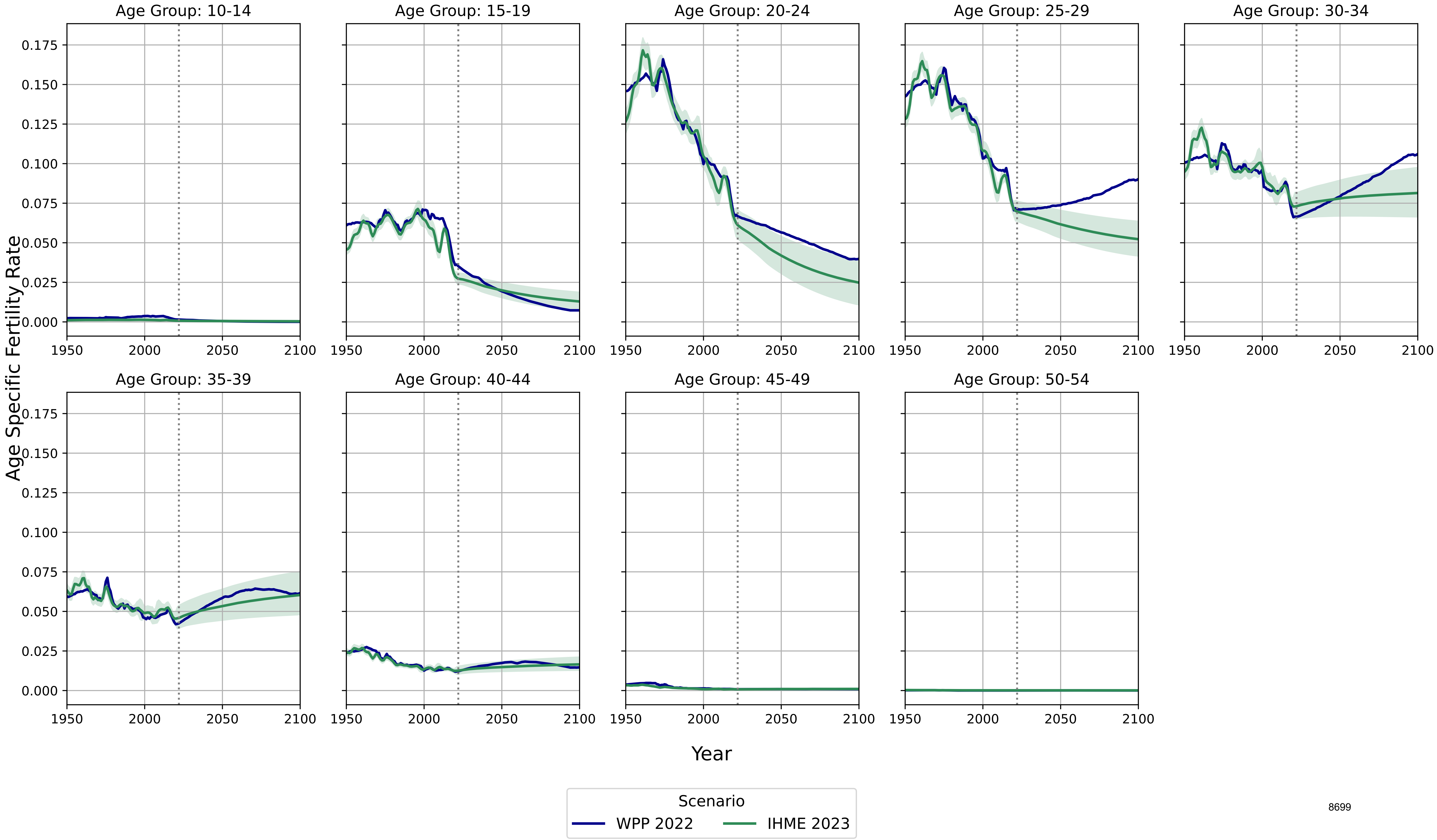

Supplemental Results Figure S8: Five-year interval ASFR from IHME reference scenario and the UNPD medium variant scenario by location, 1950-2100

Andorra

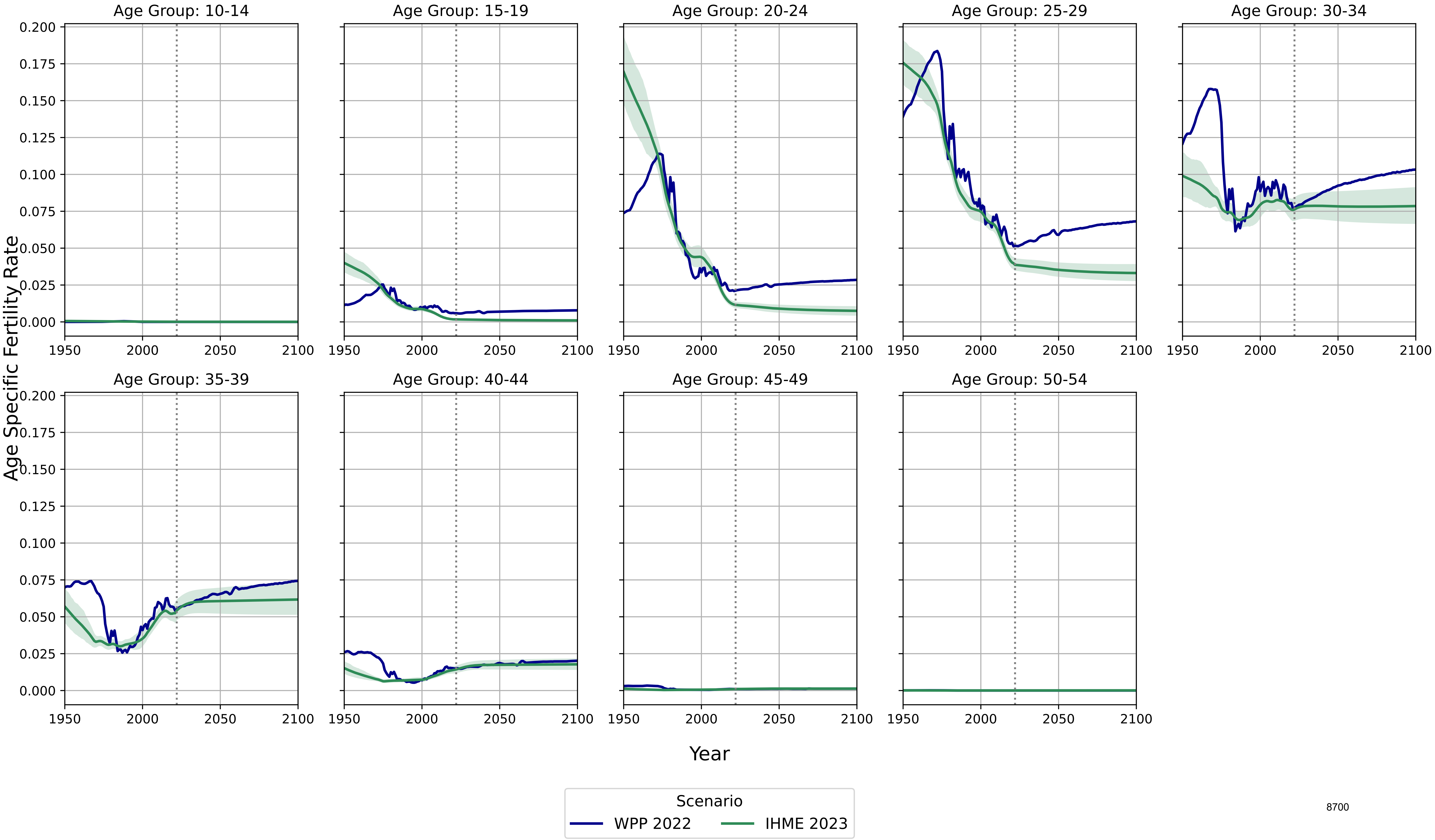

Supplemental Results Figure S8: Five-year interval ASFR from IHME reference scenario and the UNPD medium variant scenario by location, 1950-2100

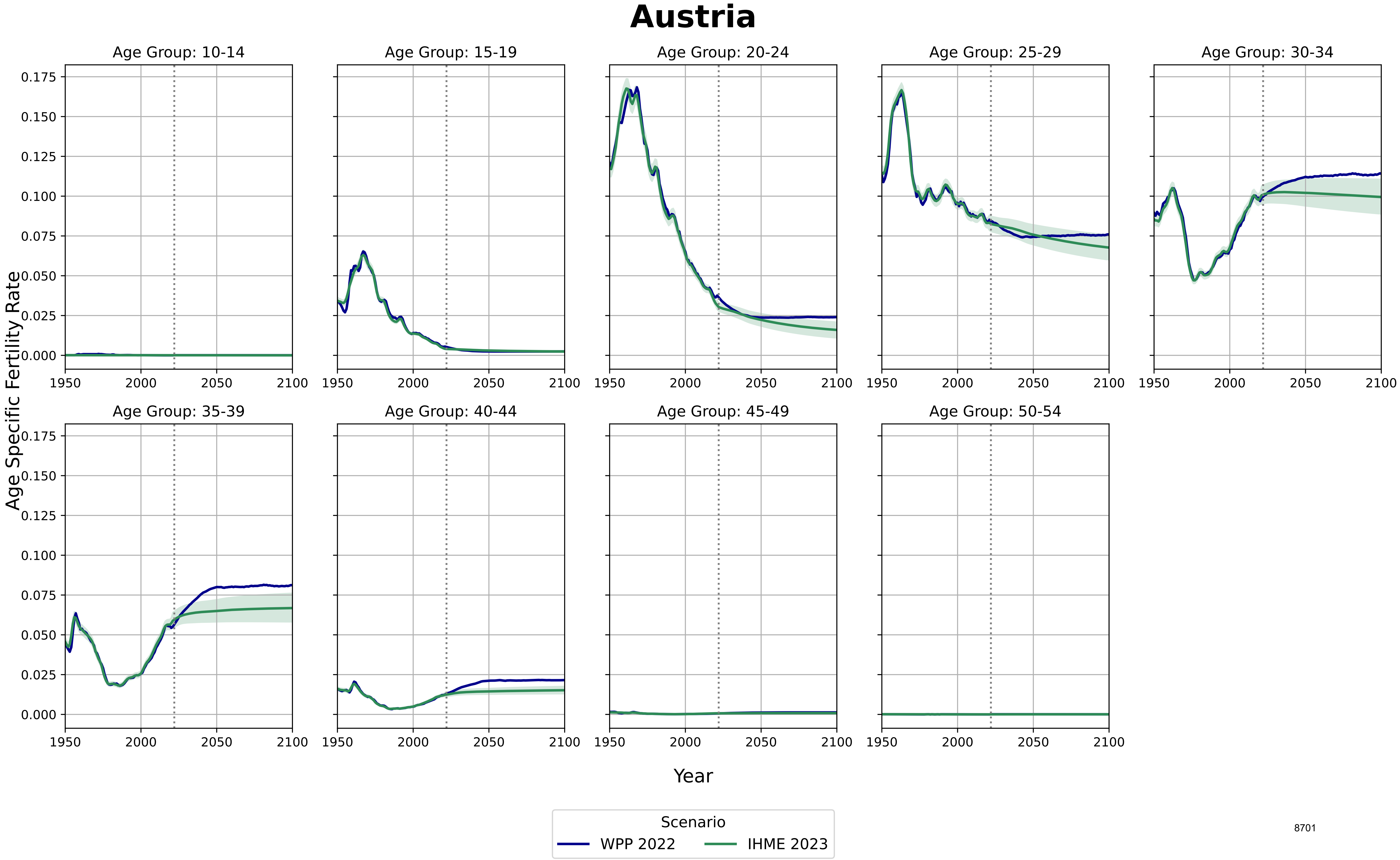

Supplemental Results Figure S8: Five-year interval ASFR from IHME reference scenario and the UNPD medium variant scenario by location, 1950-2100

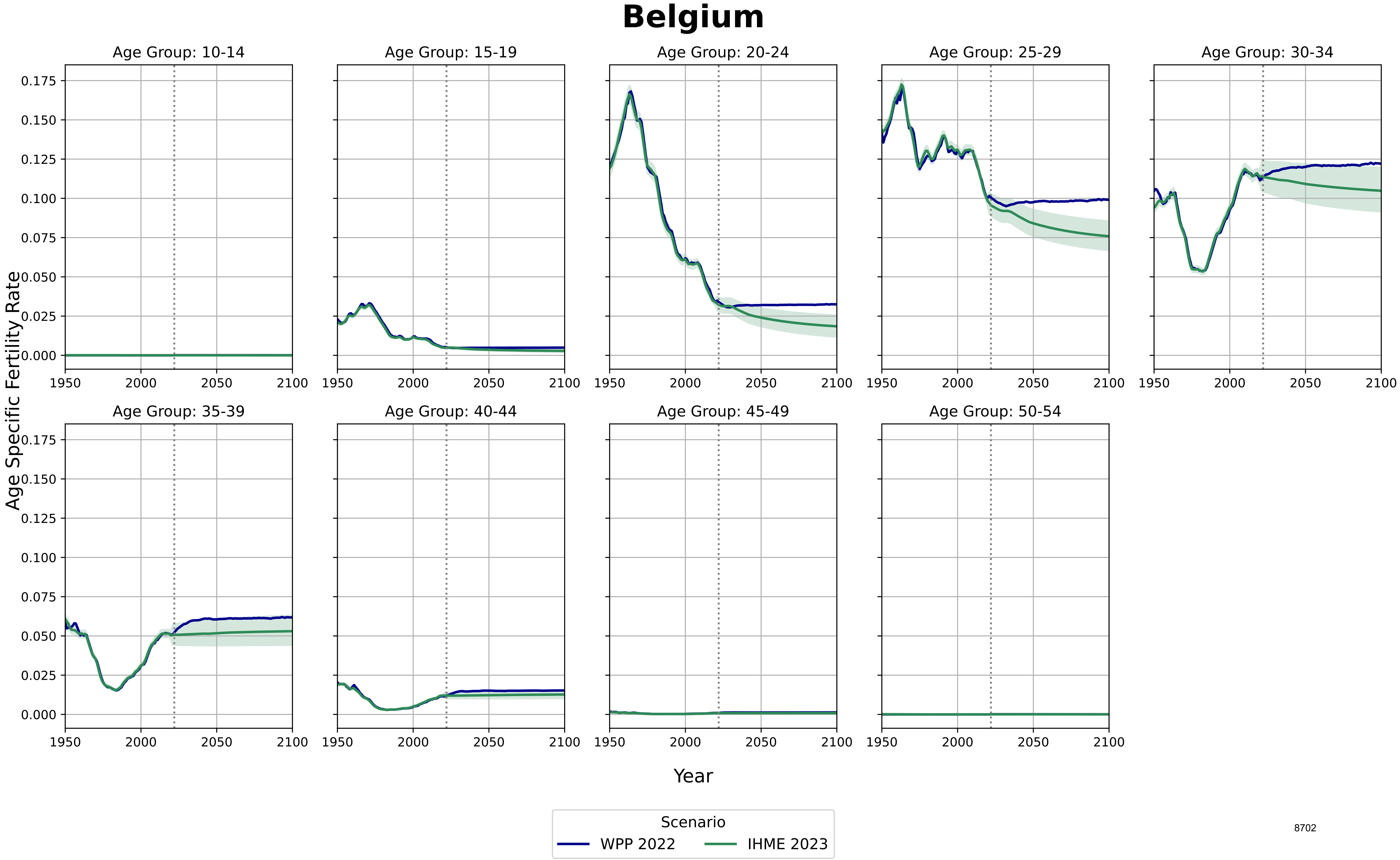

Supplemental Results Figure S8: Five-year interval ASFR from IHME reference scenario and the UNPD medium variant scenario by location, 1950-2100

Cyprus

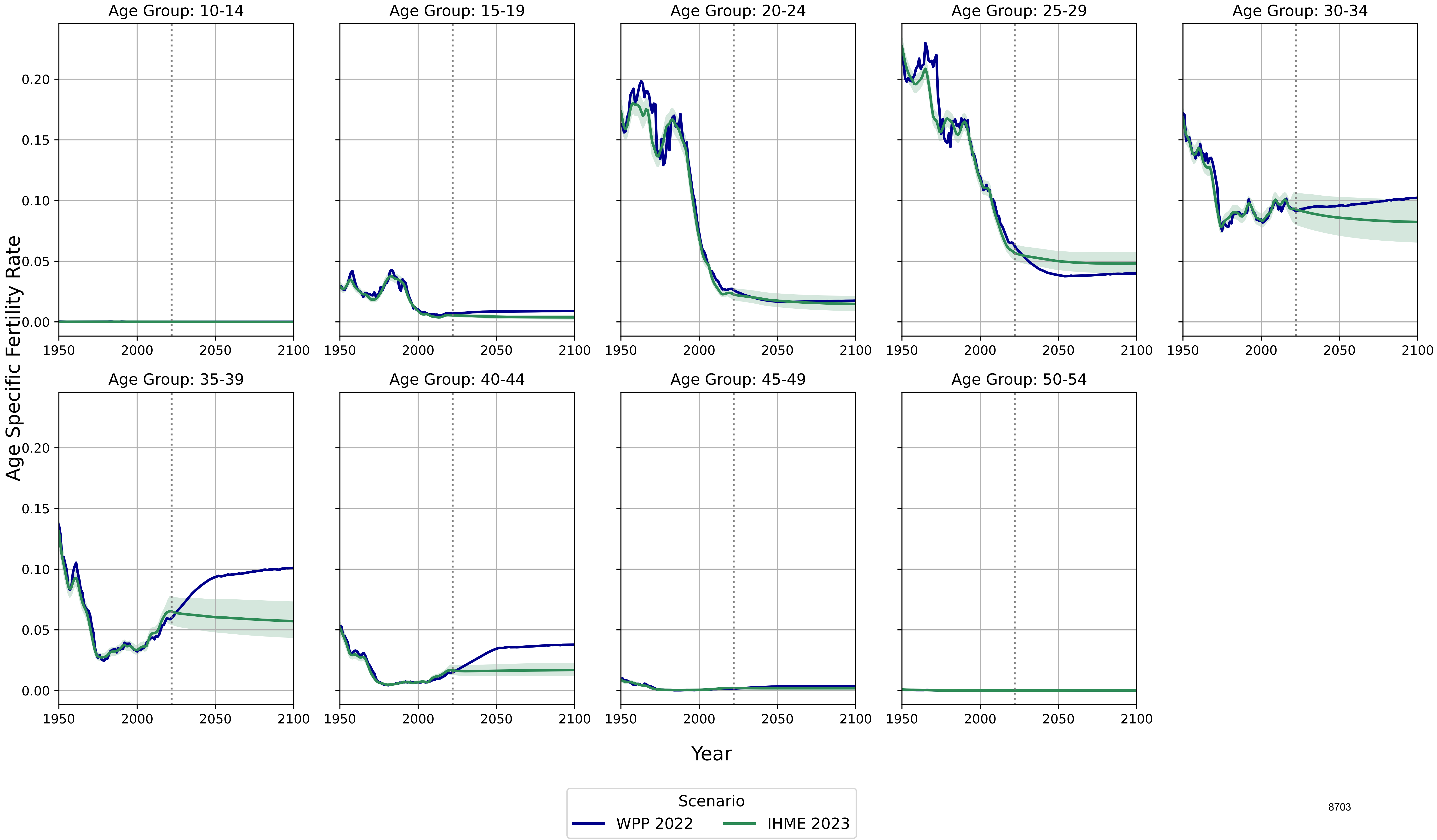

Supplemental Results Figure S8: Five-year interval ASFR from IHME reference scenario and the UNPD medium variant scenario by location, 1950-2100

Denmark

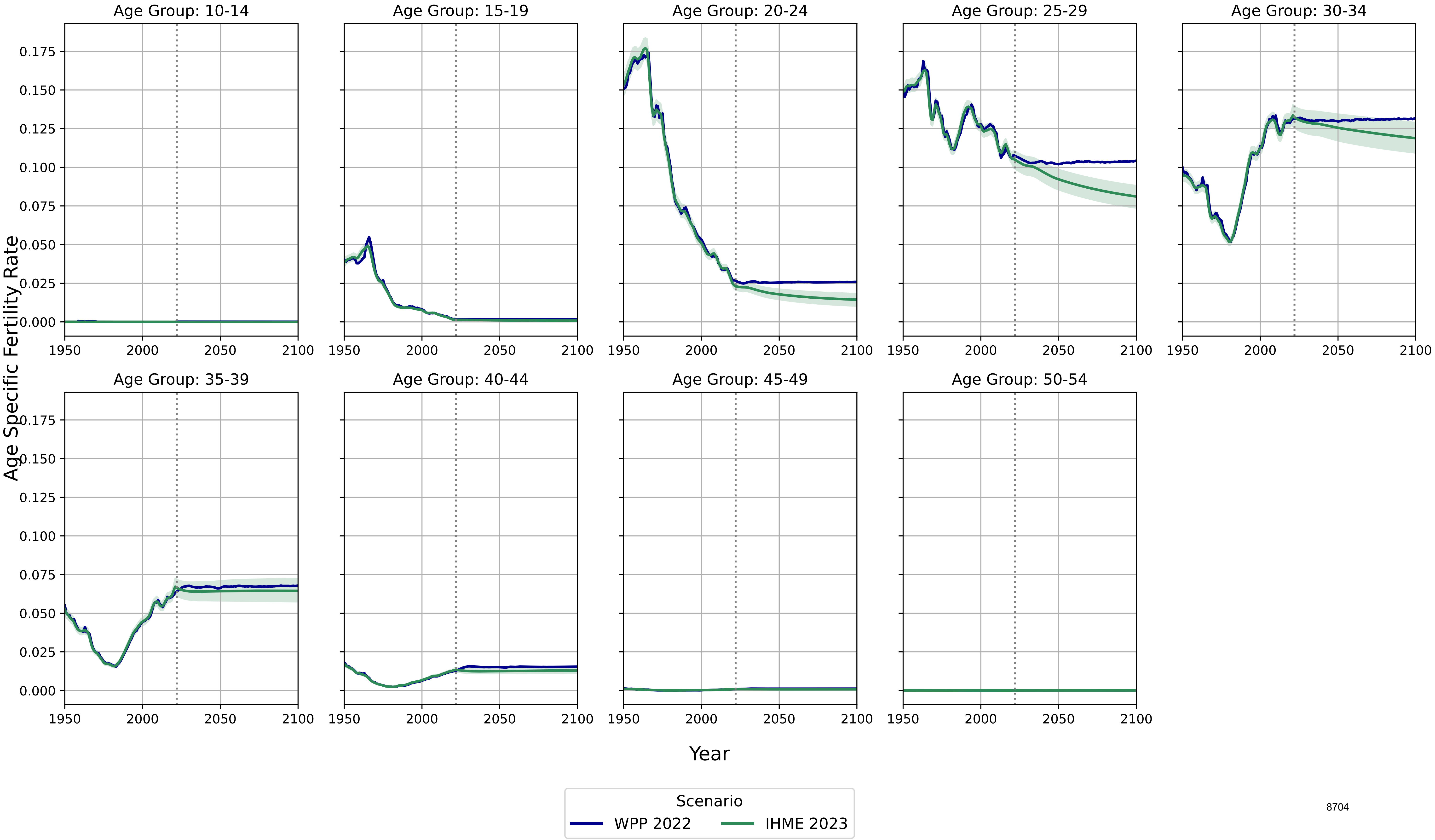

Supplemental Results Figure S8: Five-year interval ASFR from IHME reference scenario and the UNPD medium variant scenario by location, 1950-2100

Finland

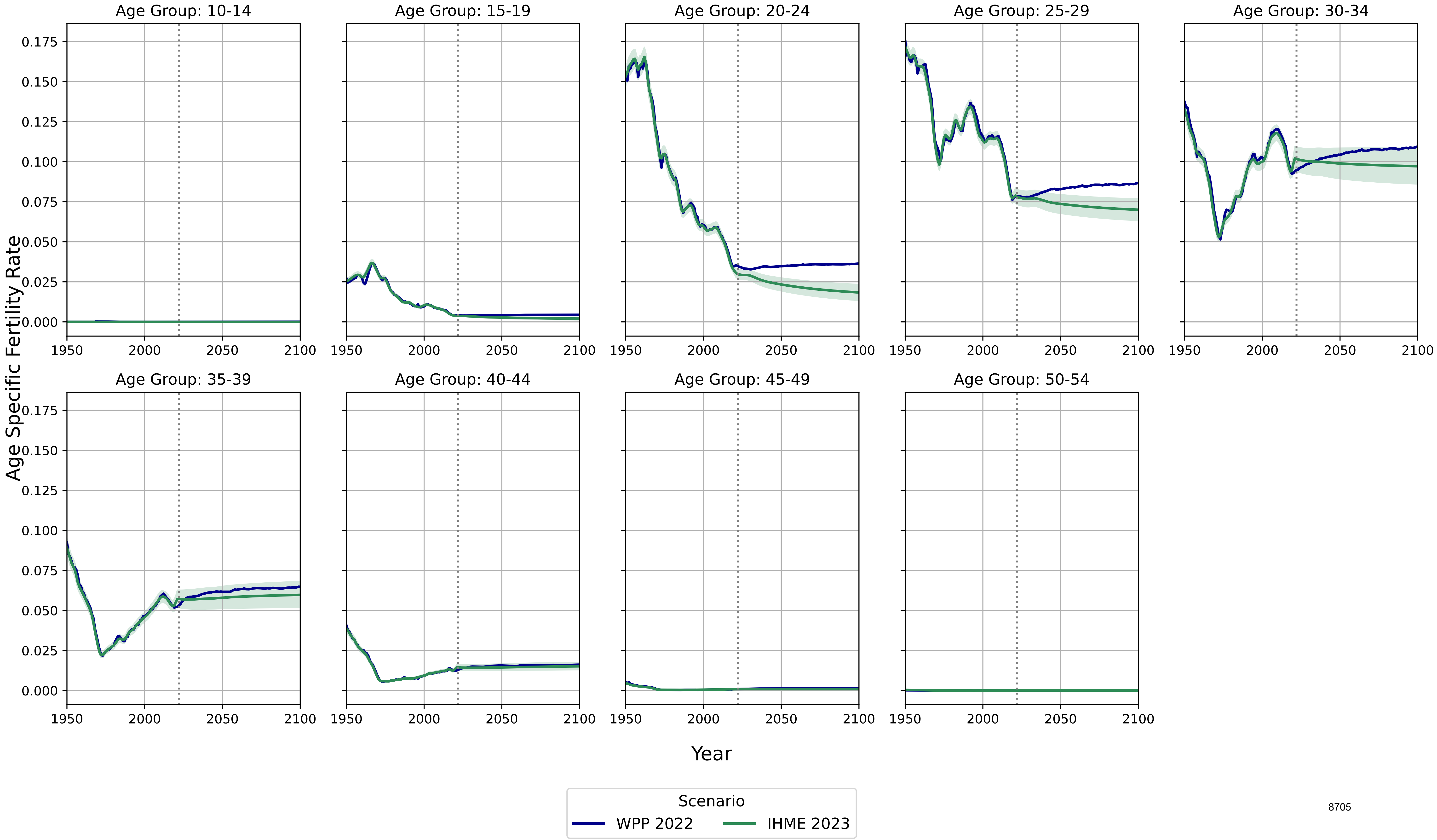

Supplemental Results Figure S8: Five-year interval ASFR from IHME reference scenario and the UNPD medium variant scenario by location, 1950-2100

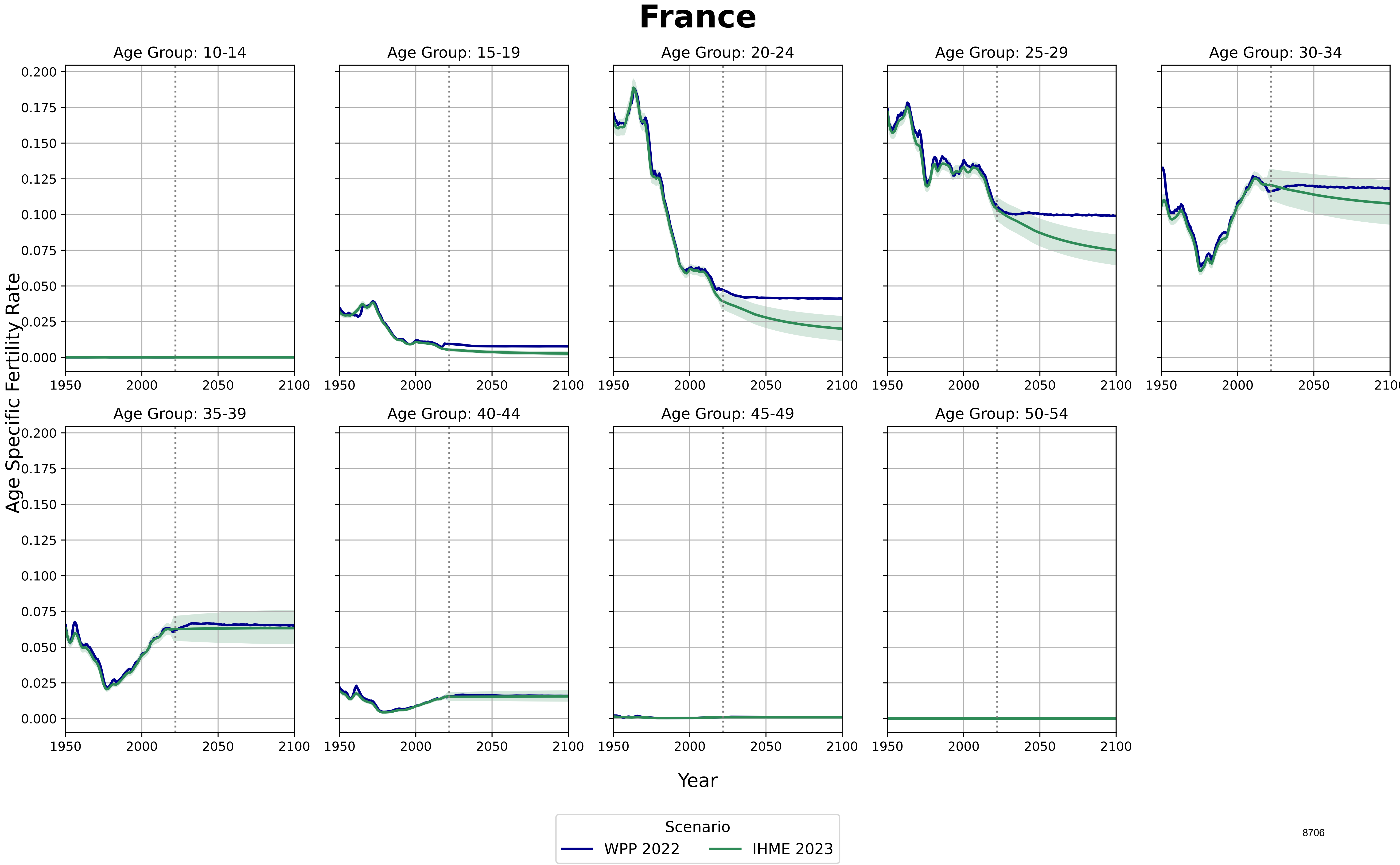

Supplemental Results Figure S8: Five-year interval ASFR from IHME reference scenario and the UNPD medium variant scenario by location, 1950-2100

Germany

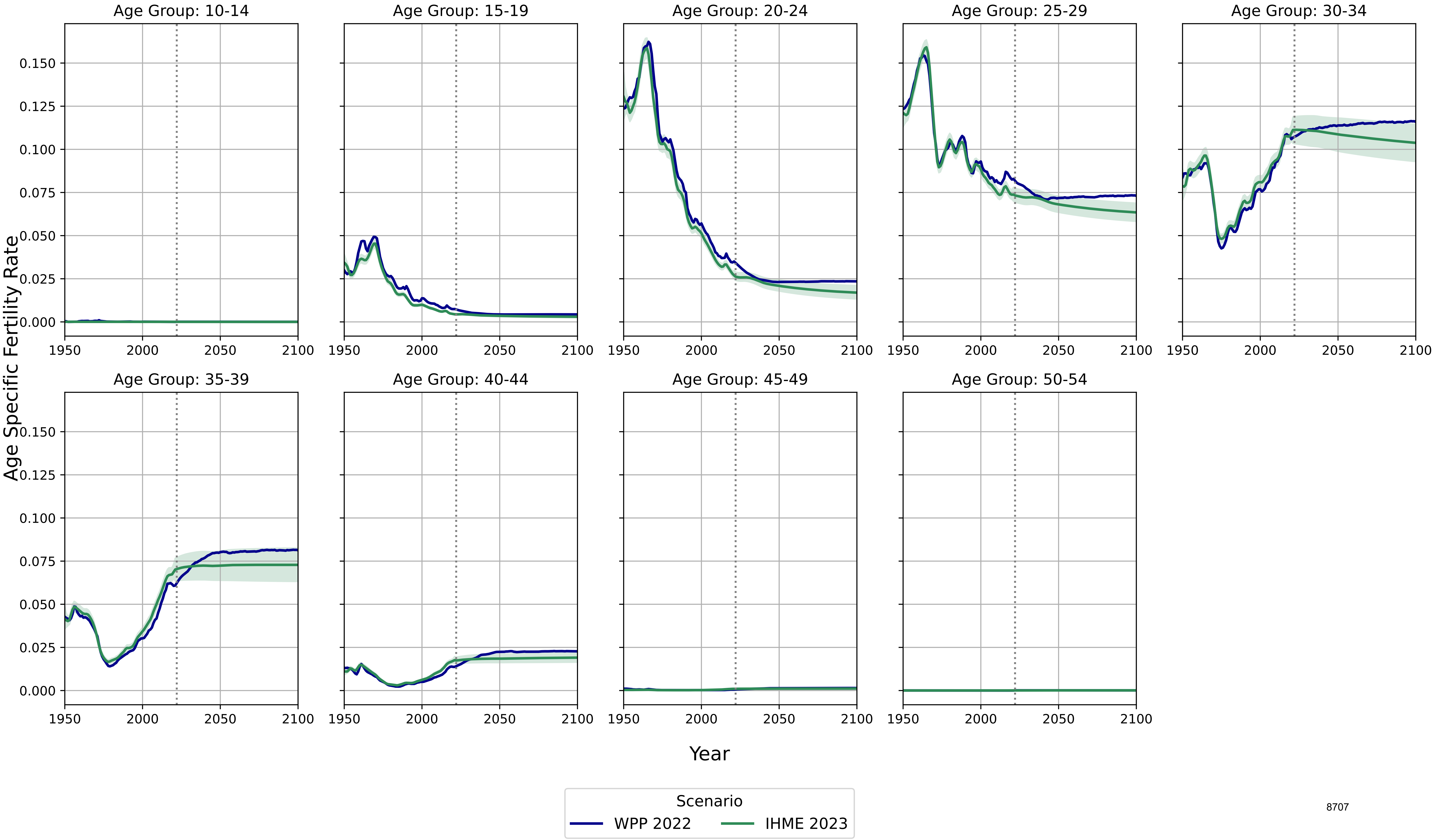

Supplemental Results Figure S8: Five-year interval ASFR from IHME reference scenario and the UNPD medium variant scenario by location, 1950-2100

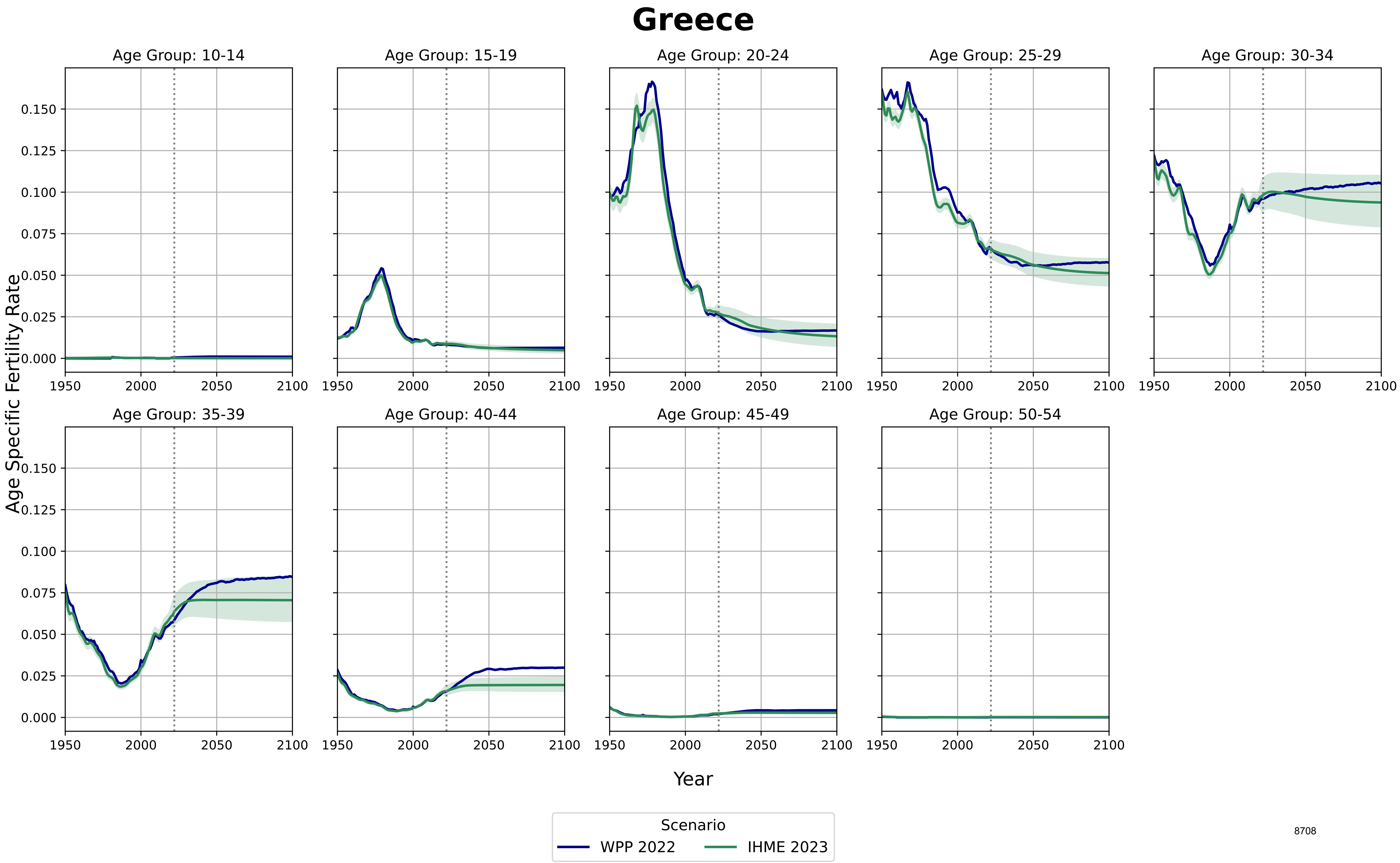

Supplemental Results Figure S8: Five-year interval ASFR from IHME reference scenario and the UNPD medium variant scenario by location, 1950-2100

Iceland

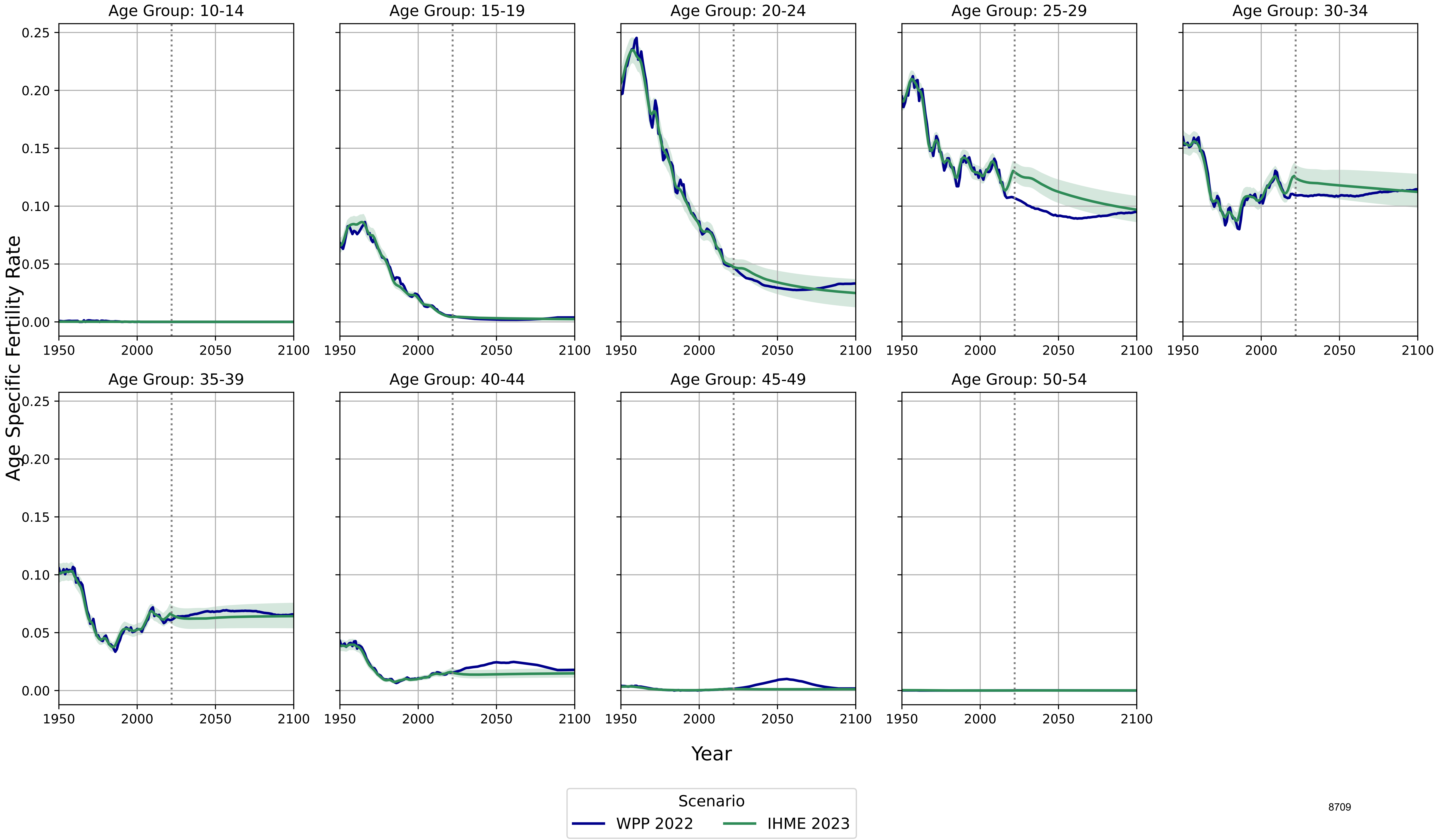

Supplemental Results Figure S8: Five-year interval ASFR from IHME reference scenario and the UNPD medium variant scenario by location, 1950-2100

Ireland

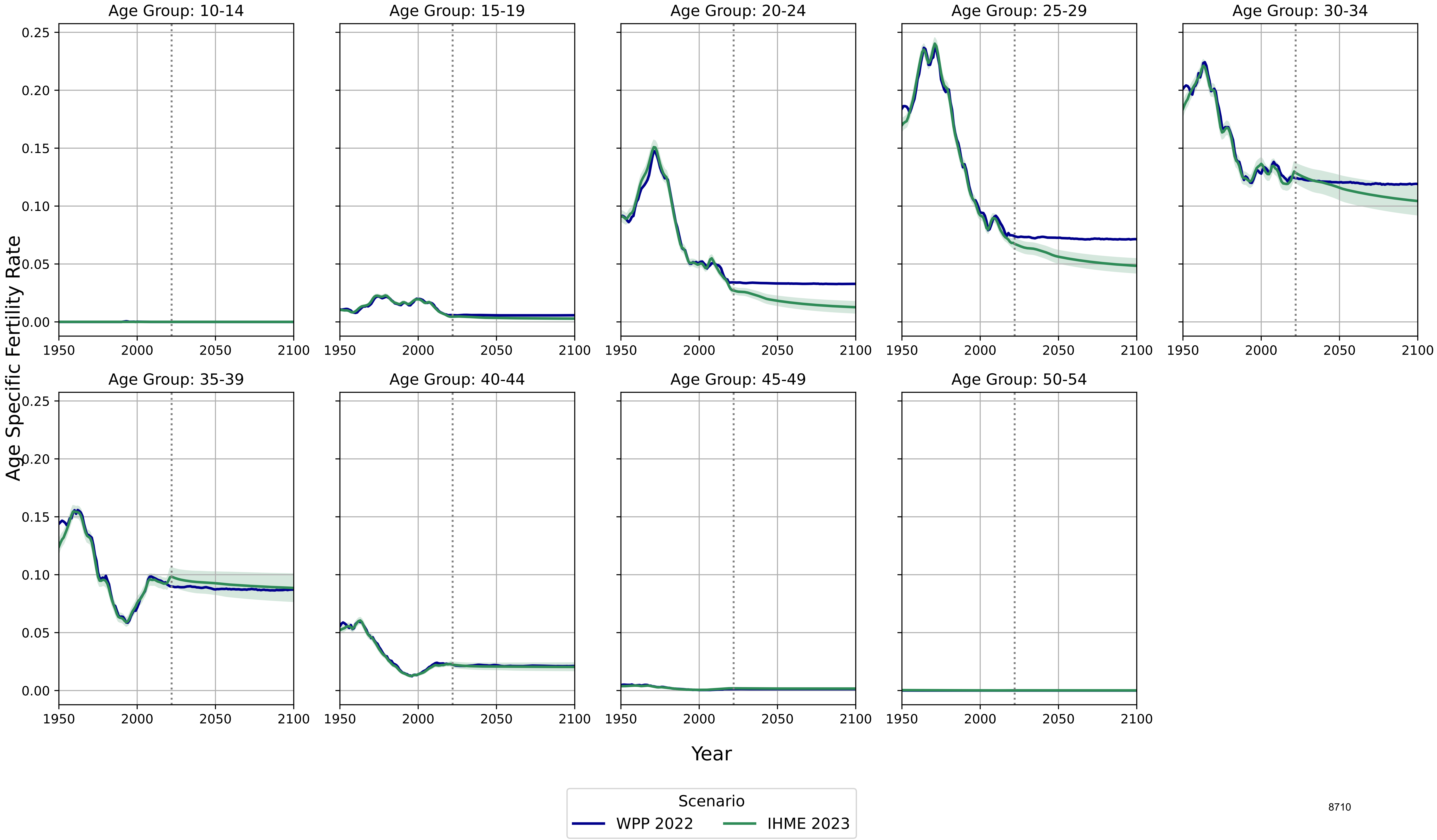

Supplemental Results Figure S8: Five-year interval ASFR from IHME reference scenario and the UNPD medium variant scenario by location, 1950-2100

Israel

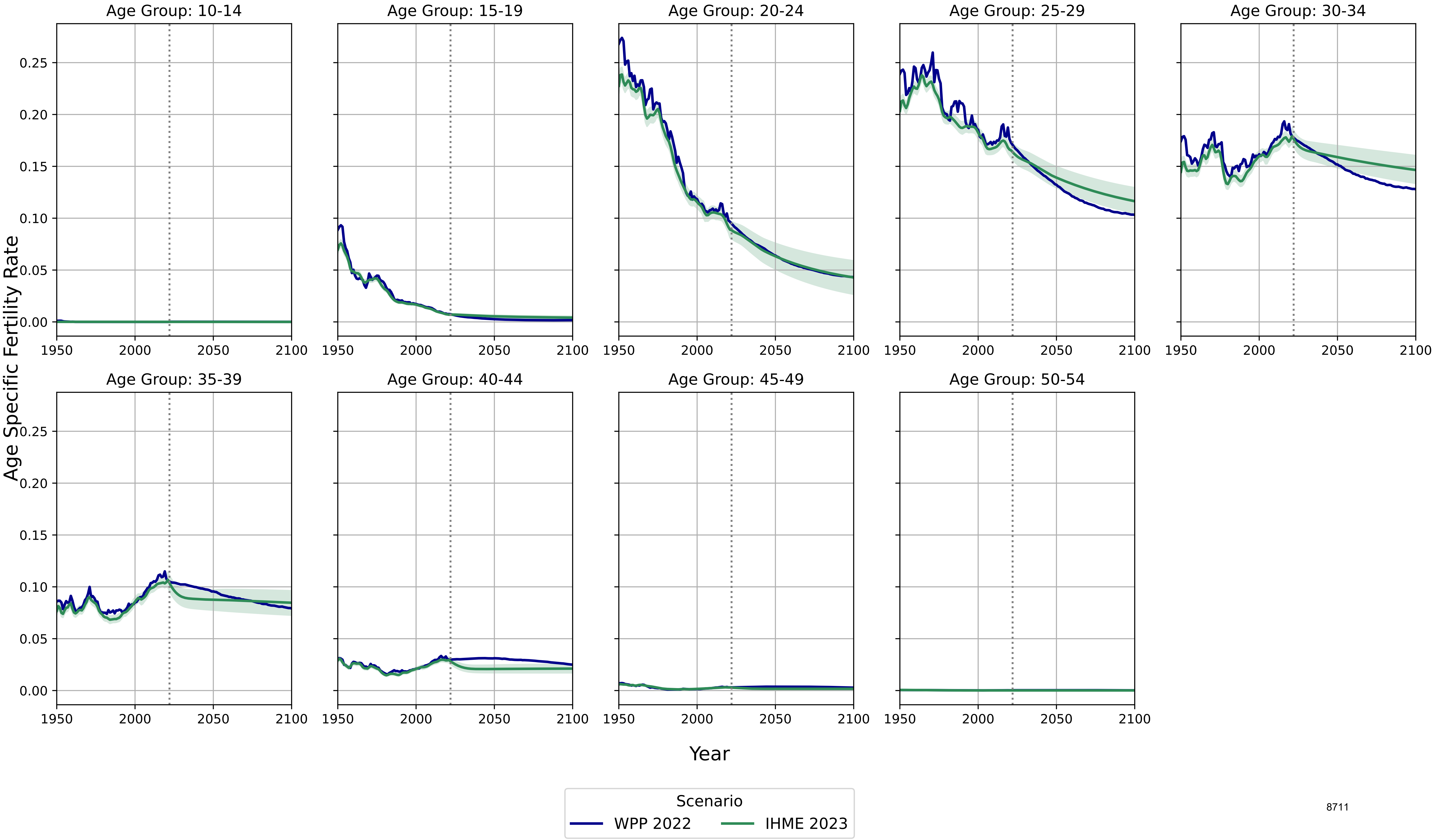

Supplemental Results Figure S8: Five-year interval ASFR from IHME reference scenario and the UNPD medium variant scenario by location, 1950-2100

Italy

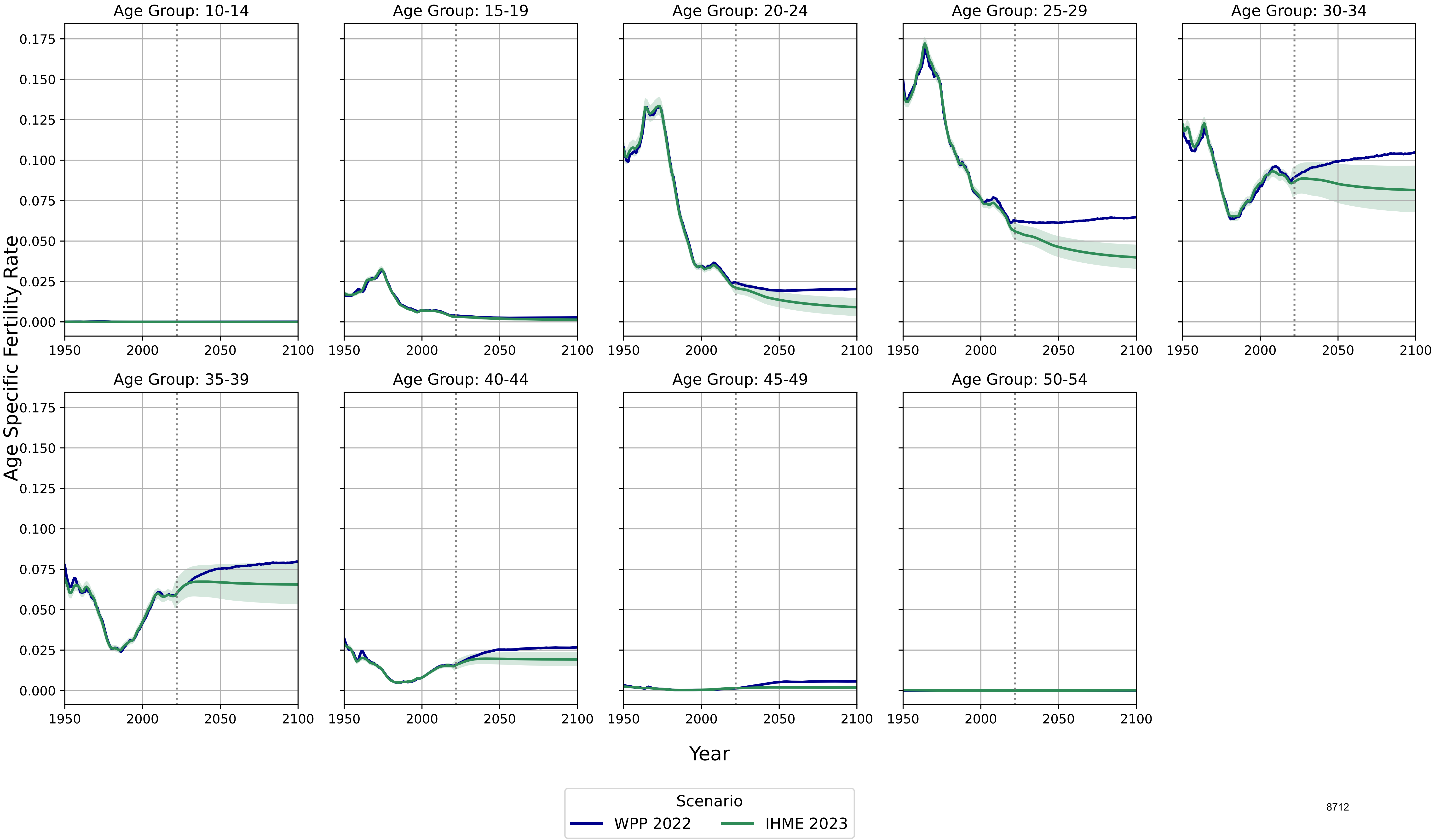

Supplemental Results Figure S8: Five-year interval ASFR from IHME reference scenario and the UNPD medium variant scenario by location, 1950-2100

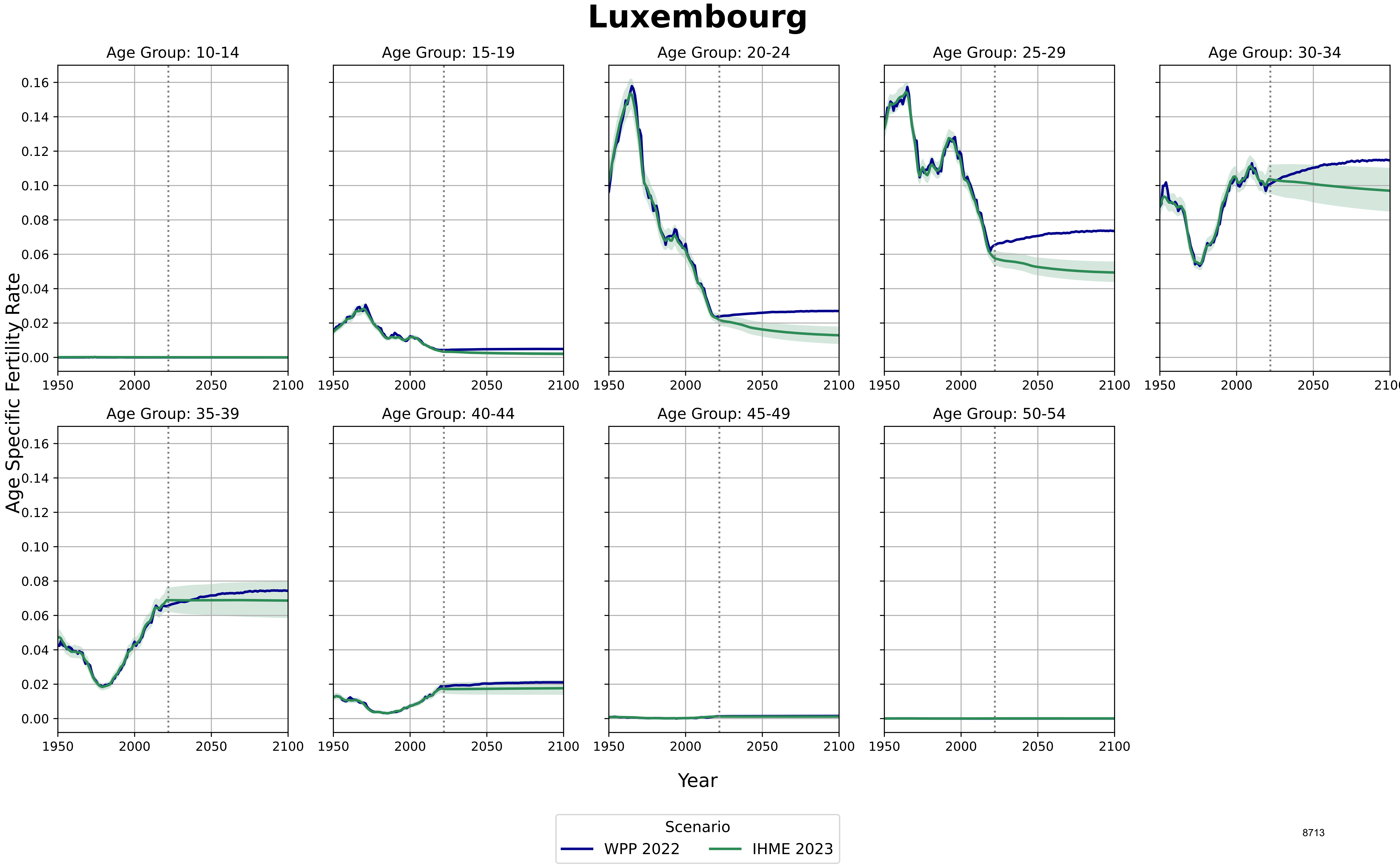

Supplemental Results Figure S8: Five-year interval ASFR from IHME reference scenario and the UNPD medium variant scenario by location, 1950-2100

Malta

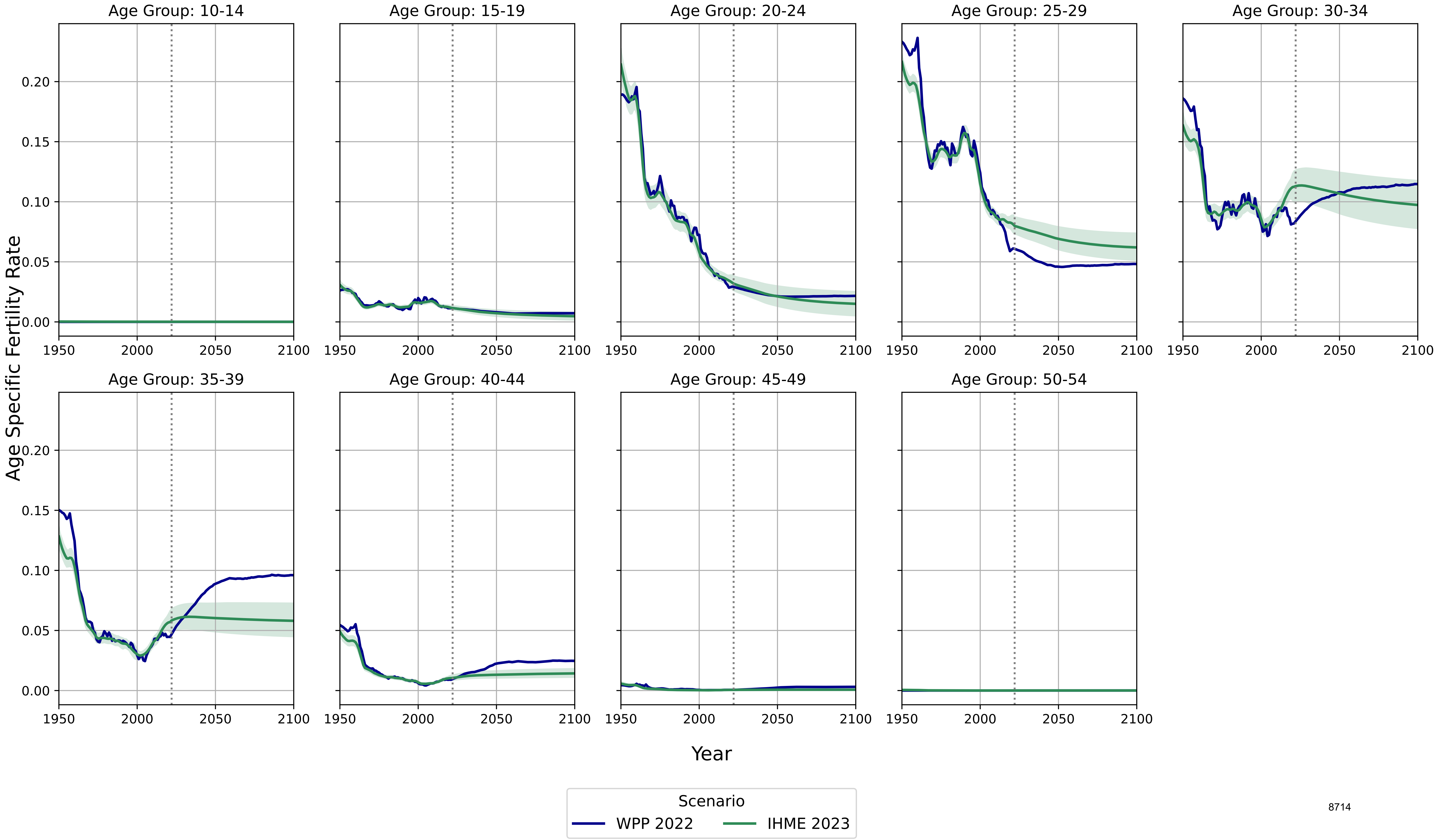

Supplemental Results Figure S8: Five-year interval ASFR from IHME reference scenario and the UNPD medium variant scenario by location, 1950-2100

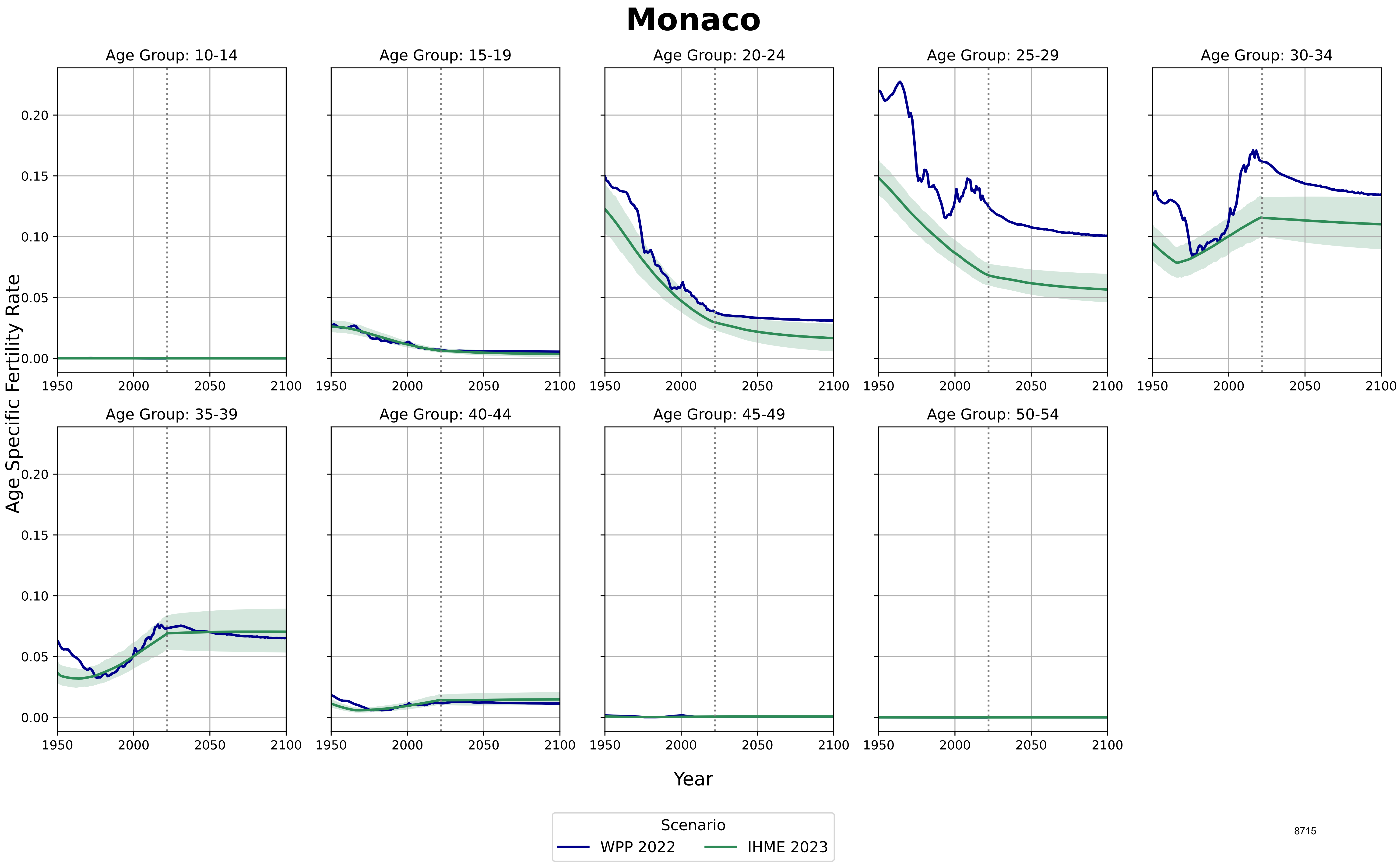

Supplemental Results Figure S8: Five-year interval ASFR from IHME reference scenario and the UNPD medium variant scenario by location, 1950-2100

Netherlands

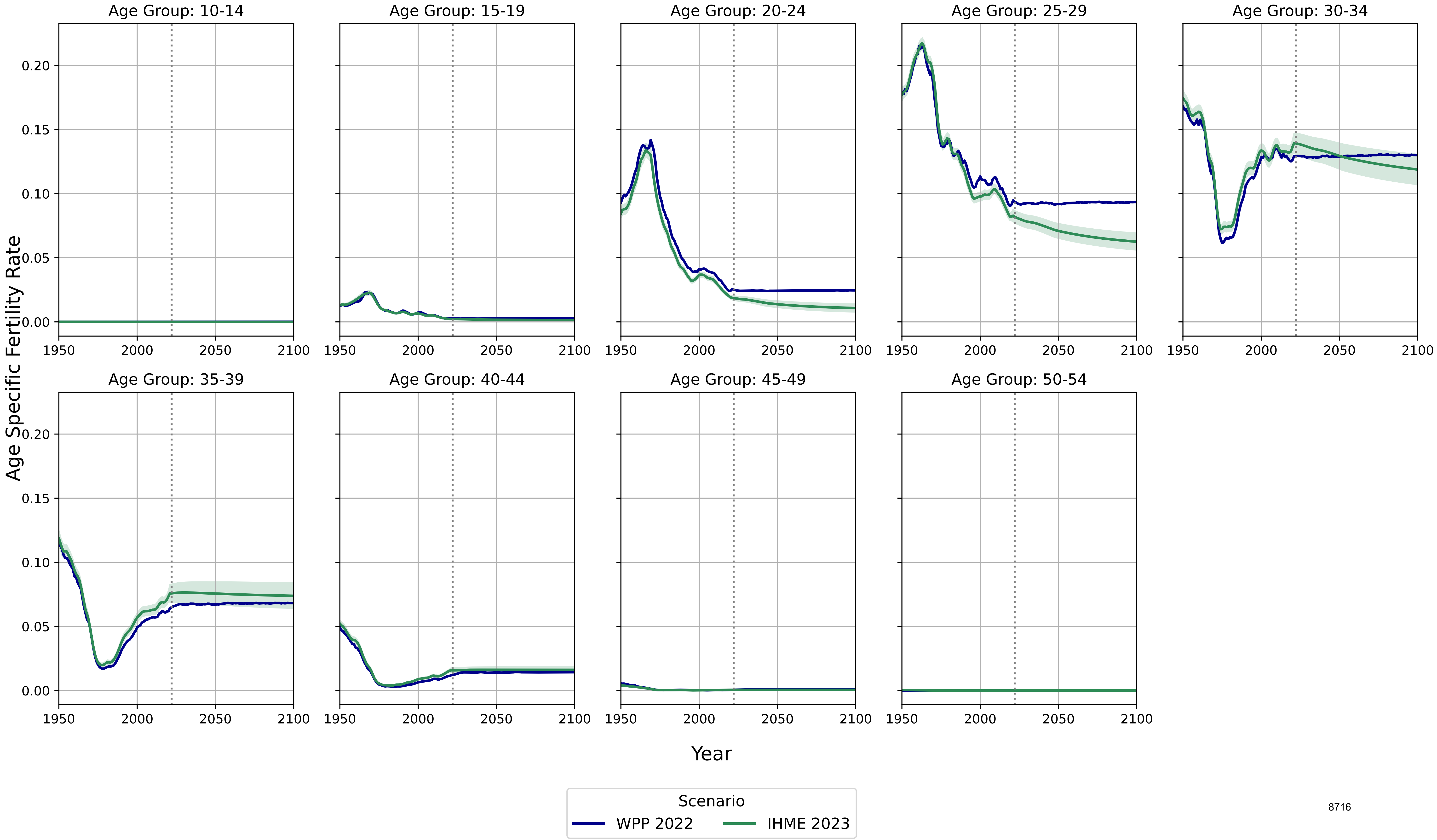

Supplemental Results Figure S8: Five-year interval ASFR from IHME reference scenario and the UNPD medium variant scenario by location, 1950-2100

Norway

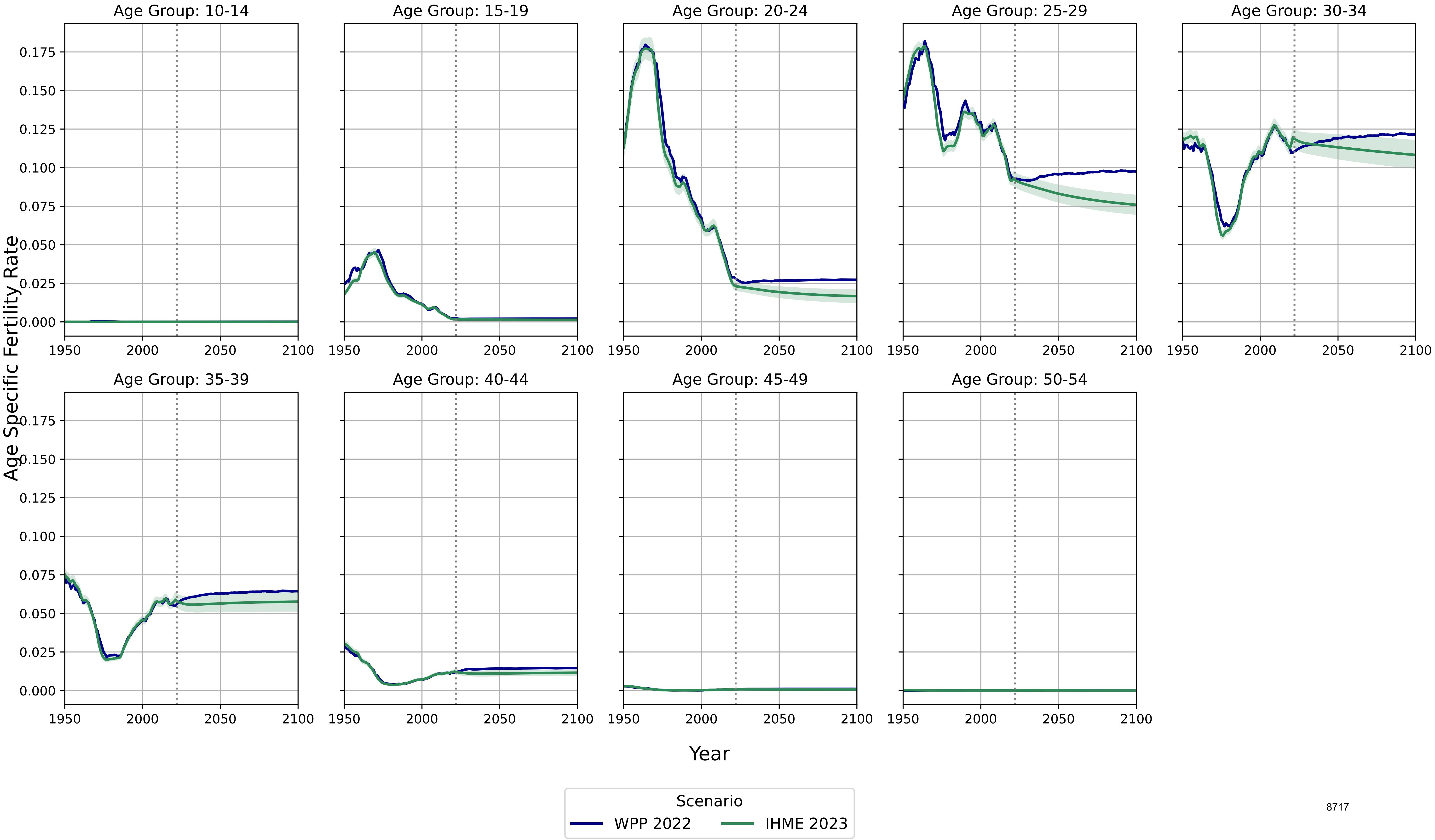

Supplemental Results Figure S8: Five-year interval ASFR from IHME reference scenario and the UNPD medium variant scenario by location, 1950-2100

Portugal

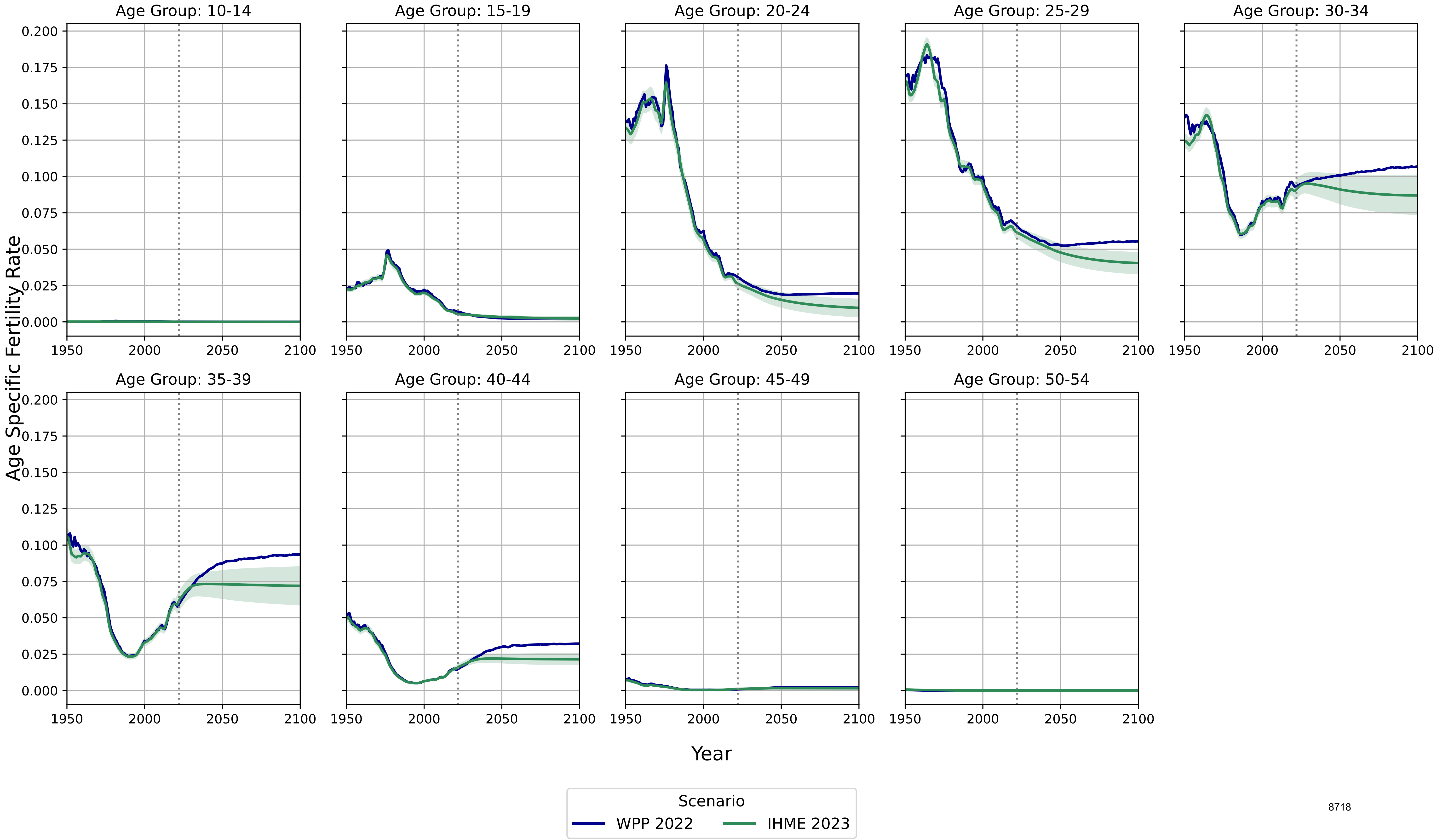

Supplemental Results Figure S8: Five-year interval ASFR from IHME reference scenario and the UNPD medium variant scenario by location, 1950-2100

San Marino

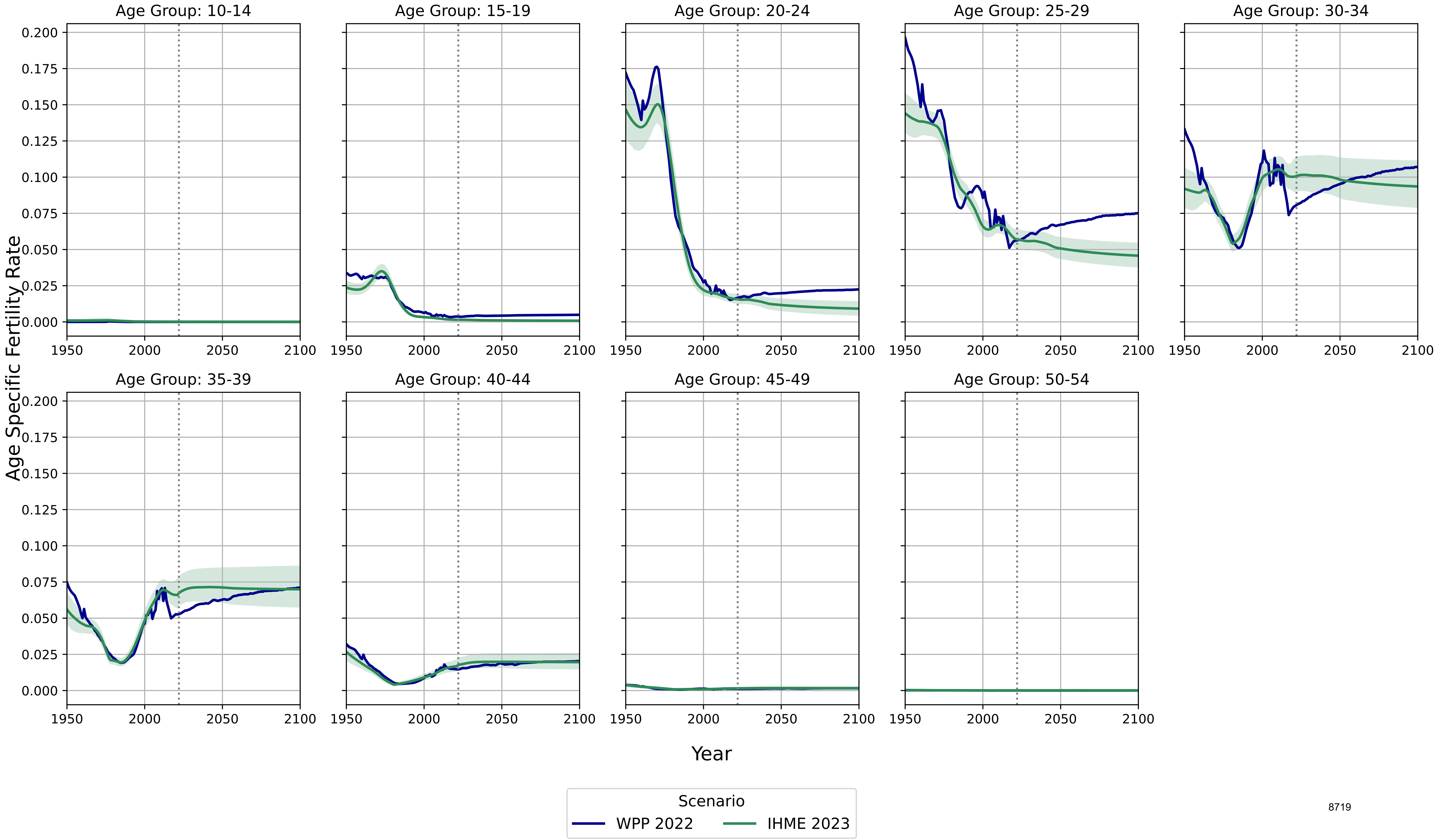

Supplemental Results Figure S8: Five-year interval ASFR from IHME reference scenario and the UNPD medium variant scenario by location, 1950-2100

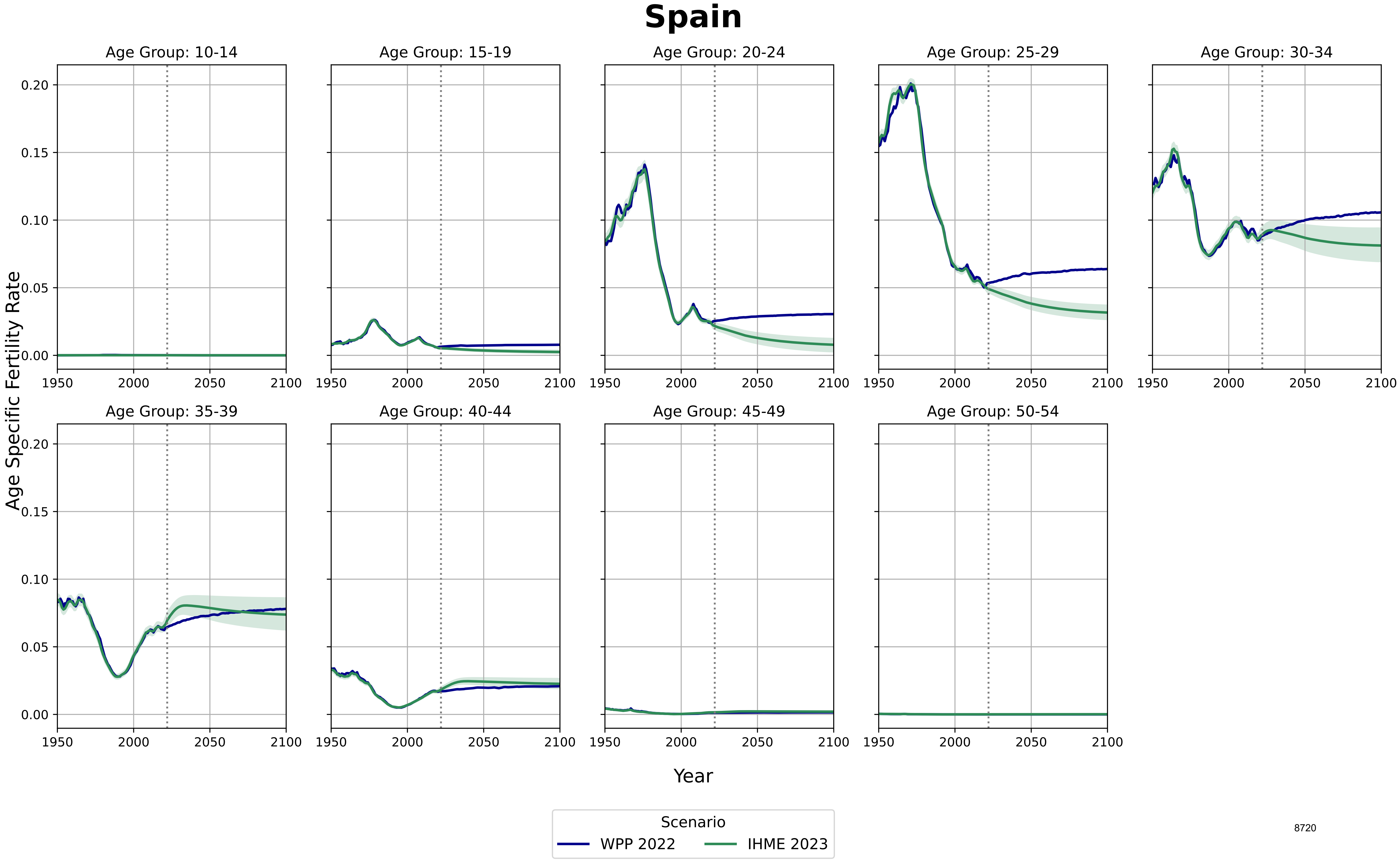

Supplemental Results Figure S8: Five-year interval ASFR from IHME reference scenario and the UNPD medium variant scenario by location, 1950-2100

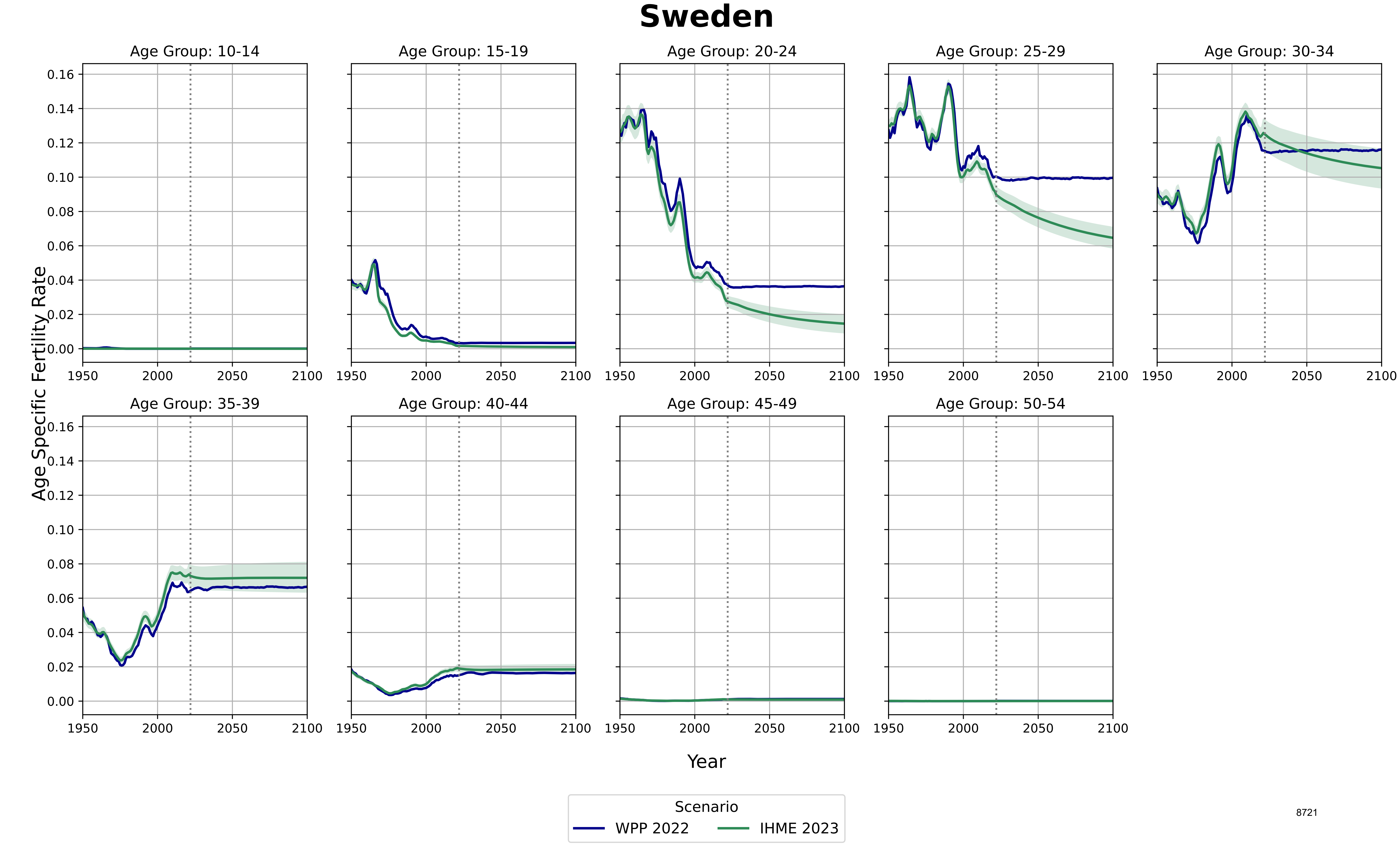

Supplemental Results Figure S8: Five-year interval ASFR from IHME reference scenario and the UNPD medium variant scenario by location, 1950-2100

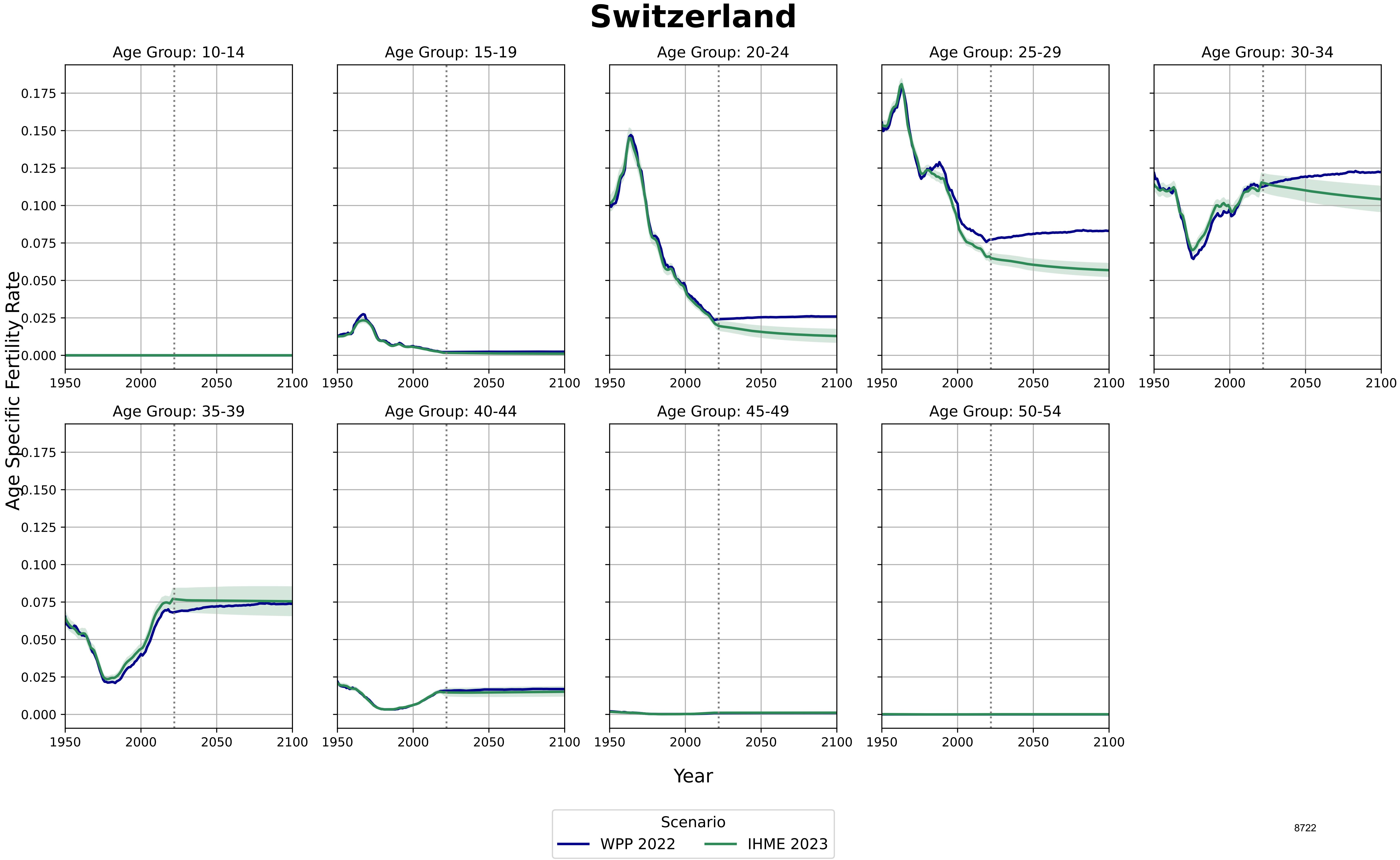

Supplemental Results Figure S8: Five-year interval ASFR from IHME reference scenario and the UNPD medium variant scenario by location, 1950-2100

UK

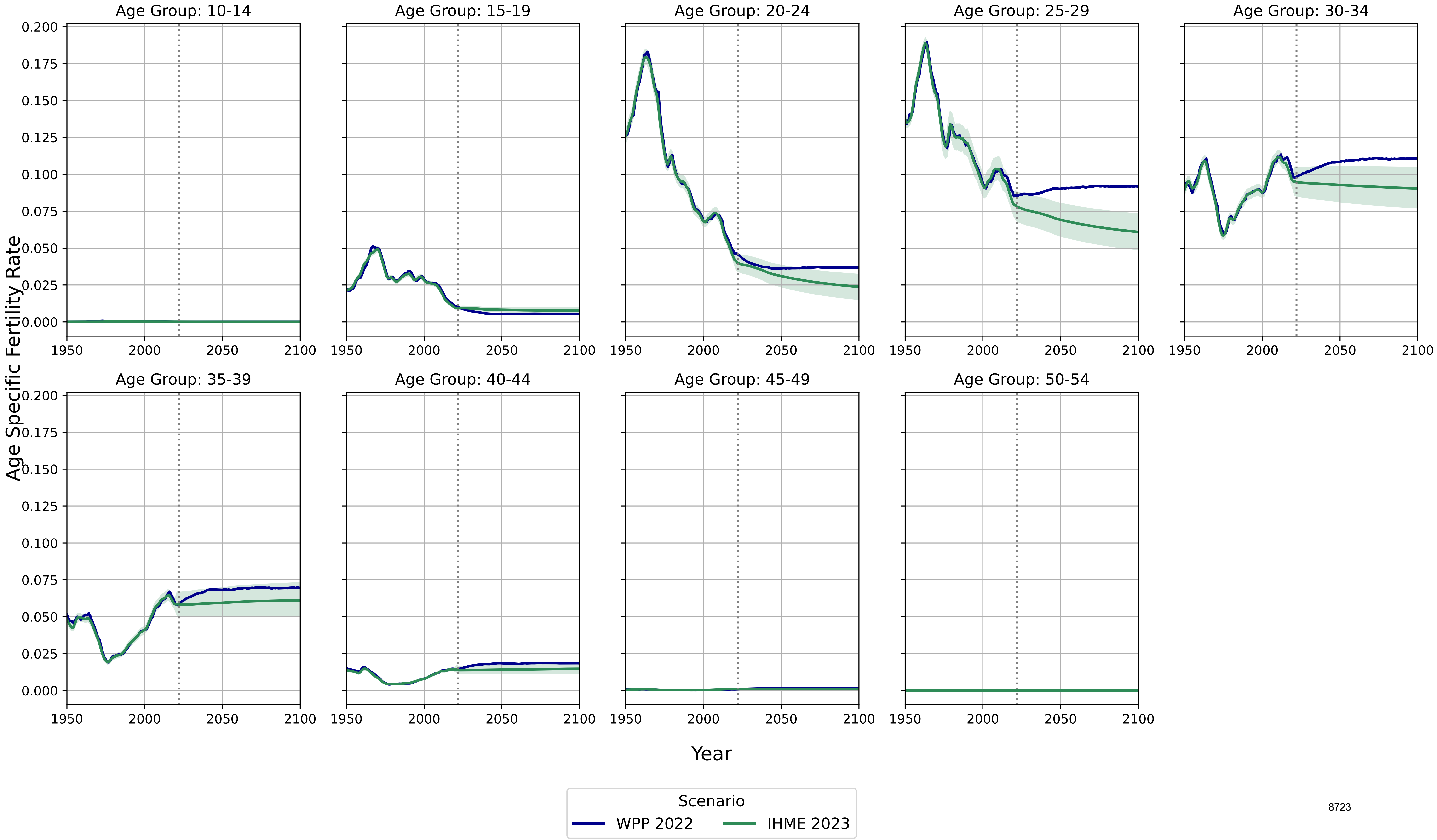

Supplemental Results Figure S8: Five-year interval ASFR from IHME reference scenario and the UNPD medium variant scenario by location, 1950-2100

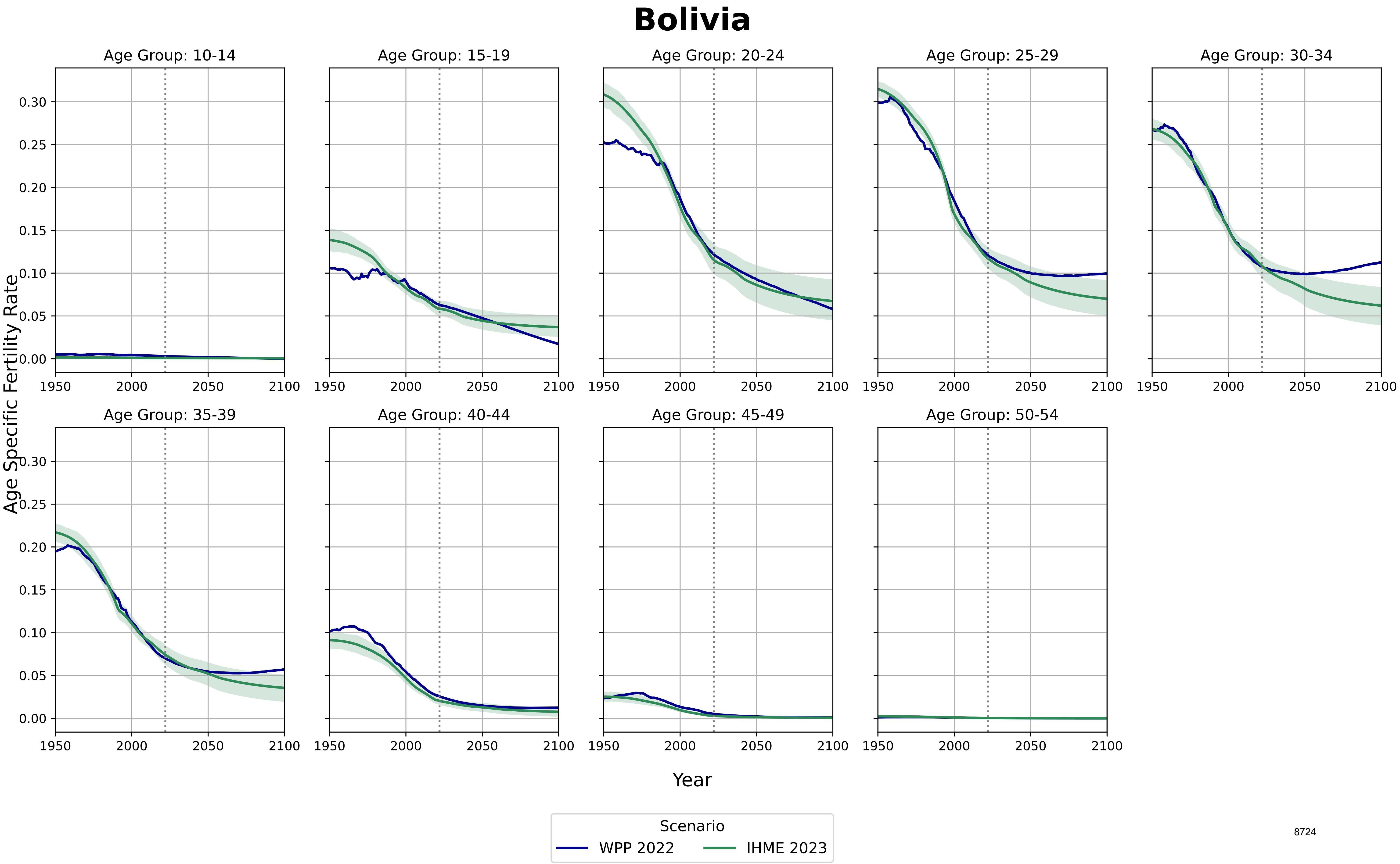

Supplemental Results Figure S8: Five-year interval ASFR from IHME reference scenario and the UNPD medium variant scenario by location, 1950-2100

Ecuador

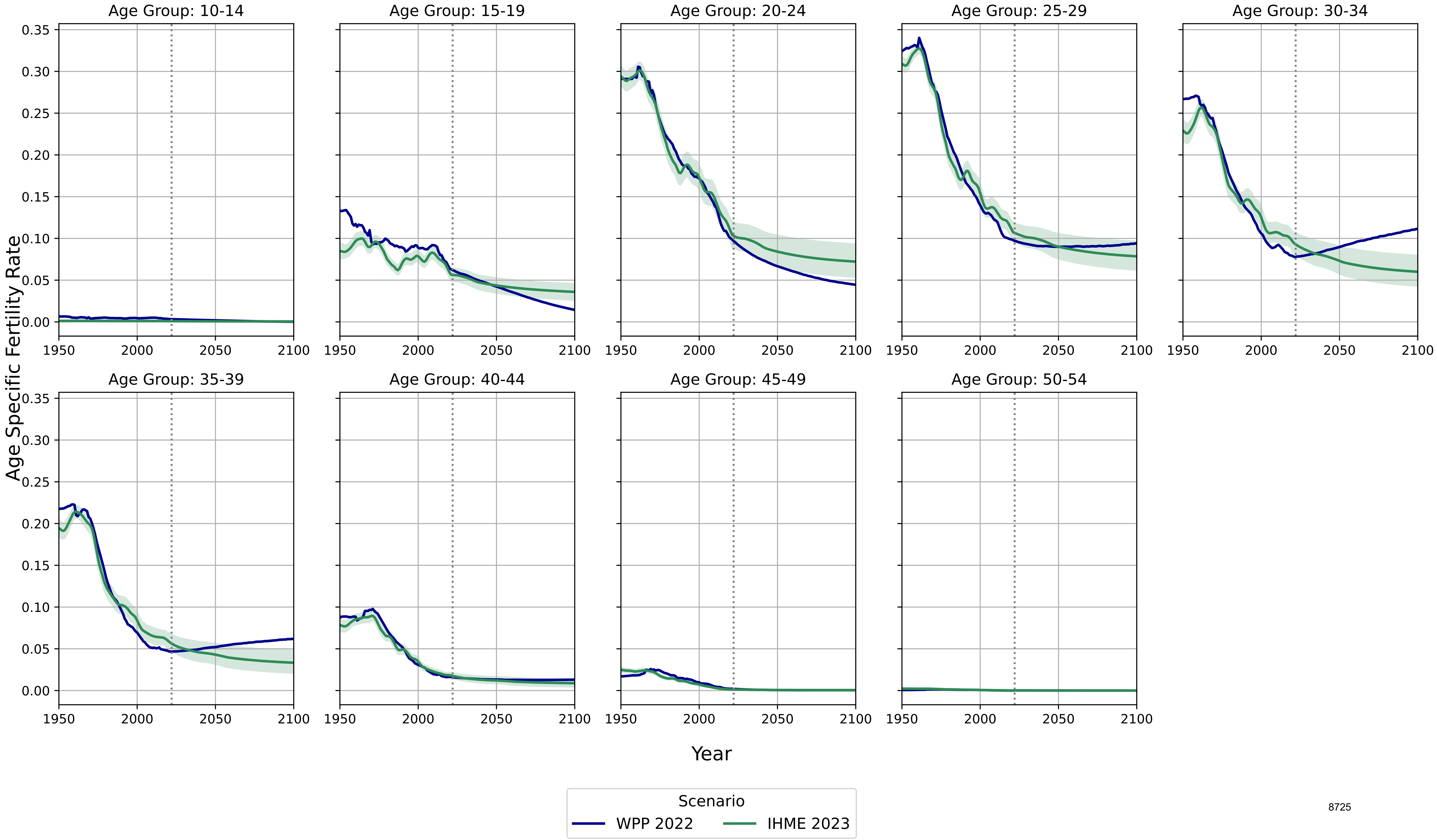

Supplemental Results Figure S8: Five-year interval ASFR from IHME reference scenario and the UNPD medium variant scenario by location, 1950-2100

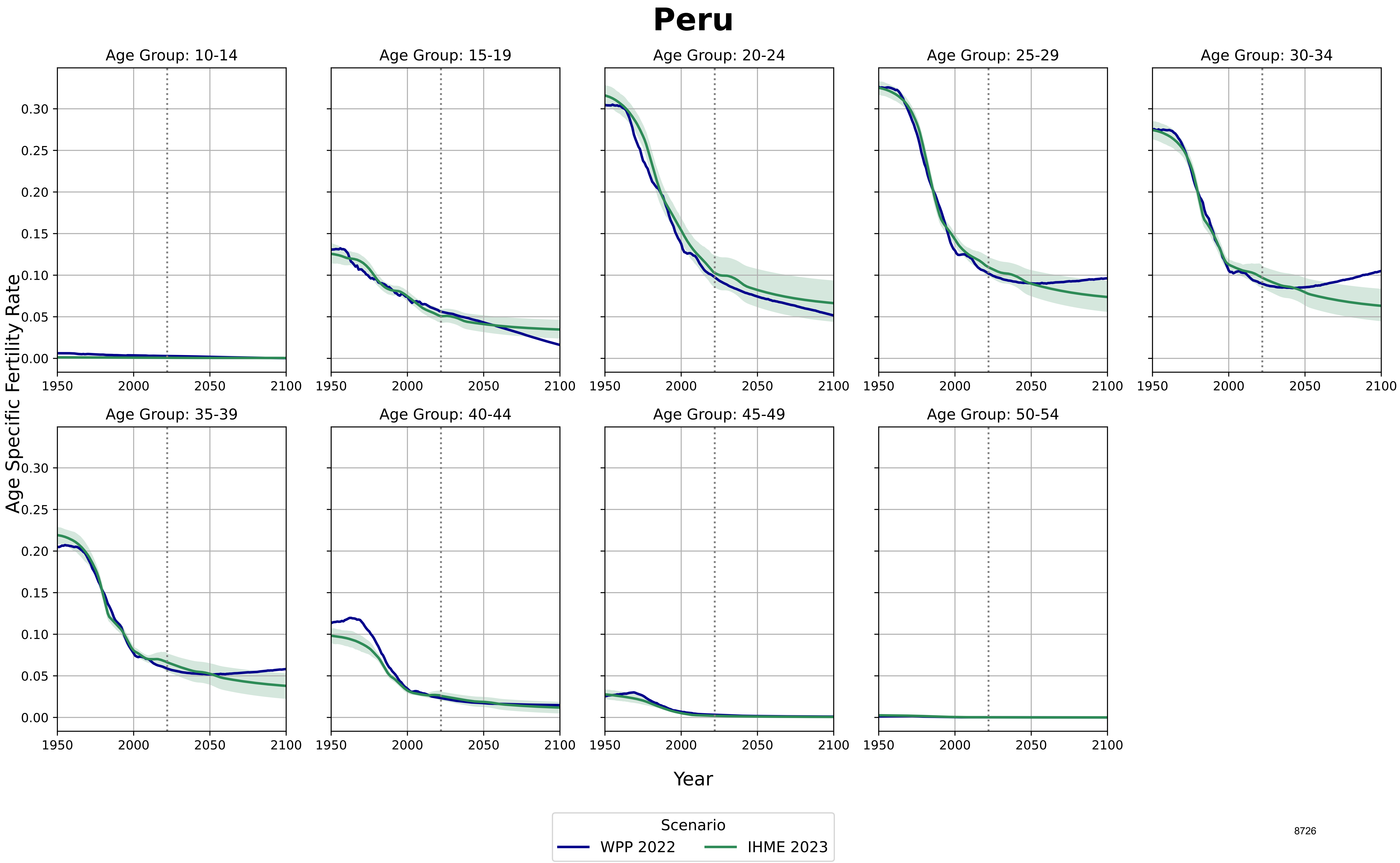

Supplemental Results Figure S8: Five-year interval ASFR from IHME reference scenario and the UNPD medium variant scenario by location, 1950-2100

Antigua and Barbuda

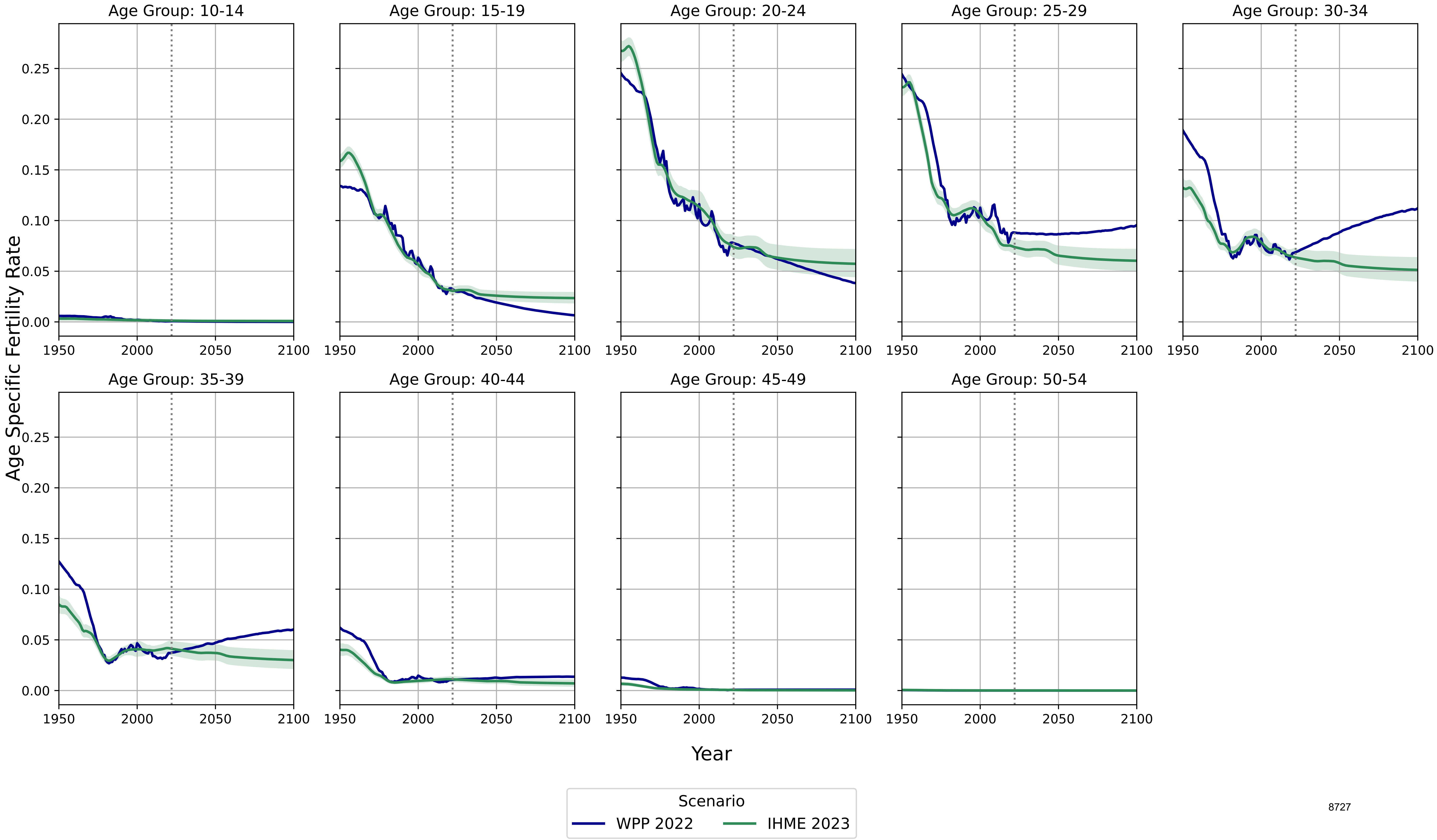

Supplemental Results Figure S8: Five-year interval ASFR from IHME reference scenario and the UNPD medium variant scenario by location, 1950-2100

The Bahamas

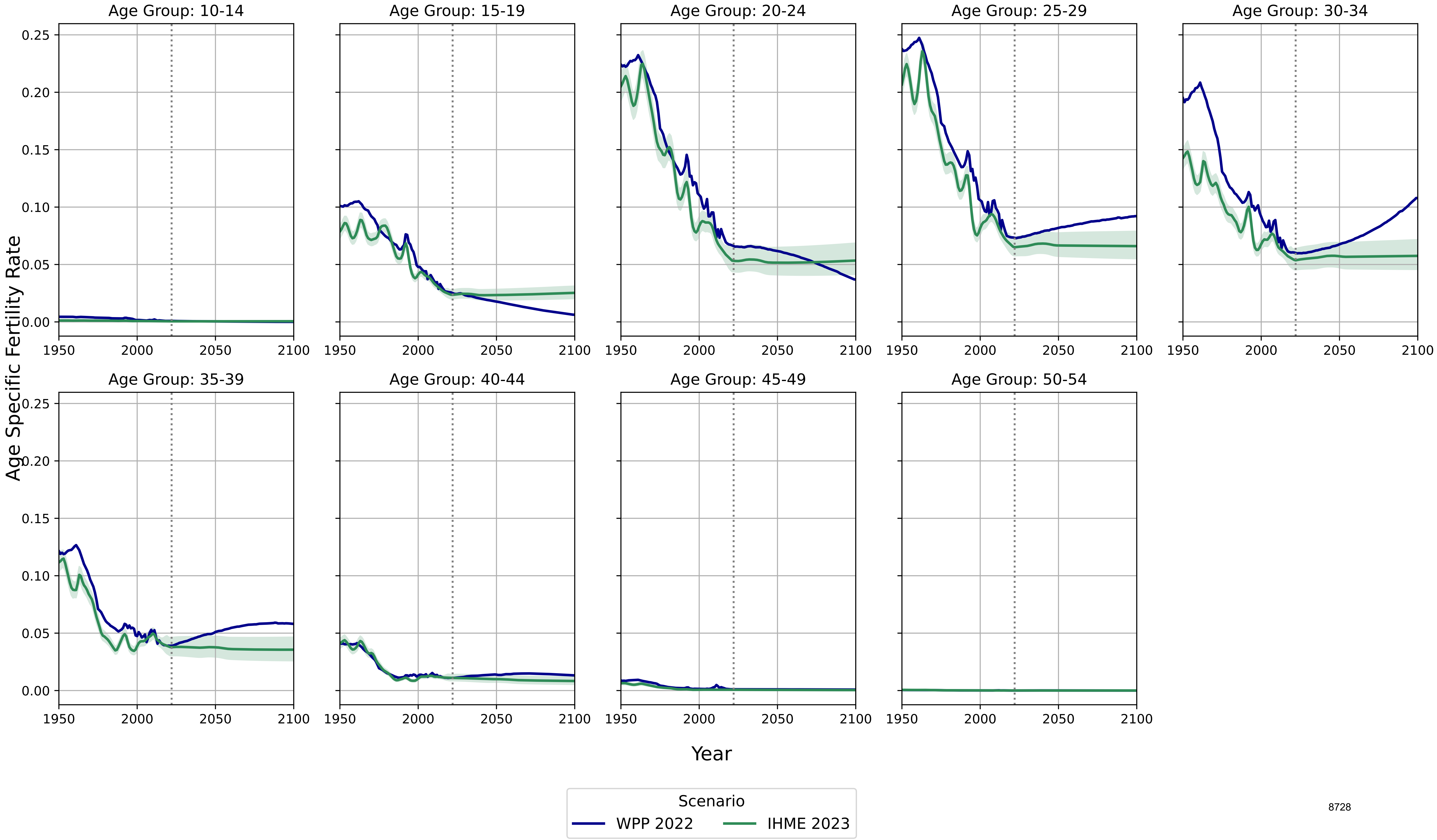

Supplemental Results Figure S8: Five-year interval ASFR from IHME reference scenario and the UNPD medium variant scenario by location, 1950-2100

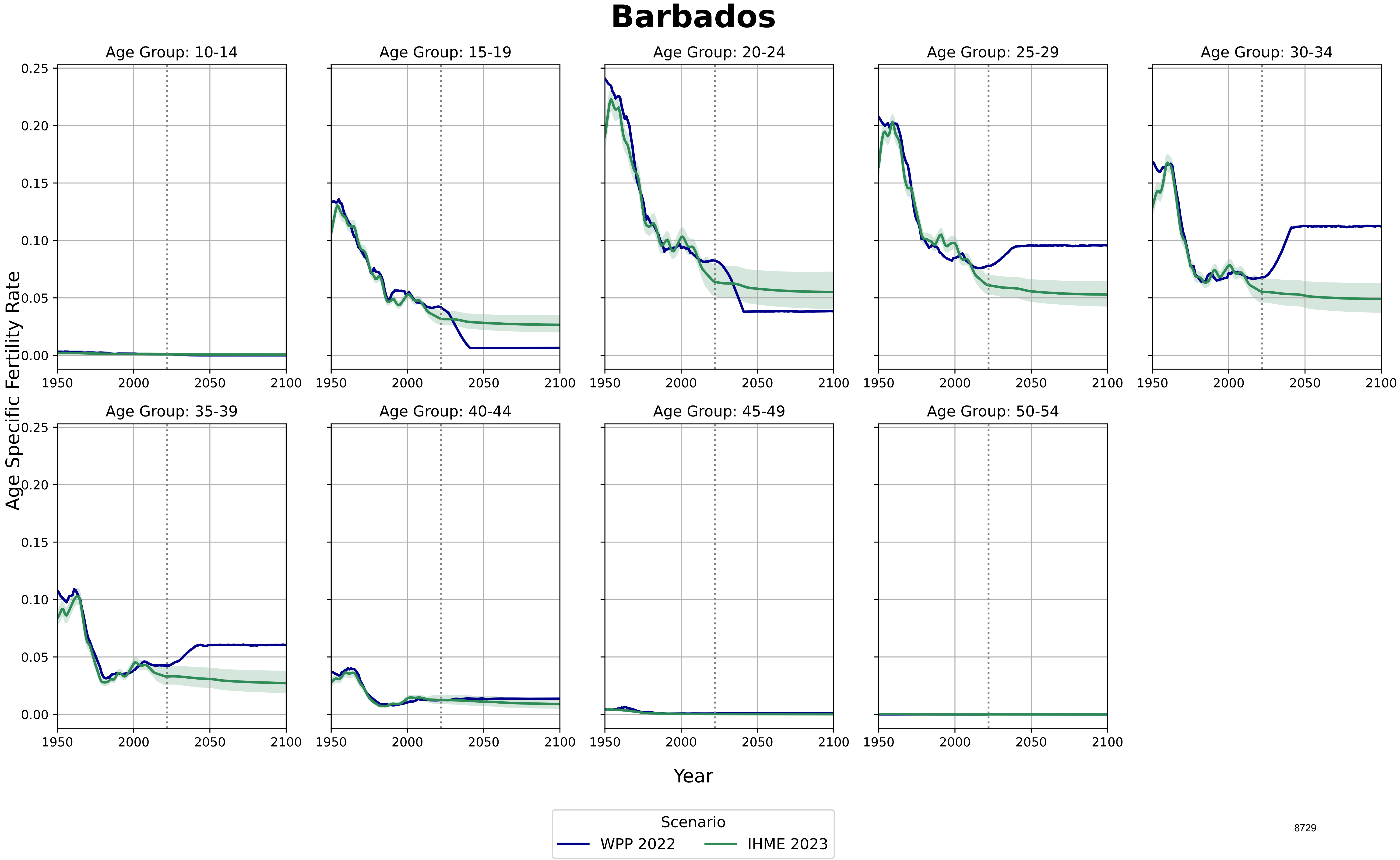

Supplemental Results Figure S8: Five-year interval ASFR from IHME reference scenario and the UNPD medium variant scenario by location, 1950-2100

Belize

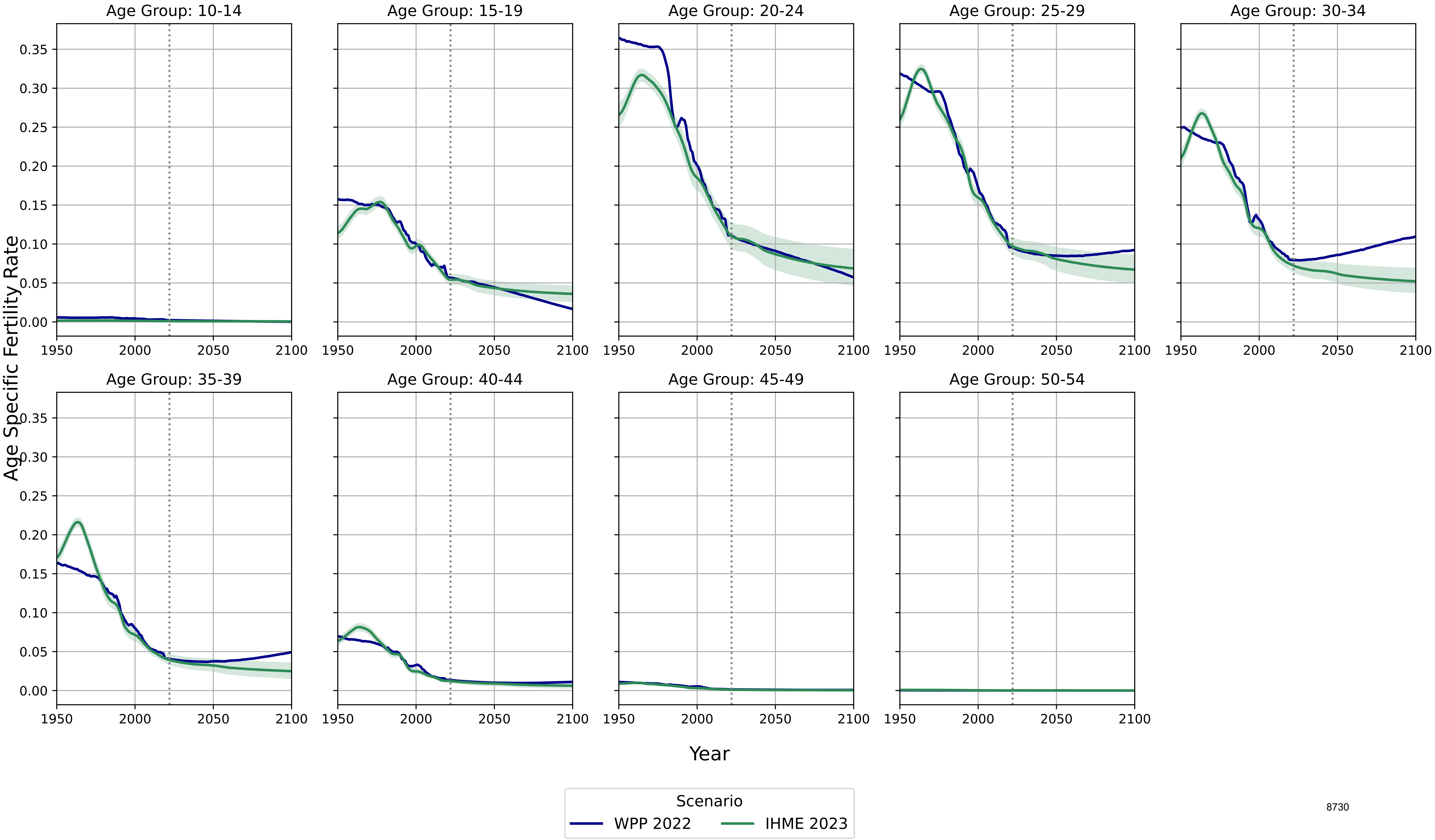

Supplemental Results Figure S8: Five-year interval ASFR from IHME reference scenario and the UNPD medium variant scenario by location, 1950-2100

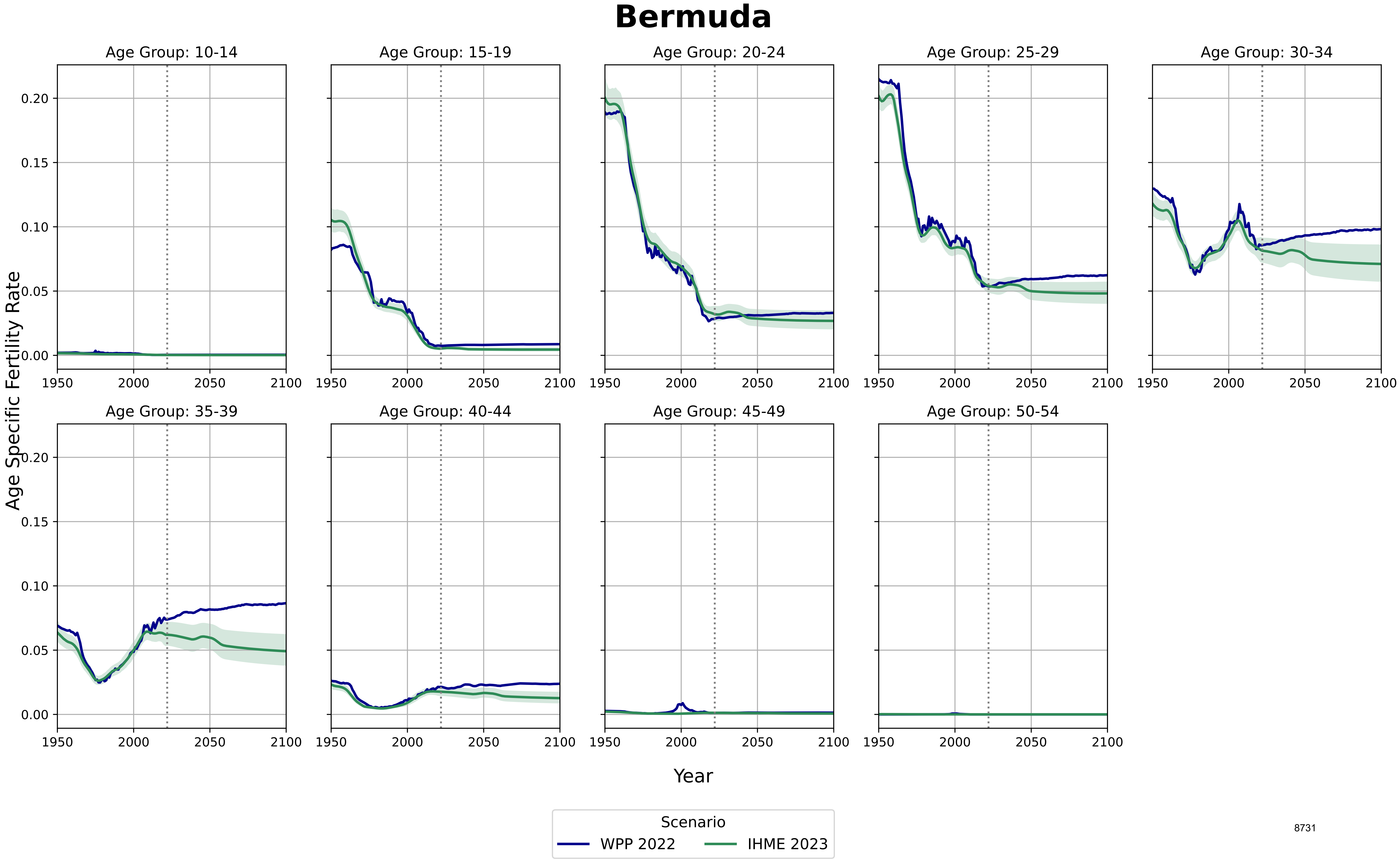

Supplemental Results Figure S8: Five-year interval ASFR from IHME reference scenario and the UNPD medium variant scenario by location, 1950-2100

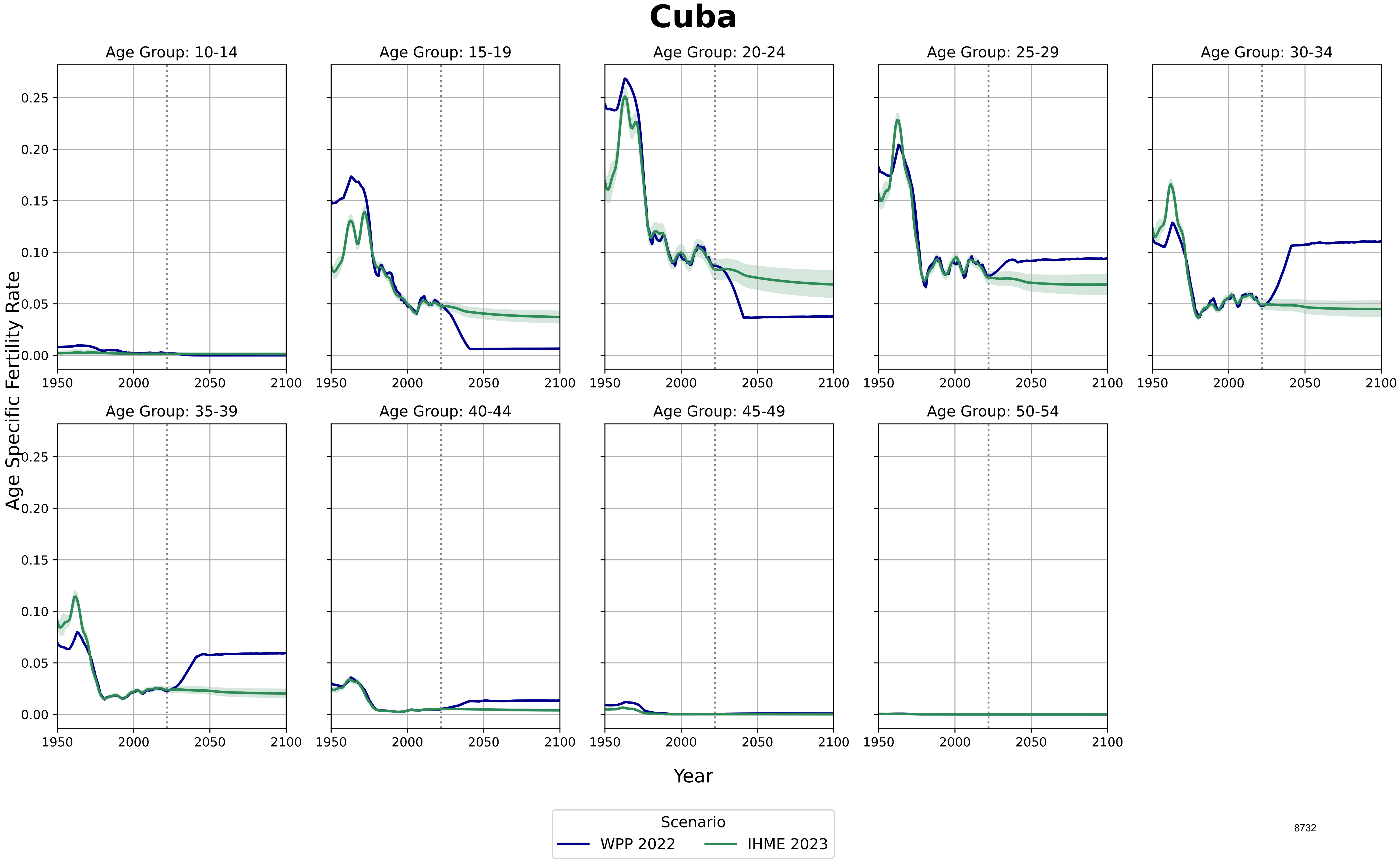

Supplemental Results Figure S8: Five-year interval ASFR from IHME reference scenario and the UNPD medium variant scenario by location, 1950-2100

Dominica

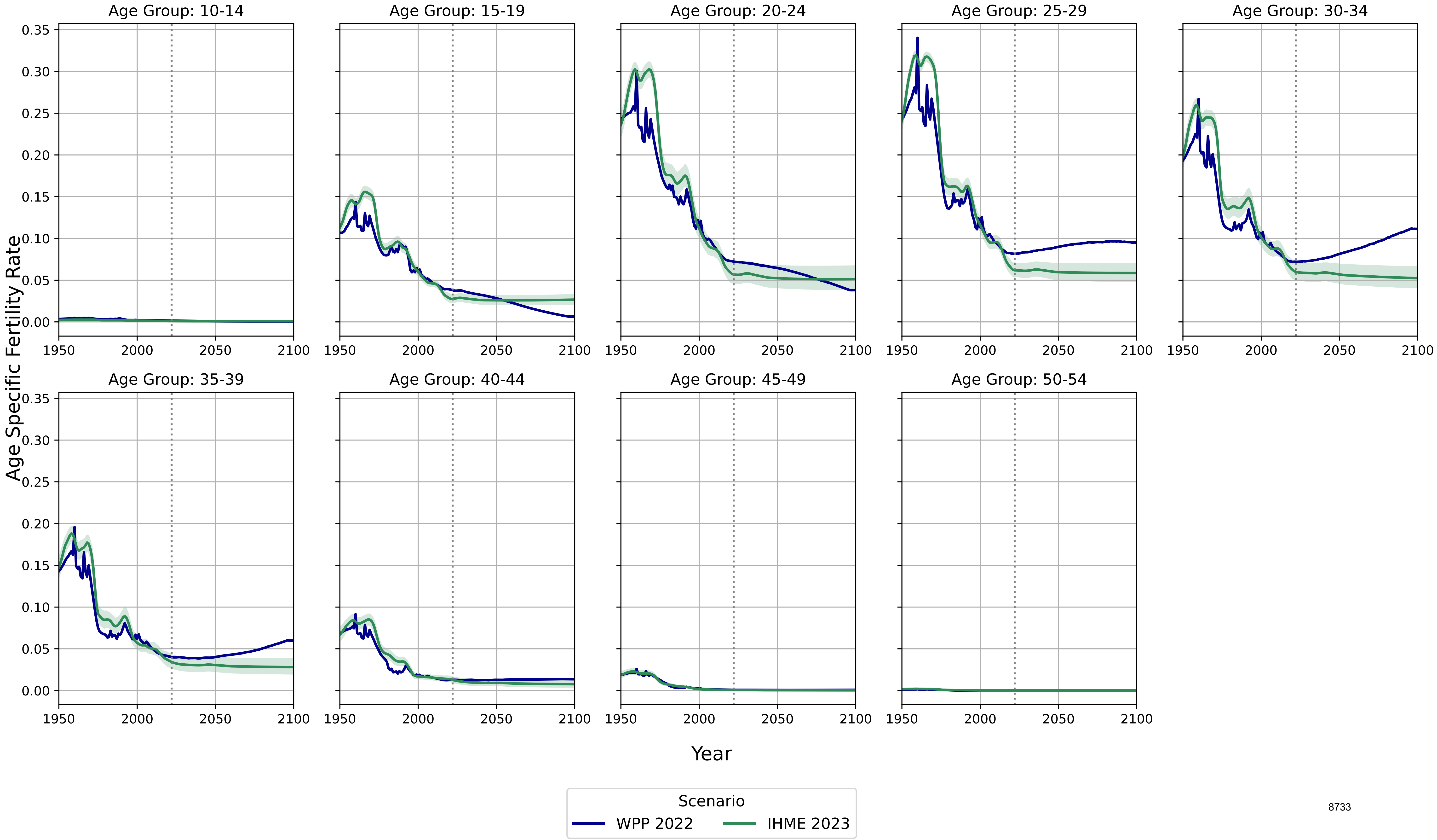

Supplemental Results Figure S8: Five-year interval ASFR from IHME reference scenario and the UNPD medium variant scenario by location, 1950-2100

Dominican Republic

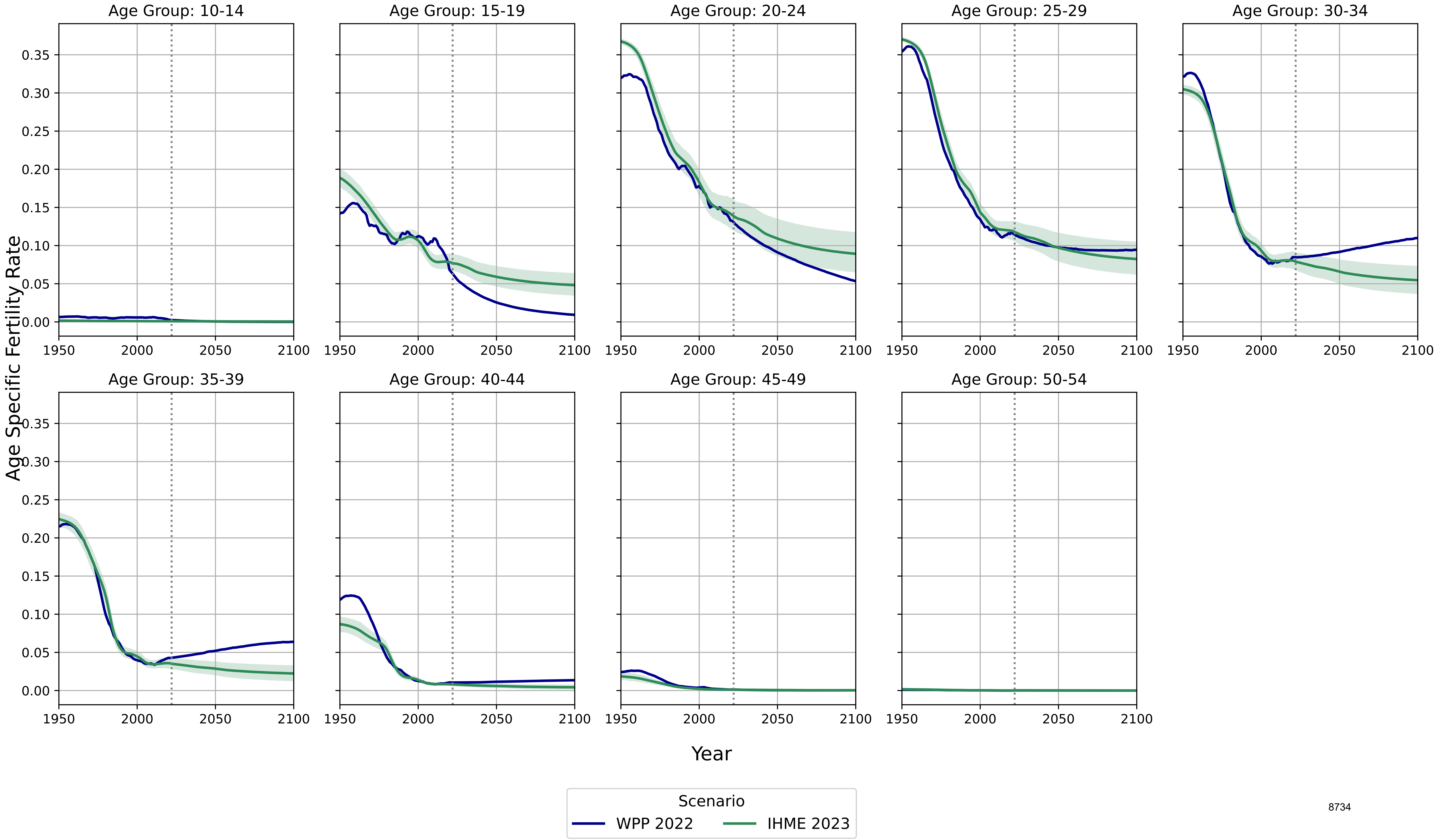

Supplemental Results Figure S8: Five-year interval ASFR from IHME reference scenario and the UNPD medium variant scenario by location, 1950-2100

Grenada

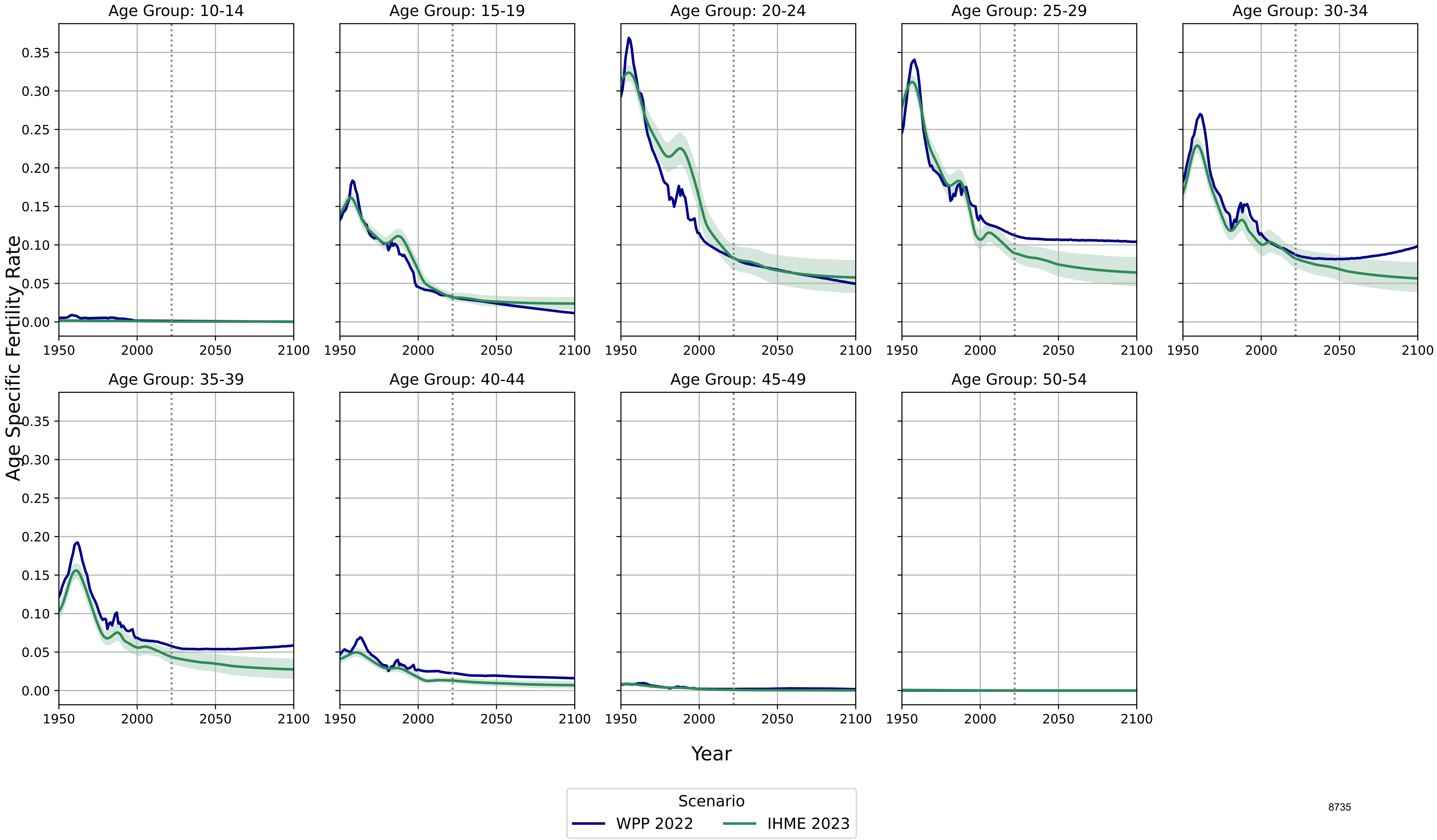

Supplemental Results Figure S8: Five-year interval ASFR from IHME reference scenario and the UNPD medium variant scenario by location, 1950-2100

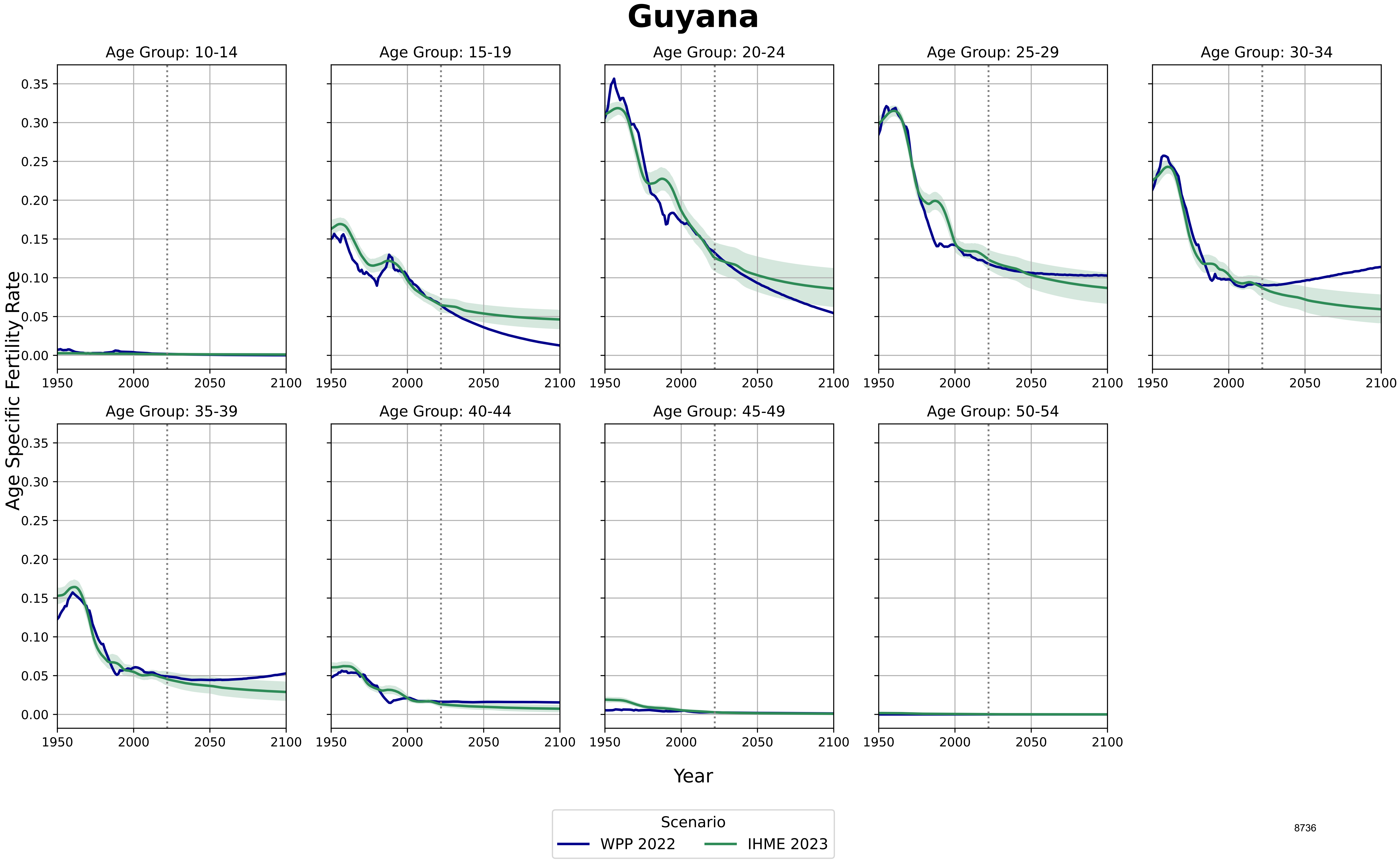

Supplemental Results Figure S8: Five-year interval ASFR from IHME reference scenario and the UNPD medium variant scenario by location, 1950-2100

Haiti

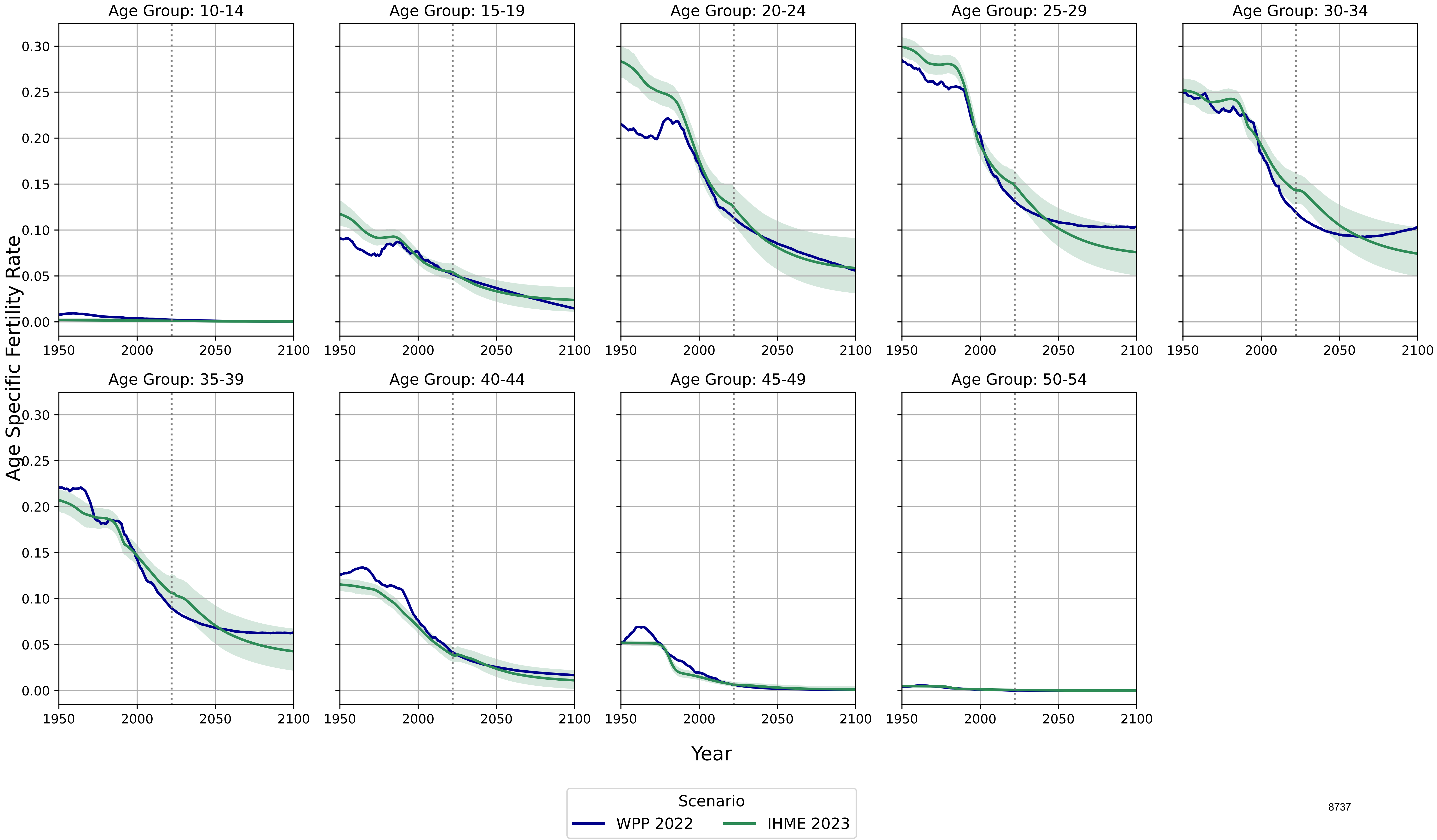

Supplemental Results Figure S8: Five-year interval ASFR from IHME reference scenario and the UNPD medium variant scenario by location, 1950-2100

Jamaica

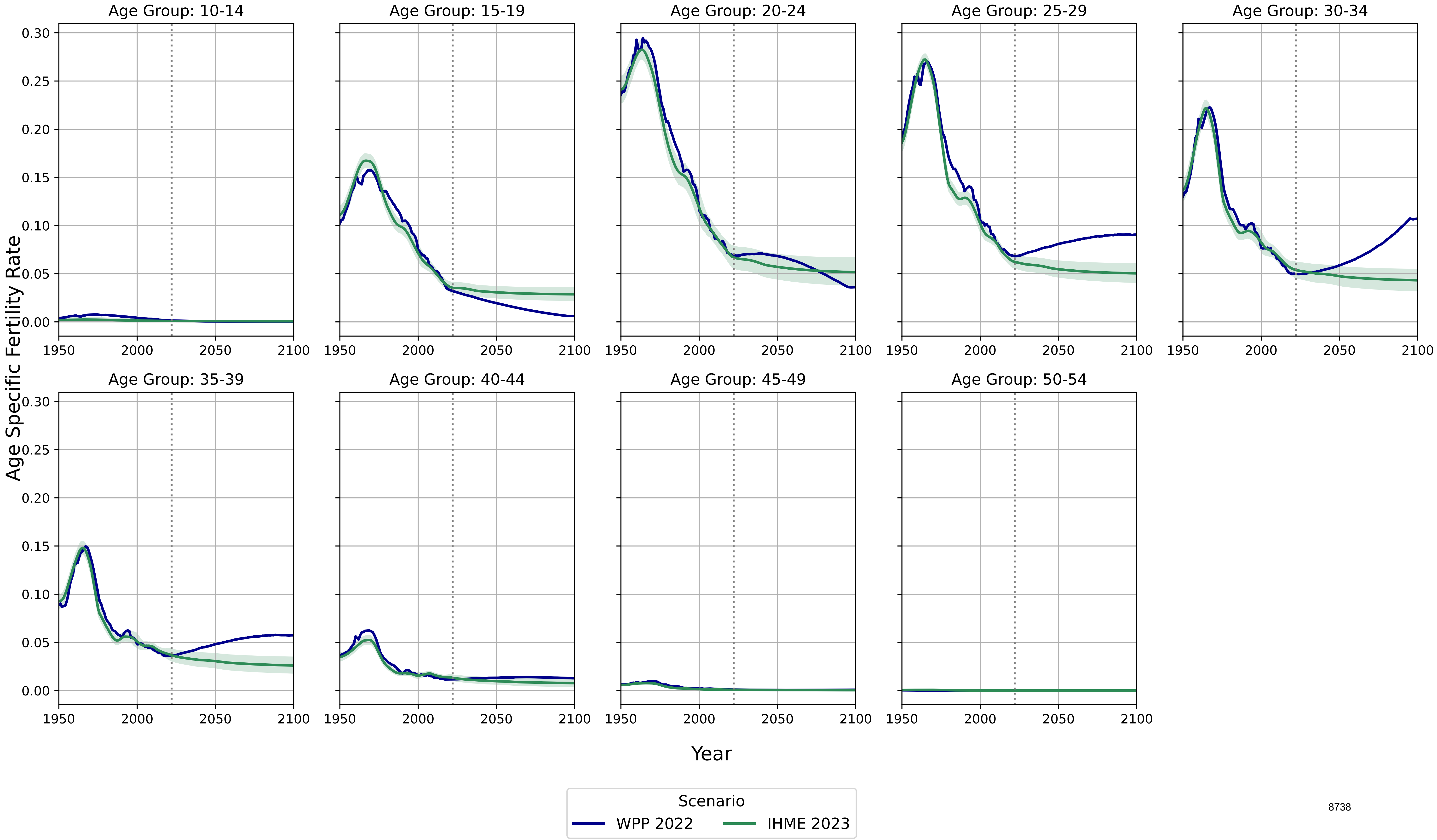

Supplemental Results Figure S8: Five-year interval ASFR from IHME reference scenario and the UNPD medium variant scenario by location, 1950-2100

Puerto Rico

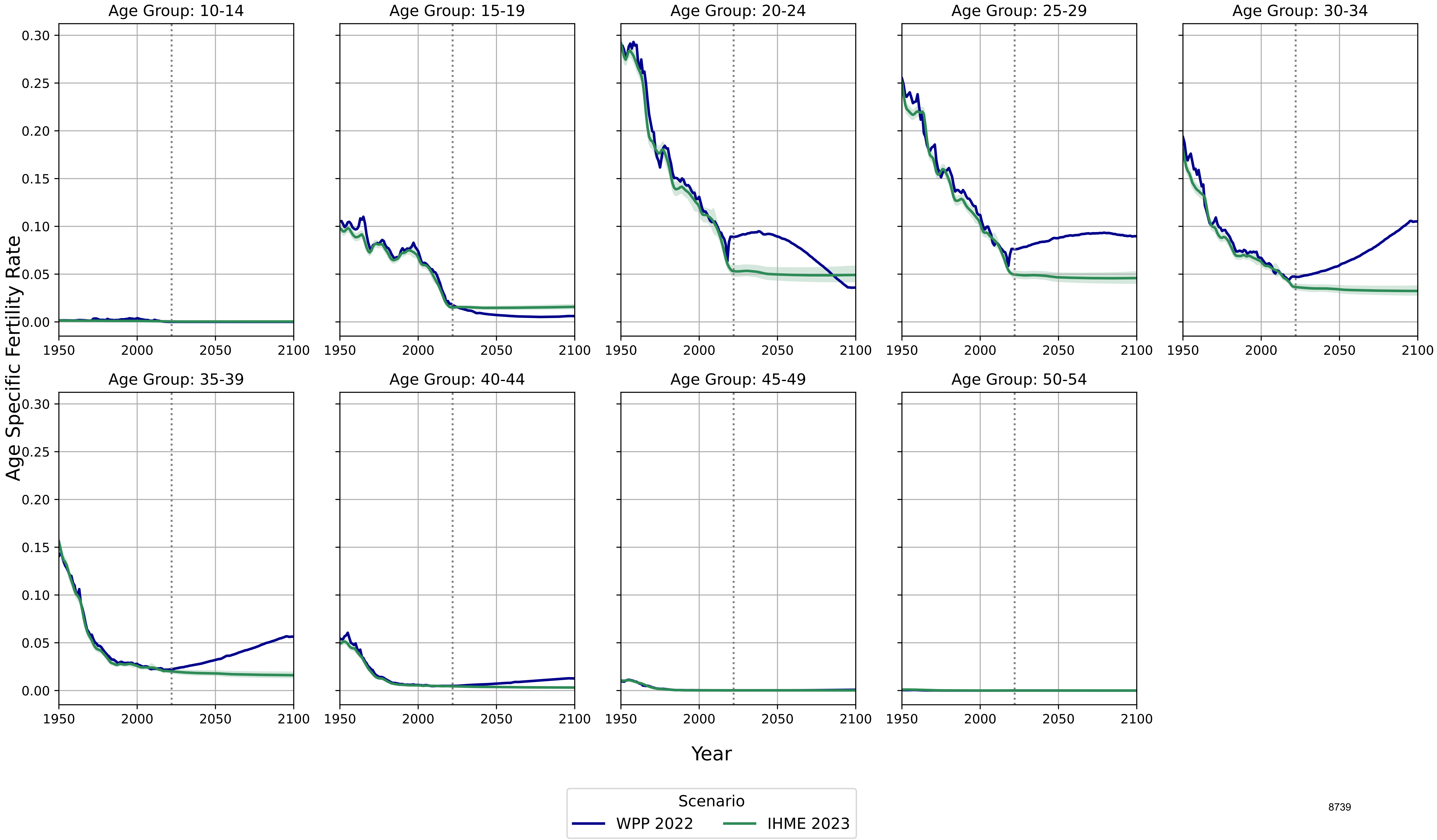

Supplemental Results Figure S8: Five-year interval ASFR from IHME reference scenario and the UNPD medium variant scenario by location, 1950-2100

Saint Kitts and Nevis

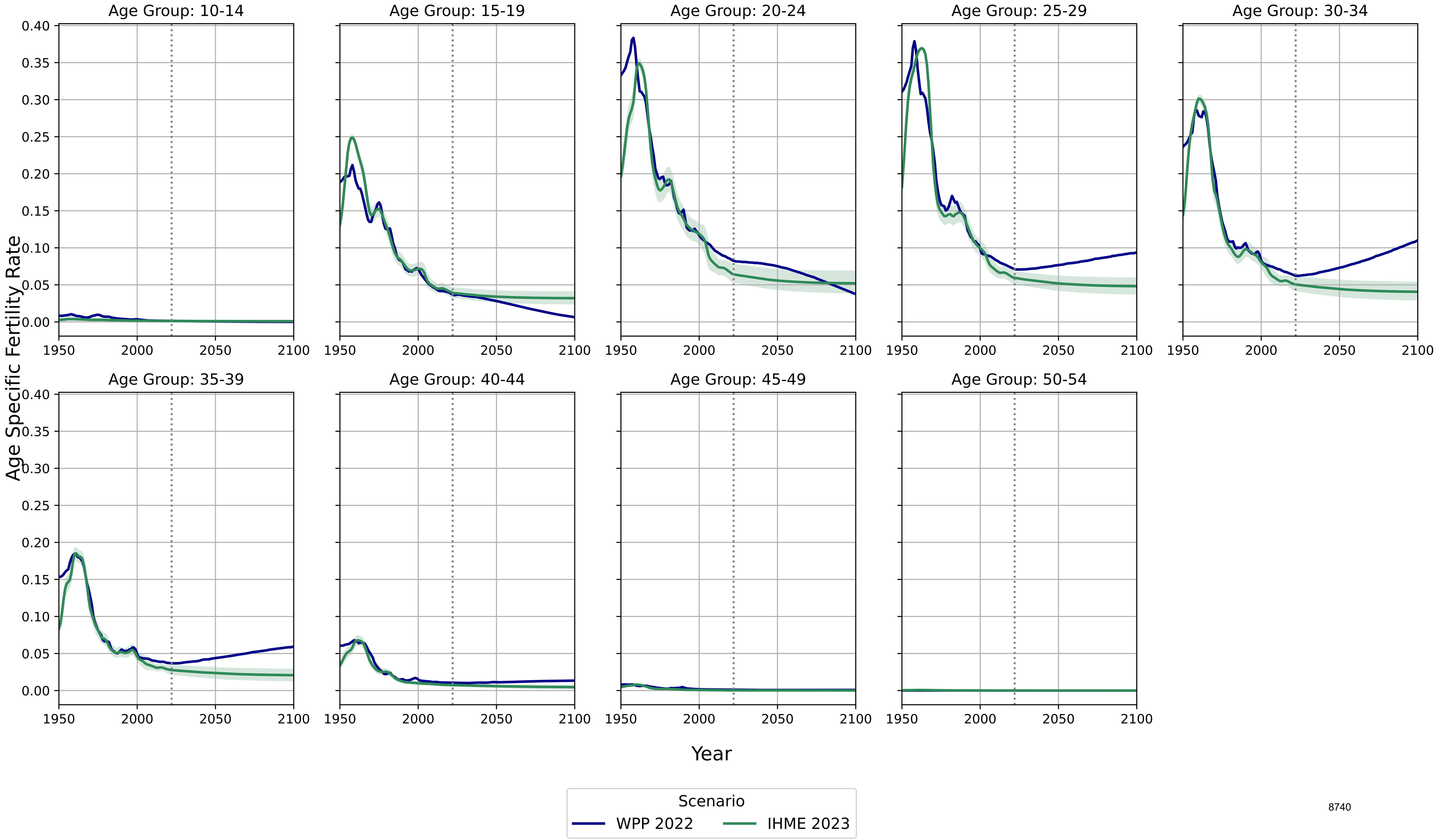

8740

Supplemental Results Figure S8: Five-year interval ASFR from IHME reference scenario and the UNPD medium variant scenario by location, 1950-2100

Saint Lucia

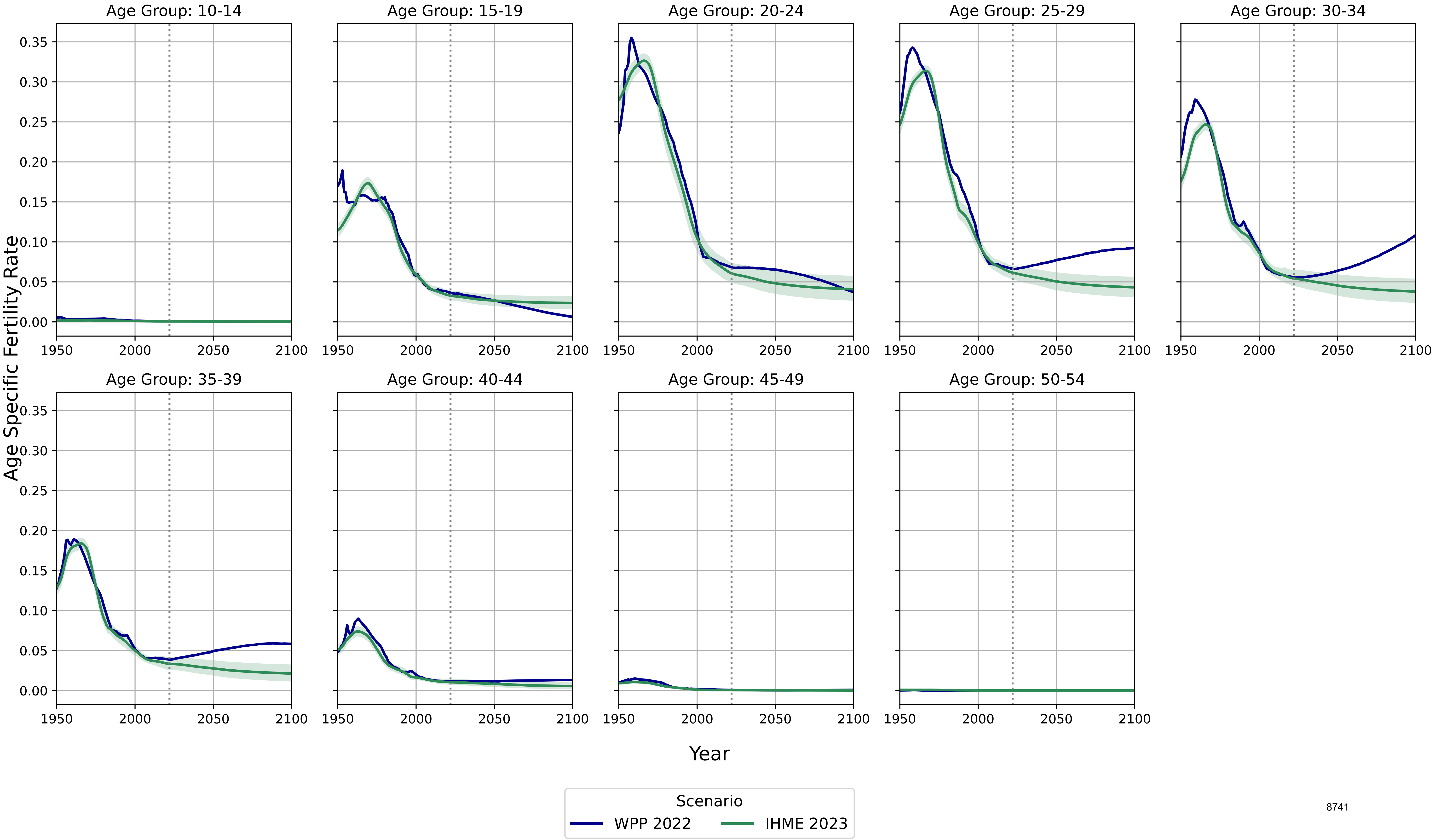

Supplemental Results Figure S8: Five-year interval ASFR from IHME reference scenario and the UNPD medium variant scenario by location, 1950-2100

Saint Vincent and the Grenadines

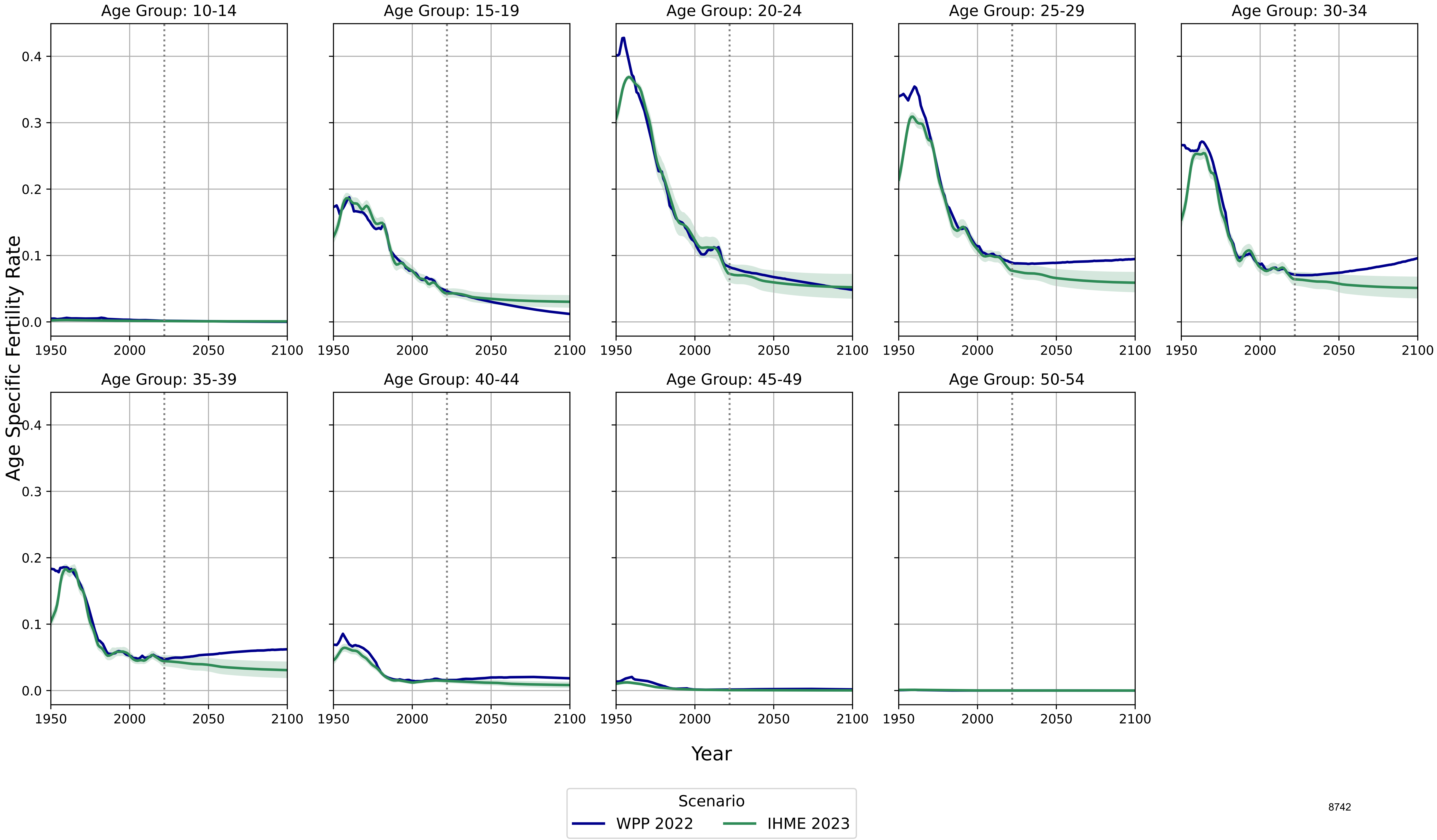

Supplemental Results Figure S8: Five-year interval ASFR from IHME reference scenario and the UNPD medium variant scenario by location, 1950-2100

Suriname

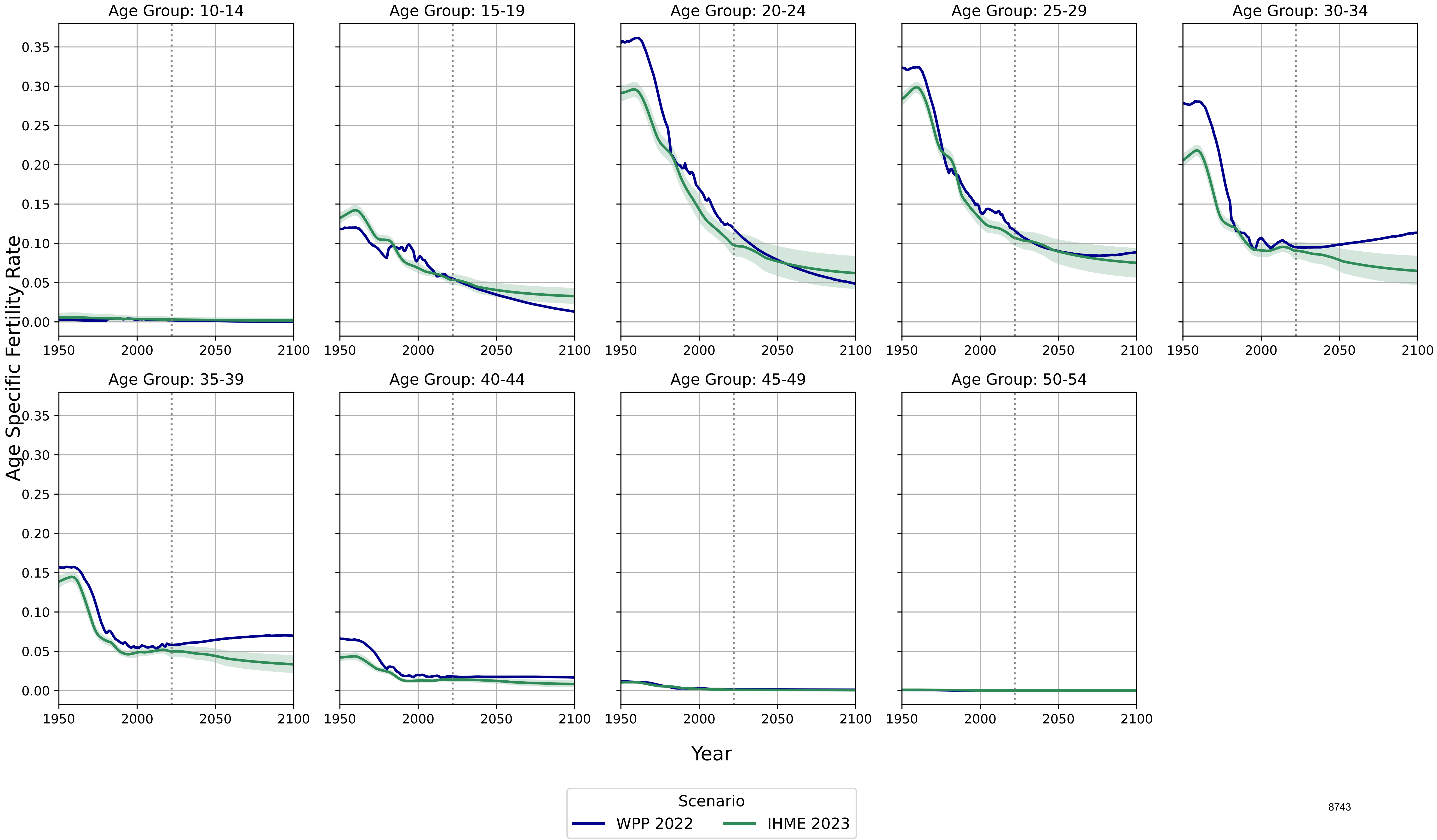

Supplemental Results Figure S8: Five-year interval ASFR from IHME reference scenario and the UNPD medium variant scenario by location, 1950-2100

Trinidad and Tobago

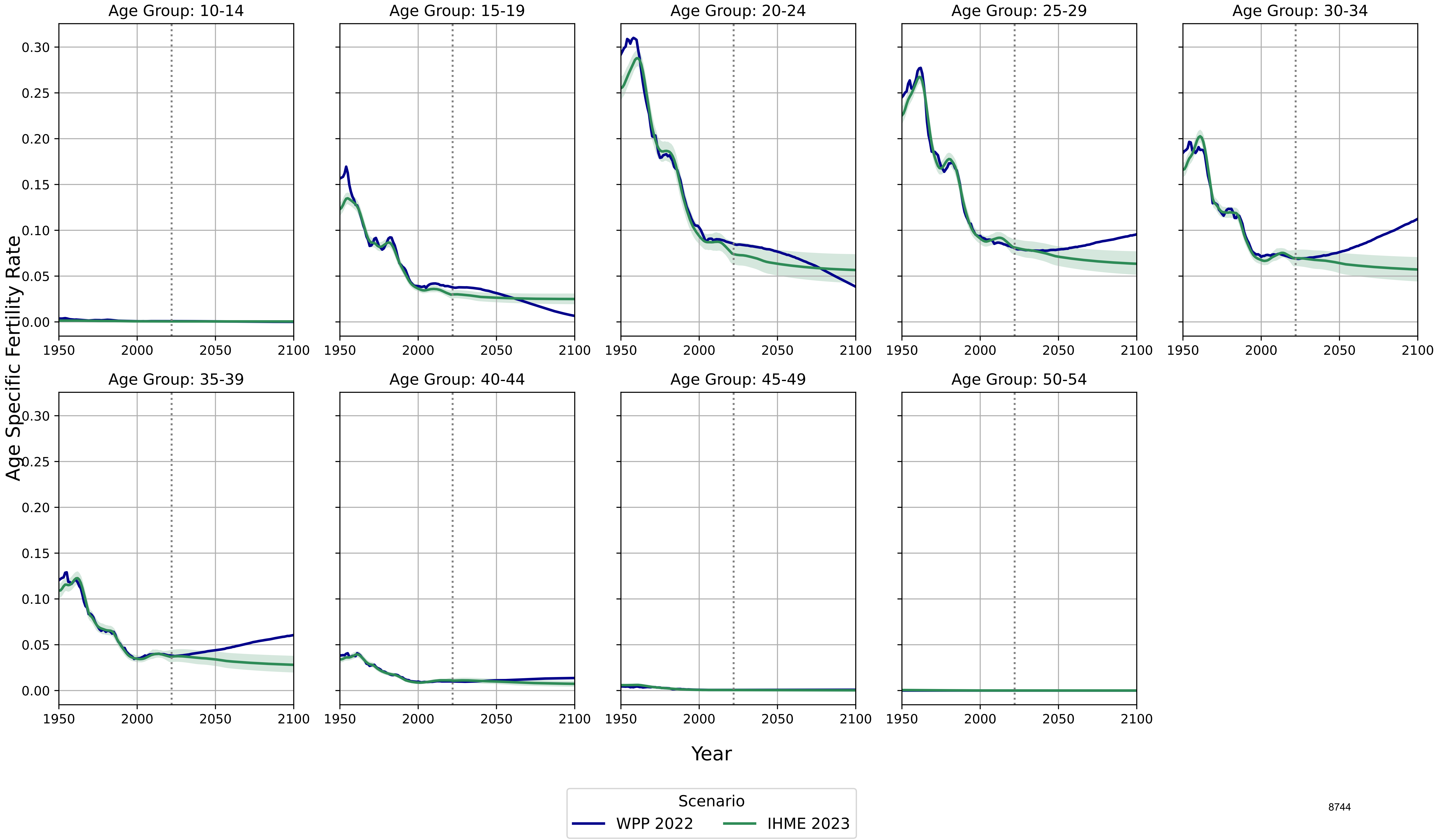

Supplemental Results Figure S8: Five-year interval ASFR from IHME reference scenario and the UNPD medium variant scenario by location, 1950-2100

Virgin Islands

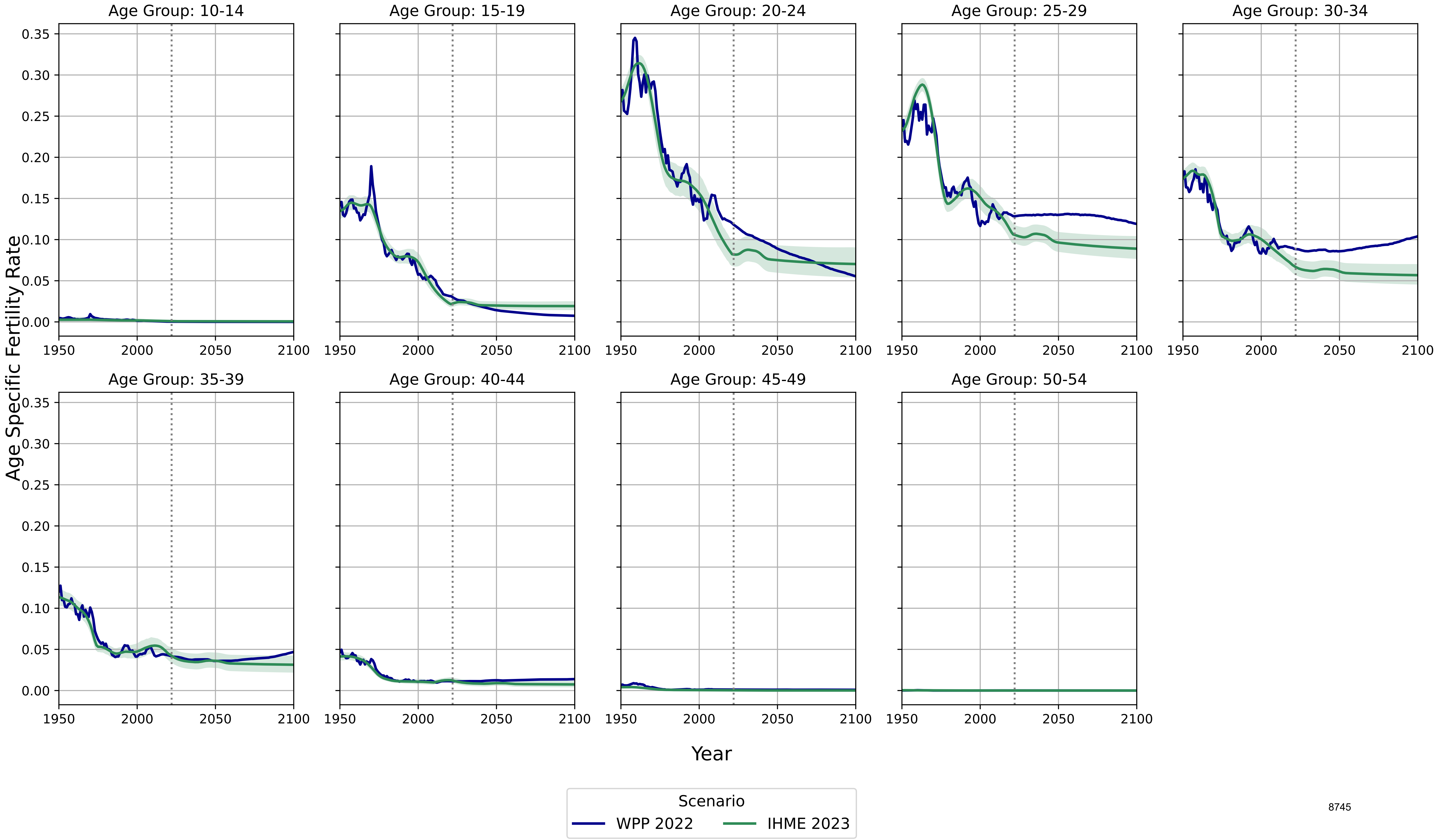

Supplemental Results Figure S8: Five-year interval ASFR from IHME reference scenario and the UNPD medium variant scenario by location, 1950-2100

Colombia

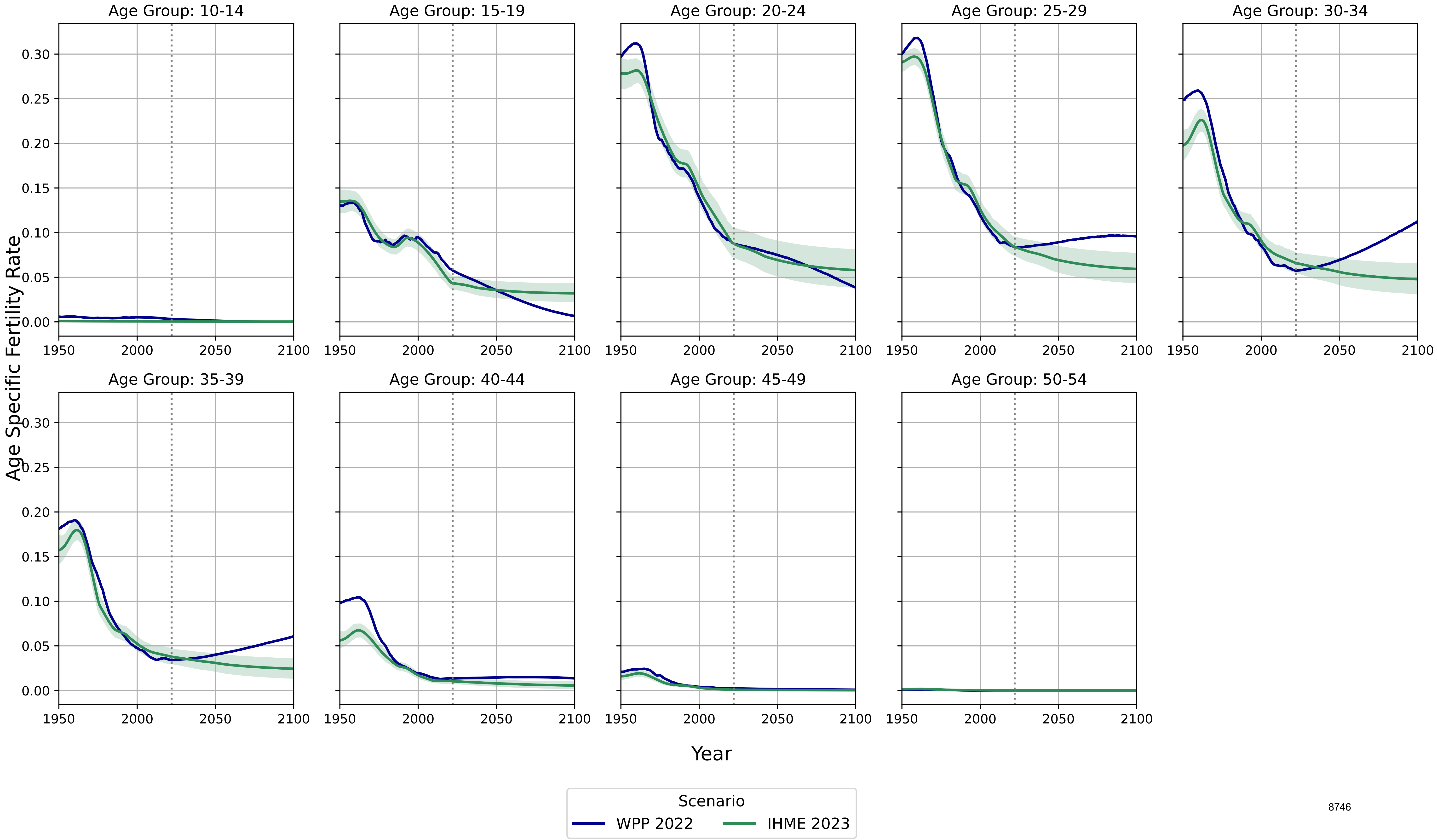

Supplemental Results Figure S8: Five-year interval ASFR from IHME reference scenario and the UNPD medium variant scenario by location, 1950-2100

Costa Rica

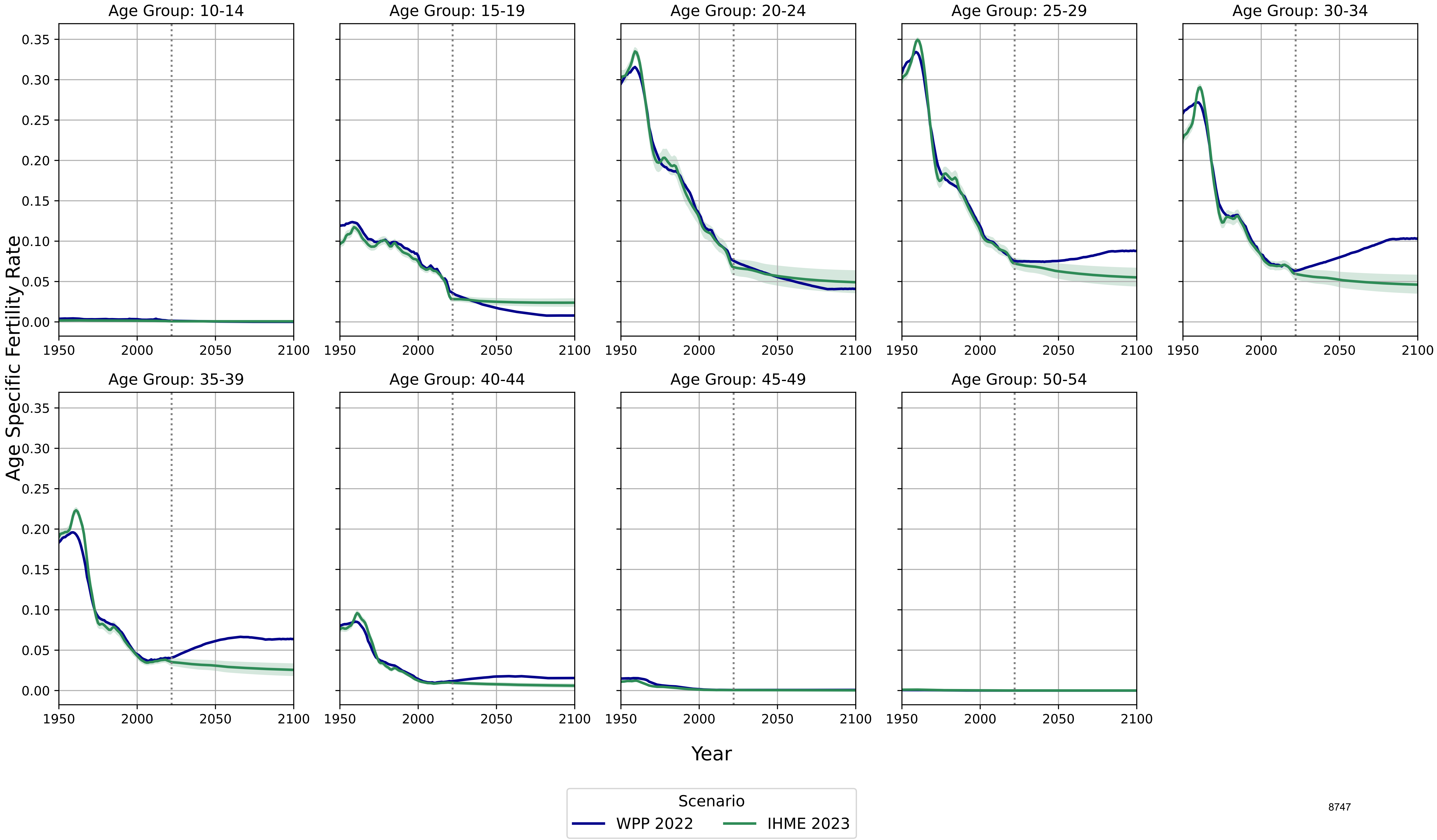

Supplemental Results Figure S8: Five-year interval ASFR from IHME reference scenario and the UNPD medium variant scenario by location, 1950-2100

El Salvador

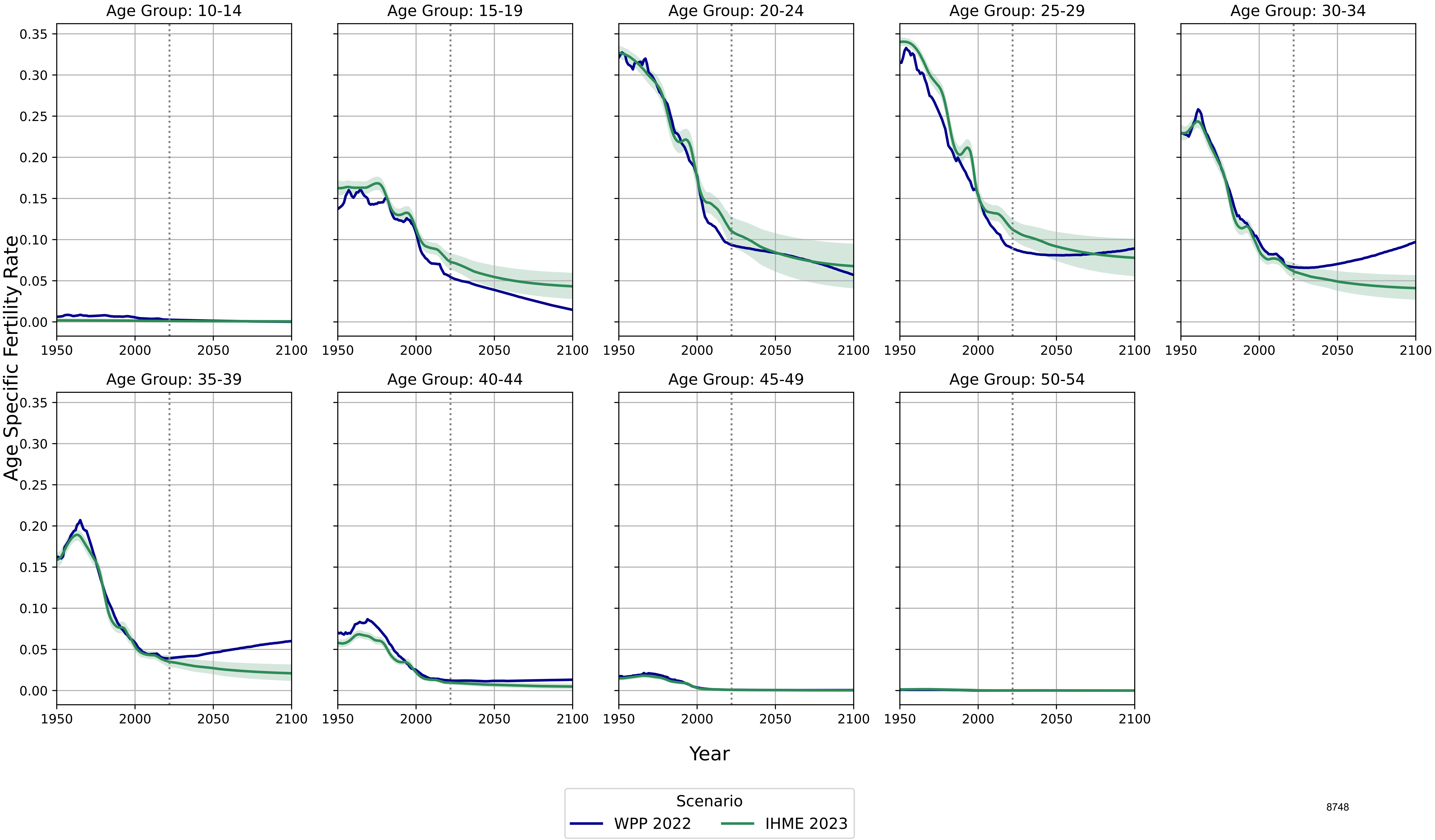

Supplemental Results Figure S8: Five-year interval ASFR from IHME reference scenario and the UNPD medium variant scenario by location, 1950-2100

Guatemala

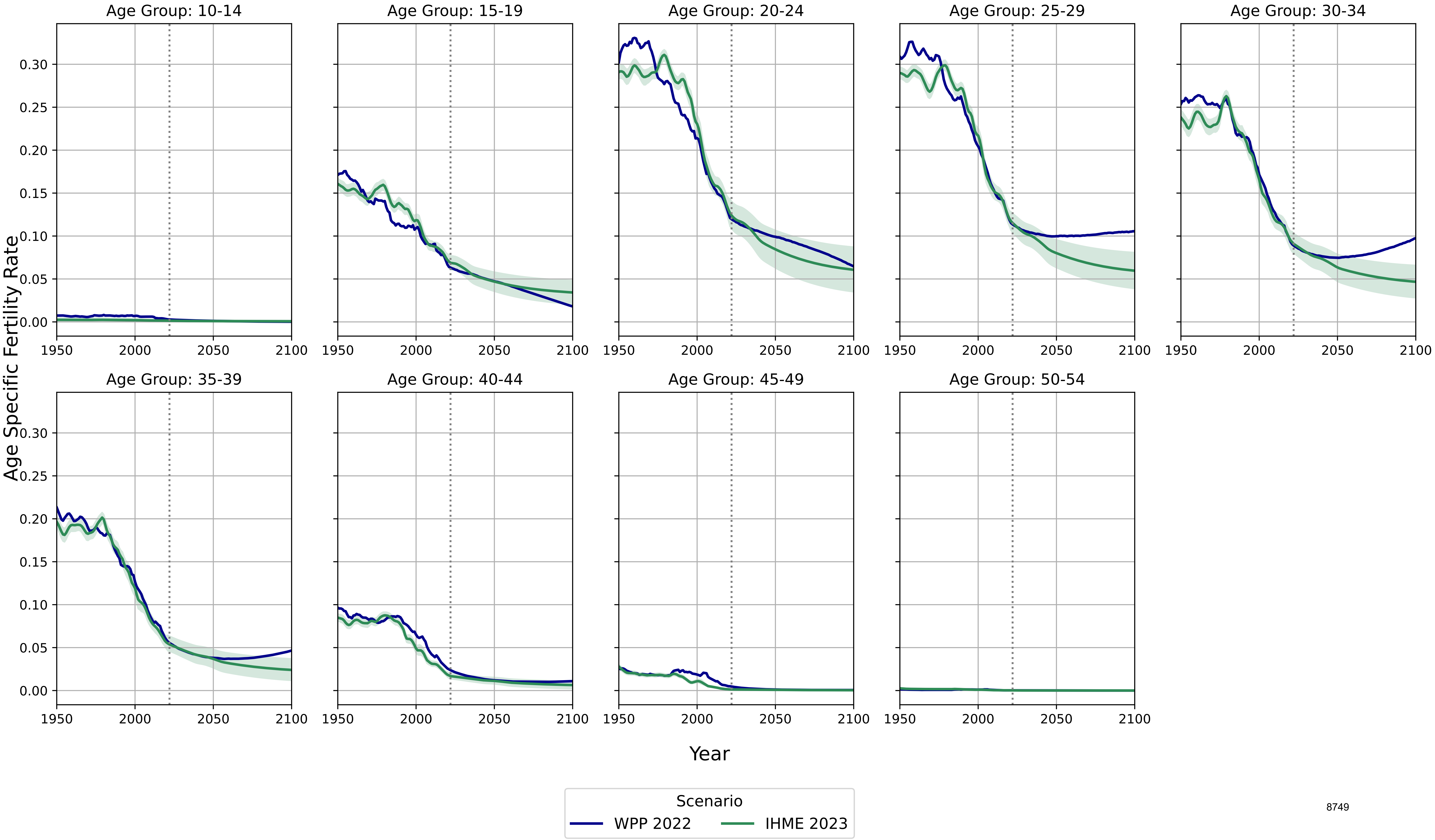

Supplemental Results Figure S8: Five-year interval ASFR from IHME reference scenario and the UNPD medium variant scenario by location, 1950-2100

Honduras

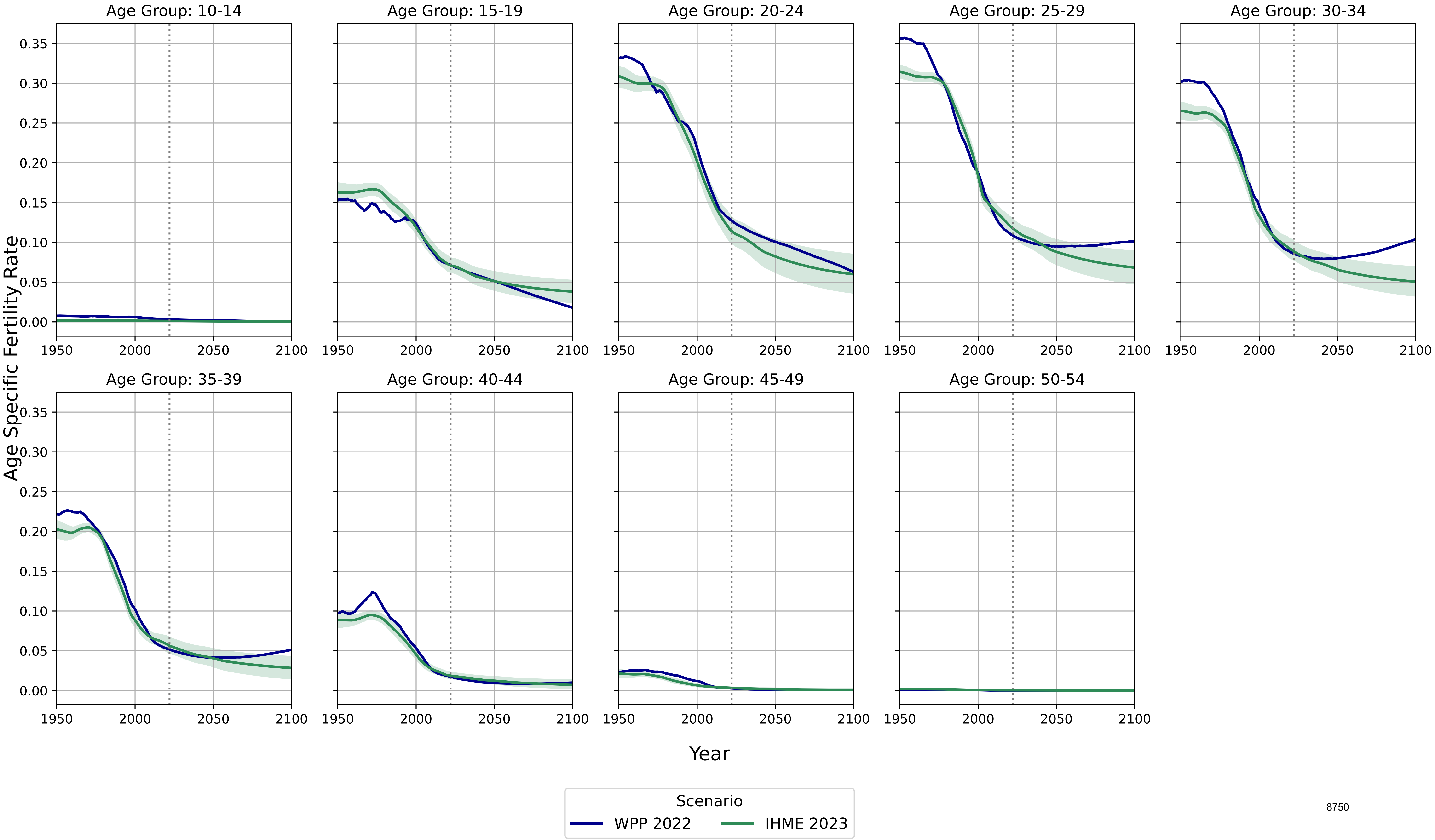

Supplemental Results Figure S8: Five-year interval ASFR from IHME reference scenario and the UNPD medium variant scenario by location, 1950-2100

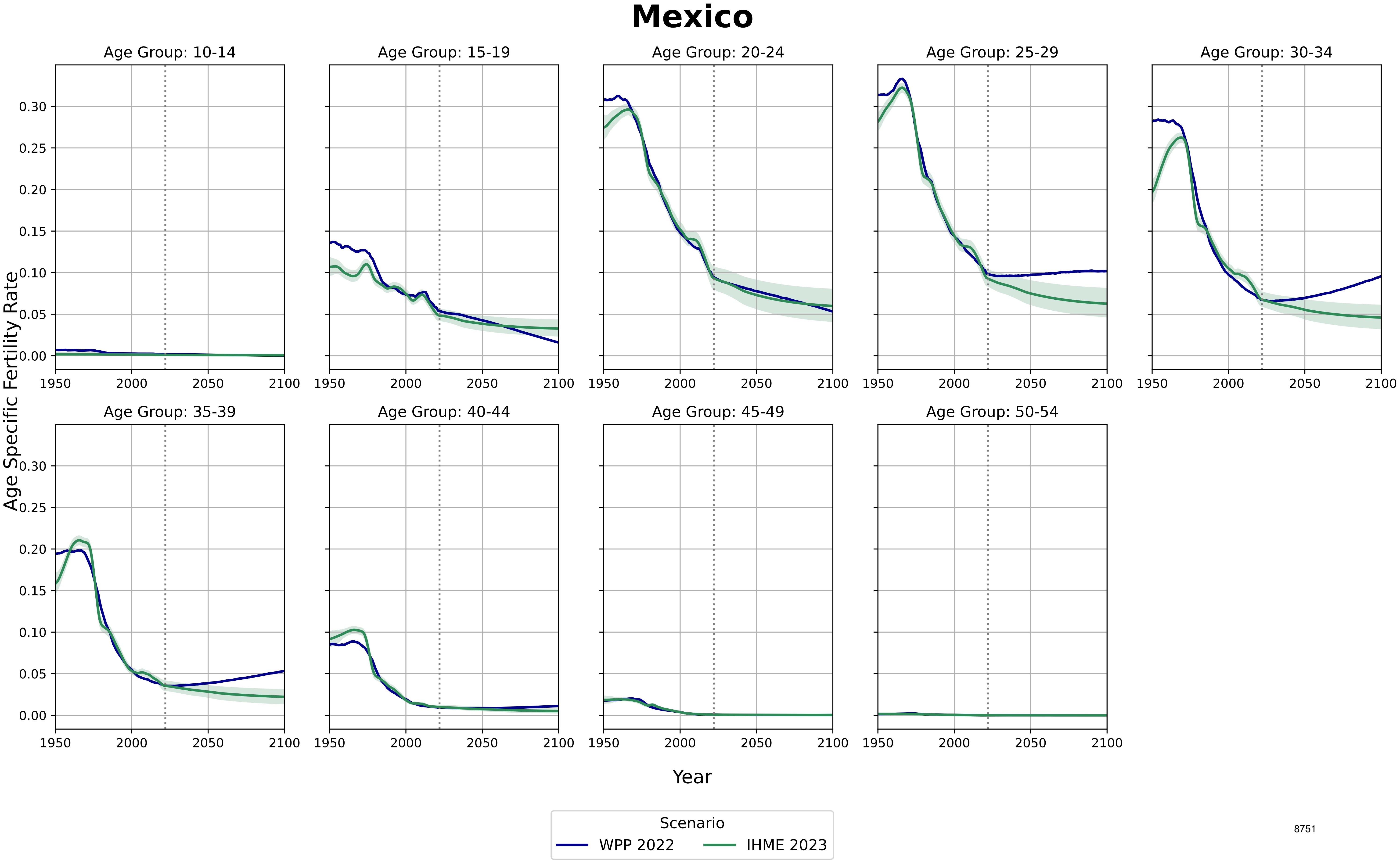

Supplemental Results Figure S8: Five-year interval ASFR from IHME reference scenario and the UNPD medium variant scenario by location, 1950-2100

Nicaragua

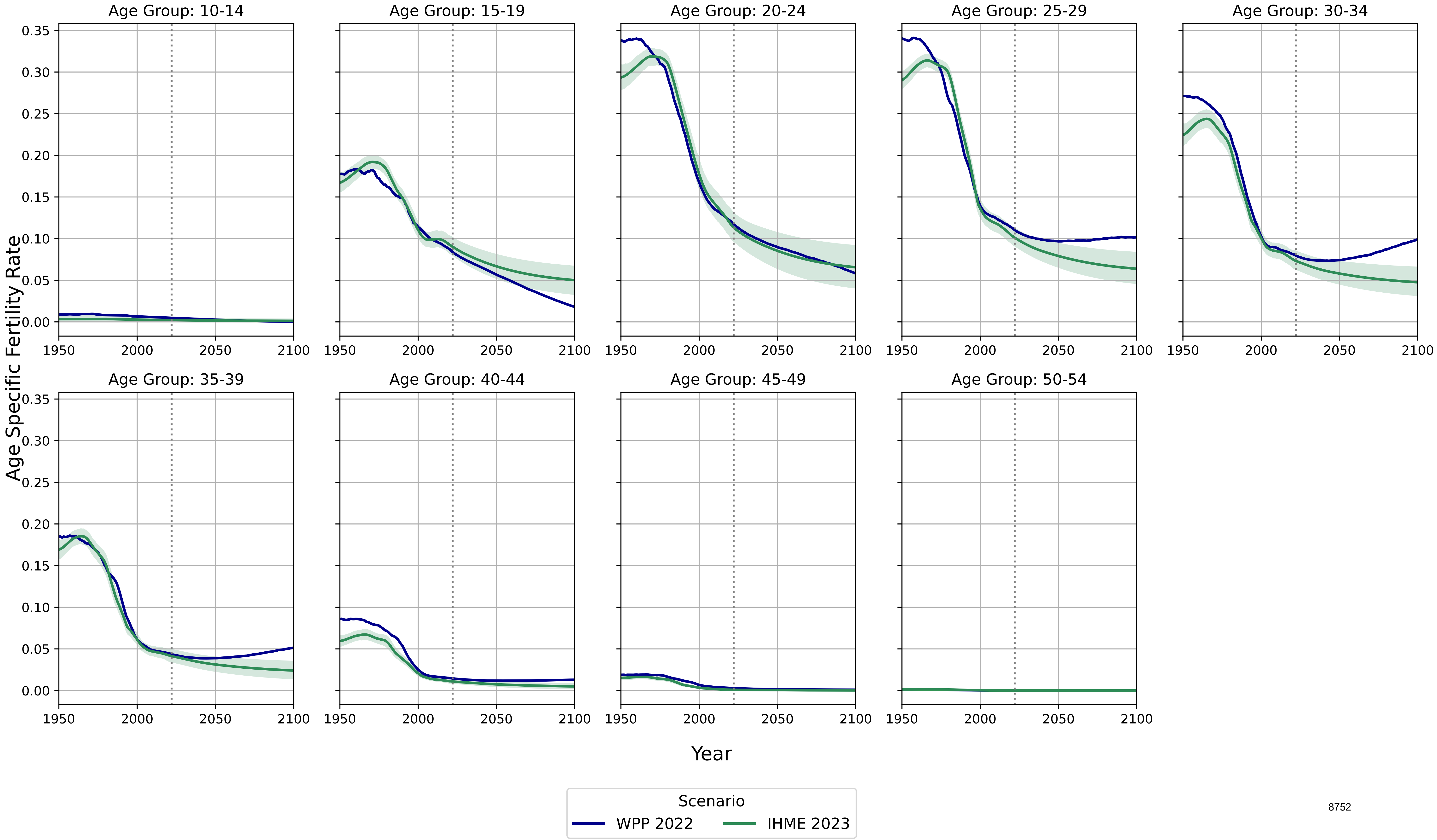

Supplemental Results Figure S8: Five-year interval ASFR from IHME reference scenario and the UNPD medium variant scenario by location, 1950-2100

Panama

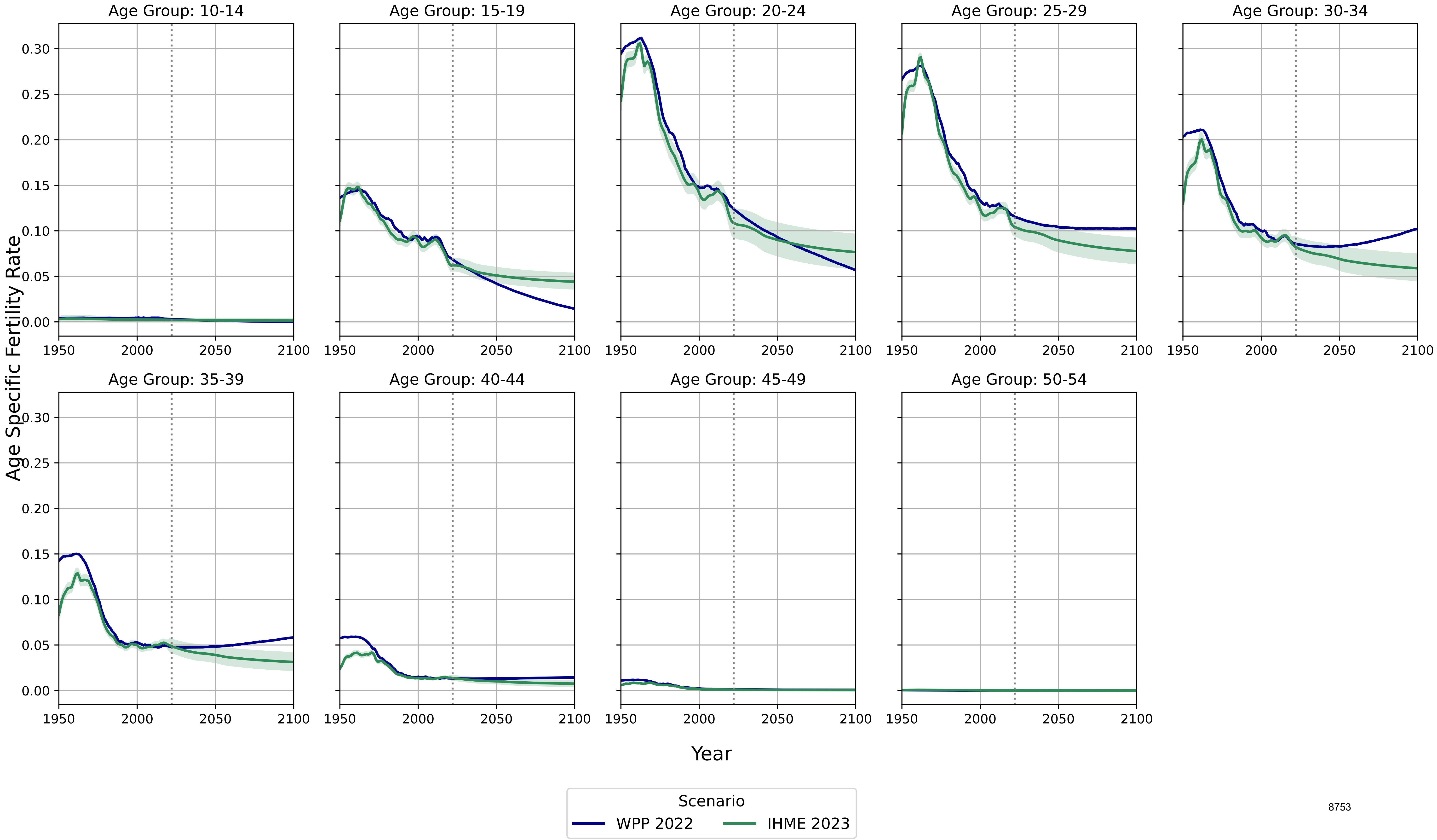

Supplemental Results Figure S8: Five-year interval ASFR from IHME reference scenario and the UNPD medium variant scenario by location, 1950-2100

Venezuela

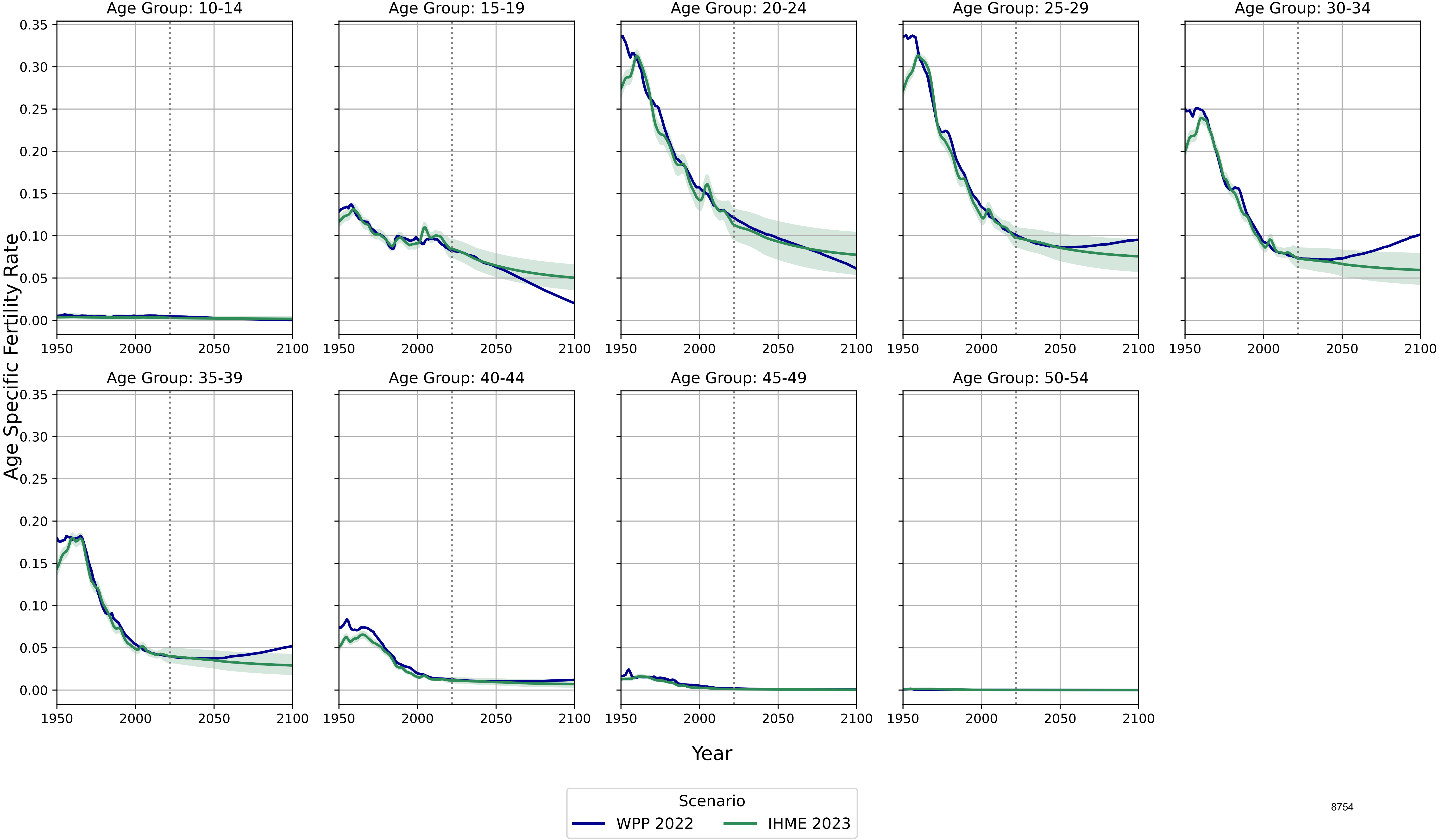

Supplemental Results Figure S8: Five-year interval ASFR from IHME reference scenario and the UNPD medium variant scenario by location, 1950-2100

Brazil

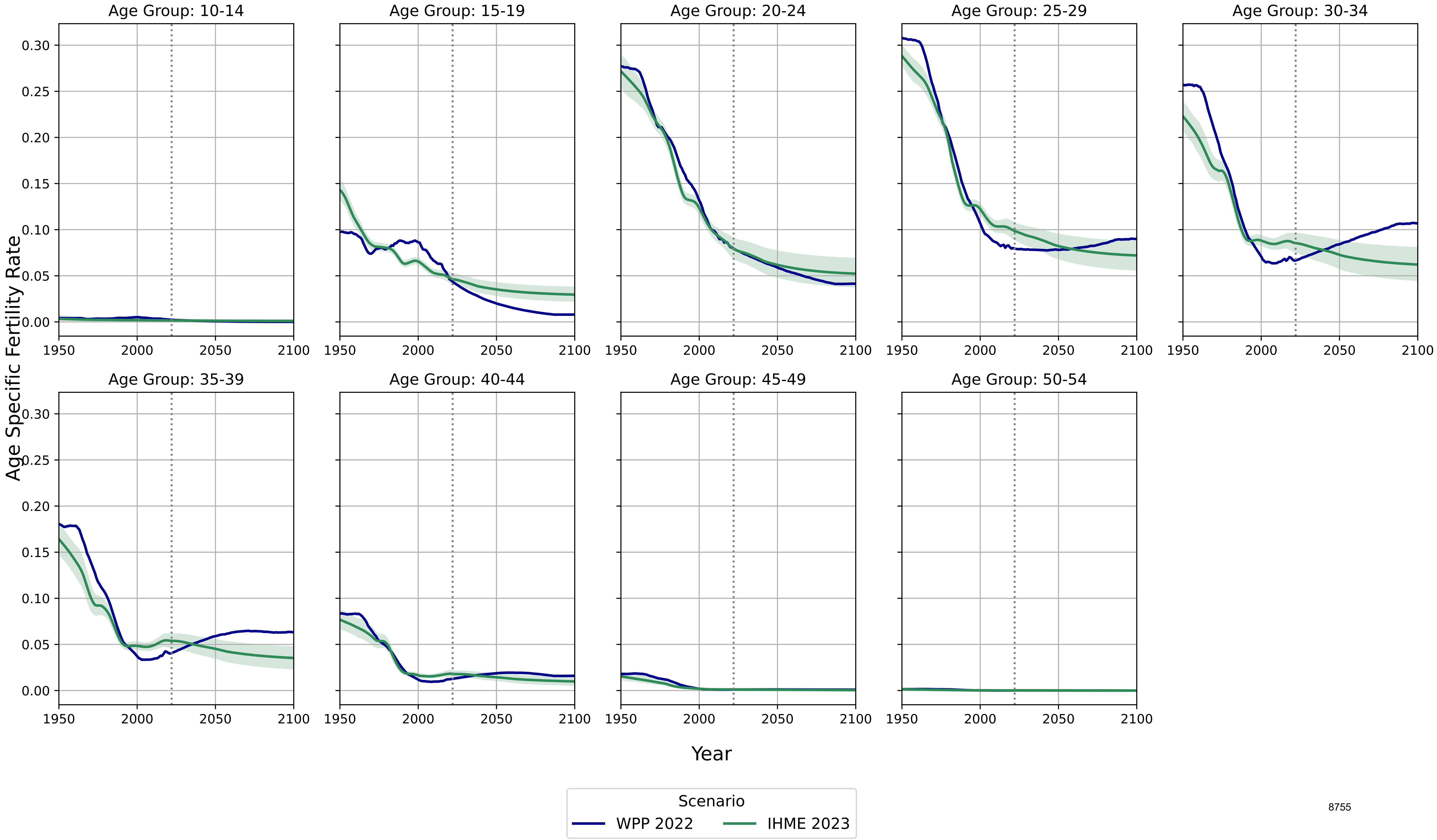

Supplemental Results Figure S8: Five-year interval ASFR from IHME reference scenario and the UNPD medium variant scenario by location, 1950-2100

Paraguay

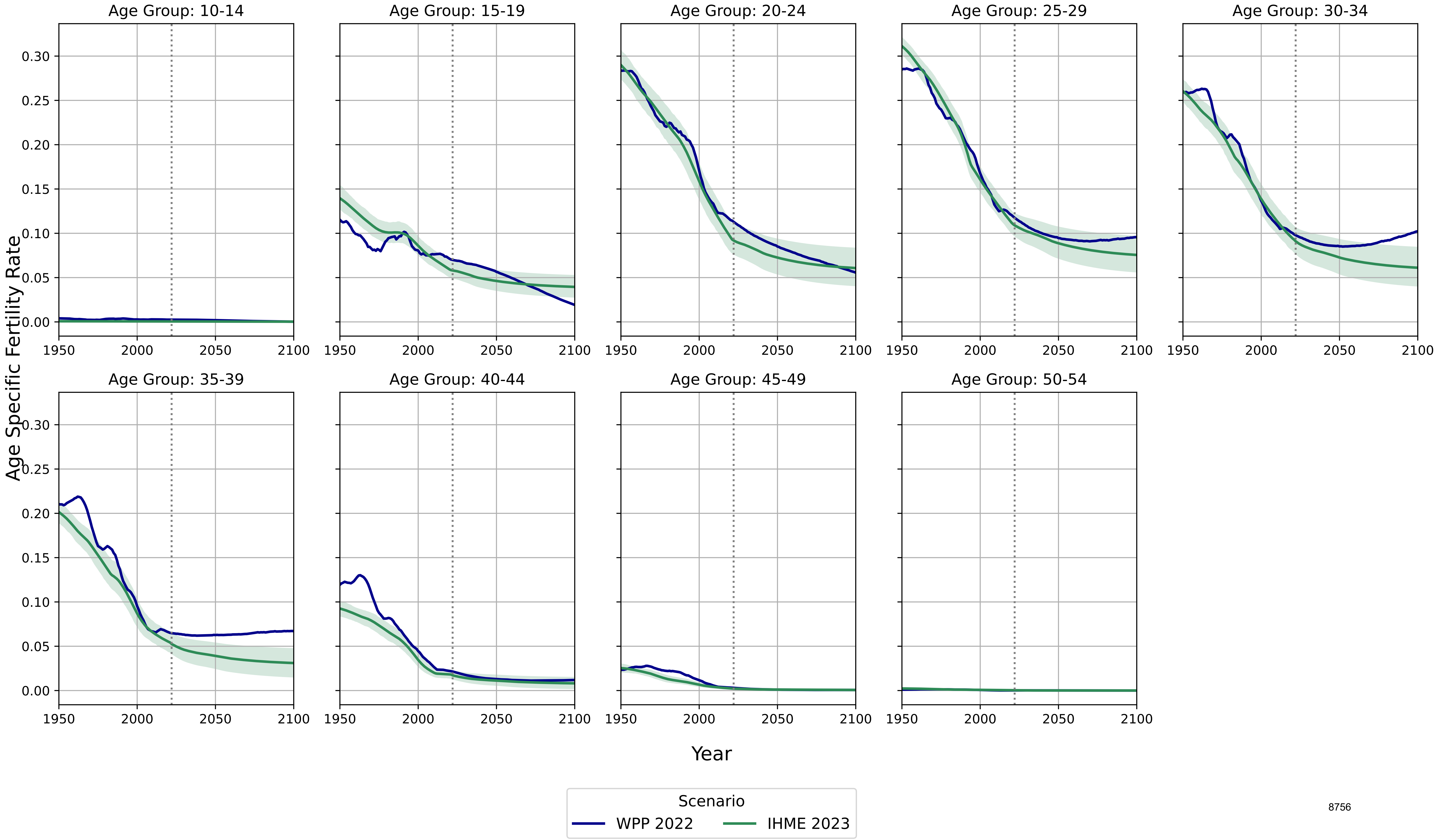

Supplemental Results Figure S8: Five-year interval ASFR from IHME reference scenario and the UNPD medium variant scenario by location, 1950-2100

Afghanistan

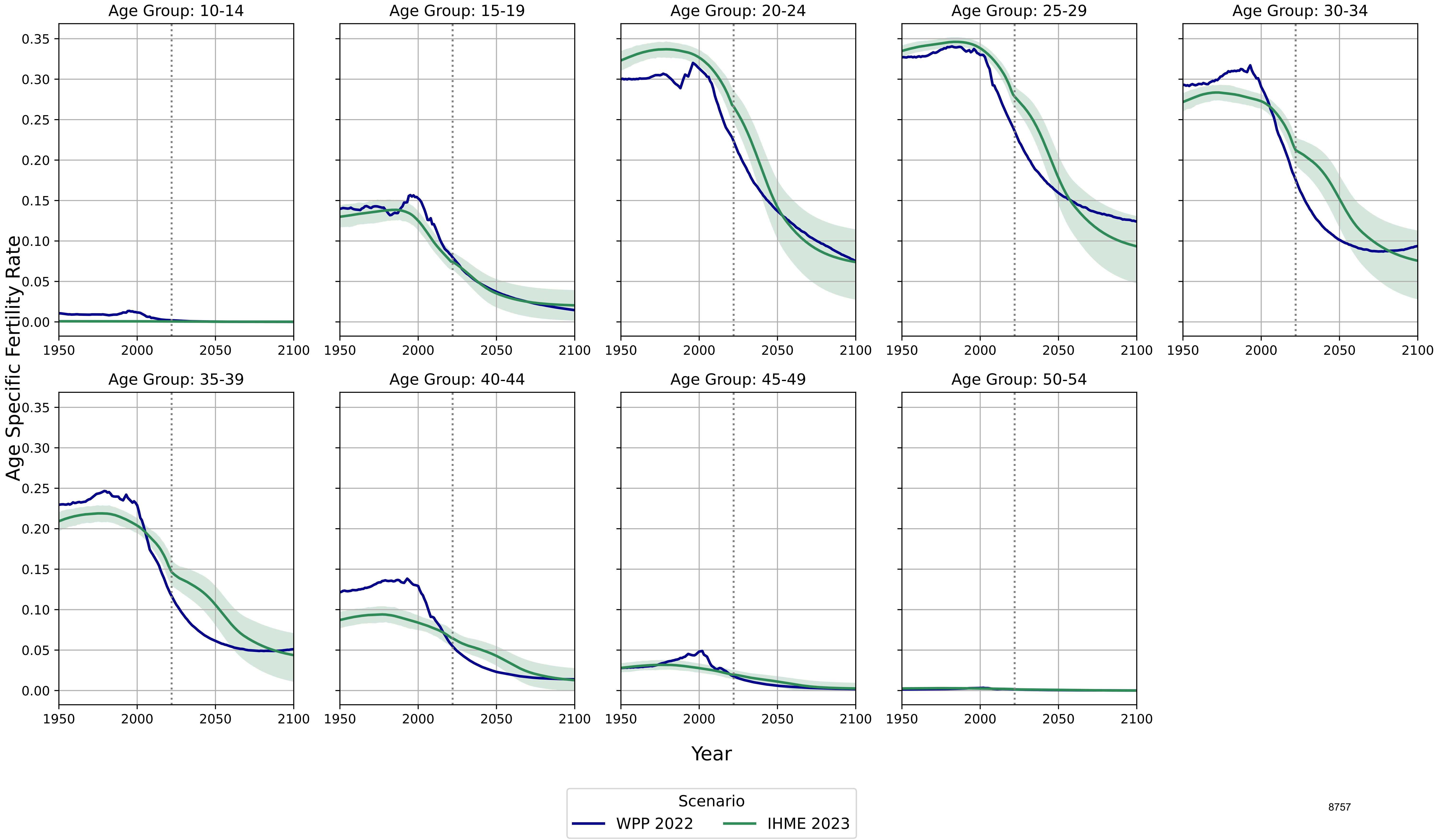

Supplemental Results Figure S8: Five-year interval ASFR from IHME reference scenario and the UNPD medium variant scenario by location, 1950-2100

Algeria

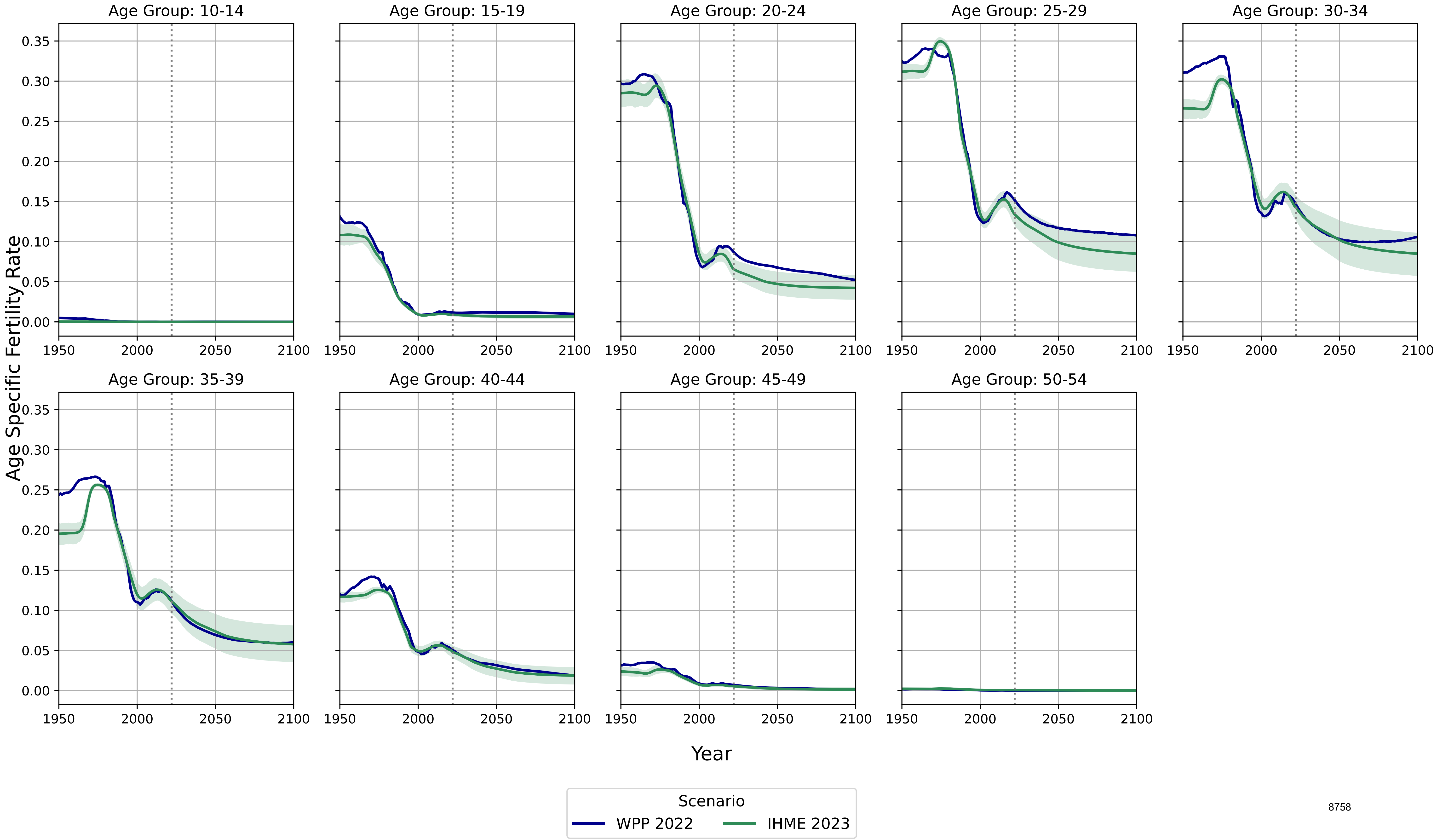

Supplemental Results Figure S8: Five-year interval ASFR from IHME reference scenario and the UNPD medium variant scenario by location, 1950-2100

Bahrain

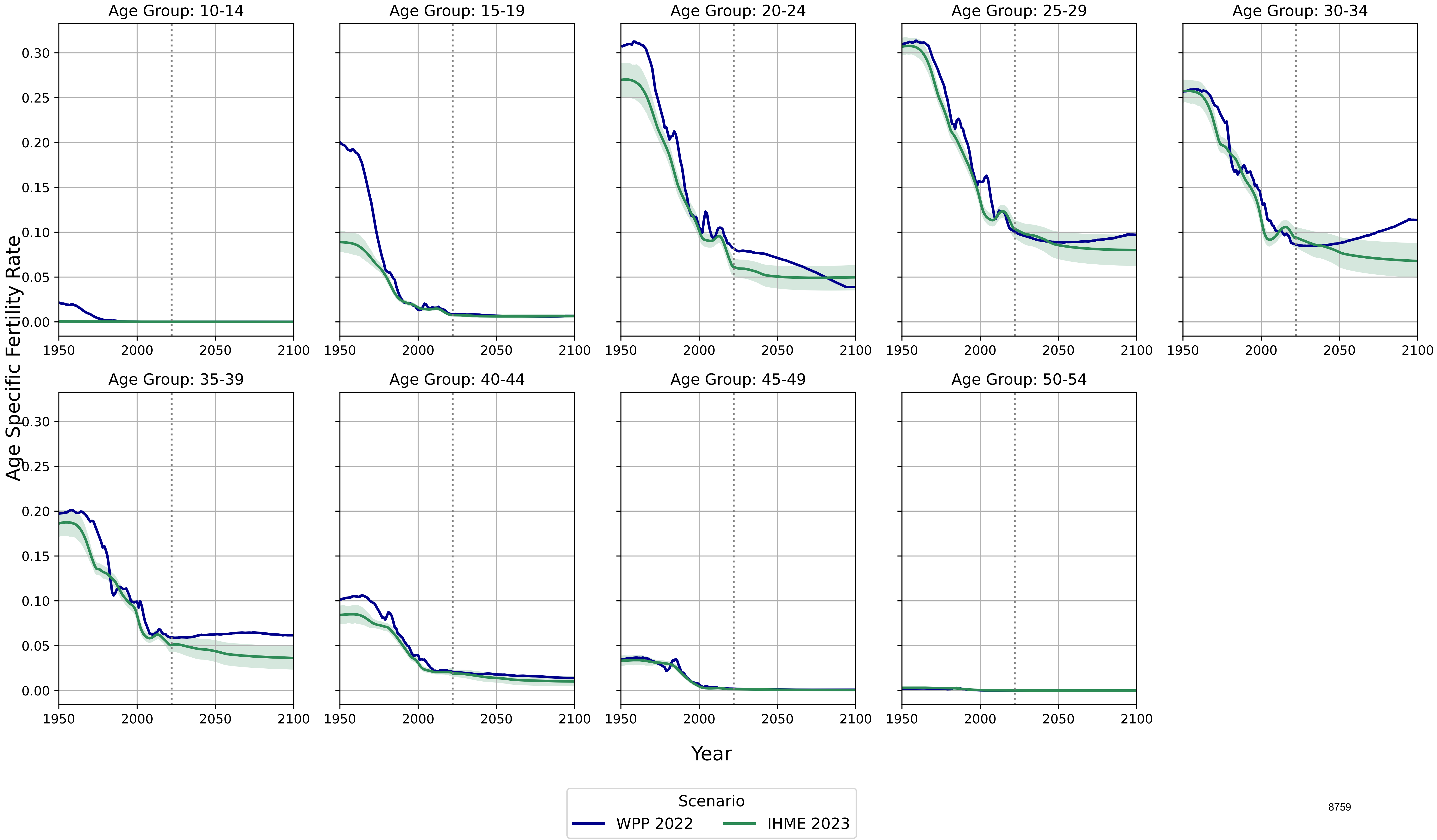

Supplemental Results Figure S8: Five-year interval ASFR from IHME reference scenario and the UNPD medium variant scenario by location, 1950-2100

Egypt

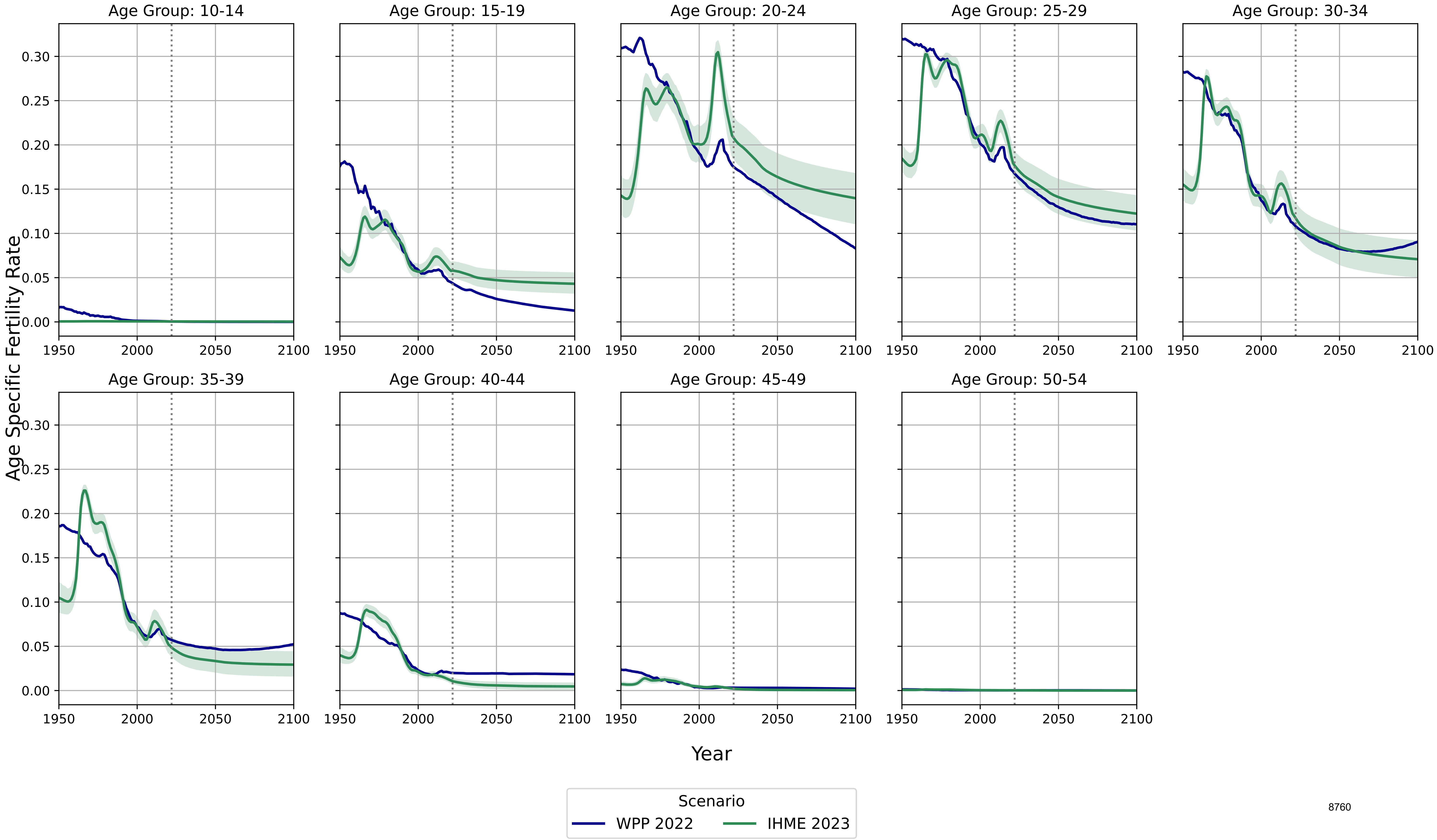

Supplemental Results Figure S8: Five-year interval ASFR from IHME reference scenario and the UNPD medium variant scenario by location, 1950-2100

Iran

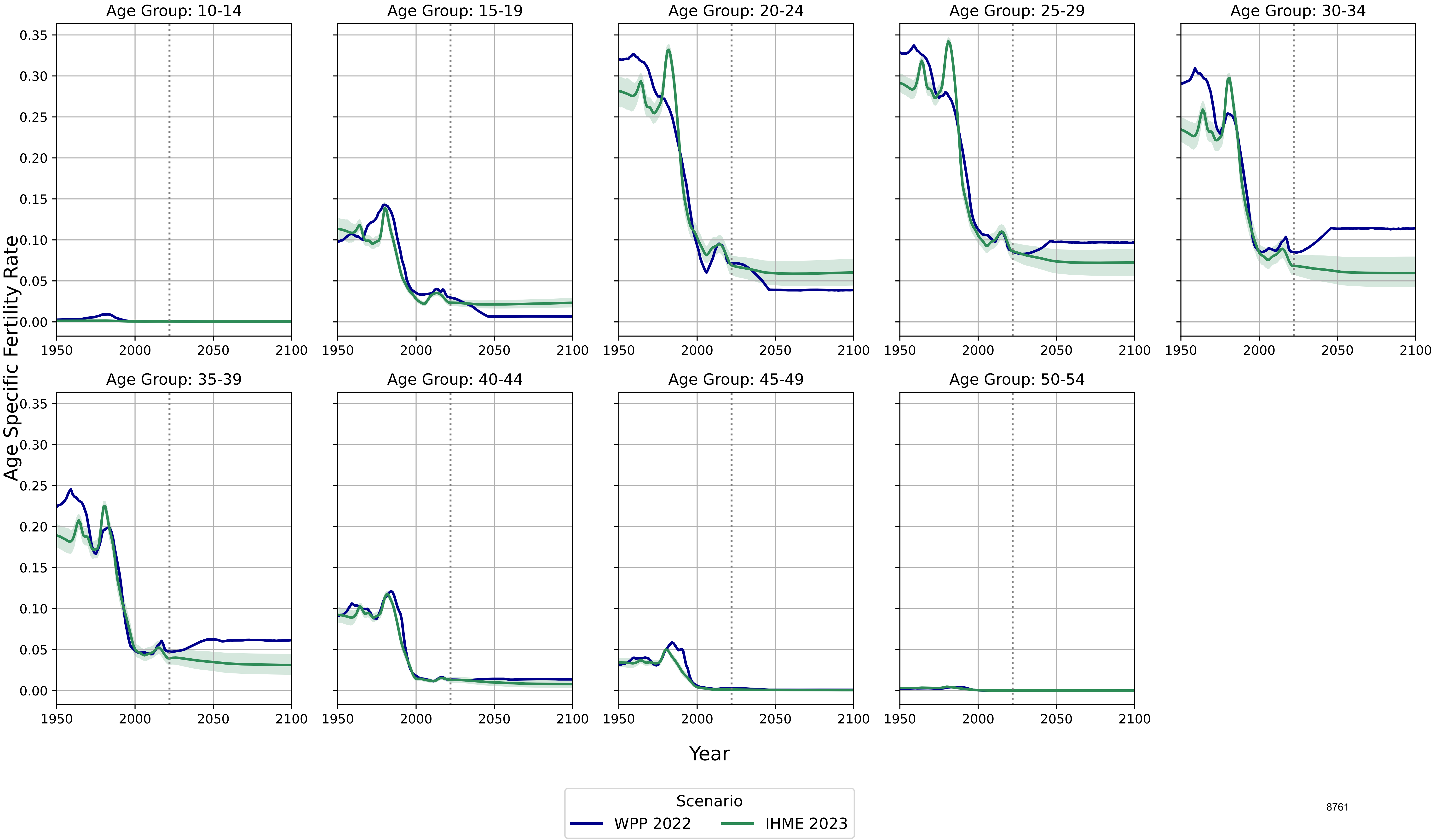

Supplemental Results Figure S8: Five-year interval ASFR from IHME reference scenario and the UNPD medium variant scenario by location, 1950-2100

Iraq

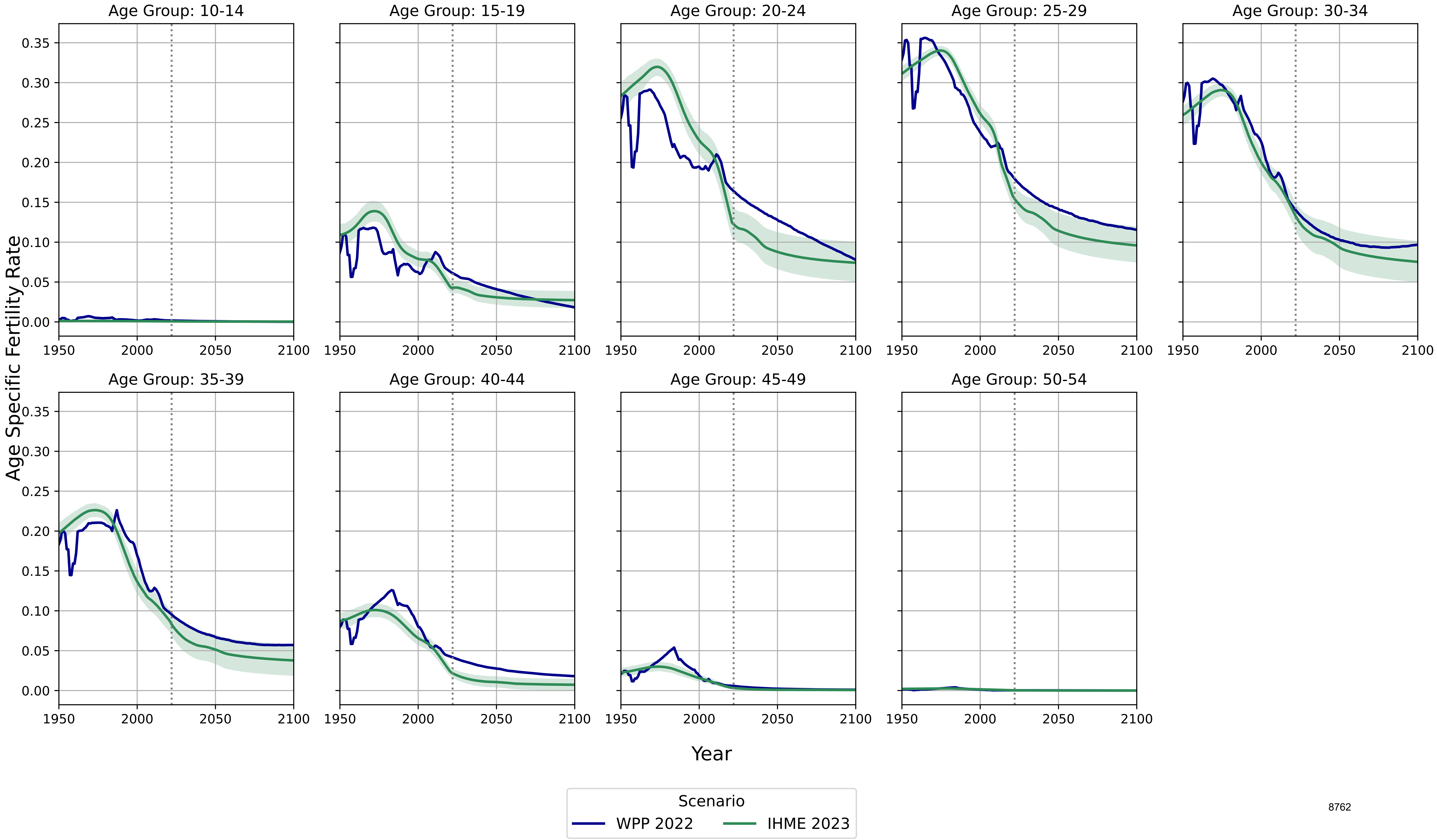

Supplemental Results Figure S8: Five-year interval ASFR from IHME reference scenario and the UNPD medium variant scenario by location, 1950-2100

Jordan

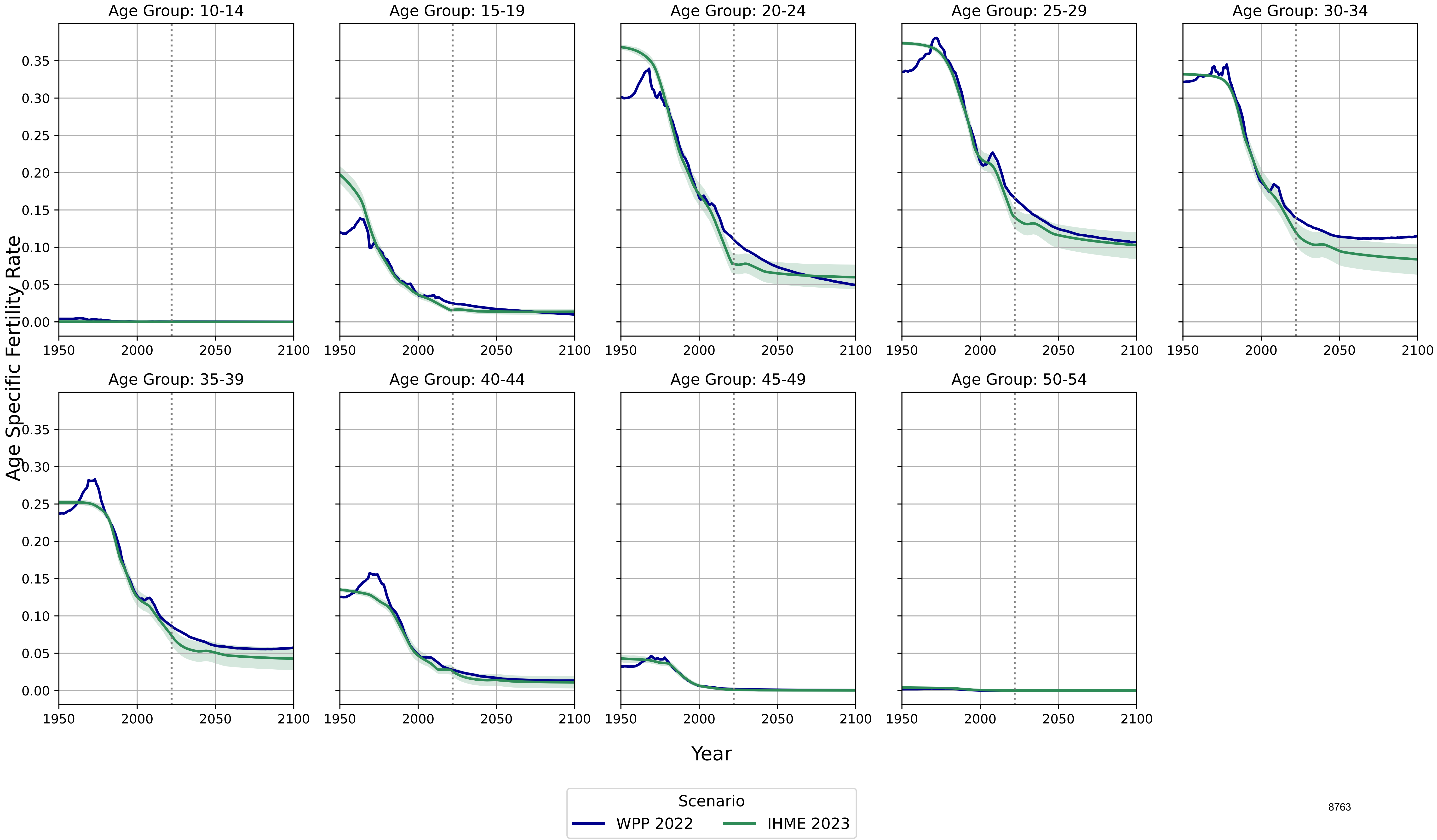

Supplemental Results Figure S8: Five-year interval ASFR from IHME reference scenario and the UNPD medium variant scenario by location, 1950-2100

Kuwait

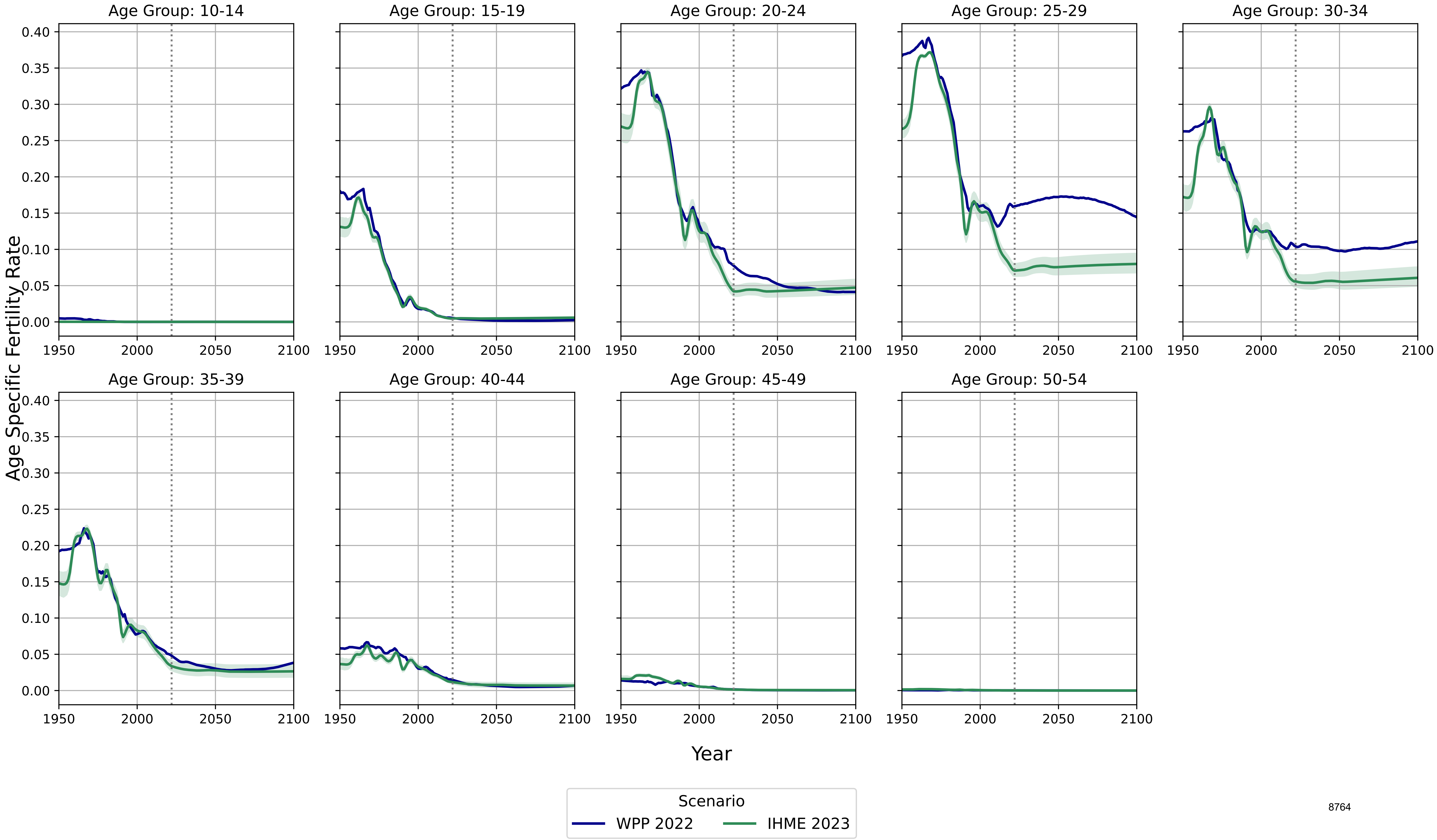

8764

Supplemental Results Figure S8: Five-year interval ASFR from IHME reference scenario and the UNPD medium variant scenario by location, 1950-2100

Lebanon

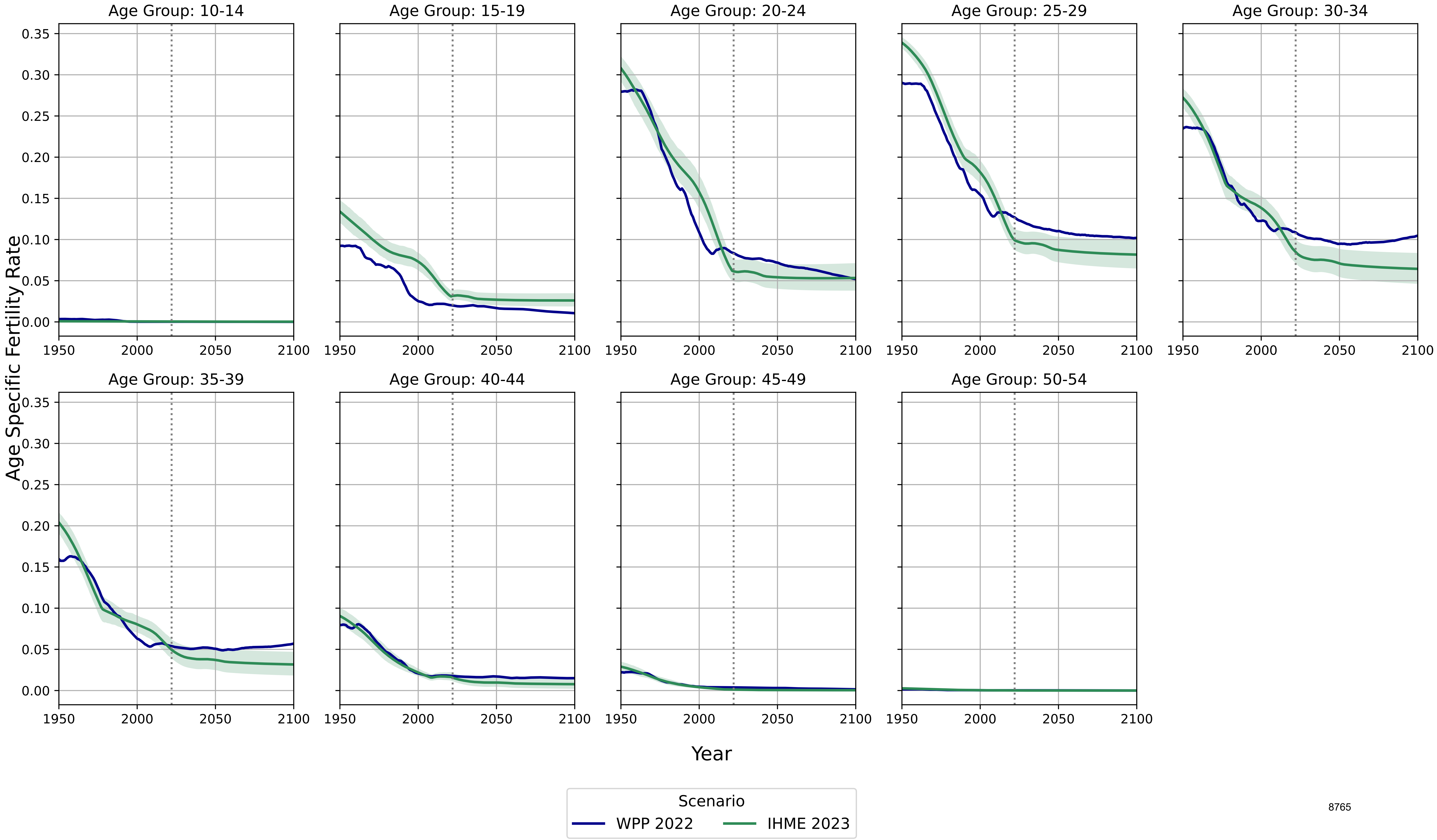

Supplemental Results Figure S8: Five-year interval ASFR from IHME reference scenario and the UNPD medium variant scenario by location, 1950-2100

Libya

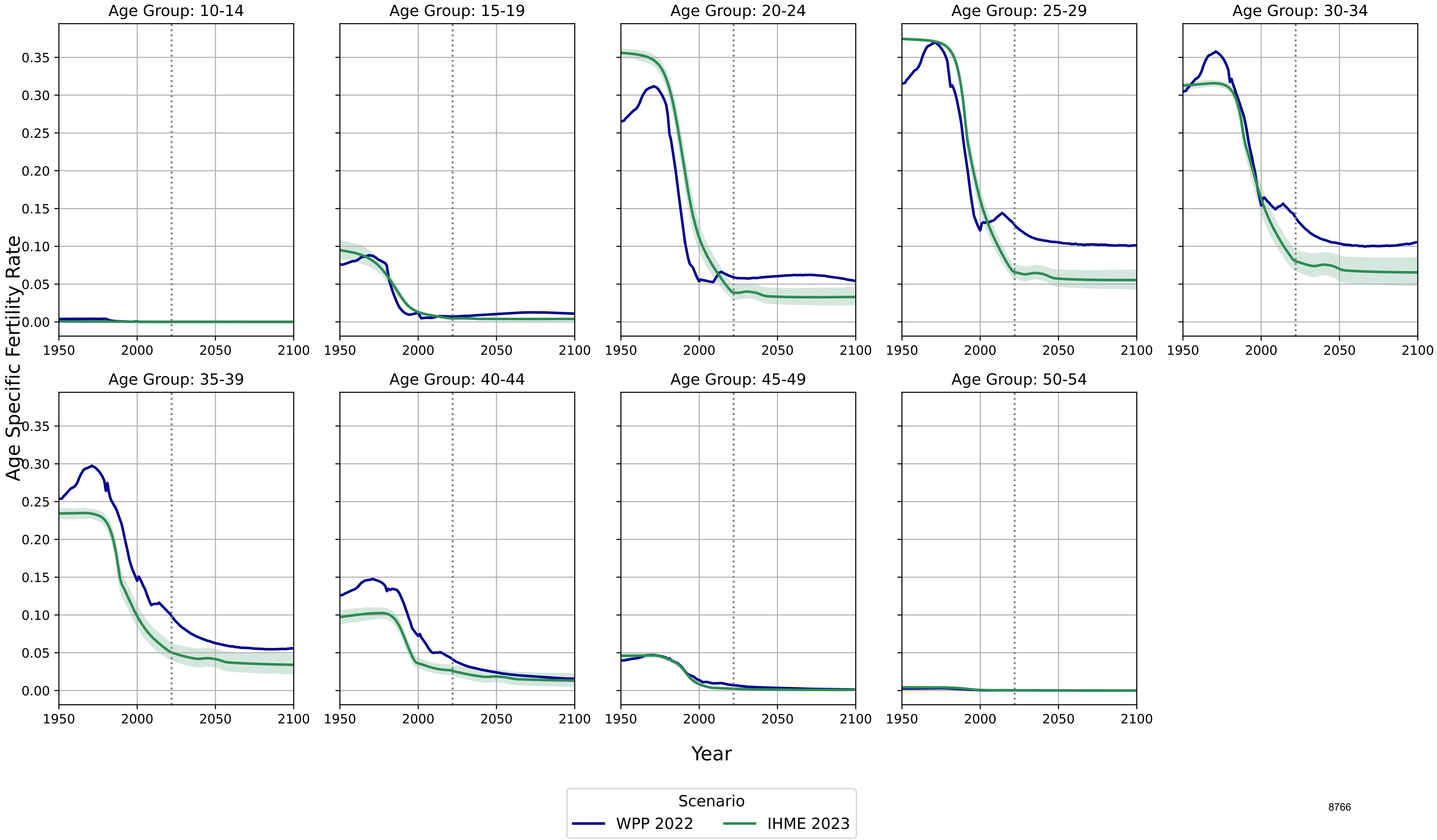

Supplemental Results Figure S8: Five-year interval ASFR from IHME reference scenario and the UNPD medium variant scenario by location, 1950-2100

Morocco

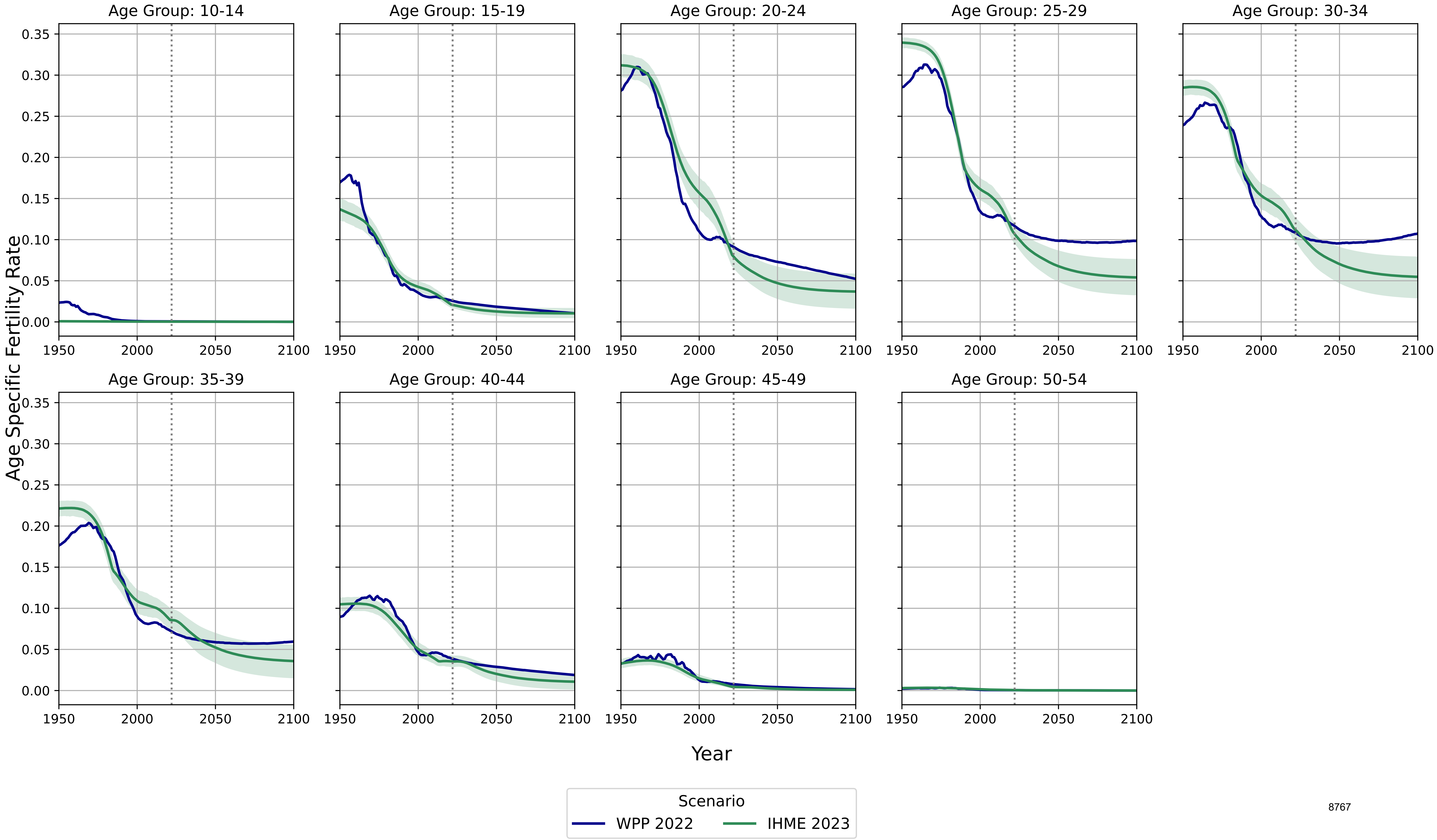

Supplemental Results Figure S8: Five-year interval ASFR from IHME reference scenario and the UNPD medium variant scenario by location, 1950-2100

Oman

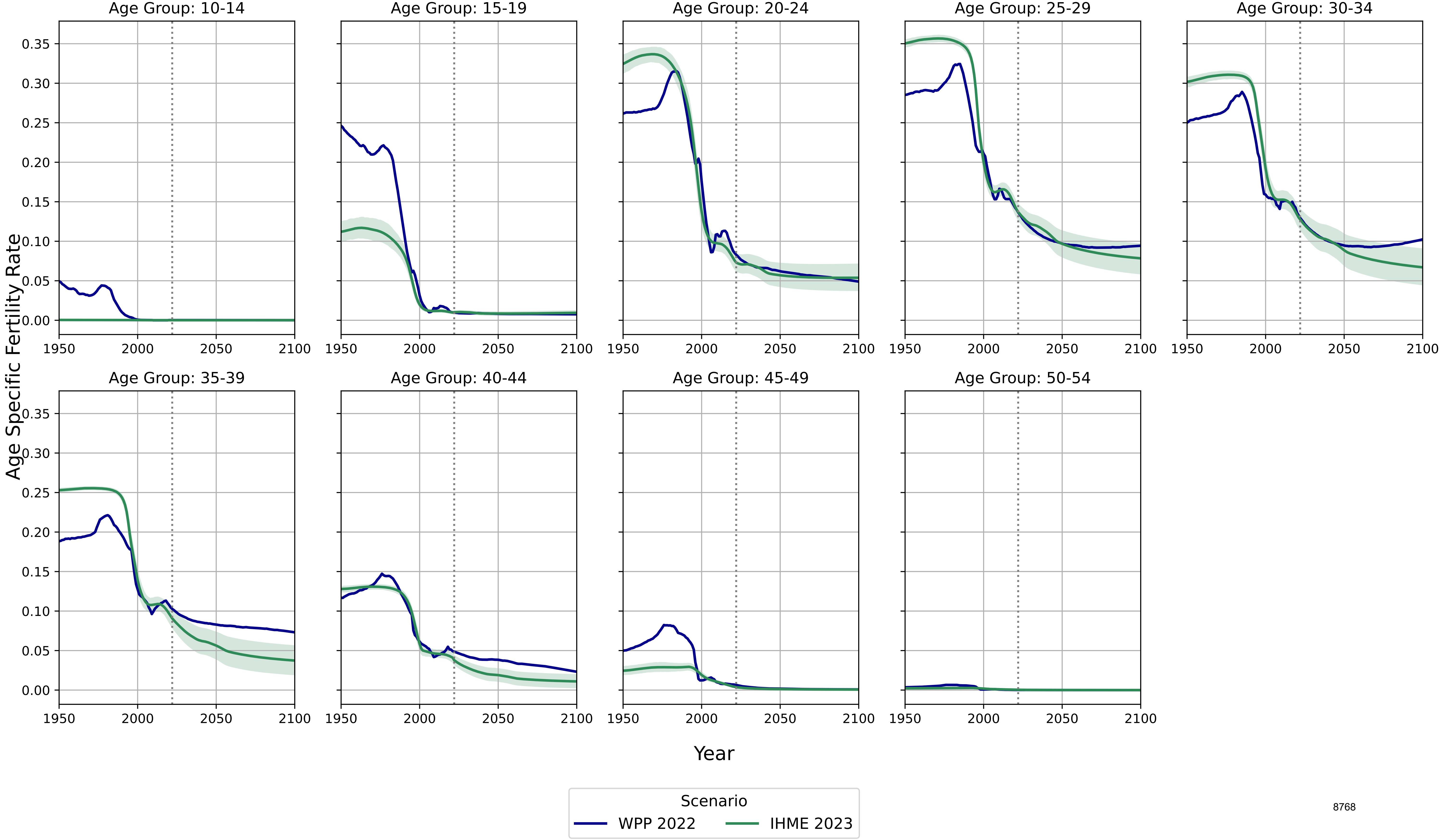

Supplemental Results Figure S8: Five-year interval ASFR from IHME reference scenario and the UNPD medium variant scenario by location, 1950-2100

Palestine

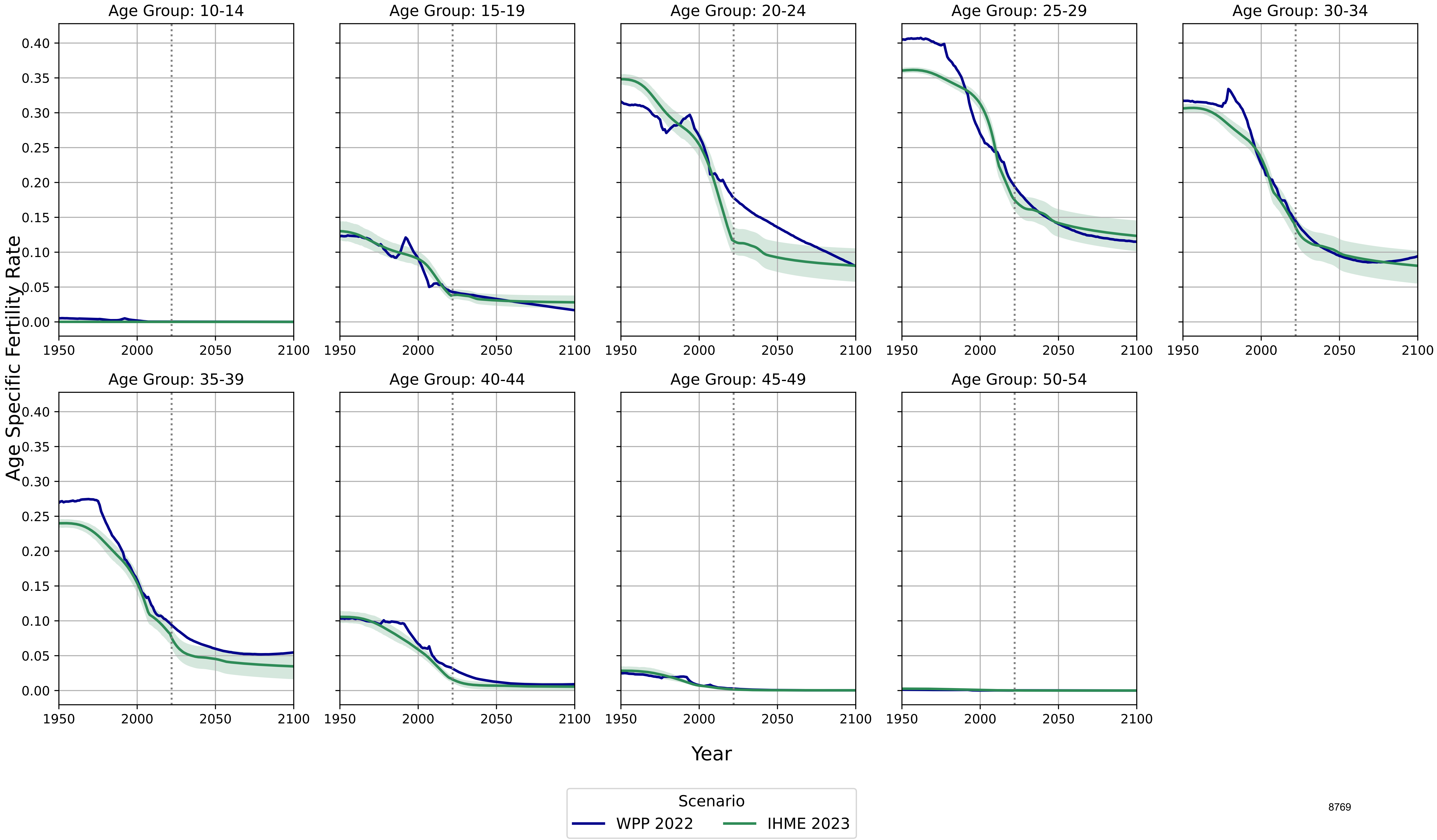

Supplemental Results Figure S8: Five-year interval ASFR from IHME reference scenario and the UNPD medium variant scenario by location, 1950-2100

Qatar

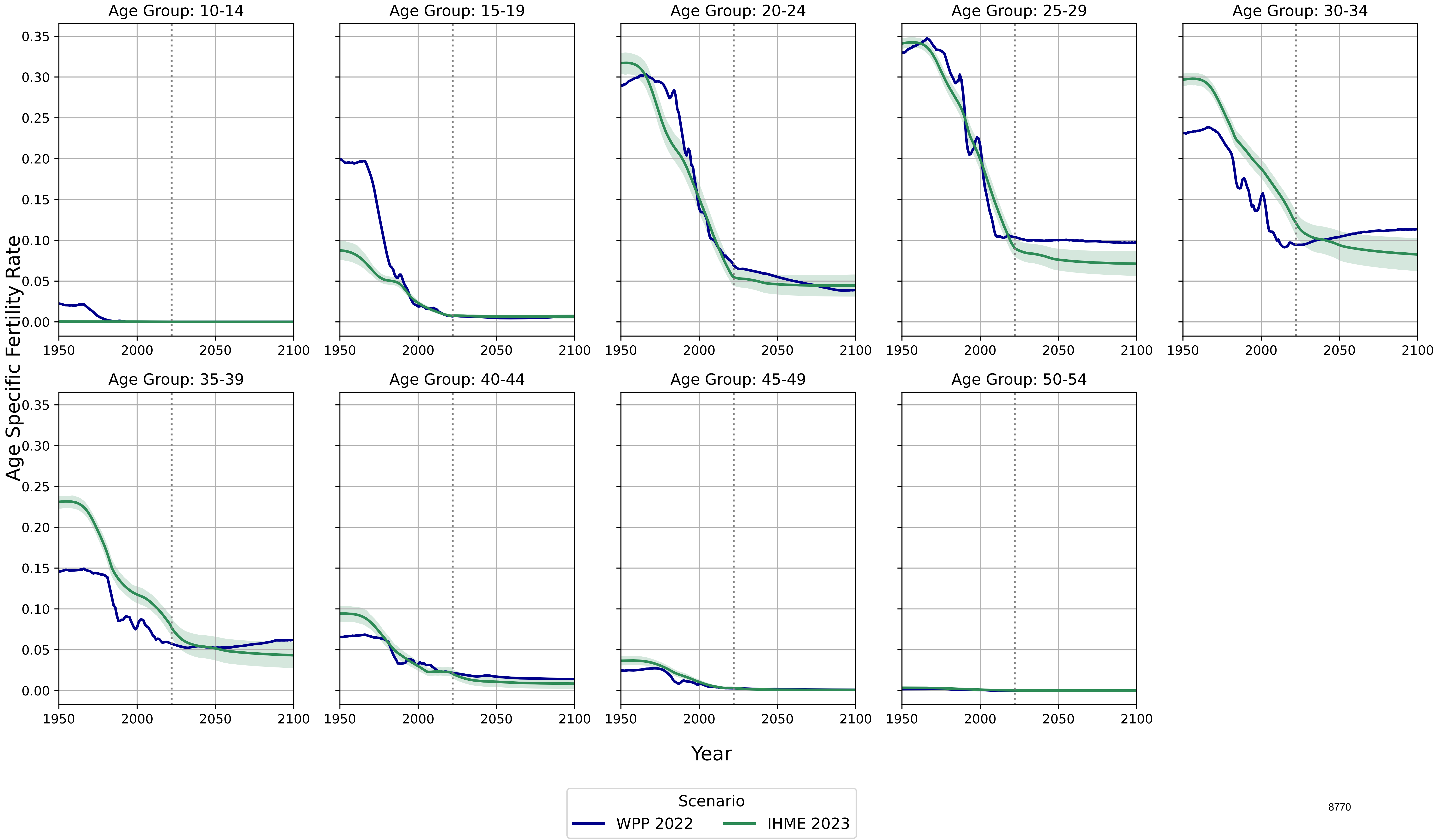

Supplemental Results Figure S8: Five-year interval ASFR from IHME reference scenario and the UNPD medium variant scenario by location, 1950-2100

Saudi Arabia

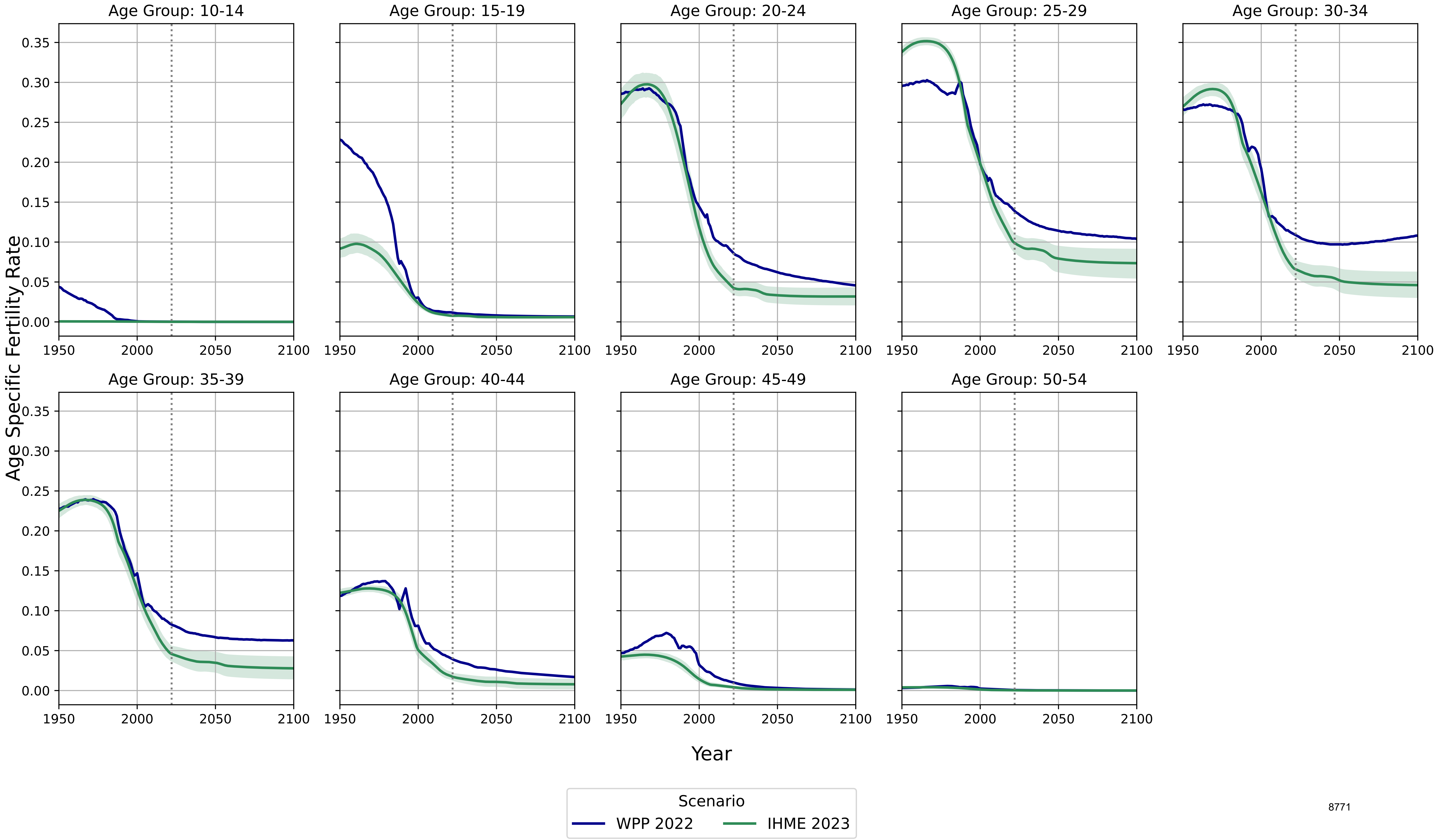

Supplemental Results Figure S8: Five-year interval ASFR from IHME reference scenario and the UNPD medium variant scenario by location, 1950-2100

Sudan

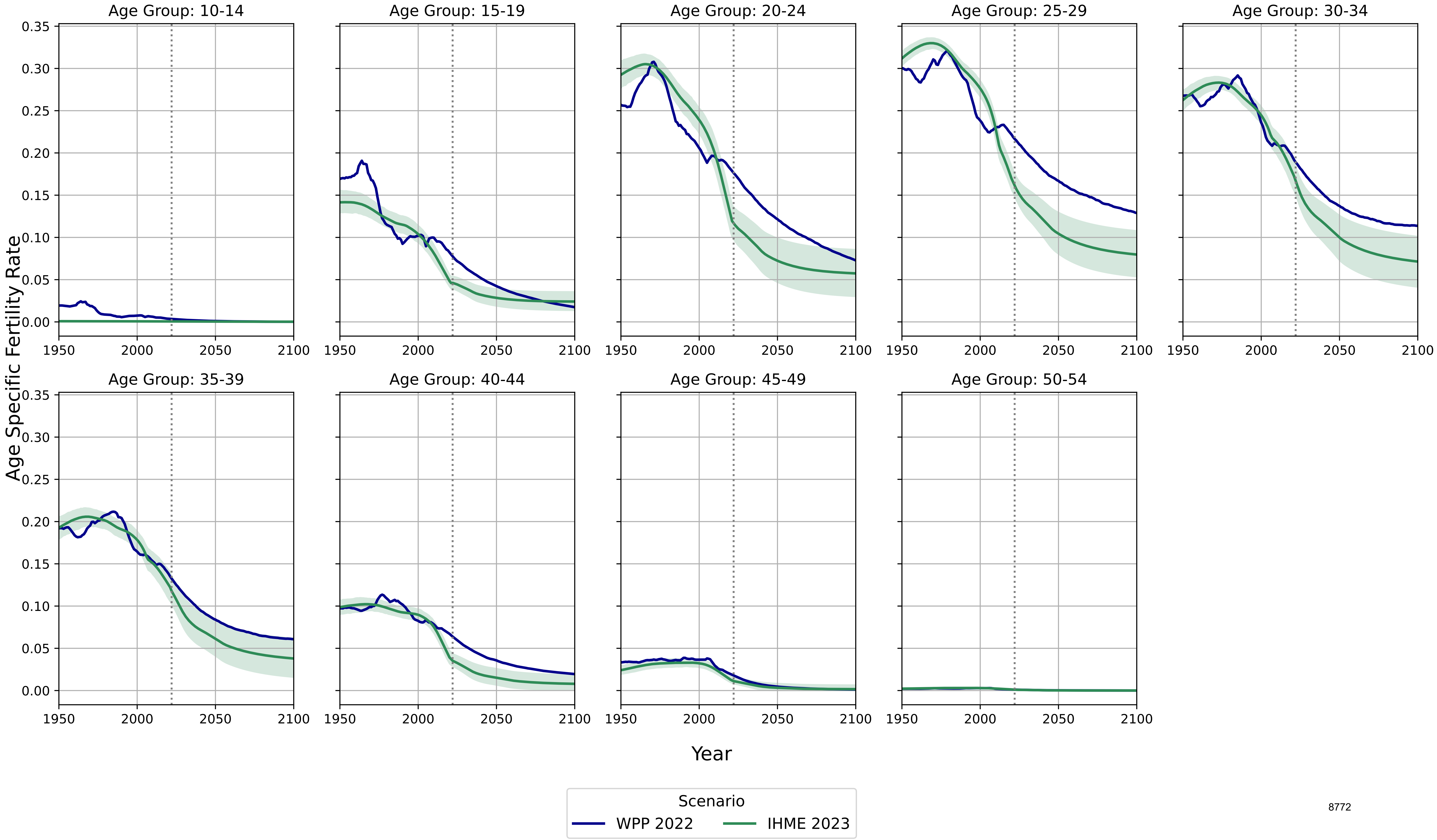

Supplemental Results Figure S8: Five-year interval ASFR from IHME reference scenario and the UNPD medium variant scenario by location, 1950-2100

Syria

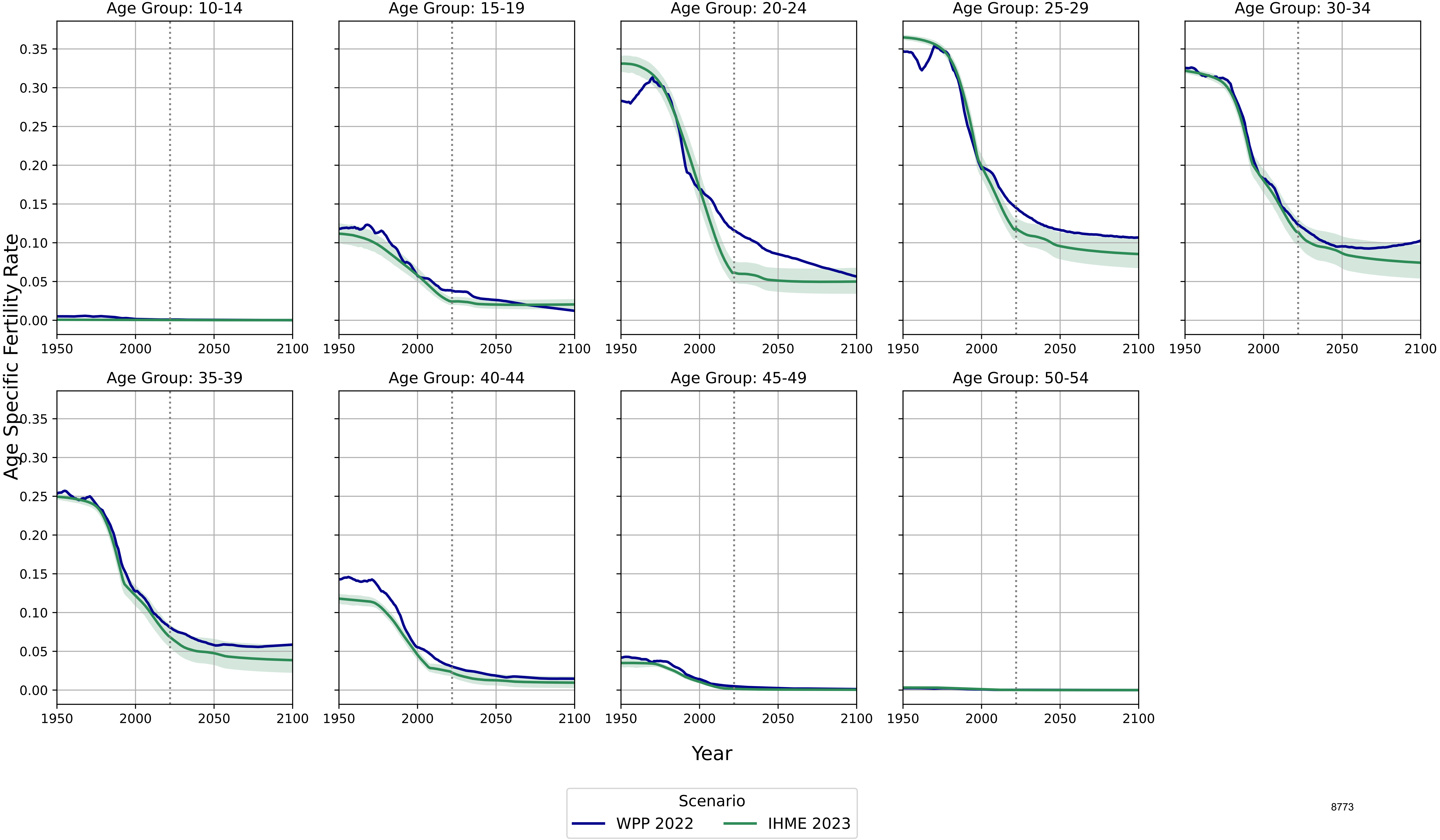

Supplemental Results Figure S8: Five-year interval ASFR from IHME reference scenario and the UNPD medium variant scenario by location, 1950-2100

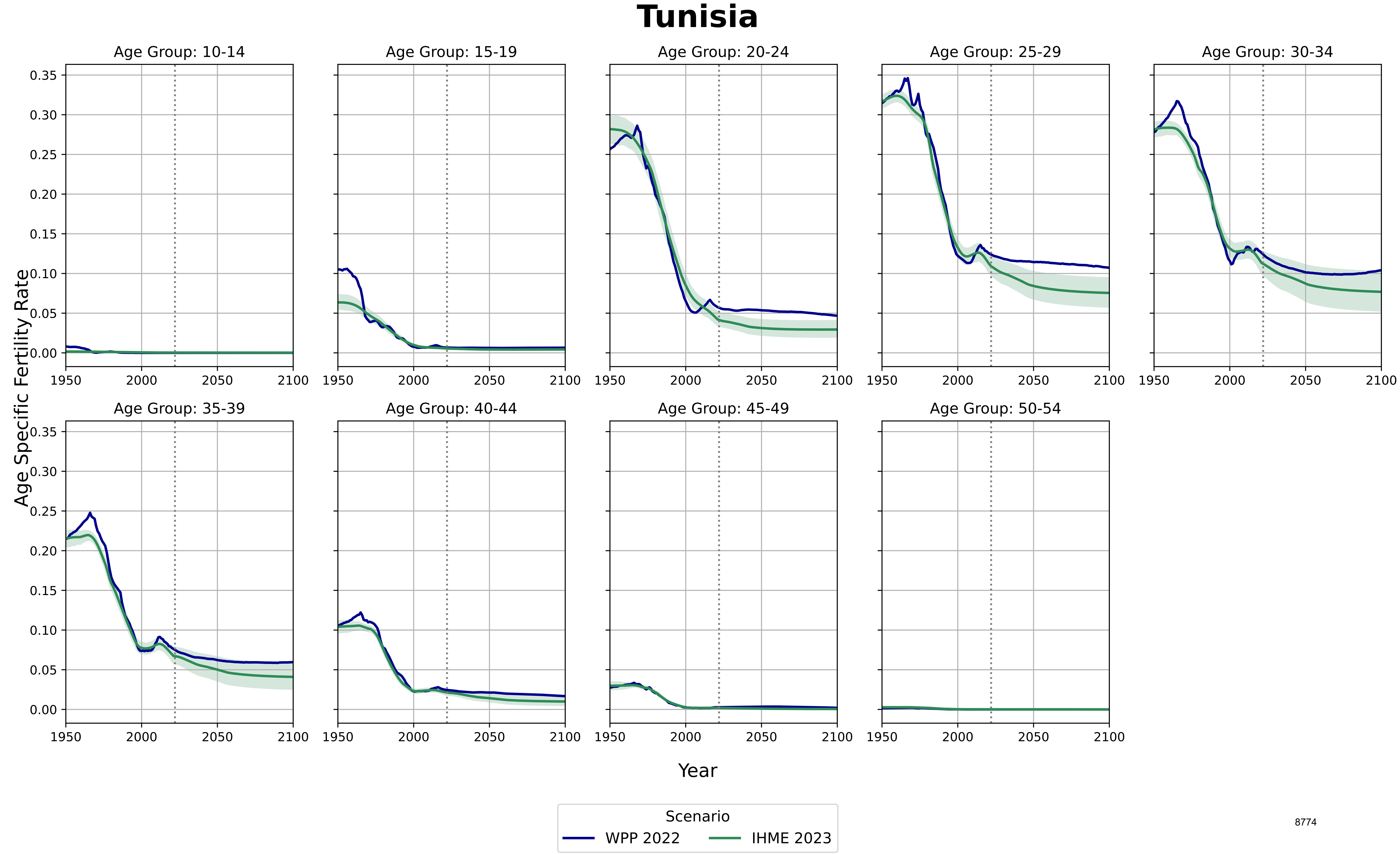

Supplemental Results Figure S8: Five-year interval ASFR from IHME reference scenario and the UNPD medium variant scenario by location, 1950-2100

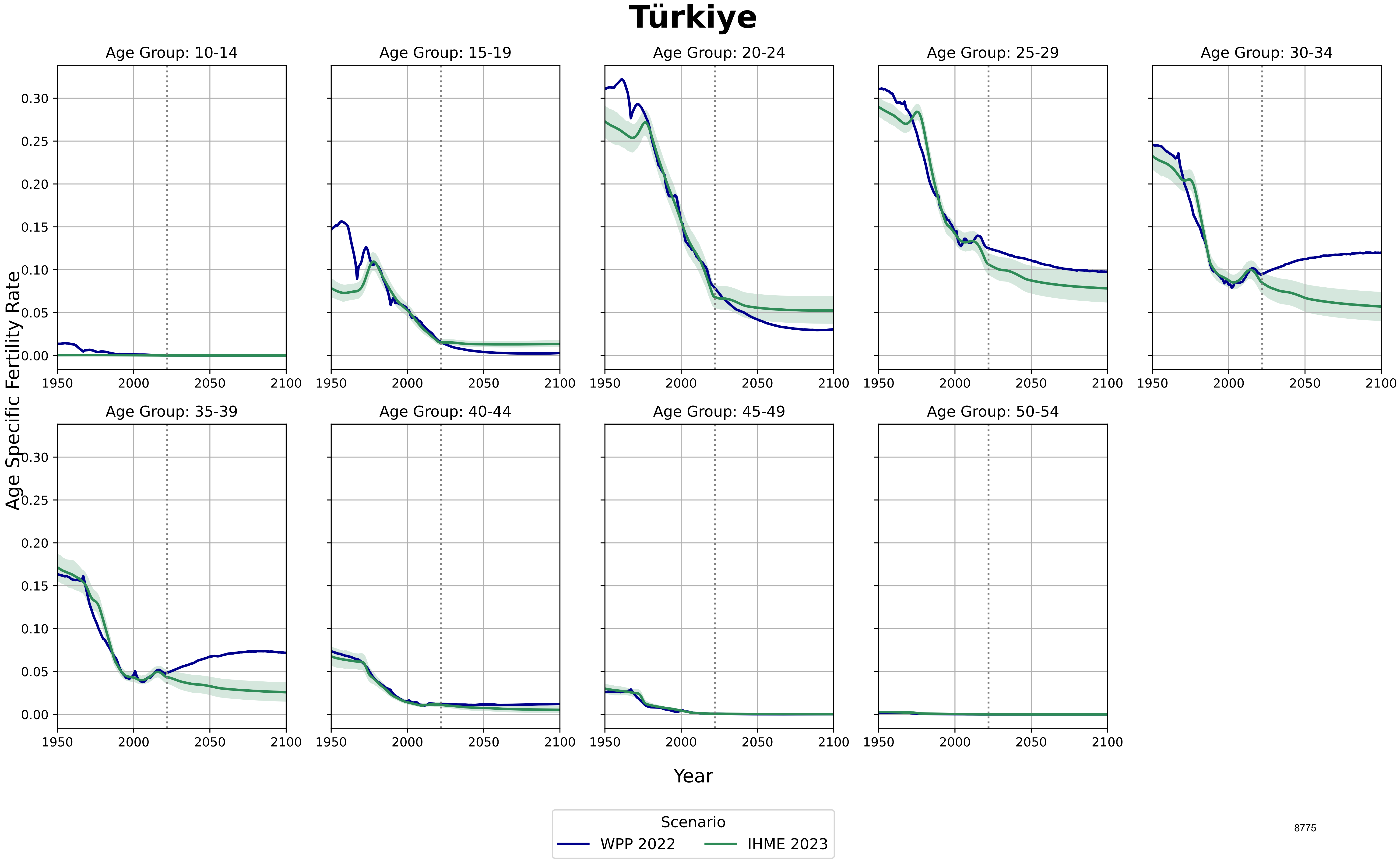

Supplemental Results Figure S8: Five-year interval ASFR from IHME reference scenario and the UNPD medium variant scenario by location, 1950-2100

United Arab Emirates

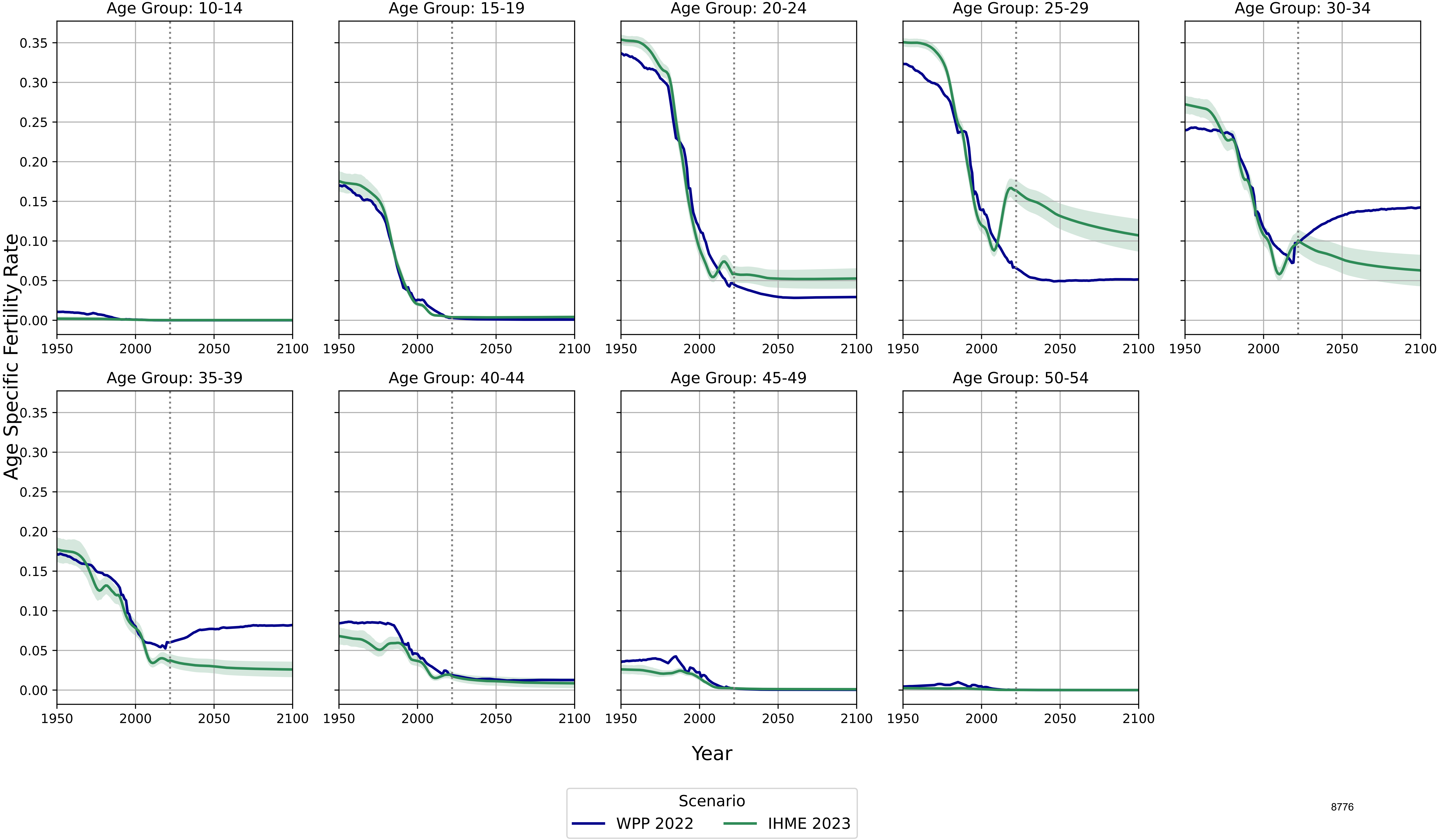

Supplemental Results Figure S8: Five-year interval ASFR from IHME reference scenario and the UNPD medium variant scenario by location, 1950-2100

Yemen

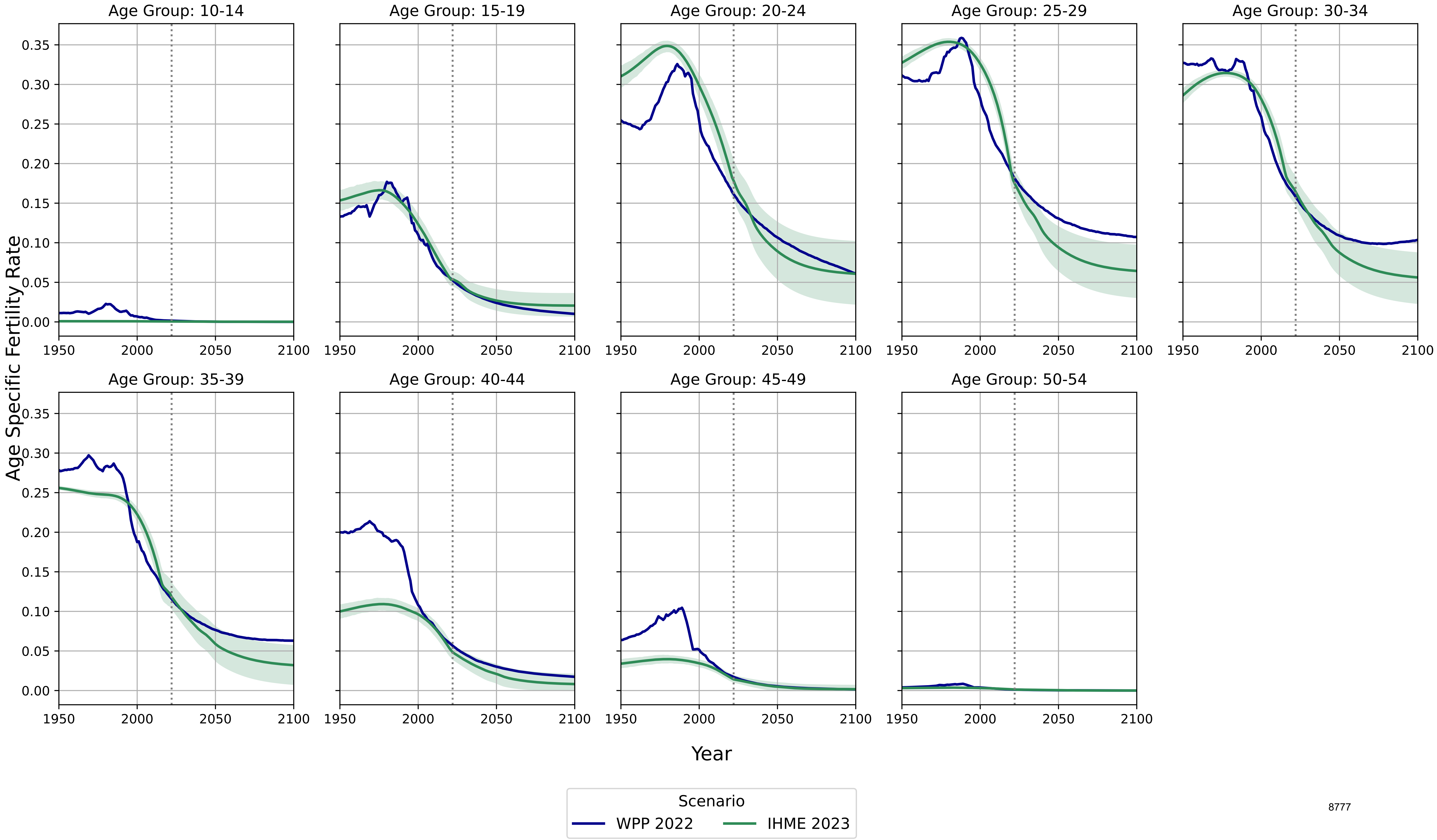

Supplemental Results Figure S8: Five-year interval ASFR from IHME reference scenario and the UNPD medium variant scenario by location, 1950-2100

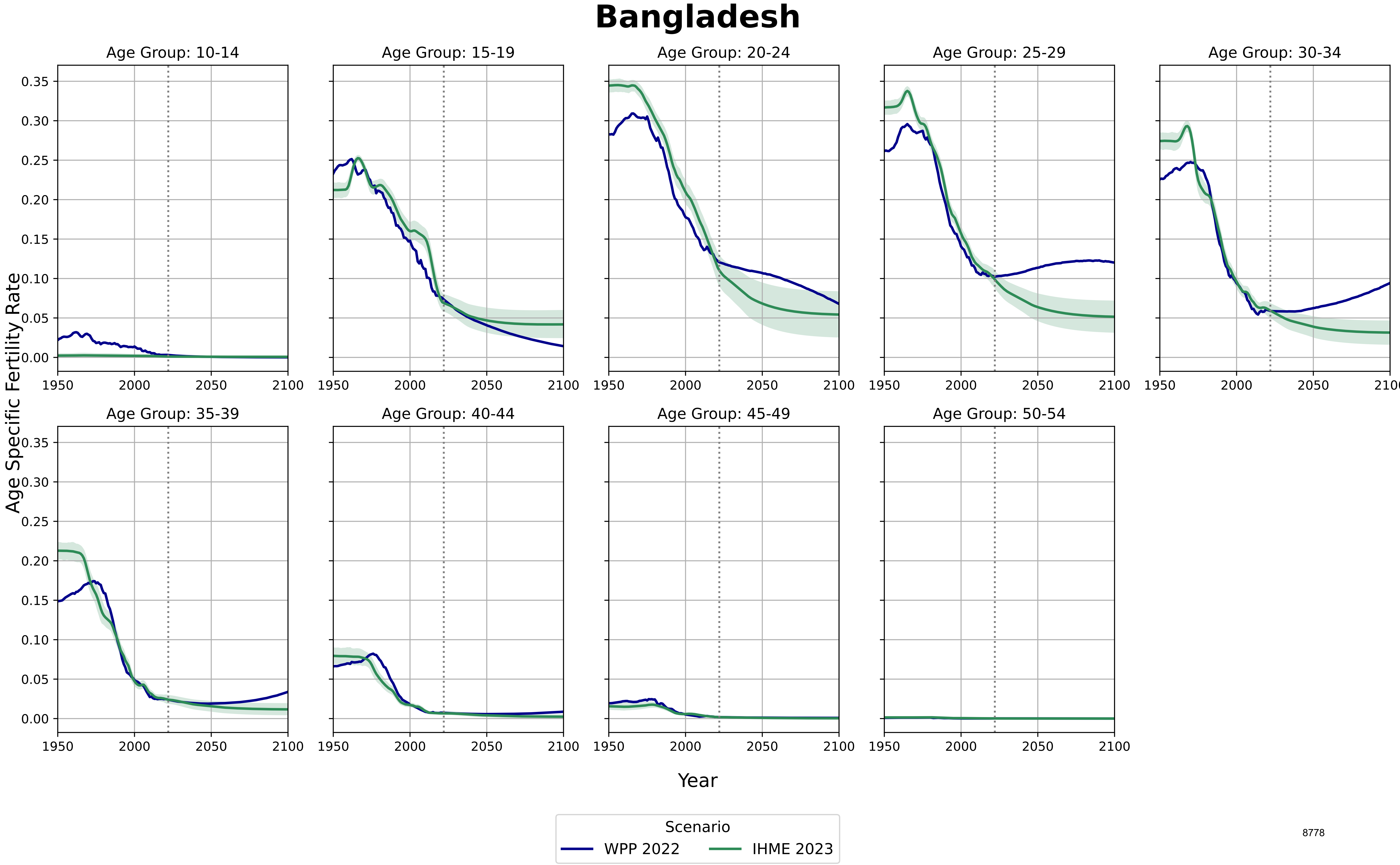

Supplemental Results Figure S8: Five-year interval ASFR from IHME reference scenario and the UNPD medium variant scenario by location, 1950-2100

Bhutan

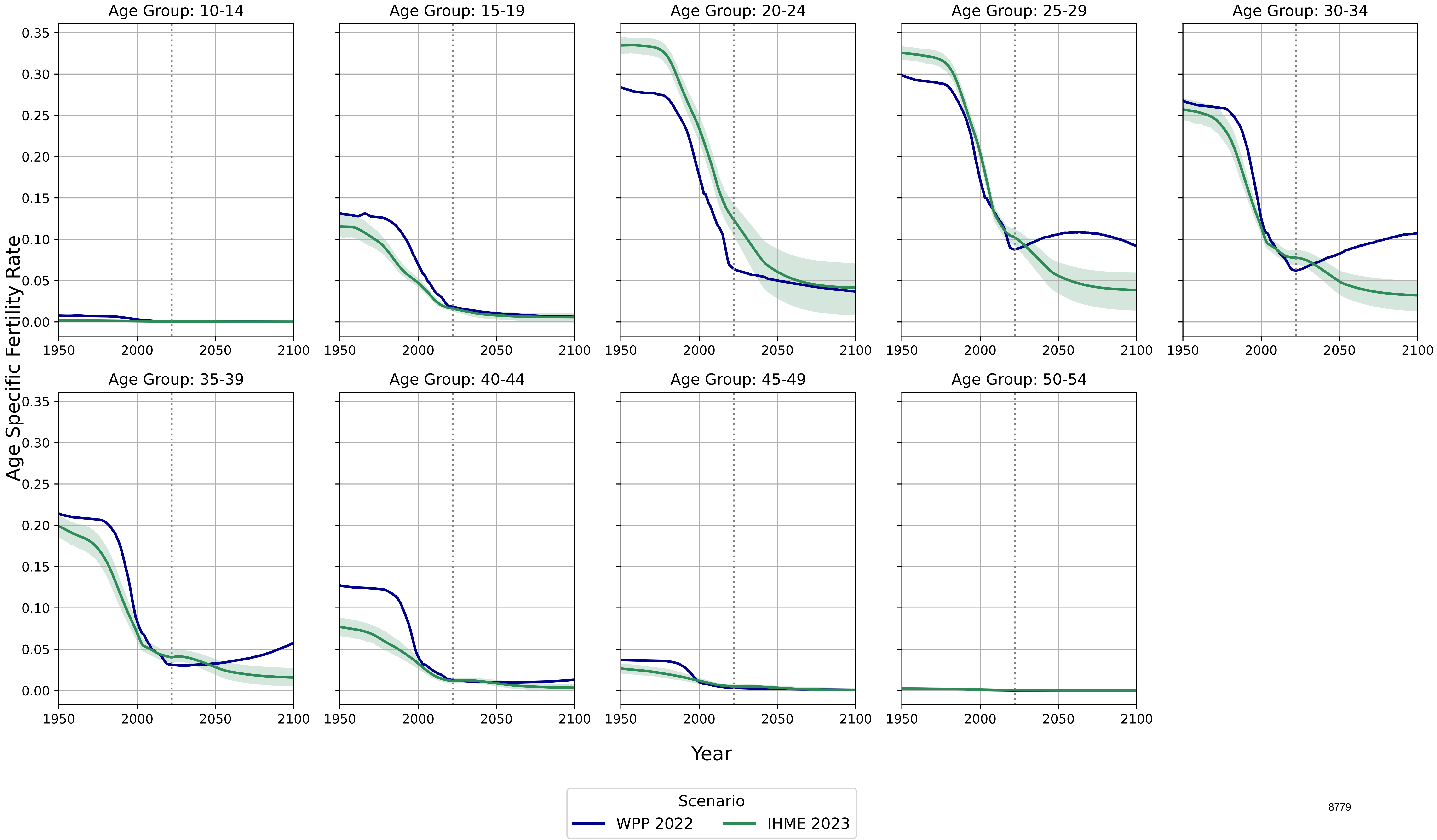

Supplemental Results Figure S8: Five-year interval ASFR from IHME reference scenario and the UNPD medium variant scenario by location, 1950-2100

India

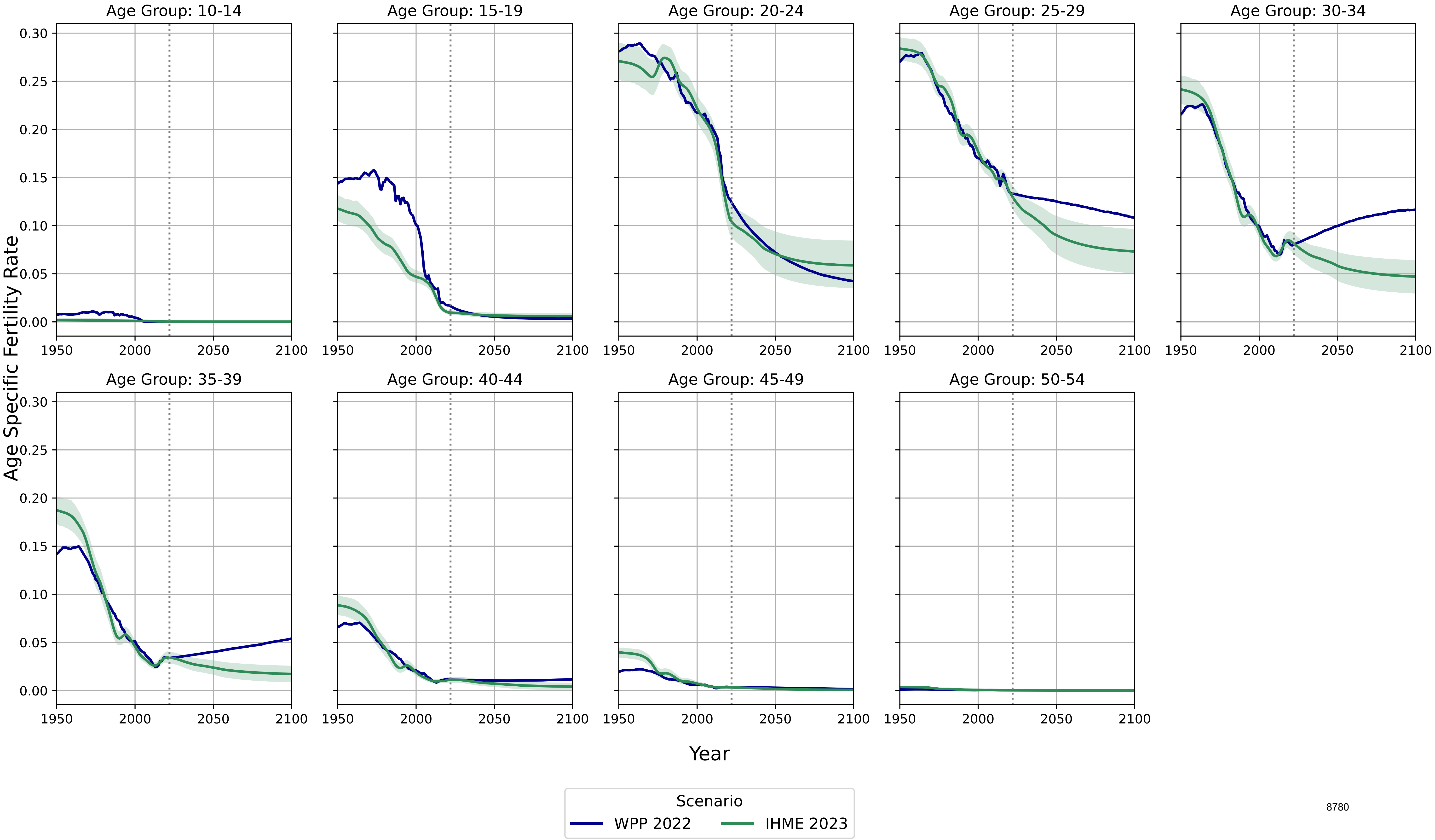

Supplemental Results Figure S8: Five-year interval ASFR from IHME reference scenario and the UNPD medium variant scenario by location, 1950-2100

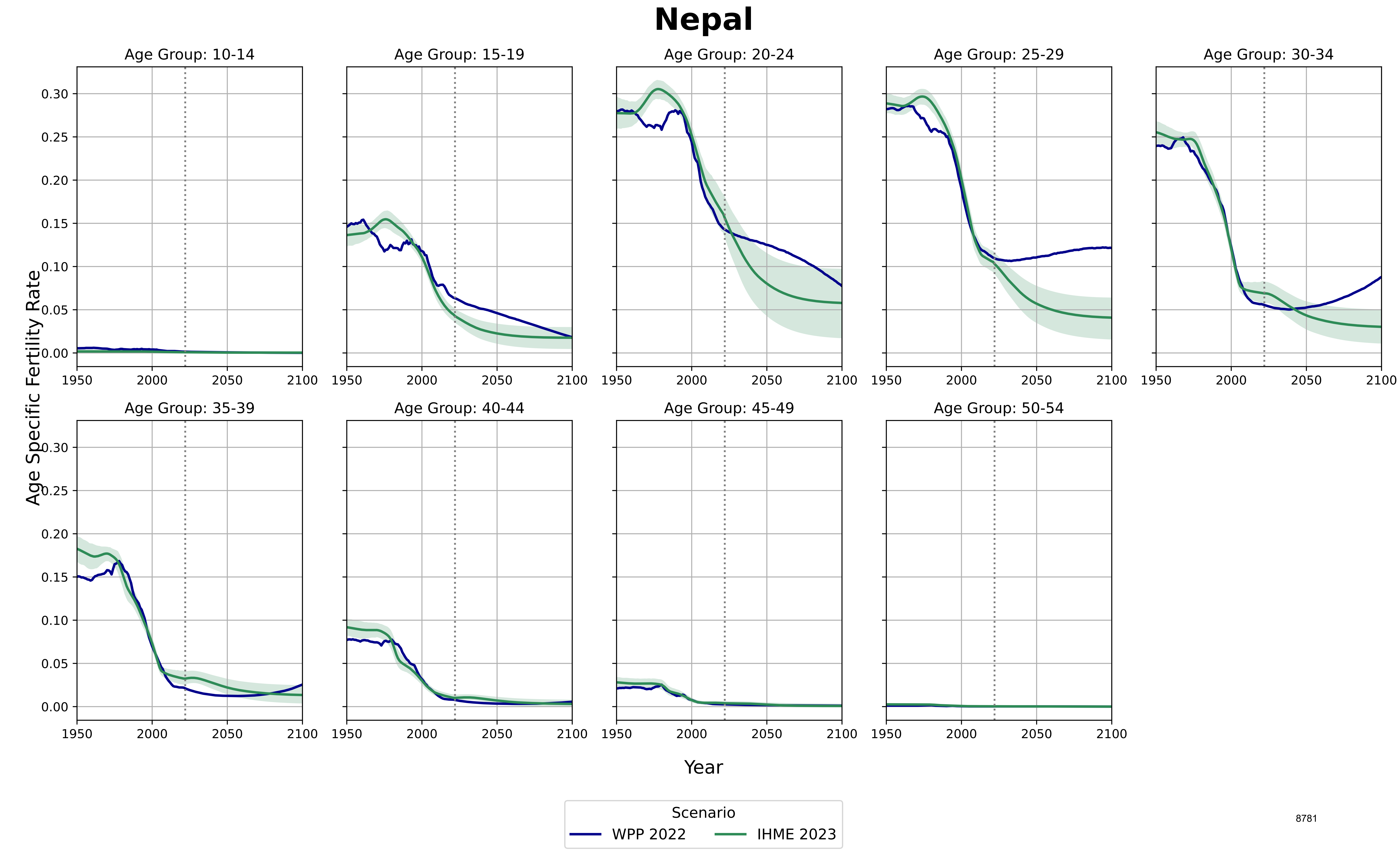

Supplemental Results Figure S8: Five-year interval ASFR from IHME reference scenario and the UNPD medium variant scenario by location, 1950-2100

Pakistan

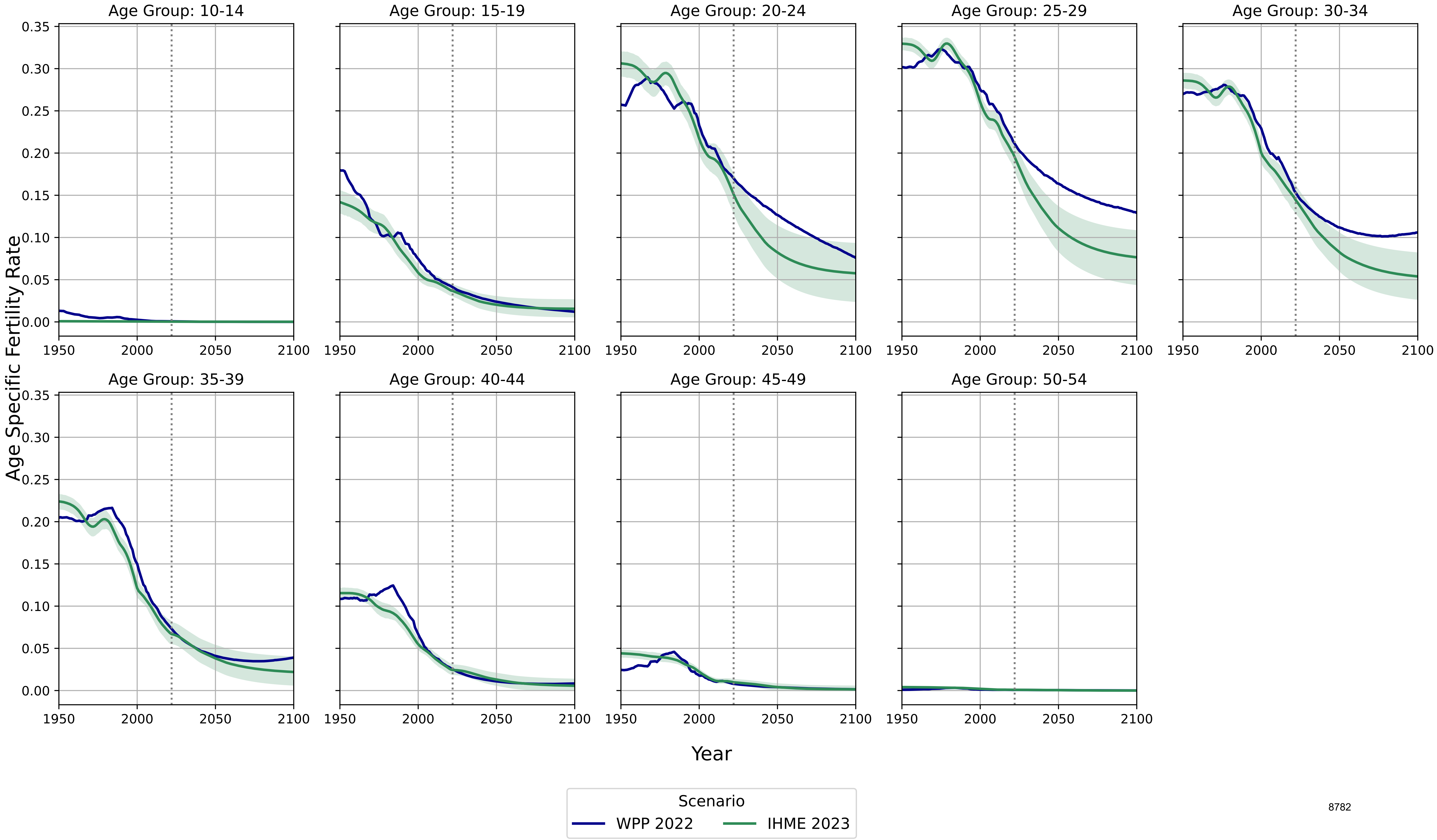

Supplemental Results Figure S8: Five-year interval ASFR from IHME reference scenario and the UNPD medium variant scenario by location, 1950-2100

China

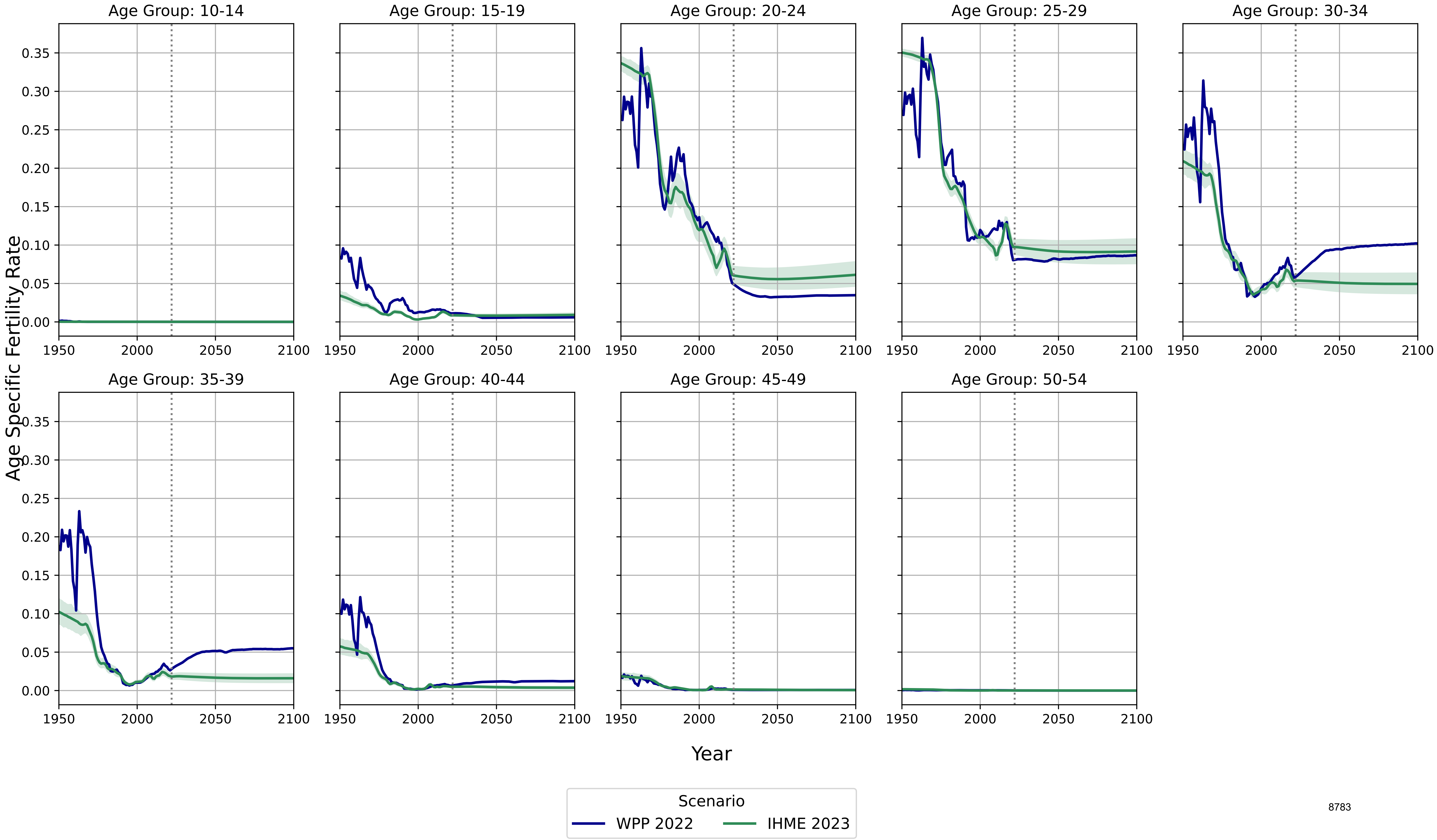

Supplemental Results Figure S8: Five-year interval ASFR from IHME reference scenario and the UNPD medium variant scenario by location, 1950-2100

North Korea

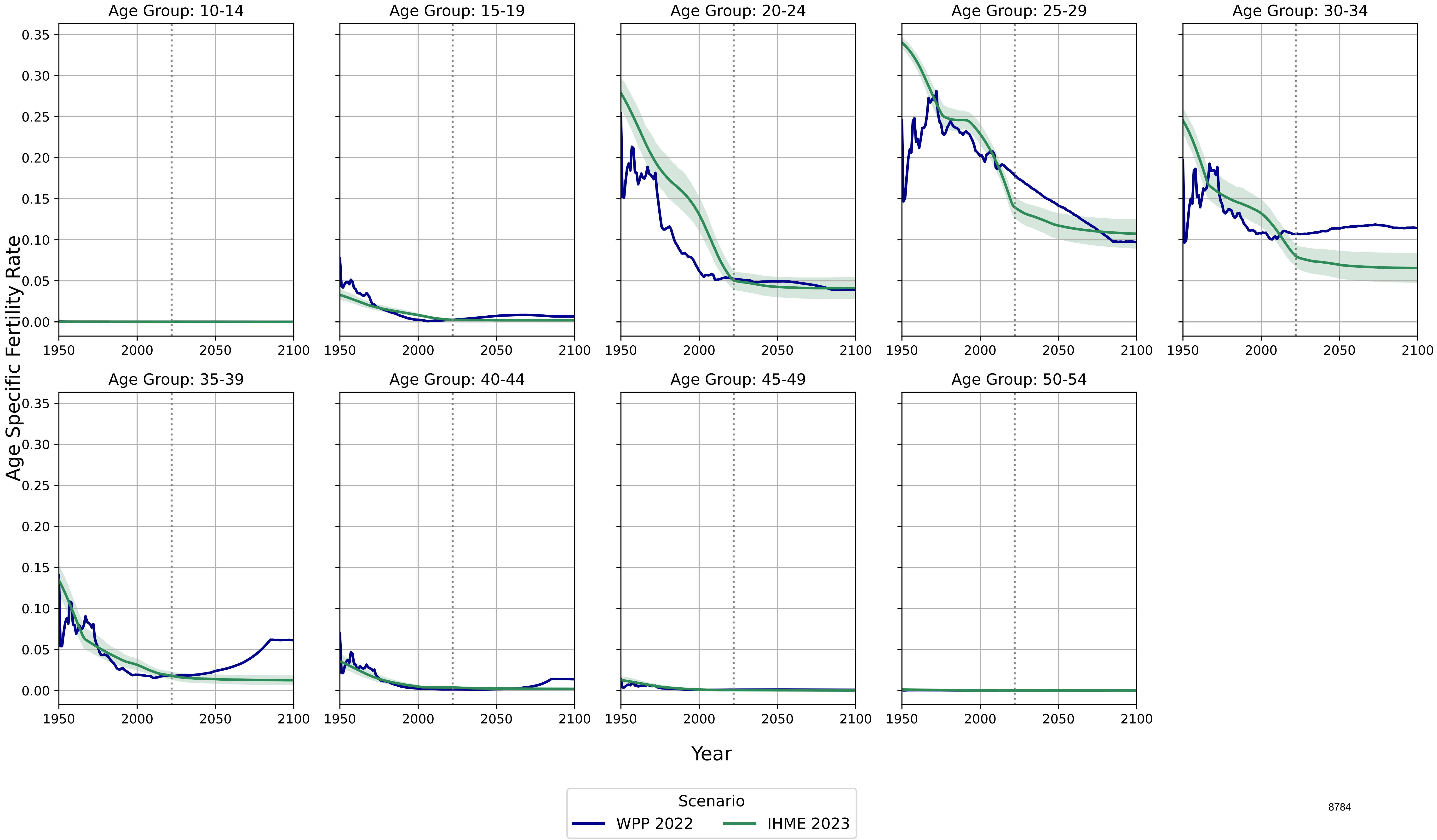

Supplemental Results Figure S8: Five-year interval ASFR from IHME reference scenario and the UNPD medium variant scenario by location, 1950-2100

Taiwan (province of China)

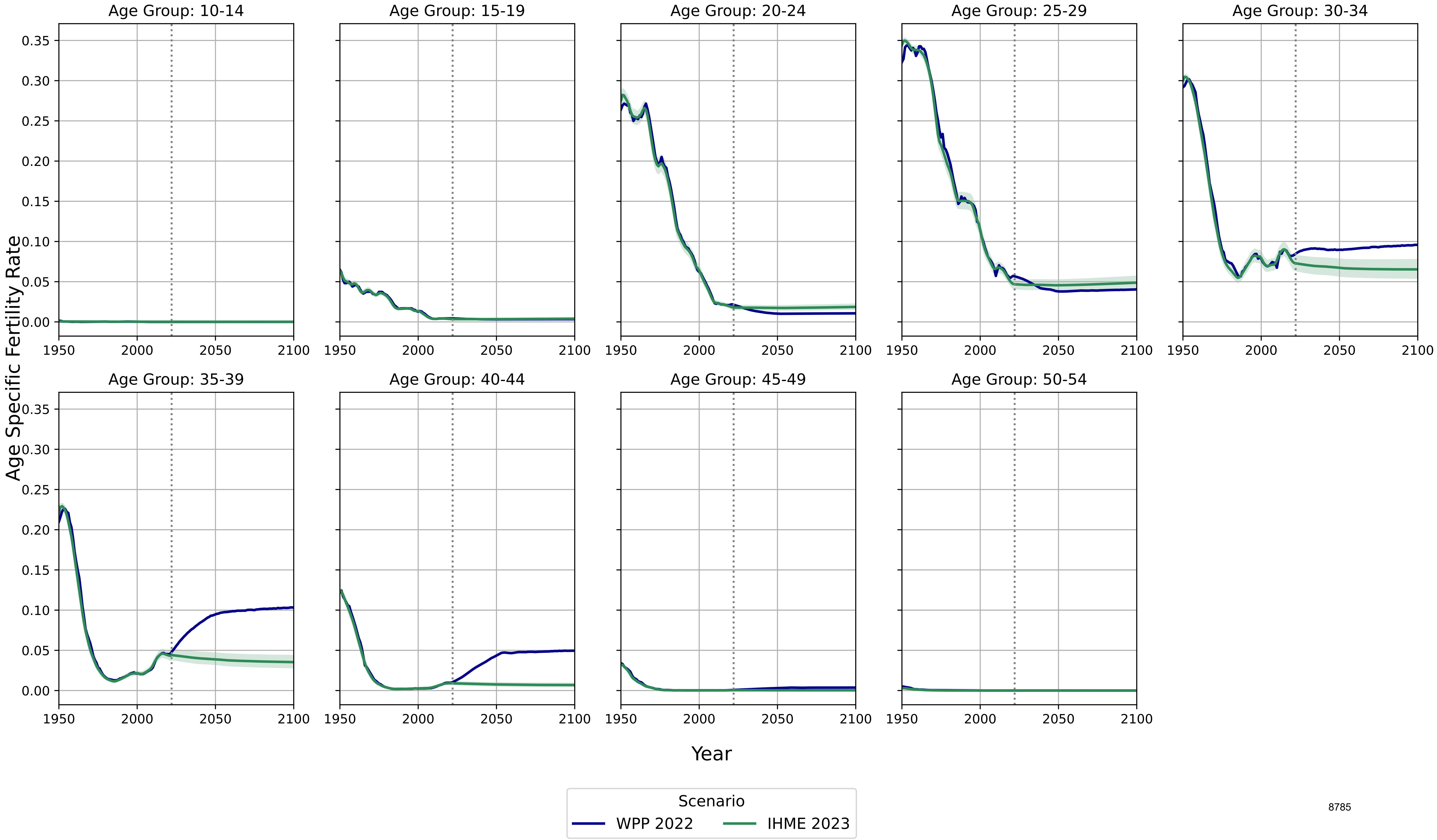

Supplemental Results Figure S8: Five-year interval ASFR from IHME reference scenario and the UNPD medium variant scenario by location, 1950-2100

American Samoa

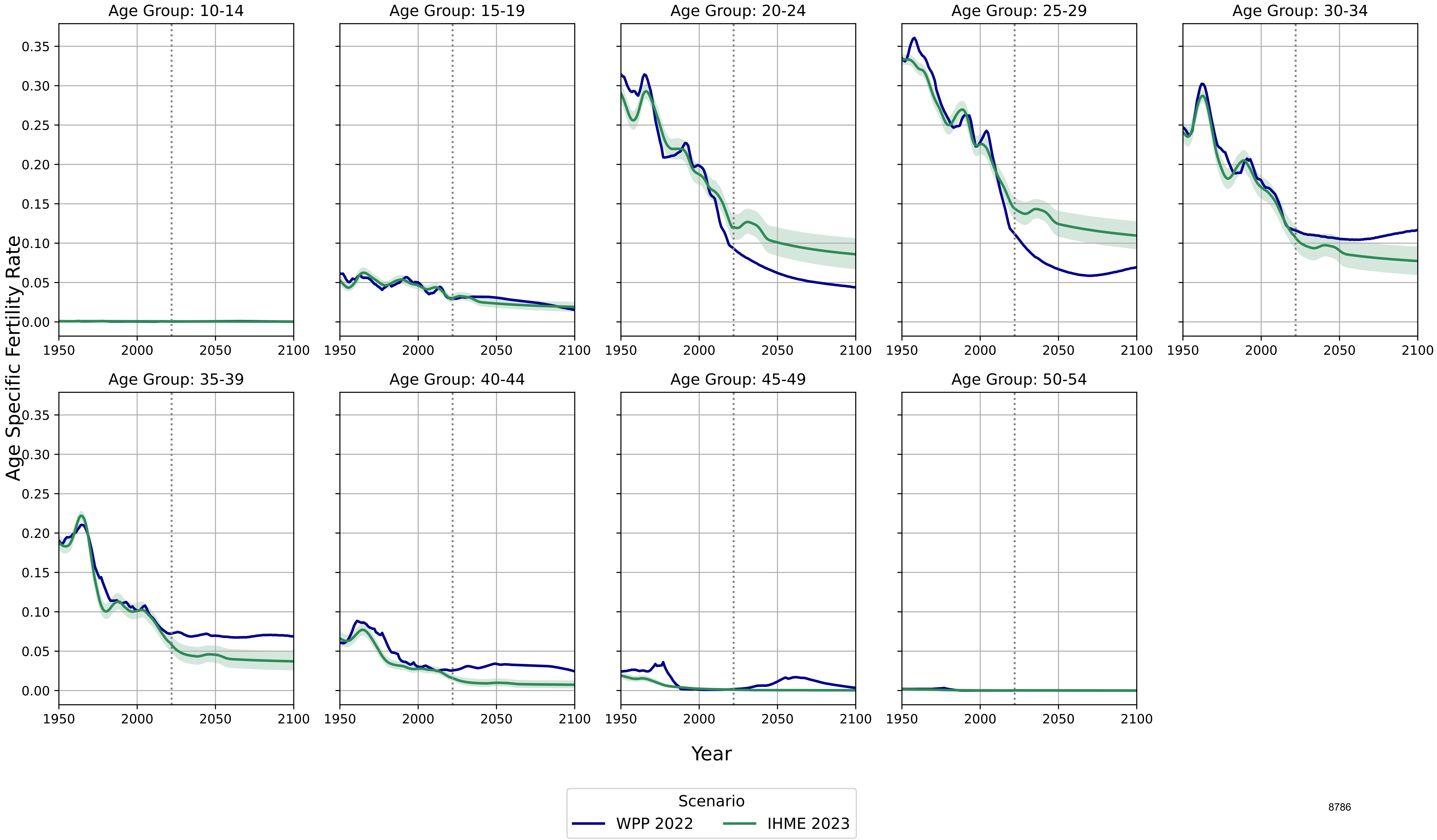

Supplemental Results Figure S8: Five-year interval ASFR from IHME reference scenario and the UNPD medium variant scenario by location, 1950-2100

Cook Islands

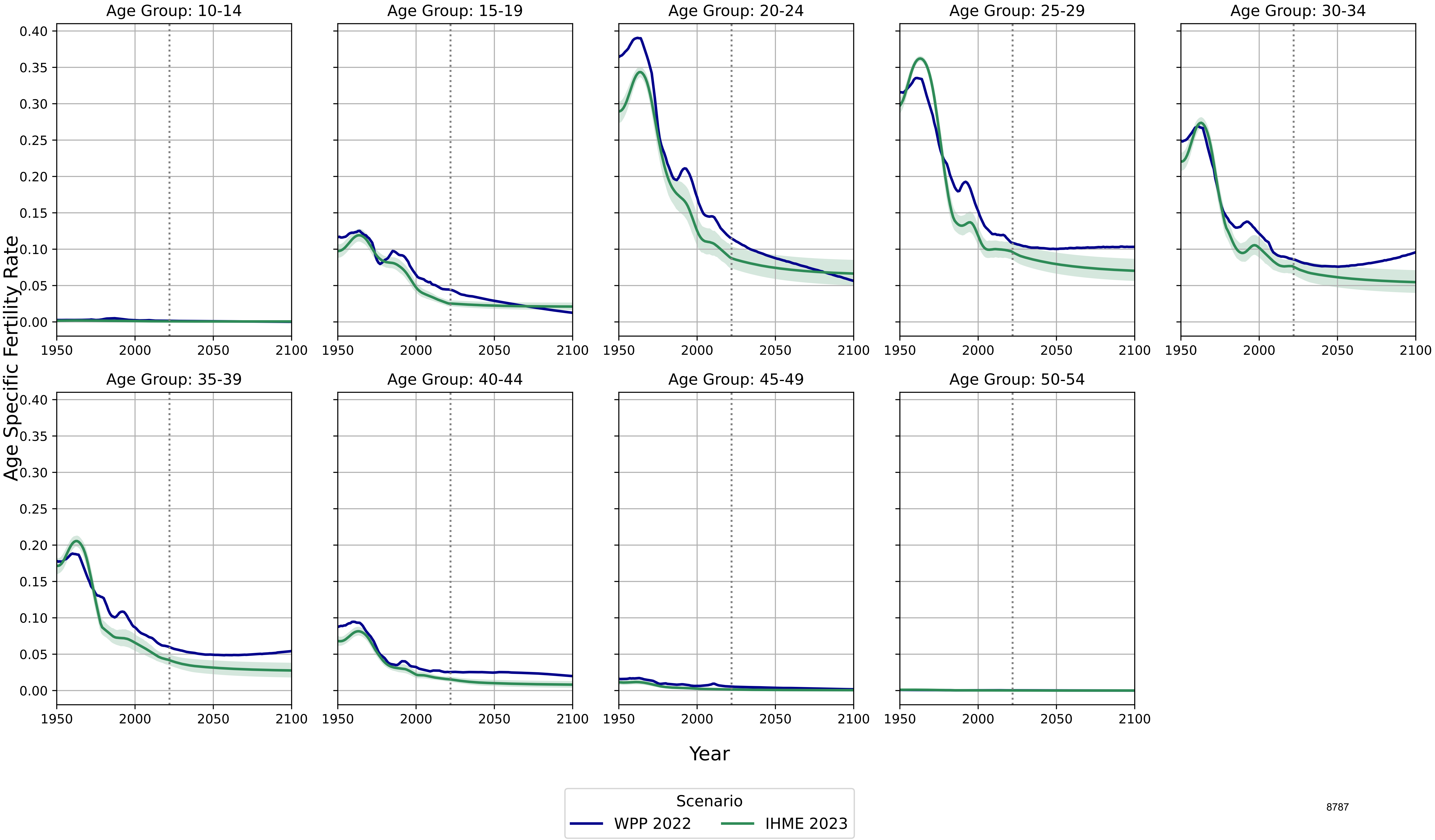

Supplemental Results Figure S8: Five-year interval ASFR from IHME reference scenario and the UNPD medium variant scenario by location, 1950-2100

Fiji

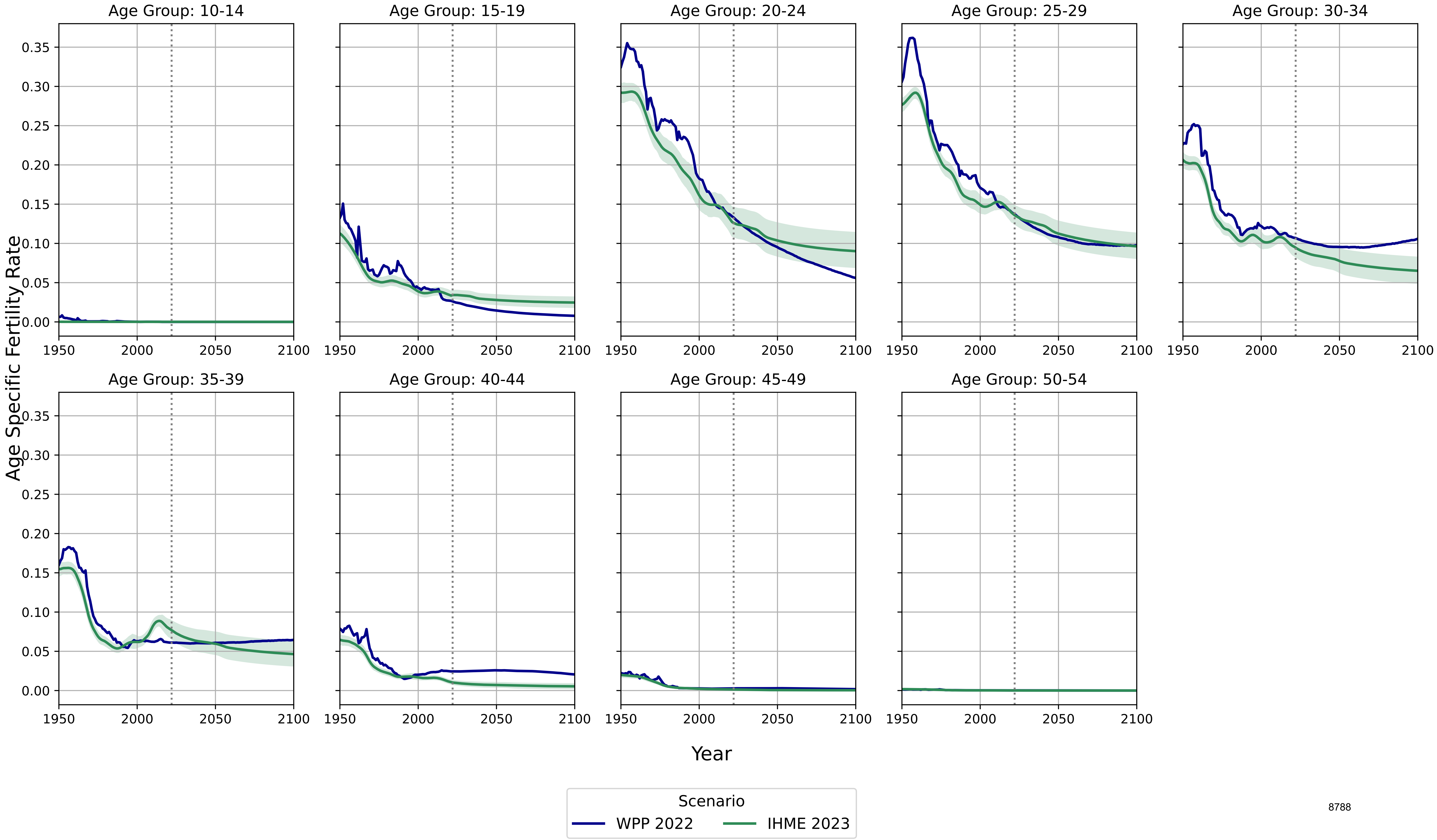

Supplemental Results Figure S8: Five-year interval ASFR from IHME reference scenario and the UNPD medium variant scenario by location, 1950-2100

Guam

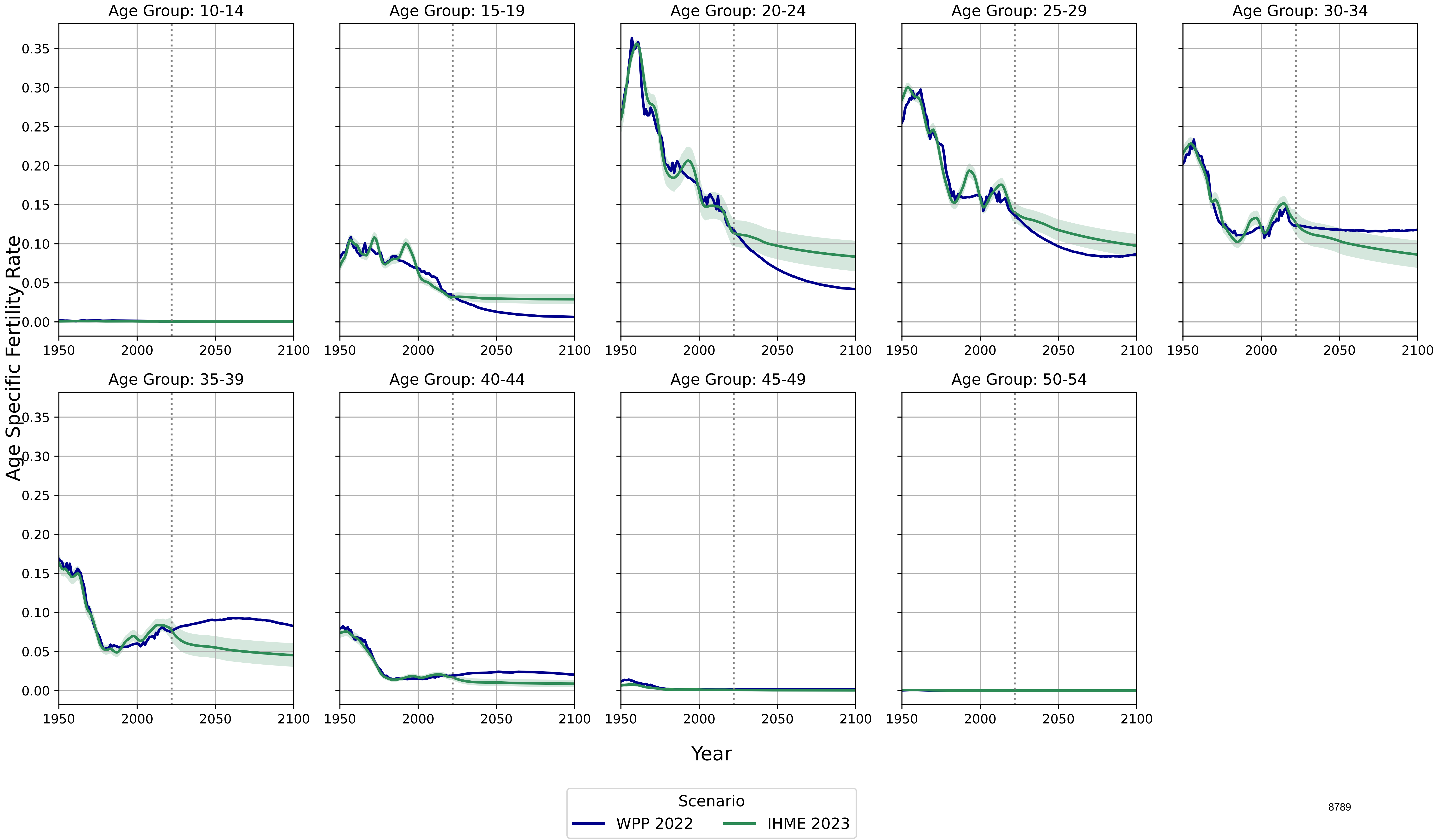

Supplemental Results Figure S8: Five-year interval ASFR from IHME reference scenario and the UNPD medium variant scenario by location, 1950-2100

Kiribati

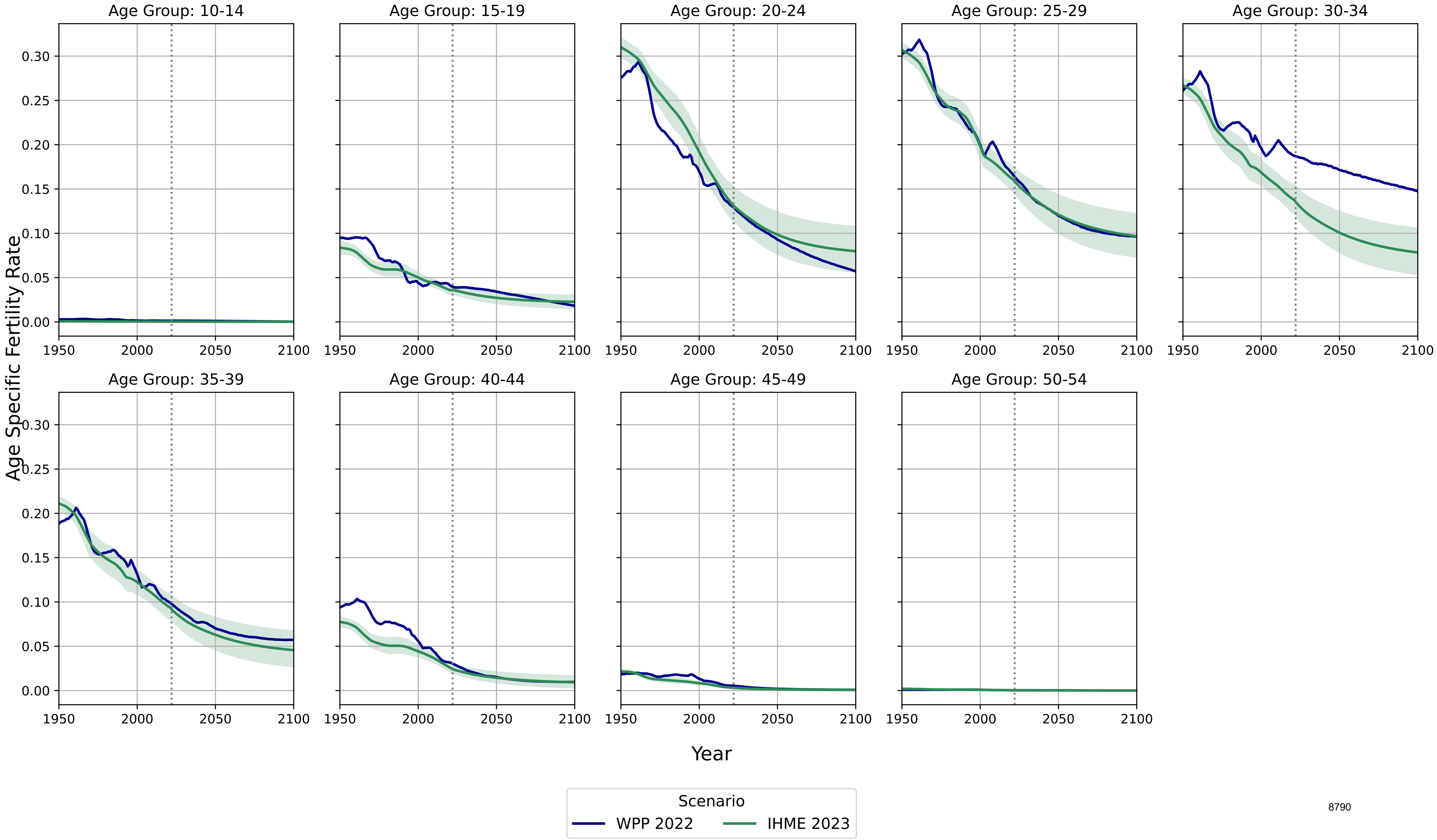

Supplemental Results Figure S8: Five-year interval ASFR from IHME reference scenario and the UNPD medium variant scenario by location, 1950-2100

Marshall Islands

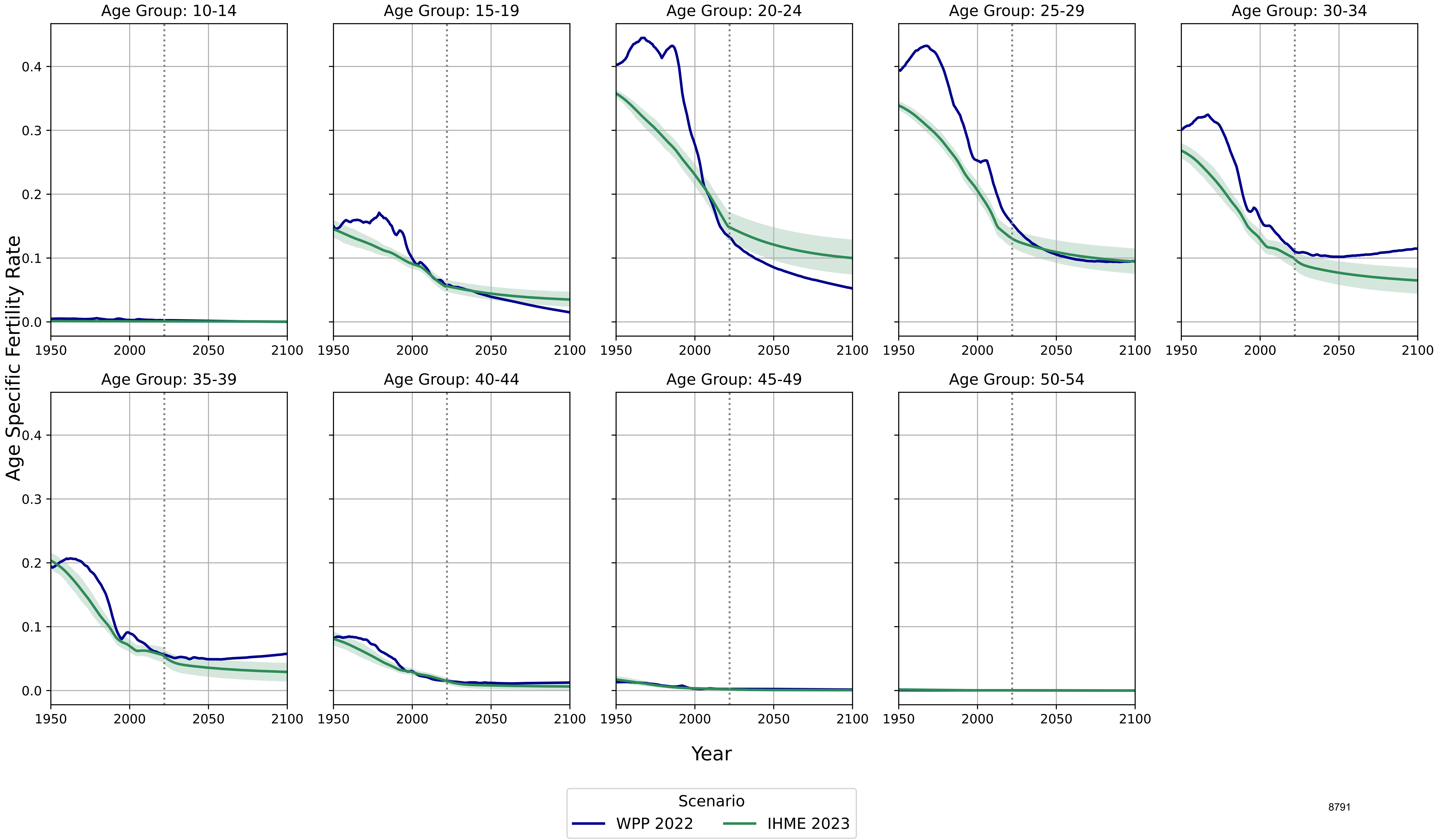

Supplemental Results Figure S8: Five-year interval ASFR from IHME reference scenario and the UNPD medium variant scenario by location, 1950-2100

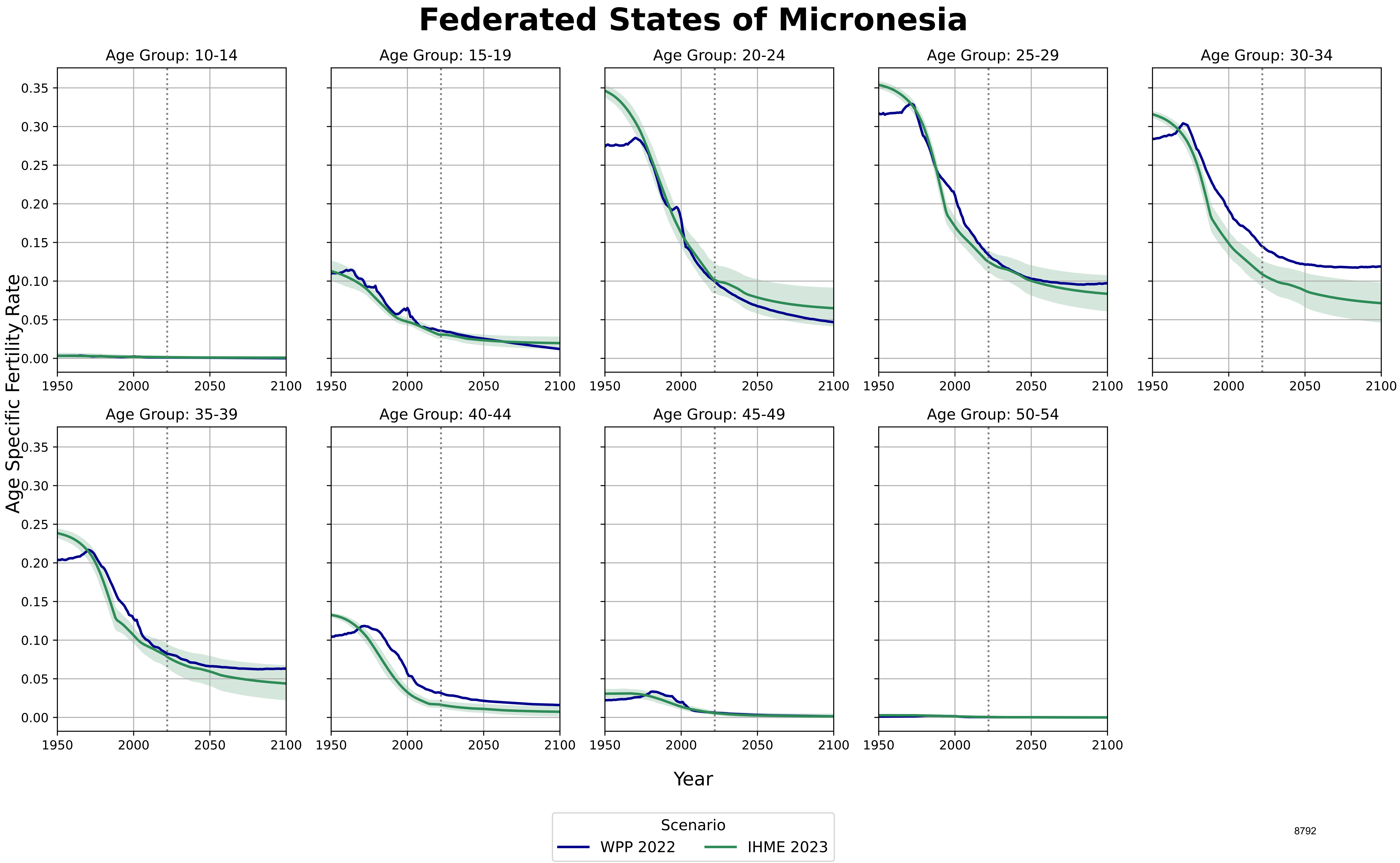

Supplemental Results Figure S8: Five-year interval ASFR from IHME reference scenario and the UNPD medium variant scenario by location, 1950-2100

Nauru

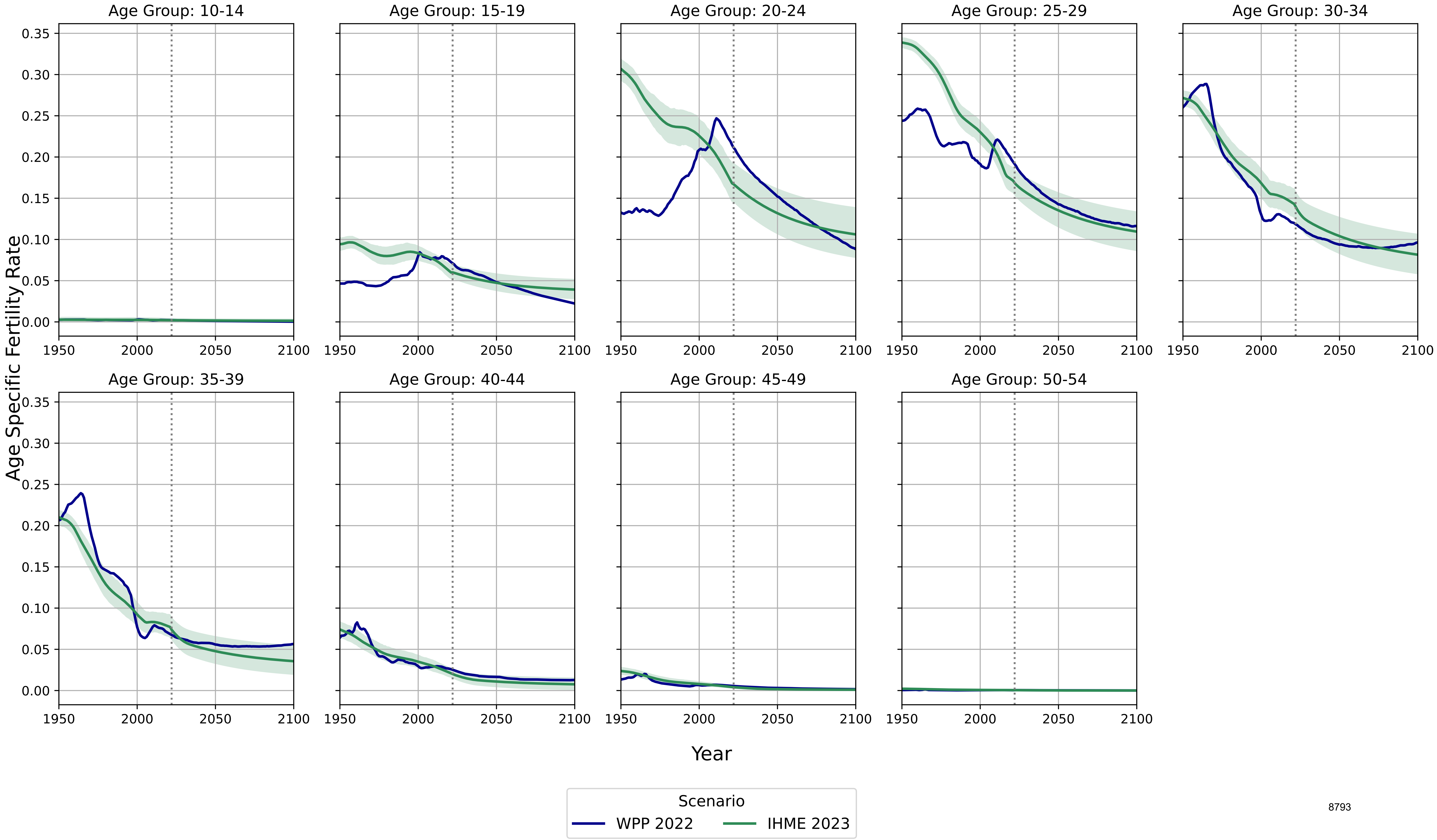

Supplemental Results Figure S8: Five-year interval ASFR from IHME reference scenario and the UNPD medium variant scenario by location, 1950-2100

Niue

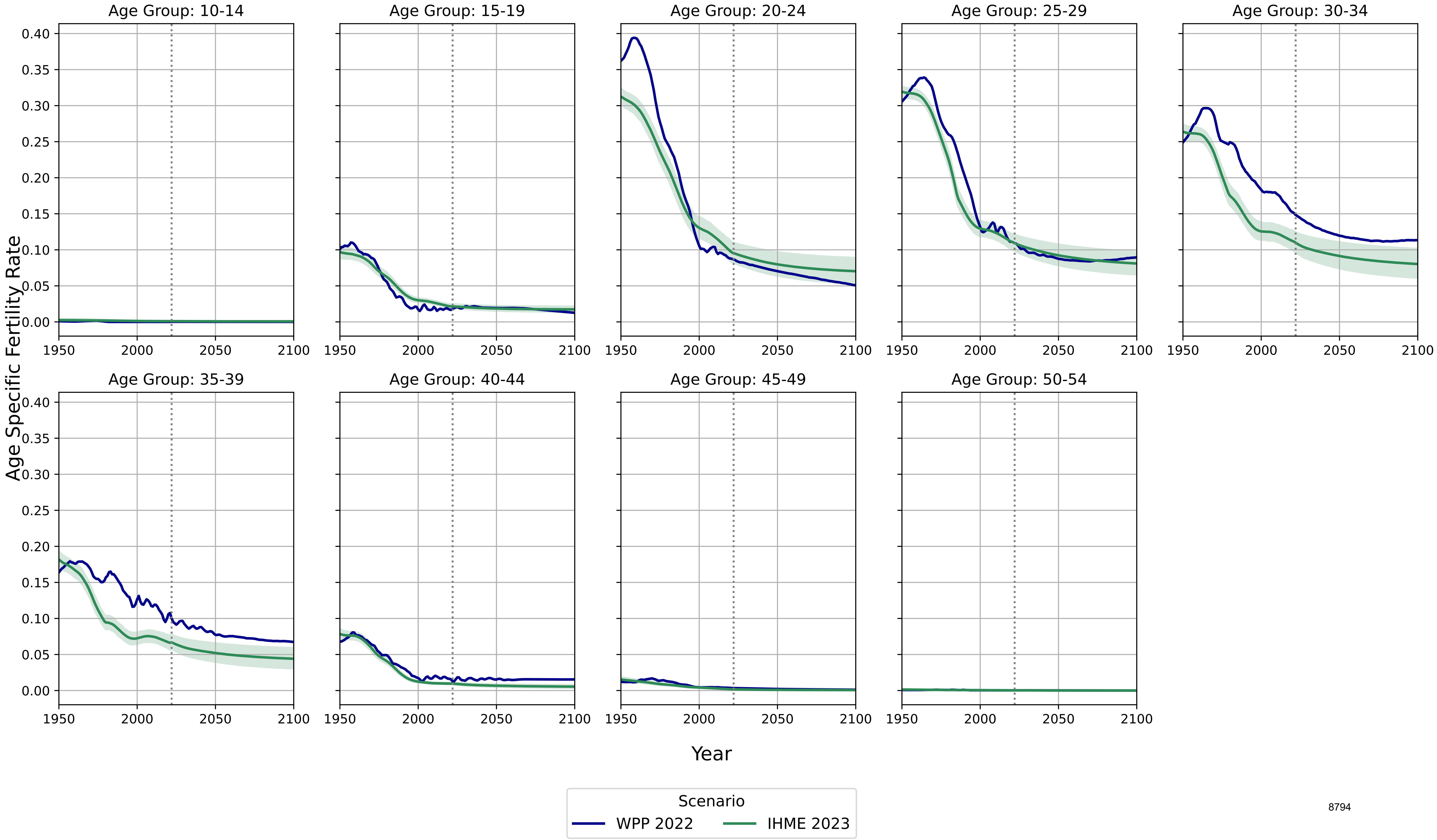

Supplemental Results Figure S8: Five-year interval ASFR from IHME reference scenario and the UNPD medium variant scenario by location, 1950-2100

Northern Mariana Islands

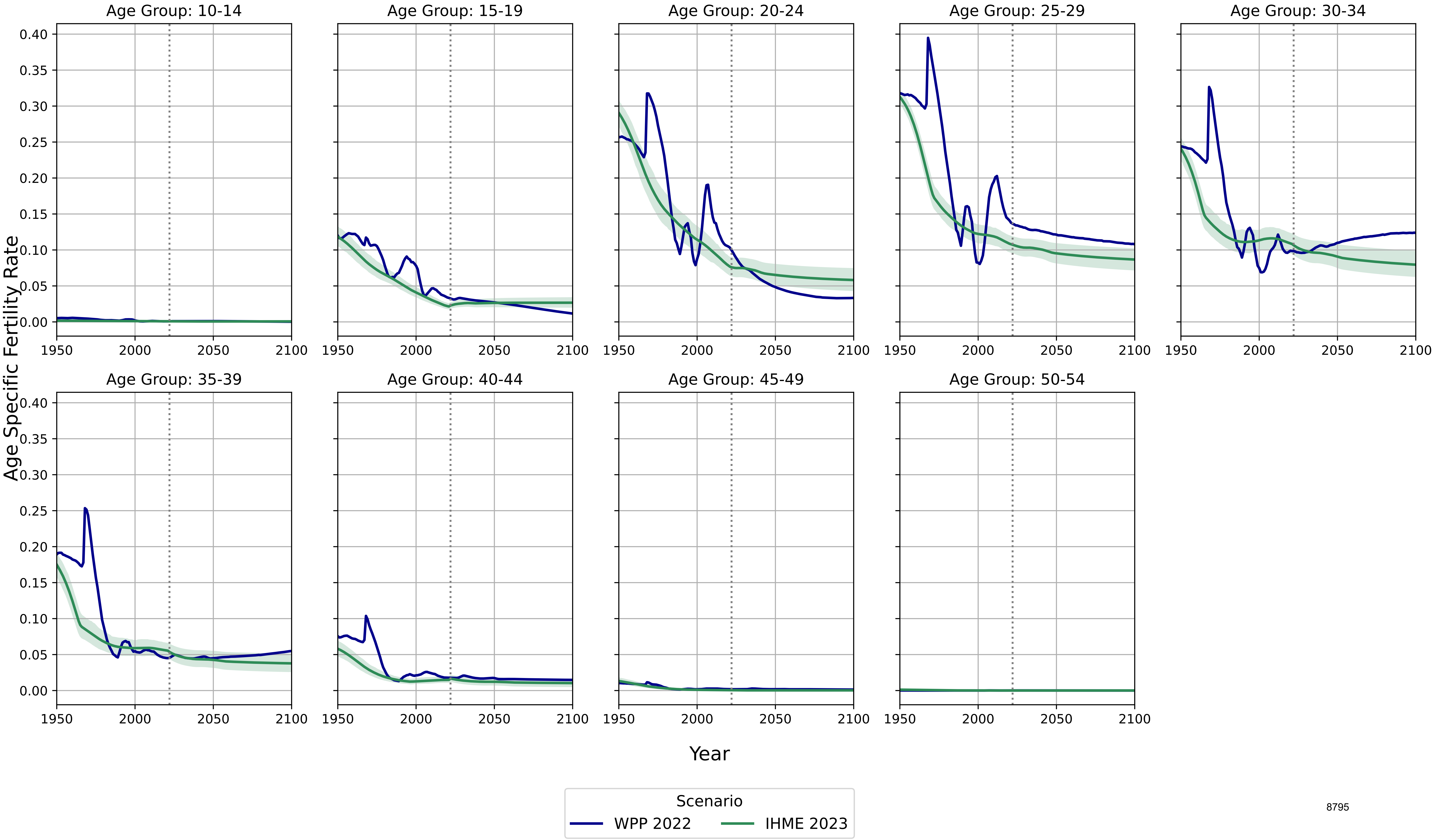

Supplemental Results Figure S8: Five-year interval ASFR from IHME reference scenario and the UNPD medium variant scenario by location, 1950-2100

Palau

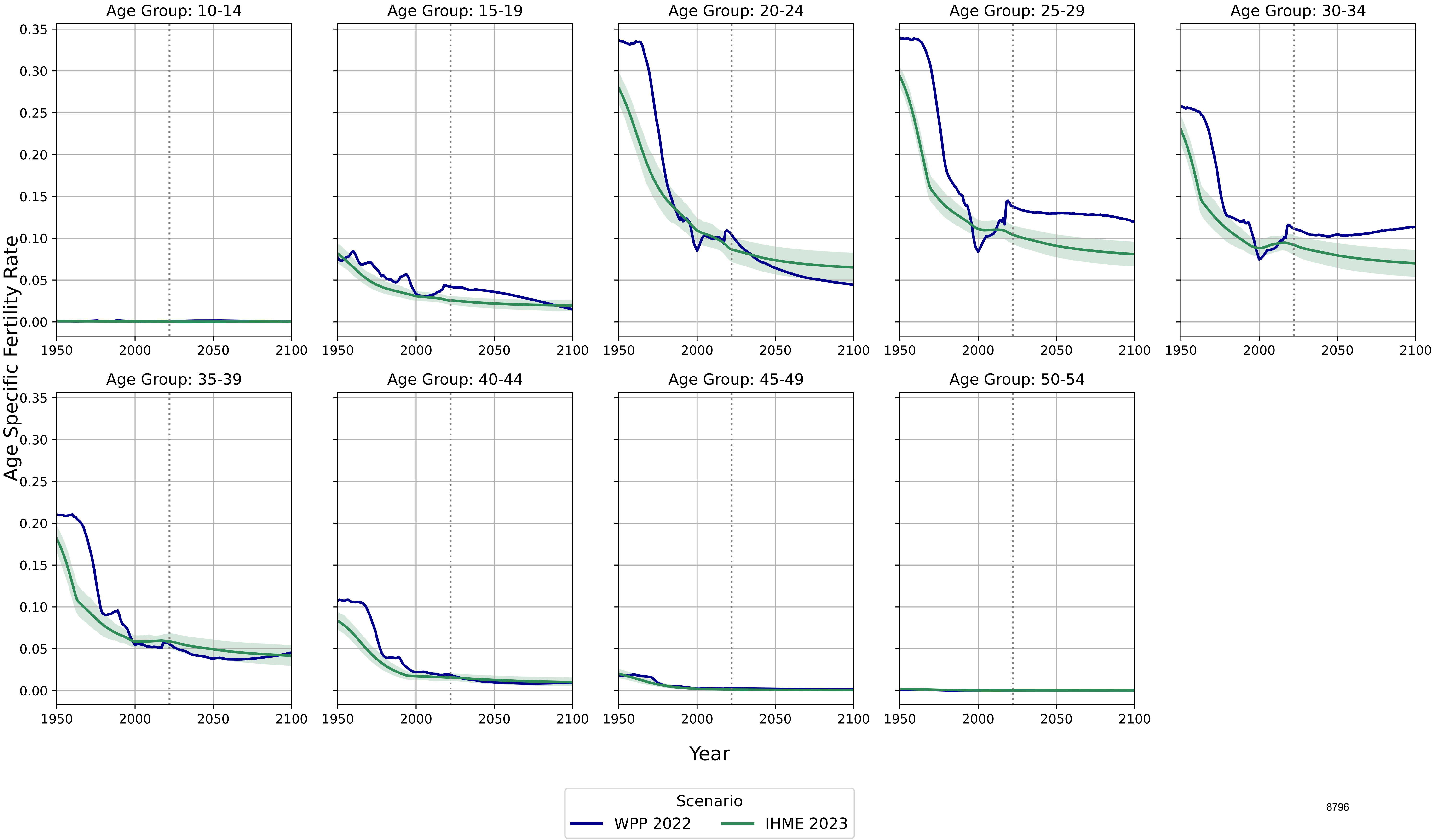

Supplemental Results Figure S8: Five-year interval ASFR from IHME reference scenario and the UNPD medium variant scenario by location, 1950-2100

Papua New Guinea

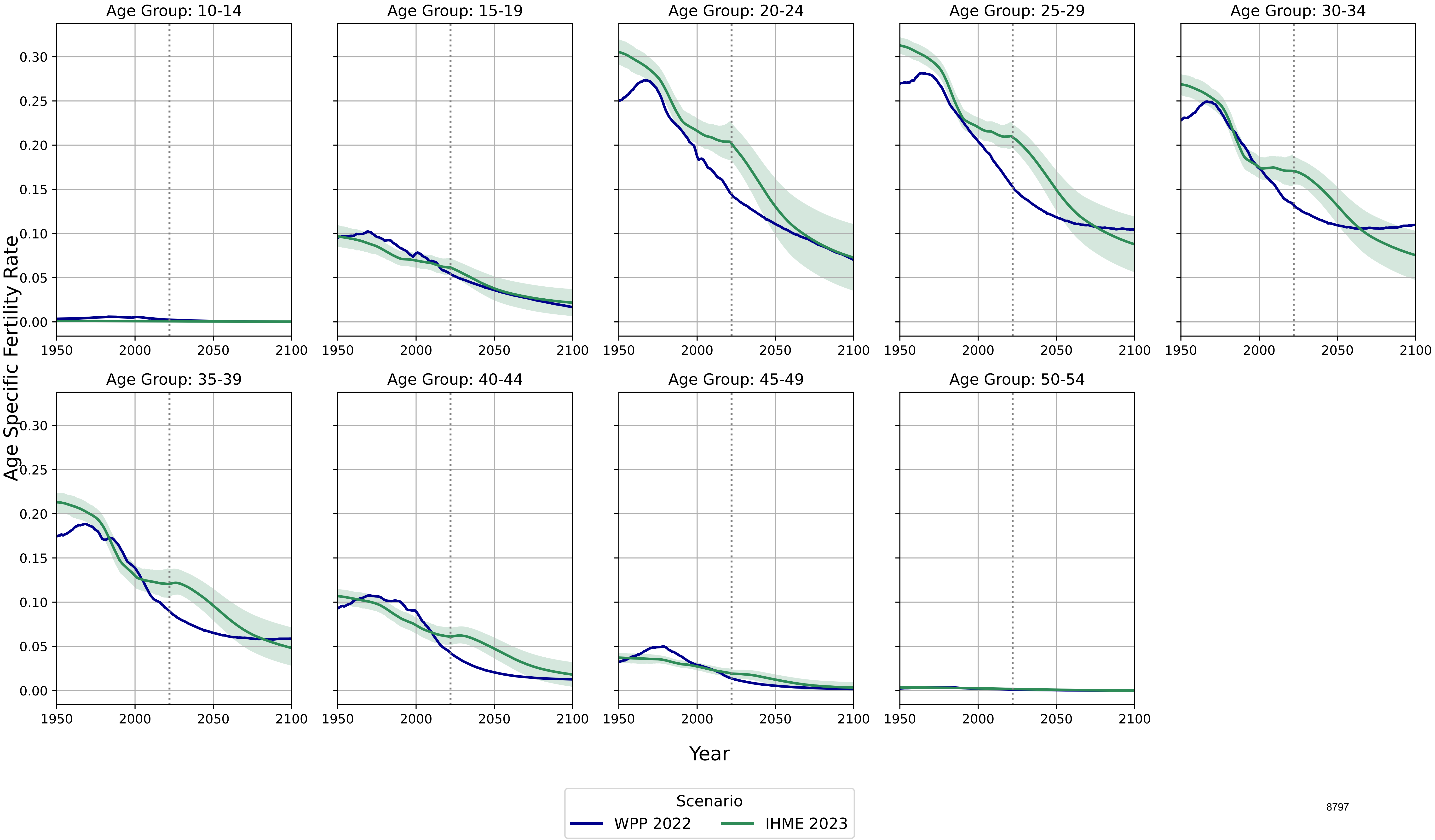

Supplemental Results Figure S8: Five-year interval ASFR from IHME reference scenario and the UNPD medium variant scenario by location, 1950-2100

Samoa

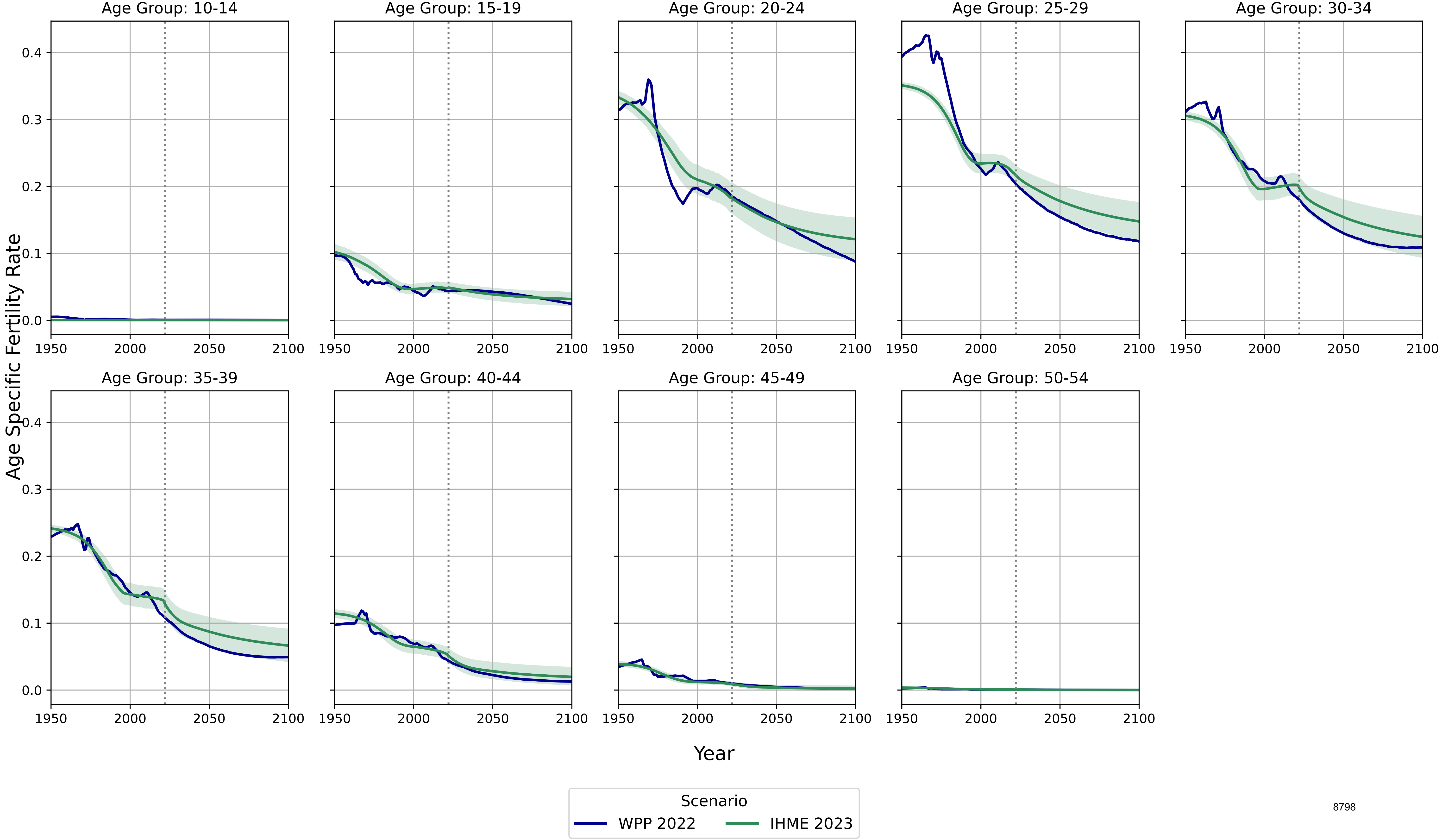

Supplemental Results Figure S8: Five-year interval ASFR from IHME reference scenario and the UNPD medium variant scenario by location, 1950-2100

Solomon Islands

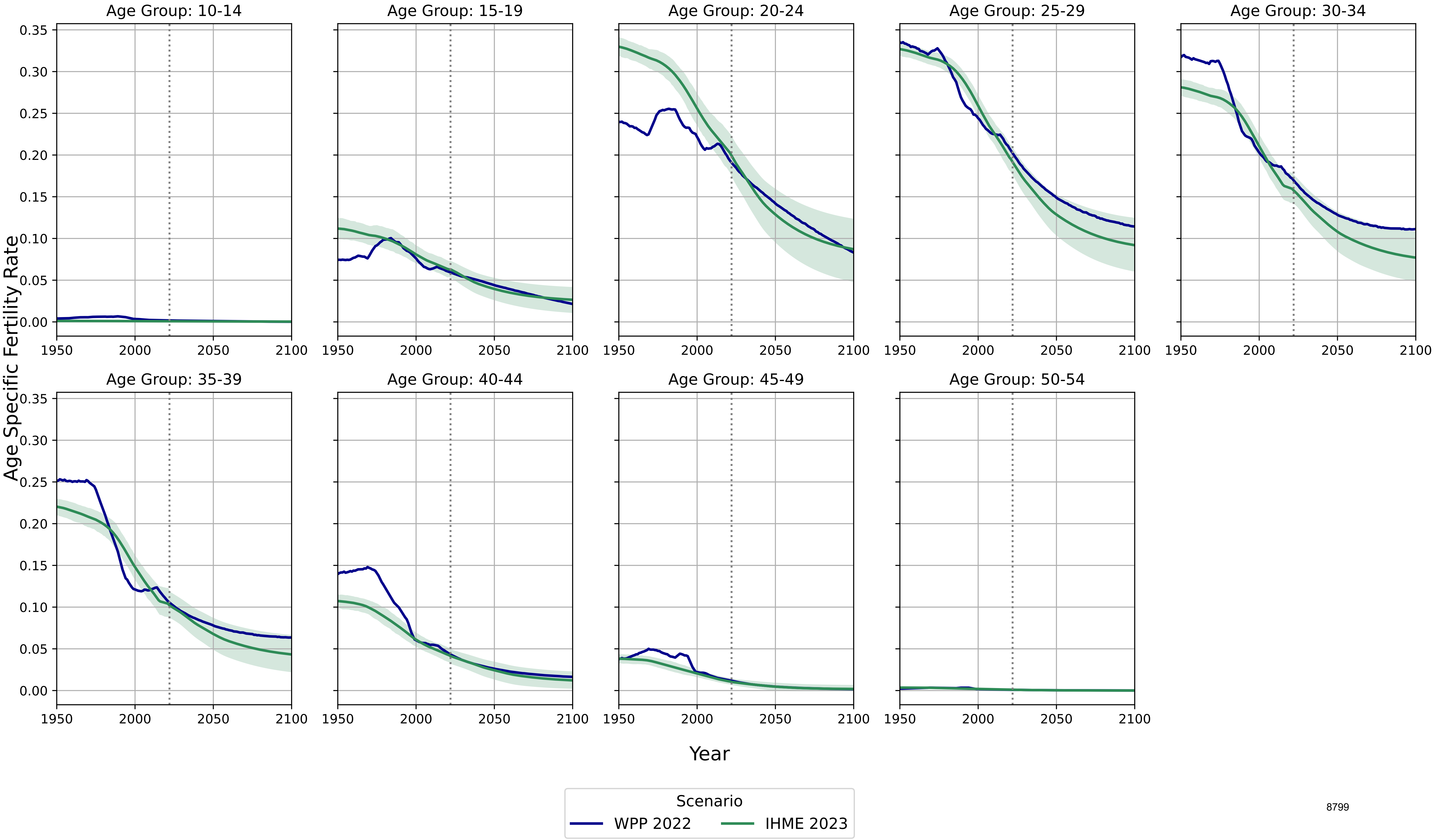

Supplemental Results Figure S8: Five-year interval ASFR from IHME reference scenario and the UNPD medium variant scenario by location, 1950-2100

Tokelau

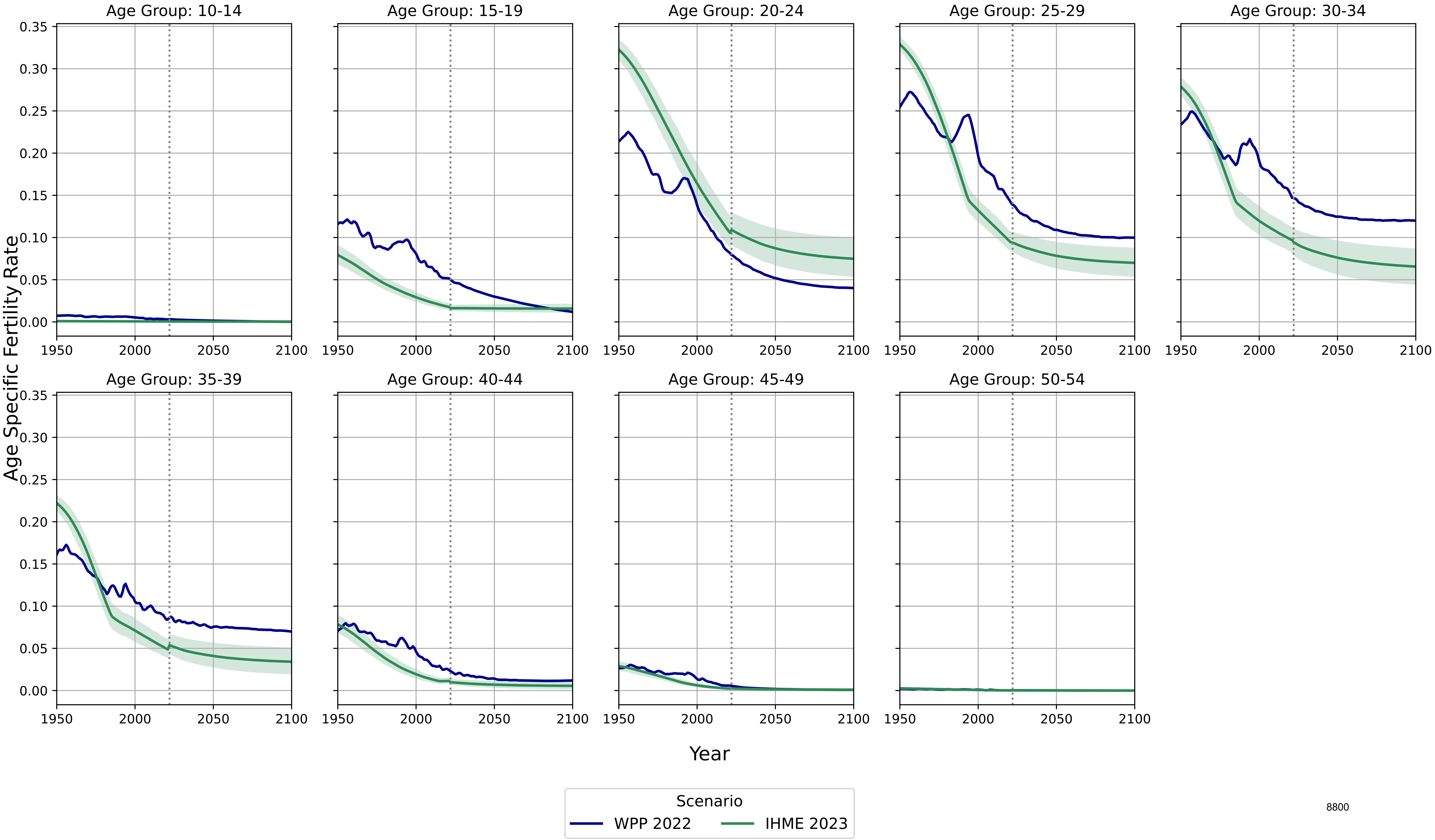

Supplemental Results Figure S8: Five-year interval ASFR from IHME reference scenario and the UNPD medium variant scenario by location, 1950-2100

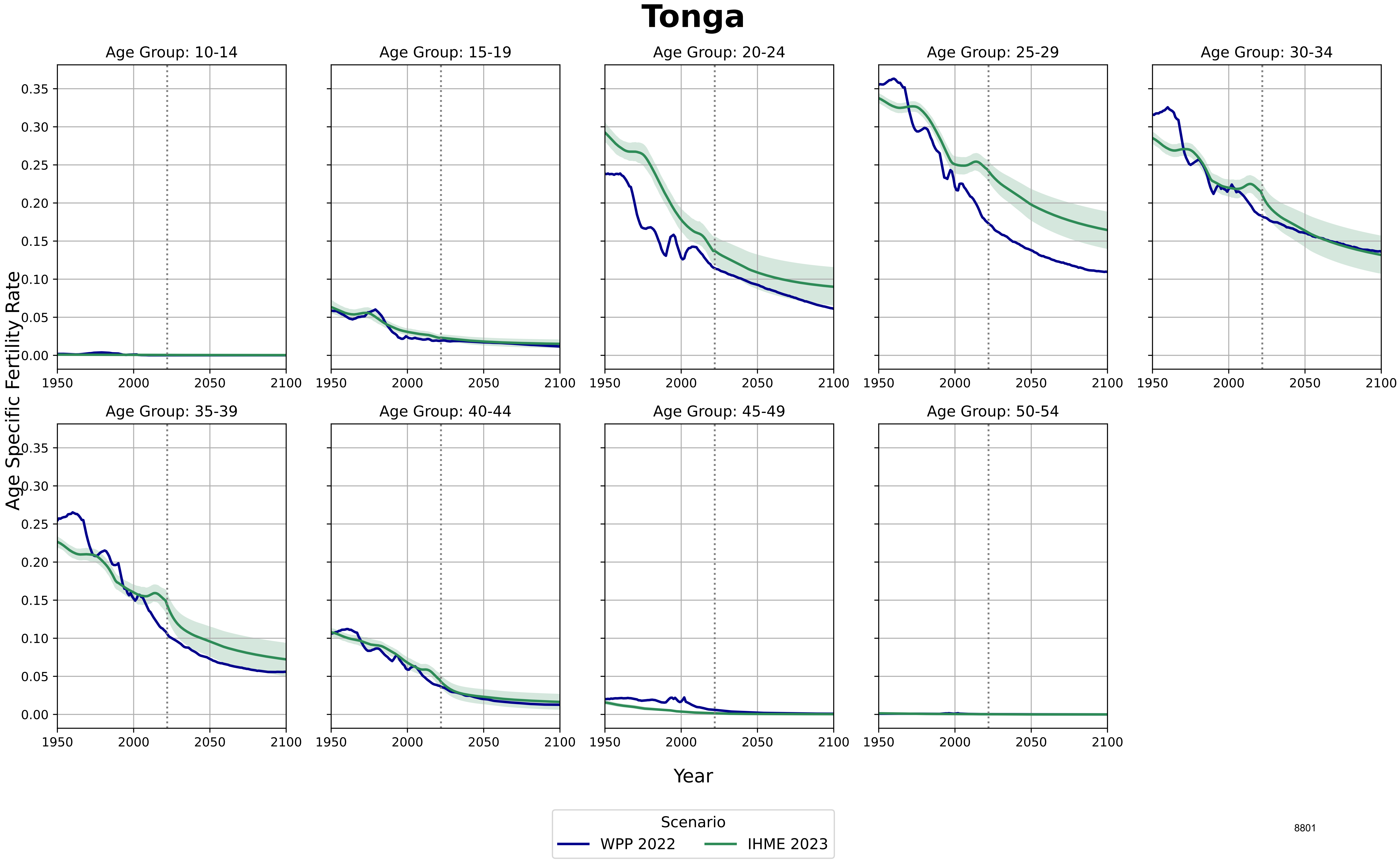

Supplemental Results Figure S8: Five-year interval ASFR from IHME reference scenario and the UNPD medium variant scenario by location, 1950-2100

Tuvalu

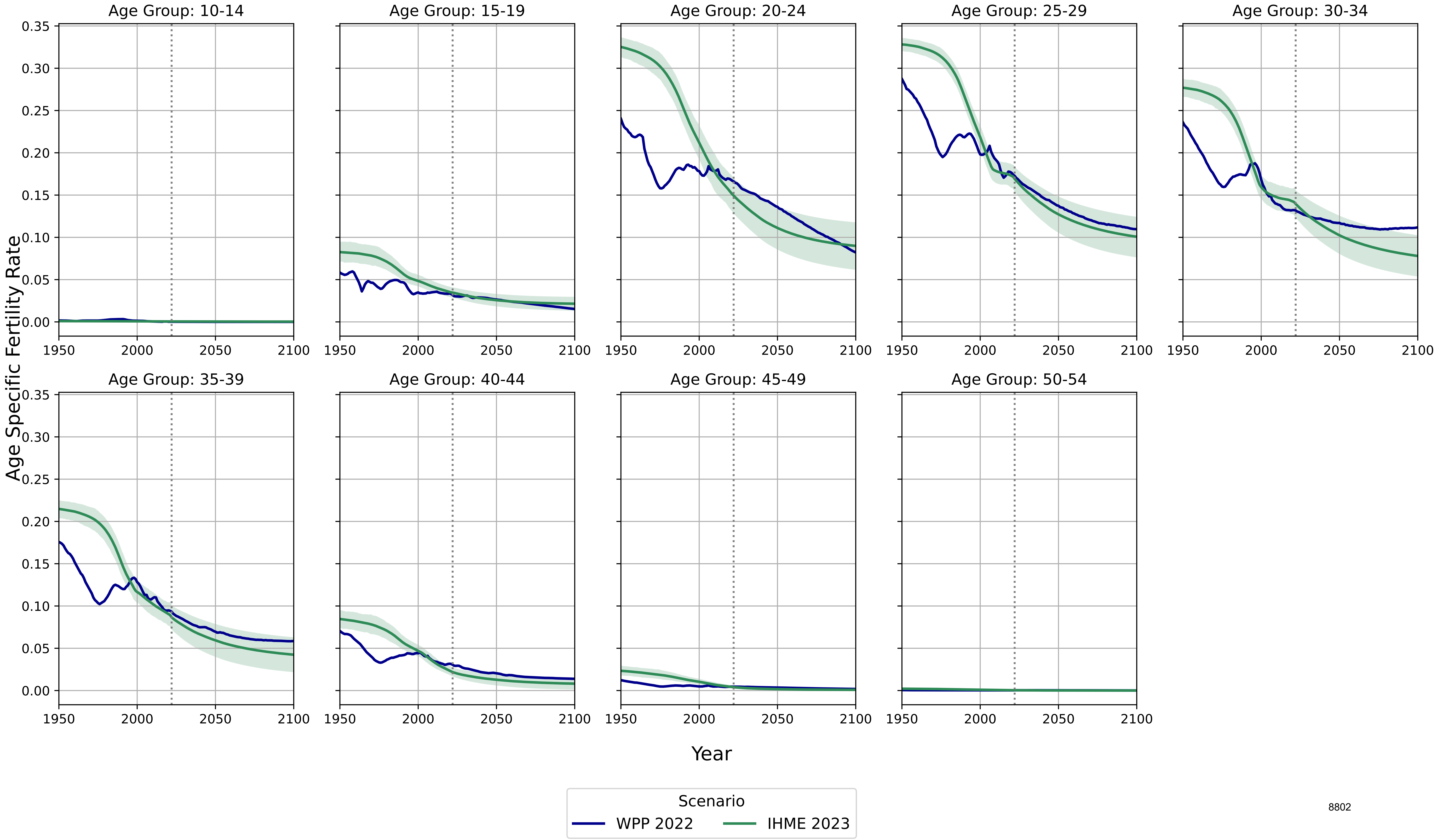

Supplemental Results Figure S8: Five-year interval ASFR from IHME reference scenario and the UNPD medium variant scenario by location, 1950-2100

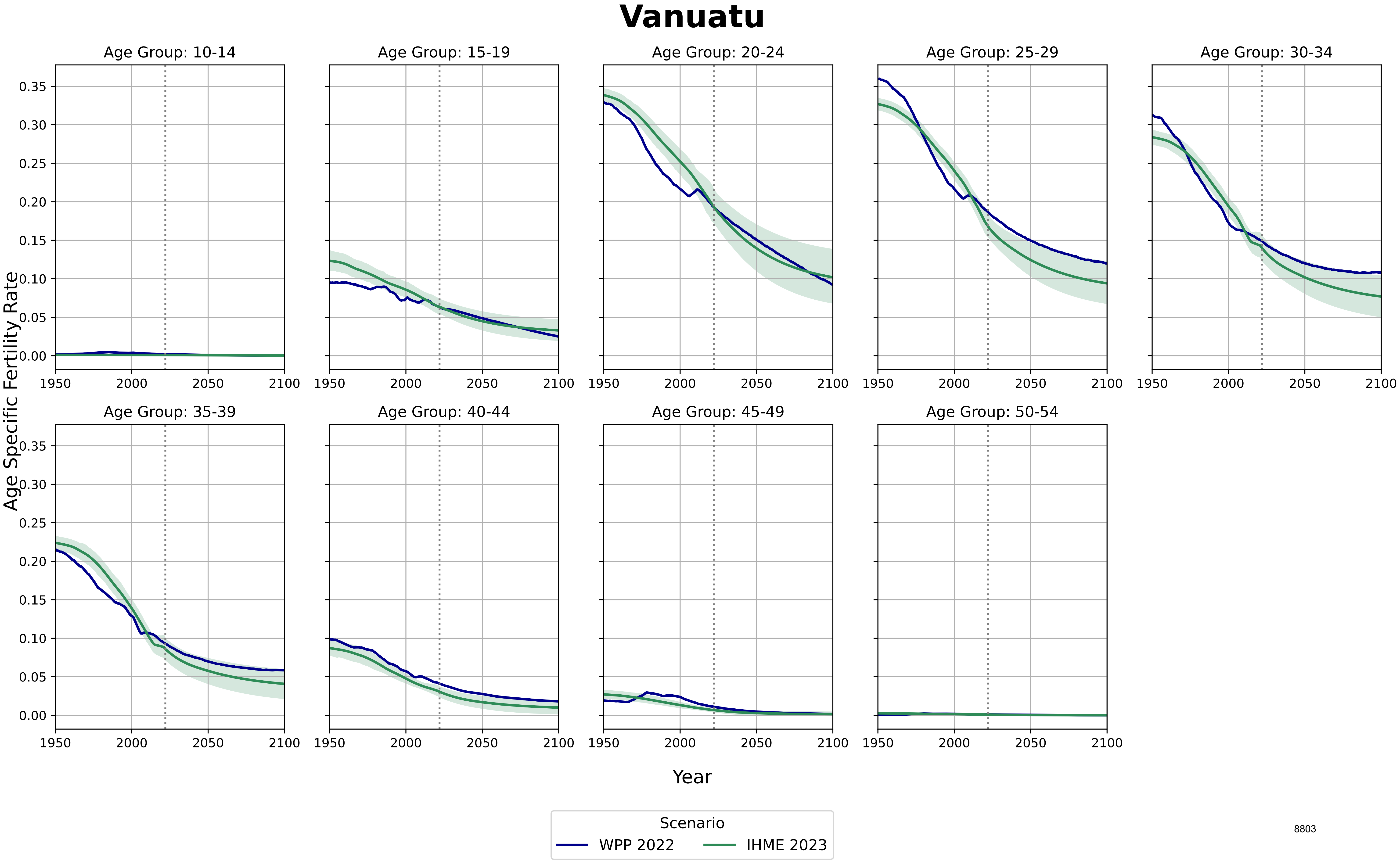

Supplemental Results Figure S8: Five-year interval ASFR from IHME reference scenario and the UNPD medium variant scenario by location, 1950-2100

Cambodia

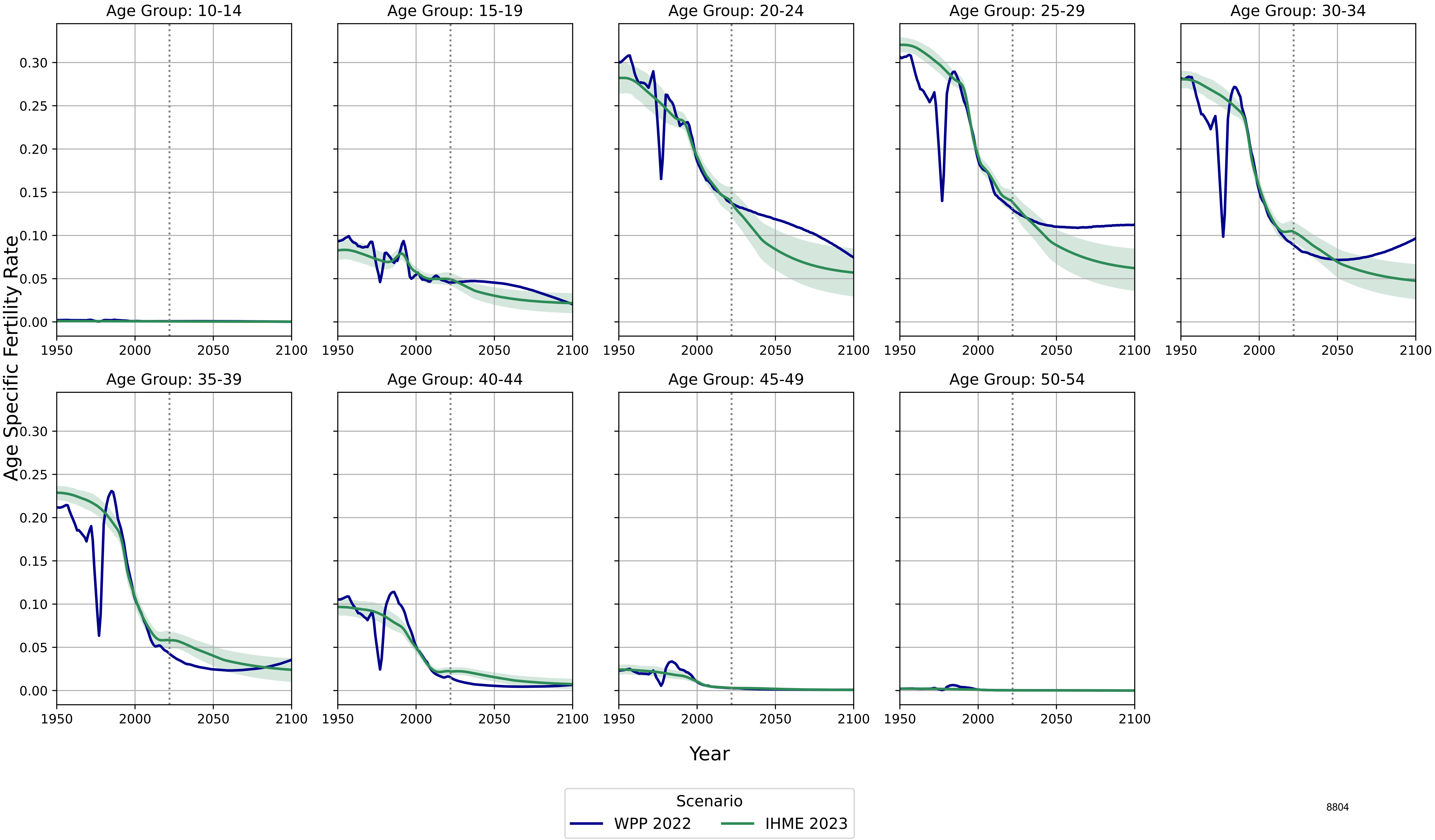

Supplemental Results Figure S8: Five-year interval ASFR from IHME reference scenario and the UNPD medium variant scenario by location, 1950-2100

Indonesia

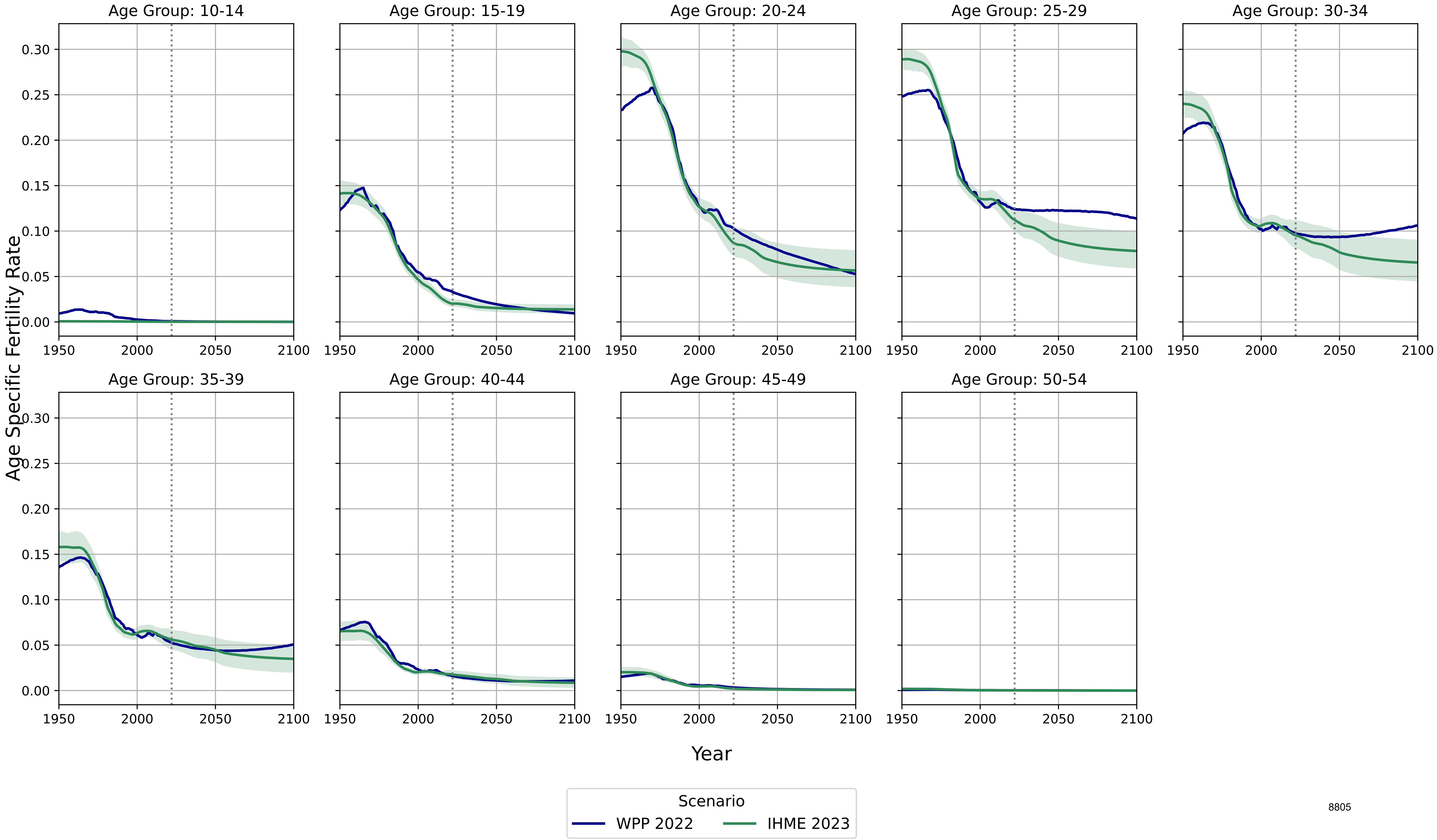

Supplemental Results Figure S8: Five-year interval ASFR from IHME reference scenario and the UNPD medium variant scenario by location, 1950-2100

Laos

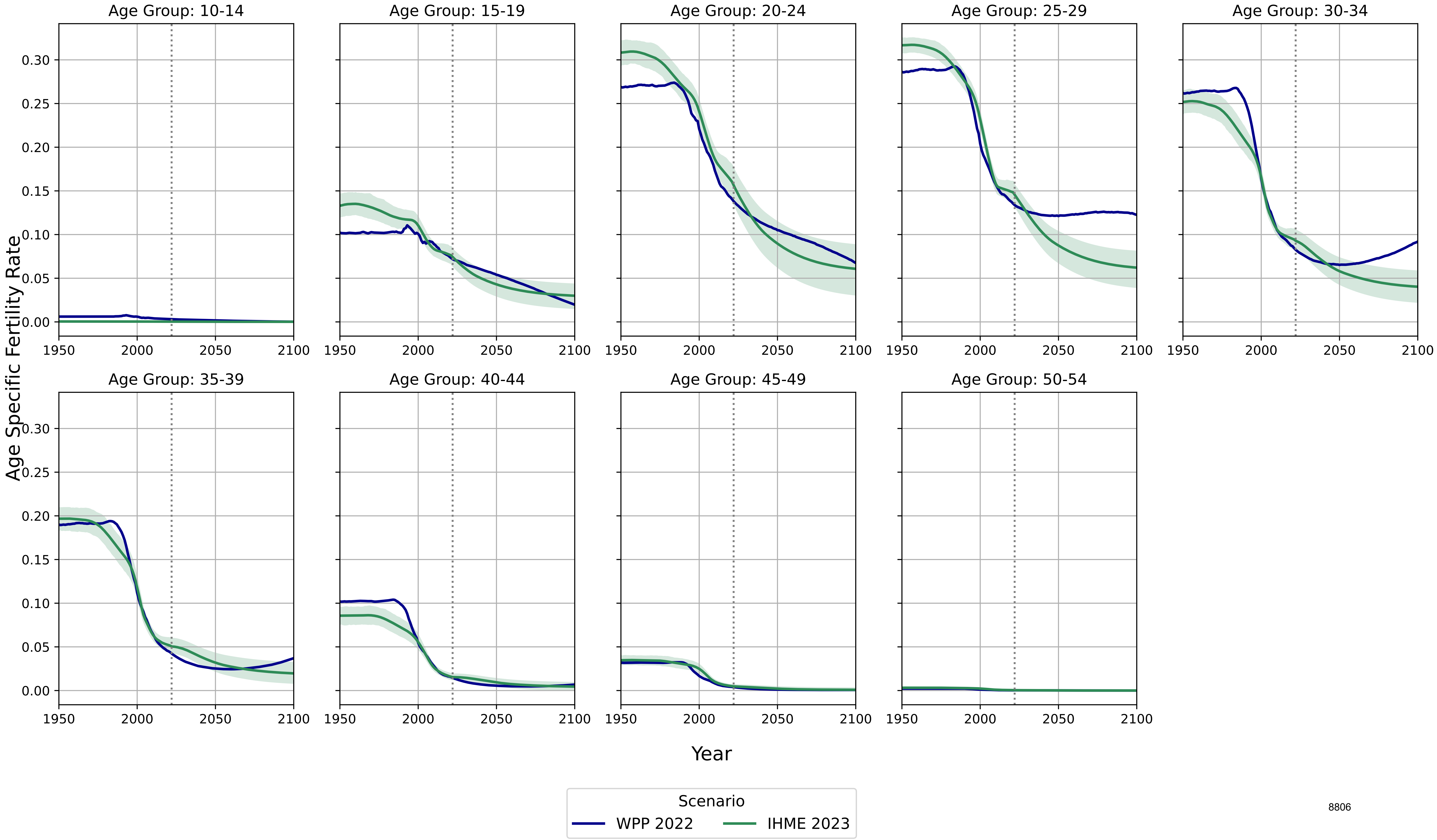

Supplemental Results Figure S8: Five-year interval ASFR from IHME reference scenario and the UNPD medium variant scenario by location, 1950-2100

Malaysia

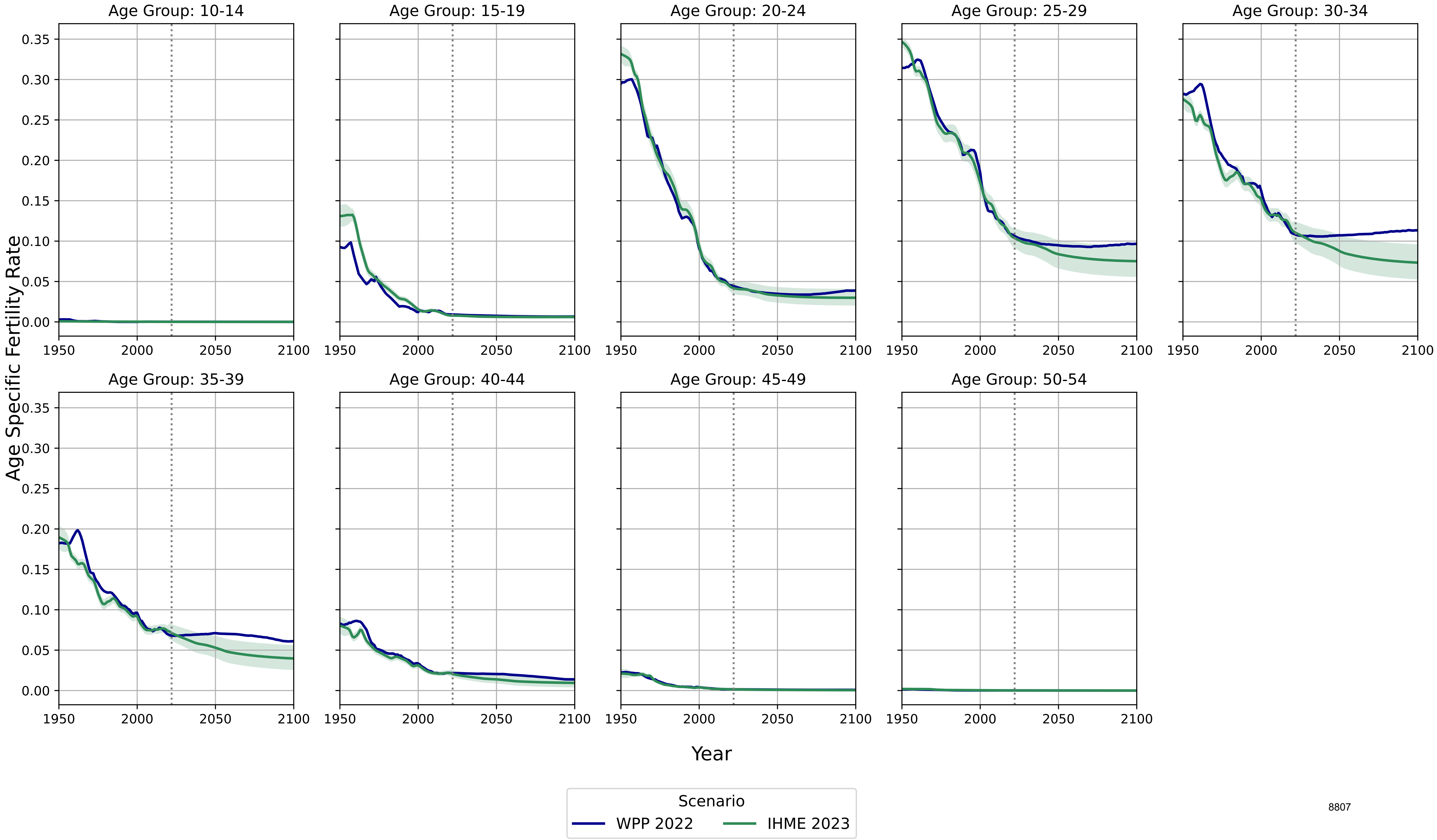

Supplemental Results Figure S8: Five-year interval ASFR from IHME reference scenario and the UNPD medium variant scenario by location, 1950-2100

Maldives

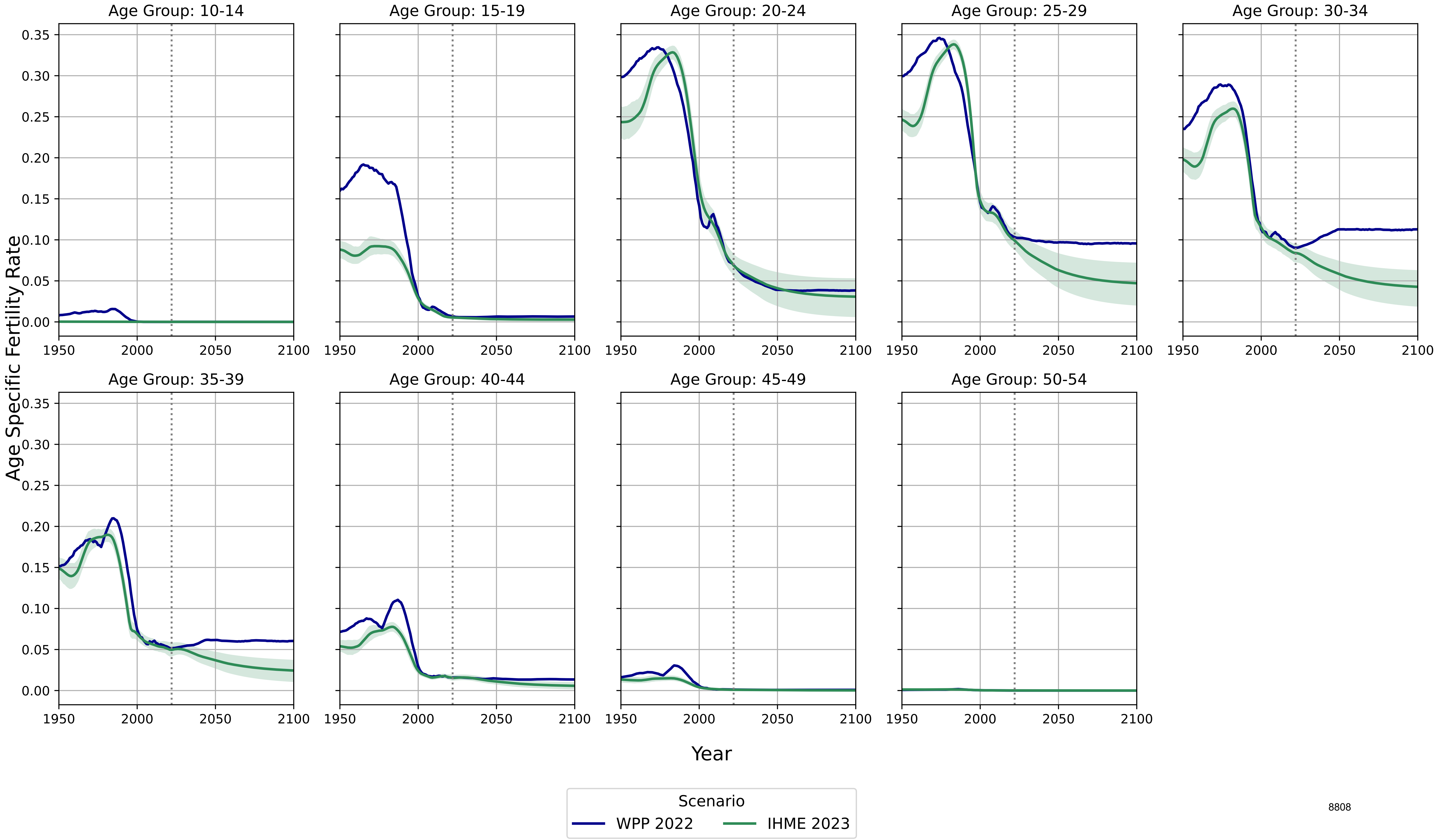

Supplemental Results Figure S8: Five-year interval ASFR from IHME reference scenario and the UNPD medium variant scenario by location, 1950-2100

Mauritius

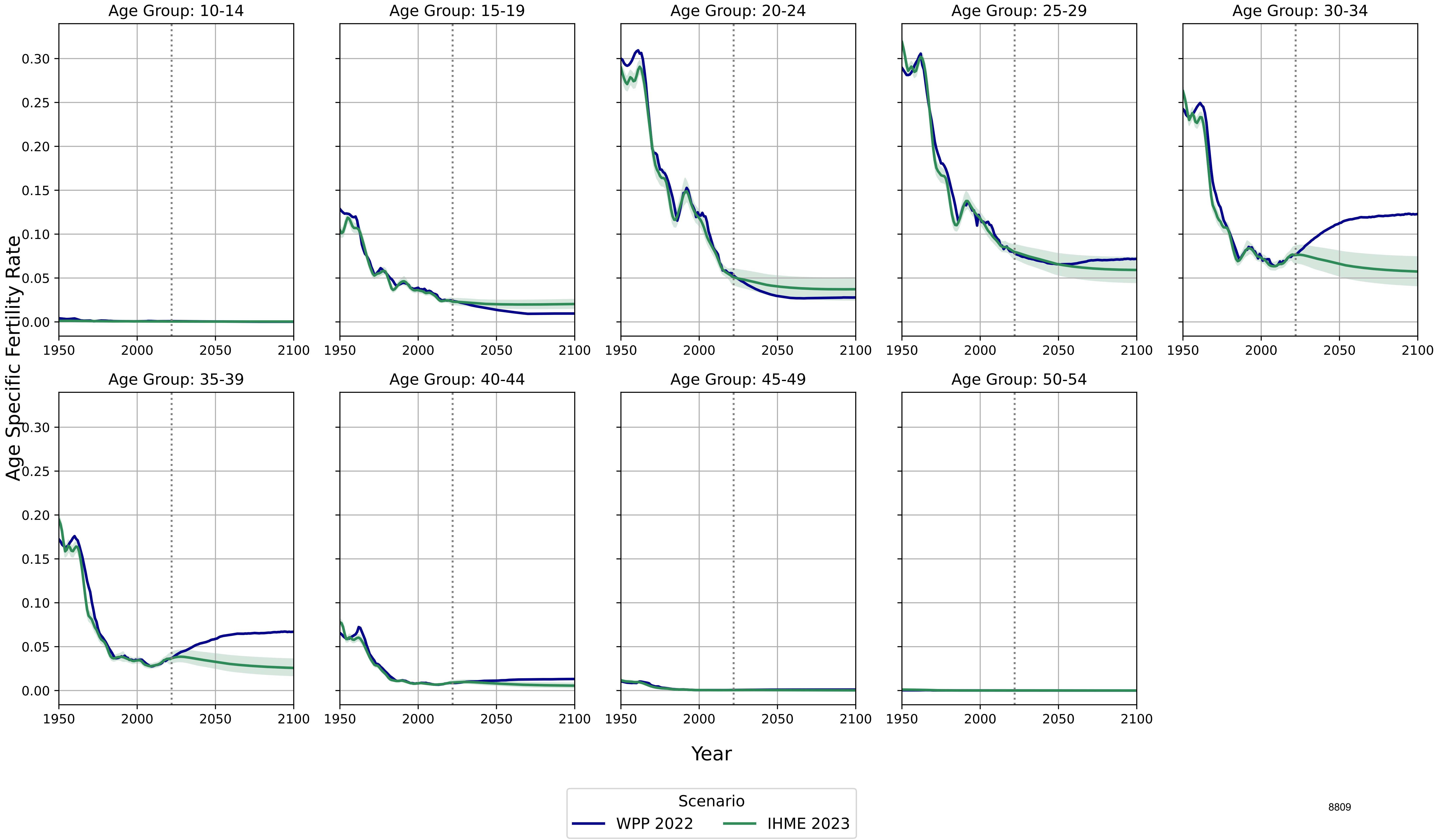

Supplemental Results Figure S8: Five-year interval ASFR from IHME reference scenario and the UNPD medium variant scenario by location, 1950-2100

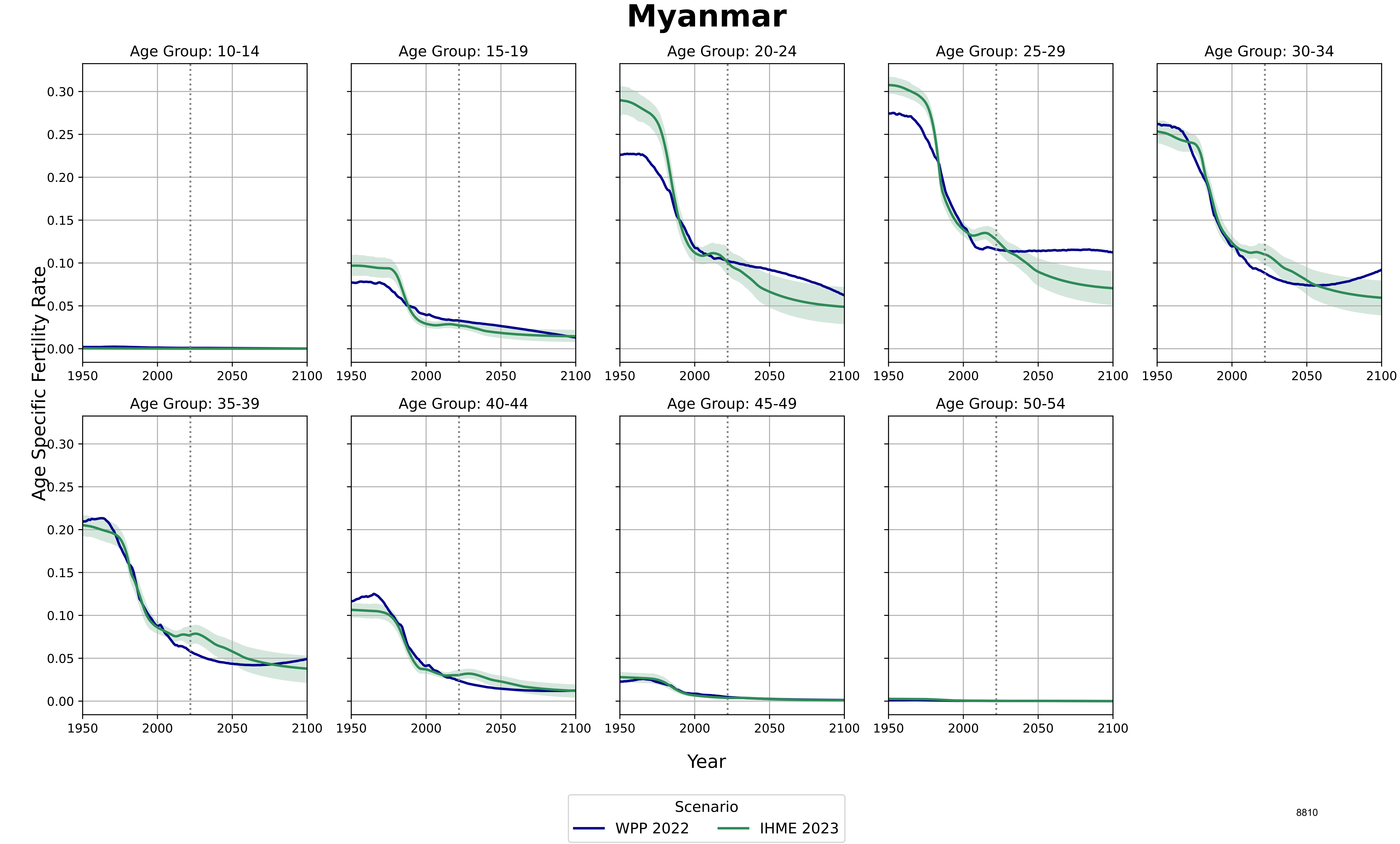

Supplemental Results Figure S8: Five-year interval ASFR from IHME reference scenario and the UNPD medium variant scenario by location, 1950-2100

Philippines

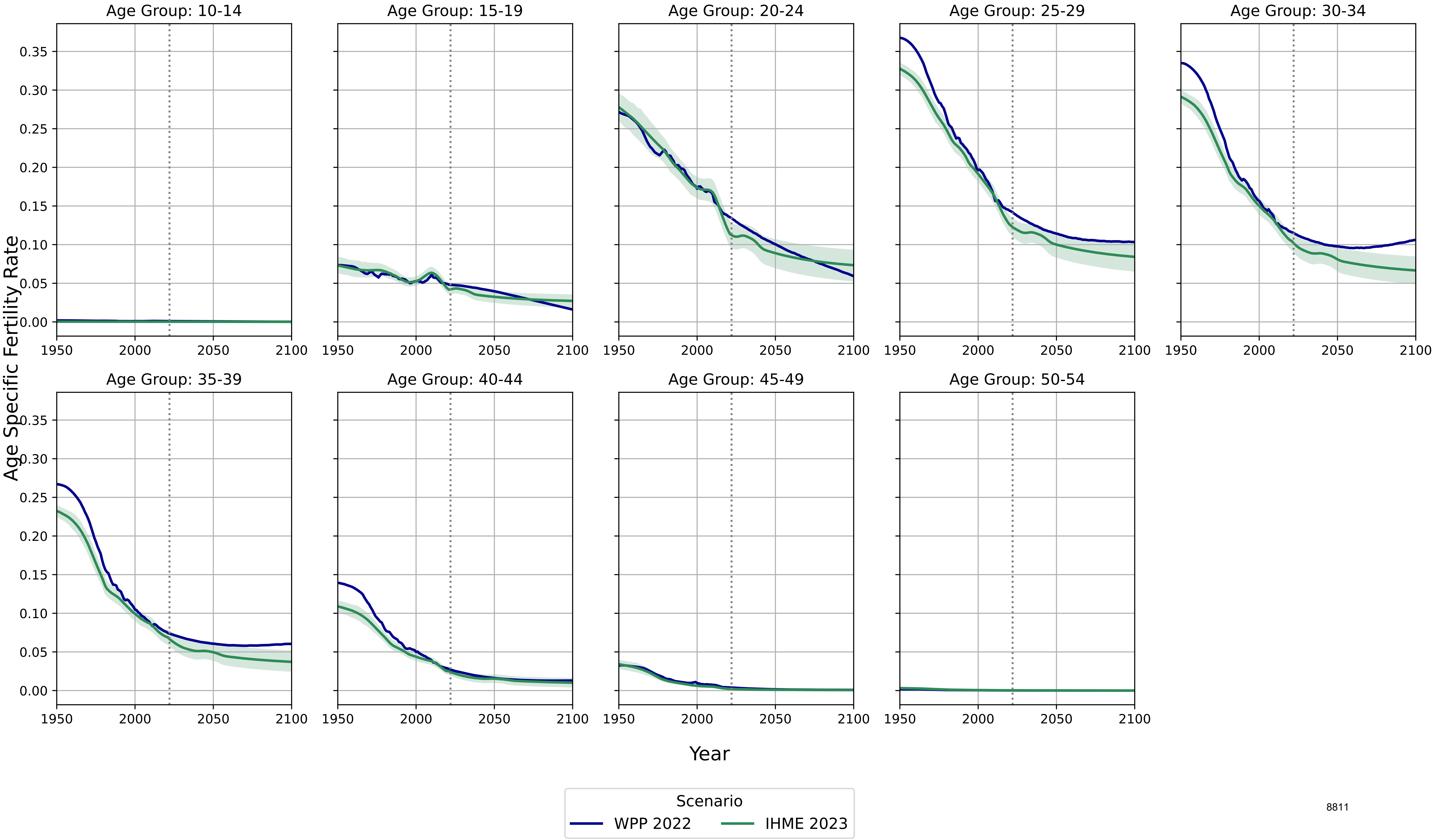

Supplemental Results Figure S8: Five-year interval ASFR from IHME reference scenario and the UNPD medium variant scenario by location, 1950-2100

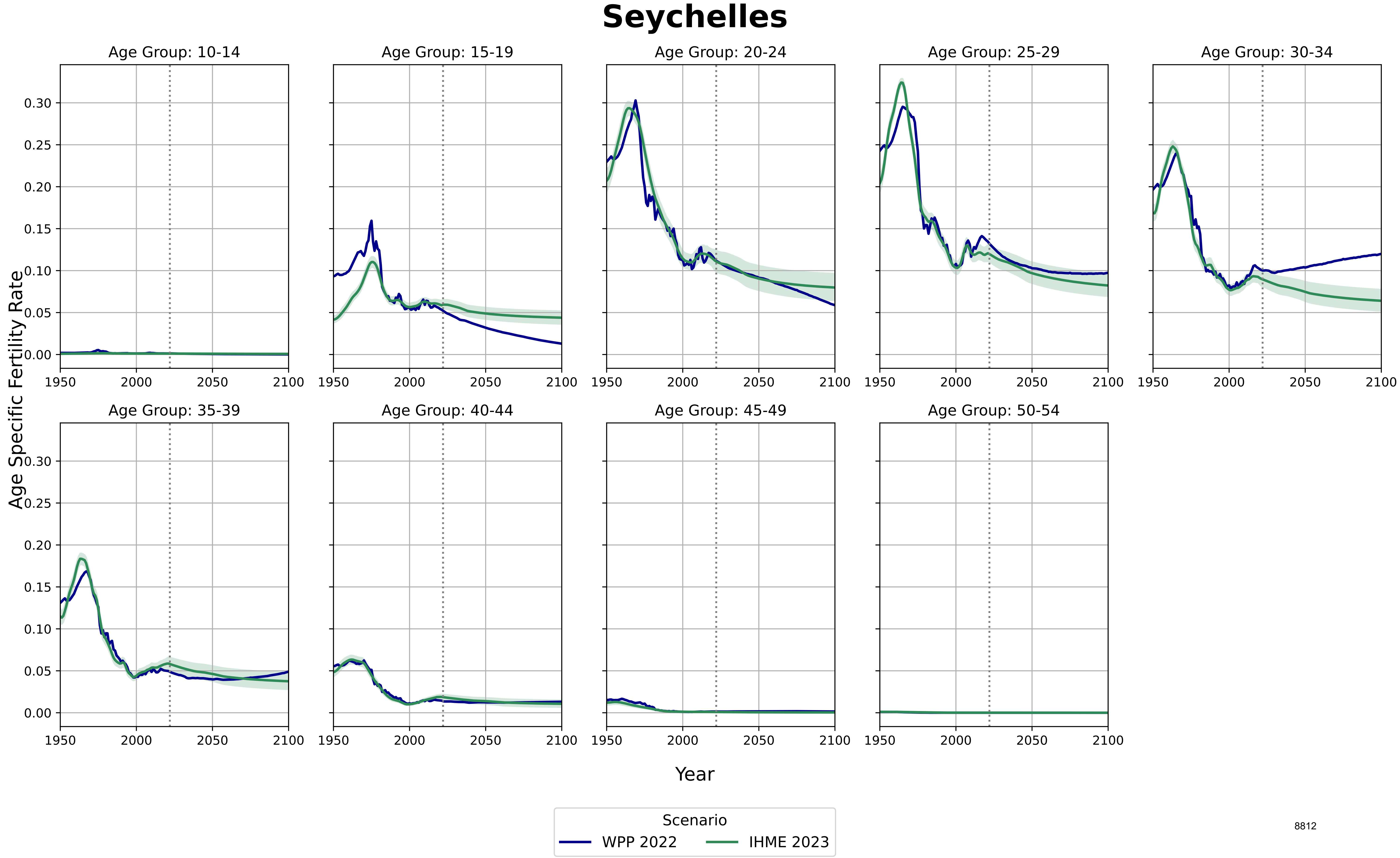

Supplemental Results Figure S8: Five-year interval ASFR from IHME reference scenario and the UNPD medium variant scenario by location, 1950-2100

Sri Lanka

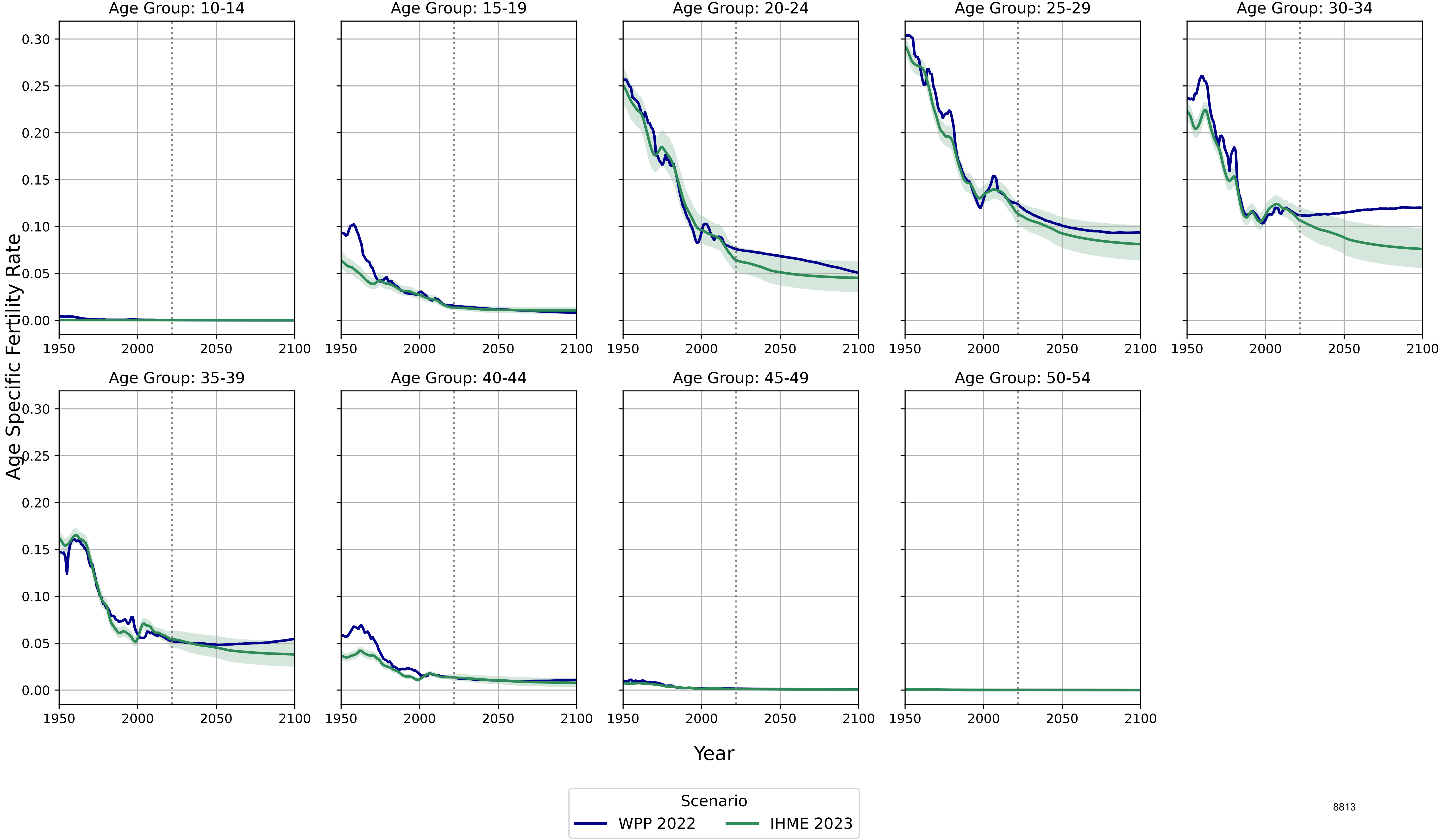

Supplemental Results Figure S8: Five-year interval ASFR from IHME reference scenario and the UNPD medium variant scenario by location, 1950-2100

Thailand

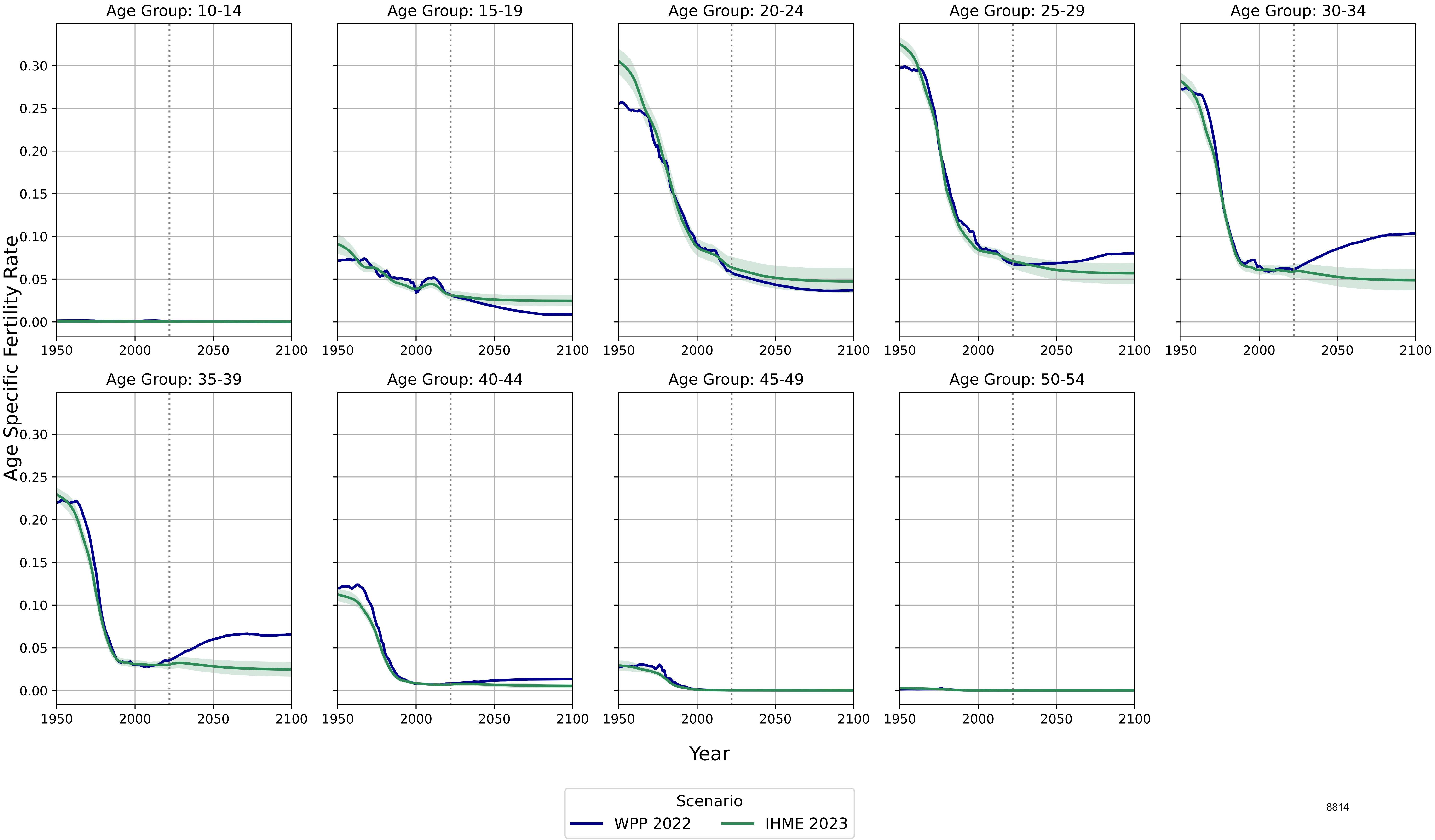

Supplemental Results Figure S8: Five-year interval ASFR from IHME reference scenario and the UNPD medium variant scenario by location, 1950-2100

Timor-Leste

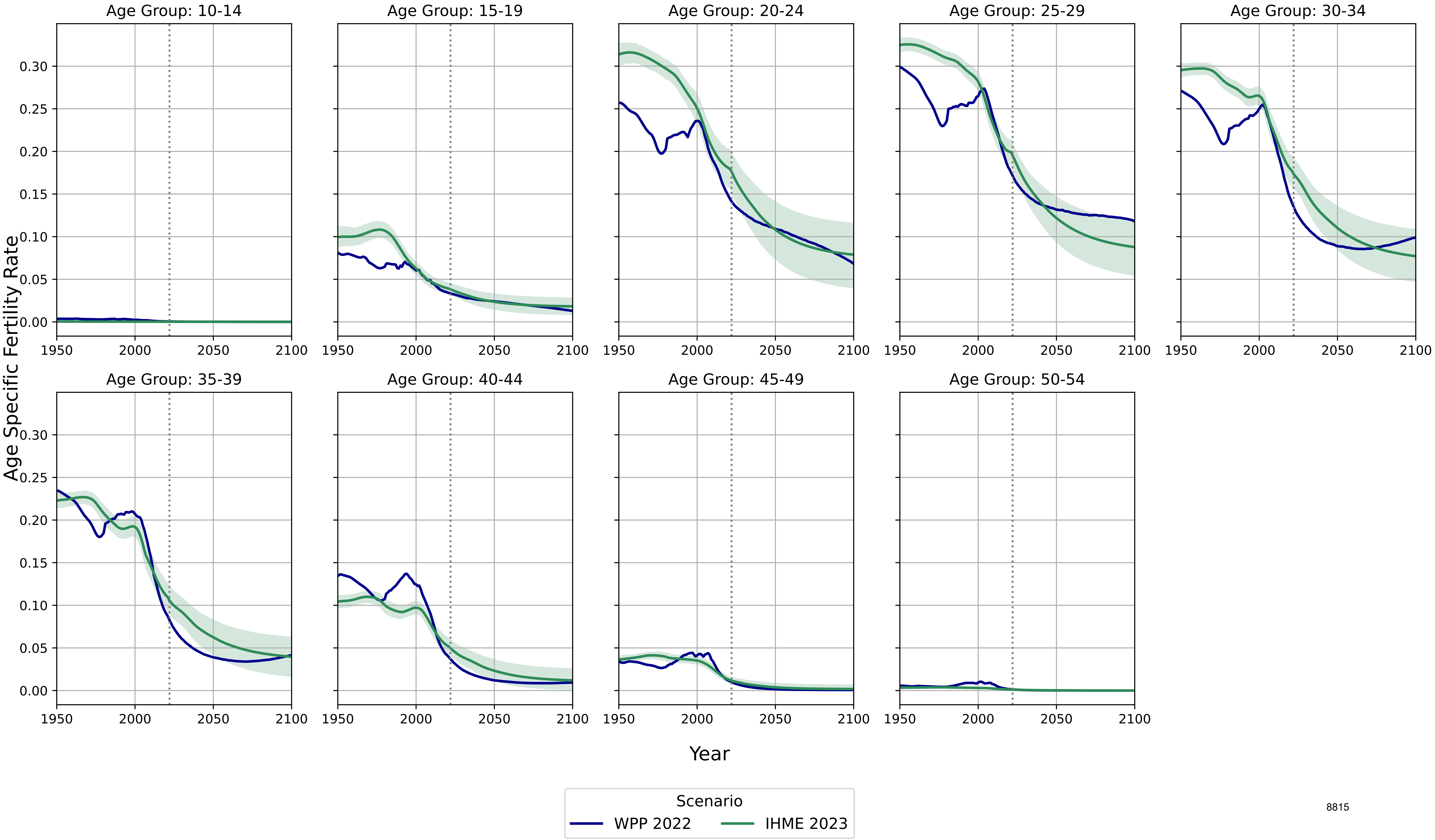

Supplemental Results Figure S8: Five-year interval ASFR from IHME reference scenario and the UNPD medium variant scenario by location, 1950-2100

Viet Nam

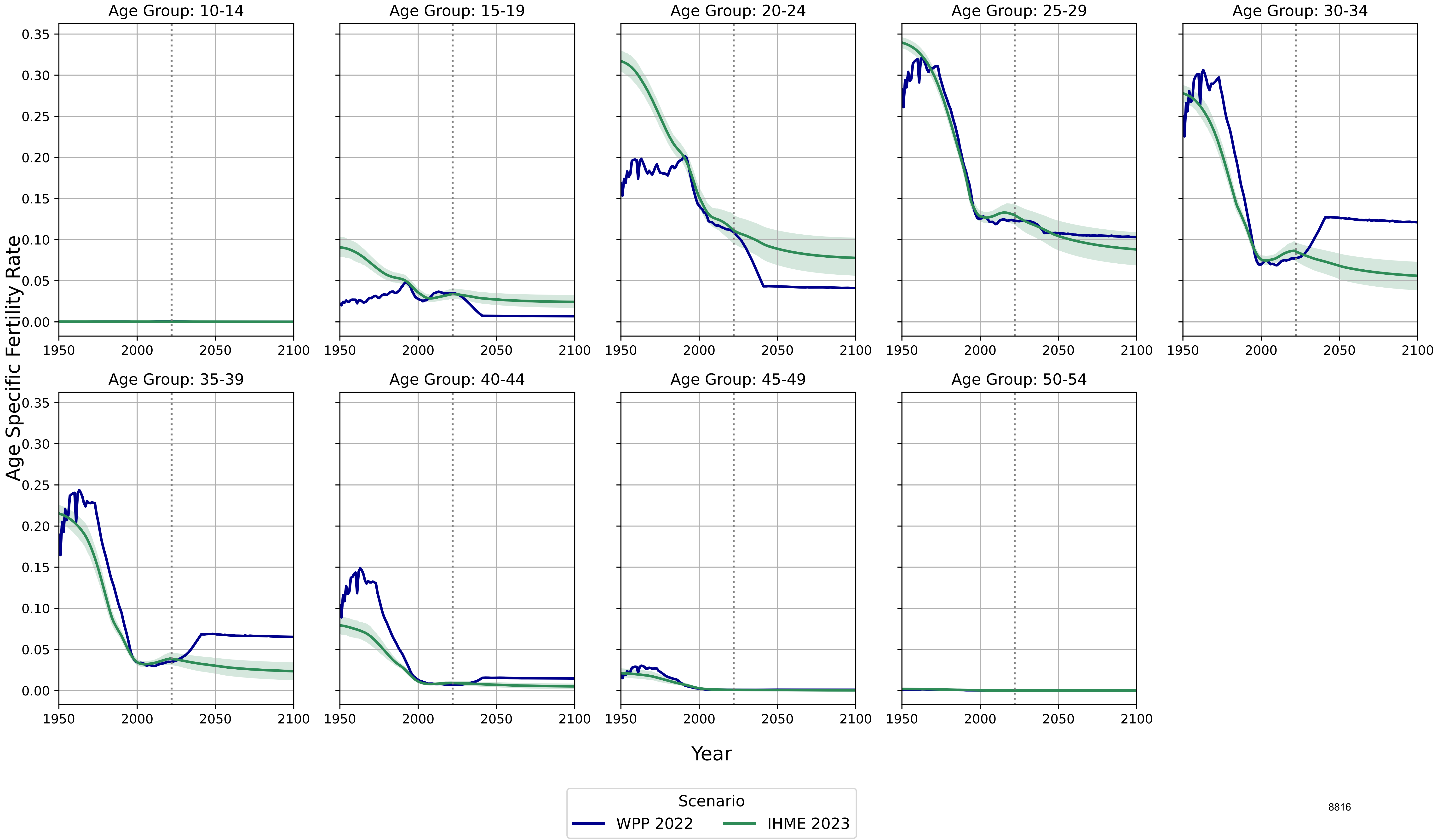

Supplemental Results Figure S8: Five-year interval ASFR from IHME reference scenario and the UNPD medium variant scenario by location, 1950-2100

Angola

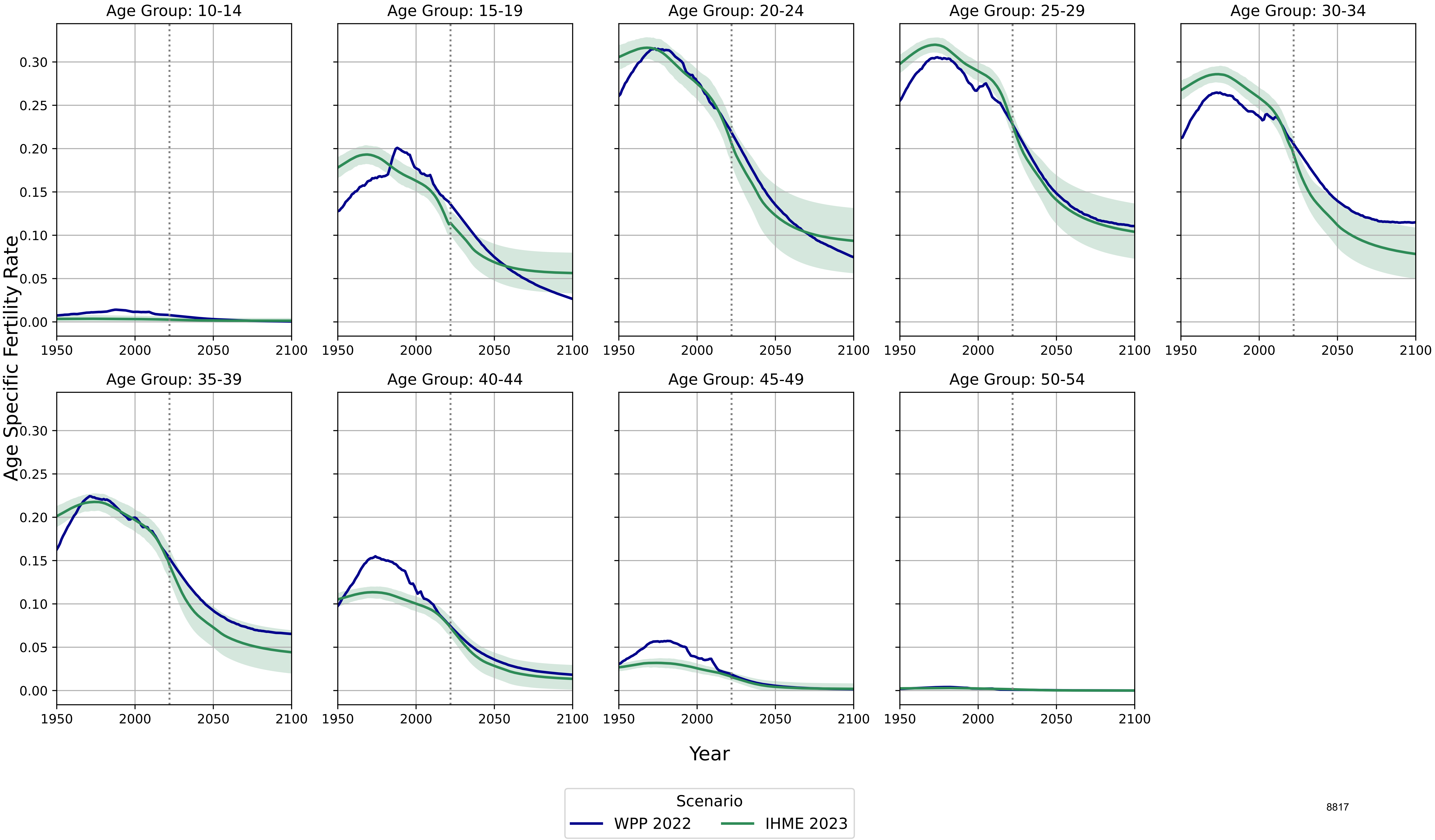

Supplemental Results Figure S8: Five-year interval ASFR from IHME reference scenario and the UNPD medium variant scenario by location, 1950-2100

Central African Republic

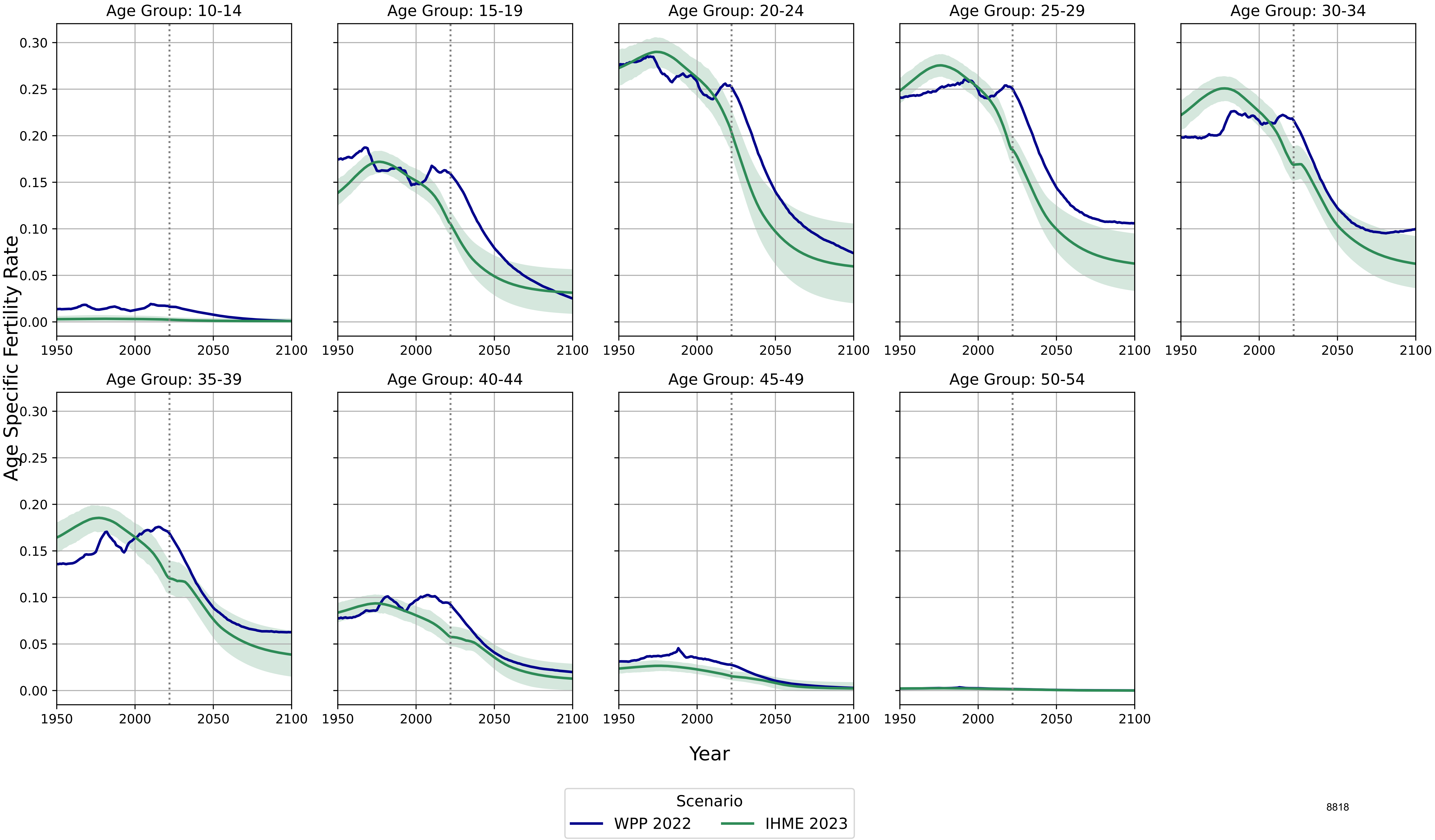

Supplemental Results Figure S8: Five-year interval ASFR from IHME reference scenario and the UNPD medium variant scenario by location, 1950-2100

Congo (Brazzaville)

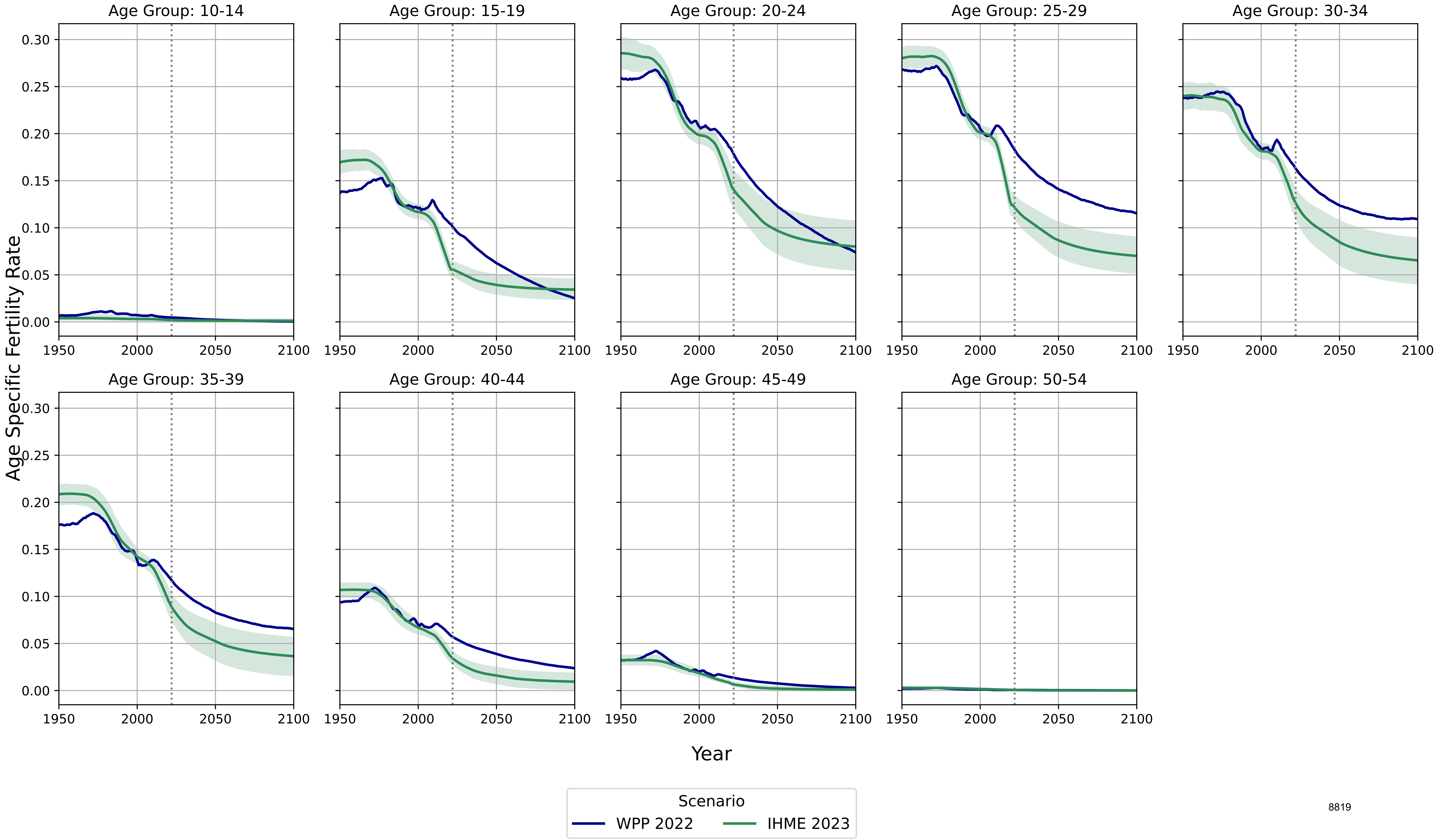

Supplemental Results Figure S8: Five-year interval ASFR from IHME reference scenario and the UNPD medium variant scenario by location, 1950-2100

DR Congo

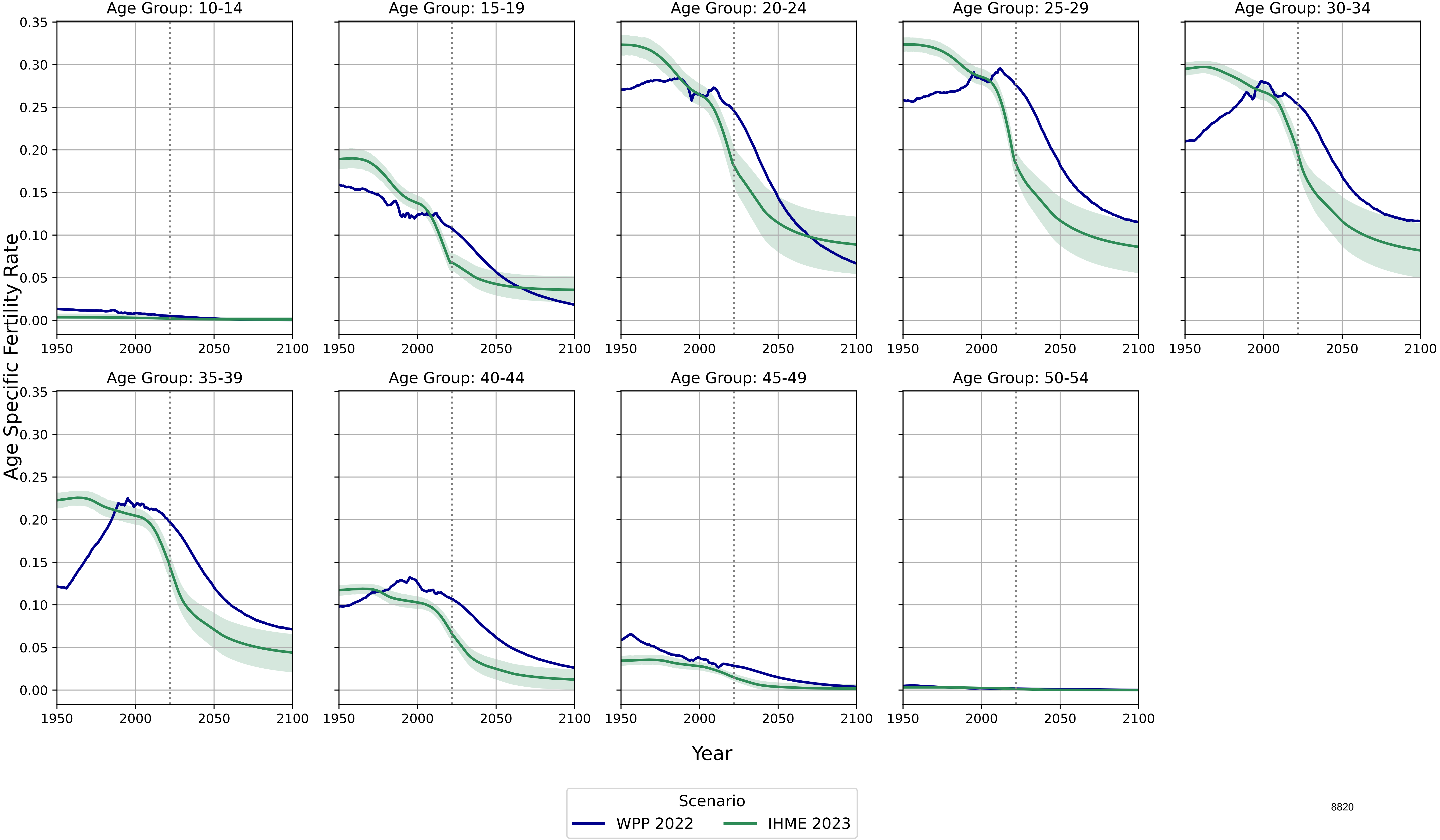

Supplemental Results Figure S8: Five-year interval ASFR from IHME reference scenario and the UNPD medium variant scenario by location, 1950-2100

Equatorial Guinea

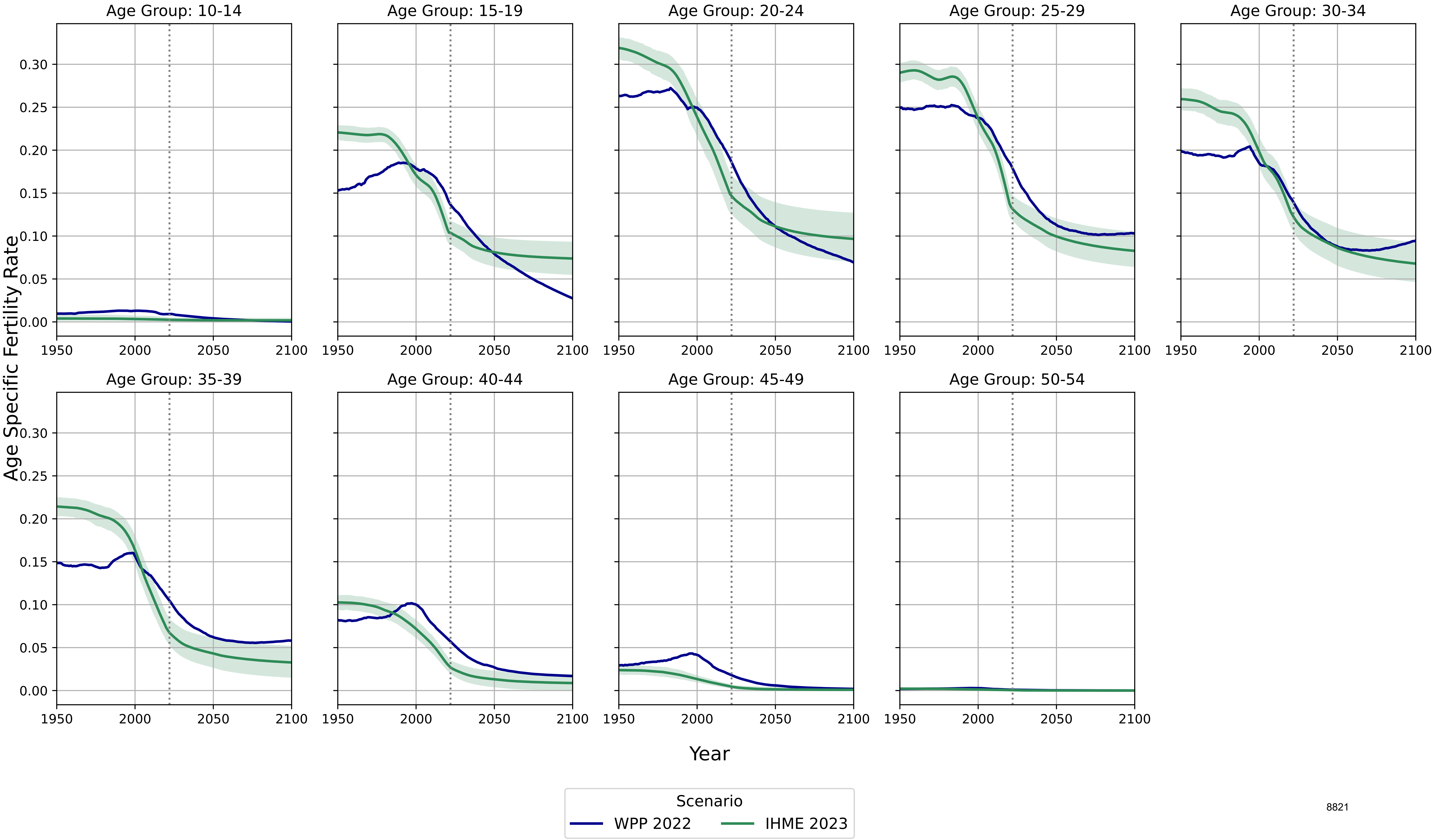

Supplemental Results Figure S8: Five-year interval ASFR from IHME reference scenario and the UNPD medium variant scenario by location, 1950-2100

Gabon

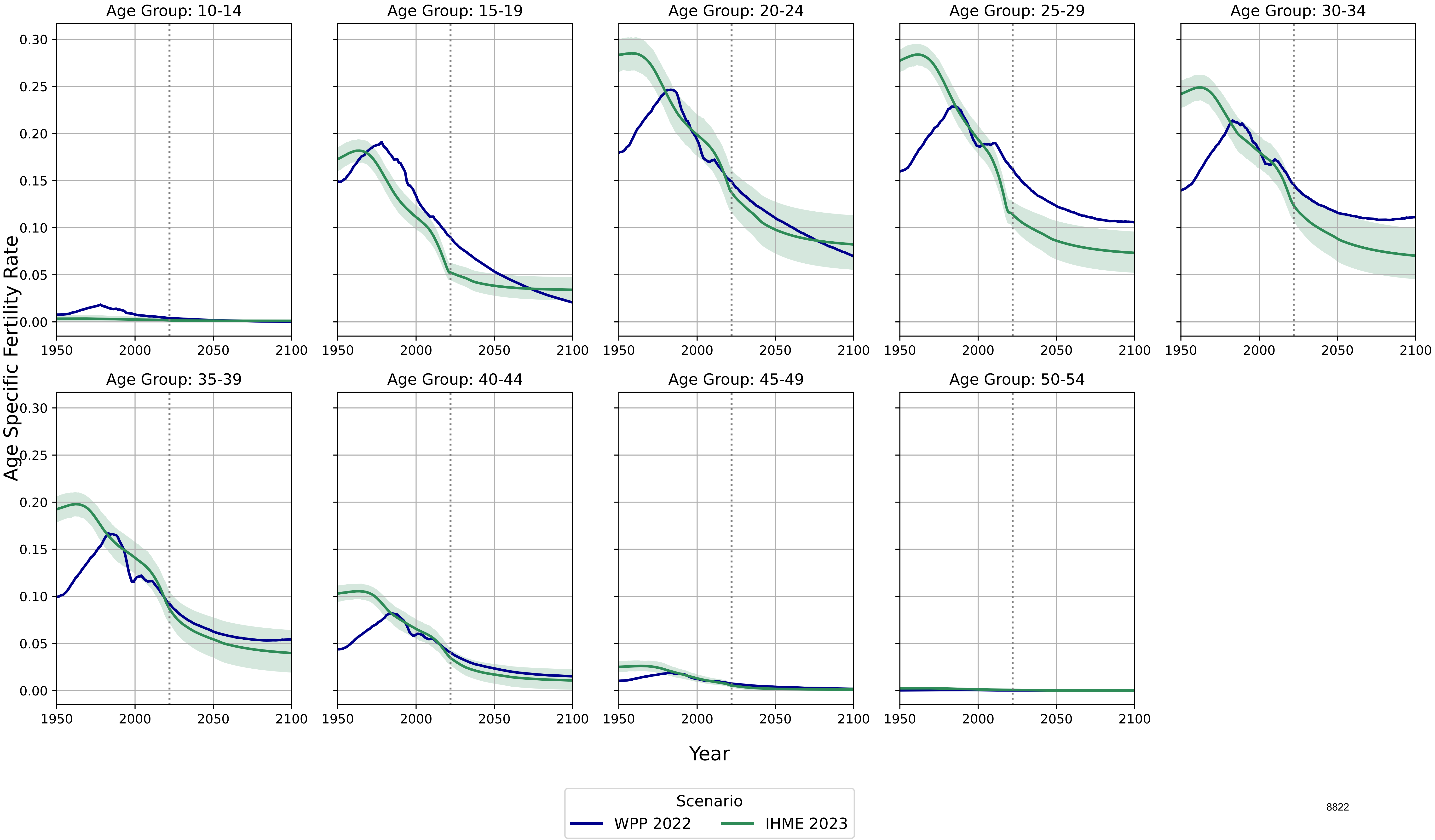

Supplemental Results Figure S8: Five-year interval ASFR from IHME reference scenario and the UNPD medium variant scenario by location, 1950-2100

Burundi

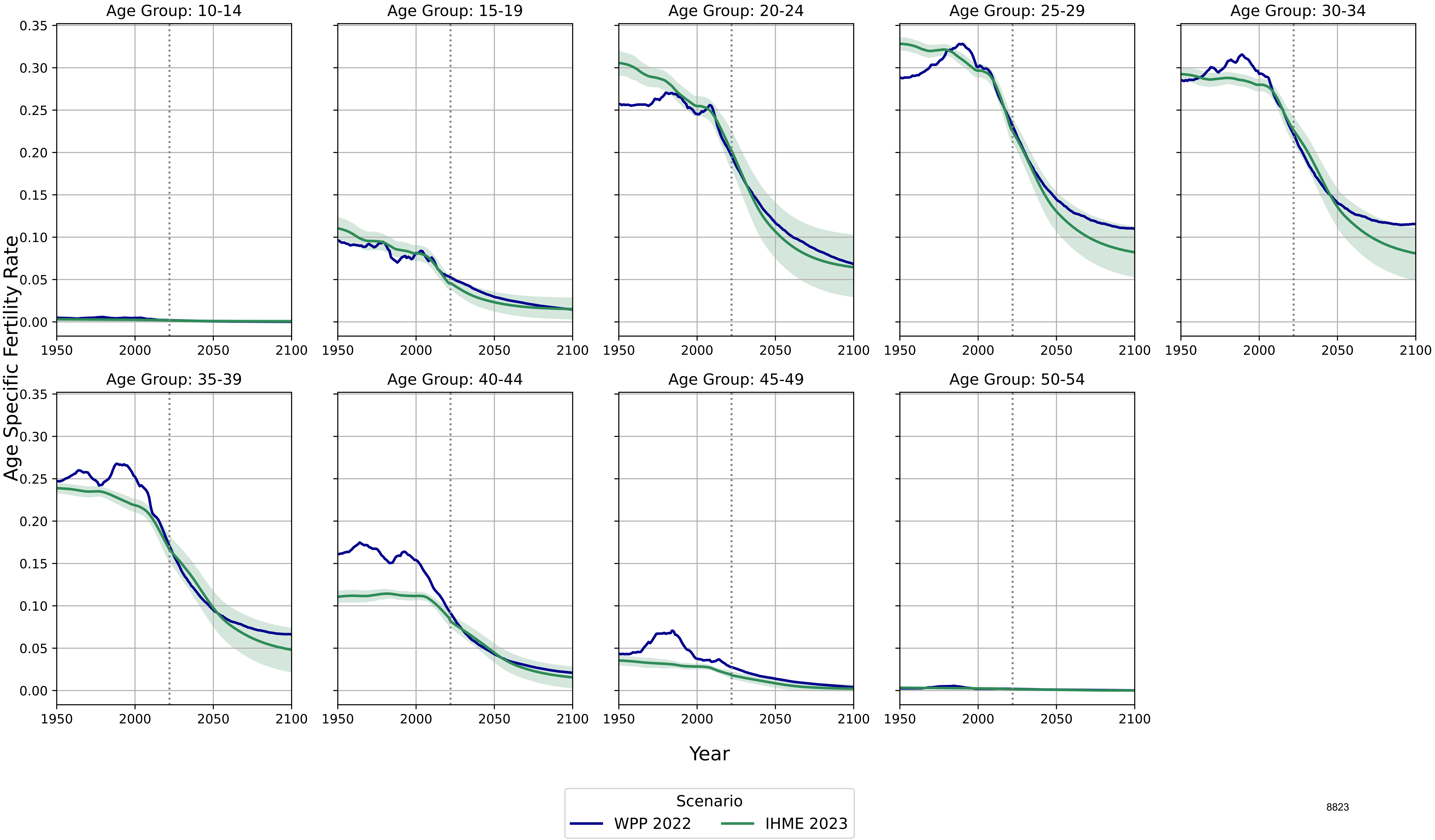

Supplemental Results Figure S8: Five-year interval ASFR from IHME reference scenario and the UNPD medium variant scenario by location, 1950-2100

Comoros

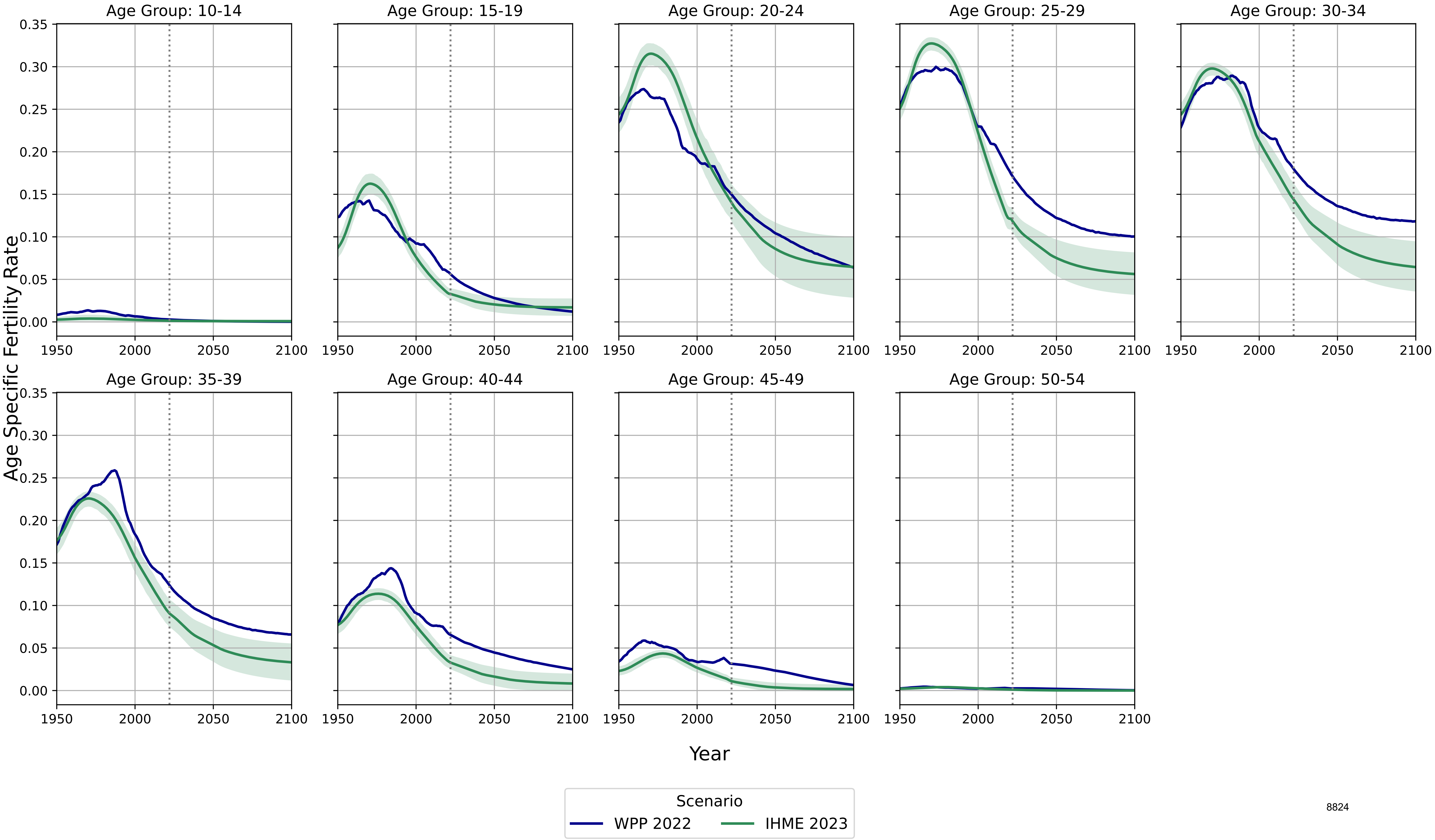

Supplemental Results Figure S8: Five-year interval ASFR from IHME reference scenario and the UNPD medium variant scenario by location, 1950-2100

Djibouti

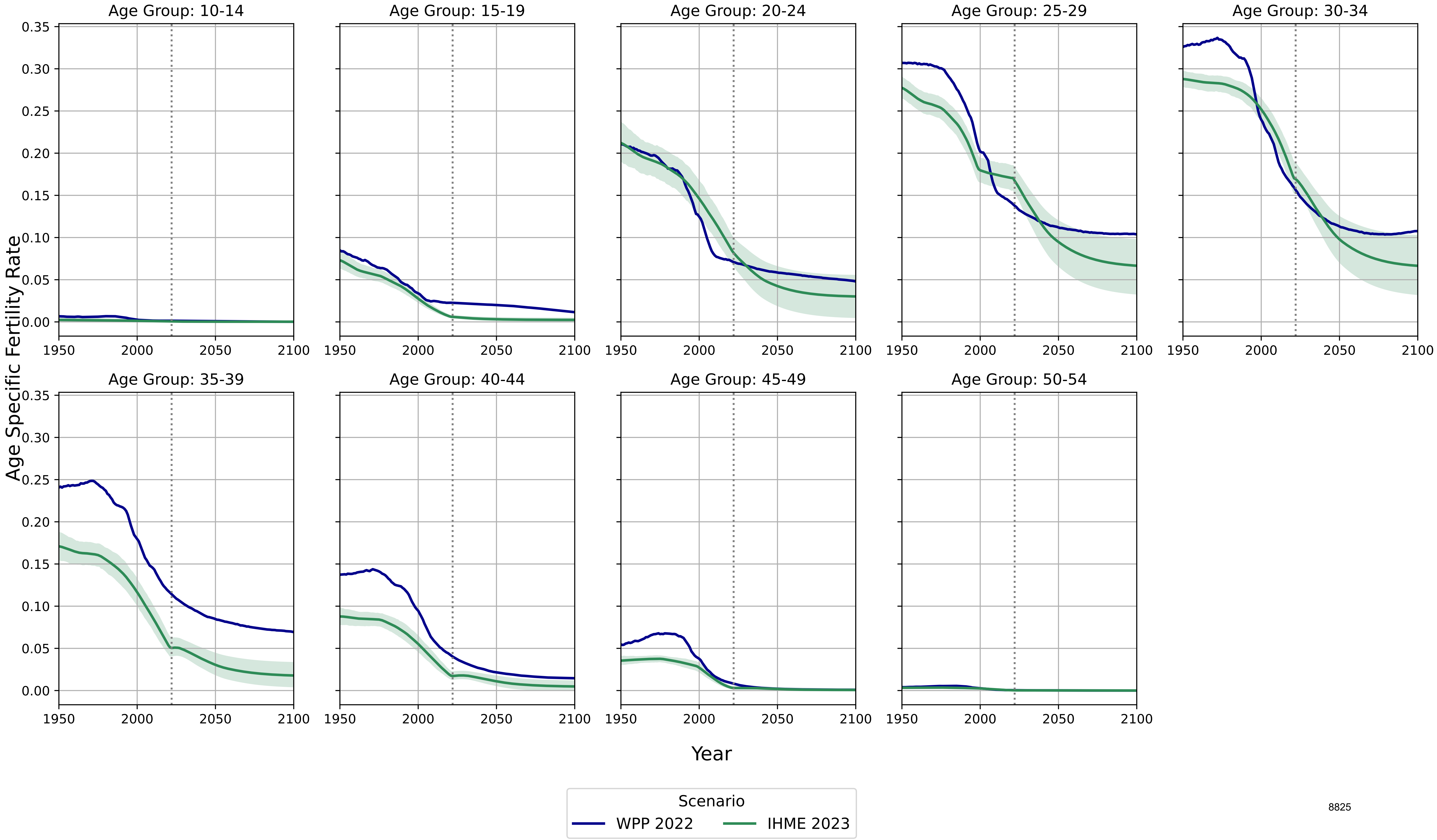

Supplemental Results Figure S8: Five-year interval ASFR from IHME reference scenario and the UNPD medium variant scenario by location, 1950-2100

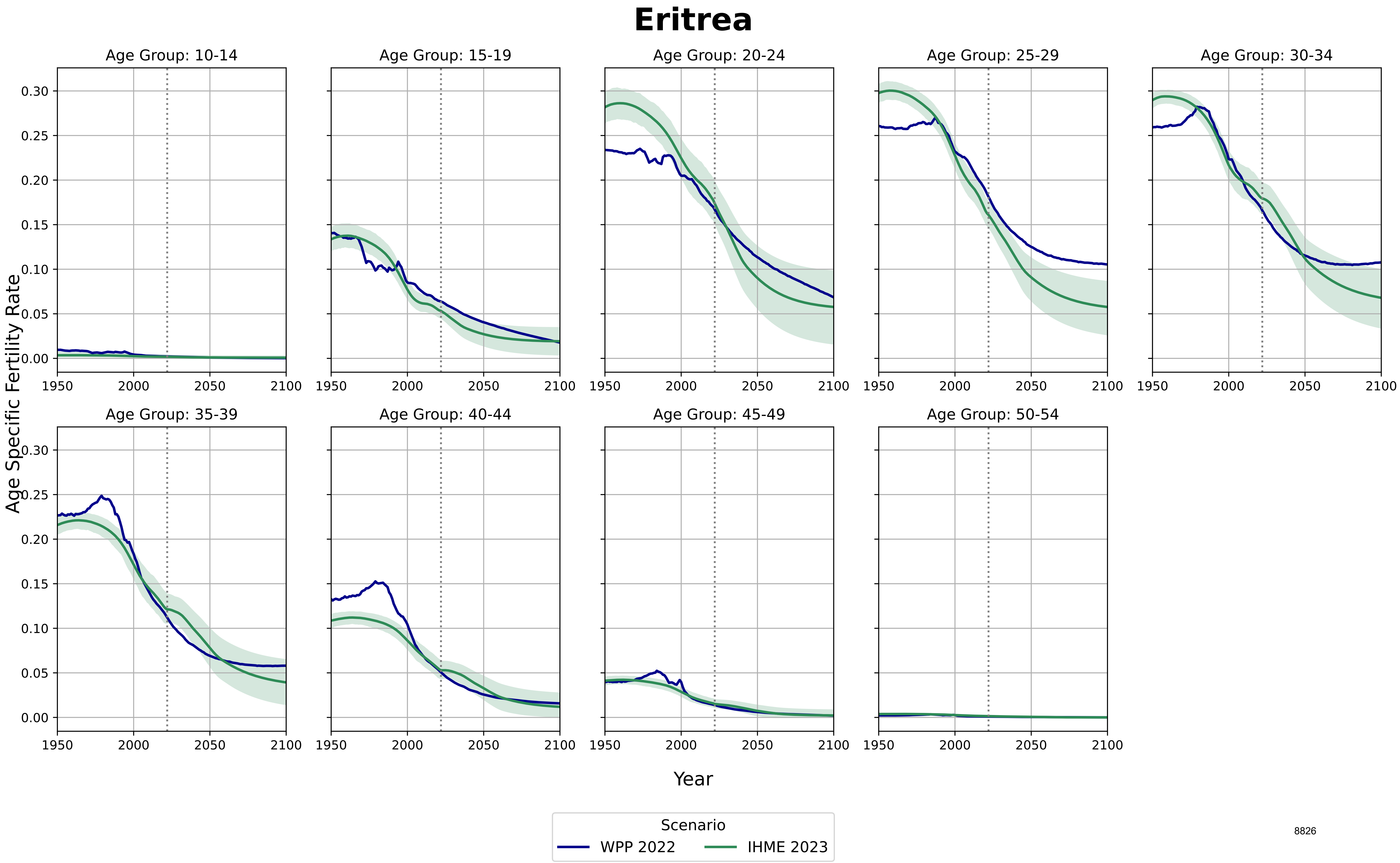

Supplemental Results Figure S8: Five-year interval ASFR from IHME reference scenario and the UNPD medium variant scenario by location, 1950-2100

Ethiopia

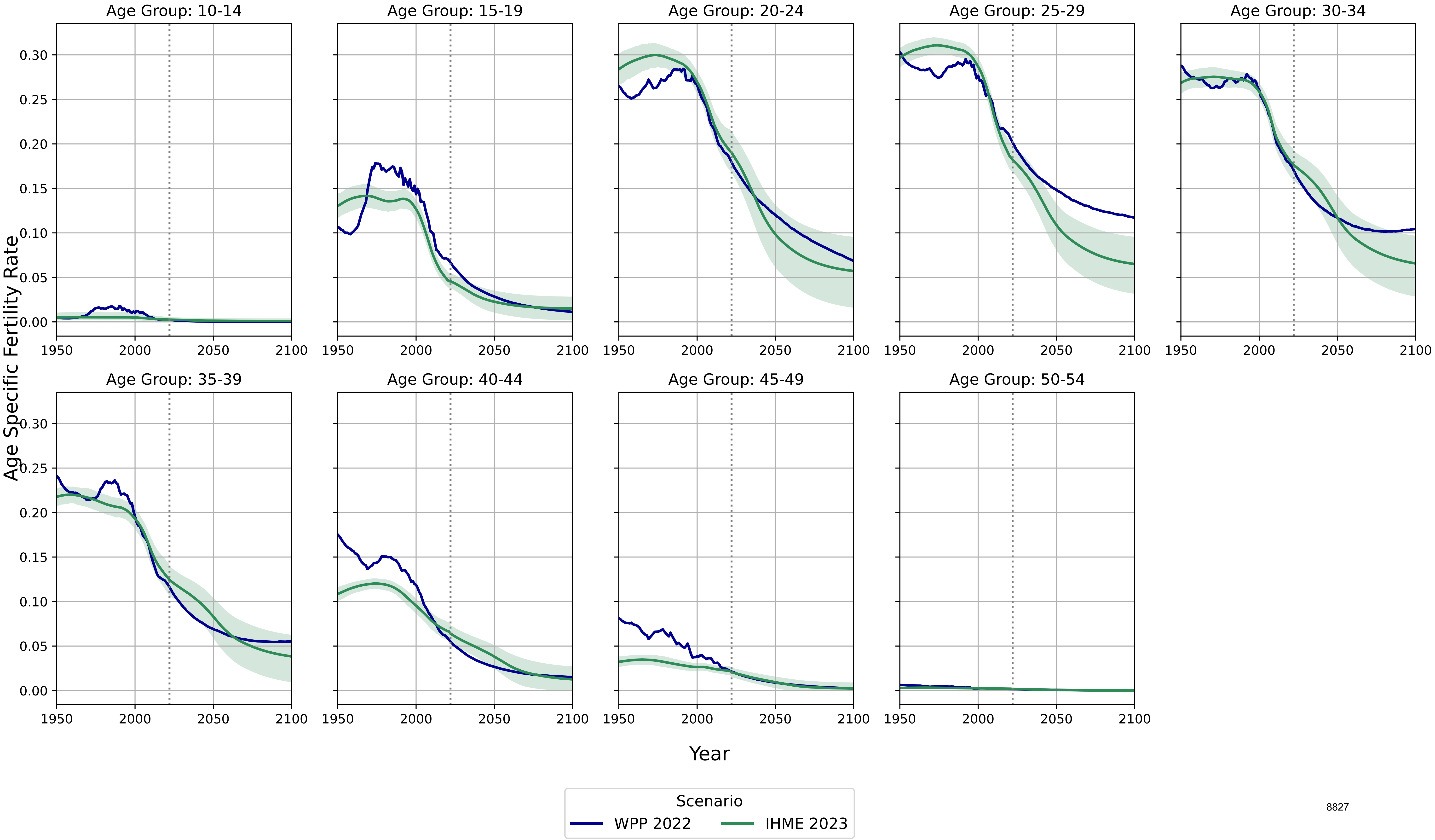

Supplemental Results Figure S8: Five-year interval ASFR from IHME reference scenario and the UNPD medium variant scenario by location, 1950-2100

Kenya

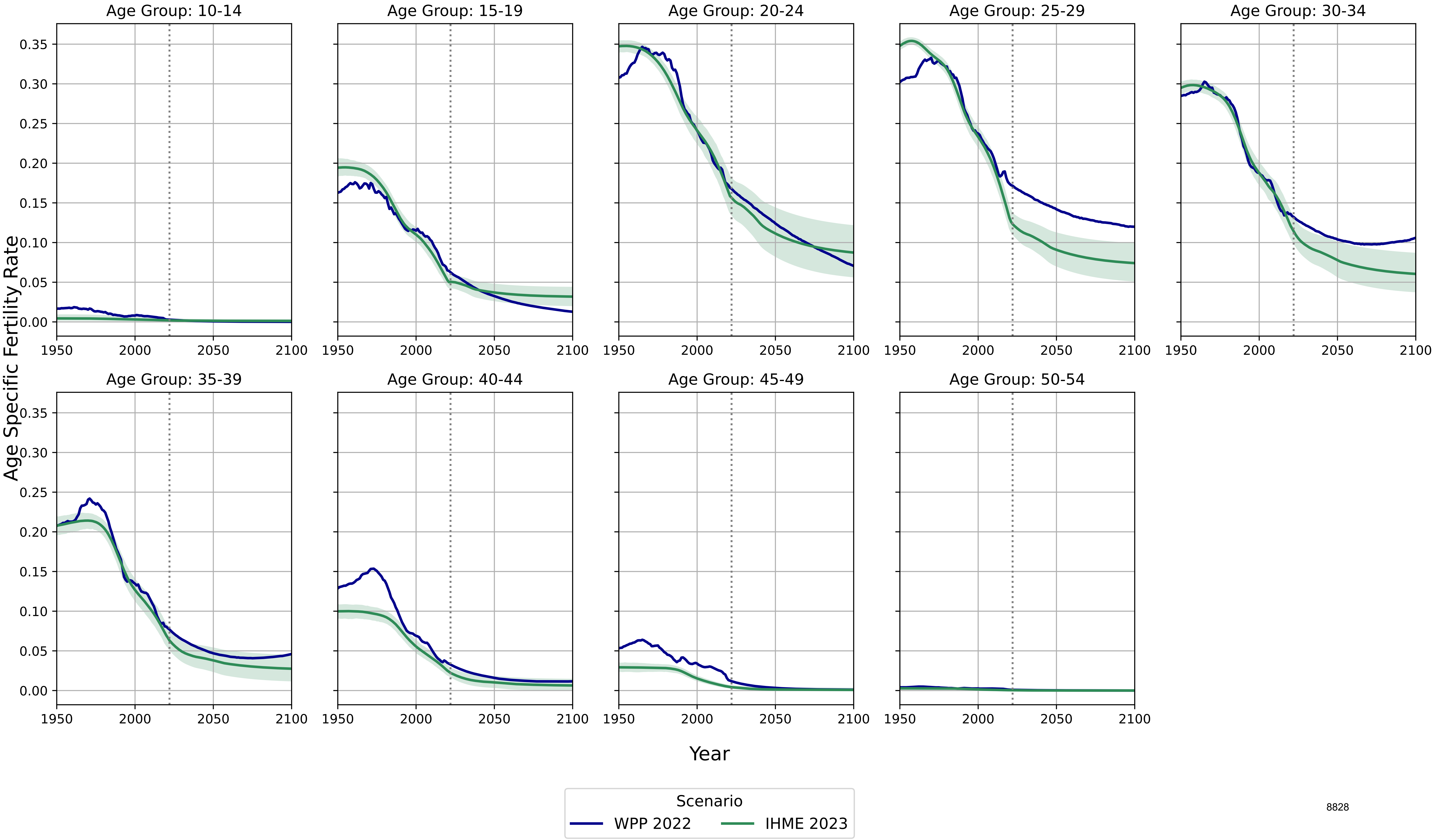

Supplemental Results Figure S8: Five-year interval ASFR from IHME reference scenario and the UNPD medium variant scenario by location, 1950-2100

Madagascar

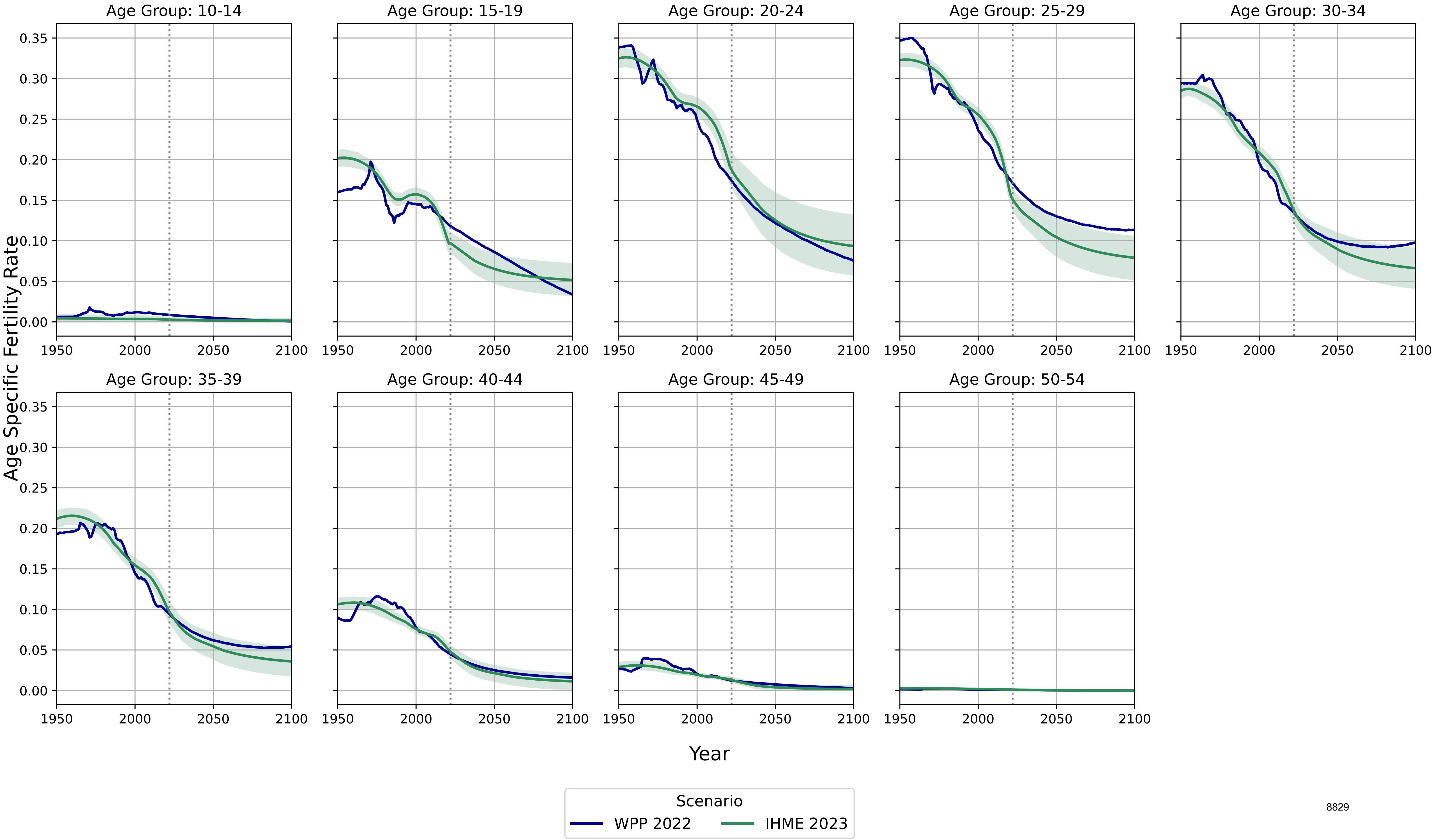

Supplemental Results Figure S8: Five-year interval ASFR from IHME reference scenario and the UNPD medium variant scenario by location, 1950-2100

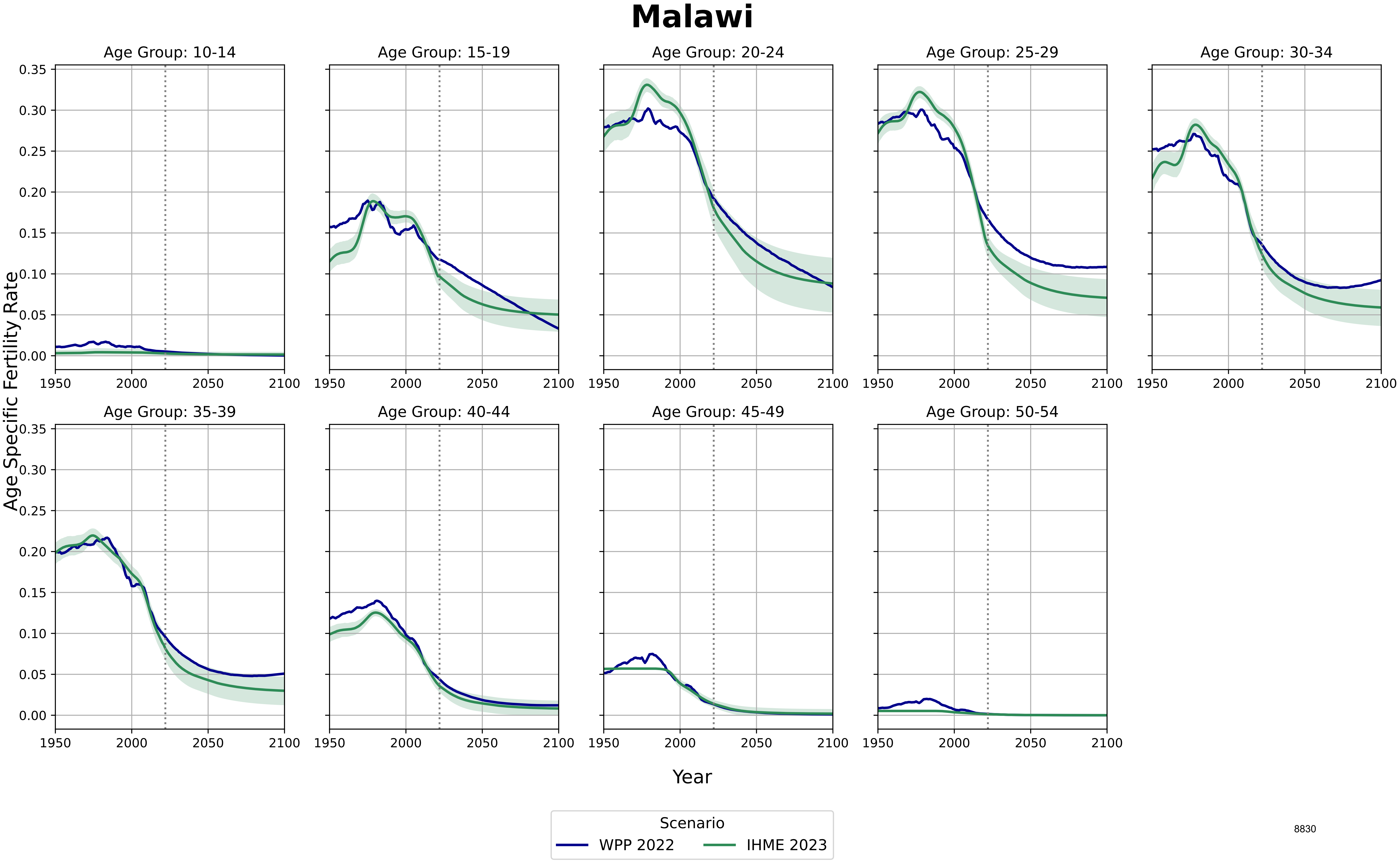

Supplemental Results Figure S8: Five-year interval ASFR from IHME reference scenario and the UNPD medium variant scenario by location, 1950-2100

Mozambique

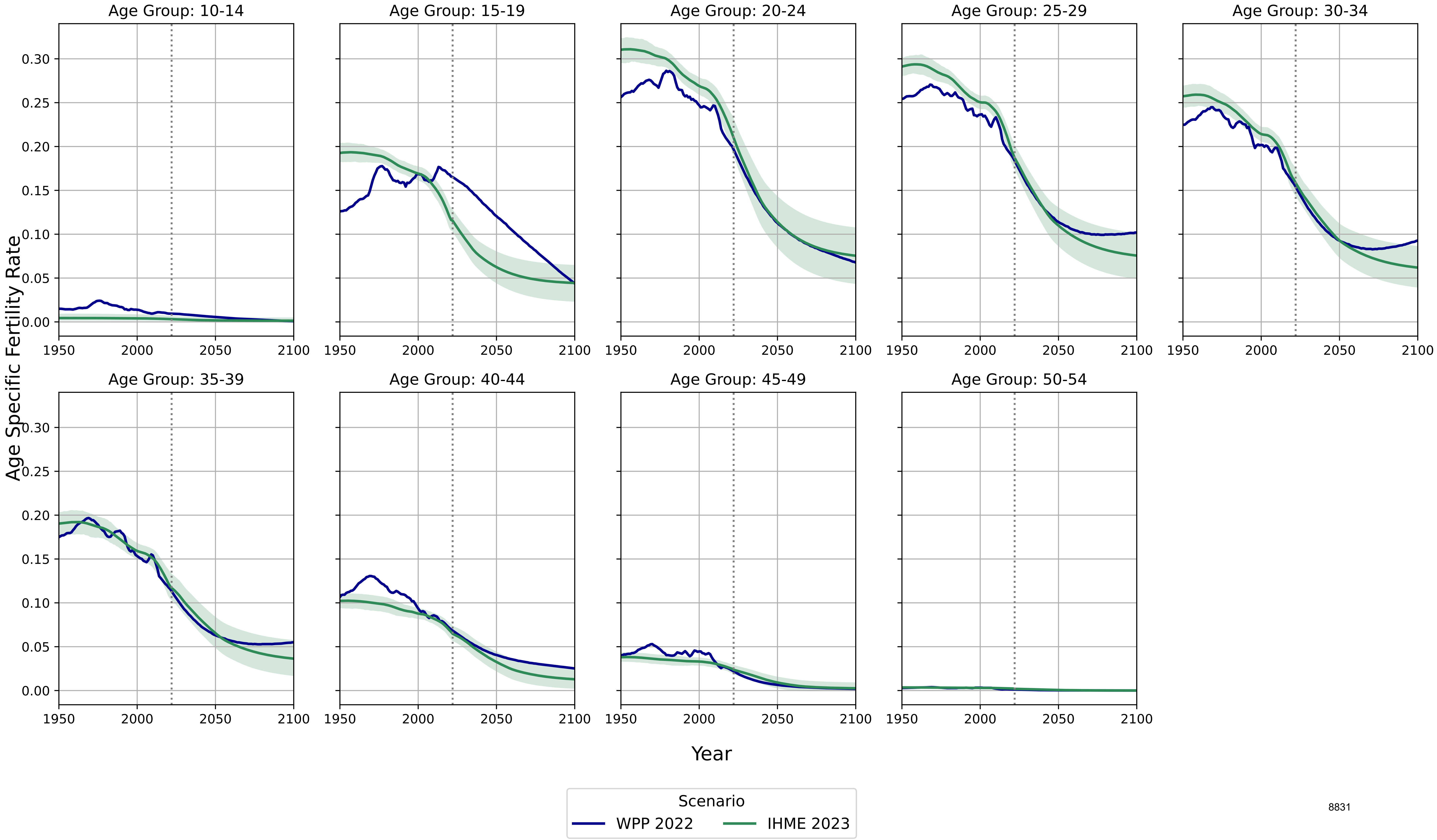

Supplemental Results Figure S8: Five-year interval ASFR from IHME reference scenario and the UNPD medium variant scenario by location, 1950-2100

Rwanda

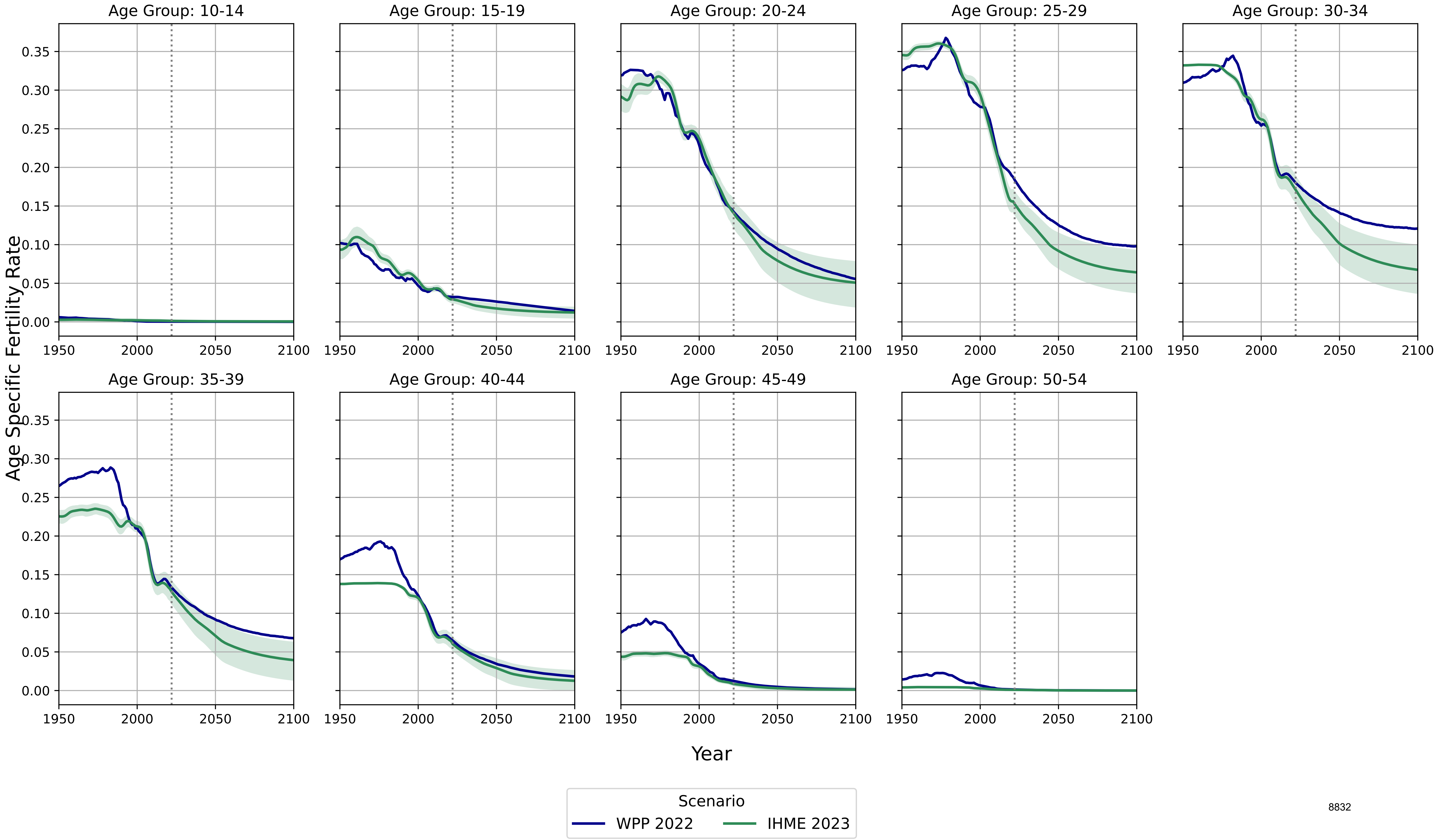

Supplemental Results Figure S8: Five-year interval ASFR from IHME reference scenario and the UNPD medium variant scenario by location, 1950-2100

Somalia

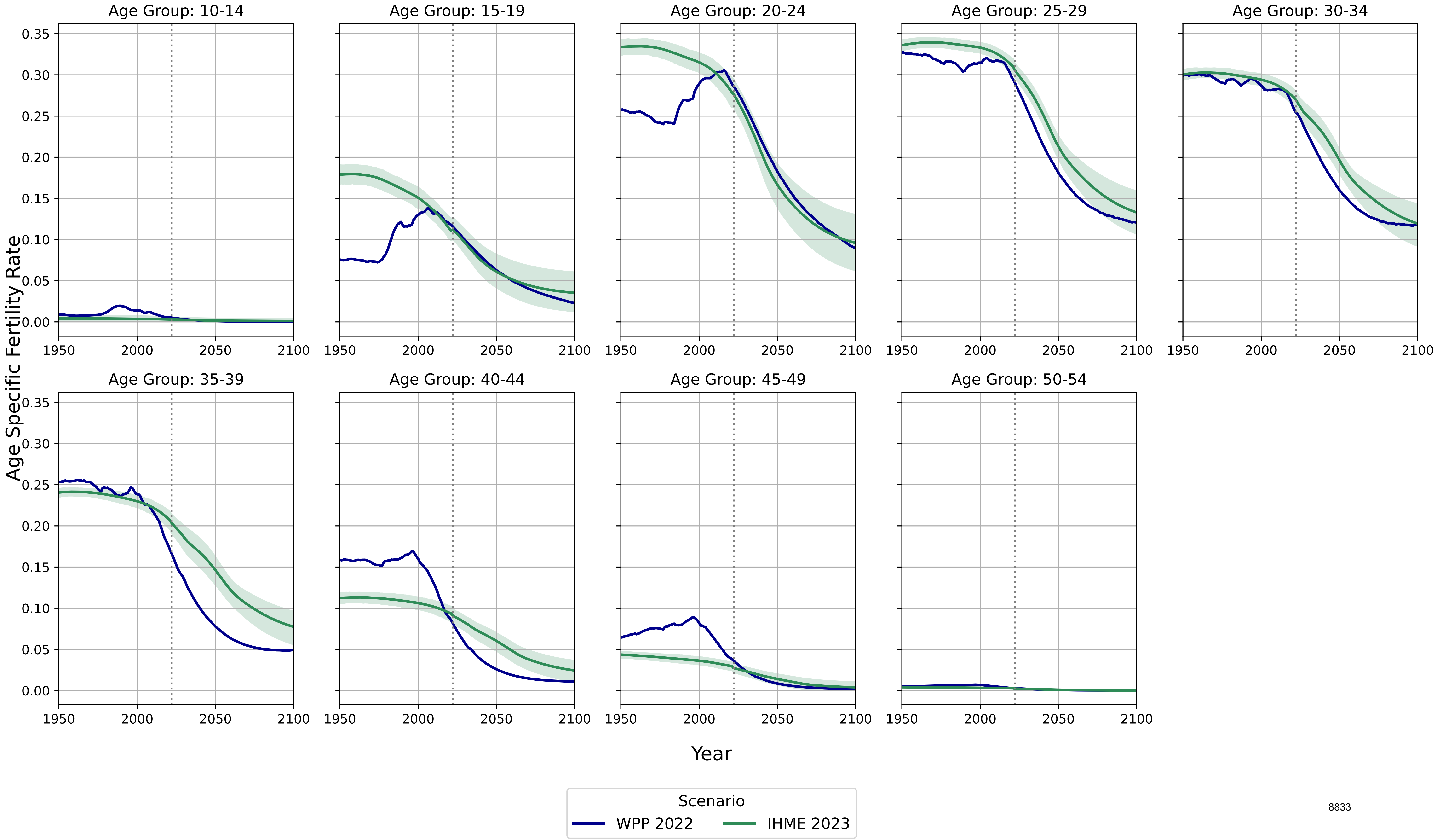

Supplemental Results Figure S8: Five-year interval ASFR from IHME reference scenario and the UNPD medium variant scenario by location, 1950-2100

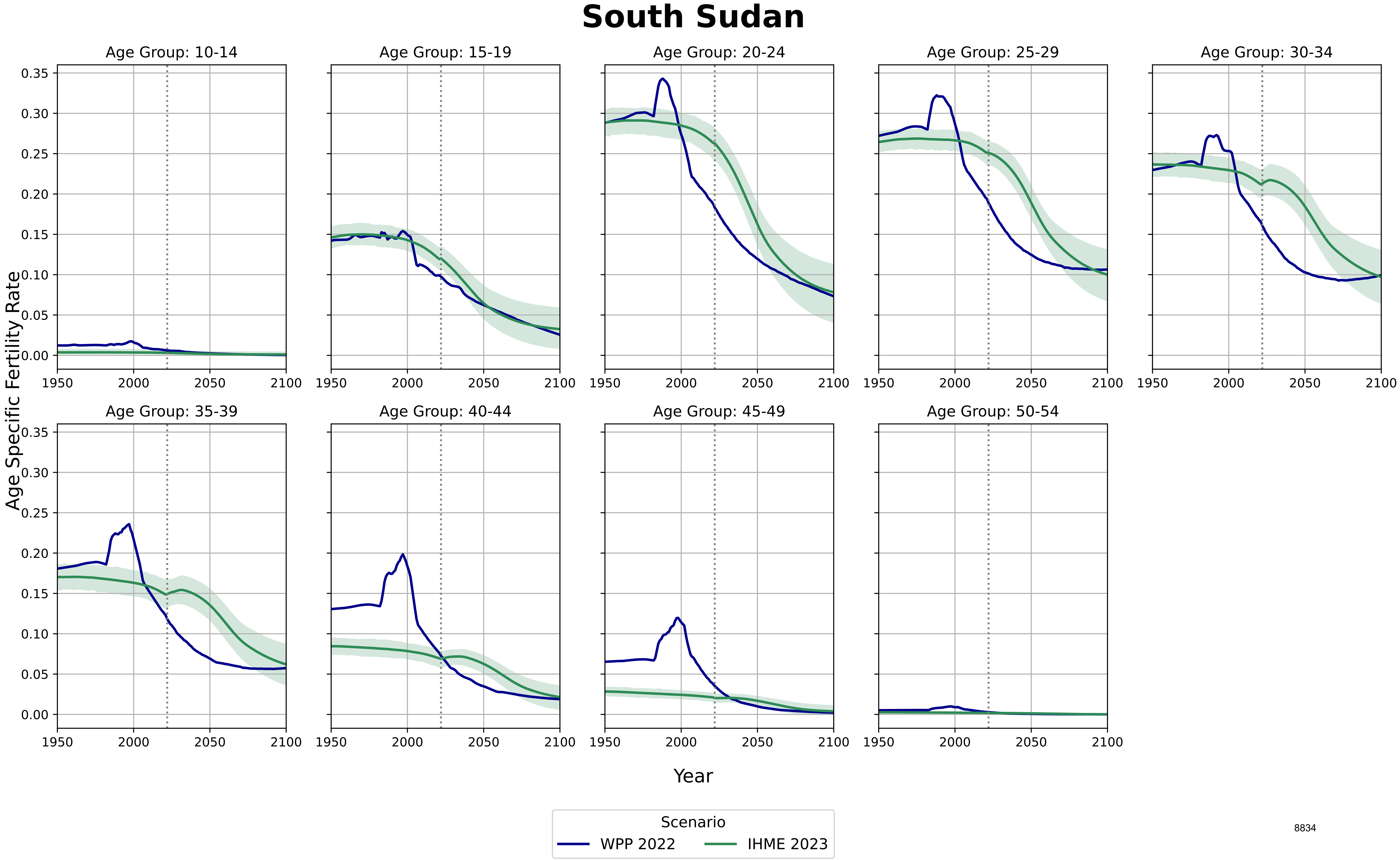

Supplemental Results Figure S8: Five-year interval ASFR from IHME reference scenario and the UNPD medium variant scenario by location, 1950-2100

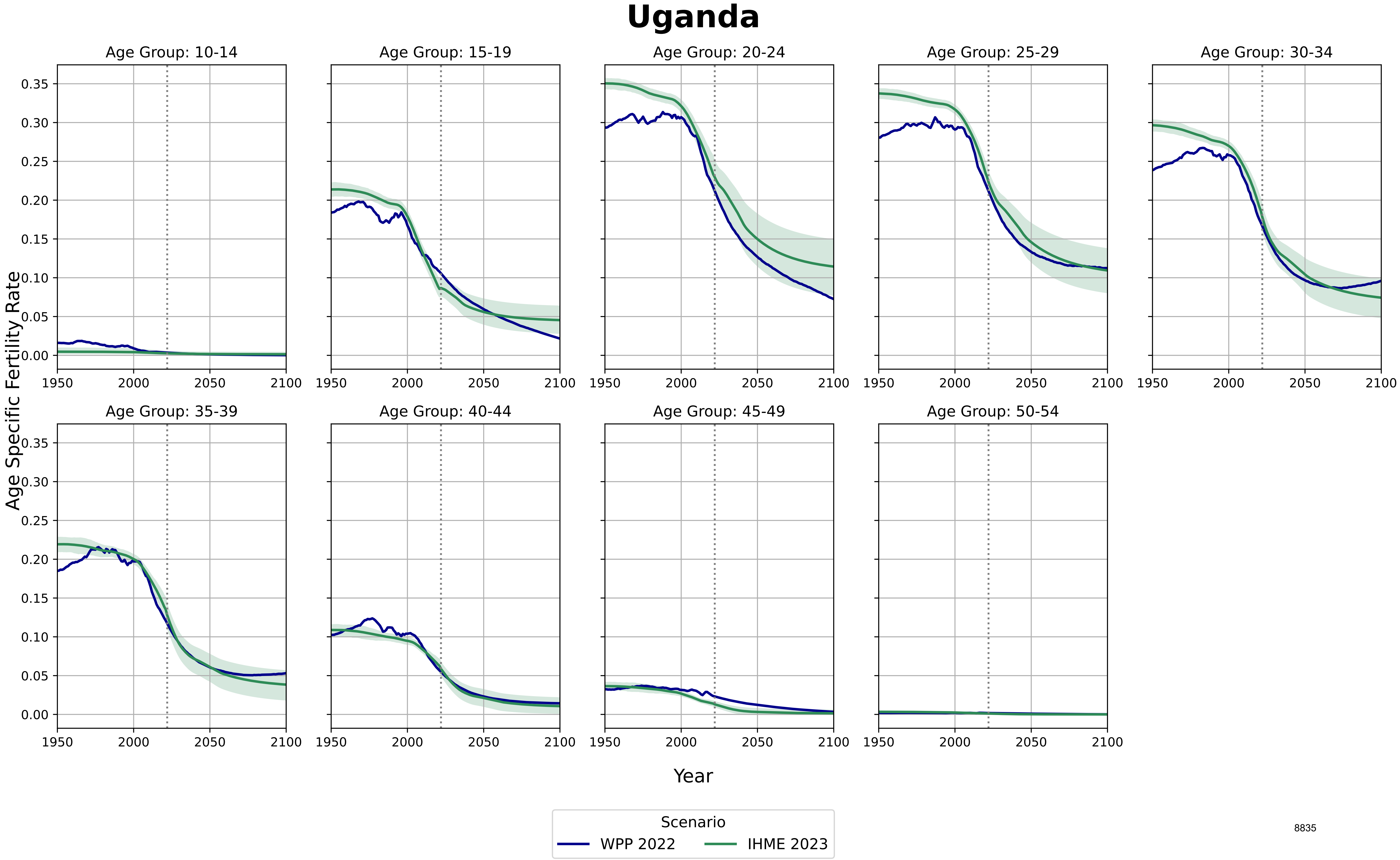

Supplemental Results Figure S8: Five-year interval ASFR from IHME reference scenario and the UNPD medium variant scenario by location, 1950-2100

Tanzania

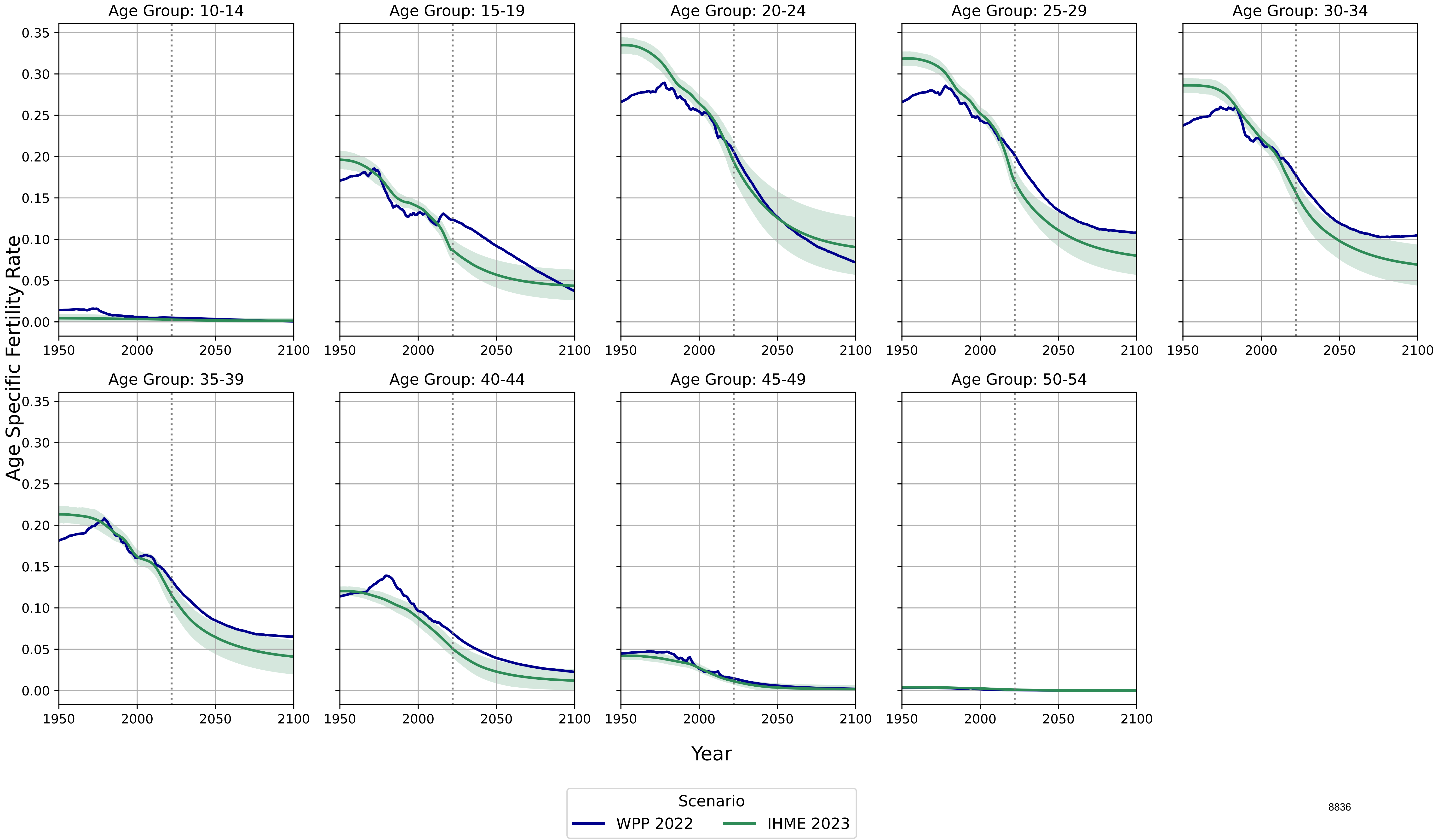

Supplemental Results Figure S8: Five-year interval ASFR from IHME reference scenario and the UNPD medium variant scenario by location, 1950-2100

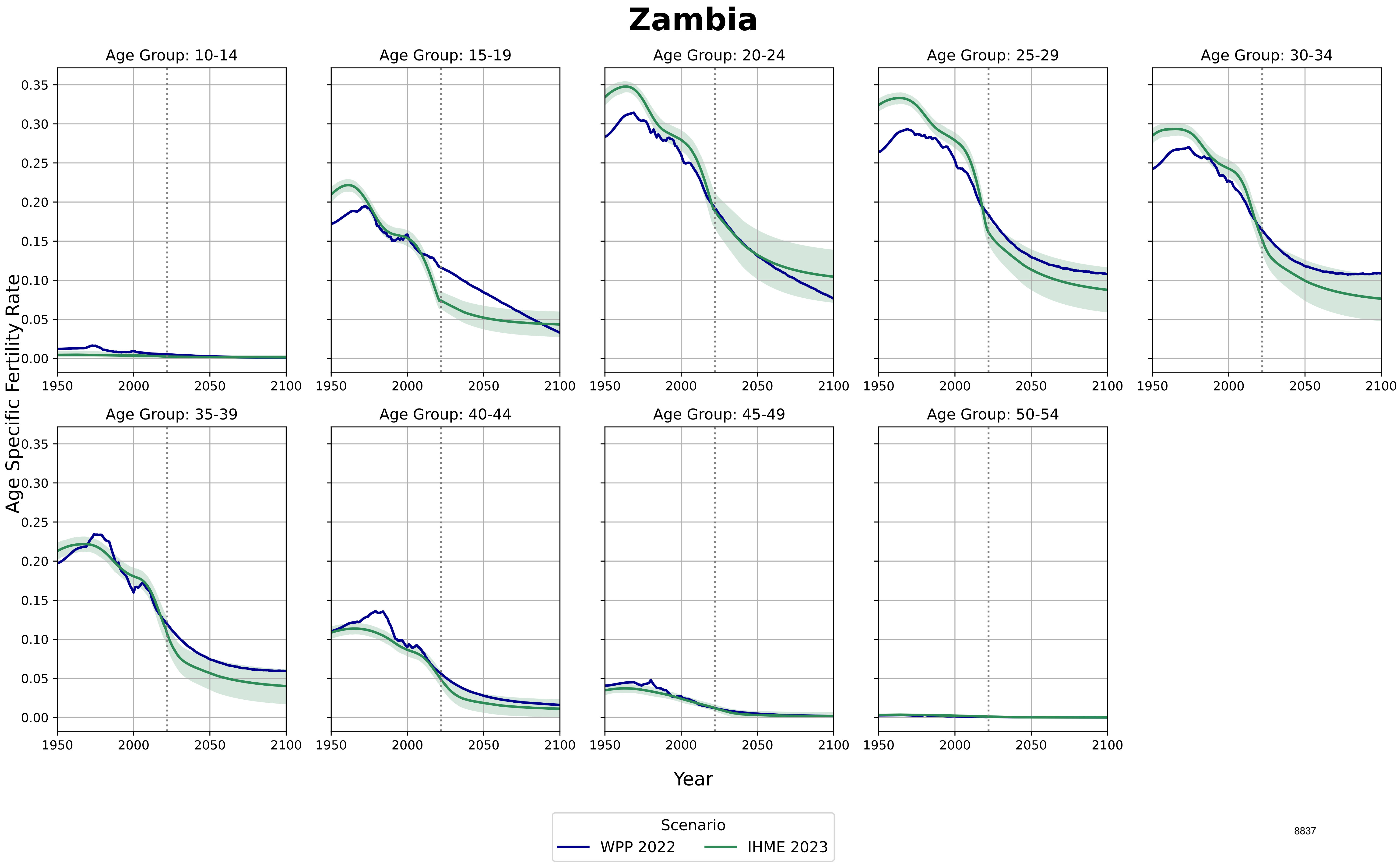

Supplemental Results Figure S8: Five-year interval ASFR from IHME reference scenario and the UNPD medium variant scenario by location, 1950-2100

Botswana

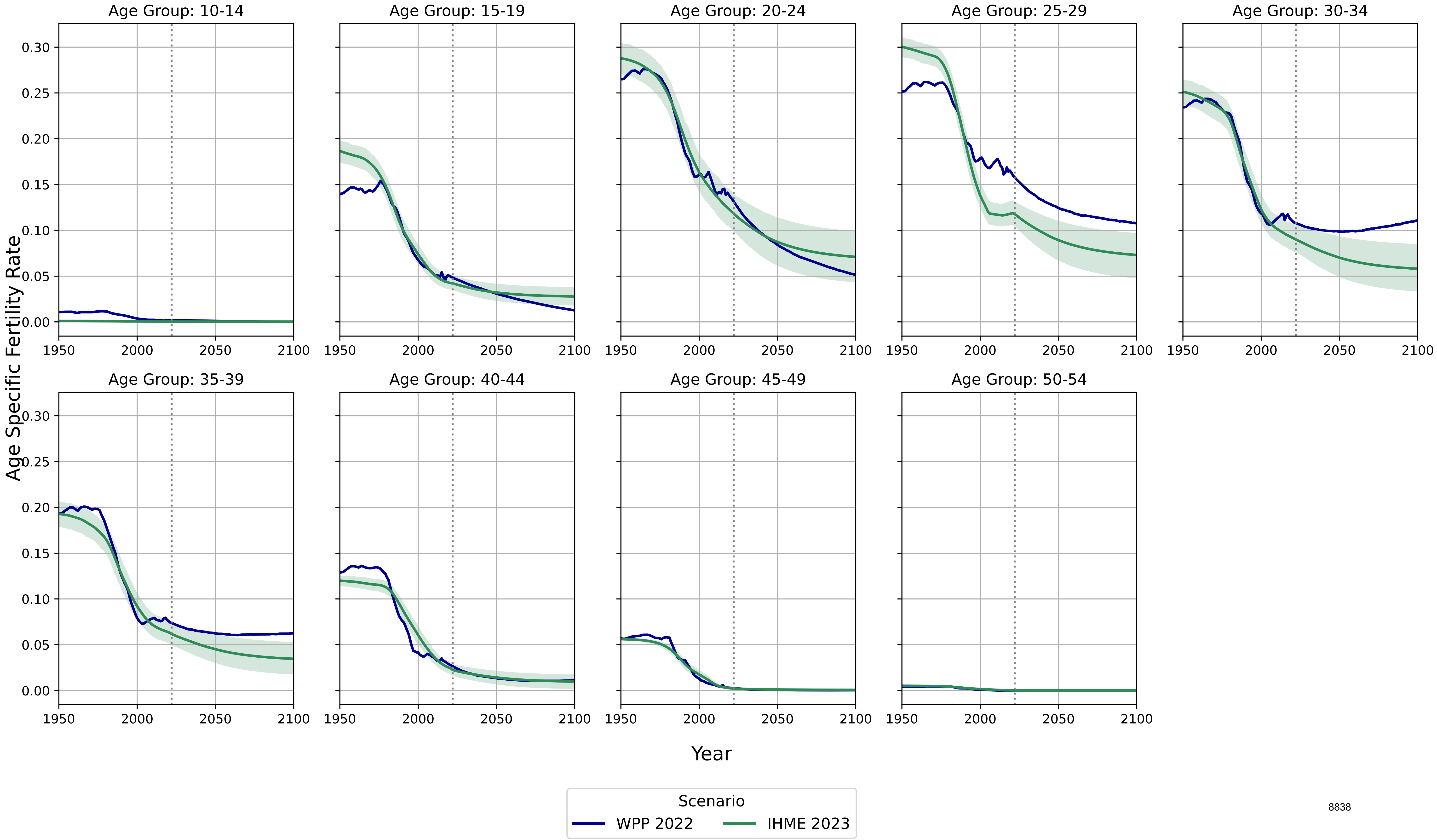

Supplemental Results Figure S8: Five-year interval ASFR from IHME reference scenario and the UNPD medium variant scenario by location, 1950-2100

Eswatini

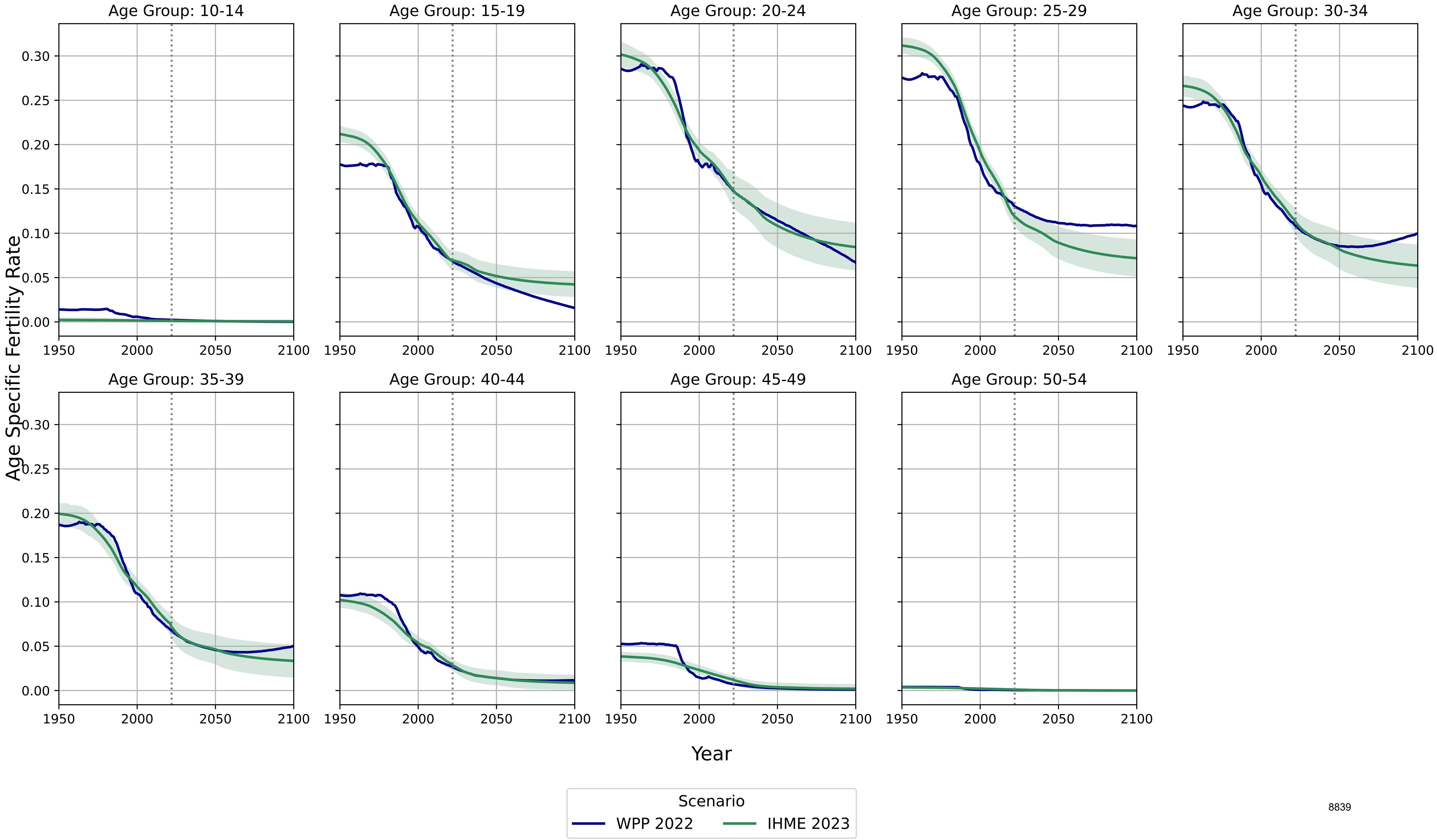

Supplemental Results Figure S8: Five-year interval ASFR from IHME reference scenario and the UNPD medium variant scenario by location, 1950-2100

Lesotho

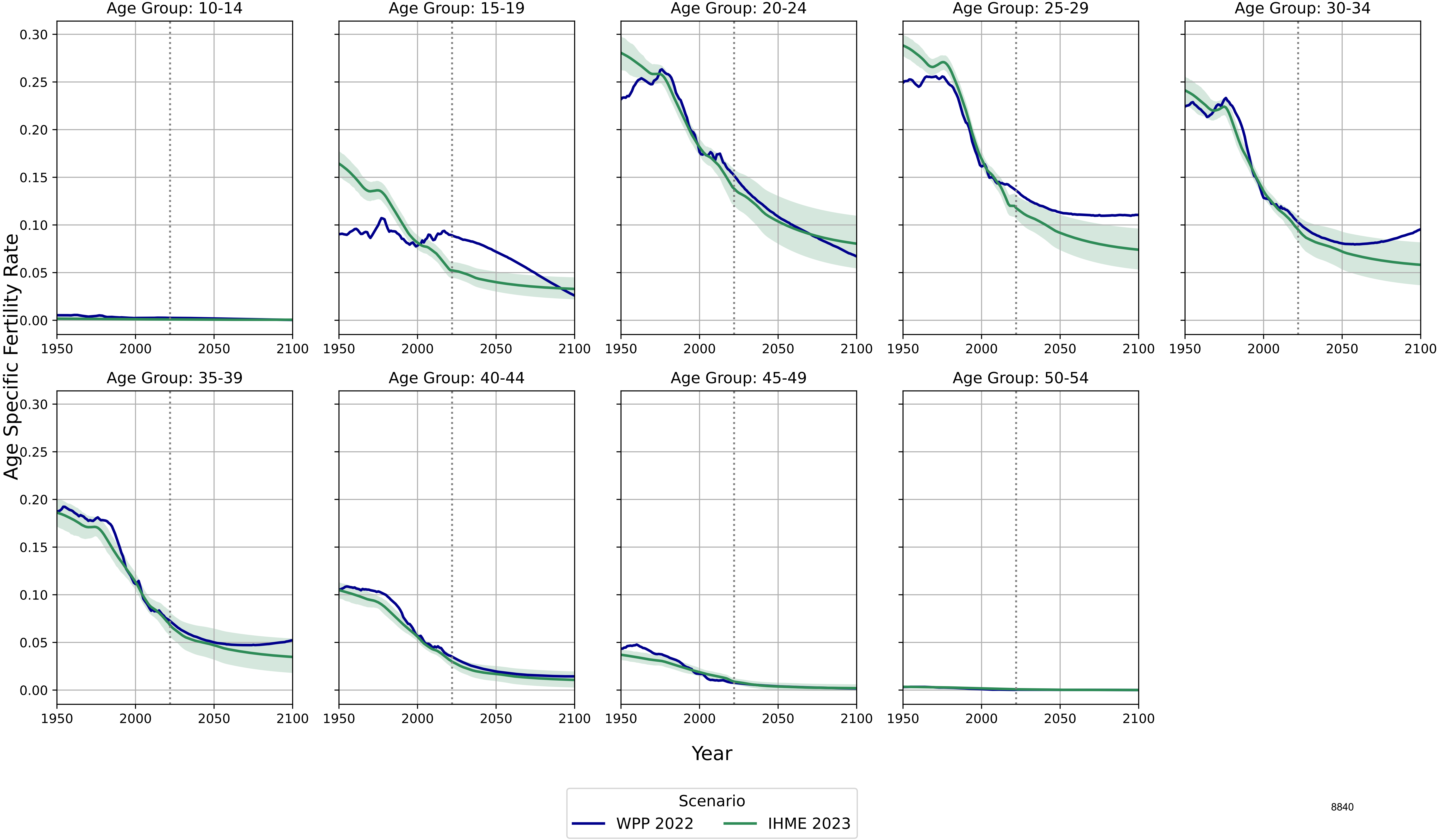

Supplemental Results Figure S8: Five-year interval ASFR from IHME reference scenario and the UNPD medium variant scenario by location, 1950-2100

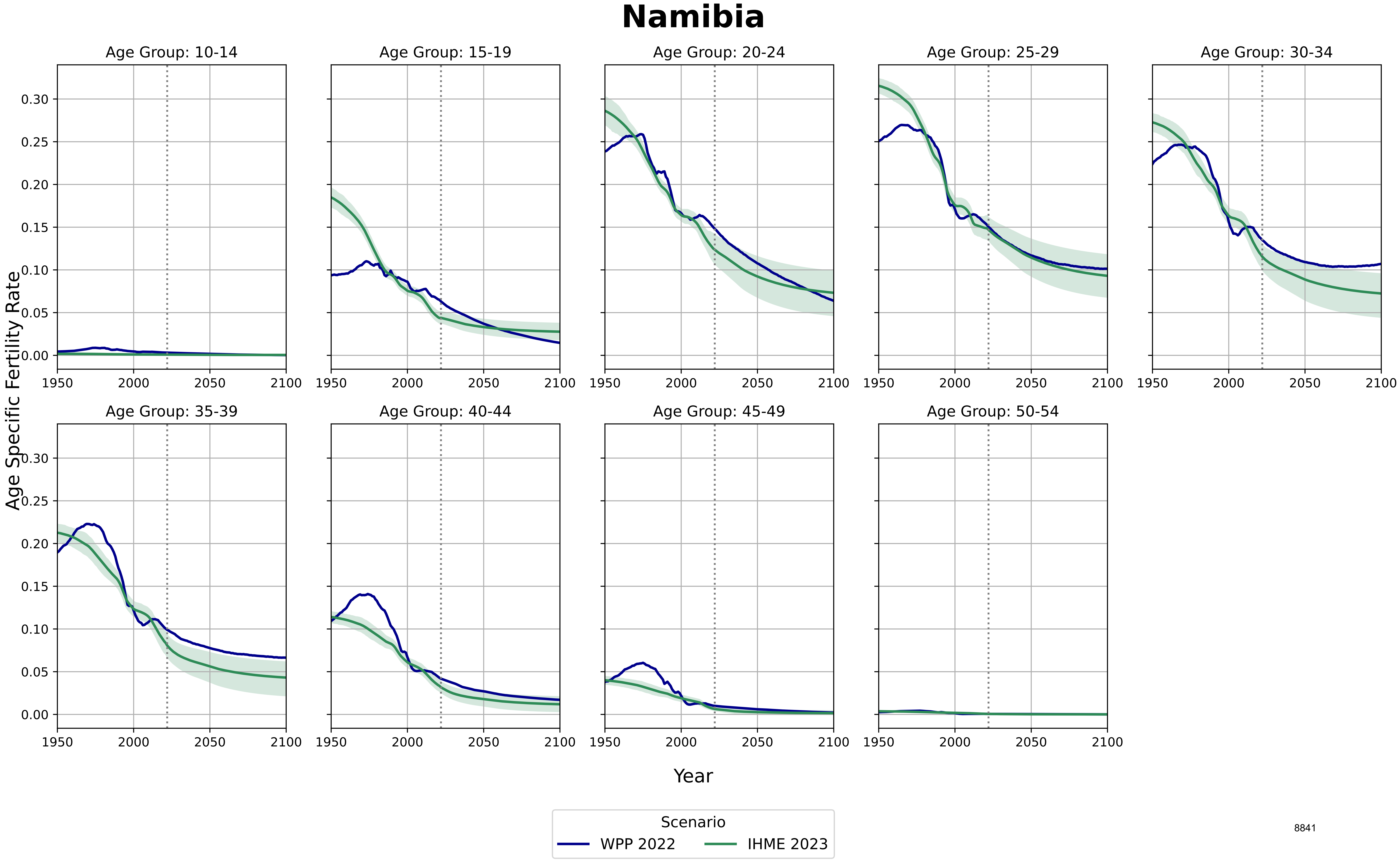

Supplemental Results Figure S8: Five-year interval ASFR from IHME reference scenario and the UNPD medium variant scenario by location, 1950-2100

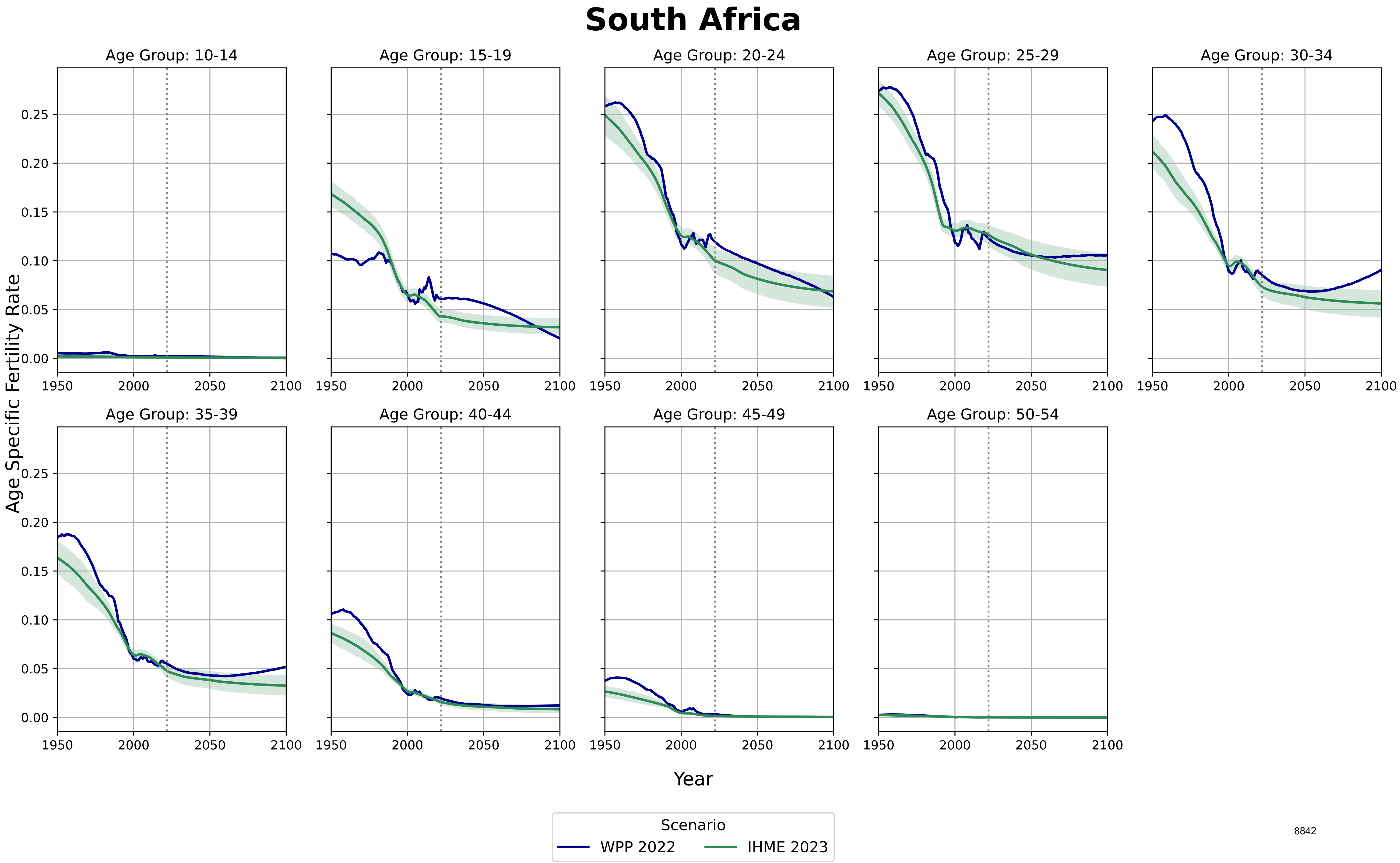

Supplemental Results Figure S8: Five-year interval ASFR from IHME reference scenario and the UNPD medium variant scenario by location, 1950-2100

Zimbabwe

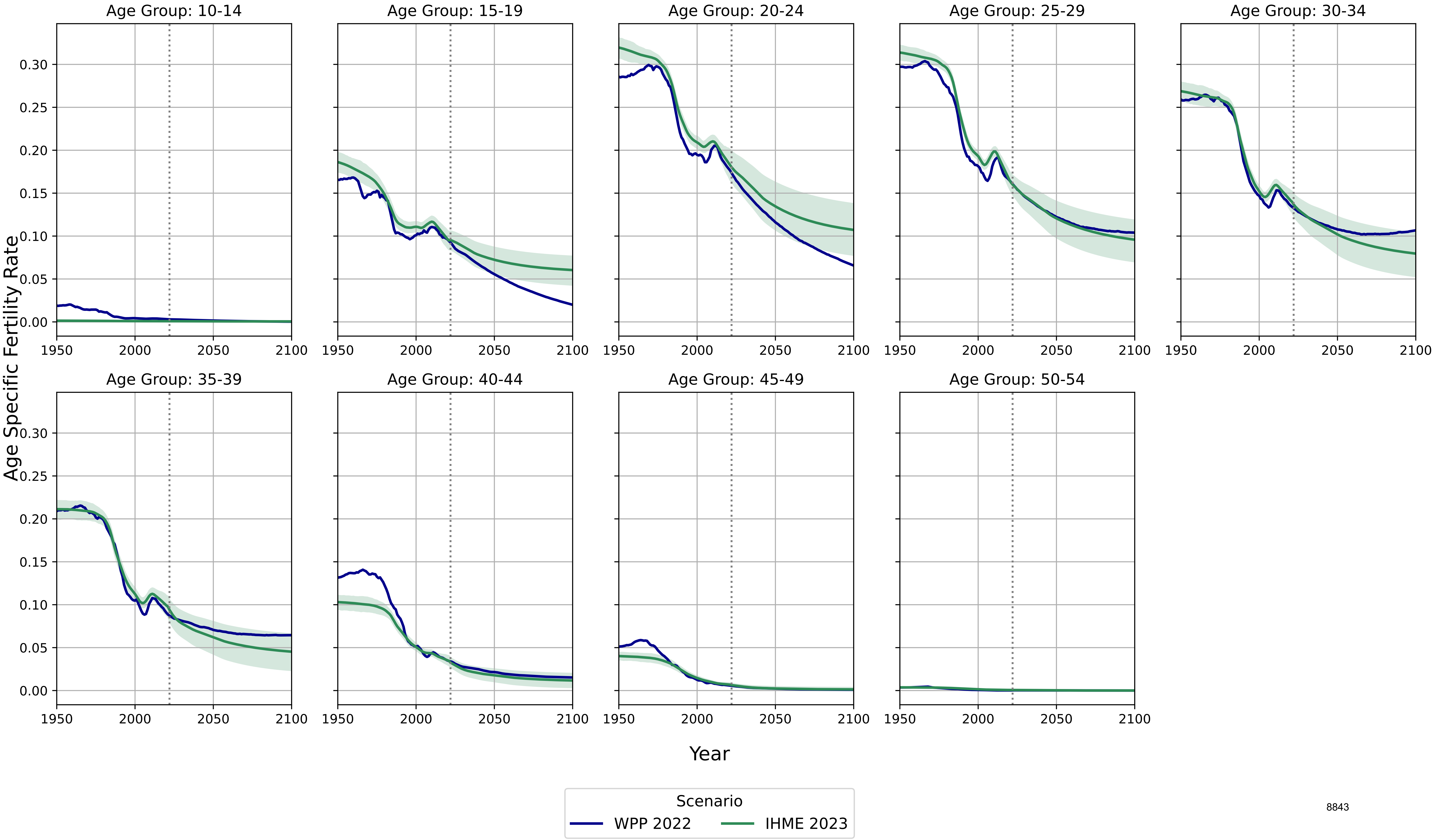

Supplemental Results Figure S8: Five-year interval ASFR from IHME reference scenario and the UNPD medium variant scenario by location, 1950-2100

Benin

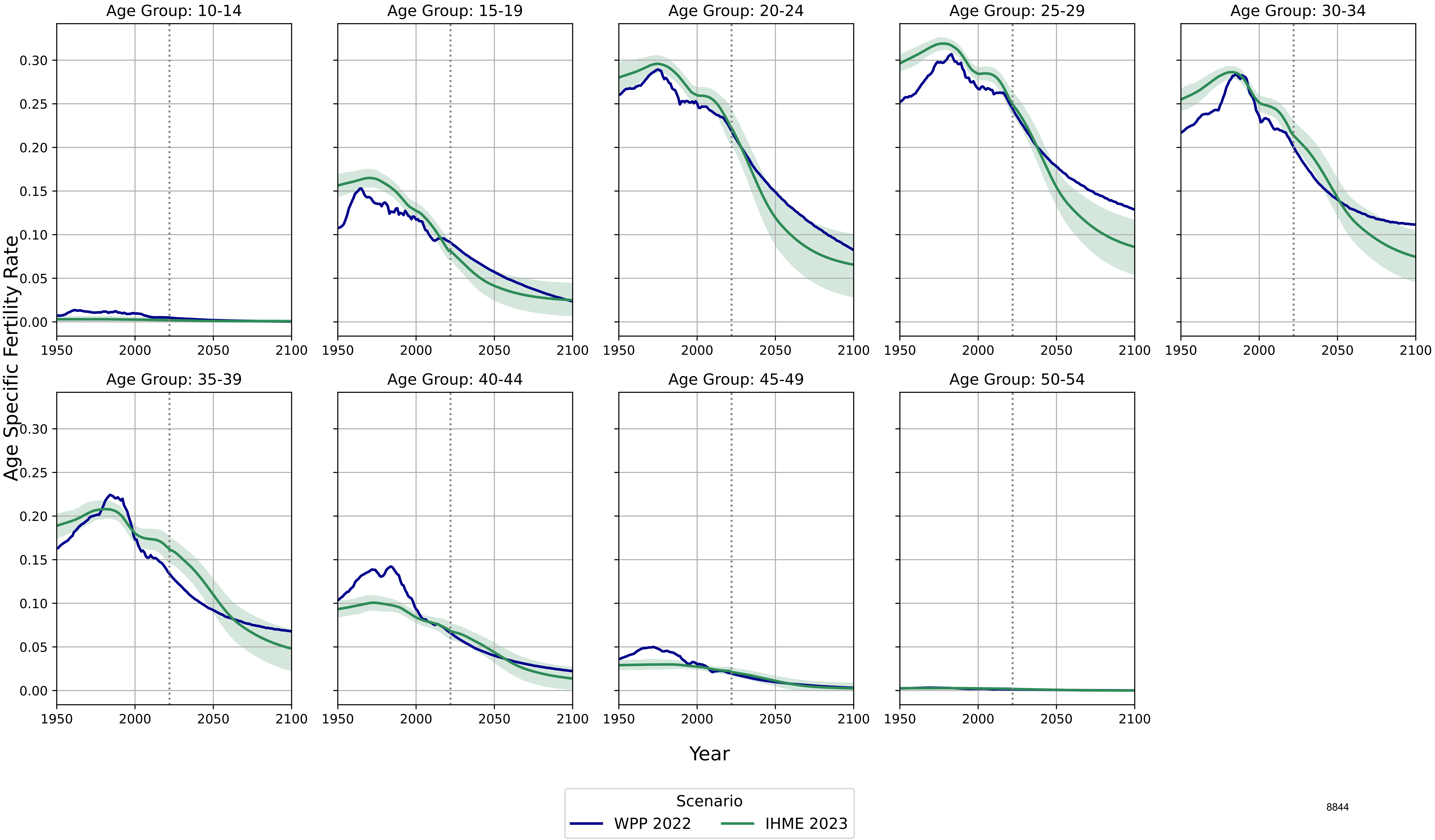

Supplemental Results Figure S8: Five-year interval ASFR from IHME reference scenario and the UNPD medium variant scenario by location, 1950-2100

Burkina Faso

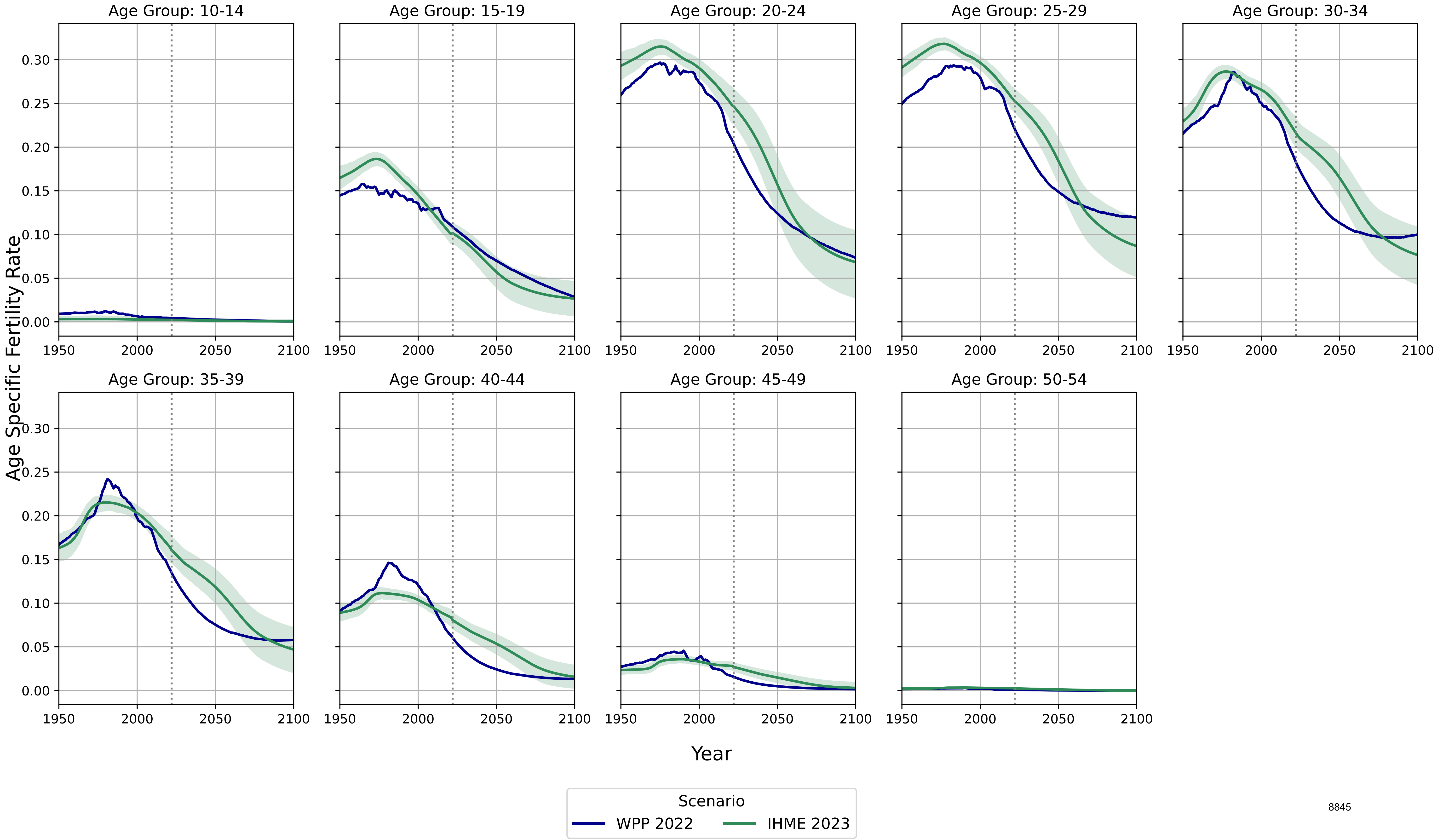

Supplemental Results Figure S8: Five-year interval ASFR from IHME reference scenario and the UNPD medium variant scenario by location, 1950-2100

Cabo Verde

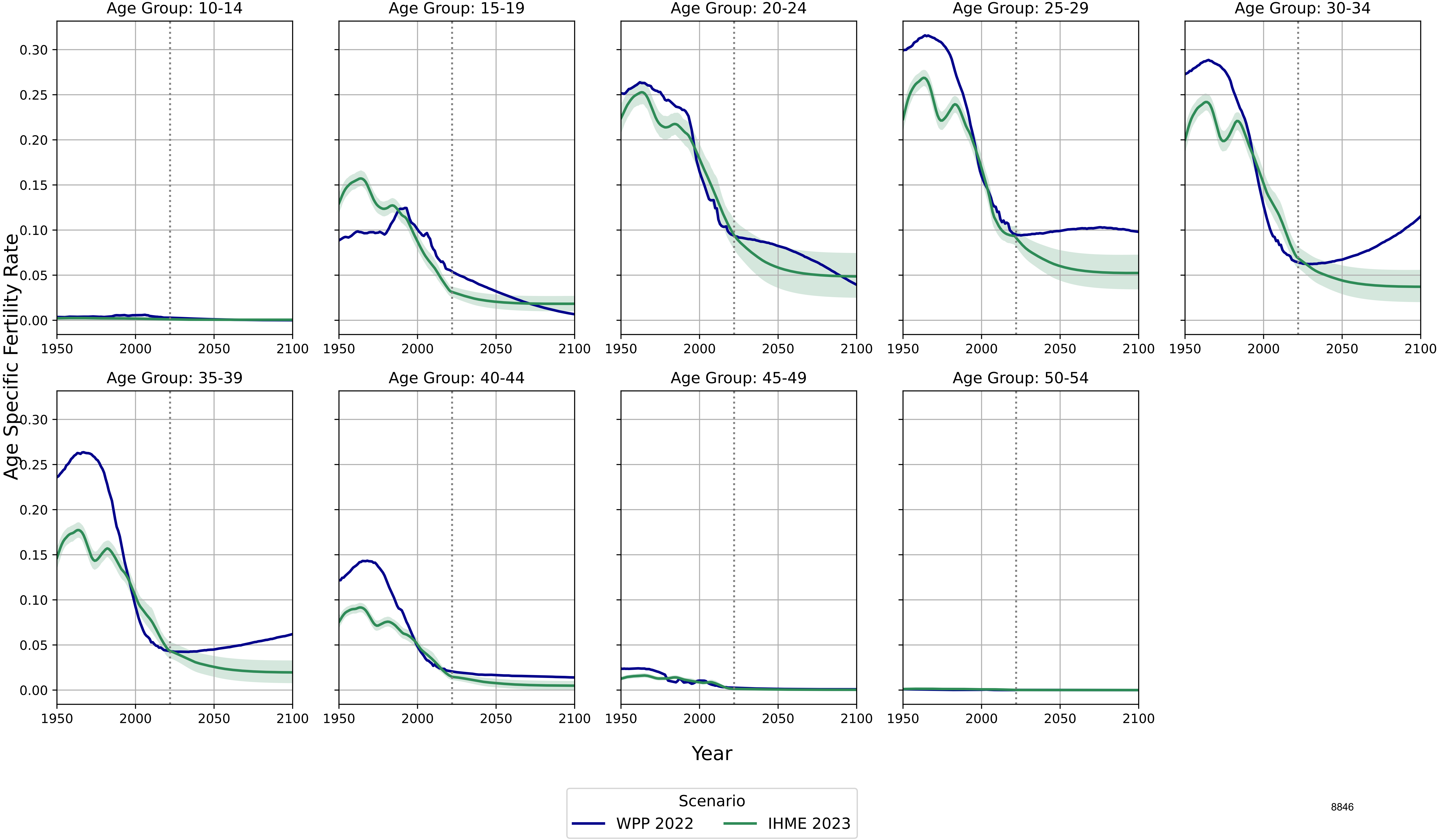

Supplemental Results Figure S8: Five-year interval ASFR from IHME reference scenario and the UNPD medium variant scenario by location, 1950-2100

Cameroon

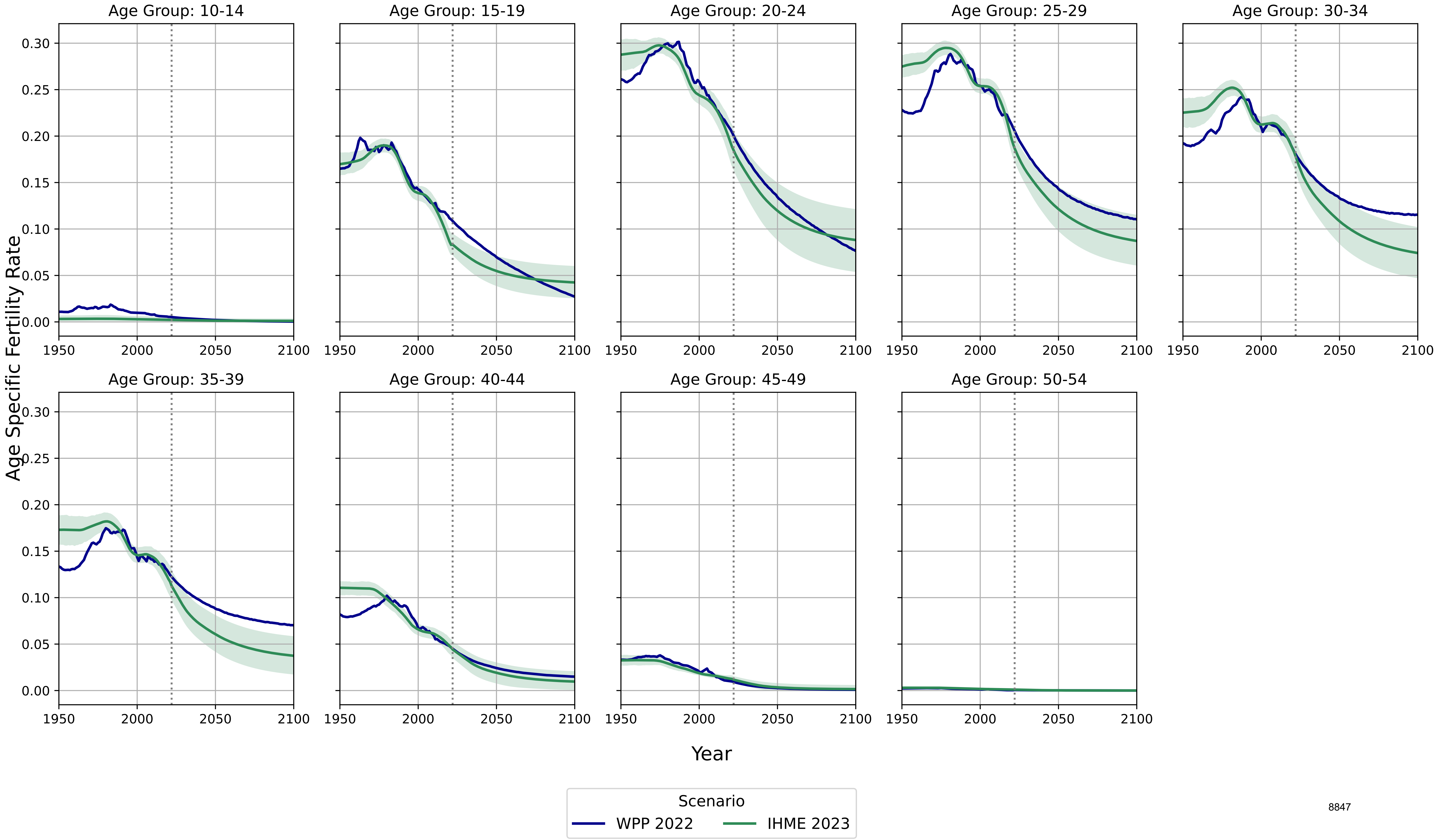

Supplemental Results Figure S8: Five-year interval ASFR from IHME reference scenario and the UNPD medium variant scenario by location, 1950-2100

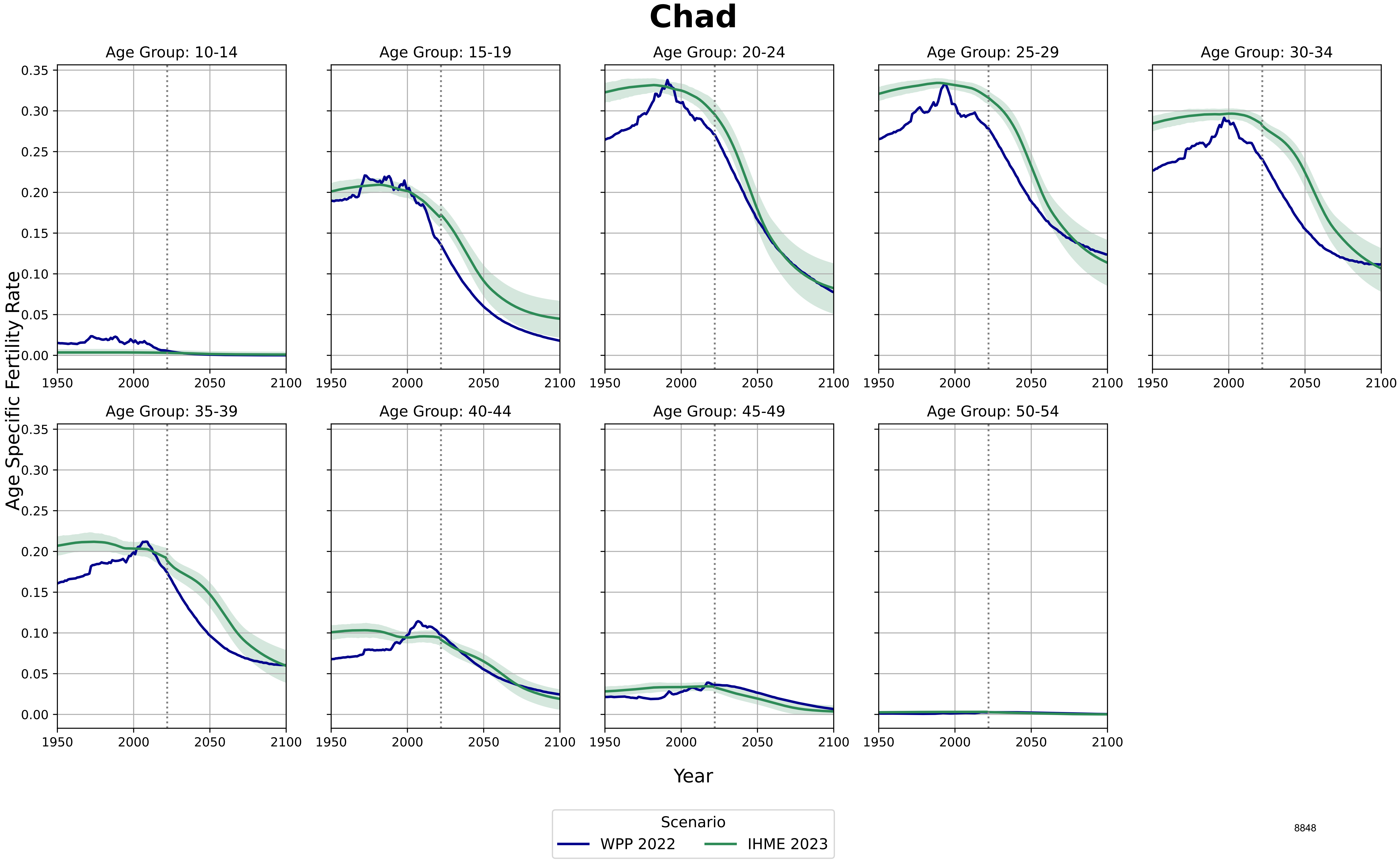

Supplemental Results Figure S8: Five-year interval ASFR from IHME reference scenario and the UNPD medium variant scenario by location, 1950-2100

Côte d'Ivoire

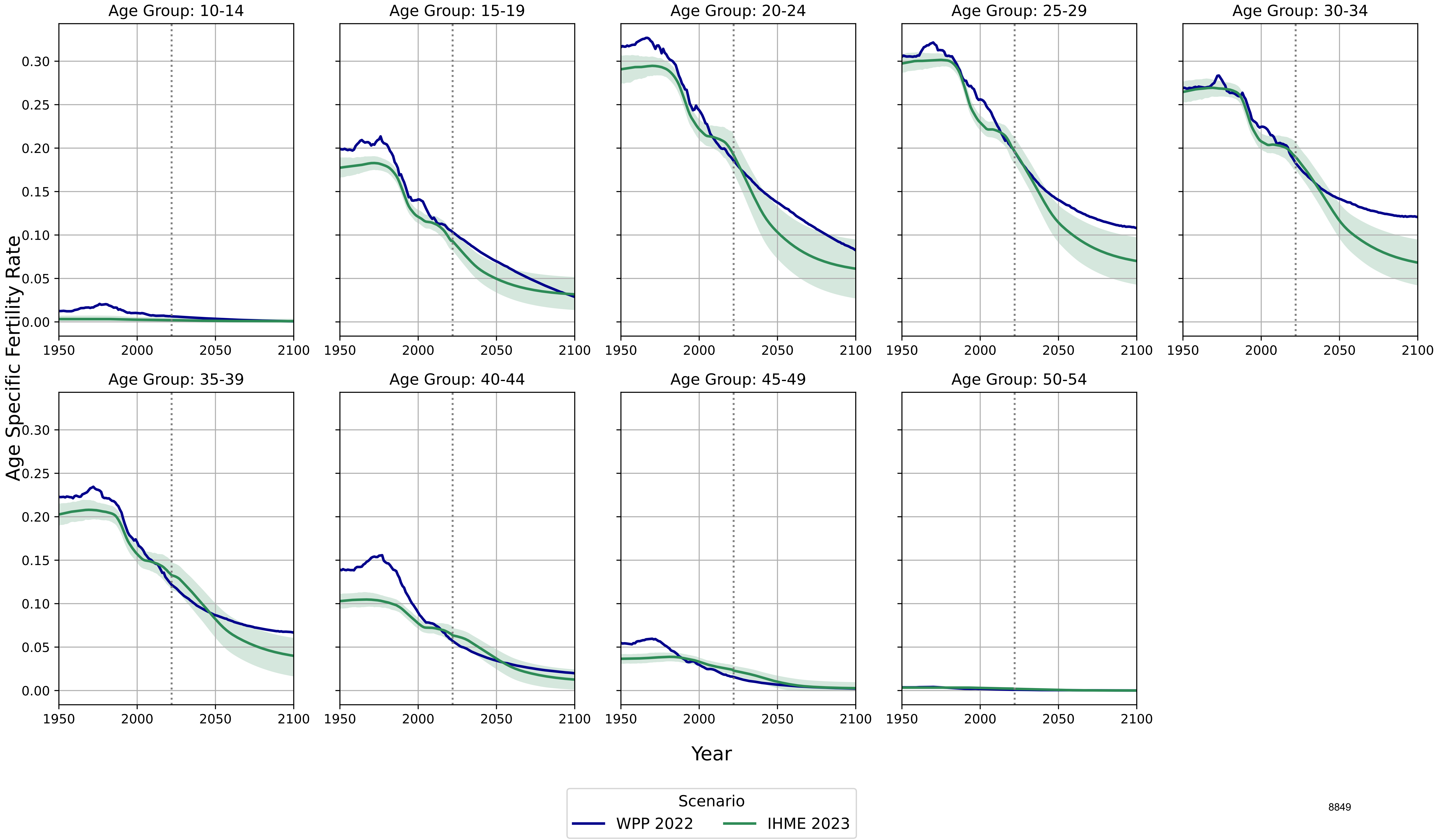

Supplemental Results Figure S8: Five-year interval ASFR from IHME reference scenario and the UNPD medium variant scenario by location, 1950-2100

The Gambia

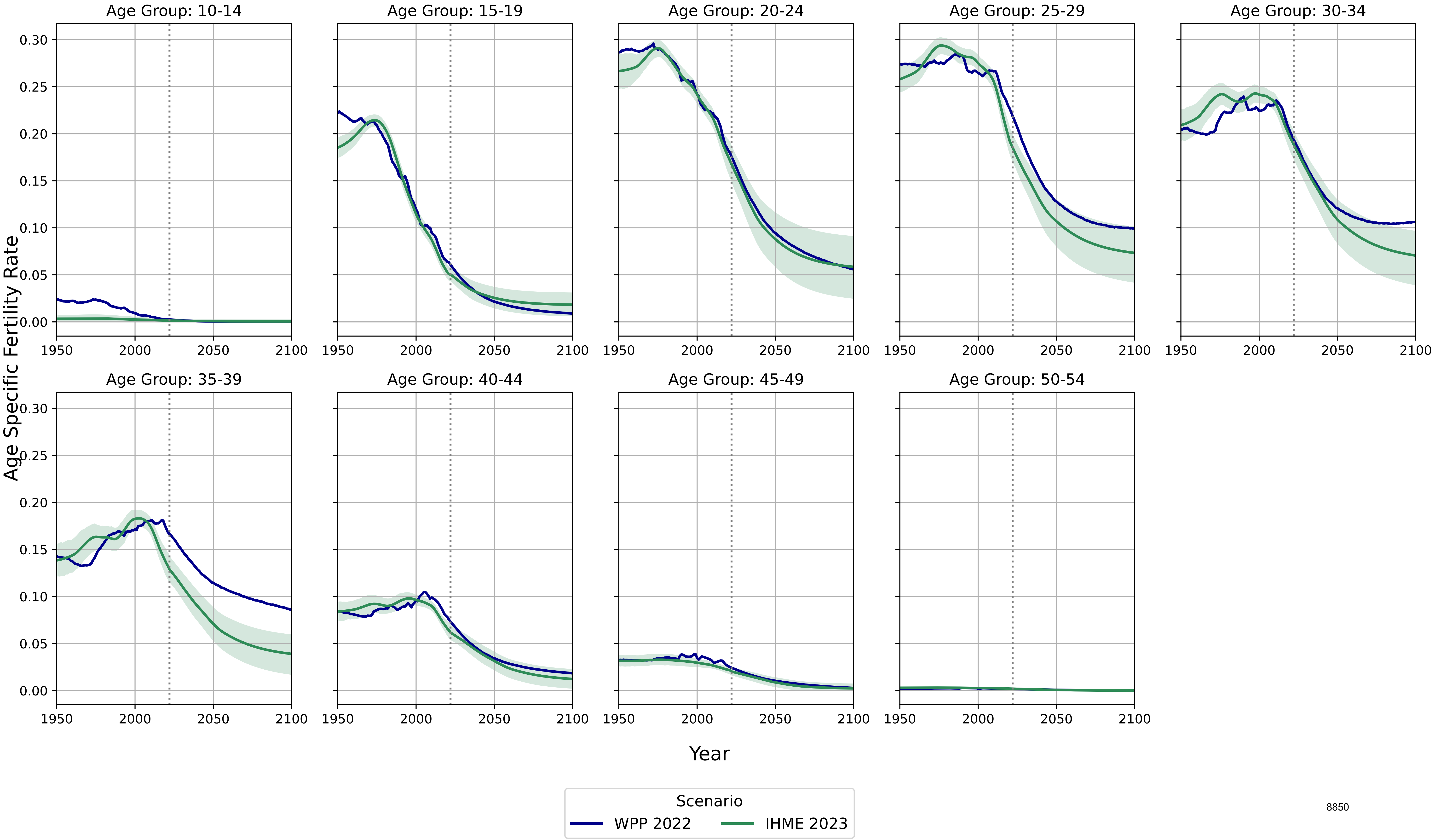

Supplemental Results Figure S8: Five-year interval ASFR from IHME reference scenario and the UNPD medium variant scenario by location, 1950-2100

Ghana

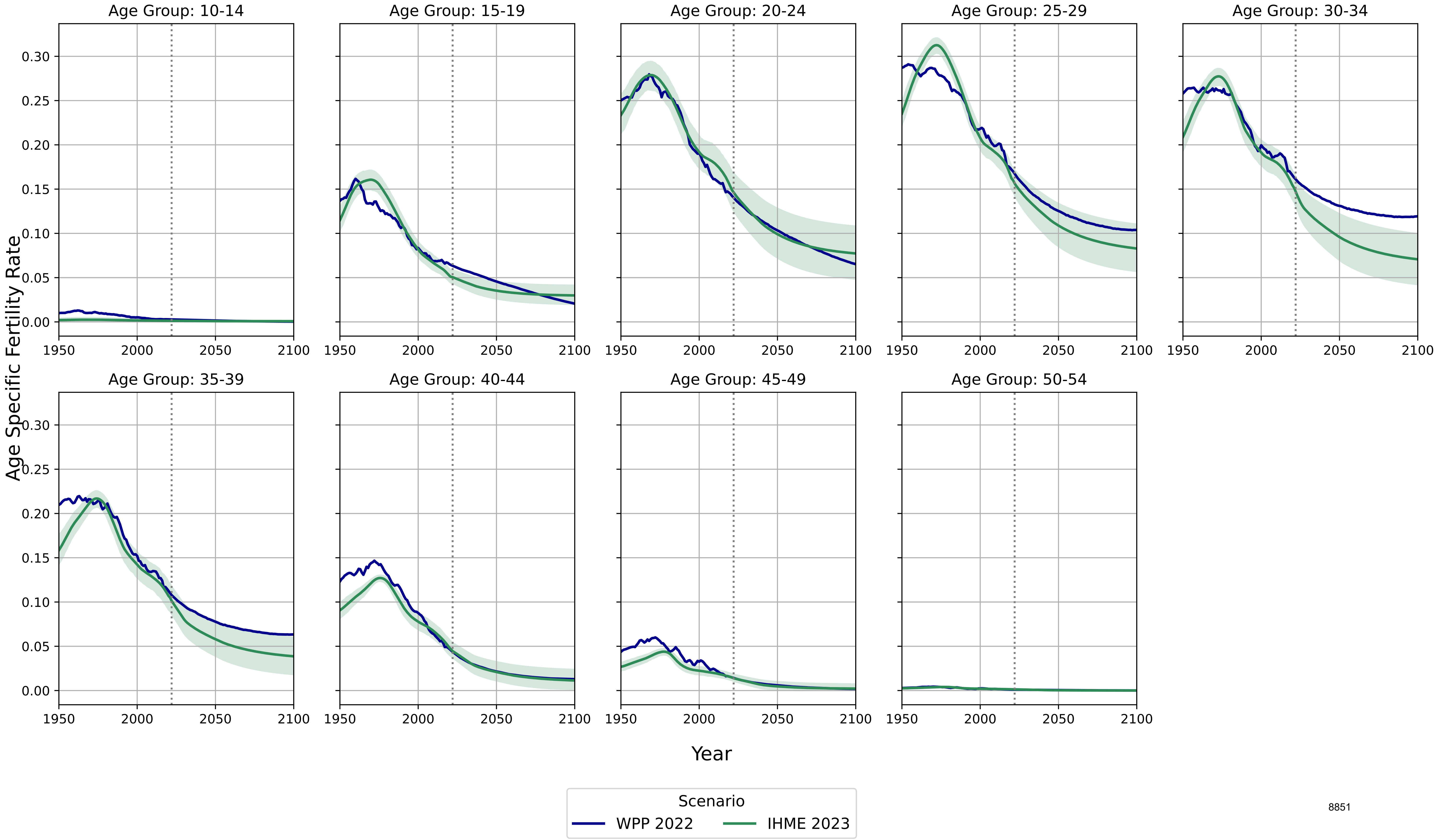

Supplemental Results Figure S8: Five-year interval ASFR from IHME reference scenario and the UNPD medium variant scenario by location, 1950-2100

Guinea

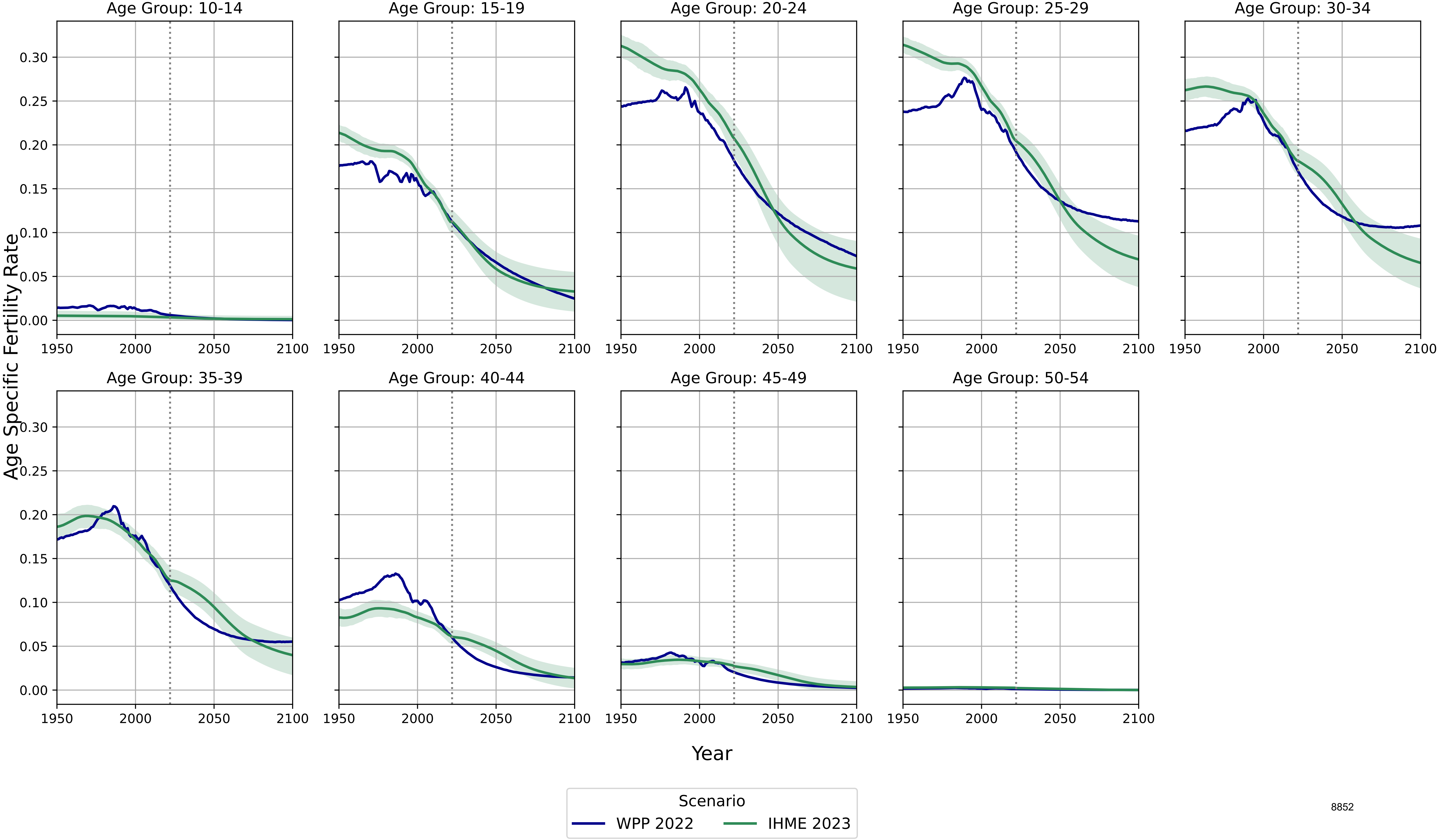

Supplemental Results Figure S8: Five-year interval ASFR from IHME reference scenario and the UNPD medium variant scenario by location, 1950-2100

Guinea-Bissau

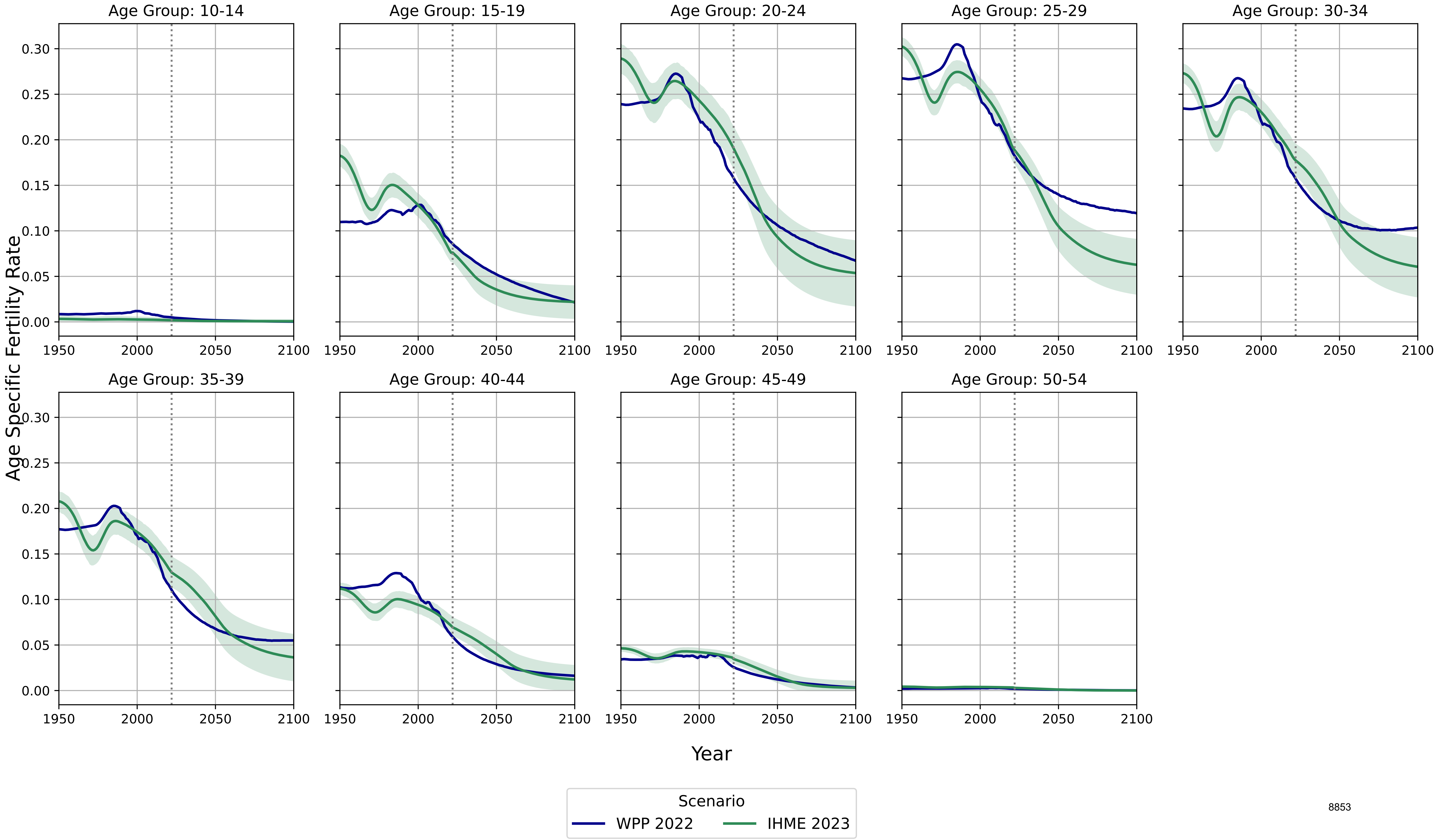

Supplemental Results Figure S8: Five-year interval ASFR from IHME reference scenario and the UNPD medium variant scenario by location, 1950-2100

Liberia

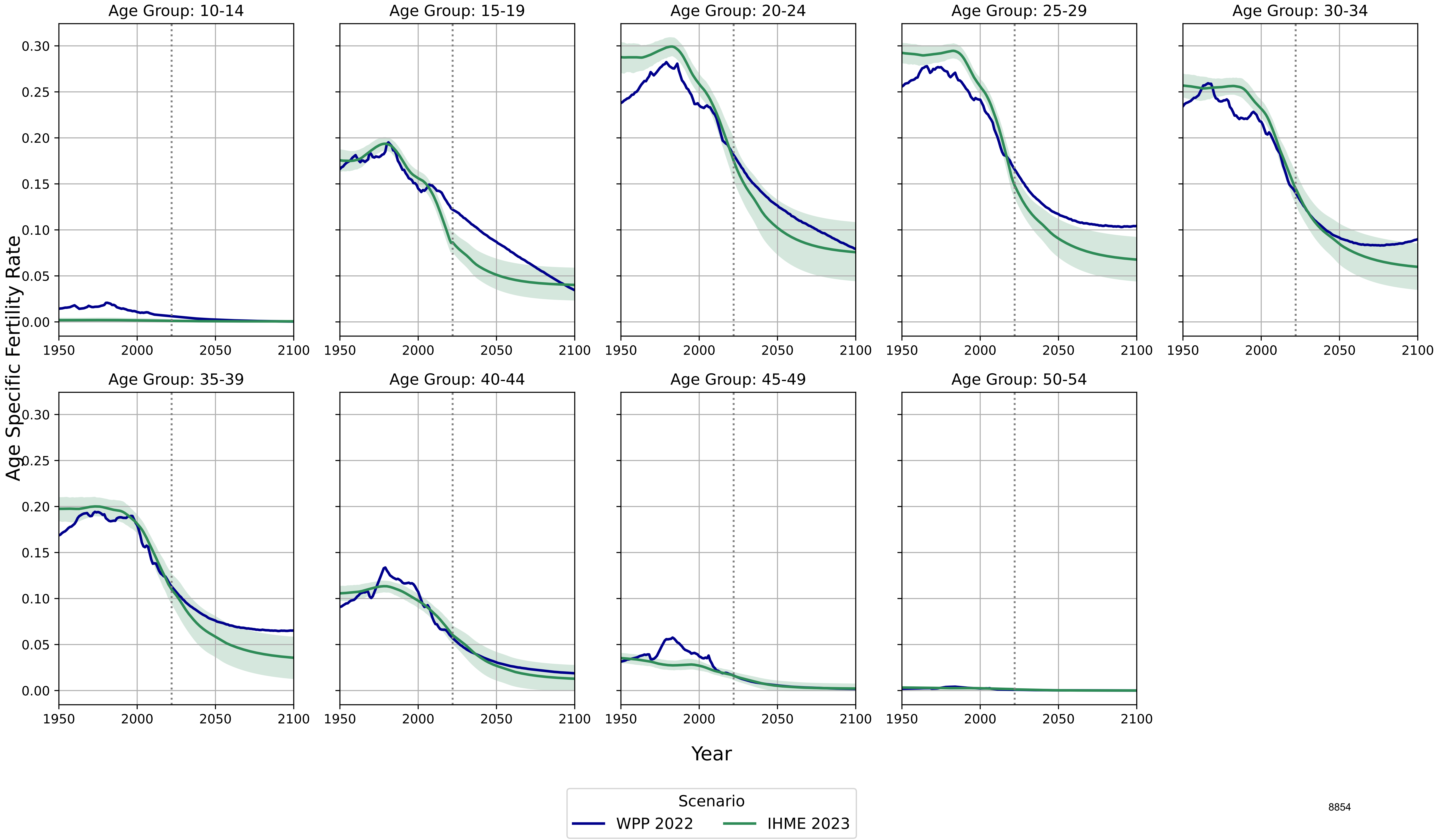

Supplemental Results Figure S8: Five-year interval ASFR from IHME reference scenario and the UNPD medium variant scenario by location, 1950-2100

Mali

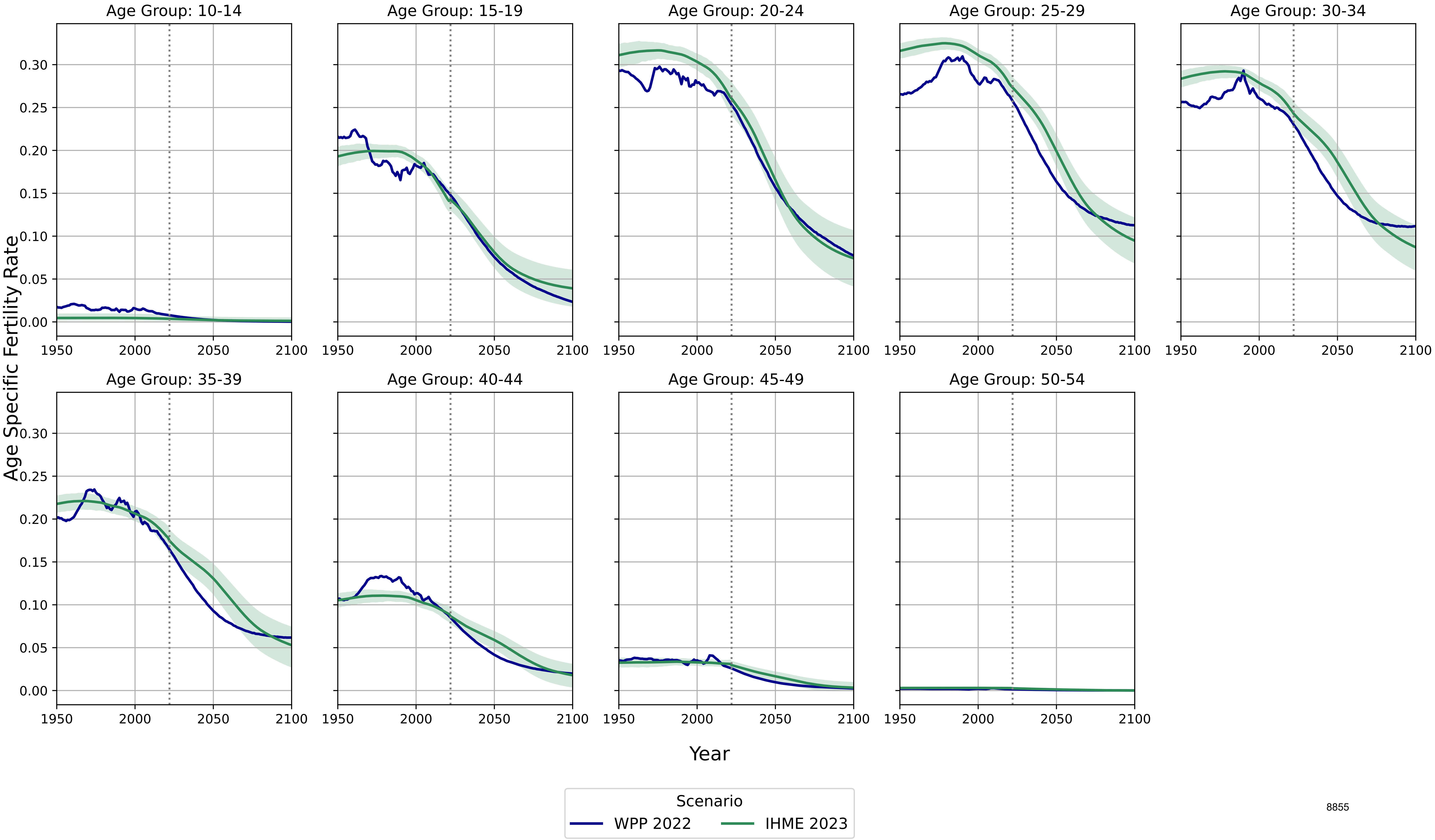

Supplemental Results Figure S8: Five-year interval ASFR from IHME reference scenario and the UNPD medium variant scenario by location, 1950-2100

Mauritania

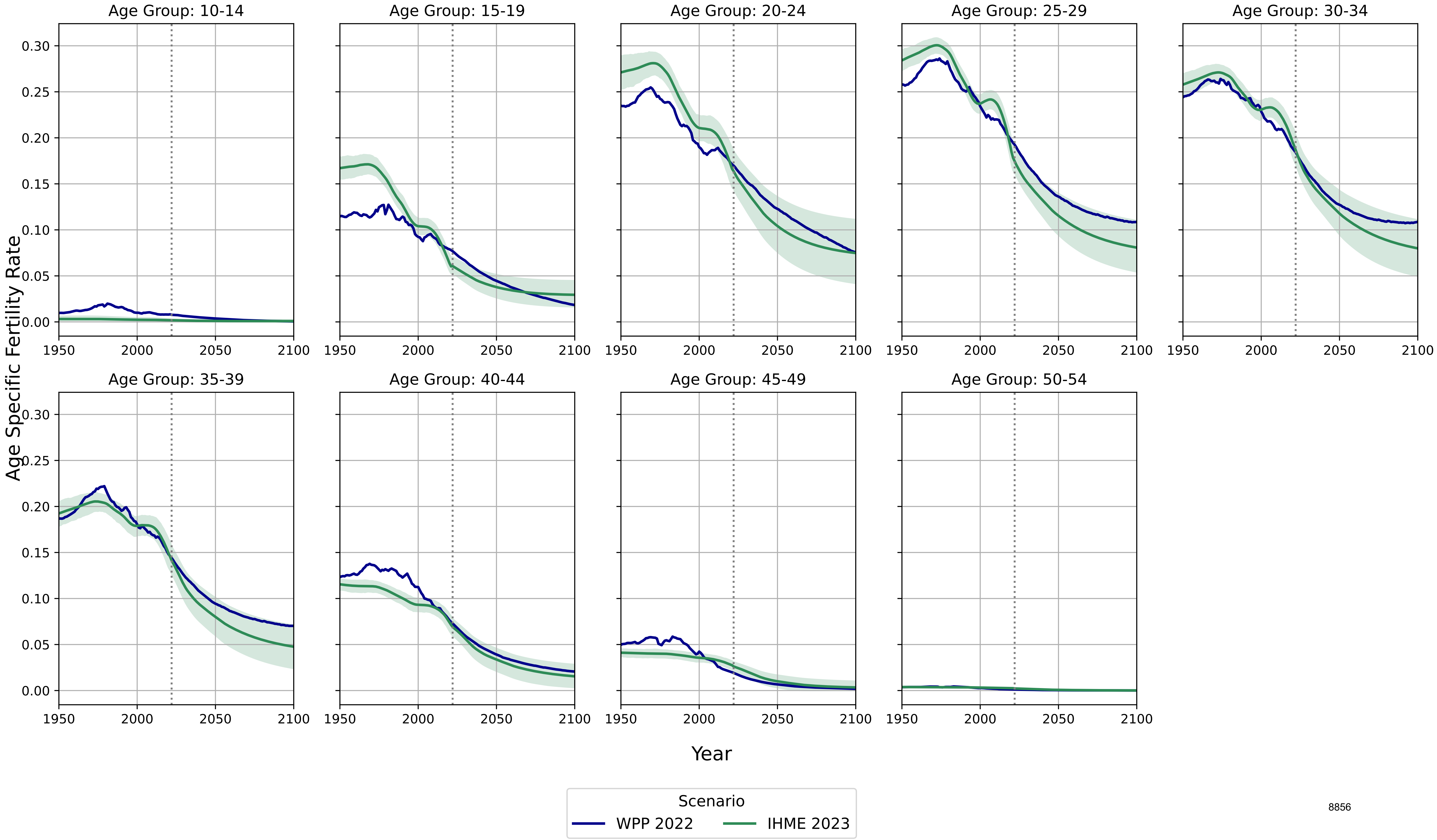

Supplemental Results Figure S8: Five-year interval ASFR from IHME reference scenario and the UNPD medium variant scenario by location, 1950-2100

Niger

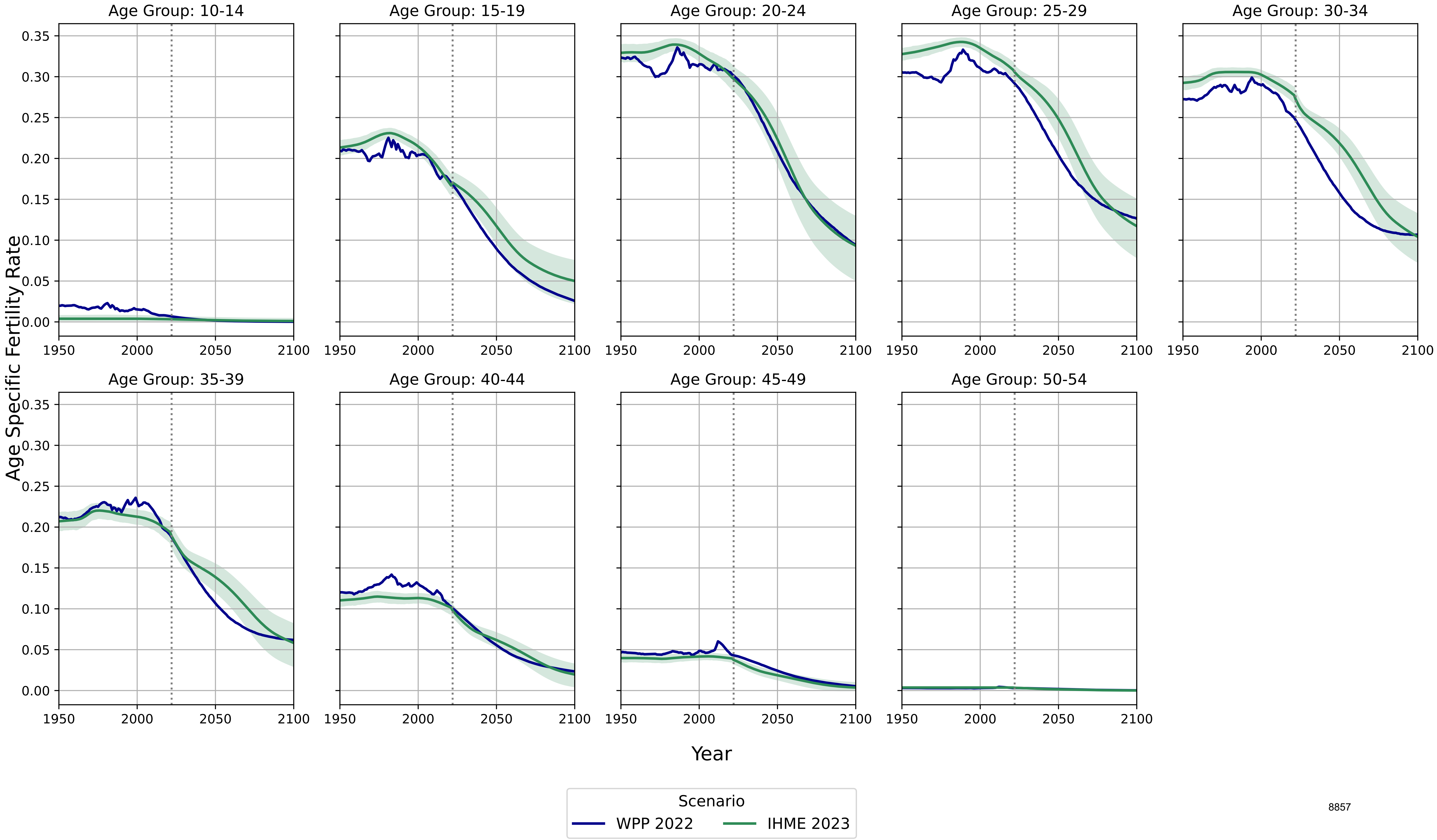

Supplemental Results Figure S8: Five-year interval ASFR from IHME reference scenario and the UNPD medium variant scenario by location, 1950-2100

Nigeria

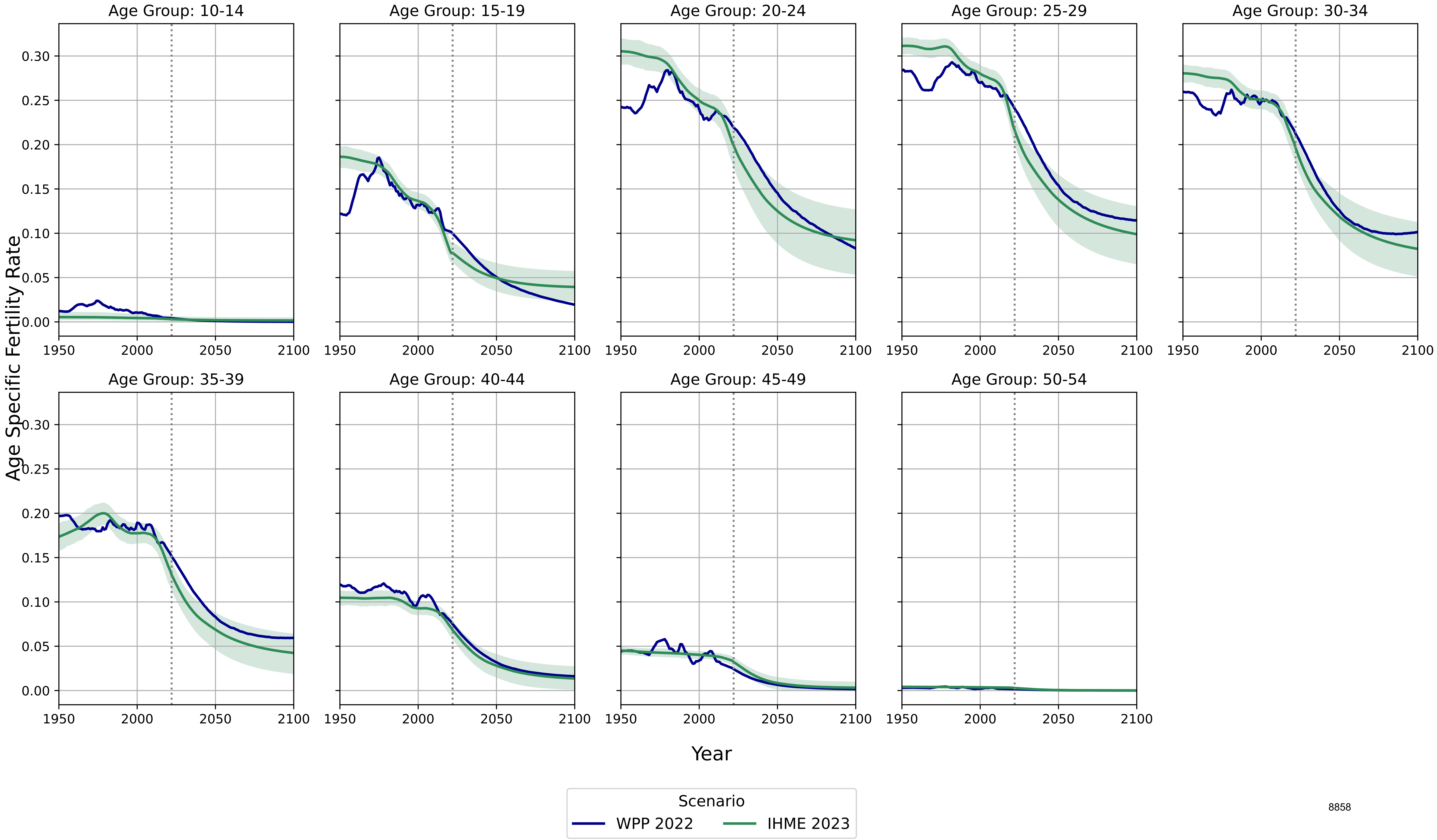

Supplemental Results Figure S8: Five-year interval ASFR from IHME reference scenario and the UNPD medium variant scenario by location, 1950-2100

São Tomé and Príncipe

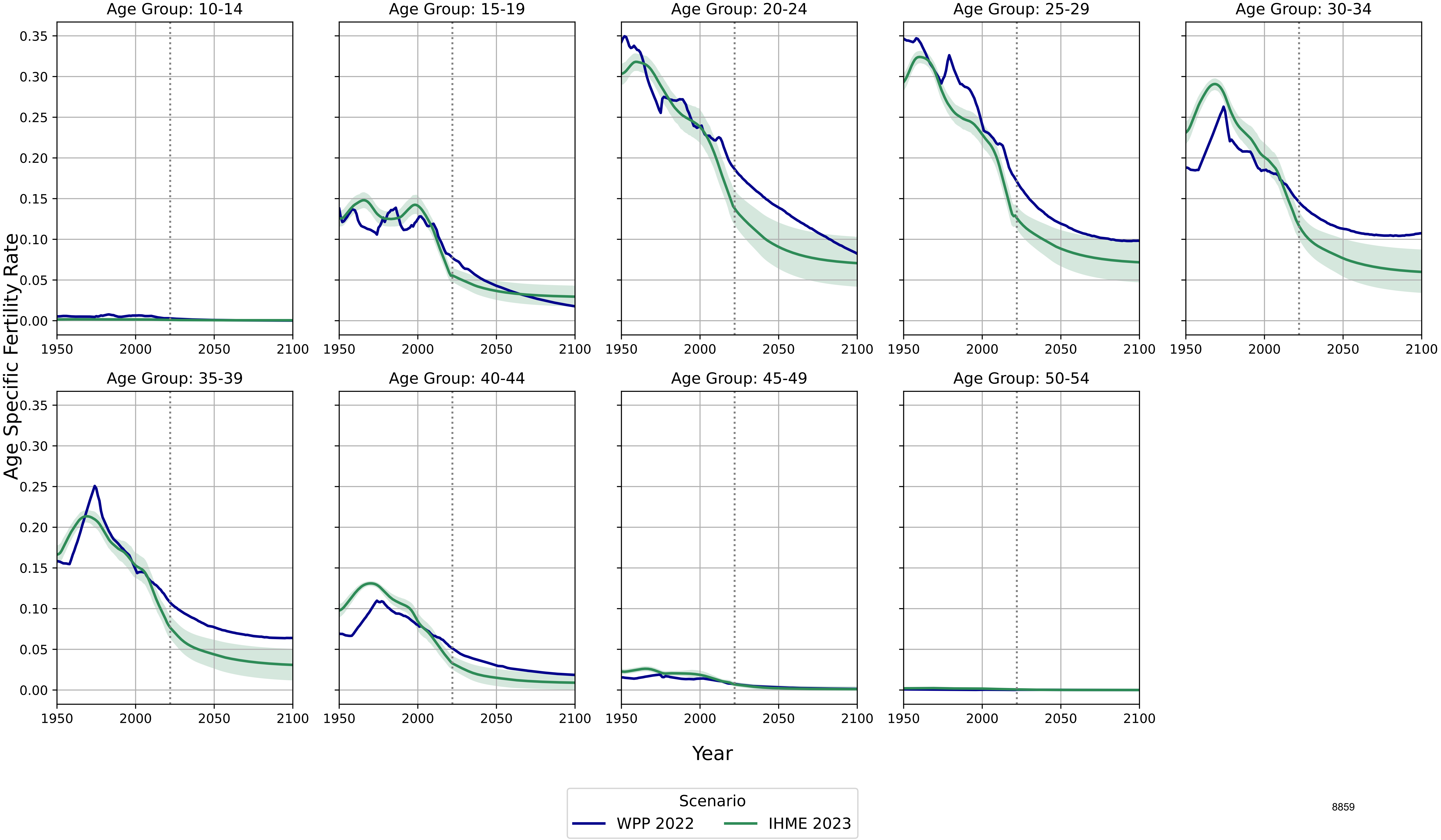

Supplemental Results Figure S8: Five-year interval ASFR from IHME reference scenario and the UNPD medium variant scenario by location, 1950-2100

Senegal

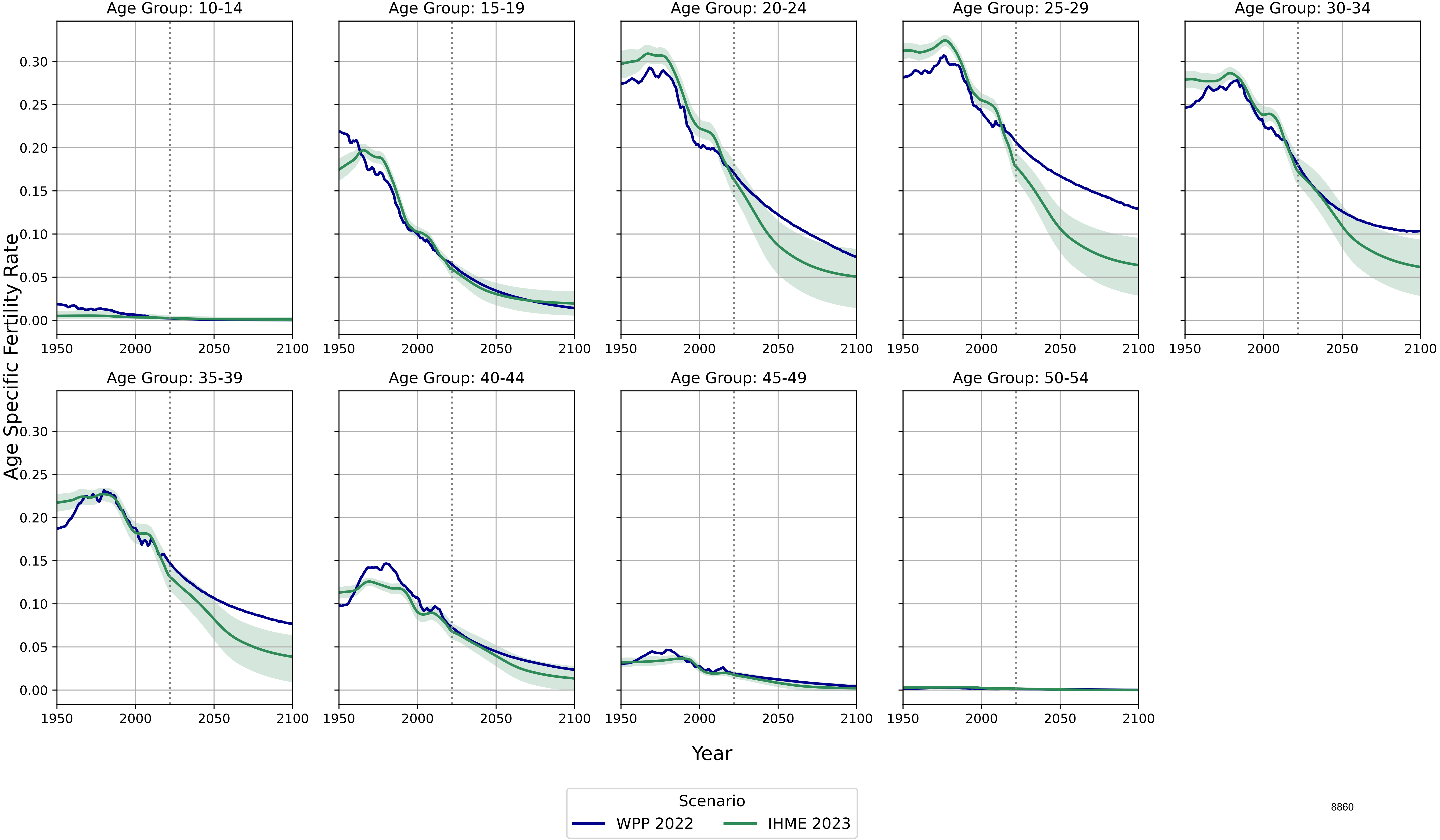

Supplemental Results Figure S8: Five-year interval ASFR from IHME reference scenario and the UNPD medium variant scenario by location, 1950-2100

Sierra Leone

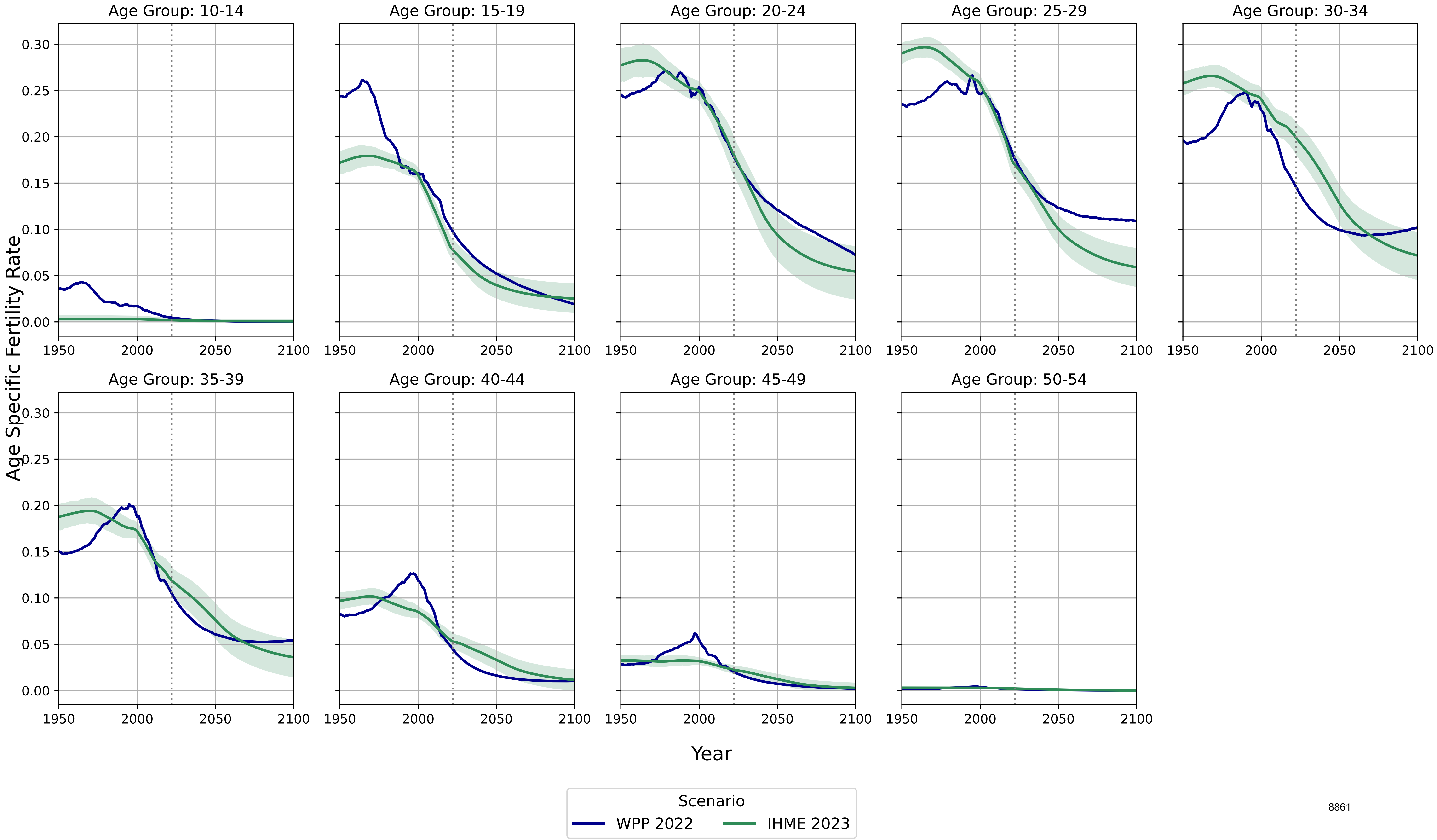

Supplemental Results Figure S8: Five-year interval ASFR from IHME reference scenario and the UNPD medium variant scenario by location, 1950-2100

Togo

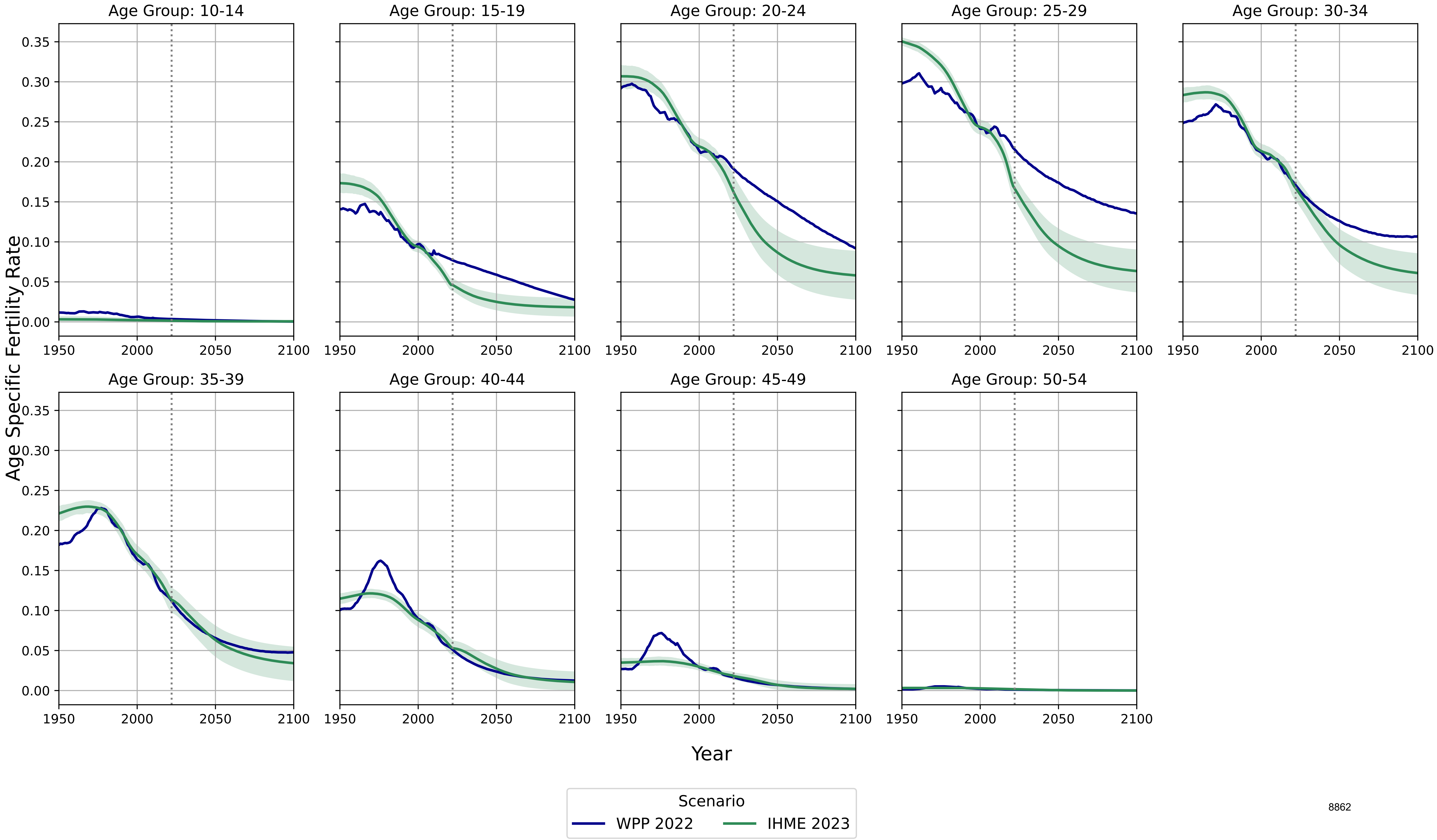

Supplemental Results Table S1a: Livebirths (in thousands) by location, both sexes combined, 1950, 1960, 1970, 1980, 1990, 2000, 2010, 2019, & 2021

Location	1950	1960	1970	1980	1990	2000	2010	2019	2021
Global	92700 (88700 to 96600)	106000 (103000 to 109000)	115000 (113000 to 118000)	122000 (119000 to 125000)	132000 (129000 to 135000)	130000 (127000 to 133000)	138000 (134000 to 141000)	134000 (128000 to 141000)	129000 (121000 to 138000)
Central Europe, eastern Europe, and central Asia	7450 (7230 to 7700)	7360 (7260 to 7470)	6260 (6130 to 6370)	7110 (7020 to 7210)	6630 (6540 to 6730)	4600 (4510 to 4680)	5660 (5550 to 5760)	5150 (5000 to 5300)	4910 (4640 to 5200)
Central Asia	1020 (986 to 1050)	1350 (1320 to 1380)	1280 (1210 to 1360)	1680 (1640 to 1710)	1970 (1940 to 2000)	1470 (1430 to 1500)	1870 (1820 to 1920)	2090 (2030 to 2160)	2070 (1990 to 2160)
Armenia	49 (46·8 to 51·1)	70·9 (68·4 to 73·5)	57 (52·8 to 61·9)	76·5 (73·2 to 79·9)	76·7 (73·5 to 79·9)	42·7 (40·8 to 44·7)	46·6 (44·1 to 49)	38·3 (35·4 to 41·1)	35 (31·6 to 38·5)
Azerbaijan	108 (100 to 115)	166 (161 to 172)	149 (142 to 156)	162 (155 to 170)	189 (181 to 196)	134 (127 to 141)	175 (165 to 185)	149 (136 to 162)	139 (124 to 155)
Georgia	84·2 (78·4 to 90·3)	102 (98·3 to 106)	86·8 (76·5 to 97·9)	91·8 (86·3 to 96·9)	85 (82·3 to 87·9)	49·6 (47·5 to 51·8)	54·1 (52·2 to 56·1)	48·9 (46·8 to 51·2)	45·2 (42·4 to 48·1)
Kazakhstan	254 (244 to 264)	359 (352 to 366)	329 (297 to 363)	365 (354 to 377)	374 (363 to 386)	222 (212 to 231)	368 (355 to 381)	416 (402 to 429)	425 (401 to 449)
Kyrgyzstan	57·9 (55·6 to 60·2)	76·1 (73·2 to 79·1)	77·6 (70 to 85·6)	112 (107 to 116)	133 (129 to 137)	108 (104 to 112)	162 (156 to 167)	166 (156 to 175)	159 (145 to 175)
Mongolia	30·2 (28·4 to 32·1)	36·6 (34·4 to 38·7)	46·2 (44·5 to 47·9)	61·9 (59·7 to 64·1)	71·7 (69·2 to 74)	53·4 (50·8 to 56·3)	73·1 (69·6 to 76·6)	82·1 (76·1 to 88·3)	80 (72·4 to 88)
Tajikistan	86·2 (82·6 to 89·9)	111 (107 to 114)	110 (105 to 114)	160 (155 to 164)	211 (206 to 217)	197 (188 to 205)	249 (237 to 260)	287 (271 to 304)	286 (268 to 307)
Turkmenistan	48·7 (46·7 to 50·5)	62·7 (60·2 to 65)	67·3 (62·9 to 72·1)	96·4 (92·9 to 99·8)	124 (120 to 129)	110 (105 to 116)	109 (103 to 116)	112 (102 to 123)	110 (99·4 to 123)
Uzbekistan	299 (281 to 317)	365 (344 to 385)	361 (338 to 387)	552 (538 to 564)	706 (691 to 720)	550 (531 to 569)	634 (600 to 670)	794 (753 to 834)	793 (734 to 854)
Central Europe	2340 (2280 to 2400)	2000 (1970 to 2030)	1950 (1920 to 1970)	2090 (2060 to 2110)	1710 (1690 to 1740)	1210 (1190 to 1230)	1210 (1190 to 1230)	1100 (1070 to 1120)	1040 (954 to 1130)
Albania	48 (45·9 to 50)	65 (63·8 to 66·2)	69·3 (66·6 to 71·9)	71·4 (68·8 to 74·3)	78·8 (76·3 to 81·4)	52 (49·5 to 54·9)	34·4 (32·4 to 36·5)	29·2 (26·7 to 31·7)	27·9 (24·7 to 31·4)
Bosnia and Herzegovina	92·6 (83·8 to 102)	113 (102 to 125)	85·9 (79 to 92·6)	79 (70·7 to 87·8)	66·5 (63·1 to 69·9)	39·6 (37·9 to 41·2)	33·3 (31·8 to 34·9)	28·2 (26·6 to 29·9)	26·7 (24·2 to 29·4)
Bulgaria	168 (160 to 176)	138 (135 to 142)	137 (134 to 141)	128 (125 to 131)	100 (97·8 to 103)	70·1 (68 to 72·4)	74·6 (72·4 to 76·8)	61·1 (58·7 to 63·8)	58·1 (53·7 to 62·4)
Croatia	90·2 (87 to 93·5)	75·3 (73·6 to 77)	62·8 (61·4 to 64·3)	67·4 (65·9 to 69·2)	53·3 (51·9 to 54·8)	42·6 (41·4 to 43·9)	42·6 (41·4 to 43·9)	36·2 (34·8 to 37·6)	34·8 (31 to 38·9)
Czechia	187 (179 to 195)	129 (126 to 132)	150 (146 to 154)	152 (149 to 156)	128 (126 to 131)	90·8 (88·1 to 93·8)	114 (111 to 117)	112 (109 to 117)	106 (95·4 to 118)
Hungary	193 (183 to 204)	143 (139 to 147)	151 (147 to 155)	147 (143 to 151)	125 (122 to 129)	96·1 (92·8 to 99·3)	91·3 (88·5 to 94·4)	91 (86·5 to 95·7)	87·9 (78·5 to 98·4)
Montenegro	12·5 (11·8 to 13·2)	12·9 (12·5 to 13·4)	10·8 (10·5 to 11·1)	10·5 (10·1 to 10·9)	9·6 (9·1 to 10)	8·7 (8·4 to 9)	7·6 (7·5 to 7·8)	7·2 (6·9 to 7·4)	7 (6·5 to 7·4)
North Macedonia	37·6 (34 to 41·5)	38·8 (34·9 to 43·1)	38·2 (35·6 to 40·8)	39·9 (36 to 44)	34·9 (34·1 to 35·6)	27·8 (27 to 28·5)	23·5 (22·7 to 24·2)	19·9 (19·3 to 20·6)	18·7 (17·7 to 19·8)
Poland	757 (737 to 777)	648 (636 to 660)	548 (536 to 559)	693 (680 to 706)	547 (535 to 559)	369 (360 to 378)	402 (394 to 411)	371 (359 to 383)	342 (305 to 381)
Romania	415 (391 to 442)	339 (327 to 351)	416 (404 to 427)	387 (376 to 399)	297 (286 to 308)	222 (213 to 230)	209 (201 to 218)	186 (177 to 195)	178 (164 to 193)
Serbia	184 (179 to 188)	162 (158 to 165)	147 (144 to 150)	158 (153 to 162)	143 (139 to 146)	104 (101 to 107)	76·8 (66·2 to 88·7)	63·4 (61 to 66·1)	61·6 (56·8 to 66·7)
Slovakia	99·6 (97·2 to 102)	87·3 (86 to 88·5)	80·8 (79·5 to 82·1)	94·6 (92·9 to 96·3)	79·1 (77·6 to 80·5)	52·8 (51·7 to 54)	60·6 (59·2 to 61·9)	57·7 (56·4 to 59)	56·3 (52·9 to 59·8)
Slovenia	32·3 (28·8 to 36·2)	28·1 (27·4 to 28·8)	27·7 (27·2 to 28·2)	29·5 (28·8 to 30·1)	22 (21·4 to 22·6)	17·8 (17·3 to 18·2)	22·2 (21·7 to 22·8)	19·3 (18·8 to 19·9)	18·8 (17·6 to 20)
Eastern Europe	4100 (3940 to 4290)	4010 (3940 to 4080)	3030 (2960 to 3090)	3350 (3290 to 3420)	2950 (2890 to 3010)	1920 (1860 to 1980)	2580 (2510 to 2640)	1960 (1870 to 2040)	1790 (1650 to 1950)
Belarus	192 (182 to 203)	197 (190 to 204)	148 (144 to 154)	156 (151 to 162)	139 (134 to 144)	92·1 (87·6 to 96·4)	110 (105 to 114)	89·7 (81·8 to 97·9)	82·5 (70·6 to 95·6)
Estonia	20·1 (19·1 to 21·2)	20·1 (19·5 to 20·7)	21·5 (21 to 22·1)	22·5 (21·9 to 23·1)	21 (20·5 to 21·5)	12·8 (12·4 to 13·2)	15·2 (14·8 to 15·7)	13·8 (13·2 to 14·4)	13·1 (12·2 to 14)
Latvia	32·9 (30·7 to 35·5)	35·7 (35 to 36·5)	35·2 (34·4 to 36·1)	35·6 (34·9 to 36·4)	36·2 (35·3 to 37·1)	20 (19·5 to 20·5)	18·8 (18·3 to 19·4)	18·4 (17·9 to 19)	16·8 (14·9 to 18·9)
Lithuania	58·6 (55·7 to 62)	61·5 (59·8 to 63·1)	55·4 (53·9 to 57·2)	51·3 (49·9 to 52·6)	55·7 (54·4 to 57·3)	33·3 (32·1 to 34·4)	31·6 (30·4 to 32·7)	26·3 (25·2 to 27·5)	23·6 (21·9 to 25·4)
Moldova	84·7 (81·4 to 88)	84·4 (82·1 to 86·8)	66 (59 to 73·4)	86·1 (83·4 to 88·9)	79·2 (76·7 to 81·8)	41·4 (40·1 to 42·8)	40·4 (38·9 to 41·8)	30·8 (28·4 to 33·6)	28·4 (25·4 to 32)
Russia	2820 (2670 to 3000)	2720 (2650 to 2780)	1930 (1870 to 1990)	2240 (2180 to 2300)	1960 (1910 to 2010)	1340 (1290 to 1380)	1850 (1790 to 1910)	1470 (1390 to 1550)	1350 (1250 to 1460)
Ukraine	891 (853 to 930)	895 (869 to 920)	768 (748 to 790)	761 (739 to 783)	662 (642 to 682)	382 (370 to 394)	510 (494 to 527)	308 (295 to 322)	278 (247 to 312)
High income	13600 (13300 to 14000)	14500 (14400 to 14700)	13900 (13700 to 14000)	12500 (12300 to 12600)	12400 (12300 to 12600)	11800 (11700 to 12000)	11900 (11800 to 12100)	10600 (10400 to 10700)	10400 (9730 to 11100)
Australasia	252 (246 to 258)	302 (299 to 306)	331 (326 to 335)	278 (273 to 284)	315 (308 to 322)	300 (295 to 306)	364 (358 to 370)	357 (350 to 365)	358 (325 to 394)
Australia	202 (197 to 208)	239 (235 to 242)	268 (264 to 273)	228 (223 to 233)	255 (249 to 261)	244 (239 to 249)	301 (295 to 306)	299 (291 to 306)	299 (268 to 332)
New Zealand	49·7 (48·7 to 50·8)	63·5 (62·6 to 64·3)	62·4 (61·5 to 63·4)	50·5 (49·4 to 51·6)	59·7 (58·4 to 60·9)	56·6 (55·5 to 57·8)	62·7 (61·3 to 64·2)	58·2 (56·9 to 59·5)	58·6 (55·2 to 62)
High-income Asia Pacific	3060 (2950 to 3170)	2670 (2590 to 2760)	3010 (2920 to 3090)	2470 (2400 to 2540)	1960 (1910 to 2020)	1820 (1770 to 1880)	1600 (1550 to 1650)	1230 (1190 to 1260)	1170 (1080 to 1280)
Brunei	2·7 (2·6 to 2·7)	4 (3·9 to 4)	4·7 (4·6 to 4·9)	5·8 (5·4 to 6·1)	7 (6·6 to 7·5)	7·2 (6·6 to 8)	6·6 (6·1 to 7·1)	6·4 (5·9 to 7)	6·4 (5·6 to 7·3)
Japan	2190 (2090 to 2290)	1590 (1540 to 1650)	1970 (1900 to 2040)	1570 (1520 to 1640)	1220 (1180 to 1270)	1180 (1130 to 1220)	1070 (1030 to 1120)	867 (832 to 900)	838 (754 to 933)
Aichi	92·2 (87·9 to 96·6)	79 (75·6 to 82·3)	117 (112 to 122)	87·2 (83·6 to 91·2)	71 (68 to 74·3)	73·2 (70 to 76·7)	69·8 (66·7 to 73)	57·5 (54·9 to 60)	55·9 (50·3 to 62·3)
Akita	30·9 (29·4 to 32·3)	21·6 (20·7 to 22·5)	19·6 (18·6 to 20·5)	15·9 (15·2 to 16·7)	11·1 (10·6 to 11·6)	8·9 (8·5 to 9·4)	6·9 (6·6 to 7·2)	4·8 (4·5 to 5)	4·4 (4 to 4·9)
Aomori	34 (32·3 to 35·8)	24·8 (23·8 to 25·9)	25·8 (24·7 to 27)	21·3 (20·5 to 22·2)	15 (14·4 to 15·7)	12·9 (12·4 to 13·5)	9·7 (9·2 to 10·2)	7·2 (6·9 to 7·5)	6·7 (6 to 7·5)
Chiba	56·8 (54·2 to 59·5)	38·1 (36·6 to 39·6)	70·4 (67·3 to 73·4)	67·3 (64·3 to 70·3)	53·4 (51 to 55·9)	54·6 (52·2 to 57·1)	51·4 (49·1 to 53·6)	41 (39·3 to 42·6)	40·3 (36·2 to 45)
Ehime	38·7 (36·9 to 40·6)	23·8 (22·8 to 24·9)	24·6 (23·6 to 25·6)	19·6 (18·8 to 20·5)	14·7 (14 to 15·3)	13 (12·4 to 13·7)	11·5 (11 to 12)	8·5 (8·2 to 8·8)	8 (7·2 to 8·9)
Fukui	19·5 (18·6 to 20·4)	12·2 (11·7 to 12·8)	13 (12·4 to 13·7)	10·5 (10 to 11)	8·6 (8·2 to 9)	7·9 (7·5 to 8·4)	6·9 (6·5 to 7·2)	5·4 (5·2 to 5·7)	5·2 (4·7 to 5·8)
Fukuoka	89·4 (85·3 to 93·7)	66·2 (63·4 to 69·2)	72·5 (69·7 to 75·4)	64·4 (62 to 67)	48·5 (46·4 to 50·5)	46·8 (44·9 to 48·8)	46·8 (44·7 to 48·8)	39·8 (38·2 to 41·4)	38·6 (34·6 to 43·2)
Fukushima	56·8 (54·1 to 59·5)	35·1 (33·6 to 36·5)	32·5 (31·1 to 33·9)	29·1 (27·9 to 30·3)	22·9 (21·8 to 23·8)	20·2 (19·4 to 21·1)	15·8 (15 to 16·6)	11·7 (11·2 to 12·2)	11·2 (10 to 12·5)
Gifu	38·8 (37 to 40·5)	28·1 (26·9 to 29·2)	33·1 (31·6 to 34·7)	25·6 (24·5 to 26·8)	20·3 (19·2 to 21·3)	19·9 (19 to 20·9)	17·1 (16·3 to 17·9)	12·7 (12·2 to 13·3)	12·1 (10·8 to 13·5)
Gunma	42·5 (40·5 to 44·4)	26·2 (24·9 to 27·5)	30·7 (28·6 to 32·9)	25·2 (24·2 to 26·2)	19·7 (18·9 to 20·6)	19·2 (18·4 to 20)	16 (15·3 to 16·6)	12 (11·5 to 12·5)	11·5 (10·3 to 12·9)
Hiroshima	54·8 (52·3 to 57·3)	36·2 (34·7 to 37·8)	46·6 (44·5 to 48·8)	37 (35·6 to 38·7)	28·6 (27·4 to 29·9)	27·2 (26 to 28·6)	25·6 (24·3 to 26·9)	20·2 (19·2 to 21·1)	19·3 (17·3 to 21·6)
Hokkaidō	107 (102 to 112)	81·4 (78·5 to 84·3)	95·5 (91·4 to 99·3)	75·4 (72·5 to 78·3)	54·3 (52 to 56·5)	46·9 (44·7 to 49)	40·2 (38·6 to 41·9)	30·7 (29·3 to 32·1)	29·2 (26 to 32·8)
Hyōgo	87·3 (83·4 to 91·3)	67·5 (65·1 to 69·8)	92·3 (88·5 to 96)	68·5 (65·6 to 71·2)	53·5 (51·3 to 55·8)	53·2 (50·9 to 55·7)	48·1 (46 to 50·2)	38 (36·3 to 39·6)	36·4 (32·8 to 40·6)
Ibaraki	55·6 (53 to 58·2)	33 (31·9 to 34·1)	38·5 (36·9 to 40)	36·4 (35 to 37·8)	28·8 (27·5 to 30)	28 (26·8 to 29·3)	23·9 (22·9 to 24·9)	18·1 (17·3 to 18·8)	17·1 (15·3 to 19·2)
Ishikawa	24·3 (23·2 to 25·4)	16 (15·4 to 16·6)	19·5 (18·5 to 20·4)	14·9 (14·3 to 15·6)	11·4 (10·9 to 11·9)	11·3 (10·7 to 11·9)	9·8 (9·4 to 10·3)	7·9 (7·5 to 8·3)	7·6 (6·8 to 8·5)
Iwate	38·6 (36·6 to 40·5)	26·5 (25·4 to 27·6)	24·6 (23·6 to 25·7)	19·2 (18·5 to 20·1)	14·3 (13·7 to 14·9)	12·3 (11·8 to 12·8)	9·7 (9·3 to 10·2)	7·1 (6·8 to 7·3)	6·5 (5·9 to 7·3)
Kagawa	23·1 (22·1 to 24·2)	14·3 (13·7 to 14·9)	15·8 (15·1 to 16·5)	12·8 (12·3 to 13·4)	9·6 (9·2 to 9·9)	9·6 (9·1 to 10·1)	8·5 (8·1 to 8·9)	6·6 (6·2 to 6·9)	6·3 (5·6 to 7)

Kagoshima	51·3 (49 to 53·7)	31·4 (30·1 to 32·7)	25·8 (24·2 to 27·4)	24·5 (23·6 to 25·6)	18·9 (18·1 to 19·7)	16 (15·3 to 16·6)	15·2 (14·6 to 15·9)	12 (11·5 to 12·5)	11·2 (10·1 to 12·6)
Kanagawa	67 (64 to 70·1)	63·7 (60·9 to 66·6)	116 (111 to 121)	94·8 (90·9 to 99)	79·4 (75·9 to 82·8)	82 (78·1 to 85·8)	78 (74·7 to 81·3)	62·9 (60·4 to 65·3)	61·3 (55·3 to 68·3)
Kōchi	22·8 (21·6 to 23·9)	12·6 (12 to 13·1)	12·8 (12·2 to 13·3)	10·1 (9·6 to 10·5)	7·4 (7·1 to 7·8)	6·7 (6·3 to 7)	5·5 (5·2 to 5·7)	4·3 (4·1 to 4·4)	4 (3·6 to 4·4)
Kumamoto	46·6 (44·5 to 48·8)	29·3 (28 to 30·6)	27 (25·9 to 28·2)	24·3 (23·3 to 25·4)	19·2 (18·1 to 20·2)	17·1 (16·4 to 17·9)	16·4 (15·6 to 17·1)	13·4 (12·9 to 13·9)	12·7 (11·4 to 14·1)
Kyōto	43·4 (41·5 to 45·4)	31·8 (30·3 to 33·2)	41·2 (39·4 to 43)	32 (30·8 to 33·4)	24 (22·9 to 25)	23·5 (22·4 to 24·6)	21·1 (20·1 to 22)	17 (16·3 to 17·6)	16·2 (14·6 to 18)
Mie	37·1 (35·3 to 38·8)	24·7 (23·6 to 25·7)	27·8 (26·6 to 29)	21·1 (20·2 to 22·1)	17·7 (17 to 18·5)	17·4 (16·6 to 18·2)	15·3 (14·7 to 16·1)	11·7 (11·1 to 12·3)	11·2 (10 to 12·5)
Miyagi	42·4 (40·5 to 44·4)	29·6 (28·3 to 30·9)	32 (30·6 to 33·4)	30·7 (29·5 to 32·2)	23·3 (22·2 to 24·3)	21·9 (20·9 to 22·8)	19·1 (18·2 to 19·9)	15 (14·4 to 15·7)	14·4 (12·9 to 16·1)
Miyazaki	30·1 (28·7 to 31·6)	19·5 (18·6 to 20·3)	18·4 (17·6 to 19·2)	17 (16·3 to 17·8)	12·4 (11·9 to 12·9)	10·9 (10·5 to 11·5)	10·2 (9·8 to 10·7)	8 (7·6 to 8·4)	7·5 (6·7 to 8·4)
Nagano	52·6 (50·4 to 54·8)	33·5 (32·2 to 34·9)	34·3 (32·9 to 35·7)	26·5 (25·4 to 27·8)	21·4 (20·4 to 22·2)	20·9 (20 to 21·8)	17·4 (16·6 to 18·2)	13·4 (12·8 to 14)	12·8 (11·5 to 14·3)
Nagasaki	47·7 (45·5 to 49·9)	29·6 (28·6 to 30·7)	26·8 (25·7 to 27·9)	22·4 (21·6 to 23·4)	16·8 (16 to 17·4)	13·9 (13·2 to 14·5)	12 (11·5 to 12·5)	9·5 (9·2 to 9·9)	8·9 (8 to 10)
Nara	18·2 (17·4 to 19)	12 (11·5 to 12·5)	16·6 (15·9 to 17·3)	16 (15·4 to 16·7)	13·2 (12·7 to 13·8)	13 (12·4 to 13·6)	10·8 (10·3 to 11·2)	8·2 (7·9 to 8·6)	7·8 (7 to 8·7)
Niigata	64·6 (61·7 to 67·5)	40·7 (39 to 42·5)	40·5 (38·8 to 42·3)	32·6 (31·4 to 34·1)	24·3 (23·2 to 25·4)	21·4 (20·5 to 22·4)	18 (17·1 to 19)	13·6 (13 to 14·2)	12·7 (11·4 to 14·2)
Ōita	30·9 (29·4 to 32·3)	19·3 (18·5 to 20·1)	19 (18·2 to 19·9)	16·1 (15·4 to 16·8)	11·9 (11·4 to 12·3)	10·8 (10·3 to 11·3)	10·1 (9·6 to 10·5)	7·8 (7·4 to 8·1)	7·3 (6·5 to 8·2)
Okayama	41·2 (39·3 to 43·1)	26·7 (25·5 to 27·8)	30·8 (29·4 to 32·3)	24·2 (23·3 to 25·4)	19 (18·2 to 19·7)	18·8 (17·8 to 19·8)	16·7 (16 to 17·5)	13·8 (13·2 to 14·4)	13·2 (11·8 to 14·8)
Okinawa	29 (27·2 to 30·8)	18·6 (17·8 to 19·4)	21·3 (20·4 to 22·2)	19·9 (19·1 to 20·7)	17·5 (16·6 to 18·3)	16·8 (16 to 17·6)	17 (16·2 to 17·8)	15·1 (14·4 to 15·7)	14·6 (13 to 16·4)
Ōsaka	105 (100 to 110)	100 (97·1 to 104)	162 (156 to 169)	111 (107 to 116)	86·6 (83·3 to 90)	86·8 (82·8 to 90·8)	75·3 (72·2 to 78·6)	62·7 (60·3 to 65·1)	61·8 (55·6 to 69)
Saga	25·3 (24·1 to 26·4)	15·4 (14·8 to 16)	13·9 (13·3 to 14·4)	12·3 (11·8 to 12·8)	9·5 (9 to 9·9)	8·5 (8·2 to 8·9)	7·6 (7·3 to 8)	6·2 (5·9 to 6·4)	5·9 (5·3 to 6·6)
Saitama	59 (56·2 to 61·7)	42·7 (40·9 to 44·4)	87·7 (84·2 to 91·4)	76·2 (73·4 to 79·3)	63·8 (61·2 to 66·3)	65·9 (63 to 69)	59·4 (56·9 to 62·1)	48·6 (46·7 to 50·5)	47·7 (42·9 to 53·1)
Shiga	23·4 (22·3 to 24·4)	14·4 (13·9 to 15)	15·4 (14·7 to 16)	15·8 (15·2 to 16·6)	13·3 (12·8 to 13·9)	14 (13·4 to 14·6)	13·4 (12·8 to 14)	10·7 (10·2 to 11·2)	10·2 (9·2 to 11·4)
Shimane	23·6 (22·5 to 24·6)	14·1 (13·5 to 14·8)	12·2 (11·4 to 13·1)	9·8 (9·4 to 10·2)	7·5 (7·1 to 7·8)	6·5 (6·1 to 6·8)	5·7 (5·5 to 6)	4·6 (4·4 to 4·9)	4·5 (4 to 5)
Shizuoka	65·9 (62·9 to 68·9)	48·3 (46·2 to 50·4)	60·6 (58 to 63·3)	47·8 (45·7 to 50·2)	37·2 (35·8 to 38·6)	35·5 (33·9 to 37·3)	31·9 (30·4 to 33·4)	23·5 (22·4 to 24·7)	22·4 (20·1 to 25·1)
Tochigi	41·8 (39·9 to 43·8)	24·9 (24 to 25·7)	28·7 (27·6 to 29·9)	25·8 (24·8 to 26·9)	19·9 (19 to 20·8)	18·8 (17·9 to 19·6)	16·5 (15·9 to 17·2)	12·5 (12 to 13)	11·7 (10·5 to 13·1)
Tokushima	20·6 (19·6 to 21·6)	12·6 (12·1 to 13·1)	12·3 (11·7 to 12·8)	10·5 (10·1 to 11)	7·8 (7·5 to 8·2)	7·1 (6·7 to 7·4)	5·9 (5·6 to 6·2)	4·7 (4·4 to 4·9)	4·3 (3·9 to 4·9)
Tōkyō	151 (144 to 158)	167 (157 to 177)	213 (203 to 223)	140 (135 to 146)	104 (99·6 to 109)	98·8 (94·7 to 103)	108 (104 to 113)	102 (98 to 107)	103 (92·7 to 114)
Tottori	15·6 (14·9 to 16·3)	9·5 (9·1 to 9·9)	9·1 (8·7 to 9·4)	8·1 (7·8 to 8·5)	6·3 (6 to 6·6)	5·5 (5·2 to 5·8)	4·9 (4·7 to 5·1)	4 (3·8 to 4·1)	3·7 (3·4 to 4·2)
Toyama	23·9 (22·8 to 25)	16·3 (15·6 to 17·1)	19·1 (18·1 to 20)	13·5 (12·9 to 14·2)	10 (9·6 to 10·4)	10 (9·6 to 10·6)	8·2 (7·8 to 8·7)	6·5 (6·2 to 6·9)	6·2 (5·6 to 7)
Wakayama	26·2 (25 to 27·5)	16·1 (15·4 to 16·8)	18 (17·1 to 18·8)	13·4 (12·7 to 14)	10·3 (9·8 to 10·7)	9·4 (8·9 to 9·9)	7·6 (7·2 to 8)	5·9 (5·6 to 6·2)	5·6 (5 to 6·2)
Yamagata	34·4 (32·8 to 35·9)	22·1 (21·2 to 23)	19·1 (18·3 to 19·9)	16·5 (15·8 to 17·2)	12·6 (12·1 to 13·2)	10·8 (10·3 to 11·4)	8·6 (8·2 to 9·1)	6·5 (6·2 to 6·8)	6 (5·4 to 6·7)
Yamaguchi	37·7 (36 to 39·4)	25·1 (24 to 26·1)	25·9 (24·8 to 27)	19·7 (18·9 to 20·6)	14 (13·4 to 14·6)	13 (12·3 to 13·6)	11·4 (10·8 to 12)	8·6 (8·2 to 8·9)	8·1 (7·2 to 9)
Yamanashi	19·7 (18·8 to 20·5)	12·3 (11·7 to 12·8)	12·3 (11·8 to 12·8)	10 (9·7 to 10·5)	8·8 (8·3 to 9·2)	8·3 (7·9 to 8·7)	6·6 (6·4 to 6·9)	5·3 (5 to 5·5)	5·1 (4·5 to 5·6)
South Korea	821 (774 to 869)	1020 (952 to 1080)	982 (930 to 1030)	847 (822 to 873)	680 (660 to 701)	581 (560 to 603)	467 (449 to 486)	296 (280 to 312)	270 (246 to 294)
Singapore	48 (45·7 to 50·3)	59·7 (58·2 to 61·2)	46·2 (43·5 to 49)	42 (39·2 to 44·8)	53·9 (50·7 to 57·3)	58·3 (54·4 to 62·2)	57 (51·9 to 62·3)	57·3 (53·4 to 61·6)	55·5 (48·5 to 64)
High-income North America	4020 (3930 to 4120)	4700 (4650 to 4760)	3930 (3860 to 4010)	3950 (3870 to 4030)	4530 (4440 to 4630)	4370 (4290 to 4440)	4370 (4290 to 4440)	4040 (3970 to 4120)	4010 (3770 to 4280)
Canada	362 (353 to 371)	459 (452 to 465)	347 (340 to 354)	358 (350 to 365)	400 (392 to 407)	332 (325 to 340)	376 (369 to 384)	364 (355 to 373)	362 (324 to 401)
Greenland	1 (0·9 to 1)	1·6 (1·5 to 1·6)	1 (1 to 1·1)	1 (0·9 to 1)	1·3 (1·2 to 1·3)	0·9 (0·9 to 1)	0·8 (0·8 to 0·9)	0·8 (0·8 to 0·9)	0·8 (0·7 to 0·9)
USA	3660 (3570 to 3760)	4240 (4190 to 4300)	3580 (3510 to 3660)	3590 (3510 to 3660)	4130 (4040 to 4220)	4030 (3960 to 4110)	3990 (3910 to 4070)	3680 (3600 to 3760)	3650 (3450 to 3880)
Alabama	79·9 (78 to 82·1)	83·1 (81 to 85)	66·2 (64·5 to 67·9)	62·1 (60 to 64·3)	63·8 (61·9 to 65·5)	61·8 (59·9 to 63·7)	58·5 (56·8 to 60·2)	59·2 (57·5 to 61)	58 (54·1 to 61·9)
Alaska	3·3 (3·2 to 3·4)	6·7 (6·5 to 7)	7·2 (7 to 7·5)	9·7 (9·4 to 10)	11·8 (11·4 to 12·1)	10 (9·6 to 10·4)	11·2 (10·9 to 11·6)	9·2 (8·8 to 9·6)	9·1 (8·5 to 9·8)
Arizona	20·5 (20 to 21·1)	35·7 (34 to 37·7)	36·6 (35·4 to 37·9)	49·1 (46 to 52·1)	67·4 (65 to 69·8)	84·5 (81·8 to 87·3)	88·5 (84·6 to 92·8)	77·9 (75 to 80·6)	77·6 (72·7 to 82·9)
Arkansas	45·3 (44·1 to 46·5)	40·4 (39·2 to 41·6)	35·2 (34·3 to 36·2)	36·6 (35·3 to 37·9)	36·2 (35·2 to 37·2)	37·4 (36·2 to 38·6)	37·6 (36·4 to 38·7)	35·5 (34·2 to 36·7)	35·7 (33·2 to 38·2)
California	253 (246 to 260)	368 (350 to 388)	332 (318 to 347)	391 (365 to 417)	596 (576 to 616)	531 (513 to 550)	514 (496 to 532)	432 (417 to 450)	423 (396 to 450)
Colorado	29·3 (28·6 to 30·1)	39·9 (38·7 to 41·1)	40·3 (39 to 41·7)	51 (49·3 to 52·6)	53·4 (51·8 to 55)	66·2 (64·5 to 67·9)	66·1 (64·1 to 68·2)	58·4 (56·1 to 60·7)	62·5 (58·2 to 67·1)
Connecticut	47·8 (46·7 to 49·1)	56·5 (54·9 to 58·2)	47·2 (45·9 to 48·6)	39·4 (38·3 to 40·4)	49·7 (48·2 to 51·2)	43·1 (41·7 to 44·5)	38 (36·8 to 39·3)	34·9 (33·9 to 36)	35·5 (33·2 to 38)
Delaware	8·2 (8 to 8·5)	11·4 (10·9 to 12)	9·6 (9·2 to 10·1)	9 (8·4 to 9·6)	10·9 (10·5 to 11·3)	11 (10·5 to 11·5)	11·8 (11·3 to 12·3)	10·8 (10·2 to 11·3)	10·7 (10 to 11·5)
Washington, DC	20·4 (19·9 to 21)	17·3 (16·4 to 18·2)	13·1 (12·3 to 14)	9·8 (9·1 to 10·5)	11·3 (10·7 to 11·9)	7·8 (7·3 to 8·4)	8·5 (7·2 to 9·8)	9 (8·4 to 9·7)	9·1 (8·2 to 10)
Florida	63·3 (61·8 to 65)	108 (105 to 112)	114 (111 to 118)	134 (130 to 138)	196 (191 to 201)	204 (198 to 210)	215 (208 to 221)	219 (213 to 226)	216 (200 to 231)
Georgia	96·6 (94·2 to 99·2)	109 (104 to 115)	91·3 (88·1 to 94·4)	88·8 (84·2 to 93·5)	110 (107 to 114)	132 (128 to 137)	137 (131 to 144)	125 (121 to 129)	124 (116 to 133)
Hawaii	14·4 (14·1 to 14·8)	17·4 (16·9 to 17·9)	16 (15·5 to 16·4)	17·9 (17·4 to 18·5)	20·4 (19·8 to 20·9)	17·4 (16·7 to 18)	18·9 (18·2 to 19·5)	16·3 (15·8 to 16·9)	15·5 (14·5 to 16·7)
Idaho	14·5 (14·2 to 14·9)	16·4 (15·9 to 16·9)	14·2 (13·9 to 14·6)	19·9 (19·3 to 20·5)	16·6 (16·1 to 17·1)	20·3 (19·6 to 21)	22·3 (21·7 to 23)	22·1 (21·2 to 23)	22·3 (20·9 to 23·9)
Illinois	208 (203 to 214)	236 (229 to 242)	198 (193 to 204)	188 (182 to 193)	196 (191 to 202)	183 (178 to 188)	159 (154 to 164)	138 (134 to 143)	132 (122 to 141)
Indiana	91 (88·8 to 93·5)	110 (108 to 112)	96·3 (93·7 to 99·1)	86·7 (84·2 to 89·2)	86·7 (84·1 to 89·5)	85·9 (83·6 to 88·2)	84·2 (81·8 to 86·7)	79·4 (77·2 to 81·7)	79·5 (74·4 to 84·7)
Iowa	58·3 (56·9 to 59·9)	61·4 (59·9 to 63·1)	46·1 (44·8 to 47·5)	47·2 (45·6 to 48·7)	39·5 (38·3 to 40·7)	37·4 (36·1 to 38·7)	38·3 (37 to 39·7)	36·7 (35·6 to 37·8)	36·5 (34·3 to 39)
Kansas	41·1 (40·1 to 42·2)	47·6 (46·3 to 49)	36·7 (35·6 to 37·8)	41 (39·7 to 42·2)	38·7 (37·5 to 39·9)	38·9 (37·9 to 39·8)	40·9 (39·6 to 42·1)	34·9 (33·9 to 36)	34·6 (32·3 to 36·9)
Kentucky	71·4 (69·6 to 73·3)	72·1 (69·9 to 74·4)	59·9 (58·4 to 61·6)	58·5 (56·8 to 60·2)	55 (53·5 to 56·5)	55·2 (53·6 to 57·1)	53·6 (52 to 55·2)	51·8 (50·2 to 53·4)	51·9 (48·4 to 55·5)
Louisiana	75·7 (73·8 to 77·8)	90·8 (88·8 to 92·7)	72·9 (70·9 to 75)	82·7 (80·2 to 85)	73·2 (71·2 to 75·1)	66·7 (64·6 to 69·1)	62·6 (60·3 to 64·7)	57·3 (55·7 to 59·2)	57·1 (53·5 to 60·8)
Maine	21·6 (21 to 22·1)	22·8 (22·4 to 23·2)	17·6 (17·1 to 18·2)	16·4 (15·9 to 17)	17·2 (16·7 to 17·8)	13·6 (13·3 to 14)	12·8 (12·4 to 13·2)	11·4 (11·1 to 11·9)	11·9 (11·1 to 12·7)
Maryland	57·4 (55·9 to 58·9)	72·7 (71·2 to 74·3)	66·5 (64·5 to 68·5)	56·5 (54·6 to 58·4)	80 (77·5 to 82·6)	74·5 (72·3 to 76·7)	71·9 (69·7 to 74·3)	71 (69 to 73·1)	68·3 (63·7 to 73·1)
Massachusetts	110 (108 to 113)	114 (111 to 116)	89·6 (87·1 to 92·1)	73·3 (71·2 to 75·5)	90·9 (88 to 94·2)	81·4 (78·8 to 84·1)	73·3 (71·5 to 75·1)	69 (67 to 71·2)	69 (64·4 to 74·1)
Michigan	158 (155 to 163)	190 (187 to 194)	165 (160 to 170)	144 (139 to 148)	153 (148 to 157)	134 (131 to 137)	113 (110 to 116)	106 (103 to 108)	104 (97·7 to 112)
Minnesota	69·7 (68 to 71·6)	77·7 (75·6 to 79·8)	64·5 (62·9 to 66·2)	67·5 (65·5 to 69·6)	67·7 (65·7 to 69·7)	66·8 (65·1 to 68·6)	67·8 (66 to 69·5)	64·3 (62·5 to 66·1)	64·1 (59·9 to 68·6)

Mississippi	62·1 (60·4 to 63·9)	60·1 (58·7 to 61·4)	48·4 (47·1 to 49·7)	47·1 (45·6 to 48·6)	44 (42·5 to 45·4)	43·1 (41·6 to 44·4)	39·4 (38·1 to 40·7)	35·5 (34·5 to 36·7)	35 (32·8 to 37·4)
Missouri	89·1 (87 to 91·6)	96·3 (93·8 to 99·2)	79 (76·6 to 81·5)	78 (75·6 to 80·7)	79·4 (77·5 to 81·5)	75·2 (72·5 to 77·6)	76·8 (74·6 to 79)	69·8 (67·8 to 72)	69·1 (64·4 to 74·3)
Montana	12·9 (12·6 to 13·3)	15 (14·7 to 15·2)	12·3 (11·9 to 12·6)	14·2 (13·7 to 14·6)	11·6 (11·3 to 11·9)	11 (10·6 to 11·4)	12 (11·6 to 12·3)	10·6 (10·3 to 10·9)	11 (10·3 to 11·8)
Nebraska	29 (28·3 to 29·8)	31·1 (30·2 to 32·1)	25·3 (24·4 to 26·1)	27·3 (26·4 to 28·1)	24·3 (23·5 to 25·1)	24·6 (23·7 to 25·5)	26·1 (25·1 to 27)	24·6 (23·8 to 25·4)	24·5 (22·9 to 26·2)
Nevada	3·7 (3·6 to 3·8)	7 (6·8 to 7·2)	9·3 (9·1 to 9·6)	13·5 (13 to 14)	21·5 (20·7 to 22·2)	31·1 (30·1 to 32·1)	34·9 (33·6 to 36·2)	35 (33·8 to 36·3)	33·7 (31·4 to 36·2)
New Hampshire	12·2 (11·9 to 12·5)	13·6 (13·2 to 14)	13·1 (12·6 to 13·6)	13·5 (13·1 to 14)	17·1 (16·5 to 17·6)	14·5 (14·1 to 15)	13·1 (12·7 to 13·5)	11·8 (11·4 to 12·2)	12·4 (11·6 to 13·2)
New Jersey	116 (113 to 119)	136 (131 to 141)	116 (112 to 120)	96·7 (93·8 to 99·5)	123 (120 to 127)	116 (113 to 119)	103 (100 to 106)	104 (101 to 107)	101 (94·4 to 109)
New Mexico	20·1 (19·6 to 20·7)	28·9 (27·5 to 30·5)	21·4 (20·7 to 22·1)	26·4 (25·4 to 27·3)	27·6 (26·9 to 28·5)	27·2 (26·3 to 28·1)	26·9 (26 to 27·8)	22 (21·1 to 23)	21·2 (19·8 to 22·6)
New York	349 (340 to 358)	383 (372 to 394)	299 (290 to 308)	241 (234 to 248)	297 (290 to 306)	258 (252 to 263)	242 (236 to 249)	226 (220 to 232)	210 (196 to 225)
North Carolina	103 (100 to 106)	114 (111 to 116)	96 (93·5 to 98·7)	84·3 (81·9 to 86·7)	104 (101 to 107)	119 (116 to 122)	123 (119 to 126)	116 (113 to 119)	119 (111 to 128)
North Dakota	16·6 (16·2 to 17·1)	16·2 (15·4 to 17)	10·3 (9·7 to 10·8)	12·1 (11·4 to 12·9)	9·4 (8·9 to 9·9)	7·8 (7·4 to 8·2)	11 (10·4 to 11·5)	10·2 (9·8 to 10·7)	10 (9·3 to 10·9)
Ohio	189 (184 to 194)	231 (225 to 236)	193 (187 to 198)	168 (164 to 173)	168 (163 to 173)	152 (148 to 156)	138 (135 to 142)	130 (127 to 134)	129 (121 to 138)
Oklahoma	48 (46·9 to 49·3)	50·4 (49·2 to 51·6)	44·4 (43·1 to 45·8)	53·4 (51·8 to 55·1)	47·9 (46·6 to 49·4)	49·8 (48·4 to 51·1)	51·4 (50 to 53)	47·3 (46 to 48·8)	48·1 (45·1 to 51·2)
Oregon	31·6 (30·8 to 32·4)	35·6 (34·6 to 36·5)	33·5 (32·6 to 34·5)	42·9 (41·5 to 44·2)	42·8 (41·5 to 44·1)	45·2 (44 to 46·5)	45·7 (44·4 to 47·1)	39·2 (38 to 40·3)	40·6 (38 to 43·2)
Pennsylvania	244 (238 to 250)	247 (241 to 252)	186 (181 to 190)	160 (156 to 165)	172 (167 to 177)	145 (141 to 148)	143 (139 to 146)	134 (130 to 138)	132 (124 to 141)
Rhode Island	19·2 (18·8 to 19·7)	19 (18·2 to 19·8)	15·2 (14·5 to 15·8)	12·3 (11·8 to 12·9)	15·1 (14·4 to 15·9)	12·6 (12 to 13·3)	11·7 (11·2 to 12·3)	10·7 (10·1 to 11·3)	10·5 (9·7 to 11·4)
South Carolina	56·2 (54·8 to 57·7)	62 (60·3 to 63·5)	52·2 (50·7 to 53·7)	51·1 (49·7 to 52·6)	58·7 (57·1 to 60·4)	55·8 (54·3 to 57·2)	55·3 (53·7 to 57)	55·4 (53·9 to 56·9)	56·9 (53·2 to 60·6)
South Dakota	15·9 (15·5 to 16·3)	16·2 (15·7 to 16·7)	11·5 (11·1 to 11·8)	12·9 (12·4 to 13·3)	11·1 (10·7 to 11·4)	10·3 (10 to 10·6)	12·4 (12 to 12·9)	11 (10·7 to 11·4)	11·2 (10·4 to 11·9)
Tennessee	80·9 (78·9 to 83·1)	86·1 (83·7 to 88·3)	71 (68·7 to 72·9)	68·2 (65·6 to 70·7)	75·2 (72·6 to 77·8)	78·7 (76·1 to 81·4)	84·6 (81·8 to 87·3)	78·7 (76·1 to 81·4)	81·3 (76·3 to 86·3)
Texas	206 (201 to 212)	258 (250 to 265)	227 (220 to 234)	278 (268 to 287)	317 (308 to 327)	364 (354 to 376)	392 (379 to 405)	364 (353 to 374)	372 (348 to 395)
Utah	19·5 (19 to 20)	26·2 (25·5 to 27)	26·6 (25·8 to 27·5)	41·5 (40·3 to 42·9)	36·3 (35·1 to 37·6)	47·2 (45·7 to 48·8)	52·6 (51·2 to 54)	45·1 (43·7 to 46·7)	46·3 (43·4 to 49·3)
Vermont	8·7 (8·5 to 9)	8·8 (8·5 to 9·1)	8 (7·7 to 8·3)	7·9 (7·6 to 8·2)	8·2 (7·9 to 8·5)	6·4 (6·2 to 6·6)	5·8 (5·6 to 6)	5·4 (5·2 to 5·5)	5·3 (5 to 5·8)
Virginia	81·5 (79·5 to 83·7)	96·5 (93·8 to 99)	83·6 (81·4 to 85·8)	77·5 (75·2 to 79·7)	98·8 (95·9 to 102)	99·7 (97·1 to 102)	101 (98·7 to 104)	97·2 (94·8 to 99·8)	95·3 (89·2 to 102)
Washington	52·5 (51·2 to 53·9)	62·4 (60·9 to 63·8)	56·3 (54·6 to 58·1)	68·4 (66 to 70·8)	79·4 (76·9 to 81·9)	79·9 (77·8 to 82)	86·8 (84·2 to 89·4)	79·8 (77·6 to 82·1)	83·1 (77·9 to 88·5)
West Virginia	47·4 (46·3 to 48·7)	41·8 (40·6 to 42·8)	30·8 (29·9 to 31·6)	28·8 (27·9 to 29·8)	22·8 (22·2 to 23·6)	20·6 (19·9 to 21·3)	20·6 (20·1 to 21·2)	17·7 (17·1 to 18·2)	17 (16 to 18·1)
Wisconsin	79·2 (77·2 to 81·3)	88·9 (86·5 to 91·2)	73·6 (71·6 to 75·3)	74·5 (72·4 to 76·6)	72·7 (70·7 to 74·8)	68·5 (66·8 to 70·3)	67·3 (65·2 to 69·5)	62·2 (60·3 to 64·1)	61·5 (57·8 to 65·3)
Wyoming	6·8 (6·6 to 6·9)	8 (7·7 to 8·3)	6·1 (5·9 to 6·3)	10·7 (10·3 to 11·1)	6·9 (6·6 to 7·2)	6·2 (5·9 to 6·4)	6·8 (6·6 to 7·1)	6·2 (5·9 to 6·4)	6·1 (5·7 to 6·5)
Southern Latin America	674 (654 to 694)	799 (787 to 810)	853 (838 to 866)	987 (973 to 1000)	1030 (1020 to 1050)	993 (968 to 1020)	1040 (1020 to 1060)	823 (800 to 847)	769 (684 to 863)
Argentina	441 (424 to 458)	475 (464 to 484)	553 (542 to 563)	683 (670 to 696)	679 (667 to 692)	688 (664 to 710)	749 (729 to 770)	583 (564 to 602)	537 (473 to 607)
Chile	188 (183 to 193)	262 (258 to 266)	244 (239 to 249)	250 (244 to 256)	298 (291 to 304)	252 (246 to 258)	248 (242 to 254)	204 (196 to 211)	197 (176 to 220)
Uruguay	44·8 (42·3 to 47·1)	62 (60·3 to 63·9)	55·6 (53·8 to 57·4)	53·9 (52·5 to 55·4)	55·4 (53·9 to 56·8)	52·7 (49·7 to 55·7)	43·4 (40 to 47·1)	37 (35·3 to 38·6)	35·4 (31·5 to 39·6)
Western Europe	5620 (5460 to 5770)	6070 (6020 to 6120)	5750 (5710 to 5800)	4800 (4750 to 4860)	4600 (4550 to 4650)	4330 (4290 to 4380)	4560 (4510 to 4610)	4130 (4080 to 4190)	4090 (3840 to 4350)
Andorra	0·1 (0·1 to 0·1)	0·2 (0·2 to 0·3)	0·4 (0·4 to 0·4)	0·5 (0·5 to 0·5)	0·6 (0·6 to 0·6)	0·7 (0·6 to 0·8)	0·8 (0·8 to 0·9)	0·6 (0·5 to 0·6)	0·5 (0·5 to 0·6)
Austria	106 (102 to 110)	128 (126 to 131)	110 (108 to 112)	91·3 (89·3 to 93·3)	92·6 (90·6 to 94·7)	77·5 (75·9 to 79·1)	78·2 (76·2 to 80·3)	84·4 (82·6 to 86·2)	85·7 (80·5 to 91·1)
Belgium	142 (138 to 147)	157 (155 to 160)	141 (138 to 143)	123 (121 to 126)	125 (123 to 127)	116 (114 to 118)	129 (127 to 131)	116 (113 to 118)	113 (102 to 125)
Cyprus	13·9 (13·4 to 14·5)	14·5 (14·1 to 14·8)	12·1 (11·7 to 12·5)	13·4 (13 to 13·8)	14·6 (14·2 to 15)	11·2 (10·7 to 11·6)	13·3 (12·9 to 13·8)	15 (13·9 to 16·4)	15·1 (13·1 to 17·4)
Denmark	78·2 (75·8 to 81·1)	76·1 (74·6 to 77·6)	72·3 (70·3 to 74·4)	55·3 (53·9 to 56·7)	63·8 (62·5 to 65·2)	66·4 (64·9 to 67·8)	61·3 (60 to 62·5)	60·8 (59·3 to 62·3)	63·2 (59·7 to 66·7)
Finland	95·6 (92·8 to 98·9)	81·2 (79·6 to 82·7)	62·8 (61·4 to 64·3)	63·9 (62·1 to 65·7)	65 (63·2 to 66·7)	55·5 (54 to 56·9)	59·9 (58·7 to 61·2)	46·4 (44·9 to 48)	48·5 (45·3 to 51·7)
France	840 (817 to 863)	830 (815 to 845)	856 (840 to 872)	795 (774 to 817)	760 (742 to 779)	772 (758 to 788)	798 (784 to 812)	707 (690 to 723)	693 (623 to 767)
Germany	1100 (1020 to 1190)	1260 (1240 to 1290)	1020 (1000 to 1040)	852 (834 to 873)	862 (842 to 881)	757 (737 to 777)	671 (655 to 687)	779 (752 to 807)	790 (742 to 838)
Greece	155 (149 to 161)	155 (151 to 158)	144 (141 to 147)	143 (140 to 147)	102 (98·9 to 105)	102 (100 to 105)	111 (109 to 114)	85·3 (82·2 to 88·1)	82·3 (73·4 to 92·1)
Iceland	4 (3·8 to 4·3)	4·6 (4·5 to 4·8)	4·2 (4·1 to 4·4)	4·3 (4·2 to 4·5)	4·6 (4·4 to 4·8)	4·1 (4 to 4·3)	4·7 (4·5 to 4·9)	4·5 (4·3 to 4·7)	4·7 (4·3 to 5·1)
Ireland	63·9 (62·1 to 66)	62·7 (61·8 to 63·5)	67·1 (66·1 to 68·1)	74·1 (72·8 to 75·4)	54 (52·7 to 55·2)	57·6 (56·3 to 58·9)	73·8 (72·1 to 75·6)	59 (57·3 to 60·7)	57·7 (54 to 61·4)
Israel	47 (45·6 to 48·4)	55·4 (54·3 to 56·5)	80·2 (78·9 to 81·6)	92·9 (91·1 to 94·7)	103 (101 to 106)	136 (133 to 139)	165 (162 to 168)	180 (176 to 185)	183 (174 to 193)
Italy	883 (855 to 913)	923 (909 to 938)	915 (900 to 930)	640 (629 to 653)	565 (555 to 576)	540 (530 to 551)	559 (545 to 573)	416 (406 to 426)	398 (354 to 445)
Luxembourg	4·5 (4·2 to 4·9)	5 (4·9 to 5·2)	4·3 (4·1 to 4·4)	4·2 (4 to 4·3)	5 (4·9 to 5·2)	5·6 (5·5 to 5·8)	5·7 (5·5 to 5·9)	6·2 (5·9 to 6·6)	6·6 (6·1 to 7·1)
Malta	9·8 (9·4 to 10·2)	8·1 (7·8 to 8·3)	5·3 (5·1 to 5·5)	5·7 (5·5 to 6)	5·4 (5·2 to 5·6)	4·2 (4 to 4·3)	4·1 (3·9 to 4·3)	4·4 (4·2 to 4·6)	4·3 (3·9 to 4·8)
Monaco	0·3 (0·3 to 0·4)	0·3 (0·2 to 0·3)	0·3 (0·2 to 0·3)	0·3 (0·3 to 0·3)	0·3 (0·3 to 0·4)	0·3 (0·3 to 0·3)	0·3 (0·2 to 0·3)	0·3 (0·2 to 0·3)	0·3 (0·2 to 0·3)
Netherlands	230 (223 to 237)	244 (240 to 249)	233 (229 to 237)	179 (175 to 183)	197 (193 to 201)	206 (201 to 210)	183 (179 to 187)	170 (166 to 174)	178 (167 to 188)
Norway	61·9 (60·1 to 63·9)	62·3 (61·3 to 63·4)	64·1 (62·7 to 65·4)	47·8 (46·8 to 48·9)	58·3 (57·1 to 59·5)	58 (56·9 to 59·2)	60·5 (59·2 to 61·9)	53·7 (52·4 to 55)	55·9 (52·8 to 59·1)
Agder	3·3 (3·2 to 3·4)	3·2 (3·1 to 3·3)	3·4 (3·3 to 3·5)	2·9 (2·8 to 3)	3·3 (3·2 to 3·4)	3·3 (3·2 to 3·3)	3·5 (3·4 to 3·6)	3 (2·9 to 3·1)	3 (2·8 to 3·2)
Innlandet	5·6 (5·4 to 5·8)	5·3 (5·2 to 5·4)	5 (4·8 to 5·1)	3·5 (3·4 to 3·6)	4·1 (4 to 4·3)	4 (3·8 to 4·1)	3·6 (3·5 to 3·7)	3 (2·9 to 3·2)	3·1 (2·9 to 3·3)
Møre og Romsdal	3·9 (3·8 to 4)	4 (3·9 to 4·2)	3·9 (3·8 to 4·1)	3 (2·9 to 3·1)	3·2 (3·1 to 3·3)	3·1 (3 to 3·2)	2·9 (2·7 to 3)	2·5 (2·4 to 2·6)	2·6 (2·5 to 2·8)
Nordland	4·3 (4·2 to 4·5)	4·5 (4·4 to 4·7)	4·2 (4·1 to 4·3)	3 (2·9 to 3·1)	3·2 (3·1 to 3·4)	2·8 (2·7 to 2·9)	2·5 (2·5 to 2·6)	2·1 (2 to 2·2)	2·2 (2·1 to 2·4)
Oslo	7·9 (7·7 to 8·2)	6·8 (6·4 to 7·1)	6·6 (6·2 to 7)	4·9 (4·7 to 5·1)	7 (6·8 to 7·3)	7·8 (7·5 to 8·1)	10·2 (9·8 to 10·6)	9·1 (8·7 to 9·5)	9·6 (8·8 to 10·5)
Rogaland	4·4 (4·3 to 4·5)	4·8 (4·5 to 5)	5 (4·8 to 5·3)	4·5 (4·3 to 4·6)	5·5 (5·3 to 5·7)	5·5 (5·3 to 5·7)	6·3 (6·1 to 6·5)	5·3 (5·1 to 5·5)	5·5 (5·1 to 6)
Troms og Finnmark	4 (3·9 to 4·1)	4·2 (4·1 to 4·4)	4·2 (4·1 to 4·3)	2·9 (2·8 to 3)	3·3 (3·2 to 3·5)	3 (3 to 3·1)	2·7 (2·6 to 2·7)	2·3 (2·2 to 2·4)	2·4 (2·2 to 2·5)
Trøndelag	5·9 (5·7 to 6·1)	5·9 (5·7 to 6)	6·1 (5·8 to 6·4)	4·4 (4·2 to 4·6)	5·2 (5 to 5·4)	5·1 (4·9 to 5·2)	5·2 (5 to 5·4)	4·7 (4·5 to 5)	4·9 (4·6 to 5·3)
Vestfold og Telemark	5 (4·9 to 5·2)	5·1 (5 to 5·2)	5 (4·8 to 5·1)	3·7 (3·6 to 3·8)	4·4 (4·2 to 4·5)	4·3 (4·1 to 4·4)	4·1 (4 to 4·3)	3·6 (3·4 to 3·7)	3·6 (3·4 to 3·9)
Vestland	8·3 (8 to 8·5)	8·3 (8·1 to 8·5)	8·5 (8·2 to 8·8)	6·3 (6·2 to 6·5)	7·5 (7·3 to 7·8)	7·4 (7·2 to 7·6)	7·5 (7·3 to 7·7)	6·6 (6·4 to 6·8)	6·8 (6·4 to 7·3)

Viken	9·4 (9·1 to 9·7)	10·2 (9·9 to 10·5)	12·2 (11·9 to 12·6)	8·8 (8·5 to 9·1)	11·4 (11·1 to 11·7)	11·9 (11·5 to 12·2)	12·1 (11·8 to 12·5)	11·5 (11·1 to 11·9)	12 (11·3 to 12·8)
Portugal	206 (199 to 214)	218 (214 to 222)	181 (178 to 184)	154 (151 to 158)	116 (113 to 119)	116 (113 to 119)	99·2 (97 to 102)	86·3 (84 to 88·9)	80·6 (75·2 to 86·2)
San Marino	0·3 (0·2 to 0·3)	0·3 (0·3 to 0·3)	0·3 (0·3 to 0·3)	0·2 (0·2 to 0·3)	0·2 (0·2 to 0·2)	0·3 (0·3 to 0·3)	0·3 (0·3 to 0·3)	0·2 (0·2 to 0·3)	0·2 (0·2 to 0·2)
Spain	560 (542 to 579)	649 (637 to 661)	660 (646 to 674)	549 (538 to 560)	397 (387 to 405)	397 (386 to 409)	481 (469 to 491)	357 (348 to 365)	337 (313 to 362)
Sweden	114 (110 to 118)	103 (101 to 105)	111 (109 to 114)	95·5 (93·4 to 97·5)	123 (120 to 126)	91·2 (88·9 to 93·2)	113 (110 to 115)	114 (111 to 116)	114 (107 to 121)
Stockholm	16·4 (15·8 to 17)	15·3 (14·9 to 15·8)	21·7 (21 to 22·4)	18·7 (18·1 to 19·3)	25 (24·3 to 25·7)	22·2 (21·3 to 23·1)	28·8 (27·7 to 29·9)	28·3 (27·4 to 29·1)	28·9 (27 to 30·8)
Sweden except Stockholm	97·5 (94·5 to 101)	87·8 (86 to 89·7)	89·5 (87·5 to 91·6)	76·8 (75·1 to 78·5)	97·8 (95·6 to 100)	69 (67·4 to 70·6)	84 (82·2 to 85·8)	85·3 (83·4 to 87·2)	85 (80·2 to 90·1)
Switzerland	83·2 (80·7 to 85·8)	96·1 (94·3 to 97·7)	101 (99 to 103)	75·8 (74·2 to 77·4)	86·9 (84·8 to 88·9)	76·5 (74·9 to 78·1)	80·5 (78·8 to 82·2)	86·1 (84·3 to 88·1)	89·1 (83·5 to 95·3)
UK	809 (786 to 832)	931 (915 to 947)	900 (885 to 914)	736 (715 to 756)	793 (769 to 818)	677 (657 to 697)	804 (785 to 828)	698 (677 to 720)	684 (608 to 762)
England	650 (630 to 670)	753 (737 to 768)	734 (720 to 748)	602 (584 to 619)	662 (643 to 683)	570 (553 to 588)	684 (667 to 704)	598 (580 to 616)	586 (521 to 654)
East Midlands	52·9 (51·3 to 54·5)	61·3 (59·9 to 62·6)	61 (59·1 to 62·9)	49·6 (48 to 51·2)	54·3 (52·6 to 56·2)	45·6 (44 to 47·2)	54·9 (53·6 to 56·6)	48 (46·3 to 49·7)	47·3 (42·2 to 52·6)
Derby	3·7 (3·5 to 3·8)	4·3 (4·1 to 4·4)	4 (3·9 to 4·1)	3·1 (3 to 3·2)	3·4 (3·3 to 3·5)	2·8 (2·7 to 3)	3·5 (3·4 to 3·6)	3 (2·8 to 3·1)	2·9 (2·6 to 3·2)
Derbyshire	9·7 (9·4 to 10)	10·9 (10·5 to 11·2)	10·7 (10·3 to 11·1)	8·3 (8·1 to 8·6)	9·1 (8·8 to 9·4)	7·5 (7·2 to 7·8)	8·5 (8·3 to 8·8)	7·2 (6·9 to 7·4)	7·1 (6·3 to 7·9)
Leicester	4·8 (4·6 to 4·9)	5·4 (5·3 to 5·6)	5·7 (5·4 to 5·9)	4·8 (4·6 to 4·9)	4·7 (4·6 to 4·9)	4 (3·8 to 4·2)	5·2 (5 to 5·4)	4·5 (4·3 to 4·7)	4·5 (3·9 to 5)
Leicestershire	6·4 (6·2 to 6·6)	7·7 (7·5 to 8)	7·8 (7·6 to 8·2)	6·6 (6·3 to 6·9)	7·3 (7·1 to 7·6)	6·3 (6·1 to 6·6)	7 (6·8 to 7·2)	6·6 (6·3 to 6·8)	6·5 (5·8 to 7·2)
Lincolnshire	7 (6·8 to 7·2)	7·9 (7·6 to 8·2)	7·9 (7·6 to 8·2)	6·6 (6·3 to 6·8)	7·1 (6·9 to 7·3)	6·2 (5·9 to 6·4)	8 (7·7 to 8·3)	6·7 (6·4 to 7)	6·6 (5·9 to 7·4)
Northamptonshire	6·8 (6·6 to 7)	7·9 (7·6 to 8·1)	8·2 (8 to 8·5)	7·2 (6·9 to 7·4)	8·3 (8·1 to 8·6)	7·5 (7·2 to 7·8)	9·4 (9·1 to 9·7)	8·3 (8 to 8·7)	8·1 (7·3 to 9·1)
Nottingham	4·6 (4·5 to 4·7)	5·4 (5·1 to 5·6)	5 (4·8 to 5·2)	3·8 (3·6 to 4)	4·4 (4·3 to 4·6)	3·3 (3·2 to 3·5)	4 (3·8 to 4·2)	3·7 (3·5 to 3·9)	3·6 (3·2 to 4·1)
Nottinghamshire	9·6 (9·3 to 9·8)	11·4 (11·2 to 11·7)	11·2 (10·9 to 11·6)	8·8 (8·6 to 9·1)	9·5 (9·2 to 9·8)	7·6 (7·3 to 7·9)	9 (8·8 to 9·3)	7·8 (7·5 to 8·1)	7·6 (6·9 to 8·5)
Rutland	0·4 (0·3 to 0·4)	0·5 (0·4 to 0·5)	0·4 (0·4 to 0·5)	0·4 (0·4 to 0·4)	0·4 (0·3 to 0·4)	0·3 (0·3 to 0·3)	0·4 (0·3 to 0·4)	0·3 (0·3 to 0·3)	0·3 (0·3 to 0·4)
East of England	61·4 (59·5 to 63·2)	73·6 (71·9 to 75·3)	72·8 (70·9 to 74·5)	62·7 (60·5 to 64·9)	68·8 (66·3 to 71·4)	61 (59 to 63·1)	72·9 (70·8 to 75·4)	65·9 (63·6 to 68·3)	64·6 (57·3 to 72·2)
Bedford	1·8 (1·8 to 1·9)	2·3 (2·2 to 2·4)	2·3 (2·2 to 2·4)	1·9 (1·8 to 2)	1·8 (1·8 to 1·9)	1·8 (1·7 to 1·8)	2·1 (2 to 2·1)	2·1 (2 to 2·1)	2 (1·8 to 2·3)
Cambridgeshire	4·7 (4·6 to 4·9)	5·7 (5·6 to 5·9)	6 (5·9 to 6·2)	5·6 (5·4 to 5·8)	6·6 (6·3 to 6·8)	6 (5·8 to 6·2)	7·5 (7·3 to 7·8)	6·5 (6·3 to 6·7)	6·4 (5·7 to 7·2)
Central Bedfordshire	2·5 (2·4 to 2·6)	3·3 (3·2 to 3·4)	3·4 (3·3 to 3·5)	3·2 (3·1 to 3·3)	3·2 (3 to 3·3)	2·8 (2·7 to 2·9)	3·2 (3·1 to 3·3)	3·3 (3·1 to 3·4)	3·2 (2·9 to 3·6)
Essex	14·7 (14·3 to 15·2)	18·4 (17·5 to 19·3)	18·3 (17·5 to 19)	15·4 (14·8 to 16)	16·4 (15·8 to 17·1)	14·2 (13·7 to 14·8)	16·3 (15·7 to 16·9)	15·4 (14·8 to 16·1)	15·1 (13·5 to 16·9)
Hertfordshire	13 (12·6 to 13·4)	15·5 (15 to 15·9)	14·1 (13·7 to 14·5)	12 (11·6 to 12·4)	13·5 (12·9 to 14·1)	12·6 (12·2 to 13·1)	14·8 (14·3 to 15·4)	13·3 (12·8 to 13·7)	13·1 (11·6 to 14·6)
Luton	2·5 (2·4 to 2·6)	3·4 (3·3 to 3·6)	3·4 (3·3 to 3·6)	2·9 (2·7 to 3)	3·3 (3·2 to 3·4)	2·9 (2·8 to 3)	3·6 (3·5 to 3·7)	3·2 (3·1 to 3·4)	3·2 (2·8 to 3·6)
Norfolk	8·3 (8·1 to 8·6)	9·4 (9·1 to 9·7)	9·5 (9·2 to 9·7)	8·2 (7·9 to 8·5)	9 (8·6 to 9·3)	7·7 (7·5 to 8)	9·2 (8·9 to 9·5)	7·9 (7·6 to 8·2)	7·7 (6·8 to 8·6)
Peterborough	1·7 (1·6 to 1·7)	2 (1·9 to 2·2)	2 (2 to 2·1)	2·1 (2 to 2·2)	2·5 (2·3 to 2·6)	2·1 (2 to 2·2)	3·2 (3·1 to 3·3)	2·8 (2·6 to 2·9)	2·7 (2·4 to 3·1)
Southend-on-Sea	2·6 (2·5 to 2·7)	2·5 (2·4 to 2·7)	2·3 (2·2 to 2·4)	1·9 (1·8 to 1·9)	2·2 (2·1 to 2·3)	1·9 (1·8 to 2)	2·3 (2·2 to 2·4)	2 (1·9 to 2·1)	1·9 (1·7 to 2·2)
Suffolk	7·7 (7·4 to 7·9)	8·8 (8·5 to 9·1)	9 (8·7 to 9·2)	7·8 (7·5 to 8)	8·4 (8·1 to 8·7)	7·1 (6·8 to 7·4)	8·3 (8·1 to 8·6)	7·1 (6·8 to 7·3)	6·9 (6·1 to 7·7)
Thurrock	1·8 (1·8 to 1·9)	2·2 (2·1 to 2·4)	2·4 (2·3 to 2·5)	1·8 (1·8 to 1·9)	2 (2 to 2·1)	1·9 (1·8 to 2)	2·4 (2·3 to 2·5)	2·4 (2·3 to 2·5)	2·3 (2 to 2·6)
Greater London	115 (111 to 119)	127 (124 to 130)	117 (114 to 119)	91·3 (88·6 to 93·9)	105 (101 to 108)	103 (99·6 to 106)	131 (127 to 136)	115 (111 to 118)	112 (99 to 126)
Barking and Dagenham	3·2 (3 to 3·3)	3·1 (3 to 3·2)	2·7 (2·6 to 2·7)	2·2 (2·1 to 2·2)	2·5 (2·4 to 2·6)	2·4 (2·3 to 2·4)	3·7 (3·6 to 3·8)	3·5 (3·4 to 3·6)	3·4 (3 to 3·8)
Barnet	4·6 (4·5 to 4·8)	4·6 (4·5 to 4·7)	4·3 (4·2 to 4·5)	3·7 (3·6 to 3·8)	4·1 (3·9 to 4·2)	4·1 (3·9 to 4·2)	5·5 (5·3 to 5·7)	4·8 (4·7 to 5)	4·7 (4·2 to 5·3)
Bexley	3·3 (3·2 to 3·4)	3·8 (3·6 to 3·9)	3·4 (3·3 to 3·5)	2·6 (2·6 to 2·7)	3·1 (3 to 3·3)	2·6 (2·5 to 2·7)	3 (2·9 to 3·1)	2·8 (2·7 to 3)	2·8 (2·5 to 3·1)
Brent	4·5 (4·3 to 4·6)	5·4 (5·1 to 5·8)	5·1 (4·9 to 5·3)	3·8 (3·6 to 4)	3·9 (3·7 to 4·1)	4 (3·8 to 4·2)	5·7 (5·4 to 5·9)	4·7 (4·5 to 5)	4·6 (4 to 5·2)
Bromley	4·5 (4·4 to 4·7)	4·6 (4·4 to 4·8)	3·9 (3·8 to 4)	3·2 (3·1 to 3·3)	3·7 (3·5 to 3·8)	3·5 (3·3 to 3·6)	4 (3·8 to 4·1)	3·8 (3·7 to 3·9)	3·7 (3·3 to 4·1)
Camden	2·2 (2·2 to 2·3)	2·7 (2·6 to 2·8)	2·9 (2·8 to 3)	2·1 (2·1 to 2·2)	2·3 (2·2 to 2·4)	2·7 (2·6 to 2·8)	2·8 (2·7 to 2·9)	2·4 (2·3 to 2·5)	2·3 (2 to 2·6)
Croydon	5·4 (5·3 to 5·6)	6 (5·9 to 6·2)	5·4 (5·3 to 5·6)	4·4 (4·2 to 4·5)	4·9 (4·7 to 5·1)	4·4 (4·2 to 4·6)	5·7 (5·5 to 5·9)	5·2 (5 to 5·4)	5·1 (4·6 to 5·7)
Ealing	5 (4·8 to 5·1)	5·5 (5·4 to 5·7)	5·3 (5·1 to 5·4)	4·3 (4·2 to 4·5)	4·5 (4·3 to 4·6)	4·3 (4·2 to 4·5)	5·8 (5·6 to 6·1)	4·6 (4·4 to 4·8)	4·4 (3·9 to 5)
Enfield	4·3 (4·1 to 4·4)	4·3 (4·2 to 4·5)	4 (3·9 to 4·1)	3·3 (3·1 to 3·4)	3·9 (3·8 to 4)	3·8 (3·7 to 4)	5·1 (4·9 to 5·3)	4·3 (4·2 to 4·5)	4·2 (3·7 to 4·7)
Greenwich	3·7 (3·6 to 3·9)	3·8 (3·6 to 3·9)	3·5 (3·4 to 3·6)	3·1 (3 to 3·2)	3·4 (3·3 to 3·6)	3·2 (3·1 to 3·3)	4·5 (4·4 to 4·7)	4·1 (3·9 to 4·2)	4 (3·6 to 4·5)
Hackney	3·9 (3·8 to 4·1)	4·7 (4·5 to 4·8)	4·4 (4·3 to 4·5)	3·3 (3·2 to 3·4)	3·8 (3·7 to 3·9)	4 (3·8 to 4·1)	4·6 (4·5 to 4·8)	4 (3·9 to 4·2)	3·9 (3·5 to 4·4)
Hammersmith and Fulham	2·3 (2·2 to 2·4)	2·7 (2·6 to 2·8)	2·7 (2·6 to 2·8)	1·9 (1·9 to 2)	2·2 (2·1 to 2·2)	2·4 (2·3 to 2·5)	2·8 (2·7 to 2·9)	2·2 (2·1 to 2·3)	2·1 (1·8 to 2·4)
Haringey	3·3 (3·2 to 3·4)	4·2 (4 to 4·3)	4 (3·9 to 4·1)	2·9 (2·8 to 3)	3·5 (3·3 to 3·6)	3·7 (3·5 to 3·8)	4·4 (4·3 to 4·6)	3·5 (3·4 to 3·6)	3·4 (3 to 3·8)
Harrow	3·2 (3·1 to 3·3)	3·3 (3·2 to 3·4)	3 (2·9 to 3)	2·5 (2·4 to 2·6)	2·8 (2·7 to 2·9)	2·6 (2·4 to 2·7)	3·4 (3·3 to 3·6)	3·5 (3·3 to 3·6)	3·4 (3 to 3·8)
Havering	3·8 (3·7 to 4)	4·3 (4·2 to 4·4)	3·6 (3·5 to 3·7)	2·7 (2·6 to 2·8)	2·9 (2·8 to 3)	2·4 (2·3 to 2·4)	2·8 (2·7 to 2·9)	3·1 (3 to 3·3)	3·1 (2·8 to 3·5)
Hillingdon	3·8 (3·7 to 3·9)	4·2 (4·1 to 4·3)	3·7 (3·6 to 3·8)	3 (2·9 to 3·1)	3·5 (3·4 to 3·7)	3·3 (3·1 to 3·4)	4·2 (4 to 4·3)	4 (3·9 to 4·2)	4 (3·5 to 4·5)
Hounslow	3·2 (3·1 to 3·3)	3·5 (3·4 to 3·7)	3·4 (3·3 to 3·5)	2·9 (2·8 to 3)	3·2 (3·1 to 3·3)	3·1 (3 to 3·3)	4·4 (4·3 to 4·6)	3·8 (3·6 to 3·9)	3·7 (3·3 to 4·1)
Islington	2·7 (2·6 to 2·8)	3·5 (3·4 to 3·6)	3·2 (3·1 to 3·3)	2·2 (2·2 to 2·3)	2·7 (2·6 to 2·8)	2·5 (2·4 to 2·6)	2·9 (2·8 to 3)	2·6 (2·5 to 2·8)	2·6 (2·3 to 2·9)
Kensington and Chelsea	1·8 (1·7 to 1·9)	2·4 (2·3 to 2·5)	3·1 (3 to 3·2)	1·8 (1·7 to 1·9)	1·8 (1·7 to 1·9)	2 (2 to 2·1)	2·1 (2 to 2·2)	1·5 (1·4 to 1·6)	1·4 (1·3 to 1·6)
Kingston upon Thames	1·9 (1·9 to 2)	1·8 (1·7 to 1·9)	1·7 (1·6 to 1·8)	1·6 (1·5 to 1·6)	1·7 (1·7 to 1·8)	1·8 (1·7 to 1·8)	2·2 (2·1 to 2·3)	1·9 (1·8 to 2)	1·9 (1·7 to 2·1)
Lambeth	4·8 (4·6 to 5)	5·8 (5·6 to 6)	5·1 (5 to 5·3)	3·7 (3·6 to 3·8)	4·3 (4·1 to 4·5)	4·3 (4·1 to 4·4)	4·8 (4·6 to 4·9)	3·7 (3·6 to 3·8)	3·6 (3·1 to 4·1)
Lewisham	4·4 (4·3 to 4·6)	5 (4·8 to 5·1)	4·3 (4·2 to 4·4)	3·2 (3·1 to 3·3)	3·9 (3·8 to 4·1)	3·8 (3·6 to 3·9)	4·9 (4·7 to 5·1)	4·2 (4 to 4·3)	4 (3·6 to 4·5)
Merton	2·9 (2·8 to 2·9)	2·8 (2·7 to 2·9)	2·5 (2·4 to 2·5)	2·1 (2 to 2·1)	2·5 (2·4 to 2·6)	2·6 (2·5 to 2·7)	3·3 (3·2 to 3·5)	2·8 (2·7 to 3)	2·7 (2·4 to 3·1)
Newham	4·4 (4·3 to 4·6)	5·1 (4·6 to 5·6)	4·8 (4·4 to 5·2)	3·8 (3·5 to 4·1)	4·4 (4·2 to 4·7)	4·7 (4·5 to 5)	6·7 (6·4 to 7)	5·5 (5·3 to 5·8)	5·4 (4·7 to 6·1)
Redbridge	3·6 (3·5 to 3·7)	3·7 (3·6 to 3·8)	3·4 (3·3 to 3·5)	2·6 (2·6 to 2·7)	3·2 (3 to 3·3)	3·1 (3 to 3·2)	4·4 (4·3 to 4·6)	4·4 (4·2 to 4·5)	4·3 (3·8 to 4·8)
Richmond upon Thames	2·3 (2·3 to 2·4)	2·2 (2·1 to 2·3)	2 (2 to 2·1)	1·8 (1·8 to 1·9)	2·1 (2·1 to 2·2)	2·4 (2·3 to 2·4)	2·8 (2·8 to 2·9)	2·1 (2 to 2·2)	2 (1·8 to 2·3)
Southwark	4·7 (4·5 to 4·8)	5·2 (5 to 5·3)	4·2 (4·1 to 4·4)	3·2 (3·1 to 3·3)	4 (3·9 to 4·1)	4 (3·9 to 4·2)	4·8 (4·7 to 5)	3·8 (3·7 to 4)	3·7 (3·3 to 4·2)

Sutton	2·6 (2·5 to 2·6)	2·7 (2·6 to 2·7)	2·4 (2·3 to 2·4)	2 (2 to 2·1)	2·4 (2·3 to 2·5)	2·2 (2·1 to 2·2)	2·7 (2·6 to 2·8)	2·4 (2·3 to 2·5)	2·4 (2·1 to 2·7)
Tower Hamlets	3·4 (3·3 to 3·5)	4 (3·8 to 4·1)	3·4 (3·3 to 3·5)	2·8 (2·7 to 2·8)	3·2 (3·1 to 3·4)	3·5 (3·4 to 3·6)	4·4 (4·2 to 4·6)	4·3 (4·1 to 4·5)	4·3 (3·8 to 4·8)
Waltham Forest	4 (3·9 to 4·2)	4·2 (3·9 to 4·5)	4·1 (3·8 to 4·4)	3·2 (2·9 to 3·4)	3·8 (3·6 to 3·9)	3·5 (3·3 to 3·7)	4·9 (4·7 to 5·1)	4·3 (4·1 to 4·5)	4·3 (3·8 to 4·8)
Wandsworth	4·4 (4·3 to 4·6)	4·7 (4·5 to 4·9)	4·4 (4·2 to 4·5)	3·5 (3·4 to 3·6)	3·9 (3·8 to 4)	4 (3·8 to 4·1)	5·2 (5 to 5·4)	4·4 (4·2 to 4·6)	4·3 (3·7 to 4·8)
Westminster	2·6 (2·5 to 2·7)	3 (2·9 to 3·2)	3·1 (3 to 3·3)	2·1 (2 to 2·2)	2·4 (2·3 to 2·5)	2·5 (2·4 to 2·6)	2·7 (2·6 to 2·8)	2·3 (2·2 to 2·4)	2·2 (1·9 to 2·5)
North East England	38·9 (37·8 to 40·1)	46·9 (44·9 to 49)	45·2 (44·1 to 46·4)	35·3 (34·2 to 36·5)	34·9 (33·7 to 36)	26·5 (25·6 to 27·4)	30·5 (29·7 to 31·4)	25·3 (24·5 to 26·1)	24·6 (22 to 27·5)
County Durham	7·3 (7·1 to 7·5)	8·7 (8·2 to 9·1)	8·4 (8·1 to 8·8)	6·6 (6·4 to 6·9)	6·5 (6·3 to 6·7)	4·9 (4·8 to 5·1)	5·7 (5·6 to 5·9)	4·7 (4·5 to 4·8)	4·6 (4·1 to 5·1)
Darlington	1·4 (1·4 to 1·5)	1·6 (1·6 to 1·7)	1·6 (1·6 to 1·7)	1·3 (1·2 to 1·3)	1·4 (1·4 to 1·5)	1·1 (1·1 to 1·2)	1·4 (1·3 to 1·4)	1·1 (1 to 1·1)	1 (0·9 to 1·2)
Gateshead	3·4 (3·3 to 3·5)	4·1 (3·9 to 4·3)	3·9 (3·8 to 4)	2·7 (2·6 to 2·8)	2·6 (2·5 to 2·8)	2 (2 to 2·1)	2·4 (2·3 to 2·5)	1·9 (1·9 to 2)	1·9 (1·7 to 2·1)
Hartlepool	1·5 (1·4 to 1·5)	1·9 (1·8 to 2)	1·8 (1·8 to 1·9)	1·3 (1·3 to 1·4)	1·3 (1·3 to 1·4)	1 (1 to 1·1)	1·2 (1·1 to 1·2)	0·9 (0·9 to 1)	0·9 (0·8 to 1)
Middlesbrough	2·4 (2·4 to 2·5)	3·1 (2·9 to 3·3)	3 (2·8 to 3·1)	2·4 (2·3 to 2·5)	2·3 (2·2 to 2·3)	1·7 (1·7 to 1·8)	2 (2 to 2·1)	1·7 (1·7 to 1·8)	1·7 (1·5 to 1·9)
Newcastle upon Tyne	4·3 (4·2 to 4·4)	4·9 (4·7 to 5·2)	4·6 (4·5 to 4·7)	3·5 (3·4 to 3·7)	3·7 (3·6 to 3·8)	2·8 (2·7 to 2·9)	3·3 (3·2 to 3·4)	3 (2·9 to 3·2)	3 (2·7 to 3·4)
North Tyneside	3·1 (3 to 3·2)	3·5 (3·3 to 3·6)	3·2 (3·1 to 3·2)	2·4 (2·3 to 2·5)	2·5 (2·4 to 2·5)	2 (1·9 to 2·1)	2·4 (2·3 to 2·5)	2·1 (2 to 2·1)	2 (1·8 to 2·2)
Northumberland	4·1 (4 to 4·2)	4·5 (4·4 to 4·7)	4·5 (4·4 to 4·6)	3·8 (3·7 to 3·9)	3·6 (3·5 to 3·8)	2·9 (2·8 to 3)	3·1 (3 to 3·2)	2·5 (2·4 to 2·6)	2·4 (2·2 to 2·7)
Redcar and Cleveland	2·1 (2 to 2·1)	2·7 (2·6 to 2·9)	2·8 (2·7 to 2·9)	2·1 (2·1 to 2·2)	2 (2 to 2·1)	1·4 (1·4 to 1·5)	1·6 (1·6 to 1·7)	1·3 (1·2 to 1·3)	1·2 (1·1 to 1·3)
South Tyneside	2·6 (2·6 to 2·7)	3·2 (3 to 3·3)	2·8 (2·8 to 2·9)	2 (1·9 to 2)	2·1 (2 to 2·2)	1·5 (1·5 to 1·6)	1·7 (1·6 to 1·7)	1·5 (1·4 to 1·5)	1·4 (1·3 to 1·6)
Stockton-on-Tees	2·4 (2·3 to 2·4)	3·1 (3 to 3·2)	3 (3 to 3·1)	2·6 (2·6 to 2·7)	2·6 (2·5 to 2·7)	2 (1·9 to 2·1)	2·5 (2·4 to 2·6)	2 (1·9 to 2·1)	1·9 (1·7 to 2·2)
Sunderland	4·4 (4·3 to 4·5)	5·5 (5·2 to 5·9)	5·6 (5·3 to 5·8)	4·4 (4·3 to 4·6)	4·2 (4 to 4·4)	3 (2·9 to 3·1)	3·1 (3 to 3·2)	2·6 (2·5 to 2·7)	2·5 (2·2 to 2·8)
North West England	102 (98·4 to 105)	121 (118 to 123)	117 (115 to 120)	91·9 (89 to 94·6)	97·6 (94 to 101)	76·3 (73·9 to 78·9)	88·8 (86·3 to 91·5)	78 (75·6 to 80·5)	76·3 (68 to 84·7)
Blackburn with Darwen	2·5 (2·4 to 2·5)	2·7 (2·6 to 2·8)	2·7 (2·6 to 2·8)	2·4 (2·3 to 2·5)	2·4 (2·3 to 2·5)	2·1 (2 to 2·2)	2·4 (2·4 to 2·5)	1·9 (1·9 to 2)	1·9 (1·7 to 2·1)
Blackpool	2·2 (2·1 to 2·2)	2·1 (2 to 2·2)	1·9 (1·8 to 1·9)	1·5 (1·4 to 1·5)	1·9 (1·8 to 2)	1·5 (1·4 to 1·6)	1·8 (1·7 to 1·8)	1·5 (1·4 to 1·6)	1·5 (1·3 to 1·6)
Bolton	4 (3·9 to 4·1)	4·6 (4·4 to 4·7)	4·7 (4·6 to 4·8)	3·8 (3·7 to 3·9)	3·9 (3·7 to 4·1)	3·2 (3·1 to 3·4)	4 (3·8 to 4·1)	3·6 (3·4 to 3·7)	3·5 (3·2 to 3·9)
Bury	2·4 (2·3 to 2·5)	2·9 (2·8 to 3)	3 (2·9 to 3·1)	2·3 (2·2 to 2·4)	2·6 (2·5 to 2·7)	2·1 (2 to 2·2)	2·5 (2·4 to 2·6)	2·2 (2·1 to 2·2)	2·1 (1·9 to 2·4)
Cheshire East	4 (3·9 to 4·1)	4·7 (4·5 to 4·9)	4·5 (4·4 to 4·7)	3·7 (3·6 to 3·8)	4·2 (4 to 4·3)	3·5 (3·4 to 3·6)	4 (3·9 to 4·2)	3·6 (3·4 to 3·7)	3·5 (3·1 to 3·9)
Cheshire West and Chester	4 (3·8 to 4·1)	5 (4·8 to 5·1)	4·8 (4·7 to 5)	3·9 (3·8 to 4)	4·2 (4 to 4·4)	3·4 (3·3 to 3·5)	3·7 (3·6 to 3·9)	3·3 (3·2 to 3·4)	3·2 (2·9 to 3·6)
Cumbria	6·5 (6·3 to 6·7)	7·3 (7·1 to 7·5)	7 (6·8 to 7·2)	5·7 (5·5 to 5·9)	6·2 (5·9 to 6·4)	4·7 (4·6 to 4·9)	5·1 (5 to 5·3)	4·2 (4·1 to 4·4)	4·1 (3·7 to 4·6)
Halton	1·4 (1·4 to 1·5)	1·7 (1·6 to 1·8)	1·8 (1·8 to 1·9)	1·9 (1·8 to 2)	2 (1·9 to 2·1)	1·4 (1·4 to 1·5)	1·6 (1·6 to 1·7)	1·3 (1·3 to 1·4)	1·3 (1·1 to 1·4)
Knowsley	2·8 (2·7 to 2·9)	4 (3·9 to 4·2)	3·7 (3·6 to 3·8)	2·9 (2·8 to 3)	2·6 (2·5 to 2·7)	1·9 (1·8 to 1·9)	1·9 (1·9 to 2)	1·9 (1·8 to 2)	1·8 (1·6 to 2)
Lancashire	14·9 (14·5 to 15·4)	16·9 (16·3 to 17·5)	16·7 (16 to 17·5)	14 (13·5 to 14·5)	15·3 (14·7 to 15·9)	12·2 (11·7 to 12·7)	13·8 (13·3 to 14·3)	11·9 (11·5 to 12·4)	11·6 (10·4 to 12·9)
Liverpool	8·8 (8·5 to 9·1)	10·5 (10 to 11·2)	9·9 (9·4 to 10·4)	7 (6·7 to 7·3)	7·1 (6·8 to 7·4)	5 (4·8 to 5·2)	5·6 (5·4 to 5·8)	5·4 (5·2 to 5·6)	5·2 (4·7 to 5·8)
Manchester	8·1 (7·8 to 8·3)	9·8 (9·4 to 10·2)	9·2 (9 to 9·5)	6·6 (6·4 to 6·8)	7·2 (6·9 to 7·5)	5·5 (5·3 to 5·7)	7·6 (7·3 to 7·9)	7 (6·8 to 7·3)	7 (6·2 to 7·8)
Oldham	3·5 (3·4 to 3·6)	4·1 (4 to 4·3)	4·3 (4·2 to 4·4)	3·3 (3·2 to 3·4)	3·4 (3·3 to 3·6)	3 (2·9 to 3·2)	3·4 (3·3 to 3·6)	3·1 (3 to 3·2)	3 (2·7 to 3·3)
Rochdale	3·2 (3·1 to 3·3)	3·7 (3·6 to 3·8)	3·7 (3·6 to 3·8)	3·2 (3·1 to 3·3)	3·3 (3·2 to 3·4)	2·6 (2·5 to 2·7)	3·1 (3·1 to 3·3)	2·8 (2·7 to 2·9)	2·7 (2·4 to 3)
Salford	4·1 (4 to 4·3)	5 (4·8 to 5·2)	4·8 (4·6 to 5)	3·3 (3·2 to 3·4)	3·5 (3·4 to 3·7)	2·6 (2·5 to 2·7)	3·4 (3·3 to 3·6)	3·4 (3·3 to 3·6)	3·4 (3 to 3·7)
Sefton	4·3 (4·1 to 4·4)	5·1 (5 to 5·3)	4·6 (4·5 to 4·7)	3·5 (3·4 to 3·7)	3·7 (3·6 to 3·9)	2·7 (2·6 to 2·8)	2·9 (2·8 to 3)	2·5 (2·4 to 2·6)	2·4 (2·1 to 2·7)
St Helens	2·8 (2·7 to 2·8)	3·4 (3·3 to 3·5)	3·4 (3·3 to 3·5)	2·5 (2·5 to 2·6)	2·4 (2·3 to 2·5)	1·9 (1·8 to 2)	2·1 (2 to 2·2)	1·8 (1·7 to 1·9)	1·7 (1·6 to 1·9)
Stockport	4·1 (4 to 4·2)	5·1 (4·9 to 5·3)	4·8 (4·7 to 4·9)	3·7 (3·6 to 3·8)	3·8 (3·7 to 4)	3 (2·9 to 3·1)	3·4 (3·3 to 3·5)	3·1 (3 to 3·2)	3 (2·7 to 3·4)
Tameside	3·3 (3·2 to 3·4)	3·8 (3·7 to 3·9)	3·9 (3·7 to 4)	3 (2·9 to 3·1)	3·3 (3·2 to 3·5)	2·5 (2·4 to 2·6)	3·1 (3 to 3·2)	2·7 (2·6 to 2·8)	2·6 (2·3 to 2·8)
Trafford	3·2 (3·1 to 3·3)	3·9 (3·8 to 4)	3·6 (3·5 to 3·7)	2·8 (2·7 to 2·9)	2·9 (2·8 to 3·1)	2·4 (2·3 to 2·5)	2·9 (2·8 to 3)	2·5 (2·4 to 2·6)	2·4 (2·2 to 2·7)
Warrington	2·2 (2·2 to 2·3)	2·8 (2·7 to 2·9)	2·8 (2·7 to 2·9)	2·2 (2·1 to 2·3)	2·6 (2·5 to 2·8)	2·2 (2·2 to 2·3)	2·5 (2·4 to 2·6)	2 (1·9 to 2·1)	2 (1·8 to 2·2)
Wigan	4·2 (4·1 to 4·3)	5·2 (5 to 5·4)	5·7 (5·6 to 5·9)	4·2 (4·1 to 4·4)	4·3 (4·2 to 4·5)	3·4 (3·3 to 3·6)	3·9 (3·8 to 4·1)	3·3 (3·2 to 3·5)	3·2 (2·9 to 3·6)
Wirral	5·3 (5·1 to 5·4)	6·2 (6 to 6·3)	5·8 (5·6 to 5·9)	4·4 (4·3 to 4·6)	4·6 (4·5 to 4·8)	3·4 (3·3 to 3·5)	3·8 (3·7 to 3·9)	3·1 (3 to 3·2)	3 (2·7 to 3·3)
South East England	82·9 (80·3 to 85·7)	93·6 (91 to 96·4)	94·4 (91·7 to 96·9)	87·8 (85·1 to 90·6)	100 (97 to 103)	89·6 (86·7 to 92·7)	106 (104 to 109)	92·3 (89·5 to 95·2)	91 (81 to 101)
Bracknell Forest	0·7 (0·7 to 0·8)	1·1 (1 to 1·1)	1·1 (1 to 1·1)	1·2 (1·2 to 1·3)	1·5 (1·4 to 1·5)	1·4 (1·4 to 1·5)	1·5 (1·5 to 1·6)	1·3 (1·3 to 1·4)	1·3 (1·2 to 1·5)
Brighton and Hove	3·1 (3 to 3·3)	2·7 (2·6 to 2·8)	2·6 (2·5 to 2·7)	2·4 (2·3 to 2·5)	2·9 (2·8 to 3)	2·8 (2·6 to 2·9)	3·2 (3·1 to 3·3)	2·4 (2·3 to 2·5)	2·4 (2·1 to 2·7)
Buckinghamshire	5 (4·9 to 5·2)	6 (5·8 to 6·3)	5·8 (5·7 to 6)	5·4 (5·2 to 5·6)	6 (5·8 to 6·2)	5·5 (5·3 to 5·7)	6·2 (6 to 6·4)	5·6 (5·4 to 5·8)	5·5 (4·9 to 6·1)
East Sussex	4·9 (4·7 to 5·1)	4·6 (4·4 to 4·8)	4·7 (4·5 to 4·8)	4·3 (4·2 to 4·5)	5·3 (5·1 to 5·5)	4·7 (4·5 to 4·9)	5·5 (5·3 to 5·6)	4·6 (4·4 to 4·8)	4·6 (4·1 to 5·1)
Hampshire	11·3 (10·9 to 11·6)	14·4 (13·9 to 14·8)	15·1 (14·6 to 15·4)	13·8 (13·4 to 14·3)	15·4 (14·9 to 15·9)	13·2 (12·7 to 13·7)	15·2 (14·8 to 15·7)	13 (12·6 to 13·4)	12·8 (11·5 to 14·2)
Isle of Wight	1·4 (1·4 to 1·5)	1·4 (1·3 to 1·4)	1·3 (1·3 to 1·4)	1·2 (1·2 to 1·3)	1·4 (1·3 to 1·4)	1·1 (1·1 to 1·2)	1·3 (1·2 to 1·3)	1 (1 to 1·1)	1 (0·9 to 1·2)
Kent	14·8 (14·3 to 15·3)	16·9 (16·3 to 17·4)	17 (16·5 to 17·4)	14·9 (14·4 to 15·3)	16·9 (16·4 to 17·5)	14·8 (14·2 to 15·4)	17·6 (17·1 to 18·1)	16·2 (15·7 to 16·7)	16 (14·3 to 17·8)
Medway	2·8 (2·7 to 2·9)	3·9 (3·7 to 4)	4·4 (4·3 to 4·5)	3·8 (3·7 to 3·9)	3·9 (3·7 to 4)	3·1 (3 to 3·2)	3·5 (3·4 to 3·7)	3·3 (3·2 to 3·4)	3·2 (2·9 to 3·6)
Milton Keynes	0·9 (0·9 to 0·9)	1 (1 to 1·1)	1·3 (1·3 to 1·4)	2·2 (2·2 to 2·3)	3 (2·8 to 3·1)	2·9 (2·7 to 3)	4 (3·8 to 4·1)	3·3 (3·1 to 3·4)	3·2 (2·8 to 3·6)
Oxfordshire	5·5 (5·3 to 5·7)	6·7 (6·5 to 7)	7 (6·8 to 7·2)	6·8 (6·6 to 7)	7·8 (7·5 to 8·1)	7·1 (6·8 to 7·3)	8·3 (8 to 8·5)	7 (6·8 to 7·3)	7 (6·2 to 7·7)
Portsmouth	2·6 (2·5 to 2·6)	2·8 (2·6 to 3)	2·9 (2·7 to 3·1)	2·4 (2·3 to 2·5)	2·7 (2·6 to 2·8)	2·2 (2·1 to 2·3)	2·5 (2·4 to 2·6)	2·3 (2·2 to 2·4)	2·3 (2 to 2·5)
Reading	1·6 (1·6 to 1·7)	2 (1·9 to 2·1)	2·2 (2·1 to 2·3)	2 (1·9 to 2·1)	2 (2 to 2·1)	2 (1·9 to 2·1)	2·6 (2·5 to 2·7)	2·1 (2 to 2·2)	2·1 (1·8 to 2·3)
Slough	1·6 (1·6 to 1·6)	1·9 (1·8 to 2)	1·9 (1·8 to 1·9)	1·7 (1·6 to 1·8)	1·9 (1·8 to 2)	1·8 (1·7 to 1·9)	2·8 (2·7 to 2·9)	2·3 (2·2 to 2·4)	2·3 (2 to 2·5)
Southampton	2·7 (2·6 to 2·8)	3·2 (3 to 3·3)	3·4 (3·2 to 3·5)	2·8 (2·7 to 2·9)	3 (2·9 to 3·1)	2·4 (2·4 to 2·5)	3·2 (3·1 to 3·3)	2·9 (2·7 to 3)	2·8 (2·5 to 3·1)
Surrey	12·6 (12·2 to 13·1)	12·8 (12·4 to 13·2)	11·7 (11·4 to 12)	10·8 (10·5 to 11·2)	12·5 (12·1 to 12·9)	11·9 (11·5 to 12·3)	14 (13·5 to 14·5)	12 (11·7 to 12·4)	11·9 (10·6 to 13·2)
West Berkshire	1·3 (1·2 to 1·3)	1·6 (1·5 to 1·6)	1·6 (1·5 to 1·8)	1·6 (1·5 to 1·7)	1·9 (1·9 to 2)	1·7 (1·6 to 1·8)	2 (1·9 to 2·1)	1·5 (1·5 to 1·6)	1·5 (1·3 to 1·7)
West Sussex	7·5 (7·2 to 7·7)	7·7 (7·3 to 8)	7·4 (7·1 to 7·6)	7·4 (7·1 to 7·6)	8·4 (8·1 to 8·7)	7·8 (7·5 to 8·1)	9·2 (8·9 to 9·5)	8·1 (7·8 to 8·4)	8 (7·1 to 8·9)

Windsor and Maidenhead	1·5 (1·5 to 1·6)	1·7 (1·6 to 1·8)	1·6 (1·6 to 1·7)	1·6 (1·5 to 1·6)	1·6 (1·6 to 1·7)	1·6 (1·5 to 1·7)	1·8 (1·8 to 1·9)	1·5 (1·4 to 1·6)	1·4 (1·3 to 1·6)
Wokingham	1 (1 to 1)	1·4 (1·3 to 1·4)	1·4 (1·3 to 1·5)	1·4 (1·3 to 1·4)	1·9 (1·8 to 2)	1·7 (1·6 to 1·7)	1·9 (1·8 to 1·9)	1·7 (1·6 to 1·8)	1·7 (1·5 to 1·8)
South West England	51·1 (49·5 to 52·8)	56·4 (54·6 to 58·3)	57·3 (55·6 to 59)	50·7 (49 to 52·4)	58·3 (56·4 to 60·3)	50·1 (48·4 to 52)	60 (58·3 to 62)	51·8 (50·2 to 53·5)	51·1 (45·7 to 57)
Bath and North East Somerset	1·9 (1·8 to 1·9)	1·8 (1·8 to 1·9)	1·7 (1·7 to 1·8)	1·7 (1·6 to 1·7)	1·9 (1·8 to 1·9)	1·7 (1·6 to 1·7)	1·8 (1·7 to 1·8)	1·7 (1·6 to 1·7)	1·7 (1·5 to 1·9)
Bournemouth	1·9 (1·8 to 1·9)	1·6 (1·5 to 1·7)	1·5 (1·4 to 1·6)	1·3 (1·2 to 1·3)	1·7 (1·6 to 1·8)	1·6 (1·5 to 1·6)	2·3 (2·2 to 2·4)	2·1 (1·9 to 2·2)	2·1 (1·9 to 2·3)
Bristol, City of	5·3 (5·2 to 5·5)	6 (5·7 to 6·2)	6 (5·8 to 6·3)	5 (4·8 to 5·2)	5·5 (5·3 to 5·8)	4·7 (4·5 to 4·9)	6·1 (5·9 to 6·3)	5·4 (5·2 to 5·7)	5·3 (4·7 to 5·9)
Cornwall	5 (4·9 to 5·2)	5·2 (5·1 to 5·4)	5·4 (5·3 to 5·6)	4·9 (4·7 to 5)	5·6 (5·4 to 5·8)	4·6 (4·4 to 4·8)	5·6 (5·5 to 5·8)	4·8 (4·6 to 4·9)	4·7 (4·2 to 5·3)
Devon	6·7 (6·5 to 6·9)	6·7 (6·2 to 7·2)	6·6 (6·3 to 7)	6·1 (5·9 to 6·3)	7·5 (7·2 to 7·8)	6·4 (6·1 to 6·6)	7·5 (7·2 to 7·7)	6·4 (6·2 to 6·6)	6·3 (5·7 to 7·1)
Dorset	3·6 (3·5 to 3·7)	3·7 (3·5 to 4)	3·7 (3·5 to 3·9)	3·5 (3·2 to 3·7)	3·9 (3·8 to 4·1)	3·4 (3·2 to 3·5)	3·9 (3·7 to 4)	2·9 (2·8 to 3)	2·8 (2·5 to 3·2)
Gloucestershire	5·9 (5·7 to 6·1)	6·7 (6·5 to 7)	6·8 (6·6 to 7)	6 (5·8 to 6·2)	6·9 (6·7 to 7·2)	6 (5·8 to 6·2)	6·9 (6·6 to 7·2)	6 (5·8 to 6·2)	5·9 (5·3 to 6·6)
North Somerset	1·8 (1·7 to 1·9)	2 (1·9 to 2)	2 (2 to 2·1)	1·9 (1·8 to 2)	2 (1·9 to 2·1)	1·9 (1·8 to 2)	2·3 (2·2 to 2·4)	2 (1·9 to 2)	1·9 (1·7 to 2·2)
Plymouth	3·2 (3·1 to 3·3)	3·9 (3·7 to 4)	4 (3·9 to 4·2)	3·5 (3·4 to 3·6)	3·5 (3·4 to 3·7)	2·6 (2·5 to 2·7)	3·1 (3 to 3·2)	2·6 (2·5 to 2·7)	2·5 (2·2 to 2·8)
Poole	1·3 (1·3 to 1·4)	1·4 (1·4 to 1·5)	1·5 (1·4 to 1·5)	1·3 (1·2 to 1·4)	1·7 (1·6 to 1·8)	1·4 (1·3 to 1·5)	1·7 (1·7 to 1·8)	1·5 (1·4 to 1·6)	1·5 (1·3 to 1·7)
Somerset	5 (4·8 to 5·1)	5·5 (5·3 to 5·7)	5·6 (5·4 to 5·7)	5 (4·9 to 5·2)	5·8 (5·5 to 6)	4·9 (4·7 to 5·1)	5·9 (5·7 to 6·1)	5 (4·9 to 5·2)	5 (4·4 to 5·6)
South Gloucestershire	2·1 (2 to 2·1)	2·9 (2·8 to 3)	2·9 (2·8 to 3)	2·5 (2·4 to 2·6)	3 (2·9 to 3·2)	2·8 (2·7 to 2·9)	3·1 (3 to 3·2)	3 (2·9 to 3·1)	3 (2·7 to 3·4)
Swindon	1·8 (1·8 to 1·9)	2·4 (2·3 to 2·5)	2·4 (2·3 to 2·4)	2·2 (2·1 to 2·2)	2·7 (2·6 to 2·8)	2·3 (2·2 to 2·4)	3 (2·9 to 3·1)	2·6 (2·5 to 2·7)	2·5 (2·3 to 2·8)
Torbay	1·3 (1·3 to 1·4)	1·2 (1·2 to 1·3)	1·2 (1·2 to 1·3)	1·1 (1 to 1·1)	1·3 (1·3 to 1·4)	1·2 (1·1 to 1·2)	1·4 (1·4 to 1·5)	1·2 (1·1 to 1·2)	1·1 (1 to 1·3)
Wiltshire	4·3 (4·1 to 4·4)	5·4 (5·2 to 5·6)	5·8 (5·6 to 6)	4·8 (4·6 to 5)	5·3 (5·1 to 5·5)	4·8 (4·6 to 5)	5·6 (5·4 to 5·8)	4·6 (4·5 to 4·8)	4·6 (4·1 to 5·1)
West Midlands	75 (72·7 to 77·2)	91·7 (89·6 to 93·9)	90·5 (88·5 to 92·5)	68·7 (66·7 to 70·8)	74·7 (72·5 to 77)	61·7 (59·8 to 63·7)	72·5 (70·5 to 74·8)	64·8 (62·6 to 66·8)	63·7 (56·8 to 70·9)
Birmingham	18·1 (17·5 to 18·6)	21·1 (20·5 to 21·7)	20·4 (19·6 to 21·2)	15·5 (15 to 16·1)	16·8 (16·3 to 17·4)	14·4 (13·9 to 14·9)	17 (16·6 to 17·6)	15·2 (14·7 to 15·7)	14·9 (13·3 to 16·5)
Coventry	5 (4·9 to 5·1)	6·2 (6·1 to 6·4)	6·1 (5·9 to 6·4)	4·5 (4·3 to 4·7)	4·6 (4·4 to 4·8)	3·6 (3·5 to 3·8)	4·5 (4·4 to 4·7)	4·1 (3·9 to 4·2)	4 (3·6 to 4·5)
Dudley	4·2 (4 to 4·3)	5·4 (5·1 to 5·7)	5·4 (5·1 to 5·6)	3·8 (3·6 to 3·9)	4·2 (4 to 4·3)	3·4 (3·3 to 3·6)	3·8 (3·7 to 4)	3·5 (3·3 to 3·6)	3·4 (3 to 3·8)
Herefordshire, County of	2 (1·9 to 2)	2·2 (2·1 to 2·3)	2·1 (2 to 2·2)	1·7 (1·6 to 1·9)	2 (2 to 2·1)	1·6 (1·6 to 1·7)	1·9 (1·8 to 2)	1·6 (1·5 to 1·7)	1·6 (1·4 to 1·8)
Sandwell	5·6 (5·4 to 5·8)	6·5 (6·2 to 6·7)	6·2 (6 to 6·5)	4·3 (4·2 to 4·5)	4·5 (4·4 to 4·7)	3·8 (3·6 to 3·9)	5 (4·8 to 5·2)	4·4 (4·2 to 4·5)	4·3 (3·8 to 4·8)
Shropshire	3·1 (3 to 3·2)	3·7 (3·5 to 3·9)	3·7 (3·5 to 3·8)	2·8 (2·7 to 2·9)	3·3 (3·1 to 3·4)	2·8 (2·6 to 2·9)	3 (2·9 to 3·1)	2·6 (2·5 to 2·8)	2·6 (2·3 to 2·9)
Solihull	2·5 (2·4 to 2·6)	3·3 (3·2 to 3·5)	3·3 (3·2 to 3·4)	2·3 (2·2 to 2·3)	2·6 (2·5 to 2·7)	2 (1·9 to 2·1)	2·2 (2·2 to 2·3)	2·1 (2·1 to 2·2)	2·1 (1·9 to 2·3)
Staffordshire	9·1 (8·9 to 9·4)	12 (11·4 to 12·6)	12·3 (11·8 to 12·9)	9·8 (9·4 to 10·2)	10·4 (10 to 10·8)	8·1 (7·8 to 8·5)	9·1 (8·8 to 9·4)	8·1 (7·8 to 8·4)	8 (7·1 to 8·9)
Stoke-on-Trent	4·3 (4·2 to 4·5)	4·9 (4·7 to 5·1)	4·8 (4·6 to 5)	3·5 (3·4 to 3·6)	3·7 (3·6 to 3·8)	2·8 (2·7 to 3)	3·7 (3·6 to 3·9)	3·2 (3 to 3·3)	3·1 (2·7 to 3·4)
Telford and Wrekin	1·3 (1·3 to 1·4)	1·7 (1·6 to 1·7)	1·9 (1·8 to 2)	1·9 (1·8 to 1·9)	2·2 (2·1 to 2·2)	1·9 (1·9 to 2)	2·3 (2·2 to 2·4)	2 (1·9 to 2·1)	2 (1·8 to 2·2)
Walsall	4·1 (4 to 4·2)	5·5 (5·3 to 5·6)	5·2 (5·1 to 5·4)	3·6 (3·4 to 3·7)	3·9 (3·7 to 4)	3·3 (3·2 to 3·4)	3·8 (3·6 to 3·9)	3·5 (3·4 to 3·7)	3·5 (3·1 to 3·8)
Warwickshire	6 (5·8 to 6·2)	7·5 (7·3 to 7·7)	7·5 (7·2 to 7·7)	5·7 (5·5 to 5·9)	6·2 (6 to 6·4)	5·3 (5·1 to 5·5)	6·2 (6·1 to 6·4)	5·8 (5·6 to 6)	5·7 (5·1 to 6·3)
Wolverhampton	4·2 (4·1 to 4·3)	5·1 (4·9 to 5·3)	4·8 (4·6 to 4·9)	3·5 (3·4 to 3·6)	3·8 (3·7 to 4)	2·9 (2·8 to 3)	3·5 (3·4 to 3·6)	3·2 (3·1 to 3·4)	3·2 (2·8 to 3·5)
Worcestershire	5·6 (5·4 to 5·7)	6·6 (6·3 to 7)	6·8 (6·5 to 7·1)	5·9 (5·7 to 6·1)	6·5 (6·3 to 6·7)	5·7 (5·5 to 5·9)	6·4 (6·2 to 6·6)	5·5 (5·3 to 5·7)	5·4 (4·8 to 6)
Yorkshire and the Humber	71·2 (69·1 to 73·4)	81·7 (79·8 to 83·6)	79 (76·7 to 81)	63·5 (61·5 to 65·6)	68·9 (66·6 to 71·2)	56·2 (54·2 to 58·2)	66·7 (65 to 68·7)	57·1 (55·1 to 59·1)	55·9 (49·6 to 62·2)
Barnsley	3·4 (3·3 to 3·5)	4 (3·9 to 4·1)	3·9 (3·8 to 4)	3 (2·9 to 3·1)	3·1 (3 to 3·2)	2·3 (2·2 to 2·5)	3 (2·9 to 3·1)	2·6 (2·4 to 2·7)	2·5 (2·2 to 2·8)
Bradford	7·9 (7·6 to 8·1)	9 (8·7 to 9·3)	8·8 (8·5 to 9·1)	7·5 (7·2 to 7·7)	7·7 (7·4 to 8)	7·1 (6·8 to 7·4)	8·6 (8·4 to 8·9)	7 (6·7 to 7·3)	6·8 (6·1 to 7·6)
Calderdale	3·1 (3 to 3·2)	3·3 (3·2 to 3·4)	3·2 (3·1 to 3·2)	2·5 (2·5 to 2·6)	2·8 (2·7 to 2·9)	2·3 (2·2 to 2·4)	2·7 (2·6 to 2·8)	2·2 (2·1 to 2·3)	2·1 (1·9 to 2·4)
Doncaster	4·2 (4·1 to 4·4)	5·2 (5·1 to 5·4)	5 (4·9 to 5·1)	4 (3·9 to 4·2)	4·3 (4·1 to 4·4)	3·2 (3·1 to 3·4)	4 (3·8 to 4·1)	3·3 (3·2 to 3·5)	3·3 (2·9 to 3·6)
East Riding of Yorkshire	3·4 (3·3 to 3·5)	3·7 (3·6 to 3·8)	3·5 (3·4 to 3·6)	2·9 (2·8 to 3)	3·3 (3·2 to 3·5)	2·8 (2·7 to 3)	3·2 (3·1 to 3·3)	2·7 (2·5 to 2·8)	2·6 (2·3 to 2·9)
Kingston upon Hull, City of	4·2 (4·1 to 4·3)	5·1 (4·9 to 5·2)	5·1 (4·9 to 5·2)	3·9 (3·8 to 4·1)	4·3 (4·1 to 4·4)	2·9 (2·8 to 3·1)	3·6 (3·4 to 3·7)	3·1 (3 to 3·3)	3 (2·7 to 3·4)
Kirklees	5·7 (5·6 to 5·9)	6·4 (6·3 to 6·6)	6·4 (6·2 to 6·6)	5·3 (5·1 to 5·5)	5·5 (5·3 to 5·7)	5 (4·8 to 5·2)	5·9 (5·7 to 6·1)	4·8 (4·6 to 5)	4·7 (4·2 to 5·2)
Leeds	10·7 (10·4 to 11·1)	12·2 (11·9 to 12·6)	11·5 (11·2 to 11·8)	8·9 (8·6 to 9·2)	9·7 (9·4 to 10·1)	7·8 (7·6 to 8·1)	9·6 (9·3 to 9·9)	9 (8·7 to 9·4)	8·8 (7·8 to 9·8)
North East Lincolnshire	2·4 (2·3 to 2·5)	3 (2·9 to 3·1)	3 (2·9 to 3·1)	2·3 (2·2 to 2·4)	2·4 (2·3 to 2·5)	1·7 (1·7 to 1·8)	2·1 (2 to 2·1)	1·6 (1·5 to 1·7)	1·5 (1·4 to 1·7)
North Lincolnshire	2·1 (2 to 2·2)	2·5 (2·4 to 2·6)	2·5 (2·4 to 2·5)	2 (1·9 to 2·1)	2·1 (2 to 2·1)	1·6 (1·5 to 1·7)	2 (1·9 to 2)	1·6 (1·5 to 1·6)	1·5 (1·4 to 1·7)
North Yorkshire	6·3 (6·1 to 6·5)	6·9 (6·6 to 7·1)	6·4 (6·3 to 6·6)	5·5 (5·3 to 5·7)	6·5 (6·3 to 6·7)	5·6 (5·4 to 5·8)	6·1 (5·9 to 6·3)	5·1 (4·9 to 5·3)	5 (4·4 to 5·5)
Rotherham	3·6 (3·5 to 3·7)	4·4 (4·3 to 4·5)	4·5 (4·3 to 4·6)	3·5 (3·4 to 3·6)	3·7 (3·6 to 3·8)	2·8 (2·7 to 2·9)	3·2 (3·1 to 3·3)	2·8 (2·7 to 2·9)	2·7 (2·4 to 3)
Sheffield	7·6 (7·3 to 7·8)	8·3 (8 to 8·7)	7·8 (7·3 to 8·3)	6 (5·7 to 6·2)	6·8 (6·5 to 7)	5·7 (5·5 to 5·9)	6·5 (6·3 to 6·7)	5·8 (5·6 to 6·1)	5·8 (5·2 to 6·5)
Wakefield	4·5 (4·4 to 4·7)	5·4 (5·2 to 5·5)	5·3 (5·2 to 5·5)	4·2 (4·1 to 4·4)	4·5 (4·4 to 4·7)	3·4 (3·3 to 3·6)	4·3 (4·1 to 4·4)	3·9 (3·7 to 4)	3·8 (3·4 to 4·2)
York	2 (2 to 2·1)	2·2 (2·1 to 2·3)	2·1 (2 to 2·2)	1·9 (1·8 to 1·9)	2·1 (2 to 2·2)	1·8 (1·7 to 1·9)	2 (1·9 to 2·1)	1·7 (1·6 to 1·8)	1·7 (1·5 to 1·9)
Northern Ireland	28·8 (27·9 to 29·7)	31·7 (31·2 to 32·2)	31·7 (31·1 to 32·5)	27·7 (26·8 to 28·6)	25·9 (24·9 to 27)	21·8 (21·1 to 22·6)	25·2 (24·2 to 26·3)	21·8 (20·9 to 22·9)	20·8 (18·5 to 23·2)
Scotland	92 (89·1 to 95·1)	102 (99·7 to 104)	88·6 (86·9 to 90·4)	68 (65·8 to 70·2)	66·1 (63·5 to 68·7)	53·2 (51·3 to 55·2)	58·7 (56·6 to 61·1)	49 (47·1 to 51·2)	48·2 (42·8 to 53·9)
Wales	38·6 (37·4 to 39·8)	45·4 (44·6 to 46·2)	45·3 (44·5 to 46·1)	38·4 (37·1 to 39·6)	38·7 (37·3 to 40·3)	31·8 (30·8 to 32·9)	36·4 (35 to 37·9)	29·3 (28·3 to 30·4)	28·4 (25·3 to 31·7)
Latin America and Caribbean	6280 (6040 to 6530)	8020 (7810 to 8230)	9400 (9200 to 9590)	10300 (10100 to 10500)	10400 (10200 to 10600)	10700 (10400 to 10900)	10500 (10100 to 10800)	9740 (9190 to 10300)	9380 (8690 to 10100)
Andean Latin America	680 (656 to 702)	860 (836 to 882)	1020 (993 to 1040)	1120 (1090 to 1140)	1190 (1150 to 1220)	1210 (1180 to 1250)	1230 (1170 to 1280)	1260 (1170 to 1350)	1240 (1150 to 1350)
Bolivia	150 (143 to 158)	170 (163 to 176)	192 (183 to 199)	216 (209 to 223)	227 (219 to 235)	240 (230 to 249)	255 (242 to 268)	249 (230 to 270)	245 (224 to 270)
Ecuador	150 (142 to 158)	202 (198 to 206)	240 (235 to 246)	250 (241 to 259)	300 (285 to 314)	335 (318 to 353)	348 (327 to 369)	334 (308 to 361)	322 (285 to 364)
Peru	380 (366 to 393)	488 (472 to 502)	585 (568 to 602)	650 (631 to 670)	658 (636 to 680)	639 (615 to 664)	623 (592 to 655)	673 (615 to 738)	676 (611 to 748)
Caribbean	700 (683 to 715)	869 (854 to 883)	900 (881 to 917)	837 (818 to 857)	906 (881 to 930)	860 (829 to 890)	831 (795 to 866)	809 (755 to 868)	797 (734 to 869)
Antigua and Barbuda	1·7 (1·6 to 1·7)	1·8 (1·8 to 1·9)	1·6 (1·5 to 1·6)	1·2 (1·2 to 1·3)	1·2 (1·1 to 1·2)	1·4 (1·3 to 1·6)	1·2 (1·2 to 1·3)	1·1 (1 to 1·2)	1 (0·9 to 1·2)
The Bahamas	2·6 (2·5 to 2·7)	3·4 (3·3 to 3·5)	4·3 (4·2 to 4·4)	5 (4·8 to 5·2)	5·4 (5·1 to 5·6)	4·4 (4·2 to 4·6)	4·8 (4·5 to 5)	3·9 (3·4 to 4·6)	3·9 (3·3 to 4·5)

Barbados	6·7 (6·4 to 7·1)	7·2 (7 to 7·4)	5·1 (4·9 to 5·2)	4·3 (4·1 to 4·5)	4·2 (4 to 4·3)	3·9 (3·7 to 4·1)	3·3 (3·2 to 3·5)	2·7 (2·3 to 3·1)	2·6 (2·2 to 3·1)
Belize	2·8 (2·7 to 3)	4·2 (4·1 to 4·3)	4·8 (4·7 to 4·9)	5·7 (5·5 to 5·8)	6·4 (6·1 to 6·6)	6·7 (6·3 to 7·2)	7·4 (7 to 7·8)	7·5 (6·9 to 8·2)	7·6 (6·7 to 8·5)
Bermuda	1·1 (1·1 to 1·2)	1·1 (1·1 to 1·2)	1 (0·9 to 1·1)	0·8 (0·8 to 0·9)	0·9 (0·9 to 0·9)	0·8 (0·8 to 0·9)	0·7 (0·7 to 0·8)	0·5 (0·5 to 0·6)	0·5 (0·4 to 0·5)
Cuba	151 (144 to 158)	225 (220 to 230)	245 (238 to 252)	141 (136 to 146)	177 (170 to 183)	149 (143 to 155)	130 (125 to 136)	108 (103 to 113)	99·6 (92·7 to 107)
Dominica	1·9 (1·8 to 2)	2·7 (2·6 to 2·8)	2·7 (2·7 to 2·8)	1·9 (1·8 to 2)	1·9 (1·8 to 2)	1·2 (1·1 to 1·3)	0·9 (0·9 to 1)	0·7 (0·6 to 0·8)	0·6 (0·5 to 0·7)
Dominican Republic	132 (129 to 136)	172 (168 to 175)	189 (180 to 197)	210 (200 to 220)	230 (218 to 241)	222 (209 to 235)	200 (186 to 215)	214 (196 to 234)	214 (193 to 236)
Grenada	3·5 (3·3 to 3·6)	3·8 (3·7 to 3·9)	2·8 (2·7 to 2·9)	2·7 (2·5 to 2·8)	2·4 (2·3 to 2·6)	2·3 (2 to 2·6)	1·9 (1·7 to 2·1)	1·4 (1·3 to 1·6)	1·4 (1·2 to 1·6)
Guyana	19·4 (18·7 to 20·2)	25·7 (25·2 to 26·2)	26·2 (25·5 to 27)	26·1 (24·8 to 27·3)	27·2 (25·6 to 28·7)	20·2 (19·4 to 21·2)	16·1 (15·2 to 17)	15·9 (14·3 to 17·7)	15·3 (13·5 to 17·4)
Haiti	175 (167 to 183)	192 (183 to 199)	201 (193 to 208)	229 (221 to 236)	255 (246 to 263)	276 (262 to 290)	317 (299 to 336)	341 (307 to 377)	344 (308 to 387)
Jamaica	50·1 (46·9 to 53·6)	67·5 (65·6 to 69·3)	65·1 (63·5 to 66·9)	58·2 (56·3 to 60·2)	57·2 (55·1 to 59·4)	49·5 (44·4 to 55·4)	41·8 (39·9 to 43·8)	34·1 (30·5 to 38)	32·8 (28·4 to 37·5)
Puerto Rico	84·9 (83·2 to 86·8)	75·2 (74 to 76·4)	68·2 (66·6 to 69·7)	71·7 (69·8 to 73·7)	65·3 (63·5 to 67·3)	57·3 (54·8 to 59·9)	42 (38·6 to 45·9)	20·9 (20·2 to 21·6)	19 (17·7 to 20·5)
Saint Kitts and Nevis	1·9 (1·8 to 1·9)	2·1 (2·1 to 2·2)	1·1 (1·1 to 1·2)	1·2 (1·1 to 1·3)	0·9 (0·9 to 1)	0·8 (0·8 to 0·9)	0·6 (0·6 to 0·7)	0·6 (0·6 to 0·6)	0·6 (0·5 to 0·6)
Saint Lucia	2·9 (2·8 to 3·1)	4 (4 to 4·1)	4·3 (4·2 to 4·3)	3·9 (3·8 to 4)	3·5 (3·3 to 3·8)	2·8 (2·6 to 2·9)	2·1 (1·9 to 2·3)	1·8 (1·5 to 2)	1·7 (1·4 to 2)
Saint Vincent and the Grenadines	2·8 (2·7 to 2·9)	3·9 (3·9 to 4)	3·4 (3·4 to 3·5)	3·2 (3·1 to 3·3)	2·6 (2·4 to 2·8)	2·1 (2 to 2·2)	1·8 (1·7 to 1·9)	1·4 (1·3 to 1·5)	1·3 (1·1 to 1·5)
Suriname	7·8 (7·5 to 8·1)	11·8 (11·6 to 12)	13·2 (12·6 to 13·8)	10·3 (10 to 10·7)	9·6 (9·2 to 9·9)	9·5 (8·7 to 10·4)	10 (9·5 to 10·4)	9·3 (8·6 to 10·2)	9 (8·1 to 10)
Trinidad and Tobago	23·7 (23 to 24·6)	32·4 (31·7 to 33)	26·2 (25·6 to 26·9)	31 (29·9 to 32·1)	23·8 (22·9 to 24·7)	17·8 (17 to 18·5)	18·9 (17·8 to 20·2)	15·8 (14·6 to 17·1)	14·7 (13 to 16·7)
Virgin Islands	0·9 (0·8 to 0·9)	1·2 (1·1 to 1·2)	2·6 (2·5 to 2·8)	2·5 (2·3 to 2·6)	2·4 (2·2 to 2·5)	2·1 (1·8 to 2·3)	1·5 (1·3 to 1·7)	0·9 (0·8 to 1)	0·8 (0·7 to 0·9)
Central Latin America	2330 (2230 to 2430)	3260 (3200 to 3310)	4130 (4070 to 4200)	4480 (4380 to 4580)	5020 (4910 to 5130)	4910 (4790 to 5050)	4840 (4670 to 5010)	4120 (3820 to 4420)	3880 (3480 to 4310)
Colombia	500 (466 to 537)	691 (660 to 720)	790 (748 to 833)	824 (775 to 876)	924 (874 to 982)	899 (826 to 971)	775 (684 to 874)	690 (597 to 795)	675 (577 to 789)
Costa Rica	38·2 (37·1 to 39·4)	59·4 (58·9 to 59·8)	55·3 (54 to 56·5)	69·7 (68 to 71·6)	80·5 (77·8 to 83·2)	76 (73·7 to 78·5)	72·3 (69·8 to 74·7)	61 (58 to 64·1)	54·6 (49·8 to 59·7)
El Salvador	97·8 (95·4 to 100)	128 (127 to 130)	160 (157 to 163)	181 (177 to 185)	177 (171 to 184)	154 (146 to 162)	133 (127 to 140)	121 (110 to 133)	116 (102 to 131)
Guatemala	146 (144 to 148)	187 (184 to 190)	220 (217 to 224)	304 (299 to 309)	342 (336 to 348)	384 (373 to 396)	364 (352 to 378)	358 (339 to 378)	345 (309 to 384)
Honduras	73·7 (70 to 77·2)	91·5 (88·6 to 94·4)	117 (114 to 120)	155 (151 to 158)	180 (172 to 187)	194 (182 to 205)	207 (192 to 222)	220 (196 to 246)	220 (195 to 252)
Mexico	1170 (1120 to 1230)	1650 (1610 to 1680)	2250 (2220 to 2280)	2280 (2220 to 2330)	2570 (2500 to 2640)	2490 (2430 to 2560)	2490 (2400 to 2580)	1990 (1850 to 2120)	1860 (1690 to 2040)
Aguascalientes	9·6 (9·1 to 10)	12·7 (12·4 to 13)	17·1 (16·9 to 17·4)	19·2 (18·7 to 19·8)	23·5 (22·8 to 24·3)	25·5 (24·7 to 26·4)	27·3 (25·7 to 28·9)	23·9 (22·2 to 25·7)	22·7 (20·5 to 25)
Baja California	10·4 (9·9 to 11)	23·9 (23·3 to 24·4)	39·7 (39·1 to 40·4)	36·6 (35·4 to 37·9)	49·5 (48·1 to 51·2)	58·1 (56·1 to 60·2)	65 (62·1 to 68)	54·8 (51·2 to 58·5)	51·3 (46·6 to 56·2)
Baja California Sur	2·5 (2·4 to 2·7)	3·6 (3·5 to 3·7)	5·6 (5·5 to 5·7)	6·8 (6·6 to 7)	8·8 (8·5 to 9·1)	10·2 (9·9 to 10·6)	13·2 (12·6 to 13·8)	11·4 (10·6 to 12·3)	10·8 (9·8 to 11·9)
Campeche	5 (4·7 to 5·2)	7·3 (7·1 to 7·5)	11·4 (11·2 to 11·7)	14·8 (14·4 to 15·3)	17·3 (16·8 to 17·9)	16·8 (16·2 to 17·4)	18·5 (17·8 to 19·1)	14·5 (13·5 to 15·6)	13·4 (12·2 to 14·8)
Chiapas	37·1 (35·1 to 39·3)	56·7 (54·8 to 58·3)	78·5 (76·7 to 80)	90·5 (87·4 to 93·3)	136 (132 to 140)	128 (124 to 134)	146 (140 to 152)	126 (117 to 135)	120 (108 to 132)
Chihuahua	40·5 (38·6 to 42·5)	58·9 (57·6 to 60·1)	75·4 (74 to 76·7)	62·5 (60·9 to 64·1)	69·6 (67·5 to 71·9)	79·4 (77·1 to 81·7)	70·4 (67·7 to 73·3)	55·2 (51·2 to 59·1)	51·6 (46·7 to 56·7)
Coahuila	33·4 (31·9 to 35·2)	43·4 (42·5 to 44·1)	52·8 (51·8 to 53·9)	50·2 (48·6 to 51·8)	57·7 (55·8 to 59·7)	55·3 (53·2 to 57·4)	60·1 (57·7 to 62·6)	51·6 (47·7 to 55·4)	48·3 (43·7 to 52·9)
Colima	5·8 (5·5 to 6·1)	8·2 (8·1 to 8·4)	11·6 (11·3 to 11·8)	11·6 (11·2 to 12)	11·9 (11·4 to 12·4)	11·4 (11 to 11·9)	13·2 (12·6 to 13·8)	10·6 (9·9 to 11·4)	9·9 (8·9 to 11)
Durango	31·1 (29·6 to 32·7)	39·1 (38·4 to 39·8)	46·5 (45·9 to 47·2)	42·8 (41·4 to 44)	46 (44·6 to 47·4)	41·7 (40·2 to 43·2)	39·3 (37·5 to 41·1)	32·5 (30·2 to 34·9)	30·6 (27·8 to 33·7)
Guanajuato	67 (63·9 to 70·3)	89·2 (87·1 to 91·4)	115 (113 to 117)	117 (114 to 120)	133 (128 to 137)	128 (124 to 132)	127 (122 to 133)	105 (97·2 to 112)	98·3 (89 to 108)
Guerrero	42·3 (40·2 to 44·5)	60·1 (58·6 to 61·5)	81·5 (80·1 to 83)	85·7 (83·1 to 88·4)	103 (99·5 to 106)	104 (101 to 108)	97·4 (92·8 to 102)	74·2 (68·7 to 79·5)	69·1 (62·4 to 76·1)
Hidalgo	35·6 (33·8 to 37·6)	44·8 (43·6 to 45·9)	55·3 (54·4 to 56·3)	55·2 (53·7 to 56·7)	64·1 (62·1 to 66·1)	59·3 (57·4 to 61·4)	59·8 (57·6 to 62·1)	45·9 (42·7 to 49·3)	43·1 (39 to 47·5)
Jalisco	86·4 (82·4 to 90·7)	118 (116 to 120)	158 (156 to 160)	149 (145 to 153)	166 (161 to 172)	158 (152 to 164)	162 (156 to 167)	135 (126 to 145)	127 (115 to 139)
México	59·7 (56·9 to 62·8)	88·4 (86·5 to 90·2)	179 (176 to 182)	254 (247 to 262)	300 (291 to 312)	322 (312 to 333)	322 (307 to 336)	244 (228 to 262)	227 (206 to 250)
Mexico City	135 (127 to 143)	209 (202 to 215)	272 (265 to 279)	232 (224 to 239)	203 (196 to 210)	162 (157 to 168)	142 (138 to 148)	97 (89·8 to 104)	87·9 (79·5 to 97·1)
Michoacán de Ocampo	70·7 (67·5 to 74·3)	93·2 (91 to 95·4)	114 (112 to 116)	114 (111 to 117)	124 (120 to 128)	107 (104 to 111)	106 (101 to 111)	85·7 (79·6 to 92·1)	80·3 (72·9 to 88·3)
Morelos	12·1 (11·5 to 12·7)	18·3 (17·9 to 18·7)	28·5 (28 to 29)	31·9 (31·1 to 32·6)	35·7 (34·6 to 36·8)	34·5 (33·3 to 35·8)	37·7 (36 to 39·3)	29·4 (27·4 to 31·3)	27·4 (24·9 to 30)
Nayarit	14·6 (13·9 to 15·4)	19·8 (19·5 to 20·1)	26·7 (26·3 to 27·2)	25·6 (25 to 26·2)	26·2 (25·4 to 26·9)	23·5 (22·8 to 24·3)	24·6 (23·6 to 25·6)	19·3 (17·9 to 20·7)	18·1 (16·3 to 19·8)
Nuevo León	34·3 (32·7 to 36)	53·3 (52·3 to 54·3)	76·4 (75·1 to 77·7)	71·2 (69·2 to 73·3)	81·2 (78·4 to 84·2)	88·8 (85·9 to 91·7)	93·9 (89·8 to 97·9)	84·7 (79·1 to 90·4)	80·9 (73·5 to 88·8)
Oaxaca	57·8 (54·3 to 61·3)	76·6 (73·9 to 79·1)	94·2 (92·6 to 95·9)	89·1 (86·6 to 91·5)	101 (98·1 to 105)	99 (95·8 to 102)	96·3 (92·2 to 101)	73·4 (68·1 to 78·8)	68·6 (62·3 to 75·6)
Puebla	72 (68·5 to 75·8)	94 (91·6 to 96·1)	124 (122 to 125)	127 (123 to 130)	142 (138 to 146)	144 (140 to 148)	146 (140 to 153)	121 (112 to 129)	114 (103 to 125)
Querétaro	13·8 (13·2 to 14·5)	17·9 (17·5 to 18·3)	23·7 (23·4 to 24·1)	27·1 (26·4 to 27·8)	36·8 (35·7 to 37·9)	37 (35·6 to 38·4)	40·3 (38·3 to 42·3)	37·2 (34·4 to 40)	35·8 (32·4 to 39·5)
Quintana Roo	1·2 (1·2 to 1·3)	2·4 (2·4 to 2·5)	4·4 (4·3 to 4·5)	8·9 (8·6 to 9·2)	16 (15·4 to 16·6)	21·8 (20·7 to 22·9)	28·8 (27·1 to 30·7)	28·7 (26·7 to 30·9)	28 (25·2 to 30·9)
San Luis Potosí	38·4 (36·6 to 40·4)	49·8 (48·6 to 50·8)	62 (61·1 to 62·8)	60·3 (58·4 to 62·2)	67·2 (65·2 to 69·2)	61·5 (59·5 to 63·6)	57·5 (54·7 to 60·4)	45·5 (42·3 to 48·8)	42·7 (38·7 to 47)
Sinaloa	30·5 (29·1 to 32)	42·4 (41·7 to 43·1)	62·6 (61·7 to 63·5)	63·6 (61·9 to 65·4)	67·7 (65·5 to 69·9)	65·4 (62·9 to 68)	58·7 (56·1 to 61·3)	46·5 (43·1 to 49·8)	43·5 (39·5 to 47·9)
Sonora	24·2 (23·1 to 25·5)	36·2 (35·5 to 36·9)	49·3 (48·5 to 50·1)	44·2 (42·9 to 45·6)	51·8 (50·3 to 53·5)	54·6 (52·9 to 56·3)	55·5 (53·4 to 57·7)	40·2 (37·3 to 43·3)	37 (33·5 to 40·5)
Tabasco	16 (15·2 to 16·8)	24·8 (24·2 to 25·2)	38·1 (37·5 to 38·7)	43·3 (42·2 to 44·3)	52·1 (50·4 to 53·8)	49·9 (47·9 to 51·9)	51·7 (49·3 to 54·1)	40·1 (37·3 to 42·9)	37 (33·7 to 40·7)
Tamaulipas	32·5 (31 to 34·2)	48·3 (47·3 to 49·3)	66·4 (65·4 to 67·5)	58·3 (56·3 to 60·2)	63·1 (61 to 65·3)	64·7 (62·5 to 67·1)	67·4 (64·5 to 70·1)	51·6 (47·9 to 55·1)	47·9 (43·4 to 52·6)
Tlaxcala	12·9 (12·3 to 13·5)	16·4 (16 to 16·7)	19·6 (19·3 to 20)	20 (19·5 to 20·5)	25 (24·3 to 25·8)	26·1 (25·3 to 27)	27 (25·8 to 28·3)	22·7 (21·1 to 24·4)	21·5 (19·4 to 23·7)
Veracruz de Ignacio de la Llave	85·9 (81·2 to 90·8)	124 (120 to 127)	177 (174 to 181)	183 (178 to 188)	202 (196 to 209)	177 (171 to 184)	164 (158 to 171)	116 (108 to 124)	106 (96·1 to 117)
Yucatán	20·4 (19·2 to 21·5)	25·9 (25·2 to 26·7)	34·7 (34·1 to 35·4)	35·9 (34·8 to 37)	41 (39·6 to 42·4)	38 (36·7 to 39·4)	38·2 (36·3 to 40·1)	32·5 (30·2 to 34·9)	30·7 (27·9 to 33·7)
Zacatecas	33·2 (31·7 to 34·8)	42·1 (41·3 to 42·9)	47·3 (46·4 to 48·3)	44·2 (43 to 45·4)	42·1 (40·6 to 43·6)	36·3 (35·1 to 37·6)	35·6 (34 to 37·2)	29·3 (27 to 31·7)	27·6 (24·8 to 30·3)
Nicaragua	54·6 (52 to 57·5)	71·5 (69·5 to 73·6)	93·9 (91·6 to 96)	128 (125 to 130)	142 (135 to 147)	132 (124 to 140)	134 (124 to 144)	130 (117 to 144)	127 (111 to 143)
Panama	26·2 (25·3 to 27·2)	45·1 (44·5 to 45·8)	53·9 (52·8 to 55)	53·9 (52·2 to 55·5)	58·5 (56·6 to 60·4)	63·2 (60·8 to 65·7)	70·5 (68·1 to 73)	71·6 (68·4 to 74·9)	69·8 (62·7 to 77·3)
Venezuela	219 (212 to 226)	334 (330 to 339)	394 (386 to 402)	487 (473 to 502)	548 (526 to 570)	520 (491 to 551)	594 (572 to 615)	483 (422 to 554)	413 (356 to 471)
Tropical Latin America	2570 (2400 to 2740)	3030 (2890 to 3180)	3350 (3210 to 3490)	3880 (3770 to 3990)	3320 (3230 to 3410)	3690 (3570 to 3800)	3550 (3410 to 3690)	3560 (3340 to 3820)	3460 (3190 to 3780)

Brazil	2500 (2340 to 2670)	2960 (2810 to 3100)	3260 (3130 to 3400)	3770 (3670 to 3880)	3190 (3100 to 3280)	3550 (3440 to 3670)	3420 (3290 to 3550)	3430 (3210 to 3690)	3330 (3060 to 3650)
Acre	5·8 (5·5 to 6)	7·5 (7·2 to 7·8)	9·5 (9·2 to 9·8)	12·6 (12·3 to 13)	11·8 (11·4 to 12·3)	16·6 (16 to 17·2)	18·9 (18·1 to 19·7)	20 (18·5 to 21·6)	20 (18·4 to 21·8)
Alagoas	59·9 (56·8 to 62·7)	64·3 (61·8 to 66·7)	74 (71·5 to 76·4)	82·6 (80·3 to 84·8)	75·9 (73·3 to 78·6)	89·7 (85·9 to 93·5)	87·4 (82·9 to 91·4)	85·3 (78·9 to 92·7)	83·8 (76·4 to 92·5)
Amapá	2 (1·9 to 2·1)	3·4 (3·2 to 3·5)	5 (4·8 to 5·2)	7 (6·8 to 7·2)	7·3 (7·1 to 7·6)	12·3 (11·8 to 12·8)	13·5 (12·8 to 14·3)	15·1 (13·9 to 16·5)	15 (13·7 to 16·6)
Amazonas	26·6 (25 to 28·2)	32·4 (30·9 to 33·9)	38·3 (36·5 to 40·1)	50·7 (49·1 to 52·3)	46·4 (44·4 to 48·3)	66·9 (63·8 to 70)	76 (72·2 to 80·1)	82 (76·1 to 88·8)	81·1 (74·4 to 88·8)
Bahia	258 (243 to 272)	293 (280 to 305)	336 (323 to 349)	375 (363 to 386)	288 (278 to 299)	327 (313 to 342)	316 (299 to 332)	301 (278 to 327)	292 (265 to 322)
Ceará	140 (133 to 148)	165 (158 to 171)	190 (182 to 197)	200 (193 to 207)	166 (159 to 172)	200 (190 to 209)	207 (195 to 219)	212 (196 to 230)	208 (189 to 229)
Distrito Federal	2 (1·9 to 2·1)	5 (4·7 to 5·3)	21·2 (20·4 to 22)	40·7 (39·6 to 41·9)	38·3 (37·1 to 39·5)	46·4 (44·9 to 47·8)	42·9 (41·3 to 44·6)	42·6 (40·2 to 45·2)	40·7 (38 to 43·9)
Espírito Santo	40·8 (38·2 to 43·2)	50·1 (47·8 to 52·5)	54·4 (52 to 56·9)	65·5 (63·6 to 67·4)	56·9 (55·1 to 58·7)	62·9 (60·3 to 65·6)	58·9 (56·4 to 61·4)	60·5 (55·7 to 65·6)	58·6 (53·5 to 64·5)
Goiás	49·8 (46·4 to 53)	70·2 (66·7 to 73·6)	86·3 (82·3 to 90·4)	96·8 (93·8 to 100)	82·8 (80·5 to 85·5)	94 (90·1 to 97·8)	86·5 (82·7 to 90·5)	93 (86·6 to 101)	90·6 (82·9 to 99·3)
Maranhão	90·2 (85·8 to 94·3)	127 (123 to 132)	148 (144 to 153)	183 (178 to 187)	157 (151 to 163)	192 (184 to 201)	215 (204 to 225)	223 (207 to 241)	222 (204 to 242)
Mato Grosso	9·2 (8·7 to 9·8)	15·1 (14·4 to 15·8)	25·2 (24·1 to 26·3)	38·5 (37·5 to 39·6)	41·2 (39·9 to 42·6)	48·2 (46·1 to 50·1)	48 (45·8 to 50·1)	55·8 (51·4 to 60·7)	55·1 (50·3 to 60·5)
Mato Grosso do Sul	15·4 (14·4 to 16·4)	23·5 (22·4 to 24·7)	33·4 (31·8 to 35·1)	44·1 (42·8 to 45·3)	39·4 (38·1 to 40·7)	42·2 (40·5 to 43·9)	42 (40·2 to 43·7)	44·4 (41·4 to 47·8)	43·3 (39·6 to 47·3)
Minas Gerais	375 (350 to 400)	414 (394 to 434)	381 (364 to 397)	392 (381 to 403)	332 (322 to 342)	339 (328 to 351)	303 (290 to 316)	298 (279 to 317)	288 (266 to 311)
Pará	57·4 (53·8 to 60·8)	68·9 (65·6 to 72·2)	86·7 (82·4 to 90·8)	123 (119 to 127)	109 (104 to 113)	145 (138 to 151)	158 (150 to 167)	164 (152 to 178)	162 (148 to 179)
Paraíba	92 (86·9 to 96·8)	98·2 (93·8 to 102)	97·7 (93·5 to 102)	88·3 (84·9 to 91·9)	71 (68 to 73·9)	83·1 (79·2 to 87)	87·2 (82·2 to 92·2)	87·1 (81 to 94·4)	85·1 (78 to 93·4)
Paraná	101 (94·3 to 108)	172 (164 to 181)	219 (209 to 230)	210 (203 to 216)	179 (173 to 185)	177 (170 to 184)	155 (148 to 162)	158 (146 to 172)	153 (140 to 168)
Pernambuco	190 (179 to 199)	212 (203 to 220)	230 (221 to 240)	234 (226 to 241)	180 (174 to 187)	208 (199 to 218)	200 (190 to 211)	192 (177 to 208)	186 (171 to 204)
Piauí	53·9 (51 to 56·7)	60 (57·5 to 62·5)	71·8 (68·8 to 74·6)	81·4 (78·7 to 84)	67 (64·5 to 69·3)	77 (73·6 to 80·3)	80·1 (75·2 to 84·7)	78·4 (72·4 to 85·1)	76·6 (69·8 to 84·6)
Rio de Janeiro	200 (182 to 218)	227 (211 to 243)	241 (228 to 254)	279 (271 to 287)	229 (223 to 235)	242 (234 to 250)	219 (211 to 228)	216 (203 to 230)	207 (192 to 224)
Rio Grande do Norte	52 (49·4 to 54·5)	57·9 (55·5 to 60·3)	66 (63·4 to 68·5)	70·5 (67·8 to 73·1)	63·2 (60·8 to 65·5)	76·1 (72·5 to 79·6)	75·3 (71·2 to 79·3)	73·7 (67·5 to 80·6)	71·8 (64·6 to 79·9)
Rio Grande do Sul	172 (157 to 186)	182 (171 to 194)	176 (168 to 184)	191 (185 to 197)	156 (152 to 161)	154 (149 to 159)	125 (121 to 130)	126 (119 to 133)	120 (112 to 128)
Rondônia	1·8 (1·7 to 1·9)	2·9 (2·8 to 3·1)	4·4 (4·3 to 4·6)	16·8 (16·4 to 17·3)	26·4 (25·5 to 27·5)	32·4 (30·9 to 33·8)	31·1 (29·1 to 33)	31·7 (29·3 to 34·3)	30·9 (28·4 to 33·7)
Roraima	0·9 (0·8 to 0·9)	1·1 (1 to 1·2)	1·5 (1·5 to 1·6)	2·7 (2·7 to 2·8)	5·1 (4·9 to 5·3)	8·6 (8·2 to 9)	10·8 (10·3 to 11·3)	13·7 (12·8 to 14·7)	14 (13 to 15·1)
Santa Catarina	66·8 (61·9 to 71·6)	76·7 (72·4 to 81·1)	78·7 (74·6 to 82·8)	91·3 (88·1 to 94·3)	81·2 (78·2 to 84·3)	85·2 (81·6 to 88·4)	81·1 (77·5 to 84·9)	95·6 (88·3 to 104)	94·1 (85·8 to 103)
São Paulo	402 (370 to 436)	483 (454 to 514)	535 (508 to 562)	740 (720 to 761)	619 (603 to 635)	649 (630 to 668)	597 (576 to 622)	580 (539 to 625)	553 (502 to 609)
Sergipe	32·8 (30·9 to 34·7)	34 (32·4 to 35·5)	35·8 (34·3 to 37·3)	38·3 (37 to 39·5)	36·1 (34·8 to 37·4)	45 (42·9 to 47)	45·2 (42·6 to 47·8)	45·2 (41·7 to 49·3)	44·3 (40·4 to 49)
Tocantins	7·6 (7·2 to 8)	11·3 (10·8 to 11·8)	16·4 (15·7 to 17·1)	20·8 (20·1 to 21·5)	24·6 (23·5 to 25·8)	34·9 (33·5 to 36·3)	36·9 (34·9 to 38·8)	37·7 (34·3 to 41·1)	37 (33·4 to 41)
Paraguay	66·5 (63·7 to 69·6)	76·2 (72·5 to 79·9)	87·5 (82·1 to 92·5)	107 (98·7 to 116)	128 (118 to 140)	131 (120 to 144)	135 (119 to 153)	130 (113 to 150)	128 (110 to 148)
North Africa and Middle East	4780 (4480 to 5080)	5950 (5760 to 6120)	8090 (7920 to 8240)	11000 (10800 to 11200)	11100 (10800 to 11400)	11600 (11100 to 12000)	13800 (13200 to 14400)	12800 (12000 to 13600)	12100 (11200 to 13200)
North Africa and Middle East	4780 (4480 to 5080)	5950 (5760 to 6120)	8090 (7920 to 8240)	11000 (10800 to 11200)	11100 (10800 to 11400)	11600 (11100 to 12000)	13800 (13200 to 14400)	12800 (12000 to 13600)	12100 (11200 to 13200)
Afghanistan	354 (337 to 370)	425 (412 to 438)	517 (503 to 530)	616 (598 to 633)	449 (436 to 462)	783 (764 to 800)	910 (875 to 946)	1160 (1100 to 1220)	1210 (1150 to 1280)
Algeria	416 (392 to 439)	485 (466 to 502)	614 (588 to 639)	794 (782 to 807)	773 (749 to 800)	653 (599 to 710)	902 (848 to 957)	963 (887 to 1040)	908 (819 to 1000)
Bahrain	4 (3·7 to 4·3)	6·5 (6·1 to 6·9)	7·2 (6·9 to 7·5)	10 (9·8 to 10·3)	13·4 (13 to 13·7)	12·4 (12 to 12·9)	19·7 (19·1 to 20·5)	18 (16·6 to 19·5)	16·9 (15 to 18·8)
Egypt	557 (491 to 630)	753 (706 to 800)	1400 (1360 to 1450)	1850 (1790 to 1910)	1900 (1820 to 1980)	1960 (1860 to 2060)	3030 (2890 to 3160)	2770 (2580 to 2970)	2610 (2340 to 2920)
Iran	772 (722 to 823)	911 (874 to 949)	1150 (1130 to 1180)	1950 (1920 to 1970)	1540 (1480 to 1600)	1110 (1040 to 1190)	1380 (1300 to 1480)	1190 (1100 to 1280)	1030 (897 to 1170)
Alborz	7·9 (7·4 to 8·4)	15·6 (15 to 16·1)	24·8 (24·1 to 25·5)	42·4 (41·2 to 43·6)	32·4 (30·5 to 34·4)	27·2 (24·8 to 29·9)	36·6 (33·5 to 39·7)	32 (29 to 35·3)	27·8 (23·9 to 32)
Ardebil	21·3 (20 to 22·5)	23·3 (22·3 to 24·3)	28·6 (27·8 to 29·4)	47 (46·2 to 47·9)	35·7 (34 to 37·4)	23·5 (21·6 to 25·3)	23·6 (21·9 to 25·3)	18·3 (17·1 to 19·6)	15·7 (14·1 to 17·5)
Bushehr	6 (5·6 to 6·4)	8·4 (8·1 to 8·8)	12·1 (11·7 to 12·4)	22·6 (22 to 23·2)	20·3 (19·4 to 21·2)	13·6 (12·7 to 14·5)	17·2 (15·1 to 19·6)	18·1 (16·8 to 19·4)	16·4 (14·7 to 18·2)
Chahar Mahaal and Bakhtiari	10 (9·3 to 10·6)	10·8 (10·3 to 11·3)	13·4 (13·1 to 13·8)	24·7 (24 to 25·3)	22·1 (21·1 to 23·2)	14·7 (13·7 to 15·8)	18·4 (16·4 to 20·7)	16·6 (15·5 to 17·8)	14·4 (12·7 to 16·3)
East Azarbayejan	66·4 (61·8 to 71)	73·7 (70·5 to 76·8)	85·4 (83·1 to 87·9)	136 (133 to 139)	95·3 (91·7 to 99·1)	62·5 (58·3 to 67)	68·2 (63·3 to 73·1)	52·2 (47·9 to 56·6)	44·7 (38·4 to 51·6)
Fars	26 (24·2 to 27·8)	41·5 (39·7 to 43·2)	63·7 (61·9 to 65·5)	118 (116 to 120)	92·4 (87·7 to 97·6)	65·2 (60·6 to 70)	78·9 (73·3 to 84·6)	63·4 (56·6 to 71·1)	54·2 (45 to 64·8)
Gilan	36·3 (33·6 to 39)	44·5 (42·4 to 46·6)	55·3 (53·6 to 56·9)	80·2 (78 to 82·7)	50·1 (47·8 to 52·3)	30·1 (27·7 to 32·4)	31·2 (28·9 to 33·6)	24·2 (22·1 to 26·5)	20·6 (17·9 to 23·8)
Golestan	21·5 (20·2 to 22·8)	22·6 (21·6 to 23·8)	27·5 (26·7 to 28·5)	50·7 (49·6 to 51·9)	42·1 (40 to 44·1)	29 (26·8 to 31·3)	37·8 (34·9 to 41)	31·9 (29·9 to 33·9)	27·3 (24·6 to 30·3)
Hamadan	28·9 (27·1 to 30·8)	33·6 (32·1 to 35·2)	39·5 (38·4 to 40·7)	62·3 (61·1 to 63·5)	48·2 (45·8 to 50·6)	30·1 (27·5 to 32·5)	31·6 (29·5 to 33·6)	23·6 (22 to 25·2)	19·7 (17·7 to 22)
Hormozgan	9·7 (9·1 to 10·3)	12·9 (12·4 to 13·4)	17 (16·5 to 17·6)	30·4 (29·7 to 31)	29·5 (28·2 to 30·9)	23·6 (22·2 to 25)	33·9 (31·4 to 36·5)	34·4 (32·2 to 36·7)	30·7 (27·6 to 33·9)
Ilam	3·9 (3·7 to 4·2)	4·8 (4·6 to 5)	7·2 (7 to 7·4)	15·1 (14·8 to 15·3)	13·4 (12·8 to 13·9)	8·7 (8·1 to 9·3)	9·3 (8·4 to 10·3)	8·6 (7·9 to 9·3)	7·5 (6·5 to 8·5)
Isfahan	36·4 (33·8 to 38·9)	47·4 (45·3 to 49·6)	60·3 (58·7 to 62·1)	108 (105 to 110)	86·7 (81·9 to 92·2)	60·2 (54·7 to 66·3)	74 (68·2 to 79·5)	61·3 (53·8 to 69·4)	52·3 (42·5 to 63·9)
Kerman	39·6 (37·2 to 42·1)	33 (31·7 to 34·3)	36·7 (35·7 to 37·6)	67·7 (65·9 to 69·5)	50·3 (47·7 to 52·8)	37·5 (34·9 to 40·1)	56·9 (50·9 to 63·5)	50·9 (47·5 to 54·3)	44·1 (39·5 to 49·2)
Kermanshah	21·7 (20·2 to 23·2)	26·7 (25·6 to 27·9)	34·8 (33·9 to 35·8)	61·8 (60·4 to 63·2)	47·6 (45·3 to 49·7)	30·8 (28·3 to 33·1)	34·3 (30·8 to 38·3)	26·8 (25 to 28·7)	22·7 (20·2 to 25·5)
Khorasan-e-Razavi	73 (69·1 to 76·8)	81·3 (78·2 to 84·5)	95·6 (93·1 to 97·8)	168 (164 to 173)	138 (131 to 145)	98·7 (91·5 to 106)	130 (115 to 146)	111 (103 to 119)	96·5 (85·6 to 109)
Khuzestan	64·3 (60·3 to 68·3)	62 (59·7 to 64·3)	78·2 (76·1 to 80·6)	129 (126 to 132)	94·9 (91·6 to 98·5)	80·6 (75·3 to 86·1)	104 (97·8 to 111)	87·3 (80·4 to 94·6)	75·1 (64·9 to 86·1)
Kohgiluyeh and Boyer-Ahmad	3·7 (3·5 to 3·9)	5·2 (5 to 5·4)	7·7 (7·5 to 7·9)	15·7 (15·4 to 15·9)	16 (15·4 to 16·7)	12·1 (11·3 to 12·9)	12·7 (11·6 to 13·9)	12·8 (11·9 to 13·9)	11·6 (10·2 to 13·3)
Kurdistan	23·7 (22·2 to 25·2)	25·1 (24·1 to 26·2)	27·6 (26·8 to 28·3)	45 (44·1 to 45·9)	36·1 (34·5 to 37·8)	25·3 (23·1 to 27·4)	27·4 (25·3 to 29·7)	24 (22·3 to 25·6)	20·9 (18·7 to 23·1)
Lorestan	31·9 (30 to 33·7)	28·1 (26·9 to 29·3)	31·8 (31 to 32·7)	57·8 (56·6 to 59)	50·6 (48·3 to 53)	32·2 (29·6 to 34·8)	34·7 (30·2 to 39·7)	27·5 (25·5 to 29·5)	23·4 (20·9 to 26·2)
Markazi	8·9 (8·2 to 9·6)	17·8 (17 to 18·7)	29·3 (28·5 to 30·1)	44·1 (43·1 to 45·1)	28·4 (26·8 to 29·9)	18 (16·6 to 19·5)	21 (18·7 to 23·4)	16·7 (15·6 to 17·8)	14·2 (12·7 to 15·9)
Mazandaran	48·3 (44·7 to 52)	45·8 (43·8 to 47·9)	51·7 (50·3 to 53·3)	90·1 (87·7 to 92·4)	62·1 (59·3 to 65)	38·5 (35·4 to 41·5)	42·9 (39·9 to 46·3)	36 (32·7 to 39·5)	30·9 (26·7 to 35·6)
North Khorasan	8·5 (8 to 9)	9·8 (9·5 to 10·2)	12·2 (11·8 to 12·6)	22·1 (21·7 to 22·6)	22·9 (21·9 to 23·9)	15·8 (14·6 to 17)	18·9 (17·1 to 20·9)	14·7 (13·7 to 15·7)	12·5 (11·3 to 13·9)
Qazvin	19·7 (18·3 to 21·2)	21·3 (20·5 to 22·3)	22·5 (21·9 to 23·1)	31·8 (31 to 32·5)	24·4 (23·3 to 25·5)	17·1 (15·9 to 18·5)	20·4 (18·3 to 22·7)	17 (15·7 to 18·3)	14·6 (12·7 to 16·6)
Qom	4 (3·7 to 4·3)	8·1 (7·7 to 8·4)	13·9 (13·7 to 14·2)	23·7 (23·2 to 24·2)	19 (18·2 to 19·8)	15·5 (14·4 to 16·6)	20·9 (19·2 to 22·8)	20·5 (18·8 to 22·2)	18·2 (15·8 to 20·8)

Semnan	4 (3·7 to 4·3)	6·1 (5·8 to 6·4)	8·6 (8·4 to 8·9)	14·1 (13·7 to 14·5)	9·8 (9·3 to 10·3)	6·8 (6·3 to 7·4)	8·8 (8·1 to 9·8)	8·8 (8·2 to 9·5)	7·9 (7 to 8·8)
Sistan and Baluchistan	20·3 (19 to 21·6)	19·3 (18·5 to 20·1)	22·9 (22·3 to 23·5)	46·9 (46 to 47·9)	54·1 (51·8 to 56·6)	53·5 (50 to 57·1)	86 (82 to 90·6)	84·6 (80·1 to 89·3)	77·1 (69·1 to 86)
South Khorasan	10·8 (10·2 to 11·5)	12·9 (12·3 to 13·4)	15·9 (15·5 to 16·4)	26·9 (26·4 to 27·5)	21·5 (20·4 to 22·6)	12·3 (11·4 to 13·3)	14·2 (12·6 to 16·1)	15·1 (14·1 to 16·1)	13·7 (12·3 to 15·2)
Tehran	53 (49·1 to 57)	95·3 (91 to 99·8)	142 (137 to 147)	228 (219 to 238)	184 (173 to 197)	144 (133 to 156)	184 (172 to 197)	158 (139 to 178)	136 (112 to 165)
West Azarbayejan	24·9 (23·1 to 26·6)	35·1 (33·6 to 36·8)	47·3 (46·2 to 48·5)	82·8 (80·9 to 84·8)	66·8 (64·1 to 69·6)	50·4 (46·2 to 54·6)	65 (61·1 to 69·2)	55 (50·9 to 59·1)	47·4 (42·5 to 52·7)
Yazd	11 (10·3 to 11·6)	11·5 (11 to 12)	12·9 (12·5 to 13·3)	20·8 (20·4 to 21·1)	18 (17·2 to 18·9)	14·6 (13·5 to 15·7)	20·8 (19·3 to 22·5)	18·4 (16·9 to 19·9)	16 (14 to 18·3)
Zanjan	26·6 (25 to 28·2)	27·9 (26·8 to 28·9)	27·4 (26·5 to 28·2)	34·7 (34·2 to 35·3)	25·8 (24·7 to 27)	17·8 (16·6 to 18·9)	19·7 (18·5 to 21)	15·2 (14 to 16·6)	13 (11·3 to 14·9)
Iraq	223 (209 to 236)	309 (295 to 321)	433 (421 to 446)	606 (587 to 624)	751 (715 to 786)	932 (885 to 985)	1030 (948 to 1110)	972 (892 to 1060)	933 (854 to 1030)
Jordan	23·4 (23 to 23·7)	39·8 (39·2 to 40·4)	62·7 (61·8 to 63·7)	91·6 (90·1 to 93·1)	133 (130 to 137)	150 (142 to 158)	198 (187 to 208)	212 (194 to 233)	212 (187 to 238)
Kuwait	2·7 (2·5 to 2·9)	11·2 (11 to 11·4)	34·8 (34·4 to 35·3)	50·2 (48·9 to 51·6)	34·4 (32·1 to 36·8)	41·1 (39 to 43·2)	56·1 (52·7 to 59·9)	53·1 (48·7 to 58)	50·9 (44·1 to 59)
Lebanon	59 (56 to 61·9)	71·4 (68·2 to 74·4)	76·8 (72·6 to 80·7)	79·4 (74·3 to 84·5)	87·7 (81 to 94·3)	91·7 (86·1 to 97·7)	89·6 (81·7 to 97·9)	89·5 (77·4 to 104)	81·3 (69·4 to 96·1)
Libya	52 (50·6 to 53·3)	68·8 (67·1 to 70·3)	84·3 (82·4 to 86·2)	122 (119 to 124)	133 (126 to 142)	124 (111 to 136)	112 (99·5 to 125)	84·8 (72·2 to 101)	79·1 (66·1 to 94·5)
Morocco	463 (443 to 483)	577 (557 to 594)	706 (686 to 724)	807 (772 to 842)	804 (752 to 855)	835 (766 to 904)	838 (762 to 914)	690 (629 to 763)	647 (587 to 714)
Oman	23·9 (23·1 to 24·7)	31·7 (30·8 to 32·5)	41 (39·8 to 42·1)	59·1 (57·5 to 60·7)	73·4 (71·3 to 75·4)	56 (51·7 to 60·4)	65·7 (63 to 68·7)	83·3 (77·2 to 89·6)	81·7 (74·1 to 89·8)
Palestine	36·8 (35·7 to 38)	44·8 (43·7 to 46)	51·7 (50·3 to 53·1)	62·8 (60·4 to 65·1)	87·7 (83·9 to 91·3)	117 (112 to 122)	133 (125 to 141)	123 (113 to 135)	120 (110 to 132)
Qatar	1·2 (1·2 to 1·3)	2·5 (2·4 to 2·5)	4·7 (4·6 to 4·9)	8·2 (8 to 8·5)	11·6 (11·1 to 12·1)	13·2 (12·4 to 14)	27 (25·5 to 28·4)	36·8 (34 to 40)	38 (34·4 to 42)
Saudi Arabia	131 (125 to 138)	172 (166 to 178)	254 (245 to 262)	397 (377 to 416)	513 (478 to 548)	494 (454 to 535)	520 (460 to 579)	486 (420 to 560)	462 (396 to 535)
Sudan	239 (229 to 248)	294 (283 to 304)	492 (475 to 509)	702 (679 to 724)	915 (883 to 946)	1140 (1100 to 1190)	1270 (1180 to 1350)	1210 (1100 to 1300)	1170 (1070 to 1280)
Syria	166 (160 to 170)	224 (218 to 230)	291 (282 to 300)	373 (360 to 386)	456 (431 to 480)	495 (459 to 532)	525 (475 to 580)	206 (176 to 241)	197 (166 to 234)
Tunisia	166 (158 to 175)	191 (183 to 199)	197 (191 to 204)	222 (214 to 230)	220 (209 to 232)	186 (174 to 200)	199 (186 to 212)	180 (163 to 199)	166 (146 to 192)
Türkiye	859 (798 to 922)	1050 (998 to 1100)	1300 (1240 to 1350)	1610 (1540 to 1680)	1520 (1440 to 1590)	1440 (1340 to 1540)	1330 (1260 to 1400)	1150 (1050 to 1250)	1050 (956 to 1160)
United Arab Emirates	3·6 (3·4 to 3·7)	4·2 (4·1 to 4·4)	9·1 (8·8 to 9·5)	35·5 (34·6 to 36·5)	48·3 (45 to 51·5)	49·4 (46·2 to 52·6)	89 (82·9 to 95·6)	84 (75·4 to 93·4)	74 (64·1 to 84·7)
Yemen	224 (215 to 233)	279 (272 to 285)	358 (350 to 365)	514 (505 to 523)	660 (647 to 674)	854 (822 to 884)	1060 (1010 to 1120)	1010 (913 to 1110)	989 (886 to 1110)
South Asia	20500 (19200 to 21700)	23700 (22300 to 25000)	26600 (25500 to 27700)	31600 (30200 to 32800)	32600 (31400 to 33800)	34800 (33600 to 36000)	35500 (34200 to 36700)	32900 (30800 to 35200)	32000 (29200 to 35200)
South Asia	20500 (19200 to 21700)	23700 (22300 to 25000)	26600 (25500 to 27700)	31600 (30200 to 32800)	32600 (31400 to 33800)	34800 (33600 to 36000)	35500 (34200 to 36700)	32900 (30800 to 35200)	32000 (29200 to 35200)
Bangladesh	2070 (1990 to 2140)	2320 (2250 to 2380)	3090 (3040 to 3140)	3640 (3560 to 3720)	4160 (4060 to 4260)	4070 (3920 to 4200)	3780 (3620 to 3950)	2970 (2750 to 3200)	2810 (2480 to 3150)
Bhutan	8·5 (8·1 to 8·9)	11 (10·6 to 11·4)	14·8 (14·1 to 15·6)	19·4 (18·5 to 20·2)	22·3 (21·4 to 23·2)	17·8 (17 to 18·6)	14·6 (13·7 to 15·5)	13·2 (12·1 to 14·2)	12·6 (11·5 to 13·8)
India	16400 (15300 to 17500)	19100 (17800 to 20400)	20400 (19400 to 21400)	23500 (22300 to 24700)	23400 (22300 to 24500)	25400 (24200 to 26400)	25400 (24300 to 26500)	23100 (21300 to 25100)	22400 (19900 to 25100)
Nepal	417 (398 to 436)	469 (452 to 486)	564 (548 to 579)	728 (702 to 752)	794 (771 to 814)	782 (757 to 808)	650 (608 to 693)	645 (580 to 712)	642 (576 to 712)
Pakistan	1610 (1540 to 1680)	1740 (1680 to 1800)	2550 (2460 to 2640)	3660 (3530 to 3770)	4210 (4070 to 4350)	4580 (4420 to 4750)	5630 (5360 to 5890)	6180 (5590 to 6810)	6190 (5530 to 6960)
Azad Jammu & Kashmir	42·1 (40·9 to 43·3)	42·3 (41 to 43·6)	57·2 (54·6 to 60)	80·1 (75·6 to 84·6)	85·3 (80·4 to 89·9)	78·5 (71·6 to 85·1)	85·6 (76·7 to 96)	82·1 (70·8 to 95)	78·7 (67·3 to 93·4)
Balochistan	53·6 (50·8 to 56·3)	44·2 (42·5 to 45·8)	82·7 (79·2 to 85·9)	168 (161 to 174)	207 (198 to 217)	240 (229 to 250)	337 (319 to 354)	443 (403 to 484)	462 (416 to 514)
Gilgit-Baltistan	11·3 (10·8 to 11·8)	11·5 (11 to 12)	17·4 (16·6 to 18·3)	26·5 (25·3 to 27·6)	31·1 (29·3 to 32·9)	36·9 (34·6 to 39·1)	48·2 (45·5 to 51)	57·7 (52·8 to 62·7)	59·3 (53·9 to 65·3)
Islamabad Capital Territory	5 (4·8 to 5·2)	5·8 (5·6 to 6·1)	8·2 (7·7 to 8·7)	12·2 (11·5 to 13)	15·6 (14·3 to 17·1)	23·1 (20·7 to 25·4)	36·7 (32·6 to 40·9)	43·4 (38·7 to 48·5)	44·1 (39 to 49·6)
Khyber Pakhtunkhwa	292 (278 to 304)	301 (291 to 311)	418 (402 to 434)	562 (542 to 582)	639 (617 to 660)	771 (741 to 802)	1030 (978 to 1080)	1160 (1050 to 1270)	1170 (1050 to 1310)
Punjab	926 (885 to 966)	1000 (965 to 1030)	1410 (1370 to 1460)	1980 (1910 to 2050)	2280 (2200 to 2350)	2380 (2290 to 2470)	2830 (2690 to 2980)	3070 (2780 to 3380)	3060 (2740 to 3440)
Sindh	284 (272 to 295)	339 (327 to 350)	553 (534 to 571)	826 (803 to 850)	957 (923 to 988)	1060 (1020 to 1100)	1260 (1200 to 1320)	1320 (1190 to 1470)	1320 (1160 to 1490)
Southeast Asia, east Asia, and Oceania	31200 (29600 to 32900)	35500 (34500 to 36500)	37200 (36400 to 37900)	31700 (30800 to 32800)	36300 (34800 to 37600)	28400 (27400 to 29400)	26200 (25100 to 27400)	25800 (24400 to 27300)	22800 (21200 to 24400)
East Asia	22400 (21200 to 23600)	24300 (23500 to 25100)	25200 (24600 to 25700)	18900 (18100 to 19600)	23800 (22400 to 25000)	16200 (15300 to 17100)	13600 (12600 to 14700)	14000 (13100 to 15100)	11200 (10200 to 12200)
China	21600 (20500 to 22800)	23400 (22600 to 24100)	24300 (23700 to 24900)	18000 (17200 to 18800)	22900 (21600 to 24100)	15400 (14500 to 16300)	13000 (12000 to 14100)	13500 (12600 to 14600)	10700 (9810 to 11800)
North Korea	425 (399 to 451)	503 (468 to 537)	486 (446 to 529)	451 (409 to 500)	552 (507 to 603)	521 (477 to 567)	388 (354 to 420)	318 (283 to 356)	299 (263 to 340)
Taiwan (province of China)	366 (358 to 376)	423 (416 to 430)	373 (362 to 384)	406 (391 to 420)	323 (310 to 337)	272 (257 to 286)	192 (182 to 205)	171 (158 to 184)	157 (140 to 176)
Oceania	121 (117 to 126)	151 (146 to 156)	170 (165 to 176)	194 (187 to 200)	225 (217 to 234)	278 (265 to 290)	348 (330 to 366)	416 (383 to 451)	431 (394 to 472)
American Samoa	0·8 (0·8 to 0·8)	0·8 (0·8 to 0·8)	1 (1 to 1·1)	1·1 (1·1 to 1·1)	1·8 (1·7 to 1·8)	1·7 (1·6 to 1·8)	1·3 (1·2 to 1·3)	0·8 (0·8 to 0·9)	0·8 (0·7 to 0·9)
Cook Islands	0·6 (0·6 to 0·6)	0·9 (0·8 to 0·9)	0·8 (0·8 to 0·8)	0·4 (0·4 to 0·5)	0·4 (0·4 to 0·5)	0·3 (0·3 to 0·4)	0·3 (0·2 to 0·3)	0·2 (0·2 to 0·2)	0·2 (0·2 to 0·3)
Fiji	11·5 (11 to 12)	15·7 (15·3 to 16·1)	16 (15·4 to 16·5)	18·8 (18 to 19·6)	18·7 (17·7 to 19·7)	17·9 (16·4 to 19·4)	19·5 (18·5 to 20·4)	17·4 (15·9 to 19·1)	16·7 (14·8 to 18·8)
Guam	1·8 (1·7 to 1·8)	2·5 (2·4 to 2·5)	2·9 (2·8 to 3)	2·9 (2·8 to 3·1)	3·9 (3·7 to 4)	3·7 (3·5 to 3·9)	3·4 (3·3 to 3·6)	2·9 (2·7 to 3)	2·7 (2·4 to 3)
Kiribati	1·3 (1·3 to 1·3)	1·5 (1·5 to 1·6)	1·7 (1·6 to 1·8)	2·3 (2·1 to 2·4)	2·7 (2·5 to 2·9)	2·6 (2·4 to 2·8)	2·9 (2·7 to 3·2)	2·9 (2·7 to 3·3)	2·9 (2·6 to 3·3)
Marshall Islands	0·6 (0·6 to 0·6)	0·7 (0·7 to 0·7)	0·9 (0·9 to 1)	1·3 (1·3 to 1·4)	1·5 (1·4 to 1·6)	1·6 (1·6 to 1·7)	1·5 (1·4 to 1·6)	1·2 (1·1 to 1·3)	1·2 (1 to 1·3)
Federated States of Micronesia	2 (2 to 2·1)	2 (2 to 2·1)	2·5 (2·4 to 2·5)	3·2 (3·1 to 3·4)	3·1 (2·9 to 3·3)	2·8 (2·5 to 3)	2·2 (2 to 2·4)	1·9 (1·6 to 2·2)	1·9 (1·6 to 2·2)
Nauru	0·1 (0·1 to 0·1)	0·1 (0·1 to 0·1)	0·3 (0·2 to 0·3)	0·3 (0·3 to 0·4)	0·4 (0·3 to 0·4)	0·4 (0·3 to 0·4)	0·3 (0·3 to 0·4)	0·3 (0·3 to 0·3)	0·3 (0·3 to 0·3)
Niue	0·2 (0·2 to 0·2)	0·2 (0·2 to 0·2)	0·2 (0·2 to 0·2)	0·1 (0·1 to 0·1)	0 (0 to 0)	0 (0 to 0)	0 (0 to 0)	0 (0 to 0)	0 (0 to 0)
Northern Mariana Islands	0·2 (0·2 to 0·2)	0·2 (0·2 to 0·2)	0·2 (0·2 to 0·3)	0·4 (0·3 to 0·4)	1·2 (1·1 to 1·4)	2·5 (2·2 to 2·7)	0·8 (0·8 to 0·9)	0·6 (0·5 to 0·7)	0·6 (0·5 to 0·7)
Palau	0·3 (0·3 to 0·3)	0·3 (0·3 to 0·3)	0·3 (0·2 to 0·3)	0·2 (0·2 to 0·3)	0·3 (0·3 to 0·3)	0·3 (0·3 to 0·4)	0·3 (0·3 to 0·3)	0·2 (0·2 to 0·2)	0·2 (0·2 to 0·2)
Papua New Guinea	81·5 (77·4 to 85·4)	99·7 (95·3 to 104)	113 (108 to 117)	125 (120 to 129)	148 (141 to 154)	194 (184 to 204)	261 (245 to 276)	330 (302 to 360)	345 (313 to 382)
Samoa	4·5 (4·4 to 4·6)	5·3 (5·2 to 5·5)	5·8 (5·6 to 6·1)	5·8 (5·5 to 6)	5·5 (5·2 to 5·9)	5·6 (5·2 to 6)	5·7 (5·2 to 6·1)	6·1 (5·7 to 6·6)	6·2 (5·7 to 6·8)
Solomon Islands	4·8 (4·7 to 4·9)	6·4 (6·2 to 6·6)	7·7 (7·4 to 7·9)	9·9 (9·5 to 10·2)	13·9 (13·3 to 14·5)	17·5 (16·7 to 18·4)	19·2 (18 to 20·4)	20·2 (18·6 to 21·7)	20·5 (19 to 22·1)
Tokelau	0·1 (0·1 to 0·1)	0·1 (0·1 to 0·1)	0·1 (0·1 to 0·1)	0 (0 to 0)	0 (0 to 0)	0 (0 to 0)	0 (0 to 0)	0 (0 to 0)	0 (0 to 0)
Tonga	2·2 (2·1 to 2·3)	2·9 (2·8 to 3)	3·5 (3·4 to 3·6)	3·6 (3·5 to 3·7)	3·3 (3·2 to 3·4)	3·2 (3·1 to 3·4)	3·2 (3·1 to 3·4)	3 (2·8 to 3·3)	3 (2·7 to 3·2)
Tuvalu	0·2 (0·2 to 0·2)	0·3 (0·2 to 0·3)	0·3 (0·3 to 0·3)	0·4 (0·4 to 0·4)	0·4 (0·4 to 0·4)	0·3 (0·3 to 0·3)	0·3 (0·2 to 0·3)	0·3 (0·2 to 0·3)	0·3 (0·2 to 0·3)

Vanuatu	2·1 (2 to 2·2)	3 (2·9 to 3·1)	3·9 (3·8 to 4)	5·1 (4·9 to 5·3)	6·2 (5·9 to 6·5)	7·3 (7 to 7·6)	8·2 (7·8 to 8·6)	8·7 (7·9 to 9·5)	8·7 (8 to 9·5)
Southeast Asia	8700 (8280 to 9100)	11000 (10700 to 11400)	11900 (11500 to 12200)	12700 (12400 to 13000)	12300 (12000 to 12600)	11900 (11600 to 12200)	12300 (11800 to 12700)	11400 (10700 to 12200)	11200 (10300 to 12100)
Cambodia	212 (200 to 222)	265 (254 to 276)	306 (288 to 324)	352 (337 to 366)	432 (419 to 444)	365 (354 to 377)	365 (347 to 382)	372 (343 to 401)	373 (342 to 404)
Indonesia	3630 (3450 to 3800)	4860 (4650 to 5080)	5190 (5020 to 5340)	5180 (5020 to 5340)	4650 (4490 to 4810)	4700 (4510 to 4910)	4830 (4590 to 5070)	4460 (4050 to 4890)	4390 (3940 to 4940)
Aceh	59·4 (56·3 to 62·5)	81·8 (78·4 to 85·2)	95·4 (92·5 to 98·2)	108 (105 to 112)	102 (97·8 to 106)	106 (101 to 110)	99·9 (95·2 to 104)	91·3 (83·2 to 100)	90·8 (81·2 to 102)
Bali	67·6 (63·9 to 71·3)	82·4 (78·9 to 86)	86·9 (83·8 to 90·3)	74·3 (71·1 to 77·6)	61·6 (58·6 to 64·6)	62·6 (59·4 to 66·1)	60·2 (56·7 to 64)	52·7 (47 to 58·7)	51·5 (45·8 to 58·6)
Bangka-Belitung Islands	15·3 (14·4 to 16·2)	19·4 (18·5 to 20·2)	21·8 (20·9 to 22·6)	25·2 (24·4 to 26)	22·6 (21·7 to 23·4)	23·4 (22·2 to 24·8)	27·1 (25·7 to 28·7)	27·1 (25·1 to 29·4)	26·9 (24·5 to 29·6)
Banten	81·8 (77·5 to 86·1)	106 (101 to 110)	117 (114 to 121)	155 (149 to 160)	238 (230 to 247)	256 (244 to 269)	242 (231 to 255)	214 (197 to 232)	209 (189 to 231)
Bengkulu	18·7 (18 to 19·4)	23·7 (22·9 to 24·6)	26·7 (25·9 to 27·4)	32·6 (31·6 to 33·6)	36·8 (35·3 to 38·3)	38·2 (36·5 to 39·9)	38·8 (36·8 to 40·7)	36·3 (33·3 to 39·7)	35·9 (32·5 to 39·9)
Gorontalo	12·1 (11·4 to 12·8)	19·1 (18·2 to 20)	23·5 (22·4 to 24·6)	20·3 (19·4 to 21·2)	17·2 (16·5 to 17·9)	19·6 (18·8 to 20·6)	23·1 (22 to 24·3)	21·9 (20·2 to 23·8)	21·6 (19·7 to 23·8)
Jakarta	99·7 (95 to 104)	168 (161 to 175)	206 (199 to 214)	230 (219 to 240)	216 (207 to 225)	198 (190 to 207)	188 (177 to 198)	142 (128 to 158)	134 (119 to 152)
Jambi	29·7 (28·1 to 31·3)	42·1 (40·3 to 43·9)	47·5 (45·8 to 49·3)	56·5 (54·8 to 58·1)	55·4 (52·9 to 57·9)	60·8 (57·8 to 64)	66·3 (63·2 to 69·4)	59·2 (53·8 to 64·6)	58·1 (52·1 to 65)
West Java	574 (547 to 599)	802 (767 to 838)	884 (856 to 912)	874 (842 to 908)	735 (706 to 766)	800 (769 to 834)	881 (832 to 931)	822 (749 to 900)	807 (729 to 899)
Central Java	669 (635 to 704)	891 (849 to 934)	899 (867 to 931)	813 (784 to 843)	666 (636 to 697)	575 (543 to 607)	551 (511 to 593)	508 (459 to 565)	500 (444 to 568)
East Java	775 (730 to 820)	999 (941 to 1060)	963 (924 to 1000)	834 (797 to 869)	658 (630 to 685)	600 (572 to 628)	569 (535 to 605)	473 (420 to 530)	456 (399 to 524)
West Kalimantan	58·8 (56·3 to 61·2)	80·9 (77·9 to 84)	95·6 (92·9 to 98·3)	110 (107 to 113)	97·1 (93·3 to 101)	98·6 (93·1 to 104)	108 (104 to 113)	112 (103 to 122)	114 (104 to 126)
South Kalimantan	54 (51·3 to 56·5)	75·6 (72·7 to 78·6)	80·3 (77·9 to 82·8)	76·2 (73·2 to 79·1)	69·8 (66 to 73·4)	77·1 (72·9 to 81·5)	80·6 (75·7 to 85·4)	68·2 (60·9 to 75·6)	66 (58·7 to 74·8)
Central Kalimantan	17·8 (16·8 to 18·9)	24·9 (23·7 to 26)	28·8 (27·7 to 29·9)	34·3 (33·2 to 35·4)	37·7 (36 to 39·2)	44 (42 to 46·3)	50·8 (48·5 to 53·2)	50·7 (46·1 to 55·2)	50·5 (45·5 to 56·4)
East Kalimantan	17·1 (16·2 to 18·1)	26 (24·9 to 27·1)	32·2 (31·2 to 33·3)	40·1 (38·8 to 41·4)	44·4 (42·6 to 46·2)	58·1 (55·4 to 61·1)	68·8 (65·7 to 71·9)	66 (60·4 to 72·2)	66·1 (59·2 to 74·1)
North Kalimantan	2·2 (2·1 to 2·3)	4·1 (4 to 4·3)	5·7 (5·5 to 5·9)	6·4 (6·1 to 6·7)	7 (6·4 to 7·5)	9·6 (8·8 to 10·5)	12·5 (11·4 to 13·6)	13·6 (12·1 to 15·1)	13·8 (12·3 to 15·7)
Riau Islands	7·1 (6·6 to 7·6)	8·6 (8·1 to 9)	9·7 (9·3 to 10)	15·1 (14·5 to 15·7)	26·3 (25·1 to 27·3)	33·6 (31·8 to 35·6)	42·7 (40·8 to 44·7)	37 (33·7 to 40·3)	35·6 (32·2 to 39·6)
Lampung	75 (71·1 to 78·8)	126 (121 to 132)	159 (154 to 163)	180 (173 to 186)	167 (159 to 175)	160 (150 to 170)	160 (150 to 170)	155 (138 to 175)	155 (136 to 178)
Maluku	25·7 (24·3 to 27)	30 (28·8 to 31·3)	31·5 (30·4 to 32·5)	35·9 (34·8 to 37)	38·6 (37·2 to 40·1)	40·7 (38·7 to 42·8)	41·2 (39·3 to 43·3)	43·5 (40 to 47)	44·5 (40·2 to 49·6)
North Maluku	13·3 (12·5 to 14·2)	15·5 (14·8 to 16·2)	17 (16·3 to 17·8)	20·3 (19·5 to 21)	23·2 (22·3 to 24·1)	25·6 (24·2 to 27·2)	26·2 (24·8 to 27·8)	27·3 (25·1 to 29·5)	27·9 (25·4 to 30·7)
West Nusa Tenggara	80·2 (76 to 84·3)	98·9 (95·2 to 103)	112 (109 to 115)	118 (115 to 122)	102 (97·9 to 106)	99·1 (93·8 to 105)	103 (96·9 to 109)	96 (86·3 to 106)	94·7 (84·6 to 106)
East Nusa Tenggara	81·6 (77·2 to 85·9)	98·7 (94·9 to 103)	106 (102 to 109)	104 (100 to 106)	103 (99 to 106)	118 (112 to 124)	122 (116 to 127)	124 (114 to 135)	127 (115 to 140)
Papua	9·4 (8·9 to 9·9)	16·1 (15·2 to 16·9)	20·5 (19·7 to 21·2)	24·9 (24 to 25·7)	31·4 (30·2 to 32·8)	50·6 (48·3 to 53·2)	81·1 (77·3 to 85·1)	92·9 (85·1 to 101)	93·5 (84·7 to 104)
West Papua	8·9 (8·4 to 9·4)	13·5 (13 to 14·1)	16·8 (16·3 to 17·3)	21·2 (20·5 to 21·8)	22·4 (21·6 to 23·2)	19·5 (18·5 to 20·5)	21 (20 to 22·1)	25·3 (23·4 to 27·4)	26·8 (24·4 to 29·5)
Riau	31·8 (30·7 to 33)	51·4 (49·6 to 53·3)	63·8 (61·9 to 65·7)	75·1 (73 to 77·2)	70·4 (67·6 to 73·3)	99·9 (94·9 to 106)	137 (130 to 143)	125 (114 to 137)	123 (111 to 137)
West Sulawesi	18·9 (17·7 to 20)	22·3 (21·3 to 23·3)	24 (23·2 to 24·8)	24·5 (23·8 to 25·3)	23·9 (23·1 to 24·8)	28 (26·6 to 29·5)	32·2 (30·9 to 33·7)	33·1 (30·7 to 35·6)	33·7 (30·8 to 36·9)
South Sulawesi	150 (142 to 158)	204 (196 to 212)	228 (221 to 234)	216 (208 to 224)	185 (177 to 193)	184 (175 to 193)	176 (166 to 185)	166 (150 to 184)	165 (148 to 186)
Central Sulawesi	26·8 (25·5 to 28·1)	37·7 (36·3 to 39·1)	44·8 (43·3 to 46·3)	50·3 (48·9 to 51·8)	49·4 (47·5 to 51·3)	60·6 (57·5 to 64)	64·6 (61·8 to 67·6)	59·3 (54 to 64·6)	58·9 (53 to 65·5)
Southeast Sulawesi	24·7 (23·5 to 25·9)	31·8 (30·7 to 32·8)	37 (35·9 to 38)	41·9 (40·7 to 43·1)	43 (41·6 to 44·4)	50·8 (48·2 to 53·6)	55·7 (52·6 to 58·8)	54·7 (49·9 to 59·7)	55·4 (50 to 61·8)
North Sulawesi	43·5 (41·7 to 45·4)	47·5 (45·5 to 49·6)	47·2 (45·6 to 48·9)	45·7 (43·7 to 47·7)	45·2 (43·2 to 47)	44·8 (42·6 to 47)	43·3 (41·2 to 45·7)	41·6 (37·7 to 45·7)	41·3 (37·1 to 46·2)
West Sumatra	114 (110 to 118)	129 (125 to 134)	127 (123 to 130)	132 (128 to 136)	114 (109 to 118)	113 (107 to 119)	108 (102 to 114)	105 (95·6 to 116)	105 (94·8 to 118)
South Sumatra	80·8 (76·8 to 84·7)	116 (111 to 120)	134 (130 to 138)	164 (158 to 169)	158 (151 to 165)	163 (155 to 171)	170 (160 to 180)	149 (135 to 165)	146 (130 to 164)
North Sumatra	191 (184 to 198)	263 (254 to 272)	301 (293 to 308)	344 (334 to 353)	332 (320 to 346)	334 (315 to 354)	335 (314 to 357)	324 (294 to 354)	324 (291 to 362)
Yogyakarta	90·8 (86·1 to 95·5)	104 (100 to 109)	94 (89·9 to 98)	74·1 (71 to 77·2)	56·2 (53·8 to 58·6)	48·2 (45·9 to 50·6)	46·2 (43·4 to 49·2)	37·6 (33·9 to 41·9)	36 (31·9 to 40·9)
Laos	84·4 (79·7 to 89)	104 (99·7 to 108)	126 (120 to 133)	147 (139 to 155)	173 (164 to 181)	193 (185 to 201)	173 (166 to 181)	179 (166 to 192)	177 (162 to 192)
Malaysia	304 (290 to 317)	343 (337 to 349)	356 (346 to 365)	421 (409 to 433)	492 (474 to 509)	525 (504 to 547)	491 (471 to 513)	482 (456 to 509)	474 (424 to 535)
Maldives	2·7 (2·5 to 2·9)	3·8 (3·6 to 4·1)	4·8 (4·6 to 5·1)	6·8 (6·6 to 6·9)	8·8 (8·5 to 9)	6·1 (5·7 to 6·5)	7 (6·6 to 7·4)	6·3 (5·8 to 6·9)	6 (5·4 to 6·7)
Mauritius	23·3 (22·7 to 23·8)	25·6 (25·2 to 26·1)	20·9 (20·1 to 21·6)	23·4 (22·6 to 24·2)	22·9 (21·5 to 24·6)	20·2 (19·4 to 21)	14·8 (14·3 to 15·4)	13·1 (12·3 to 13·8)	12·7 (11·2 to 14·3)
Myanmar	869 (819 to 919)	1040 (993 to 1080)	1160 (1120 to 1210)	1330 (1260 to 1390)	1120 (1060 to 1180)	1060 (1010 to 1100)	1060 (1020 to 1100)	1080 (1010 to 1160)	1070 (983 to 1170)
Philippines	937 (892 to 983)	1250 (1180 to 1310)	1480 (1400 to 1550)	1770 (1680 to 1850)	2020 (1940 to 2100)	2220 (2130 to 2320)	2480 (2350 to 2620)	2220 (2080 to 2380)	2190 (2000 to 2370)
Seychelles	1 (1 to 1·1)	1·7 (1·7 to 1·7)	1·7 (1·7 to 1·8)	1·7 (1·6 to 1·8)	1·7 (1·6 to 1·7)	1·5 (1·4 to 1·5)	1·6 (1·6 to 1·7)	1·6 (1·5 to 1·7)	1·6 (1·4 to 1·7)
Sri Lanka	306 (290 to 324)	353 (341 to 365)	372 (354 to 390)	421 (402 to 441)	360 (341 to 379)	346 (325 to 370)	369 (349 to 391)	312 (291 to 335)	299 (265 to 336)
Thailand	1000 (957 to 1050)	1250 (1210 to 1300)	1240 (1210 to 1260)	1180 (1130 to 1230)	1030 (983 to 1080)	869 (828 to 914)	794 (754 to 834)	601 (559 to 649)	573 (520 to 636)
Timor-Leste	22·5 (21·6 to 23·4)	26·7 (26·1 to 27·3)	25 (24·4 to 25·6)	28·8 (27·9 to 29·6)	37·1 (35·8 to 38·3)	37 (35·6 to 38·4)	34·8 (32·4 to 37)	39·1 (35·2 to 43·2)	41 (36·7 to 45·6)
Viet Nam	1290 (1230 to 1350)	1500 (1440 to 1550)	1560 (1500 to 1620)	1820 (1760 to 1880)	1920 (1860 to 1990)	1530 (1460 to 1590)	1630 (1550 to 1700)	1610 (1480 to 1740)	1550 (1420 to 1710)
Sub-Saharan Africa	8850 (8460 to 9230)	11200 (10800 to 11500)	13900 (13500 to 14300)	17900 (17400 to 18300)	22300 (21800 to 22800)	28000 (27300 to 28600)	34300 (33300 to 35200)	37300 (35400 to 39300)	37700 (35500 to 40200)
Central sub-Saharan Africa	1010 (976 to 1040)	1350 (1310 to 1390)	1660 (1620 to 1700)	2060 (2000 to 2130)	2590 (2500 to 2680)	3320 (3220 to 3410)	4210 (4030 to 4380)	4460 (4220 to 4700)	4460 (4180 to 4730)
Angola	242 (230 to 253)	270 (260 to 279)	296 (287 to 306)	375 (362 to 387)	505 (483 to 528)	711 (673 to 747)	994 (927 to 1060)	1170 (1090 to 1250)	1200 (1120 to 1290)
Central African Republic	59·7 (56·4 to 62·7)	80·6 (76·4 to 84·6)	101 (96·5 to 105)	111 (106 to 115)	128 (122 to 134)	160 (151 to 169)	192 (177 to 206)	190 (176 to 205)	191 (176 to 206)
Congo (Brazzaville)	40·8 (38·4 to 43·1)	49·1 (47·1 to 51·1)	60·3 (58·1 to 62·2)	76·4 (73·1 to 79·5)	92·2 (89·1 to 95·3)	120 (116 to 123)	150 (145 to 155)	133 (122 to 144)	129 (117 to 141)
DR Congo	640 (616 to 662)	920 (894 to 945)	1170 (1130 to 1200)	1460 (1400 to 1510)	1810 (1740 to 1870)	2260 (2190 to 2320)	2790 (2680 to 2890)	2890 (2680 to 3080)	2860 (2620 to 3090)
Equatorial Guinea	9·6 (9·1 to 10)	9·9 (9·5 to 10·4)	11·1 (10·6 to 11·5)	14·4 (13·7 to 15)	20·5 (19·4 to 21·4)	28·1 (25·9 to 30·2)	37·1 (34·1 to 40·3)	38 (33·5 to 42·7)	37·4 (32·6 to 42·9)
Gabon	18·9 (17·7 to 20)	21·9 (20·8 to 22·9)	24·7 (23·4 to 25·9)	29·6 (27·7 to 31·4)	36 (33·1 to 38·5)	42·4 (38·4 to 46·3)	47·6 (43·2 to 51·9)	44·5 (38·9 to 50·6)	43·5 (37·7 to 50·3)
Eastern sub-Saharan Africa	3380 (3240 to 3510)	4310 (4190 to 4430)	5390 (5260 to 5520)	7090 (6930 to 7240)	9040 (8840 to 9240)	11300 (11000 to 11500)	13000 (12500 to 13400)	13600 (12800 to 14500)	13800 (12800 to 14900)
Burundi	125 (119 to 130)	146 (141 to 151)	178 (173 to 184)	214 (209 to 218)	259 (253 to 265)	270 (263 to 277)	400 (387 to 413)	454 (422 to 487)	469 (432 to 510)
Comoros	6·5 (6 to 6·9)	9·2 (8·7 to 9·7)	13·3 (12·9 to 13·7)	17·2 (16·6 to 17·7)	20·3 (19·5 to 21)	20·3 (18·8 to 21·7)	19·2 (17·5 to 20·9)	17·5 (15·4 to 19·7)	17·3 (15·1 to 19·8)

Djibouti	2·4 (2·3 to 2·6)	3·7 (3·6 to 3·9)	6 (5·7 to 6·3)	9·5 (9 to 10)	13·7 (12·8 to 14·6)	18·8 (17·3 to 20·5)	25·1 (23·1 to 27·2)	25·8 (23 to 28·9)	25·2 (22·2 to 28·4)
Eritrea	54·3 (51·4 to 57·2)	74·4 (71·6 to 77·3)	94 (90·6 to 97·2)	119 (114 to 124)	148 (140 to 155)	149 (138 to 160)	180 (161 to 199)	193 (173 to 216)	196 (174 to 220)
Ethiopia	868 (819 to 915)	1080 (1040 to 1120)	1320 (1270 to 1360)	1790 (1720 to 1850)	2430 (2360 to 2500)	3050 (2950 to 3150)	3150 (3000 to 3300)	3420 (3190 to 3670)	3500 (3240 to 3790)
Addis Ababa	27·2 (25·6 to 28·8)	38·4 (36·8 to 39·9)	53·5 (51 to 55·7)	72·5 (68·4 to 76·5)	61·7 (57·8 to 65·5)	55·2 (52·4 to 58·3)	55·8 (51·8 to 60·1)	50·7 (45·5 to 56·6)	47·5 (41·9 to 53·4)
Afar	19 (18 to 20)	12·3 (11·8 to 12·7)	9·3 (8·9 to 9·7)	13·8 (13·1 to 14·4)	37 (35·5 to 38·6)	66·5 (63·6 to 69·2)	64·1 (60·8 to 67·4)	63·1 (59 to 67·6)	62·7 (58·3 to 67·8)
Amhara	256 (240 to 272)	323 (309 to 336)	388 (373 to 403)	508 (486 to 528)	652 (631 to 670)	717 (689 to 743)	617 (586 to 650)	622 (573 to 676)	631 (576 to 690)
Benishangul-Gumuz	17 (15·9 to 18)	19·9 (19 to 20·7)	20·9 (20 to 21·8)	23·6 (22·6 to 24·6)	24·8 (23·9 to 25·6)	28 (26·8 to 29·1)	34 (32 to 35·9)	38·1 (35·4 to 40·9)	38·9 (36 to 42·1)
Dire Dawa	4·8 (4·5 to 5·1)	7·9 (7·6 to 8·3)	11·4 (10·8 to 11·9)	13·4 (12·6 to 14·1)	12 (11·3 to 12·7)	11·4 (10·8 to 12·1)	12·1 (11·1 to 13·1)	11·3 (10·2 to 12·5)	11 (9·9 to 12·3)
Gambella	3·9 (3·6 to 4·2)	5·8 (5·5 to 6·1)	7 (6·7 to 7·3)	7·9 (7·5 to 8·3)	8·2 (7·8 to 8·6)	10·3 (9·8 to 10·7)	11·5 (10·8 to 12·2)	11·9 (10·7 to 13·2)	12·1 (10·9 to 13·5)
Harari	2·4 (2·2 to 2·5)	3·4 (3·3 to 3·6)	4·5 (4·4 to 4·7)	5·3 (5·1 to 5·6)	5·5 (5·3 to 5·8)	6·1 (5·8 to 6·5)	6·6 (6·2 to 7)	6·3 (5·8 to 7)	6·2 (5·6 to 6·9)
Oromia	263 (249 to 276)	309 (297 to 319)	367 (354 to 379)	518 (499 to 538)	813 (788 to 838)	1150 (1110 to 1190)	1260 (1200 to 1320)	1420 (1320 to 1520)	1470 (1360 to 1580)
Somali	43·6 (41·9 to 45·3)	47·5 (46 to 48·8)	54·9 (53 to 56·7)	84·7 (82 to 87·3)	141 (136 to 146)	206 (199 to 212)	243 (232 to 254)	265 (251 to 280)	268 (254 to 284)
Southern Nations, Nationalities, and Peoples	170 (161 to 179)	239 (228 to 248)	313 (302 to 323)	423 (406 to 437)	535 (518 to 552)	636 (615 to 656)	686 (644 to 728)	762 (702 to 822)	782 (718 to 857)
Tigray	61·1 (57·5 to 64·6)	73·9 (71 to 76·9)	91·4 (87·9 to 94·7)	116 (112 to 121)	140 (136 to 144)	167 (162 to 172)	162 (154 to 169)	169 (156 to 184)	172 (156 to 189)
Kenya	310 (303 to 317)	464 (454 to 474)	610 (597 to 622)	812 (792 to 830)	992 (957 to 1030)	1210 (1150 to 1270)	1360 (1260 to 1460)	1230 (1100 to 1370)	1190 (1050 to 1340)
Baringo	4·8 (4·7 to 4·9)	7·4 (7·3 to 7·6)	9·4 (9·2 to 9·6)	11·4 (11·1 to 11·6)	14·9 (14·4 to 15·4)	17·4 (16·4 to 18·3)	17·3 (15·8 to 18·7)	15·6 (13·8 to 17·5)	15·1 (13·2 to 17·3)
Bomet	4·8 (4·8 to 4·9)	6·7 (6·6 to 6·8)	8·9 (8·7 to 9)	11·6 (11·3 to 11·8)	15·6 (15 to 16·1)	21·6 (20·4 to 22·6)	34·9 (31·9 to 37·8)	25·6 (22·5 to 28·9)	23 (20·1 to 26·4)
Bungoma	10 (9·9 to 10·1)	14·9 (14·6 to 15·1)	20·6 (20·3 to 20·9)	29 (28·5 to 29·5)	41·9 (40·5 to 43·3)	74·8 (71·6 to 78·4)	59 (55·6 to 62·3)	60·4 (54·9 to 66·3)	60·9 (55 to 67·7)
Busia	7·6 (7·4 to 7·8)	10·5 (10·2 to 10·8)	12·4 (12·1 to 12·7)	17·9 (17·5 to 18·3)	23·7 (23 to 24·5)	25·6 (24·2 to 27·1)	28·4 (26 to 30·8)	24·9 (22·2 to 28)	24·3 (21·4 to 27·6)
Elgeyo Marakwet	5·7 (5·5 to 5·8)	9·3 (9·1 to 9·5)	10·2 (10 to 10·4)	8·2 (8 to 8·3)	11·5 (11·1 to 11·9)	19·1 (18 to 20·1)	11 (9·9 to 12·1)	9·9 (8·7 to 11·1)	9·9 (8·7 to 11·4)
Embu	4·9 (4·7 to 5)	6·7 (6·5 to 6·8)	8·8 (8·6 to 9)	12·3 (12 to 12·7)	15·1 (14·4 to 15·9)	16·6 (15·4 to 17·8)	14·3 (12·7 to 15·9)	11·2 (9·7 to 12·9)	10·6 (9·1 to 12·3)
Garissa	1·6 (1·6 to 1·7)	2·6 (2·5 to 2·7)	4·2 (4·1 to 4·3)	6·4 (6·2 to 6·6)	6·5 (6·3 to 6·7)	15·4 (14·9 to 16)	29·6 (28·3 to 30·9)	36·9 (34·4 to 39·6)	37·9 (35·1 to 40·9)
Homa Bay	18·9 (18·5 to 19·2)	23·5 (23 to 23·9)	25·2 (24·6 to 25·8)	28·2 (27·6 to 28·7)	33·7 (32·6 to 34·7)	39 (37·2 to 40·8)	39·8 (37·4 to 42·2)	33·6 (30·2 to 37·2)	31·2 (27·9 to 34·9)
Isiolo	0·9 (0·8 to 0·9)	1·4 (1·3 to 1·4)	1·8 (1·7 to 1·8)	2·5 (2·5 to 2·6)	3·5 (3·3 to 3·6)	4·5 (4·2 to 4·7)	5·4 (4·9 to 5·8)	7·3 (6·4 to 8·2)	7·5 (6·6 to 8·6)
Kajiado	2·3 (2·3 to 2·4)	3·4 (3·3 to 3·4)	4·9 (4·8 to 5)	8·4 (8·2 to 8·5)	13·6 (13·1 to 14·1)	21·6 (20·4 to 22·7)	34·6 (32·2 to 37)	45·8 (41 to 50·8)	46·9 (41·8 to 52·6)
Kakamega	14·7 (14·5 to 14·9)	23·5 (23·1 to 23·8)	31·3 (30·8 to 31·8)	41·8 (41 to 42·6)	53·1 (51·2 to 55)	41·2 (38·9 to 43·5)	59·9 (55·3 to 64·3)	46·9 (41·9 to 52·3)	44·1 (38·9 to 49·9)
Kericho	5·4 (5·3 to 5·5)	11·2 (11 to 11·4)	17·2 (16·9 to 17·4)	22·3 (21·8 to 22·7)	28·9 (28 to 29·8)	34·1 (32·4 to 35·8)	24·6 (22·9 to 26·3)	30·2 (27 to 33·5)	31·4 (28 to 35·2)
Kiambu	10·4 (10 to 10·9)	14·9 (14·3 to 15·3)	22·5 (21·9 to 23·1)	33 (31·7 to 34·2)	37·3 (35 to 39·5)	51·6 (47·9 to 55·4)	51·4 (46·2 to 56·6)	52·7 (45·1 to 61·3)	51·9 (44·2 to 61·4)
Kilifi	9·9 (9·6 to 10·2)	14·9 (14·3 to 15·3)	18·8 (18·3 to 19·3)	26·7 (26 to 27·5)	31·8 (30·3 to 33·2)	40·7 (38·5 to 43·1)	48 (44·1 to 51·8)	47·1 (41·4 to 53·1)	45·3 (39·7 to 52)
Kirinyaga	6 (5·9 to 6·2)	9·3 (9·1 to 9·5)	11·6 (11·3 to 11·9)	12·9 (12·4 to 13·3)	12·8 (12·1 to 13·6)	14·2 (12·9 to 15·5)	13·8 (12·4 to 15·2)	9·7 (8·3 to 11·3)	9 (7·7 to 10·7)
Kisii	10·8 (10·5 to 11·1)	19·8 (19·5 to 20·1)	27·3 (26·8 to 27·8)	36·8 (36 to 37·6)	42·7 (41·1 to 44·3)	51·1 (48·6 to 53·6)	43·5 (39·6 to 47·1)	30·8 (27·3 to 34·5)	27·9 (24·6 to 31·7)
Kisumu	13·9 (13·6 to 14·3)	20·8 (20·3 to 21·2)	24·6 (24·1 to 25·1)	27·1 (26·5 to 27·7)	34·3 (32·9 to 35·5)	35·1 (32·7 to 37·2)	35·2 (31·7 to 38·4)	30·8 (27·2 to 34·4)	29·2 (26 to 32·9)
Kitui	11·9 (11·6 to 12·2)	16·9 (16·4 to 17·2)	20·6 (20·2 to 21)	25·2 (24·6 to 25·8)	31 (29·7 to 32·3)	35 (33·2 to 36·9)	34·6 (31·7 to 37·4)	29·4 (26·3 to 32·8)	28 (24·9 to 31·6)
Kwale	6·4 (6·2 to 6·6)	10·6 (10·3 to 10·9)	13·4 (13 to 13·7)	16·4 (15·9 to 16·9)	18·5 (17·5 to 19·4)	22·9 (21·5 to 24·3)	27·1 (24·7 to 29·3)	27·7 (24·7 to 31)	27·2 (24 to 30·9)
Laikipia	1·4 (1·4 to 1·4)	2 (2 to 2)	3·4 (3·3 to 3·5)	6·2 (6 to 6·4)	8·7 (8·2 to 9·2)	13·3 (12·5 to 14·1)	12·5 (11·3 to 13·6)	10·2 (8·9 to 11·7)	9·7 (8·4 to 11·3)
Lamu	0·8 (0·8 to 0·9)	1 (1 to 1)	1·3 (1·3 to 1·4)	2·1 (2 to 2·1)	2·5 (2·4 to 2·7)	2·8 (2·6 to 3·1)	3·1 (2·8 to 3·4)	2·8 (2·4 to 3·3)	2·8 (2·3 to 3·3)
Machakos	8·7 (8·4 to 9)	14·8 (14·3 to 15·2)	22 (21·5 to 22·6)	32·4 (31·4 to 33·5)	38·1 (36·3 to 40)	39·3 (37 to 41·6)	31 (27·9 to 34·1)	27·9 (24·3 to 31·9)	26·7 (23 to 31·1)
Makueni	10 (9·7 to 10·2)	12 (11·7 to 12·2)	14·4 (14 to 14·7)	17·4 (17 to 17·8)	19·8 (19 to 20·6)	21·5 (20·1 to 22·8)	25·4 (22·8 to 28)	18·2 (15·8 to 21)	16·5 (14 to 19·4)
Mandera	2·7 (2·6 to 2·8)	4·2 (4·1 to 4·3)	5·4 (5·2 to 5·5)	5·7 (5·5 to 5·8)	6·9 (6·7 to 7·1)	15·5 (15·1 to 15·9)	49·9 (48·3 to 51·6)	44·6 (43 to 46·1)	41·8 (40·6 to 43·2)
Marsabit	1·1 (1·1 to 1·2)	1·6 (1·6 to 1·7)	2·8 (2·7 to 2·9)	4·9 (4·7 to 5)	5·8 (5·6 to 6·1)	7·6 (7·2 to 8)	10·9 (10·1 to 11·6)	14·2 (12·8 to 15·7)	14·6 (13 to 16·3)
Meru	14 (13·6 to 14·4)	22·7 (22·2 to 23·2)	30·2 (29·6 to 30·8)	39·5 (38·6 to 40·4)	48·7 (46·8 to 50·6)	57·6 (54·4 to 60·7)	55·8 (51·1 to 60·5)	37·9 (33·5 to 42·6)	33·9 (29·8 to 38·9)
Migori	10·8 (10·5 to 11·1)	14·9 (14·5 to 15·3)	17·6 (17·2 to 18)	21·9 (21·5 to 22·3)	29 (28·2 to 29·8)	37·9 (36·5 to 39·5)	42·8 (40·7 to 44·9)	40·2 (36·9 to 43·7)	38·8 (35·4 to 42·6)
Mombasa	5·2 (4·9 to 5·4)	9·7 (9·3 to 10)	12·5 (12 to 13)	13·8 (13·1 to 14·5)	16·2 (15·3 to 17·1)	20·5 (18·6 to 22·2)	24·7 (21·9 to 27·7)	18·5 (15·6 to 21·9)	16·8 (14 to 20·5)
Murang'a	14·3 (13·9 to 14·7)	19·1 (18·6 to 19·6)	23·5 (23·1 to 23·9)	29·4 (28·6 to 30·1)	33·5 (31·7 to 35·2)	25·1 (23·5 to 26·8)	23 (20·7 to 25·2)	16·2 (14·3 to 18·1)	15 (13·2 to 17)
Nairobi	9·4 (9·2 to 9·7)	16·3 (15·7 to 16·8)	26·9 (25·7 to 28)	52·5 (48·8 to 56)	44·9 (41·1 to 48·4)	65 (58·4 to 71·4)	83·6 (72·1 to 95·9)	67·2 (57·6 to 78·8)	62·8 (53·3 to 75·1)
Nakuru	5·6 (5·5 to 5·7)	9·6 (9·3 to 9·8)	15·8 (15·5 to 16)	25·4 (24·6 to 26·1)	35·9 (34·2 to 37·7)	43·6 (40·6 to 46·4)	41·5 (37 to 46·2)	36·1 (31 to 41·9)	34·9 (29·7 to 41·3)
Nandi	5·7 (5·5 to 5·8)	8·7 (8·5 to 8·8)	12·1 (11·9 to 12·3)	15·3 (15 to 15·6)	20·6 (19·9 to 21·4)	25·3 (24 to 26·5)	26·5 (24·1 to 28·8)	23·7 (20·9 to 26·4)	22·8 (20·1 to 25·9)
Narok	3·9 (3·8 to 4)	5·5 (5·4 to 5·6)	7·4 (7·3 to 7·6)	12·5 (12·3 to 12·7)	21·3 (20·7 to 21·9)	19·4 (18·5 to 20·2)	39·5 (36·9 to 42·1)	42 (38·3 to 45·5)	41·1 (37·5 to 44·9)
Nyamira	6·1 (6 to 6·3)	7·9 (7·7 to 8·1)	9·2 (9 to 9·5)	10·5 (10·2 to 10·8)	11·3 (10·8 to 11·8)	13·7 (12·7 to 14·5)	16·4 (14·9 to 17·8)	12·8 (11·2 to 14·7)	12·1 (10·5 to 14·1)
Nyandarua	3·9 (3·8 to 3·9)	6·3 (6·2 to 6·4)	8·6 (8·4 to 8·7)	10·4 (10·1 to 10·6)	14·4 (13·7 to 15·1)	16·7 (15·4 to 18)	13·5 (12·2 to 14·9)	8·4 (7·1 to 9·8)	7·6 (6·5 to 9·1)
Nyeri	9·6 (9·2 to 9·9)	11·5 (11·1 to 11·8)	14·6 (14·1 to 15·1)	19·5 (18·6 to 20·3)	20·5 (19·1 to 21·9)	17·9 (16·2 to 19·6)	13·4 (11·7 to 15·2)	9 (7·6 to 10·6)	8·2 (6·9 to 9·9)
Samburu	1·6 (1·6 to 1·7)	3·1 (3 to 3·2)	3·9 (3·8 to 4)	4·2 (4·1 to 4·4)	6·1 (5·9 to 6·3)	7·3 (7 to 7·7)	9·6 (9 to 10·2)	11·4 (10·5 to 12·3)	11·6 (10·6 to 12·7)
Siaya	10·9 (10·6 to 11·2)	17·3 (17 to 17·6)	21 (20·6 to 21·4)	25·7 (25·2 to 26·2)	31·2 (30·4 to 32·1)	33·9 (32·4 to 35·4)	33·6 (31·4 to 35·8)	30·2 (27·5 to 32·9)	29·3 (26·5 to 32·2)
Taita Taveta	2·9 (2·8 to 3)	4·5 (4·4 to 4·7)	5·6 (5·4 to 5·8)	6·7 (6·4 to 7)	8·7 (8·2 to 9·2)	8·8 (8·1 to 9·6)	8·3 (7·3 to 9·2)	6·6 (5·6 to 7·7)	6·3 (5·4 to 7·5)
Tana River	1·6 (1·6 to 1·7)	2·2 (2·1 to 2·3)	3 (2·9 to 3·1)	5·1 (4·9 to 5·3)	6 (5·7 to 6·3)	8·6 (8·1 to 9·1)	10·2 (9·6 to 10·8)	11·4 (10·2 to 12·5)	11·4 (10·3 to 12·7)
Tharaka Nithi	3·5 (3·4 to 3·6)	3·7 (3·6 to 3·8)	3·8 (3·7 to 3·8)	3·8 (3·7 to 3·9)	4·1 (3·9 to 4·3)	4·1 (3·8 to 4·3)	4·2 (3·8 to 4·7)	7·4 (6·3 to 8·5)	8·2 (6·9 to 9·6)
Trans Nzoia	2 (2 to 2·1)	3·4 (3·3 to 3·5)	6·6 (6·5 to 6·8)	13·9 (13·7 to 14·2)	19·1 (18·4 to 19·7)	25·2 (23·8 to 26·6)	27·7 (25·2 to 30)	22·6 (19·9 to 25·4)	21·2 (18·6 to 24·4)
Turkana	4 (3·8 to 4·1)	7·8 (7·6 to 8)	10·2 (9·9 to 10·4)	8·8 (8·6 to 9·1)	11·4 (11 to 11·9)	22·5 (21·5 to 23·6)	37·2 (35·1 to 39·4)	34·2 (30·8 to 37·7)	32·5 (29·1 to 36·4)
Uasin Gishu	3·7 (3·6 to 3·7)	6·6 (6·5 to 6·7)	10·8 (10·7 to 11)	16·1 (15·8 to 16·4)	22·1 (21·3 to 22·9)	29·7 (28·1 to 31·2)	36·2 (33 to 39·4)	35·1 (31 to 39·6)	33·9 (29·8 to 38·9)
Vihiga	10·9 (10·6 to 11·2)	13·2 (12·9 to 13·5)	13·8 (13·4 to 14·2)	13·4 (13 to 13·7)	16·2 (15·5 to 16·9)	14·1 (12·9 to 15·2)	14·9 (13·3 to 16·6)	9·3 (8 to 10·9)	8·6 (7·2 to 10·2)

Wajir	2·4 (2·3 to 2·5)	3·2 (3·1 to 3·3)	5 (4·9 to 5·2)	7·4 (7·2 to 7·6)	6·7 (6·5 to 6·9)	18·8 (18·3 to 19·3)	30·7 (29·6 to 31·7)	38·6 (36·9 to 40·1)	40 (38·4 to 41·7)
West Pokot	2·5 (2·4 to 2·5)	3·4 (3·3 to 3·5)	5·1 (5 to 5·3)	9·3 (9 to 9·5)	11·8 (11·5 to 12·2)	15 (14·3 to 15·8)	18·8 (17·4 to 20·3)	16·9 (15·1 to 18·8)	16·2 (14·4 to 18·1)
Madagascar	231 (222 to 239)	281 (274 to 289)	345 (336 to 355)	428 (417 to 438)	527 (514 to 540)	691 (671 to 712)	856 (824 to 890)	894 (837 to 953)	877 (816 to 946)
Malawi	130 (121 to 139)	180 (171 to 189)	235 (225 to 245)	348 (339 to 357)	508 (497 to 520)	555 (543 to 567)	600 (578 to 623)	577 (526 to 631)	574 (512 to 640)
Mozambique	338 (325 to 349)	382 (365 to 398)	460 (445 to 475)	596 (581 to 613)	623 (608 to 639)	824 (804 to 843)	976 (948 to 1000)	1090 (1010 to 1160)	1100 (1020 to 1200)
Rwanda	137 (133 to 142)	163 (159 to 167)	201 (197 to 204)	268 (264 to 272)	318 (311 to 325)	348 (341 to 355)	358 (343 to 373)	369 (345 to 394)	374 (345 to 404)
Somalia	110 (106 to 113)	143 (140 to 147)	197 (192 to 201)	334 (326 to 341)	391 (380 to 402)	506 (484 to 526)	716 (692 to 738)	900 (865 to 939)	960 (919 to 1000)
South Sudan	111 (104 to 119)	139 (132 to 147)	174 (164 to 183)	209 (197 to 220)	257 (243 to 270)	328 (308 to 347)	413 (387 to 437)	357 (332 to 383)	384 (354 to 415)
Uganda	339 (329 to 348)	432 (422 to 441)	550 (539 to 561)	703 (689 to 717)	935 (921 to 949)	1260 (1240 to 1280)	1490 (1450 to 1530)	1590 (1500 to 1680)	1590 (1490 to 1700)
Tanzania	482 (464 to 498)	610 (591 to 627)	746 (726 to 766)	944 (913 to 972)	1220 (1180 to 1250)	1550 (1500 to 1590)	1820 (1720 to 1900)	1910 (1770 to 2040)	1900 (1760 to 2060)
Zambia	132 (127 to 137)	201 (196 to 205)	255 (250 to 260)	300 (292 to 308)	390 (378 to 401)	472 (455 to 488)	596 (572 to 620)	618 (570 to 670)	607 (553 to 669)
Southern sub-Saharan Africa	792 (739 to 845)	960 (909 to 1010)	1210 (1160 to 1270)	1520 (1470 to 1570)	1630 (1580 to 1680)	1650 (1600 to 1700)	1810 (1760 to 1870)	1690 (1600 to 1790)	1640 (1520 to 1760)
Botswana	19·4 (18·4 to 20·4)	24·7 (23·7 to 25·7)	31·4 (30 to 32·6)	40·8 (38·8 to 42·7)	46·7 (43·5 to 49·8)	48·1 (44·1 to 52·1)	48·6 (44·2 to 53·5)	49 (43·4 to 54·8)	48·7 (43·1 to 55·2)
Eswatini	12·5 (12 to 13·1)	17·1 (16·6 to 17·6)	22·2 (21·7 to 22·7)	27·7 (26·8 to 28·5)	32·6 (31·4 to 33·6)	36·1 (35 to 37·2)	34·8 (33·3 to 36·2)	30·4 (28·2 to 32·7)	29·4 (27 to 32·1)
Lesotho	29·9 (28·1 to 31·7)	36·4 (34·3 to 38·3)	45·3 (43·8 to 46·7)	55·8 (54·2 to 57·4)	54·4 (52·7 to 56·1)	51·7 (50·3 to 53·2)	48·7 (46·4 to 50·9)	43·5 (39 to 48·3)	42·5 (37·8 to 48·1)
Namibia	22·1 (21 to 23·1)	28·2 (27·1 to 29·2)	33·9 (32·9 to 34·9)	40·6 (39·2 to 41·9)	51·3 (49·9 to 52·7)	56·8 (54·7 to 58·9)	62·8 (60·2 to 65·5)	58·6 (54·5 to 63·6)	58 (53·3 to 63·7)
South Africa	572 (530 to 614)	676 (634 to 718)	832 (788 to 877)	1010 (964 to 1050)	1050 (1010 to 1090)	1030 (990 to 1070)	1140 (1100 to 1180)	1030 (972 to 1100)	988 (913 to 1070)
Eastern Cape	153 (143 to 164)	151 (142 to 160)	144 (136 to 151)	173 (165 to 182)	178 (171 to 185)	150 (143 to 156)	143 (137 to 148)	123 (116 to 130)	117 (109 to 126)
Free State	45·1 (41·7 to 48·5)	56 (52·2 to 59·7)	62·8 (59·5 to 66·2)	70·6 (67·4 to 74·3)	62·4 (60 to 64·8)	57·3 (54·9 to 59·9)	57 (54·5 to 59·3)	45·4 (42·7 to 48·6)	42·7 (39·4 to 46·1)
Gauteng	67·2 (60·2 to 74·5)	94·8 (87·1 to 103)	126 (117 to 134)	144 (136 to 152)	159 (152 to 166)	196 (187 to 204)	272 (262 to 282)	263 (246 to 281)	250 (231 to 271)
KwaZulu-Natal	118 (110 to 126)	144 (136 to 153)	192 (182 to 202)	226 (216 to 236)	233 (224 to 243)	224 (215 to 233)	232 (223 to 241)	206 (192 to 221)	197 (182 to 213)
Limpopo	55·6 (52·4 to 58·7)	67·6 (64·4 to 70·8)	111 (106 to 116)	154 (148 to 160)	156 (150 to 162)	129 (124 to 134)	128 (123 to 134)	117 (111 to 124)	114 (106 to 122)
Mpumalanga	44·3 (41·9 to 46·6)	50·4 (48 to 52·8)	52·7 (50·4 to 55)	67·1 (64·1 to 70·2)	77·4 (74·1 to 80·7)	77·4 (73·7 to 81·3)	93·9 (90·3 to 97·6)	95 (89·2 to 102)	94 (87·3 to 102)
North West	34·1 (31·8 to 36·3)	45·2 (42·5 to 47·7)	61·4 (58·1 to 64·6)	75·9 (72·7 to 79·3)	83 (79·8 to 86·1)	84·3 (81 to 87·8)	78·6 (75·7 to 81·4)	56·9 (53·5 to 60·8)	52·9 (48·9 to 56·9)
Northern Cape	16·8 (15·5 to 18·2)	19·6 (18·4 to 20·9)	21·9 (20·7 to 23·1)	23·9 (22·8 to 25·1)	21·1 (20·3 to 21·9)	17·3 (16·6 to 18·1)	23·2 (22·2 to 24·2)	24·3 (22·8 to 26·1)	24·1 (22·1 to 26·2)
Western Cape	37·1 (33 to 41·5)	46·8 (42·8 to 50·9)	61·2 (56·6 to 66)	72·7 (68·8 to 76·7)	80·5 (77·4 to 83·8)	94·1 (90·1 to 98·2)	112 (108 to 116)	101 (94·4 to 108)	95·7 (88·1 to 104)
Zimbabwe	136 (130 to 142)	178 (172 to 184)	246 (238 to 253)	345 (339 to 351)	395 (387 to 403)	427 (418 to 436)	480 (469 to 490)	472 (444 to 502)	470 (437 to 506)
Western sub-Saharan Africa	3670 (3500 to 3830)	4540 (4390 to 4680)	5640 (5480 to 5790)	7180 (6990 to 7360)	9030 (8830 to 9210)	11800 (11500 to 12000)	15300 (14800 to 15700)	17500 (16700 to 18400)	17800 (16800 to 18900)
Benin	92·8 (87 to 98·3)	108 (103 to 112)	132 (128 to 137)	171 (166 to 176)	236 (230 to 242)	309 (301 to 316)	415 (401 to 429)	504 (474 to 534)	522 (489 to 559)
Burkina Faso	188 (178 to 198)	238 (229 to 247)	305 (296 to 313)	380 (372 to 387)	461 (450 to 471)	595 (578 to 612)	766 (739 to 792)	915 (866 to 964)	951 (894 to 1010)
Cabo Verde	7 (6·5 to 7·4)	9·1 (8·9 to 9·3)	9·5 (9·2 to 9·8)	10·9 (10·5 to 11·3)	12·6 (12·1 to 13)	12·7 (11·8 to 13·6)	11·5 (10·1 to 13·1)	9 (8·1 to 10)	8·5 (7·4 to 9·7)
Cameroon	216 (203 to 230)	274 (260 to 288)	335 (326 to 343)	394 (385 to 403)	477 (467 to 488)	634 (619 to 648)	906 (872 to 939)	1030 (947 to 1110)	1030 (940 to 1140)
Chad	127 (122 to 132)	148 (143 to 151)	184 (180 to 189)	243 (236 to 249)	322 (315 to 328)	438 (430 to 447)	600 (585 to 616)	804 (777 to 831)	860 (829 to 892)
Côte d'Ivoire	128 (121 to 134)	208 (200 to 216)	294 (286 to 301)	405 (395 to 415)	553 (542 to 565)	689 (670 to 709)	850 (818 to 879)	943 (880 to 1010)	950 (880 to 1030)
The Gambia	10·7 (10 to 11·4)	13·7 (13·1 to 14·3)	22·5 (21·8 to 23·2)	34 (33·2 to 34·9)	46·3 (45·1 to 47·5)	61·4 (59·8 to 62·8)	73·8 (72·1 to 75·5)	76·9 (72·8 to 81·2)	77·9 (73·1 to 83·1)
Ghana	204 (187 to 223)	333 (319 to 348)	448 (434 to 463)	551 (532 to 568)	606 (576 to 635)	670 (632 to 707)	852 (795 to 908)	967 (872 to 1060)	967 (856 to 1080)
Guinea	127 (122 to 132)	158 (152 to 164)	203 (196 to 210)	245 (238 to 252)	288 (282 to 295)	355 (346 to 366)	421 (407 to 435)	482 (455 to 510)	495 (463 to 529)
Guinea-Bissau	31·2 (29·9 to 32·4)	28·8 (27·5 to 30)	27·4 (25·7 to 28·9)	37·4 (35·6 to 39)	45·8 (43·3 to 48·3)	54·1 (50·5 to 57·5)	66·2 (61·8 to 70·7)	71·7 (66·1 to 77·2)	72 (66·1 to 78·3)
Liberia	44·2 (41·8 to 46·6)	57·3 (54·4 to 59·9)	73·5 (71·3 to 75·6)	101 (98·2 to 103)	120 (118 to 123)	131 (128 to 135)	163 (157 to 169)	165 (150 to 181)	164 (146 to 183)
Mali	183 (175 to 190)	245 (238 to 252)	319 (311 to 327)	389 (379 to 399)	453 (445 to 461)	556 (545 to 567)	772 (749 to 794)	1010 (958 to 1050)	1060 (1010 to 1120)
Mauritania	33·5 (31·5 to 35·4)	42·8 (40·8 to 44·6)	55 (53·2 to 56·6)	71·1 (69·1 to 72·9)	87·7 (84·5 to 90·8)	104 (100 to 109)	131 (126 to 136)	137 (125 to 148)	135 (123 to 150)
Niger	144 (139 to 149)	197 (191 to 202)	268 (262 to 274)	349 (343 to 356)	444 (436 to 452)	624 (611 to 638)	816 (791 to 838)	1090 (1050 to 1140)	1170 (1130 to 1220)
Nigeria	1830 (1740 to 1910)	2090 (2010 to 2160)	2490 (2400 to 2580)	3200 (3090 to 3310)	4150 (4040 to 4260)	5700 (5500 to 5890)	7420 (7170 to 7660)	8250 (7730 to 8780)	8330 (7670 to 8970)
São Tomé and Príncipe	2·4 (2·3 to 2·5)	3·1 (3 to 3·2)	3·2 (3·1 to 3·3)	3·7 (3·6 to 3·9)	4·7 (4·5 to 4·8)	5·7 (5·3 to 6·1)	6·1 (5·8 to 6·4)	5·1 (4·6 to 5·6)	4·9 (4·3 to 5·6)
Senegal	136 (129 to 142)	182 (177 to 187)	242 (237 to 247)	304 (299 to 309)	348 (338 to 357)	413 (403 to 422)	493 (481 to 505)	486 (456 to 516)	479 (442 to 521)
Sierra Leone	91·7 (86·2 to 97)	115 (110 to 120)	131 (125 to 136)	159 (153 to 165)	205 (199 to 212)	204 (198 to 209)	260 (249 to 270)	297 (276 to 318)	303 (278 to 328)
Togo	75·8 (72·8 to 78·7)	89·6 (87·2 to 91·8)	101 (98·5 to 104)	128 (125 to 131)	160 (156 to 164)	198 (192 to 204)	245 (235 to 255)	249 (234 to 265)	244 (226 to 263)

Supplemental Results Table S1b: Livebirths (in thousands) by location, females, 1950, 1960, 1970, 1980, 1990, 2000, 2010, 2019, & 2021

Location	1950	1960	1970	1980	1990	2000	2010	2019	2021
Global	44900 (43000 to 46900)	51600 (50200 to 53000)	55900 (54700 to 57000)	59100 (57800 to 60300)	63500 (62100 to 64800)	62500 (61100 to 63900)	66400 (64700 to 68100)	64800 (61800 to 68000)	62400 (58600 to 66700)
Central Europe, eastern Europe, and central Asia	3620 (3510 to 3740)	3580 (3530 to 3630)	3050 (2990 to 3110)	3470 (3420 to 3510)	3230 (3180 to 3270)	2220 (2190 to 2270)	2740 (2690 to 2790)	2490 (2420 to 2560)	2370 (2240 to 2510)
Central Asia	495 (480 to 509)	658 (642 to 674)	629 (592 to 666)	819 (802 to 835)	959 (944 to 975)	707 (690 to 724)	899 (874 to 923)	1000 (974 to 1040)	995 (955 to 1040)
Armenia	23·6 (22·6 to 24·7)	34·2 (33 to 35·5)	27·6 (25·5 to 29·9)	37·2 (35·6 to 38·8)	37 (35·5 to 38·5)	19·9 (19 to 20·8)	21·8 (20·7 to 23)	18·1 (16·8 to 19·5)	16·6 (15 to 18·2)
Azerbaijan	52·7 (49·1 to 56·3)	81·6 (78·8 to 84·4)	73 (69·5 to 76·6)	78·6 (75·1 to 82·2)	90·8 (87·2 to 94·3)	62·3 (59·3 to 65·6)	81 (76·5 to 85·6)	69·3 (63·6 to 75·5)	64·7 (57·6 to 72·3)
Georgia	40·6 (37·8 to 43·5)	49·2 (47·4 to 50·9)	42·3 (37·3 to 47·7)	44·7 (42 to 47·2)	40·9 (39·7 to 42·3)	23·1 (22·2 to 24·2)	25·9 (25 to 26·8)	23·6 (22·5 to 24·6)	21·8 (20·4 to 23·2)
Kazakhstan	124 (119 to 129)	175 (172 to 179)	162 (146 to 178)	178 (172 to 184)	182 (177 to 188)	108 (103 to 112)	178 (172 to 184)	201 (194 to 208)	205 (194 to 217)
Kyrgyzstan	28 (27 to 29·2)	37 (35·5 to 38·4)	38 (34·2 to 41·9)	54·7 (52·5 to 56·8)	64·9 (62·9 to 66·8)	52·4 (50·4 to 54·4)	78·3 (75·8 to 80·9)	80·3 (75·7 to 84·9)	77·2 (70·5 to 84·9)
Mongolia	14·8 (14 to 15·8)	18 (16·9 to 19)	22·8 (21·9 to 23·6)	30·4 (29·3 to 31·4)	35·1 (34 to 36·3)	25·9 (24·6 to 27·3)	35·5 (33·8 to 37·2)	39·8 (36·9 to 42·8)	38·8 (35·1 to 42·7)
Tajikistan	42·2 (40·5 to 44·1)	54·3 (52·6 to 55·9)	53·8 (51·6 to 56)	78·2 (75·8 to 80·6)	103 (100 to 105)	95·2 (91·2 to 99·3)	120 (114 to 125)	138 (130 to 146)	138 (129 to 148)
Turkmenistan	23·7 (22·7 to 24·6)	30·6 (29·3 to 31·7)	33 (30·9 to 35·4)	47·2 (45·5 to 48·9)	60·5 (58·4 to 62·6)	53·6 (50·8 to 56·2)	53·1 (50·1 to 56·2)	54·4 (49·5 to 59·7)	53·6 (48·2 to 59·6)
Uzbekistan	145 (137 to 154)	178 (168 to 188)	177 (166 to 190)	270 (263 to 276)	345 (338 to 352)	267 (257 to 276)	305 (288 to 322)	380 (360 to 399)	379 (351 to 408)
Central Europe	1130 (1100 to 1160)	970 (956 to 984)	945 (932 to 958)	1010 (1000 to 1030)	832 (820 to 844)	587 (578 to 596)	588 (578 to 598)	534 (521 to 545)	504 (464 to 548)
Albania	23·4 (22·4 to 24·4)	31·7 (31·1 to 32·3)	33·8 (32·5 to 35)	34·3 (33·1 to 35·7)	37·7 (36·5 to 39)	24·8 (23·6 to 26·1)	16·3 (15·4 to 17·3)	14 (12·8 to 15·2)	13·4 (11·8 to 15·1)
Bosnia and Herzegovina	45·2 (40·9 to 49·8)	55·3 (49·9 to 61·2)	41·9 (38·6 to 45·2)	38·4 (34·4 to 42·7)	32·2 (30·6 to 33·9)	19·3 (18·5 to 20·1)	16·1 (15·4 to 16·8)	13·6 (12·8 to 14·4)	12·9 (11·7 to 14·2)
Bulgaria	81·2 (77·7 to 85·1)	67·2 (65·5 to 68·7)	66·5 (65 to 68·2)	62·1 (60·6 to 63·5)	48·8 (47·5 to 50·2)	34 (33 to 35·1)	36·2 (35·2 to 37·3)	29·7 (28·5 to 31)	28·2 (26·1 to 30·3)
Croatia	44 (42·4 to 45·6)	36·7 (35·9 to 37·5)	30·6 (29·9 to 31·4)	32·8 (32 to 33·6)	25·9 (25·2 to 26·6)	20·7 (20·1 to 21·3)	20·7 (20·1 to 21·3)	17·6 (16·9 to 18·2)	16·9 (15·1 to 18·9)
Czechia	90·8 (86·9 to 94·8)	62·9 (61·5 to 64·4)	73 (71·3 to 74·8)	74 (72·2 to 75·7)	62·5 (61·1 to 63·9)	44·1 (42·8 to 45·5)	55·6 (54·2 to 57·1)	54·9 (53 to 56·9)	51·7 (46·5 to 57·4)
Hungary	93·3 (88·2 to 98·5)	69 (67·1 to 70·8)	73·1 (71 to 75·1)	71·6 (69·7 to 73·7)	61 (59·3 to 62·7)	46·6 (45 to 48·2)	44·4 (43 to 45·9)	44·3 (42·1 to 46·6)	42·8 (38·2 to 47·9)
Montenegro	6·1 (5·7 to 6·4)	6·3 (6·1 to 6·5)	5·3 (5·1 to 5·4)	5·1 (4·9 to 5·3)	4·6 (4·4 to 4·8)	4·2 (4 to 4·3)	3·7 (3·6 to 3·7)	3·5 (3·3 to 3·6)	3·4 (3·1 to 3·6)
North Macedonia	18·6 (16·8 to 20·5)	19 (17·1 to 21·1)	18·7 (17·4 to 20)	19·3 (17·4 to 21·3)	16·8 (16·4 to 17·1)	13·4 (13 to 13·8)	11·3 (10·9 to 11·7)	9·6 (9·3 to 9·9)	9 (8·5 to 9·5)
Poland	366 (356 to 375)	313 (307 to 319)	265 (260 to 271)	337 (330 to 343)	266 (260 to 272)	179 (175 to 184)	195 (191 to 200)	180 (175 to 186)	166 (148 to 185)
Romania	202 (190 to 215)	164 (158 to 170)	202 (196 to 208)	189 (183 to 194)	145 (140 to 150)	108 (103 to 112)	102 (97·6 to 106)	90·3 (85·9 to 94·9)	86·4 (79·6 to 93·7)
Serbia	89·4 (87·1 to 91·4)	78·7 (77 to 80·4)	71·4 (69·9 to 73·1)	76·5 (74·5 to 78·5)	69·2 (67·5 to 70·9)	50·5 (49·1 to 51·9)	37·1 (32 to 42·9)	30·7 (29·5 to 32)	29·8 (27·5 to 32·2)
Slovakia	48·1 (46·9 to 49·3)	42·4 (41·7 to 42·9)	39·4 (38·7 to 40)	46·2 (45·3 to 47)	38·5 (37·8 to 39·3)	25·7 (25·1 to 26·3)	29·5 (28·9 to 30·2)	28·1 (27·5 to 28·7)	27·4 (25·7 to 29·1)
Slovenia	15·6 (13·9 to 17·5)	13·6 (13·3 to 14)	13·4 (13·2 to 13·7)	14·3 (14 to 14·7)	10·7 (10·4 to 11)	8·6 (8·4 to 8·8)	10·8 (10·5 to 11·1)	9·4 (9·1 to 9·7)	9·1 (8·5 to 9·7)
Eastern Europe	1990 (1910 to 2080)	1950 (1920 to 1990)	1480 (1450 to 1510)	1630 (1600 to 1670)	1440 (1410 to 1460)	931 (904 to 960)	1250 (1220 to 1280)	949 (909 to 992)	871 (801 to 946)
Belarus	93·2 (88·1 to 98·5)	95·6 (92 to 99·1)	72·4 (70·1 to 75)	76·2 (73·5 to 79·1)	67·6 (65·2 to 69·9)	44·6 (42·5 to 46·7)	53·2 (50·9 to 55·4)	43·5 (39·7 to 47·5)	40 (34·3 to 46·3)
Estonia	9·7 (9·2 to 10·2)	9·7 (9·5 to 10)	10·5 (10·2 to 10·7)	10·9 (10·7 to 11·2)	10·2 (10 to 10·5)	6·2 (6 to 6·4)	7·4 (7·2 to 7·6)	6·7 (6·4 to 7)	6·4 (5·9 to 6·8)
Latvia	16 (14·9 to 17·3)	17·4 (17 to 17·7)	17·1 (16·7 to 17·5)	17·3 (17 to 17·7)	17·6 (17·2 to 18·1)	9·7 (9·5 to 10)	9·2 (8·9 to 9·4)	8·9 (8·6 to 9·2)	8·1 (7·2 to 9·1)
Lithuania	28·5 (27·1 to 30·2)	30 (29·1 to 30·8)	27·1 (26·4 to 27·9)	25 (24·3 to 25·6)	27·1 (26·5 to 27·9)	16·2 (15·6 to 16·7)	15·4 (14·8 to 15·9)	12·8 (12·3 to 13·4)	11·5 (10·7 to 12·3)
Moldova	40·8 (39·2 to 42·4)	40·8 (39·7 to 41·9)	32·2 (28·8 to 35·8)	41·9 (40·6 to 43·3)	38·5 (37·3 to 39·8)	20 (19·4 to 20·7)	19·6 (18·8 to 20·2)	14·9 (13·7 to 16·2)	13·7 (12·3 to 15·5)
Russia	1370 (1300 to 1460)	1320 (1290 to 1360)	944 (916 to 971)	1090 (1070 to 1120)	952 (929 to 976)	649 (625 to 672)	898 (867 to 928)	713 (676 to 751)	657 (608 to 711)
Ukraine	433 (415 to 452)	436 (423 to 448)	375 (364 to 385)	370 (360 to 381)	322 (312 to 331)	186 (179 to 191)	247 (239 to 255)	149 (143 to 156)	135 (120 to 151)
High income	6630 (6460 to 6790)	7080 (7020 to 7140)	6740 (6670 to 6820)	6070 (6000 to 6140)	6050 (5990 to 6130)	5750 (5680 to 5820)	5820 (5750 to 5890)	5160 (5100 to 5230)	5070 (4740 to 5420)
Australasia	123 (120 to 126)	147 (145 to 149)	161 (159 to 163)	136 (133 to 138)	153 (150 to 157)	146 (144 to 149)	177 (174 to 180)	174 (170 to 177)	174 (158 to 192)
Australia	98·4 (95·9 to 101)	116 (114 to 118)	131 (128 to 133)	111 (108 to 113)	124 (121 to 127)	119 (116 to 121)	146 (144 to 149)	145 (142 to 149)	146 (131 to 162)
New Zealand	24·2 (23·7 to 24·7)	30·9 (30·5 to 31·3)	30·4 (30 to 30·9)	24·6 (24·1 to 25·1)	29 (28·4 to 29·6)	27·6 (27 to 28·1)	30·5 (29·9 to 31·2)	28·3 (27·7 to 29)	28·5 (26·9 to 30·2)
High-income Asia Pacific	1480 (1430 to 1540)	1290 (1250 to 1340)	1450 (1410 to 1500)	1190 (1160 to 1230)	946 (919 to 972)	880 (853 to 908)	779 (755 to 804)	598 (579 to 616)	570 (524 to 621)
Brunei	1·3 (1·3 to 1·3)	2 (1·9 to 2)	2·3 (2·2 to 2·4)	2·8 (2·6 to 3)	3·4 (3·2 to 3·6)	3·5 (3·2 to 3·9)	3·2 (2·9 to 3·4)	3·1 (2·8 to 3·4)	3·1 (2·7 to 3·5)
Japan	1060 (1020 to 1110)	774 (746 to 802)	956 (923 to 989)	764 (738 to 795)	595 (572 to 618)	572 (548 to 595)	522 (501 to 543)	423 (406 to 439)	408 (368 to 455)
Aichi	45·1 (42·9 to 47·2)	38·5 (36·8 to 40·1)	56·6 (54·2 to 59·1)	42·4 (40·7 to 44·4)	34·6 (33·1 to 36·2)	35·6 (34 to 37·3)	34 (32·5 to 35·5)	28 (26·8 to 29·3)	27·3 (24·5 to 30·4)
Akita	15·1 (14·4 to 15·8)	10·5 (10 to 11)	9·5 (9 to 9·9)	7·7 (7·4 to 8·1)	5·4 (5·2 to 5·7)	4·4 (4·1 to 4·6)	3·4 (3·2 to 3·5)	2·3 (2·2 to 2·5)	2·2 (2 to 2·4)
Aomori	16·6 (15·7 to 17·4)	12·1 (11·6 to 12·6)	12·5 (12 to 13·1)	10·4 (9·9 to 10·8)	7·3 (7 to 7·6)	6·3 (6·1 to 6·6)	4·7 (4·5 to 5)	3·5 (3·4 to 3·7)	3·3 (2·9 to 3·7)
Chiba	27·7 (26·5 to 29)	18·6 (17·9 to 19·3)	34·1 (32·7 to 35·6)	32·7 (31·3 to 34·2)	26 (24·8 to 27·2)	26·6 (25·4 to 27·8)	25 (23·9 to 26·1)	20 (19·1 to 20·8)	19·6 (17·6 to 21·9)
Ehime	18·9 (18 to 19·8)	11·6 (11·1 to 12·2)	11·9 (11·5 to 12·4)	9·6 (9·2 to 10)	7·2 (6·8 to 7·5)	6·4 (6·1 to 6·7)	5·6 (5·4 to 5·9)	4·2 (4 to 4·3)	3·9 (3·5 to 4·4)
Fukui	9·5 (9 to 9·9)	5·9 (5·7 to 6·2)	6·3 (6 to 6·6)	5·1 (4·8 to 5·3)	4·2 (4 to 4·4)	3·9 (3·7 to 4·1)	3·3 (3·2 to 3·5)	2·6 (2·5 to 2·8)	2·5 (2·3 to 2·8)
Fukuoka	43·6 (41·6 to 45·7)	32·2 (30·8 to 33·6)	35·2 (33·8 to 36·6)	31·3 (30·1 to 32·6)	23·6 (22·6 to 24·6)	22·8 (21·9 to 23·7)	22·8 (21·8 to 23·8)	19·4 (18·6 to 20·2)	18·8 (16·9 to 21·1)
Fukushima	27·7 (26·4 to 29·1)	17·1 (16·4 to 17·8)	15·8 (15·1 to 16·5)	14·2 (13·6 to 14·8)	11·1 (10·6 to 11·6)	9·8 (9·4 to 10·2)	7·7 (7·3 to 8·1)	5·7 (5·5 to 6)	5·5 (4·9 to 6·1)
Gifu	18·9 (18·1 to 19·7)	13·6 (13·1 to 14·2)	16 (15·3 to 16·8)	12·5 (11·9 to 13)	9·9 (9·4 to 10·4)	9·7 (9·3 to 10·2)	8·3 (8 to 8·8)	6·2 (6 to 6·5)	5·9 (5·3 to 6·6)
Gunma	20·6 (19·7 to 21·6)	12·7 (12·1 to 13·4)	14·9 (13·9 to 16)	12·2 (11·7 to 12·7)	9·6 (9·2 to 10)	9·3 (8·9 to 9·7)	7·8 (7·5 to 8·1)	5·8 (5·6 to 6·1)	5·6 (5 to 6·3)
Hiroshima	26·5 (25·3 to 27·7)	17·5 (16·8 to 18·3)	22·6 (21·6 to 23·6)	17·9 (17·2 to 18·7)	13·9 (13·3 to 14·5)	13·2 (12·6 to 13·9)	12·5 (11·9 to 13·1)	9·9 (9·4 to 10·3)	9·4 (8·5 to 10·6)
Hokkaidō	52·1 (49·5 to 54·7)	39·5 (38·1 to 40·8)	46·3 (44·3 to 48·2)	36·7 (35·3 to 38·1)	26·5 (25·3 to 27·5)	22·9 (21·9 to 24)	19·7 (18·9 to 20·5)	15 (14·3 to 15·7)	14·3 (12·7 to 16)

Hyōgo	42·4 (40·5 to 44·3)	32·8 (31·6 to 33·9)	44·8 (43 to 46·6)	33·3 (31·9 to 34·6)	26 (24·9 to 27·1)	25·9 (24·8 to 27·1)	23·4 (22·4 to 24·4)	18·5 (17·7 to 19·3)	17·7 (16 to 19·7)
Ibaraki	27·2 (25·9 to 28·5)	16·1 (15·5 to 16·6)	18·7 (17·9 to 19·4)	17·7 (17 to 18·4)	14 (13·4 to 14·6)	13·6 (13 to 14·2)	11·6 (11·2 to 12·1)	8·8 (8·4 to 9·2)	8·3 (7·4 to 9·4)
Ishikawa	11·8 (11·2 to 12·3)	7·8 (7·5 to 8·1)	9·5 (9 to 9·9)	7·3 (7 to 7·6)	5·6 (5·3 to 5·8)	5·5 (5·3 to 5·8)	4·8 (4·6 to 5)	3·9 (3·7 to 4·1)	3·7 (3·3 to 4·2)
Iwate	18·9 (17·9 to 19·9)	12·9 (12·4 to 13·5)	12 (11·5 to 12·5)	9·4 (9 to 9·8)	7 (6·7 to 7·3)	6 (5·7 to 6·2)	4·7 (4·5 to 5)	3·5 (3·3 to 3·6)	3·2 (2·9 to 3·6)
Kagawa	11·2 (10·7 to 11·8)	6·9 (6·7 to 7·2)	7·6 (7·3 to 8)	6·2 (6 to 6·5)	4·7 (4·5 to 4·8)	4·7 (4·4 to 4·9)	4·1 (3·9 to 4·3)	3·2 (3 to 3·3)	3·1 (2·7 to 3·4)
Kagoshima	25 (23·9 to 26·1)	15·3 (14·7 to 16)	12·6 (11·8 to 13·4)	12 (11·5 to 12·5)	9·2 (8·8 to 9·6)	7·8 (7·5 to 8·1)	7·4 (7·1 to 7·8)	5·9 (5·6 to 6·1)	5·5 (4·9 to 6·1)
Kanagawa	32·6 (31·1 to 34·1)	30·9 (29·6 to 32·3)	56·2 (53·8 to 58·6)	46 (44·1 to 48)	38·6 (36·9 to 40·2)	39·9 (38 to 41·7)	38 (36·4 to 39·6)	30·6 (29·4 to 31·8)	29·9 (26·9 to 33·2)
Kōchi	11·1 (10·5 to 11·6)	6·1 (5·9 to 6·4)	6·2 (5·9 to 6·5)	4·9 (4·7 to 5·1)	3·6 (3·5 to 3·8)	3·2 (3·1 to 3·4)	2·7 (2·5 to 2·8)	2·1 (2 to 2·2)	1·9 (1·7 to 2·2)
Kumamoto	22·7 (21·6 to 23·8)	14·3 (13·6 to 14·9)	13·1 (12·6 to 13·7)	11·8 (11·3 to 12·3)	9·3 (8·8 to 9·8)	8·3 (8 to 8·7)	8 (7·6 to 8·3)	6·5 (6·3 to 6·8)	6·2 (5·6 to 6·9)
Kyōto	21 (20·1 to 22)	15·4 (14·7 to 16·1)	20 (19·1 to 20·8)	15·5 (14·9 to 16·1)	11·6 (11·1 to 12·1)	11·4 (10·9 to 12)	10·2 (9·8 to 10·7)	8·3 (7·9 to 8·6)	7·9 (7·1 to 8·8)
Mie	18 (17·2 to 18·9)	11·9 (11·4 to 12·4)	13·5 (12·9 to 14·1)	10·3 (9·8 to 10·8)	8·6 (8·2 to 9)	8·5 (8·1 to 8·9)	7·5 (7·2 to 7·9)	5·7 (5·4 to 6)	5·4 (4·9 to 6·1)
Miyagi	20·6 (19·7 to 21·6)	14·3 (13·7 to 15)	15·5 (14·8 to 16·1)	14·9 (14·3 to 15·6)	11·3 (10·8 to 11·8)	10·6 (10·1 to 11·1)	9·3 (8·9 to 9·7)	7·3 (7 to 7·6)	7 (6·3 to 7·8)
Miyazaki	14·7 (14 to 15·4)	9·5 (9·1 to 9·9)	8·9 (8·6 to 9·3)	8·3 (7·9 to 8·7)	6 (5·8 to 6·3)	5·3 (5·1 to 5·6)	5 (4·8 to 5·2)	3·9 (3·7 to 4·1)	3·7 (3·3 to 4·1)
Nagano	25·6 (24·5 to 26·7)	16·3 (15·6 to 17)	16·6 (15·9 to 17·3)	12·9 (12·3 to 13·5)	10·4 (9·9 to 10·8)	10·1 (9·7 to 10·6)	8·5 (8·1 to 8·8)	6·5 (6·2 to 6·8)	6·2 (5·6 to 7)
Nagasaki	23·2 (22·1 to 24·3)	14·5 (14 to 15)	13 (12·5 to 13·6)	10·9 (10·5 to 11·3)	8·2 (7·8 to 8·5)	6·8 (6·5 to 7·1)	5·8 (5·6 to 6·1)	4·6 (4·4 to 4·8)	4·3 (3·9 to 4·8)
Nara	8·8 (8·4 to 9·2)	5·8 (5·6 to 6)	8·1 (7·7 to 8·4)	7·8 (7·5 to 8·1)	6·4 (6·2 to 6·7)	6·3 (6 to 6·6)	5·3 (5 to 5·5)	4 (3·8 to 4·2)	3·8 (3·4 to 4·2)
Niigata	31·5 (30·1 to 32·9)	19·8 (19 to 20·7)	19·7 (18·9 to 20·6)	15·9 (15·3 to 16·6)	11·8 (11·3 to 12·4)	10·4 (10 to 10·9)	8·8 (8·3 to 9·2)	6·6 (6·3 to 6·9)	6·2 (5·5 to 6·9)
Ōita	15·1 (14·4 to 15·8)	9·4 (9·1 to 9·9)	9·2 (8·8 to 9·7)	7·8 (7·5 to 8·2)	5·8 (5·5 to 6)	5·2 (5 to 5·5)	4·9 (4·7 to 5·1)	3·8 (3·6 to 4)	3·6 (3·2 to 4)
Okayama	20·1 (19·2 to 21)	13 (12·4 to 13·5)	14·9 (14·3 to 15·6)	11·8 (11·3 to 12·3)	9·2 (8·8 to 9·6)	9·1 (8·6 to 9·6)	8·1 (7·7 to 8·5)	6·7 (6·4 to 7·1)	6·4 (5·8 to 7·2)
Okinawa	14·2 (13·3 to 15·1)	9·1 (8·7 to 9·5)	10·3 (9·9 to 10·8)	9·7 (9·3 to 10·1)	8·5 (8·1 to 8·9)	8·2 (7·8 to 8·5)	8·3 (7·9 to 8·7)	7·4 (7 to 7·7)	7·1 (6·4 to 8)
Ōsaka	50·9 (48·5 to 53·3)	48·7 (47 to 50·4)	78·6 (75·6 to 81·7)	53·9 (51·8 to 56·1)	42·1 (40·5 to 43·8)	42·2 (40·3 to 44·2)	36·8 (35·2 to 38·4)	30·6 (29·4 to 31·8)	30·2 (27·1 to 33·7)
Saga	12·3 (11·7 to 12·8)	7·5 (7·2 to 7·8)	6·7 (6·4 to 7)	6 (5·7 to 6·2)	4·6 (4·4 to 4·8)	4·2 (4 to 4·3)	3·7 (3·6 to 3·9)	3 (2·9 to 3·1)	2·9 (2·6 to 3·2)
Saitama	28·5 (27·2 to 29·9)	20·7 (19·8 to 21·5)	42·5 (40·8 to 44·3)	37 (35·6 to 38·5)	31 (29·8 to 32·3)	32·1 (30·6 to 33·6)	28·9 (27·7 to 30·2)	23·7 (22·8 to 24·6)	23·2 (20·9 to 25·9)
Shiga	11·3 (10·8 to 11·8)	7 (6·7 to 7·2)	7·4 (7·1 to 7·7)	7·6 (7·4 to 8)	6·5 (6·2 to 6·8)	6·8 (6·5 to 7·1)	6·5 (6·2 to 6·8)	5·2 (5 to 5·4)	5 (4·5 to 5·5)
Shimane	11·5 (11 to 12)	6·9 (6·6 to 7·2)	5·9 (5·5 to 6·3)	4·8 (4·6 to 5)	3·6 (3·5 to 3·8)	3·1 (3 to 3·3)	2·8 (2·7 to 2·9)	2·2 (2·1 to 2·3)	2·1 (1·9 to 2·4)
Shizuoka	32 (30·6 to 33·5)	23·3 (22·3 to 24·3)	29·4 (28·1 to 30·6)	23·2 (22·2 to 24·4)	18·1 (17·4 to 18·8)	17·2 (16·5 to 18·1)	15·5 (14·8 to 16·3)	11·5 (10·9 to 12)	10·9 (9·8 to 12·2)
Tochigi	20·4 (19·4 to 21·3)	12·1 (11·7 to 12·5)	13·9 (13·3 to 14·5)	12·5 (12 to 13)	9·7 (9·2 to 10·1)	9·1 (8·7 to 9·5)	8 (7·7 to 8·3)	6 (5·8 to 6·3)	5·7 (5·1 to 6·3)
Tokushima	10 (9·5 to 10·5)	6·1 (5·9 to 6·4)	6 (5·7 to 6·2)	5·1 (4·9 to 5·3)	3·8 (3·6 to 4)	3·4 (3·3 to 3·6)	2·9 (2·7 to 3)	2·3 (2·2 to 2·4)	2·1 (1·9 to 2·4)
Tōkyō	73·2 (69·9 to 76·6)	80·7 (76·3 to 85·6)	103 (98·5 to 108)	68·1 (65·5 to 70·9)	50·6 (48·4 to 52·8)	48 (46 to 50·2)	52·6 (50·5 to 54·8)	50 (47·8 to 52·1)	50·1 (45·2 to 55·6)
Tottori	7·6 (7·2 to 7·9)	4·6 (4·4 to 4·8)	4·4 (4·2 to 4·6)	4 (3·8 to 4·1)	3·1 (2·9 to 3·2)	2·7 (2·6 to 2·8)	2·4 (2·3 to 2·5)	1·9 (1·8 to 2)	1·8 (1·6 to 2)
Toyama	11·6 (11·1 to 12·2)	7·9 (7·6 to 8·3)	9·2 (8·7 to 9·7)	6·6 (6·2 to 6·9)	4·9 (4·7 to 5·1)	4·9 (4·6 to 5·1)	4 (3·8 to 4·2)	3·2 (3 to 3·3)	3 (2·7 to 3·4)
Wakayama	12·8 (12·2 to 13·4)	7·8 (7·5 to 8·1)	8·6 (8·2 to 9·1)	6·5 (6·2 to 6·8)	5 (4·7 to 5·2)	4·6 (4·3 to 4·8)	3·7 (3·5 to 3·9)	2·9 (2·7 to 3)	2·7 (2·4 to 3)
Yamagata	16·7 (15·9 to 17·4)	10·6 (10·2 to 11·1)	9·3 (8·9 to 9·7)	8 (7·7 to 8·3)	6·2 (5·9 to 6·4)	5·3 (5 to 5·5)	4·2 (4 to 4·5)	3·2 (3 to 3·3)	2·9 (2·7 to 3·3)
Yamaguchi	18·2 (17·4 to 19·1)	12·2 (11·6 to 12·7)	12·6 (12 to 13·1)	9·6 (9·2 to 10)	6·8 (6·5 to 7·1)	6·3 (6 to 6·6)	5·6 (5·3 to 5·8)	4·2 (4 to 4·3)	3·9 (3·5 to 4·4)
Yamanashi	9·5 (9·1 to 9·9)	5·9 (5·6 to 6·1)	6 (5·7 to 6·2)	4·9 (4·7 to 5·1)	4·3 (4·1 to 4·5)	4 (3·8 to 4·2)	3·2 (3·1 to 3·4)	2·6 (2·5 to 2·7)	2·5 (2·2 to 2·8)
South Korea	396 (373 to 419)	489 (459 to 520)	473 (448 to 498)	406 (394 to 419)	321 (312 to 331)	277 (267 to 287)	226 (217 to 235)	144 (136 to 152)	131 (120 to 143)
Singapore	23·3 (22·2 to 24·4)	29 (28·2 to 29·7)	22·4 (21·1 to 23·7)	20·2 (18·9 to 21·5)	26 (24·5 to 27·7)	28·1 (26·2 to 30)	27·6 (25·1 to 30·2)	27·8 (25·9 to 29·9)	26·9 (23·5 to 31)
High-income North America	1960 (1910 to 2010)	2290 (2260 to 2320)	1920 (1880 to 1950)	1920 (1880 to 1960)	2210 (2170 to 2260)	2130 (2090 to 2170)	2130 (2090 to 2170)	1980 (1940 to 2010)	1960 (1840 to 2090)
Canada	176 (172 to 180)	223 (220 to 226)	169 (165 to 172)	174 (170 to 178)	195 (191 to 198)	162 (158 to 165)	184 (180 to 187)	178 (174 to 182)	176 (158 to 196)
Greenland	0·5 (0·5 to 0·5)	0·7 (0·7 to 0·8)	0·5 (0·5 to 0·5)	0·5 (0·5 to 0·5)	0·6 (0·6 to 0·6)	0·5 (0·4 to 0·5)	0·4 (0·4 to 0·4)	0·4 (0·4 to 0·4)	0·4 (0·3 to 0·4)
USA	1780 (1740 to 1830)	2070 (2040 to 2100)	1750 (1710 to 1780)	1750 (1710 to 1790)	2020 (1970 to 2060)	1970 (1930 to 2000)	1950 (1910 to 1990)	1800 (1760 to 1840)	1780 (1680 to 1890)
Alabama	39 (38·1 to 40·1)	40·5 (39·5 to 41·5)	32·3 (31·4 to 33·1)	30·3 (29·3 to 31·4)	31·1 (30·2 to 32)	30·2 (29·2 to 31·1)	28·6 (27·8 to 29·4)	29 (28·1 to 29·8)	28·4 (26·5 to 30·3)
Alaska	1·6 (1·6 to 1·6)	3·3 (3·2 to 3·4)	3·5 (3·4 to 3·6)	4·7 (4·6 to 4·9)	5·7 (5·5 to 5·9)	4·9 (4·7 to 5)	5·5 (5·3 to 5·7)	4·5 (4·3 to 4·7)	4·4 (4·2 to 4·8)
Arizona	10 (9·7 to 10·3)	17·4 (16·6 to 18·4)	17·8 (17·3 to 18·5)	23·9 (22·4 to 25·4)	32·9 (31·8 to 34·1)	41·3 (40 to 42·7)	43·3 (41·3 to 45·4)	38·1 (36·7 to 39·4)	37·9 (35·5 to 40·5)
Arkansas	22·1 (21·6 to 22·7)	19·8 (19·2 to 20·4)	17·2 (16·7 to 17·7)	17·9 (17·2 to 18·5)	17·7 (17·2 to 18·2)	18·2 (17·6 to 18·8)	18·4 (17·8 to 18·9)	17·3 (16·7 to 17·9)	17·4 (16·2 to 18·6)
California	123 (120 to 126)	179 (170 to 189)	162 (155 to 169)	190 (178 to 203)	291 (281 to 301)	259 (250 to 268)	251 (242 to 260)	211 (203 to 220)	206 (193 to 220)
Colorado	14·3 (13·9 to 14·6)	19·4 (18·8 to 20)	19·6 (19 to 20·3)	24·8 (24 to 25·7)	26·1 (25·3 to 26·8)	32·3 (31·5 to 33·1)	32·3 (31·3 to 33·3)	28·5 (27·4 to 29·6)	30·5 (28·4 to 32·8)
Connecticut	23·3 (22·8 to 24)	27·6 (26·8 to 28·5)	23·1 (22·4 to 23·7)	19·2 (18·6 to 19·7)	24·2 (23·5 to 25)	21 (20·4 to 21·7)	18·6 (18 to 19·2)	17·1 (16·6 to 17·6)	17·4 (16·2 to 18·6)
Delaware	4 (3·9 to 4·1)	5·5 (5·3 to 5·8)	4·7 (4·4 to 4·9)	4·4 (4·1 to 4·7)	5·3 (5·1 to 5·5)	5·4 (5·1 to 5·6)	5·8 (5·5 to 6)	5·3 (5 to 5·6)	5·2 (4·9 to 5·6)
Washington, DC	10 (9·8 to 10·3)	8·5 (8·1 to 8·9)	6·4 (6 to 6·9)	4·8 (4·5 to 5·1)	5·5 (5·2 to 5·8)	3·8 (3·6 to 4·1)	4·1 (3·5 to 4·8)	4·4 (4·1 to 4·7)	4·4 (4 to 4·9)
Florida	30·9 (30·1 to 31·7)	52·9 (51·2 to 54·6)	55·7 (54·3 to 57·2)	65·4 (63·3 to 67·4)	95·5 (93·1 to 98)	99·5 (96·6 to 102)	105 (102 to 108)	107 (104 to 110)	105 (97·7 to 113)
Georgia	47·2 (46 to 48·5)	53·4 (50·8 to 56·3)	44·5 (43 to 46)	43·3 (41·1 to 45·6)	54 (52·4 to 55·7)	64·8 (62·7 to 66·9)	67 (64 to 70·2)	61·1 (59 to 63·2)	60·8 (56·8 to 65)
Hawaii	7 (6·8 to 7·2)	8·5 (8·2 to 8·7)	7·7 (7·5 to 8)	8·7 (8·4 to 9)	9·9 (9·6 to 10·2)	8·4 (8·1 to 8·7)	9·1 (8·8 to 9·4)	7·9 (7·7 to 8·2)	7·5 (7 to 8·1)
Idaho	7·1 (6·9 to 7·2)	8 (7·7 to 8·2)	6·9 (6·8 to 7·1)	9·7 (9·4 to 10)	8·1 (7·9 to 8·3)	9·9 (9·5 to 10·3)	10·9 (10·6 to 11·2)	10·8 (10·4 to 11·2)	10·9 (10·2 to 11·7)
Illinois	102 (99·2 to 104)	115 (112 to 118)	96·7 (94·1 to 99·3)	91·7 (89 to 94·1)	96 (93·4 to 98·7)	89·2 (86·9 to 91·7)	77·8 (75·5 to 80·1)	67·7 (65·6 to 69·8)	64·3 (59·8 to 69)

Indiana	44·3 (43·2 to 45·5)	53·5 (52·6 to 54·4)	46·9 (45·6 to 48·3)	42·2 (41 to 43·4)	42·2 (41 to 43·6)	41·9 (40·8 to 43)	41·1 (39·9 to 42·3)	38·8 (37·7 to 39·9)	38·8 (36·3 to 41·3)
Iowa	28·3 (27·6 to 29·1)	29·8 (29 to 30·6)	22·4 (21·8 to 23)	23 (22·2 to 23·7)	19·3 (18·7 to 19·9)	18·2 (17·6 to 18·9)	18·7 (18·1 to 19·4)	17·9 (17·4 to 18·5)	17·9 (16·7 to 19·1)
Kansas	20 (19·5 to 20·5)	23·2 (22·5 to 23·9)	17·9 (17·3 to 18·4)	19·9 (19·3 to 20·5)	18·8 (18·3 to 19·4)	19 (18·5 to 19·4)	20 (19·4 to 20·5)	17 (16·6 to 17·6)	16·9 (15·8 to 18)
Kentucky	34·7 (33·9 to 35·6)	35·1 (34 to 36·2)	29·2 (28·4 to 30)	28·5 (27·6 to 29·3)	26·8 (26·1 to 27·6)	26·9 (26·1 to 27·8)	26·1 (25·4 to 26·9)	25·3 (24·5 to 26·1)	25·3 (23·6 to 27·1)
Louisiana	37 (36 to 38)	44·4 (43·4 to 45·4)	35·7 (34·7 to 36·7)	40·5 (39·3 to 41·6)	35·8 (34·8 to 36·7)	32·6 (31·6 to 33·8)	30·6 (29·5 to 31·7)	28·1 (27·3 to 29)	27·9 (26·2 to 29·8)
Maine	10·5 (10·3 to 10·8)	11·1 (10·9 to 11·3)	8·6 (8·3 to 8·9)	8 (7·7 to 8·3)	8·4 (8·1 to 8·7)	6·6 (6·4 to 6·8)	6·2 (6 to 6·4)	5·6 (5·4 to 5·8)	5·8 (5·4 to 6·2)
Maryland	28 (27·3 to 28·8)	35·5 (34·7 to 36·2)	32·4 (31·4 to 33·4)	27·5 (26·6 to 28·4)	39·1 (37·8 to 40·3)	36·4 (35·3 to 37·4)	35·2 (34·1 to 36·3)	34·7 (33·8 to 35·8)	33·4 (31·1 to 35·8)
Massachusetts	53·7 (52·3 to 55·1)	55·2 (54·1 to 56·3)	43·6 (42·4 to 44·8)	35·7 (34·7 to 36·8)	44·4 (42·9 to 46)	39·7 (38·4 to 41)	35·8 (34·9 to 36·7)	33·7 (32·7 to 34·8)	33·7 (31·4 to 36·2)
Michigan	77·2 (75·3 to 79·3)	92·8 (91 to 94·7)	80·5 (78·3 to 82·8)	70·1 (67·8 to 72·3)	74·4 (72·3 to 76·6)	65·5 (64 to 67)	55·1 (53·4 to 56·6)	51·5 (50·3 to 52·8)	51 (47·7 to 54·5)
Minnesota	33·9 (33·1 to 34·8)	37·8 (36·8 to 38·8)	31·3 (30·5 to 32·2)	32·8 (31·9 to 33·9)	33 (32 to 34)	32·6 (31·8 to 33·5)	33·1 (32·3 to 34)	31·5 (30·6 to 32·3)	31·3 (29·3 to 33·5)
Mississippi	30·4 (29·6 to 31·3)	29·4 (28·7 to 30·1)	23·7 (23 to 24·3)	23·1 (22·3 to 23·8)	21·5 (20·8 to 22·2)	21·1 (20·4 to 21·7)	19·3 (18·7 to 20)	17·4 (16·9 to 18)	17·2 (16·1 to 18·3)
Missouri	43·4 (42·4 to 44·6)	47 (45·7 to 48·4)	38·6 (37·4 to 39·8)	38·1 (36·9 to 39·4)	38·8 (37·9 to 39·8)	36·7 (35·4 to 37·9)	37·5 (36·4 to 38·6)	34·1 (33·1 to 35·2)	33·7 (31·4 to 36·3)
Montana	6·3 (6·2 to 6·5)	7·3 (7·2 to 7·4)	6 (5·8 to 6·2)	6·9 (6·7 to 7·1)	5·7 (5·5 to 5·8)	5·4 (5·2 to 5·5)	5·8 (5·7 to 6)	5·2 (5·1 to 5·4)	5·4 (5·1 to 5·8)
Nebraska	14·1 (13·8 to 14·5)	15·2 (14·7 to 15·6)	12·3 (11·9 to 12·7)	13·3 (12·8 to 13·7)	11·8 (11·4 to 12·2)	12 (11·5 to 12·4)	12·7 (12·2 to 13·2)	12 (11·6 to 12·4)	11·9 (11·2 to 12·8)
Nevada	1·8 (1·8 to 1·9)	3·4 (3·4 to 3·5)	4·6 (4·4 to 4·7)	6·5 (6·3 to 6·8)	10·4 (10·1 to 10·8)	15·2 (14·7 to 15·6)	17 (16·4 to 17·6)	17·1 (16·5 to 17·7)	16·4 (15·3 to 17·7)
New Hampshire	5·9 (5·8 to 6·1)	6·6 (6·4 to 6·8)	6·4 (6·2 to 6·6)	6·6 (6·4 to 6·8)	8·3 (8·1 to 8·6)	7·1 (6·9 to 7·3)	6·4 (6·2 to 6·6)	5·8 (5·6 to 6)	6·1 (5·7 to 6·5)
New Jersey	56·6 (55·2 to 58·1)	66·5 (64 to 68·9)	56·3 (54·4 to 58·4)	47·2 (45·8 to 48·6)	60·1 (58·5 to 61·9)	56·8 (55·3 to 58·3)	50·5 (49 to 52)	51 (49·4 to 52·5)	49·5 (46·1 to 53·3)
New Mexico	9·9 (9·6 to 10·1)	14·2 (13·5 to 15)	10·5 (10·2 to 10·8)	13 (12·5 to 13·4)	13·6 (13·2 to 14)	13·3 (12·9 to 13·8)	13·2 (12·8 to 13·6)	10·8 (10·4 to 11·3)	10·4 (9·7 to 11·1)
New York	170 (166 to 175)	187 (181 to 192)	146 (141 to 150)	118 (114 to 121)	145 (141 to 149)	126 (123 to 128)	118 (115 to 122)	110 (108 to 113)	102 (95·6 to 110)
North Carolina	50·3 (49·1 to 51·6)	55·5 (54·2 to 56·8)	46·9 (45·6 to 48·2)	41·1 (40 to 42·3)	50·9 (49·4 to 52·3)	58·1 (56·7 to 59·5)	60 (58·4 to 61·6)	56·6 (55·1 to 58·2)	58·4 (54·4 to 62·4)
North Dakota	8·1 (7·9 to 8·3)	7·9 (7·5 to 8·3)	5 (4·7 to 5·3)	5·9 (5·5 to 6·3)	4·6 (4·3 to 4·8)	3·8 (3·6 to 4)	5·4 (5·1 to 5·6)	5 (4·8 to 5·2)	4·9 (4·5 to 5·3)
Ohio	91·9 (89·7 to 94·3)	112 (110 to 115)	93·9 (91·2 to 96·5)	82·1 (79·9 to 84·4)	82 (79·7 to 84·4)	74·3 (72·4 to 76)	67·7 (65·9 to 69·5)	63·7 (61·9 to 65·5)	63·2 (59·2 to 67·3)
Oklahoma	23·4 (22·8 to 24)	24·6 (24 to 25·1)	21·6 (21 to 22·3)	26·1 (25·3 to 27)	23·4 (22·8 to 24·2)	24·3 (23·6 to 24·9)	25·1 (24·4 to 25·9)	23·1 (22·4 to 23·8)	23·5 (22 to 25)
Oregon	15·4 (15 to 15·8)	17·3 (16·9 to 17·8)	16·3 (15·9 to 16·8)	20·8 (20·2 to 21·5)	20·8 (20·2 to 21·5)	22 (21·4 to 22·7)	22·3 (21·6 to 23)	19·1 (18·6 to 19·6)	19·8 (18·5 to 21·1)
Pennsylvania	119 (116 to 122)	120 (118 to 123)	90·6 (88·2 to 92·8)	78 (75·8 to 80·3)	83·9 (81·4 to 86·3)	70·5 (68·8 to 72·1)	69·6 (68 to 71·2)	65·3 (63·5 to 67·1)	64·5 (60·2 to 69)
Rhode Island	9·4 (9·1 to 9·6)	9·3 (8·9 to 9·7)	7·4 (7·1 to 7·7)	6 (5·7 to 6·3)	7·4 (7 to 7·7)	6·2 (5·9 to 6·5)	5·7 (5·5 to 6)	5·2 (4·9 to 5·5)	5·2 (4·8 to 5·6)
South Carolina	27·4 (26·8 to 28·2)	30·3 (29·5 to 31·1)	25·5 (24·8 to 26·2)	24·9 (24·2 to 25·7)	28·7 (27·9 to 29·5)	27·3 (26·6 to 28)	27·1 (26·3 to 27·9)	27·1 (26·4 to 27·8)	27·8 (26 to 29·6)
South Dakota	7·7 (7·5 to 7·9)	7·9 (7·6 to 8·1)	5·6 (5·4 to 5·7)	6·3 (6·1 to 6·5)	5·4 (5·2 to 5·6)	5 (4·9 to 5·2)	6·1 (5·9 to 6·3)	5·4 (5·2 to 5·6)	5·5 (5·1 to 5·8)
Tennessee	39·5 (38·5 to 40·6)	41·9 (40·8 to 43)	34·5 (33·4 to 35·4)	33·1 (31·9 to 34·3)	36·7 (35·4 to 37·9)	38·4 (37·1 to 39·7)	41·3 (40 to 42·7)	38·5 (37·2 to 39·8)	39·7 (37·2 to 42·2)
Texas	101 (98·1 to 103)	126 (122 to 129)	111 (107 to 114)	136 (131 to 140)	155 (150 to 160)	178 (173 to 183)	192 (185 to 198)	178 (173 to 183)	182 (170 to 193)
Utah	9·5 (9·2 to 9·7)	12·7 (12·4 to 13·1)	12·9 (12·5 to 13·3)	20·1 (19·5 to 20·8)	17·6 (17·1 to 18·3)	23 (22·2 to 23·7)	25·6 (24·9 to 26·3)	22 (21·3 to 22·7)	22·5 (21·1 to 24)
Vermont	4·2 (4·1 to 4·3)	4·3 (4·1 to 4·4)	3·9 (3·7 to 4)	3·8 (3·7 to 4)	4 (3·8 to 4·1)	3·1 (3 to 3·2)	2·8 (2·7 to 2·9)	2·6 (2·5 to 2·7)	2·6 (2·4 to 2·8)
Virginia	39·8 (38·8 to 40·8)	47 (45·7 to 48·3)	40·7 (39·6 to 41·8)	37·7 (36·6 to 38·8)	48·2 (46·8 to 49·7)	48·6 (47·4 to 49·9)	49·5 (48·2 to 50·8)	47·5 (46·3 to 48·8)	46·5 (43·5 to 49·7)
Washington	25·5 (24·9 to 26·2)	30·4 (29·7 to 31·1)	27·4 (26·6 to 28·3)	33·3 (32·2 to 34·5)	38·7 (37·5 to 39·9)	39 (37·9 to 40)	42·4 (41·1 to 43·6)	38·9 (37·9 to 40·1)	40·5 (38 to 43·2)
West Virginia	23·1 (22·5 to 23·7)	20·3 (19·7 to 20·8)	14·9 (14·5 to 15·3)	14 (13·5 to 14·5)	11·1 (10·8 to 11·5)	10 (9·7 to 10·4)	10·1 (9·8 to 10·3)	8·6 (8·4 to 8·9)	8·3 (7·8 to 8·8)
Wisconsin	38·5 (37·6 to 39·6)	43·3 (42·1 to 44·4)	35·8 (34·9 to 36·7)	36·3 (35·3 to 37·3)	35·5 (34·5 to 36·5)	33·4 (32·6 to 34·3)	32·9 (31·9 to 33·9)	30·4 (29·5 to 31·3)	30 (28·2 to 31·9)
Wyoming	3·3 (3·2 to 3·4)	3·9 (3·7 to 4)	3 (2·9 to 3·1)	5·2 (5 to 5·4)	3·4 (3·2 to 3·5)	3 (2·9 to 3·1)	3·3 (3·2 to 3·4)	3 (2·9 to 3·1)	3 (2·8 to 3·2)
Southern Latin America	330 (320 to 340)	391 (386 to 397)	417 (410 to 424)	483 (476 to 490)	505 (498 to 513)	485 (473 to 496)	508 (498 to 520)	402 (391 to 414)	376 (334 to 421)
Argentina	216 (208 to 224)	232 (227 to 237)	271 (265 to 276)	334 (328 to 340)	332 (326 to 338)	336 (324 to 347)	366 (356 to 376)	284 (275 to 294)	262 (231 to 296)
Chile	92·4 (90 to 94·9)	129 (127 to 131)	120 (117 to 122)	122 (120 to 126)	145 (142 to 148)	123 (120 to 126)	121 (118 to 124)	99·8 (96·2 to 104)	96·7 (86·2 to 108)
Uruguay	21·9 (20·8 to 23·1)	30·3 (29·5 to 31·2)	27 (26·1 to 27·9)	26·3 (25·6 to 27)	27·1 (26·3 to 27·7)	25·7 (24·3 to 27·2)	21·1 (19·5 to 23)	18 (17·2 to 18·8)	17·2 (15·3 to 19·3)
Western Europe	2730 (2650 to 2810)	2950 (2920 to 2980)	2800 (2770 to 2820)	2330 (2310 to 2360)	2240 (2210 to 2260)	2110 (2080 to 2130)	2220 (2190 to 2250)	2010 (1990 to 2040)	1990 (1870 to 2120)
Andorra	0·1 (0·1 to 0·1)	0·1 (0·1 to 0·1)	0·2 (0·2 to 0·2)	0·2 (0·2 to 0·3)	0·3 (0·3 to 0·3)	0·3 (0·3 to 0·4)	0·4 (0·4 to 0·4)	0·3 (0·3 to 0·3)	0·3 (0·2 to 0·3)
Austria	51·4 (49·8 to 53·4)	62·5 (61·5 to 63·6)	53·7 (52·8 to 54·7)	44·5 (43·5 to 45·4)	45·1 (44·1 to 46·1)	37·7 (37 to 38·5)	38 (37 to 39)	41 (40·1 to 41·9)	41·6 (39·1 to 44·2)
Belgium	69·2 (66·9 to 71·5)	76·3 (75·1 to 77·4)	68·3 (67·2 to 69·5)	59·9 (58·7 to 61·1)	60·8 (59·7 to 61·9)	56·7 (55·8 to 57·7)	62·9 (61·8 to 64)	56·5 (55·3 to 57·5)	55·3 (49·9 to 61)
Cyprus	6·8 (6·5 to 7)	7 (6·9 to 7·2)	5·9 (5·7 to 6·1)	6·5 (6·3 to 6·7)	7 (6·9 to 7·2)	5·4 (5·2 to 5·6)	6·5 (6·3 to 6·7)	7·3 (6·7 to 7·9)	7·3 (6·3 to 8·4)
Denmark	37·9 (36·7 to 39·3)	37 (36·2 to 37·7)	35·1 (34·2 to 36·2)	26·9 (26·2 to 27·6)	31·1 (30·4 to 31·7)	32·3 (31·6 to 33)	29·8 (29·2 to 30·4)	29·6 (28·9 to 30·3)	30·7 (29·1 to 32·5)
Finland	46·6 (45·2 to 48·2)	39·5 (38·7 to 40·3)	30·6 (29·9 to 31·3)	31·2 (30·3 to 32·1)	31·8 (30·9 to 32·6)	27·1 (26·4 to 27·8)	29·3 (28·7 to 29·9)	22·6 (21·9 to 23·4)	23·6 (22·1 to 25·2)
France	410 (399 to 421)	406 (398 to 413)	417 (410 to 425)	388 (377 to 398)	371 (362 to 380)	376 (369 to 384)	390 (383 to 397)	346 (338 to 354)	339 (305 to 375)
Germany	537 (498 to 578)	613 (601 to 625)	498 (487 to 508)	415 (406 to 425)	419 (410 to 429)	368 (358 to 378)	327 (319 to 335)	380 (367 to 393)	385 (362 to 408)
Greece	74·4 (71·4 to 77·3)	74·6 (72·8 to 76·4)	69·6 (68 to 71·1)	69·2 (67·7 to 70·9)	49·3 (47·9 to 50·7)	49·5 (48·4 to 50·7)	54 (52·7 to 55·3)	41·4 (39·9 to 42·7)	39·9 (35·6 to 44·7)
Iceland	1·9 (1·8 to 2·1)	2·2 (2·2 to 2·3)	2 (2 to 2·1)	2·1 (2 to 2·2)	2·2 (2·1 to 2·3)	2 (1·9 to 2·1)	2·3 (2·2 to 2·4)	2·2 (2·1 to 2·3)	2·3 (2·1 to 2·4)
Ireland	31·1 (30·2 to 32·1)	30·5 (30·1 to 30·9)	32·6 (32·1 to 33·1)	36 (35·4 to 36·6)	26·2 (25·6 to 26·8)	28 (27·3 to 28·6)	36 (35·2 to 36·9)	28·7 (27·9 to 29·6)	28·1 (26·3 to 29·9)
Israel	22·9 (22·2 to 23·5)	26·9 (26·4 to 27·4)	39 (38·4 to 39·7)	45·2 (44·3 to 46·1)	50·3 (49·2 to 51·3)	66·1 (64·6 to 67·5)	80·4 (78·8 to 82)	87·6 (85·5 to 89·8)	89·1 (84·7 to 93·7)
Italy	430 (416 to 444)	449 (442 to 456)	445 (437 to 452)	311 (305 to 317)	274 (269 to 279)	262 (257 to 267)	272 (265 to 278)	202 (198 to 207)	194 (172 to 216)

Luxembourg	2·2 (2·1 to 2·4)	2·4 (2·4 to 2·5)	2·1 (2 to 2·1)	2 (2 to 2·1)	2·4 (2·4 to 2·5)	2·7 (2·6 to 2·8)	2·8 (2·7 to 2·8)	3 (2·9 to 3·2)	3·2 (3 to 3·5)
Malta	4·7 (4·5 to 4·9)	3·9 (3·8 to 4)	2·6 (2·5 to 2·7)	2·8 (2·7 to 2·9)	2·6 (2·5 to 2·7)	2 (1·9 to 2·1)	2 (1·9 to 2·1)	2·1 (2 to 2·2)	2·1 (1·9 to 2·3)
Monaco	0·2 (0·1 to 0·2)	0·1 (0·1 to 0·1)	0·1 (0·1 to 0·1)	0·1 (0·1 to 0·2)	0·1 (0·1 to 0·2)	0·1 (0·1 to 0·2)	0·1 (0·1 to 0·2)	0·1 (0·1 to 0·2)	0·1 (0·1 to 0·2)
Netherlands	111 (108 to 115)	119 (117 to 121)	113 (111 to 115)	87·2 (85·3 to 89·1)	96·4 (94·3 to 98·2)	100 (98·1 to 102)	89·3 (87·4 to 91·2)	82·9 (80·9 to 85)	86·7 (81·5 to 91·8)
Norway	30 (29·2 to 31)	30·2 (29·7 to 30·8)	31·1 (30·5 to 31·8)	23·3 (22·7 to 23·8)	28·4 (27·8 to 28·9)	28·2 (27·7 to 28·8)	29·4 (28·8 to 30·1)	26·1 (25·5 to 26·7)	27·1 (25·6 to 28·7)
Agder	1·6 (1·5 to 1·6)	1·6 (1·5 to 1·6)	1·6 (1·6 to 1·7)	1·4 (1·4 to 1·5)	1·6 (1·6 to 1·7)	1·6 (1·5 to 1·6)	1·7 (1·6 to 1·7)	1·4 (1·4 to 1·5)	1·5 (1·4 to 1·6)
Innlandet	2·7 (2·6 to 2·8)	2·6 (2·5 to 2·6)	2·4 (2·3 to 2·5)	1·7 (1·6 to 1·8)	2 (1·9 to 2·1)	1·9 (1·9 to 2)	1·7 (1·7 to 1·8)	1·5 (1·4 to 1·5)	1·5 (1·4 to 1·6)
Møre og Romsdal	1·9 (1·8 to 1·9)	1·9 (1·9 to 2)	1·9 (1·8 to 2)	1·4 (1·4 to 1·5)	1·6 (1·5 to 1·6)	1·5 (1·4 to 1·6)	1·4 (1·3 to 1·5)	1·2 (1·2 to 1·3)	1·3 (1·2 to 1·4)
Nordland	2·1 (2 to 2·2)	2·2 (2·1 to 2·3)	2 (2 to 2·1)	1·4 (1·4 to 1·5)	1·6 (1·5 to 1·6)	1·4 (1·3 to 1·4)	1·2 (1·2 to 1·3)	1 (1 to 1)	1·1 (1 to 1·1)
Oslo	3·8 (3·7 to 4)	3·3 (3·1 to 3·4)	3·2 (3 to 3·4)	2·4 (2·3 to 2·5)	3·4 (3·3 to 3·5)	3·8 (3·6 to 4)	5 (4·8 to 5·2)	4·4 (4·2 to 4·6)	4·7 (4·3 to 5·1)
Rogaland	2·1 (2·1 to 2·2)	2·3 (2·2 to 2·5)	2·5 (2·3 to 2·6)	2·2 (2·1 to 2·3)	2·7 (2·6 to 2·8)	2·7 (2·6 to 2·8)	3·1 (3 to 3·2)	2·6 (2·5 to 2·7)	2·7 (2·5 to 2·9)
Troms og Finnmark	1·9 (1·9 to 2)	2·1 (2 to 2·1)	2 (2 to 2·1)	1·4 (1·4 to 1·5)	1·6 (1·6 to 1·7)	1·5 (1·4 to 1·5)	1·3 (1·3 to 1·3)	1·1 (1·1 to 1·2)	1·2 (1·1 to 1·2)
Trøndelag	2·9 (2·8 to 2·9)	2·9 (2·8 to 2·9)	3 (2·8 to 3·2)	2·2 (2·1 to 2·2)	2·5 (2·5 to 2·6)	2·5 (2·4 to 2·6)	2·5 (2·4 to 2·6)	2·3 (2·2 to 2·4)	2·4 (2·2 to 2·5)
Vestfold og Telemark	2·4 (2·4 to 2·5)	2·5 (2·4 to 2·5)	2·4 (2·3 to 2·5)	1·8 (1·7 to 1·9)	2·1 (2·1 to 2·2)	2·1 (2 to 2·1)	2 (1·9 to 2·1)	1·7 (1·7 to 1·8)	1·8 (1·6 to 1·9)
Vestland	4 (3·9 to 4·1)	4 (3·9 to 4·1)	4·1 (4 to 4·3)	3·1 (3 to 3·2)	3·7 (3·6 to 3·8)	3·6 (3·5 to 3·7)	3·6 (3·5 to 3·7)	3·2 (3·1 to 3·3)	3·3 (3·1 to 3·5)
Viken	4·5 (4·4 to 4·7)	5 (4·8 to 5·1)	5·9 (5·8 to 6·1)	4·3 (4·1 to 4·4)	5·5 (5·4 to 5·7)	5·8 (5·6 to 5·9)	5·9 (5·7 to 6·1)	5·6 (5·4 to 5·8)	5·8 (5·5 to 6·2)
Portugal	99·8 (96·4 to 104)	106 (103 to 108)	87·5 (85·9 to 89·2)	74·7 (73·1 to 76·5)	56·3 (54·9 to 57·8)	56·1 (54·7 to 57·5)	48·3 (47·2 to 49·5)	42·2 (41 to 43·4)	39·4 (36·7 to 42·1)
San Marino	0·1 (0·1 to 0·2)	0·1 (0·1 to 0·1)	0·1 (0·1 to 0·2)	0·1 (0·1 to 0·1)	0·1 (0·1 to 0·1)	0·1 (0·1 to 0·2)	0·1 (0·1 to 0·2)	0·1 (0·1 to 0·1)	0·1 (0·1 to 0·1)
Spain	272 (264 to 282)	316 (310 to 321)	320 (314 to 327)	265 (260 to 270)	192 (187 to 196)	192 (187 to 198)	233 (227 to 238)	173 (169 to 177)	164 (152 to 176)
Sweden	55·2 (53·4 to 57·3)	50 (49 to 51·1)	54 (52·8 to 55·2)	46·5 (45·4 to 47·4)	59·7 (58·5 to 61·1)	44·3 (43·2 to 45·3)	54·8 (53·6 to 56)	55·2 (54 to 56·4)	55·4 (52·2 to 58·7)
Stockholm	7·9 (7·7 to 8·2)	7·5 (7·2 to 7·7)	10·5 (10·2 to 10·9)	9·1 (8·8 to 9·4)	12·2 (11·9 to 12·5)	10·8 (10·3 to 11·3)	14 (13·5 to 14·5)	13·7 (13·3 to 14·1)	14 (13·1 to 15)
Sweden except Stockholm	47·2 (45·7 to 49·1)	42·6 (41·7 to 43·5)	43·5 (42·5 to 44·5)	37·4 (36·5 to 38·1)	47·6 (46·5 to 48·7)	33·5 (32·7 to 34·3)	40·8 (40 to 41·7)	41·5 (40·6 to 42·4)	41·4 (39 to 43·8)
Switzerland	40·5 (39·3 to 41·8)	46·8 (46 to 47·6)	49 (48·1 to 49·9)	36·9 (36·1 to 37·7)	42·3 (41·3 to 43·3)	37·2 (36·4 to 37·9)	39·1 (38·3 to 39·9)	41·9 (41·1 to 42·9)	43·4 (40·6 to 46·4)
UK	393 (382 to 404)	452 (444 to 460)	437 (430 to 444)	358 (348 to 368)	386 (375 to 398)	330 (320 to 340)	392 (382 to 403)	340 (330 to 351)	333 (296 to 371)
England	316 (306 to 325)	365 (358 to 373)	357 (350 to 363)	293 (284 to 301)	323 (313 to 333)	278 (269 to 286)	333 (325 to 343)	291 (283 to 300)	286 (254 to 318)
East Midlands	25·7 (24·9 to 26·5)	29·8 (29·1 to 30·4)	29·6 (28·7 to 30·5)	24·1 (23·3 to 24·9)	26·4 (25·6 to 27·3)	22·1 (21·4 to 22·9)	26·7 (26·1 to 27·5)	23·4 (22·6 to 24·2)	23 (20·5 to 25·6)
Derby	1·8 (1·7 to 1·8)	2·1 (2 to 2·1)	2 (1·9 to 2)	1·5 (1·5 to 1·6)	1·7 (1·6 to 1·7)	1·4 (1·3 to 1·4)	1·7 (1·7 to 1·8)	1·5 (1·4 to 1·5)	1·4 (1·3 to 1·6)
Derbyshire	4·7 (4·6 to 4·9)	5·3 (5·1 to 5·4)	5·2 (5 to 5·4)	4·1 (3·9 to 4·2)	4·5 (4·3 to 4·6)	3·7 (3·5 to 3·8)	4·2 (4 to 4·3)	3·5 (3·4 to 3·6)	3·5 (3·1 to 3·9)
Leicester	2·3 (2·2 to 2·4)	2·6 (2·5 to 2·7)	2·7 (2·6 to 2·9)	2·3 (2·2 to 2·4)	2·3 (2·2 to 2·4)	2 (1·9 to 2)	2·5 (2·4 to 2·6)	2·2 (2·1 to 2·3)	2·2 (1·9 to 2·5)
Leicestershire	3·1 (3 to 3·2)	3·7 (3·6 to 3·9)	3·8 (3·7 to 3·9)	3·2 (3·1 to 3·3)	3·5 (3·4 to 3·7)	3·1 (3 to 3·2)	3·4 (3·3 to 3·5)	3·2 (3·1 to 3·3)	3·1 (2·8 to 3·5)
Lincolnshire	3·4 (3·3 to 3·5)	3·8 (3·7 to 4)	3·8 (3·7 to 4)	3·2 (3·1 to 3·3)	3·5 (3·3 to 3·6)	3 (2·9 to 3·1)	3·9 (3·8 to 4)	3·3 (3·1 to 3·4)	3·2 (2·9 to 3·6)
Northamptonshire	3·3 (3·2 to 3·4)	3·8 (3·7 to 3·9)	4 (3·9 to 4·1)	3·5 (3·4 to 3·6)	4·1 (3·9 to 4·2)	3·6 (3·5 to 3·8)	4·6 (4·4 to 4·7)	4·1 (3·9 to 4·2)	4 (3·5 to 4·4)
Nottingham	2·2 (2·2 to 2·3)	2·6 (2·5 to 2·7)	2·5 (2·4 to 2·5)	1·8 (1·7 to 1·9)	2·1 (2·1 to 2·2)	1·6 (1·5 to 1·7)	1·9 (1·9 to 2)	1·8 (1·7 to 1·9)	1·7 (1·5 to 2)
Nottinghamshire	4·6 (4·5 to 4·8)	5·5 (5·4 to 5·7)	5·4 (5·3 to 5·6)	4·3 (4·2 to 4·4)	4·6 (4·5 to 4·8)	3·7 (3·6 to 3·8)	4·4 (4·3 to 4·5)	3·8 (3·7 to 3·9)	3·7 (3·3 to 4·1)
Rutland	0·2 (0·2 to 0·2)	0·2 (0·2 to 0·2)	0·2 (0·2 to 0·2)	0·2 (0·2 to 0·2)	0·2 (0·2 to 0·2)	0·2 (0·1 to 0·2)	0·2 (0·2 to 0·2)	0·2 (0·1 to 0·2)	0·2 (0·1 to 0·2)
East of England	29·8 (28·9 to 30·7)	35·7 (34·9 to 36·5)	35·3 (34·4 to 36·1)	30·5 (29·4 to 31·5)	33·5 (32·3 to 34·8)	29·7 (28·7 to 30·7)	35·5 (34·5 to 36·7)	32·1 (31 to 33·2)	31·4 (27·9 to 35·1)
Bedford	0·9 (0·9 to 0·9)	1·1 (1·1 to 1·2)	1·1 (1 to 1·1)	0·9 (0·9 to 1)	0·9 (0·9 to 0·9)	0·9 (0·8 to 0·9)	1 (1 to 1·1)	1 (1 to 1·1)	1 (0·9 to 1·1)
Cambridgeshire	2·3 (2·2 to 2·4)	2·8 (2·7 to 2·8)	2·9 (2·9 to 3)	2·7 (2·6 to 2·8)	3·2 (3·1 to 3·3)	2·9 (2·8 to 3)	3·7 (3·6 to 3·8)	3·2 (3·1 to 3·3)	3·1 (2·8 to 3·5)
Central Bedfordshire	1·2 (1·2 to 1·2)	1·6 (1·5 to 1·7)	1·7 (1·6 to 1·7)	1·6 (1·5 to 1·6)	1·5 (1·5 to 1·6)	1·4 (1·3 to 1·4)	1·6 (1·5 to 1·6)	1·6 (1·5 to 1·6)	1·6 (1·4 to 1·8)
Essex	7·2 (6·9 to 7·4)	8·9 (8·5 to 9·4)	8·9 (8·5 to 9·2)	7·5 (7·2 to 7·8)	8 (7·7 to 8·3)	6·9 (6·6 to 7·2)	7·9 (7·7 to 8·2)	7·5 (7·2 to 7·8)	7·4 (6·6 to 8·2)
Hertfordshire	6·3 (6·1 to 6·5)	7·5 (7·3 to 7·7)	6·8 (6·6 to 7)	5·8 (5·6 to 6)	6·5 (6·3 to 6·8)	6·1 (5·9 to 6·3)	7·2 (7 to 7·5)	6·4 (6·2 to 6·7)	6·3 (5·6 to 7·1)
Luton	1·2 (1·2 to 1·3)	1·7 (1·6 to 1·7)	1·7 (1·6 to 1·7)	1·4 (1·3 to 1·4)	1·6 (1·5 to 1·7)	1·4 (1·4 to 1·5)	1·8 (1·7 to 1·8)	1·6 (1·5 to 1·6)	1·6 (1·4 to 1·7)
Norfolk	4 (3·9 to 4·2)	4·6 (4·4 to 4·7)	4·6 (4·5 to 4·7)	4 (3·8 to 4·1)	4·4 (4·2 to 4·5)	3·8 (3·6 to 3·9)	4·5 (4·3 to 4·6)	3·8 (3·7 to 4)	3·7 (3·3 to 4·2)
Peterborough	0·8 (0·8 to 0·8)	1 (0·9 to 1·1)	1 (1 to 1)	1 (1 to 1·1)	1·2 (1·1 to 1·3)	1 (1 to 1·1)	1·6 (1·5 to 1·6)	1·4 (1·3 to 1·4)	1·3 (1·2 to 1·5)
Southend-on-Sea	1·3 (1·2 to 1·3)	1·2 (1·2 to 1·3)	1·1 (1·1 to 1·2)	0·9 (0·9 to 0·9)	1·1 (1 to 1·1)	0·9 (0·9 to 1)	1·1 (1·1 to 1·2)	1 (0·9 to 1)	0·9 (0·8 to 1·1)
Suffolk	3·7 (3·6 to 3·8)	4·3 (4·1 to 4·4)	4·4 (4·2 to 4·5)	3·8 (3·6 to 3·9)	4·1 (3·9 to 4·2)	3·4 (3·3 to 3·6)	4 (3·9 to 4·2)	3·4 (3·3 to 3·6)	3·3 (2·9 to 3·7)
Thurrock	0·9 (0·9 to 0·9)	1·1 (1 to 1·2)	1·2 (1·1 to 1·2)	0·9 (0·9 to 0·9)	1 (1 to 1)	0·9 (0·9 to 1)	1·2 (1·1 to 1·2)	1·2 (1·1 to 1·2)	1·1 (1 to 1·3)
Greater London	55·8 (54 to 57·7)	61·6 (60·1 to 63·2)	56·9 (55·7 to 58·1)	44·5 (43·2 to 45·8)	51·1 (49·4 to 52·7)	50·3 (48·6 to 52)	64 (62·1 to 66·2)	56 (54·2 to 57·8)	54·7 (48·3 to 61·5)
Barking and Dagenham	1·5 (1·5 to 1·6)	1·5 (1·5 to 1·6)	1·3 (1·3 to 1·3)	1 (1 to 1·1)	1·2 (1·2 to 1·3)	1·2 (1·1 to 1·2)	1·8 (1·8 to 1·9)	1·7 (1·7 to 1·8)	1·7 (1·5 to 1·9)
Barnet	2·2 (2·2 to 2·3)	2·2 (2·2 to 2·3)	2·1 (2·1 to 2·2)	1·8 (1·8 to 1·9)	2 (1·9 to 2·1)	2 (1·9 to 2·1)	2·7 (2·6 to 2·8)	2·4 (2·3 to 2·5)	2·3 (2·1 to 2·6)
Bexley	1·6 (1·6 to 1·7)	1·8 (1·8 to 1·9)	1·6 (1·6 to 1·7)	1·3 (1·2 to 1·3)	1·5 (1·5 to 1·6)	1·3 (1·2 to 1·3)	1·5 (1·4 to 1·5)	1·4 (1·3 to 1·4)	1·4 (1·2 to 1·5)
Brent	2·2 (2·1 to 2·2)	2·6 (2·4 to 2·8)	2·5 (2·4 to 2·6)	1·8 (1·7 to 1·9)	1·9 (1·8 to 2)	1·9 (1·8 to 2)	2·8 (2·6 to 2·9)	2·3 (2·2 to 2·4)	2·2 (2 to 2·5)
Bromley	2·2 (2·1 to 2·3)	2·2 (2·2 to 2·3)	1·9 (1·8 to 2)	1·6 (1·5 to 1·6)	1·8 (1·7 to 1·9)	1·7 (1·6 to 1·8)	1·9 (1·9 to 2)	1·8 (1·8 to 1·9)	1·8 (1·6 to 2)
Camden	1·1 (1 to 1·1)	1·3 (1·2 to 1·4)	1·4 (1·4 to 1·5)	1 (1 to 1·1)	1·1 (1·1 to 1·2)	1·3 (1·3 to 1·4)	1·4 (1·3 to 1·4)	1·1 (1·1 to 1·2)	1·1 (1 to 1·3)
Croydon	2·6 (2·6 to 2·7)	2·9 (2·9 to 3)	2·6 (2·6 to 2·7)	2·1 (2·1 to 2·2)	2·4 (2·3 to 2·5)	2·2 (2·1 to 2·3)	2·8 (2·7 to 2·9)	2·6 (2·5 to 2·6)	2·5 (2·2 to 2·8)

Ealing	2·4 (2·3 to 2·5)	2·7 (2·6 to 2·8)	2·6 (2·5 to 2·6)	2·1 (2 to 2·2)	2·2 (2·1 to 2·3)	2·1 (2 to 2·2)	2·8 (2·7 to 3)	2·2 (2·1 to 2·3)	2·2 (1·9 to 2·4)
Enfield	2·1 (2 to 2·1)	2·1 (2 to 2·2)	1·9 (1·9 to 2)	1·6 (1·5 to 1·6)	1·9 (1·8 to 2)	1·9 (1·8 to 1·9)	2·5 (2·4 to 2·6)	2·1 (2 to 2·2)	2·1 (1·8 to 2·3)
Greenwich	1·8 (1·8 to 1·9)	1·8 (1·8 to 1·9)	1·7 (1·7 to 1·8)	1·5 (1·4 to 1·5)	1·7 (1·6 to 1·7)	1·6 (1·5 to 1·6)	2·2 (2·1 to 2·3)	2 (1·9 to 2·1)	2 (1·7 to 2·2)
Hackney	1·9 (1·8 to 2)	2·3 (2·2 to 2·4)	2·1 (2·1 to 2·2)	1·6 (1·5 to 1·7)	1·8 (1·8 to 1·9)	1·9 (1·9 to 2)	2·3 (2·2 to 2·3)	2 (1·9 to 2·1)	1·9 (1·7 to 2·2)
Hammersmith and Fulham	1·1 (1·1 to 1·2)	1·3 (1·3 to 1·4)	1·3 (1·3 to 1·4)	0·9 (0·9 to 1)	1·1 (1 to 1·1)	1·2 (1·1 to 1·2)	1·4 (1·3 to 1·4)	1·1 (1 to 1·1)	1 (0·9 to 1·2)
Haringey	1·6 (1·6 to 1·7)	2 (2 to 2·1)	2 (1·9 to 2)	1·4 (1·4 to 1·5)	1·7 (1·6 to 1·8)	1·8 (1·7 to 1·9)	2·2 (2·1 to 2·3)	1·7 (1·6 to 1·8)	1·7 (1·5 to 1·9)
Harrow	1·5 (1·5 to 1·6)	1·6 (1·6 to 1·7)	1·4 (1·4 to 1·5)	1·2 (1·2 to 1·3)	1·4 (1·3 to 1·4)	1·2 (1·2 to 1·3)	1·7 (1·6 to 1·8)	1·7 (1·6 to 1·8)	1·7 (1·5 to 1·9)
Havering	1·9 (1·8 to 1·9)	2·1 (2 to 2·1)	1·8 (1·7 to 1·8)	1·3 (1·3 to 1·4)	1·4 (1·3 to 1·5)	1·1 (1·1 to 1·2)	1·4 (1·3 to 1·4)	1·5 (1·5 to 1·6)	1·5 (1·4 to 1·7)
Hillingdon	1·9 (1·8 to 1·9)	2 (2 to 2·1)	1·8 (1·7 to 1·8)	1·5 (1·4 to 1·5)	1·7 (1·7 to 1·8)	1·6 (1·5 to 1·6)	2 (2 to 2·1)	2 (1·9 to 2)	1·9 (1·7 to 2·2)
Hounslow	1·5 (1·5 to 1·6)	1·7 (1·7 to 1·8)	1·6 (1·6 to 1·7)	1·4 (1·3 to 1·4)	1·5 (1·5 to 1·6)	1·5 (1·5 to 1·6)	2·2 (2·1 to 2·2)	1·8 (1·8 to 1·9)	1·8 (1·6 to 2)
Islington	1·3 (1·3 to 1·4)	1·7 (1·6 to 1·8)	1·5 (1·5 to 1·6)	1·1 (1·1 to 1·1)	1·3 (1·3 to 1·4)	1·2 (1·2 to 1·3)	1·4 (1·4 to 1·5)	1·3 (1·2 to 1·3)	1·3 (1·1 to 1·4)
Kensington and Chelsea	0·9 (0·8 to 0·9)	1·2 (1·1 to 1·2)	1·5 (1·5 to 1·6)	0·9 (0·8 to 0·9)	0·9 (0·8 to 0·9)	1 (1 to 1)	1 (1 to 1·1)	0·7 (0·7 to 0·8)	0·7 (0·6 to 0·8)
Kingston upon Thames	0·9 (0·9 to 1)	0·9 (0·8 to 0·9)	0·8 (0·8 to 0·9)	0·8 (0·7 to 0·8)	0·8 (0·8 to 0·9)	0·9 (0·8 to 0·9)	1·1 (1 to 1·1)	1 (0·9 to 1)	0·9 (0·8 to 1·1)
Lambeth	2·3 (2·3 to 2·4)	2·8 (2·8 to 2·9)	2·5 (2·4 to 2·6)	1·8 (1·7 to 1·9)	2·1 (2 to 2·2)	2·1 (2 to 2·2)	2·3 (2·2 to 2·4)	1·8 (1·8 to 1·9)	1·8 (1·5 to 2)
Lewisham	2·2 (2·1 to 2·2)	2·4 (2·4 to 2·5)	2·1 (2 to 2·1)	1·5 (1·5 to 1·6)	1·9 (1·8 to 2)	1·8 (1·7 to 1·9)	2·4 (2·3 to 2·4)	2 (1·9 to 2·1)	1·9 (1·7 to 2·2)
Merton	1·4 (1·3 to 1·4)	1·4 (1·3 to 1·4)	1·2 (1·2 to 1·2)	1 (1 to 1)	1·2 (1·2 to 1·3)	1·3 (1·2 to 1·3)	1·6 (1·6 to 1·7)	1·4 (1·3 to 1·4)	1·3 (1·2 to 1·5)
Newham	2·1 (2·1 to 2·2)	2·4 (2·2 to 2·7)	2·3 (2·1 to 2·5)	1·9 (1·7 to 2)	2·2 (2·1 to 2·3)	2·3 (2·2 to 2·5)	3·3 (3·1 to 3·5)	2·7 (2·6 to 2·8)	2·7 (2·3 to 3)
Redbridge	1·8 (1·7 to 1·8)	1·8 (1·7 to 1·9)	1·7 (1·6 to 1·7)	1·3 (1·2 to 1·3)	1·5 (1·5 to 1·6)	1·5 (1·4 to 1·6)	2·1 (2·1 to 2·2)	2·1 (2 to 2·2)	2·1 (1·8 to 2·3)
Richmond upon Thames	1·1 (1·1 to 1·2)	1·1 (1 to 1·1)	1 (1 to 1)	0·9 (0·9 to 0·9)	1 (1 to 1·1)	1·2 (1·1 to 1·2)	1·4 (1·4 to 1·4)	1 (1 to 1·1)	1 (0·9 to 1·1)
Southwark	2·3 (2·2 to 2·4)	2·5 (2·4 to 2·6)	2·1 (2 to 2·1)	1·5 (1·5 to 1·6)	2 (1·9 to 2)	2 (1·9 to 2)	2·4 (2·3 to 2·5)	1·9 (1·8 to 2)	1·8 (1·6 to 2·1)
Sutton	1·2 (1·2 to 1·3)	1·3 (1·2 to 1·3)	1·1 (1·1 to 1·2)	1 (1 to 1)	1·2 (1·1 to 1·2)	1 (1 to 1·1)	1·3 (1·2 to 1·3)	1·2 (1·1 to 1·2)	1·1 (1 to 1·3)
Tower Hamlets	1·7 (1·6 to 1·7)	1·9 (1·9 to 2)	1·6 (1·6 to 1·7)	1·3 (1·3 to 1·4)	1·6 (1·5 to 1·6)	1·7 (1·6 to 1·8)	2·1 (2·1 to 2·2)	2·1 (2 to 2·2)	2·1 (1·8 to 2·3)
Waltham Forest	2 (1·9 to 2)	2 (1·9 to 2·2)	2 (1·9 to 2·2)	1·5 (1·4 to 1·6)	1·8 (1·8 to 1·9)	1·7 (1·6 to 1·8)	2·4 (2·3 to 2·5)	2·1 (2 to 2·2)	2·1 (1·8 to 2·4)
Wandsworth	2·2 (2·1 to 2·2)	2·3 (2·2 to 2·4)	2·1 (2·1 to 2·2)	1·7 (1·6 to 1·8)	1·9 (1·8 to 2)	1·9 (1·9 to 2)	2·5 (2·4 to 2·6)	2·1 (2 to 2·2)	2·1 (1·8 to 2·3)
Westminster	1·2 (1·2 to 1·3)	1·5 (1·4 to 1·6)	1·5 (1·5 to 1·6)	1 (1 to 1·1)	1·1 (1·1 to 1·2)	1·2 (1·2 to 1·2)	1·3 (1·2 to 1·3)	1·1 (1·1 to 1·1)	1·1 (0·9 to 1·2)
North East England	18·9 (18·4 to 19·5)	22·8 (21·8 to 23·8)	22 (21·4 to 22·5)	17·2 (16·6 to 17·7)	17 (16·4 to 17·5)	12·9 (12·5 to 13·3)	14·9 (14·5 to 15·3)	12·3 (11·9 to 12·7)	12 (10·7 to 13·4)
County Durham	3·5 (3·4 to 3·6)	4·2 (4 to 4·4)	4·1 (3·9 to 4·3)	3·2 (3·1 to 3·3)	3·2 (3·1 to 3·3)	2·4 (2·3 to 2·5)	2·8 (2·7 to 2·9)	2·3 (2·2 to 2·4)	2·3 (2 to 2·5)
Darlington	0·7 (0·7 to 0·7)	0·8 (0·8 to 0·8)	0·8 (0·8 to 0·8)	0·6 (0·6 to 0·6)	0·7 (0·7 to 0·7)	0·5 (0·5 to 0·6)	0·7 (0·7 to 0·7)	0·5 (0·5 to 0·5)	0·5 (0·5 to 0·6)
Gateshead	1·6 (1·6 to 1·7)	2 (1·9 to 2·1)	1·9 (1·8 to 1·9)	1·3 (1·3 to 1·4)	1·3 (1·2 to 1·3)	1 (1 to 1)	1·2 (1·1 to 1·2)	0·9 (0·9 to 1)	0·9 (0·8 to 1)
Hartlepool	0·7 (0·7 to 0·7)	0·9 (0·9 to 1)	0·9 (0·9 to 0·9)	0·7 (0·6 to 0·7)	0·7 (0·6 to 0·7)	0·5 (0·5 to 0·5)	0·6 (0·5 to 0·6)	0·5 (0·4 to 0·5)	0·4 (0·4 to 0·5)
Middlesbrough	1·2 (1·2 to 1·2)	1·5 (1·4 to 1·6)	1·4 (1·4 to 1·5)	1·2 (1·1 to 1·2)	1·1 (1·1 to 1·1)	0·8 (0·8 to 0·9)	1 (1 to 1)	0·8 (0·8 to 0·9)	0·8 (0·7 to 0·9)
Newcastle upon Tyne	2·1 (2 to 2·2)	2·4 (2·3 to 2·5)	2·2 (2·2 to 2·3)	1·7 (1·6 to 1·8)	1·8 (1·7 to 1·9)	1·4 (1·3 to 1·4)	1·6 (1·6 to 1·7)	1·5 (1·4 to 1·6)	1·5 (1·3 to 1·7)
North Tyneside	1·5 (1·5 to 1·5)	1·7 (1·6 to 1·8)	1·5 (1·5 to 1·6)	1·2 (1·1 to 1·2)	1·2 (1·2 to 1·2)	1 (0·9 to 1)	1·2 (1·1 to 1·2)	1 (1 to 1)	1 (0·9 to 1·1)
Northumberland	2 (1·9 to 2·1)	2·2 (2·1 to 2·3)	2·2 (2·1 to 2·2)	1·8 (1·8 to 1·9)	1·8 (1·7 to 1·8)	1·4 (1·4 to 1·4)	1·5 (1·5 to 1·6)	1·2 (1·2 to 1·3)	1·2 (1·1 to 1·3)
Redcar and Cleveland	1 (1 to 1)	1·3 (1·3 to 1·4)	1·4 (1·3 to 1·4)	1 (1 to 1·1)	1 (1 to 1)	0·7 (0·7 to 0·7)	0·8 (0·8 to 0·8)	0·6 (0·6 to 0·6)	0·6 (0·5 to 0·7)
South Tyneside	1·3 (1·2 to 1·3)	1·5 (1·5 to 1·6)	1·4 (1·3 to 1·4)	1 (0·9 to 1)	1 (1 to 1·1)	0·7 (0·7 to 0·8)	0·8 (0·8 to 0·8)	0·7 (0·7 to 0·7)	0·7 (0·6 to 0·8)
Stockton-on-Tees	1·2 (1·1 to 1·2)	1·5 (1·4 to 1·6)	1·5 (1·4 to 1·5)	1·3 (1·2 to 1·3)	1·3 (1·2 to 1·3)	1 (0·9 to 1)	1·2 (1·2 to 1·3)	1 (0·9 to 1)	0·9 (0·8 to 1·1)
Sunderland	2·1 (2·1 to 2·2)	2·7 (2·5 to 2·9)	2·7 (2·6 to 2·8)	2·2 (2·1 to 2·2)	2 (2 to 2·1)	1·4 (1·4 to 1·5)	1·5 (1·5 to 1·6)	1·3 (1·2 to 1·3)	1·2 (1·1 to 1·4)
North West England	49·2 (47·7 to 50·7)	58·5 (57·1 to 59·7)	56·9 (55·8 to 58·1)	44·7 (43·3 to 46)	47·6 (45·8 to 49·3)	37·2 (36 to 38·4)	43·3 (42 to 44·6)	38 (36·9 to 39·3)	37·2 (33·1 to 41·3)
Blackburn with Darwen	1·2 (1·2 to 1·2)	1·3 (1·3 to 1·4)	1·3 (1·3 to 1·4)	1·2 (1·1 to 1·2)	1·2 (1·1 to 1·2)	1 (1 to 1·1)	1·2 (1·1 to 1·2)	0·9 (0·9 to 1)	0·9 (0·8 to 1)
Blackpool	1·1 (1 to 1·1)	1 (1 to 1·1)	0·9 (0·9 to 0·9)	0·7 (0·7 to 0·8)	0·9 (0·9 to 1)	0·7 (0·7 to 0·8)	0·9 (0·8 to 0·9)	0·7 (0·7 to 0·8)	0·7 (0·6 to 0·8)
Bolton	1·9 (1·9 to 2)	2·2 (2·1 to 2·3)	2·3 (2·2 to 2·3)	1·8 (1·8 to 1·9)	1·9 (1·8 to 2)	1·6 (1·5 to 1·6)	1·9 (1·9 to 2)	1·7 (1·7 to 1·8)	1·7 (1·5 to 1·9)
Bury	1·2 (1·1 to 1·2)	1·4 (1·4 to 1·5)	1·5 (1·4 to 1·5)	1·1 (1·1 to 1·2)	1·3 (1·2 to 1·3)	1 (1 to 1·1)	1·2 (1·2 to 1·3)	1·1 (1 to 1·1)	1 (0·9 to 1·2)
Cheshire East	1·9 (1·9 to 2)	2·3 (2·2 to 2·4)	2·2 (2·1 to 2·3)	1·8 (1·7 to 1·9)	2 (2 to 2·1)	1·7 (1·7 to 1·8)	2 (1·9 to 2)	1·7 (1·7 to 1·8)	1·7 (1·5 to 1·9)
Cheshire West and Chester	1·9 (1·9 to 2)	2·4 (2·4 to 2·5)	2·4 (2·3 to 2·4)	1·9 (1·8 to 2)	2 (1·9 to 2·1)	1·6 (1·6 to 1·7)	1·8 (1·8 to 1·9)	1·6 (1·5 to 1·6)	1·6 (1·4 to 1·7)
Cumbria	3·1 (3 to 3·2)	3·5 (3·4 to 3·7)	3·4 (3·3 to 3·5)	2·8 (2·7 to 2·9)	3 (2·9 to 3·1)	2·3 (2·2 to 2·4)	2·5 (2·4 to 2·6)	2·1 (2 to 2·2)	2 (1·8 to 2·3)
Halton	0·7 (0·7 to 0·7)	0·8 (0·8 to 0·8)	0·9 (0·9 to 0·9)	0·9 (0·9 to 1)	1 (0·9 to 1)	0·7 (0·7 to 0·7)	0·8 (0·8 to 0·8)	0·6 (0·6 to 0·7)	0·6 (0·6 to 0·7)
Knowsley	1·3 (1·3 to 1·4)	2 (1·9 to 2)	1·8 (1·8 to 1·8)	1·4 (1·4 to 1·5)	1·3 (1·2 to 1·3)	0·9 (0·9 to 0·9)	0·9 (0·9 to 1)	0·9 (0·9 to 1)	0·9 (0·8 to 1)
Lancashire	7·2 (7 to 7·4)	8·2 (7·9 to 8·5)	8·1 (7·7 to 8·5)	6·8 (6·5 to 7)	7·4 (7·2 to 7·7)	5·9 (5·7 to 6·2)	6·7 (6·5 to 6·9)	5·8 (5·6 to 6)	5·7 (5·1 to 6·3)
Liverpool	4·3 (4·1 to 4·4)	5·1 (4·8 to 5·4)	4·8 (4·6 to 5)	3·4 (3·2 to 3·5)	3·4 (3·3 to 3·6)	2·4 (2·3 to 2·5)	2·7 (2·6 to 2·8)	2·6 (2·5 to 2·7)	2·5 (2·3 to 2·8)
Manchester	3·9 (3·8 to 4)	4·7 (4·5 to 4·9)	4·5 (4·3 to 4·6)	3·2 (3·1 to 3·3)	3·5 (3·4 to 3·6)	2·7 (2·6 to 2·8)	3·7 (3·6 to 3·8)	3·4 (3·3 to 3·6)	3·4 (3 to 3·8)
Oldham	1·7 (1·6 to 1·7)	2 (1·9 to 2·1)	2·1 (2 to 2·1)	1·6 (1·5 to 1·6)	1·7 (1·6 to 1·8)	1·5 (1·4 to 1·5)	1·7 (1·6 to 1·7)	1·5 (1·4 to 1·5)	1·5 (1·3 to 1·6)
Rochdale	1·6 (1·5 to 1·6)	1·8 (1·8 to 1·9)	1·8 (1·8 to 1·9)	1·6 (1·5 to 1·6)	1·6 (1·6 to 1·7)	1·3 (1·2 to 1·3)	1·5 (1·5 to 1·6)	1·4 (1·3 to 1·4)	1·3 (1·2 to 1·5)
Salford	2 (1·9 to 2·1)	2·4 (2·3 to 2·5)	2·3 (2·3 to 2·4)	1·6 (1·5 to 1·6)	1·7 (1·7 to 1·8)	1·3 (1·2 to 1·3)	1·7 (1·6 to 1·7)	1·7 (1·6 to 1·7)	1·6 (1·5 to 1·8)
Sefton	2·1 (2 to 2·1)	2·5 (2·4 to 2·6)	2·2 (2·2 to 2·3)	1·7 (1·7 to 1·8)	1·8 (1·7 to 1·9)	1·3 (1·3 to 1·4)	1·4 (1·3 to 1·4)	1·2 (1·2 to 1·2)	1·2 (1 to 1·3)

St Helens	1·3 (1·3 to 1·4)	1·6 (1·6 to 1·7)	1·6 (1·6 to 1·7)	1·2 (1·2 to 1·3)	1·2 (1·1 to 1·2)	0·9 (0·9 to 1)	1 (1 to 1·1)	0·9 (0·9 to 0·9)	0·9 (0·8 to 0·9)
Stockport	2 (1·9 to 2)	2·5 (2·4 to 2·6)	2·3 (2·3 to 2·4)	1·8 (1·7 to 1·9)	1·9 (1·8 to 1·9)	1·5 (1·4 to 1·5)	1·7 (1·6 to 1·7)	1·5 (1·5 to 1·6)	1·5 (1·3 to 1·6)
Tameside	1·6 (1·5 to 1·6)	1·8 (1·8 to 1·9)	1·9 (1·8 to 1·9)	1·4 (1·4 to 1·5)	1·6 (1·6 to 1·7)	1·2 (1·2 to 1·3)	1·5 (1·5 to 1·6)	1·3 (1·3 to 1·4)	1·3 (1·1 to 1·4)
Trafford	1·6 (1·5 to 1·6)	1·9 (1·8 to 2)	1·7 (1·7 to 1·8)	1·4 (1·3 to 1·4)	1·4 (1·4 to 1·5)	1·2 (1·1 to 1·2)	1·4 (1·4 to 1·5)	1·2 (1·2 to 1·2)	1·2 (1 to 1·3)
Warrington	1·1 (1 to 1·1)	1·4 (1·3 to 1·4)	1·3 (1·3 to 1·4)	1·1 (1 to 1·1)	1·3 (1·2 to 1·3)	1·1 (1 to 1·1)	1·2 (1·1 to 1·2)	1 (0·9 to 1)	1 (0·8 to 1·1)
Wigan	2 (2 to 2·1)	2·5 (2·5 to 2·6)	2·8 (2·7 to 2·8)	2·1 (2 to 2·1)	2·1 (2 to 2·2)	1·7 (1·6 to 1·7)	1·9 (1·8 to 2)	1·6 (1·5 to 1·7)	1·6 (1·4 to 1·7)
Wirral	2·6 (2·5 to 2·6)	3 (2·9 to 3·1)	2·8 (2·7 to 2·9)	2·2 (2·1 to 2·2)	2·3 (2·2 to 2·3)	1·7 (1·6 to 1·7)	1·9 (1·8 to 1·9)	1·5 (1·4 to 1·5)	1·4 (1·3 to 1·6)
South East England	40·2 (38·9 to 41·6)	45·4 (44·1 to 46·7)	45·8 (44·5 to 46·9)	42·7 (41·3 to 44)	48·7 (47·2 to 50·3)	43·6 (42·2 to 45·1)	51·6 (50·3 to 52·9)	44·9 (43·5 to 46·3)	44·2 (39·4 to 49)
Bracknell Forest	0·4 (0·3 to 0·4)	0·5 (0·5 to 0·5)	0·5 (0·5 to 0·5)	0·6 (0·6 to 0·6)	0·7 (0·7 to 0·8)	0·7 (0·7 to 0·7)	0·8 (0·7 to 0·8)	0·7 (0·6 to 0·7)	0·6 (0·6 to 0·7)
Brighton and Hove	1·5 (1·5 to 1·6)	1·3 (1·2 to 1·3)	1·3 (1·2 to 1·3)	1·2 (1·1 to 1·2)	1·4 (1·4 to 1·5)	1·3 (1·3 to 1·4)	1·6 (1·5 to 1·6)	1·2 (1·1 to 1·2)	1·2 (1 to 1·3)
Buckinghamshire	2·4 (2·4 to 2·5)	2·9 (2·8 to 3)	2·8 (2·7 to 2·9)	2·6 (2·5 to 2·7)	2·9 (2·8 to 3)	2·7 (2·6 to 2·8)	3 (2·9 to 3·1)	2·7 (2·6 to 2·8)	2·7 (2·4 to 3)
East Sussex	2·4 (2·3 to 2·4)	2·2 (2·1 to 2·3)	2·3 (2·2 to 2·3)	2·1 (2 to 2·2)	2·6 (2·5 to 2·7)	2·3 (2·2 to 2·4)	2·6 (2·6 to 2·7)	2·2 (2·1 to 2·3)	2·2 (1·9 to 2·4)
Hampshire	5·5 (5·3 to 5·6)	6·9 (6·7 to 7·2)	7·3 (7·1 to 7·5)	6·7 (6·5 to 6·9)	7·5 (7·2 to 7·7)	6·4 (6·2 to 6·7)	7·4 (7·2 to 7·6)	6·3 (6·1 to 6·5)	6·2 (5·6 to 6·9)
Isle of Wight	0·7 (0·7 to 0·7)	0·7 (0·6 to 0·7)	0·6 (0·6 to 0·7)	0·6 (0·6 to 0·6)	0·7 (0·6 to 0·7)	0·6 (0·5 to 0·6)	0·6 (0·6 to 0·7)	0·5 (0·5 to 0·5)	0·5 (0·4 to 0·6)
Kent	7·2 (6·9 to 7·4)	8·2 (7·9 to 8·4)	8·2 (8 to 8·4)	7·2 (7 to 7·4)	8·2 (8 to 8·5)	7·2 (6·9 to 7·5)	8·6 (8·3 to 8·8)	7·8 (7·6 to 8·1)	7·8 (7 to 8·6)
Medway	1·4 (1·3 to 1·4)	1·9 (1·8 to 1·9)	2·1 (2·1 to 2·2)	1·8 (1·8 to 1·9)	1·9 (1·8 to 1·9)	1·5 (1·4 to 1·6)	1·7 (1·7 to 1·8)	1·6 (1·5 to 1·7)	1·6 (1·4 to 1·7)
Milton Keynes	0·4 (0·4 to 0·4)	0·5 (0·5 to 0·5)	0·6 (0·6 to 0·7)	1·1 (1 to 1·1)	1·4 (1·4 to 1·5)	1·4 (1·3 to 1·5)	1·9 (1·9 to 2)	1·6 (1·5 to 1·7)	1·6 (1·4 to 1·7)
Oxfordshire	2·7 (2·6 to 2·8)	3·3 (3·2 to 3·4)	3·4 (3·3 to 3·5)	3·3 (3·2 to 3·4)	3·8 (3·7 to 3·9)	3·5 (3·3 to 3·6)	4 (3·9 to 4·2)	3·4 (3·3 to 3·5)	3·4 (3 to 3·8)
Portsmouth	1·2 (1·2 to 1·3)	1·4 (1·3 to 1·5)	1·4 (1·3 to 1·5)	1·2 (1·1 to 1·2)	1·3 (1·3 to 1·4)	1·1 (1 to 1·1)	1·2 (1·2 to 1·3)	1·1 (1·1 to 1·2)	1·1 (1 to 1·2)
Reading	0·8 (0·8 to 0·8)	1 (0·9 to 1)	1·1 (1 to 1·1)	1 (0·9 to 1)	1 (1 to 1)	1 (0·9 to 1)	1·3 (1·2 to 1·3)	1 (1 to 1·1)	1 (0·9 to 1·1)
Slough	0·8 (0·8 to 0·8)	0·9 (0·9 to 1)	0·9 (0·9 to 0·9)	0·8 (0·8 to 0·9)	0·9 (0·9 to 1)	0·9 (0·8 to 0·9)	1·3 (1·3 to 1·4)	1·1 (1·1 to 1·2)	1·1 (1 to 1·2)
Southampton	1·3 (1·3 to 1·4)	1·5 (1·5 to 1·6)	1·6 (1·6 to 1·7)	1·4 (1·3 to 1·4)	1·5 (1·4 to 1·5)	1·2 (1·1 to 1·2)	1·5 (1·5 to 1·6)	1·4 (1·3 to 1·4)	1·4 (1·2 to 1·5)
Surrey	6·2 (5·9 to 6·4)	6·2 (6 to 6·4)	5·7 (5·6 to 5·8)	5·3 (5·1 to 5·4)	6·1 (5·9 to 6·3)	5·8 (5·6 to 6)	6·8 (6·6 to 7·1)	5·9 (5·7 to 6·1)	5·8 (5·2 to 6·4)
West Berkshire	0·6 (0·6 to 0·6)	0·8 (0·7 to 0·8)	0·8 (0·7 to 0·8)	0·8 (0·8 to 0·8)	0·9 (0·9 to 1)	0·8 (0·8 to 0·9)	1 (0·9 to 1)	0·8 (0·7 to 0·8)	0·7 (0·7 to 0·8)
West Sussex	3·6 (3·5 to 3·8)	3·7 (3·6 to 3·9)	3·6 (3·5 to 3·7)	3·6 (3·5 to 3·7)	4·1 (3·9 to 4·2)	3·8 (3·6 to 3·9)	4·5 (4·3 to 4·6)	3·9 (3·8 to 4·1)	3·9 (3·4 to 4·3)
Windsor and Maidenhead	0·7 (0·7 to 0·8)	0·8 (0·8 to 0·9)	0·8 (0·8 to 0·8)	0·8 (0·7 to 0·8)	0·8 (0·8 to 0·8)	0·8 (0·7 to 0·8)	0·9 (0·9 to 0·9)	0·7 (0·7 to 0·8)	0·7 (0·6 to 0·8)
Wokingham	0·5 (0·5 to 0·5)	0·7 (0·6 to 0·7)	0·7 (0·7 to 0·7)	0·7 (0·6 to 0·7)	0·9 (0·9 to 1)	0·8 (0·8 to 0·9)	0·9 (0·9 to 0·9)	0·8 (0·8 to 0·9)	0·8 (0·7 to 0·9)
South West England	24·9 (24·1 to 25·7)	27·4 (26·5 to 28·3)	27·8 (27 to 28·6)	24·6 (23·8 to 25·5)	28·4 (27·5 to 29·3)	24·4 (23·6 to 25·3)	29·2 (28·4 to 30·2)	25·2 (24·4 to 26)	24·8 (22·2 to 27·7)
Bath and North East Somerset	0·9 (0·9 to 0·9)	0·9 (0·9 to 0·9)	0·8 (0·8 to 0·9)	0·8 (0·8 to 0·8)	0·9 (0·9 to 0·9)	0·8 (0·8 to 0·8)	0·9 (0·8 to 0·9)	0·8 (0·8 to 0·8)	0·8 (0·7 to 0·9)
Bournemouth	0·9 (0·9 to 0·9)	0·8 (0·7 to 0·8)	0·7 (0·7 to 0·8)	0·6 (0·6 to 0·6)	0·8 (0·8 to 0·9)	0·8 (0·7 to 0·8)	1·1 (1·1 to 1·1)	1 (0·9 to 1·1)	1 (0·9 to 1·1)
Bristol, City of	2·6 (2·5 to 2·7)	2·9 (2·8 to 3)	2·9 (2·8 to 3)	2·4 (2·3 to 2·5)	2·7 (2·6 to 2·8)	2·3 (2·2 to 2·4)	2·9 (2·8 to 3·1)	2·6 (2·5 to 2·7)	2·6 (2·3 to 2·9)
Cornwall	2·4 (2·4 to 2·5)	2·5 (2·5 to 2·6)	2·6 (2·6 to 2·7)	2·4 (2·3 to 2·4)	2·7 (2·6 to 2·8)	2·2 (2·1 to 2·3)	2·7 (2·6 to 2·8)	2·3 (2·2 to 2·4)	2·3 (2 to 2·5)
Devon	3·2 (3·1 to 3·3)	3·2 (3 to 3·5)	3·2 (3·1 to 3·4)	3 (2·9 to 3·1)	3·6 (3·5 to 3·8)	3·1 (3 to 3·2)	3·6 (3·5 to 3·7)	3·1 (3 to 3·2)	3·1 (2·8 to 3·4)
Dorset	1·8 (1·7 to 1·8)	1·8 (1·7 to 1·9)	1·8 (1·7 to 1·9)	1·7 (1·6 to 1·8)	1·9 (1·8 to 2)	1·6 (1·6 to 1·7)	1·9 (1·8 to 2)	1·4 (1·4 to 1·5)	1·4 (1·2 to 1·5)
Gloucestershire	2·9 (2·8 to 3)	3·3 (3·1 to 3·4)	3·3 (3·2 to 3·4)	2·9 (2·8 to 3)	3·4 (3·3 to 3·5)	2·9 (2·8 to 3)	3·4 (3·2 to 3·5)	2·9 (2·8 to 3)	2·9 (2·6 to 3·2)
North Somerset	0·9 (0·8 to 0·9)	0·9 (0·9 to 1)	1 (1 to 1)	0·9 (0·9 to 0·9)	1 (0·9 to 1)	0·9 (0·9 to 1)	1·1 (1·1 to 1·2)	1 (0·9 to 1)	0·9 (0·8 to 1·1)
Plymouth	1·6 (1·5 to 1·6)	1·9 (1·8 to 1·9)	2 (1·9 to 2)	1·7 (1·6 to 1·8)	1·7 (1·6 to 1·8)	1·3 (1·2 to 1·3)	1·5 (1·5 to 1·6)	1·2 (1·2 to 1·3)	1·2 (1·1 to 1·4)
Poole	0·6 (0·6 to 0·7)	0·7 (0·7 to 0·7)	0·7 (0·7 to 0·7)	0·6 (0·6 to 0·7)	0·8 (0·8 to 0·9)	0·7 (0·6 to 0·7)	0·8 (0·8 to 0·9)	0·7 (0·7 to 0·8)	0·7 (0·6 to 0·8)
Somerset	2·4 (2·3 to 2·5)	2·6 (2·5 to 2·7)	2·7 (2·6 to 2·8)	2·5 (2·4 to 2·5)	2·8 (2·7 to 2·9)	2·4 (2·3 to 2·5)	2·9 (2·8 to 3)	2·5 (2·4 to 2·6)	2·5 (2·2 to 2·7)
South Gloucestershire	1 (1 to 1)	1·4 (1·3 to 1·4)	1·4 (1·4 to 1·4)	1·2 (1·2 to 1·2)	1·5 (1·4 to 1·5)	1·4 (1·3 to 1·4)	1·5 (1·4 to 1·6)	1·5 (1·4 to 1·5)	1·5 (1·3 to 1·6)
Swindon	0·9 (0·9 to 0·9)	1·2 (1·1 to 1·2)	1·2 (1·1 to 1·2)	1·1 (1 to 1·1)	1·3 (1·2 to 1·3)	1·1 (1·1 to 1·2)	1·4 (1·4 to 1·5)	1·3 (1·2 to 1·3)	1·2 (1·1 to 1·4)
Torbay	0·6 (0·6 to 0·7)	0·6 (0·6 to 0·6)	0·6 (0·6 to 0·6)	0·5 (0·5 to 0·5)	0·7 (0·6 to 0·7)	0·6 (0·6 to 0·6)	0·7 (0·7 to 0·7)	0·6 (0·6 to 0·6)	0·6 (0·5 to 0·6)
Wiltshire	2·1 (2 to 2·1)	2·6 (2·5 to 2·7)	2·8 (2·7 to 2·9)	2·3 (2·3 to 2·4)	2·6 (2·5 to 2·7)	2·3 (2·2 to 2·4)	2·7 (2·6 to 2·8)	2·3 (2·2 to 2·4)	2·3 (2 to 2·5)
West Midlands	36·4 (35·3 to 37·5)	44·5 (43·5 to 45·6)	43·9 (42·9 to 44·9)	33·4 (32·4 to 34·4)	36·4 (35·3 to 37·5)	30·1 (29·1 to 31)	35·3 (34·4 to 36·4)	31·6 (30·5 to 32·6)	31 (27·7 to 34·5)
Birmingham	8·8 (8·5 to 9·1)	10·2 (9·9 to 10·5)	9·9 (9·5 to 10·3)	7·5 (7·3 to 7·8)	8·2 (7·9 to 8·5)	7 (6·8 to 7·3)	8·3 (8·1 to 8·6)	7·4 (7·2 to 7·7)	7·3 (6·5 to 8·1)
Coventry	2·4 (2·4 to 2·5)	3 (3 to 3·1)	3 (2·9 to 3·1)	2·2 (2·1 to 2·3)	2·3 (2·2 to 2·3)	1·8 (1·7 to 1·8)	2·2 (2·1 to 2·3)	2 (1·9 to 2·1)	1·9 (1·7 to 2·2)
Dudley	2 (1·9 to 2·1)	2·6 (2·5 to 2·7)	2·6 (2·5 to 2·7)	1·8 (1·8 to 1·9)	2 (1·9 to 2·1)	1·7 (1·6 to 1·7)	1·8 (1·8 to 1·9)	1·7 (1·6 to 1·8)	1·6 (1·5 to 1·8)
Herefordshire, County of	1 (0·9 to 1)	1·1 (1 to 1·1)	1 (1 to 1·1)	0·9 (0·8 to 0·9)	1 (1 to 1)	0·8 (0·8 to 0·8)	0·9 (0·9 to 1)	0·8 (0·7 to 0·8)	0·8 (0·7 to 0·9)
Sandwell	2·7 (2·6 to 2·8)	3·2 (3 to 3·3)	3 (2·9 to 3·1)	2·1 (2 to 2·2)	2·2 (2·1 to 2·3)	1·8 (1·8 to 1·9)	2·4 (2·3 to 2·5)	2·1 (2 to 2·2)	2·1 (1·9 to 2·3)
Shropshire	1·5 (1·5 to 1·5)	1·8 (1·7 to 1·9)	1·8 (1·7 to 1·9)	1·4 (1·3 to 1·4)	1·6 (1·5 to 1·6)	1·3 (1·3 to 1·4)	1·5 (1·4 to 1·5)	1·3 (1·2 to 1·3)	1·3 (1·1 to 1·4)
Solihull	1·2 (1·2 to 1·2)	1·6 (1·5 to 1·7)	1·6 (1·5 to 1·7)	1·1 (1·1 to 1·1)	1·2 (1·2 to 1·3)	1 (0·9 to 1)	1·1 (1·1 to 1·1)	1 (1 to 1·1)	1 (0·9 to 1·1)
Staffordshire	4·4 (4·3 to 4·6)	5·8 (5·5 to 6·1)	6 (5·7 to 6·2)	4·8 (4·6 to 4·9)	5·1 (4·9 to 5·2)	3·9 (3·8 to 4·1)	4·4 (4·3 to 4·6)	3·9 (3·8 to 4·1)	3·9 (3·5 to 4·3)
Stoke-on-Trent	2·1 (2 to 2·2)	2·4 (2·3 to 2·5)	2·3 (2·3 to 2·4)	1·7 (1·6 to 1·7)	1·8 (1·8 to 1·9)	1·4 (1·3 to 1·4)	1·8 (1·8 to 1·9)	1·6 (1·5 to 1·6)	1·5 (1·3 to 1·7)
Telford and Wrekin	0·6 (0·6 to 0·7)	0·8 (0·8 to 0·8)	0·9 (0·9 to 1)	0·9 (0·9 to 0·9)	1·1 (1 to 1·1)	0·9 (0·9 to 1)	1·1 (1·1 to 1·2)	1 (0·9 to 1)	1 (0·9 to 1·1)
Walsall	2 (1·9 to 2)	2·6 (2·6 to 2·7)	2·5 (2·5 to 2·6)	1·7 (1·7 to 1·8)	1·9 (1·8 to 1·9)	1·6 (1·5 to 1·7)	1·8 (1·8 to 1·9)	1·7 (1·7 to 1·8)	1·7 (1·5 to 1·9)

Warwickshire	2·9 (2·8 to 3)	3·6 (3·5 to 3·8)	3·6 (3·5 to 3·7)	2·8 (2·7 to 2·9)	3 (2·9 to 3·1)	2·6 (2·5 to 2·7)	3 (3 to 3·1)	2·8 (2·7 to 2·9)	2·8 (2·5 to 3·1)
Wolverhampton	2 (2 to 2·1)	2·5 (2·4 to 2·6)	2·3 (2·2 to 2·4)	1·7 (1·6 to 1·8)	1·9 (1·8 to 1·9)	1·4 (1·4 to 1·5)	1·7 (1·6 to 1·8)	1·6 (1·5 to 1·6)	1·5 (1·4 to 1·7)
Worcestershire	2·7 (2·6 to 2·8)	3·2 (3·1 to 3·4)	3·3 (3·2 to 3·5)	2·9 (2·8 to 3)	3·2 (3·1 to 3·3)	2·8 (2·7 to 2·9)	3·1 (3 to 3·2)	2·7 (2·6 to 2·8)	2·6 (2·4 to 2·9)
Yorkshire and the Humber	34·6 (33·6 to 35·7)	39·7 (38·8 to 40·6)	38·4 (37·3 to 39·4)	30·9 (29·9 to 31·9)	33·6 (32·5 to 34·7)	27·4 (26·4 to 28·4)	32·6 (31·7 to 33·5)	27·9 (26·9 to 28·8)	27·2 (24·2 to 30·3)
Barnsley	1·6 (1·6 to 1·7)	1·9 (1·9 to 2)	1·9 (1·9 to 2)	1·4 (1·4 to 1·5)	1·5 (1·5 to 1·6)	1·1 (1·1 to 1·2)	1·5 (1·4 to 1·5)	1·2 (1·2 to 1·3)	1·2 (1·1 to 1·3)
Bradford	3·8 (3·7 to 3·9)	4·4 (4·2 to 4·5)	4·3 (4·1 to 4·4)	3·6 (3·5 to 3·8)	3·8 (3·6 to 3·9)	3·5 (3·3 to 3·6)	4·2 (4·1 to 4·4)	3·4 (3·3 to 3·6)	3·3 (3 to 3·7)
Calderdale	1·5 (1·5 to 1·6)	1·6 (1·5 to 1·6)	1·5 (1·5 to 1·6)	1·2 (1·2 to 1·3)	1·4 (1·3 to 1·4)	1·1 (1·1 to 1·2)	1·3 (1·3 to 1·4)	1·1 (1 to 1·1)	1 (0·9 to 1·2)
Doncaster	2·1 (2 to 2·1)	2·5 (2·5 to 2·6)	2·4 (2·4 to 2·5)	2 (1·9 to 2)	2·1 (2 to 2·2)	1·6 (1·5 to 1·6)	1·9 (1·9 to 2)	1·6 (1·6 to 1·7)	1·6 (1·4 to 1·8)
East Riding of Yorkshire	1·6 (1·6 to 1·7)	1·8 (1·7 to 1·9)	1·7 (1·6 to 1·7)	1·4 (1·4 to 1·5)	1·6 (1·6 to 1·7)	1·4 (1·3 to 1·4)	1·5 (1·5 to 1·6)	1·3 (1·2 to 1·3)	1·3 (1·1 to 1·4)
Kingston upon Hull, City of	2 (2 to 2·1)	2·4 (2·4 to 2·5)	2·4 (2·4 to 2·5)	1·9 (1·8 to 2)	2·1 (2 to 2·1)	1·4 (1·4 to 1·5)	1·7 (1·7 to 1·8)	1·5 (1·4 to 1·6)	1·4 (1·3 to 1·6)
Kirklees	2·8 (2·7 to 2·9)	3·1 (3 to 3·2)	3·1 (3 to 3·2)	2·6 (2·5 to 2·7)	2·7 (2·6 to 2·8)	2·4 (2·4 to 2·5)	2·9 (2·8 to 3)	2·4 (2·3 to 2·5)	2·3 (2·1 to 2·6)
Leeds	5·2 (5·1 to 5·4)	6 (5·8 to 6·1)	5·6 (5·4 to 5·8)	4·4 (4·2 to 4·5)	4·8 (4·6 to 4·9)	3·8 (3·7 to 4)	4·7 (4·5 to 4·8)	4·4 (4·2 to 4·6)	4·3 (3·8 to 4·8)
North East Lincolnshire	1·2 (1·1 to 1·2)	1·5 (1·4 to 1·5)	1·5 (1·4 to 1·5)	1·1 (1·1 to 1·2)	1·2 (1·1 to 1·2)	0·8 (0·8 to 0·9)	1 (1 to 1)	0·8 (0·8 to 0·8)	0·7 (0·7 to 0·8)
North Lincolnshire	1 (1 to 1·1)	1·2 (1·2 to 1·3)	1·2 (1·2 to 1·2)	1 (0·9 to 1)	1 (1 to 1)	0·8 (0·8 to 0·8)	1 (0·9 to 1)	0·8 (0·7 to 0·8)	0·8 (0·7 to 0·8)
North Yorkshire	3·1 (3 to 3·2)	3·3 (3·2 to 3·4)	3·1 (3 to 3·2)	2·7 (2·6 to 2·8)	3·1 (3 to 3·3)	2·7 (2·6 to 2·8)	3 (2·9 to 3·1)	2·5 (2·4 to 2·6)	2·4 (2·2 to 2·7)
Rotherham	1·7 (1·7 to 1·8)	2·2 (2·1 to 2·2)	2·2 (2·1 to 2·2)	1·7 (1·6 to 1·8)	1·8 (1·8 to 1·9)	1·4 (1·3 to 1·4)	1·6 (1·5 to 1·6)	1·4 (1·3 to 1·4)	1·3 (1·2 to 1·5)
Sheffield	3·7 (3·6 to 3·8)	4 (3·9 to 4·2)	3·8 (3·6 to 4)	2·9 (2·8 to 3)	3·3 (3·2 to 3·4)	2·8 (2·7 to 2·9)	3·2 (3·1 to 3·3)	2·8 (2·7 to 3)	2·8 (2·5 to 3·2)
Wakefield	2·2 (2·1 to 2·3)	2·6 (2·5 to 2·7)	2·6 (2·5 to 2·7)	2·1 (2 to 2·1)	2·2 (2·1 to 2·3)	1·7 (1·6 to 1·7)	2·1 (2 to 2·2)	1·9 (1·8 to 2)	1·9 (1·7 to 2·1)
York	1 (1 to 1)	1·1 (1 to 1·1)	1 (1 to 1·1)	0·9 (0·9 to 1)	1 (1 to 1·1)	0·9 (0·8 to 0·9)	1 (0·9 to 1)	0·8 (0·8 to 0·9)	0·8 (0·7 to 0·9)
Northern Ireland	13·9 (13·5 to 14·4)	15·3 (15·1 to 15·6)	15·4 (15 to 15·7)	13·5 (13·1 to 13·9)	12·6 (12·1 to 13·1)	10·6 (10·3 to 11)	12·3 (11·8 to 12·8)	10·6 (10·2 to 11·1)	10·1 (9 to 11·3)
Scotland	44·7 (43·3 to 46·2)	49·3 (48·4 to 50·3)	43 (42·1 to 43·8)	33·1 (32 to 34·2)	32·3 (31 to 33·6)	25·9 (25 to 26·9)	28·6 (27·6 to 29·8)	23·8 (22·9 to 24·9)	23·4 (20·8 to 26·2)
Wales	18·7 (18·2 to 19·3)	22·1 (21·7 to 22·5)	22 (21·7 to 22·4)	18·7 (18·1 to 19·3)	18·9 (18·2 to 19·7)	15·5 (15 to 16)	17·7 (17 to 18·5)	14·3 (13·8 to 14·8)	13·9 (12·3 to 15·4)
Latin America and Caribbean	3060 (2940 to 3180)	3910 (3810 to 4020)	4600 (4500 to 4700)	5050 (4950 to 5150)	5110 (5010 to 5220)	5220 (5100 to 5350)	5110 (4940 to 5280)	4760 (4490 to 5050)	4580 (4250 to 4930)
Andean Latin America	330 (318 to 341)	418 (406 to 429)	497 (485 to 508)	546 (534 to 559)	581 (564 to 598)	593 (575 to 612)	599 (574 to 623)	613 (571 to 658)	607 (560 to 657)
Bolivia	72·7 (69 to 76·2)	82·2 (79 to 85·4)	93·1 (89·2 to 96·7)	105 (102 to 109)	111 (107 to 115)	117 (113 to 122)	124 (118 to 131)	122 (112 to 132)	120 (109 to 132)
Ecuador	73·6 (70 to 77·7)	99·6 (97·5 to 102)	118 (116 to 121)	123 (119 to 128)	147 (140 to 154)	164 (156 to 173)	170 (160 to 181)	163 (150 to 177)	157 (139 to 178)
Peru	184 (177 to 190)	236 (229 to 243)	285 (276 to 293)	318 (309 to 328)	323 (312 to 334)	312 (300 to 324)	304 (289 to 320)	328 (300 to 360)	330 (298 to 365)
Caribbean	342 (333 to 349)	425 (417 to 431)	440 (431 to 448)	409 (400 to 419)	443 (430 to 454)	420 (405 to 435)	406 (388 to 423)	395 (368 to 424)	389 (358 to 424)
Antigua and Barbuda	0·8 (0·8 to 0·9)	0·9 (0·9 to 0·9)	0·8 (0·7 to 0·8)	0·6 (0·6 to 0·6)	0·6 (0·5 to 0·6)	0·7 (0·6 to 0·8)	0·6 (0·6 to 0·6)	0·5 (0·5 to 0·6)	0·5 (0·5 to 0·6)
The Bahamas	1·3 (1·2 to 1·3)	1·7 (1·6 to 1·7)	2·1 (2·1 to 2·2)	2·5 (2·4 to 2·6)	2·6 (2·5 to 2·8)	2·1 (2 to 2·3)	2·3 (2·2 to 2·5)	1·9 (1·6 to 2·3)	1·9 (1·6 to 2·2)
Barbados	3·3 (3·2 to 3·5)	3·6 (3·5 to 3·6)	2·5 (2·4 to 2·6)	2·1 (2 to 2·2)	2 (2 to 2·1)	1·9 (1·8 to 2)	1·6 (1·6 to 1·7)	1·3 (1·1 to 1·5)	1·3 (1·1 to 1·5)
Belize	1·4 (1·3 to 1·5)	2·1 (2 to 2·1)	2·4 (2·3 to 2·4)	2·8 (2·7 to 2·9)	3·1 (3 to 3·2)	3·3 (3·1 to 3·5)	3·7 (3·5 to 3·9)	3·7 (3·4 to 4·1)	3·7 (3·3 to 4·2)
Bermuda	0·5 (0·5 to 0·6)	0·5 (0·5 to 0·6)	0·5 (0·5 to 0·5)	0·4 (0·4 to 0·4)	0·4 (0·4 to 0·4)	0·4 (0·4 to 0·4)	0·3 (0·3 to 0·4)	0·3 (0·2 to 0·3)	0·2 (0·2 to 0·3)
Cuba	73·8 (70·3 to 77·3)	110 (107 to 112)	119 (116 to 123)	68·4 (66 to 70·9)	85 (81·8 to 88·2)	71·9 (69 to 74·9)	62·9 (60·4 to 65·6)	52·1 (49·8 to 54·3)	48 (44·6 to 51·5)
Dominica	0·9 (0·9 to 1)	1·3 (1·3 to 1·3)	1·3 (1·3 to 1·4)	0·9 (0·9 to 1)	0·9 (0·9 to 1)	0·6 (0·6 to 0·6)	0·5 (0·4 to 0·5)	0·3 (0·3 to 0·4)	0·3 (0·3 to 0·4)
Dominican Republic	64·5 (62·8 to 66)	83·7 (81·9 to 85·5)	92·4 (88·1 to 96·4)	103 (97·9 to 108)	113 (107 to 118)	109 (102 to 115)	97·8 (90·9 to 105)	105 (95·5 to 114)	104 (94·2 to 115)
Grenada	1·7 (1·6 to 1·8)	1·9 (1·8 to 1·9)	1·4 (1·3 to 1·4)	1·3 (1·2 to 1·4)	1·2 (1·1 to 1·3)	1·1 (1 to 1·3)	0·9 (0·8 to 1)	0·7 (0·6 to 0·8)	0·7 (0·6 to 0·8)
Guyana	9·4 (9·1 to 9·8)	12·4 (12·2 to 12·7)	12·7 (12·3 to 13)	12·5 (11·9 to 13·1)	13·1 (12·3 to 13·8)	9·7 (9·3 to 10·2)	7·8 (7·3 to 8·2)	7·7 (6·9 to 8·5)	7·4 (6·5 to 8·4)
Haiti	85·5 (81·6 to 89·1)	93·7 (89·5 to 97·4)	98·3 (94·7 to 102)	112 (108 to 116)	125 (120 to 129)	136 (128 to 142)	156 (147 to 165)	167 (150 to 185)	169 (151 to 190)
Jamaica	24·7 (23·1 to 26·4)	33·3 (32·3 to 34·2)	32·1 (31·3 to 33)	28·7 (27·8 to 29·6)	28·2 (27·1 to 29·3)	24·3 (21·8 to 27·2)	20·5 (19·6 to 21·5)	16·8 (14·9 to 18·6)	16·1 (13·9 to 18·4)
Puerto Rico	41·5 (40·6 to 42·4)	36·7 (36·1 to 37·3)	33·3 (32·5 to 34)	34·9 (34 to 35·9)	31·8 (30·9 to 32·8)	27·9 (26·6 to 29·1)	20·4 (18·8 to 22·3)	10·2 (9·8 to 10·5)	9·3 (8·6 to 10)
Saint Kitts and Nevis	0·9 (0·9 to 0·9)	1 (1 to 1·1)	0·6 (0·5 to 0·6)	0·6 (0·6 to 0·6)	0·5 (0·4 to 0·5)	0·4 (0·4 to 0·4)	0·3 (0·3 to 0·3)	0·3 (0·3 to 0·3)	0·3 (0·2 to 0·3)
Saint Lucia	1·4 (1·4 to 1·5)	2 (1·9 to 2)	2·1 (2 to 2·1)	1·9 (1·8 to 2)	1·7 (1·6 to 1·8)	1·4 (1·3 to 1·4)	1 (0·9 to 1·1)	0·9 (0·7 to 1)	0·8 (0·7 to 1)
Saint Vincent and the Grenadines	1·4 (1·3 to 1·4)	1·9 (1·9 to 2)	1·7 (1·6 to 1·7)	1·6 (1·5 to 1·6)	1·3 (1·2 to 1·4)	1 (1 to 1·1)	0·9 (0·8 to 0·9)	0·7 (0·6 to 0·7)	0·6 (0·6 to 0·7)
Suriname	3·8 (3·7 to 4)	5·8 (5·7 to 5·9)	6·5 (6·2 to 6·8)	5 (4·9 to 5·2)	4·7 (4·5 to 4·9)	4·6 (4·2 to 5·1)	4·9 (4·6 to 5·1)	4·6 (4·2 to 4·9)	4·4 (3·9 to 4·9)
Trinidad and Tobago	11·7 (11·3 to 12·1)	15·9 (15·6 to 16·2)	12·8 (12·5 to 13·2)	15·2 (14·6 to 15·7)	11·7 (11·2 to 12·1)	8·7 (8·3 to 9·1)	9·2 (8·7 to 9·9)	7·7 (7·1 to 8·3)	7·2 (6·3 to 8·1)
Virgin Islands	0·4 (0·4 to 0·5)	0·6 (0·6 to 0·6)	1·3 (1·2 to 1·3)	1·2 (1·1 to 1·3)	1·1 (1·1 to 1·2)	1 (0·9 to 1·1)	0·7 (0·6 to 0·8)	0·4 (0·4 to 0·5)	0·4 (0·3 to 0·4)
Central Latin America	1130 (1090 to 1180)	1590 (1560 to 1620)	2030 (1990 to 2060)	2200 (2150 to 2250)	2470 (2410 to 2520)	2410 (2350 to 2470)	2370 (2290 to 2460)	2020 (1880 to 2170)	1900 (1710 to 2110)
Colombia	245 (228 to 263)	340 (324 to 354)	389 (368 to 410)	405 (381 to 431)	452 (428 to 480)	437 (401 to 472)	376 (332 to 424)	336 (291 to 387)	329 (281 to 384)
Costa Rica	18·6 (18·1 to 19·2)	29 (28·8 to 29·2)	27 (26·4 to 27·6)	34 (33·2 to 35)	39·3 (38 to 40·6)	37·1 (36 to 38·3)	35·3 (34·1 to 36·5)	29·9 (28·4 to 31·4)	26·7 (24·4 to 29·2)
El Salvador	48 (46·8 to 49·3)	63·1 (62·2 to 64·1)	78·9 (77·3 to 80·3)	89·1 (87·1 to 91·1)	86·9 (83·8 to 90·1)	74·7 (70·7 to 78·6)	64 (60·9 to 67·4)	58·4 (53·1 to 63·9)	55·8 (49 to 63)
Guatemala	71·2 (70·2 to 72·2)	91·1 (89·7 to 92·6)	108 (106 to 110)	149 (146 to 151)	168 (165 to 170)	189 (183 to 194)	179 (173 to 186)	176 (167 to 186)	170 (152 to 189)
Honduras	35·7 (33·9 to 37·4)	44·3 (42·9 to 45·7)	56·8 (55·5 to 58)	75·3 (73·6 to 76·9)	87·6 (84 to 91·1)	94·7 (88·9 to 100)	101 (94·1 to 108)	108 (95·9 to 121)	108 (95·4 to 124)
Mexico	570 (542 to 599)	802 (784 to 819)	1100 (1090 to 1120)	1120 (1090 to 1150)	1270 (1240 to 1300)	1230 (1200 to 1270)	1230 (1190 to 1280)	981 (916 to 1050)	917 (834 to 1010)
Aguascalientes	4·6 (4·4 to 4·9)	6·2 (6 to 6·3)	8·4 (8·3 to 8·5)	9·5 (9·2 to 9·7)	11·6 (11·2 to 12)	12·6 (12·2 to 13·1)	13·4 (12·6 to 14·2)	11·8 (10·9 to 12·7)	11·2 (10·1 to 12·3)

Baja California	5 (4·8 to 5·3)	11·6 (11·3 to 11·9)	19·4 (19·1 to 19·8)	18 (17·4 to 18·6)	24·5 (23·8 to 25·3)	28·7 (27·7 to 29·8)	32 (30·6 to 33·6)	27 (25·2 to 28·9)	25·3 (23 to 27·7)
Baja California Sur	1·2 (1·1 to 1·3)	1·7 (1·7 to 1·8)	2·7 (2·7 to 2·8)	3·3 (3·2 to 3·4)	4·3 (4·2 to 4·5)	5·1 (4·9 to 5·2)	6·5 (6·2 to 6·8)	5·6 (5·2 to 6)	5·3 (4·8 to 5·9)
Campeche	2·4 (2·2 to 2·5)	3·5 (3·4 to 3·6)	5·5 (5·4 to 5·7)	7·3 (7 to 7·5)	8·6 (8·3 to 8·8)	8·3 (8 to 8·6)	9·1 (8·8 to 9·5)	7·2 (6·7 to 7·7)	6·6 (6 to 7·3)
Chiapas	17·8 (16·9 to 18·9)	27·4 (26·5 to 28·2)	38·3 (37·5 to 39·1)	44·5 (43 to 45·9)	67·4 (65·4 to 69·5)	63·6 (61·3 to 66·1)	72·1 (69 to 75·4)	62·5 (58 to 66·9)	59·2 (53·4 to 65)
Chihuahua	19·7 (18·8 to 20·7)	28·6 (28 to 29·2)	36·9 (36·2 to 37·5)	30·7 (29·9 to 31·5)	34·4 (33·4 to 35·5)	39·3 (38·1 to 40·4)	34·7 (33·4 to 36·1)	27·2 (25·3 to 29·2)	25·5 (23 to 28)
Coahuila	16·1 (15·3 to 16·9)	20·9 (20·5 to 21·3)	25·7 (25·2 to 26·2)	24·6 (23·8 to 25·4)	28·4 (27·5 to 29·4)	27·3 (26·3 to 28·4)	29·7 (28·5 to 30·9)	25·5 (23·5 to 27·3)	23·8 (21·6 to 26·1)
Colima	2·8 (2·7 to 3)	4 (3·9 to 4·1)	5·6 (5·5 to 5·7)	5·7 (5·5 to 5·9)	5·9 (5·6 to 6·1)	5·6 (5·4 to 5·9)	6·5 (6·2 to 6·8)	5·2 (4·8 to 5·6)	4·9 (4·4 to 5·4)
Durango	15·1 (14·4 to 15·9)	19·1 (18·7 to 19·4)	22·9 (22·5 to 23·2)	21·1 (20·4 to 21·7)	22·8 (22·1 to 23·5)	20·6 (19·9 to 21·4)	19·4 (18·5 to 20·3)	16 (14·9 to 17·2)	15·1 (13·7 to 16·6)
Guanajuato	32·7 (31·2 to 34·3)	43·5 (42·5 to 44·6)	56·5 (55·7 to 57·4)	57·7 (56·1 to 59·4)	65·6 (63·5 to 67·8)	63·2 (61·5 to 65·3)	62·7 (59·9 to 65·4)	51·6 (48 to 55·4)	48·5 (43·9 to 53·4)
Guerrero	20·5 (19·5 to 21·6)	29·2 (28·5 to 29·9)	39·9 (39·3 to 40·6)	42·2 (40·9 to 43·5)	50·9 (49·2 to 52·7)	51·6 (49·9 to 53·4)	48·1 (45·9 to 50·3)	36·7 (33·9 to 39·3)	34·1 (30·8 to 37·6)
Hidalgo	17·3 (16·4 to 18·2)	21·8 (21·2 to 22·3)	27·1 (26·7 to 27·6)	27·2 (26·5 to 28)	31·8 (30·8 to 32·8)	29·4 (28·5 to 30·4)	29·6 (28·5 to 30·8)	22·8 (21·2 to 24·4)	21·4 (19·3 to 23·5)
Jalisco	41·9 (40 to 44)	57·5 (56·5 to 58·5)	77·3 (76·2 to 78·4)	73·5 (71·6 to 75·4)	82·2 (79·6 to 84·9)	77·9 (75·1 to 80·9)	79·7 (76·8 to 82·4)	66·6 (62 to 71·3)	62·5 (56·6 to 68·7)
México	29 (27·6 to 30·5)	43 (42·1 to 43·9)	87·7 (86·2 to 89·2)	125 (122 to 129)	149 (144 to 154)	160 (155 to 165)	159 (152 to 166)	121 (113 to 130)	112 (102 to 124)
Mexico City	66 (62·1 to 69·9)	102 (98·6 to 105)	133 (130 to 137)	114 (110 to 118)	100 (96·9 to 104)	80·3 (77·7 to 83·1)	70·4 (68 to 72·9)	47·9 (44·4 to 51·7)	43·5 (39·3 to 48)
Michoacán de Ocampo	34·3 (32·8 to 36·1)	45·4 (44·3 to 46·4)	56 (55·1 to 56·8)	56·1 (54·6 to 57·6)	61·6 (59·7 to 63·6)	53·1 (51·3 to 55)	52·2 (49·7 to 54·8)	42·3 (39·3 to 45·5)	39·6 (36 to 43·6)
Morelos	5·8 (5·6 to 6·2)	8·8 (8·7 to 9)	13·9 (13·7 to 14·1)	15·6 (15·2 to 16)	17·6 (17·1 to 18·2)	17·1 (16·5 to 17·7)	18·6 (17·8 to 19·4)	14·6 (13·6 to 15·5)	13·5 (12·3 to 14·8)
Nayarit	7·2 (6·8 to 7·6)	9·7 (9·6 to 9·9)	13·2 (13 to 13·4)	12·6 (12·3 to 12·9)	12·9 (12·6 to 13·3)	11·6 (11·3 to 12)	12·1 (11·6 to 12·6)	9·5 (8·8 to 10·2)	8·9 (8 to 9·8)
Nuevo León	16·4 (15·7 to 17·3)	25·6 (25·1 to 26·1)	37·1 (36·5 to 37·7)	34·8 (33·8 to 35·8)	40·1 (38·7 to 41·5)	43·8 (42·4 to 45·3)	46·3 (44·3 to 48·3)	41·8 (39 to 44·6)	39·9 (36·2 to 43·7)
Oaxaca	28·2 (26·5 to 29·9)	37·4 (36·1 to 38·6)	46·4 (45·6 to 47·2)	44·1 (42·9 to 45·3)	50·3 (48·7 to 52)	49·1 (47·6 to 50·9)	47·7 (45·7 to 49·9)	36·4 (33·7 to 39)	34 (30·8 to 37·5)
Puebla	35 (33·3 to 36·9)	45·7 (44·6 to 46·8)	60·5 (59·7 to 61·4)	62·3 (60·6 to 64·1)	70 (68·1 to 72)	71·3 (69·2 to 73·5)	72·4 (69·1 to 75·7)	59·7 (55·6 to 64)	56·2 (51 to 62)
Querétaro	6·8 (6·4 to 7·1)	8·8 (8·6 to 9)	11·7 (11·5 to 11·8)	13·4 (13 to 13·7)	18·2 (17·6 to 18·8)	18·3 (17·6 to 19)	19·9 (18·9 to 20·9)	18·3 (17 to 19·8)	17·6 (16 to 19·5)
Quintana Roo	0·6 (0·6 to 0·6)	1·2 (1·2 to 1·2)	2·2 (2·1 to 2·2)	4·4 (4·2 to 4·5)	7·9 (7·6 to 8·2)	10·7 (10·2 to 11·3)	14·2 (13·3 to 15·1)	14·2 (13·2 to 15·2)	13·8 (12·4 to 15·2)
San Luis Potosí	18·6 (17·7 to 19·6)	24·2 (23·6 to 24·7)	30·3 (29·9 to 30·8)	29·7 (28·8 to 30·6)	33·3 (32·3 to 34·3)	30·5 (29·5 to 31·5)	28·4 (27 to 29·8)	22·5 (20·9 to 24·1)	21·1 (19·1 to 23·2)
Sinaloa	14·7 (14 to 15·5)	20·5 (20·2 to 20·9)	30·6 (30·2 to 31·1)	31·3 (30·5 to 32·2)	33·5 (32·4 to 34·6)	32·3 (31·1 to 33·6)	28·9 (27·6 to 30·2)	22·9 (21·2 to 24·6)	21·4 (19·5 to 23·6)
Sonora	11·9 (11·4 to 12·5)	17·8 (17·4 to 18·1)	24·3 (23·9 to 24·7)	21·8 (21·2 to 22·5)	25·6 (24·8 to 26·5)	26·9 (26·1 to 27·8)	27·3 (26·3 to 28·4)	19·8 (18·4 to 21·3)	18·2 (16·5 to 19·9)
Tabasco	7·8 (7·4 to 8·2)	12·1 (11·9 to 12·4)	18·8 (18·5 to 19·1)	21·4 (20·8 to 21·9)	25·7 (24·9 to 26·6)	24·6 (23·6 to 25·6)	25·5 (24·3 to 26·6)	19·7 (18·4 to 21·1)	18·2 (16·6 to 20·1)
Tamaulipas	16 (15·2 to 16·8)	23·7 (23·2 to 24·2)	32·7 (32·2 to 33·3)	28·8 (27·8 to 29·7)	31·2 (30·2 to 32·3)	32 (30·9 to 33·2)	33·2 (31·8 to 34·6)	25·5 (23·6 to 27·2)	23·6 (21·4 to 25·9)
Tlaxcala	6·3 (6 to 6·6)	8 (7·8 to 8·2)	9·6 (9·4 to 9·8)	9·8 (9·6 to 10·1)	12·4 (12 to 12·8)	12·9 (12·5 to 13·4)	13·3 (12·7 to 13·9)	11·2 (10·4 to 12)	10·6 (9·6 to 11·7)
Veracruz de Ignacio de la Llave	41·8 (39·5 to 44·2)	60·3 (58·4 to 62·1)	87 (85·4 to 88·7)	90·4 (88 to 92·9)	100 (97·1 to 103)	87·6 (84·4 to 90·9)	81·1 (78 to 84·2)	57 (53·1 to 61)	52·3 (47·4 to 57·6)
Yucatán	9·9 (9·3 to 10·4)	12·6 (12·2 to 12·9)	17 (16·7 to 17·3)	17·7 (17·1 to 18·2)	20·3 (19·6 to 21)	18·8 (18·2 to 19·5)	18·9 (17·9 to 19·8)	16·1 (14·9 to 17·2)	15·2 (13·8 to 16·6)
Zacatecas	16·1 (15·4 to 16·9)	20·4 (20 to 20·8)	23·1 (22·7 to 23·6)	21·7 (21·1 to 22·3)	20·8 (20·1 to 21·5)	18 (17·4 to 18·6)	17·6 (16·8 to 18·4)	14·5 (13·3 to 15·7)	13·6 (12·3 to 15)
Nicaragua	26·2 (24·9 to 27·6)	34·5 (33·5 to 35·5)	45·5 (44·3 to 46·5)	62·3 (61 to 63·6)	68·8 (65·8 to 71·7)	63·7 (59·8 to 67·8)	64·8 (60·1 to 69·5)	63·2 (56·9 to 69·9)	61·6 (54·1 to 69·6)
Panama	12·9 (12·4 to 13·4)	22·2 (21·9 to 22·5)	26·4 (25·9 to 27)	26·3 (25·5 to 27·1)	28·5 (27·6 to 29·5)	30·8 (29·7 to 32)	34·4 (33·2 to 35·6)	34·8 (33·2 to 36·3)	33·8 (30·4 to 37·5)
Venezuela	107 (104 to 110)	164 (162 to 166)	193 (189 to 197)	238 (231 to 245)	266 (256 to 277)	251 (237 to 266)	288 (278 to 298)	234 (205 to 269)	200 (173 to 229)
Tropical Latin America	1250 (1170 to 1330)	1480 (1410 to 1550)	1640 (1570 to 1710)	1900 (1840 to 1950)	1620 (1580 to 1670)	1800 (1740 to 1860)	1730 (1660 to 1800)	1740 (1630 to 1860)	1690 (1550 to 1840)
Brazil	1220 (1140 to 1300)	1440 (1370 to 1520)	1600 (1530 to 1660)	1840 (1790 to 1890)	1560 (1520 to 1600)	1730 (1680 to 1790)	1660 (1600 to 1730)	1670 (1560 to 1800)	1620 (1490 to 1780)
Acre	2·8 (2·7 to 2·9)	3·6 (3·5 to 3·7)	4·6 (4·4 to 4·7)	6·1 (5·9 to 6·3)	5·7 (5·5 to 5·9)	8 (7·8 to 8·3)	9·1 (8·7 to 9·5)	9·7 (9 to 10·4)	9·7 (8·9 to 10·6)
Alagoas	29·6 (28·1 to 31)	31·7 (30·5 to 32·9)	36·5 (35·3 to 37·7)	40·6 (39·5 to 41·7)	37·3 (36 to 38·6)	43·9 (42·1 to 45·8)	42·7 (40·5 to 44·7)	41·7 (38·6 to 45·3)	41 (37·3 to 45·2)
Amapá	1 (0·9 to 1)	1·7 (1·6 to 1·7)	2·5 (2·4 to 2·6)	3·4 (3·3 to 3·5)	3·6 (3·5 to 3·8)	6 (5·8 to 6·3)	6·6 (6·3 to 7)	7·4 (6·8 to 8·1)	7·4 (6·7 to 8·1)
Amazonas	13 (12·2 to 13·8)	15·9 (15·1 to 16·6)	18·8 (17·9 to 19·8)	25 (24·2 to 25·8)	22·9 (21·9 to 23·8)	32·9 (31·3 to 34·4)	37·2 (35·4 to 39·2)	40·1 (37·3 to 43·5)	39·7 (36·4 to 43·4)
Bahia	126 (118 to 132)	143 (136 to 149)	164 (158 to 171)	183 (177 to 189)	141 (136 to 146)	159 (152 to 167)	154 (146 to 162)	146 (136 to 159)	142 (129 to 157)
Ceará	67·3 (63·7 to 70·8)	79·3 (75·9 to 82·5)	91·7 (88·1 to 95·2)	96·7 (93·4 to 100)	80·3 (77·2 to 83·6)	96·9 (92·2 to 102)	100 (94·6 to 106)	103 (95 to 112)	101 (91·6 to 111)
Distrito Federal	1 (0·9 to 1)	2·4 (2·3 to 2·6)	10·4 (10 to 10·8)	20 (19·4 to 20·5)	18·8 (18·2 to 19·4)	22·8 (22 to 23·4)	21 (20·2 to 21·8)	20·8 (19·7 to 22·1)	19·9 (18·6 to 21·5)
Espírito Santo	19·9 (18·6 to 21·1)	24·5 (23·3 to 25·6)	26·6 (25·4 to 27·8)	32 (31 to 32·9)	27·8 (26·9 to 28·6)	30·6 (29·3 to 31·9)	28·6 (27·4 to 29·8)	29·4 (27·1 to 31·9)	28·5 (26 to 31·3)
Goiás	24·6 (22·9 to 26·2)	34·6 (32·9 to 36·3)	42·6 (40·6 to 44·6)	47·6 (46·2 to 49·2)	40·7 (39·5 to 42)	46 (44·1 to 47·9)	42·2 (40·4 to 44·2)	45·4 (42·2 to 49·2)	44·2 (40·4 to 48·4)
Maranhão	43·7 (41·5 to 45·7)	61·9 (59·6 to 64)	72·3 (70·1 to 74·5)	89·2 (86·9 to 91·4)	76·8 (73·8 to 79·7)	94 (89·9 to 98·2)	105 (99·7 to 110)	109 (101 to 118)	108 (99·6 to 118)
Mato Grosso	4·5 (4·2 to 4·8)	7·4 (7 to 7·7)	12·3 (11·8 to 12·8)	18·8 (18·3 to 19·3)	20·1 (19·5 to 20·7)	23·4 (22·4 to 24·3)	23·3 (22·2 to 24·3)	27·1 (25 to 29·5)	26·8 (24·4 to 29·4)
Mato Grosso do Sul	7·5 (7 to 8)	11·5 (10·9 to 12)	16·3 (15·5 to 17·1)	21·4 (20·8 to 22)	19·2 (18·5 to 19·8)	20·5 (19·7 to 21·3)	20·4 (19·6 to 21·3)	21·7 (20·2 to 23·3)	21·1 (19·3 to 23·1)
Minas Gerais	183 (171 to 195)	203 (193 to 213)	187 (179 to 195)	192 (187 to 197)	163 (158 to 168)	166 (160 to 172)	148 (142 to 154)	145 (136 to 155)	141 (130 to 152)
Pará	28·1 (26·3 to 29·7)	33·8 (32·2 to 35·4)	42·6 (40·5 to 44·6)	60·3 (58·4 to 62·4)	53·3 (51·1 to 55·5)	70·7 (67·3 to 73·9)	77 (72·9 to 81·3)	80·1 (74·3 to 86·8)	79 (72·3 to 87·2)
Paraíba	45 (42·5 to 47·4)	48·1 (46 to 50·2)	47·9 (45·9 to 49·8)	43·2 (41·6 to 45)	34·7 (33·3 to 36·2)	40·5 (38·6 to 42·4)	42·4 (40 to 44·8)	42·3 (39·3 to 45·9)	41·3 (37·9 to 45·4)
Paraná	49·3 (45·9 to 52·7)	84 (79·8 to 88·1)	107 (102 to 112)	102 (99 to 106)	87·4 (84·6 to 90·2)	85·9 (82·6 to 89·3)	75·2 (72·1 to 78·5)	76·8 (71·2 to 83·4)	74·3 (67·8 to 81·6)
Pernambuco	92·3 (87·4 to 97)	104 (99·1 to 108)	113 (108 to 117)	114 (111 to 118)	88·3 (85·1 to 91·6)	102 (97·2 to 107)	97·8 (92·9 to 103)	93·4 (86·5 to 101)	90·7 (83·4 to 99·3)
Piauí	26·1 (24·7 to 27·5)	29·2 (28 to 30·4)	35·1 (33·6 to 36·5)	39·8 (38·5 to 41·1)	32·8 (31·6 to 34)	37·6 (36 to 39·2)	39 (36·7 to 41·3)	38·2 (35·2 to 41·4)	37·3 (34 to 41·2)
Rio de Janeiro	97·1 (88·3 to 106)	111 (103 to 118)	118 (111 to 124)	136 (132 to 140)	112 (109 to 115)	118 (114 to 122)	107 (103 to 111)	106 (98·9 to 112)	101 (93·4 to 109)

Rio Grande do Norte	25·2 (23·9 to 26·4)	28·2 (27 to 29·3)	32·2 (31 to 33·4)	34·4 (33·1 to 35·7)	30·9 (29·8 to 32)	37·1 (35·4 to 38·8)	36·7 (34·6 to 38·6)	35·8 (32·8 to 39·2)	34·9 (31·4 to 38·8)
Rio Grande do Sul	84·1 (76·8 to 91·4)	89·5 (83·7 to 95·4)	86·2 (82·3 to 90·1)	93·5 (90·7 to 96·3)	76·3 (74·2 to 78·7)	75·3 (72·9 to 77·7)	61 (59 to 63·1)	61·2 (57·9 to 64·8)	58·4 (54·6 to 62·6)
Rondônia	0·9 (0·8 to 0·9)	1·4 (1·4 to 1·5)	2·2 (2·1 to 2·2)	8·2 (7·9 to 8·4)	12·9 (12·4 to 13·4)	15·7 (15 to 16·4)	15·1 (14·1 to 16)	15·4 (14·3 to 16·6)	15 (13·8 to 16·4)
Roraima	0·4 (0·4 to 0·5)	0·5 (0·5 to 0·6)	0·8 (0·7 to 0·8)	1·3 (1·3 to 1·4)	2·5 (2·4 to 2·6)	4·2 (4 to 4·4)	5·3 (5 to 5·5)	6·7 (6·2 to 7·2)	6·8 (6·3 to 7·4)
Santa Catarina	32·5 (30·1 to 34·9)	37·4 (35·3 to 39·6)	38·5 (36·5 to 40·5)	44·6 (43·1 to 46·1)	39·8 (38·3 to 41·3)	41·7 (39·9 to 43·2)	39·6 (37·8 to 41·4)	46·7 (43·1 to 50·6)	46 (41·9 to 50·5)
São Paulo	196 (180 to 212)	236 (222 to 251)	262 (248 to 275)	362 (352 to 372)	303 (295 to 310)	317 (307 to 326)	291 (281 to 303)	283 (263 to 305)	270 (245 to 297)
Sergipe	16 (15 to 16·9)	16·6 (15·8 to 17·3)	17·5 (16·7 to 18·2)	18·7 (18 to 19·3)	17·6 (17 to 18·2)	21·9 (20·9 to 22·9)	22 (20·7 to 23·2)	22 (20·3 to 24)	21·6 (19·7 to 23·9)
Tocantins	3·7 (3·5 to 3·9)	5·5 (5·3 to 5·8)	8 (7·7 to 8·4)	10·2 (9·8 to 10·5)	12 (11·5 to 12·6)	17 (16·3 to 17·7)	18 (17 to 18·9)	18·4 (16·7 to 20·1)	18·1 (16·3 to 20)
Paraguay	32·5 (31·1 to 34)	37·2 (35·4 to 39)	42·6 (40 to 45·1)	52·1 (48 to 56·3)	62·4 (57·1 to 68)	63·8 (58·3 to 69·7)	65·4 (57·7 to 74·1)	63·3 (54·9 to 72·9)	62·2 (53·2 to 72·1)
North Africa and Middle East	2310 (2170 to 2460)	2880 (2790 to 2960)	3920 (3840 to 4000)	5320 (5220 to 5420)	5400 (5270 to 5540)	5610 (5410 to 5820)	6690 (6410 to 6980)	6190 (5810 to 6610)	5880 (5420 to 6420)
North Africa and Middle East	2310 (2170 to 2460)	2880 (2790 to 2960)	3920 (3840 to 4000)	5320 (5220 to 5420)	5400 (5270 to 5540)	5610 (5410 to 5820)	6690 (6410 to 6980)	6190 (5810 to 6610)	5880 (5420 to 6420)
Afghanistan	174 (165 to 182)	207 (201 to 214)	250 (244 to 257)	297 (288 to 305)	215 (209 to 222)	375 (366 to 383)	435 (418 to 452)	554 (525 to 583)	579 (548 to 613)
Algeria	201 (189 to 212)	234 (225 to 243)	297 (285 to 309)	384 (378 to 390)	373 (361 to 386)	315 (289 to 342)	435 (410 to 462)	465 (428 to 503)	439 (396 to 483)
Bahrain	1·9 (1·8 to 2·1)	3·2 (3 to 3·4)	3·5 (3·4 to 3·6)	4·9 (4·8 to 5·1)	6·6 (6·4 to 6·7)	6·1 (5·9 to 6·3)	9·6 (9·3 to 10)	8·8 (8·1 to 9·5)	8·2 (7·3 to 9·2)
Egypt	270 (238 to 305)	364 (341 to 386)	679 (656 to 702)	898 (869 to 929)	922 (882 to 961)	955 (907 to 1000)	1480 (1410 to 1540)	1350 (1260 to 1450)	1270 (1140 to 1420)
Iran	376 (352 to 401)	443 (425 to 462)	559 (547 to 573)	947 (934 to 959)	755 (728 to 783)	543 (510 to 580)	673 (632 to 720)	575 (533 to 620)	499 (435 to 567)
Alborz	3·9 (3·6 to 4·1)	7·6 (7·3 to 7·9)	12 (11·6 to 12·3)	20·6 (20 to 21·1)	15·8 (14·9 to 16·8)	13·3 (12·1 to 14·6)	17·8 (16·2 to 19·2)	15·5 (14 to 17·1)	13·4 (11·5 to 15·5)
Ardebil	10·3 (9·7 to 10·9)	11·3 (10·8 to 11·8)	13·8 (13·4 to 14·2)	22·8 (22·3 to 23·2)	17·4 (16·6 to 18·2)	11·4 (10·5 to 12·3)	11·4 (10·6 to 12·2)	8·8 (8·2 to 9·4)	7·5 (6·7 to 8·4)
Bushehr	2·9 (2·7 to 3·1)	4·1 (3·9 to 4·3)	5·9 (5·7 to 6)	11 (10·7 to 11·3)	10 (9·5 to 10·4)	6·6 (6·2 to 7·1)	8·4 (7·3 to 9·5)	8·7 (8·1 to 9·4)	7·9 (7·1 to 8·8)
Chahar Mahaal and Bakhtiari	4·9 (4·6 to 5·2)	5·3 (5 to 5·5)	6·5 (6·4 to 6·7)	12 (11·7 to 12·4)	10·9 (10·4 to 11·4)	7·2 (6·7 to 7·8)	9 (8 to 10·1)	8·1 (7·5 to 8·6)	7 (6·2 to 7·9)
East Azarbayejan	32·2 (30 to 34·5)	35·7 (34·2 to 37·2)	41·3 (40·1 to 42·5)	65·7 (64·2 to 67·2)	46·5 (44·7 to 48·4)	30·4 (28·4 to 32·6)	33 (30·6 to 35·3)	25·2 (23·1 to 27·3)	21·5 (18·5 to 24·9)
Fars	12·6 (11·7 to 13·5)	20·1 (19·3 to 21)	30·8 (29·9 to 31·7)	57 (56·1 to 57·9)	45·2 (42·9 to 47·8)	31·8 (29·6 to 34·2)	38·3 (35·6 to 41·1)	30·7 (27·4 to 34·4)	26·3 (21·8 to 31·4)
Gilan	17·8 (16·5 to 19·1)	21·7 (20·7 to 22·8)	26·9 (26·1 to 27·7)	39·1 (38 to 40·3)	24·6 (23·5 to 25·7)	14·7 (13·6 to 15·9)	15·2 (14·1 to 16·4)	11·8 (10·8 to 12·9)	10 (8·7 to 11·6)
Golestan	10·6 (9·9 to 11·2)	11·1 (10·6 to 11·6)	13·4 (13 to 13·9)	24·8 (24·2 to 25·4)	20·7 (19·7 to 21·8)	14·3 (13·2 to 15·4)	18·5 (17·1 to 20·1)	15·6 (14·6 to 16·6)	13·3 (12 to 14·8)
Hamadan	14·1 (13·2 to 15)	16·3 (15·6 to 17·1)	19·1 (18·6 to 19·7)	30·2 (29·7 to 30·8)	23·6 (22·4 to 24·8)	14·7 (13·4 to 15·9)	15·3 (14·3 to 16·3)	11·4 (10·7 to 12·2)	9·5 (8·5 to 10·6)
Hormozgan	4·8 (4·5 to 5)	6·3 (6·1 to 6·6)	8·3 (8 to 8·6)	14·8 (14·5 to 15·2)	14·5 (13·9 to 15·2)	11·6 (10·9 to 12·3)	16·5 (15·3 to 17·8)	16·8 (15·7 to 17·9)	15 (13·4 to 16·5)
Ilam	1·9 (1·8 to 2)	2·3 (2·2 to 2·4)	3·5 (3·4 to 3·6)	7·4 (7·2 to 7·5)	6·6 (6·3 to 6·9)	4·3 (4 to 4·6)	4·5 (4·1 to 5)	4·2 (3·8 to 4·5)	3·6 (3·2 to 4·1)
Isfahan	17·8 (16·5 to 19)	23·1 (22·1 to 24·1)	29·3 (28·5 to 30·2)	52·3 (51 to 53·5)	42·6 (40·2 to 45·3)	29·5 (26·8 to 32·5)	36·1 (33·3 to 38·8)	29·8 (26·2 to 33·8)	25·4 (20·7 to 31·1)
Kerman	19·4 (18·2 to 20·6)	16·1 (15·5 to 16·7)	17·8 (17·4 to 18·3)	33·1 (32·2 to 33·9)	24·8 (23·5 to 26)	18·4 (17·2 to 19·7)	27·9 (24·9 to 31·1)	24·8 (23·2 to 26·5)	21·5 (19·3 to 24)
Kermanshah	10·5 (9·8 to 11·3)	12·9 (12·3 to 13·5)	16·7 (16·3 to 17·2)	29·8 (29·1 to 30·4)	23·1 (22 to 24·1)	14·9 (13·7 to 16)	16·5 (14·8 to 18·4)	12·8 (11·9 to 13·8)	10·8 (9·7 to 12·2)
Khorasan-e-Razavi	35·8 (33·9 to 37·6)	39·6 (38·2 to 41·2)	46·4 (45·2 to 47·5)	82·1 (79·6 to 84·4)	67·8 (64·1 to 71·3)	48·3 (44·9 to 51·9)	63·3 (56·2 to 71·1)	54·1 (50·2 to 57·9)	46·9 (41·6 to 52·8)
Khuzestan	31·3 (29·3 to 33·2)	30·2 (29 to 31·3)	38 (36·9 to 39·1)	62·8 (61·5 to 64·1)	46·6 (45 to 48·4)	39·5 (36·9 to 42·2)	50·8 (47·7 to 54)	42·5 (39·1 to 46)	36·5 (31·6 to 41·9)
Kohgiluyeh and Boyer-Ahmad	1·8 (1·7 to 1·9)	2·5 (2·4 to 2·6)	3·8 (3·7 to 3·8)	7·6 (7·5 to 7·7)	7·9 (7·6 to 8·2)	5·9 (5·5 to 6·3)	6·2 (5·7 to 6·8)	6·2 (5·8 to 6·8)	5·7 (4·9 to 6·5)
Kurdistan	11·3 (10·6 to 12·1)	12 (11·5 to 12·5)	13·3 (12·9 to 13·6)	21·8 (21·4 to 22·3)	17·7 (16·9 to 18·5)	12·4 (11·3 to 13·4)	13·3 (12·3 to 14·4)	11·6 (10·8 to 12·4)	10·2 (9·1 to 11·2)
Lorestan	15·4 (14·5 to 16·3)	13·6 (13 to 14·1)	15·3 (14·9 to 15·7)	27·9 (27·3 to 28·4)	24·6 (23·5 to 25·7)	15·6 (14·3 to 16·8)	16·7 (14·5 to 19·1)	13·2 (12·2 to 14·2)	11·2 (10 to 12·6)
Markazi	4·3 (4 to 4·7)	8·7 (8·3 to 9·1)	14·2 (13·8 to 14·6)	21·5 (21 to 21·9)	13·9 (13·1 to 14·7)	8·8 (8·1 to 9·5)	10·2 (9·1 to 11·4)	8·1 (7·6 to 8·6)	6·9 (6·2 to 7·7)
Mazandaran	23·7 (21·9 to 25·5)	22·4 (21·4 to 23·4)	25·2 (24·5 to 25·9)	43·9 (42·8 to 45)	30·6 (29·2 to 32)	18·9 (17·3 to 20·3)	21 (19·4 to 22·6)	17·5 (15·9 to 19·2)	15·1 (13 to 17·3)
North Khorasan	4·2 (3·9 to 4·4)	4·8 (4·6 to 5)	5·9 (5·7 to 6·1)	10·7 (10·5 to 11)	11·2 (10·7 to 11·7)	7·7 (7·1 to 8·3)	9·2 (8·3 to 10·1)	7·1 (6·6 to 7·6)	6·1 (5·5 to 6·7)
Qazvin	9·6 (8·9 to 10·3)	10·4 (9·9 to 10·8)	10·9 (10·6 to 11·2)	15·4 (15 to 15·7)	11·9 (11·4 to 12·5)	8·4 (7·7 to 9)	9·9 (8·9 to 11)	8·2 (7·6 to 8·9)	7 (6·1 to 8·1)
Qom	1·9 (1·8 to 2·1)	3·9 (3·7 to 4·1)	6·7 (6·6 to 6·9)	11·5 (11·3 to 11·8)	9·3 (8·9 to 9·7)	7·6 (7·1 to 8·1)	10·2 (9·4 to 11·1)	10 (9·2 to 10·8)	8·9 (7·7 to 10·1)
Semnan	1·9 (1·8 to 2·1)	3 (2·8 to 3·1)	4·2 (4·1 to 4·3)	6·9 (6·7 to 7·1)	4·8 (4·6 to 5·1)	3·4 (3·1 to 3·7)	4·3 (3·9 to 4·8)	4·3 (4 to 4·6)	3·9 (3·4 to 4·3)
Sistan and Baluchistan	9·6 (8·9 to 10·2)	9·1 (8·7 to 9·5)	11 (10·7 to 11·3)	22·9 (22·4 to 23·3)	26·7 (25·5 to 27·9)	26·3 (24·5 to 28)	42 (40·1 to 44·3)	41·2 (39 to 43·5)	37·6 (33·6 to 41·9)
South Khorasan	5·3 (5 to 5·6)	6·3 (6 to 6·6)	7·8 (7·5 to 8)	13·1 (12·9 to 13·4)	10·6 (10 to 11·1)	6 (5·6 to 6·5)	6·9 (6·1 to 7·8)	7·4 (6·9 to 7·8)	6·7 (6 to 7·4)
Tehran	25·9 (24 to 27·9)	46·4 (44·3 to 48·6)	68·9 (66·5 to 71·3)	111 (106 to 116)	90·3 (84·6 to 96·5)	70·5 (65·2 to 76·4)	89·6 (83·4 to 95·9)	76·6 (67·5 to 86·3)	66·1 (54·3 to 79·9)
West Azarbayejan	12·2 (11·4 to 13·1)	17·2 (16·4 to 18)	22·9 (22·4 to 23·5)	40·1 (39·2 to 41·1)	32·7 (31·3 to 34·1)	24·6 (22·5 to 26·6)	31·5 (29·7 to 33·6)	26·6 (24·6 to 28·6)	22·9 (20·5 to 25·5)
Yazd	5·4 (5 to 5·7)	5·6 (5·4 to 5·9)	6·3 (6·1 to 6·5)	10·1 (10 to 10·3)	8·9 (8·5 to 9·3)	7·2 (6·6 to 7·7)	10·2 (9·5 to 11)	9 (8·3 to 9·8)	7·8 (6·8 to 8·9)
Zanjan	13·1 (12·3 to 13·9)	13·7 (13·2 to 14·2)	13·4 (13 to 13·8)	17 (16·7 to 17·2)	12·7 (12·2 to 13·3)	8·7 (8·2 to 9·3)	9·6 (9 to 10·2)	7·4 (6·8 to 8·1)	6·3 (5·5 to 7·3)
Iraq	105 (98·8 to 112)	147 (140 to 152)	207 (202 to 213)	292 (283 to 300)	362 (345 to 379)	451 (428 to 476)	498 (458 to 538)	470 (432 to 512)	451 (413 to 498)
Jordan	11·2 (11·1 to 11·4)	19·2 (18·9 to 19·5)	30·3 (29·8 to 30·7)	44·3 (43·6 to 45)	64·6 (62·7 to 66·5)	72·6 (68·9 to 76·5)	95·8 (90·6 to 101)	103 (94·1 to 113)	102 (90·8 to 115)
Kuwait	1·3 (1·2 to 1·4)	5·5 (5·4 to 5·6)	17·1 (16·9 to 17·3)	24·7 (24 to 25·3)	16·8 (15·7 to 18)	20·1 (19·1 to 21·1)	27·3 (25·7 to 29·2)	25·9 (23·8 to 28·3)	24·8 (21·5 to 28·7)
Lebanon	28·7 (27·2 to 30·1)	34·7 (33·2 to 36·2)	37·4 (35·3 to 39·3)	38·6 (36·1 to 41)	42·5 (39·2 to 45·7)	44·3 (41·6 to 47·2)	43·2 (39·4 to 47·1)	43 (37·2 to 49·8)	39 (33·3 to 46·2)
Libya	25·3 (24·6 to 26)	33·5 (32·7 to 34·3)	41·1 (40·2 to 42·1)	59·3 (57·9 to 60·7)	65 (61·3 to 69)	60·4 (54·3 to 66·3)	54·5 (48·5 to 61)	41·3 (35·2 to 49·1)	38·5 (32·2 to 46)
Morocco	226 (216 to 236)	281 (272 to 290)	344 (335 to 353)	394 (376 to 411)	392 (367 to 417)	407 (374 to 441)	409 (372 to 446)	336 (306 to 372)	315 (286 to 348)
Oman	11·7 (11·3 to 12·1)	15·5 (15·1 to 15·9)	20·1 (19·5 to 20·6)	28·9 (28·2 to 29·8)	36 (35 to 37)	27·4 (25·3 to 29·6)	32·2 (30·8 to 33·6)	40·8 (37·8 to 43·8)	40 (36·3 to 43·9)
Palestine	17·7 (17·2 to 18·3)	21·6 (21·1 to 22·2)	25 (24·4 to 25·7)	30·5 (29·3 to 31·6)	42·7 (40·8 to 44·4)	57·1 (54·7 to 59·6)	64·8 (60·9 to 68·8)	59·9 (55 to 65·7)	58·3 (53·3 to 64·3)

Qatar	0·6 (0·5 to 0·6)	1·2 (1·1 to 1·2)	2·3 (2·2 to 2·3)	4 (3·8 to 4·1)	5·6 (5·4 to 5·9)	6·5 (6·1 to 6·8)	13·2 (12·5 to 13·9)	18 (16·7 to 19·6)	18·6 (16·8 to 20·6)
Saudi Arabia	63·9 (60·7 to 67)	83·8 (80·7 to 86·9)	124 (119 to 128)	193 (184 to 203)	250 (233 to 266)	240 (220 to 260)	252 (224 to 281)	236 (204 to 272)	224 (192 to 260)
Sudan	116 (111 to 120)	143 (137 to 148)	239 (231 to 247)	341 (330 to 351)	443 (428 to 459)	553 (532 to 574)	614 (574 to 652)	585 (536 to 632)	567 (518 to 622)
Syria	79·8 (77·5 to 82·2)	109 (106 to 111)	141 (137 to 146)	182 (176 to 188)	222 (209 to 234)	240 (223 to 258)	255 (231 to 282)	100 (85·5 to 117)	95·6 (80·6 to 114)
Tunisia	80·8 (76·5 to 85)	92·9 (88·8 to 96·9)	95·9 (92·7 to 99)	108 (104 to 112)	106 (101 to 112)	90·1 (83·9 to 96·4)	95·9 (89·7 to 102)	86·3 (78·2 to 95·6)	79·9 (69·8 to 91·9)
Türkiye	410 (381 to 440)	505 (481 to 528)	631 (604 to 657)	784 (750 to 817)	736 (698 to 771)	699 (651 to 746)	643 (610 to 681)	558 (512 to 609)	511 (464 to 565)
United Arab Emirates	1·7 (1·7 to 1·8)	2·1 (2 to 2·1)	4·5 (4·3 to 4·6)	17·3 (16·9 to 17·8)	23·6 (22 to 25·2)	24·1 (22·6 to 25·7)	43·5 (40·5 to 46·7)	41 (36·8 to 45·6)	36·1 (31·3 to 41·3)
Yemen	108 (103 to 112)	134 (131 to 138)	173 (169 to 176)	249 (244 to 253)	320 (313 to 327)	414 (398 to 429)	517 (490 to 542)	489 (443 to 540)	480 (430 to 537)
South Asia	9890 (9270 to 10500)	11500 (10900 to 12200)	12900 (12300 to 13400)	15300 (14600 to 15800)	15800 (15200 to 16300)	16700 (16100 to 17200)	17000 (16400 to 17600)	15800 (14800 to 16800)	15300 (14000 to 16800)
South Asia	9890 (9270 to 10500)	11500 (10900 to 12200)	12900 (12300 to 13400)	15300 (14600 to 15800)	15800 (15200 to 16300)	16700 (16100 to 17200)	17000 (16400 to 17600)	15800 (14800 to 16800)	15300 (14000 to 16800)
Bangladesh	1000 (965 to 1040)	1130 (1090 to 1160)	1500 (1480 to 1530)	1770 (1730 to 1810)	2020 (1970 to 2070)	1980 (1910 to 2040)	1840 (1760 to 1920)	1440 (1330 to 1550)	1360 (1200 to 1530)
Bhutan	4·2 (4 to 4·4)	5·4 (5·2 to 5·6)	7·3 (7 to 7·7)	9·6 (9·1 to 10)	11 (10·5 to 11·4)	8·7 (8·4 to 9·1)	7·2 (6·7 to 7·6)	6·4 (5·9 to 7)	6·2 (5·6 to 6·7)
India	7910 (7370 to 8440)	9340 (8720 to 9980)	9860 (9380 to 10300)	11400 (10800 to 11900)	11300 (10800 to 11800)	12100 (11600 to 12600)	12100 (11600 to 12600)	11000 (10100 to 11900)	10600 (9480 to 11900)
Nepal	202 (192 to 211)	227 (219 to 236)	274 (266 to 281)	353 (341 to 365)	385 (374 to 395)	379 (366 to 391)	314 (294 to 335)	311 (280 to 343)	310 (278 to 343)
Pakistan	774 (740 to 807)	832 (803 to 858)	1220 (1180 to 1260)	1760 (1700 to 1820)	2040 (1970 to 2100)	2210 (2130 to 2290)	2720 (2590 to 2840)	3000 (2710 to 3300)	3000 (2680 to 3380)
Azad Jammu & Kashmir	20·2 (19·6 to 20·7)	20·2 (19·6 to 20·8)	27·5 (26·2 to 28·8)	38·9 (36·6 to 41)	41·5 (39·2 to 43·8)	38·2 (34·8 to 41·4)	41·7 (37·4 to 46·7)	40·1 (34·6 to 46·4)	38·5 (32·9 to 45·7)
Balochistan	25 (23·7 to 26·3)	20·6 (19·9 to 21·4)	38·9 (37·2 to 40·4)	79·9 (76·8 to 82·8)	99·1 (94·6 to 104)	114 (110 to 120)	161 (153 to 170)	213 (194 to 232)	222 (200 to 247)
Gilgit-Baltistan	5·3 (5 to 5·5)	5·3 (5·1 to 5·5)	8·1 (7·8 to 8·5)	12·5 (11·9 to 13)	14·7 (13·8 to 15·6)	17·4 (16·3 to 18·4)	22·8 (21·5 to 24·1)	27·4 (25 to 29·7)	28·1 (25·6 to 31)
Islamabad Capital Territory	2·4 (2·3 to 2·5)	2·8 (2·7 to 2·9)	3·9 (3·7 to 4·2)	5·9 (5·5 to 6·3)	7·6 (6·9 to 8·3)	11·1 (10 to 12·2)	17·7 (15·7 to 19·7)	20·9 (18·7 to 23·4)	21·3 (18·8 to 23·9)
Khyber Pakhtunkhwa	141 (135 to 148)	145 (140 to 149)	201 (193 to 209)	272 (263 to 282)	310 (299 to 320)	372 (358 to 388)	498 (473 to 521)	564 (510 to 616)	567 (508 to 635)
Punjab	444 (424 to 463)	476 (460 to 492)	675 (652 to 697)	954 (919 to 986)	1100 (1060 to 1130)	1150 (1110 to 1190)	1370 (1300 to 1440)	1490 (1350 to 1640)	1490 (1330 to 1670)
Sindh	136 (130 to 141)	162 (156 to 167)	265 (256 to 274)	399 (388 to 411)	464 (448 to 480)	512 (491 to 534)	610 (582 to 640)	643 (577 to 712)	639 (565 to 724)
Southeast Asia, east Asia, and Oceania	15100 (14400 to 15900)	17200 (16700 to 17700)	17900 (17600 to 18300)	15200 (14800 to 15700)	17100 (16400 to 17700)	13400 (12900 to 13800)	12400 (11800 to 12900)	12200 (11500 to 12900)	10800 (10000 to 11600)
East Asia	10900 (10300 to 11500)	11800 (11400 to 12100)	12100 (11800 to 12400)	8970 (8610 to 9340)	11000 (10400 to 11600)	7470 (7060 to 7880)	6270 (5820 to 6760)	6480 (6040 to 6950)	5180 (4730 to 5660)
China	10500 (9920 to 11100)	11300 (11000 to 11700)	11700 (11400 to 11900)	8560 (8200 to 8920)	10600 (9990 to 11200)	7080 (6670 to 7490)	5990 (5540 to 6480)	6240 (5810 to 6720)	4960 (4520 to 5430)
North Korea	207 (194 to 220)	245 (228 to 262)	237 (217 to 258)	220 (200 to 244)	269 (247 to 294)	254 (233 to 277)	190 (173 to 205)	155 (138 to 174)	146 (129 to 166)
Taiwan (province of China)	179 (175 to 184)	205 (202 to 208)	180 (175 to 185)	196 (189 to 203)	155 (148 to 161)	130 (122 to 136)	92·4 (87·3 to 98·5)	82·5 (76·5 to 89)	75·7 (67·4 to 84·7)
Oceania	58·4 (56·1 to 60·6)	72·5 (70 to 74·9)	81·9 (79·4 to 84·4)	92·8 (89·7 to 95·7)	108 (104 to 112)	133 (127 to 139)	166 (157 to 175)	198 (183 to 215)	205 (188 to 225)
American Samoa	0·5 (0·4 to 0·4)	0·4 (0·4 to 0·4)	0·5 (0·5 to 0·5)	0·5 (0·5 to 0·5)	0·9 (0·8 to 0·9)	0·8 (0·8 to 0·8)	0·6 (0·6 to 0·6)	0·4 (0·4 to 0·4)	0·4 (0·3 to 0·4)
Cook Islands	0·3 (0·3 to 0·3)	0·4 (0·4 to 0·4)	0·4 (0·4 to 0·4)	0·2 (0·2 to 0·2)	0·2 (0·2 to 0·2)	0·2 (0·1 to 0·2)	0·1 (0·1 to 0·1)	0·1 (0·1 to 0·1)	0·1 (0·1 to 0·1)
Fiji	5·6 (5·3 to 5·8)	7·6 (7·4 to 7·8)	7·8 (7·5 to 8)	9·1 (8·7 to 9·5)	9·1 (8·6 to 9·5)	8·6 (7·9 to 9·4)	9·4 (8·9 to 9·8)	8·4 (7·7 to 9·2)	8 (7·1 to 9·1)
Guam	0·8 (0·8 to 0·9)	1·2 (1·2 to 1·2)	1·4 (1·4 to 1·4)	1·4 (1·4 to 1·5)	1·9 (1·8 to 1·9)	1·8 (1·7 to 1·9)	1·7 (1·6 to 1·7)	1·4 (1·3 to 1·5)	1·3 (1·2 to 1·4)
Kiribati	0·6 (0·6 to 0·7)	0·8 (0·7 to 0·8)	0·8 (0·8 to 0·9)	1·1 (1 to 1·2)	1·3 (1·2 to 1·4)	1·3 (1·2 to 1·4)	1·4 (1·3 to 1·5)	1·4 (1·3 to 1·6)	1·4 (1·3 to 1·6)
Marshall Islands	0·3 (0·3 to 0·3)	0·3 (0·3 to 0·3)	0·4 (0·4 to 0·5)	0·6 (0·6 to 0·7)	0·7 (0·7 to 0·7)	0·8 (0·7 to 0·8)	0·7 (0·6 to 0·8)	0·6 (0·5 to 0·6)	0·6 (0·5 to 0·6)
Federated States of Micronesia	1 (1 to 1)	1 (1 to 1)	1·2 (1·1 to 1·2)	1·6 (1·5 to 1·6)	1·5 (1·4 to 1·6)	1·3 (1·2 to 1·4)	1·1 (1 to 1·2)	0·9 (0·8 to 1·1)	0·9 (0·8 to 1)
Nauru	0·1 (0 to 0·1)	0·1 (0·1 to 0·1)	0·1 (0·1 to 0·1)	0·2 (0·1 to 0·2)	0·2 (0·2 to 0·2)	0·2 (0·2 to 0·2)	0·2 (0·1 to 0·2)	0·1 (0·1 to 0·2)	0·1 (0·1 to 0·2)
Niue	0·1 (0·1 to 0·1)	0·1 (0·1 to 0·1)	0·1 (0·1 to 0·1)	0 (0 to 0)	0 (0 to 0)	0 (0 to 0)	0 (0 to 0)	0 (0 to 0)	0 (0 to 0)
Northern Mariana Islands	0·1 (0·1 to 0·1)	0·1 (0·1 to 0·1)	0·1 (0·1 to 0·1)	0·2 (0·2 to 0·2)	0·6 (0·5 to 0·7)	1·2 (1·1 to 1·3)	0·4 (0·4 to 0·5)	0·3 (0·3 to 0·3)	0·3 (0·2 to 0·3)
Palau	0·1 (0·1 to 0·1)	0·1 (0·1 to 0·1)	0·1 (0·1 to 0·1)	0·1 (0·1 to 0·1)	0·1 (0·1 to 0·2)	0·2 (0·1 to 0·2)	0·1 (0·1 to 0·1)	0·1 (0·1 to 0·1)	0·1 (0·1 to 0·1)
Papua New Guinea	39·2 (37·2 to 41·1)	47·9 (45·8 to 49·9)	54·1 (52 to 56·1)	59·9 (57·7 to 62)	70·6 (67·4 to 73·7)	92·4 (87·9 to 97·2)	124 (117 to 131)	157 (144 to 172)	164 (149 to 182)
Samoa	2·2 (2·1 to 2·2)	2·6 (2·5 to 2·6)	2·8 (2·7 to 2·9)	2·8 (2·6 to 2·9)	2·6 (2·5 to 2·8)	2·7 (2·5 to 2·9)	2·7 (2·5 to 2·9)	2·9 (2·7 to 3·2)	3 (2·7 to 3·2)
Solomon Islands	2·3 (2·2 to 2·3)	3 (2·9 to 3·1)	3·6 (3·5 to 3·8)	4·7 (4·5 to 4·9)	6·6 (6·3 to 6·9)	8·4 (8 to 8·8)	9·2 (8·6 to 9·8)	9·6 (8·9 to 10·4)	9·8 (9·1 to 10·6)
Tokelau	0 (0 to 0)	0 (0 to 0)	0 (0 to 0)	0 (0 to 0)	0 (0 to 0)	0 (0 to 0)	0 (0 to 0)	0 (0 to 0)	0 (0 to 0)
Tonga	1 (1 to 1·1)	1·4 (1·3 to 1·4)	1·7 (1·6 to 1·7)	1·8 (1·7 to 1·8)	1·6 (1·5 to 1·6)	1·5 (1·5 to 1·6)	1·5 (1·5 to 1·6)	1·4 (1·3 to 1·6)	1·4 (1·3 to 1·5)
Tuvalu	0·1 (0·1 to 0·1)	0·1 (0·1 to 0·1)	0·1 (0·1 to 0·1)	0·2 (0·2 to 0·2)	0·2 (0·2 to 0·2)	0·1 (0·1 to 0·1)	0·1 (0·1 to 0·1)	0·1 (0·1 to 0·1)	0·1 (0·1 to 0·1)
Vanuatu	1 (1 to 1·1)	1·4 (1·4 to 1·5)	1·9 (1·8 to 1·9)	2·4 (2·4 to 2·5)	3 (2·9 to 3·1)	3·5 (3·3 to 3·6)	3·9 (3·7 to 4·2)	4·2 (3·8 to 4·6)	4·2 (3·9 to 4·6)
Southeast Asia	4210 (4010 to 4400)	5340 (5160 to 5530)	5750 (5580 to 5900)	6160 (5990 to 6320)	5950 (5810 to 6110)	5750 (5600 to 5920)	5930 (5720 to 6150)	5510 (5170 to 5890)	5400 (4980 to 5870)
Cambodia	103 (97·3 to 108)	128 (123 to 134)	149 (140 to 158)	171 (164 to 178)	210 (204 to 216)	178 (173 to 184)	178 (169 to 186)	182 (168 to 196)	182 (167 to 197)
Indonesia	1760 (1680 to 1850)	2360 (2260 to 2470)	2520 (2440 to 2590)	2510 (2430 to 2590)	2260 (2180 to 2330)	2280 (2190 to 2380)	2340 (2220 to 2460)	2160 (1970 to 2380)	2130 (1910 to 2400)
Aceh	28·8 (27·3 to 30·3)	39·6 (38 to 41·3)	46·2 (44·8 to 47·6)	52·3 (50·6 to 54)	49·3 (47·2 to 51·3)	50·9 (48·8 to 53·2)	48·1 (45·8 to 50·3)	43·9 (40 to 48·2)	43·6 (39 to 49·1)
Bali	32·7 (30·9 to 34·5)	39·9 (38·2 to 41·6)	42·1 (40·6 to 43·7)	35·9 (34·4 to 37·6)	29·8 (28·4 to 31·2)	30·3 (28·7 to 32)	29·1 (27·4 to 30·9)	25·4 (22·7 to 28·3)	24·8 (22·1 to 28·3)
Bangka-Belitung Islands	7·5 (7 to 7·9)	9·5 (9 to 9·9)	10·6 (10·2 to 11·1)	12·3 (11·9 to 12·7)	11 (10·6 to 11·4)	11·4 (10·8 to 12·1)	13·3 (12·6 to 14)	13·3 (12·3 to 14·4)	13·2 (12 to 14·5)
Banten	39·6 (37·5 to 41·6)	50·9 (48·8 to 53)	56·5 (54·7 to 58·3)	74·4 (71·8 to 76·8)	114 (110 to 119)	123 (117 to 129)	117 (111 to 123)	103 (94·7 to 112)	101 (91·2 to 111)
Bengkulu	9 (8·6 to 9·3)	11·4 (11 to 11·8)	12·8 (12·5 to 13·2)	15·7 (15·2 to 16·2)	17·7 (17 to 18·5)	18·4 (17·6 to 19·3)	18·7 (17·8 to 19·7)	17·5 (16·1 to 19·2)	17·3 (15·7 to 19·3)
Gorontalo	5·9 (5·6 to 6·3)	9·3 (8·9 to 9·7)	11·4 (10·9 to 11·9)	9·8 (9·4 to 10·2)	8·3 (7·9 to 8·6)	9·5 (9·1 to 9·9)	11·2 (10·6 to 11·8)	10·6 (9·8 to 11·6)	10·5 (9·6 to 11·6)

Jakarta	48·8 (46·5 to 51)	82·2 (78·8 to 85·6)	100 (96·8 to 104)	112 (106 to 117)	105 (100 to 109)	96 (92·1 to 100)	90·9 (85·8 to 96·1)	69 (62·1 to 76·7)	64·9 (57·6 to 73·4)
Jambi	14·6 (13·8 to 15·4)	20·6 (19·7 to 21·5)	23·2 (22·3 to 24)	27·5 (26·7 to 28·3)	26·9 (25·7 to 28·1)	29·5 (28·1 to 31·1)	32·2 (30·7 to 33·7)	28·8 (26·2 to 31·4)	28·2 (25·3 to 31·6)
West Java	279 (266 to 291)	390 (373 to 408)	430 (416 to 443)	425 (409 to 441)	357 (343 to 372)	390 (374 to 406)	430 (406 to 454)	402 (366 to 440)	395 (356 to 439)
Central Java	325 (308 to 341)	432 (412 to 453)	436 (420 to 451)	394 (380 to 408)	322 (308 to 338)	279 (263 to 294)	267 (248 to 288)	247 (223 to 275)	243 (216 to 276)
East Java	378 (356 to 400)	486 (458 to 514)	468 (449 to 486)	404 (387 to 421)	318 (305 to 331)	290 (277 to 304)	276 (259 to 293)	229 (203 to 257)	221 (193 to 254)
West Kalimantan	28·6 (27·4 to 29·7)	39·3 (37·9 to 40·8)	46·5 (45·2 to 47·8)	53·6 (52·1 to 55·1)	47·2 (45·3 to 48·9)	47·9 (45·2 to 50·7)	52·6 (50·4 to 54·9)	54·6 (50·2 to 59·1)	55·4 (50·4 to 61·2)
South Kalimantan	26·1 (24·8 to 27·3)	36·5 (35·1 to 37·9)	38·7 (37·6 to 39·9)	36·7 (35·3 to 38·1)	33·6 (31·8 to 35·4)	37·1 (35·1 to 39·3)	38·8 (36·5 to 41·1)	32·9 (29·4 to 36·5)	31·8 (28·3 to 36·1)
Central Kalimantan	8·7 (8·2 to 9·2)	12·1 (11·6 to 12·7)	14·1 (13·5 to 14·6)	16·7 (16·2 to 17·3)	18·3 (17·5 to 19·1)	21·4 (20·4 to 22·5)	24·7 (23·5 to 25·9)	24·6 (22·4 to 26·8)	24·5 (22·1 to 27·4)
East Kalimantan	8·3 (7·9 to 8·8)	12·6 (12 to 13·1)	15·6 (15·1 to 16·1)	19·4 (18·8 to 20)	21·4 (20·6 to 22·4)	28·1 (26·8 to 29·6)	33·4 (31·9 to 34·9)	32·1 (29·4 to 35·1)	32·2 (28·8 to 36·1)
North Kalimantan	1·1 (1 to 1·1)	2 (1·9 to 2·1)	2·8 (2·6 to 2·9)	3·1 (3 to 3·2)	3·4 (3·1 to 3·7)	4·7 (4·3 to 5·1)	6·1 (5·6 to 6·6)	6·6 (5·9 to 7·4)	6·8 (6 to 7·7)
Riau Islands	3·5 (3·3 to 3·7)	4·2 (4 to 4·4)	4·7 (4·6 to 4·9)	7·4 (7·1 to 7·7)	12·8 (12·3 to 13·3)	16·4 (15·5 to 17·4)	20·8 (19·9 to 21·8)	18·1 (16·5 to 19·6)	17·4 (15·7 to 19·3)
Lampung	36·3 (34·4 to 38·1)	61·1 (58·4 to 63·7)	76·9 (74·8 to 78·9)	86·9 (83·8 to 90)	80·7 (77 to 84·4)	77·2 (72·8 to 82·2)	77·4 (72·7 to 82·4)	75·1 (66·6 to 84·6)	74·7 (65·6 to 86·1)
Maluku	12·5 (11·8 to 13·1)	14·6 (14·1 to 15·2)	15·3 (14·8 to 15·8)	17·5 (16·9 to 18)	18·8 (18·1 to 19·5)	19·8 (18·8 to 20·8)	20 (19·1 to 21)	21 (19·4 to 22·8)	21·6 (19·5 to 24)
North Maluku	6·6 (6·1 to 6·9)	7·6 (7·3 to 7·9)	8·3 (8 to 8·7)	9·9 (9·5 to 10·2)	11·3 (10·9 to 11·8)	12·5 (11·8 to 13·3)	12·8 (12·1 to 13·5)	13·3 (12·3 to 14·4)	13·6 (12·4 to 15)
West Nusa Tenggara	39·4 (37·3 to 41·4)	48·5 (46·7 to 50·4)	54·8 (53·4 to 56·3)	57·7 (56·1 to 59·4)	49·5 (47·6 to 51·6)	48 (45·5 to 50·7)	49·6 (46·8 to 52·6)	46·2 (41·5 to 50·9)	45·6 (40·7 to 51·1)
East Nusa Tenggara	39·8 (37·6 to 41·9)	48 (46·1 to 50)	51·2 (49·4 to 52·9)	50 (48·5 to 51·5)	49·5 (47·8 to 51·2)	57 (54·3 to 59·8)	58·7 (55·9 to 61·4)	60·1 (55·2 to 65·1)	61·2 (55·6 to 67·7)
Papua	4·6 (4·3 to 4·9)	7·8 (7·4 to 8·3)	10 (9·6 to 10·3)	12·1 (11·7 to 12·5)	15·2 (14·6 to 15·9)	24·6 (23·5 to 25·9)	39·5 (37·6 to 41·4)	45·4 (41·5 to 49·4)	45·7 (41·3 to 50·8)
West Papua	4·3 (4 to 4·5)	6·5 (6·3 to 6·8)	8·1 (7·9 to 8·4)	10·2 (9·9 to 10·5)	10·8 (10·4 to 11·2)	9·4 (8·9 to 9·9)	10·1 (9·6 to 10·7)	12·2 (11·3 to 13·2)	12·9 (11·7 to 14·2)
Riau	15·4 (14·8 to 15·9)	24·8 (23·9 to 25·7)	30·8 (29·9 to 31·7)	36·2 (35·2 to 37·2)	33·9 (32·5 to 35·3)	48·1 (45·7 to 50·9)	65·8 (62·6 to 69·1)	60·5 (55·3 to 66)	59·5 (53·8 to 66)
West Sulawesi	9·1 (8·5 to 9·7)	10·7 (10·3 to 11·2)	11·6 (11·2 to 12)	11·8 (11·5 to 12·2)	11·5 (11·2 to 12)	13·5 (12·9 to 14·3)	15·6 (14·9 to 16·3)	16 (14·9 to 17·2)	16·3 (14·9 to 17·9)
South Sulawesi	73·3 (69·3 to 77·1)	99·2 (95·3 to 103)	111 (108 to 114)	105 (102 to 109)	90·2 (86·4 to 93·9)	89·6 (85·2 to 94·2)	85·7 (81·1 to 90·6)	81·3 (73·1 to 89·8)	80·7 (72·2 to 90·9)
Central Sulawesi	12·9 (12·3 to 13·5)	18·1 (17·4 to 18·8)	21·5 (20·8 to 22·3)	24·2 (23·5 to 24·9)	23·8 (22·9 to 24·7)	29·2 (27·8 to 30·9)	31·2 (29·9 to 32·7)	28·7 (26·1 to 31·2)	28·5 (25·6 to 31·7)
Southeast Sulawesi	12 (11·4 to 12·5)	15·3 (14·8 to 15·9)	17·8 (17·3 to 18·3)	20·2 (19·6 to 20·8)	20·7 (20 to 21·4)	24·5 (23·2 to 25·9)	26·9 (25·4 to 28·4)	26·5 (24·2 to 28·9)	26·8 (24·2 to 29·9)
North Sulawesi	21·3 (20·4 to 22·2)	23·1 (22·2 to 24·2)	23 (22·2 to 23·8)	22·2 (21·2 to 23·1)	21·9 (20·9 to 22·8)	21·7 (20·6 to 22·8)	21 (19·9 to 22·1)	20·2 (18·3 to 22·2)	20 (18 to 22·4)
West Sumatra	55·6 (53·5 to 57·5)	62·7 (60·5 to 65)	61·5 (59·8 to 63·3)	63·9 (61·9 to 66)	55·1 (52·8 to 57·4)	54·7 (52·1 to 57·7)	52·3 (49·4 to 55·3)	51·1 (46·5 to 56·3)	51·3 (46·1 to 57·3)
South Sumatra	39·2 (37·3 to 41·1)	56·2 (54 to 58·4)	65 (63·2 to 66·9)	79·5 (76·9 to 82·1)	76·7 (73·4 to 80·2)	79·2 (75·5 to 83·2)	82·5 (77·9 to 87·4)	72·6 (65·6 to 80·4)	70·9 (63·2 to 79·9)
North Sumatra	93·4 (90·1 to 96·5)	128 (124 to 132)	146 (143 to 150)	167 (162 to 171)	161 (155 to 168)	162 (153 to 171)	162 (152 to 173)	157 (142 to 171)	156 (141 to 175)
Yogyakarta	44 (41·7 to 46·3)	50·6 (48·5 to 52·8)	45·6 (43·6 to 47·5)	36 (34·5 to 37·5)	27·3 (26·1 to 28·4)	23·4 (22·3 to 24·6)	22·4 (21·1 to 23·9)	18·3 (16·4 to 20·3)	17·5 (15·5 to 19·9)
Laos	40·9 (38·7 to 43·2)	50·6 (48·4 to 52·7)	61·4 (58·2 to 64·5)	71·7 (67·9 to 75·5)	84·3 (80 to 88·1)	94·1 (90·3 to 97·9)	84·4 (80·8 to 88·1)	87·2 (80·8 to 93·7)	86·6 (79·3 to 93·7)
Malaysia	148 (141 to 154)	167 (164 to 170)	173 (168 to 177)	204 (199 to 210)	238 (230 to 247)	254 (244 to 265)	238 (228 to 248)	233 (220 to 246)	229 (205 to 258)
Maldives	1·3 (1·2 to 1·4)	1·8 (1·7 to 1·9)	2·3 (2·2 to 2·4)	3·3 (3·2 to 3·3)	4·3 (4·1 to 4·4)	3 (2·8 to 3·1)	3·4 (3·2 to 3·6)	3·1 (2·8 to 3·3)	2·9 (2·6 to 3·3)
Mauritius	11·4 (11·2 to 11·7)	12·6 (12·4 to 12·8)	10·2 (9·9 to 10·6)	11·5 (11·1 to 11·9)	11·3 (10·5 to 12·1)	9·9 (9·5 to 10·3)	7·3 (7 to 7·6)	6·4 (6 to 6·8)	6·2 (5·5 to 7)
Myanmar	423 (398 to 447)	505 (484 to 526)	567 (545 to 589)	648 (616 to 678)	544 (515 to 573)	513 (492 to 535)	513 (494 to 533)	525 (490 to 563)	521 (477 to 568)
Philippines	450 (428 to 472)	597 (566 to 627)	709 (671 to 744)	849 (808 to 891)	968 (929 to 1010)	1070 (1020 to 1110)	1190 (1130 to 1260)	1060 (994 to 1140)	1050 (958 to 1130)
Seychelles	0·5 (0·5 to 0·5)	0·8 (0·8 to 0·8)	0·8 (0·8 to 0·9)	0·8 (0·8 to 0·9)	0·8 (0·8 to 0·8)	0·7 (0·7 to 0·7)	0·8 (0·8 to 0·8)	0·8 (0·7 to 0·8)	0·8 (0·7 to 0·8)
Sri Lanka	150 (142 to 159)	173 (167 to 179)	182 (174 to 191)	206 (196 to 216)	176 (167 to 185)	169 (159 to 181)	180 (171 to 192)	153 (142 to 164)	146 (130 to 164)
Thailand	472 (450 to 494)	594 (574 to 614)	596 (582 to 610)	576 (551 to 601)	503 (480 to 525)	422 (402 to 444)	386 (366 to 405)	292 (272 to 315)	278 (252 to 308)
Timor-Leste	10·6 (10·2 to 11)	12·7 (12·3 to 13)	11·9 (11·6 to 12·2)	13·7 (13·3 to 14·2)	17·8 (17·2 to 18·4)	17·8 (17·1 to 18·5)	16·7 (15·6 to 17·8)	18·8 (16·9 to 20·8)	19·8 (17·7 to 22)
Viet Nam	629 (599 to 658)	728 (700 to 754)	759 (729 to 787)	883 (854 to 910)	930 (900 to 962)	737 (708 to 770)	785 (749 to 821)	774 (713 to 839)	744 (681 to 822)
Sub-Saharan Africa	4300 (4110 to 4490)	5420 (5260 to 5580)	6760 (6580 to 6940)	8690 (8470 to 8890)	10900 (10600 to 11100)	13600 (13300 to 13900)	16700 (16200 to 17200)	18200 (17300 to 19200)	18400 (17300 to 19600)
Central sub-Saharan Africa	494 (477 to 510)	660 (641 to 679)	811 (788 to 832)	1010 (974 to 1040)	1260 (1220 to 1310)	1620 (1570 to 1670)	2060 (1970 to 2140)	2180 (2060 to 2300)	2180 (2050 to 2310)
Angola	119 (114 to 125)	133 (128 to 138)	146 (141 to 150)	184 (177 to 190)	248 (236 to 259)	349 (330 to 366)	488 (455 to 518)	576 (537 to 616)	591 (548 to 636)
Central African Republic	29·4 (27·8 to 30·9)	39·6 (37·5 to 41·6)	49·6 (47·3 to 51·5)	54·1 (51·8 to 56·4)	62·4 (59·3 to 65·4)	78 (73·7 to 82·1)	93·3 (86·2 to 100)	92·6 (85·7 to 99·6)	92·9 (85·8 to 100)
Congo (Brazzaville)	20·1 (18·9 to 21·2)	24·2 (23·2 to 25·1)	29·6 (28·6 to 30·6)	37·5 (35·9 to 39·1)	45·3 (43·8 to 46·8)	58·7 (56·8 to 60·6)	73·8 (71·3 to 76·3)	65·2 (60·1 to 70·7)	63·2 (57·6 to 69·3)
DR Congo	312 (300 to 322)	448 (435 to 460)	568 (552 to 584)	710 (684 to 734)	880 (849 to 911)	1100 (1070 to 1130)	1360 (1310 to 1410)	1410 (1310 to 1500)	1390 (1280 to 1510)
Equatorial Guinea	4·7 (4·5 to 4·9)	4·9 (4·7 to 5·1)	5·4 (5·2 to 5·6)	7 (6·7 to 7·3)	9·9 (9·4 to 10·4)	13·6 (12·6 to 14·6)	18 (16·5 to 19·5)	18·4 (16·2 to 20·7)	18·1 (15·8 to 20·8)
Gabon	9·3 (8·7 to 9·8)	10·7 (10·2 to 11·2)	12·1 (11·5 to 12·7)	14·5 (13·6 to 15·4)	17·6 (16·2 to 18·9)	20·7 (18·8 to 22·6)	23·2 (21·1 to 25·3)	21·7 (19 to 24·7)	21·2 (18·4 to 24·5)
Eastern sub-Saharan Africa	1640 (1570 to 1710)	2100 (2040 to 2150)	2620 (2560 to 2690)	3450 (3380 to 3530)	4410 (4310 to 4500)	5490 (5360 to 5620)	6330 (6120 to 6530)	6660 (6250 to 7100)	6730 (6240 to 7260)
Burundi	60·4 (57·7 to 62·9)	71·2 (68·5 to 73·5)	87 (84·2 to 89·5)	105 (102 to 107)	127 (124 to 130)	133 (129 to 136)	197 (191 to 203)	224 (208 to 240)	231 (213 to 251)
Comoros	3·2 (2·9 to 3·4)	4·5 (4·2 to 4·7)	6·5 (6·3 to 6·6)	8·3 (8·1 to 8·6)	9·8 (9·4 to 10·2)	9·8 (9·1 to 10·5)	9·3 (8·5 to 10·1)	8·5 (7·5 to 9·6)	8·4 (7·3 to 9·6)
Djibouti	1·2 (1·1 to 1·3)	1·8 (1·7 to 1·9)	2·8 (2·7 to 3)	4·5 (4·3 to 4·8)	6·6 (6·1 to 7)	9 (8·3 to 9·8)	12 (11·1 to 13)	12·4 (11 to 13·9)	12·1 (10·6 to 13·6)
Eritrea	26·2 (24·8 to 27·6)	35·9 (34·6 to 37·3)	45·4 (43·8 to 46·9)	57·4 (54·9 to 59·7)	71·3 (67·7 to 74·8)	72 (66·5 to 77·3)	86·8 (77·7 to 96·1)	93·4 (83·6 to 104)	94·5 (83·9 to 106)
Ethiopia	416 (392 to 438)	518 (497 to 537)	635 (612 to 656)	862 (827 to 894)	1170 (1140 to 1210)	1480 (1430 to 1530)	1530 (1450 to 1600)	1660 (1550 to 1780)	1700 (1570 to 1840)
Addis Ababa	13 (12·3 to 13·8)	18·4 (17·7 to 19·2)	25·7 (24·6 to 26·8)	34·9 (33 to 36·9)	29·8 (27·9 to 31·6)	26·7 (25·4 to 28·2)	27·1 (25·1 to 29·1)	24·6 (22·1 to 27·5)	23 (20·3 to 25·9)
Afar	9·2 (8·7 to 9·6)	5·9 (5·7 to 6·1)	4·5 (4·3 to 4·6)	6·6 (6·3 to 6·9)	17·8 (17 to 18·6)	31·9 (30·5 to 33·3)	30·8 (29·2 to 32·4)	30·3 (28·3 to 32·5)	30·1 (28 to 32·6)

Amhara	123 (115 to 130)	155 (148 to 161)	186 (179 to 194)	245 (234 to 254)	315 (305 to 324)	347 (334 to 360)	300 (284 to 316)	303 (279 to 329)	307 (281 to 336)
Benishangul-Gumuz	8·2 (7·7 to 8·7)	9·6 (9·2 to 10)	10·1 (9·7 to 10·5)	11·4 (10·9 to 11·9)	12 (11·6 to 12·4)	13·5 (13 to 14·1)	16·5 (15·5 to 17·4)	18·5 (17·2 to 19·8)	18·9 (17·4 to 20·4)
Dire Dawa	2·3 (2·2 to 2·5)	3·8 (3·7 to 4)	5·5 (5·3 to 5·7)	6·5 (6·1 to 6·8)	5·8 (5·5 to 6·1)	5·5 (5·2 to 5·8)	5·8 (5·4 to 6·3)	5·5 (4·9 to 6)	5·3 (4·8 to 5·9)
Gambella	1·9 (1·8 to 2·1)	2·8 (2·6 to 2·9)	3·4 (3·2 to 3·5)	3·8 (3·6 to 4)	4 (3·8 to 4·2)	5 (4·8 to 5·2)	5·6 (5·3 to 6)	5·8 (5·2 to 6·4)	5·9 (5·3 to 6·6)
Harari	1·1 (1·1 to 1·2)	1·6 (1·6 to 1·7)	2·2 (2·1 to 2·3)	2·6 (2·4 to 2·7)	2·7 (2·5 to 2·8)	3 (2·8 to 3·1)	3·2 (3 to 3·4)	3·1 (2·8 to 3·4)	3 (2·7 to 3·3)
Oromia	126 (119 to 132)	148 (142 to 153)	176 (170 to 182)	250 (240 to 259)	392 (380 to 404)	555 (535 to 574)	609 (579 to 638)	688 (641 to 739)	711 (661 to 765)
Somali	20·7 (19·8 to 21·4)	22·5 (21·8 to 23·2)	26·2 (25·2 to 27)	40·6 (39·3 to 41·8)	67·7 (65·3 to 70)	99·5 (96·1 to 103)	118 (112 to 123)	129 (122 to 136)	130 (124 to 138)
Southern Nations, Nationalities, and Peoples	81·7 (77·3 to 85·8)	115 (110 to 119)	151 (146 to 156)	205 (197 to 212)	260 (251 to 268)	309 (299 to 319)	334 (313 to 354)	371 (342 to 400)	381 (350 to 417)
Tigray	29·3 (27·6 to 31)	35·5 (34·1 to 36·9)	44 (42·3 to 45·6)	56·2 (53·9 to 58·3)	67·8 (65·9 to 69·7)	80·8 (78·5 to 83·3)	78·5 (74·9 to 82·1)	82·3 (75·6 to 89·4)	83·8 (76 to 91·8)
Kenya	152 (148 to 156)	228 (222 to 232)	299 (293 to 305)	398 (388 to 407)	486 (469 to 503)	594 (564 to 623)	664 (615 to 712)	602 (540 to 669)	580 (515 to 656)
Baringo	2·4 (2·3 to 2·4)	3·7 (3·6 to 3·7)	4·6 (4·5 to 4·7)	5·6 (5·5 to 5·7)	7·3 (7·1 to 7·5)	8·5 (8 to 8·9)	8·4 (7·7 to 9·1)	7·6 (6·7 to 8·5)	7·3 (6·4 to 8·4)
Bomet	2·4 (2·3 to 2·4)	3·3 (3·2 to 3·3)	4·4 (4·3 to 4·4)	5·7 (5·5 to 5·8)	7·6 (7·3 to 7·9)	10·5 (10 to 11)	17 (15·6 to 18·4)	12·4 (10·9 to 14)	11·2 (9·8 to 12·8)
Bungoma	4·8 (4·8 to 4·9)	7·2 (7·1 to 7·3)	10 (9·8 to 10·1)	14·1 (13·8 to 14·3)	20·4 (19·7 to 21·1)	36·5 (34·9 to 38·2)	28·9 (27·2 to 30·5)	29·6 (26·9 to 32·5)	29·9 (27 to 33·2)
Busia	3·7 (3·6 to 3·8)	5·1 (5 to 5·2)	6 (5·9 to 6·2)	8·7 (8·5 to 8·9)	11·6 (11·2 to 12)	12·6 (11·9 to 13·3)	14 (12·8 to 15·2)	12·3 (11 to 13·8)	12 (10·6 to 13·7)
Elgeyo Marakwet	2·8 (2·7 to 2·8)	4·6 (4·5 to 4·7)	5 (5 to 5·1)	4 (3·9 to 4·1)	5·7 (5·5 to 5·9)	9·4 (8·9 to 9·9)	5·4 (4·8 to 5·9)	4·8 (4·2 to 5·4)	4·8 (4·3 to 5·6)
Embu	2·4 (2·3 to 2·4)	3·3 (3·2 to 3·3)	4·3 (4·2 to 4·4)	6·1 (5·9 to 6·2)	7·4 (7·1 to 7·8)	8·1 (7·6 to 8·7)	7 (6·2 to 7·8)	5·5 (4·7 to 6·3)	5·2 (4·5 to 6)
Garissa	0·8 (0·7 to 0·8)	1·3 (1·2 to 1·3)	2 (2 to 2·1)	3·1 (3 to 3·2)	3·2 (3 to 3·3)	7·5 (7·2 to 7·8)	14·4 (13·7 to 15)	17·9 (16·7 to 19·2)	18·4 (17·1 to 19·9)
Homa Bay	9·3 (9·1 to 9·4)	11·6 (11·3 to 11·8)	12·4 (12·1 to 12·7)	13·9 (13·6 to 14·2)	16·6 (16·1 to 17·1)	19·2 (18·3 to 20·1)	19·6 (18·4 to 20·7)	16·5 (14·8 to 18·2)	15·3 (13·7 to 17·1)
Isiolo	0·4 (0·4 to 0·5)	0·7 (0·7 to 0·7)	0·9 (0·8 to 0·9)	1·2 (1·2 to 1·3)	1·7 (1·6 to 1·7)	2·2 (2·1 to 2·3)	2·6 (2·4 to 2·8)	3·5 (3·1 to 4)	3·7 (3·2 to 4·2)
Kajiado	1·2 (1·1 to 1·2)	1·7 (1·7 to 1·7)	2·5 (2·4 to 2·5)	4·2 (4·1 to 4·2)	6·7 (6·5 to 7)	10·6 (10 to 11·2)	17 (15·8 to 18·2)	22·4 (20·1 to 24·8)	22·9 (20·4 to 25·7)
Kakamega	7 (6·9 to 7·1)	11·3 (11·1 to 11·4)	15·1 (14·8 to 15·4)	20·3 (19·9 to 20·7)	25·9 (25 to 26·8)	20·2 (19·1 to 21·3)	29·4 (27·2 to 31·6)	23·1 (20·6 to 25·8)	21·7 (19·2 to 24·6)
Kericho	2·7 (2·7 to 2·7)	5·6 (5·5 to 5·6)	8·5 (8·4 to 8·6)	11 (10·8 to 11·2)	14·3 (13·8 to 14·7)	16·8 (16 to 17·6)	12·1 (11·2 to 12·9)	14·8 (13·2 to 16·4)	15·4 (13·7 to 17·2)
Kiambu	5 (4·8 to 5·3)	7·2 (6·9 to 7·4)	10·9 (10·6 to 11·2)	16 (15·4 to 16·6)	18·1 (17 to 19·2)	25·2 (23·4 to 27·1)	25·2 (22·6 to 27·7)	25·8 (22·1 to 30·1)	25·4 (21·7 to 30·1)
Kilifi	4·8 (4·6 to 5)	7·2 (6·9 to 7·4)	9·1 (8·9 to 9·4)	13 (12·6 to 13·3)	15·5 (14·7 to 16·1)	19·8 (18·7 to 21)	23·3 (21·4 to 25·1)	22·8 (20·1 to 25·8)	22 (19·3 to 25·2)
Kirinyaga	3 (2·9 to 3)	4·6 (4·5 to 4·7)	5·7 (5·6 to 5·9)	6·3 (6·1 to 6·6)	6·3 (5·9 to 6·7)	7 (6·4 to 7·6)	6·8 (6·1 to 7·5)	4·8 (4·1 to 5·6)	4·4 (3·8 to 5·2)
Kisii	5·3 (5·2 to 5·5)	9·8 (9·6 to 9·9)	13·4 (13·2 to 13·7)	18·1 (17·7 to 18·4)	20·9 (20·1 to 21·7)	25 (23·8 to 26·2)	21·2 (19·3 to 23)	15 (13·3 to 16·8)	13·6 (12 to 15·4)
Kisumu	6·8 (6·6 to 6·9)	10·2 (9·9 to 10·4)	12·1 (11·8 to 12·3)	13·4 (13·1 to 13·6)	16·9 (16·2 to 17·5)	17·3 (16·1 to 18·3)	17·3 (15·6 to 18·9)	15·1 (13·4 to 16·9)	14·4 (12·8 to 16·1)
Kitui	5·8 (5·7 to 6)	8·2 (8 to 8·4)	10 (9·8 to 10·2)	12·2 (11·9 to 12·5)	15 (14·4 to 15·7)	17 (16·1 to 17·9)	16·9 (15·4 to 18·2)	14·4 (12·8 to 16)	13·7 (12·2 to 15·5)
Kwale	3 (2·9 to 3·1)	5·1 (4·9 to 5·2)	6·4 (6·2 to 6·6)	7·9 (7·6 to 8·1)	8·9 (8·5 to 9·4)	11·1 (10·4 to 11·7)	13·1 (12 to 14·2)	13·4 (11·9 to 15)	13·1 (11·6 to 15)
Laikipia	0·7 (0·7 to 0·7)	1 (1 to 1)	1·7 (1·6 to 1·7)	3 (2·9 to 3·1)	4·3 (4 to 4·5)	6·5 (6·1 to 6·9)	6·1 (5·5 to 6·7)	5 (4·3 to 5·7)	4·7 (4·1 to 5·5)
Lamu	0·4 (0·4 to 0·4)	0·5 (0·5 to 0·5)	0·6 (0·6 to 0·7)	1 (1 to 1)	1·2 (1·2 to 1·3)	1·4 (1·3 to 1·5)	1·5 (1·4 to 1·6)	1·4 (1·2 to 1·6)	1·3 (1·1 to 1·6)
Machakos	4·3 (4·2 to 4·5)	7·3 (7·1 to 7·5)	10·9 (10·6 to 11·2)	16 (15·5 to 16·5)	18·8 (17·9 to 19·7)	19·4 (18·2 to 20·5)	15·3 (13·7 to 16·8)	13·7 (12 to 15·7)	13·1 (11·3 to 15·3)
Makueni	4·9 (4·8 to 5)	5·9 (5·8 to 6)	7·1 (6·9 to 7·2)	8·5 (8·3 to 8·7)	9·7 (9·3 to 10·1)	10·5 (9·8 to 11·2)	12·4 (11·2 to 13·7)	8·9 (7·7 to 10·3)	8·1 (6·9 to 9·5)
Mandera	1·3 (1·3 to 1·4)	2·1 (2 to 2·1)	2·7 (2·6 to 2·7)	2·8 (2·7 to 2·9)	3·4 (3·3 to 3·5)	7·7 (7·4 to 7·9)	24·6 (23·7 to 25·4)	21·9 (21·2 to 22·7)	20·6 (19·9 to 21·2)
Marsabit	0·5 (0·5 to 0·6)	0·8 (0·8 to 0·8)	1·3 (1·3 to 1·4)	2·3 (2·3 to 2·4)	2·8 (2·7 to 2·9)	3·7 (3·5 to 3·9)	5·3 (4·9 to 5·6)	6·9 (6·2 to 7·6)	7 (6·3 to 7·9)
Meru	6·9 (6·7 to 7·1)	11·2 (10·9 to 11·4)	14·9 (14·6 to 15·1)	19·4 (19 to 19·8)	23·9 (23 to 24·8)	28·3 (26·7 to 29·8)	27·4 (25·1 to 29·7)	18·6 (16·5 to 20·9)	16·6 (14·6 to 19·1)
Migori	5·3 (5·2 to 5·5)	7·3 (7·1 to 7·5)	8·7 (8·4 to 8·9)	10·7 (10·5 to 11)	14·2 (13·8 to 14·6)	18·5 (17·8 to 19·3)	20·9 (19·9 to 21·9)	19·6 (18 to 21·3)	18·9 (17·3 to 20·7)
Mombasa	2·5 (2·4 to 2·6)	4·7 (4·5 to 4·9)	6 (5·8 to 6·3)	6·6 (6·3 to 7)	7·8 (7·3 to 8·2)	9·8 (8·9 to 10·6)	11·9 (10·5 to 13·3)	8·9 (7·5 to 10·6)	8·1 (6·8 to 9·9)
Murang'a	7·1 (6·9 to 7·3)	9·5 (9·2 to 9·7)	11·6 (11·4 to 11·8)	14·5 (14·1 to 14·9)	16·5 (15·6 to 17·4)	12·3 (11·6 to 13·2)	11·3 (10·2 to 12·4)	7·9 (7 to 8·9)	7·3 (6·5 to 8·3)
Nairobi	4·7 (4·5 to 4·8)	8·1 (7·8 to 8·3)	13·3 (12·7 to 13·9)	26 (24·2 to 27·8)	22·2 (20·4 to 24)	32·2 (28·9 to 35·4)	41·3 (35·7 to 47·4)	33·2 (28·5 to 39)	31 (26·3 to 37·1)
Nakuru	2·8 (2·7 to 2·9)	4·8 (4·6 to 4·9)	7·8 (7·7 to 7·9)	12·5 (12·2 to 12·9)	17·7 (16·9 to 18·6)	21·4 (20 to 22·8)	20·4 (18·2 to 22·7)	17·7 (15·2 to 20·5)	17·1 (14·5 to 20·2)
Nandi	2·8 (2·7 to 2·9)	4·3 (4·2 to 4·4)	5·9 (5·8 to 6)	7·5 (7·3 to 7·6)	10 (9·7 to 10·4)	12·3 (11·6 to 12·9)	12·9 (11·7 to 14)	11·5 (10·2 to 12·8)	11·1 (9·8 to 12·6)
Narok	1·9 (1·8 to 1·9)	2·7 (2·7 to 2·8)	3·6 (3·6 to 3·7)	6·1 (6 to 6·2)	10·4 (10·1 to 10·7)	9·5 (9·1 to 9·9)	19·3 (18·1 to 20·6)	20·5 (18·7 to 22·2)	20·1 (18·3 to 21·9)
Nyamira	3 (3 to 3·1)	3·9 (3·8 to 4)	4·5 (4·4 to 4·6)	5·1 (5 to 5·3)	5·5 (5·3 to 5·7)	6·6 (6·2 to 7)	7·9 (7·2 to 8·6)	6·2 (5·4 to 7·1)	5·8 (5 to 6·7)
Nyandarua	1·9 (1·9 to 1·9)	3·1 (3 to 3·2)	4·2 (4·2 to 4·3)	5·1 (5 to 5·2)	7·1 (6·7 to 7·4)	8·1 (7·5 to 8·8)	6·6 (5·9 to 7·3)	4·1 (3·5 to 4·8)	3·7 (3·1 to 4·4)
Nyeri	4·7 (4·5 to 4·8)	5·6 (5·4 to 5·8)	7·1 (6·9 to 7·4)	9·5 (9·1 to 9·9)	10 (9·4 to 10·7)	8·7 (7·9 to 9·6)	6·5 (5·7 to 7·4)	4·4 (3·7 to 5·2)	4 (3·4 to 4·8)
Samburu	0·8 (0·8 to 0·8)	1·5 (1·5 to 1·6)	1·9 (1·9 to 2)	2·1 (2 to 2·1)	3 (2·9 to 3·1)	3·6 (3·4 to 3·8)	4·7 (4·4 to 5)	5·6 (5·1 to 6·1)	5·7 (5·2 to 6·2)
Siaya	5·4 (5·3 to 5·6)	8·6 (8·4 to 8·8)	10·4 (10·2 to 10·6)	12·7 (12·5 to 13)	15·4 (15 to 15·9)	16·7 (16 to 17·5)	16·6 (15·5 to 17·6)	14·9 (13·5 to 16·2)	14·4 (13 to 15·8)
Taita Taveta	1·4 (1·4 to 1·4)	2·2 (2·1 to 2·3)	2·7 (2·6 to 2·8)	3·2 (3·1 to 3·4)	4·2 (4 to 4·4)	4·3 (3·9 to 4·6)	4 (3·5 to 4·4)	3·2 (2·7 to 3·7)	3·1 (2·6 to 3·6)
Tana River	0·8 (0·7 to 0·8)	1 (1 to 1·1)	1·4 (1·4 to 1·5)	2·4 (2·3 to 2·5)	2·8 (2·7 to 3)	4·1 (3·9 to 4·3)	4·9 (4·6 to 5·2)	5·4 (4·9 to 6)	5·5 (4·9 to 6·1)
Tharaka Nithi	1·7 (1·7 to 1·8)	1·8 (1·8 to 1·9)	1·8 (1·8 to 1·9)	1·9 (1·8 to 1·9)	2 (1·9 to 2·1)	2 (1·8 to 2·1)	2·1 (1·9 to 2·3)	3·6 (3·1 to 4·2)	4 (3·4 to 4·7)
Trans Nzoia	1 (1 to 1)	1·7 (1·6 to 1·7)	3·3 (3·2 to 3·3)	6·8 (6·7 to 7)	9·4 (9 to 9·7)	12·4 (11·7 to 13·1)	13·6 (12·4 to 14·7)	11·1 (9·8 to 12·5)	10·4 (9·1 to 11·9)
Turkana	2 (1·9 to 2·1)	3·9 (3·8 to 4)	5 (4·9 to 5·2)	4·4 (4·2 to 4·5)	5·6 (5·4 to 5·8)	11 (10·5 to 11·5)	18·2 (17·1 to 19·2)	16·7 (15 to 18·4)	15·8 (14·2 to 17·7)
Uasin Gishu	1·8 (1·8 to 1·8)	3·3 (3·2 to 3·3)	5·3 (5·3 to 5·4)	8 (7·8 to 8·1)	10·9 (10·5 to 11·3)	14·6 (13·9 to 15·4)	17·8 (16·2 to 19·4)	17·3 (15·2 to 19·4)	16·7 (14·6 to 19·1)
Vihiga	5·4 (5·2 to 5·5)	6·5 (6·3 to 6·6)	6·8 (6·6 to 7)	6·6 (6·4 to 6·8)	8 (7·7 to 8·3)	6·9 (6·3 to 7·5)	7·3 (6·6 to 8·2)	4·6 (3·9 to 5·4)	4·2 (3·5 to 5)

Wajir	1·2 (1·1 to 1·2)	1·6 (1·5 to 1·6)	2·5 (2·4 to 2·5)	3·7 (3·5 to 3·7)	3·3 (3·2 to 3·4)	9·2 (9 to 9·5)	15 (14·5 to 15·5)	18·9 (18·1 to 19·7)	19·6 (18·8 to 20·4)
West Pokot	1·2 (1·2 to 1·3)	1·7 (1·7 to 1·8)	2·6 (2·5 to 2·6)	4·6 (4·5 to 4·7)	5·9 (5·7 to 6·1)	7·5 (7·1 to 7·9)	9·3 (8·6 to 10·1)	8·4 (7·5 to 9·3)	8 (7·1 to 8·9)
Madagascar	113 (108 to 117)	137 (133 to 141)	168 (163 to 173)	208 (203 to 213)	256 (250 to 263)	337 (327 to 347)	417 (402 to 434)	436 (408 to 464)	427 (397 to 461)
Malawi	63·6 (59·2 to 67·8)	88·2 (83·6 to 92·7)	115 (110 to 120)	171 (166 to 175)	250 (244 to 255)	272 (266 to 278)	295 (284 to 306)	283 (258 to 310)	282 (251 to 314)
Mozambique	166 (160 to 171)	188 (179 to 196)	226 (219 to 233)	293 (286 to 301)	306 (299 to 314)	405 (395 to 414)	480 (466 to 493)	533 (498 to 570)	543 (499 to 591)
Rwanda	67 (64·7 to 69·1)	79·9 (78 to 81·8)	98·4 (96·4 to 100)	131 (129 to 133)	156 (152 to 160)	171 (167 to 174)	175 (168 to 183)	180 (169 to 193)	183 (169 to 198)
Somalia	52·4 (50·5 to 54·1)	68·6 (66·9 to 70·1)	94·2 (91·8 to 96·4)	160 (156 to 164)	188 (182 to 193)	243 (233 to 252)	344 (333 to 355)	433 (416 to 452)	462 (442 to 483)
South Sudan	53·7 (49·9 to 57·2)	66·4 (62·9 to 70)	82·6 (78 to 86·7)	99 (93·4 to 104)	122 (116 to 129)	157 (147 to 166)	198 (186 to 210)	172 (160 to 185)	186 (171 to 200)
Uganda	167 (162 to 172)	212 (207 to 217)	270 (265 to 275)	345 (338 to 352)	458 (451 to 465)	617 (606 to 627)	730 (708 to 751)	776 (734 to 821)	779 (729 to 834)
Tanzania	235 (227 to 243)	298 (289 to 306)	365 (355 to 375)	462 (447 to 476)	598 (581 to 615)	761 (738 to 783)	894 (847 to 935)	939 (873 to 1010)	938 (868 to 1010)
Zambia	64·8 (62·4 to 67)	98·6 (96·2 to 101)	125 (123 to 128)	148 (144 to 151)	192 (186 to 198)	233 (224 to 241)	294 (282 to 306)	306 (282 to 331)	300 (273 to 330)
Southern sub-Saharan Africa	385 (359 to 411)	468 (443 to 493)	591 (564 to 618)	742 (717 to 769)	798 (776 to 822)	808 (784 to 832)	891 (865 to 916)	828 (784 to 878)	805 (747 to 868)
Botswana	9·4 (8·9 to 9·8)	12 (11·5 to 12·4)	15·3 (14·6 to 15·9)	19·9 (18·9 to 20·8)	22·8 (21·2 to 24·3)	23·5 (21·5 to 25·5)	23·8 (21·6 to 26·2)	24 (21·3 to 26·9)	23·9 (21·1 to 27·1)
Eswatini	6·1 (5·8 to 6·4)	8·4 (8·1 to 8·6)	10·9 (10·6 to 11·1)	13·6 (13·2 to 14)	16 (15·4 to 16·5)	17·7 (17·2 to 18·3)	17·1 (16·4 to 17·8)	14·9 (13·9 to 16·1)	14·4 (13·2 to 15·8)
Lesotho	14·4 (13·5 to 15·3)	17·6 (16·6 to 18·5)	22 (21·3 to 22·7)	27·1 (26·3 to 27·9)	26·5 (25·7 to 27·3)	25·2 (24·5 to 26)	23·8 (22·7 to 24·9)	21·3 (19·1 to 23·7)	20·8 (18·5 to 23·6)
Namibia	10·7 (10·2 to 11·2)	13·7 (13·2 to 14·2)	16·5 (16 to 17)	19·8 (19·2 to 20·5)	25·1 (24·5 to 25·9)	27·9 (26·9 to 28·9)	30·9 (29·7 to 32·3)	28·9 (26·8 to 31·3)	28·6 (26·3 to 31·4)
South Africa	279 (258 to 299)	330 (310 to 350)	407 (385 to 429)	493 (472 to 516)	515 (496 to 534)	504 (485 to 524)	559 (539 to 580)	507 (478 to 540)	485 (449 to 523)
Eastern Cape	74·2 (69 to 79·2)	73·3 (68·8 to 77·9)	70·1 (66·3 to 73·7)	84·6 (80·6 to 89)	87 (83·7 to 90·5)	73 (69·8 to 76·2)	69·6 (66·8 to 72·4)	60 (56·8 to 63·6)	57·2 (53·2 to 61·4)
Free State	22·3 (20·6 to 24)	27·6 (25·7 to 29·4)	30·9 (29·2 to 32·5)	34·6 (33 to 36·4)	30·5 (29·3 to 31·7)	28 (26·8 to 29·2)	27·8 (26·6 to 28·9)	22·2 (20·8 to 23·7)	20·8 (19·2 to 22·5)
Gauteng	33 (29·5 to 36·5)	46·6 (42·8 to 50·6)	61·8 (57·4 to 66·3)	71·1 (67·3 to 75·1)	78·7 (75·4 to 82·2)	96·7 (92·6 to 101)	134 (130 to 139)	130 (122 to 139)	124 (114 to 134)
KwaZulu-Natal	58·1 (54·2 to 61·9)	70·9 (66·9 to 74·9)	94·2 (89·4 to 99)	111 (106 to 116)	114 (110 to 119)	110 (106 to 114)	114 (109 to 118)	101 (94·4 to 108)	96·8 (89·3 to 104)
Limpopo	27 (25·5 to 28·5)	32·8 (31·2 to 34·3)	53·9 (51·6 to 56·1)	74·7 (71·9 to 77·9)	75·9 (73 to 78·7)	62·9 (60·5 to 65·5)	62·7 (60·1 to 65·3)	57·5 (54·4 to 60·9)	55·9 (52 to 60)
Mpumalanga	21·6 (20·4 to 22·7)	24·6 (23·4 to 25·7)	25·8 (24·6 to 26·9)	32·9 (31·4 to 34·4)	38 (36·4 to 39·6)	38 (36·2 to 39·9)	46·1 (44·4 to 48)	46·7 (43·9 to 49·9)	46·3 (43 to 50)
North West	16·6 (15·5 to 17·7)	22 (20·7 to 23·2)	30 (28·3 to 31·5)	37·1 (35·6 to 38·8)	40·6 (39·1 to 42·2)	41·4 (39·7 to 43·1)	38·6 (37·2 to 40)	28 (26·3 to 29·9)	26 (24·1 to 28)
Northern Cape	8·2 (7·5 to 8·8)	9·5 (8·9 to 10·1)	10·6 (10·1 to 11·2)	11·7 (11·1 to 12·2)	10·3 (9·9 to 10·7)	8·5 (8·1 to 8·8)	11·4 (10·9 to 11·8)	11·9 (11·2 to 12·8)	11·8 (10·8 to 12·8)
Western Cape	17·9 (15·9 to 20)	22·6 (20·7 to 24·6)	29·7 (27·4 to 32)	35·3 (33·5 to 37·3)	39·3 (37·7 to 40·9)	46 (44 to 47·9)	54·9 (52·9 to 56·8)	49·4 (46·2 to 53)	46·8 (43·1 to 50·7)
Zimbabwe	65·9 (63 to 68·7)	86·4 (83·5 to 89·3)	120 (116 to 124)	169 (166 to 172)	194 (190 to 198)	209 (205 to 214)	236 (230 to 241)	232 (219 to 247)	231 (215 to 249)
Western sub-Saharan Africa	1780 (1700 to 1860)	2200 (2130 to 2270)	2740 (2660 to 2810)	3490 (3400 to 3570)	4390 (4290 to 4480)	5720 (5570 to 5860)	7440 (7230 to 7630)	8530 (8150 to 8960)	8700 (8210 to 9220)
Benin	45·4 (42·6 to 48·1)	52·7 (50·5 to 54·7)	64·6 (62·5 to 66·6)	83·3 (81·1 to 85·5)	115 (112 to 118)	150 (147 to 154)	202 (195 to 209)	246 (231 to 260)	254 (238 to 272)
Burkina Faso	91·1 (86·4 to 95·8)	116 (111 to 120)	148 (144 to 152)	185 (181 to 189)	225 (220 to 230)	291 (282 to 299)	375 (361 to 388)	448 (424 to 472)	465 (438 to 494)
Cabo Verde	3·4 (3·2 to 3·6)	4·5 (4·4 to 4·6)	4·6 (4·5 to 4·8)	5·3 (5·1 to 5·5)	6·1 (5·9 to 6·4)	6·2 (5·8 to 6·6)	5·6 (4·9 to 6·4)	4·4 (3·9 to 4·9)	4·2 (3·6 to 4·7)
Cameroon	106 (99·1 to 112)	134 (127 to 141)	163 (159 to 168)	192 (188 to 196)	232 (228 to 238)	309 (302 to 316)	441 (425 to 457)	500 (461 to 539)	503 (458 to 554)
Chad	61·6 (58·9 to 64·1)	71·3 (69·2 to 73·2)	89·3 (87·1 to 91·4)	118 (114 to 120)	156 (153 to 159)	213 (209 to 217)	292 (284 to 299)	391 (378 to 404)	418 (403 to 434)
Côte d'Ivoire	61·7 (58·4 to 64·8)	101 (97·2 to 105)	143 (139 to 146)	197 (192 to 202)	270 (264 to 275)	336 (326 to 345)	414 (399 to 429)	460 (430 to 491)	464 (429 to 501)
The Gambia	5·2 (4·9 to 5·6)	6·7 (6·4 to 7)	11 (10·6 to 11·3)	16·5 (16·1 to 16·9)	22·5 (21·9 to 23)	29·8 (29 to 30·5)	35·9 (35·1 to 36·7)	37·4 (35·4 to 39·5)	37·9 (35·6 to 40·5)
Ghana	99·9 (91·5 to 109)	163 (156 to 170)	219 (212 to 226)	269 (259 to 277)	295 (280 to 309)	326 (307 to 344)	415 (387 to 442)	470 (424 to 517)	471 (416 to 527)
Guinea	61·7 (59·2 to 64)	76·5 (73·8 to 79·2)	98·2 (94·9 to 102)	119 (115 to 122)	140 (136 to 143)	172 (168 to 177)	204 (198 to 211)	234 (221 to 248)	241 (225 to 257)
Guinea-Bissau	15·1 (14·5 to 15·6)	14 (13·3 to 14·6)	13·4 (12·5 to 14·1)	18·2 (17·4 to 19)	22·3 (21·1 to 23·5)	26·4 (24·6 to 28)	32·2 (30·1 to 34·5)	34·9 (32·2 to 37·6)	35·1 (32·2 to 38·2)
Liberia	21·5 (20·3 to 22·6)	27·8 (26·4 to 29)	35·7 (34·6 to 36·7)	49 (47·7 to 50·2)	58·6 (57·1 to 60)	63·8 (62 to 65·5)	79·3 (76·3 to 82·2)	79·9 (72·5 to 87·6)	79·3 (70·6 to 88·7)
Mali	88·8 (84·8 to 92·5)	119 (115 to 122)	155 (151 to 159)	189 (184 to 193)	220 (216 to 224)	270 (265 to 275)	376 (364 to 386)	490 (467 to 514)	519 (491 to 547)
Mauritania	16·2 (15·2 to 17·1)	20·7 (19·7 to 21·6)	26·5 (25·7 to 27·3)	34·4 (33·4 to 35·2)	42·5 (40·9 to 44)	50·7 (48·7 to 52·8)	63·7 (61·1 to 66·1)	66·7 (61·2 to 72·3)	66 (59·8 to 73)
Niger	70·2 (67·7 to 72·5)	95·8 (93 to 98·3)	130 (128 to 133)	170 (167 to 173)	217 (213 to 221)	305 (298 to 311)	398 (386 to 409)	533 (512 to 554)	574 (550 to 598)
Nigeria	881 (837 to 922)	1010 (971 to 1040)	1200 (1160 to 1250)	1550 (1490 to 1600)	2010 (1960 to 2070)	2770 (2680 to 2860)	3610 (3490 to 3730)	4030 (3770 to 4280)	4070 (3750 to 4380)
São Tomé and Príncipe	1·2 (1·1 to 1·2)	1·5 (1·5 to 1·6)	1·6 (1·5 to 1·6)	1·8 (1·8 to 1·9)	2·3 (2·2 to 2·4)	2·8 (2·6 to 3)	3 (2·8 to 3·1)	2·5 (2·2 to 2·7)	2·4 (2·1 to 2·7)
Senegal	66·2 (63 to 69·2)	88·5 (86·1 to 91)	118 (116 to 121)	148 (146 to 151)	170 (165 to 174)	201 (197 to 206)	240 (234 to 246)	237 (222 to 252)	234 (215 to 254)
Sierra Leone	45·4 (42·6 to 48)	56·6 (54·1 to 58·9)	64 (61·3 to 66·7)	77·8 (74·7 to 80·6)	100 (97·1 to 103)	99·6 (96·8 to 102)	127 (122 to 132)	145 (135 to 155)	148 (136 to 160)
Togo	37·3 (35·8 to 38·7)	44 (42·8 to 45)	49·6 (48·3 to 50·8)	62·6 (61·2 to 64)	78·2 (76·2 to 80·2)	97·1 (93·9 to 100)	120 (115 to 125)	122 (115 to 130)	120 (111 to 129)

Supplemental Results Table S1c: Livebirths (in thousands) by location, males, 1950, 1960, 1970, 1980, 1990, 2000, 2010, 2019, & 2021

Location	1950	1960	1970	1980	1990	2000	2010	2019	2021
Global	47700 (45700 to 49800)	54600 (53100 to 56100)	59500 (58200 to 60700)	62900 (61600 to 64300)	68300 (66800 to 69800)	67300 (65900 to 68800)	71300 (69500 to 73200)	69500 (66300 to 73100)	66900 (62800 to 71500)
Central Europe, eastern Europe, and central Asia	3830 (3720 to 3960)	3780 (3730 to 3840)	3200 (3140 to 3260)	3650 (3600 to 3700)	3410 (3360 to 3460)	2370 (2330 to 2410)	2920 (2870 to 2970)	2660 (2580 to 2740)	2540 (2400 to 2690)
Central Asia	521 (506 to 537)	692 (675 to 709)	655 (616 to 694)	858 (840 to 874)	1010 (996 to 1030)	759 (742 to 778)	972 (946 to 998)	1090 (1060 to 1120)	1080 (1030 to 1120)
Armenia	25·4 (24·2 to 26·5)	36·7 (35·4 to 38)	29·5 (27·3 to 32)	39·3 (37·6 to 41)	39·7 (38·1 to 41·4)	22·8 (21·8 to 23·9)	24·8 (23·5 to 26·1)	20·2 (18·7 to 21·7)	18·4 (16·7 to 20·3)
Azerbaijan	54·8 (51·1 to 58·5)	84·7 (81·8 to 87·7)	75·9 (72·3 to 79·7)	83·6 (79·9 to 87·4)	97·8 (93·9 to 102)	71·3 (67·8 to 75·1)	94·1 (88·8 to 99·4)	79·4 (72·8 to 86·4)	74 (65·9 to 82·6)
Georgia	43·6 (40·6 to 46·8)	52·7 (50·8 to 54·6)	44·5 (39·2 to 50·2)	47·1 (44·3 to 49·7)	44 (42·7 to 45·6)	26·5 (25·4 to 27·7)	28·2 (27·2 to 29·2)	25·4 (24·2 to 26·5)	23·4 (22 to 25)
Kazakhstan	130 (125 to 136)	184 (180 to 188)	168 (151 to 185)	187 (181 to 193)	192 (186 to 198)	114 (109 to 119)	190 (183 to 196)	215 (208 to 222)	220 (207 to 232)
Kyrgyzstan	29·8 (28·7 to 31)	39·2 (37·7 to 40·7)	39·6 (35·8 to 43·7)	57·2 (54·9 to 59·4)	68·2 (66·2 to 70·2)	55·5 (53·4 to 57·7)	83·2 (80·4 to 85·9)	85·3 (80·4 to 90·3)	82 (74·9 to 90·2)
Mongolia	15·4 (14·5 to 16·3)	18·6 (17·5 to 19·7)	23·4 (22·6 to 24·3)	31·5 (30·4 to 32·6)	36·5 (35·3 to 37·7)	27·5 (26·2 to 29)	37·6 (35·8 to 39·4)	42·3 (39·2 to 45·5)	41·2 (37·3 to 45·3)
Tajikistan	44 (42·1 to 45·9)	56·5 (54·8 to 58·2)	55·8 (53·5 to 58·1)	81·5 (79 to 83·9)	109 (106 to 111)	102 (97·2 to 106)	129 (123 to 134)	149 (140 to 158)	149 (139 to 159)
Turkmenistan	25 (24 to 25·9)	32·2 (30·9 to 33·3)	34·3 (32 to 36·7)	49·2 (47·4 to 50·9)	63·7 (61·5 to 65·9)	56·7 (53·8 to 59·5)	56·3 (53·1 to 59·6)	57·6 (52·4 to 63·3)	56·8 (51·1 to 63·2)
Uzbekistan	153 (144 to 163)	187 (176 to 198)	184 (172 to 197)	282 (275 to 288)	361 (353 to 368)	283 (273 to 293)	329 (312 to 348)	414 (393 to 435)	414 (383 to 446)
Central Europe	1200 (1170 to 1240)	1030 (1020 to 1050)	1000 (987 to 1020)	1070 (1060 to 1090)	881 (868 to 894)	625 (615 to 634)	623 (612 to 634)	565 (552 to 577)	534 (491 to 581)
Albania	24·5 (23·5 to 25·6)	33·3 (32·7 to 33·9)	35·5 (34·1 to 36·8)	37·1 (35·7 to 38·6)	41·1 (39·8 to 42·5)	27·3 (26 to 28·8)	18 (17 to 19·1)	15·2 (13·9 to 16·5)	14·5 (12·9 to 16·4)
Bosnia and Herzegovina	47·4 (42·9 to 52·3)	58 (52·3 to 64·2)	43·9 (40·4 to 47·4)	40·6 (36·4 to 45·2)	34·3 (32·5 to 36·1)	20·3 (19·5 to 21·2)	17·2 (16·5 to 18)	14·6 (13·8 to 15·5)	13·8 (12·5 to 15·2)
Bulgaria	86·5 (82·8 to 90·6)	71·3 (69·6 to 73)	70·6 (68·9 to 72·4)	65·8 (64·2 to 67·3)	51·6 (50·2 to 53)	36·1 (35 to 37·3)	38·4 (37·2 to 39·5)	31·4 (30·2 to 32·8)	29·9 (27·6 to 32·1)
Croatia	46·3 (44·6 to 47·9)	38·6 (37·8 to 39·5)	32·2 (31·5 to 33)	34·7 (33·9 to 35·6)	27·4 (26·7 to 28·2)	21·9 (21·3 to 22·6)	22 (21·4 to 22·6)	18·7 (17·9 to 19·4)	17·9 (16 to 20)
Czechia	95·8 (91·7 to 100)	66·5 (65 to 68·1)	77 (75·2 to 78·9)	78·2 (76·4 to 80·1)	66 (64·5 to 67·4)	46·7 (45·3 to 48·2)	58·4 (57 to 60·1)	57·6 (55·7 to 59·7)	54·3 (48·9 to 60·3)
Hungary	99·8 (94·4 to 105)	73·9 (71·9 to 75·8)	77·9 (75·6 to 80)	75·4 (73·4 to 77·6)	64·2 (62·4 to 66)	49·5 (47·8 to 51·1)	46·9 (45·5 to 48·5)	46·7 (44·4 to 49·1)	45·1 (40·3 to 50·5)
Montenegro	6·4 (6 to 6·8)	6·6 (6·4 to 6·9)	5·6 (5·4 to 5·7)	5·4 (5·2 to 5·6)	4·9 (4·7 to 5·2)	4·5 (4·4 to 4·7)	4 (3·9 to 4·1)	3·7 (3·6 to 3·9)	3·6 (3·4 to 3·8)
North Macedonia	19 (17·2 to 21)	19·8 (17·8 to 21·9)	19·5 (18·2 to 20·8)	20·6 (18·6 to 22·7)	18·1 (17·7 to 18·5)	14·4 (14 to 14·8)	12·1 (11·7 to 12·6)	10·3 (10 to 10·7)	9·7 (9·2 to 10·2)
Poland	392 (382 to 402)	335 (329 to 341)	282 (277 to 288)	357 (350 to 363)	281 (275 to 287)	190 (185 to 195)	207 (202 to 211)	190 (184 to 196)	176 (157 to 196)
Romania	213 (201 to 227)	175 (168 to 181)	214 (208 to 220)	199 (193 to 205)	153 (147 to 158)	114 (110 to 119)	108 (103 to 112)	95·3 (90·7 to 100)	91·2 (84 to 98·9)
Serbia	94·5 (92 to 96·6)	83·2 (81·4 to 84·9)	75·5 (73·9 to 77·3)	81 (78·9 to 83·1)	73·6 (71·8 to 75·4)	53·8 (52·3 to 55·3)	39·6 (34·2 to 45·8)	32·8 (31·5 to 34·2)	31·8 (29·4 to 34·4)
Slovakia	51·5 (50·3 to 52·9)	45 (44·3 to 45·6)	41·5 (40·8 to 42·1)	48·5 (47·6 to 49·3)	40·5 (39·8 to 41·3)	27·1 (26·5 to 27·7)	31·1 (30·4 to 31·7)	29·6 (28·9 to 30·3)	28·9 (27·1 to 30·7)
Slovenia	16·7 (14·8 to 18·7)	14·5 (14·1 to 14·8)	14·2 (14 to 14·5)	15·1 (14·8 to 15·5)	11·3 (11 to 11·6)	9·2 (8·9 to 9·4)	11·4 (11·2 to 11·7)	10 (9·7 to 10·3)	9·7 (9·1 to 10·3)
Eastern Europe	2100 (2020 to 2200)	2060 (2020 to 2090)	1550 (1520 to 1580)	1720 (1690 to 1750)	1520 (1490 to 1550)	987 (958 to 1020)	1330 (1290 to 1360)	1010 (964 to 1050)	924 (850 to 1000)
Belarus	99·1 (93·7 to 105)	101 (97·6 to 105)	76 (73·6 to 78·8)	80·2 (77·4 to 83·3)	71·7 (69·1 to 74·2)	47·4 (45·1 to 49·6)	56·5 (54·1 to 58·9)	46·2 (42·1 to 50·4)	42·5 (36·4 to 49·2)
Estonia	10·4 (9·9 to 11)	10·4 (10·1 to 10·7)	11·1 (10·8 to 11·3)	11·5 (11·3 to 11·9)	10·8 (10·5 to 11·1)	6·6 (6·4 to 6·8)	7·8 (7·6 to 8·1)	7·1 (6·8 to 7·4)	6·8 (6·3 to 7·2)
Latvia	16·9 (15·7 to 18·2)	18·4 (18 to 18·8)	18·1 (17·7 to 18·5)	18·3 (17·9 to 18·7)	18·6 (18·1 to 19)	10·3 (10 to 10·5)	9·7 (9·4 to 10)	9·5 (9·2 to 9·8)	8·7 (7·7 to 9·8)
Lithuania	30·1 (28·6 to 31·8)	31·5 (30·6 to 32·4)	28·3 (27·6 to 29·2)	26·3 (25·6 to 27)	28·6 (27·9 to 29·4)	17·1 (16·5 to 17·7)	16·2 (15·6 to 16·8)	13·5 (13 to 14·1)	12·1 (11·3 to 13)
Moldova	43·9 (42·2 to 45·6)	43·6 (42·4 to 44·9)	33·8 (30·2 to 37·6)	44·2 (42·8 to 45·6)	40·7 (39·4 to 42)	21·3 (20·7 to 22·1)	20·8 (20·1 to 21·6)	15·9 (14·7 to 17·3)	14·6 (13·1 to 16·5)
Russia	1450 (1370 to 1540)	1390 (1360 to 1430)	988 (958 to 1020)	1150 (1120 to 1180)	1010 (980 to 1030)	687 (663 to 712)	952 (919 to 983)	755 (716 to 796)	696 (644 to 753)
Ukraine	458 (438 to 478)	459 (446 to 472)	394 (383 to 405)	391 (380 to 402)	340 (330 to 350)	197 (190 to 203)	263 (254 to 272)	159 (152 to 166)	143 (127 to 161)
High income	7000 (6820 to 7170)	7470 (7410 to 7540)	7130 (7050 to 7210)	6410 (6340 to 6490)	6390 (6320 to 6470)	6070 (5990 to 6140)	6120 (6050 to 6190)	5420 (5350 to 5490)	5330 (4980 to 5700)
Australasia	129 (126 to 132)	155 (153 to 157)	170 (167 to 172)	143 (140 to 146)	162 (158 to 165)	154 (152 to 157)	187 (184 to 190)	183 (179 to 187)	184 (167 to 202)
Australia	104 (101 to 107)	123 (121 to 125)	138 (135 to 140)	117 (114 to 120)	131 (128 to 134)	125 (123 to 128)	154 (152 to 157)	154 (150 to 157)	154 (138 to 171)
New Zealand	25·5 (25 to 26·1)	32·6 (32·1 to 33)	32 (31·5 to 32·5)	25·9 (25·3 to 26·5)	30·7 (30 to 31·3)	29·1 (28·5 to 29·6)	32·2 (31·5 to 32·9)	29·8 (29·2 to 30·5)	30 (28·3 to 31·8)
High-income Asia Pacific	1580 (1520 to 1630)	1380 (1330 to 1420)	1550 (1510 to 1600)	1270 (1240 to 1310)	1020 (990 to 1050)	942 (913 to 971)	823 (798 to 849)	629 (610 to 649)	600 (552 to 654)
Brunei	1·4 (1·3 to 1·4)	2 (2 to 2·1)	2·4 (2·4 to 2·5)	3 (2·8 to 3·2)	3·6 (3·4 to 3·9)	3·7 (3·4 to 4·1)	3·4 (3·2 to 3·7)	3·3 (3·1 to 3·7)	3·3 (2·9 to 3·8)
Japan	1120 (1070 to 1180)	820 (790 to 849)	1020 (980 to 1050)	809 (780 to 841)	628 (604 to 652)	603 (579 to 628)	550 (528 to 572)	444 (426 to 461)	430 (386 to 478)
Aichi	47·2 (45 to 49·4)	40·5 (38·8 to 42·2)	60 (57·5 to 62·7)	44·8 (42·9 to 46·8)	36·4 (34·9 to 38·1)	37·6 (36 to 39·4)	35·9 (34·2 to 37·5)	29·4 (28·1 to 30·7)	28·7 (25·8 to 31·9)
Akita	15·8 (15·1 to 16·6)	11·1 (10·6 to 11·6)	10·1 (9·6 to 10·6)	8·2 (7·8 to 8·6)	5·7 (5·5 to 6)	4·6 (4·3 to 4·8)	3·5 (3·3 to 3·7)	2·4 (2·3 to 2·5)	2·2 (2 to 2·5)
Aomori	17·5 (16·6 to 18·4)	12·7 (12·2 to 13·3)	13·3 (12·7 to 13·9)	11 (10·5 to 11·4)	7·7 (7·4 to 8)	6·6 (6·3 to 6·9)	4·9 (4·7 to 5·2)	3·7 (3·5 to 3·8)	3·4 (3·1 to 3·8)
Chiba	29·1 (27·7 to 30·4)	19·5 (18·8 to 20·3)	36·2 (34·7 to 37·8)	34·6 (33·1 to 36·2)	27·4 (26·2 to 28·7)	28 (26·8 to 29·3)	26·4 (25·2 to 27·5)	21 (20·2 to 21·9)	20·7 (18·6 to 23·1)
Ehime	19·9 (18·9 to 20·8)	12·2 (11·6 to 12·7)	12·6 (12·1 to 13·2)	10·1 (9·7 to 10·5)	7·5 (7·1 to 7·9)	6·7 (6·4 to 7)	5·9 (5·6 to 6·1)	4·4 (4·2 to 4·5)	4·1 (3·7 to 4·6)
Fukui	10 (9·6 to 10·5)	6·3 (6·1 to 6·6)	6·7 (6·4 to 7·1)	5·4 (5·1 to 5·6)	4·4 (4·2 to 4·6)	4·1 (3·9 to 4·3)	3·5 (3·4 to 3·7)	2·8 (2·7 to 2·9)	2·7 (2·4 to 3)
Fukuoka	45·9 (43·7 to 48·1)	34 (32·6 to 35·6)	37·3 (35·9 to 38·8)	33·1 (31·9 to 34·4)	24·9 (23·8 to 25·9)	24 (23·1 to 25)	24 (22·9 to 25)	20·4 (19·5 to 21·2)	19·7 (17·7 to 22·1)
Fukushima	29·1 (27·7 to 30·5)	18 (17·2 to 18·7)	16·7 (16 to 17·4)	14·9 (14·3 to 15·5)	11·7 (11·2 to 12·2)	10·4 (10 to 10·8)	8·1 (7·7 to 8·5)	6 (5·7 to 6·2)	5·7 (5·1 to 6·4)
Gifu	19·9 (19 to 20·7)	14·4 (13·8 to 15)	17·1 (16·3 to 17·9)	13·2 (12·6 to 13·8)	10·4 (9·9 to 10·9)	10·2 (9·7 to 10·7)	8·7 (8·3 to 9·2)	6·5 (6·2 to 6·8)	6·2 (5·5 to 6·9)
Gunma	21·8 (20·8 to 22·8)	13·5 (12·8 to 14·2)	15·8 (14·7 to 16·9)	13 (12·4 to 13·5)	10·1 (9·7 to 10·6)	9·9 (9·4 to 10·3)	8·2 (7·9 to 8·5)	6·2 (5·9 to 6·4)	5·9 (5·3 to 6·6)
Hiroshima	28·4 (27·1 to 29·7)	18·7 (17·9 to 19·5)	24 (22·9 to 25·1)	19·1 (18·3 to 19·9)	14·7 (14·1 to 15·4)	14 (13·4 to 14·7)	13·1 (12·5 to 13·8)	10·3 (9·8 to 10·8)	9·9 (8·9 to 11·1)
Hokkaidō	55 (52·4 to 57·8)	42 (40·5 to 43·4)	49·1 (47 to 51·1)	38·7 (37·2 to 40·2)	27·8 (26·6 to 28·9)	23·9 (22·9 to 25)	20·6 (19·7 to 21·4)	15·7 (15 to 16·4)	14·9 (13·3 to 16·8)

Hyōgo	45 (42·9 to 47)	34·7 (33·5 to 35·9)	47·5 (45·6 to 49·4)	35·2 (33·7 to 36·6)	27·5 (26·4 to 28·7)	27·3 (26·2 to 28·6)	24·7 (23·6 to 25·7)	19·5 (18·6 to 20·3)	18·7 (16·9 to 20·8)
Ibaraki	28·4 (27·1 to 29·8)	16·9 (16·4 to 17·5)	19·8 (19 to 20·6)	18·7 (18 to 19·4)	14·9 (14·2 to 15·5)	14·4 (13·8 to 15·1)	12·3 (11·8 to 12·8)	9·3 (8·9 to 9·6)	8·8 (7·8 to 9·8)
Ishikawa	12·5 (11·9 to 13·1)	8·2 (7·9 to 8·5)	10 (9·5 to 10·5)	7·7 (7·3 to 8)	5·8 (5·6 to 6·1)	5·8 (5·5 to 6·1)	5 (4·8 to 5·2)	4·1 (3·8 to 4·3)	3·9 (3·5 to 4·4)
Iwate	19·7 (18·7 to 20·7)	13·6 (13 to 14·1)	12·7 (12·2 to 13·2)	9·9 (9·5 to 10·3)	7·3 (7 to 7·6)	6·3 (6·1 to 6·6)	5 (4·7 to 5·2)	3·6 (3·4 to 3·7)	3·3 (3 to 3·7)
Kagawa	11·9 (11·4 to 12·5)	7·4 (7·1 to 7·7)	8·1 (7·8 to 8·5)	6·6 (6·3 to 6·9)	4·9 (4·7 to 5·1)	4·9 (4·7 to 5·2)	4·3 (4·1 to 4·6)	3·4 (3·2 to 3·5)	3·2 (2·9 to 3·6)
Kagoshima	26·3 (25·1 to 27·5)	16 (15·4 to 16·7)	13·2 (12·4 to 14)	12·6 (12·1 to 13·1)	9·7 (9·3 to 10·1)	8·2 (7·8 to 8·5)	7·8 (7·5 to 8·2)	6·1 (5·9 to 6·4)	5·8 (5·2 to 6·4)
Kanagawa	34·4 (32·9 to 36)	32·8 (31·3 to 34·3)	59·8 (57·3 to 62·4)	48·8 (46·8 to 51)	40·8 (39 to 42·6)	42·1 (40·1 to 44)	40 (38·3 to 41·7)	32·3 (31 to 33·5)	31·5 (28·4 to 35)
Kōchi	11·7 (11·1 to 12·3)	6·4 (6·2 to 6·7)	6·6 (6·3 to 6·8)	5·2 (4·9 to 5·4)	3·8 (3·6 to 4)	3·4 (3·3 to 3·6)	2·8 (2·7 to 2·9)	2·2 (2·1 to 2·3)	2 (1·8 to 2·3)
Kumamoto	23·9 (22·8 to 25·1)	15·1 (14·4 to 15·7)	13·9 (13·3 to 14·5)	12·5 (12 to 13)	9·9 (9·3 to 10·4)	8·8 (8·4 to 9·2)	8·4 (8 to 8·8)	6·8 (6·6 to 7·1)	6·5 (5·8 to 7·2)
Kyōto	22·4 (21·4 to 23·4)	16·4 (15·7 to 17·2)	21·2 (20·3 to 22·1)	16·5 (15·9 to 17·2)	12·3 (11·8 to 12·9)	12·1 (11·5 to 12·6)	10·8 (10·4 to 11·3)	8·7 (8·4 to 9·1)	8·3 (7·5 to 9·3)
Mie	19·1 (18·2 to 20)	12·7 (12·2 to 13·2)	14·3 (13·7 to 15)	10·9 (10·4 to 11·4)	9·1 (8·7 to 9·5)	8·9 (8·5 to 9·3)	7·8 (7·5 to 8·2)	6 (5·7 to 6·3)	5·7 (5·1 to 6·4)
Miyagi	21·8 (20·8 to 22·8)	15·2 (14·6 to 15·9)	16·5 (15·8 to 17·2)	15·8 (15·2 to 16·6)	12 (11·4 to 12·5)	11·3 (10·8 to 11·7)	9·8 (9·3 to 10·2)	7·7 (7·4 to 8)	7·4 (6·6 to 8·2)
Miyazaki	15·4 (14·7 to 16·2)	9·9 (9·5 to 10·3)	9·5 (9·1 to 9·9)	8·7 (8·3 to 9·1)	6·3 (6·1 to 6·6)	5·6 (5·4 to 5·9)	5·2 (5 to 5·5)	4·1 (3·9 to 4·3)	3·8 (3·4 to 4·3)
Nagano	27 (25·9 to 28·2)	17·3 (16·5 to 18)	17·7 (16·9 to 18·4)	13·7 (13·1 to 14·3)	11 (10·5 to 11·4)	10·7 (10·3 to 11·2)	8·9 (8·5 to 9·3)	6·9 (6·6 to 7·2)	6·6 (5·9 to 7·3)
Nagasaki	24·5 (23·4 to 25·7)	15·1 (14·6 to 15·7)	13·8 (13·2 to 14·3)	11·5 (11·1 to 12)	8·6 (8·2 to 8·9)	7·1 (6·8 to 7·5)	6·2 (5·9 to 6·4)	4·9 (4·7 to 5·1)	4·6 (4·1 to 5·1)
Nara	9·4 (9 to 9·8)	6·2 (5·9 to 6·4)	8·5 (8·2 to 8·9)	8·2 (7·9 to 8·6)	6·8 (6·5 to 7·1)	6·7 (6·4 to 7)	5·5 (5·3 to 5·8)	4·2 (4 to 4·4)	4 (3·6 to 4·4)
Niigata	33·1 (31·7 to 34·6)	20·8 (20 to 21·8)	20·8 (19·9 to 21·7)	16·7 (16 to 17·4)	12·4 (11·9 to 13)	11 (10·5 to 11·5)	9·3 (8·8 to 9·7)	7 (6·7 to 7·3)	6·5 (5·9 to 7·3)
Ōita	15·8 (15·1 to 16·5)	9·9 (9·5 to 10·3)	9·8 (9·3 to 10·2)	8·3 (7·9 to 8·7)	6·1 (5·9 to 6·4)	5·5 (5·3 to 5·8)	5·2 (4·9 to 5·4)	4 (3·8 to 4·1)	3·7 (3·3 to 4·2)
Okayama	21·1 (20·1 to 22·1)	13·7 (13·1 to 14·3)	15·9 (15·2 to 16·6)	12·5 (12 to 13)	9·7 (9·3 to 10·1)	9·7 (9·2 to 10·2)	8·6 (8·2 to 9)	7·1 (6·7 to 7·4)	6·7 (6 to 7·6)
Okinawa	14·8 (13·9 to 15·7)	9·6 (9·2 to 10)	10·9 (10·5 to 11·4)	10·2 (9·8 to 10·7)	9 (8·6 to 9·4)	8·6 (8·3 to 9)	8·7 (8·3 to 9·1)	7·7 (7·4 to 8)	7·5 (6·7 to 8·4)
Ōsaka	54 (51·5 to 56·6)	51·8 (50·1 to 53·6)	83·8 (80·5 to 87)	57·2 (54·9 to 59·5)	44·5 (42·8 to 46·2)	44·6 (42·5 to 46·7)	38·6 (36·9 to 40·2)	32·1 (30·8 to 33·3)	31·7 (28·5 to 35·3)
Saga	13 (12·4 to 13·6)	7·9 (7·6 to 8·2)	7·2 (6·9 to 7·4)	6·3 (6·1 to 6·6)	4·9 (4·7 to 5·1)	4·4 (4·2 to 4·6)	3·9 (3·7 to 4·1)	3·2 (3 to 3·3)	3 (2·7 to 3·4)
Saitama	30·4 (29 to 31·9)	22 (21·1 to 22·9)	45·2 (43·4 to 47·1)	39·3 (37·8 to 40·8)	32·8 (31·4 to 34·1)	33·8 (32·3 to 35·4)	30·5 (29·2 to 31·9)	24·9 (23·9 to 25·9)	24·4 (22 to 27·2)
Shiga	12·1 (11·6 to 12·6)	7·5 (7·2 to 7·8)	7·9 (7·6 to 8·3)	8·2 (7·8 to 8·5)	6·8 (6·6 to 7·1)	7·2 (6·9 to 7·5)	6·9 (6·6 to 7·2)	5·5 (5·3 to 5·7)	5·2 (4·7 to 5·9)
Shimane	12·1 (11·6 to 12·7)	7·2 (6·9 to 7·6)	6·3 (5·9 to 6·8)	5 (4·8 to 5·3)	3·8 (3·7 to 4)	3·3 (3·1 to 3·5)	2·9 (2·8 to 3·1)	2·4 (2·3 to 2·5)	2·3 (2·1 to 2·6)
Shizuoka	33·9 (32·4 to 35·4)	25 (23·9 to 26·1)	31·3 (29·9 to 32·7)	24·6 (23·5 to 25·8)	19·1 (18·4 to 19·8)	18·2 (17·4 to 19·2)	16·4 (15·6 to 17·2)	12·1 (11·5 to 12·7)	11·5 (10·3 to 12·9)
Tochigi	21·4 (20·4 to 22·4)	12·8 (12·3 to 13·2)	14·8 (14·2 to 15·4)	13·3 (12·8 to 13·9)	10·2 (9·8 to 10·7)	9·6 (9·2 to 10·1)	8·5 (8·2 to 8·9)	6·4 (6·2 to 6·7)	6·1 (5·4 to 6·8)
Tokushima	10·6 (10·1 to 11·1)	6·4 (6·2 to 6·7)	6·3 (6 to 6·6)	5·4 (5·2 to 5·6)	4 (3·8 to 4·2)	3·6 (3·5 to 3·8)	3·1 (2·9 to 3·2)	2·4 (2·3 to 2·5)	2·2 (2 to 2·5)
Tōkyō	77·5 (73·9 to 81·1)	85·8 (81·1 to 91·1)	110 (105 to 115)	72 (69·2 to 75)	53·5 (51·2 to 55·8)	50·8 (48·7 to 53·1)	55·4 (53·1 to 57·8)	52·5 (50·2 to 54·8)	52·7 (47·5 to 58·4)
Tottori	8 (7·7 to 8·4)	4·9 (4·7 to 5·1)	4·6 (4·4 to 4·8)	4·2 (4 to 4·3)	3·2 (3·1 to 3·4)	2·8 (2·7 to 3)	2·5 (2·4 to 2·6)	2 (1·9 to 2·1)	1·9 (1·7 to 2·2)
Toyama	12·3 (11·7 to 12·8)	8·4 (8·1 to 8·8)	9·9 (9·4 to 10·4)	7 (6·6 to 7·3)	5·1 (4·9 to 5·3)	5·2 (4·9 to 5·5)	4·2 (4 to 4·5)	3·4 (3·2 to 3·5)	3·2 (2·9 to 3·6)
Wakayama	13·4 (12·8 to 14·1)	8·3 (8 to 8·7)	9·3 (8·9 to 9·8)	6·9 (6·6 to 7·2)	5·3 (5 to 5·5)	4·8 (4·6 to 5·1)	3·9 (3·7 to 4·1)	3 (2·9 to 3·2)	2·8 (2·5 to 3·2)
Yamagata	17·7 (16·9 to 18·5)	11·5 (11 to 11·9)	9·8 (9·4 to 10·2)	8·5 (8·1 to 8·8)	6·5 (6·2 to 6·7)	5·5 (5·3 to 5·8)	4·4 (4·2 to 4·7)	3·3 (3·1 to 3·5)	3·1 (2·8 to 3·4)
Yamaguchi	19·5 (18·6 to 20·4)	12·9 (12·4 to 13·5)	13·3 (12·7 to 13·9)	10·1 (9·7 to 10·6)	7·2 (6·9 to 7·5)	6·7 (6·3 to 7)	5·8 (5·6 to 6·1)	4·4 (4·2 to 4·6)	4·1 (3·7 to 4·6)
Yamanashi	10·2 (9·7 to 10·6)	6·4 (6·1 to 6·6)	6·3 (6·1 to 6·6)	5·1 (4·9 to 5·4)	4·5 (4·3 to 4·7)	4·3 (4·1 to 4·5)	3·4 (3·2 to 3·5)	2·7 (2·6 to 2·8)	2·6 (2·3 to 2·9)
South Korea	425 (401 to 450)	526 (494 to 559)	510 (482 to 536)	440 (428 to 454)	359 (348 to 370)	304 (293 to 315)	241 (231 to 250)	152 (144 to 160)	138 (126 to 151)
Singapore	24·7 (23·6 to 25·9)	30·7 (30 to 31·5)	23·9 (22·4 to 25·3)	21·8 (20·3 to 23·2)	27·9 (26·3 to 29·7)	30·2 (28·2 to 32·2)	29·4 (26·8 to 32·2)	29·6 (27·6 to 31·8)	28·6 (25 to 33)
High-income North America	2060 (2010 to 2110)	2410 (2380 to 2440)	2020 (1980 to 2060)	2020 (1980 to 2060)	2320 (2280 to 2370)	2240 (2200 to 2270)	2230 (2190 to 2270)	2070 (2030 to 2110)	2050 (1930 to 2190)
Canada	186 (182 to 191)	236 (232 to 239)	178 (175 to 182)	184 (180 to 187)	205 (201 to 209)	170 (167 to 174)	193 (189 to 197)	186 (182 to 191)	185 (166 to 206)
Greenland	0·5 (0·5 to 0·5)	0·8 (0·8 to 0·8)	0·5 (0·5 to 0·6)	0·5 (0·5 to 0·5)	0·6 (0·6 to 0·7)	0·5 (0·5 to 0·5)	0·4 (0·4 to 0·5)	0·4 (0·4 to 0·5)	0·4 (0·4 to 0·4)
USA	1880 (1830 to 1930)	2170 (2140 to 2200)	1840 (1800 to 1870)	1840 (1800 to 1880)	2120 (2070 to 2160)	2070 (2030 to 2100)	2040 (2000 to 2080)	1880 (1840 to 1920)	1870 (1760 to 1980)
Alabama	40·9 (39·9 to 42)	42·6 (41·5 to 43·6)	33·9 (33·1 to 34·8)	31·8 (30·7 to 32·9)	32·7 (31·7 to 33·5)	31·6 (30·6 to 32·6)	29·9 (29 to 30·7)	30·2 (29·4 to 31·2)	29·6 (27·7 to 31·6)
Alaska	1·7 (1·7 to 1·8)	3·5 (3·4 to 3·6)	3·7 (3·6 to 3·8)	5 (4·8 to 5·2)	6·1 (5·9 to 6·2)	5·2 (5 to 5·3)	5·8 (5·6 to 6)	4·7 (4·5 to 4·9)	4·7 (4·4 to 5)
Arizona	10·5 (10·2 to 10·8)	18·3 (17·4 to 19·3)	18·7 (18·1 to 19·4)	25·1 (23·6 to 26·7)	34·5 (33·3 to 35·7)	43·3 (41·9 to 44·7)	45·3 (43·2 to 47·4)	39·8 (38·3 to 41·2)	39·7 (37·2 to 42·4)
Arkansas	23·1 (22·5 to 23·7)	20·6 (20 to 21·2)	18 (17·5 to 18·5)	18·7 (18·1 to 19·4)	18·5 (18 to 19)	19·1 (18·5 to 19·8)	19·3 (18·6 to 19·8)	18·1 (17·5 to 18·8)	18·3 (17 to 19·5)
California	130 (126 to 133)	189 (179 to 199)	170 (163 to 178)	200 (187 to 214)	305 (295 to 316)	272 (263 to 282)	263 (254 to 272)	221 (213 to 230)	217 (203 to 231)
Colorado	15·1 (14·7 to 15·5)	20·5 (19·9 to 21·1)	20·7 (20·1 to 21·4)	26·1 (25·2 to 27)	27·4 (26·5 to 28·2)	33·9 (33·1 to 34·8)	33·8 (32·8 to 34·9)	29·9 (28·7 to 31)	32 (29·7 to 34·3)
Connecticut	24·5 (23·9 to 25·1)	28·9 (28 to 29·8)	24·2 (23·5 to 24·9)	20·2 (19·6 to 20·7)	25·4 (24·7 to 26·2)	22 (21·4 to 22·8)	19·4 (18·8 to 20·1)	17·8 (17·3 to 18·4)	18·2 (16·9 to 19·4)
Delaware	4·2 (4·1 to 4·3)	5·9 (5·6 to 6·2)	5 (4·7 to 5·2)	4·6 (4·3 to 4·9)	5·6 (5·4 to 5·8)	5·6 (5·3 to 5·9)	6 (5·8 to 6·3)	5·5 (5·2 to 5·8)	5·5 (5·1 to 5·9)
Washington, DC	10·4 (10·1 to 10·7)	8·8 (8·3 to 9·3)	6·7 (6·3 to 7·2)	5 (4·7 to 5·4)	5·7 (5·4 to 6·1)	4 (3·7 to 4·3)	4·3 (3·7 to 5)	4·6 (4·3 to 4·9)	4·6 (4·2 to 5·1)
Florida	32·4 (31·6 to 33·3)	55·6 (53·8 to 57·4)	58·7 (57·1 to 60·3)	68·9 (66·6 to 71)	100 (97·7 to 103)	104 (101 to 108)	110 (107 to 113)	112 (109 to 115)	110 (102 to 118)
Georgia	49·4 (48·2 to 50·8)	55·9 (53·3 to 59)	46·8 (45·2 to 48·4)	45·5 (43·1 to 47·9)	56·5 (54·9 to 58·3)	67·8 (65·6 to 70)	69·9 (66·8 to 73·3)	63·6 (61·5 to 65·8)	63·4 (59·2 to 67·8)
Hawaii	7·4 (7·2 to 7·6)	9 (8·7 to 9·2)	8·2 (8 to 8·5)	9·2 (8·9 to 9·5)	10·5 (10·2 to 10·8)	9 (8·6 to 9·3)	9·7 (9·4 to 10)	8·4 (8·1 to 8·7)	8 (7·5 to 8·6)
Idaho	7·5 (7·3 to 7·7)	8·4 (8·2 to 8·7)	7·3 (7·1 to 7·5)	10·2 (9·9 to 10·6)	8·5 (8·3 to 8·8)	10·4 (10 to 10·8)	11·4 (11·1 to 11·7)	11·3 (10·8 to 11·7)	11·4 (10·7 to 12·2)
Illinois	107 (104 to 110)	121 (117 to 124)	102 (98·8 to 104)	96·1 (93·4 to 98·7)	100 (97·8 to 103)	93·5 (91 to 96·2)	81·4 (79 to 83·8)	70·8 (68·6 to 73)	67·4 (62·6 to 72·3)

Indiana	46·7 (45·6 to 48)	56·3 (55·3 to 57·2)	49·4 (48·1 to 50·9)	44·5 (43·3 to 45·8)	44·5 (43·2 to 45·9)	44 (42·9 to 45·2)	43·1 (41·9 to 44·4)	40·7 (39·5 to 41·8)	40·7 (38·1 to 43·4)
Iowa	30 (29·3 to 30·8)	31·6 (30·8 to 32·5)	23·7 (23·1 to 24·4)	24·2 (23·4 to 25)	20·2 (19·6 to 20·8)	19·1 (18·5 to 19·8)	19·6 (18·9 to 20·3)	18·8 (18·2 to 19·3)	18·7 (17·5 to 20)
Kansas	21·1 (20·6 to 21·6)	24·4 (23·7 to 25·1)	18·8 (18·3 to 19·4)	21·1 (20·4 to 21·7)	19·8 (19·3 to 20·5)	19·9 (19·4 to 20·4)	20·9 (20·3 to 21·5)	17·8 (17·3 to 18·4)	17·7 (16·5 to 18·9)
Kentucky	36·7 (35·8 to 37·7)	37 (35·9 to 38·2)	30·8 (30 to 31·6)	30 (29·1 to 30·9)	28·2 (27·4 to 29)	28·3 (27·5 to 29·3)	27·5 (26·7 to 28·3)	26·6 (25·7 to 27·4)	26·6 (24·8 to 28·5)
Louisiana	38·8 (37·8 to 39·8)	46·4 (45·4 to 47·4)	37·2 (36·2 to 38·3)	42·2 (41 to 43·4)	37·4 (36·4 to 38·4)	34·1 (33 to 35·3)	32 (30·8 to 33·1)	29·3 (28·4 to 30·2)	29·1 (27·3 to 31·1)
Maine	11 (10·8 to 11·3)	11·7 (11·4 to 11·9)	9 (8·8 to 9·3)	8·4 (8·1 to 8·7)	8·9 (8·6 to 9·2)	7 (6·8 to 7·2)	6·6 (6·3 to 6·8)	5·9 (5·7 to 6·1)	6·1 (5·7 to 6·5)
Maryland	29·3 (28·6 to 30·1)	37·2 (36·4 to 38)	34·1 (33·1 to 35·2)	29 (28 to 29·9)	41 (39·7 to 42·3)	38·1 (37 to 39·2)	36·8 (35·6 to 38)	36·3 (35·2 to 37·3)	34·9 (32·5 to 37·4)
Massachusetts	56·6 (55·2 to 58·1)	58·2 (57 to 59·3)	46 (44·7 to 47·2)	37·6 (36·5 to 38·7)	46·6 (45 to 48·2)	41·7 (40·4 to 43·1)	37·5 (36·6 to 38·4)	35·3 (34·3 to 36·4)	35·3 (32·9 to 37·9)
Michigan	81·3 (79·3 to 83·5)	97·5 (95·6 to 99·5)	84·5 (82·2 to 86·9)	73·4 (71·1 to 75·7)	78·1 (75·9 to 80·4)	68·9 (67·2 to 70·4)	57·7 (56 to 59·3)	54 (52·7 to 55·4)	53·5 (50 to 57·1)
Minnesota	35·7 (34·9 to 36·7)	39·9 (38·8 to 41)	33·1 (32·3 to 34)	34·7 (33·6 to 35·7)	34·7 (33·6 to 35·7)	34·2 (33·3 to 35·1)	34·6 (33·7 to 35·5)	32·9 (31·9 to 33·8)	32·8 (30·6 to 35·1)
Mississippi	31·7 (30·8 to 32·6)	30·7 (30 to 31·4)	24·7 (24·1 to 25·4)	24 (23·3 to 24·8)	22·4 (21·7 to 23·2)	22 (21·3 to 22·7)	20·1 (19·4 to 20·8)	18·1 (17·5 to 18·7)	17·9 (16·7 to 19)
Missouri	45·7 (44·6 to 47)	49·4 (48·1 to 50·8)	40·4 (39·2 to 41·7)	39·9 (38·6 to 41·2)	40·6 (39·6 to 41·7)	38·5 (37·1 to 39·8)	39·3 (38·2 to 40·5)	35·7 (34·7 to 36·9)	35·4 (33 to 38)
Montana	6·6 (6·5 to 6·8)	7·7 (7·5 to 7·8)	6·3 (6·1 to 6·5)	7·3 (7 to 7·5)	6 (5·8 to 6·1)	5·6 (5·4 to 5·8)	6·1 (5·9 to 6·3)	5·4 (5·3 to 5·6)	5·6 (5·3 to 6)
Nebraska	14·9 (14·6 to 15·3)	16 (15·5 to 16·5)	13 (12·5 to 13·4)	14 (13·6 to 14·5)	12·5 (12 to 12·9)	12·6 (12·1 to 13·1)	13·3 (12·8 to 13·8)	12·6 (12·2 to 13)	12·5 (11·7 to 13·4)
Nevada	1·9 (1·8 to 1·9)	3·6 (3·5 to 3·7)	4·8 (4·7 to 4·9)	7 (6·7 to 7·2)	11 (10·7 to 11·4)	16 (15·4 to 16·5)	17·9 (17·2 to 18·5)	17·9 (17·3 to 18·6)	17·3 (16·1 to 18·5)
New Hampshire	6·3 (6·1 to 6·4)	7 (6·8 to 7·2)	6·7 (6·5 to 7)	7 (6·7 to 7·2)	8·7 (8·5 to 9)	7·4 (7·2 to 7·7)	6·7 (6·5 to 6·9)	6 (5·8 to 6·2)	6·3 (5·9 to 6·7)
New Jersey	59·4 (58 to 61)	69·8 (67·1 to 72·3)	59·2 (57·2 to 61·3)	49·5 (48 to 50·9)	62·7 (61·1 to 64·7)	59·5 (57·9 to 61·1)	52·8 (51·3 to 54·4)	53·3 (51·6 to 54·9)	51·8 (48·3 to 55·8)
New Mexico	10·3 (10 to 10·6)	14·7 (14 to 15·6)	10·9 (10·5 to 11·3)	13·4 (12·9 to 13·9)	14·1 (13·7 to 14·5)	13·9 (13·4 to 14·3)	13·7 (13·3 to 14·2)	11·2 (10·8 to 11·7)	10·8 (10·1 to 11·5)
New York	179 (174 to 184)	196 (190 to 202)	153 (148 to 158)	123 (120 to 127)	152 (148 to 157)	132 (129 to 135)	124 (121 to 127)	116 (113 to 118)	107 (100 to 115)
North Carolina	52·7 (51·4 to 54·1)	58·2 (56·7 to 59·5)	49·2 (47·8 to 50·5)	43·1 (41·9 to 44·4)	53·2 (51·7 to 54·7)	60·9 (59·4 to 62·4)	62·8 (61·1 to 64·5)	59·2 (57·6 to 60·9)	61·1 (56·9 to 65·2)
North Dakota	8·5 (8·3 to 8·8)	8·3 (7·9 to 8·7)	5·3 (5 to 5·5)	6·2 (5·8 to 6·6)	4·8 (4·5 to 5·1)	4 (3·8 to 4·2)	5·6 (5·3 to 5·9)	5·2 (5 to 5·5)	5·1 (4·8 to 5·6)
Ohio	96·9 (94·5 to 99·4)	118 (116 to 121)	98·8 (96 to 102)	86·4 (84 to 88·8)	86 (83·6 to 88·5)	78 (75·9 to 79·8)	70·9 (69 to 72·7)	66·6 (64·7 to 68·5)	66·2 (62 to 70·4)
Oklahoma	24·6 (24 to 25·3)	25·9 (25·2 to 26·5)	22·8 (22·1 to 23·5)	27·3 (26·5 to 28·2)	24·5 (23·8 to 25·3)	25·5 (24·8 to 26·2)	26·3 (25·6 to 27·1)	24·2 (23·5 to 25)	24·6 (23·1 to 26·2)
Oregon	16·2 (15·8 to 16·6)	18·2 (17·7 to 18·7)	17·2 (16·7 to 17·7)	22 (21·3 to 22·7)	22 (21·3 to 22·6)	23·2 (22·6 to 23·9)	23·4 (22·7 to 24·1)	20·1 (19·5 to 20·6)	20·8 (19·5 to 22·1)
Pennsylvania	125 (122 to 128)	126 (123 to 129)	95·2 (92·7 to 97·5)	82 (79·6 to 84·3)	88 (85·5 to 90·6)	74·1 (72·3 to 75·8)	73 (71·3 to 74·7)	68·5 (66·6 to 70·4)	67·7 (63·3 to 72·4)
Rhode Island	9·9 (9·6 to 10·1)	9·7 (9·3 to 10·2)	7·8 (7·5 to 8·1)	6·3 (6 to 6·6)	7·7 (7·4 to 8·1)	6·5 (6·1 to 6·8)	6 (5·7 to 6·3)	5·4 (5·2 to 5·7)	5·4 (5 to 5·8)
South Carolina	28·7 (28 to 29·5)	31·7 (30·8 to 32·5)	26·7 (25·9 to 27·4)	26·1 (25·4 to 26·9)	30 (29·2 to 30·9)	28·5 (27·8 to 29·2)	28·3 (27·4 to 29·1)	28·3 (27·5 to 29)	29·1 (27·2 to 31)
South Dakota	8·2 (8 to 8·4)	8·3 (8·1 to 8·6)	5·9 (5·7 to 6)	6·6 (6·4 to 6·8)	5·7 (5·5 to 5·9)	5·3 (5·1 to 5·4)	6·4 (6·1 to 6·6)	5·6 (5·5 to 5·8)	5·7 (5·3 to 6·1)
Tennessee	41·4 (40·4 to 42·5)	44·2 (42·9 to 45·3)	36·5 (35·3 to 37·5)	35·1 (33·7 to 36·4)	38·6 (37·2 to 39·9)	40·3 (39 to 41·7)	43·3 (41·8 to 44·7)	40·3 (38·9 to 41·6)	41·6 (39 to 44·2)
Texas	106 (103 to 108)	132 (128 to 135)	116 (112 to 119)	142 (137 to 147)	162 (157 to 167)	186 (181 to 192)	200 (194 to 207)	186 (180 to 191)	190 (178 to 202)
Utah	10 (9·8 to 10·3)	13·5 (13·1 to 13·9)	13·7 (13·3 to 14·2)	21·4 (20·7 to 22·1)	18·6 (18 to 19·3)	24·3 (23·5 to 25·1)	27 (26·3 to 27·7)	23·1 (22·4 to 23·9)	23·7 (22·3 to 25·3)
Vermont	4·5 (4·4 to 4·6)	4·5 (4·4 to 4·7)	4·1 (4 to 4·3)	4·1 (3·9 to 4·2)	4·2 (4·1 to 4·4)	3·3 (3·2 to 3·4)	3 (2·9 to 3·1)	2·8 (2·7 to 2·9)	2·8 (2·6 to 3)
Virginia	41·7 (40·7 to 42·8)	49·4 (48·1 to 50·7)	42·9 (41·8 to 44)	39·8 (38·6 to 40·9)	50·6 (49·1 to 52·2)	51·1 (49·7 to 52·4)	51·9 (50·5 to 53·2)	49·7 (48·5 to 51·1)	48·8 (45·6 to 52·1)
Washington	27 (26·3 to 27·7)	32 (31·3 to 32·7)	28·9 (28 to 29·8)	35·1 (33·9 to 36·3)	40·7 (39·4 to 42)	41 (39·9 to 42)	44·5 (43·1 to 45·8)	40·9 (39·7 to 42)	42·6 (39·9 to 45·4)
West Virginia	24·4 (23·8 to 25)	21·5 (20·9 to 22)	15·8 (15·4 to 16·3)	14·9 (14·4 to 15·3)	11·7 (11·4 to 12·1)	10·5 (10·2 to 10·9)	10·5 (10·3 to 10·8)	9 (8·7 to 9·3)	8·7 (8·2 to 9·3)
Wisconsin	40·6 (39·6 to 41·7)	45·6 (44·3 to 46·7)	37·7 (36·7 to 38·6)	38·2 (37·2 to 39·3)	37·2 (36·2 to 38·3)	35·1 (34·2 to 36)	34·5 (33·4 to 35·6)	31·8 (30·9 to 32·8)	31·5 (29·6 to 33·4)
Wyoming	3·5 (3·4 to 3·6)	4·1 (4 to 4·3)	3·1 (3 to 3·3)	5·5 (5·3 to 5·7)	3·5 (3·4 to 3·7)	3·2 (3·1 to 3·3)	3·5 (3·4 to 3·6)	3·2 (3 to 3·3)	3·1 (2·9 to 3·4)
Southern Latin America	343 (333 to 354)	408 (402 to 414)	436 (428 to 442)	504 (497 to 512)	528 (520 to 536)	508 (495 to 520)	532 (520 to 544)	421 (409 to 433)	394 (350 to 441)
Argentina	225 (216 to 234)	242 (237 to 247)	283 (277 to 288)	349 (342 to 356)	347 (341 to 353)	352 (340 to 363)	383 (373 to 394)	298 (289 to 308)	275 (242 to 311)
Chile	95·7 (93·2 to 98·2)	134 (131 to 136)	124 (122 to 127)	128 (125 to 131)	152 (149 to 156)	129 (126 to 132)	126 (123 to 130)	104 (99·9 to 108)	100 (89·5 to 112)
Uruguay	22·8 (21·6 to 24)	31·7 (30·8 to 32·6)	28·6 (27·6 to 29·5)	27·6 (26·9 to 28·4)	28·3 (27·5 to 29)	26·9 (25·4 to 28·5)	22·3 (20·5 to 24·2)	19 (18·2 to 19·9)	18·2 (16·2 to 20·4)
Western Europe	2890 (2810 to 2970)	3120 (3090 to 3150)	2960 (2930 to 2980)	2470 (2440 to 2500)	2360 (2340 to 2390)	2230 (2200 to 2250)	2340 (2310 to 2370)	2120 (2090 to 2150)	2100 (1970 to 2230)
Andorra	0·1 (0·1 to 0·1)	0·1 (0·1 to 0·1)	0·2 (0·2 to 0·2)	0·3 (0·3 to 0·3)	0·3 (0·3 to 0·3)	0·4 (0·3 to 0·4)	0·4 (0·4 to 0·4)	0·3 (0·3 to 0·3)	0·3 (0·3 to 0·3)
Austria	54·3 (52·5 to 56·3)	66 (64·8 to 67·1)	56·6 (55·7 to 57·7)	46·8 (45·8 to 47·8)	47·5 (46·5 to 48·6)	39·7 (39 to 40·6)	40·2 (39·2 to 41·3)	43·4 (42·5 to 44·4)	44·1 (41·4 to 46·9)
Belgium	73·2 (70·8 to 75·7)	80·8 (79·5 to 82·1)	72·3 (71·1 to 73·6)	63·4 (62·1 to 64·6)	64 (62·9 to 65·1)	59·5 (58·6 to 60·5)	65·9 (64·8 to 67·1)	59·1 (57·9 to 60·2)	57·9 (52·3 to 63·9)
Cyprus	7·2 (6·9 to 7·4)	7·4 (7·3 to 7·6)	6·2 (6 to 6·4)	6·9 (6·7 to 7·2)	7·6 (7·4 to 7·8)	5·8 (5·5 to 6)	6·9 (6·6 to 7·1)	7·8 (7·2 to 8·5)	7·8 (6·8 to 9)
Denmark	40·3 (39 to 41·8)	39·2 (38·4 to 39·9)	37·2 (36·1 to 38·3)	28·4 (27·6 to 29·1)	32·8 (32·1 to 33·5)	34·1 (33·3 to 34·8)	31·4 (30·8 to 32)	31·2 (30·4 to 32)	32·4 (30·7 to 34·2)
Finland	49 (47·6 to 50·7)	41·7 (40·8 to 42·4)	32·2 (31·5 to 32·9)	32·7 (31·8 to 33·6)	33·2 (32·3 to 34·1)	28·4 (27·7 to 29·1)	30·7 (30 to 31·3)	23·8 (23 to 24·6)	24·9 (23·2 to 26·5)
France	430 (418 to 442)	425 (417 to 433)	439 (431 to 447)	408 (397 to 419)	390 (380 to 399)	396 (388 to 404)	408 (401 to 415)	361 (353 to 370)	354 (318 to 392)
Germany	568 (526 to 611)	648 (636 to 661)	526 (515 to 537)	438 (428 to 448)	442 (432 to 452)	389 (378 to 399)	344 (336 to 352)	399 (385 to 414)	405 (381 to 429)
Greece	80·3 (77·2 to 83·5)	80·1 (78·1 to 82)	74·4 (72·7 to 76)	74·2 (72·6 to 76)	52·6 (51·1 to 54·1)	52·8 (51·6 to 54·1)	57·4 (56 to 58·8)	43·9 (42·3 to 45·3)	42·4 (37·8 to 47·4)
Iceland	2·1 (2 to 2·2)	2·4 (2·3 to 2·5)	2·2 (2·1 to 2·2)	2·2 (2·2 to 2·3)	2·4 (2·3 to 2·5)	2·1 (2 to 2·2)	2·4 (2·3 to 2·5)	2·3 (2·2 to 2·4)	2·4 (2·2 to 2·6)
Ireland	32·8 (31·9 to 33·9)	32·2 (31·7 to 32·6)	34·5 (34 to 35)	38·1 (37·4 to 38·8)	27·8 (27·2 to 28·4)	29·6 (28·9 to 30·3)	37·8 (36·9 to 38·7)	30·2 (29·4 to 31·1)	29·6 (27·7 to 31·5)
Israel	24·1 (23·4 to 24·9)	28·5 (27·9 to 29)	41·2 (40·5 to 41·9)	47·7 (46·8 to 48·6)	53·1 (51·9 to 54·1)	69·8 (68·3 to 71·3)	84·8 (83·1 to 86·5)	92·4 (90·3 to 94·8)	94·1 (89·4 to 99)
Italy	454 (439 to 469)	474 (467 to 482)	470 (462 to 478)	330 (324 to 336)	291 (286 to 297)	278 (273 to 284)	287 (280 to 294)	214 (209 to 219)	205 (182 to 229)

Luxembourg	2·3 (2·2 to 2·5)	2·6 (2·5 to 2·7)	2·2 (2·1 to 2·3)	2·1 (2·1 to 2·2)	2·6 (2·5 to 2·6)	2·9 (2·8 to 3)	2·9 (2·8 to 3)	3·2 (3 to 3·4)	3·4 (3·1 to 3·6)
Malta	5 (4·8 to 5·3)	4·2 (4 to 4·3)	2·8 (2·6 to 2·9)	3 (2·9 to 3·1)	2·8 (2·7 to 2·9)	2·2 (2·1 to 2·2)	2·1 (2 to 2·2)	2·3 (2·2 to 2·4)	2·2 (2 to 2·5)
Monaco	0·2 (0·1 to 0·2)	0·1 (0·1 to 0·2)	0·1 (0·1 to 0·2)	0·2 (0·1 to 0·2)	0·2 (0·1 to 0·2)	0·2 (0·1 to 0·2)	0·2 (0·1 to 0·2)	0·2 (0·1 to 0·2)	0·2 (0·1 to 0·2)
Netherlands	118 (115 to 122)	126 (124 to 128)	119 (117 to 121)	91·5 (89·5 to 93·5)	101 (98·8 to 103)	105 (103 to 108)	93·7 (91·7 to 95·8)	87 (84·9 to 89·2)	91 (85·6 to 96·5)
Norway	31·9 (30·9 to 32·9)	32·1 (31·5 to 32·6)	33 (32·3 to 33·6)	24·6 (24 to 25·1)	30 (29·4 to 30·6)	29·8 (29·2 to 30·4)	31·1 (30·4 to 31·8)	27·6 (26·9 to 28·3)	28·8 (27·2 to 30·4)
Agder	1·7 (1·6 to 1·7)	1·6 (1·6 to 1·7)	1·8 (1·7 to 1·8)	1·5 (1·5 to 1·5)	1·7 (1·6 to 1·8)	1·7 (1·6 to 1·7)	1·8 (1·7 to 1·8)	1·5 (1·5 to 1·6)	1·6 (1·5 to 1·7)
Innlandet	2·9 (2·8 to 3)	2·7 (2·7 to 2·8)	2·6 (2·5 to 2·6)	1·8 (1·7 to 1·9)	2·1 (2·1 to 2·2)	2 (2 to 2·1)	1·9 (1·8 to 1·9)	1·6 (1·5 to 1·6)	1·6 (1·5 to 1·7)
Møre og Romsdal	2 (1·9 to 2·1)	2·1 (2 to 2·1)	2 (1·9 to 2·1)	1·5 (1·5 to 1·6)	1·6 (1·6 to 1·7)	1·6 (1·5 to 1·6)	1·5 (1·4 to 1·5)	1·3 (1·2 to 1·3)	1·3 (1·3 to 1·4)
Nordland	2·2 (2·2 to 2·3)	2·3 (2·3 to 2·4)	2·2 (2·1 to 2·2)	1·5 (1·5 to 1·6)	1·7 (1·6 to 1·7)	1·5 (1·4 to 1·5)	1·3 (1·3 to 1·4)	1·1 (1 to 1·1)	1·1 (1·1 to 1·2)
Oslo	4·1 (4 to 4·2)	3·5 (3·3 to 3·7)	3·4 (3·2 to 3·6)	2·5 (2·4 to 2·6)	3·6 (3·5 to 3·8)	4 (3·9 to 4·2)	5·2 (5 to 5·4)	4·7 (4·5 to 4·9)	5 (4·6 to 5·4)
Rogaland	2·2 (2·2 to 2·3)	2·4 (2·3 to 2·6)	2·6 (2·4 to 2·7)	2·3 (2·2 to 2·4)	2·8 (2·7 to 2·9)	2·8 (2·7 to 2·9)	3·2 (3·1 to 3·3)	2·7 (2·6 to 2·8)	2·8 (2·6 to 3·1)
Troms og Finnmark	2 (2 to 2·1)	2·2 (2·1 to 2·2)	2·2 (2·1 to 2·2)	1·5 (1·5 to 1·6)	1·7 (1·7 to 1·8)	1·6 (1·5 to 1·6)	1·4 (1·3 to 1·4)	1·2 (1·1 to 1·2)	1·2 (1·1 to 1·3)
Trøndelag	3 (2·9 to 3·1)	3 (2·9 to 3·1)	3·1 (3 to 3·3)	2·2 (2·2 to 2·3)	2·7 (2·6 to 2·8)	2·6 (2·5 to 2·7)	2·7 (2·6 to 2·8)	2·4 (2·3 to 2·6)	2·5 (2·4 to 2·7)
Vestfold og Telemark	2·6 (2·5 to 2·7)	2·6 (2·6 to 2·7)	2·6 (2·5 to 2·6)	1·9 (1·8 to 2)	2·2 (2·2 to 2·3)	2·2 (2·1 to 2·3)	2·1 (2·1 to 2·2)	1·8 (1·8 to 1·9)	1·9 (1·7 to 2)
Vestland	4·3 (4·2 to 4·4)	4·3 (4·2 to 4·4)	4·4 (4·2 to 4·5)	3·3 (3·2 to 3·4)	3·9 (3·8 to 4)	3·8 (3·7 to 3·9)	3·8 (3·7 to 3·9)	3·4 (3·3 to 3·5)	3·5 (3·3 to 3·7)
Viken	4·8 (4·7 to 5)	5·3 (5·1 to 5·4)	6·3 (6·1 to 6·5)	4·5 (4·4 to 4·7)	5·9 (5·7 to 6)	6·1 (5·9 to 6·3)	6·2 (6 to 6·4)	5·9 (5·7 to 6·1)	6·2 (5·8 to 6·6)
Portugal	106 (103 to 111)	112 (110 to 115)	93·4 (91·7 to 95·2)	79·7 (78 to 81·7)	59·9 (58·4 to 61·4)	59·9 (58·4 to 61·3)	50·9 (49·7 to 52·2)	44·2 (43 to 45·5)	41·2 (38·5 to 44·1)
San Marino	0·1 (0·1 to 0·2)	0·1 (0·1 to 0·2)	0·2 (0·2 to 0·2)	0·1 (0·1 to 0·1)	0·1 (0·1 to 0·1)	0·2 (0·1 to 0·2)	0·2 (0·2 to 0·2)	0·1 (0·1 to 0·1)	0·1 (0·1 to 0·1)
Spain	288 (279 to 298)	333 (327 to 339)	340 (332 to 347)	284 (279 to 290)	205 (200 to 209)	205 (199 to 211)	248 (242 to 253)	184 (179 to 188)	173 (161 to 186)
Sweden	58·8 (56·9 to 61)	53·1 (52·1 to 54·2)	57·2 (55·9 to 58·5)	49·1 (48 to 50·1)	63 (61·7 to 64·5)	46·9 (45·7 to 48)	58 (56·7 to 59·3)	58·3 (57·1 to 59·5)	58·5 (55·2 to 62)
Stockholm	8·4 (8·2 to 8·8)	7·9 (7·6 to 8·1)	11·2 (10·8 to 11·5)	9·6 (9·3 to 9·9)	12·8 (12·5 to 13·2)	11·4 (10·9 to 11·9)	14·8 (14·3 to 15·4)	14·6 (14·1 to 15)	14·9 (13·9 to 15·9)
Sweden except Stockholm	50·3 (48·7 to 52·3)	45·3 (44·3 to 46·2)	46 (45 to 47·1)	39·5 (38·6 to 40·3)	50·2 (49·1 to 51·4)	35·5 (34·7 to 36·3)	43·2 (42·2 to 44·1)	43·8 (42·8 to 44·7)	43·7 (41·2 to 46·3)
Switzerland	42·7 (41·4 to 44·1)	49·3 (48·4 to 50·1)	51·8 (50·9 to 52·8)	38·9 (38·1 to 39·7)	44·5 (43·5 to 45·6)	39·3 (38·5 to 40·1)	41·4 (40·5 to 42·2)	44·2 (43·3 to 45·2)	45·7 (42·8 to 48·9)
UK	416 (404 to 428)	479 (471 to 487)	463 (456 to 470)	378 (367 to 388)	406 (394 to 419)	347 (337 to 358)	412 (402 to 424)	358 (347 to 369)	351 (312 to 391)
England	334 (324 to 344)	387 (379 to 395)	378 (370 to 385)	309 (300 to 318)	340 (330 to 350)	292 (284 to 302)	350 (342 to 361)	306 (298 to 316)	301 (267 to 335)
East Midlands	27·2 (26·4 to 28)	31·5 (30·8 to 32·2)	31·4 (30·4 to 32·4)	25·5 (24·7 to 26·3)	27·9 (27 to 28·8)	23·4 (22·6 to 24·2)	28·2 (27·5 to 29)	24·6 (23·8 to 25·5)	24·3 (21·7 to 27)
Derby	1·9 (1·8 to 1·9)	2·2 (2·1 to 2·2)	2·1 (2 to 2·1)	1·6 (1·5 to 1·6)	1·8 (1·7 to 1·8)	1·5 (1·4 to 1·5)	1·8 (1·7 to 1·9)	1·5 (1·5 to 1·6)	1·5 (1·3 to 1·6)
Derbyshire	5 (4·9 to 5·2)	5·6 (5·4 to 5·8)	5·5 (5·3 to 5·7)	4·3 (4·2 to 4·4)	4·7 (4·5 to 4·8)	3·8 (3·7 to 4)	4·3 (4·2 to 4·5)	3·7 (3·5 to 3·8)	3·6 (3·2 to 4)
Leicester	2·5 (2·4 to 2·5)	2·8 (2·7 to 2·9)	2·9 (2·8 to 3·1)	2·5 (2·4 to 2·5)	2·4 (2·3 to 2·5)	2 (2 to 2·1)	2·6 (2·5 to 2·7)	2·3 (2·2 to 2·4)	2·3 (2 to 2·5)
Leicestershire	3·3 (3·2 to 3·4)	4 (3·9 to 4·1)	4 (3·9 to 4·2)	3·4 (3·3 to 3·5)	3·8 (3·6 to 3·9)	3·3 (3·2 to 3·4)	3·6 (3·5 to 3·7)	3·4 (3·3 to 3·5)	3·4 (3 to 3·7)
Lincolnshire	3·6 (3·5 to 3·7)	4 (3·9 to 4·2)	4·1 (3·9 to 4·2)	3·4 (3·2 to 3·5)	3·6 (3·5 to 3·8)	3·2 (3 to 3·3)	4·1 (4 to 4·2)	3·5 (3·3 to 3·6)	3·4 (3 to 3·8)
Northamptonshire	3·5 (3·4 to 3·6)	4 (3·9 to 4·2)	4·2 (4·1 to 4·4)	3·7 (3·6 to 3·8)	4·3 (4·1 to 4·4)	3·8 (3·7 to 4)	4·8 (4·7 to 4·9)	4·3 (4·1 to 4·4)	4·2 (3·7 to 4·6)
Nottingham	2·4 (2·3 to 2·4)	2·8 (2·6 to 2·9)	2·6 (2·5 to 2·7)	1·9 (1·8 to 2)	2·3 (2·2 to 2·4)	1·7 (1·7 to 1·8)	2·1 (2 to 2·2)	1·9 (1·8 to 2)	1·9 (1·7 to 2·1)
Nottinghamshire	4·9 (4·8 to 5·1)	5·9 (5·7 to 6)	5·8 (5·6 to 6)	4·5 (4·4 to 4·7)	4·9 (4·7 to 5)	3·9 (3·8 to 4·1)	4·6 (4·5 to 4·8)	4 (3·8 to 4·2)	3·9 (3·5 to 4·4)
Rutland	0·2 (0·2 to 0·2)	0·2 (0·2 to 0·3)	0·2 (0·2 to 0·2)	0·2 (0·2 to 0·2)	0·2 (0·2 to 0·2)	0·2 (0·2 to 0·2)	0·2 (0·2 to 0·2)	0·2 (0·2 to 0·2)	0·2 (0·1 to 0·2)
East of England	31·6 (30·7 to 32·5)	37·9 (37 to 38·8)	37·5 (36·5 to 38·3)	32·2 (31·1 to 33·3)	35·3 (34 to 36·6)	31·4 (30·3 to 32·4)	37·4 (36·3 to 38·7)	33·8 (32·6 to 35)	33·1 (29·4 to 37)
Bedford	1 (0·9 to 1)	1·2 (1·1 to 1·3)	1·2 (1·1 to 1·2)	1 (0·9 to 1)	0·9 (0·9 to 1)	0·9 (0·9 to 0·9)	1·1 (1 to 1·1)	1·1 (1 to 1·1)	1 (0·9 to 1·2)
Cambridgeshire	2·4 (2·3 to 2·5)	2·9 (2·9 to 3)	3·1 (3 to 3·2)	2·9 (2·8 to 3)	3·4 (3·2 to 3·5)	3·1 (3 to 3·2)	3·9 (3·7 to 4)	3·3 (3·2 to 3·5)	3·3 (2·9 to 3·7)
Central Bedfordshire	1·3 (1·2 to 1·3)	1·7 (1·6 to 1·8)	1·8 (1·7 to 1·8)	1·7 (1·6 to 1·7)	1·6 (1·6 to 1·7)	1·4 (1·4 to 1·5)	1·6 (1·6 to 1·7)	1·7 (1·6 to 1·7)	1·7 (1·5 to 1·8)
Essex	7·6 (7·4 to 7·8)	9·5 (9 to 9·9)	9·4 (9 to 9·8)	7·9 (7·6 to 8·2)	8·4 (8·1 to 8·7)	7·3 (7 to 7·6)	8·4 (8·1 to 8·7)	7·9 (7·6 to 8·3)	7·8 (6·9 to 8·7)
Hertfordshire	6·7 (6·5 to 6·9)	8 (7·7 to 8·2)	7·3 (7·1 to 7·5)	6·2 (6 to 6·4)	6·9 (6·6 to 7·2)	6·5 (6·3 to 6·7)	7·6 (7·4 to 7·9)	6·8 (6·6 to 7·1)	6·7 (6 to 7·5)
Luton	1·3 (1·3 to 1·3)	1·8 (1·7 to 1·8)	1·8 (1·7 to 1·8)	1·5 (1·4 to 1·5)	1·7 (1·6 to 1·7)	1·5 (1·4 to 1·5)	1·8 (1·8 to 1·9)	1·7 (1·6 to 1·7)	1·6 (1·4 to 1·8)
Norfolk	4·3 (4·2 to 4·4)	4·8 (4·7 to 5)	4·9 (4·7 to 5)	4·2 (4·1 to 4·3)	4·6 (4·4 to 4·8)	4 (3·8 to 4·1)	4·7 (4·5 to 4·9)	4·1 (3·9 to 4·2)	3·9 (3·5 to 4·4)
Peterborough	0·9 (0·8 to 0·9)	1 (1 to 1·1)	1 (1 to 1·1)	1·1 (1 to 1·1)	1·3 (1·2 to 1·3)	1·1 (1 to 1·1)	1·6 (1·6 to 1·7)	1·4 (1·3 to 1·5)	1·4 (1·2 to 1·6)
Southend-on-Sea	1·3 (1·3 to 1·4)	1·3 (1·2 to 1·4)	1·2 (1·1 to 1·2)	1 (0·9 to 1)	1·1 (1·1 to 1·2)	1 (1 to 1)	1·2 (1·1 to 1·2)	1 (1 to 1·1)	1 (0·9 to 1·1)
Suffolk	3·9 (3·8 to 4·1)	4·5 (4·4 to 4·7)	4·6 (4·5 to 4·7)	4 (3·8 to 4·1)	4·3 (4·2 to 4·5)	3·7 (3·5 to 3·8)	4·3 (4·2 to 4·4)	3·6 (3·5 to 3·8)	3·5 (3·1 to 4)
Thurrock	1 (0·9 to 1)	1·2 (1·1 to 1·2)	1·2 (1·2 to 1·3)	0·9 (0·9 to 1)	1·1 (1 to 1·1)	1 (0·9 to 1)	1·2 (1·2 to 1·3)	1·2 (1·2 to 1·3)	1·2 (1 to 1·3)
Greater London	59 (57 to 60·9)	65·1 (63·5 to 66·7)	60 (58·7 to 61·3)	46·8 (45·4 to 48·1)	53·5 (51·8 to 55·2)	52·7 (51 to 54·5)	67·1 (65·1 to 69·4)	58·7 (56·8 to 60·5)	57·3 (50·6 to 64·4)
Barking and Dagenham	1·6 (1·6 to 1·7)	1·6 (1·6 to 1·7)	1·4 (1·4 to 1·4)	1·1 (1·1 to 1·1)	1·3 (1·3 to 1·3)	1·2 (1·2 to 1·3)	1·9 (1·8 to 2)	1·8 (1·7 to 1·8)	1·7 (1·5 to 1·9)
Barnet	2·4 (2·3 to 2·4)	2·4 (2·3 to 2·4)	2·2 (2·2 to 2·3)	1·9 (1·8 to 2)	2·1 (2 to 2·2)	2·1 (2 to 2·1)	2·8 (2·7 to 2·9)	2·5 (2·4 to 2·6)	2·4 (2·1 to 2·7)
Bexley	1·7 (1·7 to 1·8)	1·9 (1·9 to 2)	1·7 (1·7 to 1·8)	1·4 (1·3 to 1·4)	1·6 (1·6 to 1·7)	1·3 (1·3 to 1·4)	1·5 (1·5 to 1·6)	1·5 (1·4 to 1·5)	1·4 (1·3 to 1·6)
Brent	2·3 (2·2 to 2·4)	2·8 (2·6 to 3)	2·6 (2·5 to 2·7)	1·9 (1·8 to 2·1)	2 (1·9 to 2·1)	2 (1·9 to 2·1)	2·9 (2·8 to 3)	2·4 (2·3 to 2·5)	2·4 (2·1 to 2·7)
Bromley	2·3 (2·3 to 2·4)	2·4 (2·3 to 2·5)	2 (2 to 2·1)	1·7 (1·6 to 1·7)	1·9 (1·8 to 2)	1·8 (1·7 to 1·8)	2 (2 to 2·1)	1·9 (1·9 to 2)	1·9 (1·7 to 2·1)
Camden	1·2 (1·1 to 1·2)	1·4 (1·3 to 1·5)	1·5 (1·4 to 1·6)	1·1 (1·1 to 1·1)	1·2 (1·1 to 1·2)	1·4 (1·3 to 1·4)	1·4 (1·4 to 1·5)	1·2 (1·2 to 1·3)	1·2 (1 to 1·3)
Croydon	2·8 (2·7 to 2·9)	3·1 (3 to 3·2)	2·8 (2·7 to 2·9)	2·2 (2·2 to 2·3)	2·5 (2·4 to 2·6)	2·3 (2·2 to 2·4)	2·9 (2·8 to 3)	2·7 (2·6 to 2·8)	2·6 (2·3 to 2·9)

Ealing	2·5 (2·5 to 2·6)	2·8 (2·7 to 2·9)	2·7 (2·6 to 2·8)	2·2 (2·1 to 2·3)	2·3 (2·2 to 2·4)	2·2 (2·1 to 2·3)	3 (2·9 to 3·1)	2·4 (2·3 to 2·4)	2·3 (2 to 2·6)
Enfield	2·2 (2·1 to 2·3)	2·2 (2·1 to 2·3)	2 (2 to 2·1)	1·7 (1·6 to 1·7)	2 (1·9 to 2·1)	1·9 (1·9 to 2)	2·6 (2·5 to 2·7)	2·2 (2·1 to 2·3)	2·1 (1·9 to 2·4)
Greenwich	1·9 (1·9 to 2)	1·9 (1·9 to 2)	1·8 (1·8 to 1·9)	1·6 (1·5 to 1·6)	1·8 (1·7 to 1·8)	1·6 (1·6 to 1·7)	2·3 (2·2 to 2·4)	2·1 (2 to 2·2)	2·1 (1·8 to 2·3)
Hackney	2 (1·9 to 2·1)	2·4 (2·3 to 2·5)	2·2 (2·2 to 2·3)	1·7 (1·6 to 1·7)	1·9 (1·9 to 2)	2 (2 to 2·1)	2·3 (2·3 to 2·4)	2 (2 to 2·1)	2 (1·8 to 2·3)
Hammersmith and Fulham	1·2 (1·1 to 1·2)	1·4 (1·3 to 1·4)	1·4 (1·3 to 1·4)	1 (1 to 1)	1·1 (1·1 to 1·2)	1·2 (1·2 to 1·3)	1·4 (1·4 to 1·5)	1·1 (1 to 1·2)	1·1 (0·9 to 1·2)
Haringey	1·7 (1·6 to 1·8)	2·1 (2·1 to 2·2)	2·1 (2 to 2·1)	1·5 (1·4 to 1·5)	1·8 (1·7 to 1·8)	1·9 (1·8 to 1·9)	2·3 (2·2 to 2·4)	1·8 (1·7 to 1·9)	1·7 (1·5 to 1·9)
Harrow	1·6 (1·6 to 1·7)	1·7 (1·7 to 1·8)	1·5 (1·5 to 1·6)	1·3 (1·2 to 1·3)	1·4 (1·4 to 1·5)	1·3 (1·2 to 1·4)	1·8 (1·7 to 1·8)	1·8 (1·7 to 1·8)	1·7 (1·5 to 2)
Havering	2 (1·9 to 2)	2·2 (2·2 to 2·3)	1·9 (1·8 to 1·9)	1·4 (1·4 to 1·5)	1·5 (1·4 to 1·5)	1·2 (1·2 to 1·3)	1·4 (1·4 to 1·5)	1·6 (1·6 to 1·7)	1·6 (1·4 to 1·8)
Hillingdon	2 (1·9 to 2)	2·2 (2·1 to 2·2)	1·9 (1·8 to 1·9)	1·5 (1·5 to 1·6)	1·8 (1·7 to 1·9)	1·7 (1·6 to 1·7)	2·1 (2·1 to 2·2)	2·1 (2 to 2·1)	2 (1·8 to 2·3)
Hounslow	1·6 (1·6 to 1·7)	1·8 (1·8 to 1·9)	1·7 (1·7 to 1·8)	1·5 (1·4 to 1·5)	1·6 (1·6 to 1·7)	1·6 (1·6 to 1·7)	2·3 (2·2 to 2·4)	1·9 (1·9 to 2)	1·9 (1·7 to 2·1)
Islington	1·4 (1·4 to 1·5)	1·8 (1·7 to 1·9)	1·6 (1·6 to 1·7)	1·1 (1·1 to 1·2)	1·4 (1·3 to 1·4)	1·3 (1·2 to 1·3)	1·5 (1·4 to 1·5)	1·3 (1·3 to 1·4)	1·3 (1·2 to 1·5)
Kensington and Chelsea	0·9 (0·9 to 1)	1·2 (1·2 to 1·3)	1·6 (1·5 to 1·7)	0·9 (0·9 to 1)	0·9 (0·9 to 1)	1 (1 to 1·1)	1·1 (1 to 1·1)	0·8 (0·7 to 0·8)	0·7 (0·7 to 0·9)
Kingston upon Thames	1 (1 to 1)	0·9 (0·9 to 1)	0·9 (0·8 to 0·9)	0·8 (0·7 to 0·8)	0·9 (0·9 to 0·9)	0·9 (0·9 to 0·9)	1·1 (1·1 to 1·1)	1 (0·9 to 1)	1 (0·9 to 1·1)
Lambeth	2·5 (2·4 to 2·5)	3 (2·9 to 3·1)	2·6 (2·6 to 2·7)	1·9 (1·8 to 1·9)	2·2 (2·1 to 2·3)	2·2 (2·1 to 2·3)	2·4 (2·3 to 2·5)	1·9 (1·8 to 2)	1·8 (1·6 to 2·1)
Lewisham	2·3 (2·2 to 2·3)	2·5 (2·5 to 2·6)	2·2 (2·1 to 2·3)	1·6 (1·6 to 1·7)	2 (1·9 to 2·1)	1·9 (1·9 to 2)	2·5 (2·4 to 2·6)	2·2 (2·1 to 2·2)	2·1 (1·8 to 2·3)
Merton	1·5 (1·4 to 1·5)	1·5 (1·4 to 1·5)	1·3 (1·2 to 1·3)	1·1 (1 to 1·1)	1·3 (1·3 to 1·3)	1·3 (1·3 to 1·4)	1·7 (1·6 to 1·8)	1·4 (1·4 to 1·5)	1·4 (1·2 to 1·6)
Newham	2·3 (2·2 to 2·4)	2·6 (2·4 to 2·9)	2·5 (2·3 to 2·7)	2 (1·8 to 2·1)	2·3 (2·1 to 2·4)	2·4 (2·3 to 2·5)	3·4 (3·2 to 3·6)	2·8 (2·7 to 2·9)	2·7 (2·4 to 3·1)
Redbridge	1·9 (1·8 to 1·9)	1·9 (1·8 to 2)	1·8 (1·7 to 1·8)	1·4 (1·3 to 1·4)	1·6 (1·6 to 1·7)	1·6 (1·5 to 1·7)	2·3 (2·2 to 2·4)	2·3 (2·2 to 2·4)	2·2 (2 to 2·5)
Richmond upon Thames	1·2 (1·2 to 1·2)	1·1 (1·1 to 1·2)	1·1 (1 to 1·1)	0·9 (0·9 to 1)	1·1 (1·1 to 1·1)	1·2 (1·2 to 1·2)	1·4 (1·4 to 1·5)	1·1 (1 to 1·1)	1 (0·9 to 1·2)
Southwark	2·4 (2·3 to 2·5)	2·7 (2·6 to 2·7)	2·2 (2·1 to 2·2)	1·6 (1·6 to 1·7)	2 (2 to 2·1)	2 (2 to 2·1)	2·4 (2·4 to 2·5)	1·9 (1·9 to 2)	1·9 (1·6 to 2·1)
Sutton	1·3 (1·3 to 1·4)	1·4 (1·3 to 1·4)	1·2 (1·2 to 1·3)	1 (1 to 1·1)	1·2 (1·2 to 1·3)	1·1 (1·1 to 1·2)	1·4 (1·3 to 1·4)	1·3 (1·2 to 1·3)	1·2 (1·1 to 1·4)
Tower Hamlets	1·8 (1·7 to 1·8)	2·1 (2 to 2·1)	1·7 (1·7 to 1·8)	1·4 (1·4 to 1·5)	1·7 (1·6 to 1·7)	1·8 (1·7 to 1·9)	2·3 (2·2 to 2·3)	2·2 (2·1 to 2·3)	2·2 (1·9 to 2·5)
Waltham Forest	2·1 (2 to 2·1)	2·1 (2 to 2·3)	2·1 (2 to 2·3)	1·6 (1·5 to 1·7)	1·9 (1·8 to 2)	1·8 (1·7 to 1·9)	2·5 (2·4 to 2·6)	2·2 (2·1 to 2·3)	2·2 (1·9 to 2·5)
Wandsworth	2·3 (2·2 to 2·3)	2·4 (2·3 to 2·5)	2·2 (2·2 to 2·3)	1·8 (1·7 to 1·9)	2 (1·9 to 2·1)	2 (2 to 2·1)	2·7 (2·6 to 2·8)	2·2 (2·1 to 2·4)	2·2 (1·9 to 2·5)
Westminster	1·3 (1·3 to 1·4)	1·6 (1·5 to 1·7)	1·6 (1·5 to 1·7)	1·1 (1 to 1·1)	1·2 (1·2 to 1·3)	1·3 (1·2 to 1·3)	1·4 (1·3 to 1·4)	1·2 (1·1 to 1·2)	1·1 (1 to 1·3)
North East England	20 (19·4 to 20·6)	24·1 (23·1 to 25·2)	23·3 (22·7 to 23·9)	18·1 (17·5 to 18·7)	17·9 (17·3 to 18·5)	13·6 (13·1 to 14·1)	15·6 (15·2 to 16·1)	13 (12·5 to 13·4)	12·6 (11·3 to 14·1)
County Durham	3·7 (3·6 to 3·8)	4·5 (4·2 to 4·7)	4·3 (4·2 to 4·5)	3·4 (3·3 to 3·5)	3·3 (3·2 to 3·4)	2·5 (2·4 to 2·6)	2·9 (2·8 to 3)	2·4 (2·3 to 2·5)	2·3 (2·1 to 2·6)
Darlington	0·8 (0·7 to 0·8)	0·9 (0·8 to 0·9)	0·8 (0·8 to 0·9)	0·6 (0·6 to 0·7)	0·7 (0·7 to 0·8)	0·6 (0·5 to 0·6)	0·7 (0·7 to 0·7)	0·5 (0·5 to 0·6)	0·5 (0·5 to 0·6)
Gateshead	1·7 (1·7 to 1·8)	2·1 (2 to 2·2)	2 (1·9 to 2)	1·4 (1·3 to 1·4)	1·4 (1·3 to 1·4)	1 (1 to 1·1)	1·3 (1·2 to 1·3)	1 (1 to 1)	1 (0·9 to 1·1)
Hartlepool	0·7 (0·7 to 0·8)	1 (0·9 to 1)	0·9 (0·9 to 1)	0·7 (0·7 to 0·7)	0·7 (0·7 to 0·7)	0·5 (0·5 to 0·6)	0·6 (0·6 to 0·6)	0·5 (0·5 to 0·5)	0·5 (0·4 to 0·5)
Middlesbrough	1·2 (1·2 to 1·3)	1·6 (1·5 to 1·7)	1·5 (1·4 to 1·6)	1·2 (1·2 to 1·3)	1·2 (1·1 to 1·2)	0·9 (0·9 to 0·9)	1 (1 to 1·1)	0·9 (0·9 to 0·9)	0·9 (0·8 to 1)
Newcastle upon Tyne	2·2 (2·1 to 2·3)	2·5 (2·4 to 2·6)	2·4 (2·3 to 2·4)	1·8 (1·7 to 1·9)	1·9 (1·8 to 2)	1·4 (1·4 to 1·5)	1·7 (1·6 to 1·7)	1·5 (1·5 to 1·6)	1·5 (1·4 to 1·7)
North Tyneside	1·6 (1·5 to 1·6)	1·8 (1·7 to 1·9)	1·6 (1·6 to 1·7)	1·2 (1·2 to 1·3)	1·3 (1·2 to 1·3)	1 (1 to 1·1)	1·2 (1·2 to 1·3)	1·1 (1 to 1·1)	1 (0·9 to 1·2)
Northumberland	2·1 (2·1 to 2·2)	2·3 (2·2 to 2·4)	2·3 (2·3 to 2·4)	1·9 (1·9 to 2)	1·9 (1·8 to 1·9)	1·5 (1·4 to 1·5)	1·6 (1·5 to 1·7)	1·3 (1·2 to 1·3)	1·3 (1·1 to 1·4)
Redcar and Cleveland	1·1 (1 to 1·1)	1·4 (1·3 to 1·5)	1·4 (1·4 to 1·5)	1·1 (1·1 to 1·1)	1 (1 to 1·1)	0·7 (0·7 to 0·8)	0·8 (0·8 to 0·9)	0·6 (0·6 to 0·7)	0·6 (0·5 to 0·7)
South Tyneside	1·4 (1·3 to 1·4)	1·6 (1·6 to 1·7)	1·5 (1·4 to 1·5)	1 (1 to 1·1)	1·1 (1 to 1·1)	0·8 (0·8 to 0·8)	0·9 (0·8 to 0·9)	0·8 (0·7 to 0·8)	0·7 (0·7 to 0·8)
Stockton-on-Tees	1·2 (1·2 to 1·3)	1·6 (1·5 to 1·7)	1·6 (1·5 to 1·6)	1·4 (1·3 to 1·4)	1·3 (1·3 to 1·4)	1 (1 to 1·1)	1·3 (1·2 to 1·3)	1 (1 to 1·1)	1 (0·9 to 1·1)
Sunderland	2·3 (2·2 to 2·3)	2·8 (2·7 to 3)	2·9 (2·7 to 3)	2·3 (2·2 to 2·4)	2·2 (2·1 to 2·2)	1·5 (1·5 to 1·6)	1·6 (1·6 to 1·7)	1·3 (1·3 to 1·4)	1·3 (1·2 to 1·4)
North West England	52·3 (50·7 to 53·9)	62·1 (60·6 to 63·5)	60·4 (59·1 to 61·6)	47·2 (45·7 to 48·6)	50·1 (48·2 to 51·9)	39·2 (37·9 to 40·5)	45·5 (44·2 to 46·9)	40 (38·8 to 41·3)	39·1 (34·9 to 43·4)
Blackburn with Darwen	1·3 (1·2 to 1·3)	1·4 (1·3 to 1·4)	1·4 (1·4 to 1·4)	1·2 (1·2 to 1·3)	1·2 (1·2 to 1·3)	1·1 (1 to 1·1)	1·2 (1·2 to 1·3)	1 (0·9 to 1)	1 (0·9 to 1·1)
Blackpool	1·1 (1·1 to 1·1)	1·1 (1 to 1·1)	1 (0·9 to 1)	0·8 (0·7 to 0·8)	1 (0·9 to 1)	0·8 (0·7 to 0·8)	0·9 (0·9 to 0·9)	0·8 (0·7 to 0·8)	0·7 (0·7 to 0·8)
Bolton	2·1 (2 to 2·1)	2·4 (2·3 to 2·4)	2·4 (2·4 to 2·5)	2 (1·9 to 2)	2 (1·9 to 2·1)	1·7 (1·6 to 1·7)	2 (2 to 2·1)	1·8 (1·8 to 1·9)	1·8 (1·6 to 2)
Bury	1·2 (1·2 to 1·3)	1·5 (1·5 to 1·6)	1·5 (1·5 to 1·6)	1·2 (1·1 to 1·2)	1·3 (1·3 to 1·4)	1·1 (1 to 1·1)	1·3 (1·2 to 1·3)	1·1 (1·1 to 1·1)	1·1 (1 to 1·2)
Cheshire East	2·1 (2 to 2·1)	2·4 (2·3 to 2·5)	2·3 (2·3 to 2·4)	1·9 (1·8 to 2)	2·1 (2·1 to 2·2)	1·8 (1·8 to 1·9)	2·1 (2 to 2·2)	1·8 (1·8 to 1·9)	1·8 (1·6 to 2)
Cheshire West and Chester	2 (2 to 2·1)	2·6 (2·5 to 2·6)	2·5 (2·4 to 2·5)	2 (1·9 to 2·1)	2·1 (2·1 to 2·2)	1·7 (1·7 to 1·8)	1·9 (1·9 to 2)	1·7 (1·6 to 1·8)	1·7 (1·5 to 1·9)
Cumbria	3·3 (3·2 to 3·4)	3·8 (3·7 to 3·9)	3·6 (3·5 to 3·7)	2·9 (2·8 to 3)	3·1 (3 to 3·3)	2·4 (2·3 to 2·5)	2·6 (2·5 to 2·7)	2·1 (2·1 to 2·2)	2·1 (1·9 to 2·3)
Halton	0·7 (0·7 to 0·7)	0·9 (0·8 to 0·9)	0·9 (0·9 to 1)	1 (0·9 to 1)	1 (1 to 1·1)	0·7 (0·7 to 0·8)	0·8 (0·8 to 0·9)	0·7 (0·6 to 0·7)	0·7 (0·6 to 0·7)
Knowsley	1·4 (1·4 to 1·5)	2·1 (2 to 2·1)	1·9 (1·9 to 2)	1·5 (1·5 to 1·6)	1·3 (1·3 to 1·4)	0·9 (0·9 to 1)	1 (0·9 to 1)	1 (0·9 to 1)	0·9 (0·8 to 1)
Lancashire	7·7 (7·5 to 7·9)	8·7 (8·4 to 9)	8·6 (8·2 to 9)	7·2 (6·9 to 7·5)	7·9 (7·6 to 8·2)	6·3 (6 to 6·5)	7·1 (6·8 to 7·3)	6·1 (5·9 to 6·3)	6 (5·3 to 6·6)
Liverpool	4·5 (4·4 to 4·7)	5·4 (5·1 to 5·7)	5·1 (4·9 to 5·4)	3·6 (3·5 to 3·8)	3·6 (3·5 to 3·8)	2·6 (2·5 to 2·7)	2·9 (2·8 to 3)	2·8 (2·7 to 2·9)	2·7 (2·4 to 3)
Manchester	4·2 (4 to 4·3)	5·1 (4·9 to 5·3)	4·8 (4·6 to 4·9)	3·4 (3·3 to 3·5)	3·7 (3·5 to 3·8)	2·8 (2·7 to 2·9)	3·9 (3·7 to 4)	3·6 (3·5 to 3·7)	3·6 (3·1 to 4)
Oldham	1·8 (1·7 to 1·9)	2·1 (2·1 to 2·2)	2·2 (2·1 to 2·2)	1·7 (1·6 to 1·7)	1·8 (1·7 to 1·8)	1·6 (1·5 to 1·6)	1·8 (1·7 to 1·8)	1·6 (1·5 to 1·6)	1·5 (1·4 to 1·7)
Rochdale	1·6 (1·6 to 1·7)	1·9 (1·8 to 1·9)	1·9 (1·8 to 1·9)	1·6 (1·6 to 1·7)	1·7 (1·6 to 1·8)	1·4 (1·3 to 1·4)	1·6 (1·6 to 1·7)	1·4 (1·4 to 1·5)	1·4 (1·3 to 1·6)
Salford	2·1 (2·1 to 2·2)	2·6 (2·5 to 2·6)	2·5 (2·4 to 2·6)	1·7 (1·6 to 1·7)	1·8 (1·7 to 1·9)	1·3 (1·3 to 1·4)	1·8 (1·7 to 1·8)	1·8 (1·7 to 1·8)	1·7 (1·5 to 1·9)
Sefton	2·2 (2·1 to 2·3)	2·6 (2·6 to 2·7)	2·4 (2·3 to 2·4)	1·8 (1·8 to 1·9)	1·9 (1·8 to 2)	1·4 (1·3 to 1·4)	1·5 (1·4 to 1·5)	1·3 (1·2 to 1·3)	1·3 (1·1 to 1·4)

St Helens	1·4 (1·4 to 1·5)	1·7 (1·7 to 1·8)	1·8 (1·7 to 1·8)	1·3 (1·3 to 1·3)	1·2 (1·2 to 1·3)	1 (0·9 to 1)	1·1 (1 to 1·1)	0·9 (0·9 to 1)	0·9 (0·8 to 1)
Stockport	2·1 (2 to 2·2)	2·6 (2·5 to 2·7)	2·5 (2·4 to 2·5)	1·9 (1·8 to 2)	1·9 (1·9 to 2)	1·5 (1·5 to 1·6)	1·7 (1·7 to 1·8)	1·6 (1·5 to 1·6)	1·6 (1·4 to 1·7)
Tameside	1·7 (1·7 to 1·8)	2 (1·9 to 2)	2 (1·9 to 2)	1·5 (1·5 to 1·6)	1·7 (1·6 to 1·8)	1·3 (1·2 to 1·3)	1·6 (1·5 to 1·6)	1·4 (1·3 to 1·4)	1·3 (1·2 to 1·5)
Trafford	1·7 (1·6 to 1·7)	2 (1·9 to 2·1)	1·8 (1·8 to 1·9)	1·4 (1·4 to 1·5)	1·5 (1·4 to 1·6)	1·2 (1·2 to 1·3)	1·5 (1·4 to 1·5)	1·3 (1·2 to 1·3)	1·2 (1·1 to 1·4)
Warrington	1·2 (1·1 to 1·2)	1·5 (1·4 to 1·5)	1·4 (1·4 to 1·5)	1·1 (1·1 to 1·2)	1·4 (1·3 to 1·4)	1·2 (1·1 to 1·2)	1·3 (1·2 to 1·3)	1·1 (1 to 1·1)	1 (0·9 to 1·1)
Wigan	2·2 (2·1 to 2·2)	2·7 (2·6 to 2·8)	2·9 (2·9 to 3)	2·2 (2·1 to 2·3)	2·2 (2·1 to 2·3)	1·7 (1·7 to 1·8)	2 (1·9 to 2·1)	1·7 (1·6 to 1·8)	1·7 (1·5 to 1·8)
Wirral	2·7 (2·6 to 2·8)	3·2 (3·1 to 3·2)	3 (2·9 to 3)	2·3 (2·2 to 2·3)	2·4 (2·3 to 2·5)	1·7 (1·7 to 1·8)	2 (1·9 to 2)	1·6 (1·5 to 1·6)	1·5 (1·4 to 1·7)
South East England	42·7 (41·3 to 44·1)	48·3 (46·9 to 49·7)	48·7 (47·3 to 49·9)	45·1 (43·7 to 46·6)	51·3 (49·7 to 53)	46 (44·5 to 47·6)	54·5 (53·1 to 55·9)	47·4 (46 to 48·9)	46·8 (41·6 to 51·9)
Bracknell Forest	0·4 (0·4 to 0·4)	0·5 (0·5 to 0·6)	0·6 (0·5 to 0·6)	0·6 (0·6 to 0·7)	0·8 (0·7 to 0·8)	0·7 (0·7 to 0·8)	0·8 (0·8 to 0·8)	0·7 (0·7 to 0·7)	0·7 (0·6 to 0·8)
Brighton and Hove	1·6 (1·6 to 1·7)	1·4 (1·3 to 1·4)	1·3 (1·3 to 1·4)	1·2 (1·2 to 1·3)	1·5 (1·5 to 1·6)	1·4 (1·4 to 1·5)	1·7 (1·6 to 1·7)	1·2 (1·2 to 1·3)	1·2 (1·1 to 1·4)
Buckinghamshire	2·6 (2·5 to 2·7)	3·1 (3 to 3·2)	3 (2·9 to 3·1)	2·8 (2·7 to 2·9)	3·1 (3 to 3·2)	2·8 (2·7 to 2·9)	3·2 (3·1 to 3·3)	2·9 (2·8 to 3)	2·8 (2·5 to 3·2)
East Sussex	2·5 (2·5 to 2·6)	2·4 (2·3 to 2·5)	2·4 (2·3 to 2·5)	2·2 (2·1 to 2·3)	2·7 (2·6 to 2·8)	2·4 (2·3 to 2·5)	2·8 (2·7 to 2·9)	2·4 (2·3 to 2·5)	2·4 (2·1 to 2·6)
Hampshire	5·8 (5·6 to 6)	7·4 (7·2 to 7·7)	7·8 (7·6 to 8)	7·1 (6·9 to 7·3)	7·9 (7·7 to 8·2)	6·8 (6·5 to 7·1)	7·8 (7·6 to 8·1)	6·7 (6·5 to 6·9)	6·6 (5·9 to 7·3)
Isle of Wight	0·7 (0·7 to 0·8)	0·7 (0·7 to 0·7)	0·7 (0·7 to 0·7)	0·6 (0·6 to 0·7)	0·7 (0·7 to 0·7)	0·6 (0·6 to 0·6)	0·7 (0·6 to 0·7)	0·5 (0·5 to 0·6)	0·5 (0·5 to 0·6)
Kent	7·6 (7·4 to 7·9)	8·7 (8·4 to 9)	8·8 (8·5 to 9)	7·6 (7·4 to 7·9)	8·7 (8·4 to 9)	7·6 (7·3 to 7·9)	9 (8·8 to 9·3)	8·3 (8·1 to 8·6)	8·3 (7·4 to 9·1)
Medway	1·5 (1·4 to 1·5)	2 (1·9 to 2·1)	2·3 (2·2 to 2·4)	2 (1·9 to 2)	2 (1·9 to 2·1)	1·6 (1·5 to 1·7)	1·8 (1·8 to 1·9)	1·7 (1·6 to 1·8)	1·7 (1·5 to 1·9)
Milton Keynes	0·5 (0·5 to 0·5)	0·5 (0·5 to 0·6)	0·7 (0·7 to 0·7)	1·2 (1·1 to 1·2)	1·5 (1·5 to 1·6)	1·5 (1·4 to 1·5)	2 (2 to 2·1)	1·7 (1·6 to 1·7)	1·6 (1·4 to 1·8)
Oxfordshire	2·8 (2·7 to 2·9)	3·5 (3·4 to 3·6)	3·6 (3·5 to 3·7)	3·5 (3·4 to 3·6)	4 (3·9 to 4·1)	3·6 (3·5 to 3·8)	4·2 (4·1 to 4·4)	3·6 (3·5 to 3·7)	3·6 (3·2 to 4)
Portsmouth	1·3 (1·3 to 1·4)	1·4 (1·3 to 1·6)	1·5 (1·4 to 1·6)	1·2 (1·2 to 1·3)	1·4 (1·3 to 1·4)	1·1 (1·1 to 1·2)	1·3 (1·2 to 1·3)	1·2 (1·1 to 1·2)	1·2 (1 to 1·3)
Reading	0·8 (0·8 to 0·9)	1 (1 to 1·1)	1·1 (1·1 to 1·2)	1 (1 to 1·1)	1 (1 to 1·1)	1 (1 to 1·1)	1·3 (1·3 to 1·4)	1·1 (1 to 1·1)	1·1 (0·9 to 1·2)
Slough	0·8 (0·8 to 0·8)	1 (0·9 to 1)	1 (0·9 to 1)	0·9 (0·8 to 0·9)	1 (0·9 to 1)	0·9 (0·9 to 1)	1·4 (1·4 to 1·5)	1·2 (1·1 to 1·3)	1·2 (1 to 1·3)
Southampton	1·4 (1·4 to 1·4)	1·6 (1·6 to 1·7)	1·7 (1·7 to 1·8)	1·4 (1·4 to 1·5)	1·5 (1·5 to 1·6)	1·3 (1·2 to 1·3)	1·6 (1·6 to 1·7)	1·5 (1·4 to 1·5)	1·4 (1·3 to 1·6)
Surrey	6·5 (6·3 to 6·7)	6·6 (6·4 to 6·8)	6 (5·9 to 6·2)	5·5 (5·4 to 5·7)	6·4 (6·2 to 6·6)	6·1 (5·9 to 6·3)	7·1 (6·9 to 7·4)	6·2 (6 to 6·4)	6·1 (5·4 to 6·7)
West Berkshire	0·7 (0·6 to 0·7)	0·8 (0·8 to 0·8)	0·8 (0·8 to 0·9)	0·8 (0·8 to 0·9)	1 (1 to 1)	0·9 (0·8 to 0·9)	1 (1 to 1·1)	0·8 (0·8 to 0·8)	0·8 (0·7 to 0·9)
West Sussex	3·8 (3·7 to 4)	3·9 (3·8 to 4·1)	3·8 (3·7 to 3·9)	3·8 (3·7 to 3·9)	4·3 (4·2 to 4·4)	4 (3·9 to 4·2)	4·7 (4·6 to 4·9)	4·2 (4 to 4·3)	4·1 (3·7 to 4·6)
Windsor and Maidenhead	0·8 (0·7 to 0·8)	0·9 (0·8 to 0·9)	0·8 (0·8 to 0·9)	0·8 (0·8 to 0·8)	0·8 (0·8 to 0·9)	0·8 (0·8 to 0·9)	0·9 (0·9 to 1)	0·8 (0·7 to 0·8)	0·7 (0·7 to 0·8)
Wokingham	0·5 (0·5 to 0·5)	0·7 (0·7 to 0·7)	0·7 (0·7 to 0·8)	0·7 (0·7 to 0·7)	1 (0·9 to 1)	0·9 (0·8 to 0·9)	1 (0·9 to 1)	0·9 (0·8 to 0·9)	0·8 (0·8 to 0·9)
South West England	26·3 (25·5 to 27·1)	29 (28·1 to 30)	29·5 (28·6 to 30·4)	26·1 (25·2 to 27)	29·9 (29 to 30·9)	25·7 (24·8 to 26·7)	30·8 (29·9 to 31·8)	26·6 (25·8 to 27·5)	26·2 (23·5 to 29·3)
Bath and North East Somerset	0·9 (0·9 to 1)	0·9 (0·9 to 1)	0·9 (0·9 to 0·9)	0·8 (0·8 to 0·9)	0·9 (0·9 to 1)	0·9 (0·8 to 0·9)	0·9 (0·9 to 0·9)	0·9 (0·8 to 0·9)	0·9 (0·8 to 1)
Bournemouth	1 (0·9 to 1)	0·8 (0·8 to 0·9)	0·8 (0·7 to 0·8)	0·7 (0·6 to 0·7)	0·9 (0·8 to 0·9)	0·8 (0·8 to 0·8)	1·2 (1·1 to 1·2)	1·1 (1 to 1·1)	1·1 (1 to 1·2)
Bristol, City of	2·7 (2·7 to 2·8)	3·1 (3 to 3·2)	3·1 (3 to 3·2)	2·6 (2·5 to 2·7)	2·9 (2·7 to 3)	2·4 (2·3 to 2·5)	3·1 (3 to 3·2)	2·8 (2·7 to 2·9)	2·7 (2·4 to 3·1)
Cornwall	2·6 (2·5 to 2·7)	2·7 (2·6 to 2·8)	2·8 (2·7 to 2·9)	2·5 (2·4 to 2·6)	2·9 (2·8 to 3)	2·4 (2·3 to 2·5)	2·9 (2·8 to 3)	2·5 (2·4 to 2·6)	2·5 (2·2 to 2·7)
Devon	3·4 (3·3 to 3·6)	3·5 (3·2 to 3·7)	3·4 (3·3 to 3·6)	3·2 (3 to 3·3)	3·8 (3·7 to 4)	3·3 (3·1 to 3·4)	3·8 (3·7 to 4)	3·3 (3·2 to 3·4)	3·3 (2·9 to 3·6)
Dorset	1·9 (1·8 to 1·9)	1·9 (1·8 to 2·1)	1·9 (1·8 to 2)	1·8 (1·7 to 1·9)	2 (1·9 to 2·1)	1·7 (1·6 to 1·8)	2 (1·9 to 2)	1·5 (1·4 to 1·6)	1·4 (1·3 to 1·6)
Gloucestershire	3 (2·9 to 3·1)	3·5 (3·3 to 3·6)	3·5 (3·4 to 3·6)	3·1 (3 to 3·2)	3·5 (3·4 to 3·7)	3 (2·9 to 3·2)	3·5 (3·4 to 3·7)	3·1 (3 to 3·2)	3 (2·7 to 3·4)
North Somerset	0·9 (0·9 to 1)	1 (1 to 1)	1 (1 to 1·1)	1 (0·9 to 1)	1 (1 to 1·1)	1 (0·9 to 1)	1·2 (1·1 to 1·2)	1 (1 to 1)	1 (0·9 to 1·1)
Plymouth	1·7 (1·6 to 1·7)	2 (1·9 to 2·1)	2·1 (2 to 2·1)	1·8 (1·7 to 1·9)	1·8 (1·7 to 1·9)	1·4 (1·3 to 1·4)	1·6 (1·6 to 1·7)	1·3 (1·3 to 1·4)	1·3 (1·2 to 1·5)
Poole	0·7 (0·7 to 0·7)	0·8 (0·7 to 0·8)	0·8 (0·7 to 0·8)	0·7 (0·6 to 0·7)	0·9 (0·8 to 0·9)	0·7 (0·7 to 0·8)	0·9 (0·9 to 0·9)	0·8 (0·7 to 0·8)	0·8 (0·7 to 0·9)
Somerset	2·6 (2·5 to 2·7)	2·8 (2·7 to 2·9)	2·9 (2·8 to 3)	2·6 (2·5 to 2·7)	2·9 (2·8 to 3·1)	2·5 (2·4 to 2·6)	3 (2·9 to 3·1)	2·6 (2·5 to 2·7)	2·5 (2·3 to 2·8)
South Gloucestershire	1·1 (1 to 1·1)	1·5 (1·4 to 1·5)	1·5 (1·5 to 1·5)	1·3 (1·2 to 1·3)	1·6 (1·5 to 1·6)	1·4 (1·4 to 1·5)	1·6 (1·5 to 1·6)	1·6 (1·5 to 1·6)	1·6 (1·4 to 1·7)
Swindon	0·9 (0·9 to 0·9)	1·2 (1·2 to 1·3)	1·2 (1·2 to 1·3)	1·1 (1·1 to 1·1)	1·4 (1·3 to 1·4)	1·2 (1·1 to 1·2)	1·5 (1·5 to 1·6)	1·3 (1·3 to 1·4)	1·3 (1·2 to 1·4)
Torbay	0·7 (0·7 to 0·7)	0·6 (0·6 to 0·7)	0·6 (0·6 to 0·6)	0·5 (0·5 to 0·6)	0·7 (0·7 to 0·7)	0·6 (0·6 to 0·6)	0·7 (0·7 to 0·8)	0·6 (0·6 to 0·6)	0·6 (0·5 to 0·6)
Wiltshire	2·2 (2·1 to 2·3)	2·8 (2·7 to 2·9)	3 (2·9 to 3·1)	2·5 (2·4 to 2·6)	2·7 (2·6 to 2·8)	2·4 (2·3 to 2·6)	2·9 (2·8 to 3)	2·4 (2·3 to 2·5)	2·3 (2·1 to 2·6)
West Midlands	38·6 (37·4 to 39·8)	47·2 (46·1 to 48·4)	46·6 (45·6 to 47·6)	35·3 (34·3 to 36·4)	38·3 (37·2 to 39·5)	31·7 (30·7 to 32·7)	37·2 (36·2 to 38·3)	33·2 (32·1 to 34·3)	32·7 (29·1 to 36·3)
Birmingham	9·3 (9 to 9·6)	10·8 (10·5 to 11·2)	10·5 (10·1 to 10·9)	8 (7·7 to 8·2)	8·6 (8·3 to 8·9)	7·4 (7·1 to 7·6)	8·7 (8·5 to 9)	7·8 (7·5 to 8)	7·6 (6·8 to 8·5)
Coventry	2·6 (2·5 to 2·6)	3·2 (3·1 to 3·3)	3·1 (3 to 3·3)	2·3 (2·2 to 2·4)	2·4 (2·3 to 2·4)	1·9 (1·8 to 1·9)	2·3 (2·3 to 2·4)	2·1 (2 to 2·2)	2·1 (1·8 to 2·3)
Dudley	2·2 (2·1 to 2·2)	2·8 (2·7 to 3)	2·8 (2·7 to 2·9)	2 (1·9 to 2)	2·2 (2·1 to 2·2)	1·8 (1·7 to 1·9)	2 (1·9 to 2·1)	1·8 (1·7 to 1·9)	1·8 (1·6 to 2)
Herefordshire, County of	1 (1 to 1)	1·1 (1·1 to 1·2)	1·1 (1 to 1·1)	0·9 (0·8 to 0·9)	1 (1 to 1·1)	0·8 (0·8 to 0·9)	1 (0·9 to 1)	0·8 (0·8 to 0·9)	0·8 (0·7 to 0·9)
Sandwell	2·9 (2·8 to 2·9)	3·3 (3·2 to 3·5)	3·2 (3·1 to 3·3)	2·2 (2·2 to 2·3)	2·3 (2·2 to 2·4)	1·9 (1·9 to 2)	2·6 (2·5 to 2·7)	2·2 (2·2 to 2·3)	2·2 (2 to 2·5)
Shropshire	1·6 (1·5 to 1·6)	1·9 (1·8 to 2)	1·9 (1·8 to 2)	1·5 (1·4 to 1·5)	1·7 (1·6 to 1·7)	1·4 (1·4 to 1·5)	1·6 (1·5 to 1·6)	1·4 (1·3 to 1·4)	1·4 (1·2 to 1·5)
Solihull	1·3 (1·3 to 1·3)	1·7 (1·6 to 1·8)	1·7 (1·6 to 1·8)	1·2 (1·1 to 1·2)	1·3 (1·3 to 1·4)	1 (1 to 1·1)	1·2 (1·1 to 1·2)	1·1 (1·1 to 1·1)	1·1 (1 to 1·2)
Staffordshire	4·7 (4·6 to 4·9)	6·2 (5·9 to 6·5)	6·4 (6·1 to 6·6)	5 (4·8 to 5·2)	5·3 (5·2 to 5·5)	4·2 (4 to 4·3)	4·7 (4·5 to 4·8)	4·2 (4 to 4·3)	4·1 (3·7 to 4·6)
Stoke-on-Trent	2·2 (2·2 to 2·3)	2·5 (2·4 to 2·6)	2·5 (2·4 to 2·6)	1·8 (1·7 to 1·8)	1·9 (1·8 to 2)	1·4 (1·4 to 1·5)	1·9 (1·8 to 2)	1·6 (1·5 to 1·7)	1·6 (1·4 to 1·7)
Telford and Wrekin	0·7 (0·7 to 0·7)	0·9 (0·8 to 0·9)	1 (0·9 to 1)	1 (0·9 to 1)	1·1 (1·1 to 1·2)	1 (0·9 to 1)	1·2 (1·1 to 1·2)	1 (1 to 1·1)	1 (0·9 to 1·1)
Walsall	2·1 (2 to 2·2)	2·8 (2·7 to 2·9)	2·7 (2·6 to 2·8)	1·8 (1·8 to 1·9)	2 (1·9 to 2)	1·7 (1·6 to 1·8)	1·9 (1·9 to 2)	1·8 (1·7 to 1·9)	1·8 (1·6 to 2)

Warwickshire	3·1 (3 to 3·2)	3·8 (3·7 to 4)	3·8 (3·7 to 4)	2·9 (2·8 to 3)	3·2 (3·1 to 3·3)	2·7 (2·6 to 2·8)	3·2 (3·1 to 3·3)	2·9 (2·8 to 3)	2·9 (2·6 to 3·2)
Wolverhampton	2·2 (2·1 to 2·2)	2·6 (2·6 to 2·7)	2·5 (2·4 to 2·5)	1·8 (1·7 to 1·9)	2 (1·9 to 2)	1·5 (1·4 to 1·6)	1·8 (1·7 to 1·9)	1·6 (1·6 to 1·7)	1·6 (1·4 to 1·8)
Worcestershire	2·9 (2·8 to 3)	3·4 (3·2 to 3·6)	3·5 (3·4 to 3·7)	3 (2·9 to 3·1)	3·3 (3·2 to 3·4)	2·9 (2·8 to 3)	3·3 (3·2 to 3·4)	2·8 (2·7 to 2·9)	2·8 (2·5 to 3·1)
Yorkshire and the Humber	36·6 (35·5 to 37·7)	42 (41 to 43)	40·6 (39·5 to 41·6)	32·6 (31·6 to 33·6)	35·3 (34·1 to 36·4)	28·8 (27·8 to 29·8)	34·2 (33·3 to 35·2)	29·2 (28·2 to 30·2)	28·6 (25·4 to 31·8)
Barnsley	1·7 (1·7 to 1·8)	2·1 (2 to 2·1)	2 (2 to 2·1)	1·5 (1·5 to 1·6)	1·6 (1·5 to 1·7)	1·2 (1·1 to 1·3)	1·5 (1·5 to 1·6)	1·3 (1·3 to 1·4)	1·3 (1·1 to 1·4)
Bradford	4·1 (3·9 to 4·2)	4·7 (4·5 to 4·8)	4·5 (4·4 to 4·7)	3·8 (3·7 to 4)	4 (3·8 to 4·1)	3·6 (3·5 to 3·8)	4·4 (4·3 to 4·6)	3·6 (3·4 to 3·7)	3·5 (3·1 to 3·9)
Calderdale	1·6 (1·6 to 1·7)	1·7 (1·6 to 1·7)	1·6 (1·6 to 1·7)	1·3 (1·3 to 1·4)	1·4 (1·4 to 1·5)	1·2 (1·1 to 1·2)	1·4 (1·4 to 1·5)	1·1 (1·1 to 1·2)	1·1 (1 to 1·2)
Doncaster	2·2 (2·1 to 2·3)	2·7 (2·6 to 2·8)	2·6 (2·5 to 2·6)	2·1 (2 to 2·1)	2·2 (2·1 to 2·3)	1·7 (1·6 to 1·7)	2 (2 to 2·1)	1·7 (1·6 to 1·8)	1·7 (1·5 to 1·9)
East Riding of Yorkshire	1·7 (1·7 to 1·8)	1·9 (1·9 to 2)	1·8 (1·8 to 1·9)	1·5 (1·4 to 1·5)	1·7 (1·7 to 1·8)	1·5 (1·4 to 1·5)	1·7 (1·6 to 1·7)	1·4 (1·3 to 1·4)	1·4 (1·2 to 1·5)
Kingston upon Hull, City of	2·2 (2·1 to 2·2)	2·6 (2·5 to 2·7)	2·6 (2·5 to 2·7)	2 (2 to 2·1)	2·2 (2·1 to 2·3)	1·5 (1·5 to 1·6)	1·8 (1·8 to 1·9)	1·6 (1·5 to 1·7)	1·5 (1·4 to 1·7)
Kirklees	3 (2·9 to 3)	3·3 (3·2 to 3·4)	3·3 (3·2 to 3·4)	2·7 (2·6 to 2·8)	2·8 (2·7 to 2·9)	2·5 (2·4 to 2·6)	3 (2·9 to 3·1)	2·4 (2·4 to 2·5)	2·4 (2·1 to 2·7)
Leeds	5·5 (5·3 to 5·7)	6·3 (6·1 to 6·4)	5·9 (5·7 to 6·1)	4·6 (4·4 to 4·7)	5 (4·8 to 5·2)	4 (3·9 to 4·1)	4·9 (4·8 to 5·1)	4·6 (4·4 to 4·8)	4·5 (4 to 5)
North East Lincolnshire	1·2 (1·2 to 1·3)	1·6 (1·5 to 1·6)	1·5 (1·5 to 1·6)	1·2 (1·1 to 1·2)	1·2 (1·2 to 1·3)	0·9 (0·9 to 0·9)	1·1 (1 to 1·1)	0·8 (0·8 to 0·9)	0·8 (0·7 to 0·9)
North Lincolnshire	1·1 (1 to 1·1)	1·3 (1·3 to 1·4)	1·3 (1·2 to 1·3)	1 (1 to 1·1)	1·1 (1 to 1·1)	0·8 (0·8 to 0·9)	1 (1 to 1)	0·8 (0·8 to 0·8)	0·8 (0·7 to 0·9)
North Yorkshire	3·2 (3·1 to 3·3)	3·5 (3·4 to 3·6)	3·3 (3·2 to 3·4)	2·8 (2·7 to 2·9)	3·3 (3·2 to 3·4)	2·9 (2·8 to 3)	3·2 (3·1 to 3·3)	2·6 (2·5 to 2·7)	2·5 (2·3 to 2·8)
Rotherham	1·8 (1·8 to 1·9)	2·3 (2·2 to 2·3)	2·3 (2·2 to 2·4)	1·8 (1·7 to 1·8)	1·9 (1·8 to 2)	1·4 (1·4 to 1·5)	1·7 (1·6 to 1·7)	1·4 (1·4 to 1·5)	1·4 (1·2 to 1·5)
Sheffield	3·9 (3·8 to 4)	4·3 (4·1 to 4·5)	4 (3·8 to 4·3)	3·1 (2·9 to 3·2)	3·5 (3·3 to 3·6)	2·9 (2·8 to 3)	3·3 (3·2 to 3·4)	3 (2·9 to 3·1)	3 (2·7 to 3·3)
Wakefield	2·3 (2·3 to 2·4)	2·8 (2·7 to 2·9)	2·7 (2·7 to 2·8)	2·2 (2·1 to 2·2)	2·3 (2·2 to 2·4)	1·7 (1·7 to 1·8)	2·2 (2·1 to 2·2)	2 (1·9 to 2·1)	1·9 (1·7 to 2·2)
York	1 (1 to 1·1)	1·1 (1·1 to 1·2)	1·1 (1 to 1·1)	1 (0·9 to 1)	1·1 (1 to 1·1)	0·9 (0·9 to 1)	1 (1 to 1·1)	0·9 (0·8 to 0·9)	0·9 (0·8 to 1)
Northern Ireland	14·9 (14·4 to 15·4)	16·4 (16·1 to 16·7)	16·4 (16·1 to 16·8)	14·2 (13·8 to 14·7)	13·3 (12·7 to 13·8)	11·2 (10·8 to 11·6)	12·9 (12·4 to 13·5)	11·2 (10·7 to 11·7)	10·7 (9·5 to 11·9)
Scotland	47·3 (45·8 to 48·9)	52·3 (51·3 to 53·4)	45·7 (44·8 to 46·6)	34·9 (33·8 to 36)	33·8 (32·5 to 35·2)	27·3 (26·3 to 28·3)	30·1 (29 to 31·3)	25·2 (24·2 to 26·3)	24·8 (22 to 27·7)
Wales	19·8 (19·2 to 20·4)	23·3 (22·9 to 23·7)	23·2 (22·8 to 23·6)	19·7 (19 to 20·3)	19·8 (19·1 to 20·6)	16·3 (15·8 to 16·9)	18·6 (17·9 to 19·5)	15 (14·5 to 15·6)	14·6 (12·9 to 16·2)
Latin America and Caribbean	3220 (3100 to 3350)	4110 (4000 to 4210)	4800 (4690 to 4900)	5260 (5160 to 5360)	5310 (5200 to 5430)	5450 (5330 to 5580)	5340 (5170 to 5520)	4980 (4690 to 5280)	4790 (4440 to 5160)
Andean Latin America	350 (338 to 362)	442 (429 to 453)	520 (508 to 532)	569 (556 to 583)	604 (587 to 622)	621 (601 to 640)	627 (601 to 652)	642 (597 to 688)	636 (586 to 688)
Bolivia	77·8 (73·9 to 81·6)	87·4 (83·9 to 90·8)	98·4 (94·3 to 102)	111 (107 to 115)	116 (112 to 120)	123 (118 to 128)	130 (124 to 137)	127 (118 to 138)	125 (114 to 138)
Ecuador	76·1 (72·2 to 80·2)	103 (101 to 105)	122 (119 to 125)	127 (122 to 132)	153 (145 to 160)	171 (162 to 180)	178 (167 to 188)	170 (157 to 184)	164 (146 to 186)
Peru	196 (189 to 203)	252 (244 to 259)	300 (291 to 308)	332 (322 to 342)	335 (324 to 346)	327 (315 to 340)	319 (303 to 335)	344 (315 to 378)	346 (313 to 383)
Caribbean	358 (349 to 366)	445 (437 to 452)	460 (451 to 469)	428 (418 to 438)	464 (451 to 476)	440 (424 to 455)	425 (407 to 443)	414 (386 to 444)	408 (376 to 445)
Antigua and Barbuda	0·8 (0·8 to 0·9)	0·9 (0·9 to 1)	0·8 (0·8 to 0·8)	0·6 (0·6 to 0·7)	0·6 (0·6 to 0·6)	0·7 (0·6 to 0·8)	0·6 (0·6 to 0·7)	0·5 (0·5 to 0·6)	0·5 (0·5 to 0·6)
The Bahamas	1·3 (1·3 to 1·4)	1·7 (1·7 to 1·8)	2·2 (2·1 to 2·2)	2·5 (2·5 to 2·7)	2·8 (2·6 to 2·9)	2·3 (2·1 to 2·4)	2·4 (2·3 to 2·6)	2 (1·7 to 2·4)	2 (1·7 to 2·3)
Barbados	3·4 (3·2 to 3·6)	3·7 (3·6 to 3·8)	2·6 (2·5 to 2·7)	2·2 (2·1 to 2·3)	2·1 (2 to 2·2)	2 (1·9 to 2·1)	1·7 (1·6 to 1·8)	1·4 (1·2 to 1·6)	1·3 (1·1 to 1·6)
Belize	1·4 (1·4 to 1·5)	2·2 (2·1 to 2·2)	2·5 (2·4 to 2·5)	2·9 (2·8 to 3)	3·2 (3·1 to 3·3)	3·4 (3·2 to 3·7)	3·8 (3·6 to 4)	3·8 (3·5 to 4·2)	3·8 (3·4 to 4·3)
Bermuda	0·6 (0·5 to 0·6)	0·6 (0·6 to 0·6)	0·5 (0·5 to 0·5)	0·4 (0·4 to 0·4)	0·5 (0·4 to 0·5)	0·4 (0·4 to 0·5)	0·4 (0·3 to 0·4)	0·3 (0·2 to 0·3)	0·3 (0·2 to 0·3)
Cuba	77·5 (73·7 to 81·2)	115 (113 to 118)	126 (122 to 129)	72·7 (70·2 to 75·3)	91·6 (88·1 to 95)	77·3 (74·1 to 80·5)	67·3 (64·6 to 70·1)	56 (53·5 to 58·3)	51·7 (48·1 to 55·5)
Dominica	1 (0·9 to 1)	1·4 (1·4 to 1·4)	1·4 (1·4 to 1·4)	1 (0·9 to 1)	1 (0·9 to 1)	0·6 (0·6 to 0·6)	0·5 (0·4 to 0·5)	0·3 (0·3 to 0·4)	0·3 (0·3 to 0·4)
Dominican Republic	68 (66·2 to 69·6)	87·8 (85·9 to 89·7)	96·5 (92 to 101)	107 (102 to 112)	117 (111 to 123)	113 (106 to 120)	102 (95·1 to 110)	110 (100 to 120)	109 (98·7 to 121)
Grenada	1·8 (1·7 to 1·8)	1·9 (1·9 to 2)	1·4 (1·4 to 1·5)	1·4 (1·3 to 1·4)	1·2 (1·1 to 1·3)	1·2 (1 to 1·3)	1 (0·9 to 1·1)	0·7 (0·6 to 0·8)	0·7 (0·6 to 0·8)
Guyana	10 (9·6 to 10·4)	13·3 (13 to 13·5)	13·6 (13·2 to 14)	13·5 (12·9 to 14·2)	14·1 (13·3 to 14·9)	10·5 (10·1 to 11)	8·3 (7·9 to 8·8)	8·2 (7·4 to 9·1)	7·9 (7 to 9)
Haiti	89·9 (85·8 to 93·8)	98·1 (93·7 to 102)	102 (98·7 to 106)	116 (113 to 120)	130 (125 to 134)	140 (133 to 148)	162 (152 to 171)	174 (156 to 192)	176 (157 to 197)
Jamaica	25·4 (23·7 to 27·1)	34·2 (33·3 to 35·2)	33 (32·2 to 33·9)	29·5 (28·6 to 30·5)	29 (28 to 30·2)	25·2 (22·6 to 28·2)	21·3 (20·3 to 22·3)	17·4 (15·5 to 19·3)	16·7 (14·5 to 19·1)
Puerto Rico	43·4 (42·5 to 44·4)	38·5 (37·9 to 39·1)	34·9 (34·1 to 35·7)	36·7 (35·8 to 37·8)	33·5 (32·6 to 34·5)	29·5 (28·2 to 30·8)	21·6 (19·8 to 23·6)	10·7 (10·4 to 11·1)	9·8 (9·1 to 10·5)
Saint Kitts and Nevis	0·9 (0·9 to 1)	1·1 (1·1 to 1·1)	0·6 (0·6 to 0·6)	0·6 (0·6 to 0·6)	0·5 (0·4 to 0·5)	0·4 (0·4 to 0·5)	0·3 (0·3 to 0·4)	0·3 (0·3 to 0·3)	0·3 (0·3 to 0·3)
Saint Lucia	1·5 (1·4 to 1·6)	2·1 (2 to 2·1)	2·2 (2·1 to 2·2)	2 (1·9 to 2·1)	1·8 (1·7 to 1·9)	1·4 (1·3 to 1·5)	1·1 (1 to 1·2)	0·9 (0·8 to 1)	0·9 (0·7 to 1)
Saint Vincent and the Grenadines	1·4 (1·4 to 1·5)	2 (2 to 2)	1·7 (1·7 to 1·8)	1·6 (1·6 to 1·7)	1·3 (1·2 to 1·4)	1·1 (1 to 1·1)	0·9 (0·9 to 0·9)	0·7 (0·7 to 0·8)	0·7 (0·6 to 0·8)
Suriname	4 (3·8 to 4·1)	6 (5·9 to 6·2)	6·7 (6·4 to 7·1)	5·3 (5·1 to 5·4)	4·9 (4·7 to 5·1)	4·9 (4·4 to 5·3)	5·1 (4·9 to 5·3)	4·8 (4·4 to 5·2)	4·6 (4·1 to 5·2)
Trinidad and Tobago	12·1 (11·7 to 12·5)	16·5 (16·2 to 16·8)	13·4 (13 to 13·7)	15·8 (15·2 to 16·3)	12·1 (11·6 to 12·6)	9·1 (8·7 to 9·5)	9·7 (9·1 to 10·3)	8·1 (7·5 to 8·7)	7·5 (6·7 to 8·5)
Virgin Islands	0·4 (0·4 to 0·5)	0·6 (0·6 to 0·6)	1·3 (1·3 to 1·4)	1·3 (1·2 to 1·3)	1·2 (1·1 to 1·3)	1·1 (0·9 to 1·2)	0·8 (0·7 to 0·9)	0·5 (0·4 to 0·5)	0·4 (0·4 to 0·5)
Central Latin America	1190 (1140 to 1250)	1670 (1640 to 1690)	2110 (2070 to 2140)	2280 (2230 to 2330)	2550 (2490 to 2610)	2500 (2440 to 2570)	2470 (2380 to 2560)	2100 (1950 to 2250)	1980 (1780 to 2200)
Colombia	255 (238 to 274)	351 (335 to 366)	401 (380 to 423)	419 (394 to 445)	472 (446 to 501)	462 (424 to 499)	399 (352 to 450)	354 (306 to 408)	346 (296 to 405)
Costa Rica	19·6 (19 to 20·2)	30·4 (30·2 to 30·6)	28·2 (27·6 to 28·9)	35·7 (34·8 to 36·7)	41·2 (39·8 to 42·5)	38·8 (37·7 to 40·1)	37 (35·7 to 38·2)	31·1 (29·6 to 32·7)	27·9 (25·4 to 30·5)
El Salvador	49·8 (48·6 to 51·2)	65·4 (64·4 to 66·4)	81·4 (79·7 to 82·8)	92·1 (90·1 to 94·2)	90 (86·9 to 93·4)	79·7 (75·5 to 83·9)	69·1 (65·8 to 72·7)	62·8 (57·1 to 68·8)	60·1 (52·7 to 67·8)
Guatemala	74·9 (73·9 to 76)	95·8 (94·3 to 97·3)	113 (111 to 115)	155 (152 to 157)	174 (171 to 177)	196 (190 to 202)	185 (179 to 192)	182 (172 to 192)	175 (157 to 195)
Honduras	38 (36·2 to 39·9)	47·1 (45·6 to 48·6)	60·1 (58·8 to 61·4)	79·4 (77·5 to 81·1)	92 (88·2 to 95·7)	99 (92·9 to 105)	106 (98·2 to 113)	112 (100 to 126)	113 (99·7 to 129)
Mexico	602 (573 to 634)	846 (827 to 864)	1150 (1130 to 1160)	1150 (1130 to 1180)	1300 (1260 to 1330)	1260 (1230 to 1290)	1260 (1220 to 1310)	1000 (938 to 1070)	940 (855 to 1030)
Aguascalientes	4·9 (4·7 to 5·2)	6·5 (6·4 to 6·7)	8·7 (8·6 to 8·9)	9·8 (9·5 to 10)	11·9 (11·5 to 12·3)	12·9 (12·5 to 13·4)	13·9 (13 to 14·7)	12·1 (11·3 to 13·1)	11·5 (10·4 to 12·7)

Baja California	5·4 (5·1 to 5·7)	12·3 (12 to 12·6)	20·3 (20 to 20·6)	18·6 (18 to 19·2)	25·1 (24·3 to 25·9)	29·4 (28·4 to 30·5)	32·9 (31·5 to 34·5)	27·7 (25·9 to 29·6)	26 (23·6 to 28·5)
Baja California Sur	1·3 (1·3 to 1·4)	1·9 (1·8 to 1·9)	2·9 (2·8 to 2·9)	3·5 (3·4 to 3·6)	4·4 (4·3 to 4·6)	5·2 (5 to 5·4)	6·7 (6·4 to 7)	5·8 (5·4 to 6·2)	5·5 (5 to 6)
Campeche	2·6 (2·5 to 2·8)	3·8 (3·7 to 3·9)	5·9 (5·8 to 6)	7·6 (7·3 to 7·8)	8·8 (8·5 to 9)	8·5 (8·2 to 8·8)	9·4 (9 to 9·7)	7·3 (6·8 to 7·9)	6·8 (6·2 to 7·5)
Chiapas	19·3 (18·2 to 20·4)	29·3 (28·3 to 30·1)	40·1 (39·2 to 40·9)	45·9 (44·4 to 47·4)	68·8 (66·7 to 71)	64·9 (62·5 to 67·4)	73·8 (70·6 to 77·1)	63·9 (59·4 to 68·5)	60·7 (54·7 to 66·6)
Chihuahua	20·8 (19·8 to 21·8)	30·2 (29·6 to 30·9)	38·5 (37·8 to 39·2)	31·8 (31 to 32·6)	35·2 (34·2 to 36·4)	40·2 (39 to 41·3)	35·7 (34·3 to 37·1)	27·9 (25·9 to 29·9)	26·2 (23·7 to 28·7)
Coahuila	17·4 (16·5 to 18·2)	22·5 (22 to 22·9)	27·1 (26·6 to 27·7)	25·6 (24·8 to 26·4)	29·2 (28·3 to 30·2)	28 (27 to 29·1)	30·5 (29·3 to 31·7)	26·1 (24·1 to 28·1)	24·4 (22·2 to 26·8)
Colima	3 (2·9 to 3·1)	4·2 (4·2 to 4·3)	5·9 (5·8 to 6)	5·9 (5·7 to 6·1)	6 (5·8 to 6·3)	5·8 (5·6 to 6·1)	6·7 (6·4 to 7)	5·4 (5 to 5·8)	5 (4·6 to 5·6)
Durango	15·9 (15·2 to 16·7)	20 (19·7 to 20·4)	23·6 (23·3 to 24)	21·7 (21 to 22·3)	23·3 (22·5 to 24)	21·1 (20·3 to 21·9)	19·9 (19 to 20·9)	16·5 (15·3 to 17·7)	15·5 (14·1 to 17·1)
Guanajuato	34·3 (32·8 to 36)	45·7 (44·6 to 46·8)	58·6 (57·7 to 59·5)	59·4 (57·8 to 61·1)	67 (64·9 to 69·3)	64·7 (62·8 to 66·7)	64·4 (61·5 to 67·2)	53 (49·2 to 56·9)	49·8 (45·1 to 54·9)
Guerrero	21·8 (20·7 to 22·9)	30·9 (30·1 to 31·6)	41·6 (40·9 to 42·3)	43·5 (42·2 to 44·9)	52 (50·3 to 53·8)	52·7 (50·9 to 54·5)	49·3 (47 to 51·6)	37·5 (34·7 to 40·2)	35 (31·6 to 38·5)
Hidalgo	18·3 (17·4 to 19·3)	23 (22·4 to 23·6)	28·2 (27·7 to 28·7)	28 (27·2 to 28·7)	32·3 (31·3 to 33·4)	29·9 (28·9 to 30·9)	30·2 (29·1 to 31·3)	23·2 (21·5 to 24·9)	21·8 (19·7 to 24)
Jalisco	44·5 (42·4 to 46·7)	60·8 (59·7 to 61·9)	80·5 (79·4 to 81·6)	75·8 (73·8 to 77·8)	84·1 (81·5 to 86·8)	79·8 (76·9 to 82·8)	82 (79 to 84·8)	68·5 (63·8 to 73·3)	64·4 (58·3 to 70·7)
México	30·7 (29·3 to 32·3)	45·4 (44·4 to 46·3)	91 (89·4 to 92·6)	129 (125 to 133)	152 (147 to 157)	163 (158 to 168)	162 (155 to 170)	123 (115 to 132)	115 (104 to 126)
Mexico City	69·3 (65·3 to 73·4)	107 (104 to 110)	138 (135 to 142)	118 (113 to 122)	103 (99·1 to 106)	82·1 (79·4 to 84·9)	72·1 (69·6 to 74·6)	49 (45·4 to 52·8)	44·5 (40·2 to 49·1)
Michoacán de Ocampo	36·4 (34·7 to 38·3)	47·8 (46·7 to 48·9)	58 (57·1 to 58·9)	57·6 (56 to 59·1)	62·8 (60·8 to 64·8)	54·2 (52·3 to 56·1)	53·6 (50·9 to 56·2)	43·4 (40·3 to 46·6)	40·7 (36·9 to 44·7)
Morelos	6·2 (5·9 to 6·6)	9·4 (9·2 to 9·6)	14·6 (14·4 to 14·9)	16·3 (15·9 to 16·6)	18·1 (17·5 to 18·7)	17·5 (16·8 to 18·1)	19·1 (18·2 to 19·9)	14·9 (13·9 to 15·8)	13·8 (12·6 to 15·2)
Nayarit	7·4 (7·1 to 7·8)	10·1 (9·9 to 10·2)	13·5 (13·3 to 13·8)	13 (12·7 to 13·3)	13·3 (12·9 to 13·6)	11·9 (11·6 to 12·3)	12·5 (12 to 13)	9·8 (9·1 to 10·5)	9·2 (8·3 to 10·1)
Nuevo León	17·9 (17 to 18·8)	27·7 (27·2 to 28·2)	39·3 (38·7 to 40)	36·4 (35·3 to 37·4)	41·2 (39·8 to 42·7)	45 (43·5 to 46·4)	47·6 (45·5 to 49·6)	42·9 (40·1 to 45·8)	41 (37·3 to 45)
Oaxaca	29·6 (27·9 to 31·4)	39·2 (37·8 to 40·5)	47·8 (47 to 48·7)	45 (43·8 to 46·3)	51 (49·4 to 52·7)	49·8 (48·3 to 51·6)	48·6 (46·5 to 50·9)	37 (34·4 to 39·7)	34·6 (31·4 to 38·2)
Puebla	37 (35·2 to 38·9)	48·2 (47 to 49·3)	63 (62·1 to 64)	64·3 (62·5 to 66·1)	71·6 (69·7 to 73·6)	72·8 (70·6 to 75)	74·1 (70·7 to 77·4)	60·9 (56·7 to 65·3)	57·5 (52·1 to 63·4)
Querétaro	7 (6·7 to 7·4)	9·2 (8·9 to 9·4)	12 (11·9 to 12·2)	13·7 (13·4 to 14·1)	18·6 (18 to 19·2)	18·7 (18 to 19·4)	20·4 (19·4 to 21·5)	18·8 (17·4 to 20·3)	18·1 (16·4 to 20)
Quintana Roo	0·6 (0·6 to 0·7)	1·2 (1·2 to 1·3)	2·2 (2·2 to 2·3)	4·5 (4·4 to 4·7)	8·1 (7·8 to 8·4)	11 (10·5 to 11·6)	14·6 (13·7 to 15·5)	14·6 (13·5 to 15·7)	14·2 (12·8 to 15·6)
San Luis Potosí	19·8 (18·9 to 20·8)	25·6 (25 to 26·1)	31·6 (31·2 to 32·1)	30·6 (29·7 to 31·6)	33·9 (32·9 to 34·9)	31 (30·1 to 32·1)	29·1 (27·7 to 30·5)	23 (21·4 to 24·7)	21·6 (19·6 to 23·7)
Sinaloa	15·8 (15 to 16·6)	21·9 (21·5 to 22·2)	32 (31·5 to 32·4)	32·3 (31·4 to 33·2)	34·2 (33·1 to 35·3)	33·1 (31·8 to 34·4)	29·8 (28·4 to 31·1)	23·6 (21·9 to 25·3)	22·1 (20·1 to 24·3)
Sonora	12·3 (11·7 to 12·9)	18·4 (18·1 to 18·8)	25 (24·6 to 25·4)	22·4 (21·8 to 23·1)	26·2 (25·4 to 27·1)	27·6 (26·8 to 28·5)	28·2 (27·1 to 29·3)	20·4 (18·9 to 22)	18·8 (17 to 20·6)
Tabasco	8·1 (7·7 to 8·6)	12·6 (12·4 to 12·9)	19·3 (19 to 19·6)	21·9 (21·4 to 22·5)	26·3 (25·5 to 27·2)	25·3 (24·2 to 26·3)	26·2 (25 to 27·5)	20·3 (18·9 to 21·7)	18·8 (17·1 to 20·7)
Tamaulipas	16·6 (15·8 to 17·4)	24·6 (24·1 to 25·1)	33·7 (33·2 to 34·3)	29·5 (28·5 to 30·5)	31·9 (30·8 to 33)	32·7 (31·6 to 33·9)	34·1 (32·7 to 35·5)	26·2 (24·3 to 27·9)	24·3 (22 to 26·7)
Tlaxcala	6·6 (6·3 to 6·9)	8·4 (8·2 to 8·6)	10 (9·8 to 10·2)	10·2 (9·9 to 10·4)	12·7 (12·3 to 13·1)	13·2 (12·8 to 13·7)	13·7 (13·1 to 14·3)	11·5 (10·7 to 12·3)	10·9 (9·8 to 12)
Veracruz de Ignacio de la Llave	44·1 (41·7 to 46·6)	63·4 (61·4 to 65·3)	90·2 (88·6 to 91·9)	93 (90·5 to 95·6)	102 (99·1 to 106)	89·5 (86·2 to 92·9)	83·2 (80 to 86·4)	58·5 (54·4 to 62·6)	53·7 (48·7 to 59·1)
Yucatán	10·5 (9·9 to 11·1)	13·3 (12·9 to 13·7)	17·7 (17·4 to 18·1)	18·2 (17·7 to 18·8)	20·7 (20 to 21·5)	19·2 (18·6 to 19·9)	19·4 (18·4 to 20·3)	16·5 (15·3 to 17·7)	15·6 (14·2 to 17·1)
Zacatecas	17·1 (16·3 to 18)	21·7 (21·3 to 22·1)	24·2 (23·7 to 24·7)	22·5 (21·9 to 23·1)	21·3 (20·5 to 22·1)	18·3 (17·7 to 19)	18 (17·2 to 18·8)	14·8 (13·6 to 16)	13·9 (12·6 to 15·3)
Nicaragua	28·4 (27 to 29·9)	37·1 (36 to 38·2)	48·4 (47·2 to 49·5)	65·4 (64 to 66·7)	72·6 (69·4 to 75·6)	68·1 (63·9 to 72·5)	69·3 (64·3 to 74·3)	66·9 (60·3 to 74)	65·2 (57·3 to 73·7)
Panama	13·3 (12·9 to 13·8)	22·9 (22·6 to 23·3)	27·5 (27 to 28·1)	27·6 (26·7 to 28·4)	30 (29 to 31)	32·4 (31·2 to 33·7)	36·2 (34·9 to 37·4)	36·9 (35·2 to 38·5)	36 (32·3 to 39·8)
Venezuela	112 (108 to 115)	170 (168 to 173)	201 (196 to 205)	248 (242 to 256)	282 (270 to 293)	269 (254 to 285)	306 (295 to 316)	248 (217 to 285)	212 (183 to 242)
Tropical Latin America	1320 (1230 to 1400)	1550 (1480 to 1630)	1710 (1640 to 1780)	1980 (1930 to 2040)	1700 (1650 to 1740)	1890 (1830 to 1950)	1820 (1750 to 1890)	1830 (1710 to 1960)	1770 (1630 to 1940)
Brazil	1280 (1200 to 1370)	1510 (1440 to 1590)	1670 (1600 to 1740)	1930 (1880 to 1980)	1630 (1580 to 1680)	1820 (1760 to 1880)	1750 (1690 to 1820)	1760 (1650 to 1890)	1710 (1570 to 1870)
Acre	3 (2·9 to 3·1)	3·9 (3·7 to 4)	4·9 (4·7 to 5)	6·5 (6·3 to 6·7)	6·1 (5·9 to 6·3)	8·6 (8·3 to 8·9)	9·7 (9·3 to 10·2)	10·3 (9·6 to 11·1)	10·3 (9·5 to 11·2)
Alagoas	30·3 (28·7 to 31·7)	32·5 (31·3 to 33·7)	37·5 (36·2 to 38·7)	42 (40·8 to 43·1)	38·6 (37·3 to 40)	45·8 (43·8 to 47·7)	44·7 (42·4 to 46·8)	43·6 (40·4 to 47·4)	42·9 (39 to 47·3)
Amapá	1 (1 to 1·1)	1·7 (1·6 to 1·8)	2·5 (2·4 to 2·6)	3·5 (3·4 to 3·7)	3·7 (3·6 to 3·9)	6·3 (6 to 6·5)	6·9 (6·5 to 7·3)	7·7 (7·1 to 8·4)	7·7 (7 to 8·5)
Amazonas	13·6 (12·8 to 14·4)	16·5 (15·7 to 17·3)	19·4 (18·5 to 20·4)	25·8 (25 to 26·6)	23·6 (22·5 to 24·5)	34·1 (32·5 to 35·6)	38·8 (36·8 to 40·9)	41·9 (38·9 to 45·4)	41·4 (38 to 45·3)
Bahia	132 (125 to 139)	150 (143 to 156)	172 (165 to 178)	192 (186 to 198)	147 (142 to 153)	168 (160 to 175)	162 (154 to 170)	154 (143 to 168)	150 (136 to 165)
Ceará	73·1 (69·1 to 76·8)	85·3 (81·7 to 88·8)	97·9 (94·1 to 102)	103 (99·7 to 107)	85·2 (81·9 to 88·7)	103 (98·1 to 108)	107 (101 to 113)	109 (101 to 118)	107 (97 to 118)
Distrito Federal	1 (1 to 1·1)	2·6 (2·4 to 2·7)	10·8 (10·4 to 11·2)	20·8 (20·2 to 21·4)	19·5 (18·9 to 20·1)	23·7 (22·9 to 24·4)	21·9 (21·1 to 22·8)	21·7 (20·5 to 23·1)	20·8 (19·4 to 22·4)
Espírito Santo	20·9 (19·6 to 22·2)	25·7 (24·5 to 26·8)	27·8 (26·6 to 29·1)	33·5 (32·5 to 34·5)	29·1 (28·2 to 30·1)	32·3 (31 to 33·7)	30·3 (29 to 31·6)	31·1 (28·6 to 33·7)	30·1 (27·5 to 33·2)
Goiás	25·2 (23·5 to 26·9)	35·6 (33·8 to 37·3)	43·7 (41·7 to 45·8)	49·2 (47·7 to 50·8)	42·1 (40·9 to 43·5)	48 (46 to 49·9)	44·3 (42·3 to 46·3)	47·6 (44·4 to 51·6)	46·4 (42·5 to 50·9)
Maranhão	46·5 (44·3 to 48·7)	65·4 (63 to 67·6)	75·9 (73·5 to 78·2)	93·4 (91 to 95·8)	80·1 (77 to 83·2)	98·4 (94·1 to 103)	110 (104 to 115)	114 (106 to 123)	114 (104 to 124)
Mato Grosso	4·7 (4·4 to 5)	7·7 (7·3 to 8·1)	12·9 (12·3 to 13·4)	19·8 (19·2 to 20·3)	21·2 (20·5 to 21·8)	24·8 (23·7 to 25·7)	24·7 (23·6 to 25·8)	28·7 (26·4 to 31·2)	28·3 (25·9 to 31·1)
Mato Grosso do Sul	7·9 (7·4 to 8·4)	12·1 (11·5 to 12·7)	17·1 (16·3 to 18)	22·6 (22 to 23·3)	20·2 (19·5 to 20·9)	21·7 (20·8 to 22·6)	21·6 (20·7 to 22·5)	22·8 (21·2 to 24·5)	22·2 (20·3 to 24·2)
Minas Gerais	192 (179 to 204)	212 (201 to 222)	194 (186 to 202)	200 (194 to 205)	169 (164 to 174)	174 (168 to 179)	155 (149 to 162)	152 (143 to 162)	148 (136 to 159)
Pará	29·3 (27·5 to 31·1)	35·1 (33·5 to 36·8)	44·1 (41·9 to 46·2)	62·7 (60·7 to 64·9)	55·4 (53·1 to 57·7)	73·9 (70·3 to 77·3)	80·9 (76·6 to 85·4)	84·2 (78·1 to 91·1)	83 (75·9 to 91·6)
Paraíba	46·9 (44·4 to 49·4)	50·1 (47·8 to 52·2)	49·7 (47·6 to 51·7)	45·1 (43·3 to 46·9)	36·2 (34·7 to 37·8)	42·6 (40·6 to 44·6)	44·8 (42·3 to 47·4)	44·8 (41·6 to 48·5)	43·8 (40·1 to 48)
Paraná	52 (48·4 to 55·6)	88·1 (83·7 to 92·5)	112 (107 to 118)	107 (104 to 111)	91·8 (88·7 to 94·7)	90·7 (87·2 to 94·2)	79·6 (76·3 to 83·1)	81·1 (75·2 to 88·1)	78·5 (71·6 to 86·2)
Pernambuco	97·1 (91·9 to 102)	108 (104 to 113)	118 (113 to 122)	119 (115 to 123)	92 (88·7 to 95·4)	106 (102 to 112)	103 (97·6 to 108)	98·1 (90·9 to 106)	95·3 (87·6 to 104)
Piauí	27·8 (26·3 to 29·2)	30·8 (29·5 to 32·1)	36·7 (35·2 to 38·1)	41·6 (40·2 to 42·9)	34·2 (32·9 to 35·4)	39·4 (37·7 to 41·1)	41·1 (38·6 to 43·4)	40·2 (37·1 to 43·7)	39·3 (35·8 to 43·4)
Rio de Janeiro	102 (93·2 to 112)	116 (108 to 124)	123 (116 to 130)	142 (138 to 147)	117 (114 to 120)	124 (120 to 128)	112 (108 to 117)	111 (104 to 118)	106 (98·2 to 115)

Rio Grande do Norte	26·8 (25·5 to 28·1)	29·8 (28·5 to 31)	33·8 (32·5 to 35·1)	36·1 (34·7 to 37·4)	32·3 (31·1 to 33·5)	39 (37·1 to 40·8)	38·7 (36·5 to 40·7)	37·9 (34·7 to 41·4)	36·9 (33·2 to 41)
Rio Grande do Sul	87·4 (79·8 to 95)	92·8 (86·9 to 99·1)	89·4 (85·4 to 93·4)	97·5 (94·5 to 100)	79·6 (77·4 to 82·2)	79·1 (76·5 to 81·6)	64·2 (62·2 to 66·4)	64·4 (60·9 to 68·2)	61·4 (57·4 to 65·9)
Rondônia	0·9 (0·9 to 1)	1·5 (1·5 to 1·6)	2·3 (2·2 to 2·4)	8·7 (8·4 to 8·9)	13·6 (13·1 to 14·1)	16·7 (15·9 to 17·4)	16 (15 to 17)	16·3 (15·1 to 17·6)	15·9 (14·6 to 17·3)
Roraima	0·4 (0·4 to 0·5)	0·6 (0·5 to 0·6)	0·8 (0·8 to 0·8)	1·4 (1·4 to 1·5)	2·6 (2·5 to 2·7)	4·4 (4·2 to 4·6)	5·6 (5·3 to 5·8)	7 (6·5 to 7·5)	7·2 (6·6 to 7·7)
Santa Catarina	34·3 (31·8 to 36·8)	39·3 (37·1 to 41·6)	40·2 (38·1 to 42·3)	46·7 (45 to 48·2)	41·5 (39·9 to 43·1)	43·6 (41·7 to 45·2)	41·5 (39·7 to 43·4)	48·9 (45·2 to 53)	48·2 (43·9 to 52·9)
São Paulo	206 (190 to 223)	247 (232 to 263)	273 (259 to 287)	378 (368 to 389)	316 (308 to 325)	332 (322 to 342)	306 (295 to 319)	297 (276 to 320)	284 (257 to 312)
Sergipe	16·9 (15·9 to 17·9)	17·4 (16·6 to 18·2)	18·3 (17·5 to 19·1)	19·6 (18·9 to 20·3)	18·5 (17·8 to 19·2)	23·1 (22 to 24·2)	23·2 (21·9 to 24·6)	23·2 (21·4 to 25·3)	22·7 (20·7 to 25·1)
Tocantins	3·9 (3·7 to 4·1)	5·8 (5·5 to 6)	8·4 (8 to 8·7)	10·6 (10·3 to 11)	12·6 (12 to 13·2)	17·9 (17·1 to 18·6)	18·9 (17·9 to 19·9)	19·3 (17·6 to 21·1)	19 (17·1 to 21)
Paraguay	34·1 (32·6 to 35·6)	39 (37·2 to 40·9)	44·9 (42·1 to 47·4)	55 (50·6 to 59·4)	66 (60·4 to 72)	67·6 (61·8 to 73·9)	69·4 (61·2 to 78·6)	67·2 (58·2 to 77·3)	66 (56·4 to 76·4)
North Africa and Middle East	2470 (2310 to 2620)	3070 (2970 to 3160)	4170 (4080 to 4250)	5640 (5530 to 5750)	5720 (5580 to 5870)	5950 (5740 to 6170)	7100 (6800 to 7410)	6580 (6170 to 7020)	6250 (5760 to 6820)
North Africa and Middle East	2470 (2310 to 2620)	3070 (2970 to 3160)	4170 (4080 to 4250)	5640 (5530 to 5750)	5720 (5580 to 5870)	5950 (5740 to 6170)	7100 (6800 to 7410)	6580 (6170 to 7020)	6250 (5760 to 6820)
Afghanistan	180 (171 to 188)	218 (211 to 224)	266 (259 to 273)	319 (310 to 328)	234 (227 to 240)	408 (399 to 417)	475 (457 to 494)	605 (574 to 637)	633 (599 to 670)
Algeria	215 (203 to 227)	251 (241 to 260)	317 (304 to 330)	410 (404 to 417)	400 (387 to 414)	338 (310 to 367)	466 (439 to 495)	498 (458 to 538)	469 (423 to 517)
Bahrain	2·1 (1·9 to 2·2)	3·4 (3·2 to 3·6)	3·7 (3·5 to 3·8)	5·1 (5 to 5·3)	6·8 (6·6 to 7)	6·3 (6·1 to 6·6)	10·1 (9·7 to 10·5)	9·2 (8·5 to 10)	8·6 (7·7 to 9·6)
Egypt	288 (254 to 325)	390 (365 to 414)	724 (699 to 748)	952 (920 to 984)	980 (937 to 1020)	1010 (958 to 1060)	1550 (1480 to 1620)	1420 (1320 to 1520)	1340 (1200 to 1500)
Iran	396 (370 to 422)	468 (449 to 487)	595 (581 to 609)	1000 (987 to 1010)	784 (756 to 814)	567 (533 to 606)	710 (666 to 759)	610 (565 to 658)	529 (462 to 601)
Alborz	4 (3·8 to 4·3)	8 (7·7 to 8·3)	12·8 (12·4 to 13·2)	21·8 (21·2 to 22·4)	16·5 (15·6 to 17·6)	13·9 (12·7 to 15·3)	18·9 (17·2 to 20·4)	16·5 (14·9 to 18·2)	14·3 (12·3 to 16·5)
Ardebil	10·9 (10·3 to 11·6)	12 (11·5 to 12·5)	14·8 (14·4 to 15·2)	24·3 (23·8 to 24·7)	18·3 (17·4 to 19·2)	12·1 (11·1 to 13)	12·2 (11·4 to 13·1)	9·5 (8·9 to 10·2)	8·2 (7·3 to 9·1)
Bushehr	3 (2·9 to 3·2)	4·3 (4·1 to 4·5)	6·2 (6 to 6·4)	11·6 (11·3 to 11·9)	10·3 (9·9 to 10·8)	6·9 (6·5 to 7·4)	8·8 (7·7 to 10·1)	9·3 (8·7 to 10)	8·5 (7·6 to 9·4)
Chahar Mahaal and Bakhtiari	5·1 (4·8 to 5·4)	5·5 (5·3 to 5·8)	6·9 (6·7 to 7·1)	12·6 (12·3 to 13)	11·2 (10·7 to 11·8)	7·5 (7 to 8·1)	9·5 (8·4 to 10·6)	8·6 (8 to 9·2)	7·4 (6·6 to 8·4)
East Azarbayejan	34·2 (31·8 to 36·5)	38 (36·3 to 39·6)	44·2 (42·9 to 45·5)	70 (68·4 to 71·5)	48·8 (46·9 to 50·7)	32·1 (29·9 to 34·4)	35·2 (32·7 to 37·7)	27·1 (24·8 to 29·3)	23·2 (19·9 to 26·7)
Fars	13·4 (12·4 to 14·3)	21·4 (20·5 to 22·3)	32·9 (32 to 33·8)	60·5 (59·5 to 61·5)	47·2 (44·8 to 49·9)	33·4 (31 to 35·8)	40·5 (37·7 to 43·5)	32·6 (29·2 to 36·6)	28 (23·2 to 33·4)
Gilan	18·5 (17·2 to 19·9)	22·7 (21·7 to 23·8)	28·4 (27·5 to 29·2)	41·1 (39·9 to 42·4)	25·4 (24·3 to 26·6)	15·3 (14·1 to 16·5)	16 (14·8 to 17·2)	12·4 (11·4 to 13·6)	10·6 (9·2 to 12·2)
Golestan	10·9 (10·2 to 11·6)	11·5 (11 to 12·1)	14·1 (13·7 to 14·6)	25·9 (25·3 to 26·5)	21·3 (20·3 to 22·4)	14·8 (13·6 to 15·9)	19·3 (17·9 to 21)	16·3 (15·3 to 17·4)	14 (12·6 to 15·5)
Hamadan	14·9 (13·9 to 15·8)	17·3 (16·5 to 18·1)	20·4 (19·9 to 21)	32·1 (31·5 to 32·7)	24·6 (23·4 to 25·9)	15·4 (14·1 to 16·7)	16·3 (15·2 to 17·3)	12·2 (11·4 to 13)	10·2 (9·1 to 11·4)
Hormozgan	5 (4·7 to 5·3)	6·6 (6·4 to 6·9)	8·7 (8·5 to 9)	15·5 (15·2 to 15·9)	15 (14·3 to 15·7)	12 (11·3 to 12·7)	17·3 (16·1 to 18·7)	17·7 (16·5 to 18·8)	15·8 (14·1 to 17·4)
Ilam	2 (1·9 to 2·1)	2·4 (2·3 to 2·5)	3·7 (3·6 to 3·8)	7·7 (7·6 to 7·9)	6·8 (6·5 to 7·1)	4·4 (4·1 to 4·7)	4·8 (4·3 to 5·3)	4·4 (4·1 to 4·8)	3·8 (3·4 to 4·4)
Isfahan	18·6 (17·3 to 19·9)	24·3 (23·2 to 25·4)	31·1 (30·2 to 32)	55·2 (53·8 to 56·4)	44·1 (41·6 to 46·9)	30·7 (27·9 to 33·8)	37·9 (35 to 40·7)	31·5 (27·6 to 35·6)	26·9 (21·8 to 32·8)
Kerman	20·2 (19 to 21·5)	16·9 (16·2 to 17·5)	18·8 (18·3 to 19·3)	34·6 (33·7 to 35·5)	25·5 (24·2 to 26·8)	19 (17·7 to 20·4)	29·1 (26 to 32·4)	26 (24·3 to 27·8)	22·6 (20·2 to 25·2)
Kermanshah	11·2 (10·4 to 12)	13·8 (13·2 to 14·4)	18·1 (17·6 to 18·6)	32·1 (31·3 to 32·8)	24·5 (23·3 to 25·6)	15·9 (14·6 to 17·1)	17·8 (16 to 19·9)	14 (13 to 15)	11·8 (10·6 to 13·3)
Khorasan-e-Razavi	37·3 (35·3 to 39·2)	41·6 (40 to 43·2)	49·2 (47·9 to 50·3)	86·5 (83·9 to 88·9)	70·2 (66·4 to 73·9)	50·3 (46·7 to 54)	66·5 (59·1 to 74·7)	57·1 (53 to 61·1)	49·6 (44 to 55·8)
Khuzestan	33 (31 to 35·1)	31·8 (30·6 to 33)	40·2 (39·1 to 41·5)	66·1 (64·7 to 67·5)	48·2 (46·6 to 50·1)	41·1 (38·4 to 43·9)	53·4 (50·1 to 56·7)	44·8 (41·3 to 48·6)	38·6 (33·4 to 44·2)
Kohgiluyeh and Boyer-Ahmad	1·9 (1·8 to 2)	2·7 (2·6 to 2·8)	4 (3·9 to 4·1)	8 (7·9 to 8·2)	8·2 (7·8 to 8·5)	6·1 (5·7 to 6·6)	6·5 (5·9 to 7·1)	6·6 (6·1 to 7·1)	6 (5·2 to 6·8)
Kurdistan	12·3 (11·6 to 13·1)	13·1 (12·5 to 13·7)	14·3 (13·9 to 14·7)	23·1 (22·7 to 23·6)	18·4 (17·6 to 19·2)	12·9 (11·8 to 14)	14·1 (13 to 15·2)	12·3 (11·5 to 13·2)	10·8 (9·6 to 11·9)
Lorestan	16·4 (15·5 to 17·4)	14·5 (13·9 to 15·1)	16·5 (16·1 to 17)	29·9 (29·3 to 30·5)	26 (24·8 to 27·2)	16·6 (15·3 to 17·9)	18 (15·7 to 20·6)	14·3 (13·3 to 15·4)	12·2 (10·9 to 13·6)
Markazi	4·6 (4·2 to 4·9)	9·1 (8·7 to 9·6)	15·1 (14·7 to 15·5)	22·7 (22·2 to 23·2)	14·4 (13·6 to 15·3)	9·2 (8·5 to 10)	10·8 (9·6 to 12)	8·6 (8 to 9·2)	7·3 (6·5 to 8·2)
Mazandaran	24·6 (22·8 to 26·5)	23·4 (22·4 to 24·5)	26·6 (25·8 to 27·4)	46·2 (45 to 47·3)	31·6 (30·1 to 33)	19·6 (18 to 21·1)	22 (20·4 to 23·7)	18·5 (16·8 to 20·3)	15·9 (13·7 to 18·3)
North Khorasan	4·4 (4·1 to 4·6)	5·1 (4·9 to 5·3)	6·3 (6·1 to 6·5)	11·4 (11·2 to 11·7)	11·7 (11·2 to 12·2)	8·1 (7·5 to 8·7)	9·7 (8·8 to 10·7)	7·6 (7·1 to 8·1)	6·5 (5·8 to 7·2)
Qazvin	10·1 (9·4 to 10·9)	11 (10·5 to 11·5)	11·6 (11·3 to 11·9)	16·4 (16 to 16·7)	12·5 (11·9 to 13)	8·8 (8·1 to 9·5)	10·5 (9·4 to 11·7)	8·7 (8·1 to 9·5)	7·5 (6·5 to 8·6)
Qom	2·1 (1·9 to 2·2)	4·2 (4 to 4·3)	7·2 (7·1 to 7·4)	12·2 (11·9 to 12·5)	9·7 (9·3 to 10·1)	7·9 (7·4 to 8·5)	10·7 (9·9 to 11·7)	10·5 (9·7 to 11·4)	9·3 (8·1 to 10·7)
Semnan	2 (1·9 to 2·2)	3·1 (3 to 3·3)	4·4 (4·3 to 4·6)	7·2 (7 to 7·4)	5 (4·7 to 5·2)	3·5 (3·2 to 3·8)	4·5 (4·1 to 5)	4·5 (4·2 to 4·8)	4 (3·6 to 4·5)
Sistan and Baluchistan	10·8 (10·1 to 11·4)	10·2 (9·7 to 10·6)	11·9 (11·6 to 12·3)	24·1 (23·6 to 24·6)	27·5 (26·3 to 28·7)	27·2 (25·4 to 29)	44 (41·9 to 46·4)	43·4 (41·1 to 45·8)	39·6 (35·4 to 44·1)
South Khorasan	5·5 (5·2 to 5·8)	6·6 (6·3 to 6·8)	8·2 (7·9 to 8·4)	13·8 (13·5 to 14·1)	10·9 (10·4 to 11·5)	6·3 (5·8 to 6·8)	7·3 (6·4 to 8·2)	7·8 (7·3 to 8·3)	7·1 (6·3 to 7·8)
Tehran	27·1 (25·1 to 29·1)	48·9 (46·6 to 51·2)	73·2 (70·7 to 75·8)	117 (113 to 122)	94 (88 to 100)	73·7 (68·1 to 79·9)	94·7 (88·1 to 101)	81·4 (71·7 to 91·6)	70·2 (57·7 to 84·9)
West Azarbayejan	12·6 (11·7 to 13·5)	17·9 (17·2 to 18·8)	24·4 (23·8 to 25)	42·6 (41·7 to 43·7)	34·1 (32·7 to 35·6)	25·8 (23·7 to 28)	33·5 (31·5 to 35·6)	28·4 (26·3 to 30·5)	24·5 (21·9 to 27·2)
Yazd	5·6 (5·3 to 5·9)	5·9 (5·6 to 6·1)	6·6 (6·4 to 6·8)	10·6 (10·4 to 10·8)	9·1 (8·7 to 9·5)	7·4 (6·8 to 8)	10·6 (9·9 to 11·5)	9·4 (8·6 to 10·2)	8·2 (7·1 to 9·3)
Zanjan	13·5 (12·7 to 14·3)	14·2 (13·7 to 14·7)	14 (13·6 to 14·4)	17·7 (17·5 to 18)	13·1 (12·5 to 13·7)	9 (8·5 to 9·6)	10·1 (9·5 to 10·7)	7·8 (7·2 to 8·5)	6·7 (5·8 to 7·7)
Iraq	118 (111 to 125)	162 (155 to 168)	226 (220 to 232)	315 (305 to 324)	389 (370 to 407)	482 (457 to 509)	532 (489 to 575)	502 (460 to 546)	481 (440 to 531)
Jordan	12·1 (12 to 12·3)	20·6 (20·3 to 21)	32·4 (31·9 to 32·9)	47·3 (46·5 to 48)	68·8 (66·8 to 70·8)	77·3 (73·4 to 81·4)	102 (96·5 to 107)	110 (100 to 120)	109 (96·6 to 123)
Kuwait	1·4 (1·3 to 1·5)	5·7 (5·6 to 5·8)	17·8 (17·5 to 18)	25·6 (24·9 to 26·2)	17·6 (16·4 to 18·8)	21 (20 to 22·1)	28·7 (27 to 30·7)	27·2 (25 to 29·8)	26·1 (22·6 to 30·3)
Lebanon	30·3 (28·8 to 31·8)	36·6 (35 to 38·2)	39·4 (37·3 to 41·4)	40·8 (38·2 to 43·4)	45·2 (41·7 to 48·6)	47·4 (44·5 to 50·5)	46·5 (42·4 to 50·7)	46·5 (40·2 to 53·8)	42·2 (36·1 to 50)
Libya	26·6 (25·9 to 27·4)	35·3 (34·4 to 36·1)	43·2 (42·2 to 44·2)	62·3 (60·8 to 63·7)	68·3 (64·5 to 72·6)	63·5 (57·1 to 69·8)	57·4 (51 to 64·2)	43·5 (37 to 51·7)	40·6 (33·9 to 48·5)
Morocco	237 (227 to 247)	296 (286 to 304)	361 (352 to 371)	413 (395 to 432)	412 (385 to 438)	428 (392 to 463)	430 (391 to 469)	354 (323 to 392)	332 (301 to 367)
Oman	12·2 (11·8 to 12·6)	16·2 (15·7 to 16·6)	20·9 (20·3 to 21·5)	30·1 (29·3 to 31)	37·4 (36·4 to 38·4)	28·6 (26·4 to 30·8)	33·6 (32·1 to 35·1)	42·5 (39·4 to 45·8)	41·7 (37·8 to 45·9)
Palestine	19·1 (18·5 to 19·7)	23·2 (22·6 to 23·8)	26·6 (25·9 to 27·4)	32·3 (31·1 to 33·5)	45 (43·1 to 46·9)	60·2 (57·6 to 62·8)	68·2 (64·1 to 72·5)	63·1 (58 to 69·2)	61·5 (56·2 to 67·8)

Qatar	0·6 (0·6 to 0·7)	1·3 (1·2 to 1·3)	2·5 (2·4 to 2·5)	4·3 (4·1 to 4·4)	6 (5·7 to 6·2)	6·8 (6·3 to 7·1)	13·8 (13 to 14·5)	18·8 (17·4 to 20·4)	19·4 (17·5 to 21·5)
Saudi Arabia	67·4 (64 to 70·7)	88·2 (85 to 91·5)	130 (126 to 134)	204 (194 to 214)	263 (246 to 281)	254 (233 to 275)	267 (237 to 298)	250 (216 to 289)	238 (204 to 276)
Sudan	123 (118 to 128)	151 (145 to 156)	253 (244 to 262)	362 (350 to 372)	471 (455 to 488)	589 (566 to 611)	653 (610 to 694)	620 (569 to 671)	601 (550 to 660)
Syria	85·6 (83·1 to 88·1)	116 (113 to 119)	149 (145 to 154)	191 (185 to 198)	234 (221 to 247)	255 (236 to 274)	270 (244 to 298)	106 (90·4 to 124)	101 (85·2 to 120)
Tunisia	85·7 (81·2 to 90·1)	98·3 (93·9 to 102)	101 (98 to 105)	114 (110 to 118)	113 (108 to 120)	96·5 (89·9 to 103)	103 (96·7 to 110)	93·4 (84·6 to 104)	86·6 (75·7 to 99·6)
Türkiye	449 (417 to 482)	543 (517 to 568)	668 (639 to 695)	828 (792 to 863)	780 (740 to 817)	742 (691 to 792)	682 (647 to 722)	591 (542 to 645)	542 (492 to 599)
United Arab Emirates	1·8 (1·8 to 1·9)	2·2 (2·1 to 2·3)	4·7 (4·5 to 4·9)	18·2 (17·7 to 18·7)	24·7 (23 to 26·4)	25·3 (23·6 to 26·9)	45·5 (42·4 to 48·9)	43 (38·6 to 47·8)	37·9 (32·8 to 43·4)
Yemen	116 (112 to 121)	144 (141 to 148)	185 (181 to 189)	265 (260 to 270)	340 (333 to 348)	440 (423 to 455)	548 (520 to 575)	518 (470 to 572)	509 (456 to 569)
South Asia	10600 (9920 to 11200)	12100 (11400 to 12800)	13800 (13200 to 14300)	16300 (15600 to 16900)	16800 (16200 to 17400)	18100 (17500 to 18800)	18500 (17800 to 19100)	17200 (16100 to 18400)	16700 (15200 to 18400)
South Asia	10600 (9920 to 11200)	12100 (11400 to 12800)	13800 (13200 to 14300)	16300 (15600 to 16900)	16800 (16200 to 17400)	18100 (17500 to 18800)	18500 (17800 to 19100)	17200 (16100 to 18400)	16700 (15200 to 18400)
Bangladesh	1060 (1020 to 1100)	1190 (1160 to 1230)	1590 (1560 to 1610)	1870 (1830 to 1910)	2140 (2090 to 2190)	2090 (2020 to 2160)	1950 (1860 to 2030)	1530 (1420 to 1650)	1450 (1280 to 1620)
Bhutan	4·3 (4·1 to 4·5)	5·6 (5·4 to 5·8)	7·5 (7·2 to 7·9)	9·9 (9·4 to 10·3)	11·3 (10·9 to 11·8)	9 (8·7 to 9·4)	7·4 (7 to 7·9)	6·7 (6·2 to 7·3)	6·4 (5·8 to 7)
India	8460 (7880 to 9030)	9770 (9120 to 10400)	10500 (10000 to 11000)	12200 (11500 to 12700)	12100 (11500 to 12700)	13300 (12700 to 13800)	13300 (12700 to 13800)	12100 (11200 to 13100)	11700 (10500 to 13100)
Nepal	215 (205 to 225)	242 (233 to 251)	290 (282 to 298)	374 (361 to 387)	409 (397 to 419)	403 (390 to 417)	336 (314 to 358)	334 (300 to 368)	333 (299 to 369)
Pakistan	840 (803 to 875)	913 (882 to 942)	1330 (1290 to 1380)	1890 (1830 to 1950)	2180 (2100 to 2250)	2370 (2290 to 2460)	2910 (2770 to 3040)	3180 (2880 to 3510)	3180 (2850 to 3580)
Azad Jammu & Kashmir	22 (21·3 to 22·6)	22·1 (21·5 to 22·8)	29·8 (28·4 to 31·2)	41·3 (38·9 to 43·6)	43·7 (41·3 to 46·1)	40·3 (36·8 to 43·7)	43·9 (39·4 to 49·3)	42 (36·2 to 48·6)	40·3 (34·4 to 47·8)
Balochistan	28·5 (27 to 30)	23·6 (22·7 to 24·4)	43·8 (41·9 to 45·5)	88 (84·5 to 91·2)	108 (103 to 113)	125 (120 to 131)	176 (166 to 185)	230 (209 to 252)	240 (216 to 267)
Gilgit-Baltistan	6 (5·8 to 6·3)	6·2 (5·9 to 6·4)	9·3 (8·9 to 9·8)	14 (13·4 to 14·6)	16·4 (15·5 to 17·4)	19·5 (18·3 to 20·7)	25·5 (24 to 26·9)	30·3 (27·7 to 33)	31·2 (28·3 to 34·3)
Islamabad Capital Territory	2·6 (2·5 to 2·7)	3 (2·9 to 3·2)	4·3 (4 to 4·5)	6·3 (5·9 to 6·7)	8·1 (7·4 to 8·8)	12 (10·7 to 13·2)	19·1 (16·9 to 21·2)	22·5 (20·1 to 25·1)	22·9 (20·2 to 25·7)
Khyber Pakhtunkhwa	150 (144 to 157)	156 (151 to 162)	217 (208 to 225)	290 (280 to 300)	329 (318 to 340)	398 (383 to 414)	532 (505 to 557)	598 (541 to 654)	601 (539 to 673)
Punjab	482 (461 to 503)	524 (506 to 541)	738 (713 to 762)	1030 (990 to 1060)	1180 (1140 to 1220)	1230 (1190 to 1280)	1460 (1390 to 1540)	1580 (1430 to 1740)	1570 (1410 to 1770)
Sindh	148 (142 to 154)	178 (171 to 183)	288 (278 to 297)	426 (414 to 439)	493 (475 to 509)	546 (524 to 569)	650 (620 to 682)	681 (611 to 754)	676 (598 to 766)
Southeast Asia, east Asia, and Oceania	16100 (15300 to 16900)	18300 (17800 to 18800)	19300 (18900 to 19600)	16500 (16000 to 17100)	19200 (18400 to 19900)	15000 (14500 to 15600)	13800 (13200 to 14500)	13600 (12900 to 14400)	12000 (11200 to 12900)
East Asia	11500 (10900 to 12200)	12500 (12100 to 12900)	13100 (12800 to 13400)	9880 (9480 to 10300)	12700 (12000 to 13400)	8750 (8270 to 9230)	7330 (6800 to 7910)	7550 (7040 to 8100)	6030 (5510 to 6590)
China	11100 (10500 to 11700)	12000 (11700 to 12400)	12600 (12300 to 12900)	9440 (9040 to 9840)	12300 (11600 to 12900)	8340 (7860 to 8820)	7040 (6510 to 7610)	7300 (6800 to 7850)	5790 (5280 to 6340)
North Korea	218 (205 to 231)	258 (240 to 275)	249 (229 to 271)	231 (210 to 256)	283 (260 to 309)	267 (244 to 290)	199 (181 to 215)	162 (145 to 182)	153 (135 to 174)
Taiwan (province of China)	187 (183 to 192)	218 (215 to 221)	193 (187 to 199)	210 (203 to 218)	168 (161 to 176)	142 (134 to 150)	100 (94·5 to 107)	88·5 (82 to 95·4)	81·1 (72·2 to 90·8)
Oceania	63 (60·6 to 65·5)	78·3 (75·7 to 80·9)	88·6 (85·9 to 91·3)	101 (97·2 to 104)	117 (113 to 122)	145 (138 to 152)	182 (172 to 191)	218 (200 to 236)	225 (206 to 247)
American Samoa	0·4 (0·4 to 0·4)	0·4 (0·4 to 0·4)	0·5 (0·5 to 0·6)	0·6 (0·6 to 0·6)	0·9 (0·9 to 1)	0·9 (0·8 to 0·9)	0·7 (0·6 to 0·7)	0·4 (0·4 to 0·5)	0·4 (0·4 to 0·5)
Cook Islands	0·3 (0·3 to 0·3)	0·4 (0·4 to 0·4)	0·4 (0·4 to 0·4)	0·2 (0·2 to 0·2)	0·2 (0·2 to 0·2)	0·2 (0·2 to 0·2)	0·1 (0·1 to 0·2)	0·1 (0·1 to 0·1)	0·1 (0·1 to 0·1)
Fiji	5·9 (5·6 to 6·2)	8·1 (7·9 to 8·3)	8·2 (7·9 to 8·5)	9·7 (9·3 to 10·1)	9·7 (9·2 to 10·2)	9·2 (8·5 to 10·1)	10·1 (9·6 to 10·6)	9 (8·3 to 9·9)	8·7 (7·7 to 9·8)
Guam	0·9 (0·9 to 1)	1·3 (1·3 to 1·3)	1·5 (1·4 to 1·5)	1·5 (1·5 to 1·6)	2 (1·9 to 2·1)	1·9 (1·8 to 2)	1·8 (1·7 to 1·9)	1·5 (1·4 to 1·6)	1·4 (1·3 to 1·6)
Kiribati	0·7 (0·7 to 0·7)	0·8 (0·8 to 0·8)	0·9 (0·8 to 0·9)	1·2 (1·1 to 1·2)	1·4 (1·3 to 1·5)	1·4 (1·2 to 1·5)	1·5 (1·4 to 1·6)	1·5 (1·4 to 1·7)	1·5 (1·4 to 1·7)
Marshall Islands	0·3 (0·3 to 0·3)	0·4 (0·3 to 0·4)	0·5 (0·5 to 0·5)	0·7 (0·7 to 0·7)	0·8 (0·7 to 0·8)	0·9 (0·8 to 0·9)	0·8 (0·7 to 0·8)	0·6 (0·6 to 0·7)	0·6 (0·5 to 0·7)
Federated States of Micronesia	1·1 (1 to 1·1)	1·1 (1 to 1·1)	1·3 (1·2 to 1·3)	1·7 (1·6 to 1·8)	1·6 (1·5 to 1·7)	1·4 (1·3 to 1·5)	1·2 (1 to 1·3)	1 (0·9 to 1·1)	1 (0·8 to 1·1)
Nauru	0·1 (0·1 to 0·1)	0·1 (0·1 to 0·1)	0·1 (0·1 to 0·1)	0·2 (0·2 to 0·2)	0·2 (0·2 to 0·2)	0·2 (0·2 to 0·2)	0·2 (0·2 to 0·2)	0·2 (0·1 to 0·2)	0·2 (0·1 to 0·2)
Niue	0·1 (0·1 to 0·1)	0·1 (0·1 to 0·1)	0·1 (0·1 to 0·1)	0 (0 to 0·1)	0 (0 to 0)	0 (0 to 0)	0 (0 to 0)	0 (0 to 0)	0 (0 to 0)
Northern Mariana Islands	0·1 (0·1 to 0·1)	0·1 (0·1 to 0·1)	0·1 (0·1 to 0·1)	0·2 (0·2 to 0·2)	0·6 (0·6 to 0·7)	1·3 (1·1 to 1·4)	0·4 (0·4 to 0·5)	0·3 (0·3 to 0·3)	0·3 (0·3 to 0·3)
Palau	0·1 (0·1 to 0·2)	0·1 (0·1 to 0·2)	0·1 (0·1 to 0·1)	0·1 (0·1 to 0·1)	0·2 (0·1 to 0·2)	0·2 (0·2 to 0·2)	0·1 (0·1 to 0·2)	0·1 (0·1 to 0·1)	0·1 (0·1 to 0·1)
Papua New Guinea	42·3 (40·2 to 44·4)	51·8 (49·5 to 54)	58·6 (56·3 to 60·8)	65·1 (62·7 to 67·4)	77 (73·5 to 80·4)	101 (96·2 to 106)	136 (128 to 144)	173 (158 to 189)	181 (164 to 200)
Samoa	2·3 (2·3 to 2·4)	2·8 (2·7 to 2·8)	3 (2·9 to 3·2)	3 (2·8 to 3·2)	2·9 (2·7 to 3·1)	2·9 (2·7 to 3·2)	3 (2·7 to 3·2)	3·2 (3 to 3·5)	3·3 (3 to 3·5)
Solomon Islands	2·5 (2·4 to 2·6)	3·3 (3·2 to 3·5)	4 (3·9 to 4·1)	5·2 (5 to 5·4)	7·3 (7 to 7·6)	9·2 (8·7 to 9·6)	10 (9·4 to 10·7)	10·5 (9·7 to 11·3)	10·7 (9·9 to 11·5)
Tokelau	0 (0 to 0)	0 (0 to 0)	0 (0 to 0)	0 (0 to 0)	0 (0 to 0)	0 (0 to 0)	0 (0 to 0)	0 (0 to 0)	0 (0 to 0)
Tonga	1·1 (1·1 to 1·2)	1·5 (1·5 to 1·5)	1·8 (1·8 to 1·9)	1·9 (1·8 to 1·9)	1·7 (1·7 to 1·8)	1·7 (1·6 to 1·7)	1·7 (1·6 to 1·8)	1·6 (1·5 to 1·7)	1·6 (1·4 to 1·7)
Tuvalu	0·1 (0·1 to 0·1)	0·1 (0·1 to 0·1)	0·2 (0·1 to 0·2)	0·2 (0·2 to 0·2)	0·2 (0·2 to 0·2)	0·1 (0·1 to 0·2)	0·1 (0·1 to 0·1)	0·1 (0·1 to 0·2)	0·1 (0·1 to 0·2)
Vanuatu	1·1 (1 to 1·1)	1·5 (1·5 to 1·6)	2 (1·9 to 2·1)	2·6 (2·5 to 2·7)	3·2 (3·1 to 3·4)	3·8 (3·6 to 3·9)	4·2 (4 to 4·5)	4·5 (4·1 to 4·9)	4·5 (4·2 to 4·9)
Southeast Asia	4490 (4270 to 4690)	5700 (5510 to 5900)	6110 (5930 to 6270)	6540 (6360 to 6710)	6340 (6190 to 6500)	6130 (5970 to 6320)	6330 (6100 to 6570)	5880 (5520 to 6290)	5770 (5320 to 6270)
Cambodia	109 (103 to 114)	136 (131 to 142)	157 (148 to 167)	181 (173 to 188)	221 (215 to 228)	187 (181 to 193)	187 (178 to 196)	190 (176 to 206)	191 (175 to 207)
Indonesia	1860 (1770 to 1950)	2500 (2390 to 2610)	2670 (2590 to 2750)	2660 (2580 to 2750)	2400 (2320 to 2480)	2420 (2320 to 2530)	2490 (2360 to 2610)	2290 (2080 to 2520)	2260 (2030 to 2540)
Aceh	30·6 (29 to 32·2)	42·1 (40·4 to 43·9)	49·2 (47·7 to 50·6)	55·8 (54 to 57·6)	52·8 (50·6 to 55)	54·7 (52·3 to 57·1)	51·8 (49·4 to 54·2)	47·4 (43·2 to 52·1)	47·1 (42·1 to 53)
Bali	34·9 (33 to 36·8)	42·5 (40·7 to 44·4)	44·9 (43·2 to 46·6)	38·3 (36·7 to 40·1)	31·8 (30·3 to 33·3)	32·4 (30·7 to 34·1)	31·1 (29·3 to 33·1)	27·3 (24·3 to 30·4)	26·7 (23·7 to 30·4)
Bangka-Belitung Islands	7·8 (7·4 to 8·3)	9·9 (9·5 to 10·4)	11·2 (10·7 to 11·6)	12·9 (12·5 to 13·3)	11·6 (11·1 to 12)	12 (11·4 to 12·7)	13·9 (13·1 to 14·7)	13·9 (12·8 to 15)	13·8 (12·5 to 15·1)
Banten	42·3 (40 to 44·4)	54·7 (52·4 to 56·9)	60·9 (58·9 to 62·8)	80·3 (77·5 to 82·9)	124 (119 to 128)	133 (126 to 140)	126 (120 to 132)	111 (102 to 120)	108 (98·1 to 120)
Bengkulu	9·7 (9·3 to 10·1)	12·3 (11·9 to 12·8)	13·8 (13·4 to 14·2)	16·9 (16·4 to 17·4)	19·1 (18·3 to 19·8)	19·7 (18·9 to 20·7)	20 (19 to 21·1)	18·8 (17·2 to 20·5)	18·6 (16·8 to 20·7)
Gorontalo	6·2 (5·9 to 6·6)	9·8 (9·4 to 10·3)	12·1 (11·6 to 12·7)	10·5 (10 to 10·9)	8·9 (8·5 to 9·3)	10·2 (9·7 to 10·6)	11·9 (11·3 to 12·6)	11·3 (10·4 to 12·3)	11·1 (10·1 to 12·3)

Jakarta	50·9 (48·5 to 53·2)	86·2 (82·6 to 89·8)	106 (102 to 110)	118 (113 to 124)	111 (107 to 116)	102 (98 to 107)	96·8 (91·4 to 102)	73·4 (66·1 to 81·6)	69·1 (61·3 to 78·1)
Jambi	15·1 (14·3 to 16)	21·5 (20·6 to 22·4)	24·3 (23·4 to 25·2)	29 (28·1 to 29·8)	28·5 (27·2 to 29·8)	31·3 (29·8 to 32·9)	34·1 (32·5 to 35·7)	30·4 (27·7 to 33·2)	29·9 (26·8 to 33·4)
West Java	294 (281 to 308)	412 (394 to 431)	454 (440 to 469)	450 (433 to 467)	378 (363 to 394)	411 (395 to 428)	452 (426 to 477)	420 (383 to 460)	412 (372 to 459)
Central Java	345 (327 to 362)	459 (437 to 481)	463 (447 to 479)	419 (404 to 434)	343 (328 to 359)	296 (280 to 313)	283 (263 to 305)	261 (236 to 290)	257 (228 to 292)
East Java	397 (374 to 420)	513 (483 to 543)	495 (475 to 514)	429 (411 to 448)	339 (325 to 353)	310 (295 to 324)	294 (276 to 312)	244 (216 to 273)	235 (206 to 270)
West Kalimantan	30·2 (28·9 to 31·4)	41·6 (40 to 43·2)	49·1 (47·7 to 50·5)	56·6 (55·1 to 58·2)	49·9 (48 to 51·7)	50·7 (47·9 to 53·6)	55·7 (53·4 to 58·2)	57·8 (53·1 to 62·6)	58·7 (53·4 to 64·9)
South Kalimantan	27·9 (26·6 to 29·2)	39·1 (37·6 to 40·7)	41·6 (40·3 to 42·9)	39·4 (37·9 to 40·9)	36·2 (34·2 to 38·1)	39·9 (37·8 to 42·2)	41·7 (39·2 to 44·2)	35·3 (31·5 to 39·2)	34·1 (30·4 to 38·7)
Central Kalimantan	9·1 (8·6 to 9·7)	12·7 (12·1 to 13·3)	14·8 (14·2 to 15·3)	17·6 (17 to 18·2)	19·3 (18·5 to 20·1)	22·6 (21·6 to 23·8)	26·1 (24·9 to 27·4)	26·1 (23·7 to 28·4)	26 (23·4 to 29·1)
East Kalimantan	8·8 (8·3 to 9·3)	13·4 (12·8 to 14)	16·6 (16·1 to 17·2)	20·7 (20 to 21·4)	22·9 (22 to 23·9)	30 (28·6 to 31·5)	35·4 (33·8 to 37)	33·9 (31 to 37)	33·9 (30·4 to 38)
North Kalimantan	1·1 (1·1 to 1·2)	2·1 (2 to 2·2)	2·9 (2·8 to 3·1)	3·3 (3·1 to 3·4)	3·6 (3·3 to 3·9)	4·9 (4·5 to 5·4)	6·4 (5·9 to 6·9)	6·9 (6·2 to 7·7)	7·1 (6·3 to 8)
Riau Islands	3·6 (3·4 to 3·9)	4·4 (4·1 to 4·6)	4·9 (4·8 to 5·1)	7·7 (7·4 to 8)	13·5 (12·9 to 14)	17·2 (16·3 to 18·2)	21·9 (20·9 to 22·9)	19 (17·3 to 20·6)	18·2 (16·5 to 20·3)
Lampung	38·7 (36·7 to 40·7)	65·3 (62·3 to 68·1)	82·1 (79·8 to 84·2)	92·7 (89·4 to 96)	86·2 (82·3 to 90·2)	82·5 (77·8 to 87·8)	82·8 (77·7 to 88·1)	80·3 (71·3 to 90·5)	79·9 (70·2 to 92·1)
Maluku	13·2 (12·5 to 13·9)	15·4 (14·8 to 16)	16·1 (15·6 to 16·6)	18·4 (17·8 to 19)	19·8 (19·1 to 20·6)	20·9 (19·9 to 22)	21·2 (20·2 to 22·3)	22·4 (20·6 to 24·3)	23 (20·7 to 25·6)
North Maluku	6·8 (6·4 to 7·2)	7·9 (7·6 to 8·3)	8·7 (8·3 to 9·1)	10·4 (10 to 10·7)	11·9 (11·4 to 12·4)	13·1 (12·4 to 13·9)	13·4 (12·7 to 14·2)	14 (12·9 to 15·1)	14·3 (13 to 15·7)
West Nusa Tenggara	40·8 (38·7 to 42·9)	50·5 (48·6 to 52·4)	57·3 (55·8 to 58·8)	60·6 (58·9 to 62·3)	52·4 (50·3 to 54·5)	51·1 (48·4 to 53·9)	53·2 (50·1 to 56·4)	49·8 (44·8 to 54·9)	49·2 (43·9 to 55·2)
East Nusa Tenggara	41·8 (39·5 to 44)	50·8 (48·8 to 52·9)	54·4 (52·5 to 56·3)	53·5 (51·8 to 55)	53·1 (51·2 to 54·9)	61·1 (58·2 to 64·2)	62·9 (59·9 to 65·8)	64·3 (59·1 to 69·7)	65·5 (59·5 to 72·4)
Papua	4·8 (4·5 to 5·1)	8·3 (7·8 to 8·7)	10·5 (10·1 to 10·9)	12·8 (12·4 to 13·2)	16·2 (15·5 to 16·9)	26 (24·8 to 27·4)	41·6 (39·6 to 43·6)	47·6 (43·6 to 51·8)	47·8 (43·3 to 53·2)
West Papua	4·6 (4·3 to 4·9)	7 (6·7 to 7·3)	8·7 (8·4 to 8·9)	10·9 (10·6 to 11·2)	11·6 (11·2 to 12)	10·1 (9·6 to 10·6)	10·9 (10·3 to 11·4)	13·1 (12·1 to 14·2)	13·8 (12·6 to 15·2)
Riau	16·5 (15·8 to 17)	26·6 (25·7 to 27·6)	33·1 (32·1 to 34)	38·9 (37·8 to 40)	36·5 (35 to 38)	51·8 (49·2 to 54·8)	70·7 (67·3 to 74·2)	64·8 (59·2 to 70·7)	63·7 (57·5 to 70·6)
West Sulawesi	9·8 (9·1 to 10·4)	11·5 (11 to 12)	12·4 (12 to 12·8)	12·7 (12·3 to 13·1)	12·4 (12 to 12·8)	14·5 (13·7 to 15·2)	16·6 (15·9 to 17·4)	17·1 (15·9 to 18·3)	17·4 (15·9 to 19·1)
South Sulawesi	77·2 (73 to 81·2)	105 (100 to 109)	117 (113 to 120)	111 (107 to 115)	95 (91 to 98·9)	94·1 (89·4 to 98·9)	89·8 (84·9 to 94·9)	85·1 (76·6 to 94·1)	84·6 (75·6 to 95·2)
Central Sulawesi	14 (13·3 to 14·6)	19·6 (18·9 to 20·3)	23·3 (22·5 to 24·1)	26·1 (25·4 to 26·9)	25·6 (24·6 to 26·6)	31·3 (29·7 to 33·1)	33·3 (31·9 to 34·9)	30·6 (27·9 to 33·3)	30·4 (27·3 to 33·8)
Southeast Sulawesi	12·8 (12·1 to 13·4)	16·4 (15·9 to 17)	19·1 (18·6 to 19·7)	21·7 (21·1 to 22·3)	22·3 (21·5 to 23)	26·3 (24·9 to 27·7)	28·8 (27·2 to 30·4)	28·2 (25·8 to 30·8)	28·6 (25·8 to 31·9)
North Sulawesi	22·2 (21·3 to 23·1)	24·3 (23·3 to 25·4)	24·3 (23·4 to 25·1)	23·5 (22·5 to 24·5)	23·3 (22·3 to 24·3)	23·1 (22 to 24·3)	22·4 (21·2 to 23·6)	21·5 (19·5 to 23·6)	21·3 (19·1 to 23·8)
West Sumatra	58·6 (56·4 to 60·7)	66·4 (64·1 to 68·8)	65·3 (63·4 to 67·1)	67·9 (65·7 to 70)	58·5 (56·1 to 61)	58·1 (55·2 to 61·2)	55·4 (52·3 to 58·5)	54 (49·1 to 59·5)	54·2 (48·7 to 60·6)
South Sumatra	41·6 (39·5 to 43·6)	59·5 (57·2 to 61·8)	68·8 (66·8 to 70·7)	84 (81·3 to 86·8)	81·1 (77·6 to 84·8)	83·7 (79·8 to 88)	87·1 (82·3 to 92·3)	76·7 (69·3 to 85)	74·9 (66·8 to 84·5)
North Sumatra	97·8 (94·4 to 101)	135 (130 to 139)	155 (151 to 158)	177 (172 to 182)	171 (165 to 178)	172 (163 to 182)	173 (162 to 184)	167 (152 to 183)	167 (150 to 187)
Yogyakarta	46·8 (44·4 to 49·3)	53·8 (51·6 to 56·1)	48·4 (46·3 to 50·5)	38·1 (36·6 to 39·8)	29 (27·7 to 30·2)	24·8 (23·6 to 26)	23·8 (22·3 to 25·3)	19·4 (17·4 to 21·6)	18·5 (16·4 to 21)
Laos	43·5 (41 to 45·8)	53·6 (51·3 to 55·8)	64·8 (61·4 to 68·1)	75·4 (71·4 to 79·4)	88·7 (84·1 to 92·7)	98·9 (94·9 to 103)	88·6 (84·8 to 92·5)	91·5 (84·8 to 98·4)	90·9 (83·2 to 98·4)
Malaysia	156 (149 to 163)	176 (173 to 179)	183 (178 to 187)	217 (211 to 223)	253 (244 to 262)	271 (260 to 282)	254 (243 to 265)	249 (236 to 263)	245 (219 to 276)
Maldives	1·4 (1·3 to 1·5)	2 (1·9 to 2·1)	2·5 (2·4 to 2·6)	3·5 (3·4 to 3·5)	4·5 (4·4 to 4·6)	3·1 (2·9 to 3·3)	3·6 (3·4 to 3·8)	3·2 (3 to 3·5)	3·1 (2·8 to 3·4)
Mauritius	11·8 (11·6 to 12·1)	13 (12·8 to 13·3)	10·6 (10·3 to 11)	11·9 (11·5 to 12·3)	11·7 (10·9 to 12·5)	10·3 (9·9 to 10·7)	7·5 (7·3 to 7·8)	6·7 (6·3 to 7)	6·5 (5·7 to 7·3)
Myanmar	446 (421 to 472)	532 (509 to 554)	596 (572 to 619)	681 (647 to 712)	574 (543 to 604)	542 (520 to 566)	542 (522 to 563)	555 (519 to 596)	552 (506 to 602)
Philippines	487 (463 to 511)	653 (618 to 686)	770 (729 to 809)	918 (873 to 962)	1050 (1010 to 1100)	1160 (1110 to 1210)	1290 (1220 to 1360)	1160 (1080 to 1240)	1140 (1040 to 1240)
Seychelles	0·5 (0·5 to 0·6)	0·9 (0·9 to 0·9)	0·9 (0·9 to 0·9)	0·9 (0·8 to 0·9)	0·9 (0·8 to 0·9)	0·7 (0·7 to 0·8)	0·8 (0·8 to 0·9)	0·8 (0·8 to 0·9)	0·8 (0·7 to 0·9)
Sri Lanka	156 (148 to 165)	180 (174 to 186)	190 (181 to 199)	215 (206 to 226)	184 (174 to 194)	177 (166 to 189)	188 (178 to 200)	160 (149 to 171)	153 (136 to 172)
Thailand	531 (506 to 555)	660 (637 to 682)	640 (624 to 654)	606 (580 to 632)	528 (503 to 550)	447 (426 to 470)	409 (388 to 429)	309 (288 to 334)	295 (268 to 327)
Timor-Leste	11·9 (11·4 to 12·4)	14·1 (13·7 to 14·4)	13·1 (12·8 to 13·4)	15 (14·6 to 15·5)	19·3 (18·6 to 20)	19·2 (18·5 to 20)	18 (16·8 to 19·2)	20·2 (18·2 to 22·4)	21·2 (19 to 23·6)
Viet Nam	665 (634 to 696)	771 (741 to 799)	804 (773 to 834)	937 (907 to 966)	991 (959 to 1020)	788 (757 to 823)	843 (805 to 882)	834 (769 to 904)	802 (735 to 887)
Sub-Saharan Africa	4550 (4350 to 4750)	5740 (5560 to 5900)	7140 (6940 to 7320)	9160 (8940 to 9380)	11400 (11200 to 11700)	14300 (14000 to 14700)	17500 (17000 to 18000)	19100 (18100 to 20100)	19300 (18200 to 20500)
Central sub-Saharan Africa	516 (498 to 532)	691 (671 to 711)	850 (827 to 873)	1060 (1020 to 1090)	1330 (1280 to 1370)	1700 (1650 to 1750)	2150 (2060 to 2240)	2280 (2150 to 2400)	2280 (2140 to 2420)
Angola	122 (116 to 128)	137 (132 to 142)	151 (146 to 156)	191 (185 to 197)	258 (246 to 269)	362 (343 to 381)	506 (472 to 538)	596 (555 to 637)	611 (567 to 658)
Central African Republic	30·3 (28·6 to 31·8)	41 (38·8 to 43)	51·5 (49·2 to 53·6)	56·5 (54·1 to 58·9)	65·5 (62·2 to 68·7)	82·2 (77·6 to 86·5)	98·5 (91 to 106)	97·9 (90·6 to 105)	98·2 (90·7 to 106)
Congo (Brazzaville)	20·7 (19·5 to 21·9)	25 (23·9 to 25·9)	30·6 (29·5 to 31·6)	38·8 (37·2 to 40·4)	46·9 (45·3 to 48·4)	60·8 (58·9 to 62·8)	76·4 (73·9 to 79·1)	67·6 (62·3 to 73·3)	65·5 (59·7 to 71·8)
DR Congo	328 (316 to 340)	472 (459 to 485)	599 (582 to 616)	749 (721 to 774)	928 (896 to 960)	1160 (1120 to 1190)	1430 (1380 to 1480)	1480 (1370 to 1580)	1460 (1340 to 1580)
Equatorial Guinea	4·9 (4·7 to 5·1)	5·1 (4·9 to 5·3)	5·7 (5·4 to 5·9)	7·4 (7 to 7·7)	10·5 (10 to 11)	14·5 (13·3 to 15·6)	19·1 (17·6 to 20·8)	19·6 (17·3 to 22)	19·3 (16·8 to 22·1)
Gabon	9·6 (9 to 10·2)	11·2 (10·6 to 11·7)	12·6 (11·9 to 13·2)	15·1 (14·1 to 16)	18·4 (16·9 to 19·7)	21·7 (19·6 to 23·7)	24·3 (22·1 to 26·5)	22·8 (19·9 to 25·9)	22·3 (19·3 to 25·8)
Eastern sub-Saharan Africa	1740 (1660 to 1810)	2220 (2150 to 2280)	2770 (2700 to 2830)	3640 (3560 to 3720)	4630 (4530 to 4740)	5770 (5630 to 5900)	6630 (6410 to 6850)	6980 (6550 to 7440)	7050 (6540 to 7600)
Burundi	64·5 (61·7 to 67·1)	75·4 (72·5 to 77·9)	91·4 (88·4 to 94)	109 (107 to 111)	132 (129 to 135)	137 (134 to 141)	203 (197 to 210)	230 (214 to 247)	238 (219 to 259)
Comoros	3·3 (3·1 to 3·5)	4·7 (4·5 to 5)	6·8 (6·6 to 7)	8·9 (8·6 to 9·1)	10·5 (10 to 10·8)	10·5 (9·7 to 11·2)	9·9 (9 to 10·8)	9 (7·9 to 10·1)	8·9 (7·8 to 10·1)
Djibouti	1·3 (1·2 to 1·4)	2 (1·9 to 2·1)	3·1 (3 to 3·3)	5 (4·7 to 5·2)	7·2 (6·7 to 7·6)	9·8 (9 to 10·7)	13·1 (12·1 to 14·2)	13·4 (12 to 15·1)	13·1 (11·5 to 14·8)
Eritrea	28·1 (26·6 to 29·6)	38·5 (37 to 40)	48·6 (46·9 to 50·3)	61·5 (58·8 to 64)	76·4 (72·5 to 80·1)	77·1 (71·2 to 82·8)	92·9 (83·2 to 103)	100 (89·5 to 112)	101 (89·8 to 114)
Ethiopia	452 (427 to 477)	562 (540 to 583)	685 (661 to 708)	925 (888 to 960)	1260 (1220 to 1290)	1570 (1520 to 1620)	1620 (1540 to 1700)	1760 (1640 to 1890)	1800 (1670 to 1950)
Addis Ababa	14·2 (13·3 to 15)	20 (19·2 to 20·8)	27·7 (26·5 to 28·9)	37·5 (35·4 to 39·6)	31·9 (29·9 to 33·8)	28·5 (27 to 30)	28·7 (26·7 to 30·9)	26·1 (23·4 to 29·1)	24·4 (21·5 to 27·5)
Afar	9·9 (9·3 to 10·4)	6·4 (6·1 to 6·6)	4·8 (4·6 to 5)	7·2 (6·8 to 7·5)	19·3 (18·4 to 20·1)	34·5 (33 to 36)	33·3 (31·6 to 35)	32·8 (30·6 to 35·1)	32·6 (30·3 to 35·2)

Amhara	134 (125 to 142)	168 (161 to 175)	201 (194 to 209)	263 (252 to 274)	337 (326 to 347)	370 (356 to 383)	318 (301 to 334)	319 (294 to 347)	323 (296 to 354)
Benishangul-Gumuz	8·8 (8·2 to 9·3)	10·3 (9·8 to 10·7)	10·8 (10·3 to 11·2)	12·2 (11·7 to 12·7)	12·8 (12·3 to 13·2)	14·4 (13·8 to 15)	17·5 (16·5 to 18·5)	19·6 (18·3 to 21·1)	20·1 (18·5 to 21·7)
Dire Dawa	2·5 (2·3 to 2·6)	4·1 (3·9 to 4·3)	5·9 (5·6 to 6·1)	6·9 (6·5 to 7·3)	6·2 (5·8 to 6·5)	5·9 (5·6 to 6·2)	6·2 (5·7 to 6·7)	5·8 (5·2 to 6·4)	5·7 (5·1 to 6·3)
Gambella	2 (1·9 to 2·2)	3 (2·8 to 3·1)	3·6 (3·4 to 3·8)	4·1 (3·8 to 4·3)	4·2 (4 to 4·4)	5·3 (5 to 5·5)	5·9 (5·5 to 6·3)	6·1 (5·5 to 6·7)	6·2 (5·6 to 6·9)
Harari	1·2 (1·2 to 1·3)	1·8 (1·7 to 1·9)	2·3 (2·3 to 2·4)	2·8 (2·6 to 2·9)	2·9 (2·7 to 3)	3·2 (3 to 3·3)	3·4 (3·2 to 3·6)	3·3 (3 to 3·6)	3·2 (2·9 to 3·5)
Oromia	137 (130 to 144)	161 (155 to 167)	191 (184 to 197)	269 (259 to 279)	421 (408 to 434)	592 (571 to 612)	648 (616 to 679)	731 (682 to 785)	756 (703 to 813)
Somali	23 (22·1 to 23·8)	24·9 (24·2 to 25·6)	28·7 (27·7 to 29·7)	44·1 (42·7 to 45·5)	73·1 (70·5 to 75·6)	106 (103 to 110)	125 (119 to 131)	136 (129 to 144)	138 (130 to 146)
Southern Nations, Nationalities, and Peoples	88·5 (83·8 to 92·9)	124 (118 to 128)	162 (156 to 167)	218 (210 to 225)	275 (267 to 284)	327 (316 to 337)	352 (330 to 374)	391 (360 to 422)	401 (368 to 440)
Tigray	31·8 (29·9 to 33·6)	38·4 (36·9 to 40)	47·4 (45·6 to 49·1)	60·2 (57·8 to 62·4)	72·3 (70·2 to 74·3)	85·9 (83·3 to 88·4)	83·2 (79·4 to 87)	87·1 (79·9 to 94·6)	88·6 (80·4 to 97·1)
Kenya	158 (154 to 162)	237 (231 to 241)	311 (304 to 317)	414 (404 to 423)	506 (488 to 523)	618 (588 to 649)	693 (641 to 743)	628 (564 to 698)	606 (538 to 684)
Baringo	2·4 (2·4 to 2·5)	3·8 (3·7 to 3·9)	4·8 (4·7 to 4·9)	5·8 (5·7 to 5·9)	7·6 (7·4 to 7·9)	8·9 (8·4 to 9·4)	8·9 (8·1 to 9·6)	8·1 (7·1 to 9)	7·8 (6·8 to 8·9)
Bomet	2·5 (2·4 to 2·5)	3·4 (3·4 to 3·5)	4·5 (4·5 to 4·6)	5·9 (5·8 to 6)	7·9 (7·7 to 8·2)	11 (10·5 to 11·6)	17·9 (16·4 to 19·4)	13·1 (11·5 to 14·8)	11·8 (10·3 to 13·6)
Bungoma	5·2 (5·1 to 5·2)	7·7 (7·6 to 7·8)	10·6 (10·5 to 10·8)	14·9 (14·7 to 15·2)	21·5 (20·8 to 22·2)	38·3 (36·6 to 40·2)	30·2 (28·4 to 31·8)	30·8 (27·9 to 33·8)	31 (28 to 34·4)
Busia	3·9 (3·8 to 4)	5·4 (5·3 to 5·5)	6·4 (6·2 to 6·5)	9·2 (9 to 9·4)	12·1 (11·7 to 12·5)	13 (12·3 to 13·8)	14·4 (13·2 to 15·6)	12·6 (11·2 to 14·1)	12·3 (10·8 to 14)
Elgeyo Marakwet	2·9 (2·8 to 2·9)	4·7 (4·6 to 4·8)	5·2 (5·1 to 5·3)	4·1 (4·1 to 4·2)	5·8 (5·6 to 6)	9·7 (9·2 to 10·2)	5·6 (5 to 6·1)	5 (4·4 to 5·7)	5·1 (4·4 to 5·8)
Embu	2·5 (2·4 to 2·6)	3·4 (3·3 to 3·5)	4·5 (4·4 to 4·6)	6·3 (6·1 to 6·5)	7·7 (7·3 to 8·1)	8·4 (7·9 to 9·1)	7·3 (6·5 to 8·1)	5·7 (4·9 to 6·6)	5·4 (4·6 to 6·3)
Garissa	0·8 (0·8 to 0·9)	1·3 (1·3 to 1·4)	2·2 (2·1 to 2·2)	3·3 (3·2 to 3·4)	3·4 (3·2 to 3·5)	7·9 (7·7 to 8·2)	15·2 (14·6 to 15·9)	19 (17·7 to 20·3)	19·5 (18·1 to 21)
Homa Bay	9·6 (9·4 to 9·8)	11·9 (11·7 to 12·1)	12·8 (12·5 to 13·1)	14·3 (14 to 14·6)	17·1 (16·6 to 17·6)	19·8 (18·9 to 20·7)	20·2 (19 to 21·5)	17·1 (15·4 to 18·9)	15·9 (14·2 to 17·8)
Isiolo	0·4 (0·4 to 0·5)	0·7 (0·7 to 0·7)	0·9 (0·9 to 0·9)	1·3 (1·3 to 1·3)	1·8 (1·7 to 1·8)	2·3 (2·2 to 2·4)	2·8 (2·5 to 3)	3·7 (3·3 to 4·2)	3·9 (3·4 to 4·5)
Kajiado	1·2 (1·1 to 1·2)	1·7 (1·7 to 1·7)	2·5 (2·4 to 2·5)	4·2 (4·1 to 4·3)	6·9 (6·6 to 7·1)	10·9 (10·4 to 11·5)	17·6 (16·4 to 18·9)	23·4 (20·9 to 25·9)	24 (21·4 to 26·9)
Kakamega	7·7 (7·6 to 7·8)	12·2 (12 to 12·4)	16·2 (15·9 to 16·5)	21·5 (21·1 to 21·9)	27·2 (26·3 to 28·2)	21 (19·9 to 22·2)	30·5 (28·2 to 32·7)	23·8 (21·2 to 26·5)	22·3 (19·7 to 25·3)
Kericho	2·7 (2·7 to 2·8)	5·6 (5·5 to 5·7)	8·6 (8·5 to 8·8)	11·2 (11 to 11·4)	14·6 (14·1 to 15·1)	17·3 (16·5 to 18·2)	12·5 (11·6 to 13·4)	15·4 (13·8 to 17·1)	16 (14·3 to 18)
Kiambu	5·4 (5·2 to 5·6)	7·7 (7·4 to 7·9)	11·6 (11·3 to 11·9)	17 (16·4 to 17·6)	19·2 (18 to 20·3)	26·4 (24·5 to 28·3)	26·2 (23·5 to 28·9)	26·9 (23 to 31·3)	26·5 (22·6 to 31·3)
Kilifi	5·1 (5 to 5·3)	7·7 (7·4 to 7·9)	9·7 (9·4 to 10)	13·8 (13·4 to 14·2)	16·4 (15·6 to 17·1)	21 (19·8 to 22·2)	24·7 (22·7 to 26·6)	24·2 (21·3 to 27·4)	23·3 (20·4 to 26·8)
Kirinyaga	3·1 (3 to 3·2)	4·7 (4·6 to 4·8)	5·9 (5·8 to 6·1)	6·5 (6·3 to 6·8)	6·5 (6·1 to 6·9)	7·2 (6·6 to 7·9)	7 (6·3 to 7·7)	4·9 (4·2 to 5·8)	4·6 (3·9 to 5·4)
Kisii	5·5 (5·3 to 5·6)	10 (9·9 to 10·2)	13·8 (13·6 to 14·1)	18·7 (18·3 to 19·1)	21·8 (21 to 22·6)	26·1 (24·8 to 27·4)	22·2 (20·3 to 24·1)	15·8 (14 to 17·7)	14·3 (12·6 to 16·2)
Kisumu	7·2 (7 to 7·3)	10·6 (10·4 to 10·8)	12·5 (12·3 to 12·8)	13·8 (13·5 to 14·1)	17·4 (16·7 to 18)	17·8 (16·6 to 18·9)	17·9 (16·1 to 19·5)	15·7 (13·8 to 17·5)	14·9 (13·2 to 16·7)
Kitui	6·1 (5·9 to 6·3)	8·7 (8·4 to 8·9)	10·6 (10·4 to 10·8)	13 (12·7 to 13·3)	16 (15·3 to 16·7)	18 (17 to 19)	17·8 (16·2 to 19·1)	15 (13·4 to 16·8)	14·3 (12·7 to 16·2)
Kwale	3·4 (3·3 to 3·5)	5·6 (5·4 to 5·7)	7 (6·8 to 7·2)	8·5 (8·2 to 8·8)	9·6 (9·1 to 10·1)	11·8 (11·1 to 12·6)	14 (12·8 to 15·1)	14·3 (12·7 to 16)	14 (12·4 to 16)
Laikipia	0·7 (0·7 to 0·7)	1 (1 to 1·1)	1·8 (1·7 to 1·8)	3·2 (3·1 to 3·3)	4·5 (4·2 to 4·7)	6·8 (6·4 to 7·2)	6·4 (5·8 to 7)	5·2 (4·5 to 6)	5 (4·3 to 5·8)
Lamu	0·4 (0·4 to 0·4)	0·5 (0·5 to 0·5)	0·7 (0·6 to 0·7)	1 (1 to 1·1)	1·3 (1·2 to 1·4)	1·5 (1·3 to 1·6)	1·6 (1·5 to 1·7)	1·5 (1·3 to 1·7)	1·4 (1·2 to 1·7)
Machakos	4·4 (4·3 to 4·6)	7·5 (7·3 to 7·7)	11·2 (10·9 to 11·4)	16·4 (15·9 to 17)	19·3 (18·4 to 20·3)	20 (18·8 to 21·1)	15·8 (14·2 to 17·3)	14·2 (12·4 to 16·2)	13·5 (11·7 to 15·8)
Makueni	5·1 (4·9 to 5·2)	6·1 (6 to 6·2)	7·3 (7·1 to 7·5)	8·9 (8·6 to 9·1)	10·1 (9·7 to 10·5)	11 (10·3 to 11·7)	13 (11·7 to 14·3)	9·3 (8·1 to 10·7)	8·4 (7·2 to 9·9)
Mandera	1·4 (1·3 to 1·4)	2·1 (2·1 to 2·2)	2·7 (2·6 to 2·8)	2·9 (2·8 to 3)	3·5 (3·4 to 3·6)	7·9 (7·7 to 8·1)	25·4 (24·5 to 26·2)	22·7 (21·9 to 23·4)	21·3 (20·6 to 21·9)
Marsabit	0·6 (0·6 to 0·6)	0·9 (0·8 to 0·9)	1·5 (1·4 to 1·5)	2·5 (2·4 to 2·6)	3 (2·9 to 3·1)	3·9 (3·7 to 4·1)	5·6 (5·2 to 6)	7·3 (6·6 to 8·1)	7·5 (6·7 to 8·4)
Meru	7·1 (6·9 to 7·3)	11·5 (11·3 to 11·8)	15·4 (15 to 15·7)	20·1 (19·6 to 20·6)	24·8 (23·8 to 25·7)	29·3 (27·7 to 30·9)	28·4 (26 to 30·8)	19·3 (17·1 to 21·7)	17·3 (15·2 to 19·8)
Migori	5·5 (5·3 to 5·6)	7·6 (7·3 to 7·8)	9 (8·7 to 9·2)	11·2 (10·9 to 11·4)	14·8 (14·4 to 15·2)	19·4 (18·7 to 20·2)	21·9 (20·9 to 23)	20·6 (18·9 to 22·4)	19·9 (18·2 to 21·8)
Mombasa	2·7 (2·5 to 2·8)	5 (4·8 to 5·2)	6·5 (6·2 to 6·7)	7·2 (6·8 to 7·5)	8·4 (8 to 8·9)	10·7 (9·7 to 11·6)	12·9 (11·4 to 14·4)	9·6 (8·1 to 11·4)	8·7 (7·3 to 10·6)
Murang'a	7·2 (7 to 7·4)	9·7 (9·4 to 9·9)	11·9 (11·7 to 12·1)	14·8 (14·5 to 15·2)	17 (16 to 17·8)	12·7 (11·9 to 13·6)	11·7 (10·6 to 12·9)	8·2 (7·3 to 9·3)	7·7 (6·8 to 8·7)
Nairobi	4·8 (4·6 to 4·9)	8·2 (7·9 to 8·4)	13·5 (12·9 to 14·1)	26·4 (24·6 to 28·2)	22·6 (20·7 to 24·4)	32·8 (29·5 to 36·1)	42·2 (36·4 to 48·4)	34 (29·1 to 39·9)	31·8 (27 to 38)
Nakuru	2·8 (2·8 to 2·9)	4·8 (4·7 to 5)	7·9 (7·8 to 8·1)	12·8 (12·5 to 13·2)	18·2 (17·3 to 19·1)	22·1 (20·6 to 23·6)	21·1 (18·8 to 23·5)	18·4 (15·8 to 21·4)	17·8 (15·2 to 21·1)
Nandi	2·9 (2·8 to 2·9)	4·4 (4·3 to 4·5)	6·2 (6·1 to 6·3)	7·8 (7·7 to 8)	10·6 (10·2 to 11)	13 (12·3 to 13·6)	13·6 (12·4 to 14·8)	12·2 (10·8 to 13·6)	11·7 (10·3 to 13·3)
Narok	2 (1·9 to 2)	2·8 (2·8 to 2·9)	3·8 (3·7 to 3·9)	6·4 (6·3 to 6·5)	10·9 (10·6 to 11·2)	9·9 (9·5 to 10·3)	20·2 (18·9 to 21·5)	21·5 (19·6 to 23·3)	21 (19·2 to 23)
Nyamira	3·1 (3 to 3·2)	4 (3·9 to 4·1)	4·7 (4·6 to 4·8)	5·4 (5·2 to 5·5)	5·8 (5·6 to 6·1)	7 (6·6 to 7·5)	8·5 (7·7 to 9·2)	6·7 (5·8 to 7·7)	6·3 (5·4 to 7·3)
Nyandarua	2 (1·9 to 2)	3·2 (3·1 to 3·3)	4·3 (4·3 to 4·4)	5·3 (5·1 to 5·4)	7·3 (7 to 7·7)	8·5 (7·9 to 9·2)	6·9 (6·2 to 7·7)	4·3 (3·7 to 5)	3·9 (3·3 to 4·7)
Nyeri	4·9 (4·7 to 5·1)	5·9 (5·7 to 6·1)	7·4 (7·2 to 7·7)	9·9 (9·5 to 10·4)	10·5 (9·8 to 11·2)	9·2 (8·3 to 10)	6·9 (6 to 7·8)	4·6 (3·9 to 5·5)	4·2 (3·6 to 5·1)
Samburu	0·8 (0·8 to 0·9)	1·6 (1·5 to 1·6)	2 (1·9 to 2·1)	2·2 (2·1 to 2·2)	3·1 (3 to 3·2)	3·7 (3·5 to 3·9)	4·9 (4·6 to 5·2)	5·8 (5·3 to 6·3)	5·9 (5·4 to 6·5)
Siaya	5·5 (5·3 to 5·6)	8·7 (8·5 to 8·9)	10·6 (10·4 to 10·8)	13 (12·7 to 13·2)	15·8 (15·4 to 16·2)	17·2 (16·4 to 17·9)	17 (15·9 to 18·2)	15·4 (14 to 16·7)	14·9 (13·5 to 16·4)
Taita Taveta	1·5 (1·5 to 1·6)	2·4 (2·3 to 2·4)	2·9 (2·8 to 3)	3·5 (3·3 to 3·6)	4·5 (4·2 to 4·8)	4·6 (4·2 to 5)	4·3 (3·8 to 4·7)	3·4 (2·9 to 4)	3·3 (2·8 to 3·9)
Tana River	0·8 (0·8 to 0·9)	1·2 (1·1 to 1·2)	1·6 (1·5 to 1·6)	2·7 (2·6 to 2·8)	3·1 (3 to 3·3)	4·5 (4·2 to 4·8)	5·3 (5 to 5·7)	5·9 (5·3 to 6·5)	6 (5·4 to 6·6)
Tharaka Nithi	1·8 (1·7 to 1·8)	1·9 (1·9 to 1·9)	1·9 (1·9 to 2)	1·9 (1·9 to 2)	2·1 (2 to 2·2)	2·1 (1·9 to 2·2)	2·2 (2 to 2·4)	3·8 (3·2 to 4·3)	4·2 (3·5 to 4·9)
Trans Nzoia	1 (1 to 1)	1·7 (1·7 to 1·8)	3·4 (3·3 to 3·4)	7·1 (7 to 7·2)	9·7 (9·4 to 10)	12·8 (12·1 to 13·5)	14·1 (12·8 to 15·3)	11·5 (10·1 to 13)	10·8 (9·5 to 12·4)
Turkana	2 (1·9 to 2·1)	3·9 (3·8 to 4)	5·1 (5 to 5·3)	4·5 (4·4 to 4·6)	5·8 (5·6 to 6)	11·5 (10·9 to 12)	19 (17·9 to 20·1)	17·6 (15·8 to 19·3)	16·7 (14·9 to 18·7)
Uasin Gishu	1·9 (1·8 to 1·9)	3·3 (3·3 to 3·4)	5·5 (5·4 to 5·5)	8·2 (8 to 8·3)	11·2 (10·8 to 11·6)	15·1 (14·3 to 15·8)	18·4 (16·7 to 20)	17·9 (15·8 to 20·1)	17·3 (15·2 to 19·8)
Vihiga	5·6 (5·4 to 5·7)	6·7 (6·6 to 6·9)	7 (6·8 to 7·2)	6·8 (6·6 to 7)	8·2 (7·9 to 8·6)	7·1 (6·5 to 7·7)	7·6 (6·8 to 8·4)	4·7 (4 to 5·5)	4·3 (3·6 to 5·2)

Wajir	1·2 (1·2 to 1·3)	1·6 (1·6 to 1·7)	2·5 (2·5 to 2·6)	3·8 (3·7 to 3·9)	3·4 (3·3 to 3·5)	9·6 (9·3 to 9·8)	15·7 (15·1 to 16·2)	19·7 (18·8 to 20·5)	20·4 (19·6 to 21·3)
West Pokot	1·2 (1·2 to 1·3)	1·7 (1·7 to 1·7)	2·6 (2·5 to 2·6)	4·6 (4·5 to 4·7)	5·9 (5·7 to 6·1)	7·6 (7·2 to 7·9)	9·5 (8·8 to 10·2)	8·5 (7·6 to 9·5)	8·2 (7·3 to 9·1)
Madagascar	118 (114 to 123)	144 (140 to 148)	177 (172 to 182)	219 (214 to 225)	270 (264 to 277)	354 (344 to 365)	439 (423 to 456)	458 (429 to 489)	450 (418 to 485)
Malawi	66·6 (62 to 71)	92 (87·2 to 96·7)	120 (115 to 125)	178 (173 to 182)	259 (253 to 265)	283 (276 to 289)	306 (294 to 317)	294 (268 to 321)	292 (260 to 326)
Mozambique	172 (165 to 178)	194 (186 to 203)	234 (226 to 242)	304 (296 to 312)	317 (309 to 325)	419 (409 to 429)	496 (482 to 510)	552 (515 to 590)	562 (516 to 612)
Rwanda	70·4 (67·9 to 72·7)	83·4 (81·4 to 85·5)	102 (100 to 104)	136 (134 to 138)	162 (158 to 166)	177 (174 to 181)	182 (175 to 190)	188 (176 to 201)	191 (176 to 207)
Somalia	57·4 (55·3 to 59·2)	74·8 (73 to 76·5)	102 (99·8 to 105)	174 (170 to 178)	204 (198 to 209)	263 (252 to 273)	372 (360 to 383)	467 (449 to 487)	498 (477 to 521)
South Sudan	57·6 (53·7 to 61·5)	72·6 (68·7 to 76·6)	91·6 (86·5 to 96·2)	110 (104 to 116)	135 (127 to 141)	171 (160 to 181)	214 (201 to 227)	185 (172 to 198)	199 (183 to 214)
Uganda	172 (167 to 177)	219 (214 to 224)	280 (274 to 285)	358 (351 to 365)	477 (470 to 484)	643 (631 to 653)	760 (738 to 782)	810 (766 to 856)	813 (761 to 870)
Tanzania	246 (237 to 255)	312 (302 to 321)	381 (371 to 392)	482 (466 to 496)	621 (604 to 639)	788 (764 to 810)	922 (874 to 965)	967 (899 to 1040)	965 (894 to 1040)
Zambia	67·5 (65 to 69·8)	103 (100 to 105)	130 (127 to 132)	153 (149 to 156)	198 (192 to 204)	239 (230 to 247)	302 (290 to 314)	313 (288 to 339)	307 (280 to 338)
Southern sub-Saharan Africa	407 (380 to 434)	492 (466 to 518)	620 (591 to 647)	775 (749 to 803)	832 (808 to 857)	840 (816 to 865)	924 (897 to 950)	857 (812 to 908)	832 (772 to 897)
Botswana	10·1 (9·5 to 10·6)	12·7 (12·2 to 13·2)	16·1 (15·4 to 16·8)	20·9 (19·9 to 21·9)	23·9 (22·3 to 25·5)	24·6 (22·5 to 26·7)	24·8 (22·6 to 27·3)	25 (22·1 to 27·9)	24·8 (22 to 28·1)
Eswatini	6·4 (6·1 to 6·7)	8·7 (8·4 to 9)	11·3 (11 to 11·6)	14·1 (13·7 to 14·5)	16·6 (16 to 17·1)	18·4 (17·8 to 18·9)	17·7 (16·9 to 18·4)	15·5 (14·4 to 16·6)	15 (13·7 to 16·3)
Lesotho	15·5 (14·6 to 16·5)	18·8 (17·7 to 19·8)	23·3 (22·5 to 24·1)	28·6 (27·8 to 29·5)	27·9 (27 to 28·8)	26·5 (25·7 to 27·2)	24·9 (23·7 to 26)	22·2 (19·9 to 24·6)	21·7 (19·3 to 24·5)
Namibia	11·4 (10·8 to 11·9)	14·5 (14 to 15)	17·4 (16·9 to 17·9)	20·7 (20 to 21·4)	26·1 (25·4 to 26·9)	28·9 (27·8 to 29·9)	31·9 (30·6 to 33·3)	29·7 (27·6 to 32·2)	29·4 (27 to 32·3)
South Africa	294 (272 to 315)	346 (325 to 368)	426 (402 to 448)	514 (492 to 539)	536 (517 to 556)	524 (505 to 545)	581 (560 to 602)	525 (495 to 559)	503 (465 to 542)
Eastern Cape	79·2 (73·7 to 84·6)	77·8 (73 to 82·7)	73·8 (69·9 to 77·7)	88·7 (84·6 to 93·4)	91·1 (87·7 to 94·7)	76·5 (73·2 to 79·9)	73 (70·1 to 75·9)	63·1 (59·7 to 66·8)	60·2 (55·9 to 64·6)
Free State	22·8 (21·1 to 24·6)	28·4 (26·5 to 30·3)	32 (30·3 to 33·7)	36 (34·4 to 37·9)	31·9 (30·7 to 33·1)	29·3 (28·1 to 30·7)	29·2 (27·9 to 30·4)	23·3 (21·9 to 24·9)	21·9 (20·2 to 23·6)
Gauteng	34·3 (30·7 to 38)	48·2 (44·3 to 52·3)	63·6 (59·1 to 68·2)	72·9 (69 to 77)	80·5 (77·2 to 84·2)	99 (94·7 to 103)	138 (132 to 142)	133 (124 to 142)	126 (117 to 137)
KwaZulu-Natal	60·3 (56·3 to 64·3)	73·6 (69·4 to 77·7)	97·7 (92·7 to 103)	115 (110 to 120)	119 (114 to 124)	114 (110 to 119)	118 (114 to 123)	105 (98·1 to 112)	101 (92·8 to 108)
Limpopo	28·6 (27 to 30·2)	34·8 (33·1 to 36·4)	57·2 (54·7 to 59·5)	79·1 (76·1 to 82·4)	80·1 (77·1 to 83·1)	66·2 (63·6 to 69)	65·6 (63 to 68·4)	59·8 (56·6 to 63·4)	58 (54 to 62·2)
Mpumalanga	22·8 (21·5 to 23·9)	25·9 (24·6 to 27·1)	27 (25·8 to 28·1)	34·3 (32·7 to 35·8)	39·4 (37·8 to 41·1)	39·4 (37·5 to 41·3)	47·7 (45·9 to 49·6)	48·3 (45·3 to 51·6)	47·7 (44·3 to 51·6)
North West	17·5 (16·4 to 18·7)	23·2 (21·8 to 24·5)	31·4 (29·7 to 33·1)	38·8 (37·1 to 40·5)	42·3 (40·7 to 43·9)	43 (41·3 to 44·8)	40 (38·5 to 41·4)	28·9 (27·2 to 30·9)	26·8 (24·8 to 28·9)
Northern Cape	8·7 (8 to 9·4)	10·1 (9·4 to 10·8)	11·2 (10·6 to 11·9)	12·3 (11·7 to 12·8)	10·8 (10·4 to 11·2)	8·9 (8·5 to 9·2)	11·8 (11·4 to 12·4)	12·4 (11·6 to 13·3)	12·3 (11·3 to 13·4)
Western Cape	19·3 (17·1 to 21·5)	24·2 (22·1 to 26·3)	31·6 (29·1 to 34)	37·3 (35·3 to 39·4)	41·3 (39·6 to 42·9)	48·2 (46·1 to 50·2)	57·4 (55·3 to 59·4)	51·6 (48·2 to 55·3)	48·9 (45 to 52·9)
Zimbabwe	70·1 (67 to 73·1)	91·3 (88·3 to 94·4)	126 (122 to 130)	176 (173 to 180)	202 (198 to 206)	217 (213 to 222)	244 (239 to 249)	240 (226 to 255)	239 (222 to 257)
Western sub-Saharan Africa	1890 (1800 to 1970)	2340 (2260 to 2410)	2910 (2820 to 2980)	3690 (3600 to 3780)	4640 (4540 to 4740)	6030 (5880 to 6180)	7830 (7610 to 8030)	8960 (8560 to 9410)	9140 (8620 to 9680)
Benin	47·4 (44·4 to 50·2)	55·2 (52·9 to 57·3)	67·9 (65·7 to 70)	87·8 (85·4 to 90·1)	121 (118 to 124)	159 (155 to 162)	213 (206 to 220)	259 (243 to 274)	268 (251 to 287)
Burkina Faso	96·7 (91·7 to 102)	122 (118 to 127)	156 (152 to 160)	194 (190 to 198)	236 (230 to 241)	304 (296 to 313)	391 (377 to 405)	467 (442 to 492)	486 (456 to 516)
Cabo Verde	3·6 (3·3 to 3·8)	4·7 (4·6 to 4·8)	4·9 (4·7 to 5)	5·6 (5·4 to 5·8)	6·4 (6·2 to 6·7)	6·5 (6·1 to 7)	5·9 (5·2 to 6·7)	4·6 (4·1 to 5·1)	4·3 (3·8 to 5)
Cameroon	110 (104 to 117)	140 (133 to 148)	171 (167 to 176)	202 (198 to 206)	245 (239 to 250)	325 (317 to 332)	465 (447 to 482)	526 (486 to 568)	530 (482 to 583)
Chad	65·8 (62·9 to 68·4)	76·1 (73·9 to 78·1)	95·2 (92·9 to 97·4)	125 (122 to 128)	166 (162 to 169)	226 (221 to 230)	309 (301 to 317)	413 (399 to 427)	442 (426 to 458)
Côte d'Ivoire	65·7 (62·2 to 69)	107 (103 to 111)	151 (147 to 155)	208 (203 to 213)	284 (278 to 290)	353 (343 to 363)	435 (419 to 450)	483 (451 to 515)	486 (450 to 526)
The Gambia	5·5 (5·1 to 5·9)	7 (6·7 to 7·4)	11·6 (11·2 to 11·9)	17·5 (17·1 to 17·9)	23·9 (23·2 to 24·4)	31·6 (30·7 to 32·3)	37·9 (37 to 38·8)	39·4 (37·3 to 41·6)	39·9 (37·5 to 42·6)
Ghana	104 (95·7 to 114)	171 (163 to 178)	230 (222 to 237)	283 (273 to 291)	311 (296 to 326)	344 (324 to 363)	438 (408 to 466)	496 (448 to 546)	496 (439 to 556)
Guinea	65·7 (63·1 to 68·2)	81·6 (78·7 to 84·4)	105 (101 to 108)	126 (123 to 130)	149 (145 to 152)	183 (178 to 189)	217 (210 to 224)	248 (234 to 262)	255 (238 to 272)
Guinea-Bissau	16·1 (15·5 to 16·7)	14·8 (14·1 to 15·4)	14 (13·2 to 14·8)	19·1 (18·2 to 20)	23·5 (22·2 to 24·7)	27·7 (25·9 to 29·5)	33·9 (31·7 to 36·3)	36·8 (33·9 to 39·6)	36·9 (33·9 to 40·2)
Liberia	22·8 (21·5 to 24)	29·5 (28 to 30·8)	37·8 (36·7 to 38·9)	51·9 (50·5 to 53·1)	61·9 (60·4 to 63·4)	67·5 (65·6 to 69·3)	84 (80·9 to 87·1)	84·9 (77·1 to 93·1)	84·3 (75·1 to 94·3)
Mali	94 (89·8 to 97·9)	126 (122 to 130)	164 (160 to 169)	201 (195 to 205)	233 (229 to 237)	286 (280 to 292)	397 (385 to 408)	516 (491 to 541)	546 (516 to 576)
Mauritania	17·3 (16·3 to 18·3)	22·1 (21·1 to 23·1)	28·4 (27·5 to 29·3)	36·7 (35·7 to 37·7)	45·2 (43·6 to 46·8)	53·8 (51·5 to 55·9)	67·1 (64·4 to 69·7)	70 (64·2 to 75·9)	69·3 (62·8 to 76·6)
Niger	74·2 (71·6 to 76·6)	101 (98·1 to 104)	137 (134 to 140)	179 (176 to 182)	228 (223 to 232)	320 (313 to 326)	418 (405 to 429)	559 (537 to 581)	601 (577 to 627)
Nigeria	945 (898 to 989)	1080 (1040 to 1120)	1290 (1240 to 1330)	1650 (1590 to 1710)	2140 (2080 to 2200)	2930 (2830 to 3030)	3800 (3680 to 3930)	4230 (3960 to 4490)	4260 (3930 to 4590)
São Tomé and Príncipe	1·2 (1·2 to 1·3)	1·6 (1·5 to 1·6)	1·6 (1·6 to 1·7)	1·9 (1·8 to 2)	2·4 (2·3 to 2·5)	2·9 (2·7 to 3·1)	3·1 (3 to 3·3)	2·6 (2·4 to 2·9)	2·5 (2·2 to 2·9)
Senegal	69·7 (66·3 to 72·8)	93·1 (90·5 to 95·7)	124 (121 to 127)	156 (153 to 158)	178 (173 to 183)	212 (206 to 216)	253 (246 to 259)	249 (234 to 265)	246 (226 to 267)
Sierra Leone	46·4 (43·6 to 49)	58·3 (55·7 to 60·7)	66·5 (63·8 to 69·3)	81·4 (78·2 to 84·2)	105 (102 to 108)	104 (102 to 107)	133 (127 to 138)	152 (141 to 162)	155 (142 to 167)
Togo	38·6 (37 to 40)	45·6 (44·4 to 46·8)	51·6 (50·2 to 52·8)	65·3 (63·8 to 66·7)	81·6 (79·6 to 83·6)	101 (98 to 104)	125 (120 to 130)	127 (119 to 135)	124 (115 to 134)

Supplemental Results Table S2: Replacement Total Fertility Rate in 2021

Location	2021
Afghanistan	2·33 (2·27 to 2·39)
Albania	2·12 (2·08 to 2·18)
Algeria	2·13 (2·09 to 2·18)
American Samoa	2·13 (2·08 to 2·18)
Andorra	2·08 (2·04 to 2·13)
Angola	2·24 (2·18 to 2·31)
Antigua and Barbuda	2·08 (2·04 to 2·13)
Argentina	2·09 (2·05 to 2·13)
Armenia	2·14 (2·09 to 2·19)
Australia	2·07 (2·03 to 2·11)
Austria	2·07 (2·03 to 2·11)
Azerbaijan	2·22 (2·16 to 2·28)
Bahrain	2·08 (2·04 to 2·13)
Bangladesh	2·15 (2·11 to 2·2)
Barbados	2·09 (2·05 to 2·14)
Belarus	2·09 (2·04 to 2·13)
Belgium	2·06 (2·02 to 2·1)
Belize	2·08 (2·03 to 2·13)
Benin	2·29 (2·21 to 2·39)
Bermuda	2·08 (2·04 to 2·12)
Bhutan	2·13 (2·08 to 2·18)
Bolivia	2·14 (2·1 to 2·19)
Bosnia and Herzegovina	2·09 (2·05 to 2·14)
Botswana	2·21 (2·15 to 2·29)
Brazil	2·1 (2·06 to 2·15)
Brunei	2·12 (2·08 to 2·17)

Bulgaria	2·09 (2·05 to 2·13)
Burkina Faso	2·34 (2·26 to 2·44)
Burundi	2·25 (2·17 to 2·34)
Cabo Verde	2·09 (2·05 to 2·14)
Cambodia	2·14 (2·09 to 2·22)
Cameroon	2·28 (2·21 to 2·37)
Canada	2·07 (2·03 to 2·11)
Central African Republic	2·45 (2·34 to 2·62)
Chad	2·42 (2·31 to 2·55)
Chile	2·06 (2·02 to 2·1)
Colombia	2·1 (2·06 to 2·14)
Comoros	2·22 (2·16 to 2·29)
Congo (Brazzaville)	2·21 (2·15 to 2·28)
Cook Islands	2·11 (2·07 to 2·16)
Costa Rica	2·08 (2·04 to 2·12)
Croatia	2·08 (2·04 to 2·13)
Cuba	2·1 (2·06 to 2·14)
Cyprus	2·08 (2·04 to 2·12)
Czechia	2·07 (2·03 to 2·1)
Côte d'Ivoire	2·27 (2·2 to 2·35)
DR Congo	2·25 (2·19 to 2·33)
Denmark	2·07 (2·03 to 2·11)
Djibouti	2·24 (2·16 to 2·33)
Dominica	2·12 (2·07 to 2·17)
Dominican Republic	2·12 (2·07 to 2·17)
Ecuador	2·09 (2·05 to 2·14)
Egypt	2·1 (2·06 to 2·14)
El Salvador	2·12 (2·08 to 2·16)

England	2·07<br>(2·03 to 2·11)
Equatorial Guinea	2·26<br>(2·17 to 2·38)
Eritrea	2·24<br>(2·16 to 2·35)
Estonia	2·08<br>(2·04 to 2·12)
Eswatini	2·31<br>(2·21 to 2·41)
Ethiopia	2·23<br>(2·16 to 2·3)
Federated States of Micronesia	2·16<br>(2·1 to 2·23)
Fiji	2·16<br>(2·11 to 2·21)
Finland	2·07<br>(2·03 to 2·11)
France	2·06<br>(2·02 to 2·1)
Gabon	2·18<br>(2·11 to 2·27)
Georgia	2·11<br>(2·07 to 2·16)
Germany	2·07<br>(2·03 to 2·11)
Ghana	2·21<br>(2·16 to 2·27)
Greece	2·08<br>(2·04 to 2·12)
Greenland	2·14<br>(2·1 to 2·19)
Grenada	2·11<br>(2·06 to 2·16)
Guam	2·13<br>(2·08 to 2·18)
Guatemala	2·11<br>(2·07 to 2·16)
Guinea	2·34<br>(2·25 to 2·46)
Guinea-Bissau	2·3<br>(2·22 to 2·38)
Guyana	2·16<br>(2·12 to 2·21)
Haiti	2·3<br>(2·23 to 2·39)
Honduras	2·11<br>(2·06 to 2·16)
Hungary	2·08<br>(2·04 to 2·12)
Iceland	2·08<br>(2·04 to 2·12)
India	2·21<br>(2·16 to 2·28)
Indonesia	2·14<br>(2·09 to 2·19)

Iran	2·09<br>(2·05 to 2·14)
Iraq	2·13<br>(2·08 to 2·19)
Ireland	2·07<br>(2·03 to 2·11)
Israel	2·07<br>(2·03 to 2·11)
Italy	2·07<br>(2·03 to 2·11)
Jamaica	2·09<br>(2·05 to 2·14)
Japan	2·07<br>(2·03 to 2·1)
Jordan	2·1<br>(2·06 to 2·15)
Kazakhstan	2·11<br>(2·07 to 2·16)
Kenya	2·18<br>(2·12 to 2·24)
Kiribati	2·23<br>(2·16 to 2·3)
Kuwait	2·08<br>(2·04 to 2·12)
Kyrgyzstan	2·12<br>(2·07 to 2·16)
Laos	2·17<br>(2·12 to 2·23)
Latvia	2·09<br>(2·05 to 2·13)
Lebanon	2·11<br>(2·07 to 2·17)
Lesotho	2·41<br>(2·33 to 2·49)
Liberia	2·3<br>(2·21 to 2·43)
Libya	2·13<br>(2·08 to 2·2)
Lithuania	2·08<br>(2·04 to 2·12)
Luxembourg	2·07<br>(2·03 to 2·1)
Madagascar	2·25<br>(2·19 to 2·34)
Malawi	2·24<br>(2·16 to 2·33)
Malaysia	2·1<br>(2·06 to 2·15)
Maldives	2·09<br>(2·05 to 2·15)
Mali	2·36<br>(2·28 to 2·47)
Malta	2·09<br>(2·05 to 2·13)
Marshall Islands	2·18<br>(2·12 to 2·26)

Mauritania	2·17 (2·11 to 2·24)
Mauritius	2·08 (2·05 to 2·12)
Mexico	2·08 (2·04 to 2·12)
Moldova	2·1 (2·06 to 2·16)
Monaco	2·15 (2·11 to 2·2)
Mongolia	2·12 (2·07 to 2·17)
Montenegro	2·1 (2·06 to 2·15)
Morocco	2·11 (2·06 to 2·17)
Mozambique	2·32 (2·25 to 2·42)
Myanmar	2·18 (2·12 to 2·25)
Namibia	2·18 (2·12 to 2·26)
Nauru	2·22 (2·15 to 2·31)
Nepal	2·17 (2·12 to 2·22)
Netherlands	2·07 (2·03 to 2·11)
New Zealand	2·08 (2·04 to 2·12)
Nicaragua	2·1 (2·06 to 2·14)
Niger	2·33 (2·23 to 2·45)
Nigeria	2·33 (2·26 to 2·42)
Niue	2·31 (2·27 to 2·36)
North Korea	2·09 (2·05 to 2·14)
North Macedonia	2·1 (2·06 to 2·15)
Northern Ireland	2·08 (1·99 to 2·16)
Northern Mariana Islands	2·11 (2·07 to 2·16)
Norway	2·07 (2·03 to 2·11)
Oman	2·08 (2·04 to 2·13)
Pakistan	2·25 (2·19 to 2·33)
Palau	2·21 (2·16 to 2·27)
Palestine	2·09 (2·05 to 2·14)

Panama	2·11 (2·07 to 2·15)
Papua New Guinea	2·29 (2·23 to 2·36)
Paraguay	2·11 (2·07 to 2·16)
Peru	2·11 (2·06 to 2·16)
Philippines	2·17 (2·12 to 2·21)
Poland	2·08 (2·04 to 2·12)
Portugal	2·06 (2·02 to 2·1)
Puerto Rico	2·08 (2·04 to 2·12)
Qatar	2·06 (2·02 to 2·11)
Romania	2·08 (2·04 to 2·12)
Russia	2·09 (2·05 to 2·14)
Rwanda	2·2 (2·14 to 2·25)
Saint Kitts and Nevis	2·09 (2·05 to 2·14)
Saint Lucia	2·1 (2·06 to 2·15)
Saint Vincent and the Grenadines	2·1 (2·06 to 2·15)
Samoa	2·17 (2·12 to 2·23)
San Marino	2·11 (2·06 to 2·16)
Saudi Arabia	2·1 (2·06 to 2·15)
Scotland	2·08 (2 to 2·17)
Senegal	2·19 (2·14 to 2·26)
Serbia	2·09 (2·05 to 2·13)
Seychelles	2·1 (2·06 to 2·15)
Sierra Leone	2·36 (2·28 to 2·46)
Singapore	2·08 (2·04 to 2·12)
Slovakia	2·08 (2·04 to 2·12)
Slovenia	2·08 (2·04 to 2·11)
Solomon Islands	2·19 (2·13 to 2·26)
Somalia	2·42 (2·32 to 2·54)

South Africa	2·2<br>(2·15 to 2·27)
South Korea	2·07<br>(2·03 to 2·11)
South Sudan	2·49<br>(2·36 to 2·65)
Spain	2·07<br>(2·03 to 2·11)
Sri Lanka	2·08<br>(2·04 to 2·13)
Sudan	2·18<br>(2·13 to 2·25)
Suriname	2·14<br>(2·1 to 2·19)
Sweden	2·07<br>(2·03 to 2·11)
Switzerland	2·07<br>(2·03 to 2·11)
Syria	2·11<br>(2·06 to 2·16)
São Tomé and Príncipe	2·13<br>(2·08 to 2·18)
Taiwan (province of China)	2·09<br>(2·05 to 2·13)
Tajikistan	2·17<br>(2·11 to 2·23)
Tanzania	2·21<br>(2·15 to 2·29)
Thailand	2·1<br>(2·06 to 2·14)
The Bahamas	2·1<br>(2·06 to 2·16)
The Gambia	2·23<br>(2·16 to 2·3)
Timor-Leste	2·19<br>(2·14 to 2·26)
Togo	2·23<br>(2·16 to 2·33)
Tokelau	2·35<br>(2·31 to 2·4)
Tonga	2·16<br>(2·11 to 2·21)
Trinidad and Tobago	2·1<br>(2·06 to 2·15)
Tunisia	2·13<br>(2·08 to 2·18)
Turkmenistan	2·15<br>(2·1 to 2·2)
Tuvalu	2·19<br>(2·13 to 2·25)
Türkiye	2·1<br>(2·06 to 2·14)
UK	2·07<br>(2·03 to 2·11)
USA	2·08<br>(2·04 to 2·12)

Uganda	2·26<br>(2·18 to 2·35)
Ukraine	2·1<br>(2·06 to 2·14)
United Arab Emirates	2·08<br>(2·04 to 2·12)
Uruguay	2·09<br>(2·05 to 2·13)
Uzbekistan	2·15<br>(2·1 to 2·21)
Vanuatu	2·16<br>(2·1 to 2·22)
Venezuela	2·13<br>(2·08 to 2·17)
Viet Nam	2·11<br>(2·07 to 2·17)
Virgin Islands	2·08<br>(2·04 to 2·13)
Wales	2·07<br>(2·03 to 2·11)
Yemen	2·21<br>(2·15 to 2·28)
Zambia	2·23<br>(2·16 to 2·32)
Zimbabwe	2·26<br>(2·19 to 2·35)

Supplemental Results Table S3: Total Fertility Rate in 2021 with and without Ages 10-14 and 50-54

Location	TFR	TFR without ages 10-14 and 50-54	Difference	Percent Difference
Global	2·23 (2·09 to 2·38)	2·22 (2·08 to 2·37)	-0·01 (-0·01 to 0)	-0·26 (-0·43 to -0·16)
Central Asia	2·79 (2·68 to 2·91)	2·79 (2·68 to 2·91)	0 (0 to 0)	-0·02 (-0·03 to -0·02)
Central Europe	1·48 (1·36 to 1·61)	1·48 (1·36 to 1·61)	0 (0 to 0)	-0·08 (-0·13 to -0·05)
Eastern Europe	1·38 (1·27 to 1·49)	1·38 (1·27 to 1·49)	0 (0 to 0)	-0·03 (-0·05 to -0·02)
Australasia	1·64 (1·48 to 1·8)	1·64 (1·48 to 1·8)	0 (0 to 0)	-0·06 (-0·09 to -0·05)
High-income Asia Pacific	1·12 (1·03 to 1·22)	1·12 (1·03 to 1·22)	0 (0 to 0)	-0·02 (-0·02 to -0·01)
High-income North America	1·63 (1·53 to 1·73)	1·63 (1·53 to 1·73)	0 (0 to 0)	-0·13 (-0·22 to -0·07)
Southern Latin America	1·49 (1·32 to 1·67)	1·48 (1·32 to 1·66)	-0·01 (-0·01 to 0)	-0·35 (-0·65 to -0·19)
Western Europe	1·53 (1·44 to 1·63)	1·53 (1·44 to 1·63)	0 (0 to 0)	-0·05 (-0·06 to -0·04)
Andean Latin America	2·32 (2·14 to 2·51)	2·31 (2·13 to 2·5)	0 (-0·01 to 0)	-0·21 (-0·37 to -0·12)
Caribbean	2·19 (2·02 to 2·39)	2·18 (2·01 to 2·38)	-0·01 (-0·01 to 0)	-0·29 (-0·52 to -0·16)
Central Latin America	1·87 (1·68 to 2·08)	1·87 (1·68 to 2·08)	-0·01 (-0·01 to 0)	-0·36 (-0·67 to -0·18)
Tropical Latin America	1·94 (1·79 to 2·12)	1·93 (1·78 to 2·11)	-0·01 (-0·02 to 0)	-0·44 (-0·8 to -0·22)
North Africa and Middle East	2·53 (2·33 to 2·76)	2·53 (2·33 to 2·75)	0 (-0·01 to 0)	-0·14 (-0·21 to -0·1)
South Asia	2·07 (1·89 to 2·28)	2·07 (1·88 to 2·27)	0 (-0·01 to 0)	-0·2 (-0·3 to -0·14)
East Asia	1·23 (1·12 to 1·34)	1·23 (1·12 to 1·34)	0 (0 to 0)	-0·09 (-0·13 to -0·07)
Oceania	4·02 (3·68 to 4·41)	4·01 (3·66 to 4·4)	-0·01 (-0·01 to -0·01)	-0·27 (-0·37 to -0·22)
Southeast Asia	2·05 (1·89 to 2·23)	2·05 (1·89 to 2·23)	0 (0 to 0)	-0·1 (-0·15 to -0·07)
Central sub-Saharan Africa	4·44 (4·15 to 4·72)	4·42 (4·14 to 4·7)	-0·02 (-0·03 to -0·01)	-0·39 (-0·6 to -0·27)
Eastern sub-Saharan Africa	4·09 (3·8 to 4·39)	4·07 (3·78 to 4·38)	-0·02 (-0·03 to -0·01)	-0·49 (-0·77 to -0·32)
Southern sub-Saharan Africa	2·42 (2·25 to 2·61)	2·42 (2·24 to 2·6)	-0·01 (-0·01 to 0)	-0·23 (-0·39 to -0·14)
Western sub-Saharan Africa	4·79 (4·51 to 5·09)	4·77 (4·48 to 5·07)	-0·03 (-0·04 to -0·02)	-0·56 (-0·83 to -0·41)
